# Chinook salmon and green sturgeon migrate through San Francisco Estuary despite large distortions in the local magnetic field produced by bridges

**DOI:** 10.1371/journal.pone.0169031

**Published:** 2017-06-02

**Authors:** A. Peter Klimley, Megan T. Wyman, Robert Kavet

**Affiliations:** 1 Biotelemetry Laboratory, Department of Fish, Wildlife, & Conservation Biology, University of California, Davis, Davis, California, United States of America; 2 Environment Sector, Electric Power Research Institute, Palo Alto, California, United States of America; Virginia Commonwealth University, UNITED STATES

## Abstract

Empirical evidence exists that some marine animals perceive and orient to local distortions in the earth’s main static geomagnetic field. The magnetic fields produced by undersea electric power cables that carry electricity from hydrokinetic energy sources to shore-based power stations may produce similar local distortions in the earth’s main field. Concerns exist that animals migrating along the continental shelves might orient to the magnetic field from the cables, and move either inshore or offshore away from their normal path. We have studied the effect of the Trans Bay Cable (TBC), an 85-km long, high voltage, direct current (DC) transmission line leading underwater from Pittsburg, CA to San Francisco, CA, on fishes migrating through the San Francisco Estuary. These included Chinook salmon smolts (*Oncorhynchus tshawytscha*) that migrate downstream through the San Francisco Estuary to the Pacific Ocean and adult green sturgeon (*Acipenser medirostris*), which migrate upstream from the ocean through the estuary to their spawning habitat in the upper Sacramento River and return to the ocean after spawning occurs. Based on a detailed gradiometer survey, we found that the distortions in the earth’s main field produced by bridges across the estuary were much greater than those from the Trans Bay Cable. The former anomalies exceeded the latter by an order of magnitude or more. Significant numbers of tagged Chinook salmon smolts migrated past bridges, which produced strong magnetic anomalies, to the Golden Gate Bridge, where they were recorded by dual arrays of acoustic tag-detecting monitors moored in lines across the mouth of the bay. In addition, adult green sturgeon successfully swam upstream and downstream through the estuary on the way to and from their spawning grounds. Hence, the large anomalies produced by the bridges do not appear to present a strong barrier to the natural seasonal movement patterns of salmonid smolts and adult green sturgeon.

## Introduction

The earth’s magnetic field has two components, the dipolar main field, produced by the convective movements of molten iron in the earth’s core, and distortions in this field from magnetic particles embedded in the earth’s outer crust [1]. The latter are referred to as magnetic anomalies in the earth’s field. Observational evidence exists that some marine animals perceive and orient to local distortions in the earth’s main geomagnetic field. For example, scalloped hammerhead sharks have been shown to use these magnetic gradients to guide their diurnal migrations from a seamount to and from nighttime feeding grounds [2]. The paths coincide with magnetic maxima (“ridges”) and minima (“valleys”) leading away from the seamount. Evidence exists that baleen whales strand where rotation of oceanic plates results in weakly magnetized sections of crust intersecting the western coastline of Great Britain [3] and eastern coastline of North America [4]. While suggestive, the conclusions that can be drawn from such correlational studies are limited without experimental confirmation.

**Fig 1:**
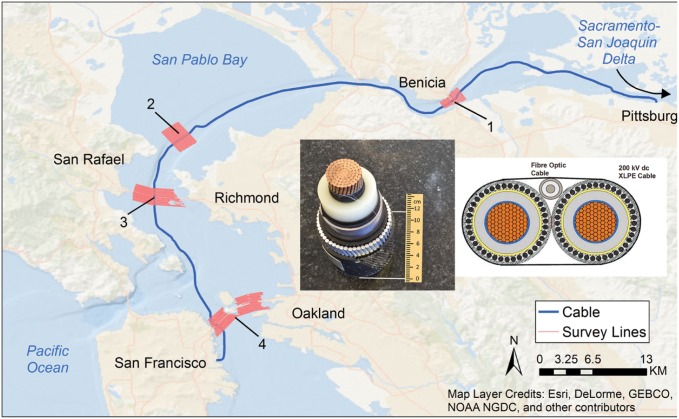
Overview of the Trans Bay Cable route and survey area. The Trans Bay Cable (dark blue line) conducts electricity from the city of Pittsburg, CA to San Francisco. Magnetic field surveys were conducted over an area with a width of 1 km (pink survey lines) at the following sites: 1) Benicia Bridge, 2) San Pablo Bay, 3) Richmond Bridge, and 4) Bay Bridge. The inset shows both a picture of the cable and its construction.

The magnetic fields produced by undersea cables that carry electricity from electric power generation sources to shore-based power stations may produce similar local distortions in the earth’s main field. Concern exists that animals that migrate along the continental shelves might orient to the magnetic fields from the cables, and move either inshore or offshore away from their normal path. We have studied the effect of the Trans Bay Cable (TBC), an 85-km long, high voltage, direct current transmission line leading from Pittsburg, CA to San Francisco, CA, on two fish species migrating through the San Francisco Estuary (Fig 1). These are Chinook salmon smolts (*Oncorhynchus tshawytscha*) that emigrate downstream through the San Francisco Estuary to the Pacific Ocean [5,6] and adult green sturgeon (*Acipenser medirostris*), which immigrate upstream to their spawning habitat in the upper Sacramento River and subsequently, back down to the ocean after spawning occurs [7,8]. Experimental [9–12] and observational studies [13] have established that species within the salmonid family orient to magnetic fields and may use them to guide their movements during migration. Anatomical studies have revealed that the sturgeons have electroreceptors, and hence are capable of using similar fields to provide guidance during migration [14].

Previously, we described the effect of the load current carried on the TBC cables on the local magnetic field [15]. The static magnetic field was measured while traversing the cable roughly perpendicular to its path at varying heights above the sea floor. Gradiometer surveys were conducted at four sites: 1) San Francisco-Oakland Bay Bridge (BB), 2) Richmond-San Rafael Bridge (RSR), 3) Benicia-Martinez Bridge (Ben), and 4) San Pablo Bay (SP). Seventy eight of 167 survey lines at these sites yielded profiles in which an anomaly in the geomagnetic field indicated the cable’s presence. The magnetic field profiles measured along survey lines that ran approximately perpendicular to the cable’s heading at the four locations together with the magnetometer’s height above the sea floor were entered into a regression model to estimate load current, the cable’s depth and the angular rotation of the cable’s two conductors from the horizontal (the ‘twist’ angle). A strong concordance was found between the measured and calculated anomalies in the geomagnetic field, as well as between the measured and calculated distance between the anomalies’ maxima and minima. This knowledge permits researchers to estimate to a first order the magnitude of the fields from the TBC cables in the future just based upon the documented (or alternatively, the assumed) line load, without having to conduct exhaustive gradiometer surveys in the future.

In this paper, we compare the magnetic field emitted by TBC to the anomalies in the geomagnetic field created by the three large bridges running perpendicular to the migration routes of these species. The strengths of the anomalies from the bridges exceed those of the cable by a magnitude of a power of ten or greater. We further analyzed the migratory movements of salmon and sturgeon migrating through the San Francisco Estuary in relation to the magnetic field anomalies from the TBC cable and bridges. In a future paper(s), we will examine in more detail the effect of the cable on the migratory path of Chinook salmon and green sturgeon.

## Methods

**Fig 2:**
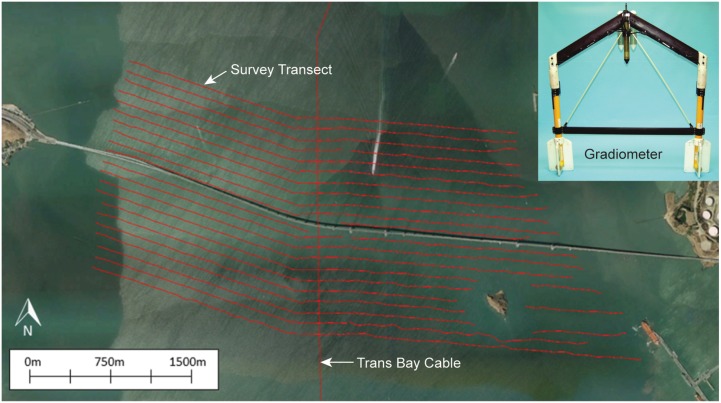
Magnetic survey transects conducted at Richmond Bridge. The survey transects (see horizontal red lines) extended one kilometer to either side of bridge. The path of the Trans Bay Cable (vertical red line) passes under the bridge in a north-south orientation. Insert: transverse gradiometer (G-882 TVG, Geometrics, Inc, San Jose, California) featuring two magnetometers mounted side by side 1.5 m apart (Photo: Geometrics).

The technology for mapping of the geomagnetic field and anomalies was described in a previous study [15] and is summarized here in brief along with the procedures for mapping the magnetic field in this study. Magnetic field surveys were conducted in four locations of the San Francisco Bay between July 10th and August 8th, 2014 (Fig 1). A cross-bay array of acoustic biotelemetry receivers (VR02, Vemco Ltd.,Halifax) were maintained at each site, the San Francisco-Oakland Bay Bridge, the Richmond-San Rafael Bridge, and the Benicia-Martinez Bridge, as well as a non-bridge location in the San Pablo Bay. These receivers were separated, based on range tests, so that they detected all fish carrying coded acoustical beacons (V7, V9, and V16, Vemco Lmt., Halifax) as the salmon and green sturgeon passed through the arrays on their migrations between the upper Sacramento River and Golden Gate. Surveys of the static magnetic field were performed with a transverse gradiometer (G-882 TVG, Geometrics, Inc., San Jose, CA) (Fig 2). This device is equipped with two cesium vapor gradiometer 'fish' separated by 1.5 m on a frame with stabilizer weights and fins. These devices measured the total magnetic field as a scalar quantity, that is, not as the resultant of three mutually-orthogonal vectors. The dual cesium sensors are synchronized to 1 ms sampling with sensitivities up to 0.004 nT/√Hz RMS or approximately 0.01 nT peak to peak at 10 Hz. A depth sensor (depth under the water surface, 0.5% accuracy) and an echo-sounder altimeter (height above the sea floor, 1% resolution) attached to the frame provided positional information to the operators. Data was transmitted through the reinforced tow line to an onboard computer (Toughbook, Panasonic), which was interfaced with a Trimble GeoExplorer XT Global Positioning System (GPS) with Hurricane LI antenna (Trimble Navigation Limited, Sunnyvale, CA) for display of the magnetic intensity measurements with sub-meter positional accuracy (after post-processing). The gradiometer was carried on a wooden platform, lowered into the water with an A-frame, and towed along survey lines pre-mapped with MagLog magnetic data acquisition software (Geometrics, Inc.). The MagLog display on the computer provided a graphic representation of the vessel’s location in relation to the pre-mapped survey lines, and this was used by the helmsperson to accurately steer the boat along the survey line.

Separate surveys were conducted to characterize the magnetic field anomalies due to the TBC and the bridges. Firstly, the gradiometer was towed perpendicular to the cable’s direction (corresponding roughly to a path parallel to the arrays of tag-detecting receivers). These lines extended along the entire span of the bridge as far towards the banks as possible and at least 1 km outwards from the fish detection array in San Pablo Bay. Transect lines started as close to the bridge/array as possible on each side and were repeated every 100 m away from the bridge/array up to 1 km (Fig 2). The purpose of these survey tracts was to verify the model of the magnetic fields produced by the cable (see Kavet et al. for details, [15]) and to provide data needed to create a local magnetic field map and a local magnetic field gradient map over the detection range of the fish detecting monitors. Secondly, the gradiometer was towed perpendicular to the bridges out to 1 km on both sides of each bridge. At least three of these transects were conducted at each bridge site. The purpose of these transects was to measure the magnetic field distortion created by the bridges. Survey tracts were conducted close to the surface (less than a meter underwater) and at greater depth in deeper locations (a meter above channel bottom). We conducted tows at these two depths in order to ensure that we sampled both the upper and lower magnetic field profiles in the water channel.

After the survey, the measurements were post-processed and mapped using MagPick magnetometer data processing software [16]. Post-processing included the following: 1) differentially correcting the GPS points to get sub-meter accuracy on survey locations using Trimble GPS Pathfinder Office software (Trimble Navigation Ltd.), 2) correcting for tidal stage based on tidal stage data downloaded from the nearest port stations operated by the National Oceanic and Atmospheric Administration, NOAA, 3) eliminating diurnal and other variations in the Earth's magnetic field by subtracting the magnetic field measures recorded at the base station at Jasper Ridge Biological Preserve, parallel to the coast of the bay, from field measures, and 4) removal of the DC offset between the two magnetometers. Stages 2 and 3 were carried out using MagPick’s universal regex parser plugin. After post-processing was complete, two types of maps were interpolated for each survey area using the survey lines that run perpendicular to the axis of the cable. The first is a total magnetic field map that illustrates the anomalies present in the local magnetic field, calculated as the average magnetic field measured by the two magnetometers minus the Earth’s total magnetic field. The second is a gradient map (also called a quasi-analytic signal map), which depicts the rate of change in the local magnetic field anomalies in nT/m. The magnitude of the quasi-analytic field was calculated using: 1) the transverse component of the gradient vector of the local magnetic field using the difference of the two magnetometers divided by their 1.5 m separation, 2) the longitudinal component of the gradient vector using the average of the two magnetometers and the data collected along the survey line, and 3) the estimated vertical derivative of the gradient vector (see [16] for details). These maps were created for the surface and deep tows at each survey location, but only the Richmond Bridge surface maps are shown here as illustrations. Graphs of the data recorded by these individual profile lines were also produced, with distance along the survey line expressed from east to west (as a convention to maintain consistency across all measured profiles) on the abscissa and the measured magnetic field in nanotelsa (nT) on the ordinate. The strength of local magnetic anomalies, such as those produced by the cable or the bridges, were quantified by taking the difference between the maxima and minima magnetic field values measured by the gradiometer along its survey route as it passed perpendicular to these objects of interest.

Records of fish movement obtained from previous biotelemetry studies carried out both before and after the Trans Bay Cable was installed in the San Francisco Estuary were analyzed in relation to the magnetic anomalies produced by the TBC and bridges. In these studies, Chinook salmon smolts were tagged with uniquely coded ultrasonic transmitters and detected throughout the San Francisco Bay as they migrated to the Pacific Ocean by receivers attached to bridges or anchored on the channel bottom. The receivers were attached to bridge supports, based on range tests, so that they detected all fish passing underneath the bridge along its entire length. The receivers were deployed at a depth of 3 m with their hydrophones facing downward. Tags were detected at all depths during range tests. Seventy five percent of the coded signals from the smaller transmitters, implanted within the body cavity of Chinook salmon smolts, were detected at a distance of 75 m from the receiver over all tidal conditions, and this was considerably farther than the greatest depth of 18 m at the channel passing under the bridge (Eric Chapman, pers. commun.). The majority of Chinook salmon smolts used in this study were tagged and tracked through the bay as part of a study to determine the reach-specific rates of movement and survival funded by CALFED and their movements relative to dredge removal and deposition sites funded by the United States Army Corps of Engineers [5–6]. Coded ultrasonic transmitters were also placed on adult green sturgeon captured throughout the estuary and northern California coast to understand the factors governing their upstream spawning migration to the headwaters of the Sacramento River, primarily funded by the California Department of Fish and Wildlife and United States Bureau of Reclamation [7–8].

The work was not carried out in a protected area, with a need to gain permission to access the area. No specific permissions were need to conduct activities. The field studies were conducted on Chinook salmon and green sturgeon. The latter is listed as 'Threatened'. However, individuals of neither species were handled, and hence no permit was required as their passage was recorded. They were tagged during different research programs, with authorization by National Marine Fisheries Service.

## Results

### Magnetic Anomaly from Cable

**Fig 3:**
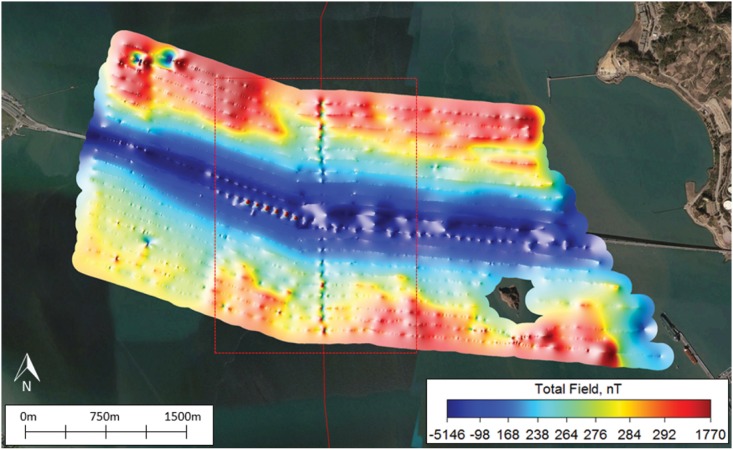
The local magnetic field anomalies existing around the Trans Bay Cable and Richmond Bridge. Note that the anomaly in the field produced by the cable is evident in the dark blue and red points along the line indicating the path of the cable that passes through the bridge along a north-south axis. The color scale uses non-linear color mapping based on the data distribution. Using this color equalization technique, each color occupies the same area on screen as any other color, ultimately increasing map resolution and visualization of smaller magnetic anomalies.

**Fig 4:**
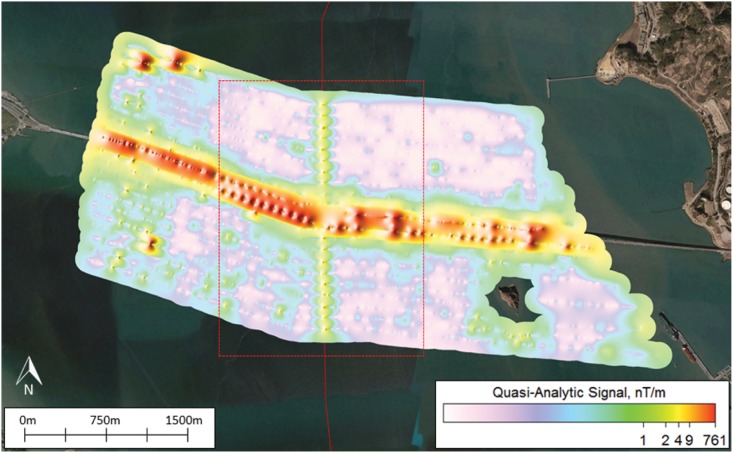
The gradient map of local magnetic fields existing around the Trans Bay Cable and Richmond Bridge. This quasi-analytic signal map illustrates the rate of change in local magnetic field anomalies, denoted as nT/m. Note that the rate of change in the local magnetic fields associated with the bridge is much greater than near the cable, and the distortion in the field extends farther from the bridge than the cable.

The DC load (or electrical demand) on the Trans Bay Cable produces a magnetic field that sums as a vector to the earth’s natural field. This vector summation leads to the appearance of a disturbance in the local background field. The anomaly in the local total field from the TBC was clearly recorded by the gradiometer during the surveys. It is apparent from the thin line with dark blue and red points with a vertical orientation on the map of the total field intensities at the Richmond Bridge (Fig 3). These were produced as the magnetometer passed over the cable at 100 m distance intervals away from the bridge on either side. The surface anomaly, based on measurements recorded by the gradiometer towed ≤ 1 m below the sea surface, is apparent on the map as hue change from blue green to red, equivalent to a difference from 238 nT to slightly over 292 nT on the color bar—a magnitude of 54 nT using the color bar to estimate the anomaly. The field distortion produced by the cable is also apparent in the map of the geomagnetic gradient, termed quasi-analytic signal (Fig 4). The geomagnetic gradients recorded as the gradiometer passed over the cable at 100 m distance intervals are apparent from a change in color from purple to yellow, a difference from a fraction of one nT/m to four nT/m. The TBC anomalies produce minute alterations in the earth’s main field.

**Fig 5:**
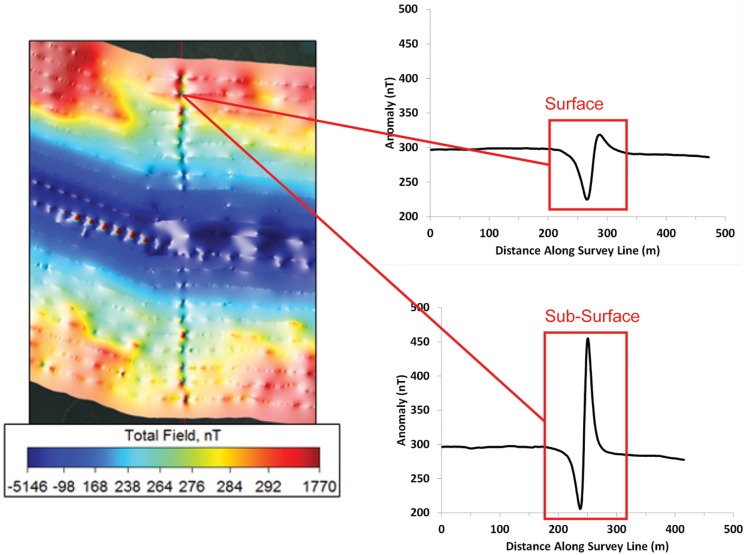
The magnetic anomaly induced by electrical current passing through the Trans Bay Cable. Profile plots of the cable’s measured magnetic anomaly are illustrated for both shallow, ≤ 1 m below surface, and deep tows, ≤ 3 m above bottom, along survey transects far away from the bridge. The anomaly is shown as the gradiometer was towed from east to west over the cable along transects orientated parallel to the bridge and perpendicular to the path of the cable. The anomaly recorded at the surface was 94 nT while the anomaly near the bottom was 245 nT. The increase in magnetic intensity in the latter profile was due to the gradiometer’s increased proximity to the cable. At ±80 meters from its centerline, the cable’s calculated contribution to the background field is about ±2 nT (~0.0042% of background) regardless of measurement depth when the cable carries its rated load of 1,000 amps. The anomaly exists over a distance of 80 meters, and consists of a negative and positive increment to the main field.

The distortion in the main field by the static magnetic field from the cable was also distinguishable in plots of the magnetic field anomaly as a function of distance traveled from east to west along the survey routes perpendicular to the cable. Profiles of magnetic intensity are shown as the gradiometer was towed across the TBC ca. 900 m north of Richmond Bridge both at the surface and sub-surface (Fig 5). The strength of the magnetic signal at the surface decreases from nearly 300 nT to a minimum of 226 nT, then increases to a maximum of 320 nT, before returning again to a relatively steady state at 290 nT, all over a distance of 150 m. The overall magnitude of the anomaly measured during this surface transect was 94 nT. As one might expect, the anomaly recorded when the gradiometer was towed closer to the channel bottom along the same transect was greater than that recorded at the surface. The signal strength decreased from 290 nT to a minimum of 206 nT, then increased to 455 nT, before dropping to its relatively steady state of 280 nT, giving an overall anomaly of 249 nT recorded over a distance of 150 m. The mean magnitudes of cable-associated anomalies measured during surface tows near the Benicia and Richmond Bridges and San Pablo Bay ranged from 93.5 to 117.0 nT, whereas the magnitude of the anomalies recorded during sub-surface tows at the same bridges varied from 235.6 to 518.0 nT (Table 1). The anomalies from the cable recorded during the sub-surface tows were significantly greater than those from the surface tows (Kruskal-Wallis Test, p<0.0001), as the field from the TBC attenuated with vertical distance in an inverse distance squared relationship (data not shown).

**Table 1:** Summary of magnetic field anomalies associated with the Trans Bay Cable (TBC) and bridges in the San Francisco Estuary. These are deviations from the earth’s natural background magnetic field. The local magnetic fields (in nanotesla, nT) of the bridges and cable were measured with both surface and deep tows during transects that ran perpendicular to the object of measure. The cable was surveyed at bridge site locations as well as a non-bridge location in San Pablo Bay. Cable data is only presented from transects where the cable anomaly was clearly identifiable. Measurements from the two magnetometers were averaged for this study.

		Benicia Bridge		San Pablo Bay	Richmond Bridge		Bay Bridge	
		TBC	Bridge	TBC	TBC	Bridge	TBC	Bridge
Surface	Transects (N)	9	2	15	11	4	0	5
	Mean	93.5	2507	117.0	95.7	2236	--	2732
	SD	42.4	490	22.1	13.4	1241	--	2184
	Median	76.4	2506	115.8	93.9	2374	--	1479
	Min	54.2	2160	68.5	72.9	728	--	901
	Max	192.9	2853	151.1	122.2	3468	--	5923
Deep	Transects (N)	7	1	20	14	3	2	4
	Mean	235.6	--	300.6	268.5	1142	518.0	4168
	SD	84.4	--	130.5	49.4	737	241.8	1997
	Median	207.9	3442	286.1	262.4	726	518.0	4363
	Min	139.8	--	144.9	197.6	707	347.0	1847
	Max	359.9	--	661.3	380.5	1993	689.0	6100

### Magnetic Anomalies from Bridges

**Fig 6:**
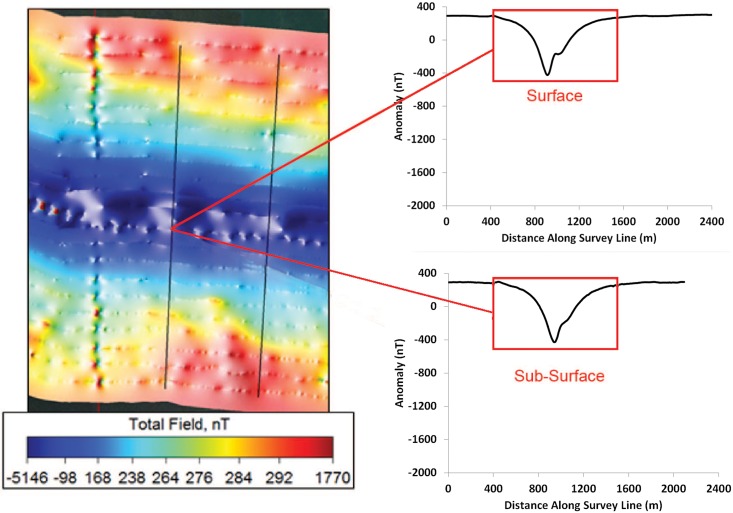
The magnetic anomaly produced by the Richmond Bridge as measured by perpendicular survey lines. Profile plots for surface and deep tows are illustrated along transects (black vertical lines) travelling perpendicular to the bridge. Note that the surface anomaly from the bridge is 728 nT and sub-surface anomaly is 726 nT, exceeding that of the cable in magnitude by a factors of 7.7 and 3.0, respectively. The anomaly occurs over a distance of 1,200 m, and does not consist of a positive and negative excursion but only a negative excursion.

**Fig 7:**
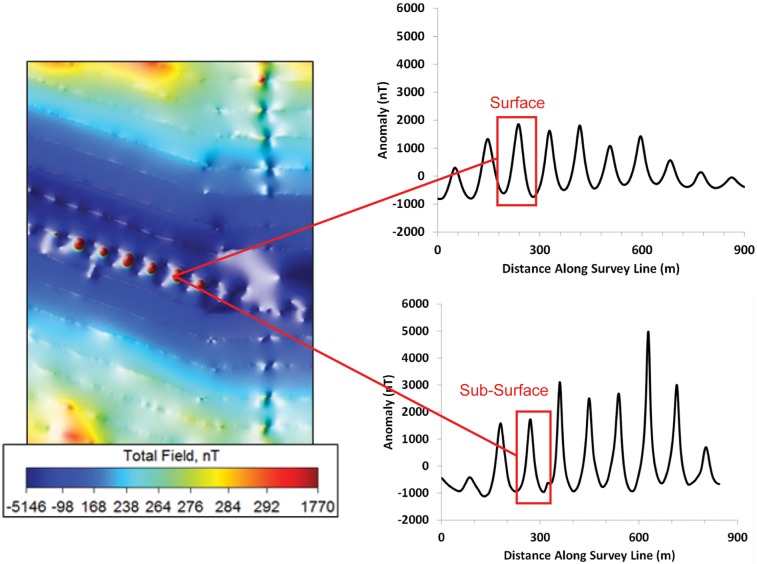
The magnetic anomaly produced by the Richmond Bridge as measured by parallel survey lines. Profile plots for surface and deep tows are shown for a survey transect located parallel to Richmond Bridge, approximately 50 m south of the bridge. Successive maxima and minima along the profile are separated by a distance of 100 m, the approximate distance between bridge supports. These observations indicate that the supports are also a strong source of anomalies, adding to the background field at each structure, and subtracting from between each structure.

The magnetic anomalies associated with the bridges differed from the cable anomaly in form, magnitude, depth distribution, and geographical extent (Fig 6). Let us first compare the anomaly from the Trans Bay Cable to that of the Richmond Bridge. The current in one conductor and an equal and opposite current in the other created a dipole magnetic field that, in this example, subtracted from the earth’s main field to the east of the cable and added to the field to the west of the cable at the Richmond Bridge. The waveform on the magnetometer records was not symmetrical with the negative excursion being smaller than the positive excursion in the case illustrated. This was due to the vectoral addition of the local main field and the dipolar field of the cable, and reinforcement of the field on one side and some cancelation on the other side. The structure of the anomaly from the bridge is more complex. The ferromagnetic materials within the bridge abutments, which are likely concrete with iron bars, concentrate the flux lines from the earth’s field leading to a deficit between the abutments. This is apparent in the scalloped pattern of alternating positive and negative peaks on the record of the total field when the boat was driven parallel to the bridge (see Fig 7). This pattern differed from the single negative peak on the record when the magnetometer was towed under the bridge (see Fig 6). The magnetometer passed within 25 m of abutments during the former transect and 50 m during the latter. For this reason, the positive anomalies from the abutments were present on the record from survey line along the length of the bridge and not on the line underneath the bridge, in which the boat was 50 m from the closest abutments. The latter survey line was farther from the abutments, and for this reason the anomaly was more negative than for transect parallel to the bridge. In conclusion, a large object with ferromagnetic materials, such as the Richmond Bridge, thus distorts the magnetic flux lines from the earth’s field, which would be essentially uniform in the bridge’s absence.

The bridges produce larger anomalies in the total field than those produced by current on the TBC. The difference in magnitude of the anomalies from these two sources of static magnetic fields was strikingly apparent both by visual inspection of the total field and gradient maps as well as by comparisons of the anomalies measured from the plots of magnetic intensity from survey transects orientated perpendicular to the cable and bridge. For instance, anomalies in the total field shown on the map varied from red to dark blue on the color scale, indicating variation on the order 1,770 to -5,146 nT (Fig 3). The anomalies revealed in the quasi-analytic signal varied from the color purple to red on the color scale, a gradient of less than one to 761 nT/m (Fig 4). Furthermore, the magnitude of anomaly produced by the Richmond Bridge was similar at the surface and sub-surface tows, with overall strengths of 728 and 726 nT, respectively. In both profiles, the measured anomaly decreased from roughly 300 nT to -400 nT before rising the same level. The mean measured strength of the surface magnetic anomalies created by the three bridges of 2,492 nT was roughly 26 times that mean strength of 94.6 nT to the anomalies produced by the TBC. The mean strength of the sub-surface anomalies of the Richmond and Bay Bridges of 2655 nT exceeded the mean anomaly of 330.6 nT of the cable near the three bridges and San Pablo Bay was less, a factor of 8.0, but still substantial.

Unlike the cable anomalies, those from the bridges varied with depth at different bridges. While the negative excursion recorded on the sub-surface transect was similar to that recorded on the surface transect at the Richmond Bridge in Fig 6, this was not the case at the other bridges or at different transect lines at Richmond Bridge. At the Benicia and Bay Bridges or at different transect lines further east on Richmond Bridge, the anomalies from deep tows were larger than those from surface tows. However, there was no significant difference in the magnitude of the negative excursions when the surface transectswere compared to the sub-surface transects near the bridges (Kruskal-Wallis Test, p<0.05). Differences in the magnitude of the bridge anomalies likely stem from variation in the construction materials of the bridge span and supports, the height of the bridge span over the water surface at the perpendicular crossing, and the volume of traffic occurring on the bridge at the time.

Finally, the bridges altered the earth’s field over a greater geographic extent than the TBC. For example, the lateral width of the anomaly from the cable sampled near Richmond Bridge was less than or equal to 150 m (Fig 3), whereas the width of the anomaly from Richmond Bridge extended outward over total distances of 1200 m (Fig 6). This lateral width from the bridge anomaly exceeded that of the lateral width of the cable anomaly by nearly an order of magnitude.

The large anomalies produced by the ferromagnetic properties of the bridges often masked the anomalies of the Trans Bay Cable. The anomaly in the total field produced by the cable could not be discriminated from the larger anomaly recorded to a distance of 300 m north and 200 m south of the Richmond Bridge (see the dark blue color on Figs 3, 5, 6 and 7). The ‘signature’ of the cable was not apparent on the four nearest transects parallel to the bridge on the north side and three transects on the south side. The anomaly in the magnetic gradient of the bridge masked that of the cable to a distance of 100 m north and south of the Richmond Bridge (see red signal in Fig 4). At the Bay Bridge, only two of 20 surface and 20 sub-surface surveys of total field revealed the presence of the Trans Bay Cable.

### Passage of Migratory Fishes through Bridge Magnetic Anomalies

The strong magnetic anomalies produced by the bridges along the migration route of Chinook salmon smolts and adult green sturgeon do not appear to present a major barrier to migration movements in the San Francisco Estuary. Many of the tagged Chinook salmon smolts were detected near the Richmond Bridge and later when they reached the Golden Gate Bridge on their migration to the sea. A total of 1025 smolts with ultrasonic beacons placed on them from 2007–2011 were detected passing under the Benicia Bridge and entering San Francisco Bay (Table 2). Of this total, 573 (56%) were detected as they passed near to the Richmond Bridge. A total of 457 (45%) reached the Golden Gate Bridge at the mouth of the bay. Hence, slightly fewer than half did not stop their outward migration upon encountering the strong anomaly at Richmond Bridge. We do not know the fate of the 452 smolts, or 44%, that did not reach Richmond Bridge after passing under Benicia Bridge as well as the fate of the 116, or 20%, that did not pass from the latter to Golden Gate Bridge.

**Table 2:** Passage of Chinook salmon smolts and adult green sturgeon through the magnetic anomalies produced by the Richmond, Benicia, and Golden Gate Bridges. These are deviations from the earth’s natural background magnetic field. Data represents the number of individual fish detected at each bridge in each year as well as the percentage of total fish detected at the first array for subsequent locations along each migration route.

Year	Chinook Salmon (Outbound Migrations)			Green Sturgeon (Outbound Migrations)			Green Sturgeon (Inbound Migrations)		
	Benicia Bridge (N)	Richmond Bridge N (%)	Golden Gate N (%)	Benicia Bridge (N)	Richmond Bridge N (%)	Golden Gate N (%)	Golden Gate (N)	Richmond Bridge N (%)	Benicia Bridge N (%)
**2007**	32	19 (59%)	25 (78%)	5	3 (60%)	4 (80%)	6	5 (83%)	6 (100%)
**2008**	143	61 (43%)	61 (43%)	7	5 (71%)	7 (100%)	4	3 (75%)	4 (100%)
**2009**	375	201 (54%)	150 (40%)	12	12 (100%)	12 (100%)	5	5 (100%)	5 (100%)
**2010**	332	215 (65%)	165 (50%)	36	34 (94%)	33 (92%)	9	9 (100%)	8 (89%)
**2011**	143	77 (54%)	56 (39%)	19	19 (100%)	19 (100%)	3	3 (100%)	3 (100%)
**2012**				45	43 (96%)	43 (96%)	17	17 (100%)	11 (65%)
**2013**				5	5 (100%)	5 (100%)	15	15 (100%)	15 (100%)
**2014**				21	21 (100%)	19 (90%)	15	15 (100%)	15 (100%)
**Total**	1025	573 (56%)	457 (45%)	150	142 (95%)	142 (95%)	74	72 (97%)	67 (91%)

Green sturgeons were not strongly deterred by the anomalies associated with the bridges. They migrate through the San Francisco Estuary to the upper reaches of the Sacramento River where they spawn and return through the estuary to the ocean after spawning concluded. A total of 74 inbound migration trips and 150 outbound migration trips by adult green sturgeon were monitored from 2007 to 2014 (Table 2). Many of the sturgeon were tagged and released upstream prior to their outbound migration. Inbound migrations were first detected at Golden Gate Bridge, then Richmond Bridge, then Benicia Bridge while outbound migrations moved from Benicia Bridge, to Richmond Bridge, to Golden Gate Bridge. Of these 74 inbound movements, 72 (97%) resulted in detections at Richmond Bridge and 67 (91%) produced detections at Benicia Bridge at the confluence of San Francisco Bay with the Sacramento-San Joaquin River Delta. Furthermore, 142 (95%) of the 150 total outbound migrations resulted in detections at the Richmond Bridge and Golden Gate Bridge. Hence, almost all of the green sturgeon entering or exiting the bay experienced the strong anomalies associated with the bridges but were not deterred from their upriver or downriver migration movements.

## Discussion

Magnetic field anomalies were detected near the TBC due to its load current and the bridge structures that span the bay within the San Francisco Estuary due to their distortion of the natural geomagnetic field. We found that the distortions in the earth’s main field produced by bridges were much greater in intensity and area than those from the cable. The former anomalies exceeded the latter by over an order of one or two magnitudes. Although the cable anomalies increased strongly with depth, the bridge anomalies were not consistently stronger at either the surface or deep tows. A salmon smolt swimming along the cable would be experiencing a small anomaly, and it might utilize this to move out of the bay. However, it would encounter a much stronger anomaly as it passed underneath the bridge. Would it ignore this increase in magnetic intensity and continue on its migration out of the bay, or would it deflect its movement east or west along the bridge?

Despite the magnitude of the anomalies produced by bridges, significant numbers of tagged Chinook salmon smolts migrated downstream past the large anomaly produced by the Richmond Bridge, to the Golden Gate Bridge, where they were detected by dual arrays of tag-detecting monitors moored in lines across the mouth of the bay. Furthermore, over 90% of adult green sturgeon that entered the mouth of the bay during inbound migrations passed Richmond Bridge and were detected at Benicia Bridge on their way upstream to spawn in the headwaters of the Sacramento River. Outbound migrations experienced similar success, with 95% of green sturgeon traveling downstream through the San Francisco Bay being detected at Richmond Bridge and Golden Gate Bridge on their way to the Pacific Ocean. Hence, salmonids and green sturgeon are not strongly deterred by these strong magnetic anomalies from bridges that run perpendicular to their migratory route.

Substantial percentages of Chinook salmon smolts migrating downstream as well as adult green sturgeon moving upstream and downstream passed the Richmond Bridge with its strong magnetic anomaly. These are species, for which evidence exists that they orient to magnetic field [9–13] or have electroreceptors capable of perceiving magnetic fields (the electroreceptors may detect motional electromotive force as a fish’s motion ‘cuts through’ the geomagnetic field’s lines of flux) [14]. Westerberg and Begout Anras [17] tracked 25 silver eels in the vicinity of a high voltage, direct current cable with a greater load than the Trans Bay Cable, off the southern coast of Sweden. Approximately 60% of the eels passed over the cable during the short tracks, indicating that the cable’s magnetic field did not obstruct their migration to any significant extent. The responses of eels were different in the presence of an alternating current. Coded acoustic beacons and stationary receivers similar to those used in this study were used to examine the effect of the magnetic field from a cable transmitting alternating current on silver eels in the Baltic Sea. The rates of movement of eels passing between arrays of monitors north and south of the cable were compared to rates of movement across the cable between arrays on either side [18]. The swimming speeds of 60 eels were significantly lower around the cable than both north and south of the cable. However, the behavior of the eels could not be monitored during passage, leading the researchers to conclude that further work is needed to understand the nature of the effect.

Some salmon smolts detected at the Richmond Bridge did not reach the Golden Gate. We do not know the fate of the smolts that did not reach Richmond Bridge after passing under Benicia Bridge as well as the fate of the smolts that did not pass from the former to the Golden Gate Bridge. There could be various reasons for this such as: 1) loss of life due to predation, 2) tag shedding, 3) low detection probability, or 4) miss-directed orientation. There is evidence to be presented elsewhere (Wyman et al., in prep.) that more smolts may be detected at the Bay Bridge after the cable was activated than before, perhaps indicating that the cable may affect the migration movements of some fish to some degree. Westerberg and Lagenfeldt argued that an intensive tracking study is necessary to identify any effect on a migratory species [18]. We recommend that an experiment be conducted to describe the response of Chinook smolts to the static magnetic field from bridges. Do they slow down when passing them? Conversely, do they move sideways along the length of the bridge? An array of monitors could be deployed that extends across the Richmond Bridge, and extends northward and southward of the bridge. Late-fall run Chinook salmon smolts could be released upstream of the bridge and tracked in two dimensions as they moved through it to the bay and continue their migration to the ocean. Alternatively, transmitters carrying a strain gauge and 3-axis accelerometer could be placed on green sturgeon to characterize the swimming behavior of the fish as they pass by the bridge and a 3-axis gradiometer to measure the strength of the static magnetic field induced by the bridge. Descriptions of their fine-scale movements in the absence of the cable have already been published [19, 20] but those studies did not consider the potential role of the geomagnetic field or the magnetic anomalies from the bridges. These experimental studies would provide a better understanding of the responses of migratory fish to static magnetic field anomalies from large bridge structures.

## Supporting Information

S1 Fig Raw Data, Anomaly Gradient Map of TBC. (PDF)

S2 Fig Raw Data, Anomaly Map and Surface and Subsurface Profiles of TBC Away From Bridge. (PDF)

S3 Fig Raw Data, Anomaly Map and Surface and Subsurface Profiles of TBC Near Bridge. (PDF)

S4 Fig Raw Data, Anomaly Map and Surface and Subsurface Profiles of TBC Across Bridge. (PDF)

S1 Table Raw Data, Summary of Anomalies Associated with TBC. (XLSX)
