# Supplementary material for: Chinook salmon and green sturgeon migrate through San Francisco Estuary despite large distortions in the local magnetic field produced by bridges
Source: PLoS One. 2017 Jun 2;12(6):e0169031. doi: 10.1371/journal.pone.0169031 (PMC5456031; doi:10.1371/journal.pone.0169031)
Supplement: S2 Fig — (PDF) [file pone.0169031.s002.pdf]

Record	Location	#Longitude1	Latitude1	Z1	ALT1	T1	Alt+Depth1	Longitude2	Latitude2	Z2	ALT2	T2	Alt+Depth2	LINE	DATE	TIME
1	RSRS19	-122.447	37.944	0.61	16.01	297.40	16.62	-122.447	37.944	0.30	16.01	295.64	16.31	2	7/10/2014	35:11.0
2	RSRS19	-122.447	37.944	0.61	16.04	297.44	16.65	-122.447	37.944	0.34	16.04	295.68	16.38	2	7/10/2014	35:11.1
3	RSRS19	-122.447	37.944	0.65	16.03	297.46	16.67	-122.447	37.944	0.22	16.03	295.64	16.25	2	7/10/2014	35:11.2
4	RSRS19	-122.447	37.944	0.61	15.97	297.47	16.58	-122.447	37.944	0.30	15.97	295.66	16.27	2	7/10/2014	35:11.2
5	RSRS19	-122.447	37.944	0.61	15.96	297.51	16.57	-122.447	37.944	0.42	15.96	295.71	16.38	2	7/10/2014	35:11.4
6	RSRS19	-122.447	37.944	0.61	15.95	297.51	16.56	-122.447	37.944	0.25	15.95	295.77	16.20	2	7/10/2014	35:11.5
7	RSRS19	-122.447	37.944	0.61	15.93	297.51	16.54	-122.447	37.944	0.42	15.93	295.79	16.35	2	7/10/2014	35:11.6
8	RSRS19	-122.447	37.944	0.61	15.95	297.49	16.56	-122.447	37.944	0.34	15.95	295.82	16.29	2	7/10/2014	35:11.6
9	RSRS19	-122.447	37.944	0.61	15.87	297.46	16.48	-122.447	37.944	0.25	15.87	295.82	16.13	2	7/10/2014	35:11.7
10	RSRS19	-122.447	37.944	0.61	15.84	297.54	16.45	-122.447	37.944	0.39	15.84	295.89	16.23	2	7/10/2014	35:11.9
11	RSRS19	-122.447	37.944	0.65	15.83	297.54	16.48	-122.447	37.944	0.34	15.83	295.85	16.17	2	7/10/2014	35:12.0
12	RSRS19	-122.447	37.944	0.61	15.80	297.56	16.41	-122.447	37.944	0.34	15.80	295.84	16.14	2	7/10/2014	35:12.0
13	RSRS19	-122.447	37.944	0.65	15.79	297.58	16.44	-122.447	37.944	0.30	15.79	295.82	16.09	2	7/10/2014	35:12.2
14	RSRS19	-122.447	37.944	0.61	15.77	297.65	16.39	-122.447	37.944	0.25	15.77	295.87	16.03	2	7/10/2014	35:12.3
15	RSRS19	-122.447	37.944	0.65	15.76	297.69	16.41	-122.447	37.944	0.39	15.76	295.89	16.15	2	7/10/2014	35:12.3
16	RSRS19	-122.447	37.944	0.61	15.82	297.71	16.43	-122.447	37.944	0.39	15.82	295.89	16.21	2	7/10/2014	35:12.4
17	RSRS19	-122.447	37.944	0.61	15.77	297.78	16.39	-122.447	37.944	0.30	15.77	295.96	16.08	2	7/10/2014	35:12.6
18	RSRS19	-122.447	37.944	0.61	15.76	297.76	16.37	-122.447	37.944	0.42	15.76	295.96	16.18	2	7/10/2014	35:12.7
19	RSRS19	-122.447	37.944	0.61	15.80	297.76	16.41	-122.447	37.944	0.39	15.80	296.00	16.18	2	7/10/2014	35:12.7
20	RSRS19	-122.447	37.944	0.61	15.78	297.74	16.39	-122.447	37.944	0.25	15.78	296.02	16.03	2	7/10/2014	35:12.8
21	RSRS19	-122.447	37.944	0.65	15.78	297.79	16.43	-122.447	37.944	0.51	15.78	296.03	16.29	2	7/10/2014	35:12.9
22	RSRS19	-122.447	37.944	0.61	15.75	297.79	16.36	-122.447	37.944	0.34	15.75	296.05	16.09	2	7/10/2014	35:13.1
23	RSRS19	-122.447	37.944	0.61	15.77	297.83	16.39	-122.447	37.944	0.25	15.77	296.05	16.03	2	7/10/2014	35:13.2
24	RSRS19	-122.447	37.944	0.61	15.74	297.85	16.35	-122.447	37.944	0.39	15.74	296.05	16.13	2	7/10/2014	35:13.2
25	RSRS19	-122.447	37.944	0.65	15.84	297.89	16.49	-122.447	37.944	0.39	15.84	296.07	16.23	2	7/10/2014	35:13.4
26	RSRS19	-122.447	37.944	0.61	15.75	297.94	16.36	-122.447	37.944	0.30	15.75	296.14	16.05	2	7/10/2014	35:13.4
27	RSRS19	-122.447	37.944	0.61	15.76	298.01	16.37	-122.447	37.944	0.46	15.76	296.21	16.21	2	7/10/2014	35:13.5
28	RSRS19	-122.447	37.944	0.61	15.73	297.99	16.35	-122.447	37.944	0.25	15.73	296.23	15.99	2	7/10/2014	35:13.6
29	RSRS19	-122.447	37.944	0.65	15.76	298.01	16.40	-122.447	37.944	0.34	15.76	296.21	16.09	2	7/10/2014	35:13.8
30	RSRS19	-122.447	37.944	0.61	15.78	298.01	16.39	-122.447	37.944	0.42	15.78	296.21	16.20	2	7/10/2014	35:13.9
31	RSRS19	-122.447	37.944	0.61	15.73	298.03	16.35	-122.447	37.944	0.22	15.73	296.14	15.95	2	7/10/2014	35:13.9
32	RSRS19	-122.447	37.944	0.61	15.81	298.01	16.42	-122.447	37.944	0.30	15.81	296.16	16.11	2	7/10/2014	35:14.0

33	RSRS19	-122.447	37.944	0.65	15.82	298.06	16.46	-122.447	37.944	0.39	15.82	296.15	16.21	2	7/10/2014	35:14.2
34	RSRS19	-122.447	37.944	0.56	15.80	298.04	16.36	-122.447	37.944	0.22	15.80	296.16	16.02	2	7/10/2014	35:14.3
35	RSRS19	-122.447	37.944	0.61	15.83	298.10	16.44	-122.447	37.944	0.34	15.83	296.21	16.16	2	7/10/2014	35:14.3
36	RSRS19	-122.447	37.944	0.61	15.86	298.06	16.48	-122.447	37.944	0.34	15.86	296.21	16.20	2	7/10/2014	35:14.4
37	RSRS19	-122.447	37.944	0.61	15.86	298.08	16.48	-122.447	37.944	0.17	15.86	296.21	16.03	2	7/10/2014	35:14.5
38	RSRS19	-122.447	37.944	0.61	16.00	298.06	16.61	-122.447	37.944	0.39	16.00	296.26	16.39	2	7/10/2014	35:14.7
39	RSRS19	-122.447	37.944	0.61	15.91	298.04	16.52	-122.447	37.944	0.25	15.91	296.21	16.17	2	7/10/2014	35:14.7
40	RSRS19	-122.447	37.944	0.61	15.92	298.02	16.53	-122.447	37.944	0.14	15.92	296.19	16.05	2	7/10/2014	35:14.8
41	RSRS19	-122.447	37.944	0.61	15.91	298.11	16.52	-122.447	37.944	0.42	15.91	296.26	16.33	2	7/10/2014	35:15.0
42	RSRS19	-122.447	37.944	0.61	15.91	298.06	16.52	-122.447	37.944	0.22	15.91	296.23	16.13	2	7/10/2014	35:15.1
43	RSRS19	-122.447	37.944	0.61	15.90	298.15	16.52	-122.447	37.944	0.22	15.90	296.24	16.12	2	7/10/2014	35:15.1
44	RSRS19	-122.447	37.944	0.61	15.93	298.13	16.54	-122.447	37.944	0.34	15.93	296.28	16.27	2	7/10/2014	35:15.2
45	RSRS19	-122.447	37.944	0.61	15.91	298.15	16.52	-122.447	37.944	0.14	15.91	296.24	16.05	2	7/10/2014	35:15.4
46	RSRS19	-122.447	37.944	0.61	15.91	298.04	16.52	-122.447	37.944	0.25	15.91	296.17	16.17	2	7/10/2014	35:15.5
47	RSRS19	-122.447	37.944	0.61	15.91	298.02	16.52	-122.447	37.944	0.30	15.91	296.20	16.22	2	7/10/2014	35:15.5
48	RSRS19	-122.447	37.944	0.61	15.93	298.11	16.54	-122.447	37.944	0.14	15.93	296.24	16.06	2	7/10/2014	35:15.6
49	RSRS19	-122.447	37.944	0.61	15.88	298.11	16.49	-122.447	37.944	0.30	15.88	296.33	16.18	2	7/10/2014	35:15.7
50	RSRS19	-122.447	37.944	0.61	15.92	298.07	16.53	-122.447	37.944	0.22	15.92	296.27	16.14	2	7/10/2014	35:15.9
51	RSRS19	-122.447	37.944	0.61	15.89	298.13	16.50	-122.447	37.944	0.14	15.89	296.33	16.02	2	7/10/2014	35:15.9
52	RSRS19	-122.447	37.944	0.61	15.90	298.16	16.51	-122.447	37.944	0.34	15.90	296.35	16.23	2	7/10/2014	35:16.0
53	RSRS19	-122.447	37.944	0.61	15.88	298.18	16.49	-122.447	37.944	0.22	15.88	296.35	16.10	2	7/10/2014	35:16.2
54	RSRS19	-122.447	37.944	0.56	15.86	298.16	16.43	-122.447	37.944	0.14	15.86	296.31	16.00	2	7/10/2014	35:16.2
55	RSRS19	-122.447	37.944	0.61	15.83	298.16	16.45	-122.447	37.944	0.34	15.83	296.33	16.17	2	7/10/2014	35:16.3
56	RSRS19	-122.447	37.944	0.56	15.85	298.14	16.41	-122.447	37.944	0.14	15.85	296.36	15.98	2	7/10/2014	35:16.4
57	RSRS19	-122.447	37.944	0.61	15.90	298.16	16.52	-122.447	37.944	0.22	15.90	296.38	16.12	2	7/10/2014	35:16.6
58	RSRS19	-122.447	37.944	0.56	15.78	298.16	16.34	-122.447	37.944	0.25	15.78	296.40	16.03	2	7/10/2014	35:16.7
59	RSRS19	-122.447	37.944	0.61	15.78	298.14	16.39	-122.447	37.944	0.08	15.78	296.38	15.86	2	7/10/2014	35:16.7
60	RSRS19	-122.447	37.944	0.56	15.76	298.14	16.32	-122.447	37.944	0.22	15.76	296.32	15.98	2	7/10/2014	35:16.8
61	RSRS19	-122.447	37.944	0.61	15.74	298.14	16.35	-122.447	37.944	0.22	15.74	296.36	15.96	2	7/10/2014	35:17.0
62	RSRS19	-122.447	37.944	0.56	15.76	298.16	16.32	-122.447	37.944	0.05	15.76	296.36	15.81	2	7/10/2014	35:17.1
63	RSRS19	-122.447	37.944	0.61	15.74	298.25	16.35	-122.447	37.944	0.34	15.74	296.43	16.08	2	7/10/2014	35:17.1
64	RSRS19	-122.447	37.944	0.56	15.74	298.32	16.30	-122.447	37.944	0.17	15.74	296.47	15.91	2	7/10/2014	35:17.2
65	RSRS19	-122.447	37.944	0.61	15.74	298.27	16.35	-122.447	37.944	0.17	15.74	296.45	15.91	2	7/10/2014	35:17.3

66	RSRS19	-122.447	37.944	0.56	15.74	298.24	16.30	-122.447	37.944	0.30	15.74	296.41	16.05	2	7/10/2014	35:17.5
67	RSRS19	-122.447	37.944	0.61	15.72	298.17	16.33	-122.447	37.944	0.14	15.72	296.41	15.85	2	7/10/2014	35:17.5
68	RSRS19	-122.447	37.944	0.56	15.72	298.15	16.28	-122.447	37.944	0.14	15.72	296.41	15.85	2	7/10/2014	35:17.6
69	RSRS19	-122.447	37.944	0.61	15.90	298.10	16.52	-122.447	37.944	0.30	15.90	296.39	16.21	2	7/10/2014	35:17.8
70	RSRS19	-122.447	37.944	0.56	15.85	298.13	16.41	-122.447	37.944	0.14	15.85	296.45	15.98	2	7/10/2014	35:17.9
71	RSRS19	-122.447	37.944	0.56	15.68	298.17	16.24	-122.447	37.944	0.22	15.68	296.48	15.90	2	7/10/2014	35:17.9
72	RSRS19	-122.447	37.944	0.56	15.93	298.15	16.50	-122.447	37.944	0.25	15.93	296.46	16.19	2	7/10/2014	35:18.0
73	RSRS19	-122.447	37.944	0.61	15.83	298.20	16.45	-122.447	37.944	0.14	15.83	296.51	15.97	2	7/10/2014	35:18.2
74	RSRS19	-122.447	37.944	0.56	15.80	298.19	16.36	-122.447	37.944	0.14	15.80	296.50	15.94	2	7/10/2014	35:18.3
75	RSRS19	-122.447	37.944	0.56	15.65	298.24	16.21	-122.447	37.944	0.30	15.65	296.52	15.95	2	7/10/2014	35:18.3
76	RSRS19	-122.447	37.944	0.56	15.81	298.33	16.37	-122.447	37.944	0.17	15.81	296.54	15.98	2	7/10/2014	35:18.4
77	RSRS19	-122.447	37.944	0.61	15.81	298.22	16.42	-122.447	37.944	0.25	15.81	296.51	16.06	2	7/10/2014	35:18.6
78	RSRS19	-122.447	37.944	0.56	15.62	298.20	16.19	-122.447	37.944	0.39	15.62	296.44	16.01	2	7/10/2014	35:18.7
79	RSRS19	-122.447	37.944	0.61	15.60	298.20	16.21	-122.447	37.944	0.17	15.60	296.40	15.76	2	7/10/2014	35:18.7
80	RSRS19	-122.447	37.944	0.56	15.61	298.15	16.17	-122.447	37.944	0.17	15.61	296.39	15.78	2	7/10/2014	35:18.8
81	RSRS19	-122.447	37.944	0.56	15.73	298.18	16.29	-122.447	37.944	0.30	15.73	296.40	16.03	2	7/10/2014	35:19.0
82	RSRS19	-122.447	37.944	0.56	15.62	298.11	16.19	-122.447	37.944	0.17	15.62	296.39	15.79	2	7/10/2014	35:19.0
83	RSRS19	-122.447	37.944	0.61	15.56	298.14	16.17	-122.447	37.944	0.39	15.56	296.38	15.94	2	7/10/2014	35:19.1
84	RSRS19	-122.447	37.944	0.56	15.57	298.16	16.13	-122.447	37.944	0.25	15.57	296.38	15.83	2	7/10/2014	35:19.2
85	RSRS19	-122.447	37.944	0.56	15.69	298.23	16.26	-122.447	37.944	0.14	15.69	296.40	15.83	2	7/10/2014	35:19.4
86	RSRS19	-122.447	37.944	0.56	15.72	298.11	16.28	-122.447	37.944	0.30	15.72	296.44	16.02	2	7/10/2014	35:19.5
87	RSRS19	-122.447	37.944	0.61	15.53	298.16	16.14	-122.447	37.944	0.34	15.53	296.42	15.86	2	7/10/2014	35:19.5
88	RSRS19	-122.447	37.944	0.56	15.77	298.12	16.33	-122.447	37.944	0.17	15.77	296.38	15.94	2	7/10/2014	35:19.6
89	RSRS19	-122.447	37.944	0.61	15.55	298.07	16.16	-122.447	37.944	0.39	15.55	296.36	15.94	2	7/10/2014	35:19.8
90	RSRS19	-122.447	37.944	0.61	15.66	298.05	16.28	-122.447	37.944	0.30	15.66	296.36	15.97	2	7/10/2014	35:19.8
91	RSRS19	-122.447	37.944	0.61	15.59	298.05	16.20	-122.447	37.944	0.25	15.59	296.32	15.84	2	7/10/2014	35:19.9
92	RSRS19	-122.447	37.944	0.56	15.60	298.05	16.16	-122.447	37.944	0.39	15.60	296.31	15.98	2	7/10/2014	35:20.0
93	RSRS19	-122.447	37.944	0.61	15.56	298.01	16.17	-122.447	37.944	0.25	15.56	296.32	15.81	2	7/10/2014	35:20.1
94	RSRS19	-122.447	37.944	0.61	15.56	298.08	16.17	-122.447	37.944	0.30	15.56	296.28	15.86	2	7/10/2014	35:20.3
95	RSRS19	-122.447	37.944	0.61	15.60	298.08	16.22	-122.447	37.944	0.39	15.60	296.30	15.99	2	7/10/2014	35:20.3
96	RSRS19	-122.447	37.944	0.56	15.66	298.03	16.22	-122.447	37.944	0.22	15.66	296.28	15.88	2	7/10/2014	35:20.4
97	RSRS19	-122.447	37.944	0.61	15.61	298.01	16.22	-122.447	37.944	0.30	15.61	296.27	15.91	2	7/10/2014	35:20.6
98	RSRS19	-122.447	37.944	0.61	15.60	297.99	16.21	-122.447	37.944	0.42	15.60	296.21	16.02	2	7/10/2014	35:20.7

99	RSRS19	-122.447	37.944	0.56	15.61	297.99	16.17	-122.447	37.944	0.25	15.61	296.25	15.86	2	7/10/2014	35:20.7
100	RSRS19	-122.447	37.944	0.61	15.61	297.93	16.22	-122.447	37.944	0.30	15.61	296.19	15.91	2	7/10/2014	35:20.8
101	RSRS19	-122.447	37.944	0.61	15.59	297.86	16.20	-122.447	37.944	0.39	15.59	296.15	15.97	2	7/10/2014	35:21.0
102	RSRS19	-122.447	37.944	0.61	15.60	297.89	16.21	-122.447	37.944	0.34	15.60	296.13	15.94	2	7/10/2014	35:21.1
103	RSRS19	-122.447	37.944	0.61	15.61	297.91	16.22	-122.447	37.944	0.42	15.61	296.17	16.03	2	7/10/2014	35:21.1
104	RSRS19	-122.447	37.944	0.61	15.60	297.88	16.21	-122.447	37.944	0.34	15.60	296.19	15.94	2	7/10/2014	35:21.2
105	RSRS19	-122.447	37.944	0.61	15.64	297.89	16.25	-122.447	37.944	0.30	15.64	296.17	15.94	2	7/10/2014	35:21.4
106	RSRS19	-122.447	37.944	0.61	15.67	297.84	16.28	-122.447	37.944	0.34	15.67	296.15	16.01	2	7/10/2014	35:21.5
107	RSRS19	-122.447	37.944	0.61	15.72	297.91	16.33	-122.447	37.944	0.42	15.72	296.19	16.14	2	7/10/2014	35:21.5
108	RSRS19	-122.447	37.944	0.61	15.67	297.91	16.28	-122.447	37.944	0.34	15.67	296.17	16.01	2	7/10/2014	35:21.6
109	RSRS19	-122.447	37.944	0.61	15.68	297.89	16.29	-122.447	37.944	0.46	15.68	296.17	16.13	2	7/10/2014	35:21.8
110	RSRS19	-122.447	37.944	0.56	15.69	297.84	16.26	-122.447	37.944	0.39	15.69	296.13	16.08	2	7/10/2014	35:21.8
111	RSRS19	-122.447	37.944	0.61	15.69	297.83	16.31	-122.447	37.944	0.34	15.69	296.11	16.03	2	7/10/2014	35:21.9
112	RSRS19	-122.447	37.944	0.61	15.70	297.83	16.32	-122.447	37.944	0.39	15.70	296.11	16.09	2	7/10/2014	35:22.0
113	RSRS19	-122.447	37.944	0.61	15.72	297.89	16.33	-122.447	37.944	0.39	15.72	296.16	16.11	2	7/10/2014	35:22.2
114	RSRS19	-122.447	37.944	0.56	15.71	297.80	16.27	-122.447	37.944	0.34	15.71	296.09	16.05	2	7/10/2014	35:22.3
115	RSRS19	-122.447	37.944	0.61	15.72	297.78	16.33	-122.447	37.944	0.51	15.72	296.09	16.22	2	7/10/2014	35:22.3
116	RSRS19	-122.447	37.944	0.56	15.71	297.81	16.27	-122.447	37.944	0.39	15.71	296.07	16.10	2	7/10/2014	35:22.4
117	RSRS19	-122.447	37.944	0.61	15.67	297.83	16.28	-122.447	37.944	0.30	15.67	296.09	15.97	2	7/10/2014	35:22.5
118	RSRS19	-122.447	37.944	0.61	15.67	297.83	16.28	-122.447	37.944	0.42	15.67	296.11	16.09	2	7/10/2014	35:22.7
119	RSRS19	-122.447	37.944	0.61	15.66	297.89	16.28	-122.447	37.944	0.30	15.66	296.12	15.97	2	7/10/2014	35:22.7
120	RSRS19	-122.447	37.944	0.61	15.70	297.92	16.32	-122.447	37.944	0.39	15.70	296.16	16.09	2	7/10/2014	35:22.8
121	RSRS19	-122.447	37.944	0.61	15.73	297.96	16.35	-122.447	37.944	0.46	15.73	296.14	16.19	2	7/10/2014	35:22.9
122	RSRS19	-122.447	37.944	0.56	15.69	297.96	16.25	-122.447	37.944	0.30	15.69	296.12	15.99	2	7/10/2014	35:23.1
123	RSRS19	-122.447	37.944	0.61	15.69	297.99	16.31	-122.447	37.944	0.34	15.69	296.14	16.03	2	7/10/2014	35:23.1
124	RSRS19	-122.447	37.944	0.61	15.66	298.03	16.27	-122.447	37.944	0.42	15.66	296.12	16.08	2	7/10/2014	35:23.2
125	RSRS19	-122.447	37.944	0.61	15.68	297.99	16.29	-122.447	37.944	0.30	15.68	296.14	15.98	2	7/10/2014	35:23.4
126	RSRS19	-122.447	37.944	0.61	15.76	298.01	16.37	-122.447	37.944	0.42	15.76	296.12	16.18	2	7/10/2014	35:23.5
127	RSRS19	-122.447	37.944	0.61	15.65	297.99	16.26	-122.447	37.944	0.42	15.65	296.10	16.07	2	7/10/2014	35:23.5
128	RSRS19	-122.447	37.944	0.56	15.64	298.01	16.20	-122.447	37.944	0.22	15.64	296.12	15.86	2	7/10/2014	35:23.6
129	RSRS19	-122.447	37.944	0.61	15.66	298.04	16.27	-122.447	37.944	0.34	15.66	296.14	15.99	2	7/10/2014	35:23.8
130	RSRS19	-122.447	37.944	0.61	15.70	298.06	16.32	-122.447	37.944	0.42	15.70	296.19	16.13	2	7/10/2014	35:23.9
131	RSRS19	-122.447	37.944	0.61	15.69	298.14	16.30	-122.447	37.944	0.25	15.69	296.21	15.94	2	7/10/2014	35:23.9

132	RSRS19	-122.447	37.944	0.61	15.64	298.10	16.25	-122.447	37.944	0.34	15.64	296.25	15.98	2	7/10/2014	35:24.0
133	RSRS19	-122.447	37.944	0.61	15.67	298.17	16.28	-122.447	37.944	0.34	15.67	296.28	16.01	2	7/10/2014	35:24.2
134	RSRS19	-122.447	37.944	0.61	15.67	298.13	16.28	-122.447	37.944	0.25	15.67	296.32	15.92	2	7/10/2014	35:24.3
135	RSRS19	-122.447	37.944	0.61	15.69	298.21	16.31	-122.447	37.944	0.39	15.69	296.35	16.08	2	7/10/2014	35:24.3
136	RSRS19	-122.447	37.944	0.61	15.73	298.17	16.34	-122.447	37.944	0.39	15.73	296.35	16.11	2	7/10/2014	35:24.4
137	RSRS19	-122.447	37.944	0.61	15.73	298.19	16.34	-122.447	37.944	0.25	15.73	296.35	15.98	2	7/10/2014	35:24.6
138	RSRS19	-122.447	37.944	0.61	15.73	298.26	16.35	-122.447	37.944	0.39	15.73	296.35	16.12	2	7/10/2014	35:24.6
139	RSRS19	-122.447	37.944	0.61	15.73	298.17	16.34	-122.447	37.944	0.25	15.73	296.39	15.98	2	7/10/2014	35:24.7
140	RSRS19	-122.447	37.944	0.61	15.80	298.22	16.41	-122.447	37.944	0.22	15.80	296.33	16.01	2	7/10/2014	35:24.9
141	RSRS19	-122.447	37.944	0.61	15.76	298.26	16.37	-122.447	37.944	0.42	15.76	296.37	16.18	2	7/10/2014	35:25.0
142	RSRS19	-122.447	37.944	0.56	15.77	298.26	16.33	-122.447	37.944	0.22	15.77	296.39	15.99	2	7/10/2014	35:25.1
143	RSRS19	-122.447	37.944	0.61	15.83	298.31	16.45	-122.447	37.944	0.25	15.83	296.42	16.09	2	7/10/2014	35:25.1
144	RSRS19	-122.447	37.944	0.56	15.80	298.31	16.36	-122.447	37.944	0.39	15.80	296.44	16.19	2	7/10/2014	35:25.3
145	RSRS19	-122.447	37.944	0.61	15.83	298.33	16.44	-122.447	37.944	0.25	15.83	296.46	16.08	2	7/10/2014	35:25.4
146	RSRS19	-122.447	37.944	0.56	15.86	298.29	16.42	-122.447	37.944	0.25	15.86	296.40	16.11	2	7/10/2014	35:25.5
147	RSRS19	-122.447	37.944	0.61	15.87	298.33	16.48	-122.447	37.944	0.39	15.87	296.40	16.26	2	7/10/2014	35:25.5
148	RSRS19	-122.447	37.944	0.61	15.89	298.33	16.50	-122.447	37.944	0.17	15.89	296.40	16.06	2	7/10/2014	35:25.7
149	RSRS19	-122.447	37.944	0.61	15.92	298.33	16.53	-122.447	37.944	0.42	15.92	296.38	16.34	2	7/10/2014	35:25.7
150	RSRS19	-122.447	37.944	0.61	15.90	298.27	16.51	-122.447	37.944	0.30	15.90	296.38	16.20	2	7/10/2014	35:25.9
151	RSRS19	-122.447	37.944	0.61	15.90	298.27	16.51	-122.447	37.944	0.22	15.90	296.40	16.12	2	7/10/2014	35:25.9
152	RSRS19	-122.447	37.944	0.61	15.94	298.29	16.55	-122.447	37.944	0.34	15.94	296.42	16.28	2	7/10/2014	35:26.1
153	RSRS19	-122.447	37.944	0.61	15.91	298.32	16.52	-122.447	37.944	0.30	15.91	296.45	16.21	2	7/10/2014	35:26.2
154	RSRS19	-122.447	37.944	0.61	15.90	298.29	16.51	-122.447	37.944	0.22	15.90	296.56	16.12	2	7/10/2014	35:26.3
155	RSRS19	-122.447	37.944	0.61	15.90	298.34	16.51	-122.447	37.944	0.39	15.90	296.54	16.28	2	7/10/2014	35:26.3
156	RSRS19	-122.447	37.944	0.56	15.86	298.32	16.43	-122.447	37.944	0.22	15.86	296.47	16.08	2	7/10/2014	35:26.5
157	RSRS19	-122.447	37.944	0.61	15.83	298.34	16.45	-122.447	37.944	0.25	15.83	296.45	16.09	2	7/10/2014	35:26.6
158	RSRS19	-122.447	37.944	0.61	15.81	298.32	16.42	-122.447	37.944	0.34	15.81	296.43	16.15	2	7/10/2014	35:26.7
159	RSRS19	-122.447	37.944	0.61	15.80	298.25	16.41	-122.447	37.944	0.30	15.80	296.38	16.10	2	7/10/2014	35:26.7
160	RSRS19	-122.447	37.944	0.61	15.77	298.27	16.38	-122.447	37.944	0.25	15.77	296.37	16.03	2	7/10/2014	35:26.9
161	RSRS19	-122.447	37.944	0.61	15.74	298.24	16.35	-122.447	37.944	0.39	15.74	296.36	16.13	2	7/10/2014	35:27.0
162	RSRS19	-122.447	37.944	0.61	15.72	298.32	16.33	-122.447	37.944	0.25	15.72	296.32	15.97	2	7/10/2014	35:27.1
163	RSRS19	-122.447	37.944	0.61	15.72	298.28	16.33	-122.447	37.944	0.25	15.72	296.32	15.97	2	7/10/2014	35:27.1
164	RSRS19	-122.447	37.944	0.61	15.70	298.32	16.32	-122.447	37.944	0.39	15.70	296.39	16.09	2	7/10/2014	35:27.3

165	RSRS19	-122.447	37.944	0.61	15.73	298.30	16.34	-122.447	37.944	0.30	15.73	296.35	16.03	2	7/10/2014	35:27.4
166	RSRS19	-122.447	37.944	0.56	15.71	298.28	16.27	-122.447	37.944	0.25	15.71	296.36	15.96	2	7/10/2014	35:27.4
167	RSRS19	-122.447	37.944	0.61	15.71	298.28	16.32	-122.447	37.944	0.42	15.71	296.35	16.13	2	7/10/2014	35:27.5
168	RSRS19	-122.447	37.944	0.61	15.73	298.28	16.34	-122.447	37.944	0.25	15.73	296.35	15.98	2	7/10/2014	35:27.7
169	RSRS19	-122.447	37.944	0.61	15.71	298.26	16.32	-122.447	37.944	0.25	15.71	296.35	15.96	2	7/10/2014	35:27.8
170	RSRS19	-122.447	37.944	0.61	15.75	298.24	16.36	-122.447	37.944	0.34	15.75	296.30	16.09	2	7/10/2014	35:27.9
171	RSRS19	-122.447	37.944	0.61	15.80	298.28	16.41	-122.447	37.944	0.22	15.80	296.30	16.02	2	7/10/2014	35:27.9
172	RSRS19	-122.447	37.944	0.61	15.71	298.33	16.32	-122.447	37.944	0.34	15.71	296.29	16.05	2	7/10/2014	35:28.1
173	RSRS19	-122.447	37.944	0.61	15.80	298.24	16.41	-122.447	37.944	0.39	15.80	296.28	16.18	2	7/10/2014	35:28.2
174	RSRS19	-122.447	37.944	0.61	15.69	298.33	16.30	-122.447	37.944	0.25	15.69	296.29	15.94	2	7/10/2014	35:28.3
175	RSRS19	-122.447	37.944	0.61	15.82	298.26	16.43	-122.447	37.944	0.30	15.82	296.24	16.12	2	7/10/2014	35:28.3
176	RSRS19	-122.447	37.944	0.61	15.94	298.24	16.55	-122.447	37.944	0.34	15.94	296.24	16.28	2	7/10/2014	35:28.5
177	RSRS19	-122.447	37.944	0.65	15.85	298.22	16.50	-122.447	37.944	0.25	15.85	296.27	16.10	2	7/10/2014	35:28.5
178	RSRS19	-122.447	37.944	0.61	15.81	298.22	16.42	-122.447	37.944	0.42	15.81	296.29	16.23	2	7/10/2014	35:28.7
179	RSRS19	-122.447	37.944	0.65	15.83	298.23	16.48	-122.447	37.944	0.34	15.83	296.29	16.17	2	7/10/2014	35:28.7
180	RSRS19	-122.447	37.944	0.61	15.83	298.31	16.44	-122.447	37.944	0.22	15.83	296.31	16.05	2	7/10/2014	35:28.9
181	RSRS19	-122.447	37.944	0.65	15.71	298.25	16.36	-122.447	37.944	0.39	15.71	296.33	16.10	2	7/10/2014	35:29.0
182	RSRS19	-122.447	37.944	0.61	15.83	298.31	16.45	-122.447	37.944	0.25	15.83	296.33	16.09	2	7/10/2014	35:29.1
183	RSRS19	-122.447	37.944	0.61	15.83	298.34	16.44	-122.447	37.944	0.42	15.83	296.34	16.25	2	7/10/2014	35:29.1
184	RSRS19	-122.447	37.944	0.61	15.89	298.36	16.50	-122.447	37.944	0.34	15.89	296.36	16.22	2	7/10/2014	35:29.3
185	RSRS19	-122.447	37.944	0.65	15.88	298.29	16.53	-122.447	37.944	0.22	15.88	296.27	16.10	2	7/10/2014	35:29.3
186	RSRS19	-122.447	37.944	0.56	15.88	298.23	16.44	-122.447	37.944	0.42	15.88	296.18	16.30	2	7/10/2014	35:29.5
187	RSRS19	-122.447	37.944	0.61	15.91	298.21	16.52	-122.447	37.944	0.34	15.91	296.21	16.25	2	7/10/2014	35:29.5
188	RSRS19	-122.447	37.944	0.61	15.93	298.23	16.54	-122.447	37.944	0.22	15.93	296.19	16.15	2	7/10/2014	35:29.7
189	RSRS19	-122.447	37.944	0.61	16.03	298.21	16.65	-122.447	37.944	0.39	16.03	296.23	16.42	2	7/10/2014	35:29.8
190	RSRS19	-122.447	37.944	0.61	16.00	298.17	16.61	-122.447	37.944	0.30	16.00	296.23	16.30	2	7/10/2014	35:29.9
191	RSRS19	-122.447	37.944	0.65	15.96	298.21	16.60	-122.447	37.944	0.30	15.96	296.26	16.26	2	7/10/2014	35:29.9
192	RSRS19	-122.447	37.944	0.61	15.77	298.21	16.38	-122.447	37.944	0.42	15.77	296.28	16.19	2	7/10/2014	35:30.1
193	RSRS19	-122.447	37.944	0.65	15.80	298.25	16.44	-122.447	37.944	0.30	15.80	296.32	16.10	2	7/10/2014	35:30.2
194	RSRS19	-122.447	37.944	0.61	15.83	298.24	16.45	-122.447	37.944	0.30	15.83	296.32	16.14	2	7/10/2014	35:30.2
195	RSRS19	-122.447	37.944	0.61	15.76	298.24	16.37	-122.447	37.944	0.46	15.76	296.26	16.22	2	7/10/2014	35:30.3
196	RSRS19	-122.447	37.944	0.61	15.71	298.19	16.32	-122.447	37.944	0.22	15.71	296.19	15.93	2	7/10/2014	35:30.5
197	RSRS19	-122.447	37.944	0.65	15.74	298.19	16.39	-122.447	37.944	0.39	15.74	296.17	16.13	2	7/10/2014	35:30.6

198	RSRS19	-122.447	37.944	0.61	15.75	298.24	16.36	-122.447	37.944	0.34	15.75	296.15	16.09	2	7/10/2014	35:30.7
199	RSRS19	-122.447	37.944	0.65	15.77	298.17	16.42	-122.447	37.944	0.17	15.77	296.13	15.94	2	7/10/2014	35:30.7
200	RSRS19	-122.447	37.944	0.61	15.82	298.22	16.43	-122.447	37.944	0.39	15.82	296.17	16.21	2	7/10/2014	35:30.9
201	RSRS19	-122.447	37.944	0.65	15.81	298.26	16.46	-122.447	37.944	0.39	15.81	296.20	16.20	2	7/10/2014	35:31.0
202	RSRS19	-122.447	37.944	0.61	15.82	298.17	16.43	-122.447	37.944	0.22	15.82	296.26	16.04	2	7/10/2014	35:31.1
203	RSRS19	-122.447	37.944	0.65	15.83	298.17	16.47	-122.447	37.944	0.34	15.83	296.24	16.16	2	7/10/2014	35:31.1
204	RSRS19	-122.447	37.944	0.61	15.88	298.11	16.49	-122.447	37.944	0.39	15.88	296.19	16.27	2	7/10/2014	35:31.3
205	RSRS19	-122.448	37.944	0.61	15.86	298.09	16.48	-122.448	37.944	0.30	15.86	296.17	16.17	2	7/10/2014	35:31.3
206	RSRS19	-122.448	37.944	0.61	15.90	298.02	16.51	-122.448	37.944	0.34	15.90	296.13	16.23	2	7/10/2014	35:31.5
207	RSRS19	-122.448	37.944	0.61	15.92	298.02	16.53	-122.448	37.944	0.39	15.92	296.14	16.31	2	7/10/2014	35:31.5
208	RSRS19	-122.448	37.944	0.61	15.90	297.98	16.51	-122.448	37.944	0.17	15.90	296.07	16.06	2	7/10/2014	35:31.7
209	RSRS19	-122.448	37.944	0.65	15.90	298.00	16.54	-122.448	37.944	0.42	15.90	296.09	16.32	2	7/10/2014	35:31.8
210	RSRS19	-122.448	37.944	0.61	15.94	298.02	16.55	-122.448	37.944	0.39	15.94	296.09	16.33	2	7/10/2014	35:31.9
211	RSRS19	-122.448	37.944	0.65	15.97	298.03	16.61	-122.448	37.944	0.22	15.97	296.12	16.18	2	7/10/2014	35:31.9
212	RSRS19	-122.448	37.944	0.61	15.93	298.05	16.54	-122.448	37.944	0.34	15.93	296.09	16.27	2	7/10/2014	35:32.1
213	RSRS19	-122.448	37.944	0.65	15.93	298.03	16.58	-122.448	37.944	0.42	15.93	296.14	16.36	2	7/10/2014	35:32.2
214	RSRS19	-122.448	37.944	0.56	15.98	298.05	16.54	-122.448	37.944	0.25	15.98	296.11	16.23	2	7/10/2014	35:32.3
215	RSRS19	-122.448	37.944	0.65	15.96	298.03	16.60	-122.448	37.944	0.34	15.96	296.12	16.29	2	7/10/2014	35:32.3
216	RSRS19	-122.448	37.944	0.61	15.99	298.03	16.60	-122.448	37.944	0.42	15.99	296.16	16.41	2	7/10/2014	35:32.5
217	RSRS19	-122.448	37.944	0.65	15.97	298.03	16.61	-122.448	37.944	0.30	15.97	296.12	16.27	2	7/10/2014	35:32.6
218	RSRS19	-122.448	37.944	0.61	15.97	297.99	16.58	-122.448	37.944	0.39	15.97	296.07	16.36	2	7/10/2014	35:32.7
219	RSRS19	-122.448	37.944	0.65	15.95	297.97	16.60	-122.448	37.944	0.42	15.95	296.03	16.37	2	7/10/2014	35:32.7
220	RSRS19	-122.448	37.944	0.61	15.97	298.01	16.58	-122.448	37.944	0.25	15.97	296.05	16.22	2	7/10/2014	35:32.9
221	RSRS19	-122.448	37.944	0.65	15.98	297.99	16.63	-122.448	37.944	0.34	15.98	296.07	16.32	2	7/10/2014	35:33.0
222	RSRS19	-122.448	37.944	0.61	15.96	298.08	16.57	-122.448	37.944	0.42	15.96	296.06	16.38	2	7/10/2014	35:33.0
223	RSRS19	-122.448	37.944	0.61	15.95	298.06	16.56	-122.448	37.944	0.30	15.95	296.08	16.25	2	7/10/2014	35:33.1
224	RSRS19	-122.448	37.944	0.61	15.90	298.06	16.51	-122.448	37.944	0.34	15.90	296.10	16.24	2	7/10/2014	35:33.3
225	RSRS19	-122.448	37.944	0.65	15.88	298.06	16.53	-122.448	37.944	0.42	15.88	296.12	16.30	2	7/10/2014	35:33.4
226	RSRS19	-122.448	37.944	0.61	15.86	298.06	16.47	-122.448	37.944	0.34	15.86	296.14	16.19	2	7/10/2014	35:33.4
227	RSRS19	-122.448	37.944	0.65	15.85	298.08	16.50	-122.448	37.944	0.39	15.85	296.14	16.24	2	7/10/2014	35:33.5
228	RSRS19	-122.448	37.944	0.61	15.82	298.06	16.43	-122.448	37.944	0.42	15.82	296.22	16.24	2	7/10/2014	35:33.7
229	RSRS19	-122.448	37.944	0.65	15.79	298.04	16.43	-122.448	37.944	0.34	15.79	296.17	16.13	2	7/10/2014	35:33.8
230	RSRS19	-122.448	37.944	0.61	15.77	298.04	16.38	-122.448	37.944	0.42	15.77	296.13	16.19	2	7/10/2014	35:33.9

231	RSRS19	-122.448	37.944	0.61	15.75	298.02	16.36	-122.448	37.944	0.46	15.75	296.08	16.20	2	7/10/2014	35:33.9
232	RSRS19	-122.448	37.944	0.61	15.76	298.06	16.37	-122.448	37.944	0.30	15.76	296.08	16.07	2	7/10/2014	35:34.1
233	RSRS19	-122.448	37.944	0.61	15.76	298.13	16.37	-122.448	37.944	0.30	15.76	296.11	16.06	2	7/10/2014	35:34.1
234	RSRS19	-122.448	37.944	0.61	15.69	298.13	16.31	-122.448	37.944	0.42	15.69	296.15	16.12	2	7/10/2014	35:34.3
235	RSRS19	-122.448	37.944	0.61	15.73	298.11	16.35	-122.448	37.944	0.34	15.73	296.18	16.07	2	7/10/2014	35:34.3
236	RSRS19	-122.448	37.944	0.61	15.71	298.02	16.32	-122.448	37.944	0.30	15.71	296.11	16.01	2	7/10/2014	35:34.5
237	RSRS19	-122.448	37.944	0.65	15.70	298.09	16.35	-122.448	37.944	0.46	15.70	296.15	16.16	2	7/10/2014	35:34.6
238	RSRS19	-122.448	37.944	0.61	15.67	298.09	16.28	-122.448	37.944	0.34	15.67	296.13	16.01	2	7/10/2014	35:34.7
239	RSRS19	-122.448	37.944	0.65	15.66	298.09	16.30	-122.448	37.944	0.34	15.66	296.09	15.99	2	7/10/2014	35:34.7
240	RSRS19	-122.448	37.944	0.61	15.68	298.07	16.29	-122.448	37.944	0.42	15.68	296.07	16.10	2	7/10/2014	35:34.9
241	RSRS19	-122.448	37.944	0.65	15.64	298.07	16.29	-122.448	37.944	0.34	15.64	296.05	15.98	2	7/10/2014	35:35.0
242	RSRS19	-122.448	37.944	0.61	15.68	298.09	16.29	-122.448	37.944	0.34	15.68	296.07	16.02	2	7/10/2014	35:35.1
243	RSRS19	-122.448	37.944	0.65	15.69	298.11	16.33	-122.448	37.944	0.46	15.69	296.11	16.14	2	7/10/2014	35:35.1
244	RSRS19	-122.448	37.944	0.61	15.97	298.11	16.58	-122.448	37.944	0.30	15.97	296.12	16.28	2	7/10/2014	35:35.3
245	RSRS19	-122.448	37.944	0.65	15.93	298.12	16.58	-122.448	37.944	0.30	15.93	296.07	16.24	2	7/10/2014	35:35.4
246	RSRS19	-122.448	37.944	0.61	16.01	298.07	16.62	-122.448	37.944	0.39	16.01	296.07	16.40	2	7/10/2014	35:35.5
247	RSRS19	-122.448	37.944	0.65	15.96	298.10	16.60	-122.448	37.944	0.34	15.96	296.10	16.29	2	7/10/2014	35:35.5
248	RSRS19	-122.448	37.944	0.61	15.95	298.10	16.56	-122.448	37.944	0.30	15.95	296.01	16.25	2	7/10/2014	35:35.7
249	RSRS19	-122.448	37.944	0.65	15.94	298.07	16.59	-122.448	37.944	0.42	15.94	296.07	16.36	2	7/10/2014	35:35.8
250	RSRS19	-122.448	37.944	0.61	15.97	298.08	16.58	-122.448	37.944	0.34	15.97	296.05	16.30	2	7/10/2014	35:35.9
251	RSRS19	-122.448	37.944	0.65	15.97	298.01	16.61	-122.448	37.944	0.34	15.97	296.01	16.30	2	7/10/2014	35:35.9
252	RSRS19	-122.448	37.944	0.61	15.94	298.03	16.55	-122.448	37.944	0.42	15.94	296.01	16.36	2	7/10/2014	35:36.1
253	RSRS19	-122.448	37.944	0.65	15.95	298.04	16.60	-122.448	37.944	0.30	15.95	296.03	16.25	2	7/10/2014	35:36.2
254	RSRS19	-122.448	37.944	0.61	15.93	297.97	16.55	-122.448	37.944	0.34	15.93	295.98	16.27	2	7/10/2014	35:36.3
255	RSRS19	-122.448	37.944	0.61	15.93	297.99	16.55	-122.448	37.944	0.46	15.93	296.01	16.39	2	7/10/2014	35:36.3
256	RSRS19	-122.448	37.944	0.61	15.95	297.90	16.56	-122.448	37.944	0.25	15.95	295.97	16.20	2	7/10/2014	35:36.5
257	RSRS19	-122.448	37.944	0.64	15.93	297.90	16.58	-122.448	37.944	0.34	15.93	295.93	16.27	2	7/10/2014	35:36.6
258	RSRS19	-122.448	37.944	0.56	15.94	297.99	16.50	-122.448	37.944	0.39	15.94	295.99	16.33	2	7/10/2014	35:36.7
259	RSRS19	-122.448	37.944	0.61	15.97	297.97	16.58	-122.448	37.944	0.25	15.97	295.97	16.22	2	7/10/2014	35:36.7
260	RSRS19	-122.448	37.944	0.61	15.81	297.95	16.42	-122.448	37.944	0.34	15.81	295.93	16.15	2	7/10/2014	35:36.9
261	RSRS19	-122.448	37.944	0.64	15.84	297.91	16.49	-122.448	37.944	0.42	15.84	295.89	16.26	2	7/10/2014	35:36.9
262	RSRS19	-122.448	37.944	0.61	15.80	297.89	16.41	-122.448	37.944	0.30	15.80	295.86	16.10	2	7/10/2014	35:37.1
263	RSRS19	-122.448	37.944	0.64	15.76	297.86	16.40	-122.448	37.944	0.34	15.76	295.87	16.09	2	7/10/2014	35:37.1

264	RSRS19	-122.448	37.944	0.61	15.79	297.91	16.40	-122.448	37.944	0.39	15.79	295.84	16.18	2	7/10/2014	35:37.3
265	RSRS19	-122.448	37.944	0.64	15.83	297.91	16.48	-122.448	37.944	0.25	15.83	295.89	16.09	2	7/10/2014	35:37.4
266	RSRS19	-122.448	37.944	0.61	15.83	297.89	16.44	-122.448	37.944	0.39	15.83	295.89	16.21	2	7/10/2014	35:37.5
267	RSRS19	-122.448	37.944	0.61	15.76	297.87	16.37	-122.448	37.944	0.42	15.76	295.83	16.18	2	7/10/2014	35:37.5
268	RSRS19	-122.448	37.944	0.61	15.77	297.89	16.38	-122.448	37.944	0.22	15.77	295.87	15.99	2	7/10/2014	35:37.7
269	RSRS19	-122.448	37.944	0.64	15.73	297.87	16.38	-122.448	37.944	0.39	15.73	295.85	16.12	2	7/10/2014	35:37.8
270	RSRS19	-122.448	37.944	0.61	15.85	297.85	16.46	-122.448	37.944	0.39	15.85	295.87	16.24	2	7/10/2014	35:37.9
271	RSRS19	-122.448	37.944	0.64	15.71	297.83	16.36	-122.448	37.944	0.25	15.71	295.83	15.96	2	7/10/2014	35:37.9
272	RSRS19	-122.448	37.944	0.61	15.72	297.87	16.33	-122.448	37.944	0.34	15.72	295.87	16.05	2	7/10/2014	35:38.1
273	RSRS19	-122.448	37.944	0.64	15.69	297.89	16.34	-122.448	37.944	0.34	15.69	295.90	16.03	2	7/10/2014	35:38.2
274	RSRS19	-122.448	37.944	0.61	15.73	297.94	16.35	-122.448	37.944	0.22	15.73	295.87	15.95	2	7/10/2014	35:38.3
275	RSRS19	-122.448	37.944	0.64	15.73	297.94	16.37	-122.448	37.944	0.34	15.73	295.85	16.06	2	7/10/2014	35:38.3
276	RSRS19	-122.448	37.944	0.61	15.73	297.98	16.35	-122.448	37.944	0.39	15.73	295.90	16.12	2	7/10/2014	35:38.5
277	RSRS19	-122.448	37.944	0.64	15.78	297.96	16.42	-122.448	37.944	0.25	15.78	295.87	16.03	2	7/10/2014	35:38.6
278	RSRS19	-122.448	37.944	0.61	15.73	297.94	16.35	-122.448	37.944	0.34	15.73	295.79	16.07	2	7/10/2014	35:38.6
279	RSRS19	-122.448	37.944	0.64	15.76	297.90	16.40	-122.448	37.944	0.34	15.76	295.79	16.09	2	7/10/2014	35:38.7
280	RSRS19	-122.448	37.944	0.61	15.93	297.94	16.55	-122.448	37.944	0.22	15.93	295.81	16.15	2	7/10/2014	35:38.9
281	RSRS19	-122.448	37.944	0.61	15.97	297.90	16.58	-122.448	37.944	0.39	15.97	295.77	16.36	2	7/10/2014	35:39.0
282	RSRS19	-122.448	37.944	0.61	15.79	297.90	16.40	-122.448	37.944	0.34	15.79	295.79	16.12	2	7/10/2014	35:39.1
283	RSRS19	-122.448	37.944	0.61	15.89	297.95	16.50	-122.448	37.944	0.25	15.89	295.86	16.14	2	7/10/2014	35:39.1
284	RSRS19	-122.448	37.944	0.61	15.92	297.88	16.53	-122.448	37.944	0.34	15.92	295.82	16.25	2	7/10/2014	35:39.3
285	RSRS19	-122.448	37.944	0.61	15.98	297.95	16.59	-122.448	37.944	0.34	15.98	295.90	16.32	2	7/10/2014	35:39.4
286	RSRS19	-122.448	37.944	0.61	16.02	297.99	16.63	-122.448	37.944	0.22	16.02	295.95	16.24	2	7/10/2014	35:39.5
287	RSRS19	-122.448	37.944	0.64	15.98	298.03	16.63	-122.448	37.944	0.39	15.98	295.97	16.37	2	7/10/2014	35:39.5
288	RSRS19	-122.448	37.944	0.61	15.97	298.06	16.58	-122.448	37.944	0.30	15.97	295.99	16.27	2	7/10/2014	35:39.7
289	RSRS19	-122.448	37.944	0.64	15.95	298.06	16.59	-122.448	37.944	0.22	15.95	295.97	16.17	2	7/10/2014	35:39.7
290	RSRS19	-122.448	37.944	0.61	16.00	298.10	16.61	-122.448	37.944	0.42	16.00	295.95	16.42	2	7/10/2014	35:39.9
291	RSRS19	-122.448	37.944	0.61	16.01	298.08	16.62	-122.448	37.944	0.34	16.01	295.93	16.35	2	7/10/2014	35:39.9
292	RSRS19	-122.448	37.944	0.61	16.00	298.13	16.61	-122.448	37.944	0.22	16.00	295.95	16.21	2	7/10/2014	35:40.1
293	RSRS19	-122.448	37.944	0.61	16.00	298.15	16.61	-122.448	37.944	0.34	16.00	296.00	16.34	2	7/10/2014	35:40.2
294	RSRS19	-122.448	37.944	0.61	16.03	298.20	16.64	-122.448	37.944	0.17	16.03	296.04	16.19	2	7/10/2014	35:40.2
295	RSRS19	-122.448	37.944	0.64	16.02	298.20	16.66	-122.448	37.944	0.39	16.02	296.11	16.41	2	7/10/2014	35:40.3
296	RSRS19	-122.448	37.944	0.61	16.01	298.22	16.62	-122.448	37.944	0.34	16.01	296.13	16.35	2	7/10/2014	35:40.5

297	RSRS19	-122.448	37.944	0.61	15.99	298.24	16.60	-122.448	37.944	0.22	15.99	296.20	16.21	2	7/10/2014	35:40.6
298	RSRS19	-122.448	37.944	0.61	16.03	298.26	16.65	-122.448	37.944	0.39	16.03	296.24	16.42	2	7/10/2014	35:40.7
299	RSRS19	-122.448	37.944	0.61	16.00	298.24	16.61	-122.448	37.944	0.30	16.00	296.24	16.31	2	7/10/2014	35:40.7
300	RSRS19	-122.448	37.944	0.61	15.97	298.24	16.58	-122.448	37.944	0.17	15.97	296.22	16.13	2	7/10/2014	35:40.9
301	RSRS19	-122.448	37.944	0.61	16.04	298.29	16.65	-122.448	37.944	0.34	16.04	296.24	16.38	2	7/10/2014	35:41.0
302	RSRS19	-122.448	37.944	0.61	15.94	298.27	16.55	-122.448	37.944	0.25	15.94	296.22	16.19	2	7/10/2014	35:41.1
303	RSRS19	-122.448	37.944	0.64	15.95	298.29	16.59	-122.448	37.944	0.30	15.95	296.22	16.25	2	7/10/2014	35:41.1
304	RSRS19	-122.448	37.944	0.61	15.95	298.33	16.56	-122.448	37.944	0.39	15.95	296.26	16.34	2	7/10/2014	35:41.3
305	RSRS19	-122.448	37.944	0.61	15.95	298.38	16.56	-122.448	37.944	0.22	15.95	296.25	16.17	2	7/10/2014	35:41.4
306	RSRS19	-122.448	37.944	0.61	15.97	298.40	16.58	-122.448	37.944	0.22	15.97	296.27	16.19	2	7/10/2014	35:41.4
307	RSRS19	-122.448	37.944	0.64	15.99	298.38	16.63	-122.448	37.944	0.39	15.99	296.27	16.37	2	7/10/2014	35:41.5
308	RSRS19	-122.448	37.944	0.61	16.00	298.40	16.61	-122.448	37.944	0.22	16.00	296.33	16.21	2	7/10/2014	35:41.7
309	RSRS19	-122.448	37.944	0.64	16.00	298.40	16.64	-122.448	37.944	0.39	16.00	296.33	16.38	2	7/10/2014	35:41.8
310	RSRS19	-122.448	37.944	0.61	16.01	298.40	16.62	-122.448	37.944	0.30	16.01	296.34	16.31	2	7/10/2014	35:41.9
311	RSRS19	-122.448	37.944	0.61	16.00	298.41	16.61	-122.448	37.944	0.17	16.00	296.36	16.17	2	7/10/2014	35:41.9
312	RSRS19	-122.448	37.944	0.61	16.00	298.38	16.61	-122.448	37.944	0.39	16.00	296.36	16.38	2	7/10/2014	35:42.1
313	RSRS19	-122.448	37.944	0.61	15.97	298.41	16.58	-122.448	37.944	0.25	15.97	296.34	16.22	2	7/10/2014	35:42.2
314	RSRS19	-122.448	37.944	0.61	15.97	298.34	16.58	-122.448	37.944	0.13	15.97	296.28	16.10	2	7/10/2014	35:42.3
315	RSRS19	-122.448	37.944	0.61	15.94	298.43	16.55	-122.448	37.944	0.30	15.94	296.32	16.24	2	7/10/2014	35:42.3
316	RSRS19	-122.448	37.944	0.61	15.96	298.39	16.57	-122.448	37.944	0.30	15.96	296.30	16.26	2	7/10/2014	35:42.5
317	RSRS19	-122.448	37.944	0.64	16.00	298.45	16.64	-122.448	37.944	0.22	16.00	296.33	16.21	2	7/10/2014	35:42.5
318	RSRS19	-122.448	37.944	0.61	15.93	298.48	16.54	-122.448	37.944	0.39	15.93	296.32	16.32	2	7/10/2014	35:42.7
319	RSRS19	-122.448	37.944	0.61	15.92	298.43	16.53	-122.448	37.944	0.25	15.92	296.32	16.17	2	7/10/2014	35:42.7
320	RSRS19	-122.448	37.944	0.61	15.91	298.52	16.52	-122.448	37.944	0.25	15.91	296.37	16.16	2	7/10/2014	35:42.9
321	RSRS19	-122.448	37.944	0.61	15.89	298.48	16.50	-122.448	37.944	0.39	15.89	296.34	16.27	2	7/10/2014	35:43.0
322	RSRS19	-122.448	37.944	0.61	15.90	298.48	16.51	-122.448	37.944	0.22	15.90	296.35	16.11	2	7/10/2014	35:43.0
323	RSRS19	-122.448	37.944	0.61	15.93	298.46	16.55	-122.448	37.944	0.25	15.93	296.35	16.19	2	7/10/2014	35:43.1
324	RSRS19	-122.448	37.944	0.61	16.17	298.44	16.78	-122.448	37.944	0.30	16.17	296.30	16.47	2	7/10/2014	35:43.3
325	RSRS19	-122.448	37.944	0.61	16.09	298.44	16.70	-122.448	37.944	0.13	16.09	296.31	16.22	2	7/10/2014	35:43.4
326	RSRS19	-122.448	37.944	0.61	16.09	298.50	16.71	-122.448	37.944	0.22	16.09	296.32	16.31	2	7/10/2014	35:43.5
327	RSRS19	-122.448	37.944	0.61	16.11	298.48	16.72	-122.448	37.944	0.34	16.11	296.28	16.45	2	7/10/2014	35:43.5
328	RSRS19	-122.448	37.944	0.61	16.07	298.53	16.68	-122.448	37.944	0.17	16.07	296.33	16.24	2	7/10/2014	35:43.7
329	RSRS19	-122.448	37.944	0.61	16.09	298.51	16.70	-122.448	37.944	0.22	16.09	296.33	16.31	2	7/10/2014	35:43.8

330	RSRS19	-122.448	37.944	0.61	16.06	298.51	16.67	-122.448	37.944	0.34	16.06	296.33	16.40	2	7/10/2014	35:43.9
331	RSRS19	-122.448	37.944	0.61	16.09	298.46	16.70	-122.448	37.944	0.17	16.09	296.38	16.26	2	7/10/2014	35:44.0
332	RSRS19	-122.448	37.944	0.56	16.07	298.46	16.63	-122.448	37.944	0.25	16.07	296.35	16.32	2	7/10/2014	35:44.1
333	RSRS19	-122.448	37.944	0.61	16.09	298.44	16.71	-122.448	37.944	0.39	16.09	296.38	16.48	2	7/10/2014	35:44.2
334	RSRS19	-122.448	37.944	0.56	16.07	298.47	16.63	-122.448	37.944	0.17	16.07	296.40	16.24	2	7/10/2014	35:44.2
335	RSRS19	-122.448	37.944	0.61	16.10	298.40	16.72	-122.448	37.944	0.25	16.10	296.33	16.36	2	7/10/2014	35:44.3
336	RSRS19	-122.448	37.944	0.61	16.11	298.42	16.72	-122.448	37.944	0.25	16.11	296.33	16.36	2	7/10/2014	35:44.5
337	RSRS19	-122.448	37.944	0.61	16.17	298.44	16.78	-122.448	37.944	0.13	16.17	296.33	16.30	2	7/10/2014	35:44.6
338	RSRS19	-122.448	37.944	0.56	16.23	298.47	16.79	-122.448	37.944	0.34	16.23	296.29	16.56	2	7/10/2014	35:44.7
339	RSRS19	-122.448	37.944	0.61	16.13	298.47	16.74	-122.448	37.944	0.25	16.13	296.32	16.38	2	7/10/2014	35:44.7
340	RSRS19	-122.448	37.944	0.61	16.15	298.47	16.76	-122.448	37.944	0.13	16.15	296.29	16.28	2	7/10/2014	35:44.9
341	RSRS19	-122.448	37.944	0.61	16.13	298.45	16.74	-122.448	37.944	0.30	16.13	296.36	16.43	2	7/10/2014	35:45.0
342	RSRS19	-122.448	37.944	0.61	16.22	298.40	16.83	-122.448	37.944	0.30	16.22	296.34	16.52	2	7/10/2014	35:45.1
343	RSRS19	-122.448	37.944	0.61	16.18	298.34	16.79	-122.448	37.944	0.17	16.18	296.34	16.35	2	7/10/2014	35:45.1
344	RSRS19	-122.448	37.944	0.61	16.15	298.41	16.76	-122.448	37.944	0.30	16.15	296.38	16.45	2	7/10/2014	35:45.3
345	RSRS19	-122.448	37.944	0.61	16.12	298.36	16.73	-122.448	37.944	0.30	16.12	296.34	16.42	2	7/10/2014	35:45.3
346	RSRS19	-122.448	37.944	0.61	16.16	298.34	16.77	-122.448	37.944	0.17	16.16	296.38	16.33	2	7/10/2014	35:45.5
347	RSRS19	-122.448	37.944	0.61	16.10	298.41	16.72	-122.448	37.944	0.39	16.10	296.34	16.49	2	7/10/2014	35:45.6
348	RSRS19	-122.448	37.944	0.61	16.09	298.30	16.71	-122.448	37.944	0.25	16.09	296.36	16.35	2	7/10/2014	35:45.7
349	RSRS19	-122.448	37.944	0.61	16.08	298.39	16.69	-122.448	37.944	0.17	16.08	296.36	16.25	2	7/10/2014	35:45.8
350	RSRS19	-122.448	37.944	0.61	16.09	298.37	16.70	-122.448	37.944	0.39	16.09	296.39	16.48	2	7/10/2014	35:45.8
351	RSRS19	-122.448	37.944	0.61	16.11	298.41	16.72	-122.448	37.944	0.30	16.11	296.39	16.41	2	7/10/2014	35:45.9
352	RSRS19	-122.448	37.944	0.61	16.11	298.43	16.72	-122.448	37.944	0.22	16.11	296.41	16.33	2	7/10/2014	35:46.1
353	RSRS19	-122.448	37.944	0.61	16.10	298.50	16.72	-122.448	37.944	0.39	16.10	296.41	16.49	2	7/10/2014	35:46.2
354	RSRS19	-122.448	37.944	0.61	16.15	298.50	16.76	-122.448	37.944	0.17	16.15	296.45	16.32	2	7/10/2014	35:46.3
355	RSRS19	-122.448	37.944	0.61	16.07	298.52	16.68	-122.448	37.944	0.30	16.07	296.46	16.37	2	7/10/2014	35:46.3
356	RSRS19	-122.448	37.944	0.61	16.10	298.55	16.72	-122.448	37.944	0.34	16.10	296.52	16.44	2	7/10/2014	35:46.5
357	RSRS19	-122.448	37.944	0.61	16.11	298.55	16.72	-122.448	37.944	0.25	16.11	296.46	16.36	2	7/10/2014	35:46.6
358	RSRS19	-122.448	37.944	0.61	16.07	298.55	16.68	-122.448	37.944	0.30	16.07	296.48	16.37	2	7/10/2014	35:46.7
359	RSRS19	-122.448	37.944	0.61	16.04	298.52	16.65	-122.448	37.944	0.34	16.04	296.46	16.38	2	7/10/2014	35:46.7
360	RSRS19	-122.448	37.944	0.61	16.02	298.57	16.63	-122.448	37.944	0.17	16.02	296.48	16.19	2	7/10/2014	35:46.9
361	RSRS19	-122.448	37.944	0.61	16.00	298.51	16.61	-122.448	37.944	0.39	16.00	296.48	16.39	2	7/10/2014	35:46.9
362	RSRS19	-122.448	37.944	0.61	15.98	298.55	16.59	-122.448	37.944	0.39	15.98	296.51	16.37	2	7/10/2014	35:47.1

363	RSRS19	-122.448	37.944	0.61	15.97	298.66	16.58	-122.448	37.944	0.22	15.97	296.51	16.18	2	7/10/2014	35:47.1
364	RSRS19	-122.448	37.944	0.61	15.97	298.55	16.58	-122.448	37.944	0.34	15.97	296.62	16.30	2	7/10/2014	35:47.3
365	RSRS19	-122.448	37.944	0.61	15.94	298.66	16.55	-122.448	37.944	0.30	15.94	296.66	16.24	2	7/10/2014	35:47.4
366	RSRS19	-122.448	37.944	0.61	15.94	298.64	16.55	-122.448	37.944	0.22	15.94	296.71	16.16	2	7/10/2014	35:47.5
367	RSRS19	-122.448	37.944	0.61	15.90	298.58	16.51	-122.448	37.944	0.46	15.90	296.66	16.36	2	7/10/2014	35:47.5
368	RSRS19	-122.448	37.944	0.61	15.90	298.66	16.51	-122.448	37.944	0.22	15.90	296.60	16.12	2	7/10/2014	35:47.7
369	RSRS19	-122.448	37.944	0.61	15.88	298.55	16.49	-122.448	37.944	0.30	15.88	296.60	16.18	2	7/10/2014	35:47.8
370	RSRS19	-122.448	37.944	0.61	15.87	298.69	16.48	-122.448	37.944	0.39	15.87	296.60	16.26	2	7/10/2014	35:47.9
371	RSRS19	-122.448	37.944	0.61	15.89	298.69	16.50	-122.448	37.944	0.25	15.89	296.64	16.14	2	7/10/2014	35:47.9
372	RSRS19	-122.448	37.944	0.61	15.82	298.67	16.43	-122.448	37.944	0.30	15.82	296.65	16.12	2	7/10/2014	35:48.1
373	RSRS19	-122.448	37.944	0.61	15.80	298.69	16.41	-122.448	37.944	0.46	15.80	296.64	16.25	2	7/10/2014	35:48.1
374	RSRS19	-122.448	37.944	0.61	15.83	298.69	16.44	-122.448	37.944	0.25	15.83	296.72	16.08	2	7/10/2014	35:48.3
375	RSRS19	-122.448	37.944	0.61	15.79	298.71	16.40	-122.448	37.944	0.39	15.79	296.71	16.18	2	7/10/2014	35:48.3
376	RSRS19	-122.448	37.944	0.61	15.78	298.73	16.39	-122.448	37.944	0.39	15.78	296.78	16.17	2	7/10/2014	35:48.5
377	RSRS19	-122.448	37.944	0.61	15.76	298.78	16.37	-122.448	37.944	0.25	15.76	296.83	16.01	2	7/10/2014	35:48.6
378	RSRS19	-122.448	37.944	0.61	15.76	298.74	16.37	-122.448	37.944	0.39	15.76	296.76	16.14	2	7/10/2014	35:48.6
379	RSRS19	-122.448	37.944	0.64	15.73	298.76	16.38	-122.448	37.944	0.39	15.73	296.72	16.12	2	7/10/2014	35:48.7
380	RSRS19	-122.448	37.944	0.61	15.72	298.65	16.33	-122.448	37.944	0.25	15.72	296.71	15.97	2	7/10/2014	35:48.9
381	RSRS19	-122.448	37.944	0.64	15.97	298.72	16.61	-122.448	37.944	0.34	15.97	296.70	16.30	2	7/10/2014	35:49.0
382	RSRS19	-122.448	37.944	0.61	15.85	298.76	16.46	-122.448	37.944	0.34	15.85	296.76	16.18	2	7/10/2014	35:49.1
383	RSRS19	-122.448	37.944	0.64	15.85	298.76	16.49	-122.448	37.944	0.34	15.85	296.76	16.18	2	7/10/2014	35:49.1
384	RSRS19	-122.448	37.944	0.61	15.86	298.83	16.47	-122.448	37.944	0.46	15.86	296.79	16.31	2	7/10/2014	35:49.3
385	RSRS19	-122.448	37.944	0.61	15.94	298.85	16.55	-122.448	37.944	0.34	15.94	296.85	16.28	2	7/10/2014	35:49.4
386	RSRS19	-122.448	37.944	0.61	15.94	298.98	16.55	-122.448	37.944	0.22	15.94	296.98	16.16	2	7/10/2014	35:49.5
387	RSRS19	-122.448	37.944	0.61	15.95	298.94	16.56	-122.448	37.944	0.42	15.95	296.96	16.37	2	7/10/2014	35:49.5
388	RSRS19	-122.448	37.944	0.61	15.92	298.94	16.53	-122.448	37.944	0.30	15.92	296.96	16.22	2	7/10/2014	35:49.7
389	RSRS19	-122.448	37.944	0.61	15.87	298.92	16.48	-122.448	37.944	0.30	15.87	296.99	16.17	2	7/10/2014	35:49.7
390	RSRS19	-122.448	37.944	0.61	15.86	298.97	16.48	-122.448	37.944	0.39	15.86	296.95	16.25	2	7/10/2014	35:49.9
391	RSRS19	-122.448	37.944	0.61	15.81	298.99	16.42	-122.448	37.944	0.30	15.81	297.01	16.11	2	7/10/2014	35:49.9
392	RSRS19	-122.448	37.944	0.61	15.81	299.05	16.42	-122.448	37.944	0.30	15.81	296.99	16.11	2	7/10/2014	35:50.1
393	RSRS19	-122.448	37.944	0.61	15.84	299.03	16.45	-122.448	37.944	0.42	15.84	296.99	16.26	2	7/10/2014	35:50.2
394	RSRS19	-122.448	37.944	0.61	15.88	299.04	16.49	-122.448	37.944	0.25	15.88	296.99	16.13	2	7/10/2014	35:50.3
395	RSRS19	-122.448	37.944	0.61	15.85	299.08	16.46	-122.448	37.944	0.25	15.85	297.08	16.10	2	7/10/2014	35:50.3

396	RSRS19	-122.448	37.944	0.61	15.84	299.10	16.45	-122.448	37.944	0.39	15.84	297.15	16.23	2	7/10/2014	35:50.5
397	RSRS19	-122.448	37.944	0.61	15.86	299.10	16.47	-122.448	37.944	0.30	15.86	297.15	16.16	2	7/10/2014	35:50.6
398	RSRS19	-122.448	37.944	0.61	15.88	299.08	16.49	-122.448	37.944	0.30	15.88	297.19	16.18	2	7/10/2014	35:50.7
399	RSRS19	-122.448	37.944	0.61	15.91	299.08	16.52	-122.448	37.944	0.39	15.91	297.19	16.30	2	7/10/2014	35:50.7
400	RSRS19	-122.448	37.944	0.61	15.87	299.09	16.48	-122.448	37.944	0.17	15.87	297.13	16.04	2	7/10/2014	35:50.9
401	RSRS19	-122.448	37.944	0.61	15.86	299.08	16.47	-122.448	37.944	0.30	15.86	297.11	16.16	2	7/10/2014	35:50.9
402	RSRS19	-122.448	37.944	0.61	16.01	299.13	16.62	-122.448	37.944	0.39	16.01	297.10	16.40	2	7/10/2014	35:51.1
403	RSRS19	-122.448	37.944	0.61	16.00	299.13	16.61	-122.448	37.944	0.25	16.00	297.04	16.25	2	7/10/2014	35:51.1
404	RSRS19	-122.448	37.944	0.61	16.05	299.13	16.66	-122.448	37.944	0.34	16.05	297.08	16.39	2	7/10/2014	35:51.3
405	RSRS19	-122.448	37.944	0.61	16.05	299.15	16.66	-122.448	37.944	0.34	16.05	297.17	16.39	2	7/10/2014	35:51.4
406	RSRS19	-122.448	37.944	0.61	16.10	299.17	16.72	-122.448	37.944	0.22	16.10	297.18	16.32	2	7/10/2014	35:51.4
407	RSRS19	-122.448	37.944	0.61	16.03	299.22	16.65	-122.448	37.944	0.39	16.03	297.22	16.42	2	7/10/2014	35:51.5
408	RSRS19	-122.448	37.944	0.61	16.01	299.18	16.62	-122.448	37.944	0.39	16.01	297.26	16.40	2	7/10/2014	35:51.7
409	RSRS19	-122.448	37.944	0.61	16.02	299.11	16.63	-122.448	37.944	0.22	16.02	297.16	16.24	2	7/10/2014	35:51.8
410	RSRS19	-122.448	37.944	0.61	16.00	299.11	16.61	-122.448	37.944	0.30	16.00	297.15	16.31	2	7/10/2014	35:51.9
411	RSRS19	-122.448	37.944	0.61	15.97	299.09	16.58	-122.448	37.944	0.30	15.97	297.13	16.27	2	7/10/2014	35:51.9
412	RSRS19	-122.448	37.944	0.61	16.00	299.16	16.61	-122.448	37.944	0.25	16.00	297.18	16.26	2	7/10/2014	35:52.0
413	RSRS19	-122.448	37.944	0.61	15.96	299.16	16.57	-122.448	37.944	0.39	15.96	297.18	16.34	2	7/10/2014	35:52.2
414	RSRS19	-122.448	37.944	0.61	16.00	299.24	16.61	-122.448	37.944	0.25	16.00	297.29	16.25	2	7/10/2014	35:52.3
415	RSRS19	-122.448	37.944	0.61	15.96	299.27	16.57	-122.448	37.944	0.22	15.96	297.33	16.17	2	7/10/2014	35:52.3
416	RSRS19	-122.448	37.944	0.56	15.96	299.29	16.52	-122.448	37.944	0.34	15.96	297.32	16.29	2	7/10/2014	35:52.5
417	RSRS19	-122.448	37.944	0.61	15.96	299.32	16.57	-122.448	37.944	0.39	15.96	297.45	16.34	2	7/10/2014	35:52.5
418	RSRS19	-122.448	37.944	0.61	15.96	299.29	16.57	-122.448	37.944	0.22	15.96	297.38	16.17	2	7/10/2014	35:52.7
419	RSRS19	-122.448	37.944	0.61	15.93	299.23	16.54	-122.448	37.944	0.39	15.93	297.32	16.32	2	7/10/2014	35:52.7
420	RSRS19	-122.448	37.944	0.61	15.93	299.23	16.54	-122.448	37.944	0.25	15.93	297.32	16.18	2	7/10/2014	35:52.9
421	RSRS19	-122.448	37.944	0.61	15.92	299.27	16.53	-122.448	37.944	0.30	15.92	297.32	16.22	2	7/10/2014	35:53.0
422	RSRS19	-122.448	37.944	0.61	15.93	299.34	16.54	-122.448	37.944	0.30	15.93	297.47	16.24	2	7/10/2014	35:53.1
423	RSRS19	-122.448	37.944	0.61	15.92	299.36	16.53	-122.448	37.944	0.22	15.92	297.52	16.14	2	7/10/2014	35:53.1
424	RSRS19	-122.448	37.944	0.61	15.97	299.41	16.58	-122.448	37.944	0.34	15.97	297.47	16.30	2	7/10/2014	35:53.3
425	RSRS19	-122.448	37.944	0.61	15.93	299.47	16.54	-122.448	37.944	0.39	15.93	297.54	16.32	2	7/10/2014	35:53.4
426	RSRS19	-122.448	37.944	0.61	15.93	299.47	16.54	-122.448	37.944	0.25	15.93	297.56	16.18	2	7/10/2014	35:53.5
427	RSRS19	-122.448	37.944	0.61	15.95	299.52	16.56	-122.448	37.944	0.25	15.95	297.65	16.20	2	7/10/2014	35:53.5
428	RSRS19	-122.448	37.944	0.61	15.97	299.48	16.58	-122.448	37.944	0.30	15.97	297.63	16.27	2	7/10/2014	35:53.7

429	RSRS19	-122.448	37.944	0.61	15.98	299.52	16.59	-122.448	37.944	0.25	15.98	297.59	16.23	2	7/10/2014	35:53.7
430	RSRS19	-122.448	37.944	0.61	15.97	299.48	16.58	-122.448	37.944	0.30	15.97	297.65	16.27	2	7/10/2014	35:53.9
431	RSRS19	-122.448	37.944	0.61	15.91	299.50	16.52	-122.448	37.944	0.39	15.91	297.65	16.30	2	7/10/2014	35:53.9
432	RSRS19	-122.448	37.944	0.61	15.91	299.59	16.52	-122.448	37.944	0.22	15.91	297.67	16.13	2	7/10/2014	35:54.1
433	RSRS19	-122.448	37.944	0.61	15.91	299.48	16.52	-122.448	37.944	0.30	15.91	297.70	16.21	2	7/10/2014	35:54.2
434	RSRS19	-122.448	37.944	0.56	15.93	299.66	16.49	-122.448	37.944	0.34	15.93	297.81	16.26	2	7/10/2014	35:54.2
435	RSRS19	-122.448	37.944	0.61	15.89	299.61	16.50	-122.448	37.944	0.22	15.89	297.72	16.10	2	7/10/2014	35:54.3
436	RSRS19	-122.448	37.944	0.56	15.88	299.61	16.44	-122.448	37.944	0.30	15.88	297.70	16.18	2	7/10/2014	35:54.5
437	RSRS19	-122.448	37.944	0.61	15.85	299.66	16.46	-122.448	37.944	0.34	15.85	297.73	16.18	2	7/10/2014	35:54.6
438	RSRS19	-122.448	37.944	0.56	15.86	299.71	16.42	-122.448	37.944	0.30	15.86	297.70	16.16	2	7/10/2014	35:54.7
439	RSRS19	-122.448	37.944	0.61	15.83	299.64	16.44	-122.448	37.944	0.39	15.83	297.70	16.22	2	7/10/2014	35:54.7
440	RSRS19	-122.448	37.944	0.61	15.82	299.68	16.43	-122.448	37.944	0.30	15.82	297.75	16.12	2	7/10/2014	35:54.8
441	RSRS19	-122.448	37.944	0.61	15.83	299.73	16.44	-122.448	37.944	0.17	15.83	297.77	16.00	2	7/10/2014	35:55.0
442	RSRS19	-122.448	37.944	0.61	15.87	299.73	16.48	-122.448	37.944	0.30	15.87	297.84	16.17	2	7/10/2014	35:55.1
443	RSRS19	-122.448	37.944	0.61	15.95	299.75	16.56	-122.448	37.944	0.34	15.95	297.84	16.28	2	7/10/2014	35:55.1
444	RSRS19	-122.448	37.944	0.61	15.85	299.73	16.46	-122.448	37.944	0.17	15.85	297.84	16.01	2	7/10/2014	35:55.3
445	RSRS19	-122.448	37.944	0.61	15.93	299.75	16.54	-122.448	37.944	0.34	15.93	297.86	16.26	2	7/10/2014	35:55.3
446	RSRS19	-122.448	37.944	0.56	15.92	299.71	16.48	-122.448	37.944	0.22	15.92	297.82	16.14	2	7/10/2014	35:55.5
447	RSRS19	-122.448	37.944	0.56	15.97	299.75	16.53	-122.448	37.944	0.22	15.97	297.82	16.19	2	7/10/2014	35:55.5
448	RSRS19	-122.448	37.944	0.56	15.93	299.71	16.49	-122.448	37.944	0.34	15.93	297.75	16.26	2	7/10/2014	35:55.7
449	RSRS19	-122.448	37.944	0.61	16.02	299.74	16.63	-122.448	37.944	0.25	16.02	297.76	16.27	2	7/10/2014	35:55.8
450	RSRS19	-122.448	37.944	0.56	16.02	299.80	16.58	-122.448	37.944	0.17	16.02	297.78	16.18	2	7/10/2014	35:55.9
451	RSRS19	-122.448	37.944	0.61	16.04	299.80	16.65	-122.448	37.944	0.34	16.04	297.80	16.38	2	7/10/2014	35:55.9
452	RSRS19	-122.448	37.944	0.56	16.06	299.76	16.62	-122.448	37.944	0.22	16.06	297.78	16.28	2	7/10/2014	35:56.1
453	RSRS19	-122.448	37.944	0.61	16.04	299.76	16.65	-122.448	37.944	0.17	16.04	297.80	16.21	2	7/10/2014	35:56.2
454	RSRS19	-122.448	37.944	0.56	16.06	299.71	16.62	-122.448	37.944	0.30	16.06	297.75	16.36	2	7/10/2014	35:56.3
455	RSRS19	-122.448	37.944	0.56	16.09	299.71	16.65	-122.448	37.944	0.17	16.09	297.74	16.26	2	7/10/2014	35:56.3
456	RSRS19	-122.448	37.944	0.56	16.07	299.56	16.63	-122.448	37.944	0.17	16.07	297.70	16.24	2	7/10/2014	35:56.4
457	RSRS19	-122.448	37.944	0.61	16.06	299.63	16.67	-122.448	37.944	0.30	16.06	297.70	16.36	2	7/10/2014	35:56.6
458	RSRS19	-122.448	37.944	0.56	16.09	299.58	16.65	-122.448	37.944	0.08	16.09	297.67	16.17	2	7/10/2014	35:56.7
459	RSRS19	-122.448	37.944	0.61	16.07	299.63	16.68	-122.448	37.944	0.30	16.07	297.69	16.37	2	7/10/2014	35:56.7
460	RSRS19	-122.448	37.944	0.56	16.09	299.61	16.65	-122.448	37.944	0.25	16.09	297.70	16.34	2	7/10/2014	35:56.9
461	RSRS19	-122.448	37.944	0.56	16.06	299.63	16.62	-122.448	37.944	0.08	16.06	297.70	16.14	2	7/10/2014	35:57.0

462	RSRS19	-122.448	37.944	0.56	16.09	299.63	16.65	-122.448	37.944	0.17	16.09	297.70	16.26	2	7/10/2014	35:57.0
463	RSRS19	-122.448	37.944	0.56	16.08	299.68	16.64	-122.448	37.944	0.34	16.08	297.72	16.41	2	7/10/2014	35:57.1
464	RSRS19	-122.448	37.944	0.56	16.10	299.59	16.66	-122.448	37.944	0.13	16.10	297.68	16.24	2	7/10/2014	35:57.3
465	RSRS19	-122.448	37.944	0.61	16.17	299.61	16.78	-122.448	37.944	0.13	16.17	297.67	16.31	2	7/10/2014	35:57.4
466	RSRS19	-122.448	37.944	0.56	16.07	299.64	16.63	-122.448	37.944	0.17	16.07	297.73	16.24	2	7/10/2014	35:57.5
467	RSRS19	-122.448	37.944	0.61	16.06	299.66	16.67	-122.448	37.944	0.13	16.06	297.74	16.20	2	7/10/2014	35:57.5
468	RSRS19	-122.448	37.944	0.56	16.08	299.64	16.64	-122.448	37.944	0.22	16.08	297.77	16.30	2	7/10/2014	35:57.6
469	RSRS19	-122.448	37.944	0.61	16.08	299.66	16.69	-122.448	37.944	0.34	16.08	297.77	16.41	2	7/10/2014	35:57.8
470	RSRS19	-122.448	37.944	0.56	16.06	299.66	16.62	-122.448	37.944	0.13	16.06	297.86	16.19	2	7/10/2014	35:57.8
471	RSRS19	-122.448	37.944	0.56	16.07	299.71	16.63	-122.448	37.944	0.13	16.07	297.90	16.20	2	7/10/2014	35:57.9
472	RSRS19	-122.448	37.944	0.56	16.05	299.70	16.61	-122.448	37.944	0.25	16.05	297.95	16.30	2	7/10/2014	35:58.1
473	RSRS19	-122.448	37.944	0.61	16.07	299.66	16.68	-122.448	37.944	0.17	16.07	297.84	16.24	2	7/10/2014	35:58.1
474	RSRS19	-122.448	37.944	0.56	15.97	299.64	16.53	-122.448	37.944	0.17	15.97	297.86	16.14	2	7/10/2014	35:58.3
475	RSRS19	-122.448	37.944	0.61	15.99	299.73	16.60	-122.448	37.944	0.30	15.99	297.88	16.29	2	7/10/2014	35:58.3
476	RSRS19	-122.448	37.944	0.56	16.00	299.71	16.56	-122.448	37.944	0.13	16.00	297.89	16.14	2	7/10/2014	35:58.5
477	RSRS19	-122.448	37.944	0.56	16.01	299.75	16.57	-122.448	37.944	0.17	16.01	297.91	16.18	2	7/10/2014	35:58.6
478	RSRS19	-122.448	37.944	0.56	16.03	299.78	16.59	-122.448	37.944	0.30	16.03	297.90	16.34	2	7/10/2014	35:58.7
479	RSRS19	-122.448	37.944	0.61	16.02	299.80	16.63	-122.448	37.944	0.22	16.02	297.93	16.24	2	7/10/2014	35:58.7
480	RSRS19	-122.448	37.944	0.56	16.01	299.75	16.57	-122.448	37.944	0.08	16.01	297.86	16.09	2	7/10/2014	35:58.9
481	RSRS19	-122.448	37.944	0.61	16.02	299.80	16.63	-122.448	37.944	0.30	16.02	297.89	16.32	2	7/10/2014	35:59.0
482	RSRS19	-122.448	37.944	0.56	16.03	299.87	16.59	-122.448	37.944	0.25	16.03	297.91	16.29	2	7/10/2014	35:59.1
483	RSRS19	-122.448	37.944	0.56	16.03	299.65	16.58	-122.448	37.944	0.13	16.03	297.87	16.16	2	7/10/2014	35:59.1
484	RSRS19	-122.448	37.944	0.56	16.01	299.65	16.57	-122.448	37.944	0.22	16.01	297.85	16.23	2	7/10/2014	35:59.2
485	RSRS19	-122.448	37.944	0.61	16.00	299.67	16.61	-122.448	37.944	0.30	16.00	297.87	16.30	2	7/10/2014	35:59.4
486	RSRS19	-122.448	37.944	0.56	16.02	299.74	16.58	-122.448	37.944	0.13	16.02	297.87	16.15	2	7/10/2014	35:59.5
487	RSRS19	-122.448	37.944	0.61	16.03	299.69	16.64	-122.448	37.944	0.30	16.03	297.89	16.34	2	7/10/2014	35:59.5
488	RSRS19	-122.448	37.944	0.56	16.03	299.70	16.58	-122.448	37.944	0.25	16.03	297.83	16.28	2	7/10/2014	35:59.7
489	RSRS19	-122.448	37.944	0.61	16.02	299.76	16.63	-122.448	37.944	0.13	16.02	297.92	16.15	2	7/10/2014	35:59.8
490	RSRS19	-122.448	37.944	0.56	16.05	299.78	16.61	-122.448	37.944	0.34	16.05	297.87	16.39	2	7/10/2014	35:59.8
491	RSRS19	-122.448	37.944	0.61	16.01	299.79	16.62	-122.448	37.944	0.25	16.01	297.92	16.26	2	7/10/2014	35:59.9
492	RSRS19	-122.448	37.944	0.56	16.00	299.76	16.56	-122.448	37.944	0.17	16.00	297.94	16.17	2	7/10/2014	36:00.1
493	RSRS19	-122.448	37.944	0.56	16.00	299.68	16.56	-122.448	37.944	0.34	16.00	297.83	16.33	2	7/10/2014	36:00.2
494	RSRS19	-122.448	37.944	0.56	15.99	299.48	16.55	-122.448	37.944	0.25	15.99	297.83	16.24	2	7/10/2014	36:00.3

495	RSRS19	-122.448	37.944	0.56	15.98	299.64	16.54	-122.448	37.944	0.17	15.98	297.90	16.15	2	7/10/2014	36:00.3
496	RSRS19	-122.448	37.944	0.56	15.97	299.61	16.52	-122.448	37.944	0.34	15.97	297.92	16.30	2	7/10/2014	36:00.4
497	RSRS19	-122.448	37.944	0.56	15.97	299.63	16.52	-122.448	37.944	0.22	15.97	297.90	16.18	2	7/10/2014	36:00.6
498	RSRS19	-122.448	37.944	0.56	15.97	299.66	16.53	-122.448	37.944	0.17	15.97	297.86	16.14	2	7/10/2014	36:00.7
499	RSRS19	-122.448	37.944	0.61	15.95	299.68	16.56	-122.448	37.944	0.42	15.95	297.88	16.37	2	7/10/2014	36:00.7
500	RSRS19	-122.448	37.944	0.56	15.93	299.70	16.49	-122.448	37.944	0.25	15.93	297.88	16.19	2	7/10/2014	36:00.9
501	RSRS19	-122.448	37.944	0.61	15.94	299.73	16.55	-122.448	37.944	0.30	15.94	297.88	16.24	2	7/10/2014	36:00.9
502	RSRS19	-122.448	37.944	0.56	15.96	299.75	16.52	-122.448	37.944	0.34	15.96	297.88	16.29	2	7/10/2014	36:01.1
503	RSRS19	-122.448	37.944	0.61	15.94	299.70	16.55	-122.448	37.944	0.17	15.94	297.92	16.11	2	7/10/2014	36:01.1
504	RSRS19	-122.448	37.944	0.56	15.91	299.73	16.47	-122.448	37.944	0.39	15.91	297.91	16.30	2	7/10/2014	36:01.3
505	RSRS19	-122.448	37.944	0.56	15.92	299.67	16.48	-122.448	37.944	0.39	15.92	297.86	16.30	2	7/10/2014	36:01.4
506	RSRS19	-122.448	37.944	0.56	15.87	299.60	16.43	-122.448	37.944	0.25	15.87	297.75	16.12	2	7/10/2014	36:01.5
507	RSRS19	-122.448	37.944	0.61	15.86	299.58	16.47	-122.448	37.944	0.34	15.86	297.73	16.19	2	7/10/2014	36:01.5
508	RSRS19	-122.448	37.944	0.56	15.86	299.58	16.42	-122.448	37.944	0.30	15.86	297.66	16.17	2	7/10/2014	36:01.7
509	RSRS19	-122.448	37.944	0.61	15.85	299.73	16.46	-122.448	37.944	0.30	15.85	297.88	16.15	2	7/10/2014	36:01.8
510	RSRS19	-122.448	37.944	0.56	15.83	299.71	16.39	-122.448	37.944	0.39	15.83	297.86	16.22	2	7/10/2014	36:01.8
511	RSRS19	-122.448	37.944	0.61	15.84	299.67	16.45	-122.448	37.944	0.30	15.84	297.91	16.14	2	7/10/2014	36:01.9
512	RSRS19	-122.448	37.944	0.56	15.83	299.71	16.39	-122.448	37.944	0.22	15.83	297.93	16.04	2	7/10/2014	36:02.0
513	RSRS19	-122.448	37.944	0.61	15.83	299.69	16.44	-122.448	37.944	0.42	15.83	297.89	16.25	2	7/10/2014	36:02.2
514	RSRS19	-122.448	37.944	0.56	15.79	299.64	16.35	-122.448	37.944	0.34	15.79	297.84	16.12	2	7/10/2014	36:02.3
515	RSRS19	-122.448	37.944	0.56	15.80	299.69	16.35	-122.448	37.944	0.39	15.80	297.85	16.18	2	7/10/2014	36:02.3
516	RSRS19	-122.448	37.944	0.56	15.81	299.69	16.37	-122.448	37.944	0.39	15.81	297.85	16.20	2	7/10/2014	36:02.5
517	RSRS19	-122.448	37.944	0.61	15.78	299.70	16.39	-122.448	37.944	0.25	15.78	297.85	16.03	2	7/10/2014	36:02.6
518	RSRS19	-122.448	37.944	0.56	15.77	299.65	16.33	-122.448	37.944	0.42	15.77	297.83	16.19	2	7/10/2014	36:02.6
519	RSRS19	-122.448	37.944	0.61	15.76	299.78	16.37	-122.448	37.944	0.42	15.76	297.87	16.18	2	7/10/2014	36:02.7
520	RSRS19	-122.448	37.944	0.56	15.75	299.65	16.31	-122.448	37.944	0.30	15.75	297.89	16.05	2	7/10/2014	36:02.9
521	RSRS19	-122.448	37.944	0.61	15.76	299.70	16.37	-122.448	37.944	0.42	15.76	297.92	16.18	2	7/10/2014	36:03.0
522	RSRS19	-122.448	37.944	0.56	15.73	299.68	16.29	-122.448	37.944	0.42	15.73	297.87	16.15	2	7/10/2014	36:03.1
523	RSRS19	-122.448	37.944	0.61	15.73	299.70	16.34	-122.448	37.944	0.30	15.73	297.94	16.03	2	7/10/2014	36:03.1
524	RSRS19	-122.448	37.944	0.61	15.73	299.72	16.34	-122.448	37.944	0.50	15.73	297.92	16.23	2	7/10/2014	36:03.2
525	RSRS19	-122.448	37.944	0.61	15.71	299.70	16.32	-122.448	37.944	0.42	15.71	297.94	16.13	2	7/10/2014	36:03.4
526	RSRS19	-122.448	37.944	0.56	15.74	299.72	16.30	-122.448	37.944	0.34	15.74	297.92	16.08	2	7/10/2014	36:03.5
527	RSRS19	-122.448	37.944	0.61	15.71	299.72	16.32	-122.448	37.944	0.42	15.71	297.86	16.13	2	7/10/2014	36:03.5

528	RSRS19	-122.448	37.944	0.56	15.71	299.74	16.27	-122.448	37.944	0.39	15.71	297.88	16.10	2	7/10/2014	36:03.7
529	RSRS19	-122.448	37.944	0.61	15.69	299.75	16.30	-122.448	37.944	0.50	15.69	297.92	16.20	2	7/10/2014	36:03.7
530	RSRS19	-122.448	37.944	0.61	15.71	299.70	16.32	-122.448	37.944	0.42	15.71	297.86	16.13	2	7/10/2014	36:03.9
531	RSRS19	-122.448	37.944	0.61	15.70	299.68	16.31	-122.448	37.944	0.39	15.70	297.83	16.09	2	7/10/2014	36:03.9
532	RSRS19	-122.448	37.944	0.56	15.71	299.71	16.27	-122.448	37.944	0.39	15.71	297.86	16.10	2	7/10/2014	36:04.1
533	RSRS19	-122.448	37.944	0.56	15.69	299.73	16.25	-122.448	37.944	0.50	15.69	297.93	16.19	2	7/10/2014	36:04.2
534	RSRS19	-122.448	37.944	0.56	15.73	299.73	16.29	-122.448	37.944	0.39	15.73	297.92	16.12	2	7/10/2014	36:04.3
535	RSRS19	-122.448	37.944	0.61	15.72	299.77	16.33	-122.448	37.944	0.50	15.72	297.97	16.22	2	7/10/2014	36:04.3
536	RSRS19	-122.448	37.944	0.61	15.69	299.73	16.30	-122.448	37.944	0.45	15.69	297.93	16.14	2	7/10/2014	36:04.5
537	RSRS19	-122.448	37.944	0.61	15.69	299.73	16.30	-122.448	37.944	0.34	15.69	297.93	16.02	2	7/10/2014	36:04.6
538	RSRS19	-122.448	37.944	0.61	15.70	299.71	16.31	-122.448	37.944	0.50	15.70	297.93	16.21	2	7/10/2014	36:04.7
539	RSRS19	-122.448	37.944	0.61	15.71	299.67	16.32	-122.448	37.944	0.45	15.71	297.86	16.16	2	7/10/2014	36:04.7
540	RSRS19	-122.448	37.944	0.61	15.67	299.71	16.28	-122.448	37.944	0.34	15.67	297.91	16.01	2	7/10/2014	36:04.9
541	RSRS19	-122.448	37.944	0.61	15.66	299.69	16.27	-122.448	37.944	0.54	15.66	297.86	16.19	2	7/10/2014	36:05.0
542	RSRS19	-122.448	37.944	0.61	15.69	299.73	16.30	-122.448	37.944	0.39	15.69	297.86	16.07	2	7/10/2014	36:05.1
543	RSRS19	-122.448	37.944	0.61	15.70	299.76	16.31	-122.448	37.944	0.45	15.70	297.89	16.16	2	7/10/2014	36:05.1
544	RSRS19	-122.448	37.944	0.56	15.71	299.76	16.27	-122.448	37.944	0.45	15.71	297.87	16.16	2	7/10/2014	36:05.3
545	RSRS19	-122.448	37.944	0.61	15.73	299.80	16.34	-122.448	37.944	0.39	15.73	297.91	16.11	2	7/10/2014	36:05.4
546	RSRS19	-122.448	37.944	0.61	15.73	299.76	16.34	-122.448	37.944	0.45	15.73	297.93	16.18	2	7/10/2014	36:05.4
547	RSRS19	-122.448	37.944	0.61	15.69	299.71	16.30	-122.448	37.944	0.42	15.69	297.80	16.11	2	7/10/2014	36:05.5
548	RSRS19	-122.448	37.944	0.61	15.69	299.65	16.30	-122.448	37.944	0.39	15.69	297.83	16.08	2	7/10/2014	36:05.7
549	RSRS19	-122.448	37.944	0.61	15.66	299.67	16.27	-122.448	37.944	0.50	15.66	297.85	16.17	2	7/10/2014	36:05.8
550	RSRS19	-122.448	37.944	0.56	15.68	299.65	16.24	-122.448	37.944	0.42	15.68	297.87	16.10	2	7/10/2014	36:05.9
551	RSRS19	-122.448	37.944	0.64	15.67	299.65	16.32	-122.448	37.944	0.50	15.67	297.80	16.18	2	7/10/2014	36:05.9
552	RSRS19	-122.448	37.944	0.61	15.67	299.67	16.28	-122.448	37.944	0.45	15.67	297.83	16.13	2	7/10/2014	36:06.1
553	RSRS19	-122.448	37.944	0.61	15.66	299.70	16.27	-122.448	37.944	0.39	15.66	297.85	16.05	2	7/10/2014	36:06.2
554	RSRS19	-122.448	37.944	0.56	15.66	299.72	16.22	-122.448	37.944	0.50	15.66	297.83	16.17	2	7/10/2014	36:06.3
555	RSRS19	-122.448	37.944	0.61	15.67	299.70	16.28	-122.448	37.944	0.54	15.67	297.81	16.21	2	7/10/2014	36:06.3
556	RSRS19	-122.448	37.944	0.61	15.65	299.72	16.26	-122.448	37.944	0.34	15.65	297.81	15.98	2	7/10/2014	36:06.5
557	RSRS19	-122.448	37.944	0.61	15.65	299.68	16.26	-122.448	37.944	0.45	15.65	297.77	16.10	2	7/10/2014	36:06.5
558	RSRS19	-122.448	37.944	0.61	15.65	299.70	16.26	-122.448	37.944	0.42	15.65	297.78	16.07	2	7/10/2014	36:06.7
559	RSRS19	-122.448	37.944	0.64	15.73	299.68	16.38	-122.448	37.944	0.42	15.73	297.77	16.15	2	7/10/2014	36:06.7
560	RSRS19	-122.448	37.944	0.61	15.66	299.70	16.27	-122.448	37.944	0.54	15.66	297.86	16.20	2	7/10/2014	36:06.9

561	RSRS19	-122.448	37.944	0.64	15.66	299.75	16.30	-122.448	37.944	0.39	15.66	297.88	16.04	2	7/10/2014	36:07.0
562	RSRS19	-122.448	37.944	0.61	15.64	299.72	16.25	-122.448	37.944	0.50	15.64	297.86	16.14	2	7/10/2014	36:07.1
563	RSRS19	-122.448	37.944	0.61	15.66	299.73	16.27	-122.448	37.944	0.50	15.66	297.86	16.16	2	7/10/2014	36:07.1
564	RSRS19	-122.448	37.944	0.61	15.66	299.73	16.27	-122.448	37.944	0.45	15.66	297.84	16.12	2	7/10/2014	36:07.3
565	RSRS19	-122.448	37.944	0.61	15.62	299.75	16.23	-122.448	37.944	0.50	15.62	297.86	16.13	2	7/10/2014	36:07.4
566	RSRS19	-122.448	37.944	0.61	15.64	299.75	16.25	-122.448	37.944	0.34	15.64	297.86	15.98	2	7/10/2014	36:07.5
567	RSRS19	-122.448	37.944	0.64	15.61	299.75	16.25	-122.448	37.944	0.45	15.61	297.89	16.06	2	7/10/2014	36:07.5
568	RSRS19	-122.448	37.944	0.61	15.63	299.80	16.24	-122.448	37.944	0.42	15.63	297.90	16.05	2	7/10/2014	36:07.6
569	RSRS19	-122.448	37.944	0.61	15.61	299.75	16.22	-122.448	37.944	0.34	15.61	297.93	15.94	2	7/10/2014	36:07.8
570	RSRS19	-122.448	37.944	0.61	15.62	299.78	16.23	-122.448	37.944	0.50	15.62	297.95	16.12	2	7/10/2014	36:07.9
571	RSRS19	-122.448	37.944	0.61	15.62	299.75	16.23	-122.448	37.944	0.45	15.62	297.93	16.08	2	7/10/2014	36:07.9
572	RSRS19	-122.448	37.944	0.61	15.60	299.78	16.21	-122.448	37.944	0.38	15.60	297.95	15.99	2	7/10/2014	36:08.1
573	RSRS19	-122.448	37.944	0.61	15.63	299.80	16.24	-122.448	37.944	0.50	15.63	297.95	16.14	2	7/10/2014	36:08.2
574	RSRS19	-122.448	37.944	0.61	15.62	299.73	16.23	-122.448	37.944	0.42	15.62	297.93	16.04	2	7/10/2014	36:08.2
575	RSRS19	-122.448	37.944	0.61	15.65	299.74	16.26	-122.448	37.944	0.42	15.65	297.93	16.07	2	7/10/2014	36:08.3
576	RSRS19	-122.448	37.944	0.61	15.64	299.78	16.25	-122.448	37.944	0.50	15.64	297.93	16.14	2	7/10/2014	36:08.5
577	RSRS19	-122.448	37.944	0.61	15.67	299.74	16.28	-122.448	37.944	0.33	15.67	297.94	16.01	2	7/10/2014	36:08.6
578	RSRS19	-122.448	37.944	0.61	15.66	299.71	16.27	-122.448	37.944	0.42	15.66	297.93	16.08	2	7/10/2014	36:08.6
579	RSRS19	-122.448	37.944	0.61	15.67	299.71	16.28	-122.448	37.944	0.50	15.67	297.94	16.17	2	7/10/2014	36:08.7
580	RSRS19	-122.448	37.944	0.61	15.69	299.69	16.30	-122.448	37.944	0.33	15.69	297.89	16.02	2	7/10/2014	36:08.9
581	RSRS19	-122.448	37.944	0.61	15.69	299.70	16.30	-122.448	37.944	0.42	15.69	297.85	16.11	2	7/10/2014	36:09.0
582	RSRS19	-122.448	37.944	0.61	15.69	299.67	16.30	-122.448	37.944	0.38	15.69	297.83	16.07	2	7/10/2014	36:09.1
583	RSRS19	-122.448	37.944	0.61	15.70	299.63	16.31	-122.448	37.944	0.33	15.70	297.78	16.04	2	7/10/2014	36:09.1
584	RSRS19	-122.448	37.944	0.56	15.70	299.65	16.26	-122.448	37.944	0.45	15.70	297.76	16.16	2	7/10/2014	36:09.3
585	RSRS19	-122.448	37.944	0.61	15.73	299.63	16.33	-122.448	37.944	0.33	15.73	297.70	16.06	2	7/10/2014	36:09.3
586	RSRS19	-122.448	37.944	0.61	15.76	299.59	16.37	-122.448	37.944	0.33	15.76	297.67	16.09	2	7/10/2014	36:09.5
587	RSRS19	-122.448	37.944	0.61	15.77	299.59	16.38	-122.448	37.944	0.45	15.77	297.59	16.23	2	7/10/2014	36:09.5
588	RSRS19	-122.448	37.944	0.56	15.78	299.63	16.34	-122.448	37.944	0.22	15.78	297.63	16.00	2	7/10/2014	36:09.7
589	RSRS19	-122.448	37.944	0.61	15.79	299.70	16.40	-122.448	37.944	0.38	15.79	297.72	16.17	2	7/10/2014	36:09.8
590	RSRS19	-122.448	37.944	0.61	15.83	299.74	16.44	-122.448	37.944	0.42	15.83	297.77	16.25	2	7/10/2014	36:09.9
591	RSRS19	-122.448	37.944	0.61	15.84	299.68	16.45	-122.448	37.944	0.25	15.84	297.70	16.09	2	7/10/2014	36:09.9
592	RSRS19	-122.448	37.944	0.56	15.81	299.66	16.37	-122.448	37.944	0.38	15.81	297.68	16.20	2	7/10/2014	36:10.1
593	RSRS19	-122.448	37.944	0.61	15.80	299.68	16.41	-122.448	37.944	0.38	15.80	297.68	16.19	2	7/10/2014	36:10.2

594	RSRS19	-122.448	37.944	0.56	15.81	299.64	16.37	-122.448	37.944	0.25	15.81	297.58	16.06	2	7/10/2014	36:10.3
595	RSRS19	-122.448	37.944	0.61	15.76	299.64	16.37	-122.448	37.944	0.38	15.76	297.66	16.15	2	7/10/2014	36:10.3
596	RSRS19	-122.448	37.944	0.56	15.79	299.64	16.35	-122.448	37.944	0.33	15.79	297.75	16.12	2	7/10/2014	36:10.5
597	RSRS19	-122.448	37.944	0.61	15.76	299.66	16.37	-122.448	37.944	0.33	15.76	297.62	16.09	2	7/10/2014	36:10.6
598	RSRS19	-122.448	37.944	0.56	15.75	299.64	16.31	-122.448	37.944	0.33	15.75	297.68	16.08	2	7/10/2014	36:10.7
599	RSRS19	-122.448	37.944	0.61	15.73	299.71	16.33	-122.448	37.944	0.17	15.73	297.71	15.89	2	7/10/2014	36:10.7
600	RSRS19	-122.448	37.944	0.56	15.74	299.73	16.30	-122.448	37.944	0.30	15.74	297.77	16.04	2	7/10/2014	36:10.9
601	RSRS19	-122.448	37.944	0.61	15.73	299.77	16.33	-122.448	37.944	0.38	15.73	297.82	16.11	2	7/10/2014	36:11.0
602	RSRS19	-122.448	37.944	0.56	15.71	299.80	16.27	-122.448	37.944	0.25	15.71	297.86	15.96	2	7/10/2014	36:11.0
603	RSRS19	-122.448	37.944	0.61	15.71	299.80	16.32	-122.448	37.944	0.33	15.71	297.91	16.04	2	7/10/2014	36:11.1
604	RSRS19	-122.448	37.944	0.61	15.70	299.80	16.31	-122.448	37.944	0.38	15.70	297.93	16.09	2	7/10/2014	36:11.3
605	RSRS19	-122.448	37.944	0.61	15.70	299.80	16.31	-122.448	37.944	0.25	15.70	297.89	15.95	2	7/10/2014	36:11.3
606	RSRS19	-122.448	37.944	0.56	15.68	299.82	16.24	-122.449	37.944	0.30	15.68	297.87	15.98	2	7/10/2014	36:11.5
607	RSRS19	-122.449	37.944	0.61	15.68	299.80	16.29	-122.449	37.944	0.38	15.68	297.82	16.06	2	7/10/2014	36:11.5
608	RSRS19	-122.449	37.944	0.56	15.68	299.80	16.24	-122.449	37.944	0.25	15.68	297.74	15.93	2	7/10/2014	36:11.7
609	RSRS19	-122.449	37.944	0.61	15.66	299.83	16.27	-122.449	37.944	0.30	15.66	297.78	15.96	2	7/10/2014	36:11.8
610	RSRS19	-122.449	37.944	0.61	15.67	299.89	16.28	-122.449	37.944	0.30	15.67	297.80	15.97	2	7/10/2014	36:11.9
611	RSRS19	-122.449	37.944	0.61	15.69	299.83	16.30	-122.449	37.944	0.22	15.69	297.78	15.90	2	7/10/2014	36:11.9
612	RSRS19	-122.449	37.944	0.56	15.68	299.83	16.24	-122.449	37.944	0.30	15.68	297.78	15.98	2	7/10/2014	36:12.1
613	RSRS19	-122.449	37.944	0.61	15.69	299.85	16.30	-122.449	37.944	0.38	15.69	297.83	16.08	2	7/10/2014	36:12.1
614	RSRS19	-122.449	37.944	0.56	15.76	299.85	16.32	-122.449	37.944	0.22	15.76	297.90	15.97	2	7/10/2014	36:12.3
615	RSRS19	-122.449	37.944	0.61	15.72	299.83	16.33	-122.449	37.944	0.30	15.72	297.87	16.02	2	7/10/2014	36:12.3
616	RSRS19	-122.449	37.944	0.56	15.72	299.85	16.28	-122.449	37.944	0.33	15.72	297.90	16.05	2	7/10/2014	36:12.5
617	RSRS19	-122.449	37.944	0.61	15.74	299.85	16.35	-122.449	37.944	0.17	15.74	297.83	15.91	2	7/10/2014	36:12.6
618	RSRS19	-122.449	37.944	0.56	15.73	299.85	16.29	-122.449	37.944	0.38	15.73	297.81	16.12	2	7/10/2014	36:12.7
619	RSRS19	-122.449	37.944	0.61	15.80	299.86	16.40	-122.449	37.944	0.25	15.80	297.75	16.05	2	7/10/2014	36:12.7
620	RSRS19	-122.449	37.944	0.56	15.73	299.83	16.29	-122.449	37.944	0.13	15.73	297.75	15.87	2	7/10/2014	36:12.9
621	RSRS19	-122.449	37.944	0.61	15.78	299.81	16.39	-122.449	37.944	0.42	15.78	297.68	16.20	2	7/10/2014	36:13.0
622	RSRS19	-122.449	37.944	0.56	15.75	299.79	16.31	-122.449	37.944	0.08	15.75	297.66	15.83	2	7/10/2014	36:13.1
623	RSRS19	-122.449	37.944	0.61	15.78	299.81	16.39	-122.449	37.944	0.30	15.78	297.75	16.08	2	7/10/2014	36:13.1
624	RSRS19	-122.449	37.944	0.56	15.76	299.83	16.32	-122.449	37.944	0.30	15.76	297.77	16.06	2	7/10/2014	36:13.2
625	RSRS19	-122.449	37.944	0.61	15.79	299.84	16.40	-122.449	37.944	0.22	15.79	297.78	16.00	2	7/10/2014	36:13.4
626	RSRS19	-122.449	37.944	0.56	15.76	299.80	16.32	-122.449	37.944	0.22	15.76	297.83	15.98	2	7/10/2014	36:13.5

627	RSRS19	-122.449	37.944	0.61	15.76	299.77	16.37	-122.449	37.944	0.33	15.76	297.82	16.10	2	7/10/2014	36:13.5
628	RSRS19	-122.449	37.944	0.56	15.77	299.75	16.33	-122.449	37.944	0.22	15.77	297.86	15.99	2	7/10/2014	36:13.7
629	RSRS19	-122.449	37.944	0.61	15.76	299.68	16.37	-122.449	37.944	0.30	15.76	297.77	16.06	2	7/10/2014	36:13.8
630	RSRS19	-122.449	37.944	0.56	15.79	299.69	16.35	-122.449	37.944	0.33	15.79	297.75	16.12	2	7/10/2014	36:13.8
631	RSRS19	-122.449	37.944	0.61	15.76	299.64	16.37	-122.449	37.944	0.22	15.76	297.69	15.98	2	7/10/2014	36:13.9
632	RSRS19	-122.449	37.944	0.56	15.75	299.69	16.31	-122.449	37.944	0.25	15.75	297.69	16.00	2	7/10/2014	36:14.0
633	RSRS19	-122.449	37.944	0.61	15.74	299.67	16.35	-122.449	37.944	0.22	15.74	297.65	15.96	2	7/10/2014	36:14.2
634	RSRS19	-122.449	37.944	0.56	15.73	299.69	16.29	-122.449	37.944	0.30	15.73	297.69	16.03	2	7/10/2014	36:14.3
635	RSRS19	-122.449	37.944	0.61	15.71	299.64	16.32	-122.449	37.944	0.30	15.71	297.67	16.01	2	7/10/2014	36:14.3
636	RSRS19	-122.449	37.944	0.56	15.73	299.67	16.29	-122.449	37.944	0.13	15.73	297.67	15.87	2	7/10/2014	36:14.5
637	RSRS19	-122.449	37.944	0.56	15.79	299.69	16.35	-122.449	37.944	0.33	15.79	297.67	16.12	2	7/10/2014	36:14.6
638	RSRS19	-122.449	37.944	0.56	15.76	299.69	16.32	-122.449	37.944	0.30	15.76	297.67	16.06	2	7/10/2014	36:14.7
639	RSRS19	-122.449	37.944	0.61	15.76	299.69	16.37	-122.449	37.944	0.13	15.76	297.69	15.89	2	7/10/2014	36:14.7
640	RSRS19	-122.449	37.944	0.56	15.81	299.67	16.37	-122.449	37.944	0.30	15.81	297.71	16.11	2	7/10/2014	36:14.9
641	RSRS19	-122.449	37.944	0.56	15.77	299.63	16.33	-122.449	37.944	0.22	15.77	297.61	15.99	2	7/10/2014	36:14.9
642	RSRS19	-122.449	37.944	0.56	15.79	299.74	16.35	-122.449	37.944	0.08	15.79	297.74	15.87	2	7/10/2014	36:15.1
643	RSRS19	-122.449	37.944	0.61	15.78	299.65	16.39	-122.449	37.944	0.42	15.78	297.67	16.20	2	7/10/2014	36:15.1
644	RSRS19	-122.449	37.944	0.56	15.77	299.63	16.33	-122.449	37.944	0.17	15.77	297.65	15.94	2	7/10/2014	36:15.3
645	RSRS19	-122.449	37.944	0.56	15.73	299.56	16.29	-122.449	37.944	0.22	15.73	297.56	15.95	2	7/10/2014	36:15.4
646	RSRS19	-122.449	37.944	0.56	15.75	299.52	16.31	-122.449	37.944	0.25	15.75	297.54	16.00	2	7/10/2014	36:15.4
647	RSRS19	-122.449	37.944	0.56	15.73	299.59	16.28	-122.449	37.944	0.30	15.73	297.59	16.03	2	7/10/2014	36:15.5
648	RSRS19	-122.449	37.944	0.56	15.77	299.66	16.33	-122.449	37.944	0.30	15.77	297.59	16.07	2	7/10/2014	36:15.7
649	RSRS19	-122.449	37.944	0.56	15.76	299.54	16.32	-122.449	37.944	0.22	15.76	297.64	15.97	2	7/10/2014	36:15.8
650	RSRS19	-122.449	37.944	0.56	15.74	299.66	16.30	-122.449	37.944	0.08	15.74	297.65	15.82	2	7/10/2014	36:15.9
651	RSRS19	-122.449	37.944	0.56	15.76	299.61	16.32	-122.449	37.944	0.33	15.76	297.68	16.09	2	7/10/2014	36:15.9
652	RSRS19	-122.449	37.944	0.56	15.80	299.70	16.35	-122.449	37.944	0.25	15.80	297.68	16.05	2	7/10/2014	36:16.0
653	RSRS19	-122.449	37.944	0.56	15.82	299.70	16.38	-122.449	37.944	0.13	15.82	297.66	15.95	2	7/10/2014	36:16.2
654	RSRS19	-122.449	37.944	0.56	15.83	299.75	16.38	-122.449	37.944	0.25	15.83	297.66	16.08	2	7/10/2014	36:16.3
655	RSRS19	-122.449	37.944	0.56	15.79	299.72	16.35	-122.449	37.944	0.25	15.79	297.61	16.04	2	7/10/2014	36:16.3
656	RSRS19	-122.449	37.944	0.56	15.83	299.70	16.38	-122.449	37.944	0.05	15.83	297.53	15.87	2	7/10/2014	36:16.5
657	RSRS19	-122.449	37.944	0.56	15.80	299.64	16.36	-122.449	37.944	0.25	15.80	297.48	16.05	2	7/10/2014	36:16.6
658	RSRS19	-122.449	37.944	0.56	15.83	299.66	16.38	-122.449	37.944	0.22	15.83	297.51	16.04	2	7/10/2014	36:16.6
659	RSRS19	-122.449	37.944	0.56	15.85	299.60	16.41	-122.449	37.944	0.13	15.85	297.51	15.98	2	7/10/2014	36:16.7

660	RSRS19	-122.449	37.944	0.56	15.83	299.62	16.39	-122.449	37.944	0.17	15.83	297.62	16.00	2	7/10/2014	36:16.9
661	RSRS19	-122.449	37.944	0.56	15.86	299.64	16.42	-122.449	37.944	0.25	15.86	297.66	16.11	2	7/10/2014	36:17.0
662	RSRS19	-122.449	37.944	0.56	15.87	299.64	16.43	-122.449	37.944	0.08	15.87	297.73	15.95	2	7/10/2014	36:17.1
663	RSRS19	-122.449	37.944	0.56	15.83	299.65	16.39	-122.449	37.944	0.30	15.83	297.71	16.13	2	7/10/2014	36:17.1
664	RSRS19	-122.449	37.944	0.56	15.84	299.64	16.40	-122.449	37.944	0.17	15.84	297.62	16.01	2	7/10/2014	36:17.3
665	RSRS19	-122.449	37.944	0.56	15.83	299.56	16.39	-122.449	37.944	0.08	15.83	297.60	15.91	2	7/10/2014	36:17.4
666	RSRS19	-122.449	37.944	0.56	15.82	299.64	16.38	-122.449	37.944	0.30	15.82	297.65	16.12	2	7/10/2014	36:17.5
667	RSRS19	-122.449	37.944	0.56	15.81	299.60	16.37	-122.449	37.944	0.13	15.81	297.60	15.94	2	7/10/2014	36:17.5
668	RSRS19	-122.449	37.944	0.56	15.80	299.56	16.35	-122.449	37.944	0.22	15.80	297.56	16.01	2	7/10/2014	36:17.7
669	RSRS19	-122.449	37.944	0.56	15.80	299.63	16.36	-122.449	37.944	0.25	15.80	297.59	16.05	2	7/10/2014	36:17.7
670	RSRS19	-122.449	37.944	0.56	15.88	299.60	16.44	-122.449	37.944	0.01	15.88	297.58	15.89	2	7/10/2014	36:17.9
671	RSRS19	-122.449	37.944	0.56	15.83	299.65	16.38	-122.449	37.944	0.25	15.83	297.65	16.08	2	7/10/2014	36:17.9
672	RSRS19	-122.449	37.944	0.56	15.85	299.76	16.41	-122.449	37.944	0.22	15.85	297.74	16.06	2	7/10/2014	36:18.1
673	RSRS19	-122.449	37.944	0.56	15.89	299.74	16.45	-122.449	37.944	0.05	15.89	297.74	15.93	2	7/10/2014	36:18.1
674	RSRS19	-122.449	37.944	0.56	15.84	299.76	16.40	-122.449	37.944	0.25	15.84	297.77	16.09	2	7/10/2014	36:18.2
675	RSRS19	-122.449	37.944	0.56	15.86	299.74	16.42	-122.449	37.944	0.25	15.86	297.74	16.11	2	7/10/2014	36:18.3
676	RSRS19	-122.449	37.944	0.56	15.88	299.79	16.44	-122.449	37.944	0.05	15.88	297.76	15.93	2	7/10/2014	36:18.5
677	RSRS19	-122.449	37.944	0.56	15.86	299.70	16.42	-122.449	37.944	0.22	15.86	297.70	16.08	2	7/10/2014	36:18.6
678	RSRS19	-122.449	37.944	0.56	15.84	299.68	16.40	-122.449	37.944	0.22	15.84	297.67	16.06	2	7/10/2014	36:18.7
679	RSRS19	-122.449	37.944	0.56	15.81	299.63	16.37	-122.449	37.944	0.08	15.81	297.70	15.89	2	7/10/2014	36:18.7
680	RSRS19	-122.449	37.944	0.52	15.82	299.61	16.34	-122.449	37.944	0.30	15.82	297.70	16.12	2	7/10/2014	36:18.9
681	RSRS19	-122.449	37.944	0.56	15.80	299.61	16.36	-122.449	37.944	0.22	15.80	297.66	16.02	2	7/10/2014	36:19.0
682	RSRS19	-122.449	37.944	0.52	15.85	299.51	16.37	-122.449	37.944	0.05	15.85	297.55	15.90	2	7/10/2014	36:19.1
683	RSRS19	-122.449	37.944	0.56	15.86	299.68	16.42	-122.449	37.944	0.25	15.86	297.61	16.11	2	7/10/2014	36:19.2
684	RSRS19	-122.449	37.944	0.52	15.87	299.86	16.40	-122.449	37.944	0.08	15.87	297.79	15.95	2	7/10/2014	36:19.3
685	RSRS19	-122.449	37.944	0.56	15.81	299.77	16.37	-122.449	37.944	0.17	15.81	297.70	15.98	2	7/10/2014	36:19.4
686	RSRS19	-122.449	37.944	0.52	15.85	299.75	16.37	-122.449	37.944	0.25	15.85	297.68	16.10	2	7/10/2014	36:19.4
687	RSRS19	-122.449	37.944	0.56	15.84	299.73	16.40	-122.449	37.944	0.08	15.84	297.60	15.92	2	7/10/2014	36:19.6
688	RSRS19	-122.449	37.944	0.56	15.83	299.70	16.39	-122.449	37.944	0.05	15.83	297.53	15.88	2	7/10/2014	36:19.7
689	RSRS19	-122.449	37.944	0.56	15.89	299.64	16.45	-122.449	37.944	0.22	15.89	297.57	16.10	2	7/10/2014	36:19.8
690	RSRS19	-122.449	37.944	0.56	15.82	299.68	16.38	-122.449	37.944	0.17	15.82	297.66	15.98	2	7/10/2014	36:19.9
691	RSRS19	-122.449	37.944	0.56	15.83	299.60	16.39	-122.449	37.944	0.08	15.83	297.54	15.91	2	7/10/2014	36:20.0
692	RSRS19	-122.449	37.944	0.56	15.83	299.64	16.39	-122.449	37.944	0.30	15.83	297.66	16.13	2	7/10/2014	36:20.1

693	RSRS19	-122.449	37.944	0.56	15.86	299.64	16.42	-122.449	37.944	0.22	15.86	297.58	16.07	2	7/10/2014	36:20.2
694	RSRS19	-122.449	37.944	0.56	15.81	299.64	16.37	-122.449	37.944	0.08	15.81	297.58	15.89	2	7/10/2014	36:20.3
695	RSRS19	-122.449	37.944	0.56	15.84	299.64	16.40	-122.449	37.944	0.30	15.84	297.55	16.14	2	7/10/2014	36:20.4
696	RSRS19	-122.449	37.944	0.56	15.83	299.64	16.38	-122.449	37.944	0.22	15.83	297.58	16.04	2	7/10/2014	36:20.5
697	RSRS19	-122.449	37.944	0.56	15.83	299.69	16.39	-122.449	37.944	0.08	15.83	297.60	15.91	2	7/10/2014	36:20.5
698	RSRS19	-122.449	37.944	0.56	15.83	299.64	16.39	-122.449	37.944	0.30	15.83	297.69	16.13	2	7/10/2014	36:20.7
699	RSRS19	-122.449	37.944	0.56	15.84	299.67	16.40	-122.449	37.944	0.22	15.84	297.67	16.06	2	7/10/2014	36:20.8
700	RSRS19	-122.449	37.944	0.56	15.80	299.67	16.35	-122.449	37.944	0.05	15.80	297.62	15.84	2	7/10/2014	36:20.9
701	RSRS19	-122.449	37.944	0.56	15.78	299.69	16.34	-122.449	37.944	0.25	15.78	297.65	16.03	2	7/10/2014	36:21.0
702	RSRS19	-122.449	37.944	0.52	15.80	299.72	16.32	-122.449	37.944	0.22	15.80	297.65	16.01	2	7/10/2014	36:21.0
703	RSRS19	-122.449	37.944	0.56	15.82	299.74	16.38	-122.449	37.944	0.13	15.82	297.71	15.95	2	7/10/2014	36:21.2
704	RSRS19	-122.449	37.944	0.56	15.77	299.76	16.33	-122.449	37.944	0.25	15.77	297.72	16.02	2	7/10/2014	36:21.3
705	RSRS19	-122.449	37.944	0.61	15.85	299.71	16.46	-122.449	37.944	0.30	15.85	297.76	16.15	2	7/10/2014	36:21.4
706	RSRS19	-122.449	37.944	0.56	15.84	299.70	16.40	-122.449	37.944	0.08	15.84	297.74	15.92	2	7/10/2014	36:21.5
707	RSRS19	-122.449	37.944	0.56	15.77	299.65	16.33	-122.449	37.944	0.33	15.77	297.72	16.11	2	7/10/2014	36:21.6
708	RSRS19	-122.449	37.944	0.56	15.83	299.70	16.38	-122.449	37.944	0.22	15.83	297.76	16.04	2	7/10/2014	36:21.6
709	RSRS19	-122.449	37.944	0.56	15.77	299.65	16.33	-122.449	37.944	0.08	15.77	297.70	15.85	2	7/10/2014	36:21.8
710	RSRS19	-122.449	37.944	0.56	15.80	299.70	16.35	-122.449	37.944	0.33	15.80	297.74	16.13	2	7/10/2014	36:21.9
711	RSRS19	-122.449	37.944	0.56	15.77	299.70	16.33	-122.449	37.944	0.17	15.77	297.72	15.94	2	7/10/2014	36:22.0
712	RSRS19	-122.449	37.944	0.56	15.80	299.75	16.35	-122.449	37.944	0.17	15.80	297.77	15.96	2	7/10/2014	36:22.1
713	RSRS19	-122.449	37.944	0.61	15.80	299.77	16.41	-122.449	37.944	0.33	15.80	297.75	16.13	2	7/10/2014	36:22.2
714	RSRS19	-122.449	37.944	0.56	15.79	299.83	16.35	-122.449	37.944	0.17	15.79	297.84	15.95	2	7/10/2014	36:22.2
715	RSRS19	-122.449	37.944	0.61	15.75	299.83	16.36	-122.449	37.944	0.22	15.75	297.79	15.96	2	7/10/2014	36:22.4
716	RSRS19	-122.449	37.944	0.56	15.76	299.88	16.32	-122.449	37.944	0.30	15.76	297.90	16.06	2	7/10/2014	36:22.5
717	RSRS19	-122.449	37.944	0.61	15.80	299.88	16.40	-122.449	37.944	0.30	15.80	297.84	16.10	2	7/10/2014	36:22.6
718	RSRS19	-122.449	37.944	0.56	15.77	299.86	16.33	-122.449	37.944	0.17	15.77	297.86	15.94	2	7/10/2014	36:22.7
719	RSRS19	-122.449	37.944	0.56	15.73	299.82	16.28	-122.449	37.944	0.42	15.73	297.86	16.14	2	7/10/2014	36:22.8
720	RSRS19	-122.449	37.944	0.56	15.74	299.80	16.30	-122.449	37.944	0.33	15.74	297.82	16.08	2	7/10/2014	36:22.9
721	RSRS19	-122.449	37.944	0.61	15.72	299.90	16.33	-122.449	37.944	0.25	15.72	297.88	15.97	2	7/10/2014	36:23.0
722	RSRS19	-122.449	37.944	0.56	15.71	299.82	16.27	-122.449	37.944	0.30	15.71	297.88	16.01	2	7/10/2014	36:23.1
723	RSRS19	-122.449	37.944	0.61	15.68	299.93	16.29	-122.449	37.944	0.38	15.68	297.93	16.06	2	7/10/2014	36:23.2
724	RSRS19	-122.449	37.944	0.56	15.72	299.95	16.28	-122.449	37.944	0.25	15.72	298.02	15.97	2	7/10/2014	36:23.3
725	RSRS19	-122.449	37.944	0.61	15.70	299.95	16.31	-122.449	37.944	0.33	15.70	298.02	16.04	2	7/10/2014	36:23.3

726	RSRS19	-122.449	37.944	0.61	15.66	299.97	16.27	-122.449	37.944	0.38	15.66	298.01	16.05	2	7/10/2014	36:23.5
727	RSRS19	-122.449	37.944	0.61	15.69	300.02	16.30	-122.449	37.944	0.30	15.69	298.09	15.99	2	7/10/2014	36:23.6
728	RSRS19	-122.449	37.944	0.56	15.65	300.04	16.21	-122.449	37.944	0.30	15.65	298.04	15.95	2	7/10/2014	36:23.7
729	RSRS19	-122.449	37.944	0.61	15.65	300.04	16.26	-122.449	37.944	0.42	15.65	298.07	16.07	2	7/10/2014	36:23.8
730	RSRS19	-122.449	37.944	0.61	15.67	300.02	16.28	-122.449	37.944	0.30	15.67	298.06	15.97	2	7/10/2014	36:23.8
731	RSRS19	-122.449	37.944	0.61	15.66	300.05	16.27	-122.449	37.944	0.33	15.66	298.09	16.00	2	7/10/2014	36:24.0
732	RSRS19	-122.449	37.944	0.61	15.64	300.02	16.25	-122.449	37.944	0.42	15.64	298.06	16.06	2	7/10/2014	36:24.1
733	RSRS19	-122.449	37.944	0.61	15.63	300.05	16.24	-122.449	37.944	0.30	15.63	298.09	15.93	2	7/10/2014	36:24.2
734	RSRS19	-122.449	37.944	0.56	15.62	300.07	16.18	-122.449	37.944	0.30	15.62	298.09	15.92	2	7/10/2014	36:24.3
735	RSRS19	-122.449	37.944	0.61	15.62	300.05	16.23	-122.449	37.944	0.42	15.62	298.12	16.04	2	7/10/2014	36:24.4
736	RSRS19	-122.449	37.944	0.61	15.68	300.07	16.29	-122.449	37.944	0.33	15.68	298.09	16.01	2	7/10/2014	36:24.5
737	RSRS19	-122.449	37.944	0.61	15.62	300.09	16.23	-122.449	37.944	0.33	15.62	298.12	15.96	2	7/10/2014	36:24.6
738	RSRS19	-122.449	37.944	0.56	15.64	300.07	16.20	-122.449	37.944	0.42	15.64	298.13	16.06	2	7/10/2014	36:24.7
739	RSRS19	-122.449	37.944	0.61	15.61	300.09	16.22	-122.449	37.944	0.33	15.61	298.18	15.94	2	7/10/2014	36:24.8
740	RSRS19	-122.449	37.944	0.61	15.65	300.05	16.26	-122.449	37.944	0.30	15.65	298.10	15.95	2	7/10/2014	36:24.9
741	RSRS19	-122.449	37.944	0.61	15.62	299.99	16.23	-122.449	37.944	0.42	15.62	298.07	16.04	2	7/10/2014	36:24.9
742	RSRS19	-122.449	37.944	0.61	15.60	300.01	16.21	-122.449	37.944	0.42	15.60	298.12	16.02	2	7/10/2014	36:25.1
743	RSRS19	-122.449	37.944	0.61	15.56	300.03	16.17	-122.449	37.944	0.38	15.56	298.05	15.95	2	7/10/2014	36:25.2
744	RSRS19	-122.449	37.944	0.61	15.60	300.05	16.20	-122.449	37.944	0.42	15.60	298.10	16.01	2	7/10/2014	36:25.3
745	RSRS19	-122.449	37.944	0.61	15.57	300.01	16.18	-122.449	37.944	0.33	15.57	298.05	15.90	2	7/10/2014	36:25.4
746	RSRS19	-122.449	37.944	0.61	15.59	300.01	16.19	-122.449	37.944	0.38	15.59	298.08	15.97	2	7/10/2014	36:25.5
747	RSRS19	-122.449	37.944	0.61	15.56	299.99	16.17	-122.449	37.944	0.50	15.56	298.01	16.07	2	7/10/2014	36:25.6
748	RSRS19	-122.449	37.944	0.61	15.56	299.90	16.16	-122.449	37.944	0.38	15.56	297.88	15.94	2	7/10/2014	36:25.7
749	RSRS19	-122.449	37.944	0.61	15.66	299.83	16.26	-122.449	37.944	0.38	15.66	297.84	16.04	2	7/10/2014	36:25.8
750	RSRS19	-122.449	37.944	0.61	15.64	299.86	16.25	-122.449	37.944	0.42	15.64	297.86	16.06	2	7/10/2014	36:25.9
751	RSRS19	-122.449	37.944	0.61	15.60	299.80	16.21	-122.449	37.944	0.33	15.60	297.84	15.94	2	7/10/2014	36:26.0
752	RSRS19	-122.449	37.944	0.61	15.55	299.82	16.16	-122.449	37.944	0.45	15.55	297.84	16.00	2	7/10/2014	36:26.1
753	RSRS19	-122.449	37.944	0.61	15.61	299.77	16.22	-122.449	37.944	0.45	15.61	297.86	16.06	2	7/10/2014	36:26.1
754	RSRS19	-122.449	37.944	0.56	15.60	299.77	16.16	-122.449	37.944	0.30	15.60	297.82	15.90	2	7/10/2014	36:26.2
755	RSRS19	-122.449	37.944	0.61	15.58	299.75	16.19	-122.449	37.944	0.50	15.58	297.84	16.08	2	7/10/2014	36:26.4
756	RSRS19	-122.449	37.944	0.61	15.59	299.71	16.19	-122.449	37.944	0.42	15.59	297.82	16.00	2	7/10/2014	36:26.5
757	RSRS19	-122.449	37.944	0.61	15.61	299.69	16.22	-122.449	37.944	0.30	15.61	297.78	15.91	2	7/10/2014	36:26.6
758	RSRS19	-122.449	37.944	0.61	15.55	299.58	16.16	-122.449	37.944	0.45	15.55	297.69	16.00	2	7/10/2014	36:26.6

759	RSRS19	-122.449	37.944	0.61	15.57	299.54	16.18	-122.449	37.944	0.33	15.57	297.60	15.90	2	7/10/2014	36:26.8
760	RSRS19	-122.449	37.944	0.61	15.55	299.51	16.16	-122.449	37.944	0.33	15.55	297.51	15.88	2	7/10/2014	36:26.9
761	RSRS19	-122.449	37.944	0.61	15.53	299.47	16.14	-122.449	37.944	0.45	15.53	297.49	15.98	2	7/10/2014	36:27.0
762	RSRS19	-122.449	37.944	0.61	15.55	299.50	16.16	-122.449	37.944	0.38	15.55	297.43	15.93	2	7/10/2014	36:27.1
763	RSRS19	-122.449	37.944	0.61	15.60	299.41	16.21	-122.449	37.944	0.42	15.60	297.41	16.02	2	7/10/2014	36:27.2
764	RSRS19	-122.449	37.944	0.61	15.56	299.39	16.16	-122.449	37.944	0.42	15.56	297.43	15.97	2	7/10/2014	36:27.2
765	RSRS19	-122.449	37.944	0.61	15.60	299.39	16.21	-122.449	37.944	0.25	15.60	297.45	15.85	2	7/10/2014	36:27.4
766	RSRS19	-122.449	37.944	0.61	15.60	299.36	16.21	-122.449	37.944	0.45	15.60	297.39	16.05	2	7/10/2014	36:27.5
767	RSRS19	-122.449	37.944	0.61	15.60	299.37	16.21	-122.449	37.944	0.38	15.60	297.52	15.99	2	7/10/2014	36:27.6
768	RSRS19	-122.449	37.944	0.56	15.61	299.41	16.17	-122.449	37.944	0.25	15.61	297.45	15.86	2	7/10/2014	36:27.6
769	RSRS19	-122.449	37.944	0.61	15.65	299.35	16.26	-122.449	37.944	0.45	15.65	297.39	16.10	2	7/10/2014	36:27.7
770	RSRS19	-122.449	37.944	0.61	15.61	299.37	16.22	-122.449	37.944	0.30	15.61	297.45	15.91	2	7/10/2014	36:27.9
771	RSRS19	-122.449	37.944	0.61	15.57	299.35	16.18	-122.449	37.944	0.30	15.57	297.39	15.87	2	7/10/2014	36:28.0
772	RSRS19	-122.449	37.944	0.61	15.62	299.32	16.23	-122.449	37.944	0.38	15.62	297.34	16.00	2	7/10/2014	36:28.1
773	RSRS19	-122.449	37.944	0.61	15.60	299.30	16.21	-122.449	37.944	0.22	15.60	297.33	15.82	2	7/10/2014	36:28.2
774	RSRS19	-122.449	37.944	0.61	15.60	299.31	16.21	-122.449	37.944	0.30	15.60	297.33	15.90	2	7/10/2014	36:28.3
775	RSRS19	-122.449	37.944	0.61	15.56	299.33	16.17	-122.449	37.944	0.38	15.56	297.33	15.95	2	7/10/2014	36:28.4
776	RSRS19	-122.449	37.944	0.61	15.56	299.31	16.17	-122.449	37.944	0.25	15.56	297.37	15.81	2	7/10/2014	36:28.5
777	RSRS19	-122.449	37.944	0.61	15.59	299.37	16.19	-122.449	37.944	0.33	15.59	297.37	15.92	2	7/10/2014	36:28.6
778	RSRS19	-122.449	37.944	0.61	15.58	299.24	16.19	-122.449	37.944	0.30	15.58	297.24	15.88	2	7/10/2014	36:28.7
779	RSRS19	-122.449	37.944	0.56	15.60	299.31	16.15	-122.449	37.944	0.25	15.60	297.27	15.84	2	7/10/2014	36:28.8
780	RSRS19	-122.449	37.944	0.61	15.65	299.44	16.26	-122.449	37.944	0.42	15.65	297.57	16.07	2	7/10/2014	36:28.9
781	RSRS19	-122.449	37.944	0.61	15.64	299.42	16.25	-122.449	37.944	0.38	15.64	297.62	16.02	2	7/10/2014	36:28.9
782	RSRS19	-122.449	37.944	0.61	15.63	299.33	16.24	-122.449	37.944	0.25	15.63	297.55	15.88	2	7/10/2014	36:29.1
783	RSRS19	-122.449	37.944	0.56	15.60	299.31	16.15	-122.449	37.944	0.33	15.60	297.51	15.93	2	7/10/2014	36:29.2
784	RSRS19	-122.449	37.944	0.61	15.61	299.25	16.22	-122.449	37.944	0.33	15.61	297.29	15.94	2	7/10/2014	36:29.3
785	RSRS19	-122.449	37.944	0.61	15.56	299.29	16.16	-122.449	37.944	0.30	15.56	297.25	15.85	2	7/10/2014	36:29.4
786	RSRS19	-122.449	37.944	0.61	15.57	299.47	16.18	-122.449	37.944	0.33	15.57	297.38	15.90	2	7/10/2014	36:29.4
787	RSRS19	-122.449	37.944	0.61	15.56	299.42	16.16	-122.449	37.944	0.25	15.56	297.36	15.80	2	7/10/2014	36:29.6
788	RSRS19	-122.449	37.944	0.61	15.62	299.38	16.23	-122.449	37.944	0.22	15.62	297.34	15.84	2	7/10/2014	36:29.7
789	RSRS19	-122.449	37.944	0.61	15.58	299.36	16.19	-122.449	37.944	0.38	15.58	297.38	15.96	2	7/10/2014	36:29.8
790	RSRS19	-122.449	37.944	0.61	15.60	299.34	16.20	-122.449	37.944	0.25	15.60	297.36	15.84	2	7/10/2014	36:29.9
791	RSRS19	-122.449	37.944	0.61	15.63	299.30	16.24	-122.449	37.944	0.25	15.63	297.34	15.88	2	7/10/2014	36:30.0

792	RSRS19	-122.449	37.944	0.56	15.62	299.36	16.18	-122.449	37.944	0.33	15.62	297.38	15.95	2	7/10/2014	36:30.0
793	RSRS19	-122.449	37.944	0.61	15.69	299.43	16.30	-122.449	37.944	0.13	15.69	297.45	15.82	2	7/10/2014	36:30.2
794	RSRS19	-122.449	37.944	0.56	15.66	299.41	16.22	-122.449	37.944	0.30	15.66	297.45	15.96	2	7/10/2014	36:30.3
795	RSRS19	-122.449	37.944	0.61	15.62	299.36	16.23	-122.449	37.944	0.33	15.62	297.43	15.95	2	7/10/2014	36:30.4
796	RSRS19	-122.449	37.944	0.56	15.62	299.43	16.18	-122.449	37.944	0.08	15.62	297.41	15.70	2	7/10/2014	36:30.5
797	RSRS19	-122.449	37.944	0.61	15.69	299.23	16.30	-122.449	37.944	0.30	15.69	297.37	15.99	2	7/10/2014	36:30.5
798	RSRS19	-122.449	37.944	0.56	15.66	299.23	16.22	-122.449	37.944	0.33	15.66	297.32	16.00	2	7/10/2014	36:30.7
799	RSRS19	-122.449	37.944	0.61	15.59	299.26	16.19	-122.449	37.944	0.16	15.59	297.32	15.75	2	7/10/2014	36:30.8
800	RSRS19	-122.449	37.944	0.56	15.73	299.13	16.29	-122.449	37.944	0.33	15.73	297.26	16.07	2	7/10/2014	36:30.9
801	RSRS19	-122.449	37.944	0.61	15.56	299.19	16.17	-122.449	37.944	0.25	15.56	297.28	15.81	2	7/10/2014	36:31.0
802	RSRS19	-122.449	37.944	0.56	15.62	299.13	16.18	-122.449	37.944	0.16	15.62	297.24	15.79	2	7/10/2014	36:31.1
803	RSRS19	-122.449	37.944	0.61	15.58	299.17	16.19	-122.449	37.944	0.42	15.58	297.28	16.00	2	7/10/2014	36:31.2
804	RSRS19	-122.449	37.944	0.56	15.61	299.19	16.17	-122.449	37.944	0.16	15.61	297.33	15.77	2	7/10/2014	36:31.3
805	RSRS19	-122.449	37.944	0.56	15.66	299.17	16.21	-122.449	37.944	0.22	15.66	297.37	15.87	2	7/10/2014	36:31.4
806	RSRS19	-122.449	37.944	0.56	15.65	299.20	16.21	-122.449	37.944	0.38	15.65	297.40	16.03	2	7/10/2014	36:31.4
807	RSRS19	-122.449	37.944	0.61	15.61	299.13	16.22	-122.449	37.944	0.22	15.61	297.35	15.82	2	7/10/2014	36:31.6
808	RSRS19	-122.449	37.944	0.56	15.65	299.13	16.21	-122.449	37.944	0.25	15.65	297.35	15.90	2	7/10/2014	36:31.7
809	RSRS19	-122.449	37.944	0.61	15.56	299.13	16.16	-122.449	37.944	0.38	15.56	297.31	15.94	2	7/10/2014	36:31.8
810	RSRS19	-122.449	37.944	0.56	15.62	299.15	16.18	-122.449	37.944	0.22	15.62	297.35	15.83	2	7/10/2014	36:31.8
811	RSRS19	-122.449	37.944	0.61	15.55	299.16	16.16	-122.449	37.944	0.30	15.55	297.33	15.85	2	7/10/2014	36:32.0
812	RSRS19	-122.449	37.944	0.56	15.53	299.16	16.09	-122.449	37.944	0.38	15.53	297.33	15.92	2	7/10/2014	36:32.1
813	RSRS19	-122.449	37.944	0.61	15.59	299.18	16.19	-122.449	37.944	0.16	15.59	297.31	15.75	2	7/10/2014	36:32.2
814	RSRS19	-122.449	37.944	0.56	15.55	299.16	16.11	-122.449	37.944	0.38	15.55	297.33	15.93	2	7/10/2014	36:32.2
815	RSRS19	-122.449	37.944	0.61	15.53	299.20	16.14	-122.449	37.944	0.42	15.53	297.36	15.95	2	7/10/2014	36:32.4
816	RSRS19	-122.449	37.944	0.61	15.53	299.16	16.13	-122.449	37.944	0.16	15.53	297.33	15.69	2	7/10/2014	36:32.5
817	RSRS19	-122.449	37.944	0.61	15.53	299.14	16.14	-122.449	37.944	0.38	15.53	297.33	15.92	2	7/10/2014	36:32.6
818	RSRS19	-122.449	37.944	0.56	15.51	299.12	16.07	-122.449	37.944	0.33	15.51	297.32	15.84	2	7/10/2014	36:32.7
819	RSRS19	-122.449	37.944	0.61	15.56	299.12	16.16	-122.449	37.944	0.25	15.56	297.27	15.80	2	7/10/2014	36:32.8
820	RSRS19	-122.449	37.944	0.61	15.55	299.12	16.16	-122.449	37.944	0.38	15.55	297.27	15.93	2	7/10/2014	36:32.8
821	RSRS19	-122.449	37.944	0.61	15.49	299.12	16.10	-122.449	37.944	0.33	15.49	297.23	15.83	2	7/10/2014	36:33.0
822	RSRS19	-122.449	37.944	0.56	15.51	299.10	16.07	-122.449	37.944	0.22	15.51	297.18	15.73	2	7/10/2014	36:33.0
823	RSRS19	-122.449	37.944	0.61	15.50	299.08	16.11	-122.449	37.944	0.42	15.50	297.19	15.92	2	7/10/2014	36:33.2
824	RSRS19	-122.449	37.944	0.56	15.54	299.08	16.10	-122.449	37.944	0.33	15.54	297.16	15.87	2	7/10/2014	36:33.3

825	RSRS19	-122.449	37.944	0.61	15.49	299.08	16.10	-122.449	37.944	0.25	15.49	297.19	15.74	2	7/10/2014	36:33.3
826	RSRS19	-122.449	37.944	0.56	15.53	299.03	16.08	-122.449	37.944	0.42	15.53	297.12	15.94	2	7/10/2014	36:33.4
827	RSRS19	-122.449	37.944	0.61	15.54	298.99	16.15	-122.449	37.944	0.30	15.54	297.13	15.84	2	7/10/2014	36:33.6
828	RSRS19	-122.449	37.944	0.61	15.55	299.04	16.16	-122.449	37.944	0.30	15.55	297.10	15.85	2	7/10/2014	36:33.7
829	RSRS19	-122.449	37.944	0.61	15.56	298.88	16.16	-122.449	37.944	0.42	15.56	297.08	15.97	2	7/10/2014	36:33.8
830	RSRS19	-122.449	37.944	0.61	15.51	298.97	16.12	-122.449	37.944	0.30	15.51	297.10	15.81	2	7/10/2014	36:33.8
831	RSRS19	-122.449	37.944	0.61	15.53	298.95	16.14	-122.449	37.944	0.30	15.53	297.11	15.83	2	7/10/2014	36:34.0
832	RSRS19	-122.449	37.944	0.56	15.56	298.95	16.11	-122.449	37.944	0.38	15.56	297.10	15.94	2	7/10/2014	36:34.1
833	RSRS19	-122.449	37.944	0.61	15.55	298.95	16.16	-122.449	37.944	0.22	15.55	297.15	15.76	2	7/10/2014	36:34.2
834	RSRS19	-122.449	37.944	0.56	15.54	298.97	16.10	-122.449	37.944	0.33	15.54	297.13	15.87	2	7/10/2014	36:34.2
835	RSRS19	-122.449	37.944	0.61	15.56	298.95	16.17	-122.449	37.944	0.38	15.56	297.15	15.95	2	7/10/2014	36:34.4
836	RSRS19	-122.449	37.944	0.56	15.58	298.98	16.14	-122.449	37.944	0.22	15.58	297.13	15.79	2	7/10/2014	36:34.4
837	RSRS19	-122.449	37.944	0.61	15.57	298.93	16.18	-122.449	37.944	0.33	15.57	297.15	15.90	2	7/10/2014	36:34.6
838	RSRS19	-122.449	37.944	0.56	15.58	298.96	16.14	-122.449	37.944	0.33	15.58	297.13	15.91	2	7/10/2014	36:34.6
839	RSRS19	-122.449	37.944	0.61	15.60	299.00	16.20	-122.449	37.944	0.16	15.60	297.22	15.76	2	7/10/2014	36:34.8
840	RSRS19	-122.449	37.944	0.56	15.60	299.09	16.16	-122.449	37.944	0.42	15.60	297.27	16.02	2	7/10/2014	36:34.9
841	RSRS19	-122.449	37.944	0.61	15.67	299.09	16.28	-122.449	37.944	0.25	15.67	297.31	15.92	2	7/10/2014	36:35.0
842	RSRS19	-122.449	37.944	0.56	15.62	299.11	16.18	-122.449	37.944	0.16	15.62	297.33	15.78	2	7/10/2014	36:35.0
843	RSRS19	-122.449	37.944	0.61	15.62	299.16	16.23	-122.449	37.944	0.42	15.62	297.35	16.04	2	7/10/2014	36:35.2
844	RSRS19	-122.449	37.944	0.56	15.62	299.16	16.18	-122.449	37.944	0.16	15.62	297.29	15.79	2	7/10/2014	36:35.3
845	RSRS19	-122.449	37.944	0.61	15.60	299.20	16.20	-122.449	37.944	0.30	15.60	297.31	15.89	2	7/10/2014	36:35.4
846	RSRS19	-122.449	37.944	0.56	15.61	299.20	16.17	-122.449	37.944	0.33	15.61	297.33	15.94	2	7/10/2014	36:35.4
847	RSRS19	-122.449	37.944	0.56	15.63	299.25	16.19	-122.449	37.944	0.13	15.63	297.33	15.76	2	7/10/2014	36:35.6
848	RSRS19	-122.449	37.944	0.56	15.65	299.29	16.21	-122.449	37.944	0.33	15.65	297.40	15.98	2	7/10/2014	36:35.6
849	RSRS19	-122.449	37.944	0.56	15.67	299.25	16.23	-122.449	37.944	0.30	15.67	297.31	15.97	2	7/10/2014	36:35.7
850	RSRS19	-122.449	37.944	0.56	15.64	299.32	16.20	-122.449	37.944	0.08	15.64	297.39	15.72	2	7/10/2014	36:35.8
851	RSRS19	-122.449	37.944	0.61	15.64	299.34	16.25	-122.449	37.944	0.30	15.64	297.45	15.94	2	7/10/2014	36:36.0
852	RSRS19	-122.449	37.944	0.56	15.65	299.21	16.21	-122.449	37.944	0.16	15.65	297.25	15.81	2	7/10/2014	36:36.1
853	RSRS19	-122.449	37.944	0.56	15.67	299.38	16.23	-122.449	37.944	0.16	15.67	297.43	15.84	2	7/10/2014	36:36.1
854	RSRS19	-122.449	37.944	0.56	15.68	299.49	16.24	-122.449	37.944	0.30	15.68	297.53	15.98	2	7/10/2014	36:36.2
855	RSRS19	-122.449	37.944	0.56	15.66	299.30	16.21	-122.449	37.944	0.13	15.66	297.52	15.79	2	7/10/2014	36:36.4
856	RSRS19	-122.449	37.944	0.52	15.67	299.36	16.19	-122.449	37.944	0.22	15.67	297.47	15.89	2	7/10/2014	36:36.5
857	RSRS19	-122.449	37.944	0.56	15.69	299.37	16.24	-122.449	37.944	0.25	15.69	297.50	15.94	2	7/10/2014	36:36.6

858	RSRS19	-122.449	37.944	0.56	15.69	299.36	16.25	-122.449	37.944	0.08	15.69	297.49	15.77	2	7/10/2014	36:36.6
859	RSRS19	-122.449	37.944	0.56	15.69	299.34	16.25	-122.449	37.944	0.25	15.69	297.48	15.94	2	7/10/2014	36:36.8
860	RSRS19	-122.449	37.944	0.52	15.70	299.35	16.23	-122.449	37.944	0.22	15.70	297.48	15.92	2	7/10/2014	36:36.9
861	RSRS19	-122.449	37.944	0.56	15.69	299.37	16.25	-122.449	37.944	0.08	15.69	297.50	15.77	2	7/10/2014	36:37.0
862	RSRS19	-122.449	37.944	0.56	15.72	299.35	16.28	-122.449	37.944	0.25	15.72	297.50	15.97	2	7/10/2014	36:37.0
863	RSRS19	-122.449	37.944	0.56	15.68	299.26	16.24	-122.449	37.944	0.16	15.68	297.50	15.84	2	7/10/2014	36:37.2
864	RSRS19	-122.449	37.944	0.56	15.66	299.24	16.22	-122.449	37.944	0.08	15.66	297.48	15.74	2	7/10/2014	36:37.2
865	RSRS19	-122.449	37.944	0.56	15.69	299.22	16.24	-122.449	37.944	0.33	15.69	297.46	16.02	2	7/10/2014	36:37.4
866	RSRS19	-122.449	37.944	0.52	15.66	299.20	16.18	-122.449	37.944	0.13	15.66	297.46	15.79	2	7/10/2014	36:37.4
867	RSRS19	-122.449	37.944	0.56	15.66	299.15	16.22	-122.449	37.944	0.25	15.66	297.42	15.91	2	7/10/2014	36:37.6
868	RSRS19	-122.449	37.944	0.56	15.66	299.11	16.21	-122.449	37.944	0.25	15.66	297.35	15.91	2	7/10/2014	36:37.7
869	RSRS19	-122.449	37.944	0.56	15.62	299.09	16.18	-122.449	37.944	0.01	15.62	297.31	15.63	2	7/10/2014	36:37.8
870	RSRS19	-122.449	37.944	0.56	15.65	299.11	16.21	-122.449	37.944	0.25	15.65	297.28	15.90	2	7/10/2014	36:37.8
871	RSRS19	-122.449	37.944	0.56	15.65	299.05	16.21	-122.449	37.944	0.25	15.65	297.22	15.90	2	7/10/2014	36:38.0
872	RSRS19	-122.449	37.944	0.52	15.63	299.07	16.16	-122.449	37.944	0.05	15.63	297.22	15.68	2	7/10/2014	36:38.1
873	RSRS19	-122.449	37.944	0.56	15.65	299.15	16.21	-122.449	37.944	0.30	15.65	297.36	15.95	2	7/10/2014	36:38.2
874	RSRS19	-122.449	37.944	0.56	15.67	299.09	16.23	-122.449	37.944	0.08	15.67	297.35	15.75	2	7/10/2014	36:38.2
875	RSRS19	-122.449	37.944	0.56	15.73	299.09	16.29	-122.449	37.944	0.22	15.73	297.33	15.95	2	7/10/2014	36:38.4
876	RSRS19	-122.449	37.944	0.52	15.68	299.07	16.20	-122.449	37.944	0.30	15.68	297.35	15.98	2	7/10/2014	36:38.4
877	RSRS19	-122.449	37.944	0.56	15.68	299.03	16.24	-122.449	37.944	0.08	15.68	297.32	15.76	2	7/10/2014	36:38.6
878	RSRS19	-122.449	37.944	0.52	15.67	298.96	16.19	-122.449	37.944	0.30	15.67	297.25	15.97	2	7/10/2014	36:38.6
879	RSRS19	-122.449	37.944	0.56	15.66	298.96	16.21	-122.449	37.944	0.22	15.66	297.16	15.87	2	7/10/2014	36:38.8
880	RSRS19	-122.449	37.944	0.52	15.67	298.85	16.19	-122.449	37.944	0.13	15.67	297.11	15.80	2	7/10/2014	36:38.9
881	RSRS19	-122.449	37.944	0.52	15.66	298.89	16.19	-122.449	37.944	0.13	15.66	297.03	15.79	2	7/10/2014	36:38.9
882	RSRS19	-122.449	37.944	0.56	15.64	298.92	16.20	-122.449	37.944	0.01	15.64	297.12	15.65	2	7/10/2014	36:39.0
883	RSRS19	-122.449	37.944	0.56	15.69	298.90	16.25	-122.449	37.944	0.16	15.69	297.12	15.86	2	7/10/2014	36:39.2
884	RSRS19	-122.449	37.944	0.52	15.70	298.90	16.23	-122.449	37.944	0.25	15.70	297.16	15.95	2	7/10/2014	36:39.3
885	RSRS19	-122.449	37.944	0.56	15.72	298.94	16.27	-122.449	37.944	-0.07	15.72	297.19	15.64	2	7/10/2014	36:39.4
886	RSRS19	-122.449	37.944	0.56	15.74	298.92	16.30	-122.449	37.944	0.25	15.74	297.18	15.99	2	7/10/2014	36:39.4
887	RSRS19	-122.449	37.944	0.56	15.73	298.86	16.28	-122.449	37.944	0.05	15.73	297.27	15.77	2	7/10/2014	36:39.6
888	RSRS19	-122.449	37.944	0.52	15.76	298.88	16.28	-122.449	37.944	0.16	15.76	297.12	15.93	2	7/10/2014	36:39.7
889	RSRS19	-122.449	37.944	0.56	15.76	298.82	16.31	-122.449	37.944	0.16	15.76	297.10	15.92	2	7/10/2014	36:39.8
890	RSRS19	-122.449	37.944	0.52	15.73	298.79	16.26	-122.449	37.944	-0.07	15.73	297.08	15.66	2	7/10/2014	36:39.8

891	RSRS19	-122.449	37.944	0.56	15.76	298.79	16.31	-122.449	37.944	0.21	15.76	297.04	15.97	2	7/10/2014	36:40.0
892	RSRS19	-122.449	37.944	0.52	15.75	298.82	16.27	-122.449	37.944	0.13	15.75	297.08	15.88	2	7/10/2014	36:40.0
893	RSRS19	-122.449	37.944	0.56	15.75	298.86	16.30	-122.449	37.944	0.01	15.75	297.09	15.76	2	7/10/2014	36:40.2
894	RSRS19	-122.449	37.944	0.52	15.76	298.87	16.28	-122.449	37.944	0.13	15.76	297.10	15.89	2	7/10/2014	36:40.2
895	RSRS19	-122.449	37.944	0.52	15.76	298.80	16.28	-122.449	37.944	0.08	15.76	297.04	15.83	2	7/10/2014	36:40.4
896	RSRS19	-122.449	37.944	0.52	15.76	298.82	16.28	-122.449	37.944	0.08	15.76	297.11	15.83	2	7/10/2014	36:40.5
897	RSRS19	-122.449	37.944	0.56	15.76	298.86	16.32	-122.449	37.944	0.21	15.76	297.11	15.98	2	7/10/2014	36:40.6
898	RSRS19	-122.449	37.944	0.52	15.79	298.80	16.31	-122.449	37.944	0.05	15.79	297.10	15.83	2	7/10/2014	36:40.6
899	RSRS19	-122.449	37.944	0.56	15.76	298.80	16.31	-122.449	37.944	0.13	15.76	297.02	15.89	2	7/10/2014	36:40.8
900	RSRS19	-122.449	37.944	0.52	15.80	298.76	16.32	-122.449	37.944	0.16	15.80	296.98	15.96	2	7/10/2014	36:40.9
901	RSRS19	-122.449	37.944	0.56	15.76	298.78	16.31	-122.449	37.944	-0.04	15.76	296.93	15.72	2	7/10/2014	36:41.0
902	RSRS19	-122.449	37.944	0.52	15.76	298.82	16.28	-122.449	37.944	0.16	15.76	297.00	15.93	2	7/10/2014	36:41.0
903	RSRS19	-122.449	37.944	0.56	15.76	298.69	16.31	-122.449	37.944	-0.04	15.76	296.91	15.72	2	7/10/2014	36:41.2
904	RSRS19	-122.449	37.944	0.52	15.80	298.61	16.32	-122.449	37.944	0.01	15.80	296.78	15.81	2	7/10/2014	36:41.3
905	RSRS19	-122.449	37.944	0.52	15.76	298.65	16.28	-122.449	37.944	0.16	15.76	296.76	15.93	2	7/10/2014	36:41.4
906	RSRS19	-122.449	37.944	0.52	15.76	298.65	16.28	-122.449	37.944	0.01	15.76	296.85	15.77	2	7/10/2014	36:41.4
907	RSRS19	-122.449	37.944	0.56	15.80	298.81	16.35	-122.449	37.944	0.01	15.80	296.96	15.81	2	7/10/2014	36:41.6
908	RSRS19	-122.449	37.944	0.52	15.76	298.94	16.28	-122.449	37.944	0.16	15.76	297.13	15.92	2	7/10/2014	36:41.7
909	RSRS19	-122.449	37.944	0.56	15.80	299.09	16.35	-122.449	37.944	0.13	15.80	297.29	15.92	2	7/10/2014	36:41.7
910	RSRS19	-122.449	37.944	0.52	15.76	299.21	16.28	-122.449	37.944	0.13	15.76	297.42	15.89	2	7/10/2014	36:41.8
911	RSRS19	-122.449	37.944	0.56	15.76	299.25	16.32	-122.449	37.944	0.21	15.76	297.43	15.98	2	7/10/2014	36:42.0
912	RSRS19	-122.449	37.944	0.52	15.77	299.36	16.29	-122.449	37.944	-0.04	15.77	297.51	15.73	2	7/10/2014	36:42.1
913	RSRS19	-122.449	37.944	0.52	15.77	299.47	16.29	-122.449	37.944	0.21	15.77	297.60	15.99	2	7/10/2014	36:42.2
914	RSRS19	-122.449	37.944	0.52	15.76	299.42	16.28	-122.449	37.944	0.13	15.76	297.58	15.89	2	7/10/2014	36:42.2
915	RSRS19	-122.449	37.944	0.52	15.80	299.47	16.32	-122.449	37.944	0.01	15.80	297.60	15.81	2	7/10/2014	36:42.4
916	RSRS19	-122.449	37.944	0.52	15.80	299.34	16.32	-122.449	37.944	0.16	15.80	297.56	15.96	2	7/10/2014	36:42.5
917	RSRS19	-122.449	37.944	0.56	15.80	299.27	16.36	-122.449	37.944	0.08	15.80	297.47	15.88	2	7/10/2014	36:42.5
918	RSRS19	-122.449	37.944	0.52	15.81	299.21	16.33	-122.449	37.944	0.01	15.81	297.41	15.82	2	7/10/2014	36:42.6
919	RSRS19	-122.449	37.944	0.52	15.80	299.21	16.32	-122.449	37.944	0.13	15.80	297.45	15.92	2	7/10/2014	36:42.8
920	RSRS19	-122.449	37.944	0.52	15.80	299.17	16.32	-122.449	37.944	0.08	15.80	297.41	15.88	2	7/10/2014	36:42.8
921	RSRS19	-122.449	37.944	0.52	15.80	299.13	16.32	-122.449	37.944	0.01	15.80	297.39	15.81	2	7/10/2014	36:43.0
922	RSRS19	-122.449	37.944	0.52	15.81	299.04	16.33	-122.449	37.944	0.16	15.81	297.28	15.97	2	7/10/2014	36:43.0
923	RSRS19	-122.449	37.944	0.52	15.83	298.95	16.36	-122.449	37.944	0.01	15.83	297.19	15.84	2	7/10/2014	36:43.2

924	RSRS19	-122.449	37.944	0.52	15.84	298.93	16.36	-122.449	37.944	0.05	15.84	297.10	15.89	2	7/10/2014	36:43.3
925	RSRS19	-122.449	37.944	0.52	15.82	298.86	16.34	-122.449	37.944	0.16	15.82	297.04	15.98	2	7/10/2014	36:43.4
926	RSRS19	-122.449	37.944	0.52	15.82	298.78	16.34	-122.449	37.944	-0.07	15.82	296.95	15.74	2	7/10/2014	36:43.4
927	RSRS19	-122.449	37.944	0.52	15.83	298.73	16.36	-122.449	37.944	0.08	15.83	296.95	15.91	2	7/10/2014	36:43.6
928	RSRS19	-122.449	37.944	0.52	15.83	298.67	16.35	-122.449	37.944	0.08	15.83	296.89	15.90	2	7/10/2014	36:43.7
929	RSRS19	-122.449	37.944	0.52	15.83	298.64	16.35	-122.449	37.944	-0.13	15.83	296.86	15.70	2	7/10/2014	36:43.8
930	RSRS19	-122.449	37.944	0.52	15.86	298.51	16.39	-122.449	37.944	0.16	15.86	296.73	16.03	2	7/10/2014	36:43.8
931	RSRS19	-122.449	37.944	0.52	15.83	298.51	16.35	-122.449	37.944	0.13	15.83	296.67	15.96	2	7/10/2014	36:44.0
932	RSRS19	-122.449	37.944	0.52	15.81	298.52	16.33	-122.449	37.944	-0.07	15.81	296.63	15.74	2	7/10/2014	36:44.1
933	RSRS19	-122.449	37.944	0.52	15.83	298.45	16.35	-122.449	37.944	0.13	15.83	296.63	15.96	2	7/10/2014	36:44.2
934	RSRS19	-122.449	37.944	0.52	15.81	298.41	16.33	-122.449	37.944	0.08	15.81	296.50	15.89	2	7/10/2014	36:44.2
935	RSRS19	-122.449	37.944	0.52	15.82	298.32	16.34	-122.449	37.944	-0.07	15.82	296.43	15.74	2	7/10/2014	36:44.4
936	RSRS19	-122.449	37.944	0.47	15.80	298.21	16.27	-122.449	37.944	0.13	15.80	296.32	15.93	2	7/10/2014	36:44.5
937	RSRS19	-122.449	37.944	0.52	15.80	298.13	16.32	-122.449	37.944	0.13	15.80	296.26	15.93	2	7/10/2014	36:44.5
938	RSRS19	-122.449	37.944	0.47	15.83	298.10	16.30	-122.449	37.944	0.01	15.83	296.19	15.84	2	7/10/2014	36:44.6
939	RSRS19	-122.449	37.944	0.52	15.80	298.13	16.32	-122.449	37.944	0.13	15.80	296.21	15.93	2	7/10/2014	36:44.8
940	RSRS19	-122.449	37.944	0.52	15.85	298.04	16.37	-122.449	37.944	0.01	15.85	296.19	15.86	2	7/10/2014	36:44.9
941	RSRS19	-122.449	37.944	0.52	15.83	297.93	16.35	-122.449	37.944	-0.04	15.83	296.11	15.79	2	7/10/2014	36:45.0
942	RSRS19	-122.449	37.944	0.52	15.81	297.89	16.33	-122.449	37.944	0.13	15.81	296.07	15.94	2	7/10/2014	36:45.0
943	RSRS19	-122.449	37.944	0.52	15.86	297.82	16.38	-122.449	37.944	0.13	15.86	296.04	15.99	2	7/10/2014	36:45.2
944	RSRS19	-122.449	37.944	0.47	15.80	297.82	16.27	-122.449	37.944	0.01	15.80	295.97	15.81	2	7/10/2014	36:45.3
945	RSRS19	-122.449	37.944	0.52	15.82	297.82	16.34	-122.449	37.944	0.13	15.82	295.93	15.95	2	7/10/2014	36:45.4
946	RSRS19	-122.449	37.944	0.52	15.83	297.76	16.35	-122.449	37.944	0.01	15.83	295.91	15.84	2	7/10/2014	36:45.4
947	RSRS19	-122.449	37.944	0.52	15.83	297.76	16.35	-122.449	37.944	-0.04	15.83	295.86	15.79	2	7/10/2014	36:45.6
948	RSRS19	-122.449	37.944	0.47	15.76	297.74	16.23	-122.449	37.944	0.16	15.76	295.91	15.93	2	7/10/2014	36:45.6
949	RSRS19	-122.449	37.944	0.56	15.76	297.69	16.32	-122.449	37.944	0.05	15.76	295.89	15.81	2	7/10/2014	36:45.8
950	RSRS19	-122.449	37.944	0.52	15.74	297.69	16.26	-122.449	37.944	-0.04	15.74	295.82	15.70	2	7/10/2014	36:45.8
951	RSRS19	-122.449	37.944	0.52	15.73	297.67	16.25	-122.449	37.944	0.13	15.73	295.85	15.85	2	7/10/2014	36:46.0
952	RSRS19	-122.449	37.944	0.52	15.71	297.67	16.23	-122.449	37.944	-0.04	15.71	295.78	15.67	2	7/10/2014	36:46.1
953	RSRS19	-122.449	37.944	0.52	15.68	297.65	16.20	-122.449	37.944	0.16	15.68	295.76	15.84	2	7/10/2014	36:46.2
954	RSRS19	-122.449	37.944	0.52	15.68	297.70	16.20	-122.449	37.944	0.08	15.68	295.76	15.76	2	7/10/2014	36:46.2
955	RSRS19	-122.449	37.944	0.52	15.64	297.68	16.16	-122.449	37.944	-0.04	15.64	295.74	15.60	2	7/10/2014	36:46.4
956	RSRS19	-122.449	37.944	0.52	15.64	297.72	16.16	-122.449	37.944	0.16	15.64	295.74	15.80	2	7/10/2014	36:46.5

957	RSRS19	-122.449	37.944	0.52	15.66	297.65	16.19	-122.449	37.944	0.05	15.66	295.72	15.71	2	7/10/2014	36:46.6
958	RSRS19	-122.449	37.944	0.47	15.62	297.68	16.09	-122.449	37.944	-0.04	15.62	295.74	15.58	2	7/10/2014	36:46.6
959	RSRS19	-122.449	37.944	0.52	15.61	297.66	16.13	-122.449	37.944	0.21	15.61	295.79	15.82	2	7/10/2014	36:46.8
960	RSRS19	-122.449	37.944	0.47	15.67	297.64	16.14	-122.449	37.944	-0.07	15.67	295.74	15.60	2	7/10/2014	36:46.9
961	RSRS19	-122.449	37.944	0.52	15.63	297.55	16.16	-122.449	37.944	0.05	15.63	295.66	15.68	2	7/10/2014	36:47.0
962	RSRS19	-122.449	37.944	0.52	15.62	297.55	16.14	-122.449	37.944	0.13	15.62	295.68	15.75	2	7/10/2014	36:47.0
963	RSRS19	-122.449	37.944	0.52	15.63	297.53	16.16	-122.449	37.944	-0.04	15.63	295.64	15.59	2	7/10/2014	36:47.2
964	RSRS19	-122.449	37.944	0.52	15.74	297.59	16.26	-122.449	37.944	0.05	15.74	295.64	15.79	2	7/10/2014	36:47.3
965	RSRS19	-122.449	37.944	0.52	15.70	297.53	16.23	-122.449	37.944	0.13	15.70	295.57	15.83	2	7/10/2014	36:47.3
966	RSRS19	-122.449	37.944	0.52	15.78	297.57	16.30	-122.449	37.944	-0.16	15.78	295.60	15.62	2	7/10/2014	36:47.4
967	RSRS19	-122.449	37.944	0.52	15.84	297.60	16.36	-122.449	37.944	0.16	15.84	295.66	16.01	2	7/10/2014	36:47.6
968	RSRS19	-122.449	37.944	0.52	15.80	297.59	16.32	-122.449	37.944	0.05	15.80	295.64	15.85	2	7/10/2014	36:47.7
969	RSRS19	-122.449	37.944	0.52	15.83	297.57	16.36	-122.449	37.944	-0.04	15.83	295.60	15.79	2	7/10/2014	36:47.8
970	RSRS19	-122.449	37.944	0.47	15.83	297.55	16.30	-122.449	37.944	0.16	15.83	295.60	15.99	2	7/10/2014	36:47.8
971	RSRS19	-122.449	37.944	0.52	15.84	297.53	16.36	-122.449	37.944	-0.04	15.84	295.60	15.80	2	7/10/2014	36:48.0
972	RSRS19	-122.449	37.944	0.52	15.83	297.51	16.36	-122.449	37.944	0.01	15.83	295.64	15.84	2	7/10/2014	36:48.1
973	RSRS19	-122.449	37.944	0.52	15.85	297.40	16.37	-122.449	37.944	0.16	15.85	295.53	16.01	2	7/10/2014	36:48.2
974	RSRS19	-122.449	37.944	0.47	15.79	297.32	16.26	-122.449	37.944	0.05	15.79	295.45	15.83	2	7/10/2014	36:48.3
975	RSRS19	-122.449	37.944	0.52	15.76	297.29	16.28	-122.449	37.944	0.08	15.76	295.40	15.83	2	7/10/2014	36:48.4
976	RSRS19	-122.449	37.944	0.52	15.76	297.32	16.28	-122.449	37.944	0.13	15.76	295.45	15.89	2	7/10/2014	36:48.4
977	RSRS19	-122.449	37.944	0.52	15.74	297.32	16.26	-122.449	37.944	0.05	15.74	295.52	15.79	2	7/10/2014	36:48.6
978	RSRS19	-122.449	37.944	0.52	15.79	297.30	16.31	-122.449	37.944	0.05	15.79	295.49	15.83	2	7/10/2014	36:48.6
979	RSRS19	-122.449	37.944	0.52	15.81	297.21	16.33	-122.449	37.944	0.16	15.81	295.36	15.97	2	7/10/2014	36:48.8
980	RSRS19	-122.449	37.944	0.52	15.70	297.14	16.23	-122.449	37.944	0.01	15.70	295.30	15.71	2	7/10/2014	36:48.9
981	RSRS19	-122.449	37.944	0.52	15.73	297.28	16.26	-122.449	37.944	0.13	15.73	295.21	15.86	2	7/10/2014	36:49.0
982	RSRS19	-122.449	37.944	0.52	15.69	297.19	16.21	-122.449	37.944	0.05	15.69	295.28	15.73	2	7/10/2014	36:49.0
983	RSRS19	-122.449	37.944	0.52	15.69	297.19	16.21	-122.449	37.944	0.08	15.69	295.17	15.77	2	7/10/2014	36:49.2
984	RSRS19	-122.449	37.944	0.52	15.70	297.13	16.23	-122.449	37.944	0.21	15.70	295.10	15.92	2	7/10/2014	36:49.3
985	RSRS19	-122.449	37.944	0.52	15.70	297.17	16.23	-122.449	37.944	-0.07	15.70	295.13	15.63	2	7/10/2014	36:49.4
986	RSRS19	-122.449	37.944	0.52	15.73	297.26	16.25	-122.449	37.944	0.05	15.73	295.23	15.77	2	7/10/2014	36:49.4
987	RSRS19	-122.449	37.944	0.52	15.69	297.23	16.22	-122.449	37.944	0.16	15.69	295.21	15.86	2	7/10/2014	36:49.6
988	RSRS19	-122.449	37.944	0.52	15.73	297.22	16.26	-122.449	37.944	-0.04	15.73	295.19	15.69	2	7/10/2014	36:49.7
989	RSRS19	-122.449	37.944	0.52	15.71	297.13	16.23	-122.449	37.944	0.05	15.71	295.15	15.76	2	7/10/2014	36:49.8

990	RSRS19	-122.449	37.944	0.52	15.73	297.06	16.26	-122.449	37.944	0.16	15.73	295.11	15.90	2	7/10/2014	36:49.8
991	RSRS19	-122.449	37.944	0.52	15.75	297.04	16.27	-122.449	37.944	0.05	15.75	295.04	15.79	2	7/10/2014	36:50.0
992	RSRS19	-122.449	37.944	0.52	15.73	296.95	16.25	-122.449	37.944	0.01	15.73	294.95	15.74	2	7/10/2014	36:50.1
993	RSRS19	-122.449	37.944	0.52	15.69	296.85	16.22	-122.449	37.944	0.08	15.69	294.91	15.77	2	7/10/2014	36:50.1
994	RSRS19	-122.449	37.944	0.52	15.73	296.87	16.26	-122.449	37.944	-0.04	15.73	294.82	15.69	2	7/10/2014	36:50.2
995	RSRS19	-122.449	37.944	0.52	15.73	296.74	16.26	-122.449	37.944	0.13	15.73	294.65	15.86	2	7/10/2014	36:50.4
996	RSRS19	-122.449	37.944	0.52	15.74	296.65	16.26	-122.449	37.944	0.08	15.74	294.65	15.82	2	7/10/2014	36:50.5
997	RSRS19	-122.449	37.944	0.52	15.76	296.69	16.28	-122.449	37.944	-0.07	15.76	294.63	15.69	2	7/10/2014	36:50.6
998	RSRS19	-122.449	37.944	0.52	15.78	296.59	16.30	-122.449	37.944	0.16	15.78	294.61	15.94	2	7/10/2014	36:50.6
999	RSRS19	-122.449	37.944	0.52	15.80	296.50	16.32	-122.450	37.944	0.08	15.80	294.48	15.87	2	7/10/2014	36:50.8
1000	RSRS19	-122.450	37.944	0.47	15.83	296.36	16.30	-122.450	37.944	-0.04	15.83	294.36	15.79	2	7/10/2014	36:50.9
1001	RSRS19	-122.450	37.944	0.52	15.83	296.26	16.35	-122.450	37.944	0.16	15.83	294.28	15.99	2	7/10/2014	36:51.0
1002	RSRS19	-122.450	37.944	0.52	15.79	296.12	16.31	-122.450	37.944	0.01	15.79	294.12	15.80	2	7/10/2014	36:51.0
1003	RSRS19	-122.450	37.944	0.52	15.82	296.02	16.34	-122.450	37.944	0.05	15.82	294.00	15.86	2	7/10/2014	36:51.2
1004	RSRS19	-122.450	37.944	0.52	15.83	295.93	16.35	-122.450	37.944	0.13	15.83	293.91	15.96	2	7/10/2014	36:51.2
1005	RSRS19	-122.450	37.944	0.52	15.79	295.84	16.31	-122.450	37.944	-0.13	15.79	293.80	15.66	2	7/10/2014	36:51.4
1006	RSRS19	-122.450	37.944	0.52	15.78	295.71	16.30	-122.450	37.944	0.08	15.78	293.71	15.86	2	7/10/2014	36:51.4
1007	RSRS19	-122.450	37.944	0.52	15.79	295.62	16.31	-122.450	37.944	0.16	15.79	293.61	15.95	2	7/10/2014	36:51.6
1008	RSRS19	-122.450	37.944	0.52	15.86	295.54	16.38	-122.450	37.944	-0.13	15.86	293.51	15.73	2	7/10/2014	36:51.7
1009	RSRS19	-122.450	37.944	0.52	15.80	295.43	16.32	-122.450	37.944	0.16	15.80	293.43	15.96	2	7/10/2014	36:51.8
1010	RSRS19	-122.450	37.944	0.52	15.83	295.34	16.36	-122.450	37.944	0.13	15.83	293.36	15.96	2	7/10/2014	36:51.8
1011	RSRS19	-122.450	37.944	0.52	15.86	295.23	16.38	-122.450	37.944	-0.07	15.86	293.21	15.78	2	7/10/2014	36:52.0
1012	RSRS19	-122.450	37.944	0.52	15.80	295.05	16.32	-122.450	37.944	0.16	15.80	293.08	15.96	2	7/10/2014	36:52.0
1013	RSRS19	-122.450	37.944	0.52	15.80	294.99	16.32	-122.450	37.944	0.13	15.80	293.01	15.92	2	7/10/2014	36:52.2
1014	RSRS19	-122.450	37.944	0.52	15.83	294.84	16.35	-122.450	37.944	-0.04	15.83	292.79	15.79	2	7/10/2014	36:52.2
1015	RSRS19	-122.450	37.944	0.52	15.74	294.70	16.26	-122.450	37.944	0.21	15.74	292.71	15.96	2	7/10/2014	36:52.4
1016	RSRS19	-122.450	37.944	0.52	15.76	294.64	16.28	-122.450	37.944	0.13	15.76	292.60	15.89	2	7/10/2014	36:52.5
1017	RSRS19	-122.450	37.944	0.52	15.73	294.47	16.26	-122.450	37.944	0.01	15.73	292.49	15.75	2	7/10/2014	36:52.6
1018	RSRS19	-122.450	37.944	0.47	15.72	294.38	16.19	-122.450	37.944	0.16	15.72	292.44	15.88	2	7/10/2014	36:52.6
1019	RSRS19	-122.450	37.944	0.52	15.70	294.27	16.23	-122.450	37.944	0.05	15.70	292.25	15.75	2	7/10/2014	36:52.8
1020	RSRS19	-122.450	37.944	0.52	15.69	294.20	16.21	-122.450	37.944	-0.04	15.69	292.25	15.65	2	7/10/2014	36:52.9
1021	RSRS19	-122.450	37.944	0.52	15.66	294.07	16.19	-122.450	37.944	0.21	15.66	292.10	15.88	2	7/10/2014	36:52.9
1022	RSRS19	-122.450	37.944	0.47	15.68	293.98	16.15	-122.450	37.944	0.05	15.68	292.03	15.72	2	7/10/2014	36:53.0

1023	RSRS19	-122.450	37.944	0.52	15.66	293.83	16.18	-122.450	37.944	0.05	15.66	291.86	15.70	2	7/10/2014	36:53.2
1024	RSRS19	-122.450	37.944	0.52	15.64	293.74	16.16	-122.450	37.944	0.21	15.64	291.81	15.85	2	7/10/2014	36:53.3
1025	RSRS19	-122.450	37.944	0.52	15.62	293.53	16.15	-122.450	37.944	0.08	15.62	291.57	15.70	2	7/10/2014	36:53.4
1026	RSRS19	-122.450	37.944	0.47	15.62	293.46	16.09	-122.450	37.944	0.16	15.62	291.48	15.78	2	7/10/2014	36:53.4
1027	RSRS19	-122.450	37.944	0.52	15.62	293.35	16.14	-122.450	37.944	0.25	15.62	291.31	15.87	2	7/10/2014	36:53.6
1028	RSRS19	-122.450	37.944	0.52	15.61	293.31	16.13	-122.450	37.944	-0.04	15.61	291.31	15.57	2	7/10/2014	36:53.7
1029	RSRS19	-122.450	37.944	0.52	15.66	293.22	16.19	-122.450	37.944	0.16	15.66	291.26	15.83	2	7/10/2014	36:53.8
1030	RSRS19	-122.450	37.944	0.47	15.63	293.05	16.10	-122.450	37.944	0.16	15.63	291.18	15.80	2	7/10/2014	36:53.8
1031	RSRS19	-122.450	37.944	0.52	15.62	292.90	16.15	-122.450	37.944	-0.04	15.62	291.00	15.58	2	7/10/2014	36:54.0
1032	RSRS19	-122.450	37.944	0.52	15.60	292.78	16.12	-122.450	37.944	0.21	15.60	290.87	15.82	2	7/10/2014	36:54.0
1033	RSRS19	-122.450	37.944	0.52	15.57	292.67	16.09	-122.450	37.944	0.21	15.57	290.74	15.79	2	7/10/2014	36:54.2
1034	RSRS19	-122.450	37.944	0.52	15.56	292.54	16.09	-122.450	37.944	0.05	15.56	290.58	15.61	2	7/10/2014	36:54.2
1035	RSRS19	-122.450	37.944	0.52	15.59	292.47	16.11	-122.450	37.944	0.13	15.59	290.45	15.72	2	7/10/2014	36:54.4
1036	RSRS19	-122.450	37.944	0.52	15.58	292.35	16.10	-122.450	37.944	0.16	15.58	290.37	15.74	2	7/10/2014	36:54.5
1037	RSRS19	-122.450	37.944	0.52	15.58	292.28	16.10	-122.450	37.944	0.05	15.58	290.26	15.62	2	7/10/2014	36:54.6
1038	RSRS19	-122.450	37.944	0.47	15.60	292.17	16.07	-122.450	37.944	0.16	15.60	290.15	15.77	2	7/10/2014	36:54.6
1039	RSRS19	-122.450	37.944	0.52	15.61	292.02	16.13	-122.450	37.944	0.25	15.61	290.02	15.86	2	7/10/2014	36:54.8
1040	RSRS19	-122.450	37.944	0.47	15.60	291.88	16.06	-122.450	37.944	0.04	15.60	289.86	15.64	2	7/10/2014	36:54.9
1041	RSRS19	-122.450	37.944	0.52	15.62	291.69	16.14	-122.450	37.944	0.13	15.62	289.67	15.75	2	7/10/2014	36:55.0
1042	RSRS19	-122.450	37.944	0.52	15.67	291.61	16.19	-122.450	37.944	0.21	15.67	289.56	15.88	2	7/10/2014	36:55.0
1043	RSRS19	-122.450	37.944	0.52	15.62	291.49	16.14	-122.450	37.944	0.08	15.62	289.43	15.70	2	7/10/2014	36:55.2
1044	RSRS19	-122.450	37.944	0.52	15.64	291.36	16.16	-122.450	37.944	0.25	15.64	289.32	15.89	2	7/10/2014	36:55.3
1045	RSRS19	-122.450	37.944	0.56	15.64	291.16	16.20	-122.450	37.944	0.21	15.64	289.14	15.85	2	7/10/2014	36:55.4
1046	RSRS19	-122.450	37.944	0.52	15.64	290.99	16.16	-122.450	37.944	0.04	15.64	288.90	15.68	2	7/10/2014	36:55.4
1047	RSRS19	-122.450	37.944	0.52	15.62	290.86	16.14	-122.450	37.944	0.30	15.62	288.82	15.92	2	7/10/2014	36:55.6
1048	RSRS19	-122.450	37.944	0.47	15.63	290.84	16.10	-122.450	37.944	0.13	15.63	288.77	15.76	2	7/10/2014	36:55.7
1049	RSRS19	-122.450	37.944	0.52	15.62	290.66	16.14	-122.450	37.944	0.13	15.62	288.57	15.75	2	7/10/2014	36:55.7
1050	RSRS19	-122.450	37.944	0.52	15.67	290.57	16.19	-122.450	37.944	0.25	15.67	288.51	15.92	2	7/10/2014	36:55.9
1051	RSRS19	-122.450	37.944	0.52	15.62	290.42	16.14	-122.450	37.944	0.01	15.62	288.38	15.63	2	7/10/2014	36:56.0
1052	RSRS19	-122.450	37.944	0.47	15.66	290.36	16.13	-122.450	37.944	0.16	15.66	288.20	15.83	2	7/10/2014	36:56.1
1053	RSRS19	-122.450	37.944	0.52	15.66	290.25	16.18	-122.450	37.944	0.25	15.66	288.12	15.90	2	7/10/2014	36:56.2
1054	RSRS19	-122.450	37.944	0.52	15.71	290.16	16.23	-122.450	37.944	0.08	15.71	288.00	15.79	2	7/10/2014	36:56.2
1055	RSRS19	-122.450	37.944	0.52	15.71	290.03	16.23	-122.450	37.944	0.16	15.71	287.88	15.87	2	7/10/2014	36:56.4

1056	RSRS19	-122.450	37.944	0.52	15.72	289.94	16.24	-122.450	37.944	0.25	15.72	287.76	15.97	2	7/10/2014	36:56.5
1057	RSRS19	-122.450	37.944	0.52	15.73	289.85	16.25	-122.450	37.944	0.04	15.73	287.61	15.77	2	7/10/2014	36:56.6
1058	RSRS19	-122.450	37.944	0.52	15.69	289.68	16.22	-122.450	37.944	0.16	15.69	287.48	15.86	2	7/10/2014	36:56.6
1059	RSRS19	-122.450	37.944	0.52	15.69	289.55	16.22	-122.450	37.944	0.25	15.69	287.39	15.94	2	7/10/2014	36:56.8
1060	RSRS19	-122.450	37.944	0.52	15.76	289.39	16.28	-122.450	37.944	0.08	15.76	287.20	15.84	2	7/10/2014	36:56.8
1061	RSRS19	-122.450	37.944	0.56	15.69	289.19	16.25	-122.450	37.944	0.25	15.69	287.09	15.94	2	7/10/2014	36:57.0
1062	RSRS19	-122.450	37.944	0.52	15.71	289.09	16.23	-122.450	37.944	0.16	15.71	286.98	15.87	2	7/10/2014	36:57.0
1063	RSRS19	-122.450	37.944	0.52	15.70	288.92	16.22	-122.450	37.944	0.08	15.70	286.83	15.78	2	7/10/2014	36:57.2
1064	RSRS19	-122.450	37.944	0.52	15.67	288.85	16.19	-122.450	37.944	0.25	15.67	286.76	15.92	2	7/10/2014	36:57.3
1065	RSRS19	-122.450	37.944	0.56	15.70	288.70	16.26	-122.450	37.944	0.08	15.70	286.60	15.78	2	7/10/2014	36:57.4
1066	RSRS19	-122.450	37.944	0.52	15.75	288.67	16.27	-122.450	37.944	0.04	15.75	286.54	15.79	2	7/10/2014	36:57.4
1067	RSRS19	-122.450	37.944	0.52	15.73	288.56	16.26	-122.450	37.944	0.30	15.73	286.41	16.03	2	7/10/2014	36:57.6
1068	RSRS19	-122.450	37.944	0.52	15.69	288.41	16.21	-122.450	37.944	0.16	15.69	286.26	15.85	2	7/10/2014	36:57.7
1069	RSRS19	-122.450	37.944	0.52	15.70	288.26	16.22	-122.450	37.944	0.16	15.70	286.11	15.87	2	7/10/2014	36:57.8
1070	RSRS19	-122.450	37.944	0.52	15.69	288.04	16.21	-122.450	37.944	0.25	15.69	285.86	15.93	2	7/10/2014	36:57.8
1071	RSRS19	-122.450	37.944	0.56	15.66	287.84	16.22	-122.450	37.944	0.08	15.66	285.64	15.74	2	7/10/2014	36:58.0
1072	RSRS19	-122.450	37.944	0.52	15.66	287.73	16.19	-122.450	37.944	0.13	15.66	285.56	15.79	2	7/10/2014	36:58.1
1073	RSRS19	-122.450	37.944	0.52	15.69	287.55	16.22	-122.450	37.944	0.21	15.69	285.40	15.91	2	7/10/2014	36:58.2
1074	RSRS19	-122.450	37.944	0.52	15.71	287.40	16.23	-122.450	37.944	0.13	15.71	285.25	15.84	2	7/10/2014	36:58.2
1075	RSRS19	-122.450	37.944	0.52	15.71	287.30	16.23	-122.450	37.944	0.30	15.71	285.12	16.01	2	7/10/2014	36:58.4
1076	RSRS19	-122.450	37.944	0.52	15.73	287.12	16.25	-122.450	37.944	0.13	15.73	284.94	15.85	2	7/10/2014	36:58.5
1077	RSRS19	-122.450	37.944	0.56	15.74	286.99	16.30	-122.450	37.944	0.13	15.74	284.73	15.87	2	7/10/2014	36:58.5
1078	RSRS19	-122.450	37.944	0.52	15.80	286.64	16.32	-122.450	37.944	0.30	15.80	284.48	16.09	2	7/10/2014	36:58.6
1079	RSRS19	-122.450	37.944	0.52	15.77	286.51	16.29	-122.450	37.944	0.08	15.77	284.20	15.85	2	7/10/2014	36:58.8
1080	RSRS19	-122.450	37.944	0.52	15.76	286.20	16.28	-122.450	37.944	0.08	15.76	283.98	15.83	2	7/10/2014	36:58.9
1081	RSRS19	-122.450	37.944	0.56	15.76	286.00	16.31	-122.450	37.944	0.21	15.76	283.72	15.97	2	7/10/2014	36:59.0
1082	RSRS19	-122.450	37.944	0.52	15.77	285.74	16.29	-122.450	37.944	0.16	15.77	283.52	15.93	2	7/10/2014	36:59.0
1083	RSRS19	-122.450	37.944	0.56	15.79	285.52	16.34	-122.450	37.944	0.30	15.79	283.26	16.09	2	7/10/2014	36:59.2
1084	RSRS19	-122.450	37.944	0.52	15.80	285.32	16.32	-122.450	37.944	0.25	15.80	283.08	16.04	2	7/10/2014	36:59.3
1085	RSRS19	-122.450	37.944	0.52	15.81	285.06	16.33	-122.450	37.944	0.08	15.81	282.82	15.89	2	7/10/2014	36:59.4
1086	RSRS19	-122.450	37.944	0.52	15.78	284.87	16.30	-122.450	37.944	0.30	15.78	282.62	16.08	2	7/10/2014	36:59.4
1087	RSRS19	-122.450	37.944	0.56	15.81	284.67	16.37	-122.450	37.944	0.25	15.81	282.36	16.06	2	7/10/2014	36:59.6
1088	RSRS19	-122.450	37.944	0.52	15.78	284.42	16.30	-122.450	37.944	0.13	15.78	282.07	15.91	2	7/10/2014	36:59.6

1089	RSRS19	-122.450	37.944	0.56	15.81	284.20	16.37	-122.450	37.944	0.33	15.81	281.79	16.14	2	7/10/2014	36:59.8
1090	RSRS19	-122.450	37.944	0.52	15.86	283.99	16.39	-122.450	37.944	0.21	15.86	281.55	16.08	2	7/10/2014	36:59.8
1091	RSRS19	-122.450	37.944	0.56	15.79	283.72	16.34	-122.450	37.944	0.13	15.79	281.28	15.92	2	7/10/2014	37:00.0
1092	RSRS19	-122.450	37.944	0.52	15.81	283.52	16.33	-122.450	37.944	0.33	15.81	281.09	16.14	2	7/10/2014	37:00.1
1093	RSRS19	-122.450	37.944	0.56	15.78	283.20	16.33	-122.450	37.944	0.21	15.78	280.82	15.99	2	7/10/2014	37:00.1
1094	RSRS19	-122.450	37.944	0.52	15.84	282.96	16.36	-122.450	37.944	0.16	15.84	280.54	16.00	2	7/10/2014	37:00.2
1095	RSRS19	-122.450	37.944	0.56	15.83	282.69	16.38	-122.450	37.944	0.33	15.83	280.27	16.16	2	7/10/2014	37:00.4
1096	RSRS19	-122.450	37.944	0.52	15.86	282.43	16.38	-122.450	37.944	0.16	15.86	280.04	16.02	2	7/10/2014	37:00.5
1097	RSRS19	-122.450	37.944	0.56	15.76	282.17	16.31	-122.450	37.944	0.16	15.76	279.75	15.92	2	7/10/2014	37:00.6
1098	RSRS19	-122.450	37.944	0.52	15.77	281.92	16.29	-122.450	37.944	0.33	15.77	279.46	16.10	2	7/10/2014	37:00.6
1099	RSRS19	-122.450	37.944	0.52	15.72	281.60	16.24	-122.450	37.944	0.16	15.72	279.18	15.88	2	7/10/2014	37:00.8
1100	RSRS19	-122.450	37.944	0.52	15.69	281.29	16.22	-122.450	37.944	0.25	15.69	278.85	15.94	2	7/10/2014	37:00.9
1101	RSRS19	-122.450	37.944	0.56	15.74	280.94	16.30	-122.450	37.944	0.30	15.74	278.48	16.04	2	7/10/2014	37:01.0
1102	RSRS19	-122.450	37.944	0.52	15.66	280.61	16.19	-122.450	37.944	0.08	15.66	278.15	15.74	2	7/10/2014	37:01.0
1103	RSRS19	-122.450	37.944	0.56	15.65	280.26	16.20	-122.450	37.944	0.21	15.65	277.79	15.86	2	7/10/2014	37:01.2
1104	RSRS19	-122.450	37.944	0.52	15.65	279.95	16.17	-122.450	37.944	0.25	15.65	277.45	15.90	2	7/10/2014	37:01.3
1105	RSRS19	-122.450	37.944	0.56	15.78	279.64	16.33	-122.450	37.944	0.16	15.78	277.16	15.94	2	7/10/2014	37:01.3
1106	RSRS19	-122.450	37.944	0.52	15.69	279.33	16.21	-122.450	37.944	0.30	15.69	276.85	15.98	2	7/10/2014	37:01.4
1107	RSRS19	-122.450	37.944	0.56	15.74	278.96	16.30	-122.450	37.944	0.30	15.74	276.55	16.04	2	7/10/2014	37:01.6
1108	RSRS19	-122.450	37.944	0.52	15.70	278.61	16.22	-122.450	37.944	0.13	15.70	276.19	15.83	2	7/10/2014	37:01.7
1109	RSRS19	-122.450	37.944	0.56	15.76	278.26	16.32	-122.450	37.944	0.25	15.76	275.87	16.01	2	7/10/2014	37:01.8
1110	RSRS19	-122.450	37.944	0.52	15.77	277.84	16.29	-122.450	37.944	0.25	15.77	275.40	16.02	2	7/10/2014	37:01.8
1111	RSRS19	-122.450	37.944	0.56	15.78	277.47	16.33	-122.450	37.944	0.13	15.78	275.01	15.91	2	7/10/2014	37:02.0
1112	RSRS19	-122.450	37.944	0.52	15.76	277.04	16.28	-122.450	37.944	0.42	15.76	274.53	16.18	2	7/10/2014	37:02.1
1113	RSRS19	-122.450	37.944	0.56	15.76	276.59	16.31	-122.450	37.944	0.21	15.76	274.09	15.97	2	7/10/2014	37:02.2
1114	RSRS19	-122.450	37.944	0.56	15.79	276.16	16.34	-122.450	37.944	0.16	15.79	273.62	15.95	2	7/10/2014	37:02.2
1115	RSRS19	-122.450	37.944	0.56	15.78	275.74	16.33	-122.450	37.944	0.38	15.78	273.19	16.16	2	7/10/2014	37:02.4
1116	RSRS19	-122.450	37.944	0.52	15.78	275.16	16.30	-122.450	37.944	0.13	15.78	272.64	15.91	2	7/10/2014	37:02.4
1117	RSRS19	-122.450	37.944	0.56	15.81	274.70	16.37	-122.450	37.944	0.30	15.81	272.14	16.11	2	7/10/2014	37:02.6
1118	RSRS19	-122.450	37.944	0.52	15.81	274.16	16.33	-122.450	37.944	0.25	15.81	271.60	16.06	2	7/10/2014	37:02.7
1119	RSRS19	-122.450	37.944	0.56	15.78	273.65	16.33	-122.450	37.944	0.13	15.78	271.08	15.91	2	7/10/2014	37:02.8
1120	RSRS19	-122.450	37.944	0.56	15.78	273.08	16.33	-122.450	37.944	0.25	15.78	270.53	16.03	2	7/10/2014	37:02.8
1121	RSRS19	-122.450	37.944	0.56	15.80	272.57	16.36	-122.450	37.944	0.25	15.80	269.98	16.05	2	7/10/2014	37:02.9

1122	RSRS19	-122.450	37.944	0.52	15.81	272.05	16.33	-122.450	37.944	0.16	15.81	269.40	15.97	2	7/10/2014	37:03.0
1123	RSRS19	-122.450	37.944	0.56	15.80	271.50	16.35	-122.450	37.944	0.33	15.80	268.89	16.13	2	7/10/2014	37:03.2
1124	RSRS19	-122.450	37.944	0.56	15.83	270.98	16.39	-122.450	37.944	0.25	15.83	268.34	16.08	2	7/10/2014	37:03.3
1125	RSRS19	-122.450	37.944	0.56	15.89	270.38	16.44	-122.450	37.944	0.16	15.89	267.78	16.05	2	7/10/2014	37:03.4
1126	RSRS19	-122.450	37.944	0.56	15.85	269.81	16.40	-122.450	37.944	0.30	15.85	267.19	16.15	2	7/10/2014	37:03.4
1127	RSRS19	-122.450	37.944	0.56	15.87	269.17	16.43	-122.450	37.944	0.16	15.87	266.60	16.03	2	7/10/2014	37:03.6
1128	RSRS19	-122.450	37.944	0.56	15.88	268.62	16.44	-122.450	37.944	0.25	15.88	265.96	16.13	2	7/10/2014	37:03.7
1129	RSRS19	-122.450	37.944	0.56	15.93	268.03	16.49	-122.450	37.944	0.30	15.93	265.42	16.23	2	7/10/2014	37:03.8
1130	RSRS19	-122.450	37.944	0.56	15.92	267.50	16.47	-122.450	37.944	0.04	15.92	264.82	15.96	2	7/10/2014	37:03.8
1131	RSRS19	-122.450	37.944	0.56	15.96	266.84	16.51	-122.450	37.944	0.30	15.96	264.20	16.25	2	7/10/2014	37:04.0
1132	RSRS19	-122.450	37.944	0.56	15.94	266.29	16.49	-122.450	37.944	0.25	15.94	263.54	16.19	2	7/10/2014	37:04.1
1133	RSRS19	-122.450	37.944	0.56	15.90	265.68	16.46	-122.450	37.944	0.08	15.90	262.91	15.98	2	7/10/2014	37:04.1
1134	RSRS19	-122.450	37.944	0.56	15.93	265.11	16.48	-122.450	37.944	0.21	15.93	262.32	16.14	2	7/10/2014	37:04.3
1135	RSRS19	-122.450	37.944	0.56	15.90	264.52	16.46	-122.450	37.944	0.30	15.90	261.70	16.20	2	7/10/2014	37:04.4
1136	RSRS19	-122.450	37.944	0.52	15.93	263.90	16.45	-122.450	37.944	0.13	15.93	261.07	16.06	2	7/10/2014	37:04.5
1137	RSRS19	-122.450	37.944	0.56	15.95	263.20	16.50	-122.450	37.944	0.25	15.95	260.41	16.20	2	7/10/2014	37:04.6
1138	RSRS19	-122.450	37.944	0.56	15.93	262.62	16.49	-122.450	37.944	0.25	15.93	259.81	16.18	2	7/10/2014	37:04.6
1139	RSRS19	-122.450	37.944	0.56	15.91	261.99	16.47	-122.450	37.944	0.13	15.91	259.29	16.04	2	7/10/2014	37:04.8
1140	RSRS19	-122.450	37.944	0.52	15.89	261.38	16.41	-122.450	37.944	0.33	15.89	258.71	16.22	2	7/10/2014	37:04.9
1141	RSRS19	-122.450	37.944	0.56	15.88	260.71	16.44	-122.450	37.944	0.08	15.88	258.05	15.96	2	7/10/2014	37:05.0
1142	RSRS19	-122.450	37.944	0.52	15.86	260.02	16.38	-122.450	37.944	0.13	15.86	257.40	15.99	2	7/10/2014	37:05.0
1143	RSRS19	-122.450	37.944	0.56	15.83	259.33	16.39	-122.450	37.944	0.33	15.83	256.65	16.16	2	7/10/2014	37:05.2
1144	RSRS19	-122.450	37.944	0.56	15.81	258.63	16.37	-122.450	37.944	0.08	15.81	255.97	15.89	2	7/10/2014	37:05.2
1145	RSRS19	-122.450	37.944	0.56	15.78	257.91	16.33	-122.450	37.944	0.16	15.78	255.21	15.94	2	7/10/2014	37:05.4
1146	RSRS19	-122.450	37.944	0.52	15.79	257.16	16.31	-122.450	37.944	0.25	15.79	254.43	16.04	2	7/10/2014	37:05.4
1147	RSRS19	-122.450	37.944	0.56	15.78	256.41	16.33	-122.450	37.944	0.13	15.78	253.69	15.91	2	7/10/2014	37:05.6
1148	RSRS19	-122.450	37.944	0.56	15.83	255.73	16.38	-122.450	37.944	0.21	15.83	252.96	16.04	2	7/10/2014	37:05.7
1149	RSRS19	-122.450	37.944	0.56	15.83	254.98	16.39	-122.450	37.944	0.30	15.83	252.28	16.13	2	7/10/2014	37:05.7
1150	RSRS19	-122.450	37.944	0.52	15.87	254.28	16.39	-122.450	37.944	0.08	15.87	251.55	15.95	2	7/10/2014	37:05.9
1151	RSRS19	-122.450	37.944	0.56	15.86	253.49	16.41	-122.450	37.944	0.30	15.86	250.85	16.15	2	7/10/2014	37:06.0
1152	RSRS19	-122.450	37.944	0.56	15.86	252.70	16.41	-122.450	37.944	0.21	15.86	250.02	16.07	2	7/10/2014	37:06.1
1153	RSRS19	-122.450	37.944	0.56	15.86	251.93	16.41	-122.450	37.944	0.13	15.86	249.30	15.99	2	7/10/2014	37:06.2
1154	RSRS19	-122.450	37.944	0.52	15.85	251.07	16.37	-122.450	37.944	0.33	15.85	248.43	16.18	2	7/10/2014	37:06.3

1155	RSRS19	-122.450	37.944	0.56	15.86	250.24	16.41	-122.450	37.944	0.21	15.86	247.56	16.07	2	7/10/2014	37:06.4
1156	RSRS19	-122.450	37.944	0.56	15.83	249.41	16.39	-122.450	37.944	0.13	15.83	246.74	15.96	2	7/10/2014	37:06.5
1157	RSRS19	-122.450	37.944	0.56	15.86	248.50	16.42	-122.450	37.944	0.33	15.86	245.85	16.20	2	7/10/2014	37:06.6
1158	RSRS19	-122.450	37.944	0.52	15.86	247.60	16.39	-122.450	37.944	0.21	15.86	244.97	16.08	2	7/10/2014	37:06.6
1159	RSRS19	-122.450	37.944	0.56	15.87	246.81	16.43	-122.450	37.944	0.21	15.87	244.17	16.08	2	7/10/2014	37:06.8
1160	RSRS19	-122.450	37.944	0.52	15.88	245.98	16.40	-122.450	37.944	0.30	15.88	243.36	16.18	2	7/10/2014	37:06.9
1161	RSRS19	-122.450	37.944	0.56	15.88	245.20	16.44	-122.450	37.944	0.21	15.88	242.57	16.09	2	7/10/2014	37:06.9
1162	RSRS19	-122.450	37.944	0.52	15.87	244.31	16.39	-122.450	37.944	0.16	15.87	241.73	16.03	2	7/10/2014	37:07.0
1163	RSRS19	-122.450	37.944	0.56	15.88	243.47	16.44	-122.450	37.944	0.33	15.88	240.88	16.21	2	7/10/2014	37:07.2
1164	RSRS19	-122.450	37.944	0.52	15.87	242.51	16.39	-122.450	37.944	0.16	15.87	239.97	16.03	2	7/10/2014	37:07.3
1165	RSRS19	-122.450	37.944	0.52	15.87	241.54	16.39	-122.450	37.944	0.38	15.87	239.05	16.25	2	7/10/2014	37:07.4
1166	RSRS19	-122.450	37.944	0.52	15.80	240.57	16.32	-122.450	37.944	0.25	15.80	238.12	16.04	2	7/10/2014	37:07.4
1167	RSRS19	-122.450	37.944	0.56	15.80	239.70	16.35	-122.450	37.944	0.13	15.80	237.28	15.92	2	7/10/2014	37:07.6
1168	RSRS19	-122.450	37.944	0.52	15.73	238.81	16.25	-122.450	37.944	0.25	15.73	236.45	15.97	2	7/10/2014	37:07.7
1169	RSRS19	-122.450	37.944	0.56	15.69	237.96	16.25	-122.450	37.944	0.30	15.69	235.59	15.99	2	7/10/2014	37:07.8
1170	RSRS19	-122.450	37.944	0.56	15.69	237.18	16.25	-122.450	37.944	0.21	15.69	234.85	15.91	2	7/10/2014	37:07.8
1171	RSRS19	-122.450	37.944	0.56	15.67	236.38	16.23	-122.450	37.944	0.33	15.67	234.05	16.00	2	7/10/2014	37:08.0
1172	RSRS19	-122.450	37.944	0.52	15.77	235.61	16.29	-122.450	37.944	0.25	15.77	233.30	16.02	2	7/10/2014	37:08.0
1173	RSRS19	-122.450	37.944	0.52	15.67	234.88	16.19	-122.450	37.944	0.21	15.67	232.62	15.88	2	7/10/2014	37:08.2
1174	RSRS19	-122.450	37.944	0.52	15.66	234.12	16.19	-122.450	37.944	0.38	15.66	231.91	16.05	2	7/10/2014	37:08.2
1175	RSRS19	-122.450	37.944	0.56	15.65	233.26	16.20	-122.450	37.944	0.30	15.65	231.08	15.95	2	7/10/2014	37:08.4
1176	RSRS19	-122.450	37.944	0.56	15.69	232.38	16.24	-122.450	37.944	0.30	15.69	230.29	15.98	2	7/10/2014	37:08.5
1177	RSRS19	-122.450	37.944	0.56	15.63	231.56	16.19	-122.450	37.944	0.30	15.63	229.52	15.93	2	7/10/2014	37:08.5
1178	RSRS19	-122.450	37.944	0.52	15.61	230.85	16.13	-122.450	37.944	0.13	15.61	228.84	15.74	2	7/10/2014	37:08.6
1179	RSRS19	-122.450	37.944	0.56	15.58	230.14	16.13	-122.450	37.944	0.33	15.58	228.14	15.91	2	7/10/2014	37:08.8
1180	RSRS19	-122.450	37.944	0.56	15.56	229.59	16.11	-122.450	37.944	0.30	15.56	227.65	15.85	2	7/10/2014	37:08.9
1181	RSRS19	-122.450	37.944	0.56	15.55	229.06	16.10	-122.450	37.944	0.21	15.55	227.13	15.76	2	7/10/2014	37:09.0
1182	RSRS19	-122.450	37.944	0.52	15.55	228.49	16.07	-122.450	37.944	0.33	15.55	226.62	15.88	2	7/10/2014	37:09.0
1183	RSRS19	-122.450	37.944	0.56	15.57	228.11	16.13	-122.450	37.944	0.30	15.57	226.28	15.87	2	7/10/2014	37:09.2
1184	RSRS19	-122.450	37.944	0.56	15.53	227.64	16.09	-122.450	37.944	0.25	15.53	225.86	15.78	2	7/10/2014	37:09.3
1185	RSRS19	-122.450	37.944	0.56	15.58	227.20	16.13	-122.450	37.944	0.25	15.58	225.47	15.83	2	7/10/2014	37:09.4
1186	RSRS19	-122.450	37.944	0.56	15.61	226.76	16.16	-122.450	37.944	0.30	15.61	225.08	15.91	2	7/10/2014	37:09.4
1187	RSRS19	-122.450	37.944	0.56	15.62	226.33	16.18	-122.450	37.944	0.25	15.62	224.75	15.87	2	7/10/2014	37:09.6

1188	RSRS19	-122.450	37.944	0.56	15.59	225.97	16.14	-122.450	37.944	0.25	15.59	224.43	15.83	2	7/10/2014	37:09.6
1189	RSRS19	-122.450	37.944	0.56	15.60	225.68	16.16	-122.450	37.944	0.21	15.60	224.21	15.82	2	7/10/2014	37:09.8
1190	RSRS19	-122.450	37.944	0.52	15.62	225.44	16.14	-122.450	37.944	0.33	15.62	224.01	15.95	2	7/10/2014	37:09.8
1191	RSRS19	-122.450	37.944	0.56	15.65	225.39	16.20	-122.450	37.944	0.21	15.65	224.04	15.86	2	7/10/2014	37:10.0
1192	RSRS19	-122.450	37.944	0.56	15.62	225.34	16.17	-122.450	37.944	0.25	15.62	224.08	15.87	2	7/10/2014	37:10.1
1193	RSRS19	-122.450	37.944	0.56	15.62	225.26	16.18	-122.450	37.944	0.33	15.62	224.19	15.95	2	7/10/2014	37:10.2
1194	RSRS19	-122.450	37.944	0.52	15.60	225.26	16.12	-122.450	37.944	0.16	15.60	224.28	15.76	2	7/10/2014	37:10.2
1195	RSRS19	-122.450	37.944	0.56	15.60	225.23	16.16	-122.450	37.944	0.21	15.60	224.36	15.82	2	7/10/2014	37:10.4
1196	RSRS19	-122.450	37.944	0.55	15.60	225.38	16.16	-122.450	37.944	0.33	15.60	224.62	15.93	2	7/10/2014	37:10.5
1197	RSRS19	-122.450	37.944	0.55	15.55	225.45	16.10	-122.450	37.944	0.16	15.55	224.79	15.71	2	7/10/2014	37:10.6
1198	RSRS19	-122.450	37.944	0.55	15.54	225.64	16.09	-122.450	37.944	0.25	15.54	225.16	15.78	2	7/10/2014	37:10.6
1199	RSRS19	-122.450	37.944	0.55	15.53	225.89	16.08	-122.450	37.944	0.33	15.53	225.49	15.86	2	7/10/2014	37:10.8
1200	RSRS19	-122.450	37.944	0.52	15.54	226.15	16.06	-122.450	37.944	0.25	15.54	225.87	15.78	2	7/10/2014	37:10.8
1201	RSRS19	-122.450	37.944	0.55	15.53	226.51	16.08	-122.450	37.944	0.30	15.53	226.31	15.82	2	7/10/2014	37:11.0
1202	RSRS19	-122.450	37.944	0.52	15.53	226.94	16.05	-122.450	37.944	0.25	15.53	226.82	15.78	2	7/10/2014	37:11.0
1203	RSRS19	-122.450	37.944	0.55	15.55	227.54	16.10	-122.450	37.944	0.16	15.55	227.48	15.71	2	7/10/2014	37:11.2
1204	RSRS19	-122.450	37.944	0.52	15.57	228.22	16.09	-122.450	37.944	0.30	15.57	228.22	15.87	2	7/10/2014	37:11.3
1205	RSRS19	-122.450	37.944	0.55	15.56	228.93	16.12	-122.450	37.944	0.21	15.56	228.99	15.78	2	7/10/2014	37:11.3
1206	RSRS19	-122.450	37.944	0.52	15.56	229.67	16.08	-122.450	37.944	0.13	15.56	229.81	15.69	2	7/10/2014	37:11.4
1207	RSRS19	-122.450	37.944	0.55	15.60	230.45	16.15	-122.450	37.944	0.30	15.60	230.68	15.89	2	7/10/2014	37:11.6
1208	RSRS19	-122.450	37.944	0.52	15.61	231.32	16.13	-122.450	37.944	0.25	15.61	231.67	15.86	2	7/10/2014	37:11.7
1209	RSRS19	-122.450	37.944	0.55	15.60	232.18	16.16	-122.450	37.944	0.25	15.60	232.71	15.85	2	7/10/2014	37:11.8
1210	RSRS19	-122.450	37.944	0.55	15.71	233.10	16.26	-122.450	37.944	0.30	15.71	233.76	16.01	2	7/10/2014	37:11.8
1211	RSRS19	-122.450	37.944	0.55	15.70	234.07	16.26	-122.450	37.944	0.25	15.70	234.88	15.95	2	7/10/2014	37:11.9
1212	RSRS19	-122.450	37.944	0.52	15.67	235.09	16.19	-122.450	37.944	0.21	15.67	236.00	15.88	2	7/10/2014	37:12.1
1213	RSRS19	-122.450	37.944	0.55	15.69	236.14	16.25	-122.450	37.944	0.33	15.69	237.14	16.02	2	7/10/2014	37:12.2
1214	RSRS19	-122.450	37.944	0.52	15.69	237.40	16.21	-122.450	37.944	0.21	15.69	238.38	15.91	2	7/10/2014	37:12.2
1215	RSRS19	-122.450	37.944	0.55	15.71	238.54	16.26	-122.450	37.944	0.25	15.71	239.60	15.96	2	7/10/2014	37:12.3
1216	RSRS19	-122.450	37.944	0.52	15.69	239.88	16.21	-122.450	37.944	0.30	15.69	240.93	15.99	2	7/10/2014	37:12.4
1217	RSRS19	-122.450	37.944	0.52	15.66	241.18	16.18	-122.450	37.944	0.30	15.66	242.28	15.96	2	7/10/2014	37:12.6
1218	RSRS19	-122.450	37.944	0.55	15.65	242.66	16.20	-122.450	37.944	0.21	15.65	243.80	15.86	2	7/10/2014	37:12.6
1219	RSRS19	-122.450	37.944	0.55	15.66	244.15	16.21	-122.450	37.944	0.38	15.66	245.34	16.04	2	7/10/2014	37:12.8
1220	RSRS19	-122.450	37.944	0.55	15.66	245.69	16.21	-122.450	37.944	0.25	15.66	246.88	15.90	2	7/10/2014	37:12.9

1221	RSRS19	-122.450	37.944	0.55	15.65	247.23	16.20	-122.450	37.944	0.21	15.65	248.46	15.86	2	7/10/2014	37:13.0
1222	RSRS19	-122.450	37.944	0.52	15.66	248.88	16.18	-122.450	37.944	0.33	15.66	250.09	15.99	2	7/10/2014	37:13.1
1223	RSRS19	-122.450	37.944	0.55	15.69	250.55	16.25	-122.450	37.944	0.21	15.69	251.78	15.91	2	7/10/2014	37:13.2
1224	RSRS19	-122.450	37.944	0.55	15.72	252.27	16.27	-122.450	37.944	0.25	15.72	253.50	15.96	2	7/10/2014	37:13.3
1225	RSRS19	-122.450	37.944	0.55	15.68	253.98	16.23	-122.450	37.944	0.38	15.68	255.21	16.06	2	7/10/2014	37:13.4
1226	RSRS19	-122.450	37.944	0.55	15.66	255.72	16.21	-122.450	37.944	0.25	15.66	256.96	15.90	2	7/10/2014	37:13.5
1227	RSRS19	-122.450	37.944	0.55	15.66	257.41	16.21	-122.450	37.944	0.25	15.66	258.64	15.90	2	7/10/2014	37:13.6
1228	RSRS19	-122.450	37.944	0.52	15.63	259.06	16.15	-122.450	37.944	0.33	15.63	260.32	15.96	2	7/10/2014	37:13.6
1229	RSRS19	-122.450	37.944	0.55	15.64	260.76	16.19	-122.450	37.944	0.30	15.64	261.97	15.94	2	7/10/2014	37:13.8
1230	RSRS19	-122.450	37.944	0.52	15.64	262.43	16.16	-122.450	37.944	0.25	15.64	263.64	15.89	2	7/10/2014	37:13.9
1231	RSRS19	-122.450	37.944	0.55	15.65	264.10	16.20	-122.450	37.944	0.33	15.65	265.31	15.98	2	7/10/2014	37:14.0
1232	RSRS19	-122.450	37.944	0.55	15.64	265.84	16.19	-122.450	37.944	0.30	15.64	266.98	15.94	2	7/10/2014	37:14.1
1233	RSRS19	-122.450	37.944	0.55	15.66	267.60	16.22	-122.450	37.944	0.30	15.66	268.74	15.96	2	7/10/2014	37:14.1
1234	RSRS19	-122.450	37.944	0.55	15.65	269.40	16.20	-122.450	37.944	0.38	15.65	270.50	16.03	2	7/10/2014	37:14.3
1235	RSRS19	-122.450	37.944	0.55	15.64	271.27	16.19	-122.450	37.944	0.30	15.64	272.35	15.94	2	7/10/2014	37:14.4
1236	RSRS19	-122.450	37.944	0.55	15.62	273.23	16.17	-122.450	37.944	0.30	15.62	274.26	15.91	2	7/10/2014	37:14.5
1237	RSRS19	-122.450	37.944	0.55	15.62	275.09	16.18	-122.450	37.944	0.41	15.62	276.07	16.04	2	7/10/2014	37:14.6
1238	RSRS19	-122.450	37.944	0.55	15.65	276.84	16.20	-122.450	37.944	0.25	15.65	277.85	15.90	2	7/10/2014	37:14.7
1239	RSRS19	-122.450	37.944	0.55	15.59	278.60	16.14	-122.450	37.944	0.33	15.59	279.52	15.92	2	7/10/2014	37:14.7
1240	RSRS19	-122.450	37.944	0.55	15.56	280.24	16.12	-122.450	37.944	0.38	15.56	281.13	15.95	2	7/10/2014	37:14.9
1241	RSRS19	-122.450	37.944	0.55	15.60	281.94	16.16	-122.450	37.944	0.33	15.60	282.75	15.93	2	7/10/2014	37:15.0
1242	RSRS19	-122.450	37.944	0.55	15.60	283.56	16.15	-122.450	37.944	0.33	15.60	284.34	15.93	2	7/10/2014	37:15.1
1243	RSRS19	-122.450	37.944	0.55	15.54	285.15	16.09	-122.450	37.944	0.41	15.54	285.87	15.95	2	7/10/2014	37:15.2
1244	RSRS19	-122.450	37.944	0.55	15.54	286.76	16.09	-122.450	37.944	0.38	15.54	287.47	15.92	2	7/10/2014	37:15.2
1245	RSRS19	-122.450	37.944	0.61	15.53	288.30	16.13	-122.450	37.944	0.38	15.53	289.02	15.91	2	7/10/2014	37:15.4
1246	RSRS19	-122.450	37.944	0.55	15.56	289.92	16.11	-122.450	37.944	0.41	15.56	290.56	15.97	2	7/10/2014	37:15.5
1247	RSRS19	-122.450	37.944	0.55	15.51	291.42	16.06	-122.450	37.944	0.38	15.51	292.08	15.89	2	7/10/2014	37:15.6
1248	RSRS19	-122.450	37.944	0.55	15.52	293.03	16.07	-122.450	37.944	0.33	15.52	293.60	15.85	2	7/10/2014	37:15.7
1249	RSRS19	-122.450	37.944	0.55	15.48	294.49	16.04	-122.450	37.944	0.45	15.48	295.05	15.93	2	7/10/2014	37:15.8
1250	RSRS19	-122.450	37.944	0.55	15.47	296.04	16.03	-122.450	37.944	0.30	15.47	296.46	15.77	2	7/10/2014	37:15.9
1251	RSRS19	-122.450	37.944	0.55	15.47	297.36	16.03	-122.450	37.944	0.30	15.47	297.73	15.77	2	7/10/2014	37:16.0
1252	RSRS19	-122.450	37.944	0.55	15.49	298.61	16.05	-122.450	37.944	0.38	15.49	299.01	15.88	2	7/10/2014	37:16.1
1253	RSRS19	-122.450	37.944	0.55	15.43	299.95	15.98	-122.450	37.944	0.38	15.43	300.24	15.81	2	7/10/2014	37:16.2

1254	RSRS19	-122.450	37.944	0.55	15.41	301.12	15.96	-122.450	37.944	0.33	15.41	301.34	15.74	2	7/10/2014	37:16.3
1255	RSRS19	-122.450	37.944	0.55	15.39	302.29	15.95	-122.450	37.944	0.45	15.39	302.46	15.84	2	7/10/2014	37:16.4
1256	RSRS19	-122.450	37.944	0.55	15.41	303.36	15.96	-122.450	37.944	0.33	15.41	303.48	15.74	2	7/10/2014	37:16.4
1257	RSRS19	-122.450	37.944	0.55	15.39	304.42	15.95	-122.450	37.944	0.38	15.39	304.49	15.78	2	7/10/2014	37:16.6
1258	RSRS19	-122.450	37.944	0.52	15.34	305.48	15.86	-122.450	37.944	0.41	15.34	305.52	15.76	2	7/10/2014	37:16.7
1259	RSRS19	-122.450	37.944	0.55	15.36	306.49	15.91	-122.450	37.944	0.38	15.36	306.47	15.74	2	7/10/2014	37:16.8
1260	RSRS19	-122.450	37.944	0.55	15.32	307.48	15.88	-122.450	37.944	0.38	15.32	307.45	15.71	2	7/10/2014	37:16.9
1261	RSRS19	-122.450	37.944	0.61	15.36	308.40	15.97	-122.450	37.944	0.45	15.36	308.34	15.81	2	7/10/2014	37:16.9
1262	RSRS19	-122.450	37.944	0.55	15.38	309.33	15.93	-122.450	37.944	0.38	15.38	309.22	15.76	2	7/10/2014	37:17.1
1263	RSRS19	-122.450	37.944	0.61	15.40	310.21	16.01	-122.450	37.944	0.45	15.40	309.97	15.85	2	7/10/2014	37:17.2
1264	RSRS19	-122.450	37.944	0.55	15.41	310.98	15.96	-122.450	37.944	0.41	15.41	310.69	15.82	2	7/10/2014	37:17.3
1265	RSRS19	-122.450	37.944	0.55	15.44	311.81	16.00	-122.450	37.944	0.41	15.44	311.40	15.86	2	7/10/2014	37:17.4
1266	RSRS19	-122.450	37.944	0.55	15.45	312.43	16.00	-122.450	37.944	0.41	15.45	311.92	15.86	2	7/10/2014	37:17.5
1267	RSRS19	-122.450	37.944	0.61	15.43	313.24	16.04	-122.450	37.944	0.38	15.43	312.63	15.81	2	7/10/2014	37:17.5
1268	RSRS19	-122.450	37.944	0.55	15.43	313.77	15.99	-122.450	37.944	0.33	15.43	313.24	15.76	2	7/10/2014	37:17.7
1269	RSRS19	-122.450	37.944	0.55	15.45	314.32	16.00	-122.450	37.944	0.45	15.45	313.75	15.90	2	7/10/2014	37:17.8
1270	RSRS19	-122.450	37.944	0.55	15.43	314.89	15.99	-122.450	37.944	0.41	15.43	314.19	15.85	2	7/10/2014	37:17.9
1271	RSRS19	-122.450	37.944	0.55	15.44	315.40	16.00	-122.450	37.944	0.38	15.44	314.74	15.82	2	7/10/2014	37:18.0
1272	RSRS19	-122.450	37.944	0.55	15.42	315.89	15.97	-122.450	37.944	0.41	15.42	315.12	15.83	2	7/10/2014	37:18.0
1273	RSRS19	-122.450	37.944	0.61	15.41	316.31	16.01	-122.450	37.944	0.41	15.41	315.54	15.82	2	7/10/2014	37:18.2
1274	RSRS19	-122.450	37.944	0.55	15.42	316.70	15.97	-122.450	37.944	0.38	15.42	315.87	15.80	2	7/10/2014	37:18.3
1275	RSRS19	-122.450	37.944	0.61	15.41	317.10	16.01	-122.450	37.944	0.50	15.41	316.24	15.91	2	7/10/2014	37:18.4
1276	RSRS19	-122.450	37.944	0.55	15.43	317.34	15.99	-122.450	37.944	0.38	15.43	316.48	15.81	2	7/10/2014	37:18.5
1277	RSRS19	-122.450	37.944	0.61	15.42	317.72	16.02	-122.450	37.944	0.50	15.42	316.73	15.92	2	7/10/2014	37:18.6
1278	RSRS19	-122.450	37.944	0.55	15.42	317.98	15.97	-122.450	37.944	0.45	15.42	316.95	15.87	2	7/10/2014	37:18.7
1279	RSRS19	-122.450	37.944	0.61	15.43	318.24	16.04	-122.450	37.944	0.41	15.43	317.06	15.85	2	7/10/2014	37:18.8
1280	RSRS19	-122.450	37.944	0.55	15.43	318.46	15.98	-122.450	37.944	0.53	15.43	317.19	15.96	2	7/10/2014	37:18.9
1281	RSRS19	-122.450	37.944	0.61	15.45	318.64	16.05	-122.450	37.944	0.45	15.45	317.28	15.90	2	7/10/2014	37:19.0
1282	RSRS19	-122.450	37.944	0.55	15.46	318.79	16.01	-122.450	37.944	0.41	15.46	317.37	15.87	2	7/10/2014	37:19.1
1283	RSRS19	-122.450	37.944	0.61	15.53	318.97	16.13	-122.450	37.944	0.45	15.53	317.52	15.98	2	7/10/2014	37:19.1
1284	RSRS19	-122.450	37.944	0.55	15.53	319.11	16.08	-122.450	37.944	0.38	15.53	317.65	15.91	2	7/10/2014	37:19.3
1285	RSRS19	-122.450	37.944	0.61	15.56	319.17	16.16	-122.450	37.944	0.38	15.56	317.68	15.94	2	7/10/2014	37:19.4
1286	RSRS19	-122.450	37.944	0.55	15.56	319.21	16.11	-122.450	37.944	0.45	15.56	317.74	16.00	2	7/10/2014	37:19.5

1287	RSRS19	-122.450	37.944	0.61	15.54	319.33	16.14	-122.450	37.944	0.41	15.54	317.74	15.95	2	7/10/2014	37:19.6
1288	RSRS19	-122.450	37.944	0.55	15.54	319.33	16.09	-122.450	37.944	0.38	15.54	317.81	15.92	2	7/10/2014	37:19.7
1289	RSRS19	-122.450	37.944	0.61	15.53	319.35	16.13	-122.450	37.944	0.45	15.53	317.73	15.98	2	7/10/2014	37:19.7
1290	RSRS19	-122.450	37.944	0.55	15.53	319.33	16.08	-122.450	37.944	0.41	15.53	317.72	15.94	2	7/10/2014	37:19.9
1291	RSRS19	-122.450	37.944	0.61	15.56	319.37	16.16	-122.450	37.944	0.41	15.56	317.72	15.97	2	7/10/2014	37:20.0
1292	RSRS19	-122.450	37.944	0.55	15.53	319.35	16.08	-122.450	37.944	0.41	15.53	317.70	15.94	2	7/10/2014	37:20.1
1293	RSRS19	-122.450	37.944	0.55	15.50	319.31	16.06	-122.450	37.944	0.41	15.50	317.66	15.92	2	7/10/2014	37:20.2
1294	RSRS19	-122.450	37.944	0.55	15.48	319.24	16.03	-122.450	37.944	0.38	15.48	317.60	15.86	2	7/10/2014	37:20.3
1295	RSRS19	-122.450	37.944	0.61	15.46	319.18	16.06	-122.450	37.944	0.45	15.46	317.49	15.91	2	7/10/2014	37:20.3
1296	RSRS19	-122.450	37.944	0.55	15.43	319.11	15.99	-122.450	37.944	0.41	15.43	317.40	15.85	2	7/10/2014	37:20.5
1297	RSRS19	-122.450	37.944	0.61	15.42	319.05	16.02	-122.450	37.944	0.41	15.42	317.36	15.83	2	7/10/2014	37:20.6
1298	RSRS19	-122.450	37.944	0.55	15.41	318.87	15.96	-122.450	37.944	0.45	15.41	317.16	15.86	2	7/10/2014	37:20.7
1299	RSRS19	-122.450	37.944	0.61	15.43	318.81	16.03	-122.450	37.944	0.45	15.43	317.12	15.87	2	7/10/2014	37:20.8
1300	RSRS19	-122.450	37.944	0.55	15.40	318.72	15.96	-122.450	37.944	0.38	15.40	316.88	15.78	2	7/10/2014	37:20.8
1301	RSRS19	-122.450	37.944	0.61	15.41	318.61	16.01	-122.450	37.944	0.50	15.41	316.79	15.91	2	7/10/2014	37:21.0
1302	RSRS19	-122.450	37.944	0.55	15.43	318.41	15.98	-122.450	37.944	0.41	15.43	316.61	15.84	2	7/10/2014	37:21.1
1303	RSRS19	-122.450	37.944	0.61	15.42	318.28	16.02	-122.450	37.944	0.41	15.42	316.39	15.83	2	7/10/2014	37:21.2
1304	RSRS19	-122.450	37.944	0.61	15.45	318.09	16.05	-122.450	37.944	0.45	15.45	316.15	15.90	2	7/10/2014	37:21.3
1305	RSRS19	-122.450	37.944	0.61	15.46	317.91	16.07	-122.450	37.944	0.41	15.46	315.96	15.88	2	7/10/2014	37:21.4
1306	RSRS19	-122.450	37.944	0.55	15.53	317.76	16.09	-122.450	37.944	0.38	15.53	315.82	15.91	2	7/10/2014	37:21.5
1307	RSRS19	-122.450	37.944	0.61	15.55	317.63	16.15	-122.450	37.944	0.45	15.55	315.65	16.00	2	7/10/2014	37:21.6
1308	RSRS19	-122.450	37.944	0.55	15.59	317.27	16.14	-122.450	37.944	0.41	15.59	315.38	16.00	2	7/10/2014	37:21.7
1309	RSRS19	-122.450	37.944	0.55	15.56	317.08	16.12	-122.450	37.944	0.38	15.56	315.14	15.95	2	7/10/2014	37:21.8
1310	RSRS19	-122.450	37.944	0.61	15.60	316.88	16.21	-122.450	37.944	0.45	15.60	314.93	16.05	2	7/10/2014	37:21.9
1311	RSRS19	-122.450	37.944	0.61	15.59	316.68	16.19	-122.450	37.944	0.41	15.59	314.66	16.00	2	7/10/2014	37:21.9
1312	RSRS19	-122.450	37.944	0.61	15.60	316.31	16.21	-122.450	37.944	0.41	15.60	314.44	16.02	2	7/10/2014	37:22.1
1313	RSRS19	-122.450	37.944	0.61	15.62	316.11	16.23	-122.450	37.944	0.45	15.62	314.11	16.07	2	7/10/2014	37:22.2
1314	RSRS19	-122.450	37.944	0.55	15.64	315.87	16.19	-122.450	37.944	0.33	15.64	313.89	15.97	2	7/10/2014	37:22.3
1315	RSRS19	-122.450	37.944	0.61	15.66	315.57	16.26	-122.450	37.944	0.45	15.66	313.72	16.11	2	7/10/2014	37:22.4
1316	RSRS19	-122.450	37.944	0.55	15.68	315.35	16.23	-122.450	37.944	0.38	15.68	313.35	16.06	2	7/10/2014	37:22.5
1317	RSRS19	-122.450	37.944	0.61	15.66	315.06	16.27	-122.450	37.944	0.33	15.66	313.13	15.99	2	7/10/2014	37:22.5
1318	RSRS19	-122.450	37.944	0.55	15.63	314.89	16.19	-122.450	37.944	0.38	15.63	312.95	16.01	2	7/10/2014	37:22.7
1319	RSRS19	-122.450	37.944	0.61	15.63	314.69	16.24	-122.450	37.944	0.33	15.63	312.67	15.96	2	7/10/2014	37:22.8

1320	RSRS19	-122.450	37.944	0.55	15.63	314.36	16.19	-122.450	37.944	0.33	15.63	312.49	15.96	2	7/10/2014	37:22.9
1321	RSRS19	-122.450	37.944	0.61	15.63	314.25	16.24	-122.450	37.944	0.41	15.63	312.27	16.05	2	7/10/2014	37:23.0
1322	RSRS19	-122.450	37.944	0.55	15.62	313.97	16.18	-122.450	37.944	0.45	15.62	312.10	16.07	2	7/10/2014	37:23.1
1323	RSRS19	-122.450	37.944	0.61	15.62	313.75	16.22	-122.450	37.944	0.38	15.62	311.84	16.00	2	7/10/2014	37:23.1
1324	RSRS19	-122.450	37.944	0.61	15.61	313.53	16.21	-122.450	37.944	0.41	15.61	311.59	16.02	2	7/10/2014	37:23.3
1325	RSRS19	-122.450	37.944	0.61	15.62	313.37	16.23	-122.450	37.944	0.41	15.62	311.39	16.04	2	7/10/2014	37:23.4
1326	RSRS19	-122.450	37.944	0.55	15.61	313.05	16.16	-122.450	37.944	0.33	15.61	311.14	15.94	2	7/10/2014	37:23.5
1327	RSRS19	-122.450	37.944	0.61	15.60	312.85	16.21	-122.450	37.944	0.45	15.60	310.89	16.05	2	7/10/2014	37:23.6
1328	RSRS19	-122.450	37.944	0.55	15.61	312.63	16.16	-122.450	37.944	0.33	15.61	310.63	15.94	2	7/10/2014	37:23.6
1329	RSRS19	-122.450	37.944	0.61	15.58	312.32	16.18	-122.450	37.944	0.30	15.58	310.30	15.88	2	7/10/2014	37:23.8
1330	RSRS19	-122.450	37.944	0.55	15.61	312.28	16.16	-122.450	37.944	0.38	15.61	310.17	15.99	2	7/10/2014	37:23.9
1331	RSRS19	-122.450	37.944	0.61	15.66	311.92	16.26	-122.450	37.944	0.41	15.66	309.92	16.07	2	7/10/2014	37:24.0
1332	RSRS19	-122.450	37.944	0.55	15.60	311.73	16.15	-122.450	37.944	0.30	15.60	309.68	15.89	2	7/10/2014	37:24.1
1333	RSRS19	-122.450	37.944	0.55	15.62	311.51	16.17	-122.450	37.944	0.38	15.62	309.49	16.00	2	7/10/2014	37:24.2
1334	RSRS19	-122.450	37.944	0.55	15.71	311.29	16.26	-122.450	37.944	0.33	15.71	309.20	16.04	2	7/10/2014	37:24.3
1335	RSRS19	-122.450	37.944	0.55	15.67	311.09	16.23	-122.450	37.944	0.33	15.67	309.05	16.00	2	7/10/2014	37:24.4
1336	RSRS19	-122.450	37.944	0.55	15.67	310.90	16.23	-122.450	37.944	0.30	15.67	308.85	15.97	2	7/10/2014	37:24.5
1337	RSRS19	-122.450	37.944	0.61	15.66	310.72	16.27	-122.450	37.944	0.41	15.66	308.70	16.08	2	7/10/2014	37:24.5
1338	RSRS19	-122.450	37.944	0.55	15.65	310.50	16.20	-122.450	37.944	0.33	15.65	308.50	15.98	2	7/10/2014	37:24.7
1339	RSRS19	-122.450	37.944	0.61	15.71	310.35	16.32	-122.450	37.944	0.38	15.71	308.30	16.09	2	7/10/2014	37:24.7
1340	RSRS19	-122.450	37.944	0.55	15.66	310.17	16.22	-122.450	37.944	0.38	15.66	308.08	16.05	2	7/10/2014	37:24.9
1341	RSRS19	-122.450	37.944	0.61	15.64	309.89	16.25	-122.450	37.944	0.33	15.64	307.89	15.97	2	7/10/2014	37:25.0
1342	RSRS19	-122.450	37.944	0.55	15.61	309.73	16.16	-122.450	37.944	0.33	15.61	307.65	15.94	2	7/10/2014	37:25.1
1343	RSRS19	-122.450	37.944	0.55	15.62	309.45	16.17	-122.450	37.944	0.38	15.62	307.39	16.00	2	7/10/2014	37:25.2
1344	RSRS19	-122.450	37.944	0.55	15.56	309.27	16.11	-122.450	37.944	0.30	15.56	307.14	15.85	2	7/10/2014	37:25.3
1345	RSRS19	-122.450	37.944	0.61	15.57	309.10	16.18	-122.450	37.944	0.38	15.57	306.95	15.95	2	7/10/2014	37:25.3
1346	RSRS19	-122.450	37.944	0.55	15.54	308.88	16.09	-122.450	37.944	0.38	15.54	306.75	15.92	2	7/10/2014	37:25.5
1347	RSRS19	-122.450	37.944	0.61	15.53	308.68	16.13	-122.450	37.944	0.33	15.53	306.55	15.86	2	7/10/2014	37:25.6
1348	RSRS19	-122.450	37.944	0.55	15.55	308.55	16.10	-122.450	37.944	0.33	15.55	306.33	15.88	2	7/10/2014	37:25.7
1349	RSRS19	-122.450	37.944	0.55	15.59	308.36	16.14	-122.450	37.944	0.38	15.59	306.22	15.97	2	7/10/2014	37:25.8
1350	RSRS19	-122.450	37.944	0.55	15.56	308.16	16.12	-122.450	37.944	0.30	15.56	306.00	15.86	2	7/10/2014	37:25.9
1351	RSRS19	-122.450	37.944	0.61	15.56	307.98	16.16	-122.450	37.944	0.33	15.56	305.83	15.89	2	7/10/2014	37:25.9
1352	RSRS19	-122.450	37.944	0.55	15.62	307.79	16.17	-122.450	37.944	0.38	15.62	305.65	16.00	2	7/10/2014	37:26.1

1353	RSRS19	-122.450	37.944	0.60	15.61	307.57	16.21	-122.450	37.944	0.38	15.61	305.46	15.99	2	7/10/2014	37:26.2
1354	RSRS19	-122.450	37.944	0.55	15.64	307.35	16.19	-122.450	37.944	0.33	15.64	305.24	15.97	2	7/10/2014	37:26.3
1355	RSRS19	-122.450	37.944	0.60	15.60	307.15	16.20	-122.450	37.944	0.41	15.60	305.04	16.01	2	7/10/2014	37:26.4
1356	RSRS19	-122.450	37.944	0.55	15.61	306.95	16.16	-122.450	37.944	0.25	15.61	304.89	15.85	2	7/10/2014	37:26.4
1357	RSRS19	-122.450	37.944	0.60	15.64	306.76	16.24	-122.450	37.944	0.33	15.64	304.62	15.97	2	7/10/2014	37:26.6
1358	RSRS19	-122.450	37.944	0.55	15.62	306.54	16.18	-122.450	37.944	0.41	15.62	304.43	16.04	2	7/10/2014	37:26.7
1359	RSRS19	-122.450	37.944	0.60	15.62	306.36	16.22	-122.450	37.944	0.33	15.62	304.21	15.95	2	7/10/2014	37:26.8
1360	RSRS19	-122.450	37.944	0.55	15.58	306.19	16.13	-122.450	37.944	0.33	15.58	304.03	15.91	2	7/10/2014	37:26.9
1361	RSRS19	-122.450	37.944	0.60	15.60	305.95	16.20	-122.450	37.944	0.38	15.60	303.84	15.98	2	7/10/2014	37:27.0
1362	RSRS19	-122.450	37.944	0.55	15.58	305.86	16.13	-122.450	37.944	0.29	15.58	303.68	15.87	2	7/10/2014	37:27.1
1363	RSRS19	-122.450	37.944	0.55	15.55	305.70	16.10	-122.450	37.944	0.33	15.55	303.55	15.88	2	7/10/2014	37:27.2
1364	RSRS19	-122.450	37.944	0.55	15.53	305.46	16.09	-122.450	37.944	0.38	15.53	303.31	15.91	2	7/10/2014	37:27.2
1365	RSRS19	-122.450	37.944	0.60	15.48	305.29	16.08	-122.450	37.944	0.33	15.48	303.16	15.81	2	7/10/2014	37:27.4
1366	RSRS19	-122.450	37.944	0.55	15.46	305.18	16.02	-122.450	37.944	0.33	15.46	302.96	15.79	2	7/10/2014	37:27.5
1367	RSRS19	-122.450	37.944	0.60	15.46	304.92	16.06	-122.450	37.944	0.41	15.46	302.79	15.87	2	7/10/2014	37:27.5
1368	RSRS19	-122.450	37.944	0.55	15.40	304.76	15.96	-122.450	37.944	0.29	15.40	302.56	15.70	2	7/10/2014	37:27.7
1369	RSRS19	-122.450	37.944	0.60	15.39	304.54	15.99	-122.450	37.944	0.29	15.39	302.35	15.68	2	7/10/2014	37:27.8
1370	RSRS19	-122.450	37.944	0.55	15.50	304.44	16.05	-122.450	37.944	0.38	15.50	302.19	15.88	2	7/10/2014	37:27.9
1371	RSRS19	-122.450	37.944	0.60	15.42	304.28	16.02	-122.450	37.944	0.29	15.42	302.04	15.71	2	7/10/2014	37:28.0
1372	RSRS19	-122.450	37.944	0.55	15.39	304.08	15.94	-122.450	37.944	0.38	15.39	301.87	15.77	2	7/10/2014	37:28.1
1373	RSRS19	-122.450	37.944	0.60	15.43	303.98	16.03	-122.450	37.944	0.33	15.43	301.78	15.75	2	7/10/2014	37:28.1
1374	RSRS19	-122.450	37.944	0.55	15.43	303.82	15.98	-122.450	37.944	0.25	15.43	301.63	15.67	2	7/10/2014	37:28.3
1375	RSRS19	-122.450	37.944	0.60	15.41	303.62	16.01	-122.450	37.944	0.33	15.41	301.43	15.74	2	7/10/2014	37:28.4
1376	RSRS19	-122.450	37.944	0.55	15.44	303.47	15.99	-122.450	37.944	0.33	15.44	301.32	15.77	2	7/10/2014	37:28.5
1377	RSRS19	-122.450	37.944	0.55	15.40	303.27	15.96	-122.450	37.944	0.29	15.40	301.10	15.70	2	7/10/2014	37:28.6
1378	RSRS19	-122.450	37.944	0.55	15.39	303.12	15.94	-122.450	37.944	0.33	15.39	300.99	15.72	2	7/10/2014	37:28.7
1379	RSRS19	-122.450	37.944	0.55	15.40	302.97	15.96	-122.450	37.944	0.33	15.40	300.81	15.73	2	7/10/2014	37:28.8
1380	RSRS19	-122.450	37.944	0.55	15.43	302.81	15.99	-122.450	37.944	0.21	15.43	300.66	15.64	2	7/10/2014	37:28.9
1381	RSRS19	-122.450	37.944	0.60	15.48	302.68	16.09	-122.450	37.944	0.29	15.48	300.53	15.78	2	7/10/2014	37:29.0
1382	RSRS19	-122.450	37.944	0.55	15.44	302.58	15.99	-122.450	37.944	0.29	15.44	300.44	15.74	2	7/10/2014	37:29.1
1383	RSRS19	-122.450	37.944	0.60	15.42	302.49	16.02	-122.450	37.944	0.25	15.42	300.33	15.66	2	7/10/2014	37:29.2
1384	RSRS19	-122.450	37.944	0.55	15.46	302.31	16.01	-122.450	37.944	0.33	15.46	300.22	15.79	2	7/10/2014	37:29.2
1385	RSRS19	-122.450	37.944	0.60	15.43	302.25	16.03	-122.450	37.944	0.29	15.43	300.05	15.72	2	7/10/2014	37:29.4

1386	RSRS19	-122.450	37.944	0.55	15.42	302.11	15.97	-122.450	37.944	0.21	15.42	299.96	15.63	2	7/10/2014	37:29.5
1387	RSRS19	-122.450	37.944	0.55	15.44	302.01	15.99	-122.450	37.944	0.33	15.44	299.81	15.77	2	7/10/2014	37:29.6
1388	RSRS19	-122.450	37.944	0.55	15.47	301.92	16.03	-122.450	37.944	0.25	15.47	299.68	15.72	2	7/10/2014	37:29.7
1389	RSRS19	-122.450	37.944	0.60	15.46	301.77	16.07	-122.450	37.944	0.29	15.46	299.55	15.76	2	7/10/2014	37:29.8
1390	RSRS19	-122.450	37.944	0.55	15.47	301.66	16.03	-122.450	37.944	0.29	15.47	299.43	15.77	2	7/10/2014	37:29.9
1391	RSRS19	-122.450	37.944	0.60	15.50	301.50	16.11	-122.450	37.944	0.25	15.50	299.30	15.75	2	7/10/2014	37:30.0
1392	RSRS19	-122.450	37.944	0.55	15.57	301.40	16.12	-122.450	37.944	0.29	15.57	299.20	15.87	2	7/10/2014	37:30.1
1393	RSRS19	-122.450	37.944	0.60	15.50	301.27	16.11	-122.450	37.944	0.33	15.50	299.09	15.83	2	7/10/2014	37:30.2
1394	RSRS19	-122.450	37.944	0.55	15.52	301.15	16.07	-122.450	37.944	0.21	15.52	298.98	15.73	2	7/10/2014	37:30.3
1395	RSRS19	-122.450	37.944	0.55	15.57	301.05	16.12	-122.450	37.944	0.33	15.57	298.85	15.90	2	7/10/2014	37:30.3
1396	RSRS19	-122.450	37.944	0.55	15.56	300.98	16.11	-122.450	37.944	0.29	15.56	298.74	15.85	2	7/10/2014	37:30.5
1397	RSRS19	-122.450	37.944	0.55	15.49	300.83	16.05	-122.450	37.944	0.21	15.49	298.60	15.71	2	7/10/2014	37:30.6
1398	RSRS19	-122.450	37.944	0.52	15.52	300.78	16.04	-122.450	37.944	0.33	15.52	298.50	15.85	2	7/10/2014	37:30.7
1399	RSRS19	-122.450	37.944	0.55	15.55	300.67	16.10	-122.450	37.944	0.29	15.55	298.43	15.84	2	7/10/2014	37:30.8
1400	RSRS19	-122.450	37.944	0.55	15.69	300.52	16.24	-122.450	37.944	0.13	15.69	298.28	15.81	2	7/10/2014	37:30.9
1401	RSRS19	-122.450	37.944	0.55	15.64	300.43	16.19	-122.451	37.944	0.29	15.64	298.17	15.94	2	7/10/2014	37:30.9
1402	RSRS19	-122.451	37.944	0.55	15.69	300.35	16.25	-122.451	37.944	0.25	15.69	298.17	15.94	2	7/10/2014	37:31.1
1403	RSRS19	-122.451	37.944	0.55	15.75	300.24	16.30	-122.451	37.944	0.21	15.75	298.04	15.96	2	7/10/2014	37:31.2
1404	RSRS19	-122.451	37.944	0.55	15.69	300.15	16.24	-122.451	37.944	0.33	15.69	297.97	16.02	2	7/10/2014	37:31.3
1405	RSRS19	-122.451	37.944	0.55	15.68	300.04	16.23	-122.451	37.944	0.16	15.68	297.89	15.84	2	7/10/2014	37:31.3
1406	RSRS19	-122.451	37.944	0.52	15.66	299.89	16.18	-122.451	37.944	0.16	15.66	297.78	15.82	2	7/10/2014	37:31.5
1407	RSRS19	-122.451	37.944	0.55	15.63	299.78	16.19	-122.451	37.944	0.25	15.63	297.64	15.88	2	7/10/2014	37:31.6
1408	RSRS19	-122.451	37.944	0.55	15.61	299.65	16.16	-122.451	37.944	0.13	15.61	297.54	15.74	2	7/10/2014	37:31.7
1409	RSRS19	-122.451	37.944	0.55	15.61	299.56	16.16	-122.451	37.944	0.21	15.61	297.40	15.82	2	7/10/2014	37:31.8
1410	RSRS19	-122.451	37.944	0.55	15.58	299.47	16.13	-122.451	37.944	0.25	15.58	297.28	15.82	2	7/10/2014	37:31.9
1411	RSRS19	-122.451	37.944	0.55	15.56	299.41	16.12	-122.451	37.944	0.13	15.56	297.21	15.69	2	7/10/2014	37:32.0
1412	RSRS19	-122.451	37.944	0.55	15.57	299.36	16.12	-122.451	37.944	0.21	15.57	297.14	15.78	2	7/10/2014	37:32.0
1413	RSRS19	-122.451	37.944	0.55	15.62	299.32	16.18	-122.451	37.944	0.25	15.62	297.06	15.87	2	7/10/2014	37:32.2
1414	RSRS19	-122.451	37.944	0.55	15.65	299.21	16.20	-122.451	37.944	0.13	15.65	296.97	15.78	2	7/10/2014	37:32.3
1415	RSRS19	-122.451	37.944	0.55	15.64	299.17	16.19	-122.451	37.944	0.21	15.64	296.93	15.85	2	7/10/2014	37:32.4
1416	RSRS19	-122.451	37.944	0.55	15.70	299.08	16.26	-122.451	37.944	0.25	15.70	296.84	15.95	2	7/10/2014	37:32.5
1417	RSRS19	-122.451	37.944	0.55	15.63	298.95	16.19	-122.451	37.944	0.08	15.63	296.75	15.71	2	7/10/2014	37:32.6
1418	RSRS19	-122.451	37.944	0.52	15.66	298.80	16.18	-122.451	37.944	0.21	15.66	296.60	15.88	2	7/10/2014	37:32.7

1419	RSRS19	-122.451	37.944	0.55	15.70	298.71	16.26	-122.451	37.944	0.29	15.70	296.49	16.00	2	7/10/2014	37:32.8
1420	RSRS19	-122.451	37.944	0.55	15.67	298.64	16.22	-122.451	37.944	0.08	15.67	296.40	15.75	2	7/10/2014	37:32.9
1421	RSRS19	-122.451	37.944	0.55	15.65	298.56	16.20	-122.451	37.944	0.25	15.65	296.36	15.89	2	7/10/2014	37:33.0
1422	RSRS19	-122.451	37.944	0.52	15.64	298.47	16.16	-122.451	37.944	0.21	15.64	296.23	15.85	2	7/10/2014	37:33.1
1423	RSRS19	-122.451	37.944	0.55	15.62	298.45	16.17	-122.451	37.944	0.13	15.62	296.23	15.74	2	7/10/2014	37:33.1
1424	RSRS19	-122.451	37.944	0.52	15.69	298.40	16.21	-122.451	37.944	0.21	15.69	296.23	15.90	2	7/10/2014	37:33.3
1425	RSRS19	-122.451	37.944	0.55	15.66	298.34	16.22	-122.451	37.944	0.16	15.66	296.08	15.82	2	7/10/2014	37:33.4
1426	RSRS19	-122.451	37.944	0.52	15.62	298.28	16.14	-122.451	37.944	0.08	15.62	296.10	15.70	2	7/10/2014	37:33.5
1427	RSRS19	-122.451	37.944	0.55	15.62	298.23	16.18	-122.451	37.944	0.25	15.62	296.05	15.87	2	7/10/2014	37:33.6
1428	RSRS19	-122.451	37.944	0.52	15.66	298.12	16.18	-122.451	37.944	0.08	15.66	295.92	15.73	2	7/10/2014	37:33.7
1429	RSRS19	-122.451	37.944	0.55	15.66	298.03	16.21	-122.451	37.944	0.13	15.66	295.86	15.78	2	7/10/2014	37:33.7
1430	RSRS19	-122.451	37.944	0.52	15.66	297.99	16.18	-122.451	37.944	0.16	15.66	295.77	15.82	2	7/10/2014	37:33.9
1431	RSRS19	-122.451	37.944	0.55	15.62	297.93	16.18	-122.451	37.944	0.08	15.62	295.73	15.70	2	7/10/2014	37:34.0
1432	RSRS19	-122.451	37.944	0.52	15.66	297.88	16.18	-122.451	37.944	0.21	15.66	295.66	15.87	2	7/10/2014	37:34.1
1433	RSRS19	-122.451	37.944	0.55	15.66	297.79	16.22	-122.451	37.944	0.16	15.66	295.51	15.82	2	7/10/2014	37:34.2
1434	RSRS19	-122.451	37.944	0.52	15.76	297.71	16.28	-122.451	37.944	0.01	15.76	295.42	15.77	2	7/10/2014	37:34.3
1435	RSRS19	-122.451	37.944	0.52	15.68	297.62	16.20	-122.451	37.944	0.25	15.68	295.34	15.92	2	7/10/2014	37:34.4
1436	RSRS19	-122.451	37.944	0.52	15.73	297.54	16.25	-122.451	37.944	0.04	15.73	295.27	15.78	2	7/10/2014	37:34.5
1437	RSRS19	-122.451	37.944	0.52	15.72	297.45	16.24	-122.451	37.944	0.08	15.72	295.14	15.79	2	7/10/2014	37:34.6
1438	RSRS19	-122.451	37.944	0.52	15.75	297.34	16.27	-122.451	37.944	0.21	15.75	295.03	15.96	2	7/10/2014	37:34.7
1439	RSRS19	-122.451	37.944	0.55	15.77	297.25	16.33	-122.451	37.944	0.04	15.77	294.96	15.81	2	7/10/2014	37:34.8
1440	RSRS19	-122.451	37.944	0.52	15.78	297.16	16.30	-122.451	37.944	0.04	15.78	294.90	15.82	2	7/10/2014	37:34.8
1441	RSRS19	-122.451	37.944	0.55	15.80	297.12	16.35	-122.451	37.944	0.13	15.80	294.83	15.92	2	7/10/2014	37:35.0
1442	RSRS19	-122.451	37.944	0.52	15.75	297.08	16.27	-122.451	37.944	0.08	15.75	294.82	15.82	2	7/10/2014	37:35.1
1443	RSRS19	-122.451	37.944	0.55	15.82	297.01	16.37	-122.451	37.944	0.16	15.82	294.73	15.98	2	7/10/2014	37:35.2
1444	RSRS19	-122.451	37.944	0.52	15.78	296.95	16.30	-122.451	37.944	0.16	15.78	294.68	15.94	2	7/10/2014	37:35.3
1445	RSRS19	-122.451	37.944	0.52	15.76	296.92	16.28	-122.451	37.944	0.08	15.76	294.62	15.84	2	7/10/2014	37:35.4
1446	RSRS19	-122.451	37.944	0.52	15.81	296.82	16.33	-122.451	37.944	0.21	15.81	294.53	16.02	2	7/10/2014	37:35.5
1447	RSRS19	-122.451	37.944	0.52	15.79	296.75	16.31	-122.451	37.944	0.08	15.79	294.47	15.86	2	7/10/2014	37:35.6
1448	RSRS19	-122.451	37.944	0.47	15.77	296.58	16.24	-122.451	37.944	0.04	15.77	294.27	15.81	2	7/10/2014	37:35.7
1449	RSRS19	-122.451	37.944	0.52	15.80	296.67	16.31	-122.451	37.944	0.21	15.80	294.29	16.01	2	7/10/2014	37:35.8
1450	RSRS19	-122.451	37.944	0.52	15.84	296.58	16.36	-122.451	37.944	0.08	15.84	294.33	15.92	2	7/10/2014	37:35.9
1451	RSRS19	-122.451	37.944	0.52	15.82	296.51	16.34	-122.451	37.944	0.04	15.82	294.29	15.86	2	7/10/2014	37:35.9

1452	RSRS19	-122.451	37.944	0.52	15.86	296.44	16.38	-122.451	37.944	0.16	15.86	294.23	16.02	2	7/10/2014	37:36.1
1453	RSRS19	-122.451	37.944	0.52	15.83	296.41	16.35	-122.451	37.944	0.13	15.83	294.21	15.96	2	7/10/2014	37:36.2
1454	RSRS19	-122.451	37.944	0.47	15.86	296.29	16.32	-122.451	37.944	0.01	15.86	294.12	15.87	2	7/10/2014	37:36.3
1455	RSRS19	-122.451	37.944	0.52	15.83	296.20	16.35	-122.451	37.944	0.16	15.83	294.05	15.99	2	7/10/2014	37:36.4
1456	RSRS19	-122.451	37.944	0.52	15.80	296.10	16.32	-122.451	37.944	0.13	15.80	293.94	15.93	2	7/10/2014	37:36.5
1457	RSRS19	-122.451	37.944	0.52	15.81	295.99	16.33	-122.451	37.944	0.08	15.81	293.86	15.89	2	7/10/2014	37:36.5
1458	RSRS19	-122.451	37.944	0.52	15.85	295.97	16.37	-122.451	37.944	0.13	15.85	293.83	15.97	2	7/10/2014	37:36.7
1459	RSRS19	-122.451	37.944	0.52	15.85	295.86	16.37	-122.451	37.944	0.13	15.85	293.64	15.97	2	7/10/2014	37:36.7
1460	RSRS19	-122.451	37.944	0.47	15.82	295.86	16.28	-122.451	37.944	0.08	15.82	293.64	15.89	2	7/10/2014	37:36.9
1461	RSRS19	-122.451	37.944	0.52	15.88	295.86	16.40	-122.451	37.944	0.16	15.88	293.68	16.04	2	7/10/2014	37:37.0
1462	RSRS19	-122.451	37.944	0.52	15.87	295.86	16.39	-122.451	37.944	-0.04	15.87	293.66	15.83	2	7/10/2014	37:37.1
1463	RSRS19	-122.451	37.944	0.52	15.88	295.79	16.40	-122.451	37.944	0.08	15.88	293.66	15.96	2	7/10/2014	37:37.2
1464	RSRS19	-122.451	37.944	0.47	15.85	295.66	16.32	-122.451	37.944	0.16	15.85	293.51	16.01	2	7/10/2014	37:37.3
1465	RSRS19	-122.451	37.944	0.47	15.88	295.58	16.35	-122.451	37.944	-0.04	15.88	293.40	15.84	2	7/10/2014	37:37.4
1466	RSRS19	-122.451	37.944	0.47	15.85	295.49	16.32	-122.451	37.944	0.04	15.85	293.22	15.89	2	7/10/2014	37:37.5
1467	RSRS19	-122.451	37.944	0.52	15.83	295.36	16.35	-122.451	37.944	0.13	15.83	293.16	15.96	2	7/10/2014	37:37.6
1468	RSRS19	-122.451	37.944	0.47	15.93	295.38	16.40	-122.451	37.944	0.04	15.93	293.07	15.98	2	7/10/2014	37:37.6
1469	RSRS19	-122.451	37.944	0.52	15.84	295.27	16.36	-122.451	37.944	0.08	15.84	293.03	15.92	2	7/10/2014	37:37.8
1470	RSRS19	-122.451	37.944	0.52	15.89	295.27	16.41	-122.451	37.944	0.13	15.89	293.01	16.01	2	7/10/2014	37:37.9
1471	RSRS19	-122.451	37.944	0.52	15.89	295.23	16.41	-122.451	37.944	0.04	15.89	293.03	15.93	2	7/10/2014	37:38.0
1472	RSRS19	-122.451	37.944	0.47	15.90	295.18	16.37	-122.451	37.944	0.04	15.90	293.03	15.94	2	7/10/2014	37:38.1
1473	RSRS19	-122.451	37.944	0.47	15.88	295.16	16.35	-122.451	37.944	0.16	15.88	292.99	16.04	2	7/10/2014	37:38.1
1474	RSRS19	-122.451	37.944	0.47	15.86	295.10	16.33	-122.451	37.944	0.01	15.86	292.96	15.87	2	7/10/2014	37:38.3
1475	RSRS19	-122.451	37.944	0.52	15.84	295.06	16.36	-122.451	37.944	0.08	15.84	292.90	15.92	2	7/10/2014	37:38.4
1476	RSRS19	-122.451	37.944	0.47	15.84	294.99	16.31	-122.451	37.944	0.13	15.84	292.81	15.97	2	7/10/2014	37:38.5
1477	RSRS19	-122.451	37.944	0.52	15.83	294.90	16.35	-122.451	37.944	-0.08	15.83	292.71	15.75	2	7/10/2014	37:38.6
1478	RSRS19	-122.451	37.944	0.47	15.83	294.82	16.29	-122.451	37.944	0.04	15.83	292.64	15.87	2	7/10/2014	37:38.7
1479	RSRS19	-122.451	37.944	0.52	15.83	294.77	16.35	-122.451	37.944	0.13	15.83	292.66	15.96	2	7/10/2014	37:38.7
1480	RSRS19	-122.451	37.944	0.47	15.83	294.73	16.30	-122.451	37.944	0.01	15.83	292.55	15.84	2	7/10/2014	37:38.9
1481	RSRS19	-122.451	37.944	0.52	15.86	294.69	16.38	-122.451	37.944	0.04	15.86	292.60	15.91	2	7/10/2014	37:39.0
1482	RSRS19	-122.451	37.944	0.47	15.85	294.68	16.32	-122.451	37.944	0.08	15.85	292.55	15.92	2	7/10/2014	37:39.1
1483	RSRS19	-122.451	37.944	0.47	15.86	294.60	16.33	-122.451	37.944	-0.04	15.86	292.51	15.82	2	7/10/2014	37:39.2
1484	RSRS19	-122.451	37.944	0.47	15.97	294.58	16.43	-122.451	37.944	0.04	15.97	292.46	16.01	2	7/10/2014	37:39.3

1485	RSRS19	-122.451	37.944	0.52	15.97	294.58	16.48	-122.451	37.944	0.13	15.97	292.45	16.09	2	7/10/2014	37:39.3
1486	RSRS19	-122.451	37.944	0.47	15.96	294.54	16.42	-122.451	37.944	0.01	15.96	292.37	15.96	2	7/10/2014	37:39.5
1487	RSRS19	-122.451	37.944	0.52	16.03	294.51	16.54	-122.451	37.944	0.13	16.03	292.31	16.15	2	7/10/2014	37:39.6
1488	RSRS19	-122.451	37.944	0.47	16.00	294.43	16.46	-122.451	37.944	0.04	16.00	292.25	16.04	2	7/10/2014	37:39.7
1489	RSRS19	-122.451	37.944	0.52	16.01	294.36	16.53	-122.451	37.944	-0.08	16.01	292.12	15.93	2	7/10/2014	37:39.8
1490	RSRS19	-122.451	37.944	0.47	16.02	294.27	16.49	-122.451	37.944	0.13	16.02	292.10	16.14	2	7/10/2014	37:39.9
1491	RSRS19	-122.451	37.944	0.52	15.97	294.23	16.49	-122.451	37.944	0.08	15.97	292.02	16.05	2	7/10/2014	37:40.0
1492	RSRS19	-122.451	37.944	0.47	15.96	294.12	16.42	-122.451	37.944	-0.04	15.96	292.06	15.91	2	7/10/2014	37:40.1
1493	RSRS19	-122.451	37.944	0.52	15.99	294.10	16.51	-122.451	37.944	0.13	15.99	291.97	16.11	2	7/10/2014	37:40.2
1494	RSRS19	-122.451	37.944	0.47	15.95	293.99	16.42	-122.451	37.944	0.04	15.95	291.88	15.99	2	7/10/2014	37:40.3
1495	RSRS19	-122.451	37.944	0.47	15.94	293.92	16.41	-122.451	37.944	-0.04	15.94	291.82	15.90	2	7/10/2014	37:40.4
1496	RSRS19	-122.451	37.944	0.47	15.90	293.88	16.36	-122.451	37.944	0.08	15.90	291.75	15.97	2	7/10/2014	37:40.4
1497	RSRS19	-122.451	37.944	0.47	15.93	293.80	16.40	-122.451	37.944	0.04	15.93	291.73	15.97	2	7/10/2014	37:40.6
1498	RSRS19	-122.451	37.944	0.47	15.88	293.75	16.35	-122.451	37.944	-0.04	15.88	291.60	15.84	2	7/10/2014	37:40.7
1499	RSRS19	-122.451	37.944	0.52	15.86	293.76	16.38	-122.451	37.944	0.16	15.86	291.58	16.02	2	7/10/2014	37:40.8
1500	RSRS19	-122.451	37.944	0.47	15.95	293.73	16.42	-122.451	37.944	0.04	15.95	291.53	15.99	2	7/10/2014	37:40.8
1501	RSRS19	-122.451	37.944	0.52	15.89	293.69	16.41	-122.451	37.944	-0.04	15.89	291.51	15.84	2	7/10/2014	37:40.9
1502	RSRS19	-122.451	37.944	0.47	15.86	293.65	16.33	-122.451	37.944	0.13	15.86	291.45	15.99	2	7/10/2014	37:41.1
1503	RSRS19	-122.451	37.944	0.47	15.85	293.54	16.32	-122.451	37.944	0.08	15.85	291.36	15.92	2	7/10/2014	37:41.2
1504	RSRS19	-122.451	37.944	0.47	15.85	293.45	16.32	-122.451	37.944	-0.08	15.85	291.27	15.77	2	7/10/2014	37:41.3
1505	RSRS19	-122.451	37.944	0.47	15.83	293.34	16.30	-122.451	37.944	0.16	15.83	291.14	15.99	2	7/10/2014	37:41.4
1506	RSRS19	-122.451	37.944	0.47	15.85	293.27	16.32	-122.451	37.944	0.08	15.85	291.05	15.92	2	7/10/2014	37:41.5
1507	RSRS19	-122.451	37.944	0.47	15.97	293.18	16.43	-122.451	37.944	-0.04	15.97	291.01	15.92	2	7/10/2014	37:41.5
1508	RSRS19	-122.451	37.944	0.47	15.90	293.10	16.37	-122.451	37.944	0.08	15.90	290.90	15.98	2	7/10/2014	37:41.7
1509	RSRS19	-122.451	37.944	0.52	15.94	293.19	16.46	-122.451	37.944	0.01	15.94	291.02	15.95	2	7/10/2014	37:41.8
1510	RSRS19	-122.451	37.944	0.47	15.93	293.19	16.40	-122.451	37.944	0.07	15.93	291.12	16.01	2	7/10/2014	37:41.9
1511	RSRS19	-122.451	37.944	0.52	15.97	293.14	16.49	-122.451	37.944	0.07	15.97	291.13	16.05	2	7/10/2014	37:42.0
1512	RSRS19	-122.451	37.944	0.47	15.90	293.10	16.37	-122.451	37.944	-0.13	15.90	291.08	15.77	2	7/10/2014	37:42.1
1513	RSRS19	-122.451	37.944	0.47	15.94	293.06	16.41	-122.451	37.944	0.16	15.94	291.06	16.10	2	7/10/2014	37:42.1
1514	RSRS19	-122.451	37.944	0.43	15.93	292.99	16.37	-122.451	37.944	0.07	15.93	290.93	16.01	2	7/10/2014	37:42.3
1515	RSRS19	-122.451	37.944	0.47	15.93	292.95	16.40	-122.451	37.944	0.04	15.93	290.86	15.98	2	7/10/2014	37:42.4
1516	RSRS19	-122.451	37.944	0.47	15.92	292.93	16.38	-122.451	37.944	0.13	15.92	290.75	16.04	2	7/10/2014	37:42.5
1517	RSRS19	-122.451	37.944	0.52	15.91	292.86	16.43	-122.451	37.944	0.07	15.91	290.73	15.99	2	7/10/2014	37:42.6

1518	RSRS19	-122.451	37.944	0.43	15.89	292.89	16.32	-122.451	37.944	0.04	15.89	290.73	15.93	2	7/10/2014	37:42.7
1519	RSRS19	-122.451	37.944	0.52	15.93	292.78	16.45	-122.451	37.944	0.16	15.93	290.67	16.09	2	7/10/2014	37:42.8
1520	RSRS19	-122.451	37.944	0.47	15.93	292.78	16.39	-122.451	37.944	-0.04	15.93	290.69	15.88	2	7/10/2014	37:42.9
1521	RSRS19	-122.451	37.944	0.47	15.93	292.85	16.40	-122.451	37.944	0.13	15.93	290.76	16.06	2	7/10/2014	37:43.0
1522	RSRS19	-122.451	37.944	0.47	15.94	292.85	16.41	-122.451	37.944	0.13	15.94	290.82	16.06	2	7/10/2014	37:43.1
1523	RSRS19	-122.451	37.944	0.52	15.96	292.82	16.47	-122.451	37.944	0.04	15.96	290.73	16.00	2	7/10/2014	37:43.2
1524	RSRS19	-122.451	37.944	0.47	15.95	292.78	16.42	-122.451	37.944	0.04	15.95	290.67	15.99	2	7/10/2014	37:43.2
1525	RSRS19	-122.451	37.944	0.52	15.97	292.74	16.49	-122.451	37.944	0.13	15.97	290.57	16.10	2	7/10/2014	37:43.4
1526	RSRS19	-122.451	37.944	0.47	15.99	292.65	16.45	-122.451	37.944	0.01	15.99	290.50	15.99	2	7/10/2014	37:43.5
1527	RSRS19	-122.451	37.944	0.47	16.00	292.67	16.46	-122.451	37.944	0.13	16.00	290.45	16.12	2	7/10/2014	37:43.6
1528	RSRS19	-122.451	37.944	0.47	16.00	292.67	16.46	-122.451	37.944	0.16	16.00	290.47	16.16	2	7/10/2014	37:43.7
1529	RSRS19	-122.451	37.944	0.52	15.96	292.67	16.47	-122.451	37.944	0.01	15.96	290.43	15.96	2	7/10/2014	37:43.7
1530	RSRS19	-122.451	37.944	0.47	15.98	292.63	16.45	-122.451	37.944	0.16	15.98	290.50	16.14	2	7/10/2014	37:43.9
1531	RSRS19	-122.451	37.944	0.52	16.02	292.57	16.54	-122.451	37.944	0.13	16.02	290.39	16.14	2	7/10/2014	37:44.0
1532	RSRS19	-122.451	37.944	0.47	16.00	292.48	16.46	-122.451	37.944	0.07	16.00	290.33	16.07	2	7/10/2014	37:44.1
1533	RSRS19	-122.451	37.944	0.52	15.94	292.46	16.46	-122.451	37.944	0.16	15.94	290.30	16.10	2	7/10/2014	37:44.2
1534	RSRS19	-122.451	37.944	0.47	15.98	292.44	16.45	-122.451	37.944	0.01	15.98	290.29	15.99	2	7/10/2014	37:44.3
1535	RSRS19	-122.451	37.944	0.52	15.93	292.42	16.45	-122.451	37.944	0.01	15.93	290.26	15.94	2	7/10/2014	37:44.3
1536	RSRS19	-122.451	37.944	0.47	15.95	292.39	16.42	-122.451	37.944	0.21	15.95	290.22	16.16	2	7/10/2014	37:44.5
1537	RSRS19	-122.451	37.944	0.47	15.96	292.37	16.42	-122.451	37.944	0.07	15.96	290.27	16.03	2	7/10/2014	37:44.6
1538	RSRS19	-122.451	37.944	0.47	15.90	292.29	16.37	-122.451	37.944	0.01	15.90	290.20	15.91	2	7/10/2014	37:44.7
1539	RSRS19	-122.451	37.944	0.52	15.93	292.31	16.45	-122.451	37.944	0.16	15.93	290.28	16.09	2	7/10/2014	37:44.8
1540	RSRS19	-122.451	37.944	0.47	15.89	292.22	16.35	-122.451	37.944	0.07	15.89	290.22	15.96	2	7/10/2014	37:44.9
1541	RSRS19	-122.451	37.944	0.47	15.86	292.22	16.33	-122.451	37.944	0.07	15.86	290.18	15.94	2	7/10/2014	37:45.0
1542	RSRS19	-122.451	37.944	0.47	15.86	292.16	16.32	-122.451	37.944	0.07	15.86	290.09	15.93	2	7/10/2014	37:45.1
1543	RSRS19	-122.451	37.944	0.52	15.83	292.16	16.34	-122.451	37.944	0.07	15.83	290.05	15.90	2	7/10/2014	37:45.2
1544	RSRS19	-122.451	37.944	0.47	15.84	292.15	16.31	-122.451	37.944	0.07	15.84	290.05	15.92	2	7/10/2014	37:45.3
1545	RSRS19	-122.451	37.944	0.52	15.93	292.14	16.45	-122.451	37.944	0.16	15.93	290.05	16.09	2	7/10/2014	37:45.4
1546	RSRS19	-122.451	37.944	0.47	15.85	292.18	16.31	-122.451	37.944	0.01	15.85	290.07	15.86	2	7/10/2014	37:45.5
1547	RSRS19	-122.451	37.944	0.52	15.88	292.18	16.40	-122.451	37.944	0.04	15.88	290.10	15.92	2	7/10/2014	37:45.6
1548	RSRS19	-122.451	37.944	0.47	15.94	292.21	16.41	-122.451	37.944	0.16	15.94	290.08	16.10	2	7/10/2014	37:45.7
1549	RSRS19	-122.451	37.944	0.52	15.91	292.14	16.43	-122.451	37.944	0.04	15.91	290.10	15.95	2	7/10/2014	37:45.8
1550	RSRS19	-122.451	37.944	0.47	15.95	292.14	16.42	-122.451	37.944	0.01	15.95	290.10	15.96	2	7/10/2014	37:45.9

1551	RSRS19	-122.451	37.944	0.47	15.93	292.12	16.40	-122.451	37.944	0.13	15.93	290.07	16.06	2	7/10/2014	37:46.0
1552	RSRS19	-122.451	37.944	0.47	15.93	292.01	16.39	-122.451	37.944	0.07	15.93	290.01	16.00	2	7/10/2014	37:46.0
1553	RSRS19	-122.451	37.944	0.52	15.89	292.01	16.41	-122.451	37.944	0.04	15.89	289.93	15.93	2	7/10/2014	37:46.2
1554	RSRS19	-122.451	37.944	0.47	15.93	291.92	16.39	-122.451	37.944	0.13	15.93	289.74	16.05	2	7/10/2014	37:46.3
1555	RSRS19	-122.451	37.944	0.52	15.86	291.82	16.38	-122.451	37.944	0.13	15.86	289.66	15.99	2	7/10/2014	37:46.4
1556	RSRS19	-122.451	37.944	0.47	15.93	291.83	16.39	-122.451	37.944	0.04	15.93	289.59	15.97	2	7/10/2014	37:46.5
1557	RSRS19	-122.451	37.944	0.47	15.93	291.73	16.40	-122.451	37.944	0.16	15.93	289.46	16.09	2	7/10/2014	37:46.5
1558	RSRS19	-122.451	37.944	0.47	15.96	291.79	16.42	-122.451	37.944	-0.04	15.96	289.51	15.91	2	7/10/2014	37:46.7
1559	RSRS19	-122.451	37.944	0.52	15.98	291.80	16.50	-122.451	37.944	0.04	15.98	289.60	16.02	2	7/10/2014	37:46.8
1560	RSRS19	-122.451	37.944	0.47	15.97	291.84	16.44	-122.451	37.944	0.16	15.97	289.64	16.13	2	7/10/2014	37:46.9
1561	RSRS19	-122.451	37.944	0.47	15.96	291.80	16.42	-122.451	37.944	-0.04	15.96	289.68	15.91	2	7/10/2014	37:47.0
1562	RSRS19	-122.451	37.944	0.47	16.00	291.75	16.47	-122.451	37.944	0.07	16.00	289.64	16.08	2	7/10/2014	37:47.1
1563	RSRS19	-122.451	37.944	0.47	16.00	291.82	16.46	-122.451	37.944	0.07	16.00	289.64	16.07	2	7/10/2014	37:47.1
1564	RSRS19	-122.451	37.944	0.47	16.00	291.65	16.46	-122.451	37.944	-0.04	16.00	289.58	15.95	2	7/10/2014	37:47.3
1565	RSRS19	-122.451	37.944	0.52	15.98	291.64	16.50	-122.451	37.944	0.04	15.98	289.54	16.02	2	7/10/2014	37:47.4
1566	RSRS19	-122.451	37.944	0.47	15.97	291.60	16.43	-122.451	37.944	0.13	15.97	289.49	16.09	2	7/10/2014	37:47.5
1567	RSRS19	-122.451	37.944	0.47	15.97	291.53	16.44	-122.451	37.944	0.01	15.97	289.43	15.98	2	7/10/2014	37:47.6
1568	RSRS19	-122.451	37.944	0.47	16.03	291.71	16.50	-122.451	37.944	0.01	16.03	289.58	16.04	2	7/10/2014	37:47.6
1569	RSRS19	-122.451	37.944	0.52	16.00	291.71	16.51	-122.451	37.944	0.07	16.00	289.56	16.07	2	7/10/2014	37:47.8
1570	RSRS19	-122.451	37.944	0.47	15.96	291.58	16.42	-122.451	37.944	0.01	15.96	289.43	15.96	2	7/10/2014	37:47.9
1571	RSRS19	-122.451	37.944	0.47	15.93	291.41	16.40	-122.451	37.944	-0.04	15.93	289.32	15.89	2	7/10/2014	37:48.0
1572	RSRS19	-122.451	37.944	0.47	15.95	291.45	16.42	-122.451	37.944	0.04	15.95	289.30	15.99	2	7/10/2014	37:48.1
1573	RSRS19	-122.451	37.944	0.47	16.01	291.58	16.48	-122.451	37.944	-0.04	16.01	289.61	15.97	2	7/10/2014	37:48.2
1574	RSRS19	-122.451	37.944	0.43	16.00	291.61	16.43	-122.451	37.944	0.04	16.00	289.61	16.04	2	7/10/2014	37:48.3
1575	RSRS19	-122.451	37.944	0.47	15.93	291.59	16.40	-122.451	37.944	0.07	15.93	289.72	16.01	2	7/10/2014	37:48.4
1576	RSRS19	-122.451	37.944	0.47	15.98	291.70	16.45	-122.451	37.944	-0.13	15.98	289.74	15.85	2	7/10/2014	37:48.5
1577	RSRS19	-122.451	37.944	0.47	15.98	291.65	16.45	-122.451	37.944	0.07	15.98	289.70	16.05	2	7/10/2014	37:48.6
1578	RSRS19	-122.451	37.944	0.47	15.95	291.63	16.42	-122.451	37.944	0.07	15.95	289.65	16.02	2	7/10/2014	37:48.7
1579	RSRS19	-122.451	37.944	0.47	15.93	291.63	16.39	-122.451	37.944	-0.04	15.93	289.67	15.88	2	7/10/2014	37:48.8
1580	RSRS19	-122.451	37.944	0.43	15.93	291.55	16.37	-122.451	37.944	0.01	15.93	289.66	15.94	2	7/10/2014	37:48.8
1581	RSRS19	-122.451	37.944	0.47	15.88	291.55	16.35	-122.451	37.944	0.07	15.88	289.72	15.95	2	7/10/2014	37:48.9
1582	RSRS19	-122.451	37.944	0.47	15.88	291.52	16.35	-122.451	37.944	-0.08	15.88	289.72	15.80	2	7/10/2014	37:49.1
1583	RSRS19	-122.451	37.944	0.47	15.87	291.50	16.34	-122.451	37.944	0.13	15.87	289.70	16.00	2	7/10/2014	37:49.2

1584	RSRS19	-122.451	37.944	0.43	15.87	291.50	16.30	-122.451	37.944	0.01	15.87	289.68	15.88	2	7/10/2014	37:49.3
1585	RSRS19	-122.451	37.944	0.47	15.85	291.51	16.31	-122.451	37.944	-0.13	15.85	289.64	15.72	2	7/10/2014	37:49.3
1586	RSRS19	-122.451	37.944	0.47	15.86	291.57	16.33	-122.451	37.944	0.13	15.86	289.73	15.99	2	7/10/2014	37:49.5
1587	RSRS19	-122.451	37.944	0.47	15.85	291.62	16.31	-122.451	37.944	-0.04	15.85	289.75	15.80	2	7/10/2014	37:49.6
1588	RSRS19	-122.451	37.944	0.43	15.88	291.59	16.31	-122.451	37.944	0.01	15.88	289.80	15.89	2	7/10/2014	37:49.7
1589	RSRS19	-122.451	37.944	0.47	15.88	291.57	16.35	-122.451	37.944	0.13	15.88	289.73	16.01	2	7/10/2014	37:49.8
1590	RSRS19	-122.451	37.944	0.43	15.93	291.51	16.36	-122.451	37.944	-0.08	15.93	289.68	15.85	2	7/10/2014	37:49.9
1591	RSRS19	-122.451	37.944	0.47	15.88	291.51	16.35	-122.451	37.944	0.13	15.88	289.67	16.01	2	7/10/2014	37:49.9
1592	RSRS19	-122.451	37.944	0.43	15.88	291.46	16.31	-122.451	37.944	0.16	15.88	289.60	16.04	2	7/10/2014	37:50.1
1593	RSRS19	-122.451	37.944	0.47	15.88	291.49	16.35	-122.451	37.944	-0.04	15.88	289.60	15.84	2	7/10/2014	37:50.2
1594	RSRS19	-122.451	37.944	0.43	15.88	291.47	16.31	-122.451	37.944	0.13	15.88	289.62	16.01	2	7/10/2014	37:50.3
1595	RSRS19	-122.451	37.944	0.47	15.88	291.47	16.35	-122.451	37.944	0.13	15.88	289.67	16.01	2	7/10/2014	37:50.3
1596	RSRS19	-122.451	37.944	0.43	15.85	291.49	16.28	-122.451	37.944	0.04	15.85	289.69	15.89	2	7/10/2014	37:50.4
1597	RSRS19	-122.451	37.944	0.47	15.83	291.45	16.30	-122.451	37.944	0.21	15.83	289.67	16.04	2	7/10/2014	37:50.5
1598	RSRS19	-122.451	37.944	0.47	15.81	291.47	16.28	-122.451	37.944	0.01	15.81	289.69	15.82	2	7/10/2014	37:50.7
1599	RSRS19	-122.451	37.944	0.47	15.80	291.47	16.27	-122.451	37.944	0.04	15.80	289.69	15.84	2	7/10/2014	37:50.8
1600	RSRS19	-122.451	37.944	0.47	15.79	291.52	16.25	-122.451	37.944	0.16	15.79	289.69	15.95	2	7/10/2014	37:50.9
1601	RSRS19	-122.451	37.944	0.47	15.78	291.54	16.25	-122.451	37.944	0.07	15.78	289.65	15.85	2	7/10/2014	37:50.9
1602	RSRS19	-122.451	37.944	0.47	15.79	291.54	16.25	-122.451	37.944	0.07	15.79	289.60	15.86	2	7/10/2014	37:51.1
1603	RSRS19	-122.451	37.944	0.43	15.76	291.56	16.19	-122.451	37.944	0.21	15.76	289.65	15.97	2	7/10/2014	37:51.2
1604	RSRS19	-122.451	37.944	0.43	15.76	291.56	16.19	-122.451	37.944	0.01	15.76	289.65	15.76	2	7/10/2014	37:51.3
1605	RSRS19	-122.451	37.944	0.47	15.73	291.54	16.20	-122.451	37.944	0.07	15.73	289.65	15.81	2	7/10/2014	37:51.3
1606	RSRS19	-122.451	37.944	0.47	15.76	291.56	16.23	-122.451	37.944	0.21	15.76	289.72	15.97	2	7/10/2014	37:51.5
1607	RSRS19	-122.451	37.944	0.47	15.75	291.63	16.21	-122.451	37.944	0.07	15.75	289.81	15.82	2	7/10/2014	37:51.6
1608	RSRS19	-122.451	37.944	0.47	15.76	291.61	16.23	-122.451	37.944	0.21	15.76	289.80	15.97	2	7/10/2014	37:51.6
1609	RSRS19	-122.451	37.944	0.47	15.77	291.66	16.24	-122.451	37.944	0.13	15.77	289.85	15.90	2	7/10/2014	37:51.7
1610	RSRS19	-122.451	37.944	0.47	15.77	291.74	16.24	-122.451	37.944	0.01	15.77	289.90	15.78	2	7/10/2014	37:51.9
1611	RSRS19	-122.451	37.944	0.47	15.77	291.70	16.24	-122.451	37.944	0.24	15.77	289.87	16.02	2	7/10/2014	37:52.0
1612	RSRS19	-122.451	37.944	0.47	15.79	291.67	16.25	-122.451	37.944	0.13	15.79	289.85	15.91	2	7/10/2014	37:52.1
1613	RSRS19	-122.451	37.944	0.47	15.78	291.73	16.25	-122.451	37.944	0.04	15.78	289.86	15.82	2	7/10/2014	37:52.1
1614	RSRS19	-122.451	37.944	0.47	15.77	291.66	16.24	-122.451	37.944	0.16	15.77	289.85	15.93	2	7/10/2014	37:52.3
1615	RSRS19	-122.451	37.944	0.47	15.77	291.68	16.24	-122.451	37.944	0.13	15.77	289.83	15.90	2	7/10/2014	37:52.4
1616	RSRS19	-122.451	37.944	0.47	15.77	291.61	16.24	-122.451	37.944	0.16	15.77	289.80	15.93	2	7/10/2014	37:52.5

1617	RSRS19	-122.451	37.944	0.47	15.81	291.68	16.28	-122.451	37.944	0.21	15.81	289.83	16.02	2	7/10/2014	37:52.5
1618	RSRS19	-122.451	37.944	0.43	15.86	291.68	16.30	-122.451	37.944	0.01	15.86	289.84	15.87	2	7/10/2014	37:52.7
1619	RSRS19	-122.451	37.944	0.47	15.83	291.66	16.30	-122.451	37.944	0.24	15.83	289.82	16.08	2	7/10/2014	37:52.7
1620	RSRS19	-122.451	37.944	0.43	15.86	291.73	16.29	-122.451	37.944	0.04	15.86	289.82	15.90	2	7/10/2014	37:52.9
1621	RSRS19	-122.451	37.944	0.47	15.84	291.73	16.31	-122.451	37.944	0.13	15.84	289.80	15.97	2	7/10/2014	37:52.9
1622	RSRS19	-122.451	37.944	0.47	15.84	291.64	16.31	-122.451	37.944	0.21	15.84	289.68	16.05	2	7/10/2014	37:53.1
1623	RSRS19	-122.451	37.944	0.47	15.89	291.60	16.35	-122.451	37.944	0.07	15.89	289.70	15.96	2	7/10/2014	37:53.2
1624	RSRS19	-122.451	37.944	0.43	15.85	291.58	16.28	-122.451	37.944	0.04	15.85	289.67	15.89	2	7/10/2014	37:53.2
1625	RSRS19	-122.451	37.944	0.47	15.88	291.56	16.35	-122.451	37.944	0.21	15.88	289.71	16.09	2	7/10/2014	37:53.3
1626	RSRS19	-122.451	37.944	0.43	15.86	291.49	16.30	-122.451	37.944	0.01	15.86	289.75	15.87	2	7/10/2014	37:53.5
1627	RSRS19	-122.451	37.944	0.47	15.83	291.43	16.29	-122.451	37.944	0.13	15.83	289.71	15.95	2	7/10/2014	37:53.6
1628	RSRS19	-122.451	37.944	0.43	15.81	291.45	16.24	-122.451	37.944	0.21	15.81	289.73	16.02	2	7/10/2014	37:53.7
1629	RSRS19	-122.451	37.944	0.47	15.80	291.39	16.27	-122.451	37.944	0.13	15.80	289.69	15.93	2	7/10/2014	37:53.7
1630	RSRS19	-122.451	37.944	0.43	15.81	291.41	16.24	-122.451	37.944	0.16	15.81	289.67	15.97	2	7/10/2014	37:53.9
1631	RSRS19	-122.451	37.944	0.47	15.80	291.41	16.26	-122.451	37.944	0.04	15.80	289.60	15.84	2	7/10/2014	37:54.0
1632	RSRS19	-122.451	37.944	0.43	15.77	291.34	16.20	-122.451	37.944	0.21	15.77	289.52	15.98	2	7/10/2014	37:54.1
1633	RSRS19	-122.451	37.944	0.47	15.80	291.43	16.27	-122.451	37.944	0.21	15.80	289.60	16.01	2	7/10/2014	37:54.1
1634	RSRS19	-122.451	37.944	0.43	15.74	291.43	16.17	-122.451	37.944	0.04	15.74	289.60	15.78	2	7/10/2014	37:54.3
1635	RSRS19	-122.451	37.944	0.47	15.75	291.37	16.21	-122.451	37.944	0.24	15.75	289.58	15.99	2	7/10/2014	37:54.3
1636	RSRS19	-122.451	37.944	0.43	15.73	291.28	16.17	-122.451	37.944	0.04	15.73	289.54	15.78	2	7/10/2014	37:54.5
1637	RSRS19	-122.451	37.944	0.47	15.80	291.24	16.26	-122.451	37.944	0.21	15.80	289.48	16.01	2	7/10/2014	37:54.5
1638	RSRS19	-122.451	37.944	0.43	15.79	291.17	16.22	-122.451	37.944	0.21	15.79	289.49	16.00	2	7/10/2014	37:54.7
1639	RSRS19	-122.451	37.944	0.47	15.76	291.10	16.23	-122.451	37.944	0.13	15.76	289.39	15.89	2	7/10/2014	37:54.8
1640	RSRS19	-122.451	37.944	0.47	15.73	291.08	16.19	-122.451	37.944	0.07	15.73	289.39	15.80	2	7/10/2014	37:54.9
1641	RSRS19	-122.451	37.944	0.47	15.73	291.11	16.20	-122.451	37.944	0.13	15.73	289.37	15.86	2	7/10/2014	37:54.9
1642	RSRS19	-122.451	37.944	0.43	15.73	291.02	16.16	-122.451	37.944	0.07	15.73	289.32	15.80	2	7/10/2014	37:55.1
1643	RSRS19	-122.451	37.944	0.47	15.73	291.06	16.19	-122.451	37.944	0.29	15.73	289.33	16.02	2	7/10/2014	37:55.2
1644	RSRS19	-122.451	37.944	0.43	15.73	291.15	16.16	-122.451	37.944	0.13	15.73	289.46	15.85	2	7/10/2014	37:55.3
1645	RSRS19	-122.451	37.944	0.43	15.73	291.11	16.16	-122.451	37.944	0.13	15.73	289.39	15.85	2	7/10/2014	37:55.4
1646	RSRS19	-122.451	37.944	0.43	15.70	291.07	16.14	-122.451	37.944	0.24	15.70	289.39	15.95	2	7/10/2014	37:55.5
1647	RSRS19	-122.451	37.944	0.47	15.70	291.00	16.17	-122.451	37.944	0.04	15.70	289.33	15.74	2	7/10/2014	37:55.5
1648	RSRS19	-122.451	37.944	0.43	15.73	291.02	16.17	-122.451	37.944	0.16	15.73	289.33	15.89	2	7/10/2014	37:55.7
1649	RSRS19	-122.451	37.944	0.47	15.72	290.98	16.18	-122.451	37.944	0.24	15.72	289.27	15.96	2	7/10/2014	37:55.7

1650	RSRS19	-122.451	37.944	0.47	15.76	290.98	16.22	-122.451	37.944	0.16	15.76	289.28	15.92	2	7/10/2014	37:55.9
1651	RSRS19	-122.451	37.944	0.47	15.75	291.05	16.21	-122.451	37.944	0.24	15.75	289.33	15.99	2	7/10/2014	37:56.0
1652	RSRS19	-122.451	37.944	0.47	15.84	290.92	16.31	-122.451	37.944	0.13	15.84	289.22	15.97	2	7/10/2014	37:56.0
1653	RSRS19	-122.451	37.944	0.47	15.83	290.94	16.29	-122.451	37.944	0.24	15.83	289.25	16.07	2	7/10/2014	37:56.1
1654	RSRS19	-122.451	37.944	0.43	15.74	291.00	16.17	-122.451	37.944	0.24	15.74	289.31	15.99	2	7/10/2014	37:56.3
1655	RSRS19	-122.451	37.944	0.47	15.79	290.94	16.25	-122.451	37.944	0.13	15.79	289.29	15.91	2	7/10/2014	37:56.4
1656	RSRS19	-122.451	37.944	0.43	15.73	290.98	16.17	-122.451	37.944	0.29	15.73	289.23	16.03	2	7/10/2014	37:56.5
1657	RSRS19	-122.451	37.944	0.47	15.73	290.94	16.19	-122.451	37.944	0.16	15.73	289.25	15.88	2	7/10/2014	37:56.5
1658	RSRS19	-122.451	37.944	0.43	15.73	290.90	16.16	-122.451	37.944	0.07	15.73	289.25	15.80	2	7/10/2014	37:56.7
1659	RSRS19	-122.451	37.944	0.47	15.73	290.92	16.20	-122.451	37.944	0.33	15.73	289.23	16.06	2	7/10/2014	37:56.8
1660	RSRS19	-122.451	37.944	0.43	15.76	290.97	16.19	-122.451	37.944	0.13	15.76	289.14	15.89	2	7/10/2014	37:56.9
1661	RSRS19	-122.451	37.944	0.47	15.77	290.81	16.24	-122.451	37.944	0.24	15.77	289.20	16.02	2	7/10/2014	37:56.9
1662	RSRS19	-122.451	37.944	0.43	15.80	290.83	16.23	-122.451	37.944	0.24	15.80	289.17	16.04	2	7/10/2014	37:57.1
1663	RSRS19	-122.451	37.944	0.47	15.76	290.88	16.22	-122.451	37.944	0.13	15.76	289.14	15.88	2	7/10/2014	37:57.1
1664	RSRS19	-122.451	37.944	0.43	15.76	290.84	16.19	-122.451	37.944	0.38	15.76	289.11	16.14	2	7/10/2014	37:57.3
1665	RSRS19	-122.451	37.944	0.47	15.75	290.88	16.21	-122.451	37.944	0.21	15.75	289.12	15.96	2	7/10/2014	37:57.4
1666	RSRS19	-122.451	37.944	0.43	15.72	290.75	16.15	-122.451	37.944	0.24	15.72	289.08	15.96	2	7/10/2014	37:57.5
1667	RSRS19	-122.451	37.944	0.47	15.72	290.81	16.18	-122.451	37.944	0.29	15.72	289.12	16.01	2	7/10/2014	37:57.6
1668	RSRS19	-122.451	37.944	0.47	15.71	290.80	16.18	-122.451	37.944	0.12	15.71	289.08	15.83	2	7/10/2014	37:57.7
1669	RSRS19	-122.451	37.944	0.47	15.71	290.80	16.18	-122.451	37.944	0.33	15.71	289.08	16.04	2	7/10/2014	37:57.7
1670	RSRS19	-122.451	37.944	0.47	15.72	290.84	16.18	-122.451	37.944	0.21	15.72	289.13	15.93	2	7/10/2014	37:57.9
1671	RSRS19	-122.451	37.944	0.47	15.73	290.86	16.19	-122.451	37.944	0.21	15.73	289.17	15.93	2	7/10/2014	37:58.0
1672	RSRS19	-122.451	37.944	0.43	15.78	290.88	16.21	-122.451	37.944	0.38	15.78	289.20	16.16	2	7/10/2014	37:58.1
1673	RSRS19	-122.451	37.944	0.47	15.81	290.89	16.28	-122.451	37.944	0.12	15.81	289.19	15.93	2	7/10/2014	37:58.1
1674	RSRS19	-122.451	37.944	0.43	15.77	290.84	16.20	-122.451	37.944	0.29	15.77	289.17	16.07	2	7/10/2014	37:58.3
1675	RSRS19	-122.451	37.944	0.47	15.74	290.85	16.21	-122.451	37.944	0.29	15.74	289.15	16.03	2	7/10/2014	37:58.3
1676	RSRS19	-122.451	37.944	0.47	15.73	290.78	16.20	-122.451	37.944	0.16	15.73	289.06	15.89	2	7/10/2014	37:58.5
1677	RSRS19	-122.451	37.944	0.47	15.74	290.73	16.21	-122.451	37.944	0.33	15.74	289.09	16.07	2	7/10/2014	37:58.5
1678	RSRS19	-122.451	37.944	0.47	15.73	290.78	16.20	-122.451	37.944	0.24	15.73	289.04	15.98	2	7/10/2014	37:58.7
1679	RSRS19	-122.451	37.944	0.47	15.76	290.72	16.23	-122.451	37.944	0.24	15.76	289.03	16.01	2	7/10/2014	37:58.8
1680	RSRS19	-122.451	37.944	0.43	15.74	290.76	16.17	-122.451	37.944	0.38	15.74	289.07	16.12	2	7/10/2014	37:58.8
1681	RSRS19	-122.451	37.944	0.47	15.75	290.83	16.21	-122.451	37.944	0.16	15.75	289.13	15.91	2	7/10/2014	37:58.9
1682	RSRS19	-122.451	37.944	0.43	15.76	290.85	16.19	-122.451	37.944	0.29	15.76	289.17	16.05	2	7/10/2014	37:59.1

1683	RSRS19	-122.451	37.944	0.47	15.78	290.89	16.24	-122.451	37.944	0.33	15.78	289.13	16.11	2	7/10/2014	37:59.2
1684	RSRS19	-122.451	37.944	0.43	15.80	290.85	16.23	-122.451	37.944	0.12	15.80	289.13	15.92	2	7/10/2014	37:59.3
1685	RSRS19	-122.451	37.944	0.47	15.79	290.83	16.25	-122.451	37.944	0.38	15.79	289.09	16.17	2	7/10/2014	37:59.4
1686	RSRS19	-122.451	37.944	0.47	15.82	290.79	16.28	-122.451	37.944	0.24	15.82	289.09	16.06	2	7/10/2014	37:59.5
1687	RSRS19	-122.451	37.944	0.47	15.84	290.79	16.31	-122.451	37.944	0.21	15.84	289.05	16.05	2	7/10/2014	37:59.6
1688	RSRS19	-122.451	37.944	0.43	15.81	290.76	16.24	-122.451	37.944	0.29	15.81	289.05	16.10	2	7/10/2014	37:59.7
1689	RSRS19	-122.451	37.944	0.47	15.80	290.76	16.27	-122.451	37.944	0.16	15.80	289.05	15.96	2	7/10/2014	37:59.8
1690	RSRS19	-122.451	37.944	0.47	15.81	290.77	16.28	-122.451	37.944	0.29	15.81	289.08	16.10	2	7/10/2014	37:59.9
1691	RSRS19	-122.451	37.944	0.47	15.82	290.79	16.28	-122.451	37.944	0.29	15.82	289.09	16.11	2	7/10/2014	37:59.9
1692	RSRS19	-122.451	37.944	0.47	15.78	290.81	16.24	-122.451	37.944	0.12	15.78	289.10	15.90	2	7/10/2014	38:00.1
1693	RSRS19	-122.451	37.944	0.47	15.76	290.81	16.23	-122.451	37.944	0.29	15.76	289.12	16.06	2	7/10/2014	38:00.1
1694	RSRS19	-122.451	37.944	0.47	15.74	290.81	16.21	-122.451	37.944	0.21	15.74	289.14	15.95	2	7/10/2014	38:00.3
1695	RSRS19	-122.451	37.944	0.47	15.77	290.77	16.24	-122.451	37.944	0.16	15.77	289.12	15.93	2	7/10/2014	38:00.4
1696	RSRS19	-122.451	37.944	0.43	15.71	290.82	16.14	-122.451	37.944	0.29	15.71	289.16	16.00	2	7/10/2014	38:00.5
1697	RSRS19	-122.451	37.944	0.47	15.72	290.77	16.18	-122.451	37.944	0.16	15.72	289.12	15.88	2	7/10/2014	38:00.5
1698	RSRS19	-122.451	37.944	0.43	15.70	290.82	16.13	-122.451	37.944	0.29	15.70	289.19	16.00	2	7/10/2014	38:00.7
1699	RSRS19	-122.451	37.944	0.47	15.74	290.82	16.21	-122.451	37.944	0.29	15.74	289.14	16.03	2	7/10/2014	38:00.8
1700	RSRS19	-122.451	37.944	0.43	15.74	290.82	16.17	-122.451	37.944	0.12	15.74	289.17	15.87	2	7/10/2014	38:00.9
1701	RSRS19	-122.451	37.944	0.47	15.72	290.82	16.18	-122.451	37.944	0.29	15.72	289.17	16.01	2	7/10/2014	38:00.9
1702	RSRS19	-122.451	37.944	0.47	15.72	290.86	16.18	-122.451	37.944	0.21	15.72	289.17	15.93	2	7/10/2014	38:01.1
1703	RSRS19	-122.451	37.944	0.47	15.74	290.84	16.21	-122.451	37.944	0.16	15.74	289.17	15.90	2	7/10/2014	38:01.1
1704	RSRS19	-122.451	37.944	0.43	15.75	290.84	16.18	-122.451	37.944	0.33	15.75	289.19	16.08	2	7/10/2014	38:01.3
1705	RSRS19	-122.451	37.944	0.47	15.78	290.80	16.24	-122.451	37.944	0.16	15.78	289.13	15.94	2	7/10/2014	38:01.4
1706	RSRS19	-122.451	37.944	0.47	15.78	290.84	16.24	-122.451	37.944	0.24	15.78	289.19	16.02	2	7/10/2014	38:01.5
1707	RSRS19	-122.451	37.944	0.47	15.80	290.91	16.26	-122.451	37.944	0.21	15.80	289.31	16.00	2	7/10/2014	38:01.6
1708	RSRS19	-122.451	37.944	0.43	15.80	290.93	16.23	-122.451	37.944	0.16	15.80	289.29	15.95	2	7/10/2014	38:01.6
1709	RSRS19	-122.451	37.944	0.47	15.84	290.91	16.31	-122.451	37.944	0.29	15.84	289.26	16.14	2	7/10/2014	38:01.8
1710	RSRS19	-122.451	37.944	0.43	15.81	290.93	16.24	-122.451	37.944	0.04	15.81	289.20	15.85	2	7/10/2014	38:01.9
1711	RSRS19	-122.451	37.944	0.47	15.80	290.91	16.26	-122.451	37.944	0.21	15.80	289.18	16.00	2	7/10/2014	38:02.0
1712	RSRS19	-122.451	37.944	0.43	15.81	290.94	16.24	-122.451	37.944	0.21	15.81	289.16	16.02	2	7/10/2014	38:02.1
1713	RSRS19	-122.451	37.944	0.47	15.79	290.94	16.25	-122.451	37.944	0.07	15.79	289.16	15.86	2	7/10/2014	38:02.1
1714	RSRS19	-122.451	37.944	0.43	15.79	290.96	16.22	-122.451	37.944	0.24	15.79	289.15	16.03	2	7/10/2014	38:02.3
1715	RSRS19	-122.451	37.944	0.47	15.77	290.94	16.24	-122.451	37.944	0.24	15.77	289.22	16.02	2	7/10/2014	38:02.4

1716	RSRS19	-122.451	37.944	0.47	15.77	291.01	16.24	-122.451	37.944	0.04	15.77	289.27	15.81	2	7/10/2014	38:02.5
1717	RSRS19	-122.451	37.944	0.47	15.76	290.96	16.23	-122.451	37.944	0.29	15.76	289.29	16.06	2	7/10/2014	38:02.5
1718	RSRS19	-122.451	37.944	0.43	15.83	290.94	16.26	-122.451	37.944	0.07	15.83	289.25	15.90	2	7/10/2014	38:02.7
1719	RSRS19	-122.451	37.944	0.47	15.77	290.94	16.24	-122.451	37.944	0.29	15.77	289.29	16.07	2	7/10/2014	38:02.7
1720	RSRS19	-122.451	37.944	0.43	15.76	290.90	16.19	-122.451	37.944	0.24	15.76	289.25	16.00	2	7/10/2014	38:02.9
1721	RSRS19	-122.451	37.944	0.43	15.73	290.88	16.16	-122.451	37.944	0.07	15.73	289.25	15.80	2	7/10/2014	38:02.9
1722	RSRS19	-122.451	37.944	0.47	15.73	290.88	16.20	-122.451	37.944	0.21	15.73	289.23	15.94	2	7/10/2014	38:03.1
1723	RSRS19	-122.451	37.944	0.47	15.76	290.83	16.22	-122.451	37.944	0.21	15.76	289.23	15.97	2	7/10/2014	38:03.2
1724	RSRS19	-122.451	37.944	0.47	15.82	290.86	16.28	-122.451	37.944	0.07	15.82	289.21	15.89	2	7/10/2014	38:03.3
1725	RSRS19	-122.451	37.944	0.47	15.77	290.88	16.24	-122.451	37.944	0.24	15.77	289.21	16.02	2	7/10/2014	38:03.3
1726	RSRS19	-122.451	37.944	0.43	15.86	290.79	16.30	-122.451	37.944	0.16	15.86	289.14	16.02	2	7/10/2014	38:03.5
1727	RSRS19	-122.451	37.944	0.47	15.86	290.88	16.32	-122.451	37.944	0.12	15.86	289.16	15.98	2	7/10/2014	38:03.6
1728	RSRS19	-122.451	37.944	0.47	15.91	290.94	16.38	-122.451	37.944	0.24	15.91	289.23	16.15	2	7/10/2014	38:03.7
1729	RSRS19	-122.451	37.944	0.47	15.93	290.93	16.40	-122.451	37.944	0.04	15.93	289.30	15.97	2	7/10/2014	38:03.8
1730	RSRS19	-122.451	37.944	0.43	15.86	290.97	16.30	-122.451	37.944	0.29	15.86	289.32	16.16	2	7/10/2014	38:03.9
1731	RSRS19	-122.451	37.944	0.47	15.97	290.95	16.43	-122.451	37.944	0.16	15.97	289.28	16.12	2	7/10/2014	38:04.0
1732	RSRS19	-122.451	37.944	0.43	15.89	290.93	16.32	-122.451	37.944	0.04	15.89	289.23	15.93	2	7/10/2014	38:04.1
1733	RSRS19	-122.451	37.944	0.43	15.89	290.86	16.32	-122.451	37.944	0.29	15.89	289.17	16.18	2	7/10/2014	38:04.1
1734	RSRS19	-122.451	37.944	0.43	15.90	290.86	16.33	-122.451	37.944	0.04	15.90	289.13	15.94	2	7/10/2014	38:04.3
1735	RSRS19	-122.451	37.944	0.47	15.93	290.89	16.40	-122.451	37.944	0.21	15.93	289.08	16.14	2	7/10/2014	38:04.4
1736	RSRS19	-122.451	37.944	0.43	15.93	290.86	16.36	-122.451	37.944	0.21	15.93	289.10	16.14	2	7/10/2014	38:04.4
1737	RSRS19	-122.451	37.944	0.47	16.00	290.89	16.46	-122.451	37.944	0.01	16.00	289.08	16.00	2	7/10/2014	38:04.5
1738	RSRS19	-122.451	37.944	0.43	15.97	290.86	16.40	-122.451	37.944	0.21	15.97	289.06	16.18	2	7/10/2014	38:04.7
1739	RSRS19	-122.451	37.944	0.47	16.01	290.91	16.48	-122.451	37.944	-0.05	16.01	289.13	15.97	2	7/10/2014	38:04.8
1740	RSRS19	-122.451	37.944	0.43	16.00	290.89	16.43	-122.451	37.944	0.16	16.00	289.13	16.15	2	7/10/2014	38:04.9
1741	RSRS19	-122.451	37.944	0.47	15.98	290.85	16.45	-122.451	37.944	0.21	15.98	289.11	16.19	2	7/10/2014	38:04.9
1742	RSRS19	-122.451	37.944	0.43	15.97	290.76	16.40	-122.451	37.944	-0.05	15.97	289.09	15.93	2	7/10/2014	38:05.1
1743	RSRS19	-122.451	37.944	0.43	15.98	290.74	16.41	-122.451	37.944	0.04	15.98	289.07	16.02	2	7/10/2014	38:05.2
1744	RSRS19	-122.451	37.944	0.43	15.95	290.74	16.38	-122.451	37.944	0.12	15.95	289.09	16.07	2	7/10/2014	38:05.2
1745	RSRS19	-122.451	37.944	0.47	16.01	290.78	16.48	-122.451	37.944	0.07	16.01	289.07	16.08	2	7/10/2014	38:05.4
1746	RSRS19	-122.451	37.944	0.43	15.96	290.83	16.39	-122.451	37.944	0.16	15.96	289.07	16.11	2	7/10/2014	38:05.5
1747	RSRS19	-122.451	37.944	0.47	15.96	290.83	16.42	-122.451	37.944	0.04	15.96	289.09	16.00	2	7/10/2014	38:05.5
1748	RSRS19	-122.451	37.944	0.43	16.00	290.83	16.43	-122.451	37.944	0.04	16.00	289.07	16.04	2	7/10/2014	38:05.7

1749	RSRS19	-122.451	37.944	0.47	15.97	290.85	16.44	-122.451	37.944	0.21	15.97	289.05	16.18	2	7/10/2014	38:05.8
1750	RSRS19	-122.451	37.944	0.43	16.00	290.83	16.43	-122.451	37.944	-0.05	16.00	289.09	15.95	2	7/10/2014	38:05.9
1751	RSRS19	-122.451	37.944	0.43	16.03	290.77	16.46	-122.451	37.944	0.21	16.03	289.01	16.23	2	7/10/2014	38:06.0
1752	RSRS19	-122.451	37.944	0.43	16.08	290.73	16.51	-122.451	37.944	-0.05	16.08	289.05	16.03	2	7/10/2014	38:06.1
1753	RSRS19	-122.451	37.944	0.43	16.05	290.77	16.48	-122.451	37.944	0.12	16.05	289.07	16.18	2	7/10/2014	38:06.1
1754	RSRS19	-122.451	37.944	0.43	15.99	290.81	16.42	-122.451	37.944	0.07	15.99	289.21	16.06	2	7/10/2014	38:06.3
1755	RSRS19	-122.451	37.944	0.43	15.99	290.81	16.42	-122.451	37.944	-0.08	15.99	289.21	15.91	2	7/10/2014	38:06.4
1756	RSRS19	-122.451	37.944	0.43	15.98	290.74	16.41	-122.451	37.944	0.16	15.98	289.18	16.14	2	7/10/2014	38:06.5
1757	RSRS19	-122.451	37.944	0.47	16.03	290.73	16.49	-122.451	37.944	0.07	16.03	289.21	16.10	2	7/10/2014	38:06.5
1758	RSRS19	-122.451	37.944	0.40	16.03	290.70	16.43	-122.451	37.944	-0.08	16.03	289.17	15.96	2	7/10/2014	38:06.7
1759	RSRS19	-122.451	37.944	0.47	15.97	290.71	16.44	-122.451	37.944	0.07	15.97	289.19	16.05	2	7/10/2014	38:06.8
1760	RSRS19	-122.451	37.944	0.43	15.99	290.70	16.42	-122.451	37.944	0.12	15.99	289.16	16.11	2	7/10/2014	38:06.9
1761	RSRS19	-122.451	37.944	0.43	15.98	290.68	16.41	-122.451	37.944	0.04	15.98	289.08	16.02	2	7/10/2014	38:06.9
1762	RSRS19	-122.451	37.944	0.43	15.97	290.75	16.40	-122.451	37.944	0.16	15.97	289.08	16.13	2	7/10/2014	38:07.1
1763	RSRS19	-122.451	37.944	0.43	15.98	290.66	16.41	-122.451	37.944	0.07	15.98	289.06	16.05	2	7/10/2014	38:07.2
1764	RSRS19	-122.451	37.944	0.43	15.98	290.71	16.41	-122.451	37.944	0.01	15.98	289.02	15.99	2	7/10/2014	38:07.2
1765	RSRS19	-122.451	37.944	0.47	15.99	290.75	16.45	-122.451	37.944	0.16	15.99	289.06	16.15	2	7/10/2014	38:07.4
1766	RSRS19	-122.451	37.944	0.43	15.98	290.77	16.41	-122.451	37.944	-0.16	15.98	289.06	15.82	2	7/10/2014	38:07.5
1767	RSRS19	-122.451	37.944	0.43	16.01	290.69	16.44	-122.451	37.944	0.21	16.01	289.08	16.22	2	7/10/2014	38:07.6
1768	RSRS19	-122.451	37.944	0.43	16.06	290.67	16.49	-122.451	37.944	0.12	16.06	289.02	16.18	2	7/10/2014	38:07.7
1769	RSRS19	-122.451	37.944	0.43	15.99	290.60	16.42	-122.451	37.944	-0.05	15.99	288.98	15.94	2	7/10/2014	38:07.8
1770	RSRS19	-122.451	37.944	0.43	16.00	290.58	16.43	-122.451	37.944	0.07	16.00	288.93	16.07	2	7/10/2014	38:07.9
1771	RSRS19	-122.451	37.944	0.43	15.98	290.60	16.41	-122.451	37.944	0.07	15.98	288.93	16.05	2	7/10/2014	38:08.0
1772	RSRS19	-122.451	37.944	0.43	16.01	290.60	16.44	-122.451	37.944	0.01	16.01	288.93	16.02	2	7/10/2014	38:08.1
1773	RSRS19	-122.451	37.944	0.47	16.00	290.63	16.47	-122.451	37.944	0.21	16.00	288.91	16.21	2	7/10/2014	38:08.2
1774	RSRS19	-122.451	37.944	0.43	16.02	290.58	16.45	-122.451	37.944	0.16	16.02	288.89	16.18	2	7/10/2014	38:08.3
1775	RSRS19	-122.451	37.944	0.47	16.06	290.65	16.53	-122.451	37.944	0.01	16.06	288.91	16.07	2	7/10/2014	38:08.3
1776	RSRS19	-122.451	37.944	0.43	16.05	290.58	16.48	-122.451	37.944	0.12	16.05	288.92	16.18	2	7/10/2014	38:08.5
1777	RSRS19	-122.451	37.944	0.43	16.07	290.63	16.50	-122.451	37.944	0.12	16.07	288.91	16.20	2	7/10/2014	38:08.6
1778	RSRS19	-122.451	37.944	0.43	16.03	290.63	16.47	-122.451	37.944	0.12	16.03	288.98	16.16	2	7/10/2014	38:08.7
1779	RSRS19	-122.451	37.944	0.43	16.03	290.61	16.47	-122.451	37.944	0.21	16.03	289.05	16.24	2	7/10/2014	38:08.8
1780	RSRS19	-122.451	37.944	0.43	16.03	290.63	16.46	-122.451	37.944	-0.05	16.03	289.05	15.98	2	7/10/2014	38:08.9
1781	RSRS19	-122.451	37.944	0.43	15.97	290.63	16.40	-122.451	37.944	0.12	15.97	289.12	16.09	2	7/10/2014	38:09.0

1782	RSRS19	-122.451	37.944	0.43	15.93	290.63	16.37	-122.451	37.944	0.12	15.93	289.11	16.06	2	7/10/2014	38:09.1
1783	RSRS19	-122.451	37.944	0.47	15.97	290.70	16.43	-122.451	37.944	0.07	15.97	289.14	16.04	2	7/10/2014	38:09.2
1784	RSRS19	-122.451	37.944	0.43	15.97	290.72	16.40	-122.451	37.944	0.21	15.97	289.20	16.17	2	7/10/2014	38:09.3
1785	RSRS19	-122.452	37.944	0.43	15.93	290.79	16.36	-122.451	37.944	0.16	15.93	289.23	16.09	2	7/10/2014	38:09.4
1786	RSRS19	-122.452	37.944	0.43	15.96	290.86	16.39	-122.452	37.944	0.04	15.96	289.26	16.00	2	7/10/2014	38:09.5
1787	RSRS19	-122.452	37.944	0.43	15.98	290.88	16.41	-122.452	37.944	0.24	15.98	289.25	16.22	2	7/10/2014	38:09.6
1788	RSRS19	-122.452	37.944	0.43	15.95	290.88	16.38	-122.452	37.944	0.04	15.95	289.23	15.99	2	7/10/2014	38:09.7
1789	RSRS19	-122.452	37.944	0.47	16.02	290.88	16.48	-122.452	37.944	0.24	16.02	289.26	16.26	2	7/10/2014	38:09.8
1790	RSRS19	-122.452	37.944	0.43	15.99	290.84	16.42	-122.452	37.944	0.21	15.99	289.23	16.20	2	7/10/2014	38:09.9
1791	RSRS19	-122.452	37.944	0.47	15.96	290.77	16.42	-122.452	37.944	0.12	15.96	289.26	16.08	2	7/10/2014	38:10.0
1792	RSRS19	-122.452	37.944	0.43	15.94	290.75	16.37	-122.452	37.944	0.16	15.94	289.23	16.10	2	7/10/2014	38:10.0
1793	RSRS19	-122.452	37.944	0.47	15.94	290.77	16.40	-122.452	37.944	0.12	15.94	289.26	16.06	2	7/10/2014	38:10.2
1794	RSRS19	-122.452	37.944	0.43	15.90	290.75	16.33	-122.452	37.944	0.16	15.90	289.26	16.06	2	7/10/2014	38:10.3
1795	RSRS19	-122.452	37.944	0.43	15.86	290.75	16.30	-122.452	37.944	0.33	15.86	289.28	16.19	2	7/10/2014	38:10.4
1796	RSRS19	-122.452	37.944	0.43	15.85	290.80	16.28	-122.452	37.944	0.12	15.85	289.28	15.97	2	7/10/2014	38:10.5
1797	RSRS19	-122.452	37.944	0.47	15.86	290.78	16.33	-122.452	37.944	0.21	15.86	289.26	16.07	2	7/10/2014	38:10.6
1798	RSRS19	-122.452	37.944	0.43	15.88	290.78	16.31	-122.452	37.944	0.29	15.88	289.23	16.17	2	7/10/2014	38:10.7
1799	RSRS19	-122.452	37.944	0.47	15.83	290.78	16.29	-122.452	37.944	0.29	15.83	289.17	16.12	2	7/10/2014	38:10.8
1800	RSRS19	-122.452	37.944	0.43	15.90	290.75	16.33	-122.452	37.944	0.21	15.90	289.17	16.11	2	7/10/2014	38:10.9
1801	RSRS19	-122.452	37.944	0.47	15.87	290.71	16.34	-122.452	37.944	0.12	15.87	289.15	16.00	2	7/10/2014	38:11.0
1802	RSRS19	-122.452	37.944	0.47	15.88	290.76	16.35	-122.452	37.944	0.33	15.88	289.14	16.21	2	7/10/2014	38:11.1
1803	RSRS19	-122.452	37.944	0.47	15.88	290.71	16.35	-122.452	37.944	0.21	15.88	289.11	16.09	2	7/10/2014	38:11.1
1804	RSRS19	-122.452	37.944	0.43	15.88	290.67	16.31	-122.452	37.944	0.04	15.88	289.06	15.92	2	7/10/2014	38:11.3
1805	RSRS19	-122.452	37.944	0.47	15.87	290.63	16.34	-122.452	37.944	0.16	15.87	289.07	16.03	2	7/10/2014	38:11.4
1806	RSRS19	-122.452	37.944	0.43	15.86	290.63	16.29	-122.452	37.944	0.24	15.86	289.11	16.10	2	7/10/2014	38:11.5
1807	RSRS19	-122.452	37.944	0.47	15.92	290.59	16.38	-122.452	37.944	0.29	15.92	289.07	16.21	2	7/10/2014	38:11.6
1808	RSRS19	-122.452	37.944	0.47	15.90	290.59	16.37	-122.452	37.944	0.21	15.90	289.07	16.11	2	7/10/2014	38:11.7
1809	RSRS19	-122.452	37.944	0.47	15.89	290.54	16.35	-122.452	37.944	0.16	15.89	289.03	16.05	2	7/10/2014	38:11.8
1810	RSRS19	-122.452	37.944	0.43	15.88	290.56	16.31	-122.452	37.944	0.29	15.88	289.00	16.17	2	7/10/2014	38:11.9
1811	RSRS19	-122.452	37.944	0.47	15.95	290.59	16.41	-122.452	37.944	0.12	15.95	289.01	16.07	2	7/10/2014	38:12.0
1812	RSRS19	-122.452	37.944	0.47	15.90	290.63	16.37	-122.452	37.944	0.29	15.90	289.05	16.20	2	7/10/2014	38:12.1
1813	RSRS19	-122.452	37.944	0.47	15.92	290.63	16.38	-122.452	37.944	0.21	15.92	289.07	16.13	2	7/10/2014	38:12.2
1814	RSRS19	-122.452	37.944	0.43	15.93	290.59	16.37	-122.452	37.944	0.16	15.93	289.03	16.09	2	7/10/2014	38:12.3

1815	RSRS19	-122.452	37.944	0.43	15.93	290.57	16.37	-122.452	37.944	0.29	15.93	288.96	16.23	2	7/10/2014	38:12.4
1816	RSRS19	-122.452	37.944	0.43	15.94	290.52	16.37	-122.452	37.944	0.16	15.94	288.94	16.10	2	7/10/2014	38:12.5
1817	RSRS19	-122.452	37.944	0.47	16.00	290.50	16.46	-122.452	37.944	0.07	16.00	288.94	16.07	2	7/10/2014	38:12.6
1818	RSRS19	-122.452	37.944	0.43	15.96	290.55	16.39	-122.452	37.944	0.21	15.96	288.96	16.17	2	7/10/2014	38:12.7
1819	RSRS19	-122.452	37.944	0.47	15.91	290.46	16.38	-122.452	37.944	0.21	15.91	288.95	16.12	2	7/10/2014	38:12.8
1820	RSRS19	-122.452	37.944	0.43	15.93	290.42	16.36	-122.452	37.944	0.24	15.93	288.90	16.17	2	7/10/2014	38:12.8
1821	RSRS19	-122.452	37.944	0.43	15.94	290.37	16.37	-122.452	37.944	0.16	15.94	288.84	16.10	2	7/10/2014	38:13.0
1822	RSRS19	-122.452	37.944	0.43	16.04	290.42	16.47	-122.452	37.944	0.16	16.04	288.83	16.20	2	7/10/2014	38:13.1
1823	RSRS19	-122.452	37.944	0.46	15.95	290.44	16.41	-122.452	37.944	0.24	15.95	288.82	16.19	2	7/10/2014	38:13.2
1824	RSRS19	-122.452	37.944	0.43	15.94	290.42	16.37	-122.452	37.944	0.21	15.94	288.77	16.15	2	7/10/2014	38:13.3
1825	RSRS19	-122.452	37.944	0.43	16.03	290.42	16.46	-122.452	37.944	0.29	16.03	288.77	16.32	2	7/10/2014	38:13.4
1826	RSRS19	-122.452	37.944	0.43	15.96	290.45	16.39	-122.452	37.944	0.12	15.96	288.75	16.08	2	7/10/2014	38:13.5
1827	RSRS19	-122.452	37.944	0.46	16.02	290.42	16.48	-122.452	37.944	0.12	16.02	288.75	16.14	2	7/10/2014	38:13.6
1828	RSRS19	-122.452	37.944	0.46	16.08	290.42	16.54	-122.452	37.944	0.24	16.08	288.73	16.32	2	7/10/2014	38:13.7
1829	RSRS19	-122.452	37.944	0.43	16.02	290.40	16.45	-122.452	37.944	0.16	16.02	288.78	16.18	2	7/10/2014	38:13.8
1830	RSRS19	-122.452	37.944	0.43	16.03	290.40	16.46	-122.452	37.944	0.12	16.03	288.76	16.16	2	7/10/2014	38:13.9
1831	RSRS19	-122.452	37.944	0.43	16.06	290.36	16.49	-122.452	37.944	0.21	16.06	288.71	16.26	2	7/10/2014	38:13.9
1832	RSRS19	-122.452	37.944	0.43	16.15	290.32	16.58	-122.452	37.944	0.04	16.15	288.67	16.19	2	7/10/2014	38:14.1
1833	RSRS19	-122.452	37.944	0.46	16.09	290.27	16.56	-122.452	37.944	0.12	16.09	288.62	16.22	2	7/10/2014	38:14.2
1834	RSRS19	-122.452	37.944	0.43	16.13	290.27	16.56	-122.452	37.944	0.24	16.13	288.64	16.37	2	7/10/2014	38:14.3
1835	RSRS19	-122.452	37.944	0.46	16.16	290.30	16.62	-122.452	37.944	0.12	16.16	288.65	16.28	2	7/10/2014	38:14.4
1836	RSRS19	-122.452	37.944	0.43	16.22	290.25	16.65	-122.452	37.944	0.21	16.22	288.63	16.43	2	7/10/2014	38:14.5
1837	RSRS19	-122.452	37.944	0.46	16.17	290.25	16.63	-122.452	37.944	0.21	16.17	288.63	16.37	2	7/10/2014	38:14.6
1838	RSRS19	-122.452	37.944	0.43	16.15	290.21	16.58	-122.452	37.944	0.04	16.15	288.60	16.19	2	7/10/2014	38:14.7
1839	RSRS19	-122.452	37.944	0.46	16.15	290.12	16.61	-122.452	37.944	0.24	16.15	288.48	16.39	2	7/10/2014	38:14.7
1840	RSRS19	-122.452	37.944	0.43	16.21	290.10	16.64	-122.452	37.944	0.12	16.21	288.41	16.33	2	7/10/2014	38:14.9
1841	RSRS19	-122.452	37.944	0.46	16.15	290.10	16.61	-122.452	37.944	0.07	16.15	288.50	16.22	2	7/10/2014	38:15.0
1842	RSRS19	-122.452	37.944	0.43	16.19	290.15	16.62	-122.452	37.944	0.21	16.19	288.54	16.40	2	7/10/2014	38:15.1
1843	RSRS19	-122.452	37.944	0.43	16.15	290.10	16.58	-122.452	37.944	0.01	16.15	288.48	16.15	2	7/10/2014	38:15.2
1844	RSRS19	-122.452	37.944	0.43	16.15	290.08	16.58	-122.452	37.944	0.12	16.15	288.43	16.27	2	7/10/2014	38:15.3
1845	RSRS19	-122.452	37.944	0.46	16.14	289.97	16.61	-122.452	37.944	0.16	16.14	288.39	16.30	2	7/10/2014	38:15.4
1846	RSRS19	-122.452	37.944	0.43	16.10	289.95	16.53	-122.452	37.944	0.07	16.10	288.30	16.18	2	7/10/2014	38:15.5
1847	RSRS19	-122.452	37.944	0.43	16.13	289.93	16.56	-122.452	37.944	0.12	16.13	288.26	16.25	2	7/10/2014	38:15.6

1848	RSRS19	-122.452	37.944	0.43	16.09	289.91	16.52	-122.452	37.944	0.12	16.09	288.27	16.21	2	7/10/2014	38:15.6
1849	RSRS19	-122.452	37.944	0.43	16.15	289.87	16.58	-122.452	37.944	0.04	16.15	288.28	16.19	2	7/10/2014	38:15.8
1850	RSRS19	-122.452	37.944	0.43	16.12	289.89	16.55	-122.452	37.944	0.07	16.12	288.30	16.19	2	7/10/2014	38:15.9
1851	RSRS19	-122.452	37.944	0.46	16.04	289.82	16.51	-122.452	37.944	0.21	16.04	288.31	16.25	2	7/10/2014	38:16.0
1852	RSRS19	-122.452	37.944	0.43	16.03	289.89	16.46	-122.452	37.944	0.07	16.03	288.35	16.11	2	7/10/2014	38:16.0
1853	RSRS19	-122.452	37.944	0.46	16.12	289.85	16.59	-122.452	37.944	0.24	16.12	288.22	16.36	2	7/10/2014	38:16.2
1854	RSRS19	-122.452	37.944	0.43	16.04	289.74	16.47	-122.452	37.944	0.01	16.04	288.13	16.05	2	7/10/2014	38:16.3
1855	RSRS19	-122.452	37.944	0.43	16.03	289.76	16.46	-122.452	37.944	0.24	16.03	288.09	16.28	2	7/10/2014	38:16.4
1856	RSRS19	-122.452	37.944	0.43	16.06	289.87	16.49	-122.452	37.944	0.07	16.06	288.20	16.14	2	7/10/2014	38:16.5
1857	RSRS19	-122.452	37.944	0.46	16.11	289.85	16.58	-122.452	37.944	0.04	16.11	288.23	16.15	2	7/10/2014	38:16.6
1858	RSRS19	-122.452	37.944	0.43	16.07	289.83	16.50	-122.452	37.944	0.12	16.07	288.20	16.20	2	7/10/2014	38:16.7
1859	RSRS19	-122.452	37.944	0.43	16.10	289.85	16.53	-122.452	37.944	0.04	16.10	288.20	16.14	2	7/10/2014	38:16.7
1860	RSRS19	-122.452	37.944	0.43	16.11	289.81	16.54	-122.452	37.944	0.04	16.11	288.18	16.15	2	7/10/2014	38:16.9
1861	RSRS19	-122.452	37.944	0.43	16.09	289.78	16.52	-122.452	37.944	0.21	16.09	288.16	16.30	2	7/10/2014	38:17.0
1862	RSRS19	-122.452	37.944	0.43	16.11	289.77	16.54	-122.452	37.944	0.04	16.11	288.23	16.15	2	7/10/2014	38:17.1
1863	RSRS19	-122.452	37.944	0.43	16.09	289.77	16.52	-122.452	37.944	0.07	16.09	288.27	16.17	2	7/10/2014	38:17.2
1864	RSRS19	-122.452	37.944	0.40	16.11	289.75	16.51	-122.452	37.944	0.12	16.11	288.27	16.23	2	7/10/2014	38:17.3
1865	RSRS19	-122.452	37.944	0.43	16.06	289.72	16.49	-122.452	37.944	0.01	16.06	288.21	16.06	2	7/10/2014	38:17.4
1866	RSRS19	-122.452	37.944	0.43	16.08	289.75	16.51	-122.452	37.944	0.21	16.08	288.18	16.29	2	7/10/2014	38:17.5
1867	RSRS19	-122.452	37.944	0.43	16.04	289.75	16.47	-122.452	37.944	0.04	16.04	288.16	16.08	2	7/10/2014	38:17.6
1868	RSRS19	-122.452	37.944	0.43	16.03	289.82	16.46	-122.452	37.944	0.07	16.03	288.23	16.11	2	7/10/2014	38:17.7
1869	RSRS19	-122.452	37.944	0.43	16.09	289.77	16.52	-122.452	37.944	0.16	16.09	288.21	16.25	2	7/10/2014	38:17.8
1870	RSRS19	-122.452	37.944	0.43	16.09	289.79	16.52	-122.452	37.944	0.01	16.09	288.19	16.09	2	7/10/2014	38:17.9
1871	RSRS19	-122.452	37.944	0.43	16.06	289.84	16.49	-122.452	37.944	0.12	16.06	288.26	16.18	2	7/10/2014	38:18.0
1872	RSRS19	-122.452	37.944	0.43	16.07	289.84	16.50	-122.452	37.944	0.12	16.07	288.21	16.20	2	7/10/2014	38:18.1
1873	RSRS19	-122.452	37.944	0.43	16.05	289.82	16.48	-122.452	37.944	-0.08	16.05	288.21	15.97	2	7/10/2014	38:18.2
1874	RSRS19	-122.452	37.944	0.43	16.09	289.84	16.52	-122.452	37.944	0.21	16.09	288.28	16.30	2	7/10/2014	38:18.3
1875	RSRS19	-122.452	37.944	0.43	16.06	289.84	16.49	-122.452	37.944	0.07	16.06	288.29	16.13	2	7/10/2014	38:18.4
1876	RSRS19	-122.452	37.944	0.40	16.12	289.77	16.52	-122.452	37.944	0.07	16.12	288.28	16.19	2	7/10/2014	38:18.4
1877	RSRS19	-122.452	37.944	0.43	16.05	289.82	16.48	-122.452	37.944	0.16	16.05	288.26	16.21	2	7/10/2014	38:18.6
1878	RSRS19	-122.452	37.944	0.43	16.06	289.80	16.49	-122.452	37.944	0.01	16.06	288.26	16.07	2	7/10/2014	38:18.7
1879	RSRS19	-122.452	37.944	0.43	16.04	289.85	16.47	-122.452	37.944	0.21	16.04	288.24	16.25	2	7/10/2014	38:18.7
1880	RSRS19	-122.452	37.944	0.43	16.04	289.84	16.47	-122.452	37.944	0.04	16.04	288.24	16.08	2	7/10/2014	38:18.9

1881	RSRS19	-122.452	37.944	0.46	16.03	289.82	16.50	-122.452	37.944	0.16	16.03	288.18	16.19	2	7/10/2014	38:19.0
1882	RSRS19	-122.452	37.944	0.43	16.06	289.93	16.49	-122.452	37.944	0.04	16.06	288.26	16.10	2	7/10/2014	38:19.1
1883	RSRS19	-122.452	37.944	0.43	16.05	289.87	16.48	-122.452	37.944	0.04	16.05	288.20	16.09	2	7/10/2014	38:19.2
1884	RSRS19	-122.452	37.944	0.43	16.04	289.85	16.47	-122.452	37.944	0.12	16.04	288.26	16.16	2	7/10/2014	38:19.3
1885	RSRS19	-122.452	37.944	0.43	16.15	289.80	16.58	-122.452	37.944	0.01	16.15	288.18	16.15	2	7/10/2014	38:19.4
1886	RSRS19	-122.452	37.944	0.43	16.15	289.78	16.58	-122.452	37.944	0.04	16.15	288.22	16.19	2	7/10/2014	38:19.5
1887	RSRS19	-122.452	37.944	0.43	16.10	289.76	16.53	-122.452	37.944	0.16	16.10	288.20	16.26	2	7/10/2014	38:19.5
1888	RSRS19	-122.452	37.944	0.43	16.11	289.70	16.54	-122.452	37.944	0.04	16.11	288.15	16.15	2	7/10/2014	38:19.7
1889	RSRS19	-122.452	37.944	0.43	16.16	289.67	16.59	-122.452	37.944	0.16	16.16	288.11	16.32	2	7/10/2014	38:19.8
1890	RSRS19	-122.452	37.944	0.43	16.09	289.79	16.52	-122.452	37.944	0.12	16.09	288.16	16.22	2	7/10/2014	38:19.9
1891	RSRS19	-122.452	37.944	0.43	16.16	289.79	16.59	-122.452	37.944	-0.05	16.16	288.08	16.11	2	7/10/2014	38:20.0
1892	RSRS19	-122.452	37.944	0.43	16.13	289.81	16.56	-122.452	37.944	0.16	16.13	288.05	16.29	2	7/10/2014	38:20.1
1893	RSRS19	-122.452	37.944	0.43	16.18	289.88	16.61	-122.452	37.944	0.21	16.18	288.14	16.39	2	7/10/2014	38:20.2
1894	RSRS19	-122.452	37.944	0.40	16.23	289.87	16.62	-122.452	37.944	0.07	16.23	288.09	16.30	2	7/10/2014	38:20.3
1895	RSRS19	-122.452	37.944	0.43	16.23	289.79	16.66	-122.452	37.944	0.16	16.23	288.05	16.39	2	7/10/2014	38:20.4
1896	RSRS19	-122.452	37.944	0.43	16.21	289.73	16.64	-122.452	37.944	-0.05	16.21	288.05	16.17	2	7/10/2014	38:20.5
1897	RSRS19	-122.452	37.944	0.43	16.18	289.79	16.61	-122.452	37.944	0.16	16.18	288.12	16.34	2	7/10/2014	38:20.6
1898	RSRS19	-122.452	37.944	0.43	16.22	289.79	16.65	-122.452	37.944	0.12	16.22	288.14	16.34	2	7/10/2014	38:20.7
1899	RSRS19	-122.452	37.944	0.43	16.20	289.83	16.63	-122.452	37.944	-0.08	16.20	288.08	16.12	2	7/10/2014	38:20.8
1900	RSRS19	-122.452	37.944	0.43	16.20	289.75	16.63	-122.452	37.944	0.12	16.20	288.08	16.33	2	7/10/2014	38:20.9
1901	RSRS19	-122.452	37.944	0.43	16.23	289.73	16.66	-122.452	37.944	0.12	16.23	288.05	16.36	2	7/10/2014	38:21.0
1902	RSRS19	-122.452	37.944	0.43	16.24	289.75	16.67	-122.452	37.944	0.04	16.24	288.08	16.28	2	7/10/2014	38:21.1
1903	RSRS19	-122.452	37.944	0.43	16.21	289.75	16.64	-122.452	37.944	0.07	16.21	288.13	16.28	2	7/10/2014	38:21.2
1904	RSRS19	-122.452	37.944	0.43	16.17	289.75	16.60	-122.452	37.944	-0.13	16.17	288.10	16.03	2	7/10/2014	38:21.2
1905	RSRS19	-122.452	37.944	0.43	16.15	289.73	16.58	-122.452	37.944	0.12	16.15	288.06	16.27	2	7/10/2014	38:21.4
1906	RSRS19	-122.452	37.944	0.43	16.15	289.73	16.58	-122.452	37.944	0.04	16.15	287.99	16.19	2	7/10/2014	38:21.5
1907	RSRS19	-122.452	37.944	0.43	16.17	289.82	16.60	-122.452	37.944	-0.13	16.17	288.02	16.03	2	7/10/2014	38:21.6
1908	RSRS19	-122.452	37.944	0.43	16.14	289.82	16.57	-122.452	37.944	0.07	16.14	288.04	16.21	2	7/10/2014	38:21.7
1909	RSRS19	-122.452	37.944	0.43	16.18	289.89	16.61	-122.452	37.944	0.07	16.18	288.04	16.25	2	7/10/2014	38:21.8
1910	RSRS19	-122.452	37.944	0.43	16.23	289.84	16.66	-122.452	37.944	-0.05	16.23	288.04	16.19	2	7/10/2014	38:21.9
1911	RSRS19	-122.452	37.944	0.43	16.23	289.89	16.66	-122.452	37.944	0.12	16.23	288.09	16.36	2	7/10/2014	38:22.0
1912	RSRS19	-122.452	37.944	0.43	16.17	289.80	16.60	-122.452	37.944	-0.05	16.17	288.06	16.13	2	7/10/2014	38:22.1
1913	RSRS19	-122.452	37.944	0.43	16.19	289.78	16.62	-122.452	37.944	0.04	16.19	288.02	16.23	2	7/10/2014	38:22.2

1914	RSRS19	-122.452	37.944	0.40	16.20	289.72	16.59	-122.452	37.944	0.07	16.20	288.06	16.27	2	7/10/2014	38:22.3
1915	RSRS19	-122.452	37.944	0.43	16.19	289.74	16.62	-122.452	37.944	0.01	16.19	288.05	16.20	2	7/10/2014	38:22.3
1916	RSRS19	-122.452	37.944	0.40	16.20	289.73	16.60	-122.452	37.944	-0.05	16.20	288.11	16.16	2	7/10/2014	38:22.5
1917	RSRS19	-122.452	37.944	0.43	16.25	289.69	16.68	-122.452	37.944	0.01	16.25	288.04	16.26	2	7/10/2014	38:22.6
1918	RSRS19	-122.452	37.944	0.43	16.24	289.76	16.67	-122.452	37.944	-0.08	16.24	288.14	16.16	2	7/10/2014	38:22.7
1919	RSRS19	-122.452	37.944	0.43	16.27	289.85	16.70	-122.452	37.944	0.12	16.27	288.21	16.39	2	7/10/2014	38:22.8
1920	RSRS19	-122.452	37.944	0.43	16.27	289.76	16.70	-122.452	37.944	-0.05	16.27	288.16	16.23	2	7/10/2014	38:22.8
1921	RSRS19	-122.452	37.944	0.43	16.29	289.74	16.72	-122.452	37.944	0.01	16.29	288.07	16.29	2	7/10/2014	38:23.0
1922	RSRS19	-122.452	37.944	0.40	16.28	289.70	16.68	-122.452	37.944	0.07	16.28	287.94	16.35	2	7/10/2014	38:23.1
1923	RSRS19	-122.452	37.944	0.43	16.30	289.79	16.73	-122.452	37.944	0.01	16.30	287.98	16.31	2	7/10/2014	38:23.2
1924	RSRS19	-122.452	37.944	0.43	16.31	289.79	16.74	-122.452	37.944	0.04	16.31	287.99	16.35	2	7/10/2014	38:23.3
1925	RSRS19	-122.452	37.944	0.43	16.31	289.88	16.74	-122.452	37.944	0.12	16.31	287.96	16.44	2	7/10/2014	38:23.4
1926	RSRS19	-122.452	37.944	0.40	16.36	289.77	16.75	-122.452	37.944	0.01	16.36	287.96	16.36	2	7/10/2014	38:23.5
1927	RSRS19	-122.452	37.944	0.43	16.34	289.77	16.77	-122.452	37.944	0.01	16.34	287.99	16.35	2	7/10/2014	38:23.6
1928	RSRS19	-122.452	37.944	0.43	16.32	289.72	16.75	-122.452	37.944	0.04	16.32	287.94	16.36	2	7/10/2014	38:23.7
1929	RSRS19	-122.452	37.944	0.43	16.27	289.64	16.70	-122.452	37.944	0.04	16.27	287.90	16.31	2	7/10/2014	38:23.8
1930	RSRS19	-122.452	37.944	0.43	16.26	289.60	16.69	-122.452	37.944	-0.05	16.26	287.88	16.21	2	7/10/2014	38:23.9
1931	RSRS19	-122.452	37.944	0.43	16.25	289.64	16.68	-122.452	37.944	-0.08	16.25	287.88	16.17	2	7/10/2014	38:24.0
1932	RSRS19	-122.452	37.944	0.40	16.26	289.61	16.66	-122.452	37.944	0.07	16.26	287.91	16.33	2	7/10/2014	38:24.0
1933	RSRS19	-122.452	37.944	0.43	16.26	289.68	16.69	-122.452	37.944	0.12	16.26	287.90	16.38	2	7/10/2014	38:24.2
1934	RSRS19	-122.452	37.944	0.43	16.20	289.64	16.63	-122.452	37.944	0.07	16.20	287.88	16.27	2	7/10/2014	38:24.3
1935	RSRS19	-122.452	37.944	0.43	16.23	289.64	16.66	-122.452	37.944	0.04	16.23	287.89	16.27	2	7/10/2014	38:24.4
1936	RSRS19	-122.452	37.944	0.43	16.23	289.62	16.66	-122.452	37.944	-0.05	16.23	287.84	16.19	2	7/10/2014	38:24.5
1937	RSRS19	-122.452	37.944	0.40	16.17	289.58	16.56	-122.452	37.944	0.04	16.17	287.85	16.20	2	7/10/2014	38:24.6
1938	RSRS19	-122.452	37.944	0.43	16.16	289.62	16.59	-122.452	37.944	0.16	16.16	287.92	16.32	2	7/10/2014	38:24.7
1939	RSRS19	-122.452	37.944	0.43	16.17	289.64	16.60	-122.452	37.944	0.07	16.17	287.91	16.25	2	7/10/2014	38:24.8
1940	RSRS19	-122.452	37.944	0.43	16.14	289.64	16.57	-122.452	37.944	0.04	16.14	287.95	16.18	2	7/10/2014	38:24.9
1941	RSRS19	-122.452	37.944	0.43	16.15	289.69	16.58	-122.452	37.944	0.04	16.15	287.91	16.19	2	7/10/2014	38:25.0
1942	RSRS19	-122.452	37.944	0.43	16.17	289.67	16.60	-122.452	37.944	0.16	16.17	287.93	16.33	2	7/10/2014	38:25.1
1943	RSRS19	-122.452	37.944	0.43	16.17	289.71	16.60	-122.452	37.944	0.04	16.17	287.89	16.21	2	7/10/2014	38:25.1
1944	RSRS19	-122.452	37.944	0.40	16.17	289.65	16.56	-122.452	37.944	-0.05	16.17	287.80	16.12	2	7/10/2014	38:25.3
1945	RSRS19	-122.452	37.944	0.43	16.24	289.56	16.67	-122.452	37.944	0.16	16.24	287.73	16.40	2	7/10/2014	38:25.4
1946	RSRS19	-122.452	37.944	0.40	16.19	289.54	16.59	-122.452	37.944	0.07	16.19	287.69	16.26	2	7/10/2014	38:25.5

1947	RSRS19	-122.452	37.944	0.43	16.23	289.48	16.66	-122.452	37.944	0.01	16.23	287.71	16.24	2	7/10/2014	38:25.5
1948	RSRS19	-122.452	37.944	0.43	16.23	289.43	16.66	-122.452	37.944	0.04	16.23	287.71	16.27	2	7/10/2014	38:25.6
1949	RSRS19	-122.452	37.944	0.43	16.25	289.43	16.68	-122.452	37.944	-0.08	16.25	287.63	16.17	2	7/10/2014	38:25.8
1950	RSRS19	-122.452	37.944	0.40	16.28	289.43	16.68	-122.452	37.944	0.12	16.28	287.61	16.40	2	7/10/2014	38:25.9
1951	RSRS19	-122.452	37.944	0.43	16.25	289.45	16.68	-122.452	37.944	0.12	16.25	287.61	16.37	2	7/10/2014	38:26.0
1952	RSRS19	-122.452	37.944	0.43	16.29	289.52	16.72	-122.452	37.944	-0.13	16.29	287.63	16.16	2	7/10/2014	38:26.1
1953	RSRS19	-122.452	37.944	0.43	16.31	289.48	16.74	-122.452	37.944	0.12	16.31	287.55	16.44	2	7/10/2014	38:26.2
1954	RSRS19	-122.452	37.944	0.40	16.43	289.48	16.82	-122.452	37.944	0.07	16.43	287.52	16.50	2	7/10/2014	38:26.3
1955	RSRS19	-122.452	37.944	0.43	16.34	289.39	16.77	-122.452	37.944	-0.05	16.34	287.45	16.30	2	7/10/2014	38:26.4
1956	RSRS19	-122.452	37.944	0.43	16.42	289.33	16.85	-122.452	37.944	0.12	16.42	287.37	16.54	2	7/10/2014	38:26.5
1957	RSRS19	-122.452	37.944	0.43	16.38	289.23	16.81	-122.452	37.944	0.07	16.38	287.37	16.45	2	7/10/2014	38:26.6
1958	RSRS19	-122.452	37.944	0.43	16.39	289.22	16.82	-122.452	37.944	-0.08	16.39	287.35	16.31	2	7/10/2014	38:26.7
1959	RSRS19	-122.452	37.944	0.43	16.42	289.15	16.85	-122.452	37.944	0.04	16.42	287.33	16.46	2	7/10/2014	38:26.8
1960	RSRS19	-122.452	37.944	0.40	16.36	289.15	16.75	-122.452	37.944	0.07	16.36	287.37	16.43	2	7/10/2014	38:26.8
1961	RSRS19	-122.452	37.944	0.43	16.41	289.06	16.84	-122.452	37.944	0.12	16.41	287.33	16.53	2	7/10/2014	38:27.0
1962	RSRS19	-122.452	37.944	0.40	16.47	289.11	16.86	-122.452	37.944	0.12	16.47	287.29	16.59	2	7/10/2014	38:27.1
1963	RSRS19	-122.452	37.944	0.43	16.51	289.09	16.94	-122.452	37.944	-0.08	16.51	287.29	16.43	2	7/10/2014	38:27.2
1964	RSRS19	-122.452	37.944	0.43	16.40	289.11	16.83	-122.452	37.944	0.16	16.40	287.24	16.55	2	7/10/2014	38:27.3
1965	RSRS19	-122.452	37.944	0.43	16.47	289.15	16.90	-122.452	37.944	0.16	16.47	287.32	16.63	2	7/10/2014	38:27.4
1966	RSRS19	-122.452	37.944	0.40	16.41	289.20	16.80	-122.452	37.944	-0.08	16.41	287.33	16.33	2	7/10/2014	38:27.5
1967	RSRS19	-122.452	37.944	0.43	16.43	289.14	16.86	-122.452	37.944	0.16	16.43	287.31	16.58	2	7/10/2014	38:27.6
1968	RSRS19	-122.452	37.944	0.43	16.39	289.11	16.82	-122.452	37.944	0.16	16.39	287.31	16.55	2	7/10/2014	38:27.7
1969	RSRS19	-122.452	37.944	0.43	16.39	289.10	16.82	-122.452	37.944	0.07	16.39	287.29	16.46	2	7/10/2014	38:27.8
1970	RSRS19	-122.452	37.944	0.40	16.40	289.03	16.79	-122.452	37.944	0.07	16.40	287.27	16.47	2	7/10/2014	38:27.9
1971	RSRS19	-122.452	37.944	0.43	16.37	289.03	16.80	-122.452	37.944	-0.05	16.37	287.27	16.33	2	7/10/2014	38:27.9
1972	RSRS19	-122.452	37.944	0.43	16.27	289.01	16.70	-122.452	37.944	-0.05	16.27	287.20	16.23	2	7/10/2014	38:28.1
1973	RSRS19	-122.452	37.944	0.43	16.24	289.05	16.67	-122.452	37.944	0.12	16.24	287.21	16.37	2	7/10/2014	38:28.2
1974	RSRS19	-122.452	37.944	0.40	16.25	289.03	16.65	-122.452	37.944	0.12	16.25	287.23	16.37	2	7/10/2014	38:28.3
1975	RSRS19	-122.452	37.944	0.43	16.23	289.08	16.66	-122.452	37.944	0.16	16.23	287.16	16.39	2	7/10/2014	38:28.4
1976	RSRS19	-122.452	37.944	0.43	16.27	289.18	16.70	-122.452	37.944	0.04	16.27	287.32	16.31	2	7/10/2014	38:28.4
1977	RSRS19	-122.452	37.944	0.43	16.33	289.21	16.76	-122.452	37.944	0.07	16.33	287.36	16.41	2	7/10/2014	38:28.6
1978	RSRS19	-122.452	37.944	0.43	16.34	289.10	16.77	-122.452	37.944	0.16	16.34	287.26	16.50	2	7/10/2014	38:28.7
1979	RSRS19	-122.452	37.944	0.43	16.30	289.17	16.73	-122.452	37.944	0.04	16.30	287.30	16.34	2	7/10/2014	38:28.8

1980	RSRS19	-122.452	37.944	0.43	16.37	289.06	16.80	-122.452	37.944	0.04	16.37	287.21	16.41	2	7/10/2014	38:28.9
1981	RSRS19	-122.452	37.944	0.43	16.27	289.10	16.70	-122.452	37.944	0.21	16.27	287.21	16.48	2	7/10/2014	38:29.0
1982	RSRS19	-122.452	37.944	0.43	16.35	289.08	16.78	-122.452	37.944	0.16	16.35	287.21	16.51	2	7/10/2014	38:29.1
1983	RSRS19	-122.452	37.944	0.43	16.27	289.10	16.70	-122.452	37.944	0.12	16.27	287.23	16.39	2	7/10/2014	38:29.2
1984	RSRS19	-122.452	37.944	0.43	16.25	289.08	16.68	-122.452	37.944	0.00	16.25	287.21	16.25	2	7/10/2014	38:29.3
1985	RSRS19	-122.452	37.944	0.43	16.26	289.02	16.69	-122.452	37.944	0.04	16.26	287.17	16.30	2	7/10/2014	38:29.4
1986	RSRS19	-122.452	37.944	0.43	16.28	289.04	16.71	-122.452	37.944	0.21	16.28	287.15	16.49	2	7/10/2014	38:29.5
1987	RSRS19	-122.452	37.944	0.43	16.26	289.06	16.69	-122.452	37.944	0.21	16.26	287.19	16.47	2	7/10/2014	38:29.6
1988	RSRS19	-122.452	37.944	0.43	16.20	289.06	16.63	-122.452	37.944	0.04	16.20	287.17	16.24	2	7/10/2014	38:29.6
1989	RSRS19	-122.452	37.944	0.43	16.26	289.04	16.69	-122.452	37.944	0.12	16.26	287.17	16.38	2	7/10/2014	38:29.8
1990	RSRS19	-122.452	37.944	0.43	16.26	289.06	16.69	-122.452	37.944	0.07	16.26	287.21	16.33	2	7/10/2014	38:29.9
1991	RSRS19	-122.452	37.944	0.43	16.30	289.05	16.73	-122.452	37.944	0.21	16.30	287.20	16.51	2	7/10/2014	38:30.0
1992	RSRS19	-122.452	37.944	0.43	16.23	289.02	16.66	-122.452	37.944	0.12	16.23	287.13	16.36	2	7/10/2014	38:30.1
1993	RSRS19	-122.452	37.944	0.43	16.35	289.05	16.78	-122.452	37.944	0.04	16.35	287.15	16.39	2	7/10/2014	38:30.2
1994	RSRS19	-122.452	37.944	0.40	16.28	289.06	16.68	-122.452	37.944	0.16	16.28	287.15	16.44	2	7/10/2014	38:30.3
1995	RSRS19	-122.452	37.944	0.43	16.22	289.02	16.65	-122.452	37.944	0.00	16.22	287.11	16.22	2	7/10/2014	38:30.4
1996	RSRS19	-122.452	37.944	0.43	16.22	289.06	16.65	-122.452	37.944	0.16	16.22	287.11	16.37	2	7/10/2014	38:30.5
1997	RSRS19	-122.452	37.944	0.43	16.31	288.96	16.74	-122.452	37.944	0.16	16.31	287.05	16.47	2	7/10/2014	38:30.6
1998	RSRS19	-122.452	37.944	0.43	16.23	288.94	16.66	-122.452	37.944	0.00	16.23	287.07	16.24	2	7/10/2014	38:30.7
1999	RSRS19	-122.452	37.944	0.43	16.33	288.94	16.76	-122.452	37.944	0.07	16.33	287.05	16.40	2	7/10/2014	38:30.7
2000	RSRS19	-122.452	37.944	0.43	16.26	288.94	16.69	-122.452	37.944	0.12	16.26	287.11	16.38	2	7/10/2014	38:30.9
2001	RSRS19	-122.452	37.944	0.43	16.24	288.96	16.67	-122.452	37.944	0.07	16.24	287.12	16.31	2	7/10/2014	38:31.0
2002	RSRS19	-122.452	37.944	0.40	16.22	288.90	16.61	-122.452	37.944	0.16	16.22	287.09	16.37	2	7/10/2014	38:31.1
2003	RSRS19	-122.452	37.944	0.46	16.29	288.90	16.75	-122.452	37.944	0.07	16.29	287.05	16.36	2	7/10/2014	38:31.2
2004	RSRS19	-122.452	37.944	0.43	16.23	288.92	16.66	-122.452	37.944	0.04	16.23	287.05	16.27	2	7/10/2014	38:31.2
2005	RSRS19	-122.452	37.944	0.43	16.30	288.96	16.73	-122.452	37.944	0.21	16.30	287.05	16.50	2	7/10/2014	38:31.4
2006	RSRS19	-122.452	37.944	0.43	16.32	288.92	16.75	-122.452	37.944	0.07	16.32	287.01	16.39	2	7/10/2014	38:31.5
2007	RSRS19	-122.452	37.944	0.46	16.29	288.92	16.75	-122.452	37.944	0.21	16.29	287.01	16.50	2	7/10/2014	38:31.6
2008	RSRS19	-122.452	37.944	0.43	16.31	288.88	16.74	-122.452	37.944	0.12	16.31	286.95	16.43	2	7/10/2014	38:31.7
2009	RSRS19	-122.452	37.944	0.46	16.34	288.92	16.80	-122.452	37.944	-0.05	16.34	286.99	16.29	2	7/10/2014	38:31.8
2010	RSRS19	-122.452	37.944	0.43	16.36	288.88	16.79	-122.452	37.944	0.24	16.36	286.99	16.60	2	7/10/2014	38:31.8
2011	RSRS19	-122.452	37.944	0.43	16.36	288.86	16.79	-122.452	37.944	0.16	16.36	286.95	16.51	2	7/10/2014	38:32.0
2012	RSRS19	-122.452	37.944	0.43	16.35	288.81	16.78	-122.452	37.944	-0.05	16.35	286.99	16.31	2	7/10/2014	38:32.1

2013	RSRS19	-122.452	37.944	0.46	16.35	288.79	16.81	-122.452	37.944	0.12	16.35	286.99	16.47	2	7/10/2014	38:32.2
2014	RSRS19	-122.452	37.944	0.43	16.35	288.77	16.78	-122.452	37.944	0.07	16.35	287.02	16.42	2	7/10/2014	38:32.3
2015	RSRS19	-122.452	37.944	0.46	16.34	288.79	16.81	-122.452	37.944	0.16	16.34	287.02	16.50	2	7/10/2014	38:32.3
2016	RSRS19	-122.452	37.944	0.43	16.34	288.77	16.77	-122.452	37.944	0.21	16.34	287.06	16.55	2	7/10/2014	38:32.5
2017	RSRS19	-122.452	37.944	0.46	16.34	288.79	16.80	-122.452	37.944	0.00	16.34	287.06	16.34	2	7/10/2014	38:32.6
2018	RSRS19	-122.452	37.944	0.43	16.33	288.80	16.76	-122.452	37.944	0.24	16.33	286.99	16.58	2	7/10/2014	38:32.7
2019	RSRS19	-122.452	37.944	0.43	16.33	288.78	16.76	-122.452	37.944	0.12	16.33	286.96	16.45	2	7/10/2014	38:32.8
2020	RSRS19	-122.452	37.944	0.43	16.33	288.73	16.76	-122.452	37.944	0.04	16.33	286.91	16.37	2	7/10/2014	38:32.9
2021	RSRS19	-122.452	37.944	0.46	16.51	288.78	16.97	-122.452	37.944	0.24	16.51	286.92	16.75	2	7/10/2014	38:33.0
2022	RSRS19	-122.452	37.944	0.43	16.36	288.71	16.79	-122.452	37.944	0.12	16.36	286.86	16.48	2	7/10/2014	38:33.1
2023	RSRS19	-122.452	37.944	0.43	16.33	288.69	16.76	-122.452	37.944	0.07	16.33	286.82	16.40	2	7/10/2014	38:33.2
2024	RSRS19	-122.452	37.944	0.43	16.35	288.69	16.78	-122.452	37.944	0.12	16.35	286.83	16.47	2	7/10/2014	38:33.3
2025	RSRS19	-122.452	37.944	0.46	16.39	288.65	16.85	-122.452	37.944	0.04	16.39	286.83	16.43	2	7/10/2014	38:33.4
2026	RSRS19	-122.452	37.944	0.43	16.41	288.63	16.84	-122.452	37.944	0.16	16.41	286.83	16.57	2	7/10/2014	38:33.5
2027	RSRS19	-122.452	37.944	0.46	16.36	288.56	16.82	-122.452	37.944	0.24	16.36	286.76	16.60	2	7/10/2014	38:33.5
2028	RSRS19	-122.452	37.944	0.43	16.41	288.56	16.83	-122.452	37.944	0.12	16.41	286.76	16.53	2	7/10/2014	38:33.7
2029	RSRS19	-122.452	37.944	0.46	16.34	288.48	16.80	-122.452	37.944	0.12	16.34	286.72	16.46	2	7/10/2014	38:33.8
2030	RSRS19	-122.452	37.944	0.43	16.31	288.49	16.74	-122.452	37.944	0.12	16.31	286.72	16.43	2	7/10/2014	38:33.9
2031	RSRS19	-122.452	37.944	0.46	16.31	288.50	16.78	-122.452	37.944	0.04	16.31	286.65	16.35	2	7/10/2014	38:34.0
2032	RSRS19	-122.452	37.944	0.43	16.33	288.48	16.76	-122.452	37.944	0.24	16.33	286.67	16.58	2	7/10/2014	38:34.0
2033	RSRS19	-122.452	37.944	0.43	16.37	288.50	16.80	-122.452	37.944	0.16	16.37	286.61	16.53	2	7/10/2014	38:34.2
2034	RSRS19	-122.452	37.944	0.43	16.32	288.48	16.75	-122.452	37.944	0.07	16.32	286.65	16.39	2	7/10/2014	38:34.3
2035	RSRS19	-122.452	37.944	0.46	16.32	288.48	16.78	-122.452	37.944	0.24	16.32	286.61	16.56	2	7/10/2014	38:34.4
2036	RSRS19	-122.452	37.944	0.43	16.35	288.46	16.78	-122.452	37.944	0.16	16.35	286.68	16.51	2	7/10/2014	38:34.5
2037	RSRS19	-122.452	37.944	0.46	16.43	288.39	16.89	-122.452	37.944	0.16	16.43	286.61	16.58	2	7/10/2014	38:34.6
2038	RSRS19	-122.452	37.944	0.43	16.40	288.33	16.82	-122.452	37.944	0.21	16.40	286.53	16.60	2	7/10/2014	38:34.6
2039	RSRS19	-122.452	37.944	0.46	16.43	288.26	16.89	-122.452	37.944	0.04	16.43	286.50	16.47	2	7/10/2014	38:34.8
2040	RSRS19	-122.452	37.944	0.43	16.34	288.24	16.77	-122.452	37.944	0.21	16.34	286.46	16.55	2	7/10/2014	38:34.9
2041	RSRS19	-122.452	37.944	0.46	16.33	288.20	16.79	-122.452	37.944	0.24	16.33	286.42	16.57	2	7/10/2014	38:35.0
2042	RSRS19	-122.452	37.944	0.43	16.37	288.18	16.80	-122.452	37.944	0.16	16.37	286.35	16.52	2	7/10/2014	38:35.0
2043	RSRS19	-122.452	37.944	0.46	16.33	288.09	16.80	-122.452	37.944	0.24	16.33	286.33	16.58	2	7/10/2014	38:35.1
2044	RSRS19	-122.452	37.944	0.43	16.39	288.11	16.82	-122.452	37.944	0.16	16.39	286.29	16.54	2	7/10/2014	38:35.3
2045	RSRS19	-122.452	37.944	0.46	16.44	288.09	16.91	-122.452	37.944	0.12	16.44	286.33	16.57	2	7/10/2014	38:35.4

2046	RSRS19	-122.452	37.944	0.43	16.34	288.05	16.77	-122.452	37.944	0.32	16.34	286.27	16.67	2	7/10/2014	38:35.5
2047	RSRS19	-122.452	37.944	0.46	16.36	288.02	16.82	-122.452	37.944	0.16	16.36	286.31	16.51	2	7/10/2014	38:35.6
2048	RSRS19	-122.452	37.944	0.43	16.30	288.01	16.73	-122.452	37.944	0.12	16.30	286.27	16.42	2	7/10/2014	38:35.7
2049	RSRS19	-122.452	37.944	0.46	16.31	287.94	16.78	-122.452	37.944	0.29	16.31	286.23	16.60	2	7/10/2014	38:35.8
2050	RSRS19	-122.452	37.944	0.43	16.34	287.94	16.77	-122.452	37.944	0.12	16.34	286.18	16.46	2	7/10/2014	38:35.9
2051	RSRS19	-122.452	37.944	0.46	16.31	287.96	16.78	-122.452	37.944	0.24	16.31	286.18	16.55	2	7/10/2014	38:36.0
2052	RSRS19	-122.452	37.944	0.43	16.30	287.90	16.73	-122.452	37.944	0.24	16.30	286.11	16.54	2	7/10/2014	38:36.1
2053	RSRS19	-122.452	37.944	0.46	16.30	287.88	16.77	-122.452	37.944	0.07	16.30	286.08	16.38	2	7/10/2014	38:36.2
2054	RSRS19	-122.452	37.944	0.46	16.30	287.88	16.77	-122.452	37.944	0.24	16.30	286.04	16.55	2	7/10/2014	38:36.3
2055	RSRS19	-122.452	37.944	0.46	16.30	287.86	16.76	-122.452	37.944	0.24	16.30	286.10	16.54	2	7/10/2014	38:36.3
2056	RSRS19	-122.452	37.944	0.43	16.27	287.83	16.70	-122.452	37.944	0.16	16.27	286.05	16.42	2	7/10/2014	38:36.5
2057	RSRS19	-122.452	37.944	0.46	16.31	287.79	16.78	-122.452	37.944	0.29	16.31	286.05	16.60	2	7/10/2014	38:36.6
2058	RSRS19	-122.452	37.944	0.43	16.31	287.79	16.74	-122.452	37.944	0.16	16.31	286.05	16.47	2	7/10/2014	38:36.7
2059	RSRS19	-122.452	37.944	0.46	16.34	287.79	16.80	-122.452	37.944	0.29	16.34	286.01	16.63	2	7/10/2014	38:36.8
2060	RSRS19	-122.452	37.944	0.43	16.30	287.82	16.73	-122.452	37.944	0.24	16.30	286.01	16.54	2	7/10/2014	38:36.8
2061	RSRS19	-122.452	37.944	0.43	16.30	287.86	16.73	-122.452	37.944	0.12	16.30	286.01	16.42	2	7/10/2014	38:37.0
2062	RSRS19	-122.452	37.944	0.43	16.28	287.82	16.71	-122.452	37.944	0.32	16.28	285.99	16.61	2	7/10/2014	38:37.1
2063	RSRS19	-122.452	37.944	0.43	16.30	287.80	16.73	-122.452	37.944	0.21	16.30	286.04	16.50	2	7/10/2014	38:37.2
2064	RSRS19	-122.452	37.944	0.43	16.24	287.75	16.67	-122.452	37.944	0.12	16.24	285.99	16.37	2	7/10/2014	38:37.3
2065	RSRS19	-122.452	37.944	0.46	16.24	287.71	16.71	-122.452	37.944	0.32	16.24	285.95	16.57	2	7/10/2014	38:37.4
2066	RSRS19	-122.452	37.944	0.46	16.22	287.71	16.68	-122.452	37.944	0.21	16.22	286.00	16.43	2	7/10/2014	38:37.4
2067	RSRS19	-122.452	37.944	0.46	16.27	287.80	16.73	-122.452	37.944	0.29	16.27	286.07	16.56	2	7/10/2014	38:37.6
2068	RSRS19	-122.452	37.944	0.43	16.23	287.73	16.66	-122.452	37.944	0.16	16.23	286.04	16.39	2	7/10/2014	38:37.7
2069	RSRS19	-122.452	37.944	0.46	16.21	287.71	16.67	-122.452	37.944	0.29	16.21	285.95	16.50	2	7/10/2014	38:37.8
2070	RSRS19	-122.452	37.944	0.46	16.20	287.71	16.67	-122.452	37.944	0.32	16.20	285.89	16.53	2	7/10/2014	38:37.9
2071	RSRS19	-122.452	37.944	0.46	16.22	287.71	16.68	-122.452	37.944	0.21	16.22	285.84	16.43	2	7/10/2014	38:37.9
2072	RSRS19	-122.452	37.944	0.43	16.36	287.63	16.79	-122.452	37.944	0.07	16.36	285.82	16.43	2	7/10/2014	38:38.1
2073	RSRS19	-122.452	37.944	0.46	16.23	287.71	16.70	-122.452	37.944	0.21	16.23	285.80	16.44	2	7/10/2014	38:38.2
2074	RSRS19	-122.452	37.944	0.43	16.26	287.69	16.69	-122.452	37.944	0.32	16.26	285.84	16.59	2	7/10/2014	38:38.3
2075	RSRS19	-122.452	37.944	0.46	16.27	287.69	16.73	-122.452	37.944	0.24	16.27	285.80	16.51	2	7/10/2014	38:38.4
2076	RSRS19	-122.452	37.944	0.43	16.33	287.63	16.76	-122.452	37.944	0.12	16.33	285.76	16.46	2	7/10/2014	38:38.5
2077	RSRS19	-122.452	37.944	0.46	16.31	287.61	16.78	-122.452	37.944	0.29	16.31	285.76	16.60	2	7/10/2014	38:38.6
2078	RSRS19	-122.452	37.944	0.43	16.33	287.54	16.76	-122.452	37.944	0.32	16.33	285.67	16.65	2	7/10/2014	38:38.7

2079	RSRS19	-122.452	37.944	0.46	16.30	287.54	16.77	-122.452	37.944	0.21	16.30	285.63	16.51	2	7/10/2014	38:38.8
2080	RSRS19	-122.452	37.944	0.43	16.30	287.50	16.73	-122.452	37.944	0.21	16.30	285.56	16.51	2	7/10/2014	38:38.9
2081	RSRS19	-122.452	37.944	0.46	16.32	287.30	16.78	-122.452	37.944	0.21	16.32	285.58	16.53	2	7/10/2014	38:39.0
2082	RSRS19	-122.452	37.944	0.46	16.36	287.39	16.82	-122.452	37.944	0.29	16.36	285.57	16.65	2	7/10/2014	38:39.1
2083	RSRS19	-122.452	37.944	0.46	16.37	287.33	16.83	-122.452	37.944	0.12	16.37	285.52	16.49	2	7/10/2014	38:39.2
2084	RSRS19	-122.452	37.944	0.43	16.34	287.35	16.77	-122.452	37.944	0.24	16.34	285.54	16.58	2	7/10/2014	38:39.3
2085	RSRS19	-122.452	37.944	0.46	16.36	287.26	16.82	-122.452	37.944	0.29	16.36	285.46	16.65	2	7/10/2014	38:39.4
2086	RSRS19	-122.452	37.944	0.43	16.39	287.17	16.82	-122.452	37.944	0.12	16.39	285.35	16.51	2	7/10/2014	38:39.5
2087	RSRS19	-122.452	37.944	0.46	16.40	287.28	16.86	-122.452	37.944	0.21	16.40	285.48	16.60	2	7/10/2014	38:39.6
2088	RSRS19	-122.452	37.944	0.43	16.41	287.29	16.84	-122.452	37.944	0.29	16.41	285.48	16.70	2	7/10/2014	38:39.6
2089	RSRS19	-122.452	37.944	0.46	16.41	287.22	16.87	-122.452	37.944	0.12	16.41	285.44	16.53	2	7/10/2014	38:39.8
2090	RSRS19	-122.452	37.944	0.46	16.47	287.15	16.94	-122.452	37.944	0.21	16.47	285.42	16.68	2	7/10/2014	38:39.9
2091	RSRS19	-122.452	37.944	0.46	16.49	287.11	16.95	-122.452	37.944	0.21	16.49	285.37	16.70	2	7/10/2014	38:40.0
2092	RSRS19	-122.452	37.944	0.46	16.44	287.02	16.91	-122.452	37.944	0.12	16.44	285.33	16.57	2	7/10/2014	38:40.1
2093	RSRS19	-122.452	37.944	0.46	16.43	287.00	16.89	-122.452	37.944	0.32	16.43	285.31	16.75	2	7/10/2014	38:40.2
2094	RSRS19	-122.452	37.944	0.46	16.41	286.96	16.87	-122.452	37.944	0.04	16.41	285.23	16.45	2	7/10/2014	38:40.2
2095	RSRS19	-122.452	37.944	0.46	16.39	286.94	16.85	-122.452	37.944	0.24	16.39	285.27	16.63	2	7/10/2014	38:40.4
2096	RSRS19	-122.452	37.944	0.43	16.44	286.91	16.87	-122.452	37.944	0.24	16.44	285.20	16.68	2	7/10/2014	38:40.4
2097	RSRS19	-122.452	37.944	0.43	16.37	286.87	16.80	-122.452	37.944	0.21	16.37	285.20	16.58	2	7/10/2014	38:40.6
2098	RSRS19	-122.452	37.944	0.46	16.37	286.83	16.84	-122.452	37.944	0.21	16.37	285.20	16.58	2	7/10/2014	38:40.7
1	RSRD19	-122.447	37.944	5.65	9.78	296.36	15.43	-122.447	37.944	6.16	9.78	295.95	15.94	1	8/1/2014	8:24:39.48
2	RSRD19	-122.447	37.944	5.74	9.76	296.36	15.50	-122.447	37.944	6.22	9.76	295.99	15.98	1	8/1/2014	8:24:39.57
3	RSRD19	-122.447	37.944	5.74	9.75	296.39	15.49	-122.447	37.944	6.22	9.75	295.99	15.96	1	8/1/2014	8:24:39.67
4	RSRD19	-122.447	37.944	5.74	9.73	296.41	15.47	-122.447	37.944	6.22	9.73	295.99	15.95	1	8/1/2014	8:24:39.76
5	RSRD19	-122.447	37.944	5.70	9.68	296.50	15.39	-122.447	37.944	6.22	9.68	296.06	15.90	1	8/1/2014	8:24:39.88
6	RSRD19	-122.447	37.944	5.77	9.65	296.50	15.42	-122.447	37.944	6.22	9.65	296.04	15.86	1	8/1/2014	8:24:39.97
7	RSRD19	-122.447	37.944	5.74	9.62	296.50	15.36	-122.447	37.944	6.22	9.62	296.09	15.84	1	8/1/2014	8:24:40.07
8	RSRD19	-122.447	37.944	5.77	9.60	296.57	15.38	-122.447	37.944	6.25	9.60	296.13	15.85	1	8/1/2014	8:24:40.16
9	RSRD19	-122.447	37.944	5.77	9.55	296.64	15.32	-122.447	37.944	6.22	9.55	296.16	15.76	1	8/1/2014	8:24:40.28
10	RSRD19	-122.447	37.944	5.77	9.56	296.64	15.33	-122.447	37.944	6.25	9.56	296.18	15.81	1	8/1/2014	8:24:40.37
11	RSRD19	-122.447	37.944	5.74	9.54	296.69	15.28	-122.447	37.944	6.25	9.54	296.25	15.79	1	8/1/2014	8:24:40.47
12	RSRD19	-122.447	37.944	5.82	9.52	296.68	15.35	-122.447	37.944	6.25	9.52	296.23	15.77	1	8/1/2014	8:24:40.56
13	RSRD19	-122.447	37.944	5.77	9.51	296.73	15.28	-122.447	37.944	6.25	9.51	296.27	15.76	1	8/1/2014	8:24:40.68

14	RSRD19	-122.447	37.944	5.82	9.52	296.78	15.35	-122.447	37.944	6.25	9.52	296.29	15.77	1	8/1/2014	8:24:40.77
15	RSRD19	-122.447	37.944	5.82	9.51	296.78	15.33	-122.447	37.944	6.25	9.51	296.32	15.76	1	8/1/2014	8:24:40.87
16	RSRD19	-122.447	37.944	5.82	9.50	296.81	15.32	-122.447	37.944	6.25	9.50	296.30	15.75	1	8/1/2014	8:24:40.96
17	RSRD19	-122.447	37.944	5.82	9.52	296.83	15.34	-122.447	37.944	6.25	9.52	296.39	15.76	1	8/1/2014	8:24:41.08
18	RSRD19	-122.447	37.944	5.86	9.56	296.81	15.42	-122.447	37.944	6.25	9.56	296.35	15.81	1	8/1/2014	8:24:41.17
19	RSRD19	-122.447	37.944	5.82	9.58	296.84	15.40	-122.447	37.944	6.22	9.58	296.35	15.79	1	8/1/2014	8:24:41.27
20	RSRD19	-122.447	37.944	5.82	9.53	296.86	15.36	-122.447	37.944	6.25	9.53	296.38	15.78	1	8/1/2014	8:24:41.36
21	RSRD19	-122.447	37.944	5.86	9.54	296.86	15.40	-122.447	37.944	6.22	9.54	296.40	15.75	1	8/1/2014	8:24:41.48
22	RSRD19	-122.447	37.944	5.86	9.53	296.82	15.39	-122.447	37.944	6.30	9.53	296.36	15.83	1	8/1/2014	8:24:41.57
23	RSRD19	-122.447	37.944	5.86	9.55	296.83	15.41	-122.447	37.944	6.25	9.55	296.37	15.80	1	8/1/2014	8:24:41.67
24	RSRD19	-122.447	37.944	5.86	9.54	296.83	15.40	-122.447	37.944	6.30	9.54	296.36	15.84	1	8/1/2014	8:24:41.76
25	RSRD19	-122.447	37.944	5.86	9.55	296.79	15.41	-122.447	37.944	6.30	9.55	296.35	15.85	1	8/1/2014	8:24:41.88
26	RSRD19	-122.447	37.944	5.91	9.50	296.79	15.41	-122.447	37.944	6.33	9.50	296.35	15.83	1	8/1/2014	8:24:41.97
27	RSRD19	-122.447	37.944	5.91	9.52	296.82	15.43	-122.447	37.944	6.33	9.52	296.33	15.85	1	8/1/2014	8:24:42.07
28	RSRD19	-122.447	37.944	5.91	9.51	296.80	15.42	-122.447	37.944	6.37	9.51	296.33	15.87	1	8/1/2014	8:24:42.16
29	RSRD19	-122.447	37.944	5.91	9.46	296.78	15.37	-122.447	37.944	6.37	9.46	296.36	15.83	1	8/1/2014	8:24:42.28
30	RSRD19	-122.447	37.944	5.95	9.46	296.81	15.41	-122.447	37.944	6.42	9.46	296.34	15.88	1	8/1/2014	8:24:42.37
31	RSRD19	-122.447	37.944	5.95	9.46	296.81	15.41	-122.447	37.944	6.37	9.46	296.35	15.83	1	8/1/2014	8:24:42.47
32	RSRD19	-122.447	37.944	5.95	9.45	296.88	15.40	-122.447	37.944	6.42	9.45	296.37	15.87	1	8/1/2014	8:24:42.56
33	RSRD19	-122.447	37.944	5.95	9.46	296.84	15.41	-122.447	37.944	6.37	9.46	296.37	15.83	1	8/1/2014	8:24:42.67
34	RSRD19	-122.447	37.944	5.95	9.47	296.88	15.41	-122.447	37.944	6.37	9.47	296.40	15.84	1	8/1/2014	8:24:42.77
35	RSRD19	-122.447	37.944	5.95	9.48	296.89	15.43	-122.447	37.944	6.37	9.48	296.40	15.85	1	8/1/2014	8:24:42.87
36	RSRD19	-122.447	37.944	5.95	9.48	296.89	15.42	-122.447	37.944	6.37	9.48	296.41	15.84	1	8/1/2014	8:24:42.96
37	RSRD19	-122.447	37.944	5.95	9.48	296.96	15.42	-122.447	37.944	6.37	9.48	296.41	15.84	1	8/1/2014	8:24:43.08
38	RSRD19	-122.447	37.944	6.00	9.47	296.88	15.46	-122.447	37.944	6.42	9.47	296.42	15.89	1	8/1/2014	8:24:43.17
39	RSRD19	-122.447	37.944	6.00	9.47	296.95	15.46	-122.447	37.944	6.37	9.47	296.42	15.84	1	8/1/2014	8:24:43.27
40	RSRD19	-122.447	37.944	6.00	9.50	296.93	15.50	-122.447	37.944	6.42	9.50	296.43	15.92	1	8/1/2014	8:24:43.36
41	RSRD19	-122.447	37.944	6.00	9.50	296.93	15.50	-122.447	37.944	6.37	9.50	296.39	15.87	1	8/1/2014	8:24:43.48
42	RSRD19	-122.447	37.944	6.08	9.53	296.94	15.61	-122.447	37.944	6.37	9.53	296.41	15.90	1	8/1/2014	8:24:43.57
43	RSRD19	-122.447	37.944	6.08	9.49	296.99	15.57	-122.447	37.944	6.37	9.49	296.42	15.86	1	8/1/2014	8:24:43.67
44	RSRD19	-122.447	37.944	6.08	9.52	296.95	15.60	-122.447	37.944	6.42	9.52	296.42	15.94	1	8/1/2014	8:24:43.76
45	RSRD19	-122.447	37.944	6.03	9.52	296.98	15.55	-122.447	37.944	6.37	9.52	296.42	15.88	1	8/1/2014	8:24:43.88
46	RSRD19	-122.447	37.944	6.03	9.53	297.00	15.56	-122.447	37.944	6.42	9.53	296.45	15.95	1	8/1/2014	8:24:43.97

47	RSRD19	-122.447	37.944	6.03	9.54	297.01	15.57	-122.447	37.944	6.42	9.54	296.48	15.96	1	8/1/2014 8:24:44.07
48	RSRD19	-122.447	37.944	6.08	9.53	297.01	15.61	-122.447	37.944	6.42	9.53	296.46	15.95	1	8/1/2014 8:24:44.16
49	RSRD19	-122.447	37.944	6.03	9.53	296.99	15.56	-122.447	37.944	6.37	9.53	296.46	15.90	1	8/1/2014 8:24:44.28
50	RSRD19	-122.447	37.944	6.08	9.58	297.02	15.66	-122.447	37.944	6.42	9.58	296.51	15.99	1	8/1/2014 8:24:44.37
51	RSRD19	-122.447	37.944	6.03	9.55	297.00	15.58	-122.447	37.944	6.37	9.55	296.47	15.92	1	8/1/2014 8:24:44.47
52	RSRD19	-122.447	37.944	6.08	9.55	297.03	15.63	-122.447	37.944	6.37	9.55	296.48	15.92	1	8/1/2014 8:24:44.56
53	RSRD19	-122.447	37.944	6.03	9.55	297.01	15.58	-122.447	37.944	6.37	9.55	296.48	15.92	1	8/1/2014 8:24:44.67
54	RSRD19	-122.447	37.944	6.08	9.55	297.06	15.64	-122.447	37.944	6.42	9.55	296.51	15.97	1	8/1/2014 8:24:44.77
55	RSRD19	-122.447	37.944	6.08	9.55	297.04	15.64	-122.447	37.944	6.37	9.55	296.51	15.92	1	8/1/2014 8:24:44.87
56	RSRD19	-122.447	37.944	6.08	9.57	297.04	15.65	-122.447	37.944	6.37	9.57	296.47	15.94	1	8/1/2014 8:24:44.96
57	RSRD19	-122.447	37.944	6.08	9.58	297.03	15.66	-122.447	37.944	6.37	9.58	296.45	15.94	1	8/1/2014 8:24:45.07
58	RSRD19	-122.447	37.944	6.08	9.59	297.03	15.67	-122.447	37.944	6.37	9.59	296.46	15.96	1	8/1/2014 8:24:45.17
59	RSRD19	-122.447	37.944	6.08	9.61	297.01	15.70	-122.447	37.944	6.37	9.61	296.44	15.98	1	8/1/2014 8:24:45.27
60	RSRD19	-122.447	37.944	6.08	9.59	297.00	15.67	-122.447	37.944	6.37	9.59	296.45	15.96	1	8/1/2014 8:24:45.36
61	RSRD19	-122.447	37.944	6.03	9.59	296.98	15.62	-122.447	37.944	6.33	9.59	296.41	15.92	1	8/1/2014 8:24:45.47
62	RSRD19	-122.447	37.944	6.08	9.59	296.96	15.67	-122.447	37.944	6.37	9.59	296.41	15.96	1	8/1/2014 8:24:45.57
63	RSRD19	-122.447	37.944	6.08	9.59	296.96	15.67	-122.447	37.944	6.33	9.59	296.42	15.93	1	8/1/2014 8:24:45.67
64	RSRD19	-122.447	37.944	6.08	9.61	296.92	15.69	-122.447	37.944	6.33	9.61	296.33	15.94	1	8/1/2014 8:24:45.76
65	RSRD19	-122.447	37.944	6.03	9.64	296.89	15.67	-122.447	37.944	6.33	9.64	296.31	15.97	1	8/1/2014 8:24:45.88
66	RSRD19	-122.447	37.944	6.08	9.62	296.91	15.71	-122.447	37.944	6.37	9.62	296.32	15.99	1	8/1/2014 8:24:45.97
67	RSRD19	-122.447	37.944	6.03	9.65	296.92	15.68	-122.447	37.944	6.30	9.65	296.30	15.95	1	8/1/2014 8:24:46.07
68	RSRD19	-122.447	37.944	6.08	9.66	296.90	15.74	-122.447	37.944	6.33	9.66	296.32	16.00	1	8/1/2014 8:24:46.16
69	RSRD19	-122.447	37.944	6.03	9.65	296.90	15.68	-122.447	37.944	6.30	9.65	296.26	15.95	1	8/1/2014 8:24:46.28
70	RSRD19	-122.447	37.944	6.08	9.64	296.91	15.72	-122.447	37.944	6.33	9.64	296.29	15.97	1	8/1/2014 8:24:46.37
71	RSRD19	-122.447	37.944	6.03	9.65	296.89	15.68	-122.447	37.944	6.30	9.65	296.25	15.95	1	8/1/2014 8:24:46.47
72	RSRD19	-122.447	37.944	6.08	9.69	296.87	15.77	-122.447	37.944	6.30	9.69	296.24	15.99	1	8/1/2014 8:24:46.56
73	RSRD19	-122.447	37.944	6.00	9.65	296.88	15.64	-122.447	37.944	6.25	9.65	296.21	15.89	1	8/1/2014 8:24:46.68
74	RSRD19	-122.447	37.944	6.03	9.65	296.92	15.68	-122.447	37.944	6.30	9.65	296.24	15.95	1	8/1/2014 8:24:46.77
75	RSRD19	-122.447	37.944	6.03	9.64	296.86	15.67	-122.447	37.944	6.30	9.64	296.24	15.94	1	8/1/2014 8:24:46.87
76	RSRD19	-122.447	37.944	6.08	9.63	296.84	15.71	-122.447	37.944	6.25	9.63	296.23	15.88	1	8/1/2014 8:24:46.96
77	RSRD19	-122.447	37.944	6.03	9.65	296.91	15.68	-122.447	37.944	6.25	9.65	296.25	15.89	1	8/1/2014 8:24:47.08
78	RSRD19	-122.447	37.944	6.08	9.61	296.76	15.70	-122.447	37.944	6.30	9.61	296.19	15.91	1	8/1/2014 8:24:47.17
79	RSRD19	-122.447	37.944	6.03	9.62	296.79	15.65	-122.447	37.944	6.30	9.62	296.24	15.92	1	8/1/2014 8:24:47.27

80	RSRD19	-122.447	37.944	6.08	9.61	296.77	15.69	-122.447	37.944	6.30	9.61	296.22	15.91	1	8/1/2014 8:24:47.36
81	RSRD19	-122.447	37.944	6.03	9.59	296.75	15.62	-122.447	37.944	6.33	9.59	296.22	15.92	1	8/1/2014 8:24:47.47
82	RSRD19	-122.447	37.944	6.08	9.55	296.73	15.64	-122.447	37.944	6.30	9.55	296.19	15.85	1	8/1/2014 8:24:47.57
83	RSRD19	-122.447	37.944	6.08	9.54	296.74	15.62	-122.447	37.944	6.30	9.54	296.21	15.84	1	8/1/2014 8:24:47.67
84	RSRD19	-122.447	37.944	6.08	9.53	296.81	15.61	-122.447	37.944	6.33	9.53	296.21	15.87	1	8/1/2014 8:24:47.76
85	RSRD19	-122.447	37.944	6.03	9.55	296.63	15.58	-122.447	37.944	6.30	9.55	296.26	15.85	1	8/1/2014 8:24:47.87
86	RSRD19	-122.447	37.944	6.08	9.55	296.68	15.63	-122.447	37.944	6.30	9.55	296.18	15.85	1	8/1/2014 8:24:47.97
87	RSRD19	-122.447	37.944	6.03	9.57	296.73	15.60	-122.447	37.944	6.25	9.57	296.22	15.82	1	8/1/2014 8:24:48.07
88	RSRD19	-122.447	37.944	6.08	9.52	296.69	15.60	-122.447	37.944	6.30	9.52	296.23	15.82	1	8/1/2014 8:24:48.16
89	RSRD19	-122.447	37.944	6.03	9.55	296.71	15.58	-122.447	37.944	6.25	9.55	296.21	15.80	1	8/1/2014 8:24:48.28
90	RSRD19	-122.447	37.944	6.03	9.57	296.76	15.60	-122.447	37.944	6.30	9.57	296.21	15.87	1	8/1/2014 8:24:48.37
91	RSRD19	-122.447	37.944	6.03	9.59	296.74	15.62	-122.447	37.944	6.25	9.59	296.24	15.84	1	8/1/2014 8:24:48.47
92	RSRD19	-122.447	37.944	6.08	9.55	296.75	15.63	-122.447	37.944	6.30	9.55	296.22	15.85	1	8/1/2014 8:24:48.56
93	RSRD19	-122.447	37.944	6.03	9.54	296.75	15.57	-122.447	37.944	6.25	9.54	296.24	15.79	1	8/1/2014 8:24:48.68
94	RSRD19	-122.447	37.944	6.03	9.56	296.80	15.59	-122.447	37.944	6.30	9.56	296.27	15.86	1	8/1/2014 8:24:48.77
95	RSRD19	-122.447	37.944	6.03	9.58	296.78	15.61	-122.447	37.944	6.25	9.58	296.30	15.83	1	8/1/2014 8:24:48.87
96	RSRD19	-122.447	37.944	6.08	9.59	296.80	15.67	-122.447	37.944	6.30	9.59	296.32	15.89	1	8/1/2014 8:24:48.96
97	RSRD19	-122.447	37.944	6.03	9.59	296.87	15.62	-122.447	37.944	6.25	9.59	296.37	15.84	1	8/1/2014 8:24:49.07
98	RSRD19	-122.447	37.944	6.08	9.61	296.85	15.70	-122.447	37.944	6.30	9.61	296.39	15.91	1	8/1/2014 8:24:49.17
99	RSRD19	-122.447	37.944	6.03	9.64	296.86	15.67	-122.447	37.944	6.25	9.64	296.40	15.89	1	8/1/2014 8:24:49.27
100	RSRD19	-122.447	37.944	6.08	9.68	296.92	15.76	-122.447	37.944	6.25	9.68	296.44	15.93	1	8/1/2014 8:24:49.36
101	RSRD19	-122.447	37.944	6.03	9.67	296.93	15.70	-122.447	37.944	6.25	9.67	296.47	15.92	1	8/1/2014 8:24:49.48
102	RSRD19	-122.447	37.944	6.03	9.69	296.93	15.72	-122.447	37.944	6.25	9.69	296.49	15.94	1	8/1/2014 8:24:49.57
103	RSRD19	-122.447	37.944	6.03	9.72	296.96	15.75	-122.447	37.944	6.22	9.72	296.52	15.93	1	8/1/2014 8:24:49.67
104	RSRD19	-122.447	37.944	6.03	9.73	296.94	15.76	-122.447	37.944	6.25	9.73	296.52	15.97	1	8/1/2014 8:24:49.76
105	RSRD19	-122.447	37.944	6.03	9.74	296.98	15.77	-122.447	37.944	6.22	9.74	296.54	15.95	1	8/1/2014 8:24:49.88
106	RSRD19	-122.447	37.944	6.03	9.75	296.96	15.78	-122.447	37.944	6.22	9.75	296.55	15.96	1	8/1/2014 8:24:49.97
107	RSRD19	-122.447	37.944	6.00	9.73	296.99	15.73	-122.447	37.944	6.16	9.73	296.55	15.89	1	8/1/2014 8:24:50.07
108	RSRD19	-122.447	37.944	6.03	9.76	296.97	15.79	-122.447	37.944	6.22	9.76	296.55	15.98	1	8/1/2014 8:24:50.16
109	RSRD19	-122.447	37.944	6.00	9.73	296.97	15.73	-122.447	37.944	6.22	9.73	296.56	15.95	1	8/1/2014 8:24:50.27
110	RSRD19	-122.447	37.944	6.03	9.72	296.98	15.75	-122.447	37.944	6.22	9.72	296.49	15.93	1	8/1/2014 8:24:50.37
111	RSRD19	-122.447	37.944	6.00	9.71	296.98	15.70	-122.447	37.944	6.16	9.71	296.52	15.87	1	8/1/2014 8:24:50.47
112	RSRD19	-122.447	37.944	6.03	9.71	296.98	15.74	-122.447	37.944	6.16	9.71	296.47	15.87	1	8/1/2014 8:24:50.56

113	RSRD19	-122.447	37.944	6.00	9.72	296.96	15.71	-122.447	37.944	6.16	9.72	296.41	15.88	1	8/1/2014	8:24:50.67
114	RSRD19	-122.447	37.944	6.03	9.71	297.01	15.74	-122.447	37.944	6.16	9.71	296.43	15.87	1	8/1/2014	8:24:50.77
115	RSRD19	-122.447	37.944	6.00	9.72	296.96	15.71	-122.447	37.944	6.16	9.72	296.44	15.88	1	8/1/2014	8:24:50.87
116	RSRD19	-122.447	37.944	6.03	9.73	296.97	15.76	-122.447	37.944	6.16	9.73	296.39	15.89	1	8/1/2014	8:24:50.96
117	RSRD19	-122.447	37.944	6.00	9.74	296.99	15.74	-122.447	37.944	6.16	9.74	296.40	15.90	1	8/1/2014	8:24:51.08
118	RSRD19	-122.447	37.944	6.00	9.75	296.97	15.74	-122.447	37.944	6.13	9.75	296.38	15.88	1	8/1/2014	8:24:51.17
119	RSRD19	-122.447	37.944	6.00	9.78	296.93	15.77	-122.447	37.944	6.13	9.78	296.40	15.91	1	8/1/2014	8:24:51.27
120	RSRD19	-122.447	37.944	6.00	9.79	296.91	15.79	-122.447	37.944	6.16	9.79	296.36	15.96	1	8/1/2014	8:24:51.36
121	RSRD19	-122.447	37.944	6.00	9.81	296.93	15.81	-122.447	37.944	6.08	9.81	296.38	15.89	1	8/1/2014	8:24:51.48
122	RSRD19	-122.447	37.944	6.00	9.82	296.89	15.81	-122.447	37.944	6.13	9.82	296.37	15.95	1	8/1/2014	8:24:51.57
123	RSRD19	-122.447	37.944	6.00	9.83	296.87	15.83	-122.447	37.944	6.13	9.83	296.36	15.96	1	8/1/2014	8:24:51.67
124	RSRD19	-122.447	37.944	6.00	9.84	296.87	15.83	-122.447	37.944	6.13	9.84	296.37	15.97	1	8/1/2014	8:24:51.76
125	RSRD19	-122.447	37.944	5.95	9.86	296.83	15.81	-122.447	37.944	6.08	9.86	296.37	15.94	1	8/1/2014	8:24:51.87
126	RSRD19	-122.447	37.944	6.00	9.90	296.81	15.90	-122.447	37.944	6.13	9.90	296.35	16.03	1	8/1/2014	8:24:51.97
127	RSRD19	-122.447	37.944	6.00	9.90	296.86	15.90	-122.447	37.944	6.08	9.90	296.37	15.98	1	8/1/2014	8:24:52.07
128	RSRD19	-122.447	37.944	6.00	9.89	296.79	15.89	-122.447	37.944	6.13	9.89	296.37	16.02	1	8/1/2014	8:24:52.16
129	RSRD19	-122.447	37.944	5.95	9.89	296.82	15.83	-122.447	37.944	6.08	9.89	296.39	15.97	1	8/1/2014	8:24:52.27
130	RSRD19	-122.447	37.944	6.00	9.89	296.82	15.88	-122.447	37.944	6.13	9.89	296.40	16.02	1	8/1/2014	8:24:52.37
131	RSRD19	-122.447	37.944	5.95	9.91	296.80	15.85	-122.447	37.944	6.08	9.91	296.32	15.99	1	8/1/2014	8:24:52.47
132	RSRD19	-122.447	37.944	6.00	9.92	296.80	15.91	-122.447	37.944	6.08	9.92	296.36	16.00	1	8/1/2014	8:24:52.56
133	RSRD19	-122.447	37.944	6.00	9.90	296.84	15.90	-122.447	37.944	6.08	9.90	296.39	15.98	1	8/1/2014	8:24:52.67
134	RSRD19	-122.447	37.944	5.95	9.91	296.83	15.85	-122.447	37.944	6.13	9.91	296.38	16.04	1	8/1/2014	8:24:52.77
135	RSRD19	-122.447	37.944	5.95	9.95	296.80	15.89	-122.447	37.944	6.08	9.95	296.41	16.03	1	8/1/2014	8:24:52.87
136	RSRD19	-122.447	37.944	6.00	9.94	296.85	15.94	-122.447	37.944	6.08	9.94	296.34	16.02	1	8/1/2014	8:24:52.96
137	RSRD19	-122.447	37.944	5.95	9.93	296.83	15.88	-122.447	37.944	6.05	9.93	296.41	15.98	1	8/1/2014	8:24:53.07
138	RSRD19	-122.447	37.944	5.95	9.94	296.83	15.88	-122.447	37.944	6.05	9.94	296.33	15.99	1	8/1/2014	8:24:53.17
139	RSRD19	-122.447	37.944	5.91	9.94	296.81	15.85	-122.447	37.944	6.05	9.94	296.39	15.99	1	8/1/2014	8:24:53.27
140	RSRD19	-122.447	37.944	5.95	9.95	296.81	15.89	-122.447	37.944	6.05	9.95	296.37	15.99	1	8/1/2014	8:24:53.36
141	RSRD19	-122.447	37.944	5.95	9.99	296.81	15.93	-122.447	37.944	6.05	9.99	296.33	16.03	1	8/1/2014	8:24:53.47
142	RSRD19	-122.447	37.944	5.95	9.99	296.81	15.93	-122.447	37.944	6.05	9.99	296.35	16.03	1	8/1/2014	8:24:53.57
143	RSRD19	-122.447	37.944	5.91	9.99	296.79	15.90	-122.447	37.944	6.01	9.99	296.35	16.01	1	8/1/2014	8:24:53.67
144	RSRD19	-122.447	37.944	5.91	10.02	296.77	15.93	-122.447	37.944	6.01	10.02	296.33	16.03	1	8/1/2014	8:24:53.76
145	RSRD19	-122.447	37.944	5.86	10.02	296.84	15.88	-122.447	37.944	6.01	10.02	296.40	16.04	1	8/1/2014	8:24:53.87

146	RSRD19	-122.447	37.944	5.91	10.13	296.84	16.04	-122.447	37.944	5.96	10.13	296.36	16.09	1	8/1/2014	8:24:53.97
147	RSRD19	-122.447	37.944	5.86	10.08	296.80	15.94	-122.447	37.944	5.96	10.08	296.38	16.04	1	8/1/2014	8:24:54.07
148	RSRD19	-122.447	37.944	5.91	10.08	296.84	15.99	-122.447	37.944	5.96	10.08	296.38	16.04	1	8/1/2014	8:24:54.16
149	RSRD19	-122.447	37.944	5.86	10.13	296.80	15.99	-122.447	37.944	5.93	10.13	296.29	16.05	1	8/1/2014	8:24:54.28
150	RSRD19	-122.447	37.944	5.86	10.13	296.80	15.99	-122.447	37.944	5.93	10.13	296.29	16.06	1	8/1/2014	8:24:54.37
151	RSRD19	-122.447	37.944	5.86	10.13	296.80	15.99	-122.447	37.944	5.88	10.13	296.30	16.01	1	8/1/2014	8:24:54.47
152	RSRD19	-122.447	37.944	5.86	10.15	296.78	16.01	-122.447	37.944	5.88	10.15	296.23	16.03	1	8/1/2014	8:24:54.56
153	RSRD19	-122.447	37.944	5.82	10.16	296.76	15.99	-122.447	37.944	5.84	10.16	296.23	16.01	1	8/1/2014	8:24:54.67
154	RSRD19	-122.447	37.944	5.82	10.19	296.78	16.01	-122.447	37.944	5.84	10.19	296.24	16.03	1	8/1/2014	8:24:54.77
155	RSRD19	-122.447	37.944	5.82	10.22	296.76	16.04	-122.447	37.944	5.79	10.22	296.20	16.01	1	8/1/2014	8:24:54.87
156	RSRD19	-122.447	37.944	5.82	10.20	296.79	16.02	-122.447	37.944	5.84	10.20	296.21	16.04	1	8/1/2014	8:24:54.96
157	RSRD19	-122.447	37.944	5.82	10.25	296.74	16.07	-122.447	37.944	5.79	10.25	296.24	16.04	1	8/1/2014	8:24:55.08
158	RSRD19	-122.447	37.944	5.82	10.21	296.74	16.03	-122.447	37.944	5.79	10.21	296.19	16.00	1	8/1/2014	8:24:55.17
159	RSRD19	-122.447	37.944	5.77	10.24	296.72	16.01	-122.447	37.944	5.79	10.24	296.19	16.03	1	8/1/2014	8:24:55.27
160	RSRD19	-122.447	37.944	5.82	10.24	296.70	16.06	-122.447	37.944	5.79	10.24	296.17	16.03	1	8/1/2014	8:24:55.36
161	RSRD19	-122.447	37.944	5.77	10.22	296.72	16.00	-122.447	37.944	5.79	10.22	296.19	16.02	1	8/1/2014	8:24:55.48
162	RSRD19	-122.447	37.944	5.82	10.18	296.66	16.01	-122.447	37.944	5.84	10.18	296.15	16.02	1	8/1/2014	8:24:55.57
163	RSRD19	-122.447	37.944	5.77	10.25	296.66	16.02	-122.447	37.944	5.79	10.25	296.17	16.04	1	8/1/2014	8:24:55.67
164	RSRD19	-122.447	37.944	5.77	10.18	296.64	15.95	-122.447	37.944	5.84	10.18	296.13	16.02	1	8/1/2014	8:24:55.76
165	RSRD19	-122.447	37.944	5.77	10.13	296.59	15.91	-122.447	37.944	5.79	10.13	296.11	15.92	1	8/1/2014	8:24:55.88
166	RSRD19	-122.447	37.944	5.77	10.09	296.60	15.87	-122.447	37.944	5.84	10.09	296.09	15.94	1	8/1/2014	8:24:55.97
167	RSRD19	-122.447	37.944	5.77	10.09	296.58	15.87	-122.447	37.944	5.79	10.09	296.11	15.89	1	8/1/2014	8:24:56.07
168	RSRD19	-122.447	37.944	5.77	10.11	296.60	15.88	-122.447	37.944	5.79	10.11	296.09	15.90	1	8/1/2014	8:24:56.16
169	RSRD19	-122.447	37.944	5.74	10.06	296.49	15.79	-122.447	37.944	5.79	10.06	296.11	15.85	1	8/1/2014	8:24:56.27
170	RSRD19	-122.447	37.944	5.74	10.00	296.53	15.74	-122.447	37.944	5.79	10.00	296.11	15.80	1	8/1/2014	8:24:56.37
171	RSRD19	-122.447	37.944	5.74	10.03	296.53	15.77	-122.447	37.944	5.79	10.03	296.09	15.82	1	8/1/2014	8:24:56.47
172	RSRD19	-122.447	37.944	5.77	10.02	296.47	15.80	-122.447	37.944	5.79	10.02	296.09	15.82	1	8/1/2014	8:24:56.56
173	RSRD19	-122.447	37.944	5.74	9.99	296.49	15.73	-122.447	37.944	5.76	9.99	296.09	15.75	1	8/1/2014	8:24:56.67
174	RSRD19	-122.447	37.944	5.77	10.04	296.42	15.82	-122.447	37.944	5.79	10.04	296.03	15.83	1	8/1/2014	8:24:56.77
175	RSRD19	-122.447	37.944	5.74	10.09	296.45	15.83	-122.447	37.944	5.79	10.09	296.05	15.89	1	8/1/2014	8:24:56.87
176	RSRD19	-122.447	37.944	5.74	10.00	296.40	15.74	-122.447	37.944	5.76	10.00	296.03	15.76	1	8/1/2014	8:24:56.96
177	RSRD19	-122.447	37.944	5.74	9.97	296.42	15.71	-122.447	37.944	5.79	9.97	296.03	15.76	1	8/1/2014	8:24:57.08
178	RSRD19	-122.447	37.944	5.74	9.98	296.40	15.72	-122.448	37.944	5.79	9.98	295.99	15.77	1	8/1/2014	8:24:57.17

179	RSRD19	-122.447	37.944	5.74	10.01	296.34	15.75	-122.448	37.944	5.76	10.01	295.99	15.77	1	8/1/2014	8:24:57.27
180	RSRD19	-122.448	37.944	5.74	9.99	296.38	15.73	-122.448	37.944	5.79	9.99	295.90	15.78	1	8/1/2014	8:24:57.36
181	RSRD19	-122.448	37.944	5.70	10.02	296.32	15.73	-122.448	37.944	5.76	10.02	295.92	15.78	1	8/1/2014	8:24:57.47
182	RSRD19	-122.448	37.944	5.74	10.00	296.34	15.74	-122.448	37.944	5.79	10.00	295.88	15.80	1	8/1/2014	8:24:57.57
183	RSRD19	-122.448	37.944	5.74	10.00	296.34	15.74	-122.448	37.944	5.79	10.00	295.86	15.80	1	8/1/2014	8:24:57.67
184	RSRD19	-122.448	37.944	5.74	9.99	296.32	15.73	-122.448	37.944	5.79	9.99	295.86	15.78	1	8/1/2014	8:24:57.76
185	RSRD19	-122.448	37.944	5.74	10.02	296.32	15.76	-122.448	37.944	5.79	10.02	295.83	15.82	1	8/1/2014	8:24:57.88
186	RSRD19	-122.448	37.944	5.74	10.02	296.34	15.76	-122.448	37.944	5.79	10.02	295.79	15.82	1	8/1/2014	8:24:57.97
187	RSRD19	-122.448	37.944	5.74	10.02	296.30	15.76	-122.448	37.944	5.79	10.02	295.79	15.82	1	8/1/2014	8:24:58.07
188	RSRD19	-122.448	37.944	5.77	10.02	296.30	15.80	-122.448	37.944	5.84	10.02	295.82	15.87	1	8/1/2014	8:24:58.16
189	RSRD19	-122.448	37.944	5.74	10.03	296.30	15.77	-122.448	37.944	5.79	10.03	295.79	15.82	1	8/1/2014	8:24:58.28
190	RSRD19	-122.448	37.944	5.74	10.06	296.30	15.80	-122.448	37.944	5.79	10.06	295.75	15.85	1	8/1/2014	8:24:58.37
191	RSRD19	-122.448	37.944	5.74	10.11	296.30	15.85	-122.448	37.944	5.79	10.11	295.82	15.90	1	8/1/2014	8:24:58.47
192	RSRD19	-122.448	37.944	5.74	10.15	296.32	15.89	-122.448	37.944	5.79	10.15	295.79	15.94	1	8/1/2014	8:24:58.56
193	RSRD19	-122.448	37.944	5.74	10.16	296.26	15.90	-122.448	37.944	5.79	10.16	295.82	15.96	1	8/1/2014	8:24:58.68
194	RSRD19	-122.448	37.944	5.74	10.20	296.32	15.94	-122.448	37.944	5.76	10.20	295.80	15.96	1	8/1/2014	8:24:58.77
195	RSRD19	-122.448	37.944	5.70	10.21	296.26	15.91	-122.448	37.944	5.76	10.21	295.82	15.97	1	8/1/2014	8:24:58.87
196	RSRD19	-122.448	37.944	5.74	10.20	296.26	15.94	-122.448	37.944	5.76	10.20	295.79	15.96	1	8/1/2014	8:24:58.96
197	RSRD19	-122.448	37.944	5.74	10.22	296.28	15.96	-122.448	37.944	5.76	10.22	295.77	15.98	1	8/1/2014	8:24:59.07
198	RSRD19	-122.448	37.944	5.74	10.25	296.26	15.99	-122.448	37.944	5.79	10.25	295.79	16.04	1	8/1/2014	8:24:59.17
199	RSRD19	-122.448	37.944	5.74	10.23	296.26	15.97	-122.448	37.944	5.84	10.23	295.75	16.08	1	8/1/2014	8:24:59.27
200	RSRD19	-122.448	37.944	5.74	10.24	296.28	15.98	-122.448	37.944	5.79	10.24	295.75	16.03	1	8/1/2014	8:24:59.36
201	RSRD19	-122.448	37.944	5.74	10.24	296.26	15.98	-122.448	37.944	5.76	10.24	295.73	16.00	1	8/1/2014	8:24:59.47
202	RSRD19	-122.448	37.944	5.74	10.24	296.19	15.98	-122.448	37.944	5.79	10.24	295.73	16.03	1	8/1/2014	8:24:59.57
203	RSRD19	-122.448	37.944	5.74	10.24	296.24	15.98	-122.448	37.944	5.79	10.24	295.77	16.03	1	8/1/2014	8:24:59.67
204	RSRD19	-122.448	37.944	5.74	10.22	296.24	15.96	-122.448	37.944	5.84	10.22	295.75	16.07	1	8/1/2014	8:24:59.76
205	RSRD19	-122.448	37.944	5.74	10.24	296.21	15.98	-122.448	37.944	5.79	10.24	295.75	16.03	1	8/1/2014	8:24:59.88
206	RSRD19	-122.448	37.944	5.74	10.22	296.17	15.96	-122.448	37.944	5.84	10.22	295.69	16.06	1	8/1/2014	8:24:59.97
207	RSRD19	-122.448	37.944	5.74	10.20	296.19	15.94	-122.448	37.944	5.79	10.20	295.71	15.99	1	8/1/2014	8:25:00.07
208	RSRD19	-122.448	37.944	5.74	10.20	296.20	15.94	-122.448	37.944	5.79	10.20	295.69	15.99	1	8/1/2014	8:25:00.16
209	RSRD19	-122.448	37.944	5.74	10.20	296.21	15.94	-122.448	37.944	5.84	10.20	295.69	16.04	1	8/1/2014	8:25:00.27
210	RSRD19	-122.448	37.944	5.74	10.17	296.20	15.91	-122.448	37.944	5.79	10.17	295.69	15.96	1	8/1/2014	8:25:00.37
211	RSRD19	-122.448	37.944	5.70	10.15	296.22	15.86	-122.448	37.944	5.79	10.15	295.69	15.95	1	8/1/2014	8:25:00.47

212	RSRD19	-122.448	37.944	5.74	10.16	296.19	15.90	-122.448	37.944	5.79	10.16	295.64	15.96	1	8/1/2014	8:25:00.56
213	RSRD19	-122.448	37.944	5.74	10.15	296.17	15.89	-122.448	37.944	5.79	10.15	295.67	15.95	1	8/1/2014	8:25:00.67
214	RSRD19	-122.448	37.944	5.74	10.15	296.15	15.89	-122.448	37.944	5.79	10.15	295.62	15.95	1	8/1/2014	8:25:00.77
215	RSRD19	-122.448	37.944	5.74	10.17	296.13	15.91	-122.448	37.944	5.76	10.17	295.62	15.93	1	8/1/2014	8:25:00.87
216	RSRD19	-122.448	37.944	5.74	10.18	296.08	15.92	-122.448	37.944	5.79	10.18	295.60	15.97	1	8/1/2014	8:25:00.96
217	RSRD19	-122.448	37.944	5.70	10.18	296.09	15.89	-122.448	37.944	5.76	10.18	295.62	15.94	1	8/1/2014	8:25:01.07
218	RSRD19	-122.448	37.944	5.74	10.21	296.06	15.95	-122.448	37.944	5.76	10.21	295.63	15.97	1	8/1/2014	8:25:01.17
219	RSRD19	-122.448	37.944	5.74	10.29	296.09	16.03	-122.448	37.944	5.76	10.29	295.62	16.05	1	8/1/2014	8:25:01.27
220	RSRD19	-122.448	37.944	5.74	10.26	296.02	15.99	-122.448	37.944	5.76	10.26	295.60	16.01	1	8/1/2014	8:25:01.36
221	RSRD19	-122.448	37.944	5.70	10.28	295.95	15.98	-122.448	37.944	5.76	10.28	295.56	16.04	1	8/1/2014	8:25:01.47
222	RSRD19	-122.448	37.944	5.70	10.30	295.97	16.01	-122.448	37.944	5.76	10.30	295.53	16.06	1	8/1/2014	8:25:01.57
223	RSRD19	-122.448	37.944	5.70	10.27	295.91	15.97	-122.448	37.944	5.76	10.27	295.49	16.02	1	8/1/2014	8:25:01.67
224	RSRD19	-122.448	37.944	5.74	10.25	295.86	15.99	-122.448	37.944	5.79	10.25	295.47	16.04	1	8/1/2014	8:25:01.76
225	RSRD19	-122.448	37.944	5.70	10.25	295.80	15.95	-122.448	37.944	5.76	10.25	295.42	16.01	1	8/1/2014	8:25:01.87
226	RSRD19	-122.448	37.944	5.70	10.22	295.80	15.93	-122.448	37.944	5.79	10.22	295.40	16.02	1	8/1/2014	8:25:01.97
227	RSRD19	-122.448	37.944	5.74	10.20	295.78	15.93	-122.448	37.944	5.79	10.20	295.36	15.99	1	8/1/2014	8:25:02.07
228	RSRD19	-122.448	37.944	5.74	10.18	295.69	15.92	-122.448	37.944	5.84	10.18	295.34	16.02	1	8/1/2014	8:25:02.16
229	RSRD19	-122.448	37.944	5.74	10.17	295.64	15.91	-122.448	37.944	5.84	10.17	295.30	16.01	1	8/1/2014	8:25:02.27
230	RSRD19	-122.448	37.944	5.74	10.14	295.62	15.88	-122.448	37.944	5.84	10.14	295.29	15.98	1	8/1/2014	8:25:02.37
231	RSRD19	-122.448	37.944	5.74	10.13	295.60	15.87	-122.448	37.944	5.84	10.13	295.20	15.97	1	8/1/2014	8:25:02.47
232	RSRD19	-122.448	37.944	5.74	10.07	295.53	15.81	-122.448	37.944	5.88	10.07	295.20	15.95	1	8/1/2014	8:25:02.56
233	RSRD19	-122.448	37.944	5.74	10.06	295.53	15.80	-122.448	37.944	5.88	10.06	295.18	15.94	1	8/1/2014	8:25:02.67
234	RSRD19	-122.448	37.944	5.74	10.06	295.49	15.79	-122.448	37.944	5.93	10.06	295.16	15.98	1	8/1/2014	8:25:02.77
235	RSRD19	-122.448	37.944	5.74	10.03	295.49	15.77	-122.448	37.944	5.88	10.03	295.18	15.91	1	8/1/2014	8:25:02.87
236	RSRD19	-122.448	37.944	5.77	10.02	295.47	15.79	-122.448	37.944	5.93	10.02	295.13	15.94	1	8/1/2014	8:25:02.96
237	RSRD19	-122.448	37.944	5.74	10.09	295.42	15.82	-122.448	37.944	5.88	10.09	295.12	15.96	1	8/1/2014	8:25:03.07
238	RSRD19	-122.448	37.944	5.77	10.03	295.42	15.81	-122.448	37.944	5.93	10.03	295.09	15.96	1	8/1/2014	8:25:03.17
239	RSRD19	-122.448	37.944	5.74	10.10	295.35	15.84	-122.448	37.944	5.88	10.10	295.07	15.98	1	8/1/2014	8:25:03.27
240	RSRD19	-122.448	37.944	5.77	10.06	295.38	15.83	-122.448	37.944	5.93	10.06	295.01	15.98	1	8/1/2014	8:25:03.36
241	RSRD19	-122.448	37.944	5.74	10.12	295.38	15.86	-122.448	37.944	5.88	10.12	295.00	15.99	1	8/1/2014	8:25:03.47
242	RSRD19	-122.448	37.944	5.77	10.09	295.33	15.86	-122.448	37.944	5.88	10.09	294.96	15.96	1	8/1/2014	8:25:03.57
243	RSRD19	-122.448	37.944	5.74	10.13	295.33	15.87	-122.448	37.944	5.88	10.13	294.93	16.00	1	8/1/2014	8:25:03.67
244	RSRD19	-122.448	37.944	5.77	10.08	295.29	15.85	-122.448	37.944	5.93	10.08	294.89	16.01	1	8/1/2014	8:25:03.76

245	RSRD19	-122.448	37.944	5.74	10.11	295.27	15.85	-122.448	37.944	5.93	10.11	294.87	16.04	1	8/1/2014	8:25:03.87
246	RSRD19	-122.448	37.944	5.77	10.08	295.24	15.85	-122.448	37.944	5.88	10.08	294.85	15.96	1	8/1/2014	8:25:03.97
247	RSRD19	-122.448	37.944	5.74	10.16	295.18	15.90	-122.448	37.944	5.88	10.16	294.74	16.04	1	8/1/2014	8:25:04.07
248	RSRD19	-122.448	37.944	5.77	10.14	295.18	15.91	-122.448	37.944	5.88	10.14	294.76	16.02	1	8/1/2014	8:25:04.16
249	RSRD19	-122.448	37.944	5.74	10.11	295.11	15.85	-122.448	37.944	5.84	10.11	294.69	15.95	1	8/1/2014	8:25:04.27
250	RSRD19	-122.448	37.944	5.77	10.14	295.07	15.91	-122.448	37.944	5.88	10.14	294.63	16.02	1	8/1/2014	8:25:04.37
251	RSRD19	-122.448	37.944	5.74	10.13	294.98	15.87	-122.448	37.944	5.88	10.13	294.58	16.00	1	8/1/2014	8:25:04.47
252	RSRD19	-122.448	37.944	5.77	10.13	294.98	15.91	-122.448	37.944	5.88	10.13	294.52	16.01	1	8/1/2014	8:25:04.56
253	RSRD19	-122.448	37.944	5.74	10.16	294.89	15.90	-122.448	37.944	5.88	10.16	294.45	16.04	1	8/1/2014	8:25:04.67
254	RSRD19	-122.448	37.944	5.77	10.22	294.85	16.00	-122.448	37.944	5.88	10.22	294.41	16.10	1	8/1/2014	8:25:04.77
255	RSRD19	-122.448	37.944	5.77	10.20	294.83	15.97	-122.448	37.944	5.84	10.20	294.36	16.04	1	8/1/2014	8:25:04.87
256	RSRD19	-122.448	37.944	5.77	10.21	294.81	15.98	-122.448	37.944	5.88	10.21	294.34	16.09	1	8/1/2014	8:25:04.96
257	RSRD19	-122.448	37.944	5.77	10.25	294.78	16.02	-122.448	37.944	5.84	10.25	294.34	16.09	1	8/1/2014	8:25:05.07
258	RSRD19	-122.448	37.944	5.77	10.31	294.72	16.08	-122.448	37.944	5.84	10.31	294.32	16.15	1	8/1/2014	8:25:05.17
259	RSRD19	-122.448	37.944	5.74	10.29	294.70	16.03	-122.448	37.944	5.84	10.29	294.28	16.14	1	8/1/2014	8:25:05.27
260	RSRD19	-122.448	37.944	5.74	10.37	294.70	16.11	-122.448	37.944	5.84	10.37	294.28	16.21	1	8/1/2014	8:25:05.36
261	RSRD19	-122.448	37.944	5.74	10.34	294.61	16.08	-122.448	37.944	5.79	10.34	294.17	16.13	1	8/1/2014	8:25:05.47
262	RSRD19	-122.448	37.944	5.74	10.34	294.61	16.08	-122.448	37.944	5.79	10.34	294.21	16.13	1	8/1/2014	8:25:05.57
263	RSRD19	-122.448	37.944	5.70	10.41	294.56	16.11	-122.448	37.944	5.76	10.41	294.17	16.17	1	8/1/2014	8:25:05.67
264	RSRD19	-122.448	37.944	5.74	10.38	294.54	16.12	-122.448	37.944	5.79	10.38	294.17	16.17	1	8/1/2014	8:25:05.76
265	RSRD19	-122.448	37.944	5.70	10.37	294.50	16.08	-122.448	37.944	5.76	10.37	294.12	16.13	1	8/1/2014	8:25:05.87
266	RSRD19	-122.448	37.944	5.74	10.41	294.48	16.15	-122.448	37.944	5.76	10.41	294.08	16.17	1	8/1/2014	8:25:05.97
267	RSRD19	-122.448	37.944	5.70	10.41	294.43	16.11	-122.448	37.944	5.76	10.41	294.01	16.17	1	8/1/2014	8:25:06.07
268	RSRD19	-122.448	37.944	5.70	10.44	294.43	16.15	-122.448	37.944	5.76	10.44	294.01	16.20	1	8/1/2014	8:25:06.16
269	RSRD19	-122.448	37.944	5.70	10.48	294.43	16.18	-122.448	37.944	5.71	10.48	293.99	16.19	1	8/1/2014	8:25:06.27
270	RSRD19	-122.448	37.944	5.70	10.46	294.38	16.17	-122.448	37.944	5.71	10.46	293.92	16.17	1	8/1/2014	8:25:06.37
271	RSRD19	-122.448	37.944	5.65	10.46	294.38	16.12	-122.448	37.944	5.67	10.46	293.92	16.14	1	8/1/2014	8:25:06.47
272	RSRD19	-122.448	37.944	5.70	10.48	294.36	16.18	-122.448	37.944	5.67	10.48	293.90	16.15	1	8/1/2014	8:25:06.56
273	RSRD19	-122.448	37.944	5.65	10.50	294.36	16.15	-122.448	37.944	5.67	10.50	293.85	16.17	1	8/1/2014	8:25:06.67
274	RSRD19	-122.448	37.944	5.70	10.50	294.34	16.21	-122.448	37.944	5.67	10.50	293.86	16.18	1	8/1/2014	8:25:06.77
275	RSRD19	-122.448	37.944	5.65	10.52	294.36	16.17	-122.448	37.944	5.67	10.52	293.83	16.19	1	8/1/2014	8:25:06.87
276	RSRD19	-122.448	37.944	5.70	10.58	294.36	16.29	-122.448	37.944	5.67	10.58	293.90	16.26	1	8/1/2014	8:25:06.96
277	RSRD19	-122.448	37.944	5.65	10.53	294.27	16.18	-122.448	37.944	5.64	10.53	293.81	16.17	1	8/1/2014	8:25:07.07

278	RSRD19	-122.448	37.944	5.70	10.53	294.32	16.24	-122.448	37.944	5.67	10.53	293.88	16.21	1	8/1/2014	8:25:07.17
279	RSRD19	-122.448	37.944	5.65	10.53	294.27	16.18	-122.448	37.944	5.64	10.53	293.83	16.17	1	8/1/2014	8:25:07.27
280	RSRD19	-122.448	37.944	5.65	10.55	294.25	16.20	-122.448	37.944	5.67	10.55	293.88	16.22	1	8/1/2014	8:25:07.36
281	RSRD19	-122.448	37.944	5.65	10.54	294.27	16.19	-122.448	37.944	5.64	10.54	293.85	16.18	1	8/1/2014	8:25:07.47
282	RSRD19	-122.448	37.944	5.65	10.53	294.23	16.18	-122.448	37.944	5.64	10.53	293.86	16.17	1	8/1/2014	8:25:07.57
283	RSRD19	-122.448	37.944	5.65	10.54	294.27	16.19	-122.448	37.944	5.64	10.54	293.85	16.18	1	8/1/2014	8:25:07.67
284	RSRD19	-122.448	37.944	5.65	10.51	294.23	16.17	-122.448	37.944	5.67	10.51	293.94	16.19	1	8/1/2014	8:25:07.76
285	RSRD19	-122.448	37.944	5.65	10.50	294.25	16.15	-122.448	37.944	5.64	10.50	293.90	16.14	1	8/1/2014	8:25:07.87
286	RSRD19	-122.448	37.944	5.65	10.53	294.20	16.19	-122.448	37.944	5.67	10.53	293.89	16.21	1	8/1/2014	8:25:07.97
287	RSRD19	-122.448	37.944	5.65	10.50	294.23	16.15	-122.448	37.944	5.67	10.50	293.94	16.17	1	8/1/2014	8:25:08.07
288	RSRD19	-122.448	37.944	5.65	10.50	294.23	16.15	-122.448	37.944	5.67	10.50	293.94	16.17	1	8/1/2014	8:25:08.16
289	RSRD19	-122.448	37.944	5.65	10.52	294.20	16.17	-122.448	37.944	5.64	10.52	293.96	16.16	1	8/1/2014	8:25:08.27
290	RSRD19	-122.448	37.944	5.65	10.50	294.22	16.15	-122.448	37.944	5.67	10.50	293.98	16.17	1	8/1/2014	8:25:08.37
291	RSRD19	-122.448	37.944	5.65	10.49	294.22	16.14	-122.448	37.944	5.67	10.49	293.98	16.16	1	8/1/2014	8:25:08.47
292	RSRD19	-122.448	37.944	5.65	10.50	294.22	16.16	-122.448	37.944	5.67	10.50	293.93	16.18	1	8/1/2014	8:25:08.56
293	RSRD19	-122.448	37.944	5.62	10.47	294.24	16.09	-122.448	37.944	5.67	10.47	293.98	16.14	1	8/1/2014	8:25:08.67
294	RSRD19	-122.448	37.944	5.65	10.46	294.20	16.12	-122.448	37.944	5.71	10.46	293.91	16.17	1	8/1/2014	8:25:08.77
295	RSRD19	-122.448	37.944	5.65	10.49	294.22	16.14	-122.448	37.944	5.67	10.49	293.96	16.16	1	8/1/2014	8:25:08.87
296	RSRD19	-122.448	37.944	5.65	10.46	294.22	16.12	-122.448	37.944	5.71	10.46	293.91	16.17	1	8/1/2014	8:25:08.96
297	RSRD19	-122.448	37.944	5.65	10.43	294.21	16.09	-122.448	37.944	5.71	10.43	293.93	16.14	1	8/1/2014	8:25:09.07
298	RSRD19	-122.448	37.944	5.65	10.41	294.20	16.06	-122.448	37.944	5.76	10.41	293.98	16.17	1	8/1/2014	8:25:09.17
299	RSRD19	-122.448	37.944	5.65	10.40	294.24	16.06	-122.448	37.944	5.71	10.40	293.97	16.11	1	8/1/2014	8:25:09.27
300	RSRD19	-122.448	37.944	5.65	10.40	294.22	16.06	-122.448	37.944	5.71	10.40	293.98	16.11	1	8/1/2014	8:25:09.36
301	RSRD19	-122.448	37.944	5.65	10.43	294.24	16.09	-122.448	37.944	5.71	10.43	293.97	16.14	1	8/1/2014	8:25:09.47
302	RSRD19	-122.448	37.944	5.65	10.42	294.28	16.07	-122.448	37.944	5.71	10.42	294.00	16.13	1	8/1/2014	8:25:09.57
303	RSRD19	-122.448	37.944	5.65	10.40	294.19	16.06	-122.448	37.944	5.71	10.40	293.99	16.11	1	8/1/2014	8:25:09.66
304	RSRD19	-122.448	37.944	5.65	10.42	294.26	16.07	-122.448	37.944	5.71	10.42	294.04	16.13	1	8/1/2014	8:25:09.76
305	RSRD19	-122.448	37.944	5.65	10.40	294.23	16.06	-122.448	37.944	5.71	10.40	294.06	16.11	1	8/1/2014	8:25:09.87
306	RSRD19	-122.448	37.944	5.65	10.40	294.28	16.05	-122.448	37.944	5.71	10.40	294.08	16.10	1	8/1/2014	8:25:09.97
307	RSRD19	-122.448	37.944	5.65	10.43	294.27	16.08	-122.448	37.944	5.71	10.43	294.04	16.13	1	8/1/2014	8:25:10.06
308	RSRD19	-122.448	37.944	5.65	10.44	294.32	16.10	-122.448	37.944	5.71	10.44	294.12	16.15	1	8/1/2014	8:25:10.16
309	RSRD19	-122.448	37.944	5.65	10.41	294.34	16.06	-122.448	37.944	5.71	10.41	294.23	16.12	1	8/1/2014	8:25:10.27
310	RSRD19	-122.448	37.944	5.65	10.43	294.38	16.08	-122.448	37.944	5.76	10.43	294.12	16.18	1	8/1/2014	8:25:10.37

311	RSRD19	-122.448	37.944	5.62	10.43	294.38	16.05	-122.448	37.944	5.71	10.43	294.20	16.14	1	8/1/2014	8:25:10.47
312	RSRD19	-122.448	37.944	5.65	10.43	294.42	16.09	-122.448	37.944	5.71	10.43	294.25	16.14	1	8/1/2014	8:25:10.56
313	RSRD19	-122.448	37.944	5.62	10.43	294.49	16.05	-122.448	37.944	5.71	10.43	294.27	16.14	1	8/1/2014	8:25:10.67
314	RSRD19	-122.448	37.944	5.65	10.45	294.51	16.10	-122.448	37.944	5.67	10.45	294.29	16.12	1	8/1/2014	8:25:10.77
315	RSRD19	-122.448	37.944	5.62	10.50	294.53	16.12	-122.448	37.944	5.67	10.50	294.36	16.18	1	8/1/2014	8:25:10.87
316	RSRD19	-122.448	37.944	5.62	10.50	294.53	16.12	-122.448	37.944	5.67	10.50	294.35	16.18	1	8/1/2014	8:25:10.96
317	RSRD19	-122.448	37.944	5.62	10.46	294.61	16.08	-122.448	37.944	5.67	10.46	294.42	16.14	1	8/1/2014	8:25:11.07
318	RSRD19	-122.448	37.944	5.65	10.48	294.66	16.13	-122.448	37.944	5.67	10.48	294.46	16.15	1	8/1/2014	8:25:11.17
319	RSRD19	-122.448	37.944	5.62	10.50	294.66	16.12	-122.448	37.944	5.64	10.50	294.46	16.14	1	8/1/2014	8:25:11.27
320	RSRD19	-122.448	37.944	5.62	10.49	294.74	16.11	-122.448	37.944	5.64	10.49	294.50	16.13	1	8/1/2014	8:25:11.36
321	RSRD19	-122.448	37.944	5.62	10.53	294.74	16.15	-122.448	37.944	5.64	10.53	294.52	16.17	1	8/1/2014	8:25:11.47
322	RSRD19	-122.448	37.944	5.62	10.50	294.74	16.12	-122.448	37.944	5.67	10.50	294.56	16.18	1	8/1/2014	8:25:11.57
323	RSRD19	-122.448	37.944	5.62	10.51	294.80	16.13	-122.448	37.944	5.59	10.51	294.56	16.10	1	8/1/2014	8:25:11.67
324	RSRD19	-122.448	37.944	5.62	10.56	294.82	16.18	-122.448	37.944	5.64	10.56	294.63	16.20	1	8/1/2014	8:25:11.76
325	RSRD19	-122.448	37.944	5.57	10.51	294.85	16.08	-122.448	37.944	5.64	10.51	294.64	16.15	1	8/1/2014	8:25:11.87
326	RSRD19	-122.448	37.944	5.62	10.52	294.89	16.14	-122.448	37.944	5.64	10.52	294.67	16.16	1	8/1/2014	8:25:11.97
327	RSRD19	-122.448	37.944	5.62	10.51	294.91	16.13	-122.448	37.944	5.64	10.51	294.71	16.15	1	8/1/2014	8:25:12.07
328	RSRD19	-122.448	37.944	5.62	10.50	294.88	16.12	-122.448	37.944	5.64	10.50	294.71	16.14	1	8/1/2014	8:25:12.16
329	RSRD19	-122.448	37.944	5.62	10.50	294.95	16.12	-122.448	37.944	5.64	10.50	294.73	16.14	1	8/1/2014	8:25:12.27
330	RSRD19	-122.448	37.944	5.62	10.47	294.94	16.09	-122.448	37.944	5.64	10.47	294.75	16.11	1	8/1/2014	8:25:12.37
331	RSRD19	-122.448	37.944	5.62	10.47	294.94	16.09	-122.448	37.944	5.64	10.47	294.76	16.11	1	8/1/2014	8:25:12.47
332	RSRD19	-122.448	37.944	5.62	10.46	294.96	16.08	-122.448	37.944	5.67	10.46	294.77	16.14	1	8/1/2014	8:25:12.56
333	RSRD19	-122.448	37.944	5.62	10.46	294.96	16.08	-122.448	37.944	5.67	10.46	294.83	16.13	1	8/1/2014	8:25:12.67
334	RSRD19	-122.448	37.944	5.62	10.43	294.96	16.05	-122.448	37.944	5.67	10.43	294.85	16.10	1	8/1/2014	8:25:12.77
335	RSRD19	-122.448	37.944	5.62	10.40	294.98	16.02	-122.448	37.944	5.71	10.40	294.84	16.10	1	8/1/2014	8:25:12.87
336	RSRD19	-122.448	37.944	5.62	10.38	294.95	16.00	-122.448	37.944	5.71	10.38	294.83	16.09	1	8/1/2014	8:25:12.96
337	RSRD19	-122.448	37.944	5.62	10.38	294.95	16.00	-122.448	37.944	5.71	10.38	294.84	16.09	1	8/1/2014	8:25:13.07
338	RSRD19	-122.448	37.944	5.65	10.35	294.99	16.00	-122.448	37.944	5.76	10.35	294.86	16.11	1	8/1/2014	8:25:13.17
339	RSRD19	-122.448	37.944	5.62	10.34	294.97	15.95	-122.448	37.944	5.76	10.34	294.86	16.09	1	8/1/2014	8:25:13.27
340	RSRD19	-122.448	37.944	5.65	10.30	295.06	15.96	-122.448	37.944	5.79	10.30	294.86	16.10	1	8/1/2014	8:25:13.36
341	RSRD19	-122.448	37.944	5.62	10.29	295.05	15.91	-122.448	37.944	5.76	10.29	294.88	16.05	1	8/1/2014	8:25:13.47
342	RSRD19	-122.448	37.944	5.65	10.29	295.01	15.95	-122.448	37.944	5.79	10.29	294.92	16.09	1	8/1/2014	8:25:13.57
343	RSRD19	-122.448	37.944	5.65	10.29	295.07	15.95	-122.448	37.944	5.79	10.29	294.94	16.09	1	8/1/2014	8:25:13.67

344	RSRD19	-122.448	37.944	5.65	10.27	295.10	15.92	-122.448	37.944	5.79	10.27	294.96	16.06	1	8/1/2014	8:25:13.76
345	RSRD19	-122.448	37.944	5.65	10.27	295.09	15.92	-122.448	37.944	5.79	10.27	294.96	16.06	1	8/1/2014	8:25:13.87
346	RSRD19	-122.448	37.944	5.70	10.24	295.13	15.94	-122.448	37.944	5.84	10.24	295.02	16.08	1	8/1/2014	8:25:13.97
347	RSRD19	-122.448	37.944	5.65	10.25	295.15	15.90	-122.448	37.944	5.79	10.25	295.07	16.04	1	8/1/2014	8:25:14.07
348	RSRD19	-122.448	37.944	5.70	10.27	295.19	15.98	-122.448	37.944	5.84	10.27	295.06	16.11	1	8/1/2014	8:25:14.16
349	RSRD19	-122.448	37.944	5.65	10.22	295.22	15.87	-122.448	37.944	5.79	10.22	295.11	16.01	1	8/1/2014	8:25:14.27
350	RSRD19	-122.448	37.944	5.70	10.23	295.23	15.94	-122.448	37.944	5.84	10.23	295.17	16.08	1	8/1/2014	8:25:14.37
351	RSRD19	-122.448	37.944	5.70	10.22	295.23	15.92	-122.448	37.944	5.84	10.22	295.16	16.06	1	8/1/2014	8:25:14.47
352	RSRD19	-122.448	37.944	5.70	10.20	295.28	15.91	-122.448	37.944	5.88	10.20	295.21	16.08	1	8/1/2014	8:25:14.56
353	RSRD19	-122.448	37.944	5.70	10.20	295.29	15.90	-122.448	37.944	5.84	10.20	295.25	16.04	1	8/1/2014	8:25:14.67
354	RSRD19	-122.448	37.944	5.70	10.20	295.32	15.91	-122.448	37.944	5.88	10.20	295.21	16.08	1	8/1/2014	8:25:14.77
355	RSRD19	-122.448	37.944	5.70	10.17	295.36	15.88	-122.448	37.944	5.88	10.17	295.27	16.05	1	8/1/2014	8:25:14.87
356	RSRD19	-122.448	37.944	5.74	10.17	295.36	15.91	-122.448	37.944	5.93	10.17	295.27	16.10	1	8/1/2014	8:25:14.96
357	RSRD19	-122.448	37.944	5.70	10.16	295.40	15.87	-122.448	37.944	5.88	10.16	295.31	16.04	1	8/1/2014	8:25:15.07
358	RSRD19	-122.448	37.944	5.74	10.14	295.40	15.88	-122.448	37.944	5.93	10.14	295.31	16.07	1	8/1/2014	8:25:15.17
359	RSRD19	-122.448	37.944	5.74	10.15	295.42	15.89	-122.448	37.944	5.93	10.15	295.31	16.08	1	8/1/2014	8:25:15.27
360	RSRD19	-122.448	37.944	5.74	10.13	295.42	15.87	-122.448	37.944	5.96	10.13	295.33	16.09	1	8/1/2014	8:25:15.36
361	RSRD19	-122.448	37.944	5.74	10.12	295.41	15.86	-122.448	37.944	5.93	10.12	295.35	16.04	1	8/1/2014	8:25:15.47
362	RSRD19	-122.448	37.944	5.74	10.12	295.39	15.86	-122.448	37.944	6.01	10.12	295.37	16.13	1	8/1/2014	8:25:15.57
363	RSRD19	-122.448	37.944	5.74	10.09	295.41	15.82	-122.448	37.944	5.96	10.09	295.33	16.05	1	8/1/2014	8:25:15.67
364	RSRD19	-122.448	37.944	5.74	10.09	295.41	15.83	-122.448	37.944	6.01	10.09	295.38	16.11	1	8/1/2014	8:25:15.76
365	RSRD19	-122.448	37.944	5.74	10.06	295.36	15.80	-122.448	37.944	6.01	10.06	295.36	16.07	1	8/1/2014	8:25:15.87
366	RSRD19	-122.448	37.944	5.77	10.03	295.38	15.81	-122.448	37.944	6.05	10.03	295.38	16.08	1	8/1/2014	8:25:15.97
367	RSRD19	-122.448	37.944	5.77	10.04	295.36	15.82	-122.448	37.944	6.05	10.04	295.36	16.09	1	8/1/2014	8:25:16.07
368	RSRD19	-122.448	37.944	5.77	10.01	295.34	15.78	-122.448	37.944	6.08	10.01	295.38	16.09	1	8/1/2014	8:25:16.16
369	RSRD19	-122.448	37.944	5.77	10.01	295.36	15.78	-122.448	37.944	6.05	10.01	295.42	16.05	1	8/1/2014	8:25:16.27
370	RSRD19	-122.448	37.944	5.82	9.98	295.31	15.80	-122.448	37.944	6.08	9.98	295.40	16.06	1	8/1/2014	8:25:16.37
371	RSRD19	-122.448	37.944	5.77	10.01	295.33	15.78	-122.448	37.944	6.08	10.01	295.35	16.09	1	8/1/2014	8:25:16.47
372	RSRD19	-122.448	37.944	5.82	9.95	295.35	15.78	-122.448	37.944	6.08	9.95	295.38	16.03	1	8/1/2014	8:25:16.56
373	RSRD19	-122.448	37.944	5.77	9.92	295.33	15.70	-122.448	37.944	6.08	9.92	295.37	16.00	1	8/1/2014	8:25:16.67
374	RSRD19	-122.448	37.944	5.82	9.91	295.35	15.73	-122.448	37.944	6.13	9.91	295.37	16.04	1	8/1/2014	8:25:16.77
375	RSRD19	-122.448	37.944	5.82	9.87	295.33	15.69	-122.448	37.944	6.16	9.87	295.39	16.03	1	8/1/2014	8:25:16.87
376	RSRD19	-122.448	37.944	5.86	9.85	295.33	15.71	-122.448	37.944	6.16	9.85	295.37	16.02	1	8/1/2014	8:25:16.96

377	RSRD19	-122.448	37.944	5.86	9.84	295.33	15.70	-122.448	37.944	6.16	9.84	295.35	16.00	1	8/1/2014 8:25:17.07
378	RSRD19	-122.448	37.944	5.86	9.78	295.26	15.64	-122.448	37.944	6.22	9.78	295.35	16.00	1	8/1/2014 8:25:17.17
379	RSRD19	-122.448	37.944	5.86	9.79	295.28	15.65	-122.448	37.944	6.22	9.79	295.30	16.01	1	8/1/2014 8:25:17.27
380	RSRD19	-122.448	37.944	5.91	9.76	295.23	15.67	-122.448	37.944	6.25	9.76	295.30	16.01	1	8/1/2014 8:25:17.36
381	RSRD19	-122.448	37.944	5.86	9.68	295.19	15.54	-122.448	37.944	6.25	9.68	295.28	15.93	1	8/1/2014 8:25:17.47
382	RSRD19	-122.448	37.944	5.95	9.68	295.19	15.62	-122.448	37.944	6.30	9.68	295.23	15.98	1	8/1/2014 8:25:17.57
383	RSRD19	-122.448	37.944	5.91	9.62	295.21	15.54	-122.448	37.944	6.30	9.62	295.25	15.92	1	8/1/2014 8:25:17.67
384	RSRD19	-122.448	37.944	5.95	9.60	295.17	15.55	-122.448	37.944	6.34	9.60	295.21	15.94	1	8/1/2014 8:25:17.76
385	RSRD19	-122.448	37.944	5.95	9.55	295.12	15.49	-122.448	37.944	6.34	9.55	295.19	15.88	1	8/1/2014 8:25:17.87
386	RSRD19	-122.448	37.944	6.00	9.51	295.18	15.50	-122.448	37.944	6.37	9.51	295.21	15.88	1	8/1/2014 8:25:17.97
387	RSRD19	-122.448	37.944	5.95	9.48	295.12	15.43	-122.448	37.944	6.37	9.48	295.18	15.85	1	8/1/2014 8:25:18.07
388	RSRD19	-122.448	37.944	6.00	9.48	295.12	15.48	-122.448	37.944	6.42	9.48	295.18	15.90	1	8/1/2014 8:25:18.16
389	RSRD19	-122.448	37.944	6.00	9.40	295.10	15.40	-122.448	37.944	6.42	9.40	295.16	15.82	1	8/1/2014 8:25:18.27
390	RSRD19	-122.448	37.944	6.03	9.38	295.14	15.41	-122.448	37.944	6.45	9.38	295.16	15.83	1	8/1/2014 8:25:18.37
391	RSRD19	-122.448	37.944	6.03	9.37	295.12	15.40	-122.448	37.944	6.45	9.37	295.16	15.82	1	8/1/2014 8:25:18.47
392	RSRD19	-122.448	37.944	6.03	9.35	295.10	15.38	-122.448	37.944	6.45	9.35	295.16	15.80	1	8/1/2014 8:25:18.56
393	RSRD19	-122.448	37.944	6.03	9.32	295.16	15.35	-122.448	37.944	6.45	9.32	295.23	15.78	1	8/1/2014 8:25:18.67
394	RSRD19	-122.448	37.944	6.08	9.32	295.08	15.41	-122.448	37.944	6.50	9.32	295.18	15.83	1	8/1/2014 8:25:18.77
395	RSRD19	-122.448	37.944	6.03	9.33	295.12	15.36	-122.448	37.944	6.50	9.33	295.19	15.83	1	8/1/2014 8:25:18.87
396	RSRD19	-122.448	37.944	6.08	9.36	295.16	15.44	-122.448	37.944	6.50	9.36	295.25	15.87	1	8/1/2014 8:25:18.96
397	RSRD19	-122.448	37.944	6.03	9.29	295.17	15.32	-122.448	37.944	6.50	9.29	295.23	15.80	1	8/1/2014 8:25:19.07
398	RSRD19	-122.448	37.944	6.12	9.38	295.14	15.50	-122.448	37.944	6.54	9.38	295.23	15.92	1	8/1/2014 8:25:19.17
399	RSRD19	-122.448	37.944	6.08	9.29	295.21	15.38	-122.448	37.944	6.50	9.29	295.25	15.80	1	8/1/2014 8:25:19.27
400	RSRD19	-122.448	37.944	6.12	9.29	295.21	15.41	-122.448	37.944	6.54	9.29	295.27	15.83	1	8/1/2014 8:25:19.36
401	RSRD19	-122.448	37.944	6.20	9.28	295.21	15.48	-122.448	37.944	6.54	9.28	295.23	15.82	1	8/1/2014 8:25:19.47
402	RSRD19	-122.448	37.944	6.20	9.27	295.21	15.47	-122.448	37.944	6.59	9.27	295.27	15.86	1	8/1/2014 8:25:19.57
403	RSRD19	-122.448	37.944	6.15	9.31	295.23	15.46	-122.448	37.944	6.59	9.31	295.25	15.90	1	8/1/2014 8:25:19.66
404	RSRD19	-122.448	37.944	6.20	9.27	295.27	15.47	-122.448	37.944	6.62	9.27	295.29	15.89	1	8/1/2014 8:25:19.76
405	RSRD19	-122.448	37.944	6.20	9.27	295.25	15.47	-122.448	37.944	6.59	9.27	295.27	15.86	1	8/1/2014 8:25:19.87
406	RSRD19	-122.448	37.944	6.24	9.28	295.27	15.51	-122.448	37.944	6.62	9.28	295.23	15.90	1	8/1/2014 8:25:19.97
407	RSRD19	-122.448	37.944	6.20	9.27	295.25	15.47	-122.448	37.944	6.62	9.27	295.25	15.89	1	8/1/2014 8:25:20.07
408	RSRD19	-122.448	37.944	6.24	9.25	295.28	15.49	-122.448	37.944	6.67	9.25	295.26	15.93	1	8/1/2014 8:25:20.16
409	RSRD19	-122.448	37.944	6.24	9.26	295.28	15.50	-122.448	37.944	6.67	9.26	295.23	15.93	1	8/1/2014 8:25:20.27

410	RSRD19	-122.448	37.944	6.24	9.26	295.25	15.50	-122.448	37.944	6.71	9.26	295.28	15.97	1	8/1/2014	8:25:20.37
411	RSRD19	-122.448	37.944	6.24	9.24	295.28	15.48	-122.448	37.944	6.71	9.24	295.26	15.95	1	8/1/2014	8:25:20.47
412	RSRD19	-122.448	37.944	6.29	9.25	295.26	15.54	-122.448	37.944	6.74	9.25	295.25	15.99	1	8/1/2014	8:25:20.56
413	RSRD19	-122.448	37.944	6.24	9.24	295.28	15.47	-122.448	37.944	6.71	9.24	295.28	15.94	1	8/1/2014	8:25:20.67
414	RSRD19	-122.448	37.944	6.32	9.24	295.26	15.57	-122.448	37.944	6.74	9.24	295.30	15.99	1	8/1/2014	8:25:20.77
415	RSRD19	-122.448	37.944	6.37	9.24	295.26	15.61	-122.448	37.944	6.74	9.24	295.32	15.98	1	8/1/2014	8:25:20.87
416	RSRD19	-122.448	37.944	6.41	9.24	295.30	15.65	-122.448	37.944	6.79	9.24	295.32	16.04	1	8/1/2014	8:25:20.96
417	RSRD19	-122.448	37.944	6.37	9.23	295.28	15.60	-122.448	37.944	6.79	9.23	295.35	16.02	1	8/1/2014	8:25:21.07
418	RSRD19	-122.448	37.944	6.41	9.22	295.33	15.63	-122.448	37.944	6.79	9.22	295.33	16.01	1	8/1/2014	8:25:21.17
419	RSRD19	-122.448	37.944	6.41	9.22	295.33	15.63	-122.448	37.944	6.79	9.22	295.37	16.01	1	8/1/2014	8:25:21.27
420	RSRD19	-122.448	37.944	6.41	9.21	295.30	15.61	-122.448	37.944	6.82	9.21	295.35	16.03	1	8/1/2014	8:25:21.36
421	RSRD19	-122.448	37.944	6.41	9.21	295.31	15.61	-122.448	37.944	6.82	9.21	295.35	16.03	1	8/1/2014	8:25:21.47
422	RSRD19	-122.448	37.944	6.41	9.19	295.38	15.60	-122.448	37.944	6.88	9.19	295.40	16.07	1	8/1/2014	8:25:21.57
423	RSRD19	-122.448	37.944	6.41	9.18	295.35	15.58	-122.448	37.944	6.88	9.18	295.39	16.05	1	8/1/2014	8:25:21.66
424	RSRD19	-122.448	37.944	6.41	9.19	295.40	15.60	-122.448	37.944	6.88	9.19	295.40	16.07	1	8/1/2014	8:25:21.76
425	RSRD19	-122.448	37.944	6.41	9.18	295.40	15.58	-122.448	37.944	6.88	9.18	295.40	16.05	1	8/1/2014	8:25:21.87
426	RSRD19	-122.448	37.944	6.44	9.18	295.51	15.63	-122.448	37.944	6.91	9.18	295.42	16.09	1	8/1/2014	8:25:21.97
427	RSRD19	-122.448	37.944	6.44	9.15	295.40	15.59	-122.448	37.944	6.91	9.15	295.45	16.06	1	8/1/2014	8:25:22.06
428	RSRD19	-122.448	37.944	6.44	9.15	295.47	15.59	-122.448	37.944	6.91	9.15	295.47	16.06	1	8/1/2014	8:25:22.16
429	RSRD19	-122.448	37.944	6.44	9.12	295.54	15.56	-122.448	37.944	6.91	9.12	295.47	16.03	1	8/1/2014	8:25:22.27
430	RSRD19	-122.448	37.944	6.44	9.11	295.54	15.56	-122.448	37.944	6.96	9.11	295.47	16.08	1	8/1/2014	8:25:22.37
431	RSRD19	-122.448	37.944	6.44	9.10	295.57	15.54	-122.448	37.944	6.96	9.10	295.54	16.06	1	8/1/2014	8:25:22.46
432	RSRD19	-122.448	37.944	6.49	9.11	295.65	15.61	-122.448	37.944	6.96	9.11	295.56	16.08	1	8/1/2014	8:25:22.58
433	RSRD19	-122.448	37.944	6.44	9.08	295.63	15.52	-122.448	37.944	6.96	9.08	295.57	16.04	1	8/1/2014	8:25:22.67
434	RSRD19	-122.448	37.944	6.53	9.06	295.68	15.59	-122.448	37.944	6.96	9.06	295.61	16.02	1	8/1/2014	8:25:22.77
435	RSRD19	-122.448	37.944	6.58	9.05	295.70	15.62	-122.448	37.944	6.99	9.05	295.70	16.04	1	8/1/2014	8:25:22.87
436	RSRD19	-122.448	37.944	6.61	9.01	295.73	15.63	-122.448	37.944	6.99	9.01	295.68	16.01	1	8/1/2014	8:25:22.98
437	RSRD19	-122.448	37.944	6.61	9.00	295.77	15.61	-122.448	37.944	6.99	9.00	295.72	15.99	1	8/1/2014	8:25:23.07
438	RSRD19	-122.448	37.944	6.61	8.98	295.79	15.60	-122.448	37.944	7.05	8.98	295.75	16.03	1	8/1/2014	8:25:23.17
439	RSRD19	-122.448	37.944	6.58	8.97	295.81	15.54	-122.448	37.944	7.05	8.97	295.77	16.01	1	8/1/2014	8:25:23.27
440	RSRD19	-122.448	37.944	6.61	8.94	295.86	15.55	-122.448	37.944	7.05	8.94	295.80	15.98	1	8/1/2014	8:25:23.38
441	RSRD19	-122.448	37.944	6.58	8.94	295.90	15.52	-122.448	37.944	7.05	8.94	295.77	15.99	1	8/1/2014	8:25:23.47
442	RSRD19	-122.448	37.944	6.61	8.88	295.91	15.49	-122.448	37.944	7.08	8.88	295.82	15.95	1	8/1/2014	8:25:23.57

443	RSRD19	-122.448	37.944	6.61	8.88	295.93	15.49	-122.448	37.944	7.08	8.88	295.80	15.95	1	8/1/2014	8:25:23.67
444	RSRD19	-122.448	37.944	6.61	8.84	296.00	15.45	-122.448	37.944	7.08	8.84	295.82	15.92	1	8/1/2014	8:25:23.78
445	RSRD19	-122.448	37.944	6.61	8.82	295.91	15.43	-122.448	37.944	7.08	8.82	295.82	15.90	1	8/1/2014	8:25:23.87
446	RSRD19	-122.448	37.944	6.66	8.81	296.00	15.48	-122.448	37.944	7.11	8.81	295.83	15.93	1	8/1/2014	8:25:23.97
447	RSRD19	-122.448	37.944	6.66	8.77	296.00	15.44	-122.448	37.944	7.08	8.77	295.82	15.85	1	8/1/2014	8:25:24.07
448	RSRD19	-122.448	37.944	6.70	8.78	295.98	15.48	-122.448	37.944	7.11	8.78	295.87	15.90	1	8/1/2014	8:25:24.18
449	RSRD19	-122.448	37.944	6.70	8.75	296.00	15.45	-122.448	37.944	7.11	8.75	295.85	15.86	1	8/1/2014	8:25:24.27
450	RSRD19	-122.448	37.944	6.75	8.75	296.01	15.50	-122.448	37.944	7.16	8.75	295.85	15.92	1	8/1/2014	8:25:24.37
451	RSRD19	-122.448	37.944	6.70	8.70	296.05	15.40	-122.448	37.944	7.16	8.70	295.85	15.86	1	8/1/2014	8:25:24.46
452	RSRD19	-122.448	37.944	6.75	8.70	296.01	15.45	-122.448	37.944	7.16	8.70	295.86	15.86	1	8/1/2014	8:25:24.58
453	RSRD19	-122.448	37.944	6.75	8.69	296.01	15.44	-122.448	37.944	7.20	8.69	295.85	15.89	1	8/1/2014	8:25:24.67
454	RSRD19	-122.448	37.944	6.75	8.64	296.05	15.39	-122.448	37.944	7.20	8.64	295.85	15.84	1	8/1/2014	8:25:24.77
455	RSRD19	-122.448	37.944	6.75	8.62	296.01	15.37	-122.448	37.944	7.25	8.62	295.88	15.87	1	8/1/2014	8:25:24.86
456	RSRD19	-122.448	37.944	6.75	8.61	296.05	15.36	-122.448	37.944	7.25	8.61	295.86	15.85	1	8/1/2014	8:25:24.98
457	RSRD19	-122.448	37.944	6.75	8.57	296.01	15.32	-122.448	37.944	7.25	8.57	295.90	15.82	1	8/1/2014	8:25:25.07
458	RSRD19	-122.448	37.944	6.78	8.56	296.05	15.34	-122.448	37.944	7.28	8.56	295.92	15.84	1	8/1/2014	8:25:25.17
459	RSRD19	-122.448	37.944	6.75	8.56	296.06	15.31	-122.448	37.944	7.25	8.56	295.92	15.81	1	8/1/2014	8:25:25.27
460	RSRD19	-122.448	37.944	6.78	8.58	296.08	15.37	-122.448	37.944	7.28	8.58	295.95	15.86	1	8/1/2014	8:25:25.38
461	RSRD19	-122.448	37.944	6.78	8.54	296.11	15.33	-122.448	37.944	7.28	8.54	295.97	15.83	1	8/1/2014	8:25:25.47
462	RSRD19	-122.448	37.944	6.82	8.55	296.13	15.37	-122.448	37.944	7.28	8.55	296.04	15.83	1	8/1/2014	8:25:25.57
463	RSRD19	-122.448	37.944	6.78	8.61	296.15	15.39	-122.448	37.944	7.25	8.61	295.97	15.85	1	8/1/2014	8:25:25.67
464	RSRD19	-122.448	37.944	6.82	8.58	296.15	15.40	-122.448	37.944	7.28	8.58	296.02	15.86	1	8/1/2014	8:25:25.78
465	RSRD19	-122.448	37.944	6.78	8.56	296.19	15.34	-122.448	37.944	7.25	8.56	296.06	15.81	1	8/1/2014	8:25:25.87
466	RSRD19	-122.448	37.944	6.82	8.70	296.19	15.52	-122.448	37.944	7.28	8.70	296.06	15.99	1	8/1/2014	8:25:25.97
467	RSRD19	-122.448	37.944	6.82	8.56	296.24	15.38	-122.448	37.944	7.25	8.56	296.08	15.81	1	8/1/2014	8:25:26.06
468	RSRD19	-122.448	37.944	6.82	8.54	296.22	15.35	-122.448	37.944	7.28	8.54	296.04	15.82	1	8/1/2014	8:25:26.18
469	RSRD19	-122.448	37.944	6.87	8.52	296.22	15.39	-122.448	37.944	7.28	8.52	296.11	15.80	1	8/1/2014	8:25:26.27
470	RSRD19	-122.448	37.944	6.87	8.52	296.22	15.39	-122.448	37.944	7.33	8.52	296.06	15.85	1	8/1/2014	8:25:26.37
471	RSRD19	-122.448	37.944	6.87	8.51	296.17	15.38	-122.448	37.944	7.28	8.51	296.09	15.79	1	8/1/2014	8:25:26.47
472	RSRD19	-122.448	37.944	6.90	8.49	296.20	15.39	-122.448	37.944	7.33	8.49	296.11	15.82	1	8/1/2014	8:25:26.58
473	RSRD19	-122.448	37.944	6.90	8.49	296.17	15.40	-122.448	37.944	7.33	8.49	296.11	15.83	1	8/1/2014	8:25:26.67
474	RSRD19	-122.448	37.944	6.95	8.48	296.17	15.44	-122.448	37.944	7.33	8.48	296.11	15.82	1	8/1/2014	8:25:26.77
475	RSRD19	-122.448	37.944	6.90	8.48	296.24	15.39	-122.448	37.944	7.33	8.48	296.20	15.82	1	8/1/2014	8:25:26.87

476	RSRD19	-122.448	37.944	6.95	8.43	296.20	15.38	-122.448	37.944	7.37	8.43	296.17	15.80	1	8/1/2014	8:25:26.98
477	RSRD19	-122.448	37.944	6.95	8.44	296.26	15.40	-122.448	37.944	7.33	8.44	296.22	15.78	1	8/1/2014	8:25:27.07
478	RSRD19	-122.448	37.944	6.95	8.46	296.26	15.41	-122.448	37.944	7.42	8.46	296.22	15.88	1	8/1/2014	8:25:27.17
479	RSRD19	-122.448	37.944	6.95	8.41	296.31	15.37	-122.448	37.944	7.37	8.41	296.24	15.78	1	8/1/2014	8:25:27.26
480	RSRD19	-122.448	37.944	6.95	8.44	296.30	15.40	-122.448	37.944	7.37	8.44	296.24	15.81	1	8/1/2014	8:25:27.38
481	RSRD19	-122.448	37.944	6.95	8.47	296.33	15.42	-122.448	37.944	7.37	8.47	296.24	15.83	1	8/1/2014	8:25:27.47
482	RSRD19	-122.448	37.944	6.95	8.44	296.29	15.40	-122.448	37.944	7.37	8.44	296.22	15.81	1	8/1/2014	8:25:27.57
483	RSRD19	-122.448	37.944	6.95	8.47	296.30	15.42	-122.448	37.944	7.37	8.47	296.24	15.83	1	8/1/2014	8:25:27.66
484	RSRD19	-122.448	37.944	6.99	8.46	296.31	15.45	-122.448	37.944	7.42	8.46	296.24	15.88	1	8/1/2014	8:25:27.78
485	RSRD19	-122.448	37.944	6.95	8.49	296.28	15.44	-122.448	37.944	7.37	8.49	296.20	15.86	1	8/1/2014	8:25:27.87
486	RSRD19	-122.448	37.944	6.99	8.49	296.31	15.48	-122.448	37.944	7.42	8.49	296.22	15.91	1	8/1/2014	8:25:27.97
487	RSRD19	-122.448	37.944	6.95	8.51	296.26	15.47	-122.448	37.944	7.37	8.51	296.22	15.88	1	8/1/2014	8:25:28.07
488	RSRD19	-122.448	37.944	6.99	8.51	296.30	15.49	-122.448	37.944	7.42	8.51	296.24	15.92	1	8/1/2014	8:25:28.18
489	RSRD19	-122.448	37.944	6.95	8.51	296.28	15.47	-122.448	37.944	7.42	8.51	296.22	15.93	1	8/1/2014	8:25:28.27
490	RSRD19	-122.448	37.944	6.99	8.54	296.24	15.53	-122.448	37.944	7.42	8.54	296.21	15.95	1	8/1/2014	8:25:28.37
491	RSRD19	-122.448	37.944	6.99	8.54	296.30	15.53	-122.448	37.944	7.42	8.54	296.24	15.95	1	8/1/2014	8:25:28.47
492	RSRD19	-122.448	37.944	6.99	8.60	296.30	15.59	-122.448	37.944	7.45	8.60	296.24	16.05	1	8/1/2014	8:25:28.58
493	RSRD19	-122.448	37.944	6.99	8.58	296.32	15.57	-122.448	37.944	7.42	8.58	296.26	16.00	1	8/1/2014	8:25:28.67
494	RSRD19	-122.448	37.944	7.04	8.60	296.33	15.64	-122.448	37.944	7.45	8.60	296.28	16.05	1	8/1/2014	8:25:28.77
495	RSRD19	-122.448	37.944	6.99	8.58	296.33	15.57	-122.448	37.944	7.45	8.58	296.26	16.03	1	8/1/2014	8:25:28.86
496	RSRD19	-122.448	37.944	7.04	8.60	296.35	15.64	-122.448	37.944	7.49	8.60	296.26	16.08	1	8/1/2014	8:25:28.98
497	RSRD19	-122.448	37.944	6.99	8.59	296.35	15.58	-122.448	37.944	7.45	8.59	296.24	16.04	1	8/1/2014	8:25:29.07
498	RSRD19	-122.448	37.944	7.04	8.60	296.37	15.64	-122.448	37.944	7.49	8.60	296.25	16.08	1	8/1/2014	8:25:29.17
499	RSRD19	-122.448	37.944	6.99	8.60	296.34	15.59	-122.448	37.944	7.49	8.60	296.25	16.08	1	8/1/2014	8:25:29.27
500	RSRD19	-122.448	37.944	7.04	8.59	296.36	15.63	-122.448	37.944	7.49	8.59	296.23	16.08	1	8/1/2014	8:25:29.38
501	RSRD19	-122.448	37.944	7.04	8.59	296.34	15.63	-122.448	37.944	7.45	8.59	296.23	16.04	1	8/1/2014	8:25:29.47
502	RSRD19	-122.448	37.944	7.04	8.58	296.36	15.62	-122.448	37.944	7.45	8.58	296.26	16.03	1	8/1/2014	8:25:29.57
503	RSRD19	-122.448	37.944	7.04	8.59	296.39	15.63	-122.448	37.944	7.49	8.59	296.21	16.08	1	8/1/2014	8:25:29.67
504	RSRD19	-122.448	37.944	7.04	8.60	296.39	15.64	-122.448	37.944	7.49	8.60	296.23	16.08	1	8/1/2014	8:25:29.78
505	RSRD19	-122.448	37.944	7.04	8.61	296.36	15.65	-122.448	37.944	7.45	8.61	296.21	16.06	1	8/1/2014	8:25:29.87
506	RSRD19	-122.448	37.944	7.04	8.61	296.36	15.65	-122.448	37.944	7.45	8.61	296.21	16.06	1	8/1/2014	8:25:29.97
507	RSRD19	-122.448	37.944	7.04	8.62	296.39	15.66	-122.448	37.944	7.45	8.62	296.18	16.07	1	8/1/2014	8:25:30.07
508	RSRD19	-122.448	37.944	7.07	8.60	296.34	15.67	-122.448	37.944	7.45	8.60	296.21	16.05	1	8/1/2014	8:25:30.18

509	RSRD19	-122.448	37.944	7.04	8.62	296.39	15.66	-122.448	37.944	7.45	8.62	296.21	16.07	1	8/1/2014	8:25:30.28
510	RSRD19	-122.448	37.944	7.07	8.61	296.39	15.69	-122.448	37.944	7.45	8.61	296.19	16.06	1	8/1/2014	8:25:30.37
511	RSRD19	-122.448	37.944	7.04	8.61	296.39	15.65	-122.448	37.944	7.45	8.61	296.25	16.06	1	8/1/2014	8:25:30.46
512	RSRD19	-122.448	37.944	7.07	8.61	296.36	15.68	-122.448	37.944	7.49	8.61	296.19	16.09	1	8/1/2014	8:25:30.58
513	RSRD19	-122.448	37.944	7.07	8.61	296.39	15.69	-122.448	37.944	7.45	8.61	296.21	16.06	1	8/1/2014	8:25:30.67
514	RSRD19	-122.448	37.944	7.07	8.62	296.34	15.70	-122.448	37.944	7.45	8.62	296.21	16.07	1	8/1/2014	8:25:30.77
515	RSRD19	-122.448	37.944	7.07	8.60	296.36	15.67	-122.448	37.944	7.45	8.60	296.21	16.05	1	8/1/2014	8:25:30.86
516	RSRD19	-122.448	37.944	7.07	8.55	296.34	15.63	-122.448	37.944	7.49	8.55	296.21	16.04	1	8/1/2014	8:25:30.98
517	RSRD19	-122.448	37.944	7.07	8.56	296.32	15.64	-122.448	37.944	7.49	8.56	296.21	16.05	1	8/1/2014	8:25:31.07
518	RSRD19	-122.448	37.944	7.13	8.52	296.33	15.65	-122.448	37.944	7.49	8.52	296.22	16.01	1	8/1/2014	8:25:31.17
519	RSRD19	-122.448	37.944	7.13	8.51	296.32	15.64	-122.448	37.944	7.49	8.51	296.21	16.00	1	8/1/2014	8:25:31.27
520	RSRD19	-122.448	37.944	7.16	8.48	296.30	15.64	-122.448	37.944	7.54	8.48	296.19	16.02	1	8/1/2014	8:25:31.38
521	RSRD19	-122.448	37.944	7.13	8.45	296.30	15.58	-122.448	37.944	7.49	8.45	296.22	15.94	1	8/1/2014	8:25:31.47
522	RSRD19	-122.448	37.944	7.16	8.43	296.28	15.59	-122.448	37.944	7.54	8.43	296.19	15.96	1	8/1/2014	8:25:31.57
523	RSRD19	-122.448	37.944	7.16	8.40	296.31	15.57	-122.448	37.944	7.54	8.40	296.16	15.94	1	8/1/2014	8:25:31.66
524	RSRD19	-122.448	37.944	7.20	8.37	296.33	15.56	-122.448	37.944	7.57	8.37	296.22	15.94	1	8/1/2014	8:25:31.78
525	RSRD19	-122.448	37.944	7.20	8.41	296.26	15.61	-122.448	37.944	7.54	8.41	296.15	15.95	1	8/1/2014	8:25:31.87
526	RSRD19	-122.448	37.944	7.20	8.39	296.31	15.58	-122.448	37.944	7.57	8.39	296.18	15.96	1	8/1/2014	8:25:31.97
527	RSRD19	-122.448	37.944	7.16	8.35	296.35	15.51	-122.448	37.944	7.57	8.35	296.18	15.92	1	8/1/2014	8:25:32.07
528	RSRD19	-122.448	37.944	7.20	8.36	296.25	15.55	-122.448	37.944	7.57	8.36	296.18	15.93	1	8/1/2014	8:25:32.18
529	RSRD19	-122.448	37.944	7.20	8.38	296.34	15.58	-122.448	37.944	7.54	8.38	296.16	15.92	1	8/1/2014	8:25:32.27
530	RSRD19	-122.448	37.944	7.20	8.40	296.29	15.60	-122.448	37.944	7.57	8.40	296.14	15.97	1	8/1/2014	8:25:32.37
531	RSRD19	-122.448	37.944	7.20	8.47	296.34	15.66	-122.448	37.944	7.54	8.47	296.12	16.00	1	8/1/2014	8:25:32.47
532	RSRD19	-122.448	37.944	7.20	8.43	296.34	15.62	-122.448	37.944	7.54	8.43	296.14	15.96	1	8/1/2014	8:25:32.58
533	RSRD19	-122.448	37.944	7.16	8.41	296.32	15.57	-122.448	37.944	7.49	8.41	296.15	15.90	1	8/1/2014	8:25:32.67
534	RSRD19	-122.448	37.944	7.16	8.44	296.30	15.60	-122.448	37.944	7.49	8.44	296.15	15.93	1	8/1/2014	8:25:32.77
535	RSRD19	-122.448	37.944	7.16	8.49	296.30	15.66	-122.448	37.944	7.49	8.49	296.13	15.98	1	8/1/2014	8:25:32.87
536	RSRD19	-122.448	37.944	7.20	8.45	296.31	15.65	-122.448	37.944	7.49	8.45	296.16	15.94	1	8/1/2014	8:25:32.98
537	RSRD19	-122.448	37.944	7.16	8.44	296.31	15.60	-122.448	37.944	7.49	8.44	296.14	15.92	1	8/1/2014	8:25:33.07
538	RSRD19	-122.448	37.944	7.20	8.49	296.27	15.68	-122.448	37.944	7.49	8.49	296.12	15.97	1	8/1/2014	8:25:33.17
539	RSRD19	-122.448	37.944	7.16	8.45	296.27	15.61	-122.448	37.944	7.49	8.45	296.14	15.94	1	8/1/2014	8:25:33.26
540	RSRD19	-122.448	37.944	7.20	8.47	296.28	15.67	-122.448	37.944	7.54	8.47	296.15	16.01	1	8/1/2014	8:25:33.38
541	RSRD19	-122.448	37.944	7.16	8.46	296.21	15.62	-122.448	37.944	7.54	8.46	296.13	15.99	1	8/1/2014	8:25:33.47

542	RSRD19	-122.448	37.944	7.20	8.45	296.27	15.65	-122.448	37.944	7.54	8.45	296.16	15.99	1	8/1/2014 8:25:33.57
543	RSRD19	-122.448	37.944	7.20	8.47	296.24	15.67	-122.448	37.944	7.54	8.47	296.18	16.01	1	8/1/2014 8:25:33.66
544	RSRD19	-122.448	37.944	7.20	8.46	296.23	15.65	-122.448	37.944	7.54	8.46	296.16	15.99	1	8/1/2014 8:25:33.78
545	RSRD19	-122.448	37.944	7.20	8.45	296.21	15.65	-122.448	37.944	7.54	8.45	296.16	15.99	1	8/1/2014 8:25:33.87
546	RSRD19	-122.448	37.944	7.20	8.46	296.21	15.65	-122.448	37.944	7.54	8.46	296.15	15.99	1	8/1/2014 8:25:33.97
547	RSRD19	-122.448	37.944	7.16	8.47	296.22	15.63	-122.448	37.944	7.54	8.47	296.17	16.00	1	8/1/2014 8:25:34.07
548	RSRD19	-122.448	37.944	7.20	8.49	296.20	15.68	-122.448	37.944	7.57	8.49	296.18	16.06	1	8/1/2014 8:25:34.18
549	RSRD19	-122.448	37.944	7.20	8.47	296.25	15.67	-122.448	37.944	7.54	8.47	296.16	16.01	1	8/1/2014 8:25:34.27
550	RSRD19	-122.448	37.944	7.20	8.47	296.21	15.66	-122.448	37.944	7.54	8.47	296.17	16.00	1	8/1/2014 8:25:34.37
551	RSRD19	-122.448	37.944	7.20	8.48	296.24	15.68	-122.448	37.944	7.54	8.48	296.19	16.02	1	8/1/2014 8:25:34.46
552	RSRD19	-122.448	37.944	7.20	8.47	296.20	15.66	-122.448	37.944	7.54	8.47	296.18	16.00	1	8/1/2014 8:25:34.58
553	RSRD19	-122.448	37.944	7.20	8.47	296.18	15.67	-122.448	37.944	7.49	8.47	296.14	15.96	1	8/1/2014 8:25:34.67
554	RSRD19	-122.448	37.944	7.20	8.46	296.19	15.65	-122.448	37.944	7.54	8.46	296.17	15.99	1	8/1/2014 8:25:34.77
555	RSRD19	-122.448	37.944	7.20	8.49	296.22	15.68	-122.448	37.944	7.54	8.49	296.17	16.03	1	8/1/2014 8:25:34.87
556	RSRD19	-122.448	37.944	7.20	8.51	296.18	15.71	-122.448	37.944	7.57	8.51	296.16	16.08	1	8/1/2014 8:25:34.98
557	RSRD19	-122.448	37.944	7.16	8.47	296.21	15.63	-122.448	37.944	7.54	8.47	296.16	16.00	1	8/1/2014 8:25:35.07
558	RSRD19	-122.448	37.944	7.20	8.53	296.20	15.72	-122.448	37.944	7.57	8.53	296.19	16.10	1	8/1/2014 8:25:35.17
559	RSRD19	-122.448	37.944	7.20	8.49	296.26	15.68	-122.448	37.944	7.57	8.49	296.20	16.06	1	8/1/2014 8:25:35.27
560	RSRD19	-122.448	37.944	7.25	8.47	296.23	15.72	-122.448	37.944	7.57	8.47	296.18	16.04	1	8/1/2014 8:25:35.38
561	RSRD19	-122.448	37.944	7.20	8.49	296.23	15.68	-122.448	37.944	7.57	8.49	296.21	16.06	1	8/1/2014 8:25:35.47
562	RSRD19	-122.448	37.944	7.25	8.49	296.24	15.74	-122.448	37.944	7.57	8.49	296.25	16.07	1	8/1/2014 8:25:35.57
563	RSRD19	-122.448	37.944	7.20	8.47	296.23	15.67	-122.448	37.944	7.57	8.47	296.19	16.04	1	8/1/2014 8:25:35.67
564	RSRD19	-122.448	37.944	7.25	8.47	296.24	15.71	-122.448	37.944	7.62	8.47	296.24	16.09	1	8/1/2014 8:25:35.78
565	RSRD19	-122.448	37.944	7.25	8.48	296.22	15.73	-122.448	37.944	7.57	8.48	296.25	16.05	1	8/1/2014 8:25:35.87
566	RSRD19	-122.448	37.944	7.25	8.46	296.21	15.70	-122.448	37.944	7.62	8.46	296.23	16.08	1	8/1/2014 8:25:35.97
567	RSRD19	-122.448	37.944	7.25	8.45	296.26	15.70	-122.448	37.944	7.57	8.45	296.24	16.02	1	8/1/2014 8:25:36.06
568	RSRD19	-122.448	37.944	7.25	8.45	296.25	15.70	-122.448	37.944	7.62	8.45	296.25	16.07	1	8/1/2014 8:25:36.18
569	RSRD19	-122.448	37.944	7.20	8.47	296.24	15.67	-122.448	37.944	7.57	8.47	296.22	16.04	1	8/1/2014 8:25:36.27
570	RSRD19	-122.448	37.944	7.25	8.47	296.29	15.72	-122.448	37.944	7.62	8.47	296.24	16.09	1	8/1/2014 8:25:36.37
571	RSRD19	-122.448	37.944	7.25	8.47	296.28	15.71	-122.448	37.944	7.62	8.47	296.23	16.09	1	8/1/2014 8:25:36.46
572	RSRD19	-122.448	37.944	7.25	8.44	296.29	15.69	-122.448	37.944	7.62	8.44	296.18	16.06	1	8/1/2014 8:25:36.58
573	RSRD19	-122.448	37.944	7.20	8.47	296.30	15.66	-122.448	37.944	7.62	8.47	296.21	16.09	1	8/1/2014 8:25:36.67
574	RSRD19	-122.448	37.944	7.25	8.46	296.31	15.70	-122.448	37.944	7.62	8.46	296.20	16.08	1	8/1/2014 8:25:36.77

575	RSRD19	-122.448	37.944	7.25	8.46	296.34	15.70	-122.448	37.944	7.57	8.46	296.20	16.03	1	8/1/2014	8:25:36.87
576	RSRD19	-122.448	37.944	7.25	8.46	296.35	15.70	-122.448	37.944	7.62	8.46	296.22	16.08	1	8/1/2014	8:25:36.98
577	RSRD19	-122.448	37.944	7.25	8.45	296.42	15.70	-122.448	37.944	7.57	8.45	296.18	16.02	1	8/1/2014	8:25:37.07
578	RSRD19	-122.448	37.944	7.25	8.44	296.30	15.69	-122.448	37.944	7.57	8.44	296.21	16.01	1	8/1/2014	8:25:37.17
579	RSRD19	-122.448	37.944	7.20	8.47	296.33	15.66	-122.448	37.944	7.57	8.47	296.20	16.04	1	8/1/2014	8:25:37.26
580	RSRD19	-122.448	37.944	7.25	8.45	296.35	15.70	-122.448	37.944	7.62	8.45	296.20	16.07	1	8/1/2014	8:25:37.38
581	RSRD19	-122.448	37.944	7.25	8.44	296.40	15.69	-122.448	37.944	7.57	8.44	296.21	16.01	1	8/1/2014	8:25:37.47
582	RSRD19	-122.448	37.944	7.25	8.44	296.30	15.69	-122.448	37.944	7.57	8.44	296.17	16.01	1	8/1/2014	8:25:37.57
583	RSRD19	-122.448	37.944	7.25	8.44	296.42	15.69	-122.448	37.944	7.57	8.44	296.16	16.01	1	8/1/2014	8:25:37.67
584	RSRD19	-122.448	37.944	7.25	8.49	296.26	15.74	-122.448	37.944	7.62	8.49	296.17	16.11	1	8/1/2014	8:25:37.78
585	RSRD19	-122.448	37.944	7.25	8.45	296.31	15.70	-122.448	37.944	7.57	8.45	296.18	16.02	1	8/1/2014	8:25:37.87
586	RSRD19	-122.448	37.944	7.28	8.42	296.33	15.70	-122.448	37.944	7.62	8.42	296.15	16.04	1	8/1/2014	8:25:37.97
587	RSRD19	-122.448	37.944	7.25	8.40	296.32	15.65	-122.448	37.944	7.62	8.40	296.19	16.03	1	8/1/2014	8:25:38.07
588	RSRD19	-122.448	37.944	7.28	8.40	296.33	15.68	-122.448	37.944	7.62	8.40	296.22	16.02	1	8/1/2014	8:25:38.18
589	RSRD19	-122.448	37.944	7.25	8.40	296.32	15.64	-122.448	37.944	7.62	8.40	296.19	16.02	1	8/1/2014	8:25:38.27
590	RSRD19	-122.448	37.944	7.28	8.40	296.40	15.68	-122.448	37.944	7.62	8.40	296.20	16.02	1	8/1/2014	8:25:38.37
591	RSRD19	-122.448	37.944	7.25	8.38	296.28	15.63	-122.448	37.944	7.62	8.38	296.17	16.00	1	8/1/2014	8:25:38.46
592	RSRD19	-122.448	37.944	7.28	8.35	296.36	15.63	-122.448	37.944	7.66	8.35	296.18	16.01	1	8/1/2014	8:25:38.58
593	RSRD19	-122.448	37.944	7.28	8.37	296.37	15.65	-122.448	37.944	7.66	8.37	296.17	16.02	1	8/1/2014	8:25:38.67
594	RSRD19	-122.448	37.944	7.28	8.36	296.35	15.64	-122.448	37.944	7.66	8.36	296.21	16.01	1	8/1/2014	8:25:38.77
595	RSRD19	-122.448	37.944	7.28	8.33	296.40	15.61	-122.448	37.944	7.66	8.33	296.20	15.98	1	8/1/2014	8:25:38.86
596	RSRD19	-122.448	37.944	7.33	8.33	296.37	15.67	-122.448	37.944	7.70	8.33	296.21	16.04	1	8/1/2014	8:25:38.98
597	RSRD19	-122.448	37.944	7.28	8.33	296.45	15.61	-122.448	37.944	7.70	8.33	296.23	16.04	1	8/1/2014	8:25:39.07
598	RSRD19	-122.448	37.944	7.33	8.32	296.38	15.65	-122.448	37.944	7.74	8.32	296.22	16.06	1	8/1/2014	8:25:39.17
599	RSRD19	-122.448	37.944	7.28	8.33	296.43	15.61	-122.448	37.944	7.70	8.33	296.23	16.03	1	8/1/2014	8:25:39.26
600	RSRD19	-122.448	37.944	7.33	8.35	296.49	15.68	-122.448	37.944	7.70	8.35	296.25	16.06	1	8/1/2014	8:25:39.38
601	RSRD19	-122.448	37.944	7.33	8.32	296.48	15.65	-122.448	37.944	7.70	8.32	296.26	16.02	1	8/1/2014	8:25:39.47
602	RSRD19	-122.448	37.944	7.33	8.32	296.47	15.65	-122.448	37.944	7.70	8.32	296.27	16.02	1	8/1/2014	8:25:39.57
603	RSRD19	-122.448	37.944	7.28	8.35	296.50	15.63	-122.448	37.944	7.66	8.35	296.26	16.01	1	8/1/2014	8:25:39.66
604	RSRD19	-122.448	37.944	7.33	8.34	296.52	15.68	-122.448	37.944	7.66	8.34	296.33	16.00	1	8/1/2014	8:25:39.78
605	RSRD19	-122.448	37.944	7.28	8.34	296.55	15.62	-122.448	37.944	7.62	8.34	296.33	15.96	1	8/1/2014	8:25:39.87
606	RSRD19	-122.448	37.944	7.33	8.33	296.55	15.67	-122.448	37.944	7.70	8.33	296.35	16.04	1	8/1/2014	8:25:39.97
607	RSRD19	-122.448	37.944	7.33	8.33	296.61	15.67	-122.448	37.944	7.66	8.33	296.38	15.99	1	8/1/2014	8:25:40.06

608	RSRD19	-122.448	37.944	7.28	8.33	296.60	15.61	-122.448	37.944	7.66	8.33	296.38	15.99	1	8/1/2014	8:25:40.18
609	RSRD19	-122.448	37.944	7.33	8.32	296.63	15.65	-122.448	37.944	7.66	8.32	296.37	15.97	1	8/1/2014	8:25:40.27
610	RSRD19	-122.448	37.944	7.33	8.36	296.65	15.69	-122.448	37.944	7.66	8.36	296.42	16.01	1	8/1/2014	8:25:40.37
611	RSRD19	-122.448	37.944	7.28	8.33	296.64	15.61	-122.448	37.944	7.62	8.33	296.39	15.95	1	8/1/2014	8:25:40.46
612	RSRD19	-122.448	37.944	7.33	8.33	296.67	15.67	-122.448	37.944	7.66	8.33	296.44	15.99	1	8/1/2014	8:25:40.58
613	RSRD19	-122.448	37.944	7.28	8.35	296.71	15.63	-122.448	37.944	7.62	8.35	296.47	15.97	1	8/1/2014	8:25:40.67
614	RSRD19	-122.448	37.944	7.33	8.33	296.77	15.66	-122.448	37.944	7.62	8.33	296.46	15.95	1	8/1/2014	8:25:40.77
615	RSRD19	-122.448	37.944	7.28	8.33	296.69	15.61	-122.448	37.944	7.57	8.33	296.51	15.90	1	8/1/2014	8:25:40.86
616	RSRD19	-122.448	37.944	7.33	8.35	296.79	15.68	-122.448	37.944	7.62	8.35	296.48	15.97	1	8/1/2014	8:25:40.98
617	RSRD19	-122.448	37.944	7.33	8.33	296.76	15.67	-122.448	37.944	7.62	8.33	296.52	15.95	1	8/1/2014	8:25:41.07
618	RSRD19	-122.448	37.944	7.28	8.33	296.84	15.61	-122.448	37.944	7.62	8.33	296.51	15.95	1	8/1/2014	8:25:41.17
619	RSRD19	-122.448	37.944	7.33	8.33	296.81	15.67	-122.448	37.944	7.57	8.33	296.54	15.90	1	8/1/2014	8:25:41.26
620	RSRD19	-122.448	37.944	7.33	8.35	296.83	15.68	-122.448	37.944	7.57	8.35	296.56	15.92	1	8/1/2014	8:25:41.38
621	RSRD19	-122.448	37.944	7.28	8.37	296.86	15.65	-122.448	37.944	7.57	8.37	296.59	15.94	1	8/1/2014	8:25:41.47
622	RSRD19	-122.448	37.944	7.28	8.35	296.85	15.63	-122.448	37.944	7.54	8.35	296.61	15.89	1	8/1/2014	8:25:41.57
623	RSRD19	-122.448	37.944	7.28	8.35	296.88	15.63	-122.448	37.944	7.54	8.35	296.62	15.89	1	8/1/2014	8:25:41.66
624	RSRD19	-122.448	37.944	7.28	8.37	296.89	15.65	-122.448	37.944	7.54	8.37	296.65	15.90	1	8/1/2014	8:25:41.78
625	RSRD19	-122.448	37.944	7.28	8.33	296.90	15.61	-122.448	37.944	7.54	8.33	296.69	15.87	1	8/1/2014	8:25:41.87
626	RSRD19	-122.448	37.944	7.28	8.34	296.92	15.62	-122.448	37.944	7.57	8.34	296.69	15.91	1	8/1/2014	8:25:41.97
627	RSRD19	-122.448	37.944	7.28	8.35	296.88	15.63	-122.448	37.944	7.54	8.35	296.69	15.89	1	8/1/2014	8:25:42.06
628	RSRD19	-122.448	37.944	7.33	8.33	296.90	15.67	-122.448	37.944	7.54	8.33	296.72	15.87	1	8/1/2014	8:25:42.18
629	RSRD19	-122.448	37.944	7.25	8.35	296.95	15.60	-122.448	37.944	7.49	8.35	296.80	15.84	1	8/1/2014	8:25:42.27
630	RSRD19	-122.448	37.944	7.28	8.36	296.99	15.64	-122.448	37.944	7.49	8.36	296.77	15.84	1	8/1/2014	8:25:42.37
631	RSRD19	-122.448	37.944	7.28	8.40	296.99	15.68	-122.448	37.944	7.49	8.40	296.82	15.89	1	8/1/2014	8:25:42.46
632	RSRD19	-122.448	37.944	7.28	8.44	297.03	15.72	-122.448	37.944	7.54	8.44	296.85	15.97	1	8/1/2014	8:25:42.58
633	RSRD19	-122.448	37.944	7.25	8.46	297.06	15.70	-122.448	37.944	7.45	8.46	296.91	15.91	1	8/1/2014	8:25:42.67
634	RSRD19	-122.448	37.944	7.25	8.44	297.08	15.69	-122.448	37.944	7.45	8.44	296.88	15.89	1	8/1/2014	8:25:42.77
635	RSRD19	-122.448	37.944	7.25	8.46	297.13	15.70	-122.448	37.944	7.42	8.46	296.93	15.88	1	8/1/2014	8:25:42.86
636	RSRD19	-122.448	37.944	7.25	8.47	297.14	15.72	-122.448	37.944	7.42	8.47	296.94	15.89	1	8/1/2014	8:25:42.98
637	RSRD19	-122.448	37.944	7.20	8.51	297.20	15.71	-122.448	37.944	7.42	8.51	296.95	15.93	1	8/1/2014	8:25:43.07
638	RSRD19	-122.448	37.944	7.25	8.52	297.21	15.77	-122.448	37.944	7.42	8.52	296.96	15.94	1	8/1/2014	8:25:43.17
639	RSRD19	-122.448	37.944	7.20	8.51	297.24	15.71	-122.448	37.944	7.37	8.51	297.04	15.88	1	8/1/2014	8:25:43.26
640	RSRD19	-122.448	37.944	7.25	8.54	297.23	15.79	-122.448	37.944	7.37	8.54	296.99	15.91	1	8/1/2014	8:25:43.38

641	RSRD19	-122.448	37.944	7.20	8.56	297.26	15.75	-122.448	37.944	7.33	8.56	297.06	15.89	1	8/1/2014 8:25:43.47
642	RSRD19	-122.448	37.944	7.25	8.56	297.23	15.80	-122.448	37.944	7.37	8.56	297.05	15.93	1	8/1/2014 8:25:43.57
643	RSRD19	-122.448	37.944	7.20	8.56	297.32	15.75	-122.448	37.944	7.37	8.56	297.06	15.93	1	8/1/2014 8:25:43.66
644	RSRD19	-122.448	37.944	7.25	8.56	297.22	15.80	-122.448	37.944	7.33	8.56	297.11	15.89	1	8/1/2014 8:25:43.78
645	RSRD19	-122.448	37.944	7.20	8.59	297.30	15.79	-122.448	37.944	7.33	8.59	297.10	15.92	1	8/1/2014 8:25:43.87
646	RSRD19	-122.448	37.944	7.20	8.56	297.24	15.76	-122.448	37.944	7.33	8.56	297.13	15.90	1	8/1/2014 8:25:43.97
647	RSRD19	-122.448	37.944	7.16	8.61	297.32	15.77	-122.448	37.944	7.28	8.61	297.14	15.89	1	8/1/2014 8:25:44.06
648	RSRD19	-122.448	37.944	7.16	8.60	297.28	15.76	-122.448	37.944	7.33	8.60	297.13	15.93	1	8/1/2014 8:25:44.18
649	RSRD19	-122.448	37.944	7.16	8.61	297.31	15.77	-122.448	37.944	7.25	8.61	297.16	15.86	1	8/1/2014 8:25:44.27
650	RSRD19	-122.448	37.944	7.16	8.63	297.32	15.79	-122.448	37.944	7.28	8.63	297.19	15.92	1	8/1/2014 8:25:44.37
651	RSRD19	-122.448	37.944	7.16	8.64	297.35	15.80	-122.448	37.944	7.25	8.64	297.22	15.89	1	8/1/2014 8:25:44.46
652	RSRD19	-122.448	37.944	7.16	8.68	297.34	15.84	-122.448	37.944	7.25	8.68	297.23	15.92	1	8/1/2014 8:25:44.58
653	RSRD19	-122.448	37.944	7.13	8.66	297.37	15.79	-122.448	37.944	7.25	8.66	297.28	15.91	1	8/1/2014 8:25:44.67
654	RSRD19	-122.448	37.944	7.13	8.68	297.35	15.80	-122.448	37.944	7.25	8.68	297.29	15.92	1	8/1/2014 8:25:44.77
655	RSRD19	-122.448	37.944	7.13	8.66	297.36	15.79	-122.448	37.944	7.20	8.66	297.34	15.86	1	8/1/2014 8:25:44.86
656	RSRD19	-122.448	37.944	7.16	8.68	297.33	15.84	-122.448	37.944	7.20	8.68	297.30	15.87	1	8/1/2014 8:25:44.98
657	RSRD19	-122.448	37.944	7.13	8.69	297.44	15.82	-122.448	37.944	7.20	8.69	297.33	15.89	1	8/1/2014 8:25:45.07
658	RSRD19	-122.448	37.944	7.13	8.68	297.27	15.81	-122.448	37.944	7.20	8.68	297.34	15.88	1	8/1/2014 8:25:45.17
659	RSRD19	-122.448	37.944	7.13	8.71	297.39	15.84	-122.448	37.944	7.16	8.71	297.35	15.88	1	8/1/2014 8:25:45.26
660	RSRD19	-122.448	37.944	7.13	8.69	297.40	15.82	-122.448	37.944	7.20	8.69	297.40	15.89	1	8/1/2014 8:25:45.38
661	RSRD19	-122.448	37.944	7.13	8.71	297.40	15.84	-122.448	37.944	7.16	8.71	297.38	15.88	1	8/1/2014 8:25:45.47
662	RSRD19	-122.448	37.944	7.13	8.71	297.43	15.84	-122.448	37.944	7.20	8.71	297.41	15.91	1	8/1/2014 8:25:45.57
663	RSRD19	-122.448	37.944	7.13	8.73	297.44	15.86	-122.448	37.944	7.16	8.73	297.40	15.89	1	8/1/2014 8:25:45.67
664	RSRD19	-122.448	37.944	7.13	8.72	297.47	15.85	-122.448	37.944	7.16	8.72	297.38	15.88	1	8/1/2014 8:25:45.78
665	RSRD19	-122.448	37.944	7.13	8.72	297.48	15.85	-122.448	37.944	7.16	8.72	297.43	15.88	1	8/1/2014 8:25:45.87
666	RSRD19	-122.448	37.944	7.13	8.74	297.48	15.87	-122.448	37.944	7.16	8.74	297.48	15.91	1	8/1/2014 8:25:45.97
667	RSRD19	-122.448	37.944	7.07	8.74	297.51	15.82	-122.448	37.944	7.11	8.74	297.49	15.86	1	8/1/2014 8:25:46.07
668	RSRD19	-122.448	37.944	7.07	8.77	297.54	15.85	-122.448	37.944	7.11	8.77	297.50	15.89	1	8/1/2014 8:25:46.18
669	RSRD19	-122.448	37.944	7.07	8.81	297.54	15.88	-122.448	37.944	7.08	8.81	297.52	15.88	1	8/1/2014 8:25:46.27
670	RSRD19	-122.448	37.944	7.07	8.81	297.57	15.89	-122.448	37.944	7.08	8.81	297.51	15.89	1	8/1/2014 8:25:46.37
671	RSRD19	-122.448	37.944	7.04	8.84	297.58	15.88	-122.448	37.944	7.05	8.84	297.58	15.88	1	8/1/2014 8:25:46.46
672	RSRD19	-122.448	37.944	7.04	8.86	297.61	15.90	-122.448	37.944	7.05	8.86	297.56	15.90	1	8/1/2014 8:25:46.58
673	RSRD19	-122.448	37.944	6.99	8.87	297.61	15.86	-122.448	37.944	7.05	8.87	297.57	15.91	1	8/1/2014 8:25:46.67

674	RSRD19	-122.448	37.944	7.04	8.93	297.61	15.97	-122.448	37.944	6.99	8.93	297.55	15.92	1	8/1/2014	8:25:46.77
675	RSRD19	-122.448	37.944	6.99	8.89	297.64	15.88	-122.448	37.944	6.96	8.89	297.55	15.85	1	8/1/2014	8:25:46.86
676	RSRD19	-122.448	37.944	6.99	8.91	297.63	15.90	-122.448	37.944	6.96	8.91	297.54	15.87	1	8/1/2014	8:25:46.98
677	RSRD19	-122.448	37.944	6.99	8.94	297.63	15.93	-122.448	37.944	6.96	8.94	297.56	15.91	1	8/1/2014	8:25:47.07
678	RSRD19	-122.448	37.944	6.99	8.99	297.61	15.98	-122.448	37.944	6.96	8.99	297.52	15.95	1	8/1/2014	8:25:47.17
679	RSRD19	-122.448	37.944	6.95	8.97	297.61	15.92	-122.448	37.944	6.96	8.97	297.51	15.93	1	8/1/2014	8:25:47.26
680	RSRD19	-122.448	37.944	6.99	8.98	297.58	15.97	-122.448	37.944	6.91	8.98	297.49	15.89	1	8/1/2014	8:25:47.38
681	RSRD19	-122.448	37.944	6.95	8.98	297.56	15.93	-122.448	37.944	6.91	8.98	297.49	15.89	1	8/1/2014	8:25:47.47
682	RSRD19	-122.448	37.944	6.95	9.03	297.58	15.98	-122.448	37.944	6.91	9.03	297.47	15.94	1	8/1/2014	8:25:47.57
683	RSRD19	-122.448	37.944	6.95	9.01	297.54	15.96	-122.448	37.944	6.88	9.01	297.43	15.88	1	8/1/2014	8:25:47.66
684	RSRD19	-122.448	37.944	6.95	9.01	297.54	15.97	-122.448	37.944	6.88	9.01	297.41	15.89	1	8/1/2014	8:25:47.78
685	RSRD19	-122.448	37.944	6.90	9.01	297.55	15.91	-122.448	37.944	6.88	9.01	297.44	15.88	1	8/1/2014	8:25:47.87
686	RSRD19	-122.448	37.944	6.90	9.05	297.59	15.96	-122.448	37.944	6.88	9.05	297.39	15.93	1	8/1/2014	8:25:47.97
687	RSRD19	-122.448	37.944	6.90	9.04	297.57	15.94	-122.448	37.944	6.82	9.04	297.44	15.86	1	8/1/2014	8:25:48.06
688	RSRD19	-122.448	37.944	6.90	9.10	297.53	16.00	-122.448	37.944	6.82	9.10	297.38	15.92	1	8/1/2014	8:25:48.18
689	RSRD19	-122.448	37.944	6.87	9.07	297.55	15.94	-122.448	37.944	6.82	9.07	297.43	15.89	1	8/1/2014	8:25:48.27
690	RSRD19	-122.448	37.944	6.90	9.12	297.51	16.03	-122.448	37.944	6.82	9.12	297.40	15.95	1	8/1/2014	8:25:48.37
691	RSRD19	-122.448	37.944	6.87	9.08	297.52	15.95	-122.448	37.944	6.82	9.08	297.43	15.91	1	8/1/2014	8:25:48.46
692	RSRD19	-122.448	37.944	6.87	9.08	297.49	15.95	-122.448	37.944	6.82	9.08	297.39	15.90	1	8/1/2014	8:25:48.58
693	RSRD19	-122.448	37.944	6.87	9.08	297.47	15.95	-122.448	37.944	6.82	9.08	297.41	15.91	1	8/1/2014	8:25:48.67
694	RSRD19	-122.448	37.944	6.87	9.08	297.47	15.95	-122.448	37.944	6.82	9.08	297.38	15.91	1	8/1/2014	8:25:48.77
695	RSRD19	-122.448	37.944	6.82	9.09	297.45	15.91	-122.448	37.944	6.82	9.09	297.45	15.91	1	8/1/2014	8:25:48.86
696	RSRD19	-122.448	37.944	6.87	9.08	297.52	15.95	-122.448	37.944	6.82	9.08	297.36	15.90	1	8/1/2014	8:25:48.98
697	RSRD19	-122.448	37.944	6.82	9.08	297.41	15.90	-122.448	37.944	6.82	9.08	297.37	15.91	1	8/1/2014	8:25:49.07
698	RSRD19	-122.448	37.944	6.87	9.10	297.45	15.97	-122.448	37.944	6.82	9.10	297.39	15.92	1	8/1/2014	8:25:49.17
699	RSRD19	-122.448	37.944	6.82	9.11	297.43	15.93	-122.448	37.944	6.79	9.11	297.38	15.91	1	8/1/2014	8:25:49.26
700	RSRD19	-122.448	37.944	6.87	9.10	297.41	15.97	-122.448	37.944	6.79	9.10	297.37	15.89	1	8/1/2014	8:25:49.38
701	RSRD19	-122.448	37.944	6.82	9.09	297.41	15.91	-122.448	37.944	6.79	9.09	297.38	15.88	1	8/1/2014	8:25:49.47
702	RSRD19	-122.448	37.944	6.82	9.10	297.36	15.92	-122.448	37.944	6.82	9.10	297.36	15.92	1	8/1/2014	8:25:49.57
703	RSRD19	-122.448	37.944	6.82	9.07	297.36	15.89	-122.448	37.944	6.79	9.07	297.34	15.86	1	8/1/2014	8:25:49.66
704	RSRD19	-122.449	37.944	6.82	9.08	297.38	15.90	-122.449	37.944	6.82	9.08	297.36	15.91	1	8/1/2014	8:25:49.78
705	RSRD19	-122.449	37.944	6.78	9.08	297.36	15.87	-122.449	37.944	6.79	9.08	297.34	15.88	1	8/1/2014	8:25:49.87
706	RSRD19	-122.449	37.944	6.82	9.08	297.40	15.90	-122.449	37.944	6.79	9.08	297.29	15.88	1	8/1/2014	8:25:49.97

707	RSRD19	-122.449	37.944	6.82	9.08	297.37	15.90	-122.449	37.944	6.79	9.08	297.35	15.88	1	8/1/2014	8:25:50.06
708	RSRD19	-122.449	37.944	6.82	9.14	297.37	15.95	-122.449	37.944	6.79	9.14	297.31	15.93	1	8/1/2014	8:25:50.18
709	RSRD19	-122.449	37.944	6.78	9.13	297.36	15.91	-122.449	37.944	6.74	9.13	297.30	15.87	1	8/1/2014	8:25:50.27
710	RSRD19	-122.449	37.944	6.78	9.11	297.39	15.90	-122.449	37.944	6.74	9.11	297.27	15.86	1	8/1/2014	8:25:50.37
711	RSRD19	-122.449	37.944	6.78	9.12	297.36	15.91	-122.449	37.944	6.74	9.12	297.29	15.86	1	8/1/2014	8:25:50.46
712	RSRD19	-122.449	37.944	6.78	9.13	297.40	15.91	-122.449	37.944	6.74	9.13	297.25	15.87	1	8/1/2014	8:25:50.58
713	RSRD19	-122.449	37.944	6.75	9.16	297.37	15.91	-122.449	37.944	6.67	9.16	297.26	15.83	1	8/1/2014	8:25:50.67
714	RSRD19	-122.449	37.944	6.78	9.18	297.41	15.97	-122.449	37.944	6.71	9.18	297.26	15.89	1	8/1/2014	8:25:50.77
715	RSRD19	-122.449	37.944	6.75	9.20	297.34	15.95	-122.449	37.944	6.67	9.20	297.23	15.87	1	8/1/2014	8:25:50.86
716	RSRD19	-122.449	37.944	6.78	9.19	297.36	15.97	-122.449	37.944	6.67	9.19	297.23	15.86	1	8/1/2014	8:25:50.98
717	RSRD19	-122.449	37.944	6.75	9.22	297.33	15.97	-122.449	37.944	6.62	9.22	297.20	15.84	1	8/1/2014	8:25:51.07
718	RSRD19	-122.449	37.944	6.75	9.23	297.31	15.98	-122.449	37.944	6.62	9.23	297.15	15.85	1	8/1/2014	8:25:51.17
719	RSRD19	-122.449	37.944	6.70	9.32	297.30	16.02	-122.449	37.944	6.62	9.32	297.13	15.95	1	8/1/2014	8:25:51.26
720	RSRD19	-122.449	37.944	6.70	9.28	297.25	15.97	-122.449	37.944	6.62	9.28	297.12	15.90	1	8/1/2014	8:25:51.38
721	RSRD19	-122.449	37.944	6.70	9.28	297.20	15.98	-122.449	37.944	6.54	9.28	297.00	15.82	1	8/1/2014	8:25:51.47
722	RSRD19	-122.449	37.944	6.70	9.28	297.20	15.98	-122.449	37.944	6.59	9.28	297.00	15.87	1	8/1/2014	8:25:51.57
723	RSRD19	-122.449	37.944	6.70	9.32	297.17	16.02	-122.449	37.944	6.54	9.32	296.95	15.86	1	8/1/2014	8:25:51.66
724	RSRD19	-122.449	37.944	6.70	9.34	297.10	16.04	-122.449	37.944	6.54	9.34	296.87	15.88	1	8/1/2014	8:25:51.78
725	RSRD19	-122.449	37.944	6.66	9.35	297.09	16.02	-122.449	37.944	6.50	9.35	296.89	15.86	1	8/1/2014	8:25:51.87
726	RSRD19	-122.449	37.944	6.70	9.33	297.04	16.03	-122.449	37.944	6.50	9.33	296.84	15.83	1	8/1/2014	8:25:51.97
727	RSRD19	-122.449	37.944	6.66	9.35	297.03	16.01	-122.449	37.944	6.50	9.35	296.77	15.85	1	8/1/2014	8:25:52.06
728	RSRD19	-122.449	37.944	6.66	9.38	296.91	16.05	-122.449	37.944	6.50	9.38	296.78	15.89	1	8/1/2014	8:25:52.18
729	RSRD19	-122.449	37.944	6.61	9.36	296.97	15.97	-122.449	37.944	6.45	9.36	296.72	15.81	1	8/1/2014	8:25:52.27
730	RSRD19	-122.449	37.944	6.66	9.38	296.83	16.05	-122.449	37.944	6.45	9.38	296.68	15.84	1	8/1/2014	8:25:52.37
731	RSRD19	-122.449	37.944	6.61	9.38	296.82	15.99	-122.449	37.944	6.42	9.38	296.66	15.80	1	8/1/2014	8:25:52.46
732	RSRD19	-122.449	37.944	6.61	9.39	296.83	16.01	-122.449	37.944	6.45	9.39	296.62	15.85	1	8/1/2014	8:25:52.58
733	RSRD19	-122.449	37.944	6.61	9.39	296.76	16.01	-122.449	37.944	6.45	9.39	296.63	15.85	1	8/1/2014	8:25:52.67
734	RSRD19	-122.449	37.944	6.61	9.39	296.73	16.01	-122.449	37.944	6.45	9.39	296.57	15.85	1	8/1/2014	8:25:52.77
735	RSRD19	-122.449	37.944	6.58	9.39	296.70	15.97	-122.449	37.944	6.50	9.39	296.55	15.90	1	8/1/2014	8:25:52.86
736	RSRD19	-122.449	37.944	6.61	9.38	296.71	15.99	-122.449	37.944	6.45	9.38	296.51	15.83	1	8/1/2014	8:25:52.98
737	RSRD19	-122.449	37.944	6.58	9.39	296.63	15.97	-122.449	37.944	6.45	9.39	296.50	15.85	1	8/1/2014	8:25:53.07
738	RSRD19	-122.449	37.944	6.61	9.35	296.63	15.96	-122.449	37.944	6.45	9.35	296.47	15.80	1	8/1/2014	8:25:53.17
739	RSRD19	-122.449	37.944	6.58	9.35	296.55	15.92	-122.449	37.944	6.45	9.35	296.42	15.80	1	8/1/2014	8:25:53.26

740	RSRD19	-122.449	37.944	6.61	9.34	296.54	15.95	-122.449	37.944	6.50	9.34	296.46	15.84	1	8/1/2014	8:25:53.38
741	RSRD19	-122.449	37.944	6.58	9.31	296.53	15.89	-122.449	37.944	6.45	9.31	296.40	15.76	1	8/1/2014	8:25:53.47
742	RSRD19	-122.449	37.944	6.61	9.31	296.50	15.92	-122.449	37.944	6.50	9.31	296.41	15.81	1	8/1/2014	8:25:53.57
743	RSRD19	-122.449	37.944	6.58	9.29	296.49	15.87	-122.449	37.944	6.50	9.29	296.40	15.80	1	8/1/2014	8:25:53.66
744	RSRD19	-122.449	37.944	6.61	9.27	296.45	15.88	-122.449	37.944	6.50	9.27	296.39	15.77	1	8/1/2014	8:25:53.78
745	RSRD19	-122.449	37.944	6.58	9.25	296.42	15.83	-122.449	37.944	6.50	9.25	296.36	15.76	1	8/1/2014	8:25:53.87
746	RSRD19	-122.449	37.944	6.58	9.26	296.44	15.84	-122.449	37.944	6.50	9.26	296.41	15.76	1	8/1/2014	8:25:53.97
747	RSRD19	-122.449	37.944	6.58	9.23	296.38	15.81	-122.449	37.944	6.54	9.23	296.36	15.77	1	8/1/2014	8:25:54.06
748	RSRD19	-122.449	37.944	6.61	9.24	296.41	15.85	-122.449	37.944	6.54	9.24	296.33	15.78	1	8/1/2014	8:25:54.18
749	RSRD19	-122.449	37.944	6.58	9.24	296.40	15.82	-122.449	37.944	6.54	9.24	296.34	15.78	1	8/1/2014	8:25:54.27
750	RSRD19	-122.449	37.944	6.58	9.21	296.37	15.79	-122.449	37.944	6.54	9.21	296.28	15.74	1	8/1/2014	8:25:54.37
751	RSRD19	-122.449	37.944	6.58	9.19	296.40	15.77	-122.449	37.944	6.54	9.19	296.30	15.73	1	8/1/2014	8:25:54.46
752	RSRD19	-122.449	37.944	6.58	9.18	296.39	15.76	-122.449	37.944	6.54	9.18	296.28	15.72	1	8/1/2014	8:25:54.58
753	RSRD19	-122.449	37.944	6.58	9.19	296.38	15.77	-122.449	37.944	6.54	9.19	296.25	15.73	1	8/1/2014	8:25:54.67
754	RSRD19	-122.449	37.944	6.58	9.19	296.39	15.77	-122.449	37.944	6.59	9.19	296.26	15.78	1	8/1/2014	8:25:54.77
755	RSRD19	-122.449	37.944	6.58	9.18	296.40	15.75	-122.449	37.944	6.50	9.18	296.25	15.68	1	8/1/2014	8:25:54.86
756	RSRD19	-122.449	37.944	6.58	9.20	296.37	15.78	-122.449	37.944	6.54	9.20	296.24	15.74	1	8/1/2014	8:25:54.98
757	RSRD19	-122.449	37.944	6.58	9.21	296.42	15.79	-122.449	37.944	6.50	9.21	296.25	15.71	1	8/1/2014	8:25:55.07
758	RSRD19	-122.449	37.944	6.58	9.18	296.43	15.76	-122.449	37.944	6.54	9.18	296.26	15.72	1	8/1/2014	8:25:55.17
759	RSRD19	-122.449	37.944	6.58	9.18	296.38	15.76	-122.449	37.944	6.54	9.18	296.23	15.72	1	8/1/2014	8:25:55.26
760	RSRD19	-122.449	37.944	6.58	9.18	296.44	15.76	-122.449	37.944	6.54	9.18	296.24	15.72	1	8/1/2014	8:25:55.38
761	RSRD19	-122.449	37.944	6.58	9.21	296.40	15.79	-122.449	37.944	6.50	9.21	296.25	15.71	1	8/1/2014	8:25:55.47
762	RSRD19	-122.449	37.944	6.58	9.16	296.42	15.74	-122.449	37.944	6.54	9.16	296.24	15.70	1	8/1/2014	8:25:55.57
763	RSRD19	-122.449	37.944	6.53	9.16	296.40	15.69	-122.449	37.944	6.50	9.16	296.23	15.66	1	8/1/2014	8:25:55.66
764	RSRD19	-122.449	37.944	6.58	9.15	296.39	15.72	-122.449	37.944	6.54	9.15	296.26	15.68	1	8/1/2014	8:25:55.77
765	RSRD19	-122.449	37.944	6.58	9.12	296.43	15.70	-122.449	37.944	6.50	9.12	296.27	15.63	1	8/1/2014	8:25:55.87
766	RSRD19	-122.449	37.944	6.58	9.13	296.42	15.71	-122.449	37.944	6.54	9.13	296.28	15.67	1	8/1/2014	8:25:55.97
767	RSRD19	-122.449	37.944	6.53	9.13	296.43	15.66	-122.449	37.944	6.50	9.13	296.27	15.63	1	8/1/2014	8:25:56.06
768	RSRD19	-122.449	37.944	6.58	9.13	296.46	15.71	-122.449	37.944	6.54	9.13	296.35	15.67	1	8/1/2014	8:25:56.18
769	RSRD19	-122.449	37.944	6.53	9.12	296.45	15.65	-122.449	37.944	6.54	9.12	296.30	15.66	1	8/1/2014	8:25:56.27
770	RSRD19	-122.449	37.944	6.58	9.15	296.46	15.72	-122.449	37.944	6.54	9.15	296.31	15.68	1	8/1/2014	8:25:56.37
771	RSRD19	-122.449	37.944	6.53	9.12	296.47	15.65	-122.449	37.944	6.54	9.12	296.29	15.66	1	8/1/2014	8:25:56.46
772	RSRD19	-122.449	37.944	6.58	9.19	296.48	15.77	-122.449	37.944	6.59	9.19	296.31	15.78	1	8/1/2014	8:25:56.58

773	RSRD19	-122.449	37.944	6.53	9.13	296.47	15.66	-122.449	37.944	6.54	9.13	296.32	15.67	1	8/1/2014 8:25:56.67
774	RSRD19	-122.449	37.944	6.58	9.12	296.49	15.70	-122.449	37.944	6.54	9.12	296.27	15.66	1	8/1/2014 8:25:56.77
775	RSRD19	-122.449	37.944	6.53	9.12	296.52	15.65	-122.449	37.944	6.50	9.12	296.35	15.63	1	8/1/2014 8:25:56.86
776	RSRD19	-122.449	37.944	6.58	9.14	296.51	15.72	-122.449	37.944	6.54	9.14	296.31	15.68	1	8/1/2014 8:25:56.98
777	RSRD19	-122.449	37.944	6.53	9.11	296.48	15.64	-122.449	37.944	6.54	9.11	296.30	15.65	1	8/1/2014 8:25:57.07
778	RSRD19	-122.449	37.944	6.58	9.12	296.51	15.70	-122.449	37.944	6.54	9.12	296.29	15.66	1	8/1/2014 8:25:57.17
779	RSRD19	-122.449	37.944	6.53	9.10	296.52	15.63	-122.449	37.944	6.54	9.10	296.35	15.64	1	8/1/2014 8:25:57.26
780	RSRD19	-122.449	37.944	6.53	9.10	296.51	15.63	-122.449	37.944	6.54	9.10	296.32	15.64	1	8/1/2014 8:25:57.38
781	RSRD19	-122.449	37.944	6.53	9.10	296.50	15.63	-122.449	37.944	6.54	9.10	296.35	15.64	1	8/1/2014 8:25:57.47
782	RSRD19	-122.449	37.944	6.53	9.09	296.50	15.62	-122.449	37.944	6.54	9.09	296.34	15.63	1	8/1/2014 8:25:57.57
783	RSRD19	-122.449	37.944	6.53	9.08	296.49	15.61	-122.449	37.944	6.54	9.08	296.31	15.62	1	8/1/2014 8:25:57.66
784	RSRD19	-122.449	37.944	6.58	9.07	296.48	15.65	-122.449	37.944	6.59	9.07	296.34	15.66	1	8/1/2014 8:25:57.78
785	RSRD19	-122.449	37.944	6.58	9.04	296.51	15.62	-122.449	37.944	6.59	9.04	296.31	15.62	1	8/1/2014 8:25:57.87
786	RSRD19	-122.449	37.944	6.58	9.01	296.44	15.59	-122.449	37.944	6.59	9.01	296.35	15.60	1	8/1/2014 8:25:57.97
787	RSRD19	-122.449	37.944	6.58	9.00	296.47	15.58	-122.449	37.944	6.62	9.00	296.36	15.62	1	8/1/2014 8:25:58.06
788	RSRD19	-122.449	37.944	6.58	8.97	296.44	15.54	-122.449	37.944	6.67	8.97	296.34	15.64	1	8/1/2014 8:25:58.18
789	RSRD19	-122.449	37.944	6.58	8.94	296.37	15.52	-122.449	37.944	6.62	8.94	296.26	15.57	1	8/1/2014 8:25:58.27
790	RSRD19	-122.449	37.944	6.61	8.92	296.34	15.53	-122.449	37.944	6.71	8.92	296.23	15.63	1	8/1/2014 8:25:58.37
791	RSRD19	-122.449	37.944	6.58	8.93	296.40	15.51	-122.449	37.944	6.71	8.93	296.29	15.64	1	8/1/2014 8:25:58.46
792	RSRD19	-122.449	37.944	6.61	8.92	296.40	15.53	-122.449	37.944	6.71	8.92	296.32	15.63	1	8/1/2014 8:25:58.57
793	RSRD19	-122.449	37.944	6.61	8.89	296.42	15.50	-122.449	37.944	6.71	8.89	296.33	15.60	1	8/1/2014 8:25:58.67
794	RSRD19	-122.449	37.944	6.66	8.88	296.43	15.55	-122.449	37.944	6.74	8.88	296.35	15.63	1	8/1/2014 8:25:58.77
795	RSRD19	-122.449	37.944	6.61	8.87	296.39	15.48	-122.449	37.944	6.74	8.87	296.29	15.61	1	8/1/2014 8:25:58.86
796	RSRD19	-122.449	37.944	6.66	8.85	296.38	15.52	-122.449	37.944	6.79	8.85	296.29	15.64	1	8/1/2014 8:25:58.97
797	RSRD19	-122.449	37.944	6.66	8.86	296.39	15.52	-122.449	37.944	6.79	8.86	296.30	15.65	1	8/1/2014 8:25:59.07
798	RSRD19	-122.449	37.944	6.66	8.84	296.34	15.50	-122.449	37.944	6.79	8.84	296.30	15.63	1	8/1/2014 8:25:59.17
799	RSRD19	-122.449	37.944	6.66	8.84	296.38	15.50	-122.449	37.944	6.82	8.84	296.29	15.66	1	8/1/2014 8:25:59.26
800	RSRD19	-122.449	37.944	6.70	8.81	296.37	15.51	-122.449	37.944	6.82	8.81	296.30	15.64	1	8/1/2014 8:25:59.38
801	RSRD19	-122.449	37.944	6.66	8.81	296.36	15.48	-122.449	37.944	6.82	8.81	296.29	15.64	1	8/1/2014 8:25:59.47
802	RSRD19	-122.449	37.944	6.70	8.81	296.36	15.51	-122.449	37.944	6.82	8.81	296.27	15.64	1	8/1/2014 8:25:59.57
803	RSRD19	-122.449	37.944	6.66	8.83	296.33	15.49	-122.449	37.944	6.82	8.83	296.24	15.65	1	8/1/2014 8:25:59.66
804	RSRD19	-122.449	37.944	6.70	8.84	296.34	15.54	-122.449	37.944	6.88	8.84	296.25	15.71	1	8/1/2014 8:25:59.77
805	RSRD19	-122.449	37.944	6.66	8.81	296.33	15.48	-122.449	37.944	6.82	8.81	296.18	15.64	1	8/1/2014 8:25:59.87

806	RSRD19	-122.449	37.944	6.70	8.84	296.31	15.54	-122.449	37.944	6.88	8.84	296.20	15.72	1	8/1/2014	8:25:59.97
807	RSRD19	-122.449	37.944	6.70	8.82	296.30	15.52	-122.449	37.944	6.88	8.82	296.21	15.70	1	8/1/2014	8:26:00.06
808	RSRD19	-122.449	37.944	6.75	8.83	296.27	15.58	-122.449	37.944	6.88	8.83	296.16	15.70	1	8/1/2014	8:26:00.18
809	RSRD19	-122.449	37.944	6.70	8.80	296.27	15.50	-122.449	37.944	6.88	8.80	296.14	15.67	1	8/1/2014	8:26:00.27
810	RSRD19	-122.449	37.944	6.75	8.79	296.24	15.54	-122.449	37.944	6.88	8.79	296.15	15.66	1	8/1/2014	8:26:00.37
811	RSRD19	-122.449	37.944	6.70	8.82	296.24	15.52	-122.449	37.944	6.88	8.82	296.13	15.70	1	8/1/2014	8:26:00.46
812	RSRD19	-122.449	37.944	6.75	8.83	296.18	15.58	-122.449	37.944	6.91	8.83	296.10	15.74	1	8/1/2014	8:26:00.58
813	RSRD19	-122.449	37.944	6.70	8.79	296.18	15.49	-122.449	37.944	6.91	8.79	296.09	15.70	1	8/1/2014	8:26:00.67
814	RSRD19	-122.449	37.944	6.75	8.81	296.22	15.56	-122.449	37.944	6.91	8.81	296.11	15.72	1	8/1/2014	8:26:00.77
815	RSRD19	-122.449	37.944	6.75	8.77	296.17	15.52	-122.449	37.944	6.91	8.77	296.06	15.68	1	8/1/2014	8:26:00.86
816	RSRD19	-122.449	37.944	6.75	8.80	296.17	15.55	-122.449	37.944	6.91	8.80	296.05	15.71	1	8/1/2014	8:26:00.98
817	RSRD19	-122.449	37.944	6.75	8.75	296.14	15.50	-122.449	37.944	6.96	8.75	296.05	15.71	1	8/1/2014	8:26:01.07
818	RSRD19	-122.449	37.944	6.78	8.74	296.11	15.52	-122.449	37.944	6.99	8.74	296.05	15.73	1	8/1/2014	8:26:01.17
819	RSRD19	-122.449	37.944	6.78	8.74	296.13	15.53	-122.449	37.944	6.96	8.74	296.04	15.71	1	8/1/2014	8:26:01.26
820	RSRD19	-122.449	37.944	6.78	8.73	296.08	15.51	-122.449	37.944	6.99	8.73	296.02	15.72	1	8/1/2014	8:26:01.37
821	RSRD19	-122.449	37.944	6.78	8.70	296.05	15.49	-122.449	37.944	6.99	8.70	295.99	15.70	1	8/1/2014	8:26:01.47
822	RSRD19	-122.449	37.944	6.78	8.69	296.03	15.47	-122.449	37.944	7.05	8.69	296.03	15.74	1	8/1/2014	8:26:01.57
823	RSRD19	-122.449	37.944	6.78	8.69	296.02	15.47	-122.449	37.944	7.05	8.69	295.98	15.74	1	8/1/2014	8:26:01.66
824	RSRD19	-122.449	37.944	6.82	8.68	296.02	15.49	-122.449	37.944	7.05	8.68	296.00	15.72	1	8/1/2014	8:26:01.77
825	RSRD19	-122.449	37.944	6.82	8.68	295.99	15.49	-122.449	37.944	7.05	8.68	295.95	15.72	1	8/1/2014	8:26:01.87
826	RSRD19	-122.449	37.944	6.82	8.67	295.99	15.48	-122.449	37.944	7.08	8.67	295.95	15.75	1	8/1/2014	8:26:01.97
827	RSRD19	-122.449	37.944	6.82	8.64	295.99	15.46	-122.449	37.944	7.05	8.64	295.94	15.69	1	8/1/2014	8:26:02.06
828	RSRD19	-122.449	37.944	6.82	8.63	295.98	15.45	-122.449	37.944	7.08	8.63	295.94	15.71	1	8/1/2014	8:26:02.18
829	RSRD19	-122.449	37.944	6.82	8.63	295.96	15.45	-122.449	37.944	7.08	8.63	295.93	15.71	1	8/1/2014	8:26:02.27
830	RSRD19	-122.449	37.944	6.82	8.63	295.95	15.45	-122.449	37.944	7.08	8.63	295.91	15.71	1	8/1/2014	8:26:02.37
831	RSRD19	-122.449	37.944	6.87	8.62	295.93	15.49	-122.449	37.944	7.08	8.62	295.88	15.70	1	8/1/2014	8:26:02.46
832	RSRD19	-122.449	37.944	6.87	8.61	295.90	15.48	-122.449	37.944	7.11	8.61	295.88	15.72	1	8/1/2014	8:26:02.57
833	RSRD19	-122.449	37.944	6.87	8.63	295.88	15.50	-122.449	37.944	7.11	8.63	295.86	15.75	1	8/1/2014	8:26:02.67
834	RSRD19	-122.449	37.944	6.90	8.60	295.85	15.50	-122.449	37.944	7.16	8.60	295.85	15.76	1	8/1/2014	8:26:02.77
835	RSRD19	-122.449	37.944	6.87	8.56	295.83	15.43	-122.449	37.944	7.16	8.56	295.85	15.72	1	8/1/2014	8:26:02.86
836	RSRD19	-122.449	37.944	6.90	8.61	295.80	15.51	-122.449	37.944	7.16	8.61	295.82	15.77	1	8/1/2014	8:26:02.98
837	RSRD19	-122.449	37.944	6.87	8.54	295.80	15.41	-122.449	37.944	7.20	8.54	295.85	15.73	1	8/1/2014	8:26:03.07
838	RSRD19	-122.449	37.944	6.90	8.53	295.80	15.43	-122.449	37.944	7.20	8.53	295.88	15.73	1	8/1/2014	8:26:03.17

839	RSRD19	-122.449	37.944	6.90	8.52	295.75	15.42	-122.449	37.944	7.20	8.52	295.81	15.72	1	8/1/2014 8:26:03.26
840	RSRD19	-122.449	37.944	6.95	8.54	295.77	15.49	-122.449	37.944	7.25	8.54	295.86	15.78	1	8/1/2014 8:26:03.38
841	RSRD19	-122.449	37.944	6.90	8.53	295.77	15.43	-122.449	37.944	7.25	8.53	295.85	15.78	1	8/1/2014 8:26:03.47
842	RSRD19	-122.449	37.944	6.95	8.52	295.81	15.48	-122.449	37.944	7.25	8.52	295.83	15.77	1	8/1/2014 8:26:03.57
843	RSRD19	-122.449	37.944	6.95	8.56	295.76	15.51	-122.449	37.944	7.25	8.56	295.78	15.81	1	8/1/2014 8:26:03.66
844	RSRD19	-122.449	37.944	6.95	8.53	295.78	15.48	-122.449	37.944	7.25	8.53	295.82	15.78	1	8/1/2014 8:26:03.78
845	RSRD19	-122.449	37.944	6.95	8.54	295.80	15.49	-122.449	37.944	7.25	8.54	295.77	15.78	1	8/1/2014 8:26:03.87
846	RSRD19	-122.449	37.944	6.95	8.53	295.77	15.48	-122.449	37.944	7.25	8.53	295.78	15.78	1	8/1/2014 8:26:03.97
847	RSRD19	-122.449	37.944	6.95	8.52	295.75	15.48	-122.449	37.944	7.25	8.52	295.75	15.77	1	8/1/2014 8:26:04.06
848	RSRD19	-122.449	37.944	6.99	8.54	295.77	15.53	-122.449	37.944	7.25	8.54	295.75	15.78	1	8/1/2014 8:26:04.18
849	RSRD19	-122.449	37.944	6.95	8.54	295.79	15.50	-122.449	37.944	7.25	8.54	295.75	15.79	1	8/1/2014 8:26:04.27
850	RSRD19	-122.449	37.944	6.99	8.55	295.74	15.54	-122.449	37.944	7.25	8.55	295.70	15.80	1	8/1/2014 8:26:04.37
851	RSRD19	-122.449	37.944	6.95	8.56	295.72	15.52	-122.449	37.944	7.25	8.56	295.70	15.81	1	8/1/2014 8:26:04.46
852	RSRD19	-122.449	37.944	6.99	8.55	295.74	15.54	-122.449	37.944	7.25	8.55	295.70	15.80	1	8/1/2014 8:26:04.58
853	RSRD19	-122.449	37.944	6.95	8.56	295.72	15.52	-122.449	37.944	7.25	8.56	295.65	15.81	1	8/1/2014 8:26:04.67
854	RSRD19	-122.449	37.944	6.99	8.56	295.70	15.55	-122.449	37.944	7.25	8.56	295.63	15.81	1	8/1/2014 8:26:04.77
855	RSRD19	-122.449	37.944	6.95	8.58	295.69	15.54	-122.449	37.944	7.25	8.58	295.62	15.83	1	8/1/2014 8:26:04.86
856	RSRD19	-122.449	37.944	6.99	8.61	295.67	15.60	-122.449	37.944	7.25	8.61	295.62	15.86	1	8/1/2014 8:26:04.98
857	RSRD19	-122.449	37.944	6.95	8.60	295.67	15.55	-122.449	37.944	7.20	8.60	295.60	15.80	1	8/1/2014 8:26:05.07
858	RSRD19	-122.449	37.944	6.99	8.60	295.64	15.59	-122.449	37.944	7.25	8.60	295.55	15.85	1	8/1/2014 8:26:05.17
859	RSRD19	-122.449	37.944	6.95	8.58	295.62	15.54	-122.449	37.944	7.25	8.58	295.53	15.83	1	8/1/2014 8:26:05.26
860	RSRD19	-122.449	37.944	6.99	8.61	295.57	15.60	-122.449	37.944	7.25	8.61	295.51	15.85	1	8/1/2014 8:26:05.38
861	RSRD19	-122.449	37.944	6.99	8.58	295.57	15.57	-122.449	37.944	7.25	8.58	295.51	15.83	1	8/1/2014 8:26:05.47
862	RSRD19	-122.449	37.944	6.99	8.59	295.58	15.58	-122.449	37.944	7.25	8.59	295.48	15.84	1	8/1/2014 8:26:05.57
863	RSRD19	-122.449	37.944	6.99	8.56	295.53	15.55	-122.449	37.944	7.25	8.56	295.46	15.81	1	8/1/2014 8:26:05.66
864	RSRD19	-122.449	37.944	7.04	8.56	295.52	15.60	-122.449	37.944	7.28	8.56	295.43	15.84	1	8/1/2014 8:26:05.78
865	RSRD19	-122.449	37.944	6.99	8.54	295.52	15.53	-122.449	37.944	7.25	8.54	295.46	15.79	1	8/1/2014 8:26:05.87
866	RSRD19	-122.449	37.944	6.99	8.54	295.54	15.53	-122.449	37.944	7.28	8.54	295.46	15.83	1	8/1/2014 8:26:05.97
867	RSRD19	-122.449	37.944	6.99	8.60	295.48	15.59	-122.449	37.944	7.25	8.60	295.45	15.85	1	8/1/2014 8:26:06.06
868	RSRD19	-122.449	37.944	7.04	8.53	295.52	15.57	-122.449	37.944	7.28	8.53	295.45	15.81	1	8/1/2014 8:26:06.18
869	RSRD19	-122.449	37.944	6.99	8.54	295.49	15.53	-122.449	37.944	7.25	8.54	295.43	15.78	1	8/1/2014 8:26:06.27
870	RSRD19	-122.449	37.944	6.99	8.56	295.50	15.55	-122.449	37.944	7.25	8.56	295.45	15.81	1	8/1/2014 8:26:06.37
871	RSRD19	-122.449	37.944	6.99	8.53	295.49	15.52	-122.449	37.944	7.25	8.53	295.47	15.78	1	8/1/2014 8:26:06.46

872	RSRD19	-122.449	37.944	7.04	8.54	295.42	15.58	-122.449	37.944	7.28	8.54	295.42	15.82	1	8/1/2014	8:26:06.58
873	RSRD19	-122.449	37.944	6.99	8.54	295.49	15.53	-122.449	37.944	7.25	8.54	295.47	15.78	1	8/1/2014	8:26:06.67
874	RSRD19	-122.449	37.944	7.04	8.52	295.42	15.56	-122.449	37.944	7.28	8.52	295.44	15.80	1	8/1/2014	8:26:06.77
875	RSRD19	-122.449	37.944	6.99	8.51	295.40	15.49	-122.449	37.944	7.28	8.51	295.35	15.79	1	8/1/2014	8:26:06.86
876	RSRD19	-122.449	37.944	7.04	8.51	295.38	15.55	-122.449	37.944	7.33	8.51	295.35	15.84	1	8/1/2014	8:26:06.98
877	RSRD19	-122.449	37.944	6.99	8.49	295.37	15.48	-122.449	37.944	7.33	8.49	295.38	15.83	1	8/1/2014	8:26:07.07
878	RSRD19	-122.449	37.944	7.04	8.48	295.37	15.52	-122.449	37.944	7.33	8.48	295.37	15.82	1	8/1/2014	8:26:07.17
879	RSRD19	-122.449	37.944	7.04	8.48	295.44	15.52	-122.449	37.944	7.28	8.48	295.40	15.76	1	8/1/2014	8:26:07.26
880	RSRD19	-122.449	37.944	7.04	8.47	295.48	15.51	-122.449	37.944	7.37	8.47	295.46	15.84	1	8/1/2014	8:26:07.37
881	RSRD19	-122.449	37.944	7.04	8.47	295.54	15.51	-122.449	37.944	7.28	8.47	295.46	15.75	1	8/1/2014	8:26:07.47
882	RSRD19	-122.449	37.944	7.04	8.45	295.59	15.49	-122.449	37.944	7.33	8.45	295.50	15.78	1	8/1/2014	8:26:07.57
883	RSRD19	-122.449	37.944	6.99	8.48	295.67	15.47	-122.449	37.944	7.33	8.48	295.56	15.82	1	8/1/2014	8:26:07.66
884	RSRD19	-122.449	37.944	7.04	8.51	295.74	15.55	-122.449	37.944	7.28	8.51	295.63	15.79	1	8/1/2014	8:26:07.77
885	RSRD19	-122.449	37.944	7.04	8.47	295.80	15.51	-122.449	37.944	7.28	8.47	295.76	15.75	1	8/1/2014	8:26:07.87
886	RSRD19	-122.449	37.944	7.07	8.45	295.89	15.53	-122.449	37.944	7.28	8.45	295.69	15.73	1	8/1/2014	8:26:07.97
887	RSRD19	-122.449	37.944	6.99	8.47	295.95	15.46	-122.449	37.944	7.28	8.47	295.89	15.75	1	8/1/2014	8:26:08.06
888	RSRD19	-122.449	37.944	7.04	8.47	296.02	15.51	-122.449	37.944	7.28	8.47	295.84	15.75	1	8/1/2014	8:26:08.17
889	RSRD19	-122.449	37.944	7.04	8.47	296.10	15.51	-122.449	37.944	7.28	8.47	295.93	15.75	1	8/1/2014	8:26:08.27
890	RSRD19	-122.449	37.944	7.04	8.49	296.14	15.53	-122.449	37.944	7.28	8.49	295.99	15.77	1	8/1/2014	8:26:08.37
891	RSRD19	-122.449	37.944	7.04	8.51	296.21	15.55	-122.449	37.944	7.25	8.51	296.06	15.75	1	8/1/2014	8:26:08.46
892	RSRD19	-122.449	37.944	7.04	8.51	296.25	15.55	-122.449	37.944	7.25	8.51	296.12	15.75	1	8/1/2014	8:26:08.58
893	RSRD19	-122.449	37.944	7.04	8.49	296.32	15.53	-122.449	37.944	7.25	8.49	296.18	15.74	1	8/1/2014	8:26:08.67
894	RSRD19	-122.449	37.944	7.04	8.51	296.38	15.55	-122.449	37.944	7.28	8.51	296.23	15.79	1	8/1/2014	8:26:08.77
895	RSRD19	-122.449	37.944	7.04	8.49	296.45	15.54	-122.449	37.944	7.25	8.49	296.29	15.74	1	8/1/2014	8:26:08.86
896	RSRD19	-122.449	37.944	7.04	8.51	296.51	15.55	-122.449	37.944	7.20	8.51	296.33	15.71	1	8/1/2014	8:26:08.98
897	RSRD19	-122.449	37.944	7.04	8.49	296.53	15.54	-122.449	37.944	7.20	8.49	296.39	15.69	1	8/1/2014	8:26:09.07
898	RSRD19	-122.449	37.944	7.04	8.49	296.64	15.54	-122.449	37.944	7.20	8.49	296.44	15.69	1	8/1/2014	8:26:09.17
899	RSRD19	-122.449	37.944	7.04	8.47	296.59	15.51	-122.449	37.944	7.20	8.47	296.46	15.67	1	8/1/2014	8:26:09.26
900	RSRD19	-122.449	37.944	7.04	8.48	296.70	15.52	-122.449	37.944	7.20	8.48	296.50	15.68	1	8/1/2014	8:26:09.38
901	RSRD19	-122.449	37.944	7.04	8.49	296.70	15.53	-122.449	37.944	7.20	8.49	296.54	15.69	1	8/1/2014	8:26:09.47
902	RSRD19	-122.449	37.944	7.04	8.49	296.78	15.54	-122.449	37.944	7.20	8.49	296.59	15.69	1	8/1/2014	8:26:09.57
903	RSRD19	-122.449	37.944	7.04	8.47	296.86	15.51	-122.449	37.944	7.16	8.47	296.65	15.63	1	8/1/2014	8:26:09.66
904	RSRD19	-122.449	37.944	7.04	8.48	296.76	15.52	-122.449	37.944	7.20	8.48	296.67	15.68	1	8/1/2014	8:26:09.78

905	RSRD19	-122.449	37.944	6.99	8.51	296.84	15.50	-122.449	37.944	7.16	8.51	296.69	15.68	1	8/1/2014 8:26:09.87
906	RSRD19	-122.449	37.944	7.04	8.48	296.88	15.52	-122.449	37.944	7.20	8.48	296.71	15.68	1	8/1/2014 8:26:09.97
907	RSRD19	-122.449	37.944	6.99	8.49	296.90	15.48	-122.449	37.944	7.20	8.49	296.72	15.69	1	8/1/2014 8:26:10.06
908	RSRD19	-122.449	37.944	7.04	8.49	296.86	15.54	-122.449	37.944	7.16	8.49	296.70	15.66	1	8/1/2014 8:26:10.17
909	RSRD19	-122.449	37.944	6.99	8.49	296.90	15.49	-122.449	37.944	7.16	8.49	296.74	15.66	1	8/1/2014 8:26:10.27
910	RSRD19	-122.449	37.944	7.04	8.47	296.89	15.51	-122.449	37.944	7.20	8.47	296.72	15.67	1	8/1/2014 8:26:10.37
911	RSRD19	-122.449	37.944	6.99	8.49	296.85	15.48	-122.449	37.944	7.20	8.49	296.70	15.69	1	8/1/2014 8:26:10.46
912	RSRD19	-122.449	37.944	7.04	8.48	296.84	15.52	-122.449	37.944	7.25	8.48	296.67	15.73	1	8/1/2014 8:26:10.57
913	RSRD19	-122.449	37.944	6.99	8.49	296.80	15.48	-122.449	37.944	7.20	8.49	296.64	15.69	1	8/1/2014 8:26:10.67
914	RSRD19	-122.449	37.944	7.04	8.47	296.77	15.51	-122.449	37.944	7.20	8.47	296.64	15.67	1	8/1/2014 8:26:10.77
915	RSRD19	-122.449	37.944	7.04	8.48	296.75	15.52	-122.449	37.944	7.20	8.48	296.62	15.68	1	8/1/2014 8:26:10.86
916	RSRD19	-122.449	37.944	7.04	8.47	296.74	15.51	-122.449	37.944	7.20	8.47	296.61	15.67	1	8/1/2014 8:26:10.98
917	RSRD19	-122.449	37.944	7.04	8.47	296.72	15.51	-122.449	37.944	7.16	8.47	296.59	15.64	1	8/1/2014 8:26:11.07
918	RSRD19	-122.449	37.944	7.04	8.51	296.70	15.55	-122.449	37.944	7.20	8.51	296.61	15.70	1	8/1/2014 8:26:11.17
919	RSRD19	-122.449	37.944	6.99	8.47	296.67	15.46	-122.449	37.944	7.20	8.47	296.56	15.67	1	8/1/2014 8:26:11.26
920	RSRD19	-122.449	37.944	7.04	8.44	296.62	15.48	-122.449	37.944	7.20	8.44	296.53	15.64	1	8/1/2014 8:26:11.38
921	RSRD19	-122.449	37.944	7.04	8.44	296.64	15.48	-122.449	37.944	7.20	8.44	296.55	15.64	1	8/1/2014 8:26:11.47
922	RSRD19	-122.449	37.944	7.04	8.44	296.61	15.48	-122.449	37.944	7.20	8.44	296.53	15.63	1	8/1/2014 8:26:11.57
923	RSRD19	-122.449	37.944	7.04	8.41	296.57	15.45	-122.449	37.944	7.20	8.41	296.53	15.61	1	8/1/2014 8:26:11.66
924	RSRD19	-122.449	37.944	7.04	8.40	296.61	15.44	-122.449	37.944	7.20	8.40	296.52	15.60	1	8/1/2014 8:26:11.77
925	RSRD19	-122.449	37.944	7.04	8.40	296.56	15.44	-122.449	37.944	7.20	8.40	296.50	15.60	1	8/1/2014 8:26:11.87
926	RSRD19	-122.449	37.944	7.04	8.40	296.58	15.44	-122.449	37.944	7.20	8.40	296.53	15.60	1	8/1/2014 8:26:11.97
927	RSRD19	-122.449	37.944	7.04	8.39	296.56	15.43	-122.449	37.944	7.20	8.39	296.47	15.59	1	8/1/2014 8:26:12.06
928	RSRD19	-122.449	37.944	7.04	8.38	296.57	15.42	-122.449	37.944	7.25	8.38	296.49	15.63	1	8/1/2014 8:26:12.18
929	RSRD19	-122.449	37.944	7.04	8.37	296.57	15.41	-122.449	37.944	7.20	8.37	296.59	15.57	1	8/1/2014 8:26:12.27
930	RSRD19	-122.449	37.944	7.04	8.37	296.52	15.41	-122.449	37.944	7.20	8.37	296.37	15.57	1	8/1/2014 8:26:12.37
931	RSRD19	-122.449	37.944	6.99	8.37	296.52	15.36	-122.449	37.944	7.20	8.37	296.43	15.57	1	8/1/2014 8:26:12.46
932	RSRD19	-122.449	37.944	7.04	8.37	296.49	15.41	-122.449	37.944	7.20	8.37	296.40	15.57	1	8/1/2014 8:26:12.58
933	RSRD19	-122.449	37.944	7.04	8.37	296.47	15.41	-122.449	37.944	7.20	8.37	296.35	15.57	1	8/1/2014 8:26:12.67
934	RSRD19	-122.449	37.944	7.04	8.37	296.42	15.41	-122.449	37.944	7.25	8.37	296.37	15.62	1	8/1/2014 8:26:12.77
935	RSRD19	-122.449	37.944	7.04	8.37	296.39	15.41	-122.449	37.944	7.20	8.37	296.32	15.57	1	8/1/2014 8:26:12.86
936	RSRD19	-122.449	37.944	7.04	8.36	296.37	15.40	-122.449	37.944	7.25	8.36	296.30	15.61	1	8/1/2014 8:26:12.97
937	RSRD19	-122.449	37.944	7.04	8.36	296.36	15.40	-122.449	37.944	7.25	8.36	296.32	15.61	1	8/1/2014 8:26:13.07

938	RSRD19	-122.449	37.944	7.04	8.33	296.32	15.38	-122.449	37.944	7.25	8.33	296.25	15.58	1	8/1/2014 8:26:13.17
939	RSRD19	-122.449	37.944	7.04	8.35	296.31	15.39	-122.449	37.944	7.25	8.35	296.27	15.60	1	8/1/2014 8:26:13.26
940	RSRD19	-122.449	37.944	7.04	8.33	296.27	15.38	-122.449	37.944	7.25	8.33	296.20	15.58	1	8/1/2014 8:26:13.37
941	RSRD19	-122.449	37.944	7.04	8.33	296.30	15.38	-122.449	37.944	7.25	8.33	296.24	15.58	1	8/1/2014 8:26:13.47
942	RSRD19	-122.449	37.944	7.08	8.33	296.30	15.41	-122.449	37.944	7.28	8.33	296.26	15.62	1	8/1/2014 8:26:13.57
943	RSRD19	-122.449	37.944	7.04	8.33	296.28	15.38	-122.449	37.944	7.25	8.33	296.21	15.58	1	8/1/2014 8:26:13.66
944	RSRD19	-122.449	37.944	7.08	8.35	296.34	15.43	-122.449	37.944	7.28	8.35	296.27	15.63	1	8/1/2014 8:26:13.78
945	RSRD19	-122.449	37.944	7.04	8.35	296.31	15.39	-122.449	37.944	7.25	8.35	296.22	15.60	1	8/1/2014 8:26:13.87
946	RSRD19	-122.449	37.944	7.08	8.32	296.33	15.39	-122.449	37.944	7.25	8.32	296.24	15.57	1	8/1/2014 8:26:13.97
947	RSRD19	-122.449	37.944	7.04	8.35	296.35	15.39	-122.449	37.944	7.25	8.35	296.30	15.60	1	8/1/2014 8:26:14.06
948	RSRD19	-122.449	37.944	7.08	8.32	296.35	15.39	-122.449	37.944	7.28	8.32	296.26	15.60	1	8/1/2014 8:26:14.18
949	RSRD19	-122.449	37.944	7.04	8.36	296.36	15.40	-122.449	37.944	7.25	8.36	296.32	15.61	1	8/1/2014 8:26:14.27
950	RSRD19	-122.449	37.944	7.08	8.34	296.36	15.42	-122.449	37.944	7.28	8.34	296.27	15.63	1	8/1/2014 8:26:14.37
951	RSRD19	-122.449	37.944	7.08	8.33	296.42	15.41	-122.449	37.944	7.28	8.33	296.31	15.62	1	8/1/2014 8:26:14.46
952	RSRD19	-122.449	37.944	7.08	8.31	296.42	15.39	-122.449	37.944	7.28	8.31	296.33	15.60	1	8/1/2014 8:26:14.57
953	RSRD19	-122.449	37.944	7.08	8.36	296.40	15.44	-122.449	37.944	7.28	8.36	296.39	15.64	1	8/1/2014 8:26:14.67
954	RSRD19	-122.449	37.944	7.08	8.33	296.41	15.40	-122.449	37.944	7.28	8.33	296.35	15.61	1	8/1/2014 8:26:14.77
955	RSRD19	-122.449	37.944	7.08	8.32	296.43	15.39	-122.449	37.944	7.28	8.32	296.41	15.60	1	8/1/2014 8:26:14.88
956	RSRD19	-122.449	37.944	7.08	8.32	296.43	15.39	-122.449	37.944	7.28	8.32	296.36	15.60	1	8/1/2014 8:26:14.98
957	RSRD19	-122.449	37.944	7.08	8.33	296.54	15.40	-122.449	37.944	7.28	8.33	296.50	15.61	1	8/1/2014 8:26:15.07
958	RSRD19	-122.449	37.944	7.08	8.36	296.56	15.44	-122.449	37.944	7.28	8.36	296.53	15.64	1	8/1/2014 8:26:15.17
959	RSRD19	-122.449	37.944	7.08	8.34	296.55	15.42	-122.449	37.944	7.28	8.34	296.53	15.63	1	8/1/2014 8:26:15.28
960	RSRD19	-122.449	37.944	7.08	8.35	296.59	15.43	-122.449	37.944	7.28	8.35	296.53	15.63	1	8/1/2014 8:26:15.38
961	RSRD19	-122.449	37.944	7.08	8.33	296.61	15.41	-122.449	37.944	7.28	8.33	296.55	15.62	1	8/1/2014 8:26:15.47
962	RSRD19	-122.449	37.944	7.08	8.34	296.57	15.42	-122.449	37.944	7.28	8.34	296.50	15.63	1	8/1/2014 8:26:15.57
963	RSRD19	-122.449	37.944	7.08	8.33	296.61	15.41	-122.449	37.944	7.28	8.33	296.56	15.62	1	8/1/2014 8:26:15.68
964	RSRD19	-122.449	37.944	7.13	8.33	296.58	15.45	-122.449	37.944	7.28	8.33	296.51	15.61	1	8/1/2014 8:26:15.78
965	RSRD19	-122.449	37.944	7.08	8.33	296.58	15.40	-122.449	37.944	7.28	8.33	296.49	15.61	1	8/1/2014 8:26:15.87
966	RSRD19	-122.449	37.944	7.13	8.32	296.55	15.44	-122.449	37.944	7.28	8.32	296.53	15.60	1	8/1/2014 8:26:15.97
967	RSRD19	-122.449	37.944	7.08	8.31	296.57	15.39	-122.449	37.944	7.28	8.31	296.53	15.60	1	8/1/2014 8:26:16.08
968	RSRD19	-122.449	37.944	7.13	8.33	296.51	15.45	-122.449	37.944	7.37	8.33	296.51	15.70	1	8/1/2014 8:26:16.17
969	RSRD19	-122.449	37.944	7.08	8.29	296.52	15.37	-122.449	37.944	7.33	8.29	296.48	15.62	1	8/1/2014 8:26:16.27
970	RSRD19	-122.449	37.944	7.13	8.28	296.52	15.41	-122.449	37.944	7.37	8.28	296.50	15.65	1	8/1/2014 8:26:16.37

971	RSRD19	-122.449	37.944	7.08	8.30	296.52	15.37	-122.449	37.944	7.33	8.30	296.52	15.63	1	8/1/2014	8:26:16.48
972	RSRD19	-122.449	37.944	7.13	8.27	296.52	15.39	-122.449	37.944	7.33	8.27	296.50	15.60	1	8/1/2014	8:26:16.57
973	RSRD19	-122.449	37.944	7.08	8.27	296.58	15.34	-122.449	37.944	7.37	8.27	296.52	15.63	1	8/1/2014	8:26:16.67
974	RSRD19	-122.449	37.944	7.13	8.26	296.56	15.39	-122.449	37.944	7.37	8.26	296.54	15.63	1	8/1/2014	8:26:16.77
975	RSRD19	-122.449	37.944	7.08	8.27	296.60	15.34	-122.449	37.944	7.33	8.27	296.56	15.60	1	8/1/2014	8:26:16.88
976	RSRD19	-122.449	37.944	7.13	8.25	296.58	15.38	-122.449	37.944	7.37	8.25	296.56	15.62	1	8/1/2014	8:26:16.97
977	RSRD19	-122.449	37.944	7.13	8.27	296.60	15.40	-122.449	37.944	7.33	8.27	296.55	15.61	1	8/1/2014	8:26:17.07
978	RSRD19	-122.449	37.944	7.13	8.23	296.60	15.35	-122.449	37.944	7.37	8.23	296.53	15.60	1	8/1/2014	8:26:17.17
979	RSRD19	-122.449	37.944	7.13	8.23	296.60	15.35	-122.449	37.944	7.33	8.23	296.51	15.56	1	8/1/2014	8:26:17.28
980	RSRD19	-122.449	37.944	7.16	8.21	296.59	15.37	-122.449	37.944	7.37	8.21	296.51	15.58	1	8/1/2014	8:26:17.37
981	RSRD19	-122.449	37.944	7.13	8.21	296.57	15.34	-122.449	37.944	7.37	8.21	296.53	15.58	1	8/1/2014	8:26:17.47
982	RSRD19	-122.449	37.944	7.13	8.19	296.60	15.32	-122.449	37.944	7.37	8.19	296.53	15.56	1	8/1/2014	8:26:17.57
983	RSRD19	-122.449	37.944	7.13	8.18	296.57	15.31	-122.449	37.944	7.42	8.18	296.46	15.60	1	8/1/2014	8:26:17.68
984	RSRD19	-122.449	37.944	7.13	8.17	296.59	15.30	-122.449	37.944	7.42	8.17	296.52	15.59	1	8/1/2014	8:26:17.77
985	RSRD19	-122.449	37.944	7.13	8.16	296.61	15.28	-122.449	37.944	7.42	8.16	296.50	15.58	1	8/1/2014	8:26:17.87
986	RSRD19	-122.449	37.944	7.16	8.14	296.57	15.30	-122.449	37.944	7.42	8.14	296.48	15.56	1	8/1/2014	8:26:17.97
987	RSRD19	-122.449	37.944	7.16	8.14	296.59	15.30	-122.449	37.944	7.42	8.14	296.52	15.55	1	8/1/2014	8:26:18.08
988	RSRD19	-122.449	37.944	7.16	8.11	296.61	15.27	-122.449	37.944	7.45	8.11	296.53	15.56	1	8/1/2014	8:26:18.17
989	RSRD19	-122.449	37.944	7.16	8.15	296.61	15.31	-122.449	37.944	7.42	8.15	296.55	15.57	1	8/1/2014	8:26:18.27
990	RSRD19	-122.449	37.944	7.16	8.13	296.66	15.29	-122.449	37.944	7.45	8.13	296.55	15.58	1	8/1/2014	8:26:18.37
991	RSRD19	-122.449	37.944	7.16	8.13	296.63	15.29	-122.449	37.944	7.45	8.13	296.57	15.58	1	8/1/2014	8:26:18.48
992	RSRD19	-122.449	37.944	7.16	8.13	296.66	15.29	-122.449	37.944	7.42	8.13	296.55	15.55	1	8/1/2014	8:26:18.57
993	RSRD19	-122.449	37.944	7.16	8.13	296.68	15.29	-122.449	37.944	7.42	8.13	296.57	15.55	1	8/1/2014	8:26:18.67
994	RSRD19	-122.449	37.944	7.20	8.14	296.66	15.33	-122.449	37.944	7.42	8.14	296.57	15.55	1	8/1/2014	8:26:18.77
995	RSRD19	-122.449	37.944	7.16	8.13	296.65	15.29	-122.449	37.944	7.42	8.13	296.57	15.55	1	8/1/2014	8:26:18.88
996	RSRD19	-122.449	37.944	7.20	8.14	296.64	15.34	-122.449	37.944	7.45	8.14	296.55	15.60	1	8/1/2014	8:26:18.97
997	RSRD19	-122.449	37.944	7.16	8.15	296.64	15.31	-122.449	37.944	7.42	8.15	296.55	15.57	1	8/1/2014	8:26:19.07
998	RSRD19	-122.449	37.944	7.16	8.14	296.61	15.30	-122.449	37.944	7.45	8.14	296.55	15.59	1	8/1/2014	8:26:19.17
999	RSRD19	-122.449	37.944	7.16	8.13	296.61	15.29	-122.449	37.944	7.45	8.13	296.52	15.58	1	8/1/2014	8:26:19.28
1000	RSRD19	-122.449	37.944	7.16	8.14	296.60	15.30	-122.449	37.944	7.45	8.14	296.53	15.59	1	8/1/2014	8:26:19.37
1001	RSRD19	-122.449	37.944	7.16	8.13	296.60	15.29	-122.449	37.944	7.45	8.13	296.55	15.58	1	8/1/2014	8:26:19.47
1002	RSRD19	-122.449	37.944	7.20	8.12	296.59	15.32	-122.449	37.944	7.45	8.12	296.51	15.57	1	8/1/2014	8:26:19.57
1003	RSRD19	-122.449	37.944	7.16	8.12	296.60	15.28	-122.449	37.944	7.45	8.12	296.53	15.57	1	8/1/2014	8:26:19.68

1004	RSRD19	-122.449	37.944	7.20	8.14	296.60	15.33	-122.449	37.944	7.45	8.14	296.51	15.59	1	8/1/2014	8:26:19.77
1005	RSRD19	-122.449	37.944	7.16	8.14	296.55	15.30	-122.449	37.944	7.45	8.14	296.51	15.59	1	8/1/2014	8:26:19.87
1006	RSRD19	-122.449	37.944	7.20	8.12	296.55	15.32	-122.449	37.944	7.49	8.12	296.51	15.61	1	8/1/2014	8:26:19.97
1007	RSRD19	-122.449	37.944	7.20	8.12	296.56	15.32	-122.449	37.944	7.45	8.12	296.44	15.57	1	8/1/2014	8:26:20.08
1008	RSRD19	-122.449	37.944	7.20	8.12	296.53	15.32	-122.449	37.944	7.45	8.12	296.47	15.57	1	8/1/2014	8:26:20.17
1009	RSRD19	-122.449	37.944	7.20	8.10	296.54	15.30	-122.449	37.944	7.45	8.10	296.45	15.56	1	8/1/2014	8:26:20.27
1010	RSRD19	-122.449	37.944	7.25	8.09	296.54	15.34	-122.449	37.944	7.49	8.09	296.43	15.57	1	8/1/2014	8:26:20.37
1011	RSRD19	-122.449	37.944	7.20	8.10	296.45	15.29	-122.449	37.944	7.45	8.10	296.43	15.55	1	8/1/2014	8:26:20.48
1012	RSRD19	-122.449	37.944	7.20	8.08	296.43	15.28	-122.449	37.944	7.49	8.08	296.38	15.57	1	8/1/2014	8:26:20.57
1013	RSRD19	-122.449	37.944	7.20	8.09	296.43	15.28	-122.449	37.944	7.49	8.09	296.36	15.57	1	8/1/2014	8:26:20.67
1014	RSRD19	-122.449	37.944	7.25	8.10	296.32	15.34	-122.449	37.944	7.49	8.10	296.37	15.58	1	8/1/2014	8:26:20.77
1015	RSRD19	-122.449	37.944	7.20	8.06	296.32	15.25	-122.449	37.944	7.49	8.06	296.34	15.54	1	8/1/2014	8:26:20.88
1016	RSRD19	-122.449	37.944	7.25	8.05	296.30	15.30	-122.449	37.944	7.54	8.05	296.39	15.59	1	8/1/2014	8:26:20.98
1017	RSRD19	-122.449	37.944	7.20	8.03	296.26	15.22	-122.449	37.944	7.54	8.03	296.37	15.57	1	8/1/2014	8:26:21.07
1018	RSRD19	-122.449	37.944	7.25	8.00	296.24	15.25	-122.449	37.944	7.54	8.00	296.28	15.54	1	8/1/2014	8:26:21.17
1019	RSRD19	-122.449	37.944	7.20	8.02	296.24	15.21	-122.449	37.944	7.54	8.02	296.32	15.55	1	8/1/2014	8:26:21.28
1020	RSRD19	-122.449	37.944	7.25	8.00	296.17	15.25	-122.449	37.944	7.57	8.00	296.22	15.57	1	8/1/2014	8:26:21.38
1021	RSRD19	-122.449	37.944	7.20	8.00	296.17	15.19	-122.449	37.944	7.57	8.00	296.26	15.57	1	8/1/2014	8:26:21.47
1022	RSRD19	-122.449	37.944	7.25	7.97	296.15	15.22	-122.449	37.944	7.57	7.97	296.22	15.54	1	8/1/2014	8:26:21.57
1023	RSRD19	-122.449	37.944	7.25	7.97	296.11	15.21	-122.449	37.944	7.57	7.97	296.15	15.54	1	8/1/2014	8:26:21.68
1024	RSRD19	-122.449	37.944	7.28	7.97	296.13	15.25	-122.449	37.944	7.57	7.97	296.18	15.54	1	8/1/2014	8:26:21.78
1025	RSRD19	-122.449	37.944	7.25	7.95	296.09	15.20	-122.449	37.944	7.57	7.95	296.11	15.52	1	8/1/2014	8:26:21.87
1026	RSRD19	-122.449	37.944	7.28	7.95	296.05	15.23	-122.449	37.944	7.62	7.95	296.07	15.57	1	8/1/2014	8:26:21.97
1027	RSRD19	-122.449	37.944	7.25	7.95	296.05	15.20	-122.449	37.944	7.57	7.95	296.07	15.52	1	8/1/2014	8:26:22.08
1028	RSRD19	-122.449	37.944	7.28	7.94	296.03	15.22	-122.449	37.944	7.62	7.94	296.03	15.56	1	8/1/2014	8:26:22.17
1029	RSRD19	-122.449	37.944	7.25	7.93	295.96	15.18	-122.449	37.944	7.62	7.93	296.01	15.56	1	8/1/2014	8:26:22.27
1030	RSRD19	-122.449	37.944	7.28	7.94	295.98	15.22	-122.449	37.944	7.62	7.94	296.01	15.56	1	8/1/2014	8:26:22.37
1031	RSRD19	-122.449	37.944	7.25	7.94	295.95	15.19	-122.449	37.944	7.62	7.94	295.94	15.56	1	8/1/2014	8:26:22.48
1032	RSRD19	-122.449	37.944	7.28	7.94	295.94	15.22	-122.449	37.944	7.62	7.94	295.95	15.56	1	8/1/2014	8:26:22.58
1033	RSRD19	-122.449	37.944	7.28	7.92	295.92	15.20	-122.449	37.944	7.62	7.92	295.92	15.54	1	8/1/2014	8:26:22.67
1034	RSRD19	-122.449	37.944	7.33	7.93	295.90	15.26	-122.449	37.944	7.62	7.93	295.86	15.55	1	8/1/2014	8:26:22.77
1035	RSRD19	-122.449	37.944	7.33	7.94	295.86	15.28	-122.449	37.944	7.62	7.94	295.86	15.56	1	8/1/2014	8:26:22.88
1036	RSRD19	-122.449	37.944	7.33	7.92	295.88	15.25	-122.449	37.944	7.62	7.92	295.88	15.54	1	8/1/2014	8:26:22.97

1037	RSRD19	-122.449	37.944	7.28	7.97	295.84	15.25	-122.449	37.944	7.57	7.97	295.84	15.54	1	8/1/2014 8:26:23.07
1038	RSRD19	-122.449	37.944	7.33	7.93	295.82	15.26	-122.449	37.944	7.62	7.93	295.79	15.55	1	8/1/2014 8:26:23.17
1039	RSRD19	-122.449	37.944	7.33	7.92	295.82	15.25	-122.449	37.944	7.62	7.92	295.77	15.54	1	8/1/2014 8:26:23.28
1040	RSRD19	-122.449	37.944	7.37	7.92	295.77	15.28	-122.449	37.944	7.62	7.92	295.75	15.54	1	8/1/2014 8:26:23.38
1041	RSRD19	-122.449	37.944	7.33	7.93	295.75	15.26	-122.449	37.944	7.57	7.93	295.71	15.50	1	8/1/2014 8:26:23.47
1042	RSRD19	-122.449	37.944	7.37	7.94	295.71	15.31	-122.449	37.944	7.62	7.94	295.71	15.56	1	8/1/2014 8:26:23.57
1043	RSRD19	-122.449	37.944	7.33	7.92	295.71	15.25	-122.449	37.944	7.62	7.92	295.64	15.54	1	8/1/2014 8:26:23.68
1044	RSRD19	-122.449	37.944	7.37	7.93	295.64	15.29	-122.449	37.944	7.62	7.93	295.62	15.55	1	8/1/2014 8:26:23.78
1045	RSRD19	-122.449	37.944	7.33	7.91	295.62	15.24	-122.449	37.944	7.62	7.91	295.56	15.53	1	8/1/2014 8:26:23.87
1046	RSRD19	-122.449	37.944	7.37	7.91	295.60	15.28	-122.449	37.944	7.66	7.91	295.53	15.57	1	8/1/2014 8:26:23.97
1047	RSRD19	-122.449	37.944	7.33	7.92	295.56	15.25	-122.449	37.944	7.62	7.92	295.51	15.54	1	8/1/2014 8:26:24.08
1048	RSRD19	-122.449	37.944	7.37	7.92	295.56	15.28	-122.449	37.944	7.62	7.92	295.51	15.54	1	8/1/2014 8:26:24.18
1049	RSRD19	-122.449	37.944	7.33	7.89	295.49	15.23	-122.449	37.944	7.62	7.89	295.42	15.52	1	8/1/2014 8:26:24.27
1050	RSRD19	-122.449	37.944	7.37	7.92	295.47	15.28	-122.449	37.944	7.66	7.92	295.45	15.57	1	8/1/2014 8:26:24.37
1051	RSRD19	-122.449	37.944	7.37	7.92	295.45	15.28	-122.449	37.944	7.62	7.92	295.43	15.54	1	8/1/2014 8:26:24.48
1052	RSRD19	-122.449	37.944	7.37	7.93	295.42	15.29	-122.449	37.944	7.66	7.93	295.36	15.58	1	8/1/2014 8:26:24.58
1053	RSRD19	-122.449	37.944	7.37	7.95	295.40	15.32	-122.449	37.944	7.66	7.95	295.35	15.61	1	8/1/2014 8:26:24.67
1054	RSRD19	-122.449	37.944	7.37	7.92	295.38	15.28	-122.449	37.944	7.66	7.92	295.33	15.57	1	8/1/2014 8:26:24.77
1055	RSRD19	-122.449	37.944	7.37	7.92	295.31	15.28	-122.449	37.944	7.66	7.92	295.29	15.57	1	8/1/2014 8:26:24.88
1056	RSRD19	-122.449	37.944	7.42	7.92	295.31	15.34	-122.449	37.944	7.66	7.92	295.27	15.57	1	8/1/2014 8:26:24.97
1057	RSRD19	-122.449	37.944	7.37	7.91	295.27	15.28	-122.449	37.944	7.66	7.91	295.26	15.57	1	8/1/2014 8:26:25.07
1058	RSRD19	-122.449	37.944	7.42	7.93	295.27	15.35	-122.449	37.944	7.66	7.93	295.22	15.58	1	8/1/2014 8:26:25.17
1059	RSRD19	-122.449	37.944	7.37	7.92	295.23	15.28	-122.449	37.944	7.66	7.92	295.20	15.57	1	8/1/2014 8:26:25.28
1060	RSRD19	-122.449	37.944	7.42	7.93	295.22	15.35	-122.449	37.944	7.71	7.93	295.20	15.63	1	8/1/2014 8:26:25.37
1061	RSRD19	-122.449	37.944	7.37	7.95	295.22	15.32	-122.449	37.944	7.66	7.95	295.15	15.61	1	8/1/2014 8:26:25.47
1062	RSRD19	-122.449	37.944	7.42	7.93	295.17	15.35	-122.449	37.944	7.71	7.93	295.11	15.63	1	8/1/2014 8:26:25.57
1063	RSRD19	-122.449	37.944	7.37	7.91	295.15	15.28	-122.449	37.944	7.66	7.91	295.13	15.57	1	8/1/2014 8:26:25.68
1064	RSRD19	-122.449	37.944	7.37	7.93	295.17	15.30	-122.449	37.944	7.71	7.93	295.13	15.64	1	8/1/2014 8:26:25.77
1065	RSRD19	-122.449	37.944	7.37	7.91	295.19	15.28	-122.449	37.944	7.66	7.91	295.08	15.57	1	8/1/2014 8:26:25.87
1066	RSRD19	-122.449	37.944	7.37	7.91	295.04	15.28	-122.449	37.944	7.71	7.91	295.10	15.62	1	8/1/2014 8:26:25.97
1067	RSRD19	-122.449	37.944	7.37	7.92	295.13	15.28	-122.449	37.944	7.66	7.92	295.08	15.57	1	8/1/2014 8:26:26.08
1068	RSRD19	-122.449	37.944	7.42	7.93	295.11	15.35	-122.449	37.944	7.71	7.93	295.06	15.63	1	8/1/2014 8:26:26.17
1069	RSRD19	-122.449	37.944	7.37	7.92	295.08	15.28	-122.449	37.944	7.71	7.92	295.10	15.62	1	8/1/2014 8:26:26.27

1070	RSRD19	-122.449	37.944	7.37	7.93	295.06	15.29	-122.449	37.944	7.71	7.93	295.03	15.63	1	8/1/2014	8:26:26.37
1071	RSRD19	-122.449	37.944	7.37	7.94	295.04	15.31	-122.449	37.944	7.66	7.94	294.99	15.60	1	8/1/2014	8:26:26.48
1072	RSRD19	-122.449	37.944	7.42	7.93	295.03	15.35	-122.449	37.944	7.71	7.93	295.01	15.63	1	8/1/2014	8:26:26.57
1073	RSRD19	-122.449	37.944	7.37	7.93	295.01	15.30	-122.449	37.944	7.66	7.93	295.01	15.59	1	8/1/2014	8:26:26.67
1074	RSRD19	-122.449	37.944	7.42	7.91	294.97	15.33	-122.449	37.944	7.71	7.91	294.99	15.62	1	8/1/2014	8:26:26.77
1075	RSRD19	-122.449	37.944	7.42	7.90	294.96	15.32	-122.449	37.944	7.71	7.90	294.94	15.61	1	8/1/2014	8:26:26.88
1076	RSRD19	-122.449	37.944	7.42	7.92	294.94	15.34	-122.449	37.944	7.74	7.92	294.96	15.66	1	8/1/2014	8:26:26.97
1077	RSRD19	-122.449	37.944	7.37	7.94	294.87	15.31	-122.449	37.944	7.71	7.94	294.92	15.65	1	8/1/2014	8:26:27.07
1078	RSRD19	-122.449	37.944	7.42	7.90	294.89	15.32	-122.449	37.944	7.71	7.90	294.91	15.61	1	8/1/2014	8:26:27.17
1079	RSRD19	-122.449	37.944	7.37	7.92	294.80	15.28	-122.449	37.944	7.71	7.92	294.89	15.62	1	8/1/2014	8:26:27.28
1080	RSRD19	-122.449	37.944	7.42	7.90	294.85	15.32	-122.449	37.944	7.74	7.90	294.89	15.64	1	8/1/2014	8:26:27.37
1081	RSRD19	-122.449	37.944	7.42	7.92	294.78	15.34	-122.449	37.944	7.74	7.92	294.89	15.66	1	8/1/2014	8:26:27.47
1082	RSRD19	-122.449	37.944	7.42	7.91	294.75	15.33	-122.449	37.944	7.74	7.91	294.84	15.65	1	8/1/2014	8:26:27.57
1083	RSRD19	-122.449	37.944	7.42	7.94	294.73	15.36	-122.449	37.944	7.71	7.94	294.84	15.65	1	8/1/2014	8:26:27.68
1084	RSRD19	-122.449	37.944	7.42	7.92	294.75	15.34	-122.449	37.944	7.74	7.92	294.75	15.66	1	8/1/2014	8:26:27.77
1085	RSRD19	-122.449	37.944	7.42	7.93	294.60	15.35	-122.449	37.944	7.74	7.93	294.75	15.67	1	8/1/2014	8:26:27.87
1086	RSRD19	-122.449	37.944	7.42	7.93	294.66	15.35	-122.449	37.944	7.74	7.93	294.74	15.67	1	8/1/2014	8:26:27.97
1087	RSRD19	-122.449	37.944	7.42	7.94	294.63	15.36	-122.449	37.944	7.71	7.94	294.70	15.65	1	8/1/2014	8:26:28.08
1088	RSRD19	-122.449	37.944	7.45	7.95	294.61	15.40	-122.449	37.944	7.74	7.95	294.65	15.69	1	8/1/2014	8:26:28.17
1089	RSRD19	-122.449	37.944	7.42	7.93	294.54	15.35	-122.449	37.944	7.71	7.93	294.63	15.63	1	8/1/2014	8:26:28.27
1090	RSRD19	-122.449	37.944	7.45	8.00	294.56	15.45	-122.449	37.944	7.74	8.00	294.61	15.74	1	8/1/2014	8:26:28.37
1091	RSRD19	-122.449	37.944	7.42	7.95	294.58	15.37	-122.449	37.944	7.74	7.95	294.58	15.69	1	8/1/2014	8:26:28.48
1092	RSRD19	-122.449	37.944	7.45	7.93	294.40	15.39	-122.449	37.944	7.77	7.93	294.51	15.71	1	8/1/2014	8:26:28.57
1093	RSRD19	-122.449	37.944	7.42	7.93	294.47	15.35	-122.449	37.944	7.74	7.93	294.47	15.67	1	8/1/2014	8:26:28.67
1094	RSRD19	-122.449	37.944	7.45	7.93	294.35	15.39	-122.449	37.944	7.77	7.93	294.44	15.71	1	8/1/2014	8:26:28.77
1095	RSRD19	-122.449	37.944	7.42	7.98	294.38	15.40	-122.449	37.944	7.74	7.98	294.40	15.72	1	8/1/2014	8:26:28.88
1096	RSRD19	-122.449	37.944	7.45	7.94	294.35	15.39	-122.449	37.944	7.77	7.94	294.37	15.72	1	8/1/2014	8:26:28.97
1097	RSRD19	-122.449	37.944	7.42	7.93	294.29	15.35	-122.449	37.944	7.77	7.93	294.35	15.71	1	8/1/2014	8:26:29.07
1098	RSRD19	-122.449	37.944	7.42	7.93	294.26	15.35	-122.449	37.944	7.77	7.93	294.28	15.71	1	8/1/2014	8:26:29.17
1099	RSRD19	-122.449	37.944	7.42	7.94	294.24	15.36	-122.449	37.944	7.77	7.94	294.30	15.72	1	8/1/2014	8:26:29.28
1100	RSRD19	-122.449	37.944	7.45	7.97	294.21	15.42	-122.449	37.944	7.82	7.97	294.26	15.79	1	8/1/2014	8:26:29.37
1101	RSRD19	-122.449	37.944	7.42	7.97	294.21	15.38	-122.449	37.944	7.77	7.97	294.25	15.74	1	8/1/2014	8:26:29.47
1102	RSRD19	-122.449	37.944	7.45	7.96	294.14	15.41	-122.449	37.944	7.77	7.96	294.21	15.73	1	8/1/2014	8:26:29.57

1103	RSRD19	-122.449	37.944	7.45	7.95	294.16	15.40	-122.449	37.944	7.77	7.95	294.18	15.72	1	8/1/2014	8:26:29.68
1104	RSRD19	-122.449	37.944	7.45	7.96	294.09	15.41	-122.449	37.944	7.82	7.96	294.12	15.78	1	8/1/2014	8:26:29.77
1105	RSRD19	-122.449	37.944	7.45	7.96	294.07	15.41	-122.449	37.944	7.77	7.96	294.09	15.73	1	8/1/2014	8:26:29.87
1106	RSRD19	-122.449	37.944	7.50	7.97	294.05	15.47	-122.449	37.944	7.77	7.97	294.11	15.74	1	8/1/2014	8:26:29.97
1107	RSRD19	-122.449	37.944	7.45	7.97	294.04	15.42	-122.449	37.944	7.82	7.97	294.04	15.79	1	8/1/2014	8:26:30.08
1108	RSRD19	-122.449	37.944	7.50	7.97	294.04	15.47	-122.449	37.944	7.82	7.97	294.04	15.79	1	8/1/2014	8:26:30.17
1109	RSRD19	-122.449	37.944	7.45	7.97	293.99	15.42	-122.449	37.944	7.82	7.97	294.04	15.79	1	8/1/2014	8:26:30.27
1110	RSRD19	-122.449	37.944	7.50	7.97	293.97	15.47	-122.449	37.944	7.82	7.97	293.99	15.79	1	8/1/2014	8:26:30.37
1111	RSRD19	-122.449	37.944	7.45	7.95	293.94	15.40	-122.449	37.944	7.82	7.95	293.97	15.77	1	8/1/2014	8:26:30.48
1112	RSRD19	-122.449	37.944	7.50	7.94	293.92	15.45	-122.449	37.944	7.86	7.94	293.94	15.80	1	8/1/2014	8:26:30.57
1113	RSRD19	-122.449	37.944	7.50	7.94	293.88	15.45	-122.449	37.944	7.82	7.94	293.94	15.77	1	8/1/2014	8:26:30.67
1114	RSRD19	-122.449	37.944	7.50	7.98	293.90	15.48	-122.449	37.944	7.86	7.98	293.92	15.84	1	8/1/2014	8:26:30.77
1115	RSRD19	-122.449	37.944	7.50	7.93	293.80	15.44	-122.449	37.944	7.86	7.93	293.87	15.79	1	8/1/2014	8:26:30.88
1116	RSRD19	-122.449	37.944	7.57	7.94	293.82	15.51	-122.449	37.944	7.91	7.94	293.84	15.85	1	8/1/2014	8:26:30.97
1117	RSRD19	-122.449	37.944	7.57	7.93	293.76	15.51	-122.449	37.944	7.86	7.93	293.82	15.79	1	8/1/2014	8:26:31.07
1118	RSRD19	-122.449	37.944	7.57	7.93	293.73	15.50	-122.449	37.944	7.86	7.93	293.76	15.79	1	8/1/2014	8:26:31.17
1119	RSRD19	-122.449	37.944	7.57	7.93	293.71	15.50	-122.449	37.944	7.86	7.93	293.75	15.79	1	8/1/2014	8:26:31.28
1120	RSRD19	-122.449	37.944	7.62	7.98	293.68	15.60	-122.449	37.944	7.91	7.98	293.73	15.89	1	8/1/2014	8:26:31.37
1121	RSRD19	-122.449	37.944	7.57	7.97	293.64	15.54	-122.449	37.944	7.86	7.97	293.66	15.83	1	8/1/2014	8:26:31.47
1122	RSRD19	-122.449	37.944	7.57	7.94	293.64	15.51	-122.449	37.944	7.91	7.94	293.65	15.85	1	8/1/2014	8:26:31.57
1123	RSRD19	-122.449	37.944	7.62	7.93	293.63	15.55	-122.449	37.944	7.91	7.93	293.66	15.84	1	8/1/2014	8:26:31.68
1124	RSRD19	-122.449	37.944	7.62	7.94	293.54	15.57	-122.449	37.944	7.91	7.94	293.56	15.85	1	8/1/2014	8:26:31.77
1125	RSRD19	-122.449	37.944	7.62	7.93	293.56	15.55	-122.449	37.944	7.91	7.93	293.61	15.84	1	8/1/2014	8:26:31.87
1126	RSRD19	-122.449	37.944	7.62	7.94	293.49	15.57	-122.449	37.944	7.91	7.94	293.54	15.85	1	8/1/2014	8:26:31.97
1127	RSRD19	-122.449	37.944	7.57	7.97	293.49	15.54	-122.449	37.944	7.91	7.97	293.54	15.87	1	8/1/2014	8:26:32.08
1128	RSRD19	-122.449	37.944	7.62	7.97	293.42	15.59	-122.449	37.944	7.94	7.97	293.51	15.91	1	8/1/2014	8:26:32.17
1129	RSRD19	-122.449	37.944	7.57	7.97	293.44	15.54	-122.449	37.944	7.91	7.97	293.44	15.88	1	8/1/2014	8:26:32.27
1130	RSRD19	-122.449	37.944	7.62	8.04	293.38	15.66	-122.449	37.944	7.94	8.04	293.47	15.98	1	8/1/2014	8:26:32.37
1131	RSRD19	-122.449	37.944	7.62	8.00	293.39	15.63	-122.449	37.944	7.91	8.00	293.46	15.91	1	8/1/2014	8:26:32.48
1132	RSRD19	-122.449	37.944	7.62	7.99	293.37	15.61	-122.449	37.944	7.91	7.99	293.41	15.90	1	8/1/2014	8:26:32.57
1133	RSRD19	-122.449	37.944	7.62	8.03	293.35	15.65	-122.449	37.944	7.91	8.03	293.44	15.94	1	8/1/2014	8:26:32.67
1134	RSRD19	-122.449	37.944	7.62	8.00	293.30	15.63	-122.449	37.944	7.94	8.00	293.44	15.94	1	8/1/2014	8:26:32.77
1135	RSRD19	-122.449	37.944	7.57	8.00	293.30	15.57	-122.449	37.944	7.91	8.00	293.34	15.91	1	8/1/2014	8:26:32.88

1136	RSRD19	-122.449	37.944	7.62	8.04	293.28	15.66	-122.449	37.944	7.91	8.04	293.41	15.95	1	8/1/2014	8:26:32.97
1137	RSRD19	-122.449	37.944	7.57	8.03	293.26	15.61	-122.449	37.944	7.91	8.03	293.36	15.94	1	8/1/2014	8:26:33.07
1138	RSRD19	-122.449	37.944	7.62	8.02	293.21	15.64	-122.449	37.944	7.91	8.02	293.34	15.93	1	8/1/2014	8:26:33.16
1139	RSRD19	-122.449	37.944	7.62	8.04	293.20	15.66	-122.449	37.944	7.91	8.04	293.32	15.95	1	8/1/2014	8:26:33.28
1140	RSRD19	-122.449	37.944	7.62	8.03	293.20	15.66	-122.449	37.944	7.94	8.03	293.29	15.98	1	8/1/2014	8:26:33.37
1141	RSRD19	-122.449	37.944	7.62	8.03	293.14	15.65	-122.449	37.944	7.91	8.03	293.33	15.94	1	8/1/2014	8:26:33.47
1142	RSRD19	-122.449	37.944	7.62	8.08	293.11	15.71	-122.449	37.944	7.91	8.08	293.22	15.99	1	8/1/2014	8:26:33.57
1143	RSRD19	-122.449	37.944	7.62	8.02	293.09	15.64	-122.449	37.944	7.91	8.02	293.22	15.93	1	8/1/2014	8:26:33.68
1144	RSRD19	-122.449	37.944	7.62	8.02	293.07	15.64	-122.449	37.944	7.94	8.02	293.24	15.96	1	8/1/2014	8:26:33.77
1145	RSRD19	-122.449	37.944	7.62	8.07	293.02	15.69	-122.449	37.944	7.94	8.07	293.17	16.01	1	8/1/2014	8:26:33.87
1146	RSRD19	-122.449	37.944	7.62	8.00	292.98	15.62	-122.449	37.944	7.99	8.00	293.11	15.99	1	8/1/2014	8:26:33.97
1147	RSRD19	-122.449	37.944	7.62	8.01	292.91	15.64	-122.449	37.944	7.99	8.01	293.08	16.01	1	8/1/2014	8:26:34.08
1148	RSRD19	-122.449	37.944	7.62	8.00	292.84	15.63	-122.449	37.944	8.03	8.00	293.01	16.03	1	8/1/2014	8:26:34.17
1149	RSRD19	-122.449	37.944	7.62	7.98	292.82	15.60	-122.449	37.944	7.99	7.98	292.97	15.97	1	8/1/2014	8:26:34.27
1150	RSRD19	-122.449	37.944	7.66	7.97	292.75	15.62	-122.449	37.944	8.03	7.97	292.95	15.99	1	8/1/2014	8:26:34.37
1151	RSRD19	-122.449	37.944	7.66	7.95	292.70	15.61	-122.449	37.944	8.03	7.95	292.90	15.98	1	8/1/2014	8:26:34.48
1152	RSRD19	-122.449	37.944	7.66	7.93	292.66	15.59	-122.449	37.944	8.08	7.93	292.90	16.01	1	8/1/2014	8:26:34.57
1153	RSRD19	-122.449	37.944	7.66	7.93	292.59	15.59	-122.449	37.944	8.03	7.93	292.81	15.96	1	8/1/2014	8:26:34.67
1154	RSRD19	-122.449	37.944	7.71	7.97	292.55	15.67	-122.449	37.944	8.08	7.97	292.81	16.04	1	8/1/2014	8:26:34.77
1155	RSRD19	-122.449	37.944	7.66	7.93	292.55	15.59	-122.449	37.944	8.08	7.93	292.76	16.01	1	8/1/2014	8:26:34.88
1156	RSRD19	-122.449	37.944	7.71	7.93	292.52	15.64	-122.449	37.944	8.11	7.93	292.74	16.04	1	8/1/2014	8:26:34.97
1157	RSRD19	-122.449	37.944	7.66	7.92	292.48	15.57	-122.449	37.944	8.08	7.92	292.70	16.00	1	8/1/2014	8:26:35.07
1158	RSRD19	-122.449	37.944	7.71	7.91	292.41	15.62	-122.449	37.944	8.11	7.91	292.68	16.02	1	8/1/2014	8:26:35.17
1159	RSRD19	-122.449	37.944	7.66	7.93	292.45	15.59	-122.449	37.944	8.08	7.93	292.65	16.01	1	8/1/2014	8:26:35.28
1160	RSRD19	-122.449	37.944	7.71	7.93	292.43	15.64	-122.449	37.944	8.08	7.93	292.59	16.01	1	8/1/2014	8:26:35.37
1161	RSRD19	-122.449	37.944	7.66	7.94	292.36	15.60	-122.449	37.944	8.08	7.94	292.56	16.02	1	8/1/2014	8:26:35.47
1162	RSRD19	-122.449	37.944	7.71	7.93	292.39	15.64	-122.449	37.944	8.08	7.93	292.56	16.01	1	8/1/2014	8:26:35.57
1163	RSRD19	-122.449	37.944	7.66	7.95	292.32	15.61	-122.449	37.944	8.08	7.95	292.48	16.03	1	8/1/2014	8:26:35.68
1164	RSRD19	-122.449	37.944	7.71	7.95	292.27	15.66	-122.449	37.944	8.08	7.95	292.47	16.03	1	8/1/2014	8:26:35.77
1165	RSRD19	-122.449	37.944	7.66	7.96	292.25	15.62	-122.449	37.944	8.08	7.96	292.43	16.04	1	8/1/2014	8:26:35.87
1166	RSRD19	-122.449	37.944	7.66	7.94	292.23	15.60	-122.449	37.944	8.08	7.94	292.38	16.02	1	8/1/2014	8:26:35.96
1167	RSRD19	-122.449	37.944	7.66	7.94	292.16	15.60	-122.449	37.944	8.08	7.94	292.36	16.02	1	8/1/2014	8:26:36.08
1168	RSRD19	-122.449	37.944	7.71	7.96	292.10	15.67	-122.449	37.944	8.11	7.96	292.30	16.07	1	8/1/2014	8:26:36.17

1169	RSRD19	-122.449	37.944	7.71	7.97	292.07	15.67	-122.449	37.944	8.11	7.97	292.27	16.08	1	8/1/2014	8:26:36.27
1170	RSRD19	-122.449	37.944	7.71	7.93	291.99	15.64	-122.449	37.944	8.11	7.93	292.20	16.04	1	8/1/2014	8:26:36.37
1171	RSRD19	-122.449	37.944	7.71	7.93	291.96	15.64	-122.449	37.944	8.11	7.93	292.14	16.05	1	8/1/2014	8:26:36.48
1172	RSRD19	-122.449	37.944	7.71	7.95	291.90	15.66	-122.449	37.944	8.11	7.95	292.10	16.06	1	8/1/2014	8:26:36.57
1173	RSRD19	-122.449	37.944	7.71	7.93	291.81	15.64	-122.449	37.944	8.11	7.93	292.01	16.04	1	8/1/2014	8:26:36.67
1174	RSRD19	-122.449	37.944	7.74	7.91	291.79	15.65	-122.449	37.944	8.15	7.91	291.97	16.06	1	8/1/2014	8:26:36.76
1175	RSRD19	-122.449	37.944	7.71	7.92	291.74	15.63	-122.449	37.944	8.11	7.92	291.94	16.03	1	8/1/2014	8:26:36.88
1176	RSRD19	-122.449	37.944	7.71	7.93	291.68	15.64	-122.449	37.944	8.15	7.93	291.88	16.07	1	8/1/2014	8:26:36.97
1177	RSRD19	-122.449	37.944	7.71	7.89	291.64	15.60	-122.449	37.944	8.15	7.89	291.81	16.04	1	8/1/2014	8:26:37.07
1178	RSRD19	-122.449	37.944	7.74	7.89	291.57	15.63	-122.449	37.944	8.15	7.89	291.74	16.03	1	8/1/2014	8:26:37.17
1179	RSRD19	-122.449	37.944	7.74	7.89	291.52	15.63	-122.449	37.944	8.15	7.89	291.73	16.03	1	8/1/2014	8:26:37.28
1180	RSRD19	-122.449	37.944	7.74	7.89	291.46	15.63	-122.449	37.944	8.20	7.89	291.65	16.09	1	8/1/2014	8:26:37.37
1181	RSRD19	-122.449	37.944	7.74	7.89	291.42	15.63	-122.449	37.944	8.20	7.89	291.59	16.09	1	8/1/2014	8:26:37.47
1182	RSRD19	-122.449	37.944	7.74	7.87	291.35	15.62	-122.449	37.944	8.20	7.87	291.57	16.07	1	8/1/2014	8:26:37.57
1183	RSRD19	-122.449	37.944	7.83	7.88	291.30	15.71	-122.449	37.944	8.15	7.88	291.52	16.03	1	8/1/2014	8:26:37.68
1184	RSRD19	-122.449	37.944	7.86	7.88	291.24	15.74	-122.449	37.944	8.20	7.88	291.50	16.08	1	8/1/2014	8:26:37.77
1185	RSRD19	-122.449	37.944	7.83	7.87	291.24	15.70	-122.449	37.944	8.20	7.87	291.46	16.07	1	8/1/2014	8:26:37.87
1186	RSRD19	-122.449	37.944	7.86	7.87	291.24	15.74	-122.449	37.944	8.23	7.87	291.46	16.10	1	8/1/2014	8:26:37.97
1187	RSRD19	-122.449	37.944	7.83	7.86	291.20	15.69	-122.449	37.944	8.20	7.86	291.46	16.06	1	8/1/2014	8:26:38.08
1188	RSRD19	-122.449	37.944	7.86	7.84	291.15	15.70	-122.449	37.944	8.23	7.84	291.40	16.07	1	8/1/2014	8:26:38.17
1189	RSRD19	-122.449	37.944	7.86	7.84	291.11	15.70	-122.449	37.944	8.20	7.84	291.41	16.04	1	8/1/2014	8:26:38.27
1190	RSRD19	-122.449	37.944	7.86	7.84	291.09	15.70	-122.449	37.944	8.23	7.84	291.37	16.07	1	8/1/2014	8:26:38.37
1191	RSRD19	-122.449	37.944	7.86	7.83	291.00	15.69	-122.449	37.944	8.23	7.83	291.33	16.06	1	8/1/2014	8:26:38.48
1192	RSRD19	-122.449	37.944	7.91	7.81	290.96	15.72	-122.449	37.944	8.28	7.81	291.29	16.09	1	8/1/2014	8:26:38.57
1193	RSRD19	-122.449	37.944	7.86	7.81	290.89	15.67	-122.449	37.944	8.23	7.81	291.24	16.04	1	8/1/2014	8:26:38.67
1194	RSRD19	-122.449	37.944	7.86	7.82	290.89	15.68	-122.449	37.944	8.28	7.82	291.22	16.10	1	8/1/2014	8:26:38.77
1195	RSRD19	-122.449	37.944	7.86	7.80	290.80	15.67	-122.449	37.944	8.23	7.80	291.15	16.03	1	8/1/2014	8:26:38.88
1196	RSRD19	-122.449	37.944	7.86	7.82	290.76	15.68	-122.449	37.944	8.28	7.82	291.13	16.10	1	8/1/2014	8:26:38.97
1197	RSRD19	-122.449	37.944	7.83	7.79	290.74	15.62	-122.449	37.944	8.28	7.79	291.09	16.08	1	8/1/2014	8:26:39.07
1198	RSRD19	-122.449	37.944	7.86	7.79	290.72	15.66	-122.449	37.944	8.28	7.79	291.07	16.08	1	8/1/2014	8:26:39.17
1199	RSRD19	-122.449	37.944	7.86	7.79	290.61	15.66	-122.449	37.944	8.28	7.79	290.96	16.08	1	8/1/2014	8:26:39.28
1200	RSRD19	-122.449	37.944	7.91	7.79	290.61	15.71	-122.449	37.944	8.32	7.79	290.94	16.11	1	8/1/2014	8:26:39.37
1201	RSRD19	-122.449	37.944	7.86	7.80	290.55	15.67	-122.449	37.944	8.28	7.80	290.90	16.08	1	8/1/2014	8:26:39.47

1202	RSRD19	-122.449	37.944	7.91	7.82	290.53	15.73	-122.449	37.944	8.28	7.82	290.83	16.10	1	8/1/2014	8:26:39.57
1203	RSRD19	-122.449	37.944	7.86	7.79	290.41	15.66	-122.449	37.944	8.32	7.79	290.77	16.11	1	8/1/2014	8:26:39.68
1204	RSRD19	-122.449	37.944	7.91	7.79	290.42	15.71	-122.449	37.944	8.32	7.79	290.72	16.11	1	8/1/2014	8:26:39.77
1205	RSRD19	-122.449	37.944	7.86	7.81	290.35	15.67	-122.449	37.944	8.32	7.81	290.66	16.13	1	8/1/2014	8:26:39.87
1206	RSRD19	-122.449	37.944	7.86	7.78	290.31	15.64	-122.449	37.944	8.37	7.78	290.60	16.14	1	8/1/2014	8:26:39.97
1207	RSRD19	-122.449	37.944	7.86	7.78	290.26	15.64	-122.449	37.944	8.32	7.78	290.53	16.09	1	8/1/2014	8:26:40.08
1208	RSRD19	-122.449	37.944	7.91	7.76	290.20	15.68	-122.449	37.944	8.32	7.76	290.46	16.08	1	8/1/2014	8:26:40.17
1209	RSRD19	-122.449	37.944	7.91	7.76	290.18	15.67	-122.449	37.944	8.32	7.76	290.42	16.07	1	8/1/2014	8:26:40.27
1210	RSRD19	-122.449	37.944	7.91	7.76	290.13	15.67	-122.449	37.944	8.32	7.76	290.35	16.07	1	8/1/2014	8:26:40.37
1211	RSRD19	-122.449	37.944	7.91	7.75	290.09	15.66	-122.449	37.944	8.32	7.75	290.31	16.07	1	8/1/2014	8:26:40.48
1212	RSRD19	-122.449	37.944	7.91	7.73	290.07	15.65	-122.449	37.944	8.37	7.73	290.27	16.10	1	8/1/2014	8:26:40.57
1213	RSRD19	-122.449	37.944	7.91	7.72	290.03	15.63	-122.449	37.944	8.32	7.72	290.20	16.03	1	8/1/2014	8:26:40.67
1214	RSRD19	-122.449	37.944	7.91	7.71	290.01	15.62	-122.449	37.944	8.32	7.71	290.16	16.03	1	8/1/2014	8:26:40.77
1215	RSRD19	-122.449	37.944	7.91	7.72	289.96	15.64	-122.449	37.944	8.32	7.72	290.14	16.04	1	8/1/2014	8:26:40.88
1216	RSRD19	-122.449	37.944	7.91	7.72	289.86	15.63	-122.449	37.944	8.32	7.72	290.07	16.03	1	8/1/2014	8:26:40.97
1217	RSRD19	-122.449	37.944	7.91	7.73	289.83	15.65	-122.449	37.944	8.32	7.73	290.06	16.05	1	8/1/2014	8:26:41.07
1218	RSRD19	-122.449	37.944	7.91	7.70	289.79	15.61	-122.449	37.944	8.32	7.70	289.99	16.01	1	8/1/2014	8:26:41.17
1219	RSRD19	-122.449	37.944	7.91	7.71	289.70	15.62	-122.449	37.944	8.32	7.71	289.90	16.03	1	8/1/2014	8:26:41.28
1220	RSRD19	-122.449	37.944	7.95	7.66	289.62	15.61	-122.449	37.944	8.37	7.66	289.88	16.03	1	8/1/2014	8:26:41.37
1221	RSRD19	-122.449	37.944	7.91	7.66	289.53	15.58	-122.449	37.944	8.32	7.66	289.79	15.98	1	8/1/2014	8:26:41.47
1222	RSRD19	-122.449	37.944	7.95	7.63	289.47	15.58	-122.449	37.944	8.37	7.63	289.73	16.00	1	8/1/2014	8:26:41.57
1223	RSRD19	-122.449	37.944	7.95	7.64	289.40	15.59	-122.449	37.944	8.37	7.64	289.66	16.01	1	8/1/2014	8:26:41.68
1224	RSRD19	-122.449	37.944	7.95	7.63	289.31	15.57	-122.449	37.944	8.37	7.63	289.55	15.99	1	8/1/2014	8:26:41.77
1225	RSRD19	-122.449	37.944	7.95	7.61	289.25	15.56	-122.449	37.944	8.32	7.61	289.51	15.93	1	8/1/2014	8:26:41.87
1226	RSRD19	-122.449	37.944	7.95	7.59	289.16	15.54	-122.449	37.944	8.40	7.59	289.40	15.99	1	8/1/2014	8:26:41.97
1227	RSRD19	-122.449	37.944	7.91	7.62	289.08	15.53	-122.449	37.944	8.37	7.62	289.34	15.98	1	8/1/2014	8:26:42.08
1228	RSRD19	-122.449	37.944	7.95	7.58	289.03	15.53	-122.449	37.944	8.37	7.58	289.29	15.95	1	8/1/2014	8:26:42.17
1229	RSRD19	-122.449	37.944	7.91	7.59	288.96	15.50	-122.449	37.944	8.37	7.59	289.23	15.95	1	8/1/2014	8:26:42.27
1230	RSRD19	-122.449	37.944	7.95	7.59	288.94	15.54	-122.449	37.944	8.37	7.59	289.19	15.96	1	8/1/2014	8:26:42.36
1231	RSRD19	-122.449	37.944	7.95	7.53	288.86	15.48	-122.449	37.944	8.37	7.53	289.12	15.90	1	8/1/2014	8:26:42.48
1232	RSRD19	-122.449	37.944	7.95	7.53	288.81	15.48	-122.449	37.944	8.40	7.53	289.05	15.93	1	8/1/2014	8:26:42.57
1233	RSRD19	-122.449	37.944	7.95	7.55	288.75	15.50	-122.449	37.944	8.37	7.55	288.99	15.91	1	8/1/2014	8:26:42.67
1234	RSRD19	-122.449	37.944	7.95	7.56	288.71	15.51	-122.449	37.944	8.40	7.56	288.95	15.96	1	8/1/2014	8:26:42.77

1235	RSRD19	-122.449	37.944	7.95	7.53	288.62	15.47	-122.449	37.944	8.37	7.53	288.86	15.89	1	8/1/2014	8:26:42.88
1236	RSRD19	-122.449	37.944	7.95	7.53	288.55	15.48	-122.449	37.944	8.37	7.53	288.79	15.90	1	8/1/2014	8:26:42.97
1237	RSRD19	-122.449	37.944	7.95	7.59	288.53	15.54	-122.449	37.944	8.37	7.59	288.75	15.96	1	8/1/2014	8:26:43.07
1238	RSRD19	-122.449	37.944	7.95	7.52	288.45	15.46	-122.449	37.944	8.37	7.52	288.69	15.88	1	8/1/2014	8:26:43.17
1239	RSRD19	-122.449	37.944	7.95	7.53	288.38	15.48	-122.449	37.944	8.37	7.53	288.60	15.90	1	8/1/2014	8:26:43.28
1240	RSRD19	-122.449	37.944	8.00	7.52	288.27	15.52	-122.449	37.944	8.37	7.52	288.53	15.88	1	8/1/2014	8:26:43.37
1241	RSRD19	-122.449	37.944	7.95	7.49	288.23	15.44	-122.449	37.944	8.32	7.49	288.47	15.81	1	8/1/2014	8:26:43.47
1242	RSRD19	-122.449	37.944	7.95	7.49	288.14	15.44	-122.449	37.944	8.37	7.49	288.36	15.86	1	8/1/2014	8:26:43.57
1243	RSRD19	-122.449	37.944	7.95	7.49	288.05	15.44	-122.449	37.944	8.37	7.49	288.32	15.85	1	8/1/2014	8:26:43.68
1244	RSRD19	-122.449	37.944	8.00	7.49	287.94	15.49	-122.449	37.944	8.37	7.49	288.19	15.85	1	8/1/2014	8:26:43.77
1245	RSRD19	-122.449	37.944	7.95	7.48	287.88	15.43	-122.449	37.944	8.32	7.48	288.12	15.79	1	8/1/2014	8:26:43.87
1246	RSRD19	-122.449	37.944	8.00	7.49	287.77	15.49	-122.449	37.944	8.37	7.49	287.99	15.85	1	8/1/2014	8:26:43.97
1247	RSRD19	-122.449	37.944	7.95	7.48	287.70	15.43	-122.449	37.944	8.37	7.48	287.95	15.84	1	8/1/2014	8:26:44.08
1248	RSRD19	-122.449	37.944	7.95	7.44	287.60	15.39	-122.449	37.944	8.37	7.44	287.84	15.81	1	8/1/2014	8:26:44.17
1249	RSRD19	-122.449	37.944	7.95	7.42	287.53	15.37	-122.449	37.944	8.37	7.42	287.75	15.78	1	8/1/2014	8:26:44.27
1250	RSRD19	-122.449	37.944	7.95	7.41	287.42	15.36	-122.449	37.944	8.40	7.41	287.66	15.81	1	8/1/2014	8:26:44.36
1251	RSRD19	-122.449	37.944	8.00	7.41	287.36	15.41	-122.449	37.944	8.37	7.41	287.60	15.77	1	8/1/2014	8:26:44.48
1252	RSRD19	-122.449	37.944	8.00	7.39	287.25	15.39	-122.449	37.944	8.40	7.39	287.49	15.79	1	8/1/2014	8:26:44.57
1253	RSRD19	-122.449	37.944	8.00	7.37	287.16	15.37	-122.449	37.944	8.40	7.37	287.40	15.77	1	8/1/2014	8:26:44.67
1254	RSRD19	-122.449	37.944	8.00	7.38	287.07	15.38	-122.449	37.944	8.40	7.38	287.34	15.78	1	8/1/2014	8:26:44.76
1255	RSRD19	-122.449	37.944	8.00	7.38	286.96	15.38	-122.449	37.944	8.40	7.38	287.25	15.78	1	8/1/2014	8:26:44.88
1256	RSRD19	-122.449	37.944	8.00	7.37	286.86	15.37	-122.449	37.944	8.40	7.37	287.16	15.77	1	8/1/2014	8:26:44.97
1257	RSRD19	-122.449	37.944	8.00	7.38	286.76	15.38	-122.449	37.944	8.40	7.38	287.07	15.78	1	8/1/2014	8:26:45.07
1258	RSRD19	-122.449	37.944	8.00	7.36	286.65	15.36	-122.449	37.944	8.40	7.36	286.98	15.76	1	8/1/2014	8:26:45.16
1259	RSRD19	-122.449	37.944	8.00	7.39	286.57	15.39	-122.449	37.944	8.40	7.39	286.90	15.79	1	8/1/2014	8:26:45.28
1260	RSRD19	-122.449	37.944	8.03	7.38	286.44	15.41	-122.449	37.944	8.40	7.38	286.77	15.78	1	8/1/2014	8:26:45.37
1261	RSRD19	-122.449	37.944	8.00	7.42	286.39	15.42	-122.449	37.944	8.40	7.42	286.70	15.82	1	8/1/2014	8:26:45.47
1262	RSRD19	-122.449	37.944	8.00	7.44	286.29	15.44	-122.449	37.944	8.40	7.44	286.60	15.84	1	8/1/2014	8:26:45.57
1263	RSRD19	-122.449	37.944	8.00	7.46	286.20	15.46	-122.449	37.944	8.40	7.46	286.46	15.86	1	8/1/2014	8:26:45.68
1264	RSRD19	-122.449	37.944	8.03	7.45	286.09	15.48	-122.449	37.944	8.40	7.45	286.40	15.85	1	8/1/2014	8:26:45.77
1265	RSRD19	-122.450	37.944	8.00	7.45	285.98	15.45	-122.450	37.944	8.40	7.45	286.31	15.85	1	8/1/2014	8:26:45.87
1266	RSRD19	-122.450	37.944	8.03	7.46	285.87	15.50	-122.450	37.944	8.40	7.46	286.20	15.86	1	8/1/2014	8:26:45.97
1267	RSRD19	-122.450	37.944	8.00	7.47	285.80	15.47	-122.450	37.944	8.37	7.47	286.09	15.84	1	8/1/2014	8:26:46.08

1268	RSRD19	-122.450	37.944	8.03	7.48	285.67	15.51	-122.450	37.944	8.37	7.48	286.00	15.84	1	8/1/2014	8:26:46.17
1269	RSRD19	-122.450	37.944	8.00	7.46	285.56	15.46	-122.450	37.944	8.37	7.46	285.89	15.82	1	8/1/2014	8:26:46.27
1270	RSRD19	-122.450	37.944	8.03	7.46	285.47	15.49	-122.450	37.944	8.40	7.46	285.80	15.86	1	8/1/2014	8:26:46.37
1271	RSRD19	-122.450	37.944	8.03	7.55	285.41	15.58	-122.450	37.944	8.37	7.55	285.76	15.91	1	8/1/2014	8:26:46.48
1272	RSRD19	-122.450	37.944	8.03	7.52	285.25	15.55	-122.450	37.944	8.40	7.52	285.63	15.92	1	8/1/2014	8:26:46.57
1273	RSRD19	-122.450	37.944	8.03	7.45	285.19	15.48	-122.450	37.944	8.37	7.45	285.51	15.81	1	8/1/2014	8:26:46.67
1274	RSRD19	-122.450	37.944	8.09	7.45	285.03	15.53	-122.450	37.944	8.40	7.45	285.45	15.85	1	8/1/2014	8:26:46.77
1275	RSRD19	-122.450	37.944	8.09	7.47	284.95	15.56	-122.450	37.944	8.37	7.47	285.32	15.84	1	8/1/2014	8:26:46.88
1276	RSRD19	-122.450	37.944	8.09	7.44	284.88	15.53	-122.450	37.944	8.40	7.44	285.25	15.84	1	8/1/2014	8:26:46.97
1277	RSRD19	-122.450	37.944	8.09	7.46	284.81	15.54	-122.450	37.944	8.37	7.46	285.16	15.82	1	8/1/2014	8:26:47.07
1278	RSRD19	-122.450	37.944	8.12	7.46	284.61	15.58	-122.450	37.944	8.40	7.46	285.10	15.86	1	8/1/2014	8:26:47.16
1279	RSRD19	-122.450	37.944	8.09	7.49	284.61	15.58	-122.450	37.944	8.37	7.49	284.96	15.86	1	8/1/2014	8:26:47.28
1280	RSRD19	-122.450	37.944	8.12	7.45	284.48	15.57	-122.450	37.944	8.40	7.45	284.85	15.85	1	8/1/2014	8:26:47.37
1281	RSRD19	-122.450	37.944	8.09	7.47	284.37	15.56	-122.450	37.944	8.40	7.47	284.78	15.87	1	8/1/2014	8:26:47.47
1282	RSRD19	-122.450	37.944	8.12	7.46	284.28	15.58	-122.450	37.944	8.40	7.46	284.66	15.86	1	8/1/2014	8:26:47.56
1283	RSRD19	-122.450	37.944	8.09	7.46	284.17	15.54	-122.450	37.944	8.40	7.46	284.59	15.86	1	8/1/2014	8:26:47.68
1284	RSRD19	-122.450	37.944	8.09	7.46	284.08	15.54	-122.450	37.944	8.40	7.46	284.46	15.86	1	8/1/2014	8:26:47.77
1285	RSRD19	-122.450	37.944	8.09	7.47	283.93	15.56	-122.450	37.944	8.40	7.47	284.35	15.87	1	8/1/2014	8:26:47.87
1286	RSRD19	-122.450	37.944	8.09	7.46	283.86	15.55	-122.450	37.944	8.40	7.46	284.26	15.86	1	8/1/2014	8:26:47.97
1287	RSRD19	-122.450	37.944	8.09	7.46	283.73	15.55	-122.450	37.944	8.37	7.46	284.17	15.83	1	8/1/2014	8:26:48.08
1288	RSRD19	-122.450	37.944	8.12	7.52	283.62	15.64	-122.450	37.944	8.40	7.52	284.03	15.92	1	8/1/2014	8:26:48.17
1289	RSRD19	-122.450	37.944	8.09	7.48	283.51	15.56	-122.450	37.944	8.37	7.48	283.97	15.84	1	8/1/2014	8:26:48.27
1290	RSRD19	-122.450	37.944	8.09	7.47	283.42	15.56	-122.450	37.944	8.40	7.47	283.88	15.87	1	8/1/2014	8:26:48.37
1291	RSRD19	-122.450	37.944	8.09	7.48	283.33	15.56	-122.450	37.944	8.40	7.48	283.79	15.88	1	8/1/2014	8:26:48.48
1292	RSRD19	-122.450	37.944	8.12	7.46	283.20	15.58	-122.450	37.944	8.40	7.46	283.68	15.86	1	8/1/2014	8:26:48.57
1293	RSRD19	-122.450	37.944	8.09	7.44	283.06	15.53	-122.450	37.944	8.40	7.44	283.55	15.84	1	8/1/2014	8:26:48.67
1294	RSRD19	-122.450	37.944	8.09	7.46	282.95	15.55	-122.450	37.944	8.40	7.46	283.46	15.86	1	8/1/2014	8:26:48.76
1295	RSRD19	-122.450	37.944	8.09	7.42	282.82	15.50	-122.450	37.944	8.37	7.42	283.30	15.78	1	8/1/2014	8:26:48.88
1296	RSRD19	-122.450	37.944	8.09	7.40	282.69	15.49	-122.450	37.944	8.45	7.40	283.24	15.85	1	8/1/2014	8:26:48.97
1297	RSRD19	-122.450	37.944	8.09	7.42	282.57	15.51	-122.450	37.944	8.40	7.42	283.08	15.82	1	8/1/2014	8:26:49.07
1298	RSRD19	-122.450	37.944	8.12	7.36	282.44	15.47	-122.450	37.944	8.40	7.36	282.95	15.75	1	8/1/2014	8:26:49.17
1299	RSRD19	-122.450	37.944	8.09	7.32	282.31	15.40	-122.450	37.944	8.37	7.32	282.79	15.68	1	8/1/2014	8:26:49.28
1300	RSRD19	-122.450	37.944	8.12	7.31	282.11	15.43	-122.450	37.944	8.37	7.31	282.64	15.67	1	8/1/2014	8:26:49.37

1301	RSRD19	-122.450	37.944	8.09	7.28	281.93	15.37	-122.450	37.944	8.37	7.28	282.48	15.65	1	8/1/2014	8:26:49.47
1302	RSRD19	-122.450	37.944	8.09	7.31	281.73	15.39	-122.450	37.944	8.40	7.31	282.31	15.71	1	8/1/2014	8:26:49.57
1303	RSRD19	-122.450	37.944	8.09	7.32	281.53	15.40	-122.450	37.944	8.32	7.32	282.13	15.63	1	8/1/2014	8:26:49.68
1304	RSRD19	-122.450	37.944	8.09	7.32	281.34	15.41	-122.450	37.944	8.37	7.32	281.88	15.69	1	8/1/2014	8:26:49.77
1305	RSRD19	-122.450	37.944	8.09	7.33	281.11	15.42	-122.450	37.944	8.32	7.33	281.75	15.65	1	8/1/2014	8:26:49.87
1306	RSRD19	-122.450	37.944	8.09	7.32	280.91	15.40	-122.450	37.944	8.32	7.32	281.51	15.63	1	8/1/2014	8:26:49.96
1307	RSRD19	-122.450	37.944	8.09	7.34	280.70	15.43	-122.450	37.944	8.32	7.34	281.31	15.66	1	8/1/2014	8:26:50.08
1308	RSRD19	-122.450	37.944	8.09	7.36	280.49	15.45	-122.450	37.944	8.32	7.36	281.16	15.68	1	8/1/2014	8:26:50.18
1309	RSRD19	-122.450	37.944	8.09	7.37	280.29	15.46	-122.450	37.944	8.32	7.37	280.91	15.69	1	8/1/2014	8:26:50.27
1310	RSRD19	-122.450	37.944	8.09	7.39	280.03	15.48	-122.450	37.944	8.32	7.39	280.72	15.71	1	8/1/2014	8:26:50.36
1311	RSRD19	-122.450	37.944	8.03	7.39	279.92	15.42	-122.450	37.944	8.28	7.39	280.53	15.67	1	8/1/2014	8:26:50.48
1312	RSRD19	-122.450	37.944	8.09	7.39	279.65	15.47	-122.450	37.944	8.32	7.39	280.33	15.70	1	8/1/2014	8:26:50.57
1313	RSRD19	-122.450	37.944	8.09	7.40	279.50	15.49	-122.450	37.944	8.28	7.40	280.15	15.68	1	8/1/2014	8:26:50.67
1314	RSRD19	-122.450	37.944	8.09	7.40	279.28	15.49	-122.450	37.944	8.32	7.40	279.96	15.72	1	8/1/2014	8:26:50.77
1315	RSRD19	-122.450	37.944	8.03	7.39	279.10	15.43	-122.450	37.944	8.28	7.39	279.78	15.67	1	8/1/2014	8:26:50.88
1316	RSRD19	-122.450	37.944	8.09	7.39	278.88	15.48	-122.450	37.944	8.32	7.39	279.60	15.71	1	8/1/2014	8:26:50.97
1317	RSRD19	-122.450	37.944	8.03	7.40	278.64	15.44	-122.450	37.944	8.28	7.40	279.36	15.68	1	8/1/2014	8:26:51.07
1318	RSRD19	-122.450	37.944	8.09	7.42	278.43	15.51	-122.450	37.944	8.32	7.42	279.18	15.74	1	8/1/2014	8:26:51.17
1319	RSRD19	-122.450	37.944	8.03	7.42	278.23	15.46	-122.450	37.944	8.28	7.42	278.98	15.71	1	8/1/2014	8:26:51.28
1320	RSRD19	-122.450	37.944	8.09	7.42	278.00	15.50	-122.450	37.944	8.28	7.42	278.76	15.70	1	8/1/2014	8:26:51.37
1321	RSRD19	-122.450	37.944	8.03	7.44	277.75	15.47	-122.450	37.944	8.28	7.44	278.54	15.72	1	8/1/2014	8:26:51.47
1322	RSRD19	-122.450	37.944	8.03	7.46	277.55	15.49	-122.450	37.944	8.28	7.46	278.32	15.74	1	8/1/2014	8:26:51.56
1323	RSRD19	-122.450	37.944	8.03	7.51	277.26	15.54	-122.450	37.944	8.28	7.51	278.07	15.79	1	8/1/2014	8:26:51.68
1324	RSRD19	-122.450	37.944	8.09	7.49	277.04	15.58	-122.450	37.944	8.28	7.49	277.88	15.78	1	8/1/2014	8:26:51.77
1325	RSRD19	-122.450	37.944	8.03	7.52	276.80	15.55	-122.450	37.944	8.28	7.52	277.60	15.80	1	8/1/2014	8:26:51.87
1326	RSRD19	-122.450	37.944	8.09	7.51	276.53	15.60	-122.450	37.944	8.28	7.51	277.42	15.79	1	8/1/2014	8:26:51.97
1327	RSRD19	-122.450	37.944	8.03	7.52	276.27	15.55	-122.450	37.944	8.28	7.52	277.15	15.80	1	8/1/2014	8:26:52.08
1328	RSRD19	-122.450	37.944	8.09	7.50	276.05	15.59	-122.450	37.944	8.32	7.50	276.97	15.82	1	8/1/2014	8:26:52.17
1329	RSRD19	-122.450	37.944	8.03	7.53	275.80	15.57	-122.450	37.944	8.28	7.53	276.73	15.81	1	8/1/2014	8:26:52.27
1330	RSRD19	-122.450	37.944	8.09	7.49	275.52	15.57	-122.450	37.944	8.28	7.49	276.46	15.77	1	8/1/2014	8:26:52.37
1331	RSRD19	-122.450	37.944	8.03	7.52	275.28	15.55	-122.450	37.944	8.28	7.52	276.24	15.80	1	8/1/2014	8:26:52.48
1332	RSRD19	-122.450	37.944	8.09	7.50	275.03	15.59	-122.450	37.944	8.28	7.50	276.00	15.78	1	8/1/2014	8:26:52.57
1333	RSRD19	-122.450	37.944	8.03	7.52	274.75	15.55	-122.450	37.944	8.23	7.52	275.74	15.75	1	8/1/2014	8:26:52.67

1334	RSRD19	-122.450	37.944	8.09	7.52	274.48	15.60	-122.450	37.944	8.28	7.52	275.52	15.80	1	8/1/2014	8:26:52.77
1335	RSRD19	-122.450	37.944	8.03	7.50	274.24	15.54	-122.450	37.944	8.28	7.50	275.27	15.78	1	8/1/2014	8:26:52.88
1336	RSRD19	-122.450	37.944	8.03	7.51	273.98	15.54	-122.450	37.944	8.28	7.51	275.03	15.79	1	8/1/2014	8:26:52.97
1337	RSRD19	-122.450	37.944	8.03	7.53	273.69	15.56	-122.450	37.944	8.23	7.53	274.77	15.76	1	8/1/2014	8:26:53.07
1338	RSRD19	-122.450	37.944	8.09	7.52	273.42	15.60	-122.450	37.944	8.23	7.52	274.50	15.75	1	8/1/2014	8:26:53.16
1339	RSRD19	-122.450	37.944	8.03	7.52	273.16	15.55	-122.450	37.944	8.23	7.52	274.26	15.75	1	8/1/2014	8:26:53.28
1340	RSRD19	-122.450	37.944	8.03	7.52	272.83	15.55	-122.450	37.944	8.28	7.52	273.98	15.80	1	8/1/2014	8:26:53.37
1341	RSRD19	-122.450	37.944	8.03	7.51	272.48	15.54	-122.450	37.944	8.23	7.51	273.65	15.74	1	8/1/2014	8:26:53.47
1342	RSRD19	-122.450	37.944	8.03	7.52	272.15	15.55	-122.450	37.944	8.23	7.52	273.34	15.75	1	8/1/2014	8:26:53.56
1343	RSRD19	-122.450	37.944	8.03	7.55	271.75	15.58	-122.450	37.944	8.23	7.55	272.97	15.78	1	8/1/2014	8:26:53.68
1344	RSRD19	-122.450	37.944	8.03	7.51	271.40	15.54	-122.450	37.944	8.23	7.51	272.66	15.74	1	8/1/2014	8:26:53.77
1345	RSRD19	-122.450	37.944	8.03	7.53	271.03	15.57	-122.450	37.944	8.23	7.53	272.28	15.76	1	8/1/2014	8:26:53.87
1346	RSRD19	-122.450	37.944	8.03	7.53	270.66	15.56	-122.450	37.944	8.23	7.53	271.93	15.76	1	8/1/2014	8:26:53.96
1347	RSRD19	-122.450	37.944	8.03	7.53	270.30	15.56	-122.450	37.944	8.23	7.53	271.60	15.76	1	8/1/2014	8:26:54.08
1348	RSRD19	-122.450	37.944	8.03	7.53	269.93	15.57	-122.450	37.944	8.28	7.53	271.24	15.81	1	8/1/2014	8:26:54.17
1349	RSRD19	-122.450	37.944	8.03	7.53	269.52	15.56	-122.450	37.944	8.23	7.53	270.86	15.76	1	8/1/2014	8:26:54.27
1350	RSRD19	-122.450	37.944	8.03	7.53	269.12	15.56	-122.450	37.944	8.23	7.53	270.48	15.76	1	8/1/2014	8:26:54.36
1351	RSRD19	-122.450	37.944	8.03	7.55	268.70	15.58	-122.450	37.944	8.23	7.55	270.14	15.78	1	8/1/2014	8:26:54.48
1352	RSRD19	-122.450	37.944	8.03	7.57	268.27	15.61	-122.450	37.944	8.23	7.57	269.72	15.80	1	8/1/2014	8:26:54.57
1353	RSRD19	-122.450	37.944	8.00	7.59	267.82	15.59	-122.450	37.944	8.20	7.59	269.30	15.78	1	8/1/2014	8:26:54.67
1354	RSRD19	-122.450	37.944	8.03	7.59	267.41	15.63	-122.450	37.944	8.20	7.59	268.93	15.79	1	8/1/2014	8:26:54.76
1355	RSRD19	-122.450	37.944	8.00	7.61	267.01	15.61	-122.450	37.944	8.20	7.61	268.57	15.81	1	8/1/2014	8:26:54.88
1356	RSRD19	-122.450	37.944	8.03	7.59	266.56	15.62	-122.450	37.944	8.23	7.59	268.14	15.82	1	8/1/2014	8:26:54.97
1357	RSRD19	-122.450	37.944	8.00	7.60	266.14	15.60	-122.450	37.944	8.20	7.60	267.76	15.80	1	8/1/2014	8:26:55.07
1358	RSRD19	-122.450	37.944	8.03	7.58	265.70	15.61	-122.450	37.944	8.20	7.58	267.35	15.78	1	8/1/2014	8:26:55.16
1359	RSRD19	-122.450	37.944	8.00	7.59	265.24	15.59	-122.450	37.944	8.20	7.59	266.93	15.78	1	8/1/2014	8:26:55.28
1360	RSRD19	-122.450	37.944	8.00	7.59	264.69	15.59	-122.450	37.944	8.20	7.59	266.45	15.79	1	8/1/2014	8:26:55.37
1361	RSRD19	-122.450	37.944	8.00	7.60	264.25	15.60	-122.450	37.944	8.15	7.60	266.03	15.75	1	8/1/2014	8:26:55.47
1362	RSRD19	-122.450	37.944	8.00	7.62	263.75	15.62	-122.450	37.944	8.20	7.62	265.57	15.81	1	8/1/2014	8:26:55.57
1363	RSRD19	-122.450	37.944	8.00	7.62	263.24	15.62	-122.450	37.944	8.15	7.62	265.09	15.76	1	8/1/2014	8:26:55.68
1364	RSRD19	-122.450	37.944	8.00	7.63	262.72	15.63	-122.450	37.944	8.15	7.63	264.61	15.77	1	8/1/2014	8:26:55.77
1365	RSRD19	-122.450	37.944	8.00	7.67	262.24	15.67	-122.450	37.944	8.11	7.67	264.15	15.78	1	8/1/2014	8:26:55.87
1366	RSRD19	-122.450	37.944	8.03	7.64	261.71	15.67	-122.450	37.944	8.15	7.64	263.69	15.78	1	8/1/2014	8:26:55.96

1367	RSRD19	-122.450	37.944	8.00	7.70	261.23	15.70	-122.450	37.944	8.11	7.70	263.21	15.82	1	8/1/2014 8:26:56.08
1368	RSRD19	-122.450	37.944	8.00	7.66	260.70	15.66	-122.450	37.944	8.11	7.66	262.71	15.78	1	8/1/2014 8:26:56.17
1369	RSRD19	-122.450	37.944	8.00	7.65	260.20	15.65	-122.450	37.944	8.11	7.65	262.22	15.76	1	8/1/2014 8:26:56.27
1370	RSRD19	-122.450	37.944	8.00	7.65	259.70	15.65	-122.450	37.944	8.11	7.65	261.77	15.76	1	8/1/2014 8:26:56.36
1371	RSRD19	-122.450	37.944	7.95	7.68	259.17	15.63	-122.450	37.944	8.11	7.68	261.28	15.79	1	8/1/2014 8:26:56.48
1372	RSRD19	-122.450	37.944	8.00	7.66	258.69	15.66	-122.450	37.944	8.11	7.66	260.80	15.78	1	8/1/2014 8:26:56.57
1373	RSRD19	-122.450	37.944	7.95	7.65	258.18	15.60	-122.450	37.944	8.11	7.65	260.34	15.76	1	8/1/2014 8:26:56.67
1374	RSRD19	-122.450	37.944	8.00	7.67	257.66	15.67	-122.450	37.944	8.11	7.67	259.86	15.78	1	8/1/2014 8:26:56.77
1375	RSRD19	-122.450	37.944	7.95	7.64	257.11	15.59	-122.450	37.944	8.11	7.64	259.34	15.75	1	8/1/2014 8:26:56.88
1376	RSRD19	-122.450	37.944	7.95	7.64	256.57	15.59	-122.450	37.944	8.11	7.64	258.83	15.75	1	8/1/2014 8:26:56.97
1377	RSRD19	-122.450	37.944	7.95	7.64	256.00	15.59	-122.450	37.944	8.08	7.64	258.33	15.72	1	8/1/2014 8:26:57.07
1378	RSRD19	-122.450	37.944	8.00	7.62	255.41	15.62	-122.450	37.944	8.11	7.62	257.76	15.73	1	8/1/2014 8:26:57.16
1379	RSRD19	-122.450	37.944	7.95	7.62	254.79	15.57	-122.450	37.944	8.11	7.62	257.21	15.73	1	8/1/2014 8:26:57.28
1380	RSRD19	-122.450	37.944	8.00	7.66	254.18	15.66	-122.450	37.944	8.11	7.66	256.67	15.78	1	8/1/2014 8:26:57.37
1381	RSRD19	-122.450	37.944	7.95	7.61	253.53	15.56	-122.450	37.944	8.08	7.61	256.01	15.69	1	8/1/2014 8:26:57.47
1382	RSRD19	-122.450	37.944	8.00	7.60	252.87	15.60	-122.450	37.944	8.11	7.60	255.41	15.71	1	8/1/2014 8:26:57.57
1383	RSRD19	-122.450	37.944	7.95	7.60	252.21	15.55	-122.450	37.944	8.11	7.60	254.82	15.71	1	8/1/2014 8:26:57.68
1384	RSRD19	-122.450	37.944	7.95	7.60	251.56	15.55	-122.450	37.944	8.11	7.60	254.21	15.71	1	8/1/2014 8:26:57.77
1385	RSRD19	-122.450	37.944	7.95	7.62	250.88	15.57	-122.450	37.944	8.08	7.62	253.59	15.70	1	8/1/2014 8:26:57.87
1386	RSRD19	-122.450	37.944	8.00	7.63	250.17	15.63	-122.450	37.944	8.11	7.63	252.92	15.75	1	8/1/2014 8:26:57.97
1387	RSRD19	-122.450	37.944	7.95	7.63	249.41	15.58	-122.450	37.944	8.11	7.63	252.29	15.75	1	8/1/2014 8:26:58.08
1388	RSRD19	-122.450	37.944	7.95	7.65	248.69	15.60	-122.450	37.944	8.11	7.65	251.60	15.76	1	8/1/2014 8:26:58.17
1389	RSRD19	-122.450	37.944	7.95	7.64	247.96	15.59	-122.450	37.944	8.08	7.64	250.89	15.72	1	8/1/2014 8:26:58.27
1390	RSRD19	-122.450	37.944	7.95	7.63	247.18	15.58	-122.450	37.944	8.11	7.63	250.17	15.75	1	8/1/2014 8:26:58.37
1391	RSRD19	-122.450	37.944	7.95	7.65	246.39	15.60	-122.450	37.944	8.11	7.65	249.45	15.76	1	8/1/2014 8:26:58.48
1392	RSRD19	-122.450	37.944	7.95	7.64	245.58	15.59	-122.450	37.944	8.08	7.64	248.66	15.72	1	8/1/2014 8:26:58.57
1393	RSRD19	-122.450	37.944	7.95	7.65	244.77	15.60	-122.450	37.944	8.08	7.65	247.89	15.73	1	8/1/2014 8:26:58.67
1394	RSRD19	-122.450	37.944	7.95	7.66	243.98	15.61	-122.450	37.944	8.08	7.66	247.17	15.74	1	8/1/2014 8:26:58.76
1395	RSRD19	-122.450	37.944	7.95	7.66	243.15	15.61	-122.450	37.944	8.08	7.66	246.40	15.74	1	8/1/2014 8:26:58.88
1396	RSRD19	-122.450	37.944	7.95	7.70	242.36	15.65	-122.450	37.944	8.08	7.70	245.66	15.78	1	8/1/2014 8:26:58.97
1397	RSRD19	-122.450	37.944	7.95	7.68	241.55	15.63	-122.450	37.944	8.03	7.68	244.91	15.71	1	8/1/2014 8:26:59.07
1398	RSRD19	-122.450	37.944	7.95	7.72	240.74	15.67	-122.450	37.944	8.08	7.72	244.13	15.80	1	8/1/2014 8:26:59.16
1399	RSRD19	-122.450	37.944	7.95	7.71	239.95	15.66	-122.450	37.944	8.03	7.71	243.41	15.74	1	8/1/2014 8:26:59.28

1400	RSRD19	-122.450	37.944	7.95	7.73	239.12	15.68	-122.450	37.944	8.03	7.73	242.64	15.76	1	8/1/2014	8:26:59.37
1401	RSRD19	-122.450	37.944	7.95	7.75	238.29	15.70	-122.450	37.944	8.03	7.75	241.85	15.78	1	8/1/2014	8:26:59.47
1402	RSRD19	-122.450	37.944	7.95	7.76	237.44	15.70	-122.450	37.944	8.08	7.76	241.04	15.84	1	8/1/2014	8:26:59.57
1403	RSRD19	-122.450	37.944	7.91	7.83	236.59	15.74	-122.450	37.944	7.99	7.83	240.26	15.82	1	8/1/2014	8:26:59.68
1404	RSRD19	-122.450	37.944	7.91	7.80	235.75	15.72	-122.450	37.944	8.03	7.80	239.44	15.83	1	8/1/2014	8:26:59.77
1405	RSRD19	-122.450	37.944	7.91	7.79	234.88	15.70	-122.450	37.944	7.99	7.79	238.63	15.78	1	8/1/2014	8:26:59.87
1406	RSRD19	-122.450	37.944	7.91	7.80	234.04	15.72	-122.450	37.944	7.99	7.80	237.87	15.80	1	8/1/2014	8:26:59.96
1407	RSRD19	-122.450	37.944	7.91	7.79	233.15	15.71	-122.450	37.944	7.99	7.79	236.99	15.79	1	8/1/2014	8:27:00.08
1408	RSRD19	-122.450	37.944	7.91	7.81	232.27	15.72	-122.450	37.944	7.99	7.81	236.18	15.80	1	8/1/2014	8:27:00.17
1409	RSRD19	-122.450	37.944	7.86	7.83	231.40	15.69	-122.450	37.944	7.94	7.83	235.35	15.77	1	8/1/2014	8:27:00.27
1410	RSRD19	-122.450	37.944	7.91	7.86	230.58	15.77	-122.450	37.944	7.94	7.86	234.55	15.80	1	8/1/2014	8:27:00.37
1411	RSRD19	-122.450	37.944	7.86	7.89	229.78	15.75	-122.450	37.944	7.91	7.89	233.75	15.80	1	8/1/2014	8:27:00.48
1412	RSRD19	-122.450	37.944	7.91	7.88	228.90	15.79	-122.450	37.944	7.94	7.88	232.99	15.82	1	8/1/2014	8:27:00.57
1413	RSRD19	-122.450	37.944	7.91	7.93	228.16	15.85	-122.450	37.944	7.91	7.93	232.20	15.84	1	8/1/2014	8:27:00.67
1414	RSRD19	-122.450	37.944	7.91	7.93	227.37	15.84	-122.450	37.944	7.91	7.93	231.50	15.84	1	8/1/2014	8:27:00.77
1415	RSRD19	-122.450	37.944	7.86	7.94	226.56	15.81	-122.450	37.944	7.86	7.94	230.72	15.80	1	8/1/2014	8:27:00.88
1416	RSRD19	-122.450	37.944	7.86	7.94	225.78	15.81	-122.450	37.944	7.91	7.94	230.00	15.85	1	8/1/2014	8:27:00.97
1417	RSRD19	-122.450	37.944	7.83	7.98	225.01	15.81	-122.450	37.944	7.91	7.98	229.23	15.89	1	8/1/2014	8:27:01.07
1418	RSRD19	-122.450	37.944	7.86	7.98	224.24	15.84	-122.450	37.944	7.91	7.98	228.48	15.89	1	8/1/2014	8:27:01.17
1419	RSRD19	-122.450	37.944	7.86	7.95	223.48	15.81	-122.450	37.944	7.91	7.95	227.72	15.86	1	8/1/2014	8:27:01.28
1420	RSRD19	-122.450	37.944	7.86	7.96	222.63	15.82	-122.450	37.944	7.91	7.96	226.90	15.87	1	8/1/2014	8:27:01.37
1421	RSRD19	-122.450	37.944	7.86	7.96	221.84	15.82	-122.450	37.944	7.91	7.96	226.14	15.87	1	8/1/2014	8:27:01.47
1422	RSRD19	-122.450	37.944	7.86	7.96	220.98	15.82	-122.450	37.944	7.91	7.96	225.32	15.87	1	8/1/2014	8:27:01.57
1423	RSRD19	-122.450	37.944	7.83	7.93	220.24	15.76	-122.450	37.944	7.91	7.93	224.56	15.84	1	8/1/2014	8:27:01.68
1424	RSRD19	-122.450	37.944	7.86	7.93	219.48	15.80	-122.450	37.944	7.94	7.93	223.83	15.88	1	8/1/2014	8:27:01.77
1425	RSRD19	-122.450	37.944	7.83	7.90	218.71	15.73	-122.450	37.944	7.91	7.90	223.05	15.81	1	8/1/2014	8:27:01.87
1426	RSRD19	-122.450	37.944	7.86	7.89	217.88	15.75	-122.450	37.944	7.94	7.89	222.26	15.83	1	8/1/2014	8:27:01.96
1427	RSRD19	-122.450	37.944	7.83	7.86	217.12	15.69	-122.450	37.944	7.91	7.86	221.49	15.77	1	8/1/2014	8:27:02.08
1428	RSRD19	-122.450	37.944	7.86	7.86	216.33	15.73	-122.450	37.944	7.91	7.86	220.71	15.77	1	8/1/2014	8:27:02.17
1429	RSRD19	-122.450	37.944	7.83	7.86	215.48	15.69	-122.450	37.944	7.91	7.86	219.87	15.77	1	8/1/2014	8:27:02.27
1430	RSRD19	-122.450	37.944	7.86	7.86	214.65	15.72	-122.450	37.944	7.94	7.86	219.05	15.80	1	8/1/2014	8:27:02.36
1431	RSRD19	-122.450	37.944	7.83	7.82	213.86	15.65	-122.450	37.944	7.94	7.82	218.26	15.76	1	8/1/2014	8:27:02.48
1432	RSRD19	-122.450	37.944	7.86	7.84	213.00	15.70	-122.450	37.944	7.94	7.84	217.41	15.78	1	8/1/2014	8:27:02.57

1433	RSRD19	-122.450	37.944	7.83	7.80	212.35	15.63	-122.450	37.944	7.91	7.80	216.66	15.71	1	8/1/2014	8:27:02.67
1434	RSRD19	-122.450	37.944	7.86	7.78	211.61	15.64	-122.450	37.944	7.94	7.78	215.84	15.72	1	8/1/2014	8:27:02.76
1435	RSRD19	-122.450	37.944	7.83	7.79	210.89	15.62	-122.450	37.944	7.91	7.79	215.06	15.70	1	8/1/2014	8:27:02.88
1436	RSRD19	-122.450	37.944	7.86	7.75	210.23	15.61	-122.450	37.944	7.94	7.75	214.27	15.69	1	8/1/2014	8:27:02.97
1437	RSRD19	-122.450	37.944	7.83	7.76	209.59	15.59	-122.450	37.944	7.91	7.76	213.56	15.67	1	8/1/2014	8:27:03.07
1438	RSRD19	-122.450	37.944	7.86	7.81	208.99	15.67	-122.450	37.944	7.94	7.81	212.86	15.75	1	8/1/2014	8:27:03.16
1439	RSRD19	-122.450	37.944	7.83	7.77	208.40	15.60	-122.450	37.944	7.91	7.77	212.17	15.68	1	8/1/2014	8:27:03.28
1440	RSRD19	-122.450	37.944	7.86	7.78	207.86	15.64	-122.450	37.944	7.91	7.78	211.50	15.69	1	8/1/2014	8:27:03.37
1441	RSRD19	-122.450	37.944	7.83	7.79	207.36	15.62	-122.450	37.944	7.91	7.79	210.87	15.70	1	8/1/2014	8:27:03.47
1442	RSRD19	-122.450	37.944	7.86	7.84	206.88	15.70	-122.450	37.944	7.94	7.84	210.23	15.78	1	8/1/2014	8:27:03.56
1443	RSRD19	-122.450	37.944	7.83	7.82	206.45	15.65	-122.450	37.944	7.91	7.82	209.63	15.73	1	8/1/2014	8:27:03.68
1444	RSRD19	-122.450	37.944	7.83	7.85	206.10	15.68	-122.450	37.944	7.91	7.85	209.11	15.76	1	8/1/2014	8:27:03.77
1445	RSRD19	-122.450	37.944	7.83	7.88	205.62	15.71	-122.450	37.944	7.91	7.88	208.50	15.79	1	8/1/2014	8:27:03.87
1446	RSRD19	-122.450	37.944	7.83	7.89	205.36	15.72	-122.450	37.944	7.94	7.89	208.05	15.84	1	8/1/2014	8:27:03.96
1447	RSRD19	-122.450	37.944	7.83	7.91	205.08	15.74	-122.450	37.944	7.91	7.91	207.62	15.82	1	8/1/2014	8:27:04.08
1448	RSRD19	-122.450	37.944	7.86	7.89	204.87	15.76	-122.450	37.944	7.94	7.89	207.16	15.84	1	8/1/2014	8:27:04.17
1449	RSRD19	-122.450	37.944	7.83	7.88	204.76	15.71	-122.450	37.944	7.91	7.88	206.84	15.79	1	8/1/2014	8:27:04.27
1450	RSRD19	-122.450	37.944	7.86	7.88	204.68	15.74	-122.450	37.944	7.94	7.88	206.53	15.82	1	8/1/2014	8:27:04.36
1451	RSRD19	-122.450	37.944	7.83	7.88	204.73	15.71	-122.450	37.944	7.91	7.88	206.29	15.79	1	8/1/2014	8:27:04.48
1452	RSRD19	-122.450	37.944	7.83	7.86	204.96	15.69	-122.450	37.944	7.94	7.86	206.16	15.81	1	8/1/2014	8:27:04.57
1453	RSRD19	-122.450	37.944	7.83	7.88	205.13	15.71	-122.450	37.944	7.91	7.88	206.04	15.79	1	8/1/2014	8:27:04.67
1454	RSRD19	-122.450	37.944	7.83	7.88	205.39	15.71	-122.450	37.944	7.94	7.88	205.94	15.82	1	8/1/2014	8:27:04.76
1455	RSRD19	-122.450	37.944	7.83	7.87	205.77	15.70	-122.450	37.944	7.94	7.87	205.97	15.82	1	8/1/2014	8:27:04.88
1456	RSRD19	-122.450	37.944	7.86	7.87	206.21	15.74	-122.450	37.944	7.94	7.87	206.03	15.82	1	8/1/2014	8:27:04.97
1457	RSRD19	-122.450	37.944	7.83	7.89	206.70	15.72	-122.450	37.944	7.94	7.89	206.15	15.83	1	8/1/2014	8:27:05.07
1458	RSRD19	-122.450	37.944	7.86	7.89	207.34	15.75	-122.450	37.944	7.94	7.89	206.34	15.83	1	8/1/2014	8:27:05.16
1459	RSRD19	-122.450	37.944	7.83	7.91	207.93	15.74	-122.450	37.944	7.94	7.91	206.61	15.85	1	8/1/2014	8:27:05.28
1460	RSRD19	-122.450	37.944	7.83	7.93	208.73	15.76	-122.450	37.944	7.99	7.93	206.94	15.93	1	8/1/2014	8:27:05.37
1461	RSRD19	-122.450	37.944	7.83	7.91	209.45	15.74	-122.450	37.944	7.99	7.91	207.30	15.91	1	8/1/2014	8:27:05.47
1462	RSRD19	-122.450	37.944	7.83	7.89	210.27	15.72	-122.450	37.944	7.99	7.89	207.66	15.89	1	8/1/2014	8:27:05.56
1463	RSRD19	-122.450	37.944	7.83	7.89	211.16	15.72	-122.450	37.944	7.99	7.89	208.18	15.89	1	8/1/2014	8:27:05.68
1464	RSRD19	-122.450	37.944	7.86	7.89	212.11	15.75	-122.450	37.944	8.03	7.89	208.69	15.92	1	8/1/2014	8:27:05.77
1465	RSRD19	-122.450	37.944	7.86	7.86	213.13	15.73	-122.450	37.944	8.03	7.86	209.25	15.89	1	8/1/2014	8:27:05.87

1466	RSRD19	-122.450	37.944	7.86	7.87	214.30	15.74	-122.450	37.944	8.03	7.87	209.96	15.90	1	8/1/2014 8:27:05.96
1467	RSRD19	-122.450	37.944	7.86	7.86	215.61	15.73	-122.450	37.944	8.03	7.86	210.75	15.89	1	8/1/2014 8:27:06.08
1468	RSRD19	-122.450	37.944	7.86	7.86	217.11	15.72	-122.450	37.944	8.08	7.86	211.70	15.94	1	8/1/2014 8:27:06.17
1469	RSRD19	-122.450	37.944	7.86	7.84	218.85	15.70	-122.450	37.944	8.08	7.84	212.78	15.92	1	8/1/2014 8:27:06.27
1470	RSRD19	-122.450	37.944	7.86	7.82	220.71	15.68	-122.450	37.944	8.08	7.82	213.98	15.90	1	8/1/2014 8:27:06.36
1471	RSRD19	-122.450	37.944	7.86	7.83	222.91	15.69	-122.450	37.944	8.08	7.83	215.42	15.91	1	8/1/2014 8:27:06.48
1472	RSRD19	-122.450	37.944	7.86	7.81	225.27	15.67	-122.450	37.944	8.08	7.81	217.03	15.89	1	8/1/2014 8:27:06.57
1473	RSRD19	-122.450	37.944	7.86	7.79	227.90	15.66	-122.450	37.944	8.03	7.79	218.84	15.82	1	8/1/2014 8:27:06.67
1474	RSRD19	-122.450	37.944	7.86	7.79	230.76	15.65	-122.450	37.944	8.08	7.79	220.90	15.87	1	8/1/2014 8:27:06.76
1475	RSRD19	-122.450	37.944	7.86	7.77	233.80	15.64	-122.450	37.944	8.03	7.77	223.05	15.80	1	8/1/2014 8:27:06.88
1476	RSRD19	-122.450	37.944	7.86	7.79	236.89	15.65	-122.450	37.944	8.08	7.79	225.32	15.87	1	8/1/2014 8:27:06.97
1477	RSRD19	-122.450	37.944	7.86	7.79	240.11	15.65	-122.450	37.944	8.08	7.79	227.80	15.87	1	8/1/2014 8:27:07.07
1478	RSRD19	-122.450	37.944	7.86	7.78	243.41	15.64	-122.450	37.944	8.08	7.78	230.31	15.86	1	8/1/2014 8:27:07.16
1479	RSRD19	-122.450	37.944	7.86	7.79	246.81	15.65	-122.450	37.944	8.08	7.79	232.99	15.87	1	8/1/2014 8:27:07.28
1480	RSRD19	-122.450	37.944	7.91	7.78	250.38	15.69	-122.450	37.944	8.11	7.78	235.78	15.89	1	8/1/2014 8:27:07.37
1481	RSRD19	-122.450	37.944	7.86	7.82	254.04	15.68	-122.450	37.944	8.08	7.82	238.75	15.90	1	8/1/2014 8:27:07.47
1482	RSRD19	-122.450	37.944	7.91	7.80	257.89	15.72	-122.450	37.944	8.11	7.80	241.88	15.92	1	8/1/2014 8:27:07.56
1483	RSRD19	-122.450	37.944	7.86	7.85	261.96	15.71	-122.450	37.944	8.11	7.85	245.23	15.96	1	8/1/2014 8:27:07.68
1484	RSRD19	-122.450	37.944	7.91	7.86	266.24	15.77	-122.450	37.944	8.11	7.86	248.80	15.97	1	8/1/2014 8:27:07.77
1485	RSRD19	-122.450	37.944	7.86	7.86	270.73	15.73	-122.450	37.944	8.11	7.86	252.59	15.98	1	8/1/2014 8:27:07.87
1486	RSRD19	-122.450	37.944	7.91	7.86	275.46	15.77	-122.450	37.944	8.11	7.86	256.63	15.97	1	8/1/2014 8:27:07.96
1487	RSRD19	-122.450	37.944	7.86	7.86	280.49	15.73	-122.450	37.944	8.08	7.86	260.94	15.94	1	8/1/2014 8:27:08.08
1488	RSRD19	-122.450	37.944	7.86	7.86	285.71	15.72	-122.450	37.944	8.11	7.86	265.50	15.97	1	8/1/2014 8:27:08.17
1489	RSRD19	-122.450	37.944	7.86	7.82	291.16	15.68	-122.450	37.944	8.11	7.82	270.30	15.93	1	8/1/2014 8:27:08.27
1490	RSRD19	-122.450	37.944	7.91	7.81	296.82	15.72	-122.450	37.944	8.11	7.81	275.30	15.92	1	8/1/2014 8:27:08.36
1491	RSRD19	-122.450	37.944	7.86	7.79	302.68	15.65	-122.450	37.944	8.08	7.79	280.58	15.87	1	8/1/2014 8:27:08.48
1492	RSRD19	-122.450	37.944	7.91	7.81	308.58	15.72	-122.450	37.944	8.08	7.81	285.96	15.89	1	8/1/2014 8:27:08.57
1493	RSRD19	-122.450	37.944	7.86	7.82	314.66	15.68	-122.450	37.944	8.08	7.82	291.55	15.90	1	8/1/2014 8:27:08.67
1494	RSRD19	-122.450	37.944	7.86	7.80	320.67	15.67	-122.450	37.944	8.11	7.80	297.16	15.92	1	8/1/2014 8:27:08.78
1495	RSRD19	-122.450	37.944	7.86	7.79	326.70	15.66	-122.450	37.944	8.08	7.79	302.87	15.87	1	8/1/2014 8:27:08.88
1496	RSRD19	-122.450	37.944	7.91	7.79	332.71	15.71	-122.450	37.944	8.08	7.79	308.61	15.87	1	8/1/2014 8:27:08.97
1497	RSRD19	-122.450	37.944	7.86	7.83	338.59	15.69	-122.450	37.944	8.03	7.83	314.31	15.86	1	8/1/2014 8:27:09.07
1498	RSRD19	-122.450	37.944	7.91	7.87	344.47	15.79	-122.450	37.944	8.08	7.87	320.11	15.95	1	8/1/2014 8:27:09.18

1499	RSRD19	-122.450	37.944	7.86	7.84	350.37	15.70	-122.450	37.944	8.03	7.84	325.85	15.86	1	8/1/2014	8:27:09.28
1500	RSRD19	-122.450	37.944	7.91	7.86	356.18	15.77	-122.450	37.944	8.08	7.86	331.62	15.94	1	8/1/2014	8:27:09.37
1501	RSRD19	-122.450	37.944	7.86	7.93	361.99	15.80	-122.450	37.944	8.03	7.93	337.48	15.96	1	8/1/2014	8:27:09.47
1502	RSRD19	-122.450	37.944	7.91	7.99	367.83	15.90	-122.450	37.944	8.08	7.99	343.22	16.07	1	8/1/2014	8:27:09.58
1503	RSRD19	-122.450	37.944	7.86	8.01	373.46	15.88	-122.450	37.944	8.03	8.01	349.06	16.04	1	8/1/2014	8:27:09.68
1504	RSRD19	-122.450	37.944	7.86	7.97	379.01	15.84	-122.450	37.944	8.08	7.97	354.71	16.05	1	8/1/2014	8:27:09.77
1505	RSRD19	-122.450	37.944	7.86	8.00	384.36	15.86	-122.450	37.944	8.08	8.00	360.33	16.08	1	8/1/2014	8:27:09.87
1506	RSRD19	-122.450	37.944	7.91	8.03	389.56	15.94	-122.450	37.944	8.03	8.03	365.83	16.06	1	8/1/2014	8:27:09.98
1507	RSRD19	-122.450	37.944	7.86	8.00	394.53	15.86	-122.450	37.944	8.03	8.00	371.20	16.03	1	8/1/2014	8:27:10.08
1508	RSRD19	-122.450	37.944	7.86	8.01	399.20	15.88	-122.450	37.944	8.03	8.01	376.47	16.04	1	8/1/2014	8:27:10.17
1509	RSRD19	-122.450	37.944	7.86	8.03	403.74	15.90	-122.450	37.944	8.03	8.03	381.68	16.06	1	8/1/2014	8:27:10.27
1510	RSRD19	-122.450	37.944	7.86	8.10	408.03	15.96	-122.450	37.944	8.08	8.10	386.77	16.18	1	8/1/2014	8:27:10.38
1511	RSRD19	-122.450	37.944	7.86	8.05	412.20	15.91	-122.450	37.944	8.03	8.05	391.77	16.08	1	8/1/2014	8:27:10.48
1512	RSRD19	-122.450	37.944	7.91	8.03	416.29	15.95	-122.450	37.944	8.08	8.03	396.78	16.11	1	8/1/2014	8:27:10.57
1513	RSRD19	-122.450	37.944	7.86	8.05	420.25	15.91	-122.450	37.944	8.03	8.05	401.65	16.08	1	8/1/2014	8:27:10.67
1514	RSRD19	-122.450	37.944	7.91	8.03	424.07	15.95	-122.450	37.944	8.03	8.03	406.45	16.06	1	8/1/2014	8:27:10.78
1515	RSRD19	-122.450	37.944	7.86	8.09	427.84	15.95	-122.450	37.944	8.03	8.09	411.21	16.12	1	8/1/2014	8:27:10.88
1516	RSRD19	-122.450	37.944	7.86	8.08	431.14	15.95	-122.450	37.944	8.03	8.08	415.61	16.11	1	8/1/2014	8:27:10.97
1517	RSRD19	-122.450	37.944	7.86	8.09	434.40	15.95	-122.450	37.944	8.03	8.09	420.01	16.12	1	8/1/2014	8:27:11.07
1518	RSRD19	-122.450	37.944	7.86	8.10	437.42	15.97	-122.450	37.944	8.03	8.10	424.14	16.13	1	8/1/2014	8:27:11.18
1519	RSRD19	-122.450	37.944	7.83	8.12	440.17	15.95	-122.450	37.944	8.03	8.12	428.08	16.15	1	8/1/2014	8:27:11.28
1520	RSRD19	-122.450	37.944	7.86	8.13	442.55	15.99	-122.450	37.944	8.03	8.13	431.55	16.16	1	8/1/2014	8:27:11.37
1521	RSRD19	-122.450	37.944	7.83	8.10	444.87	15.93	-122.450	37.944	7.99	8.10	435.04	16.10	1	8/1/2014	8:27:11.47
1522	RSRD19	-122.450	37.944	7.86	8.10	446.79	15.96	-122.450	37.944	8.03	8.10	438.11	16.13	1	8/1/2014	8:27:11.58
1523	RSRD19	-122.450	37.944	7.83	8.07	448.50	15.90	-122.450	37.944	7.99	8.07	440.92	16.06	1	8/1/2014	8:27:11.68
1524	RSRD19	-122.450	37.944	7.86	8.06	450.01	15.92	-122.450	37.944	8.03	8.06	443.61	16.09	1	8/1/2014	8:27:11.77
1525	RSRD19	-122.450	37.944	7.83	8.04	451.20	15.87	-122.450	37.944	7.99	8.04	445.92	16.03	1	8/1/2014	8:27:11.87
1526	RSRD19	-122.450	37.944	7.86	8.04	452.26	15.90	-122.450	37.944	8.03	8.04	448.10	16.07	1	8/1/2014	8:27:11.98
1527	RSRD19	-122.450	37.944	7.86	8.02	453.08	15.88	-122.450	37.944	7.99	8.02	449.96	16.01	1	8/1/2014	8:27:12.08
1528	RSRD19	-122.450	37.944	7.86	8.01	453.61	15.88	-122.450	37.944	8.03	8.01	451.58	16.04	1	8/1/2014	8:27:12.17
1529	RSRD19	-122.450	37.944	7.86	8.00	453.97	15.87	-122.450	37.944	7.99	8.00	453.03	16.00	1	8/1/2014	8:27:12.27
1530	RSRD19	-122.450	37.944	7.86	8.04	454.19	15.90	-122.450	37.944	8.03	8.04	454.33	16.07	1	8/1/2014	8:27:12.38
1531	RSRD19	-122.450	37.944	7.86	7.99	454.18	15.85	-122.450	37.944	7.99	7.99	455.25	15.98	1	8/1/2014	8:27:12.47

1532	RSRD19	-122.450	37.944	7.86	8.00	453.94	15.87	-122.450	37.944	8.03	8.00	456.09	16.03	1	8/1/2014	8:27:12.57
1533	RSRD19	-122.450	37.944	7.83	7.99	453.55	15.82	-122.450	37.944	8.03	7.99	456.63	16.02	1	8/1/2014	8:27:12.67
1534	RSRD19	-122.450	37.944	7.86	7.97	453.03	15.84	-122.450	37.944	7.99	7.97	457.05	15.97	1	8/1/2014	8:27:12.78
1535	RSRD19	-122.450	37.944	7.83	8.00	452.31	15.83	-122.450	37.944	7.99	8.00	457.21	16.00	1	8/1/2014	8:27:12.87
1536	RSRD19	-122.450	37.944	7.86	8.00	451.27	15.86	-122.450	37.944	7.99	8.00	457.01	15.99	1	8/1/2014	8:27:12.97
1537	RSRD19	-122.450	37.944	7.86	7.99	450.27	15.85	-122.450	37.944	7.99	7.99	456.78	15.98	1	8/1/2014	8:27:13.07
1538	RSRD19	-122.450	37.944	7.86	7.99	448.98	15.85	-122.450	37.944	7.99	7.99	456.30	15.98	1	8/1/2014	8:27:13.18
1539	RSRD19	-122.450	37.944	7.83	8.00	447.60	15.83	-122.450	37.944	7.99	8.00	455.64	15.99	1	8/1/2014	8:27:13.28
1540	RSRD19	-122.450	37.944	7.86	8.01	445.90	15.88	-122.450	37.944	7.94	8.01	454.59	15.96	1	8/1/2014	8:27:13.37
1541	RSRD19	-122.450	37.944	7.83	8.04	444.17	15.87	-122.450	37.944	7.94	8.04	453.52	15.98	1	8/1/2014	8:27:13.47
1542	RSRD19	-122.450	37.944	7.83	8.03	442.29	15.86	-122.450	37.944	7.94	8.03	452.11	15.98	1	8/1/2014	8:27:13.58
1543	RSRD19	-122.450	37.944	7.83	8.07	440.26	15.90	-122.450	37.944	7.91	8.07	450.65	15.98	1	8/1/2014	8:27:13.68
1544	RSRD19	-122.450	37.944	7.83	8.05	438.20	15.88	-122.450	37.944	7.94	8.05	449.04	15.99	1	8/1/2014	8:27:13.77
1545	RSRD19	-122.450	37.944	7.83	8.06	436.03	15.89	-122.450	37.944	7.91	8.06	447.30	15.97	1	8/1/2014	8:27:13.87
1546	RSRD19	-122.450	37.944	7.83	8.08	433.94	15.91	-122.450	37.944	7.91	8.08	445.53	15.99	1	8/1/2014	8:27:13.98
1547	RSRD19	-122.450	37.944	7.80	8.07	431.75	15.86	-122.450	37.944	7.91	8.07	443.68	15.98	1	8/1/2014	8:27:14.08
1548	RSRD19	-122.450	37.944	7.83	8.07	429.60	15.90	-122.450	37.944	7.91	8.07	441.77	15.98	1	8/1/2014	8:27:14.17
1549	RSRD19	-122.450	37.944	7.80	8.04	427.42	15.84	-122.450	37.944	7.91	8.04	439.84	15.95	1	8/1/2014	8:27:14.27
1550	RSRD19	-122.450	37.944	7.83	8.07	425.11	15.90	-122.450	37.944	7.91	8.07	437.74	15.98	1	8/1/2014	8:27:14.38
1551	RSRD19	-122.450	37.944	7.83	8.04	422.97	15.87	-122.450	37.944	7.86	8.04	435.69	15.90	1	8/1/2014	8:27:14.48
1552	RSRD19	-122.450	37.944	7.83	8.04	420.72	15.87	-122.450	37.944	7.91	8.04	433.64	15.95	1	8/1/2014	8:27:14.57
1553	RSRD19	-122.450	37.944	7.83	8.03	418.53	15.86	-122.450	37.944	7.86	8.03	431.52	15.89	1	8/1/2014	8:27:14.67
1554	RSRD19	-122.450	37.944	7.83	8.07	416.39	15.90	-122.450	37.944	7.91	8.07	429.56	15.98	1	8/1/2014	8:27:14.78
1555	RSRD19	-122.450	37.944	7.83	8.06	414.14	15.89	-122.450	37.944	7.91	8.06	427.36	15.97	1	8/1/2014	8:27:14.88
1556	RSRD19	-122.450	37.944	7.83	8.01	412.00	15.84	-122.450	37.944	7.91	8.01	425.35	15.92	1	8/1/2014	8:27:14.97
1557	RSRD19	-122.450	37.944	7.83	8.03	409.79	15.86	-122.450	37.944	7.91	8.03	423.24	15.94	1	8/1/2014	8:27:15.07
1558	RSRD19	-122.450	37.944	7.83	8.03	407.61	15.86	-122.450	37.944	7.91	8.03	421.11	15.94	1	8/1/2014	8:27:15.18
1559	RSRD19	-122.450	37.944	7.83	8.03	405.43	15.86	-122.450	37.944	7.91	8.03	418.97	15.94	1	8/1/2014	8:27:15.28
1560	RSRD19	-122.450	37.944	7.83	8.01	403.24	15.84	-122.450	37.944	7.91	8.01	416.79	15.92	1	8/1/2014	8:27:15.37
1561	RSRD19	-122.450	37.944	7.83	8.03	401.04	15.86	-122.450	37.944	7.91	8.03	414.61	15.94	1	8/1/2014	8:27:15.47
1562	RSRD19	-122.450	37.944	7.83	8.10	398.78	15.93	-122.450	37.944	7.91	8.10	412.32	16.01	1	8/1/2014	8:27:15.58
1563	RSRD19	-122.450	37.944	7.80	8.05	396.47	15.85	-122.450	37.944	7.91	8.05	409.96	15.96	1	8/1/2014	8:27:15.68
1564	RSRD19	-122.450	37.944	7.83	8.03	394.14	15.86	-122.450	37.944	7.91	8.03	407.57	15.94	1	8/1/2014	8:27:15.77

1565	RSRD19	-122.450	37.944	7.80	8.03	391.81	15.82	-122.450	37.944	7.86	8.03	405.09	15.89	1	8/1/2014	8:27:15.87
1566	RSRD19	-122.450	37.944	7.83	8.10	389.48	15.93	-122.450	37.944	7.91	8.10	402.63	16.01	1	8/1/2014	8:27:15.98
1567	RSRD19	-122.450	37.944	7.80	8.06	387.17	15.85	-122.450	37.944	7.86	8.06	400.16	15.92	1	8/1/2014	8:27:16.08
1568	RSRD19	-122.450	37.944	7.80	8.06	384.86	15.85	-122.450	37.944	7.86	8.06	397.67	15.92	1	8/1/2014	8:27:16.17
1569	RSRD19	-122.450	37.944	7.80	8.06	382.64	15.85	-122.450	37.944	7.83	8.06	395.17	15.88	1	8/1/2014	8:27:16.27
1570	RSRD19	-122.450	37.944	7.80	8.06	380.42	15.85	-122.450	37.944	7.86	8.06	392.78	15.92	1	8/1/2014	8:27:16.38
1571	RSRD19	-122.450	37.944	7.80	8.08	378.22	15.88	-122.450	37.944	7.83	8.08	390.25	15.91	1	8/1/2014	8:27:16.48
1572	RSRD19	-122.450	37.944	7.80	8.10	376.12	15.90	-122.450	37.944	7.83	8.10	387.94	15.93	1	8/1/2014	8:27:16.57
1573	RSRD19	-122.450	37.944	7.80	8.10	373.96	15.89	-122.450	37.944	7.78	8.10	385.53	15.87	1	8/1/2014	8:27:16.67
1574	RSRD19	-122.450	37.944	7.80	8.10	371.98	15.90	-122.450	37.944	7.83	8.10	383.30	15.93	1	8/1/2014	8:27:16.78
1575	RSRD19	-122.450	37.944	7.80	8.10	369.94	15.90	-122.450	37.944	7.78	8.10	381.05	15.88	1	8/1/2014	8:27:16.88
1576	RSRD19	-122.450	37.944	7.80	8.10	368.00	15.90	-122.450	37.944	7.78	8.10	378.91	15.88	1	8/1/2014	8:27:16.97
1577	RSRD19	-122.450	37.944	7.74	8.09	366.07	15.83	-122.450	37.944	7.78	8.09	376.75	15.86	1	8/1/2014	8:27:17.07
1578	RSRD19	-122.450	37.944	7.80	8.13	364.22	15.92	-122.450	37.944	7.78	8.13	374.75	15.90	1	8/1/2014	8:27:17.18
1579	RSRD19	-122.450	37.944	7.74	8.16	362.35	15.90	-122.450	37.944	7.74	8.16	372.71	15.90	1	8/1/2014	8:27:17.28
1580	RSRD19	-122.450	37.944	7.74	8.14	360.68	15.88	-122.450	37.944	7.78	8.14	370.79	15.91	1	8/1/2014	8:27:17.37
1581	RSRD19	-122.450	37.944	7.74	8.15	358.96	15.89	-122.450	37.944	7.74	8.15	368.95	15.89	1	8/1/2014	8:27:17.47
1582	RSRD19	-122.450	37.944	7.74	8.16	357.29	15.91	-122.450	37.944	7.78	8.16	367.08	15.94	1	8/1/2014	8:27:17.58
1583	RSRD19	-122.450	37.944	7.74	8.19	355.71	15.93	-122.450	37.944	7.74	8.19	365.27	15.93	1	8/1/2014	8:27:17.68
1584	RSRD19	-122.450	37.944	7.74	8.21	354.17	15.96	-122.450	37.944	7.74	8.21	363.54	15.95	1	8/1/2014	8:27:17.77
1585	RSRD19	-122.450	37.944	7.71	8.21	352.61	15.92	-122.450	37.944	7.71	8.21	361.78	15.92	1	8/1/2014	8:27:17.87
1586	RSRD19	-122.450	37.944	7.74	8.24	351.11	15.99	-122.450	37.944	7.74	8.24	360.06	15.98	1	8/1/2014	8:27:17.98
1587	RSRD19	-122.450	37.944	7.71	8.25	349.68	15.96	-122.450	37.944	7.71	8.25	358.39	15.96	1	8/1/2014	8:27:18.08
1588	RSRD19	-122.450	37.944	7.74	8.26	348.19	16.00	-122.450	37.944	7.74	8.26	356.74	16.00	1	8/1/2014	8:27:18.17
1589	RSRD19	-122.450	37.944	7.71	8.26	346.73	15.97	-122.450	37.944	7.71	8.26	355.04	15.97	1	8/1/2014	8:27:18.27
1590	RSRD19	-122.450	37.944	7.74	8.25	345.32	15.99	-122.450	37.944	7.74	8.25	353.53	15.99	1	8/1/2014	8:27:18.38
1591	RSRD19	-122.450	37.944	7.71	8.25	343.89	15.96	-122.450	37.944	7.71	8.25	351.81	15.96	1	8/1/2014	8:27:18.48
1592	RSRD19	-122.450	37.944	7.74	8.24	342.48	15.99	-122.450	37.944	7.71	8.24	350.27	15.95	1	8/1/2014	8:27:18.57
1593	RSRD19	-122.450	37.944	7.71	8.25	341.10	15.96	-122.450	37.944	7.71	8.25	348.66	15.96	1	8/1/2014	8:27:18.67
1594	RSRD19	-122.450	37.944	7.71	8.29	339.69	16.00	-122.450	37.944	7.71	8.29	347.14	16.00	1	8/1/2014	8:27:18.78
1595	RSRD19	-122.450	37.944	7.71	8.27	338.35	15.98	-122.450	37.944	7.66	8.27	345.60	15.93	1	8/1/2014	8:27:18.88
1596	RSRD19	-122.450	37.944	7.71	8.31	336.94	16.02	-122.450	37.944	7.66	8.31	344.06	15.97	1	8/1/2014	8:27:18.97
1597	RSRD19	-122.450	37.944	7.71	8.31	335.66	16.02	-122.450	37.944	7.66	8.31	342.54	15.97	1	8/1/2014	8:27:19.07

1598	RSRD19	-122.450	37.944	7.71	8.33	334.34	16.04	-122.450	37.944	7.66	8.33	341.13	15.99	1	8/1/2014	8:27:19.18
1599	RSRD19	-122.450	37.944	7.71	8.37	333.15	16.08	-122.450	37.944	7.62	8.37	339.68	15.99	1	8/1/2014	8:27:19.28
1600	RSRD19	-122.450	37.944	7.71	8.40	331.89	16.11	-122.450	37.944	7.62	8.40	338.42	16.02	1	8/1/2014	8:27:19.37
1601	RSRD19	-122.450	37.944	7.66	8.39	330.84	16.05	-122.450	37.944	7.62	8.39	337.10	16.01	1	8/1/2014	8:27:19.47
1602	RSRD19	-122.450	37.944	7.66	8.44	329.71	16.10	-122.450	37.944	7.62	8.44	335.87	16.07	1	8/1/2014	8:27:19.58
1603	RSRD19	-122.450	37.944	7.66	8.40	328.70	16.06	-122.450	37.944	7.57	8.40	334.68	15.98	1	8/1/2014	8:27:19.67
1604	RSRD19	-122.450	37.944	7.71	8.40	327.64	16.11	-122.450	37.944	7.62	8.40	333.51	16.02	1	8/1/2014	8:27:19.77
1605	RSRD19	-122.450	37.944	7.66	8.40	326.58	16.06	-122.450	37.944	7.57	8.40	332.32	15.98	1	8/1/2014	8:27:19.87
1606	RSRD19	-122.450	37.944	7.66	8.41	325.66	16.07	-122.450	37.944	7.57	8.41	331.17	15.99	1	8/1/2014	8:27:19.98
1607	RSRD19	-122.450	37.944	7.66	8.44	324.75	16.09	-122.450	37.944	7.57	8.44	330.16	16.01	1	8/1/2014	8:27:20.08
1608	RSRD19	-122.450	37.944	7.66	8.45	323.82	16.11	-122.450	37.944	7.57	8.45	329.16	16.02	1	8/1/2014	8:27:20.17
1609	RSRD19	-122.450	37.944	7.66	8.47	322.94	16.13	-122.450	37.944	7.57	8.47	328.15	16.04	1	8/1/2014	8:27:20.27
1610	RSRD19	-122.450	37.944	7.66	8.47	322.08	16.13	-122.450	37.944	7.57	8.47	327.22	16.04	1	8/1/2014	8:27:20.38
1611	RSRD19	-122.450	37.944	7.66	8.49	321.29	16.15	-122.450	37.944	7.57	8.49	326.21	16.06	1	8/1/2014	8:27:20.48
1612	RSRD19	-122.450	37.944	7.66	8.49	320.34	16.15	-122.450	37.944	7.57	8.49	325.24	16.06	1	8/1/2014	8:27:20.57
1613	RSRD19	-122.450	37.944	7.66	8.49	319.59	16.15	-122.450	37.944	7.57	8.49	324.31	16.06	1	8/1/2014	8:27:20.67
1614	RSRD19	-122.450	37.944	7.66	8.49	318.81	16.15	-122.450	37.944	7.57	8.49	323.43	16.06	1	8/1/2014	8:27:20.78
1615	RSRD19	-122.450	37.944	7.63	8.48	317.95	16.11	-122.450	37.944	7.54	8.48	322.54	16.02	1	8/1/2014	8:27:20.88
1616	RSRD19	-122.450	37.944	7.63	8.49	317.24	16.12	-122.450	37.944	7.57	8.49	321.55	16.07	1	8/1/2014	8:27:20.97
1617	RSRD19	-122.450	37.944	7.63	8.49	316.49	16.11	-122.450	37.944	7.49	8.49	320.75	15.98	1	8/1/2014	8:27:21.07
1618	RSRD19	-122.450	37.944	7.63	8.52	315.76	16.15	-122.450	37.944	7.49	8.52	319.91	16.01	1	8/1/2014	8:27:21.18
1619	RSRD19	-122.450	37.944	7.63	8.53	315.07	16.15	-122.450	37.944	7.49	8.53	319.12	16.01	1	8/1/2014	8:27:21.27
1620	RSRD19	-122.450	37.944	7.63	8.55	314.41	16.18	-122.450	37.944	7.49	8.55	318.30	16.04	1	8/1/2014	8:27:21.37
1621	RSRD19	-122.450	37.944	7.57	8.56	313.70	16.13	-122.450	37.944	7.45	8.56	317.55	16.01	1	8/1/2014	8:27:21.47
1622	RSRD19	-122.450	37.944	7.63	8.58	313.08	16.21	-122.450	37.944	7.49	8.58	316.84	16.07	1	8/1/2014	8:27:21.58
1623	RSRD19	-122.450	37.944	7.57	8.58	312.42	16.15	-122.450	37.944	7.45	8.58	316.11	16.04	1	8/1/2014	8:27:21.67
1624	RSRD19	-122.450	37.944	7.63	8.63	311.82	16.26	-122.450	37.944	7.49	8.63	315.38	16.12	1	8/1/2014	8:27:21.77
1625	RSRD19	-122.450	37.944	7.57	8.58	311.20	16.15	-122.450	37.944	7.45	8.58	314.74	16.04	1	8/1/2014	8:27:21.87
1626	RSRD19	-122.450	37.944	7.57	8.59	310.64	16.16	-122.450	37.944	7.45	8.59	314.03	16.04	1	8/1/2014	8:27:21.98
1627	RSRD19	-122.450	37.944	7.57	8.60	310.11	16.17	-122.450	37.944	7.45	8.60	313.38	16.05	1	8/1/2014	8:27:22.08
1628	RSRD19	-122.450	37.944	7.57	8.57	309.51	16.15	-122.450	37.944	7.45	8.57	312.81	16.03	1	8/1/2014	8:27:22.17
1629	RSRD19	-122.450	37.944	7.57	8.59	308.93	16.16	-122.450	37.944	7.42	8.59	312.16	16.01	1	8/1/2014	8:27:22.27
1630	RSRD19	-122.450	37.944	7.57	8.59	308.42	16.16	-122.450	37.944	7.45	8.59	311.56	16.04	1	8/1/2014	8:27:22.38

1631	RSRD19	-122.450	37.944	7.57	8.57	307.91	16.15	-122.450	37.944	7.42	8.57	311.01	15.99	1	8/1/2014 8:27:22.47
1632	RSRD19	-122.450	37.944	7.57	8.57	307.40	16.15	-122.450	37.944	7.42	8.57	310.41	15.99	1	8/1/2014 8:27:22.57
1633	RSRD19	-122.450	37.944	7.54	8.56	306.95	16.10	-122.450	37.944	7.37	8.56	309.90	15.93	1	8/1/2014 8:27:22.67
1634	RSRD19	-122.450	37.944	7.57	8.56	306.48	16.13	-122.450	37.944	7.42	8.56	309.36	15.98	1	8/1/2014 8:27:22.78
1635	RSRD19	-122.450	37.944	7.54	8.51	306.02	16.05	-122.450	37.944	7.37	8.51	308.81	15.88	1	8/1/2014 8:27:22.88
1636	RSRD19	-122.450	37.944	7.57	8.53	305.57	16.10	-122.450	37.944	7.42	8.53	308.36	15.95	1	8/1/2014 8:27:22.97
1637	RSRD19	-122.450	37.944	7.54	8.51	305.12	16.05	-122.450	37.944	7.42	8.51	307.81	15.93	1	8/1/2014 8:27:23.07
1638	RSRD19	-122.450	37.944	7.54	8.51	304.70	16.04	-122.450	37.944	7.42	8.51	307.34	15.93	1	8/1/2014 8:27:23.18
1639	RSRD19	-122.450	37.944	7.54	8.51	304.30	16.04	-122.450	37.944	7.37	8.51	306.87	15.87	1	8/1/2014 8:27:23.28
1640	RSRD19	-122.450	37.944	7.54	8.53	303.89	16.07	-122.450	37.944	7.37	8.53	306.35	15.90	1	8/1/2014 8:27:23.37
1641	RSRD19	-122.450	37.944	7.51	8.51	303.47	16.02	-122.450	37.944	7.42	8.51	305.91	15.93	1	8/1/2014 8:27:23.47
1642	RSRD19	-122.450	37.944	7.54	8.51	303.05	16.04	-122.450	37.944	7.42	8.51	305.46	15.93	1	8/1/2014 8:27:23.58
1643	RSRD19	-122.450	37.944	7.54	8.51	302.75	16.04	-122.450	37.944	7.37	8.51	305.06	15.87	1	8/1/2014 8:27:23.68
1644	RSRD19	-122.450	37.944	7.54	8.56	302.33	16.10	-122.450	37.944	7.42	8.56	304.62	15.98	1	8/1/2014 8:27:23.77
1645	RSRD19	-122.450	37.944	7.51	8.53	301.97	16.03	-122.450	37.944	7.37	8.53	304.24	15.90	1	8/1/2014 8:27:23.87
1646	RSRD19	-122.450	37.944	7.54	8.56	301.65	16.10	-122.450	37.944	7.42	8.56	303.81	15.98	1	8/1/2014 8:27:23.98
1647	RSRD19	-122.450	37.944	7.51	8.54	301.30	16.05	-122.450	37.944	7.37	8.54	303.43	15.91	1	8/1/2014 8:27:24.07
1648	RSRD19	-122.450	37.944	7.51	8.59	300.96	16.10	-122.450	37.944	7.37	8.59	303.05	15.96	1	8/1/2014 8:27:24.17
1649	RSRD19	-122.450	37.944	7.51	8.57	300.67	16.08	-122.450	37.944	7.37	8.57	302.67	15.94	1	8/1/2014 8:27:24.27
1650	RSRD19	-122.450	37.944	7.51	8.60	300.36	16.10	-122.450	37.944	7.37	8.60	302.33	15.97	1	8/1/2014 8:27:24.38
1651	RSRD19	-122.450	37.944	7.51	8.60	300.04	16.10	-122.450	37.944	7.34	8.60	301.98	15.93	1	8/1/2014 8:27:24.47
1652	RSRD19	-122.450	37.944	7.51	8.60	299.81	16.10	-122.450	37.944	7.34	8.60	301.64	15.93	1	8/1/2014 8:27:24.57
1653	RSRD19	-122.450	37.944	7.45	8.59	299.43	16.04	-122.450	37.944	7.28	8.59	301.30	15.87	1	8/1/2014 8:27:24.67
1654	RSRD19	-122.450	37.944	7.51	8.60	299.19	16.10	-122.450	37.944	7.34	8.60	300.95	15.93	1	8/1/2014 8:27:24.78
1655	RSRD19	-122.450	37.944	7.45	8.61	299.02	16.06	-122.450	37.944	7.28	8.61	300.67	15.89	1	8/1/2014 8:27:24.88
1656	RSRD19	-122.450	37.944	7.51	8.61	298.67	16.11	-122.450	37.944	7.34	8.61	300.36	15.94	1	8/1/2014 8:27:24.97
1657	RSRD19	-122.450	37.944	7.45	8.61	298.40	16.07	-122.450	37.944	7.28	8.61	300.09	15.90	1	8/1/2014 8:27:25.07
1658	RSRD19	-122.450	37.944	7.45	8.63	298.21	16.08	-122.450	37.944	7.28	8.63	299.82	15.91	1	8/1/2014 8:27:25.18
1659	RSRD19	-122.450	37.944	7.45	8.61	297.97	16.06	-122.450	37.944	7.34	8.61	299.55	15.94	1	8/1/2014 8:27:25.27
1660	RSRD19	-122.450	37.944	7.45	8.62	297.81	16.07	-122.450	37.944	7.34	8.62	299.32	15.96	1	8/1/2014 8:27:25.37
1661	RSRD19	-122.450	37.944	7.45	8.62	297.54	16.07	-122.450	37.944	7.34	8.62	299.05	15.96	1	8/1/2014 8:27:25.47
1662	RSRD19	-122.450	37.944	7.45	8.60	297.35	16.05	-122.450	37.944	7.28	8.60	298.85	15.88	1	8/1/2014 8:27:25.58
1663	RSRD19	-122.450	37.944	7.45	8.59	297.15	16.04	-122.450	37.944	7.28	8.59	298.60	15.87	1	8/1/2014 8:27:25.68

1664	RSRD19	-122.450	37.944	7.45	8.59	296.94	16.04	-122.450	37.944	7.28	8.59	298.37	15.87	1	8/1/2014 8:27:25.77
1665	RSRD19	-122.450	37.944	7.42	8.61	296.76	16.03	-122.450	37.944	7.28	8.61	298.15	15.89	1	8/1/2014 8:27:25.87
1666	RSRD19	-122.450	37.944	7.45	8.61	296.49	16.06	-122.450	37.944	7.28	8.61	297.83	15.89	1	8/1/2014 8:27:25.98
1667	RSRD19	-122.450	37.944	7.42	8.61	296.35	16.03	-122.450	37.944	7.28	8.61	297.67	15.90	1	8/1/2014 8:27:26.08
1668	RSRD19	-122.450	37.944	7.45	8.60	296.15	16.05	-122.450	37.944	7.34	8.60	297.47	15.93	1	8/1/2014 8:27:26.17
1669	RSRD19	-122.450	37.944	7.45	8.60	295.95	16.05	-122.450	37.944	7.28	8.60	297.24	15.88	1	8/1/2014 8:27:26.27
1670	RSRD19	-122.450	37.944	7.45	8.60	295.74	16.05	-122.450	37.944	7.34	8.60	297.02	15.93	1	8/1/2014 8:27:26.38
1671	RSRD19	-122.450	37.944	7.42	8.58	295.54	16.00	-122.450	37.944	7.28	8.58	296.84	15.87	1	8/1/2014 8:27:26.48
1672	RSRD19	-122.450	37.944	7.45	8.57	295.36	16.03	-122.450	37.944	7.28	8.57	296.57	15.86	1	8/1/2014 8:27:26.57
1673	RSRD19	-122.450	37.944	7.45	8.59	295.11	16.04	-122.450	37.944	7.28	8.59	296.39	15.87	1	8/1/2014 8:27:26.67
1674	RSRD19	-122.450	37.944	7.45	8.63	294.95	16.08	-122.450	37.944	7.34	8.63	296.16	15.96	1	8/1/2014 8:27:26.78
1675	RSRD19	-122.450	37.944	7.42	8.59	294.73	16.01	-122.450	37.944	7.28	8.59	295.93	15.87	1	8/1/2014 8:27:26.88
1676	RSRD19	-122.450	37.944	7.42	8.57	294.57	15.99	-122.450	37.944	7.34	8.57	295.76	15.91	1	8/1/2014 8:27:26.97
1677	RSRD19	-122.450	37.944	7.42	8.61	294.41	16.03	-122.450	37.944	7.28	8.61	295.55	15.90	1	8/1/2014 8:27:27.07
1678	RSRD19	-122.450	37.944	7.45	8.56	294.18	16.01	-122.450	37.944	7.34	8.56	295.35	15.89	1	8/1/2014 8:27:27.18
1679	RSRD19	-122.450	37.944	7.42	8.56	294.02	15.98	-122.450	37.944	7.28	8.56	295.16	15.84	1	8/1/2014 8:27:27.27
1680	RSRD19	-122.450	37.944	7.42	8.56	293.84	15.98	-122.450	37.944	7.28	8.56	294.96	15.85	1	8/1/2014 8:27:27.37
1681	RSRD19	-122.450	37.944	7.42	8.57	293.68	15.99	-122.450	37.944	7.28	8.57	294.79	15.86	1	8/1/2014 8:27:27.47
1682	RSRD19	-122.450	37.944	7.42	8.61	293.50	16.03	-122.450	37.944	7.34	8.61	294.58	15.95	1	8/1/2014 8:27:27.58
1683	RSRD19	-122.450	37.944	7.42	8.61	293.34	16.03	-122.450	37.944	7.28	8.61	294.40	15.89	1	8/1/2014 8:27:27.67
1684	RSRD19	-122.450	37.944	7.42	8.63	293.16	16.05	-122.450	37.944	7.34	8.63	294.24	15.96	1	8/1/2014 8:27:27.77
1685	RSRD19	-122.450	37.944	7.42	8.63	293.07	16.05	-122.450	37.944	7.28	8.63	294.08	15.92	1	8/1/2014 8:27:27.87
1686	RSRD19	-122.450	37.944	7.42	8.61	292.89	16.03	-122.450	37.944	7.28	8.61	293.86	15.89	1	8/1/2014 8:27:27.98
1687	RSRD19	-122.450	37.944	7.37	8.60	292.73	15.97	-122.450	37.944	7.28	8.60	293.72	15.88	1	8/1/2014 8:27:28.07
1688	RSRD19	-122.450	37.944	7.42	8.61	292.57	16.03	-122.450	37.944	7.28	8.61	293.50	15.89	1	8/1/2014 8:27:28.17
1689	RSRD19	-122.450	37.944	7.37	8.57	292.48	15.94	-122.450	37.944	7.28	8.57	293.41	15.86	1	8/1/2014 8:27:28.27
1690	RSRD19	-122.450	37.944	7.42	8.58	292.32	16.00	-122.450	37.944	7.28	8.58	293.25	15.87	1	8/1/2014 8:27:28.38
1691	RSRD19	-122.450	37.944	7.37	8.60	292.19	15.97	-122.450	37.944	7.28	8.60	293.07	15.88	1	8/1/2014 8:27:28.48
1692	RSRD19	-122.450	37.944	7.42	8.59	292.05	16.01	-122.450	37.944	7.28	8.59	292.96	15.87	1	8/1/2014 8:27:28.57
1693	RSRD19	-122.450	37.944	7.37	8.60	291.94	15.97	-122.450	37.944	7.28	8.60	292.80	15.88	1	8/1/2014 8:27:28.67
1694	RSRD19	-122.450	37.944	7.37	8.61	291.80	15.97	-122.450	37.944	7.25	8.61	292.66	15.86	1	8/1/2014 8:27:28.78
1695	RSRD19	-122.450	37.944	7.37	8.64	291.69	16.01	-122.450	37.944	7.25	8.64	292.50	15.89	1	8/1/2014 8:27:28.88
1696	RSRD19	-122.450	37.944	7.37	8.63	291.55	16.00	-122.450	37.944	7.28	8.63	292.39	15.91	1	8/1/2014 8:27:28.97

1697	RSRD19	-122.450	37.944	7.37	8.64	291.48	16.01	-122.450	37.944	7.25	8.64	292.23	15.89	1	8/1/2014	8:27:29.07
1698	RSRD19	-122.450	37.944	7.42	8.61	291.26	16.03	-122.450	37.944	7.28	8.61	292.12	15.90	1	8/1/2014	8:27:29.18
1699	RSRD19	-122.450	37.944	7.37	8.61	291.22	15.97	-122.450	37.944	7.25	8.61	291.98	15.86	1	8/1/2014	8:27:29.28
1700	RSRD19	-122.450	37.944	7.42	8.61	291.08	16.03	-122.450	37.944	7.34	8.61	291.87	15.95	1	8/1/2014	8:27:29.37
1701	RSRD19	-122.450	37.944	7.37	8.63	290.99	16.00	-122.450	37.944	7.28	8.63	291.76	15.91	1	8/1/2014	8:27:29.47
1702	RSRD19	-122.450	37.944	7.37	8.61	290.88	15.97	-122.450	37.944	7.28	8.61	291.63	15.89	1	8/1/2014	8:27:29.58
1703	RSRD19	-122.450	37.944	7.37	8.60	290.76	15.97	-122.450	37.944	7.28	8.60	291.53	15.88	1	8/1/2014	8:27:29.68
1704	RSRD19	-122.450	37.944	7.37	8.63	290.70	16.00	-122.450	37.944	7.34	8.63	291.42	15.97	1	8/1/2014	8:27:29.77
1705	RSRD19	-122.450	37.944	7.37	8.62	290.60	15.99	-122.450	37.944	7.28	8.62	291.33	15.91	1	8/1/2014	8:27:29.87
1706	RSRD19	-122.450	37.944	7.37	8.60	290.49	15.97	-122.450	37.944	7.28	8.60	291.24	15.88	1	8/1/2014	8:27:29.98
1707	RSRD19	-122.450	37.944	7.37	8.58	290.42	15.95	-122.450	37.944	7.28	8.58	291.12	15.87	1	8/1/2014	8:27:30.07
1708	RSRD19	-122.450	37.944	7.37	8.61	290.31	15.97	-122.450	37.944	7.28	8.61	291.04	15.89	1	8/1/2014	8:27:30.17
1709	RSRD19	-122.450	37.944	7.37	8.60	290.22	15.97	-122.450	37.944	7.28	8.60	290.92	15.88	1	8/1/2014	8:27:30.27
1710	RSRD19	-122.450	37.944	7.42	8.57	290.15	15.99	-122.450	37.944	7.34	8.57	290.86	15.91	1	8/1/2014	8:27:30.38
1711	RSRD19	-122.450	37.944	7.37	8.58	290.00	15.95	-122.450	37.944	7.28	8.58	290.70	15.87	1	8/1/2014	8:27:30.47
1712	RSRD19	-122.450	37.944	7.42	8.56	289.97	15.98	-122.450	37.944	7.34	8.56	290.63	15.90	1	8/1/2014	8:27:30.57
1713	RSRD19	-122.450	37.944	7.37	8.57	289.84	15.94	-122.450	37.944	7.34	8.57	290.52	15.91	1	8/1/2014	8:27:30.67
1714	RSRD19	-122.450	37.944	7.42	8.59	289.80	16.01	-122.450	37.944	7.34	8.59	290.41	15.93	1	8/1/2014	8:27:30.78
1715	RSRD19	-122.450	37.944	7.37	8.56	289.71	15.93	-122.450	37.944	7.34	8.56	290.37	15.90	1	8/1/2014	8:27:30.87
1716	RSRD19	-122.450	37.944	7.42	8.56	289.61	15.98	-122.450	37.944	7.34	8.56	290.26	15.89	1	8/1/2014	8:27:30.97
1717	RSRD19	-122.450	37.944	7.37	8.49	289.53	15.86	-122.450	37.944	7.37	8.49	290.16	15.86	1	8/1/2014	8:27:31.07
1718	RSRD19	-122.450	37.944	7.37	8.53	289.39	15.90	-122.450	37.944	7.37	8.53	290.01	15.90	1	8/1/2014	8:27:31.18
1719	RSRD19	-122.450	37.944	7.37	8.46	289.33	15.83	-122.450	37.944	7.37	8.46	289.96	15.83	1	8/1/2014	8:27:31.28
1720	RSRD19	-122.450	37.944	7.37	8.45	289.28	15.82	-122.450	37.944	7.42	8.45	289.87	15.87	1	8/1/2014	8:27:31.37
1721	RSRD19	-122.450	37.944	7.37	8.42	289.17	15.79	-122.450	37.944	7.37	8.42	289.81	15.79	1	8/1/2014	8:27:31.47
1722	RSRD19	-122.450	37.944	7.42	8.42	289.12	15.84	-122.450	37.944	7.42	8.42	289.72	15.84	1	8/1/2014	8:27:31.58
1723	RSRD19	-122.450	37.944	7.37	8.42	289.04	15.79	-122.450	37.944	7.42	8.42	289.65	15.84	1	8/1/2014	8:27:31.68
1724	RSRD19	-122.450	37.944	7.42	8.42	289.01	15.84	-122.450	37.944	7.42	8.42	289.58	15.84	1	8/1/2014	8:27:31.77
1725	RSRD19	-122.450	37.944	7.37	8.41	288.90	15.78	-122.450	37.944	7.42	8.41	289.52	15.83	1	8/1/2014	8:27:31.87
1726	RSRD19	-122.450	37.944	7.42	8.40	288.90	15.82	-122.450	37.944	7.42	8.40	289.48	15.82	1	8/1/2014	8:27:31.98
1727	RSRD19	-122.450	37.944	7.42	8.42	288.82	15.84	-122.450	37.944	7.42	8.42	289.38	15.84	1	8/1/2014	8:27:32.08
1728	RSRD19	-122.450	37.944	7.37	8.43	288.75	15.80	-122.450	37.944	7.42	8.43	289.32	15.85	1	8/1/2014	8:27:32.17
1729	RSRD19	-122.450	37.944	7.42	8.44	288.73	15.86	-122.450	37.944	7.42	8.44	289.28	15.86	1	8/1/2014	8:27:32.27

1730	RSRD19	-122.450	37.944	7.42	8.48	288.66	15.90	-122.450	37.944	7.42	8.48	289.19	15.90	1	8/1/2014 8:27:32.38
1731	RSRD19	-122.450	37.944	7.42	8.47	288.63	15.89	-122.450	37.944	7.42	8.47	289.18	15.89	1	8/1/2014 8:27:32.48
1732	RSRD19	-122.450	37.944	7.42	8.48	288.57	15.90	-122.450	37.944	7.42	8.48	289.12	15.90	1	8/1/2014 8:27:32.57
1733	RSRD19	-122.450	37.944	7.37	8.47	288.50	15.84	-122.450	37.944	7.37	8.47	289.05	15.84	1	8/1/2014 8:27:32.67
1734	RSRD19	-122.450	37.944	7.42	8.49	288.50	15.91	-122.450	37.944	7.42	8.49	289.01	15.91	1	8/1/2014 8:27:32.78
1735	RSRD19	-122.450	37.944	7.42	8.47	288.46	15.89	-122.450	37.944	7.37	8.47	288.97	15.84	1	8/1/2014 8:27:32.87
1736	RSRD19	-122.450	37.944	7.42	8.47	288.39	15.89	-122.450	37.944	7.42	8.47	288.88	15.89	1	8/1/2014 8:27:32.97
1737	RSRD19	-122.450	37.944	7.37	8.47	288.33	15.84	-122.450	37.944	7.42	8.47	288.84	15.89	1	8/1/2014 8:27:33.07
1738	RSRD19	-122.450	37.944	7.42	8.49	288.31	15.91	-122.450	37.944	7.42	8.49	288.77	15.91	1	8/1/2014 8:27:33.18
1739	RSRD19	-122.450	37.944	7.37	8.47	288.26	15.84	-122.450	37.944	7.42	8.47	288.77	15.89	1	8/1/2014 8:27:33.27
1740	RSRD19	-122.450	37.944	7.42	8.48	288.22	15.90	-122.450	37.944	7.42	8.48	288.67	15.90	1	8/1/2014 8:27:33.37
1741	RSRD19	-122.450	37.944	7.42	8.42	288.20	15.84	-122.450	37.944	7.45	8.42	288.62	15.87	1	8/1/2014 8:27:33.47
1742	RSRD19	-122.450	37.944	7.42	8.40	288.09	15.82	-122.450	37.944	7.45	8.40	288.57	15.86	1	8/1/2014 8:27:33.58
1743	RSRD19	-122.450	37.944	7.42	8.40	288.07	15.82	-122.450	37.944	7.42	8.40	288.55	15.82	1	8/1/2014 8:27:33.68
1744	RSRD19	-122.450	37.944	7.42	8.44	288.07	15.86	-122.450	37.944	7.45	8.44	288.53	15.90	1	8/1/2014 8:27:33.77
1745	RSRD19	-122.450	37.944	7.37	8.39	288.03	15.76	-122.450	37.944	7.45	8.39	288.47	15.84	1	8/1/2014 8:27:33.87
1746	RSRD19	-122.450	37.944	7.42	8.37	287.96	15.79	-122.450	37.944	7.45	8.37	288.40	15.83	1	8/1/2014 8:27:33.98
1747	RSRD19	-122.450	37.944	7.42	8.41	287.99	15.83	-122.450	37.944	7.42	8.41	288.38	15.83	1	8/1/2014 8:27:34.08
1748	RSRD19	-122.450	37.944	7.42	8.37	287.92	15.79	-122.450	37.944	7.45	8.37	288.32	15.82	1	8/1/2014 8:27:34.17
1749	RSRD19	-122.450	37.944	7.37	8.38	287.88	15.75	-122.450	37.944	7.42	8.38	288.27	15.80	1	8/1/2014 8:27:34.27
1750	RSRD19	-122.450	37.944	7.42	8.36	287.88	15.78	-122.450	37.944	7.45	8.36	288.28	15.81	1	8/1/2014 8:27:34.38
1751	RSRD19	-122.450	37.944	7.42	8.37	287.82	15.79	-122.450	37.944	7.42	8.37	288.21	15.79	1	8/1/2014 8:27:34.47
1752	RSRD19	-122.450	37.944	7.42	8.34	287.81	15.76	-122.450	37.944	7.45	8.34	288.19	15.80	1	8/1/2014 8:27:34.57
1753	RSRD19	-122.450	37.944	7.42	8.33	287.77	15.75	-122.450	37.944	7.45	8.33	288.17	15.79	1	8/1/2014 8:27:34.67
1754	RSRD19	-122.450	37.944	7.42	8.33	287.73	15.75	-122.450	37.944	7.45	8.33	288.13	15.79	1	8/1/2014 8:27:34.78
1755	RSRD19	-122.450	37.944	7.42	8.36	287.71	15.78	-122.450	37.944	7.45	8.36	288.11	15.81	1	8/1/2014 8:27:34.88
1756	RSRD19	-122.450	37.944	7.45	8.37	287.67	15.82	-122.450	37.944	7.45	8.37	288.04	15.82	1	8/1/2014 8:27:34.97
1757	RSRD19	-122.450	37.944	7.42	8.35	287.65	15.77	-122.450	37.944	7.45	8.35	288.11	15.80	1	8/1/2014 8:27:35.07
1758	RSRD19	-122.450	37.944	7.45	8.37	287.60	15.82	-122.450	37.944	7.45	8.37	287.92	15.83	1	8/1/2014 8:27:35.18
1759	RSRD19	-122.450	37.944	7.42	8.39	287.56	15.81	-122.450	37.944	7.45	8.39	287.98	15.84	1	8/1/2014 8:27:35.28
1760	RSRD19	-122.450	37.944	7.42	8.36	287.56	15.78	-122.450	37.944	7.49	8.36	287.96	15.85	1	8/1/2014 8:27:35.37
1761	RSRD19	-122.450	37.944	7.42	8.36	287.50	15.78	-122.450	37.944	7.49	8.36	287.90	15.85	1	8/1/2014 8:27:35.47
1762	RSRD19	-122.450	37.944	7.45	8.37	287.48	15.82	-122.450	37.944	7.49	8.37	287.85	15.85	1	8/1/2014 8:27:35.58

1763	RSRD19	-122.450	37.944	7.42	8.39	287.44	15.81	-122.450	37.944	7.49	8.39	287.83	15.87	1	8/1/2014	8:27:35.68
1764	RSRD19	-122.450	37.944	7.45	8.37	287.42	15.82	-122.450	37.944	7.54	8.37	287.80	15.91	1	8/1/2014	8:27:35.77
1765	RSRD19	-122.450	37.944	7.42	8.39	287.40	15.81	-122.450	37.944	7.49	8.39	287.77	15.87	1	8/1/2014	8:27:35.87
1766	RSRD19	-122.450	37.944	7.45	8.38	287.40	15.84	-122.450	37.944	7.54	8.38	287.75	15.92	1	8/1/2014	8:27:35.98
1767	RSRD19	-122.450	37.944	7.42	8.39	287.36	15.81	-122.450	37.944	7.49	8.39	287.69	15.87	1	8/1/2014	8:27:36.07
1768	RSRD19	-122.450	37.944	7.45	8.37	287.34	15.82	-122.450	37.944	7.54	8.37	287.67	15.90	1	8/1/2014	8:27:36.17
1769	RSRD19	-122.450	37.944	7.42	8.37	287.36	15.79	-122.450	37.944	7.54	8.37	287.68	15.90	1	8/1/2014	8:27:36.27
1770	RSRD19	-122.450	37.944	7.45	8.34	287.28	15.80	-122.450	37.944	7.54	8.34	287.63	15.88	1	8/1/2014	8:27:36.38
1771	RSRD19	-122.450	37.944	7.42	8.32	287.33	15.74	-122.450	37.944	7.49	8.32	287.59	15.80	1	8/1/2014	8:27:36.47
1772	RSRD19	-122.450	37.944	7.45	8.31	287.29	15.77	-122.450	37.944	7.54	8.31	287.57	15.85	1	8/1/2014	8:27:36.57
1773	RSRD19	-122.450	37.944	7.45	8.30	287.26	15.76	-122.450	37.944	7.49	8.30	287.55	15.79	1	8/1/2014	8:27:36.67
1774	RSRD19	-122.450	37.944	7.45	8.30	287.24	15.75	-122.450	37.944	7.54	8.30	287.55	15.83	1	8/1/2014	8:27:36.78
1775	RSRD19	-122.450	37.944	7.45	8.30	287.23	15.75	-122.450	37.944	7.54	8.30	287.49	15.83	1	8/1/2014	8:27:36.88
1776	RSRD19	-122.450	37.944	7.45	8.29	287.18	15.74	-122.450	37.944	7.49	8.29	287.47	15.78	1	8/1/2014	8:27:36.97
1777	RSRD19	-122.450	37.944	7.42	8.29	287.12	15.71	-122.450	37.944	7.49	8.29	287.43	15.78	1	8/1/2014	8:27:37.07
1778	RSRD19	-122.450	37.944	7.45	8.28	287.10	15.73	-122.450	37.944	7.54	8.28	287.38	15.82	1	8/1/2014	8:27:37.18
1779	RSRD19	-122.450	37.944	7.45	8.28	287.04	15.73	-122.450	37.944	7.54	8.28	287.32	15.82	1	8/1/2014	8:27:37.27
1780	RSRD19	-122.450	37.944	7.45	8.30	287.01	15.75	-122.450	37.944	7.54	8.30	287.28	15.83	1	8/1/2014	8:27:37.37
1781	RSRD19	-122.450	37.944	7.45	8.30	286.95	15.75	-122.450	37.944	7.54	8.30	287.22	15.83	1	8/1/2014	8:27:37.47
1782	RSRD19	-122.450	37.944	7.45	8.29	286.91	15.74	-122.450	37.944	7.54	8.29	287.19	15.83	1	8/1/2014	8:27:37.58
1783	RSRD19	-122.450	37.944	7.45	8.30	286.87	15.75	-122.450	37.944	7.54	8.30	287.14	15.83	1	8/1/2014	8:27:37.68
1784	RSRD19	-122.450	37.944	7.45	8.30	286.85	15.76	-122.450	37.944	7.54	8.30	287.11	15.84	1	8/1/2014	8:27:37.77
1785	RSRD19	-122.450	37.944	7.45	8.29	286.76	15.74	-122.450	37.944	7.54	8.29	287.05	15.83	1	8/1/2014	8:27:37.87
1786	RSRD19	-122.450	37.944	7.45	8.28	286.79	15.73	-122.450	37.944	7.57	8.28	287.03	15.85	1	8/1/2014	8:27:37.98
1787	RSRD19	-122.450	37.944	7.45	8.25	286.75	15.70	-122.450	37.944	7.54	8.25	286.98	15.79	1	8/1/2014	8:27:38.08
1788	RSRD19	-122.450	37.944	7.45	8.24	286.70	15.70	-122.450	37.944	7.54	8.24	286.92	15.78	1	8/1/2014	8:27:38.17
1789	RSRD19	-122.450	37.944	7.45	8.24	286.64	15.70	-122.450	37.944	7.54	8.24	286.88	15.78	1	8/1/2014	8:27:38.27
1790	RSRD19	-122.450	37.944	7.45	8.22	286.62	15.67	-122.450	37.944	7.57	8.22	286.84	15.79	1	8/1/2014	8:27:38.38
1791	RSRD19	-122.450	37.944	7.45	8.22	286.61	15.67	-122.450	37.944	7.54	8.22	286.80	15.76	1	8/1/2014	8:27:38.48
1792	RSRD19	-122.450	37.944	7.45	8.19	286.54	15.65	-122.450	37.944	7.57	8.19	286.76	15.77	1	8/1/2014	8:27:38.57
1793	RSRD19	-122.450	37.944	7.45	8.19	286.58	15.64	-122.450	37.944	7.57	8.19	286.67	15.76	1	8/1/2014	8:27:38.67
1794	RSRD19	-122.450	37.944	7.51	8.16	286.39	15.67	-122.450	37.944	7.57	8.16	286.65	15.74	1	8/1/2014	8:27:38.78
1795	RSRD19	-122.450	37.944	7.45	8.15	286.41	15.60	-122.450	37.944	7.57	8.15	286.61	15.72	1	8/1/2014	8:27:38.87

1796	RSRD19	-122.450	37.944	7.51	8.14	286.37	15.65	-122.450	37.944	7.57	8.14	286.57	15.71	1	8/1/2014	8:27:38.97
1797	RSRD19	-122.450	37.944	7.45	8.14	286.35	15.60	-122.450	37.944	7.57	8.14	286.55	15.71	1	8/1/2014	8:27:39.07
1798	RSRD19	-122.450	37.944	7.45	8.14	286.31	15.60	-122.450	37.944	7.62	8.14	286.51	15.77	1	8/1/2014	8:27:39.18
1799	RSRD19	-122.450	37.944	7.45	8.10	286.27	15.56	-122.450	37.944	7.57	8.10	286.42	15.68	1	8/1/2014	8:27:39.27
1800	RSRD19	-122.450	37.944	7.51	8.10	286.23	15.61	-122.450	37.944	7.62	8.10	286.44	15.73	1	8/1/2014	8:27:39.37
1801	RSRD19	-122.450	37.944	7.51	8.08	286.21	15.59	-122.450	37.944	7.62	8.08	286.40	15.70	1	8/1/2014	8:27:39.47
1802	RSRD19	-122.450	37.944	7.51	8.07	286.18	15.58	-122.450	37.944	7.62	8.07	286.36	15.69	1	8/1/2014	8:27:39.58
1803	RSRD19	-122.450	37.944	7.51	8.07	286.19	15.57	-122.450	37.944	7.62	8.07	286.36	15.69	1	8/1/2014	8:27:39.67
1804	RSRD19	-122.450	37.944	7.51	8.04	286.15	15.55	-122.450	37.944	7.62	8.04	286.32	15.66	1	8/1/2014	8:27:39.77
1805	RSRD19	-122.450	37.944	7.51	8.04	286.15	15.55	-122.450	37.944	7.62	8.04	286.32	15.66	1	8/1/2014	8:27:39.87
1806	RSRD19	-122.450	37.944	7.51	8.05	286.11	15.56	-122.450	37.944	7.66	8.05	286.31	15.71	1	8/1/2014	8:27:39.98
1807	RSRD19	-122.450	37.944	7.45	8.07	286.09	15.52	-122.450	37.944	7.62	8.07	286.27	15.69	1	8/1/2014	8:27:40.07
1808	RSRD19	-122.450	37.944	7.51	8.09	286.09	15.59	-122.450	37.944	7.62	8.09	286.26	15.71	1	8/1/2014	8:27:40.17
1809	RSRD19	-122.451	37.944	7.45	8.06	286.05	15.51	-122.450	37.944	7.62	8.06	286.23	15.68	1	8/1/2014	8:27:40.27
1810	RSRD19	-122.451	37.944	7.51	8.04	286.05	15.55	-122.451	37.944	7.62	8.04	286.16	15.66	1	8/1/2014	8:27:40.38
1811	RSRD19	-122.451	37.944	7.45	8.08	286.07	15.53	-122.451	37.944	7.57	8.08	286.20	15.65	1	8/1/2014	8:27:40.47
1812	RSRD19	-122.451	37.944	7.51	8.03	286.03	15.53	-122.451	37.944	7.57	8.03	286.14	15.60	1	8/1/2014	8:27:40.57
1813	RSRD19	-122.451	37.944	7.51	8.02	286.01	15.52	-122.451	37.944	7.57	8.02	286.16	15.59	1	8/1/2014	8:27:40.67
1814	RSRD19	-122.451	37.944	7.51	8.01	286.01	15.52	-122.451	37.944	7.62	8.01	286.10	15.64	1	8/1/2014	8:27:40.78
1815	RSRD19	-122.451	37.944	7.45	8.00	286.01	15.45	-122.451	37.944	7.57	8.00	286.10	15.57	1	8/1/2014	8:27:40.87
1816	RSRD19	-122.451	37.944	7.51	8.00	285.97	15.50	-122.451	37.944	7.62	8.00	286.08	15.62	1	8/1/2014	8:27:40.97
1817	RSRD19	-122.451	37.944	7.51	8.00	285.97	15.50	-122.451	37.944	7.57	8.00	286.06	15.57	1	8/1/2014	8:27:41.07
1818	RSRD19	-122.451	37.944	7.51	7.97	285.95	15.47	-122.451	37.944	7.57	7.97	286.06	15.54	1	8/1/2014	8:27:41.18
1819	RSRD19	-122.451	37.944	7.45	7.97	285.97	15.42	-122.451	37.944	7.62	7.97	286.04	15.59	1	8/1/2014	8:27:41.27
1820	RSRD19	-122.451	37.944	7.51	7.95	285.91	15.45	-122.451	37.944	7.62	7.95	286.00	15.57	1	8/1/2014	8:27:41.37
1821	RSRD19	-122.451	37.944	7.45	7.97	285.96	15.42	-122.451	37.944	7.57	7.97	286.04	15.54	1	8/1/2014	8:27:41.47
1822	RSRD19	-122.451	37.944	7.51	7.97	285.89	15.48	-122.451	37.944	7.62	7.97	286.00	15.59	1	8/1/2014	8:27:41.58
1823	RSRD19	-122.451	37.944	7.51	7.95	285.90	15.45	-122.451	37.944	7.62	7.95	285.99	15.57	1	8/1/2014	8:27:41.67
1824	RSRD19	-122.451	37.944	7.51	7.95	285.94	15.45	-122.451	37.944	7.62	7.95	286.03	15.57	1	8/1/2014	8:27:41.77
1825	RSRD19	-122.451	37.944	7.51	7.96	285.92	15.46	-122.451	37.944	7.66	7.96	286.01	15.62	1	8/1/2014	8:27:41.87
1826	RSRD19	-122.451	37.944	7.54	7.97	285.90	15.50	-122.451	37.944	7.66	7.97	286.01	15.62	1	8/1/2014	8:27:41.98
1827	RSRD19	-122.451	37.944	7.51	7.98	285.92	15.49	-122.451	37.944	7.66	7.98	286.01	15.64	1	8/1/2014	8:27:42.07
1828	RSRD19	-122.451	37.944	7.54	7.95	285.88	15.49	-122.451	37.944	7.66	7.95	285.99	15.61	1	8/1/2014	8:27:42.17

1829	RSRD19	-122.451	37.944	7.51	7.97	285.88	15.47	-122.451	37.944	7.62	7.97	285.97	15.59	1	8/1/2014 8:27:42.27
1830	RSRD19	-122.451	37.944	7.54	7.99	285.92	15.53	-122.451	37.944	7.66	7.99	285.99	15.64	1	8/1/2014 8:27:42.38
1831	RSRD19	-122.451	37.944	7.51	7.97	285.89	15.47	-122.451	37.944	7.66	7.97	285.99	15.62	1	8/1/2014 8:27:42.47
1832	RSRD19	-122.451	37.944	7.51	8.00	285.89	15.50	-122.451	37.944	7.62	8.00	285.95	15.62	1	8/1/2014 8:27:42.57
1833	RSRD19	-122.451	37.944	7.51	8.00	285.87	15.50	-122.451	37.944	7.62	8.00	285.95	15.62	1	8/1/2014 8:27:42.67
1834	RSRD19	-122.451	37.944	7.54	8.03	285.85	15.57	-122.451	37.944	7.62	8.03	285.96	15.66	1	8/1/2014 8:27:42.78
1835	RSRD19	-122.451	37.944	7.51	8.03	285.82	15.54	-122.451	37.944	7.62	8.03	285.89	15.66	1	8/1/2014 8:27:42.87
1836	RSRD19	-122.451	37.944	7.54	8.03	285.83	15.57	-122.451	37.944	7.62	8.03	285.92	15.65	1	8/1/2014 8:27:42.97
1837	RSRD19	-122.451	37.944	7.51	8.03	285.83	15.54	-122.451	37.944	7.62	8.03	285.90	15.66	1	8/1/2014 8:27:43.07
1838	RSRD19	-122.451	37.944	7.51	8.03	285.79	15.54	-122.451	37.944	7.62	8.03	285.89	15.66	1	8/1/2014 8:27:43.18
1839	RSRD19	-122.451	37.944	7.51	8.03	285.77	15.54	-122.451	37.944	7.62	8.03	285.85	15.66	1	8/1/2014 8:27:43.27
1840	RSRD19	-122.451	37.944	7.54	8.04	285.77	15.58	-122.451	37.944	7.66	8.04	285.86	15.70	1	8/1/2014 8:27:43.37
1841	RSRD19	-122.451	37.944	7.51	8.02	285.75	15.52	-122.451	37.944	7.62	8.02	285.83	15.64	1	8/1/2014 8:27:43.47
1842	RSRD19	-122.451	37.944	7.54	8.03	285.75	15.57	-122.451	37.944	7.66	8.03	285.81	15.69	1	8/1/2014 8:27:43.58
1843	RSRD19	-122.451	37.944	7.54	8.00	285.75	15.54	-122.451	37.944	7.66	8.00	285.83	15.66	1	8/1/2014 8:27:43.67
1844	RSRD19	-122.451	37.944	7.54	7.98	285.73	15.52	-122.451	37.944	7.71	7.98	285.81	15.69	1	8/1/2014 8:27:43.77
1845	RSRD19	-122.451	37.944	7.54	7.98	285.73	15.52	-122.451	37.944	7.66	7.98	285.81	15.64	1	8/1/2014 8:27:43.87
1846	RSRD19	-122.451	37.944	7.54	7.97	285.73	15.51	-122.451	37.944	7.71	7.97	285.78	15.68	1	8/1/2014 8:27:43.98
1847	RSRD19	-122.451	37.944	7.54	7.97	285.73	15.50	-122.451	37.944	7.66	7.97	285.78	15.62	1	8/1/2014 8:27:44.07
1848	RSRD19	-122.451	37.944	7.51	7.97	285.73	15.48	-122.451	37.944	7.66	7.97	285.73	15.63	1	8/1/2014 8:27:44.17
1849	RSRD19	-122.451	37.944	7.51	7.98	285.71	15.49	-122.451	37.944	7.66	7.98	285.75	15.64	1	8/1/2014 8:27:44.27
1850	RSRD19	-122.451	37.944	7.51	7.99	285.72	15.49	-122.451	37.944	7.71	7.99	285.76	15.69	1	8/1/2014 8:27:44.38
1851	RSRD19	-122.451	37.944	7.54	7.97	285.71	15.51	-122.451	37.944	7.66	7.97	285.72	15.63	1	8/1/2014 8:27:44.47
1852	RSRD19	-122.451	37.944	7.54	7.97	285.72	15.50	-122.451	37.944	7.66	7.97	285.76	15.62	1	8/1/2014 8:27:44.57
1853	RSRD19	-122.451	37.944	7.51	7.97	285.70	15.48	-122.451	37.944	7.62	7.97	285.71	15.59	1	8/1/2014 8:27:44.67
1854	RSRD19	-122.451	37.944	7.54	7.98	285.70	15.52	-122.451	37.944	7.66	7.98	285.72	15.64	1	8/1/2014 8:27:44.78
1855	RSRD19	-122.451	37.944	7.51	7.98	285.70	15.49	-122.451	37.944	7.66	7.98	285.70	15.64	1	8/1/2014 8:27:44.87
1856	RSRD19	-122.451	37.944	7.54	7.96	285.70	15.50	-122.451	37.944	7.66	7.96	285.70	15.62	1	8/1/2014 8:27:44.97
1857	RSRD19	-122.451	37.944	7.54	7.95	285.66	15.49	-122.451	37.944	7.62	7.95	285.70	15.57	1	8/1/2014 8:27:45.07
1858	RSRD19	-122.451	37.944	7.54	7.94	285.66	15.48	-122.451	37.944	7.66	7.94	285.66	15.60	1	8/1/2014 8:27:45.18
1859	RSRD19	-122.451	37.944	7.51	7.95	285.66	15.45	-122.451	37.944	7.62	7.95	285.66	15.57	1	8/1/2014 8:27:45.27
1860	RSRD19	-122.451	37.944	7.51	7.95	285.64	15.45	-122.451	37.944	7.62	7.95	285.64	15.57	1	8/1/2014 8:27:45.37
1861	RSRD19	-122.451	37.944	7.51	7.97	285.62	15.48	-122.451	37.944	7.62	7.97	285.62	15.59	1	8/1/2014 8:27:45.47

1862	RSRD19	-122.451	37.944	7.54	7.95	285.62	15.49	-122.451	37.944	7.62	7.95	285.59	15.57	1	8/1/2014	8:27:45.58
1863	RSRD19	-122.451	37.944	7.51	7.96	285.58	15.46	-122.451	37.944	7.62	7.96	285.58	15.58	1	8/1/2014	8:27:45.67
1864	RSRD19	-122.451	37.944	7.51	7.96	285.58	15.46	-122.451	37.944	7.62	7.96	285.53	15.58	1	8/1/2014	8:27:45.77
1865	RSRD19	-122.451	37.944	7.51	8.00	285.51	15.50	-122.451	37.944	7.62	8.00	285.49	15.62	1	8/1/2014	8:27:45.87
1866	RSRD19	-122.451	37.944	7.54	7.97	285.58	15.50	-122.451	37.944	7.62	7.97	285.54	15.59	1	8/1/2014	8:27:45.98
1867	RSRD19	-122.451	37.944	7.51	7.99	285.54	15.49	-122.451	37.944	7.62	7.99	285.49	15.61	1	8/1/2014	8:27:46.07
1868	RSRD19	-122.451	37.944	7.54	8.01	285.51	15.55	-122.451	37.944	7.66	8.01	285.49	15.67	1	8/1/2014	8:27:46.17
1869	RSRD19	-122.451	37.944	7.54	8.03	285.52	15.57	-122.451	37.944	7.62	8.03	285.51	15.66	1	8/1/2014	8:27:46.27
1870	RSRD19	-122.451	37.944	7.54	8.01	285.49	15.55	-122.451	37.944	7.62	8.01	285.47	15.64	1	8/1/2014	8:27:46.38
1871	RSRD19	-122.451	37.944	7.54	8.07	285.47	15.61	-122.451	37.944	7.62	8.07	285.49	15.69	1	8/1/2014	8:27:46.47
1872	RSRD19	-122.451	37.944	7.54	8.08	285.48	15.62	-122.451	37.944	7.62	8.08	285.47	15.70	1	8/1/2014	8:27:46.57
1873	RSRD19	-122.451	37.944	7.54	8.10	285.45	15.64	-122.451	37.944	7.62	8.10	285.43	15.73	1	8/1/2014	8:27:46.67
1874	RSRD19	-122.451	37.944	7.54	8.08	285.47	15.62	-122.451	37.944	7.66	8.08	285.46	15.74	1	8/1/2014	8:27:46.78
1875	RSRD19	-122.451	37.944	7.51	8.10	285.39	15.61	-122.451	37.944	7.62	8.10	285.41	15.73	1	8/1/2014	8:27:46.87
1876	RSRD19	-122.451	37.944	7.54	8.10	285.39	15.64	-122.451	37.944	7.62	8.10	285.41	15.72	1	8/1/2014	8:27:46.97
1877	RSRD19	-122.451	37.944	7.51	8.10	285.37	15.61	-122.451	37.944	7.62	8.10	285.35	15.73	1	8/1/2014	8:27:47.07
1878	RSRD19	-122.451	37.944	7.54	8.11	285.33	15.65	-122.451	37.944	7.62	8.11	285.35	15.73	1	8/1/2014	8:27:47.18
1879	RSRD19	-122.451	37.944	7.51	8.12	285.30	15.62	-122.451	37.944	7.62	8.12	285.30	15.74	1	8/1/2014	8:27:47.27
1880	RSRD19	-122.451	37.944	7.54	8.15	285.29	15.69	-122.451	37.944	7.62	8.15	285.29	15.77	1	8/1/2014	8:27:47.37
1881	RSRD19	-122.451	37.944	7.51	8.11	285.26	15.61	-122.451	37.944	7.57	8.11	285.26	15.68	1	8/1/2014	8:27:47.47
1882	RSRD19	-122.451	37.944	7.54	8.11	285.22	15.65	-122.451	37.944	7.57	8.11	285.20	15.68	1	8/1/2014	8:27:47.58
1883	RSRD19	-122.451	37.944	7.51	8.14	285.20	15.65	-122.451	37.944	7.57	8.14	285.20	15.71	1	8/1/2014	8:27:47.67
1884	RSRD19	-122.451	37.944	7.54	8.15	285.20	15.69	-122.451	37.944	7.57	8.15	285.17	15.72	1	8/1/2014	8:27:47.77
1885	RSRD19	-122.451	37.944	7.51	8.15	285.13	15.66	-122.451	37.944	7.57	8.15	285.15	15.72	1	8/1/2014	8:27:47.87
1886	RSRD19	-122.451	37.944	7.51	8.22	285.13	15.73	-122.451	37.944	7.57	8.22	285.13	15.79	1	8/1/2014	8:27:47.98
1887	RSRD19	-122.451	37.944	7.51	8.19	285.09	15.70	-122.451	37.944	7.57	8.19	285.11	15.77	1	8/1/2014	8:27:48.07
1888	RSRD19	-122.451	37.944	7.51	8.25	285.07	15.75	-122.451	37.944	7.57	8.25	285.09	15.82	1	8/1/2014	8:27:48.17
1889	RSRD19	-122.451	37.944	7.51	8.21	285.07	15.72	-122.451	37.944	7.54	8.21	285.07	15.75	1	8/1/2014	8:27:48.27
1890	RSRD19	-122.451	37.944	7.51	8.27	285.02	15.77	-122.451	37.944	7.57	8.27	285.03	15.84	1	8/1/2014	8:27:48.38
1891	RSRD19	-122.451	37.944	7.51	8.24	285.03	15.75	-122.451	37.944	7.54	8.24	285.00	15.78	1	8/1/2014	8:27:48.47
1892	RSRD19	-122.451	37.944	7.51	8.26	284.98	15.76	-122.451	37.944	7.57	8.26	285.01	15.83	1	8/1/2014	8:27:48.57
1893	RSRD19	-122.451	37.944	7.51	8.27	284.92	15.78	-122.451	37.944	7.54	8.27	284.94	15.81	1	8/1/2014	8:27:48.67
1894	RSRD19	-122.451	37.944	7.51	8.28	284.96	15.79	-122.451	37.944	7.54	8.28	284.94	15.82	1	8/1/2014	8:27:48.78

1895	RSRD19	-122.451	37.944	7.45	8.29	284.92	15.74	-122.451	37.944	7.54	8.29	284.90	15.83	1	8/1/2014	8:27:48.87
1896	RSRD19	-122.451	37.944	7.51	8.30	284.91	15.80	-122.451	37.944	7.57	8.30	284.94	15.87	1	8/1/2014	8:27:48.97
1897	RSRD19	-122.451	37.944	7.51	8.30	284.89	15.80	-122.451	37.944	7.54	8.30	284.87	15.83	1	8/1/2014	8:27:49.07
1898	RSRD19	-122.451	37.944	7.51	8.29	284.87	15.80	-122.451	37.944	7.54	8.29	284.87	15.83	1	8/1/2014	8:27:49.18
1899	RSRD19	-122.451	37.944	7.45	8.27	284.89	15.73	-122.451	37.944	7.54	8.27	284.87	15.81	1	8/1/2014	8:27:49.27
1900	RSRD19	-122.451	37.944	7.51	8.27	284.83	15.77	-122.451	37.944	7.54	8.27	284.85	15.80	1	8/1/2014	8:27:49.37
1901	RSRD19	-122.451	37.944	7.45	8.25	284.85	15.70	-122.451	37.944	7.49	8.25	284.83	15.74	1	8/1/2014	8:27:49.47
1902	RSRD19	-122.451	37.944	7.45	8.24	284.81	15.70	-122.451	37.944	7.49	8.24	284.83	15.73	1	8/1/2014	8:27:49.58
1903	RSRD19	-122.451	37.944	7.45	8.25	284.85	15.70	-122.451	37.944	7.49	8.25	284.83	15.74	1	8/1/2014	8:27:49.67
1904	RSRD19	-122.451	37.944	7.51	8.23	284.78	15.74	-122.451	37.944	7.54	8.23	284.78	15.77	1	8/1/2014	8:27:49.77
1905	RSRD19	-122.451	37.944	7.45	8.25	284.80	15.70	-122.451	37.944	7.49	8.25	284.82	15.74	1	8/1/2014	8:27:49.87
1906	RSRD19	-122.451	37.944	7.45	8.26	284.80	15.71	-122.451	37.944	7.49	8.26	284.83	15.75	1	8/1/2014	8:27:49.98
1907	RSRD19	-122.451	37.944	7.45	8.26	284.78	15.71	-122.451	37.944	7.49	8.26	284.82	15.75	1	8/1/2014	8:27:50.07
1908	RSRD19	-122.451	37.944	7.45	8.27	284.78	15.72	-122.451	37.944	7.49	8.27	284.78	15.75	1	8/1/2014	8:27:50.17
1909	RSRD19	-122.451	37.944	7.45	8.27	284.76	15.73	-122.451	37.944	7.49	8.27	284.76	15.76	1	8/1/2014	8:27:50.27
1910	RSRD19	-122.451	37.944	7.51	8.27	284.78	15.77	-122.451	37.944	7.49	8.27	284.80	15.75	1	8/1/2014	8:27:50.38
1911	RSRD19	-122.451	37.944	7.45	8.27	284.78	15.73	-122.451	37.944	7.49	8.27	284.76	15.76	1	8/1/2014	8:27:50.47
1912	RSRD19	-122.451	37.944	7.45	8.30	284.82	15.76	-122.451	37.944	7.54	8.30	284.78	15.84	1	8/1/2014	8:27:50.57
1913	RSRD19	-122.451	37.944	7.45	8.27	284.78	15.72	-122.451	37.944	7.49	8.27	284.80	15.75	1	8/1/2014	8:27:50.67
1914	RSRD19	-122.451	37.944	7.45	8.27	284.82	15.72	-122.451	37.944	7.49	8.27	284.82	15.75	1	8/1/2014	8:27:50.78
1915	RSRD19	-122.451	37.944	7.45	8.27	284.80	15.73	-122.451	37.944	7.49	8.27	284.77	15.76	1	8/1/2014	8:27:50.87
1916	RSRD19	-122.451	37.944	7.51	8.29	284.82	15.80	-122.451	37.944	7.54	8.29	284.82	15.83	1	8/1/2014	8:27:50.97
1917	RSRD19	-122.451	37.944	7.45	8.28	284.84	15.73	-122.451	37.944	7.49	8.28	284.83	15.77	1	8/1/2014	8:27:51.07
1918	RSRD19	-122.451	37.944	7.51	8.30	284.86	15.81	-122.451	37.944	7.49	8.30	284.86	15.79	1	8/1/2014	8:27:51.18
1919	RSRD19	-122.451	37.944	7.45	8.32	284.84	15.77	-122.451	37.944	7.45	8.32	284.86	15.77	1	8/1/2014	8:27:51.27
1920	RSRD19	-122.451	37.944	7.45	8.30	284.83	15.75	-122.451	37.944	7.49	8.30	284.83	15.78	1	8/1/2014	8:27:51.37
1921	RSRD19	-122.451	37.944	7.45	8.33	284.83	15.78	-122.451	37.944	7.49	8.33	284.86	15.82	1	8/1/2014	8:27:51.47
1922	RSRD19	-122.451	37.944	7.45	8.28	284.86	15.74	-122.451	37.944	7.49	8.28	284.83	15.77	1	8/1/2014	8:27:51.58
1923	RSRD19	-122.451	37.944	7.45	8.28	284.79	15.74	-122.451	37.944	7.49	8.28	284.83	15.77	1	8/1/2014	8:27:51.67
1924	RSRD19	-122.451	37.944	7.51	8.27	284.81	15.77	-122.451	37.944	7.49	8.27	284.83	15.75	1	8/1/2014	8:27:51.77
1925	RSRD19	-122.451	37.944	7.42	8.27	284.80	15.69	-122.451	37.944	7.45	8.27	284.82	15.73	1	8/1/2014	8:27:51.87
1926	RSRD19	-122.451	37.944	7.45	8.29	284.78	15.74	-122.451	37.944	7.49	8.29	284.83	15.78	1	8/1/2014	8:27:51.98
1927	RSRD19	-122.451	37.944	7.45	8.29	284.74	15.74	-122.451	37.944	7.49	8.29	284.78	15.78	1	8/1/2014	8:27:52.07

1928	RSRD19	-122.451	37.944	7.45	8.30	284.76	15.76	-122.451	37.944	7.49	8.30	284.78	15.79	1	8/1/2014	8:27:52.17
1929	RSRD19	-122.451	37.944	7.45	8.28	284.76	15.74	-122.451	37.944	7.45	8.28	284.78	15.74	1	8/1/2014	8:27:52.27
1930	RSRD19	-122.451	37.944	7.45	8.29	284.69	15.74	-122.451	37.944	7.49	8.29	284.73	15.78	1	8/1/2014	8:27:52.38
1931	RSRD19	-122.451	37.944	7.42	8.30	284.71	15.72	-122.451	37.944	7.49	8.30	284.71	15.78	1	8/1/2014	8:27:52.47
1932	RSRD19	-122.451	37.944	7.45	8.33	284.69	15.78	-122.451	37.944	7.49	8.33	284.73	15.82	1	8/1/2014	8:27:52.57
1933	RSRD19	-122.451	37.944	7.42	8.35	284.73	15.77	-122.451	37.944	7.45	8.35	284.66	15.81	1	8/1/2014	8:27:52.67
1934	RSRD19	-122.451	37.944	7.45	8.36	284.61	15.81	-122.451	37.944	7.49	8.36	284.63	15.85	1	8/1/2014	8:27:52.78
1935	RSRD19	-122.451	37.944	7.42	8.37	284.63	15.79	-122.451	37.944	7.45	8.37	284.66	15.82	1	8/1/2014	8:27:52.87
1936	RSRD19	-122.451	37.944	7.45	8.35	284.63	15.80	-122.451	37.944	7.45	8.35	284.63	15.81	1	8/1/2014	8:27:52.97
1937	RSRD19	-122.451	37.944	7.42	8.34	284.63	15.76	-122.451	37.944	7.45	8.34	284.63	15.80	1	8/1/2014	8:27:53.07
1938	RSRD19	-122.451	37.944	7.45	8.34	284.61	15.80	-122.451	37.944	7.45	8.34	284.61	15.80	1	8/1/2014	8:27:53.18
1939	RSRD19	-122.451	37.944	7.42	8.36	284.60	15.78	-122.451	37.944	7.45	8.36	284.62	15.81	1	8/1/2014	8:27:53.27
1940	RSRD19	-122.451	37.944	7.42	8.33	284.63	15.75	-122.451	37.944	7.49	8.33	284.60	15.82	1	8/1/2014	8:27:53.37
1941	RSRD19	-122.451	37.944	7.42	8.33	284.60	15.75	-122.451	37.944	7.45	8.33	284.63	15.79	1	8/1/2014	8:27:53.47
1942	RSRD19	-122.451	37.944	7.45	8.33	284.62	15.79	-122.451	37.944	7.49	8.33	284.64	15.82	1	8/1/2014	8:27:53.58
1943	RSRD19	-122.451	37.944	7.42	8.34	284.67	15.76	-122.451	37.944	7.45	8.34	284.64	15.80	1	8/1/2014	8:27:53.67
1944	RSRD19	-122.451	37.944	7.42	8.37	284.59	15.79	-122.451	37.944	7.45	8.37	284.62	15.82	1	8/1/2014	8:27:53.77
1945	RSRD19	-122.451	37.944	7.42	8.35	284.66	15.77	-122.451	37.944	7.45	8.35	284.64	15.81	1	8/1/2014	8:27:53.87
1946	RSRD19	-122.451	37.944	7.45	8.38	284.61	15.84	-122.451	37.944	7.49	8.38	284.64	15.87	1	8/1/2014	8:27:53.98
1947	RSRD19	-122.451	37.944	7.42	8.39	284.59	15.81	-122.451	37.944	7.45	8.39	284.63	15.84	1	8/1/2014	8:27:54.07
1948	RSRD19	-122.451	37.944	7.45	8.39	284.59	15.84	-122.451	37.944	7.49	8.39	284.59	15.88	1	8/1/2014	8:27:54.17
1949	RSRD19	-122.451	37.944	7.42	8.39	284.60	15.81	-122.451	37.944	7.45	8.39	284.63	15.84	1	8/1/2014	8:27:54.27
1950	RSRD19	-122.451	37.944	7.45	8.39	284.58	15.84	-122.451	37.944	7.54	8.39	284.62	15.93	1	8/1/2014	8:27:54.38
1951	RSRD19	-122.451	37.944	7.42	8.39	284.60	15.81	-122.451	37.944	7.49	8.39	284.60	15.88	1	8/1/2014	8:27:54.47
1952	RSRD19	-122.451	37.944	7.45	8.40	284.60	15.85	-122.451	37.944	7.49	8.40	284.64	15.89	1	8/1/2014	8:27:54.57
1953	RSRD19	-122.451	37.944	7.42	8.38	284.57	15.80	-122.451	37.944	7.49	8.38	284.60	15.87	1	8/1/2014	8:27:54.67
1954	RSRD19	-122.451	37.944	7.45	8.35	284.58	15.80	-122.451	37.944	7.54	8.35	284.62	15.89	1	8/1/2014	8:27:54.78
1955	RSRD19	-122.451	37.944	7.42	8.33	284.55	15.75	-122.451	37.944	7.54	8.33	284.59	15.87	1	8/1/2014	8:27:54.87
1956	RSRD19	-122.451	37.944	7.45	8.35	284.52	15.80	-122.451	37.944	7.54	8.35	284.61	15.89	1	8/1/2014	8:27:54.97
1957	RSRD19	-122.451	37.944	7.45	8.31	284.50	15.77	-122.451	37.944	7.57	8.31	284.54	15.89	1	8/1/2014	8:27:55.07
1958	RSRD19	-122.451	37.944	7.45	8.30	284.52	15.76	-122.451	37.944	7.57	8.30	284.58	15.88	1	8/1/2014	8:27:55.18
1959	RSRD19	-122.451	37.944	7.45	8.28	284.43	15.74	-122.451	37.944	7.54	8.28	284.52	15.82	1	8/1/2014	8:27:55.27
1960	RSRD19	-122.451	37.944	7.51	8.32	284.47	15.82	-122.451	37.944	7.57	8.32	284.54	15.89	1	8/1/2014	8:27:55.37

1961	RSRD19	-122.451	37.944	7.45	8.26	284.45	15.71	-122.451	37.944	7.62	8.26	284.49	15.88	1	8/1/2014	8:27:55.47
1962	RSRD19	-122.451	37.944	7.51	8.25	284.42	15.75	-122.451	37.944	7.62	8.25	284.51	15.87	1	8/1/2014	8:27:55.58
1963	RSRD19	-122.451	37.944	7.45	8.23	284.44	15.69	-122.451	37.944	7.62	8.23	284.50	15.86	1	8/1/2014	8:27:55.67
1964	RSRD19	-122.451	37.944	7.51	8.23	284.44	15.74	-122.451	37.944	7.62	8.23	284.49	15.86	1	8/1/2014	8:27:55.77
1965	RSRD19	-122.451	37.944	7.51	8.24	284.44	15.75	-122.451	37.944	7.62	8.24	284.52	15.87	1	8/1/2014	8:27:55.87
1966	RSRD19	-122.451	37.944	7.51	8.25	284.41	15.75	-122.451	37.944	7.62	8.25	284.48	15.87	1	8/1/2014	8:27:55.98
1967	RSRD19	-122.451	37.944	7.51	8.26	284.43	15.76	-122.451	37.944	7.62	8.26	284.47	15.88	1	8/1/2014	8:27:56.07
1968	RSRD19	-122.451	37.944	7.51	8.25	284.41	15.75	-122.451	37.944	7.66	8.25	284.50	15.91	1	8/1/2014	8:27:56.17
1969	RSRD19	-122.451	37.944	7.51	8.27	284.38	15.78	-122.451	37.944	7.66	8.27	284.45	15.93	1	8/1/2014	8:27:56.27
1970	RSRD19	-122.451	37.944	7.51	8.25	284.36	15.75	-122.451	37.944	7.71	8.25	284.43	15.96	1	8/1/2014	8:27:56.38
1971	RSRD19	-122.451	37.944	7.51	8.23	284.36	15.73	-122.451	37.944	7.66	8.23	284.44	15.89	1	8/1/2014	8:27:56.47
1972	RSRD19	-122.451	37.944	7.51	8.23	284.38	15.73	-122.451	37.944	7.66	8.23	284.40	15.89	1	8/1/2014	8:27:56.57
1973	RSRD19	-122.451	37.944	7.51	8.26	284.33	15.76	-122.451	37.944	7.66	8.26	284.37	15.92	1	8/1/2014	8:27:56.67
1974	RSRD19	-122.451	37.944	7.51	8.23	284.35	15.73	-122.451	37.944	7.71	8.23	284.42	15.94	1	8/1/2014	8:27:56.78
1975	RSRD19	-122.451	37.944	7.51	8.24	284.33	15.75	-122.451	37.944	7.66	8.24	284.37	15.90	1	8/1/2014	8:27:56.87
1976	RSRD19	-122.451	37.944	7.54	8.23	284.32	15.77	-122.451	37.944	7.66	8.23	284.37	15.89	1	8/1/2014	8:27:56.97
1977	RSRD19	-122.451	37.944	7.51	8.19	284.35	15.70	-122.451	37.944	7.66	8.19	284.39	15.85	1	8/1/2014	8:27:57.07
1978	RSRD19	-122.451	37.944	7.54	8.20	284.30	15.74	-122.451	37.944	7.66	8.20	284.30	15.86	1	8/1/2014	8:27:57.18
1979	RSRD19	-122.451	37.944	7.51	8.17	284.34	15.68	-122.451	37.944	7.66	8.17	284.31	15.83	1	8/1/2014	8:27:57.27
1980	RSRD19	-122.451	37.944	7.51	8.19	284.29	15.70	-122.451	37.944	7.71	8.19	284.29	15.90	1	8/1/2014	8:27:57.37
1981	RSRD19	-122.451	37.944	7.51	8.18	284.29	15.68	-122.451	37.944	7.66	8.18	284.27	15.84	1	8/1/2014	8:27:57.47
1982	RSRD19	-122.451	37.944	7.54	8.17	284.29	15.71	-122.451	37.944	7.71	8.17	284.27	15.88	1	8/1/2014	8:27:57.58
1983	RSRD19	-122.451	37.944	7.54	8.17	284.31	15.71	-122.451	37.944	7.66	8.17	284.26	15.83	1	8/1/2014	8:27:57.67
1984	RSRD19	-122.451	37.944	7.54	8.17	284.26	15.71	-122.451	37.944	7.71	8.17	284.22	15.88	1	8/1/2014	8:27:57.77
1985	RSRD19	-122.451	37.944	7.54	8.15	284.28	15.69	-122.451	37.944	7.66	8.15	284.24	15.81	1	8/1/2014	8:27:57.87
1986	RSRD19	-122.451	37.944	7.54	8.14	284.30	15.68	-122.451	37.944	7.71	8.14	284.21	15.85	1	8/1/2014	8:27:57.98
1987	RSRD19	-122.451	37.944	7.51	8.14	284.25	15.64	-122.451	37.944	7.71	8.14	284.19	15.84	1	8/1/2014	8:27:58.07
1988	RSRD19	-122.451	37.944	7.54	8.15	284.28	15.69	-122.451	37.944	7.71	8.15	284.21	15.86	1	8/1/2014	8:27:58.17
1989	RSRD19	-122.451	37.944	7.54	8.14	284.25	15.68	-122.451	37.944	7.66	8.14	284.20	15.80	1	8/1/2014	8:27:58.26
1990	RSRD19	-122.451	37.944	7.54	8.12	284.24	15.66	-122.451	37.944	7.71	8.12	284.20	15.83	1	8/1/2014	8:27:58.38
1991	RSRD19	-122.451	37.944	7.51	8.12	284.22	15.63	-122.451	37.944	7.71	8.12	284.18	15.83	1	8/1/2014	8:27:58.47
1992	RSRD19	-122.451	37.944	7.57	8.12	284.22	15.69	-122.451	37.944	7.74	8.12	284.18	15.86	1	8/1/2014	8:27:58.57
1993	RSRD19	-122.451	37.944	7.54	8.08	284.19	15.62	-122.451	37.944	7.71	8.08	284.13	15.79	1	8/1/2014	8:27:58.66

1994	RSRD19	-122.451	37.944	7.57	8.07	284.19	15.64	-122.451	37.944	7.74	8.07	284.16	15.81	1	8/1/2014	8:27:58.78
1995	RSRD19	-122.451	37.944	7.54	8.06	284.13	15.60	-122.451	37.944	7.71	8.06	284.14	15.76	1	8/1/2014	8:27:58.87
1996	RSRD19	-122.451	37.944	7.54	8.04	284.10	15.58	-122.451	37.944	7.74	8.04	284.06	15.78	1	8/1/2014	8:27:58.97
1997	RSRD19	-122.451	37.944	7.54	8.03	284.12	15.57	-122.451	37.944	7.74	8.03	284.12	15.78	1	8/1/2014	8:27:59.07
1998	RSRD19	-122.451	37.944	7.57	8.05	284.05	15.62	-122.451	37.944	7.78	8.05	284.07	15.83	1	8/1/2014	8:27:59.18
1999	RSRD19	-122.451	37.944	7.57	8.05	284.05	15.62	-122.451	37.944	7.78	8.05	284.04	15.83	1	8/1/2014	8:27:59.27
2000	RSRD19	-122.451	37.944	7.57	8.03	284.07	15.61	-122.451	37.944	7.78	8.03	284.05	15.81	1	8/1/2014	8:27:59.37
2001	RSRD19	-122.451	37.944	7.57	8.03	284.04	15.61	-122.451	37.944	7.78	8.03	284.04	15.81	1	8/1/2014	8:27:59.46
2002	RSRD19	-122.451	37.944	7.57	8.01	284.04	15.59	-122.451	37.944	7.78	8.01	284.02	15.79	1	8/1/2014	8:27:59.58
2003	RSRD19	-122.451	37.944	7.57	8.00	284.02	15.58	-122.451	37.944	7.78	8.00	283.97	15.78	1	8/1/2014	8:27:59.67
2004	RSRD19	-122.451	37.944	7.57	8.02	284.01	15.59	-122.451	37.944	7.78	8.02	284.01	15.79	1	8/1/2014	8:27:59.77
2005	RSRD19	-122.451	37.944	7.57	8.00	284.01	15.57	-122.451	37.944	7.78	8.00	283.94	15.77	1	8/1/2014	8:27:59.87
2006	RSRD19	-122.451	37.944	7.57	8.00	283.96	15.57	-122.451	37.944	7.78	8.00	283.92	15.77	1	8/1/2014	8:27:59.98
2007	RSRD19	-122.451	37.944	7.57	8.00	283.98	15.58	-122.451	37.944	7.78	8.00	283.92	15.78	1	8/1/2014	8:28:00.07
2008	RSRD19	-122.451	37.944	7.57	8.02	284.00	15.59	-122.451	37.944	7.78	8.02	283.91	15.79	1	8/1/2014	8:28:00.17
2009	RSRD19	-122.451	37.944	7.57	8.06	283.96	15.63	-122.451	37.944	7.78	8.06	283.91	15.83	1	8/1/2014	8:28:00.27
2010	RSRD19	-122.451	37.944	7.63	8.01	283.96	15.64	-122.451	37.944	7.78	8.01	283.91	15.79	1	8/1/2014	8:28:00.38
2011	RSRD19	-122.451	37.944	7.57	8.00	283.93	15.57	-122.451	37.944	7.78	8.00	283.89	15.77	1	8/1/2014	8:28:00.47
2012	RSRD19	-122.451	37.944	7.57	8.01	283.93	15.59	-122.451	37.944	7.78	8.01	283.84	15.79	1	8/1/2014	8:28:00.57
2013	RSRD19	-122.451	37.944	7.57	8.04	283.95	15.61	-122.451	37.944	7.74	8.04	283.88	15.78	1	8/1/2014	8:28:00.67
2014	RSRD19	-122.451	37.944	7.57	8.03	283.95	15.61	-122.451	37.944	7.78	8.03	283.86	15.81	1	8/1/2014	8:28:00.78
2015	RSRD19	-122.451	37.944	7.57	8.05	283.93	15.62	-122.451	37.944	7.74	8.05	283.88	15.79	1	8/1/2014	8:28:00.87
2016	RSRD19	-122.451	37.944	7.57	8.06	283.92	15.63	-122.451	37.944	7.78	8.06	283.85	15.83	1	8/1/2014	8:28:00.97
2017	RSRD19	-122.451	37.944	7.57	8.09	283.87	15.66	-122.451	37.944	7.74	8.09	283.85	15.83	1	8/1/2014	8:28:01.08
2018	RSRD19	-122.451	37.944	7.57	8.03	283.94	15.61	-122.451	37.944	7.74	8.03	283.83	15.78	1	8/1/2014	8:28:01.18
2019	RSRD19	-122.451	37.944	7.57	8.04	283.89	15.61	-122.451	37.944	7.74	8.04	283.85	15.78	1	8/1/2014	8:28:01.27
2020	RSRD19	-122.451	37.944	7.57	8.06	283.87	15.63	-122.451	37.944	7.74	8.06	283.82	15.80	1	8/1/2014	8:28:01.37
2021	RSRD19	-122.451	37.944	7.57	8.07	283.87	15.65	-122.451	37.944	7.74	8.07	283.85	15.81	1	8/1/2014	8:28:01.48
2022	RSRD19	-122.451	37.944	7.57	8.07	283.89	15.65	-122.451	37.944	7.74	8.07	283.84	15.81	1	8/1/2014	8:28:01.58
2023	RSRD19	-122.451	37.944	7.54	8.08	283.86	15.62	-122.451	37.944	7.74	8.08	283.82	15.82	1	8/1/2014	8:28:01.67
2024	RSRD19	-122.451	37.944	7.57	8.09	283.82	15.66	-122.451	37.944	7.74	8.09	283.82	15.83	1	8/1/2014	8:28:01.77
2025	RSRD19	-122.451	37.944	7.54	8.10	283.83	15.64	-122.451	37.944	7.71	8.10	283.83	15.81	1	8/1/2014	8:28:01.88
2026	RSRD19	-122.451	37.944	7.57	8.11	283.83	15.68	-122.451	37.944	7.74	8.11	283.80	15.85	1	8/1/2014	8:28:01.98

2027	RSRD19	-122.451	37.944	7.57	8.10	283.83	15.67	-122.451	37.944	7.74	8.10	283.81	15.84	1	8/1/2014	8:28:02.07
2028	RSRD19	-122.451	37.944	7.63	8.10	283.81	15.73	-122.451	37.944	7.74	8.10	283.81	15.85	1	8/1/2014	8:28:02.17
2029	RSRD19	-122.451	37.944	7.57	8.14	283.80	15.72	-122.451	37.944	7.74	8.14	283.77	15.88	1	8/1/2014	8:28:02.28
2030	RSRD19	-122.451	37.944	7.57	8.12	283.80	15.69	-122.451	37.944	7.78	8.12	283.78	15.90	1	8/1/2014	8:28:02.38
2031	RSRD19	-122.451	37.944	7.57	8.15	283.76	15.72	-122.451	37.944	7.74	8.15	283.78	15.89	1	8/1/2014	8:28:02.47
2032	RSRD19	-122.451	37.944	7.57	8.09	283.78	15.66	-122.451	37.944	7.78	8.09	283.74	15.86	1	8/1/2014	8:28:02.57
2033	RSRD19	-122.451	37.944	7.57	8.10	283.73	15.68	-122.451	37.944	7.74	8.10	283.75	15.85	1	8/1/2014	8:28:02.68
2034	RSRD19	-122.451	37.944	7.57	8.15	283.71	15.72	-122.451	37.944	7.78	8.15	283.74	15.93	1	8/1/2014	8:28:02.78
2035	RSRD19	-122.451	37.944	7.57	8.11	283.71	15.68	-122.451	37.944	7.74	8.11	283.72	15.85	1	8/1/2014	8:28:02.87
2036	RSRD19	-122.451	37.944	7.57	8.10	283.70	15.67	-122.451	37.944	7.78	8.10	283.75	15.87	1	8/1/2014	8:28:02.97
2037	RSRD19	-122.451	37.944	7.57	8.12	283.70	15.69	-122.451	37.944	7.78	8.12	283.73	15.90	1	8/1/2014	8:28:03.08
2038	RSRD19	-122.451	37.944	7.57	8.10	283.68	15.67	-122.451	37.944	7.78	8.10	283.72	15.87	1	8/1/2014	8:28:03.18
2039	RSRD19	-122.451	37.944	7.57	8.16	283.68	15.73	-122.451	37.944	7.78	8.16	283.72	15.93	1	8/1/2014	8:28:03.27
2040	RSRD19	-122.451	37.944	7.63	8.08	283.68	15.71	-122.451	37.944	7.78	8.08	283.72	15.86	1	8/1/2014	8:28:03.37
2041	RSRD19	-122.451	37.944	7.57	8.07	283.70	15.65	-122.451	37.944	7.78	8.07	283.72	15.85	1	8/1/2014	8:28:03.48
2042	RSRD19	-122.451	37.944	7.63	8.07	283.72	15.70	-122.451	37.944	7.78	8.07	283.74	15.85	1	8/1/2014	8:28:03.58
2043	RSRD19	-122.451	37.944	7.57	8.13	283.67	15.70	-122.451	37.944	7.74	8.13	283.69	15.87	1	8/1/2014	8:28:03.67
2044	RSRD19	-122.451	37.944	7.63	8.06	283.72	15.68	-122.451	37.944	7.78	8.06	283.72	15.83	1	8/1/2014	8:28:03.77
2045	RSRD19	-122.451	37.944	7.57	8.07	283.69	15.64	-122.451	37.944	7.74	8.07	283.76	15.81	1	8/1/2014	8:28:03.88
2046	RSRD19	-122.451	37.944	7.63	8.06	283.71	15.68	-122.451	37.944	7.78	8.06	283.71	15.83	1	8/1/2014	8:28:03.98
2047	RSRD19	-122.451	37.944	7.57	8.03	283.69	15.61	-122.451	37.944	7.74	8.03	283.69	15.78	1	8/1/2014	8:28:04.07
2048	RSRD19	-122.451	37.944	7.57	8.03	283.71	15.61	-122.451	37.944	7.74	8.03	283.74	15.78	1	8/1/2014	8:28:04.17
2049	RSRD19	-122.451	37.944	7.57	8.07	283.71	15.65	-122.451	37.944	7.74	8.07	283.68	15.81	1	8/1/2014	8:28:04.28
2050	RSRD19	-122.451	37.944	7.63	8.05	283.66	15.68	-122.451	37.944	7.74	8.05	283.66	15.79	1	8/1/2014	8:28:04.38
2051	RSRD19	-122.451	37.944	7.54	8.07	283.68	15.61	-122.451	37.944	7.74	8.07	283.66	15.81	1	8/1/2014	8:28:04.47
2052	RSRD19	-122.451	37.944	7.57	8.03	283.66	15.60	-122.451	37.944	7.74	8.03	283.62	15.77	1	8/1/2014	8:28:04.57
2053	RSRD19	-122.451	37.944	7.57	8.04	283.68	15.61	-122.451	37.944	7.74	8.04	283.64	15.78	1	8/1/2014	8:28:04.68
2054	RSRD19	-122.451	37.944	7.63	8.10	283.61	15.73	-122.451	37.944	7.74	8.10	283.59	15.85	1	8/1/2014	8:28:04.78
2055	RSRD19	-122.451	37.944	7.57	8.07	283.66	15.64	-122.451	37.944	7.74	8.07	283.62	15.81	1	8/1/2014	8:28:04.87
2056	RSRD19	-122.451	37.944	7.63	8.06	283.61	15.68	-122.451	37.944	7.74	8.06	283.57	15.80	1	8/1/2014	8:28:04.97
2057	RSRD19	-122.451	37.944	7.57	8.07	283.61	15.64	-122.451	37.944	7.71	8.07	283.57	15.77	1	8/1/2014	8:28:05.08
2058	RSRD19	-122.451	37.944	7.63	8.07	283.59	15.70	-122.451	37.944	7.74	8.07	283.62	15.81	1	8/1/2014	8:28:05.18
2059	RSRD19	-122.451	37.944	7.57	8.10	283.59	15.67	-122.451	37.944	7.71	8.10	283.62	15.81	1	8/1/2014	8:28:05.27

2060	RSRD19	-122.451	37.944	7.57	8.07	283.57	15.65	-122.451	37.944	7.74	8.07	283.57	15.81	1	8/1/2014	8:28:05.37
2061	RSRD19	-122.451	37.944	7.57	8.08	283.52	15.66	-122.451	37.944	7.74	8.08	283.55	15.82	1	8/1/2014	8:28:05.48
2062	RSRD19	-122.451	37.944	7.57	8.07	283.55	15.65	-122.451	37.944	7.74	8.07	283.54	15.81	1	8/1/2014	8:28:05.58
2063	RSRD19	-122.451	37.944	7.57	8.06	283.53	15.63	-122.451	37.944	7.74	8.06	283.52	15.80	1	8/1/2014	8:28:05.67
2064	RSRD19	-122.451	37.944	7.63	8.06	283.45	15.68	-122.451	37.944	7.74	8.06	283.50	15.80	1	8/1/2014	8:28:05.77
2065	RSRD19	-122.451	37.944	7.57	8.07	283.46	15.64	-122.451	37.944	7.71	8.07	283.46	15.77	1	8/1/2014	8:28:05.88
2066	RSRD19	-122.451	37.944	7.57	8.06	283.43	15.63	-122.451	37.944	7.74	8.06	283.46	15.80	1	8/1/2014	8:28:05.98
2067	RSRD19	-122.451	37.944	7.57	8.04	283.41	15.61	-122.451	37.944	7.74	8.04	283.46	15.78	1	8/1/2014	8:28:06.07
2068	RSRD19	-122.451	37.944	7.63	8.02	283.41	15.64	-122.451	37.944	7.74	8.02	283.43	15.76	1	8/1/2014	8:28:06.17
2069	RSRD19	-122.451	37.944	7.57	8.03	283.39	15.61	-122.451	37.944	7.74	8.03	283.44	15.78	1	8/1/2014	8:28:06.28
2070	RSRD19	-122.451	37.944	7.63	8.03	283.37	15.65	-122.451	37.944	7.74	8.03	283.44	15.77	1	8/1/2014	8:28:06.38
2071	RSRD19	-122.451	37.944	7.57	8.00	283.33	15.58	-122.451	37.944	7.74	8.00	283.37	15.74	1	8/1/2014	8:28:06.47
2072	RSRD19	-122.451	37.944	7.63	7.98	283.37	15.61	-122.451	37.944	7.78	7.98	283.42	15.76	1	8/1/2014	8:28:06.57
2073	RSRD19	-122.451	37.944	7.63	7.97	283.37	15.59	-122.451	37.944	7.78	7.97	283.42	15.74	1	8/1/2014	8:28:06.68
2074	RSRD19	-122.451	37.944	7.63	7.96	283.35	15.58	-122.451	37.944	7.78	7.96	283.42	15.73	1	8/1/2014	8:28:06.78
2075	RSRD19	-122.451	37.944	7.63	7.96	283.37	15.58	-122.451	37.944	7.78	7.96	283.42	15.73	1	8/1/2014	8:28:06.87
2076	RSRD19	-122.451	37.944	7.63	7.94	283.35	15.57	-122.451	37.944	7.78	7.94	283.44	15.72	1	8/1/2014	8:28:06.97
2077	RSRD19	-122.451	37.944	7.63	7.94	283.33	15.57	-122.451	37.944	7.78	7.94	283.45	15.72	1	8/1/2014	8:28:07.08
2078	RSRD19	-122.451	37.944	7.63	7.98	283.38	15.61	-122.451	37.944	7.83	7.98	283.42	15.81	1	8/1/2014	8:28:07.18
2079	RSRD19	-122.451	37.944	7.63	7.94	283.33	15.57	-122.451	37.944	7.78	7.94	283.47	15.72	1	8/1/2014	8:28:07.27
2080	RSRD19	-122.451	37.944	7.63	7.97	283.36	15.59	-122.451	37.944	7.83	7.97	283.45	15.79	1	8/1/2014	8:28:07.37
2081	RSRD19	-122.451	37.944	7.63	7.97	283.36	15.59	-122.451	37.944	7.78	7.97	283.47	15.74	1	8/1/2014	8:28:07.48
2082	RSRD19	-122.451	37.944	7.63	7.96	283.34	15.58	-122.451	37.944	7.83	7.96	283.47	15.79	1	8/1/2014	8:28:07.58
2083	RSRD19	-122.451	37.944	7.63	7.96	283.36	15.58	-122.451	37.944	7.83	7.96	283.48	15.79	1	8/1/2014	8:28:07.67
2084	RSRD19	-122.451	37.944	7.63	7.95	283.32	15.57	-122.451	37.944	7.83	7.95	283.45	15.78	1	8/1/2014	8:28:07.77
2085	RSRD19	-122.451	37.944	7.63	7.93	283.37	15.55	-122.451	37.944	7.83	7.93	283.45	15.75	1	8/1/2014	8:28:07.88
2086	RSRD19	-122.451	37.944	7.63	7.93	283.35	15.56	-122.451	37.944	7.86	7.93	283.44	15.79	1	8/1/2014	8:28:07.98
2087	RSRD19	-122.451	37.944	7.63	7.93	283.35	15.55	-122.451	37.944	7.86	7.93	283.50	15.79	1	8/1/2014	8:28:08.07
2088	RSRD19	-122.451	37.944	7.66	7.89	283.35	15.55	-122.451	37.944	7.86	7.89	283.45	15.76	1	8/1/2014	8:28:08.17
2089	RSRD19	-122.451	37.944	7.66	7.89	283.37	15.55	-122.451	37.944	7.86	7.89	283.44	15.76	1	8/1/2014	8:28:08.28
2090	RSRD19	-122.451	37.944	7.66	7.88	283.35	15.54	-122.451	37.944	7.86	7.88	283.48	15.74	1	8/1/2014	8:28:08.38
2091	RSRD19	-122.451	37.944	7.63	7.91	283.40	15.54	-122.451	37.944	7.86	7.91	283.46	15.77	1	8/1/2014	8:28:08.47
2092	RSRD19	-122.451	37.944	7.66	7.91	283.36	15.57	-122.451	37.944	7.86	7.91	283.47	15.77	1	8/1/2014	8:28:08.57

2093	RSRD19	-122.451	37.944	7.66	7.89	283.40	15.55	-122.451	37.944	7.86	7.89	283.49	15.76	1	8/1/2014 8:28:08.68
2094	RSRD19	-122.451	37.944	7.66	7.92	283.38	15.58	-122.451	37.944	7.86	7.92	283.47	15.78	1	8/1/2014 8:28:08.78
2095	RSRD19	-122.451	37.944	7.66	7.91	283.39	15.57	-122.451	37.944	7.86	7.91	283.47	15.77	1	8/1/2014 8:28:08.87
2096	RSRD19	-122.451	37.944	7.66	7.97	283.39	15.63	-122.451	37.944	7.86	7.97	283.45	15.83	1	8/1/2014 8:28:08.97
2097	RSRD19	-122.451	37.944	7.66	7.93	283.37	15.59	-122.451	37.944	7.86	7.93	283.47	15.79	1	8/1/2014 8:28:09.08
2098	RSRD19	-122.451	37.944	7.66	7.95	283.37	15.61	-122.451	37.944	7.86	7.95	283.46	15.81	1	8/1/2014 8:28:09.18
2099	RSRD19	-122.451	37.944	7.66	7.96	283.35	15.62	-122.451	37.944	7.91	7.96	283.45	15.87	1	8/1/2014 8:28:09.27
2100	RSRD19	-122.451	37.944	7.66	7.98	283.33	15.64	-122.451	37.944	7.91	7.98	283.42	15.89	1	8/1/2014 8:28:09.37
2101	RSRD19	-122.451	37.944	7.66	7.95	283.31	15.61	-122.451	37.944	7.91	7.95	283.44	15.86	1	8/1/2014 8:28:09.48
2102	RSRD19	-122.451	37.944	7.66	7.94	283.32	15.60	-122.451	37.944	7.91	7.94	283.42	15.85	1	8/1/2014 8:28:09.58
2103	RSRD19	-122.451	37.944	7.66	7.93	283.34	15.59	-122.451	37.944	7.91	7.93	283.47	15.84	1	8/1/2014 8:28:09.67
2104	RSRD19	-122.451	37.944	7.71	7.93	283.30	15.64	-122.451	37.944	7.91	7.93	283.36	15.84	1	8/1/2014 8:28:09.77
2105	RSRD19	-122.451	37.944	7.66	7.92	283.30	15.58	-122.451	37.944	7.91	7.92	283.46	15.83	1	8/1/2014 8:28:09.88
2106	RSRD19	-122.451	37.944	7.71	7.93	283.35	15.64	-122.451	37.944	7.91	7.93	283.42	15.84	1	8/1/2014 8:28:09.98
2107	RSRD19	-122.451	37.944	7.66	7.92	283.29	15.58	-122.451	37.944	7.91	7.92	283.40	15.83	1	8/1/2014 8:28:10.07
2108	RSRD19	-122.451	37.944	7.71	7.93	283.31	15.64	-122.451	37.944	7.91	7.93	283.42	15.84	1	8/1/2014 8:28:10.17
2109	RSRD19	-122.451	37.944	7.66	7.93	283.34	15.59	-122.451	37.944	7.86	7.93	283.41	15.79	1	8/1/2014 8:28:10.28
2110	RSRD19	-122.451	37.944	7.66	7.94	283.30	15.60	-122.451	37.944	7.91	7.94	283.40	15.85	1	8/1/2014 8:28:10.38
2111	RSRD19	-122.451	37.944	7.66	7.97	283.34	15.63	-122.451	37.944	7.91	7.97	283.41	15.88	1	8/1/2014 8:28:10.47
2112	RSRD19	-122.451	37.944	7.71	7.94	283.33	15.65	-122.451	37.944	7.91	7.94	283.39	15.85	1	8/1/2014 8:28:10.57
2113	RSRD19	-122.451	37.944	7.66	7.93	283.31	15.59	-122.451	37.944	7.86	7.93	283.37	15.79	1	8/1/2014 8:28:10.68
2114	RSRD19	-122.451	37.944	7.71	7.93	283.29	15.64	-122.451	37.944	7.91	7.93	283.35	15.84	1	8/1/2014 8:28:10.78
2115	RSRD19	-122.451	37.944	7.71	7.93	283.27	15.64	-122.451	37.944	7.91	7.93	283.36	15.84	1	8/1/2014 8:28:10.87
2116	RSRD19	-122.451	37.944	7.66	7.92	283.27	15.58	-122.451	37.944	7.95	7.92	283.36	15.86	1	8/1/2014 8:28:10.97
2117	RSRD19	-122.451	37.944	7.71	7.91	283.28	15.62	-122.451	37.944	7.91	7.91	283.37	15.82	1	8/1/2014 8:28:11.08
2118	RSRD19	-122.451	37.944	7.71	7.92	283.28	15.63	-122.451	37.944	7.91	7.92	283.37	15.83	1	8/1/2014 8:28:11.18
2119	RSRD19	-122.451	37.944	7.66	7.91	283.26	15.57	-122.451	37.944	7.95	7.91	283.37	15.86	1	8/1/2014 8:28:11.27
2120	RSRD19	-122.451	37.944	7.71	7.90	283.24	15.61	-122.451	37.944	7.95	7.90	283.35	15.85	1	8/1/2014 8:28:11.37
2121	RSRD19	-122.451	37.944	7.71	7.89	283.25	15.61	-122.451	37.944	7.91	7.89	283.35	15.81	1	8/1/2014 8:28:11.48
2122	RSRD19	-122.451	37.944	7.71	7.89	283.27	15.61	-122.451	37.944	7.95	7.89	283.36	15.84	1	8/1/2014 8:28:11.58
2123	RSRD19	-122.451	37.944	7.71	7.89	283.21	15.61	-122.451	37.944	7.91	7.89	283.33	15.81	1	8/1/2014 8:28:11.67
2124	RSRD19	-122.451	37.944	7.71	7.89	283.22	15.60	-122.451	37.944	7.95	7.89	283.33	15.83	1	8/1/2014 8:28:11.77
2125	RSRD19	-122.451	37.944	7.71	7.87	283.24	15.58	-122.451	37.944	7.95	7.87	283.33	15.82	1	8/1/2014 8:28:11.88

2126	RSRD19	-122.451	37.944	7.71	7.96	283.21	15.67	-122.451	37.944	8.00	7.96	283.34	15.95	1	8/1/2014	8:28:11.98
2127	RSRD19	-122.451	37.944	7.71	7.86	283.19	15.57	-122.451	37.944	7.95	7.86	283.32	15.80	1	8/1/2014	8:28:12.07
2128	RSRD19	-122.451	37.944	7.71	7.84	283.21	15.55	-122.451	37.944	8.00	7.84	283.32	15.83	1	8/1/2014	8:28:12.17
2129	RSRD19	-122.451	37.944	7.71	7.88	283.20	15.59	-122.451	37.944	7.95	7.88	283.32	15.82	1	8/1/2014	8:28:12.28
2130	RSRD19	-122.451	37.944	7.74	7.82	283.22	15.56	-122.451	37.944	8.00	7.82	283.33	15.81	1	8/1/2014	8:28:12.38
2131	RSRD19	-122.451	37.944	7.71	7.80	283.25	15.51	-122.451	37.944	8.00	7.80	283.33	15.80	1	8/1/2014	8:28:12.47
2132	RSRD19	-122.451	37.944	7.74	7.84	283.23	15.58	-122.451	37.944	8.00	7.84	283.34	15.83	1	8/1/2014	8:28:12.57
2133	RSRD19	-122.451	37.944	7.74	7.83	283.28	15.57	-122.451	37.944	8.00	7.83	283.36	15.82	1	8/1/2014	8:28:12.68
2134	RSRD19	-122.451	37.944	7.74	7.85	283.24	15.59	-122.451	37.944	8.00	7.85	283.37	15.84	1	8/1/2014	8:28:12.78
2135	RSRD19	-122.451	37.944	7.71	7.86	283.26	15.57	-122.451	37.944	8.00	7.86	283.39	15.86	1	8/1/2014	8:28:12.87
2136	RSRD19	-122.451	37.944	7.74	7.85	283.27	15.59	-122.451	37.944	8.00	7.85	283.40	15.84	1	8/1/2014	8:28:12.97
2137	RSRD19	-122.451	37.944	7.74	7.85	283.30	15.59	-122.451	37.944	8.00	7.85	283.36	15.84	1	8/1/2014	8:28:13.08
2138	RSRD19	-122.451	37.944	7.74	7.84	283.28	15.59	-122.451	37.944	8.00	7.84	283.37	15.84	1	8/1/2014	8:28:13.18
2139	RSRD19	-122.451	37.944	7.74	7.86	283.30	15.60	-122.451	37.944	8.00	7.86	283.39	15.85	1	8/1/2014	8:28:13.27
2140	RSRD19	-122.451	37.944	7.74	7.88	283.31	15.62	-122.451	37.944	8.00	7.88	283.37	15.88	1	8/1/2014	8:28:13.37
2141	RSRD19	-122.451	37.944	7.71	7.87	283.29	15.58	-122.451	37.944	8.00	7.87	283.38	15.87	1	8/1/2014	8:28:13.48
2142	RSRD19	-122.451	37.944	7.74	7.85	283.34	15.59	-122.451	37.944	8.00	7.85	283.40	15.84	1	8/1/2014	8:28:13.58
2143	RSRD19	-122.451	37.944	7.71	7.84	283.32	15.55	-122.451	37.944	8.00	7.84	283.39	15.83	1	8/1/2014	8:28:13.67
2144	RSRD19	-122.451	37.944	7.80	7.84	283.32	15.64	-122.451	37.944	8.03	7.84	283.41	15.87	1	8/1/2014	8:28:13.77
2145	RSRD19	-122.451	37.944	7.74	7.81	283.31	15.55	-122.451	37.944	8.00	7.81	283.41	15.81	1	8/1/2014	8:28:13.88
2146	RSRD19	-122.451	37.944	7.74	7.80	283.31	15.55	-122.451	37.944	8.03	7.80	283.40	15.83	1	8/1/2014	8:28:13.98
2147	RSRD19	-122.451	37.944	7.74	7.82	283.32	15.56	-122.451	37.944	8.03	7.82	283.43	15.85	1	8/1/2014	8:28:14.07
2148	RSRD19	-122.451	37.944	7.80	7.79	283.32	15.59	-122.451	37.944	8.03	7.79	283.41	15.82	1	8/1/2014	8:28:14.17
2149	RSRD19	-122.451	37.944	7.74	7.81	283.33	15.55	-122.451	37.944	8.03	7.81	283.41	15.84	1	8/1/2014	8:28:14.28
2150	RSRD19	-122.451	37.944	7.80	7.83	283.33	15.62	-122.451	37.944	8.08	7.83	283.46	15.91	1	8/1/2014	8:28:14.38
2151	RSRD19	-122.451	37.944	7.74	7.80	283.33	15.55	-122.451	37.944	8.03	7.80	283.40	15.83	1	8/1/2014	8:28:14.47
2152	RSRD19	-122.451	37.944	7.80	7.80	283.32	15.60	-122.451	37.944	8.03	7.80	283.40	15.83	1	8/1/2014	8:28:14.57
2153	RSRD19	-122.451	37.944	7.74	7.84	283.30	15.58	-122.451	37.944	8.00	7.84	283.43	15.83	1	8/1/2014	8:28:14.68
2154	RSRD19	-122.451	37.944	7.80	7.84	283.37	15.63	-122.451	37.944	8.03	7.84	283.43	15.86	1	8/1/2014	8:28:14.78
2155	RSRD19	-122.451	37.944	7.74	7.85	283.35	15.59	-122.451	37.944	8.00	7.85	283.44	15.84	1	8/1/2014	8:28:14.87
2156	RSRD19	-122.451	37.944	7.74	7.85	283.36	15.59	-122.451	37.944	8.03	7.85	283.44	15.88	1	8/1/2014	8:28:14.97
2157	RSRD19	-122.451	37.944	7.74	7.84	283.38	15.59	-122.451	37.944	8.00	7.84	283.45	15.84	1	8/1/2014	8:28:15.08
2158	RSRD19	-122.451	37.944	7.80	7.86	283.39	15.66	-122.451	37.944	8.00	7.86	283.46	15.86	1	8/1/2014	8:28:15.18

2159	RSRD19	-122.451	37.944	7.74	7.87	283.35	15.62	-122.451	37.944	8.00	7.87	283.43	15.87	1	8/1/2014	8:28:15.27
2160	RSRD19	-122.451	37.944	7.80	7.89	283.35	15.69	-122.451	37.944	8.00	7.89	283.42	15.88	1	8/1/2014	8:28:15.37
2161	RSRD19	-122.451	37.944	7.74	7.86	283.33	15.60	-122.451	37.944	8.00	7.86	283.39	15.85	1	8/1/2014	8:28:15.48
2162	RSRD19	-122.451	37.944	7.80	7.85	283.34	15.64	-122.451	37.944	8.03	7.85	283.40	15.88	1	8/1/2014	8:28:15.58
2163	RSRD19	-122.451	37.944	7.74	7.86	283.34	15.60	-122.451	37.944	8.03	7.86	283.39	15.89	1	8/1/2014	8:28:15.67
2164	RSRD19	-122.451	37.944	7.80	7.86	283.32	15.65	-122.451	37.944	8.03	7.86	283.41	15.89	1	8/1/2014	8:28:15.77
2165	RSRD19	-122.451	37.944	7.74	7.86	283.28	15.60	-122.451	37.944	8.03	7.86	283.35	15.89	1	8/1/2014	8:28:15.88
2166	RSRD19	-122.451	37.944	7.80	7.82	283.31	15.62	-122.451	37.944	8.03	7.82	283.37	15.85	1	8/1/2014	8:28:15.98
2167	RSRD19	-122.451	37.944	7.74	7.81	283.27	15.55	-122.451	37.944	8.08	7.81	283.35	15.89	1	8/1/2014	8:28:16.07
2168	RSRD19	-122.451	37.944	7.80	7.81	283.25	15.61	-122.451	37.944	8.08	7.81	283.34	15.89	1	8/1/2014	8:28:16.17
2169	RSRD19	-122.451	37.944	7.80	7.81	283.30	15.61	-122.451	37.944	8.08	7.81	283.34	15.89	1	8/1/2014	8:28:16.28
2170	RSRD19	-122.451	37.944	7.80	7.79	283.26	15.59	-122.451	37.944	8.08	7.79	283.39	15.88	1	8/1/2014	8:28:16.38
2171	RSRD19	-122.451	37.944	7.80	7.80	283.30	15.60	-122.451	37.944	8.08	7.80	283.37	15.88	1	8/1/2014	8:28:16.47
2172	RSRD19	-122.451	37.944	7.80	7.79	283.26	15.59	-122.451	37.944	8.08	7.79	283.38	15.88	1	8/1/2014	8:28:16.57
2173	RSRD19	-122.451	37.944	7.80	7.82	283.29	15.62	-122.451	37.944	8.08	7.82	283.40	15.90	1	8/1/2014	8:28:16.68
2174	RSRD19	-122.451	37.944	7.83	7.80	283.27	15.63	-122.451	37.944	8.08	7.80	283.38	15.88	1	8/1/2014	8:28:16.78
2175	RSRD19	-122.451	37.944	7.80	7.79	283.28	15.59	-122.451	37.944	8.08	7.79	283.41	15.88	1	8/1/2014	8:28:16.87
2176	RSRD19	-122.451	37.944	7.83	7.79	283.26	15.62	-122.451	37.944	8.11	7.79	283.37	15.90	1	8/1/2014	8:28:16.97
2177	RSRD19	-122.451	37.944	7.80	7.79	283.24	15.59	-122.451	37.944	8.11	7.79	283.37	15.90	1	8/1/2014	8:28:17.08
2178	RSRD19	-122.451	37.944	7.83	7.79	283.27	15.63	-122.451	37.944	8.11	7.79	283.40	15.91	1	8/1/2014	8:28:17.18
2179	RSRD19	-122.451	37.944	7.83	7.75	283.22	15.58	-122.451	37.944	8.11	7.75	283.38	15.86	1	8/1/2014	8:28:17.27
2180	RSRD19	-122.451	37.944	7.83	7.73	283.21	15.57	-122.451	37.944	8.15	7.73	283.40	15.88	1	8/1/2014	8:28:17.37
2181	RSRD19	-122.451	37.944	7.80	7.72	283.25	15.51	-122.451	37.944	8.15	7.72	283.40	15.87	1	8/1/2014	8:28:17.48
2182	RSRD19	-122.451	37.944	7.83	7.71	283.21	15.54	-122.451	37.944	8.15	7.71	283.37	15.86	1	8/1/2014	8:28:17.58
2183	RSRD19	-122.451	37.944	7.83	7.72	283.26	15.55	-122.451	37.944	8.15	7.72	283.37	15.87	1	8/1/2014	8:28:17.67
2184	RSRD19	-122.451	37.944	7.86	7.73	283.26	15.60	-122.451	37.944	8.15	7.73	283.40	15.88	1	8/1/2014	8:28:17.77
2185	RSRD19	-122.451	37.944	7.80	7.72	283.27	15.52	-122.451	37.944	8.15	7.72	283.40	15.87	1	8/1/2014	8:28:17.88
2186	RSRD19	-122.451	37.944	7.83	7.72	283.29	15.55	-122.451	37.944	8.15	7.72	283.38	15.87	1	8/1/2014	8:28:17.98
2187	RSRD19	-122.451	37.944	7.83	7.72	283.29	15.55	-122.451	37.944	8.15	7.72	283.40	15.87	1	8/1/2014	8:28:18.07
2188	RSRD19	-122.451	37.944	7.83	7.71	283.32	15.54	-122.451	37.944	8.15	7.71	283.39	15.86	1	8/1/2014	8:28:18.17
2189	RSRD19	-122.451	37.944	7.83	7.71	283.35	15.54	-122.451	37.944	8.11	7.71	283.41	15.82	1	8/1/2014	8:28:18.28
2190	RSRD19	-122.451	37.944	7.86	7.72	283.33	15.58	-122.451	37.944	8.15	7.72	283.42	15.87	1	8/1/2014	8:28:18.38
2191	RSRD19	-122.451	37.944	7.83	7.70	283.35	15.53	-122.451	37.944	8.11	7.70	283.41	15.81	1	8/1/2014	8:28:18.47

2192	RSRD19	-122.451	37.944	7.83	7.72	283.38	15.55	-122.451	37.944	8.15	7.72	283.42	15.87	1	8/1/2014	8:28:18.57
2193	RSRD19	-122.451	37.944	7.83	7.70	283.36	15.53	-122.451	37.944	8.11	7.70	283.45	15.82	1	8/1/2014	8:28:18.68
2194	RSRD19	-122.451	37.944	7.86	7.67	283.36	15.54	-122.451	37.944	8.15	7.67	283.40	15.82	1	8/1/2014	8:28:18.78
2195	RSRD19	-122.451	37.944	7.83	7.71	283.36	15.54	-122.451	37.944	8.11	7.71	283.41	15.82	1	8/1/2014	8:28:18.87
2196	RSRD19	-122.451	37.944	7.86	7.67	283.36	15.54	-122.451	37.944	8.15	7.67	283.41	15.82	1	8/1/2014	8:28:18.97
2197	RSRD19	-122.451	37.944	7.83	7.66	283.34	15.49	-122.451	37.944	8.15	7.66	283.41	15.81	1	8/1/2014	8:28:19.08
2198	RSRD19	-122.451	37.944	7.86	7.66	283.34	15.53	-122.451	37.944	8.15	7.66	283.37	15.81	1	8/1/2014	8:28:19.18
2199	RSRD19	-122.451	37.944	7.83	7.67	283.33	15.50	-122.451	37.944	8.15	7.67	283.37	15.82	1	8/1/2014	8:28:19.27
2200	RSRD19	-122.451	37.944	7.83	7.68	283.35	15.51	-122.451	37.944	8.15	7.68	283.35	15.83	1	8/1/2014	8:28:19.37
2201	RSRD19	-122.451	37.944	7.83	7.68	283.31	15.51	-122.451	37.944	8.11	7.68	283.38	15.79	1	8/1/2014	8:28:19.48
2202	RSRD19	-122.451	37.944	7.86	7.71	283.31	15.57	-122.451	37.944	8.11	7.71	283.33	15.82	1	8/1/2014	8:28:19.58
2203	RSRD19	-122.451	37.944	7.83	7.70	283.32	15.53	-122.451	37.944	8.11	7.70	283.34	15.82	1	8/1/2014	8:28:19.67
2204	RSRD19	-122.451	37.944	7.86	7.72	283.32	15.58	-122.451	37.944	8.15	7.72	283.34	15.87	1	8/1/2014	8:28:19.77
2205	RSRD19	-122.451	37.944	7.83	7.68	283.32	15.51	-122.451	37.944	8.11	7.68	283.34	15.79	1	8/1/2014	8:28:19.88
2206	RSRD19	-122.451	37.944	7.86	7.65	283.28	15.51	-122.451	37.944	8.15	7.65	283.30	15.80	1	8/1/2014	8:28:19.98
2207	RSRD19	-122.451	37.944	7.83	7.64	283.30	15.47	-122.451	37.944	8.15	7.64	283.34	15.79	1	8/1/2014	8:28:20.07
2208	RSRD19	-122.451	37.944	7.86	7.60	283.28	15.47	-122.451	37.944	8.15	7.60	283.33	15.75	1	8/1/2014	8:28:20.17
2209	RSRD19	-122.451	37.944	7.86	7.59	283.28	15.45	-122.451	37.944	8.15	7.59	283.35	15.73	1	8/1/2014	8:28:20.28
2210	RSRD19	-122.451	37.944	7.86	7.55	283.28	15.41	-122.451	37.944	8.20	7.55	283.35	15.75	1	8/1/2014	8:28:20.38
2211	RSRD19	-122.451	37.944	7.86	7.53	283.31	15.40	-122.451	37.944	8.15	7.53	283.36	15.68	1	8/1/2014	8:28:20.47
2212	RSRD19	-122.451	37.944	7.86	7.51	283.29	15.38	-122.451	37.944	8.20	7.51	283.33	15.71	1	8/1/2014	8:28:20.57
2213	RSRD19	-122.451	37.944	7.83	7.50	283.29	15.33	-122.451	37.944	8.15	7.50	283.35	15.65	1	8/1/2014	8:28:20.68
2214	RSRD19	-122.451	37.944	7.86	7.53	283.29	15.39	-122.451	37.944	8.15	7.53	283.35	15.67	1	8/1/2014	8:28:20.78
2215	RSRD19	-122.451	37.944	7.83	7.52	283.31	15.35	-122.451	37.944	8.15	7.52	283.38	15.66	1	8/1/2014	8:28:20.87
2216	RSRD19	-122.451	37.944	7.92	7.53	283.29	15.44	-122.451	37.944	8.15	7.53	283.36	15.67	1	8/1/2014	8:28:20.97
2217	RSRD19	-122.451	37.944	7.86	7.56	283.32	15.43	-122.451	37.944	8.15	7.56	283.36	15.71	1	8/1/2014	8:28:21.08
2218	RSRD19	-122.451	37.944	7.86	7.56	283.27	15.43	-122.451	37.944	8.15	7.56	283.34	15.71	1	8/1/2014	8:28:21.18
2219	RSRD19	-122.451	37.944	7.86	7.65	283.25	15.51	-122.451	37.944	8.15	7.65	283.34	15.80	1	8/1/2014	8:28:21.27
2220	RSRD19	-122.451	37.944	7.86	7.59	283.28	15.46	-122.451	37.944	8.15	7.59	283.34	15.74	1	8/1/2014	8:28:21.37
2221	RSRD19	-122.451	37.944	7.86	7.59	283.21	15.46	-122.451	37.944	8.20	7.59	283.27	15.79	1	8/1/2014	8:28:21.48
2222	RSRD19	-122.451	37.944	7.92	7.59	283.21	15.51	-122.451	37.944	8.20	7.59	283.28	15.79	1	8/1/2014	8:28:21.58
2223	RSRD19	-122.451	37.944	7.86	7.64	283.21	15.50	-122.451	37.944	8.20	7.64	283.30	15.84	1	8/1/2014	8:28:21.67
2224	RSRD19	-122.451	37.944	7.86	7.58	283.19	15.44	-122.451	37.944	8.23	7.58	283.26	15.81	1	8/1/2014	8:28:21.77

2225	RSRD19	-122.451	37.944	7.86	7.58	283.19	15.44	-122.451	37.944	8.20	7.58	283.28	15.78	1	8/1/2014	8:28:21.88
2226	RSRD19	-122.451	37.944	7.92	7.57	283.19	15.49	-122.451	37.944	8.23	7.57	283.28	15.81	1	8/1/2014	8:28:21.98
2227	RSRD19	-122.451	37.944	7.86	7.59	283.15	15.45	-122.451	37.944	8.20	7.59	283.24	15.78	1	8/1/2014	8:28:22.07
2228	RSRD19	-122.451	37.944	7.92	7.56	283.19	15.48	-122.451	37.944	8.20	7.56	283.28	15.76	1	8/1/2014	8:28:22.17
2229	RSRD19	-122.451	37.944	7.92	7.64	283.15	15.55	-122.451	37.944	8.20	7.64	283.21	15.84	1	8/1/2014	8:28:22.28
2230	RSRD19	-122.451	37.944	7.92	7.55	283.13	15.46	-122.451	37.944	8.20	7.55	283.24	15.75	1	8/1/2014	8:28:22.38
2231	RSRD19	-122.451	37.944	7.92	7.55	283.17	15.46	-122.451	37.944	8.20	7.55	283.22	15.75	1	8/1/2014	8:28:22.47
2232	RSRD19	-122.451	37.944	7.92	7.56	283.15	15.47	-122.451	37.944	8.20	7.56	283.19	15.76	1	8/1/2014	8:28:22.57
2233	RSRD19	-122.451	37.944	7.92	7.54	283.11	15.46	-122.451	37.944	8.20	7.54	283.20	15.74	1	8/1/2014	8:28:22.68
2234	RSRD19	-122.451	37.944	7.92	7.54	283.15	15.46	-122.451	37.944	8.20	7.54	283.20	15.74	1	8/1/2014	8:28:22.78
2235	RSRD19	-122.451	37.944	7.92	7.54	283.13	15.46	-122.451	37.944	8.20	7.54	283.18	15.74	1	8/1/2014	8:28:22.87
2236	RSRD19	-122.451	37.944	7.92	7.54	283.11	15.46	-122.451	37.944	8.23	7.54	283.17	15.77	1	8/1/2014	8:28:22.97
2237	RSRD19	-122.451	37.944	7.86	7.56	283.13	15.42	-122.451	37.944	8.20	7.56	283.15	15.76	1	8/1/2014	8:28:23.08
2238	RSRD19	-122.451	37.944	7.92	7.56	283.13	15.48	-122.451	37.944	8.20	7.56	283.15	15.76	1	8/1/2014	8:28:23.18
2239	RSRD19	-122.451	37.944	7.86	7.59	283.15	15.45	-122.451	37.944	8.20	7.59	283.18	15.78	1	8/1/2014	8:28:23.27
2240	RSRD19	-122.451	37.944	7.92	7.63	283.11	15.54	-122.451	37.944	8.20	7.63	283.17	15.83	1	8/1/2014	8:28:23.37
2241	RSRD19	-122.451	37.944	7.86	7.65	283.15	15.51	-122.451	37.944	8.15	7.65	283.18	15.80	1	8/1/2014	8:28:23.48
2242	RSRD19	-122.451	37.944	7.92	7.65	283.13	15.57	-122.451	37.944	8.20	7.65	283.15	15.85	1	8/1/2014	8:28:23.58
2243	RSRD19	-122.451	37.944	7.92	7.67	283.15	15.59	-122.451	37.944	8.15	7.67	283.18	15.82	1	8/1/2014	8:28:23.67
2244	RSRD19	-122.451	37.944	7.92	7.70	283.17	15.62	-122.451	37.944	8.20	7.70	283.19	15.90	1	8/1/2014	8:28:23.77
2245	RSRD19	-122.451	37.944	7.92	7.69	283.13	15.60	-122.451	37.944	8.15	7.69	283.17	15.83	1	8/1/2014	8:28:23.88
2246	RSRD19	-122.451	37.944	7.92	7.69	283.15	15.60	-122.451	37.944	8.20	7.69	283.20	15.89	1	8/1/2014	8:28:23.98
2247	RSRD19	-122.451	37.944	7.92	7.69	283.11	15.60	-122.451	37.944	8.15	7.69	283.15	15.83	1	8/1/2014	8:28:24.07
2248	RSRD19	-122.451	37.944	7.95	7.70	283.15	15.65	-122.451	37.944	8.20	7.70	283.21	15.90	1	8/1/2014	8:28:24.17
2249	RSRD19	-122.451	37.944	7.92	7.68	283.11	15.60	-122.451	37.944	8.23	7.68	283.19	15.91	1	8/1/2014	8:28:24.28
2250	RSRD19	-122.451	37.944	7.95	7.66	283.15	15.62	-122.451	37.944	8.23	7.66	283.19	15.90	1	8/1/2014	8:28:24.38
2251	RSRD19	-122.451	37.944	7.92	7.66	283.11	15.58	-122.451	37.944	8.20	7.66	283.22	15.86	1	8/1/2014	8:28:24.47
2252	RSRD19	-122.451	37.944	7.95	7.64	283.15	15.59	-122.451	37.944	8.23	7.64	283.19	15.87	1	8/1/2014	8:28:24.57
2253	RSRD19	-122.451	37.944	7.92	7.66	283.12	15.57	-122.451	37.944	8.23	7.66	283.24	15.89	1	8/1/2014	8:28:24.68
2254	RSRD19	-122.451	37.944	7.95	7.64	283.14	15.59	-122.451	37.944	8.23	7.64	283.21	15.87	1	8/1/2014	8:28:24.78
2255	RSRD19	-122.451	37.944	7.95	7.64	283.10	15.59	-122.451	37.944	8.23	7.64	283.21	15.87	1	8/1/2014	8:28:24.87
2256	RSRD19	-122.451	37.944	7.95	7.64	283.12	15.59	-122.451	37.944	8.23	7.64	283.21	15.87	1	8/1/2014	8:28:24.97
2257	RSRD19	-122.451	37.944	7.95	7.63	283.14	15.58	-122.451	37.944	8.23	7.63	283.23	15.87	1	8/1/2014	8:28:25.08

2258	RSRD19	-122.451	37.944	7.95	7.64	283.12	15.59	-122.451	37.944	8.23	7.64	283.21	15.87	1	8/1/2014	8:28:25.18
2259	RSRD19	-122.451	37.944	7.95	7.66	283.10	15.62	-122.451	37.944	8.23	7.66	283.20	15.90	1	8/1/2014	8:28:25.27
2260	RSRD19	-122.451	37.944	7.95	7.62	283.12	15.57	-122.451	37.944	8.23	7.62	283.23	15.85	1	8/1/2014	8:28:25.37
2261	RSRD19	-122.451	37.944	7.95	7.68	283.12	15.63	-122.451	37.944	8.23	7.68	283.20	15.91	1	8/1/2014	8:28:25.48
2262	RSRD19	-122.451	37.944	7.95	7.63	283.07	15.58	-122.451	37.944	8.28	7.63	283.18	15.91	1	8/1/2014	8:28:25.58
2263	RSRD19	-122.451	37.944	7.95	7.62	283.09	15.57	-122.451	37.944	8.23	7.62	283.20	15.85	1	8/1/2014	8:28:25.67
2264	RSRD19	-122.451	37.944	7.95	7.63	283.07	15.58	-122.451	37.944	8.23	7.63	283.20	15.87	1	8/1/2014	8:28:25.77
2265	RSRD19	-122.451	37.944	7.95	7.63	283.07	15.58	-122.451	37.944	8.23	7.63	283.22	15.86	1	8/1/2014	8:28:25.88
2266	RSRD19	-122.451	37.944	7.95	7.62	283.07	15.57	-122.451	37.944	8.23	7.62	283.18	15.85	1	8/1/2014	8:28:25.98
2267	RSRD19	-122.451	37.944	7.95	7.61	283.00	15.56	-122.451	37.944	8.23	7.61	283.15	15.84	1	8/1/2014	8:28:26.07
2268	RSRD19	-122.451	37.944	7.95	7.64	283.07	15.59	-122.451	37.944	8.23	7.64	283.18	15.87	1	8/1/2014	8:28:26.17
2269	RSRD19	-122.451	37.944	7.95	7.63	283.02	15.58	-122.451	37.944	8.23	7.63	283.15	15.87	1	8/1/2014	8:28:26.28
2270	RSRD19	-122.451	37.944	7.95	7.63	283.02	15.58	-122.451	37.944	8.28	7.63	283.13	15.92	1	8/1/2014	8:28:26.38
2271	RSRD19	-122.451	37.944	7.95	7.68	282.99	15.63	-122.451	37.944	8.23	7.68	283.12	15.91	1	8/1/2014	8:28:26.47
2272	RSRD19	-122.451	37.944	8.00	7.70	282.99	15.70	-122.451	37.944	8.28	7.70	283.10	15.99	1	8/1/2014	8:28:26.57
2273	RSRD19	-122.451	37.944	7.95	7.66	282.95	15.62	-122.451	37.944	8.23	7.66	283.10	15.90	1	8/1/2014	8:28:26.68
2274	RSRD19	-122.451	37.944	7.95	7.66	282.93	15.61	-122.451	37.944	8.23	7.66	283.08	15.89	1	8/1/2014	8:28:26.78
2275	RSRD19	-122.451	37.944	7.95	7.66	282.90	15.61	-122.451	37.944	8.23	7.66	283.06	15.89	1	8/1/2014	8:28:26.87
2276	RSRD19	-122.451	37.944	7.95	7.65	282.86	15.60	-122.451	37.944	8.23	7.65	283.03	15.88	1	8/1/2014	8:28:26.97
2277	RSRD19	-122.451	37.944	7.95	7.64	282.86	15.59	-122.451	37.944	8.20	7.64	283.03	15.84	1	8/1/2014	8:28:27.08
2278	RSRD19	-122.451	37.944	7.95	7.66	282.83	15.61	-122.451	37.944	8.23	7.66	283.01	15.89	1	8/1/2014	8:28:27.18
2279	RSRD19	-122.451	37.944	7.95	7.66	282.81	15.62	-122.451	37.944	8.23	7.66	282.96	15.90	1	8/1/2014	8:28:27.27
2280	RSRD19	-122.451	37.944	7.95	7.67	282.79	15.62	-122.451	37.944	8.23	7.67	282.89	15.90	1	8/1/2014	8:28:27.37
2281	RSRD19	-122.451	37.944	7.95	7.68	282.78	15.63	-122.451	37.944	8.20	7.68	282.85	15.88	1	8/1/2014	8:28:27.48
2282	RSRD19	-122.451	37.944	7.95	7.72	282.76	15.68	-122.451	37.944	8.20	7.72	282.85	15.92	1	8/1/2014	8:28:27.58
2283	RSRD19	-122.451	37.944	7.95	7.71	282.76	15.66	-122.451	37.944	8.20	7.71	282.81	15.91	1	8/1/2014	8:28:27.67
2284	RSRD19	-122.451	37.944	7.95	7.67	282.67	15.62	-122.451	37.944	8.20	7.67	282.76	15.87	1	8/1/2014	8:28:27.77
2285	RSRD19	-122.451	37.944	7.92	7.70	282.69	15.62	-122.451	37.944	8.15	7.70	282.75	15.85	1	8/1/2014	8:28:27.88
2286	RSRD19	-122.451	37.944	7.95	7.69	282.62	15.64	-122.451	37.944	8.20	7.69	282.69	15.89	1	8/1/2014	8:28:27.98
2287	RSRD19	-122.451	37.944	7.92	7.73	282.60	15.65	-122.451	37.944	8.20	7.73	282.71	15.93	1	8/1/2014	8:28:28.07
2288	RSRD19	-122.451	37.944	7.95	7.67	282.58	15.62	-122.451	37.944	8.20	7.67	282.66	15.87	1	8/1/2014	8:28:28.17
2289	RSRD19	-122.451	37.944	7.95	7.68	282.53	15.63	-122.451	37.944	8.15	7.68	282.60	15.83	1	8/1/2014	8:28:28.28
2290	RSRD19	-122.451	37.944	7.95	7.66	282.51	15.62	-122.451	37.944	8.20	7.66	282.59	15.86	1	8/1/2014	8:28:28.38

2291	RSRD19	-122.451	37.944	7.95	7.72	282.48	15.67	-122.451	37.944	8.20	7.72	282.55	15.92	1	8/1/2014	8:28:28.47
2292	RSRD19	-122.451	37.944	7.95	7.69	282.42	15.64	-122.451	37.944	8.20	7.69	282.51	15.89	1	8/1/2014	8:28:28.57
2293	RSRD19	-122.451	37.944	7.95	7.64	282.39	15.59	-122.451	37.944	8.20	7.64	282.48	15.84	1	8/1/2014	8:28:28.68
2294	RSRD19	-122.451	37.944	7.95	7.64	282.37	15.59	-122.451	37.944	8.20	7.64	282.44	15.84	1	8/1/2014	8:28:28.78
2295	RSRD19	-122.451	37.944	7.95	7.63	282.34	15.58	-122.451	37.944	8.20	7.63	282.43	15.83	1	8/1/2014	8:28:28.87
2296	RSRD19	-122.451	37.944	8.00	7.67	282.30	15.67	-122.451	37.944	8.23	7.67	282.39	15.90	1	8/1/2014	8:28:28.97
2297	RSRD19	-122.451	37.944	7.95	7.60	282.28	15.55	-122.451	37.944	8.20	7.60	282.36	15.80	1	8/1/2014	8:28:29.08
2298	RSRD19	-122.451	37.944	7.95	7.59	282.25	15.55	-122.451	37.944	8.23	7.59	282.34	15.83	1	8/1/2014	8:28:29.18
2299	RSRD19	-122.451	37.944	7.95	7.61	282.21	15.56	-122.451	37.944	8.20	7.61	282.29	15.81	1	8/1/2014	8:28:29.27
2300	RSRD19	-122.451	37.944	8.00	7.60	282.18	15.60	-122.451	37.944	8.23	7.60	282.31	15.83	1	8/1/2014	8:28:29.37
2301	RSRD19	-122.451	37.944	7.95	7.61	282.16	15.56	-122.451	37.944	8.20	7.61	282.22	15.81	1	8/1/2014	8:28:29.48
2302	RSRD19	-122.451	37.944	8.00	7.63	282.14	15.63	-122.451	37.944	8.23	7.63	282.27	15.86	1	8/1/2014	8:28:29.58
2303	RSRD19	-122.451	37.944	7.95	7.63	282.13	15.58	-122.451	37.944	8.20	7.63	282.24	15.83	1	8/1/2014	8:28:29.67
2304	RSRD19	-122.451	37.944	8.00	7.63	282.09	15.63	-122.451	37.944	8.23	7.63	282.22	15.86	1	8/1/2014	8:28:29.77
2305	RSRD19	-122.451	37.944	7.95	7.65	282.08	15.60	-122.451	37.944	8.23	7.65	282.19	15.88	1	8/1/2014	8:28:29.88
2306	RSRD19	-122.451	37.944	8.00	7.69	282.07	15.69	-122.451	37.944	8.23	7.69	282.20	15.92	1	8/1/2014	8:28:29.98
2307	RSRD19	-122.451	37.944	7.95	7.64	282.02	15.59	-122.451	37.944	8.20	7.64	282.15	15.84	1	8/1/2014	8:28:30.07
2308	RSRD19	-122.451	37.944	7.95	7.63	281.99	15.58	-122.451	37.944	8.23	7.63	282.15	15.87	1	8/1/2014	8:28:30.17
2309	RSRD19	-122.451	37.944	7.95	7.63	281.97	15.58	-122.451	37.944	8.20	7.63	282.10	15.83	1	8/1/2014	8:28:30.28
2310	RSRD19	-122.451	37.944	8.00	7.63	281.93	15.63	-122.451	37.944	8.23	7.63	282.06	15.87	1	8/1/2014	8:28:30.38
2311	RSRD19	-122.451	37.944	7.95	7.63	281.90	15.58	-122.451	37.944	8.20	7.63	282.03	15.83	1	8/1/2014	8:28:30.47
2312	RSRD19	-122.451	37.944	8.00	7.63	281.92	15.63	-122.451	37.944	8.23	7.63	282.01	15.86	1	8/1/2014	8:28:30.57
2313	RSRD19	-122.451	37.944	7.95	7.62	281.88	15.57	-122.451	37.944	8.20	7.62	281.97	15.82	1	8/1/2014	8:28:30.68
2314	RSRD19	-122.451	37.944	8.00	7.62	281.88	15.62	-122.451	37.944	8.20	7.62	281.94	15.82	1	8/1/2014	8:28:30.78
2315	RSRD19	-122.451	37.944	7.95	7.63	281.81	15.58	-122.451	37.944	8.20	7.63	281.92	15.83	1	8/1/2014	8:28:30.87
2316	RSRD19	-122.451	37.944	7.95	7.64	281.81	15.59	-122.451	37.944	8.20	7.64	281.90	15.84	1	8/1/2014	8:28:30.97
2317	RSRD19	-122.451	37.944	7.95	7.66	281.81	15.61	-122.451	37.944	8.20	7.66	281.87	15.85	1	8/1/2014	8:28:31.08
2318	RSRD19	-122.451	37.944	7.95	7.67	281.74	15.62	-122.451	37.944	8.20	7.67	281.87	15.87	1	8/1/2014	8:28:31.18
2319	RSRD19	-122.451	37.944	7.95	7.66	281.72	15.61	-122.451	37.944	8.15	7.66	281.80	15.80	1	8/1/2014	8:28:31.27
2320	RSRD19	-122.451	37.944	8.00	7.66	281.69	15.66	-122.451	37.944	8.20	7.66	281.80	15.85	1	8/1/2014	8:28:31.37
2321	RSRD19	-122.451	37.944	7.95	7.65	281.61	15.60	-122.451	37.944	8.20	7.65	281.70	15.85	1	8/1/2014	8:28:31.48
2322	RSRD19	-122.451	37.944	8.00	7.66	281.65	15.66	-122.451	37.944	8.20	7.66	281.74	15.85	1	8/1/2014	8:28:31.58
2323	RSRD19	-122.451	37.944	7.95	7.66	281.60	15.61	-122.451	37.944	8.20	7.66	281.71	15.85	1	8/1/2014	8:28:31.67

2324	RSRD19	-122.451	37.944	8.00	7.66	281.58	15.67	-122.451	37.944	8.20	7.66	281.65	15.86	1	8/1/2014 8:28:31.77
2325	RSRD19	-122.451	37.944	8.00	7.66	281.58	15.66	-122.451	37.944	8.15	7.66	281.64	15.80	1	8/1/2014 8:28:31.88
2326	RSRD19	-122.451	37.944	8.00	7.68	281.51	15.68	-122.451	37.944	8.20	7.68	281.60	15.88	1	8/1/2014 8:28:31.98
2327	RSRD19	-122.451	37.944	7.95	7.64	281.49	15.59	-122.451	37.944	8.20	7.64	281.53	15.84	1	8/1/2014 8:28:32.07
2328	RSRD19	-122.451	37.944	8.00	7.63	281.49	15.63	-122.451	37.944	8.20	7.63	281.51	15.83	1	8/1/2014 8:28:32.17
2329	RSRD19	-122.451	37.944	7.95	7.64	281.44	15.59	-122.451	37.944	8.20	7.64	281.49	15.84	1	8/1/2014 8:28:32.28
2330	RSRD19	-122.451	37.944	8.00	7.66	281.42	15.66	-122.451	37.944	8.20	7.66	281.44	15.85	1	8/1/2014 8:28:32.38
2331	RSRD19	-122.451	37.944	7.95	7.66	281.40	15.62	-122.451	37.944	8.20	7.66	281.44	15.86	1	8/1/2014 8:28:32.47
2332	RSRD19	-122.451	37.944	8.00	7.63	281.33	15.63	-122.451	37.944	8.20	7.63	281.36	15.83	1	8/1/2014 8:28:32.57
2333	RSRD19	-122.451	37.944	7.95	7.66	281.33	15.61	-122.451	37.944	8.20	7.66	281.37	15.85	1	8/1/2014 8:28:32.68
2334	RSRD19	-122.451	37.944	8.00	7.68	281.31	15.68	-122.451	37.944	8.23	7.68	281.33	15.91	1	8/1/2014 8:28:32.78
2335	RSRD19	-122.451	37.944	8.00	7.71	281.27	15.71	-122.451	37.944	8.20	7.71	281.31	15.91	1	8/1/2014 8:28:32.87
2336	RSRD19	-122.451	37.944	8.00	7.66	281.26	15.67	-122.451	37.944	8.20	7.66	281.28	15.86	1	8/1/2014 8:28:32.97
2337	RSRD19	-122.451	37.944	8.00	7.70	281.17	15.70	-122.451	37.944	8.20	7.70	281.22	15.90	1	8/1/2014 8:28:33.08
2338	RSRD19	-122.451	37.944	8.00	7.72	281.19	15.73	-122.451	37.944	8.20	7.72	281.21	15.92	1	8/1/2014 8:28:33.18
2339	RSRD19	-122.451	37.944	8.00	7.72	281.10	15.72	-122.451	37.944	8.20	7.72	281.19	15.92	1	8/1/2014 8:28:33.27
2340	RSRD19	-122.451	37.944	8.00	7.71	281.08	15.71	-122.452	37.944	8.20	7.71	281.13	15.91	1	8/1/2014 8:28:33.37
2341	RSRD19	-122.452	37.944	8.00	7.76	281.06	15.77	-122.452	37.944	8.20	7.76	281.13	15.96	1	8/1/2014 8:28:33.48
2342	RSRD19	-122.452	37.944	8.00	7.76	281.01	15.77	-122.452	37.944	8.20	7.76	281.10	15.96	1	8/1/2014 8:28:33.58
2343	RSRD19	-122.452	37.944	7.95	7.75	281.01	15.70	-122.452	37.944	8.20	7.75	281.10	15.95	1	8/1/2014 8:28:33.67
2344	RSRD19	-122.452	37.944	8.00	7.75	280.99	15.75	-122.452	37.944	8.20	7.75	281.10	15.95	1	8/1/2014 8:28:33.77
2345	RSRD19	-122.452	37.944	8.00	7.76	280.97	15.77	-122.452	37.944	8.20	7.76	281.08	15.96	1	8/1/2014 8:28:33.88
2346	RSRD19	-122.452	37.944	8.00	7.75	280.90	15.75	-122.452	37.944	8.20	7.75	281.01	15.95	1	8/1/2014 8:28:33.98
2347	RSRD19	-122.452	37.944	8.00	7.79	280.93	15.79	-122.452	37.944	8.20	7.79	281.03	15.99	1	8/1/2014 8:28:34.07
2348	RSRD19	-122.452	37.944	8.00	7.75	280.90	15.75	-122.452	37.944	8.20	7.75	280.97	15.95	1	8/1/2014 8:28:34.17
2349	RSRD19	-122.452	37.944	7.95	7.78	280.86	15.73	-122.452	37.944	8.15	7.78	280.94	15.93	1	8/1/2014 8:28:34.28
2350	RSRD19	-122.452	37.944	8.00	7.78	280.83	15.78	-122.452	37.944	8.20	7.78	280.96	15.98	1	8/1/2014 8:28:34.38
2351	RSRD19	-122.452	37.944	7.95	7.80	280.83	15.75	-122.452	37.944	8.15	7.80	280.90	15.95	1	8/1/2014 8:28:34.47
2352	RSRD19	-122.452	37.944	8.00	7.76	280.77	15.77	-122.452	37.944	8.20	7.76	280.90	15.96	1	8/1/2014 8:28:34.57
2353	RSRD19	-122.452	37.944	7.95	7.76	280.77	15.71	-122.452	37.944	8.15	7.76	280.87	15.90	1	8/1/2014 8:28:34.68
2354	RSRD19	-122.452	37.944	8.00	7.75	280.74	15.75	-122.452	37.944	8.20	7.75	280.86	15.95	1	8/1/2014 8:28:34.78
2355	RSRD19	-122.452	37.944	7.95	7.77	280.74	15.72	-122.452	37.944	8.15	7.77	280.81	15.92	1	8/1/2014 8:28:34.87
2356	RSRD19	-122.452	37.944	7.95	7.77	280.70	15.72	-122.452	37.944	8.20	7.77	280.79	15.97	1	8/1/2014 8:28:34.97

2357	RSRD19	-122.452	37.944	7.95	7.76	280.65	15.72	-122.452	37.944	8.15	7.76	280.74	15.91	1	8/1/2014	8:28:35.08
2358	RSRD19	-122.452	37.944	8.00	7.77	280.67	15.77	-122.452	37.944	8.15	7.77	280.79	15.92	1	8/1/2014	8:28:35.17
2359	RSRD19	-122.452	37.944	7.95	7.84	280.63	15.79	-122.452	37.944	8.15	7.84	280.72	15.98	1	8/1/2014	8:28:35.27
2360	RSRD19	-122.452	37.944	8.00	7.82	280.61	15.82	-122.452	37.944	8.15	7.82	280.69	15.97	1	8/1/2014	8:28:35.37
2361	RSRD19	-122.452	37.944	7.95	7.85	280.57	15.80	-122.452	37.944	8.11	7.85	280.68	15.96	1	8/1/2014	8:28:35.48
2362	RSRD19	-122.452	37.944	8.00	7.81	280.54	15.81	-122.452	37.944	8.15	7.81	280.65	15.96	1	8/1/2014	8:28:35.57
2363	RSRD19	-122.452	37.944	7.95	7.82	280.50	15.77	-122.452	37.944	8.15	7.82	280.63	15.97	1	8/1/2014	8:28:35.67
2364	RSRD19	-122.452	37.944	7.95	7.81	280.45	15.76	-122.452	37.944	8.15	7.81	280.61	15.96	1	8/1/2014	8:28:35.77
2365	RSRD19	-122.452	37.944	7.95	7.81	280.45	15.76	-122.452	37.944	8.11	7.81	280.54	15.92	1	8/1/2014	8:28:35.88
2366	RSRD19	-122.452	37.944	7.95	7.84	280.43	15.79	-122.452	37.944	8.15	7.84	280.54	15.98	1	8/1/2014	8:28:35.98
2367	RSRD19	-122.452	37.944	7.92	7.89	280.41	15.81	-122.452	37.944	8.11	7.89	280.50	16.01	1	8/1/2014	8:28:36.07
2368	RSRD19	-122.452	37.944	7.95	7.86	280.34	15.81	-122.452	37.944	8.15	7.86	280.45	16.00	1	8/1/2014	8:28:36.17
2369	RSRD19	-122.452	37.944	7.92	7.86	280.36	15.77	-122.452	37.944	8.11	7.86	280.45	15.97	1	8/1/2014	8:28:36.28
2370	RSRD19	-122.452	37.944	8.00	7.86	280.34	15.86	-122.452	37.944	8.15	7.86	280.43	16.01	1	8/1/2014	8:28:36.38
2371	RSRD19	-122.452	37.944	7.95	7.93	280.29	15.88	-122.452	37.944	8.11	7.93	280.38	16.05	1	8/1/2014	8:28:36.47
2372	RSRD19	-122.452	37.944	7.95	7.86	280.27	15.81	-122.452	37.944	8.15	7.86	280.36	16.01	1	8/1/2014	8:28:36.57
2373	RSRD19	-122.452	37.944	7.95	7.86	280.29	15.81	-122.452	37.944	8.11	7.86	280.32	15.97	1	8/1/2014	8:28:36.68
2374	RSRD19	-122.452	37.944	7.95	7.85	280.18	15.80	-122.452	37.944	8.11	7.85	280.31	15.96	1	8/1/2014	8:28:36.77
2375	RSRD19	-122.452	37.944	7.95	7.85	280.18	15.80	-122.452	37.944	8.11	7.85	280.25	15.96	1	8/1/2014	8:28:36.87
2376	RSRD19	-122.452	37.944	7.95	7.84	280.18	15.79	-122.452	37.944	8.11	7.84	280.23	15.95	1	8/1/2014	8:28:36.97
2377	RSRD19	-122.452	37.944	7.95	7.85	280.16	15.80	-122.452	37.944	8.11	7.85	280.25	15.96	1	8/1/2014	8:28:37.08
2378	RSRD19	-122.452	37.944	7.95	7.84	280.11	15.79	-122.452	37.944	8.11	7.84	280.18	15.96	1	8/1/2014	8:28:37.18
2379	RSRD19	-122.452	37.944	7.95	7.87	280.14	15.82	-122.452	37.944	8.11	7.87	280.20	15.99	1	8/1/2014	8:28:37.27
2380	RSRD19	-122.452	37.944	7.95	7.85	280.07	15.80	-122.452	37.944	8.11	7.85	280.19	15.96	1	8/1/2014	8:28:37.37
2381	RSRD19	-122.452	37.944	7.95	7.87	280.11	15.82	-122.452	37.944	8.11	7.87	280.15	15.99	1	8/1/2014	8:28:37.48
2382	RSRD19	-122.452	37.944	7.95	7.85	280.05	15.80	-122.452	37.944	8.11	7.85	280.13	15.96	1	8/1/2014	8:28:37.58
2383	RSRD19	-122.452	37.944	7.92	7.86	280.02	15.78	-122.452	37.944	8.11	7.86	280.07	15.98	1	8/1/2014	8:28:37.67
2384	RSRD19	-122.452	37.944	7.95	7.85	280.04	15.80	-122.452	37.944	8.15	7.85	280.11	16.00	1	8/1/2014	8:28:37.77
2385	RSRD19	-122.452	37.944	7.95	7.87	280.02	15.82	-122.452	37.944	8.11	7.87	280.08	15.99	1	8/1/2014	8:28:37.88
2386	RSRD19	-122.452	37.944	7.95	7.87	280.00	15.82	-122.452	37.944	8.15	7.87	280.07	16.02	1	8/1/2014	8:28:37.97
2387	RSRD19	-122.452	37.944	7.95	7.86	279.98	15.81	-122.452	37.944	8.11	7.86	280.02	15.98	1	8/1/2014	8:28:38.07
2388	RSRD19	-122.452	37.944	7.95	7.87	279.95	15.82	-122.452	37.944	8.11	7.87	280.02	15.99	1	8/1/2014	8:28:38.17
2389	RSRD19	-122.452	37.944	7.95	7.90	279.91	15.85	-122.452	37.944	8.11	7.90	280.02	16.02	1	8/1/2014	8:28:38.28

2390	RSRD19	-122.452	37.944	7.95	7.88	279.95	15.83	-122.452	37.944	8.11	7.88	280.04	15.99	1	8/1/2014	8:28:38.37
2391	RSRD19	-122.452	37.944	7.95	7.89	279.93	15.85	-122.452	37.944	8.11	7.89	280.02	16.01	1	8/1/2014	8:28:38.47
2392	RSRD19	-122.452	37.944	7.95	7.89	279.91	15.85	-122.452	37.944	8.11	7.89	280.00	16.01	1	8/1/2014	8:28:38.57
2393	RSRD19	-122.452	37.944	7.95	7.92	279.91	15.87	-122.452	37.944	8.08	7.92	280.02	16.00	1	8/1/2014	8:28:38.68
2394	RSRD19	-122.452	37.944	7.95	7.93	279.88	15.88	-122.452	37.944	8.11	7.93	279.95	16.04	1	8/1/2014	8:28:38.78
2395	RSRD19	-122.452	37.944	7.95	7.92	279.86	15.87	-122.452	37.944	8.08	7.92	279.97	16.00	1	8/1/2014	8:28:38.87
2396	RSRD19	-122.452	37.944	7.95	7.94	279.86	15.89	-122.452	37.944	8.08	7.94	279.93	16.02	1	8/1/2014	8:28:38.97
2397	RSRD19	-122.452	37.944	7.92	7.94	279.84	15.86	-122.452	37.944	8.08	7.94	279.90	16.02	1	8/1/2014	8:28:39.08
2398	RSRD19	-122.452	37.944	7.95	7.95	279.84	15.90	-122.452	37.944	8.08	7.95	279.90	16.03	1	8/1/2014	8:28:39.18
2399	RSRD19	-122.452	37.944	7.95	8.00	279.79	15.95	-122.452	37.944	8.08	8.00	279.86	16.08	1	8/1/2014	8:28:39.27
2400	RSRD19	-122.452	37.944	7.92	7.97	279.79	15.89	-122.452	37.944	8.08	7.97	279.85	16.05	1	8/1/2014	8:28:39.37
2401	RSRD19	-122.452	37.944	7.92	7.99	279.79	15.90	-122.452	37.944	8.08	7.99	279.81	16.07	1	8/1/2014	8:28:39.48
2402	RSRD19	-122.452	37.944	7.95	7.99	279.75	15.94	-122.452	37.944	8.08	7.99	279.81	16.07	1	8/1/2014	8:28:39.57
2403	RSRD19	-122.452	37.944	7.92	8.00	279.70	15.92	-122.452	37.944	8.08	8.00	279.77	16.08	1	8/1/2014	8:28:39.67
2404	RSRD19	-122.452	37.944	7.95	8.03	279.70	15.98	-122.452	37.944	8.03	8.03	279.75	16.06	1	8/1/2014	8:28:39.77
2405	RSRD19	-122.452	37.944	7.92	8.07	279.67	15.98	-122.452	37.944	8.03	8.07	279.74	16.10	1	8/1/2014	8:28:39.88
2406	RSRD19	-122.452	37.944	7.92	8.02	279.66	15.93	-122.452	37.944	8.08	8.02	279.72	16.10	1	8/1/2014	8:28:39.98
2407	RSRD19	-122.452	37.944	7.92	8.01	279.63	15.93	-122.452	37.944	8.03	8.01	279.72	16.04	1	8/1/2014	8:28:40.07
2408	RSRD19	-122.452	37.944	7.95	8.03	279.65	15.98	-122.452	37.944	8.08	8.03	279.68	16.11	1	8/1/2014	8:28:40.17
2409	RSRD19	-122.452	37.944	7.92	8.03	279.63	15.94	-122.452	37.944	8.03	8.03	279.68	16.06	1	8/1/2014	8:28:40.28
2410	RSRD19	-122.452	37.944	7.95	8.02	279.61	15.97	-122.452	37.944	8.08	8.02	279.67	16.10	1	8/1/2014	8:28:40.38
2411	RSRD19	-122.452	37.944	7.92	8.03	279.61	15.95	-122.452	37.944	8.03	8.03	279.67	16.06	1	8/1/2014	8:28:40.47
2412	RSRD19	-122.452	37.944	7.95	8.01	279.61	15.96	-122.452	37.944	8.03	8.01	279.68	16.04	1	8/1/2014	8:28:40.57
2413	RSRD19	-122.452	37.944	7.92	8.03	279.63	15.95	-122.452	37.944	8.03	8.03	279.67	16.06	1	8/1/2014	8:28:40.68
2414	RSRD19	-122.452	37.944	7.92	8.01	279.58	15.93	-122.452	37.944	8.03	8.01	279.65	16.04	1	8/1/2014	8:28:40.78
2415	RSRD19	-122.452	37.944	7.92	8.01	279.62	15.93	-122.452	37.944	8.03	8.01	279.65	16.04	1	8/1/2014	8:28:40.87
2416	RSRD19	-122.452	37.944	7.92	8.00	279.60	15.92	-122.452	37.944	8.03	8.00	279.67	16.03	1	8/1/2014	8:28:40.97
2417	RSRD19	-122.452	37.944	7.92	8.01	279.65	15.93	-122.452	37.944	8.00	8.01	279.69	16.01	1	8/1/2014	8:28:41.08
2418	RSRD19	-122.452	37.944	7.92	8.00	279.62	15.92	-122.452	37.944	8.03	8.00	279.67	16.03	1	8/1/2014	8:28:41.18
2419	RSRD19	-122.452	37.944	7.92	8.03	279.60	15.95	-122.452	37.944	8.00	8.03	279.67	16.03	1	8/1/2014	8:28:41.27
2420	RSRD19	-122.452	37.944	7.92	8.01	279.58	15.93	-122.452	37.944	8.03	8.01	279.67	16.04	1	8/1/2014	8:28:41.37
2421	RSRD19	-122.452	37.944	7.92	8.02	279.58	15.93	-122.452	37.944	8.03	8.02	279.64	16.05	1	8/1/2014	8:28:41.48
2422	RSRD19	-122.452	37.944	7.92	8.06	279.60	15.97	-122.452	37.944	8.03	8.06	279.64	16.09	1	8/1/2014	8:28:41.58

2423	RSRD19	-122.452	37.944	7.92	7.99	279.56	15.90	-122.452	37.944	8.03	7.99	279.64	16.02	1	8/1/2014 8:28:41.67
2424	RSRD19	-122.452	37.944	7.92	7.97	279.53	15.88	-122.452	37.944	8.08	7.97	279.60	16.05	1	8/1/2014 8:28:41.77
2425	RSRD19	-122.452	37.944	7.86	7.95	279.53	15.81	-122.452	37.944	8.03	7.95	279.65	15.98	1	8/1/2014 8:28:41.88
2426	RSRD19	-122.452	37.944	7.92	7.96	279.51	15.87	-122.452	37.944	8.08	7.96	279.58	16.04	1	8/1/2014 8:28:41.98
2427	RSRD19	-122.452	37.944	7.92	7.96	279.53	15.87	-122.452	37.944	8.08	7.96	279.62	16.04	1	8/1/2014 8:28:42.07
2428	RSRD19	-122.452	37.944	7.92	7.93	279.49	15.84	-122.452	37.944	8.08	7.93	279.58	16.01	1	8/1/2014 8:28:42.17
2429	RSRD19	-122.452	37.944	7.92	7.92	279.51	15.83	-122.452	37.944	8.03	7.92	279.57	15.95	1	8/1/2014 8:28:42.28
2430	RSRD19	-122.452	37.944	7.92	7.94	279.49	15.86	-122.452	37.944	8.08	7.94	279.57	16.02	1	8/1/2014 8:28:42.38
2431	RSRD19	-122.452	37.944	7.92	7.95	279.48	15.86	-122.452	37.944	8.03	7.95	279.59	15.98	1	8/1/2014 8:28:42.47
2432	RSRD19	-122.452	37.944	7.92	7.97	279.49	15.89	-122.452	37.944	8.08	7.97	279.59	16.05	1	8/1/2014 8:28:42.57
2433	RSRD19	-122.452	37.944	7.92	8.03	279.51	15.95	-122.452	37.944	8.03	8.03	279.59	16.06	1	8/1/2014 8:28:42.68
2434	RSRD19	-122.452	37.944	7.92	8.01	279.48	15.93	-122.452	37.944	8.08	8.01	279.55	16.09	1	8/1/2014 8:28:42.78
2435	RSRD19	-122.452	37.944	7.92	8.03	279.49	15.94	-122.452	37.944	8.03	8.03	279.59	16.06	1	8/1/2014 8:28:42.87
2436	RSRD19	-122.452	37.944	7.92	8.06	279.51	15.97	-122.452	37.944	8.03	8.06	279.55	16.09	1	8/1/2014 8:28:42.97
2437	RSRD19	-122.452	37.944	7.87	8.07	279.49	15.93	-122.452	37.944	8.03	8.07	279.57	16.10	1	8/1/2014 8:28:43.08
2438	RSRD19	-122.452	37.944	7.92	8.07	279.46	15.98	-122.452	37.944	8.00	8.07	279.53	16.06	1	8/1/2014 8:28:43.18
2439	RSRD19	-122.452	37.944	7.87	8.07	279.44	15.93	-122.452	37.944	8.00	8.07	279.53	16.06	1	8/1/2014 8:28:43.27
2440	RSRD19	-122.452	37.944	7.92	8.07	279.42	15.98	-122.452	37.944	8.00	8.07	279.48	16.06	1	8/1/2014 8:28:43.37
2441	RSRD19	-122.452	37.944	7.87	8.08	279.38	15.95	-122.452	37.944	8.00	8.08	279.46	16.08	1	8/1/2014 8:28:43.48
2442	RSRD19	-122.452	37.944	7.92	8.08	279.33	16.00	-122.452	37.944	8.00	8.08	279.40	16.08	1	8/1/2014 8:28:43.58
2443	RSRD19	-122.452	37.944	7.87	8.08	279.31	15.95	-122.452	37.944	7.95	8.08	279.42	16.03	1	8/1/2014 8:28:43.67
2444	RSRD19	-122.452	37.944	7.92	8.11	279.29	16.03	-122.452	37.944	8.00	8.11	279.35	16.11	1	8/1/2014 8:28:43.77
2445	RSRD19	-122.452	37.944	7.87	8.10	279.24	15.96	-122.452	37.944	7.95	8.10	279.35	16.04	1	8/1/2014 8:28:43.88
2446	RSRD19	-122.452	37.944	7.87	8.08	279.22	15.95	-122.452	37.944	8.00	8.08	279.33	16.08	1	8/1/2014 8:28:43.98
2447	RSRD19	-122.452	37.944	7.87	8.10	279.18	15.96	-122.452	37.944	7.95	8.10	279.29	16.04	1	8/1/2014 8:28:44.07
2448	RSRD19	-122.452	37.944	7.87	8.10	279.15	15.96	-122.452	37.944	7.95	8.10	279.24	16.04	1	8/1/2014 8:28:44.17
2449	RSRD19	-122.452	37.944	7.87	8.11	279.14	15.98	-122.452	37.944	7.95	8.11	279.27	16.06	1	8/1/2014 8:28:44.28
2450	RSRD19	-122.452	37.944	7.87	8.15	279.09	16.02	-122.452	37.944	7.95	8.15	279.21	16.10	1	8/1/2014 8:28:44.37
2451	RSRD19	-122.452	37.944	7.87	8.13	279.07	15.99	-122.452	37.944	7.95	8.13	279.16	16.07	1	8/1/2014 8:28:44.47
2452	RSRD19	-122.452	37.944	7.87	8.16	279.03	16.02	-122.452	37.944	7.95	8.16	279.12	16.10	1	8/1/2014 8:28:44.57
2453	RSRD19	-122.452	37.944	7.87	8.15	279.00	16.02	-122.452	37.944	7.91	8.15	279.11	16.06	1	8/1/2014 8:28:44.68
2454	RSRD19	-122.452	37.944	7.87	8.18	278.96	16.05	-122.452	37.944	7.91	8.18	279.10	16.09	1	8/1/2014 8:28:44.78
2455	RSRD19	-122.452	37.944	7.83	8.23	278.95	16.06	-122.452	37.944	7.91	8.23	279.03	16.14	1	8/1/2014 8:28:44.87

2456	RSRD19	-122.452	37.944	7.87	8.23	278.94	16.09	-122.452	37.944	7.95	8.23	279.03	16.17	1	8/1/2014 8:28:44.97
2457	RSRD19	-122.452	37.944	7.83	8.21	278.88	16.04	-122.452	37.944	7.91	8.21	278.94	16.12	1	8/1/2014 8:28:45.08
2458	RSRD19	-122.452	37.944	7.87	8.21	278.86	16.08	-122.452	37.944	7.91	8.21	278.95	16.12	1	8/1/2014 8:28:45.17
2459	RSRD19	-122.452	37.944	7.83	8.20	278.81	16.04	-122.452	37.944	7.91	8.20	278.90	16.12	1	8/1/2014 8:28:45.27
2460	RSRD19	-122.452	37.944	7.87	8.21	278.77	16.08	-122.452	37.944	7.91	8.21	278.87	16.12	1	8/1/2014 8:28:45.37
2461	RSRD19	-122.452	37.944	7.83	8.23	278.75	16.06	-122.452	37.944	7.91	8.23	278.81	16.14	1	8/1/2014 8:28:45.48
2462	RSRD19	-122.452	37.944	7.83	8.23	278.73	16.06	-122.452	37.944	7.91	8.23	278.84	16.14	1	8/1/2014 8:28:45.58
2463	RSRD19	-122.452	37.944	7.83	8.22	278.66	16.05	-122.452	37.944	7.91	8.22	278.78	16.13	1	8/1/2014 8:28:45.67
2464	RSRD19	-122.452	37.944	7.83	8.24	278.64	16.07	-122.452	37.944	7.95	8.24	278.75	16.19	1	8/1/2014 8:28:45.77
2465	RSRD19	-122.452	37.944	7.83	8.25	278.63	16.08	-122.452	37.944	7.91	8.25	278.73	16.16	1	8/1/2014 8:28:45.88
2466	RSRD19	-122.452	37.944	7.87	8.23	278.60	16.09	-122.452	37.944	7.95	8.23	278.71	16.17	1	8/1/2014 8:28:45.98
2467	RSRD19	-122.452	37.944	7.83	8.25	278.56	16.08	-122.452	37.944	7.91	8.25	278.67	16.16	1	8/1/2014 8:28:46.07
2468	RSRD19	-122.452	37.944	7.87	8.27	278.56	16.13	-122.452	37.944	7.95	8.27	278.69	16.21	1	8/1/2014 8:28:46.17
2469	RSRD19	-122.452	37.944	7.83	8.30	278.52	16.14	-122.452	37.944	7.91	8.30	278.65	16.22	1	8/1/2014 8:28:46.28
2470	RSRD19	-122.452	37.944	7.87	8.29	278.48	16.16	-122.452	37.944	7.95	8.29	278.61	16.24	1	8/1/2014 8:28:46.38
2471	RSRD19	-122.452	37.944	7.83	8.32	278.46	16.15	-122.452	37.944	7.95	8.32	278.61	16.26	1	8/1/2014 8:28:46.47
2472	RSRD19	-122.452	37.944	7.87	8.31	278.44	16.18	-122.452	37.944	7.95	8.31	278.57	16.26	1	8/1/2014 8:28:46.57
2473	RSRD19	-122.452	37.944	7.83	8.32	278.42	16.15	-122.452	37.944	7.95	8.32	278.57	16.26	1	8/1/2014 8:28:46.68
2474	RSRD19	-122.452	37.944	7.87	8.31	278.40	16.18	-122.452	37.944	7.95	8.31	278.49	16.26	1	8/1/2014 8:28:46.77
2475	RSRD19	-122.452	37.944	7.83	8.31	278.38	16.14	-122.452	37.944	7.91	8.31	278.51	16.22	1	8/1/2014 8:28:46.87
2476	RSRD19	-122.452	37.944	7.83	8.33	278.36	16.17	-122.452	37.944	7.95	8.33	278.46	16.28	1	8/1/2014 8:28:46.97
2477	RSRD19	-122.452	37.944	7.87	8.32	278.34	16.18	-122.452	37.944	7.91	8.32	278.47	16.23	1	8/1/2014 8:28:47.08
2478	RSRD19	-122.452	37.944	7.87	8.32	278.27	16.18	-122.452	37.944	7.95	8.32	278.38	16.26	1	8/1/2014 8:28:47.17
2479	RSRD19	-122.452	37.944	7.83	8.33	278.27	16.17	-122.452	37.944	7.91	8.33	278.43	16.25	1	8/1/2014 8:28:47.27
2480	RSRD19	-122.452	37.944	7.87	8.33	278.25	16.19	-122.452	37.944	7.95	8.33	278.41	16.27	1	8/1/2014 8:28:47.37
2481	RSRD19	-122.452	37.944	7.83	8.34	278.24	16.18	-122.452	37.944	7.91	8.34	278.39	16.26	1	8/1/2014 8:28:47.48
2482	RSRD19	-122.452	37.944	7.87	8.33	278.26	16.20	-122.452	37.944	7.91	8.33	278.37	16.25	1	8/1/2014 8:28:47.58
2483	RSRD19	-122.452	37.944	7.83	8.33	278.20	16.16	-122.452	37.944	7.91	8.33	278.33	16.24	1	8/1/2014 8:28:47.67
2484	RSRD19	-122.452	37.944	7.83	8.35	278.22	16.18	-122.452	37.944	7.91	8.35	278.35	16.26	1	8/1/2014 8:28:47.77
2485	RSRD19	-122.452	37.944	7.83	8.33	278.16	16.17	-122.452	37.944	7.91	8.33	278.29	16.25	1	8/1/2014 8:28:47.88
2486	RSRD19	-122.452	37.944	7.83	8.38	278.16	16.21	-122.452	37.944	7.91	8.38	278.29	16.29	1	8/1/2014 8:28:47.97
2487	RSRD19	-122.452	37.944	7.83	8.35	278.12	16.18	-122.452	37.944	7.91	8.35	278.27	16.26	1	8/1/2014 8:28:48.07
2488	RSRD19	-122.452	37.944	7.87	8.38	278.10	16.25	-122.452	37.944	7.91	8.38	278.25	16.29	1	8/1/2014 8:28:48.17

2489	RSRD19	-122.452	37.944	7.83	8.37	278.10	16.20	-122.452	37.944	7.91	8.37	278.23	16.28	1	8/1/2014	8:28:48.28
2490	RSRD19	-122.452	37.944	7.87	8.36	278.08	16.23	-122.452	37.944	7.91	8.36	278.24	16.27	1	8/1/2014	8:28:48.38
2491	RSRD19	-122.452	37.944	7.83	8.37	278.08	16.20	-122.452	37.944	7.91	8.37	278.21	16.28	1	8/1/2014	8:28:48.47
2492	RSRD19	-122.452	37.944	7.83	8.37	278.04	16.20	-122.452	37.944	7.91	8.37	278.18	16.28	1	8/1/2014	8:28:48.57
2493	RSRD19	-122.452	37.944	7.83	8.33	278.07	16.17	-122.452	37.944	7.86	8.33	278.17	16.20	1	8/1/2014	8:28:48.68
2494	RSRD19	-122.452	37.944	7.83	8.35	278.00	16.18	-122.452	37.944	7.91	8.35	278.14	16.26	1	8/1/2014	8:28:48.78
2495	RSRD19	-122.452	37.944	7.83	8.35	278.05	16.18	-122.452	37.944	7.91	8.35	278.14	16.26	1	8/1/2014	8:28:48.87
2496	RSRD19	-122.452	37.944	7.83	8.37	277.99	16.20	-122.452	37.944	7.91	8.37	278.14	16.28	1	8/1/2014	8:28:48.97
2497	RSRD19	-122.452	37.944	7.83	8.35	277.99	16.18	-122.452	37.944	7.91	8.35	278.10	16.26	1	8/1/2014	8:28:49.08
2498	RSRD19	-122.452	37.944	7.83	8.33	277.97	16.17	-122.452	37.944	7.91	8.33	278.12	16.25	1	8/1/2014	8:28:49.18
2499	RSRD19	-122.452	37.944	7.83	8.33	277.97	16.16	-122.452	37.944	7.91	8.33	278.08	16.24	1	8/1/2014	8:28:49.27
2500	RSRD19	-122.452	37.944	7.83	8.33	277.91	16.17	-122.452	37.944	7.91	8.33	278.07	16.25	1	8/1/2014	8:28:49.37
2501	RSRD19	-122.452	37.944	7.80	8.32	277.91	16.12	-122.452	37.944	7.91	8.32	278.02	16.23	1	8/1/2014	8:28:49.49
2502	RSRD19	-122.452	37.944	7.83	8.31	277.87	16.14	-122.452	37.944	7.91	8.31	278.02	16.22	1	8/1/2014	8:28:49.58
2503	RSRD19	-122.452	37.944	7.83	8.30	277.87	16.14	-122.452	37.944	7.91	8.30	278.01	16.22	1	8/1/2014	8:28:49.67
2504	RSRD19	-122.452	37.944	7.83	8.29	277.83	16.12	-122.452	37.944	7.95	8.29	278.01	16.24	1	8/1/2014	8:28:49.77
2505	RSRD19	-122.452	37.944	7.83	8.29	277.81	16.12	-122.452	37.944	7.95	8.29	277.96	16.24	1	8/1/2014	8:28:49.88
2506	RSRD19	-122.452	37.944	7.83	8.27	277.80	16.11	-122.452	37.944	8.00	8.27	277.97	16.27	1	8/1/2014	8:28:49.97
2507	RSRD19	-122.452	37.944	7.83	8.26	277.75	16.09	-122.452	37.944	7.95	8.26	277.92	16.20	1	8/1/2014	8:28:50.07
2508	RSRD19	-122.452	37.944	7.83	8.24	277.78	16.07	-122.452	37.944	8.00	8.24	277.93	16.24	1	8/1/2014	8:28:50.17
2509	RSRD19	-122.452	37.944	7.83	8.23	277.71	16.06	-122.452	37.944	8.00	8.23	277.96	16.23	1	8/1/2014	8:28:50.28
2510	RSRD19	-122.452	37.944	7.87	8.24	277.74	16.11	-122.452	37.944	8.00	8.24	277.90	16.24	1	8/1/2014	8:28:50.37
2511	RSRD19	-122.452	37.944	7.83	8.22	277.72	16.05	-122.452	37.944	8.00	8.22	277.89	16.22	1	8/1/2014	8:28:50.47
2512	RSRD19	-122.452	37.944	7.87	8.22	277.67	16.09	-122.452	37.944	8.00	8.22	277.89	16.22	1	8/1/2014	8:28:50.57
2513	RSRD19	-122.452	37.944	7.83	8.23	277.68	16.06	-122.452	37.944	8.00	8.23	277.85	16.22	1	8/1/2014	8:28:50.68
2514	RSRD19	-122.452	37.944	7.87	8.23	277.68	16.09	-122.452	37.944	8.00	8.23	277.86	16.22	1	8/1/2014	8:28:50.77
2515	RSRD19	-122.452	37.944	7.83	8.22	277.66	16.05	-122.452	37.944	7.95	8.22	277.86	16.17	1	8/1/2014	8:28:50.87
2516	RSRD19	-122.452	37.944	7.87	8.25	277.64	16.11	-122.452	37.944	8.00	8.25	277.82	16.25	1	8/1/2014	8:28:50.97
2517	RSRD19	-122.452	37.944	7.83	8.22	277.62	16.05	-122.452	37.944	7.95	8.22	277.77	16.17	1	8/1/2014	8:28:51.08
2518	RSRD19	-122.452	37.944	7.87	8.21	277.62	16.08	-122.452	37.944	7.95	8.21	277.80	16.16	1	8/1/2014	8:28:51.18
2519	RSRD19	-122.452	37.944	7.83	8.19	277.58	16.03	-122.452	37.944	7.95	8.19	277.73	16.14	1	8/1/2014	8:28:51.27
2520	RSRD19	-122.452	37.944	7.87	8.21	277.54	16.08	-122.452	37.944	7.95	8.21	277.74	16.16	1	8/1/2014	8:28:51.37
2521	RSRD19	-122.452	37.944	7.83	8.20	277.54	16.04	-122.452	37.944	7.95	8.20	277.72	16.15	1	8/1/2014	8:28:51.48

2522	RSRD19	-122.452	37.944	7.87	8.21	277.52	16.08	-122.452	37.944	8.00	8.21	277.67	16.21	1	8/1/2014	8:28:51.58
2523	RSRD19	-122.452	37.944	7.83	8.19	277.46	16.03	-122.452	37.944	7.95	8.19	277.68	16.14	1	8/1/2014	8:28:51.67
2524	RSRD19	-122.452	37.944	7.87	8.19	277.49	16.06	-122.452	37.944	8.00	8.19	277.64	16.19	1	8/1/2014	8:28:51.77
