# Supplementary material for: Chinook salmon and green sturgeon migrate through San Francisco Estuary despite large distortions in the local magnetic field produced by bridges
Source: PLoS One. 2017 Jun 2;12(6):e0169031. doi: 10.1371/journal.pone.0169031 (PMC5456031; doi:10.1371/journal.pone.0169031)
Supplement: S3 Fig — (PDF) [file pone.0169031.s003.pdf]

Record	Set	Longitude1	Latitude1	Z1	ALT1	T1	Alt+Depth1	Longitude2	Latitude2	Z2	ALT2	T2	Alt+Depth2	LINE	DATE	TIME
1	RSPe_2	-122.445	37.927	-0.34	15.09	291.59	14.75	-122.445	37.927	-0.32	15.09	291.81	14.77	9	7/14/2014	50:24.9
2	RSPe_2	-122.445	37.927	-0.39	15.19	291.55	14.80	-122.445	37.927	-0.36	15.19	291.85	14.84	9	7/14/2014	50:24.8
3	RSPe_2	-122.445	37.927	-0.34	15.23	291.57	14.89	-122.445	37.927	-0.36	15.23	291.92	14.88	9	7/14/2014	50:24.7
4	RSPe_2	-122.445	37.927	-0.39	15.32	291.64	14.93	-122.445	37.927	-0.41	15.32	291.99	14.92	9	7/14/2014	50:24.6
5	RSPe_2	-122.445	37.927	-0.39	15.32	291.60	14.93	-122.445	37.927	-0.41	15.32	291.94	14.92	9	7/14/2014	50:24.5
6	RSPe_2	-122.445	37.927	-0.39	15.31	291.53	14.91	-122.445	37.927	-0.44	15.31	291.85	14.87	9	7/14/2014	50:24.4
7	RSPe_2	-122.445	37.927	-0.39	15.30	291.51	14.91	-122.445	37.927	-0.44	15.30	291.77	14.86	9	7/14/2014	50:24.3
8	RSPe_2	-122.445	37.927	-0.43	15.35	291.53	14.92	-122.445	37.927	-0.49	15.35	291.66	14.85	9	7/14/2014	50:24.2
9	RSPe_2	-122.445	37.927	-0.39	15.31	291.60	14.91	-122.445	37.927	-0.36	15.31	291.57	14.95	9	7/14/2014	50:24.1
10	RSPe_2	-122.445	37.927	-0.43	15.27	291.67	14.84	-122.445	37.927	-0.49	15.27	291.57	14.78	9	7/14/2014	50:24.0
11	RSPe_2	-122.445	37.927	-0.39	15.28	291.75	14.88	-122.445	37.927	-0.53	15.28	291.53	14.75	9	7/14/2014	50:23.9
12	RSPe_2	-122.445	37.927	-0.43	15.29	291.86	14.86	-122.445	37.927	-0.44	15.29	291.57	14.85	9	7/14/2014	50:23.8
13	RSPe_2	-122.445	37.927	-0.39	15.26	291.91	14.87	-122.445	37.927	-0.58	15.26	291.66	14.69	9	7/14/2014	50:23.7
14	RSPe_2	-122.445	37.927	-0.43	15.24	291.88	14.81	-122.445	37.927	-0.36	15.24	291.67	14.88	9	7/14/2014	50:23.6
15	RSPe_2	-122.445	37.927	-0.43	15.25	291.80	14.82	-122.445	37.927	-0.44	15.25	291.64	14.81	9	7/14/2014	50:23.5
16	RSPe_2	-122.445	37.927	-0.48	15.27	291.80	14.79	-122.445	37.927	-0.53	15.27	291.71	14.74	9	7/14/2014	50:23.4
17	RSPe_2	-122.445	37.927	-0.43	15.22	291.71	14.79	-122.445	37.927	-0.49	15.22	291.75	14.73	9	7/14/2014	50:23.3
18	RSPe_2	-122.445	37.927	-0.48	15.28	291.69	14.80	-122.445	37.927	-0.58	15.28	291.86	14.70	9	7/14/2014	50:23.2
19	RSPe_2	-122.445	37.927	-0.43	15.31	291.78	14.88	-122.445	37.927	-0.58	15.31	292.02	14.73	9	7/14/2014	50:23.1
20	RSPe_2	-122.445	37.927	-0.48	15.42	291.73	14.94	-122.445	37.927	-0.61	15.42	291.93	14.81	9	7/14/2014	50:23.0
21	RSPe_2	-122.445	37.927	-0.48	15.36	291.69	14.88	-122.445	37.927	-0.53	15.36	291.80	14.83	9	7/14/2014	50:22.9
22	RSPe_2	-122.445	37.927	-0.51	15.43	291.69	14.92	-122.445	37.927	-0.61	15.43	291.65	14.82	9	7/14/2014	50:22.8
23	RSPe_2	-122.445	37.927	-0.48	15.39	291.85	14.91	-122.445	37.927	-0.61	15.39	291.66	14.78	9	7/14/2014	50:22.7
24	RSPe_2	-122.445	37.927	-0.48	15.40	291.85	14.92	-122.445	37.927	-0.58	15.40	291.62	14.83	9	7/14/2014	50:22.6
25	RSPe_2	-122.445	37.927	-0.51	15.50	291.85	14.99	-122.445	37.927	-0.61	15.50	291.58	14.89	9	7/14/2014	50:22.5
26	RSPe_2	-122.445	37.927	-0.48	15.52	291.80	15.04	-122.445	37.927	-0.58	15.52	291.62	14.94	9	7/14/2014	50:22.4
27	RSPe_2	-122.445	37.927	-0.48	15.53	291.80	15.05	-122.445	37.927	-0.64	15.53	291.67	14.89	9	7/14/2014	50:22.3
28	RSPe_2	-122.445	37.927	-0.51	15.56	291.65	15.05	-122.445	37.927	-0.61	15.56	291.69	14.95	9	7/14/2014	50:22.2
29	RSPe_2	-122.445	37.927	-0.48	15.55	291.67	15.07	-122.445	37.927	-0.61	15.55	291.75	14.94	9	7/14/2014	50:22.1
30	RSPe_2	-122.445	37.927	-0.51	15.58	291.70	15.07	-122.445	37.927	-0.58	15.58	291.81	15.00	9	7/14/2014	50:22.0
31	RSPe_2	-122.445	37.927	-0.48	15.59	291.65	15.11	-122.445	37.927	-0.61	15.59	291.62	14.98	9	7/14/2014	50:21.9
32	RSPe_2	-122.445	37.927	-0.51	15.54	291.54	15.02	-122.445	37.927	-0.64	15.54	291.58	14.89	9	7/14/2014	50:21.8

33	RSPe_2	-122.445	37.927	-0.48	15.59	291.48	15.11	-122.445	37.927	-0.64	15.59	291.54	14.94	9	7/14/2014	50:21.7
34	RSPe_2	-122.445	37.927	-0.51	15.60	291.52	15.09	-122.445	37.927	-0.64	15.60	291.61	14.96	9	7/14/2014	50:21.6
35	RSPe_2	-122.445	37.927	-0.48	15.61	291.57	15.13	-122.445	37.927	-0.61	15.61	291.63	15.00	9	7/14/2014	50:21.5
36	RSPe_2	-122.445	37.927	-0.51	15.62	291.61	15.10	-122.445	37.927	-0.69	15.62	291.54	14.92	9	7/14/2014	50:21.4
37	RSPe_2	-122.445	37.927	-0.51	15.53	291.65	15.02	-122.445	37.927	-0.64	15.53	291.48	14.89	9	7/14/2014	50:21.3
38	RSPe_2	-122.445	37.927	-0.51	15.51	291.61	15.00	-122.445	37.927	-0.64	15.51	291.47	14.87	9	7/14/2014	50:21.2
39	RSPe_2	-122.445	37.927	-0.51	15.52	291.70	15.00	-122.445	37.927	-0.69	15.52	291.50	14.82	9	7/14/2014	50:21.1
40	RSPe_2	-122.445	37.927	-0.51	15.51	291.57	15.00	-122.445	37.927	-0.69	15.51	291.52	14.82	9	7/14/2014	50:21.0
41	RSPe_2	-122.445	37.927	-0.48	15.50	291.63	15.02	-122.445	37.927	-0.69	15.50	291.58	14.81	9	7/14/2014	50:20.9
42	RSPe_2	-122.445	37.927	-0.51	15.49	291.59	14.98	-122.445	37.927	-0.73	15.49	291.58	14.77	9	7/14/2014	50:20.8
43	RSPe_2	-122.445	37.927	-0.51	15.51	291.57	15.00	-122.445	37.927	-0.69	15.51	291.63	14.82	9	7/14/2014	50:20.7
44	RSPe_2	-122.445	37.927	-0.51	15.49	291.53	14.98	-122.445	37.927	-0.73	15.49	291.52	14.77	9	7/14/2014	50:20.6
45	RSPe_2	-122.445	37.927	-0.51	15.58	291.44	15.07	-122.445	37.927	-0.73	15.58	291.50	14.85	9	7/14/2014	50:20.5
46	RSPe_2	-122.445	37.927	-0.51	15.49	291.42	14.98	-122.445	37.927	-0.73	15.49	291.48	14.77	9	7/14/2014	50:20.4
47	RSPe_2	-122.445	37.927	-0.48	15.52	291.44	15.04	-122.445	37.927	-0.73	15.52	291.46	14.79	9	7/14/2014	50:20.3
48	RSPe_2	-122.445	37.927	-0.51	15.56	291.48	15.05	-122.445	37.927	-0.73	15.56	291.48	14.84	9	7/14/2014	50:20.2
49	RSPe_2	-122.445	37.927	-0.48	15.60	291.48	15.12	-122.445	37.927	-0.69	15.60	291.55	14.91	9	7/14/2014	50:20.1
50	RSPe_2	-122.445	37.927	-0.51	15.64	291.51	15.13	-122.445	37.927	-0.73	15.64	291.52	14.91	9	7/14/2014	50:20.0
51	RSPe_2	-122.445	37.927	-0.48	15.66	291.53	15.18	-122.445	37.927	-0.64	15.66	291.50	15.02	9	7/14/2014	50:19.9
52	RSPe_2	-122.445	37.927	-0.48	15.75	291.46	15.27	-122.445	37.927	-0.69	15.75	291.48	15.05	9	7/14/2014	50:19.8
53	RSPe_2	-122.445	37.927	-0.48	15.70	291.40	15.22	-122.445	37.927	-0.73	15.70	291.35	14.97	9	7/14/2014	50:19.7
54	RSPe_2	-122.445	37.927	-0.51	15.72	291.36	15.20	-122.445	37.927	-0.73	15.72	291.35	14.99	9	7/14/2014	50:19.6
55	RSPe_2	-122.445	37.927	-0.48	15.78	291.37	15.30	-122.445	37.927	-0.69	15.78	291.30	15.08	9	7/14/2014	50:19.5
56	RSPe_2	-122.445	37.927	-0.51	15.76	291.31	15.25	-122.445	37.927	-0.78	15.76	291.26	14.98	9	7/14/2014	50:19.4
57	RSPe_2	-122.445	37.927	-0.48	15.75	291.31	15.27	-122.445	37.927	-0.69	15.75	291.25	15.05	9	7/14/2014	50:19.3
58	RSPe_2	-122.445	37.927	-0.51	15.72	291.38	15.20	-122.445	37.927	-0.69	15.72	291.24	15.02	9	7/14/2014	50:19.2
59	RSPe_2	-122.445	37.927	-0.48	15.69	291.34	15.21	-122.445	37.927	-0.69	15.69	291.25	14.99	9	7/14/2014	50:19.1
60	RSPe_2	-122.445	37.927	-0.51	15.72	291.36	15.20	-122.445	37.927	-0.73	15.72	291.24	14.99	9	7/14/2014	50:19.0
61	RSPe_2	-122.445	37.927	-0.48	15.73	291.29	15.25	-122.445	37.927	-0.73	15.73	291.24	15.00	9	7/14/2014	50:18.9
62	RSPe_2	-122.445	37.927	-0.51	15.66	291.29	15.15	-122.445	37.927	-0.73	15.66	291.23	14.94	9	7/14/2014	50:18.8
63	RSPe_2	-122.445	37.927	-0.48	15.66	291.23	15.18	-122.445	37.927	-0.69	15.66	291.22	14.96	9	7/14/2014	50:18.7
64	RSPe_2	-122.445	37.927	-0.51	15.63	291.31	15.12	-122.445	37.927	-0.78	15.63	291.22	14.85	9	7/14/2014	50:18.6
65	RSPe_2	-122.445	37.927	-0.51	15.63	291.32	15.12	-122.445	37.927	-0.69	15.63	291.25	14.94	9	7/14/2014	50:18.5

66	RSPe_2	-122.445	37.927	-0.51	15.62	291.36	15.10	-122.445	37.927	-0.73	15.62	291.29	14.89	9	7/14/2014	50:18.4
67	RSPe_2	-122.445	37.927	-0.48	15.62	291.47	15.14	-122.445	37.927	-0.73	15.62	291.34	14.89	9	7/14/2014	50:18.3
68	RSPe_2	-122.445	37.927	-0.48	15.62	291.49	15.14	-122.445	37.927	-0.78	15.62	291.33	14.84	9	7/14/2014	50:18.2
69	RSPe_2	-122.445	37.927	-0.48	15.60	291.61	15.12	-122.445	37.927	-0.73	15.60	291.34	14.87	9	7/14/2014	50:18.1
70	RSPe_2	-122.445	37.927	-0.51	15.60	291.54	15.09	-122.445	37.927	-0.73	15.60	291.25	14.87	9	7/14/2014	50:18.0
71	RSPe_2	-122.445	37.927	-0.48	15.59	291.45	15.11	-122.445	37.927	-0.73	15.59	291.27	14.86	9	7/14/2014	50:17.9
72	RSPe_2	-122.445	37.927	-0.48	15.58	291.28	15.10	-122.445	37.927	-0.73	15.58	291.25	14.85	9	7/14/2014	50:17.8
73	RSPe_2	-122.445	37.927	-0.48	15.63	291.14	15.15	-122.445	37.927	-0.73	15.63	291.30	14.90	9	7/14/2014	50:17.7
74	RSPe_2	-122.445	37.927	-0.48	15.66	291.21	15.18	-122.445	37.927	-0.73	15.66	291.40	14.93	9	7/14/2014	50:17.6
75	RSPe_2	-122.445	37.927	-0.48	15.65	291.25	15.17	-122.445	37.927	-0.73	15.65	291.49	14.92	9	7/14/2014	50:17.5
76	RSPe_2	-122.445	37.927	-0.51	15.71	291.32	15.20	-122.445	37.927	-0.78	15.71	291.49	14.93	9	7/14/2014	50:17.4
77	RSPe_2	-122.445	37.927	-0.48	15.76	291.41	15.28	-122.445	37.927	-0.69	15.76	291.45	15.07	9	7/14/2014	50:17.3
78	RSPe_2	-122.445	37.927	-0.51	15.81	291.43	15.30	-122.445	37.927	-0.69	15.81	291.41	15.12	9	7/14/2014	50:17.2
79	RSPe_2	-122.445	37.927	-0.48	15.80	291.41	15.32	-122.445	37.927	-0.78	15.80	291.32	15.02	9	7/14/2014	50:17.1
80	RSPe_2	-122.445	37.927	-0.51	15.94	291.34	15.43	-122.445	37.927	-0.81	15.94	291.21	15.13	9	7/14/2014	50:17.0
81	RSPe_2	-122.445	37.927	-0.51	15.90	291.28	15.39	-122.445	37.927	-0.78	15.90	291.12	15.12	9	7/14/2014	50:16.9
82	RSPe_2	-122.445	37.927	-0.51	15.83	291.19	15.32	-122.445	37.927	-0.73	15.83	291.05	15.10	9	7/14/2014	50:16.8
83	RSPe_2	-122.445	37.927	-0.48	15.72	291.21	15.24	-122.445	37.927	-0.78	15.72	291.03	14.94	9	7/14/2014	50:16.7
84	RSPe_2	-122.445	37.927	-0.48	15.78	291.34	15.30	-122.445	37.927	-0.81	15.78	291.10	14.97	9	7/14/2014	50:16.6
85	RSPe_2	-122.445	37.927	-0.48	15.76	291.32	15.28	-122.445	37.927	-0.81	15.76	291.16	14.94	9	7/14/2014	50:16.5
86	RSPe_2	-122.445	37.927	-0.51	15.71	291.28	15.20	-122.445	37.927	-0.78	15.71	291.25	14.93	9	7/14/2014	50:16.4
87	RSPe_2	-122.445	37.927	-0.48	15.72	291.33	15.24	-122.445	37.927	-0.81	15.72	291.26	14.90	9	7/14/2014	50:16.3
88	RSPe_2	-122.445	37.927	-0.48	15.76	291.37	15.28	-122.445	37.927	-0.87	15.76	291.32	14.89	9	7/14/2014	50:16.2
89	RSPe_2	-122.445	37.927	-0.48	15.87	291.41	15.39	-122.445	37.927	-0.81	15.87	291.34	15.06	9	7/14/2014	50:16.1
90	RSPe_2	-122.445	37.927	-0.48	15.79	291.45	15.31	-122.445	37.927	-0.78	15.79	291.32	15.01	9	7/14/2014	50:16.0
91	RSPe_2	-122.445	37.927	-0.48	15.89	291.39	15.41	-122.445	37.927	-0.78	15.89	291.30	15.11	9	7/14/2014	50:15.9
92	RSPe_2	-122.445	37.927	-0.51	15.80	291.28	15.28	-122.445	37.927	-0.73	15.80	291.23	15.07	9	7/14/2014	50:15.8
93	RSPe_2	-122.445	37.927	-0.48	15.90	291.11	15.42	-122.445	37.927	-0.78	15.90	291.13	15.12	9	7/14/2014	50:15.7
94	RSPe_2	-122.445	37.927	-0.48	15.89	291.00	15.41	-122.445	37.927	-0.81	15.89	291.10	15.07	9	7/14/2014	50:15.6
95	RSPe_2	-122.445	37.927	-0.48	15.90	290.96	15.42	-122.445	37.927	-0.78	15.90	291.06	15.12	9	7/14/2014	50:15.5
96	RSPe_2	-122.445	37.927	-0.51	15.97	290.96	15.45	-122.445	37.927	-0.81	15.97	291.02	15.15	9	7/14/2014	50:15.4
97	RSPe_2	-122.445	37.927	-0.48	15.97	291.04	15.49	-122.445	37.927	-0.81	15.97	291.00	15.16	9	7/14/2014	50:15.3
98	RSPe_2	-122.445	37.927	-0.48	15.97	291.15	15.49	-122.445	37.927	-0.87	15.97	291.00	15.10	9	7/14/2014	50:15.2

99	RSPe_2	-122.445	37.927	-0.48	16.01	291.20	15.53	-122.445	37.927	-0.78	16.01	290.95	15.23	9	7/14/2014	50:15.1
100	RSPe_2	-122.445	37.927	-0.51	15.93	291.22	15.42	-122.445	37.927	-0.81	15.93	290.98	15.12	9	7/14/2014	50:15.0
101	RSPe_2	-122.445	37.927	-0.48	15.96	291.11	15.48	-122.445	37.927	-0.78	15.96	290.90	15.18	9	7/14/2014	50:14.9
102	RSPe_2	-122.445	37.927	-0.48	15.91	291.00	15.43	-122.445	37.927	-0.87	15.91	290.91	15.05	9	7/14/2014	50:14.8
103	RSPe_2	-122.445	37.927	-0.48	15.89	290.92	15.41	-122.445	37.927	-0.81	15.89	290.91	15.07	9	7/14/2014	50:14.7
104	RSPe_2	-122.445	37.927	-0.51	15.96	290.96	15.44	-122.445	37.927	-0.90	15.96	290.93	15.06	9	7/14/2014	50:14.6
105	RSPe_2	-122.445	37.927	-0.48	15.89	291.01	15.41	-122.445	37.927	-0.90	15.89	290.96	14.99	9	7/14/2014	50:14.5
106	RSPe_2	-122.445	37.927	-0.51	15.95	291.05	15.44	-122.445	37.927	-0.87	15.95	290.89	15.08	9	7/14/2014	50:14.4
107	RSPe_2	-122.445	37.927	-0.51	15.80	291.18	15.29	-122.445	37.927	-0.78	15.80	290.93	15.02	9	7/14/2014	50:14.3
108	RSPe_2	-122.445	37.927	-0.51	15.79	291.27	15.27	-122.445	37.927	-0.87	15.79	290.94	14.92	9	7/14/2014	50:14.2
109	RSPe_2	-122.445	37.927	-0.48	15.77	291.31	15.29	-122.445	37.927	-0.81	15.77	290.91	14.96	9	7/14/2014	50:14.1
110	RSPe_2	-122.445	37.927	-0.51	15.73	291.31	15.22	-122.445	37.927	-0.87	15.73	290.89	14.87	9	7/14/2014	50:14.0
111	RSPe_2	-122.445	37.927	-0.51	15.65	291.29	15.14	-122.445	37.927	-0.81	15.65	290.91	14.84	9	7/14/2014	50:13.9
112	RSPe_2	-122.445	37.927	-0.51	15.61	291.31	15.10	-122.445	37.927	-0.81	15.61	290.91	14.80	9	7/14/2014	50:13.8
113	RSPe_2	-122.445	37.927	-0.51	15.60	291.32	15.09	-122.445	37.927	-0.78	15.60	290.99	14.82	9	7/14/2014	50:13.7
114	RSPe_2	-122.445	37.927	-0.51	15.60	291.30	15.08	-122.445	37.927	-0.87	15.60	290.96	14.73	9	7/14/2014	50:13.6
115	RSPe_2	-122.445	37.927	-0.48	15.55	291.27	15.07	-122.445	37.927	-0.78	15.55	291.03	14.77	9	7/14/2014	50:13.5
116	RSPe_2	-122.445	37.927	-0.51	15.57	291.16	15.06	-122.445	37.927	-0.81	15.57	290.98	14.76	9	7/14/2014	50:13.4
117	RSPe_2	-122.445	37.927	-0.48	15.55	291.05	15.07	-122.445	37.927	-0.78	15.55	291.05	14.77	9	7/14/2014	50:13.3
118	RSPe_2	-122.445	37.927	-0.51	15.59	291.01	15.07	-122.445	37.927	-0.81	15.59	291.03	14.77	9	7/14/2014	50:13.2
119	RSPe_2	-122.445	37.927	-0.51	15.69	291.08	15.17	-122.445	37.927	-0.78	15.69	291.18	14.91	9	7/14/2014	50:13.1
120	RSPe_2	-122.445	37.927	-0.48	15.74	291.29	15.26	-122.445	37.927	-0.78	15.74	291.33	14.96	9	7/14/2014	50:13.0
121	RSPe_2	-122.445	37.927	-0.48	15.88	291.39	15.40	-122.445	37.927	-0.81	15.88	291.36	15.07	9	7/14/2014	50:12.9
122	RSPe_2	-122.445	37.927	-0.48	15.90	291.43	15.42	-122.445	37.927	-0.73	15.90	291.38	15.17	9	7/14/2014	50:12.8
123	RSPe_2	-122.445	37.927	-0.51	15.88	291.36	15.37	-122.445	37.927	-0.87	15.88	291.29	15.02	9	7/14/2014	50:12.7
124	RSPe_2	-122.445	37.927	-0.48	15.88	291.34	15.40	-122.445	37.927	-0.73	15.88	291.25	15.15	9	7/14/2014	50:12.6
125	RSPe_2	-122.445	37.927	-0.48	15.93	291.38	15.45	-122.445	37.927	-0.78	15.93	291.23	15.15	9	7/14/2014	50:12.5
126	RSPe_2	-122.445	37.927	-0.48	15.85	291.34	15.37	-122.445	37.927	-0.81	15.85	291.16	15.03	9	7/14/2014	50:12.4
127	RSPe_2	-122.445	37.927	-0.48	15.86	291.41	15.38	-122.445	37.927	-0.81	15.86	291.18	15.05	9	7/14/2014	50:12.3
128	RSPe_2	-122.445	37.927	-0.48	15.89	291.39	15.41	-122.445	37.927	-0.78	15.89	291.20	15.11	9	7/14/2014	50:12.2
129	RSPe_2	-122.445	37.927	-0.48	15.91	291.41	15.43	-122.445	37.927	-0.78	15.91	291.23	15.13	9	7/14/2014	50:12.1
130	RSPe_2	-122.445	37.927	-0.51	15.84	291.32	15.33	-122.445	37.927	-0.78	15.84	291.23	15.06	9	7/14/2014	50:12.0
131	RSPe_2	-122.445	37.927	-0.48	15.92	291.39	15.44	-122.445	37.927	-0.78	15.92	291.30	15.14	9	7/14/2014	50:11.9

132	RSPe_2	-122.445	37.927	-0.48	15.90	291.48	15.42	-122.445	37.927	-0.78	15.90	291.41	15.12	9	7/14/2014	50:11.8
133	RSPe_2	-122.445	37.927	-0.48	15.92	291.54	15.44	-122.445	37.927	-0.78	15.92	291.39	15.14	9	7/14/2014	50:11.7
134	RSPe_2	-122.445	37.927	-0.48	15.91	291.61	15.43	-122.445	37.927	-0.78	15.91	291.38	15.13	9	7/14/2014	50:11.6
135	RSPe_2	-122.445	37.927	-0.48	15.96	291.65	15.48	-122.445	37.927	-0.78	15.96	291.39	15.18	9	7/14/2014	50:11.5
136	RSPe_2	-122.445	37.927	-0.48	15.70	291.72	15.22	-122.445	37.927	-0.78	15.70	291.41	14.92	9	7/14/2014	50:11.4
137	RSPe_2	-122.445	37.927	-0.48	15.70	291.63	15.22	-122.445	37.927	-0.73	15.70	291.36	14.98	9	7/14/2014	50:11.3
138	RSPe_2	-122.445	37.927	-0.48	15.66	291.61	15.19	-122.445	37.927	-0.78	15.66	291.41	14.89	9	7/14/2014	50:11.2
139	RSPe_2	-122.445	37.927	-0.43	15.65	291.53	15.22	-122.445	37.927	-0.78	15.65	291.46	14.87	9	7/14/2014	50:11.1
140	RSPe_2	-122.445	37.927	-0.51	15.60	291.48	15.08	-122.445	37.927	-0.78	15.60	291.56	14.82	9	7/14/2014	50:11.0
141	RSPe_2	-122.445	37.927	-0.43	15.58	291.50	15.15	-122.445	37.927	-0.73	15.58	291.64	14.85	9	7/14/2014	50:10.9
142	RSPe_2	-122.445	37.927	-0.48	15.58	291.46	15.10	-122.445	37.927	-0.86	15.58	291.64	14.72	9	7/14/2014	50:10.8
143	RSPe_2	-122.445	37.927	-0.43	15.58	291.41	15.15	-122.445	37.927	-0.78	15.58	291.65	14.80	9	7/14/2014	50:10.7
144	RSPe_2	-122.445	37.927	-0.48	15.69	291.39	15.21	-122.445	37.927	-0.78	15.69	291.54	14.91	9	7/14/2014	50:10.6
145	RSPe_2	-122.445	37.927	-0.43	15.61	291.46	15.18	-122.445	37.927	-0.73	15.61	291.57	14.88	9	7/14/2014	50:10.5
146	RSPe_2	-122.445	37.927	-0.48	15.63	291.48	15.15	-122.445	37.927	-0.73	15.63	291.59	14.91	9	7/14/2014	50:10.4
147	RSPe_2	-122.445	37.927	-0.43	15.69	291.53	15.26	-122.445	37.927	-0.78	15.69	291.54	14.91	9	7/14/2014	50:10.3
148	RSPe_2	-122.445	37.927	-0.48	15.71	291.55	15.23	-122.445	37.927	-0.78	15.71	291.46	14.93	9	7/14/2014	50:10.2
149	RSPe_2	-122.445	37.927	-0.43	15.63	291.62	15.21	-122.445	37.927	-0.78	15.63	291.50	14.85	9	7/14/2014	50:10.1
150	RSPe_2	-122.445	37.927	-0.43	15.73	291.61	15.30	-122.445	37.927	-0.73	15.73	291.41	15.00	9	7/14/2014	50:10.0
151	RSPe_2	-122.445	37.927	-0.43	15.84	291.57	15.41	-122.445	37.927	-0.73	15.84	291.40	15.11	9	7/14/2014	50:09.9
152	RSPe_2	-122.445	37.927	-0.48	15.70	291.53	15.22	-122.445	37.927	-0.69	15.70	291.37	15.01	9	7/14/2014	50:09.8
153	RSPe_2	-122.445	37.927	-0.43	15.70	291.55	15.28	-122.445	37.927	-0.73	15.70	291.35	14.98	9	7/14/2014	50:09.7
154	RSPe_2	-122.445	37.927	-0.43	15.78	291.64	15.35	-122.445	37.927	-0.78	15.78	291.48	15.00	9	7/14/2014	50:09.6
155	RSPe_2	-122.445	37.927	-0.43	15.73	291.80	15.30	-122.445	37.927	-0.73	15.73	291.55	15.00	9	7/14/2014	50:09.5
156	RSPe_2	-122.445	37.927	-0.48	15.69	291.93	15.22	-122.445	37.927	-0.78	15.69	291.67	14.92	9	7/14/2014	50:09.4
157	RSPe_2	-122.445	37.927	-0.48	15.66	291.82	15.19	-122.445	37.927	-0.73	15.66	291.46	14.94	9	7/14/2014	50:09.3
158	RSPe_2	-122.445	37.927	-0.48	15.72	291.86	15.24	-122.445	37.927	-0.69	15.72	291.51	15.02	9	7/14/2014	50:09.2
159	RSPe_2	-122.445	37.927	-0.48	15.62	291.97	15.14	-122.445	37.927	-0.69	15.62	291.62	14.92	9	7/14/2014	50:09.1
160	RSPe_2	-122.445	37.927	-0.48	15.60	291.75	15.12	-122.445	37.927	-0.61	15.60	291.53	14.99	9	7/14/2014	50:09.0
161	RSPe_2	-122.445	37.927	-0.43	15.51	291.75	15.08	-122.445	37.927	-0.64	15.51	291.53	14.87	9	7/14/2014	50:08.9
162	RSPe_2	-122.445	37.927	-0.48	15.49	291.84	15.02	-122.445	37.927	-0.69	15.49	291.64	14.80	9	7/14/2014	50:08.8
163	RSPe_2	-122.445	37.927	-0.43	15.49	291.89	15.07	-122.445	37.927	-0.73	15.49	291.77	14.77	9	7/14/2014	50:08.7
164	RSPe_2	-122.445	37.927	-0.48	15.48	292.00	15.00	-122.445	37.927	-0.73	15.48	291.98	14.75	9	7/14/2014	50:08.6

165	RSPe_2	-122.445	37.927	-0.43	15.51	291.95	15.08	-122.445	37.927	-0.73	15.51	292.02	14.78	9	7/14/2014	50:08.5
166	RSPe_2	-122.445	37.927	-0.48	15.52	291.89	15.04	-122.445	37.927	-0.78	15.52	292.04	14.74	9	7/14/2014	50:08.4
167	RSPe_2	-122.445	37.927	-0.43	15.55	291.89	15.12	-122.445	37.927	-0.69	15.55	292.02	14.85	9	7/14/2014	50:08.3
168	RSPe_2	-122.445	37.927	-0.48	15.52	291.82	15.04	-122.445	37.927	-0.69	15.52	291.97	14.82	9	7/14/2014	50:08.2
169	RSPe_2	-122.445	37.927	-0.43	15.57	291.80	15.14	-122.445	37.927	-0.73	15.57	291.93	14.84	9	7/14/2014	50:08.1
170	RSPe_2	-122.445	37.927	-0.43	15.61	291.87	15.18	-122.445	37.927	-0.69	15.61	291.93	14.92	9	7/14/2014	50:08.0
171	RSPe_2	-122.445	37.927	-0.43	15.64	291.89	15.21	-122.445	37.927	-0.78	15.64	291.95	14.86	9	7/14/2014	50:07.9
172	RSPe_2	-122.445	37.927	-0.48	15.80	291.91	15.32	-122.445	37.927	-0.73	15.80	291.97	15.07	9	7/14/2014	50:07.8
173	RSPe_2	-122.445	37.927	-0.43	15.82	291.78	15.39	-122.445	37.927	-0.58	15.82	291.93	15.24	9	7/14/2014	50:07.7
174	RSPe_2	-122.445	37.927	-0.43	15.86	291.61	15.44	-122.445	37.927	-0.78	15.86	291.89	15.09	9	7/14/2014	50:07.6
175	RSPe_2	-122.445	37.927	-0.43	15.80	291.61	15.37	-122.445	37.927	-0.78	15.80	291.89	15.02	9	7/14/2014	50:07.5
176	RSPe_2	-122.445	37.927	-0.43	15.84	291.63	15.41	-122.445	37.927	-0.78	15.84	291.89	15.06	9	7/14/2014	50:07.4
177	RSPe_2	-122.445	37.927	-0.43	15.85	291.70	15.42	-122.445	37.927	-0.78	15.85	291.91	15.07	9	7/14/2014	50:07.3
178	RSPe_2	-122.445	37.927	-0.43	15.81	291.80	15.38	-122.445	37.927	-0.90	15.81	291.91	14.91	9	7/14/2014	50:07.2
179	RSPe_2	-122.445	37.927	-0.43	15.84	291.98	15.41	-122.445	37.927	-0.81	15.84	291.91	15.03	9	7/14/2014	50:07.1
180	RSPe_2	-122.445	37.927	-0.43	15.82	292.09	15.39	-122.445	37.927	-0.64	15.82	291.89	15.17	9	7/14/2014	50:07.0
181	RSPe_2	-122.445	37.927	-0.43	15.92	292.05	15.49	-122.445	37.927	-0.69	15.92	291.80	15.22	9	7/14/2014	50:06.9
182	RSPe_2	-122.445	37.927	-0.43	15.73	292.03	15.31	-122.445	37.927	-0.73	15.73	291.80	15.01	9	7/14/2014	50:06.8
183	RSPe_2	-122.445	37.927	-0.43	15.71	291.98	15.28	-122.445	37.927	-0.69	15.71	291.72	15.02	9	7/14/2014	50:06.7
184	RSPe_2	-122.445	37.927	-0.43	15.66	291.90	15.24	-122.445	37.927	-0.73	15.66	291.63	14.94	9	7/14/2014	50:06.6
185	RSPe_2	-122.445	37.927	-0.43	15.62	291.83	15.19	-122.445	37.927	-0.64	15.62	291.63	14.97	9	7/14/2014	50:06.5
186	RSPe_2	-122.445	37.927	-0.43	15.70	291.81	15.28	-122.445	37.927	-0.78	15.70	291.69	14.92	9	7/14/2014	50:06.4
187	RSPe_2	-122.445	37.927	-0.43	15.73	291.83	15.30	-122.445	37.927	-0.78	15.73	291.76	14.95	9	7/14/2014	50:06.3
188	RSPe_2	-122.445	37.927	-0.48	15.75	291.81	15.27	-122.445	37.927	-0.73	15.75	291.80	15.02	9	7/14/2014	50:06.2
189	RSPe_2	-122.445	37.927	-0.43	15.80	291.83	15.37	-122.445	37.927	-0.73	15.80	291.94	15.07	9	7/14/2014	50:06.1
190	RSPe_2	-122.445	37.927	-0.43	15.89	291.90	15.46	-122.445	37.927	-0.81	15.89	292.03	15.07	9	7/14/2014	50:06.0
191	RSPe_2	-122.445	37.927	-0.43	15.90	291.97	15.47	-122.445	37.927	-0.81	15.90	292.10	15.08	9	7/14/2014	50:05.9
192	RSPe_2	-122.445	37.927	-0.43	15.93	291.99	15.50	-122.445	37.927	-0.81	15.93	292.09	15.12	9	7/14/2014	50:05.8
193	RSPe_2	-122.445	37.927	-0.43	15.97	292.12	15.54	-122.445	37.927	-0.86	15.97	292.09	15.10	9	7/14/2014	50:05.7
194	RSPe_2	-122.445	37.927	-0.48	15.95	292.21	15.47	-122.445	37.927	-0.69	15.95	291.99	15.26	9	7/14/2014	50:05.6
195	RSPe_2	-122.445	37.927	-0.43	15.93	292.38	15.50	-122.445	37.927	-0.81	15.93	291.98	15.12	9	7/14/2014	50:05.5
196	RSPe_2	-122.445	37.927	-0.48	15.97	292.43	15.49	-122.445	37.927	-0.61	15.97	291.94	15.36	9	7/14/2014	50:05.4
197	RSPe_2	-122.445	37.927	-0.43	15.86	292.43	15.43	-122.445	37.927	-0.69	15.86	291.92	15.16	9	7/14/2014	50:05.3

198	RSPe_2	-122.445	37.927	-0.48	15.67	292.41	15.19	-122.445	37.927	-0.81	15.67	291.96	14.86	9	7/14/2014	50:05.2
199	RSPe_2	-122.445	37.927	-0.48	15.66	292.19	15.19	-122.445	37.927	-0.81	15.66	291.95	14.85	9	7/14/2014	50:05.1
200	RSPe_2	-122.445	37.927	-0.48	15.57	292.06	15.09	-122.445	37.927	-0.78	15.57	292.01	14.79	9	7/14/2014	50:05.0
201	RSPe_2	-122.445	37.927	-0.43	15.58	291.97	15.15	-122.445	37.927	-0.86	15.58	292.10	14.72	9	7/14/2014	50:04.9
202	RSPe_2	-122.445	37.927	-0.48	15.69	291.90	15.22	-122.445	37.927	-0.81	15.69	292.19	14.88	9	7/14/2014	50:04.8
203	RSPe_2	-122.445	37.927	-0.43	15.78	291.93	15.35	-122.445	37.927	-0.86	15.78	292.21	14.92	9	7/14/2014	50:04.7
204	RSPe_2	-122.445	37.927	-0.48	15.79	291.99	15.31	-122.445	37.927	-0.81	15.79	292.28	14.98	9	7/14/2014	50:04.6
205	RSPe_2	-122.445	37.927	-0.48	15.83	292.06	15.35	-122.445	37.927	-0.90	15.83	292.23	14.94	9	7/14/2014	50:04.5
206	RSPe_2	-122.445	37.927	-0.43	15.86	292.15	15.44	-122.445	37.927	-0.81	15.86	292.23	15.05	9	7/14/2014	50:04.4
207	RSPe_2	-122.445	37.927	-0.43	15.90	292.23	15.47	-122.445	37.927	-0.98	15.90	292.24	14.91	9	7/14/2014	50:04.3
208	RSPe_2	-122.445	37.927	-0.48	15.91	292.33	15.43	-122.445	37.927	-0.90	15.91	292.21	15.01	9	7/14/2014	50:04.2
209	RSPe_2	-122.445	37.927	-0.43	15.94	292.28	15.51	-122.445	37.927	-0.81	15.94	292.17	15.13	9	7/14/2014	50:04.1
210	RSPe_2	-122.445	37.927	-0.43	15.97	292.28	15.54	-122.445	37.927	-0.81	15.97	292.12	15.16	9	7/14/2014	50:04.0
211	RSPe_2	-122.445	37.927	-0.43	15.92	292.26	15.49	-122.445	37.927	-0.78	15.92	292.19	15.14	9	7/14/2014	50:03.9
212	RSPe_2	-122.445	37.927	-0.48	15.92	292.19	15.44	-122.445	37.927	-0.81	15.92	292.21	15.11	9	7/14/2014	50:03.8
213	RSPe_2	-122.445	37.927	-0.43	15.95	292.24	15.52	-122.445	37.927	-0.78	15.95	292.24	15.17	9	7/14/2014	50:03.7
214	RSPe_2	-122.445	37.927	-0.43	15.96	292.28	15.53	-122.445	37.927	-0.98	15.96	292.32	14.97	9	7/14/2014	50:03.6
215	RSPe_2	-122.445	37.927	-0.39	15.94	292.30	15.55	-122.445	37.927	-0.90	15.94	292.32	15.04	9	7/14/2014	50:03.5
216	RSPe_2	-122.445	37.927	-0.48	15.91	292.20	15.43	-122.445	37.927	-0.98	15.91	292.28	14.93	9	7/14/2014	50:03.4
217	RSPe_2	-122.445	37.927	-0.43	15.93	292.20	15.51	-122.445	37.927	-0.86	15.93	292.22	15.07	9	7/14/2014	50:03.3
218	RSPe_2	-122.445	37.927	-0.43	15.95	292.15	15.52	-122.445	37.927	-0.86	15.95	292.11	15.09	9	7/14/2014	50:03.2
219	RSPe_2	-122.445	37.927	-0.39	15.95	292.13	15.56	-122.445	37.927	-0.95	15.95	292.15	15.00	9	7/14/2014	50:03.1
220	RSPe_2	-122.445	37.927	-0.43	16.08	292.22	15.65	-122.445	37.927	-0.69	16.08	292.17	15.39	9	7/14/2014	50:03.0
221	RSPe_2	-122.445	37.927	-0.39	16.04	292.37	15.65	-122.445	37.927	-0.86	16.04	292.15	15.18	9	7/14/2014	50:02.9
222	RSPe_2	-122.445	37.927	-0.39	15.88	292.35	15.49	-122.445	37.927	-0.78	15.88	292.10	15.10	9	7/14/2014	50:02.8
223	RSPe_2	-122.445	37.927	-0.39	15.85	292.33	15.46	-122.445	37.927	-0.64	15.85	292.15	15.20	9	7/14/2014	50:02.7
224	RSPe_2	-122.445	37.927	-0.39	15.82	292.33	15.42	-122.445	37.927	-0.73	15.82	292.10	15.09	9	7/14/2014	50:02.6
225	RSPe_2	-122.445	37.927	-0.34	15.82	292.29	15.48	-122.445	37.927	-0.73	15.82	292.11	15.09	9	7/14/2014	50:02.5
226	RSPe_2	-122.445	37.927	-0.39	15.75	292.33	15.36	-122.445	37.927	-0.69	15.75	292.11	15.05	9	7/14/2014	50:02.4
227	RSPe_2	-122.445	37.927	-0.34	15.82	292.36	15.48	-122.445	37.927	-0.73	15.82	292.16	15.09	9	7/14/2014	50:02.3
228	RSPe_2	-122.445	37.927	-0.34	15.70	292.36	15.36	-122.445	37.927	-0.78	15.70	292.22	14.92	9	7/14/2014	50:02.2
229	RSPe_2	-122.445	37.927	-0.34	15.80	292.36	15.46	-122.445	37.927	-0.64	15.80	292.22	15.16	9	7/14/2014	50:02.1
230	RSPe_2	-122.445	37.927	-0.34	15.80	292.36	15.45	-122.445	37.927	-0.73	15.80	292.24	15.07	9	7/14/2014	50:02.0

231	RSPe_2	-122.445	37.927	-0.31	15.83	292.36	15.52	-122.445	37.927	-0.78	15.83	292.29	15.05	9	7/14/2014	50:01.9
232	RSPe_2	-122.445	37.927	-0.31	15.83	292.29	15.52	-122.445	37.927	-0.61	15.83	292.24	15.22	9	7/14/2014	50:01.8
233	RSPe_2	-122.445	37.927	-0.31	15.84	292.20	15.53	-122.445	37.927	-0.64	15.84	292.24	15.20	9	7/14/2014	50:01.7
234	RSPe_2	-122.445	37.927	-0.34	15.90	292.29	15.56	-122.445	37.927	-0.64	15.90	292.23	15.26	9	7/14/2014	50:01.6
235	RSPe_2	-122.445	37.927	-0.31	15.92	292.32	15.61	-122.445	37.927	-0.44	15.92	292.14	15.48	9	7/14/2014	50:01.5
236	RSPe_2	-122.445	37.927	-0.31	15.76	292.36	15.45	-122.445	37.927	-0.64	15.76	292.20	15.12	9	7/14/2014	50:01.4
237	RSPe_2	-122.445	37.927	-0.26	15.76	292.32	15.51	-122.445	37.927	-0.58	15.76	292.14	15.19	9	7/14/2014	50:01.3
238	RSPe_2	-122.445	37.927	-0.31	15.73	292.43	15.42	-122.445	37.927	-0.58	15.73	292.11	15.15	9	7/14/2014	50:01.2
239	RSPe_2	-122.445	37.927	-0.26	15.71	292.47	15.45	-122.445	37.927	-0.58	15.71	292.14	15.13	9	7/14/2014	50:01.1
240	RSPe_2	-122.445	37.927	-0.31	15.66	292.51	15.36	-122.445	37.927	-0.44	15.66	292.11	15.22	9	7/14/2014	50:01.0
241	RSPe_2	-122.445	37.927	-0.26	15.64	292.45	15.38	-122.445	37.927	-0.58	15.64	292.09	15.06	9	7/14/2014	50:00.9
242	RSPe_2	-122.445	37.927	-0.26	15.60	292.43	15.34	-122.445	37.927	-0.44	15.60	292.08	15.15	9	7/14/2014	50:00.8
243	RSPe_2	-122.445	37.927	-0.26	15.54	292.39	15.28	-122.445	37.927	-0.52	15.54	292.12	15.01	9	7/14/2014	50:00.7
244	RSPe_2	-122.445	37.927	-0.26	15.48	292.34	15.22	-122.445	37.927	-0.41	15.48	292.14	15.07	9	7/14/2014	50:00.6
245	RSPe_2	-122.445	37.927	-0.26	15.37	292.19	15.11	-122.445	37.927	-0.44	15.37	292.12	14.93	9	7/14/2014	50:00.5
246	RSPe_2	-122.445	37.927	-0.26	15.31	292.12	15.05	-122.445	37.927	-0.44	15.31	292.16	14.87	9	7/14/2014	50:00.4
247	RSPe_2	-122.445	37.927	-0.22	15.36	292.21	15.14	-122.445	37.927	-0.36	15.36	292.32	15.01	9	7/14/2014	50:00.3
248	RSPe_2	-122.445	37.927	-0.22	15.45	292.28	15.22	-122.445	37.927	-0.58	15.45	292.32	14.87	9	7/14/2014	50:00.2
249	RSPe_2	-122.445	37.927	-0.22	15.44	292.25	15.22	-122.445	37.927	-0.61	15.44	292.34	14.83	9	7/14/2014	50:00.1
250	RSPe_2	-122.445	37.927	-0.22	15.46	292.30	15.24	-122.445	37.927	-0.58	15.46	292.36	14.89	9	7/14/2014	50:00.0
251	RSPe_2	-122.445	37.927	-0.22	15.46	292.39	15.23	-122.445	37.927	-0.36	15.46	292.32	15.10	9	7/14/2014	49:59.9
252	RSPe_2	-122.445	37.927	-0.22	15.44	292.37	15.22	-122.445	37.927	-0.44	15.44	292.21	15.00	9	7/14/2014	49:59.8
253	RSPe_2	-122.445	37.927	-0.22	15.53	292.39	15.30	-122.445	37.927	-0.32	15.53	292.18	15.20	9	7/14/2014	49:59.7
254	RSPe_2	-122.445	37.927	-0.22	15.49	292.41	15.27	-122.445	37.927	-0.44	15.49	292.10	15.05	9	7/14/2014	49:59.6
255	RSPe_2	-122.445	37.927	-0.22	15.40	292.35	15.18	-122.445	37.927	-0.24	15.40	292.08	15.17	9	7/14/2014	49:59.5
256	RSPe_2	-122.445	37.927	-0.22	15.42	292.28	15.19	-122.445	37.927	-0.32	15.42	292.10	15.09	9	7/14/2014	49:59.4
257	RSPe_2	-122.445	37.927	-0.19	15.47	292.24	15.28	-122.445	37.927	-0.32	15.47	292.10	15.15	9	7/14/2014	49:59.3
258	RSPe_2	-122.445	37.927	-0.22	15.44	292.22	15.22	-122.445	37.927	-0.41	15.44	292.12	15.03	9	7/14/2014	49:59.2
259	RSPe_2	-122.445	37.927	-0.22	15.51	292.24	15.29	-122.445	37.927	-0.36	15.51	292.17	15.15	9	7/14/2014	49:59.1
260	RSPe_2	-122.445	37.927	-0.22	15.49	292.28	15.27	-122.445	37.927	-0.41	15.49	292.24	15.09	9	7/14/2014	49:59.0
261	RSPe_2	-122.445	37.927	-0.22	15.48	292.26	15.26	-122.445	37.927	-0.24	15.48	292.32	15.25	9	7/14/2014	49:58.9
262	RSPe_2	-122.445	37.927	-0.22	15.56	292.44	15.33	-122.445	37.927	-0.41	15.56	292.32	15.15	9	7/14/2014	49:58.8
263	RSPe_2	-122.445	37.927	-0.22	15.62	292.46	15.40	-122.445	37.927	-0.41	15.62	292.32	15.22	9	7/14/2014	49:58.7

264	RSPe_2	-122.445	37.927	-0.22	15.54	292.42	15.32	-122.445	37.927	-0.49	15.54	292.28	15.05	9	7/14/2014	49:58.6
265	RSPe_2	-122.445	37.927	-0.22	15.60	292.37	15.37	-122.445	37.927	-0.27	15.60	292.19	15.32	9	7/14/2014	49:58.5
266	RSPe_2	-122.445	37.927	-0.22	15.50	292.24	15.28	-122.445	37.927	-0.36	15.50	292.08	15.15	9	7/14/2014	49:58.4
267	RSPe_2	-122.445	37.927	-0.22	15.47	292.20	15.25	-122.445	37.927	-0.41	15.47	292.06	15.07	9	7/14/2014	49:58.3
268	RSPe_2	-122.445	37.927	-0.26	15.54	292.17	15.28	-122.445	37.927	-0.41	15.54	292.06	15.13	9	7/14/2014	49:58.2
269	RSPe_2	-122.445	37.927	-0.22	15.46	292.18	15.23	-122.445	37.927	-0.36	15.46	292.11	15.10	9	7/14/2014	49:58.1
270	RSPe_2	-122.445	37.927	-0.26	15.46	292.18	15.21	-122.445	37.927	-0.32	15.46	292.18	15.14	9	7/14/2014	49:58.0
271	RSPe_2	-122.445	37.927	-0.26	15.43	292.18	15.18	-122.445	37.927	-0.49	15.43	292.22	14.94	9	7/14/2014	49:57.9
272	RSPe_2	-122.445	37.927	-0.26	15.49	292.22	15.24	-122.445	37.927	-0.49	15.49	292.22	15.00	9	7/14/2014	49:57.8
273	RSPe_2	-122.445	37.927	-0.26	15.47	292.24	15.22	-122.445	37.927	-0.49	15.47	292.26	14.98	9	7/14/2014	49:57.7
274	RSPe_2	-122.445	37.927	-0.31	15.44	292.33	15.13	-122.445	37.927	-0.52	15.44	292.24	14.92	9	7/14/2014	49:57.6
275	RSPe_2	-122.445	37.927	-0.26	15.44	292.46	15.18	-122.445	37.927	-0.52	15.44	292.30	14.92	9	7/14/2014	49:57.5
276	RSPe_2	-122.445	37.927	-0.31	15.42	292.51	15.11	-122.445	37.927	-0.27	15.42	292.30	15.15	9	7/14/2014	49:57.4
277	RSPe_2	-122.445	37.927	-0.31	15.40	292.47	15.10	-122.445	37.927	-0.49	15.40	292.18	14.91	9	7/14/2014	49:57.3
278	RSPe_2	-122.445	37.927	-0.31	15.39	292.53	15.08	-122.445	37.927	-0.52	15.39	292.13	14.86	9	7/14/2014	49:57.2
279	RSPe_2	-122.445	37.927	-0.31	15.48	292.69	15.17	-122.445	37.927	-0.20	15.48	292.20	15.28	9	7/14/2014	49:57.1
280	RSPe_2	-122.445	37.927	-0.34	15.36	292.71	15.02	-122.445	37.927	-0.52	15.36	292.18	14.84	9	7/14/2014	49:57.0
281	RSPe_2	-122.445	37.927	-0.34	15.34	292.64	15.00	-122.445	37.927	-0.35	15.34	292.11	14.99	9	7/14/2014	49:56.9
282	RSPe_2	-122.445	37.927	-0.34	15.19	292.49	14.84	-122.445	37.927	-0.41	15.19	292.07	14.78	9	7/14/2014	49:56.8
283	RSPe_2	-122.445	37.927	-0.31	15.17	292.45	14.86	-122.445	37.927	-0.61	15.17	292.13	14.56	9	7/14/2014	49:56.7
284	RSPe_2	-122.445	37.927	-0.34	15.23	292.42	14.89	-122.445	37.927	-0.49	15.23	292.24	14.74	9	7/14/2014	49:56.6
285	RSPe_2	-122.445	37.927	-0.31	15.47	292.40	15.17	-122.445	37.927	-0.52	15.47	292.36	14.95	9	7/14/2014	49:56.5
286	RSPe_2	-122.445	37.927	-0.39	15.47	292.34	15.08	-122.445	37.927	-0.52	15.47	292.40	14.95	9	7/14/2014	49:56.4
287	RSPe_2	-122.445	37.927	-0.34	15.60	292.40	15.26	-122.445	37.927	-0.57	15.60	292.58	15.03	9	7/14/2014	49:56.3
288	RSPe_2	-122.445	37.927	-0.39	15.56	292.58	15.16	-122.445	37.927	-0.41	15.56	292.73	15.15	9	7/14/2014	49:56.2
289	RSPe_2	-122.445	37.927	-0.34	15.69	292.63	15.35	-122.445	37.927	-0.52	15.69	292.71	15.17	9	7/14/2014	49:56.1
290	RSPe_2	-122.445	37.927	-0.34	15.72	292.65	15.38	-122.445	37.927	-0.35	15.72	292.80	15.36	9	7/14/2014	49:56.0
291	RSPe_2	-122.445	37.927	-0.34	15.70	292.49	15.36	-122.445	37.927	-0.49	15.70	292.51	15.21	9	7/14/2014	49:55.9
292	RSPe_2	-122.445	37.927	-0.39	15.74	292.34	15.35	-122.445	37.927	-0.52	15.74	292.40	15.22	9	7/14/2014	49:55.8
293	RSPe_2	-122.445	37.927	-0.39	15.71	292.54	15.32	-122.445	37.927	-0.49	15.71	292.49	15.22	9	7/14/2014	49:55.7
294	RSPe_2	-122.445	37.927	-0.39	15.78	292.50	15.39	-122.445	37.927	-0.52	15.78	292.38	15.26	9	7/14/2014	49:55.6
295	RSPe_2	-122.445	37.927	-0.39	15.69	292.52	15.30	-122.445	37.927	-0.44	15.69	292.32	15.25	9	7/14/2014	49:55.5
296	RSPe_2	-122.445	37.927	-0.39	15.73	292.63	15.34	-122.445	37.927	-0.57	15.73	292.38	15.16	9	7/14/2014	49:55.4

297	RSPe_2	-122.445	37.927	-0.39	15.68	292.69	15.29	-122.445	37.927	-0.61	15.68	292.41	15.07	9	7/14/2014	49:55.3
298	RSPe_2	-122.445	37.927	-0.43	15.69	292.65	15.26	-122.445	37.927	-0.64	15.69	292.40	15.04	9	7/14/2014	49:55.2
299	RSPe_2	-122.445	37.927	-0.43	15.64	292.65	15.21	-122.445	37.927	-0.49	15.64	292.34	15.15	9	7/14/2014	49:55.1
300	RSPe_2	-122.445	37.927	-0.43	15.62	292.67	15.19	-122.445	37.927	-0.64	15.62	292.40	14.98	9	7/14/2014	49:55.0
301	RSPe_2	-122.445	37.927	-0.39	15.62	292.67	15.23	-122.445	37.927	-0.81	15.62	292.47	14.81	9	7/14/2014	49:54.9
302	RSPe_2	-122.445	37.927	-0.43	15.56	292.63	15.14	-122.445	37.927	-0.52	15.56	292.54	15.04	9	7/14/2014	49:54.8
303	RSPe_2	-122.445	37.927	-0.39	15.53	292.59	15.14	-122.445	37.927	-0.44	15.53	292.49	15.09	9	7/14/2014	49:54.7
304	RSPe_2	-122.445	37.927	-0.43	15.56	292.57	15.13	-122.445	37.927	-0.64	15.56	292.56	14.91	9	7/14/2014	49:54.6
305	RSPe_2	-122.445	37.927	-0.39	15.49	292.59	15.10	-122.445	37.927	-0.52	15.49	292.63	14.97	9	7/14/2014	49:54.5
306	RSPe_2	-122.445	37.927	-0.43	15.48	292.57	15.06	-122.445	37.927	-0.61	15.48	292.63	14.88	9	7/14/2014	49:54.4
307	RSPe_2	-122.445	37.927	-0.39	15.59	292.55	15.19	-122.445	37.927	-0.49	15.59	292.52	15.10	9	7/14/2014	49:54.3
308	RSPe_2	-122.445	37.927	-0.39	15.72	292.64	15.33	-122.445	37.927	-0.64	15.72	292.47	15.08	9	7/14/2014	49:54.2
309	RSPe_2	-122.445	37.927	-0.39	15.49	292.48	15.10	-122.445	37.927	-0.52	15.49	292.35	14.97	9	7/14/2014	49:54.1
310	RSPe_2	-122.445	37.927	-0.43	15.34	292.52	14.91	-122.445	37.927	-0.57	15.34	292.23	14.77	9	7/14/2014	49:54.0
311	RSPe_2	-122.445	37.927	-0.39	15.35	292.48	14.95	-122.445	37.927	-0.52	15.35	292.23	14.82	9	7/14/2014	49:53.9
312	RSPe_2	-122.445	37.927	-0.43	15.31	292.57	14.88	-122.445	37.927	-0.64	15.31	292.37	14.66	9	7/14/2014	49:53.8
313	RSPe_2	-122.445	37.927	-0.39	15.32	292.46	14.93	-122.445	37.927	-0.61	15.32	292.46	14.72	9	7/14/2014	49:53.7
314	RSPe_2	-122.445	37.927	-0.43	15.35	292.51	14.92	-122.445	37.927	-0.57	15.35	292.59	14.77	9	7/14/2014	49:53.6
315	RSPe_2	-122.445	37.927	-0.39	15.39	292.61	15.00	-122.445	37.927	-0.49	15.39	292.65	14.90	9	7/14/2014	49:53.5
316	RSPe_2	-122.445	37.927	-0.43	15.43	292.46	15.01	-122.445	37.927	-0.49	15.43	292.54	14.94	9	7/14/2014	49:53.4
317	RSPe_2	-122.445	37.927	-0.39	15.50	292.46	15.11	-122.445	37.927	-0.64	15.50	292.64	14.86	9	7/14/2014	49:53.3
318	RSPe_2	-122.445	37.927	-0.43	15.53	292.46	15.10	-122.445	37.927	-0.57	15.53	292.57	14.95	9	7/14/2014	49:53.2
319	RSPe_2	-122.445	37.927	-0.43	15.54	292.33	15.11	-122.445	37.927	-0.61	15.54	292.52	14.93	9	7/14/2014	49:53.1
320	RSPe_2	-122.445	37.927	-0.43	15.56	292.33	15.13	-122.445	37.927	-0.57	15.56	292.49	14.98	9	7/14/2014	49:53.0
321	RSPe_2	-122.445	37.927	-0.39	15.54	292.29	15.15	-122.445	37.927	-0.49	15.54	292.37	15.05	9	7/14/2014	49:52.9
322	RSPe_2	-122.445	37.927	-0.43	15.58	292.44	15.15	-122.445	37.927	-0.64	15.58	292.46	14.94	9	7/14/2014	49:52.8
323	RSPe_2	-122.445	37.927	-0.39	15.56	292.53	15.16	-122.445	37.927	-0.57	15.56	292.35	14.98	9	7/14/2014	49:52.7
324	RSPe_2	-122.445	37.927	-0.43	15.61	292.44	15.18	-122.445	37.927	-0.64	15.61	292.31	14.97	9	7/14/2014	49:52.6
325	RSPe_2	-122.445	37.927	-0.43	15.63	292.49	15.21	-122.445	37.927	-0.61	15.63	292.31	15.02	9	7/14/2014	49:52.5
326	RSPe_2	-122.445	37.927	-0.48	15.60	292.44	15.12	-122.445	37.927	-0.61	15.60	292.31	14.99	9	7/14/2014	49:52.4
327	RSPe_2	-122.445	37.927	-0.43	15.65	292.36	15.22	-122.445	37.927	-0.61	15.65	292.33	15.04	9	7/14/2014	49:52.3
328	RSPe_2	-122.445	37.927	-0.48	15.62	292.31	15.15	-122.445	37.927	-0.61	15.62	292.34	15.01	9	7/14/2014	49:52.2
329	RSPe_2	-122.445	37.927	-0.43	15.60	292.14	15.17	-122.445	37.927	-0.61	15.60	292.31	14.99	9	7/14/2014	49:52.1

330	RSPe_2	-122.445	37.927	-0.48	15.59	292.01	15.11	-122.445	37.927	-0.69	15.59	292.29	14.89	9	7/14/2014	49:52.0
331	RSPe_2	-122.445	37.927	-0.43	15.56	292.25	15.13	-122.445	37.927	-0.69	15.56	292.58	14.86	9	7/14/2014	49:51.9
332	RSPe_2	-122.445	37.927	-0.48	15.62	292.25	15.14	-122.445	37.927	-0.81	15.62	292.33	14.81	9	7/14/2014	49:51.8
333	RSPe_2	-122.445	37.927	-0.48	15.63	292.40	15.16	-122.445	37.927	-0.52	15.63	292.40	15.11	9	7/14/2014	49:51.7
334	RSPe_2	-122.445	37.927	-0.51	15.66	292.62	15.14	-122.445	37.927	-0.73	15.66	292.40	14.93	9	7/14/2014	49:51.6
335	RSPe_2	-122.445	37.927	-0.51	15.64	292.69	15.13	-122.445	37.927	-0.61	15.64	292.38	15.03	9	7/14/2014	49:51.5
336	RSPe_2	-122.445	37.927	-0.51	15.67	292.62	15.16	-122.445	37.927	-0.73	15.67	292.25	14.94	9	7/14/2014	49:51.4
337	RSPe_2	-122.445	37.927	-0.48	15.58	292.74	15.10	-122.445	37.927	-0.52	15.58	292.24	15.06	9	7/14/2014	49:51.3
338	RSPe_2	-122.445	37.927	-0.51	15.55	292.74	15.04	-122.445	37.927	-0.69	15.55	292.18	14.86	9	7/14/2014	49:51.2
339	RSPe_2	-122.445	37.927	-0.51	15.54	292.56	15.03	-122.445	37.927	-0.61	15.54	292.07	14.93	9	7/14/2014	49:51.1
340	RSPe_2	-122.445	37.927	-0.56	15.73	292.36	15.17	-122.445	37.927	-0.78	15.73	292.03	14.96	9	7/14/2014	49:51.0
341	RSPe_2	-122.445	37.927	-0.51	15.73	292.32	15.21	-122.445	37.927	-0.81	15.73	292.25	14.91	9	7/14/2014	49:50.9
342	RSPe_2	-122.445	37.927	-0.56	15.75	292.27	15.18	-122.445	37.927	-0.90	15.75	292.47	14.85	9	7/14/2014	49:50.8
343	RSPe_2	-122.445	37.927	-0.56	15.78	292.10	15.22	-122.445	37.927	-0.86	15.78	292.56	14.92	9	7/14/2014	49:50.7
344	RSPe_2	-122.445	37.927	-0.60	15.85	292.03	15.25	-122.445	37.927	-0.95	15.85	292.80	14.90	9	7/14/2014	49:50.6
345	RSPe_2	-122.445	37.927	-0.56	15.86	292.15	15.30	-122.445	37.927	-0.73	15.86	292.91	15.14	9	7/14/2014	49:50.5
346	RSPe_2	-122.445	37.927	-0.56	15.93	292.19	15.36	-122.445	37.927	-0.98	15.93	292.95	14.95	9	7/14/2014	49:50.4
347	RSPe_2	-122.445	37.927	-0.56	16.00	292.23	15.44	-122.445	37.927	-0.95	16.00	292.83	15.06	9	7/14/2014	49:50.3
348	RSPe_2	-122.445	37.927	-0.60	15.99	292.28	15.39	-122.445	37.927	-0.98	15.99	292.56	15.01	9	7/14/2014	49:50.2
349	RSPe_2	-122.445	37.927	-0.56	15.94	292.37	15.38	-122.445	37.927	-0.73	15.94	292.54	15.21	9	7/14/2014	49:50.1
350	RSPe_2	-122.445	37.927	-0.60	15.94	292.45	15.34	-122.445	37.927	-0.81	15.94	292.34	15.13	9	7/14/2014	49:50.0
351	RSPe_2	-122.445	37.927	-0.56	15.90	292.52	15.33	-122.445	37.927	-0.95	15.90	292.36	14.95	9	7/14/2014	49:49.9
352	RSPe_2	-122.445	37.927	-0.60	15.87	292.48	15.27	-122.445	37.927	-0.98	15.87	292.47	14.89	9	7/14/2014	49:49.8
353	RSPe_2	-122.445	37.927	-0.60	15.86	292.32	15.27	-122.445	37.927	-1.10	15.86	292.49	14.76	9	7/14/2014	49:49.7
354	RSPe_2	-122.445	37.927	-0.60	15.91	292.17	15.31	-122.445	37.927	-1.01	15.91	292.63	14.90	9	7/14/2014	49:49.6
355	RSPe_2	-122.445	37.927	-0.60	15.97	292.22	15.37	-122.445	37.927	-1.10	15.97	292.80	14.87	9	7/14/2014	49:49.5
356	RSPe_2	-122.445	37.927	-0.60	16.00	292.28	15.41	-122.445	37.927	-1.10	16.00	292.87	14.90	9	7/14/2014	49:49.4
357	RSPe_2	-122.445	37.927	-0.56	16.09	292.61	15.53	-122.445	37.927	-1.10	16.09	293.06	14.99	9	7/14/2014	49:49.3
358	RSPe_2	-122.445	37.927	-0.60	16.00	292.57	15.41	-122.445	37.927	-1.01	16.00	292.74	14.99	9	7/14/2014	49:49.2
359	RSPe_2	-122.445	37.927	-0.60	16.15	292.55	15.55	-122.445	37.927	-1.10	16.15	292.58	15.05	9	7/14/2014	49:49.1
360	RSPe_2	-122.445	37.927	-0.60	16.02	292.30	15.42	-122.445	37.927	-1.15	16.02	292.27	14.87	9	7/14/2014	49:49.0
361	RSPe_2	-122.445	37.927	-0.56	16.05	292.28	15.49	-122.445	37.927	-0.98	16.05	292.17	15.07	9	7/14/2014	49:48.9
362	RSPe_2	-122.445	37.927	-0.56	16.06	292.66	15.50	-122.445	37.927	-1.18	16.06	292.36	14.88	9	7/14/2014	49:48.8

363	RSPe_2	-122.445	37.927	-0.56	16.09	292.70	15.53	-122.445	37.927	-0.98	16.09	292.50	15.11	9	7/14/2014	49:48.7
364	RSPe_2	-122.445	37.927	-0.56	16.09	292.75	15.53	-122.445	37.927	-1.24	16.09	292.57	14.85	9	7/14/2014	49:48.6
365	RSPe_2	-122.445	37.927	-0.56	16.13	292.75	15.57	-122.445	37.927	-1.10	16.13	292.79	15.03	9	7/14/2014	49:48.5
366	RSPe_2	-122.445	37.927	-0.56	16.22	292.77	15.65	-122.445	37.927	-1.07	16.22	292.92	15.15	9	7/14/2014	49:48.4
367	RSPe_2	-122.445	37.927	-0.51	16.23	292.64	15.72	-122.445	37.927	-1.15	16.23	293.03	15.08	9	7/14/2014	49:48.3
368	RSPe_2	-122.445	37.927	-0.56	16.23	292.42	15.67	-122.445	37.927	-1.15	16.23	292.95	15.08	9	7/14/2014	49:48.2
369	RSPe_2	-122.445	37.927	-0.51	16.23	292.07	15.72	-122.445	37.927	-1.10	16.23	292.70	15.13	9	7/14/2014	49:48.1
370	RSPe_2	-122.445	37.927	-0.56	16.25	292.24	15.69	-122.445	37.927	-1.18	16.25	292.83	15.07	9	7/14/2014	49:48.0
371	RSPe_2	-122.445	37.927	-0.51	16.30	292.37	15.79	-122.445	37.927	-1.10	16.30	292.83	15.20	9	7/14/2014	49:47.9
372	RSPe_2	-122.445	37.927	-0.51	16.39	292.37	15.88	-122.445	37.927	-1.10	16.39	292.70	15.29	9	7/14/2014	49:47.8
373	RSPe_2	-122.445	37.927	-0.51	16.37	292.36	15.86	-122.445	37.927	-1.15	16.37	292.48	15.22	9	7/14/2014	49:47.7
374	RSPe_2	-122.445	37.927	-0.51	16.34	292.58	15.83	-122.445	37.927	-1.18	16.34	292.53	15.16	9	7/14/2014	49:47.6
375	RSPe_2	-122.445	37.927	-0.51	16.40	292.69	15.88	-122.445	37.927	-1.07	16.40	292.51	15.33	9	7/14/2014	49:47.5
376	RSPe_2	-122.445	37.927	-0.48	16.35	292.73	15.87	-122.445	37.927	-1.07	16.35	292.46	15.28	9	7/14/2014	49:47.4
377	RSPe_2	-122.445	37.927	-0.48	16.29	292.68	15.81	-122.445	37.927	-1.01	16.29	292.33	15.27	9	7/14/2014	49:47.3
378	RSPe_2	-122.445	37.927	-0.48	16.29	292.66	15.81	-122.445	37.927	-1.01	16.29	292.31	15.27	9	7/14/2014	49:47.2
379	RSPe_2	-122.445	37.927	-0.48	16.27	292.66	15.79	-122.445	37.927	-1.10	16.27	292.31	15.17	9	7/14/2014	49:47.1
380	RSPe_2	-122.445	37.927	-0.48	16.23	292.54	15.75	-122.445	37.927	-0.98	16.23	292.35	15.25	9	7/14/2014	49:47.0
381	RSPe_2	-122.445	37.927	-0.43	16.13	292.42	15.71	-122.445	37.927	-1.01	16.13	292.46	15.12	9	7/14/2014	49:46.9
382	RSPe_2	-122.445	37.927	-0.43	16.17	292.36	15.75	-122.445	37.927	-1.07	16.17	292.46	15.11	9	7/14/2014	49:46.8
383	RSPe_2	-122.445	37.927	-0.43	16.15	292.36	15.72	-122.445	37.927	-0.95	16.15	292.55	15.20	9	7/14/2014	49:46.7
384	RSPe_2	-122.445	37.927	-0.43	16.09	292.38	15.66	-122.445	37.927	-0.98	16.09	292.49	15.11	9	7/14/2014	49:46.6
385	RSPe_2	-122.445	37.927	-0.39	16.02	292.43	15.63	-122.445	37.927	-1.10	16.02	292.53	14.92	9	7/14/2014	49:46.5
386	RSPe_2	-122.445	37.927	-0.39	16.00	292.43	15.60	-122.445	37.927	-0.95	16.00	292.51	15.05	9	7/14/2014	49:46.4
387	RSPe_2	-122.445	37.927	-0.39	15.89	292.32	15.50	-122.445	37.927	-1.07	15.89	292.44	14.82	9	7/14/2014	49:46.3
388	RSPe_2	-122.445	37.927	-0.39	15.88	292.34	15.49	-122.445	37.927	-0.98	15.88	292.46	14.90	9	7/14/2014	49:46.2
389	RSPe_2	-122.445	37.927	-0.34	15.84	292.34	15.50	-122.445	37.927	-0.90	15.84	292.36	14.95	9	7/14/2014	49:46.1
390	RSPe_2	-122.445	37.927	-0.39	15.83	292.36	15.44	-122.445	37.927	-0.90	15.83	292.29	14.94	9	7/14/2014	49:46.0
391	RSPe_2	-122.445	37.927	-0.34	15.79	292.39	15.45	-122.445	37.927	-0.86	15.79	292.17	14.93	9	7/14/2014	49:45.9
392	RSPe_2	-122.445	37.927	-0.34	15.81	292.34	15.47	-122.445	37.927	-0.86	15.81	292.18	14.95	9	7/14/2014	49:45.8
393	RSPe_2	-122.445	37.927	-0.34	15.73	292.23	15.38	-122.445	37.927	-0.78	15.73	292.08	14.95	9	7/14/2014	49:45.7
394	RSPe_2	-122.445	37.927	-0.34	15.77	292.28	15.43	-122.445	37.927	-0.86	15.77	292.18	14.91	9	7/14/2014	49:45.6
395	RSPe_2	-122.445	37.927	-0.34	15.84	292.30	15.50	-122.445	37.927	-0.95	15.84	292.30	14.89	9	7/14/2014	49:45.5

396	RSPe_2	-122.445	37.927	-0.34	15.93	292.34	15.59	-122.445	37.927	-0.90	15.93	292.38	15.04	9	7/14/2014	49:45.4
397	RSPe_2	-122.445	37.927	-0.31	15.90	292.39	15.59	-122.445	37.927	-0.69	15.90	292.38	15.20	9	7/14/2014	49:45.3
398	RSPe_2	-122.445	37.927	-0.34	15.88	292.56	15.54	-122.445	37.927	-0.81	15.88	292.40	15.07	9	7/14/2014	49:45.2
399	RSPe_2	-122.445	37.927	-0.31	15.85	292.63	15.54	-122.445	37.927	-0.81	15.85	292.37	15.04	9	7/14/2014	49:45.1
400	RSPe_2	-122.445	37.927	-0.31	15.82	292.52	15.51	-122.445	37.927	-0.90	15.82	292.43	14.92	9	7/14/2014	49:45.0
401	RSPe_2	-122.445	37.927	-0.31	15.85	292.48	15.54	-122.445	37.927	-0.69	15.85	292.45	15.16	9	7/14/2014	49:44.9
402	RSPe_2	-122.445	37.927	-0.31	15.79	292.30	15.48	-122.445	37.927	-0.69	15.79	292.43	15.10	9	7/14/2014	49:44.8
403	RSPe_2	-122.445	37.927	-0.26	15.83	292.30	15.58	-122.445	37.927	-0.69	15.83	292.48	15.14	9	7/14/2014	49:44.7
404	RSPe_2	-122.445	37.927	-0.26	15.78	292.24	15.52	-122.445	37.927	-0.78	15.78	292.43	15.00	9	7/14/2014	49:44.6
405	RSPe_2	-122.445	37.927	-0.26	15.76	292.28	15.50	-122.445	37.927	-0.69	15.76	292.49	15.06	9	7/14/2014	49:44.5
406	RSPe_2	-122.445	37.927	-0.26	15.76	292.32	15.50	-122.445	37.927	-0.64	15.76	292.52	15.11	9	7/14/2014	49:44.4
407	RSPe_2	-122.445	37.927	-0.22	15.71	292.24	15.49	-122.445	37.927	-0.73	15.71	292.54	14.98	9	7/14/2014	49:44.3
408	RSPe_2	-122.445	37.927	-0.22	15.73	292.20	15.51	-122.445	37.927	-0.64	15.73	292.63	15.09	9	7/14/2014	49:44.2
409	RSPe_2	-122.445	37.927	-0.22	15.70	292.09	15.48	-122.445	37.927	-0.69	15.70	292.61	15.01	9	7/14/2014	49:44.1
410	RSPe_2	-122.445	37.927	-0.22	15.69	292.02	15.47	-122.445	37.927	-0.69	15.69	292.61	15.00	9	7/14/2014	49:44.0
411	RSPe_2	-122.445	37.927	-0.19	15.70	291.95	15.52	-122.445	37.927	-0.69	15.70	292.59	15.01	9	7/14/2014	49:43.9
412	RSPe_2	-122.445	37.927	-0.22	15.75	291.91	15.53	-122.445	37.927	-0.69	15.75	292.54	15.06	9	7/14/2014	49:43.8
413	RSPe_2	-122.445	37.927	-0.19	15.69	291.93	15.50	-122.445	37.927	-0.64	15.69	292.48	15.05	9	7/14/2014	49:43.7
414	RSPe_2	-122.445	37.927	-0.19	15.83	291.98	15.65	-122.445	37.927	-0.69	15.83	292.52	15.14	9	7/14/2014	49:43.6
415	RSPe_2	-122.445	37.927	-0.19	15.83	292.22	15.64	-122.445	37.927	-0.73	15.83	292.50	15.10	9	7/14/2014	49:43.5
416	RSPe_2	-122.445	37.927	-0.19	15.84	292.40	15.66	-122.445	37.927	-0.73	15.84	292.44	15.12	9	7/14/2014	49:43.4
417	RSPe_2	-122.445	37.927	-0.14	15.79	292.40	15.65	-122.445	37.927	-0.52	15.79	292.36	15.27	9	7/14/2014	49:43.3
418	RSPe_2	-122.445	37.927	-0.19	15.76	292.40	15.57	-122.445	37.927	-0.64	15.76	292.24	15.11	9	7/14/2014	49:43.2
419	RSPe_2	-122.445	37.927	-0.14	15.66	292.35	15.52	-122.445	37.927	-0.57	15.66	292.15	15.08	9	7/14/2014	49:43.1
420	RSPe_2	-122.445	37.927	-0.14	15.71	292.27	15.58	-122.445	37.927	-0.52	15.71	292.11	15.19	9	7/14/2014	49:43.0
421	RSPe_2	-122.445	37.927	-0.10	15.66	292.24	15.56	-122.445	37.927	-0.61	15.66	292.15	15.06	9	7/14/2014	49:42.9
422	RSPe_2	-122.445	37.927	-0.10	15.58	292.24	15.48	-122.445	37.927	-0.64	15.58	292.18	14.94	9	7/14/2014	49:42.8
423	RSPe_2	-122.445	37.927	-0.05	15.61	292.31	15.56	-122.445	37.927	-0.49	15.61	292.28	15.12	9	7/14/2014	49:42.7
424	RSPe_2	-122.445	37.927	-0.10	15.57	292.35	15.47	-122.445	37.927	-0.61	15.57	292.33	14.96	9	7/14/2014	49:42.6
425	RSPe_2	-122.445	37.927	-0.05	15.57	292.33	15.52	-122.445	37.927	-0.57	15.57	292.37	15.00	9	7/14/2014	49:42.5
426	RSPe_2	-122.445	37.927	-0.10	15.59	292.38	15.49	-122.445	37.927	-0.52	15.59	292.35	15.06	9	7/14/2014	49:42.4
427	RSPe_2	-122.445	37.927	-0.02	15.56	292.40	15.54	-122.445	37.927	-0.44	15.56	292.33	15.12	9	7/14/2014	49:42.3
428	RSPe_2	-122.445	37.927	-0.02	15.56	292.45	15.54	-122.445	37.927	-0.44	15.56	292.40	15.12	9	7/14/2014	49:42.2

429	RSPe_2	-122.445	37.927	-0.02	15.50	292.47	15.49	-122.445	37.927	-0.44	15.50	292.42	15.06	9	7/14/2014 49:42.1
430	RSPe_2	-122.445	37.927	-0.02	15.43	292.40	15.41	-122.445	37.927	-0.44	15.43	292.35	14.99	9	7/14/2014 49:42.0
431	RSPe_2	-122.445	37.927	0.04	15.55	292.38	15.58	-122.445	37.927	-0.35	15.55	292.35	15.19	9	7/14/2014 49:41.9
432	RSPe_2	-122.445	37.927	-0.02	15.39	292.23	15.38	-122.445	37.927	-0.41	15.39	292.24	14.99	9	7/14/2014 49:41.8
433	RSPe_2	-122.445	37.927	0.07	15.34	292.36	15.41	-122.445	37.927	-0.24	15.34	292.32	15.11	9	7/14/2014 49:41.7
434	RSPe_2	-122.445	37.927	0.04	15.22	292.45	15.25	-122.445	37.927	-0.32	15.22	292.38	14.90	9	7/14/2014 49:41.6
435	RSPe_2	-122.445	37.927	0.07	15.18	292.45	15.25	-122.445	37.927	-0.27	15.18	292.34	14.91	9	7/14/2014 49:41.5
436	RSPe_2	-122.445	37.927	0.07	15.19	292.34	15.27	-122.445	37.927	-0.20	15.19	292.33	14.99	9	7/14/2014 49:41.4
437	RSPe_2	-122.445	37.927	0.12	15.15	292.25	15.27	-122.445	37.927	-0.27	15.15	292.34	14.88	9	7/14/2014 49:41.3
438	RSPe_2	-122.445	37.927	0.07	15.09	292.21	15.16	-122.445	37.927	-0.20	15.09	292.36	14.89	9	7/14/2014 49:41.2
439	RSPe_2	-122.445	37.927	0.12	15.14	292.12	15.26	-122.445	37.927	-0.20	15.14	292.34	14.94	9	7/14/2014 49:41.1
440	RSPe_2	-122.445	37.927	0.07	15.07	292.12	15.14	-122.445	37.927	-0.20	15.07	292.42	14.87	9	7/14/2014 49:41.0
441	RSPe_2	-122.445	37.927	0.12	15.10	292.12	15.22	-122.445	37.927	-0.20	15.10	292.45	14.90	9	7/14/2014 49:40.9
442	RSPe_2	-122.445	37.927	0.12	15.08	292.17	15.21	-122.445	37.927	-0.24	15.08	292.52	14.85	9	7/14/2014 49:40.8
443	RSPe_2	-122.445	37.927	0.16	15.10	292.17	15.26	-122.445	37.927	-0.15	15.10	292.52	14.95	9	7/14/2014 49:40.7
444	RSPe_2	-122.445	37.927	0.16	15.10	292.23	15.26	-122.445	37.927	-0.20	15.10	292.58	14.90	9	7/14/2014 49:40.6
445	RSPe_2	-122.445	37.927	0.16	15.20	292.26	15.36	-122.445	37.927	-0.15	15.20	292.54	15.05	9	7/14/2014 49:40.5
446	RSPe_2	-122.445	37.927	0.16	15.13	292.23	15.29	-122.445	37.927	-0.20	15.13	292.48	14.93	9	7/14/2014 49:40.4
447	RSPe_2	-122.445	37.927	0.16	15.13	292.30	15.29	-122.445	37.927	-0.07	15.13	292.47	15.06	9	7/14/2014 49:40.3
448	RSPe_2	-122.445	37.927	0.16	15.13	292.21	15.28	-122.445	37.927	-0.07	15.13	292.36	15.06	9	7/14/2014 49:40.2
449	RSPe_2	-122.445	37.927	0.19	15.15	292.21	15.34	-122.445	37.927	0.02	15.15	292.33	15.17	9	7/14/2014 49:40.1
450	RSPe_2	-122.445	37.927	0.19	15.11	292.19	15.30	-122.445	37.927	0.02	15.11	292.27	15.13	9	7/14/2014 49:40.0
451	RSPe_2	-122.445	37.927	0.19	15.15	292.17	15.34	-122.445	37.927	0.02	15.15	292.24	15.17	9	7/14/2014 49:39.9
452	RSPe_2	-122.445	37.927	0.19	15.08	292.13	15.27	-122.445	37.927	-0.03	15.08	292.23	15.05	9	7/14/2014 49:39.8
453	RSPe_2	-122.445	37.927	0.19	15.07	292.15	15.26	-122.445	37.927	0.02	15.07	292.27	15.09	9	7/14/2014 49:39.7
454	RSPe_2	-122.445	37.927	0.19	15.05	292.13	15.24	-122.445	37.927	0.02	15.05	292.28	15.06	9	7/14/2014 49:39.6
455	RSPe_2	-122.445	37.927	0.24	15.03	292.15	15.27	-122.445	37.927	0.02	15.03	292.30	15.05	9	7/14/2014 49:39.5
456	RSPe_2	-122.445	37.927	0.24	15.00	292.15	15.24	-122.445	37.927	0.05	15.00	292.30	15.05	9	7/14/2014 49:39.4
457	RSPe_2	-122.445	37.927	0.24	15.02	292.19	15.26	-122.445	37.927	0.05	15.02	292.37	15.07	9	7/14/2014 49:39.3
458	RSPe_2	-122.445	37.927	0.24	15.03	292.20	15.27	-122.445	37.927	0.10	15.03	292.36	15.13	9	7/14/2014 49:39.2
459	RSPe_2	-122.445	37.927	0.28	15.02	292.24	15.29	-122.445	37.927	0.17	15.02	292.43	15.19	9	7/14/2014 49:39.1
460	RSPe_2	-122.445	37.927	0.24	14.99	292.29	15.23	-122.445	37.927	0.05	14.99	292.50	15.04	9	7/14/2014 49:39.0
461	RSPe_2	-122.445	37.927	0.28	15.00	292.24	15.28	-122.445	37.927	0.10	15.00	292.46	15.10	9	7/14/2014 49:38.9

462	RSPe_2	-122.445	37.927	0.24	14.97	292.26	15.21	-122.445	37.927	0.14	14.97	292.48	15.11	9	7/14/2014	49:38.8
463	RSPe_2	-122.445	37.927	0.28	14.93	292.24	15.21	-122.445	37.927	0.14	14.93	292.48	15.07	9	7/14/2014	49:38.7
464	RSPe_2	-122.445	37.927	0.28	14.92	292.26	15.19	-122.445	37.927	0.14	14.92	292.48	15.05	9	7/14/2014	49:38.6
465	RSPe_2	-122.445	37.927	0.28	14.88	292.22	15.15	-122.445	37.927	0.22	14.88	292.49	15.10	9	7/14/2014	49:38.5
466	RSPe_2	-122.445	37.927	0.28	14.82	292.22	15.10	-122.445	37.927	0.10	14.82	292.46	14.92	9	7/14/2014	49:38.4
467	RSPe_2	-122.445	37.927	0.33	14.77	292.13	15.09	-122.445	37.927	0.17	14.77	292.43	14.94	9	7/14/2014	49:38.3
468	RSPe_2	-122.445	37.927	0.33	14.74	292.09	15.07	-122.445	37.927	0.17	14.74	292.39	14.91	9	7/14/2014	49:38.2
469	RSPe_2	-122.445	37.927	0.33	14.70	292.14	15.03	-122.445	37.927	0.14	14.70	292.51	14.84	9	7/14/2014	49:38.1
470	RSPe_2	-122.445	37.927	0.33	14.71	292.16	15.04	-122.445	37.928	0.14	14.71	292.55	14.85	9	7/14/2014	49:38.0
471	RSPe_2	-122.445	37.928	0.33	14.67	292.18	15.00	-122.445	37.928	0.17	14.67	292.64	14.84	9	7/14/2014	49:37.9
472	RSPe_2	-122.445	37.928	0.33	14.67	292.24	15.00	-122.445	37.928	0.17	14.67	292.68	14.84	9	7/14/2014	49:37.8
473	RSPe_2	-122.445	37.928	0.36	14.68	292.31	15.04	-122.445	37.928	0.17	14.68	292.68	14.85	9	7/14/2014	49:37.7
474	RSPe_2	-122.445	37.928	0.36	14.70	292.33	15.06	-122.445	37.928	0.17	14.70	292.70	14.87	9	7/14/2014	49:37.6
475	RSPe_2	-122.445	37.928	0.36	14.67	292.40	15.03	-122.445	37.928	0.22	14.67	292.68	14.89	9	7/14/2014	49:37.5
476	RSPe_2	-122.445	37.928	0.36	14.71	292.40	15.07	-122.445	37.928	0.22	14.71	292.66	14.93	9	7/14/2014	49:37.4
477	RSPe_2	-122.445	37.928	0.36	14.67	292.43	15.03	-122.445	37.928	0.31	14.67	292.64	14.98	9	7/14/2014	49:37.3
478	RSPe_2	-122.445	37.928	0.36	14.68	292.36	15.04	-122.445	37.928	0.26	14.68	292.60	14.94	9	7/14/2014	49:37.2
479	RSPe_2	-122.445	37.928	0.36	14.69	292.32	15.05	-122.445	37.928	0.31	14.69	292.57	15.00	9	7/14/2014	49:37.1
480	RSPe_2	-122.445	37.928	0.36	14.69	292.25	15.05	-122.445	37.928	0.31	14.69	292.55	15.00	9	7/14/2014	49:37.0
481	RSPe_2	-122.445	37.928	0.41	14.69	292.23	15.11	-122.445	37.928	0.26	14.69	292.62	14.95	9	7/14/2014	49:36.9
482	RSPe_2	-122.445	37.928	0.41	14.73	292.18	15.14	-122.445	37.928	0.26	14.73	292.59	14.98	9	7/14/2014	49:36.8
483	RSPe_2	-122.445	37.928	0.41	14.84	292.18	15.25	-122.445	37.928	0.31	14.84	292.64	15.14	9	7/14/2014	49:36.7
484	RSPe_2	-122.445	37.928	0.41	14.87	292.16	15.28	-122.445	37.928	0.22	14.87	292.60	15.09	9	7/14/2014	49:36.6
485	RSPe_2	-122.445	37.928	0.41	14.81	292.25	15.23	-122.445	37.928	0.31	14.81	292.71	15.12	9	7/14/2014	49:36.5
486	RSPe_2	-122.445	37.928	0.41	14.82	292.30	15.23	-122.445	37.928	0.26	14.82	292.64	15.08	9	7/14/2014	49:36.4
487	RSPe_2	-122.445	37.928	0.41	14.85	292.39	15.27	-122.445	37.928	0.31	14.85	292.67	15.16	9	7/14/2014	49:36.3
488	RSPe_2	-122.445	37.928	0.41	14.86	292.49	15.28	-122.445	37.928	0.26	14.86	292.64	15.12	9	7/14/2014	49:36.2
489	RSPe_2	-122.445	37.928	0.41	14.81	292.54	15.22	-122.445	37.928	0.31	14.81	292.63	15.12	9	7/14/2014	49:36.1
490	RSPe_2	-122.445	37.928	0.41	14.82	292.50	15.23	-122.445	37.928	0.26	14.82	292.62	15.08	9	7/14/2014	49:36.0
491	RSPe_2	-122.445	37.928	0.45	14.79	292.56	15.24	-122.445	37.928	0.34	14.79	292.62	15.13	9	7/14/2014	49:35.9
492	RSPe_2	-122.445	37.928	0.41	14.78	292.52	15.20	-122.445	37.928	0.31	14.78	292.63	15.09	9	7/14/2014	49:35.8
493	RSPe_2	-122.445	37.928	0.45	14.76	292.52	15.21	-122.445	37.928	0.34	14.76	292.67	15.10	9	7/14/2014	49:35.7
494	RSPe_2	-122.445	37.928	0.41	14.72	292.54	15.14	-122.445	37.928	0.34	14.72	292.70	15.06	9	7/14/2014	49:35.6

495	RSPe_2	-122.445	37.928	0.45	14.70	292.52	15.15	-122.445	37.928	0.43	14.70	292.76	15.12	9	7/14/2014	49:35.5
496	RSPe_2	-122.445	37.928	0.41	14.65	292.45	15.06	-122.445	37.928	0.34	14.65	292.69	14.99	9	7/14/2014	49:35.4
497	RSPe_2	-122.445	37.928	0.45	14.61	292.39	15.05	-122.445	37.928	0.39	14.61	292.72	15.00	9	7/14/2014	49:35.3
498	RSPe_2	-122.445	37.928	0.45	14.59	292.39	15.03	-122.445	37.928	0.39	14.59	292.71	14.98	9	7/14/2014	49:35.2
499	RSPe_2	-122.445	37.928	0.48	14.57	292.32	15.06	-122.445	37.928	0.46	14.57	292.69	15.03	9	7/14/2014	49:35.1
500	RSPe_2	-122.445	37.928	0.45	14.55	292.28	14.99	-122.445	37.928	0.43	14.55	292.70	14.97	9	7/14/2014	49:35.0
501	RSPe_2	-122.445	37.928	0.45	14.55	292.32	15.00	-122.445	37.928	0.39	14.55	292.67	14.94	9	7/14/2014	49:34.9
502	RSPe_2	-122.445	37.928	0.45	14.57	292.35	15.02	-122.445	37.928	0.43	14.57	292.70	14.99	9	7/14/2014	49:34.8
503	RSPe_2	-122.445	37.928	0.48	14.56	292.39	15.04	-122.445	37.928	0.43	14.56	292.77	14.98	9	7/14/2014	49:34.7
504	RSPe_2	-122.445	37.928	0.45	14.55	292.37	15.00	-122.445	37.928	0.39	14.55	292.70	14.94	9	7/14/2014	49:34.6
505	RSPe_2	-122.445	37.928	0.48	14.55	292.46	15.03	-122.445	37.928	0.43	14.55	292.74	14.98	9	7/14/2014	49:34.5
506	RSPe_2	-122.445	37.928	0.45	14.55	292.46	15.00	-122.445	37.928	0.39	14.55	292.66	14.94	9	7/14/2014	49:34.4
507	RSPe_2	-122.445	37.928	0.48	14.55	292.42	15.03	-122.445	37.928	0.39	14.55	292.63	14.94	9	7/14/2014	49:34.3
508	RSPe_2	-122.445	37.928	0.48	14.55	292.48	15.03	-122.445	37.928	0.43	14.55	292.61	14.97	9	7/14/2014	49:34.2
509	RSPe_2	-122.445	37.928	0.48	14.54	292.42	15.02	-122.445	37.928	0.51	14.54	292.54	15.05	9	7/14/2014	49:34.1
510	RSPe_2	-122.445	37.928	0.48	14.53	292.42	15.01	-122.445	37.928	0.46	14.53	292.52	14.99	9	7/14/2014	49:34.0
511	RSPe_2	-122.445	37.928	0.53	14.48	292.37	15.02	-122.445	37.928	0.46	14.48	292.55	14.94	9	7/14/2014	49:33.9
512	RSPe_2	-122.445	37.928	0.48	14.45	292.38	14.93	-122.445	37.928	0.51	14.45	292.59	14.96	9	7/14/2014	49:33.8
513	RSPe_2	-122.445	37.928	0.53	14.43	292.44	14.96	-122.445	37.928	0.54	14.43	292.57	14.97	9	7/14/2014	49:33.7
514	RSPe_2	-122.445	37.928	0.53	14.37	292.35	14.90	-122.445	37.928	0.46	14.37	292.59	14.82	9	7/14/2014	49:33.6
515	RSPe_2	-122.445	37.928	0.53	14.48	292.31	15.01	-122.445	37.928	0.51	14.48	292.61	14.99	9	7/14/2014	49:33.5
516	RSPe_2	-122.445	37.928	0.53	14.38	292.27	14.92	-122.445	37.928	0.51	14.38	292.59	14.89	9	7/14/2014	49:33.4
517	RSPe_2	-122.445	37.928	0.57	14.43	292.27	15.00	-122.445	37.928	0.59	14.43	292.64	15.02	9	7/14/2014	49:33.3
518	RSPe_2	-122.445	37.928	0.48	14.39	292.27	14.87	-122.445	37.928	0.51	14.39	292.63	14.90	9	7/14/2014	49:33.2
519	RSPe_2	-122.445	37.928	0.53	14.42	292.23	14.95	-122.445	37.928	0.51	14.42	292.57	14.93	9	7/14/2014	49:33.1
520	RSPe_2	-122.445	37.928	0.53	14.41	292.31	14.94	-122.445	37.928	0.54	14.41	292.59	14.95	9	7/14/2014	49:33.0
521	RSPe_2	-122.445	37.928	0.57	14.38	292.33	14.94	-122.445	37.928	0.54	14.38	292.57	14.92	9	7/14/2014	49:32.9
522	RSPe_2	-122.445	37.928	0.53	14.45	292.40	14.98	-122.445	37.928	0.54	14.45	292.57	14.99	9	7/14/2014	49:32.8
523	RSPe_2	-122.445	37.928	0.57	14.41	292.40	14.97	-122.445	37.928	0.59	14.41	292.55	15.00	9	7/14/2014	49:32.7
524	RSPe_2	-122.445	37.928	0.57	14.41	292.42	14.97	-122.445	37.928	0.63	14.41	292.53	15.04	9	7/14/2014	49:32.6
525	RSPe_2	-122.445	37.928	0.57	14.39	292.38	14.96	-122.445	37.928	0.63	14.39	292.48	15.02	9	7/14/2014	49:32.5
526	RSPe_2	-122.445	37.928	0.53	14.38	292.33	14.92	-122.445	37.928	0.63	14.38	292.46	15.01	9	7/14/2014	49:32.4
527	RSPe_2	-122.445	37.928	0.57	14.47	292.29	15.04	-122.445	37.928	0.68	14.47	292.49	15.15	9	7/14/2014	49:32.3

528	RSPe_2	-122.445	37.928	0.57	14.43	292.23	15.00	-122.445	37.928	0.71	14.43	292.51	15.14	9	7/14/2014	49:32.2
529	RSPe_2	-122.445	37.928	0.62	14.42	292.18	15.04	-122.445	37.928	0.68	14.42	292.47	15.10	9	7/14/2014	49:32.1
530	RSPe_2	-122.445	37.928	0.57	14.43	292.12	15.00	-122.445	37.928	0.63	14.43	292.53	15.06	9	7/14/2014	49:32.0
531	RSPe_2	-122.445	37.928	0.62	14.47	292.14	15.09	-122.445	37.928	0.68	14.47	292.58	15.15	9	7/14/2014	49:31.9
532	RSPe_2	-122.445	37.928	0.57	14.46	292.12	15.03	-122.445	37.928	0.63	14.46	292.60	15.09	9	7/14/2014	49:31.8
533	RSPe_2	-122.445	37.928	0.62	14.45	292.16	15.06	-122.445	37.928	0.68	14.45	292.62	15.12	9	7/14/2014	49:31.7
534	RSPe_2	-122.445	37.928	0.62	14.47	292.16	15.09	-122.445	37.928	0.68	14.47	292.53	15.15	9	7/14/2014	49:31.6
535	RSPe_2	-122.445	37.928	0.62	14.47	292.23	15.09	-122.445	37.928	0.68	14.47	292.51	15.15	9	7/14/2014	49:31.5
536	RSPe_2	-122.445	37.928	0.57	14.50	292.27	15.07	-122.445	37.928	0.68	14.50	292.47	15.18	9	7/14/2014	49:31.4
537	RSPe_2	-122.445	37.928	0.62	14.48	292.25	15.10	-122.445	37.928	0.71	14.48	292.41	15.19	9	7/14/2014	49:31.3
538	RSPe_2	-122.445	37.928	0.62	14.43	292.23	15.05	-122.445	37.928	0.76	14.43	292.34	15.19	9	7/14/2014	49:31.2
539	RSPe_2	-122.445	37.928	0.62	14.42	292.28	15.04	-122.445	37.928	0.76	14.42	292.32	15.18	9	7/14/2014	49:31.1
540	RSPe_2	-122.445	37.928	0.62	14.38	292.28	14.99	-122.445	37.928	0.76	14.38	292.25	15.14	9	7/14/2014	49:31.0
541	RSPe_2	-122.445	37.928	0.65	14.38	292.23	15.03	-122.445	37.928	0.80	14.38	292.23	15.17	9	7/14/2014	49:30.9
542	RSPe_2	-122.445	37.928	0.62	14.28	292.21	14.90	-122.445	37.928	0.76	14.28	292.18	15.05	9	7/14/2014	49:30.8
543	RSPe_2	-122.445	37.928	0.65	14.22	292.19	14.87	-122.445	37.928	0.83	14.22	292.14	15.05	9	7/14/2014	49:30.7
544	RSPe_2	-122.445	37.928	0.62	14.24	292.17	14.86	-122.445	37.928	0.83	14.24	292.25	15.07	9	7/14/2014	49:30.6
545	RSPe_2	-122.445	37.928	0.65	14.17	292.17	14.83	-122.445	37.928	0.80	14.17	292.23	14.97	9	7/14/2014	49:30.5
546	RSPe_2	-122.445	37.928	0.62	14.17	292.15	14.79	-122.445	37.928	0.80	14.17	292.28	14.97	9	7/14/2014	49:30.4
547	RSPe_2	-122.445	37.928	0.65	14.15	292.13	14.80	-122.445	37.928	0.80	14.15	292.32	14.94	9	7/14/2014	49:30.3
548	RSPe_2	-122.445	37.928	0.62	14.17	292.13	14.79	-122.445	37.928	0.76	14.17	292.35	14.94	9	7/14/2014	49:30.2
549	RSPe_2	-122.445	37.928	0.62	14.12	292.08	14.74	-122.445	37.928	0.80	14.12	292.32	14.92	9	7/14/2014	49:30.1
550	RSPe_2	-122.445	37.928	0.62	14.15	292.04	14.77	-122.445	37.928	0.80	14.15	292.32	14.95	9	7/14/2014	49:30.0
551	RSPe_2	-122.445	37.928	0.62	14.17	292.04	14.79	-122.445	37.928	0.80	14.17	292.30	14.97	9	7/14/2014	49:29.9
552	RSPe_2	-122.445	37.928	0.62	14.17	291.97	14.79	-122.445	37.928	0.80	14.17	292.25	14.97	9	7/14/2014	49:29.8
553	RSPe_2	-122.445	37.928	0.62	14.14	291.95	14.75	-122.445	37.928	0.83	14.14	292.25	14.97	9	7/14/2014	49:29.7
554	RSPe_2	-122.445	37.928	0.62	14.15	291.93	14.77	-122.445	37.928	0.80	14.15	292.21	14.95	9	7/14/2014	49:29.6
555	RSPe_2	-122.445	37.928	0.65	14.15	291.89	14.80	-122.445	37.928	0.83	14.15	292.15	14.98	9	7/14/2014	49:29.5
556	RSPe_2	-122.445	37.928	0.62	14.17	291.80	14.79	-122.445	37.928	0.83	14.17	292.06	15.01	9	7/14/2014	49:29.4
557	RSPe_2	-122.445	37.928	0.62	14.16	291.78	14.78	-122.445	37.928	0.92	14.16	291.97	15.07	9	7/14/2014	49:29.3
558	RSPe_2	-122.445	37.928	0.62	14.16	291.80	14.78	-122.445	37.928	0.88	14.16	291.99	15.04	9	7/14/2014	49:29.2
559	RSPe_2	-122.445	37.928	0.62	14.18	291.80	14.80	-122.445	37.928	0.92	14.18	292.02	15.10	9	7/14/2014	49:29.1
560	RSPe_2	-122.445	37.928	0.57	14.17	291.78	14.74	-122.445	37.928	0.83	14.17	291.95	15.01	9	7/14/2014	49:29.0

561	RSPe_2	-122.445	37.928	0.62	14.19	291.76	14.81	-122.445	37.928	0.83	14.19	291.97	15.02	9	7/14/2014	49:28.9
562	RSPe_2	-122.445	37.928	0.62	14.26	291.69	14.88	-122.445	37.928	0.83	14.26	291.90	15.09	9	7/14/2014	49:28.8
563	RSPe_2	-122.445	37.928	0.62	14.22	291.69	14.83	-122.445	37.928	0.83	14.22	291.89	15.05	9	7/14/2014	49:28.7
564	RSPe_2	-122.445	37.928	0.57	14.25	291.74	14.82	-122.445	37.928	0.80	14.25	291.89	15.05	9	7/14/2014	49:28.6
565	RSPe_2	-122.445	37.928	0.62	14.24	291.65	14.86	-122.445	37.928	0.83	14.24	291.85	15.07	9	7/14/2014	49:28.5
566	RSPe_2	-122.445	37.928	0.57	14.27	291.70	14.84	-122.445	37.928	0.80	14.27	291.84	15.07	9	7/14/2014	49:28.4
567	RSPe_2	-122.445	37.928	0.57	14.26	291.72	14.83	-122.445	37.928	0.83	14.26	291.78	15.09	9	7/14/2014	49:28.3
568	RSPe_2	-122.445	37.928	0.57	14.21	291.78	14.77	-122.445	37.928	0.83	14.21	291.80	15.04	9	7/14/2014	49:28.2
569	RSPe_2	-122.445	37.928	0.57	14.17	291.74	14.74	-122.445	37.928	0.83	14.17	291.80	15.01	9	7/14/2014	49:28.1
570	RSPe_2	-122.445	37.928	0.57	14.20	291.78	14.77	-122.445	37.928	0.88	14.20	291.80	15.08	9	7/14/2014	49:28.0
571	RSPe_2	-122.445	37.928	0.57	14.27	291.76	14.84	-122.445	37.928	0.88	14.27	291.83	15.15	9	7/14/2014	49:27.9
572	RSPe_2	-122.445	37.928	0.57	14.15	291.76	14.72	-122.445	37.928	0.88	14.15	291.83	15.03	9	7/14/2014	49:27.8
573	RSPe_2	-122.445	37.928	0.57	14.19	291.79	14.76	-122.445	37.928	0.92	14.19	291.85	15.11	9	7/14/2014	49:27.7
574	RSPe_2	-122.445	37.928	0.57	14.15	291.79	14.72	-122.445	37.928	0.92	14.15	291.85	15.07	9	7/14/2014	49:27.6
575	RSPe_2	-122.445	37.928	0.57	14.18	291.77	14.75	-122.445	37.928	0.92	14.18	291.90	15.10	9	7/14/2014	49:27.5
576	RSPe_2	-122.445	37.928	0.53	14.16	291.77	14.69	-122.445	37.928	0.88	14.16	291.89	15.04	9	7/14/2014	49:27.4
577	RSPe_2	-122.445	37.928	0.57	14.18	291.76	14.75	-122.445	37.928	0.88	14.18	291.94	15.06	9	7/14/2014	49:27.3
578	RSPe_2	-122.445	37.928	0.53	14.18	291.72	14.72	-122.445	37.928	0.88	14.18	291.97	15.06	9	7/14/2014	49:27.2
579	RSPe_2	-122.445	37.928	0.53	14.18	291.72	14.72	-122.445	37.928	0.88	14.18	292.00	15.06	9	7/14/2014	49:27.1
580	RSPe_2	-122.445	37.928	0.48	14.22	291.70	14.70	-122.445	37.928	0.83	14.22	292.01	15.05	9	7/14/2014	49:27.0
581	RSPe_2	-122.445	37.928	0.53	14.24	291.66	14.77	-122.445	37.928	0.83	14.24	292.01	15.07	9	7/14/2014	49:26.9
582	RSPe_2	-122.445	37.928	0.48	14.25	291.70	14.74	-122.445	37.928	0.80	14.25	292.03	15.05	9	7/14/2014	49:26.8
583	RSPe_2	-122.445	37.928	0.48	14.27	291.61	14.75	-122.445	37.928	0.80	14.27	291.96	15.07	9	7/14/2014	49:26.7
584	RSPe_2	-122.445	37.928	0.48	14.31	291.68	14.79	-122.445	37.928	0.80	14.31	291.94	15.11	9	7/14/2014	49:26.6
585	RSPe_2	-122.445	37.928	0.48	14.31	291.64	14.79	-122.445	37.928	0.80	14.31	291.94	15.11	9	7/14/2014	49:26.5
586	RSPe_2	-122.445	37.928	0.45	14.33	291.68	14.78	-122.445	37.928	0.76	14.33	291.90	15.09	9	7/14/2014	49:26.4
587	RSPe_2	-122.445	37.928	0.48	14.34	291.73	14.82	-122.445	37.928	0.80	14.34	291.90	15.14	9	7/14/2014	49:26.3
588	RSPe_2	-122.445	37.928	0.45	14.31	291.70	14.76	-122.445	37.928	0.76	14.31	291.85	15.07	9	7/14/2014	49:26.2
589	RSPe_2	-122.445	37.928	0.48	14.31	291.68	14.79	-122.445	37.928	0.76	14.31	291.81	15.08	9	7/14/2014	49:26.1
590	RSPe_2	-122.445	37.928	0.45	14.31	291.62	14.76	-122.445	37.928	0.80	14.31	291.77	15.11	9	7/14/2014	49:26.0
591	RSPe_2	-122.445	37.928	0.48	14.31	291.60	14.79	-122.445	37.928	0.80	14.31	291.73	15.11	9	7/14/2014	49:25.9
592	RSPe_2	-122.445	37.928	0.45	14.33	291.58	14.78	-122.445	37.928	0.76	14.33	291.77	15.09	9	7/14/2014	49:25.8
593	RSPe_2	-122.445	37.928	0.45	14.31	291.58	14.76	-122.445	37.928	0.80	14.31	291.79	15.11	9	7/14/2014	49:25.7

594	RSPe_2	-122.445	37.928	0.41	14.29	291.55	14.71	-122.445	37.928	0.76	14.29	291.86	15.06	9	7/14/2014 49:25.6
595	RSPe_2	-122.445	37.928	0.45	14.30	291.51	14.74	-122.445	37.928	0.76	14.30	291.81	15.06	9	7/14/2014 49:25.5
596	RSPe_2	-122.445	37.928	0.41	14.31	291.49	14.73	-122.445	37.928	0.71	14.31	291.83	15.03	9	7/14/2014 49:25.4
597	RSPe_2	-122.445	37.928	0.41	14.35	291.45	14.77	-122.445	37.928	0.68	14.35	291.79	15.03	9	7/14/2014 49:25.3
598	RSPe_2	-122.445	37.928	0.36	14.35	291.40	14.71	-122.445	37.928	0.63	14.35	291.82	14.97	9	7/14/2014 49:25.2
599	RSPe_2	-122.445	37.928	0.41	14.41	291.36	14.82	-122.445	37.928	0.63	14.41	291.80	15.04	9	7/14/2014 49:25.1
600	RSPe_2	-122.445	37.928	0.36	14.41	291.34	14.77	-122.445	37.928	0.60	14.41	291.77	15.00	9	7/14/2014 49:25.0
601	RSPe_2	-122.445	37.928	0.36	14.41	291.27	14.77	-122.445	37.928	0.60	14.41	291.75	15.00	9	7/14/2014 49:24.9
602	RSPe_2	-122.445	37.928	0.36	14.45	291.27	14.81	-122.445	37.928	0.54	14.45	291.70	14.99	9	7/14/2014 49:24.8
603	RSPe_2	-122.445	37.928	0.36	14.52	291.23	14.88	-122.445	37.928	0.54	14.52	291.66	15.06	9	7/14/2014 49:24.7
604	RSPe_2	-122.445	37.928	0.33	14.56	291.23	14.89	-122.445	37.928	0.54	14.56	291.62	15.10	9	7/14/2014 49:24.6
605	RSPe_2	-122.445	37.928	0.36	14.57	291.20	14.93	-122.445	37.928	0.54	14.57	291.53	15.11	9	7/14/2014 49:24.5
606	RSPe_2	-122.445	37.928	0.33	14.64	291.19	14.97	-122.445	37.928	0.54	14.64	291.45	15.18	9	7/14/2014 49:24.4
607	RSPe_2	-122.445	37.928	0.33	14.65	291.19	14.97	-122.445	37.928	0.51	14.65	291.40	15.16	9	7/14/2014 49:24.3
608	RSPe_2	-122.445	37.928	0.33	14.65	291.05	14.98	-122.445	37.928	0.51	14.65	291.29	15.16	9	7/14/2014 49:24.2
609	RSPe_2	-122.445	37.928	0.33	14.68	291.03	15.00	-122.445	37.928	0.54	14.68	291.21	15.22	9	7/14/2014 49:24.1
610	RSPe_2	-122.445	37.928	0.33	14.68	291.01	15.01	-122.445	37.928	0.60	14.68	291.27	15.28	9	7/14/2014 49:24.0
611	RSPe_2	-122.445	37.928	0.33	14.73	291.01	15.06	-122.445	37.928	0.54	14.73	291.29	15.27	9	7/14/2014 49:23.9
612	RSPe_2	-122.445	37.928	0.28	14.72	290.99	15.00	-122.445	37.928	0.54	14.72	291.33	15.27	9	7/14/2014 49:23.8
613	RSPe_2	-122.445	37.928	0.33	14.75	290.96	15.07	-122.445	37.928	0.51	14.75	291.32	15.26	9	7/14/2014 49:23.7
614	RSPe_2	-122.445	37.928	0.28	14.78	290.97	15.05	-122.445	37.928	0.46	14.78	291.31	15.24	9	7/14/2014 49:23.6
615	RSPe_2	-122.445	37.928	0.28	14.80	290.91	15.08	-122.445	37.928	0.43	14.80	291.30	15.23	9	7/14/2014 49:23.5
616	RSPe_2	-122.445	37.928	0.24	14.81	290.92	15.06	-122.445	37.928	0.39	14.81	291.27	15.21	9	7/14/2014 49:23.4
617	RSPe_2	-122.445	37.928	0.24	14.85	290.93	15.09	-122.445	37.928	0.39	14.85	291.32	15.24	9	7/14/2014 49:23.3
618	RSPe_2	-122.445	37.928	0.24	14.85	290.97	15.09	-122.445	37.928	0.34	14.85	291.27	15.19	9	7/14/2014 49:23.2
619	RSPe_2	-122.445	37.928	0.24	14.92	290.95	15.17	-122.445	37.928	0.34	14.92	291.25	15.26	9	7/14/2014 49:23.1
620	RSPe_2	-122.445	37.928	0.19	14.89	290.99	15.08	-122.445	37.928	0.31	14.89	291.25	15.19	9	7/14/2014 49:23.0
621	RSPe_2	-122.445	37.928	0.19	14.90	290.99	15.09	-122.445	37.928	0.34	14.90	291.25	15.24	9	7/14/2014 49:22.9
622	RSPe_2	-122.445	37.928	0.19	14.90	290.99	15.09	-122.445	37.928	0.31	14.90	291.23	15.21	9	7/14/2014 49:22.8
623	RSPe_2	-122.445	37.928	0.19	14.90	291.06	15.09	-122.445	37.928	0.31	14.90	291.24	15.21	9	7/14/2014 49:22.7
624	RSPe_2	-122.445	37.928	0.19	14.89	291.09	15.08	-122.445	37.928	0.26	14.89	291.28	15.15	9	7/14/2014 49:22.6
625	RSPe_2	-122.445	37.928	0.19	14.88	291.08	15.07	-122.445	37.928	0.31	14.88	291.25	15.19	9	7/14/2014 49:22.5
626	RSPe_2	-122.445	37.928	0.19	14.87	290.95	15.06	-122.445	37.928	0.26	14.87	291.17	15.13	9	7/14/2014 49:22.4

627	RSPe_2	-122.445	37.928	0.19	14.83	290.93	15.02	-122.445	37.928	0.34	14.83	291.15	15.17	9	7/14/2014	49:22.3
628	RSPe_2	-122.445	37.928	0.19	14.81	290.93	15.01	-122.445	37.928	0.34	14.81	291.23	15.16	9	7/14/2014	49:22.2
629	RSPe_2	-122.445	37.928	0.19	14.83	290.89	15.02	-122.445	37.928	0.39	14.83	291.28	15.22	9	7/14/2014	49:22.1
630	RSPe_2	-122.445	37.928	0.16	14.81	290.89	14.97	-122.445	37.928	0.31	14.81	291.28	15.12	9	7/14/2014	49:22.0
631	RSPe_2	-122.445	37.928	0.16	14.81	290.91	14.97	-122.445	37.928	0.31	14.81	291.30	15.12	9	7/14/2014	49:21.9
632	RSPe_2	-122.445	37.928	0.12	14.89	290.89	15.01	-122.445	37.928	0.22	14.89	291.24	15.11	9	7/14/2014	49:21.8
633	RSPe_2	-122.445	37.928	0.16	14.83	290.85	14.99	-122.445	37.928	0.22	14.83	291.20	15.05	9	7/14/2014	49:21.7
634	RSPe_2	-122.445	37.928	0.12	14.84	290.84	14.96	-122.445	37.928	0.17	14.84	291.13	15.01	9	7/14/2014	49:21.6
635	RSPe_2	-122.445	37.928	0.16	14.87	290.83	15.03	-122.445	37.928	0.17	14.87	291.10	15.04	9	7/14/2014	49:21.5
636	RSPe_2	-122.445	37.928	0.12	14.89	290.83	15.01	-122.445	37.928	0.14	14.89	291.08	15.02	9	7/14/2014	49:21.4
637	RSPe_2	-122.445	37.928	0.12	14.88	290.78	15.00	-122.445	37.928	0.14	14.88	291.02	15.02	9	7/14/2014	49:21.3
638	RSPe_2	-122.445	37.928	0.07	14.90	290.78	14.97	-122.445	37.928	0.10	14.90	291.02	15.01	9	7/14/2014	49:21.2
639	RSPe_2	-122.445	37.928	0.12	14.90	290.78	15.02	-122.445	37.928	0.14	14.90	290.98	15.04	9	7/14/2014	49:21.1
640	RSPe_2	-122.445	37.928	0.12	14.92	290.80	15.04	-122.445	37.928	0.14	14.92	290.99	15.05	9	7/14/2014	49:21.0
641	RSPe_2	-122.445	37.928	0.12	14.93	290.78	15.06	-122.445	37.928	0.10	14.93	290.96	15.04	9	7/14/2014	49:20.9
642	RSPe_2	-122.445	37.928	0.12	14.92	290.83	15.05	-122.445	37.928	0.10	14.92	290.98	15.03	9	7/14/2014	49:20.8
643	RSPe_2	-122.445	37.928	0.12	14.92	290.91	15.05	-122.445	37.928	0.14	14.92	291.02	15.06	9	7/14/2014	49:20.7
644	RSPe_2	-122.445	37.928	0.07	14.92	290.96	14.99	-122.445	37.928	0.14	14.92	290.95	15.05	9	7/14/2014	49:20.6
645	RSPe_2	-122.445	37.928	0.12	14.95	290.94	15.08	-122.445	37.928	0.17	14.95	290.89	15.13	9	7/14/2014	49:20.5
646	RSPe_2	-122.445	37.928	0.07	14.90	291.01	14.97	-122.445	37.928	0.17	14.90	290.89	15.07	9	7/14/2014	49:20.4
647	RSPe_2	-122.445	37.928	0.12	14.95	291.07	15.08	-122.445	37.928	0.26	14.95	290.87	15.21	9	7/14/2014	49:20.3
648	RSPe_2	-122.445	37.928	0.07	14.91	291.11	14.98	-122.445	37.928	0.22	14.91	290.93	15.13	9	7/14/2014	49:20.2
649	RSPe_2	-122.445	37.928	0.12	14.92	291.16	15.04	-122.445	37.928	0.17	14.92	291.00	15.09	9	7/14/2014	49:20.1
650	RSPe_2	-122.445	37.928	0.07	14.94	291.16	15.01	-122.445	37.928	0.14	14.94	291.02	15.08	9	7/14/2014	49:20.0
651	RSPe_2	-122.445	37.928	0.07	14.94	291.16	15.01	-122.445	37.928	0.17	14.94	291.03	15.11	9	7/14/2014	49:19.9
652	RSPe_2	-122.445	37.928	0.07	14.99	291.18	15.06	-122.445	37.928	0.14	14.99	291.13	15.13	9	7/14/2014	49:19.8
653	RSPe_2	-122.445	37.928	0.07	15.00	291.23	15.07	-122.445	37.928	0.14	15.00	291.20	15.14	9	7/14/2014	49:19.7
654	RSPe_2	-122.445	37.928	0.04	14.99	291.25	15.03	-122.445	37.928	0.10	14.99	291.29	15.10	9	7/14/2014	49:19.6
655	RSPe_2	-122.445	37.928	0.04	15.02	291.36	15.06	-122.445	37.928	0.14	15.02	291.38	15.16	9	7/14/2014	49:19.5
656	RSPe_2	-122.445	37.928	0.04	15.08	291.38	15.12	-122.445	37.928	0.05	15.08	291.42	15.13	9	7/14/2014	49:19.4
657	RSPe_2	-122.445	37.928	0.04	15.04	291.36	15.08	-122.445	37.928	0.10	15.04	291.36	15.14	9	7/14/2014	49:19.3
658	RSPe_2	-122.445	37.928	0.04	15.06	291.43	15.10	-122.445	37.928	0.10	15.06	291.47	15.17	9	7/14/2014	49:19.2
659	RSPe_2	-122.445	37.928	0.07	15.12	291.52	15.19	-122.445	37.928	0.10	15.12	291.47	15.22	9	7/14/2014	49:19.1

660	RSPe_2	-122.445	37.928	0.04	14.96	291.58	15.00	-122.445	37.928	0.14	14.96	291.54	15.10	9	7/14/2014	49:19.0
661	RSPe_2	-122.445	37.928	0.07	14.94	291.52	15.02	-122.445	37.928	0.14	14.94	291.54	15.08	9	7/14/2014	49:18.9
662	RSPe_2	-122.445	37.928	0.04	14.92	291.56	14.96	-122.445	37.928	0.14	14.92	291.62	15.06	9	7/14/2014	49:18.8
663	RSPe_2	-122.445	37.928	0.04	14.97	291.61	15.01	-122.445	37.928	0.22	14.97	291.69	15.19	9	7/14/2014	49:18.7
664	RSPe_2	-122.445	37.928	0.04	14.94	291.67	14.98	-122.445	37.928	0.22	14.94	291.82	15.17	9	7/14/2014	49:18.6
665	RSPe_2	-122.445	37.928	0.07	14.96	291.69	15.03	-122.445	37.928	0.22	14.96	291.89	15.18	9	7/14/2014	49:18.5
666	RSPe_2	-122.445	37.928	-0.01	14.94	291.76	14.93	-122.445	37.928	0.17	14.94	291.94	15.11	9	7/14/2014	49:18.4
667	RSPe_2	-122.445	37.928	0.04	14.92	291.74	14.96	-122.445	37.928	0.14	14.92	291.89	15.06	9	7/14/2014	49:18.3
668	RSPe_2	-122.445	37.928	-0.01	14.94	291.74	14.93	-122.445	37.928	0.14	14.94	291.93	15.08	9	7/14/2014	49:18.2
669	RSPe_2	-122.445	37.928	0.04	14.94	291.79	14.98	-122.445	37.928	0.14	14.94	292.01	15.08	9	7/14/2014	49:18.1
670	RSPe_2	-122.445	37.928	-0.01	14.95	291.79	14.94	-122.445	37.928	0.14	14.95	291.98	15.09	9	7/14/2014	49:18.0
671	RSPe_2	-122.445	37.928	-0.01	14.94	291.79	14.93	-122.445	37.928	0.10	14.94	292.00	15.05	9	7/14/2014	49:17.9
672	RSPe_2	-122.445	37.928	-0.01	15.03	291.81	15.02	-122.445	37.928	0.05	15.03	292.01	15.08	9	7/14/2014	49:17.8
673	RSPe_2	-122.445	37.928	-0.01	14.98	291.85	14.96	-122.445	37.928	0.10	14.98	292.02	15.08	9	7/14/2014	49:17.7
674	RSPe_2	-122.445	37.928	-0.01	15.02	291.94	15.01	-122.445	37.928	0.05	15.02	292.15	15.08	9	7/14/2014	49:17.6
675	RSPe_2	-122.445	37.928	-0.01	15.02	292.01	15.00	-122.445	37.928	0.05	15.02	292.23	15.07	9	7/14/2014	49:17.5
676	RSPe_2	-122.445	37.928	-0.01	15.01	292.05	14.99	-122.445	37.928	0.02	15.01	292.23	15.03	9	7/14/2014	49:17.4
677	RSPe_2	-122.445	37.928	-0.01	15.02	291.99	15.01	-122.445	37.928	0.05	15.02	292.16	15.08	9	7/14/2014	49:17.3
678	RSPe_2	-122.445	37.928	-0.01	15.06	291.97	15.05	-122.445	37.928	0.14	15.06	292.18	15.20	9	7/14/2014	49:17.2
679	RSPe_2	-122.445	37.928	-0.01	15.08	291.90	15.07	-122.445	37.928	0.14	15.08	292.11	15.22	9	7/14/2014	49:17.1
680	RSPe_2	-122.445	37.928	-0.01	15.09	291.77	15.08	-122.445	37.928	0.14	15.09	292.01	15.23	9	7/14/2014	49:17.0
681	RSPe_2	-122.445	37.928	0.04	15.10	291.79	15.14	-122.445	37.928	0.17	15.10	292.02	15.27	9	7/14/2014	49:16.9
682	RSPe_2	-122.445	37.928	-0.01	15.13	291.79	15.12	-122.445	37.928	0.10	15.13	292.05	15.23	9	7/14/2014	49:16.8
683	RSPe_2	-122.445	37.928	-0.01	15.16	291.83	15.15	-122.445	37.928	0.14	15.16	292.03	15.30	9	7/14/2014	49:16.7
684	RSPe_2	-122.445	37.928	-0.01	15.19	291.75	15.17	-122.445	37.928	0.10	15.19	291.90	15.29	9	7/14/2014	49:16.6
685	RSPe_2	-122.445	37.928	-0.01	15.20	291.90	15.19	-122.445	37.928	0.10	15.20	292.12	15.31	9	7/14/2014	49:16.5
686	RSPe_2	-122.445	37.928	-0.05	15.22	291.99	15.17	-122.445	37.928	0.02	15.22	292.18	15.24	9	7/14/2014	49:16.4
687	RSPe_2	-122.445	37.928	-0.01	15.26	291.97	15.24	-122.445	37.928	0.05	15.26	292.16	15.31	9	7/14/2014	49:16.3
688	RSPe_2	-122.445	37.928	-0.10	15.32	292.08	15.22	-122.445	37.928	0.02	15.32	292.23	15.34	9	7/14/2014	49:16.2
689	RSPe_2	-122.445	37.928	-0.05	15.29	292.06	15.25	-122.445	37.928	0.02	15.29	292.30	15.31	9	7/14/2014	49:16.1
690	RSPe_2	-122.445	37.928	-0.10	15.28	292.15	15.18	-122.445	37.928	0.02	15.28	292.34	15.30	9	7/14/2014	49:16.0
691	RSPe_2	-122.445	37.928	-0.05	15.37	292.21	15.32	-122.445	37.928	0.02	15.37	292.34	15.39	9	7/14/2014	49:15.9
692	RSPe_2	-122.445	37.928	-0.10	15.29	292.26	15.19	-122.445	37.928	0.02	15.29	292.32	15.31	9	7/14/2014	49:15.8

693	RSPe_2	-122.445	37.928	-0.05	15.29	292.24	15.25	-122.445	37.928	-0.03	15.29	292.28	15.26	9	7/14/2014	49:15.7
694	RSPe_2	-122.445	37.928	-0.10	15.26	292.19	15.16	-122.445	37.928	-0.03	15.26	292.27	15.23	9	7/14/2014	49:15.6
695	RSPe_2	-122.445	37.928	-0.10	15.26	292.17	15.16	-122.445	37.928	0.05	15.26	292.19	15.31	9	7/14/2014	49:15.5
696	RSPe_2	-122.445	37.928	-0.10	15.29	292.04	15.19	-122.445	37.928	0.05	15.29	292.04	15.34	9	7/14/2014	49:15.4
697	RSPe_2	-122.445	37.928	-0.10	15.23	292.04	15.13	-122.445	37.928	0.05	15.23	292.06	15.29	9	7/14/2014	49:15.3
698	RSPe_2	-122.445	37.928	-0.10	15.24	292.13	15.14	-122.445	37.928	0.05	15.24	292.17	15.29	9	7/14/2014	49:15.2
699	RSPe_2	-122.445	37.928	-0.10	15.26	292.13	15.16	-122.445	37.928	0.05	15.26	292.23	15.31	9	7/14/2014	49:15.1
700	RSPe_2	-122.445	37.928	-0.10	15.26	292.19	15.16	-122.445	37.928	0.02	15.26	292.36	15.28	9	7/14/2014	49:15.0
701	RSPe_2	-122.445	37.928	-0.10	15.34	292.24	15.24	-122.445	37.928	0.02	15.34	292.39	15.36	9	7/14/2014	49:14.9
702	RSPe_2	-122.445	37.928	-0.13	15.32	292.26	15.19	-122.445	37.928	-0.06	15.32	292.51	15.26	9	7/14/2014	49:14.8
703	RSPe_2	-122.445	37.928	-0.13	15.37	292.26	15.24	-122.445	37.928	-0.06	15.37	292.52	15.31	9	7/14/2014	49:14.7
704	RSPe_2	-122.445	37.928	-0.13	15.39	292.35	15.25	-122.445	37.928	-0.12	15.39	292.68	15.27	9	7/14/2014	49:14.6
705	RSPe_2	-122.445	37.928	-0.13	15.39	292.33	15.26	-122.445	37.928	-0.12	15.39	292.68	15.28	9	7/14/2014	49:14.5
706	RSPe_2	-122.445	37.928	-0.18	15.41	292.37	15.23	-122.445	37.928	-0.12	15.41	292.74	15.29	9	7/14/2014	49:14.4
707	RSPe_2	-122.445	37.928	-0.13	15.43	292.39	15.30	-122.445	37.928	-0.12	15.43	292.73	15.32	9	7/14/2014	49:14.3
708	RSPe_2	-122.445	37.928	-0.18	15.43	292.44	15.25	-122.445	37.928	-0.12	15.43	292.76	15.32	9	7/14/2014	49:14.2
709	RSPe_2	-122.445	37.928	-0.18	15.44	292.55	15.26	-122.445	37.928	-0.12	15.44	292.77	15.33	9	7/14/2014	49:14.1
710	RSPe_2	-122.445	37.928	-0.18	15.44	292.62	15.26	-122.445	37.928	-0.15	15.44	292.72	15.29	9	7/14/2014	49:14.0
711	RSPe_2	-122.445	37.928	-0.18	15.43	292.60	15.25	-122.445	37.928	-0.12	15.43	292.59	15.32	9	7/14/2014	49:13.9
712	RSPe_2	-122.445	37.928	-0.18	15.42	292.58	15.23	-122.445	37.928	-0.12	15.42	292.62	15.30	9	7/14/2014	49:13.8
713	RSPe_2	-122.445	37.928	-0.18	15.40	292.62	15.22	-122.445	37.928	-0.03	15.40	292.61	15.37	9	7/14/2014	49:13.7
714	RSPe_2	-122.445	37.928	-0.18	15.39	292.64	15.20	-122.445	37.928	-0.12	15.39	292.64	15.27	9	7/14/2014	49:13.6
715	RSPe_2	-122.445	37.928	-0.18	15.34	292.62	15.16	-122.445	37.928	-0.12	15.34	292.68	15.23	9	7/14/2014	49:13.5
716	RSPe_2	-122.445	37.928	-0.22	15.39	292.71	15.18	-122.445	37.928	-0.15	15.39	292.72	15.25	9	7/14/2014	49:13.4
717	RSPe_2	-122.445	37.928	-0.18	15.36	292.71	15.18	-122.445	37.928	-0.15	15.36	292.72	15.21	9	7/14/2014	49:13.3
718	RSPe_2	-122.445	37.928	-0.22	15.29	292.71	15.08	-122.445	37.928	-0.20	15.29	292.71	15.09	9	7/14/2014	49:13.2
719	RSPe_2	-122.445	37.928	-0.22	15.31	292.71	15.09	-122.445	37.928	-0.15	15.31	292.77	15.16	9	7/14/2014	49:13.1
720	RSPe_2	-122.445	37.928	-0.25	15.29	292.71	15.03	-122.445	37.928	-0.23	15.29	292.82	15.05	9	7/14/2014	49:13.0
721	RSPe_2	-122.445	37.928	-0.25	15.31	292.69	15.05	-122.445	37.928	-0.23	15.31	292.93	15.07	9	7/14/2014	49:12.9
722	RSPe_2	-122.445	37.928	-0.25	15.32	292.69	15.07	-122.445	37.928	-0.32	15.32	293.01	15.01	9	7/14/2014	49:12.8
723	RSPe_2	-122.445	37.928	-0.22	15.38	292.67	15.16	-122.445	37.928	-0.27	15.38	293.06	15.11	9	7/14/2014	49:12.7
724	RSPe_2	-122.445	37.928	-0.25	15.40	292.75	15.15	-122.445	37.928	-0.27	15.40	293.21	15.14	9	7/14/2014	49:12.6
725	RSPe_2	-122.445	37.928	-0.25	15.42	292.84	15.16	-122.445	37.928	-0.27	15.42	293.21	15.15	9	7/14/2014	49:12.5

726	RSPe_2	-122.445	37.928	-0.30	15.46	292.91	15.16	-122.445	37.928	-0.35	15.46	293.21	15.11	9	7/14/2014	49:12.4
727	RSPe_2	-122.445	37.928	-0.25	15.41	292.93	15.16	-122.445	37.928	-0.32	15.41	293.08	15.09	9	7/14/2014	49:12.3
728	RSPe_2	-122.445	37.928	-0.30	15.46	292.92	15.15	-122.445	37.928	-0.32	15.46	292.99	15.14	9	7/14/2014	49:12.2
729	RSPe_2	-122.445	37.928	-0.25	15.39	292.91	15.14	-122.445	37.928	-0.32	15.39	292.93	15.08	9	7/14/2014	49:12.1
730	RSPe_2	-122.445	37.928	-0.30	15.39	292.85	15.09	-122.445	37.928	-0.27	15.39	292.86	15.13	9	7/14/2014	49:12.0
731	RSPe_2	-122.445	37.928	-0.25	15.39	292.91	15.14	-122.445	37.928	-0.27	15.39	292.91	15.13	9	7/14/2014	49:11.9
732	RSPe_2	-122.445	37.928	-0.30	15.42	292.89	15.11	-122.445	37.928	-0.27	15.42	292.86	15.15	9	7/14/2014	49:11.8
733	RSPe_2	-122.445	37.928	-0.25	15.39	292.89	15.14	-122.445	37.928	-0.32	15.39	292.89	15.08	9	7/14/2014	49:11.7
734	RSPe_2	-122.445	37.928	-0.30	15.45	292.89	15.14	-122.445	37.928	-0.35	15.45	292.91	15.09	9	7/14/2014	49:11.6
735	RSPe_2	-122.445	37.928	-0.30	15.44	292.85	15.14	-122.445	37.928	-0.35	15.44	292.96	15.09	9	7/14/2014	49:11.5
736	RSPe_2	-122.445	37.928	-0.30	15.52	292.88	15.21	-122.445	37.928	-0.35	15.52	293.02	15.17	9	7/14/2014	49:11.4
737	RSPe_2	-122.445	37.928	-0.30	15.52	292.85	15.21	-122.445	37.928	-0.40	15.52	293.02	15.11	9	7/14/2014	49:11.3
738	RSPe_2	-122.445	37.928	-0.30	15.58	292.87	15.28	-122.445	37.928	-0.40	15.58	293.11	15.18	9	7/14/2014	49:11.2
739	RSPe_2	-122.445	37.928	-0.30	15.60	292.80	15.29	-122.445	37.928	-0.44	15.60	293.16	15.16	9	7/14/2014	49:11.1
740	RSPe_2	-122.445	37.928	-0.34	15.67	292.80	15.33	-122.445	37.928	-0.44	15.67	293.15	15.23	9	7/14/2014	49:11.0
741	RSPe_2	-122.445	37.928	-0.30	15.77	292.88	15.47	-122.445	37.928	-0.40	15.77	293.26	15.37	9	7/14/2014	49:10.9
742	RSPe_2	-122.445	37.928	-0.34	15.76	292.96	15.42	-122.445	37.928	-0.44	15.76	293.24	15.32	9	7/14/2014	49:10.8
743	RSPe_2	-122.445	37.928	-0.30	15.78	292.94	15.48	-122.445	37.928	-0.44	15.78	293.16	15.34	9	7/14/2014	49:10.7
744	RSPe_2	-122.445	37.928	-0.34	15.82	292.88	15.48	-122.445	37.928	-0.49	15.82	293.03	15.33	9	7/14/2014	49:10.6
745	RSPe_2	-122.445	37.928	-0.34	15.81	292.83	15.47	-122.445	37.928	-0.44	15.81	292.94	15.37	9	7/14/2014	49:10.5
746	RSPe_2	-122.445	37.928	-0.34	15.81	292.83	15.47	-122.445	37.928	-0.52	15.81	292.97	15.29	9	7/14/2014	49:10.4
747	RSPe_2	-122.445	37.928	-0.30	15.86	292.90	15.56	-122.445	37.928	-0.49	15.86	292.99	15.38	9	7/14/2014	49:10.3
748	RSPe_2	-122.445	37.928	-0.34	15.83	292.96	15.49	-122.445	37.928	-0.52	15.83	293.05	15.31	9	7/14/2014	49:10.2
749	RSPe_2	-122.445	37.928	-0.30	15.78	293.05	15.48	-122.445	37.928	-0.49	15.78	293.18	15.29	9	7/14/2014	49:10.1
750	RSPe_2	-122.445	37.928	-0.34	15.85	293.14	15.51	-122.445	37.928	-0.44	15.85	293.14	15.41	9	7/14/2014	49:10.0
751	RSPe_2	-122.445	37.928	-0.34	15.82	293.20	15.48	-122.445	37.928	-0.44	15.82	293.13	15.38	9	7/14/2014	49:09.9
752	RSPe_2	-122.445	37.928	-0.34	15.83	293.08	15.50	-122.445	37.928	-0.49	15.83	293.01	15.35	9	7/14/2014	49:09.8
753	RSPe_2	-122.445	37.928	-0.34	15.71	292.99	15.37	-122.445	37.928	-0.52	15.71	292.96	15.19	9	7/14/2014	49:09.7
754	RSPe_2	-122.445	37.928	-0.34	15.70	293.01	15.37	-122.445	37.928	-0.52	15.70	292.99	15.18	9	7/14/2014	49:09.6
755	RSPe_2	-122.445	37.928	-0.34	15.71	293.04	15.37	-122.445	37.928	-0.52	15.71	293.01	15.19	9	7/14/2014	49:09.5
756	RSPe_2	-122.445	37.928	-0.34	15.75	293.05	15.41	-122.445	37.928	-0.57	15.75	293.07	15.18	9	7/14/2014	49:09.4
757	RSPe_2	-122.445	37.928	-0.34	15.76	293.01	15.42	-122.445	37.928	-0.57	15.76	293.07	15.19	9	7/14/2014	49:09.3
758	RSPe_2	-122.445	37.928	-0.39	15.76	293.01	15.37	-122.445	37.928	-0.57	15.76	293.16	15.19	9	7/14/2014	49:09.2

759	RSPe_2	-122.445	37.928	-0.34	15.76	293.06	15.42	-122.445	37.928	-0.61	15.76	293.27	15.15	9	7/14/2014	49:09.1
760	RSPe_2	-122.445	37.928	-0.39	15.76	293.10	15.37	-122.445	37.928	-0.64	15.76	293.25	15.12	9	7/14/2014	49:09.0
761	RSPe_2	-122.445	37.928	-0.39	15.76	292.99	15.37	-122.445	37.928	-0.52	15.76	293.21	15.24	9	7/14/2014	49:08.9
762	RSPe_2	-122.445	37.928	-0.39	15.78	292.84	15.39	-122.445	37.928	-0.61	15.78	293.17	15.17	9	7/14/2014	49:08.8
763	RSPe_2	-122.445	37.928	-0.39	15.77	292.93	15.38	-122.445	37.928	-0.49	15.77	293.12	15.29	9	7/14/2014	49:08.7
764	RSPe_2	-122.445	37.928	-0.34	15.79	292.88	15.45	-122.445	37.928	-0.61	15.79	293.12	15.18	9	7/14/2014	49:08.6
765	RSPe_2	-122.445	37.928	-0.34	15.80	292.75	15.46	-122.445	37.928	-0.57	15.80	293.06	15.22	9	7/14/2014	49:08.5
766	RSPe_2	-122.445	37.928	-0.34	15.88	292.60	15.54	-122.445	37.928	-0.61	15.88	292.81	15.27	9	7/14/2014	49:08.4
767	RSPe_2	-122.445	37.928	-0.34	15.86	292.53	15.53	-122.445	37.928	-0.57	15.86	292.77	15.29	9	7/14/2014	49:08.3
768	RSPe_2	-122.445	37.928	-0.39	15.89	292.71	15.50	-122.445	37.928	-0.61	15.89	292.84	15.28	9	7/14/2014	49:08.2
769	RSPe_2	-122.445	37.928	-0.34	15.90	292.82	15.56	-122.445	37.928	-0.57	15.90	292.88	15.33	9	7/14/2014	49:08.1
770	RSPe_2	-122.445	37.928	-0.39	15.97	292.89	15.58	-122.445	37.928	-0.61	15.97	292.73	15.36	9	7/14/2014	49:08.0
771	RSPe_2	-122.445	37.928	-0.39	16.00	292.98	15.61	-122.445	37.928	-0.64	16.00	292.71	15.36	9	7/14/2014	49:07.9
772	RSPe_2	-122.445	37.928	-0.39	16.03	293.17	15.64	-122.445	37.928	-0.61	16.03	292.86	15.42	9	7/14/2014	49:07.8
773	RSPe_2	-122.445	37.928	-0.39	16.03	293.18	15.65	-122.445	37.928	-0.61	16.03	292.84	15.43	9	7/14/2014	49:07.7
774	RSPe_2	-122.445	37.928	-0.39	16.04	293.17	15.65	-122.445	37.928	-0.64	16.04	292.84	15.40	9	7/14/2014	49:07.6
775	RSPe_2	-122.445	37.928	-0.39	16.06	293.07	15.67	-122.445	37.928	-0.64	16.06	292.84	15.42	9	7/14/2014	49:07.5
776	RSPe_2	-122.445	37.928	-0.39	16.08	293.06	15.69	-122.445	37.928	-0.72	16.08	292.93	15.36	9	7/14/2014	49:07.4
777	RSPe_2	-122.445	37.928	-0.39	16.04	293.15	15.65	-122.445	37.928	-0.61	16.04	293.04	15.44	9	7/14/2014	49:07.3
778	RSPe_2	-122.445	37.928	-0.39	16.03	293.18	15.64	-122.445	37.928	-0.69	16.03	293.13	15.34	9	7/14/2014	49:07.2
779	RSPe_2	-122.445	37.928	-0.34	16.04	293.05	15.70	-122.445	37.928	-0.57	16.04	293.08	15.47	9	7/14/2014	49:07.1
780	RSPe_2	-122.445	37.928	-0.39	16.05	293.02	15.66	-122.445	37.928	-0.64	16.05	293.15	15.41	9	7/14/2014	49:07.0
781	RSPe_2	-122.445	37.928	-0.34	16.04	293.09	15.70	-122.445	37.928	-0.57	16.04	293.04	15.47	9	7/14/2014	49:06.9
782	RSPe_2	-122.445	37.928	-0.34	16.03	293.11	15.70	-122.445	37.928	-0.52	16.03	293.06	15.51	9	7/14/2014	49:06.8
783	RSPe_2	-122.445	37.928	-0.34	16.02	292.96	15.68	-122.445	37.928	-0.49	16.02	292.95	15.53	9	7/14/2014	49:06.7
784	RSPe_2	-122.445	37.928	-0.34	15.95	292.98	15.61	-122.445	37.928	-0.57	15.95	292.98	15.38	9	7/14/2014	49:06.6
785	RSPe_2	-122.445	37.928	-0.34	15.92	292.87	15.58	-122.445	37.928	-0.57	15.92	293.00	15.35	9	7/14/2014	49:06.5
786	RSPe_2	-122.445	37.928	-0.39	15.92	292.67	15.53	-122.445	37.928	-0.61	15.92	292.96	15.31	9	7/14/2014	49:06.4
787	RSPe_2	-122.445	37.928	-0.34	15.94	292.67	15.60	-122.445	37.928	-0.61	15.94	293.05	15.33	9	7/14/2014	49:06.3
788	RSPe_2	-122.445	37.928	-0.39	15.98	292.92	15.59	-122.445	37.928	-0.61	15.98	293.36	15.37	9	7/14/2014	49:06.2
789	RSPe_2	-122.445	37.928	-0.34	15.96	293.05	15.62	-122.445	37.928	-0.61	15.96	293.37	15.35	9	7/14/2014	49:06.1
790	RSPe_2	-122.445	37.928	-0.39	16.00	293.03	15.61	-122.445	37.928	-0.64	16.00	293.22	15.36	9	7/14/2014	49:06.0
791	RSPe_2	-122.445	37.928	-0.34	15.97	293.05	15.63	-122.445	37.928	-0.61	15.97	293.13	15.36	9	7/14/2014	49:05.9

792	RSPe_2	-122.445	37.928	-0.34	16.00	293.01	15.67	-122.445	37.928	-0.61	16.00	293.02	15.40	9	7/14/2014	49:05.8
793	RSPe_2	-122.445	37.928	-0.34	15.90	292.92	15.56	-122.445	37.928	-0.64	15.90	292.83	15.26	9	7/14/2014	49:05.7
794	RSPe_2	-122.445	37.928	-0.34	15.89	292.68	15.55	-122.445	37.928	-0.69	15.89	292.67	15.20	9	7/14/2014	49:05.6
795	RSPe_2	-122.445	37.928	-0.34	15.88	292.74	15.54	-122.445	37.928	-0.61	15.88	292.70	15.27	9	7/14/2014	49:05.5
796	RSPe_2	-122.445	37.928	-0.34	15.86	292.66	15.53	-122.445	37.928	-0.61	15.86	292.70	15.26	9	7/14/2014	49:05.4
797	RSPe_2	-122.445	37.928	-0.30	15.86	292.77	15.56	-122.445	37.928	-0.61	15.86	292.87	15.26	9	7/14/2014	49:05.3
798	RSPe_2	-122.445	37.928	-0.34	15.90	292.88	15.56	-122.445	37.928	-0.57	15.90	293.05	15.32	9	7/14/2014	49:05.2
799	RSPe_2	-122.445	37.928	-0.30	15.92	292.86	15.61	-122.445	37.928	-0.61	15.92	293.05	15.31	9	7/14/2014	49:05.1
800	RSPe_2	-122.445	37.928	-0.30	15.91	292.79	15.61	-122.445	37.928	-0.64	15.91	292.94	15.27	9	7/14/2014	49:05.0
801	RSPe_2	-122.445	37.928	-0.30	15.91	292.73	15.61	-122.445	37.928	-0.57	15.91	292.81	15.34	9	7/14/2014	49:04.9
802	RSPe_2	-122.445	37.928	-0.30	15.92	292.64	15.61	-122.445	37.928	-0.64	15.92	292.74	15.28	9	7/14/2014	49:04.8
803	RSPe_2	-122.445	37.928	-0.30	15.90	292.59	15.60	-122.445	37.928	-0.61	15.90	292.72	15.30	9	7/14/2014	49:04.7
804	RSPe_2	-122.445	37.928	-0.30	15.93	292.60	15.62	-122.445	37.928	-0.64	15.93	292.70	15.29	9	7/14/2014	49:04.6
805	RSPe_2	-122.445	37.928	-0.30	15.96	292.64	15.65	-122.445	37.928	-0.61	15.96	292.77	15.35	9	7/14/2014	49:04.5
806	RSPe_2	-122.445	37.928	-0.30	15.99	292.75	15.68	-122.445	37.928	-0.57	15.99	292.79	15.42	9	7/14/2014	49:04.4
807	RSPe_2	-122.445	37.928	-0.25	15.99	292.80	15.73	-122.445	37.928	-0.61	15.99	292.79	15.38	9	7/14/2014	49:04.3
808	RSPe_2	-122.445	37.928	-0.25	15.99	292.73	15.73	-122.445	37.928	-0.64	15.99	292.72	15.35	9	7/14/2014	49:04.2
809	RSPe_2	-122.445	37.928	-0.25	16.00	292.73	15.75	-122.445	37.928	-0.44	16.00	292.62	15.57	9	7/14/2014	49:04.1
810	RSPe_2	-122.445	37.928	-0.25	16.02	292.73	15.77	-122.445	37.928	-0.49	16.02	292.59	15.53	9	7/14/2014	49:04.0
811	RSPe_2	-122.445	37.928	-0.25	16.06	292.86	15.81	-122.445	37.928	-0.52	16.06	292.66	15.54	9	7/14/2014	49:03.9
812	RSPe_2	-122.445	37.928	-0.25	16.00	292.86	15.74	-122.445	37.928	-0.35	16.00	292.61	15.64	9	7/14/2014	49:03.8
813	RSPe_2	-122.445	37.928	-0.22	16.01	292.75	15.79	-122.445	37.928	-0.49	16.01	292.66	15.52	9	7/14/2014	49:03.7
814	RSPe_2	-122.445	37.928	-0.25	15.96	292.78	15.70	-122.445	37.928	-0.40	15.96	292.60	15.55	9	7/14/2014	49:03.6
815	RSPe_2	-122.445	37.928	-0.22	15.97	292.64	15.75	-122.445	37.928	-0.52	15.97	292.55	15.44	9	7/14/2014	49:03.5
816	RSPe_2	-122.445	37.928	-0.25	15.87	292.56	15.62	-122.445	37.928	-0.49	15.87	292.53	15.38	9	7/14/2014	49:03.4
817	RSPe_2	-122.445	37.928	-0.22	15.86	292.47	15.65	-122.445	37.928	-0.49	15.86	292.51	15.38	9	7/14/2014	49:03.3
818	RSPe_2	-122.445	37.928	-0.22	15.83	292.51	15.61	-122.445	37.928	-0.52	15.83	292.60	15.31	9	7/14/2014	49:03.2
819	RSPe_2	-122.445	37.928	-0.18	15.83	292.45	15.65	-122.445	37.928	-0.49	15.83	292.57	15.35	9	7/14/2014	49:03.1
820	RSPe_2	-122.445	37.928	-0.22	15.83	292.31	15.62	-122.445	37.928	-0.49	15.83	292.51	15.35	9	7/14/2014	49:03.0
821	RSPe_2	-122.445	37.928	-0.18	15.84	292.49	15.66	-122.445	37.928	-0.44	15.84	292.64	15.41	9	7/14/2014	49:02.9
822	RSPe_2	-122.445	37.928	-0.22	15.82	292.58	15.60	-122.445	37.928	-0.49	15.82	292.69	15.33	9	7/14/2014	49:02.8
823	RSPe_2	-122.445	37.928	-0.18	15.82	292.56	15.63	-122.445	37.928	-0.49	15.82	292.58	15.33	9	7/14/2014	49:02.7
824	RSPe_2	-122.445	37.928	-0.22	15.84	292.69	15.62	-122.445	37.928	-0.44	15.84	292.53	15.41	9	7/14/2014	49:02.6

825	RSPe_2	-122.445	37.928	-0.18	15.78	292.69	15.60	-122.445	37.928	-0.44	15.78	292.47	15.34	9	7/14/2014	49:02.5
826	RSPe_2	-122.445	37.928	-0.22	15.76	292.60	15.54	-122.445	37.928	-0.52	15.76	292.35	15.24	9	7/14/2014	49:02.4
827	RSPe_2	-122.445	37.928	-0.18	15.74	292.56	15.56	-122.445	37.928	-0.35	15.74	292.29	15.39	9	7/14/2014	49:02.3
828	RSPe_2	-122.445	37.928	-0.18	15.73	292.47	15.54	-122.445	37.928	-0.44	15.73	292.29	15.29	9	7/14/2014	49:02.2
829	RSPe_2	-122.445	37.928	-0.18	15.74	292.45	15.56	-122.445	37.928	-0.44	15.74	292.34	15.31	9	7/14/2014	49:02.1
830	RSPe_2	-122.445	37.928	-0.22	15.73	292.36	15.51	-122.445	37.928	-0.49	15.73	292.36	15.24	9	7/14/2014	49:02.0
831	RSPe_2	-122.445	37.928	-0.18	15.76	292.32	15.58	-122.445	37.928	-0.35	15.76	292.40	15.41	9	7/14/2014	49:01.9
832	RSPe_2	-122.445	37.928	-0.18	15.79	292.21	15.61	-122.445	37.928	-0.40	15.79	292.36	15.39	9	7/14/2014	49:01.8
833	RSPe_2	-122.445	37.928	-0.13	15.97	292.21	15.83	-122.445	37.928	-0.35	15.97	292.40	15.61	9	7/14/2014	49:01.7
834	RSPe_2	-122.445	37.928	-0.18	15.86	292.26	15.68	-122.445	37.928	-0.35	15.86	292.40	15.51	9	7/14/2014	49:01.6
835	RSPe_2	-122.445	37.928	-0.13	15.93	292.10	15.80	-122.445	37.928	-0.40	15.93	292.30	15.53	9	7/14/2014	49:01.5
836	RSPe_2	-122.445	37.928	-0.18	15.93	292.04	15.75	-122.445	37.928	-0.44	15.93	292.26	15.49	9	7/14/2014	49:01.4
837	RSPe_2	-122.445	37.928	-0.13	15.95	292.12	15.82	-122.445	37.928	-0.35	15.95	292.32	15.60	9	7/14/2014	49:01.3
838	RSPe_2	-122.445	37.928	-0.18	15.98	292.17	15.80	-122.445	37.928	-0.40	15.98	292.34	15.58	9	7/14/2014	49:01.2
839	RSPe_2	-122.445	37.928	-0.13	16.00	292.11	15.86	-122.445	37.928	-0.40	16.00	292.28	15.59	9	7/14/2014	49:01.1
840	RSPe_2	-122.445	37.928	-0.18	16.01	292.13	15.83	-122.445	37.928	-0.40	16.01	292.32	15.61	9	7/14/2014	49:01.0
841	RSPe_2	-122.445	37.928	-0.13	15.99	292.11	15.86	-122.445	37.928	-0.44	15.99	292.24	15.55	9	7/14/2014	49:00.9
842	RSPe_2	-122.445	37.928	-0.18	16.00	292.02	15.81	-122.445	37.928	-0.44	16.00	292.08	15.56	9	7/14/2014	49:00.8
843	RSPe_2	-122.445	37.928	-0.13	16.01	292.11	15.88	-122.445	37.928	-0.40	16.01	292.17	15.61	9	7/14/2014	49:00.7
844	RSPe_2	-122.445	37.928	-0.18	15.99	292.33	15.80	-122.445	37.928	-0.27	15.99	292.15	15.72	9	7/14/2014	49:00.6
845	RSPe_2	-122.445	37.928	-0.13	15.94	292.30	15.81	-122.445	37.928	-0.35	15.94	292.10	15.59	9	7/14/2014	49:00.5
846	RSPe_2	-122.445	37.928	-0.13	15.92	292.19	15.79	-122.445	37.928	-0.32	15.92	291.99	15.60	9	7/14/2014	49:00.4
847	RSPe_2	-122.445	37.928	-0.13	15.93	292.04	15.80	-122.445	37.928	-0.35	15.93	291.89	15.58	9	7/14/2014	49:00.3
848	RSPe_2	-122.445	37.928	-0.18	15.83	291.98	15.65	-122.445	37.928	-0.35	15.83	291.82	15.48	9	7/14/2014	49:00.2
849	RSPe_2	-122.445	37.928	-0.10	15.80	291.89	15.70	-122.445	37.928	-0.35	15.80	291.85	15.44	9	7/14/2014	49:00.1
850	RSPe_2	-122.445	37.928	-0.13	15.69	291.91	15.56	-122.445	37.928	-0.40	15.69	291.91	15.29	9	7/14/2014	49:00.0
851	RSPe_2	-122.445	37.928	-0.13	15.70	292.02	15.57	-122.445	37.928	-0.40	15.70	292.04	15.30	9	7/14/2014	48:59.9
852	RSPe_2	-122.445	37.928	-0.13	15.82	292.04	15.69	-122.445	37.928	-0.40	15.82	292.24	15.42	9	7/14/2014	48:59.8
853	RSPe_2	-122.445	37.928	-0.10	15.84	292.00	15.74	-122.445	37.928	-0.35	15.84	292.26	15.49	9	7/14/2014	48:59.7
854	RSPe_2	-122.445	37.928	-0.13	15.78	291.98	15.65	-122.445	37.928	-0.40	15.78	292.26	15.38	9	7/14/2014	48:59.6
855	RSPe_2	-122.445	37.928	-0.10	15.83	291.93	15.73	-122.445	37.928	-0.44	15.83	292.26	15.39	9	7/14/2014	48:59.5
856	RSPe_2	-122.445	37.928	-0.10	15.78	291.96	15.68	-122.445	37.928	-0.44	15.78	292.15	15.34	9	7/14/2014	48:59.4
857	RSPe_2	-122.445	37.928	-0.13	15.86	291.96	15.73	-122.445	37.928	-0.35	15.86	292.04	15.51	9	7/14/2014	48:59.3

858	RSPe_2	-122.445	37.928	-0.13	15.99	291.96	15.86	-122.445	37.928	-0.52	15.99	291.97	15.47	9	7/14/2014	48:59.2
859	RSPe_2	-122.445	37.928	-0.10	16.04	292.05	15.94	-122.445	37.928	-0.44	16.04	291.96	15.61	9	7/14/2014	48:59.1
860	RSPe_2	-122.445	37.928	-0.10	16.01	292.11	15.91	-122.445	37.928	-0.32	16.01	291.87	15.69	9	7/14/2014	48:59.0
861	RSPe_2	-122.445	37.928	-0.10	15.84	292.13	15.74	-122.445	37.928	-0.35	15.84	291.87	15.49	9	7/14/2014	48:58.9
862	RSPe_2	-122.445	37.928	-0.10	15.80	292.12	15.70	-122.445	37.928	-0.35	15.80	291.84	15.44	9	7/14/2014	48:58.8
863	RSPe_2	-122.445	37.928	-0.05	15.80	292.11	15.75	-122.445	37.928	-0.27	15.80	291.87	15.53	9	7/14/2014	48:58.7
864	RSPe_2	-122.445	37.928	-0.10	15.80	292.03	15.70	-122.445	37.928	-0.27	15.80	291.87	15.53	9	7/14/2014	48:58.6
865	RSPe_2	-122.445	37.928	-0.10	15.84	291.88	15.74	-122.445	37.928	-0.27	15.84	291.83	15.58	9	7/14/2014	48:58.5
866	RSPe_2	-122.445	37.928	-0.10	15.80	291.79	15.70	-122.445	37.928	-0.35	15.80	291.74	15.45	9	7/14/2014	48:58.4
867	RSPe_2	-122.445	37.928	-0.05	15.88	291.81	15.83	-122.445	37.928	-0.32	15.88	291.87	15.56	9	7/14/2014	48:58.3
868	RSPe_2	-122.445	37.928	-0.10	15.86	291.88	15.77	-122.445	37.928	-0.32	15.86	291.96	15.55	9	7/14/2014	48:58.2
869	RSPe_2	-122.445	37.928	-0.05	15.86	291.88	15.82	-122.445	37.928	-0.32	15.86	291.87	15.55	9	7/14/2014	48:58.1
870	RSPe_2	-122.445	37.928	-0.10	15.87	291.99	15.77	-122.445	37.928	-0.32	15.87	291.96	15.55	9	7/14/2014	48:58.0
871	RSPe_2	-122.445	37.928	-0.10	15.89	292.11	15.79	-122.445	37.928	-0.32	15.89	291.98	15.57	9	7/14/2014	48:57.9
872	RSPe_2	-122.445	37.928	-0.13	15.88	292.10	15.75	-122.445	37.928	-0.23	15.88	292.01	15.65	9	7/14/2014	48:57.8
873	RSPe_2	-122.445	37.928	-0.10	15.86	292.10	15.76	-122.445	37.928	-0.32	15.86	291.92	15.54	9	7/14/2014	48:57.7
874	RSPe_2	-122.445	37.928	-0.10	15.86	292.03	15.76	-122.445	37.928	-0.35	15.86	291.85	15.51	9	7/14/2014	48:57.6
875	RSPe_2	-122.445	37.928	-0.05	15.83	292.01	15.79	-122.445	37.928	-0.27	15.83	291.83	15.57	9	7/14/2014	48:57.5
876	RSPe_2	-122.445	37.928	-0.10	15.79	291.97	15.69	-122.445	37.928	-0.32	15.79	291.86	15.47	9	7/14/2014	48:57.4
877	RSPe_2	-122.445	37.928	-0.10	15.79	291.99	15.69	-122.445	37.928	-0.32	15.79	291.92	15.47	9	7/14/2014	48:57.3
878	RSPe_2	-122.445	37.928	-0.10	15.75	292.01	15.65	-122.445	37.928	-0.35	15.75	291.92	15.40	9	7/14/2014	48:57.2
879	RSPe_2	-122.445	37.928	-0.10	15.83	292.03	15.73	-122.445	37.928	-0.32	15.83	291.96	15.51	9	7/14/2014	48:57.1
880	RSPe_2	-122.445	37.928	-0.10	15.77	292.03	15.67	-122.445	37.928	-0.35	15.77	291.99	15.42	9	7/14/2014	48:57.0
881	RSPe_2	-122.445	37.928	-0.05	15.74	292.01	15.70	-122.445	37.928	-0.32	15.74	291.95	15.42	9	7/14/2014	48:56.9
882	RSPe_2	-122.445	37.928	-0.10	15.73	292.06	15.64	-122.445	37.928	-0.35	15.73	292.01	15.38	9	7/14/2014	48:56.8
883	RSPe_2	-122.445	37.928	-0.10	15.74	292.10	15.64	-122.445	37.928	-0.32	15.74	292.03	15.42	9	7/14/2014	48:56.7
884	RSPe_2	-122.445	37.928	-0.10	15.81	292.10	15.71	-122.445	37.928	-0.23	15.81	292.04	15.58	9	7/14/2014	48:56.6
885	RSPe_2	-122.445	37.928	-0.10	15.80	292.08	15.70	-122.445	37.928	-0.27	15.80	292.03	15.53	9	7/14/2014	48:56.5
886	RSPe_2	-122.445	37.928	-0.10	15.81	292.02	15.71	-122.445	37.928	-0.32	15.81	292.01	15.49	9	7/14/2014	48:56.4
887	RSPe_2	-122.445	37.928	-0.10	15.89	291.99	15.79	-122.445	37.928	-0.27	15.89	291.95	15.62	9	7/14/2014	48:56.3
888	RSPe_2	-122.445	37.928	-0.10	15.84	292.04	15.74	-122.445	37.928	-0.32	15.84	291.99	15.53	9	7/14/2014	48:56.2
889	RSPe_2	-122.445	37.928	-0.05	15.92	292.08	15.87	-122.445	37.928	-0.27	15.92	291.97	15.65	9	7/14/2014	48:56.1
890	RSPe_2	-122.445	37.928	-0.10	15.86	292.04	15.76	-122.445	37.928	-0.27	15.86	291.91	15.59	9	7/14/2014	48:56.0

891	RSPe_2	-122.445	37.928	-0.10	15.90	292.11	15.80	-122.445	37.928	-0.27	15.90	291.95	15.63	9	7/14/2014	48:55.9
892	RSPe_2	-122.445	37.928	-0.10	15.88	292.04	15.78	-122.445	37.928	-0.32	15.88	291.93	15.56	9	7/14/2014	48:55.8
893	RSPe_2	-122.445	37.928	-0.10	15.88	292.04	15.78	-122.445	37.928	-0.27	15.88	291.97	15.61	9	7/14/2014	48:55.7
894	RSPe_2	-122.445	37.928	-0.10	15.90	292.09	15.80	-122.445	37.928	-0.32	15.90	291.99	15.58	9	7/14/2014	48:55.6
895	RSPe_2	-122.445	37.928	-0.10	15.86	292.06	15.76	-122.445	37.928	-0.35	15.86	291.98	15.51	9	7/14/2014	48:55.5
896	RSPe_2	-122.445	37.928	-0.10	15.85	291.97	15.75	-122.445	37.928	-0.27	15.85	291.88	15.58	9	7/14/2014	48:55.4
897	RSPe_2	-122.445	37.928	-0.05	15.90	292.13	15.85	-122.445	37.928	-0.23	15.90	291.97	15.66	9	7/14/2014	48:55.3
898	RSPe_2	-122.445	37.928	-0.10	15.83	292.04	15.74	-122.445	37.928	-0.20	15.83	291.93	15.63	9	7/14/2014	48:55.2
899	RSPe_2	-122.445	37.928	-0.10	15.83	292.00	15.74	-122.445	37.928	-0.23	15.83	291.93	15.60	9	7/14/2014	48:55.1
900	RSPe_2	-122.445	37.928	-0.05	15.80	291.89	15.75	-122.445	37.928	-0.27	15.80	291.87	15.53	9	7/14/2014	48:55.0
901	RSPe_2	-122.445	37.928	-0.05	15.80	291.85	15.75	-122.445	37.928	-0.23	15.80	291.84	15.56	9	7/14/2014	48:54.9
902	RSPe_2	-122.445	37.928	-0.10	15.80	291.94	15.70	-122.445	37.928	-0.23	15.80	291.82	15.56	9	7/14/2014	48:54.8
903	RSPe_2	-122.445	37.928	-0.05	15.88	291.98	15.83	-122.445	37.928	-0.23	15.88	292.04	15.65	9	7/14/2014	48:54.7
904	RSPe_2	-122.445	37.928	-0.05	15.78	292.05	15.73	-122.445	37.928	-0.23	15.78	292.15	15.55	9	7/14/2014	48:54.6
905	RSPe_2	-122.445	37.928	-0.05	15.77	291.98	15.73	-122.445	37.928	-0.23	15.77	292.02	15.54	9	7/14/2014	48:54.5
906	RSPe_2	-122.445	37.928	-0.10	15.73	292.00	15.63	-122.445	37.928	-0.27	15.73	291.93	15.46	9	7/14/2014	48:54.4
907	RSPe_2	-122.445	37.928	-0.10	15.75	292.05	15.65	-122.445	37.928	-0.23	15.75	291.87	15.52	9	7/14/2014	48:54.3
908	RSPe_2	-122.445	37.928	-0.10	15.69	292.03	15.59	-122.445	37.928	-0.27	15.69	291.87	15.42	9	7/14/2014	48:54.2
909	RSPe_2	-122.445	37.928	-0.10	15.66	292.05	15.57	-122.445	37.928	-0.27	15.66	291.89	15.40	9	7/14/2014	48:54.1
910	RSPe_2	-122.445	37.928	-0.10	15.65	292.01	15.55	-122.445	37.928	-0.27	15.65	291.84	15.38	9	7/14/2014	48:54.0
911	RSPe_2	-122.445	37.928	-0.05	15.66	291.92	15.61	-122.445	37.928	-0.27	15.66	291.85	15.39	9	7/14/2014	48:53.9
912	RSPe_2	-122.445	37.928	-0.05	15.62	291.89	15.57	-122.445	37.928	-0.32	15.62	291.84	15.30	9	7/14/2014	48:53.8
913	RSPe_2	-122.445	37.928	-0.05	15.62	291.83	15.58	-122.445	37.928	-0.15	15.62	291.87	15.48	9	7/14/2014	48:53.7
914	RSPe_2	-122.445	37.928	-0.10	15.63	291.85	15.54	-122.445	37.928	-0.27	15.63	291.95	15.37	9	7/14/2014	48:53.6
915	RSPe_2	-122.445	37.928	-0.01	15.69	291.89	15.68	-122.445	37.928	-0.23	15.69	292.00	15.46	9	7/14/2014	48:53.5
916	RSPe_2	-122.445	37.928	-0.05	15.77	291.86	15.73	-122.445	37.928	-0.23	15.77	292.02	15.54	9	7/14/2014	48:53.4
917	RSPe_2	-122.445	37.928	-0.01	15.78	291.92	15.77	-122.445	37.928	-0.23	15.78	292.05	15.55	9	7/14/2014	48:53.3
918	RSPe_2	-122.445	37.928	-0.05	15.95	291.90	15.90	-122.445	37.928	-0.27	15.95	291.96	15.68	9	7/14/2014	48:53.2
919	RSPe_2	-122.445	37.928	-0.01	15.98	291.90	15.97	-122.445	37.928	-0.23	15.98	291.94	15.75	9	7/14/2014	48:53.1
920	RSPe_2	-122.445	37.928	-0.05	15.94	291.85	15.89	-122.445	37.928	-0.20	15.94	291.89	15.74	9	7/14/2014	48:53.0
921	RSPe_2	-122.445	37.928	-0.01	15.93	291.86	15.92	-122.445	37.928	-0.20	15.93	291.85	15.74	9	7/14/2014	48:52.9
922	RSPe_2	-122.445	37.928	-0.05	15.93	291.81	15.88	-122.445	37.928	-0.20	15.93	291.81	15.73	9	7/14/2014	48:52.8
923	RSPe_2	-122.445	37.928	-0.01	15.93	291.86	15.92	-122.445	37.928	-0.20	15.93	291.83	15.73	9	7/14/2014	48:52.7

924	RSPe_2	-122.445	37.928	-0.01	15.94	291.81	15.93	-122.445	37.928	-0.27	15.94	291.84	15.67	9	7/14/2014	48:52.6
925	RSPe_2	-122.445	37.928	-0.01	15.91	291.86	15.90	-122.445	37.928	-0.20	15.91	291.90	15.71	9	7/14/2014	48:52.5
926	RSPe_2	-122.445	37.928	-0.01	15.92	291.79	15.91	-122.445	37.928	-0.27	15.92	291.85	15.65	9	7/14/2014	48:52.4
927	RSPe_2	-122.445	37.928	-0.01	15.93	291.75	15.92	-122.445	37.928	-0.23	15.93	291.79	15.70	9	7/14/2014	48:52.3
928	RSPe_2	-122.445	37.928	-0.01	15.91	291.68	15.90	-122.445	37.928	-0.20	15.91	291.76	15.71	9	7/14/2014	48:52.2
929	RSPe_2	-122.445	37.928	-0.01	15.94	291.73	15.93	-122.445	37.928	-0.11	15.94	291.74	15.83	9	7/14/2014	48:52.1
930	RSPe_2	-122.445	37.928	-0.01	15.93	291.77	15.92	-122.445	37.928	-0.23	15.93	291.74	15.70	9	7/14/2014	48:52.0
931	RSPe_2	-122.445	37.928	0.04	15.92	291.79	15.96	-122.445	37.928	-0.15	15.92	291.74	15.77	9	7/14/2014	48:51.9
932	RSPe_2	-122.445	37.928	-0.01	15.91	291.92	15.90	-122.445	37.928	-0.23	15.91	291.74	15.68	9	7/14/2014	48:51.8
933	RSPe_2	-122.445	37.928	0.04	15.90	291.97	15.94	-122.445	37.928	-0.23	15.90	291.75	15.67	9	7/14/2014	48:51.7
934	RSPe_2	-122.445	37.928	0.04	15.85	291.95	15.89	-122.445	37.928	-0.27	15.85	291.71	15.58	9	7/14/2014	48:51.6
935	RSPe_2	-122.445	37.928	0.04	15.78	291.93	15.82	-122.445	37.928	-0.03	15.78	291.66	15.75	9	7/14/2014	48:51.5
936	RSPe_2	-122.445	37.928	0.04	15.74	291.86	15.78	-122.445	37.928	-0.20	15.74	291.68	15.54	9	7/14/2014	48:51.4
937	RSPe_2	-122.445	37.928	0.04	15.65	291.84	15.69	-122.445	37.928	-0.15	15.65	291.64	15.50	9	7/14/2014	48:51.3
938	RSPe_2	-122.445	37.928	0.04	15.62	291.77	15.66	-122.445	37.928	-0.15	15.62	291.69	15.48	9	7/14/2014	48:51.2
939	RSPe_2	-122.445	37.928	0.07	15.62	291.84	15.70	-122.445	37.928	-0.06	15.62	291.74	15.56	9	7/14/2014	48:51.1
940	RSPe_2	-122.445	37.928	0.04	15.60	291.75	15.64	-122.445	37.928	-0.15	15.60	291.71	15.45	9	7/14/2014	48:51.0
941	RSPe_2	-122.445	37.928	0.04	15.58	291.89	15.62	-122.445	37.928	-0.11	15.58	291.84	15.47	9	7/14/2014	48:50.9
942	RSPe_2	-122.445	37.928	0.04	15.61	291.84	15.65	-122.445	37.928	-0.03	15.61	291.82	15.58	9	7/14/2014	48:50.8
943	RSPe_2	-122.445	37.928	0.04	15.58	291.85	15.62	-122.445	37.928	-0.06	15.58	291.84	15.52	9	7/14/2014	48:50.7
944	RSPe_2	-122.445	37.928	0.04	15.60	291.91	15.64	-122.445	37.928	-0.11	15.60	291.86	15.49	9	7/14/2014	48:50.6
945	RSPe_2	-122.445	37.928	0.07	15.69	291.80	15.76	-122.445	37.928	-0.06	15.69	291.73	15.62	9	7/14/2014	48:50.5
946	RSPe_2	-122.445	37.928	0.04	15.60	291.86	15.64	-122.445	37.928	-0.15	15.60	291.79	15.45	9	7/14/2014	48:50.4
947	RSPe_2	-122.445	37.928	0.07	15.62	291.82	15.70	-122.445	37.928	-0.06	15.62	291.77	15.56	9	7/14/2014	48:50.3
948	RSPe_2	-122.445	37.928	0.04	15.58	291.85	15.62	-122.445	37.928	-0.15	15.58	291.75	15.43	9	7/14/2014	48:50.2
949	RSPe_2	-122.445	37.928	0.04	15.65	291.76	15.69	-122.445	37.928	-0.11	15.65	291.75	15.54	9	7/14/2014	48:50.1
950	RSPe_2	-122.445	37.928	0.04	15.75	291.67	15.79	-122.445	37.928	-0.06	15.75	291.67	15.69	9	7/14/2014	48:50.0
951	RSPe_2	-122.445	37.928	0.07	15.65	291.67	15.72	-122.445	37.928	-0.03	15.65	291.63	15.62	9	7/14/2014	48:49.9
952	RSPe_2	-122.445	37.928	0.04	15.71	291.70	15.75	-122.445	37.928	-0.06	15.71	291.67	15.65	9	7/14/2014	48:49.8
953	RSPe_2	-122.445	37.928	0.04	15.81	291.67	15.85	-122.445	37.928	-0.11	15.81	291.60	15.70	9	7/14/2014	48:49.7
954	RSPe_2	-122.445	37.928	0.04	15.77	291.65	15.81	-122.445	37.928	-0.15	15.77	291.58	15.62	9	7/14/2014	48:49.6
955	RSPe_2	-122.445	37.928	0.07	15.80	291.63	15.87	-122.445	37.928	-0.06	15.80	291.58	15.73	9	7/14/2014	48:49.5
956	RSPe_2	-122.445	37.928	0.04	15.80	291.63	15.84	-122.445	37.928	-0.06	15.80	291.51	15.74	9	7/14/2014	48:49.4

957	RSPe_2	-122.445	37.928	0.07	15.83	291.56	15.91	-122.445	37.928	-0.06	15.83	291.54	15.77	9	7/14/2014	48:49.3
958	RSPe_2	-122.445	37.928	0.07	15.87	291.56	15.95	-122.445	37.928	-0.03	15.87	291.54	15.84	9	7/14/2014	48:49.2
959	RSPe_2	-122.445	37.928	0.07	15.90	291.54	15.97	-122.445	37.928	-0.03	15.90	291.58	15.87	9	7/14/2014	48:49.1
960	RSPe_2	-122.445	37.928	0.07	15.90	291.59	15.98	-122.445	37.928	-0.06	15.90	291.52	15.84	9	7/14/2014	48:49.0
961	RSPe_2	-122.445	37.928	0.07	15.90	291.52	15.98	-122.445	37.928	-0.06	15.90	291.49	15.84	9	7/14/2014	48:48.9
962	RSPe_2	-122.445	37.928	0.04	15.94	291.59	15.98	-122.445	37.928	-0.15	15.94	291.59	15.79	9	7/14/2014	48:48.8
963	RSPe_2	-122.445	37.928	0.07	15.91	291.54	15.99	-122.445	37.928	-0.11	15.91	291.56	15.80	9	7/14/2014	48:48.7
964	RSPe_2	-122.445	37.928	0.04	15.90	291.48	15.94	-122.445	37.928	-0.06	15.90	291.50	15.84	9	7/14/2014	48:48.6
965	RSPe_2	-122.445	37.928	0.07	15.91	291.50	15.99	-122.445	37.928	-0.03	15.91	291.52	15.88	9	7/14/2014	48:48.5
966	RSPe_2	-122.445	37.928	0.04	15.91	291.54	15.95	-122.445	37.928	-0.11	15.91	291.54	15.80	9	7/14/2014	48:48.4
967	RSPe_2	-122.445	37.928	0.08	15.92	291.51	15.99	-122.445	37.928	-0.11	15.92	291.52	15.81	9	7/14/2014	48:48.3
968	RSPe_2	-122.445	37.928	0.04	15.92	291.53	15.96	-122.445	37.928	-0.15	15.92	291.54	15.77	9	7/14/2014	48:48.2
969	RSPe_2	-122.445	37.928	0.08	15.90	291.46	15.98	-122.445	37.928	-0.03	15.90	291.57	15.87	9	7/14/2014	48:48.1
970	RSPe_2	-122.445	37.928	0.04	15.89	291.55	15.93	-122.445	37.928	-0.20	15.89	291.50	15.69	9	7/14/2014	48:48.0
971	RSPe_2	-122.445	37.928	0.08	15.91	291.51	15.99	-122.445	37.928	-0.11	15.91	291.52	15.80	9	7/14/2014	48:47.9
972	RSPe_2	-122.445	37.928	0.08	15.89	291.48	15.96	-122.445	37.928	-0.15	15.89	291.46	15.74	9	7/14/2014	48:47.8
973	RSPe_2	-122.445	37.928	0.08	15.92	291.48	15.99	-122.445	37.928	-0.11	15.92	291.44	15.81	9	7/14/2014	48:47.7
974	RSPe_2	-122.445	37.928	0.08	15.89	291.44	15.96	-122.445	37.928	-0.15	15.89	291.39	15.74	9	7/14/2014	48:47.6
975	RSPe_2	-122.445	37.928	0.08	15.90	291.40	15.98	-122.445	37.928	-0.06	15.90	291.42	15.84	9	7/14/2014	48:47.5
976	RSPe_2	-122.445	37.928	0.08	15.93	291.36	16.01	-122.445	37.928	-0.03	15.93	291.37	15.91	9	7/14/2014	48:47.4
977	RSPe_2	-122.445	37.928	0.08	15.92	291.35	15.99	-122.445	37.928	-0.06	15.92	291.40	15.86	9	7/14/2014	48:47.3
978	RSPe_2	-122.445	37.928	0.08	15.94	291.36	16.01	-122.445	37.928	-0.06	15.94	291.40	15.88	9	7/14/2014	48:47.2
979	RSPe_2	-122.445	37.928	0.08	15.95	291.40	16.02	-122.445	37.928	-0.11	15.95	291.42	15.84	9	7/14/2014	48:47.1
980	RSPe_2	-122.445	37.928	0.08	15.96	291.40	16.03	-122.445	37.928	-0.15	15.96	291.35	15.81	9	7/14/2014	48:47.0
981	RSPe_2	-122.445	37.928	0.08	16.00	291.40	16.07	-122.445	37.928	-0.06	16.00	291.27	15.93	9	7/14/2014	48:46.9
982	RSPe_2	-122.445	37.928	0.08	15.92	291.42	15.99	-122.445	37.928	-0.11	15.92	291.19	15.81	9	7/14/2014	48:46.8
983	RSPe_2	-122.445	37.928	0.08	15.94	291.44	16.01	-122.445	37.928	-0.15	15.94	291.15	15.79	9	7/14/2014	48:46.7
984	RSPe_2	-122.445	37.928	0.08	15.86	291.42	15.93	-122.445	37.928	-0.15	15.86	291.13	15.71	9	7/14/2014	48:46.6
985	RSPe_2	-122.445	37.928	0.08	15.83	291.43	15.90	-122.445	37.928	-0.11	15.83	291.13	15.71	9	7/14/2014	48:46.5
986	RSPe_2	-122.445	37.928	0.08	15.78	291.42	15.85	-122.445	37.928	-0.03	15.78	291.18	15.75	9	7/14/2014	48:46.4
987	RSPe_2	-122.445	37.928	0.13	15.70	291.38	15.83	-122.445	37.928	-0.06	15.70	291.25	15.64	9	7/14/2014	48:46.3
988	RSPe_2	-122.445	37.928	0.08	15.66	291.45	15.74	-122.445	37.928	-0.15	15.66	291.33	15.52	9	7/14/2014	48:46.2
989	RSPe_2	-122.445	37.928	0.08	15.66	291.36	15.73	-122.445	37.928	-0.06	15.66	291.38	15.59	9	7/14/2014	48:46.1

990	RSPe_2	-122.445	37.928	0.08	15.69	291.40	15.77	-122.445	37.928	-0.11	15.69	291.44	15.58	9	7/14/2014 48:46.0
991	RSPe_2	-122.445	37.928	0.08	15.67	291.29	15.75	-122.445	37.928	-0.11	15.67	291.33	15.56	9	7/14/2014 48:45.9
992	RSPe_2	-122.445	37.928	0.08	15.71	291.25	15.79	-122.445	37.928	-0.11	15.71	291.35	15.60	9	7/14/2014 48:45.8
993	RSPe_2	-122.445	37.928	0.08	15.66	291.28	15.74	-122.445	37.928	-0.11	15.66	291.29	15.55	9	7/14/2014 48:45.7
994	RSPe_2	-122.445	37.928	0.04	15.66	291.32	15.70	-122.445	37.928	-0.15	15.66	291.29	15.51	9	7/14/2014 48:45.6
995	RSPe_2	-122.445	37.928	0.08	15.62	291.47	15.70	-122.445	37.928	-0.06	15.62	291.31	15.56	9	7/14/2014 48:45.5
996	RSPe_2	-122.445	37.928	0.04	15.64	291.45	15.68	-122.445	37.928	-0.11	15.64	291.23	15.53	9	7/14/2014 48:45.4
997	RSPe_2	-122.445	37.928	0.08	15.63	291.50	15.71	-122.445	37.928	-0.03	15.63	291.27	15.61	9	7/14/2014 48:45.3
998	RSPe_2	-122.445	37.928	0.04	15.63	291.49	15.67	-122.445	37.928	-0.03	15.63	291.23	15.61	9	7/14/2014 48:45.2
999	RSPe_2	-122.445	37.928	0.13	15.68	291.50	15.80	-122.445	37.928	-0.03	15.68	291.29	15.65	9	7/14/2014 48:45.1
1000	RSPe_2	-122.445	37.928	0.08	15.65	291.43	15.72	-122.445	37.928	-0.11	15.65	291.25	15.54	9	7/14/2014 48:45.0
1001	RSPe_2	-122.445	37.928	0.13	15.66	291.47	15.79	-122.445	37.928	-0.03	15.66	291.29	15.64	9	7/14/2014 48:44.9
1002	RSPe_2	-122.445	37.928	0.08	15.69	291.43	15.76	-122.445	37.928	-0.03	15.69	291.30	15.66	9	7/14/2014 48:44.8
1003	RSPe_2	-122.445	37.928	0.13	15.70	291.48	15.83	-122.445	37.928	-0.03	15.70	291.34	15.68	9	7/14/2014 48:44.7
1004	RSPe_2	-122.445	37.928	0.08	15.75	291.39	15.82	-122.445	37.928	-0.03	15.75	291.31	15.72	9	7/14/2014 48:44.6
1005	RSPe_2	-122.445	37.928	0.08	15.74	291.41	15.82	-122.445	37.928	-0.06	15.74	291.31	15.68	9	7/14/2014 48:44.5
1006	RSPe_2	-122.445	37.928	0.08	15.80	291.43	15.87	-122.445	37.928	-0.03	15.80	291.36	15.77	9	7/14/2014 48:44.4
1007	RSPe_2	-122.445	37.928	0.13	15.81	291.41	15.94	-122.445	37.928	-0.03	15.81	291.34	15.78	9	7/14/2014 48:44.3
1008	RSPe_2	-122.445	37.928	0.08	15.83	291.28	15.90	-122.445	37.928	-0.03	15.83	291.26	15.80	9	7/14/2014 48:44.2
1009	RSPe_2	-122.445	37.928	0.08	15.85	291.32	15.92	-122.445	37.928	0.02	15.85	291.29	15.87	9	7/14/2014 48:44.1
1010	RSPe_2	-122.445	37.928	0.08	15.83	291.30	15.91	-122.445	37.928	0.02	15.83	291.28	15.86	9	7/14/2014 48:44.0
1011	RSPe_2	-122.445	37.928	0.13	15.79	291.33	15.91	-122.445	37.928	-0.03	15.79	291.24	15.76	9	7/14/2014 48:43.9
1012	RSPe_2	-122.445	37.928	0.08	15.77	291.37	15.85	-122.445	37.928	-0.03	15.77	291.21	15.74	9	7/14/2014 48:43.8
1013	RSPe_2	-122.445	37.928	0.08	15.75	291.32	15.82	-122.445	37.928	0.06	15.75	291.20	15.80	9	7/14/2014 48:43.7
1014	RSPe_2	-122.445	37.928	0.08	15.74	291.26	15.82	-122.445	37.928	-0.03	15.74	291.06	15.71	9	7/14/2014 48:43.6
1015	RSPe_2	-122.445	37.928	0.13	15.76	291.20	15.88	-122.445	37.928	-0.03	15.76	291.08	15.73	9	7/14/2014 48:43.5
1016	RSPe_2	-122.445	37.928	0.08	15.77	291.26	15.85	-122.445	37.928	-0.03	15.77	291.13	15.74	9	7/14/2014 48:43.4
1017	RSPe_2	-122.445	37.928	0.13	15.76	291.42	15.88	-122.445	37.928	-0.03	15.76	291.28	15.73	9	7/14/2014 48:43.3
1018	RSPe_2	-122.445	37.928	0.08	15.83	291.57	15.90	-122.445	37.928	-0.03	15.83	291.39	15.80	9	7/14/2014 48:43.2
1019	RSPe_2	-122.445	37.928	0.08	15.88	291.50	15.96	-122.445	37.928	-0.03	15.88	291.24	15.85	9	7/14/2014 48:43.1
1020	RSPe_2	-122.445	37.928	0.04	15.85	291.51	15.89	-122.445	37.928	-0.11	15.85	291.19	15.74	9	7/14/2014 48:43.0
1021	RSPe_2	-122.445	37.928	0.08	15.88	291.55	15.96	-122.445	37.928	-0.06	15.88	291.29	15.82	9	7/14/2014 48:42.9
1022	RSPe_2	-122.445	37.928	0.08	15.85	291.51	15.92	-122.445	37.928	-0.06	15.85	291.28	15.79	9	7/14/2014 48:42.8

1023	RSPe_2	-122.445	37.928	0.08	15.88	291.52	15.96	-122.445	37.928	-0.06	15.88	291.28	15.82	9	7/14/2014	48:42.7
1024	RSPe_2	-122.445	37.928	0.08	15.88	291.60	15.96	-122.445	37.928	-0.06	15.88	291.37	15.82	9	7/14/2014	48:42.6
1025	RSPe_2	-122.445	37.928	0.13	15.86	291.53	15.99	-122.445	37.928	-0.03	15.86	291.44	15.84	9	7/14/2014	48:42.5
1026	RSPe_2	-122.445	37.928	0.08	15.85	291.50	15.92	-122.445	37.928	0.02	15.85	291.46	15.87	9	7/14/2014	48:42.4
1027	RSPe_2	-122.445	37.928	0.13	15.87	291.47	16.00	-122.445	37.928	0.02	15.87	291.44	15.89	9	7/14/2014	48:42.3
1028	RSPe_2	-122.445	37.928	0.08	15.86	291.47	15.93	-122.445	37.928	0.02	15.86	291.44	15.88	9	7/14/2014	48:42.2
1029	RSPe_2	-122.445	37.928	0.13	15.83	291.45	15.96	-122.445	37.928	0.06	15.83	291.42	15.89	9	7/14/2014	48:42.1
1030	RSPe_2	-122.445	37.928	0.08	15.81	291.38	15.89	-122.445	37.928	0.02	15.81	291.28	15.83	9	7/14/2014	48:42.0
1031	RSPe_2	-122.445	37.928	0.13	15.83	291.36	15.95	-122.445	37.928	0.11	15.83	291.38	15.93	9	7/14/2014	48:41.9
1032	RSPe_2	-122.445	37.928	0.08	15.80	291.35	15.88	-122.445	37.928	0.06	15.80	291.42	15.86	9	7/14/2014	48:41.8
1033	RSPe_2	-122.445	37.928	0.13	15.81	291.40	15.94	-122.445	37.928	0.06	15.81	291.44	15.87	9	7/14/2014	48:41.7
1034	RSPe_2	-122.445	37.928	0.08	15.77	291.40	15.85	-122.445	37.928	-0.03	15.77	291.44	15.74	9	7/14/2014	48:41.6
1035	RSPe_2	-122.445	37.928	0.13	15.75	291.38	15.87	-122.445	37.928	0.06	15.75	291.40	15.80	9	7/14/2014	48:41.5
1036	RSPe_2	-122.445	37.928	0.08	15.75	291.38	15.82	-122.445	37.928	0.02	15.75	291.37	15.77	9	7/14/2014	48:41.4
1037	RSPe_2	-122.445	37.928	0.13	15.73	291.40	15.86	-122.445	37.928	0.06	15.73	291.31	15.79	9	7/14/2014	48:41.3
1038	RSPe_2	-122.445	37.928	0.08	15.69	291.52	15.77	-122.445	37.928	0.11	15.69	291.33	15.80	9	7/14/2014	48:41.2
1039	RSPe_2	-122.445	37.928	0.13	15.68	291.49	15.80	-122.445	37.928	0.11	15.68	291.29	15.79	9	7/14/2014	48:41.1
1040	RSPe_2	-122.445	37.928	0.08	15.65	291.60	15.72	-122.445	37.928	0.11	15.65	291.29	15.76	9	7/14/2014	48:41.0
1041	RSPe_2	-122.445	37.928	0.13	15.65	291.54	15.78	-122.445	37.928	0.14	15.65	291.25	15.79	9	7/14/2014	48:40.9
1042	RSPe_2	-122.445	37.928	0.13	15.60	291.51	15.73	-122.445	37.928	0.14	15.60	291.22	15.74	9	7/14/2014	48:40.8
1043	RSPe_2	-122.445	37.928	0.16	15.60	291.38	15.76	-122.445	37.928	0.14	15.60	291.23	15.74	9	7/14/2014	48:40.7
1044	RSPe_2	-122.445	37.928	0.08	15.66	291.38	15.73	-122.445	37.928	0.11	15.66	291.30	15.76	9	7/14/2014	48:40.6
1045	RSPe_2	-122.445	37.928	0.16	15.60	291.47	15.76	-122.445	37.928	0.11	15.60	291.50	15.71	9	7/14/2014	48:40.5
1046	RSPe_2	-122.445	37.928	0.13	15.62	291.48	15.74	-122.445	37.928	0.11	15.62	291.60	15.73	9	7/14/2014	48:40.4
1047	RSPe_2	-122.445	37.928	0.13	15.63	291.43	15.76	-122.445	37.928	0.14	15.63	291.60	15.77	9	7/14/2014	48:40.3
1048	RSPe_2	-122.445	37.928	0.13	15.62	291.38	15.74	-122.445	37.928	0.06	15.62	291.52	15.67	9	7/14/2014	48:40.2
1049	RSPe_2	-122.445	37.928	0.16	15.65	291.26	15.81	-122.445	37.928	0.11	15.65	291.49	15.76	9	7/14/2014	48:40.1
1050	RSPe_2	-122.445	37.928	0.13	15.65	291.32	15.78	-122.445	37.928	0.11	15.65	291.54	15.76	9	7/14/2014	48:40.0
1051	RSPe_2	-122.445	37.928	0.13	15.66	291.25	15.79	-122.445	37.928	0.11	15.66	291.49	15.77	9	7/14/2014	48:39.9
1052	RSPe_2	-122.445	37.928	0.13	15.68	291.28	15.80	-122.445	37.928	0.06	15.68	291.49	15.73	9	7/14/2014	48:39.8
1053	RSPe_2	-122.445	37.928	0.13	15.68	291.28	15.80	-122.445	37.928	0.06	15.68	291.48	15.73	9	7/14/2014	48:39.7
1054	RSPe_2	-122.445	37.928	0.13	15.71	291.30	15.84	-122.445	37.928	0.06	15.71	291.40	15.77	9	7/14/2014	48:39.6
1055	RSPe_2	-122.445	37.928	0.13	15.73	291.26	15.85	-122.445	37.928	0.14	15.73	291.30	15.87	9	7/14/2014	48:39.5

1056	RSPe_2	-122.445	37.928	0.13	15.81	291.26	15.94	-122.445	37.928	0.11	15.81	291.30	15.92	9	7/14/2014 48:39.4
1057	RSPe_2	-122.445	37.928	0.13	15.79	291.17	15.91	-122.445	37.928	0.11	15.79	291.19	15.90	9	7/14/2014 48:39.3
1058	RSPe_2	-122.445	37.928	0.08	15.81	291.17	15.89	-122.445	37.928	0.14	15.81	291.23	15.95	9	7/14/2014 48:39.2
1059	RSPe_2	-122.445	37.928	0.13	15.82	291.26	15.94	-122.445	37.928	0.11	15.82	291.23	15.92	9	7/14/2014 48:39.1
1060	RSPe_2	-122.445	37.928	0.08	15.86	291.26	15.93	-122.445	37.928	0.06	15.86	291.28	15.91	9	7/14/2014 48:39.0
1061	RSPe_2	-122.445	37.928	0.13	15.86	291.28	15.99	-122.445	37.928	0.11	15.86	291.30	15.97	9	7/14/2014 48:38.9
1062	RSPe_2	-122.445	37.928	0.08	15.90	291.20	15.98	-122.445	37.928	0.06	15.90	291.30	15.96	9	7/14/2014 48:38.8
1063	RSPe_2	-122.445	37.928	0.13	15.90	291.20	16.02	-122.445	37.928	0.11	15.90	291.26	16.00	9	7/14/2014 48:38.7
1064	RSPe_2	-122.445	37.928	0.13	15.89	291.22	16.01	-122.445	37.928	0.11	15.89	291.28	15.99	9	7/14/2014 48:38.6
1065	RSPe_2	-122.445	37.928	0.16	15.93	291.22	16.09	-122.445	37.928	0.11	15.93	291.26	16.04	9	7/14/2014 48:38.5
1066	RSPe_2	-122.445	37.928	0.13	15.90	291.19	16.02	-122.445	37.928	0.06	15.90	291.15	15.95	9	7/14/2014 48:38.4
1067	RSPe_2	-122.445	37.928	0.16	15.89	291.20	16.05	-122.445	37.928	0.11	15.89	291.15	15.99	9	7/14/2014 48:38.3
1068	RSPe_2	-122.445	37.928	0.13	15.90	291.13	16.02	-122.445	37.928	0.06	15.90	291.04	15.95	9	7/14/2014 48:38.2
1069	RSPe_2	-122.445	37.928	0.16	15.89	291.11	16.05	-122.445	37.928	0.11	15.89	291.04	15.99	9	7/14/2014 48:38.1
1070	RSPe_2	-122.445	37.928	0.13	15.90	291.14	16.02	-122.445	37.928	0.11	15.90	291.11	16.00	9	7/14/2014 48:38.0
1071	RSPe_2	-122.445	37.928	0.16	15.90	291.09	16.06	-122.445	37.928	0.06	15.90	291.11	15.95	9	7/14/2014 48:37.9
1072	RSPe_2	-122.445	37.928	0.13	15.86	291.07	15.98	-122.445	37.928	0.06	15.86	291.15	15.91	9	7/14/2014 48:37.8
1073	RSPe_2	-122.445	37.928	0.13	15.86	291.04	15.98	-122.445	37.928	0.06	15.86	291.22	15.91	9	7/14/2014 48:37.7
1074	RSPe_2	-122.445	37.928	0.13	15.84	291.00	15.97	-122.445	37.928	0.02	15.84	291.21	15.86	9	7/14/2014 48:37.6
1075	RSPe_2	-122.445	37.928	0.13	15.83	290.98	15.96	-122.445	37.928	0.02	15.83	291.22	15.86	9	7/14/2014 48:37.5
1076	RSPe_2	-122.445	37.928	0.13	15.83	290.96	15.95	-122.445	37.928	0.06	15.83	291.28	15.88	9	7/14/2014 48:37.4
1077	RSPe_2	-122.445	37.928	0.13	15.83	290.93	15.95	-122.445	37.928	0.06	15.83	291.22	15.88	9	7/14/2014 48:37.3
1078	RSPe_2	-122.445	37.928	0.13	15.85	290.98	15.97	-122.445	37.928	0.06	15.85	291.24	15.90	9	7/14/2014 48:37.2
1079	RSPe_2	-122.445	37.928	0.16	15.83	290.98	15.99	-122.445	37.928	0.06	15.83	291.13	15.88	9	7/14/2014 48:37.1
1080	RSPe_2	-122.445	37.928	0.13	15.82	290.99	15.94	-122.445	37.928	0.06	15.82	291.11	15.87	9	7/14/2014 48:37.0
1081	RSPe_2	-122.445	37.928	0.16	15.79	291.00	15.95	-122.445	37.928	0.06	15.79	291.05	15.84	9	7/14/2014 48:36.9
1082	RSPe_2	-122.445	37.928	0.13	15.83	290.96	15.96	-122.445	37.928	0.06	15.83	291.02	15.89	9	7/14/2014 48:36.8
1083	RSPe_2	-122.445	37.928	0.13	15.80	290.92	15.92	-122.445	37.928	0.11	15.80	290.93	15.90	9	7/14/2014 48:36.7
1084	RSPe_2	-122.445	37.928	0.13	15.73	290.88	15.86	-122.445	37.928	0.02	15.73	290.91	15.76	9	7/14/2014 48:36.6
1085	RSPe_2	-122.445	37.928	0.13	15.69	290.87	15.82	-122.445	37.928	0.02	15.69	290.87	15.72	9	7/14/2014 48:36.5
1086	RSPe_2	-122.445	37.928	0.13	15.73	290.85	15.85	-122.445	37.928	0.02	15.73	290.87	15.75	9	7/14/2014 48:36.4
1087	RSPe_2	-122.445	37.928	0.16	15.74	290.81	15.90	-122.445	37.928	0.02	15.74	290.86	15.76	9	7/14/2014 48:36.3
1088	RSPe_2	-122.445	37.928	0.08	15.67	290.75	15.75	-122.445	37.929	-0.03	15.67	290.79	15.64	9	7/14/2014 48:36.2

1089	RSPe_2	-122.445	37.929	0.16	15.75	290.70	15.91	-122.445	37.929	0.02	15.75	290.83	15.77	9	7/14/2014	48:36.1
1090	RSPe_2	-122.445	37.929	0.13	15.69	290.68	15.82	-122.445	37.929	-0.03	15.69	290.81	15.67	9	7/14/2014	48:36.0
1091	RSPe_2	-122.445	37.929	0.13	15.70	290.60	15.83	-122.445	37.929	0.02	15.70	290.79	15.73	9	7/14/2014	48:35.9
1092	RSPe_2	-122.445	37.929	0.13	15.73	290.61	15.85	-122.445	37.929	-0.03	15.73	290.76	15.70	9	7/14/2014	48:35.8
1093	RSPe_2	-122.445	37.929	0.16	15.73	290.64	15.89	-122.445	37.929	0.06	15.73	290.72	15.79	9	7/14/2014	48:35.7
1094	RSPe_2	-122.445	37.929	0.13	15.74	290.64	15.87	-122.445	37.929	0.02	15.74	290.75	15.76	9	7/14/2014	48:35.6
1095	RSPe_2	-122.445	37.929	0.13	15.74	290.66	15.87	-122.445	37.929	0.02	15.74	290.74	15.76	9	7/14/2014	48:35.5
1096	RSPe_2	-122.445	37.929	0.13	15.74	290.64	15.87	-122.445	37.929	0.02	15.74	290.70	15.76	9	7/14/2014	48:35.4
1097	RSPe_2	-122.445	37.929	0.13	15.78	290.66	15.91	-122.445	37.929	0.02	15.78	290.63	15.80	9	7/14/2014	48:35.3
1098	RSPe_2	-122.445	37.929	0.08	15.76	290.64	15.84	-122.445	37.929	0.02	15.76	290.55	15.78	9	7/14/2014	48:35.2
1099	RSPe_2	-122.445	37.929	0.13	15.72	290.62	15.84	-122.445	37.929	0.02	15.72	290.46	15.74	9	7/14/2014	48:35.1
1100	RSPe_2	-122.445	37.929	0.13	15.72	290.62	15.84	-122.445	37.929	0.02	15.72	290.48	15.74	9	7/14/2014	48:35.0
1101	RSPe_2	-122.445	37.929	0.13	15.73	290.62	15.86	-122.445	37.929	0.02	15.73	290.48	15.76	9	7/14/2014	48:34.9
1102	RSPe_2	-122.445	37.929	0.13	15.76	290.64	15.88	-122.445	37.929	0.02	15.76	290.46	15.78	9	7/14/2014	48:34.8
1103	RSPe_2	-122.445	37.929	0.13	15.78	290.62	15.91	-122.445	37.929	0.06	15.78	290.46	15.84	9	7/14/2014	48:34.7
1104	RSPe_2	-122.445	37.929	0.13	15.81	290.58	15.94	-122.445	37.929	0.02	15.81	290.53	15.83	9	7/14/2014	48:34.6
1105	RSPe_2	-122.445	37.929	0.13	15.83	290.53	15.96	-122.445	37.929	-0.03	15.83	290.55	15.81	9	7/14/2014	48:34.5
1106	RSPe_2	-122.445	37.929	0.13	15.86	290.45	15.98	-122.445	37.929	-0.03	15.86	290.53	15.83	9	7/14/2014	48:34.4
1107	RSPe_2	-122.445	37.929	0.16	15.90	290.40	16.06	-122.445	37.929	0.02	15.90	290.58	15.92	9	7/14/2014	48:34.3
1108	RSPe_2	-122.445	37.929	0.13	15.97	290.42	16.10	-122.445	37.929	0.02	15.97	290.64	16.00	9	7/14/2014	48:34.2
1109	RSPe_2	-122.445	37.929	0.16	15.97	290.40	16.13	-122.445	37.929	0.06	15.97	290.62	16.02	9	7/14/2014	48:34.1
1110	RSPe_2	-122.445	37.929	0.13	16.00	290.36	16.13	-122.445	37.929	0.06	16.00	290.62	16.06	9	7/14/2014	48:34.0
1111	RSPe_2	-122.445	37.929	0.16	16.06	290.31	16.22	-122.445	37.929	0.02	16.06	290.55	16.08	9	7/14/2014	48:33.9
1112	RSPe_2	-122.445	37.929	0.13	16.02	290.31	16.15	-122.445	37.929	0.02	16.02	290.52	16.04	9	7/14/2014	48:33.8
1113	RSPe_2	-122.445	37.929	0.16	16.03	290.30	16.20	-122.445	37.929	0.02	16.03	290.56	16.06	9	7/14/2014	48:33.7
1114	RSPe_2	-122.445	37.929	0.13	16.06	290.27	16.19	-122.445	37.929	-0.03	16.06	290.53	16.04	9	7/14/2014	48:33.6
1115	RSPe_2	-122.445	37.929	0.13	16.09	290.30	16.22	-122.445	37.929	0.06	16.09	290.55	16.15	9	7/14/2014	48:33.5
1116	RSPe_2	-122.445	37.929	0.13	16.06	290.32	16.19	-122.445	37.929	-0.03	16.06	290.55	16.04	9	7/14/2014	48:33.4
1117	RSPe_2	-122.445	37.929	0.13	16.14	290.28	16.27	-122.445	37.929	-0.03	16.14	290.47	16.12	9	7/14/2014	48:33.3
1118	RSPe_2	-122.445	37.929	0.08	16.10	290.27	16.18	-122.445	37.929	-0.06	16.10	290.44	16.04	9	7/14/2014	48:33.2
1119	RSPe_2	-122.445	37.929	0.13	16.10	290.30	16.23	-122.445	37.929	-0.03	16.10	290.36	16.08	9	7/14/2014	48:33.1
1120	RSPe_2	-122.445	37.929	0.08	16.10	290.25	16.18	-122.445	37.929	-0.03	16.10	290.33	16.08	9	7/14/2014	48:33.0
1121	RSPe_2	-122.445	37.929	0.13	16.09	290.21	16.22	-122.445	37.929	-0.06	16.09	290.25	16.03	9	7/14/2014	48:32.9

1122	RSPe_2	-122.445	37.929	0.13	16.15	290.28	16.28	-122.445	37.929	-0.06	16.15	290.29	16.09	9	7/14/2014	48:32.8
1123	RSPe_2	-122.445	37.929	0.13	16.13	290.19	16.26	-122.445	37.929	-0.06	16.13	290.23	16.07	9	7/14/2014	48:32.7
1124	RSPe_2	-122.445	37.929	0.08	16.11	290.17	16.19	-122.445	37.929	-0.06	16.11	290.21	16.05	9	7/14/2014	48:32.6
1125	RSPe_2	-122.445	37.929	0.13	16.12	290.26	16.25	-122.445	37.929	-0.06	16.12	290.25	16.06	9	7/14/2014	48:32.5
1126	RSPe_2	-122.445	37.929	0.08	16.12	290.28	16.20	-122.445	37.929	-0.06	16.12	290.31	16.06	9	7/14/2014	48:32.4
1127	RSPe_2	-122.445	37.929	0.13	16.12	290.32	16.25	-122.445	37.929	-0.11	16.12	290.32	16.01	9	7/14/2014	48:32.3
1128	RSPe_2	-122.445	37.929	0.08	16.09	290.21	16.17	-122.445	37.929	-0.06	16.09	290.25	16.03	9	7/14/2014	48:32.2
1129	RSPe_2	-122.445	37.929	0.13	16.11	290.23	16.24	-122.445	37.929	-0.06	16.11	290.25	16.05	9	7/14/2014	48:32.1
1130	RSPe_2	-122.445	37.929	0.08	16.14	290.19	16.22	-122.445	37.929	-0.06	16.14	290.20	16.08	9	7/14/2014	48:32.0
1131	RSPe_2	-122.445	37.929	0.08	16.06	290.11	16.14	-122.445	37.929	-0.11	16.06	290.13	15.95	9	7/14/2014	48:31.9
1132	RSPe_2	-122.445	37.929	0.08	16.09	290.08	16.17	-122.445	37.929	-0.11	16.09	290.12	15.98	9	7/14/2014	48:31.8
1133	RSPe_2	-122.445	37.929	0.13	16.06	290.07	16.18	-122.445	37.929	-0.11	16.06	290.12	15.94	9	7/14/2014	48:31.7
1134	RSPe_2	-122.445	37.929	0.08	16.07	289.99	16.15	-122.445	37.929	-0.11	16.07	290.08	15.96	9	7/14/2014	48:31.6
1135	RSPe_2	-122.445	37.929	0.13	16.09	289.91	16.22	-122.445	37.929	-0.06	16.09	290.06	16.03	9	7/14/2014	48:31.5
1136	RSPe_2	-122.445	37.929	0.08	16.11	289.95	16.19	-122.445	37.929	-0.11	16.11	290.10	16.00	9	7/14/2014	48:31.4
1137	RSPe_2	-122.445	37.929	0.13	16.12	290.02	16.25	-122.445	37.929	-0.11	16.12	290.21	16.01	9	7/14/2014	48:31.3
1138	RSPe_2	-122.445	37.929	0.08	16.11	290.02	16.19	-122.445	37.929	-0.11	16.11	290.17	16.00	9	7/14/2014	48:31.2
1139	RSPe_2	-122.445	37.929	0.13	16.14	290.04	16.27	-122.445	37.929	-0.06	16.14	290.13	16.08	9	7/14/2014	48:31.1
1140	RSPe_2	-122.445	37.929	0.08	16.14	290.00	16.22	-122.445	37.929	-0.11	16.14	290.08	16.03	9	7/14/2014	48:31.0
1141	RSPe_2	-122.445	37.929	0.08	16.16	289.98	16.23	-122.445	37.929	-0.11	16.16	290.04	16.05	9	7/14/2014	48:30.9
1142	RSPe_2	-122.445	37.929	0.08	16.16	289.98	16.23	-122.445	37.929	-0.11	16.16	290.00	16.05	9	7/14/2014	48:30.8
1143	RSPe_2	-122.445	37.929	0.08	16.14	290.00	16.22	-122.445	37.929	-0.11	16.14	290.02	16.03	9	7/14/2014	48:30.7
1144	RSPe_2	-122.445	37.929	0.08	16.14	290.00	16.22	-122.445	37.929	-0.11	16.14	290.04	16.03	9	7/14/2014	48:30.6
1145	RSPe_2	-122.445	37.929	0.08	16.10	290.07	16.18	-122.445	37.929	-0.15	16.10	290.02	15.96	9	7/14/2014	48:30.5
1146	RSPe_2	-122.445	37.929	0.08	16.13	290.00	16.20	-122.445	37.929	-0.15	16.13	289.97	15.98	9	7/14/2014	48:30.4
1147	RSPe_2	-122.445	37.929	0.08	16.09	290.05	16.17	-122.445	37.929	-0.11	16.09	289.98	15.98	9	7/14/2014	48:30.3
1148	RSPe_2	-122.445	37.929	0.08	16.12	289.96	16.20	-122.445	37.929	-0.15	16.12	289.91	15.98	9	7/14/2014	48:30.2
1149	RSPe_2	-122.445	37.929	0.08	16.00	289.87	16.07	-122.445	37.929	-0.11	16.00	289.87	15.88	9	7/14/2014	48:30.1
1150	RSPe_2	-122.445	37.929	0.08	15.95	289.85	16.03	-122.445	37.929	-0.15	15.95	289.87	15.80	9	7/14/2014	48:30.0
1151	RSPe_2	-122.445	37.929	0.13	15.92	289.87	16.05	-122.445	37.929	-0.11	15.92	289.96	15.81	9	7/14/2014	48:29.9
1152	RSPe_2	-122.445	37.929	0.08	15.92	289.87	15.99	-122.445	37.929	-0.15	15.92	290.02	15.77	9	7/14/2014	48:29.8
1153	RSPe_2	-122.445	37.929	0.13	15.91	289.83	16.04	-122.445	37.929	-0.11	15.91	290.05	15.80	9	7/14/2014	48:29.7
1154	RSPe_2	-122.445	37.929	0.08	15.97	289.83	16.05	-122.445	37.929	-0.15	15.97	290.09	15.83	9	7/14/2014	48:29.6

1155	RSPe_2	-122.445	37.929	0.13	15.99	289.85	16.11	-122.445	37.929	-0.11	15.99	290.13	15.88	9	7/14/2014	48:29.5
1156	RSPe_2	-122.445	37.929	0.08	16.00	289.94	16.07	-122.445	37.929	-0.15	16.00	290.11	15.85	9	7/14/2014	48:29.4
1157	RSPe_2	-122.445	37.929	0.08	15.96	289.98	16.03	-122.445	37.929	-0.20	15.96	290.09	15.76	9	7/14/2014	48:29.3
1158	RSPe_2	-122.445	37.929	0.08	15.95	289.98	16.03	-122.445	37.929	-0.15	15.95	290.02	15.80	9	7/14/2014	48:29.2
1159	RSPe_2	-122.445	37.929	0.08	15.93	290.03	16.01	-122.445	37.929	-0.20	15.93	290.00	15.74	9	7/14/2014	48:29.1
1160	RSPe_2	-122.445	37.929	0.08	15.93	289.99	16.01	-122.445	37.929	-0.20	15.93	289.96	15.74	9	7/14/2014	48:29.0
1161	RSPe_2	-122.445	37.929	0.13	15.94	290.03	16.07	-122.445	37.929	-0.15	15.94	289.92	15.79	9	7/14/2014	48:28.9
1162	RSPe_2	-122.445	37.929	0.08	15.97	289.98	16.04	-122.445	37.929	-0.15	15.97	289.89	15.82	9	7/14/2014	48:28.8
1163	RSPe_2	-122.445	37.929	0.13	15.96	290.01	16.08	-122.445	37.929	-0.20	15.96	289.98	15.76	9	7/14/2014	48:28.7
1164	RSPe_2	-122.445	37.929	0.08	15.99	289.94	16.06	-122.445	37.929	-0.15	15.99	289.96	15.84	9	7/14/2014	48:28.6
1165	RSPe_2	-122.445	37.929	0.13	16.06	289.99	16.18	-122.445	37.929	-0.15	16.06	290.01	15.91	9	7/14/2014	48:28.5
1166	RSPe_2	-122.445	37.929	0.13	16.08	290.04	16.21	-122.445	37.929	-0.15	16.08	290.10	15.93	9	7/14/2014	48:28.4
1167	RSPe_2	-122.445	37.929	0.13	16.06	290.10	16.18	-122.445	37.929	-0.15	16.06	290.03	15.91	9	7/14/2014	48:28.3
1168	RSPe_2	-122.445	37.929	0.13	16.16	290.01	16.29	-122.445	37.929	-0.20	16.16	290.05	15.96	9	7/14/2014	48:28.2
1169	RSPe_2	-122.445	37.929	0.13	16.17	289.99	16.30	-122.445	37.929	-0.15	16.17	290.07	16.03	9	7/14/2014	48:28.1
1170	RSPe_2	-122.445	37.929	0.08	16.25	289.97	16.33	-122.445	37.929	-0.26	16.25	290.10	15.99	9	7/14/2014	48:28.0
1171	RSPe_2	-122.445	37.929	0.13	16.23	289.99	16.36	-122.445	37.929	-0.20	16.23	290.12	16.04	9	7/14/2014	48:27.9
1172	RSPe_2	-122.445	37.929	0.13	16.26	290.01	16.39	-122.445	37.929	-0.20	16.26	290.14	16.06	9	7/14/2014	48:27.8
1173	RSPe_2	-122.445	37.929	0.13	16.26	290.02	16.39	-122.445	37.929	-0.15	16.26	290.13	16.11	9	7/14/2014	48:27.7
1174	RSPe_2	-122.445	37.929	0.13	16.30	290.13	16.42	-122.445	37.929	-0.15	16.30	290.12	16.15	9	7/14/2014	48:27.6
1175	RSPe_2	-122.445	37.929	0.13	16.28	290.17	16.41	-122.445	37.929	-0.11	16.28	290.12	16.17	9	7/14/2014	48:27.5
1176	RSPe_2	-122.445	37.929	0.13	16.24	290.19	16.37	-122.445	37.929	-0.20	16.24	290.07	16.05	9	7/14/2014	48:27.4
1177	RSPe_2	-122.445	37.929	0.16	16.26	290.21	16.42	-122.445	37.929	-0.11	16.26	290.04	16.15	9	7/14/2014	48:27.3
1178	RSPe_2	-122.445	37.929	0.13	16.23	290.20	16.36	-122.445	37.929	-0.15	16.23	289.92	16.08	9	7/14/2014	48:27.2
1179	RSPe_2	-122.445	37.929	0.13	16.23	290.15	16.36	-122.445	37.929	-0.15	16.23	289.95	16.08	9	7/14/2014	48:27.1
1180	RSPe_2	-122.445	37.929	0.13	16.23	290.17	16.36	-122.445	37.929	-0.15	16.23	289.93	16.08	9	7/14/2014	48:27.0
1181	RSPe_2	-122.445	37.929	0.16	16.23	290.11	16.39	-122.445	37.929	-0.11	16.23	289.99	16.12	9	7/14/2014	48:26.9
1182	RSPe_2	-122.445	37.929	0.16	16.23	290.05	16.39	-122.445	37.929	-0.15	16.23	290.00	16.08	9	7/14/2014	48:26.8
1183	RSPe_2	-122.445	37.929	0.16	16.27	289.91	16.43	-122.445	37.929	-0.11	16.27	289.90	16.16	9	7/14/2014	48:26.7
1184	RSPe_2	-122.445	37.929	0.16	16.25	289.91	16.41	-122.445	37.929	-0.11	16.25	289.90	16.14	9	7/14/2014	48:26.6
1185	RSPe_2	-122.445	37.929	0.16	16.27	289.89	16.43	-122.445	37.929	-0.06	16.27	289.97	16.21	9	7/14/2014	48:26.5
1186	RSPe_2	-122.445	37.929	0.16	16.28	289.91	16.44	-122.445	37.929	-0.11	16.28	290.00	16.17	9	7/14/2014	48:26.4
1187	RSPe_2	-122.445	37.929	0.16	16.32	289.91	16.48	-122.445	37.929	-0.11	16.32	289.97	16.21	9	7/14/2014	48:26.3

1188	RSPe_2	-122.445	37.929	0.16	16.31	289.91	16.47	-122.445	37.929	-0.11	16.31	290.00	16.20	9	7/14/2014	48:26.2
1189	RSPe_2	-122.445	37.929	0.16	16.32	289.91	16.48	-122.445	37.929	-0.06	16.32	289.95	16.26	9	7/14/2014	48:26.1
1190	RSPe_2	-122.445	37.929	0.16	16.27	289.91	16.43	-122.445	37.929	-0.15	16.27	289.94	16.12	9	7/14/2014	48:26.0
1191	RSPe_2	-122.445	37.929	0.19	16.32	289.87	16.51	-122.445	37.929	-0.11	16.32	289.91	16.21	9	7/14/2014	48:25.9
1192	RSPe_2	-122.445	37.929	0.16	16.27	289.80	16.43	-122.445	37.929	-0.15	16.27	289.80	16.13	9	7/14/2014	48:25.8
1193	RSPe_2	-122.445	37.929	0.19	16.17	289.78	16.37	-122.445	37.929	-0.11	16.17	289.82	16.06	9	7/14/2014	48:25.7
1194	RSPe_2	-122.445	37.929	0.19	16.23	289.71	16.43	-122.445	37.929	-0.06	16.23	289.73	16.17	9	7/14/2014	48:25.6
1195	RSPe_2	-122.445	37.929	0.19	16.18	289.67	16.38	-122.445	37.929	-0.03	16.18	289.69	16.15	9	7/14/2014	48:25.5
1196	RSPe_2	-122.445	37.929	0.19	16.08	289.67	16.27	-122.445	37.929	-0.03	16.08	289.63	16.05	9	7/14/2014	48:25.4
1197	RSPe_2	-122.445	37.929	0.25	16.09	289.74	16.34	-122.445	37.929	-0.03	16.09	289.60	16.06	9	7/14/2014	48:25.3
1198	RSPe_2	-122.445	37.929	0.19	15.99	289.74	16.18	-122.445	37.929	-0.06	15.99	289.61	15.93	9	7/14/2014	48:25.2
1199	RSPe_2	-122.445	37.929	0.25	16.00	289.72	16.24	-122.445	37.929	-0.03	16.00	289.52	15.97	9	7/14/2014	48:25.1
1200	RSPe_2	-122.445	37.929	0.19	15.88	289.70	16.08	-122.445	37.929	-0.03	15.88	289.51	15.85	9	7/14/2014	48:25.0
1201	RSPe_2	-122.445	37.929	0.25	15.85	289.61	16.10	-122.445	37.929	-0.03	15.85	289.46	15.82	9	7/14/2014	48:24.9
1202	RSPe_2	-122.445	37.929	0.25	15.84	289.48	16.09	-122.445	37.929	-0.06	15.84	289.42	15.78	9	7/14/2014	48:24.8
1203	RSPe_2	-122.445	37.929	0.25	15.86	289.45	16.11	-122.445	37.929	-0.03	15.86	289.49	15.84	9	7/14/2014	48:24.7
1204	RSPe_2	-122.445	37.929	0.25	15.90	289.57	16.14	-122.445	37.929	-0.03	15.90	289.54	15.87	9	7/14/2014	48:24.6
1205	RSPe_2	-122.445	37.929	0.25	15.92	289.61	16.17	-122.445	37.929	-0.03	15.92	289.58	15.89	9	7/14/2014	48:24.5
1206	RSPe_2	-122.445	37.929	0.25	15.95	289.67	16.20	-122.445	37.929	-0.06	15.95	289.61	15.89	9	7/14/2014	48:24.4
1207	RSPe_2	-122.445	37.929	0.28	16.02	289.66	16.30	-122.445	37.929	0.02	16.02	289.50	16.04	9	7/14/2014	48:24.3
1208	RSPe_2	-122.445	37.929	0.25	16.06	289.70	16.31	-122.445	37.929	-0.03	16.06	289.54	16.04	9	7/14/2014	48:24.2
1209	RSPe_2	-122.445	37.929	0.28	16.00	289.68	16.28	-122.445	37.929	0.06	16.00	289.50	16.05	9	7/14/2014	48:24.1
1210	RSPe_2	-122.445	37.929	0.25	16.06	289.64	16.31	-122.445	37.929	0.11	16.06	289.52	16.17	9	7/14/2014	48:24.0
1211	RSPe_2	-122.445	37.929	0.28	16.10	289.48	16.39	-122.445	37.929	0.06	16.10	289.46	16.16	9	7/14/2014	48:23.9
1212	RSPe_2	-122.445	37.929	0.25	16.17	289.48	16.42	-122.445	37.929	0.02	16.17	289.61	16.20	9	7/14/2014	48:23.8
1213	RSPe_2	-122.445	37.929	0.25	16.14	289.55	16.39	-122.445	37.929	0.06	16.14	289.66	16.20	9	7/14/2014	48:23.7
1214	RSPe_2	-122.445	37.929	0.25	16.16	289.57	16.41	-122.445	37.929	0.06	16.16	289.67	16.22	9	7/14/2014	48:23.6
1215	RSPe_2	-122.445	37.929	0.28	16.21	289.52	16.49	-122.445	37.929	0.11	16.21	289.67	16.32	9	7/14/2014	48:23.5
1216	RSPe_2	-122.445	37.929	0.28	16.20	289.53	16.48	-122.445	37.929	0.02	16.20	289.71	16.22	9	7/14/2014	48:23.4
1217	RSPe_2	-122.445	37.929	0.28	16.19	289.53	16.47	-122.445	37.929	0.02	16.19	289.62	16.21	9	7/14/2014	48:23.3
1218	RSPe_2	-122.445	37.929	0.28	16.17	289.60	16.45	-122.445	37.929	0.02	16.17	289.64	16.20	9	7/14/2014	48:23.2
1219	RSPe_2	-122.445	37.929	0.33	16.17	289.44	16.51	-122.445	37.929	0.11	16.17	289.50	16.28	9	7/14/2014	48:23.1
1220	RSPe_2	-122.445	37.929	0.28	16.15	289.44	16.43	-122.445	37.929	0.06	16.15	289.44	16.21	9	7/14/2014	48:23.0

1221	RSPe_2	-122.445	37.929	0.28	16.17	289.55	16.45	-122.445	37.929	0.11	16.17	289.57	16.28	9	7/14/2014	48:22.9
1222	RSPe_2	-122.445	37.929	0.28	16.18	289.60	16.46	-122.445	37.929	0.06	16.18	289.48	16.24	9	7/14/2014	48:22.8
1223	RSPe_2	-122.445	37.929	0.33	16.19	289.60	16.52	-122.445	37.929	0.06	16.19	289.49	16.25	9	7/14/2014	48:22.7
1224	RSPe_2	-122.445	37.929	0.28	16.16	289.60	16.44	-122.445	37.929	0.06	16.16	289.46	16.22	9	7/14/2014	48:22.6
1225	RSPe_2	-122.445	37.929	0.28	16.13	289.58	16.41	-122.445	37.929	0.06	16.13	289.42	16.19	9	7/14/2014	48:22.5
1226	RSPe_2	-122.445	37.929	0.28	16.13	289.62	16.41	-122.445	37.929	0.06	16.13	289.50	16.18	9	7/14/2014	48:22.4
1227	RSPe_2	-122.445	37.929	0.33	16.17	289.64	16.50	-122.445	37.929	0.06	16.17	289.53	16.22	9	7/14/2014	48:22.3
1228	RSPe_2	-122.445	37.929	0.28	16.14	289.67	16.42	-122.445	37.929	0.11	16.14	289.53	16.25	9	7/14/2014	48:22.2
1229	RSPe_2	-122.445	37.929	0.33	16.13	289.67	16.46	-122.445	37.929	0.06	16.13	289.57	16.19	9	7/14/2014	48:22.1
1230	RSPe_2	-122.445	37.929	0.33	16.17	289.75	16.50	-122.445	37.929	0.06	16.17	289.57	16.22	9	7/14/2014	48:22.0
1231	RSPe_2	-122.445	37.929	0.33	16.13	289.73	16.46	-122.445	37.929	0.11	16.13	289.58	16.24	9	7/14/2014	48:21.9
1232	RSPe_2	-122.445	37.929	0.33	16.13	289.75	16.46	-122.445	37.929	0.14	16.13	289.60	16.27	9	7/14/2014	48:21.8
1233	RSPe_2	-122.445	37.929	0.37	16.07	289.67	16.44	-122.445	37.929	0.18	16.07	289.56	16.25	9	7/14/2014	48:21.7
1234	RSPe_2	-122.445	37.929	0.37	16.06	289.56	16.42	-122.445	37.929	0.18	16.06	289.57	16.23	9	7/14/2014	48:21.6
1235	RSPe_2	-122.445	37.929	0.37	16.04	289.40	16.41	-122.445	37.929	0.23	16.04	289.49	16.27	9	7/14/2014	48:21.5
1236	RSPe_2	-122.445	37.929	0.33	16.09	289.32	16.43	-122.445	37.929	0.18	16.09	289.47	16.27	9	7/14/2014	48:21.4
1237	RSPe_2	-122.445	37.929	0.37	16.02	289.38	16.38	-122.445	37.929	0.23	16.02	289.51	16.24	9	7/14/2014	48:21.3
1238	RSPe_2	-122.445	37.929	0.37	16.00	289.47	16.37	-122.445	37.929	0.18	16.00	289.60	16.18	9	7/14/2014	48:21.2
1239	RSPe_2	-122.445	37.929	0.37	16.02	289.47	16.38	-122.445	37.929	0.18	16.02	289.58	16.19	9	7/14/2014	48:21.1
1240	RSPe_2	-122.445	37.929	0.33	16.02	289.45	16.35	-122.445	37.929	0.18	16.02	289.42	16.19	9	7/14/2014	48:21.0
1241	RSPe_2	-122.445	37.929	0.37	16.02	289.50	16.38	-122.445	37.929	0.14	16.02	289.47	16.16	9	7/14/2014	48:20.9
1242	RSPe_2	-122.445	37.929	0.37	16.04	289.58	16.41	-122.445	37.929	0.11	16.04	289.49	16.15	9	7/14/2014	48:20.8
1243	RSPe_2	-122.445	37.929	0.37	16.00	289.54	16.37	-122.445	37.929	0.14	16.00	289.40	16.15	9	7/14/2014	48:20.7
1244	RSPe_2	-122.445	37.929	0.37	16.02	289.45	16.39	-122.445	37.929	0.14	16.02	289.38	16.16	9	7/14/2014	48:20.6
1245	RSPe_2	-122.445	37.929	0.37	16.00	289.41	16.37	-122.445	37.929	0.18	16.00	289.36	16.18	9	7/14/2014	48:20.5
1246	RSPe_2	-122.445	37.929	0.37	15.97	289.26	16.34	-122.445	37.929	0.14	15.97	289.33	16.12	9	7/14/2014	48:20.4
1247	RSPe_2	-122.445	37.929	0.42	15.97	289.17	16.39	-122.445	37.929	0.18	15.97	289.21	16.15	9	7/14/2014	48:20.3
1248	RSPe_2	-122.445	37.929	0.37	15.97	289.19	16.33	-122.445	37.929	0.23	15.97	289.30	16.19	9	7/14/2014	48:20.2
1249	RSPe_2	-122.445	37.929	0.37	15.96	289.15	16.32	-122.445	37.929	0.18	15.96	289.27	16.13	9	7/14/2014	48:20.1
1250	RSPe_2	-122.445	37.929	0.42	15.95	289.17	16.37	-122.445	37.929	0.18	15.95	289.27	16.13	9	7/14/2014	48:20.0
1251	RSPe_2	-122.445	37.929	0.42	15.95	289.17	16.37	-122.445	37.929	0.18	15.95	289.23	16.13	9	7/14/2014	48:19.9
1252	RSPe_2	-122.445	37.929	0.37	15.96	289.11	16.32	-122.445	37.929	0.18	15.96	289.12	16.13	9	7/14/2014	48:19.8
1253	RSPe_2	-122.445	37.929	0.42	15.97	289.13	16.39	-122.445	37.929	0.18	15.97	289.19	16.15	9	7/14/2014	48:19.7

1254	RSPe_2	-122.445	37.929	0.37	15.98	289.15	16.35	-122.445	37.929	0.11	15.98	289.14	16.09	9	7/14/2014	48:19.6
1255	RSPe_2	-122.445	37.929	0.42	15.94	289.17	16.36	-122.445	37.929	0.18	15.94	289.17	16.12	9	7/14/2014	48:19.5
1256	RSPe_2	-122.445	37.929	0.37	15.96	289.28	16.32	-122.445	37.929	0.14	15.96	289.19	16.10	9	7/14/2014	48:19.4
1257	RSPe_2	-122.445	37.929	0.37	15.99	289.33	16.35	-122.445	37.929	0.18	15.99	289.19	16.16	9	7/14/2014	48:19.3
1258	RSPe_2	-122.445	37.929	0.37	15.93	289.33	16.30	-122.445	37.929	0.14	15.93	289.14	16.07	9	7/14/2014	48:19.2
1259	RSPe_2	-122.445	37.929	0.42	15.95	289.26	16.37	-122.445	37.929	0.14	15.95	289.07	16.09	9	7/14/2014	48:19.1
1260	RSPe_2	-122.445	37.929	0.42	15.89	289.18	16.31	-122.445	37.929	0.23	15.89	289.02	16.11	9	7/14/2014	48:19.0
1261	RSPe_2	-122.445	37.929	0.42	15.90	289.15	16.32	-122.445	37.929	0.23	15.90	289.04	16.13	9	7/14/2014	48:18.9
1262	RSPe_2	-122.445	37.929	0.42	15.90	289.09	16.31	-122.445	37.929	0.18	15.90	289.04	16.07	9	7/14/2014	48:18.8
1263	RSPe_2	-122.445	37.929	0.45	15.88	289.11	16.33	-122.445	37.929	0.23	15.88	289.11	16.11	9	7/14/2014	48:18.7
1264	RSPe_2	-122.445	37.929	0.42	15.89	289.07	16.31	-122.445	37.929	0.23	15.89	289.11	16.11	9	7/14/2014	48:18.6
1265	RSPe_2	-122.445	37.929	0.45	15.90	289.04	16.36	-122.445	37.929	0.23	15.90	289.13	16.13	9	7/14/2014	48:18.5
1266	RSPe_2	-122.445	37.929	0.42	15.96	289.07	16.38	-122.445	37.929	0.18	15.96	289.13	16.13	9	7/14/2014	48:18.4
1267	RSPe_2	-122.445	37.929	0.45	15.96	289.09	16.41	-122.445	37.929	0.23	15.96	289.17	16.18	9	7/14/2014	48:18.3
1268	RSPe_2	-122.445	37.929	0.42	16.16	289.07	16.58	-122.445	37.929	0.18	16.16	289.11	16.34	9	7/14/2014	48:18.2
1269	RSPe_2	-122.445	37.929	0.45	16.12	289.00	16.57	-122.445	37.929	0.23	16.12	289.02	16.35	9	7/14/2014	48:18.1
1270	RSPe_2	-122.445	37.929	0.45	16.12	288.98	16.57	-122.445	37.929	0.23	16.12	289.00	16.35	9	7/14/2014	48:18.0
1271	RSPe_2	-122.445	37.929	0.45	16.13	288.94	16.59	-122.445	37.929	0.23	16.13	288.94	16.36	9	7/14/2014	48:17.9
1272	RSPe_2	-122.445	37.929	0.42	16.06	288.96	16.48	-122.445	37.929	0.18	16.06	288.93	16.24	9	7/14/2014	48:17.8
1273	RSPe_2	-122.445	37.929	0.45	16.07	288.96	16.53	-122.445	37.929	0.23	16.07	288.91	16.30	9	7/14/2014	48:17.7
1274	RSPe_2	-122.445	37.929	0.45	16.03	289.00	16.49	-122.445	37.929	0.23	16.03	288.93	16.26	9	7/14/2014	48:17.6
1275	RSPe_2	-122.445	37.929	0.49	16.03	288.94	16.51	-122.445	37.929	0.18	16.03	288.94	16.20	9	7/14/2014	48:17.5
1276	RSPe_2	-122.445	37.929	0.45	16.00	288.94	16.46	-122.445	37.929	0.23	16.00	288.92	16.23	9	7/14/2014	48:17.4
1277	RSPe_2	-122.445	37.929	0.49	15.97	288.89	16.46	-122.445	37.929	0.26	15.97	288.92	16.23	9	7/14/2014	48:17.3
1278	RSPe_2	-122.445	37.929	0.45	15.97	288.92	16.43	-122.445	37.929	0.23	15.97	288.89	16.20	9	7/14/2014	48:17.2
1279	RSPe_2	-122.445	37.929	0.49	15.96	288.90	16.44	-122.445	37.929	0.26	15.96	288.88	16.22	9	7/14/2014	48:17.1
1280	RSPe_2	-122.445	37.929	0.49	15.95	288.83	16.44	-122.445	37.929	0.26	15.95	288.87	16.21	9	7/14/2014	48:17.0
1281	RSPe_2	-122.445	37.929	0.49	15.90	288.85	16.39	-122.445	37.929	0.26	15.90	288.89	16.16	9	7/14/2014	48:16.9
1282	RSPe_2	-122.445	37.929	0.49	15.90	288.81	16.38	-122.445	37.929	0.31	15.90	288.80	16.21	9	7/14/2014	48:16.8
1283	RSPe_2	-122.445	37.929	0.54	15.88	288.74	16.42	-122.445	37.929	0.26	15.88	288.80	16.14	9	7/14/2014	48:16.7
1284	RSPe_2	-122.445	37.929	0.49	15.87	288.81	16.36	-122.445	37.929	0.26	15.87	288.78	16.13	9	7/14/2014	48:16.6
1285	RSPe_2	-122.445	37.929	0.49	15.92	288.79	16.41	-122.445	37.929	0.31	15.92	288.77	16.23	9	7/14/2014	48:16.5
1286	RSPe_2	-122.445	37.929	0.49	15.88	288.81	16.37	-122.445	37.929	0.31	15.88	288.78	16.19	9	7/14/2014	48:16.4

1287	RSPe_2	-122.445	37.929	0.54	15.90	288.77	16.44	-122.445	37.929	0.35	15.90	288.72	16.25	9	7/14/2014	48:16.3
1288	RSPe_2	-122.445	37.929	0.49	15.93	288.77	16.42	-122.445	37.929	0.31	15.93	288.78	16.24	9	7/14/2014	48:16.2
1289	RSPe_2	-122.445	37.929	0.54	15.98	288.81	16.52	-122.445	37.929	0.31	15.98	288.79	16.29	9	7/14/2014	48:16.1
1290	RSPe_2	-122.445	37.929	0.49	15.98	288.77	16.47	-122.445	37.929	0.26	15.98	288.78	16.24	9	7/14/2014	48:16.0
1291	RSPe_2	-122.445	37.929	0.57	16.03	288.75	16.60	-122.445	37.929	0.35	16.03	288.77	16.37	9	7/14/2014	48:15.9
1292	RSPe_2	-122.445	37.929	0.54	16.00	288.81	16.54	-122.445	37.929	0.35	16.00	288.79	16.34	9	7/14/2014	48:15.8
1293	RSPe_2	-122.445	37.929	0.57	15.99	288.80	16.56	-122.445	37.929	0.31	15.99	288.75	16.30	9	7/14/2014	48:15.7
1294	RSPe_2	-122.445	37.929	0.54	15.96	288.82	16.50	-122.445	37.929	0.31	15.96	288.74	16.27	9	7/14/2014	48:15.6
1295	RSPe_2	-122.445	37.929	0.57	15.95	288.84	16.52	-122.445	37.929	0.40	15.95	288.72	16.35	9	7/14/2014	48:15.5
1296	RSPe_2	-122.445	37.929	0.54	15.97	288.82	16.50	-122.445	37.929	0.35	15.97	288.70	16.31	9	7/14/2014	48:15.4
1297	RSPe_2	-122.445	37.929	0.57	15.96	288.80	16.53	-122.445	37.929	0.40	15.96	288.74	16.35	9	7/14/2014	48:15.3
1298	RSPe_2	-122.445	37.929	0.57	15.95	288.82	16.52	-122.445	37.929	0.35	15.95	288.75	16.30	9	7/14/2014	48:15.2
1299	RSPe_2	-122.445	37.929	0.57	15.91	288.81	16.48	-122.445	37.929	0.40	15.91	288.79	16.31	9	7/14/2014	48:15.1
1300	RSPe_2	-122.445	37.929	0.57	15.93	288.77	16.50	-122.445	37.929	0.35	15.93	288.81	16.27	9	7/14/2014	48:15.0
1301	RSPe_2	-122.445	37.929	0.57	15.91	288.82	16.48	-122.445	37.929	0.35	15.91	288.80	16.26	9	7/14/2014	48:14.9
1302	RSPe_2	-122.445	37.929	0.57	15.93	288.80	16.50	-122.445	37.929	0.40	15.93	288.80	16.33	9	7/14/2014	48:14.8
1303	RSPe_2	-122.445	37.929	0.62	15.98	288.76	16.60	-122.445	37.929	0.43	15.98	288.76	16.41	9	7/14/2014	48:14.7
1304	RSPe_2	-122.445	37.929	0.57	15.93	288.76	16.51	-122.445	37.929	0.40	15.93	288.70	16.33	9	7/14/2014	48:14.6
1305	RSPe_2	-122.445	37.929	0.62	15.91	288.80	16.54	-122.445	37.929	0.43	15.91	288.71	16.34	9	7/14/2014	48:14.5
1306	RSPe_2	-122.445	37.929	0.62	15.99	288.75	16.61	-122.445	37.929	0.43	15.99	288.66	16.42	9	7/14/2014	48:14.4
1307	RSPe_2	-122.445	37.929	0.66	15.96	288.76	16.61	-122.445	37.929	0.46	15.96	288.64	16.42	9	7/14/2014	48:14.3
1308	RSPe_2	-122.445	37.929	0.62	15.93	288.76	16.55	-122.445	37.929	0.43	15.93	288.62	16.36	9	7/14/2014	48:14.2
1309	RSPe_2	-122.445	37.929	0.66	16.02	288.73	16.68	-122.445	37.929	0.43	16.02	288.61	16.45	9	7/14/2014	48:14.1
1310	RSPe_2	-122.445	37.929	0.62	15.90	288.67	16.52	-122.445	37.929	0.43	15.90	288.55	16.33	9	7/14/2014	48:14.0
1311	RSPe_2	-122.445	37.929	0.66	15.95	288.63	16.61	-122.445	37.929	0.46	15.95	288.58	16.41	9	7/14/2014	48:13.9
1312	RSPe_2	-122.445	37.929	0.66	15.82	288.60	16.48	-122.445	37.929	0.46	15.82	288.58	16.28	9	7/14/2014	48:13.8
1313	RSPe_2	-122.445	37.929	0.66	15.86	288.61	16.52	-122.445	37.929	0.46	15.86	288.60	16.33	9	7/14/2014	48:13.7
1314	RSPe_2	-122.445	37.929	0.66	15.77	288.58	16.43	-122.445	37.929	0.46	15.77	288.58	16.24	9	7/14/2014	48:13.6
1315	RSPe_2	-122.445	37.929	0.71	15.77	288.56	16.48	-122.445	37.929	0.51	15.77	288.58	16.29	9	7/14/2014	48:13.5
1316	RSPe_2	-122.445	37.929	0.71	15.76	288.54	16.47	-122.445	37.929	0.46	15.76	288.58	16.22	9	7/14/2014	48:13.4
1317	RSPe_2	-122.445	37.929	0.71	15.73	288.54	16.44	-122.445	37.929	0.51	15.73	288.54	16.24	9	7/14/2014	48:13.3
1318	RSPe_2	-122.445	37.929	0.71	15.73	288.52	16.44	-122.445	37.929	0.51	15.73	288.51	16.24	9	7/14/2014	48:13.2
1319	RSPe_2	-122.445	37.929	0.74	15.76	288.54	16.51	-122.445	37.929	0.55	15.76	288.48	16.31	9	7/14/2014	48:13.1

1320	RSPe_2	-122.445	37.929	0.71	15.66	288.52	16.37	-122.445	37.929	0.55	15.66	288.47	16.21	9	7/14/2014	48:13.0
1321	RSPe_2	-122.445	37.929	0.74	15.62	288.50	16.37	-122.445	37.929	0.55	15.62	288.43	16.17	9	7/14/2014	48:12.9
1322	RSPe_2	-122.445	37.929	0.74	15.59	288.43	16.33	-122.445	37.929	0.55	15.59	288.34	16.14	9	7/14/2014	48:12.8
1323	RSPe_2	-122.445	37.929	0.74	15.57	288.48	16.32	-122.445	37.929	0.63	15.57	288.30	16.21	9	7/14/2014	48:12.7
1324	RSPe_2	-122.445	37.929	0.74	15.56	288.35	16.30	-122.445	37.929	0.60	15.56	288.23	16.16	9	7/14/2014	48:12.6
1325	RSPe_2	-122.445	37.929	0.80	15.53	288.32	16.32	-122.445	37.929	0.60	15.53	288.17	16.13	9	7/14/2014	48:12.5
1326	RSPe_2	-122.445	37.929	0.80	15.52	288.28	16.31	-122.445	37.929	0.68	15.52	288.17	16.20	9	7/14/2014	48:12.4
1327	RSPe_2	-122.445	37.929	0.80	15.54	288.26	16.33	-122.445	37.929	0.68	15.54	288.15	16.22	9	7/14/2014	48:12.3
1328	RSPe_2	-122.445	37.929	0.80	15.51	288.24	16.31	-122.445	37.929	0.68	15.51	288.14	16.19	9	7/14/2014	48:12.2
1329	RSPe_2	-122.445	37.929	0.80	15.53	288.21	16.32	-122.445	37.929	0.68	15.53	288.10	16.21	9	7/14/2014	48:12.1
1330	RSPe_2	-122.445	37.929	0.80	15.53	288.15	16.32	-122.445	37.929	0.68	15.53	288.08	16.21	9	7/14/2014	48:12.0
1331	RSPe_2	-122.445	37.929	0.83	15.53	288.17	16.36	-122.445	37.929	0.72	15.53	288.11	16.24	9	7/14/2014	48:11.9
1332	RSPe_2	-122.445	37.929	0.80	15.56	288.15	16.35	-122.445	37.929	0.72	15.56	288.10	16.27	9	7/14/2014	48:11.8
1333	RSPe_2	-122.445	37.929	0.83	15.56	288.13	16.38	-122.445	37.929	0.72	15.56	288.04	16.27	9	7/14/2014	48:11.7
1334	RSPe_2	-122.445	37.929	0.83	15.62	288.07	16.45	-122.445	37.929	0.72	15.62	288.01	16.34	9	7/14/2014	48:11.6
1335	RSPe_2	-122.445	37.929	0.83	15.65	288.09	16.48	-122.445	37.929	0.77	15.65	288.02	16.42	9	7/14/2014	48:11.5
1336	RSPe_2	-122.445	37.929	0.83	15.66	288.07	16.49	-122.445	37.929	0.80	15.66	287.93	16.46	9	7/14/2014	48:11.4
1337	RSPe_2	-122.445	37.929	0.86	15.67	288.04	16.54	-122.445	37.929	0.77	15.67	287.97	16.44	9	7/14/2014	48:11.3
1338	RSPe_2	-122.445	37.929	0.86	15.65	287.96	16.51	-122.445	37.929	0.77	15.65	287.89	16.42	9	7/14/2014	48:11.2
1339	RSPe_2	-122.445	37.929	0.92	15.65	287.98	16.56	-122.445	37.929	0.89	15.65	287.93	16.54	9	7/14/2014	48:11.1
1340	RSPe_2	-122.445	37.929	0.92	15.60	288.00	16.52	-122.445	37.929	0.80	15.60	287.89	16.41	9	7/14/2014	48:11.0
1341	RSPe_2	-122.445	37.929	0.92	15.63	287.96	16.55	-122.445	37.929	0.84	15.63	287.86	16.47	9	7/14/2014	48:10.9
1342	RSPe_2	-122.445	37.929	0.86	15.59	287.93	16.45	-122.445	37.929	0.89	15.59	287.89	16.47	9	7/14/2014	48:10.8
1343	RSPe_2	-122.445	37.929	0.92	15.56	287.90	16.48	-122.445	37.929	0.92	15.56	287.83	16.49	9	7/14/2014	48:10.7
1344	RSPe_2	-122.445	37.929	0.92	15.56	287.87	16.48	-122.445	37.929	0.92	15.56	287.80	16.49	9	7/14/2014	48:10.6
1345	RSPe_2	-122.445	37.929	0.95	15.53	287.89	16.48	-122.445	37.929	0.89	15.53	287.79	16.41	9	7/14/2014	48:10.5
1346	RSPe_2	-122.445	37.929	0.92	15.55	287.92	16.46	-122.445	37.929	0.97	15.55	287.78	16.52	9	7/14/2014	48:10.4
1347	RSPe_2	-122.445	37.929	0.95	15.47	287.92	16.42	-122.445	37.929	0.92	15.47	287.80	16.39	9	7/14/2014	48:10.3
1348	RSPe_2	-122.445	37.929	0.92	15.46	287.94	16.37	-122.445	37.929	0.89	15.46	287.76	16.34	9	7/14/2014	48:10.2
1349	RSPe_2	-122.445	37.929	0.95	15.41	287.92	16.36	-122.445	37.929	0.97	15.41	287.74	16.38	9	7/14/2014	48:10.1
1350	RSPe_2	-122.445	37.929	0.95	15.32	287.98	16.27	-122.445	37.929	0.84	15.32	287.74	16.16	9	7/14/2014	48:10.0
1351	RSPe_2	-122.445	37.929	1.00	15.25	287.96	16.25	-122.445	37.929	0.92	15.25	287.76	16.17	9	7/14/2014	48:09.9
1352	RSPe_2	-122.445	37.929	1.00	15.22	287.96	16.22	-122.445	37.929	1.01	15.22	287.79	16.23	9	7/14/2014	48:09.8

1353	RSPe_2	-122.445	37.929	1.00	15.18	287.92	16.18	-122.445	37.929	1.14	15.18	287.76	16.32	9	7/14/2014	48:09.7
1354	RSPe_2	-122.445	37.929	1.00	15.15	287.92	16.15	-122.445	37.929	1.06	15.15	287.74	16.20	9	7/14/2014	48:09.6
1355	RSPe_2	-122.445	37.929	1.03	15.13	287.94	16.17	-122.445	37.929	1.14	15.13	287.76	16.27	9	7/14/2014	48:09.5
1356	RSPe_2	-122.445	37.929	1.00	15.13	287.99	16.13	-122.445	37.929	1.14	15.13	287.80	16.27	9	7/14/2014	48:09.4
1357	RSPe_2	-122.445	37.929	1.03	15.13	287.96	16.16	-122.445	37.929	1.06	15.13	287.83	16.18	9	7/14/2014	48:09.3
1358	RSPe_2	-122.445	37.929	1.03	15.10	287.99	16.14	-122.445	37.929	1.14	15.10	287.83	16.24	9	7/14/2014	48:09.2
1359	RSPe_2	-122.445	37.929	1.09	15.12	287.94	16.20	-122.445	37.929	1.21	15.12	287.83	16.33	9	7/14/2014	48:09.1
1360	RSPe_2	-122.445	37.929	1.03	15.11	287.94	16.14	-122.445	37.929	1.14	15.11	287.77	16.25	9	7/14/2014	48:09.0
1361	RSPe_2	-122.445	37.929	1.09	15.09	287.95	16.18	-122.445	37.929	1.21	15.09	287.81	16.30	9	7/14/2014	48:08.9
1362	RSPe_2	-122.445	37.929	1.09	15.14	287.97	16.23	-122.445	37.929	1.21	15.14	287.83	16.35	9	7/14/2014	48:08.8
1363	RSPe_2	-122.445	37.929	1.09	15.11	288.04	16.20	-122.445	37.929	1.14	15.11	287.85	16.25	9	7/14/2014	48:08.7
1364	RSPe_2	-122.445	37.929	1.09	15.14	288.03	16.23	-122.445	37.929	1.26	15.14	287.85	16.40	9	7/14/2014	48:08.6
1365	RSPe_2	-122.445	37.929	1.12	15.15	288.04	16.27	-122.445	37.929	1.26	15.15	287.83	16.41	9	7/14/2014	48:08.5
1366	RSPe_2	-122.445	37.929	1.09	15.22	288.08	16.30	-122.445	37.929	1.21	15.22	287.83	16.43	9	7/14/2014	48:08.4
1367	RSPe_2	-122.445	37.929	1.12	15.17	287.99	16.29	-122.445	37.929	1.29	15.17	287.79	16.46	9	7/14/2014	48:08.3
1368	RSPe_2	-122.445	37.929	1.12	15.22	288.02	16.34	-122.445	37.929	1.29	15.22	287.79	16.51	9	7/14/2014	48:08.2
1369	RSPe_2	-122.445	37.929	1.12	15.15	288.04	16.27	-122.445	37.929	1.38	15.15	287.82	16.53	9	7/14/2014	48:08.1
1370	RSPe_2	-122.445	37.929	1.12	15.15	287.95	16.27	-122.445	37.929	1.34	15.15	287.81	16.50	9	7/14/2014	48:08.0
1371	RSPe_2	-122.445	37.929	1.12	15.14	287.90	16.26	-122.445	37.929	1.34	15.14	287.82	16.48	9	7/14/2014	48:07.9
1372	RSPe_2	-122.445	37.929	1.12	15.13	287.91	16.25	-122.445	37.929	1.34	15.13	287.75	16.47	9	7/14/2014	48:07.8
1373	RSPe_2	-122.445	37.929	1.17	15.13	287.84	16.30	-122.445	37.929	1.38	15.13	287.73	16.50	9	7/14/2014	48:07.7
1374	RSPe_2	-122.445	37.929	1.12	15.19	287.82	16.31	-122.445	37.929	1.34	15.19	287.70	16.54	9	7/14/2014	48:07.6
1375	RSPe_2	-122.445	37.929	1.17	15.10	287.82	16.27	-122.445	37.929	1.46	15.10	287.73	16.56	9	7/14/2014	48:07.5
1376	RSPe_2	-122.445	37.929	1.17	15.10	287.75	16.27	-122.445	37.929	1.38	15.10	287.68	16.48	9	7/14/2014	48:07.4
1377	RSPe_2	-122.445	37.929	1.17	15.07	287.71	16.24	-122.445	37.929	1.43	15.07	287.66	16.50	9	7/14/2014	48:07.3
1378	RSPe_2	-122.445	37.929	1.17	15.08	287.71	16.25	-122.445	37.929	1.46	15.08	287.62	16.54	9	7/14/2014	48:07.2
1379	RSPe_2	-122.445	37.929	1.17	15.06	287.67	16.23	-122.445	37.929	1.46	15.06	287.55	16.53	9	7/14/2014	48:07.1
1380	RSPe_2	-122.445	37.929	1.17	15.06	287.64	16.23	-122.445	37.929	1.43	15.06	287.53	16.48	9	7/14/2014	48:07.0
1381	RSPe_2	-122.445	37.929	1.17	15.04	287.67	16.21	-122.445	37.929	1.51	15.04	287.53	16.55	9	7/14/2014	48:06.9
1382	RSPe_2	-122.445	37.929	1.12	15.03	287.67	16.15	-122.445	37.929	1.51	15.03	287.51	16.54	9	7/14/2014	48:06.8
1383	RSPe_2	-122.445	37.929	1.17	15.03	287.67	16.20	-122.445	37.929	1.55	15.03	287.51	16.58	9	7/14/2014	48:06.7
1384	RSPe_2	-122.445	37.929	1.17	15.01	287.64	16.18	-122.445	37.929	1.55	15.01	287.46	16.56	9	7/14/2014	48:06.6
1385	RSPe_2	-122.445	37.929	1.17	14.99	287.56	16.16	-122.445	37.929	1.51	14.99	287.42	16.50	9	7/14/2014	48:06.5

1386	RSPe_2	-122.445	37.929	1.17	14.99	287.56	16.16	-122.445	37.929	1.51	14.99	287.42	16.50	9	7/14/2014	48:06.4
1387	RSPe_2	-122.445	37.929	1.17	14.99	287.58	16.16	-122.445	37.929	1.58	14.99	287.45	16.57	9	7/14/2014	48:06.3
1388	RSPe_2	-122.445	37.929	1.12	14.91	287.54	16.03	-122.445	37.929	1.55	14.91	287.43	16.45	9	7/14/2014	48:06.2
1389	RSPe_2	-122.445	37.929	1.17	14.85	287.52	16.03	-122.445	37.929	1.58	14.85	287.45	16.43	9	7/14/2014	48:06.1
1390	RSPe_2	-122.445	37.929	1.12	14.86	287.52	15.98	-122.445	37.929	1.55	14.86	287.49	16.41	9	7/14/2014	48:06.0
1391	RSPe_2	-122.445	37.929	1.12	14.81	287.52	15.93	-122.445	37.929	1.55	14.81	287.49	16.36	9	7/14/2014	48:05.9
1392	RSPe_2	-122.445	37.929	1.12	14.81	287.47	15.93	-122.445	37.929	1.55	14.81	287.50	16.36	9	7/14/2014	48:05.8
1393	RSPe_2	-122.445	37.929	1.17	14.80	287.48	15.97	-122.445	37.929	1.58	14.80	287.49	16.38	9	7/14/2014	48:05.7
1394	RSPe_2	-122.445	37.929	1.12	14.78	287.45	15.90	-122.445	37.929	1.58	14.78	287.42	16.36	9	7/14/2014	48:05.6
1395	RSPe_2	-122.445	37.929	1.12	14.78	287.45	15.90	-122.445	37.929	1.58	14.78	287.45	16.36	9	7/14/2014	48:05.5
1396	RSPe_2	-122.445	37.929	1.09	14.75	287.43	15.83	-122.445	37.929	1.55	14.75	287.45	16.29	9	7/14/2014	48:05.4
1397	RSPe_2	-122.445	37.929	1.12	14.71	287.44	15.83	-122.445	37.929	1.58	14.71	287.41	16.30	9	7/14/2014	48:05.3
1398	RSPe_2	-122.445	37.929	1.12	14.73	287.41	15.85	-122.445	37.929	1.58	14.73	287.39	16.31	9	7/14/2014	48:05.2
1399	RSPe_2	-122.445	37.929	1.12	14.71	287.43	15.83	-122.445	37.929	1.58	14.71	287.41	16.30	9	7/14/2014	48:05.1
1400	RSPe_2	-122.445	37.929	1.09	14.71	287.35	15.80	-122.445	37.929	1.58	14.71	287.39	16.29	9	7/14/2014	48:05.0
1401	RSPe_2	-122.445	37.929	1.12	14.69	287.39	15.81	-122.445	37.929	1.58	14.69	287.39	16.27	9	7/14/2014	48:04.9
1402	RSPe_2	-122.445	37.929	1.09	14.71	287.30	15.80	-122.445	37.929	1.55	14.71	287.43	16.26	9	7/14/2014	48:04.8
1403	RSPe_2	-122.445	37.929	1.09	14.71	287.28	15.80	-122.445	37.929	1.58	14.71	287.39	16.30	9	7/14/2014	48:04.7
1404	RSPe_2	-122.445	37.929	1.09	14.71	287.28	15.80	-122.445	37.929	1.55	14.71	287.39	16.26	9	7/14/2014	48:04.6
1405	RSPe_2	-122.445	37.929	1.04	14.71	287.31	15.75	-122.445	37.929	1.58	14.71	287.42	16.30	9	7/14/2014	48:04.5
1406	RSPe_2	-122.445	37.929	1.04	14.75	287.22	15.79	-122.445	37.929	1.51	14.75	287.41	16.27	9	7/14/2014	48:04.4
1407	RSPe_2	-122.445	37.929	1.04	14.77	287.24	15.80	-122.445	37.929	1.51	14.77	287.46	16.28	9	7/14/2014	48:04.3
1408	RSPe_2	-122.445	37.929	1.00	14.79	287.20	15.79	-122.445	37.929	1.51	14.79	287.46	16.31	9	7/14/2014	48:04.2
1409	RSPe_2	-122.445	37.929	1.04	14.81	287.17	15.85	-122.445	37.929	1.51	14.81	287.43	16.33	9	7/14/2014	48:04.1
1410	RSPe_2	-122.445	37.929	1.00	14.82	287.17	15.82	-122.445	37.929	1.51	14.82	287.50	16.34	9	7/14/2014	48:04.0
1411	RSPe_2	-122.445	37.929	1.04	14.88	287.11	15.91	-122.445	37.929	1.51	14.88	287.46	16.39	9	7/14/2014	48:03.9
1412	RSPe_2	-122.445	37.929	1.00	14.86	287.09	15.86	-122.445	37.929	1.46	14.86	287.44	16.33	9	7/14/2014	48:03.8
1413	RSPe_2	-122.445	37.929	1.00	14.90	287.11	15.90	-122.445	37.929	1.46	14.90	287.46	16.37	9	7/14/2014	48:03.7
1414	RSPe_2	-122.445	37.929	0.95	14.94	287.07	15.89	-122.445	37.929	1.46	14.94	287.41	16.41	9	7/14/2014	48:03.6
1415	RSPe_2	-122.445	37.929	1.00	14.97	287.09	15.97	-122.445	37.929	1.46	14.97	287.42	16.43	9	7/14/2014	48:03.5
1416	RSPe_2	-122.445	37.929	0.95	15.02	287.07	15.97	-122.445	37.929	1.38	15.02	287.35	16.39	9	7/14/2014	48:03.4
1417	RSPe_2	-122.445	37.929	0.95	14.96	287.13	15.91	-122.445	37.929	1.43	14.96	287.37	16.39	9	7/14/2014	48:03.3
1418	RSPe_2	-122.445	37.929	0.95	15.03	287.13	15.98	-122.445	37.929	1.38	15.03	287.33	16.41	9	7/14/2014	48:03.2

1419	RSPe_2	-122.445	37.929	0.95	15.03	287.20	15.98	-122.445	37.929	1.38	15.03	287.31	16.41	9	7/14/2014	48:03.1
1420	RSPe_2	-122.445	37.929	0.92	15.08	287.16	15.99	-122.445	37.929	1.35	15.08	287.33	16.42	9	7/14/2014	48:03.0
1421	RSPe_2	-122.445	37.929	0.92	15.09	287.16	16.01	-122.445	37.929	1.35	15.09	287.24	16.44	9	7/14/2014	48:02.9
1422	RSPe_2	-122.445	37.929	0.92	15.10	287.16	16.02	-122.445	37.929	1.35	15.10	287.29	16.45	9	7/14/2014	48:02.8
1423	RSPe_2	-122.445	37.929	0.92	15.16	287.14	16.08	-122.445	37.929	1.29	15.16	287.20	16.46	9	7/14/2014	48:02.7
1424	RSPe_2	-122.445	37.929	0.92	15.20	287.09	16.12	-122.445	37.929	1.29	15.20	287.18	16.50	9	7/14/2014	48:02.6
1425	RSPe_2	-122.445	37.929	0.92	15.19	287.08	16.11	-122.445	37.929	1.29	15.19	287.18	16.49	9	7/14/2014	48:02.5
1426	RSPe_2	-122.445	37.929	0.87	15.25	287.05	16.11	-122.445	37.929	1.29	15.25	287.15	16.54	9	7/14/2014	48:02.4
1427	RSPe_2	-122.445	37.929	0.87	15.26	287.03	16.12	-122.445	37.929	1.29	15.26	287.11	16.55	9	7/14/2014	48:02.3
1428	RSPe_2	-122.445	37.929	0.87	15.29	287.01	16.15	-122.445	37.929	1.26	15.29	287.09	16.55	9	7/14/2014	48:02.2
1429	RSPe_2	-122.445	37.929	0.87	15.30	286.94	16.17	-122.445	37.929	1.29	15.30	287.09	16.60	9	7/14/2014	48:02.1
1430	RSPe_2	-122.445	37.929	0.83	15.31	286.92	16.14	-122.445	37.929	1.26	15.31	287.07	16.57	9	7/14/2014	48:02.0
1431	RSPe_2	-122.445	37.929	0.87	15.29	286.97	16.16	-122.445	37.929	1.29	15.29	287.12	16.59	9	7/14/2014	48:01.9
1432	RSPe_2	-122.445	37.929	0.83	15.36	286.99	16.19	-122.445	37.929	1.26	15.36	287.12	16.62	9	7/14/2014	48:01.8
1433	RSPe_2	-122.445	37.929	0.83	15.39	286.92	16.22	-122.445	37.929	1.26	15.39	287.14	16.66	9	7/14/2014	48:01.7
1434	RSPe_2	-122.445	37.929	0.83	15.32	287.01	16.15	-122.445	37.929	1.21	15.32	287.18	16.53	9	7/14/2014	48:01.6
1435	RSPe_2	-122.445	37.929	0.83	15.34	286.99	16.17	-122.445	37.929	1.21	15.34	287.23	16.55	9	7/14/2014	48:01.5
1436	RSPe_2	-122.445	37.929	0.80	15.40	286.97	16.20	-122.445	37.929	1.21	15.40	287.20	16.61	9	7/14/2014	48:01.4
1437	RSPe_2	-122.445	37.929	0.83	15.30	286.97	16.13	-122.445	37.929	1.21	15.30	287.21	16.51	9	7/14/2014	48:01.3
1438	RSPe_2	-122.445	37.929	0.80	15.32	286.99	16.12	-122.445	37.929	1.18	15.32	287.27	16.50	9	7/14/2014	48:01.2
1439	RSPe_2	-122.445	37.929	0.80	15.39	286.99	16.19	-122.445	37.929	1.18	15.39	287.25	16.57	9	7/14/2014	48:01.1
1440	RSPe_2	-122.445	37.929	0.75	15.38	286.99	16.12	-122.445	37.929	1.14	15.38	287.26	16.52	9	7/14/2014	48:01.0
1441	RSPe_2	-122.445	37.929	0.80	15.35	287.00	16.14	-122.445	37.929	1.14	15.35	287.27	16.49	9	7/14/2014	48:00.9
1442	RSPe_2	-122.445	37.929	0.75	15.36	286.97	16.10	-122.445	37.929	1.14	15.36	287.23	16.50	9	7/14/2014	48:00.8
1443	RSPe_2	-122.445	37.929	0.75	15.30	286.97	16.05	-122.445	37.929	1.18	15.30	287.23	16.48	9	7/14/2014	48:00.7
1444	RSPe_2	-122.445	37.929	0.75	15.36	287.01	16.10	-122.445	37.929	1.09	15.36	287.23	16.45	9	7/14/2014	48:00.6
1445	RSPe_2	-122.445	37.929	0.75	15.31	287.01	16.05	-122.445	37.929	1.14	15.31	287.21	16.45	9	7/14/2014	48:00.5
1446	RSPe_2	-122.445	37.929	0.75	15.29	287.04	16.03	-122.445	37.929	1.14	15.29	287.21	16.43	9	7/14/2014	48:00.4
1447	RSPe_2	-122.445	37.929	0.75	15.32	287.02	16.06	-122.445	37.929	1.14	15.32	287.19	16.46	9	7/14/2014	48:00.3
1448	RSPe_2	-122.445	37.929	0.75	15.30	287.08	16.05	-122.445	37.929	1.09	15.30	287.26	16.39	9	7/14/2014	48:00.2
1449	RSPe_2	-122.445	37.929	0.75	15.30	287.06	16.05	-122.445	37.929	1.09	15.30	287.23	16.39	9	7/14/2014	48:00.1
1450	RSPe_2	-122.445	37.929	0.75	15.31	287.11	16.05	-122.445	37.929	1.09	15.31	287.26	16.40	9	7/14/2014	48:00.0
1451	RSPe_2	-122.445	37.929	0.71	15.37	287.08	16.08	-122.445	37.929	1.09	15.37	287.33	16.46	9	7/14/2014	47:59.9

1452	RSPe_2	-122.445	37.929	0.66	15.36	287.11	16.01	-122.445	37.929	1.06	15.36	287.32	16.41	9	7/14/2014	47:59.8
1453	RSPe_2	-122.445	37.929	0.71	15.36	287.11	16.07	-122.445	37.929	1.06	15.36	287.36	16.42	9	7/14/2014	47:59.7
1454	RSPe_2	-122.445	37.929	0.66	15.39	287.11	16.05	-122.445	37.929	1.06	15.39	287.42	16.45	9	7/14/2014	47:59.6
1455	RSPe_2	-122.445	37.929	0.71	15.39	287.15	16.11	-122.445	37.929	1.06	15.39	287.43	16.45	9	7/14/2014	47:59.5
1456	RSPe_2	-122.445	37.929	0.66	15.46	287.13	16.12	-122.445	37.929	1.01	15.46	287.43	16.47	9	7/14/2014	47:59.4
1457	RSPe_2	-122.445	37.929	0.66	15.43	287.11	16.08	-122.445	37.929	1.01	15.43	287.43	16.43	9	7/14/2014	47:59.3
1458	RSPe_2	-122.445	37.929	0.66	15.44	287.15	16.10	-122.445	37.929	1.01	15.44	287.41	16.45	9	7/14/2014	47:59.2
1459	RSPe_2	-122.445	37.929	0.66	15.51	287.13	16.17	-122.445	37.929	1.01	15.51	287.37	16.52	9	7/14/2014	47:59.1
1460	RSPe_2	-122.445	37.929	0.66	15.49	287.13	16.15	-122.445	37.929	0.97	15.49	287.37	16.47	9	7/14/2014	47:59.0
1461	RSPe_2	-122.445	37.929	0.66	15.49	287.15	16.15	-122.445	37.929	0.97	15.49	287.37	16.47	9	7/14/2014	47:58.9
1462	RSPe_2	-122.445	37.929	0.63	15.50	287.13	16.13	-122.445	37.929	0.97	15.50	287.31	16.47	9	7/14/2014	47:58.8
1463	RSPe_2	-122.445	37.929	0.66	15.56	287.09	16.21	-122.445	37.929	0.97	15.56	287.29	16.53	9	7/14/2014	47:58.7
1464	RSPe_2	-122.445	37.929	0.57	15.56	287.12	16.13	-122.445	37.929	0.97	15.56	287.28	16.53	9	7/14/2014	47:58.6
1465	RSPe_2	-122.445	37.929	0.63	15.76	287.09	16.38	-122.445	37.929	0.92	15.76	287.29	16.68	9	7/14/2014	47:58.5
1466	RSPe_2	-122.445	37.929	0.63	15.73	287.05	16.35	-122.445	37.929	0.92	15.73	287.28	16.65	9	7/14/2014	47:58.4
1467	RSPe_2	-122.445	37.929	0.63	15.76	287.07	16.39	-122.445	37.929	0.92	15.76	287.27	16.68	9	7/14/2014	47:58.3
1468	RSPe_2	-122.445	37.929	0.57	15.63	287.02	16.21	-122.445	37.929	0.92	15.63	287.21	16.56	9	7/14/2014	47:58.2
1469	RSPe_2	-122.445	37.929	0.63	15.73	287.00	16.36	-122.445	37.929	0.92	15.73	287.24	16.66	9	7/14/2014	47:58.1
1470	RSPe_2	-122.445	37.929	0.57	15.77	287.02	16.35	-122.445	37.929	0.84	15.77	287.22	16.61	9	7/14/2014	47:58.0
1471	RSPe_2	-122.445	37.929	0.57	15.81	286.99	16.38	-122.445	37.929	0.92	15.81	287.20	16.73	9	7/14/2014	47:57.9
1472	RSPe_2	-122.445	37.929	0.54	15.84	286.94	16.38	-122.445	37.929	0.84	15.84	287.18	16.68	9	7/14/2014	47:57.8
1473	RSPe_2	-122.445	37.929	0.57	15.80	286.94	16.37	-122.445	37.929	0.89	15.80	287.14	16.69	9	7/14/2014	47:57.7
1474	RSPe_2	-122.445	37.929	0.54	15.86	286.92	16.40	-122.445	37.929	0.89	15.86	287.10	16.75	9	7/14/2014	47:57.6
1475	RSPe_2	-122.445	37.929	0.57	15.90	286.88	16.48	-122.445	37.929	0.89	15.90	287.05	16.79	9	7/14/2014	47:57.5
1476	RSPe_2	-122.445	37.929	0.54	15.86	286.90	16.40	-122.445	37.929	0.89	15.86	287.14	16.75	9	7/14/2014	47:57.4
1477	RSPe_2	-122.445	37.929	0.57	15.90	286.90	16.47	-122.445	37.929	0.89	15.90	287.09	16.78	9	7/14/2014	47:57.3
1478	RSPe_2	-122.445	37.929	0.54	15.86	286.85	16.40	-122.445	37.929	0.80	15.86	287.10	16.66	9	7/14/2014	47:57.2
1479	RSPe_2	-122.445	37.929	0.54	15.96	286.90	16.50	-122.445	37.929	0.89	15.96	287.14	16.84	9	7/14/2014	47:57.1
1480	RSPe_2	-122.445	37.929	0.49	15.93	286.90	16.42	-122.445	37.929	0.80	15.93	287.10	16.74	9	7/14/2014	47:57.0
1481	RSPe_2	-122.445	37.929	0.54	15.90	286.84	16.44	-122.445	37.929	0.80	15.90	287.12	16.71	9	7/14/2014	47:56.9
1482	RSPe_2	-122.445	37.929	0.45	15.94	286.88	16.39	-122.445	37.929	0.80	15.94	287.10	16.74	9	7/14/2014	47:56.8
1483	RSPe_2	-122.445	37.929	0.49	15.90	286.81	16.39	-122.445	37.929	0.80	15.90	287.10	16.71	9	7/14/2014	47:56.7
1484	RSPe_2	-122.445	37.929	0.45	15.94	286.81	16.39	-122.445	37.929	0.72	15.94	287.07	16.66	9	7/14/2014	47:56.6

1485	RSPe_2	-122.445	37.929	0.49	15.95	286.77	16.44	-122.445	37.929	0.77	15.95	287.05	16.72	9	7/14/2014	47:56.5
1486	RSPe_2	-122.445	37.929	0.45	15.96	286.70	16.41	-122.445	37.929	0.72	15.96	286.99	16.68	9	7/14/2014	47:56.4
1487	RSPe_2	-122.445	37.929	0.49	15.98	286.73	16.47	-122.445	37.929	0.72	15.98	287.01	16.70	9	7/14/2014	47:56.3
1488	RSPe_2	-122.445	37.929	0.45	15.96	286.69	16.41	-122.445	37.929	0.72	15.96	286.97	16.68	9	7/14/2014	47:56.2
1489	RSPe_2	-122.445	37.929	0.49	15.99	286.66	16.48	-122.445	37.929	0.72	15.99	286.91	16.71	9	7/14/2014	47:56.1
1490	RSPe_2	-122.445	37.929	0.45	15.95	286.64	16.40	-122.445	37.929	0.69	15.95	286.88	16.63	9	7/14/2014	47:56.0
1491	RSPe_2	-122.445	37.929	0.45	15.95	286.58	16.40	-122.445	37.929	0.72	15.95	286.83	16.67	9	7/14/2014	47:55.9
1492	RSPe_2	-122.445	37.929	0.45	15.97	286.58	16.42	-122.445	37.929	0.69	15.97	286.77	16.65	9	7/14/2014	47:55.8
1493	RSPe_2	-122.445	37.929	0.45	15.93	286.55	16.39	-122.445	37.929	0.72	15.93	286.75	16.65	9	7/14/2014	47:55.7
1494	RSPe_2	-122.445	37.929	0.42	15.93	286.60	16.35	-122.445	37.929	0.69	15.93	286.75	16.61	9	7/14/2014	47:55.6
1495	RSPe_2	-122.445	37.929	0.45	15.92	286.53	16.37	-122.445	37.929	0.69	15.92	286.70	16.60	9	7/14/2014	47:55.5
1496	RSPe_2	-122.445	37.929	0.42	15.93	286.51	16.35	-122.445	37.929	0.63	15.93	286.71	16.56	9	7/14/2014	47:55.4
1497	RSPe_2	-122.445	37.929	0.42	15.95	286.47	16.37	-122.445	37.929	0.69	15.95	286.69	16.63	9	7/14/2014	47:55.3
1498	RSPe_2	-122.445	37.929	0.37	15.95	286.49	16.32	-122.445	37.929	0.63	15.95	286.69	16.58	9	7/14/2014	47:55.2
1499	RSPe_2	-122.445	37.929	0.42	15.95	286.45	16.37	-122.445	37.929	0.63	15.95	286.66	16.58	9	7/14/2014	47:55.1
1500	RSPe_2	-122.445	37.929	0.37	15.95	286.43	16.32	-122.445	37.929	0.60	15.95	286.64	16.55	9	7/14/2014	47:55.0
1501	RSPe_2	-122.445	37.929	0.37	16.01	286.43	16.38	-122.445	37.929	0.60	16.01	286.64	16.61	9	7/14/2014	47:54.9
1502	RSPe_2	-122.445	37.929	0.37	15.97	286.40	16.33	-122.445	37.929	0.55	15.97	286.64	16.52	9	7/14/2014	47:54.8
1503	RSPe_2	-122.445	37.929	0.37	15.97	286.38	16.34	-122.445	37.929	0.60	15.97	286.62	16.57	9	7/14/2014	47:54.7
1504	RSPe_2	-122.445	37.929	0.37	15.98	286.34	16.35	-122.445	37.929	0.60	15.98	286.62	16.58	9	7/14/2014	47:54.6
1505	RSPe_2	-122.445	37.929	0.37	15.99	286.36	16.36	-122.445	37.929	0.60	15.99	286.66	16.59	9	7/14/2014	47:54.5
1506	RSPe_2	-122.445	37.929	0.33	16.00	286.32	16.33	-122.445	37.929	0.55	16.00	286.69	16.55	9	7/14/2014	47:54.4
1507	RSPe_2	-122.445	37.929	0.37	16.02	286.34	16.39	-122.445	37.929	0.55	16.02	286.67	16.57	9	7/14/2014	47:54.3
1508	RSPe_2	-122.445	37.929	0.33	16.01	286.28	16.34	-122.445	37.929	0.52	16.01	286.62	16.53	9	7/14/2014	47:54.2
1509	RSPe_2	-122.445	37.929	0.33	16.03	286.32	16.36	-122.445	37.929	0.52	16.03	286.65	16.54	9	7/14/2014	47:54.1
1510	RSPe_2	-122.445	37.929	0.28	16.02	286.23	16.30	-122.445	37.929	0.47	16.02	286.64	16.48	9	7/14/2014	47:54.0
1511	RSPe_2	-122.445	37.929	0.28	16.03	286.26	16.31	-122.445	37.929	0.47	16.03	286.58	16.49	9	7/14/2014	47:53.9
1512	RSPe_2	-122.445	37.929	0.28	16.04	286.21	16.32	-122.445	37.929	0.43	16.04	286.56	16.47	9	7/14/2014	47:53.8
1513	RSPe_2	-122.445	37.929	0.28	16.05	286.10	16.33	-122.445	37.929	0.43	16.05	286.47	16.48	9	7/14/2014	47:53.7
1514	RSPe_2	-122.445	37.929	0.28	16.06	286.06	16.35	-122.445	37.929	0.43	16.06	286.47	16.50	9	7/14/2014	47:53.6
1515	RSPe_2	-122.445	37.929	0.28	16.07	286.04	16.36	-122.445	37.929	0.40	16.07	286.39	16.47	9	7/14/2014	47:53.5
1516	RSPe_2	-122.445	37.929	0.25	16.10	285.97	16.35	-122.445	37.929	0.40	16.10	286.36	16.50	9	7/14/2014	47:53.4
1517	RSPe_2	-122.445	37.929	0.28	16.11	285.97	16.39	-122.445	37.929	0.40	16.11	286.34	16.51	9	7/14/2014	47:53.3

1518	RSPe_2	-122.445	37.929	0.28	16.14	285.99	16.43	-122.445	37.929	0.35	16.14	286.31	16.49	9	7/14/2014	47:53.2
1519	RSPe_2	-122.445	37.929	0.28	16.20	285.95	16.49	-122.445	37.929	0.40	16.20	286.21	16.60	9	7/14/2014	47:53.1
1520	RSPe_2	-122.445	37.929	0.25	16.20	285.95	16.45	-122.445	37.929	0.35	16.20	286.17	16.55	9	7/14/2014	47:53.0
1521	RSPe_2	-122.445	37.929	0.28	16.20	285.95	16.49	-122.445	37.929	0.35	16.20	286.12	16.55	9	7/14/2014	47:52.9
1522	RSPe_2	-122.445	37.929	0.28	16.23	285.95	16.51	-122.445	37.929	0.40	16.23	286.08	16.63	9	7/14/2014	47:52.8
1523	RSPe_2	-122.445	37.929	0.25	16.21	285.97	16.46	-122.445	37.929	0.35	16.21	286.08	16.56	9	7/14/2014	47:52.7
1524	RSPe_2	-122.445	37.929	0.25	16.23	285.98	16.48	-122.445	37.929	0.35	16.23	286.04	16.58	9	7/14/2014	47:52.6
1525	RSPe_2	-122.445	37.929	0.25	16.24	285.93	16.49	-122.445	37.929	0.35	16.24	286.02	16.59	9	7/14/2014	47:52.5
1526	RSPe_2	-122.445	37.929	0.25	16.27	285.91	16.52	-122.445	37.929	0.31	16.27	286.02	16.59	9	7/14/2014	47:52.4
1527	RSPe_2	-122.445	37.929	0.25	16.24	285.91	16.49	-122.445	37.929	0.35	16.24	285.99	16.59	9	7/14/2014	47:52.3
1528	RSPe_2	-122.445	37.929	0.20	16.27	285.89	16.46	-122.445	37.929	0.31	16.27	286.04	16.58	9	7/14/2014	47:52.2
1529	RSPe_2	-122.445	37.929	0.25	16.29	285.89	16.54	-122.445	37.929	0.31	16.29	286.02	16.60	9	7/14/2014	47:52.1
1530	RSPe_2	-122.445	37.929	0.20	16.28	285.85	16.48	-122.445	37.929	0.26	16.28	286.08	16.54	9	7/14/2014	47:52.0
1531	RSPe_2	-122.445	37.929	0.20	16.31	285.87	16.51	-122.445	37.929	0.31	16.31	286.09	16.63	9	7/14/2014	47:51.9
1532	RSPe_2	-122.445	37.929	0.20	16.29	285.82	16.49	-122.445	37.929	0.26	16.29	286.09	16.55	9	7/14/2014	47:51.8
1533	RSPe_2	-122.445	37.929	0.20	16.34	285.81	16.54	-122.445	37.929	0.26	16.34	286.07	16.60	9	7/14/2014	47:51.7
1534	RSPe_2	-122.445	37.929	0.20	16.35	285.81	16.55	-122.445	37.929	0.26	16.35	286.00	16.61	9	7/14/2014	47:51.6
1535	RSPe_2	-122.445	37.929	0.25	16.36	285.76	16.61	-122.445	37.929	0.31	16.36	285.98	16.67	9	7/14/2014	47:51.5
1536	RSPe_2	-122.445	37.929	0.20	16.41	285.74	16.60	-122.445	37.929	0.26	16.41	285.95	16.67	9	7/14/2014	47:51.4
1537	RSPe_2	-122.445	37.929	0.20	16.47	285.74	16.67	-122.445	37.929	0.31	16.47	285.92	16.79	9	7/14/2014	47:51.3
1538	RSPe_2	-122.445	37.929	0.20	16.43	285.67	16.63	-122.445	37.929	0.31	16.43	285.80	16.74	9	7/14/2014	47:51.2
1539	RSPe_2	-122.445	37.929	0.20	16.49	285.70	16.69	-122.445	37.929	0.26	16.49	285.84	16.75	9	7/14/2014	47:51.1
1540	RSPe_2	-122.445	37.929	0.20	16.48	285.74	16.68	-122.445	37.929	0.26	16.48	285.83	16.74	9	7/14/2014	47:51.0
1541	RSPe_2	-122.445	37.929	0.20	16.46	285.61	16.66	-122.445	37.929	0.26	16.46	285.69	16.72	9	7/14/2014	47:50.9
1542	RSPe_2	-122.445	37.929	0.20	16.44	285.63	16.64	-122.445	37.929	0.26	16.44	285.70	16.71	9	7/14/2014	47:50.8
1543	RSPe_2	-122.445	37.929	0.20	16.43	285.72	16.63	-122.445	37.929	0.26	16.43	285.65	16.69	9	7/14/2014	47:50.7
1544	RSPe_2	-122.445	37.929	0.20	16.54	285.61	16.74	-122.445	37.929	0.23	16.54	285.65	16.77	9	7/14/2014	47:50.6
1545	RSPe_2	-122.445	37.929	0.20	16.50	285.74	16.69	-122.445	37.929	0.26	16.50	285.67	16.76	9	7/14/2014	47:50.5
1546	RSPe_2	-122.445	37.929	0.16	16.47	285.72	16.64	-122.445	37.929	0.31	16.47	285.71	16.79	9	7/14/2014	47:50.4
1547	RSPe_2	-122.445	37.929	0.20	16.51	285.68	16.71	-122.445	37.929	0.26	16.51	285.68	16.77	9	7/14/2014	47:50.3
1548	RSPe_2	-122.445	37.929	0.16	16.38	285.64	16.55	-122.445	37.929	0.26	16.38	285.65	16.65	9	7/14/2014	47:50.2
1549	RSPe_2	-122.445	37.929	0.20	16.40	285.61	16.59	-122.445	37.929	0.26	16.40	285.61	16.66	9	7/14/2014	47:50.1
1550	RSPe_2	-122.445	37.929	0.16	16.46	285.59	16.62	-122.445	37.929	0.26	16.46	285.61	16.72	9	7/14/2014	47:50.0

1551	RSPe_2	-122.445	37.929	0.20	16.45	285.53	16.65	-122.445	37.929	0.26	16.45	285.61	16.71	9	7/14/2014	47:49.9
1552	RSPe_2	-122.445	37.929	0.16	16.46	285.48	16.62	-122.445	37.929	0.26	16.46	285.61	16.72	9	7/14/2014	47:49.8
1553	RSPe_2	-122.445	37.929	0.20	16.44	285.44	16.64	-122.445	37.929	0.26	16.44	285.55	16.70	9	7/14/2014	47:49.7
1554	RSPe_2	-122.445	37.929	0.16	16.45	285.37	16.62	-122.445	37.929	0.23	16.45	285.61	16.68	9	7/14/2014	47:49.6
1555	RSPe_2	-122.445	37.929	0.20	16.43	285.33	16.63	-122.445	37.929	0.23	16.43	285.55	16.66	9	7/14/2014	47:49.5
1556	RSPe_2	-122.445	37.929	0.16	16.46	285.29	16.62	-122.445	37.929	0.23	16.46	285.55	16.69	9	7/14/2014	47:49.4
1557	RSPe_2	-122.445	37.929	0.16	16.44	285.33	16.61	-122.445	37.929	0.26	16.44	285.51	16.71	9	7/14/2014	47:49.3
1558	RSPe_2	-122.445	37.929	0.16	16.42	285.27	16.58	-122.445	37.929	0.23	16.42	285.48	16.65	9	7/14/2014	47:49.2
1559	RSPe_2	-122.445	37.929	0.16	16.43	285.31	16.59	-122.445	37.929	0.26	16.43	285.44	16.69	9	7/14/2014	47:49.1
1560	RSPe_2	-122.445	37.929	0.13	16.46	285.33	16.59	-122.445	37.929	0.23	16.46	285.46	16.69	9	7/14/2014	47:49.0
1561	RSPe_2	-122.445	37.929	0.16	16.44	285.42	16.60	-122.445	37.929	0.23	16.44	285.44	16.67	9	7/14/2014	47:48.9
1562	RSPe_2	-122.445	37.929	0.16	16.46	285.38	16.62	-122.445	37.929	0.23	16.46	285.37	16.69	9	7/14/2014	47:48.8
1563	RSPe_2	-122.445	37.929	0.16	16.44	285.35	16.60	-122.445	37.929	0.26	16.44	285.33	16.70	9	7/14/2014	47:48.7
1564	RSPe_2	-122.445	37.929	0.13	16.43	285.34	16.56	-122.445	37.929	0.31	16.43	285.33	16.74	9	7/14/2014	47:48.6
1565	RSPe_2	-122.445	37.929	0.16	16.41	285.36	16.57	-122.445	37.929	0.26	16.41	285.31	16.67	9	7/14/2014	47:48.5
1566	RSPe_2	-122.445	37.929	0.13	16.40	285.34	16.53	-122.445	37.929	0.23	16.40	285.31	16.62	9	7/14/2014	47:48.4
1567	RSPe_2	-122.445	37.929	0.16	16.38	285.33	16.55	-122.445	37.929	0.26	16.38	285.33	16.65	9	7/14/2014	47:48.3
1568	RSPe_2	-122.445	37.929	0.16	16.38	285.29	16.55	-122.445	37.929	0.23	16.38	285.31	16.61	9	7/14/2014	47:48.2
1569	RSPe_2	-122.445	37.929	0.16	16.40	285.27	16.56	-122.445	37.929	0.26	16.40	285.36	16.66	9	7/14/2014	47:48.1
1570	RSPe_2	-122.445	37.929	0.13	16.43	285.27	16.56	-122.445	37.929	0.18	16.43	285.39	16.61	9	7/14/2014	47:48.0
1571	RSPe_2	-122.445	37.929	0.13	16.41	285.25	16.54	-122.445	37.929	0.18	16.41	285.37	16.58	9	7/14/2014	47:47.9
1572	RSPe_2	-122.445	37.929	0.13	16.41	285.20	16.54	-122.445	37.929	0.18	16.41	285.36	16.59	9	7/14/2014	47:47.8
1573	RSPe_2	-122.445	37.929	0.16	16.44	285.17	16.61	-122.445	37.929	0.23	16.44	285.40	16.67	9	7/14/2014	47:47.7
1574	RSPe_2	-122.445	37.929	0.13	16.44	285.21	16.57	-122.445	37.929	0.23	16.44	285.36	16.67	9	7/14/2014	47:47.6
1575	RSPe_2	-122.445	37.929	0.13	16.44	285.16	16.57	-122.445	37.929	0.23	16.44	285.34	16.67	9	7/14/2014	47:47.5
1576	RSPe_2	-122.445	37.929	0.08	16.45	285.17	16.53	-122.445	37.929	0.23	16.45	285.32	16.68	9	7/14/2014	47:47.4
1577	RSPe_2	-122.445	37.929	0.13	16.46	285.14	16.59	-122.445	37.929	0.18	16.46	285.27	16.64	9	7/14/2014	47:47.3
1578	RSPe_2	-122.445	37.929	0.13	16.50	285.12	16.63	-122.445	37.929	0.18	16.50	285.25	16.68	9	7/14/2014	47:47.2
1579	RSPe_2	-122.445	37.929	0.13	16.51	285.10	16.64	-122.445	37.929	0.18	16.51	285.22	16.69	9	7/14/2014	47:47.1
1580	RSPe_2	-122.445	37.929	0.13	16.53	285.07	16.66	-122.445	37.929	0.15	16.53	285.21	16.68	9	7/14/2014	47:47.0
1581	RSPe_2	-122.445	37.929	0.13	16.55	285.06	16.68	-122.445	37.929	0.18	16.55	285.18	16.73	9	7/14/2014	47:46.9
1582	RSPe_2	-122.445	37.929	0.13	16.62	284.95	16.75	-122.445	37.929	0.15	16.62	285.10	16.77	9	7/14/2014	47:46.8
1583	RSPe_2	-122.445	37.929	0.13	16.61	284.92	16.74	-122.445	37.929	0.15	16.61	285.10	16.76	9	7/14/2014	47:46.7

1584	RSPe_2	-122.445	37.929	0.08	16.80	284.91	16.88	-122.445	37.929	0.15	16.80	285.07	16.94	9	7/14/2014	47:46.6
1585	RSPe_2	-122.445	37.929	0.13	16.56	284.88	16.69	-122.445	37.929	0.15	16.56	285.05	16.70	9	7/14/2014	47:46.5
1586	RSPe_2	-122.445	37.929	0.08	16.69	284.86	16.77	-122.445	37.929	0.15	16.69	285.05	16.83	9	7/14/2014	47:46.4
1587	RSPe_2	-122.445	37.929	0.13	16.77	284.84	16.90	-122.445	37.929	0.15	16.77	285.01	16.92	9	7/14/2014	47:46.3
1588	RSPe_2	-122.445	37.929	0.08	16.68	284.84	16.76	-122.445	37.929	0.11	16.68	285.05	16.79	9	7/14/2014	47:46.2
1589	RSPe_2	-122.445	37.929	0.08	16.72	284.86	16.79	-122.445	37.929	0.15	16.72	285.05	16.86	9	7/14/2014	47:46.1
1590	RSPe_2	-122.445	37.929	0.08	16.67	284.91	16.75	-122.445	37.929	0.11	16.67	285.03	16.78	9	7/14/2014	47:46.0
1591	RSPe_2	-122.445	37.929	0.08	16.69	284.91	16.77	-122.445	37.929	0.15	16.69	284.99	16.83	9	7/14/2014	47:45.9
1592	RSPe_2	-122.445	37.929	0.08	16.66	284.89	16.74	-122.445	37.929	0.06	16.66	284.96	16.72	9	7/14/2014	47:45.8
1593	RSPe_2	-122.445	37.929	0.08	16.74	284.91	16.82	-122.445	37.929	0.11	16.74	284.95	16.85	9	7/14/2014	47:45.7
1594	RSPe_2	-122.445	37.929	0.08	16.70	284.85	16.78	-122.445	37.929	0.06	16.70	284.90	16.76	9	7/14/2014	47:45.6
1595	RSPe_2	-122.445	37.929	0.08	16.68	284.84	16.76	-122.445	37.929	0.06	16.68	284.90	16.74	9	7/14/2014	47:45.5
1596	RSPe_2	-122.445	37.929	0.08	16.68	284.86	16.76	-122.445	37.929	0.06	16.68	284.90	16.74	9	7/14/2014	47:45.4
1597	RSPe_2	-122.445	37.929	0.08	16.70	284.87	16.78	-122.445	37.929	0.03	16.70	284.91	16.72	9	7/14/2014	47:45.3
1598	RSPe_2	-122.445	37.929	0.04	16.67	284.87	16.71	-122.445	37.929	0.03	16.67	284.92	16.69	9	7/14/2014	47:45.2
1599	RSPe_2	-122.445	37.929	0.08	16.67	284.93	16.75	-122.445	37.929	0.06	16.67	284.91	16.73	9	7/14/2014	47:45.1
1600	RSPe_2	-122.445	37.929	0.04	16.70	284.95	16.75	-122.445	37.929	0.03	16.70	284.97	16.73	9	7/14/2014	47:45.0
1601	RSPe_2	-122.445	37.929	0.08	16.70	284.96	16.78	-122.445	37.929	0.06	16.70	284.93	16.76	9	7/14/2014	47:44.9
1602	RSPe_2	-122.445	37.929	0.04	16.70	284.89	16.74	-122.445	37.929	-0.02	16.70	284.91	16.67	9	7/14/2014	47:44.8
1603	RSPe_2	-122.445	37.929	0.08	16.68	284.89	16.76	-122.445	37.929	0.06	16.68	284.87	16.74	9	7/14/2014	47:44.7
1604	RSPe_2	-122.445	37.929	0.04	16.74	284.85	16.78	-122.445	37.929	0.03	16.74	284.80	16.76	9	7/14/2014	47:44.6
1605	RSPe_2	-122.445	37.929	0.08	16.70	284.80	16.78	-122.445	37.929	0.06	16.70	284.80	16.76	9	7/14/2014	47:44.5
1606	RSPe_2	-122.445	37.929	0.04	16.76	284.76	16.80	-122.445	37.929	0.03	16.76	284.80	16.79	9	7/14/2014	47:44.4
1607	RSPe_2	-122.445	37.929	0.04	16.74	284.74	16.79	-122.445	37.929	0.03	16.74	284.80	16.77	9	7/14/2014	47:44.3
1608	RSPe_2	-122.445	37.929	0.04	16.77	284.70	16.82	-122.445	37.929	-0.02	16.77	284.80	16.75	9	7/14/2014	47:44.2
1609	RSPe_2	-122.445	37.929	0.08	16.81	284.72	16.88	-122.445	37.929	0.03	16.81	284.83	16.83	9	7/14/2014	47:44.1
1610	RSPe_2	-122.445	37.929	0.04	16.82	284.67	16.87	-122.445	37.929	-0.02	16.82	284.78	16.80	9	7/14/2014	47:44.0
1611	RSPe_2	-122.445	37.929	0.08	16.85	284.70	16.93	-122.445	37.929	0.06	16.85	284.83	16.91	9	7/14/2014	47:43.9
1612	RSPe_2	-122.445	37.929	0.08	16.89	284.70	16.97	-122.445	37.929	0.06	16.89	284.78	16.95	9	7/14/2014	47:43.8
1613	RSPe_2	-122.445	37.929	0.08	16.90	284.76	16.98	-122.445	37.929	0.06	16.90	284.74	16.96	9	7/14/2014	47:43.7
1614	RSPe_2	-122.445	37.929	0.08	16.97	284.74	17.05	-122.445	37.929	0.06	16.97	284.67	17.03	9	7/14/2014	47:43.6
1615	RSPe_2	-122.445	37.929	0.08	16.93	284.76	17.01	-122.445	37.929	0.06	16.93	284.63	16.99	9	7/14/2014	47:43.5
1616	RSPe_2	-122.445	37.929	0.08	16.95	284.75	17.03	-122.445	37.929	0.06	16.95	284.57	17.01	9	7/14/2014	47:43.4

1617	RSPe_2	-122.445	37.929	0.08	16.91	284.72	16.99	-122.445	37.929	0.06	16.91	284.48	16.97	9	7/14/2014 47:43.3
1618	RSPe_2	-122.445	37.929	0.04	16.90	284.61	16.94	-122.445	37.929	0.06	16.90	284.41	16.96	9	7/14/2014 47:43.2
1619	RSPe_2	-122.445	37.929	0.08	16.87	284.57	16.94	-122.445	37.929	0.06	16.87	284.43	16.93	9	7/14/2014 47:43.1
1620	RSPe_2	-122.445	37.929	0.04	16.88	284.59	16.93	-122.445	37.929	0.03	16.88	284.43	16.91	9	7/14/2014 47:43.0
1621	RSPe_2	-122.445	37.929	0.08	16.86	284.50	16.94	-122.445	37.929	0.06	16.86	284.45	16.92	9	7/14/2014 47:42.9
1622	RSPe_2	-122.445	37.929	0.04	16.86	284.46	16.90	-122.445	37.929	0.03	16.86	284.54	16.89	9	7/14/2014 47:42.8
1623	RSPe_2	-122.445	37.929	0.04	16.88	284.46	16.93	-122.445	37.929	0.03	16.88	284.57	16.91	9	7/14/2014 47:42.7
1624	RSPe_2	-122.445	37.929	0.04	16.94	284.51	16.99	-122.445	37.929	-0.02	16.94	284.70	16.92	9	7/14/2014 47:42.6
1625	RSPe_2	-122.445	37.929	0.08	16.92	284.44	17.00	-122.445	37.929	0.03	16.92	284.63	16.95	9	7/14/2014 47:42.5
1626	RSPe_2	-122.445	37.929	0.04	16.89	284.35	16.94	-122.445	37.929	0.03	16.89	284.57	16.92	9	7/14/2014 47:42.4
1627	RSPe_2	-122.445	37.929	0.08	16.88	284.33	16.95	-122.445	37.929	0.06	16.88	284.55	16.94	9	7/14/2014 47:42.3
1628	RSPe_2	-122.445	37.929	0.04	16.98	284.35	17.03	-122.445	37.929	0.03	16.98	284.48	17.01	9	7/14/2014 47:42.2
1629	RSPe_2	-122.445	37.929	0.08	16.87	284.33	16.94	-122.445	37.929	0.03	16.87	284.43	16.89	9	7/14/2014 47:42.1
1630	RSPe_2	-122.445	37.929	0.04	16.91	284.33	16.96	-122.445	37.929	0.03	16.91	284.37	16.94	9	7/14/2014 47:42.0
1631	RSPe_2	-122.445	37.929	0.04	16.81	284.35	16.86	-122.445	37.929	0.03	16.81	284.31	16.84	9	7/14/2014 47:41.9
1632	RSPe_2	-122.445	37.929	0.04	16.79	284.38	16.83	-122.445	37.929	0.03	16.79	284.30	16.82	9	7/14/2014 47:41.8
1633	RSPe_2	-122.445	37.929	0.08	16.73	284.38	16.81	-122.445	37.929	0.03	16.73	284.24	16.75	9	7/14/2014 47:41.7
1634	RSPe_2	-122.445	37.929	-0.01	16.74	284.40	16.74	-122.445	37.929	0.03	16.74	284.22	16.77	9	7/14/2014 47:41.6
1635	RSPe_2	-122.445	37.929	0.04	16.65	284.38	16.69	-122.445	37.929	0.03	16.65	284.22	16.68	9	7/14/2014 47:41.5
1636	RSPe_2	-122.445	37.929	0.04	16.68	284.36	16.73	-122.445	37.929	-0.02	16.68	284.17	16.66	9	7/14/2014 47:41.4
1637	RSPe_2	-122.445	37.929	0.08	16.65	284.36	16.72	-122.445	37.929	0.06	16.65	284.13	16.71	9	7/14/2014 47:41.3
1638	RSPe_2	-122.445	37.929	0.04	16.68	284.32	16.73	-122.445	37.929	-0.02	16.68	284.18	16.66	9	7/14/2014 47:41.2
1639	RSPe_2	-122.445	37.929	0.08	16.68	284.33	16.76	-122.445	37.929	0.06	16.68	284.15	16.74	9	7/14/2014 47:41.1
1640	RSPe_2	-122.445	37.929	0.04	16.67	284.32	16.72	-122.445	37.929	-0.02	16.67	284.20	16.65	9	7/14/2014 47:41.0
1641	RSPe_2	-122.445	37.929	0.04	16.72	284.32	16.76	-122.445	37.929	0.03	16.72	284.22	16.74	9	7/14/2014 47:40.9
1642	RSPe_2	-122.445	37.929	0.04	16.77	284.34	16.81	-122.445	37.929	-0.02	16.77	284.27	16.74	9	7/14/2014 47:40.8
1643	RSPe_2	-122.445	37.929	0.04	16.75	284.34	16.80	-122.445	37.929	0.03	16.75	284.22	16.78	9	7/14/2014 47:40.7
1644	RSPe_2	-122.445	37.929	-0.01	16.83	284.36	16.82	-122.445	37.929	-0.02	16.83	284.24	16.80	9	7/14/2014 47:40.6
1645	RSPe_2	-122.445	37.929	0.04	16.81	284.45	16.86	-122.445	37.929	0.06	16.81	284.27	16.87	9	7/14/2014 47:40.5
1646	RSPe_2	-122.445	37.929	0.04	16.87	284.42	16.91	-122.445	37.929	0.03	16.87	284.21	16.89	9	7/14/2014 47:40.4
1647	RSPe_2	-122.445	37.929	0.04	16.85	284.39	16.90	-122.445	37.929	0.03	16.85	284.21	16.88	9	7/14/2014 47:40.3
1648	RSPe_2	-122.445	37.929	0.04	16.90	284.32	16.94	-122.445	37.929	0.06	16.90	284.16	16.96	9	7/14/2014 47:40.2
1649	RSPe_2	-122.445	37.929	-0.01	16.80	284.25	16.79	-122.445	37.929	0.06	16.80	284.16	16.86	9	7/14/2014 47:40.1

1650	RSPe_2	-122.445	37.929	0.04	16.77	284.14	16.81	-122.445	37.929	0.06	16.77	284.17	16.83	9	7/14/2014	47:40.0
1651	RSPe_2	-122.445	37.929	0.04	16.79	284.12	16.83	-122.445	37.929	0.11	16.79	284.18	16.90	9	7/14/2014	47:39.9
1652	RSPe_2	-122.445	37.929	0.04	16.82	284.01	16.87	-122.445	37.929	0.06	16.82	284.16	16.88	9	7/14/2014	47:39.8
1653	RSPe_2	-122.445	37.929	0.04	16.85	284.06	16.90	-122.445	37.929	0.06	16.85	284.27	16.91	9	7/14/2014	47:39.7
1654	RSPe_2	-122.445	37.929	-0.01	16.91	284.10	16.91	-122.445	37.929	0.03	16.91	284.34	16.94	9	7/14/2014	47:39.6
1655	RSPe_2	-122.445	37.929	0.04	16.96	284.17	17.01	-122.445	37.929	0.06	16.96	284.32	17.02	9	7/14/2014	47:39.5
1656	RSPe_2	-122.445	37.929	-0.01	17.02	284.25	17.01	-122.445	37.929	-0.02	17.02	284.25	17.00	9	7/14/2014	47:39.4
1657	RSPe_2	-122.445	37.929	0.04	16.98	284.23	17.03	-122.445	37.929	0.03	16.98	284.16	17.01	9	7/14/2014	47:39.3
1658	RSPe_2	-122.445	37.929	-0.01	16.98	284.30	16.98	-122.445	37.929	-0.02	16.98	284.11	16.96	9	7/14/2014	47:39.2
1659	RSPe_2	-122.445	37.929	0.05	17.05	284.28	17.09	-122.445	37.929	0.03	17.05	284.03	17.07	9	7/14/2014	47:39.1
1660	RSPe_2	-122.445	37.929	-0.01	17.06	284.28	17.05	-122.445	37.929	0.03	17.06	284.01	17.09	9	7/14/2014	47:39.0
1661	RSPe_2	-122.445	37.929	0.05	16.94	284.26	16.99	-122.445	37.929	0.03	16.94	283.97	16.97	9	7/14/2014	47:38.9
1662	RSPe_2	-122.445	37.929	-0.01	16.94	284.23	16.94	-122.445	37.929	-0.02	16.94	284.03	16.92	9	7/14/2014	47:38.8
1663	RSPe_2	-122.445	37.929	0.05	16.90	284.21	16.94	-122.445	37.929	0.06	16.90	284.06	16.96	9	7/14/2014	47:38.7
1664	RSPe_2	-122.445	37.929	-0.01	16.84	284.19	16.84	-122.445	37.929	0.03	16.84	284.10	16.87	9	7/14/2014	47:38.6
1665	RSPe_2	-122.445	37.929	0.05	16.86	284.11	16.91	-122.445	37.929	-0.02	16.86	284.08	16.84	9	7/14/2014	47:38.5
1666	RSPe_2	-122.445	37.929	0.05	16.81	284.17	16.86	-122.445	37.929	0.03	16.81	284.15	16.84	9	7/14/2014	47:38.4
1667	RSPe_2	-122.445	37.929	0.05	16.81	284.13	16.85	-122.445	37.929	0.06	16.81	284.17	16.87	9	7/14/2014	47:38.3
1668	RSPe_2	-122.445	37.929	0.05	16.82	284.11	16.87	-122.445	37.929	0.03	16.82	284.10	16.85	9	7/14/2014	47:38.2
1669	RSPe_2	-122.445	37.929	0.05	16.84	284.08	16.88	-122.445	37.929	0.03	16.84	284.10	16.86	9	7/14/2014	47:38.1
1670	RSPe_2	-122.445	37.929	-0.01	16.80	284.06	16.79	-122.445	37.929	0.03	16.80	284.08	16.82	9	7/14/2014	47:38.0
1671	RSPe_2	-122.445	37.929	0.05	16.77	284.06	16.81	-122.445	37.929	0.06	16.77	284.08	16.83	9	7/14/2014	47:37.9
1672	RSPe_2	-122.445	37.929	-0.01	16.79	284.06	16.78	-122.445	37.929	0.03	16.79	284.08	16.82	9	7/14/2014	47:37.8
1673	RSPe_2	-122.445	37.929	0.05	16.77	284.11	16.82	-122.445	37.929	-0.02	16.77	284.08	16.75	9	7/14/2014	47:37.7
1674	RSPe_2	-122.445	37.929	-0.01	16.74	284.13	16.73	-122.445	37.929	-0.02	16.74	284.06	16.71	9	7/14/2014	47:37.6
1675	RSPe_2	-122.445	37.929	0.05	16.77	284.11	16.82	-122.445	37.929	-0.02	16.77	284.04	16.75	9	7/14/2014	47:37.5
1676	RSPe_2	-122.445	37.929	-0.01	16.72	284.11	16.72	-122.445	37.929	-0.02	16.72	283.98	16.70	9	7/14/2014	47:37.4
1677	RSPe_2	-122.445	37.929	0.05	16.81	284.05	16.85	-122.445	37.929	0.03	16.81	283.93	16.83	9	7/14/2014	47:37.3
1678	RSPe_2	-122.445	37.929	-0.01	16.79	284.09	16.78	-122.445	37.929	-0.02	16.79	283.91	16.77	9	7/14/2014	47:37.2
1679	RSPe_2	-122.445	37.929	0.05	16.76	284.06	16.80	-122.445	37.929	0.03	16.76	283.98	16.79	9	7/14/2014	47:37.1
1680	RSPe_2	-122.445	37.929	-0.01	16.77	284.14	16.76	-122.445	37.929	0.03	16.77	284.02	16.79	9	7/14/2014	47:37.0
1681	RSPe_2	-122.445	37.929	0.05	16.78	284.15	16.83	-122.445	37.929	-0.02	16.78	284.04	16.76	9	7/14/2014	47:36.9
1682	RSPe_2	-122.445	37.929	-0.01	16.81	284.18	16.81	-122.445	37.929	-0.02	16.81	284.00	16.79	9	7/14/2014	47:36.8

1683	RSPe_2	-122.445	37.929	0.05	16.80	284.11	16.84	-122.445	37.929	0.06	16.80	284.05	16.86	9	7/14/2014	47:36.7
1684	RSPe_2	-122.445	37.929	-0.01	16.84	284.16	16.83	-122.445	37.929	0.03	16.84	284.02	16.86	9	7/14/2014	47:36.6
1685	RSPe_2	-122.445	37.929	0.05	16.91	284.11	16.95	-122.445	37.929	0.06	16.91	284.05	16.97	9	7/14/2014	47:36.5
1686	RSPe_2	-122.445	37.929	-0.01	17.00	284.09	17.00	-122.445	37.929	0.03	17.00	284.04	17.03	9	7/14/2014	47:36.4
1687	RSPe_2	-122.445	37.929	-0.01	16.98	284.09	16.97	-122.445	37.929	0.06	16.98	284.00	17.04	9	7/14/2014	47:36.3
1688	RSPe_2	-122.445	37.929	-0.01	17.00	284.09	16.99	-122.445	37.929	0.03	17.00	284.05	17.03	9	7/14/2014	47:36.2
1689	RSPe_2	-122.445	37.929	-0.01	17.03	284.09	17.03	-122.445	37.929	0.11	17.03	284.09	17.14	9	7/14/2014	47:36.1
1690	RSPe_2	-122.445	37.929	-0.01	17.07	284.14	17.06	-122.445	37.929	-0.02	17.07	284.10	17.05	9	7/14/2014	47:36.0
1691	RSPe_2	-122.445	37.929	0.05	17.04	284.18	17.08	-122.445	37.929	0.03	17.04	284.14	17.06	9	7/14/2014	47:35.9
1692	RSPe_2	-122.445	37.929	-0.01	17.06	284.27	17.05	-122.445	37.929	-0.02	17.06	284.18	17.04	9	7/14/2014	47:35.8
1693	RSPe_2	-122.445	37.929	-0.01	17.09	284.21	17.08	-122.445	37.929	-0.02	17.09	284.11	17.07	9	7/14/2014	47:35.7
1694	RSPe_2	-122.445	37.929	-0.01	17.06	284.23	17.05	-122.445	37.929	0.03	17.06	284.18	17.09	9	7/14/2014	47:35.6
1695	RSPe_2	-122.445	37.929	0.05	17.08	284.21	17.12	-122.445	37.929	0.06	17.08	284.18	17.14	9	7/14/2014	47:35.5
1696	RSPe_2	-122.445	37.929	-0.01	17.08	284.21	17.08	-122.445	37.929	0.03	17.08	284.18	17.11	9	7/14/2014	47:35.4
1697	RSPe_2	-122.445	37.929	0.05	17.06	284.14	17.10	-122.445	37.929	0.03	17.06	284.14	17.09	9	7/14/2014	47:35.3
1698	RSPe_2	-122.445	37.929	-0.01	17.05	284.14	17.04	-122.445	37.929	-0.02	17.05	284.12	17.02	9	7/14/2014	47:35.2
1699	RSPe_2	-122.445	37.929	-0.01	17.02	284.10	17.01	-122.445	37.929	-0.02	17.02	284.10	17.00	9	7/14/2014	47:35.1
1700	RSPe_2	-122.445	37.929	-0.01	17.03	284.10	17.03	-122.445	37.929	-0.02	17.03	284.12	17.01	9	7/14/2014	47:35.0
1701	RSPe_2	-122.445	37.929	0.05	17.05	284.10	17.10	-122.445	37.929	0.06	17.05	284.12	17.11	9	7/14/2014	47:34.9
1702	RSPe_2	-122.445	37.929	-0.01	17.09	284.10	17.08	-122.445	37.929	-0.02	17.09	284.12	17.07	9	7/14/2014	47:34.8
1703	RSPe_2	-122.445	37.929	0.05	17.01	284.14	17.06	-122.445	37.929	0.03	17.01	284.14	17.04	9	7/14/2014	47:34.7
1704	RSPe_2	-122.445	37.929	-0.01	17.03	284.21	17.03	-122.445	37.929	-0.02	17.03	284.14	17.01	9	7/14/2014	47:34.6
1705	RSPe_2	-122.445	37.929	-0.01	16.99	284.26	16.98	-122.445	37.929	-0.02	16.99	284.16	16.97	9	7/14/2014	47:34.5
1706	RSPe_2	-122.445	37.929	-0.01	16.98	284.28	16.97	-122.445	37.929	-0.02	16.98	284.12	16.95	9	7/14/2014	47:34.4
1707	RSPe_2	-122.445	37.929	-0.01	17.01	284.28	17.01	-122.445	37.929	0.03	17.01	284.12	17.04	9	7/14/2014	47:34.3
1708	RSPe_2	-122.445	37.929	-0.01	16.94	284.26	16.93	-122.445	37.929	-0.02	16.94	284.08	16.91	9	7/14/2014	47:34.2
1709	RSPe_2	-122.445	37.929	0.05	16.94	284.20	16.98	-122.445	37.929	-0.02	16.94	284.02	16.91	9	7/14/2014	47:34.1
1710	RSPe_2	-122.445	37.929	-0.01	16.91	284.15	16.90	-122.445	37.929	-0.02	16.91	284.08	16.88	9	7/14/2014	47:34.0
1711	RSPe_2	-122.445	37.929	-0.01	16.90	284.11	16.89	-122.445	37.929	-0.02	16.90	284.06	16.88	9	7/14/2014	47:33.9
1712	RSPe_2	-122.445	37.929	-0.01	16.90	284.13	16.89	-122.445	37.929	-0.02	16.90	284.08	16.88	9	7/14/2014	47:33.8
1713	RSPe_2	-122.445	37.929	-0.01	16.89	284.06	16.89	-122.445	37.929	0.03	16.89	284.10	16.92	9	7/14/2014	47:33.7
1714	RSPe_2	-122.445	37.929	-0.01	16.89	284.11	16.89	-122.445	37.929	0.03	16.89	284.19	16.92	9	7/14/2014	47:33.6
1715	RSPe_2	-122.445	37.929	0.05	16.91	284.13	16.96	-122.445	37.929	0.03	16.91	284.14	16.94	9	7/14/2014	47:33.5

1716	RSPe_2	-122.445	37.929	-0.01	16.91	284.13	16.90	-122.445	37.929	0.03	16.91	284.13	16.93	9	7/14/2014	47:33.4
1717	RSPe_2	-122.445	37.929	0.05	16.97	284.20	17.01	-122.445	37.929	-0.02	16.97	284.17	16.94	9	7/14/2014	47:33.3
1718	RSPe_2	-122.445	37.929	-0.01	16.94	284.13	16.93	-122.445	37.929	-0.02	16.94	284.06	16.91	9	7/14/2014	47:33.2
1719	RSPe_2	-122.445	37.929	-0.01	16.94	284.15	16.93	-122.445	37.929	0.03	16.94	284.04	16.96	9	7/14/2014	47:33.1
1720	RSPe_2	-122.445	37.929	-0.04	16.93	284.17	16.89	-122.445	37.929	-0.02	16.93	284.02	16.91	9	7/14/2014	47:33.0
1721	RSPe_2	-122.445	37.929	0.05	16.94	284.11	16.99	-122.445	37.929	0.03	16.94	283.97	16.97	9	7/14/2014	47:32.9
1722	RSPe_2	-122.445	37.929	-0.01	16.98	284.11	16.98	-122.445	37.929	0.03	16.98	284.01	17.01	9	7/14/2014	47:32.8
1723	RSPe_2	-122.445	37.929	-0.01	16.93	284.04	16.92	-122.445	37.929	0.06	16.93	283.99	16.99	9	7/14/2014	47:32.7
1724	RSPe_2	-122.445	37.929	-0.01	16.96	284.05	16.96	-122.445	37.929	-0.02	16.96	284.11	16.94	9	7/14/2014	47:32.6
1725	RSPe_2	-122.445	37.929	-0.01	16.96	284.09	16.96	-122.445	37.929	0.03	16.96	284.15	16.99	9	7/14/2014	47:32.5
1726	RSPe_2	-122.445	37.929	-0.01	16.99	284.05	16.98	-122.445	37.929	-0.02	16.99	284.20	16.97	9	7/14/2014	47:32.4
1727	RSPe_2	-122.445	37.929	-0.01	17.12	284.11	17.12	-122.445	37.929	0.03	17.12	284.20	17.15	9	7/14/2014	47:32.3
1728	RSPe_2	-122.445	37.929	-0.01	17.16	284.14	17.15	-122.445	37.929	-0.06	17.16	284.19	17.10	9	7/14/2014	47:32.2
1729	RSPe_2	-122.445	37.929	-0.01	17.18	284.09	17.17	-122.445	37.929	-0.06	17.18	284.11	17.12	9	7/14/2014	47:32.1
1730	RSPe_2	-122.445	37.929	-0.01	17.24	284.05	17.23	-122.445	37.929	-0.06	17.24	284.08	17.18	9	7/14/2014	47:32.0
1731	RSPe_2	-122.445	37.929	-0.01	17.23	284.05	17.22	-122.445	37.929	-0.02	17.23	284.02	17.21	9	7/14/2014	47:31.9
1732	RSPe_2	-122.445	37.929	-0.01	17.18	284.00	17.18	-122.445	37.929	-0.02	17.18	283.94	17.16	9	7/14/2014	47:31.8
1733	RSPe_2	-122.445	37.929	-0.01	17.24	283.98	17.23	-122.445	37.929	-0.02	17.24	283.92	17.22	9	7/14/2014	47:31.7
1734	RSPe_2	-122.445	37.929	-0.04	17.16	283.98	17.12	-122.445	37.929	-0.06	17.16	283.93	17.10	9	7/14/2014	47:31.6
1735	RSPe_2	-122.445	37.929	-0.01	17.18	284.07	17.18	-122.445	37.929	-0.06	17.18	283.96	17.12	9	7/14/2014	47:31.5
1736	RSPe_2	-122.445	37.929	-0.04	17.20	284.05	17.16	-122.445	37.929	-0.06	17.20	283.98	17.14	9	7/14/2014	47:31.4
1737	RSPe_2	-122.445	37.929	-0.01	17.21	284.09	17.21	-122.445	37.929	-0.06	17.21	284.02	17.16	9	7/14/2014	47:31.3
1738	RSPe_2	-122.445	37.929	-0.01	17.18	284.08	17.18	-122.445	37.929	-0.02	17.18	284.02	17.16	9	7/14/2014	47:31.2
1739	RSPe_2	-122.445	37.929	-0.01	17.23	284.09	17.22	-122.445	37.929	-0.02	17.23	284.03	17.21	9	7/14/2014	47:31.1
1740	RSPe_2	-122.445	37.929	-0.01	17.21	284.10	17.20	-122.445	37.929	-0.06	17.21	284.00	17.15	9	7/14/2014	47:31.0
1741	RSPe_2	-122.445	37.929	-0.01	17.23	284.07	17.22	-122.445	37.929	0.03	17.23	284.01	17.26	9	7/14/2014	47:30.9
1742	RSPe_2	-122.445	37.929	-0.04	17.16	284.16	17.12	-122.445	37.929	-0.02	17.16	284.07	17.14	9	7/14/2014	47:30.8
1743	RSPe_2	-122.445	37.929	-0.01	17.17	284.17	17.16	-122.445	37.929	-0.06	17.17	284.03	17.11	9	7/14/2014	47:30.7
1744	RSPe_2	-122.445	37.929	-0.04	17.18	284.14	17.14	-122.445	37.929	-0.06	17.18	284.01	17.12	9	7/14/2014	47:30.6
1745	RSPe_2	-122.445	37.929	-0.04	17.09	284.30	17.05	-122.445	37.929	-0.11	17.09	284.05	16.98	9	7/14/2014	47:30.5
1746	RSPe_2	-122.445	37.929	-0.04	17.12	284.27	17.08	-122.445	37.929	-0.11	17.12	284.09	17.01	9	7/14/2014	47:30.4
1747	RSPe_2	-122.445	37.929	-0.04	17.14	284.25	17.10	-122.445	37.929	-0.06	17.14	284.10	17.08	9	7/14/2014	47:30.3
1748	RSPe_2	-122.445	37.929	-0.04	17.09	284.23	17.05	-122.445	37.929	-0.14	17.09	284.16	16.95	9	7/14/2014	47:30.2

1749	RSPe_2	-122.445	37.929	-0.04	17.10	284.25	17.06	-122.445	37.929	-0.11	17.10	284.25	16.99	9	7/14/2014	47:30.1
1750	RSPe_2	-122.445	37.929	-0.04	17.12	284.28	17.08	-122.445	37.929	-0.11	17.12	284.30	17.01	9	7/14/2014	47:30.0
1751	RSPe_2	-122.445	37.929	-0.04	17.14	284.26	17.10	-122.445	37.929	-0.06	17.14	284.32	17.09	9	7/14/2014	47:29.9
1752	RSPe_2	-122.445	37.929	-0.04	17.18	284.23	17.14	-122.445	37.929	-0.06	17.18	284.23	17.12	9	7/14/2014	47:29.8
1753	RSPe_2	-122.445	37.929	-0.04	17.25	284.26	17.21	-122.445	37.929	-0.02	17.25	284.27	17.22	9	7/14/2014	47:29.7
1754	RSPe_2	-122.445	37.929	-0.09	17.18	284.28	17.08	-122.445	37.929	-0.06	17.18	284.27	17.12	9	7/14/2014	47:29.6
1755	RSPe_2	-122.445	37.929	-0.04	17.21	284.30	17.17	-122.445	37.929	-0.06	17.21	284.26	17.16	9	7/14/2014	47:29.5
1756	RSPe_2	-122.445	37.929	-0.09	17.18	284.30	17.09	-122.445	37.929	-0.11	17.18	284.21	17.07	9	7/14/2014	47:29.4
1757	RSPe_2	-122.445	37.929	-0.04	17.21	284.28	17.17	-122.445	37.929	-0.11	17.21	284.23	17.10	9	7/14/2014	47:29.3
1758	RSPe_2	-122.445	37.929	-0.09	17.22	284.30	17.13	-122.445	37.929	-0.14	17.22	284.27	17.08	9	7/14/2014	47:29.2
1759	RSPe_2	-122.445	37.929	-0.09	17.24	284.33	17.15	-122.445	37.929	-0.11	17.24	284.30	17.13	9	7/14/2014	47:29.1
1760	RSPe_2	-122.445	37.929	-0.09	17.27	284.24	17.18	-122.445	37.929	-0.14	17.27	284.30	17.13	9	7/14/2014	47:29.0
1761	RSPe_2	-122.445	37.929	-0.09	17.28	284.26	17.19	-122.445	37.929	-0.14	17.28	284.28	17.14	9	7/14/2014	47:28.9
1762	RSPe_2	-122.445	37.929	-0.13	17.28	284.30	17.16	-122.445	37.929	-0.19	17.28	284.34	17.09	9	7/14/2014	47:28.8
1763	RSPe_2	-122.445	37.929	-0.13	17.29	284.39	17.17	-122.445	37.929	-0.14	17.29	284.33	17.15	9	7/14/2014	47:28.7
1764	RSPe_2	-122.445	37.929	-0.13	17.33	284.39	17.20	-122.445	37.929	-0.14	17.33	284.27	17.19	9	7/14/2014	47:28.6
1765	RSPe_2	-122.445	37.929	-0.09	17.35	284.44	17.26	-122.445	37.929	-0.14	17.35	284.26	17.21	9	7/14/2014	47:28.5
1766	RSPe_2	-122.445	37.929	-0.09	17.32	284.44	17.23	-122.445	37.929	-0.14	17.32	284.23	17.18	9	7/14/2014	47:28.4
1767	RSPe_2	-122.445	37.929	-0.09	17.35	284.37	17.26	-122.445	37.929	-0.14	17.35	284.17	17.21	9	7/14/2014	47:28.3
1768	RSPe_2	-122.445	37.929	-0.13	17.32	284.31	17.19	-122.445	37.929	-0.14	17.32	284.19	17.17	9	7/14/2014	47:28.2
1769	RSPe_2	-122.445	37.929	-0.09	17.27	284.31	17.18	-122.445	37.929	-0.14	17.27	284.17	17.13	9	7/14/2014	47:28.1
1770	RSPe_2	-122.445	37.929	-0.13	17.25	284.29	17.12	-122.445	37.929	-0.19	17.25	284.19	17.05	9	7/14/2014	47:28.0
1771	RSPe_2	-122.445	37.929	-0.13	17.27	284.22	17.14	-122.445	37.929	-0.14	17.27	284.17	17.13	9	7/14/2014	47:27.9
1772	RSPe_2	-122.445	37.929	-0.18	17.33	284.22	17.15	-122.445	37.929	-0.14	17.33	284.28	17.19	9	7/14/2014	47:27.8
1773	RSPe_2	-122.445	37.929	-0.13	17.27	284.31	17.14	-122.445	37.929	-0.19	17.27	284.40	17.07	9	7/14/2014	47:27.7
1774	RSPe_2	-122.445	37.929	-0.18	17.31	284.31	17.13	-122.445	37.929	-0.19	17.31	284.43	17.12	9	7/14/2014	47:27.6
1775	RSPe_2	-122.445	37.929	-0.13	17.25	284.36	17.12	-122.445	37.929	-0.19	17.25	284.45	17.05	9	7/14/2014	47:27.5
1776	RSPe_2	-122.445	37.930	-0.18	17.34	284.38	17.16	-122.445	37.930	-0.19	17.34	284.51	17.14	9	7/14/2014	47:27.4
1777	RSPe_2	-122.445	37.930	-0.13	17.36	284.40	17.24	-122.445	37.930	-0.19	17.36	284.53	17.17	9	7/14/2014	47:27.3
1778	RSPe_2	-122.445	37.930	-0.18	17.31	284.44	17.13	-122.445	37.930	-0.23	17.31	284.53	17.08	9	7/14/2014	47:27.2
1779	RSPe_2	-122.445	37.930	-0.13	17.29	284.49	17.17	-122.445	37.930	-0.23	17.29	284.44	17.07	9	7/14/2014	47:27.1
1780	RSPe_2	-122.445	37.930	-0.18	17.35	284.47	17.17	-122.445	37.930	-0.23	17.35	284.33	17.12	9	7/14/2014	47:27.0
1781	RSPe_2	-122.445	37.930	-0.13	17.18	284.51	17.06	-122.445	37.930	-0.19	17.18	284.26	16.99	9	7/14/2014	47:26.9

1782	RSPe_2	-122.445	37.930	-0.18	17.24	284.49	17.06	-122.445	37.930	-0.14	17.24	284.22	17.10	9	7/14/2014	47:26.8
1783	RSPe_2	-122.445	37.930	-0.13	17.14	284.54	17.01	-122.445	37.930	-0.19	17.14	284.27	16.95	9	7/14/2014	47:26.7
1784	RSPe_2	-122.445	37.930	-0.18	17.15	284.49	16.98	-122.445	37.930	-0.23	17.15	284.22	16.93	9	7/14/2014	47:26.6
1785	RSPe_2	-122.445	37.930	-0.13	17.14	284.54	17.01	-122.445	37.930	-0.19	17.14	284.27	16.95	9	7/14/2014	47:26.5
1786	RSPe_2	-122.445	37.930	-0.18	17.13	284.43	16.95	-122.445	37.930	-0.23	17.13	284.18	16.90	9	7/14/2014	47:26.4
1787	RSPe_2	-122.445	37.930	-0.18	17.12	284.43	16.94	-122.445	37.930	-0.23	17.12	284.28	16.89	9	7/14/2014	47:26.3
1788	RSPe_2	-122.445	37.930	-0.18	17.14	284.47	16.96	-122.445	37.930	-0.19	17.14	284.31	16.95	9	7/14/2014	47:26.2
1789	RSPe_2	-122.445	37.930	-0.18	17.13	284.40	16.95	-122.445	37.930	-0.14	17.13	284.29	16.99	9	7/14/2014	47:26.1
1790	RSPe_2	-122.445	37.930	-0.18	17.17	284.36	16.99	-122.445	37.930	-0.19	17.17	284.32	16.98	9	7/14/2014	47:26.0
1791	RSPe_2	-122.445	37.930	-0.13	17.19	284.32	17.07	-122.445	37.930	-0.14	17.19	284.23	17.05	9	7/14/2014	47:25.9
1792	RSPe_2	-122.445	37.930	-0.18	17.21	284.45	17.04	-122.445	37.930	-0.19	17.21	284.43	17.02	9	7/14/2014	47:25.8
1793	RSPe_2	-122.445	37.930	-0.13	17.25	284.45	17.13	-122.445	37.930	-0.19	17.25	284.49	17.06	9	7/14/2014	47:25.7
1794	RSPe_2	-122.445	37.930	-0.18	17.30	284.43	17.12	-122.445	37.930	-0.19	17.30	284.49	17.11	9	7/14/2014	47:25.6
1795	RSPe_2	-122.445	37.930	-0.18	17.35	284.45	17.18	-122.445	37.930	-0.14	17.35	284.56	17.21	9	7/14/2014	47:25.5
1796	RSPe_2	-122.445	37.930	-0.18	17.34	284.52	17.16	-122.445	37.930	-0.14	17.34	284.54	17.19	9	7/14/2014	47:25.4
1797	RSPe_2	-122.445	37.930	-0.18	17.37	284.52	17.19	-122.445	37.930	-0.19	17.37	284.58	17.18	9	7/14/2014	47:25.3
1798	RSPe_2	-122.445	37.930	-0.18	17.35	284.54	17.18	-122.445	37.930	-0.19	17.35	284.58	17.16	9	7/14/2014	47:25.2
1799	RSPe_2	-122.445	37.930	-0.18	17.38	284.57	17.20	-122.445	37.930	-0.19	17.38	284.51	17.19	9	7/14/2014	47:25.1
1800	RSPe_2	-122.445	37.930	-0.21	17.42	284.54	17.21	-122.445	37.930	-0.22	17.42	284.52	17.20	9	7/14/2014	47:25.0
1801	RSPe_2	-122.445	37.930	-0.18	17.42	284.46	17.24	-122.445	37.930	-0.19	17.42	284.43	17.22	9	7/14/2014	47:24.9
1802	RSPe_2	-122.445	37.930	-0.18	17.42	284.46	17.25	-122.445	37.930	-0.22	17.42	284.45	17.20	9	7/14/2014	47:24.8
1803	RSPe_2	-122.445	37.930	-0.18	17.44	284.37	17.26	-122.445	37.930	-0.19	17.44	284.39	17.25	9	7/14/2014	47:24.7
1804	RSPe_2	-122.445	37.930	-0.18	17.44	284.32	17.26	-122.445	37.930	-0.22	17.44	284.39	17.21	9	7/14/2014	47:24.6
1805	RSPe_2	-122.445	37.930	-0.18	17.46	284.35	17.28	-122.445	37.930	-0.19	17.46	284.45	17.27	9	7/14/2014	47:24.5
1806	RSPe_2	-122.445	37.930	-0.18	17.49	284.32	17.31	-122.445	37.930	-0.22	17.49	284.45	17.26	9	7/14/2014	47:24.4
1807	RSPe_2	-122.445	37.930	-0.18	17.51	284.39	17.33	-122.445	37.930	-0.19	17.51	284.48	17.32	9	7/14/2014	47:24.3
1808	RSPe_2	-122.445	37.930	-0.21	17.51	284.46	17.30	-122.445	37.930	-0.22	17.51	284.54	17.29	9	7/14/2014	47:24.2
1809	RSPe_2	-122.445	37.930	-0.18	17.53	284.46	17.35	-122.445	37.930	-0.22	17.53	284.52	17.31	9	7/14/2014	47:24.1
1810	RSPe_2	-122.445	37.930	-0.18	17.52	284.37	17.35	-122.445	37.930	-0.22	17.52	284.41	17.30	9	7/14/2014	47:24.0
1811	RSPe_2	-122.445	37.930	-0.18	17.55	284.42	17.37	-122.445	37.930	-0.19	17.55	284.48	17.35	9	7/14/2014	47:23.9
1812	RSPe_2	-122.445	37.930	-0.18	17.56	284.46	17.38	-122.445	37.930	-0.22	17.56	284.43	17.33	9	7/14/2014	47:23.8
1813	RSPe_2	-122.445	37.930	-0.21	17.55	284.42	17.34	-122.445	37.930	-0.22	17.55	284.41	17.32	9	7/14/2014	47:23.7
1814	RSPe_2	-122.445	37.930	-0.21	17.51	284.46	17.30	-122.445	37.930	-0.26	17.51	284.46	17.25	9	7/14/2014	47:23.6

1815	RSPe_2	-122.445	37.930	-0.18	17.47	284.48	17.29	-122.445	37.930	-0.26	17.47	284.43	17.21	9	7/14/2014 47:23.5
1816	RSPe_2	-122.445	37.930	-0.21	17.45	284.44	17.24	-122.445	37.930	-0.26	17.45	284.37	17.20	9	7/14/2014 47:23.4
1817	RSPe_2	-122.445	37.930	-0.18	17.42	284.48	17.24	-122.445	37.930	-0.22	17.42	284.33	17.19	9	7/14/2014 47:23.3
1818	RSPe_2	-122.445	37.930	-0.18	17.42	284.45	17.24	-122.445	37.930	-0.19	17.42	284.33	17.22	9	7/14/2014 47:23.2
1819	RSPe_2	-122.445	37.930	-0.18	17.38	284.51	17.20	-122.445	37.930	-0.26	17.38	284.41	17.12	9	7/14/2014 47:23.1
1820	RSPe_2	-122.445	37.930	-0.18	17.37	284.44	17.19	-122.445	37.930	-0.22	17.37	284.33	17.14	9	7/14/2014 47:23.0
1821	RSPe_2	-122.445	37.930	-0.18	17.35	284.44	17.17	-122.445	37.930	-0.22	17.35	284.37	17.12	9	7/14/2014 47:22.9
1822	RSPe_2	-122.445	37.930	-0.18	17.35	284.38	17.18	-122.445	37.930	-0.19	17.35	284.42	17.16	9	7/14/2014 47:22.8
1823	RSPe_2	-122.445	37.930	-0.13	17.35	284.42	17.23	-122.445	37.930	-0.26	17.35	284.48	17.09	9	7/14/2014 47:22.7
1824	RSPe_2	-122.445	37.930	-0.18	17.35	284.35	17.18	-122.445	37.930	-0.26	17.35	284.52	17.09	9	7/14/2014 47:22.6
1825	RSPe_2	-122.445	37.930	-0.18	17.36	284.33	17.19	-122.445	37.930	-0.22	17.36	284.55	17.14	9	7/14/2014 47:22.5
1826	RSPe_2	-122.445	37.930	-0.18	17.41	284.29	17.23	-122.445	37.930	-0.19	17.41	284.53	17.21	9	7/14/2014 47:22.4
1827	RSPe_2	-122.445	37.930	-0.18	17.36	284.27	17.19	-122.445	37.930	-0.22	17.36	284.55	17.14	9	7/14/2014 47:22.3
1828	RSPe_2	-122.445	37.930	-0.18	17.40	284.33	17.22	-122.445	37.930	-0.22	17.40	284.57	17.17	9	7/14/2014 47:22.2
1829	RSPe_2	-122.445	37.930	-0.13	17.42	284.40	17.30	-122.445	37.930	-0.26	17.42	284.59	17.16	9	7/14/2014 47:22.1
1830	RSPe_2	-122.445	37.930	-0.18	17.44	284.49	17.26	-122.445	37.930	-0.26	17.44	284.53	17.18	9	7/14/2014 47:22.0
1831	RSPe_2	-122.445	37.930	-0.18	17.46	284.45	17.28	-122.445	37.930	-0.22	17.46	284.40	17.23	9	7/14/2014 47:21.9
1832	RSPe_2	-122.445	37.930	-0.18	17.42	284.42	17.25	-122.445	37.930	-0.19	17.42	284.31	17.23	9	7/14/2014 47:21.8
1833	RSPe_2	-122.445	37.930	-0.18	17.42	284.51	17.25	-122.445	37.930	-0.22	17.42	284.29	17.20	9	7/14/2014 47:21.7
1834	RSPe_2	-122.445	37.930	-0.18	17.43	284.45	17.26	-122.445	37.930	-0.26	17.43	284.20	17.17	9	7/14/2014 47:21.6
1835	RSPe_2	-122.445	37.930	-0.13	17.49	284.49	17.36	-122.445	37.930	-0.14	17.49	284.30	17.35	9	7/14/2014 47:21.5
1836	RSPe_2	-122.445	37.930	-0.18	17.39	284.56	17.22	-122.445	37.930	-0.19	17.39	284.36	17.20	9	7/14/2014 47:21.4
1837	RSPe_2	-122.445	37.930	-0.13	17.39	284.52	17.27	-122.445	37.930	-0.22	17.39	284.40	17.17	9	7/14/2014 47:21.3
1838	RSPe_2	-122.445	37.930	-0.18	17.45	284.34	17.27	-122.445	37.930	-0.31	17.45	284.34	17.14	9	7/14/2014 47:21.2
1839	RSPe_2	-122.445	37.930	-0.13	17.40	284.36	17.27	-122.445	37.930	-0.19	17.40	284.46	17.21	9	7/14/2014 47:21.1
1840	RSPe_2	-122.445	37.930	-0.18	17.60	284.38	17.42	-122.445	37.930	-0.19	17.60	284.56	17.41	9	7/14/2014 47:21.0
1841	RSPe_2	-122.445	37.930	-0.13	17.58	284.40	17.45	-122.445	37.930	-0.26	17.58	284.66	17.32	9	7/14/2014 47:20.9
1842	RSPe_2	-122.445	37.930	-0.13	17.67	284.39	17.54	-122.445	37.930	-0.19	17.67	284.76	17.48	9	7/14/2014 47:20.8
1843	RSPe_2	-122.445	37.930	-0.09	17.72	284.34	17.62	-122.445	37.930	-0.26	17.72	284.69	17.46	9	7/14/2014 47:20.7
1844	RSPe_2	-122.445	37.930	-0.13	17.68	284.36	17.56	-122.445	37.930	-0.19	17.68	284.69	17.49	9	7/14/2014 47:20.6
1845	RSPe_2	-122.445	37.930	-0.09	17.68	284.45	17.59	-122.445	37.930	-0.14	17.68	284.67	17.54	9	7/14/2014 47:20.5
1846	RSPe_2	-122.445	37.930	-0.13	17.72	284.50	17.59	-122.445	37.930	-0.11	17.72	284.58	17.61	9	7/14/2014 47:20.4
1847	RSPe_2	-122.445	37.930	-0.09	17.72	284.58	17.62	-122.445	37.930	-0.11	17.72	284.47	17.61	9	7/14/2014 47:20.3

1848	RSPe_2	-122.445	37.930	-0.13	17.68	284.63	17.56	-122.445	37.930	-0.14	17.68	284.40	17.54	9	7/14/2014 47:20.2
1849	RSPe_2	-122.445	37.930	-0.13	17.63	284.68	17.51	-122.445	37.930	-0.14	17.63	284.31	17.49	9	7/14/2014 47:20.1
1850	RSPe_2	-122.445	37.930	-0.13	17.61	284.74	17.48	-122.445	37.930	-0.14	17.61	284.32	17.47	9	7/14/2014 47:20.0
1851	RSPe_2	-122.445	37.930	-0.09	17.63	284.61	17.53	-122.445	37.930	-0.14	17.63	284.19	17.48	9	7/14/2014 47:19.9
1852	RSPe_2	-122.445	37.930	-0.13	17.59	284.61	17.47	-122.445	37.930	-0.19	17.59	284.23	17.40	9	7/14/2014 47:19.8
1853	RSPe_2	-122.445	37.930	-0.09	17.60	284.59	17.51	-122.445	37.930	-0.14	17.60	284.30	17.46	9	7/14/2014 47:19.7
1854	RSPe_2	-122.445	37.930	-0.09	17.60	284.57	17.51	-122.445	37.930	-0.14	17.60	284.43	17.46	9	7/14/2014 47:19.6
1855	RSPe_2	-122.445	37.930	-0.09	17.60	284.59	17.51	-122.445	37.930	-0.19	17.60	284.56	17.41	9	7/14/2014 47:19.5
1856	RSPe_2	-122.445	37.930	-0.09	17.61	284.50	17.52	-122.445	37.930	-0.19	17.61	284.67	17.42	9	7/14/2014 47:19.4
1857	RSPe_2	-122.445	37.930	-0.09	17.65	284.52	17.56	-122.445	37.930	-0.14	17.65	284.76	17.51	9	7/14/2014 47:19.3
1858	RSPe_2	-122.445	37.930	-0.09	17.67	284.44	17.58	-122.445	37.930	-0.14	17.67	284.70	17.53	9	7/14/2014 47:19.2
1859	RSPe_2	-122.445	37.930	-0.04	17.68	284.44	17.65	-122.445	37.930	-0.19	17.68	284.65	17.49	9	7/14/2014 47:19.1
1860	RSPe_2	-122.445	37.930	-0.09	17.68	284.46	17.59	-122.445	37.930	-0.14	17.68	284.54	17.54	9	7/14/2014 47:19.0
1861	RSPe_2	-122.445	37.930	-0.04	17.65	284.48	17.61	-122.445	37.930	-0.11	17.65	284.40	17.55	9	7/14/2014 47:18.9
1862	RSPe_2	-122.445	37.930	-0.09	17.65	284.48	17.56	-122.445	37.930	-0.11	17.65	284.24	17.54	9	7/14/2014 47:18.8
1863	RSPe_2	-122.445	37.930	-0.09	17.61	284.38	17.52	-122.445	37.930	-0.06	17.61	284.15	17.56	9	7/14/2014 47:18.7
1864	RSPe_2	-122.445	37.930	-0.09	17.59	284.39	17.50	-122.445	37.930	-0.11	17.59	284.12	17.49	9	7/14/2014 47:18.6
1865	RSPe_2	-122.445	37.930	-0.09	17.59	284.35	17.50	-122.445	37.930	-0.06	17.59	284.09	17.54	9	7/14/2014 47:18.5
1866	RSPe_2	-122.445	37.930	-0.13	17.57	284.31	17.45	-122.445	37.930	-0.11	17.57	284.11	17.46	9	7/14/2014 47:18.4
1867	RSPe_2	-122.445	37.930	-0.09	17.58	284.24	17.49	-122.445	37.930	-0.11	17.58	284.17	17.48	9	7/14/2014 47:18.3
1868	RSPe_2	-122.445	37.930	-0.09	17.59	284.20	17.50	-122.445	37.930	-0.11	17.59	284.18	17.49	9	7/14/2014 47:18.2
1869	RSPe_2	-122.445	37.930	-0.09	17.59	284.20	17.50	-122.445	37.930	-0.14	17.59	284.26	17.45	9	7/14/2014 47:18.1
1870	RSPe_2	-122.445	37.930	-0.09	17.61	284.14	17.52	-122.445	37.930	-0.11	17.61	284.24	17.50	9	7/14/2014 47:18.0
1871	RSPe_2	-122.445	37.930	-0.04	17.57	284.16	17.53	-122.445	37.930	-0.14	17.57	284.22	17.43	9	7/14/2014 47:17.9
1872	RSPe_2	-122.445	37.930	-0.09	17.56	284.09	17.47	-122.445	37.930	-0.11	17.56	284.17	17.45	9	7/14/2014 47:17.8
1873	RSPe_2	-122.445	37.930	-0.09	17.54	284.05	17.45	-122.445	37.930	-0.19	17.54	284.11	17.35	9	7/14/2014 47:17.7
1874	RSPe_2	-122.445	37.930	-0.13	17.56	284.03	17.43	-122.445	37.930	-0.19	17.56	284.07	17.36	9	7/14/2014 47:17.6
1875	RSPe_2	-122.445	37.930	-0.09	17.53	284.12	17.44	-122.445	37.930	-0.06	17.53	284.11	17.47	9	7/14/2014 47:17.5
1876	RSPe_2	-122.445	37.930	-0.09	17.51	284.18	17.42	-122.445	37.930	-0.14	17.51	284.10	17.37	9	7/14/2014 47:17.4
1877	RSPe_2	-122.445	37.930	-0.09	17.45	284.16	17.36	-122.445	37.930	-0.11	17.45	284.07	17.35	9	7/14/2014 47:17.3
1878	RSPe_2	-122.445	37.930	-0.13	17.42	284.18	17.29	-122.445	37.930	-0.11	17.42	284.03	17.31	9	7/14/2014 47:17.2
1879	RSPe_2	-122.445	37.930	-0.09	17.40	284.10	17.31	-122.445	37.930	-0.02	17.40	283.98	17.38	9	7/14/2014 47:17.1
1880	RSPe_2	-122.445	37.930	-0.13	17.41	284.05	17.28	-122.445	37.930	-0.14	17.41	284.00	17.27	9	7/14/2014 47:17.0

1881	RSPe_2	-122.445	37.930	-0.09	17.39	284.05	17.29	-122.445	37.930	-0.14	17.39	284.05	17.24	9	7/14/2014	47:16.9
1882	RSPe_2	-122.445	37.930	-0.13	17.42	283.99	17.29	-122.445	37.930	-0.19	17.42	284.05	17.22	9	7/14/2014	47:16.8
1883	RSPe_2	-122.445	37.930	-0.09	17.37	283.90	17.28	-122.445	37.930	-0.19	17.37	284.00	17.18	9	7/14/2014	47:16.7
1884	RSPe_2	-122.445	37.930	-0.13	17.38	283.81	17.25	-122.445	37.930	-0.11	17.38	283.97	17.27	9	7/14/2014	47:16.6
1885	RSPe_2	-122.445	37.930	-0.13	17.40	283.84	17.27	-122.445	37.930	-0.06	17.40	284.01	17.34	9	7/14/2014	47:16.5
1886	RSPe_2	-122.445	37.930	-0.18	17.39	283.90	17.21	-122.445	37.930	-0.26	17.39	284.01	17.13	9	7/14/2014	47:16.4
1887	RSPe_2	-122.445	37.930	-0.13	17.42	283.90	17.29	-122.445	37.930	-0.19	17.42	284.03	17.22	9	7/14/2014	47:16.3
1888	RSPe_2	-122.445	37.930	-0.13	17.45	283.95	17.32	-122.445	37.930	-0.22	17.45	284.00	17.22	9	7/14/2014	47:16.2
1889	RSPe_2	-122.445	37.930	-0.13	17.44	283.95	17.31	-122.445	37.930	-0.19	17.44	283.96	17.25	9	7/14/2014	47:16.1
1890	RSPe_2	-122.445	37.930	-0.13	17.42	283.90	17.30	-122.445	37.930	-0.26	17.42	283.99	17.16	9	7/14/2014	47:16.0
1891	RSPe_2	-122.445	37.930	-0.18	17.47	283.90	17.29	-122.445	37.930	-0.22	17.47	283.94	17.24	9	7/14/2014	47:15.9
1892	RSPe_2	-122.445	37.930	-0.18	17.47	283.90	17.29	-122.445	37.930	-0.22	17.47	284.01	17.24	9	7/14/2014	47:15.8
1893	RSPe_2	-122.445	37.930	-0.13	17.48	284.02	17.35	-122.445	37.930	-0.19	17.48	284.10	17.28	9	7/14/2014	47:15.7
1894	RSPe_2	-122.445	37.930	-0.18	17.49	283.99	17.32	-122.445	37.930	-0.26	17.49	284.10	17.23	9	7/14/2014	47:15.6
1895	RSPe_2	-122.445	37.930	-0.13	17.51	283.99	17.38	-122.445	37.930	-0.31	17.51	283.97	17.20	9	7/14/2014	47:15.5
1896	RSPe_2	-122.445	37.930	-0.21	17.45	283.99	17.24	-122.445	37.930	-0.26	17.45	283.92	17.20	9	7/14/2014	47:15.4
1897	RSPe_2	-122.445	37.930	-0.18	17.50	283.97	17.32	-122.445	37.930	-0.31	17.50	283.81	17.19	9	7/14/2014	47:15.3
1898	RSPe_2	-122.445	37.930	-0.21	17.45	283.91	17.24	-122.445	37.930	-0.31	17.45	283.74	17.14	9	7/14/2014	47:15.2
1899	RSPe_2	-122.445	37.930	-0.18	17.52	283.91	17.35	-122.445	37.930	-0.26	17.52	283.70	17.27	9	7/14/2014	47:15.1
1900	RSPe_2	-122.445	37.930	-0.21	17.49	283.91	17.28	-122.445	37.930	-0.31	17.49	283.86	17.18	9	7/14/2014	47:15.0
1901	RSPe_2	-122.445	37.930	-0.18	17.51	283.82	17.34	-122.445	37.930	-0.31	17.51	283.82	17.20	9	7/14/2014	47:14.9
1902	RSPe_2	-122.445	37.930	-0.21	17.54	283.84	17.33	-122.445	37.930	-0.34	17.54	283.92	17.19	9	7/14/2014	47:14.8
1903	RSPe_2	-122.445	37.930	-0.21	17.61	283.89	17.40	-122.445	37.930	-0.34	17.61	283.99	17.27	9	7/14/2014	47:14.7
1904	RSPe_2	-122.445	37.930	-0.21	17.68	283.95	17.47	-122.445	37.930	-0.34	17.68	284.04	17.34	9	7/14/2014	47:14.6
1905	RSPe_2	-122.445	37.930	-0.18	17.75	283.99	17.57	-122.445	37.930	-0.40	17.75	284.06	17.35	9	7/14/2014	47:14.5
1906	RSPe_2	-122.445	37.930	-0.25	17.77	284.09	17.53	-122.445	37.930	-0.40	17.77	284.14	17.38	9	7/14/2014	47:14.4
1907	RSPe_2	-122.445	37.930	-0.21	17.77	284.08	17.56	-122.445	37.930	-0.40	17.77	284.15	17.38	9	7/14/2014	47:14.3
1908	RSPe_2	-122.445	37.930	-0.21	17.79	283.98	17.58	-122.445	37.930	-0.40	17.79	284.05	17.40	9	7/14/2014	47:14.2
1909	RSPe_2	-122.445	37.930	-0.21	17.86	283.96	17.65	-122.445	37.930	-0.43	17.86	284.04	17.43	9	7/14/2014	47:14.1
1910	RSPe_2	-122.445	37.930	-0.21	17.97	283.96	17.76	-122.445	37.930	-0.48	17.97	283.91	17.49	9	7/14/2014	47:14.0
1911	RSPe_2	-122.445	37.930	-0.21	17.99	283.80	17.78	-122.445	37.930	-0.43	17.99	283.82	17.56	9	7/14/2014	47:13.9
1912	RSPe_2	-122.445	37.930	-0.25	18.03	283.78	17.78	-122.445	37.930	-0.43	18.03	283.76	17.60	9	7/14/2014	47:13.8
1913	RSPe_2	-122.445	37.930	-0.21	18.06	283.80	17.85	-122.445	37.930	-0.43	18.06	283.83	17.63	9	7/14/2014	47:13.7

1914	RSPe_2	-122.445	37.930	-0.25	18.17	283.80	17.93	-122.445	37.930	-0.40	18.17	283.93	17.78	9	7/14/2014	47:13.6
1915	RSPe_2	-122.445	37.930	-0.21	18.24	283.76	18.03	-122.445	37.930	-0.43	18.24	284.00	17.81	9	7/14/2014	47:13.5
1916	RSPe_2	-122.445	37.930	-0.21	18.24	283.80	18.03	-122.445	37.930	-0.51	18.24	284.09	17.73	9	7/14/2014	47:13.4
1917	RSPe_2	-122.445	37.930	-0.21	18.29	283.94	18.08	-122.445	37.930	-0.43	18.29	284.11	17.86	9	7/14/2014	47:13.3
1918	RSPe_2	-122.445	37.930	-0.21	18.32	284.01	18.11	-122.445	37.930	-0.51	18.32	284.11	17.80	9	7/14/2014	47:13.2
1919	RSPe_2	-122.445	37.930	-0.21	18.31	284.05	18.10	-122.445	37.930	-0.51	18.31	283.94	17.80	9	7/14/2014	47:13.1
1920	RSPe_2	-122.445	37.930	-0.25	18.33	284.05	18.08	-122.445	37.930	-0.48	18.33	283.83	17.85	9	7/14/2014	47:13.0
1921	RSPe_2	-122.445	37.930	-0.21	18.33	283.98	18.12	-122.445	37.930	-0.51	18.33	283.69	17.81	9	7/14/2014	47:12.9
1922	RSPe_2	-122.445	37.930	-0.25	18.23	283.87	17.98	-122.445	37.930	-0.48	18.23	283.54	17.75	9	7/14/2014	47:12.8
1923	RSPe_2	-122.445	37.930	-0.25	18.18	283.92	17.93	-122.445	37.930	-0.43	18.18	283.58	17.75	9	7/14/2014	47:12.7
1924	RSPe_2	-122.445	37.930	-0.25	18.13	283.96	17.89	-122.445	37.930	-0.48	18.13	283.66	17.65	9	7/14/2014	47:12.6
1925	RSPe_2	-122.445	37.930	-0.21	18.00	283.90	17.79	-122.445	37.930	-0.51	18.00	283.67	17.49	9	7/14/2014	47:12.5
1926	RSPe_2	-122.445	37.930	-0.25	18.00	283.86	17.76	-122.445	37.930	-0.56	18.00	283.79	17.44	9	7/14/2014	47:12.4
1927	RSPe_2	-122.445	37.930	-0.21	17.98	283.88	17.77	-122.445	37.930	-0.56	17.98	283.98	17.42	9	7/14/2014	47:12.3
1928	RSPe_2	-122.445	37.930	-0.25	18.02	283.92	17.77	-122.445	37.930	-0.56	18.02	284.22	17.45	9	7/14/2014	47:12.2
1929	RSPe_2	-122.445	37.930	-0.21	18.05	283.96	17.84	-122.445	37.930	-0.48	18.05	284.25	17.57	9	7/14/2014	47:12.1
1930	RSPe_2	-122.445	37.930	-0.25	18.06	283.94	17.82	-122.445	37.930	-0.48	18.06	284.25	17.58	9	7/14/2014	47:12.0
1931	RSPe_2	-122.445	37.930	-0.21	18.11	284.01	17.90	-122.445	37.930	-0.48	18.11	284.11	17.63	9	7/14/2014	47:11.9
1932	RSPe_2	-122.445	37.930	-0.25	18.12	283.99	17.87	-122.445	37.930	-0.48	18.12	284.02	17.64	9	7/14/2014	47:11.8
1933	RSPe_2	-122.445	37.930	-0.21	18.07	284.03	17.86	-122.445	37.930	-0.51	18.07	283.92	17.56	9	7/14/2014	47:11.7
1934	RSPe_2	-122.445	37.930	-0.25	18.08	284.12	17.83	-122.445	37.930	-0.48	18.08	283.83	17.60	9	7/14/2014	47:11.6
1935	RSPe_2	-122.445	37.930	-0.21	17.98	284.17	17.77	-122.445	37.930	-0.39	17.98	283.86	17.59	9	7/14/2014	47:11.5
1936	RSPe_2	-122.445	37.930	-0.21	17.94	284.30	17.73	-122.445	37.930	-0.48	17.94	283.85	17.46	9	7/14/2014	47:11.4
1937	RSPe_2	-122.445	37.930	-0.21	17.91	284.32	17.70	-122.445	37.930	-0.43	17.91	283.86	17.48	9	7/14/2014	47:11.3
1938	RSPe_2	-122.445	37.930	-0.21	17.85	284.30	17.65	-122.445	37.930	-0.39	17.85	283.86	17.46	9	7/14/2014	47:11.2
1939	RSPe_2	-122.445	37.930	-0.21	17.81	284.23	17.60	-122.445	37.930	-0.34	17.81	283.88	17.47	9	7/14/2014	47:11.1
1940	RSPe_2	-122.445	37.930	-0.21	17.80	284.08	17.59	-122.445	37.930	-0.48	17.80	283.85	17.32	9	7/14/2014	47:11.0
1941	RSPe_2	-122.445	37.930	-0.18	17.78	284.08	17.60	-122.445	37.930	-0.43	17.78	283.96	17.35	9	7/14/2014	47:10.9
1942	RSPe_2	-122.445	37.930	-0.18	17.81	284.02	17.63	-122.445	37.930	-0.43	17.81	284.03	17.38	9	7/14/2014	47:10.8
1943	RSPe_2	-122.445	37.930	-0.18	17.81	284.06	17.63	-122.445	37.930	-0.48	17.81	284.12	17.33	9	7/14/2014	47:10.7
1944	RSPe_2	-122.445	37.930	-0.21	17.82	284.11	17.61	-122.445	37.930	-0.39	17.82	284.19	17.43	9	7/14/2014	47:10.6
1945	RSPe_2	-122.445	37.930	-0.12	18.05	284.17	17.93	-122.445	37.930	-0.43	18.05	284.28	17.63	9	7/14/2014	47:10.5
1946	RSPe_2	-122.445	37.930	-0.21	17.95	284.19	17.74	-122.445	37.930	-0.31	17.95	284.21	17.64	9	7/14/2014	47:10.4

1947	RSPe_2	-122.445	37.930	-0.18	18.09	284.17	17.91	-122.445	37.930	-0.39	18.09	284.20	17.69	9	7/14/2014	47:10.3
1948	RSPe_2	-122.445	37.930	-0.18	18.02	284.13	17.84	-122.445	37.930	-0.48	18.02	284.13	17.54	9	7/14/2014	47:10.2
1949	RSPe_2	-122.445	37.930	-0.18	18.14	284.17	17.96	-122.445	37.930	-0.39	18.14	284.10	17.75	9	7/14/2014	47:10.1
1950	RSPe_2	-122.445	37.930	-0.18	18.17	284.15	17.99	-122.445	37.930	-0.31	18.17	284.10	17.86	9	7/14/2014	47:10.0
1951	RSPe_2	-122.445	37.930	-0.12	18.27	284.20	18.15	-122.445	37.930	-0.31	18.27	284.13	17.96	9	7/14/2014	47:09.9
1952	RSPe_2	-122.445	37.930	-0.18	18.26	284.15	18.08	-122.445	37.930	-0.31	18.26	284.14	17.95	9	7/14/2014	47:09.8
1953	RSPe_2	-122.445	37.930	-0.12	18.26	284.20	18.14	-122.445	37.930	-0.31	18.26	284.19	17.95	9	7/14/2014	47:09.7
1954	RSPe_2	-122.445	37.930	-0.18	18.24	284.20	18.07	-122.445	37.930	-0.34	18.24	284.17	17.90	9	7/14/2014	47:09.6
1955	RSPe_2	-122.445	37.930	-0.12	18.21	284.17	18.08	-122.445	37.930	-0.34	18.21	284.19	17.87	9	7/14/2014	47:09.5
1956	RSPe_2	-122.445	37.930	-0.12	18.20	284.22	18.08	-122.445	37.930	-0.34	18.20	284.20	17.86	9	7/14/2014	47:09.4
1957	RSPe_2	-122.445	37.930	-0.12	18.21	284.22	18.08	-122.445	37.930	-0.31	18.21	284.15	17.90	9	7/14/2014	47:09.3
1958	RSPe_2	-122.445	37.930	-0.12	18.21	284.24	18.08	-122.445	37.930	-0.31	18.21	284.20	17.90	9	7/14/2014	47:09.2
1959	RSPe_2	-122.445	37.930	-0.12	18.21	284.14	18.08	-122.445	37.930	-0.39	18.21	284.16	17.81	9	7/14/2014	47:09.1
1960	RSPe_2	-122.445	37.930	-0.12	18.24	284.16	18.12	-122.445	37.930	-0.34	18.24	284.19	17.90	9	7/14/2014	47:09.0
1961	RSPe_2	-122.445	37.930	-0.12	18.25	284.07	18.12	-122.445	37.930	-0.26	18.25	284.13	17.99	9	7/14/2014	47:08.9
1962	RSPe_2	-122.445	37.930	-0.12	18.27	284.09	18.15	-122.445	37.930	-0.39	18.27	284.16	17.88	9	7/14/2014	47:08.8
1963	RSPe_2	-122.445	37.930	-0.12	18.24	284.16	18.12	-122.445	37.930	-0.34	18.24	284.17	17.90	9	7/14/2014	47:08.7
1964	RSPe_2	-122.445	37.930	-0.12	18.30	284.20	18.17	-122.445	37.930	-0.39	18.30	284.20	17.90	9	7/14/2014	47:08.6
1965	RSPe_2	-122.445	37.930	-0.12	18.24	284.18	18.12	-122.445	37.930	-0.43	18.24	284.20	17.81	9	7/14/2014	47:08.5
1966	RSPe_2	-122.445	37.930	-0.12	18.24	284.12	18.12	-122.445	37.930	-0.39	18.24	284.09	17.85	9	7/14/2014	47:08.4
1967	RSPe_2	-122.445	37.930	-0.12	18.26	284.16	18.13	-122.445	37.930	-0.34	18.26	284.16	17.91	9	7/14/2014	47:08.3
1968	RSPe_2	-122.445	37.930	-0.18	18.23	284.29	18.05	-122.445	37.930	-0.39	18.23	284.17	17.83	9	7/14/2014	47:08.2
1969	RSPe_2	-122.445	37.930	-0.12	18.22	284.31	18.09	-122.445	37.930	-0.39	18.22	284.18	17.82	9	7/14/2014	47:08.1
1970	RSPe_2	-122.445	37.930	-0.18	18.24	284.36	18.06	-122.445	37.930	-0.34	18.24	284.25	17.89	9	7/14/2014	47:08.0
1971	RSPe_2	-122.445	37.930	-0.12	18.23	284.36	18.10	-122.445	37.930	-0.34	18.23	284.27	17.88	9	7/14/2014	47:07.9
1972	RSPe_2	-122.445	37.930	-0.18	18.24	284.36	18.06	-122.445	37.930	-0.34	18.24	284.33	17.89	9	7/14/2014	47:07.8
1973	RSPe_2	-122.445	37.930	-0.18	18.22	284.38	18.04	-122.445	37.930	-0.39	18.22	284.33	17.82	9	7/14/2014	47:07.7
1974	RSPe_2	-122.445	37.930	-0.18	18.26	284.36	18.09	-122.445	37.930	-0.39	18.26	284.31	17.87	9	7/14/2014	47:07.6
1975	RSPe_2	-122.445	37.930	-0.18	18.19	284.34	18.02	-122.445	37.930	-0.43	18.19	284.23	17.77	9	7/14/2014	47:07.5
1976	RSPe_2	-122.445	37.930	-0.21	18.14	284.30	17.93	-122.445	37.930	-0.43	18.14	284.18	17.71	9	7/14/2014	47:07.4
1977	RSPe_2	-122.445	37.930	-0.18	18.18	284.27	18.00	-122.445	37.930	-0.43	18.18	284.09	17.75	9	7/14/2014	47:07.3
1978	RSPe_2	-122.445	37.930	-0.21	18.11	284.21	17.90	-122.445	37.930	-0.48	18.11	284.07	17.63	9	7/14/2014	47:07.2
1979	RSPe_2	-122.445	37.930	-0.18	18.12	284.27	17.94	-122.445	37.930	-0.43	18.12	284.05	17.69	9	7/14/2014	47:07.1

1980	RSPe_2	-122.445	37.930	-0.21	18.11	284.25	17.90	-122.445	37.930	-0.43	18.11	284.11	17.68	9	7/14/2014	47:07.0
1981	RSPe_2	-122.445	37.930	-0.18	18.10	284.38	17.93	-122.445	37.930	-0.43	18.10	284.25	17.67	9	7/14/2014	47:06.9
1982	RSPe_2	-122.445	37.930	-0.21	18.13	284.43	17.92	-122.445	37.930	-0.48	18.13	284.33	17.65	9	7/14/2014	47:06.8
1983	RSPe_2	-122.445	37.930	-0.21	18.05	284.52	17.84	-122.445	37.930	-0.51	18.05	284.45	17.54	9	7/14/2014	47:06.7
1984	RSPe_2	-122.445	37.930	-0.21	18.17	284.48	17.96	-122.445	37.930	-0.48	18.17	284.47	17.69	9	7/14/2014	47:06.6
1985	RSPe_2	-122.445	37.930	-0.18	18.11	284.37	17.93	-122.445	37.930	-0.51	18.11	284.43	17.60	9	7/14/2014	47:06.5
1986	RSPe_2	-122.445	37.930	-0.21	18.14	284.28	17.93	-122.445	37.930	-0.51	18.14	284.34	17.63	9	7/14/2014	47:06.4
1987	RSPe_2	-122.445	37.930	-0.18	18.16	284.39	17.98	-122.445	37.930	-0.48	18.16	284.38	17.68	9	7/14/2014	47:06.3
1988	RSPe_2	-122.445	37.930	-0.24	18.17	284.17	17.92	-122.445	37.930	-0.43	18.17	284.21	17.74	9	7/14/2014	47:06.2
1989	RSPe_2	-122.445	37.930	-0.21	18.17	284.21	17.96	-122.445	37.930	-0.39	18.17	284.32	17.78	9	7/14/2014	47:06.1
1990	RSPe_2	-122.445	37.930	-0.21	18.29	284.34	18.08	-122.445	37.930	-0.48	18.29	284.28	17.81	9	7/14/2014	47:06.0
1991	RSPe_2	-122.445	37.930	-0.24	18.47	284.46	18.23	-122.445	37.930	-0.51	18.47	284.35	17.96	9	7/14/2014	47:05.9
1992	RSPe_2	-122.445	37.930	-0.24	18.19	284.50	17.95	-122.445	37.930	-0.51	18.19	284.32	17.68	9	7/14/2014	47:05.8
1993	RSPe_2	-122.445	37.930	-0.21	18.13	284.52	17.92	-122.445	37.930	-0.56	18.13	284.32	17.57	9	7/14/2014	47:05.7
1994	RSPe_2	-122.445	37.930	-0.24	18.22	284.52	17.97	-122.445	37.930	-0.56	18.22	284.33	17.66	9	7/14/2014	47:05.6
1995	RSPe_2	-122.445	37.930	-0.24	18.06	284.30	17.82	-122.445	37.930	-0.56	18.06	284.18	17.50	9	7/14/2014	47:05.5
1996	RSPe_2	-122.445	37.930	-0.24	18.07	284.28	17.83	-122.445	37.930	-0.60	18.07	284.23	17.47	9	7/14/2014	47:05.4
1997	RSPe_2	-122.445	37.930	-0.24	18.14	284.35	17.90	-122.445	37.930	-0.56	18.14	284.29	17.58	9	7/14/2014	47:05.3
1998	RSPe_2	-122.445	37.930	-0.29	18.20	284.35	17.91	-122.445	37.930	-0.56	18.20	284.30	17.64	9	7/14/2014	47:05.2
1999	RSPe_2	-122.445	37.930	-0.24	18.13	284.44	17.89	-122.445	37.930	-0.60	18.13	284.30	17.54	9	7/14/2014	47:05.1
2000	RSPe_2	-122.445	37.930	-0.24	18.10	284.37	17.86	-122.445	37.930	-0.63	18.10	284.24	17.47	9	7/14/2014	47:05.0
2001	RSPe_2	-122.445	37.930	-0.24	18.26	284.35	18.01	-122.445	37.930	-0.60	18.26	284.21	17.66	9	7/14/2014	47:04.9
2002	RSPe_2	-122.445	37.930	-0.24	18.25	284.29	18.00	-122.445	37.930	-0.56	18.25	284.13	17.68	9	7/14/2014	47:04.8
2003	RSPe_2	-122.445	37.930	-0.24	18.25	284.18	18.00	-122.445	37.930	-0.56	18.25	284.06	17.68	9	7/14/2014	47:04.7
2004	RSPe_2	-122.445	37.930	-0.24	18.28	284.13	18.03	-122.445	37.930	-0.60	18.28	284.04	17.68	9	7/14/2014	47:04.6
2005	RSPe_2	-122.445	37.930	-0.24	18.40	284.22	18.15	-122.445	37.930	-0.56	18.40	284.16	17.83	9	7/14/2014	47:04.5
2006	RSPe_2	-122.445	37.930	-0.29	18.38	284.29	18.08	-122.445	37.930	-0.51	18.38	284.19	17.87	9	7/14/2014	47:04.4
2007	RSPe_2	-122.445	37.930	-0.24	18.36	284.42	18.11	-122.445	37.930	-0.56	18.36	284.15	17.79	9	7/14/2014	47:04.3
2008	RSPe_2	-122.445	37.930	-0.29	18.44	284.49	18.15	-122.445	37.930	-0.56	18.44	284.13	17.88	9	7/14/2014	47:04.2
2009	RSPe_2	-122.445	37.930	-0.24	18.45	284.62	18.20	-122.445	37.930	-0.56	18.45	284.22	17.89	9	7/14/2014	47:04.1
2010	RSPe_2	-122.445	37.930	-0.33	18.36	284.68	18.04	-122.445	37.930	-0.51	18.36	284.18	17.85	9	7/14/2014	47:04.0
2011	RSPe_2	-122.445	37.930	-0.29	18.36	284.64	18.06	-122.445	37.930	-0.56	18.36	284.28	17.79	9	7/14/2014	47:03.9
2012	RSPe_2	-122.445	37.930	-0.29	18.32	284.62	18.02	-122.445	37.930	-0.48	18.32	284.29	17.84	9	7/14/2014	47:03.8

2013	RSPe_2	-122.445	37.930	-0.29	18.33	284.79	18.04	-122.445	37.930	-0.60	18.33	284.34	17.74	9	7/14/2014	47:03.7
2014	RSPe_2	-122.445	37.930	-0.29	18.30	284.84	18.01	-122.445	37.930	-0.56	18.30	284.46	17.74	9	7/14/2014	47:03.6
2015	RSPe_2	-122.445	37.930	-0.29	18.26	284.79	17.96	-122.445	37.930	-0.56	18.26	284.49	17.69	9	7/14/2014	47:03.5
2016	RSPe_2	-122.445	37.930	-0.29	18.30	284.76	18.00	-122.445	37.930	-0.60	18.30	284.48	17.70	9	7/14/2014	47:03.4
2017	RSPe_2	-122.445	37.930	-0.29	18.29	284.71	17.99	-122.445	37.930	-0.56	18.29	284.49	17.73	9	7/14/2014	47:03.3
2018	RSPe_2	-122.445	37.930	-0.29	18.29	284.69	17.99	-122.445	37.930	-0.51	18.29	284.55	17.78	9	7/14/2014	47:03.2
2019	RSPe_2	-122.445	37.930	-0.29	18.27	284.61	17.98	-122.445	37.930	-0.51	18.27	284.51	17.76	9	7/14/2014	47:03.1
2020	RSPe_2	-122.445	37.930	-0.29	18.28	284.64	17.98	-122.445	37.930	-0.51	18.28	284.66	17.77	9	7/14/2014	47:03.0
2021	RSPe_2	-122.445	37.930	-0.29	18.30	284.69	18.01	-122.445	37.930	-0.51	18.30	284.73	17.79	9	7/14/2014	47:02.9
2022	RSPe_2	-122.445	37.930	-0.29	18.32	284.67	18.02	-122.445	37.930	-0.51	18.32	284.77	17.80	9	7/14/2014	47:02.8
2023	RSPe_2	-122.445	37.930	-0.29	18.36	284.64	18.07	-122.445	37.930	-0.56	18.36	284.75	17.80	9	7/14/2014	47:02.7
2024	RSPe_2	-122.445	37.930	-0.29	18.42	284.76	18.12	-122.445	37.930	-0.56	18.42	284.84	17.85	9	7/14/2014	47:02.6
2025	RSPe_2	-122.445	37.930	-0.29	18.48	284.80	18.19	-122.445	37.930	-0.60	18.48	284.84	17.88	9	7/14/2014	47:02.5
2026	RSPe_2	-122.445	37.930	-0.29	18.42	284.92	18.12	-122.445	37.930	-0.56	18.42	284.82	17.85	9	7/14/2014	47:02.4
2027	RSPe_2	-122.445	37.930	-0.24	18.42	284.91	18.17	-122.445	37.930	-0.51	18.42	284.71	17.91	9	7/14/2014	47:02.3
2028	RSPe_2	-122.445	37.930	-0.29	18.40	285.00	18.10	-122.445	37.930	-0.43	18.40	284.77	17.97	9	7/14/2014	47:02.2
2029	RSPe_2	-122.445	37.930	-0.29	18.34	285.03	18.05	-122.445	37.930	-0.56	18.34	284.69	17.78	9	7/14/2014	47:02.1
2030	RSPe_2	-122.445	37.930	-0.29	18.35	284.98	18.05	-122.445	37.930	-0.48	18.35	284.71	17.87	9	7/14/2014	47:02.0
2031	RSPe_2	-122.445	37.930	-0.24	18.15	284.98	17.91	-122.445	37.930	-0.51	18.15	284.67	17.64	9	7/14/2014	47:01.9
2032	RSPe_2	-122.445	37.930	-0.29	18.06	284.92	17.77	-122.445	37.930	-0.51	18.06	284.69	17.55	9	7/14/2014	47:01.8
2033	RSPe_2	-122.445	37.930	-0.24	17.93	284.94	17.69	-122.445	37.930	-0.51	17.93	284.72	17.42	9	7/14/2014	47:01.7
2034	RSPe_2	-122.445	37.930	-0.29	17.94	284.98	17.65	-122.445	37.930	-0.51	17.94	284.80	17.43	9	7/14/2014	47:01.6
2035	RSPe_2	-122.445	37.930	-0.29	17.84	285.07	17.55	-122.445	37.930	-0.51	17.84	284.90	17.33	9	7/14/2014	47:01.5
2036	RSPe_2	-122.445	37.930	-0.29	17.85	285.18	17.55	-122.445	37.930	-0.51	17.85	285.02	17.33	9	7/14/2014	47:01.4
2037	RSPe_2	-122.445	37.930	-0.24	17.85	285.18	17.60	-122.445	37.930	-0.51	17.85	285.05	17.33	9	7/14/2014	47:01.3
2038	RSPe_2	-122.445	37.930	-0.24	17.78	285.22	17.53	-122.445	37.930	-0.51	17.78	285.09	17.26	9	7/14/2014	47:01.2
2039	RSPe_2	-122.445	37.930	-0.24	17.79	285.16	17.54	-122.445	37.930	-0.43	17.79	285.09	17.36	9	7/14/2014	47:01.1
2040	RSPe_2	-122.445	37.930	-0.29	17.70	285.14	17.41	-122.445	37.930	-0.51	17.70	285.03	17.19	9	7/14/2014	47:01.0
2041	RSPe_2	-122.445	37.930	-0.29	17.65	285.16	17.35	-122.445	37.930	-0.51	17.65	285.02	17.13	9	7/14/2014	47:00.9
2042	RSPe_2	-122.445	37.930	-0.24	17.65	285.21	17.41	-122.445	37.930	-0.48	17.65	285.03	17.17	9	7/14/2014	47:00.8
2043	RSPe_2	-122.445	37.930	-0.24	17.63	285.21	17.39	-122.445	37.930	-0.43	17.63	285.03	17.20	9	7/14/2014	47:00.7
2044	RSPe_2	-122.445	37.930	-0.29	17.65	285.25	17.36	-122.445	37.930	-0.48	17.65	285.07	17.17	9	7/14/2014	47:00.6
2045	RSPe_2	-122.445	37.930	-0.24	17.63	285.34	17.39	-122.445	37.930	-0.43	17.63	285.12	17.20	9	7/14/2014	47:00.5

2046	RSPe_2	-122.445	37.930	-0.24	17.67	285.32	17.43	-122.445	37.930	-0.48	17.67	285.16	17.19	9	7/14/2014	47:00.4
2047	RSPe_2	-122.445	37.930	-0.24	17.64	285.40	17.40	-122.445	37.930	-0.43	17.64	285.18	17.21	9	7/14/2014	47:00.3
2048	RSPe_2	-122.445	37.930	-0.24	17.72	285.45	17.47	-122.445	37.930	-0.43	17.72	285.23	17.29	9	7/14/2014	47:00.2
2049	RSPe_2	-122.445	37.930	-0.24	17.73	285.38	17.49	-122.445	37.930	-0.39	17.73	285.16	17.34	9	7/14/2014	47:00.1
2050	RSPe_2	-122.445	37.930	-0.24	17.70	285.41	17.46	-122.445	37.930	-0.39	17.70	285.25	17.31	9	7/14/2014	47:00.0
2051	RSPe_2	-122.445	37.930	-0.24	17.69	285.43	17.45	-122.445	37.930	-0.48	17.69	285.27	17.22	9	7/14/2014	46:59.9
2052	RSPe_2	-122.445	37.930	-0.24	17.72	285.49	17.48	-122.445	37.930	-0.39	17.72	285.29	17.33	9	7/14/2014	46:59.8
2053	RSPe_2	-122.445	37.930	-0.21	17.79	285.50	17.58	-122.445	37.930	-0.39	17.79	285.32	17.39	9	7/14/2014	46:59.7
2054	RSPe_2	-122.445	37.930	-0.21	17.80	285.56	17.59	-122.445	37.930	-0.43	17.80	285.38	17.37	9	7/14/2014	46:59.6
2055	RSPe_2	-122.445	37.930	-0.21	17.80	285.57	17.59	-122.445	37.930	-0.39	17.80	285.45	17.41	9	7/14/2014	46:59.5
2056	RSPe_2	-122.445	37.930	-0.24	17.82	285.58	17.57	-122.445	37.930	-0.34	17.82	285.49	17.47	9	7/14/2014	46:59.4
2057	RSPe_2	-122.445	37.930	-0.21	17.85	285.59	17.65	-122.445	37.930	-0.34	17.85	285.52	17.51	9	7/14/2014	46:59.3
2058	RSPe_2	-122.445	37.930	-0.21	17.89	285.63	17.68	-122.445	37.930	-0.39	17.89	285.56	17.50	9	7/14/2014	46:59.2
2059	RSPe_2	-122.445	37.930	-0.21	17.85	285.66	17.64	-122.445	37.930	-0.34	17.85	285.57	17.50	9	7/14/2014	46:59.1
2060	RSPe_2	-122.445	37.930	-0.21	17.88	285.65	17.67	-122.445	37.930	-0.34	17.88	285.56	17.53	9	7/14/2014	46:59.0
2061	RSPe_2	-122.445	37.930	-0.21	17.87	285.72	17.66	-122.445	37.930	-0.34	17.87	285.63	17.53	9	7/14/2014	46:58.9
2062	RSPe_2	-122.445	37.930	-0.24	17.91	285.67	17.67	-122.445	37.930	-0.34	17.91	285.58	17.57	9	7/14/2014	46:58.8
2063	RSPe_2	-122.445	37.930	-0.21	17.90	285.77	17.69	-122.445	37.930	-0.34	17.90	285.63	17.56	9	7/14/2014	46:58.7
2064	RSPe_2	-122.445	37.930	-0.21	17.92	285.81	17.72	-122.445	37.930	-0.34	17.92	285.65	17.58	9	7/14/2014	46:58.6
2065	RSPe_2	-122.445	37.930	-0.21	17.94	285.77	17.73	-122.445	37.930	-0.39	17.94	285.68	17.55	9	7/14/2014	46:58.5
2066	RSPe_2	-122.445	37.930	-0.21	17.95	285.77	17.74	-122.445	37.930	-0.43	17.95	285.67	17.52	9	7/14/2014	46:58.4
2067	RSPe_2	-122.445	37.930	-0.18	17.99	285.79	17.82	-122.445	37.930	-0.26	17.99	285.70	17.74	9	7/14/2014	46:58.3
2068	RSPe_2	-122.445	37.930	-0.21	18.01	285.79	17.80	-122.445	37.930	-0.31	18.01	285.71	17.70	9	7/14/2014	46:58.2
2069	RSPe_2	-122.445	37.930	-0.18	18.04	285.84	17.86	-122.445	37.930	-0.34	18.04	285.83	17.70	9	7/14/2014	46:58.1
2070	RSPe_2	-122.445	37.930	-0.21	18.09	285.88	17.88	-122.445	37.930	-0.31	18.09	285.81	17.78	9	7/14/2014	46:58.0
2071	RSPe_2	-122.445	37.930	-0.18	18.11	285.88	17.93	-122.445	37.930	-0.26	18.11	285.85	17.85	9	7/14/2014	46:57.9
2072	RSPe_2	-122.445	37.930	-0.21	18.13	285.92	17.92	-122.445	37.930	-0.34	18.13	285.87	17.79	9	7/14/2014	46:57.8
2073	RSPe_2	-122.445	37.930	-0.18	18.13	285.99	17.96	-122.445	37.930	-0.31	18.13	285.92	17.82	9	7/14/2014	46:57.7
2074	RSPe_2	-122.445	37.930	-0.17	18.11	286.10	17.93	-122.445	37.930	-0.31	18.11	286.01	17.80	9	7/14/2014	46:57.6
2075	RSPe_2	-122.445	37.930	-0.17	18.13	286.13	17.96	-122.445	37.930	-0.34	18.13	286.03	17.79	9	7/14/2014	46:57.5
2076	RSPe_2	-122.445	37.930	-0.17	18.12	286.21	17.94	-122.445	37.930	-0.31	18.12	286.12	17.81	9	7/14/2014	46:57.4
2077	RSPe_2	-122.445	37.930	-0.17	18.07	286.24	17.90	-122.445	37.930	-0.34	18.07	286.09	17.73	9	7/14/2014	46:57.3
2078	RSPe_2	-122.445	37.930	-0.17	18.05	286.24	17.87	-122.445	37.930	-0.31	18.05	286.16	17.74	9	7/14/2014	46:57.2

2079	RSPe_2	-122.445	37.930	-0.17	18.04	286.23	17.86	-122.445	37.930	-0.31	18.04	286.17	17.73	9	7/14/2014	46:57.1
2080	RSPe_2	-122.445	37.930	-0.17	18.05	286.24	17.88	-122.445	37.930	-0.34	18.05	286.23	17.71	9	7/14/2014	46:57.0
2081	RSPe_2	-122.445	37.930	-0.17	18.03	286.32	17.86	-122.445	37.930	-0.22	18.03	286.32	17.81	9	7/14/2014	46:56.9
2082	RSPe_2	-122.445	37.930	-0.17	18.04	286.37	17.86	-122.445	37.930	-0.26	18.04	286.32	17.78	9	7/14/2014	46:56.8
2083	RSPe_2	-122.445	37.930	-0.12	18.05	286.44	17.93	-122.445	37.930	-0.31	18.05	286.37	17.74	9	7/14/2014	46:56.7
2084	RSPe_2	-122.445	37.930	-0.17	18.14	286.39	17.97	-122.445	37.930	-0.31	18.14	286.34	17.83	9	7/14/2014	46:56.6
2085	RSPe_2	-122.445	37.930	-0.17	18.12	286.48	17.94	-122.445	37.930	-0.22	18.12	286.37	17.90	9	7/14/2014	46:56.5
2086	RSPe_2	-122.445	37.930	-0.17	18.14	286.48	17.97	-122.445	37.930	-0.31	18.14	286.39	17.83	9	7/14/2014	46:56.4
2087	RSPe_2	-122.445	37.930	-0.17	18.05	286.50	17.88	-122.445	37.930	-0.34	18.05	286.41	17.71	9	7/14/2014	46:56.3
2088	RSPe_2	-122.445	37.930	-0.17	18.00	286.57	17.83	-122.445	37.930	-0.31	18.00	286.46	17.69	9	7/14/2014	46:56.2
2089	RSPe_2	-122.445	37.930	-0.17	17.96	286.66	17.78	-122.445	37.930	-0.31	17.96	286.50	17.65	9	7/14/2014	46:56.1
2090	RSPe_2	-122.445	37.930	-0.17	17.93	286.77	17.76	-122.445	37.930	-0.26	17.93	286.54	17.67	9	7/14/2014	46:56.0
2091	RSPe_2	-122.445	37.930	-0.17	17.90	286.97	17.73	-122.445	37.930	-0.22	17.90	286.66	17.68	9	7/14/2014	46:55.9
2092	RSPe_2	-122.445	37.930	-0.17	17.87	287.10	17.70	-122.445	37.930	-0.22	17.87	286.72	17.65	9	7/14/2014	46:55.8
2093	RSPe_2	-122.445	37.930	-0.17	17.92	287.14	17.75	-122.445	37.930	-0.31	17.92	286.75	17.62	9	7/14/2014	46:55.7
2094	RSPe_2	-122.445	37.930	-0.17	17.84	287.19	17.67	-122.445	37.930	-0.26	17.84	286.80	17.58	9	7/14/2014	46:55.6
2095	RSPe_2	-122.445	37.930	-0.12	17.83	287.21	17.71	-122.445	37.930	-0.26	17.83	286.92	17.58	9	7/14/2014	46:55.5
2096	RSPe_2	-122.445	37.930	-0.17	17.72	287.14	17.55	-122.445	37.930	-0.31	17.72	286.90	17.41	9	7/14/2014	46:55.4
2097	RSPe_2	-122.445	37.930	-0.12	17.70	287.21	17.58	-122.445	37.930	-0.26	17.70	287.01	17.44	9	7/14/2014	46:55.3
2098	RSPe_2	-122.445	37.930	-0.17	17.74	287.26	17.56	-122.445	37.930	-0.31	17.74	287.10	17.43	9	7/14/2014	46:55.2
2099	RSPe_2	-122.445	37.930	-0.17	17.68	287.23	17.50	-122.445	37.930	-0.26	17.68	287.17	17.42	9	7/14/2014	46:55.1
2100	RSPe_2	-122.445	37.930	-0.17	17.65	287.32	17.48	-122.445	37.930	-0.31	17.65	287.23	17.34	9	7/14/2014	46:55.0
2101	RSPe_2	-122.445	37.930	-0.12	17.66	287.43	17.54	-122.445	37.930	-0.26	17.66	287.32	17.41	9	7/14/2014	46:54.9
2102	RSPe_2	-122.445	37.930	-0.17	17.64	287.41	17.47	-122.445	37.930	-0.26	17.64	287.32	17.38	9	7/14/2014	46:54.8
2103	RSPe_2	-122.445	37.930	-0.12	17.66	287.52	17.54	-122.445	37.930	-0.26	17.66	287.36	17.41	9	7/14/2014	46:54.7
2104	RSPe_2	-122.445	37.930	-0.17	17.64	287.54	17.47	-122.445	37.930	-0.31	17.64	287.34	17.33	9	7/14/2014	46:54.6
2105	RSPe_2	-122.445	37.930	-0.12	17.64	287.63	17.52	-122.445	37.930	-0.22	17.64	287.43	17.42	9	7/14/2014	46:54.5
2106	RSPe_2	-122.445	37.930	-0.17	17.68	287.61	17.50	-122.445	37.930	-0.26	17.68	287.43	17.42	9	7/14/2014	46:54.4
2107	RSPe_2	-122.445	37.930	-0.17	17.64	287.57	17.47	-122.445	37.930	-0.31	17.64	287.44	17.33	9	7/14/2014	46:54.3
2108	RSPe_2	-122.445	37.930	-0.17	17.65	287.59	17.47	-122.445	37.930	-0.31	17.65	287.49	17.34	9	7/14/2014	46:54.2
2109	RSPe_2	-122.445	37.930	-0.12	17.66	287.59	17.54	-122.445	37.930	-0.22	17.66	287.57	17.44	9	7/14/2014	46:54.1
2110	RSPe_2	-122.445	37.930	-0.17	17.72	287.63	17.54	-122.445	37.930	-0.31	17.72	287.65	17.41	9	7/14/2014	46:54.0
2111	RSPe_2	-122.445	37.930	-0.12	17.75	287.66	17.63	-122.445	37.930	-0.26	17.75	287.68	17.50	9	7/14/2014	46:53.9

2112	RSPe_2	-122.445	37.930	-0.17	17.84	287.68	17.67	-122.445	37.930	-0.26	17.84	287.69	17.58	9	7/14/2014 46:53.8
2113	RSPe_2	-122.445	37.930	-0.12	17.88	287.88	17.75	-122.445	37.930	-0.26	17.88	287.79	17.62	9	7/14/2014 46:53.7
2114	RSPe_2	-122.445	37.930	-0.17	17.85	287.97	17.67	-122.445	37.930	-0.31	17.85	287.83	17.54	9	7/14/2014 46:53.6
2115	RSPe_2	-122.445	37.930	-0.12	17.85	288.15	17.73	-122.445	37.930	-0.22	17.85	287.92	17.63	9	7/14/2014 46:53.5
2116	RSPe_2	-122.445	37.930	-0.12	17.87	288.21	17.75	-122.445	37.930	-0.31	17.87	288.01	17.56	9	7/14/2014 46:53.4
2117	RSPe_2	-122.445	37.930	-0.12	17.85	288.32	17.73	-122.445	37.930	-0.19	17.85	288.03	17.66	9	7/14/2014 46:53.3
2118	RSPe_2	-122.445	37.930	-0.12	17.87	288.32	17.75	-122.445	37.930	-0.26	17.87	288.01	17.61	9	7/14/2014 46:53.2
2119	RSPe_2	-122.445	37.930	-0.12	17.83	288.32	17.71	-122.445	37.930	-0.22	17.83	288.02	17.61	9	7/14/2014 46:53.1
2120	RSPe_2	-122.445	37.930	-0.17	17.85	288.34	17.67	-122.445	37.930	-0.26	17.85	288.05	17.59	9	7/14/2014 46:53.0
2121	RSPe_2	-122.445	37.930	-0.12	17.86	288.30	17.74	-122.445	37.930	-0.19	17.86	288.03	17.67	9	7/14/2014 46:52.9
2122	RSPe_2	-122.445	37.930	-0.12	17.86	288.43	17.74	-122.445	37.930	-0.22	17.86	288.12	17.64	9	7/14/2014 46:52.8
2123	RSPe_2	-122.445	37.930	-0.12	17.89	288.56	17.77	-122.445	37.930	-0.26	17.89	288.23	17.64	9	7/14/2014 46:52.7
2124	RSPe_2	-122.445	37.930	-0.12	17.92	288.79	17.80	-122.445	37.930	-0.26	17.92	288.36	17.67	9	7/14/2014 46:52.6
2125	RSPe_2	-122.445	37.930	-0.12	17.93	288.92	17.81	-122.445	37.930	-0.19	17.93	288.52	17.74	9	7/14/2014 46:52.5
2126	RSPe_2	-122.445	37.930	-0.17	17.96	289.05	17.78	-122.445	37.930	-0.19	17.96	288.63	17.77	9	7/14/2014 46:52.4
2127	RSPe_2	-122.445	37.930	-0.12	17.99	289.01	17.86	-122.445	37.930	-0.22	17.99	288.71	17.76	9	7/14/2014 46:52.3
2128	RSPe_2	-122.445	37.930	-0.12	18.03	288.96	17.90	-122.445	37.930	-0.22	18.03	288.78	17.80	9	7/14/2014 46:52.2
2129	RSPe_2	-122.445	37.930	-0.12	18.09	288.96	17.96	-122.445	37.930	-0.22	18.09	288.85	17.86	9	7/14/2014 46:52.1
2130	RSPe_2	-122.445	37.930	-0.12	18.08	289.01	17.96	-122.445	37.930	-0.22	18.08	288.93	17.86	9	7/14/2014 46:52.0
2131	RSPe_2	-122.445	37.930	-0.09	18.13	289.10	18.04	-122.445	37.930	-0.14	18.13	289.01	17.99	9	7/14/2014 46:51.9
2132	RSPe_2	-122.445	37.930	-0.12	18.09	289.14	17.96	-122.445	37.930	-0.19	18.09	289.05	17.90	9	7/14/2014 46:51.8
2133	RSPe_2	-122.445	37.930	-0.09	18.09	289.19	18.00	-122.445	37.930	-0.11	18.09	289.03	17.98	9	7/14/2014 46:51.7
2134	RSPe_2	-122.445	37.930	-0.12	18.08	289.32	17.96	-122.445	37.930	-0.14	18.08	289.05	17.94	9	7/14/2014 46:51.6
2135	RSPe_2	-122.445	37.930	-0.09	18.07	289.36	17.98	-122.445	37.930	-0.11	18.07	289.07	17.96	9	7/14/2014 46:51.5
2136	RSPe_2	-122.445	37.930	-0.09	18.07	289.54	17.98	-122.445	37.930	-0.05	18.07	289.21	18.02	9	7/14/2014 46:51.4
2137	RSPe_2	-122.445	37.930	-0.09	18.06	289.58	17.97	-122.445	37.930	-0.11	18.06	289.27	17.96	9	7/14/2014 46:51.3
2138	RSPe_2	-122.445	37.930	-0.12	18.05	289.69	17.93	-122.445	37.930	-0.14	18.05	289.34	17.91	9	7/14/2014 46:51.2
2139	RSPe_2	-122.445	37.930	-0.09	18.03	289.72	17.94	-122.445	37.930	-0.11	18.03	289.36	17.93	9	7/14/2014 46:51.1
2140	RSPe_2	-122.445	37.930	-0.09	18.01	289.72	17.92	-122.445	37.930	-0.05	18.01	289.43	17.96	9	7/14/2014 46:51.0
2141	RSPe_2	-122.445	37.930	-0.09	18.00	289.81	17.91	-122.445	37.930	-0.11	18.00	289.58	17.90	9	7/14/2014 46:50.9
2142	RSPe_2	-122.445	37.930	-0.12	18.00	289.87	17.88	-122.445	37.930	-0.05	18.00	289.72	17.95	9	7/14/2014 46:50.8
2143	RSPe_2	-122.445	37.930	-0.09	18.01	289.94	17.92	-122.445	37.930	-0.11	18.01	289.84	17.91	9	7/14/2014 46:50.7
2144	RSPe_2	-122.445	37.930	-0.09	18.00	289.93	17.91	-122.445	37.930	-0.11	18.00	289.93	17.90	9	7/14/2014 46:50.6

2145	RSPe_2	-122.445	37.930	-0.09	17.97	289.94	17.88	-122.445	37.930	-0.11	17.97	290.00	17.87	9	7/14/2014 46:50.5
2146	RSPe_2	-122.445	37.930	-0.09	17.98	290.00	17.89	-122.445	37.930	-0.14	17.98	290.13	17.84	9	7/14/2014 46:50.4
2147	RSPe_2	-122.445	37.930	-0.09	17.96	289.96	17.87	-122.445	37.930	-0.11	17.96	290.16	17.85	9	7/14/2014 46:50.3
2148	RSPe_2	-122.445	37.930	-0.09	17.96	290.01	17.87	-122.445	37.930	-0.11	17.96	290.24	17.85	9	7/14/2014 46:50.2
2149	RSPe_2	-122.445	37.930	-0.09	17.89	290.05	17.80	-122.445	37.930	-0.05	17.89	290.29	17.83	9	7/14/2014 46:50.1
2150	RSPe_2	-122.445	37.930	-0.09	17.87	290.14	17.78	-122.445	37.930	-0.14	17.87	290.31	17.73	9	7/14/2014 46:50.0
2151	RSPe_2	-122.445	37.930	-0.09	17.87	290.18	17.78	-122.445	37.930	-0.14	17.87	290.33	17.73	9	7/14/2014 46:49.9
2152	RSPe_2	-122.445	37.930	-0.09	17.87	290.34	17.78	-122.445	37.930	-0.19	17.87	290.33	17.68	9	7/14/2014 46:49.8
2153	RSPe_2	-122.445	37.930	-0.09	17.85	290.47	17.77	-122.445	37.930	-0.14	17.85	290.42	17.72	9	7/14/2014 46:49.7
2154	RSPe_2	-122.445	37.930	-0.12	17.89	290.60	17.76	-122.445	37.930	-0.14	17.89	290.42	17.75	9	7/14/2014 46:49.6
2155	RSPe_2	-122.445	37.930	-0.09	17.85	290.69	17.77	-122.445	37.930	-0.14	17.85	290.41	17.72	9	7/14/2014 46:49.5
2156	RSPe_2	-122.445	37.930	-0.09	17.79	290.71	17.70	-122.445	37.930	-0.19	17.79	290.42	17.60	9	7/14/2014 46:49.4
2157	RSPe_2	-122.445	37.930	-0.09	17.75	290.80	17.66	-122.445	37.930	-0.19	17.75	290.47	17.56	9	7/14/2014 46:49.3
2158	RSPe_2	-122.445	37.930	-0.09	17.68	290.71	17.60	-122.445	37.930	-0.14	17.68	290.36	17.55	9	7/14/2014 46:49.2
2159	RSPe_2	-122.445	37.930	-0.09	17.72	290.71	17.63	-122.445	37.930	-0.05	17.72	290.49	17.66	9	7/14/2014 46:49.1
2160	RSPe_2	-122.445	37.930	-0.09	17.67	290.67	17.58	-122.445	37.930	-0.14	17.67	290.53	17.53	9	7/14/2014 46:49.0
2161	RSPe_2	-122.445	37.930	-0.09	17.68	290.72	17.60	-122.445	37.930	-0.05	17.68	290.58	17.63	9	7/14/2014 46:48.9
2162	RSPe_2	-122.445	37.930	-0.09	17.70	290.74	17.61	-122.445	37.930	-0.11	17.70	290.76	17.60	9	7/14/2014 46:48.8
2163	RSPe_2	-122.445	37.930	-0.04	17.69	290.65	17.66	-122.445	37.930	-0.11	17.69	290.71	17.59	9	7/14/2014 46:48.7
2164	RSPe_2	-122.445	37.930	-0.09	17.73	290.72	17.64	-122.445	37.930	-0.11	17.73	290.87	17.63	9	7/14/2014 46:48.6
2165	RSPe_2	-122.445	37.930	-0.09	17.77	290.87	17.68	-122.445	37.930	-0.14	17.77	291.11	17.63	9	7/14/2014 46:48.5
2166	RSPe_2	-122.445	37.930	-0.09	17.79	290.85	17.70	-122.445	37.930	-0.19	17.79	291.16	17.60	9	7/14/2014 46:48.4
2167	RSPe_2	-122.445	37.930	-0.09	17.80	290.96	17.71	-122.445	37.930	-0.19	17.80	291.28	17.61	9	7/14/2014 46:48.3
2168	RSPe_2	-122.445	37.930	-0.12	17.81	290.98	17.69	-122.445	37.930	-0.22	17.81	291.31	17.59	9	7/14/2014 46:48.2
2169	RSPe_2	-122.445	37.930	-0.09	17.82	291.00	17.73	-122.445	37.930	-0.22	17.82	291.31	17.60	9	7/14/2014 46:48.1
2170	RSPe_2	-122.445	37.930	-0.12	17.85	291.02	17.72	-122.445	37.930	-0.19	17.85	291.22	17.66	9	7/14/2014 46:48.0
2171	RSPe_2	-122.445	37.930	-0.12	17.82	291.10	17.70	-122.445	37.930	-0.22	17.82	291.25	17.60	9	7/14/2014 46:47.9
2172	RSPe_2	-122.445	37.930	-0.12	17.85	291.18	17.73	-122.445	37.930	-0.22	17.85	291.22	17.63	9	7/14/2014 46:47.8
2173	RSPe_2	-122.445	37.930	-0.09	17.89	291.21	17.80	-122.445	37.930	-0.19	17.89	291.16	17.70	9	7/14/2014 46:47.7
2174	RSPe_2	-122.445	37.930	-0.12	17.98	291.27	17.86	-122.445	37.930	-0.22	17.98	291.14	17.76	9	7/14/2014 46:47.6
2175	RSPe_2	-122.445	37.930	-0.12	17.91	291.34	17.79	-122.445	37.930	-0.22	17.91	291.13	17.69	9	7/14/2014 46:47.5
2176	RSPe_2	-122.445	37.930	-0.12	17.90	291.40	17.78	-122.445	37.930	-0.19	17.90	291.09	17.71	9	7/14/2014 46:47.4
2177	RSPe_2	-122.445	37.930	-0.09	17.96	291.49	17.87	-122.445	37.930	-0.22	17.96	291.14	17.74	9	7/14/2014 46:47.3

2178	RSPe_2	-122.445	37.930	-0.09	18.05	291.54	17.96	-122.445	37.930	-0.22	18.05	291.20	17.83	9	7/14/2014	46:47.2
2179	RSPe_2	-122.445	37.930	-0.09	18.06	291.56	17.97	-122.445	37.930	-0.19	18.06	291.27	17.87	9	7/14/2014	46:47.1
2180	RSPe_2	-122.445	37.930	-0.12	18.07	291.63	17.95	-122.445	37.930	-0.22	18.07	291.33	17.85	9	7/14/2014	46:47.0
2181	RSPe_2	-122.445	37.930	-0.09	18.05	291.63	17.97	-122.445	37.930	-0.22	18.05	291.40	17.83	9	7/14/2014	46:46.9
2182	RSPe_2	-122.445	37.930	-0.12	18.07	291.78	17.95	-122.445	37.930	-0.22	18.07	291.52	17.85	9	7/14/2014	46:46.8
2183	RSPe_2	-122.445	37.930	-0.09	18.07	291.87	17.98	-122.445	37.930	-0.22	18.07	291.67	17.85	9	7/14/2014	46:46.7
2184	RSPe_2	-122.445	37.930	-0.12	18.12	291.94	18.00	-122.445	37.930	-0.22	18.12	291.67	17.90	9	7/14/2014	46:46.6
2185	RSPe_2	-122.445	37.930	-0.09	18.11	291.98	18.02	-122.445	37.930	-0.22	18.11	291.71	17.88	9	7/14/2014	46:46.5
2186	RSPe_2	-122.445	37.930	-0.12	18.12	291.87	18.00	-122.445	37.930	-0.26	18.12	291.67	17.87	9	7/14/2014	46:46.4
2187	RSPe_2	-122.445	37.930	-0.12	18.15	291.89	18.03	-122.445	37.930	-0.22	18.15	291.65	17.93	9	7/14/2014	46:46.3
2188	RSPe_2	-122.445	37.930	-0.12	18.13	291.98	18.01	-122.445	37.930	-0.31	18.13	291.76	17.82	9	7/14/2014	46:46.2
2189	RSPe_2	-122.445	37.930	-0.09	18.17	292.14	18.08	-122.445	37.930	-0.19	18.17	291.89	17.98	9	7/14/2014	46:46.1
2190	RSPe_2	-122.445	37.930	-0.12	18.17	292.16	18.04	-122.445	37.930	-0.19	18.17	291.93	17.98	9	7/14/2014	46:46.0
2191	RSPe_2	-122.445	37.930	-0.09	18.12	292.14	18.04	-122.445	37.930	-0.19	18.12	291.96	17.93	9	7/14/2014	46:45.9
2192	RSPe_2	-122.445	37.930	-0.12	18.16	292.11	18.03	-122.445	37.930	-0.22	18.16	292.02	17.93	9	7/14/2014	46:45.8
2193	RSPe_2	-122.445	37.930	-0.09	18.19	292.12	18.10	-122.445	37.930	-0.22	18.19	292.06	17.96	9	7/14/2014	46:45.7
2194	RSPe_2	-122.445	37.930	-0.12	18.16	292.09	18.03	-122.445	37.930	-0.22	18.16	292.13	17.93	9	7/14/2014	46:45.6
2195	RSPe_2	-122.445	37.930	-0.09	18.19	291.98	18.10	-122.445	37.930	-0.26	18.19	292.07	17.94	9	7/14/2014	46:45.5
2196	RSPe_2	-122.445	37.930	-0.12	18.24	292.01	18.11	-122.445	37.930	-0.22	18.24	292.13	18.01	9	7/14/2014	46:45.4
2197	RSPe_2	-122.445	37.930	-0.09	18.24	292.01	18.15	-122.445	37.930	-0.19	18.24	292.14	18.05	9	7/14/2014	46:45.3
2198	RSPe_2	-122.445	37.930	-0.12	18.24	292.08	18.11	-122.445	37.930	-0.19	18.24	292.16	18.05	9	7/14/2014	46:45.2
2199	RSPe_2	-122.445	37.930	-0.09	18.24	292.07	18.15	-122.445	37.930	-0.22	18.24	292.05	18.01	9	7/14/2014	46:45.1
2200	RSPe_2	-122.445	37.930	-0.12	18.24	292.10	18.12	-122.445	37.930	-0.22	18.24	291.98	18.02	9	7/14/2014	46:45.0
2201	RSPe_2	-122.445	37.930	-0.09	18.24	292.05	18.15	-122.445	37.930	-0.19	18.24	291.94	18.05	9	7/14/2014	46:44.9
2202	RSPe_2	-122.445	37.930	-0.09	18.23	292.12	18.14	-122.445	37.930	-0.19	18.23	291.94	18.04	9	7/14/2014	46:44.8
2203	RSPe_2	-122.445	37.930	-0.09	18.24	292.05	18.15	-122.445	37.930	-0.19	18.24	291.85	18.05	9	7/14/2014	46:44.7
2204	RSPe_2	-122.445	37.930	-0.09	18.25	292.10	18.16	-122.445	37.930	-0.19	18.25	291.89	18.06	9	7/14/2014	46:44.6
2205	RSPe_2	-122.445	37.930	-0.09	18.23	292.06	18.14	-122.445	37.930	-0.19	18.23	291.82	18.04	9	7/14/2014	46:44.5
2206	RSPe_2	-122.445	37.930	-0.09	18.25	292.05	18.16	-122.445	37.930	-0.22	18.25	291.86	18.02	9	7/14/2014	46:44.4
2207	RSPe_2	-122.445	37.930	-0.04	18.24	292.08	18.20	-122.445	37.930	-0.19	18.24	291.87	18.05	9	7/14/2014	46:44.3
2208	RSPe_2	-122.445	37.930	-0.09	18.25	292.17	18.16	-122.445	37.930	-0.19	18.25	291.92	18.06	9	7/14/2014	46:44.2
2209	RSPe_2	-122.445	37.930	-0.04	18.26	292.16	18.22	-122.445	37.930	-0.14	18.26	292.03	18.12	9	7/14/2014	46:44.1
2210	RSPe_2	-122.445	37.930	-0.09	18.24	292.23	18.15	-122.445	37.930	-0.19	18.24	292.10	18.05	9	7/14/2014	46:44.0

2211	RSPe_2	-122.445	37.930	-0.04	18.24	292.34	18.20	-122.445	37.930	-0.19	18.24	292.18	18.05	9	7/14/2014	46:43.9
2212	RSPe_2	-122.445	37.930	-0.04	18.25	292.43	18.21	-122.445	37.930	-0.14	18.25	292.25	18.11	9	7/14/2014	46:43.8
2213	RSPe_2	-122.445	37.930	-0.04	18.21	292.43	18.17	-122.445	37.930	-0.14	18.21	292.23	18.07	9	7/14/2014	46:43.7
2214	RSPe_2	-122.445	37.930	-0.04	18.20	292.46	18.17	-122.445	37.930	-0.10	18.20	292.30	18.10	9	7/14/2014	46:43.6
2215	RSPe_2	-122.445	37.930	0.00	18.22	292.48	18.22	-122.445	37.930	-0.10	18.22	292.26	18.11	9	7/14/2014	46:43.5
2216	RSPe_2	-122.445	37.930	-0.04	18.17	292.55	18.13	-122.445	37.930	-0.10	18.17	292.30	18.06	9	7/14/2014	46:43.4
2217	RSPe_2	-122.445	37.930	0.00	18.15	292.63	18.15	-122.445	37.930	-0.05	18.15	292.34	18.10	9	7/14/2014	46:43.3
2218	RSPe_2	-122.445	37.930	-0.04	18.12	292.68	18.09	-122.445	37.930	-0.05	18.12	292.36	18.07	9	7/14/2014	46:43.2
2219	RSPe_2	-122.445	37.930	0.00	18.05	292.74	18.05	-122.445	37.930	-0.05	18.05	292.41	18.00	9	7/14/2014	46:43.1
2220	RSPe_2	-122.445	37.930	-0.04	18.01	292.77	17.97	-122.445	37.930	-0.05	18.01	292.50	17.96	9	7/14/2014	46:43.0
2221	RSPe_2	-122.445	37.930	0.00	17.83	292.79	17.83	-122.445	37.930	-0.05	17.83	292.55	17.78	9	7/14/2014	46:42.9
2222	RSPe_2	-122.445	37.930	0.00	17.88	292.75	17.88	-122.445	37.930	-0.05	17.88	292.66	17.82	9	7/14/2014	46:42.8
2223	RSPe_2	-122.445	37.930	0.00	17.75	292.75	17.75	-122.445	37.930	-0.05	17.75	292.74	17.69	9	7/14/2014	46:42.7
2224	RSPe_2	-122.445	37.930	0.00	17.69	292.77	17.69	-122.445	37.930	-0.05	17.69	292.85	17.64	9	7/14/2014	46:42.6
2225	RSPe_2	-122.445	37.930	0.05	17.70	292.77	17.75	-122.445	37.930	-0.05	17.70	292.92	17.65	9	7/14/2014	46:42.5
2226	RSPe_2	-122.445	37.930	0.00	17.63	292.83	17.62	-122.445	37.930	-0.02	17.63	293.03	17.61	9	7/14/2014	46:42.4
2227	RSPe_2	-122.445	37.930	0.05	17.70	292.83	17.75	-122.445	37.930	-0.02	17.70	292.94	17.68	9	7/14/2014	46:42.3
2228	RSPe_2	-122.445	37.930	0.00	17.68	292.88	17.68	-122.445	37.930	-0.02	17.68	292.90	17.66	9	7/14/2014	46:42.2
2229	RSPe_2	-122.445	37.930	0.05	17.74	292.84	17.79	-122.445	37.930	0.03	17.74	292.81	17.77	9	7/14/2014	46:42.1
2230	RSPe_2	-122.445	37.930	0.00	17.58	292.84	17.58	-122.445	37.930	0.07	17.58	292.70	17.65	9	7/14/2014	46:42.0
2231	RSPe_2	-122.445	37.930	0.00	17.61	292.99	17.61	-122.445	37.930	0.12	17.61	292.72	17.72	9	7/14/2014	46:41.9
2232	RSPe_2	-122.445	37.930	0.05	17.54	292.95	17.59	-122.445	37.930	0.12	17.54	292.66	17.65	9	7/14/2014	46:41.8
2233	RSPe_2	-122.445	37.930	0.05	17.53	292.97	17.58	-122.445	37.930	0.12	17.53	292.70	17.65	9	7/14/2014	46:41.7
2234	RSPe_2	-122.445	37.930	0.00	17.54	292.93	17.54	-122.445	37.930	0.07	17.54	292.66	17.60	9	7/14/2014	46:41.6
2235	RSPe_2	-122.445	37.930	0.05	17.54	292.93	17.59	-122.445	37.930	0.07	17.54	292.72	17.60	9	7/14/2014	46:41.5
2236	RSPe_2	-122.445	37.930	0.05	17.61	292.89	17.66	-122.445	37.930	0.07	17.61	292.77	17.68	9	7/14/2014	46:41.4
2237	RSPe_2	-122.445	37.930	0.05	17.57	292.89	17.62	-122.445	37.930	0.12	17.57	292.84	17.69	9	7/14/2014	46:41.3
2238	RSPe_2	-122.445	37.930	0.00	17.64	292.84	17.64	-122.445	37.930	0.03	17.64	292.90	17.67	9	7/14/2014	46:41.2
2239	RSPe_2	-122.445	37.930	0.05	17.63	292.86	17.68	-122.445	37.930	0.07	17.63	292.90	17.70	9	7/14/2014	46:41.1
2240	RSPe_2	-122.445	37.930	0.00	17.64	292.84	17.64	-122.445	37.930	0.07	17.64	292.90	17.70	9	7/14/2014	46:41.0
2241	RSPe_2	-122.445	37.930	0.05	17.66	292.89	17.71	-122.445	37.930	0.07	17.66	292.93	17.73	9	7/14/2014	46:40.9
2242	RSPe_2	-122.445	37.930	0.00	17.75	292.89	17.75	-122.445	37.930	0.03	17.75	292.92	17.78	9	7/14/2014	46:40.8
2243	RSPe_2	-122.445	37.930	0.00	17.75	292.91	17.75	-122.445	37.930	0.07	17.75	292.86	17.81	9	7/14/2014	46:40.7

2244	RSPe_2	-122.445	37.930	0.00	17.77	292.95	17.77	-122.445	37.930	0.03	17.77	292.86	17.80	9	7/14/2014	46:40.6
2245	RSPe_2	-122.445	37.930	0.05	17.86	292.98	17.91	-122.445	37.930	0.03	17.86	292.84	17.89	9	7/14/2014	46:40.5
2246	RSPe_2	-122.445	37.930	0.00	17.80	292.96	17.80	-122.445	37.930	0.07	17.80	292.80	17.87	9	7/14/2014	46:40.4
2247	RSPe_2	-122.445	37.930	0.05	17.84	292.98	17.89	-122.445	37.930	0.07	17.84	292.82	17.91	9	7/14/2014	46:40.3
2248	RSPe_2	-122.445	37.930	0.00	17.89	293.02	17.88	-122.445	37.930	0.07	17.89	292.78	17.95	9	7/14/2014	46:40.2
2249	RSPe_2	-122.445	37.930	0.05	17.91	292.93	17.96	-122.445	37.930	0.07	17.91	292.76	17.97	9	7/14/2014	46:40.1
2250	RSPe_2	-122.445	37.930	0.00	17.93	292.91	17.93	-122.445	37.930	0.12	17.93	292.80	18.05	9	7/14/2014	46:40.0
2251	RSPe_2	-122.445	37.930	0.05	17.93	292.87	17.98	-122.445	37.930	0.12	17.93	292.89	18.05	9	7/14/2014	46:39.9
2252	RSPe_2	-122.445	37.930	0.00	17.93	292.85	17.93	-122.445	37.930	0.07	17.93	292.91	18.00	9	7/14/2014	46:39.8
2253	RSPe_2	-122.445	37.930	0.05	17.93	292.81	17.98	-122.445	37.930	0.07	17.93	292.93	18.00	9	7/14/2014	46:39.7
2254	RSPe_2	-122.445	37.930	0.00	17.95	292.70	17.95	-122.445	37.930	0.03	17.95	292.89	17.98	9	7/14/2014	46:39.6
2255	RSPe_2	-122.445	37.930	0.00	17.98	292.63	17.98	-122.445	37.930	0.07	17.98	292.85	18.04	9	7/14/2014	46:39.5
2256	RSPe_2	-122.445	37.930	-0.04	17.97	292.67	17.94	-122.445	37.930	-0.02	17.97	292.82	17.95	9	7/14/2014	46:39.4
2257	RSPe_2	-122.445	37.930	0.00	17.99	292.70	17.99	-122.445	37.930	-0.02	17.99	292.89	17.97	9	7/14/2014	46:39.3
2258	RSPe_2	-122.445	37.930	-0.04	18.02	292.76	17.98	-122.445	37.930	-0.02	18.02	292.85	18.00	9	7/14/2014	46:39.2
2259	RSPe_2	-122.445	37.930	-0.04	18.03	292.76	18.00	-122.445	37.930	-0.02	18.03	292.80	18.01	9	7/14/2014	46:39.1
2260	RSPe_2	-122.445	37.930	-0.04	18.03	292.81	17.99	-122.445	37.930	-0.05	18.03	292.85	17.97	9	7/14/2014	46:39.0
2261	RSPe_2	-122.445	37.930	0.00	18.03	292.90	18.03	-122.445	37.930	-0.02	18.03	292.78	18.01	9	7/14/2014	46:38.9
2262	RSPe_2	-122.445	37.930	-0.04	18.05	292.88	18.02	-122.445	37.930	-0.02	18.05	292.83	18.04	9	7/14/2014	46:38.8
2263	RSPe_2	-122.445	37.930	0.00	18.03	292.90	18.02	-122.445	37.930	-0.02	18.03	292.81	18.01	9	7/14/2014	46:38.7
2264	RSPe_2	-122.445	37.930	-0.04	18.03	292.88	17.99	-122.445	37.930	-0.05	18.03	292.80	17.97	9	7/14/2014	46:38.6
2265	RSPe_2	-122.445	37.930	-0.04	18.03	292.83	17.99	-122.445	37.930	-0.05	18.03	292.83	17.97	9	7/14/2014	46:38.5
2266	RSPe_2	-122.445	37.930	-0.04	18.05	292.81	18.02	-122.445	37.930	-0.05	18.05	292.80	18.00	9	7/14/2014	46:38.4
2267	RSPe_2	-122.445	37.930	-0.04	18.04	292.79	18.00	-122.445	37.930	-0.02	18.04	292.83	18.02	9	7/14/2014	46:38.3
2268	RSPe_2	-122.445	37.930	-0.04	18.02	292.77	17.98	-122.445	37.930	-0.05	18.02	292.77	17.96	9	7/14/2014	46:38.2
2269	RSPe_2	-122.445	37.930	-0.04	18.05	292.79	18.02	-122.445	37.930	-0.10	18.05	292.77	17.95	9	7/14/2014	46:38.1
2270	RSPe_2	-122.445	37.930	-0.04	18.05	292.81	18.02	-122.445	37.930	-0.05	18.05	292.76	18.00	9	7/14/2014	46:38.0
2271	RSPe_2	-122.445	37.930	-0.04	18.06	292.81	18.03	-122.445	37.930	-0.10	18.06	292.75	17.96	9	7/14/2014	46:37.9
2272	RSPe_2	-122.445	37.930	-0.04	18.08	292.75	18.04	-122.445	37.930	-0.05	18.08	292.68	18.03	9	7/14/2014	46:37.8
2273	RSPe_2	-122.445	37.930	-0.04	18.08	292.66	18.04	-122.445	37.930	-0.05	18.08	292.61	18.03	9	7/14/2014	46:37.7
2274	RSPe_2	-122.445	37.930	-0.04	18.12	292.66	18.08	-122.445	37.930	-0.05	18.12	292.63	18.07	9	7/14/2014	46:37.6
2275	RSPe_2	-122.445	37.930	-0.04	18.14	292.62	18.10	-122.445	37.930	-0.05	18.14	292.63	18.09	9	7/14/2014	46:37.5
2276	RSPe_2	-122.445	37.930	-0.04	18.14	292.62	18.10	-122.445	37.930	-0.10	18.14	292.66	18.04	9	7/14/2014	46:37.4

2277	RSPe_2	-122.445	37.930	-0.04	18.17	292.60	18.13	-122.445	37.930	-0.10	18.17	292.70	18.07	9	7/14/2014 46:37.3
2278	RSPe_2	-122.445	37.930	-0.04	18.17	292.64	18.13	-122.445	37.930	-0.10	18.17	292.68	18.07	9	7/14/2014 46:37.2
2279	RSPe_2	-122.445	37.930	-0.09	18.24	292.62	18.15	-122.445	37.930	-0.10	18.24	292.68	18.13	9	7/14/2014 46:37.1
2280	RSPe_2	-122.445	37.930	-0.04	18.20	292.58	18.17	-122.445	37.930	-0.14	18.20	292.55	18.06	9	7/14/2014 46:37.0
2281	RSPe_2	-122.445	37.930	-0.04	18.19	292.58	18.15	-122.445	37.930	-0.10	18.19	292.51	18.08	9	7/14/2014 46:36.9
2282	RSPe_2	-122.445	37.930	-0.09	18.22	292.49	18.13	-122.445	37.930	-0.10	18.22	292.44	18.11	9	7/14/2014 46:36.8
2283	RSPe_2	-122.445	37.930	-0.04	18.12	292.44	18.09	-122.445	37.930	-0.10	18.12	292.37	18.02	9	7/14/2014 46:36.7
2284	RSPe_2	-122.445	37.930	-0.04	18.07	292.38	18.03	-122.445	37.930	-0.10	18.07	292.33	17.97	9	7/14/2014 46:36.6
2285	RSPe_2	-122.445	37.930	-0.04	18.03	292.32	18.00	-122.445	37.930	-0.05	18.03	292.27	17.98	9	7/14/2014 46:36.5
2286	RSPe_2	-122.445	37.930	-0.04	17.92	292.25	17.88	-122.445	37.930	-0.10	17.92	292.18	17.81	9	7/14/2014 46:36.4
2287	RSPe_2	-122.445	37.930	-0.04	17.98	292.25	17.94	-122.445	37.930	-0.05	17.98	292.20	17.93	9	7/14/2014 46:36.3
2288	RSPe_2	-122.445	37.930	-0.04	17.90	292.25	17.87	-122.445	37.930	-0.10	17.90	292.20	17.80	9	7/14/2014 46:36.2
2289	RSPe_2	-122.445	37.930	-0.04	17.91	292.27	17.87	-122.445	37.930	-0.05	17.91	292.20	17.86	9	7/14/2014 46:36.1
2290	RSPe_2	-122.445	37.930	-0.09	17.90	292.30	17.81	-122.445	37.930	-0.10	17.90	292.20	17.80	9	7/14/2014 46:36.0
2291	RSPe_2	-122.445	37.930	-0.04	17.90	292.25	17.87	-122.445	37.930	-0.10	17.90	292.16	17.80	9	7/14/2014 46:35.9
2292	RSPe_2	-122.445	37.930	-0.09	17.89	292.25	17.80	-122.445	37.930	-0.14	17.89	292.07	17.75	9	7/14/2014 46:35.8
2293	RSPe_2	-122.445	37.930	-0.09	17.88	292.25	17.79	-122.445	37.930	-0.10	17.88	292.02	17.77	9	7/14/2014 46:35.7
2294	RSPe_2	-122.445	37.930	-0.09	17.85	292.21	17.77	-122.445	37.930	-0.14	17.85	292.01	17.72	9	7/14/2014 46:35.6
2295	RSPe_2	-122.445	37.930	-0.09	17.85	292.21	17.77	-122.445	37.930	-0.10	17.85	291.99	17.75	9	7/14/2014 46:35.5
2296	RSPe_2	-122.445	37.930	-0.09	17.82	292.12	17.73	-122.445	37.930	-0.14	17.82	291.94	17.68	9	7/14/2014 46:35.4
2297	RSPe_2	-122.445	37.930	-0.04	17.79	292.01	17.76	-122.445	37.930	-0.10	17.79	291.94	17.69	9	7/14/2014 46:35.3
2298	RSPe_2	-122.445	37.930	-0.09	17.81	291.99	17.72	-122.445	37.930	-0.10	17.81	291.97	17.70	9	7/14/2014 46:35.2
2299	RSPe_2	-122.445	37.930	-0.09	17.92	291.92	17.83	-122.445	37.930	-0.10	17.92	291.96	17.81	9	7/14/2014 46:35.1
2300	RSPe_2	-122.445	37.930	-0.09	17.82	291.84	17.73	-122.445	37.930	-0.19	17.82	291.95	17.63	9	7/14/2014 46:35.0
2301	RSPe_2	-122.445	37.930	-0.09	17.90	291.75	17.81	-122.445	37.930	-0.14	17.90	291.86	17.76	9	7/14/2014 46:34.9
2302	RSPe_2	-122.445	37.930	-0.09	17.91	291.73	17.82	-122.445	37.930	-0.14	17.91	291.83	17.77	9	7/14/2014 46:34.8
2303	RSPe_2	-122.445	37.930	-0.04	18.03	291.64	17.99	-122.445	37.930	-0.14	18.03	291.75	17.89	9	7/14/2014 46:34.7
2304	RSPe_2	-122.445	37.930	-0.09	18.01	291.62	17.92	-122.445	37.930	-0.14	18.01	291.62	17.87	9	7/14/2014 46:34.6
2305	RSPe_2	-122.445	37.930	-0.09	18.06	291.62	17.98	-122.445	37.930	-0.14	18.06	291.57	17.93	9	7/14/2014 46:34.5
2306	RSPe_2	-122.445	37.930	-0.12	18.03	291.62	17.91	-122.445	37.930	-0.19	18.03	291.42	17.84	9	7/14/2014 46:34.4
2307	RSPe_2	-122.445	37.930	-0.09	18.07	291.62	17.98	-122.445	37.930	-0.14	18.07	291.35	17.93	9	7/14/2014 46:34.3
2308	RSPe_2	-122.445	37.930	-0.09	18.02	291.58	17.93	-122.445	37.930	-0.19	18.02	291.29	17.83	9	7/14/2014 46:34.2
2309	RSPe_2	-122.445	37.930	-0.09	18.11	291.52	18.02	-122.445	37.930	-0.14	18.11	291.20	17.97	9	7/14/2014 46:34.1

2310	RSPe_2	-122.445	37.930	-0.09	18.13	291.34	18.04	-122.445	37.930	-0.14	18.13	291.09	17.99	9	7/14/2014	46:34.0
2311	RSPe_2	-122.445	37.930	-0.09	18.07	291.21	17.98	-122.445	37.930	-0.14	18.07	291.08	17.93	9	7/14/2014	46:33.9
2312	RSPe_2	-122.445	37.930	-0.12	18.12	291.16	18.00	-122.445	37.930	-0.19	18.12	291.02	17.93	9	7/14/2014	46:33.8
2313	RSPe_2	-122.445	37.930	-0.09	18.10	291.05	18.01	-122.445	37.930	-0.10	18.10	291.02	17.99	9	7/14/2014	46:33.7
2314	RSPe_2	-122.445	37.930	-0.12	18.20	290.96	18.08	-122.445	37.930	-0.22	18.20	291.01	17.98	9	7/14/2014	46:33.6
2315	RSPe_2	-122.445	37.930	-0.12	18.15	290.88	18.03	-122.445	37.930	-0.22	18.15	290.89	17.93	9	7/14/2014	46:33.5
2316	RSPe_2	-122.445	37.930	-0.12	18.14	290.83	18.02	-122.445	37.930	-0.22	18.14	290.83	17.92	9	7/14/2014	46:33.4
2317	RSPe_2	-122.445	37.930	-0.09	18.17	290.82	18.08	-122.445	37.930	-0.19	18.17	290.77	17.98	9	7/14/2014	46:33.3
2318	RSPe_2	-122.445	37.930	-0.12	18.27	290.70	18.15	-122.445	37.930	-0.22	18.27	290.65	18.05	9	7/14/2014	46:33.2
2319	RSPe_2	-122.445	37.930	-0.12	18.35	290.66	18.23	-122.445	37.930	-0.19	18.35	290.53	18.16	9	7/14/2014	46:33.1
2320	RSPe_2	-122.445	37.930	-0.12	18.29	290.57	18.17	-122.445	37.930	-0.22	18.29	290.39	18.07	9	7/14/2014	46:33.0
2321	RSPe_2	-122.445	37.930	-0.09	18.29	290.57	18.20	-122.445	37.930	-0.22	18.29	290.37	18.07	9	7/14/2014	46:32.9
2322	RSPe_2	-122.445	37.930	-0.12	18.30	290.51	18.18	-122.445	37.930	-0.19	18.30	290.35	18.12	9	7/14/2014	46:32.8
2323	RSPe_2	-122.445	37.930	-0.12	18.33	290.45	18.20	-122.445	37.930	-0.14	18.33	290.26	18.19	9	7/14/2014	46:32.7
2324	RSPe_2	-122.445	37.930	-0.12	18.31	290.33	18.19	-122.445	37.930	-0.19	18.31	290.22	18.12	9	7/14/2014	46:32.6
2325	RSPe_2	-122.445	37.930	-0.12	18.36	290.25	18.24	-122.445	37.930	-0.19	18.36	290.18	18.18	9	7/14/2014	46:32.5
2326	RSPe_2	-122.445	37.930	-0.12	18.36	290.09	18.24	-122.445	37.930	-0.26	18.36	290.11	18.10	9	7/14/2014	46:32.4
2327	RSPe_2	-122.445	37.930	-0.12	18.36	290.05	18.24	-122.445	37.930	-0.22	18.36	290.08	18.14	9	7/14/2014	46:32.3
2328	RSPe_2	-122.445	37.930	-0.12	18.40	289.98	18.28	-122.445	37.930	-0.22	18.40	290.00	18.18	9	7/14/2014	46:32.2
2329	RSPe_2	-122.445	37.930	-0.17	18.43	289.98	18.26	-122.445	37.930	-0.22	18.43	289.93	18.21	9	7/14/2014	46:32.1
2330	RSPe_2	-122.445	37.930	-0.17	18.43	289.90	18.25	-122.445	37.930	-0.22	18.43	289.87	18.21	9	7/14/2014	46:32.0
2331	RSPe_2	-122.445	37.930	-0.17	18.50	289.92	18.32	-122.445	37.930	-0.22	18.50	289.76	18.27	9	7/14/2014	46:31.9
2332	RSPe_2	-122.445	37.930	-0.17	18.48	289.83	18.31	-122.445	37.930	-0.26	18.48	289.69	18.23	9	7/14/2014	46:31.8
2333	RSPe_2	-122.445	37.930	-0.12	18.51	289.77	18.39	-122.445	37.930	-0.31	18.51	289.54	18.20	9	7/14/2014	46:31.7
2334	RSPe_2	-122.445	37.930	-0.17	18.52	289.66	18.35	-122.445	37.930	-0.26	18.52	289.50	18.26	9	7/14/2014	46:31.6
2335	RSPe_2	-122.445	37.930	-0.12	18.57	289.50	18.45	-122.445	37.930	-0.31	18.57	289.40	18.27	9	7/14/2014	46:31.5
2336	RSPe_2	-122.445	37.930	-0.17	18.50	289.33	18.33	-122.445	37.930	-0.26	18.50	289.32	18.25	9	7/14/2014	46:31.4
2337	RSPe_2	-122.445	37.930	-0.17	18.56	289.26	18.38	-122.445	37.930	-0.31	18.56	289.35	18.25	9	7/14/2014	46:31.3
2338	RSPe_2	-122.445	37.930	-0.17	18.57	289.24	18.40	-122.445	37.930	-0.22	18.57	289.39	18.35	9	7/14/2014	46:31.2
2339	RSPe_2	-122.445	37.930	-0.12	18.59	289.16	18.47	-122.445	37.930	-0.22	18.59	289.30	18.37	9	7/14/2014	46:31.1
2340	RSPe_2	-122.445	37.930	-0.17	18.63	289.15	18.45	-122.445	37.930	-0.26	18.63	289.21	18.37	9	7/14/2014	46:31.0
2341	RSPe_2	-122.445	37.930	-0.12	18.62	289.11	18.50	-122.445	37.930	-0.31	18.62	289.09	18.31	9	7/14/2014	46:30.9
2342	RSPe_2	-122.445	37.930	-0.17	18.67	289.09	18.50	-122.445	37.930	-0.31	18.67	288.98	18.37	9	7/14/2014	46:30.8

2343	RSPe_2	-122.445	37.930	-0.17	18.63	289.02	18.45	-122.445	37.930	-0.26	18.63	288.80	18.37	9	7/14/2014	46:30.7
2344	RSPe_2	-122.445	37.930	-0.17	18.58	288.96	18.41	-122.445	37.930	-0.26	18.58	288.67	18.32	9	7/14/2014	46:30.6
2345	RSPe_2	-122.445	37.930	-0.17	18.56	288.85	18.38	-122.445	37.930	-0.26	18.56	288.49	18.30	9	7/14/2014	46:30.5
2346	RSPe_2	-122.445	37.930	-0.17	18.54	288.73	18.37	-122.445	37.930	-0.31	18.54	288.36	18.23	9	7/14/2014	46:30.4
2347	RSPe_2	-122.445	37.930	-0.17	18.54	288.68	18.37	-122.445	37.930	-0.26	18.54	288.30	18.29	9	7/14/2014	46:30.3
2348	RSPe_2	-122.445	37.930	-0.17	18.50	288.61	18.32	-122.445	37.930	-0.26	18.50	288.25	18.24	9	7/14/2014	46:30.2
2349	RSPe_2	-122.445	37.930	-0.12	18.55	288.58	18.43	-122.445	37.930	-0.22	18.55	288.34	18.33	9	7/14/2014	46:30.1
2350	RSPe_2	-122.445	37.930	-0.17	18.47	288.52	18.30	-122.445	37.930	-0.26	18.47	288.32	18.22	9	7/14/2014	46:30.0
2351	RSPe_2	-122.445	37.930	-0.12	18.53	288.45	18.41	-122.445	37.930	-0.26	18.53	288.37	18.28	9	7/14/2014	46:29.9
2352	RSPe_2	-122.445	37.930	-0.17	18.59	288.35	18.42	-122.445	37.930	-0.31	18.59	288.36	18.29	9	7/14/2014	46:29.8
2353	RSPe_2	-122.445	37.930	-0.12	18.60	288.26	18.48	-122.445	37.931	-0.22	18.60	288.34	18.38	9	7/14/2014	46:29.7
2354	RSPe_2	-122.445	37.931	-0.17	18.63	288.17	18.45	-122.445	37.931	-0.26	18.63	288.28	18.37	9	7/14/2014	46:29.6
2355	RSPe_2	-122.445	37.931	-0.12	18.59	288.09	18.47	-122.445	37.931	-0.26	18.59	288.21	18.33	9	7/14/2014	46:29.5
2356	RSPe_2	-122.445	37.931	-0.17	18.64	287.95	18.47	-122.445	37.931	-0.31	18.64	288.06	18.34	9	7/14/2014	46:29.4
2357	RSPe_2	-122.445	37.931	-0.12	18.61	287.86	18.49	-122.445	37.931	-0.26	18.61	287.95	18.36	9	7/14/2014	46:29.3
2358	RSPe_2	-122.445	37.931	-0.17	18.61	287.76	18.44	-122.445	37.931	-0.31	18.61	287.86	18.31	9	7/14/2014	46:29.2
2359	RSPe_2	-122.445	37.931	-0.12	18.62	287.71	18.50	-122.445	37.931	-0.26	18.62	287.80	18.37	9	7/14/2014	46:29.1
2360	RSPe_2	-122.445	37.931	-0.17	18.63	287.62	18.45	-122.445	37.931	-0.26	18.63	287.68	18.37	9	7/14/2014	46:29.0
2361	RSPe_2	-122.445	37.931	-0.12	18.58	287.56	18.46	-122.445	37.931	-0.26	18.58	287.53	18.32	9	7/14/2014	46:28.9
2362	RSPe_2	-122.445	37.931	-0.12	18.57	287.51	18.45	-122.445	37.931	-0.26	18.57	287.42	18.32	9	7/14/2014	46:28.8
2363	RSPe_2	-122.445	37.931	-0.12	18.56	287.43	18.44	-122.445	37.931	-0.19	18.56	287.34	18.37	9	7/14/2014	46:28.7
2364	RSPe_2	-122.445	37.931	-0.17	18.52	287.47	18.35	-122.445	37.931	-0.22	18.52	287.29	18.30	9	7/14/2014	46:28.6
2365	RSPe_2	-122.445	37.931	-0.12	18.47	287.43	18.35	-122.445	37.931	-0.22	18.47	287.29	18.25	9	7/14/2014	46:28.5
2366	RSPe_2	-122.445	37.931	-0.12	18.47	287.43	18.35	-122.445	37.931	-0.22	18.47	287.27	18.25	9	7/14/2014	46:28.4
2367	RSPe_2	-122.445	37.931	-0.12	18.48	287.34	18.36	-122.445	37.931	-0.22	18.48	287.25	18.26	9	7/14/2014	46:28.3
2368	RSPe_2	-122.445	37.931	-0.12	18.45	287.28	18.33	-122.445	37.931	-0.22	18.45	287.22	18.23	9	7/14/2014	46:28.2
2369	RSPe_2	-122.445	37.931	-0.12	18.38	287.21	18.26	-122.445	37.931	-0.19	18.38	287.16	18.19	9	7/14/2014	46:28.1
2370	RSPe_2	-122.445	37.931	-0.12	18.36	287.08	18.24	-122.445	37.931	-0.19	18.36	287.07	18.17	9	7/14/2014	46:28.0
2371	RSPe_2	-122.445	37.931	-0.09	18.33	287.02	18.25	-122.445	37.931	-0.19	18.33	287.04	18.15	9	7/14/2014	46:27.9
2372	RSPe_2	-122.445	37.931	-0.12	18.30	286.99	18.18	-122.445	37.931	-0.22	18.30	286.94	18.08	9	7/14/2014	46:27.8
2373	RSPe_2	-122.445	37.931	-0.12	18.30	286.95	18.17	-122.445	37.931	-0.19	18.30	286.84	18.11	9	7/14/2014	46:27.7
2374	RSPe_2	-122.445	37.931	-0.17	18.24	286.93	18.07	-122.445	37.931	-0.22	18.24	286.77	18.02	9	7/14/2014	46:27.6
2375	RSPe_2	-122.445	37.931	-0.12	18.21	286.91	18.09	-122.445	37.931	-0.19	18.21	286.68	18.02	9	7/14/2014	46:27.5

2376	RSPe_2	-122.445	37.931	-0.17	18.21	286.86	18.04	-122.445	37.931	-0.14	18.21	286.70	18.07	9	7/14/2014	46:27.4
2377	RSPe_2	-122.445	37.931	-0.12	18.21	286.77	18.09	-122.445	37.931	-0.19	18.21	286.60	18.02	9	7/14/2014	46:27.3
2378	RSPe_2	-122.445	37.931	-0.12	18.18	286.78	18.06	-122.445	37.931	-0.19	18.18	286.64	17.99	9	7/14/2014	46:27.2
2379	RSPe_2	-122.445	37.931	-0.12	18.17	286.71	18.04	-122.445	37.931	-0.19	18.17	286.62	17.98	9	7/14/2014	46:27.1
2380	RSPe_2	-122.445	37.931	-0.12	18.17	286.60	18.04	-122.445	37.931	-0.22	18.17	286.62	17.94	9	7/14/2014	46:27.0
2381	RSPe_2	-122.445	37.931	-0.12	18.14	286.53	18.02	-122.445	37.931	-0.19	18.14	286.60	17.95	9	7/14/2014	46:26.9
2382	RSPe_2	-122.445	37.931	-0.12	18.15	286.34	18.03	-122.445	37.931	-0.22	18.15	286.53	17.93	9	7/14/2014	46:26.8
2383	RSPe_2	-122.445	37.931	-0.12	18.16	286.25	18.04	-122.445	37.931	-0.22	18.16	286.49	17.94	9	7/14/2014	46:26.7
2384	RSPe_2	-122.445	37.931	-0.17	18.14	286.23	17.97	-122.445	37.931	-0.22	18.14	286.43	17.92	9	7/14/2014	46:26.6
2385	RSPe_2	-122.445	37.931	-0.09	18.14	286.14	18.05	-122.445	37.931	-0.19	18.14	286.31	17.95	9	7/14/2014	46:26.5
2386	RSPe_2	-122.445	37.931	-0.12	18.12	286.12	18.00	-122.445	37.931	-0.19	18.12	286.23	17.93	9	7/14/2014	46:26.4
2387	RSPe_2	-122.445	37.931	-0.12	18.12	286.05	18.00	-122.445	37.931	-0.19	18.12	286.12	17.94	9	7/14/2014	46:26.3
2388	RSPe_2	-122.445	37.931	-0.12	18.14	285.97	18.02	-122.445	37.931	-0.19	18.14	285.97	17.95	9	7/14/2014	46:26.2
2389	RSPe_2	-122.445	37.931	-0.12	18.13	285.90	18.01	-122.445	37.931	-0.19	18.13	285.90	17.94	9	7/14/2014	46:26.1
2390	RSPe_2	-122.445	37.931	-0.17	18.19	285.84	18.02	-122.445	37.931	-0.19	18.19	285.92	18.01	9	7/14/2014	46:26.0
2391	RSPe_2	-122.445	37.931	-0.12	18.16	285.83	18.04	-122.445	37.931	-0.19	18.16	285.88	17.97	9	7/14/2014	46:25.9
2392	RSPe_2	-122.445	37.931	-0.12	18.12	285.80	18.00	-122.445	37.931	-0.19	18.12	285.88	17.94	9	7/14/2014	46:25.8
2393	RSPe_2	-122.445	37.931	-0.09	18.17	285.66	18.08	-122.445	37.931	-0.19	18.17	285.85	17.98	9	7/14/2014	46:25.7
2394	RSPe_2	-122.445	37.931	-0.12	18.18	285.49	18.06	-122.445	37.931	-0.19	18.18	285.75	17.99	9	7/14/2014	46:25.6
2395	RSPe_2	-122.445	37.931	-0.12	18.21	285.38	18.09	-122.445	37.931	-0.14	18.21	285.68	18.07	9	7/14/2014	46:25.5
2396	RSPe_2	-122.445	37.931	-0.12	18.21	285.29	18.09	-122.445	37.931	-0.19	18.21	285.60	18.02	9	7/14/2014	46:25.4
2397	RSPe_2	-122.445	37.931	-0.12	18.26	285.22	18.14	-122.445	37.931	-0.19	18.26	285.53	18.07	9	7/14/2014	46:25.3
2398	RSPe_2	-122.445	37.931	-0.12	18.27	285.16	18.15	-122.445	37.931	-0.22	18.27	285.44	18.05	9	7/14/2014	46:25.2
2399	RSPe_2	-122.445	37.931	-0.12	18.39	285.18	18.26	-122.445	37.931	-0.19	18.39	285.33	18.20	9	7/14/2014	46:25.1
2400	RSPe_2	-122.445	37.931	-0.12	18.47	285.14	18.34	-122.445	37.931	-0.26	18.47	285.25	18.21	9	7/14/2014	46:25.0
2401	RSPe_2	-122.445	37.931	-0.12	18.37	285.10	18.25	-122.445	37.931	-0.22	18.37	285.14	18.15	9	7/14/2014	46:24.9
2402	RSPe_2	-122.445	37.931	-0.12	18.62	285.12	18.50	-122.445	37.931	-0.22	18.62	285.03	18.40	9	7/14/2014	46:24.8
2403	RSPe_2	-122.445	37.931	-0.12	18.42	284.96	18.30	-122.445	37.931	-0.26	18.42	284.94	18.16	9	7/14/2014	46:24.7
2404	RSPe_2	-122.445	37.931	-0.12	18.47	285.03	18.34	-122.445	37.931	-0.26	18.47	284.89	18.21	9	7/14/2014	46:24.6
2405	RSPe_2	-122.445	37.931	-0.12	18.51	284.92	18.39	-122.445	37.931	-0.26	18.51	284.87	18.25	9	7/14/2014	46:24.5
2406	RSPe_2	-122.445	37.931	-0.12	18.46	284.81	18.33	-122.445	37.931	-0.26	18.46	284.74	18.20	9	7/14/2014	46:24.4
2407	RSPe_2	-122.445	37.931	-0.12	18.56	284.64	18.44	-122.445	37.931	-0.22	18.56	284.64	18.34	9	7/14/2014	46:24.3
2408	RSPe_2	-122.445	37.931	-0.12	18.47	284.50	18.34	-122.445	37.931	-0.26	18.47	284.52	18.21	9	7/14/2014	46:24.2

2409	RSPe_2	-122.445	37.931	-0.12	18.50	284.38	18.38	-122.445	37.931	-0.22	18.50	284.43	18.28	9	7/14/2014	46:24.1
2410	RSPe_2	-122.445	37.931	-0.12	18.50	284.29	18.38	-122.445	37.931	-0.22	18.50	284.30	18.28	9	7/14/2014	46:24.0
2411	RSPe_2	-122.445	37.931	-0.12	18.52	284.22	18.40	-122.445	37.931	-0.22	18.52	284.24	18.30	9	7/14/2014	46:23.9
2412	RSPe_2	-122.445	37.931	-0.17	18.63	284.13	18.46	-122.445	37.931	-0.26	18.63	284.19	18.38	9	7/14/2014	46:23.8
2413	RSPe_2	-122.445	37.931	-0.17	18.56	284.20	18.38	-122.445	37.931	-0.26	18.56	284.22	18.30	9	7/14/2014	46:23.7
2414	RSPe_2	-122.445	37.931	-0.17	18.52	284.16	18.35	-122.445	37.931	-0.26	18.52	284.19	18.27	9	7/14/2014	46:23.6
2415	RSPe_2	-122.445	37.931	-0.17	18.52	284.05	18.35	-122.445	37.931	-0.31	18.52	284.06	18.22	9	7/14/2014	46:23.5
2416	RSPe_2	-122.445	37.931	-0.17	18.57	283.98	18.39	-122.445	37.931	-0.31	18.57	284.02	18.26	9	7/14/2014	46:23.4
2417	RSPe_2	-122.445	37.931	-0.17	18.53	283.90	18.36	-122.445	37.931	-0.26	18.53	283.96	18.28	9	7/14/2014	46:23.3
2418	RSPe_2	-122.445	37.931	-0.21	18.57	283.80	18.36	-122.445	37.931	-0.31	18.57	283.85	18.26	9	7/14/2014	46:23.2
2419	RSPe_2	-122.445	37.931	-0.17	18.71	283.70	18.54	-122.445	37.931	-0.31	18.71	283.73	18.41	9	7/14/2014	46:23.1
2420	RSPe_2	-122.445	37.931	-0.21	18.70	283.59	18.50	-122.445	37.931	-0.31	18.70	283.60	18.40	9	7/14/2014	46:23.0
2421	RSPe_2	-122.445	37.931	-0.17	18.71	283.39	18.54	-122.445	37.931	-0.31	18.71	283.45	18.41	9	7/14/2014	46:22.9
2422	RSPe_2	-122.445	37.931	-0.21	18.86	283.34	18.65	-122.445	37.931	-0.31	18.86	283.36	18.55	9	7/14/2014	46:22.8
2423	RSPe_2	-122.445	37.931	-0.21	18.82	283.28	18.61	-122.445	37.931	-0.26	18.82	283.32	18.56	9	7/14/2014	46:22.7
2424	RSPe_2	-122.445	37.931	-0.21	18.74	283.26	18.54	-122.445	37.931	-0.31	18.74	283.26	18.44	9	7/14/2014	46:22.6
2425	RSPe_2	-122.445	37.931	-0.21	18.80	283.22	18.60	-122.445	37.931	-0.34	18.80	283.19	18.46	9	7/14/2014	46:22.5
2426	RSPe_2	-122.445	37.931	-0.24	18.89	283.11	18.65	-122.445	37.931	-0.31	18.89	283.17	18.58	9	7/14/2014	46:22.4
2427	RSPe_2	-122.445	37.931	-0.21	18.82	283.00	18.61	-122.445	37.931	-0.34	18.82	283.06	18.48	9	7/14/2014	46:22.3
2428	RSPe_2	-122.445	37.931	-0.24	18.84	282.91	18.60	-122.445	37.931	-0.43	18.84	283.04	18.42	9	7/14/2014	46:22.2
2429	RSPe_2	-122.445	37.931	-0.24	18.80	282.85	18.56	-122.445	37.931	-0.39	18.80	283.02	18.41	9	7/14/2014	46:22.1
2430	RSPe_2	-122.445	37.931	-0.29	18.84	282.84	18.55	-122.445	37.931	-0.43	18.84	282.95	18.42	9	7/14/2014	46:22.0
2431	RSPe_2	-122.445	37.931	-0.24	18.94	282.95	18.70	-122.445	37.931	-0.43	18.94	282.99	18.51	9	7/14/2014	46:21.9
2432	RSPe_2	-122.445	37.931	-0.29	18.94	282.93	18.65	-122.445	37.931	-0.47	18.94	282.89	18.47	9	7/14/2014	46:21.8
2433	RSPe_2	-122.445	37.931	-0.24	18.85	282.83	18.61	-122.445	37.931	-0.47	18.85	282.71	18.38	9	7/14/2014	46:21.7
2434	RSPe_2	-122.445	37.931	-0.29	18.88	282.52	18.59	-122.445	37.931	-0.47	18.88	282.49	18.41	9	7/14/2014	46:21.6
2435	RSPe_2	-122.445	37.931	-0.29	18.91	282.36	18.62	-122.445	37.931	-0.43	18.91	282.36	18.49	9	7/14/2014	46:21.5
2436	RSPe_2	-122.445	37.931	-0.29	18.83	282.32	18.54	-122.445	37.931	-0.43	18.83	282.38	18.40	9	7/14/2014	46:21.4
2437	RSPe_2	-122.445	37.931	-0.29	18.84	282.19	18.55	-122.445	37.931	-0.43	18.84	282.27	18.42	9	7/14/2014	46:21.3
2438	RSPe_2	-122.445	37.931	-0.33	18.92	282.03	18.59	-122.445	37.931	-0.51	18.92	282.12	18.41	9	7/14/2014	46:21.2
2439	RSPe_2	-122.445	37.931	-0.29	18.94	282.01	18.64	-122.445	37.931	-0.51	18.94	282.10	18.43	9	7/14/2014	46:21.1
2440	RSPe_2	-122.445	37.931	-0.29	18.94	281.95	18.65	-122.445	37.931	-0.51	18.94	282.05	18.43	9	7/14/2014	46:21.0
2441	RSPe_2	-122.445	37.931	-0.29	18.95	281.84	18.66	-122.445	37.931	-0.47	18.95	281.97	18.48	9	7/14/2014	46:20.9

2442	RSPe_2	-122.445	37.931	-0.33	19.03	281.80	18.71	-122.445	37.931	-0.51	19.03	281.95	18.53	9	7/14/2014	46:20.8
2443	RSPe_2	-122.445	37.931	-0.33	19.04	281.67	18.72	-122.445	37.931	-0.56	19.04	281.90	18.48	9	7/14/2014	46:20.7
2444	RSPe_2	-122.445	37.931	-0.33	19.11	281.60	18.79	-122.445	37.931	-0.56	19.11	281.83	18.55	9	7/14/2014	46:20.6
2445	RSPe_2	-122.445	37.931	-0.33	19.16	281.51	18.83	-122.445	37.931	-0.59	19.16	281.79	18.56	9	7/14/2014	46:20.5
2446	RSPe_2	-122.445	37.931	-0.38	19.20	281.42	18.82	-122.445	37.931	-0.63	19.20	281.70	18.57	9	7/14/2014	46:20.4
2447	RSPe_2	-122.445	37.931	-0.33	19.17	281.33	18.84	-122.445	37.931	-0.59	19.17	281.57	18.57	9	7/14/2014	46:20.3
2448	RSPe_2	-122.445	37.931	-0.38	19.15	281.25	18.77	-122.445	37.931	-0.63	19.15	281.48	18.52	9	7/14/2014	46:20.2
2449	RSPe_2	-122.445	37.931	-0.33	19.20	281.10	18.88	-122.445	37.931	-0.63	19.20	281.29	18.58	9	7/14/2014	46:20.1
2450	RSPe_2	-122.445	37.931	-0.38	19.20	280.99	18.82	-122.445	37.931	-0.63	19.20	281.09	18.57	9	7/14/2014	46:20.0
2451	RSPe_2	-122.445	37.931	-0.33	19.26	280.88	18.93	-122.445	37.931	-0.63	19.26	280.98	18.63	9	7/14/2014	46:19.9
2452	RSPe_2	-122.445	37.931	-0.33	19.18	280.70	18.86	-122.445	37.931	-0.63	19.18	280.80	18.56	9	7/14/2014	46:19.8
2453	RSPe_2	-122.445	37.931	-0.33	19.22	280.57	18.89	-122.445	37.931	-0.68	19.22	280.74	18.54	9	7/14/2014	46:19.7
2454	RSPe_2	-122.445	37.931	-0.38	19.29	280.35	18.91	-122.445	37.931	-0.68	19.29	280.61	18.61	9	7/14/2014	46:19.6
2455	RSPe_2	-122.445	37.931	-0.33	19.25	280.24	18.93	-122.445	37.931	-0.68	19.25	280.54	18.57	9	7/14/2014	46:19.5
2456	RSPe_2	-122.445	37.931	-0.38	19.32	280.22	18.95	-122.445	37.931	-0.71	19.32	280.63	18.61	9	7/14/2014	46:19.4
2457	RSPe_2	-122.445	37.931	-0.38	19.45	280.40	19.07	-122.445	37.931	-0.76	19.45	280.68	18.69	9	7/14/2014	46:19.3
2458	RSPe_2	-122.445	37.931	-0.38	19.38	280.38	19.00	-122.445	37.931	-0.76	19.38	280.59	18.61	9	7/14/2014	46:19.2
2459	RSPe_2	-122.445	37.931	-0.38	19.51	280.42	19.14	-122.445	37.931	-0.76	19.51	280.46	18.75	9	7/14/2014	46:19.1
2460	RSPe_2	-122.445	37.931	-0.38	19.51	280.38	19.14	-122.445	37.931	-0.76	19.51	280.30	18.75	9	7/14/2014	46:19.0
2461	RSPe_2	-122.445	37.931	-0.38	19.31	280.35	18.93	-122.445	37.931	-0.76	19.31	280.30	18.54	9	7/14/2014	46:18.9
2462	RSPe_2	-122.445	37.931	-0.38	19.32	280.27	18.95	-122.445	37.931	-0.85	19.32	280.20	18.47	9	7/14/2014	46:18.8
2463	RSPe_2	-122.445	37.931	-0.38	19.28	280.11	18.91	-122.445	37.931	-0.71	19.28	280.11	18.57	9	7/14/2014	46:18.7
2464	RSPe_2	-122.445	37.931	-0.38	19.23	279.98	18.85	-122.445	37.931	-0.76	19.23	280.02	18.46	9	7/14/2014	46:18.6
2465	RSPe_2	-122.445	37.931	-0.38	19.20	279.85	18.82	-122.445	37.931	-0.76	19.20	279.96	18.43	9	7/14/2014	46:18.5
2466	RSPe_2	-122.445	37.931	-0.38	19.15	279.79	18.77	-122.445	37.931	-0.80	19.15	279.93	18.35	9	7/14/2014	46:18.4
2467	RSPe_2	-122.445	37.931	-0.38	19.11	279.39	18.74	-122.445	37.931	-0.76	19.11	279.67	18.35	9	7/14/2014	46:18.3
2468	RSPe_2	-122.445	37.931	-0.41	19.15	279.15	18.73	-122.445	37.931	-0.71	19.15	279.41	18.43	9	7/14/2014	46:18.2
2469	RSPe_2	-122.445	37.931	-0.38	19.10	279.04	18.73	-122.445	37.931	-0.76	19.10	279.23	18.34	9	7/14/2014	46:18.1
2470	RSPe_2	-122.445	37.931	-0.38	19.18	279.06	18.81	-122.445	37.931	-0.80	19.18	279.32	18.39	9	7/14/2014	46:18.0
2471	RSPe_2	-122.445	37.931	-0.38	19.17	279.26	18.80	-122.445	37.931	-0.76	19.17	279.39	18.41	9	7/14/2014	46:17.9
2472	RSPe_2	-122.445	37.931	-0.41	19.16	279.35	18.74	-122.445	37.931	-0.80	19.16	279.42	18.36	9	7/14/2014	46:17.8
2473	RSPe_2	-122.445	37.931	-0.38	19.15	279.30	18.77	-122.445	37.931	-0.80	19.15	279.37	18.35	9	7/14/2014	46:17.7
2474	RSPe_2	-122.445	37.931	-0.38	19.21	279.24	18.84	-122.445	37.931	-0.85	19.21	279.32	18.37	9	7/14/2014	46:17.6

2475	RSPe_2	-122.445	37.931	-0.38	19.20	279.04	18.83	-122.445	37.931	-0.85	19.20	279.17	18.36	9	7/14/2014	46:17.5
2476	RSPe_2	-122.445	37.931	-0.38	19.27	278.91	18.89	-122.445	37.931	-0.88	19.27	279.06	18.39	9	7/14/2014	46:17.4
2477	RSPe_2	-122.445	37.931	-0.38	19.24	278.62	18.86	-122.445	37.931	-0.85	19.24	278.86	18.39	9	7/14/2014	46:17.3
2478	RSPe_2	-122.445	37.931	-0.41	19.26	278.51	18.85	-122.445	37.931	-0.88	19.26	278.66	18.38	9	7/14/2014	46:17.2
2479	RSPe_2	-122.445	37.931	-0.38	19.10	278.30	18.73	-122.445	37.931	-0.85	19.10	278.47	18.26	9	7/14/2014	46:17.1
2480	RSPe_2	-122.445	37.931	-0.38	19.09	278.26	18.71	-122.445	37.931	-0.88	19.09	278.34	18.21	9	7/14/2014	46:17.0
2481	RSPe_2	-122.445	37.931	-0.33	19.15	278.36	18.82	-122.445	37.931	-0.88	19.15	278.34	18.26	9	7/14/2014	46:16.9
2482	RSPe_2	-122.445	37.931	-0.38	19.10	278.32	18.73	-122.445	37.931	-0.88	19.10	278.30	18.22	9	7/14/2014	46:16.8
2483	RSPe_2	-122.445	37.931	-0.33	19.24	278.27	18.92	-122.445	37.931	-0.85	19.24	278.25	18.40	9	7/14/2014	46:16.7
2484	RSPe_2	-122.445	37.931	-0.38	19.24	278.23	18.87	-122.445	37.931	-0.93	19.24	278.18	18.31	9	7/14/2014	46:16.6
2485	RSPe_2	-122.445	37.931	-0.33	19.23	278.19	18.90	-122.445	37.931	-0.85	19.23	278.25	18.38	9	7/14/2014	46:16.5
2486	RSPe_2	-122.445	37.931	-0.33	19.18	278.06	18.86	-122.445	37.931	-0.88	19.18	278.18	18.30	9	7/14/2014	46:16.4
2487	RSPe_2	-122.445	37.931	-0.33	19.24	277.86	18.92	-122.445	37.931	-0.85	19.24	278.12	18.40	9	7/14/2014	46:16.3
2488	RSPe_2	-122.445	37.931	-0.38	19.24	277.73	18.86	-122.445	37.931	-0.93	19.24	277.99	18.30	9	7/14/2014	46:16.2
2489	RSPe_2	-122.445	37.931	-0.32	19.21	277.60	18.89	-122.445	37.931	-0.85	19.21	277.79	18.37	9	7/14/2014	46:16.1
2490	RSPe_2	-122.445	37.931	-0.32	19.18	277.51	18.86	-122.445	37.931	-0.88	19.18	277.68	18.30	9	7/14/2014	46:16.0
2491	RSPe_2	-122.445	37.931	-0.32	19.31	277.47	18.98	-122.445	37.931	-0.88	19.31	277.51	18.43	9	7/14/2014	46:15.9
2492	RSPe_2	-122.445	37.931	-0.32	19.20	277.38	18.88	-122.445	37.931	-0.88	19.20	277.37	18.32	9	7/14/2014	46:15.8
2493	RSPe_2	-122.445	37.931	-0.32	19.15	277.29	18.82	-122.445	37.931	-0.85	19.15	277.31	18.30	9	7/14/2014	46:15.7
2494	RSPe_2	-122.445	37.931	-0.32	19.12	277.29	18.79	-122.445	37.931	-0.88	19.12	277.27	18.24	9	7/14/2014	46:15.6
2495	RSPe_2	-122.445	37.931	-0.29	19.17	277.41	18.88	-122.445	37.931	-0.85	19.17	277.25	18.32	9	7/14/2014	46:15.5
2496	RSPe_2	-122.445	37.931	-0.29	19.32	277.34	19.03	-122.445	37.931	-0.80	19.32	277.17	18.53	9	7/14/2014	46:15.4
2497	RSPe_2	-122.445	37.931	-0.29	19.12	277.25	18.83	-122.445	37.931	-0.85	19.12	277.10	18.27	9	7/14/2014	46:15.3
2498	RSPe_2	-122.445	37.931	-0.29	19.31	277.14	19.02	-122.445	37.931	-0.88	19.31	276.96	18.43	9	7/14/2014	46:15.2
2499	RSPe_2	-122.445	37.931	-0.24	19.02	276.92	18.78	-122.445	37.931	-0.76	19.02	276.90	18.26	9	7/14/2014	46:15.1
2500	RSPe_2	-122.445	37.931	-0.24	19.26	276.70	19.02	-122.445	37.931	-0.68	19.26	276.76	18.58	9	7/14/2014	46:15.0
2501	RSPe_2	-122.445	37.931	-0.24	19.11	276.50	18.87	-122.445	37.931	-0.76	19.11	276.59	18.35	9	7/14/2014	46:14.9
2502	RSPe_2	-122.445	37.931	-0.24	19.34	276.33	19.10	-122.445	37.931	-0.71	19.34	276.47	18.63	9	7/14/2014	46:14.8
2503	RSPe_2	-122.445	37.931	-0.21	19.39	276.13	19.19	-122.445	37.931	-0.59	19.39	276.31	18.80	9	7/14/2014	46:14.7
2504	RSPe_2	-122.445	37.931	-0.24	19.41	276.18	19.17	-122.445	37.931	-0.63	19.41	276.14	18.79	9	7/14/2014	46:14.6
2505	RSPe_2	-122.445	37.931	-0.21	19.43	276.40	19.22	-122.445	37.931	-0.63	19.43	276.24	18.80	9	7/14/2014	46:14.5
2506	RSPe_2	-122.445	37.931	-0.21	19.43	276.64	19.22	-122.445	37.931	-0.68	19.43	276.33	18.75	9	7/14/2014	46:14.4
2507	RSPe_2	-122.445	37.931	-0.21	19.18	276.42	18.98	-122.445	37.931	-0.63	19.18	276.27	18.56	9	7/14/2014	46:14.3

2508	RSPe_2	-122.445	37.931	-0.24	19.26	276.31	19.02	-122.445	37.931	-0.59	19.26	276.15	18.66	9	7/14/2014	46:14.2
2509	RSPe_2	-122.445	37.931	-0.21	19.35	276.15	19.15	-122.445	37.931	-0.59	19.35	275.98	18.76	9	7/14/2014	46:14.1
2510	RSPe_2	-122.445	37.931	-0.24	19.23	276.03	18.99	-122.445	37.931	-0.59	19.23	275.89	18.63	9	7/14/2014	46:14.0
2511	RSPe_2	-122.445	37.931	-0.21	19.20	275.87	18.99	-122.445	37.931	-0.59	19.20	275.80	18.60	9	7/14/2014	46:13.9
2512	RSPe_2	-122.445	37.931	-0.24	19.21	275.76	18.97	-122.445	37.931	-0.68	19.21	275.67	18.54	9	7/14/2014	46:13.8
2513	RSPe_2	-122.445	37.931	-0.21	19.26	275.67	19.05	-122.445	37.931	-0.59	19.26	275.59	18.66	9	7/14/2014	46:13.7
2514	RSPe_2	-122.445	37.931	-0.21	19.27	275.52	19.06	-122.445	37.931	-0.59	19.27	275.47	18.67	9	7/14/2014	46:13.6
2515	RSPe_2	-122.445	37.931	-0.17	19.29	275.41	19.12	-122.445	37.931	-0.59	19.29	275.37	18.70	9	7/14/2014	46:13.5
2516	RSPe_2	-122.445	37.931	-0.21	19.24	275.24	19.04	-122.445	37.931	-0.59	19.24	275.23	18.65	9	7/14/2014	46:13.4
2517	RSPe_2	-122.445	37.931	-0.17	19.26	275.15	19.09	-122.445	37.931	-0.51	19.26	275.15	18.75	9	7/14/2014	46:13.3
2518	RSPe_2	-122.445	37.931	-0.17	19.20	275.09	19.03	-122.445	37.931	-0.71	19.20	275.15	18.49	9	7/14/2014	46:13.2
2519	RSPe_2	-122.445	37.931	-0.17	19.24	274.95	19.07	-122.445	37.931	-0.47	19.24	275.02	18.76	9	7/14/2014	46:13.1
2520	RSPe_2	-122.445	37.931	-0.17	19.30	274.89	19.12	-122.445	37.931	-0.42	19.30	274.95	18.87	9	7/14/2014	46:13.0
2521	RSPe_2	-122.445	37.931	-0.12	19.28	274.73	19.16	-122.445	37.931	-0.51	19.28	274.85	18.78	9	7/14/2014	46:12.9
2522	RSPe_2	-122.445	37.931	-0.17	19.22	274.58	19.05	-122.445	37.931	-0.51	19.22	274.64	18.71	9	7/14/2014	46:12.8
2523	RSPe_2	-122.445	37.931	-0.12	19.23	274.42	19.11	-122.445	37.931	-0.47	19.23	274.53	18.75	9	7/14/2014	46:12.7
2524	RSPe_2	-122.445	37.931	-0.17	19.26	274.36	19.09	-122.445	37.931	-0.51	19.26	274.46	18.75	9	7/14/2014	46:12.6
2525	RSPe_2	-122.445	37.931	-0.12	19.20	274.30	19.08	-122.445	37.931	-0.47	19.20	274.36	18.72	9	7/14/2014	46:12.5
2526	RSPe_2	-122.445	37.931	-0.12	19.13	274.23	19.01	-122.445	37.931	-0.47	19.13	274.30	18.65	9	7/14/2014	46:12.4
2527	RSPe_2	-122.445	37.931	-0.12	19.14	274.10	19.02	-122.445	37.931	-0.51	19.14	274.18	18.63	9	7/14/2014	46:12.3
2528	RSPe_2	-122.445	37.931	-0.12	19.08	274.08	18.96	-122.445	37.931	-0.47	19.08	274.07	18.60	9	7/14/2014	46:12.2
2529	RSPe_2	-122.445	37.931	-0.12	18.97	273.92	18.85	-122.445	37.931	-0.47	18.97	273.99	18.50	9	7/14/2014	46:12.1
2530	RSPe_2	-122.445	37.931	-0.12	18.99	273.84	18.87	-122.445	37.931	-0.39	18.99	273.92	18.60	9	7/14/2014	46:12.0
2531	RSPe_2	-122.445	37.931	-0.12	18.83	273.73	18.71	-122.445	37.931	-0.42	18.83	273.74	18.41	9	7/14/2014	46:11.9
2532	RSPe_2	-122.445	37.931	-0.12	18.83	273.71	18.71	-122.445	37.931	-0.47	18.83	273.74	18.36	9	7/14/2014	46:11.8
2533	RSPe_2	-122.445	37.931	-0.09	18.87	273.55	18.79	-122.445	37.931	-0.39	18.87	273.55	18.48	9	7/14/2014	46:11.7
2534	RSPe_2	-122.445	37.931	-0.12	18.79	273.40	18.67	-122.445	37.931	-0.47	18.79	273.48	18.31	9	7/14/2014	46:11.6
2535	RSPe_2	-122.445	37.931	-0.09	18.76	273.25	18.67	-122.445	37.931	-0.31	18.76	273.37	18.45	9	7/14/2014	46:11.5
2536	RSPe_2	-122.445	37.931	-0.09	18.65	273.07	18.56	-122.445	37.931	-0.34	18.65	273.22	18.31	9	7/14/2014	46:11.4
2537	RSPe_2	-122.445	37.931	-0.09	18.73	272.89	18.64	-122.445	37.931	-0.34	18.73	273.11	18.39	9	7/14/2014	46:11.3
2538	RSPe_2	-122.445	37.931	-0.12	18.74	272.85	18.62	-122.445	37.931	-0.42	18.74	273.06	18.32	9	7/14/2014	46:11.2
2539	RSPe_2	-122.445	37.931	-0.09	18.74	272.87	18.66	-122.445	37.931	-0.39	18.74	273.03	18.35	9	7/14/2014	46:11.1
2540	RSPe_2	-122.445	37.931	-0.12	18.74	272.79	18.62	-122.445	37.931	-0.39	18.74	272.89	18.35	9	7/14/2014	46:11.0

2541	RSPe_2	-122.445	37.931	-0.12	18.62	272.72	18.50	-122.445	37.931	-0.42	18.62	272.79	18.20	9	7/14/2014	46:10.9
2542	RSPe_2	-122.445	37.931	-0.12	18.64	272.70	18.52	-122.445	37.931	-0.47	18.64	272.76	18.17	9	7/14/2014	46:10.8
2543	RSPe_2	-122.445	37.931	-0.09	18.62	272.66	18.53	-122.445	37.931	-0.39	18.62	272.70	18.23	9	7/14/2014	46:10.7
2544	RSPe_2	-122.445	37.931	-0.12	18.75	272.64	18.63	-122.445	37.931	-0.42	18.75	272.61	18.33	9	7/14/2014	46:10.6
2545	RSPe_2	-122.445	37.931	-0.09	18.77	272.48	18.69	-122.445	37.931	-0.47	18.77	272.50	18.30	9	7/14/2014	46:10.5
2546	RSPe_2	-122.445	37.931	-0.12	18.81	272.35	18.69	-122.445	37.931	-0.47	18.81	272.39	18.34	9	7/14/2014	46:10.4
2547	RSPe_2	-122.445	37.931	-0.09	18.76	272.20	18.68	-122.445	37.931	-0.34	18.76	272.22	18.43	9	7/14/2014	46:10.3
2548	RSPe_2	-122.445	37.931	-0.12	18.73	272.00	18.61	-122.445	37.931	-0.39	18.73	272.17	18.34	9	7/14/2014	46:10.2
2549	RSPe_2	-122.445	37.931	-0.09	18.72	271.87	18.63	-122.445	37.931	-0.39	18.72	272.02	18.33	9	7/14/2014	46:10.1
2550	RSPe_2	-122.445	37.931	-0.12	18.83	271.80	18.71	-122.445	37.931	-0.39	18.83	271.95	18.44	9	7/14/2014	46:10.0
2551	RSPe_2	-122.445	37.931	-0.09	18.73	271.89	18.65	-122.445	37.931	-0.42	18.73	271.98	18.31	9	7/14/2014	46:09.9
2552	RSPe_2	-122.445	37.931	-0.09	18.78	271.89	18.70	-122.445	37.931	-0.42	18.78	271.97	18.36	9	7/14/2014	46:09.8
2553	RSPe_2	-122.445	37.931	-0.09	18.79	271.80	18.70	-122.445	37.931	-0.34	18.79	271.89	18.45	9	7/14/2014	46:09.7
2554	RSPe_2	-122.445	37.931	-0.12	18.85	271.65	18.73	-122.445	37.931	-0.42	18.85	271.77	18.43	9	7/14/2014	46:09.6
2555	RSPe_2	-122.445	37.931	-0.12	18.81	271.57	18.69	-122.445	37.931	-0.34	18.81	271.74	18.47	9	7/14/2014	46:09.5
2556	RSPe_2	-122.445	37.931	-0.12	18.92	271.50	18.80	-122.445	37.931	-0.51	18.92	271.58	18.41	9	7/14/2014	46:09.4
2557	RSPe_2	-122.445	37.931	-0.09	18.93	271.39	18.84	-122.445	37.931	-0.39	18.93	271.47	18.54	9	7/14/2014	46:09.3
2558	RSPe_2	-122.445	37.931	-0.09	18.96	271.30	18.87	-122.445	37.931	-0.47	18.96	271.34	18.48	9	7/14/2014	46:09.2
2559	RSPe_2	-122.445	37.931	-0.09	18.94	271.08	18.85	-122.445	37.931	-0.42	18.94	271.16	18.51	9	7/14/2014	46:09.1
2560	RSPe_2	-122.445	37.931	-0.12	19.16	271.01	19.04	-122.445	37.931	-0.42	19.16	271.06	18.73	9	7/14/2014	46:09.0
2561	RSPe_2	-122.445	37.931	-0.09	19.21	270.98	19.13	-122.445	37.931	-0.39	19.21	270.95	18.82	9	7/14/2014	46:08.9
2562	RSPe_2	-122.445	37.931	-0.09	19.19	270.84	19.10	-122.445	37.931	-0.47	19.19	270.90	18.71	9	7/14/2014	46:08.8
2563	RSPe_2	-122.445	37.931	-0.09	19.20	270.86	19.11	-122.445	37.931	-0.39	19.20	270.84	18.81	9	7/14/2014	46:08.7
2564	RSPe_2	-122.445	37.931	-0.12	19.20	270.78	19.08	-122.445	37.931	-0.51	19.20	270.76	18.70	9	7/14/2014	46:08.6
2565	RSPe_2	-122.445	37.931	-0.09	19.20	270.58	19.12	-122.445	37.931	-0.39	19.20	270.60	18.81	9	7/14/2014	46:08.5
2566	RSPe_2	-122.445	37.931	-0.09	19.24	270.63	19.16	-122.445	37.931	-0.34	19.24	270.58	18.90	9	7/14/2014	46:08.4
2567	RSPe_2	-122.445	37.931	-0.09	19.19	270.56	19.10	-122.445	37.931	-0.34	19.19	270.49	18.85	9	7/14/2014	46:08.3
2568	RSPe_2	-122.445	37.931	-0.09	19.15	270.36	19.06	-122.445	37.931	-0.42	19.15	270.33	18.72	9	7/14/2014	46:08.2
2569	RSPe_2	-122.445	37.931	-0.09	19.16	270.29	19.07	-122.445	37.931	-0.39	19.16	270.30	18.77	9	7/14/2014	46:08.1
2570	RSPe_2	-122.445	37.931	-0.09	19.18	270.25	19.10	-122.445	37.931	-0.34	19.18	270.20	18.84	9	7/14/2014	46:08.0
2571	RSPe_2	-122.445	37.931	-0.09	19.20	270.17	19.11	-122.445	37.931	-0.34	19.20	270.14	18.86	9	7/14/2014	46:07.9
2572	RSPe_2	-122.445	37.931	-0.09	19.08	270.01	18.99	-122.445	37.931	-0.39	19.08	270.07	18.69	9	7/14/2014	46:07.8
2573	RSPe_2	-122.445	37.931	-0.09	19.10	269.88	19.02	-122.445	37.931	-0.39	19.10	269.92	18.71	9	7/14/2014	46:07.7

2574	RSPe_2	-122.445	37.931	-0.09	19.11	269.79	19.03	-122.445	37.931	-0.39	19.11	269.83	18.72	9	7/14/2014	46:07.6
2575	RSPe_2	-122.445	37.931	-0.09	19.13	269.64	19.04	-122.445	37.931	-0.47	19.13	269.68	18.65	9	7/14/2014	46:07.5
2576	RSPe_2	-122.445	37.931	-0.09	19.10	269.53	19.01	-122.445	37.931	-0.34	19.10	269.52	18.76	9	7/14/2014	46:07.4
2577	RSPe_2	-122.445	37.931	-0.03	19.07	269.46	19.03	-122.445	37.931	-0.34	19.07	269.46	18.73	9	7/14/2014	46:07.3
2578	RSPe_2	-122.445	37.931	-0.09	19.04	269.40	18.96	-122.445	37.931	-0.39	19.04	269.37	18.65	9	7/14/2014	46:07.2
2579	RSPe_2	-122.445	37.931	-0.03	18.99	269.34	18.96	-122.445	37.931	-0.31	18.99	269.27	18.68	9	7/14/2014	46:07.1
2580	RSPe_2	-122.445	37.931	-0.09	19.01	269.29	18.92	-122.445	37.931	-0.39	19.01	269.22	18.62	9	7/14/2014	46:07.0
2581	RSPe_2	-122.445	37.931	-0.09	18.97	269.23	18.88	-122.445	37.931	-0.25	18.97	269.16	18.71	9	7/14/2014	46:06.9
2582	RSPe_2	-122.445	37.931	-0.09	18.94	269.07	18.86	-122.445	37.931	-0.25	18.94	269.04	18.69	9	7/14/2014	46:06.8
2583	RSPe_2	-122.445	37.931	-0.03	18.94	269.01	18.91	-122.445	37.931	-0.34	18.94	268.92	18.60	9	7/14/2014	46:06.7
2584	RSPe_2	-122.445	37.931	-0.09	18.95	268.87	18.87	-122.445	37.931	-0.25	18.95	268.87	18.70	9	7/14/2014	46:06.6
2585	RSPe_2	-122.445	37.931	-0.03	18.90	268.77	18.87	-122.445	37.931	-0.19	18.90	268.76	18.72	9	7/14/2014	46:06.5
2586	RSPe_2	-122.445	37.931	-0.09	18.90	268.65	18.82	-122.445	37.931	-0.31	18.90	268.69	18.60	9	7/14/2014	46:06.4
2587	RSPe_2	-122.445	37.931	-0.03	18.93	268.59	18.89	-122.445	37.931	-0.22	18.93	268.67	18.71	9	7/14/2014	46:06.3
2588	RSPe_2	-122.445	37.931	-0.03	18.97	268.50	18.94	-122.445	37.931	-0.25	18.97	268.61	18.72	9	7/14/2014	46:06.2
2589	RSPe_2	-122.445	37.931	-0.03	18.88	268.32	18.85	-122.445	37.931	-0.31	18.88	268.48	18.58	9	7/14/2014	46:06.1
2590	RSPe_2	-122.445	37.931	-0.09	18.91	268.13	18.82	-122.445	37.931	-0.22	18.91	268.36	18.69	9	7/14/2014	46:06.0
2591	RSPe_2	-122.445	37.931	-0.03	18.94	268.11	18.90	-122.445	37.931	-0.14	18.94	268.30	18.80	9	7/14/2014	46:05.9
2592	RSPe_2	-122.445	37.931	-0.09	18.93	268.11	18.84	-122.445	37.931	-0.22	18.93	268.24	18.71	9	7/14/2014	46:05.8
2593	RSPe_2	-122.445	37.931	-0.03	18.88	268.07	18.85	-122.445	37.931	-0.22	18.88	268.15	18.66	9	7/14/2014	46:05.7
2594	RSPe_2	-122.445	37.931	-0.03	18.80	267.96	18.76	-122.445	37.931	-0.22	18.80	267.99	18.58	9	7/14/2014	46:05.6
2595	RSPe_2	-122.445	37.931	-0.03	18.68	267.91	18.65	-122.445	37.931	-0.22	18.68	267.89	18.46	9	7/14/2014	46:05.5
2596	RSPe_2	-122.445	37.931	-0.03	18.70	267.78	18.66	-122.445	37.931	-0.25	18.70	267.75	18.44	9	7/14/2014	46:05.4
2597	RSPe_2	-122.445	37.931	-0.03	18.61	267.56	18.58	-122.445	37.931	-0.14	18.61	267.60	18.48	9	7/14/2014	46:05.3
2598	RSPe_2	-122.445	37.931	-0.03	18.54	267.47	18.51	-122.445	37.931	-0.19	18.54	267.56	18.35	9	7/14/2014	46:05.2
2599	RSPe_2	-122.445	37.931	0.00	18.47	267.43	18.47	-122.445	37.931	-0.19	18.47	267.58	18.28	9	7/14/2014	46:05.1
2600	RSPe_2	-122.445	37.931	-0.03	18.59	267.26	18.56	-122.445	37.931	-0.22	18.59	267.47	18.37	9	7/14/2014	46:05.0
2601	RSPe_2	-122.445	37.931	-0.03	18.43	267.21	18.39	-122.445	37.931	-0.19	18.43	267.46	18.24	9	7/14/2014	46:04.9
2602	RSPe_2	-122.445	37.931	-0.03	18.40	267.19	18.37	-122.445	37.931	-0.22	18.40	267.41	18.18	9	7/14/2014	46:04.8
2603	RSPe_2	-122.445	37.931	0.00	18.38	267.17	18.38	-122.445	37.931	-0.22	18.38	267.43	18.16	9	7/14/2014	46:04.7
2604	RSPe_2	-122.445	37.931	-0.03	18.39	267.17	18.35	-122.445	37.931	-0.19	18.39	267.35	18.20	9	7/14/2014	46:04.6
2605	RSPe_2	-122.445	37.931	-0.03	18.43	267.10	18.39	-122.445	37.931	-0.22	18.43	267.21	18.21	9	7/14/2014	46:04.5
2606	RSPe_2	-122.445	37.931	-0.03	18.35	267.04	18.31	-122.445	37.931	-0.22	18.35	267.07	18.13	9	7/14/2014	46:04.4

2607	RSPe_2	-122.445	37.931	0.00	18.32	266.97	18.32	-122.445	37.931	-0.19	18.32	266.91	18.13	9	7/14/2014	46:04.3
2608	RSPe_2	-122.445	37.931	0.00	18.34	266.84	18.34	-122.445	37.931	-0.10	18.34	266.79	18.24	9	7/14/2014	46:04.2
2609	RSPe_2	-122.445	37.931	0.00	18.30	266.76	18.30	-122.445	37.931	-0.14	18.30	266.65	18.17	9	7/14/2014	46:04.1
2610	RSPe_2	-122.445	37.931	0.00	18.21	266.60	18.21	-122.445	37.931	-0.14	18.21	266.51	18.07	9	7/14/2014	46:04.0
2611	RSPe_2	-122.445	37.931	0.00	18.18	266.38	18.18	-122.445	37.931	-0.14	18.18	266.25	18.04	9	7/14/2014	46:03.9
2612	RSPe_2	-122.445	37.931	0.00	18.16	266.32	18.16	-122.445	37.931	-0.14	18.16	266.18	18.02	9	7/14/2014	46:03.8
2613	RSPe_2	-122.445	37.931	0.00	18.19	266.36	18.19	-122.445	37.931	-0.14	18.19	266.20	18.06	9	7/14/2014	46:03.7
2614	RSPe_2	-122.445	37.931	0.00	18.17	266.29	18.17	-122.445	37.931	-0.14	18.17	266.18	18.03	9	7/14/2014	46:03.6
2615	RSPe_2	-122.445	37.931	0.00	18.21	266.28	18.21	-122.445	37.931	-0.10	18.21	266.18	18.11	9	7/14/2014	46:03.5
2616	RSPe_2	-122.445	37.931	0.00	18.16	266.12	18.16	-122.445	37.931	-0.14	18.16	266.14	18.02	9	7/14/2014	46:03.4
2617	RSPe_2	-122.445	37.931	0.00	18.16	266.01	18.16	-122.445	37.931	-0.14	18.16	266.10	18.02	9	7/14/2014	46:03.3
2618	RSPe_2	-122.445	37.931	0.00	18.26	265.95	18.26	-122.445	37.931	-0.19	18.26	266.10	18.07	9	7/14/2014	46:03.2
2619	RSPe_2	-122.445	37.931	0.00	18.26	265.79	18.26	-122.445	37.931	-0.10	18.26	266.01	18.16	9	7/14/2014	46:03.1
2620	RSPe_2	-122.445	37.931	-0.03	18.24	265.64	18.20	-122.445	37.931	-0.10	18.24	265.85	18.13	9	7/14/2014	46:03.0
2621	RSPe_2	-122.445	37.931	-0.03	18.27	265.38	18.24	-122.445	37.931	-0.14	18.27	265.66	18.14	9	7/14/2014	46:02.9
2622	RSPe_2	-122.445	37.931	-0.03	18.27	265.24	18.24	-122.445	37.931	-0.14	18.27	265.57	18.14	9	7/14/2014	46:02.8
2623	RSPe_2	-122.445	37.931	0.00	18.38	265.22	18.38	-122.445	37.931	-0.14	18.38	265.53	18.24	9	7/14/2014	46:02.7
2624	RSPe_2	-122.445	37.931	-0.03	18.33	265.16	18.30	-122.445	37.931	-0.14	18.33	265.44	18.20	9	7/14/2014	46:02.6
2625	RSPe_2	-122.445	37.931	0.00	18.47	264.99	18.47	-122.445	37.931	-0.22	18.47	265.26	18.25	9	7/14/2014	46:02.5
2626	RSPe_2	-122.445	37.931	0.00	18.40	264.92	18.40	-122.445	37.931	-0.14	18.40	265.20	18.26	9	7/14/2014	46:02.4
2627	RSPe_2	-122.445	37.931	0.00	18.59	264.83	18.59	-122.445	37.931	-0.14	18.59	265.07	18.46	9	7/14/2014	46:02.3
2628	RSPe_2	-122.445	37.931	0.00	18.50	264.67	18.50	-122.445	37.931	-0.19	18.50	264.89	18.31	9	7/14/2014	46:02.2
2629	RSPe_2	-122.445	37.931	0.00	18.56	264.56	18.56	-122.445	37.931	-0.05	18.56	264.82	18.51	9	7/14/2014	46:02.1
2630	RSPe_2	-122.445	37.931	0.00	18.58	264.52	18.58	-122.445	37.931	-0.10	18.58	264.65	18.48	9	7/14/2014	46:02.0
2631	RSPe_2	-122.445	37.931	0.00	18.62	264.48	18.62	-122.445	37.931	-0.14	18.62	264.52	18.49	9	7/14/2014	46:01.9
2632	RSPe_2	-122.445	37.931	0.00	18.59	264.33	18.59	-122.445	37.931	-0.10	18.59	264.36	18.49	9	7/14/2014	46:01.8
2633	RSPe_2	-122.445	37.931	0.00	18.73	264.24	18.73	-122.445	37.931	-0.10	18.73	264.26	18.63	9	7/14/2014	46:01.7
2634	RSPe_2	-122.445	37.931	-0.03	18.69	264.24	18.66	-122.445	37.931	-0.10	18.69	264.16	18.59	9	7/14/2014	46:01.6
2635	RSPe_2	-122.445	37.931	0.00	18.67	264.08	18.67	-122.445	37.931	-0.14	18.67	264.06	18.54	9	7/14/2014	46:01.5
2636	RSPe_2	-122.445	37.931	-0.03	18.73	264.04	18.70	-122.445	37.931	-0.14	18.73	264.02	18.60	9	7/14/2014	46:01.4
2637	RSPe_2	-122.445	37.931	0.00	18.74	263.89	18.74	-122.445	37.931	-0.19	18.74	263.91	18.56	9	7/14/2014	46:01.3
2638	RSPe_2	-122.445	37.931	-0.03	18.76	263.83	18.73	-122.445	37.931	-0.14	18.76	263.91	18.62	9	7/14/2014	46:01.2
2639	RSPe_2	-122.445	37.931	0.00	18.83	263.67	18.83	-122.445	37.931	-0.19	18.83	263.78	18.64	9	7/14/2014	46:01.1

2640	RSPe_2	-122.445	37.931	0.00	18.78	263.50	18.78	-122.445	37.931	-0.19	18.78	263.73	18.60	9	7/14/2014	46:01.0
2641	RSPe_2	-122.445	37.931	0.00	18.81	263.50	18.81	-122.445	37.931	-0.14	18.81	263.67	18.68	9	7/14/2014	46:00.9
2642	RSPe_2	-122.445	37.931	0.00	18.86	263.40	18.86	-122.445	37.931	-0.19	18.86	263.56	18.67	9	7/14/2014	46:00.8
2643	RSPe_2	-122.445	37.931	0.00	18.84	263.34	18.84	-122.445	37.931	-0.10	18.84	263.47	18.74	9	7/14/2014	46:00.7
2644	RSPe_2	-122.445	37.931	0.00	18.90	263.27	18.90	-122.445	37.931	-0.14	18.90	263.36	18.76	9	7/14/2014	46:00.6
2645	RSPe_2	-122.445	37.931	-0.03	18.73	263.24	18.70	-122.445	37.931	-0.10	18.73	263.25	18.63	9	7/14/2014	46:00.5
2646	RSPe_2	-122.445	37.931	-0.03	18.73	263.15	18.70	-122.445	37.931	-0.14	18.73	263.12	18.59	9	7/14/2014	46:00.4
2647	RSPe_2	-122.445	37.931	-0.03	18.70	263.06	18.67	-122.445	37.931	-0.14	18.70	263.08	18.57	9	7/14/2014	46:00.3
2648	RSPe_2	-122.445	37.931	-0.03	18.70	262.95	18.66	-122.445	37.931	-0.14	18.70	262.94	18.56	9	7/14/2014	46:00.2
2649	RSPe_2	-122.445	37.931	0.00	18.70	262.89	18.70	-122.445	37.931	-0.14	18.70	262.88	18.56	9	7/14/2014	46:00.1
2650	RSPe_2	-122.445	37.931	-0.03	18.73	262.82	18.70	-122.445	37.931	-0.14	18.73	262.80	18.60	9	7/14/2014	46:00.0
2651	RSPe_2	-122.445	37.931	0.00	18.71	262.67	18.71	-122.445	37.931	-0.19	18.71	262.74	18.53	9	7/14/2014	45:59.9
2652	RSPe_2	-122.445	37.931	-0.03	18.76	262.51	18.73	-122.445	37.931	-0.14	18.76	262.64	18.63	9	7/14/2014	45:59.8
2653	RSPe_2	-122.445	37.931	0.00	18.72	262.53	18.72	-122.445	37.931	-0.19	18.72	262.58	18.53	9	7/14/2014	45:59.7
2654	RSPe_2	-122.445	37.931	-0.03	18.77	262.45	18.74	-122.445	37.931	-0.19	18.77	262.53	18.59	9	7/14/2014	45:59.6
2655	RSPe_2	-122.445	37.931	0.00	18.77	262.34	18.77	-122.445	37.931	-0.05	18.77	262.45	18.72	9	7/14/2014	45:59.5
2656	RSPe_2	-122.445	37.931	-0.03	18.76	262.25	18.73	-122.445	37.931	-0.10	18.76	262.31	18.66	9	7/14/2014	45:59.4
2657	RSPe_2	-122.445	37.931	0.00	18.78	262.14	18.78	-122.445	37.931	-0.14	18.78	262.20	18.65	9	7/14/2014	45:59.3
2658	RSPe_2	-122.445	37.931	0.00	18.80	261.98	18.80	-122.445	37.931	-0.10	18.80	262.12	18.70	9	7/14/2014	45:59.2
2659	RSPe_2	-122.445	37.931	0.00	18.83	261.92	18.84	-122.445	37.931	-0.19	18.83	262.12	18.65	9	7/14/2014	45:59.1
2660	RSPe_2	-122.445	37.931	0.00	18.86	261.75	18.86	-122.445	37.931	-0.14	18.86	261.99	18.72	9	7/14/2014	45:59.0
2661	RSPe_2	-122.445	37.931	0.00	18.87	261.66	18.87	-122.445	37.931	-0.19	18.87	261.88	18.69	9	7/14/2014	45:58.9
2662	RSPe_2	-122.445	37.931	0.00	18.84	261.55	18.84	-122.445	37.931	-0.19	18.84	261.79	18.65	9	7/14/2014	45:58.8
2663	RSPe_2	-122.445	37.931	0.00	18.90	261.42	18.90	-122.445	37.931	-0.14	18.90	261.66	18.76	9	7/14/2014	45:58.7
2664	RSPe_2	-122.445	37.931	0.00	18.90	261.31	18.90	-122.445	37.931	-0.19	18.90	261.56	18.72	9	7/14/2014	45:58.6
2665	RSPe_2	-122.445	37.931	0.00	18.90	261.22	18.90	-122.445	37.931	-0.14	18.90	261.46	18.76	9	7/14/2014	45:58.5
2666	RSPe_2	-122.445	37.931	-0.03	18.86	261.09	18.82	-122.445	37.931	-0.22	18.86	261.34	18.64	9	7/14/2014	45:58.4
2667	RSPe_2	-122.445	37.931	0.00	18.84	261.07	18.84	-122.445	37.931	-0.14	18.84	261.29	18.71	9	7/14/2014	45:58.3
2668	RSPe_2	-122.445	37.931	0.00	18.80	260.89	18.80	-122.445	37.931	-0.22	18.80	261.08	18.58	9	7/14/2014	45:58.2
2669	RSPe_2	-122.445	37.931	0.00	18.76	260.74	18.77	-122.445	37.931	-0.10	18.76	260.96	18.66	9	7/14/2014	45:58.1
2670	RSPe_2	-122.445	37.931	0.00	18.73	260.81	18.73	-122.445	37.931	-0.19	18.73	260.95	18.55	9	7/14/2014	45:58.0
2671	RSPe_2	-122.445	37.931	0.00	18.71	260.76	18.71	-122.445	37.931	-0.14	18.71	260.91	18.58	9	7/14/2014	45:57.9
2672	RSPe_2	-122.445	37.931	0.00	18.66	260.70	18.66	-122.445	37.931	-0.10	18.66	260.84	18.56	9	7/14/2014	45:57.8

2673	RSPe_2	-122.445	37.931	0.05	18.68	260.63	18.73	-122.445	37.931	-0.19	18.68	260.80	18.49	9	7/14/2014 45:57.7
2674	RSPe_2	-122.445	37.931	0.00	18.70	260.48	18.70	-122.445	37.931	-0.19	18.70	260.71	18.51	9	7/14/2014 45:57.6
2675	RSPe_2	-122.445	37.931	0.00	18.71	260.44	18.71	-122.445	37.931	-0.14	18.71	260.65	18.58	9	7/14/2014 45:57.5
2676	RSPe_2	-122.445	37.931	0.00	18.62	260.30	18.62	-122.445	37.931	-0.22	18.62	260.50	18.40	9	7/14/2014 45:57.4
2677	RSPe_2	-122.445	37.931	0.05	18.54	260.17	18.59	-122.445	37.931	-0.19	18.54	260.41	18.36	9	7/14/2014 45:57.3
2678	RSPe_2	-122.445	37.931	0.00	18.51	260.15	18.51	-122.445	37.931	-0.25	18.51	260.25	18.26	9	7/14/2014 45:57.2
2679	RSPe_2	-122.445	37.931	0.05	18.45	260.02	18.50	-122.445	37.931	-0.19	18.45	260.17	18.26	9	7/14/2014 45:57.1
2680	RSPe_2	-122.445	37.931	0.00	18.48	259.88	18.48	-122.445	37.931	-0.19	18.48	259.99	18.30	9	7/14/2014 45:57.0
2681	RSPe_2	-122.445	37.931	0.05	18.47	259.84	18.52	-122.445	37.931	-0.10	18.47	259.88	18.37	9	7/14/2014 45:56.9
2682	RSPe_2	-122.445	37.931	0.00	18.43	259.76	18.43	-122.445	37.931	-0.19	18.43	259.96	18.24	9	7/14/2014 45:56.8
2683	RSPe_2	-122.445	37.931	0.00	18.46	259.69	18.46	-122.445	37.931	-0.14	18.46	259.87	18.32	9	7/14/2014 45:56.7
2684	RSPe_2	-122.445	37.931	0.00	18.51	259.67	18.51	-122.445	37.931	-0.22	18.51	259.91	18.29	9	7/14/2014 45:56.6
2685	RSPe_2	-122.445	37.931	0.05	18.58	259.56	18.63	-122.445	37.931	-0.22	18.58	259.80	18.36	9	7/14/2014 45:56.5
2686	RSPe_2	-122.445	37.931	0.00	18.62	259.48	18.62	-122.445	37.931	-0.14	18.62	259.77	18.49	9	7/14/2014 45:56.4
2687	RSPe_2	-122.445	37.931	0.00	18.63	259.38	18.63	-122.445	37.931	-0.14	18.63	259.63	18.49	9	7/14/2014 45:56.3
2688	RSPe_2	-122.445	37.931	0.00	18.70	259.32	18.70	-122.445	37.931	-0.19	18.70	259.58	18.51	9	7/14/2014 45:56.2
2689	RSPe_2	-122.445	37.931	0.05	18.74	259.21	18.80	-122.445	37.931	-0.14	18.74	259.40	18.61	9	7/14/2014 45:56.1
2690	RSPe_2	-122.445	37.931	0.00	18.79	259.19	18.79	-122.445	37.931	-0.22	18.79	259.31	18.57	9	7/14/2014 45:56.0
2691	RSPe_2	-122.445	37.931	0.05	18.76	259.15	18.82	-122.445	37.931	-0.19	18.76	259.19	18.58	9	7/14/2014 45:55.9
2692	RSPe_2	-122.445	37.931	0.00	18.70	259.08	18.70	-122.445	37.931	-0.25	18.70	259.08	18.44	9	7/14/2014 45:55.8
2693	RSPe_2	-122.445	37.931	0.05	18.83	259.06	18.88	-122.445	37.931	-0.25	18.83	258.99	18.58	9	7/14/2014 45:55.7
2694	RSPe_2	-122.445	37.931	0.00	18.72	259.01	18.72	-122.445	37.931	-0.22	18.72	258.94	18.50	9	7/14/2014 45:55.6
2695	RSPe_2	-122.445	37.931	0.05	18.67	258.93	18.73	-122.445	37.931	-0.22	18.67	258.88	18.45	9	7/14/2014 45:55.5
2696	RSPe_2	-122.445	37.931	0.00	18.64	258.81	18.64	-122.445	37.931	-0.22	18.64	258.76	18.42	9	7/14/2014 45:55.4
2697	RSPe_2	-122.445	37.931	0.05	18.67	258.67	18.73	-122.445	37.931	-0.19	18.67	258.64	18.49	9	7/14/2014 45:55.3
2698	RSPe_2	-122.445	37.931	0.00	18.64	258.62	18.64	-122.445	37.931	-0.22	18.64	258.60	18.42	9	7/14/2014 45:55.2
2699	RSPe_2	-122.445	37.931	0.05	18.64	258.51	18.69	-122.445	37.931	-0.19	18.64	258.55	18.46	9	7/14/2014 45:55.1
2700	RSPe_2	-122.445	37.931	0.00	18.59	258.45	18.60	-122.445	37.931	-0.19	18.59	258.50	18.41	9	7/14/2014 45:55.0
2701	RSPe_2	-122.445	37.931	0.05	18.63	258.29	18.68	-122.445	37.931	-0.19	18.63	258.40	18.45	9	7/14/2014 45:54.9
2702	RSPe_2	-122.445	37.931	0.05	18.63	258.18	18.68	-122.445	37.931	-0.19	18.63	258.28	18.44	9	7/14/2014 45:54.8
2703	RSPe_2	-122.445	37.931	0.05	18.69	257.96	18.74	-122.445	37.931	-0.19	18.69	258.21	18.50	9	7/14/2014 45:54.7
2704	RSPe_2	-122.445	37.931	0.05	18.71	257.77	18.76	-122.445	37.931	-0.19	18.71	258.03	18.53	9	7/14/2014 45:54.6
2705	RSPe_2	-122.445	37.931	0.05	18.71	257.71	18.76	-122.445	37.931	-0.14	18.71	257.97	18.58	9	7/14/2014 45:54.5

2706	RSPe_2	-122.445	37.931	0.05	18.75	257.68	18.80	-122.445	37.931	-0.19	18.75	257.93	18.56	9	7/14/2014	45:54.4
2707	RSPe_2	-122.445	37.931	0.05	18.97	257.57	19.03	-122.445	37.931	-0.14	18.97	257.81	18.84	9	7/14/2014	45:54.3
2708	RSPe_2	-122.445	37.931	0.05	18.94	257.50	18.99	-122.445	37.931	-0.19	18.94	257.79	18.76	9	7/14/2014	45:54.2
2709	RSPe_2	-122.445	37.931	0.09	19.01	257.46	19.10	-122.445	37.931	-0.22	19.01	257.65	18.79	9	7/14/2014	45:54.1
2710	RSPe_2	-122.445	37.931	0.05	19.06	257.35	19.11	-122.445	37.931	-0.25	19.06	257.57	18.81	9	7/14/2014	45:54.0
2711	RSPe_2	-122.445	37.931	0.09	19.11	257.29	19.20	-122.445	37.931	-0.14	19.11	257.46	18.98	9	7/14/2014	45:53.9
2712	RSPe_2	-122.445	37.931	0.09	19.16	257.16	19.24	-122.445	37.931	-0.19	19.16	257.33	18.97	9	7/14/2014	45:53.8
2713	RSPe_2	-122.445	37.931	0.09	19.12	257.05	19.21	-122.445	37.931	-0.05	19.12	257.15	19.07	9	7/14/2014	45:53.7
2714	RSPe_2	-122.445	37.931	0.05	19.09	256.89	19.14	-122.445	37.931	-0.10	19.09	257.00	18.99	9	7/14/2014	45:53.6
2715	RSPe_2	-122.445	37.931	0.09	19.08	256.85	19.16	-122.445	37.931	-0.14	19.08	256.92	18.94	9	7/14/2014	45:53.5
2716	RSPe_2	-122.445	37.931	0.09	19.04	256.65	19.13	-122.445	37.931	-0.19	19.04	256.69	18.86	9	7/14/2014	45:53.4
2717	RSPe_2	-122.445	37.931	0.14	19.09	256.63	19.23	-122.445	37.931	-0.14	19.09	256.65	18.96	9	7/14/2014	45:53.3
2718	RSPe_2	-122.445	37.931	0.09	19.08	256.61	19.17	-122.445	37.931	-0.14	19.08	256.61	18.95	9	7/14/2014	45:53.2
2719	RSPe_2	-122.445	37.931	0.14	19.08	256.55	19.22	-122.445	37.931	-0.14	19.08	256.56	18.95	9	7/14/2014	45:53.1
2720	RSPe_2	-122.445	37.931	0.09	19.06	256.57	19.15	-122.445	37.931	-0.14	19.06	256.56	18.92	9	7/14/2014	45:53.0
2721	RSPe_2	-122.445	37.931	0.14	19.02	256.43	19.16	-122.445	37.931	-0.10	19.02	256.43	18.92	9	7/14/2014	45:52.9
2722	RSPe_2	-122.445	37.931	0.09	19.09	256.28	19.18	-122.445	37.931	-0.10	19.09	256.39	18.99	9	7/14/2014	45:52.8
2723	RSPe_2	-122.445	37.931	0.14	19.05	256.17	19.19	-122.445	37.931	-0.14	19.05	256.23	18.91	9	7/14/2014	45:52.7
2724	RSPe_2	-122.445	37.931	0.14	19.01	256.07	19.14	-122.445	37.931	-0.19	19.01	256.19	18.82	9	7/14/2014	45:52.6
2725	RSPe_2	-122.445	37.931	0.14	19.00	255.93	19.13	-122.445	37.931	-0.14	19.00	256.13	18.86	9	7/14/2014	45:52.5
2726	RSPe_2	-122.445	37.931	0.14	19.00	255.81	19.13	-122.445	37.931	-0.19	19.00	256.00	18.81	9	7/14/2014	45:52.4
2727	RSPe_2	-122.445	37.931	0.14	19.03	255.69	19.17	-122.445	37.931	-0.14	19.03	255.90	18.90	9	7/14/2014	45:52.3
2728	RSPe_2	-122.445	37.931	0.14	19.04	255.63	19.18	-122.445	37.931	-0.14	19.04	255.76	18.91	9	7/14/2014	45:52.2
2729	RSPe_2	-122.445	37.931	0.14	19.02	255.41	19.16	-122.445	37.931	-0.05	19.02	255.60	18.97	9	7/14/2014	45:52.1
2730	RSPe_2	-122.445	37.931	0.14	19.03	255.34	19.17	-122.445	37.931	-0.14	19.03	255.49	18.89	9	7/14/2014	45:52.0
2731	RSPe_2	-122.445	37.931	0.17	18.94	255.34	19.11	-122.445	37.931	-0.05	18.94	255.33	18.89	9	7/14/2014	45:51.9
2732	RSPe_2	-122.445	37.931	0.14	18.77	255.27	18.91	-122.445	37.931	-0.14	18.77	255.27	18.64	9	7/14/2014	45:51.8
2733	RSPe_2	-122.445	37.931	0.17	18.76	255.28	18.94	-122.445	37.931	-0.10	18.76	255.17	18.66	9	7/14/2014	45:51.7
2734	RSPe_2	-122.445	37.931	0.14	18.76	255.24	18.90	-122.445	37.931	-0.10	18.76	255.10	18.66	9	7/14/2014	45:51.6
2735	RSPe_2	-122.445	37.931	0.14	18.66	255.19	18.80	-122.445	37.931	-0.02	18.66	255.08	18.65	9	7/14/2014	45:51.5
2736	RSPe_2	-122.445	37.931	0.09	18.65	255.05	18.74	-122.445	37.931	-0.02	18.65	254.97	18.63	9	7/14/2014	45:51.4
2737	RSPe_2	-122.445	37.931	0.14	18.61	254.86	18.75	-122.445	37.931	-0.05	18.61	254.88	18.56	9	7/14/2014	45:51.3
2738	RSPe_2	-122.445	37.931	0.14	18.44	254.70	18.58	-122.445	37.931	-0.10	18.44	254.77	18.34	9	7/14/2014	45:51.2

2739	RSPe_2	-122.445	37.931	0.17	18.50	254.60	18.68	-122.445	37.931	-0.02	18.50	254.76	18.49	9	7/14/2014	45:51.1
2740	RSPe_2	-122.445	37.931	0.14	18.48	254.55	18.62	-122.445	37.931	-0.14	18.48	254.77	18.35	9	7/14/2014	45:51.0
2741	RSPe_2	-122.445	37.931	0.14	18.42	254.52	18.56	-122.445	37.931	-0.14	18.42	254.64	18.28	9	7/14/2014	45:50.9
2742	RSPe_2	-122.445	37.931	0.14	18.44	254.53	18.58	-122.445	37.931	-0.14	18.44	254.62	18.31	9	7/14/2014	45:50.8
2743	RSPe_2	-122.445	37.931	0.17	18.42	254.41	18.59	-122.445	37.931	-0.05	18.42	254.46	18.37	9	7/14/2014	45:50.7
2744	RSPe_2	-122.445	37.931	0.14	18.41	254.31	18.55	-122.445	37.931	-0.05	18.41	254.31	18.36	9	7/14/2014	45:50.6
2745	RSPe_2	-122.445	37.931	0.17	18.42	254.19	18.59	-122.445	37.931	-0.05	18.42	254.13	18.37	9	7/14/2014	45:50.5
2746	RSPe_2	-122.445	37.931	0.14	18.34	254.05	18.48	-122.445	37.931	-0.10	18.34	254.09	18.24	9	7/14/2014	45:50.4
2747	RSPe_2	-122.445	37.931	0.17	18.36	253.96	18.54	-122.445	37.931	-0.02	18.36	254.03	18.35	9	7/14/2014	45:50.3
2748	RSPe_2	-122.445	37.931	0.14	18.46	253.85	18.59	-122.445	37.931	-0.10	18.46	253.92	18.35	9	7/14/2014	45:50.2
2749	RSPe_2	-122.445	37.931	0.17	18.31	253.77	18.48	-122.445	37.931	0.07	18.31	253.85	18.38	9	7/14/2014	45:50.1
2750	RSPe_2	-122.445	37.931	0.17	18.38	253.70	18.55	-122.445	37.931	0.03	18.38	253.81	18.41	9	7/14/2014	45:50.0
2751	RSPe_2	-122.445	37.931	0.17	18.41	253.64	18.58	-122.445	37.931	-0.10	18.41	253.65	18.31	9	7/14/2014	45:49.9
2752	RSPe_2	-122.445	37.931	0.14	18.43	253.55	18.57	-122.445	37.931	0.07	18.43	253.57	18.50	9	7/14/2014	45:49.8
2753	RSPe_2	-122.445	37.931	0.17	18.48	253.44	18.65	-122.445	37.931	-0.10	18.48	253.39	18.38	9	7/14/2014	45:49.7
2754	RSPe_2	-122.445	37.931	0.17	18.53	253.22	18.71	-122.445	37.931	-0.14	18.53	253.26	18.40	9	7/14/2014	45:49.6
2755	RSPe_2	-122.445	37.931	0.21	18.51	253.16	18.72	-122.445	37.931	-0.02	18.51	253.11	18.49	9	7/14/2014	45:49.5
2756	RSPe_2	-122.445	37.931	0.17	18.58	253.02	18.75	-122.445	37.931	-0.05	18.58	252.99	18.53	9	7/14/2014	45:49.4
2757	RSPe_2	-122.445	37.931	0.21	18.57	252.92	18.78	-122.445	37.931	-0.02	18.57	252.92	18.56	9	7/14/2014	45:49.3
2758	RSPe_2	-122.445	37.931	0.21	18.56	252.81	18.76	-122.445	37.931	-0.14	18.56	252.88	18.42	9	7/14/2014	45:49.2
2759	RSPe_2	-122.445	37.931	0.21	18.63	252.74	18.83	-122.445	37.931	-0.02	18.63	252.85	18.61	9	7/14/2014	45:49.1
2760	RSPe_2	-122.445	37.931	0.17	18.59	252.70	18.77	-122.445	37.931	0.03	18.59	252.73	18.63	9	7/14/2014	45:49.0
2761	RSPe_2	-122.445	37.931	0.26	18.73	252.61	18.99	-122.445	37.931	-0.02	18.73	252.72	18.71	9	7/14/2014	45:48.9
2762	RSPe_2	-122.445	37.931	0.21	18.66	252.57	18.86	-122.445	37.931	0.03	18.66	252.57	18.69	9	7/14/2014	45:48.8
2763	RSPe_2	-122.445	37.931	0.26	18.68	252.44	18.94	-122.445	37.931	-0.02	18.68	252.47	18.66	9	7/14/2014	45:48.7
2764	RSPe_2	-122.445	37.931	0.26	18.75	252.39	19.01	-122.445	37.931	0.07	18.75	252.37	18.82	9	7/14/2014	45:48.6
2765	RSPe_2	-122.445	37.931	0.26	18.74	252.30	19.00	-122.445	37.931	0.07	18.74	252.21	18.81	9	7/14/2014	45:48.5
2766	RSPe_2	-122.445	37.931	0.26	18.79	252.26	19.05	-122.445	37.931	0.12	18.79	252.16	18.91	9	7/14/2014	45:48.4
2767	RSPe_2	-122.445	37.931	0.26	18.77	252.26	19.03	-122.445	37.931	0.07	18.77	252.14	18.84	9	7/14/2014	45:48.3
2768	RSPe_2	-122.445	37.931	0.26	18.76	252.26	19.02	-122.445	37.931	0.19	18.76	252.14	18.95	9	7/14/2014	45:48.2
2769	RSPe_2	-122.445	37.931	0.26	18.70	252.18	18.96	-122.445	37.931	0.15	18.70	252.06	18.86	9	7/14/2014	45:48.1
2770	RSPe_2	-122.445	37.931	0.26	18.66	252.07	18.92	-122.445	37.931	0.12	18.66	252.04	18.78	9	7/14/2014	45:48.0
2771	RSPe_2	-122.445	37.931	0.29	18.59	252.02	18.89	-122.445	37.931	0.24	18.59	251.98	18.83	9	7/14/2014	45:47.9

2772	RSPe_2	-122.445	37.931	0.26	18.59	252.02	18.85	-122.445	37.931	0.19	18.59	251.97	18.78	9	7/14/2014	45:47.8
2773	RSPe_2	-122.445	37.931	0.29	18.57	251.89	18.87	-122.445	37.931	0.15	18.57	251.95	18.73	9	7/14/2014	45:47.7
2774	RSPe_2	-122.445	37.931	0.29	18.57	251.83	18.87	-122.445	37.931	0.15	18.57	251.92	18.73	9	7/14/2014	45:47.6
2775	RSPe_2	-122.445	37.931	0.29	18.51	251.63	18.80	-122.445	37.931	0.24	18.51	251.76	18.75	9	7/14/2014	45:47.5
2776	RSPe_2	-122.445	37.931	0.29	18.50	251.54	18.79	-122.445	37.931	0.19	18.50	251.70	18.68	9	7/14/2014	45:47.4
2777	RSPe_2	-122.445	37.931	0.29	18.48	251.43	18.77	-122.445	37.931	0.24	18.48	251.62	18.72	9	7/14/2014	45:47.3
2778	RSPe_2	-122.445	37.931	0.29	18.47	251.35	18.77	-122.445	37.931	0.24	18.47	251.50	18.71	9	7/14/2014	45:47.2
2779	RSPe_2	-122.445	37.931	0.34	18.43	251.32	18.77	-122.445	37.931	0.27	18.43	251.44	18.70	9	7/14/2014	45:47.1
2780	RSPe_2	-122.445	37.931	0.34	18.39	251.23	18.73	-122.445	37.931	0.24	18.39	251.29	18.62	9	7/14/2014	45:47.0
2781	RSPe_2	-122.445	37.931	0.34	18.39	251.24	18.73	-122.445	37.931	0.27	18.39	251.25	18.66	9	7/14/2014	45:46.9
2782	RSPe_2	-122.445	37.931	0.34	18.35	251.13	18.69	-122.445	37.931	0.27	18.35	251.13	18.62	9	7/14/2014	45:46.8
2783	RSPe_2	-122.445	37.931	0.34	18.38	250.99	18.72	-122.445	37.931	0.32	18.38	251.01	18.70	9	7/14/2014	45:46.7
2784	RSPe_2	-122.445	37.931	0.29	18.36	250.95	18.66	-122.445	37.931	0.32	18.36	250.91	18.69	9	7/14/2014	45:46.6
2785	RSPe_2	-122.445	37.931	0.34	18.30	250.85	18.64	-122.445	37.931	0.27	18.30	250.82	18.57	9	7/14/2014	45:46.5
2786	RSPe_2	-122.445	37.931	0.34	18.28	250.69	18.62	-122.445	37.931	0.36	18.28	250.71	18.64	9	7/14/2014	45:46.4
2787	RSPe_2	-122.445	37.931	0.38	18.30	250.60	18.68	-122.445	37.931	0.32	18.30	250.62	18.63	9	7/14/2014	45:46.3
2788	RSPe_2	-122.445	37.931	0.38	18.30	250.53	18.67	-122.445	37.931	0.32	18.30	250.57	18.62	9	7/14/2014	45:46.2
2789	RSPe_2	-122.445	37.931	0.38	18.22	250.34	18.60	-122.445	37.931	0.32	18.22	250.40	18.54	9	7/14/2014	45:46.1
2790	RSPe_2	-122.445	37.931	0.34	18.28	250.38	18.62	-122.445	37.931	0.36	18.28	250.45	18.64	9	7/14/2014	45:46.0
2791	RSPe_2	-122.445	37.931	0.38	18.27	250.30	18.65	-122.445	37.931	0.27	18.27	250.45	18.54	9	7/14/2014	45:45.9
2792	RSPe_2	-122.445	37.931	0.34	18.40	250.23	18.75	-122.445	37.931	0.36	18.40	250.36	18.76	9	7/14/2014	45:45.8
2793	RSPe_2	-122.445	37.931	0.38	18.40	250.21	18.78	-122.445	37.931	0.36	18.40	250.34	18.76	9	7/14/2014	45:45.7
2794	RSPe_2	-122.445	37.931	0.38	18.39	250.06	18.76	-122.445	37.931	0.32	18.39	250.24	18.71	9	7/14/2014	45:45.6
2795	RSPe_2	-122.445	37.931	0.38	18.44	250.01	18.82	-122.445	37.931	0.36	18.44	250.16	18.80	9	7/14/2014	45:45.5
2796	RSPe_2	-122.445	37.931	0.38	18.45	249.90	18.83	-122.445	37.931	0.41	18.45	250.07	18.86	9	7/14/2014	45:45.4
2797	RSPe_2	-122.445	37.931	0.38	18.41	249.77	18.79	-122.445	37.931	0.41	18.41	249.97	18.82	9	7/14/2014	45:45.3
2798	RSPe_2	-122.445	37.931	0.38	18.40	249.75	18.77	-122.445	37.931	0.36	18.40	249.83	18.75	9	7/14/2014	45:45.2
2799	RSPe_2	-122.445	37.931	0.38	18.33	249.64	18.71	-122.445	37.931	0.36	18.33	249.75	18.69	9	7/14/2014	45:45.1
2800	RSPe_2	-122.445	37.931	0.38	18.33	249.58	18.70	-122.445	37.931	0.36	18.33	249.63	18.68	9	7/14/2014	45:45.0
2801	RSPe_2	-122.445	37.931	0.43	18.30	249.42	18.73	-122.445	37.931	0.44	18.30	249.46	18.74	9	7/14/2014	45:44.9
2802	RSPe_2	-122.445	37.931	0.43	18.25	249.38	18.68	-122.445	37.931	0.47	18.25	249.41	18.72	9	7/14/2014	45:44.8
2803	RSPe_2	-122.445	37.931	0.43	18.22	249.27	18.65	-122.445	37.931	0.47	18.22	249.24	18.69	9	7/14/2014	45:44.7
2804	RSPe_2	-122.445	37.931	0.43	18.21	249.27	18.64	-122.445	37.931	0.41	18.21	249.22	18.62	9	7/14/2014	45:44.6

2805	RSPe_2	-122.445	37.931	0.46	18.19	249.16	18.65	-122.445	37.931	0.44	18.19	249.12	18.63	9	7/14/2014	45:44.5
2806	RSPe_2	-122.445	37.931	0.43	18.21	249.16	18.64	-122.445	37.931	0.44	18.21	249.13	18.65	9	7/14/2014	45:44.4
2807	RSPe_2	-122.445	37.931	0.46	18.13	249.08	18.60	-122.445	37.931	0.47	18.13	249.01	18.61	9	7/14/2014	45:44.3
2808	RSPe_2	-122.445	37.931	0.43	18.10	249.01	18.53	-122.445	37.931	0.47	18.10	249.01	18.58	9	7/14/2014	45:44.2
2809	RSPe_2	-122.445	37.931	0.43	18.07	248.94	18.50	-122.445	37.931	0.47	18.07	248.96	18.54	9	7/14/2014	45:44.1
2810	RSPe_2	-122.445	37.931	0.43	18.05	248.84	18.48	-122.445	37.931	0.44	18.05	248.89	18.49	9	7/14/2014	45:44.0
2811	RSPe_2	-122.445	37.931	0.46	18.03	248.83	18.50	-122.445	37.931	0.53	18.03	248.87	18.56	9	7/14/2014	45:43.9
2812	RSPe_2	-122.445	37.931	0.43	18.11	248.72	18.54	-122.445	37.931	0.53	18.11	248.79	18.63	9	7/14/2014	45:43.8
2813	RSPe_2	-122.445	37.931	0.43	18.05	248.66	18.48	-122.445	37.931	0.53	18.05	248.79	18.58	9	7/14/2014	45:43.7
2814	RSPe_2	-122.445	37.931	0.43	18.00	248.57	18.43	-122.445	37.931	0.53	18.00	248.66	18.53	9	7/14/2014	45:43.6
2815	RSPe_2	-122.445	37.931	0.43	17.98	248.42	18.41	-122.445	37.931	0.56	17.98	248.52	18.54	9	7/14/2014	45:43.5
2816	RSPe_2	-122.445	37.931	0.43	17.99	248.46	18.42	-122.445	37.931	0.53	17.99	248.54	18.52	9	7/14/2014	45:43.4
2817	RSPe_2	-122.445	37.931	0.46	17.96	248.42	18.43	-122.445	37.931	0.53	17.96	248.53	18.49	9	7/14/2014	45:43.3
2818	RSPe_2	-122.445	37.931	0.43	17.96	248.29	18.39	-122.445	37.931	0.53	17.96	248.42	18.49	9	7/14/2014	45:43.2
2819	RSPe_2	-122.445	37.931	0.43	17.96	248.24	18.39	-122.445	37.931	0.56	17.96	248.33	18.52	9	7/14/2014	45:43.1
2820	RSPe_2	-122.445	37.931	0.43	17.95	248.13	18.38	-122.445	37.931	0.53	17.95	248.29	18.47	9	7/14/2014	45:43.0
2821	RSPe_2	-122.445	37.931	0.46	17.99	248.02	18.46	-122.445	37.931	0.53	17.99	248.22	18.52	9	7/14/2014	45:42.9
2822	RSPe_2	-122.445	37.931	0.43	17.94	247.98	18.37	-122.445	37.931	0.53	17.94	248.18	18.47	9	7/14/2014	45:42.8
2823	RSPe_2	-122.445	37.931	0.46	17.96	247.88	18.43	-122.445	37.931	0.53	17.96	248.13	18.49	9	7/14/2014	45:42.7
2824	RSPe_2	-122.445	37.931	0.43	17.96	247.78	18.39	-122.445	37.931	0.47	17.96	248.04	18.44	9	7/14/2014	45:42.6
2825	RSPe_2	-122.445	37.931	0.43	18.05	247.74	18.48	-122.445	37.931	0.47	18.05	247.94	18.53	9	7/14/2014	45:42.5
2826	RSPe_2	-122.445	37.931	0.43	18.03	247.65	18.46	-122.445	37.931	0.47	18.03	247.90	18.50	9	7/14/2014	45:42.4
2827	RSPe_2	-122.445	37.931	0.46	17.99	247.59	18.46	-122.445	37.931	0.47	17.99	247.81	18.47	9	7/14/2014	45:42.3
2828	RSPe_2	-122.445	37.931	0.43	17.99	247.50	18.42	-122.445	37.931	0.47	17.99	247.72	18.47	9	7/14/2014	45:42.2
2829	RSPe_2	-122.445	37.931	0.43	18.05	247.50	18.48	-122.445	37.931	0.53	18.05	247.67	18.57	9	7/14/2014	45:42.1
2830	RSPe_2	-122.445	37.931	0.43	18.12	247.44	18.55	-122.445	37.931	0.53	18.12	247.65	18.65	9	7/14/2014	45:42.0
2831	RSPe_2	-122.445	37.931	0.43	18.19	247.41	18.62	-122.445	37.931	0.47	18.19	247.56	18.66	9	7/14/2014	45:41.9
2832	RSPe_2	-122.445	37.931	0.43	18.18	247.33	18.61	-122.445	37.931	0.47	18.18	247.54	18.65	9	7/14/2014	45:41.8
2833	RSPe_2	-122.445	37.931	0.43	18.33	247.26	18.76	-122.445	37.931	0.47	18.33	247.46	18.80	9	7/14/2014	45:41.7
2834	RSPe_2	-122.445	37.931	0.38	18.36	247.22	18.74	-122.445	37.931	0.47	18.36	247.48	18.83	9	7/14/2014	45:41.6
2835	RSPe_2	-122.445	37.931	0.43	18.31	247.19	18.74	-122.445	37.931	0.53	18.31	247.41	18.84	9	7/14/2014	45:41.5
2836	RSPe_2	-122.445	37.931	0.43	18.32	247.13	18.75	-122.445	37.931	0.47	18.32	247.36	18.79	9	7/14/2014	45:41.4
2837	RSPe_2	-122.445	37.931	0.43	18.34	247.11	18.77	-122.445	37.931	0.53	18.34	247.31	18.87	9	7/14/2014	45:41.3

2838	RSPe_2	-122.445	37.931	0.38	18.36	247.07	18.74	-122.445	37.931	0.47	18.36	247.25	18.84	9	7/14/2014	45:41.2
2839	RSPe_2	-122.445	37.931	0.43	18.36	247.05	18.79	-122.445	37.931	0.47	18.36	247.29	18.84	9	7/14/2014	45:41.1
2840	RSPe_2	-122.445	37.931	0.43	18.41	246.98	18.84	-122.445	37.931	0.44	18.41	247.18	18.85	9	7/14/2014	45:41.0
2841	RSPe_2	-122.445	37.931	0.43	18.37	246.89	18.80	-122.445	37.931	0.47	18.37	247.09	18.85	9	7/14/2014	45:40.9
2842	RSPe_2	-122.445	37.931	0.43	18.40	246.85	18.83	-122.445	37.931	0.44	18.40	247.02	18.84	9	7/14/2014	45:40.8
2843	RSPe_2	-122.445	37.931	0.43	18.37	246.85	18.80	-122.445	37.931	0.44	18.37	246.96	18.81	9	7/14/2014	45:40.7
2844	RSPe_2	-122.445	37.931	0.38	18.36	246.79	18.74	-122.445	37.931	0.44	18.36	246.86	18.81	9	7/14/2014	45:40.6
2845	RSPe_2	-122.445	37.931	0.43	18.36	246.79	18.79	-122.445	37.931	0.47	18.36	246.82	18.84	9	7/14/2014	45:40.5
2846	RSPe_2	-122.445	37.931	0.43	18.37	246.65	18.80	-122.445	37.931	0.44	18.37	246.76	18.81	9	7/14/2014	45:40.4
2847	RSPe_2	-122.445	37.931	0.43	18.37	246.61	18.80	-122.445	37.931	0.47	18.37	246.74	18.85	9	7/14/2014	45:40.3
2848	RSPe_2	-122.445	37.931	0.43	18.36	246.50	18.79	-122.445	37.931	0.44	18.36	246.68	18.81	9	7/14/2014	45:40.2
2849	RSPe_2	-122.445	37.931	0.38	18.36	246.39	18.74	-122.445	37.931	0.44	18.36	246.60	18.80	9	7/14/2014	45:40.1
2850	RSPe_2	-122.445	37.931	0.43	18.40	246.33	18.83	-122.445	37.931	0.44	18.40	246.54	18.84	9	7/14/2014	45:40.0
2851	RSPe_2	-122.445	37.931	0.43	18.38	246.26	18.81	-122.445	37.931	0.44	18.38	246.45	18.82	9	7/14/2014	45:39.9
2852	RSPe_2	-122.445	37.931	0.43	18.40	246.20	18.83	-122.445	37.931	0.44	18.40	246.36	18.84	9	7/14/2014	45:39.8
2853	RSPe_2	-122.445	37.931	0.43	18.38	246.13	18.81	-122.445	37.931	0.44	18.38	246.26	18.82	9	7/14/2014	45:39.7
2854	RSPe_2	-122.445	37.931	0.38	18.36	246.09	18.74	-122.445	37.931	0.47	18.36	246.22	18.84	9	7/14/2014	45:39.6
2855	RSPe_2	-122.445	37.931	0.43	18.41	246.03	18.84	-122.445	37.931	0.47	18.41	246.09	18.89	9	7/14/2014	45:39.5
2856	RSPe_2	-122.445	37.931	0.43	18.41	246.00	18.84	-122.445	37.931	0.44	18.41	246.04	18.85	9	7/14/2014	45:39.4
2857	RSPe_2	-122.445	37.931	0.43	18.39	245.95	18.82	-122.445	37.931	0.47	18.39	246.00	18.86	9	7/14/2014	45:39.3
2858	RSPe_2	-122.445	37.931	0.43	18.36	245.87	18.79	-122.445	37.931	0.53	18.36	245.96	18.89	9	7/14/2014	45:39.2
2859	RSPe_2	-122.445	37.931	0.43	18.35	245.87	18.78	-122.445	37.931	0.53	18.35	245.97	18.87	9	7/14/2014	45:39.1
2860	RSPe_2	-122.445	37.931	0.43	18.33	245.90	18.76	-122.445	37.931	0.47	18.33	246.01	18.80	9	7/14/2014	45:39.0
2861	RSPe_2	-122.445	37.931	0.43	18.33	245.87	18.76	-122.445	37.931	0.47	18.33	246.00	18.81	9	7/14/2014	45:38.9
2862	RSPe_2	-122.445	37.931	0.43	18.33	245.81	18.76	-122.445	37.931	0.44	18.33	246.03	18.77	9	7/14/2014	45:38.8
2863	RSPe_2	-122.445	37.931	0.46	18.33	245.76	18.79	-122.445	37.931	0.44	18.33	245.96	18.77	9	7/14/2014	45:38.7
2864	RSPe_2	-122.445	37.931	0.43	18.30	245.72	18.73	-122.445	37.931	0.44	18.30	245.98	18.75	9	7/14/2014	45:38.6
2865	RSPe_2	-122.445	37.931	0.43	18.30	245.66	18.73	-122.445	37.931	0.47	18.30	245.89	18.77	9	7/14/2014	45:38.5
2866	RSPe_2	-122.445	37.931	0.43	18.28	245.54	18.71	-122.445	37.931	0.47	18.28	245.87	18.75	9	7/14/2014	45:38.4
2867	RSPe_2	-122.445	37.931	0.43	18.26	245.48	18.69	-122.445	37.931	0.47	18.26	245.76	18.74	9	7/14/2014	45:38.3
2868	RSPe_2	-122.445	37.931	0.43	18.28	245.39	18.71	-122.445	37.931	0.47	18.28	245.66	18.75	9	7/14/2014	45:38.2
2869	RSPe_2	-122.445	37.931	0.43	18.21	245.38	18.64	-122.445	37.931	0.53	18.21	245.61	18.73	9	7/14/2014	45:38.1
2870	RSPe_2	-122.445	37.931	0.43	18.19	245.35	18.62	-122.445	37.931	0.47	18.19	245.50	18.67	9	7/14/2014	45:38.0

2871	RSPe_2	-122.445	37.931	0.43	18.13	245.35	18.56	-122.445	37.931	0.53	18.13	245.44	18.66	9	7/14/2014	45:37.9
2872	RSPe_2	-122.445	37.931	0.43	18.12	245.27	18.55	-122.445	37.931	0.47	18.12	245.37	18.60	9	7/14/2014	45:37.8
2873	RSPe_2	-122.445	37.931	0.46	18.12	245.24	18.59	-122.445	37.931	0.56	18.12	245.33	18.68	9	7/14/2014	45:37.7
2874	RSPe_2	-122.445	37.931	0.43	18.05	245.13	18.48	-122.445	37.931	0.53	18.05	245.22	18.57	9	7/14/2014	45:37.6
2875	RSPe_2	-122.445	37.931	0.46	18.04	245.02	18.50	-122.445	37.931	0.53	18.04	245.19	18.56	9	7/14/2014	45:37.5
2876	RSPe_2	-122.445	37.931	0.43	17.97	244.91	18.40	-122.445	37.931	0.53	17.97	245.13	18.50	9	7/14/2014	45:37.4
2877	RSPe_2	-122.445	37.931	0.43	17.96	244.85	18.39	-122.445	37.931	0.53	17.96	245.08	18.48	9	7/14/2014	45:37.3
2878	RSPe_2	-122.445	37.931	0.43	17.95	244.76	18.38	-122.445	37.931	0.44	17.95	245.02	18.39	9	7/14/2014	45:37.2
2879	RSPe_2	-122.445	37.931	0.43	17.93	244.65	18.36	-122.445	37.931	0.47	17.93	244.89	18.41	9	7/14/2014	45:37.1
2880	RSPe_2	-122.445	37.931	0.43	17.96	244.65	18.39	-122.445	37.931	0.47	17.96	244.91	18.44	9	7/14/2014	45:37.0
2881	RSPe_2	-122.445	37.931	0.43	17.93	244.59	18.36	-122.445	37.931	0.47	17.93	244.85	18.41	9	7/14/2014	45:36.9
2882	RSPe_2	-122.445	37.931	0.43	17.92	244.54	18.35	-122.445	37.931	0.47	17.92	244.71	18.39	9	7/14/2014	45:36.8
2883	RSPe_2	-122.445	37.931	0.43	17.94	244.48	18.37	-122.445	37.931	0.47	17.94	244.67	18.42	9	7/14/2014	45:36.7
2884	RSPe_2	-122.445	37.931	0.43	17.88	244.41	18.31	-122.445	37.931	0.47	17.88	244.56	18.35	9	7/14/2014	45:36.6
2885	RSPe_2	-122.445	37.931	0.43	17.87	244.37	18.30	-122.445	37.931	0.53	17.87	244.50	18.40	9	7/14/2014	45:36.5
2886	RSPe_2	-122.445	37.931	0.43	17.87	244.32	18.30	-122.445	37.931	0.53	17.87	244.43	18.40	9	7/14/2014	45:36.4
2887	RSPe_2	-122.445	37.931	0.43	17.83	244.26	18.26	-122.445	37.931	0.53	17.83	244.36	18.36	9	7/14/2014	45:36.3
2888	RSPe_2	-122.445	37.931	0.43	17.85	244.24	18.28	-122.445	37.931	0.53	17.85	244.29	18.38	9	7/14/2014	45:36.2
2889	RSPe_2	-122.445	37.931	0.43	17.85	244.15	18.28	-122.445	37.931	0.53	17.85	244.24	18.38	9	7/14/2014	45:36.1
2890	RSPe_2	-122.445	37.931	0.43	17.83	244.11	18.26	-122.445	37.931	0.56	17.83	244.21	18.39	9	7/14/2014	45:36.0
2891	RSPe_2	-122.445	37.931	0.46	17.82	243.96	18.28	-122.445	37.931	0.56	17.82	244.04	18.38	9	7/14/2014	45:35.9
2892	RSPe_2	-122.445	37.931	0.46	17.81	243.87	18.27	-122.445	37.931	0.56	17.81	243.97	18.37	9	7/14/2014	45:35.8
2893	RSPe_2	-122.445	37.931	0.43	17.83	243.88	18.26	-122.445	37.931	0.56	17.83	243.97	18.39	9	7/14/2014	45:35.7
2894	RSPe_2	-122.445	37.931	0.43	17.85	243.85	18.28	-122.445	37.931	0.56	17.85	244.02	18.41	9	7/14/2014	45:35.6
2895	RSPe_2	-122.445	37.931	0.43	17.80	243.85	18.23	-122.445	37.931	0.56	17.80	244.03	18.36	9	7/14/2014	45:35.5
2896	RSPe_2	-122.445	37.931	0.43	17.85	243.80	18.28	-122.445	37.931	0.53	17.85	243.99	18.38	9	7/14/2014	45:35.4
2897	RSPe_2	-122.445	37.931	0.46	17.89	243.75	18.35	-122.445	37.931	0.56	17.89	243.98	18.45	9	7/14/2014	45:35.3
2898	RSPe_2	-122.445	37.931	0.43	17.82	243.72	18.25	-122.445	37.931	0.53	17.82	243.98	18.35	9	7/14/2014	45:35.2
2899	RSPe_2	-122.445	37.931	0.46	17.83	243.69	18.30	-122.445	37.931	0.56	17.83	243.98	18.39	9	7/14/2014	45:35.1
2900	RSPe_2	-122.445	37.931	0.43	17.85	243.61	18.28	-122.445	37.931	0.53	17.85	243.90	18.37	9	7/14/2014	45:35.0
2901	RSPe_2	-122.445	37.931	0.46	17.89	243.59	18.36	-122.445	37.931	0.56	17.89	243.88	18.45	9	7/14/2014	45:34.9
2902	RSPe_2	-122.445	37.931	0.43	17.89	243.54	18.32	-122.445	37.931	0.53	17.89	243.85	18.42	9	7/14/2014	45:34.8
2903	RSPe_2	-122.445	37.931	0.46	17.92	243.50	18.38	-122.445	37.931	0.56	17.92	243.81	18.48	9	7/14/2014	45:34.7

2904	RSPe_2	-122.445	37.931	0.43	17.91	243.45	18.34	-122.445	37.931	0.53	17.91	243.76	18.44	9	7/14/2014	45:34.6
2905	RSPe_2	-122.445	37.931	0.43	18.03	243.46	18.46	-122.445	37.931	0.56	18.03	243.74	18.59	9	7/14/2014	45:34.5
2906	RSPe_2	-122.445	37.931	0.47	18.04	243.41	18.50	-122.445	37.931	0.53	18.04	243.65	18.57	9	7/14/2014	45:34.4
2907	RSPe_2	-122.445	37.931	0.43	18.07	243.45	18.50	-122.445	37.931	0.56	18.07	243.71	18.63	9	7/14/2014	45:34.3
2908	RSPe_2	-122.445	37.931	0.43	18.04	243.44	18.47	-122.445	37.931	0.53	18.04	243.59	18.57	9	7/14/2014	45:34.2
2909	RSPe_2	-122.445	37.931	0.47	18.11	243.48	18.57	-122.445	37.931	0.56	18.11	243.68	18.67	9	7/14/2014	45:34.1
2910	RSPe_2	-122.445	37.931	0.43	18.11	243.53	18.54	-122.445	37.931	0.53	18.11	243.67	18.63	9	7/14/2014	45:34.0
2911	RSPe_2	-122.445	37.931	0.47	18.09	243.55	18.55	-122.445	37.931	0.56	18.09	243.73	18.65	9	7/14/2014	45:33.9
2912	RSPe_2	-122.445	37.931	0.47	18.09	243.57	18.55	-122.445	37.931	0.53	18.09	243.65	18.61	9	7/14/2014	45:33.8
2913	RSPe_2	-122.445	37.931	0.47	18.06	243.40	18.53	-122.445	37.931	0.56	18.06	243.59	18.62	9	7/14/2014	45:33.7
2914	RSPe_2	-122.445	37.931	0.47	18.10	243.32	18.57	-122.445	37.931	0.56	18.10	243.52	18.66	9	7/14/2014	45:33.6
2915	RSPe_2	-122.445	37.931	0.47	18.10	243.29	18.57	-122.445	37.931	0.56	18.10	243.52	18.66	9	7/14/2014	45:33.5
2916	RSPe_2	-122.445	37.931	0.43	18.07	243.22	18.50	-122.445	37.931	0.53	18.07	243.50	18.60	9	7/14/2014	45:33.4
2917	RSPe_2	-122.445	37.931	0.47	18.11	243.16	18.57	-122.445	37.931	0.56	18.11	243.39	18.67	9	7/14/2014	45:33.3
2918	RSPe_2	-122.445	37.931	0.47	18.11	243.09	18.57	-122.445	37.931	0.56	18.11	243.32	18.67	9	7/14/2014	45:33.2
2919	RSPe_2	-122.445	37.931	0.47	18.10	243.05	18.57	-122.445	37.931	0.61	18.10	243.30	18.71	9	7/14/2014	45:33.1
2920	RSPe_2	-122.445	37.931	0.47	18.12	242.96	18.58	-122.445	37.931	0.56	18.12	243.19	18.68	9	7/14/2014	45:33.0
2921	RSPe_2	-122.445	37.931	0.47	18.12	243.01	18.59	-122.445	37.931	0.61	18.12	243.20	18.74	9	7/14/2014	45:32.9
2922	RSPe_2	-122.445	37.931	0.47	18.12	242.89	18.59	-122.445	37.931	0.56	18.12	243.15	18.69	9	7/14/2014	45:32.8
2923	RSPe_2	-122.445	37.931	0.47	18.11	242.91	18.57	-122.445	37.931	0.56	18.11	243.15	18.67	9	7/14/2014	45:32.7
2924	RSPe_2	-122.445	37.931	0.43	18.23	242.90	18.66	-122.445	37.931	0.53	18.23	243.12	18.75	9	7/14/2014	45:32.6
2925	RSPe_2	-122.445	37.931	0.43	18.19	242.85	18.62	-122.445	37.931	0.53	18.19	243.11	18.71	9	7/14/2014	45:32.5
2926	RSPe_2	-122.445	37.931	0.43	18.24	242.83	18.67	-122.445	37.931	0.53	18.24	243.09	18.76	9	7/14/2014	45:32.4
2927	RSPe_2	-122.445	37.931	0.43	18.29	242.81	18.72	-122.445	37.931	0.53	18.29	243.12	18.82	9	7/14/2014	45:32.3
2928	RSPe_2	-122.445	37.931	0.43	18.30	242.78	18.74	-122.445	37.931	0.53	18.30	243.13	18.83	9	7/14/2014	45:32.2
2929	RSPe_2	-122.445	37.931	0.43	18.30	242.85	18.73	-122.445	37.931	0.56	18.30	243.13	18.86	9	7/14/2014	45:32.1
2930	RSPe_2	-122.445	37.931	0.43	18.31	242.84	18.74	-122.445	37.931	0.48	18.31	243.11	18.79	9	7/14/2014	45:32.0
2931	RSPe_2	-122.445	37.931	0.47	18.32	242.79	18.78	-122.445	37.931	0.48	18.32	243.12	18.79	9	7/14/2014	45:31.9
2932	RSPe_2	-122.445	37.931	0.43	18.37	242.74	18.80	-122.445	37.931	0.53	18.37	243.00	18.90	9	7/14/2014	45:31.8
2933	RSPe_2	-122.445	37.931	0.43	18.30	242.77	18.73	-122.445	37.931	0.56	18.30	242.98	18.86	9	7/14/2014	45:31.7
2934	RSPe_2	-122.445	37.931	0.43	18.30	242.76	18.73	-122.445	37.931	0.56	18.30	242.92	18.86	9	7/14/2014	45:31.6
2935	RSPe_2	-122.445	37.931	0.47	18.30	242.74	18.77	-122.445	37.931	0.56	18.30	242.87	18.87	9	7/14/2014	45:31.5
2936	RSPe_2	-122.445	37.931	0.43	18.26	242.71	18.69	-122.445	37.931	0.53	18.26	242.81	18.79	9	7/14/2014	45:31.4

2937	RSPe_2	-122.445	37.931	0.47	18.25	242.75	18.71	-122.445	37.931	0.56	18.25	242.82	18.81	9	7/14/2014	45:31.3
2938	RSPe_2	-122.445	37.931	0.43	18.24	242.73	18.67	-122.445	37.931	0.53	18.24	242.82	18.76	9	7/14/2014	45:31.2
2939	RSPe_2	-122.445	37.931	0.43	18.24	242.69	18.67	-122.445	37.931	0.53	18.24	242.78	18.76	9	7/14/2014	45:31.1
2940	RSPe_2	-122.445	37.931	0.38	18.22	242.68	18.60	-122.445	37.931	0.48	18.22	242.74	18.69	9	7/14/2014	45:31.0
2941	RSPe_2	-122.445	37.931	0.43	18.25	242.64	18.68	-122.445	37.931	0.53	18.25	242.81	18.77	9	7/14/2014	45:30.9
2942	RSPe_2	-122.445	37.932	0.38	18.24	242.60	18.62	-122.445	37.932	0.48	18.24	242.76	18.72	9	7/14/2014	45:30.8
2943	RSPe_2	-122.445	37.932	0.43	18.23	242.55	18.66	-122.445	37.932	0.53	18.23	242.77	18.75	9	7/14/2014	45:30.7
2944	RSPe_2	-122.445	37.932	0.38	18.23	242.49	18.60	-122.445	37.932	0.44	18.23	242.78	18.67	9	7/14/2014	45:30.6
2945	RSPe_2	-122.445	37.932	0.43	18.24	242.47	18.67	-122.445	37.932	0.53	18.24	242.75	18.77	9	7/14/2014	45:30.5
2946	RSPe_2	-122.445	37.932	0.38	18.26	242.38	18.64	-122.445	37.932	0.53	18.26	242.73	18.78	9	7/14/2014	45:30.4
2947	RSPe_2	-122.445	37.932	0.43	18.26	242.32	18.69	-122.445	37.932	0.56	18.26	242.64	18.82	9	7/14/2014	45:30.3
2948	RSPe_2	-122.445	37.932	0.38	18.26	242.20	18.64	-122.445	37.932	0.53	18.26	242.55	18.78	9	7/14/2014	45:30.2
2949	RSPe_2	-122.445	37.932	0.43	18.26	242.18	18.69	-122.445	37.932	0.56	18.26	242.48	18.82	9	7/14/2014	45:30.1
2950	RSPe_2	-122.445	37.932	0.43	18.24	242.12	18.67	-122.445	37.932	0.56	18.24	242.46	18.80	9	7/14/2014	45:30.0
2951	RSPe_2	-122.445	37.932	0.43	18.25	242.12	18.68	-122.445	37.932	0.56	18.25	242.41	18.81	9	7/14/2014	45:29.9
2952	RSPe_2	-122.445	37.932	0.38	18.24	242.07	18.62	-122.445	37.932	0.56	18.24	242.33	18.80	9	7/14/2014	45:29.8
2953	RSPe_2	-122.445	37.932	0.43	18.24	242.03	18.67	-122.445	37.932	0.56	18.24	242.24	18.80	9	7/14/2014	45:29.7
2954	RSPe_2	-122.445	37.932	0.38	18.26	241.99	18.64	-122.445	37.932	0.53	18.26	242.19	18.78	9	7/14/2014	45:29.6
2955	RSPe_2	-122.445	37.932	0.38	18.24	241.97	18.62	-122.445	37.932	0.56	18.24	242.20	18.80	9	7/14/2014	45:29.5
2956	RSPe_2	-122.445	37.932	0.38	18.24	241.94	18.61	-122.445	37.932	0.48	18.24	242.09	18.71	9	7/14/2014	45:29.4
2957	RSPe_2	-122.445	37.932	0.38	18.24	241.88	18.62	-122.445	37.932	0.48	18.24	242.07	18.72	9	7/14/2014	45:29.3
2958	RSPe_2	-122.445	37.932	0.38	18.25	241.86	18.63	-122.445	37.932	0.48	18.25	242.05	18.72	9	7/14/2014	45:29.2
2959	RSPe_2	-122.445	37.932	0.35	18.25	241.82	18.59	-122.445	37.932	0.48	18.25	242.03	18.72	9	7/14/2014	45:29.1
2960	RSPe_2	-122.445	37.932	0.35	18.26	241.77	18.60	-122.445	37.932	0.48	18.26	241.99	18.73	9	7/14/2014	45:29.0
2961	RSPe_2	-122.445	37.932	0.35	18.26	241.75	18.61	-122.445	37.932	0.48	18.26	241.97	18.74	9	7/14/2014	45:28.9
2962	RSPe_2	-122.445	37.932	0.35	18.29	241.68	18.63	-122.445	37.932	0.48	18.29	241.92	18.76	9	7/14/2014	45:28.8
2963	RSPe_2	-122.445	37.932	0.35	18.31	241.57	18.65	-122.445	37.932	0.44	18.31	241.88	18.75	9	7/14/2014	45:28.7
2964	RSPe_2	-122.445	37.932	0.35	18.28	241.58	18.62	-122.445	37.932	0.44	18.28	241.81	18.72	9	7/14/2014	45:28.6
2965	RSPe_2	-122.445	37.932	0.35	18.30	241.49	18.64	-122.445	37.932	0.48	18.30	241.84	18.77	9	7/14/2014	45:28.5
2966	RSPe_2	-122.445	37.932	0.35	18.31	241.45	18.65	-122.445	37.932	0.48	18.31	241.74	18.79	9	7/14/2014	45:28.4
2967	RSPe_2	-122.445	37.932	0.35	18.32	241.43	18.66	-122.445	37.932	0.48	18.32	241.71	18.79	9	7/14/2014	45:28.3
2968	RSPe_2	-122.445	37.932	0.35	18.35	241.35	18.69	-122.445	37.932	0.44	18.35	241.63	18.79	9	7/14/2014	45:28.2
2969	RSPe_2	-122.445	37.932	0.35	18.35	241.29	18.69	-122.445	37.932	0.44	18.35	241.60	18.79	9	7/14/2014	45:28.1

2970	RSPe_2	-122.445	37.932	0.35	18.30	241.38	18.65	-122.445	37.932	0.48	18.30	241.65	18.78	9	7/14/2014	45:28.0
2971	RSPe_2	-122.445	37.932	0.29	18.26	241.32	18.56	-122.445	37.932	0.48	18.26	241.58	18.74	9	7/14/2014	45:27.9
2972	RSPe_2	-122.445	37.932	0.29	18.30	241.27	18.60	-122.445	37.932	0.44	18.30	241.53	18.75	9	7/14/2014	45:27.8
2973	RSPe_2	-122.445	37.932	0.35	18.33	241.27	18.67	-122.445	37.932	0.44	18.33	241.51	18.77	9	7/14/2014	45:27.7
2974	RSPe_2	-122.445	37.932	0.29	18.24	241.20	18.53	-122.445	37.932	0.41	18.24	241.46	18.65	9	7/14/2014	45:27.6
2975	RSPe_2	-122.445	37.932	0.29	18.23	241.21	18.52	-122.445	37.932	0.41	18.23	241.42	18.63	9	7/14/2014	45:27.5
2976	RSPe_2	-122.445	37.932	0.26	18.19	241.21	18.45	-122.445	37.932	0.41	18.19	241.38	18.60	9	7/14/2014	45:27.4
2977	RSPe_2	-122.445	37.932	0.29	18.15	241.16	18.44	-122.445	37.932	0.41	18.15	241.40	18.56	9	7/14/2014	45:27.3
2978	RSPe_2	-122.445	37.932	0.29	18.16	241.14	18.45	-122.445	37.932	0.36	18.16	241.34	18.51	9	7/14/2014	45:27.2
2979	RSPe_2	-122.445	37.932	0.29	18.11	241.08	18.40	-122.445	37.932	0.41	18.11	241.30	18.52	9	7/14/2014	45:27.1
2980	RSPe_2	-122.445	37.932	0.29	18.07	241.04	18.36	-122.445	37.932	0.36	18.07	241.26	18.43	9	7/14/2014	45:27.0
2981	RSPe_2	-122.445	37.932	0.29	18.06	240.92	18.36	-122.445	37.932	0.36	18.06	241.15	18.42	9	7/14/2014	45:26.9
2982	RSPe_2	-122.445	37.932	0.29	18.09	240.86	18.38	-122.445	37.932	0.36	18.09	241.19	18.44	9	7/14/2014	45:26.8
2983	RSPe_2	-122.445	37.932	0.29	18.04	240.88	18.33	-122.445	37.932	0.44	18.04	241.18	18.48	9	7/14/2014	45:26.7
2984	RSPe_2	-122.445	37.932	0.26	17.99	240.81	18.25	-122.445	37.932	0.41	17.99	241.13	18.40	9	7/14/2014	45:26.6
2985	RSPe_2	-122.445	37.932	0.29	18.03	240.80	18.32	-122.445	37.932	0.41	18.03	241.12	18.43	9	7/14/2014	45:26.5
2986	RSPe_2	-122.445	37.932	0.26	17.98	240.76	18.24	-122.445	37.932	0.41	17.98	241.09	18.39	9	7/14/2014	45:26.4
2987	RSPe_2	-122.445	37.932	0.29	17.99	240.73	18.29	-122.445	37.932	0.36	17.99	241.07	18.35	9	7/14/2014	45:26.3
2988	RSPe_2	-122.445	37.932	0.26	18.04	240.67	18.30	-122.445	37.932	0.36	18.04	241.01	18.40	9	7/14/2014	45:26.2
2989	RSPe_2	-122.445	37.932	0.26	18.00	240.64	18.26	-122.445	37.932	0.32	18.00	240.93	18.32	9	7/14/2014	45:26.1
2990	RSPe_2	-122.445	37.932	0.26	17.96	240.60	18.22	-122.445	37.932	0.32	17.96	240.84	18.29	9	7/14/2014	45:26.0
2991	RSPe_2	-122.445	37.932	0.26	18.03	240.60	18.29	-122.445	37.932	0.32	18.03	240.79	18.35	9	7/14/2014	45:25.9
2992	RSPe_2	-122.445	37.932	0.26	17.92	240.52	18.18	-122.445	37.932	0.27	17.92	240.73	18.19	9	7/14/2014	45:25.8
2993	RSPe_2	-122.445	37.932	0.21	17.96	240.52	18.17	-122.445	37.932	0.27	17.96	240.69	18.24	9	7/14/2014	45:25.7
2994	RSPe_2	-122.445	37.932	0.21	17.95	240.47	18.16	-122.445	37.932	0.32	17.95	240.63	18.27	9	7/14/2014	45:25.6
2995	RSPe_2	-122.445	37.932	0.26	17.89	240.48	18.15	-122.445	37.932	0.32	17.89	240.62	18.22	9	7/14/2014	45:25.5
2996	RSPe_2	-122.445	37.932	0.21	17.95	240.38	18.16	-122.445	37.932	0.32	17.95	240.51	18.27	9	7/14/2014	45:25.4
2997	RSPe_2	-122.445	37.932	0.26	17.87	240.30	18.13	-122.445	37.932	0.32	17.87	240.48	18.20	9	7/14/2014	45:25.3
2998	RSPe_2	-122.445	37.932	0.21	17.85	240.23	18.06	-122.445	37.932	0.32	17.85	240.42	18.18	9	7/14/2014	45:25.2
2999	RSPe_2	-122.445	37.932	0.26	17.88	240.21	18.14	-122.445	37.932	0.32	17.88	240.47	18.20	9	7/14/2014	45:25.1
3000	RSPe_2	-122.445	37.932	0.21	17.87	240.13	18.08	-122.445	37.932	0.32	17.87	240.44	18.20	9	7/14/2014	45:25.0
3001	RSPe_2	-122.445	37.932	0.26	17.89	240.06	18.15	-122.445	37.932	0.27	17.89	240.45	18.17	9	7/14/2014	45:24.9
3002	RSPe_2	-122.445	37.932	0.21	17.90	240.02	18.11	-122.445	37.932	0.27	17.90	240.47	18.18	9	7/14/2014	45:24.8

3003	RSPe_2	-122.445	37.932	0.21	17.94	240.03	18.15	-122.445	37.932	0.27	17.94	240.43	18.21	9	7/14/2014	45:24.7
3004	RSPe_2	-122.445	37.932	0.21	17.94	239.97	18.15	-122.445	37.932	0.24	17.94	240.43	18.18	9	7/14/2014	45:24.6
3005	RSPe_2	-122.445	37.932	0.21	18.00	240.02	18.21	-122.445	37.932	0.24	18.00	240.47	18.24	9	7/14/2014	45:24.5
3006	RSPe_2	-122.445	37.932	0.17	18.15	240.02	18.32	-122.445	37.932	0.24	18.15	240.43	18.39	9	7/14/2014	45:24.4
3007	RSPe_2	-122.445	37.932	0.21	18.03	239.95	18.24	-122.445	37.932	0.19	18.03	240.32	18.22	9	7/14/2014	45:24.3
3008	RSPe_2	-122.445	37.932	0.17	18.10	239.93	18.28	-122.445	37.932	0.19	18.10	240.25	18.29	9	7/14/2014	45:24.2
3009	RSPe_2	-122.445	37.932	0.21	18.09	239.93	18.29	-122.445	37.932	0.19	18.09	240.19	18.28	9	7/14/2014	45:24.1
3010	RSPe_2	-122.445	37.932	0.17	18.13	239.86	18.31	-122.445	37.932	0.19	18.13	240.06	18.32	9	7/14/2014	45:24.0
3011	RSPe_2	-122.445	37.932	0.17	18.23	239.82	18.40	-122.445	37.932	0.19	18.23	239.99	18.41	9	7/14/2014	45:23.9
3012	RSPe_2	-122.445	37.932	0.17	18.17	239.81	18.34	-122.445	37.932	0.19	18.17	239.93	18.35	9	7/14/2014	45:23.8
3013	RSPe_2	-122.445	37.932	0.17	18.12	239.80	18.30	-122.445	37.932	0.19	18.12	239.91	18.31	9	7/14/2014	45:23.7
3014	RSPe_2	-122.445	37.932	0.17	18.21	239.78	18.38	-122.445	37.932	0.19	18.21	239.88	18.40	9	7/14/2014	45:23.6
3015	RSPe_2	-122.445	37.932	0.21	18.17	239.79	18.37	-122.445	37.932	0.24	18.17	239.89	18.40	9	7/14/2014	45:23.5
3016	RSPe_2	-122.445	37.932	0.17	18.17	239.78	18.34	-122.445	37.932	0.19	18.17	239.91	18.35	9	7/14/2014	45:23.4
3017	RSPe_2	-122.445	37.932	0.17	18.15	239.69	18.32	-122.445	37.932	0.24	18.15	239.88	18.39	9	7/14/2014	45:23.3
3018	RSPe_2	-122.445	37.932	0.17	18.16	239.65	18.33	-122.445	37.932	0.19	18.16	239.93	18.35	9	7/14/2014	45:23.2
3019	RSPe_2	-122.445	37.932	0.17	18.18	239.61	18.35	-122.445	37.932	0.19	18.18	239.98	18.37	9	7/14/2014	45:23.1
3020	RSPe_2	-122.445	37.932	0.14	18.21	239.61	18.35	-122.445	37.932	0.16	18.21	240.02	18.36	9	7/14/2014	45:23.0
3021	RSPe_2	-122.445	37.932	0.17	18.31	239.59	18.48	-122.445	37.932	0.16	18.31	240.00	18.46	9	7/14/2014	45:22.9
3022	RSPe_2	-122.445	37.932	0.14	18.26	239.56	18.40	-122.445	37.932	0.12	18.26	240.00	18.38	9	7/14/2014	45:22.8
3023	RSPe_2	-122.445	37.932	0.14	18.27	239.61	18.41	-122.445	37.932	0.12	18.27	239.92	18.39	9	7/14/2014	45:22.7
3024	RSPe_2	-122.445	37.932	0.14	18.38	239.61	18.52	-122.445	37.932	0.16	18.38	239.92	18.53	9	7/14/2014	45:22.6
3025	RSPe_2	-122.445	37.932	0.17	18.36	239.61	18.54	-122.445	37.932	0.16	18.36	239.83	18.52	9	7/14/2014	45:22.5
3026	RSPe_2	-122.445	37.932	0.14	18.33	239.54	18.47	-122.445	37.932	0.16	18.33	239.74	18.48	9	7/14/2014	45:22.4
3027	RSPe_2	-122.445	37.932	0.17	18.34	239.43	18.52	-122.445	37.932	0.16	18.34	239.66	18.50	9	7/14/2014	45:22.3
3028	RSPe_2	-122.445	37.932	0.14	18.33	239.39	18.47	-122.445	37.932	0.12	18.33	239.59	18.45	9	7/14/2014	45:22.2
3029	RSPe_2	-122.445	37.932	0.17	18.49	239.35	18.66	-122.445	37.932	0.16	18.49	239.64	18.64	9	7/14/2014	45:22.1
3030	RSPe_2	-122.445	37.932	0.14	18.39	239.24	18.53	-122.445	37.932	0.12	18.39	239.54	18.51	9	7/14/2014	45:22.0
3031	RSPe_2	-122.445	37.932	0.17	18.40	239.18	18.58	-122.445	37.932	0.12	18.40	239.54	18.52	9	7/14/2014	45:21.9
3032	RSPe_2	-122.445	37.932	0.14	18.51	239.13	18.65	-122.445	37.932	0.16	18.51	239.55	18.66	9	7/14/2014	45:21.8
3033	RSPe_2	-122.445	37.932	0.14	18.45	239.11	18.59	-122.445	37.932	0.12	18.45	239.59	18.57	9	7/14/2014	45:21.7
3034	RSPe_2	-122.445	37.932	0.14	18.49	239.11	18.63	-122.445	37.932	0.12	18.49	239.57	18.61	9	7/14/2014	45:21.6
3035	RSPe_2	-122.445	37.932	0.14	18.54	239.15	18.68	-122.445	37.932	0.07	18.54	239.60	18.61	9	7/14/2014	45:21.5

3036	RSPe_2	-122.445	37.932	0.14	18.58	239.13	18.72	-122.445	37.932	0.04	18.58	239.54	18.62	9	7/14/2014	45:21.4
3037	RSPe_2	-122.445	37.932	0.14	18.65	239.20	18.79	-122.445	37.932	0.07	18.65	239.57	18.72	9	7/14/2014	45:21.3
3038	RSPe_2	-122.445	37.932	0.14	18.61	239.20	18.75	-122.445	37.932	0.04	18.61	239.53	18.65	9	7/14/2014	45:21.2
3039	RSPe_2	-122.445	37.932	0.14	18.67	239.18	18.81	-122.445	37.932	0.07	18.67	239.48	18.74	9	7/14/2014	45:21.1
3040	RSPe_2	-122.445	37.932	0.09	18.65	239.13	18.74	-122.445	37.932	0.04	18.65	239.42	18.69	9	7/14/2014	45:21.0
3041	RSPe_2	-122.445	37.932	0.14	18.70	239.03	18.84	-122.445	37.932	0.07	18.70	239.35	18.77	9	7/14/2014	45:20.9
3042	RSPe_2	-122.445	37.932	0.14	18.66	239.03	18.80	-122.445	37.932	0.04	18.66	239.31	18.70	9	7/14/2014	45:20.8
3043	RSPe_2	-122.445	37.932	0.14	18.71	239.00	18.85	-122.445	37.932	0.07	18.71	239.27	18.78	9	7/14/2014	45:20.7
3044	RSPe_2	-122.445	37.932	0.09	18.74	238.98	18.83	-122.445	37.932	0.04	18.74	239.22	18.78	9	7/14/2014	45:20.6
3045	RSPe_2	-122.445	37.932	0.14	18.71	238.94	18.85	-122.445	37.932	0.07	18.71	239.20	18.78	9	7/14/2014	45:20.5
3046	RSPe_2	-122.445	37.932	0.14	18.70	238.90	18.84	-122.445	37.932	0.04	18.70	239.10	18.73	9	7/14/2014	45:20.4
3047	RSPe_2	-122.445	37.932	0.14	18.73	238.85	18.87	-122.445	37.932	0.07	18.73	239.05	18.80	9	7/14/2014	45:20.3
3048	RSPe_2	-122.445	37.932	0.14	18.71	238.85	18.85	-122.445	37.932	0.07	18.71	239.03	18.78	9	7/14/2014	45:20.2
3049	RSPe_2	-122.445	37.932	0.14	18.70	238.81	18.84	-122.445	37.932	0.07	18.70	238.96	18.77	9	7/14/2014	45:20.1
3050	RSPe_2	-122.445	37.932	0.14	18.70	238.88	18.84	-122.445	37.932	0.07	18.70	238.98	18.77	9	7/14/2014	45:20.0
3051	RSPe_2	-122.445	37.932	0.14	18.74	238.87	18.88	-122.445	37.932	0.07	18.74	238.98	18.81	9	7/14/2014	45:19.9
3052	RSPe_2	-122.445	37.932	0.14	18.71	238.84	18.85	-122.445	37.932	-0.01	18.71	238.92	18.70	9	7/14/2014	45:19.8
3053	RSPe_2	-122.445	37.932	0.14	18.68	238.84	18.82	-122.445	37.932	0.04	18.68	238.90	18.72	9	7/14/2014	45:19.7
3054	RSPe_2	-122.445	37.932	0.14	18.74	238.79	18.88	-122.445	37.932	0.04	18.74	238.87	18.78	9	7/14/2014	45:19.6
3055	RSPe_2	-122.445	37.932	0.14	18.69	238.68	18.83	-122.445	37.932	0.07	18.69	238.81	18.76	9	7/14/2014	45:19.5
3056	RSPe_2	-122.445	37.932	0.14	18.68	238.71	18.82	-122.445	37.932	0.04	18.68	238.79	18.72	9	7/14/2014	45:19.4
3057	RSPe_2	-122.445	37.932	0.14	18.68	238.64	18.82	-122.445	37.932	0.04	18.68	238.77	18.72	9	7/14/2014	45:19.3
3058	RSPe_2	-122.445	37.932	0.14	18.69	238.55	18.83	-122.445	37.932	0.04	18.69	238.74	18.73	9	7/14/2014	45:19.2
3059	RSPe_2	-122.445	37.932	0.14	18.69	238.55	18.83	-122.445	37.932	0.07	18.69	238.66	18.76	9	7/14/2014	45:19.1
3060	RSPe_2	-122.445	37.932	0.09	18.66	238.40	18.75	-122.445	37.932	0.04	18.66	238.57	18.70	9	7/14/2014	45:19.0
3061	RSPe_2	-122.445	37.932	0.14	18.68	238.34	18.82	-122.445	37.932	0.12	18.68	238.55	18.80	9	7/14/2014	45:18.9
3062	RSPe_2	-122.445	37.932	0.14	18.68	238.23	18.82	-122.445	37.932	0.07	18.68	238.48	18.75	9	7/14/2014	45:18.8
3063	RSPe_2	-122.445	37.932	0.18	18.66	238.16	18.84	-122.445	37.932	0.04	18.66	238.42	18.70	9	7/14/2014	45:18.7
3064	RSPe_2	-122.445	37.932	0.14	18.70	238.11	18.84	-122.445	37.932	0.07	18.70	238.41	18.77	9	7/14/2014	45:18.6
3065	RSPe_2	-122.445	37.932	0.18	18.70	238.07	18.88	-122.445	37.932	0.07	18.70	238.40	18.77	9	7/14/2014	45:18.5
3066	RSPe_2	-122.445	37.932	0.14	18.71	237.96	18.85	-122.445	37.932	0.07	18.71	238.37	18.78	9	7/14/2014	45:18.4
3067	RSPe_2	-122.445	37.932	0.18	18.70	237.92	18.88	-122.445	37.932	0.12	18.70	238.33	18.82	9	7/14/2014	45:18.3
3068	RSPe_2	-122.445	37.932	0.14	18.65	237.86	18.79	-122.445	37.932	0.12	18.65	238.22	18.77	9	7/14/2014	45:18.2

3069	RSPe_2	-122.445	37.932	0.18	18.67	237.83	18.85	-122.445	37.932	0.12	18.67	238.17	18.80	9	7/14/2014	45:18.1
3070	RSPe_2	-122.445	37.932	0.14	18.63	237.81	18.77	-122.445	37.932	0.07	18.63	238.05	18.70	9	7/14/2014	45:18.0
3071	RSPe_2	-122.445	37.932	0.18	18.69	237.70	18.87	-122.445	37.932	0.12	18.69	238.02	18.81	9	7/14/2014	45:17.9
3072	RSPe_2	-122.445	37.932	0.18	18.59	237.66	18.77	-122.445	37.932	0.07	18.59	237.92	18.67	9	7/14/2014	45:17.8
3073	RSPe_2	-122.445	37.932	0.18	18.56	237.59	18.73	-122.445	37.932	0.12	18.56	237.81	18.68	9	7/14/2014	45:17.7
3074	RSPe_2	-122.445	37.932	0.18	18.57	237.47	18.75	-122.445	37.932	0.07	18.57	237.73	18.64	9	7/14/2014	45:17.6
3075	RSPe_2	-122.445	37.932	0.18	18.52	237.44	18.69	-122.445	37.932	0.12	18.52	237.68	18.64	9	7/14/2014	45:17.5
3076	RSPe_2	-122.445	37.932	0.18	18.46	237.31	18.63	-122.445	37.932	0.07	18.46	237.52	18.53	9	7/14/2014	45:17.4
3077	RSPe_2	-122.445	37.932	0.18	18.44	237.24	18.62	-122.445	37.932	0.12	18.44	237.50	18.56	9	7/14/2014	45:17.3
3078	RSPe_2	-122.445	37.932	0.18	18.42	237.17	18.59	-122.445	37.932	0.07	18.42	237.40	18.49	9	7/14/2014	45:17.2
3079	RSPe_2	-122.445	37.932	0.18	18.37	237.16	18.55	-122.445	37.932	0.12	18.37	237.36	18.49	9	7/14/2014	45:17.1
3080	RSPe_2	-122.445	37.932	0.18	18.40	237.07	18.58	-122.445	37.932	0.12	18.40	237.28	18.52	9	7/14/2014	45:17.0
3081	RSPe_2	-122.445	37.932	0.18	18.33	237.00	18.50	-122.445	37.932	0.12	18.33	237.20	18.45	9	7/14/2014	45:16.9
3082	RSPe_2	-122.445	37.932	0.18	18.34	236.97	18.52	-122.445	37.932	0.12	18.34	237.15	18.46	9	7/14/2014	45:16.8
3083	RSPe_2	-122.445	37.932	0.21	18.36	236.91	18.57	-122.445	37.932	0.16	18.36	237.07	18.51	9	7/14/2014	45:16.7
3084	RSPe_2	-122.445	37.932	0.21	18.30	236.85	18.51	-122.445	37.932	0.12	18.30	237.00	18.43	9	7/14/2014	45:16.6
3085	RSPe_2	-122.445	37.932	0.21	18.26	236.78	18.47	-122.445	37.932	0.12	18.26	236.93	18.39	9	7/14/2014	45:16.5
3086	RSPe_2	-122.445	37.932	0.21	18.27	236.74	18.48	-122.445	37.932	0.16	18.27	236.86	18.43	9	7/14/2014	45:16.4
3087	RSPe_2	-122.445	37.932	0.21	18.24	236.63	18.45	-122.445	37.932	0.12	18.24	236.81	18.36	9	7/14/2014	45:16.3
3088	RSPe_2	-122.445	37.932	0.21	18.23	236.57	18.43	-122.445	37.932	0.16	18.23	236.74	18.38	9	7/14/2014	45:16.2
3089	RSPe_2	-122.445	37.932	0.21	18.26	236.46	18.47	-122.445	37.932	0.12	18.26	236.64	18.38	9	7/14/2014	45:16.1
3090	RSPe_2	-122.445	37.932	0.18	18.24	236.39	18.41	-122.445	37.932	0.16	18.24	236.63	18.39	9	7/14/2014	45:16.0
3091	RSPe_2	-122.445	37.932	0.21	18.24	236.37	18.45	-122.445	37.932	0.16	18.24	236.54	18.40	9	7/14/2014	45:15.9
3092	RSPe_2	-122.445	37.932	0.18	18.18	236.31	18.35	-122.445	37.932	0.12	18.18	236.52	18.30	9	7/14/2014	45:15.8
3093	RSPe_2	-122.445	37.932	0.21	18.19	236.26	18.40	-122.445	37.932	0.16	18.19	236.44	18.34	9	7/14/2014	45:15.7
3094	RSPe_2	-122.445	37.932	0.21	18.17	236.20	18.37	-122.445	37.932	0.19	18.17	236.32	18.36	9	7/14/2014	45:15.6
3095	RSPe_2	-122.445	37.932	0.21	18.14	236.15	18.35	-122.445	37.932	0.19	18.14	236.28	18.33	9	7/14/2014	45:15.5
3096	RSPe_2	-122.445	37.932	0.21	18.17	236.05	18.38	-122.445	37.932	0.19	18.17	236.17	18.36	9	7/14/2014	45:15.4
3097	RSPe_2	-122.445	37.932	0.21	18.23	235.96	18.43	-122.445	37.932	0.19	18.23	236.09	18.42	9	7/14/2014	45:15.3
3098	RSPe_2	-122.445	37.932	0.21	18.10	235.78	18.31	-122.445	37.932	0.16	18.10	235.97	18.25	9	7/14/2014	45:15.2
3099	RSPe_2	-122.445	37.932	0.21	18.10	235.76	18.31	-122.445	37.932	0.16	18.10	235.99	18.25	9	7/14/2014	45:15.1
3100	RSPe_2	-122.445	37.932	0.21	18.05	235.68	18.26	-122.445	37.932	0.16	18.05	235.95	18.21	9	7/14/2014	45:15.0
3101	RSPe_2	-122.445	37.932	0.21	18.10	235.59	18.31	-122.445	37.932	0.19	18.10	235.91	18.29	9	7/14/2014	45:14.9

3102	RSPe_2	-122.445	37.932	0.21	18.07	235.52	18.28	-122.445	37.932	0.16	18.07	235.91	18.23	9	7/14/2014	45:14.8
3103	RSPe_2	-122.445	37.932	0.21	18.03	235.52	18.24	-122.445	37.932	0.19	18.03	235.81	18.22	9	7/14/2014	45:14.7
3104	RSPe_2	-122.445	37.932	0.21	18.12	235.45	18.33	-122.445	37.932	0.16	18.12	235.75	18.28	9	7/14/2014	45:14.6
3105	RSPe_2	-122.445	37.932	0.21	18.13	235.37	18.34	-122.445	37.932	0.19	18.13	235.63	18.32	9	7/14/2014	45:14.5
3106	RSPe_2	-122.445	37.932	0.21	18.09	235.35	18.30	-122.445	37.932	0.24	18.09	235.49	18.33	9	7/14/2014	45:14.4
3107	RSPe_2	-122.445	37.932	0.26	18.19	235.28	18.45	-122.445	37.932	0.27	18.19	235.41	18.46	9	7/14/2014	45:14.3
3108	RSPe_2	-122.445	37.932	0.26	18.07	235.24	18.33	-122.445	37.932	0.24	18.07	235.34	18.31	9	7/14/2014	45:14.2
3109	RSPe_2	-122.445	37.932	0.21	18.10	235.15	18.31	-122.445	37.932	0.24	18.10	235.26	18.34	9	7/14/2014	45:14.1
3110	RSPe_2	-122.445	37.932	0.21	18.10	235.06	18.31	-122.445	37.932	0.24	18.10	235.17	18.34	9	7/14/2014	45:14.0
3111	RSPe_2	-122.445	37.932	0.26	18.09	235.02	18.35	-122.445	37.932	0.27	18.09	235.19	18.36	9	7/14/2014	45:13.9
3112	RSPe_2	-122.445	37.932	0.21	18.11	234.96	18.32	-122.445	37.932	0.24	18.11	235.11	18.35	9	7/14/2014	45:13.8
3113	RSPe_2	-122.445	37.932	0.26	18.00	234.89	18.26	-122.445	37.932	0.24	18.00	235.13	18.24	9	7/14/2014	45:13.7
3114	RSPe_2	-122.445	37.932	0.21	18.03	234.85	18.23	-122.445	37.932	0.24	18.03	235.13	18.27	9	7/14/2014	45:13.6
3115	RSPe_2	-122.445	37.932	0.26	18.15	234.84	18.41	-122.445	37.932	0.19	18.15	235.15	18.34	9	7/14/2014	45:13.5
3116	RSPe_2	-122.445	37.932	0.21	18.19	234.81	18.40	-122.445	37.932	0.19	18.19	235.11	18.38	9	7/14/2014	45:13.4
3117	RSPe_2	-122.445	37.932	0.26	18.22	234.76	18.48	-122.445	37.932	0.19	18.22	235.08	18.41	9	7/14/2014	45:13.3
3118	RSPe_2	-122.445	37.932	0.21	18.25	234.74	18.46	-122.445	37.932	0.24	18.25	235.08	18.49	9	7/14/2014	45:13.2
3119	RSPe_2	-122.445	37.932	0.26	18.24	234.69	18.50	-122.445	37.932	0.24	18.24	235.00	18.48	9	7/14/2014	45:13.1
3120	RSPe_2	-122.445	37.932	0.21	18.24	234.67	18.44	-122.445	37.932	0.24	18.24	234.91	18.48	9	7/14/2014	45:13.0
3121	RSPe_2	-122.445	37.932	0.26	18.34	234.63	18.60	-122.445	37.932	0.24	18.34	234.85	18.58	9	7/14/2014	45:12.9
3122	RSPe_2	-122.445	37.932	0.21	18.26	234.65	18.47	-122.445	37.932	0.24	18.26	234.79	18.50	9	7/14/2014	45:12.8
3123	RSPe_2	-122.445	37.932	0.26	18.24	234.59	18.50	-122.445	37.932	0.27	18.24	234.71	18.51	9	7/14/2014	45:12.7
3124	RSPe_2	-122.445	37.932	0.21	18.24	234.52	18.45	-122.445	37.932	0.24	18.24	234.70	18.48	9	7/14/2014	45:12.6
3125	RSPe_2	-122.445	37.932	0.26	18.24	234.46	18.50	-122.445	37.932	0.27	18.24	234.63	18.51	9	7/14/2014	45:12.5
3126	RSPe_2	-122.445	37.932	0.21	18.26	234.41	18.47	-122.445	37.932	0.24	18.26	234.54	18.50	9	7/14/2014	45:12.4
3127	RSPe_2	-122.445	37.932	0.26	18.26	234.32	18.52	-122.445	37.932	0.24	18.26	234.56	18.50	9	7/14/2014	45:12.3
3128	RSPe_2	-122.445	37.932	0.26	18.26	234.24	18.52	-122.445	37.932	0.24	18.26	234.52	18.50	9	7/14/2014	45:12.2
3129	RSPe_2	-122.445	37.932	0.26	18.27	234.19	18.53	-122.445	37.932	0.27	18.27	234.50	18.55	9	7/14/2014	45:12.1
3130	RSPe_2	-122.445	37.932	0.21	18.28	234.11	18.49	-122.445	37.932	0.24	18.28	234.41	18.52	9	7/14/2014	45:12.0
3131	RSPe_2	-122.445	37.932	0.26	18.34	234.06	18.60	-122.445	37.932	0.27	18.34	234.39	18.62	9	7/14/2014	45:11.9
3132	RSPe_2	-122.445	37.932	0.21	18.35	234.01	18.56	-122.445	37.932	0.24	18.35	234.33	18.59	9	7/14/2014	45:11.8
3133	RSPe_2	-122.445	37.932	0.26	18.44	234.00	18.70	-122.445	37.932	0.24	18.44	234.32	18.68	9	7/14/2014	45:11.7
3134	RSPe_2	-122.445	37.932	0.26	18.44	233.93	18.70	-122.445	37.932	0.27	18.44	234.17	18.71	9	7/14/2014	45:11.6

3135	RSPe_2	-122.445	37.932	0.26	18.44	233.89	18.70	-122.445	37.932	0.24	18.44	234.07	18.68	9	7/14/2014	45:11.5
3136	RSPe_2	-122.445	37.932	0.26	18.41	233.85	18.67	-122.445	37.932	0.24	18.41	233.99	18.65	9	7/14/2014	45:11.4
3137	RSPe_2	-122.445	37.932	0.26	18.48	233.85	18.74	-122.445	37.932	0.27	18.48	233.93	18.76	9	7/14/2014	45:11.3
3138	RSPe_2	-122.445	37.932	0.26	18.44	233.69	18.70	-122.445	37.932	0.24	18.44	233.76	18.68	9	7/14/2014	45:11.2
3139	RSPe_2	-122.445	37.932	0.26	18.45	233.63	18.71	-122.445	37.932	0.32	18.45	233.65	18.77	9	7/14/2014	45:11.1
3140	RSPe_2	-122.445	37.932	0.26	18.46	233.57	18.72	-122.445	37.932	0.27	18.46	233.63	18.73	9	7/14/2014	45:11.0
3141	RSPe_2	-122.445	37.932	0.26	18.49	233.52	18.75	-122.445	37.932	0.32	18.49	233.60	18.81	9	7/14/2014	45:10.9
3142	RSPe_2	-122.445	37.932	0.26	18.47	233.39	18.73	-122.445	37.932	0.32	18.47	233.54	18.80	9	7/14/2014	45:10.8
3143	RSPe_2	-122.445	37.932	0.26	18.53	233.28	18.80	-122.445	37.932	0.32	18.53	233.47	18.86	9	7/14/2014	45:10.7
3144	RSPe_2	-122.445	37.932	0.26	18.50	233.19	18.76	-122.445	37.932	0.27	18.50	233.43	18.78	9	7/14/2014	45:10.6
3145	RSPe_2	-122.445	37.932	0.29	18.50	233.18	18.80	-122.445	37.932	0.27	18.50	233.46	18.78	9	7/14/2014	45:10.5
3146	RSPe_2	-122.445	37.932	0.26	18.51	233.07	18.77	-122.445	37.932	0.24	18.51	233.36	18.75	9	7/14/2014	45:10.4
3147	RSPe_2	-122.445	37.932	0.29	18.52	232.96	18.82	-122.445	37.932	0.27	18.52	233.28	18.80	9	7/14/2014	45:10.3
3148	RSPe_2	-122.445	37.932	0.26	18.53	232.86	18.80	-122.445	37.932	0.32	18.53	233.13	18.86	9	7/14/2014	45:10.2
3149	RSPe_2	-122.445	37.932	0.29	18.54	232.80	18.84	-122.445	37.932	0.27	18.54	233.05	18.82	9	7/14/2014	45:10.1
3150	RSPe_2	-122.445	37.932	0.26	18.57	232.71	18.83	-122.445	37.932	0.27	18.57	232.93	18.84	9	7/14/2014	45:10.0
3151	RSPe_2	-122.445	37.932	0.29	18.55	232.65	18.85	-122.445	37.932	0.32	18.55	232.82	18.88	9	7/14/2014	45:09.9
3152	RSPe_2	-122.445	37.932	0.26	18.52	232.58	18.79	-122.445	37.932	0.32	18.52	232.68	18.85	9	7/14/2014	45:09.8
3153	RSPe_2	-122.445	37.932	0.26	18.52	232.52	18.79	-122.445	37.932	0.32	18.52	232.60	18.85	9	7/14/2014	45:09.7
3154	RSPe_2	-122.445	37.932	0.26	18.51	232.46	18.77	-122.445	37.932	0.36	18.51	232.49	18.87	9	7/14/2014	45:09.6
3155	RSPe_2	-122.445	37.932	0.29	18.47	232.36	18.77	-122.445	37.932	0.36	18.47	232.38	18.83	9	7/14/2014	45:09.5
3156	RSPe_2	-122.445	37.932	0.26	18.48	232.26	18.74	-122.445	37.932	0.32	18.48	232.25	18.81	9	7/14/2014	45:09.4
3157	RSPe_2	-122.445	37.932	0.29	18.47	232.15	18.77	-122.445	37.932	0.36	18.47	232.19	18.83	9	7/14/2014	45:09.3
3158	RSPe_2	-122.445	37.932	0.26	18.45	232.12	18.71	-122.445	37.932	0.36	18.45	232.16	18.81	9	7/14/2014	45:09.2
3159	RSPe_2	-122.445	37.932	0.29	18.47	232.02	18.76	-122.445	37.932	0.36	18.47	232.11	18.82	9	7/14/2014	45:09.1
3160	RSPe_2	-122.445	37.932	0.26	18.47	231.95	18.73	-122.445	37.932	0.32	18.47	232.08	18.79	9	7/14/2014	45:09.0
3161	RSPe_2	-122.445	37.932	0.29	18.50	231.87	18.79	-122.445	37.932	0.36	18.50	232.10	18.86	9	7/14/2014	45:08.9
3162	RSPe_2	-122.445	37.932	0.21	18.50	231.79	18.71	-122.445	37.932	0.27	18.50	232.06	18.77	9	7/14/2014	45:08.8
3163	RSPe_2	-122.445	37.932	0.26	18.50	231.76	18.76	-122.445	37.932	0.32	18.50	232.04	18.83	9	7/14/2014	45:08.7
3164	RSPe_2	-122.445	37.932	0.26	18.52	231.66	18.78	-122.445	37.932	0.27	18.52	231.97	18.79	9	7/14/2014	45:08.6
3165	RSPe_2	-122.445	37.932	0.26	18.51	231.58	18.77	-122.445	37.932	0.32	18.51	231.90	18.83	9	7/14/2014	45:08.5
3166	RSPe_2	-122.445	37.932	0.26	18.52	231.45	18.78	-122.445	37.932	0.32	18.52	231.79	18.84	9	7/14/2014	45:08.4
3167	RSPe_2	-122.445	37.932	0.26	18.52	231.43	18.79	-122.445	37.932	0.32	18.52	231.72	18.85	9	7/14/2014	45:08.3

3168	RSPe_2	-122.445	37.932	0.26	18.55	231.36	18.81	-122.445	37.932	0.27	18.55	231.62	18.83	9	7/14/2014	45:08.2
3169	RSPe_2	-122.445	37.932	0.26	18.52	231.30	18.79	-122.445	37.932	0.32	18.52	231.52	18.85	9	7/14/2014	45:08.1
3170	RSPe_2	-122.445	37.932	0.26	18.54	231.21	18.80	-122.445	37.932	0.32	18.54	231.41	18.87	9	7/14/2014	45:08.0
3171	RSPe_2	-122.445	37.932	0.26	18.50	231.07	18.76	-122.445	37.932	0.32	18.50	231.35	18.83	9	7/14/2014	45:07.9
3172	RSPe_2	-122.445	37.932	0.26	18.54	231.10	18.80	-122.445	37.932	0.32	18.54	231.26	18.87	9	7/14/2014	45:07.8
3173	RSPe_2	-122.445	37.932	0.21	18.50	230.95	18.71	-122.445	37.932	0.32	18.50	231.18	18.83	9	7/14/2014	45:07.7
3174	RSPe_2	-122.445	37.932	0.21	18.50	230.86	18.71	-122.445	37.932	0.27	18.50	231.12	18.77	9	7/14/2014	45:07.6
3175	RSPe_2	-122.445	37.932	0.26	18.43	230.84	18.70	-122.445	37.932	0.32	18.43	231.08	18.76	9	7/14/2014	45:07.5
3176	RSPe_2	-122.445	37.932	0.26	18.43	230.79	18.70	-122.445	37.932	0.27	18.43	231.05	18.71	9	7/14/2014	45:07.4
3177	RSPe_2	-122.445	37.932	0.26	18.42	230.75	18.68	-122.445	37.932	0.27	18.42	230.97	18.69	9	7/14/2014	45:07.3
3178	RSPe_2	-122.445	37.932	0.26	18.45	230.66	18.71	-122.445	37.932	0.27	18.45	230.89	18.72	9	7/14/2014	45:07.2
3179	RSPe_2	-122.445	37.932	0.21	18.48	230.64	18.69	-122.445	37.932	0.27	18.48	230.92	18.76	9	7/14/2014	45:07.1
3180	RSPe_2	-122.445	37.932	0.21	18.40	230.58	18.61	-122.445	37.932	0.27	18.40	230.81	18.68	9	7/14/2014	45:07.0
3181	RSPe_2	-122.445	37.932	0.26	18.36	230.51	18.63	-122.445	37.932	0.28	18.36	230.75	18.64	9	7/14/2014	45:06.9
3182	RSPe_2	-122.445	37.932	0.21	18.35	230.45	18.56	-122.445	37.932	0.33	18.35	230.66	18.67	9	7/14/2014	45:06.8
3183	RSPe_2	-122.445	37.932	0.26	18.33	230.34	18.59	-122.445	37.932	0.28	18.33	230.57	18.60	9	7/14/2014	45:06.7
3184	RSPe_2	-122.445	37.932	0.21	18.31	230.18	18.52	-122.445	37.932	0.36	18.31	230.41	18.67	9	7/14/2014	45:06.6
3185	RSPe_2	-122.445	37.932	0.26	18.31	230.09	18.57	-122.445	37.932	0.36	18.31	230.28	18.67	9	7/14/2014	45:06.5
3186	RSPe_2	-122.445	37.932	0.21	18.26	230.10	18.47	-122.445	37.932	0.33	18.26	230.24	18.59	9	7/14/2014	45:06.4
3187	RSPe_2	-122.445	37.932	0.26	18.24	230.01	18.50	-122.445	37.932	0.33	18.24	230.12	18.57	9	7/14/2014	45:06.3
3188	RSPe_2	-122.445	37.932	0.26	18.23	229.97	18.49	-122.445	37.932	0.28	18.23	230.04	18.50	9	7/14/2014	45:06.2
3189	RSPe_2	-122.445	37.932	0.21	18.24	229.94	18.45	-122.445	37.932	0.33	18.24	229.94	18.56	9	7/14/2014	45:06.1
3190	RSPe_2	-122.445	37.932	0.21	18.19	229.81	18.40	-122.445	37.932	0.28	18.19	229.87	18.46	9	7/14/2014	45:06.0
3191	RSPe_2	-122.445	37.932	0.26	18.17	229.82	18.43	-122.445	37.932	0.36	18.17	229.83	18.53	9	7/14/2014	45:05.9
3192	RSPe_2	-122.445	37.932	0.21	18.17	229.77	18.38	-122.445	37.932	0.33	18.17	229.79	18.49	9	7/14/2014	45:05.8
3193	RSPe_2	-122.445	37.932	0.21	18.13	229.64	18.34	-122.445	37.932	0.33	18.13	229.73	18.46	9	7/14/2014	45:05.7
3194	RSPe_2	-122.445	37.932	0.21	18.17	229.59	18.38	-122.445	37.932	0.33	18.17	229.67	18.50	9	7/14/2014	45:05.6
3195	RSPe_2	-122.445	37.932	0.21	18.11	229.47	18.32	-122.445	37.932	0.33	18.11	229.65	18.43	9	7/14/2014	45:05.5
3196	RSPe_2	-122.445	37.932	0.18	18.13	229.37	18.31	-122.445	37.932	0.28	18.13	229.55	18.41	9	7/14/2014	45:05.4
3197	RSPe_2	-122.445	37.932	0.21	18.08	229.25	18.29	-122.445	37.932	0.33	18.08	229.57	18.41	9	7/14/2014	45:05.3
3198	RSPe_2	-122.445	37.932	0.18	18.10	229.12	18.28	-122.445	37.932	0.28	18.10	229.49	18.38	9	7/14/2014	45:05.2
3199	RSPe_2	-122.445	37.932	0.18	18.10	229.02	18.28	-122.445	37.932	0.28	18.10	229.45	18.38	9	7/14/2014	45:05.1
3200	RSPe_2	-122.445	37.932	0.18	18.08	228.89	18.26	-122.445	37.932	0.28	18.08	229.37	18.35	9	7/14/2014	45:05.0

3201	RSPe_2	-122.445	37.932	0.21	18.05	228.83	18.26	-122.445	37.932	0.28	18.05	229.33	18.33	9	7/14/2014	45:04.9
3202	RSPe_2	-122.445	37.932	0.18	18.11	228.70	18.28	-122.445	37.932	0.24	18.11	229.19	18.35	9	7/14/2014	45:04.8
3203	RSPe_2	-122.445	37.932	0.18	18.06	228.67	18.24	-122.445	37.932	0.24	18.06	229.11	18.30	9	7/14/2014	45:04.7
3204	RSPe_2	-122.445	37.932	0.18	18.09	228.53	18.26	-122.445	37.932	0.19	18.09	228.96	18.28	9	7/14/2014	45:04.6
3205	RSPe_2	-122.445	37.932	0.18	18.11	228.48	18.28	-122.445	37.932	0.24	18.11	228.90	18.35	9	7/14/2014	45:04.5
3206	RSPe_2	-122.445	37.932	0.14	18.10	228.50	18.24	-122.445	37.932	0.19	18.10	228.79	18.29	9	7/14/2014	45:04.4
3207	RSPe_2	-122.445	37.932	0.18	18.16	228.46	18.33	-122.445	37.932	0.19	18.16	228.70	18.35	9	7/14/2014	45:04.3
3208	RSPe_2	-122.445	37.932	0.14	18.11	228.44	18.25	-122.445	37.932	0.19	18.11	228.66	18.30	9	7/14/2014	45:04.2
3209	RSPe_2	-122.445	37.932	0.14	18.18	228.41	18.32	-122.445	37.932	0.16	18.18	228.61	18.33	9	7/14/2014	45:04.1
3210	RSPe_2	-122.445	37.932	0.14	18.10	228.35	18.24	-122.445	37.932	0.12	18.10	228.51	18.22	9	7/14/2014	45:04.0
3211	RSPe_2	-122.445	37.932	0.14	18.10	228.25	18.24	-122.445	37.932	0.16	18.10	228.46	18.25	9	7/14/2014	45:03.9
3212	RSPe_2	-122.445	37.932	0.14	18.12	228.22	18.27	-122.445	37.932	0.16	18.12	228.44	18.28	9	7/14/2014	45:03.8
3213	RSPe_2	-122.445	37.932	0.14	18.11	228.11	18.25	-122.445	37.932	0.16	18.11	228.42	18.26	9	7/14/2014	45:03.7
3214	RSPe_2	-122.445	37.932	0.09	18.13	228.04	18.22	-122.445	37.932	0.12	18.13	228.35	18.26	9	7/14/2014	45:03.6
3215	RSPe_2	-122.445	37.932	0.14	18.18	227.91	18.32	-122.445	37.932	0.16	18.18	228.28	18.33	9	7/14/2014	45:03.5
3216	RSPe_2	-122.445	37.932	0.09	18.18	227.83	18.27	-122.445	37.932	0.12	18.18	228.20	18.30	9	7/14/2014	45:03.4
3217	RSPe_2	-122.445	37.932	0.14	18.19	227.79	18.33	-122.445	37.932	0.12	18.19	228.15	18.31	9	7/14/2014	45:03.3
3218	RSPe_2	-122.445	37.932	0.14	18.27	227.72	18.41	-122.445	37.932	0.12	18.27	228.09	18.39	9	7/14/2014	45:03.2
3219	RSPe_2	-122.445	37.932	0.14	18.22	227.65	18.36	-122.445	37.932	0.12	18.22	228.02	18.34	9	7/14/2014	45:03.1
3220	RSPe_2	-122.445	37.932	0.09	18.27	227.61	18.36	-122.445	37.932	0.07	18.27	227.98	18.34	9	7/14/2014	45:03.0
3221	RSPe_2	-122.445	37.932	0.14	18.30	227.57	18.44	-122.445	37.932	0.07	18.30	227.91	18.37	9	7/14/2014	45:02.9
3222	RSPe_2	-122.445	37.932	0.09	18.31	227.54	18.40	-122.445	37.932	-0.01	18.31	227.83	18.30	9	7/14/2014	45:02.8
3223	RSPe_2	-122.445	37.932	0.09	18.28	227.52	18.37	-122.445	37.932	0.04	18.28	227.74	18.32	9	7/14/2014	45:02.7
3224	RSPe_2	-122.445	37.932	0.09	18.30	227.48	18.40	-122.445	37.932	-0.01	18.30	227.65	18.29	9	7/14/2014	45:02.6
3225	RSPe_2	-122.445	37.932	0.09	18.36	227.42	18.45	-122.445	37.932	0.07	18.36	227.57	18.43	9	7/14/2014	45:02.5
3226	RSPe_2	-122.445	37.932	0.09	18.35	227.37	18.44	-122.445	37.932	-0.01	18.35	227.52	18.34	9	7/14/2014	45:02.4
3227	RSPe_2	-122.445	37.932	0.09	18.32	227.35	18.41	-122.445	37.932	-0.01	18.32	227.43	18.30	9	7/14/2014	45:02.3
3228	RSPe_2	-122.445	37.932	0.09	18.33	227.21	18.42	-122.445	37.932	0.04	18.33	227.37	18.37	9	7/14/2014	45:02.2
3229	RSPe_2	-122.445	37.932	0.09	18.34	227.18	18.43	-122.445	37.932	0.04	18.34	227.35	18.38	9	7/14/2014	45:02.1
3230	RSPe_2	-122.445	37.932	0.09	18.35	227.07	18.44	-122.445	37.932	-0.01	18.35	227.28	18.34	9	7/14/2014	45:02.0
3231	RSPe_2	-122.445	37.932	0.06	18.36	226.94	18.42	-122.445	37.932	-0.01	18.36	227.15	18.35	9	7/14/2014	45:01.9
3232	RSPe_2	-122.445	37.932	0.06	18.40	226.76	18.46	-122.445	37.932	-0.01	18.40	227.04	18.39	9	7/14/2014	45:01.8
3233	RSPe_2	-122.445	37.932	0.09	18.59	226.68	18.68	-122.445	37.932	-0.01	18.59	227.01	18.58	9	7/14/2014	45:01.7

3234	RSPe_2	-122.445	37.932	0.06	18.48	226.59	18.54	-122.445	37.932	-0.01	18.48	226.96	18.47	9	7/14/2014 45:01.6
3235	RSPe_2	-122.445	37.932	0.06	18.58	226.49	18.64	-122.445	37.932	0.04	18.58	226.93	18.62	9	7/14/2014 45:01.5
3236	RSPe_2	-122.445	37.932	0.06	18.57	226.41	18.63	-122.445	37.932	0.04	18.57	226.87	18.61	9	7/14/2014 45:01.4
3237	RSPe_2	-122.445	37.932	0.09	18.65	226.32	18.74	-122.445	37.932	-0.01	18.65	226.78	18.64	9	7/14/2014 45:01.3
3238	RSPe_2	-122.445	37.932	0.06	18.70	226.24	18.76	-122.445	37.932	-0.05	18.70	226.65	18.66	9	7/14/2014 45:01.2
3239	RSPe_2	-122.445	37.932	0.06	18.72	226.21	18.77	-122.445	37.932	-0.01	18.72	226.54	18.71	9	7/14/2014 45:01.1
3240	RSPe_2	-122.445	37.932	0.06	18.68	226.11	18.74	-122.445	37.932	-0.05	18.68	226.31	18.63	9	7/14/2014 45:01.0
3241	RSPe_2	-122.445	37.932	0.09	18.78	226.01	18.87	-122.445	37.932	-0.01	18.78	226.19	18.77	9	7/14/2014 45:00.9
3242	RSPe_2	-122.445	37.932	0.06	18.70	225.99	18.75	-122.445	37.932	-0.01	18.70	226.00	18.68	9	7/14/2014 45:00.8
3243	RSPe_2	-122.445	37.932	0.06	18.69	225.80	18.75	-122.445	37.932	-0.01	18.69	225.77	18.68	9	7/14/2014 45:00.7
3244	RSPe_2	-122.445	37.932	0.06	18.69	225.73	18.75	-122.445	37.932	0.04	18.69	225.66	18.73	9	7/14/2014 45:00.6
3245	RSPe_2	-122.445	37.932	0.09	18.65	225.71	18.74	-122.445	37.932	0.04	18.65	225.58	18.69	9	7/14/2014 45:00.5
3246	RSPe_2	-122.445	37.932	0.06	18.66	225.65	18.72	-122.445	37.932	-0.01	18.66	225.53	18.65	9	7/14/2014 45:00.4
3247	RSPe_2	-122.445	37.932	0.09	18.64	225.54	18.73	-122.445	37.932	0.04	18.64	225.47	18.68	9	7/14/2014 45:00.3
3248	RSPe_2	-122.445	37.932	0.06	18.60	225.51	18.66	-122.445	37.932	0.04	18.60	225.46	18.64	9	7/14/2014 45:00.2
3249	RSPe_2	-122.445	37.932	0.09	18.64	225.38	18.73	-122.445	37.932	0.04	18.64	225.47	18.68	9	7/14/2014 45:00.1
3250	RSPe_2	-122.445	37.932	0.06	18.63	225.19	18.69	-122.445	37.932	-0.01	18.63	225.43	18.62	9	7/14/2014 45:00.0
3251	RSPe_2	-122.445	37.932	0.06	18.63	225.03	18.68	-122.445	37.932	0.04	18.63	225.35	18.66	9	7/14/2014 44:59.9
3252	RSPe_2	-122.445	37.932	0.01	18.66	224.91	18.66	-122.445	37.932	-0.01	18.66	225.29	18.65	9	7/14/2014 44:59.8
3253	RSPe_2	-122.445	37.932	0.06	18.66	224.72	18.71	-122.445	37.932	-0.01	18.66	225.19	18.65	9	7/14/2014 44:59.7
3254	RSPe_2	-122.445	37.932	0.06	18.72	224.66	18.77	-122.445	37.932	-0.05	18.72	225.12	18.67	9	7/14/2014 44:59.6
3255	RSPe_2	-122.445	37.932	0.06	18.76	224.53	18.82	-122.445	37.932	-0.01	18.76	225.01	18.75	9	7/14/2014 44:59.5
3256	RSPe_2	-122.445	37.932	0.06	18.88	224.51	18.94	-122.445	37.932	-0.01	18.88	224.85	18.87	9	7/14/2014 44:59.4
3257	RSPe_2	-122.445	37.932	0.06	18.92	224.40	18.98	-122.445	37.932	0.04	18.92	224.73	18.96	9	7/14/2014 44:59.3
3258	RSPe_2	-122.445	37.932	0.06	18.92	224.32	18.98	-122.445	37.932	-0.05	18.92	224.57	18.87	9	7/14/2014 44:59.2
3259	RSPe_2	-122.445	37.932	0.06	18.93	224.23	18.98	-122.445	37.932	0.04	18.93	224.44	18.97	9	7/14/2014 44:59.1
3260	RSPe_2	-122.445	37.932	0.01	18.90	224.13	18.91	-122.445	37.932	-0.01	18.90	224.29	18.89	9	7/14/2014 44:59.0
3261	RSPe_2	-122.445	37.932	0.06	18.92	223.99	18.98	-122.445	37.932	-0.05	18.92	224.22	18.87	9	7/14/2014 44:58.9
3262	RSPe_2	-122.445	37.932	0.01	18.94	223.97	18.95	-122.445	37.932	-0.01	18.94	224.16	18.93	9	7/14/2014 44:58.8
3263	RSPe_2	-122.445	37.932	0.06	18.98	223.85	19.04	-122.445	37.932	-0.01	18.98	224.13	18.97	9	7/14/2014 44:58.7
3264	RSPe_2	-122.445	37.932	0.06	18.99	223.84	19.05	-122.445	37.932	-0.05	18.99	224.12	18.94	9	7/14/2014 44:58.6
3265	RSPe_2	-122.445	37.932	0.06	19.01	223.66	19.07	-122.445	37.932	-0.05	19.01	224.03	18.97	9	7/14/2014 44:58.5
3266	RSPe_2	-122.445	37.932	0.01	19.03	223.57	19.03	-122.445	37.932	-0.05	19.03	223.96	18.98	9	7/14/2014 44:58.4

3267	RSPe_2	-122.445	37.932	0.06	19.04	223.46	19.10	-122.445	37.932	-0.01	19.04	223.83	19.03	9	7/14/2014	44:58.3
3268	RSPe_2	-122.445	37.932	0.01	19.03	223.28	19.04	-122.445	37.932	-0.01	19.03	223.67	19.02	9	7/14/2014	44:58.2
3269	RSPe_2	-122.445	37.932	0.06	19.04	223.18	19.10	-122.445	37.932	-0.05	19.04	223.57	19.00	9	7/14/2014	44:58.1
3270	RSPe_2	-122.445	37.932	0.01	19.03	223.10	19.04	-122.445	37.932	-0.10	19.03	223.41	18.94	9	7/14/2014	44:58.0
3271	RSPe_2	-122.445	37.932	0.06	19.05	222.89	19.11	-122.445	37.932	-0.10	19.05	223.28	18.95	9	7/14/2014	44:57.9
3272	RSPe_2	-122.445	37.932	0.01	19.03	222.82	19.03	-122.445	37.932	-0.10	19.03	223.13	18.93	9	7/14/2014	44:57.8
3273	RSPe_2	-122.445	37.932	0.06	19.01	222.79	19.06	-122.445	37.932	-0.01	19.01	223.09	18.99	9	7/14/2014	44:57.7
3274	RSPe_2	-122.445	37.932	0.01	19.01	222.67	19.02	-122.445	37.932	-0.05	19.01	223.02	18.97	9	7/14/2014	44:57.6
3275	RSPe_2	-122.445	37.932	0.06	19.01	222.63	19.07	-122.445	37.932	-0.10	19.01	222.92	18.91	9	7/14/2014	44:57.5
3276	RSPe_2	-122.445	37.932	0.01	19.02	222.52	19.03	-122.445	37.932	-0.10	19.02	222.84	18.92	9	7/14/2014	44:57.4
3277	RSPe_2	-122.445	37.932	0.09	19.04	222.48	19.14	-122.445	37.932	-0.05	19.04	222.77	19.00	9	7/14/2014	44:57.3
3278	RSPe_2	-122.445	37.932	0.06	19.02	222.33	19.08	-122.445	37.932	-0.05	19.02	222.64	18.97	9	7/14/2014	44:57.2
3279	RSPe_2	-122.445	37.932	0.09	19.02	222.26	19.11	-122.445	37.932	-0.01	19.02	222.60	19.01	9	7/14/2014	44:57.1
3280	RSPe_2	-122.445	37.932	0.06	19.05	222.24	19.11	-122.445	37.932	-0.05	19.05	222.54	19.00	9	7/14/2014	44:57.0
3281	RSPe_2	-122.445	37.932	0.06	19.03	222.02	19.08	-122.445	37.932	-0.05	19.03	222.46	18.98	9	7/14/2014	44:56.9
3282	RSPe_2	-122.445	37.932	0.01	19.07	221.96	19.07	-122.445	37.932	-0.05	19.07	222.31	19.02	9	7/14/2014	44:56.8
3283	RSPe_2	-122.445	37.932	0.06	19.12	221.87	19.17	-122.445	37.932	-0.10	19.12	222.24	19.02	9	7/14/2014	44:56.7
3284	RSPe_2	-122.445	37.932	0.06	19.05	221.74	19.11	-122.445	37.932	-0.10	19.05	222.13	18.95	9	7/14/2014	44:56.6
3285	RSPe_2	-122.445	37.932	0.06	19.10	221.66	19.16	-122.445	37.932	-0.10	19.10	222.06	19.01	9	7/14/2014	44:56.5
3286	RSPe_2	-122.445	37.932	0.06	19.06	221.65	19.12	-122.445	37.932	-0.10	19.06	221.97	18.96	9	7/14/2014	44:56.4
3287	RSPe_2	-122.445	37.932	0.06	19.10	221.56	19.16	-122.445	37.932	-0.05	19.10	221.86	19.06	9	7/14/2014	44:56.3
3288	RSPe_2	-122.445	37.932	0.06	19.08	221.48	19.13	-122.445	37.932	-0.05	19.08	221.75	19.03	9	7/14/2014	44:56.2
3289	RSPe_2	-122.445	37.932	0.09	19.07	221.41	19.16	-122.445	37.932	-0.05	19.07	221.62	19.02	9	7/14/2014	44:56.1
3290	RSPe_2	-122.445	37.932	0.06	19.02	221.29	19.08	-122.445	37.932	-0.05	19.02	221.52	18.97	9	7/14/2014	44:56.0
3291	RSPe_2	-122.445	37.932	0.06	19.03	221.17	19.09	-122.445	37.932	-0.01	19.03	221.44	19.02	9	7/14/2014	44:55.9
3292	RSPe_2	-122.445	37.932	0.06	19.03	221.06	19.09	-122.445	37.932	-0.01	19.03	221.40	19.02	9	7/14/2014	44:55.8
3293	RSPe_2	-122.445	37.932	0.09	19.01	220.99	19.10	-122.445	37.932	-0.05	19.01	221.32	18.97	9	7/14/2014	44:55.7
3294	RSPe_2	-122.445	37.932	0.06	19.04	220.98	19.10	-122.445	37.932	-0.05	19.04	221.34	19.00	9	7/14/2014	44:55.6
3295	RSPe_2	-122.445	37.932	0.09	19.05	220.93	19.14	-122.445	37.932	-0.05	19.05	221.35	19.00	9	7/14/2014	44:55.5
3296	RSPe_2	-122.445	37.932	0.06	19.03	220.84	19.08	-122.445	37.932	-0.10	19.03	221.24	18.93	9	7/14/2014	44:55.4
3297	RSPe_2	-122.445	37.932	0.06	19.06	220.76	19.12	-122.445	37.932	-0.10	19.06	221.19	18.96	9	7/14/2014	44:55.3
3298	RSPe_2	-122.445	37.932	0.01	19.09	220.73	19.10	-122.445	37.932	-0.13	19.09	221.12	18.96	9	7/14/2014	44:55.2
3299	RSPe_2	-122.445	37.932	0.06	18.98	220.63	19.04	-122.445	37.932	-0.10	18.98	220.97	18.88	9	7/14/2014	44:55.1

3300	RSPe_2	-122.445	37.932	0.06	18.91	220.53	18.97	-122.445	37.932	-0.13	18.91	220.84	18.78	9	7/14/2014	44:55.0
3301	RSPe_2	-122.445	37.932	0.06	18.94	220.50	19.00	-122.445	37.932	-0.10	18.94	220.75	18.84	9	7/14/2014	44:54.9
3302	RSPe_2	-122.445	37.932	0.06	18.75	220.36	18.81	-122.445	37.932	-0.05	18.75	220.60	18.70	9	7/14/2014	44:54.8
3303	RSPe_2	-122.445	37.932	0.09	18.66	220.17	18.75	-122.445	37.932	-0.05	18.66	220.43	18.62	9	7/14/2014	44:54.7
3304	RSPe_2	-122.445	37.932	0.06	18.64	220.05	18.70	-122.445	37.932	-0.01	18.64	220.28	18.63	9	7/14/2014	44:54.6
3305	RSPe_2	-122.445	37.932	0.09	18.66	219.92	18.75	-122.445	37.932	-0.01	18.66	220.17	18.65	9	7/14/2014	44:54.5
3306	RSPe_2	-122.445	37.932	0.09	18.52	219.79	18.61	-122.445	37.932	-0.01	18.52	220.09	18.51	9	7/14/2014	44:54.4
3307	RSPe_2	-122.445	37.932	0.09	18.52	219.56	18.61	-122.445	37.932	-0.01	18.52	219.95	18.51	9	7/14/2014	44:54.3
3308	RSPe_2	-122.445	37.932	0.09	18.51	219.49	18.60	-122.445	37.932	-0.01	18.51	219.85	18.50	9	7/14/2014	44:54.2
3309	RSPe_2	-122.445	37.932	0.09	18.37	219.40	18.46	-122.445	37.932	-0.01	18.37	219.80	18.36	9	7/14/2014	44:54.1
3310	RSPe_2	-122.445	37.932	0.09	18.38	219.33	18.47	-122.445	37.932	-0.01	18.38	219.78	18.37	9	7/14/2014	44:54.0
3311	RSPe_2	-122.445	37.932	0.09	18.30	219.22	18.40	-122.445	37.932	-0.01	18.30	219.67	18.29	9	7/14/2014	44:53.9
3312	RSPe_2	-122.445	37.932	0.09	18.29	219.22	18.38	-122.445	37.932	-0.05	18.29	219.68	18.24	9	7/14/2014	44:53.8
3313	RSPe_2	-122.445	37.932	0.09	18.40	219.12	18.49	-122.445	37.932	-0.05	18.40	219.59	18.35	9	7/14/2014	44:53.7
3314	RSPe_2	-122.445	37.932	0.09	18.39	219.20	18.48	-122.445	37.932	-0.05	18.39	219.52	18.34	9	7/14/2014	44:53.6
3315	RSPe_2	-122.445	37.932	0.09	18.35	219.16	18.44	-122.445	37.932	-0.05	18.35	219.49	18.30	9	7/14/2014	44:53.5
3316	RSPe_2	-122.445	37.932	0.06	18.29	219.07	18.34	-122.445	37.932	-0.10	18.29	219.32	18.19	9	7/14/2014	44:53.4
3317	RSPe_2	-122.445	37.932	0.09	18.29	219.05	18.38	-122.445	37.932	-0.10	18.29	219.21	18.19	9	7/14/2014	44:53.3
3318	RSPe_2	-122.445	37.932	0.06	18.30	218.92	18.35	-122.445	37.932	-0.05	18.30	219.08	18.25	9	7/14/2014	44:53.2
3319	RSPe_2	-122.445	37.932	0.09	18.24	218.81	18.33	-122.445	37.932	-0.01	18.24	218.92	18.23	9	7/14/2014	44:53.1
3320	RSPe_2	-122.445	37.932	0.06	18.25	218.70	18.30	-122.445	37.932	0.04	18.25	218.82	18.29	9	7/14/2014	44:53.0
3321	RSPe_2	-122.445	37.932	0.09	18.23	218.64	18.32	-122.445	37.932	0.04	18.23	218.74	18.26	9	7/14/2014	44:52.9
3322	RSPe_2	-122.445	37.932	0.06	18.14	218.57	18.20	-122.445	37.932	-0.01	18.14	218.68	18.13	9	7/14/2014	44:52.8
3323	RSPe_2	-122.445	37.932	0.09	18.15	218.37	18.24	-122.445	37.932	0.04	18.15	218.55	18.19	9	7/14/2014	44:52.7
3324	RSPe_2	-122.445	37.932	0.09	18.16	218.19	18.25	-122.445	37.932	-0.01	18.16	218.43	18.15	9	7/14/2014	44:52.6
3325	RSPe_2	-122.445	37.932	0.09	18.16	218.02	18.25	-122.445	37.932	0.04	18.16	218.37	18.20	9	7/14/2014	44:52.5
3326	RSPe_2	-122.445	37.932	0.09	18.21	217.93	18.30	-122.445	37.932	0.04	18.21	218.32	18.25	9	7/14/2014	44:52.4
3327	RSPe_2	-122.445	37.932	0.09	18.25	217.85	18.34	-122.445	37.932	-0.01	18.25	218.34	18.24	9	7/14/2014	44:52.3
3328	RSPe_2	-122.445	37.932	0.09	18.34	217.67	18.43	-122.445	37.932	0.04	18.34	218.19	18.38	9	7/14/2014	44:52.2
3329	RSPe_2	-122.445	37.932	0.09	18.31	217.68	18.40	-122.445	37.932	-0.05	18.31	218.23	18.26	9	7/14/2014	44:52.1
3330	RSPe_2	-122.445	37.932	0.06	18.36	217.65	18.41	-122.445	37.932	-0.01	18.36	218.13	18.35	9	7/14/2014	44:52.0
3331	RSPe_2	-122.445	37.932	0.09	18.36	217.61	18.45	-122.445	37.932	-0.01	18.36	218.07	18.35	9	7/14/2014	44:51.9
3332	RSPe_2	-122.445	37.932	0.06	18.32	217.58	18.37	-122.445	37.932	-0.01	18.32	218.02	18.31	9	7/14/2014	44:51.8

3333	RSPe_2	-122.445	37.932	0.09	18.38	217.57	18.47	-122.445	37.932	-0.01	18.38	217.90	18.37	9	7/14/2014	44:51.7
3334	RSPe_2	-122.445	37.932	0.06	18.38	217.45	18.44	-122.445	37.932	-0.05	18.38	217.74	18.33	9	7/14/2014	44:51.6
3335	RSPe_2	-122.445	37.932	0.09	18.40	217.37	18.49	-122.445	37.932	-0.01	18.40	217.63	18.38	9	7/14/2014	44:51.5
3336	RSPe_2	-122.445	37.932	0.06	18.33	217.24	18.38	-122.445	37.932	-0.01	18.33	217.47	18.32	9	7/14/2014	44:51.4
3337	RSPe_2	-122.445	37.932	0.06	18.30	217.19	18.36	-122.445	37.932	-0.01	18.30	217.38	18.29	9	7/14/2014	44:51.3
3338	RSPe_2	-122.445	37.932	0.06	18.28	217.12	18.34	-122.445	37.932	-0.01	18.28	217.30	18.27	9	7/14/2014	44:51.2
3339	RSPe_2	-122.445	37.932	0.09	18.29	217.13	18.38	-122.445	37.932	-0.01	18.29	217.24	18.28	9	7/14/2014	44:51.1
3340	RSPe_2	-122.445	37.932	0.06	18.32	217.06	18.37	-122.445	37.932	0.04	18.32	217.20	18.36	9	7/14/2014	44:51.0
3341	RSPe_2	-122.445	37.932	0.09	18.30	216.98	18.39	-122.445	37.932	-0.01	18.30	217.13	18.28	9	7/14/2014	44:50.9
3342	RSPe_2	-122.445	37.932	0.01	18.33	216.87	18.33	-122.445	37.932	-0.01	18.33	217.09	18.32	9	7/14/2014	44:50.8
3343	RSPe_2	-122.445	37.932	0.06	18.31	216.76	18.37	-122.445	37.932	-0.01	18.31	216.97	18.30	9	7/14/2014	44:50.7
3344	RSPe_2	-122.445	37.932	0.06	18.40	216.63	18.46	-122.445	37.932	-0.01	18.40	216.95	18.39	9	7/14/2014	44:50.6
3345	RSPe_2	-122.445	37.932	0.06	18.40	216.39	18.46	-122.445	37.932	-0.01	18.40	216.87	18.39	9	7/14/2014	44:50.5
3346	RSPe_2	-122.445	37.932	0.06	18.46	216.26	18.51	-122.445	37.932	-0.01	18.46	216.84	18.44	9	7/14/2014	44:50.4
3347	RSPe_2	-122.445	37.932	0.06	18.50	216.18	18.55	-122.445	37.932	-0.01	18.50	216.78	18.49	9	7/14/2014	44:50.3
3348	RSPe_2	-122.445	37.932	0.01	18.51	216.04	18.52	-122.445	37.932	-0.05	18.51	216.72	18.46	9	7/14/2014	44:50.2
3349	RSPe_2	-122.445	37.932	0.06	18.62	216.01	18.68	-122.445	37.932	-0.01	18.62	216.67	18.61	9	7/14/2014	44:50.1
3350	RSPe_2	-122.445	37.932	0.06	18.70	215.97	18.75	-122.445	37.932	-0.01	18.70	216.56	18.68	9	7/14/2014	44:50.0
3351	RSPe_2	-122.445	37.932	0.06	18.70	215.84	18.76	-122.445	37.932	-0.01	18.70	216.36	18.69	9	7/14/2014	44:49.9
3352	RSPe_2	-122.445	37.932	0.06	18.74	215.84	18.80	-122.445	37.932	-0.05	18.74	216.29	18.70	9	7/14/2014	44:49.8
3353	RSPe_2	-122.445	37.932	0.06	18.75	215.78	18.81	-122.445	37.932	-0.05	18.75	216.15	18.71	9	7/14/2014	44:49.7
3354	RSPe_2	-122.445	37.932	0.01	18.73	215.70	18.73	-122.445	37.932	-0.05	18.73	216.06	18.68	9	7/14/2014	44:49.6
3355	RSPe_2	-122.445	37.932	0.06	18.74	215.67	18.80	-122.445	37.932	-0.01	18.74	215.90	18.73	9	7/14/2014	44:49.5
3356	RSPe_2	-122.445	37.932	0.06	18.74	215.58	18.80	-122.445	37.932	-0.05	18.74	215.79	18.70	9	7/14/2014	44:49.4
3357	RSPe_2	-122.445	37.932	0.06	18.67	215.51	18.73	-122.445	37.932	-0.01	18.67	215.64	18.66	9	7/14/2014	44:49.3
3358	RSPe_2	-122.445	37.932	0.01	18.68	215.44	18.69	-122.445	37.932	-0.05	18.68	215.57	18.64	9	7/14/2014	44:49.2
3359	RSPe_2	-122.445	37.932	0.06	18.71	215.34	18.77	-122.445	37.932	-0.01	18.71	215.47	18.70	9	7/14/2014	44:49.1
3360	RSPe_2	-122.445	37.932	0.01	18.70	215.27	18.70	-122.445	37.932	-0.10	18.70	215.40	18.60	9	7/14/2014	44:49.0
3361	RSPe_2	-122.445	37.932	0.01	18.84	215.12	18.85	-122.445	37.932	-0.01	18.84	215.35	18.83	9	7/14/2014	44:48.9
3362	RSPe_2	-122.445	37.932	0.01	18.75	215.08	18.76	-122.445	37.932	-0.05	18.75	215.30	18.71	9	7/14/2014	44:48.8
3363	RSPe_2	-122.445	37.932	0.06	18.72	215.01	18.78	-122.445	37.932	-0.01	18.72	215.32	18.71	9	7/14/2014	44:48.7
3364	RSPe_2	-122.445	37.932	0.01	18.76	214.93	18.77	-122.445	37.932	-0.05	18.76	215.25	18.71	9	7/14/2014	44:48.6
3365	RSPe_2	-122.445	37.932	0.01	18.82	214.86	18.83	-122.445	37.932	-0.01	18.82	215.21	18.81	9	7/14/2014	44:48.5

3366	RSPe_2	-122.445	37.932	0.01	18.84	214.76	18.85	-122.445	37.932	-0.05	18.84	215.14	18.80	9	7/14/2014	44:48.4
3367	RSPe_2	-122.445	37.932	0.01	18.80	214.64	18.81	-122.445	37.932	-0.05	18.80	215.10	18.76	9	7/14/2014	44:48.3
3368	RSPe_2	-122.445	37.932	0.01	18.83	214.53	18.84	-122.445	37.932	-0.05	18.83	215.01	18.78	9	7/14/2014	44:48.2
3369	RSPe_2	-122.445	37.932	0.01	18.83	214.47	18.84	-122.445	37.932	-0.10	18.83	214.98	18.73	9	7/14/2014	44:48.1
3370	RSPe_2	-122.445	37.932	-0.03	18.87	214.40	18.84	-122.445	37.932	-0.10	18.87	214.90	18.78	9	7/14/2014	44:48.0
3371	RSPe_2	-122.445	37.932	0.01	18.93	214.33	18.93	-122.445	37.932	-0.05	18.93	214.81	18.88	9	7/14/2014	44:47.9
3372	RSPe_2	-122.445	37.932	-0.03	18.94	214.28	18.91	-122.445	37.932	-0.10	18.94	214.76	18.84	9	7/14/2014	44:47.8
3373	RSPe_2	-122.445	37.932	0.01	18.93	214.31	18.93	-122.445	37.932	-0.10	18.93	214.62	18.83	9	7/14/2014	44:47.7
3374	RSPe_2	-122.445	37.932	-0.03	18.93	214.25	18.90	-122.445	37.932	-0.13	18.93	214.57	18.80	9	7/14/2014	44:47.6
3375	RSPe_2	-122.445	37.932	0.01	18.94	214.22	18.94	-122.445	37.932	-0.05	18.94	214.49	18.89	9	7/14/2014	44:47.5
3376	RSPe_2	-122.445	37.932	-0.03	18.97	214.12	18.95	-122.445	37.932	-0.13	18.97	214.46	18.84	9	7/14/2014	44:47.4
3377	RSPe_2	-122.445	37.932	-0.03	18.98	214.01	18.95	-122.445	37.932	-0.10	18.98	214.35	18.88	9	7/14/2014	44:47.3
3378	RSPe_2	-122.445	37.932	-0.03	18.97	213.96	18.94	-122.445	37.932	-0.13	18.97	214.27	18.84	9	7/14/2014	44:47.2
3379	RSPe_2	-122.445	37.932	-0.03	18.97	213.81	18.94	-122.445	37.932	-0.10	18.97	214.16	18.87	9	7/14/2014	44:47.1
3380	RSPe_2	-122.445	37.932	0.01	19.02	213.79	19.03	-122.445	37.932	-0.13	19.02	214.12	18.89	9	7/14/2014	44:47.0
3381	RSPe_2	-122.445	37.932	0.01	18.98	213.66	18.99	-122.445	37.932	-0.05	18.98	214.03	18.93	9	7/14/2014	44:46.9
3382	RSPe_2	-122.445	37.932	-0.03	19.01	213.61	18.98	-122.445	37.932	-0.13	19.01	213.96	18.88	9	7/14/2014	44:46.8
3383	RSPe_2	-122.445	37.932	0.01	19.03	213.51	19.03	-122.445	37.932	-0.05	19.03	213.91	18.98	9	7/14/2014	44:46.7
3384	RSPe_2	-122.445	37.932	0.01	19.02	213.44	19.03	-122.445	37.932	-0.10	19.02	213.88	18.92	9	7/14/2014	44:46.6
3385	RSPe_2	-122.445	37.932	0.01	19.03	213.40	19.03	-122.445	37.932	-0.10	19.03	213.84	18.93	9	7/14/2014	44:46.5
3386	RSPe_2	-122.445	37.932	0.01	19.04	213.33	19.05	-122.445	37.932	-0.05	19.04	213.77	19.00	9	7/14/2014	44:46.4
3387	RSPe_2	-122.445	37.932	0.01	19.05	213.40	19.06	-122.445	37.932	-0.10	19.05	213.75	18.95	9	7/14/2014	44:46.3
3388	RSPe_2	-122.445	37.932	-0.03	19.03	213.33	19.01	-122.445	37.932	-0.13	19.03	213.67	18.90	9	7/14/2014	44:46.2
3389	RSPe_2	-122.445	37.932	0.01	19.05	213.31	19.06	-122.445	37.932	-0.10	19.05	213.60	18.95	9	7/14/2014	44:46.1
3390	RSPe_2	-122.445	37.932	-0.03	19.08	213.31	19.05	-122.445	37.932	-0.10	19.08	213.55	18.98	9	7/14/2014	44:46.0
3391	RSPe_2	-122.445	37.932	0.01	19.10	213.29	19.11	-122.445	37.932	-0.10	19.10	213.49	19.01	9	7/14/2014	44:45.9
3392	RSPe_2	-122.445	37.932	-0.03	19.03	213.27	19.01	-122.445	37.932	-0.13	19.03	213.49	18.90	9	7/14/2014	44:45.8
3393	RSPe_2	-122.445	37.932	-0.03	19.07	213.27	19.04	-122.445	37.932	-0.10	19.07	213.45	18.97	9	7/14/2014	44:45.7
3394	RSPe_2	-122.445	37.932	-0.03	19.01	213.20	18.98	-122.445	37.932	-0.10	19.01	213.39	18.91	9	7/14/2014	44:45.6
3395	RSPe_2	-122.445	37.932	0.01	19.06	213.16	19.07	-122.445	37.932	-0.10	19.06	213.35	18.96	9	7/14/2014	44:45.5
3396	RSPe_2	-122.445	37.932	-0.03	18.97	213.03	18.95	-122.445	37.932	-0.10	18.97	213.24	18.88	9	7/14/2014	44:45.4
3397	RSPe_2	-122.445	37.932	0.01	18.94	212.92	18.95	-122.445	37.932	-0.10	18.94	213.14	18.85	9	7/14/2014	44:45.3
3398	RSPe_2	-122.445	37.932	-0.03	18.94	212.81	18.91	-122.445	37.932	-0.10	18.94	213.07	18.85	9	7/14/2014	44:45.2

3399	RSPe_2	-122.445	37.932	0.01	18.76	212.76	18.77	-122.445	37.932	-0.10	18.76	212.99	18.66	9	7/14/2014	44:45.1
3400	RSPe_2	-122.445	37.932	-0.03	18.76	212.66	18.74	-122.445	37.932	-0.10	18.76	212.96	18.67	9	7/14/2014	44:45.0
3401	RSPe_2	-122.445	37.932	0.01	18.63	212.68	18.63	-122.445	37.932	-0.05	18.63	213.05	18.58	9	7/14/2014	44:44.9
3402	RSPe_2	-122.445	37.932	-0.03	18.59	212.71	18.56	-122.445	37.932	-0.05	18.59	213.05	18.54	9	7/14/2014	44:44.8
3403	RSPe_2	-122.445	37.932	0.01	18.55	212.66	18.56	-122.445	37.932	-0.10	18.55	213.01	18.46	9	7/14/2014	44:44.7
3404	RSPe_2	-122.445	37.932	0.01	18.59	212.66	18.59	-122.445	37.932	-0.05	18.59	213.03	18.54	9	7/14/2014	44:44.6
3405	RSPe_2	-122.445	37.932	0.01	18.59	212.66	18.59	-122.445	37.932	-0.10	18.59	212.99	18.49	9	7/14/2014	44:44.5
3406	RSPe_2	-122.445	37.932	0.01	18.58	212.52	18.59	-122.445	37.932	-0.10	18.58	212.94	18.48	9	7/14/2014	44:44.4
3407	RSPe_2	-122.445	37.932	0.01	18.47	212.53	18.47	-122.445	37.932	-0.05	18.47	212.86	18.42	9	7/14/2014	44:44.3
3408	RSPe_2	-122.445	37.932	0.01	18.43	212.36	18.43	-122.445	37.932	-0.10	18.43	212.79	18.33	9	7/14/2014	44:44.2
3409	RSPe_2	-122.445	37.932	0.01	18.36	212.27	18.37	-122.445	37.932	-0.01	18.36	212.64	18.35	9	7/14/2014	44:44.1
3410	RSPe_2	-122.445	37.932	0.01	18.32	212.16	18.32	-122.445	37.932	-0.05	18.32	212.57	18.27	9	7/14/2014	44:44.0
3411	RSPe_2	-122.445	37.932	0.01	18.30	212.13	18.30	-122.445	37.932	-0.05	18.30	212.45	18.25	9	7/14/2014	44:43.9
3412	RSPe_2	-122.445	37.932	0.01	18.29	212.03	18.29	-122.445	37.932	-0.01	18.29	212.34	18.28	9	7/14/2014	44:43.8
3413	RSPe_2	-122.445	37.932	0.01	18.27	211.98	18.28	-122.445	37.932	-0.01	18.27	212.21	18.26	9	7/14/2014	44:43.7
3414	RSPe_2	-122.445	37.932	-0.03	18.24	211.95	18.21	-122.445	37.932	-0.01	18.24	212.14	18.23	9	7/14/2014	44:43.6
3415	RSPe_2	-122.445	37.932	0.01	18.30	211.85	18.31	-122.445	37.932	-0.05	18.30	212.03	18.26	9	7/14/2014	44:43.5
3416	RSPe_2	-122.445	37.932	0.01	18.26	211.79	18.26	-122.445	37.932	-0.05	18.26	211.96	18.21	9	7/14/2014	44:43.4
3417	RSPe_2	-122.445	37.932	0.06	18.25	211.71	18.30	-122.445	37.932	0.04	18.25	211.88	18.29	9	7/14/2014	44:43.3
3418	RSPe_2	-122.445	37.932	0.01	18.32	211.66	18.32	-122.445	37.932	0.04	18.32	211.87	18.36	9	7/14/2014	44:43.2
3419	RSPe_2	-122.445	37.932	0.01	18.30	211.63	18.30	-122.445	37.932	-0.05	18.30	211.76	18.25	9	7/14/2014	44:43.1
3420	RSPe_2	-122.445	37.932	0.01	18.29	211.53	18.29	-122.445	37.932	-0.01	18.29	211.75	18.28	9	7/14/2014	44:43.0
3421	RSPe_2	-122.445	37.932	0.06	18.31	211.49	18.37	-122.445	37.932	0.07	18.31	211.68	18.38	9	7/14/2014	44:42.9
3422	RSPe_2	-122.445	37.932	0.01	18.31	211.35	18.32	-122.445	37.932	0.04	18.31	211.66	18.35	9	7/14/2014	44:42.8
3423	RSPe_2	-122.445	37.932	0.06	18.30	211.29	18.36	-122.445	37.932	0.04	18.30	211.60	18.34	9	7/14/2014	44:42.7
3424	RSPe_2	-122.445	37.932	0.06	18.30	211.21	18.35	-122.445	37.932	-0.05	18.30	211.55	18.25	9	7/14/2014	44:42.6
3425	RSPe_2	-122.445	37.932	0.06	18.27	211.00	18.33	-122.445	37.932	-0.01	18.27	211.50	18.26	9	7/14/2014	44:42.5
3426	RSPe_2	-122.445	37.932	0.01	18.35	210.85	18.35	-122.445	37.932	-0.01	18.35	211.37	18.34	9	7/14/2014	44:42.4
3427	RSPe_2	-122.445	37.932	0.06	18.31	210.81	18.37	-122.445	37.932	-0.01	18.31	211.35	18.30	9	7/14/2014	44:42.3
3428	RSPe_2	-122.445	37.932	0.06	18.34	210.72	18.40	-122.445	37.932	-0.01	18.34	211.29	18.33	9	7/14/2014	44:42.2
3429	RSPe_2	-122.445	37.932	0.06	18.33	210.70	18.38	-122.445	37.932	0.04	18.33	211.21	18.37	9	7/14/2014	44:42.1
3430	RSPe_2	-122.445	37.932	0.06	18.37	210.63	18.43	-122.445	37.932	-0.01	18.37	211.05	18.36	9	7/14/2014	44:42.0
3431	RSPe_2	-122.445	37.932	0.06	18.35	210.57	18.40	-122.445	37.932	-0.01	18.35	210.90	18.34	9	7/14/2014	44:41.9

3432	RSPe_2	-122.445	37.932	0.06	18.35	210.57	18.40	-122.445	37.932	-0.05	18.35	210.83	18.30	9	7/14/2014	44:41.8
3433	RSPe_2	-122.445	37.932	0.06	18.34	210.48	18.40	-122.445	37.932	-0.01	18.34	210.72	18.33	9	7/14/2014	44:41.7
3434	RSPe_2	-122.445	37.932	0.06	18.33	210.38	18.39	-122.445	37.932	-0.01	18.33	210.66	18.32	9	7/14/2014	44:41.6
3435	RSPe_2	-122.445	37.932	0.06	18.35	210.26	18.40	-122.445	37.932	-0.01	18.35	210.57	18.34	9	7/14/2014	44:41.5
3436	RSPe_2	-122.445	37.932	0.06	18.32	210.15	18.37	-122.445	37.932	-0.05	18.32	210.52	18.27	9	7/14/2014	44:41.4
3437	RSPe_2	-122.445	37.932	0.09	18.38	209.98	18.47	-122.445	37.932	0.04	18.38	210.47	18.42	9	7/14/2014	44:41.3
3438	RSPe_2	-122.445	37.932	0.06	18.40	209.91	18.46	-122.445	37.932	-0.01	18.40	210.41	18.39	9	7/14/2014	44:41.2
3439	RSPe_2	-122.445	37.932	0.09	18.49	209.82	18.58	-122.445	37.932	-0.01	18.49	210.35	18.48	9	7/14/2014	44:41.1
3440	RSPe_2	-122.445	37.932	0.06	18.52	209.68	18.58	-122.445	37.932	0.04	18.52	210.24	18.56	9	7/14/2014	44:41.0
3441	RSPe_2	-122.445	37.932	0.09	18.55	209.70	18.64	-122.445	37.932	-0.01	18.55	210.23	18.54	9	7/14/2014	44:40.9
3442	RSPe_2	-122.445	37.932	0.06	18.58	209.67	18.64	-122.445	37.932	-0.05	18.58	210.17	18.53	9	7/14/2014	44:40.8
3443	RSPe_2	-122.445	37.932	0.09	18.66	209.72	18.76	-122.445	37.932	-0.01	18.66	210.16	18.65	9	7/14/2014	44:40.7
3444	RSPe_2	-122.445	37.932	0.06	18.70	209.65	18.75	-122.445	37.932	-0.05	18.70	209.99	18.65	9	7/14/2014	44:40.6
3445	RSPe_2	-122.445	37.932	0.09	18.67	209.69	18.77	-122.445	37.932	-0.01	18.67	209.94	18.66	9	7/14/2014	44:40.5
3446	RSPe_2	-122.445	37.932	0.06	18.69	209.57	18.75	-122.445	37.932	-0.01	18.69	209.82	18.68	9	7/14/2014	44:40.4
3447	RSPe_2	-122.445	37.932	0.06	18.66	209.57	18.72	-122.445	37.932	-0.01	18.66	209.72	18.65	9	7/14/2014	44:40.3
3448	RSPe_2	-122.445	37.932	0.09	18.66	209.45	18.75	-122.445	37.932	-0.01	18.66	209.54	18.65	9	7/14/2014	44:40.2
3449	RSPe_2	-122.445	37.932	0.09	18.65	209.35	18.74	-122.445	37.932	0.04	18.65	209.46	18.69	9	7/14/2014	44:40.1
3450	RSPe_2	-122.445	37.932	0.09	18.66	209.21	18.75	-122.445	37.932	-0.01	18.66	209.32	18.65	9	7/14/2014	44:40.0
3451	RSPe_2	-122.445	37.932	0.09	18.63	209.13	18.72	-122.445	37.932	0.04	18.63	209.21	18.67	9	7/14/2014	44:39.9
3452	RSPe_2	-122.445	37.932	0.09	18.66	209.00	18.75	-122.445	37.932	-0.01	18.66	209.15	18.65	9	7/14/2014	44:39.8
3453	RSPe_2	-122.445	37.932	0.09	18.58	208.91	18.67	-122.445	37.932	0.04	18.58	209.10	18.62	9	7/14/2014	44:39.7
3454	RSPe_2	-122.445	37.932	0.09	18.57	208.83	18.66	-122.445	37.932	0.04	18.57	209.04	18.60	9	7/14/2014	44:39.6
3455	RSPe_2	-122.445	37.932	0.14	18.52	208.78	18.67	-122.445	37.932	0.04	18.52	209.02	18.56	9	7/14/2014	44:39.5
3456	RSPe_2	-122.445	37.932	0.09	18.57	208.73	18.66	-122.445	37.932	0.04	18.57	209.00	18.60	9	7/14/2014	44:39.4
3457	RSPe_2	-122.445	37.932	0.09	18.58	208.65	18.67	-122.445	37.932	0.04	18.58	208.92	18.62	9	7/14/2014	44:39.3
3458	RSPe_2	-122.445	37.932	0.09	18.54	208.63	18.63	-122.445	37.932	0.04	18.54	208.89	18.58	9	7/14/2014	44:39.2
3459	RSPe_2	-122.445	37.932	0.14	18.54	208.56	18.69	-122.445	37.932	0.07	18.54	208.84	18.62	9	7/14/2014	44:39.1
3460	RSPe_2	-122.445	37.932	0.09	18.53	208.52	18.63	-122.445	37.932	0.07	18.53	208.74	18.61	9	7/14/2014	44:39.0
3461	RSPe_2	-122.445	37.932	0.14	18.52	208.44	18.66	-122.445	37.932	0.07	18.52	208.65	18.59	9	7/14/2014	44:38.9
3462	RSPe_2	-122.445	37.932	0.09	18.50	208.39	18.60	-122.445	37.932	0.13	18.50	208.58	18.63	9	7/14/2014	44:38.8
3463	RSPe_2	-122.445	37.932	0.14	18.51	208.28	18.65	-122.445	37.932	0.13	18.51	208.48	18.63	9	7/14/2014	44:38.7
3464	RSPe_2	-122.445	37.932	0.09	18.47	208.21	18.57	-122.445	37.932	0.07	18.47	208.37	18.55	9	7/14/2014	44:38.6

3465	RSPe_2	-122.445	37.932	0.09	18.47	208.08	18.56	-122.445	37.932	0.07	18.47	208.27	18.54	9	7/14/2014	44:38.5
3466	RSPe_2	-122.445	37.932	0.09	18.43	207.97	18.53	-122.445	37.932	0.07	18.43	208.14	18.51	9	7/14/2014	44:38.4
3467	RSPe_2	-122.445	37.932	0.14	18.46	207.86	18.60	-122.445	37.932	0.13	18.46	208.01	18.58	9	7/14/2014	44:38.3
3468	RSPe_2	-122.445	37.932	0.14	18.41	207.67	18.56	-122.445	37.932	0.07	18.41	207.93	18.49	9	7/14/2014	44:38.2
3469	RSPe_2	-122.445	37.932	0.14	18.40	207.58	18.54	-122.445	37.932	0.13	18.40	207.78	18.52	9	7/14/2014	44:38.1
3470	RSPe_2	-122.445	37.932	0.09	18.43	207.47	18.52	-122.445	37.932	0.07	18.43	207.64	18.50	9	7/14/2014	44:38.0
3471	RSPe_2	-122.445	37.932	0.14	18.38	207.30	18.52	-122.445	37.932	0.07	18.38	207.56	18.45	9	7/14/2014	44:37.9
3472	RSPe_2	-122.445	37.932	0.14	18.42	207.15	18.56	-122.445	37.932	0.07	18.42	207.40	18.49	9	7/14/2014	44:37.8
3473	RSPe_2	-122.445	37.932	0.14	18.38	207.03	18.52	-122.445	37.932	0.13	18.38	207.38	18.50	9	7/14/2014	44:37.7
3474	RSPe_2	-122.445	37.932	0.09	18.41	206.86	18.51	-122.445	37.932	0.07	18.41	207.25	18.49	9	7/14/2014	44:37.6
3475	RSPe_2	-122.445	37.932	0.14	18.39	206.75	18.53	-122.445	37.932	0.13	18.39	207.22	18.51	9	7/14/2014	44:37.5
3476	RSPe_2	-122.445	37.932	0.14	18.39	206.59	18.53	-122.445	37.932	0.04	18.39	207.07	18.43	9	7/14/2014	44:37.4
3477	RSPe_2	-122.445	37.932	0.14	18.40	206.45	18.55	-122.445	37.932	0.13	18.40	206.95	18.53	9	7/14/2014	44:37.3
3478	RSPe_2	-122.445	37.932	0.09	18.42	206.33	18.51	-122.445	37.932	0.07	18.42	206.87	18.49	9	7/14/2014	44:37.2
3479	RSPe_2	-122.445	37.932	0.14	18.45	206.23	18.59	-122.445	37.932	0.07	18.45	206.73	18.52	9	7/14/2014	44:37.1
3480	RSPe_2	-122.445	37.932	0.09	18.40	206.09	18.50	-122.445	37.932	0.04	18.40	206.57	18.44	9	7/14/2014	44:37.0
3481	RSPe_2	-122.445	37.932	0.14	18.43	205.91	18.57	-122.445	37.932	0.07	18.43	206.33	18.50	9	7/14/2014	44:36.9
3482	RSPe_2	-122.445	37.932	0.14	18.48	205.89	18.63	-122.445	37.932	0.04	18.48	206.26	18.52	9	7/14/2014	44:36.8
3483	RSPe_2	-122.445	37.932	0.14	18.42	205.86	18.56	-122.445	37.932	0.07	18.42	206.07	18.49	9	7/14/2014	44:36.7
3484	RSPe_2	-122.445	37.932	0.14	18.43	205.87	18.58	-122.445	37.932	0.07	18.43	205.96	18.51	9	7/14/2014	44:36.6
3485	RSPe_2	-122.445	37.932	0.18	18.52	205.74	18.70	-122.445	37.932	0.13	18.52	205.86	18.64	9	7/14/2014	44:36.5
3486	RSPe_2	-122.445	37.932	0.14	18.48	205.73	18.63	-122.445	37.932	0.04	18.48	205.74	18.52	9	7/14/2014	44:36.4
3487	RSPe_2	-122.445	37.932	0.18	18.41	205.54	18.59	-122.445	37.932	0.07	18.41	205.56	18.49	9	7/14/2014	44:36.3
3488	RSPe_2	-122.445	37.932	0.14	18.44	205.43	18.58	-122.445	37.932	0.13	18.44	205.44	18.57	9	7/14/2014	44:36.2
3489	RSPe_2	-122.445	37.932	0.14	18.47	205.31	18.61	-122.445	37.932	0.13	18.47	205.38	18.59	9	7/14/2014	44:36.1
3490	RSPe_2	-122.445	37.932	0.14	18.40	205.13	18.54	-122.445	37.932	0.07	18.40	205.26	18.47	9	7/14/2014	44:36.0
3491	RSPe_2	-122.445	37.932	0.14	18.45	205.04	18.59	-122.445	37.932	0.07	18.45	205.21	18.52	9	7/14/2014	44:35.9
3492	RSPe_2	-122.445	37.932	0.09	18.44	204.91	18.53	-122.445	37.932	0.07	18.44	205.18	18.51	9	7/14/2014	44:35.8
3493	RSPe_2	-122.445	37.932	0.14	18.45	204.89	18.59	-122.445	37.932	0.13	18.45	205.16	18.57	9	7/14/2014	44:35.7
3494	RSPe_2	-122.445	37.932	0.14	18.53	204.76	18.68	-122.445	37.932	0.07	18.53	205.07	18.61	9	7/14/2014	44:35.6
3495	RSPe_2	-122.445	37.932	0.14	18.57	204.63	18.71	-122.445	37.932	0.07	18.57	204.89	18.64	9	7/14/2014	44:35.5
3496	RSPe_2	-122.445	37.932	0.14	18.58	204.50	18.72	-122.445	37.932	0.07	18.58	204.76	18.65	9	7/14/2014	44:35.4
3497	RSPe_2	-122.445	37.932	0.14	18.67	204.34	18.82	-122.445	37.932	0.13	18.67	204.54	18.80	9	7/14/2014	44:35.3

3498	RSPe_2	-122.445	37.932	0.14	18.60	204.19	18.75	-122.445	37.932	0.07	18.60	204.44	18.68	9	7/14/2014	44:35.2
3499	RSPe_2	-122.445	37.932	0.14	18.67	204.13	18.82	-122.445	37.932	0.07	18.67	204.32	18.75	9	7/14/2014	44:35.1
3500	RSPe_2	-122.445	37.932	0.09	18.64	204.03	18.74	-122.445	37.932	0.07	18.64	204.18	18.72	9	7/14/2014	44:35.0
3501	RSPe_2	-122.445	37.932	0.14	18.63	203.96	18.77	-122.445	37.932	0.04	18.63	204.11	18.67	9	7/14/2014	44:34.9
3502	RSPe_2	-122.445	37.932	0.09	18.64	203.85	18.74	-122.445	37.932	0.04	18.64	204.01	18.68	9	7/14/2014	44:34.8
3503	RSPe_2	-122.445	37.932	0.14	18.61	203.75	18.76	-122.445	37.932	0.04	18.61	203.95	18.65	9	7/14/2014	44:34.7
3504	RSPe_2	-122.445	37.932	0.09	18.65	203.63	18.74	-122.445	37.932	0.04	18.65	203.86	18.69	9	7/14/2014	44:34.6
3505	RSPe_2	-122.445	37.932	0.14	18.61	203.51	18.76	-122.445	37.932	0.04	18.61	203.77	18.65	9	7/14/2014	44:34.5
3506	RSPe_2	-122.445	37.932	0.09	18.72	203.41	18.81	-122.445	37.932	-0.01	18.72	203.65	18.71	9	7/14/2014	44:34.4
3507	RSPe_2	-122.445	37.932	0.14	18.61	203.25	18.76	-122.445	37.932	0.04	18.61	203.57	18.65	9	7/14/2014	44:34.3
3508	RSPe_2	-122.445	37.932	0.14	18.57	203.10	18.72	-122.445	37.932	0.04	18.57	203.38	18.61	9	7/14/2014	44:34.2
3509	RSPe_2	-122.445	37.932	0.14	18.66	202.99	18.80	-122.445	37.932	0.07	18.66	203.31	18.73	9	7/14/2014	44:34.1
3510	RSPe_2	-122.445	37.932	0.09	18.56	202.94	18.65	-122.445	37.932	0.04	18.56	203.19	18.60	9	7/14/2014	44:34.0
3511	RSPe_2	-122.445	37.932	0.14	18.52	202.88	18.66	-122.445	37.932	0.04	18.52	203.11	18.56	9	7/14/2014	44:33.9
3512	RSPe_2	-122.445	37.932	0.14	18.59	202.77	18.74	-122.445	37.932	0.07	18.59	202.93	18.67	9	7/14/2014	44:33.8
3513	RSPe_2	-122.445	37.932	0.14	18.52	202.71	18.67	-122.445	37.932	0.04	18.52	202.79	18.56	9	7/14/2014	44:33.7
3514	RSPe_2	-122.445	37.932	0.09	18.46	202.60	18.55	-122.445	37.932	0.04	18.46	202.68	18.50	9	7/14/2014	44:33.6
3515	RSPe_2	-122.445	37.932	0.14	18.37	202.51	18.51	-122.445	37.932	0.04	18.37	202.57	18.41	9	7/14/2014	44:33.5
3516	RSPe_2	-122.445	37.932	0.09	18.33	202.35	18.42	-122.445	37.932	0.04	18.33	202.40	18.37	9	7/14/2014	44:33.4
3517	RSPe_2	-122.445	37.932	0.09	18.24	202.29	18.33	-122.445	37.932	0.04	18.24	202.29	18.28	9	7/14/2014	44:33.3
3518	RSPe_2	-122.445	37.932	0.09	18.23	202.16	18.32	-122.445	37.932	-0.01	18.23	202.20	18.22	9	7/14/2014	44:33.2
3519	RSPe_2	-122.445	37.932	0.14	18.33	202.11	18.48	-122.445	37.932	0.04	18.33	202.11	18.37	9	7/14/2014	44:33.1
3520	RSPe_2	-122.445	37.932	0.09	18.22	201.94	18.31	-122.445	37.932	-0.01	18.22	201.93	18.21	9	7/14/2014	44:33.0
3521	RSPe_2	-122.445	37.932	0.14	18.19	201.83	18.33	-122.445	37.932	0.04	18.19	201.81	18.23	9	7/14/2014	44:32.9
3522	RSPe_2	-122.445	37.932	0.09	18.16	201.68	18.25	-122.445	37.932	-0.01	18.16	201.67	18.15	9	7/14/2014	44:32.8
3523	RSPe_2	-122.445	37.932	0.09	18.02	201.54	18.11	-122.445	37.932	0.04	18.02	201.54	18.06	9	7/14/2014	44:32.7
3524	RSPe_2	-122.445	37.932	0.09	18.01	201.33	18.10	-122.445	37.932	-0.01	18.01	201.43	18.00	9	7/14/2014	44:32.6
3525	RSPe_2	-122.445	37.932	0.09	17.99	201.15	18.09	-122.445	37.932	0.04	17.99	201.29	18.03	9	7/14/2014	44:32.5
3526	RSPe_2	-122.445	37.932	0.09	17.97	200.91	18.07	-122.445	37.932	0.04	17.97	201.10	18.01	9	7/14/2014	44:32.4
3527	RSPe_2	-122.445	37.932	0.14	17.99	200.72	18.14	-122.445	37.932	0.07	17.99	200.95	18.07	9	7/14/2014	44:32.3
3528	RSPe_2	-122.445	37.932	0.09	17.96	200.49	18.06	-122.445	37.932	0.04	17.96	200.75	18.00	9	7/14/2014	44:32.2
3529	RSPe_2	-122.445	37.932	0.14	17.97	200.32	18.12	-122.445	37.932	0.07	17.97	200.56	18.05	9	7/14/2014	44:32.1
3530	RSPe_2	-122.445	37.932	0.09	17.99	200.16	18.09	-122.445	37.932	0.04	17.99	200.44	18.03	9	7/14/2014	44:32.0

3531	RSPe_2	-122.445	37.932	0.14	17.99	200.05	18.14	-122.445	37.932	0.04	17.99	200.24	18.03	9	7/14/2014	44:31.9
3532	RSPe_2	-122.445	37.932	0.09	18.00	199.95	18.09	-122.445	37.932	0.04	18.00	200.07	18.04	9	7/14/2014	44:31.8
3533	RSPe_2	-122.445	37.932	0.14	18.03	199.78	18.18	-122.445	37.932	0.07	18.03	199.90	18.11	9	7/14/2014	44:31.7
3534	RSPe_2	-122.445	37.932	0.09	17.97	199.64	18.07	-122.445	37.932	0.04	17.97	199.74	18.01	9	7/14/2014	44:31.6
3535	RSPe_2	-122.445	37.932	0.14	17.99	199.44	18.13	-122.445	37.932	0.04	17.99	199.53	18.03	9	7/14/2014	44:31.5
3536	RSPe_2	-122.445	37.932	0.09	17.96	199.29	18.06	-122.445	37.932	0.04	17.96	199.37	18.00	9	7/14/2014	44:31.4
3537	RSPe_2	-122.445	37.932	0.14	17.97	199.07	18.12	-122.445	37.932	0.04	17.97	199.18	18.01	9	7/14/2014	44:31.3
3538	RSPe_2	-122.445	37.932	0.14	17.95	198.94	18.09	-122.445	37.932	-0.01	17.95	199.04	17.94	9	7/14/2014	44:31.2
3539	RSPe_2	-122.445	37.932	0.18	17.94	198.74	18.12	-122.445	37.932	0.07	17.94	198.85	18.01	9	7/14/2014	44:31.1
3540	RSPe_2	-122.445	37.932	0.14	17.98	198.55	18.12	-122.445	37.932	0.07	17.98	198.70	18.05	9	7/14/2014	44:31.0
3541	RSPe_2	-122.445	37.932	0.18	17.94	198.42	18.12	-122.445	37.932	0.07	17.94	198.62	18.01	9	7/14/2014	44:30.9
3542	RSPe_2	-122.445	37.932	0.14	17.97	198.27	18.12	-122.445	37.932	0.04	17.97	198.46	18.01	9	7/14/2014	44:30.8
3543	RSPe_2	-122.445	37.932	0.18	17.93	198.22	18.11	-122.445	37.932	0.13	17.93	198.36	18.06	9	7/14/2014	44:30.7
3544	RSPe_2	-122.445	37.932	0.14	17.93	198.09	18.07	-122.445	37.932	0.13	17.93	198.26	18.06	9	7/14/2014	44:30.6
3545	RSPe_2	-122.445	37.932	0.18	17.94	197.95	18.12	-122.445	37.932	0.16	17.94	198.15	18.10	9	7/14/2014	44:30.5
3546	RSPe_2	-122.445	37.932	0.18	17.90	197.89	18.08	-122.445	37.932	0.07	17.90	197.99	17.98	9	7/14/2014	44:30.4
3547	RSPe_2	-122.445	37.932	0.21	17.89	197.66	18.10	-122.445	37.932	0.13	17.89	197.80	18.02	9	7/14/2014	44:30.3
3548	RSPe_2	-122.445	37.932	0.18	17.89	197.49	18.06	-122.445	37.932	0.13	17.89	197.57	18.01	9	7/14/2014	44:30.2
3549	RSPe_2	-122.445	37.932	0.21	17.88	197.30	18.09	-122.445	37.932	0.16	17.88	197.42	18.04	9	7/14/2014	44:30.1
3550	RSPe_2	-122.445	37.932	0.18	17.86	197.12	18.04	-122.445	37.932	0.16	17.86	197.26	18.02	9	7/14/2014	44:30.0
3551	RSPe_2	-122.445	37.932	0.21	17.89	196.97	18.10	-122.445	37.932	0.19	17.89	197.10	18.08	9	7/14/2014	44:29.9
3552	RSPe_2	-122.445	37.932	0.21	17.92	196.90	18.14	-122.445	37.932	0.13	17.92	196.99	18.05	9	7/14/2014	44:29.8
3553	RSPe_2	-122.445	37.932	0.21	17.88	196.82	18.09	-122.445	37.932	0.16	17.88	196.95	18.04	9	7/14/2014	44:29.7
3554	RSPe_2	-122.445	37.932	0.21	17.89	196.71	18.10	-122.445	37.932	0.13	17.89	196.83	18.02	9	7/14/2014	44:29.6
3555	RSPe_2	-122.445	37.932	0.21	17.93	196.56	18.14	-122.445	37.932	0.16	17.93	196.71	18.09	9	7/14/2014	44:29.5
3556	RSPe_2	-122.445	37.932	0.21	17.93	196.44	18.14	-122.445	37.932	0.13	17.93	196.59	18.06	9	7/14/2014	44:29.4
3557	RSPe_2	-122.445	37.932	0.21	17.93	196.26	18.14	-122.445	37.932	0.16	17.93	196.46	18.09	9	7/14/2014	44:29.3
3558	RSPe_2	-122.445	37.932	0.18	17.99	196.14	18.17	-122.445	37.932	0.13	17.99	196.32	18.12	9	7/14/2014	44:29.2
3559	RSPe_2	-122.445	37.932	0.21	17.99	195.99	18.20	-122.445	37.932	0.16	17.99	196.13	18.14	9	7/14/2014	44:29.1
3560	RSPe_2	-122.445	37.932	0.21	18.02	195.92	18.23	-122.445	37.932	0.16	18.02	196.00	18.18	9	7/14/2014	44:29.0
3561	RSPe_2	-122.445	37.932	0.21	18.02	195.74	18.23	-122.445	37.932	0.16	18.02	195.82	18.18	9	7/14/2014	44:28.9
3562	RSPe_2	-122.445	37.932	0.21	18.04	195.58	18.25	-122.445	37.932	0.16	18.04	195.60	18.20	9	7/14/2014	44:28.8
3563	RSPe_2	-122.445	37.932	0.21	18.06	195.55	18.28	-122.445	37.932	0.16	18.06	195.56	18.22	9	7/14/2014	44:28.7

3564	RSPe_2	-122.445	37.932	0.21	18.09	195.44	18.30	-122.445	37.932	0.16	18.09	195.43	18.25	9	7/14/2014	44:28.6
3565	RSPe_2	-122.445	37.932	0.21	18.11	195.28	18.32	-122.445	37.932	0.16	18.11	195.33	18.27	9	7/14/2014	44:28.5
3566	RSPe_2	-122.445	37.932	0.21	18.12	195.18	18.34	-122.445	37.932	0.16	18.12	195.25	18.28	9	7/14/2014	44:28.4
3567	RSPe_2	-122.445	37.932	0.21	18.14	195.06	18.35	-122.445	37.932	0.19	18.14	195.15	18.33	9	7/14/2014	44:28.3
3568	RSPe_2	-122.445	37.932	0.21	18.16	194.89	18.37	-122.445	37.932	0.16	18.16	195.04	18.32	9	7/14/2014	44:28.2
3569	RSPe_2	-122.445	37.932	0.21	18.19	194.80	18.40	-122.445	37.932	0.16	18.19	194.95	18.35	9	7/14/2014	44:28.1
3570	RSPe_2	-122.445	37.932	0.21	18.21	194.68	18.42	-122.445	37.932	0.16	18.21	194.83	18.37	9	7/14/2014	44:28.0
3571	RSPe_2	-122.445	37.932	0.21	18.24	194.52	18.45	-122.445	37.932	0.19	18.24	194.73	18.43	9	7/14/2014	44:27.9
3572	RSPe_2	-122.445	37.932	0.18	18.24	194.43	18.41	-122.445	37.932	0.16	18.24	194.60	18.39	9	7/14/2014	44:27.8
3573	RSPe_2	-122.445	37.932	0.26	18.26	194.25	18.53	-122.445	37.932	0.16	18.26	194.47	18.42	9	7/14/2014	44:27.7
3574	RSPe_2	-122.445	37.932	0.26	18.27	194.15	18.54	-122.445	37.932	0.19	18.27	194.34	18.46	9	7/14/2014	44:27.6
3575	RSPe_2	-122.445	37.932	0.26	18.30	194.01	18.57	-122.445	37.932	0.19	18.30	194.21	18.50	9	7/14/2014	44:27.5
3576	RSPe_2	-122.445	37.932	0.21	18.33	193.91	18.54	-122.445	37.932	0.24	18.33	194.08	18.57	9	7/14/2014	44:27.4
3577	RSPe_2	-122.445	37.932	0.26	18.33	193.82	18.59	-122.445	37.932	0.24	18.33	193.97	18.57	9	7/14/2014	44:27.3
3578	RSPe_2	-122.445	37.932	0.21	18.33	193.75	18.55	-122.445	37.932	0.16	18.33	193.77	18.49	9	7/14/2014	44:27.2
3579	RSPe_2	-122.445	37.932	0.26	18.39	193.56	18.65	-122.445	37.932	0.19	18.39	193.61	18.58	9	7/14/2014	44:27.1
3580	RSPe_2	-122.445	37.932	0.26	18.39	193.45	18.65	-122.445	37.932	0.19	18.39	193.46	18.58	9	7/14/2014	44:27.0
3581	RSPe_2	-122.445	37.932	0.26	18.37	193.34	18.63	-122.445	37.932	0.24	18.37	193.27	18.61	9	7/14/2014	44:26.9
3582	RSPe_2	-122.445	37.932	0.26	18.43	193.19	18.69	-122.445	37.932	0.19	18.43	193.09	18.62	9	7/14/2014	44:26.8
3583	RSPe_2	-122.445	37.932	0.26	18.45	193.05	18.71	-122.445	37.932	0.24	18.45	192.99	18.69	9	7/14/2014	44:26.7
3584	RSPe_2	-122.445	37.932	0.26	18.48	192.95	18.75	-122.445	37.932	0.19	18.48	192.90	18.67	9	7/14/2014	44:26.6
3585	RSPe_2	-122.445	37.932	0.26	18.50	192.79	18.77	-122.445	37.932	0.24	18.50	192.77	18.75	9	7/14/2014	44:26.5
3586	RSPe_2	-122.445	37.932	0.26	18.51	192.68	18.77	-122.445	37.932	0.24	18.51	192.75	18.75	9	7/14/2014	44:26.4
3587	RSPe_2	-122.445	37.932	0.30	18.53	192.59	18.83	-122.445	37.932	0.24	18.53	192.70	18.78	9	7/14/2014	44:26.3
3588	RSPe_2	-122.445	37.932	0.26	18.54	192.40	18.81	-122.445	37.932	0.19	18.54	192.61	18.73	9	7/14/2014	44:26.2
3589	RSPe_2	-122.445	37.932	0.30	18.58	192.26	18.88	-122.445	37.932	0.19	18.58	192.52	18.77	9	7/14/2014	44:26.1
3590	RSPe_2	-122.445	37.932	0.26	18.57	192.16	18.83	-122.445	37.932	0.19	18.57	192.44	18.76	9	7/14/2014	44:26.0
3591	RSPe_2	-122.445	37.932	0.30	18.57	192.00	18.86	-122.445	37.932	0.19	18.57	192.27	18.76	9	7/14/2014	44:25.9
3592	RSPe_2	-122.445	37.932	0.26	18.57	191.84	18.83	-122.445	37.932	0.19	18.57	192.02	18.76	9	7/14/2014	44:25.8
3593	RSPe_2	-122.445	37.932	0.30	18.57	191.68	18.86	-122.445	37.932	0.24	18.57	191.83	18.81	9	7/14/2014	44:25.7
3594	RSPe_2	-122.445	37.932	0.26	18.58	191.54	18.84	-122.445	37.932	0.19	18.58	191.65	18.77	9	7/14/2014	44:25.6
3595	RSPe_2	-122.445	37.932	0.30	18.60	191.37	18.90	-122.445	37.932	0.24	18.60	191.38	18.85	9	7/14/2014	44:25.5
3596	RSPe_2	-122.445	37.932	0.26	18.59	191.29	18.85	-122.445	37.932	0.19	18.59	191.25	18.78	9	7/14/2014	44:25.4

3597	RSPe_2	-122.445	37.932	0.30	18.60	191.10	18.90	-122.445	37.932	0.24	18.60	191.10	18.85	9	7/14/2014 44:25.3
3598	RSPe_2	-122.445	37.932	0.26	18.62	190.97	18.89	-122.445	37.932	0.24	18.62	190.97	18.86	9	7/14/2014 44:25.2
3599	RSPe_2	-122.445	37.932	0.30	18.63	190.86	18.93	-122.445	37.932	0.24	18.63	190.84	18.88	9	7/14/2014 44:25.1
3600	RSPe_2	-122.445	37.932	0.30	18.65	190.76	18.95	-122.445	37.932	0.24	18.65	190.71	18.89	9	7/14/2014 44:25.0
3601	RSPe_2	-122.445	37.932	0.30	18.66	190.60	18.96	-122.445	37.932	0.24	18.66	190.61	18.91	9	7/14/2014 44:24.9
3602	RSPe_2	-122.445	37.932	0.26	18.59	190.49	18.86	-122.445	37.932	0.19	18.59	190.49	18.79	9	7/14/2014 44:24.8
3603	RSPe_2	-122.445	37.932	0.30	18.57	190.35	18.87	-122.445	37.932	0.24	18.57	190.35	18.82	9	7/14/2014 44:24.7
3604	RSPe_2	-122.445	37.932	0.30	18.58	190.21	18.88	-122.445	37.932	0.24	18.58	190.28	18.82	9	7/14/2014 44:24.6
3605	RSPe_2	-122.445	37.932	0.30	18.59	190.14	18.89	-122.445	37.932	0.24	18.59	190.16	18.83	9	7/14/2014 44:24.5
3606	RSPe_2	-122.445	37.932	0.30	18.49	190.06	18.79	-122.445	37.932	0.28	18.49	190.03	18.76	9	7/14/2014 44:24.4
3607	RSPe_2	-122.445	37.932	0.30	18.47	189.87	18.76	-122.445	37.932	0.28	18.47	189.86	18.74	9	7/14/2014 44:24.3
3608	RSPe_2	-122.445	37.932	0.30	18.43	189.80	18.73	-122.445	37.932	0.24	18.43	189.76	18.68	9	7/14/2014 44:24.2
3609	RSPe_2	-122.445	37.932	0.30	18.36	189.62	18.66	-122.445	37.932	0.28	18.36	189.55	18.64	9	7/14/2014 44:24.1
3610	RSPe_2	-122.445	37.932	0.26	18.34	189.46	18.61	-122.445	37.932	0.28	18.34	189.45	18.62	9	7/14/2014 44:24.0
3611	RSPe_2	-122.445	37.932	0.30	18.21	189.31	18.51	-122.445	37.932	0.33	18.21	189.29	18.54	9	7/14/2014 44:23.9
3612	RSPe_2	-122.445	37.932	0.30	18.18	189.11	18.48	-122.445	37.932	0.28	18.18	189.15	18.46	9	7/14/2014 44:23.8
3613	RSPe_2	-122.445	37.932	0.30	18.18	188.94	18.48	-122.445	37.932	0.33	18.18	189.00	18.51	9	7/14/2014 44:23.7
3614	RSPe_2	-122.445	37.932	0.30	18.17	188.81	18.47	-122.445	37.932	0.28	18.17	188.95	18.45	9	7/14/2014 44:23.6
3615	RSPe_2	-122.445	37.932	0.35	18.16	188.70	18.51	-122.445	37.932	0.28	18.16	188.80	18.43	9	7/14/2014 44:23.5
3616	RSPe_2	-122.445	37.932	0.30	18.10	188.56	18.40	-122.445	37.932	0.33	18.10	188.67	18.43	9	7/14/2014 44:23.4
3617	RSPe_2	-122.445	37.932	0.30	18.08	188.41	18.38	-122.445	37.932	0.33	18.08	188.56	18.41	9	7/14/2014 44:23.3
3618	RSPe_2	-122.445	37.932	0.30	18.05	188.29	18.35	-122.445	37.932	0.28	18.05	188.43	18.33	9	7/14/2014 44:23.2
3619	RSPe_2	-122.445	37.932	0.35	18.07	188.20	18.42	-122.445	37.932	0.33	18.07	188.31	18.40	9	7/14/2014 44:23.1
3620	RSPe_2	-122.445	37.932	0.30	18.05	188.11	18.35	-122.445	37.932	0.28	18.05	188.18	18.33	9	7/14/2014 44:23.0
3621	RSPe_2	-122.445	37.932	0.35	18.06	187.95	18.41	-122.445	37.932	0.28	18.06	187.94	18.34	9	7/14/2014 44:22.9
3622	RSPe_2	-122.445	37.932	0.30	18.02	187.89	18.32	-122.445	37.932	0.33	18.02	187.85	18.35	9	7/14/2014 44:22.8
3623	RSPe_2	-122.445	37.932	0.35	18.05	187.82	18.40	-122.445	37.932	0.33	18.05	187.71	18.38	9	7/14/2014 44:22.7
3624	RSPe_2	-122.445	37.932	0.30	18.04	187.74	18.34	-122.445	37.932	0.28	18.04	187.62	18.32	9	7/14/2014 44:22.6
3625	RSPe_2	-122.445	37.932	0.35	17.99	187.61	18.34	-122.445	37.932	0.33	17.99	187.39	18.32	9	7/14/2014 44:22.5
3626	RSPe_2	-122.445	37.932	0.30	18.00	187.42	18.30	-122.445	37.932	0.28	18.00	187.23	18.28	9	7/14/2014 44:22.4
3627	RSPe_2	-122.445	37.932	0.35	18.05	187.26	18.40	-122.445	37.932	0.33	18.05	187.09	18.38	9	7/14/2014 44:22.3
3628	RSPe_2	-122.445	37.932	0.30	17.93	187.08	18.23	-122.445	37.932	0.33	17.93	186.97	18.26	9	7/14/2014 44:22.2
3629	RSPe_2	-122.445	37.932	0.35	17.95	186.96	18.30	-122.445	37.932	0.41	17.95	186.90	18.36	9	7/14/2014 44:22.1

3630	RSPe_2	-122.445	37.932	0.35	17.92	186.78	18.27	-122.445	37.932	0.36	17.92	186.81	18.29	9	7/14/2014	44:22.0
3631	RSPe_2	-122.445	37.932	0.35	18.02	186.69	18.37	-122.445	37.932	0.33	18.02	186.80	18.35	9	7/14/2014	44:21.9
3632	RSPe_2	-122.445	37.932	0.30	17.99	186.53	18.28	-122.445	37.932	0.28	17.99	186.67	18.26	9	7/14/2014	44:21.8
3633	RSPe_2	-122.445	37.932	0.35	18.08	186.45	18.43	-122.445	37.932	0.33	18.08	186.63	18.41	9	7/14/2014	44:21.7
3634	RSPe_2	-122.445	37.932	0.30	18.16	186.33	18.46	-122.445	37.932	0.28	18.16	186.49	18.43	9	7/14/2014	44:21.6
3635	RSPe_2	-122.445	37.932	0.30	18.11	186.20	18.41	-122.445	37.932	0.33	18.11	186.35	18.44	9	7/14/2014	44:21.5
3636	RSPe_2	-122.445	37.932	0.30	18.26	186.13	18.56	-122.445	37.932	0.33	18.26	186.20	18.59	9	7/14/2014	44:21.4
3637	RSPe_2	-122.445	37.932	0.30	18.33	186.05	18.63	-122.445	37.932	0.28	18.33	186.01	18.60	9	7/14/2014	44:21.3
3638	RSPe_2	-122.445	37.932	0.30	18.26	186.01	18.56	-122.445	37.932	0.28	18.26	185.83	18.53	9	7/14/2014	44:21.2
3639	RSPe_2	-122.445	37.932	0.30	18.33	185.88	18.63	-122.445	37.932	0.33	18.33	185.68	18.66	9	7/14/2014	44:21.1
3640	RSPe_2	-122.445	37.932	0.30	18.34	185.79	18.64	-122.445	37.932	0.28	18.34	185.52	18.62	9	7/14/2014	44:21.0
3641	RSPe_2	-122.445	37.932	0.35	18.23	185.70	18.58	-122.445	37.932	0.33	18.23	185.43	18.55	9	7/14/2014	44:20.9
3642	RSPe_2	-122.445	37.932	0.30	18.24	185.55	18.53	-122.445	37.932	0.41	18.24	185.26	18.65	9	7/14/2014	44:20.8
3643	RSPe_2	-122.445	37.932	0.35	18.20	185.42	18.55	-122.445	37.932	0.36	18.20	185.20	18.57	9	7/14/2014	44:20.7
3644	RSPe_2	-122.445	37.932	0.30	18.24	185.22	18.53	-122.445	37.932	0.36	18.24	185.06	18.60	9	7/14/2014	44:20.6
3645	RSPe_2	-122.445	37.932	0.35	18.22	185.08	18.57	-122.445	37.932	0.36	18.22	184.99	18.58	9	7/14/2014	44:20.5
3646	RSPe_2	-122.445	37.932	0.30	18.20	184.90	18.50	-122.445	37.932	0.33	18.20	184.89	18.53	9	7/14/2014	44:20.4
3647	RSPe_2	-122.445	37.932	0.35	18.20	184.78	18.55	-122.445	37.932	0.33	18.20	184.85	18.53	9	7/14/2014	44:20.3
3648	RSPe_2	-122.445	37.932	0.30	18.24	184.68	18.53	-122.445	37.932	0.33	18.24	184.76	18.56	9	7/14/2014	44:20.2
3649	RSPe_2	-122.445	37.932	0.35	18.27	184.48	18.62	-122.445	37.932	0.45	18.27	184.60	18.72	9	7/14/2014	44:20.1
3650	RSPe_2	-122.445	37.932	0.35	18.25	184.39	18.60	-122.445	37.932	0.36	18.25	184.46	18.61	9	7/14/2014	44:20.0
3651	RSPe_2	-122.445	37.932	0.35	18.27	184.21	18.62	-122.445	37.932	0.36	18.27	184.27	18.63	9	7/14/2014	44:19.9
3652	RSPe_2	-122.445	37.932	0.35	18.25	184.11	18.60	-122.445	37.932	0.36	18.25	184.10	18.61	9	7/14/2014	44:19.8
3653	RSPe_2	-122.445	37.932	0.35	18.26	183.93	18.61	-122.445	37.932	0.41	18.26	183.92	18.68	9	7/14/2014	44:19.7
3654	RSPe_2	-122.445	37.932	0.35	18.27	183.84	18.62	-122.445	37.932	0.36	18.27	183.79	18.63	9	7/14/2014	44:19.6
3655	RSPe_2	-122.445	37.932	0.35	18.26	183.67	18.61	-122.445	37.932	0.36	18.26	183.63	18.63	9	7/14/2014	44:19.5
3656	RSPe_2	-122.445	37.932	0.35	18.30	183.53	18.65	-122.445	37.932	0.36	18.30	183.48	18.66	9	7/14/2014	44:19.4
3657	RSPe_2	-122.445	37.932	0.35	18.30	183.37	18.65	-122.445	37.932	0.41	18.30	183.38	18.71	9	7/14/2014	44:19.3
3658	RSPe_2	-122.445	37.932	0.35	18.33	183.20	18.68	-122.445	37.932	0.36	18.33	183.24	18.69	9	7/14/2014	44:19.2
3659	RSPe_2	-122.445	37.932	0.38	18.32	183.12	18.70	-122.445	37.933	0.45	18.32	183.11	18.76	9	7/14/2014	44:19.1
3660	RSPe_2	-122.445	37.932	0.35	18.34	183.03	18.69	-122.445	37.933	0.41	18.34	183.01	18.76	9	7/14/2014	44:19.0
3661	RSPe_2	-122.445	37.933	0.35	18.39	182.89	18.74	-122.445	37.933	0.36	18.39	182.91	18.75	9	7/14/2014	44:18.9
3662	RSPe_2	-122.445	37.933	0.35	18.40	182.81	18.75	-122.445	37.933	0.41	18.40	182.84	18.81	9	7/14/2014	44:18.8

3663	RSPe_2	-122.445	37.933	0.38	18.40	182.70	18.78	-122.445	37.933	0.41	18.40	182.72	18.81	9	7/14/2014	44:18.7
3664	RSPe_2	-122.445	37.933	0.38	18.44	182.55	18.82	-122.445	37.933	0.41	18.44	182.61	18.85	9	7/14/2014	44:18.6
3665	RSPe_2	-122.445	37.933	0.38	18.43	182.48	18.81	-122.445	37.933	0.45	18.43	182.52	18.88	9	7/14/2014	44:18.5
3666	RSPe_2	-122.445	37.933	0.38	18.49	182.44	18.87	-122.445	37.933	0.41	18.49	182.48	18.90	9	7/14/2014	44:18.4
3667	RSPe_2	-122.445	37.933	0.38	18.46	182.31	18.84	-122.445	37.933	0.45	18.46	182.37	18.90	9	7/14/2014	44:18.3
3668	RSPe_2	-122.445	37.933	0.35	18.49	182.25	18.84	-122.445	37.933	0.45	18.49	182.26	18.93	9	7/14/2014	44:18.2
3669	RSPe_2	-122.445	37.933	0.38	18.50	182.20	18.89	-122.445	37.933	0.45	18.50	182.20	18.95	9	7/14/2014	44:18.1
3670	RSPe_2	-122.445	37.933	0.35	18.50	182.12	18.85	-122.445	37.933	0.48	18.50	182.16	18.98	9	7/14/2014	44:18.0
3671	RSPe_2	-122.445	37.933	0.38	18.53	182.01	18.92	-122.445	37.933	0.45	18.53	181.99	18.98	9	7/14/2014	44:17.9
3672	RSPe_2	-122.445	37.933	0.38	18.51	181.88	18.89	-122.445	37.933	0.41	18.51	181.90	18.92	9	7/14/2014	44:17.8
3673	RSPe_2	-122.445	37.933	0.38	18.48	181.81	18.87	-122.445	37.933	0.45	18.48	181.78	18.93	9	7/14/2014	44:17.7
3674	RSPe_2	-122.445	37.933	0.35	18.47	181.62	18.82	-122.445	37.933	0.45	18.47	181.62	18.91	9	7/14/2014	44:17.6
3675	RSPe_2	-122.445	37.933	0.38	18.51	181.49	18.89	-122.445	37.933	0.45	18.51	181.47	18.96	9	7/14/2014	44:17.5
3676	RSPe_2	-122.445	37.933	0.38	18.46	181.35	18.84	-122.445	37.933	0.45	18.46	181.37	18.90	9	7/14/2014	44:17.4
3677	RSPe_2	-122.445	37.933	0.38	18.47	181.29	18.85	-122.445	37.933	0.48	18.47	181.27	18.95	9	7/14/2014	44:17.3
3678	RSPe_2	-122.445	37.933	0.38	18.52	181.15	18.91	-122.445	37.933	0.45	18.52	181.15	18.97	9	7/14/2014	44:17.2
3679	RSPe_2	-122.445	37.933	0.44	18.47	181.05	18.90	-122.445	37.933	0.53	18.47	181.08	19.00	9	7/14/2014	44:17.1
3680	RSPe_2	-122.445	37.933	0.44	18.44	180.90	18.88	-122.445	37.933	0.48	18.44	180.96	18.92	9	7/14/2014	44:17.0
3681	RSPe_2	-122.445	37.933	0.44	18.47	180.76	18.90	-122.445	37.933	0.53	18.47	180.79	19.00	9	7/14/2014	44:16.9
3682	RSPe_2	-122.445	37.933	0.38	18.40	180.62	18.78	-122.445	37.933	0.45	18.40	180.73	18.84	9	7/14/2014	44:16.8
3683	RSPe_2	-122.445	37.933	0.44	18.39	180.48	18.82	-122.445	37.933	0.48	18.39	180.58	18.87	9	7/14/2014	44:16.7
3684	RSPe_2	-122.445	37.933	0.44	18.41	180.36	18.85	-122.445	37.933	0.48	18.41	180.45	18.89	9	7/14/2014	44:16.6
3685	RSPe_2	-122.445	37.933	0.44	18.36	180.26	18.79	-122.445	37.933	0.48	18.36	180.36	18.84	9	7/14/2014	44:16.5
3686	RSPe_2	-122.445	37.933	0.44	18.36	180.09	18.80	-122.445	37.933	0.45	18.36	180.25	18.81	9	7/14/2014	44:16.4
3687	RSPe_2	-122.445	37.933	0.44	18.31	179.97	18.75	-122.445	37.933	0.48	18.31	180.08	18.79	9	7/14/2014	44:16.3
3688	RSPe_2	-122.445	37.933	0.44	18.33	179.84	18.76	-122.445	37.933	0.53	18.33	179.96	18.86	9	7/14/2014	44:16.2
3689	RSPe_2	-122.445	37.933	0.44	18.27	179.68	18.71	-122.445	37.933	0.57	18.27	179.75	18.84	9	7/14/2014	44:16.1
3690	RSPe_2	-122.445	37.933	0.44	18.24	179.58	18.68	-122.445	37.933	0.53	18.24	179.61	18.77	9	7/14/2014	44:16.0
3691	RSPe_2	-122.445	37.933	0.47	18.28	179.40	18.75	-122.445	37.933	0.57	18.28	179.42	18.84	9	7/14/2014	44:15.9
3692	RSPe_2	-122.445	37.933	0.47	18.25	179.16	18.72	-122.445	37.933	0.57	18.25	179.21	18.81	9	7/14/2014	44:15.8
3693	RSPe_2	-122.445	37.933	0.47	18.18	179.10	18.65	-122.445	37.933	0.57	18.18	179.08	18.74	9	7/14/2014	44:15.7
3694	RSPe_2	-122.445	37.933	0.47	18.17	179.03	18.64	-122.445	37.933	0.57	18.17	179.01	18.74	9	7/14/2014	44:15.6
3695	RSPe_2	-122.445	37.933	0.47	18.17	178.88	18.64	-122.445	37.933	0.57	18.17	178.88	18.73	9	7/14/2014	44:15.5

3696	RSPe_2	-122.445	37.933	0.44	18.16	178.81	18.59	-122.445	37.933	0.53	18.16	178.80	18.69	9	7/14/2014	44:15.4
3697	RSPe_2	-122.445	37.933	0.47	18.13	178.68	18.60	-122.445	37.933	0.57	18.13	178.64	18.70	9	7/14/2014	44:15.3
3698	RSPe_2	-122.445	37.933	0.44	18.14	178.58	18.58	-122.445	37.933	0.57	18.14	178.56	18.71	9	7/14/2014	44:15.2
3699	RSPe_2	-122.445	37.933	0.47	18.12	178.41	18.59	-122.445	37.933	0.57	18.12	178.42	18.68	9	7/14/2014	44:15.1
3700	RSPe_2	-122.445	37.933	0.47	18.10	178.31	18.57	-122.445	37.933	0.57	18.10	178.32	18.66	9	7/14/2014	44:15.0
3701	RSPe_2	-122.445	37.933	0.47	18.07	178.14	18.54	-122.445	37.933	0.62	18.07	178.16	18.69	9	7/14/2014	44:14.9
3702	RSPe_2	-122.445	37.933	0.44	18.07	178.00	18.51	-122.445	37.933	0.53	18.07	178.08	18.60	9	7/14/2014	44:14.8
3703	RSPe_2	-122.445	37.933	0.47	17.99	177.83	18.46	-122.445	37.933	0.62	17.99	177.93	18.60	9	7/14/2014	44:14.7
3704	RSPe_2	-122.445	37.933	0.44	17.98	177.60	18.42	-122.445	37.933	0.57	17.98	177.72	18.55	9	7/14/2014	44:14.6
3705	RSPe_2	-122.445	37.933	0.50	17.87	177.36	18.38	-122.445	37.933	0.65	17.87	177.56	18.52	9	7/14/2014	44:14.5
3706	RSPe_2	-122.445	37.933	0.47	17.90	177.29	18.37	-122.445	37.933	0.62	17.90	177.47	18.52	9	7/14/2014	44:14.4
3707	RSPe_2	-122.445	37.933	0.50	17.77	177.10	18.27	-122.445	37.933	0.65	17.77	177.27	18.42	9	7/14/2014	44:14.3
3708	RSPe_2	-122.445	37.933	0.47	17.74	176.93	18.21	-122.445	37.933	0.62	17.74	177.10	18.36	9	7/14/2014	44:14.2
3709	RSPe_2	-122.445	37.933	0.47	17.72	176.80	18.19	-122.445	37.933	0.65	17.72	176.97	18.37	9	7/14/2014	44:14.1
3710	RSPe_2	-122.445	37.933	0.47	17.72	176.65	18.19	-122.445	37.933	0.65	17.72	176.82	18.37	9	7/14/2014	44:14.0
3711	RSPe_2	-122.445	37.933	0.50	17.71	176.56	18.21	-122.445	37.933	0.70	17.71	176.66	18.41	9	7/14/2014	44:13.9
3712	RSPe_2	-122.445	37.933	0.47	17.65	176.43	18.12	-122.445	37.933	0.65	17.65	176.57	18.30	9	7/14/2014	44:13.8
3713	RSPe_2	-122.445	37.933	0.50	17.67	176.32	18.17	-122.445	37.933	0.65	17.67	176.46	18.32	9	7/14/2014	44:13.7
3714	RSPe_2	-122.445	37.933	0.47	17.65	176.16	18.12	-122.445	37.933	0.65	17.65	176.30	18.30	9	7/14/2014	44:13.6
3715	RSPe_2	-122.445	37.933	0.47	17.65	176.04	18.12	-122.445	37.933	0.65	17.65	176.17	18.30	9	7/14/2014	44:13.5
3716	RSPe_2	-122.445	37.933	0.47	17.63	175.98	18.10	-122.445	37.933	0.62	17.63	176.08	18.24	9	7/14/2014	44:13.4
3717	RSPe_2	-122.445	37.933	0.50	17.56	175.79	18.06	-122.445	37.933	0.65	17.56	175.90	18.21	9	7/14/2014	44:13.3
3718	RSPe_2	-122.445	37.933	0.47	17.58	175.71	18.05	-122.445	37.933	0.65	17.58	175.82	18.23	9	7/14/2014	44:13.2
3719	RSPe_2	-122.445	37.933	0.50	17.50	175.52	18.00	-122.445	37.933	0.65	17.50	175.70	18.15	9	7/14/2014	44:13.1
3720	RSPe_2	-122.445	37.933	0.47	17.51	175.49	17.98	-122.445	37.933	0.70	17.51	175.59	18.21	9	7/14/2014	44:13.0
3721	RSPe_2	-122.445	37.933	0.50	17.40	175.34	17.90	-122.445	37.933	0.65	17.40	175.47	18.05	9	7/14/2014	44:12.9
3722	RSPe_2	-122.445	37.933	0.47	17.39	175.20	17.86	-122.445	37.933	0.65	17.39	175.32	18.04	9	7/14/2014	44:12.8
3723	RSPe_2	-122.445	37.933	0.50	17.49	175.07	18.00	-122.445	37.933	0.70	17.49	175.25	18.19	9	7/14/2014	44:12.7
3724	RSPe_2	-122.445	37.933	0.47	17.36	175.00	17.83	-122.445	37.933	0.70	17.36	175.19	18.06	9	7/14/2014	44:12.6
3725	RSPe_2	-122.445	37.933	0.50	17.36	174.92	17.87	-122.445	37.933	0.70	17.36	175.16	18.06	9	7/14/2014	44:12.5
3726	RSPe_2	-122.445	37.933	0.47	17.43	174.87	17.90	-122.445	37.933	0.70	17.43	175.10	18.13	9	7/14/2014	44:12.4
3727	RSPe_2	-122.445	37.933	0.50	17.41	174.85	17.91	-122.445	37.933	0.70	17.41	175.04	18.11	9	7/14/2014	44:12.3
3728	RSPe_2	-122.445	37.933	0.47	17.36	174.75	17.83	-122.445	37.933	0.65	17.36	174.97	18.01	9	7/14/2014	44:12.2

3729	RSPe_2	-122.445	37.933	0.47	17.45	174.70	17.92	-122.445	37.933	0.65	17.45	174.90	18.10	9	7/14/2014	44:12.1
3730	RSPe_2	-122.445	37.933	0.47	17.40	174.65	17.87	-122.445	37.933	0.65	17.40	174.85	18.05	9	7/14/2014	44:12.0
3731	RSPe_2	-122.445	37.933	0.47	17.42	174.55	17.89	-122.445	37.933	0.70	17.42	174.77	18.12	9	7/14/2014	44:11.9
3732	RSPe_2	-122.445	37.933	0.47	17.46	174.41	17.93	-122.445	37.933	0.65	17.46	174.60	18.11	9	7/14/2014	44:11.8
3733	RSPe_2	-122.445	37.933	0.47	17.48	174.31	17.95	-122.445	37.933	0.70	17.48	174.52	18.18	9	7/14/2014	44:11.7
3734	RSPe_2	-122.445	37.933	0.47	17.51	174.24	17.98	-122.445	37.933	0.62	17.51	174.41	18.13	9	7/14/2014	44:11.6
3735	RSPe_2	-122.445	37.933	0.47	17.46	174.04	17.93	-122.445	37.933	0.70	17.46	174.21	18.16	9	7/14/2014	44:11.5
3736	RSPe_2	-122.445	37.933	0.47	17.52	173.87	18.00	-122.445	37.933	0.70	17.52	174.09	18.23	9	7/14/2014	44:11.4
3737	RSPe_2	-122.445	37.933	0.47	17.56	173.67	18.03	-122.445	37.933	0.70	17.56	173.97	18.26	9	7/14/2014	44:11.3
3738	RSPe_2	-122.445	37.933	0.47	17.58	173.54	18.05	-122.445	37.933	0.70	17.58	173.79	18.28	9	7/14/2014	44:11.2
3739	RSPe_2	-122.445	37.933	0.47	17.60	173.35	18.07	-122.445	37.933	0.70	17.60	173.63	18.30	9	7/14/2014	44:11.1
3740	RSPe_2	-122.445	37.933	0.47	17.56	173.24	18.03	-122.445	37.933	0.65	17.56	173.50	18.21	9	7/14/2014	44:11.0
3741	RSPe_2	-122.445	37.933	0.47	17.58	173.10	18.05	-122.445	37.933	0.70	17.58	173.38	18.28	9	7/14/2014	44:10.9
3742	RSPe_2	-122.445	37.933	0.44	17.56	172.97	17.99	-122.445	37.933	0.65	17.56	173.21	18.21	9	7/14/2014	44:10.8
3743	RSPe_2	-122.445	37.933	0.47	17.59	172.85	18.06	-122.445	37.933	0.74	17.59	173.12	18.33	9	7/14/2014	44:10.7
3744	RSPe_2	-122.445	37.933	0.44	17.56	172.69	17.99	-122.445	37.933	0.65	17.56	172.89	18.21	9	7/14/2014	44:10.6
3745	RSPe_2	-122.445	37.933	0.44	17.52	172.60	17.96	-122.445	37.933	0.70	17.52	172.82	18.23	9	7/14/2014	44:10.5
3746	RSPe_2	-122.445	37.933	0.44	17.55	172.46	17.98	-122.445	37.933	0.65	17.55	172.61	18.20	9	7/14/2014	44:10.4
3747	RSPe_2	-122.445	37.933	0.44	17.52	172.34	17.96	-122.445	37.933	0.65	17.52	172.54	18.18	9	7/14/2014	44:10.3
3748	RSPe_2	-122.445	37.933	0.44	17.63	172.27	18.06	-122.445	37.933	0.62	17.63	172.37	18.24	9	7/14/2014	44:10.2
3749	RSPe_2	-122.445	37.933	0.44	17.66	172.15	18.10	-122.445	37.933	0.65	17.66	172.29	18.32	9	7/14/2014	44:10.1
3750	RSPe_2	-122.445	37.933	0.44	17.56	172.05	18.00	-122.445	37.933	0.65	17.56	172.13	18.21	9	7/14/2014	44:10.0
3751	RSPe_2	-122.445	37.933	0.44	17.59	171.90	18.03	-122.445	37.933	0.70	17.59	172.00	18.30	9	7/14/2014	44:09.9
3752	RSPe_2	-122.445	37.933	0.38	17.52	171.82	17.91	-122.445	37.933	0.65	17.52	171.87	18.18	9	7/14/2014	44:09.8
3753	RSPe_2	-122.445	37.933	0.44	17.57	171.74	18.01	-122.445	37.933	0.70	17.57	171.77	18.27	9	7/14/2014	44:09.7
3754	RSPe_2	-122.445	37.933	0.38	17.55	171.59	17.93	-122.445	37.933	0.62	17.55	171.60	18.16	9	7/14/2014	44:09.6
3755	RSPe_2	-122.445	37.933	0.44	17.51	171.42	17.95	-122.445	37.933	0.65	17.51	171.50	18.17	9	7/14/2014	44:09.5
3756	RSPe_2	-122.445	37.933	0.38	17.58	171.29	17.97	-122.445	37.933	0.62	17.58	171.37	18.20	9	7/14/2014	44:09.4
3757	RSPe_2	-122.445	37.933	0.44	17.52	171.20	17.96	-122.445	37.933	0.70	17.52	171.25	18.23	9	7/14/2014	44:09.3
3758	RSPe_2	-122.445	37.933	0.38	17.50	170.96	17.89	-122.445	37.933	0.65	17.50	171.05	18.15	9	7/14/2014	44:09.2
3759	RSPe_2	-122.445	37.933	0.44	17.58	170.83	18.01	-122.445	37.933	0.74	17.58	170.98	18.31	9	7/14/2014	44:09.1
3760	RSPe_2	-122.445	37.933	0.38	17.58	170.70	17.97	-122.445	37.933	0.70	17.58	170.86	18.29	9	7/14/2014	44:09.0
3761	RSPe_2	-122.445	37.933	0.38	17.58	170.60	17.97	-122.445	37.933	0.74	17.58	170.81	18.32	9	7/14/2014	44:08.9

3762	RSPe_2	-122.445	37.933	0.38	17.63	170.52	18.01	-122.445	37.933	0.65	17.63	170.74	18.28	9	7/14/2014	44:08.8
3763	RSPe_2	-122.445	37.933	0.38	17.68	170.44	18.06	-122.445	37.933	0.70	17.68	170.65	18.38	9	7/14/2014	44:08.7
3764	RSPe_2	-122.445	37.933	0.35	17.66	170.33	18.01	-122.445	37.933	0.65	17.66	170.52	18.32	9	7/14/2014	44:08.6
3765	RSPe_2	-122.445	37.933	0.38	17.68	170.18	18.07	-122.445	37.933	0.70	17.68	170.41	18.39	9	7/14/2014	44:08.5
3766	RSPe_2	-122.445	37.933	0.35	17.68	170.08	18.04	-122.445	37.933	0.62	17.68	170.29	18.30	9	7/14/2014	44:08.4
3767	RSPe_2	-122.445	37.933	0.35	17.78	169.96	18.13	-122.445	37.933	0.65	17.78	170.19	18.43	9	7/14/2014	44:08.3
3768	RSPe_2	-122.445	37.933	0.35	17.81	169.87	18.16	-122.445	37.933	0.65	17.81	170.07	18.46	9	7/14/2014	44:08.2
3769	RSPe_2	-122.445	37.933	0.30	17.80	169.73	18.10	-122.445	37.933	0.62	17.80	169.95	18.42	9	7/14/2014	44:08.1
3770	RSPe_2	-122.445	37.933	0.30	17.86	169.57	18.16	-122.445	37.933	0.62	17.86	169.75	18.48	9	7/14/2014	44:08.0
3771	RSPe_2	-122.445	37.933	0.35	17.87	169.39	18.22	-122.445	37.933	0.65	17.87	169.63	18.52	9	7/14/2014	44:07.9
3772	RSPe_2	-122.445	37.933	0.30	17.83	169.29	18.13	-122.445	37.933	0.62	17.83	169.52	18.45	9	7/14/2014	44:07.8
3773	RSPe_2	-122.445	37.933	0.35	17.88	169.16	18.23	-122.445	37.933	0.65	17.88	169.44	18.53	9	7/14/2014	44:07.7
3774	RSPe_2	-122.445	37.933	0.30	17.93	169.06	18.23	-122.445	37.933	0.62	17.93	169.33	18.55	9	7/14/2014	44:07.6
3775	RSPe_2	-122.445	37.933	0.30	17.98	168.97	18.28	-122.445	37.933	0.62	17.98	169.27	18.60	9	7/14/2014	44:07.5
3776	RSPe_2	-122.445	37.933	0.30	17.98	168.86	18.28	-122.445	37.933	0.57	17.98	169.16	18.55	9	7/14/2014	44:07.4
3777	RSPe_2	-122.445	37.933	0.30	18.03	168.74	18.33	-122.445	37.933	0.57	18.03	169.05	18.59	9	7/14/2014	44:07.3
3778	RSPe_2	-122.445	37.933	0.27	18.00	168.64	18.27	-122.445	37.933	0.57	18.00	168.99	18.57	9	7/14/2014	44:07.2
3779	RSPe_2	-122.445	37.933	0.27	18.09	168.62	18.35	-122.445	37.933	0.53	18.09	168.94	18.62	9	7/14/2014	44:07.1
3780	RSPe_2	-122.445	37.933	0.27	18.14	168.51	18.41	-122.445	37.933	0.53	18.14	168.87	18.67	9	7/14/2014	44:07.0
3781	RSPe_2	-122.445	37.933	0.27	18.12	168.42	18.38	-122.445	37.933	0.48	18.12	168.75	18.60	9	7/14/2014	44:06.9
3782	RSPe_2	-122.445	37.933	0.21	18.30	168.38	18.51	-122.445	37.933	0.48	18.30	168.68	18.78	9	7/14/2014	44:06.8
3783	RSPe_2	-122.445	37.933	0.27	18.21	168.23	18.47	-122.445	37.933	0.48	18.21	168.57	18.69	9	7/14/2014	44:06.7
3784	RSPe_2	-122.445	37.933	0.21	18.25	168.08	18.46	-122.445	37.933	0.45	18.25	168.42	18.70	9	7/14/2014	44:06.6
3785	RSPe_2	-122.445	37.933	0.27	18.30	167.94	18.57	-122.445	37.933	0.48	18.30	168.24	18.79	9	7/14/2014	44:06.5
3786	RSPe_2	-122.445	37.933	0.21	18.40	167.80	18.61	-122.445	37.933	0.45	18.40	168.09	18.84	9	7/14/2014	44:06.4
3787	RSPe_2	-122.445	37.933	0.21	18.33	167.64	18.55	-122.445	37.933	0.45	18.33	167.92	18.78	9	7/14/2014	44:06.3
3788	RSPe_2	-122.445	37.933	0.21	18.39	167.55	18.60	-122.445	37.933	0.45	18.39	167.83	18.83	9	7/14/2014	44:06.2
3789	RSPe_2	-122.445	37.933	0.21	18.39	167.38	18.60	-122.445	37.933	0.45	18.39	167.62	18.83	9	7/14/2014	44:06.1
3790	RSPe_2	-122.445	37.933	0.18	18.41	167.31	18.59	-122.445	37.933	0.45	18.41	167.50	18.86	9	7/14/2014	44:06.0
3791	RSPe_2	-122.445	37.933	0.21	18.41	167.15	18.63	-122.445	37.933	0.45	18.41	167.33	18.86	9	7/14/2014	44:05.9
3792	RSPe_2	-122.445	37.933	0.18	18.43	167.05	18.61	-122.445	37.933	0.45	18.43	167.23	18.88	9	7/14/2014	44:05.8
3793	RSPe_2	-122.445	37.933	0.18	18.43	166.99	18.61	-122.445	37.933	0.45	18.43	167.16	18.88	9	7/14/2014	44:05.7
3794	RSPe_2	-122.445	37.933	0.18	18.47	166.87	18.65	-122.445	37.933	0.45	18.47	167.08	18.91	9	7/14/2014	44:05.6

3795	RSPe_2	-122.445	37.933	0.18	18.46	166.74	18.64	-122.445	37.933	0.41	18.46	166.94	18.87	9	7/14/2014 44:05.5
3796	RSPe_2	-122.445	37.933	0.15	18.46	166.60	18.60	-122.445	37.933	0.36	18.46	166.80	18.82	9	7/14/2014 44:05.4
3797	RSPe_2	-122.445	37.933	0.15	18.50	166.35	18.64	-122.445	37.933	0.36	18.50	166.65	18.86	9	7/14/2014 44:05.3
3798	RSPe_2	-122.445	37.933	0.15	18.46	166.15	18.60	-122.445	37.933	0.33	18.46	166.45	18.79	9	7/14/2014 44:05.2
3799	RSPe_2	-122.445	37.933	0.15	18.50	165.95	18.65	-122.445	37.933	0.33	18.50	166.32	18.83	9	7/14/2014 44:05.1
3800	RSPe_2	-122.445	37.933	0.10	18.41	165.77	18.51	-122.445	37.933	0.28	18.41	166.17	18.69	9	7/14/2014 44:05.0
3801	RSPe_2	-122.445	37.933	0.15	18.39	165.59	18.53	-122.445	37.933	0.33	18.39	165.98	18.72	9	7/14/2014 44:04.9
3802	RSPe_2	-122.445	37.933	0.10	18.37	165.40	18.47	-122.445	37.933	0.28	18.37	165.79	18.65	9	7/14/2014 44:04.8
3803	RSPe_2	-122.445	37.933	0.10	18.35	165.23	18.44	-122.445	37.933	0.28	18.35	165.60	18.63	9	7/14/2014 44:04.7
3804	RSPe_2	-122.445	37.933	0.10	18.33	165.05	18.43	-122.445	37.933	0.28	18.33	165.37	18.61	9	7/14/2014 44:04.6
3805	RSPe_2	-122.445	37.933	0.10	18.38	164.95	18.47	-122.445	37.933	0.33	18.38	165.16	18.71	9	7/14/2014 44:04.5
3806	RSPe_2	-122.445	37.933	0.10	18.31	164.76	18.40	-122.445	37.933	0.28	18.31	165.00	18.59	9	7/14/2014 44:04.4
3807	RSPe_2	-122.445	37.933	0.10	18.27	164.60	18.37	-122.445	37.933	0.28	18.27	164.85	18.55	9	7/14/2014 44:04.3
3808	RSPe_2	-122.445	37.933	0.06	18.26	164.46	18.32	-122.445	37.933	0.28	18.26	164.65	18.54	9	7/14/2014 44:04.2
3809	RSPe_2	-122.445	37.933	0.06	18.26	164.29	18.32	-122.445	37.933	0.28	18.26	164.47	18.54	9	7/14/2014 44:04.1
3810	RSPe_2	-122.445	37.933	0.06	18.24	164.19	18.30	-122.445	37.933	0.25	18.24	164.39	18.48	9	7/14/2014 44:04.0
3811	RSPe_2	-122.445	37.933	0.06	18.24	164.05	18.30	-122.445	37.933	0.25	18.24	164.27	18.49	9	7/14/2014 44:03.9
3812	RSPe_2	-122.445	37.933	0.06	18.25	163.98	18.31	-122.445	37.933	0.19	18.25	164.11	18.44	9	7/14/2014 44:03.8
3813	RSPe_2	-122.445	37.933	0.06	18.22	163.83	18.28	-122.445	37.933	0.19	18.22	164.07	18.41	9	7/14/2014 44:03.7
3814	RSPe_2	-122.445	37.933	0.01	18.23	163.71	18.23	-122.445	37.933	0.16	18.23	163.95	18.39	9	7/14/2014 44:03.6
3815	RSPe_2	-122.445	37.933	0.06	18.21	163.59	18.27	-122.445	37.933	0.19	18.21	163.86	18.40	9	7/14/2014 44:03.5
3816	RSPe_2	-122.445	37.933	0.01	18.31	163.54	18.32	-122.445	37.933	0.16	18.31	163.85	18.47	9	7/14/2014 44:03.4
3817	RSPe_2	-122.445	37.933	0.01	18.23	163.44	18.23	-122.445	37.933	0.16	18.23	163.77	18.39	9	7/14/2014 44:03.3
3818	RSPe_2	-122.445	37.933	-0.03	18.22	163.30	18.19	-122.445	37.933	0.13	18.22	163.69	18.35	9	7/14/2014 44:03.2
3819	RSPe_2	-122.445	37.933	0.01	18.21	163.21	18.22	-122.445	37.933	0.16	18.21	163.52	18.37	9	7/14/2014 44:03.1
3820	RSPe_2	-122.445	37.933	-0.03	18.23	163.09	18.20	-122.445	37.933	0.16	18.23	163.48	18.39	9	7/14/2014 44:03.0
3821	RSPe_2	-122.445	37.933	0.01	18.26	162.99	18.27	-122.445	37.933	0.16	18.26	163.28	18.42	9	7/14/2014 44:02.9
3822	RSPe_2	-122.445	37.933	-0.03	18.26	162.89	18.23	-122.445	37.933	0.13	18.26	163.18	18.38	9	7/14/2014 44:02.8
3823	RSPe_2	-122.445	37.933	-0.03	18.24	162.82	18.21	-122.445	37.933	0.13	18.24	163.03	18.36	9	7/14/2014 44:02.7
3824	RSPe_2	-122.445	37.933	-0.03	18.21	162.69	18.18	-122.445	37.933	0.13	18.21	162.89	18.34	9	7/14/2014 44:02.6
3825	RSPe_2	-122.445	37.933	-0.03	18.24	162.58	18.22	-122.445	37.933	0.13	18.24	162.75	18.37	9	7/14/2014 44:02.5
3826	RSPe_2	-122.445	37.933	-0.03	18.20	162.43	18.18	-122.445	37.933	0.08	18.20	162.67	18.28	9	7/14/2014 44:02.4
3827	RSPe_2	-122.445	37.933	-0.03	18.20	162.32	18.18	-122.445	37.933	0.08	18.20	162.51	18.28	9	7/14/2014 44:02.3

3828	RSPe_2	-122.445	37.933	-0.03	18.27	162.13	18.25	-122.445	37.933	0.04	18.27	162.43	18.31	9	7/14/2014	44:02.2
3829	RSPe_2	-122.445	37.933	-0.03	18.18	161.97	18.15	-122.445	37.933	0.04	18.18	162.30	18.22	9	7/14/2014	44:02.1
3830	RSPe_2	-122.445	37.933	-0.08	18.19	161.82	18.12	-122.445	37.933	-0.01	18.19	162.22	18.19	9	7/14/2014	44:02.0
3831	RSPe_2	-122.445	37.933	-0.03	18.25	161.69	18.22	-122.445	37.933	-0.01	18.25	162.12	18.24	9	7/14/2014	44:01.9
3832	RSPe_2	-122.445	37.933	-0.08	18.23	161.52	18.15	-122.445	37.933	0.04	18.23	161.99	18.27	9	7/14/2014	44:01.8
3833	RSPe_2	-122.445	37.933	-0.08	18.23	161.47	18.15	-122.445	37.933	-0.01	18.23	161.96	18.22	9	7/14/2014	44:01.7
3834	RSPe_2	-122.445	37.933	-0.11	18.23	161.36	18.11	-122.445	37.933	-0.04	18.23	161.88	18.18	9	7/14/2014	44:01.6
3835	RSPe_2	-122.445	37.933	-0.08	18.24	161.34	18.16	-122.445	37.933	-0.01	18.24	161.82	18.23	9	7/14/2014	44:01.5
3836	RSPe_2	-122.445	37.933	-0.08	18.28	161.24	18.20	-122.445	37.933	-0.04	18.28	161.68	18.24	9	7/14/2014	44:01.4
3837	RSPe_2	-122.445	37.933	-0.08	18.26	161.19	18.19	-122.445	37.933	-0.04	18.26	161.60	18.22	9	7/14/2014	44:01.3
3838	RSPe_2	-122.445	37.933	-0.11	18.26	161.09	18.15	-122.445	37.933	-0.04	18.26	161.44	18.22	9	7/14/2014	44:01.2
3839	RSPe_2	-122.445	37.933	-0.08	18.25	161.03	18.17	-122.445	37.933	-0.01	18.25	161.29	18.24	9	7/14/2014	44:01.1
3840	RSPe_2	-122.445	37.933	-0.11	18.24	160.96	18.12	-122.445	37.933	-0.09	18.24	161.15	18.14	9	7/14/2014	44:01.0
3841	RSPe_2	-122.445	37.933	-0.08	18.24	160.86	18.16	-122.445	37.933	-0.04	18.24	161.06	18.19	9	7/14/2014	44:00.9
3842	RSPe_2	-122.445	37.933	-0.11	18.20	160.79	18.09	-122.445	37.933	-0.09	18.20	160.89	18.11	9	7/14/2014	44:00.8
3843	RSPe_2	-122.445	37.933	-0.11	18.19	160.62	18.08	-122.445	37.933	-0.09	18.19	160.74	18.10	9	7/14/2014	44:00.7
3844	RSPe_2	-122.445	37.933	-0.16	18.23	160.48	18.06	-122.445	37.933	-0.09	18.23	160.61	18.13	9	7/14/2014	44:00.6
3845	RSPe_2	-122.445	37.933	-0.11	18.17	160.32	18.06	-122.445	37.933	-0.13	18.17	160.52	18.04	9	7/14/2014	44:00.5
3846	RSPe_2	-122.445	37.933	-0.16	18.17	160.21	18.01	-122.445	37.933	-0.18	18.17	160.46	17.99	9	7/14/2014	44:00.4
3847	RSPe_2	-122.445	37.933	-0.11	18.15	160.04	18.04	-122.445	37.933	-0.18	18.15	160.36	17.97	9	7/14/2014	44:00.3
3848	RSPe_2	-122.445	37.933	-0.16	18.17	159.91	18.01	-122.445	37.933	-0.18	18.17	160.29	17.99	9	7/14/2014	44:00.2
3849	RSPe_2	-122.445	37.933	-0.16	18.22	159.78	18.06	-122.445	37.933	-0.18	18.22	160.24	18.04	9	7/14/2014	44:00.1
3850	RSPe_2	-122.445	37.933	-0.16	18.24	159.65	18.07	-122.445	37.933	-0.18	18.24	160.16	18.06	9	7/14/2014	44:00.0
3851	RSPe_2	-122.445	37.933	-0.16	18.26	159.54	18.10	-122.445	37.933	-0.18	18.26	160.04	18.09	9	7/14/2014	43:59.9
3852	RSPe_2	-122.445	37.933	-0.16	18.32	159.41	18.15	-122.445	37.933	-0.18	18.32	159.94	18.14	9	7/14/2014	43:59.8
3853	RSPe_2	-122.445	37.933	-0.16	18.35	159.26	18.19	-122.445	37.933	-0.18	18.35	159.78	18.17	9	7/14/2014	43:59.7
3854	RSPe_2	-122.445	37.933	-0.20	18.36	159.13	18.17	-122.445	37.933	-0.21	18.36	159.54	18.15	9	7/14/2014	43:59.6
3855	RSPe_2	-122.445	37.933	-0.16	18.37	159.00	18.21	-122.445	37.933	-0.18	18.37	159.35	18.19	9	7/14/2014	43:59.5
3856	RSPe_2	-122.445	37.933	-0.20	18.33	158.88	18.13	-122.445	37.933	-0.21	18.33	159.11	18.12	9	7/14/2014	43:59.4
3857	RSPe_2	-122.445	37.933	-0.16	18.41	158.78	18.25	-122.445	37.933	-0.21	18.41	158.95	18.20	9	7/14/2014	43:59.3
3858	RSPe_2	-122.445	37.933	-0.20	18.31	158.67	18.11	-122.445	37.933	-0.25	18.31	158.78	18.06	9	7/14/2014	43:59.2
3859	RSPe_2	-122.445	37.933	-0.16	18.30	158.59	18.14	-122.445	37.933	-0.21	18.30	158.65	18.09	9	7/14/2014	43:59.1
3860	RSPe_2	-122.445	37.933	-0.20	18.30	158.49	18.10	-122.445	37.933	-0.25	18.30	158.56	18.05	9	7/14/2014	43:59.0

3861	RSPe_2	-122.445	37.933	-0.16	18.26	158.45	18.10	-122.445	37.933	-0.25	18.26	158.52	18.02	9	7/14/2014	43:58.9
3862	RSPe_2	-122.445	37.933	-0.20	18.28	158.29	18.08	-122.445	37.933	-0.25	18.28	158.44	18.03	9	7/14/2014	43:58.8
3863	RSPe_2	-122.445	37.933	-0.16	18.26	158.19	18.09	-122.445	37.933	-0.21	18.26	158.40	18.05	9	7/14/2014	43:58.7
3864	RSPe_2	-122.445	37.933	-0.20	18.26	158.12	18.06	-122.445	37.933	-0.30	18.26	158.39	17.96	9	7/14/2014	43:58.6
3865	RSPe_2	-122.445	37.933	-0.16	18.29	158.04	18.13	-122.445	37.933	-0.30	18.29	158.29	17.99	9	7/14/2014	43:58.5
3866	RSPe_2	-122.445	37.933	-0.20	18.37	157.92	18.17	-122.445	37.933	-0.30	18.37	158.19	18.07	9	7/14/2014	43:58.4
3867	RSPe_2	-122.445	37.933	-0.16	18.30	157.85	18.14	-122.445	37.933	-0.25	18.30	158.13	18.06	9	7/14/2014	43:58.3
3868	RSPe_2	-122.445	37.933	-0.23	18.33	157.75	18.10	-122.445	37.933	-0.30	18.33	157.98	18.04	9	7/14/2014	43:58.2
3869	RSPe_2	-122.445	37.933	-0.20	18.37	157.58	18.17	-122.445	37.933	-0.25	18.37	157.84	18.13	9	7/14/2014	43:58.1
3870	RSPe_2	-122.445	37.933	-0.20	18.40	157.44	18.21	-122.445	37.933	-0.30	18.40	157.71	18.11	9	7/14/2014	43:58.0
3871	RSPe_2	-122.445	37.933	-0.20	18.36	157.29	18.17	-122.445	37.933	-0.30	18.36	157.52	18.07	9	7/14/2014	43:57.9
3872	RSPe_2	-122.445	37.933	-0.23	18.47	157.12	18.23	-122.445	37.933	-0.30	18.47	157.42	18.17	9	7/14/2014	43:57.8
3873	RSPe_2	-122.445	37.933	-0.20	18.43	156.85	18.24	-122.445	37.933	-0.30	18.43	157.22	18.14	9	7/14/2014	43:57.7
3874	RSPe_2	-122.445	37.933	-0.23	18.46	156.76	18.23	-122.445	37.933	-0.33	18.46	157.07	18.13	9	7/14/2014	43:57.6
3875	RSPe_2	-122.445	37.933	-0.19	18.41	156.65	18.22	-122.445	37.933	-0.29	18.41	156.96	18.12	9	7/14/2014	43:57.5
3876	RSPe_2	-122.445	37.933	-0.19	18.50	156.57	18.30	-122.445	37.933	-0.33	18.50	156.86	18.17	9	7/14/2014	43:57.4
3877	RSPe_2	-122.445	37.933	-0.19	18.52	156.46	18.32	-122.445	37.933	-0.29	18.52	156.73	18.22	9	7/14/2014	43:57.3
3878	RSPe_2	-122.445	37.933	-0.19	18.60	156.37	18.41	-122.445	37.933	-0.29	18.60	156.56	18.31	9	7/14/2014	43:57.2
3879	RSPe_2	-122.445	37.933	-0.19	18.50	156.15	18.30	-122.445	37.933	-0.24	18.50	156.35	18.25	9	7/14/2014	43:57.1
3880	RSPe_2	-122.445	37.933	-0.19	18.50	156.00	18.30	-122.445	37.933	-0.29	18.50	156.17	18.20	9	7/14/2014	43:57.0
3881	RSPe_2	-122.445	37.933	-0.19	18.62	155.84	18.43	-122.445	37.933	-0.33	18.62	156.04	18.29	9	7/14/2014	43:56.9
3882	RSPe_2	-122.445	37.933	-0.19	18.63	155.65	18.44	-122.445	37.933	-0.33	18.63	155.86	18.30	9	7/14/2014	43:56.8
3883	RSPe_2	-122.445	37.933	-0.19	18.59	155.50	18.40	-122.445	37.933	-0.29	18.59	155.76	18.30	9	7/14/2014	43:56.7
3884	RSPe_2	-122.445	37.933	-0.19	18.63	155.32	18.43	-122.445	37.933	-0.38	18.63	155.53	18.25	9	7/14/2014	43:56.6
3885	RSPe_2	-122.445	37.933	-0.19	18.66	155.17	18.47	-122.445	37.933	-0.33	18.66	155.30	18.33	9	7/14/2014	43:56.5
3886	RSPe_2	-122.445	37.933	-0.19	18.63	155.05	18.44	-122.445	37.933	-0.29	18.63	155.12	18.34	9	7/14/2014	43:56.4
3887	RSPe_2	-122.445	37.933	-0.19	18.65	154.99	18.45	-122.445	37.933	-0.29	18.65	154.94	18.35	9	7/14/2014	43:56.3
3888	RSPe_2	-122.445	37.933	-0.23	18.62	154.89	18.39	-122.445	37.933	-0.33	18.62	154.79	18.29	9	7/14/2014	43:56.2
3889	RSPe_2	-122.445	37.933	-0.19	18.64	154.77	18.45	-122.445	37.933	-0.29	18.64	154.66	18.35	9	7/14/2014	43:56.1
3890	RSPe_2	-122.445	37.933	-0.19	18.59	154.59	18.39	-122.445	37.933	-0.33	18.59	154.47	18.26	9	7/14/2014	43:56.0
3891	RSPe_2	-122.445	37.933	-0.19	18.63	154.29	18.43	-122.445	37.933	-0.38	18.63	154.31	18.25	9	7/14/2014	43:55.9
3892	RSPe_2	-122.445	37.933	-0.19	18.56	154.03	18.36	-122.445	37.933	-0.41	18.56	154.13	18.14	9	7/14/2014	43:55.8
3893	RSPe_2	-122.445	37.933	-0.19	18.53	153.85	18.34	-122.445	37.933	-0.38	18.53	154.01	18.15	9	7/14/2014	43:55.7

3894	RSPe_2	-122.445	37.933	-0.19	18.51	153.57	18.31	-122.445	37.933	-0.38	18.51	153.83	18.13	9	7/14/2014	43:55.6
3895	RSPe_2	-122.445	37.933	-0.19	18.51	153.35	18.31	-122.445	37.933	-0.38	18.51	153.62	18.13	9	7/14/2014	43:55.5
3896	RSPe_2	-122.445	37.933	-0.19	18.51	153.19	18.31	-122.445	37.933	-0.33	18.51	153.42	18.18	9	7/14/2014	43:55.4
3897	RSPe_2	-122.445	37.933	-0.19	18.50	152.91	18.30	-122.445	37.933	-0.33	18.50	153.24	18.17	9	7/14/2014	43:55.3
3898	RSPe_2	-122.445	37.933	-0.19	18.50	152.70	18.30	-122.445	37.933	-0.38	18.50	153.02	18.12	9	7/14/2014	43:55.2
3899	RSPe_2	-122.445	37.933	-0.16	18.49	152.53	18.33	-122.445	37.933	-0.38	18.49	152.88	18.11	9	7/14/2014	43:55.1
3900	RSPe_2	-122.445	37.933	-0.19	18.49	152.33	18.29	-122.445	37.933	-0.33	18.49	152.65	18.16	9	7/14/2014	43:55.0
3901	RSPe_2	-122.445	37.933	-0.16	18.49	152.07	18.33	-122.445	37.933	-0.33	18.49	152.43	18.16	9	7/14/2014	43:54.9
3902	RSPe_2	-122.445	37.933	-0.19	18.50	151.90	18.30	-122.445	37.933	-0.33	18.50	152.05	18.17	9	7/14/2014	43:54.8
3903	RSPe_2	-122.445	37.933	-0.16	18.52	151.69	18.36	-122.445	37.933	-0.38	18.52	151.83	18.14	9	7/14/2014	43:54.7
3904	RSPe_2	-122.445	37.933	-0.16	18.53	151.44	18.37	-122.445	37.933	-0.38	18.53	151.59	18.15	9	7/14/2014	43:54.6
3905	RSPe_2	-122.445	37.933	-0.16	18.52	151.20	18.36	-122.445	37.933	-0.33	18.52	151.35	18.19	9	7/14/2014	43:54.5
3906	RSPe_2	-122.445	37.933	-0.19	18.52	150.90	18.33	-122.445	37.933	-0.24	18.52	151.11	18.28	9	7/14/2014	43:54.4
3907	RSPe_2	-122.445	37.933	-0.16	18.58	150.68	18.42	-122.445	37.933	-0.33	18.58	150.79	18.25	9	7/14/2014	43:54.3
3908	RSPe_2	-122.445	37.933	-0.16	18.55	150.43	18.39	-122.445	37.933	-0.29	18.55	150.51	18.26	9	7/14/2014	43:54.2
3909	RSPe_2	-122.445	37.933	-0.16	18.59	150.29	18.43	-122.445	37.933	-0.29	18.59	150.29	18.29	9	7/14/2014	43:54.1
3910	RSPe_2	-122.445	37.933	-0.16	18.57	150.13	18.40	-122.445	37.933	-0.38	18.57	150.07	18.19	9	7/14/2014	43:54.0
3911	RSPe_2	-122.445	37.933	-0.11	18.57	149.89	18.46	-122.445	37.933	-0.33	18.57	149.87	18.24	9	7/14/2014	43:53.9
3912	RSPe_2	-122.445	37.933	-0.16	18.59	149.73	18.43	-122.445	37.933	-0.24	18.59	149.57	18.35	9	7/14/2014	43:53.8
3913	RSPe_2	-122.445	37.933	-0.11	18.56	149.48	18.45	-122.445	37.933	-0.29	18.56	149.34	18.26	9	7/14/2014	43:53.7
3914	RSPe_2	-122.445	37.933	-0.16	18.56	149.22	18.40	-122.445	37.933	-0.29	18.56	149.08	18.26	9	7/14/2014	43:53.6
3915	RSPe_2	-122.445	37.933	-0.11	18.49	149.00	18.38	-122.445	37.933	-0.33	18.49	148.88	18.16	9	7/14/2014	43:53.5
3916	RSPe_2	-122.445	37.933	-0.11	18.45	148.71	18.34	-122.445	37.933	-0.29	18.45	148.72	18.15	9	7/14/2014	43:53.4
3917	RSPe_2	-122.445	37.933	-0.11	18.40	148.48	18.29	-122.445	37.933	-0.24	18.40	148.48	18.15	9	7/14/2014	43:53.3
3918	RSPe_2	-122.445	37.933	-0.11	18.38	148.26	18.27	-122.445	37.933	-0.33	18.38	148.21	18.05	9	7/14/2014	43:53.2
3919	RSPe_2	-122.445	37.933	-0.11	18.38	147.98	18.27	-122.445	37.933	-0.24	18.38	147.95	18.14	9	7/14/2014	43:53.1
3920	RSPe_2	-122.445	37.933	-0.16	18.29	147.74	18.13	-122.445	37.933	-0.24	18.29	147.72	18.04	9	7/14/2014	43:53.0
3921	RSPe_2	-122.445	37.933	-0.11	18.29	147.51	18.18	-122.445	37.933	-0.29	18.29	147.52	17.99	9	7/14/2014	43:52.9
3922	RSPe_2	-122.445	37.933	-0.16	18.37	147.30	18.21	-122.445	37.933	-0.21	18.37	147.26	18.16	9	7/14/2014	43:52.8
3923	RSPe_2	-122.445	37.933	-0.08	18.35	147.13	18.27	-122.445	37.933	-0.18	18.35	147.10	18.17	9	7/14/2014	43:52.7
3924	RSPe_2	-122.445	37.933	-0.11	18.27	146.90	18.16	-122.445	37.933	-0.18	18.27	146.90	18.09	9	7/14/2014	43:52.6
3925	RSPe_2	-122.445	37.933	-0.08	18.32	146.71	18.24	-122.445	37.933	-0.21	18.32	146.69	18.11	9	7/14/2014	43:52.5
3926	RSPe_2	-122.445	37.933	-0.11	18.24	146.46	18.13	-122.445	37.933	-0.21	18.24	146.44	18.03	9	7/14/2014	43:52.4

3927	RSPe_2	-122.445	37.933	-0.08	18.27	146.13	18.20	-122.445	37.933	-0.24	18.27	146.15	18.03	9	7/14/2014 43:52.3
3928	RSPe_2	-122.445	37.933	-0.11	18.14	145.86	18.03	-122.445	37.933	-0.24	18.14	145.95	17.90	9	7/14/2014 43:52.2
3929	RSPe_2	-122.445	37.933	-0.11	18.30	145.57	18.19	-122.445	37.933	-0.21	18.30	145.72	18.09	9	7/14/2014 43:52.1
3930	RSPe_2	-122.445	37.933	-0.11	18.19	145.33	18.08	-122.445	37.933	-0.29	18.19	145.50	17.89	9	7/14/2014 43:52.0
3931	RSPe_2	-122.445	37.933	-0.08	18.24	145.09	18.16	-122.445	37.933	-0.33	18.24	145.28	17.91	9	7/14/2014 43:51.9
3932	RSPe_2	-122.445	37.933	-0.11	18.40	144.89	18.29	-122.445	37.933	-0.21	18.40	145.08	18.19	9	7/14/2014 43:51.8
3933	RSPe_2	-122.445	37.933	-0.11	18.37	144.76	18.26	-122.445	37.933	-0.29	18.37	144.88	18.08	9	7/14/2014 43:51.7
3934	RSPe_2	-122.445	37.933	-0.11	18.59	144.57	18.48	-122.445	37.933	-0.38	18.59	144.65	18.21	9	7/14/2014 43:51.6
3935	RSPe_2	-122.445	37.933	-0.11	18.50	144.37	18.39	-122.445	37.933	-0.21	18.50	144.35	18.29	9	7/14/2014 43:51.5
3936	RSPe_2	-122.445	37.933	-0.08	18.63	144.19	18.56	-122.445	37.933	-0.33	18.63	144.16	18.30	9	7/14/2014 43:51.4
3937	RSPe_2	-122.445	37.933	-0.08	18.57	143.85	18.50	-122.445	37.933	-0.29	18.57	143.73	18.28	9	7/14/2014 43:51.3
3938	RSPe_2	-122.445	37.933	-0.11	18.59	143.62	18.48	-122.445	37.933	-0.29	18.59	143.50	18.30	9	7/14/2014 43:51.2
3939	RSPe_2	-122.445	37.933	-0.08	18.46	143.37	18.38	-122.445	37.933	-0.18	18.46	143.20	18.28	9	7/14/2014 43:51.1
3940	RSPe_2	-122.445	37.933	-0.08	18.42	143.00	18.34	-122.445	37.933	-0.29	18.42	142.86	18.12	9	7/14/2014 43:51.0
3941	RSPe_2	-122.445	37.933	-0.08	18.50	142.67	18.42	-122.445	37.933	-0.21	18.50	142.60	18.29	9	7/14/2014 43:50.9
3942	RSPe_2	-122.445	37.933	-0.11	18.36	142.41	18.25	-122.445	37.933	-0.21	18.36	142.39	18.15	9	7/14/2014 43:50.8
3943	RSPe_2	-122.445	37.933	-0.08	18.44	142.21	18.36	-122.445	37.933	-0.13	18.44	142.19	18.31	9	7/14/2014 43:50.7
3944	RSPe_2	-122.445	37.933	-0.11	18.38	142.08	18.27	-122.445	37.933	-0.21	18.38	142.00	18.17	9	7/14/2014 43:50.6
3945	RSPe_2	-122.445	37.933	-0.08	18.36	141.93	18.29	-122.445	37.933	-0.24	18.36	141.82	18.12	9	7/14/2014 43:50.5
3946	RSPe_2	-122.445	37.933	-0.11	18.34	141.73	18.23	-122.445	37.933	-0.24	18.34	141.53	18.10	9	7/14/2014 43:50.4
3947	RSPe_2	-122.445	37.933	-0.08	18.36	141.57	18.28	-122.445	37.933	-0.18	18.36	141.29	18.18	9	7/14/2014 43:50.3
3948	RSPe_2	-122.445	37.933	-0.11	18.33	141.41	18.22	-122.445	37.933	-0.18	18.33	141.11	18.15	9	7/14/2014 43:50.2
3949	RSPe_2	-122.445	37.933	-0.11	18.31	141.12	18.20	-122.445	37.933	-0.21	18.31	140.79	18.10	9	7/14/2014 43:50.1
3950	RSPe_2	-122.445	37.933	-0.08	18.23	140.74	18.15	-122.445	37.933	-0.21	18.23	140.49	18.02	9	7/14/2014 43:50.0
3951	RSPe_2	-122.445	37.933	-0.08	18.19	140.45	18.12	-122.445	37.933	-0.24	18.19	140.19	17.95	9	7/14/2014 43:49.9
3952	RSPe_2	-122.445	37.933	-0.08	18.19	140.26	18.11	-122.445	37.933	-0.18	18.19	140.01	18.01	9	7/14/2014 43:49.8
3953	RSPe_2	-122.445	37.933	-0.08	18.16	140.01	18.08	-122.445	37.933	-0.29	18.16	139.79	17.86	9	7/14/2014 43:49.7
3954	RSPe_2	-122.445	37.933	-0.11	18.19	139.76	18.08	-122.445	37.933	-0.13	18.19	139.56	18.06	9	7/14/2014 43:49.6
3955	RSPe_2	-122.445	37.933	-0.08	18.19	139.60	18.12	-122.445	37.933	-0.04	18.19	139.43	18.15	9	7/14/2014 43:49.5
3956	RSPe_2	-122.445	37.933	-0.11	18.18	139.36	18.07	-122.445	37.933	-0.18	18.18	139.15	18.00	9	7/14/2014 43:49.4
3957	RSPe_2	-122.445	37.933	-0.08	18.13	139.02	18.06	-122.445	37.933	-0.13	18.13	138.86	18.01	9	7/14/2014 43:49.3
3958	RSPe_2	-122.445	37.933	-0.11	18.12	138.77	18.01	-122.445	37.933	-0.24	18.12	138.58	17.88	9	7/14/2014 43:49.2
3959	RSPe_2	-122.445	37.933	-0.08	18.12	138.49	18.04	-122.445	37.933	-0.13	18.12	138.29	17.99	9	7/14/2014 43:49.1

3960	RSPe_2	-122.445	37.933	-0.11	18.10	138.04	17.99	-122.445	37.933	-0.21	18.10	137.97	17.89	9	7/14/2014	43:49.0
3961	RSPe_2	-122.445	37.933	-0.08	18.11	137.83	18.03	-122.445	37.933	-0.01	18.11	137.71	18.10	9	7/14/2014	43:48.9
3962	RSPe_2	-122.445	37.933	-0.08	18.09	137.51	18.01	-122.445	37.933	-0.18	18.09	137.45	17.91	9	7/14/2014	43:48.8
3963	RSPe_2	-122.445	37.933	-0.08	18.05	137.27	17.97	-122.445	37.933	-0.21	18.05	137.21	17.84	9	7/14/2014	43:48.7
3964	RSPe_2	-122.445	37.933	-0.08	18.05	136.90	17.98	-122.445	37.933	-0.18	18.05	136.92	17.88	9	7/14/2014	43:48.6
3965	RSPe_2	-122.445	37.933	-0.08	18.03	136.71	17.96	-122.445	37.933	-0.21	18.03	136.62	17.82	9	7/14/2014	43:48.5
3966	RSPe_2	-122.445	37.933	-0.08	18.03	136.43	17.96	-122.445	37.933	-0.24	18.03	136.23	17.79	9	7/14/2014	43:48.4
3967	RSPe_2	-122.445	37.933	-0.08	18.01	136.23	17.93	-122.445	37.933	-0.18	18.01	136.02	17.83	9	7/14/2014	43:48.3
3968	RSPe_2	-122.445	37.933	-0.08	17.98	135.99	17.90	-122.445	37.933	-0.13	17.98	135.67	17.85	9	7/14/2014	43:48.2
3969	RSPe_2	-122.445	37.933	-0.02	17.43	135.69	17.41	-122.445	37.933	-0.04	17.43	135.31	17.39	9	7/14/2014	43:48.1
3970	RSPe_2	-122.445	37.933	-0.08	17.39	135.48	17.32	-122.445	37.933	-0.18	17.39	135.05	17.21	9	7/14/2014	43:48.0
3971	RSPe_2	-122.445	37.933	-0.02	17.94	135.17	17.92	-122.445	37.933	-0.09	17.94	134.71	17.85	9	7/14/2014	43:47.9
3972	RSPe_2	-122.445	37.933	-0.08	17.92	134.85	17.85	-122.445	37.933	-0.04	17.92	134.33	17.88	9	7/14/2014	43:47.8
3973	RSPe_2	-122.445	37.933	-0.08	17.94	134.60	17.86	-122.445	37.933	-0.09	17.94	134.05	17.85	9	7/14/2014	43:47.7
3974	RSPe_2	-122.445	37.933	-0.08	17.93	134.36	17.85	-122.445	37.933	-0.01	17.93	133.88	17.92	9	7/14/2014	43:47.6
3975	RSPe_2	-122.445	37.933	-0.02	17.92	134.14	17.89	-122.445	37.933	-0.09	17.92	133.61	17.82	9	7/14/2014	43:47.5
3976	RSPe_2	-122.445	37.933	-0.02	17.96	133.85	17.93	-122.445	37.933	-0.09	17.96	133.34	17.86	9	7/14/2014	43:47.4
3977	RSPe_2	-122.445	37.933	-0.02	17.97	133.49	17.95	-122.445	37.933	-0.04	17.97	133.13	17.93	9	7/14/2014	43:47.3
3978	RSPe_2	-122.445	37.933	-0.08	17.98	133.14	17.90	-122.445	37.933	-0.01	17.98	132.86	17.97	9	7/14/2014	43:47.2
3979	RSPe_2	-122.445	37.933	-0.02	17.97	132.83	17.95	-122.445	37.933	-0.01	17.97	132.57	17.97	9	7/14/2014	43:47.1
3980	RSPe_2	-122.445	37.933	-0.08	17.95	132.51	17.87	-122.445	37.933	-0.01	17.95	132.35	17.94	9	7/14/2014	43:47.0
3981	RSPe_2	-122.445	37.933	-0.02	17.97	132.25	17.95	-122.445	37.933	-0.04	17.97	132.06	17.93	9	7/14/2014	43:46.9
3982	RSPe_2	-122.445	37.933	-0.02	17.97	131.98	17.95	-122.445	37.933	-0.01	17.97	131.82	17.97	9	7/14/2014	43:46.8
3983	RSPe_2	-122.445	37.933	-0.02	17.99	131.73	17.96	-122.445	37.933	0.08	17.99	131.55	18.06	9	7/14/2014	43:46.7
3984	RSPe_2	-122.445	37.933	-0.02	17.96	131.46	17.94	-122.445	37.933	-0.09	17.96	131.18	17.87	9	7/14/2014	43:46.6
3985	RSPe_2	-122.445	37.933	-0.02	17.94	131.16	17.92	-122.445	37.933	0.04	17.94	130.88	17.98	9	7/14/2014	43:46.5
3986	RSPe_2	-122.445	37.933	-0.02	17.99	130.94	17.96	-122.445	37.933	-0.01	17.99	130.58	17.98	9	7/14/2014	43:46.4
3987	RSPe_2	-122.445	37.933	-0.02	17.90	130.65	17.88	-122.445	37.933	-0.01	17.90	130.31	17.90	9	7/14/2014	43:46.3
3988	RSPe_2	-122.445	37.933	-0.08	17.95	130.31	17.87	-122.445	37.933	-0.01	17.95	130.01	17.94	9	7/14/2014	43:46.2
3989	RSPe_2	-122.445	37.933	-0.02	17.89	130.07	17.86	-122.445	37.933	0.04	17.89	129.72	17.93	9	7/14/2014	43:46.1
3990	RSPe_2	-122.445	37.933	-0.02	17.93	129.81	17.91	-122.445	37.933	0.08	17.93	129.47	18.01	9	7/14/2014	43:46.0
3991	RSPe_2	-122.445	37.933	-0.02	17.92	129.61	17.89	-122.445	37.933	0.04	17.92	129.20	17.96	9	7/14/2014	43:45.9
3992	RSPe_2	-122.445	37.933	-0.02	17.92	129.31	17.90	-122.445	37.933	-0.01	17.92	128.93	17.92	9	7/14/2014	43:45.8

3993	RSPe_2	-122.445	37.933	-0.02	17.92	129.02	17.90	-122.445	37.933	0.08	17.92	128.60	18.00	9	7/14/2014	43:45.7
3994	RSPe_2	-122.445	37.933	-0.08	17.98	128.82	17.90	-122.445	37.933	0.04	17.98	128.39	18.02	9	7/14/2014	43:45.6
3995	RSPe_2	-122.445	37.933	-0.02	17.96	128.55	17.93	-122.445	37.933	0.04	17.96	128.11	18.00	9	7/14/2014	43:45.5
3996	RSPe_2	-122.445	37.933	-0.02	18.00	128.31	17.98	-122.445	37.933	0.13	18.00	127.84	18.13	9	7/14/2014	43:45.4
3997	RSPe_2	-122.445	37.933	-0.02	17.98	128.11	17.96	-122.445	37.933	0.08	17.98	127.60	18.06	9	7/14/2014	43:45.3
3998	RSPe_2	-122.445	37.933	-0.02	17.99	127.91	17.96	-122.445	37.933	0.08	17.99	127.39	18.06	9	7/14/2014	43:45.2
3999	RSPe_2	-122.445	37.933	-0.02	17.99	127.65	17.96	-122.445	37.933	0.08	17.99	127.17	18.06	9	7/14/2014	43:45.1
4000	RSPe_2	-122.445	37.933	-0.02	18.02	127.42	17.99	-122.445	37.933	0.08	18.02	126.94	18.09	9	7/14/2014	43:45.0
4001	RSPe_2	-122.445	37.933	-0.02	17.99	127.24	17.97	-122.445	37.933	0.08	17.99	126.86	18.07	9	7/14/2014	43:44.9
4002	RSPe_2	-122.445	37.933	-0.08	18.00	127.01	17.92	-122.445	37.933	0.08	18.00	126.70	18.08	9	7/14/2014	43:44.8
4003	RSPe_2	-122.445	37.933	-0.02	17.99	126.74	17.96	-122.445	37.933	0.08	17.99	126.56	18.06	9	7/14/2014	43:44.7
4004	RSPe_2	-122.445	37.933	-0.08	17.99	126.53	17.92	-122.445	37.933	0.04	17.99	126.41	18.04	9	7/14/2014	43:44.6
4005	RSPe_2	-122.445	37.933	-0.08	17.95	126.36	17.87	-122.445	37.933	0.08	17.95	126.24	18.02	9	7/14/2014	43:44.5
4006	RSPe_2	-122.445	37.933	-0.08	17.87	126.11	17.80	-122.445	37.933	0.04	17.87	126.04	17.91	9	7/14/2014	43:44.4
4007	RSPe_2	-122.445	37.933	-0.02	17.92	125.96	17.89	-122.445	37.933	0.08	17.92	125.80	17.99	9	7/14/2014	43:44.3
4008	RSPe_2	-122.445	37.933	-0.08	17.71	125.73	17.63	-122.445	37.933	-0.01	17.71	125.57	17.70	9	7/14/2014	43:44.2
4009	RSPe_2	-122.445	37.933	-0.08	17.73	125.59	17.66	-122.445	37.933	0.04	17.73	125.40	17.78	9	7/14/2014	43:44.1
4010	RSPe_2	-122.445	37.933	-0.08	17.78	125.41	17.70	-122.445	37.933	0.04	17.78	125.24	17.82	9	7/14/2014	43:44.0
4011	RSPe_2	-122.445	37.933	-0.08	17.65	125.22	17.58	-122.445	37.933	-0.01	17.65	125.03	17.65	9	7/14/2014	43:43.9
4012	RSPe_2	-122.445	37.933	-0.08	17.57	125.00	17.50	-122.445	37.933	-0.01	17.57	124.90	17.56	9	7/14/2014	43:43.8
4013	RSPe_2	-122.445	37.933	-0.08	17.63	124.90	17.56	-122.445	37.933	0.04	17.63	124.76	17.68	9	7/14/2014	43:43.7
4014	RSPe_2	-122.445	37.933	-0.11	17.54	124.74	17.43	-122.445	37.933	0.04	17.54	124.50	17.58	9	7/14/2014	43:43.6
4015	RSPe_2	-122.445	37.933	-0.11	17.58	124.52	17.47	-122.445	37.933	-0.01	17.58	124.25	17.58	9	7/14/2014	43:43.5
4016	RSPe_2	-122.445	37.933	-0.11	17.51	124.34	17.40	-122.445	37.933	-0.01	17.51	124.04	17.51	9	7/14/2014	43:43.4
4017	RSPe_2	-122.445	37.933	-0.08	17.51	124.11	17.43	-122.445	37.933	-0.01	17.51	123.72	17.50	9	7/14/2014	43:43.3
4018	RSPe_2	-122.445	37.933	-0.11	17.45	123.91	17.35	-122.445	37.933	-0.04	17.45	123.43	17.41	9	7/14/2014	43:43.2
4019	RSPe_2	-122.445	37.933	-0.11	17.41	123.68	17.30	-122.445	37.933	-0.01	17.41	123.26	17.40	9	7/14/2014	43:43.1
4020	RSPe_2	-122.445	37.933	-0.11	17.40	123.48	17.29	-122.445	37.933	-0.04	17.40	122.94	17.36	9	7/14/2014	43:43.0
4021	RSPe_2	-122.445	37.933	-0.11	17.39	123.25	17.28	-122.445	37.933	-0.01	17.39	122.74	17.38	9	7/14/2014	43:42.9
4022	RSPe_2	-122.445	37.933	-0.11	17.36	123.07	17.25	-122.445	37.933	-0.04	17.36	122.49	17.32	9	7/14/2014	43:42.8
4023	RSPe_2	-122.445	37.933	-0.11	17.37	122.82	17.26	-122.445	37.933	-0.04	17.37	122.34	17.33	9	7/14/2014	43:42.7
4024	RSPe_2	-122.445	37.933	-0.16	17.39	122.63	17.23	-122.445	37.933	-0.04	17.39	122.13	17.35	9	7/14/2014	43:42.6
4025	RSPe_2	-122.445	37.933	-0.11	17.37	122.41	17.26	-122.445	37.933	-0.01	17.37	122.02	17.36	9	7/14/2014	43:42.5

4026	RSPe_2	-122.445	37.933	-0.16	17.40	122.26	17.24	-122.445	37.933	-0.09	17.40	121.88	17.31	9	7/14/2014	43:42.4
4027	RSPe_2	-122.445	37.933	-0.11	17.33	122.02	17.22	-122.445	37.933	-0.04	17.33	121.70	17.29	9	7/14/2014	43:42.3
4028	RSPe_2	-122.445	37.933	-0.16	17.31	121.76	17.15	-122.445	37.933	-0.09	17.31	121.57	17.22	9	7/14/2014	43:42.2
4029	RSPe_2	-122.445	37.933	-0.16	17.30	121.57	17.14	-122.445	37.933	-0.09	17.30	121.35	17.21	9	7/14/2014	43:42.1
4030	RSPe_2	-122.445	37.933	-0.16	17.28	121.26	17.12	-122.445	37.933	-0.13	17.28	121.19	17.16	9	7/14/2014	43:42.0
4031	RSPe_2	-122.445	37.933	-0.16	17.28	120.96	17.12	-122.445	37.933	-0.04	17.28	120.95	17.24	9	7/14/2014	43:41.9
4032	RSPe_2	-122.445	37.933	-0.16	17.30	120.71	17.14	-122.445	37.933	-0.13	17.30	120.78	17.17	9	7/14/2014	43:41.8
4033	RSPe_2	-122.445	37.933	-0.16	17.31	120.48	17.15	-122.445	37.933	-0.09	17.31	120.63	17.22	9	7/14/2014	43:41.7
4034	RSPe_2	-122.445	37.933	-0.16	17.36	120.28	17.20	-122.445	37.933	-0.13	17.36	120.44	17.24	9	7/14/2014	43:41.6
4035	RSPe_2	-122.445	37.933	-0.16	17.39	120.08	17.23	-122.445	37.933	-0.09	17.39	120.15	17.29	9	7/14/2014	43:41.5
4036	RSPe_2	-122.445	37.933	-0.16	17.38	119.90	17.22	-122.445	37.933	-0.13	17.38	119.81	17.25	9	7/14/2014	43:41.4
4037	RSPe_2	-122.445	37.933	-0.16	17.30	119.71	17.14	-122.445	37.933	-0.09	17.30	119.45	17.21	9	7/14/2014	43:41.3
4038	RSPe_2	-122.445	37.933	-0.19	17.30	119.58	17.10	-122.445	37.933	-0.09	17.30	119.11	17.21	9	7/14/2014	43:41.2
4039	RSPe_2	-122.445	37.933	-0.16	17.25	119.34	17.09	-122.445	37.933	-0.09	17.25	118.83	17.16	9	7/14/2014	43:41.1
4040	RSPe_2	-122.445	37.933	-0.19	17.21	119.13	17.02	-122.445	37.933	-0.13	17.21	118.54	17.09	9	7/14/2014	43:41.0
4041	RSPe_2	-122.445	37.933	-0.16	17.25	118.88	17.09	-122.445	37.933	-0.13	17.25	118.30	17.12	9	7/14/2014	43:40.9
4042	RSPe_2	-122.445	37.933	-0.19	17.25	118.69	17.05	-122.445	37.933	-0.18	17.25	118.16	17.07	9	7/14/2014	43:40.8
4043	RSPe_2	-122.445	37.933	-0.16	17.24	118.44	17.08	-122.445	37.933	-0.13	17.24	118.08	17.11	9	7/14/2014	43:40.7
4044	RSPe_2	-122.445	37.933	-0.16	17.24	118.23	17.08	-122.445	37.933	-0.21	17.24	117.93	17.03	9	7/14/2014	43:40.6
4045	RSPe_2	-122.445	37.933	-0.16	17.27	118.00	17.11	-122.445	37.933	-0.21	17.27	117.76	17.06	9	7/14/2014	43:40.5
4046	RSPe_2	-122.445	37.933	-0.19	17.26	117.71	17.07	-122.445	37.933	-0.18	17.26	117.49	17.08	9	7/14/2014	43:40.4
4047	RSPe_2	-122.445	37.933	-0.19	17.28	117.45	17.08	-122.445	37.933	-0.18	17.28	117.25	17.10	9	7/14/2014	43:40.3
4048	RSPe_2	-122.445	37.933	-0.19	17.28	117.21	17.09	-122.445	37.933	-0.18	17.28	117.01	17.11	9	7/14/2014	43:40.2
4049	RSPe_2	-122.445	37.933	-0.19	17.28	116.95	17.08	-122.445	37.933	-0.21	17.28	116.77	17.07	9	7/14/2014	43:40.1
4050	RSPe_2	-122.445	37.933	-0.19	17.32	116.71	17.13	-122.445	37.933	-0.21	17.32	116.53	17.11	9	7/14/2014	43:40.0
4051	RSPe_2	-122.445	37.933	-0.16	17.32	116.47	17.16	-122.445	37.933	-0.18	17.32	116.30	17.14	9	7/14/2014	43:39.9
4052	RSPe_2	-122.445	37.933	-0.19	17.35	116.25	17.15	-122.445	37.933	-0.18	17.35	116.10	17.17	9	7/14/2014	43:39.8
4053	RSPe_2	-122.445	37.933	-0.16	17.37	115.99	17.21	-122.445	37.933	-0.18	17.37	115.77	17.19	9	7/14/2014	43:39.7
4054	RSPe_2	-122.445	37.933	-0.19	17.35	115.67	17.16	-122.445	37.933	-0.18	17.35	115.50	17.18	9	7/14/2014	43:39.6
4055	RSPe_2	-122.445	37.933	-0.16	17.39	115.47	17.23	-122.445	37.933	-0.13	17.39	115.26	17.26	9	7/14/2014	43:39.5
4056	RSPe_2	-122.445	37.933	-0.19	17.41	115.21	17.21	-122.445	37.933	-0.21	17.41	115.04	17.20	9	7/14/2014	43:39.4
4057	RSPe_2	-122.445	37.933	-0.19	17.39	114.92	17.19	-122.445	37.933	-0.18	17.39	114.81	17.21	9	7/14/2014	43:39.3
4058	RSPe_2	-122.445	37.933	-0.19	17.39	114.71	17.20	-122.445	37.933	-0.21	17.39	114.52	17.18	9	7/14/2014	43:39.2

4059	RSPe_2	-122.445	37.933	-0.16	17.45	114.50	17.29	-122.445	37.933	-0.18	17.45	114.31	17.28	9	7/14/2014	43:39.1
4060	RSPe_2	-122.445	37.933	-0.19	17.45	114.28	17.26	-122.445	37.933	-0.21	17.45	114.07	17.25	9	7/14/2014	43:39.0
4061	RSPe_2	-122.445	37.933	-0.19	17.44	114.06	17.24	-122.445	37.933	-0.21	17.44	113.81	17.23	9	7/14/2014	43:38.9
4062	RSPe_2	-122.445	37.933	-0.19	17.46	113.82	17.27	-122.445	37.933	-0.18	17.46	113.53	17.28	9	7/14/2014	43:38.8
4063	RSPe_2	-122.445	37.933	-0.16	17.51	113.56	17.35	-122.445	37.933	-0.21	17.51	113.32	17.30	9	7/14/2014	43:38.7
4064	RSPe_2	-122.445	37.933	-0.19	17.52	113.34	17.33	-122.445	37.933	-0.18	17.52	113.06	17.35	9	7/14/2014	43:38.6
4065	RSPe_2	-122.445	37.933	-0.19	17.50	113.10	17.31	-122.445	37.933	-0.21	17.50	112.78	17.29	9	7/14/2014	43:38.5
4066	RSPe_2	-122.445	37.933	-0.19	17.50	112.80	17.31	-122.445	37.933	-0.18	17.50	112.52	17.32	9	7/14/2014	43:38.4
4067	RSPe_2	-122.445	37.933	-0.16	17.52	112.51	17.36	-122.445	37.933	-0.18	17.52	112.23	17.35	9	7/14/2014	43:38.3
4068	RSPe_2	-122.445	37.933	-0.19	17.58	112.23	17.39	-122.445	37.933	-0.18	17.58	111.92	17.41	9	7/14/2014	43:38.2
4069	RSPe_2	-122.445	37.933	-0.16	17.59	111.90	17.43	-122.445	37.933	-0.18	17.59	111.64	17.42	9	7/14/2014	43:38.1
4070	RSPe_2	-122.445	37.933	-0.16	17.59	111.72	17.43	-122.445	37.933	-0.18	17.59	111.44	17.42	9	7/14/2014	43:38.0
4071	RSPe_2	-122.445	37.933	-0.16	17.55	111.47	17.39	-122.445	37.933	-0.18	17.55	111.27	17.37	9	7/14/2014	43:37.9
4072	RSPe_2	-122.445	37.933	-0.19	17.57	111.23	17.38	-122.445	37.933	-0.18	17.57	111.04	17.39	9	7/14/2014	43:37.8
4073	RSPe_2	-122.445	37.933	-0.16	17.58	110.99	17.42	-122.445	37.933	-0.18	17.58	110.79	17.41	9	7/14/2014	43:37.7
4074	RSPe_2	-122.445	37.933	-0.19	17.64	110.79	17.45	-122.445	37.933	-0.21	17.64	110.58	17.43	9	7/14/2014	43:37.6
4075	RSPe_2	-122.445	37.933	-0.16	17.60	110.55	17.44	-122.445	37.933	-0.13	17.60	110.31	17.47	9	7/14/2014	43:37.5
4076	RSPe_2	-122.445	37.933	-0.19	17.61	110.34	17.41	-122.445	37.933	-0.18	17.61	110.05	17.43	9	7/14/2014	43:37.4
4077	RSPe_2	-122.445	37.933	-0.19	17.64	110.14	17.45	-122.445	37.933	-0.18	17.64	109.79	17.46	9	7/14/2014	43:37.3
4078	RSPe_2	-122.445	37.933	-0.19	17.63	109.94	17.44	-122.445	37.933	-0.18	17.63	109.55	17.46	9	7/14/2014	43:37.2
4079	RSPe_2	-122.445	37.933	-0.19	17.66	109.73	17.47	-122.445	37.933	-0.18	17.66	109.33	17.49	9	7/14/2014	43:37.1
4080	RSPe_2	-122.445	37.933	-0.19	17.68	109.48	17.49	-122.445	37.933	-0.13	17.68	109.07	17.56	9	7/14/2014	43:37.0
4081	RSPe_2	-122.445	37.933	-0.16	17.71	109.27	17.55	-122.445	37.933	-0.09	17.71	108.90	17.62	9	7/14/2014	43:36.9
4082	RSPe_2	-122.445	37.933	-0.19	17.70	109.10	17.51	-122.445	37.933	-0.13	17.70	108.66	17.58	9	7/14/2014	43:36.8
4083	RSPe_2	-122.445	37.933	-0.19	17.72	108.87	17.52	-122.445	37.933	-0.18	17.72	108.54	17.54	9	7/14/2014	43:36.7
4084	RSPe_2	-122.445	37.933	-0.19	17.72	108.71	17.53	-122.445	37.933	-0.18	17.72	108.36	17.55	9	7/14/2014	43:36.6
4085	RSPe_2	-122.445	37.933	-0.19	17.72	108.49	17.53	-122.445	37.933	-0.13	17.72	108.20	17.60	9	7/14/2014	43:36.5
4086	RSPe_2	-122.445	37.933	-0.19	17.74	108.25	17.54	-122.445	37.933	-0.18	17.74	107.97	17.56	9	7/14/2014	43:36.4
4087	RSPe_2	-122.445	37.933	-0.19	17.75	107.96	17.55	-122.445	37.933	-0.18	17.75	107.80	17.57	9	7/14/2014	43:36.3
4088	RSPe_2	-122.445	37.933	-0.19	17.75	107.75	17.55	-122.445	37.933	-0.21	17.75	107.60	17.54	9	7/14/2014	43:36.2
4089	RSPe_2	-122.445	37.933	-0.19	17.79	107.45	17.60	-122.445	37.933	-0.21	17.79	107.40	17.58	9	7/14/2014	43:36.1
4090	RSPe_2	-122.445	37.933	-0.23	17.82	107.25	17.59	-122.445	37.933	-0.24	17.82	107.27	17.57	9	7/14/2014	43:36.0
4091	RSPe_2	-122.445	37.933	-0.19	17.73	106.99	17.54	-122.445	37.933	-0.18	17.73	107.05	17.56	9	7/14/2014	43:35.9

4092	RSPe_2	-122.445	37.933	-0.23	17.79	106.79	17.56	-122.445	37.933	-0.21	17.79	106.86	17.58	9	7/14/2014	43:35.8
4093	RSPe_2	-122.445	37.933	-0.19	17.75	106.49	17.56	-122.445	37.933	-0.21	17.75	106.59	17.54	9	7/14/2014	43:35.7
4094	RSPe_2	-122.445	37.933	-0.19	17.79	106.31	17.60	-122.445	37.933	-0.21	17.79	106.38	17.58	9	7/14/2014	43:35.6
4095	RSPe_2	-122.445	37.933	-0.19	17.82	106.12	17.63	-122.445	37.933	-0.21	17.82	106.13	17.61	9	7/14/2014	43:35.5
4096	RSPe_2	-122.445	37.933	-0.23	17.80	106.01	17.57	-122.445	37.933	-0.21	17.80	105.95	17.59	9	7/14/2014	43:35.4
4097	RSPe_2	-122.445	37.933	-0.19	17.80	105.86	17.61	-122.445	37.933	-0.21	17.80	105.72	17.59	9	7/14/2014	43:35.3
4098	RSPe_2	-122.445	37.933	-0.23	17.75	105.74	17.52	-122.445	37.933	-0.24	17.75	105.50	17.50	9	7/14/2014	43:35.2
4099	RSPe_2	-122.445	37.933	-0.23	17.78	105.53	17.55	-122.445	37.933	-0.21	17.78	105.29	17.57	9	7/14/2014	43:35.1
4100	RSPe_2	-122.445	37.933	-0.23	17.73	105.40	17.50	-122.445	37.933	-0.21	17.73	105.09	17.52	9	7/14/2014	43:35.0
4101	RSPe_2	-122.445	37.933	-0.23	17.68	105.22	17.45	-122.445	37.933	-0.29	17.68	104.92	17.38	9	7/14/2014	43:34.9
4102	RSPe_2	-122.445	37.933	-0.23	17.70	105.10	17.47	-122.445	37.933	-0.24	17.70	104.79	17.46	9	7/14/2014	43:34.8
4103	RSPe_2	-122.445	37.933	-0.23	17.70	104.95	17.47	-122.445	37.933	-0.24	17.70	104.61	17.46	9	7/14/2014	43:34.7
4104	RSPe_2	-122.445	37.933	-0.23	17.67	104.75	17.44	-122.445	37.933	-0.21	17.67	104.47	17.46	9	7/14/2014	43:34.6
4105	RSPe_2	-122.445	37.933	-0.19	17.68	104.57	17.48	-122.445	37.933	-0.21	17.68	104.33	17.47	9	7/14/2014	43:34.5
4106	RSPe_2	-122.445	37.933	-0.23	17.66	104.39	17.43	-122.445	37.933	-0.24	17.66	104.19	17.42	9	7/14/2014	43:34.4
4107	RSPe_2	-122.445	37.933	-0.23	17.74	104.25	17.51	-122.445	37.933	-0.21	17.74	104.06	17.53	9	7/14/2014	43:34.3
4108	RSPe_2	-122.445	37.933	-0.23	17.71	104.14	17.48	-122.445	37.933	-0.24	17.71	103.86	17.47	9	7/14/2014	43:34.2
4109	RSPe_2	-122.445	37.933	-0.23	17.68	103.90	17.46	-122.445	37.933	-0.18	17.68	103.65	17.51	9	7/14/2014	43:34.1
4110	RSPe_2	-122.445	37.933	-0.23	17.68	103.72	17.46	-122.445	37.933	-0.21	17.68	103.41	17.48	9	7/14/2014	43:34.0
4111	RSPe_2	-122.445	37.933	-0.23	17.70	103.55	17.47	-122.445	37.933	-0.21	17.70	103.27	17.49	9	7/14/2014	43:33.9
4112	RSPe_2	-122.445	37.933	-0.23	17.66	103.23	17.43	-122.445	37.933	-0.24	17.66	103.03	17.42	9	7/14/2014	43:33.8
4113	RSPe_2	-122.445	37.933	-0.23	17.68	103.01	17.46	-122.445	37.933	-0.21	17.68	102.83	17.48	9	7/14/2014	43:33.7
4114	RSPe_2	-122.445	37.933	-0.23	17.66	102.76	17.43	-122.445	37.933	-0.29	17.66	102.75	17.37	9	7/14/2014	43:33.6
4115	RSPe_2	-122.445	37.933	-0.23	17.67	102.57	17.44	-122.445	37.933	-0.24	17.67	102.56	17.43	9	7/14/2014	43:33.5
4116	RSPe_2	-122.445	37.933	-0.23	17.67	102.29	17.44	-122.445	37.933	-0.29	17.67	102.42	17.38	9	7/14/2014	43:33.4
4117	RSPe_2	-122.445	37.933	-0.23	17.69	102.22	17.47	-122.445	37.933	-0.24	17.69	102.38	17.45	9	7/14/2014	43:33.3
4118	RSPe_2	-122.445	37.933	-0.23	17.70	102.03	17.47	-122.445	37.933	-0.29	17.70	102.17	17.41	9	7/14/2014	43:33.2
4119	RSPe_2	-122.445	37.933	-0.19	17.72	101.83	17.53	-122.445	37.933	-0.24	17.72	101.98	17.48	9	7/14/2014	43:33.1
4120	RSPe_2	-122.445	37.933	-0.23	17.70	101.57	17.47	-122.445	37.933	-0.29	17.70	101.59	17.41	9	7/14/2014	43:33.0
4121	RSPe_2	-122.445	37.933	-0.23	17.79	101.29	17.56	-122.445	37.933	-0.24	17.79	101.29	17.55	9	7/14/2014	43:32.9
4122	RSPe_2	-122.445	37.933	-0.23	17.70	101.00	17.47	-122.445	37.933	-0.24	17.70	100.87	17.46	9	7/14/2014	43:32.8
4123	RSPe_2	-122.445	37.933	-0.23	17.73	100.75	17.50	-122.445	37.933	-0.21	17.73	100.56	17.52	9	7/14/2014	43:32.7
4124	RSPe_2	-122.445	37.933	-0.23	17.70	100.51	17.47	-122.445	37.933	-0.21	17.70	100.29	17.49	9	7/14/2014	43:32.6

4125	RSPe_2	-122.445	37.933	-0.23	17.69	100.33	17.47	-122.445	37.933	-0.24	17.69	100.05	17.45	9	7/14/2014	43:32.5
4126	RSPe_2	-122.445	37.933	-0.23	17.63	100.14	17.40	-122.445	37.933	-0.29	17.63	99.81	17.34	9	7/14/2014	43:32.4
4127	RSPe_2	-122.445	37.933	-0.19	17.57	99.92	17.38	-122.445	37.933	-0.21	17.57	99.57	17.36	9	7/14/2014	43:32.3
4128	RSPe_2	-122.445	37.933	-0.23	17.61	99.72	17.39	-122.445	37.933	-0.24	17.61	99.33	17.37	9	7/14/2014	43:32.2
4129	RSPe_2	-122.445	37.933	-0.23	17.56	99.53	17.33	-122.445	37.933	-0.24	17.56	99.22	17.32	9	7/14/2014	43:32.1
4130	RSPe_2	-122.445	37.933	-0.23	17.55	99.27	17.32	-122.445	37.933	-0.24	17.55	98.94	17.30	9	7/14/2014	43:32.0
4131	RSPe_2	-122.445	37.933	-0.23	17.56	99.03	17.33	-122.445	37.933	-0.24	17.56	98.76	17.31	9	7/14/2014	43:31.9
4132	RSPe_2	-122.445	37.933	-0.23	17.44	98.79	17.21	-122.445	37.933	-0.29	17.44	98.62	17.14	9	7/14/2014	43:31.8
4133	RSPe_2	-122.445	37.933	-0.23	17.36	98.54	17.13	-122.445	37.933	-0.24	17.36	98.39	17.12	9	7/14/2014	43:31.7
4134	RSPe_2	-122.445	37.933	-0.23	17.39	98.31	17.16	-122.445	37.933	-0.24	17.39	98.22	17.15	9	7/14/2014	43:31.6
4135	RSPe_2	-122.445	37.933	-0.23	17.34	98.06	17.11	-122.445	37.933	-0.21	17.34	97.98	17.13	9	7/14/2014	43:31.5
4136	RSPe_2	-122.445	37.933	-0.23	17.32	97.88	17.09	-122.445	37.933	-0.21	17.32	97.72	17.11	9	7/14/2014	43:31.4
4137	RSPe_2	-122.445	37.933	-0.19	17.30	97.59	17.10	-122.445	37.933	-0.18	17.30	97.37	17.12	9	7/14/2014	43:31.3
4138	RSPe_2	-122.445	37.933	-0.23	17.29	97.32	17.06	-122.445	37.933	-0.21	17.29	97.04	17.08	9	7/14/2014	43:31.2
4139	RSPe_2	-122.445	37.933	-0.23	17.21	97.00	16.98	-122.445	37.933	-0.21	17.21	96.78	17.00	9	7/14/2014	43:31.1
4140	RSPe_2	-122.445	37.933	-0.23	17.20	96.65	16.97	-122.445	37.933	-0.21	17.20	96.46	16.99	9	7/14/2014	43:31.0
4141	RSPe_2	-122.445	37.933	-0.19	17.09	96.38	16.90	-122.445	37.933	-0.21	17.09	96.25	16.88	9	7/14/2014	43:30.9
4142	RSPe_2	-122.445	37.933	-0.23	17.08	96.02	16.85	-122.445	37.933	-0.24	17.08	95.96	16.83	9	7/14/2014	43:30.8
4143	RSPe_2	-122.445	37.933	-0.23	17.05	95.74	16.82	-122.445	37.933	-0.21	17.05	95.81	16.84	9	7/14/2014	43:30.7
4144	RSPe_2	-122.445	37.933	-0.23	17.08	95.42	16.85	-122.445	37.933	-0.24	17.08	95.63	16.84	9	7/14/2014	43:30.6
4145	RSPe_2	-122.445	37.933	-0.19	17.05	95.08	16.86	-122.445	37.933	-0.21	17.05	95.32	16.84	9	7/14/2014	43:30.5
4146	RSPe_2	-122.445	37.933	-0.23	17.01	94.83	16.79	-122.445	37.933	-0.29	17.01	95.21	16.72	9	7/14/2014	43:30.4
4147	RSPe_2	-122.445	37.933	-0.19	16.97	94.67	16.77	-122.445	37.933	-0.24	16.97	95.01	16.72	9	7/14/2014	43:30.3
4148	RSPe_2	-122.445	37.933	-0.19	17.05	94.44	16.86	-122.445	37.933	-0.24	17.05	94.72	16.81	9	7/14/2014	43:30.2
4149	RSPe_2	-122.445	37.933	-0.19	17.02	94.12	16.83	-122.445	37.933	-0.21	17.02	94.37	16.81	9	7/14/2014	43:30.1
4150	RSPe_2	-122.445	37.933	-0.23	17.00	93.88	16.78	-122.445	37.933	-0.24	17.00	94.01	16.76	9	7/14/2014	43:30.0
4151	RSPe_2	-122.445	37.933	-0.19	16.95	93.63	16.76	-122.445	37.933	-0.24	16.95	93.63	16.71	9	7/14/2014	43:29.9
4152	RSPe_2	-122.445	37.933	-0.19	16.95	93.39	16.76	-122.445	37.933	-0.21	16.95	93.28	16.74	9	7/14/2014	43:29.8
4153	RSPe_2	-122.445	37.933	-0.16	16.92	93.08	16.76	-122.445	37.933	-0.21	16.92	92.95	16.71	9	7/14/2014	43:29.7
4154	RSPe_2	-122.445	37.933	-0.19	16.86	92.81	16.67	-122.445	37.933	-0.21	16.86	92.62	16.65	9	7/14/2014	43:29.6
4155	RSPe_2	-122.445	37.933	-0.16	16.81	92.52	16.65	-122.445	37.933	-0.18	16.81	92.37	16.63	9	7/14/2014	43:29.5
4156	RSPe_2	-122.445	37.933	-0.16	16.78	92.24	16.63	-122.445	37.933	-0.18	16.78	92.09	16.61	9	7/14/2014	43:29.4
4157	RSPe_2	-122.445	37.933	-0.16	16.77	91.88	16.61	-122.445	37.933	-0.18	16.77	91.84	16.59	9	7/14/2014	43:29.3

4158	RSPe_2	-122.445	37.933	-0.19	16.80	91.65	16.60	-122.445	37.933	-0.21	16.80	91.60	16.59	9	7/14/2014	43:29.2
4159	RSPe_2	-122.445	37.933	-0.16	16.74	91.36	16.58	-122.445	37.933	-0.21	16.74	91.34	16.53	9	7/14/2014	43:29.1
4160	RSPe_2	-122.445	37.933	-0.19	16.75	91.04	16.56	-122.445	37.933	-0.21	16.75	91.08	16.55	9	7/14/2014	43:29.0
4161	RSPe_2	-122.445	37.933	-0.16	16.72	90.80	16.56	-122.445	37.933	-0.21	16.72	90.81	16.51	9	7/14/2014	43:28.9
4162	RSPe_2	-122.445	37.933	-0.19	16.65	90.47	16.45	-122.445	37.933	-0.21	16.65	90.47	16.44	9	7/14/2014	43:28.8
4163	RSPe_2	-122.445	37.933	-0.16	16.66	90.17	16.50	-122.445	37.933	-0.12	16.66	90.16	16.53	9	7/14/2014	43:28.7
4164	RSPe_2	-122.445	37.933	-0.19	16.70	89.88	16.51	-122.445	37.933	-0.21	16.70	89.83	16.49	9	7/14/2014	43:28.6
4165	RSPe_2	-122.445	37.933	-0.16	16.58	89.56	16.42	-122.445	37.933	-0.18	16.58	89.49	16.41	9	7/14/2014	43:28.5
4166	RSPe_2	-122.445	37.933	-0.16	16.55	89.12	16.39	-122.445	37.933	-0.18	16.55	89.12	16.37	9	7/14/2014	43:28.4
4167	RSPe_2	-122.445	37.933	-0.16	16.47	88.75	16.32	-122.445	37.933	-0.12	16.47	88.75	16.35	9	7/14/2014	43:28.3
4168	RSPe_2	-122.445	37.933	-0.16	16.53	88.43	16.37	-122.445	37.933	-0.12	16.53	88.42	16.40	9	7/14/2014	43:28.2
4169	RSPe_2	-122.445	37.933	-0.16	16.46	88.01	16.30	-122.445	37.933	-0.12	16.46	88.11	16.33	9	7/14/2014	43:28.1
4170	RSPe_2	-122.445	37.933	-0.16	16.43	87.70	16.27	-122.445	37.933	-0.12	16.43	87.85	16.30	9	7/14/2014	43:28.0
4171	RSPe_2	-122.445	37.933	-0.11	16.44	87.36	16.34	-122.445	37.933	-0.09	16.44	87.62	16.35	9	7/14/2014	43:27.9
4172	RSPe_2	-122.445	37.933	-0.16	16.47	87.07	16.31	-122.445	37.933	-0.12	16.47	87.33	16.34	9	7/14/2014	43:27.8
4173	RSPe_2	-122.445	37.933	-0.11	16.47	86.77	16.36	-122.445	37.933	-0.18	16.47	87.03	16.29	9	7/14/2014	43:27.7
4174	RSPe_2	-122.445	37.933	-0.16	16.50	86.53	16.34	-122.445	37.933	-0.12	16.50	86.78	16.37	9	7/14/2014	43:27.6
4175	RSPe_2	-122.445	37.933	-0.16	16.50	86.16	16.34	-122.445	37.933	-0.09	16.50	86.48	16.41	9	7/14/2014	43:27.5
4176	RSPe_2	-122.445	37.933	-0.16	16.51	85.83	16.35	-122.445	37.933	-0.18	16.51	86.16	16.34	9	7/14/2014	43:27.4
4177	RSPe_2	-122.445	37.933	-0.16	16.49	85.53	16.33	-122.445	37.933	-0.12	16.49	85.88	16.37	9	7/14/2014	43:27.3
4178	RSPe_2	-122.445	37.933	-0.16	16.56	85.16	16.40	-122.445	37.933	-0.12	16.56	85.51	16.44	9	7/14/2014	43:27.2
4179	RSPe_2	-122.445	37.933	-0.16	16.53	84.92	16.38	-122.445	37.933	-0.18	16.53	85.24	16.36	9	7/14/2014	43:27.1
4180	RSPe_2	-122.445	37.933	-0.19	16.57	84.66	16.37	-122.445	37.933	-0.18	16.57	84.94	16.39	9	7/14/2014	43:27.0
4181	RSPe_2	-122.445	37.933	-0.11	16.61	84.36	16.51	-122.445	37.933	-0.12	16.61	84.59	16.49	9	7/14/2014	43:26.9
4182	RSPe_2	-122.445	37.933	-0.16	16.59	84.02	16.43	-122.445	37.933	-0.12	16.59	84.27	16.47	9	7/14/2014	43:26.8
4183	RSPe_2	-122.445	37.933	-0.16	16.63	83.70	16.47	-122.445	37.933	-0.12	16.63	83.98	16.50	9	7/14/2014	43:26.7
4184	RSPe_2	-122.445	37.933	-0.16	16.62	83.31	16.46	-122.445	37.933	-0.12	16.62	83.64	16.50	9	7/14/2014	43:26.6
4185	RSPe_2	-122.445	37.933	-0.11	16.70	83.00	16.60	-122.445	37.933	-0.12	16.70	83.29	16.58	9	7/14/2014	43:26.5
4186	RSPe_2	-122.445	37.933	-0.16	16.62	82.65	16.46	-122.445	37.933	-0.18	16.62	82.98	16.45	9	7/14/2014	43:26.4
4187	RSPe_2	-122.445	37.933	-0.11	16.61	82.33	16.51	-122.445	37.933	-0.18	16.61	82.69	16.44	9	7/14/2014	43:26.3
4188	RSPe_2	-122.445	37.933	-0.16	16.58	82.03	16.42	-122.445	37.933	-0.12	16.58	82.31	16.45	9	7/14/2014	43:26.2
4189	RSPe_2	-122.445	37.933	-0.16	16.60	81.81	16.44	-122.445	37.933	-0.12	16.60	82.02	16.47	9	7/14/2014	43:26.1
4190	RSPe_2	-122.445	37.933	-0.16	16.66	81.52	16.50	-122.445	37.933	-0.12	16.66	81.68	16.53	9	7/14/2014	43:26.0

4191	RSPe_2	-122.445	37.933	-0.16	16.65	81.27	16.49	-122.445	37.933	-0.09	16.65	81.35	16.56	9	7/14/2014	43:25.9
4192	RSPe_2	-122.445	37.933	-0.16	16.58	80.89	16.42	-122.445	37.933	-0.12	16.58	80.91	16.45	9	7/14/2014	43:25.8
4193	RSPe_2	-122.445	37.933	-0.11	16.53	80.48	16.42	-122.445	37.933	-0.09	16.53	80.45	16.44	9	7/14/2014	43:25.7
4194	RSPe_2	-122.445	37.933	-0.16	16.55	80.15	16.39	-122.445	37.933	-0.12	16.55	80.06	16.43	9	7/14/2014	43:25.6
4195	RSPe_2	-122.445	37.933	-0.11	16.51	79.58	16.40	-122.445	37.933	-0.09	16.51	79.65	16.42	9	7/14/2014	43:25.5
4196	RSPe_2	-122.445	37.933	-0.16	16.51	79.08	16.35	-122.445	37.933	-0.12	16.51	79.23	16.38	9	7/14/2014	43:25.4
4197	RSPe_2	-122.445	37.933	-0.11	16.58	78.80	16.47	-122.445	37.933	-0.09	16.58	78.97	16.49	9	7/14/2014	43:25.3
4198	RSPe_2	-122.445	37.933	-0.16	16.58	78.46	16.42	-122.445	37.933	-0.12	16.58	78.80	16.46	9	7/14/2014	43:25.2
4199	RSPe_2	-122.445	37.933	-0.11	16.61	78.03	16.51	-122.445	37.933	-0.01	16.61	78.56	16.61	9	7/14/2014	43:25.1
4200	RSPe_2	-122.445	37.933	-0.16	16.65	77.65	16.49	-122.445	37.933	-0.12	16.65	78.23	16.53	9	7/14/2014	43:25.0
4201	RSPe_2	-122.445	37.933	-0.11	16.73	77.28	16.62	-122.445	37.933	-0.09	16.73	77.87	16.64	9	7/14/2014	43:24.9
4202	RSPe_2	-122.445	37.933	-0.11	16.74	76.91	16.64	-122.445	37.933	-0.29	16.74	77.47	16.45	9	7/14/2014	43:24.8
4203	RSPe_2	-122.445	37.933	-0.11	16.84	76.47	16.73	-122.445	37.933	-0.12	16.84	77.08	16.71	9	7/14/2014	43:24.7
4204	RSPe_2	-122.445	37.933	-0.16	16.78	76.01	16.63	-122.445	37.933	-0.01	16.78	76.65	16.78	9	7/14/2014	43:24.6
4205	RSPe_2	-122.445	37.933	-0.11	16.92	75.56	16.81	-122.445	37.933	-0.09	16.92	76.15	16.83	9	7/14/2014	43:24.5
4206	RSPe_2	-122.445	37.933	-0.16	16.84	75.09	16.68	-122.445	37.933	-0.01	16.84	75.68	16.84	9	7/14/2014	43:24.4
4207	RSPe_2	-122.445	37.933	-0.11	16.92	74.79	16.81	-122.445	37.933	-0.12	16.92	75.33	16.80	9	7/14/2014	43:24.3
4208	RSPe_2	-122.445	37.933	-0.11	17.00	74.36	16.90	-122.445	37.933	-0.04	17.00	74.90	16.97	9	7/14/2014	43:24.2
4209	RSPe_2	-122.445	37.933	-0.11	17.04	73.96	16.93	-122.445	37.933	-0.04	17.04	74.51	17.00	9	7/14/2014	43:24.1
4210	RSPe_2	-122.445	37.933	-0.11	17.00	73.48	16.89	-122.445	37.933	-0.01	17.00	74.05	16.99	9	7/14/2014	43:24.0
4211	RSPe_2	-122.445	37.933	-0.11	17.05	73.11	16.94	-122.445	37.933	0.05	17.05	73.71	17.09	9	7/14/2014	43:23.9
4212	RSPe_2	-122.445	37.933	-0.11	17.05	72.81	16.94	-122.445	37.933	-0.04	17.05	73.38	17.01	9	7/14/2014	43:23.8
4213	RSPe_2	-122.445	37.933	-0.11	17.14	72.44	17.04	-122.445	37.933	-0.01	17.14	73.05	17.14	9	7/14/2014	43:23.7
4214	RSPe_2	-122.445	37.933	-0.16	17.18	72.03	17.02	-122.445	37.933	-0.01	17.18	72.70	17.17	9	7/14/2014	43:23.6
4215	RSPe_2	-122.445	37.933	-0.11	17.22	71.77	17.11	-122.445	37.933	-0.09	17.22	72.38	17.13	9	7/14/2014	43:23.5
4216	RSPe_2	-122.445	37.933	-0.11	17.22	71.42	17.11	-122.445	37.933	-0.04	17.22	72.00	17.18	9	7/14/2014	43:23.4
4217	RSPe_2	-122.445	37.933	-0.11	17.21	71.04	17.10	-122.445	37.933	0.13	17.21	71.69	17.34	9	7/14/2014	43:23.3
4218	RSPe_2	-122.445	37.933	-0.16	17.24	70.59	17.08	-122.445	37.933	0.05	17.24	71.31	17.28	9	7/14/2014	43:23.2
4219	RSPe_2	-122.445	37.933	-0.11	17.27	70.15	17.16	-122.445	37.933	-0.01	17.27	70.85	17.26	9	7/14/2014	43:23.1
4220	RSPe_2	-122.445	37.933	-0.11	17.24	69.72	17.13	-122.445	37.933	0.05	17.24	70.50	17.28	9	7/14/2014	43:23.0
4221	RSPe_2	-122.445	37.933	-0.11	17.18	69.30	17.07	-122.445	37.933	0.05	17.18	70.11	17.23	9	7/14/2014	43:22.9
4222	RSPe_2	-122.445	37.933	-0.11	17.18	69.04	17.07	-122.445	37.933	-0.01	17.18	69.73	17.17	9	7/14/2014	43:22.8
4223	RSPe_2	-122.445	37.933	-0.11	17.20	68.71	17.09	-122.445	37.933	0.05	17.20	69.46	17.24	9	7/14/2014	43:22.7

4224	RSPe_2	-122.445	37.933	-0.16	17.17	68.41	17.01	-122.445	37.933	0.05	17.17	69.11	17.22	9	7/14/2014	43:22.6
4225	RSPe_2	-122.445	37.933	-0.11	17.21	68.11	17.11	-122.445	37.933	0.08	17.21	68.87	17.29	9	7/14/2014	43:22.5
4226	RSPe_2	-122.445	37.933	-0.11	17.17	67.78	17.06	-122.445	37.933	0.05	17.17	68.50	17.22	9	7/14/2014	43:22.4
4227	RSPe_2	-122.445	37.933	-0.11	17.18	67.50	17.07	-122.445	37.933	0.05	17.18	68.24	17.22	9	7/14/2014	43:22.3
4228	RSPe_2	-122.445	37.933	-0.16	17.21	67.17	17.06	-122.445	37.933	-0.01	17.21	67.98	17.21	9	7/14/2014	43:22.2
4229	RSPe_2	-122.445	37.933	-0.16	17.18	66.86	17.02	-122.445	37.933	-0.01	17.18	67.73	17.17	9	7/14/2014	43:22.1
4230	RSPe_2	-122.445	37.933	-0.16	17.21	66.47	17.05	-122.445	37.933	-0.04	17.21	67.39	17.17	9	7/14/2014	43:22.0
4231	RSPe_2	-122.445	37.933	-0.11	17.18	66.10	17.07	-122.445	37.933	-0.01	17.18	67.04	17.17	9	7/14/2014	43:21.9
4232	RSPe_2	-122.445	37.933	-0.16	17.08	65.69	16.92	-122.445	37.933	-0.01	17.08	66.65	17.08	9	7/14/2014	43:21.8
4233	RSPe_2	-122.445	37.933	-0.11	17.00	65.36	16.89	-122.445	37.933	0.05	17.00	66.35	17.04	9	7/14/2014	43:21.7
4234	RSPe_2	-122.445	37.933	-0.16	16.95	65.10	16.79	-122.445	37.933	-0.01	16.95	66.05	16.95	9	7/14/2014	43:21.6
4235	RSPe_2	-122.445	37.933	-0.16	16.97	64.86	16.81	-122.445	37.933	0.05	16.97	65.75	17.01	9	7/14/2014	43:21.5
4236	RSPe_2	-122.445	37.933	-0.16	16.83	64.55	16.67	-122.445	37.933	-0.01	16.83	65.47	16.82	9	7/14/2014	43:21.4
4237	RSPe_2	-122.445	37.933	-0.16	16.83	64.25	16.67	-122.445	37.933	-0.01	16.83	65.12	16.82	9	7/14/2014	43:21.3
4238	RSPe_2	-122.445	37.933	-0.16	16.73	63.99	16.57	-122.445	37.933	-0.01	16.73	64.84	16.72	9	7/14/2014	43:21.2
4239	RSPe_2	-122.445	37.933	-0.16	16.65	63.75	16.49	-122.445	37.933	0.05	16.65	64.55	16.69	9	7/14/2014	43:21.1
4240	RSPe_2	-122.445	37.933	-0.19	16.72	63.47	16.52	-122.445	37.933	-0.01	16.72	64.21	16.71	9	7/14/2014	43:21.0
4241	RSPe_2	-122.445	37.933	-0.19	16.60	63.14	16.40	-122.445	37.933	-0.04	16.60	63.93	16.56	9	7/14/2014	43:20.9
4242	RSPe_2	-122.445	37.933	-0.19	16.51	62.82	16.32	-122.445	37.933	-0.09	16.51	63.62	16.42	9	7/14/2014	43:20.8
4243	RSPe_2	-122.445	37.933	-0.23	16.49	62.51	16.26	-122.445	37.933	-0.01	16.49	63.36	16.48	9	7/14/2014	43:20.7
4244	RSPe_2	-122.445	37.933	-0.23	16.46	62.29	16.23	-122.445	37.933	-0.04	16.46	63.06	16.42	9	7/14/2014	43:20.6
4245	RSPe_2	-122.445	37.933	-0.23	16.46	61.90	16.23	-122.445	37.933	-0.04	16.46	62.74	16.42	9	7/14/2014	43:20.5
4246	RSPe_2	-122.445	37.933	-0.23	16.48	61.59	16.25	-122.445	37.933	-0.09	16.48	62.42	16.39	9	7/14/2014	43:20.4
4247	RSPe_2	-122.445	37.933	-0.23	16.45	61.31	16.22	-122.445	37.933	-0.04	16.45	62.12	16.41	9	7/14/2014	43:20.3
4248	RSPe_2	-122.445	37.933	-0.23	16.44	61.05	16.22	-122.445	37.933	0.05	16.44	61.85	16.49	9	7/14/2014	43:20.2
4249	RSPe_2	-122.445	37.933	-0.23	16.46	60.75	16.23	-122.445	37.933	-0.04	16.46	61.60	16.42	9	7/14/2014	43:20.1
4250	RSPe_2	-122.445	37.933	-0.28	16.55	60.33	16.27	-122.445	37.933	-0.04	16.55	61.22	16.51	9	7/14/2014	43:20.0
4251	RSPe_2	-122.445	37.933	-0.23	16.57	59.98	16.34	-122.445	37.933	-0.09	16.57	60.90	16.48	9	7/14/2014	43:19.9
4252	RSPe_2	-122.445	37.933	-0.28	16.51	59.57	16.23	-122.445	37.933	-0.12	16.51	60.53	16.39	9	7/14/2014	43:19.8
4253	RSPe_2	-122.445	37.933	-0.28	16.52	59.18	16.24	-122.445	37.933	-0.04	16.52	60.15	16.48	9	7/14/2014	43:19.7
4254	RSPe_2	-122.445	37.933	-0.28	16.55	58.81	16.27	-122.445	37.933	-0.09	16.55	59.79	16.46	9	7/14/2014	43:19.6
4255	RSPe_2	-122.445	37.933	-0.28	16.56	58.46	16.28	-122.445	37.933	-0.04	16.56	59.46	16.52	9	7/14/2014	43:19.5
4256	RSPe_2	-122.445	37.933	-0.31	16.58	58.18	16.27	-122.445	37.933	-0.04	16.58	59.11	16.54	9	7/14/2014	43:19.4

4257	RSPe_2	-122.445	37.933	-0.31	16.60	57.85	16.29	-122.445	37.933	-0.04	16.60	58.78	16.57	9	7/14/2014	43:19.3
4258	RSPe_2	-122.445	37.933	-0.36	16.62	57.58	16.26	-122.445	37.933	-0.12	16.62	58.51	16.50	9	7/14/2014	43:19.2
4259	RSPe_2	-122.445	37.933	-0.31	16.68	57.31	16.37	-122.445	37.933	-0.12	16.68	58.28	16.56	9	7/14/2014	43:19.1
4260	RSPe_2	-122.445	37.933	-0.36	16.66	57.05	16.29	-122.445	37.933	-0.12	16.66	58.05	16.53	9	7/14/2014	43:19.0
4261	RSPe_2	-122.445	37.933	-0.36	16.73	56.78	16.36	-122.445	37.933	-0.18	16.73	57.83	16.55	9	7/14/2014	43:18.9
4262	RSPe_2	-122.445	37.933	-0.40	16.76	56.48	16.36	-122.445	37.933	-0.18	16.76	57.60	16.58	9	7/14/2014	43:18.8
4263	RSPe_2	-122.445	37.933	-0.40	16.78	56.20	16.39	-122.445	37.933	-0.21	16.78	57.35	16.58	9	7/14/2014	43:18.7
4264	RSPe_2	-122.445	37.933	-0.40	16.88	55.87	16.48	-122.445	37.933	-0.24	16.88	57.07	16.63	9	7/14/2014	43:18.6
4265	RSPe_2	-122.445	37.933	-0.40	16.91	55.58	16.51	-122.445	37.933	-0.21	16.91	56.80	16.70	9	7/14/2014	43:18.5
4266	RSPe_2	-122.445	37.933	-0.45	16.95	55.26	16.50	-122.445	37.933	-0.24	16.95	56.48	16.71	9	7/14/2014	43:18.4
4267	RSPe_2	-122.445	37.933	-0.45	17.03	54.94	16.58	-122.445	37.933	-0.24	17.03	56.11	16.79	9	7/14/2014	43:18.3
4268	RSPe_2	-122.445	37.933	-0.48	17.13	54.73	16.64	-122.445	37.933	-0.33	17.13	55.76	16.80	9	7/14/2014	43:18.2
4269	RSPe_2	-122.445	37.933	-0.45	17.17	54.35	16.72	-122.445	37.933	-0.33	17.17	55.41	16.84	9	7/14/2014	43:18.1
4270	RSPe_2	-122.445	37.933	-0.48	17.24	54.08	16.76	-122.445	37.933	-0.29	17.24	55.09	16.95	9	7/14/2014	43:18.0
4271	RSPe_2	-122.445	37.933	-0.48	17.39	53.82	16.90	-122.445	37.933	-0.29	17.39	54.78	17.09	9	7/14/2014	43:17.9
4272	RSPe_2	-122.445	37.933	-0.48	17.29	53.45	16.81	-122.445	37.933	-0.41	17.29	54.44	16.88	9	7/14/2014	43:17.8
4273	RSPe_2	-122.445	37.933	-0.48	17.46	53.13	16.98	-122.445	37.933	-0.41	17.46	54.12	17.05	9	7/14/2014	43:17.7
4274	RSPe_2	-122.445	37.933	-0.57	17.39	52.73	16.82	-122.445	37.933	-0.38	17.39	53.74	17.01	9	7/14/2014	43:17.6
4275	RSPe_2	-122.445	37.933	-0.54	17.48	52.32	16.94	-122.445	37.933	-0.38	17.48	53.34	17.10	9	7/14/2014	43:17.5
4276	RSPe_2	-122.445	37.933	-0.57	17.41	51.90	16.84	-122.445	37.933	-0.41	17.41	52.89	16.99	9	7/14/2014	43:17.4
4277	RSPe_2	-122.445	37.933	-0.57	17.45	51.51	16.88	-122.445	37.933	-0.41	17.45	52.52	17.04	9	7/14/2014	43:17.3
4278	RSPe_2	-122.445	37.933	-0.57	17.53	51.23	16.96	-122.445	37.933	-0.50	17.53	52.21	17.03	9	7/14/2014	43:17.2
4279	RSPe_2	-122.445	37.933	-0.57	17.63	50.80	17.06	-122.445	37.933	-0.55	17.63	51.84	17.08	9	7/14/2014	43:17.1
4280	RSPe_2	-122.445	37.933	-0.60	17.72	50.32	17.11	-122.445	37.933	-0.55	17.72	51.45	17.17	9	7/14/2014	43:17.0
4281	RSPe_2	-122.445	37.933	-0.60	17.80	49.97	17.20	-122.445	37.933	-0.55	17.80	51.08	17.26	9	7/14/2014	43:16.9
4282	RSPe_2	-122.445	37.933	-0.60	17.85	49.90	17.24	-122.445	37.933	-0.62	17.85	51.12	17.23	9	7/14/2014	43:16.8
4283	RSPe_2	-122.445	37.933	-0.60	17.86	49.66	17.26	-122.445	37.933	-0.58	17.86	50.72	17.28	9	7/14/2014	43:16.7
4284	RSPe_2	-122.445	37.933	-0.65	17.86	49.27	17.21	-122.445	37.933	-0.62	17.86	50.21	17.25	9	7/14/2014	43:16.6
4285	RSPe_2	-122.445	37.933	-0.65	17.90	48.92	17.25	-122.445	37.933	-0.58	17.90	49.70	17.32	9	7/14/2014	43:16.5
4286	RSPe_2	-122.445	37.933	-0.69	17.72	48.51	17.03	-122.445	37.933	-0.62	17.72	49.21	17.11	9	7/14/2014	43:16.4
4287	RSPe_2	-122.445	37.933	-0.65	17.60	48.12	16.94	-122.445	37.933	-0.58	17.60	48.70	17.02	9	7/14/2014	43:16.3
4288	RSPe_2	-122.445	37.933	-0.69	17.30	47.63	16.61	-122.445	37.933	-0.67	17.30	48.26	16.63	9	7/14/2014	43:16.2
4289	RSPe_2	-122.445	37.933	-0.69	17.22	47.24	16.53	-122.445	37.933	-0.70	17.22	47.98	16.52	9	7/14/2014	43:16.1

4290	RSPe_2	-122.445	37.933	-0.74	17.21	46.79	16.47	-122.445	37.933	-0.75	17.21	47.51	16.47	9	7/14/2014	43:16.0
4291	RSPe_2	-122.445	37.933	-0.69	17.12	46.48	16.43	-122.445	37.933	-0.70	17.12	47.35	16.42	9	7/14/2014	43:15.9
4292	RSPe_2	-122.445	37.933	-0.74	16.95	46.07	16.21	-122.445	37.933	-0.75	16.95	46.85	16.20	9	7/14/2014	43:15.8
4293	RSPe_2	-122.445	37.933	-0.74	16.96	45.74	16.22	-122.445	37.933	-0.78	16.96	46.68	16.18	9	7/14/2014	43:15.7
4294	RSPe_2	-122.445	37.933	-0.74	16.96	45.61	16.22	-122.445	37.933	-0.78	16.96	46.54	16.18	9	7/14/2014	43:15.6
4295	RSPe_2	-122.445	37.933	-0.74	16.93	45.13	16.19	-122.445	37.933	-0.78	16.93	46.26	16.15	9	7/14/2014	43:15.5
4296	RSPe_2	-122.445	37.933	-0.77	16.97	44.74	16.19	-122.445	37.933	-0.78	16.97	45.85	16.18	9	7/14/2014	43:15.4
4297	RSPe_2	-122.445	37.933	-0.74	17.11	44.48	16.37	-122.445	37.933	-0.83	17.11	45.81	16.27	9	7/14/2014	43:15.3
4298	RSPe_2	-122.445	37.933	-0.82	17.12	44.15	16.30	-122.445	37.933	-0.87	17.12	45.61	16.25	9	7/14/2014	43:15.2
4299	RSPe_2	-122.445	37.933	-0.77	17.24	43.76	16.47	-122.445	37.933	-0.87	17.24	45.22	16.37	9	7/14/2014	43:15.1
4300	RSPe_2	-122.445	37.933	-0.77	17.23	43.49	16.46	-122.445	37.933	-0.87	17.23	44.83	16.36	9	7/14/2014	43:15.0
4301	RSPe_2	-122.445	37.933	-0.77	17.34	43.11	16.56	-122.445	37.933	-0.87	17.34	44.31	16.47	9	7/14/2014	43:14.9
4302	RSPe_2	-122.445	37.933	-0.82	17.29	42.72	16.47	-122.445	37.933	-0.87	17.29	43.76	16.43	9	7/14/2014	43:14.8
4303	RSPe_2	-122.445	37.933	-0.77	17.24	42.21	16.47	-122.445	37.933	-0.87	17.24	43.15	16.37	9	7/14/2014	43:14.7
4304	RSPe_2	-122.445	37.934	-0.82	17.02	41.69	16.20	-122.445	37.933	-0.99	17.02	42.76	16.03	9	7/14/2014	43:14.6
4305	RSPe_2	-122.445	37.934	-0.82	16.98	41.03	16.15	-122.445	37.934	-0.95	16.98	42.32	16.02	9	7/14/2014	43:14.5
4306	RSPe_2	-122.445	37.934	-0.82	17.00	40.75	16.18	-122.445	37.934	-1.04	17.00	42.19	15.97	9	7/14/2014	43:14.4
4307	RSPe_2	-122.445	37.934	-0.77	17.05	40.53	16.27	-122.445	37.934	-0.99	17.05	42.13	16.06	9	7/14/2014	43:14.3
4308	RSPe_2	-122.445	37.934	-0.82	17.21	40.10	16.38	-122.445	37.934	-1.04	17.21	41.85	16.17	9	7/14/2014	43:14.2
4309	RSPe_2	-122.445	37.934	-0.77	17.29	39.97	16.52	-122.445	37.934	-1.04	17.29	41.67	16.26	9	7/14/2014	43:14.1
4310	RSPe_2	-122.445	37.934	-0.82	17.42	39.88	16.59	-122.445	37.934	-1.12	17.42	41.41	16.30	9	7/14/2014	43:14.0
4311	RSPe_2	-122.445	37.934	-0.77	17.44	39.62	16.66	-122.445	37.934	-1.04	17.44	40.97	16.40	9	7/14/2014	43:13.9
4312	RSPe_2	-122.445	37.934	-0.86	17.45	39.21	16.59	-122.445	37.934	-1.04	17.45	40.36	16.41	9	7/14/2014	43:13.8
4313	RSPe_2	-122.445	37.934	-0.82	17.42	38.79	16.59	-122.445	37.934	-1.15	17.42	39.84	16.26	9	7/14/2014	43:13.7
4314	RSPe_2	-122.445	37.934	-0.86	17.42	38.47	16.56	-122.445	37.934	-1.04	17.42	39.36	16.38	9	7/14/2014	43:13.6
4315	RSPe_2	-122.445	37.934	-0.82	17.42	37.95	16.60	-122.445	37.934	-1.12	17.42	38.97	16.30	9	7/14/2014	43:13.5
4316	RSPe_2	-122.445	37.934	-0.82	17.37	37.38	16.54	-122.445	37.934	-1.12	17.37	38.47	16.25	9	7/14/2014	43:13.4
4317	RSPe_2	-122.445	37.934	-0.82	17.45	36.77	16.63	-122.445	37.934	-1.12	17.45	38.10	16.33	9	7/14/2014	43:13.3
4318	RSPe_2	-122.445	37.934	-0.86	17.42	36.26	16.56	-122.445	37.934	-1.21	17.42	37.75	16.21	9	7/14/2014	43:13.2
4319	RSPe_2	-122.445	37.934	-0.82	17.53	35.90	16.71	-122.445	37.934	-1.15	17.53	37.56	16.38	9	7/14/2014	43:13.1
4320	RSPe_2	-122.445	37.934	-0.86	17.71	35.51	16.85	-122.445	37.934	-1.21	17.71	37.17	16.50	9	7/14/2014	43:13.0
4321	RSPe_2	-122.445	37.934	-0.82	17.81	35.18	16.98	-122.445	37.934	-1.15	17.81	36.74	16.65	9	7/14/2014	43:12.9
4322	RSPe_2	-122.445	37.934	-0.86	17.73	34.81	16.87	-122.445	37.934	-1.15	17.73	36.20	16.58	9	7/14/2014	43:12.8

4323	RSPe_2	-122.445	37.934	-0.86	17.75	34.42	16.89	-122.445	37.934	-1.04	17.75	35.68	16.71	9	7/14/2014	43:12.7
4324	RSPe_2	-122.445	37.934	-0.86	17.68	34.02	16.82	-122.445	37.934	-1.12	17.68	35.11	16.56	9	7/14/2014	43:12.6
4325	RSPe_2	-122.445	37.934	-0.82	17.69	33.57	16.87	-122.445	37.934	-1.07	17.69	34.59	16.62	9	7/14/2014	43:12.5
4326	RSPe_2	-122.445	37.934	-0.86	17.68	33.16	16.82	-122.445	37.934	-1.15	17.68	34.11	16.53	9	7/14/2014	43:12.4
4327	RSPe_2	-122.445	37.934	-0.86	17.61	32.98	16.75	-122.445	37.934	-1.12	17.61	33.89	16.49	9	7/14/2014	43:12.3
4328	RSPe_2	-122.445	37.934	-0.82	17.65	32.74	16.82	-122.445	37.934	-1.15	17.65	33.57	16.49	9	7/14/2014	43:12.2
4329	RSPe_2	-122.445	37.934	-0.82	17.60	32.39	16.77	-122.445	37.934	-1.12	17.60	33.17	16.48	9	7/14/2014	43:12.1
4330	RSPe_2	-122.445	37.934	-0.86	17.60	32.06	16.74	-122.445	37.934	-1.07	17.60	32.72	16.53	9	7/14/2014	43:12.0
4331	RSPe_2	-122.445	37.934	-0.82	17.49	31.76	16.67	-122.445	37.934	-1.04	17.49	32.46	16.46	9	7/14/2014	43:11.9
4332	RSPe_2	-122.445	37.934	-0.86	17.47	31.35	16.61	-122.445	37.934	-1.12	17.47	32.04	16.35	9	7/14/2014	43:11.8
4333	RSPe_2	-122.445	37.934	-0.82	17.51	30.80	16.69	-122.445	37.934	-1.07	17.51	31.67	16.44	9	7/14/2014	43:11.7
4334	RSPe_2	-122.445	37.934	-0.86	17.59	30.43	16.73	-122.445	37.934	-1.07	17.59	31.41	16.52	9	7/14/2014	43:11.6
4335	RSPe_2	-122.445	37.934	-0.82	17.56	30.04	16.74	-122.445	37.934	-1.12	17.56	31.15	16.44	9	7/14/2014	43:11.5
4336	RSPe_2	-122.445	37.934	-0.86	17.67	29.67	16.81	-122.445	37.934	-1.12	17.67	30.85	16.55	9	7/14/2014	43:11.4
4337	RSPe_2	-122.445	37.934	-0.82	17.69	29.32	16.87	-122.445	37.934	-1.12	17.69	30.47	16.57	9	7/14/2014	43:11.3
4338	RSPe_2	-122.445	37.934	-0.86	17.76	28.80	16.90	-122.445	37.934	-1.12	17.76	30.04	16.64	9	7/14/2014	43:11.2
4339	RSPe_2	-122.445	37.934	-0.82	17.87	28.30	17.05	-122.445	37.934	-0.99	17.87	29.56	16.89	9	7/14/2014	43:11.1
4340	RSPe_2	-122.445	37.934	-0.86	17.89	27.75	17.03	-122.445	37.934	-0.99	17.89	29.01	16.91	9	7/14/2014	43:11.0
4341	RSPe_2	-122.445	37.934	-0.82	17.95	27.20	17.12	-122.445	37.934	-1.04	17.95	28.34	16.91	9	7/14/2014	43:10.9
4342	RSPe_2	-122.445	37.934	-0.82	18.02	26.88	17.19	-122.445	37.934	-0.95	18.02	27.94	17.07	9	7/14/2014	43:10.8
4343	RSPe_2	-122.445	37.934	-0.82	18.04	26.55	17.21	-122.445	37.934	-0.95	18.04	27.47	17.09	9	7/14/2014	43:10.7
4344	RSPe_2	-122.445	37.934	-0.86	17.98	26.05	17.12	-122.445	37.934	-0.99	17.98	26.97	16.99	9	7/14/2014	43:10.6
4345	RSPe_2	-122.445	37.934	-0.82	17.99	25.35	17.17	-122.445	37.934	-0.95	17.99	26.47	17.04	9	7/14/2014	43:10.5
4346	RSPe_2	-122.445	37.934	-0.82	17.91	24.76	17.08	-122.445	37.934	-1.04	17.91	25.96	16.87	9	7/14/2014	43:10.4
4347	RSPe_2	-122.445	37.934	-0.82	17.79	24.13	16.97	-122.445	37.934	-1.04	17.79	25.53	16.75	9	7/14/2014	43:10.3
4348	RSPe_2	-122.445	37.934	-0.82	17.64	23.74	16.81	-122.445	37.934	-1.04	17.64	25.13	16.60	9	7/14/2014	43:10.2
4349	RSPe_2	-122.445	37.934	-0.82	17.68	23.26	16.86	-122.445	37.934	-0.99	17.68	24.64	16.70	9	7/14/2014	43:10.1
4350	RSPe_2	-122.445	37.934	-0.82	17.57	22.83	16.75	-122.445	37.934	-1.15	17.57	24.25	16.42	9	7/14/2014	43:10.0
4351	RSPe_2	-122.445	37.934	-0.82	17.68	22.48	16.86	-122.445	37.934	-1.04	17.68	23.74	16.65	9	7/14/2014	43:09.9
4352	RSPe_2	-122.445	37.934	-0.82	17.63	22.08	16.80	-122.445	37.934	-1.07	17.63	23.17	16.55	9	7/14/2014	43:09.8
4353	RSPe_2	-122.445	37.934	-0.82	17.51	21.58	16.68	-122.445	37.934	-0.99	17.51	22.59	16.52	9	7/14/2014	43:09.7
4354	RSPe_2	-122.445	37.934	-0.86	17.45	21.19	16.59	-122.445	37.934	-0.99	17.45	22.11	16.47	9	7/14/2014	43:09.6
4355	RSPe_2	-122.445	37.934	-0.82	17.45	20.61	16.62	-122.445	37.934	-0.87	17.45	21.54	16.58	9	7/14/2014	43:09.5

4356	RSPe_2	-122.445	37.934	-0.86	17.45	20.21	16.59	-122.445	37.934	-1.04	17.45	21.19	16.42	9	7/14/2014	43:09.4
4357	RSPe_2	-122.445	37.934	-0.86	17.47	19.80	16.61	-122.445	37.934	-1.07	17.47	20.97	16.40	9	7/14/2014	43:09.3
4358	RSPe_2	-122.445	37.934	-0.86	17.56	19.67	16.70	-122.445	37.934	-1.07	17.56	21.00	16.48	9	7/14/2014	43:09.2
4359	RSPe_2	-122.445	37.934	-0.86	17.56	19.43	16.70	-122.445	37.934	-1.12	17.56	20.89	16.44	9	7/14/2014	43:09.1
4360	RSPe_2	-122.445	37.934	-0.91	17.59	18.97	16.68	-122.445	37.934	-1.12	17.59	20.43	16.47	9	7/14/2014	43:09.0
4361	RSPe_2	-122.445	37.934	-0.91	17.65	18.56	16.74	-122.445	37.934	-1.21	17.65	20.02	16.45	9	7/14/2014	43:08.9
4362	RSPe_2	-122.445	37.934	-0.91	17.68	18.12	16.77	-122.445	37.934	-1.21	17.68	19.56	16.47	9	7/14/2014	43:08.8
4363	RSPe_2	-122.445	37.934	-0.91	17.76	17.58	16.85	-122.445	37.934	-1.21	17.76	19.04	16.56	9	7/14/2014	43:08.7
4364	RSPe_2	-122.445	37.934	-0.94	17.76	16.77	16.82	-122.445	37.934	-1.32	17.76	18.32	16.44	9	7/14/2014	43:08.6
4365	RSPe_2	-122.445	37.934	-0.91	17.89	16.29	16.98	-122.445	37.934	-1.32	17.89	17.84	16.56	9	7/14/2014	43:08.5
4366	RSPe_2	-122.445	37.934	-0.94	18.50	16.01	17.56	-122.445	37.934	-1.36	18.50	17.36	17.15	9	7/14/2014	43:08.4
4367	RSPe_2	-122.445	37.934	-0.91	18.43	15.77	17.52	-122.445	37.934	-1.36	18.43	16.86	17.07	9	7/14/2014	43:08.3
4368	RSPe_2	-122.445	37.934	-0.94	17.96	15.94	17.02	-122.445	37.934	-1.27	17.96	16.51	16.69	9	7/14/2014	43:08.2
4369	RSPe_2	-122.445	37.934	-0.94	17.96	15.88	17.02	-122.445	37.934	-1.41	17.96	16.09	16.55	9	7/14/2014	43:08.1
4370	RSPe_2	-122.445	37.934	-0.98	17.94	15.48	16.96	-122.445	37.934	-1.27	17.94	15.53	16.67	9	7/14/2014	43:08.0
4371	RSPe_2	-122.445	37.934	-0.94	17.93	14.81	16.99	-122.445	37.934	-1.36	17.93	15.00	16.57	9	7/14/2014	43:07.9
4372	RSPe_2	-122.445	37.934	-0.98	17.92	13.76	16.94	-122.445	37.934	-1.49	17.92	14.42	16.42	9	7/14/2014	43:07.8
4373	RSPe_2	-122.445	37.934	-0.98	17.91	13.20	16.93	-122.445	37.934	-1.44	17.91	14.37	16.46	9	7/14/2014	43:07.7
4374	RSPe_2	-122.445	37.934	-0.98	17.90	12.48	16.92	-122.445	37.934	-1.49	17.90	14.22	16.40	9	7/14/2014	43:07.6
4375	RSPe_2	-122.445	37.934	-0.98	17.88	12.09	16.91	-122.445	37.934	-1.49	17.88	14.22	16.39	9	7/14/2014	43:07.5
4376	RSPe_2	-122.445	37.934	-1.03	17.87	11.91	16.84	-122.445	37.934	-1.49	17.87	13.74	16.38	9	7/14/2014	43:07.4
4377	RSPe_2	-122.445	37.934	-0.98	17.86	11.67	16.88	-122.445	37.934	-1.44	17.86	13.28	16.42	9	7/14/2014	43:07.3
4378	RSPe_2	-122.445	37.934	-1.03	17.85	11.32	16.82	-122.445	37.934	-1.44	17.85	12.89	16.41	9	7/14/2014	43:07.2
4379	RSPe_2	-122.445	37.934	-0.98	17.84	11.19	16.86	-122.445	37.934	-1.36	17.84	12.41	16.48	9	7/14/2014	43:07.1
4380	RSPe_2	-122.445	37.934	-1.03	17.83	10.69	16.80	-122.445	37.934	-1.53	17.83	11.96	16.30	9	7/14/2014	43:07.0
4381	RSPe_2	-122.445	37.934	-0.98	17.82	9.97	16.84	-122.445	37.934	-1.53	17.82	11.33	16.29	9	7/14/2014	43:06.9
4382	RSPe_2	-122.445	37.934	-1.03	17.80	9.27	16.77	-122.445	37.934	-1.49	17.80	10.58	16.31	9	7/14/2014	43:06.8
4383	RSPe_2	-122.445	37.934	-0.98	17.79	8.38	16.81	-122.445	37.934	-1.44	17.79	9.99	16.35	9	7/14/2014	43:06.7
4384	RSPe_2	-122.445	37.934	-0.98	17.78	7.79	16.80	-122.445	37.934	-1.53	17.78	9.60	16.25	9	7/14/2014	43:06.6
4385	RSPe_2	-122.445	37.934	-0.98	17.77	7.45	16.79	-122.445	37.934	-1.49	17.77	9.14	16.28	9	7/14/2014	43:06.5
4386	RSPe_2	-122.445	37.934	-0.98	17.76	7.12	16.78	-122.445	37.934	-1.49	17.76	8.67	16.27	9	7/14/2014	43:06.4
4387	RSPe_2	-122.445	37.934	-0.98	17.75	6.57	16.77	-122.445	37.934	-1.53	17.75	8.19	16.22	9	7/14/2014	43:06.3
4388	RSPe_2	-122.445	37.934	-0.98	17.74	6.09	16.76	-122.445	37.934	-1.53	17.74	7.56	16.21	9	7/14/2014	43:06.2

4389	RSPe_2	-122.445	37.934	-0.94	17.72	5.53	16.78	-122.445	37.934	-1.61	17.72	6.92	16.11	9	7/14/2014 43:06.1
4390	RSPe_2	-122.445	37.934	-0.98	17.71	5.16	16.73	-122.445	37.934	-1.53	17.71	6.38	16.19	9	7/14/2014 43:06.0
4391	RSPe_2	-122.445	37.934	-0.98	17.70	4.79	16.72	-122.445	37.934	-1.53	17.70	6.10	16.17	9	7/14/2014 43:05.9
4392	RSPe_2	-122.445	37.934	-0.98	17.69	4.18	16.71	-122.445	37.934	-1.58	17.69	5.77	16.11	9	7/14/2014 43:05.8
4393	RSPe_2	-122.445	37.934	-0.94	17.68	3.22	16.73	-122.445	37.934	-1.49	17.68	5.38	16.19	9	7/14/2014 43:05.7
4394	RSPe_2	-122.445	37.934	-0.98	17.67	2.61	16.69	-122.445	37.934	-1.44	17.67	4.70	16.22	9	7/14/2014 43:05.6
4395	RSPe_2	-122.445	37.934	-0.94	17.66	2.00	16.71	-122.445	37.934	-1.49	17.66	3.85	16.16	9	7/14/2014 43:05.5
4396	RSPe_2	-122.445	37.934	-0.98	17.65	1.41	16.67	-122.445	37.934	-1.44	17.65	2.80	16.20	9	7/14/2014 43:05.4
4397	RSPe_2	-122.445	37.934	-0.94	17.63	1.28	16.69	-122.445	37.934	-1.49	17.63	2.30	16.14	9	7/14/2014 43:05.3
4398	RSPe_2	-122.445	37.934	-0.98	17.62	1.13	16.64	-122.445	37.934	-1.53	17.62	1.56	16.09	9	7/14/2014 43:05.2
4399	RSPe_2	-122.445	37.934	-0.94	17.61	0.76	16.67	-122.445	37.934	-1.49	17.61	1.17	16.12	9	7/14/2014 43:05.1
4400	RSPe_2	-122.445	37.934	-0.98	17.60	0.52	16.62	-122.445	37.934	-1.49	17.60	1.04	16.11	9	7/14/2014 43:05.0
4401	RSPe_2	-122.445	37.934	-0.94	17.61	0.04	16.66	-122.445	37.934	-1.49	17.61	0.47	16.11	9	7/14/2014 43:04.9
4402	RSPe_2	-122.445	37.934	-0.98	17.59	-0.18	16.61	-122.445	37.934	-1.44	17.59	0.17	16.15	9	7/14/2014 43:04.8
4403	RSPe_2	-122.445	37.934	-0.94	17.61	-0.79	16.67	-122.445	37.934	-1.49	17.61	-0.27	16.12	9	7/14/2014 43:04.7
4404	RSPe_2	-122.445	37.934	-0.98	17.64	-1.73	16.66	-122.445	37.934	-1.49	17.64	-0.92	16.15	9	7/14/2014 43:04.6
4405	RSPe_2	-122.445	37.934	-0.94	17.64	-2.69	16.69	-122.445	37.934	-1.36	17.64	-1.66	16.28	9	7/14/2014 43:04.5
4406	RSPe_2	-122.445	37.934	-0.98	17.63	-3.60	16.65	-122.445	37.934	-1.44	17.63	-2.32	16.19	9	7/14/2014 43:04.4
4407	RSPe_2	-122.445	37.934	-0.94	17.63	-3.84	16.68	-122.445	37.934	-1.49	17.63	-2.78	16.13	9	7/14/2014 43:04.3
4408	RSPe_2	-122.445	37.934	-0.94	17.62	-4.06	16.68	-122.445	37.934	-1.49	17.62	-3.17	16.13	9	7/14/2014 43:04.2
4409	RSPe_2	-122.445	37.934	-0.94	17.62	-4.19	16.67	-122.445	37.934	-1.44	17.62	-3.58	16.18	9	7/14/2014 43:04.1
4410	RSPe_2	-122.445	37.934	-0.98	17.61	-4.82	16.64	-122.445	37.934	-1.41	17.61	-4.21	16.21	9	7/14/2014 43:04.0
4411	RSPe_2	-122.445	37.934	-0.94	17.61	-5.65	16.67	-122.445	37.934	-1.44	17.61	-4.98	16.17	9	7/14/2014 43:03.9
4412	RSPe_2	-122.445	37.934	-0.94	17.66	-6.46	16.72	-122.445	37.934	-1.36	17.66	-5.52	16.31	9	7/14/2014 43:03.8
4413	RSPe_2	-122.445	37.934	-0.91	17.81	-7.33	16.90	-122.445	37.934	-1.36	17.81	-5.85	16.45	9	7/14/2014 43:03.7
4414	RSPe_2	-122.445	37.934	-0.94	17.90	-8.25	16.96	-122.445	37.934	-1.58	17.90	-6.22	16.32	9	7/14/2014 43:03.6
4415	RSPe_2	-122.445	37.934	-0.91	17.90	-8.88	16.99	-122.445	37.934	-1.53	17.90	-6.78	16.38	9	7/14/2014 43:03.5
4416	RSPe_2	-122.445	37.934	-0.94	17.90	-9.31	16.96	-122.445	37.934	-1.49	17.90	-7.26	16.41	9	7/14/2014 43:03.4
4417	RSPe_2	-122.445	37.934	-0.91	18.03	-9.73	17.11	-122.445	37.934	-1.58	18.03	-7.98	16.45	9	7/14/2014 43:03.3
4418	RSPe_2	-122.445	37.934	-0.94	18.12	-10.08	17.17	-122.445	37.934	-1.61	18.12	-8.27	16.51	9	7/14/2014 43:03.2
4419	RSPe_2	-122.445	37.934	-0.91	18.78	-10.40	17.87	-122.445	37.934	-1.41	18.78	-8.88	17.37	9	7/14/2014 43:03.1
4420	RSPe_2	-122.445	37.934	-0.91	18.30	-10.84	17.38	-122.445	37.934	-1.61	18.30	-9.21	16.68	9	7/14/2014 43:03.0
4421	RSPe_2	-122.445	37.934	-0.91	18.57	-11.56	17.65	-122.445	37.934	-1.58	18.57	-9.70	16.99	9	7/14/2014 43:02.9

4422	RSPe_2	-122.445	37.934	-0.91	18.81	-12.85	17.90	-122.445	37.934	-1.44	18.81	-10.79	17.37	9	7/14/2014	43:02.8
4423	RSPe_2	-122.445	37.934	-0.91	18.85	-13.04	17.94	-122.445	37.934	-1.78	18.85	-10.93	17.07	9	7/14/2014	43:02.7
4424	RSPe_2	-122.445	37.934	-0.91	18.85	-13.43	17.94	-122.445	37.934	-1.53	18.85	-11.62	17.32	9	7/14/2014	43:02.6
4425	RSPe_2	-122.445	37.934	-0.91	18.47	-13.65	17.55	-122.445	37.934	-1.41	18.47	-12.32	17.06	9	7/14/2014	43:02.5
4426	RSPe_2	-122.445	37.934	-0.94	18.47	-14.20	17.53	-122.445	37.934	-1.53	18.47	-13.24	16.95	9	7/14/2014	43:02.4
4427	RSPe_2	-122.445	37.934	-0.91	18.48	-14.54	17.57	-122.445	37.934	-1.53	18.48	-14.06	16.95	9	7/14/2014	43:02.3
4428	RSPe_2	-122.445	37.934	-0.94	18.49	-15.37	17.54	-122.445	37.934	-1.44	18.49	-14.87	17.05	9	7/14/2014	43:02.2
4429	RSPe_2	-122.445	37.934	-0.91	18.50	-15.74	17.59	-122.445	37.934	-1.41	18.50	-15.39	17.09	9	7/14/2014	43:02.1
4430	RSPe_2	-122.445	37.934	-0.91	18.50	-16.64	17.59	-122.445	37.934	-1.44	18.50	-16.09	17.06	9	7/14/2014	43:02.0
4431	RSPe_2	-122.445	37.934	-0.86	18.50	-17.31	17.64	-122.445	37.934	-1.41	18.50	-16.64	17.10	9	7/14/2014	43:01.9
4432	RSPe_2	-122.445	37.934	-0.91	18.06	-17.71	17.15	-122.445	37.934	-1.53	18.06	-17.29	16.54	9	7/14/2014	43:01.8
4433	RSPe_2	-122.445	37.934	-0.86	18.05	-18.79	17.20	-122.445	37.934	-1.44	18.05	-17.92	16.61	9	7/14/2014	43:01.7
4434	RSPe_2	-122.445	37.934	-0.91	18.05	-19.58	17.14	-122.445	37.934	-1.44	18.05	-19.03	16.61	9	7/14/2014	43:01.6
4435	RSPe_2	-122.445	37.934	-0.86	17.97	-19.82	17.11	-122.445	37.934	-1.41	17.97	-19.40	16.56	9	7/14/2014	43:01.5
4436	RSPe_2	-122.445	37.934	-0.91	17.96	-20.25	17.05	-122.445	37.934	-1.49	17.96	-19.91	16.47	9	7/14/2014	43:01.4
4437	RSPe_2	-122.445	37.934	-0.86	17.98	-20.67	17.12	-122.445	37.934	-1.21	17.98	-20.63	16.77	9	7/14/2014	43:01.3
4438	RSPe_2	-122.445	37.934	-0.91	18.03	-21.17	17.12	-122.445	37.934	-1.44	18.03	-21.09	16.59	9	7/14/2014	43:01.2
4439	RSPe_2	-122.445	37.934	-0.86	18.05	-21.82	17.20	-122.445	37.934	-1.41	18.05	-21.84	16.65	9	7/14/2014	43:01.1
4440	RSPe_2	-122.445	37.934	-0.86	18.10	-22.61	17.24	-122.445	37.934	-1.32	18.10	-22.59	16.78	9	7/14/2014	43:01.0
4441	RSPe_2	-122.445	37.934	-0.86	18.16	-23.00	17.30	-122.445	37.934	-1.32	18.16	-22.83	16.83	9	7/14/2014	43:00.9
4442	RSPe_2	-122.445	37.934	-0.91	18.01	-23.46	17.10	-122.445	37.934	-1.32	18.01	-23.20	16.69	9	7/14/2014	43:00.8
4443	RSPe_2	-122.445	37.934	-0.86	18.12	-24.44	17.26	-122.445	37.934	-1.36	18.12	-24.25	16.76	9	7/14/2014	43:00.7
4444	RSPe_2	-122.445	37.934	-0.91	17.96	-24.99	17.05	-122.445	37.934	-1.27	17.96	-24.92	16.69	9	7/14/2014	43:00.6
4445	RSPe_2	-122.445	37.934	-0.86	17.95	-25.22	17.09	-122.445	37.934	-1.27	17.95	-25.22	16.67	9	7/14/2014	43:00.5
4446	RSPe_2	-122.445	37.934	-0.91	17.97	-25.81	17.06	-122.445	37.934	-1.32	17.97	-25.83	16.65	9	7/14/2014	43:00.4
4447	RSPe_2	-122.445	37.934	-0.86	17.99	-26.40	17.13	-122.445	37.934	-1.24	17.99	-26.16	16.75	9	7/14/2014	43:00.3
4448	RSPe_2	-122.445	37.934	-0.86	18.02	-27.29	17.16	-122.445	37.934	-1.32	18.02	-26.44	16.69	9	7/14/2014	43:00.2
4449	RSPe_2	-122.445	37.934	-0.86	18.02	-27.84	17.16	-122.445	37.934	-1.32	18.02	-26.81	16.69	9	7/14/2014	43:00.1
4450	RSPe_2	-122.445	37.934	-0.86	18.04	-28.23	17.18	-122.445	37.934	-1.36	18.04	-27.32	16.68	9	7/14/2014	43:00.0
4451	RSPe_2	-122.445	37.934	-0.86	18.03	-28.69	17.17	-122.445	37.934	-1.32	18.03	-28.03	16.71	9	7/14/2014	42:59.9
4452	RSPe_2	-122.445	37.934	-0.86	18.04	-29.30	17.18	-122.445	37.934	-1.24	18.04	-28.71	16.80	9	7/14/2014	42:59.8
4453	RSPe_2	-122.445	37.934	-0.82	18.00	-29.80	17.18	-122.445	37.934	-1.24	18.00	-29.41	16.76	9	7/14/2014	42:59.7
4454	RSPe_2	-122.445	37.934	-0.86	17.98	-30.45	17.12	-122.445	37.934	-1.27	17.98	-30.02	16.71	9	7/14/2014	42:59.6

4455	RSPe_2	-122.445	37.934	-0.82	17.94	-31.28	17.12	-122.445	37.934	-1.27	17.94	-30.52	16.67	9	7/14/2014 42:59.5
4456	RSPe_2	-122.445	37.934	-0.82	17.96	-32.29	17.14	-122.445	37.934	-1.32	17.96	-31.24	16.64	9	7/14/2014 42:59.4
4457	RSPe_2	-122.445	37.934	-0.77	17.96	-33.05	17.18	-122.445	37.934	-1.24	17.96	-31.83	16.72	9	7/14/2014 42:59.3
4458	RSPe_2	-122.445	37.934	-0.82	17.99	-33.83	17.16	-122.445	37.934	-1.32	17.99	-32.48	16.66	9	7/14/2014 42:59.2
4459	RSPe_2	-122.445	37.934	-0.77	18.05	-34.22	17.28	-122.445	37.934	-1.27	18.05	-32.98	16.78	9	7/14/2014 42:59.1
4460	RSPe_2	-122.445	37.934	-0.77	18.17	-34.84	17.40	-122.445	37.934	-1.32	18.17	-33.66	16.85	9	7/14/2014 42:59.0
4461	RSPe_2	-122.445	37.934	-0.77	18.21	-35.49	17.44	-122.445	37.934	-1.27	18.21	-34.42	16.94	9	7/14/2014 42:58.9
4462	RSPe_2	-122.445	37.934	-0.77	18.27	-36.06	17.50	-122.445	37.934	-1.32	18.27	-35.16	16.95	9	7/14/2014 42:58.8
4463	RSPe_2	-122.445	37.934	-0.74	18.30	-36.45	17.57	-122.445	37.934	-1.32	18.30	-35.73	16.98	9	7/14/2014 42:58.7
4464	RSPe_2	-122.445	37.934	-0.77	18.36	-36.80	17.58	-122.445	37.934	-1.21	18.36	-36.36	17.15	9	7/14/2014 42:58.6
4465	RSPe_2	-122.445	37.934	-0.69	18.26	-37.28	17.57	-122.445	37.934	-1.27	18.26	-36.93	16.98	9	7/14/2014 42:58.5
4466	RSPe_2	-122.445	37.934	-0.74	18.21	-37.84	17.47	-122.445	37.934	-1.12	18.21	-37.65	17.09	9	7/14/2014 42:58.4
4467	RSPe_2	-122.445	37.934	-0.69	18.19	-38.52	17.51	-122.445	37.934	-1.24	18.19	-38.39	16.95	9	7/14/2014 42:58.3
4468	RSPe_2	-122.445	37.934	-0.69	18.13	-39.22	17.44	-122.445	37.934	-1.12	18.13	-39.02	17.01	9	7/14/2014 42:58.2
4469	RSPe_2	-122.445	37.934	-0.69	18.12	-39.87	17.43	-122.445	37.934	-1.15	18.12	-39.56	16.96	9	7/14/2014 42:58.1
4470	RSPe_2	-122.445	37.934	-0.69	18.13	-40.59	17.44	-122.445	37.934	-1.15	18.13	-40.28	16.98	9	7/14/2014 42:58.0
4471	RSPe_2	-122.445	37.934	-0.69	17.99	-41.35	17.30	-122.445	37.934	-1.12	17.99	-41.05	16.87	9	7/14/2014 42:57.9
4472	RSPe_2	-122.445	37.934	-0.69	17.79	-41.85	17.10	-122.445	37.934	-1.15	17.79	-41.81	16.64	9	7/14/2014 42:57.8
4473	RSPe_2	-122.445	37.934	-0.69	17.61	-42.73	16.93	-122.445	37.934	-1.07	17.61	-42.79	16.54	9	7/14/2014 42:57.7
4474	RSPe_2	-122.445	37.934	-0.69	17.56	-43.01	16.87	-122.445	37.934	-1.04	17.56	-43.34	16.52	9	7/14/2014 42:57.6
4475	RSPe_2	-122.445	37.934	-0.65	17.56	-43.18	16.90	-122.445	37.934	-1.04	17.56	-43.68	16.52	9	7/14/2014 42:57.5
4476	RSPe_2	-122.445	37.934	-0.69	17.50	-43.66	16.81	-122.445	37.934	-1.04	17.50	-44.23	16.46	9	7/14/2014 42:57.4
4477	RSPe_2	-122.445	37.934	-0.65	17.45	-44.29	16.80	-122.445	37.934	-1.04	17.45	-44.77	16.42	9	7/14/2014 42:57.3
4478	RSPe_2	-122.445	37.934	-0.65	17.45	-44.99	16.80	-122.445	37.934	-1.04	17.45	-45.24	16.42	9	7/14/2014 42:57.2
4479	RSPe_2	-122.445	37.934	-0.65	17.45	-45.60	16.80	-122.445	37.934	-0.95	17.45	-45.69	16.50	9	7/14/2014 42:57.1
4480	RSPe_2	-122.445	37.934	-0.65	17.47	-46.52	16.82	-122.445	37.934	-0.92	17.47	-46.28	16.55	9	7/14/2014 42:57.0
4481	RSPe_2	-122.445	37.934	-0.60	17.49	-47.17	16.89	-122.445	37.934	-0.99	17.49	-46.89	16.51	9	7/14/2014 42:56.9
4482	RSPe_2	-122.445	37.934	-0.65	17.52	-47.87	16.87	-122.445	37.934	-0.92	17.52	-47.43	16.61	9	7/14/2014 42:56.8
4483	RSPe_2	-122.445	37.934	-0.60	17.54	-48.63	16.94	-122.445	37.934	-0.95	17.54	-48.18	16.59	9	7/14/2014 42:56.7
4484	RSPe_2	-122.445	37.934	-0.60	17.56	-49.24	16.96	-122.445	37.934	-1.07	17.56	-48.92	16.49	9	7/14/2014 42:56.6
4485	RSPe_2	-122.445	37.934	-0.60	17.51	-49.85	16.91	-122.445	37.934	-0.78	17.51	-49.68	16.73	9	7/14/2014 42:56.5
4486	RSPe_2	-122.445	37.934	-0.60	17.49	-50.49	16.89	-122.445	37.934	-0.83	17.49	-50.53	16.66	9	7/14/2014 42:56.4
4487	RSPe_2	-122.445	37.934	-0.57	17.48	-51.33	16.91	-122.445	37.934	-0.78	17.48	-51.44	16.69	9	7/14/2014 42:56.3

4488	RSPe_2	-122.445	37.934	-0.60	17.43	-52.01	16.83	-122.445	37.934	-0.78	17.43	-52.03	16.65	9	7/14/2014	42:56.2
4489	RSPe_2	-122.445	37.934	-0.57	17.38	-52.82	16.81	-122.445	37.934	-0.75	17.38	-52.82	16.63	9	7/14/2014	42:56.1
4490	RSPe_2	-122.445	37.934	-0.60	17.41	-53.76	16.80	-122.445	37.934	-0.83	17.41	-53.74	16.57	9	7/14/2014	42:56.0
4491	RSPe_2	-122.445	37.934	-0.57	17.42	-54.54	16.85	-122.445	37.934	-0.70	17.42	-54.39	16.72	9	7/14/2014	42:55.9
4492	RSPe_2	-122.445	37.934	-0.57	17.38	-55.09	16.81	-122.445	37.934	-0.87	17.38	-55.05	16.51	9	7/14/2014	42:55.8
4493	RSPe_2	-122.445	37.934	-0.57	17.37	-55.72	16.80	-122.445	37.934	-0.78	17.37	-55.80	16.59	9	7/14/2014	42:55.7
4494	RSPe_2	-122.445	37.934	-0.57	17.40	-56.11	16.83	-122.445	37.934	-0.66	17.40	-56.37	16.74	9	7/14/2014	42:55.6
4495	RSPe_2	-122.445	37.934	-0.57	17.38	-56.48	16.81	-122.445	37.934	-0.75	17.38	-56.89	16.63	9	7/14/2014	42:55.5
4496	RSPe_2	-122.445	37.934	-0.60	17.38	-57.31	16.78	-122.445	37.934	-0.78	17.38	-57.68	16.60	9	7/14/2014	42:55.4
4497	RSPe_2	-122.445	37.934	-0.57	17.39	-58.18	16.82	-122.445	37.934	-0.70	17.39	-58.49	16.69	9	7/14/2014	42:55.3
4498	RSPe_2	-122.445	37.934	-0.57	17.19	-58.92	16.62	-122.445	37.934	-0.75	17.19	-59.16	16.44	9	7/14/2014	42:55.2
4499	RSPe_2	-122.445	37.934	-0.57	17.19	-59.71	16.62	-122.445	37.934	-0.61	17.19	-59.95	16.58	9	7/14/2014	42:55.1
4500	RSPe_2	-122.445	37.934	-0.57	17.05	-60.21	16.48	-122.445	37.934	-0.66	17.05	-60.58	16.38	9	7/14/2014	42:55.0
4501	RSPe_2	-122.445	37.934	-0.57	17.00	-60.45	16.43	-122.445	37.934	-0.66	17.00	-60.99	16.34	9	7/14/2014	42:54.9
4502	RSPe_2	-122.445	37.934	-0.60	17.00	-61.01	16.40	-122.445	37.934	-0.70	17.00	-61.76	16.30	9	7/14/2014	42:54.8
4503	RSPe_2	-122.445	37.934	-0.60	16.96	-61.54	16.36	-122.445	37.934	-0.70	16.96	-62.22	16.26	9	7/14/2014	42:54.7
4504	RSPe_2	-122.445	37.934	-0.60	16.91	-62.06	16.31	-122.445	37.934	-0.75	16.91	-62.69	16.17	9	7/14/2014	42:54.6
4505	RSPe_2	-122.445	37.934	-0.57	16.88	-62.78	16.31	-122.445	37.934	-0.75	16.88	-63.15	16.13	9	7/14/2014	42:54.5
4506	RSPe_2	-122.445	37.934	-0.60	16.84	-63.52	16.24	-122.445	37.934	-0.66	16.84	-63.74	16.17	9	7/14/2014	42:54.4
4507	RSPe_2	-122.445	37.934	-0.57	16.81	-64.31	16.24	-122.445	37.934	-0.75	16.81	-64.30	16.06	9	7/14/2014	42:54.3
4508	RSPe_2	-122.445	37.934	-0.60	16.77	-64.96	16.17	-122.445	37.934	-0.75	16.77	-64.96	16.02	9	7/14/2014	42:54.2
4509	RSPe_2	-122.445	37.934	-0.57	16.77	-65.72	16.21	-122.445	37.934	-0.70	16.77	-65.61	16.08	9	7/14/2014	42:54.1
4510	RSPe_2	-122.445	37.934	-0.60	16.70	-66.62	16.10	-122.445	37.934	-0.66	16.70	-66.40	16.04	9	7/14/2014	42:54.0
4511	RSPe_2	-122.445	37.934	-0.60	16.72	-67.25	16.12	-122.445	37.934	-0.66	16.72	-67.01	16.06	9	7/14/2014	42:53.9
4512	RSPe_2	-122.445	37.934	-0.60	16.69	-67.71	16.09	-122.445	37.934	-0.75	16.69	-67.59	15.94	9	7/14/2014	42:53.8
4513	RSPe_2	-122.445	37.934	-0.60	16.70	-68.25	16.10	-122.445	37.934	-0.70	16.70	-68.27	16.01	9	7/14/2014	42:53.7
4514	RSPe_2	-122.445	37.934	-0.60	16.69	-68.77	16.09	-122.445	37.934	-0.78	16.69	-68.93	15.91	9	7/14/2014	42:53.6
4515	RSPe_2	-122.445	37.934	-0.60	16.70	-69.36	16.10	-122.445	37.934	-0.78	16.70	-69.71	15.92	9	7/14/2014	42:53.5
4516	RSPe_2	-122.445	37.934	-0.60	16.70	-70.10	16.10	-122.445	37.934	-0.75	16.70	-70.35	15.95	9	7/14/2014	42:53.4
4517	RSPe_2	-122.445	37.934	-0.60	16.72	-70.65	16.11	-122.445	37.934	-0.75	16.72	-70.88	15.97	9	7/14/2014	42:53.3
4518	RSPe_2	-122.445	37.934	-0.60	16.74	-71.34	16.13	-122.445	37.934	-0.83	16.74	-71.48	15.90	9	7/14/2014	42:53.2
4519	RSPe_2	-122.445	37.934	-0.60	16.74	-71.85	16.13	-122.445	37.934	-0.78	16.74	-71.96	15.95	9	7/14/2014	42:53.1
4520	RSPe_2	-122.445	37.934	-0.60	16.76	-72.17	16.16	-122.445	37.934	-0.78	16.76	-72.43	15.98	9	7/14/2014	42:53.0

4521	RSPe_2	-122.445	37.934	-0.60	16.73	-72.70	16.12	-122.445	37.934	-0.78	16.73	-73.11	15.94	9	7/14/2014 42:52.9
4522	RSPe_2	-122.445	37.934	-0.60	16.76	-73.35	16.16	-122.445	37.934	-0.83	16.76	-73.85	15.93	9	7/14/2014 42:52.8
4523	RSPe_2	-122.445	37.934	-0.60	16.68	-73.96	16.08	-122.445	37.934	-0.78	16.68	-74.72	15.90	9	7/14/2014 42:52.7
4524	RSPe_2	-122.445	37.934	-0.60	16.65	-74.66	16.04	-122.445	37.934	-0.78	16.65	-75.66	15.86	9	7/14/2014 42:52.6
4525	RSPe_2	-122.445	37.934	-0.60	16.66	-75.35	16.06	-122.445	37.934	-0.83	16.66	-76.40	15.83	9	7/14/2014 42:52.5
4526	RSPe_2	-122.445	37.934	-0.60	16.70	-75.97	16.10	-122.445	37.934	-0.75	16.70	-77.00	15.95	9	7/14/2014 42:52.4
4527	RSPe_2	-122.445	37.934	-0.60	16.77	-76.56	16.17	-122.445	37.934	-0.78	16.77	-77.49	15.99	9	7/14/2014 42:52.3
4528	RSPe_2	-122.445	37.934	-0.60	16.77	-77.19	16.17	-122.445	37.934	-0.78	16.77	-78.02	15.99	9	7/14/2014 42:52.2
4529	RSPe_2	-122.445	37.934	-0.60	16.74	-77.60	16.14	-122.445	37.934	-0.75	16.74	-78.32	16.00	9	7/14/2014 42:52.1
4530	RSPe_2	-122.445	37.934	-0.65	16.84	-78.14	16.18	-122.445	37.934	-0.78	16.84	-78.78	16.06	9	7/14/2014 42:52.0
4531	RSPe_2	-122.445	37.934	-0.60	16.86	-78.89	16.26	-122.445	37.934	-0.75	16.86	-79.41	16.11	9	7/14/2014 42:51.9
4532	RSPe_2	-122.445	37.934	-0.65	16.87	-79.45	16.21	-122.445	37.934	-0.83	16.87	-79.92	16.03	9	7/14/2014 42:51.8
4533	RSPe_2	-122.445	37.934	-0.60	16.88	-80.13	16.28	-122.445	37.934	-0.75	16.88	-80.46	16.13	9	7/14/2014 42:51.7
4534	RSPe_2	-122.445	37.934	-0.65	16.94	-80.74	16.28	-122.445	37.934	-0.78	16.94	-80.98	16.15	9	7/14/2014 42:51.6
4535	RSPe_2	-122.445	37.934	-0.60	16.90	-81.72	16.30	-122.445	37.934	-0.75	16.90	-81.76	16.15	9	7/14/2014 42:51.5
4536	RSPe_2	-122.445	37.934	-0.60	17.00	-82.22	16.40	-122.445	37.934	-0.83	17.00	-82.27	16.17	9	7/14/2014 42:51.4
4537	RSPe_2	-122.445	37.934	-0.65	17.05	-82.77	16.39	-122.445	37.934	-0.78	17.05	-82.94	16.26	9	7/14/2014 42:51.3
4538	RSPe_2	-122.445	37.934	-0.65	17.05	-83.75	16.40	-122.445	37.934	-0.87	17.05	-83.92	16.19	9	7/14/2014 42:51.2
4539	RSPe_2	-122.445	37.934	-0.65	17.12	-84.45	16.47	-122.445	37.934	-0.83	17.12	-84.79	16.29	9	7/14/2014 42:51.1
4540	RSPe_2	-122.445	37.934	-0.65	17.14	-85.03	16.49	-122.445	37.934	-0.83	17.14	-85.52	16.31	9	7/14/2014 42:51.0
4541	RSPe_2	-122.445	37.934	-0.65	17.26	-85.71	16.61	-122.445	37.934	-0.95	17.26	-86.13	16.31	9	7/14/2014 42:50.9
4542	RSPe_2	-122.445	37.934	-0.65	17.25	-86.51	16.59	-122.445	37.934	-0.87	17.25	-86.79	16.38	9	7/14/2014 42:50.8
4543	RSPe_2	-122.445	37.934	-0.65	17.15	-87.24	16.50	-122.445	37.934	-0.87	17.15	-87.46	16.29	9	7/14/2014 42:50.7
4544	RSPe_2	-122.445	37.934	-0.65	17.12	-87.98	16.47	-122.445	37.934	-0.92	17.12	-88.21	16.20	9	7/14/2014 42:50.6
4545	RSPe_2	-122.445	37.934	-0.65	17.10	-88.78	16.45	-122.445	37.934	-0.95	17.10	-89.01	16.15	9	7/14/2014 42:50.5
4546	RSPe_2	-122.445	37.934	-0.65	17.07	-89.31	16.42	-122.445	37.934	-0.99	17.07	-89.66	16.08	9	7/14/2014 42:50.4
4547	RSPe_2	-122.445	37.934	-0.65	17.05	-89.74	16.39	-122.445	37.934	-0.92	17.05	-90.31	16.13	9	7/14/2014 42:50.3
4548	RSPe_2	-122.445	37.934	-0.69	17.01	-90.46	16.33	-122.445	37.934	-0.95	17.01	-91.03	16.06	9	7/14/2014 42:50.2
4549	RSPe_2	-122.445	37.934	-0.65	16.98	-91.18	16.33	-122.445	37.934	-0.95	16.98	-91.77	16.03	9	7/14/2014 42:50.1
4550	RSPe_2	-122.445	37.934	-0.69	16.96	-91.92	16.27	-122.445	37.934	-0.99	16.96	-92.49	15.98	9	7/14/2014 42:50.0
4551	RSPe_2	-122.445	37.934	-0.65	16.94	-93.01	16.28	-122.445	37.934	-0.99	16.94	-93.47	15.95	9	7/14/2014 42:49.9
4552	RSPe_2	-122.445	37.934	-0.69	16.94	-93.43	16.26	-122.445	37.934	-0.99	16.94	-93.94	15.96	9	7/14/2014 42:49.8
4553	RSPe_2	-122.445	37.934	-0.65	16.94	-93.91	16.29	-122.445	37.934	-1.07	16.94	-94.19	15.87	9	7/14/2014 42:49.7

4554	RSPe_2	-122.445	37.934	-0.69	16.98	-94.34	16.29	-122.445	37.934	-0.99	16.98	-94.60	15.99	9	7/14/2014 42:49.6
4555	RSPe_2	-122.445	37.934	-0.65	16.98	-95.13	16.33	-122.445	37.934	-0.95	16.98	-95.30	16.03	9	7/14/2014 42:49.5
4556	RSPe_2	-122.445	37.934	-0.69	16.97	-95.86	16.28	-122.445	37.934	-1.04	16.97	-96.28	15.93	9	7/14/2014 42:49.4
4557	RSPe_2	-122.445	37.934	-0.65	16.92	-96.83	16.27	-122.445	37.934	-1.07	16.92	-97.42	15.85	9	7/14/2014 42:49.3
4558	RSPe_2	-122.445	37.934	-0.69	16.88	-97.81	16.19	-122.445	37.934	-0.99	16.88	-98.49	15.89	9	7/14/2014 42:49.2
4559	RSPe_2	-122.445	37.934	-0.65	16.78	-98.85	16.13	-122.445	37.934	-1.04	16.78	-99.49	15.75	9	7/14/2014 42:49.1
4560	RSPe_2	-122.445	37.934	-0.65	16.81	-99.64	16.15	-122.445	37.934	-1.07	16.81	-100.51	15.74	9	7/14/2014 42:49.0
4561	RSPe_2	-122.445	37.934	-0.65	16.83	-99.88	16.17	-122.445	37.934	-1.07	16.83	-100.79	15.76	9	7/14/2014 42:48.9
4562	RSPe_2	-122.445	37.934	-0.65	16.88	-100.16	16.22	-122.445	37.934	-1.12	16.88	-101.13	15.76	9	7/14/2014 42:48.8
4563	RSPe_2	-122.445	37.934	-0.65	16.89	-100.60	16.24	-122.445	37.934	-1.12	16.89	-101.64	15.77	9	7/14/2014 42:48.7
4564	RSPe_2	-122.445	37.934	-0.65	16.95	-101.21	16.30	-122.445	37.934	-1.07	16.95	-101.98	15.88	9	7/14/2014 42:48.6
4565	RSPe_2	-122.445	37.934	-0.65	16.98	-101.86	16.32	-122.445	37.934	-1.07	16.98	-102.58	15.91	9	7/14/2014 42:48.5
4566	RSPe_2	-122.445	37.934	-0.69	16.99	-102.58	16.30	-122.445	37.934	-1.15	16.99	-103.41	15.84	9	7/14/2014 42:48.4
4567	RSPe_2	-122.445	37.934	-0.65	16.96	-103.47	16.31	-122.445	37.934	-1.20	16.96	-104.41	15.76	9	7/14/2014 42:48.3
4568	RSPe_2	-122.445	37.934	-0.65	16.93	-104.31	16.28	-122.445	37.934	-1.15	16.93	-105.31	15.78	9	7/14/2014 42:48.2
4569	RSPe_2	-122.445	37.934	-0.60	16.91	-105.17	16.31	-122.445	37.934	-1.12	16.91	-106.05	15.79	9	7/14/2014 42:48.1
4570	RSPe_2	-122.445	37.934	-0.60	16.87	-106.14	16.26	-122.445	37.934	-1.20	16.87	-106.73	15.66	9	7/14/2014 42:48.0
4571	RSPe_2	-122.445	37.934	-0.60	16.85	-107.12	16.25	-122.445	37.934	-1.12	16.85	-107.40	15.74	9	7/14/2014 42:47.9
4572	RSPe_2	-122.445	37.934	-0.60	16.84	-107.90	16.24	-122.445	37.934	-1.12	16.84	-108.10	15.72	9	7/14/2014 42:47.8
4573	RSPe_2	-122.445	37.934	-0.60	16.91	-108.75	16.31	-122.445	37.934	-1.07	16.91	-108.93	15.84	9	7/14/2014 42:47.7
4574	RSPe_2	-122.445	37.934	-0.60	16.86	-109.39	16.26	-122.445	37.934	-1.07	16.86	-109.93	15.79	9	7/14/2014 42:47.6
4575	RSPe_2	-122.445	37.934	-0.60	16.90	-110.08	16.30	-122.445	37.934	-1.03	16.90	-110.71	15.86	9	7/14/2014 42:47.5
4576	RSPe_2	-122.445	37.934	-0.60	16.86	-110.67	16.26	-122.445	37.934	-1.07	16.86	-111.38	15.79	9	7/14/2014 42:47.4
4577	RSPe_2	-122.445	37.934	-0.57	16.84	-111.43	16.28	-122.445	37.934	-1.03	16.84	-112.04	15.81	9	7/14/2014 42:47.3
4578	RSPe_2	-122.445	37.934	-0.60	16.82	-112.21	16.22	-122.445	37.934	-1.07	16.82	-112.76	15.75	9	7/14/2014 42:47.2
4579	RSPe_2	-122.445	37.934	-0.57	16.83	-113.07	16.26	-122.445	37.934	-0.98	16.83	-113.48	15.84	9	7/14/2014 42:47.1
4580	RSPe_2	-122.445	37.934	-0.57	16.83	-114.01	16.26	-122.445	37.934	-1.07	16.83	-114.27	15.76	9	7/14/2014 42:47.0
4581	RSPe_2	-122.445	37.934	-0.53	16.81	-114.68	16.28	-122.445	37.934	-1.03	16.81	-114.77	15.78	9	7/14/2014 42:46.9
4582	RSPe_2	-122.445	37.934	-0.57	16.80	-115.51	16.23	-122.445	37.934	-1.12	16.80	-115.33	15.68	9	7/14/2014 42:46.8
4583	RSPe_2	-122.445	37.934	-0.53	16.80	-116.62	16.26	-122.445	37.934	-1.07	16.80	-116.10	15.73	9	7/14/2014 42:46.7
4584	RSPe_2	-122.445	37.934	-0.53	16.80	-117.47	16.26	-122.445	37.934	-1.12	16.80	-116.86	15.68	9	7/14/2014 42:46.6
4585	RSPe_2	-122.445	37.934	-0.48	16.88	-118.28	16.39	-122.445	37.934	-0.98	16.88	-117.68	15.89	9	7/14/2014 42:46.5
4586	RSPe_2	-122.445	37.934	-0.48	16.98	-119.04	16.50	-122.445	37.934	-1.15	16.98	-118.60	15.83	9	7/14/2014 42:46.4

4587	RSPe_2	-122.445	37.934	-0.48	17.04	-120.04	16.56	-122.445	37.934	-0.92	17.04	-119.71	16.12	9	7/14/2014	42:46.3
4588	RSPe_2	-122.445	37.934	-0.48	17.06	-121.13	16.58	-122.445	37.934	-0.98	17.06	-120.94	16.07	9	7/14/2014	42:46.2
4589	RSPe_2	-122.445	37.934	-0.45	17.05	-122.14	16.60	-122.445	37.934	-0.95	17.05	-122.01	16.10	9	7/14/2014	42:46.1
4590	RSPe_2	-122.445	37.934	-0.48	17.05	-122.90	16.56	-122.445	37.934	-0.98	17.05	-122.94	16.06	9	7/14/2014	42:46.0
4591	RSPe_2	-122.445	37.934	-0.45	17.03	-123.44	16.58	-122.445	37.934	-0.92	17.03	-123.64	16.12	9	7/14/2014	42:45.9
4592	RSPe_2	-122.445	37.934	-0.45	17.02	-124.05	16.57	-122.445	37.934	-0.98	17.02	-124.34	16.04	9	7/14/2014	42:45.8
4593	RSPe_2	-122.445	37.934	-0.45	17.04	-124.67	16.59	-122.445	37.934	-0.95	17.04	-125.01	16.09	9	7/14/2014	42:45.7
4594	RSPe_2	-122.445	37.934	-0.45	16.93	-125.43	16.48	-122.445	37.934	-1.03	16.93	-125.71	15.89	9	7/14/2014	42:45.6
4595	RSPe_2	-122.445	37.934	-0.40	16.93	-126.32	16.53	-122.445	37.934	-0.95	16.93	-126.54	15.98	9	7/14/2014	42:45.5
4596	RSPe_2	-122.445	37.934	-0.45	16.91	-127.32	16.46	-122.445	37.934	-0.95	16.91	-127.48	15.96	9	7/14/2014	42:45.4
4597	RSPe_2	-122.445	37.934	-0.45	16.83	-128.20	16.38	-122.445	37.934	-0.92	16.83	-128.40	15.91	9	7/14/2014	42:45.3
4598	RSPe_2	-122.445	37.934	-0.45	16.83	-129.03	16.38	-122.445	37.934	-0.98	16.83	-129.24	15.84	9	7/14/2014	42:45.2
4599	RSPe_2	-122.445	37.934	-0.40	16.84	-129.77	16.44	-122.445	37.934	-1.03	16.84	-129.97	15.80	9	7/14/2014	42:45.1
4600	RSPe_2	-122.445	37.934	-0.45	16.78	-130.72	16.34	-122.445	37.934	-0.95	16.78	-130.86	15.83	9	7/14/2014	42:45.0
4601	RSPe_2	-122.445	37.934	-0.40	16.81	-131.51	16.42	-122.445	37.934	-0.98	16.81	-131.59	15.83	9	7/14/2014	42:44.9
4602	RSPe_2	-122.445	37.934	-0.40	16.87	-132.26	16.47	-122.445	37.934	-0.98	16.87	-132.21	15.88	9	7/14/2014	42:44.8
4603	RSPe_2	-122.445	37.934	-0.40	17.00	-133.27	16.60	-122.445	37.934	-0.92	17.00	-133.04	16.08	9	7/14/2014	42:44.7
4604	RSPe_2	-122.445	37.934	-0.40	16.94	-134.00	16.55	-122.445	37.934	-0.92	16.94	-133.78	16.03	9	7/14/2014	42:44.6
4605	RSPe_2	-122.445	37.934	-0.40	17.09	-134.72	16.69	-122.445	37.934	-0.92	17.09	-134.49	16.17	9	7/14/2014	42:44.5
4606	RSPe_2	-122.445	37.934	-0.40	17.02	-135.42	16.62	-122.445	37.934	-0.92	17.02	-135.32	16.10	9	7/14/2014	42:44.4
4607	RSPe_2	-122.445	37.934	-0.36	17.12	-136.32	16.75	-122.445	37.934	-0.95	17.12	-136.17	16.17	9	7/14/2014	42:44.3
4608	RSPe_2	-122.445	37.934	-0.36	17.15	-137.18	16.79	-122.445	37.934	-0.92	17.15	-137.14	16.24	9	7/14/2014	42:44.2
4609	RSPe_2	-122.445	37.934	-0.36	17.11	-137.94	16.75	-122.445	37.934	-0.95	17.11	-138.07	16.16	9	7/14/2014	42:44.1
4610	RSPe_2	-122.445	37.934	-0.36	17.11	-138.62	16.75	-122.445	37.934	-0.87	17.11	-138.91	16.24	9	7/14/2014	42:44.0
4611	RSPe_2	-122.445	37.934	-0.36	16.99	-139.52	16.63	-122.445	37.934	-0.87	16.99	-139.93	16.12	9	7/14/2014	42:43.9
4612	RSPe_2	-122.445	37.934	-0.36	16.91	-140.28	16.55	-122.445	37.934	-0.92	16.91	-140.83	15.99	9	7/14/2014	42:43.8
4613	RSPe_2	-122.445	37.934	-0.36	16.91	-141.21	16.55	-122.445	37.934	-0.78	16.91	-141.76	16.13	9	7/14/2014	42:43.7
4614	RSPe_2	-122.445	37.934	-0.36	16.80	-142.13	16.44	-122.445	37.934	-0.83	16.80	-142.52	15.97	9	7/14/2014	42:43.6
4615	RSPe_2	-122.445	37.934	-0.36	16.80	-143.07	16.44	-122.445	37.934	-0.78	16.80	-143.40	16.02	9	7/14/2014	42:43.5
4616	RSPe_2	-122.445	37.934	-0.36	16.72	-143.96	16.35	-122.445	37.934	-0.78	16.72	-144.32	15.93	9	7/14/2014	42:43.4
4617	RSPe_2	-122.445	37.934	-0.36	16.65	-144.82	16.29	-122.445	37.934	-0.78	16.65	-145.18	15.87	9	7/14/2014	42:43.3
4618	RSPe_2	-122.445	37.934	-0.36	16.61	-145.64	16.25	-122.445	37.934	-0.87	16.61	-146.11	15.75	9	7/14/2014	42:43.2
4619	RSPe_2	-122.445	37.934	-0.36	16.67	-146.47	16.31	-122.445	37.934	-0.78	16.67	-146.95	15.89	9	7/14/2014	42:43.1

4620	RSPe_2	-122.445	37.934	-0.36	16.67	-147.29	16.31	-122.445	37.934	-0.75	16.67	-147.65	15.93	9	7/14/2014	42:43.0
4621	RSPe_2	-122.445	37.934	-0.31	16.70	-148.15	16.39	-122.445	37.934	-0.70	16.70	-148.41	16.00	9	7/14/2014	42:42.9
4622	RSPe_2	-122.445	37.934	-0.31	16.69	-149.00	16.38	-122.445	37.934	-0.83	16.69	-149.07	15.86	9	7/14/2014	42:42.8
4623	RSPe_2	-122.445	37.934	-0.31	16.74	-149.92	16.43	-122.445	37.934	-0.70	16.74	-149.98	16.04	9	7/14/2014	42:42.7
4624	RSPe_2	-122.445	37.934	-0.36	16.76	-151.05	16.40	-122.445	37.934	-0.75	16.76	-150.97	16.01	9	7/14/2014	42:42.6
4625	RSPe_2	-122.445	37.934	-0.28	17.11	-152.05	16.83	-122.445	37.934	-0.70	17.11	-151.97	16.41	9	7/14/2014	42:42.5
4626	RSPe_2	-122.445	37.934	-0.28	17.00	-152.61	16.72	-122.445	37.934	-0.75	17.00	-152.64	16.25	9	7/14/2014	42:42.4
4627	RSPe_2	-122.445	37.934	-0.28	16.99	-153.29	16.71	-122.445	37.934	-0.61	16.99	-153.40	16.38	9	7/14/2014	42:42.3
4628	RSPe_2	-122.445	37.934	-0.28	17.08	-154.10	16.80	-122.445	37.934	-0.66	17.08	-154.23	16.41	9	7/14/2014	42:42.2
4629	RSPe_2	-122.445	37.934	-0.28	17.03	-154.93	16.76	-122.445	37.934	-0.61	17.03	-155.10	16.42	9	7/14/2014	42:42.1
4630	RSPe_2	-122.445	37.934	-0.31	17.09	-155.67	16.78	-122.445	37.934	-0.61	17.09	-155.84	16.48	9	7/14/2014	42:42.0
4631	RSPe_2	-122.445	37.934	-0.28	16.97	-156.41	16.69	-122.445	37.934	-0.58	16.97	-156.63	16.39	9	7/14/2014	42:41.9
4632	RSPe_2	-122.445	37.934	-0.31	17.16	-157.22	16.85	-122.445	37.934	-0.61	17.16	-157.50	16.55	9	7/14/2014	42:41.8
4633	RSPe_2	-122.445	37.934	-0.28	17.01	-158.02	16.74	-122.445	37.934	-0.54	17.01	-158.38	16.47	9	7/14/2014	42:41.7
4634	RSPe_2	-122.445	37.934	-0.28	16.98	-158.81	16.70	-122.445	37.934	-0.61	16.98	-159.36	16.36	9	7/14/2014	42:41.6
4635	RSPe_2	-122.445	37.934	-0.28	16.89	-159.66	16.62	-122.445	37.934	-0.54	16.89	-160.27	16.35	9	7/14/2014	42:41.5
4636	RSPe_2	-122.445	37.934	-0.28	16.87	-160.51	16.59	-122.445	37.934	-0.54	16.87	-161.18	16.32	9	7/14/2014	42:41.4
4637	RSPe_2	-122.445	37.934	-0.22	16.81	-161.16	16.59	-122.445	37.934	-0.54	16.81	-161.89	16.27	9	7/14/2014	42:41.3
4638	RSPe_2	-122.445	37.934	-0.28	16.76	-162.08	16.48	-122.445	37.934	-0.61	16.76	-162.71	16.15	9	7/14/2014	42:41.2
4639	RSPe_2	-122.445	37.934	-0.22	16.59	-162.99	16.37	-122.445	37.934	-0.49	16.59	-163.46	16.10	9	7/14/2014	42:41.1
4640	RSPe_2	-122.445	37.934	-0.28	16.38	-163.80	16.11	-122.445	37.934	-0.54	16.38	-164.07	15.84	9	7/14/2014	42:41.0
4641	RSPe_2	-122.445	37.934	-0.22	16.28	-164.63	16.06	-122.445	37.934	-0.54	16.28	-164.75	15.74	9	7/14/2014	42:40.9
4642	RSPe_2	-122.445	37.934	-0.28	16.31	-165.48	16.04	-122.445	37.934	-0.49	16.31	-165.53	15.82	9	7/14/2014	42:40.8
4643	RSPe_2	-122.445	37.934	-0.22	16.33	-166.26	16.10	-122.445	37.934	-0.46	16.33	-166.37	15.87	9	7/14/2014	42:40.7
4644	RSPe_2	-122.445	37.934	-0.28	16.27	-167.09	15.99	-122.445	37.934	-0.49	16.27	-167.22	15.77	9	7/14/2014	42:40.6
4645	RSPe_2	-122.445	37.934	-0.22	16.11	-167.95	15.89	-122.445	37.934	-0.54	16.11	-168.14	15.57	9	7/14/2014	42:40.5
4646	RSPe_2	-122.445	37.934	-0.22	16.11	-168.71	15.89	-122.445	37.934	-0.54	16.11	-169.03	15.57	9	7/14/2014	42:40.4
4647	RSPe_2	-122.445	37.934	-0.22	15.99	-169.60	15.76	-122.445	37.934	-0.46	15.99	-169.93	15.53	9	7/14/2014	42:40.3
4648	RSPe_2	-122.445	37.934	-0.22	15.93	-170.39	15.70	-122.445	37.934	-0.66	15.93	-170.80	15.27	9	7/14/2014	42:40.2
4649	RSPe_2	-122.445	37.934	-0.22	15.86	-171.30	15.63	-122.445	37.934	-0.32	15.86	-171.74	15.53	9	7/14/2014	42:40.1
4650	RSPe_2	-122.445	37.934	-0.22	15.84	-172.24	15.62	-122.445	37.934	-0.54	15.84	-172.59	15.30	9	7/14/2014	42:40.0
4651	RSPe_2	-122.445	37.934	-0.19	15.81	-173.14	15.62	-122.445	37.934	-0.46	15.81	-173.54	15.35	9	7/14/2014	42:39.9
4652	RSPe_2	-122.445	37.934	-0.22	15.80	-174.07	15.57	-122.445	37.934	-0.46	15.80	-174.41	15.34	9	7/14/2014	42:39.8

4653	RSPe_2	-122.445	37.934	-0.19	15.83	-174.96	15.64	-122.445	37.934	-0.46	15.83	-175.33	15.37	9	7/14/2014	42:39.7
4654	RSPe_2	-122.445	37.934	-0.22	15.85	-175.96	15.62	-122.445	37.934	-0.49	15.85	-176.26	15.36	9	7/14/2014	42:39.6
4655	RSPe_2	-122.445	37.934	-0.22	15.76	-176.89	15.53	-122.445	37.934	-0.46	15.76	-177.28	15.30	9	7/14/2014	42:39.5
4656	RSPe_2	-122.445	37.934	-0.22	15.75	-177.77	15.52	-122.445	37.934	-0.41	15.75	-178.18	15.34	9	7/14/2014	42:39.4
4657	RSPe_2	-122.445	37.934	-0.22	15.74	-178.54	15.52	-122.445	37.934	-0.46	15.74	-179.04	15.28	9	7/14/2014	42:39.3
4658	RSPe_2	-122.445	37.934	-0.22	15.71	-179.25	15.49	-122.445	37.934	-0.46	15.71	-179.88	15.25	9	7/14/2014	42:39.2
4659	RSPe_2	-122.445	37.934	-0.22	15.69	-180.11	15.47	-122.445	37.934	-0.46	15.69	-180.77	15.24	9	7/14/2014	42:39.1
4660	RSPe_2	-122.445	37.934	-0.22	15.67	-180.92	15.45	-122.445	37.934	-0.46	15.67	-181.65	15.21	9	7/14/2014	42:39.0
4661	RSPe_2	-122.445	37.934	-0.22	15.66	-181.76	15.43	-122.445	37.934	-0.46	15.66	-182.57	15.20	9	7/14/2014	42:38.9
4662	RSPe_2	-122.445	37.934	-0.22	15.66	-182.66	15.43	-122.445	37.934	-0.46	15.66	-183.43	15.20	9	7/14/2014	42:38.8
4663	RSPe_2	-122.445	37.934	-0.19	15.63	-183.62	15.44	-122.445	37.934	-0.46	15.63	-184.31	15.17	9	7/14/2014	42:38.7
4664	RSPe_2	-122.445	37.934	-0.22	15.64	-184.54	15.42	-122.445	37.934	-0.49	15.64	-185.20	15.15	9	7/14/2014	42:38.6
4665	RSPe_2	-122.445	37.934	-0.19	15.67	-185.52	15.48	-122.445	37.934	-0.41	15.67	-186.06	15.26	9	7/14/2014	42:38.5
4666	RSPe_2	-122.445	37.934	-0.28	15.70	-186.52	15.43	-122.445	37.934	-0.38	15.70	-186.99	15.33	9	7/14/2014	42:38.4
4667	RSPe_2	-122.445	37.934	-0.22	15.68	-187.38	15.45	-122.445	37.934	-0.41	15.68	-187.75	15.27	9	7/14/2014	42:38.3
4668	RSPe_2	-122.445	37.934	-0.22	15.73	-188.33	15.50	-122.445	37.934	-0.46	15.73	-188.60	15.27	9	7/14/2014	42:38.2
4669	RSPe_2	-122.445	37.934	-0.22	15.73	-189.18	15.50	-122.445	37.934	-0.41	15.73	-189.38	15.32	9	7/14/2014	42:38.1
4670	RSPe_2	-122.445	37.934	-0.22	15.75	-190.07	15.52	-122.445	37.934	-0.46	15.75	-190.18	15.29	9	7/14/2014	42:38.0
4671	RSPe_2	-122.445	37.934	-0.22	15.77	-191.01	15.55	-122.445	37.934	-0.46	15.77	-191.04	15.31	9	7/14/2014	42:37.9
4672	RSPe_2	-122.445	37.934	-0.28	15.80	-191.95	15.52	-122.445	37.934	-0.49	15.80	-191.90	15.31	9	7/14/2014	42:37.8
4673	RSPe_2	-122.445	37.934	-0.22	15.82	-192.85	15.59	-122.445	37.934	-0.49	15.82	-192.81	15.32	9	7/14/2014	42:37.7
4674	RSPe_2	-122.445	37.934	-0.28	15.85	-193.82	15.57	-122.445	37.934	-0.46	15.85	-193.83	15.39	9	7/14/2014	42:37.6
4675	RSPe_2	-122.445	37.934	-0.22	15.86	-194.66	15.63	-122.445	37.934	-0.61	15.86	-194.76	15.25	9	7/14/2014	42:37.5
4676	RSPe_2	-122.445	37.934	-0.22	15.88	-195.59	15.66	-122.445	37.934	-0.49	15.88	-195.80	15.39	9	7/14/2014	42:37.4
4677	RSPe_2	-122.445	37.934	-0.22	15.92	-196.55	15.69	-122.445	37.934	-0.46	15.92	-196.86	15.46	9	7/14/2014	42:37.3
4678	RSPe_2	-122.445	37.934	-0.22	15.85	-197.55	15.62	-122.445	37.934	-0.49	15.85	-197.83	15.36	9	7/14/2014	42:37.2
4679	RSPe_2	-122.445	37.934	-0.22	15.93	-198.55	15.71	-122.445	37.934	-0.46	15.93	-198.92	15.48	9	7/14/2014	42:37.1
4680	RSPe_2	-122.445	37.934	-0.22	15.84	-199.54	15.62	-122.445	37.934	-0.54	15.84	-199.87	15.30	9	7/14/2014	42:37.0
4681	RSPe_2	-122.445	37.934	-0.22	15.84	-200.47	15.62	-122.445	37.934	-0.54	15.84	-200.88	15.30	9	7/14/2014	42:36.9
4682	RSPe_2	-122.445	37.934	-0.28	15.85	-201.40	15.57	-122.445	37.934	-0.49	15.85	-201.83	15.36	9	7/14/2014	42:36.8
4683	RSPe_2	-122.445	37.934	-0.22	15.86	-202.26	15.63	-122.445	37.934	-0.54	15.86	-202.70	15.31	9	7/14/2014	42:36.7
4684	RSPe_2	-122.445	37.934	-0.22	15.86	-203.11	15.63	-122.445	37.934	-0.61	15.86	-203.61	15.25	9	7/14/2014	42:36.6
4685	RSPe_2	-122.445	37.934	-0.22	15.90	-203.88	15.67	-122.445	37.934	-0.54	15.90	-204.48	15.35	9	7/14/2014	42:36.5

4686	RSPe_2	-122.445	37.934	-0.28	15.95	-204.72	15.67	-122.445	37.934	-0.58	15.95	-205.30	15.37	9	7/14/2014	42:36.4
4687	RSPe_2	-122.445	37.934	-0.22	16.02	-205.40	15.79	-122.445	37.934	-0.54	16.02	-206.01	15.47	9	7/14/2014	42:36.3
4688	RSPe_2	-122.445	37.934	-0.22	16.03	-206.14	15.80	-122.445	37.934	-0.54	16.03	-206.64	15.48	9	7/14/2014	42:36.2
4689	RSPe_2	-122.445	37.934	-0.22	16.05	-206.93	15.83	-122.445	37.934	-0.46	16.05	-207.37	15.59	9	7/14/2014	42:36.1
4690	RSPe_2	-122.445	37.934	-0.28	16.09	-207.77	15.81	-122.445	37.934	-0.58	16.09	-208.24	15.51	9	7/14/2014	42:36.0
4691	RSPe_2	-122.445	37.934	-0.22	16.08	-208.67	15.85	-122.445	37.934	-0.54	16.08	-209.09	15.54	9	7/14/2014	42:35.9
4692	RSPe_2	-122.445	37.934	-0.28	16.13	-209.55	15.85	-122.445	37.934	-0.54	16.13	-209.97	15.58	9	7/14/2014	42:35.8
4693	RSPe_2	-122.445	37.934	-0.22	16.13	-210.50	15.90	-122.445	37.934	-0.54	16.13	-210.93	15.58	9	7/14/2014	42:35.7
4694	RSPe_2	-122.445	37.934	-0.28	16.13	-211.40	15.85	-122.445	37.934	-0.58	16.13	-211.93	15.55	9	7/14/2014	42:35.6
4695	RSPe_2	-122.445	37.934	-0.22	16.13	-212.34	15.90	-122.445	37.934	-0.58	16.13	-212.94	15.55	9	7/14/2014	42:35.5
4696	RSPe_2	-122.445	37.934	-0.28	16.14	-213.31	15.87	-122.445	37.934	-0.54	16.14	-213.99	15.60	9	7/14/2014	42:35.4
4697	RSPe_2	-122.445	37.934	-0.22	16.17	-214.23	15.94	-122.445	37.934	-0.54	16.17	-214.98	15.62	9	7/14/2014	42:35.3
4698	RSPe_2	-122.445	37.934	-0.22	16.13	-215.19	15.91	-122.445	37.934	-0.58	16.13	-215.91	15.55	9	7/14/2014	42:35.2
4699	RSPe_2	-122.445	37.934	-0.22	16.17	-216.09	15.94	-122.445	37.934	-0.49	16.17	-216.75	15.67	9	7/14/2014	42:35.1
4700	RSPe_2	-122.445	37.934	-0.22	16.14	-216.94	15.92	-122.445	37.934	-0.54	16.14	-217.49	15.60	9	7/14/2014	42:35.0
4701	RSPe_2	-122.445	37.934	-0.22	16.11	-217.74	15.89	-122.445	37.934	-0.49	16.11	-218.24	15.62	9	7/14/2014	42:34.9
4702	RSPe_2	-122.445	37.934	-0.22	16.20	-218.51	15.98	-122.445	37.934	-0.49	16.20	-219.06	15.71	9	7/14/2014	42:34.8
4703	RSPe_2	-122.445	37.934	-0.22	16.11	-219.33	15.89	-122.445	37.934	-0.49	16.11	-219.90	15.62	9	7/14/2014	42:34.7
4704	RSPe_2	-122.445	37.934	-0.28	16.06	-220.19	15.79	-122.445	37.934	-0.54	16.06	-220.83	15.52	9	7/14/2014	42:34.6
4705	RSPe_2	-122.445	37.934	-0.22	16.13	-221.11	15.90	-122.445	37.934	-0.49	16.13	-221.77	15.64	9	7/14/2014	42:34.5
4706	RSPe_2	-122.445	37.934	-0.22	15.94	-222.14	15.72	-122.445	37.934	-0.54	15.94	-222.81	15.40	9	7/14/2014	42:34.4
4707	RSPe_2	-122.445	37.934	-0.22	15.83	-222.98	15.60	-122.445	37.934	-0.49	15.83	-223.67	15.33	9	7/14/2014	42:34.3
4708	RSPe_2	-122.445	37.934	-0.22	15.78	-223.88	15.56	-122.445	37.934	-0.49	15.78	-224.63	15.29	9	7/14/2014	42:34.2
4709	RSPe_2	-122.445	37.934	-0.22	15.81	-224.96	15.59	-122.445	37.934	-0.54	15.81	-225.71	15.27	9	7/14/2014	42:34.1
4710	RSPe_2	-122.445	37.934	-0.22	15.67	-226.03	15.45	-122.445	37.934	-0.49	15.67	-226.79	15.18	9	7/14/2014	42:34.0
4711	RSPe_2	-122.445	37.934	-0.19	15.68	-227.16	15.49	-122.445	37.934	-0.49	15.68	-227.86	15.19	9	7/14/2014	42:33.9
4712	RSPe_2	-122.445	37.934	-0.19	15.61	-228.12	15.42	-122.445	37.934	-0.41	15.61	-228.86	15.20	9	7/14/2014	42:33.8
4713	RSPe_2	-122.445	37.934	-0.19	15.63	-229.01	15.44	-122.445	37.934	-0.46	15.63	-229.75	15.18	9	7/14/2014	42:33.7
4714	RSPe_2	-122.445	37.934	-0.19	15.57	-229.82	15.38	-122.445	37.934	-0.49	15.57	-230.56	15.08	9	7/14/2014	42:33.6
4715	RSPe_2	-122.445	37.934	-0.19	15.56	-230.70	15.37	-122.445	37.934	-0.41	15.56	-231.39	15.15	9	7/14/2014	42:33.5
4716	RSPe_2	-122.445	37.934	-0.19	15.53	-231.55	15.34	-122.445	37.934	-0.46	15.53	-232.19	15.07	9	7/14/2014	42:33.4
4717	RSPe_2	-122.445	37.934	-0.19	15.49	-232.43	15.31	-122.445	37.934	-0.46	15.49	-233.04	15.04	9	7/14/2014	42:33.3
4718	RSPe_2	-122.445	37.934	-0.19	15.51	-233.38	15.32	-122.445	37.934	-0.49	15.51	-233.96	15.02	9	7/14/2014	42:33.2

4719	RSPe_2	-122.445	37.934	-0.19	15.43	-234.36	15.24	-122.445	37.934	-0.46	15.43	-234.92	14.97	9	7/14/2014	42:33.1
4720	RSPe_2	-122.445	37.934	-0.19	15.50	-235.38	15.31	-122.445	37.934	-0.54	15.50	-235.94	14.96	9	7/14/2014	42:33.0
4721	RSPe_2	-122.445	37.934	-0.19	15.48	-236.43	15.30	-122.445	37.934	-0.41	15.48	-237.02	15.08	9	7/14/2014	42:32.9
4722	RSPe_2	-122.445	37.934	-0.19	15.36	-237.39	15.17	-122.445	37.934	-0.37	15.36	-238.11	14.99	9	7/14/2014	42:32.8
4723	RSPe_2	-122.445	37.934	-0.16	15.35	-238.42	15.19	-122.445	37.934	-0.37	15.35	-239.12	14.97	9	7/14/2014	42:32.7
4724	RSPe_2	-122.445	37.934	-0.19	15.43	-239.30	15.24	-122.445	37.934	-0.37	15.43	-240.13	15.05	9	7/14/2014	42:32.6
4725	RSPe_2	-122.445	37.934	-0.16	15.37	-240.22	15.22	-122.445	37.934	-0.37	15.37	-241.07	15.00	9	7/14/2014	42:32.5
4726	RSPe_2	-122.445	37.934	-0.16	15.42	-241.13	15.26	-122.445	37.934	-0.37	15.42	-241.94	15.04	9	7/14/2014	42:32.4
4727	RSPe_2	-122.445	37.934	-0.16	15.47	-242.00	15.32	-122.445	37.934	-0.32	15.47	-242.83	15.15	9	7/14/2014	42:32.3
4728	RSPe_2	-122.445	37.934	-0.16	15.46	-242.91	15.30	-122.445	37.934	-0.41	15.46	-243.67	15.05	9	7/14/2014	42:32.2
4729	RSPe_2	-122.445	37.934	-0.10	15.43	-243.74	15.32	-122.445	37.934	-0.37	15.43	-244.57	15.05	9	7/14/2014	42:32.1
4730	RSPe_2	-122.445	37.934	-0.16	15.50	-244.62	15.35	-122.445	37.934	-0.32	15.50	-245.45	15.18	9	7/14/2014	42:32.0
4731	RSPe_2	-122.445	37.934	-0.16	15.41	-245.57	15.25	-122.445	37.934	-0.37	15.41	-246.47	15.04	9	7/14/2014	42:31.9
4732	RSPe_2	-122.445	37.934	-0.16	15.40	-246.59	15.25	-122.445	37.934	-0.37	15.40	-247.48	15.03	9	7/14/2014	42:31.8
4733	RSPe_2	-122.445	37.934	-0.10	15.38	-247.58	15.27	-122.445	37.934	-0.37	15.38	-248.55	15.00	9	7/14/2014	42:31.7
4734	RSPe_2	-122.445	37.934	-0.16	15.35	-248.66	15.19	-122.445	37.934	-0.37	15.35	-249.60	14.97	9	7/14/2014	42:31.6
4735	RSPe_2	-122.445	37.934	-0.10	15.32	-249.55	15.21	-122.445	37.934	-0.29	15.32	-250.48	15.03	9	7/14/2014	42:31.5
4736	RSPe_2	-122.445	37.934	-0.10	15.32	-250.48	15.22	-122.445	37.934	-0.32	15.32	-251.43	15.00	9	7/14/2014	42:31.4
4737	RSPe_2	-122.445	37.934	-0.10	15.35	-251.43	15.24	-122.445	37.934	-0.29	15.35	-252.36	15.06	9	7/14/2014	42:31.3
4738	RSPe_2	-122.445	37.934	-0.10	15.39	-252.30	15.28	-122.445	37.934	-0.37	15.39	-253.22	15.01	9	7/14/2014	42:31.2
4739	RSPe_2	-122.445	37.934	-0.10	15.43	-253.23	15.32	-122.445	37.934	-0.41	15.43	-254.14	15.02	9	7/14/2014	42:31.1
4740	RSPe_2	-122.445	37.934	-0.10	15.39	-254.12	15.28	-122.445	37.934	-0.41	15.39	-255.01	14.98	9	7/14/2014	42:31.0
4741	RSPe_2	-122.445	37.934	-0.07	15.43	-254.85	15.36	-122.445	37.934	-0.37	15.43	-255.79	15.06	9	7/14/2014	42:30.9
4742	RSPe_2	-122.445	37.934	-0.10	15.44	-255.68	15.34	-122.445	37.934	-0.32	15.44	-256.59	15.12	9	7/14/2014	42:30.8
4743	RSPe_2	-122.445	37.934	-0.07	15.43	-256.51	15.36	-122.445	37.934	-0.29	15.43	-257.58	15.14	9	7/14/2014	42:30.7
4744	RSPe_2	-122.445	37.934	-0.10	15.43	-257.45	15.32	-122.445	37.934	-0.32	15.43	-258.52	15.10	9	7/14/2014	42:30.6
4745	RSPe_2	-122.445	37.934	-0.07	15.42	-258.35	15.35	-122.445	37.934	-0.32	15.42	-259.57	15.09	9	7/14/2014	42:30.5
4746	RSPe_2	-122.445	37.934	-0.10	15.40	-259.45	15.30	-122.445	37.934	-0.32	15.40	-260.64	15.08	9	7/14/2014	42:30.4
4747	RSPe_2	-122.445	37.934	-0.07	15.43	-260.48	15.36	-122.445	37.934	-0.20	15.43	-261.69	15.22	9	7/14/2014	42:30.3
4748	RSPe_2	-122.445	37.934	-0.10	15.41	-261.60	15.31	-122.445	37.934	-0.24	15.41	-262.83	15.17	9	7/14/2014	42:30.2
4749	RSPe_2	-122.445	37.934	-0.07	15.40	-262.62	15.33	-122.445	37.934	-0.29	15.40	-263.83	15.11	9	7/14/2014	42:30.1
4750	RSPe_2	-122.445	37.934	-0.07	15.43	-263.70	15.36	-122.445	37.934	-0.29	15.43	-264.93	15.14	9	7/14/2014	42:30.0
4751	RSPe_2	-122.445	37.934	-0.07	15.54	-264.80	15.47	-122.445	37.934	-0.24	15.54	-266.06	15.30	9	7/14/2014	42:29.9

4752	RSPe_2	-122.445	37.934	-0.07	15.49	-265.98	15.42	-122.445	37.934	-0.32	15.49	-267.18	15.17	9	7/14/2014 42:29.8
4753	RSPe_2	-122.445	37.934	-0.07	15.48	-267.10	15.41	-122.445	37.934	-0.17	15.48	-268.29	15.31	9	7/14/2014 42:29.7
4754	RSPe_2	-122.445	37.934	-0.07	15.48	-268.20	15.41	-122.445	37.934	-0.24	15.48	-269.38	15.24	9	7/14/2014 42:29.6
4755	RSPe_2	-122.445	37.934	-0.07	15.53	-269.31	15.46	-122.445	37.934	-0.20	15.53	-270.47	15.33	9	7/14/2014 42:29.5
4756	RSPe_2	-122.445	37.934	-0.07	15.50	-270.32	15.43	-122.445	37.934	-0.20	15.50	-271.49	15.30	9	7/14/2014 42:29.4
4757	RSPe_2	-122.445	37.934	-0.07	15.50	-271.38	15.43	-122.445	37.934	-0.29	15.50	-272.55	15.21	9	7/14/2014 42:29.3
4758	RSPe_2	-122.445	37.934	-0.07	15.45	-272.40	15.38	-122.445	37.934	-0.20	15.45	-273.63	15.24	9	7/14/2014 42:29.2
4759	RSPe_2	-122.445	37.934	-0.02	15.50	-273.35	15.48	-122.445	37.934	-0.20	15.50	-274.57	15.30	9	7/14/2014 42:29.1
4760	RSPe_2	-122.445	37.934	-0.07	15.51	-274.33	15.44	-122.445	37.934	-0.17	15.51	-275.52	15.34	9	7/14/2014 42:29.0
4761	RSPe_2	-122.445	37.934	-0.02	15.56	-275.28	15.55	-122.445	37.934	-0.17	15.56	-276.53	15.39	9	7/14/2014 42:28.9
4762	RSPe_2	-122.445	37.934	-0.07	15.53	-276.29	15.46	-122.445	37.934	-0.17	15.53	-277.53	15.36	9	7/14/2014 42:28.8
4763	RSPe_2	-122.445	37.934	-0.02	15.59	-277.27	15.57	-122.445	37.934	-0.17	15.59	-278.49	15.42	9	7/14/2014 42:28.7
4764	RSPe_2	-122.445	37.934	-0.07	15.63	-278.18	15.56	-122.445	37.934	-0.20	15.63	-279.48	15.43	9	7/14/2014 42:28.6
4765	RSPe_2	-122.445	37.934	-0.02	15.62	-279.15	15.61	-122.445	37.934	-0.12	15.62	-280.43	15.50	9	7/14/2014 42:28.5
4766	RSPe_2	-122.445	37.934	-0.07	15.70	-280.13	15.63	-122.445	37.934	-0.24	15.70	-281.45	15.47	9	7/14/2014 42:28.4
4767	RSPe_2	-122.445	37.934	-0.02	15.63	-280.97	15.62	-122.445	37.934	-0.20	15.63	-282.35	15.43	9	7/14/2014 42:28.3
4768	RSPe_2	-122.445	37.934	-0.07	15.70	-281.94	15.63	-122.445	37.934	-0.20	15.70	-283.42	15.50	9	7/14/2014 42:28.2
4769	RSPe_2	-122.445	37.934	-0.02	15.72	-282.90	15.70	-122.445	37.934	-0.20	15.72	-284.45	15.51	9	7/14/2014 42:28.1
4770	RSPe_2	-122.445	37.934	-0.07	15.70	-283.85	15.63	-122.445	37.934	-0.17	15.70	-285.54	15.53	9	7/14/2014 42:28.0
4771	RSPe_2	-122.445	37.934	-0.02	15.70	-284.82	15.69	-122.445	37.934	-0.20	15.70	-286.47	15.50	9	7/14/2014 42:27.9
4772	RSPe_2	-122.445	37.934	-0.07	15.69	-285.75	15.62	-122.445	37.934	-0.20	15.69	-287.49	15.49	9	7/14/2014 42:27.8
4773	RSPe_2	-122.445	37.934	-0.07	15.73	-286.77	15.66	-122.445	37.934	-0.12	15.73	-288.52	15.61	9	7/14/2014 42:27.7
4774	RSPe_2	-122.445	37.934	-0.07	15.69	-287.74	15.62	-122.445	37.934	-0.20	15.69	-289.50	15.49	9	7/14/2014 42:27.6
4775	RSPe_2	-122.445	37.934	-0.02	15.81	-288.80	15.79	-122.445	37.934	-0.17	15.81	-290.51	15.64	9	7/14/2014 42:27.5
4776	RSPe_2	-122.445	37.934	-0.07	15.87	-289.82	15.80	-122.445	37.934	-0.17	15.87	-291.48	15.70	9	7/14/2014 42:27.4
4777	RSPe_2	-122.445	37.934	-0.02	15.89	-290.75	15.87	-122.445	37.934	-0.12	15.89	-292.33	15.77	9	7/14/2014 42:27.3
4778	RSPe_2	-122.445	37.934	-0.10	15.93	-291.62	15.82	-122.445	37.934	-0.20	15.93	-293.24	15.72	9	7/14/2014 42:27.2
4779	RSPe_2	-122.445	37.934	-0.07	16.05	-292.42	15.98	-122.445	37.934	-0.20	16.05	-294.08	15.85	9	7/14/2014 42:27.1
4780	RSPe_2	-122.445	37.934	-0.07	15.99	-293.28	15.92	-122.445	37.934	-0.20	15.99	-294.97	15.78	9	7/14/2014 42:27.0
4781	RSPe_2	-122.445	37.934	-0.07	16.14	-294.17	16.07	-122.445	37.934	-0.20	16.14	-295.92	15.94	9	7/14/2014 42:26.9
4782	RSPe_2	-122.445	37.934	-0.07	16.03	-295.08	15.96	-122.445	37.934	-0.20	16.03	-296.96	15.82	9	7/14/2014 42:26.8
4783	RSPe_2	-122.445	37.934	-0.07	16.06	-296.03	15.99	-122.445	37.934	-0.12	16.06	-297.94	15.94	9	7/14/2014 42:26.7
4784	RSPe_2	-122.445	37.934	-0.07	16.07	-296.92	16.00	-122.445	37.934	-0.24	16.07	-298.89	15.83	9	7/14/2014 42:26.6

4785	RSPe_2	-122.445	37.934	-0.07	16.10	-297.91	16.03	-122.445	37.934	-0.09	16.10	-299.86	16.02	9	7/14/2014	42:26.5
4786	RSPe_2	-122.445	37.934	-0.07	16.12	-298.87	16.05	-122.445	37.934	-0.09	16.12	-300.73	16.04	9	7/14/2014	42:26.4
4787	RSPe_2	-122.445	37.934	-0.02	16.17	-299.77	16.15	-122.445	37.934	-0.24	16.17	-301.57	15.93	9	7/14/2014	42:26.3
4788	RSPe_2	-122.445	37.934	-0.07	16.21	-300.85	16.14	-122.445	37.934	-0.24	16.21	-302.57	15.97	9	7/14/2014	42:26.2
4789	RSPe_2	-122.445	37.934	-0.07	16.22	-301.96	16.15	-122.445	37.934	-0.09	16.22	-303.76	16.13	9	7/14/2014	42:26.1
4790	RSPe_2	-122.445	37.934	-0.07	16.13	-302.78	16.06	-122.445	37.934	-0.17	16.13	-304.45	15.96	9	7/14/2014	42:26.0
4791	RSPe_2	-122.445	37.934	-0.02	16.07	-303.43	16.05	-122.445	37.934	-0.17	16.07	-305.03	15.90	9	7/14/2014	42:25.9
4792	RSPe_2	-122.445	37.934	-0.07	16.13	-304.32	16.06	-122.445	37.934	-0.17	16.13	-305.90	15.96	9	7/14/2014	42:25.8
4793	RSPe_2	-122.445	37.934	-0.07	16.02	-305.36	15.95	-122.445	37.934	-0.20	16.02	-306.85	15.81	9	7/14/2014	42:25.7
4794	RSPe_2	-122.445	37.934	-0.07	15.90	-306.27	15.83	-122.445	37.934	-0.20	15.90	-307.75	15.69	9	7/14/2014	42:25.6
4795	RSPe_2	-122.445	37.934	-0.10	15.80	-307.05	15.69	-122.445	37.934	-0.20	15.80	-308.50	15.59	9	7/14/2014	42:25.5
4796	RSPe_2	-122.445	37.934	-0.10	15.80	-307.89	15.69	-122.445	37.934	-0.29	15.80	-309.32	15.51	9	7/14/2014	42:25.4
4797	RSPe_2	-122.445	37.934	-0.10	15.80	-308.63	15.70	-122.445	37.934	-0.20	15.80	-310.23	15.60	9	7/14/2014	42:25.3
4798	RSPe_2	-122.445	37.934	-0.10	15.90	-309.49	15.79	-122.445	37.934	-0.24	15.90	-311.07	15.66	9	7/14/2014	42:25.2
4799	RSPe_2	-122.445	37.934	-0.10	15.73	-310.18	15.62	-122.445	37.934	-0.29	15.73	-311.91	15.44	9	7/14/2014	42:25.1
4800	RSPe_2	-122.445	37.934	-0.10	15.67	-310.94	15.57	-122.445	37.934	-0.32	15.67	-312.67	15.35	9	7/14/2014	42:25.0
4801	RSPe_2	-122.445	37.934	-0.10	15.62	-311.74	15.51	-122.445	37.934	-0.24	15.62	-313.50	15.38	9	7/14/2014	42:24.9
4802	RSPe_2	-122.445	37.934	-0.10	15.71	-312.72	15.61	-122.445	37.934	-0.32	15.71	-314.49	15.39	9	7/14/2014	42:24.8
4803	RSPe_2	-122.445	37.934	-0.07	15.57	-313.56	15.50	-122.445	37.934	-0.20	15.57	-315.42	15.37	9	7/14/2014	42:24.7
4804	RSPe_2	-122.445	37.934	-0.10	15.53	-314.45	15.43	-122.445	37.934	-0.24	15.53	-316.31	15.29	9	7/14/2014	42:24.6
4805	RSPe_2	-122.445	37.934	-0.07	15.46	-315.29	15.39	-122.445	37.934	-0.20	15.46	-317.34	15.26	9	7/14/2014	42:24.5
4806	RSPe_2	-122.445	37.934	-0.10	15.48	-316.05	15.38	-122.445	37.934	-0.32	15.48	-318.21	15.16	9	7/14/2014	42:24.4
4807	RSPe_2	-122.445	37.934	-0.10	15.45	-316.83	15.34	-122.445	37.934	-0.20	15.45	-319.12	15.24	9	7/14/2014	42:24.3
4808	RSPe_2	-122.445	37.934	-0.10	15.36	-317.58	15.25	-122.445	37.934	-0.29	15.36	-319.89	15.07	9	7/14/2014	42:24.2
4809	RSPe_2	-122.445	37.934	-0.10	15.45	-318.26	15.34	-122.445	37.934	-0.29	15.45	-320.64	15.16	9	7/14/2014	42:24.1
4810	RSPe_2	-122.445	37.934	-0.16	15.32	-319.14	15.16	-122.445	37.934	-0.24	15.32	-321.41	15.08	9	7/14/2014	42:24.0
4811	RSPe_2	-122.445	37.934	-0.10	15.29	-319.92	15.19	-122.445	37.934	-0.24	15.29	-322.15	15.06	9	7/14/2014	42:23.9
4812	RSPe_2	-122.445	37.934	-0.10	15.33	-320.48	15.23	-122.445	37.934	-0.29	15.33	-322.67	15.04	9	7/14/2014	42:23.8
4813	RSPe_2	-122.445	37.934	-0.10	15.39	-321.15	15.28	-122.445	37.934	-0.29	15.39	-323.19	15.10	9	7/14/2014	42:23.7
4814	RSPe_2	-122.445	37.934	-0.16	15.36	-321.97	15.20	-122.445	37.934	-0.32	15.36	-323.99	15.03	9	7/14/2014	42:23.6
4815	RSPe_2	-122.445	37.934	-0.10	15.35	-322.80	15.24	-122.445	37.934	-0.24	15.35	-324.77	15.11	9	7/14/2014	42:23.5
4816	RSPe_2	-122.445	37.934	-0.16	15.39	-323.56	15.24	-122.445	37.934	-0.37	15.39	-325.48	15.02	9	7/14/2014	42:23.4
4817	RSPe_2	-122.445	37.934	-0.10	15.42	-324.33	15.31	-122.445	37.934	-0.37	15.42	-326.31	15.04	9	7/14/2014	42:23.3

4818	RSPe_2	-122.445	37.934	-0.16	15.41	-325.20	15.25	-122.445	37.934	-0.29	15.41	-327.21	15.12	9	7/14/2014	42:23.2
4819	RSPe_2	-122.445	37.934	-0.16	15.47	-326.09	15.32	-122.445	37.934	-0.29	15.47	-328.10	15.18	9	7/14/2014	42:23.1
4820	RSPe_2	-122.445	37.934	-0.16	15.48	-326.91	15.32	-122.445	37.934	-0.29	15.48	-329.01	15.19	9	7/14/2014	42:23.0
4821	RSPe_2	-122.445	37.934	-0.10	15.50	-327.71	15.40	-122.445	37.934	-0.37	15.50	-329.92	15.13	9	7/14/2014	42:22.9
4822	RSPe_2	-122.445	37.934	-0.16	15.45	-328.49	15.29	-122.445	37.934	-0.32	15.45	-330.65	15.12	9	7/14/2014	42:22.8
4823	RSPe_2	-122.445	37.934	-0.10	15.48	-329.18	15.38	-122.445	37.934	-0.37	15.48	-331.45	15.11	9	7/14/2014	42:22.7
4824	RSPe_2	-122.445	37.934	-0.16	15.52	-329.94	15.36	-122.445	37.934	-0.32	15.52	-332.25	15.19	9	7/14/2014	42:22.6
4825	RSPe_2	-122.445	37.934	-0.10	15.55	-330.69	15.44	-122.445	37.934	-0.29	15.55	-333.01	15.26	9	7/14/2014	42:22.5
4826	RSPe_2	-122.445	37.934	-0.16	15.54	-331.47	15.38	-122.445	37.934	-0.32	15.54	-333.85	15.22	9	7/14/2014	42:22.4
4827	RSPe_2	-122.445	37.934	-0.10	15.53	-332.21	15.43	-122.445	37.934	-0.32	15.53	-334.55	15.21	9	7/14/2014	42:22.3
4828	RSPe_2	-122.445	37.934	-0.16	15.55	-332.99	15.39	-122.445	37.934	-0.32	15.55	-335.35	15.23	9	7/14/2014	42:22.2
4829	RSPe_2	-122.445	37.934	-0.16	15.53	-333.70	15.38	-122.445	37.934	-0.32	15.53	-336.06	15.21	9	7/14/2014	42:22.1
4830	RSPe_2	-122.445	37.934	-0.16	15.56	-334.48	15.40	-122.445	37.934	-0.32	15.56	-336.82	15.23	9	7/14/2014	42:22.0
4831	RSPe_2	-122.445	37.934	-0.10	15.57	-335.22	15.47	-122.445	37.934	-0.37	15.57	-337.59	15.20	9	7/14/2014	42:21.9
4832	RSPe_2	-122.445	37.934	-0.16	15.61	-336.06	15.45	-122.445	37.934	-0.37	15.61	-338.46	15.24	9	7/14/2014	42:21.8
4833	RSPe_2	-122.445	37.934	-0.16	15.60	-336.95	15.45	-122.445	37.935	-0.29	15.60	-339.35	15.31	9	7/14/2014	42:21.7
4834	RSPe_2	-122.445	37.935	-0.19	15.63	-337.81	15.44	-122.445	37.935	-0.41	15.63	-340.32	15.23	9	7/14/2014	42:21.6
4835	RSPe_2	-122.445	37.935	-0.16	15.62	-338.74	15.47	-122.445	37.935	-0.32	15.62	-341.21	15.30	9	7/14/2014	42:21.5
4836	RSPe_2	-122.445	37.935	-0.16	15.64	-339.63	15.49	-122.445	37.935	-0.29	15.64	-342.07	15.35	9	7/14/2014	42:21.4
4837	RSPe_2	-122.445	37.935	-0.16	15.67	-340.50	15.52	-122.445	37.935	-0.32	15.67	-342.90	15.35	9	7/14/2014	42:21.3
4838	RSPe_2	-122.445	37.935	-0.16	15.70	-341.27	15.55	-122.445	37.935	-0.37	15.70	-343.59	15.33	9	7/14/2014	42:21.2
4839	RSPe_2	-122.445	37.935	-0.16	15.78	-341.97	15.62	-122.445	37.935	-0.37	15.78	-344.18	15.41	9	7/14/2014	42:21.1
4840	RSPe_2	-122.445	37.935	-0.16	15.80	-342.64	15.64	-122.445	37.935	-0.37	15.80	-344.78	15.42	9	7/14/2014	42:21.0
4841	RSPe_2	-122.445	37.935	-0.16	15.83	-343.29	15.68	-122.445	37.935	-0.32	15.83	-345.41	15.51	9	7/14/2014	42:20.9
4842	RSPe_2	-122.445	37.935	-0.16	15.89	-343.91	15.73	-122.445	37.935	-0.37	15.89	-346.06	15.51	9	7/14/2014	42:20.8
4843	RSPe_2	-122.445	37.935	-0.10	15.92	-344.61	15.82	-122.445	37.935	-0.37	15.92	-346.86	15.55	9	7/14/2014	42:20.7
4844	RSPe_2	-122.445	37.935	-0.15	15.94	-345.36	15.79	-122.445	37.935	-0.46	15.94	-347.62	15.48	9	7/14/2014	42:20.6
4845	RSPe_2	-122.445	37.935	-0.10	15.98	-346.14	15.88	-122.445	37.935	-0.37	15.98	-348.39	15.61	9	7/14/2014	42:20.5
4846	RSPe_2	-122.445	37.935	-0.15	16.03	-346.96	15.88	-122.445	37.935	-0.46	16.03	-349.21	15.58	9	7/14/2014	42:20.4
4847	RSPe_2	-122.445	37.935	-0.10	16.02	-347.73	15.92	-122.445	37.935	-0.46	16.02	-349.93	15.56	9	7/14/2014	42:20.3
4848	RSPe_2	-122.445	37.935	-0.10	16.11	-348.57	16.01	-122.445	37.935	-0.37	16.11	-350.75	15.74	9	7/14/2014	42:20.2
4849	RSPe_2	-122.445	37.935	-0.10	16.09	-349.37	15.99	-122.445	37.935	-0.41	16.09	-351.59	15.68	9	7/14/2014	42:20.1
4850	RSPe_2	-122.445	37.935	-0.10	16.08	-350.17	15.98	-122.445	37.935	-0.41	16.08	-352.42	15.67	9	7/14/2014	42:20.0

4851	RSPe_2	-122.445	37.935	-0.10	16.07	-350.92	15.97	-122.445	37.935	-0.32	16.07	-353.22	15.75	9	7/14/2014	42:19.9
4852	RSPe_2	-122.445	37.935	-0.15	16.05	-351.73	15.90	-122.445	37.935	-0.41	16.05	-354.04	15.64	9	7/14/2014	42:19.8
4853	RSPe_2	-122.445	37.935	-0.10	16.04	-352.42	15.94	-122.445	37.935	-0.41	16.04	-354.91	15.63	9	7/14/2014	42:19.7
4854	RSPe_2	-122.445	37.935	-0.15	16.03	-353.17	15.88	-122.445	37.935	-0.32	16.03	-355.68	15.71	9	7/14/2014	42:19.6
4855	RSPe_2	-122.445	37.935	-0.10	16.03	-354.02	15.93	-122.445	37.935	-0.41	16.03	-356.57	15.63	9	7/14/2014	42:19.5
4856	RSPe_2	-122.445	37.935	-0.10	16.01	-354.80	15.91	-122.445	37.935	-0.29	16.01	-357.39	15.72	9	7/14/2014	42:19.4
4857	RSPe_2	-122.445	37.935	-0.10	16.06	-355.68	15.95	-122.445	37.935	-0.32	16.06	-358.13	15.73	9	7/14/2014	42:19.3
4858	RSPe_2	-122.445	37.935	-0.10	16.01	-356.31	15.91	-122.445	37.935	-0.32	16.01	-358.87	15.69	9	7/14/2014	42:19.2
4859	RSPe_2	-122.445	37.935	-0.10	15.98	-357.14	15.88	-122.445	37.935	-0.37	15.98	-359.62	15.61	9	7/14/2014	42:19.1
4860	RSPe_2	-122.445	37.935	-0.10	15.97	-357.94	15.86	-122.445	37.935	-0.37	15.97	-360.45	15.59	9	7/14/2014	42:19.0
4861	RSPe_2	-122.445	37.935	-0.07	15.94	-358.76	15.87	-122.445	37.935	-0.32	15.94	-361.40	15.62	9	7/14/2014	42:18.9
4862	RSPe_2	-122.445	37.935	-0.10	15.93	-359.56	15.83	-122.445	37.935	-0.41	15.93	-362.11	15.52	9	7/14/2014	42:18.8
4863	RSPe_2	-122.445	37.935	-0.07	15.90	-360.44	15.83	-122.445	37.935	-0.24	15.90	-363.04	15.67	9	7/14/2014	42:18.7
4864	RSPe_2	-122.445	37.935	-0.10	15.79	-361.35	15.69	-122.445	37.935	-0.46	15.79	-363.91	15.33	9	7/14/2014	42:18.6
4865	RSPe_2	-122.445	37.935	-0.07	15.76	-362.35	15.69	-122.445	37.935	-0.32	15.76	-364.84	15.44	9	7/14/2014	42:18.5
4866	RSPe_2	-122.445	37.935	-0.10	15.65	-363.35	15.55	-122.445	37.935	-0.32	15.65	-365.75	15.33	9	7/14/2014	42:18.4
4867	RSPe_2	-122.445	37.935	-0.07	15.66	-364.21	15.59	-122.445	37.935	-0.24	15.66	-366.55	15.42	9	7/14/2014	42:18.3
4868	RSPe_2	-122.445	37.935	-0.10	15.70	-365.08	15.60	-122.445	37.935	-0.29	15.70	-367.45	15.42	9	7/14/2014	42:18.2
4869	RSPe_2	-122.445	37.935	-0.07	15.73	-365.83	15.67	-122.445	37.935	-0.37	15.73	-368.17	15.36	9	7/14/2014	42:18.1
4870	RSPe_2	-122.445	37.935	-0.07	15.82	-366.57	15.75	-122.445	37.935	-0.24	15.82	-369.01	15.58	9	7/14/2014	42:18.0
4871	RSPe_2	-122.445	37.935	-0.07	15.77	-367.46	15.70	-122.445	37.935	-0.12	15.77	-369.88	15.65	9	7/14/2014	42:17.9
4872	RSPe_2	-122.445	37.935	-0.07	15.77	-368.28	15.70	-122.445	37.935	-0.37	15.77	-370.75	15.40	9	7/14/2014	42:17.8
4873	RSPe_2	-122.445	37.935	-0.07	15.76	-369.08	15.69	-122.445	37.935	-0.24	15.76	-371.65	15.53	9	7/14/2014	42:17.7
4874	RSPe_2	-122.445	37.935	-0.07	15.88	-369.88	15.81	-122.445	37.935	-0.24	15.88	-372.41	15.64	9	7/14/2014	42:17.6
4875	RSPe_2	-122.445	37.935	-0.07	15.93	-370.61	15.86	-122.445	37.935	-0.24	15.93	-373.19	15.69	9	7/14/2014	42:17.5
4876	RSPe_2	-122.445	37.935	-0.07	15.94	-371.33	15.87	-122.445	37.935	-0.29	15.94	-373.97	15.65	9	7/14/2014	42:17.4
4877	RSPe_2	-122.445	37.935	-0.07	15.88	-372.09	15.81	-122.445	37.935	-0.24	15.88	-374.68	15.64	9	7/14/2014	42:17.3
4878	RSPe_2	-122.445	37.935	-0.07	15.90	-372.80	15.83	-122.445	37.935	-0.29	15.90	-375.42	15.61	9	7/14/2014	42:17.2
4879	RSPe_2	-122.445	37.935	-0.02	15.93	-373.58	15.92	-122.445	37.935	-0.20	15.93	-376.07	15.73	9	7/14/2014	42:17.1
4880	RSPe_2	-122.445	37.935	-0.07	16.00	-374.14	15.93	-122.445	37.935	-0.32	16.00	-376.61	15.67	9	7/14/2014	42:17.0
4881	RSPe_2	-122.445	37.935	-0.02	16.04	-374.92	16.02	-122.445	37.935	-0.17	16.04	-377.43	15.87	9	7/14/2014	42:16.9
4882	RSPe_2	-122.445	37.935	-0.02	16.03	-375.68	16.02	-122.445	37.935	-0.20	16.03	-378.21	15.83	9	7/14/2014	42:16.8
4883	RSPe_2	-122.445	37.935	-0.02	16.08	-376.46	16.06	-122.445	37.935	-0.20	16.08	-378.88	15.88	9	7/14/2014	42:16.7

4884	RSPe_2	-122.445	37.935	-0.07	16.03	-377.30	15.96	-122.445	37.935	-0.37	16.03	-379.73	15.65	9	7/14/2014	42:16.6
4885	RSPe_2	-122.445	37.935	-0.02	15.97	-378.04	15.95	-122.445	37.935	-0.09	15.97	-380.57	15.88	9	7/14/2014	42:16.5
4886	RSPe_2	-122.445	37.935	-0.02	15.88	-378.94	15.86	-122.445	37.935	-0.32	15.88	-381.39	15.56	9	7/14/2014	42:16.4
4887	RSPe_2	-122.445	37.935	-0.02	15.82	-379.70	15.80	-122.445	37.935	-0.20	15.82	-382.30	15.61	9	7/14/2014	42:16.3
4888	RSPe_2	-122.445	37.935	-0.02	15.71	-380.52	15.69	-122.445	37.935	-0.24	15.71	-383.01	15.47	9	7/14/2014	42:16.2
4889	RSPe_2	-122.445	37.935	-0.02	15.71	-381.37	15.69	-122.445	37.935	-0.24	15.71	-383.81	15.47	9	7/14/2014	42:16.1
4890	RSPe_2	-122.445	37.935	-0.07	15.69	-382.06	15.62	-122.445	37.935	-0.20	15.69	-384.59	15.48	9	7/14/2014	42:16.0
4891	RSPe_2	-122.445	37.935	-0.02	15.71	-382.80	15.69	-122.445	37.935	-0.17	15.71	-385.37	15.54	9	7/14/2014	42:15.9
4892	RSPe_2	-122.445	37.935	-0.02	15.65	-383.64	15.63	-122.445	37.935	-0.29	15.65	-386.19	15.36	9	7/14/2014	42:15.8
4893	RSPe_2	-122.445	37.935	-0.02	15.56	-384.40	15.55	-122.445	37.935	-0.17	15.56	-386.93	15.39	9	7/14/2014	42:15.7
4894	RSPe_2	-122.445	37.935	-0.02	15.53	-385.17	15.52	-122.445	37.935	-0.20	15.53	-387.82	15.33	9	7/14/2014	42:15.6
4895	RSPe_2	-122.445	37.935	0.02	15.50	-386.11	15.52	-122.445	37.935	-0.20	15.50	-388.68	15.30	9	7/14/2014	42:15.5
4896	RSPe_2	-122.445	37.935	-0.02	15.51	-386.89	15.49	-122.445	37.935	-0.17	15.51	-389.57	15.34	9	7/14/2014	42:15.4
4897	RSPe_2	-122.445	37.935	0.02	15.48	-387.90	15.50	-122.445	37.935	-0.32	15.48	-390.39	15.16	9	7/14/2014	42:15.3
4898	RSPe_2	-122.445	37.935	0.02	15.46	-388.64	15.47	-122.445	37.935	-0.03	15.46	-391.26	15.42	9	7/14/2014	42:15.2
4899	RSPe_2	-122.445	37.935	0.02	15.43	-389.55	15.45	-122.445	37.935	-0.12	15.43	-392.16	15.31	9	7/14/2014	42:15.1
4900	RSPe_2	-122.445	37.935	0.02	15.41	-390.35	15.43	-122.445	37.935	-0.17	15.41	-393.03	15.24	9	7/14/2014	42:15.0
4901	RSPe_2	-122.445	37.935	0.02	15.39	-391.28	15.41	-122.445	37.935	-0.12	15.39	-393.87	15.28	9	7/14/2014	42:14.9
4902	RSPe_2	-122.445	37.935	0.02	15.40	-391.99	15.42	-122.445	37.935	-0.12	15.40	-394.76	15.28	9	7/14/2014	42:14.8
4903	RSPe_2	-122.445	37.935	0.02	15.42	-392.84	15.43	-122.445	37.935	-0.09	15.42	-395.43	15.33	9	7/14/2014	42:14.7
4904	RSPe_2	-122.445	37.935	0.02	15.45	-393.66	15.46	-122.445	37.935	-0.12	15.45	-396.23	15.33	9	7/14/2014	42:14.6
4905	RSPe_2	-122.445	37.935	0.07	15.53	-394.37	15.59	-122.445	37.935	-0.12	15.53	-396.95	15.41	9	7/14/2014	42:14.5
4906	RSPe_2	-122.445	37.935	0.02	15.58	-395.08	15.60	-122.445	37.935	-0.09	15.58	-397.68	15.49	9	7/14/2014	42:14.4
4907	RSPe_2	-122.445	37.935	0.07	15.57	-395.71	15.64	-122.445	37.935	-0.09	15.57	-398.22	15.49	9	7/14/2014	42:14.3
4908	RSPe_2	-122.445	37.935	0.02	15.58	-396.32	15.60	-122.445	37.935	-0.09	15.58	-398.92	15.49	9	7/14/2014	42:14.2
4909	RSPe_2	-122.445	37.935	0.07	15.52	-396.84	15.59	-122.445	37.935	-0.03	15.52	-399.59	15.48	9	7/14/2014	42:14.1
4910	RSPe_2	-122.445	37.935	0.07	15.50	-397.45	15.57	-122.445	37.935	-0.09	15.50	-400.09	15.42	9	7/14/2014	42:14.0
4911	RSPe_2	-122.445	37.935	0.07	15.50	-397.94	15.57	-122.445	37.935	0.00	15.50	-400.75	15.50	9	7/14/2014	42:13.9
4912	RSPe_2	-122.445	37.935	0.07	15.37	-398.55	15.44	-122.445	37.935	0.00	15.37	-401.23	15.37	9	7/14/2014	42:13.8
4913	RSPe_2	-122.445	37.935	0.07	15.31	-399.15	15.38	-122.445	37.935	0.00	15.31	-401.88	15.31	9	7/14/2014	42:13.7
4914	RSPe_2	-122.445	37.935	0.07	15.35	-399.78	15.41	-122.445	37.935	-0.03	15.35	-402.46	15.31	9	7/14/2014	42:13.6
4915	RSPe_2	-122.445	37.935	0.10	15.24	-400.34	15.34	-122.445	37.935	0.00	15.24	-403.09	15.24	9	7/14/2014	42:13.5
4916	RSPe_2	-122.445	37.935	0.07	15.19	-400.97	15.26	-122.445	37.935	-0.03	15.19	-403.67	15.16	9	7/14/2014	42:13.4

4917	RSPe_2	-122.445	37.935	0.07	15.21	-401.53	15.27	-122.445	37.935	0.00	15.21	-404.17	15.21	9	7/14/2014	42:13.3
4918	RSPe_2	-122.445	37.935	0.07	15.16	-402.05	15.23	-122.445	37.935	0.00	15.16	-404.73	15.16	9	7/14/2014	42:13.2
4919	RSPe_2	-122.445	37.935	0.10	15.17	-402.48	15.27	-122.445	37.935	0.05	15.17	-405.21	15.22	9	7/14/2014	42:13.1
4920	RSPe_2	-122.445	37.935	0.07	15.17	-403.02	15.24	-122.445	37.935	0.00	15.17	-405.69	15.17	9	7/14/2014	42:13.0
4921	RSPe_2	-122.445	37.935	0.10	15.15	-403.50	15.26	-122.445	37.935	0.05	15.15	-406.20	15.20	9	7/14/2014	42:12.9
4922	RSPe_2	-122.445	37.935	0.07	15.16	-404.04	15.23	-122.445	37.935	0.08	15.16	-406.73	15.25	9	7/14/2014	42:12.8
4923	RSPe_2	-122.445	37.935	0.07	15.15	-404.54	15.22	-122.445	37.935	0.00	15.15	-407.16	15.15	9	7/14/2014	42:12.7
4924	RSPe_2	-122.445	37.935	0.07	15.16	-405.04	15.23	-122.445	37.935	-0.03	15.16	-407.73	15.13	9	7/14/2014	42:12.6
4925	RSPe_2	-122.445	37.935	0.10	15.22	-405.58	15.33	-122.445	37.935	0.05	15.22	-408.15	15.27	9	7/14/2014	42:12.5
4926	RSPe_2	-122.445	37.935	0.07	15.19	-406.07	15.25	-122.445	37.935	0.05	15.19	-408.67	15.24	9	7/14/2014	42:12.4
4927	RSPe_2	-122.445	37.935	0.10	15.18	-406.59	15.28	-122.445	37.935	0.00	15.18	-409.14	15.18	9	7/14/2014	42:12.3
4928	RSPe_2	-122.445	37.935	0.07	15.19	-406.98	15.25	-122.445	37.935	0.08	15.19	-409.58	15.27	9	7/14/2014	42:12.2
4929	RSPe_2	-122.445	37.935	0.10	15.24	-407.50	15.34	-122.445	37.935	0.08	15.24	-410.10	15.32	9	7/14/2014	42:12.1
4930	RSPe_2	-122.445	37.935	0.10	15.32	-407.89	15.43	-122.445	37.935	0.05	15.32	-410.57	15.37	9	7/14/2014	42:12.0
4931	RSPe_2	-122.445	37.935	0.10	15.35	-408.28	15.45	-122.445	37.935	0.17	15.35	-410.98	15.51	9	7/14/2014	42:11.9
4932	RSPe_2	-122.445	37.935	0.07	15.36	-408.65	15.43	-122.445	37.935	0.08	15.36	-411.37	15.45	9	7/14/2014	42:11.8
4933	RSPe_2	-122.445	37.935	0.10	15.39	-409.17	15.50	-122.445	37.935	0.08	15.39	-411.80	15.48	9	7/14/2014	42:11.7
4934	RSPe_2	-122.445	37.935	0.07	15.42	-409.51	15.48	-122.445	37.935	0.05	15.42	-412.26	15.47	9	7/14/2014	42:11.6
4935	RSPe_2	-122.445	37.935	0.07	15.44	-410.05	15.51	-122.445	37.935	0.08	15.44	-412.70	15.52	9	7/14/2014	42:11.5
4936	RSPe_2	-122.445	37.935	0.07	15.48	-410.49	15.55	-122.445	37.935	0.00	15.48	-413.10	15.48	9	7/14/2014	42:11.4
4937	RSPe_2	-122.445	37.935	0.07	15.49	-410.98	15.56	-122.445	37.935	0.00	15.49	-413.64	15.49	9	7/14/2014	42:11.3
4938	RSPe_2	-122.445	37.935	0.07	15.55	-411.46	15.62	-122.445	37.935	0.05	15.55	-414.08	15.60	9	7/14/2014	42:11.2
4939	RSPe_2	-122.445	37.935	0.10	15.64	-411.89	15.74	-122.445	37.935	0.05	15.64	-414.49	15.69	9	7/14/2014	42:11.1
4940	RSPe_2	-122.445	37.935	0.07	15.60	-412.37	15.67	-122.445	37.935	0.05	15.60	-414.88	15.65	9	7/14/2014	42:11.0
4941	RSPe_2	-122.445	37.935	0.07	15.68	-412.73	15.75	-122.445	37.935	0.05	15.68	-415.33	15.73	9	7/14/2014	42:10.9
4942	RSPe_2	-122.445	37.935	0.07	15.74	-413.21	15.81	-122.445	37.935	0.00	15.74	-415.77	15.74	9	7/14/2014	42:10.8
4943	RSPe_2	-122.445	37.935	0.07	15.72	-413.56	15.79	-122.445	37.935	-0.09	15.72	-416.20	15.63	9	7/14/2014	42:10.7
4944	RSPe_2	-122.445	37.935	0.02	15.78	-413.96	15.80	-122.445	37.935	0.05	15.78	-416.63	15.83	9	7/14/2014	42:10.6
4945	RSPe_2	-122.445	37.935	0.07	15.81	-414.34	15.88	-122.445	37.935	0.05	15.81	-417.06	15.86	9	7/14/2014	42:10.5
4946	RSPe_2	-122.445	37.935	0.07	15.79	-414.81	15.86	-122.445	37.935	0.05	15.79	-417.56	15.84	9	7/14/2014	42:10.4
4947	RSPe_2	-122.445	37.935	0.07	15.90	-415.09	15.96	-122.445	37.935	0.00	15.90	-417.90	15.90	9	7/14/2014	42:10.3
4948	RSPe_2	-122.445	37.935	0.07	15.70	-415.42	15.77	-122.445	37.935	0.05	15.70	-418.23	15.75	9	7/14/2014	42:10.2
4949	RSPe_2	-122.445	37.935	0.07	15.64	-415.61	15.71	-122.445	37.935	0.00	15.64	-418.51	15.64	9	7/14/2014	42:10.1

4950	RSPe_2	-122.445	37.935	0.07	15.61	-415.95	15.68	-122.445	37.935	-0.03	15.61	-418.81	15.58	9	7/14/2014 42:10.0
4951	RSPe_2	-122.445	37.935	0.07	15.57	-416.28	15.64	-122.445	37.935	0.05	15.57	-419.11	15.62	9	7/14/2014 42:09.9
4952	RSPe_2	-122.445	37.935	0.02	15.51	-416.60	15.53	-122.445	37.935	-0.12	15.51	-419.44	15.39	9	7/14/2014 42:09.8
4953	RSPe_2	-122.445	37.935	0.07	15.58	-416.95	15.65	-122.445	37.935	0.00	15.58	-419.70	15.58	9	7/14/2014 42:09.7
4954	RSPe_2	-122.445	37.935	0.02	15.55	-417.23	15.57	-122.445	37.935	-0.09	15.55	-420.00	15.46	9	7/14/2014 42:09.6
4955	RSPe_2	-122.445	37.935	0.02	15.45	-417.49	15.46	-122.445	37.935	-0.03	15.45	-420.24	15.41	9	7/14/2014 42:09.5
4956	RSPe_2	-122.445	37.935	0.02	15.42	-417.73	15.43	-122.445	37.935	-0.09	15.42	-420.50	15.33	9	7/14/2014 42:09.4
4957	RSPe_2	-122.445	37.935	0.02	15.41	-417.94	15.43	-122.445	37.935	-0.03	15.41	-420.75	15.38	9	7/14/2014 42:09.3
4958	RSPe_2	-122.445	37.935	0.07	15.38	-418.18	15.45	-122.445	37.935	0.00	15.38	-420.97	15.38	9	7/14/2014 42:09.2
4959	RSPe_2	-122.445	37.935	0.07	15.41	-418.37	15.48	-122.445	37.935	-0.03	15.41	-421.21	15.38	9	7/14/2014 42:09.1
4960	RSPe_2	-122.445	37.935	0.02	15.37	-418.55	15.39	-122.445	37.935	-0.03	15.37	-421.41	15.34	9	7/14/2014 42:09.0
4961	RSPe_2	-122.445	37.935	0.07	15.30	-418.83	15.37	-122.445	37.935	-0.03	15.30	-421.67	15.27	9	7/14/2014 42:08.9
4962	RSPe_2	-122.445	37.935	0.02	15.35	-418.96	15.36	-122.445	37.935	-0.03	15.35	-421.90	15.31	9	7/14/2014 42:08.8
4963	RSPe_2	-122.445	37.935	0.07	15.25	-419.30	15.32	-122.445	37.935	-0.03	15.25	-422.22	15.21	9	7/14/2014 42:08.7
4964	RSPe_2	-122.445	37.935	0.02	15.24	-419.56	15.26	-122.445	37.935	-0.09	15.24	-422.44	15.15	9	7/14/2014 42:08.6
4965	RSPe_2	-122.445	37.935	0.07	15.20	-419.82	15.27	-122.445	37.935	0.00	15.20	-422.66	15.20	9	7/14/2014 42:08.5
4966	RSPe_2	-122.445	37.935	0.07	15.18	-420.04	15.25	-122.445	37.935	-0.09	15.18	-422.87	15.09	9	7/14/2014 42:08.4
4967	RSPe_2	-122.445	37.935	0.07	15.22	-420.32	15.29	-122.445	37.935	-0.03	15.22	-423.11	15.18	9	7/14/2014 42:08.3
4968	RSPe_2	-122.445	37.935	0.07	15.25	-420.56	15.32	-122.445	37.935	0.00	15.25	-423.37	15.25	9	7/14/2014 42:08.2
4969	RSPe_2	-122.445	37.935	0.07	15.36	-420.73	15.43	-122.445	37.935	0.00	15.36	-423.52	15.36	9	7/14/2014 42:08.1
4970	RSPe_2	-122.445	37.935	0.02	15.33	-420.75	15.35	-122.445	37.935	-0.03	15.33	-423.56	15.30	9	7/14/2014 42:08.0
4971	RSPe_2	-122.445	37.935	0.07	15.41	-420.84	15.48	-122.445	37.935	-0.03	15.41	-423.69	15.38	9	7/14/2014 42:07.9
4972	RSPe_2	-122.445	37.935	0.07	15.40	-420.99	15.47	-122.445	37.935	-0.03	15.40	-423.87	15.37	9	7/14/2014 42:07.8
4973	RSPe_2	-122.445	37.935	0.07	15.44	-421.10	15.51	-122.445	37.935	0.05	15.44	-424.06	15.49	9	7/14/2014 42:07.7
4974	RSPe_2	-122.445	37.935	0.07	15.46	-421.12	15.52	-122.445	37.935	0.00	15.46	-424.12	15.46	9	7/14/2014 42:07.6
4975	RSPe_2	-122.445	37.935	0.10	15.46	-421.20	15.57	-122.445	37.935	-0.03	15.46	-424.23	15.43	9	7/14/2014 42:07.5
4976	RSPe_2	-122.445	37.935	0.07	15.45	-421.22	15.51	-122.445	37.935	-0.03	15.45	-424.38	15.41	9	7/14/2014 42:07.4
4977	RSPe_2	-122.445	37.935	0.07	15.44	-421.35	15.51	-122.445	37.935	0.00	15.44	-424.51	15.44	9	7/14/2014 42:07.3
4978	RSPe_2	-122.445	37.935	0.07	15.43	-421.44	15.50	-122.445	37.935	0.05	15.43	-424.62	15.48	9	7/14/2014 42:07.2
4979	RSPe_2	-122.445	37.935	0.07	15.39	-421.53	15.46	-122.445	37.935	0.00	15.39	-424.75	15.39	9	7/14/2014 42:07.1
4980	RSPe_2	-122.445	37.935	0.07	15.39	-421.70	15.46	-122.445	37.935	0.00	15.39	-424.86	15.39	9	7/14/2014 42:07.0
4981	RSPe_2	-122.445	37.935	0.07	15.37	-421.69	15.44	-122.445	37.935	-0.03	15.37	-424.81	15.34	9	7/14/2014 42:06.9
4982	RSPe_2	-122.445	37.935	0.07	15.41	-421.61	15.48	-122.445	37.935	-0.03	15.41	-424.79	15.38	9	7/14/2014 42:06.8

4983	RSPe_2	-122.445	37.935	0.07	15.48	-421.61	15.55	-122.445	37.935	0.00	15.48	-424.70	15.48	9	7/14/2014	42:06.7
4984	RSPe_2	-122.445	37.935	0.07	15.48	-421.57	15.55	-122.445	37.935	0.00	15.48	-424.64	15.49	9	7/14/2014	42:06.6
4985	RSPe_2	-122.445	37.935	0.07	15.52	-421.54	15.59	-122.445	37.935	0.00	15.52	-424.70	15.52	9	7/14/2014	42:06.5
4986	RSPe_2	-122.445	37.935	0.07	15.59	-421.46	15.66	-122.445	37.935	0.00	15.59	-424.53	15.59	9	7/14/2014	42:06.4
4987	RSPe_2	-122.445	37.935	0.07	15.62	-421.37	15.69	-122.445	37.935	0.05	15.62	-424.42	15.67	9	7/14/2014	42:06.3
4988	RSPe_2	-122.445	37.935	0.02	15.64	-421.24	15.66	-122.445	37.935	0.09	15.64	-424.38	15.73	9	7/14/2014	42:06.2
4989	RSPe_2	-122.445	37.935	0.02	15.69	-421.19	15.71	-122.445	37.935	0.00	15.69	-424.30	15.69	9	7/14/2014	42:06.1
4990	RSPe_2	-122.445	37.935	0.02	15.74	-421.08	15.76	-122.445	37.935	0.00	15.74	-424.18	15.74	9	7/14/2014	42:06.0
4991	RSPe_2	-122.445	37.935	0.07	15.77	-421.11	15.84	-122.445	37.935	-0.03	15.77	-424.29	15.74	9	7/14/2014	42:05.9
4992	RSPe_2	-122.445	37.935	0.02	15.78	-420.97	15.80	-122.445	37.935	-0.03	15.78	-424.07	15.75	9	7/14/2014	42:05.8
4993	RSPe_2	-122.445	37.935	0.02	15.93	-420.93	15.95	-122.445	37.935	-0.03	15.93	-424.07	15.90	9	7/14/2014	42:05.7
4994	RSPe_2	-122.445	37.935	0.02	15.96	-420.97	15.97	-122.445	37.935	-0.03	15.96	-424.07	15.92	9	7/14/2014	42:05.6
4995	RSPe_2	-122.445	37.935	0.02	15.98	-420.97	16.00	-122.445	37.935	-0.03	15.98	-424.07	15.95	9	7/14/2014	42:05.5
4996	RSPe_2	-122.445	37.935	0.02	15.97	-420.86	15.99	-122.445	37.935	-0.12	15.97	-424.00	15.85	9	7/14/2014	42:05.4
4997	RSPe_2	-122.445	37.935	0.07	16.03	-420.75	16.09	-122.445	37.935	-0.03	16.03	-423.94	15.99	9	7/14/2014	42:05.3
4998	RSPe_2	-122.445	37.935	0.02	16.06	-420.71	16.08	-122.445	37.935	-0.03	16.06	-423.93	16.03	9	7/14/2014	42:05.2
4999	RSPe_2	-122.445	37.935	0.02	15.93	-420.65	15.95	-122.445	37.935	0.00	15.93	-423.94	15.93	9	7/14/2014	42:05.1
5000	RSPe_2	-122.445	37.935	0.02	15.90	-420.60	15.92	-122.445	37.935	-0.08	15.90	-423.92	15.82	9	7/14/2014	42:05.0
5001	RSPe_2	-122.445	37.935	0.02	15.83	-420.54	15.85	-122.445	37.935	-0.03	15.83	-423.89	15.80	9	7/14/2014	42:04.9
5002	RSPe_2	-122.445	37.935	0.02	15.76	-420.38	15.78	-122.445	37.935	0.00	15.76	-423.74	15.76	9	7/14/2014	42:04.8
5003	RSPe_2	-122.445	37.935	0.02	15.66	-420.23	15.67	-122.445	37.935	-0.08	15.66	-423.65	15.57	9	7/14/2014	42:04.7
5004	RSPe_2	-122.445	37.935	0.02	15.66	-419.95	15.67	-122.445	37.935	-0.12	15.66	-423.48	15.54	9	7/14/2014	42:04.6
5005	RSPe_2	-122.445	37.935	0.02	15.57	-419.71	15.59	-122.445	37.935	-0.08	15.57	-423.32	15.49	9	7/14/2014	42:04.5
5006	RSPe_2	-122.445	37.935	-0.02	15.57	-419.50	15.56	-122.445	37.935	-0.08	15.57	-423.20	15.49	9	7/14/2014	42:04.4
5007	RSPe_2	-122.445	37.935	0.02	15.48	-419.34	15.50	-122.445	37.935	-0.08	15.48	-423.02	15.40	9	7/14/2014	42:04.3
5008	RSPe_2	-122.445	37.935	-0.02	15.50	-419.04	15.49	-122.445	37.935	-0.12	15.50	-422.82	15.38	9	7/14/2014	42:04.2
5009	RSPe_2	-122.445	37.935	0.02	15.48	-418.97	15.50	-122.445	37.935	-0.08	15.48	-422.78	15.40	9	7/14/2014	42:04.1
5010	RSPe_2	-122.445	37.935	-0.02	15.35	-418.78	15.33	-122.445	37.935	-0.08	15.35	-422.57	15.26	9	7/14/2014	42:04.0
5011	RSPe_2	-122.445	37.935	0.02	15.32	-418.54	15.34	-122.445	37.935	-0.12	15.32	-422.32	15.20	9	7/14/2014	42:03.9
5012	RSPe_2	-122.445	37.935	-0.02	15.29	-418.26	15.28	-122.445	37.935	-0.08	15.29	-422.05	15.21	9	7/14/2014	42:03.8
5013	RSPe_2	-122.445	37.935	-0.02	15.25	-418.04	15.23	-122.445	37.935	-0.03	15.25	-421.79	15.22	9	7/14/2014	42:03.7
5014	RSPe_2	-122.445	37.935	-0.02	15.24	-417.84	15.22	-122.445	37.935	-0.17	15.24	-421.56	15.07	9	7/14/2014	42:03.6
5015	RSPe_2	-122.445	37.935	0.02	15.22	-417.63	15.24	-122.445	37.935	-0.12	15.22	-421.37	15.10	9	7/14/2014	42:03.5

5016	RSPe_2	-122.445	37.935	-0.02	15.22	-417.41	15.20	-122.445	37.935	-0.08	15.22	-421.15	15.13	9	7/14/2014	42:03.4
5017	RSPe_2	-122.445	37.935	0.02	15.19	-417.26	15.21	-122.445	37.935	-0.03	15.19	-420.94	15.16	9	7/14/2014	42:03.3
5018	RSPe_2	-122.445	37.935	-0.02	15.26	-417.13	15.25	-122.445	37.935	-0.12	15.26	-420.74	15.15	9	7/14/2014	42:03.2
5019	RSPe_2	-122.445	37.935	0.02	15.19	-416.91	15.20	-122.445	37.935	-0.08	15.19	-420.55	15.10	9	7/14/2014	42:03.1
5020	RSPe_2	-122.445	37.935	-0.02	15.22	-416.76	15.20	-122.445	37.935	-0.12	15.22	-420.30	15.10	9	7/14/2014	42:03.0
5021	RSPe_2	-122.445	37.935	0.02	15.32	-416.41	15.34	-122.445	37.935	-0.12	15.32	-419.98	15.20	9	7/14/2014	42:02.9
5022	RSPe_2	-122.445	37.935	-0.02	15.22	-416.04	15.21	-122.445	37.935	-0.17	15.22	-419.67	15.05	9	7/14/2014	42:02.8
5023	RSPe_2	-122.445	37.935	-0.02	15.32	-415.70	15.31	-122.445	37.935	-0.12	15.32	-419.39	15.21	9	7/14/2014	42:02.7
5024	RSPe_2	-122.445	37.935	-0.02	15.38	-415.37	15.36	-122.445	37.935	-0.17	15.38	-419.13	15.21	9	7/14/2014	42:02.6
5025	RSPe_2	-122.445	37.935	0.02	15.44	-415.02	15.46	-122.445	37.935	-0.12	15.44	-418.85	15.32	9	7/14/2014	42:02.5
5026	RSPe_2	-122.445	37.935	-0.02	15.37	-414.76	15.36	-122.445	37.935	-0.12	15.37	-418.62	15.25	9	7/14/2014	42:02.4
5027	RSPe_2	-122.445	37.935	-0.02	15.49	-414.39	15.48	-122.445	37.935	-0.08	15.49	-418.24	15.41	9	7/14/2014	42:02.3
5028	RSPe_2	-122.445	37.935	-0.02	15.53	-414.05	15.52	-122.445	37.935	-0.12	15.53	-417.96	15.41	9	7/14/2014	42:02.2
5029	RSPe_2	-122.445	37.935	0.02	15.55	-413.70	15.57	-122.445	37.935	-0.12	15.55	-417.62	15.43	9	7/14/2014	42:02.1
5030	RSPe_2	-122.445	37.935	-0.02	15.60	-413.37	15.59	-122.445	37.935	-0.12	15.60	-417.24	15.48	9	7/14/2014	42:02.0
5031	RSPe_2	-122.445	37.935	0.02	15.62	-413.05	15.64	-122.445	37.935	-0.17	15.62	-416.88	15.45	9	7/14/2014	42:01.9
5032	RSPe_2	-122.445	37.935	-0.02	15.65	-412.51	15.63	-122.445	37.935	-0.17	15.65	-416.51	15.48	9	7/14/2014	42:01.8
5033	RSPe_2	-122.445	37.935	0.02	15.75	-412.22	15.77	-122.445	37.935	-0.12	15.75	-416.20	15.63	9	7/14/2014	42:01.7
5034	RSPe_2	-122.445	37.935	-0.02	15.69	-411.64	15.67	-122.445	37.935	-0.17	15.69	-415.82	15.52	9	7/14/2014	42:01.6
5035	RSPe_2	-122.445	37.935	0.02	15.67	-411.16	15.69	-122.445	37.935	-0.12	15.67	-415.45	15.55	9	7/14/2014	42:01.5
5036	RSPe_2	-122.445	37.935	-0.02	15.73	-410.53	15.72	-122.445	37.935	-0.17	15.73	-414.93	15.57	9	7/14/2014	42:01.4
5037	RSPe_2	-122.445	37.935	-0.02	15.72	-410.01	15.70	-122.445	37.935	-0.12	15.72	-414.47	15.60	9	7/14/2014	42:01.3
5038	RSPe_2	-122.445	37.935	-0.02	15.69	-409.58	15.68	-122.445	37.935	-0.12	15.69	-414.02	15.58	9	7/14/2014	42:01.2
5039	RSPe_2	-122.445	37.935	0.02	15.72	-409.17	15.74	-122.445	37.935	-0.08	15.72	-413.63	15.63	9	7/14/2014	42:01.1
5040	RSPe_2	-122.445	37.935	-0.02	15.82	-408.69	15.80	-122.445	37.935	-0.12	15.82	-413.11	15.70	9	7/14/2014	42:01.0
5041	RSPe_2	-122.445	37.935	0.02	15.72	-408.39	15.74	-122.445	37.935	-0.20	15.72	-412.65	15.52	9	7/14/2014	42:00.9
5042	RSPe_2	-122.445	37.935	-0.02	15.76	-407.78	15.75	-122.445	37.935	-0.12	15.76	-412.13	15.64	9	7/14/2014	42:00.8
5043	RSPe_2	-122.445	37.935	-0.02	15.82	-407.39	15.80	-122.445	37.935	-0.12	15.82	-411.63	15.70	9	7/14/2014	42:00.7
5044	RSPe_2	-122.445	37.935	-0.02	15.85	-407.02	15.83	-122.445	37.935	-0.17	15.85	-411.26	15.68	9	7/14/2014	42:00.6
5045	RSPe_2	-122.445	37.935	-0.02	15.86	-406.80	15.84	-122.445	37.935	-0.20	15.86	-411.00	15.66	9	7/14/2014	42:00.5
5046	RSPe_2	-122.445	37.935	-0.02	15.84	-406.39	15.83	-122.445	37.935	-0.20	15.84	-410.70	15.64	9	7/14/2014	42:00.4
5047	RSPe_2	-122.445	37.935	-0.02	15.86	-405.96	15.85	-122.445	37.935	-0.17	15.86	-410.22	15.70	9	7/14/2014	42:00.3
5048	RSPe_2	-122.445	37.935	-0.02	15.85	-405.43	15.83	-122.445	37.935	-0.17	15.85	-409.85	15.68	9	7/14/2014	42:00.2

5049	RSPe_2	-122.445	37.935	-0.02	15.76	-405.02	15.74	-122.445	37.935	-0.20	15.76	-409.42	15.55	9	7/14/2014	42:00.1
5050	RSPe_2	-122.445	37.935	-0.02	15.74	-404.31	15.73	-122.445	37.935	-0.20	15.74	-408.90	15.54	9	7/14/2014	42:00.0
5051	RSPe_2	-122.445	37.935	-0.02	15.77	-403.68	15.76	-122.445	37.935	-0.17	15.77	-408.25	15.60	9	7/14/2014	41:59.9
5052	RSPe_2	-122.445	37.935	-0.02	15.68	-403.10	15.66	-122.445	37.935	-0.17	15.68	-407.75	15.51	9	7/14/2014	41:59.8
5053	RSPe_2	-122.445	37.935	-0.02	15.66	-402.68	15.65	-122.445	37.935	-0.17	15.66	-407.21	15.50	9	7/14/2014	41:59.7
5054	RSPe_2	-122.445	37.935	-0.02	15.61	-402.16	15.59	-122.445	37.935	-0.12	15.61	-406.76	15.49	9	7/14/2014	41:59.6
5055	RSPe_2	-122.445	37.935	-0.02	15.62	-401.77	15.60	-122.445	37.935	-0.20	15.62	-406.30	15.42	9	7/14/2014	41:59.5
5056	RSPe_2	-122.445	37.935	-0.07	15.58	-401.19	15.51	-122.445	37.935	-0.17	15.58	-405.76	15.41	9	7/14/2014	41:59.4
5057	RSPe_2	-122.445	37.935	-0.02	15.60	-400.65	15.59	-122.445	37.935	-0.17	15.60	-405.26	15.43	9	7/14/2014	41:59.3
5058	RSPe_2	-122.445	37.935	-0.02	15.57	-400.11	15.56	-122.445	37.935	-0.20	15.57	-404.78	15.37	9	7/14/2014	41:59.2
5059	RSPe_2	-122.445	37.935	-0.02	15.56	-399.58	15.54	-122.445	37.935	-0.20	15.56	-404.21	15.35	9	7/14/2014	41:59.1
5060	RSPe_2	-122.445	37.935	-0.02	15.56	-398.95	15.55	-122.445	37.935	-0.20	15.56	-403.69	15.36	9	7/14/2014	41:59.0
5061	RSPe_2	-122.445	37.935	-0.02	15.58	-398.39	15.56	-122.445	37.935	-0.20	15.58	-403.13	15.38	9	7/14/2014	41:58.9
5062	RSPe_2	-122.445	37.935	-0.07	15.56	-397.81	15.50	-122.445	37.935	-0.20	15.56	-402.52	15.36	9	7/14/2014	41:58.8
5063	RSPe_2	-122.445	37.935	-0.02	15.59	-397.05	15.57	-122.445	37.935	-0.20	15.59	-401.90	15.38	9	7/14/2014	41:58.7
5064	RSPe_2	-122.445	37.935	-0.02	15.57	-396.51	15.56	-122.445	37.935	-0.17	15.57	-401.31	15.40	9	7/14/2014	41:58.6
5065	RSPe_2	-122.445	37.935	-0.02	15.53	-395.99	15.51	-122.445	37.935	-0.17	15.53	-400.79	15.36	9	7/14/2014	41:58.5
5066	RSPe_2	-122.445	37.935	-0.02	15.53	-395.33	15.51	-122.445	37.935	-0.20	15.53	-400.23	15.32	9	7/14/2014	41:58.4
5067	RSPe_2	-122.445	37.935	-0.02	15.54	-394.68	15.52	-122.445	37.935	-0.17	15.54	-399.58	15.37	9	7/14/2014	41:58.3
5068	RSPe_2	-122.445	37.935	-0.02	15.53	-394.16	15.51	-122.445	37.935	-0.17	15.53	-399.10	15.36	9	7/14/2014	41:58.2
5069	RSPe_2	-122.445	37.935	-0.02	15.53	-393.56	15.52	-122.445	37.935	-0.12	15.53	-398.51	15.41	9	7/14/2014	41:58.1
5070	RSPe_2	-122.445	37.935	-0.02	15.53	-392.99	15.51	-122.445	37.935	-0.17	15.53	-397.97	15.36	9	7/14/2014	41:58.0
5071	RSPe_2	-122.445	37.935	-0.02	15.53	-392.41	15.51	-122.445	37.935	-0.12	15.53	-397.39	15.41	9	7/14/2014	41:57.9
5072	RSPe_2	-122.445	37.935	-0.07	15.54	-391.67	15.47	-122.445	37.935	-0.20	15.54	-396.82	15.34	9	7/14/2014	41:57.8
5073	RSPe_2	-122.445	37.935	-0.02	15.57	-391.02	15.56	-122.445	37.935	-0.17	15.57	-396.17	15.40	9	7/14/2014	41:57.7
5074	RSPe_2	-122.445	37.935	-0.02	15.57	-390.35	15.56	-122.445	37.935	-0.17	15.57	-395.48	15.40	9	7/14/2014	41:57.6
5075	RSPe_2	-122.445	37.935	-0.02	15.64	-389.59	15.62	-122.445	37.935	-0.12	15.64	-394.70	15.52	9	7/14/2014	41:57.5
5076	RSPe_2	-122.445	37.935	-0.07	15.64	-388.81	15.57	-122.445	37.935	-0.12	15.64	-393.92	15.52	9	7/14/2014	41:57.4
5077	RSPe_2	-122.445	37.935	-0.07	15.66	-387.99	15.59	-122.445	37.935	-0.12	15.66	-393.08	15.54	9	7/14/2014	41:57.3
5078	RSPe_2	-122.445	37.935	-0.07	15.68	-387.27	15.61	-122.445	37.935	-0.12	15.68	-392.36	15.56	9	7/14/2014	41:57.2
5079	RSPe_2	-122.445	37.935	-0.02	15.70	-386.64	15.69	-122.445	37.935	-0.12	15.70	-391.60	15.59	9	7/14/2014	41:57.1
5080	RSPe_2	-122.445	37.935	-0.07	15.71	-385.84	15.64	-122.445	37.935	-0.29	15.71	-390.73	15.42	9	7/14/2014	41:57.0
5081	RSPe_2	-122.445	37.935	-0.02	15.75	-385.00	15.73	-122.445	37.935	-0.17	15.75	-390.04	15.58	9	7/14/2014	41:56.9

5082	RSPe_2	-122.445	37.935	-0.07	15.76	-384.09	15.69	-122.445	37.935	-0.17	15.76	-389.17	15.59	9	7/14/2014	41:56.8
5083	RSPe_2	-122.445	37.935	-0.02	15.75	-383.44	15.73	-122.445	37.935	-0.17	15.75	-388.48	15.58	9	7/14/2014	41:56.7
5084	RSPe_2	-122.445	37.935	-0.07	15.80	-382.64	15.73	-122.445	37.935	-0.20	15.80	-387.83	15.60	9	7/14/2014	41:56.6
5085	RSPe_2	-122.445	37.935	-0.07	15.76	-381.94	15.69	-122.445	37.935	-0.17	15.76	-387.07	15.59	9	7/14/2014	41:56.5
5086	RSPe_2	-122.445	37.935	-0.07	15.77	-381.07	15.70	-122.445	37.935	-0.20	15.77	-386.42	15.57	9	7/14/2014	41:56.4
5087	RSPe_2	-122.445	37.935	-0.02	15.82	-380.30	15.80	-122.445	37.935	-0.17	15.82	-385.49	15.65	9	7/14/2014	41:56.3
5088	RSPe_2	-122.445	37.935	-0.07	15.82	-379.45	15.75	-122.445	37.935	-0.17	15.82	-384.82	15.65	9	7/14/2014	41:56.2
5089	RSPe_2	-122.445	37.935	-0.07	15.83	-378.58	15.77	-122.445	37.935	-0.17	15.83	-384.06	15.66	9	7/14/2014	41:56.1
5090	RSPe_2	-122.445	37.935	-0.07	15.90	-377.72	15.83	-122.445	37.935	-0.20	15.90	-383.19	15.70	9	7/14/2014	41:56.0
5091	RSPe_2	-122.445	37.935	-0.07	15.93	-376.89	15.87	-122.445	37.935	-0.20	15.93	-382.44	15.73	9	7/14/2014	41:55.9
5092	RSPe_2	-122.445	37.935	-0.07	16.03	-376.03	15.96	-122.445	37.935	-0.20	16.03	-381.55	15.82	9	7/14/2014	41:55.8
5093	RSPe_2	-122.445	37.935	-0.07	15.97	-375.12	15.90	-122.445	37.935	-0.17	15.97	-380.64	15.80	9	7/14/2014	41:55.7
5094	RSPe_2	-122.445	37.935	-0.10	16.00	-374.25	15.89	-122.445	37.935	-0.20	16.00	-379.77	15.79	9	7/14/2014	41:55.6
5095	RSPe_2	-122.445	37.935	-0.07	16.00	-373.49	15.94	-122.445	37.935	-0.20	16.00	-379.05	15.80	9	7/14/2014	41:55.5
5096	RSPe_2	-122.445	37.935	-0.10	16.04	-372.82	15.94	-122.445	37.935	-0.20	16.04	-378.23	15.84	9	7/14/2014	41:55.4
5097	RSPe_2	-122.445	37.935	-0.07	16.13	-372.06	16.06	-122.445	37.935	-0.24	16.13	-377.54	15.90	9	7/14/2014	41:55.3
5098	RSPe_2	-122.445	37.935	-0.10	16.10	-371.17	16.00	-122.445	37.935	-0.20	16.10	-376.54	15.90	9	7/14/2014	41:55.2
5099	RSPe_2	-122.445	37.935	-0.07	16.09	-370.28	16.03	-122.445	37.935	-0.17	16.09	-375.70	15.93	9	7/14/2014	41:55.1
5100	RSPe_2	-122.445	37.935	-0.10	16.04	-369.37	15.94	-122.445	37.935	-0.24	16.04	-374.79	15.81	9	7/14/2014	41:55.0
5101	RSPe_2	-122.445	37.935	-0.07	15.93	-368.61	15.87	-122.445	37.935	-0.20	15.93	-374.07	15.73	9	7/14/2014	41:54.9
5102	RSPe_2	-122.445	37.935	-0.10	15.94	-367.75	15.84	-122.445	37.935	-0.20	15.94	-373.20	15.74	9	7/14/2014	41:54.8
5103	RSPe_2	-122.445	37.935	-0.07	15.88	-366.82	15.81	-122.445	37.935	-0.20	15.88	-372.27	15.68	9	7/14/2014	41:54.7
5104	RSPe_2	-122.445	37.935	-0.10	15.83	-365.97	15.72	-122.445	37.935	-0.29	15.83	-371.45	15.54	9	7/14/2014	41:54.6
5105	RSPe_2	-122.445	37.935	-0.07	15.78	-365.17	15.71	-122.445	37.935	-0.17	15.78	-370.64	15.61	9	7/14/2014	41:54.5
5106	RSPe_2	-122.445	37.935	-0.10	15.79	-364.45	15.69	-122.445	37.935	-0.17	15.79	-369.84	15.62	9	7/14/2014	41:54.4
5107	RSPe_2	-122.445	37.935	-0.07	15.73	-363.80	15.66	-122.445	37.935	-0.20	15.73	-369.11	15.52	9	7/14/2014	41:54.3
5108	RSPe_2	-122.445	37.935	-0.10	15.74	-362.94	15.64	-122.445	37.935	-0.24	15.74	-368.22	15.51	9	7/14/2014	41:54.2
5109	RSPe_2	-122.445	37.935	-0.07	15.75	-362.09	15.68	-122.445	37.935	-0.20	15.75	-367.29	15.55	9	7/14/2014	41:54.1
5110	RSPe_2	-122.445	37.935	-0.10	15.73	-361.14	15.63	-122.445	37.935	-0.29	15.73	-366.42	15.45	9	7/14/2014	41:54.0
5111	RSPe_2	-122.445	37.935	-0.10	15.73	-360.12	15.62	-122.445	37.935	-0.24	15.73	-365.49	15.49	9	7/14/2014	41:53.9
5112	RSPe_2	-122.445	37.935	-0.10	15.80	-359.32	15.69	-122.445	37.935	-0.29	15.80	-364.69	15.51	9	7/14/2014	41:53.8
5113	RSPe_2	-122.445	37.935	-0.10	15.73	-358.37	15.62	-122.445	37.935	-0.24	15.73	-363.84	15.49	9	7/14/2014	41:53.7
5114	RSPe_2	-122.445	37.935	-0.10	15.69	-357.46	15.59	-122.445	37.935	-0.24	15.69	-363.00	15.46	9	7/14/2014	41:53.6

5115	RSPe_2	-122.445	37.935	-0.10	15.68	-356.52	15.58	-122.445	37.935	-0.24	15.68	-362.20	15.44	9	7/14/2014	41:53.5
5116	RSPe_2	-122.445	37.935	-0.10	15.67	-355.55	15.57	-122.445	37.935	-0.24	15.67	-361.26	15.44	9	7/14/2014	41:53.4
5117	RSPe_2	-122.445	37.935	-0.10	15.63	-354.53	15.53	-122.445	37.935	-0.24	15.63	-360.40	15.40	9	7/14/2014	41:53.3
5118	RSPe_2	-122.445	37.935	-0.10	15.62	-353.51	15.52	-122.445	37.935	-0.29	15.62	-359.42	15.33	9	7/14/2014	41:53.2
5119	RSPe_2	-122.445	37.935	-0.10	15.61	-352.47	15.51	-122.445	37.935	-0.24	15.61	-358.34	15.37	9	7/14/2014	41:53.1
5120	RSPe_2	-122.445	37.935	-0.10	15.61	-351.54	15.51	-122.445	37.935	-0.24	15.61	-357.43	15.37	9	7/14/2014	41:53.0
5121	RSPe_2	-122.445	37.935	-0.07	15.61	-350.52	15.54	-122.445	37.935	-0.29	15.61	-356.28	15.32	9	7/14/2014	41:52.9
5122	RSPe_2	-122.445	37.935	-0.10	15.62	-349.64	15.52	-122.445	37.935	-0.29	15.62	-355.31	15.34	9	7/14/2014	41:52.8
5123	RSPe_2	-122.445	37.935	-0.10	15.63	-348.75	15.53	-122.445	37.935	-0.24	15.63	-354.31	15.40	9	7/14/2014	41:52.7
5124	RSPe_2	-122.445	37.935	-0.10	15.66	-347.86	15.56	-122.445	37.935	-0.32	15.66	-353.38	15.34	9	7/14/2014	41:52.6
5125	RSPe_2	-122.445	37.935	-0.10	15.68	-346.88	15.58	-122.445	37.935	-0.29	15.68	-352.36	15.39	9	7/14/2014	41:52.5
5126	RSPe_2	-122.445	37.935	-0.10	15.69	-346.04	15.59	-122.445	37.935	-0.29	15.69	-351.56	15.41	9	7/14/2014	41:52.4
5127	RSPe_2	-122.445	37.935	-0.10	15.71	-345.06	15.61	-122.445	37.935	-0.29	15.71	-350.60	15.42	9	7/14/2014	41:52.3
5128	RSPe_2	-122.445	37.935	-0.15	15.75	-344.09	15.60	-122.445	37.935	-0.29	15.75	-349.69	15.46	9	7/14/2014	41:52.2
5129	RSPe_2	-122.445	37.935	-0.10	15.77	-343.18	15.67	-122.445	37.935	-0.29	15.77	-348.91	15.49	9	7/14/2014	41:52.1
5130	RSPe_2	-122.445	37.935	-0.15	15.78	-342.12	15.63	-122.445	37.935	-0.29	15.78	-347.94	15.49	9	7/14/2014	41:52.0
5131	RSPe_2	-122.445	37.935	-0.10	15.87	-341.10	15.77	-122.445	37.935	-0.29	15.87	-346.99	15.59	9	7/14/2014	41:51.9
5132	RSPe_2	-122.445	37.935	-0.15	15.86	-339.99	15.71	-122.445	37.935	-0.29	15.86	-346.01	15.57	9	7/14/2014	41:51.8
5133	RSPe_2	-122.445	37.935	-0.10	15.86	-338.97	15.76	-122.445	37.935	-0.24	15.86	-345.01	15.63	9	7/14/2014	41:51.7
5134	RSPe_2	-122.445	37.935	-0.10	15.90	-337.93	15.79	-122.445	37.935	-0.29	15.90	-344.02	15.61	9	7/14/2014	41:51.6
5135	RSPe_2	-122.445	37.935	-0.10	15.90	-336.98	15.79	-122.445	37.935	-0.29	15.90	-343.06	15.61	9	7/14/2014	41:51.5
5136	RSPe_2	-122.445	37.935	-0.10	15.89	-335.98	15.79	-122.445	37.935	-0.29	15.89	-341.96	15.60	9	7/14/2014	41:51.4
5137	RSPe_2	-122.445	37.935	-0.10	15.92	-334.99	15.82	-122.445	37.935	-0.29	15.92	-340.96	15.63	9	7/14/2014	41:51.3
5138	RSPe_2	-122.445	37.935	-0.10	15.94	-334.05	15.84	-122.445	37.935	-0.32	15.94	-339.98	15.62	9	7/14/2014	41:51.2
5139	RSPe_2	-122.445	37.935	-0.10	15.99	-332.99	15.89	-122.445	37.935	-0.29	15.99	-338.86	15.70	9	7/14/2014	41:51.1
5140	RSPe_2	-122.445	37.935	-0.15	16.01	-332.02	15.86	-122.445	37.935	-0.32	16.01	-337.84	15.69	9	7/14/2014	41:51.0
5141	RSPe_2	-122.445	37.935	-0.07	16.09	-331.00	16.03	-122.445	37.935	-0.29	16.09	-336.74	15.81	9	7/14/2014	41:50.9
5142	RSPe_2	-122.445	37.935	-0.10	16.13	-329.98	16.03	-122.445	37.935	-0.29	16.13	-335.76	15.85	9	7/14/2014	41:50.8
5143	RSPe_2	-122.445	37.935	-0.10	16.12	-329.11	16.02	-122.445	37.935	-0.29	16.12	-334.88	15.84	9	7/14/2014	41:50.7
5144	RSPe_2	-122.445	37.935	-0.15	16.16	-328.23	16.01	-122.445	37.935	-0.29	16.16	-334.05	15.87	9	7/14/2014	41:50.6
5145	RSPe_2	-122.445	37.935	-0.10	16.15	-327.38	16.05	-122.445	37.935	-0.24	16.15	-333.23	15.91	9	7/14/2014	41:50.5
5146	RSPe_2	-122.445	37.935	-0.10	16.15	-326.60	16.05	-122.445	37.935	-0.29	16.15	-332.49	15.86	9	7/14/2014	41:50.4
5147	RSPe_2	-122.445	37.935	-0.10	16.15	-325.71	16.05	-122.445	37.935	-0.29	16.15	-331.62	15.86	9	7/14/2014	41:50.3

5148	RSPe_2	-122.445	37.935	-0.10	16.12	-324.83	16.02	-122.445	37.935	-0.29	16.12	-330.76	15.84	9	7/14/2014	41:50.2
5149	RSPe_2	-122.445	37.935	-0.10	16.08	-323.87	15.98	-122.445	37.935	-0.29	16.08	-329.85	15.79	9	7/14/2014	41:50.1
5150	RSPe_2	-122.445	37.935	-0.10	16.06	-322.96	15.96	-122.445	37.935	-0.32	16.06	-328.96	15.74	9	7/14/2014	41:50.0
5151	RSPe_2	-122.445	37.935	-0.07	16.05	-322.03	15.98	-122.445	37.935	-0.29	16.05	-328.01	15.77	9	7/14/2014	41:49.9
5152	RSPe_2	-122.445	37.935	-0.10	16.02	-321.06	15.92	-122.445	37.935	-0.32	16.02	-327.03	15.70	9	7/14/2014	41:49.8
5153	RSPe_2	-122.445	37.935	-0.10	15.99	-320.12	15.89	-122.445	37.935	-0.32	15.99	-326.03	15.67	9	7/14/2014	41:49.7
5154	RSPe_2	-122.445	37.935	-0.10	15.95	-319.15	15.85	-122.445	37.935	-0.29	15.95	-325.06	15.66	9	7/14/2014	41:49.6
5155	RSPe_2	-122.445	37.935	-0.10	15.96	-318.13	15.86	-122.445	37.935	-0.29	15.96	-323.93	15.67	9	7/14/2014	41:49.5
5156	RSPe_2	-122.445	37.935	-0.10	15.93	-317.11	15.83	-122.445	37.935	-0.32	15.93	-322.92	15.61	9	7/14/2014	41:49.4
5157	RSPe_2	-122.445	37.935	-0.07	15.92	-316.09	15.85	-122.445	37.935	-0.29	15.92	-321.90	15.63	9	7/14/2014	41:49.3
5158	RSPe_2	-122.445	37.935	-0.10	15.91	-315.12	15.81	-122.445	37.935	-0.32	15.91	-320.89	15.59	9	7/14/2014	41:49.2
5159	RSPe_2	-122.445	37.935	-0.10	15.93	-314.21	15.83	-122.445	37.935	-0.24	15.93	-319.95	15.70	9	7/14/2014	41:49.1
5160	RSPe_2	-122.445	37.935	-0.10	15.90	-313.34	15.80	-122.445	37.935	-0.29	15.90	-319.14	15.62	9	7/14/2014	41:49.0
5161	RSPe_2	-122.445	37.935	-0.07	15.90	-312.56	15.83	-122.445	37.935	-0.32	15.90	-318.30	15.58	9	7/14/2014	41:48.9
5162	RSPe_2	-122.445	37.935	-0.10	15.87	-311.70	15.77	-122.445	37.935	-0.32	15.87	-317.50	15.55	9	7/14/2014	41:48.8
5163	RSPe_2	-122.445	37.935	-0.07	15.85	-310.87	15.78	-122.445	37.935	-0.24	15.85	-316.70	15.61	9	7/14/2014	41:48.7
5164	RSPe_2	-122.445	37.935	-0.10	15.87	-310.03	15.77	-122.445	37.935	-0.29	15.87	-315.81	15.59	9	7/14/2014	41:48.6
5165	RSPe_2	-122.445	37.935	-0.10	15.78	-309.07	15.68	-122.445	37.935	-0.32	15.78	-314.90	15.46	9	7/14/2014	41:48.5
5166	RSPe_2	-122.445	37.935	-0.10	15.78	-308.18	15.68	-122.445	37.935	-0.29	15.78	-313.96	15.49	9	7/14/2014	41:48.4
5167	RSPe_2	-122.445	37.935	-0.10	15.80	-307.14	15.70	-122.445	37.935	-0.32	15.80	-312.90	15.48	9	7/14/2014	41:48.3
5168	RSPe_2	-122.445	37.935	-0.10	15.73	-306.02	15.62	-122.445	37.935	-0.37	15.73	-311.82	15.35	9	7/14/2014	41:48.2
5169	RSPe_2	-122.445	37.935	-0.10	15.73	-304.87	15.63	-122.445	37.935	-0.37	15.73	-310.74	15.36	9	7/14/2014	41:48.1
5170	RSPe_2	-122.445	37.935	-0.10	15.72	-303.73	15.62	-122.445	37.935	-0.37	15.72	-309.67	15.35	9	7/14/2014	41:48.0
5171	RSPe_2	-122.445	37.935	-0.07	15.65	-302.66	15.58	-122.445	37.935	-0.29	15.65	-308.76	15.36	9	7/14/2014	41:47.9
5172	RSPe_2	-122.445	37.935	-0.10	15.60	-301.53	15.50	-122.445	37.935	-0.32	15.60	-307.77	15.28	9	7/14/2014	41:47.8
5173	RSPe_2	-122.445	37.935	-0.07	15.56	-300.49	15.49	-122.445	37.935	-0.29	15.56	-306.84	15.27	9	7/14/2014	41:47.7
5174	RSPe_2	-122.445	37.935	-0.10	15.51	-299.52	15.41	-122.445	37.935	-0.32	15.51	-305.95	15.19	9	7/14/2014	41:47.6
5175	RSPe_2	-122.445	37.935	-0.10	15.48	-298.52	15.38	-122.445	37.935	-0.29	15.48	-304.99	15.20	9	7/14/2014	41:47.5
5176	RSPe_2	-122.445	37.935	-0.10	15.48	-297.61	15.38	-122.445	37.935	-0.32	15.48	-304.02	15.16	9	7/14/2014	41:47.4
5177	RSPe_2	-122.445	37.935	-0.10	15.43	-296.56	15.33	-122.445	37.935	-0.29	15.43	-302.94	15.15	9	7/14/2014	41:47.3
5178	RSPe_2	-122.445	37.935	-0.10	15.43	-295.55	15.32	-122.445	37.935	-0.29	15.43	-301.83	15.14	9	7/14/2014	41:47.2
5179	RSPe_2	-122.445	37.935	-0.10	15.46	-294.62	15.36	-122.445	37.935	-0.24	15.46	-300.79	15.22	9	7/14/2014	41:47.1
5180	RSPe_2	-122.445	37.935	-0.10	15.47	-293.60	15.37	-122.445	37.935	-0.37	15.47	-299.72	15.10	9	7/14/2014	41:47.0

5181	RSPe_2	-122.445	37.935	-0.10	15.54	-292.78	15.44	-122.445	37.935	-0.32	15.54	-298.76	15.22	9	7/14/2014	41:46.9
5182	RSPe_2	-122.445	37.935	-0.15	15.65	-291.82	15.50	-122.445	37.935	-0.32	15.65	-297.71	15.33	9	7/14/2014	41:46.8
5183	RSPe_2	-122.445	37.935	-0.10	15.56	-290.97	15.46	-122.445	37.935	-0.32	15.56	-296.74	15.24	9	7/14/2014	41:46.7
5184	RSPe_2	-122.445	37.935	-0.15	15.61	-289.98	15.46	-122.445	37.935	-0.32	15.61	-295.70	15.29	9	7/14/2014	41:46.6
5185	RSPe_2	-122.445	37.935	-0.10	15.71	-289.01	15.61	-122.445	37.935	-0.32	15.71	-294.70	15.39	9	7/14/2014	41:46.5
5186	RSPe_2	-122.445	37.935	-0.10	15.90	-288.08	15.79	-122.445	37.935	-0.32	15.90	-293.71	15.58	9	7/14/2014	41:46.4
5187	RSPe_2	-122.445	37.935	-0.07	15.83	-286.99	15.76	-122.445	37.935	-0.37	15.83	-292.58	15.46	9	7/14/2014	41:46.3
5188	RSPe_2	-122.445	37.935	-0.10	15.86	-286.02	15.76	-122.445	37.935	-0.29	15.86	-291.56	15.58	9	7/14/2014	41:46.2
5189	RSPe_2	-122.445	37.935	-0.07	15.88	-285.02	15.81	-122.445	37.935	-0.29	15.88	-290.57	15.59	9	7/14/2014	41:46.1
5190	RSPe_2	-122.445	37.935	-0.07	15.79	-284.29	15.72	-122.445	37.935	-0.32	15.79	-289.80	15.47	9	7/14/2014	41:46.0
5191	RSPe_2	-122.445	37.935	-0.07	15.80	-283.38	15.73	-122.445	37.935	-0.29	15.80	-288.93	15.51	9	7/14/2014	41:45.9
5192	RSPe_2	-122.445	37.935	-0.10	15.79	-282.46	15.69	-122.445	37.935	-0.32	15.79	-288.07	15.47	9	7/14/2014	41:45.8
5193	RSPe_2	-122.445	37.935	-0.07	15.86	-281.38	15.80	-122.445	37.935	-0.32	15.86	-287.01	15.54	9	7/14/2014	41:45.7
5194	RSPe_2	-122.445	37.935	-0.10	15.81	-280.21	15.71	-122.445	37.935	-0.32	15.81	-285.91	15.49	9	7/14/2014	41:45.6
5195	RSPe_2	-122.445	37.935	-0.07	15.80	-278.95	15.73	-122.445	37.935	-0.32	15.80	-284.73	15.48	9	7/14/2014	41:45.5
5196	RSPe_2	-122.445	37.935	-0.10	15.86	-277.79	15.76	-122.445	37.935	-0.32	15.86	-283.55	15.54	9	7/14/2014	41:45.4
5197	RSPe_2	-122.445	37.935	-0.07	15.83	-276.66	15.76	-122.445	37.935	-0.29	15.83	-282.42	15.54	9	7/14/2014	41:45.3
5198	RSPe_2	-122.445	37.935	-0.10	15.86	-275.56	15.76	-122.445	37.935	-0.32	15.86	-281.31	15.54	9	7/14/2014	41:45.2
5199	RSPe_2	-122.445	37.935	-0.07	15.88	-274.53	15.81	-122.445	37.935	-0.29	15.88	-280.17	15.59	9	7/14/2014	41:45.1
5200	RSPe_2	-122.445	37.935	-0.07	15.93	-273.41	15.87	-122.445	37.935	-0.32	15.93	-279.02	15.61	9	7/14/2014	41:45.0
5201	RSPe_2	-122.445	37.935	-0.07	15.92	-272.37	15.85	-122.445	37.935	-0.24	15.92	-277.78	15.68	9	7/14/2014	41:44.9
5202	RSPe_2	-122.445	37.935	-0.07	16.03	-271.24	15.97	-122.445	37.935	-0.32	16.03	-276.55	15.71	9	7/14/2014	41:44.8
5203	RSPe_2	-122.445	37.935	-0.07	16.05	-270.12	15.98	-122.445	37.935	-0.29	16.05	-275.33	15.77	9	7/14/2014	41:44.7
5204	RSPe_2	-122.445	37.935	-0.07	16.05	-268.94	15.98	-122.445	37.935	-0.29	16.05	-274.08	15.77	9	7/14/2014	41:44.6
5205	RSPe_2	-122.445	37.935	-0.07	16.05	-267.78	15.98	-122.445	37.935	-0.24	16.05	-272.82	15.82	9	7/14/2014	41:44.5
5206	RSPe_2	-122.445	37.935	-0.07	16.08	-266.56	16.01	-122.445	37.935	-0.29	16.08	-271.61	15.79	9	7/14/2014	41:44.4
5207	RSPe_2	-122.445	37.935	-0.02	16.06	-265.48	16.04	-122.445	37.935	-0.29	16.06	-270.52	15.77	9	7/14/2014	41:44.3
5208	RSPe_2	-122.445	37.935	-0.07	16.04	-264.40	15.97	-122.445	37.935	-0.32	16.04	-269.47	15.72	9	7/14/2014	41:44.2
5209	RSPe_2	-122.445	37.935	-0.07	16.05	-263.33	15.98	-122.445	37.935	-0.32	16.05	-268.42	15.73	9	7/14/2014	41:44.1
5210	RSPe_2	-122.445	37.935	-0.07	16.04	-262.26	15.97	-122.445	37.935	-0.29	16.04	-267.33	15.76	9	7/14/2014	41:44.0
5211	RSPe_2	-122.445	37.935	-0.02	16.06	-261.21	16.04	-122.445	37.935	-0.24	16.06	-266.26	15.82	9	7/14/2014	41:43.9
5212	RSPe_2	-122.445	37.935	-0.02	16.03	-260.13	16.01	-122.445	37.935	-0.32	16.03	-265.20	15.71	9	7/14/2014	41:43.8
5213	RSPe_2	-122.445	37.935	-0.02	15.96	-259.07	15.94	-122.445	37.935	-0.32	15.96	-264.11	15.64	9	7/14/2014	41:43.7

5214	RSPe_2	-122.445	37.935	-0.07	15.91	-258.07	15.84	-122.445	37.935	-0.32	15.91	-263.09	15.59	9	7/14/2014	41:43.6
5215	RSPe_2	-122.445	37.935	-0.02	16.00	-257.12	15.99	-122.445	37.935	-0.24	16.00	-262.06	15.77	9	7/14/2014	41:43.5
5216	RSPe_2	-122.445	37.935	-0.02	15.86	-256.25	15.85	-122.445	37.935	-0.32	15.86	-261.14	15.54	9	7/14/2014	41:43.4
5217	RSPe_2	-122.445	37.935	-0.02	15.90	-255.39	15.89	-122.445	37.935	-0.24	15.90	-260.28	15.67	9	7/14/2014	41:43.3
5218	RSPe_2	-122.445	37.935	-0.07	15.84	-254.62	15.78	-122.445	37.935	-0.24	15.84	-259.40	15.61	9	7/14/2014	41:43.2
5219	RSPe_2	-122.445	37.935	-0.02	15.77	-253.81	15.76	-122.445	37.935	-0.29	15.77	-258.55	15.49	9	7/14/2014	41:43.1
5220	RSPe_2	-122.445	37.935	-0.02	15.73	-252.91	15.71	-122.445	37.935	-0.29	15.73	-257.67	15.44	9	7/14/2014	41:43.0
5221	RSPe_2	-122.445	37.935	-0.02	15.66	-251.85	15.64	-122.445	37.935	-0.29	15.66	-256.68	15.37	9	7/14/2014	41:42.9
5222	RSPe_2	-122.445	37.935	-0.02	15.61	-250.64	15.59	-122.445	37.935	-0.24	15.61	-255.54	15.37	9	7/14/2014	41:42.8
5223	RSPe_2	-122.445	37.935	-0.02	15.66	-249.49	15.64	-122.445	37.935	-0.24	15.66	-254.45	15.42	9	7/14/2014	41:42.7
5224	RSPe_2	-122.445	37.935	-0.02	15.60	-248.37	15.59	-122.445	37.935	-0.29	15.60	-253.29	15.32	9	7/14/2014	41:42.6
5225	RSPe_2	-122.445	37.935	-0.02	15.61	-247.15	15.59	-122.445	37.935	-0.20	15.61	-252.03	15.41	9	7/14/2014	41:42.5
5226	RSPe_2	-122.445	37.935	-0.02	15.60	-246.15	15.59	-122.445	37.935	-0.24	15.60	-250.92	15.37	9	7/14/2014	41:42.4
5227	RSPe_2	-122.445	37.935	0.02	15.60	-245.20	15.62	-122.445	37.935	-0.20	15.60	-249.88	15.40	9	7/14/2014	41:42.3
5228	RSPe_2	-122.445	37.935	-0.02	15.60	-244.48	15.59	-122.445	37.935	-0.24	15.60	-249.05	15.37	9	7/14/2014	41:42.2
5229	RSPe_2	-122.445	37.935	0.02	15.67	-243.75	15.69	-122.445	37.935	-0.24	15.67	-248.28	15.44	9	7/14/2014	41:42.1
5230	RSPe_2	-122.445	37.935	-0.02	15.64	-243.17	15.63	-122.445	37.935	-0.24	15.64	-247.67	15.41	9	7/14/2014	41:42.0
5231	RSPe_2	-122.445	37.935	0.02	15.59	-242.54	15.61	-122.445	37.935	-0.17	15.59	-247.09	15.42	9	7/14/2014	41:41.9
5232	RSPe_2	-122.445	37.935	-0.01	15.62	-241.78	15.60	-122.445	37.935	-0.17	15.62	-246.44	15.45	9	7/14/2014	41:41.8
5233	RSPe_2	-122.445	37.935	0.02	15.59	-241.08	15.61	-122.445	37.935	-0.20	15.59	-245.74	15.39	9	7/14/2014	41:41.7
5234	RSPe_2	-122.445	37.935	0.02	15.63	-240.13	15.65	-122.445	37.935	-0.17	15.63	-244.85	15.47	9	7/14/2014	41:41.6
5235	RSPe_2	-122.445	37.935	0.02	15.68	-239.01	15.70	-122.445	37.935	-0.20	15.68	-243.81	15.48	9	7/14/2014	41:41.5
5236	RSPe_2	-122.445	37.935	0.02	15.63	-237.94	15.65	-122.445	37.935	-0.20	15.63	-242.86	15.43	9	7/14/2014	41:41.4
5237	RSPe_2	-122.445	37.935	0.02	15.62	-236.90	15.64	-122.445	37.935	-0.17	15.62	-241.86	15.46	9	7/14/2014	41:41.3
5238	RSPe_2	-122.445	37.935	0.02	15.66	-235.91	15.68	-122.445	37.935	-0.17	15.66	-240.85	15.49	9	7/14/2014	41:41.2
5239	RSPe_2	-122.445	37.935	0.02	15.68	-234.86	15.70	-122.445	37.935	-0.17	15.68	-239.85	15.51	9	7/14/2014	41:41.1
5240	RSPe_2	-122.445	37.935	0.02	15.69	-234.00	15.71	-122.445	37.935	-0.17	15.69	-238.88	15.53	9	7/14/2014	41:41.0
5241	RSPe_2	-122.445	37.935	0.02	15.78	-233.18	15.80	-122.445	37.935	-0.17	15.78	-238.04	15.61	9	7/14/2014	41:40.9
5242	RSPe_2	-122.445	37.935	0.02	15.71	-232.39	15.73	-122.445	37.935	-0.17	15.71	-237.33	15.54	9	7/14/2014	41:40.8
5243	RSPe_2	-122.445	37.935	0.02	15.79	-231.73	15.81	-122.445	37.935	-0.17	15.79	-236.72	15.62	9	7/14/2014	41:40.7
5244	RSPe_2	-122.445	37.935	0.02	15.73	-231.01	15.75	-122.445	37.935	-0.17	15.73	-235.99	15.57	9	7/14/2014	41:40.6
5245	RSPe_2	-122.445	37.935	0.02	15.74	-230.26	15.76	-122.445	37.935	-0.12	15.74	-235.33	15.63	9	7/14/2014	41:40.5
5246	RSPe_2	-122.445	37.935	0.02	15.73	-229.42	15.75	-122.445	37.935	-0.12	15.73	-234.52	15.61	9	7/14/2014	41:40.4

5247	RSPe_2	-122.445	37.935	0.07	15.73	-228.54	15.81	-122.445	37.935	-0.12	15.73	-233.62	15.62	9	7/14/2014	41:40.3
5248	RSPe_2	-122.445	37.935	0.07	15.73	-227.60	15.80	-122.445	37.935	-0.12	15.73	-232.73	15.61	9	7/14/2014	41:40.2
5249	RSPe_2	-122.445	37.935	0.02	15.75	-226.74	15.77	-122.445	37.935	-0.08	15.75	-231.82	15.67	9	7/14/2014	41:40.1
5250	RSPe_2	-122.445	37.935	0.07	15.77	-225.90	15.84	-122.445	37.935	-0.12	15.77	-230.90	15.66	9	7/14/2014	41:40.0
5251	RSPe_2	-122.445	37.935	0.07	15.78	-225.14	15.85	-122.445	37.935	-0.12	15.78	-230.03	15.66	9	7/14/2014	41:39.9
5252	RSPe_2	-122.445	37.935	0.07	15.82	-224.40	15.89	-122.445	37.935	-0.08	15.82	-229.09	15.74	9	7/14/2014	41:39.8
5253	RSPe_2	-122.445	37.935	0.11	15.87	-223.53	15.98	-122.445	37.935	-0.08	15.87	-228.19	15.79	9	7/14/2014	41:39.7
5254	RSPe_2	-122.445	37.935	0.07	15.88	-222.95	15.95	-122.445	37.935	-0.08	15.88	-227.52	15.80	9	7/14/2014	41:39.6
5255	RSPe_2	-122.445	37.935	0.11	15.88	-222.38	15.99	-122.445	37.935	-0.08	15.88	-226.83	15.80	9	7/14/2014	41:39.5
5256	RSPe_2	-122.445	37.935	0.07	15.88	-221.84	15.95	-122.445	37.935	-0.08	15.88	-226.32	15.80	9	7/14/2014	41:39.4
5257	RSPe_2	-122.445	37.935	0.11	15.90	-220.98	16.00	-122.445	37.935	-0.08	15.90	-225.57	15.81	9	7/14/2014	41:39.3
5258	RSPe_2	-122.445	37.935	0.07	15.90	-220.07	15.97	-122.445	37.935	-0.03	15.90	-224.74	15.86	9	7/14/2014	41:39.2
5259	RSPe_2	-122.445	37.935	0.11	15.90	-219.29	16.01	-122.445	37.935	0.00	15.90	-224.09	15.91	9	7/14/2014	41:39.1
5260	RSPe_2	-122.445	37.935	0.07	15.90	-218.46	15.97	-122.445	37.935	-0.03	15.90	-223.36	15.86	9	7/14/2014	41:39.0
5261	RSPe_2	-122.445	37.935	0.11	15.90	-217.60	16.01	-122.445	37.935	0.00	15.90	-222.60	15.91	9	7/14/2014	41:38.9
5262	RSPe_2	-122.445	37.935	0.07	15.93	-216.84	16.00	-122.445	37.935	0.00	15.93	-221.88	15.93	9	7/14/2014	41:38.8
5263	RSPe_2	-122.445	37.935	0.11	15.92	-215.99	16.02	-122.445	37.935	0.00	15.92	-221.06	15.92	9	7/14/2014	41:38.7
5264	RSPe_2	-122.445	37.935	0.11	15.93	-215.22	16.03	-122.445	37.935	-0.03	15.93	-220.29	15.90	9	7/14/2014	41:38.6
5265	RSPe_2	-122.445	37.935	0.11	15.94	-214.52	16.05	-122.445	37.935	0.05	15.94	-219.56	15.99	9	7/14/2014	41:38.5
5266	RSPe_2	-122.445	37.935	0.11	15.93	-213.87	16.04	-122.445	37.935	0.00	15.93	-218.89	15.94	9	7/14/2014	41:38.4
5267	RSPe_2	-122.445	37.935	0.16	15.96	-213.15	16.11	-122.445	37.935	0.09	15.96	-218.07	16.04	9	7/14/2014	41:38.3
5268	RSPe_2	-122.445	37.935	0.11	15.98	-212.40	16.09	-122.445	37.935	-0.03	15.98	-217.30	15.95	9	7/14/2014	41:38.2
5269	RSPe_2	-122.445	37.935	0.11	15.96	-211.72	16.06	-122.445	37.935	0.05	15.96	-216.57	16.01	9	7/14/2014	41:38.1
5270	RSPe_2	-122.445	37.935	0.11	15.98	-211.05	16.09	-122.445	37.935	0.09	15.98	-215.88	16.07	9	7/14/2014	41:38.0
5271	RSPe_2	-122.445	37.935	0.16	15.98	-210.53	16.14	-122.445	37.935	0.09	15.98	-215.32	16.07	9	7/14/2014	41:37.9
5272	RSPe_2	-122.445	37.935	0.16	15.99	-209.85	16.14	-122.445	37.935	0.09	15.99	-214.64	16.07	9	7/14/2014	41:37.8
5273	RSPe_2	-122.445	37.935	0.16	16.01	-209.21	16.17	-122.445	37.935	0.09	16.01	-214.01	16.10	9	7/14/2014	41:37.7
5274	RSPe_2	-122.445	37.935	0.11	16.02	-208.56	16.12	-122.445	37.935	0.14	16.02	-213.30	16.16	9	7/14/2014	41:37.6
5275	RSPe_2	-122.445	37.935	0.16	16.06	-207.90	16.21	-122.445	37.935	0.14	16.06	-212.65	16.19	9	7/14/2014	41:37.5
5276	RSPe_2	-122.445	37.935	0.11	16.03	-207.30	16.14	-122.445	37.935	0.09	16.03	-212.01	16.12	9	7/14/2014	41:37.4
5277	RSPe_2	-122.445	37.935	0.16	15.99	-206.65	16.14	-122.445	37.935	0.14	15.99	-211.33	16.13	9	7/14/2014	41:37.3
5278	RSPe_2	-122.445	37.935	0.16	15.99	-205.94	16.14	-122.445	37.935	0.09	15.99	-210.58	16.07	9	7/14/2014	41:37.2
5279	RSPe_2	-122.445	37.935	0.16	15.81	-205.26	15.97	-122.445	37.935	0.17	15.81	-209.76	15.98	9	7/14/2014	41:37.1

5280	RSPe_2	-122.445	37.935	0.16	15.78	-204.52	15.94	-122.445	37.935	0.14	15.78	-209.03	15.92	9	7/14/2014	41:37.0
5281	RSPe_2	-122.445	37.935	0.16	15.75	-203.86	15.91	-122.445	37.935	0.14	15.75	-208.30	15.89	9	7/14/2014	41:36.9
5282	RSPe_2	-122.445	37.935	0.16	15.68	-203.20	15.84	-122.445	37.935	0.14	15.68	-207.58	15.82	9	7/14/2014	41:36.8
5283	RSPe_2	-122.445	37.935	0.16	15.64	-202.50	15.80	-122.445	37.935	0.17	15.64	-206.91	15.81	9	7/14/2014	41:36.7
5284	RSPe_2	-122.445	37.935	0.16	15.62	-201.88	15.78	-122.445	37.935	0.14	15.62	-206.25	15.76	9	7/14/2014	41:36.6
5285	RSPe_2	-122.445	37.935	0.19	15.58	-201.34	15.77	-122.445	37.935	0.14	15.58	-205.74	15.72	9	7/14/2014	41:36.5
5286	RSPe_2	-122.445	37.935	0.16	15.57	-200.67	15.73	-122.445	37.935	0.14	15.57	-205.10	15.71	9	7/14/2014	41:36.4
5287	RSPe_2	-122.445	37.935	0.16	15.54	-200.17	15.70	-122.445	37.935	0.17	15.54	-204.56	15.71	9	7/14/2014	41:36.3
5288	RSPe_2	-122.445	37.935	0.16	15.48	-199.52	15.64	-122.445	37.935	0.14	15.48	-203.89	15.62	9	7/14/2014	41:36.2
5289	RSPe_2	-122.445	37.935	0.19	15.50	-198.95	15.69	-122.445	37.935	0.21	15.50	-203.29	15.71	9	7/14/2014	41:36.1
5290	RSPe_2	-122.445	37.935	0.16	15.42	-198.36	15.57	-122.445	37.935	0.17	15.42	-202.74	15.59	9	7/14/2014	41:36.0
5291	RSPe_2	-122.445	37.935	0.19	15.44	-197.67	15.63	-122.445	37.935	0.17	15.44	-202.09	15.61	9	7/14/2014	41:35.9
5292	RSPe_2	-122.445	37.935	0.19	15.40	-197.12	15.59	-122.445	37.935	0.17	15.40	-201.47	15.57	9	7/14/2014	41:35.8
5293	RSPe_2	-122.445	37.935	0.19	15.33	-196.42	15.52	-122.445	37.935	0.14	15.33	-200.82	15.47	9	7/14/2014	41:35.7
5294	RSPe_2	-122.445	37.935	0.19	15.29	-195.89	15.48	-122.445	37.935	0.14	15.29	-200.23	15.43	9	7/14/2014	41:35.6
5295	RSPe_2	-122.445	37.935	0.19	15.27	-195.40	15.46	-122.445	37.935	0.17	15.27	-199.68	15.44	9	7/14/2014	41:35.5
5296	RSPe_2	-122.445	37.935	0.19	15.25	-194.82	15.44	-122.445	37.935	0.14	15.25	-199.08	15.39	9	7/14/2014	41:35.4
5297	RSPe_2	-122.445	37.935	0.19	15.24	-194.26	15.43	-122.445	37.935	0.17	15.24	-198.52	15.41	9	7/14/2014	41:35.3
5298	RSPe_2	-122.445	37.935	0.16	15.23	-193.73	15.39	-122.445	37.935	0.14	15.23	-197.93	15.37	9	7/14/2014	41:35.2
5299	RSPe_2	-122.445	37.935	0.19	15.22	-193.17	15.41	-122.445	37.935	0.17	15.22	-197.37	15.39	9	7/14/2014	41:35.1
5300	RSPe_2	-122.445	37.935	0.16	15.25	-192.61	15.41	-122.445	37.935	0.17	15.25	-196.72	15.42	9	7/14/2014	41:35.0
5301	RSPe_2	-122.445	37.935	0.19	15.28	-192.02	15.47	-122.445	37.935	0.14	15.28	-196.07	15.42	9	7/14/2014	41:34.9
5302	RSPe_2	-122.445	37.935	0.19	15.25	-191.40	15.44	-122.445	37.935	0.14	15.25	-195.50	15.39	9	7/14/2014	41:34.8
5303	RSPe_2	-122.445	37.935	0.19	15.30	-190.94	15.49	-122.445	37.935	0.14	15.30	-194.90	15.44	9	7/14/2014	41:34.7
5304	RSPe_2	-122.445	37.935	0.19	15.33	-190.38	15.52	-122.445	37.935	0.09	15.33	-194.35	15.42	9	7/14/2014	41:34.6
5305	RSPe_2	-122.445	37.935	0.19	15.35	-189.91	15.54	-122.445	37.935	0.17	15.35	-193.88	15.52	9	7/14/2014	41:34.5
5306	RSPe_2	-122.445	37.935	0.19	15.32	-189.51	15.51	-122.445	37.935	0.14	15.32	-193.42	15.46	9	7/14/2014	41:34.4
5307	RSPe_2	-122.445	37.935	0.19	15.48	-189.06	15.68	-122.445	37.935	0.17	15.48	-193.02	15.66	9	7/14/2014	41:34.3
5308	RSPe_2	-122.445	37.935	0.19	15.46	-188.63	15.65	-122.445	37.935	0.21	15.46	-192.57	15.67	9	7/14/2014	41:34.2
5309	RSPe_2	-122.445	37.935	0.19	15.55	-188.14	15.74	-122.445	37.935	0.17	15.55	-192.06	15.72	9	7/14/2014	41:34.1
5310	RSPe_2	-122.445	37.935	0.19	15.47	-187.63	15.66	-122.445	37.935	0.14	15.47	-191.55	15.61	9	7/14/2014	41:34.0
5311	RSPe_2	-122.445	37.935	0.19	15.50	-187.18	15.69	-122.445	37.935	0.17	15.50	-191.02	15.67	9	7/14/2014	41:33.9
5312	RSPe_2	-122.445	37.935	0.16	15.47	-186.78	15.63	-122.445	37.935	0.14	15.47	-190.52	15.61	9	7/14/2014	41:33.8

5313	RSPe_2	-122.445	37.935	0.19	15.49	-186.36	15.69	-122.445	37.935	0.14	15.49	-189.99	15.63	9	7/14/2014	41:33.7
5314	RSPe_2	-122.445	37.935	0.16	15.52	-185.91	15.67	-122.445	37.935	0.09	15.52	-189.51	15.60	9	7/14/2014	41:33.6
5315	RSPe_2	-122.445	37.935	0.19	15.54	-185.63	15.73	-122.445	37.935	0.14	15.54	-189.12	15.68	9	7/14/2014	41:33.5
5316	RSPe_2	-122.445	37.935	0.16	15.81	-185.23	15.97	-122.445	37.935	0.09	15.81	-188.76	15.90	9	7/14/2014	41:33.4
5317	RSPe_2	-122.445	37.935	0.19	15.59	-184.84	15.78	-122.445	37.935	0.14	15.59	-188.37	15.72	9	7/14/2014	41:33.3
5318	RSPe_2	-122.445	37.935	0.16	15.82	-184.33	15.97	-122.445	37.935	0.09	15.82	-187.90	15.90	9	7/14/2014	41:33.2
5319	RSPe_2	-122.445	37.935	0.19	15.81	-183.86	16.00	-122.445	37.935	0.14	15.81	-187.43	15.95	9	7/14/2014	41:33.1
5320	RSPe_2	-122.445	37.935	0.16	15.78	-183.33	15.94	-122.445	37.935	0.14	15.78	-186.98	15.92	9	7/14/2014	41:33.0
5321	RSPe_2	-122.445	37.935	0.19	15.73	-182.90	15.92	-122.445	37.935	0.09	15.73	-186.57	15.81	9	7/14/2014	41:32.9
5322	RSPe_2	-122.445	37.935	0.16	15.69	-182.41	15.84	-122.445	37.935	0.05	15.69	-186.16	15.74	9	7/14/2014	41:32.8
5323	RSPe_2	-122.445	37.935	0.16	15.70	-181.94	15.86	-122.445	37.935	0.09	15.70	-185.72	15.79	9	7/14/2014	41:32.7
5324	RSPe_2	-122.445	37.935	0.16	15.78	-181.34	15.94	-122.445	37.935	0.09	15.78	-185.11	15.87	9	7/14/2014	41:32.6
5325	RSPe_2	-122.445	37.935	0.16	15.84	-180.70	16.00	-122.445	37.935	0.05	15.84	-184.51	15.90	9	7/14/2014	41:32.5
5326	RSPe_2	-122.445	37.935	0.16	15.71	-180.27	15.87	-122.445	37.935	0.05	15.71	-184.00	15.76	9	7/14/2014	41:32.4
5327	RSPe_2	-122.445	37.935	0.16	15.76	-179.82	15.92	-122.445	37.935	0.05	15.76	-183.48	15.82	9	7/14/2014	41:32.3
5328	RSPe_2	-122.445	37.935	0.11	15.80	-179.23	15.91	-122.445	37.935	0.00	15.80	-182.85	15.80	9	7/14/2014	41:32.2
5329	RSPe_2	-122.445	37.935	0.16	15.77	-178.65	15.93	-122.445	37.935	0.05	15.77	-182.29	15.83	9	7/14/2014	41:32.1
5330	RSPe_2	-122.445	37.935	0.11	15.79	-178.16	15.89	-122.445	37.935	0.05	15.79	-181.80	15.84	9	7/14/2014	41:32.0
5331	RSPe_2	-122.445	37.935	0.16	15.75	-177.82	15.91	-122.445	37.935	0.09	15.75	-181.51	15.84	9	7/14/2014	41:31.9
5332	RSPe_2	-122.445	37.935	0.11	15.69	-177.43	15.80	-122.445	37.935	0.05	15.69	-181.16	15.75	9	7/14/2014	41:31.8
5333	RSPe_2	-122.445	37.935	0.16	15.66	-177.03	15.81	-122.445	37.935	0.05	15.66	-180.84	15.71	9	7/14/2014	41:31.7
5334	RSPe_2	-122.445	37.935	0.11	15.63	-176.64	15.74	-122.445	37.935	0.05	15.63	-180.57	15.69	9	7/14/2014	41:31.6
5335	RSPe_2	-122.445	37.935	0.16	15.62	-176.17	15.78	-122.445	37.935	0.09	15.62	-180.12	15.71	9	7/14/2014	41:31.5
5336	RSPe_2	-122.445	37.935	0.11	15.60	-175.73	15.70	-122.445	37.935	0.05	15.60	-179.74	15.65	9	7/14/2014	41:31.4
5337	RSPe_2	-122.445	37.935	0.16	15.60	-175.38	15.75	-122.445	37.935	0.00	15.60	-179.40	15.60	9	7/14/2014	41:31.3
5338	RSPe_2	-122.445	37.935	0.11	15.62	-174.95	15.72	-122.445	37.935	0.00	15.62	-178.94	15.62	9	7/14/2014	41:31.2
5339	RSPe_2	-122.445	37.935	0.11	15.57	-174.51	15.68	-122.445	37.935	0.05	15.57	-178.45	15.62	9	7/14/2014	41:31.1
5340	RSPe_2	-122.445	37.935	0.11	15.62	-174.07	15.73	-122.445	37.935	0.00	15.62	-177.98	15.63	9	7/14/2014	41:31.0
5341	RSPe_2	-122.445	37.935	0.11	15.60	-173.63	15.71	-122.445	37.935	0.00	15.60	-177.49	15.61	9	7/14/2014	41:30.9
5342	RSPe_2	-122.445	37.935	0.11	15.61	-173.20	15.72	-122.445	37.935	-0.03	15.61	-177.09	15.58	9	7/14/2014	41:30.8
5343	RSPe_2	-122.445	37.935	0.11	15.61	-172.82	15.72	-122.445	37.935	0.00	15.61	-176.74	15.61	9	7/14/2014	41:30.7
5344	RSPe_2	-122.445	37.935	0.11	15.63	-172.39	15.74	-122.445	37.935	0.00	15.63	-176.35	15.64	9	7/14/2014	41:30.6
5345	RSPe_2	-122.445	37.935	0.11	15.62	-171.97	15.72	-122.445	37.935	0.05	15.62	-175.98	15.67	9	7/14/2014	41:30.5

5346	RSPe_2	-122.445	37.935	0.07	15.64	-171.47	15.71	-122.445	37.935	-0.08	15.64	-175.55	15.56	9	7/14/2014	41:30.4
5347	RSPe_2	-122.445	37.935	0.11	15.64	-171.16	15.75	-122.445	37.935	0.00	15.64	-175.29	15.64	9	7/14/2014	41:30.3
5348	RSPe_2	-122.445	37.935	0.07	15.62	-170.86	15.69	-122.445	37.935	-0.03	15.62	-175.07	15.59	9	7/14/2014	41:30.2
5349	RSPe_2	-122.445	37.935	0.11	15.61	-170.66	15.72	-122.445	37.935	-0.03	15.61	-174.83	15.58	9	7/14/2014	41:30.1
5350	RSPe_2	-122.445	37.935	0.07	15.60	-170.55	15.67	-122.445	37.935	-0.08	15.60	-174.64	15.51	9	7/14/2014	41:30.0
5351	RSPe_2	-122.445	37.935	0.11	15.60	-170.32	15.71	-122.445	37.935	-0.03	15.60	-174.39	15.57	9	7/14/2014	41:29.9
5352	RSPe_2	-122.445	37.935	0.07	15.61	-170.01	15.68	-122.445	37.935	-0.03	15.61	-174.01	15.58	9	7/14/2014	41:29.8
5353	RSPe_2	-122.445	37.935	0.11	15.59	-169.85	15.69	-122.445	37.935	-0.03	15.59	-173.78	15.56	9	7/14/2014	41:29.7
5354	RSPe_2	-122.445	37.935	0.07	15.60	-169.57	15.67	-122.445	37.935	-0.08	15.60	-173.52	15.51	9	7/14/2014	41:29.6
5355	RSPe_2	-122.445	37.935	0.11	15.62	-169.35	15.73	-122.445	37.935	-0.08	15.62	-173.29	15.54	9	7/14/2014	41:29.5
5356	RSPe_2	-122.445	37.935	0.07	15.62	-169.05	15.69	-122.445	37.935	-0.08	15.62	-173.01	15.54	9	7/14/2014	41:29.4
5357	RSPe_2	-122.445	37.935	0.11	15.64	-168.65	15.75	-122.445	37.935	-0.03	15.64	-172.71	15.61	9	7/14/2014	41:29.3
5358	RSPe_2	-122.445	37.935	0.07	15.66	-168.19	15.73	-122.445	37.935	-0.03	15.66	-172.33	15.63	9	7/14/2014	41:29.2
5359	RSPe_2	-122.445	37.935	0.11	15.67	-167.86	15.78	-122.445	37.935	-0.03	15.67	-172.03	15.64	9	7/14/2014	41:29.1
5360	RSPe_2	-122.445	37.935	0.07	15.69	-167.57	15.76	-122.445	37.935	-0.12	15.69	-171.83	15.57	9	7/14/2014	41:29.0
5361	RSPe_2	-122.445	37.935	0.07	15.71	-167.38	15.78	-122.445	37.935	-0.03	15.71	-171.59	15.68	9	7/14/2014	41:28.9
5362	RSPe_2	-122.445	37.935	0.07	15.73	-167.19	15.81	-122.445	37.935	-0.08	15.73	-171.47	15.65	9	7/14/2014	41:28.8
5363	RSPe_2	-122.445	37.935	0.11	15.73	-167.05	15.83	-122.445	37.935	-0.12	15.73	-171.24	15.61	9	7/14/2014	41:28.7
5364	RSPe_2	-122.445	37.935	0.07	15.73	-167.01	15.80	-122.445	37.935	-0.08	15.73	-171.13	15.64	9	7/14/2014	41:28.6
5365	RSPe_2	-122.445	37.935	0.11	15.74	-166.98	15.85	-122.445	37.935	-0.08	15.74	-171.09	15.66	9	7/14/2014	41:28.5
5366	RSPe_2	-122.445	37.935	0.07	15.76	-166.97	15.83	-122.445	37.935	-0.03	15.76	-171.07	15.73	9	7/14/2014	41:28.4
5367	RSPe_2	-122.445	37.935	0.11	15.80	-166.94	15.91	-122.445	37.935	0.00	15.80	-171.01	15.80	9	7/14/2014	41:28.3
5368	RSPe_2	-122.445	37.935	0.11	15.81	-166.84	15.92	-122.445	37.935	0.00	15.81	-170.87	15.81	9	7/14/2014	41:28.2
5369	RSPe_2	-122.445	37.935	0.11	15.90	-166.70	16.01	-122.445	37.935	-0.03	15.90	-170.76	15.87	9	7/14/2014	41:28.1
5370	RSPe_2	-122.445	37.935	0.07	15.92	-166.55	15.99	-122.445	37.935	-0.08	15.92	-170.64	15.84	9	7/14/2014	41:28.0
5371	RSPe_2	-122.445	37.935	0.11	15.90	-166.43	16.01	-122.445	37.935	-0.03	15.90	-170.58	15.87	9	7/14/2014	41:27.9
5372	RSPe_2	-122.445	37.935	0.11	15.90	-166.23	16.01	-122.445	37.935	-0.03	15.90	-170.45	15.87	9	7/14/2014	41:27.8
5373	RSPe_2	-122.445	37.935	0.11	15.93	-166.15	16.04	-122.445	37.935	0.00	15.93	-170.31	15.94	9	7/14/2014	41:27.7
5374	RSPe_2	-122.445	37.935	0.11	15.91	-166.04	16.02	-122.445	37.935	-0.03	15.91	-170.14	15.88	9	7/14/2014	41:27.6
5375	RSPe_2	-122.445	37.935	0.16	15.93	-166.04	16.09	-122.445	37.935	0.00	15.93	-170.02	15.93	9	7/14/2014	41:27.5
5376	RSPe_2	-122.445	37.935	0.11	15.93	-166.04	16.04	-122.445	37.935	-0.08	15.93	-169.88	15.85	9	7/14/2014	41:27.4
5377	RSPe_2	-122.445	37.935	0.16	15.93	-166.12	16.09	-122.445	37.935	-0.03	15.93	-169.76	15.90	9	7/14/2014	41:27.3
5378	RSPe_2	-122.445	37.935	0.11	15.94	-166.10	16.05	-122.445	37.935	-0.03	15.94	-169.67	15.91	9	7/14/2014	41:27.2

5379	RSPe_2	-122.445	37.935	0.11	15.96	-166.08	16.06	-122.445	37.935	-0.03	15.96	-169.53	15.93	9	7/14/2014 41:27.1
5380	RSPe_2	-122.445	37.935	0.11	15.97	-165.99	16.07	-122.445	37.935	0.00	15.97	-169.46	15.97	9	7/14/2014 41:27.0
5381	RSPe_2	-122.445	37.935	0.16	15.99	-165.92	16.15	-122.445	37.935	0.00	15.99	-169.39	15.99	9	7/14/2014 41:26.9
5382	RSPe_2	-122.445	37.935	0.11	16.01	-165.78	16.12	-122.445	37.935	0.00	16.01	-169.33	16.01	9	7/14/2014 41:26.8
5383	RSPe_2	-122.445	37.935	0.16	16.00	-165.63	16.16	-122.445	37.935	-0.03	16.00	-169.27	15.97	9	7/14/2014 41:26.7
5384	RSPe_2	-122.445	37.935	0.11	16.01	-165.52	16.12	-122.445	37.935	-0.03	16.01	-169.34	15.98	9	7/14/2014 41:26.6
5385	RSPe_2	-122.445	37.935	0.16	16.03	-165.43	16.19	-122.445	37.935	0.09	16.03	-169.30	16.12	9	7/14/2014 41:26.5
5386	RSPe_2	-122.445	37.935	0.16	16.08	-165.34	16.24	-122.445	37.935	0.05	16.08	-169.32	16.13	9	7/14/2014 41:26.4
5387	RSPe_2	-122.445	37.935	0.19	16.06	-165.34	16.25	-122.445	37.935	0.00	16.06	-169.28	16.06	9	7/14/2014 41:26.3
5388	RSPe_2	-122.445	37.935	0.16	16.06	-165.27	16.21	-122.445	37.935	0.09	16.06	-169.25	16.14	9	7/14/2014 41:26.2
5389	RSPe_2	-122.445	37.935	0.19	16.13	-165.27	16.32	-122.445	37.935	0.14	16.13	-169.20	16.27	9	7/14/2014 41:26.1
5390	RSPe_2	-122.445	37.935	0.16	15.94	-165.16	16.10	-122.445	37.935	0.05	15.94	-169.03	15.99	9	7/14/2014 41:26.0
5391	RSPe_2	-122.445	37.935	0.19	15.93	-165.14	16.13	-122.445	37.935	0.05	15.93	-168.98	15.99	9	7/14/2014 41:25.9
5392	RSPe_2	-122.445	37.935	0.19	15.89	-165.14	16.08	-122.445	37.935	0.00	15.89	-168.96	15.89	9	7/14/2014 41:25.8
5393	RSPe_2	-122.445	37.935	0.19	15.80	-165.13	15.99	-122.445	37.935	0.00	15.80	-168.89	15.80	9	7/14/2014 41:25.7
5394	RSPe_2	-122.445	37.935	0.19	15.76	-165.14	15.95	-122.445	37.935	0.09	15.76	-168.91	15.84	9	7/14/2014 41:25.6
5395	RSPe_2	-122.445	37.935	0.23	15.74	-165.23	15.97	-122.445	37.935	0.09	15.74	-168.96	15.83	9	7/14/2014 41:25.5
5396	RSPe_2	-122.445	37.935	0.19	15.60	-165.31	15.79	-122.445	37.935	0.05	15.60	-169.06	15.66	9	7/14/2014 41:25.4
5397	RSPe_2	-122.445	37.935	0.23	15.71	-165.40	15.94	-122.445	37.935	0.09	15.71	-169.13	15.80	9	7/14/2014 41:25.3
5398	RSPe_2	-122.445	37.935	0.19	15.55	-165.36	15.74	-122.445	37.935	0.09	15.55	-169.11	15.64	9	7/14/2014 41:25.2
5399	RSPe_2	-122.445	37.935	0.19	15.47	-165.40	15.66	-122.445	37.935	0.05	15.47	-169.08	15.53	9	7/14/2014 41:25.1
5400	RSPe_2	-122.445	37.935	0.19	15.47	-165.37	15.66	-122.445	37.935	0.14	15.47	-169.07	15.61	9	7/14/2014 41:25.0
5401	RSPe_2	-122.445	37.935	0.23	15.48	-165.31	15.71	-122.445	37.935	0.09	15.48	-169.00	15.57	9	7/14/2014 41:24.9
5402	RSPe_2	-122.445	37.935	0.19	15.44	-165.26	15.63	-122.445	37.935	0.09	15.44	-168.98	15.53	9	7/14/2014 41:24.8
5403	RSPe_2	-122.445	37.935	0.19	15.42	-165.24	15.61	-122.445	37.935	0.09	15.42	-168.91	15.50	9	7/14/2014 41:24.7
5404	RSPe_2	-122.445	37.935	0.19	15.38	-165.24	15.57	-122.445	37.935	0.09	15.38	-168.99	15.47	9	7/14/2014 41:24.6
5405	RSPe_2	-122.445	37.935	0.23	15.36	-165.33	15.58	-122.445	37.935	0.09	15.36	-168.99	15.44	9	7/14/2014 41:24.5
5406	RSPe_2	-122.445	37.935	0.19	15.35	-165.28	15.54	-122.445	37.935	0.09	15.35	-168.95	15.43	9	7/14/2014 41:24.4
5407	RSPe_2	-122.445	37.935	0.23	15.32	-165.24	15.54	-122.445	37.935	0.09	15.32	-168.95	15.41	9	7/14/2014 41:24.3
5408	RSPe_2	-122.445	37.935	0.23	15.41	-165.24	15.64	-122.445	37.935	0.09	15.41	-168.89	15.50	9	7/14/2014 41:24.2
5409	RSPe_2	-122.445	37.935	0.23	15.31	-165.17	15.53	-122.445	37.935	0.09	15.31	-168.82	15.40	9	7/14/2014 41:24.1
5410	RSPe_2	-122.445	37.935	0.19	15.28	-165.09	15.47	-122.445	37.935	0.17	15.28	-168.80	15.45	9	7/14/2014 41:24.0
5411	RSPe_2	-122.445	37.935	0.23	15.32	-165.04	15.54	-122.445	37.935	0.17	15.32	-168.71	15.49	9	7/14/2014 41:23.9

5412	RSPe_2	-122.445	37.935	0.23	15.34	-165.02	15.57	-122.445	37.935	0.14	15.34	-168.71	15.48	9	7/14/2014	41:23.8
5413	RSPe_2	-122.445	37.935	0.23	15.34	-164.99	15.57	-122.445	37.935	0.09	15.34	-168.77	15.43	9	7/14/2014	41:23.7
5414	RSPe_2	-122.445	37.935	0.23	15.45	-165.04	15.67	-122.445	37.935	0.09	15.45	-168.83	15.53	9	7/14/2014	41:23.6
5415	RSPe_2	-122.445	37.935	0.23	15.45	-165.08	15.67	-122.445	37.935	0.14	15.45	-168.93	15.59	9	7/14/2014	41:23.5
5416	RSPe_2	-122.445	37.935	0.19	15.35	-165.17	15.54	-122.445	37.935	0.14	15.35	-169.04	15.48	9	7/14/2014	41:23.4
5417	RSPe_2	-122.445	37.935	0.23	15.39	-165.24	15.61	-122.445	37.935	0.14	15.39	-169.12	15.53	9	7/14/2014	41:23.3
5418	RSPe_2	-122.445	37.935	0.23	15.48	-165.26	15.71	-122.445	37.935	0.14	15.48	-169.21	15.62	9	7/14/2014	41:23.2
5419	RSPe_2	-122.445	37.935	0.23	15.42	-165.37	15.64	-122.445	37.935	0.14	15.42	-169.28	15.56	9	7/14/2014	41:23.1
5420	RSPe_2	-122.445	37.935	0.23	15.47	-165.41	15.70	-122.445	37.935	0.14	15.47	-169.33	15.61	9	7/14/2014	41:23.0
5421	RSPe_2	-122.445	37.935	0.23	15.50	-165.36	15.73	-122.445	37.935	0.17	15.50	-169.29	15.67	9	7/14/2014	41:22.9
5422	RSPe_2	-122.445	37.935	0.23	15.48	-165.36	15.71	-122.445	37.935	0.09	15.48	-169.25	15.57	9	7/14/2014	41:22.8
5423	RSPe_2	-122.445	37.935	0.23	15.52	-165.34	15.74	-122.445	37.935	0.21	15.52	-169.26	15.72	9	7/14/2014	41:22.7
5424	RSPe_2	-122.445	37.935	0.23	15.53	-165.27	15.76	-122.445	37.935	0.14	15.53	-169.21	15.67	9	7/14/2014	41:22.6
5425	RSPe_2	-122.445	37.935	0.28	15.59	-165.30	15.86	-122.445	37.935	0.17	15.59	-169.29	15.76	9	7/14/2014	41:22.5
5426	RSPe_2	-122.445	37.935	0.23	15.55	-165.36	15.77	-122.445	37.936	0.14	15.55	-169.34	15.69	9	7/14/2014	41:22.4
5427	RSPe_2	-122.445	37.935	0.28	15.66	-165.34	15.94	-122.445	37.936	0.17	15.66	-169.44	15.84	9	7/14/2014	41:22.3
5428	RSPe_2	-122.445	37.936	0.23	15.64	-165.36	15.87	-122.445	37.936	0.21	15.64	-169.47	15.85	9	7/14/2014	41:22.2
5429	RSPe_2	-122.445	37.936	0.28	15.66	-165.44	15.94	-122.445	37.936	0.21	15.66	-169.55	15.87	9	7/14/2014	41:22.1
5430	RSPe_2	-122.445	37.936	0.23	15.71	-165.47	15.94	-122.445	37.936	0.17	15.71	-169.63	15.88	9	7/14/2014	41:22.0
5431	RSPe_2	-122.445	37.936	0.28	15.63	-165.57	15.91	-122.445	37.936	0.26	15.63	-169.77	15.89	9	7/14/2014	41:21.9
5432	RSPe_2	-122.445	37.936	0.28	15.69	-165.57	15.97	-122.445	37.936	0.21	15.69	-169.66	15.90	9	7/14/2014	41:21.8
5433	RSPe_2	-122.445	37.936	0.23	15.77	-165.64	16.00	-122.445	37.936	0.21	15.77	-169.78	15.98	9	7/14/2014	41:21.7
5434	RSPe_2	-122.445	37.936	0.28	15.72	-165.64	16.00	-122.445	37.936	0.26	15.72	-169.77	15.98	9	7/14/2014	41:21.6
5435	RSPe_2	-122.445	37.936	0.28	15.72	-165.62	16.00	-122.445	37.936	0.26	15.72	-169.72	15.98	9	7/14/2014	41:21.5
5436	RSPe_2	-122.445	37.936	0.23	15.74	-165.60	15.97	-122.445	37.936	0.17	15.74	-169.74	15.91	9	7/14/2014	41:21.4
5437	RSPe_2	-122.445	37.936	0.28	15.66	-165.55	15.94	-122.445	37.936	0.21	15.66	-169.66	15.87	9	7/14/2014	41:21.3
5438	RSPe_2	-122.445	37.936	0.23	15.65	-165.57	15.88	-122.445	37.936	0.17	15.65	-169.68	15.82	9	7/14/2014	41:21.2
5439	RSPe_2	-122.445	37.936	0.28	15.69	-165.59	15.97	-122.445	37.936	0.26	15.69	-169.76	15.94	9	7/14/2014	41:21.1
5440	RSPe_2	-122.445	37.936	0.23	15.63	-165.68	15.86	-122.445	37.936	0.21	15.63	-169.83	15.84	9	7/14/2014	41:21.0
5441	RSPe_2	-122.445	37.936	0.28	15.60	-165.75	15.87	-122.445	37.936	0.26	15.60	-169.96	15.85	9	7/14/2014	41:20.9
5442	RSPe_2	-122.445	37.936	0.23	15.53	-165.81	15.76	-122.445	37.936	0.26	15.53	-170.08	15.79	9	7/14/2014	41:20.8
5443	RSPe_2	-122.445	37.936	0.28	15.46	-165.89	15.73	-122.445	37.936	0.29	15.46	-170.14	15.75	9	7/14/2014	41:20.7
5444	RSPe_2	-122.445	37.936	0.28	15.43	-165.95	15.71	-122.445	37.936	0.26	15.43	-170.21	15.69	9	7/14/2014	41:20.6

5445	RSPe_2	-122.445	37.936	0.28	15.41	-166.00	15.69	-122.445	37.936	0.26	15.41	-170.22	15.67	9	7/14/2014	41:20.5
5446	RSPe_2	-122.445	37.936	0.28	15.39	-166.09	15.67	-122.445	37.936	0.29	15.39	-170.25	15.69	9	7/14/2014	41:20.4
5447	RSPe_2	-122.445	37.936	0.28	15.39	-166.15	15.67	-122.445	37.936	0.26	15.39	-170.31	15.64	9	7/14/2014	41:20.3
5448	RSPe_2	-122.445	37.936	0.28	15.32	-166.19	15.60	-122.445	37.936	0.26	15.32	-170.25	15.57	9	7/14/2014	41:20.2
5449	RSPe_2	-122.445	37.936	0.28	15.28	-166.26	15.56	-122.445	37.936	0.26	15.28	-170.33	15.53	9	7/14/2014	41:20.1
5450	RSPe_2	-122.445	37.936	0.23	15.27	-166.28	15.50	-122.445	37.936	0.26	15.27	-170.30	15.53	9	7/14/2014	41:20.0
5451	RSPe_2	-122.445	37.936	0.28	15.26	-166.33	15.54	-122.445	37.936	0.26	15.26	-170.39	15.52	9	7/14/2014	41:19.9
5452	RSPe_2	-122.445	37.936	0.28	15.29	-166.43	15.56	-122.445	37.936	0.29	15.29	-170.45	15.58	9	7/14/2014	41:19.8
5453	RSPe_2	-122.445	37.936	0.31	15.32	-166.56	15.64	-122.445	37.936	0.26	15.32	-170.52	15.58	9	7/14/2014	41:19.7
5454	RSPe_2	-122.445	37.936	0.28	15.21	-166.60	15.48	-122.445	37.936	0.26	15.21	-170.63	15.46	9	7/14/2014	41:19.6
5455	RSPe_2	-122.445	37.936	0.31	15.17	-166.76	15.48	-122.445	37.936	0.26	15.17	-170.71	15.43	9	7/14/2014	41:19.5
5456	RSPe_2	-122.445	37.936	0.28	15.16	-166.90	15.44	-122.445	37.936	0.21	15.16	-170.90	15.37	9	7/14/2014	41:19.4
5457	RSPe_2	-122.445	37.936	0.31	15.15	-167.00	15.46	-122.445	37.936	0.29	15.15	-171.04	15.44	9	7/14/2014	41:19.3
5458	RSPe_2	-122.445	37.936	0.28	15.15	-167.08	15.43	-122.445	37.936	0.26	15.15	-171.06	15.40	9	7/14/2014	41:19.2
5459	RSPe_2	-122.445	37.936	0.31	15.10	-167.11	15.41	-122.445	37.936	0.26	15.10	-171.14	15.36	9	7/14/2014	41:19.1
5460	RSPe_2	-122.445	37.936	0.28	15.11	-167.10	15.39	-122.445	37.936	0.29	15.11	-171.15	15.40	9	7/14/2014	41:19.0
5461	RSPe_2	-122.445	37.936	0.31	15.13	-167.11	15.44	-122.445	37.936	0.34	15.13	-171.17	15.47	9	7/14/2014	41:18.9
5462	RSPe_2	-122.445	37.936	0.31	15.15	-167.13	15.46	-122.445	37.936	0.34	15.15	-171.20	15.49	9	7/14/2014	41:18.8
5463	RSPe_2	-122.445	37.936	0.31	15.13	-167.13	15.44	-122.445	37.936	0.34	15.13	-171.20	15.47	9	7/14/2014	41:18.7
5464	RSPe_2	-122.445	37.936	0.31	15.13	-167.13	15.44	-122.445	37.936	0.29	15.13	-171.20	15.42	9	7/14/2014	41:18.6
5465	RSPe_2	-122.445	37.936	0.31	15.15	-167.13	15.46	-122.445	37.936	0.34	15.15	-171.21	15.49	9	7/14/2014	41:18.5
5466	RSPe_2	-122.445	37.936	0.28	15.21	-167.14	15.48	-122.445	37.936	0.29	15.21	-171.26	15.50	9	7/14/2014	41:18.4
5467	RSPe_2	-122.445	37.936	0.31	15.15	-167.24	15.46	-122.445	37.936	0.29	15.15	-171.32	15.44	9	7/14/2014	41:18.3
5468	RSPe_2	-122.445	37.936	0.28	15.17	-167.23	15.45	-122.445	37.936	0.29	15.17	-171.34	15.46	9	7/14/2014	41:18.2
5469	RSPe_2	-122.445	37.936	0.31	15.18	-167.28	15.49	-122.445	37.936	0.29	15.18	-171.34	15.47	9	7/14/2014	41:18.1
5470	RSPe_2	-122.445	37.936	0.28	15.20	-167.26	15.48	-122.445	37.936	0.26	15.20	-171.37	15.46	9	7/14/2014	41:18.0
5471	RSPe_2	-122.445	37.936	0.31	15.23	-167.29	15.55	-122.445	37.936	0.29	15.23	-171.33	15.52	9	7/14/2014	41:17.9
5472	RSPe_2	-122.445	37.936	0.28	15.26	-167.39	15.54	-122.445	37.936	0.29	15.26	-171.44	15.56	9	7/14/2014	41:17.8
5473	RSPe_2	-122.445	37.936	0.31	15.27	-167.45	15.58	-122.445	37.936	0.29	15.27	-171.55	15.56	9	7/14/2014	41:17.7
5474	RSPe_2	-122.445	37.936	0.28	15.31	-167.54	15.59	-122.445	37.936	0.29	15.31	-171.58	15.60	9	7/14/2014	41:17.6
5475	RSPe_2	-122.445	37.936	0.31	15.38	-167.60	15.69	-122.445	37.936	0.34	15.38	-171.64	15.72	9	7/14/2014	41:17.5
5476	RSPe_2	-122.445	37.936	0.28	15.36	-167.66	15.64	-122.445	37.936	0.29	15.36	-171.71	15.65	9	7/14/2014	41:17.4
5477	RSPe_2	-122.445	37.936	0.31	15.36	-167.76	15.68	-122.445	37.936	0.29	15.36	-171.81	15.65	9	7/14/2014	41:17.3

5478	RSPe_2	-122.445	37.936	0.28	15.37	-167.91	15.65	-122.445	37.936	0.26	15.37	-171.96	15.63	9	7/14/2014	41:17.2
5479	RSPe_2	-122.445	37.936	0.31	15.36	-168.08	15.68	-122.445	37.936	0.34	15.36	-172.06	15.71	9	7/14/2014	41:17.1
5480	RSPe_2	-122.445	37.936	0.28	15.37	-168.17	15.65	-122.445	37.936	0.29	15.37	-172.16	15.66	9	7/14/2014	41:17.0
5481	RSPe_2	-122.445	37.936	0.31	15.39	-168.30	15.70	-122.445	37.936	0.34	15.39	-172.26	15.73	9	7/14/2014	41:16.9
5482	RSPe_2	-122.445	37.936	0.28	15.37	-168.38	15.65	-122.445	37.936	0.26	15.37	-172.35	15.63	9	7/14/2014	41:16.8
5483	RSPe_2	-122.445	37.936	0.31	15.36	-168.47	15.68	-122.445	37.936	0.34	15.36	-172.40	15.71	9	7/14/2014	41:16.7
5484	RSPe_2	-122.445	37.936	0.28	15.39	-168.49	15.67	-122.445	37.936	0.29	15.39	-172.47	15.69	9	7/14/2014	41:16.6
5485	RSPe_2	-122.445	37.936	0.31	15.41	-168.62	15.72	-122.445	37.936	0.34	15.41	-172.59	15.75	9	7/14/2014	41:16.5
5486	RSPe_2	-122.445	37.936	0.31	15.43	-168.67	15.75	-122.445	37.936	0.34	15.43	-172.62	15.78	9	7/14/2014	41:16.4
5487	RSPe_2	-122.445	37.936	0.31	15.44	-168.71	15.75	-122.445	37.936	0.43	15.44	-172.69	15.87	9	7/14/2014	41:16.3
5488	RSPe_2	-122.445	37.936	0.31	15.47	-168.73	15.79	-122.445	37.936	0.34	15.47	-172.73	15.82	9	7/14/2014	41:16.2
5489	RSPe_2	-122.445	37.936	0.31	15.45	-168.75	15.76	-122.445	37.936	0.38	15.45	-172.78	15.82	9	7/14/2014	41:16.1
5490	RSPe_2	-122.445	37.936	0.31	15.48	-168.73	15.79	-122.445	37.936	0.34	15.48	-172.81	15.82	9	7/14/2014	41:16.0
5491	RSPe_2	-122.445	37.936	0.31	15.42	-168.80	15.73	-122.445	37.936	0.38	15.42	-172.88	15.79	9	7/14/2014	41:15.9
5492	RSPe_2	-122.445	37.936	0.28	15.36	-168.82	15.64	-122.445	37.936	0.34	15.36	-172.97	15.71	9	7/14/2014	41:15.8
5493	RSPe_2	-122.445	37.936	0.31	15.39	-168.88	15.70	-122.445	37.936	0.38	15.39	-173.04	15.76	9	7/14/2014	41:15.7
5494	RSPe_2	-122.445	37.936	0.31	15.36	-168.94	15.68	-122.445	37.936	0.34	15.36	-173.06	15.71	9	7/14/2014	41:15.6
5495	RSPe_2	-122.445	37.936	0.31	15.39	-168.95	15.71	-122.445	37.936	0.34	15.39	-173.14	15.74	9	7/14/2014	41:15.5
5496	RSPe_2	-122.445	37.936	0.28	15.37	-168.99	15.65	-122.445	37.936	0.29	15.37	-173.18	15.66	9	7/14/2014	41:15.4
5497	RSPe_2	-122.445	37.936	0.31	15.28	-169.12	15.59	-122.445	37.936	0.34	15.28	-173.26	15.62	9	7/14/2014	41:15.3
5498	RSPe_2	-122.445	37.936	0.28	15.29	-169.19	15.57	-122.445	37.936	0.38	15.29	-173.31	15.67	9	7/14/2014	41:15.2
5499	RSPe_2	-122.445	37.936	0.31	15.32	-169.25	15.64	-122.445	37.936	0.38	15.32	-173.41	15.70	9	7/14/2014	41:15.1
5500	RSPe_2	-122.445	37.936	0.31	15.25	-169.42	15.56	-122.445	37.936	0.34	15.25	-173.52	15.59	9	7/14/2014	41:15.0
5501	RSPe_2	-122.445	37.936	0.31	15.30	-169.53	15.62	-122.445	37.936	0.38	15.30	-173.70	15.68	9	7/14/2014	41:14.9
5502	RSPe_2	-122.445	37.936	0.31	15.29	-169.66	15.61	-122.445	37.936	0.38	15.29	-173.77	15.67	9	7/14/2014	41:14.8
5503	RSPe_2	-122.445	37.936	0.31	15.30	-169.75	15.62	-122.445	37.936	0.38	15.30	-173.85	15.68	9	7/14/2014	41:14.7
5504	RSPe_2	-122.445	37.936	0.31	15.29	-169.82	15.60	-122.445	37.936	0.34	15.29	-173.87	15.63	9	7/14/2014	41:14.6
5505	RSPe_2	-122.445	37.936	0.31	15.26	-169.87	15.57	-122.445	37.936	0.34	15.26	-173.96	15.60	9	7/14/2014	41:14.5
5506	RSPe_2	-122.445	37.936	0.28	15.28	-169.90	15.56	-122.445	37.936	0.34	15.28	-173.91	15.62	9	7/14/2014	41:14.4
5507	RSPe_2	-122.445	37.936	0.31	15.35	-169.94	15.66	-122.445	37.936	0.34	15.35	-173.96	15.69	9	7/14/2014	41:14.3
5508	RSPe_2	-122.445	37.936	0.28	15.36	-169.88	15.64	-122.445	37.936	0.29	15.36	-173.90	15.65	9	7/14/2014	41:14.2
5509	RSPe_2	-122.445	37.936	0.31	15.37	-169.90	15.68	-122.445	37.936	0.29	15.37	-173.89	15.66	9	7/14/2014	41:14.1
5510	RSPe_2	-122.445	37.936	0.28	15.39	-169.91	15.67	-122.445	37.936	0.34	15.39	-173.94	15.73	9	7/14/2014	41:14.0

5511	RSPe_2	-122.445	37.936	0.31	15.36	-169.94	15.67	-122.445	37.936	0.38	15.36	-174.02	15.73	9	7/14/2014	41:13.9
5512	RSPe_2	-122.445	37.936	0.28	15.36	-170.02	15.64	-122.445	37.936	0.34	15.36	-174.11	15.71	9	7/14/2014	41:13.8
5513	RSPe_2	-122.445	37.936	0.31	15.37	-170.16	15.68	-122.445	37.936	0.34	15.37	-174.21	15.71	9	7/14/2014	41:13.7
5514	RSPe_2	-122.445	37.936	0.28	15.36	-170.27	15.63	-122.445	37.936	0.34	15.36	-174.33	15.70	9	7/14/2014	41:13.6
5515	RSPe_2	-122.445	37.936	0.31	15.35	-170.38	15.66	-122.445	37.936	0.38	15.35	-174.45	15.72	9	7/14/2014	41:13.5
5516	RSPe_2	-122.445	37.936	0.28	15.31	-170.45	15.59	-122.445	37.936	0.38	15.31	-174.54	15.68	9	7/14/2014	41:13.4
5517	RSPe_2	-122.445	37.936	0.31	15.34	-170.62	15.65	-122.445	37.936	0.34	15.34	-174.66	15.68	9	7/14/2014	41:13.3
5518	RSPe_2	-122.445	37.936	0.28	15.30	-170.70	15.58	-122.445	37.936	0.34	15.30	-174.76	15.65	9	7/14/2014	41:13.2
5519	RSPe_2	-122.445	37.936	0.31	15.34	-170.85	15.65	-122.445	37.936	0.34	15.34	-174.83	15.68	9	7/14/2014	41:13.1
5520	RSPe_2	-122.445	37.936	0.28	15.32	-170.90	15.60	-122.445	37.936	0.29	15.32	-174.84	15.61	9	7/14/2014	41:13.0
5521	RSPe_2	-122.445	37.936	0.28	15.34	-170.88	15.62	-122.445	37.936	0.34	15.34	-174.82	15.68	9	7/14/2014	41:12.9
5522	RSPe_2	-122.445	37.936	0.28	15.35	-170.92	15.62	-122.445	37.936	0.29	15.35	-174.85	15.64	9	7/14/2014	41:12.8
5523	RSPe_2	-122.445	37.936	0.28	15.40	-171.02	15.68	-122.445	37.936	0.34	15.40	-174.93	15.75	9	7/14/2014	41:12.7
5524	RSPe_2	-122.445	37.936	0.28	15.37	-171.05	15.65	-122.445	37.936	0.34	15.37	-175.00	15.71	9	7/14/2014	41:12.6
5525	RSPe_2	-122.445	37.936	0.31	15.41	-171.00	15.72	-122.445	37.936	0.34	15.41	-175.00	15.75	9	7/14/2014	41:12.5
5526	RSPe_2	-122.445	37.936	0.28	15.41	-171.05	15.69	-122.445	37.936	0.34	15.41	-175.04	15.75	9	7/14/2014	41:12.4
5527	RSPe_2	-122.445	37.936	0.31	15.43	-171.05	15.75	-122.445	37.936	0.29	15.43	-175.04	15.72	9	7/14/2014	41:12.3
5528	RSPe_2	-122.445	37.936	0.28	15.43	-171.13	15.71	-122.445	37.936	0.34	15.43	-175.15	15.78	9	7/14/2014	41:12.2
5529	RSPe_2	-122.445	37.936	0.31	15.44	-171.22	15.75	-122.445	37.936	0.34	15.44	-175.18	15.78	9	7/14/2014	41:12.1
5530	RSPe_2	-122.445	37.936	0.28	15.46	-171.26	15.74	-122.445	37.936	0.34	15.46	-175.25	15.80	9	7/14/2014	41:12.0
5531	RSPe_2	-122.445	37.936	0.31	15.46	-171.35	15.78	-122.445	37.936	0.34	15.46	-175.25	15.81	9	7/14/2014	41:11.9
5532	RSPe_2	-122.445	37.936	0.28	15.50	-171.28	15.78	-122.445	37.936	0.34	15.50	-175.18	15.84	9	7/14/2014	41:11.8
5533	RSPe_2	-122.445	37.936	0.28	15.50	-171.32	15.78	-122.445	37.936	0.34	15.50	-175.21	15.84	9	7/14/2014	41:11.7
5534	RSPe_2	-122.445	37.936	0.28	15.52	-171.35	15.80	-122.445	37.936	0.34	15.52	-175.21	15.86	9	7/14/2014	41:11.6
5535	RSPe_2	-122.445	37.936	0.31	15.62	-171.30	15.93	-122.445	37.936	0.34	15.62	-175.13	15.96	9	7/14/2014	41:11.5
5536	RSPe_2	-122.445	37.936	0.28	15.64	-171.15	15.92	-122.445	37.936	0.29	15.64	-174.99	15.93	9	7/14/2014	41:11.4
5537	RSPe_2	-122.445	37.936	0.31	15.64	-170.97	15.95	-122.445	37.936	0.34	15.64	-174.81	15.98	9	7/14/2014	41:11.3
5538	RSPe_2	-122.445	37.936	0.28	15.75	-170.80	16.03	-122.445	37.936	0.29	15.75	-174.66	16.04	9	7/14/2014	41:11.2
5539	RSPe_2	-122.445	37.936	0.28	15.66	-170.78	15.94	-122.445	37.936	0.34	15.66	-174.56	16.01	9	7/14/2014	41:11.1
5540	RSPe_2	-122.445	37.936	0.28	15.72	-170.72	16.00	-122.445	37.936	0.34	15.72	-174.49	16.06	9	7/14/2014	41:11.0
5541	RSPe_2	-122.445	37.936	0.28	15.73	-170.67	16.00	-122.445	37.936	0.34	15.73	-174.43	16.07	9	7/14/2014	41:10.9
5542	RSPe_2	-122.445	37.936	0.28	15.77	-170.54	16.05	-122.445	37.936	0.29	15.77	-174.29	16.06	9	7/14/2014	41:10.8
5543	RSPe_2	-122.445	37.936	0.28	15.78	-170.41	16.06	-122.445	37.936	0.34	15.78	-174.22	16.12	9	7/14/2014	41:10.7

5544	RSPe_2	-122.445	37.936	0.28	15.80	-170.41	16.07	-122.445	37.936	0.34	15.80	-174.24	16.14	9	7/14/2014 41:10.6
5545	RSPe_2	-122.445	37.936	0.28	15.84	-170.36	16.12	-122.445	37.936	0.34	15.84	-174.21	16.19	9	7/14/2014 41:10.5
5546	RSPe_2	-122.445	37.936	0.28	15.75	-170.20	16.03	-122.445	37.936	0.34	15.75	-174.14	16.09	9	7/14/2014 41:10.4
5547	RSPe_2	-122.445	37.936	0.28	15.64	-169.92	15.92	-122.445	37.936	0.34	15.64	-173.83	15.98	9	7/14/2014 41:10.3
5548	RSPe_2	-122.445	37.936	0.23	15.68	-169.68	15.91	-122.445	37.936	0.29	15.68	-173.61	15.97	9	7/14/2014 41:10.2
5549	RSPe_2	-122.445	37.936	0.28	15.56	-169.51	15.83	-122.445	37.936	0.34	15.56	-173.46	15.90	9	7/14/2014 41:10.1
5550	RSPe_2	-122.445	37.936	0.23	15.48	-169.35	15.71	-122.445	37.936	0.34	15.48	-173.24	15.82	9	7/14/2014 41:10.0
5551	RSPe_2	-122.445	37.936	0.28	15.48	-169.27	15.76	-122.445	37.936	0.29	15.48	-173.14	15.78	9	7/14/2014 41:09.9
5552	RSPe_2	-122.445	37.936	0.23	15.52	-169.00	15.74	-122.445	37.936	0.34	15.52	-172.84	15.86	9	7/14/2014 41:09.8
5553	RSPe_2	-122.445	37.936	0.23	15.51	-168.87	15.74	-122.445	37.936	0.29	15.51	-172.68	15.80	9	7/14/2014 41:09.7
5554	RSPe_2	-122.445	37.936	0.23	15.44	-168.90	15.67	-122.445	37.936	0.29	15.44	-172.70	15.73	9	7/14/2014 41:09.6
5555	RSPe_2	-122.445	37.936	0.23	15.39	-168.89	15.62	-122.445	37.936	0.34	15.39	-172.68	15.74	9	7/14/2014 41:09.5
5556	RSPe_2	-122.445	37.936	0.23	15.39	-168.85	15.61	-122.445	37.936	0.29	15.39	-172.67	15.68	9	7/14/2014 41:09.4
5557	RSPe_2	-122.445	37.936	0.28	15.47	-168.71	15.75	-122.445	37.936	0.34	15.47	-172.52	15.82	9	7/14/2014 41:09.3
5558	RSPe_2	-122.445	37.936	0.23	15.37	-168.48	15.60	-122.445	37.936	0.34	15.37	-172.36	15.71	9	7/14/2014 41:09.2
5559	RSPe_2	-122.445	37.936	0.23	15.33	-168.41	15.56	-122.445	37.936	0.29	15.33	-172.30	15.63	9	7/14/2014 41:09.1
5560	RSPe_2	-122.445	37.936	0.23	15.34	-168.36	15.57	-122.445	37.936	0.29	15.34	-172.26	15.63	9	7/14/2014 41:09.0
5561	RSPe_2	-122.445	37.936	0.23	15.32	-168.35	15.54	-122.445	37.936	0.29	15.32	-172.27	15.61	9	7/14/2014 41:08.9
5562	RSPe_2	-122.445	37.936	0.19	15.29	-168.28	15.49	-122.445	37.936	0.29	15.29	-172.21	15.59	9	7/14/2014 41:08.8
5563	RSPe_2	-122.445	37.936	0.23	15.28	-168.15	15.50	-122.445	37.936	0.29	15.28	-172.07	15.57	9	7/14/2014 41:08.7
5564	RSPe_2	-122.445	37.936	0.19	15.27	-168.06	15.46	-122.445	37.936	0.26	15.27	-171.93	15.53	9	7/14/2014 41:08.6
5565	RSPe_2	-122.445	37.936	0.23	15.35	-167.99	15.57	-122.445	37.936	0.26	15.35	-171.81	15.60	9	7/14/2014 41:08.5
5566	RSPe_2	-122.445	37.936	0.23	15.32	-167.97	15.54	-122.445	37.936	0.21	15.32	-171.73	15.53	9	7/14/2014 41:08.4
5567	RSPe_2	-122.445	37.936	0.23	15.39	-167.90	15.62	-122.445	37.936	0.29	15.39	-171.64	15.69	9	7/14/2014 41:08.3
5568	RSPe_2	-122.445	37.936	0.19	15.36	-167.82	15.56	-122.445	37.936	0.26	15.36	-171.52	15.62	9	7/14/2014 41:08.2
5569	RSPe_2	-122.445	37.936	0.19	15.51	-167.75	15.70	-122.445	37.936	0.26	15.51	-171.43	15.77	9	7/14/2014 41:08.1
5570	RSPe_2	-122.445	37.936	0.19	15.46	-167.68	15.65	-122.445	37.936	0.21	15.46	-171.40	15.66	9	7/14/2014 41:08.0
5571	RSPe_2	-122.445	37.936	0.19	15.45	-167.75	15.64	-122.445	37.936	0.26	15.45	-171.48	15.70	9	7/14/2014 41:07.9
5572	RSPe_2	-122.445	37.936	0.19	15.53	-167.84	15.73	-122.445	37.936	0.21	15.53	-171.54	15.74	9	7/14/2014 41:07.8
5573	RSPe_2	-122.445	37.936	0.19	15.56	-167.84	15.76	-122.445	37.936	0.26	15.56	-171.59	15.82	9	7/14/2014 41:07.7
5574	RSPe_2	-122.445	37.936	0.19	15.53	-167.86	15.72	-122.445	37.936	0.21	15.53	-171.57	15.73	9	7/14/2014 41:07.6
5575	RSPe_2	-122.445	37.936	0.19	15.48	-167.82	15.68	-122.445	37.936	0.21	15.48	-171.54	15.69	9	7/14/2014 41:07.5
5576	RSPe_2	-122.445	37.936	0.19	15.65	-167.85	15.84	-122.445	37.936	0.21	15.65	-171.60	15.86	9	7/14/2014 41:07.4

5577	RSPe_2	-122.445	37.936	0.16	15.68	-167.88	15.84	-122.445	37.936	0.21	15.68	-171.54	15.89	9	7/14/2014	41:07.3
5578	RSPe_2	-122.445	37.936	0.19	15.69	-167.81	15.89	-122.445	37.936	0.17	15.69	-171.50	15.87	9	7/14/2014	41:07.2
5579	RSPe_2	-122.445	37.936	0.19	15.72	-167.75	15.91	-122.445	37.936	0.17	15.72	-171.43	15.89	9	7/14/2014	41:07.1
5580	RSPe_2	-122.445	37.936	0.16	15.73	-167.70	15.89	-122.445	37.936	0.17	15.73	-171.38	15.91	9	7/14/2014	41:07.0
5581	RSPe_2	-122.445	37.936	0.19	15.75	-167.68	15.94	-122.445	37.936	0.21	15.75	-171.32	15.96	9	7/14/2014	41:06.9
5582	RSPe_2	-122.445	37.936	0.16	15.78	-167.65	15.94	-122.445	37.936	0.17	15.78	-171.25	15.95	9	7/14/2014	41:06.8
5583	RSPe_2	-122.445	37.936	0.16	15.80	-167.59	15.95	-122.445	37.936	0.17	15.80	-171.23	15.97	9	7/14/2014	41:06.7
5584	RSPe_2	-122.445	37.936	0.16	15.81	-167.57	15.97	-122.445	37.936	0.14	15.81	-171.17	15.95	9	7/14/2014	41:06.6
5585	RSPe_2	-122.445	37.936	0.16	15.84	-167.61	16.00	-122.445	37.936	0.14	15.84	-171.14	15.98	9	7/14/2014	41:06.5
5586	RSPe_2	-122.445	37.936	0.16	15.82	-167.65	15.98	-122.445	37.936	0.09	15.82	-171.21	15.91	9	7/14/2014	41:06.4
5587	RSPe_2	-122.445	37.936	0.16	15.90	-167.65	16.05	-122.445	37.936	0.14	15.90	-171.19	16.04	9	7/14/2014	41:06.3
5588	RSPe_2	-122.445	37.936	0.16	15.86	-167.62	16.02	-122.445	37.936	0.14	15.86	-171.13	16.00	9	7/14/2014	41:06.2
5589	RSPe_2	-122.445	37.936	0.16	15.91	-167.63	16.07	-122.445	37.936	0.14	15.91	-171.19	16.05	9	7/14/2014	41:06.1
5590	RSPe_2	-122.445	37.936	0.16	15.87	-167.61	16.03	-122.445	37.936	0.09	15.87	-171.16	15.96	9	7/14/2014	41:06.0
5591	RSPe_2	-122.445	37.936	0.16	15.88	-167.59	16.04	-122.445	37.936	0.14	15.88	-171.20	16.02	9	7/14/2014	41:05.9
5592	RSPe_2	-122.445	37.936	0.11	15.90	-167.61	16.00	-122.445	37.936	0.09	15.90	-171.24	15.99	9	7/14/2014	41:05.8
5593	RSPe_2	-122.445	37.936	0.16	15.90	-167.61	16.06	-122.445	37.936	0.09	15.90	-171.25	15.99	9	7/14/2014	41:05.7
5594	RSPe_2	-122.445	37.936	0.11	15.90	-167.56	16.00	-122.445	37.936	0.09	15.90	-171.30	15.99	9	7/14/2014	41:05.6
5595	RSPe_2	-122.445	37.936	0.11	15.88	-167.57	15.99	-122.445	37.936	0.09	15.88	-171.30	15.97	9	7/14/2014	41:05.5
5596	RSPe_2	-122.445	37.936	0.11	15.90	-167.52	16.00	-122.445	37.936	0.09	15.90	-171.27	15.99	9	7/14/2014	41:05.4
5597	RSPe_2	-122.445	37.936	0.11	15.91	-167.39	16.02	-122.445	37.936	0.06	15.91	-171.16	15.97	9	7/14/2014	41:05.3
5598	RSPe_2	-122.445	37.936	0.11	15.91	-167.27	16.02	-122.445	37.936	0.06	15.91	-171.05	15.97	9	7/14/2014	41:05.2
5599	RSPe_2	-122.445	37.936	0.11	15.93	-167.26	16.04	-122.445	37.936	0.09	15.93	-170.96	16.02	9	7/14/2014	41:05.1
5600	RSPe_2	-122.445	37.936	0.11	15.95	-167.21	16.06	-122.445	37.936	0.09	15.95	-170.88	16.04	9	7/14/2014	41:05.0
5601	RSPe_2	-122.445	37.936	0.11	15.93	-167.05	16.04	-122.445	37.936	0.06	15.93	-170.74	15.99	9	7/14/2014	41:04.9
5602	RSPe_2	-122.445	37.936	0.07	15.93	-167.04	16.01	-122.445	37.936	0.06	15.93	-170.60	15.99	9	7/14/2014	41:04.8
5603	RSPe_2	-122.445	37.936	0.11	15.96	-166.96	16.07	-122.445	37.936	0.06	15.96	-170.53	16.01	9	7/14/2014	41:04.7
5604	RSPe_2	-122.445	37.936	0.11	15.93	-166.97	16.04	-122.445	37.936	0.06	15.93	-170.53	15.98	9	7/14/2014	41:04.6
5605	RSPe_2	-122.445	37.936	0.11	15.91	-167.04	16.02	-122.445	37.936	0.09	15.91	-170.54	16.00	9	7/14/2014	41:04.5
5606	RSPe_2	-122.445	37.936	0.07	15.90	-167.05	15.98	-122.445	37.936	0.06	15.90	-170.53	15.96	9	7/14/2014	41:04.4
5607	RSPe_2	-122.445	37.936	0.11	15.90	-166.93	16.01	-122.445	37.936	0.06	15.90	-170.43	15.96	9	7/14/2014	41:04.3
5608	RSPe_2	-122.445	37.936	0.07	15.89	-166.92	15.96	-122.445	37.936	0.06	15.89	-170.45	15.94	9	7/14/2014	41:04.2
5609	RSPe_2	-122.445	37.936	0.07	15.86	-166.88	15.94	-122.445	37.936	0.01	15.86	-170.43	15.87	9	7/14/2014	41:04.1

5610	RSPe_2	-122.445	37.936	0.02	15.88	-166.82	15.90	-122.445	37.936	0.01	15.88	-170.37	15.89	9	7/14/2014	41:04.0
5611	RSPe_2	-122.445	37.936	0.07	15.88	-166.72	15.95	-122.445	37.936	0.01	15.88	-170.26	15.89	9	7/14/2014	41:03.9
5612	RSPe_2	-122.445	37.936	0.07	15.88	-166.57	15.95	-122.445	37.936	-0.03	15.88	-170.14	15.85	9	7/14/2014	41:03.8
5613	RSPe_2	-122.445	37.936	0.07	15.88	-166.37	15.95	-122.445	37.936	0.01	15.88	-169.92	15.89	9	7/14/2014	41:03.7
5614	RSPe_2	-122.445	37.936	0.02	15.90	-166.28	15.92	-122.445	37.936	0.01	15.90	-169.82	15.90	9	7/14/2014	41:03.6
5615	RSPe_2	-122.445	37.936	0.07	15.91	-166.18	15.99	-122.445	37.936	0.01	15.91	-169.64	15.92	9	7/14/2014	41:03.5
5616	RSPe_2	-122.445	37.936	0.07	15.93	-166.07	16.01	-122.445	37.936	-0.08	15.93	-169.53	15.86	9	7/14/2014	41:03.4
5617	RSPe_2	-122.445	37.936	0.07	15.93	-165.98	16.00	-122.445	37.936	-0.03	15.93	-169.41	15.90	9	7/14/2014	41:03.3
5618	RSPe_2	-122.445	37.936	0.07	15.93	-165.96	16.01	-122.445	37.936	-0.03	15.93	-169.35	15.91	9	7/14/2014	41:03.2
5619	RSPe_2	-122.445	37.936	0.07	15.92	-165.84	15.99	-122.445	37.936	0.01	15.92	-169.24	15.92	9	7/14/2014	41:03.1
5620	RSPe_2	-122.445	37.936	0.02	15.93	-165.71	15.96	-122.445	37.936	-0.03	15.93	-169.04	15.91	9	7/14/2014	41:03.0
5621	RSPe_2	-122.445	37.936	0.07	15.97	-165.65	16.05	-122.445	37.936	-0.03	15.97	-169.01	15.94	9	7/14/2014	41:02.9
5622	RSPe_2	-122.445	37.936	0.02	15.94	-165.60	15.96	-122.445	37.936	-0.08	15.94	-168.97	15.86	9	7/14/2014	41:02.8
5623	RSPe_2	-122.445	37.936	0.07	15.97	-165.56	16.05	-122.445	37.936	-0.08	15.97	-168.90	15.89	9	7/14/2014	41:02.7
5624	RSPe_2	-122.445	37.936	0.02	15.94	-165.37	15.96	-122.445	37.936	-0.08	15.94	-168.72	15.86	9	7/14/2014	41:02.6
5625	RSPe_2	-122.445	37.936	0.02	16.07	-165.30	16.10	-122.445	37.936	-0.08	16.07	-168.61	15.99	9	7/14/2014	41:02.5
5626	RSPe_2	-122.445	37.936	0.07	15.97	-165.32	16.05	-122.445	37.936	-0.11	15.97	-168.64	15.86	9	7/14/2014	41:02.4
5627	RSPe_2	-122.445	37.936	0.07	16.01	-165.39	16.08	-122.445	37.936	-0.08	16.01	-168.66	15.93	9	7/14/2014	41:02.3
5628	RSPe_2	-122.445	37.936	0.02	16.00	-165.30	16.02	-122.445	37.936	-0.08	16.00	-168.63	15.92	9	7/14/2014	41:02.2
5629	RSPe_2	-122.445	37.936	0.07	16.12	-165.14	16.20	-122.445	37.936	-0.11	16.12	-168.58	16.01	9	7/14/2014	41:02.1
5630	RSPe_2	-122.445	37.936	0.02	16.09	-165.01	16.12	-122.445	37.936	-0.11	16.09	-168.52	15.98	9	7/14/2014	41:02.0
5631	RSPe_2	-122.445	37.936	0.07	16.08	-165.01	16.15	-122.445	37.936	-0.08	16.08	-168.49	16.00	9	7/14/2014	41:01.9
5632	RSPe_2	-122.445	37.936	0.02	16.09	-164.95	16.12	-122.445	37.936	-0.16	16.09	-168.41	15.93	9	7/14/2014	41:01.8
5633	RSPe_2	-122.445	37.936	0.07	16.10	-164.86	16.18	-122.445	37.936	-0.11	16.10	-168.26	15.99	9	7/14/2014	41:01.7
5634	RSPe_2	-122.445	37.936	0.02	16.13	-164.75	16.15	-122.445	37.936	-0.16	16.13	-168.15	15.96	9	7/14/2014	41:01.6
5635	RSPe_2	-122.445	37.936	0.02	16.13	-164.65	16.16	-122.445	37.936	-0.16	16.13	-167.98	15.97	9	7/14/2014	41:01.5
5636	RSPe_2	-122.445	37.936	-0.01	16.16	-164.51	16.15	-122.445	37.936	-0.16	16.16	-167.85	15.99	9	7/14/2014	41:01.4
5637	RSPe_2	-122.445	37.936	0.02	16.16	-164.44	16.18	-122.445	37.936	-0.16	16.16	-167.78	15.99	9	7/14/2014	41:01.3
5638	RSPe_2	-122.445	37.936	0.02	16.17	-164.42	16.19	-122.445	37.936	-0.20	16.17	-167.72	15.97	9	7/14/2014	41:01.2
5639	RSPe_2	-122.445	37.936	0.02	16.17	-164.41	16.19	-122.445	37.936	-0.16	16.17	-167.69	16.00	9	7/14/2014	41:01.1
5640	RSPe_2	-122.445	37.936	0.02	16.21	-164.42	16.23	-122.445	37.936	-0.20	16.21	-167.67	16.01	9	7/14/2014	41:01.0
5641	RSPe_2	-122.445	37.936	0.02	16.18	-164.38	16.20	-122.445	37.936	-0.20	16.18	-167.63	15.98	9	7/14/2014	41:00.9
5642	RSPe_2	-122.445	37.936	-0.01	16.21	-164.30	16.20	-122.445	37.936	-0.20	16.21	-167.58	16.01	9	7/14/2014	41:00.8

5643	RSPe_2	-122.445	37.936	0.02	16.18	-164.29	16.20	-122.445	37.936	-0.16	16.18	-167.62	16.02	9	7/14/2014 41:00.7
5644	RSPe_2	-122.445	37.936	0.02	16.20	-164.26	16.22	-122.445	37.936	-0.16	16.20	-167.61	16.03	9	7/14/2014 41:00.6
5645	RSPe_2	-122.445	37.936	0.02	16.22	-164.28	16.24	-122.445	37.936	-0.16	16.22	-167.61	16.05	9	7/14/2014 41:00.5
5646	RSPe_2	-122.445	37.936	0.02	16.25	-164.24	16.27	-122.445	37.936	-0.20	16.25	-167.63	16.05	9	7/14/2014 41:00.4
5647	RSPe_2	-122.445	37.936	0.02	16.19	-164.22	16.21	-122.445	37.936	-0.16	16.19	-167.62	16.03	9	7/14/2014 41:00.3
5648	RSPe_2	-122.445	37.936	0.02	16.21	-164.17	16.23	-122.445	37.936	-0.16	16.21	-167.60	16.05	9	7/14/2014 41:00.2
5649	RSPe_2	-122.445	37.936	0.07	16.20	-164.00	16.28	-122.445	37.936	-0.11	16.20	-167.40	16.09	9	7/14/2014 41:00.1
5650	RSPe_2	-122.445	37.936	0.02	16.21	-163.78	16.23	-122.445	37.936	-0.16	16.21	-167.19	16.05	9	7/14/2014 41:00.0
5651	RSPe_2	-122.445	37.936	0.07	16.25	-163.54	16.32	-122.445	37.936	-0.16	16.25	-166.95	16.09	9	7/14/2014 40:59.9
5652	RSPe_2	-122.445	37.936	0.02	16.31	-163.38	16.34	-122.445	37.936	-0.20	16.31	-166.82	16.12	9	7/14/2014 40:59.8
5653	RSPe_2	-122.445	37.936	0.07	16.28	-163.34	16.36	-122.445	37.936	-0.20	16.28	-166.76	16.08	9	7/14/2014 40:59.7
5654	RSPe_2	-122.445	37.936	0.02	16.23	-163.22	16.25	-122.445	37.936	-0.20	16.23	-166.64	16.03	9	7/14/2014 40:59.6
5655	RSPe_2	-122.445	37.936	0.07	16.27	-163.19	16.35	-122.445	37.936	-0.20	16.27	-166.68	16.07	9	7/14/2014 40:59.5
5656	RSPe_2	-122.445	37.936	0.02	16.24	-163.14	16.27	-122.445	37.936	-0.20	16.24	-166.61	16.05	9	7/14/2014 40:59.4
5657	RSPe_2	-122.445	37.936	0.07	16.18	-163.09	16.25	-122.445	37.936	-0.11	16.18	-166.55	16.07	9	7/14/2014 40:59.3
5658	RSPe_2	-122.445	37.936	0.02	16.17	-163.07	16.19	-122.445	37.936	-0.16	16.17	-166.48	16.00	9	7/14/2014 40:59.2
5659	RSPe_2	-122.445	37.936	0.07	16.17	-163.05	16.25	-122.445	37.936	-0.16	16.17	-166.43	16.01	9	7/14/2014 40:59.1
5660	RSPe_2	-122.445	37.936	0.02	16.23	-163.05	16.26	-122.445	37.936	-0.20	16.23	-166.38	16.04	9	7/14/2014 40:59.0
5661	RSPe_2	-122.445	37.936	0.07	16.20	-163.02	16.28	-122.445	37.936	-0.11	16.20	-166.28	16.09	9	7/14/2014 40:58.9
5662	RSPe_2	-122.445	37.936	0.02	16.32	-162.90	16.34	-122.445	37.936	-0.16	16.32	-166.14	16.16	9	7/14/2014 40:58.8
5663	RSPe_2	-122.445	37.936	0.07	16.36	-162.73	16.43	-122.445	37.936	-0.11	16.36	-165.98	16.24	9	7/14/2014 40:58.7
5664	RSPe_2	-122.445	37.936	0.02	16.36	-162.53	16.38	-122.445	37.936	-0.11	16.36	-165.89	16.24	9	7/14/2014 40:58.6
5665	RSPe_2	-122.445	37.936	0.07	16.37	-162.42	16.45	-122.445	37.936	-0.08	16.37	-165.76	16.29	9	7/14/2014 40:58.5
5666	RSPe_2	-122.445	37.936	0.02	16.37	-162.27	16.40	-122.445	37.936	-0.11	16.37	-165.76	16.26	9	7/14/2014 40:58.4
5667	RSPe_2	-122.445	37.936	0.07	16.41	-162.20	16.48	-122.445	37.936	-0.16	16.41	-165.62	16.24	9	7/14/2014 40:58.3
5668	RSPe_2	-122.445	37.936	0.02	16.42	-162.09	16.44	-122.445	37.936	-0.11	16.42	-165.60	16.31	9	7/14/2014 40:58.2
5669	RSPe_2	-122.445	37.936	0.02	16.44	-162.07	16.47	-122.445	37.936	-0.11	16.44	-165.55	16.33	9	7/14/2014 40:58.1
5670	RSPe_2	-122.445	37.936	0.07	16.30	-162.02	16.38	-122.445	37.936	-0.16	16.30	-165.47	16.14	9	7/14/2014 40:58.0
5671	RSPe_2	-122.445	37.936	0.02	16.25	-161.92	16.27	-122.445	37.936	-0.11	16.25	-165.32	16.14	9	7/14/2014 40:57.9
5672	RSPe_2	-122.445	37.936	0.07	16.17	-161.92	16.25	-122.445	37.936	-0.16	16.17	-165.27	16.01	9	7/14/2014 40:57.8
5673	RSPe_2	-122.445	37.936	0.07	16.16	-161.85	16.23	-122.445	37.936	-0.16	16.16	-165.15	15.99	9	7/14/2014 40:57.7
5674	RSPe_2	-122.445	37.936	0.02	16.20	-161.65	16.22	-122.445	37.936	-0.16	16.20	-164.99	16.03	9	7/14/2014 40:57.6
5675	RSPe_2	-122.445	37.936	0.07	16.16	-161.58	16.23	-122.445	37.936	-0.16	16.16	-164.87	15.99	9	7/14/2014 40:57.5

5676	RSPe_2	-122.445	37.936	0.07	16.13	-161.35	16.21	-122.445	37.936	-0.16	16.13	-164.73	15.97	9	7/14/2014	40:57.4
5677	RSPe_2	-122.445	37.936	0.07	16.16	-161.24	16.23	-122.445	37.936	-0.11	16.16	-164.60	16.05	9	7/14/2014	40:57.3
5678	RSPe_2	-122.445	37.936	0.02	16.16	-161.14	16.18	-122.445	37.936	-0.11	16.16	-164.52	16.05	9	7/14/2014	40:57.2
5679	RSPe_2	-122.445	37.936	0.02	16.14	-161.00	16.17	-122.445	37.936	-0.16	16.14	-164.45	15.98	9	7/14/2014	40:57.1
5680	RSPe_2	-122.445	37.936	0.07	16.06	-161.04	16.14	-122.445	37.936	-0.16	16.06	-164.39	15.90	9	7/14/2014	40:57.0
5681	RSPe_2	-122.445	37.936	0.07	16.13	-160.98	16.20	-122.445	37.936	-0.11	16.13	-164.38	16.01	9	7/14/2014	40:56.9
5682	RSPe_2	-122.445	37.936	0.07	16.06	-160.86	16.13	-122.445	37.936	-0.11	16.06	-164.33	15.94	9	7/14/2014	40:56.8
5683	RSPe_2	-122.445	37.936	0.07	16.00	-160.78	16.08	-122.445	37.936	-0.08	16.00	-164.26	15.92	9	7/14/2014	40:56.7
5684	RSPe_2	-122.445	37.936	0.02	15.94	-160.77	15.96	-122.445	37.936	-0.16	15.94	-164.33	15.78	9	7/14/2014	40:56.6
5685	RSPe_2	-122.445	37.936	0.07	15.93	-160.67	16.01	-122.445	37.936	-0.11	15.93	-164.22	15.82	9	7/14/2014	40:56.5
5686	RSPe_2	-122.445	37.936	0.02	15.89	-160.57	15.91	-122.445	37.936	-0.16	15.89	-164.09	15.72	9	7/14/2014	40:56.4
5687	RSPe_2	-122.445	37.936	0.02	15.83	-160.42	15.86	-122.445	37.936	-0.11	15.83	-163.96	15.72	9	7/14/2014	40:56.3
5688	RSPe_2	-122.445	37.936	0.02	15.83	-160.27	15.86	-122.445	37.936	-0.11	15.83	-163.76	15.72	9	7/14/2014	40:56.2
5689	RSPe_2	-122.445	37.936	0.07	15.85	-160.14	15.92	-122.445	37.936	-0.11	15.85	-163.57	15.74	9	7/14/2014	40:56.1
5690	RSPe_2	-122.445	37.936	0.02	15.84	-159.96	15.87	-122.445	37.936	-0.16	15.84	-163.35	15.68	9	7/14/2014	40:56.0
5691	RSPe_2	-122.445	37.936	0.02	15.84	-159.85	15.87	-122.445	37.936	-0.16	15.84	-163.26	15.68	9	7/14/2014	40:55.9
5692	RSPe_2	-122.445	37.936	0.02	15.84	-159.72	15.87	-122.445	37.936	-0.16	15.84	-163.12	15.68	9	7/14/2014	40:55.8
5693	RSPe_2	-122.445	37.936	0.02	15.92	-159.63	15.94	-122.445	37.936	-0.16	15.92	-163.00	15.75	9	7/14/2014	40:55.7
5694	RSPe_2	-122.445	37.936	0.02	15.82	-159.55	15.84	-122.445	37.936	-0.16	15.82	-162.93	15.65	9	7/14/2014	40:55.6
5695	RSPe_2	-122.445	37.936	0.07	15.84	-159.46	15.92	-122.445	37.936	-0.11	15.84	-162.88	15.73	9	7/14/2014	40:55.5
5696	RSPe_2	-122.445	37.936	0.02	15.82	-159.44	15.84	-122.445	37.936	-0.16	15.82	-162.81	15.65	9	7/14/2014	40:55.4
5697	RSPe_2	-122.445	37.936	0.02	15.80	-159.39	15.82	-122.445	37.936	-0.08	15.80	-162.71	15.72	9	7/14/2014	40:55.3
5698	RSPe_2	-122.445	37.936	0.02	15.81	-159.28	15.83	-122.445	37.936	-0.11	15.81	-162.62	15.70	9	7/14/2014	40:55.2
5699	RSPe_2	-122.445	37.936	0.07	15.80	-159.20	15.87	-122.445	37.936	-0.11	15.80	-162.45	15.68	9	7/14/2014	40:55.1
5700	RSPe_2	-122.445	37.936	0.02	15.82	-159.07	15.84	-122.445	37.936	-0.11	15.82	-162.39	15.70	9	7/14/2014	40:55.0
5701	RSPe_2	-122.445	37.936	0.07	15.82	-158.98	15.89	-122.445	37.936	-0.11	15.82	-162.29	15.70	9	7/14/2014	40:54.9
5702	RSPe_2	-122.445	37.936	0.02	15.86	-158.88	15.88	-122.445	37.936	-0.11	15.86	-162.20	15.74	9	7/14/2014	40:54.8
5703	RSPe_2	-122.445	37.936	0.07	15.85	-158.74	15.92	-122.445	37.936	-0.11	15.85	-162.01	15.74	9	7/14/2014	40:54.7
5704	RSPe_2	-122.445	37.936	0.07	15.85	-158.59	15.92	-122.445	37.936	-0.11	15.85	-161.89	15.74	9	7/14/2014	40:54.6
5705	RSPe_2	-122.445	37.936	0.07	15.87	-158.51	15.95	-122.445	37.936	-0.16	15.87	-161.72	15.71	9	7/14/2014	40:54.5
5706	RSPe_2	-122.445	37.936	0.02	15.89	-158.41	15.91	-122.445	37.936	-0.16	15.89	-161.68	15.72	9	7/14/2014	40:54.4
5707	RSPe_2	-122.445	37.936	0.07	15.91	-158.39	15.99	-122.445	37.936	-0.16	15.91	-161.63	15.75	9	7/14/2014	40:54.3
5708	RSPe_2	-122.445	37.936	0.07	15.93	-158.25	16.01	-122.445	37.936	-0.20	15.93	-161.49	15.74	9	7/14/2014	40:54.2

5709	RSPe_2	-122.445	37.936	0.07	15.95	-158.10	16.02	-122.445	37.936	-0.20	15.95	-161.33	15.75	9	7/14/2014	40:54.1
5710	RSPe_2	-122.445	37.936	0.07	16.09	-157.94	16.16	-122.445	37.936	-0.23	16.09	-161.27	15.86	9	7/14/2014	40:54.0
5711	RSPe_2	-122.445	37.936	0.08	16.16	-157.87	16.23	-122.445	37.936	-0.16	16.16	-161.26	16.00	9	7/14/2014	40:53.9
5712	RSPe_2	-122.445	37.936	0.08	16.14	-157.66	16.22	-122.445	37.936	-0.20	16.14	-161.14	15.95	9	7/14/2014	40:53.8
5713	RSPe_2	-122.445	37.936	0.08	16.14	-157.42	16.22	-122.445	37.936	-0.16	16.14	-160.97	15.98	9	7/14/2014	40:53.7
5714	RSPe_2	-122.445	37.936	0.08	16.13	-157.25	16.21	-122.445	37.936	-0.16	16.13	-160.85	15.97	9	7/14/2014	40:53.6
5715	RSPe_2	-122.445	37.936	0.11	16.15	-157.07	16.26	-122.445	37.936	-0.11	16.15	-160.72	16.04	9	7/14/2014	40:53.5
5716	RSPe_2	-122.445	37.936	0.08	16.19	-156.96	16.27	-122.445	37.936	-0.16	16.19	-160.52	16.03	9	7/14/2014	40:53.4
5717	RSPe_2	-122.445	37.936	0.11	16.17	-156.83	16.28	-122.445	37.936	-0.16	16.17	-160.38	16.00	9	7/14/2014	40:53.3
5718	RSPe_2	-122.445	37.936	0.08	16.21	-156.70	16.29	-122.445	37.936	-0.16	16.21	-160.18	16.05	9	7/14/2014	40:53.2
5719	RSPe_2	-122.445	37.936	0.11	16.17	-156.55	16.28	-122.445	37.936	-0.16	16.17	-160.00	16.00	9	7/14/2014	40:53.1
5720	RSPe_2	-122.445	37.936	0.08	16.23	-156.40	16.30	-122.445	37.936	-0.16	16.23	-159.86	16.07	9	7/14/2014	40:53.0
5721	RSPe_2	-122.445	37.936	0.11	16.20	-156.20	16.31	-122.445	37.936	-0.16	16.20	-159.72	16.04	9	7/14/2014	40:52.9
5722	RSPe_2	-122.445	37.936	0.08	16.23	-155.97	16.30	-122.445	37.936	-0.11	16.23	-159.53	16.12	9	7/14/2014	40:52.8
5723	RSPe_2	-122.445	37.936	0.11	16.21	-155.72	16.32	-122.445	37.936	-0.11	16.21	-159.34	16.10	9	7/14/2014	40:52.7
5724	RSPe_2	-122.445	37.936	0.11	16.20	-155.52	16.31	-122.445	37.936	-0.16	16.20	-159.16	16.03	9	7/14/2014	40:52.6
5725	RSPe_2	-122.445	37.936	0.11	16.22	-155.29	16.33	-122.445	37.936	-0.08	16.22	-158.92	16.14	9	7/14/2014	40:52.5
5726	RSPe_2	-122.445	37.936	0.11	16.21	-155.13	16.32	-122.445	37.936	-0.03	16.21	-158.68	16.18	9	7/14/2014	40:52.4
5727	RSPe_2	-122.445	37.936	0.11	16.21	-154.86	16.32	-122.445	37.936	-0.08	16.21	-158.41	16.13	9	7/14/2014	40:52.3
5728	RSPe_2	-122.445	37.936	0.11	16.21	-154.64	16.32	-122.445	37.936	-0.11	16.21	-158.18	16.10	9	7/14/2014	40:52.2
5729	RSPe_2	-122.445	37.936	0.11	16.23	-154.45	16.34	-122.445	37.936	-0.08	16.23	-157.97	16.15	9	7/14/2014	40:52.1
5730	RSPe_2	-122.445	37.936	0.11	16.21	-154.33	16.32	-122.445	37.936	-0.08	16.21	-157.85	16.13	9	7/14/2014	40:52.0
5731	RSPe_2	-122.445	37.936	0.11	16.20	-154.18	16.31	-122.445	37.936	-0.08	16.20	-157.72	16.12	9	7/14/2014	40:51.9
5732	RSPe_2	-122.445	37.936	0.11	16.18	-154.14	16.29	-122.445	37.936	-0.03	16.18	-157.58	16.15	9	7/14/2014	40:51.8
5733	RSPe_2	-122.445	37.936	0.11	16.16	-154.01	16.27	-122.445	37.936	-0.08	16.16	-157.46	16.08	9	7/14/2014	40:51.7
5734	RSPe_2	-122.445	37.936	0.11	16.17	-153.85	16.28	-122.445	37.936	-0.11	16.17	-157.28	16.06	9	7/14/2014	40:51.6
5735	RSPe_2	-122.445	37.936	0.16	16.18	-153.68	16.34	-122.445	37.936	-0.03	16.18	-157.13	16.15	9	7/14/2014	40:51.5
5736	RSPe_2	-122.445	37.936	0.11	16.17	-153.44	16.28	-122.445	37.936	-0.03	16.17	-156.94	16.15	9	7/14/2014	40:51.4
5737	RSPe_2	-122.445	37.936	0.16	16.23	-153.18	16.39	-122.445	37.936	-0.08	16.23	-156.64	16.15	9	7/14/2014	40:51.3
5738	RSPe_2	-122.445	37.936	0.16	16.21	-152.91	16.37	-122.445	37.936	-0.03	16.21	-156.40	16.18	9	7/14/2014	40:51.2
5739	RSPe_2	-122.445	37.936	0.16	16.30	-152.68	16.46	-122.445	37.936	-0.03	16.30	-156.17	16.27	9	7/14/2014	40:51.1
5740	RSPe_2	-122.445	37.936	0.16	16.24	-152.48	16.40	-122.445	37.936	0.09	16.24	-155.97	16.33	9	7/14/2014	40:51.0
5741	RSPe_2	-122.445	37.936	0.16	16.23	-152.26	16.39	-122.445	37.936	0.06	16.23	-155.77	16.29	9	7/14/2014	40:50.9

5742	RSPe_2	-122.445	37.936	0.16	16.26	-152.01	16.42	-122.445	37.936	0.01	16.26	-155.54	16.27	9	7/14/2014	40:50.8
5743	RSPe_2	-122.445	37.936	0.19	16.23	-151.76	16.43	-122.445	37.936	0.09	16.23	-155.29	16.32	9	7/14/2014	40:50.7
5744	RSPe_2	-122.445	37.936	0.19	16.30	-151.62	16.49	-122.445	37.936	0.06	16.30	-155.12	16.35	9	7/14/2014	40:50.6
5745	RSPe_2	-122.445	37.936	0.19	16.22	-151.45	16.41	-122.445	37.936	0.01	16.22	-154.93	16.23	9	7/14/2014	40:50.5
5746	RSPe_2	-122.445	37.936	0.19	16.22	-151.29	16.41	-122.445	37.936	0.01	16.22	-154.80	16.23	9	7/14/2014	40:50.4
5747	RSPe_2	-122.445	37.936	0.19	16.17	-151.10	16.37	-122.445	37.936	0.01	16.17	-154.66	16.18	9	7/14/2014	40:50.3
5748	RSPe_2	-122.445	37.936	0.16	16.15	-150.87	16.31	-122.445	37.936	0.06	16.15	-154.43	16.21	9	7/14/2014	40:50.2
5749	RSPe_2	-122.445	37.936	0.19	16.05	-150.73	16.25	-122.445	37.936	0.06	16.05	-154.30	16.11	9	7/14/2014	40:50.1
5750	RSPe_2	-122.445	37.936	0.19	16.03	-150.51	16.23	-122.445	37.936	0.06	16.03	-154.10	16.09	9	7/14/2014	40:50.0
5751	RSPe_2	-122.445	37.936	0.19	15.97	-150.18	16.17	-122.445	37.936	0.09	15.97	-153.83	16.06	9	7/14/2014	40:49.9
5752	RSPe_2	-122.445	37.936	0.19	15.92	-149.92	16.11	-122.445	37.936	0.09	15.92	-153.52	16.01	9	7/14/2014	40:49.8
5753	RSPe_2	-122.445	37.936	0.23	15.92	-149.65	16.15	-122.445	37.936	0.14	15.92	-153.23	16.06	9	7/14/2014	40:49.7
5754	RSPe_2	-122.445	37.936	0.19	15.84	-149.33	16.04	-122.445	37.936	0.14	15.84	-152.90	15.98	9	7/14/2014	40:49.6
5755	RSPe_2	-122.445	37.936	0.23	15.80	-149.06	16.03	-122.445	37.936	0.14	15.80	-152.60	15.94	9	7/14/2014	40:49.5
5756	RSPe_2	-122.445	37.936	0.23	15.80	-148.82	16.03	-122.445	37.936	0.18	15.80	-152.31	15.98	9	7/14/2014	40:49.4
5757	RSPe_2	-122.445	37.936	0.23	15.72	-148.54	15.95	-122.445	37.936	0.14	15.72	-152.06	15.86	9	7/14/2014	40:49.3
5758	RSPe_2	-122.445	37.936	0.23	15.73	-148.42	15.95	-122.445	37.936	0.09	15.73	-151.82	15.82	9	7/14/2014	40:49.2
5759	RSPe_2	-122.445	37.936	0.23	15.73	-148.17	15.96	-122.445	37.936	0.14	15.73	-151.56	15.88	9	7/14/2014	40:49.1
5760	RSPe_2	-122.445	37.936	0.19	15.68	-147.91	15.87	-122.445	37.936	0.09	15.68	-151.33	15.77	9	7/14/2014	40:49.0
5761	RSPe_2	-122.445	37.936	0.23	15.70	-147.65	15.93	-122.445	37.936	0.21	15.70	-151.02	15.91	9	7/14/2014	40:48.9
5762	RSPe_2	-122.445	37.936	0.19	15.63	-147.38	15.83	-122.445	37.936	0.09	15.63	-150.81	15.72	9	7/14/2014	40:48.8
5763	RSPe_2	-122.445	37.936	0.19	15.62	-147.14	15.81	-122.445	37.936	0.14	15.62	-150.62	15.76	9	7/14/2014	40:48.7
5764	RSPe_2	-122.445	37.936	0.19	15.63	-146.85	15.83	-122.445	37.936	0.09	15.63	-150.41	15.72	9	7/14/2014	40:48.6
5765	RSPe_2	-122.445	37.936	0.23	15.64	-146.56	15.87	-122.445	37.936	0.09	15.64	-150.11	15.73	9	7/14/2014	40:48.5
5766	RSPe_2	-122.445	37.936	0.19	15.56	-146.29	15.76	-122.445	37.936	0.09	15.56	-149.86	15.66	9	7/14/2014	40:48.4
5767	RSPe_2	-122.445	37.936	0.23	15.56	-146.06	15.78	-122.445	37.936	0.18	15.56	-149.63	15.73	9	7/14/2014	40:48.3
5768	RSPe_2	-122.445	37.936	0.19	15.52	-145.80	15.71	-122.445	37.936	0.18	15.52	-149.32	15.69	9	7/14/2014	40:48.2
5769	RSPe_2	-122.445	37.936	0.19	15.55	-145.52	15.74	-122.445	37.936	0.14	15.55	-149.05	15.69	9	7/14/2014	40:48.1
5770	RSPe_2	-122.445	37.936	0.23	15.55	-145.20	15.78	-122.445	37.936	0.14	15.55	-148.71	15.69	9	7/14/2014	40:48.0
5771	RSPe_2	-122.445	37.936	0.23	15.53	-145.04	15.76	-122.445	37.936	0.14	15.53	-148.53	15.67	9	7/14/2014	40:47.9
5772	RSPe_2	-122.445	37.936	0.19	15.50	-144.87	15.70	-122.445	37.936	0.14	15.50	-148.37	15.64	9	7/14/2014	40:47.8
5773	RSPe_2	-122.445	37.936	0.23	15.66	-144.63	15.89	-122.445	37.936	0.18	15.66	-148.12	15.84	9	7/14/2014	40:47.7
5774	RSPe_2	-122.445	37.936	0.19	15.60	-144.43	15.79	-122.445	37.936	0.14	15.60	-147.86	15.74	9	7/14/2014	40:47.6

5775	RSPe_2	-122.445	37.936	0.23	15.63	-144.17	15.86	-122.445	37.936	0.14	15.63	-147.58	15.78	9	7/14/2014 40:47.5
5776	RSPe_2	-122.445	37.936	0.19	15.70	-143.86	15.90	-122.445	37.936	0.14	15.70	-147.27	15.85	9	7/14/2014 40:47.4
5777	RSPe_2	-122.445	37.936	0.23	15.77	-143.60	16.00	-122.445	37.936	0.09	15.77	-146.98	15.86	9	7/14/2014 40:47.3
5778	RSPe_2	-122.445	37.936	0.19	15.78	-143.29	15.97	-122.445	37.936	0.18	15.78	-146.72	15.96	9	7/14/2014 40:47.2
5779	RSPe_2	-122.445	37.936	0.23	15.87	-142.95	16.10	-122.445	37.936	0.18	15.87	-146.39	16.05	9	7/14/2014 40:47.1
5780	RSPe_2	-122.445	37.936	0.23	15.81	-142.62	16.04	-122.445	37.936	0.14	15.81	-146.09	15.95	9	7/14/2014 40:47.0
5781	RSPe_2	-122.445	37.936	0.23	15.81	-142.31	16.04	-122.445	37.936	0.14	15.81	-145.87	15.95	9	7/14/2014 40:46.9
5782	RSPe_2	-122.445	37.936	0.23	15.82	-142.05	16.05	-122.445	37.936	0.09	15.82	-145.61	15.91	9	7/14/2014 40:46.8
5783	RSPe_2	-122.445	37.936	0.23	15.78	-141.76	16.01	-122.445	37.936	0.14	15.78	-145.31	15.92	9	7/14/2014 40:46.7
5784	RSPe_2	-122.445	37.936	0.23	15.79	-141.46	16.02	-122.445	37.936	0.14	15.79	-144.99	15.93	9	7/14/2014 40:46.6
5785	RSPe_2	-122.445	37.936	0.23	15.73	-141.15	15.96	-122.445	37.936	0.18	15.73	-144.68	15.91	9	7/14/2014 40:46.5
5786	RSPe_2	-122.445	37.936	0.19	15.76	-140.99	15.95	-122.445	37.936	0.14	15.76	-144.43	15.90	9	7/14/2014 40:46.4
5787	RSPe_2	-122.445	37.936	0.23	15.78	-140.74	16.01	-122.445	37.936	0.18	15.78	-144.09	15.96	9	7/14/2014 40:46.3
5788	RSPe_2	-122.445	37.936	0.23	15.75	-140.52	15.98	-122.445	37.936	0.18	15.75	-143.82	15.92	9	7/14/2014 40:46.2
5789	RSPe_2	-122.445	37.936	0.23	15.79	-140.21	16.02	-122.445	37.936	0.14	15.79	-143.42	15.93	9	7/14/2014 40:46.1
5790	RSPe_2	-122.445	37.936	0.23	15.86	-139.96	16.09	-122.445	37.936	0.18	15.86	-143.14	16.04	9	7/14/2014 40:46.0
5791	RSPe_2	-122.445	37.936	0.23	15.80	-139.73	16.03	-122.445	37.936	0.18	15.80	-142.82	15.98	9	7/14/2014 40:45.9
5792	RSPe_2	-122.445	37.936	0.23	15.86	-139.42	16.09	-122.445	37.936	0.18	15.86	-142.54	16.03	9	7/14/2014 40:45.8
5793	RSPe_2	-122.445	37.936	0.28	15.90	-139.23	16.18	-122.445	37.936	0.18	15.90	-142.30	16.07	9	7/14/2014 40:45.7
5794	RSPe_2	-122.445	37.936	0.23	15.98	-138.94	16.21	-122.445	37.936	0.18	15.98	-142.07	16.16	9	7/14/2014 40:45.6
5795	RSPe_2	-122.445	37.936	0.28	15.93	-138.73	16.22	-122.445	37.936	0.21	15.93	-141.89	16.14	9	7/14/2014 40:45.5
5796	RSPe_2	-122.445	37.936	0.23	15.96	-138.51	16.19	-122.445	37.936	0.14	15.96	-141.69	16.10	9	7/14/2014 40:45.4
5797	RSPe_2	-122.445	37.936	0.23	15.96	-138.20	16.19	-122.445	37.936	0.21	15.96	-141.48	16.17	9	7/14/2014 40:45.3
5798	RSPe_2	-122.445	37.936	0.23	15.94	-137.96	16.17	-122.445	37.936	0.14	15.94	-141.24	16.08	9	7/14/2014 40:45.2
5799	RSPe_2	-122.445	37.936	0.23	15.94	-137.68	16.17	-122.445	37.936	0.18	15.94	-140.94	16.12	9	7/14/2014 40:45.1
5800	RSPe_2	-122.445	37.936	0.23	15.94	-137.42	16.17	-122.445	37.936	0.18	15.94	-140.66	16.12	9	7/14/2014 40:45.0
5801	RSPe_2	-122.445	37.936	0.28	15.95	-137.13	16.23	-122.445	37.936	0.18	15.95	-140.36	16.13	9	7/14/2014 40:44.9
5802	RSPe_2	-122.445	37.936	0.23	15.96	-136.93	16.19	-122.445	37.936	0.18	15.96	-140.13	16.13	9	7/14/2014 40:44.8
5803	RSPe_2	-122.445	37.936	0.28	16.01	-136.63	16.29	-122.445	37.936	0.21	16.01	-139.82	16.22	9	7/14/2014 40:44.7
5804	RSPe_2	-122.445	37.936	0.23	16.00	-136.30	16.23	-122.445	37.936	0.18	16.00	-139.48	16.18	9	7/14/2014 40:44.6
5805	RSPe_2	-122.445	37.936	0.23	16.03	-136.02	16.26	-122.445	37.936	0.18	16.03	-139.20	16.21	9	7/14/2014 40:44.5
5806	RSPe_2	-122.445	37.936	0.28	16.03	-135.73	16.31	-122.445	37.936	0.21	16.03	-138.85	16.24	9	7/14/2014 40:44.4
5807	RSPe_2	-122.445	37.936	0.28	16.04	-135.45	16.32	-122.445	37.936	0.21	16.04	-138.56	16.25	9	7/14/2014 40:44.3

5808	RSPe_2	-122.445	37.936	0.23	16.07	-135.14	16.30	-122.445	37.936	0.21	16.07	-138.27	16.28	9	7/14/2014	40:44.2
5809	RSPe_2	-122.445	37.936	0.28	16.11	-134.90	16.39	-122.445	37.936	0.21	16.11	-138.02	16.32	9	7/14/2014	40:44.1
5810	RSPe_2	-122.445	37.936	0.28	16.08	-134.63	16.36	-122.445	37.936	0.18	16.08	-137.78	16.26	9	7/14/2014	40:44.0
5811	RSPe_2	-122.445	37.936	0.28	16.06	-134.31	16.34	-122.445	37.936	0.18	16.06	-137.54	16.24	9	7/14/2014	40:43.9
5812	RSPe_2	-122.445	37.936	0.23	16.03	-134.03	16.26	-122.445	37.936	0.18	16.03	-137.28	16.21	9	7/14/2014	40:43.8
5813	RSPe_2	-122.445	37.936	0.23	16.00	-133.75	16.23	-122.445	37.936	0.18	16.00	-137.02	16.18	9	7/14/2014	40:43.7
5814	RSPe_2	-122.445	37.936	0.23	15.95	-133.41	16.18	-122.445	37.936	0.18	15.95	-136.69	16.13	9	7/14/2014	40:43.6
5815	RSPe_2	-122.445	37.936	0.28	15.94	-133.11	16.22	-122.445	37.936	0.21	15.94	-136.43	16.15	9	7/14/2014	40:43.5
5816	RSPe_2	-122.445	37.936	0.23	15.90	-132.81	16.12	-122.445	37.936	0.18	15.90	-136.06	16.07	9	7/14/2014	40:43.4
5817	RSPe_2	-122.445	37.936	0.28	15.85	-132.54	16.13	-122.445	37.936	0.21	15.85	-135.71	16.06	9	7/14/2014	40:43.3
5818	RSPe_2	-122.445	37.936	0.23	15.84	-132.35	16.07	-122.445	37.936	0.18	15.84	-135.47	16.02	9	7/14/2014	40:43.2
5819	RSPe_2	-122.445	37.936	0.28	15.80	-132.10	16.08	-122.445	37.936	0.18	15.80	-135.11	15.97	9	7/14/2014	40:43.1
5820	RSPe_2	-122.445	37.936	0.23	15.79	-131.78	16.02	-122.445	37.936	0.18	15.79	-134.75	15.96	9	7/14/2014	40:43.0
5821	RSPe_2	-122.445	37.936	0.28	15.80	-131.45	16.08	-122.445	37.936	0.18	15.80	-134.37	15.97	9	7/14/2014	40:42.9
5822	RSPe_2	-122.445	37.936	0.28	15.80	-131.10	16.08	-122.445	37.936	0.18	15.80	-134.01	15.98	9	7/14/2014	40:42.8
5823	RSPe_2	-122.445	37.936	0.28	15.75	-130.77	16.03	-122.445	37.936	0.26	15.75	-133.75	16.01	9	7/14/2014	40:42.7
5824	RSPe_2	-122.445	37.936	0.28	15.75	-130.49	16.03	-122.445	37.936	0.21	15.75	-133.53	15.96	9	7/14/2014	40:42.6
5825	RSPe_2	-122.445	37.936	0.28	15.71	-130.20	15.99	-122.445	37.936	0.21	15.71	-133.28	15.92	9	7/14/2014	40:42.5
5826	RSPe_2	-122.445	37.936	0.28	15.68	-129.82	15.96	-122.445	37.936	0.21	15.68	-132.95	15.89	9	7/14/2014	40:42.4
5827	RSPe_2	-122.445	37.936	0.28	15.69	-129.46	15.97	-122.445	37.936	0.26	15.69	-132.68	15.95	9	7/14/2014	40:42.3
5828	RSPe_2	-122.445	37.936	0.23	15.70	-129.21	15.93	-122.445	37.936	0.21	15.70	-132.43	15.91	9	7/14/2014	40:42.2
5829	RSPe_2	-122.445	37.936	0.28	15.70	-128.93	15.98	-122.445	37.936	0.26	15.70	-132.12	15.96	9	7/14/2014	40:42.1
5830	RSPe_2	-122.445	37.936	0.28	15.55	-128.59	15.83	-122.445	37.936	0.26	15.55	-131.79	15.81	9	7/14/2014	40:42.0
5831	RSPe_2	-122.445	37.936	0.28	15.54	-128.28	15.82	-122.445	37.936	0.26	15.54	-131.36	15.80	9	7/14/2014	40:41.9
5832	RSPe_2	-122.445	37.936	0.28	15.52	-127.97	15.80	-122.445	37.936	0.26	15.52	-130.97	15.78	9	7/14/2014	40:41.8
5833	RSPe_2	-122.445	37.936	0.28	15.50	-127.56	15.78	-122.445	37.936	0.26	15.50	-130.55	15.76	9	7/14/2014	40:41.7
5834	RSPe_2	-122.445	37.936	0.28	15.49	-127.29	15.78	-122.445	37.936	0.26	15.49	-130.26	15.76	9	7/14/2014	40:41.6
5835	RSPe_2	-122.445	37.936	0.32	15.46	-126.95	15.77	-122.445	37.936	0.26	15.46	-129.89	15.72	9	7/14/2014	40:41.5
5836	RSPe_2	-122.445	37.936	0.28	15.44	-126.64	15.72	-122.445	37.936	0.21	15.44	-129.56	15.65	9	7/14/2014	40:41.4
5837	RSPe_2	-122.445	37.936	0.28	15.44	-126.24	15.72	-122.445	37.936	0.29	15.44	-129.19	15.74	9	7/14/2014	40:41.3
5838	RSPe_2	-122.445	37.936	0.28	15.42	-125.94	15.70	-122.445	37.936	0.29	15.42	-128.87	15.71	9	7/14/2014	40:41.2
5839	RSPe_2	-122.445	37.936	0.32	15.43	-125.59	15.74	-122.445	37.936	0.29	15.43	-128.50	15.72	9	7/14/2014	40:41.1
5840	RSPe_2	-122.445	37.936	0.28	15.39	-125.29	15.68	-122.445	37.936	0.29	15.39	-128.19	15.69	9	7/14/2014	40:41.0

5841	RSPe_2	-122.445	37.936	0.32	15.41	-124.93	15.73	-122.445	37.936	0.29	15.41	-127.80	15.70	9	7/14/2014	40:40.9
5842	RSPe_2	-122.445	37.936	0.28	15.44	-124.63	15.72	-122.445	37.936	0.29	15.44	-127.46	15.74	9	7/14/2014	40:40.8
5843	RSPe_2	-122.445	37.936	0.32	15.39	-124.26	15.71	-122.445	37.936	0.35	15.39	-127.07	15.74	9	7/14/2014	40:40.7
5844	RSPe_2	-122.445	37.936	0.32	15.39	-123.99	15.70	-122.445	37.936	0.35	15.39	-126.70	15.73	9	7/14/2014	40:40.6
5845	RSPe_2	-122.445	37.936	0.32	15.41	-123.67	15.73	-122.445	37.936	0.35	15.41	-126.33	15.76	9	7/14/2014	40:40.5
5846	RSPe_2	-122.445	37.936	0.32	15.36	-123.43	15.68	-122.445	37.936	0.29	15.36	-126.03	15.66	9	7/14/2014	40:40.4
5847	RSPe_2	-122.445	37.936	0.32	15.40	-123.15	15.72	-122.445	37.936	0.35	15.40	-125.73	15.75	9	7/14/2014	40:40.3
5848	RSPe_2	-122.445	37.936	0.32	15.40	-122.86	15.72	-122.445	37.936	0.35	15.40	-125.37	15.75	9	7/14/2014	40:40.2
5849	RSPe_2	-122.445	37.936	0.32	15.44	-122.58	15.76	-122.445	37.936	0.35	15.44	-125.12	15.79	9	7/14/2014	40:40.1
5850	RSPe_2	-122.445	37.936	0.28	15.46	-122.23	15.75	-122.445	37.936	0.29	15.46	-124.79	15.76	9	7/14/2014	40:40.0
5851	RSPe_2	-122.445	37.936	0.32	15.56	-121.86	15.88	-122.445	37.936	0.29	15.56	-124.47	15.86	9	7/14/2014	40:39.9
5852	RSPe_2	-122.445	37.936	0.28	15.55	-121.55	15.83	-122.445	37.936	0.35	15.55	-124.13	15.89	9	7/14/2014	40:39.8
5853	RSPe_2	-122.445	37.936	0.32	15.58	-121.20	15.90	-122.445	37.936	0.35	15.58	-123.79	15.93	9	7/14/2014	40:39.7
5854	RSPe_2	-122.445	37.936	0.32	15.58	-120.83	15.90	-122.445	37.936	0.35	15.58	-123.49	15.93	9	7/14/2014	40:39.6
5855	RSPe_2	-122.445	37.936	0.32	15.54	-120.52	15.85	-122.445	37.936	0.35	15.54	-123.18	15.88	9	7/14/2014	40:39.5
5856	RSPe_2	-122.445	37.936	0.32	15.55	-120.28	15.86	-122.445	37.936	0.38	15.55	-122.91	15.93	9	7/14/2014	40:39.4
5857	RSPe_2	-122.445	37.936	0.37	15.54	-119.94	15.91	-122.445	37.936	0.38	15.54	-122.60	15.92	9	7/14/2014	40:39.3
5858	RSPe_2	-122.445	37.936	0.32	15.56	-119.60	15.87	-122.445	37.936	0.35	15.56	-122.24	15.90	9	7/14/2014	40:39.2
5859	RSPe_2	-122.445	37.936	0.37	15.55	-119.28	15.92	-122.445	37.936	0.35	15.55	-121.92	15.89	9	7/14/2014	40:39.1
5860	RSPe_2	-122.445	37.936	0.32	15.56	-119.01	15.88	-122.445	37.936	0.35	15.56	-121.68	15.91	9	7/14/2014	40:39.0
5861	RSPe_2	-122.445	37.936	0.37	15.56	-118.84	15.93	-122.445	37.936	0.35	15.56	-121.50	15.91	9	7/14/2014	40:38.9
5862	RSPe_2	-122.445	37.936	0.32	15.59	-118.45	15.90	-122.445	37.936	0.38	15.59	-121.07	15.97	9	7/14/2014	40:38.8
5863	RSPe_2	-122.445	37.936	0.32	15.61	-118.12	15.93	-122.445	37.936	0.35	15.61	-120.71	15.96	9	7/14/2014	40:38.7
5864	RSPe_2	-122.445	37.936	0.32	15.59	-117.79	15.90	-122.445	37.936	0.29	15.59	-120.39	15.88	9	7/14/2014	40:38.6
5865	RSPe_2	-122.445	37.936	0.32	15.60	-117.51	15.91	-122.445	37.936	0.29	15.60	-120.08	15.89	9	7/14/2014	40:38.5
5866	RSPe_2	-122.445	37.936	0.32	15.61	-117.26	15.93	-122.445	37.936	0.35	15.61	-119.80	15.96	9	7/14/2014	40:38.4
5867	RSPe_2	-122.445	37.936	0.32	15.62	-116.94	15.94	-122.445	37.936	0.35	15.62	-119.51	15.97	9	7/14/2014	40:38.3
5868	RSPe_2	-122.445	37.936	0.32	15.60	-116.63	15.92	-122.445	37.936	0.35	15.60	-119.13	15.95	9	7/14/2014	40:38.2
5869	RSPe_2	-122.445	37.936	0.37	15.62	-116.33	15.99	-122.445	37.936	0.38	15.62	-118.88	16.00	9	7/14/2014	40:38.1
5870	RSPe_2	-122.445	37.936	0.32	15.68	-116.04	15.99	-122.445	37.936	0.35	15.68	-118.55	16.02	9	7/14/2014	40:38.0
5871	RSPe_2	-122.445	37.936	0.37	15.62	-115.72	15.99	-122.445	37.936	0.35	15.62	-118.16	15.96	9	7/14/2014	40:37.9
5872	RSPe_2	-122.445	37.936	0.32	15.66	-115.46	15.97	-122.445	37.936	0.38	15.66	-117.90	16.04	9	7/14/2014	40:37.8
5873	RSPe_2	-122.445	37.936	0.37	15.65	-115.19	16.02	-122.445	37.936	0.38	15.65	-117.59	16.03	9	7/14/2014	40:37.7

5874	RSPe_2	-122.445	37.936	0.32	15.67	-114.82	15.99	-122.445	37.936	0.35	15.67	-117.23	16.02	9	7/14/2014	40:37.6
5875	RSPe_2	-122.445	37.936	0.37	15.68	-114.51	16.05	-122.445	37.936	0.38	15.68	-116.91	16.06	9	7/14/2014	40:37.5
5876	RSPe_2	-122.445	37.936	0.32	15.75	-114.23	16.06	-122.445	37.936	0.35	15.75	-116.63	16.09	9	7/14/2014	40:37.4
5877	RSPe_2	-122.445	37.936	0.37	15.78	-113.86	16.15	-122.445	37.936	0.35	15.78	-116.32	16.13	9	7/14/2014	40:37.3
5878	RSPe_2	-122.445	37.936	0.32	15.73	-113.57	16.04	-122.445	37.936	0.29	15.73	-116.04	16.02	9	7/14/2014	40:37.2
5879	RSPe_2	-122.445	37.936	0.37	15.78	-113.22	16.15	-122.445	37.936	0.29	15.78	-115.74	16.07	9	7/14/2014	40:37.1
5880	RSPe_2	-122.445	37.936	0.32	15.85	-112.88	16.16	-122.445	37.936	0.35	15.85	-115.42	16.19	9	7/14/2014	40:37.0
5881	RSPe_2	-122.445	37.936	0.37	15.74	-112.54	16.11	-122.445	37.936	0.29	15.74	-115.05	16.04	9	7/14/2014	40:36.9
5882	RSPe_2	-122.445	37.936	0.32	15.77	-112.16	16.09	-122.445	37.936	0.29	15.77	-114.71	16.07	9	7/14/2014	40:36.8
5883	RSPe_2	-122.445	37.936	0.37	15.83	-111.84	16.19	-122.445	37.936	0.35	15.83	-114.36	16.17	9	7/14/2014	40:36.7
5884	RSPe_2	-122.445	37.936	0.32	15.79	-111.52	16.10	-122.445	37.936	0.29	15.79	-114.05	16.08	9	7/14/2014	40:36.6
5885	RSPe_2	-122.445	37.936	0.37	15.83	-111.17	16.20	-122.445	37.936	0.35	15.83	-113.69	16.18	9	7/14/2014	40:36.5
5886	RSPe_2	-122.445	37.936	0.37	15.82	-110.87	16.18	-122.445	37.936	0.35	15.82	-113.35	16.16	9	7/14/2014	40:36.4
5887	RSPe_2	-122.445	37.936	0.37	15.77	-110.51	16.14	-122.445	37.936	0.35	15.77	-113.02	16.12	9	7/14/2014	40:36.3
5888	RSPe_2	-122.445	37.936	0.32	15.78	-110.21	16.10	-122.445	37.936	0.29	15.78	-112.69	16.07	9	7/14/2014	40:36.2
5889	RSPe_2	-122.445	37.936	0.37	15.86	-109.90	16.22	-122.445	37.936	0.29	15.86	-112.42	16.15	9	7/14/2014	40:36.1
5890	RSPe_2	-122.445	37.936	0.32	15.80	-109.56	16.12	-122.445	37.936	0.29	15.80	-112.08	16.10	9	7/14/2014	40:36.0
5891	RSPe_2	-122.445	37.936	0.37	15.86	-109.22	16.23	-122.445	37.936	0.35	15.86	-111.71	16.21	9	7/14/2014	40:35.9
5892	RSPe_2	-122.445	37.936	0.32	15.87	-108.92	16.19	-122.445	37.936	0.29	15.87	-111.43	16.17	9	7/14/2014	40:35.8
5893	RSPe_2	-122.445	37.936	0.37	15.90	-108.57	16.26	-122.445	37.936	0.38	15.90	-111.09	16.28	9	7/14/2014	40:35.7
5894	RSPe_2	-122.445	37.936	0.32	15.91	-108.20	16.23	-122.445	37.936	0.35	15.91	-110.71	16.26	9	7/14/2014	40:35.6
5895	RSPe_2	-122.445	37.936	0.37	15.97	-107.83	16.33	-122.445	37.936	0.38	15.97	-110.31	16.35	9	7/14/2014	40:35.5
5896	RSPe_2	-122.445	37.936	0.32	15.99	-107.47	16.30	-122.445	37.936	0.29	15.99	-109.94	16.28	9	7/14/2014	40:35.4
5897	RSPe_2	-122.445	37.936	0.37	16.02	-107.09	16.39	-122.445	37.936	0.35	16.02	-109.50	16.36	9	7/14/2014	40:35.3
5898	RSPe_2	-122.445	37.936	0.32	16.05	-106.74	16.37	-122.445	37.936	0.29	16.05	-109.18	16.35	9	7/14/2014	40:35.2
5899	RSPe_2	-122.445	37.936	0.32	15.97	-106.38	16.29	-122.445	37.936	0.38	15.97	-108.78	16.35	9	7/14/2014	40:35.1
5900	RSPe_2	-122.445	37.936	0.32	15.95	-106.05	16.27	-122.445	37.936	0.35	15.95	-108.44	16.30	9	7/14/2014	40:35.0
5901	RSPe_2	-122.445	37.936	0.37	15.96	-105.71	16.32	-122.445	37.936	0.29	15.96	-108.15	16.25	9	7/14/2014	40:34.9
5902	RSPe_2	-122.445	37.936	0.32	15.92	-105.40	16.23	-122.445	37.936	0.43	15.92	-107.80	16.35	9	7/14/2014	40:34.8
5903	RSPe_2	-122.445	37.936	0.37	15.86	-105.10	16.23	-122.445	37.936	0.35	15.86	-107.45	16.21	9	7/14/2014	40:34.7
5904	RSPe_2	-122.445	37.936	0.32	15.81	-104.77	16.13	-122.445	37.936	0.29	15.81	-107.15	16.11	9	7/14/2014	40:34.6
5905	RSPe_2	-122.445	37.936	0.37	15.75	-104.50	16.12	-122.445	37.936	0.35	15.75	-106.84	16.09	9	7/14/2014	40:34.5
5906	RSPe_2	-122.445	37.936	0.32	15.73	-104.14	16.04	-122.445	37.936	0.35	15.73	-106.48	16.07	9	7/14/2014	40:34.4

5907	RSPe_2	-122.445	37.936	0.37	15.70	-103.83	16.07	-122.445	37.936	0.35	15.70	-106.12	16.05	9	7/14/2014	40:34.3
5908	RSPe_2	-122.445	37.936	0.32	15.66	-103.52	15.97	-122.445	37.936	0.35	15.66	-105.74	16.00	9	7/14/2014	40:34.2
5909	RSPe_2	-122.445	37.936	0.32	15.59	-103.21	15.90	-122.445	37.936	0.43	15.59	-105.40	16.02	9	7/14/2014	40:34.1
5910	RSPe_2	-122.445	37.936	0.32	15.59	-102.85	15.90	-122.445	37.936	0.38	15.59	-105.01	15.97	9	7/14/2014	40:34.0
5911	RSPe_2	-122.445	37.936	0.37	15.53	-102.49	15.89	-122.445	37.936	0.35	15.53	-104.66	15.87	9	7/14/2014	40:33.9
5912	RSPe_2	-122.445	37.936	0.32	15.51	-102.12	15.83	-122.445	37.936	0.35	15.51	-104.28	15.86	9	7/14/2014	40:33.8
5913	RSPe_2	-122.445	37.936	0.37	15.53	-101.81	15.89	-122.445	37.936	0.35	15.53	-103.96	15.87	9	7/14/2014	40:33.7
5914	RSPe_2	-122.445	37.936	0.32	15.42	-101.45	15.73	-122.445	37.936	0.29	15.42	-103.65	15.71	9	7/14/2014	40:33.6
5915	RSPe_2	-122.445	37.936	0.37	15.44	-101.12	15.81	-122.445	37.936	0.35	15.44	-103.32	15.79	9	7/14/2014	40:33.5
5916	RSPe_2	-122.445	37.936	0.32	15.39	-100.79	15.71	-122.445	37.936	0.35	15.39	-103.02	15.74	9	7/14/2014	40:33.4
5917	RSPe_2	-122.445	37.936	0.32	15.36	-100.46	15.67	-122.445	37.936	0.29	15.36	-102.69	15.65	9	7/14/2014	40:33.3
5918	RSPe_2	-122.445	37.936	0.32	15.29	-100.07	15.61	-122.445	37.936	0.26	15.29	-102.32	15.55	9	7/14/2014	40:33.2
5919	RSPe_2	-122.445	37.936	0.32	15.31	-99.76	15.62	-122.445	37.936	0.29	15.31	-101.99	15.60	9	7/14/2014	40:33.1
5920	RSPe_2	-122.445	37.936	0.32	15.23	-99.44	15.55	-122.445	37.936	0.29	15.23	-101.70	15.53	9	7/14/2014	40:33.0
5921	RSPe_2	-122.445	37.936	0.32	15.19	-99.17	15.51	-122.445	37.936	0.26	15.19	-101.36	15.46	9	7/14/2014	40:32.9
5922	RSPe_2	-122.445	37.936	0.32	15.19	-98.78	15.51	-122.445	37.936	0.35	15.19	-100.99	15.54	9	7/14/2014	40:32.8
5923	RSPe_2	-122.445	37.936	0.32	15.23	-98.43	15.55	-122.445	37.936	0.29	15.23	-100.59	15.53	9	7/14/2014	40:32.7
5924	RSPe_2	-122.445	37.936	0.28	15.22	-98.06	15.50	-122.445	37.936	0.29	15.22	-100.22	15.51	9	7/14/2014	40:32.6
5925	RSPe_2	-122.445	37.936	0.32	15.23	-97.73	15.55	-122.445	37.936	0.29	15.23	-99.82	15.53	9	7/14/2014	40:32.5
5926	RSPe_2	-122.445	37.936	0.28	15.23	-97.32	15.51	-122.445	37.936	0.29	15.23	-99.41	15.53	9	7/14/2014	40:32.4
5927	RSPe_2	-122.445	37.936	0.28	15.27	-96.99	15.55	-122.445	37.936	0.26	15.27	-99.06	15.53	9	7/14/2014	40:32.3
5928	RSPe_2	-122.445	37.936	0.28	15.25	-96.62	15.53	-122.445	37.936	0.26	15.25	-98.68	15.51	9	7/14/2014	40:32.2
5929	RSPe_2	-122.445	37.936	0.28	15.28	-96.25	15.56	-122.445	37.936	0.21	15.28	-98.30	15.49	9	7/14/2014	40:32.1
5930	RSPe_2	-122.445	37.936	0.28	15.28	-95.92	15.56	-122.445	37.936	0.26	15.28	-97.93	15.54	9	7/14/2014	40:32.0
5931	RSPe_2	-122.445	37.936	0.28	15.29	-95.52	15.58	-122.445	37.936	0.26	15.29	-97.53	15.55	9	7/14/2014	40:31.9
5932	RSPe_2	-122.445	37.936	0.28	15.29	-95.16	15.58	-122.445	37.936	0.26	15.29	-97.17	15.55	9	7/14/2014	40:31.8
5933	RSPe_2	-122.445	37.936	0.28	15.29	-94.76	15.57	-122.445	37.936	0.21	15.29	-96.77	15.50	9	7/14/2014	40:31.7
5934	RSPe_2	-122.445	37.936	0.23	15.49	-94.41	15.72	-122.445	37.936	0.21	15.49	-96.41	15.71	9	7/14/2014	40:31.6
5935	RSPe_2	-122.445	37.936	0.28	15.32	-94.04	15.61	-122.445	37.936	0.21	15.32	-96.07	15.54	9	7/14/2014	40:31.5
5936	RSPe_2	-122.445	37.936	0.23	15.52	-93.71	15.75	-122.445	37.936	0.26	15.52	-95.74	15.78	9	7/14/2014	40:31.4
5937	RSPe_2	-122.445	37.936	0.28	15.49	-93.34	15.78	-122.445	37.936	0.21	15.49	-95.39	15.71	9	7/14/2014	40:31.3
5938	RSPe_2	-122.445	37.936	0.23	15.55	-93.01	15.78	-122.445	37.936	0.18	15.55	-95.09	15.73	9	7/14/2014	40:31.2
5939	RSPe_2	-122.445	37.936	0.23	15.59	-92.67	15.82	-122.445	37.936	0.18	15.59	-94.69	15.76	9	7/14/2014	40:31.1

5940	RSPe_2	-122.445	37.936	0.23	15.60	-92.32	15.83	-122.445	37.936	0.14	15.60	-94.41	15.75	9	7/14/2014	40:31.0
5941	RSPe_2	-122.445	37.936	0.23	15.67	-92.03	15.90	-122.445	37.936	0.14	15.67	-94.13	15.81	9	7/14/2014	40:30.9
5942	RSPe_2	-122.445	37.936	0.23	15.71	-91.66	15.94	-122.445	37.936	0.14	15.71	-93.67	15.85	9	7/14/2014	40:30.8
5943	RSPe_2	-122.445	37.936	0.23	15.68	-91.22	15.91	-122.445	37.936	0.14	15.68	-93.29	15.82	9	7/14/2014	40:30.7
5944	RSPe_2	-122.445	37.936	0.20	15.74	-90.89	15.94	-122.445	37.936	0.14	15.74	-92.88	15.88	9	7/14/2014	40:30.6
5945	RSPe_2	-122.445	37.936	0.23	15.73	-90.45	15.96	-122.445	37.936	0.14	15.73	-92.47	15.88	9	7/14/2014	40:30.5
5946	RSPe_2	-122.445	37.936	0.20	15.74	-90.09	15.94	-122.445	37.936	0.06	15.74	-92.12	15.80	9	7/14/2014	40:30.4
5947	RSPe_2	-122.445	37.936	0.23	15.76	-89.76	15.99	-122.445	37.936	0.14	15.76	-91.76	15.91	9	7/14/2014	40:30.3
5948	RSPe_2	-122.445	37.936	0.20	15.77	-89.39	15.97	-122.445	37.936	0.09	15.77	-91.46	15.86	9	7/14/2014	40:30.2
5949	RSPe_2	-122.445	37.936	0.23	15.77	-89.06	16.00	-122.445	37.936	0.18	15.77	-91.11	15.95	9	7/14/2014	40:30.1
5950	RSPe_2	-122.445	37.936	0.23	15.76	-88.62	15.99	-122.445	37.936	0.14	15.76	-90.76	15.91	9	7/14/2014	40:30.0
5951	RSPe_2	-122.445	37.936	0.23	15.80	-88.31	16.03	-122.445	37.936	0.14	15.80	-90.45	15.94	9	7/14/2014	40:29.9
5952	RSPe_2	-122.445	37.936	0.20	15.83	-87.99	16.03	-122.445	37.936	0.09	15.83	-90.15	15.93	9	7/14/2014	40:29.8
5953	RSPe_2	-122.445	37.936	0.23	15.82	-87.68	16.05	-122.445	37.936	0.09	15.82	-89.80	15.91	9	7/14/2014	40:29.7
5954	RSPe_2	-122.445	37.936	0.20	15.83	-87.36	16.02	-122.445	37.936	0.09	15.83	-89.45	15.92	9	7/14/2014	40:29.6
5955	RSPe_2	-122.445	37.936	0.23	15.86	-87.03	16.10	-122.445	37.936	0.14	15.86	-89.06	16.01	9	7/14/2014	40:29.5
5956	RSPe_2	-122.445	37.936	0.16	15.87	-86.68	16.03	-122.445	37.936	0.06	15.87	-88.69	15.93	9	7/14/2014	40:29.4
5957	RSPe_2	-122.445	37.936	0.20	15.93	-86.35	16.13	-122.445	37.936	0.06	15.93	-88.23	15.99	9	7/14/2014	40:29.3
5958	RSPe_2	-122.445	37.936	0.20	15.97	-85.98	16.16	-122.445	37.936	0.06	15.97	-87.88	16.02	9	7/14/2014	40:29.2
5959	RSPe_2	-122.445	37.936	0.20	16.00	-85.60	16.19	-122.445	37.936	0.09	16.00	-87.48	16.09	9	7/14/2014	40:29.1
5960	RSPe_2	-122.445	37.936	0.20	16.05	-85.21	16.25	-122.445	37.936	0.06	16.05	-87.07	16.11	9	7/14/2014	40:29.0
5961	RSPe_2	-122.445	37.936	0.23	16.06	-84.86	16.29	-122.445	37.936	0.18	16.06	-86.76	16.24	9	7/14/2014	40:28.9
5962	RSPe_2	-122.445	37.936	0.20	16.06	-84.45	16.25	-122.445	37.936	0.09	16.06	-86.41	16.15	9	7/14/2014	40:28.8
5963	RSPe_2	-122.445	37.936	0.23	16.01	-84.05	16.24	-122.445	37.936	0.09	16.01	-86.02	16.10	9	7/14/2014	40:28.7
5964	RSPe_2	-122.445	37.936	0.20	16.06	-83.75	16.25	-122.445	37.936	0.09	16.06	-85.74	16.15	9	7/14/2014	40:28.6
5965	RSPe_2	-122.445	37.936	0.23	15.97	-83.38	16.20	-122.445	37.936	0.14	15.97	-85.37	16.12	9	7/14/2014	40:28.5
5966	RSPe_2	-122.445	37.936	0.20	15.98	-83.08	16.18	-122.445	37.936	0.09	15.98	-85.03	16.07	9	7/14/2014	40:28.4
5967	RSPe_2	-122.445	37.936	0.23	16.00	-82.79	16.23	-122.445	37.936	0.09	16.00	-84.67	16.10	9	7/14/2014	40:28.3
5968	RSPe_2	-122.445	37.936	0.20	15.96	-82.46	16.15	-122.445	37.936	0.14	15.96	-84.29	16.10	9	7/14/2014	40:28.2
5969	RSPe_2	-122.445	37.936	0.20	15.97	-82.15	16.17	-122.445	37.936	0.18	15.97	-83.88	16.15	9	7/14/2014	40:28.1
5970	RSPe_2	-122.445	37.936	0.20	16.02	-81.83	16.22	-122.445	37.936	0.14	16.02	-83.51	16.16	9	7/14/2014	40:28.0
5971	RSPe_2	-122.445	37.936	0.23	16.00	-81.48	16.23	-122.445	37.936	0.14	16.00	-83.13	16.15	9	7/14/2014	40:27.9
5972	RSPe_2	-122.445	37.936	0.20	16.02	-81.08	16.22	-122.445	37.936	0.09	16.02	-82.70	16.11	9	7/14/2014	40:27.8

5973	RSPe_2	-122.445	37.936	0.23	16.00	-80.74	16.23	-122.445	37.936	0.14	16.00	-82.33	16.15	9	7/14/2014 40:27.7
5974	RSPe_2	-122.445	37.936	0.23	15.98	-80.36	16.21	-122.445	37.936	0.09	15.98	-82.02	16.07	9	7/14/2014 40:27.6
5975	RSPe_2	-122.445	37.936	0.23	16.01	-79.97	16.24	-122.445	37.936	0.18	16.01	-81.63	16.19	9	7/14/2014 40:27.5
5976	RSPe_2	-122.445	37.936	0.20	15.94	-79.62	16.14	-122.445	37.936	0.09	15.94	-81.34	16.03	9	7/14/2014 40:27.4
5977	RSPe_2	-122.445	37.936	0.23	15.98	-79.31	16.21	-122.445	37.936	0.14	15.98	-81.03	16.12	9	7/14/2014 40:27.3
5978	RSPe_2	-122.445	37.936	0.23	15.91	-78.92	16.14	-122.445	37.936	0.18	15.91	-80.76	16.09	9	7/14/2014 40:27.2
5979	RSPe_2	-122.445	37.936	0.23	15.90	-78.57	16.13	-122.445	37.936	0.14	15.90	-80.36	16.04	9	7/14/2014 40:27.1
5980	RSPe_2	-122.445	37.936	0.23	15.88	-78.25	16.11	-122.445	37.936	0.09	15.88	-80.06	15.97	9	7/14/2014 40:27.0
5981	RSPe_2	-122.445	37.936	0.23	15.87	-77.85	16.10	-122.445	37.936	0.14	15.87	-79.71	16.02	9	7/14/2014 40:26.9
5982	RSPe_2	-122.445	37.936	0.23	15.85	-77.54	16.08	-122.445	37.936	0.09	15.85	-79.35	15.94	9	7/14/2014 40:26.8
5983	RSPe_2	-122.445	37.936	0.23	15.85	-77.18	16.08	-122.445	37.936	0.14	15.85	-78.99	15.99	9	7/14/2014 40:26.7
5984	RSPe_2	-122.445	37.936	0.20	15.81	-76.89	16.01	-122.445	37.936	0.14	15.81	-78.66	15.95	9	7/14/2014 40:26.6
5985	RSPe_2	-122.445	37.936	0.23	15.83	-76.58	16.06	-122.445	37.936	0.14	15.83	-78.29	15.98	9	7/14/2014 40:26.5
5986	RSPe_2	-122.445	37.936	0.20	15.80	-76.23	16.00	-122.445	37.936	0.09	15.80	-77.96	15.89	9	7/14/2014 40:26.4
5987	RSPe_2	-122.445	37.936	0.23	15.79	-75.86	16.02	-122.445	37.936	0.14	15.79	-77.65	15.93	9	7/14/2014 40:26.3
5988	RSPe_2	-122.445	37.936	0.23	15.76	-75.49	15.99	-122.445	37.936	0.09	15.76	-77.28	15.86	9	7/14/2014 40:26.2
5989	RSPe_2	-122.445	37.936	0.28	15.76	-75.12	16.05	-122.445	37.936	0.14	15.76	-76.93	15.91	9	7/14/2014 40:26.1
5990	RSPe_2	-122.445	37.936	0.23	15.76	-74.77	15.99	-122.445	37.936	0.14	15.76	-76.60	15.90	9	7/14/2014 40:26.0
5991	RSPe_2	-122.445	37.936	0.23	15.76	-74.46	15.99	-122.445	37.936	0.18	15.76	-76.32	15.94	9	7/14/2014 40:25.9
5992	RSPe_2	-122.445	37.936	0.23	15.76	-74.10	15.99	-122.445	37.936	0.14	15.76	-75.95	15.91	9	7/14/2014 40:25.8
5993	RSPe_2	-122.445	37.936	0.23	15.77	-73.81	16.00	-122.445	37.936	0.18	15.77	-75.67	15.95	9	7/14/2014 40:25.7
5994	RSPe_2	-122.445	37.936	0.20	15.73	-73.50	15.93	-122.445	37.936	0.14	15.73	-75.32	15.88	9	7/14/2014 40:25.6
5995	RSPe_2	-122.445	37.936	0.23	15.73	-73.17	15.97	-122.445	37.936	0.21	15.73	-74.98	15.95	9	7/14/2014 40:25.5
5996	RSPe_2	-122.445	37.936	0.23	15.74	-72.91	15.97	-122.445	37.936	0.18	15.74	-74.70	15.92	9	7/14/2014 40:25.4
5997	RSPe_2	-122.445	37.936	0.23	15.73	-72.74	15.97	-122.445	37.936	0.18	15.73	-74.44	15.91	9	7/14/2014 40:25.3
5998	RSPe_2	-122.445	37.936	0.23	15.74	-72.45	15.97	-122.445	37.936	0.18	15.74	-74.05	15.92	9	7/14/2014 40:25.2
5999	RSPe_2	-122.445	37.936	0.28	15.80	-72.13	16.08	-122.445	37.936	0.18	15.80	-73.68	15.97	9	7/14/2014 40:25.1
6000	RSPe_2	-122.445	37.936	0.20	15.78	-71.80	15.98	-122.445	37.936	0.18	15.78	-73.31	15.96	9	7/14/2014 40:25.0
6001	RSPe_2	-122.445	37.936	0.23	15.78	-71.45	16.01	-122.445	37.936	0.18	15.78	-72.87	15.96	9	7/14/2014 40:24.9
6002	RSPe_2	-122.445	37.936	0.23	15.78	-71.14	16.01	-122.445	37.936	0.14	15.78	-72.56	15.92	9	7/14/2014 40:24.8
6003	RSPe_2	-122.445	37.936	0.23	15.82	-70.92	16.05	-122.445	37.936	0.18	15.82	-72.32	16.00	9	7/14/2014 40:24.7
6004	RSPe_2	-122.445	37.936	0.23	15.83	-70.62	16.06	-122.445	37.936	0.14	15.83	-71.99	15.97	9	7/14/2014 40:24.6
6005	RSPe_2	-122.445	37.936	0.23	15.81	-70.27	16.04	-122.445	37.936	0.14	15.81	-71.80	15.95	9	7/14/2014 40:24.5

6006	RSPe_2	-122.445	37.936	0.23	15.83	-70.07	16.06	-122.445	37.936	0.14	15.83	-71.55	15.97	9	7/14/2014	40:24.4
6007	RSPe_2	-122.445	37.936	0.23	15.83	-69.72	16.06	-122.445	37.936	0.14	15.83	-71.27	15.97	9	7/14/2014	40:24.3
6008	RSPe_2	-122.445	37.936	0.23	15.84	-69.44	16.07	-122.445	37.936	0.14	15.84	-71.08	15.99	9	7/14/2014	40:24.2
6009	RSPe_2	-122.445	37.936	0.23	15.83	-69.18	16.06	-122.445	37.936	0.14	15.83	-70.83	15.98	9	7/14/2014	40:24.1
6010	RSPe_2	-122.445	37.936	0.23	15.84	-68.85	16.07	-122.445	37.936	0.14	15.84	-70.55	15.99	9	7/14/2014	40:24.0
6011	RSPe_2	-122.445	37.936	0.28	15.84	-68.52	16.13	-122.445	37.936	0.14	15.84	-70.24	15.99	9	7/14/2014	40:23.9
6012	RSPe_2	-122.445	37.936	0.23	15.86	-68.17	16.09	-122.445	37.936	0.09	15.86	-69.94	15.95	9	7/14/2014	40:23.8
6013	RSPe_2	-122.445	37.936	0.28	15.84	-67.82	16.13	-122.445	37.936	0.18	15.84	-69.59	16.02	9	7/14/2014	40:23.7
6014	RSPe_2	-122.445	37.936	0.28	15.89	-67.49	16.17	-122.445	37.936	0.18	15.89	-69.28	16.07	9	7/14/2014	40:23.6
6015	RSPe_2	-122.445	37.936	0.28	15.84	-67.12	16.13	-122.445	37.936	0.18	15.84	-68.98	16.02	9	7/14/2014	40:23.5
6016	RSPe_2	-122.445	37.936	0.28	15.87	-66.86	16.15	-122.445	37.936	0.18	15.87	-68.67	16.05	9	7/14/2014	40:23.4
6017	RSPe_2	-122.445	37.936	0.28	15.87	-66.57	16.15	-122.445	37.936	0.21	15.87	-68.43	16.08	9	7/14/2014	40:23.3
6018	RSPe_2	-122.445	37.936	0.28	15.91	-66.27	16.19	-122.445	37.936	0.21	15.91	-68.08	16.12	9	7/14/2014	40:23.2
6019	RSPe_2	-122.445	37.936	0.32	15.93	-65.92	16.25	-122.445	37.936	0.26	15.93	-67.73	16.20	9	7/14/2014	40:23.1
6020	RSPe_2	-122.445	37.936	0.28	16.01	-65.70	16.29	-122.445	37.936	0.21	16.01	-67.47	16.22	9	7/14/2014	40:23.0
6021	RSPe_2	-122.445	37.936	0.32	16.01	-65.40	16.33	-122.445	37.936	0.26	16.01	-67.18	16.27	9	7/14/2014	40:22.9
6022	RSPe_2	-122.445	37.936	0.28	16.04	-65.11	16.32	-122.445	37.936	0.21	16.04	-66.88	16.25	9	7/14/2014	40:22.8
6023	RSPe_2	-122.445	37.936	0.32	16.04	-64.89	16.36	-122.445	37.936	0.21	16.04	-66.62	16.25	9	7/14/2014	40:22.7
6024	RSPe_2	-122.445	37.936	0.28	16.06	-64.59	16.34	-122.445	37.936	0.21	16.06	-66.33	16.27	9	7/14/2014	40:22.6
6025	RSPe_2	-122.445	37.936	0.28	16.04	-64.30	16.32	-122.445	37.936	0.21	16.04	-66.11	16.25	9	7/14/2014	40:22.5
6026	RSPe_2	-122.445	37.936	0.28	16.03	-63.96	16.32	-122.445	37.936	0.21	16.03	-65.88	16.25	9	7/14/2014	40:22.4
6027	RSPe_2	-122.445	37.936	0.32	16.05	-63.69	16.37	-122.445	37.936	0.26	16.05	-65.59	16.31	9	7/14/2014	40:22.3
6028	RSPe_2	-122.445	37.936	0.23	16.06	-63.39	16.29	-122.445	37.936	0.21	16.06	-65.35	16.27	9	7/14/2014	40:22.2
6029	RSPe_2	-122.445	37.936	0.28	16.05	-63.08	16.33	-122.445	37.936	0.30	16.05	-65.04	16.35	9	7/14/2014	40:22.1
6030	RSPe_2	-122.445	37.936	0.28	16.04	-62.82	16.32	-122.445	37.936	0.26	16.04	-64.72	16.30	9	7/14/2014	40:22.0
6031	RSPe_2	-122.445	37.936	0.32	16.06	-62.42	16.38	-122.445	37.936	0.30	16.06	-64.21	16.36	9	7/14/2014	40:21.9
6032	RSPe_2	-122.445	37.936	0.28	16.06	-62.08	16.35	-122.445	37.936	0.26	16.06	-63.80	16.33	9	7/14/2014	40:21.8
6033	RSPe_2	-122.445	37.936	0.28	16.06	-61.77	16.35	-122.445	37.936	0.30	16.06	-63.47	16.36	9	7/14/2014	40:21.7
6034	RSPe_2	-122.445	37.936	0.28	16.09	-61.42	16.37	-122.445	37.936	0.30	16.09	-63.08	16.39	9	7/14/2014	40:21.6
6035	RSPe_2	-122.445	37.936	0.32	16.13	-61.12	16.44	-122.445	37.936	0.26	16.13	-62.73	16.39	9	7/14/2014	40:21.5
6036	RSPe_2	-122.445	37.936	0.28	16.16	-60.83	16.44	-122.445	37.936	0.21	16.16	-62.45	16.37	9	7/14/2014	40:21.4
6037	RSPe_2	-122.445	37.936	0.28	16.18	-60.64	16.46	-122.445	37.936	0.21	16.18	-62.25	16.39	9	7/14/2014	40:21.3
6038	RSPe_2	-122.445	37.936	0.28	16.20	-60.28	16.49	-122.445	37.936	0.21	16.20	-61.94	16.41	9	7/14/2014	40:21.2

6039	RSPe_2	-122.445	37.936	0.28	16.21	-59.98	16.49	-122.445	37.936	0.21	16.21	-61.72	16.42	9	7/14/2014	40:21.1
6040	RSPe_2	-122.445	37.936	0.23	16.22	-59.74	16.45	-122.445	37.936	0.21	16.22	-61.50	16.43	9	7/14/2014	40:21.0
6041	RSPe_2	-122.445	37.936	0.28	16.21	-59.43	16.49	-122.445	37.936	0.26	16.21	-61.26	16.47	9	7/14/2014	40:20.9
6042	RSPe_2	-122.445	37.936	0.23	16.24	-59.10	16.47	-122.445	37.936	0.21	16.24	-60.96	16.46	9	7/14/2014	40:20.8
6043	RSPe_2	-122.445	37.936	0.28	16.21	-58.76	16.49	-122.445	37.936	0.21	16.21	-60.63	16.42	9	7/14/2014	40:20.7
6044	RSPe_2	-122.445	37.936	0.23	16.21	-58.51	16.44	-122.445	37.936	0.18	16.21	-60.35	16.39	9	7/14/2014	40:20.6
6045	RSPe_2	-122.445	37.936	0.28	16.22	-58.14	16.50	-122.445	37.936	0.21	16.22	-59.98	16.43	9	7/14/2014	40:20.5
6046	RSPe_2	-122.445	37.936	0.23	16.24	-57.88	16.47	-122.445	37.936	0.26	16.24	-59.65	16.51	9	7/14/2014	40:20.4
6047	RSPe_2	-122.445	37.936	0.28	16.23	-57.55	16.51	-122.445	37.936	0.26	16.23	-59.27	16.49	9	7/14/2014	40:20.3
6048	RSPe_2	-122.445	37.936	0.23	16.24	-57.22	16.47	-122.445	37.936	0.21	16.24	-58.90	16.46	9	7/14/2014	40:20.2
6049	RSPe_2	-122.445	37.936	0.28	16.29	-56.96	16.57	-122.445	37.936	0.26	16.29	-58.57	16.55	9	7/14/2014	40:20.1
6050	RSPe_2	-122.445	37.936	0.28	16.29	-56.63	16.57	-122.445	37.936	0.21	16.29	-58.21	16.50	9	7/14/2014	40:20.0
6051	RSPe_2	-122.445	37.936	0.28	16.30	-56.37	16.58	-122.445	37.936	0.26	16.30	-57.90	16.56	9	7/14/2014	40:19.9
6052	RSPe_2	-122.445	37.936	0.23	16.22	-56.07	16.45	-122.445	37.936	0.21	16.22	-57.57	16.43	9	7/14/2014	40:19.8
6053	RSPe_2	-122.445	37.936	0.23	16.27	-55.78	16.50	-122.445	37.936	0.21	16.27	-57.31	16.48	9	7/14/2014	40:19.7
6054	RSPe_2	-122.445	37.936	0.23	16.24	-55.54	16.47	-122.445	37.936	0.21	16.24	-57.07	16.46	9	7/14/2014	40:19.6
6055	RSPe_2	-122.445	37.936	0.23	16.16	-55.30	16.39	-122.445	37.936	0.18	16.16	-56.83	16.34	9	7/14/2014	40:19.5
6056	RSPe_2	-122.445	37.936	0.23	16.14	-55.08	16.37	-122.445	37.936	0.18	16.14	-56.61	16.32	9	7/14/2014	40:19.4
6057	RSPe_2	-122.445	37.936	0.23	16.18	-54.86	16.41	-122.445	37.936	0.18	16.18	-56.39	16.36	9	7/14/2014	40:19.3
6058	RSPe_2	-122.445	37.936	0.20	16.09	-54.58	16.29	-122.445	37.936	0.18	16.09	-56.13	16.27	9	7/14/2014	40:19.2
6059	RSPe_2	-122.445	37.936	0.20	16.01	-54.25	16.21	-122.445	37.936	0.18	16.01	-55.87	16.19	9	7/14/2014	40:19.1
6060	RSPe_2	-122.445	37.936	0.20	15.98	-54.03	16.18	-122.445	37.936	0.18	15.98	-55.60	16.16	9	7/14/2014	40:19.0
6061	RSPe_2	-122.445	37.936	0.23	15.98	-53.75	16.21	-122.445	37.936	0.14	15.98	-55.34	16.12	9	7/14/2014	40:18.9
6062	RSPe_2	-122.445	37.936	0.20	15.97	-53.47	16.16	-122.445	37.936	0.14	15.97	-55.04	16.11	9	7/14/2014	40:18.8
6063	RSPe_2	-122.445	37.936	0.23	15.93	-53.16	16.17	-122.445	37.936	0.14	15.93	-54.71	16.08	9	7/14/2014	40:18.7
6064	RSPe_2	-122.445	37.936	0.23	15.87	-52.83	16.10	-122.445	37.936	0.14	15.87	-54.42	16.02	9	7/14/2014	40:18.6
6065	RSPe_2	-122.445	37.936	0.23	15.89	-52.55	16.12	-122.445	37.936	0.14	15.89	-54.11	16.03	9	7/14/2014	40:18.5
6066	RSPe_2	-122.445	37.936	0.20	15.86	-52.24	16.05	-122.445	37.936	0.14	15.86	-53.86	16.00	9	7/14/2014	40:18.4
6067	RSPe_2	-122.445	37.936	0.23	15.96	-51.94	16.19	-122.445	37.936	0.14	15.96	-53.59	16.10	9	7/14/2014	40:18.3
6068	RSPe_2	-122.445	37.936	0.20	15.86	-51.72	16.06	-122.445	37.936	0.09	15.86	-53.37	15.96	9	7/14/2014	40:18.2
6069	RSPe_2	-122.445	37.936	0.20	15.83	-51.54	16.02	-122.445	37.936	0.09	15.83	-53.16	15.92	9	7/14/2014	40:18.1
6070	RSPe_2	-122.445	37.936	0.20	15.90	-51.17	16.09	-122.445	37.936	0.09	15.90	-52.90	15.99	9	7/14/2014	40:18.0
6071	RSPe_2	-122.445	37.936	0.20	15.77	-51.00	15.97	-122.445	37.936	0.09	15.77	-52.61	15.87	9	7/14/2014	40:17.9

6072	RSPe_2	-122.445	37.936	0.20	15.73	-50.71	15.92	-122.445	37.936	0.09	15.73	-52.35	15.82	9	7/14/2014	40:17.8
6073	RSPe_2	-122.445	37.936	0.23	15.66	-50.47	15.90	-122.445	37.936	0.09	15.66	-52.06	15.76	9	7/14/2014	40:17.7
6074	RSPe_2	-122.445	37.936	0.20	15.64	-50.23	15.84	-122.445	37.936	0.06	15.64	-51.82	15.70	9	7/14/2014	40:17.6
6075	RSPe_2	-122.445	37.936	0.20	15.69	-49.92	15.89	-122.445	37.936	0.09	15.69	-51.45	15.78	9	7/14/2014	40:17.5
6076	RSPe_2	-122.445	37.936	0.20	15.69	-49.62	15.89	-122.445	37.936	0.06	15.69	-51.12	15.75	9	7/14/2014	40:17.4
6077	RSPe_2	-122.445	37.936	0.23	15.68	-49.25	15.91	-122.445	37.936	0.06	15.68	-50.82	15.74	9	7/14/2014	40:17.3
6078	RSPe_2	-122.445	37.936	0.20	15.66	-48.88	15.86	-122.445	37.936	0.01	15.66	-50.49	15.67	9	7/14/2014	40:17.2
6079	RSPe_2	-122.445	37.936	0.23	15.70	-48.53	15.94	-122.445	37.936	0.06	15.70	-50.14	15.76	9	7/14/2014	40:17.1
6080	RSPe_2	-122.445	37.936	0.20	15.70	-48.13	15.90	-122.445	37.936	0.06	15.70	-49.79	15.76	9	7/14/2014	40:17.0
6081	RSPe_2	-122.445	37.936	0.23	15.71	-47.76	15.94	-122.445	37.936	0.09	15.71	-49.44	15.80	9	7/14/2014	40:16.9
6082	RSPe_2	-122.445	37.936	0.20	15.67	-47.41	15.87	-122.445	37.936	0.06	15.67	-49.14	15.73	9	7/14/2014	40:16.8
6083	RSPe_2	-122.445	37.936	0.23	15.66	-47.13	15.90	-122.445	37.936	0.15	15.66	-48.87	15.81	9	7/14/2014	40:16.7
6084	RSPe_2	-122.445	37.936	0.20	15.60	-46.82	15.80	-122.445	37.936	0.09	15.60	-48.55	15.70	9	7/14/2014	40:16.6
6085	RSPe_2	-122.445	37.936	0.23	15.66	-46.52	15.90	-122.445	37.936	0.09	15.66	-48.26	15.76	9	7/14/2014	40:16.5
6086	RSPe_2	-122.445	37.936	0.20	15.59	-46.21	15.78	-122.445	37.936	0.09	15.59	-48.00	15.68	9	7/14/2014	40:16.4
6087	RSPe_2	-122.445	37.936	0.23	15.59	-45.88	15.82	-122.445	37.936	0.09	15.59	-47.67	15.68	9	7/14/2014	40:16.3
6088	RSPe_2	-122.445	37.936	0.20	15.59	-45.53	15.78	-122.445	37.936	0.06	15.59	-47.38	15.65	9	7/14/2014	40:16.2
6089	RSPe_2	-122.445	37.936	0.23	15.73	-45.25	15.97	-122.445	37.936	0.06	15.73	-47.04	15.79	9	7/14/2014	40:16.1
6090	RSPe_2	-122.445	37.936	0.20	15.72	-44.97	15.92	-122.445	37.936	0.06	15.72	-46.69	15.78	9	7/14/2014	40:16.0
6091	RSPe_2	-122.445	37.936	0.23	15.64	-44.61	15.87	-122.445	37.936	0.09	15.64	-46.32	15.73	9	7/14/2014	40:15.9
6092	RSPe_2	-122.445	37.936	0.20	15.73	-44.31	15.93	-122.445	37.936	0.06	15.73	-45.97	15.79	9	7/14/2014	40:15.8
6093	RSPe_2	-122.445	37.936	0.23	15.79	-43.96	16.02	-122.445	37.936	0.09	15.79	-45.62	15.88	9	7/14/2014	40:15.7
6094	RSPe_2	-122.445	37.936	0.20	15.78	-43.72	15.98	-122.445	37.936	0.09	15.78	-45.29	15.87	9	7/14/2014	40:15.6
6095	RSPe_2	-122.445	37.936	0.23	15.96	-43.35	16.19	-122.445	37.936	0.09	15.96	-44.99	16.05	9	7/14/2014	40:15.5
6096	RSPe_2	-122.445	37.936	0.20	15.81	-43.11	16.01	-122.445	37.936	0.09	15.81	-44.66	15.90	9	7/14/2014	40:15.4
6097	RSPe_2	-122.445	37.936	0.23	15.83	-42.80	16.06	-122.445	37.936	0.09	15.83	-44.39	15.92	9	7/14/2014	40:15.3
6098	RSPe_2	-122.445	37.936	0.20	15.83	-42.46	16.02	-122.445	37.936	0.09	15.83	-44.00	15.92	9	7/14/2014	40:15.2
6099	RSPe_2	-122.445	37.936	0.23	15.94	-42.17	16.17	-122.445	37.936	0.09	15.94	-43.63	16.03	9	7/14/2014	40:15.1
6100	RSPe_2	-122.445	37.936	0.20	15.87	-41.75	16.07	-122.445	37.936	0.09	15.87	-43.30	15.97	9	7/14/2014	40:15.0
6101	RSPe_2	-122.445	37.936	0.23	15.90	-41.49	16.13	-122.445	37.936	0.09	15.90	-43.00	16.00	9	7/14/2014	40:14.9
6102	RSPe_2	-122.445	37.936	0.20	15.90	-41.21	16.10	-122.445	37.936	0.06	15.90	-42.67	15.96	9	7/14/2014	40:14.8
6103	RSPe_2	-122.445	37.936	0.23	15.89	-40.94	16.12	-122.445	37.936	0.15	15.89	-42.38	16.03	9	7/14/2014	40:14.7
6104	RSPe_2	-122.445	37.936	0.23	15.97	-40.53	16.20	-122.445	37.936	0.06	15.97	-42.04	16.03	9	7/14/2014	40:14.6

6105	RSPe_2	-122.445	37.936	0.23	15.90	-40.24	16.13	-122.445	37.936	0.09	15.90	-41.79	16.00	9	7/14/2014 40:14.5
6106	RSPe_2	-122.445	37.936	0.23	16.00	-39.96	16.23	-122.445	37.936	0.09	16.00	-41.49	16.09	9	7/14/2014 40:14.4
6107	RSPe_2	-122.445	37.936	0.28	15.95	-39.61	16.23	-122.445	37.936	0.09	15.95	-41.25	16.04	9	7/14/2014 40:14.3
6108	RSPe_2	-122.445	37.936	0.23	15.94	-39.26	16.17	-122.445	37.936	0.09	15.94	-40.90	16.03	9	7/14/2014 40:14.2
6109	RSPe_2	-122.445	37.936	0.28	15.95	-38.92	16.23	-122.445	37.936	0.18	15.95	-40.57	16.13	9	7/14/2014 40:14.1
6110	RSPe_2	-122.445	37.936	0.23	15.87	-38.60	16.10	-122.445	37.936	0.15	15.87	-40.27	16.02	9	7/14/2014 40:14.0
6111	RSPe_2	-122.445	37.936	0.23	15.96	-38.26	16.19	-122.445	37.936	0.21	15.96	-39.94	16.17	9	7/14/2014 40:13.9
6112	RSPe_2	-122.445	37.936	0.23	15.88	-37.95	16.11	-122.445	37.936	0.15	15.88	-39.57	16.03	9	7/14/2014 40:13.8
6113	RSPe_2	-122.445	37.936	0.28	15.90	-37.73	16.18	-122.445	37.936	0.15	15.90	-39.26	16.04	9	7/14/2014 40:13.7
6114	RSPe_2	-122.445	37.936	0.23	15.86	-37.34	16.10	-122.445	37.936	0.09	15.86	-38.93	15.96	9	7/14/2014 40:13.6
6115	RSPe_2	-122.445	37.936	0.28	15.96	-37.12	16.24	-122.445	37.936	0.15	15.96	-38.63	16.10	9	7/14/2014 40:13.5
6116	RSPe_2	-122.445	37.936	0.23	15.90	-36.79	16.13	-122.445	37.936	0.15	15.90	-38.26	16.05	9	7/14/2014 40:13.4
6117	RSPe_2	-122.445	37.936	0.28	15.91	-36.49	16.20	-122.445	37.936	0.15	15.91	-37.91	16.06	9	7/14/2014 40:13.3
6118	RSPe_2	-122.445	37.936	0.23	15.94	-36.21	16.17	-122.445	37.936	0.15	15.94	-37.65	16.08	9	7/14/2014 40:13.2
6119	RSPe_2	-122.445	37.936	0.28	16.02	-35.94	16.30	-122.445	37.936	0.15	16.02	-37.32	16.16	9	7/14/2014 40:13.1
6120	RSPe_2	-122.445	37.936	0.28	16.01	-35.59	16.29	-122.445	37.936	0.21	16.01	-36.97	16.22	9	7/14/2014 40:13.0
6121	RSPe_2	-122.445	37.936	0.28	16.11	-35.18	16.40	-122.445	37.936	0.18	16.11	-36.62	16.29	9	7/14/2014 40:12.9
6122	RSPe_2	-122.445	37.936	0.28	16.12	-34.80	16.41	-122.445	37.936	0.18	16.12	-36.27	16.30	9	7/14/2014 40:12.8
6123	RSPe_2	-122.445	37.936	0.28	16.03	-34.46	16.32	-122.445	37.936	0.18	16.03	-35.92	16.21	9	7/14/2014 40:12.7
6124	RSPe_2	-122.445	37.936	0.28	16.11	-34.17	16.40	-122.445	37.936	0.18	16.11	-35.66	16.29	9	7/14/2014 40:12.6
6125	RSPe_2	-122.445	37.936	0.32	16.08	-33.84	16.40	-122.445	37.936	0.21	16.08	-35.29	16.29	9	7/14/2014 40:12.5
6126	RSPe_2	-122.445	37.936	0.28	16.05	-33.60	16.34	-122.445	37.936	0.21	16.05	-35.05	16.26	9	7/14/2014 40:12.4
6127	RSPe_2	-122.445	37.936	0.28	16.07	-33.34	16.36	-122.445	37.936	0.21	16.07	-34.83	16.29	9	7/14/2014 40:12.3
6128	RSPe_2	-122.445	37.936	0.28	16.06	-33.03	16.35	-122.445	37.936	0.21	16.06	-34.50	16.28	9	7/14/2014 40:12.2
6129	RSPe_2	-122.445	37.936	0.32	16.04	-32.79	16.36	-122.445	37.936	0.21	16.04	-34.23	16.25	9	7/14/2014 40:12.1
6130	RSPe_2	-122.445	37.936	0.28	16.03	-32.53	16.32	-122.445	37.936	0.21	16.03	-33.89	16.25	9	7/14/2014 40:12.0
6131	RSPe_2	-122.445	37.936	0.32	16.07	-32.16	16.39	-122.445	37.936	0.21	16.07	-33.55	16.29	9	7/14/2014 40:11.9
6132	RSPe_2	-122.445	37.936	0.28	16.10	-31.86	16.39	-122.445	37.936	0.18	16.10	-33.23	16.28	9	7/14/2014 40:11.8
6133	RSPe_2	-122.445	37.936	0.32	16.13	-31.55	16.45	-122.445	37.936	0.21	16.13	-32.82	16.34	9	7/14/2014 40:11.7
6134	RSPe_2	-122.445	37.936	0.28	16.17	-31.16	16.45	-122.445	37.936	0.18	16.17	-32.47	16.34	9	7/14/2014 40:11.6
6135	RSPe_2	-122.445	37.936	0.32	16.16	-30.92	16.48	-122.445	37.936	0.18	16.16	-32.16	16.34	9	7/14/2014 40:11.5
6136	RSPe_2	-122.445	37.936	0.28	16.13	-30.63	16.41	-122.445	37.936	0.21	16.13	-31.83	16.34	9	7/14/2014 40:11.4
6137	RSPe_2	-122.445	37.936	0.32	16.06	-30.33	16.37	-122.445	37.936	0.21	16.06	-31.46	16.27	9	7/14/2014 40:11.3

6138	RSPe_2	-122.445	37.936	0.32	16.00	-30.04	16.31	-122.445	37.936	0.21	16.00	-31.13	16.21	9	7/14/2014	40:11.2
6139	RSPe_2	-122.445	37.936	0.32	16.09	-29.78	16.41	-122.445	37.936	0.21	16.09	-30.85	16.31	9	7/14/2014	40:11.1
6140	RSPe_2	-122.445	37.936	0.28	15.96	-29.43	16.24	-122.445	37.936	0.21	15.96	-30.46	16.17	9	7/14/2014	40:11.0
6141	RSPe_2	-122.445	37.936	0.32	16.00	-29.13	16.31	-122.445	37.936	0.21	16.00	-30.11	16.21	9	7/14/2014	40:10.9
6142	RSPe_2	-122.445	37.936	0.32	15.99	-28.80	16.31	-122.445	37.936	0.21	15.99	-29.85	16.20	9	7/14/2014	40:10.8
6143	RSPe_2	-122.445	37.936	0.32	15.97	-28.43	16.28	-122.445	37.936	0.21	15.97	-29.52	16.18	9	7/14/2014	40:10.7
6144	RSPe_2	-122.445	37.936	0.32	15.94	-28.12	16.26	-122.445	37.936	0.18	15.94	-29.26	16.12	9	7/14/2014	40:10.6
6145	RSPe_2	-122.445	37.936	0.37	15.85	-27.77	16.22	-122.445	37.936	0.18	15.85	-28.95	16.03	9	7/14/2014	40:10.5
6146	RSPe_2	-122.445	37.936	0.28	15.83	-27.49	16.11	-122.445	37.936	0.18	15.83	-28.66	16.01	9	7/14/2014	40:10.4
6147	RSPe_2	-122.445	37.936	0.32	15.81	-27.16	16.13	-122.445	37.936	0.21	15.81	-28.38	16.02	9	7/14/2014	40:10.3
6148	RSPe_2	-122.445	37.936	0.32	15.81	-26.94	16.13	-122.445	37.936	0.18	15.81	-28.14	15.99	9	7/14/2014	40:10.2
6149	RSPe_2	-122.445	37.936	0.32	15.76	-26.52	16.08	-122.445	37.936	0.21	15.76	-27.83	15.98	9	7/14/2014	40:10.1
6150	RSPe_2	-122.445	37.936	0.32	15.71	-26.33	16.03	-122.445	37.936	0.18	15.71	-27.55	15.89	9	7/14/2014	40:10.0
6151	RSPe_2	-122.445	37.936	0.37	15.64	-25.96	16.01	-122.445	37.936	0.21	15.64	-27.24	15.85	9	7/14/2014	40:09.9
6152	RSPe_2	-122.445	37.936	0.32	15.62	-25.61	15.94	-122.445	37.936	0.18	15.62	-26.90	15.80	9	7/14/2014	40:09.8
6153	RSPe_2	-122.445	37.936	0.37	15.62	-25.19	15.99	-122.445	37.936	0.21	15.62	-26.55	15.83	9	7/14/2014	40:09.7
6154	RSPe_2	-122.445	37.936	0.37	15.59	-24.74	15.96	-122.445	37.936	0.18	15.59	-26.20	15.77	9	7/14/2014	40:09.6
6155	RSPe_2	-122.445	37.936	0.37	15.59	-24.32	15.96	-122.445	37.936	0.30	15.59	-25.89	15.88	9	7/14/2014	40:09.5
6156	RSPe_2	-122.445	37.936	0.37	15.57	-23.90	15.94	-122.445	37.936	0.30	15.57	-25.52	15.87	9	7/14/2014	40:09.4
6157	RSPe_2	-122.445	37.936	0.37	15.54	-23.53	15.91	-122.445	37.936	0.30	15.54	-25.10	15.84	9	7/14/2014	40:09.3
6158	RSPe_2	-122.445	37.936	0.37	15.52	-23.29	15.89	-122.445	37.936	0.26	15.52	-24.82	15.78	9	7/14/2014	40:09.2
6159	RSPe_2	-122.445	37.936	0.40	15.50	-23.09	15.90	-122.445	37.936	0.30	15.50	-24.51	15.80	9	7/14/2014	40:09.1
6160	RSPe_2	-122.445	37.936	0.37	15.53	-22.85	15.90	-122.445	37.936	0.30	15.53	-24.19	15.82	9	7/14/2014	40:09.0
6161	RSPe_2	-122.445	37.936	0.40	15.47	-22.55	15.88	-122.445	37.936	0.30	15.47	-23.84	15.77	9	7/14/2014	40:08.9
6162	RSPe_2	-122.445	37.936	0.37	15.47	-22.33	15.84	-122.445	37.936	0.35	15.47	-23.44	15.82	9	7/14/2014	40:08.8
6163	RSPe_2	-122.445	37.936	0.37	15.46	-21.89	15.83	-122.445	37.936	0.30	15.46	-23.10	15.76	9	7/14/2014	40:08.7
6164	RSPe_2	-122.445	37.936	0.37	15.51	-21.68	15.88	-122.445	37.936	0.30	15.51	-22.77	15.81	9	7/14/2014	40:08.6
6165	RSPe_2	-122.445	37.936	0.40	15.46	-21.35	15.87	-122.445	37.936	0.26	15.46	-22.44	15.73	9	7/14/2014	40:08.5
6166	RSPe_2	-122.445	37.936	0.37	15.48	-21.02	15.85	-122.445	37.936	0.30	15.48	-22.11	15.78	9	7/14/2014	40:08.4
6167	RSPe_2	-122.445	37.936	0.37	15.56	-20.67	15.93	-122.445	37.936	0.30	15.56	-21.70	15.86	9	7/14/2014	40:08.3
6168	RSPe_2	-122.445	37.936	0.37	15.55	-20.30	15.92	-122.445	37.936	0.35	15.55	-21.48	15.90	9	7/14/2014	40:08.2
6169	RSPe_2	-122.445	37.936	0.40	15.62	-20.04	16.02	-122.445	37.936	0.30	15.62	-21.06	15.92	9	7/14/2014	40:08.1
6170	RSPe_2	-122.445	37.936	0.37	15.60	-19.56	15.96	-122.445	37.936	0.26	15.60	-20.74	15.86	9	7/14/2014	40:08.0

6171	RSPe_2	-122.445	37.937	0.40	15.61	-19.18	16.01	-122.445	37.937	0.30	15.61	-20.32	15.91	9	7/14/2014	40:07.9
6172	RSPe_2	-122.445	37.937	0.37	15.60	-18.84	15.96	-122.445	37.937	0.30	15.60	-20.10	15.89	9	7/14/2014	40:07.8
6173	RSPe_2	-122.445	37.937	0.40	15.64	-18.53	16.04	-122.445	37.937	0.35	15.64	-19.77	15.99	9	7/14/2014	40:07.7
6174	RSPe_2	-122.445	37.937	0.37	15.61	-18.16	15.98	-122.445	37.937	0.35	15.61	-19.31	15.96	9	7/14/2014	40:07.6
6175	RSPe_2	-122.445	37.937	0.37	15.57	-17.85	15.94	-122.445	37.937	0.35	15.57	-19.07	15.92	9	7/14/2014	40:07.5
6176	RSPe_2	-122.445	37.937	0.40	15.56	-17.54	15.96	-122.445	37.937	0.26	15.56	-18.70	15.82	9	7/14/2014	40:07.4
6177	RSPe_2	-122.445	37.937	0.40	15.54	-17.26	15.94	-122.445	37.937	0.35	15.54	-18.40	15.89	9	7/14/2014	40:07.3
6178	RSPe_2	-122.445	37.937	0.40	15.60	-16.93	16.00	-122.445	37.937	0.30	15.60	-18.13	15.89	9	7/14/2014	40:07.2
6179	RSPe_2	-122.445	37.937	0.46	15.53	-16.67	15.98	-122.445	37.937	0.38	15.53	-17.79	15.91	9	7/14/2014	40:07.1
6180	RSPe_2	-122.445	37.937	0.40	15.51	-16.41	15.91	-122.445	37.937	0.35	15.51	-17.48	15.86	9	7/14/2014	40:07.0
6181	RSPe_2	-122.445	37.937	0.46	15.53	-16.08	15.98	-122.445	37.937	0.43	15.53	-17.13	15.96	9	7/14/2014	40:06.9
6182	RSPe_2	-122.445	37.937	0.40	15.49	-15.69	15.90	-122.445	37.937	0.38	15.49	-16.74	15.88	9	7/14/2014	40:06.8
6183	RSPe_2	-122.445	37.937	0.46	15.50	-15.40	15.96	-122.445	37.937	0.38	15.50	-16.52	15.88	9	7/14/2014	40:06.7
6184	RSPe_2	-122.445	37.937	0.40	15.47	-15.12	15.88	-122.445	37.937	0.35	15.47	-16.25	15.82	9	7/14/2014	40:06.6
6185	RSPe_2	-122.445	37.937	0.46	15.48	-14.79	15.93	-122.445	37.937	0.38	15.48	-15.95	15.86	9	7/14/2014	40:06.5
6186	RSPe_2	-122.445	37.937	0.40	15.47	-14.53	15.88	-122.445	37.937	0.38	15.47	-15.71	15.85	9	7/14/2014	40:06.4
6187	RSPe_2	-122.445	37.937	0.46	15.48	-14.27	15.93	-122.445	37.937	0.38	15.48	-15.43	15.86	9	7/14/2014	40:06.3
6188	RSPe_2	-122.445	37.937	0.46	15.46	-13.92	15.91	-122.445	37.937	0.38	15.46	-15.10	15.84	9	7/14/2014	40:06.2
6189	RSPe_2	-122.445	37.937	0.46	15.45	-13.64	15.90	-122.445	37.937	0.38	15.45	-14.88	15.83	9	7/14/2014	40:06.1
6190	RSPe_2	-122.445	37.937	0.40	15.53	-13.35	15.94	-122.445	37.937	0.38	15.53	-14.44	15.91	9	7/14/2014	40:06.0
6191	RSPe_2	-122.445	37.937	0.46	15.48	-13.13	15.94	-122.445	37.937	0.38	15.48	-14.18	15.87	9	7/14/2014	40:05.9
6192	RSPe_2	-122.445	37.937	0.40	15.48	-12.87	15.89	-122.445	37.937	0.38	15.48	-13.98	15.87	9	7/14/2014	40:05.8
6193	RSPe_2	-122.445	37.937	0.46	15.51	-12.74	15.97	-122.445	37.937	0.43	15.51	-13.68	15.94	9	7/14/2014	40:05.7
6194	RSPe_2	-122.445	37.937	0.46	15.54	-12.43	15.99	-122.445	37.937	0.35	15.54	-13.39	15.89	9	7/14/2014	40:05.6
6195	RSPe_2	-122.445	37.937	0.46	15.48	-12.17	15.94	-122.445	37.937	0.38	15.48	-13.15	15.86	9	7/14/2014	40:05.5
6196	RSPe_2	-122.445	37.937	0.40	15.54	-11.89	15.94	-122.445	37.937	0.38	15.54	-12.89	15.92	9	7/14/2014	40:05.4
6197	RSPe_2	-122.445	37.937	0.40	15.50	-11.65	15.91	-122.445	37.937	0.38	15.50	-12.69	15.88	9	7/14/2014	40:05.3
6198	RSPe_2	-122.445	37.937	0.46	15.58	-11.43	16.04	-122.445	37.937	0.38	15.58	-12.43	15.96	9	7/14/2014	40:05.2
6199	RSPe_2	-122.445	37.937	0.46	15.59	-11.25	16.04	-122.445	37.937	0.43	15.59	-12.19	16.02	9	7/14/2014	40:05.1
6200	RSPe_2	-122.445	37.937	0.40	15.61	-10.95	16.01	-122.445	37.937	0.38	15.61	-12.04	15.99	9	7/14/2014	40:05.0
6201	RSPe_2	-122.445	37.937	0.46	15.62	-10.79	16.07	-122.445	37.937	0.38	15.62	-11.82	16.00	9	7/14/2014	40:04.9
6202	RSPe_2	-122.445	37.937	0.40	15.62	-10.49	16.03	-122.445	37.937	0.35	15.62	-11.62	15.97	9	7/14/2014	40:04.8
6203	RSPe_2	-122.445	37.937	0.46	15.63	-10.33	16.09	-122.445	37.937	0.38	15.63	-11.45	16.02	9	7/14/2014	40:04.7

6204	RSPe_2	-122.445	37.937	0.40	15.63	-9.90	16.04	-122.445	37.937	0.38	15.63	-11.08	16.02	9	7/14/2014	40:04.6
6205	RSPe_2	-122.445	37.937	0.40	15.64	-9.66	16.04	-122.445	37.937	0.38	15.64	-10.81	16.02	9	7/14/2014	40:04.5
6206	RSPe_2	-122.445	37.937	0.40	15.66	-9.26	16.07	-122.445	37.937	0.38	15.66	-10.46	16.05	9	7/14/2014	40:04.4
6207	RSPe_2	-122.445	37.937	0.40	15.79	-9.09	16.19	-122.445	37.937	0.38	15.79	-10.22	16.17	9	7/14/2014	40:04.3
6208	RSPe_2	-122.445	37.937	0.40	15.75	-8.76	16.15	-122.445	37.937	0.35	15.75	-9.87	16.10	9	7/14/2014	40:04.2
6209	RSPe_2	-122.445	37.937	0.40	15.73	-8.48	16.13	-122.445	37.937	0.43	15.73	-9.68	16.16	9	7/14/2014	40:04.1
6210	RSPe_2	-122.445	37.937	0.37	15.77	-8.22	16.14	-122.445	37.937	0.35	15.77	-9.31	16.12	9	7/14/2014	40:04.0
6211	RSPe_2	-122.445	37.937	0.40	15.80	-7.93	16.20	-122.445	37.937	0.38	15.80	-8.96	16.18	9	7/14/2014	40:03.9
6212	RSPe_2	-122.445	37.937	0.40	15.76	-7.60	16.16	-122.445	37.937	0.30	15.76	-8.61	16.05	9	7/14/2014	40:03.8
6213	RSPe_2	-122.445	37.937	0.40	15.78	-7.32	16.18	-122.445	37.937	0.38	15.78	-8.23	16.16	9	7/14/2014	40:03.7
6214	RSPe_2	-122.445	37.937	0.40	15.82	-6.95	16.22	-122.445	37.937	0.35	15.82	-7.91	16.17	9	7/14/2014	40:03.6
6215	RSPe_2	-122.445	37.937	0.40	15.89	-6.64	16.29	-122.445	37.937	0.38	15.89	-7.51	16.27	9	7/14/2014	40:03.5
6216	RSPe_2	-122.445	37.937	0.40	15.83	-6.31	16.24	-122.445	37.937	0.38	15.83	-7.21	16.22	9	7/14/2014	40:03.4
6217	RSPe_2	-122.445	37.937	0.46	15.86	-6.05	16.32	-122.445	37.937	0.38	15.86	-6.90	16.25	9	7/14/2014	40:03.3
6218	RSPe_2	-122.445	37.937	0.40	15.90	-5.70	16.31	-122.445	37.937	0.38	15.90	-6.65	16.29	9	7/14/2014	40:03.2
6219	RSPe_2	-122.445	37.937	0.46	15.95	-5.51	16.41	-122.445	37.937	0.43	15.95	-6.27	16.38	9	7/14/2014	40:03.1
6220	RSPe_2	-122.445	37.937	0.40	15.93	-5.16	16.33	-122.445	37.937	0.38	15.93	-6.05	16.31	9	7/14/2014	40:03.0
6221	RSPe_2	-122.445	37.937	0.46	15.95	-4.91	16.41	-122.445	37.937	0.38	15.95	-5.72	16.33	9	7/14/2014	40:02.9
6222	RSPe_2	-122.445	37.937	0.40	15.97	-4.59	16.38	-122.445	37.937	0.38	15.97	-5.37	16.36	9	7/14/2014	40:02.8
6223	RSPe_2	-122.445	37.937	0.46	15.97	-4.28	16.42	-122.445	37.937	0.43	15.97	-5.11	16.40	9	7/14/2014	40:02.7
6224	RSPe_2	-122.445	37.937	0.40	15.94	-4.00	16.34	-122.445	37.937	0.38	15.94	-4.74	16.32	9	7/14/2014	40:02.6
6225	RSPe_2	-122.445	37.937	0.46	15.92	-3.65	16.37	-122.445	37.937	0.38	15.92	-4.43	16.30	9	7/14/2014	40:02.5
6226	RSPe_2	-122.445	37.937	0.40	15.91	-3.23	16.32	-122.445	37.937	0.38	15.91	-4.07	16.29	9	7/14/2014	40:02.4
6227	RSPe_2	-122.445	37.937	0.46	15.86	-2.91	16.32	-122.445	37.937	0.43	15.86	-3.78	16.30	9	7/14/2014	40:02.3
6228	RSPe_2	-122.445	37.937	0.46	15.90	-2.49	16.35	-122.445	37.937	0.43	15.90	-3.39	16.33	9	7/14/2014	40:02.2
6229	RSPe_2	-122.445	37.937	0.46	15.85	-2.23	16.30	-122.445	37.937	0.47	15.85	-3.08	16.32	9	7/14/2014	40:02.1
6230	RSPe_2	-122.445	37.937	0.46	15.86	-1.88	16.32	-122.445	37.937	0.47	15.86	-2.78	16.33	9	7/14/2014	40:02.0
6231	RSPe_2	-122.445	37.937	0.49	15.81	-1.64	16.30	-122.445	37.937	0.47	15.81	-2.40	16.28	9	7/14/2014	40:01.9
6232	RSPe_2	-122.445	37.937	0.46	15.79	-1.40	16.24	-122.445	37.937	0.43	15.79	-2.21	16.22	9	7/14/2014	40:01.8
6233	RSPe_2	-122.445	37.937	0.46	15.81	-1.09	16.27	-122.445	37.937	0.47	15.81	-1.90	16.28	9	7/14/2014	40:01.7
6234	RSPe_2	-122.445	37.937	0.46	15.75	-0.90	16.20	-122.445	37.937	0.47	15.75	-1.64	16.22	9	7/14/2014	40:01.6
6235	RSPe_2	-122.445	37.937	0.49	15.76	-0.57	16.25	-122.445	37.937	0.43	15.76	-1.42	16.19	9	7/14/2014	40:01.5
6236	RSPe_2	-122.445	37.937	0.46	15.72	-0.37	16.17	-122.445	37.937	0.43	15.72	-1.22	16.15	9	7/14/2014	40:01.4

6237	RSPe_2	-122.445	37.937	0.49	15.73	-0.24	16.22	-122.445	37.937	0.47	15.73	-1.03	16.19	9	7/14/2014	40:01.3
6238	RSPe_2	-122.445	37.937	0.46	15.69	0.09	16.15	-122.445	37.937	0.47	15.69	-0.79	16.16	9	7/14/2014	40:01.2
6239	RSPe_2	-122.445	37.937	0.49	15.67	0.37	16.16	-122.445	37.937	0.47	15.67	-0.57	16.14	9	7/14/2014	40:01.1
6240	RSPe_2	-122.445	37.937	0.46	15.67	0.64	16.13	-122.445	37.937	0.47	15.67	-0.22	16.14	9	7/14/2014	40:01.0
6241	RSPe_2	-122.445	37.937	0.49	15.66	0.88	16.15	-122.445	37.937	0.47	15.66	0.00	16.13	9	7/14/2014	40:00.9
6242	RSPe_2	-122.445	37.937	0.46	15.60	1.20	16.05	-122.445	37.937	0.47	15.60	0.37	16.06	9	7/14/2014	40:00.8
6243	RSPe_2	-122.445	37.937	0.49	15.61	1.49	16.10	-122.445	37.937	0.50	15.61	0.68	16.11	9	7/14/2014	40:00.7
6244	RSPe_2	-122.445	37.937	0.46	15.59	1.82	16.04	-122.445	37.937	0.47	15.59	1.03	16.05	9	7/14/2014	40:00.6
6245	RSPe_2	-122.445	37.937	0.49	15.56	2.10	16.05	-122.445	37.937	0.50	15.56	1.29	16.07	9	7/14/2014	40:00.5
6246	RSPe_2	-122.445	37.937	0.49	15.57	2.34	16.06	-122.445	37.937	0.47	15.57	1.64	16.04	9	7/14/2014	40:00.4
6247	RSPe_2	-122.445	37.937	0.49	15.55	2.69	16.04	-122.445	37.937	0.55	15.55	1.97	16.10	9	7/14/2014	40:00.3
6248	RSPe_2	-122.445	37.937	0.49	15.55	2.97	16.04	-122.445	37.937	0.47	15.55	2.27	16.02	9	7/14/2014	40:00.2
6249	RSPe_2	-122.445	37.937	0.49	15.55	3.14	16.04	-122.445	37.937	0.50	15.55	2.45	16.05	9	7/14/2014	40:00.1
6250	RSPe_2	-122.445	37.937	0.49	15.48	3.45	15.97	-122.445	37.937	0.47	15.48	2.73	15.95	9	7/14/2014	40:00.0
6251	RSPe_2	-122.445	37.937	0.52	15.48	3.72	16.00	-122.445	37.937	0.50	15.48	3.00	15.98	9	7/14/2014	39:59.9
6252	RSPe_2	-122.445	37.937	0.49	15.46	4.00	15.95	-122.445	37.937	0.50	15.46	3.26	15.96	9	7/14/2014	39:59.8
6253	RSPe_2	-122.445	37.937	0.52	15.48	4.24	16.01	-122.445	37.937	0.50	15.48	3.52	15.99	9	7/14/2014	39:59.7
6254	RSPe_2	-122.445	37.937	0.49	15.43	4.55	15.92	-122.445	37.937	0.50	15.43	3.82	15.93	9	7/14/2014	39:59.6
6255	RSPe_2	-122.445	37.937	0.52	15.46	4.83	15.98	-122.445	37.937	0.55	15.46	4.06	16.01	9	7/14/2014	39:59.5
6256	RSPe_2	-122.445	37.937	0.49	15.36	5.00	15.85	-122.445	37.937	0.55	15.36	4.30	15.91	9	7/14/2014	39:59.4
6257	RSPe_2	-122.445	37.937	0.49	15.37	5.38	15.86	-122.445	37.937	0.50	15.37	4.61	15.87	9	7/14/2014	39:59.3
6258	RSPe_2	-122.445	37.937	0.49	15.39	5.64	15.88	-122.445	37.937	0.47	15.39	4.87	15.86	9	7/14/2014	39:59.2
6259	RSPe_2	-122.445	37.937	0.52	15.36	5.96	15.89	-122.445	37.937	0.50	15.36	5.13	15.86	9	7/14/2014	39:59.1
6260	RSPe_2	-122.445	37.937	0.52	15.32	6.25	15.85	-122.445	37.937	0.50	15.32	5.39	15.83	9	7/14/2014	39:59.0
6261	RSPe_2	-122.445	37.937	0.52	15.32	6.45	15.85	-122.445	37.937	0.55	15.32	5.77	15.88	9	7/14/2014	39:58.9
6262	RSPe_2	-122.445	37.937	0.52	15.31	6.84	15.83	-122.445	37.937	0.50	15.31	6.01	15.81	9	7/14/2014	39:58.8
6263	RSPe_2	-122.445	37.937	0.52	15.32	7.04	15.84	-122.445	37.937	0.55	15.32	6.34	15.87	9	7/14/2014	39:58.7
6264	RSPe_2	-122.445	37.937	0.52	15.32	7.36	15.85	-122.445	37.937	0.50	15.32	6.64	15.83	9	7/14/2014	39:58.6
6265	RSPe_2	-122.445	37.937	0.52	15.36	7.69	15.89	-122.445	37.937	0.50	15.36	6.97	15.86	9	7/14/2014	39:58.5
6266	RSPe_2	-122.445	37.937	0.52	15.32	8.02	15.85	-122.445	37.937	0.50	15.32	7.23	15.83	9	7/14/2014	39:58.4
6267	RSPe_2	-122.445	37.937	0.58	15.30	8.32	15.88	-122.445	37.937	0.55	15.30	7.51	15.85	9	7/14/2014	39:58.3
6268	RSPe_2	-122.445	37.937	0.52	15.32	8.63	15.84	-122.445	37.937	0.55	15.32	7.78	15.87	9	7/14/2014	39:58.2
6269	RSPe_2	-122.445	37.937	0.58	15.29	8.92	15.87	-122.445	37.937	0.55	15.29	8.04	15.85	9	7/14/2014	39:58.1

6270	RSPe_2	-122.445	37.937	0.52	15.42	9.18	15.94	-122.445	37.937	0.55	15.42	8.26	15.97	9	7/14/2014	39:58.0
6271	RSPe_2	-122.445	37.937	0.58	15.43	9.42	16.01	-122.445	37.937	0.55	15.43	8.46	15.98	9	7/14/2014	39:57.9
6272	RSPe_2	-122.445	37.937	0.52	15.27	9.66	15.79	-122.445	37.937	0.55	15.27	8.72	15.82	9	7/14/2014	39:57.8
6273	RSPe_2	-122.445	37.937	0.52	15.40	9.88	15.93	-122.445	37.937	0.59	15.40	9.00	15.99	9	7/14/2014	39:57.7
6274	RSPe_2	-122.445	37.937	0.52	15.32	10.14	15.85	-122.445	37.937	0.55	15.32	9.27	15.88	9	7/14/2014	39:57.6
6275	RSPe_2	-122.445	37.937	0.58	15.37	10.36	15.95	-122.445	37.937	0.55	15.37	9.57	15.92	9	7/14/2014	39:57.5
6276	RSPe_2	-122.445	37.937	0.52	15.34	10.66	15.86	-122.445	37.937	0.59	15.34	9.90	15.93	9	7/14/2014	39:57.4
6277	RSPe_2	-122.445	37.937	0.52	15.39	10.92	15.92	-122.445	37.937	0.59	15.39	10.18	15.98	9	7/14/2014	39:57.3
6278	RSPe_2	-122.445	37.937	0.58	15.43	11.25	16.00	-122.445	37.937	0.55	15.43	10.46	15.98	9	7/14/2014	39:57.2
6279	RSPe_2	-122.445	37.937	0.58	15.41	11.56	15.99	-122.445	37.937	0.55	15.41	10.78	15.96	9	7/14/2014	39:57.1
6280	RSPe_2	-122.445	37.937	0.52	15.36	11.85	15.88	-122.445	37.937	0.59	15.36	11.04	15.94	9	7/14/2014	39:57.0
6281	RSPe_2	-122.445	37.937	0.52	15.36	12.06	15.89	-122.445	37.937	0.59	15.36	11.30	15.95	9	7/14/2014	39:56.9
6282	RSPe_2	-122.445	37.937	0.58	15.34	12.24	15.92	-122.445	37.937	0.59	15.34	11.54	15.93	9	7/14/2014	39:56.8
6283	RSPe_2	-122.445	37.937	0.52	15.40	12.50	15.93	-122.445	37.937	0.59	15.40	11.78	15.99	9	7/14/2014	39:56.7
6284	RSPe_2	-122.445	37.937	0.52	15.43	12.72	15.95	-122.445	37.937	0.59	15.43	12.06	16.01	9	7/14/2014	39:56.6
6285	RSPe_2	-122.445	37.937	0.58	15.34	12.89	15.92	-122.445	37.937	0.55	15.34	12.26	15.89	9	7/14/2014	39:56.5
6286	RSPe_2	-122.445	37.937	0.52	15.39	13.24	15.92	-122.445	37.937	0.55	15.39	12.61	15.95	9	7/14/2014	39:56.4
6287	RSPe_2	-122.445	37.937	0.58	15.49	13.37	16.07	-122.445	37.937	0.55	15.49	12.80	16.05	9	7/14/2014	39:56.3
6288	RSPe_2	-122.445	37.937	0.52	15.46	13.55	15.99	-122.445	37.937	0.55	15.46	12.98	16.02	9	7/14/2014	39:56.2
6289	RSPe_2	-122.445	37.937	0.52	15.48	13.75	16.00	-122.445	37.937	0.59	15.48	13.22	16.07	9	7/14/2014	39:56.1
6290	RSPe_2	-122.445	37.937	0.52	15.48	14.01	16.00	-122.445	37.937	0.55	15.48	13.42	16.03	9	7/14/2014	39:56.0
6291	RSPe_2	-122.445	37.937	0.58	15.52	14.31	16.09	-122.445	37.937	0.59	15.52	13.72	16.10	9	7/14/2014	39:55.9
6292	RSPe_2	-122.445	37.937	0.58	15.54	14.60	16.11	-122.445	37.937	0.55	15.54	13.94	16.09	9	7/14/2014	39:55.8
6293	RSPe_2	-122.445	37.937	0.58	15.49	14.77	16.07	-122.445	37.937	0.55	15.49	14.16	16.05	9	7/14/2014	39:55.7
6294	RSPe_2	-122.445	37.937	0.52	15.54	15.06	16.06	-122.445	37.937	0.59	15.54	14.38	16.12	9	7/14/2014	39:55.6
6295	RSPe_2	-122.445	37.937	0.58	15.53	15.25	16.11	-122.445	37.937	0.59	15.53	14.49	16.12	9	7/14/2014	39:55.5
6296	RSPe_2	-122.445	37.937	0.58	15.48	15.41	16.06	-122.445	37.937	0.55	15.48	14.69	16.03	9	7/14/2014	39:55.4
6297	RSPe_2	-122.445	37.937	0.58	15.54	15.62	16.11	-122.445	37.937	0.64	15.54	14.86	16.18	9	7/14/2014	39:55.3
6298	RSPe_2	-122.445	37.937	0.58	15.49	15.84	16.07	-122.445	37.937	0.55	15.49	15.08	16.05	9	7/14/2014	39:55.2
6299	RSPe_2	-122.445	37.937	0.61	15.56	16.04	16.17	-122.445	37.937	0.64	15.56	15.32	16.19	9	7/14/2014	39:55.1
6300	RSPe_2	-122.445	37.937	0.58	15.54	16.26	16.11	-122.445	37.937	0.59	15.54	15.47	16.12	9	7/14/2014	39:55.0
6301	RSPe_2	-122.445	37.937	0.61	15.55	16.48	16.16	-122.445	37.937	0.59	15.55	15.71	16.13	9	7/14/2014	39:54.9
6302	RSPe_2	-122.445	37.937	0.58	15.54	16.76	16.11	-122.445	37.937	0.59	15.54	15.95	16.12	9	7/14/2014	39:54.8

6303	RSPe_2	-122.445	37.937	0.58	15.62	16.98	16.19	-122.445	37.937	0.64	15.62	16.24	16.26	9	7/14/2014	39:54.7
6304	RSPe_2	-122.445	37.937	0.58	15.59	17.22	16.16	-122.445	37.937	0.59	15.59	16.31	16.17	9	7/14/2014	39:54.6
6305	RSPe_2	-122.445	37.937	0.58	15.60	17.48	16.18	-122.445	37.937	0.59	15.60	16.58	16.19	9	7/14/2014	39:54.5
6306	RSPe_2	-122.445	37.937	0.58	15.59	17.77	16.16	-122.445	37.937	0.59	15.59	16.89	16.17	9	7/14/2014	39:54.4
6307	RSPe_2	-122.445	37.937	0.58	15.60	18.01	16.17	-122.445	37.937	0.67	15.60	17.09	16.27	9	7/14/2014	39:54.3
6308	RSPe_2	-122.445	37.937	0.58	15.61	18.29	16.19	-122.445	37.937	0.59	15.61	17.33	16.20	9	7/14/2014	39:54.2
6309	RSPe_2	-122.445	37.937	0.58	15.61	18.49	16.19	-122.445	37.937	0.64	15.61	17.66	16.25	9	7/14/2014	39:54.1
6310	RSPe_2	-122.445	37.937	0.52	15.58	18.82	16.10	-122.445	37.937	0.64	15.58	17.97	16.22	9	7/14/2014	39:54.0
6311	RSPe_2	-122.445	37.937	0.58	15.59	19.06	16.16	-122.445	37.937	0.64	15.59	18.23	16.22	9	7/14/2014	39:53.9
6312	RSPe_2	-122.445	37.937	0.52	15.61	19.30	16.13	-122.445	37.937	0.59	15.61	18.51	16.20	9	7/14/2014	39:53.8
6313	RSPe_2	-122.445	37.937	0.58	15.66	19.60	16.24	-122.445	37.937	0.67	15.66	18.86	16.34	9	7/14/2014	39:53.7
6314	RSPe_2	-122.445	37.937	0.58	15.62	19.89	16.20	-122.445	37.937	0.64	15.62	19.14	16.26	9	7/14/2014	39:53.6
6315	RSPe_2	-122.445	37.937	0.58	15.60	20.19	16.18	-122.445	37.937	0.72	15.60	19.56	16.32	9	7/14/2014	39:53.5
6316	RSPe_2	-122.445	37.937	0.58	15.62	20.45	16.20	-122.445	37.937	0.67	15.62	19.71	16.30	9	7/14/2014	39:53.4
6317	RSPe_2	-122.445	37.937	0.58	15.62	20.76	16.20	-122.445	37.937	0.67	15.62	20.06	16.29	9	7/14/2014	39:53.3
6318	RSPe_2	-122.445	37.937	0.58	15.60	21.05	16.17	-122.445	37.937	0.72	15.60	20.39	16.32	9	7/14/2014	39:53.2
6319	RSPe_2	-122.445	37.937	0.58	15.58	21.33	16.16	-122.445	37.937	0.67	15.58	20.59	16.25	9	7/14/2014	39:53.1
6320	RSPe_2	-122.445	37.937	0.58	15.57	21.57	16.15	-122.445	37.937	0.67	15.57	20.87	16.24	9	7/14/2014	39:53.0
6321	RSPe_2	-122.445	37.937	0.58	15.59	21.74	16.16	-122.445	37.937	0.67	15.59	21.07	16.26	9	7/14/2014	39:52.9
6322	RSPe_2	-122.445	37.937	0.58	15.57	21.99	16.15	-122.445	37.937	0.67	15.57	21.24	16.24	9	7/14/2014	39:52.8
6323	RSPe_2	-122.445	37.937	0.58	15.62	22.11	16.20	-122.445	37.937	0.67	15.62	21.42	16.29	9	7/14/2014	39:52.7
6324	RSPe_2	-122.445	37.937	0.52	15.59	22.31	16.11	-122.445	37.937	0.67	15.59	21.67	16.26	9	7/14/2014	39:52.6
6325	RSPe_2	-122.445	37.937	0.52	15.59	22.55	16.11	-122.445	37.937	0.64	15.59	21.94	16.22	9	7/14/2014	39:52.5
6326	RSPe_2	-122.445	37.937	0.52	15.60	22.68	16.13	-122.445	37.937	0.64	15.60	22.09	16.24	9	7/14/2014	39:52.4
6327	RSPe_2	-122.445	37.937	0.52	15.57	22.92	16.10	-122.445	37.937	0.64	15.57	22.38	16.21	9	7/14/2014	39:52.3
6328	RSPe_2	-122.445	37.937	0.52	15.60	23.14	16.12	-122.445	37.937	0.64	15.60	22.60	16.23	9	7/14/2014	39:52.2
6329	RSPe_2	-122.445	37.937	0.52	15.60	23.27	16.13	-122.445	37.937	0.67	15.60	22.82	16.27	9	7/14/2014	39:52.1
6330	RSPe_2	-122.445	37.937	0.52	15.62	23.54	16.14	-122.445	37.937	0.67	15.62	22.97	16.29	9	7/14/2014	39:52.0
6331	RSPe_2	-122.445	37.937	0.52	15.64	23.76	16.17	-122.445	37.937	0.67	15.64	23.27	16.31	9	7/14/2014	39:51.9
6332	RSPe_2	-122.445	37.937	0.52	15.66	24.04	16.18	-122.445	37.937	0.67	15.66	23.45	16.33	9	7/14/2014	39:51.8
6333	RSPe_2	-122.445	37.937	0.58	15.65	24.17	16.23	-122.445	37.937	0.67	15.65	23.65	16.32	9	7/14/2014	39:51.7
6334	RSPe_2	-122.445	37.937	0.52	15.67	24.39	16.20	-122.445	37.937	0.67	15.67	23.80	16.34	9	7/14/2014	39:51.6
6335	RSPe_2	-122.445	37.937	0.52	15.66	24.58	16.18	-122.445	37.937	0.67	15.66	23.99	16.33	9	7/14/2014	39:51.5

6336	RSPe_2	-122.445	37.937	0.52	15.65	24.67	16.17	-122.445	37.937	0.64	15.65	24.11	16.29	9	7/14/2014 39:51.4
6337	RSPe_2	-122.445	37.937	0.52	15.67	24.85	16.20	-122.445	37.937	0.64	15.67	24.25	16.31	9	7/14/2014 39:51.3
6338	RSPe_2	-122.445	37.937	0.52	15.68	25.05	16.20	-122.445	37.937	0.64	15.68	24.50	16.32	9	7/14/2014 39:51.2
6339	RSPe_2	-122.445	37.937	0.52	15.69	25.22	16.22	-122.445	37.937	0.64	15.69	24.67	16.33	9	7/14/2014 39:51.1
6340	RSPe_2	-122.445	37.937	0.52	15.71	25.44	16.24	-122.445	37.937	0.64	15.71	24.87	16.35	9	7/14/2014 39:51.0
6341	RSPe_2	-122.445	37.937	0.52	15.72	25.66	16.24	-122.445	37.937	0.64	15.72	25.13	16.36	9	7/14/2014 39:50.9
6342	RSPe_2	-122.445	37.937	0.52	15.73	25.94	16.25	-122.445	37.937	0.59	15.73	25.33	16.31	9	7/14/2014 39:50.8
6343	RSPe_2	-122.445	37.937	0.52	15.74	26.16	16.27	-122.445	37.937	0.64	15.74	25.53	16.38	9	7/14/2014 39:50.7
6344	RSPe_2	-122.445	37.937	0.52	15.74	26.36	16.27	-122.445	37.937	0.59	15.74	25.74	16.33	9	7/14/2014 39:50.6
6345	RSPe_2	-122.445	37.937	0.52	15.78	26.57	16.30	-122.445	37.937	0.67	15.78	25.94	16.45	9	7/14/2014 39:50.5
6346	RSPe_2	-122.445	37.937	0.49	15.77	26.75	16.26	-122.445	37.937	0.64	15.77	26.07	16.41	9	7/14/2014 39:50.4
6347	RSPe_2	-122.445	37.937	0.52	15.79	26.95	16.31	-122.445	37.937	0.67	15.79	26.29	16.46	9	7/14/2014 39:50.3
6348	RSPe_2	-122.445	37.937	0.49	15.83	27.19	16.32	-122.445	37.937	0.64	15.83	26.51	16.46	9	7/14/2014 39:50.2
6349	RSPe_2	-122.445	37.937	0.52	15.82	27.34	16.34	-122.445	37.937	0.64	15.82	26.66	16.46	9	7/14/2014 39:50.1
6350	RSPe_2	-122.445	37.937	0.49	15.82	27.54	16.31	-122.445	37.937	0.64	15.82	26.91	16.46	9	7/14/2014 39:50.0
6351	RSPe_2	-122.445	37.937	0.49	15.85	27.69	16.34	-122.445	37.937	0.64	15.85	27.08	16.49	9	7/14/2014 39:49.9
6352	RSPe_2	-122.445	37.937	0.49	15.86	27.86	16.36	-122.445	37.937	0.59	15.86	27.30	16.45	9	7/14/2014 39:49.8
6353	RSPe_2	-122.445	37.937	0.49	15.90	28.00	16.39	-122.445	37.937	0.59	15.90	27.55	16.48	9	7/14/2014 39:49.7
6354	RSPe_2	-122.445	37.937	0.49	15.90	28.19	16.39	-122.445	37.937	0.59	15.90	27.78	16.49	9	7/14/2014 39:49.6
6355	RSPe_2	-122.445	37.937	0.49	15.93	28.34	16.43	-122.445	37.937	0.55	15.93	28.08	16.49	9	7/14/2014 39:49.5
6356	RSPe_2	-122.445	37.937	0.49	15.95	28.46	16.44	-122.445	37.937	0.55	15.95	28.21	16.50	9	7/14/2014 39:49.4
6357	RSPe_2	-122.445	37.937	0.49	16.01	28.67	16.50	-122.445	37.937	0.59	16.01	28.43	16.60	9	7/14/2014 39:49.3
6358	RSPe_2	-122.445	37.937	0.49	16.00	28.81	16.49	-122.445	37.937	0.55	16.00	28.55	16.56	9	7/14/2014 39:49.2
6359	RSPe_2	-122.445	37.937	0.52	16.04	29.00	16.57	-122.445	37.937	0.59	16.04	28.76	16.63	9	7/14/2014 39:49.1
6360	RSPe_2	-122.445	37.937	0.49	16.06	29.24	16.55	-122.445	37.937	0.55	16.06	28.98	16.61	9	7/14/2014 39:49.0
6361	RSPe_2	-122.445	37.937	0.52	16.09	29.49	16.61	-122.445	37.937	0.55	16.09	29.17	16.64	9	7/14/2014 39:48.9
6362	RSPe_2	-122.445	37.937	0.49	16.14	29.68	16.63	-122.445	37.937	0.55	16.14	29.33	16.70	9	7/14/2014 39:48.8
6363	RSPe_2	-122.445	37.937	0.49	16.16	29.90	16.65	-122.445	37.937	0.55	16.16	29.42	16.71	9	7/14/2014 39:48.7
6364	RSPe_2	-122.445	37.937	0.49	16.17	30.07	16.66	-122.445	37.937	0.50	16.17	29.57	16.67	9	7/14/2014 39:48.6
6365	RSPe_2	-122.445	37.937	0.49	16.21	30.23	16.70	-122.445	37.937	0.50	16.21	29.64	16.71	9	7/14/2014 39:48.5
6366	RSPe_2	-122.445	37.937	0.49	16.12	30.44	16.61	-122.445	37.937	0.50	16.12	29.79	16.62	9	7/14/2014 39:48.4
6367	RSPe_2	-122.445	37.937	0.49	16.14	30.60	16.63	-122.445	37.937	0.50	16.14	29.87	16.64	9	7/14/2014 39:48.3
6368	RSPe_2	-122.445	37.937	0.46	16.09	30.73	16.55	-122.445	37.937	0.47	16.09	30.03	16.56	9	7/14/2014 39:48.2

6369	RSPe_2	-122.445	37.937	0.49	16.09	30.93	16.59	-122.445	37.937	0.50	16.09	30.21	16.60	9	7/14/2014	39:48.1
6370	RSPe_2	-122.445	37.937	0.46	15.97	31.15	16.42	-122.445	37.937	0.50	15.97	30.38	16.47	9	7/14/2014	39:48.0
6371	RSPe_2	-122.445	37.937	0.49	15.93	31.28	16.42	-122.445	37.937	0.50	15.93	30.54	16.43	9	7/14/2014	39:47.9
6372	RSPe_2	-122.445	37.937	0.49	16.00	31.50	16.49	-122.445	37.937	0.50	16.00	30.73	16.50	9	7/14/2014	39:47.8
6373	RSPe_2	-122.445	37.937	0.49	15.90	31.76	16.39	-122.445	37.937	0.50	15.90	31.02	16.40	9	7/14/2014	39:47.7
6374	RSPe_2	-122.445	37.937	0.49	15.92	32.00	16.41	-122.445	37.937	0.50	15.92	31.21	16.42	9	7/14/2014	39:47.6
6375	RSPe_2	-122.445	37.937	0.49	15.77	32.15	16.26	-122.445	37.937	0.50	15.77	31.41	16.27	9	7/14/2014	39:47.5
6376	RSPe_2	-122.445	37.937	0.49	15.72	32.41	16.21	-122.445	37.937	0.50	15.72	31.61	16.22	9	7/14/2014	39:47.4
6377	RSPe_2	-122.445	37.937	0.49	15.74	32.61	16.23	-122.445	37.937	0.50	15.74	31.84	16.24	9	7/14/2014	39:47.3
6378	RSPe_2	-122.445	37.937	0.49	15.73	32.79	16.23	-122.445	37.937	0.50	15.73	32.11	16.24	9	7/14/2014	39:47.2
6379	RSPe_2	-122.445	37.937	0.49	15.74	32.94	16.23	-122.445	37.937	0.50	15.74	32.28	16.24	9	7/14/2014	39:47.1
6380	RSPe_2	-122.445	37.937	0.46	15.66	33.09	16.11	-122.445	37.937	0.47	15.66	32.46	16.13	9	7/14/2014	39:47.0
6381	RSPe_2	-122.445	37.937	0.49	15.66	33.29	16.15	-122.445	37.937	0.47	15.66	32.68	16.13	9	7/14/2014	39:46.9
6382	RSPe_2	-122.445	37.937	0.46	15.61	33.40	16.07	-122.445	37.937	0.47	15.61	32.76	16.08	9	7/14/2014	39:46.8
6383	RSPe_2	-122.445	37.937	0.46	15.62	33.59	16.08	-122.445	37.937	0.47	15.62	32.98	16.09	9	7/14/2014	39:46.7
6384	RSPe_2	-122.445	37.937	0.46	15.60	33.77	16.06	-122.445	37.937	0.44	15.60	33.14	16.04	9	7/14/2014	39:46.6
6385	RSPe_2	-122.445	37.937	0.49	15.53	33.96	16.02	-122.445	37.937	0.47	15.53	33.33	16.00	9	7/14/2014	39:46.5
6386	RSPe_2	-122.445	37.937	0.46	15.53	34.16	15.98	-122.445	37.937	0.47	15.53	33.53	16.00	9	7/14/2014	39:46.4
6387	RSPe_2	-122.445	37.937	0.49	15.46	34.33	15.95	-122.445	37.937	0.47	15.46	33.66	15.93	9	7/14/2014	39:46.3
6388	RSPe_2	-122.445	37.937	0.46	15.44	34.53	15.90	-122.445	37.937	0.47	15.44	33.90	15.91	9	7/14/2014	39:46.2
6389	RSPe_2	-122.445	37.937	0.46	15.43	34.71	15.88	-122.445	37.937	0.47	15.43	34.04	15.89	9	7/14/2014	39:46.1
6390	RSPe_2	-122.445	37.937	0.46	15.41	34.86	15.87	-122.445	37.937	0.47	15.41	34.23	15.88	9	7/14/2014	39:46.0
6391	RSPe_2	-122.445	37.937	0.49	15.36	35.02	15.85	-122.445	37.937	0.50	15.36	34.36	15.86	9	7/14/2014	39:45.9
6392	RSPe_2	-122.445	37.937	0.46	15.42	35.17	15.87	-122.445	37.937	0.47	15.42	34.51	15.89	9	7/14/2014	39:45.8
6393	RSPe_2	-122.445	37.937	0.49	15.37	35.36	15.86	-122.445	37.937	0.44	15.37	34.67	15.81	9	7/14/2014	39:45.7
6394	RSPe_2	-122.445	37.937	0.46	15.36	35.50	15.82	-122.445	37.937	0.38	15.36	34.77	15.75	9	7/14/2014	39:45.6
6395	RSPe_2	-122.445	37.937	0.46	15.35	35.74	15.80	-122.445	37.937	0.44	15.35	34.95	15.78	9	7/14/2014	39:45.5
6396	RSPe_2	-122.445	37.937	0.46	15.36	35.89	15.82	-122.445	37.937	0.38	15.36	35.15	15.75	9	7/14/2014	39:45.4
6397	RSPe_2	-122.445	37.937	0.46	15.31	36.08	15.76	-122.445	37.937	0.47	15.31	35.30	15.78	9	7/14/2014	39:45.3
6398	RSPe_2	-122.445	37.937	0.46	15.30	36.33	15.76	-122.445	37.937	0.44	15.30	35.52	15.74	9	7/14/2014	39:45.2
6399	RSPe_2	-122.445	37.937	0.46	15.26	36.53	15.71	-122.445	37.937	0.44	15.26	35.71	15.69	9	7/14/2014	39:45.1
6400	RSPe_2	-122.445	37.937	0.46	15.25	36.74	15.71	-122.445	37.937	0.44	15.25	35.91	15.68	9	7/14/2014	39:45.0
6401	RSPe_2	-122.445	37.937	0.46	15.29	36.97	15.75	-122.445	37.937	0.44	15.29	36.15	15.73	9	7/14/2014	39:44.9

6402	RSPe_2	-122.445	37.937	0.46	15.28	37.16	15.73	-122.445	37.937	0.44	15.28	36.37	15.71	9	7/14/2014	39:44.8
6403	RSPe_2	-122.445	37.937	0.46	15.20	37.38	15.66	-122.445	37.937	0.44	15.20	36.61	15.64	9	7/14/2014	39:44.7
6404	RSPe_2	-122.445	37.937	0.46	15.22	37.53	15.67	-122.445	37.937	0.44	15.22	36.81	15.65	9	7/14/2014	39:44.6
6405	RSPe_2	-122.445	37.937	0.46	15.23	37.79	15.69	-122.445	37.937	0.44	15.23	37.04	15.67	9	7/14/2014	39:44.5
6406	RSPe_2	-122.445	37.937	0.46	15.25	38.05	15.71	-122.445	37.937	0.47	15.25	37.33	15.72	9	7/14/2014	39:44.4
6407	RSPe_2	-122.445	37.937	0.46	15.30	38.32	15.76	-122.445	37.937	0.47	15.30	37.61	15.77	9	7/14/2014	39:44.3
6408	RSPe_2	-122.445	37.937	0.46	15.26	38.56	15.72	-122.445	37.937	0.44	15.26	37.79	15.70	9	7/14/2014	39:44.2
6409	RSPe_2	-122.445	37.937	0.46	15.34	38.73	15.80	-122.445	37.937	0.44	15.34	37.97	15.78	9	7/14/2014	39:44.1
6410	RSPe_2	-122.445	37.937	0.46	15.29	39.01	15.74	-122.445	37.937	0.44	15.29	38.21	15.72	9	7/14/2014	39:44.0
6411	RSPe_2	-122.445	37.937	0.46	15.32	39.26	15.77	-122.445	37.937	0.47	15.32	38.43	15.79	9	7/14/2014	39:43.9
6412	RSPe_2	-122.445	37.937	0.46	15.44	39.43	15.90	-122.445	37.937	0.44	15.44	38.65	15.88	9	7/14/2014	39:43.8
6413	RSPe_2	-122.445	37.937	0.46	15.47	39.67	15.93	-122.445	37.937	0.47	15.47	38.86	15.94	9	7/14/2014	39:43.7
6414	RSPe_2	-122.445	37.937	0.46	15.47	39.93	15.93	-122.445	37.937	0.47	15.47	39.17	15.94	9	7/14/2014	39:43.6
6415	RSPe_2	-122.445	37.937	0.49	15.50	40.19	15.99	-122.445	37.937	0.50	15.50	39.48	16.00	9	7/14/2014	39:43.5
6416	RSPe_2	-122.445	37.937	0.46	15.53	40.42	15.98	-122.445	37.937	0.47	15.53	39.78	16.00	9	7/14/2014	39:43.4
6417	RSPe_2	-122.445	37.937	0.49	15.53	40.67	16.02	-122.445	37.937	0.50	15.53	40.04	16.03	9	7/14/2014	39:43.3
6418	RSPe_2	-122.445	37.937	0.46	15.53	40.94	15.99	-122.445	37.937	0.44	15.53	40.33	15.97	9	7/14/2014	39:43.2
6419	RSPe_2	-122.445	37.937	0.49	15.56	41.18	16.05	-122.445	37.937	0.50	15.56	40.61	16.06	9	7/14/2014	39:43.1
6420	RSPe_2	-122.445	37.937	0.46	15.57	41.46	16.03	-122.445	37.937	0.47	15.57	40.90	16.04	9	7/14/2014	39:43.0
6421	RSPe_2	-122.445	37.937	0.46	15.57	41.71	16.03	-122.445	37.937	0.44	15.57	41.07	16.01	9	7/14/2014	39:42.9
6422	RSPe_2	-122.445	37.937	0.46	15.61	41.97	16.07	-122.445	37.937	0.50	15.61	41.25	16.11	9	7/14/2014	39:42.8
6423	RSPe_2	-122.445	37.937	0.49	15.62	42.27	16.11	-122.445	37.937	0.47	15.62	41.51	16.09	9	7/14/2014	39:42.7
6424	RSPe_2	-122.445	37.937	0.49	15.58	42.62	16.07	-122.445	37.937	0.50	15.58	41.79	16.08	9	7/14/2014	39:42.6
6425	RSPe_2	-122.445	37.937	0.49	15.59	42.86	16.08	-122.445	37.937	0.50	15.59	42.03	16.09	9	7/14/2014	39:42.5
6426	RSPe_2	-122.445	37.937	0.52	15.61	43.12	16.13	-122.445	37.937	0.50	15.61	42.27	16.11	9	7/14/2014	39:42.4
6427	RSPe_2	-122.445	37.937	0.52	15.56	43.45	16.09	-122.445	37.937	0.59	15.56	42.62	16.15	9	7/14/2014	39:42.3
6428	RSPe_2	-122.445	37.937	0.52	15.57	43.73	16.10	-122.445	37.937	0.59	15.57	42.95	16.16	9	7/14/2014	39:42.2
6429	RSPe_2	-122.445	37.937	0.58	15.55	44.04	16.13	-122.445	37.937	0.55	15.55	43.26	16.10	9	7/14/2014	39:42.1
6430	RSPe_2	-122.445	37.937	0.52	15.59	44.26	16.11	-122.445	37.937	0.50	15.59	43.52	16.09	9	7/14/2014	39:42.0
6431	RSPe_2	-122.445	37.937	0.58	15.60	44.42	16.18	-122.445	37.937	0.55	15.60	43.74	16.16	9	7/14/2014	39:41.9
6432	RSPe_2	-122.445	37.937	0.53	15.61	44.67	16.14	-122.445	37.937	0.55	15.61	43.95	16.16	9	7/14/2014	39:41.8
6433	RSPe_2	-122.445	37.937	0.58	15.58	44.87	16.16	-122.445	37.937	0.55	15.58	44.20	16.13	9	7/14/2014	39:41.7
6434	RSPe_2	-122.445	37.937	0.53	15.66	45.09	16.19	-122.445	37.937	0.55	15.66	44.41	16.22	9	7/14/2014	39:41.6

6435	RSPe_2	-122.445	37.937	0.58	15.61	45.35	16.19	-122.445	37.937	0.55	15.61	44.67	16.16	9	7/14/2014 39:41.5
6436	RSPe_2	-122.445	37.937	0.53	15.63	45.57	16.16	-122.445	37.937	0.50	15.63	44.87	16.14	9	7/14/2014 39:41.4
6437	RSPe_2	-122.445	37.937	0.58	15.65	45.90	16.23	-122.445	37.937	0.64	15.65	45.17	16.29	9	7/14/2014 39:41.3
6438	RSPe_2	-122.445	37.937	0.53	15.67	46.16	16.20	-122.445	37.937	0.55	15.67	45.40	16.23	9	7/14/2014 39:41.2
6439	RSPe_2	-122.445	37.937	0.58	15.73	46.44	16.30	-122.445	37.937	0.64	15.73	45.68	16.36	9	7/14/2014 39:41.1
6440	RSPe_2	-122.445	37.937	0.53	15.75	46.73	16.27	-122.445	37.937	0.55	15.75	45.94	16.30	9	7/14/2014 39:41.0
6441	RSPe_2	-122.445	37.937	0.58	15.69	47.06	16.27	-122.445	37.937	0.64	15.69	46.29	16.33	9	7/14/2014 39:40.9
6442	RSPe_2	-122.445	37.937	0.58	15.74	47.29	16.32	-122.445	37.937	0.67	15.74	46.55	16.41	9	7/14/2014 39:40.8
6443	RSPe_2	-122.445	37.937	0.61	15.70	47.54	16.32	-122.445	37.937	0.67	15.70	46.77	16.38	9	7/14/2014 39:40.7
6444	RSPe_2	-122.445	37.937	0.58	15.73	47.76	16.31	-122.445	37.937	0.67	15.73	47.01	16.41	9	7/14/2014 39:40.6
6445	RSPe_2	-122.445	37.937	0.61	15.72	47.95	16.33	-122.445	37.937	0.67	15.72	47.23	16.39	9	7/14/2014 39:40.5
6446	RSPe_2	-122.445	37.937	0.58	15.69	48.13	16.27	-122.445	37.937	0.64	15.69	47.38	16.33	9	7/14/2014 39:40.4
6447	RSPe_2	-122.445	37.937	0.58	15.73	48.30	16.31	-122.445	37.937	0.72	15.73	47.60	16.46	9	7/14/2014 39:40.3
6448	RSPe_2	-122.445	37.937	0.58	15.76	48.52	16.34	-122.445	37.937	0.64	15.76	47.77	16.40	9	7/14/2014 39:40.2
6449	RSPe_2	-122.445	37.937	0.58	15.76	48.72	16.33	-122.445	37.937	0.67	15.76	47.97	16.43	9	7/14/2014 39:40.1
6450	RSPe_2	-122.445	37.937	0.58	15.80	48.94	16.38	-122.445	37.937	0.64	15.80	48.18	16.44	9	7/14/2014 39:40.0
6451	RSPe_2	-122.445	37.937	0.58	15.82	49.16	16.40	-122.445	37.937	0.67	15.82	48.41	16.49	9	7/14/2014 39:39.9
6452	RSPe_2	-122.445	37.937	0.58	15.83	49.40	16.41	-122.445	37.937	0.67	15.83	48.57	16.51	9	7/14/2014 39:39.8
6453	RSPe_2	-122.445	37.937	0.61	15.86	49.59	16.48	-122.445	37.937	0.72	15.86	48.82	16.59	9	7/14/2014 39:39.7
6454	RSPe_2	-122.445	37.937	0.58	15.93	49.88	16.51	-122.445	37.937	0.67	15.93	49.07	16.61	9	7/14/2014 39:39.6
6455	RSPe_2	-122.445	37.937	0.58	15.92	50.14	16.50	-122.445	37.937	0.72	15.92	49.35	16.64	9	7/14/2014 39:39.5
6456	RSPe_2	-122.445	37.937	0.58	15.93	50.29	16.51	-122.445	37.937	0.72	15.93	49.59	16.65	9	7/14/2014 39:39.4
6457	RSPe_2	-122.445	37.937	0.61	15.96	50.56	16.57	-122.445	37.937	0.72	15.96	49.87	16.68	9	7/14/2014 39:39.3
6458	RSPe_2	-122.445	37.937	0.58	15.97	50.82	16.55	-122.445	37.937	0.72	15.97	50.14	16.70	9	7/14/2014 39:39.2
6459	RSPe_2	-122.445	37.937	0.61	16.00	51.01	16.62	-122.445	37.937	0.76	16.00	50.40	16.76	9	7/14/2014 39:39.1
6460	RSPe_2	-122.445	37.937	0.58	15.93	51.19	16.51	-122.445	37.937	0.72	15.93	50.60	16.66	9	7/14/2014 39:39.0
6461	RSPe_2	-122.445	37.937	0.58	15.88	51.43	16.46	-122.445	37.937	0.72	15.88	50.86	16.60	9	7/14/2014 39:38.9
6462	RSPe_2	-122.445	37.937	0.58	15.86	51.65	16.44	-122.445	37.937	0.76	15.86	51.09	16.62	9	7/14/2014 39:38.8
6463	RSPe_2	-122.445	37.937	0.61	15.86	51.87	16.47	-122.445	37.937	0.72	15.86	51.27	16.58	9	7/14/2014 39:38.7
6464	RSPe_2	-122.445	37.937	0.58	15.85	52.06	16.43	-122.445	37.937	0.72	15.85	51.47	16.57	9	7/14/2014 39:38.6
6465	RSPe_2	-122.445	37.937	0.58	15.84	52.28	16.42	-122.445	37.937	0.72	15.84	51.69	16.57	9	7/14/2014 39:38.5
6466	RSPe_2	-122.445	37.937	0.58	15.73	52.39	16.30	-122.445	37.937	0.72	15.73	51.84	16.45	9	7/14/2014 39:38.4
6467	RSPe_2	-122.445	37.937	0.58	15.71	52.63	16.29	-122.445	37.937	0.76	15.71	52.08	16.47	9	7/14/2014 39:38.3

6468	RSPe_2	-122.445	37.937	0.58	15.73	52.83	16.31	-122.445	37.937	0.72	15.73	52.24	16.46	9	7/14/2014	39:38.2
6469	RSPe_2	-122.445	37.937	0.58	15.69	53.02	16.27	-122.445	37.937	0.72	15.69	52.45	16.42	9	7/14/2014	39:38.1
6470	RSPe_2	-122.445	37.937	0.53	15.64	53.22	16.17	-122.445	37.937	0.67	15.64	52.65	16.31	9	7/14/2014	39:38.0
6471	RSPe_2	-122.445	37.937	0.58	15.61	53.37	16.19	-122.445	37.937	0.76	15.61	52.83	16.37	9	7/14/2014	39:37.9
6472	RSPe_2	-122.445	37.937	0.53	15.60	53.57	16.12	-122.445	37.937	0.67	15.60	53.00	16.27	9	7/14/2014	39:37.8
6473	RSPe_2	-122.445	37.937	0.53	15.59	53.77	16.11	-122.445	37.937	0.67	15.59	53.24	16.26	9	7/14/2014	39:37.7
6474	RSPe_2	-122.445	37.937	0.53	15.55	53.97	16.07	-122.445	37.937	0.67	15.55	53.44	16.22	9	7/14/2014	39:37.6
6475	RSPe_2	-122.445	37.937	0.53	15.59	54.12	16.11	-122.445	37.937	0.72	15.59	53.62	16.31	9	7/14/2014	39:37.5
6476	RSPe_2	-122.445	37.937	0.53	15.60	54.33	16.13	-122.445	37.937	0.67	15.60	53.81	16.28	9	7/14/2014	39:37.4
6477	RSPe_2	-122.445	37.937	0.53	15.53	54.47	16.05	-122.445	37.937	0.67	15.53	54.00	16.20	9	7/14/2014	39:37.3
6478	RSPe_2	-122.445	37.937	0.53	15.53	54.60	16.06	-122.445	37.937	0.64	15.53	54.15	16.17	9	7/14/2014	39:37.2
6479	RSPe_2	-122.445	37.937	0.53	15.53	54.71	16.05	-122.445	37.937	0.64	15.53	54.29	16.17	9	7/14/2014	39:37.1
6480	RSPe_2	-122.445	37.937	0.53	15.56	54.88	16.08	-122.445	37.937	0.64	15.56	54.49	16.19	9	7/14/2014	39:37.0
6481	RSPe_2	-122.445	37.937	0.53	15.52	55.06	16.04	-122.445	37.937	0.67	15.52	54.71	16.19	9	7/14/2014	39:36.9
6482	RSPe_2	-122.445	37.937	0.49	15.49	55.32	15.99	-122.445	37.937	0.64	15.49	54.90	16.13	9	7/14/2014	39:36.8
6483	RSPe_2	-122.445	37.937	0.53	15.52	55.52	16.04	-122.445	37.937	0.64	15.52	55.08	16.16	9	7/14/2014	39:36.7
6484	RSPe_2	-122.445	37.937	0.49	15.52	55.72	16.01	-122.445	37.937	0.64	15.52	55.21	16.16	9	7/14/2014	39:36.6
6485	RSPe_2	-122.445	37.937	0.49	15.50	55.89	15.99	-122.445	37.937	0.64	15.50	55.39	16.14	9	7/14/2014	39:36.5
6486	RSPe_2	-122.445	37.937	0.49	15.54	56.04	16.03	-122.445	37.937	0.59	15.54	55.49	16.13	9	7/14/2014	39:36.4
6487	RSPe_2	-122.445	37.937	0.49	15.51	56.20	16.00	-122.445	37.937	0.64	15.51	55.58	16.15	9	7/14/2014	39:36.3
6488	RSPe_2	-122.445	37.937	0.49	15.46	56.31	15.95	-122.445	37.937	0.59	15.46	55.74	16.04	9	7/14/2014	39:36.2
6489	RSPe_2	-122.445	37.937	0.53	15.43	56.45	15.96	-122.445	37.937	0.64	15.43	55.93	16.07	9	7/14/2014	39:36.1
6490	RSPe_2	-122.445	37.937	0.49	15.43	56.65	15.92	-122.445	37.937	0.64	15.43	56.11	16.07	9	7/14/2014	39:36.0
6491	RSPe_2	-122.445	37.937	0.49	15.45	56.76	15.94	-122.445	37.937	0.64	15.45	56.28	16.09	9	7/14/2014	39:35.9
6492	RSPe_2	-122.445	37.937	0.49	15.48	56.92	15.98	-122.445	37.937	0.64	15.48	56.50	16.12	9	7/14/2014	39:35.8
6493	RSPe_2	-122.445	37.937	0.49	15.44	57.07	15.93	-122.445	37.937	0.59	15.44	56.59	16.03	9	7/14/2014	39:35.7
6494	RSPe_2	-122.445	37.937	0.46	15.46	57.27	15.91	-122.445	37.937	0.64	15.46	56.85	16.10	9	7/14/2014	39:35.6
6495	RSPe_2	-122.445	37.937	0.49	15.54	57.38	16.03	-122.445	37.937	0.59	15.54	56.98	16.13	9	7/14/2014	39:35.5
6496	RSPe_2	-122.445	37.937	0.46	15.49	57.53	15.95	-122.445	37.937	0.59	15.49	57.18	16.08	9	7/14/2014	39:35.4
6497	RSPe_2	-122.445	37.937	0.49	15.48	57.75	15.98	-122.445	37.937	0.59	15.48	57.39	16.07	9	7/14/2014	39:35.3
6498	RSPe_2	-122.445	37.937	0.46	15.50	57.88	15.96	-122.445	37.937	0.59	15.50	57.57	16.09	9	7/14/2014	39:35.2
6499	RSPe_2	-122.445	37.937	0.46	15.52	57.99	15.98	-122.445	37.937	0.59	15.52	57.72	16.11	9	7/14/2014	39:35.1
6500	RSPe_2	-122.445	37.937	0.46	15.53	58.10	15.99	-122.445	37.937	0.59	15.53	57.88	16.12	9	7/14/2014	39:35.0

6501	RSPe_2	-122.445	37.937	0.46	15.55	58.27	16.01	-122.445	37.937	0.55	15.55	58.03	16.10	9	7/14/2014	39:34.9
6502	RSPe_2	-122.445	37.937	0.46	15.59	58.42	16.04	-122.445	37.937	0.55	15.59	58.20	16.14	9	7/14/2014	39:34.8
6503	RSPe_2	-122.445	37.937	0.46	15.64	58.58	16.10	-122.445	37.937	0.55	15.64	58.32	16.19	9	7/14/2014	39:34.7
6504	RSPe_2	-122.445	37.937	0.46	15.65	58.80	16.11	-122.445	37.937	0.50	15.65	58.56	16.15	9	7/14/2014	39:34.6
6505	RSPe_2	-122.445	37.937	0.46	15.62	59.00	16.08	-122.445	37.937	0.50	15.62	58.71	16.13	9	7/14/2014	39:34.5
6506	RSPe_2	-122.445	37.937	0.41	15.69	59.23	16.09	-122.445	37.937	0.50	15.69	58.93	16.19	9	7/14/2014	39:34.4
6507	RSPe_2	-122.445	37.937	0.46	15.66	59.41	16.12	-122.445	37.937	0.55	15.66	59.10	16.22	9	7/14/2014	39:34.3
6508	RSPe_2	-122.445	37.937	0.46	15.82	59.56	16.28	-122.445	37.937	0.50	15.82	59.24	16.32	9	7/14/2014	39:34.2
6509	RSPe_2	-122.445	37.937	0.46	15.76	59.72	16.22	-122.445	37.937	0.50	15.76	59.41	16.27	9	7/14/2014	39:34.1
6510	RSPe_2	-122.445	37.937	0.41	15.75	59.91	16.15	-122.445	37.937	0.50	15.75	59.56	16.25	9	7/14/2014	39:34.0
6511	RSPe_2	-122.445	37.937	0.46	15.75	60.07	16.21	-122.445	37.937	0.55	15.75	59.73	16.30	9	7/14/2014	39:33.9
6512	RSPe_2	-122.445	37.937	0.41	15.76	60.22	16.16	-122.445	37.937	0.50	15.76	59.85	16.26	9	7/14/2014	39:33.8
6513	RSPe_2	-122.445	37.937	0.46	15.76	60.31	16.22	-122.445	37.937	0.55	15.76	60.02	16.32	9	7/14/2014	39:33.7
6514	RSPe_2	-122.445	37.937	0.41	15.74	60.50	16.15	-122.445	37.937	0.50	15.74	60.16	16.24	9	7/14/2014	39:33.6
6515	RSPe_2	-122.445	37.937	0.46	15.80	60.66	16.26	-122.445	37.937	0.50	15.80	60.33	16.30	9	7/14/2014	39:33.5
6516	RSPe_2	-122.445	37.937	0.41	15.77	60.81	16.18	-122.445	37.937	0.47	15.77	60.48	16.24	9	7/14/2014	39:33.4
6517	RSPe_2	-122.445	37.937	0.41	15.77	60.92	16.18	-122.445	37.937	0.50	15.77	60.56	16.28	9	7/14/2014	39:33.3
6518	RSPe_2	-122.445	37.937	0.41	15.80	61.20	16.20	-122.445	37.937	0.44	15.80	60.81	16.23	9	7/14/2014	39:33.2
6519	RSPe_2	-122.445	37.937	0.41	15.82	61.33	16.22	-122.445	37.937	0.44	15.82	61.00	16.25	9	7/14/2014	39:33.1
6520	RSPe_2	-122.445	37.937	0.37	15.83	61.53	16.20	-122.445	37.937	0.44	15.83	61.17	16.26	9	7/14/2014	39:33.0
6521	RSPe_2	-122.445	37.937	0.41	15.85	61.77	16.25	-122.445	37.937	0.47	15.85	61.44	16.32	9	7/14/2014	39:32.9
6522	RSPe_2	-122.445	37.937	0.37	15.80	61.94	16.17	-122.445	37.937	0.44	15.80	61.63	16.24	9	7/14/2014	39:32.8
6523	RSPe_2	-122.445	37.937	0.41	15.85	62.19	16.25	-122.445	37.937	0.50	15.85	61.86	16.35	9	7/14/2014	39:32.7
6524	RSPe_2	-122.445	37.937	0.37	15.83	62.29	16.20	-122.445	37.937	0.44	15.83	62.05	16.26	9	7/14/2014	39:32.6
6525	RSPe_2	-122.445	37.937	0.41	15.91	62.47	16.32	-122.445	37.937	0.47	15.91	62.23	16.38	9	7/14/2014	39:32.5
6526	RSPe_2	-122.445	37.937	0.37	15.83	62.62	16.20	-122.445	37.937	0.44	15.83	62.42	16.26	9	7/14/2014	39:32.4
6527	RSPe_2	-122.445	37.937	0.41	15.83	62.78	16.24	-122.445	37.937	0.47	15.83	62.56	16.30	9	7/14/2014	39:32.3
6528	RSPe_2	-122.445	37.937	0.32	15.87	62.97	16.19	-122.445	37.937	0.44	15.87	62.77	16.31	9	7/14/2014	39:32.2
6529	RSPe_2	-122.445	37.937	0.37	15.83	63.17	16.20	-122.445	37.937	0.44	15.83	62.95	16.26	9	7/14/2014	39:32.1
6530	RSPe_2	-122.445	37.937	0.37	15.88	63.39	16.25	-122.445	37.937	0.38	15.88	63.08	16.27	9	7/14/2014	39:32.0
6531	RSPe_2	-122.445	37.937	0.37	15.83	63.58	16.21	-122.445	37.937	0.44	15.83	63.28	16.27	9	7/14/2014	39:31.9
6532	RSPe_2	-122.445	37.937	0.32	15.86	63.76	16.18	-122.445	37.937	0.38	15.86	63.40	16.24	9	7/14/2014	39:31.8
6533	RSPe_2	-122.445	37.937	0.37	15.86	63.96	16.24	-122.445	37.937	0.38	15.86	63.58	16.25	9	7/14/2014	39:31.7

6534	RSPe_2	-122.445	37.937	0.32	15.86	64.02	16.19	-122.445	37.937	0.38	15.86	63.69	16.25	9	7/14/2014	39:31.6
6535	RSPe_2	-122.445	37.937	0.37	15.90	64.20	16.27	-122.445	37.937	0.38	15.90	63.85	16.28	9	7/14/2014	39:31.5
6536	RSPe_2	-122.445	37.937	0.32	15.88	64.42	16.20	-122.445	37.937	0.38	15.88	64.05	16.27	9	7/14/2014	39:31.4
6537	RSPe_2	-122.445	37.937	0.37	15.90	64.51	16.27	-122.445	37.937	0.38	15.90	64.17	16.29	9	7/14/2014	39:31.3
6538	RSPe_2	-122.445	37.937	0.32	15.90	64.68	16.22	-122.445	37.937	0.44	15.90	64.29	16.33	9	7/14/2014	39:31.2
6539	RSPe_2	-122.445	37.937	0.37	15.91	64.86	16.28	-122.445	37.937	0.44	15.91	64.51	16.35	9	7/14/2014	39:31.1
6540	RSPe_2	-122.445	37.937	0.32	15.96	65.05	16.28	-122.445	37.937	0.44	15.96	64.65	16.39	9	7/14/2014	39:31.0
6541	RSPe_2	-122.445	37.937	0.37	15.95	65.27	16.32	-122.445	37.937	0.44	15.95	64.90	16.39	9	7/14/2014	39:30.9
6542	RSPe_2	-122.445	37.937	0.29	15.97	65.44	16.25	-122.445	37.937	0.38	15.97	65.03	16.35	9	7/14/2014	39:30.8
6543	RSPe_2	-122.445	37.937	0.32	16.04	65.56	16.36	-122.445	37.937	0.44	16.04	65.09	16.48	9	7/14/2014	39:30.7
6544	RSPe_2	-122.445	37.937	0.32	15.94	65.68	16.26	-122.445	37.937	0.38	15.94	65.23	16.32	9	7/14/2014	39:30.6
6545	RSPe_2	-122.445	37.937	0.32	15.98	65.75	16.30	-122.445	37.937	0.38	15.98	65.33	16.37	9	7/14/2014	39:30.5
6546	RSPe_2	-122.445	37.937	0.29	16.00	65.84	16.28	-122.445	37.937	0.35	16.00	65.45	16.35	9	7/14/2014	39:30.4
6547	RSPe_2	-122.445	37.937	0.32	16.00	65.95	16.32	-122.445	37.937	0.38	16.00	65.62	16.39	9	7/14/2014	39:30.3
6548	RSPe_2	-122.445	37.937	0.29	16.04	66.04	16.33	-122.445	37.937	0.38	16.04	65.73	16.43	9	7/14/2014	39:30.2
6549	RSPe_2	-122.445	37.937	0.29	16.13	66.12	16.41	-122.445	37.937	0.35	16.13	65.90	16.48	9	7/14/2014	39:30.1
6550	RSPe_2	-122.445	37.937	0.29	16.09	66.26	16.38	-122.445	37.937	0.35	16.09	66.04	16.44	9	7/14/2014	39:30.0
6551	RSPe_2	-122.445	37.937	0.29	16.09	66.36	16.38	-122.445	37.937	0.39	16.09	66.25	16.48	9	7/14/2014	39:29.9
6552	RSPe_2	-122.445	37.937	0.29	16.16	66.60	16.45	-122.445	37.937	0.35	16.16	66.43	16.51	9	7/14/2014	39:29.8
6553	RSPe_2	-122.445	37.937	0.29	16.13	66.80	16.42	-122.445	37.937	0.35	16.13	66.62	16.48	9	7/14/2014	39:29.7
6554	RSPe_2	-122.445	37.937	0.24	16.14	67.02	16.38	-122.445	37.937	0.35	16.14	66.82	16.49	9	7/14/2014	39:29.6
6555	RSPe_2	-122.445	37.937	0.29	16.17	67.21	16.46	-122.445	37.937	0.35	16.17	66.95	16.53	9	7/14/2014	39:29.5
6556	RSPe_2	-122.445	37.937	0.24	16.17	67.37	16.41	-122.445	37.937	0.30	16.17	67.11	16.47	9	7/14/2014	39:29.4
6557	RSPe_2	-122.445	37.937	0.24	16.18	67.55	16.42	-122.445	37.937	0.30	16.18	67.23	16.48	9	7/14/2014	39:29.3
6558	RSPe_2	-122.445	37.937	0.24	16.17	67.63	16.41	-122.445	37.937	0.30	16.17	67.38	16.47	9	7/14/2014	39:29.2
6559	RSPe_2	-122.445	37.937	0.29	16.18	67.81	16.47	-122.445	37.937	0.30	16.18	67.52	16.48	9	7/14/2014	39:29.1
6560	RSPe_2	-122.445	37.937	0.24	16.19	67.96	16.43	-122.445	37.937	0.30	16.19	67.72	16.49	9	7/14/2014	39:29.0
6561	RSPe_2	-122.445	37.937	0.29	16.20	68.13	16.48	-122.445	37.937	0.30	16.20	67.89	16.50	9	7/14/2014	39:28.9
6562	RSPe_2	-122.445	37.937	0.24	16.22	68.29	16.45	-122.445	37.937	0.30	16.22	68.14	16.52	9	7/14/2014	39:28.8
6563	RSPe_2	-122.445	37.937	0.29	16.27	68.53	16.55	-122.445	37.937	0.30	16.27	68.37	16.57	9	7/14/2014	39:28.7
6564	RSPe_2	-122.445	37.937	0.24	16.27	68.77	16.50	-122.445	37.937	0.30	16.27	68.59	16.57	9	7/14/2014	39:28.6
6565	RSPe_2	-122.445	37.937	0.24	16.28	68.99	16.52	-122.445	37.937	0.35	16.28	68.76	16.63	9	7/14/2014	39:28.5
6566	RSPe_2	-122.445	37.937	0.24	16.27	69.18	16.51	-122.445	37.937	0.30	16.27	68.99	16.57	9	7/14/2014	39:28.4

6567	RSPe_2	-122.445	37.937	0.24	16.28	69.32	16.52	-122.445	37.937	0.35	16.28	69.15	16.63	9	7/14/2014 39:28.3
6568	RSPe_2	-122.445	37.937	0.24	16.29	69.49	16.52	-122.445	37.937	0.30	16.29	69.34	16.59	9	7/14/2014 39:28.2
6569	RSPe_2	-122.445	37.937	0.29	16.32	69.62	16.61	-122.445	37.937	0.35	16.32	69.51	16.67	9	7/14/2014 39:28.1
6570	RSPe_2	-122.445	37.937	0.24	16.33	69.78	16.57	-122.445	37.937	0.30	16.33	69.68	16.64	9	7/14/2014 39:28.0
6571	RSPe_2	-122.445	37.937	0.24	16.30	69.92	16.53	-122.445	37.937	0.35	16.30	69.88	16.65	9	7/14/2014 39:27.9
6572	RSPe_2	-122.445	37.937	0.24	16.33	70.10	16.57	-122.445	37.937	0.35	16.33	70.07	16.68	9	7/14/2014 39:27.8
6573	RSPe_2	-122.445	37.937	0.24	16.33	70.34	16.57	-122.445	37.937	0.35	16.33	70.23	16.69	9	7/14/2014 39:27.7
6574	RSPe_2	-122.445	37.937	0.24	16.41	70.51	16.65	-122.445	37.937	0.30	16.41	70.41	16.71	9	7/14/2014 39:27.6
6575	RSPe_2	-122.445	37.937	0.29	16.37	70.74	16.66	-122.445	37.937	0.35	16.37	70.57	16.72	9	7/14/2014 39:27.5
6576	RSPe_2	-122.445	37.937	0.24	16.46	70.98	16.69	-122.445	37.937	0.35	16.46	70.80	16.81	9	7/14/2014 39:27.4
6577	RSPe_2	-122.445	37.937	0.24	16.36	71.21	16.59	-122.445	37.937	0.44	16.36	71.06	16.79	9	7/14/2014 39:27.3
6578	RSPe_2	-122.445	37.937	0.24	16.33	71.50	16.57	-122.445	37.937	0.44	16.33	71.36	16.77	9	7/14/2014 39:27.2
6579	RSPe_2	-122.445	37.937	0.24	16.33	71.66	16.57	-122.445	37.937	0.44	16.33	71.57	16.77	9	7/14/2014 39:27.1
6580	RSPe_2	-122.445	37.937	0.24	16.33	71.89	16.57	-122.445	37.937	0.44	16.33	71.82	16.77	9	7/14/2014 39:27.0
6581	RSPe_2	-122.445	37.937	0.29	16.33	72.07	16.62	-122.445	37.937	0.39	16.33	72.05	16.72	9	7/14/2014 39:26.9
6582	RSPe_2	-122.445	37.937	0.24	16.23	72.22	16.47	-122.445	37.937	0.39	16.23	72.23	16.62	9	7/14/2014 39:26.8
6583	RSPe_2	-122.445	37.937	0.29	16.20	72.40	16.49	-122.445	37.937	0.39	16.20	72.47	16.59	9	7/14/2014 39:26.7
6584	RSPe_2	-122.445	37.937	0.20	16.22	72.55	16.42	-122.445	37.937	0.35	16.22	72.64	16.57	9	7/14/2014 39:26.6
6585	RSPe_2	-122.445	37.937	0.24	16.11	72.64	16.35	-122.445	37.937	0.35	16.11	72.79	16.46	9	7/14/2014 39:26.5
6586	RSPe_2	-122.445	37.937	0.20	16.12	72.86	16.32	-122.445	37.937	0.35	16.12	73.01	16.47	9	7/14/2014 39:26.4
6587	RSPe_2	-122.445	37.937	0.24	16.04	73.03	16.28	-122.445	37.937	0.39	16.04	73.15	16.43	9	7/14/2014 39:26.3
6588	RSPe_2	-122.445	37.937	0.24	16.06	73.16	16.29	-122.445	37.937	0.39	16.06	73.29	16.44	9	7/14/2014 39:26.2
6589	RSPe_2	-122.445	37.937	0.24	15.97	73.38	16.21	-122.445	37.937	0.39	15.97	73.51	16.36	9	7/14/2014 39:26.1
6590	RSPe_2	-122.445	37.937	0.24	15.93	73.53	16.17	-122.445	37.937	0.39	15.93	73.67	16.32	9	7/14/2014 39:26.0
6591	RSPe_2	-122.445	37.937	0.24	15.94	73.71	16.18	-122.445	37.937	0.39	15.94	73.82	16.33	9	7/14/2014 39:25.9
6592	RSPe_2	-122.445	37.937	0.24	15.93	73.95	16.16	-122.445	37.937	0.35	15.93	74.10	16.28	9	7/14/2014 39:25.8
6593	RSPe_2	-122.445	37.937	0.29	15.91	74.13	16.20	-122.445	37.937	0.35	15.91	74.28	16.26	9	7/14/2014 39:25.7
6594	RSPe_2	-122.445	37.937	0.24	15.89	74.34	16.12	-122.445	37.937	0.35	15.89	74.47	16.24	9	7/14/2014 39:25.6
6595	RSPe_2	-122.445	37.937	0.24	15.88	74.58	16.12	-122.445	37.937	0.35	15.88	74.67	16.23	9	7/14/2014 39:25.5
6596	RSPe_2	-122.445	37.937	0.24	15.87	74.84	16.11	-122.445	37.937	0.30	15.87	74.85	16.17	9	7/14/2014 39:25.4
6597	RSPe_2	-122.445	37.937	0.24	15.85	75.09	16.08	-122.445	37.937	0.35	15.85	75.03	16.20	9	7/14/2014 39:25.3
6598	RSPe_2	-122.445	37.937	0.20	15.85	75.30	16.05	-122.445	37.937	0.35	15.85	75.28	16.20	9	7/14/2014 39:25.2
6599	RSPe_2	-122.445	37.937	0.24	15.84	75.50	16.08	-122.445	37.937	0.35	15.84	75.41	16.19	9	7/14/2014 39:25.1

6600	RSPe_2	-122.445	37.937	0.24	15.81	75.70	16.05	-122.445	37.937	0.35	15.81	75.63	16.16	9	7/14/2014 39:25.0
6601	RSPe_2	-122.445	37.937	0.24	15.79	75.90	16.02	-122.445	37.937	0.39	15.79	75.87	16.17	9	7/14/2014 39:24.9
6602	RSPe_2	-122.445	37.937	0.24	15.74	76.09	15.98	-122.445	37.937	0.35	15.74	76.11	16.09	9	7/14/2014 39:24.8
6603	RSPe_2	-122.445	37.937	0.24	15.75	76.24	15.98	-122.445	37.937	0.35	15.75	76.25	16.10	9	7/14/2014 39:24.7
6604	RSPe_2	-122.445	37.937	0.20	15.74	76.38	15.94	-122.445	37.937	0.30	15.74	76.40	16.04	9	7/14/2014 39:24.6
6605	RSPe_2	-122.445	37.937	0.24	15.74	76.51	15.98	-122.445	37.937	0.35	15.74	76.59	16.09	9	7/14/2014 39:24.5
6606	RSPe_2	-122.445	37.937	0.24	15.77	76.70	16.01	-122.445	37.937	0.35	15.77	76.81	16.12	9	7/14/2014 39:24.4
6607	RSPe_2	-122.445	37.937	0.24	15.75	76.88	15.98	-122.445	37.937	0.35	15.75	77.02	16.10	9	7/14/2014 39:24.3
6608	RSPe_2	-122.445	37.937	0.20	15.80	77.04	16.00	-122.445	37.937	0.30	15.80	77.23	16.10	9	7/14/2014 39:24.2
6609	RSPe_2	-122.445	37.937	0.24	15.78	77.27	16.02	-122.445	37.937	0.30	15.78	77.42	16.08	9	7/14/2014 39:24.1
6610	RSPe_2	-122.445	37.937	0.24	15.79	77.53	16.02	-122.445	37.937	0.30	15.79	77.64	16.09	9	7/14/2014 39:24.0
6611	RSPe_2	-122.445	37.937	0.24	15.78	77.72	16.02	-122.445	37.937	0.35	15.78	77.80	16.13	9	7/14/2014 39:23.9
6612	RSPe_2	-122.445	37.937	0.20	15.89	78.02	16.09	-122.445	37.937	0.39	15.89	77.97	16.27	9	7/14/2014 39:23.8
6613	RSPe_2	-122.445	37.937	0.20	15.80	78.13	16.00	-122.445	37.937	0.30	15.80	78.12	16.10	9	7/14/2014 39:23.7
6614	RSPe_2	-122.445	37.937	0.20	15.84	78.30	16.04	-122.445	37.937	0.30	15.84	78.21	16.14	9	7/14/2014 39:23.6
6615	RSPe_2	-122.445	37.937	0.24	15.83	78.45	16.07	-122.445	37.937	0.35	15.83	78.39	16.19	9	7/14/2014 39:23.5
6616	RSPe_2	-122.445	37.937	0.20	15.81	78.61	16.01	-122.445	37.937	0.30	15.81	78.53	16.11	9	7/14/2014 39:23.4
6617	RSPe_2	-122.445	37.937	0.20	15.84	78.83	16.04	-122.445	37.937	0.30	15.84	78.71	16.14	9	7/14/2014 39:23.3
6618	RSPe_2	-122.445	37.937	0.20	15.88	78.98	16.08	-122.445	37.937	0.30	15.88	78.88	16.18	9	7/14/2014 39:23.2
6619	RSPe_2	-122.445	37.937	0.20	15.85	79.24	16.05	-122.445	37.937	0.30	15.85	79.11	16.15	9	7/14/2014 39:23.1
6620	RSPe_2	-122.445	37.937	0.17	15.87	79.35	16.04	-122.445	37.937	0.22	15.87	79.26	16.09	9	7/14/2014 39:23.0
6621	RSPe_2	-122.445	37.937	0.20	15.93	79.54	16.13	-122.445	37.937	0.27	15.93	79.45	16.20	9	7/14/2014 39:22.9
6622	RSPe_2	-122.445	37.937	0.17	15.91	79.70	16.08	-122.445	37.937	0.22	15.91	79.66	16.13	9	7/14/2014 39:22.8
6623	RSPe_2	-122.445	37.937	0.17	15.90	79.89	16.06	-122.445	37.937	0.30	15.90	79.90	16.20	9	7/14/2014 39:22.7
6624	RSPe_2	-122.445	37.937	0.17	15.86	80.05	16.03	-122.445	37.937	0.22	15.86	80.10	16.07	9	7/14/2014 39:22.6
6625	RSPe_2	-122.445	37.937	0.17	15.90	80.22	16.07	-122.445	37.937	0.27	15.90	80.31	16.17	9	7/14/2014 39:22.5
6626	RSPe_2	-122.445	37.937	0.17	15.84	80.46	16.01	-122.445	37.937	0.22	15.84	80.50	16.06	9	7/14/2014 39:22.4
6627	RSPe_2	-122.445	37.937	0.17	15.88	80.68	16.05	-122.445	37.937	0.22	15.88	80.73	16.10	9	7/14/2014 39:22.3
6628	RSPe_2	-122.445	37.937	0.12	15.92	80.81	16.04	-122.445	37.937	0.22	15.92	80.88	16.14	9	7/14/2014 39:22.2
6629	RSPe_2	-122.445	37.937	0.17	15.93	81.01	16.10	-122.445	37.937	0.27	15.93	81.13	16.20	9	7/14/2014 39:22.1
6630	RSPe_2	-122.445	37.937	0.12	15.93	81.23	16.05	-122.445	37.937	0.27	15.93	81.38	16.20	9	7/14/2014 39:22.0
6631	RSPe_2	-122.445	37.937	0.12	15.90	81.40	16.01	-122.445	37.937	0.22	15.90	81.58	16.11	9	7/14/2014 39:21.9
6632	RSPe_2	-122.445	37.937	0.12	15.99	81.56	16.10	-122.445	37.937	0.18	15.99	81.80	16.17	9	7/14/2014 39:21.8

6633	RSPe_2	-122.445	37.937	0.12	16.03	81.71	16.14	-122.445	37.937	0.18	16.03	81.97	16.21	9	7/14/2014 39:21.7
6634	RSPe_2	-122.445	37.937	0.08	16.07	81.89	16.15	-122.445	37.937	0.15	16.07	82.21	16.22	9	7/14/2014 39:21.6
6635	RSPe_2	-122.445	37.937	0.12	16.09	82.06	16.21	-122.445	37.937	0.15	16.09	82.43	16.24	9	7/14/2014 39:21.5
6636	RSPe_2	-122.445	37.937	0.08	16.17	82.25	16.25	-122.445	37.937	0.10	16.17	82.67	16.26	9	7/14/2014 39:21.4
6637	RSPe_2	-122.445	37.937	0.12	16.21	82.41	16.33	-122.445	37.937	0.15	16.21	82.83	16.36	9	7/14/2014 39:21.3
6638	RSPe_2	-122.445	37.937	0.08	16.26	82.58	16.34	-122.445	37.937	0.10	16.26	83.00	16.36	9	7/14/2014 39:21.2
6639	RSPe_2	-122.445	37.937	0.12	16.27	82.81	16.39	-122.445	37.937	0.10	16.27	83.18	16.37	9	7/14/2014 39:21.1
6640	RSPe_2	-122.445	37.937	0.08	16.33	83.02	16.41	-122.445	37.937	0.06	16.33	83.35	16.39	9	7/14/2014 39:21.0
6641	RSPe_2	-122.445	37.937	0.08	16.37	83.24	16.45	-122.445	37.937	0.06	16.37	83.52	16.43	9	7/14/2014 39:20.9
6642	RSPe_2	-122.445	37.937	0.03	16.36	83.50	16.39	-122.445	37.937	0.06	16.36	83.70	16.42	9	7/14/2014 39:20.8
6643	RSPe_2	-122.445	37.937	0.08	16.41	83.68	16.49	-122.445	37.937	0.06	16.41	83.82	16.47	9	7/14/2014 39:20.7
6644	RSPe_2	-122.445	37.937	0.03	16.37	83.92	16.40	-122.445	37.937	0.01	16.37	83.99	16.39	9	7/14/2014 39:20.6
6645	RSPe_2	-122.445	37.937	0.08	16.44	84.10	16.52	-122.445	37.937	0.01	16.44	84.14	16.45	9	7/14/2014 39:20.5
6646	RSPe_2	-122.445	37.937	0.03	16.42	84.31	16.45	-122.445	37.937	0.01	16.42	84.31	16.43	9	7/14/2014 39:20.4
6647	RSPe_2	-122.445	37.937	0.08	16.42	84.49	16.50	-122.445	37.937	0.01	16.42	84.46	16.43	9	7/14/2014 39:20.3
6648	RSPe_2	-122.445	37.937	0.03	16.43	84.62	16.46	-122.445	37.937	0.01	16.43	84.65	16.44	9	7/14/2014 39:20.2
6649	RSPe_2	-122.445	37.937	0.03	16.43	84.85	16.46	-122.445	37.937	0.01	16.43	84.81	16.44	9	7/14/2014 39:20.1
6650	RSPe_2	-122.445	37.937	0.03	16.44	85.01	16.47	-122.445	37.937	-0.02	16.44	85.05	16.42	9	7/14/2014 39:20.0
6651	RSPe_2	-122.445	37.937	0.03	16.48	85.23	16.51	-122.445	37.937	-0.07	16.48	85.36	16.41	9	7/14/2014 39:19.9
6652	RSPe_2	-122.445	37.937	0.00	16.51	85.43	16.51	-122.445	37.937	-0.07	16.51	85.51	16.44	9	7/14/2014 39:19.8
6653	RSPe_2	-122.445	37.937	0.03	16.53	85.60	16.56	-122.445	37.937	-0.07	16.53	85.72	16.46	9	7/14/2014 39:19.7
6654	RSPe_2	-122.445	37.937	0.00	16.55	85.79	16.55	-122.445	37.937	-0.11	16.55	85.95	16.44	9	7/14/2014 39:19.6
6655	RSPe_2	-122.445	37.937	0.00	16.60	86.00	16.59	-122.445	37.937	-0.02	16.60	86.14	16.58	9	7/14/2014 39:19.5
6656	RSPe_2	-122.445	37.937	0.00	16.64	86.17	16.63	-122.445	37.937	-0.11	16.64	86.34	16.53	9	7/14/2014 39:19.4
6657	RSPe_2	-122.445	37.937	0.03	16.63	86.41	16.66	-122.445	37.937	-0.07	16.63	86.61	16.56	9	7/14/2014 39:19.3
6658	RSPe_2	-122.445	37.937	0.03	16.67	86.74	16.70	-122.445	37.937	-0.11	16.67	86.87	16.57	9	7/14/2014 39:19.2
6659	RSPe_2	-122.445	37.937	0.03	16.72	86.96	16.75	-122.445	37.937	-0.02	16.72	87.10	16.70	9	7/14/2014 39:19.1
6660	RSPe_2	-122.445	37.937	0.03	16.72	87.11	16.75	-122.445	37.937	-0.07	16.72	87.28	16.64	9	7/14/2014 39:19.0
6661	RSPe_2	-122.445	37.937	0.03	16.73	87.39	16.76	-122.445	37.937	-0.07	16.73	87.50	16.66	9	7/14/2014 39:18.9
6662	RSPe_2	-122.445	37.937	0.00	16.72	87.61	16.72	-122.445	37.937	-0.07	16.72	87.71	16.65	9	7/14/2014 39:18.8
6663	RSPe_2	-122.445	37.937	0.03	16.74	87.85	16.77	-122.445	37.937	-0.07	16.74	87.94	16.67	9	7/14/2014 39:18.7
6664	RSPe_2	-122.445	37.937	0.00	16.76	88.00	16.76	-122.445	37.937	-0.11	16.76	88.10	16.65	9	7/14/2014 39:18.6
6665	RSPe_2	-122.445	37.937	0.03	16.78	88.23	16.82	-122.445	37.937	-0.11	16.78	88.30	16.68	9	7/14/2014 39:18.5

6666	RSPe_2	-122.445	37.937	0.00	16.76	88.44	16.76	-122.445	37.937	-0.11	16.76	88.53	16.65	9	7/14/2014	39:18.4
6667	RSPe_2	-122.445	37.937	0.00	16.76	88.62	16.76	-122.445	37.937	-0.11	16.76	88.70	16.65	9	7/14/2014	39:18.3
6668	RSPe_2	-122.445	37.937	0.00	16.77	88.77	16.76	-122.445	37.937	-0.11	16.77	88.83	16.66	9	7/14/2014	39:18.2
6669	RSPe_2	-122.445	37.937	0.00	16.79	88.97	16.79	-122.445	37.937	-0.16	16.79	89.03	16.63	9	7/14/2014	39:18.1
6670	RSPe_2	-122.445	37.937	0.00	16.81	89.25	16.81	-122.445	37.937	-0.11	16.81	89.36	16.71	9	7/14/2014	39:18.0
6671	RSPe_2	-122.445	37.937	0.00	16.84	89.45	16.84	-122.445	37.937	-0.10	16.84	89.58	16.74	9	7/14/2014	39:17.9
6672	RSPe_2	-122.445	37.937	0.00	16.86	89.58	16.86	-122.445	37.937	-0.16	16.86	89.77	16.71	9	7/14/2014	39:17.8
6673	RSPe_2	-122.445	37.937	0.00	16.92	89.82	16.92	-122.445	37.937	-0.16	16.92	90.01	16.77	9	7/14/2014	39:17.7
6674	RSPe_2	-122.445	37.937	0.00	16.92	90.06	16.92	-122.445	37.937	-0.16	16.92	90.23	16.77	9	7/14/2014	39:17.6
6675	RSPe_2	-122.445	37.937	0.00	16.92	90.28	16.92	-122.445	37.937	-0.16	16.92	90.45	16.77	9	7/14/2014	39:17.5
6676	RSPe_2	-122.445	37.937	0.00	16.91	90.53	16.91	-122.445	37.937	-0.10	16.91	90.69	16.81	9	7/14/2014	39:17.4
6677	RSPe_2	-122.445	37.937	0.00	16.89	90.82	16.89	-122.445	37.937	-0.10	16.89	90.95	16.79	9	7/14/2014	39:17.3
6678	RSPe_2	-122.445	37.937	-0.05	16.97	91.03	16.91	-122.445	37.937	-0.16	16.97	91.11	16.81	9	7/14/2014	39:17.2
6679	RSPe_2	-122.445	37.937	0.00	16.88	91.26	16.88	-122.445	37.937	-0.16	16.88	91.35	16.73	9	7/14/2014	39:17.1
6680	RSPe_2	-122.445	37.937	-0.05	16.94	91.43	16.89	-122.445	37.937	-0.19	16.94	91.50	16.76	9	7/14/2014	39:17.0
6681	RSPe_2	-122.445	37.937	0.00	16.77	91.53	16.77	-122.445	37.937	-0.19	16.77	91.66	16.59	9	7/14/2014	39:16.9
6682	RSPe_2	-122.445	37.937	-0.05	16.81	91.76	16.76	-122.445	37.937	-0.19	16.81	91.83	16.62	9	7/14/2014	39:16.8
6683	RSPe_2	-122.445	37.937	0.00	16.76	91.94	16.76	-122.445	37.937	-0.19	16.76	92.04	16.57	9	7/14/2014	39:16.7
6684	RSPe_2	-122.445	37.937	0.00	16.70	92.13	16.70	-122.445	37.937	-0.22	16.70	92.26	16.48	9	7/14/2014	39:16.6
6685	RSPe_2	-122.445	37.937	0.00	16.70	92.33	16.70	-122.445	37.937	-0.22	16.70	92.48	16.48	9	7/14/2014	39:16.5
6686	RSPe_2	-122.445	37.937	-0.05	16.68	92.53	16.63	-122.445	37.937	-0.27	16.68	92.71	16.41	9	7/14/2014	39:16.4
6687	RSPe_2	-122.445	37.937	0.00	16.70	92.77	16.70	-122.445	37.937	-0.22	16.70	92.96	16.48	9	7/14/2014	39:16.3
6688	RSPe_2	-122.445	37.937	-0.05	16.70	93.03	16.64	-122.445	37.937	-0.22	16.70	93.23	16.48	9	7/14/2014	39:16.2
6689	RSPe_2	-122.445	37.937	0.00	16.72	93.29	16.71	-122.445	37.937	-0.22	16.72	93.44	16.49	9	7/14/2014	39:16.1
6690	RSPe_2	-122.445	37.937	-0.05	16.65	93.53	16.60	-122.445	37.937	-0.22	16.65	93.67	16.43	9	7/14/2014	39:16.0
6691	RSPe_2	-122.445	37.937	0.00	16.64	93.74	16.63	-122.445	37.937	-0.22	16.64	93.90	16.41	9	7/14/2014	39:15.9
6692	RSPe_2	-122.445	37.937	-0.05	16.62	93.90	16.57	-122.445	37.937	-0.19	16.62	94.04	16.43	9	7/14/2014	39:15.8
6693	RSPe_2	-122.445	37.937	0.00	16.60	94.06	16.60	-122.445	37.937	-0.22	16.60	94.21	16.38	9	7/14/2014	39:15.7
6694	RSPe_2	-122.445	37.937	-0.05	16.58	94.21	16.52	-122.445	37.937	-0.22	16.58	94.39	16.35	9	7/14/2014	39:15.6
6695	RSPe_2	-122.445	37.937	0.00	16.55	94.38	16.55	-122.445	37.937	-0.22	16.55	94.56	16.33	9	7/14/2014	39:15.5
6696	RSPe_2	-122.445	37.937	-0.05	16.51	94.63	16.46	-122.445	37.937	-0.22	16.51	94.76	16.29	9	7/14/2014	39:15.4
6697	RSPe_2	-122.445	37.937	0.00	16.48	94.82	16.48	-122.445	37.937	-0.19	16.48	94.93	16.29	9	7/14/2014	39:15.3
6698	RSPe_2	-122.445	37.937	-0.05	16.52	94.95	16.47	-122.445	37.937	-0.22	16.52	95.06	16.30	9	7/14/2014	39:15.2

6699	RSPe_2	-122.445	37.937	0.00	16.46	95.13	16.46	-122.445	37.937	-0.27	16.46	95.21	16.19	9	7/14/2014	39:15.1
6700	RSPe_2	-122.445	37.937	-0.05	16.44	95.32	16.39	-122.445	37.937	-0.27	16.44	95.44	16.17	9	7/14/2014	39:15.0
6701	RSPe_2	-122.445	37.937	-0.05	16.41	95.57	16.36	-122.445	37.937	-0.27	16.41	95.60	16.14	9	7/14/2014	39:14.9
6702	RSPe_2	-122.445	37.937	-0.05	16.41	95.72	16.36	-122.445	37.937	-0.27	16.41	95.83	16.14	9	7/14/2014	39:14.8
6703	RSPe_2	-122.445	37.937	-0.05	16.45	95.96	16.40	-122.445	37.937	-0.27	16.45	96.05	16.18	9	7/14/2014	39:14.7
6704	RSPe_2	-122.445	37.937	-0.05	16.46	96.18	16.40	-122.445	37.937	-0.27	16.46	96.26	16.19	9	7/14/2014	39:14.6
6705	RSPe_2	-122.445	37.937	0.00	16.43	96.38	16.43	-122.445	37.937	-0.22	16.43	96.52	16.21	9	7/14/2014	39:14.5
6706	RSPe_2	-122.445	37.937	-0.05	16.47	96.61	16.42	-122.445	37.937	-0.27	16.47	96.77	16.20	9	7/14/2014	39:14.4
6707	RSPe_2	-122.445	37.937	-0.05	16.42	96.79	16.37	-122.445	37.937	-0.31	16.42	96.97	16.11	9	7/14/2014	39:14.3
6708	RSPe_2	-122.445	37.937	-0.05	16.44	97.03	16.39	-122.445	37.937	-0.31	16.44	97.17	16.14	9	7/14/2014	39:14.2
6709	RSPe_2	-122.445	37.937	0.00	16.44	97.18	16.44	-122.445	37.937	-0.27	16.44	97.32	16.17	9	7/14/2014	39:14.1
6710	RSPe_2	-122.445	37.937	-0.09	16.46	97.47	16.37	-122.445	37.937	-0.27	16.46	97.54	16.19	9	7/14/2014	39:14.0
6711	RSPe_2	-122.445	37.937	0.00	16.44	97.71	16.44	-122.445	37.937	-0.31	16.44	97.73	16.14	9	7/14/2014	39:13.9
6712	RSPe_2	-122.445	37.937	-0.05	16.44	97.91	16.39	-122.445	37.937	-0.31	16.44	97.90	16.14	9	7/14/2014	39:13.8
6713	RSPe_2	-122.445	37.937	-0.05	16.44	98.10	16.38	-122.445	37.937	-0.27	16.44	98.09	16.16	9	7/14/2014	39:13.7
6714	RSPe_2	-122.445	37.937	-0.05	16.41	98.21	16.35	-122.445	37.937	-0.27	16.41	98.19	16.13	9	7/14/2014	39:13.6
6715	RSPe_2	-122.445	37.937	-0.05	16.44	98.37	16.38	-122.445	37.937	-0.27	16.44	98.39	16.16	9	7/14/2014	39:13.5
6716	RSPe_2	-122.445	37.937	-0.05	16.42	98.65	16.37	-122.445	37.937	-0.27	16.42	98.69	16.15	9	7/14/2014	39:13.4
6717	RSPe_2	-122.445	37.937	-0.05	16.48	98.93	16.43	-122.445	37.937	-0.27	16.48	98.95	16.21	9	7/14/2014	39:13.3
6718	RSPe_2	-122.445	37.937	-0.05	16.49	99.08	16.44	-122.445	37.937	-0.31	16.49	99.19	16.18	9	7/14/2014	39:13.2
6719	RSPe_2	-122.445	37.937	0.00	16.55	99.22	16.55	-122.445	37.937	-0.31	16.55	99.32	16.24	9	7/14/2014	39:13.1
6720	RSPe_2	-122.445	37.937	-0.05	16.53	99.34	16.47	-122.445	37.937	-0.27	16.53	99.50	16.25	9	7/14/2014	39:13.0
6721	RSPe_2	-122.445	37.937	0.00	16.51	99.48	16.51	-122.445	37.937	-0.27	16.51	99.63	16.24	9	7/14/2014	39:12.9
6722	RSPe_2	-122.445	37.937	-0.05	16.52	99.67	16.47	-122.445	37.937	-0.27	16.52	99.79	16.25	9	7/14/2014	39:12.8
6723	RSPe_2	-122.445	37.937	0.00	16.55	99.88	16.55	-122.445	37.937	-0.27	16.55	100.02	16.28	9	7/14/2014	39:12.7
6724	RSPe_2	-122.445	37.937	-0.05	16.56	100.07	16.51	-122.445	37.937	-0.31	16.56	100.22	16.25	9	7/14/2014	39:12.6
6725	RSPe_2	-122.445	37.937	0.00	16.60	100.35	16.60	-122.445	37.937	-0.27	16.60	100.47	16.32	9	7/14/2014	39:12.5
6726	RSPe_2	-122.445	37.937	-0.05	16.62	100.58	16.57	-122.445	37.937	-0.27	16.62	100.68	16.35	9	7/14/2014	39:12.4
6727	RSPe_2	-122.445	37.937	0.00	16.63	100.81	16.63	-122.445	37.937	-0.31	16.63	100.90	16.32	9	7/14/2014	39:12.3
6728	RSPe_2	-122.445	37.937	0.00	16.63	101.03	16.63	-122.445	37.937	-0.31	16.63	101.14	16.32	9	7/14/2014	39:12.2
6729	RSPe_2	-122.445	37.937	0.00	16.66	101.25	16.66	-122.445	37.937	-0.31	16.66	101.31	16.35	9	7/14/2014	39:12.1
6730	RSPe_2	-122.445	37.937	-0.05	16.68	101.40	16.63	-122.445	37.937	-0.27	16.68	101.51	16.41	9	7/14/2014	39:12.0
6731	RSPe_2	-122.445	37.937	0.00	16.67	101.60	16.67	-122.445	37.937	-0.31	16.67	101.64	16.37	9	7/14/2014	39:11.9

6732	RSPe_2	-122.445	37.937	-0.05	16.65	101.75	16.59	-122.445	37.937	-0.27	16.65	101.77	16.37	9	7/14/2014 39:11.8
6733	RSPe_2	-122.445	37.937	0.00	16.65	101.93	16.65	-122.445	37.937	-0.27	16.65	101.97	16.38	9	7/14/2014 39:11.7
6734	RSPe_2	-122.445	37.937	0.00	16.70	102.12	16.70	-122.445	37.937	-0.31	16.70	102.10	16.40	9	7/14/2014 39:11.6
6735	RSPe_2	-122.445	37.937	0.00	16.69	102.33	16.69	-122.445	37.937	-0.27	16.69	102.32	16.42	9	7/14/2014 39:11.5
6736	RSPe_2	-122.445	37.937	0.00	16.66	102.52	16.66	-122.445	37.937	-0.27	16.66	102.51	16.39	9	7/14/2014 39:11.4
6737	RSPe_2	-122.445	37.937	0.00	16.72	102.74	16.72	-122.445	37.937	-0.27	16.72	102.75	16.45	9	7/14/2014 39:11.3
6738	RSPe_2	-122.445	37.937	0.00	16.70	102.84	16.70	-122.445	37.937	-0.27	16.70	102.82	16.43	9	7/14/2014 39:11.2
6739	RSPe_2	-122.445	37.937	0.03	16.67	103.02	16.71	-122.445	37.937	-0.27	16.67	102.98	16.40	9	7/14/2014 39:11.1
6740	RSPe_2	-122.445	37.937	0.00	16.72	103.27	16.71	-122.445	37.937	-0.22	16.72	103.16	16.49	9	7/14/2014 39:11.0
6741	RSPe_2	-122.445	37.937	0.03	16.73	103.39	16.76	-122.445	37.937	-0.27	16.73	103.28	16.45	9	7/14/2014 39:10.9
6742	RSPe_2	-122.445	37.937	0.00	16.72	103.57	16.72	-122.445	37.937	-0.31	16.72	103.48	16.41	9	7/14/2014 39:10.8
6743	RSPe_2	-122.445	37.937	0.03	16.76	103.85	16.79	-122.445	37.937	-0.27	16.76	103.71	16.49	9	7/14/2014 39:10.7
6744	RSPe_2	-122.445	37.937	0.00	16.78	104.07	16.78	-122.445	37.937	-0.36	16.78	103.98	16.43	9	7/14/2014 39:10.6
6745	RSPe_2	-122.445	37.937	0.03	16.78	104.38	16.82	-122.445	37.937	-0.31	16.78	104.23	16.48	9	7/14/2014 39:10.5
6746	RSPe_2	-122.445	37.937	0.00	16.92	104.53	16.92	-122.445	37.937	-0.31	16.92	104.39	16.61	9	7/14/2014 39:10.4
6747	RSPe_2	-122.445	37.937	0.03	16.84	104.76	16.87	-122.445	37.937	-0.31	16.84	104.64	16.53	9	7/14/2014 39:10.3
6748	RSPe_2	-122.445	37.937	0.00	16.87	105.06	16.86	-122.445	37.937	-0.31	16.87	104.79	16.56	9	7/14/2014 39:10.2
6749	RSPe_2	-122.445	37.937	0.03	16.83	105.22	16.86	-122.445	37.937	-0.27	16.83	104.98	16.55	9	7/14/2014 39:10.1
6750	RSPe_2	-122.445	37.937	0.00	16.81	105.36	16.80	-122.445	37.937	-0.27	16.81	105.05	16.53	9	7/14/2014 39:10.0
6751	RSPe_2	-122.445	37.937	0.03	16.80	105.60	16.83	-122.445	37.937	-0.27	16.80	105.29	16.52	9	7/14/2014 39:09.9
6752	RSPe_2	-122.445	37.937	0.00	16.83	105.82	16.83	-122.445	37.937	-0.27	16.83	105.47	16.55	9	7/14/2014 39:09.8
6753	RSPe_2	-122.445	37.937	0.03	16.81	106.01	16.84	-122.445	37.937	-0.22	16.81	105.67	16.59	9	7/14/2014 39:09.7
6754	RSPe_2	-122.445	37.937	0.00	16.83	106.21	16.83	-122.445	37.937	-0.27	16.83	105.86	16.55	9	7/14/2014 39:09.6
6755	RSPe_2	-122.445	37.937	0.03	16.78	106.45	16.82	-122.445	37.937	-0.22	16.78	106.10	16.56	9	7/14/2014 39:09.5
6756	RSPe_2	-122.445	37.937	0.03	16.77	106.71	16.81	-122.445	37.937	-0.22	16.77	106.32	16.55	9	7/14/2014 39:09.4
6757	RSPe_2	-122.445	37.937	0.03	16.80	106.93	16.83	-122.445	37.937	-0.22	16.80	106.56	16.58	9	7/14/2014 39:09.3
6758	RSPe_2	-122.445	37.937	0.03	16.96	107.17	16.99	-122.445	37.937	-0.22	16.96	106.82	16.74	9	7/14/2014 39:09.2
6759	RSPe_2	-122.445	37.937	0.08	16.84	107.31	16.92	-122.445	37.937	-0.22	16.84	107.02	16.62	9	7/14/2014 39:09.1
6760	RSPe_2	-122.445	37.937	0.03	16.83	107.52	16.86	-122.445	37.937	-0.27	16.83	107.19	16.55	9	7/14/2014 39:09.0
6761	RSPe_2	-122.445	37.937	0.08	16.81	107.79	16.90	-122.445	37.937	-0.19	16.81	107.44	16.62	9	7/14/2014 39:08.9
6762	RSPe_2	-122.445	37.937	0.03	16.84	108.01	16.88	-122.445	37.937	-0.16	16.84	107.72	16.69	9	7/14/2014 39:08.8
6763	RSPe_2	-122.445	37.937	0.08	16.78	108.18	16.87	-122.445	37.937	-0.16	16.78	107.89	16.63	9	7/14/2014 39:08.7
6764	RSPe_2	-122.445	37.937	0.08	16.82	108.40	16.91	-122.445	37.937	-0.19	16.82	108.12	16.63	9	7/14/2014 39:08.6

6765	RSPe_2	-122.445	37.937	0.08	16.79	108.61	16.87	-122.445	37.937	-0.02	16.79	108.39	16.77	9	7/14/2014	39:08.5
6766	RSPe_2	-122.445	37.937	0.08	16.81	108.85	16.90	-122.445	37.937	-0.19	16.81	108.64	16.62	9	7/14/2014	39:08.4
6767	RSPe_2	-122.445	37.937	0.12	16.98	109.01	17.09	-122.445	37.937	-0.07	16.98	108.87	16.91	9	7/14/2014	39:08.3
6768	RSPe_2	-122.445	37.937	0.12	16.84	109.25	16.96	-122.445	37.937	-0.16	16.84	109.03	16.69	9	7/14/2014	39:08.2
6769	RSPe_2	-122.445	37.937	0.12	16.91	109.40	17.02	-122.445	37.937	-0.10	16.91	109.21	16.80	9	7/14/2014	39:08.1
6770	RSPe_2	-122.445	37.937	0.12	16.85	109.58	16.97	-122.445	37.937	-0.07	16.85	109.36	16.78	9	7/14/2014	39:08.0
6771	RSPe_2	-122.445	37.937	0.12	16.91	109.80	17.03	-122.445	37.937	-0.10	16.91	109.51	16.81	9	7/14/2014	39:07.9
6772	RSPe_2	-122.445	37.937	0.12	16.89	110.10	17.01	-122.445	37.937	-0.07	16.89	109.73	16.82	9	7/14/2014	39:07.8
6773	RSPe_2	-122.445	37.937	0.17	16.94	110.30	17.11	-122.445	37.937	-0.07	16.94	109.85	16.87	9	7/14/2014	39:07.7
6774	RSPe_2	-122.445	37.937	0.17	16.96	110.65	17.13	-122.445	37.937	-0.07	16.96	110.08	16.89	9	7/14/2014	39:07.6
6775	RSPe_2	-122.445	37.937	0.17	16.84	110.88	17.01	-122.445	37.937	0.02	16.84	110.29	16.86	9	7/14/2014	39:07.5
6776	RSPe_2	-122.445	37.937	0.17	16.98	111.13	17.15	-122.445	37.937	-0.02	16.98	110.47	16.96	9	7/14/2014	39:07.4
6777	RSPe_2	-122.445	37.937	0.17	16.82	111.28	16.99	-122.445	37.937	-0.02	16.82	110.74	16.80	9	7/14/2014	39:07.3
6778	RSPe_2	-122.445	37.937	0.17	16.78	111.53	16.95	-122.445	37.937	0.07	16.78	111.04	16.85	9	7/14/2014	39:07.2
6779	RSPe_2	-122.445	37.937	0.20	16.80	111.63	17.00	-122.445	37.937	0.10	16.80	111.25	16.90	9	7/14/2014	39:07.1
6780	RSPe_2	-122.445	37.937	0.17	16.63	111.78	16.80	-122.445	37.937	0.07	16.63	111.50	16.69	9	7/14/2014	39:07.0
6781	RSPe_2	-122.445	37.937	0.20	16.63	111.91	16.83	-122.445	37.937	0.07	16.63	111.78	16.69	9	7/14/2014	39:06.9
6782	RSPe_2	-122.445	37.937	0.20	16.63	112.09	16.83	-122.445	37.937	0.02	16.63	112.00	16.64	9	7/14/2014	39:06.8
6783	RSPe_2	-122.445	37.937	0.20	16.56	112.30	16.76	-122.445	37.937	0.07	16.56	112.24	16.62	9	7/14/2014	39:06.7
6784	RSPe_2	-122.445	37.937	0.20	16.58	112.65	16.79	-122.445	37.937	0.10	16.58	112.50	16.68	9	7/14/2014	39:06.6
6785	RSPe_2	-122.445	37.937	0.20	16.57	112.90	16.77	-122.445	37.937	0.10	16.57	112.70	16.66	9	7/14/2014	39:06.5
6786	RSPe_2	-122.445	37.937	0.20	16.68	113.16	16.89	-122.445	37.937	0.10	16.68	112.89	16.78	9	7/14/2014	39:06.4
6787	RSPe_2	-122.445	37.937	0.20	16.65	113.34	16.85	-122.445	37.937	0.10	16.65	112.96	16.75	9	7/14/2014	39:06.3
6788	RSPe_2	-122.445	37.937	0.17	16.59	113.48	16.76	-122.445	37.937	0.10	16.59	113.09	16.69	9	7/14/2014	39:06.2
6789	RSPe_2	-122.445	37.937	0.17	16.53	113.67	16.70	-122.445	37.937	0.15	16.53	113.28	16.68	9	7/14/2014	39:06.1
6790	RSPe_2	-122.445	37.937	0.20	16.51	113.89	16.71	-122.445	37.937	0.07	16.51	113.51	16.58	9	7/14/2014	39:06.0
6791	RSPe_2	-122.445	37.937	0.20	16.47	113.96	16.68	-122.445	37.937	0.10	16.47	113.63	16.57	9	7/14/2014	39:05.9
6792	RSPe_2	-122.445	37.937	0.20	16.47	114.18	16.67	-122.445	37.937	0.07	16.47	113.86	16.53	9	7/14/2014	39:05.8
6793	RSPe_2	-122.445	37.937	0.20	16.33	114.39	16.54	-122.445	37.937	0.10	16.33	114.07	16.43	9	7/14/2014	39:05.7
6794	RSPe_2	-122.445	37.937	0.20	16.30	114.54	16.51	-122.445	37.937	0.10	16.30	114.31	16.40	9	7/14/2014	39:05.6
6795	RSPe_2	-122.445	37.937	0.24	16.36	114.69	16.60	-122.445	37.937	0.07	16.36	114.50	16.42	9	7/14/2014	39:05.5
6796	RSPe_2	-122.445	37.937	0.20	16.30	114.95	16.51	-122.445	37.937	0.10	16.30	114.73	16.40	9	7/14/2014	39:05.4
6797	RSPe_2	-122.445	37.937	0.24	16.21	115.21	16.45	-122.445	37.937	0.15	16.21	115.01	16.36	9	7/14/2014	39:05.3

6798	RSPe_2	-122.445	37.937	0.20	16.19	115.43	16.39	-122.445	37.937	0.15	16.19	115.15	16.34	9	7/14/2014 39:05.2
6799	RSPe_2	-122.445	37.937	0.20	16.17	115.59	16.38	-122.445	37.937	0.15	16.17	115.31	16.32	9	7/14/2014 39:05.1
6800	RSPe_2	-122.445	37.937	0.20	16.17	115.83	16.37	-122.445	37.937	0.10	16.17	115.48	16.27	9	7/14/2014 39:05.0
6801	RSPe_2	-122.445	37.937	0.24	16.22	116.00	16.46	-122.445	37.937	0.10	16.22	115.58	16.32	9	7/14/2014 39:04.9
6802	RSPe_2	-122.445	37.937	0.20	16.13	116.13	16.34	-122.445	37.937	0.10	16.13	115.69	16.23	9	7/14/2014 39:04.8
6803	RSPe_2	-122.445	37.937	0.20	16.06	116.41	16.26	-122.445	37.937	0.10	16.06	115.88	16.16	9	7/14/2014 39:04.7
6804	RSPe_2	-122.445	37.937	0.20	16.11	116.51	16.32	-122.445	37.937	0.10	16.11	116.07	16.21	9	7/14/2014 39:04.6
6805	RSPe_2	-122.445	37.937	0.24	16.00	116.76	16.23	-122.445	37.937	0.15	16.00	116.26	16.15	9	7/14/2014 39:04.5
6806	RSPe_2	-122.445	37.937	0.20	15.97	116.84	16.17	-122.445	37.937	0.10	15.97	116.44	16.07	9	7/14/2014 39:04.4
6807	RSPe_2	-122.445	37.937	0.24	15.90	116.96	16.13	-122.445	37.937	0.15	15.90	116.70	16.05	9	7/14/2014 39:04.3
6808	RSPe_2	-122.445	37.937	0.24	15.84	117.23	16.08	-122.445	37.937	0.15	15.84	116.99	15.99	9	7/14/2014 39:04.2
6809	RSPe_2	-122.445	37.937	0.29	15.90	117.42	16.19	-122.445	37.937	0.19	15.90	117.24	16.08	9	7/14/2014 39:04.1
6810	RSPe_2	-122.445	37.937	0.24	15.90	117.60	16.14	-122.445	37.937	0.15	15.90	117.45	16.05	9	7/14/2014 39:04.0
6811	RSPe_2	-122.445	37.937	0.29	15.95	117.79	16.24	-122.445	37.937	0.22	15.95	117.66	16.17	9	7/14/2014 39:03.9
6812	RSPe_2	-122.445	37.937	0.24	15.93	118.01	16.17	-122.445	37.937	0.19	15.93	117.82	16.12	9	7/14/2014 39:03.8
6813	RSPe_2	-122.445	37.937	0.29	16.02	118.23	16.31	-122.445	37.937	0.22	16.02	117.99	16.24	9	7/14/2014 39:03.7
6814	RSPe_2	-122.445	37.937	0.24	15.90	118.45	16.14	-122.445	37.937	0.22	15.90	118.12	16.12	9	7/14/2014 39:03.6
6815	RSPe_2	-122.445	37.937	0.24	15.90	118.58	16.14	-122.445	37.937	0.22	15.90	118.14	16.12	9	7/14/2014 39:03.5
6816	RSPe_2	-122.445	37.937	0.24	15.92	118.69	16.16	-122.445	37.937	0.19	15.92	118.29	16.10	9	7/14/2014 39:03.4
6817	RSPe_2	-122.445	37.937	0.24	15.89	118.86	16.13	-122.445	37.937	0.22	15.89	118.42	16.11	9	7/14/2014 39:03.3
6818	RSPe_2	-122.445	37.937	0.24	15.84	119.10	16.08	-122.445	37.937	0.15	15.84	118.68	15.99	9	7/14/2014 39:03.2
6819	RSPe_2	-122.445	37.937	0.29	15.86	119.28	16.15	-122.445	37.937	0.19	15.86	118.79	16.05	9	7/14/2014 39:03.1
6820	RSPe_2	-122.445	37.937	0.24	15.88	119.48	16.12	-122.445	37.937	0.22	15.88	119.06	16.10	9	7/14/2014 39:03.0
6821	RSPe_2	-122.445	37.937	0.24	15.91	119.74	16.15	-122.445	37.937	0.27	15.91	119.28	16.18	9	7/14/2014 39:02.9
6822	RSPe_2	-122.445	37.937	0.24	15.84	119.91	16.08	-122.445	37.937	0.19	15.84	119.46	16.03	9	7/14/2014 39:02.8
6823	RSPe_2	-122.445	37.937	0.29	15.95	120.16	16.24	-122.445	37.937	0.27	15.95	119.76	16.22	9	7/14/2014 39:02.7
6824	RSPe_2	-122.445	37.937	0.24	15.91	120.35	16.15	-122.445	37.937	0.27	15.91	119.96	16.18	9	7/14/2014 39:02.6
6825	RSPe_2	-122.445	37.937	0.29	15.91	120.52	16.20	-122.445	37.937	0.27	15.91	120.17	16.18	9	7/14/2014 39:02.5
6826	RSPe_2	-122.445	37.937	0.24	15.95	120.65	16.19	-122.445	37.937	0.19	15.95	120.33	16.13	9	7/14/2014 39:02.4
6827	RSPe_2	-122.445	37.937	0.29	15.97	120.79	16.26	-122.445	37.937	0.22	15.97	120.51	16.19	9	7/14/2014 39:02.3
6828	RSPe_2	-122.445	37.937	0.29	15.93	121.00	16.22	-122.445	37.937	0.22	15.93	120.70	16.15	9	7/14/2014 39:02.2
6829	RSPe_2	-122.445	37.937	0.24	15.93	121.21	16.17	-122.445	37.937	0.22	15.93	120.89	16.15	9	7/14/2014 39:02.1
6830	RSPe_2	-122.445	37.937	0.24	15.97	121.37	16.20	-122.445	37.937	0.22	15.97	121.04	16.18	9	7/14/2014 39:02.0

6831	RSPe_2	-122.445	37.937	0.29	15.97	121.49	16.26	-122.445	37.937	0.30	15.97	121.12	16.27	9	7/14/2014 39:01.9
6832	RSPe_2	-122.445	37.937	0.24	15.96	121.67	16.19	-122.445	37.937	0.27	15.96	121.26	16.23	9	7/14/2014 39:01.8
6833	RSPe_2	-122.445	37.937	0.29	15.97	121.80	16.26	-122.445	37.937	0.27	15.97	121.38	16.23	9	7/14/2014 39:01.7
6834	RSPe_2	-122.445	37.937	0.24	15.97	122.01	16.20	-122.445	37.937	0.27	15.97	121.58	16.23	9	7/14/2014 39:01.6
6835	RSPe_2	-122.445	37.937	0.29	16.00	122.19	16.29	-122.445	37.937	0.27	16.00	121.73	16.27	9	7/14/2014 39:01.5
6836	RSPe_2	-122.445	37.937	0.29	16.00	122.32	16.29	-122.445	37.937	0.22	16.00	121.90	16.22	9	7/14/2014 39:01.4
6837	RSPe_2	-122.445	37.937	0.29	16.06	122.51	16.35	-122.445	37.937	0.27	16.06	122.09	16.33	9	7/14/2014 39:01.3
6838	RSPe_2	-122.445	37.937	0.29	16.04	122.65	16.33	-122.445	37.937	0.27	16.04	122.30	16.31	9	7/14/2014 39:01.2
6839	RSPe_2	-122.445	37.937	0.29	16.04	122.86	16.33	-122.445	37.937	0.35	16.04	122.47	16.40	9	7/14/2014 39:01.1
6840	RSPe_2	-122.445	37.937	0.29	16.08	122.97	16.37	-122.445	37.937	0.27	16.08	122.68	16.35	9	7/14/2014 39:01.0
6841	RSPe_2	-122.445	37.937	0.29	16.07	123.13	16.36	-122.445	37.937	0.30	16.07	122.82	16.38	9	7/14/2014 39:00.9
6842	RSPe_2	-122.445	37.937	0.24	16.13	123.32	16.37	-122.445	37.937	0.27	16.13	122.99	16.40	9	7/14/2014 39:00.8
6843	RSPe_2	-122.445	37.937	0.29	16.10	123.41	16.39	-122.445	37.937	0.30	16.10	123.09	16.41	9	7/14/2014 39:00.7
6844	RSPe_2	-122.445	37.937	0.29	16.11	123.59	16.40	-122.445	37.937	0.27	16.11	123.24	16.38	9	7/14/2014 39:00.6
6845	RSPe_2	-122.445	37.937	0.29	16.17	123.81	16.46	-122.445	37.937	0.30	16.17	123.40	16.47	9	7/14/2014 39:00.5
6846	RSPe_2	-122.445	37.937	0.29	16.18	124.00	16.47	-122.445	37.937	0.27	16.18	123.59	16.45	9	7/14/2014 39:00.4
6847	RSPe_2	-122.445	37.937	0.29	16.21	124.24	16.50	-122.445	37.937	0.30	16.21	123.76	16.51	9	7/14/2014 39:00.3
6848	RSPe_2	-122.445	37.937	0.24	16.23	124.44	16.47	-122.445	37.937	0.27	16.23	123.91	16.50	9	7/14/2014 39:00.2
6849	RSPe_2	-122.445	37.937	0.29	16.23	124.66	16.52	-122.445	37.937	0.30	16.23	124.07	16.54	9	7/14/2014 39:00.1
6850	RSPe_2	-122.445	37.937	0.24	16.25	124.83	16.49	-122.445	37.937	0.30	16.25	124.22	16.55	9	7/14/2014 39:00.0
6851	RSPe_2	-122.445	37.937	0.29	16.25	124.96	16.54	-122.445	37.937	0.35	16.25	124.37	16.60	9	7/14/2014 38:59.9
6852	RSPe_2	-122.445	37.937	0.24	16.27	125.12	16.50	-122.445	37.937	0.35	16.27	124.55	16.62	9	7/14/2014 38:59.8
6853	RSPe_2	-122.445	37.937	0.29	16.27	125.31	16.56	-122.445	37.937	0.35	16.27	124.68	16.62	9	7/14/2014 38:59.7
6854	RSPe_2	-122.445	37.937	0.24	16.32	125.49	16.56	-122.445	37.937	0.35	16.32	124.92	16.67	9	7/14/2014 38:59.6
6855	RSPe_2	-122.445	37.937	0.24	16.30	125.65	16.54	-122.445	37.937	0.30	16.30	125.19	16.60	9	7/14/2014 38:59.5
6856	RSPe_2	-122.445	37.937	0.24	16.29	125.88	16.53	-122.445	37.937	0.35	16.29	125.44	16.64	9	7/14/2014 38:59.4
6857	RSPe_2	-122.445	37.937	0.24	16.31	126.01	16.55	-122.445	37.937	0.35	16.31	125.67	16.67	9	7/14/2014 38:59.3
6858	RSPe_2	-122.445	37.937	0.24	16.35	126.11	16.59	-122.445	37.937	0.35	16.35	125.83	16.70	9	7/14/2014 38:59.2
6859	RSPe_2	-122.445	37.937	0.24	16.37	126.25	16.61	-122.445	37.937	0.35	16.37	125.99	16.72	9	7/14/2014 38:59.1
6860	RSPe_2	-122.445	37.937	0.24	16.47	126.34	16.71	-122.445	37.937	0.35	16.47	126.13	16.83	9	7/14/2014 38:59.0
6861	RSPe_2	-122.445	37.937	0.24	16.41	126.60	16.65	-122.445	37.937	0.35	16.41	126.37	16.76	9	7/14/2014 38:58.9
6862	RSPe_2	-122.445	37.937	0.24	16.44	126.80	16.68	-122.445	37.937	0.39	16.44	126.60	16.83	9	7/14/2014 38:58.8
6863	RSPe_2	-122.445	37.937	0.24	16.50	126.97	16.73	-122.445	37.937	0.35	16.50	126.71	16.85	9	7/14/2014 38:58.7

6864	RSPe_2	-122.445	37.937	0.24	16.49	127.17	16.73	-122.445	37.937	0.30	16.49	126.91	16.79	9	7/14/2014	38:58.6
6865	RSPe_2	-122.445	37.937	0.24	16.53	127.32	16.77	-122.445	37.937	0.35	16.53	127.01	16.89	9	7/14/2014	38:58.5
6866	RSPe_2	-122.445	37.937	0.24	16.53	127.50	16.77	-122.445	37.937	0.35	16.53	127.17	16.89	9	7/14/2014	38:58.4
6867	RSPe_2	-122.445	37.937	0.24	16.56	127.70	16.80	-122.445	37.937	0.39	16.56	127.36	16.95	9	7/14/2014	38:58.3
6868	RSPe_2	-122.445	37.937	0.20	16.54	127.90	16.75	-122.445	37.937	0.35	16.54	127.57	16.90	9	7/14/2014	38:58.2
6869	RSPe_2	-122.445	37.937	0.24	16.54	128.04	16.78	-122.445	37.937	0.35	16.54	127.73	16.90	9	7/14/2014	38:58.1
6870	RSPe_2	-122.445	37.937	0.24	16.56	128.16	16.80	-122.445	37.937	0.35	16.56	127.86	16.91	9	7/14/2014	38:58.0
6871	RSPe_2	-122.445	37.937	0.24	16.53	128.35	16.77	-122.445	37.937	0.35	16.53	128.07	16.89	9	7/14/2014	38:57.9
6872	RSPe_2	-122.445	37.937	0.24	16.54	128.45	16.78	-122.445	37.937	0.35	16.54	128.24	16.90	9	7/14/2014	38:57.8
6873	RSPe_2	-122.445	37.937	0.24	16.54	128.52	16.78	-122.445	37.937	0.35	16.54	128.40	16.90	9	7/14/2014	38:57.7
6874	RSPe_2	-122.445	37.937	0.24	16.53	128.72	16.77	-122.445	37.937	0.35	16.53	128.52	16.89	9	7/14/2014	38:57.6
6875	RSPe_2	-122.445	37.937	0.24	16.59	128.82	16.83	-122.445	37.937	0.35	16.59	128.62	16.94	9	7/14/2014	38:57.5
6876	RSPe_2	-122.445	37.937	0.20	16.56	128.94	16.76	-122.445	37.937	0.35	16.56	128.75	16.91	9	7/14/2014	38:57.4
6877	RSPe_2	-122.445	37.937	0.24	16.53	129.09	16.77	-122.445	37.937	0.35	16.53	128.87	16.88	9	7/14/2014	38:57.3
6878	RSPe_2	-122.445	37.937	0.20	16.49	129.21	16.69	-122.445	37.937	0.35	16.49	128.96	16.84	9	7/14/2014	38:57.2
6879	RSPe_2	-122.445	37.937	0.24	16.50	129.32	16.73	-122.445	37.937	0.35	16.50	129.05	16.85	9	7/14/2014	38:57.1
6880	RSPe_2	-122.445	37.937	0.20	16.48	129.43	16.69	-122.445	37.937	0.30	16.48	129.20	16.78	9	7/14/2014	38:57.0
6881	RSPe_2	-122.445	37.937	0.20	16.60	129.58	16.81	-122.445	37.937	0.39	16.60	129.36	16.99	9	7/14/2014	38:56.9
6882	RSPe_2	-122.445	37.937	0.20	16.49	129.64	16.69	-122.445	37.937	0.30	16.49	129.49	16.79	9	7/14/2014	38:56.8
6883	RSPe_2	-122.445	37.937	0.24	16.37	129.82	16.61	-122.445	37.937	0.35	16.37	129.61	16.73	9	7/14/2014	38:56.7
6884	RSPe_2	-122.445	37.937	0.20	16.41	129.90	16.61	-122.445	37.937	0.30	16.41	129.71	16.71	9	7/14/2014	38:56.6
6885	RSPe_2	-122.445	37.937	0.20	16.41	129.97	16.61	-122.445	37.937	0.35	16.41	129.82	16.76	9	7/14/2014	38:56.5
6886	RSPe_2	-122.445	37.937	0.17	16.31	130.10	16.48	-122.445	37.937	0.27	16.31	129.92	16.58	9	7/14/2014	38:56.4
6887	RSPe_2	-122.445	37.937	0.20	16.45	130.24	16.66	-122.445	37.937	0.27	16.45	130.06	16.72	9	7/14/2014	38:56.3
6888	RSPe_2	-122.445	37.937	0.20	16.29	130.39	16.49	-122.445	37.937	0.27	16.29	130.17	16.56	9	7/14/2014	38:56.2
6889	RSPe_2	-122.445	37.937	0.20	16.27	130.45	16.47	-122.445	37.937	0.27	16.27	130.20	16.54	9	7/14/2014	38:56.1
6890	RSPe_2	-122.445	37.937	0.17	16.23	130.62	16.40	-122.445	37.937	0.27	16.23	130.37	16.50	9	7/14/2014	38:56.0
6891	RSPe_2	-122.445	37.937	0.17	16.19	130.85	16.36	-122.445	37.937	0.27	16.19	130.47	16.46	9	7/14/2014	38:55.9
6892	RSPe_2	-122.445	37.937	0.17	16.17	131.00	16.34	-122.445	37.937	0.27	16.17	130.68	16.44	9	7/14/2014	38:55.8
6893	RSPe_2	-122.445	37.937	0.17	16.17	131.16	16.34	-122.445	37.937	0.27	16.17	130.82	16.43	9	7/14/2014	38:55.7
6894	RSPe_2	-122.445	37.937	0.17	16.17	131.26	16.34	-122.445	37.937	0.22	16.17	130.98	16.38	9	7/14/2014	38:55.6
6895	RSPe_2	-122.445	37.937	0.17	16.20	131.42	16.37	-122.445	37.937	0.30	16.20	131.13	16.51	9	7/14/2014	38:55.5
6896	RSPe_2	-122.445	37.937	0.12	16.17	131.54	16.28	-122.445	37.937	0.22	16.17	131.33	16.38	9	7/14/2014	38:55.4

6897	RSPe_2	-122.445	37.937	0.17	16.13	131.61	16.30	-122.445	37.937	0.27	16.13	131.43	16.40	9	7/14/2014	38:55.3
6898	RSPe_2	-122.445	37.937	0.12	16.16	131.71	16.28	-122.445	37.937	0.22	16.16	131.56	16.38	9	7/14/2014	38:55.2
6899	RSPe_2	-122.445	37.937	0.17	16.13	131.70	16.30	-122.445	37.937	0.22	16.13	131.64	16.35	9	7/14/2014	38:55.1
6900	RSPe_2	-122.445	37.937	0.12	16.14	131.81	16.26	-122.445	37.937	0.22	16.14	131.76	16.36	9	7/14/2014	38:55.0
6901	RSPe_2	-122.445	37.937	0.12	16.17	131.91	16.28	-122.445	37.937	0.19	16.17	131.87	16.35	9	7/14/2014	38:54.9
6902	RSPe_2	-122.445	37.937	0.12	16.15	132.07	16.27	-122.445	37.937	0.19	16.15	132.03	16.33	9	7/14/2014	38:54.8
6903	RSPe_2	-122.445	37.937	0.12	16.16	132.21	16.28	-122.445	37.937	0.19	16.16	132.11	16.34	9	7/14/2014	38:54.7
6904	RSPe_2	-122.445	37.937	0.12	16.19	132.29	16.31	-122.445	37.937	0.15	16.19	132.19	16.34	9	7/14/2014	38:54.6
6905	RSPe_2	-122.445	37.937	0.12	16.16	132.47	16.28	-122.445	37.937	0.19	16.16	132.30	16.34	9	7/14/2014	38:54.5
6906	RSPe_2	-122.445	37.937	0.12	16.17	132.60	16.29	-122.445	37.937	0.19	16.17	132.45	16.36	9	7/14/2014	38:54.4
6907	RSPe_2	-122.445	37.937	0.12	16.16	132.79	16.28	-122.445	37.937	0.22	16.16	132.57	16.38	9	7/14/2014	38:54.3
6908	RSPe_2	-122.445	37.937	0.12	16.22	132.88	16.34	-122.445	37.937	0.15	16.22	132.73	16.37	9	7/14/2014	38:54.2
6909	RSPe_2	-122.445	37.937	0.12	16.17	132.98	16.29	-122.445	37.937	0.19	16.17	132.76	16.36	9	7/14/2014	38:54.1
6910	RSPe_2	-122.445	37.937	0.12	16.18	133.06	16.30	-122.445	37.937	0.19	16.18	132.91	16.37	9	7/14/2014	38:54.0
6911	RSPe_2	-122.445	37.937	0.12	16.19	133.10	16.31	-122.445	37.937	0.19	16.19	132.95	16.38	9	7/14/2014	38:53.9
6912	RSPe_2	-122.445	37.937	0.09	16.19	133.08	16.28	-122.445	37.937	0.15	16.19	132.99	16.34	9	7/14/2014	38:53.8
6913	RSPe_2	-122.445	37.937	0.12	16.19	133.17	16.31	-122.445	37.937	0.10	16.19	133.04	16.29	9	7/14/2014	38:53.7
6914	RSPe_2	-122.445	37.937	0.09	16.23	133.27	16.32	-122.445	37.937	0.10	16.23	133.15	16.33	9	7/14/2014	38:53.6
6915	RSPe_2	-122.445	37.937	0.12	16.23	133.45	16.35	-122.445	37.937	0.10	16.23	133.30	16.33	9	7/14/2014	38:53.5
6916	RSPe_2	-122.445	37.937	0.09	16.27	133.67	16.35	-122.445	37.937	0.10	16.27	133.48	16.37	9	7/14/2014	38:53.4
6917	RSPe_2	-122.445	37.937	0.09	16.30	133.82	16.38	-122.445	37.937	0.10	16.30	133.58	16.40	9	7/14/2014	38:53.3
6918	RSPe_2	-122.445	37.937	0.09	16.29	134.00	16.37	-122.445	37.937	0.07	16.29	133.73	16.36	9	7/14/2014	38:53.2
6919	RSPe_2	-122.445	37.937	0.09	16.29	134.12	16.37	-122.445	37.937	0.10	16.29	133.80	16.39	9	7/14/2014	38:53.1
6920	RSPe_2	-122.445	37.937	0.09	16.27	134.21	16.36	-122.445	37.937	0.07	16.27	133.96	16.34	9	7/14/2014	38:53.0
6921	RSPe_2	-122.445	37.937	0.09	16.26	134.42	16.35	-122.445	37.937	0.15	16.26	134.11	16.41	9	7/14/2014	38:52.9
6922	RSPe_2	-122.445	37.937	0.09	16.24	134.54	16.33	-122.445	37.937	0.10	16.24	134.28	16.34	9	7/14/2014	38:52.8
6923	RSPe_2	-122.445	37.937	0.09	16.25	134.69	16.34	-122.445	37.937	0.07	16.25	134.47	16.32	9	7/14/2014	38:52.7
6924	RSPe_2	-122.445	37.937	0.09	16.27	134.79	16.36	-122.445	37.937	0.07	16.27	134.57	16.34	9	7/14/2014	38:52.6
6925	RSPe_2	-122.445	37.937	0.03	16.27	134.89	16.30	-122.445	37.937	0.07	16.27	134.79	16.33	9	7/14/2014	38:52.5
6926	RSPe_2	-122.445	37.937	0.03	16.27	135.04	16.31	-122.445	37.937	0.02	16.27	134.90	16.29	9	7/14/2014	38:52.4
6927	RSPe_2	-122.445	37.937	0.09	16.29	135.16	16.37	-122.445	37.937	0.02	16.29	135.09	16.31	9	7/14/2014	38:52.3
6928	RSPe_2	-122.445	37.937	0.03	16.31	135.31	16.35	-122.445	37.937	0.02	16.31	135.16	16.33	9	7/14/2014	38:52.2
6929	RSPe_2	-122.445	37.937	0.03	16.31	135.44	16.35	-122.445	37.937	0.07	16.31	135.34	16.38	9	7/14/2014	38:52.1

6930	RSPe_2	-122.445	37.937	0.03	16.35	135.62	16.38	-122.445	37.937	-0.02	16.35	135.44	16.33	9	7/14/2014	38:52.0
6931	RSPe_2	-122.445	37.937	0.03	16.33	135.68	16.36	-122.445	37.937	0.07	16.33	135.48	16.40	9	7/14/2014	38:51.9
6932	RSPe_2	-122.445	37.937	0.03	16.40	135.75	16.43	-122.445	37.937	-0.02	16.40	135.54	16.38	9	7/14/2014	38:51.8
6933	RSPe_2	-122.445	37.937	0.03	16.35	135.91	16.38	-122.445	37.937	-0.02	16.35	135.66	16.33	9	7/14/2014	38:51.7
6934	RSPe_2	-122.445	37.937	0.00	16.36	136.08	16.36	-122.445	37.937	-0.02	16.36	135.81	16.34	9	7/14/2014	38:51.6
6935	RSPe_2	-122.445	37.937	0.03	16.32	136.25	16.35	-122.445	37.937	0.02	16.32	135.94	16.34	9	7/14/2014	38:51.5
6936	RSPe_2	-122.445	37.937	0.03	16.37	136.32	16.41	-122.445	37.937	0.02	16.37	135.99	16.39	9	7/14/2014	38:51.4
6937	RSPe_2	-122.445	37.937	0.03	16.32	136.40	16.35	-122.445	37.937	0.02	16.32	136.07	16.34	9	7/14/2014	38:51.3
6938	RSPe_2	-122.445	37.937	0.03	16.32	136.51	16.35	-122.445	37.937	0.02	16.32	136.20	16.34	9	7/14/2014	38:51.2
6939	RSPe_2	-122.445	37.937	0.00	16.33	136.68	16.33	-122.445	37.937	0.02	16.33	136.36	16.35	9	7/14/2014	38:51.1
6940	RSPe_2	-122.445	37.937	0.00	16.34	136.76	16.34	-122.445	37.937	0.02	16.34	136.49	16.36	9	7/14/2014	38:51.0
6941	RSPe_2	-122.445	37.937	0.03	16.40	136.88	16.43	-122.445	37.937	0.07	16.40	136.70	16.46	9	7/14/2014	38:50.9
6942	RSPe_2	-122.445	37.937	0.00	16.37	137.00	16.37	-122.445	37.937	-0.02	16.37	136.84	16.35	9	7/14/2014	38:50.8
6943	RSPe_2	-122.445	37.937	0.03	16.41	137.08	16.44	-122.445	37.937	0.02	16.41	136.99	16.43	9	7/14/2014	38:50.7
6944	RSPe_2	-122.445	37.937	0.00	16.44	137.22	16.44	-122.445	37.937	-0.02	16.44	137.15	16.42	9	7/14/2014	38:50.6
6945	RSPe_2	-122.445	37.937	0.03	16.53	137.23	16.56	-122.445	37.937	-0.02	16.53	137.23	16.51	9	7/14/2014	38:50.5
6946	RSPe_2	-122.445	37.937	0.00	16.59	137.34	16.59	-122.445	37.937	-0.07	16.59	137.37	16.52	9	7/14/2014	38:50.4
6947	RSPe_2	-122.445	37.937	0.00	16.62	137.46	16.62	-122.445	37.937	-0.07	16.62	137.51	16.55	9	7/14/2014	38:50.3
6948	RSPe_2	-122.445	37.937	0.00	16.62	137.56	16.62	-122.445	37.937	-0.07	16.62	137.68	16.55	9	7/14/2014	38:50.2
6949	RSPe_2	-122.445	37.937	0.00	16.64	137.63	16.64	-122.445	37.937	-0.07	16.64	137.80	16.57	9	7/14/2014	38:50.1
6950	RSPe_2	-122.445	37.937	0.00	16.67	137.80	16.67	-122.445	37.937	-0.07	16.67	137.94	16.60	9	7/14/2014	38:50.0
6951	RSPe_2	-122.445	37.937	0.00	16.70	137.98	16.70	-122.445	37.937	-0.02	16.70	138.13	16.69	9	7/14/2014	38:49.9
6952	RSPe_2	-122.445	37.937	0.00	16.70	138.07	16.70	-122.445	37.937	-0.10	16.70	138.19	16.60	9	7/14/2014	38:49.8
6953	RSPe_2	-122.445	37.937	0.03	16.76	138.20	16.79	-122.445	37.937	-0.07	16.76	138.32	16.69	9	7/14/2014	38:49.7
6954	RSPe_2	-122.445	37.937	0.00	16.76	138.33	16.76	-122.445	37.937	-0.07	16.76	138.39	16.69	9	7/14/2014	38:49.6
6955	RSPe_2	-122.445	37.937	0.03	16.77	138.48	16.81	-122.445	37.937	-0.10	16.77	138.48	16.67	9	7/14/2014	38:49.5
6956	RSPe_2	-122.445	37.937	0.00	16.77	138.61	16.77	-122.445	37.937	-0.10	16.77	138.58	16.67	9	7/14/2014	38:49.4
6957	RSPe_2	-122.445	37.937	0.00	16.80	138.79	16.80	-122.445	37.937	-0.15	16.80	138.68	16.64	9	7/14/2014	38:49.3
6958	RSPe_2	-122.445	37.937	0.00	16.77	138.90	16.77	-122.445	37.937	-0.15	16.77	138.74	16.62	9	7/14/2014	38:49.2
6959	RSPe_2	-122.445	37.937	0.03	16.81	138.96	16.84	-122.445	37.937	-0.15	16.81	138.80	16.65	9	7/14/2014	38:49.1
6960	RSPe_2	-122.445	37.937	0.00	16.78	139.04	16.78	-122.445	37.937	-0.15	16.78	138.92	16.63	9	7/14/2014	38:49.0
6961	RSPe_2	-122.445	37.937	0.03	16.81	139.25	16.84	-122.445	37.937	-0.10	16.81	139.03	16.70	9	7/14/2014	38:48.9
6962	RSPe_2	-122.445	37.937	0.00	16.83	139.31	16.83	-122.445	37.937	-0.19	16.83	139.17	16.64	9	7/14/2014	38:48.8

6963	RSPe_2	-122.445	37.937	0.03	16.83	139.50	16.86	-122.445	37.937	-0.15	16.83	139.33	16.67	9	7/14/2014	38:48.7
6964	RSPe_2	-122.445	37.937	0.00	16.78	139.65	16.78	-122.445	37.937	-0.15	16.78	139.47	16.63	9	7/14/2014	38:48.6
6965	RSPe_2	-122.445	37.938	0.00	16.81	139.83	16.81	-122.445	37.938	-0.10	16.81	139.57	16.70	9	7/14/2014	38:48.5
6966	RSPe_2	-122.445	37.938	0.00	16.74	140.00	16.74	-122.445	37.938	-0.10	16.74	139.71	16.64	9	7/14/2014	38:48.4
6967	RSPe_2	-122.445	37.938	0.03	16.77	140.14	16.81	-122.445	37.938	-0.07	16.77	139.85	16.71	9	7/14/2014	38:48.3
6968	RSPe_2	-122.445	37.938	0.00	16.76	140.21	16.76	-122.445	37.938	-0.10	16.76	139.94	16.66	9	7/14/2014	38:48.2
6969	RSPe_2	-122.445	37.938	0.00	16.73	140.27	16.73	-122.445	37.938	-0.10	16.73	140.02	16.62	9	7/14/2014	38:48.1
6970	RSPe_2	-122.445	37.938	0.00	16.75	140.35	16.75	-122.445	37.938	-0.15	16.75	140.12	16.60	9	7/14/2014	38:48.0
6971	RSPe_2	-122.445	37.938	0.03	16.76	140.40	16.79	-122.445	37.938	-0.15	16.76	140.18	16.61	9	7/14/2014	38:47.9
6972	RSPe_2	-122.445	37.938	0.00	16.78	140.58	16.78	-122.445	37.938	-0.15	16.78	140.41	16.63	9	7/14/2014	38:47.8
6973	RSPe_2	-122.445	37.938	0.03	16.74	140.71	16.77	-122.445	37.938	-0.10	16.74	140.49	16.63	9	7/14/2014	38:47.7
6974	RSPe_2	-122.445	37.938	0.00	16.77	140.86	16.77	-122.445	37.938	-0.10	16.77	140.65	16.67	9	7/14/2014	38:47.6
6975	RSPe_2	-122.445	37.938	0.03	16.74	140.91	16.78	-122.445	37.938	-0.15	16.74	140.72	16.59	9	7/14/2014	38:47.5
6976	RSPe_2	-122.445	37.938	0.03	16.80	141.06	16.83	-122.445	37.938	-0.15	16.80	140.93	16.64	9	7/14/2014	38:47.4
6977	RSPe_2	-122.445	37.938	0.03	16.80	141.27	16.83	-122.445	37.938	-0.10	16.80	141.13	16.70	9	7/14/2014	38:47.3
6978	RSPe_2	-122.445	37.938	0.00	16.79	141.45	16.79	-122.445	37.938	-0.15	16.79	141.33	16.64	9	7/14/2014	38:47.2
6979	RSPe_2	-122.445	37.938	0.03	16.90	141.57	16.93	-122.445	37.938	-0.10	16.90	141.45	16.80	9	7/14/2014	38:47.1
6980	RSPe_2	-122.445	37.938	0.00	16.88	141.72	16.88	-122.445	37.938	-0.10	16.88	141.55	16.78	9	7/14/2014	38:47.0
6981	RSPe_2	-122.445	37.938	0.03	16.92	141.85	16.95	-122.445	37.938	-0.10	16.92	141.65	16.82	9	7/14/2014	38:46.9
6982	RSPe_2	-122.445	37.938	0.00	16.90	141.95	16.90	-122.445	37.938	-0.15	16.90	141.67	16.75	9	7/14/2014	38:46.8
6983	RSPe_2	-122.445	37.938	0.03	16.92	142.07	16.95	-122.445	37.938	-0.10	16.92	141.77	16.82	9	7/14/2014	38:46.7
6984	RSPe_2	-122.445	37.938	0.00	16.88	142.18	16.88	-122.445	37.938	-0.15	16.88	141.85	16.72	9	7/14/2014	38:46.6
6985	RSPe_2	-122.445	37.938	0.03	16.88	142.30	16.91	-122.445	37.938	-0.15	16.88	141.92	16.72	9	7/14/2014	38:46.5
6986	RSPe_2	-122.445	37.938	0.00	16.99	142.36	16.99	-122.445	37.938	-0.15	16.99	142.02	16.84	9	7/14/2014	38:46.4
6987	RSPe_2	-122.445	37.938	0.00	16.89	142.54	16.89	-122.445	37.938	-0.10	16.89	142.20	16.79	9	7/14/2014	38:46.3
6988	RSPe_2	-122.445	37.938	0.00	16.88	142.80	16.88	-122.445	37.938	-0.10	16.88	142.44	16.78	9	7/14/2014	38:46.2
6989	RSPe_2	-122.445	37.938	0.03	16.94	142.92	16.97	-122.445	37.938	-0.15	16.94	142.59	16.78	9	7/14/2014	38:46.1
6990	RSPe_2	-122.445	37.938	0.00	16.98	143.08	16.98	-122.445	37.938	-0.15	16.98	142.73	16.83	9	7/14/2014	38:46.0
6991	RSPe_2	-122.445	37.938	0.03	16.95	143.23	16.99	-122.445	37.938	-0.15	16.95	142.85	16.80	9	7/14/2014	38:45.9
6992	RSPe_2	-122.445	37.938	0.03	16.95	143.36	16.99	-122.445	37.938	-0.10	16.95	143.01	16.85	9	7/14/2014	38:45.8
6993	RSPe_2	-122.445	37.938	0.03	16.96	143.47	17.00	-122.445	37.938	-0.07	16.96	143.09	16.89	9	7/14/2014	38:45.7
6994	RSPe_2	-122.445	37.938	0.00	16.98	143.58	16.98	-122.445	37.938	-0.10	16.98	143.25	16.87	9	7/14/2014	38:45.6
6995	RSPe_2	-122.445	37.938	0.03	17.03	143.73	17.07	-122.445	37.938	-0.10	17.03	143.38	16.93	9	7/14/2014	38:45.5

6996	RSPe_2	-122.445	37.938	0.00	17.05	143.80	17.05	-122.445	37.938	-0.10	17.05	143.46	16.94	9	7/14/2014 38:45.4
6997	RSPe_2	-122.445	37.938	0.00	17.01	143.88	17.01	-122.445	37.938	-0.02	17.01	143.52	17.00	9	7/14/2014 38:45.3
6998	RSPe_2	-122.445	37.938	0.00	17.05	143.95	17.05	-122.445	37.938	-0.07	17.05	143.60	16.98	9	7/14/2014 38:45.2
6999	RSPe_2	-122.445	37.938	0.03	17.07	144.14	17.10	-122.445	37.938	-0.07	17.07	143.81	17.00	9	7/14/2014 38:45.1
7000	RSPe_2	-122.445	37.938	-0.05	17.24	144.30	17.19	-122.445	37.938	-0.07	17.24	144.02	17.17	9	7/14/2014 38:45.0
7001	RSPe_2	-122.445	37.938	0.00	17.27	144.48	17.27	-122.445	37.938	-0.10	17.27	144.21	17.17	9	7/14/2014 38:44.9
7002	RSPe_2	-122.445	37.938	-0.05	17.34	144.57	17.28	-122.445	37.938	-0.07	17.34	144.40	17.27	9	7/14/2014 38:44.8
7003	RSPe_2	-122.445	37.938	0.00	17.33	144.63	17.33	-122.445	37.938	-0.07	17.33	144.48	17.26	9	7/14/2014 38:44.7
7004	RSPe_2	-122.445	37.938	-0.05	17.35	144.78	17.30	-122.445	37.938	-0.10	17.35	144.63	17.25	9	7/14/2014 38:44.6
7005	RSPe_2	-122.445	37.938	0.00	17.38	144.95	17.38	-122.445	37.938	-0.07	17.38	144.85	17.31	9	7/14/2014 38:44.5
7006	RSPe_2	-122.445	37.938	-0.05	17.42	145.00	17.37	-122.445	37.938	-0.10	17.42	144.98	17.32	9	7/14/2014 38:44.4
7007	RSPe_2	-122.445	37.938	0.00	17.31	145.09	17.31	-122.445	37.938	-0.10	17.31	145.07	17.21	9	7/14/2014 38:44.3
7008	RSPe_2	-122.445	37.938	-0.05	17.31	145.21	17.26	-122.445	37.938	-0.10	17.31	145.25	17.21	9	7/14/2014 38:44.2
7009	RSPe_2	-122.445	37.938	-0.05	17.18	145.35	17.12	-122.445	37.938	-0.10	17.18	145.41	17.07	9	7/14/2014 38:44.1
7010	RSPe_2	-122.445	37.938	-0.09	17.11	145.50	17.02	-122.445	37.938	-0.15	17.11	145.63	16.95	9	7/14/2014 38:44.0
7011	RSPe_2	-122.445	37.938	-0.05	17.12	145.69	17.06	-122.445	37.938	-0.19	17.12	145.76	16.93	9	7/14/2014 38:43.9
7012	RSPe_2	-122.445	37.938	-0.09	17.13	145.86	17.04	-122.445	37.938	-0.19	17.13	145.90	16.94	9	7/14/2014 38:43.8
7013	RSPe_2	-122.445	37.938	-0.05	17.09	146.07	17.04	-122.445	37.938	-0.19	17.09	145.97	16.90	9	7/14/2014 38:43.7
7014	RSPe_2	-122.445	37.938	-0.09	17.05	146.20	16.97	-122.445	37.938	-0.22	17.05	146.07	16.83	9	7/14/2014 38:43.6
7015	RSPe_2	-122.445	37.938	-0.05	17.11	146.38	17.05	-122.445	37.938	-0.19	17.11	146.23	16.92	9	7/14/2014 38:43.5
7016	RSPe_2	-122.445	37.938	-0.09	16.98	146.58	16.89	-122.445	37.938	-0.22	16.98	146.36	16.76	9	7/14/2014 38:43.4
7017	RSPe_2	-122.445	37.938	-0.05	16.88	146.75	16.82	-122.445	37.938	-0.19	16.88	146.57	16.69	9	7/14/2014 38:43.3
7018	RSPe_2	-122.445	37.938	-0.09	16.88	146.92	16.79	-122.445	37.938	-0.22	16.88	146.72	16.66	9	7/14/2014 38:43.2
7019	RSPe_2	-122.445	37.938	-0.09	16.96	147.10	16.88	-122.445	37.938	-0.19	16.96	146.92	16.78	9	7/14/2014 38:43.1
7020	RSPe_2	-122.445	37.938	-0.09	16.79	147.37	16.70	-122.445	37.938	-0.22	16.79	147.10	16.57	9	7/14/2014 38:43.0
7021	RSPe_2	-122.445	37.938	-0.09	16.74	147.54	16.66	-122.445	37.938	-0.19	16.74	147.33	16.56	9	7/14/2014 38:42.9
7022	RSPe_2	-122.445	37.938	-0.09	16.76	147.73	16.67	-122.445	37.938	-0.22	16.76	147.41	16.54	9	7/14/2014 38:42.8
7023	RSPe_2	-122.445	37.938	-0.09	16.75	147.88	16.67	-122.445	37.938	-0.22	16.75	147.51	16.53	9	7/14/2014 38:42.7
7024	RSPe_2	-122.445	37.938	-0.09	16.68	148.00	16.60	-122.445	37.938	-0.27	16.68	147.71	16.41	9	7/14/2014 38:42.6
7025	RSPe_2	-122.445	37.938	-0.14	16.59	148.15	16.45	-122.445	37.938	-0.19	16.59	147.81	16.40	9	7/14/2014 38:42.5
7026	RSPe_2	-122.445	37.938	-0.14	16.53	148.27	16.39	-122.445	37.938	-0.27	16.53	147.98	16.26	9	7/14/2014 38:42.4
7027	RSPe_2	-122.445	37.938	-0.09	16.50	148.42	16.41	-122.445	37.938	-0.27	16.50	148.13	16.23	9	7/14/2014 38:42.3
7028	RSPe_2	-122.445	37.938	-0.14	16.51	148.52	16.37	-122.445	37.938	-0.27	16.51	148.28	16.24	9	7/14/2014 38:42.2

7029	RSPe_2	-122.445	37.938	-0.09	16.45	148.60	16.37	-122.445	37.938	-0.27	16.45	148.40	16.18	9	7/14/2014	38:42.1
7030	RSPe_2	-122.445	37.938	-0.14	16.46	148.59	16.32	-122.445	37.938	-0.31	16.46	148.57	16.15	9	7/14/2014	38:42.0
7031	RSPe_2	-122.445	37.938	-0.14	16.48	148.76	16.35	-122.445	37.938	-0.27	16.48	148.80	16.21	9	7/14/2014	38:41.9
7032	RSPe_2	-122.445	37.938	-0.17	16.51	148.80	16.34	-122.445	37.938	-0.30	16.51	148.96	16.21	9	7/14/2014	38:41.8
7033	RSPe_2	-122.445	37.938	-0.09	16.53	149.01	16.45	-122.445	37.938	-0.27	16.53	149.17	16.26	9	7/14/2014	38:41.7
7034	RSPe_2	-122.445	37.938	-0.14	16.56	149.13	16.42	-122.445	37.938	-0.30	16.56	149.35	16.26	9	7/14/2014	38:41.6
7035	RSPe_2	-122.445	37.938	-0.09	16.57	149.37	16.48	-122.445	37.938	-0.30	16.57	149.46	16.26	9	7/14/2014	38:41.5
7036	RSPe_2	-122.445	37.938	-0.14	16.55	149.55	16.41	-122.445	37.938	-0.27	16.55	149.55	16.28	9	7/14/2014	38:41.4
7037	RSPe_2	-122.445	37.938	-0.09	16.60	149.76	16.52	-122.445	37.938	-0.27	16.60	149.59	16.33	9	7/14/2014	38:41.3
7038	RSPe_2	-122.445	37.938	-0.14	16.57	149.87	16.43	-122.445	37.938	-0.30	16.57	149.72	16.26	9	7/14/2014	38:41.2
7039	RSPe_2	-122.445	37.938	-0.09	16.56	149.96	16.47	-122.445	37.938	-0.27	16.56	149.80	16.29	9	7/14/2014	38:41.1
7040	RSPe_2	-122.445	37.938	-0.09	16.59	150.03	16.51	-122.445	37.938	-0.27	16.59	149.93	16.32	9	7/14/2014	38:41.0
7041	RSPe_2	-122.445	37.938	-0.09	16.58	150.10	16.49	-122.445	37.938	-0.27	16.58	150.04	16.31	9	7/14/2014	38:40.9
7042	RSPe_2	-122.445	37.938	-0.09	16.56	150.24	16.47	-122.445	37.938	-0.30	16.56	150.24	16.26	9	7/14/2014	38:40.8
7043	RSPe_2	-122.445	37.938	-0.09	16.65	150.38	16.56	-122.445	37.938	-0.27	16.65	150.36	16.38	9	7/14/2014	38:40.7
7044	RSPe_2	-122.445	37.938	-0.09	16.64	150.54	16.55	-122.445	37.938	-0.30	16.64	150.54	16.33	9	7/14/2014	38:40.6
7045	RSPe_2	-122.445	37.938	-0.09	16.67	150.67	16.59	-122.445	37.938	-0.27	16.67	150.63	16.40	9	7/14/2014	38:40.5
7046	RSPe_2	-122.445	37.938	-0.09	16.70	150.74	16.61	-122.445	37.938	-0.27	16.70	150.72	16.43	9	7/14/2014	38:40.4
7047	RSPe_2	-122.445	37.938	-0.09	16.70	150.95	16.61	-122.445	37.938	-0.27	16.70	150.84	16.43	9	7/14/2014	38:40.3
7048	RSPe_2	-122.445	37.938	-0.09	16.72	151.14	16.63	-122.445	37.938	-0.30	16.72	150.98	16.41	9	7/14/2014	38:40.2
7049	RSPe_2	-122.445	37.938	-0.05	16.67	151.28	16.62	-122.445	37.938	-0.27	16.67	151.06	16.40	9	7/14/2014	38:40.1
7050	RSPe_2	-122.445	37.938	-0.09	16.70	151.38	16.61	-122.445	37.938	-0.27	16.70	151.11	16.43	9	7/14/2014	38:40.0
7051	RSPe_2	-122.445	37.938	-0.09	16.70	151.48	16.62	-122.445	37.938	-0.27	16.70	151.18	16.43	9	7/14/2014	38:39.9
7052	RSPe_2	-122.445	37.938	-0.09	16.72	151.60	16.63	-122.445	37.938	-0.30	16.72	151.28	16.41	9	7/14/2014	38:39.8
7053	RSPe_2	-122.445	37.938	-0.05	16.73	151.75	16.68	-122.445	37.938	-0.27	16.73	151.39	16.46	9	7/14/2014	38:39.7
7054	RSPe_2	-122.445	37.938	-0.09	16.77	151.90	16.68	-122.445	37.938	-0.30	16.77	151.53	16.46	9	7/14/2014	38:39.6
7055	RSPe_2	-122.445	37.938	-0.05	16.76	152.06	16.71	-122.445	37.938	-0.27	16.76	151.76	16.49	9	7/14/2014	38:39.5
7056	RSPe_2	-122.445	37.938	-0.09	16.78	152.24	16.70	-122.445	37.938	-0.30	16.78	151.96	16.48	9	7/14/2014	38:39.4
7057	RSPe_2	-122.445	37.938	-0.09	16.78	152.30	16.70	-122.445	37.938	-0.27	16.78	152.09	16.51	9	7/14/2014	38:39.3
7058	RSPe_2	-122.445	37.938	-0.09	16.82	152.41	16.74	-122.445	37.938	-0.35	16.82	152.23	16.47	9	7/14/2014	38:39.2
7059	RSPe_2	-122.445	37.938	-0.09	16.87	152.54	16.78	-122.445	37.938	-0.30	16.87	152.35	16.56	9	7/14/2014	38:39.1
7060	RSPe_2	-122.445	37.938	-0.09	16.84	152.67	16.75	-122.445	37.938	-0.27	16.84	152.51	16.57	9	7/14/2014	38:39.0
7061	RSPe_2	-122.445	37.938	-0.09	16.85	152.83	16.77	-122.445	37.938	-0.30	16.85	152.60	16.55	9	7/14/2014	38:38.9

7062	RSPe_2	-122.445	37.938	-0.09	16.90	152.98	16.81	-122.445	37.938	-0.30	16.90	152.73	16.59	9	7/14/2014 38:38.8
7063	RSPe_2	-122.445	37.938	-0.09	16.87	153.11	16.78	-122.445	37.938	-0.27	16.87	152.86	16.60	9	7/14/2014 38:38.7
7064	RSPe_2	-122.445	37.938	-0.09	16.91	153.28	16.82	-122.445	37.938	-0.30	16.91	152.97	16.60	9	7/14/2014 38:38.6
7065	RSPe_2	-122.445	37.938	-0.09	16.91	153.44	16.83	-122.445	37.938	-0.27	16.91	153.15	16.64	9	7/14/2014 38:38.5
7066	RSPe_2	-122.445	37.938	-0.09	16.92	153.57	16.84	-122.445	37.938	-0.30	16.92	153.23	16.62	9	7/14/2014 38:38.4
7067	RSPe_2	-122.445	37.938	-0.05	16.91	153.70	16.86	-122.445	37.938	-0.27	16.91	153.45	16.64	9	7/14/2014 38:38.3
7068	RSPe_2	-122.445	37.938	-0.09	16.90	153.79	16.81	-122.445	37.938	-0.30	16.90	153.51	16.59	9	7/14/2014 38:38.2
7069	RSPe_2	-122.445	37.938	-0.09	16.96	154.00	16.88	-122.445	37.938	-0.27	16.96	153.73	16.69	9	7/14/2014 38:38.1
7070	RSPe_2	-122.445	37.938	-0.09	16.94	154.12	16.85	-122.445	37.938	-0.30	16.94	153.90	16.63	9	7/14/2014 38:38.0
7071	RSPe_2	-122.445	37.938	-0.05	16.97	154.35	16.92	-122.445	37.938	-0.22	16.97	154.13	16.75	9	7/14/2014 38:37.9
7072	RSPe_2	-122.445	37.938	-0.05	16.97	154.51	16.92	-122.445	37.938	-0.27	16.97	154.25	16.70	9	7/14/2014 38:37.8
7073	RSPe_2	-122.445	37.938	-0.05	16.98	154.67	16.93	-122.445	37.938	-0.27	16.98	154.39	16.71	9	7/14/2014 38:37.7
7074	RSPe_2	-122.445	37.938	-0.05	16.99	154.84	16.94	-122.445	37.938	-0.22	16.99	154.60	16.77	9	7/14/2014 38:37.6
7075	RSPe_2	-122.445	37.938	0.00	17.00	155.01	17.00	-122.445	37.938	-0.27	17.00	154.74	16.73	9	7/14/2014 38:37.5
7076	RSPe_2	-122.445	37.938	-0.05	17.00	155.25	16.95	-122.445	37.938	-0.27	17.00	154.93	16.73	9	7/14/2014 38:37.4
7077	RSPe_2	-122.445	37.938	0.00	16.99	155.45	16.99	-122.445	37.938	-0.22	16.99	155.08	16.77	9	7/14/2014 38:37.3
7078	RSPe_2	-122.445	37.938	-0.05	17.01	155.63	16.96	-122.445	37.938	-0.22	17.01	155.25	16.80	9	7/14/2014 38:37.2
7079	RSPe_2	-122.445	37.938	-0.05	17.01	155.85	16.96	-122.445	37.938	-0.19	17.01	155.40	16.83	9	7/14/2014 38:37.1
7080	RSPe_2	-122.445	37.938	-0.05	17.03	156.11	16.98	-122.445	37.938	-0.22	17.03	155.58	16.81	9	7/14/2014 38:37.0
7081	RSPe_2	-122.445	37.938	0.00	17.01	156.27	17.02	-122.445	37.938	-0.19	17.01	155.69	16.83	9	7/14/2014 38:36.9
7082	RSPe_2	-122.445	37.938	-0.05	17.02	156.48	16.97	-122.445	37.938	-0.19	17.02	155.90	16.84	9	7/14/2014 38:36.8
7083	RSPe_2	-122.445	37.938	0.00	17.04	156.68	17.04	-122.445	37.938	-0.10	17.04	156.10	16.94	9	7/14/2014 38:36.7
7084	RSPe_2	-122.445	37.938	-0.05	17.06	156.84	17.01	-122.445	37.938	-0.15	17.06	156.28	16.91	9	7/14/2014 38:36.6
7085	RSPe_2	-122.445	37.938	-0.05	17.09	157.01	17.04	-122.445	37.938	-0.15	17.09	156.48	16.94	9	7/14/2014 38:36.5
7086	RSPe_2	-122.445	37.938	-0.05	16.87	157.13	16.81	-122.445	37.938	-0.15	16.87	156.61	16.71	9	7/14/2014 38:36.4
7087	RSPe_2	-122.445	37.938	0.00	17.10	157.31	17.10	-122.445	37.938	-0.15	17.10	156.86	16.95	9	7/14/2014 38:36.3
7088	RSPe_2	-122.445	37.938	0.00	17.12	157.48	17.12	-122.445	37.938	-0.10	17.12	157.03	17.01	9	7/14/2014 38:36.2
7089	RSPe_2	-122.445	37.938	0.00	17.13	157.61	17.13	-122.445	37.938	-0.10	17.13	157.24	17.03	9	7/14/2014 38:36.1
7090	RSPe_2	-122.445	37.938	0.00	17.15	157.79	17.15	-122.445	37.938	-0.15	17.15	157.44	17.00	9	7/14/2014 38:36.0
7091	RSPe_2	-122.445	37.938	0.00	17.16	157.88	17.16	-122.445	37.938	-0.10	17.16	157.68	17.06	9	7/14/2014 38:35.9
7092	RSPe_2	-122.445	37.938	0.00	17.19	157.98	17.19	-122.445	37.938	-0.10	17.19	157.83	17.09	9	7/14/2014 38:35.8
7093	RSPe_2	-122.445	37.938	0.00	17.19	158.03	17.19	-122.445	37.938	-0.10	17.19	158.03	17.09	9	7/14/2014 38:35.7
7094	RSPe_2	-122.445	37.938	0.00	17.21	158.14	17.21	-122.445	37.938	-0.10	17.21	158.17	17.11	9	7/14/2014 38:35.6

7095	RSPe_2	-122.445	37.938	0.04	17.21	158.25	17.24	-122.445	37.938	-0.07	17.21	158.31	17.14	9	7/14/2014	38:35.5
7096	RSPe_2	-122.445	37.938	0.00	17.25	158.36	17.25	-122.445	37.938	-0.07	17.25	158.44	17.18	9	7/14/2014	38:35.4
7097	RSPe_2	-122.445	37.938	0.04	17.27	158.47	17.30	-122.445	37.938	-0.10	17.27	158.59	17.17	9	7/14/2014	38:35.3
7098	RSPe_2	-122.445	37.938	0.00	17.24	158.59	17.24	-122.445	37.938	-0.10	17.24	158.60	17.14	9	7/14/2014	38:35.2
7099	RSPe_2	-122.445	37.938	0.04	17.25	158.64	17.29	-122.445	37.938	-0.10	17.25	158.67	17.15	9	7/14/2014	38:35.1
7100	RSPe_2	-122.445	37.938	0.04	17.25	158.80	17.28	-122.445	37.938	-0.10	17.25	158.77	17.14	9	7/14/2014	38:35.0
7101	RSPe_2	-122.445	37.938	0.04	17.30	158.91	17.33	-122.445	37.938	-0.10	17.30	158.82	17.20	9	7/14/2014	38:34.9
7102	RSPe_2	-122.445	37.938	0.00	17.28	159.11	17.28	-122.445	37.938	-0.10	17.28	158.90	17.18	9	7/14/2014	38:34.8
7103	RSPe_2	-122.445	37.938	0.04	17.27	159.32	17.30	-122.445	37.938	-0.02	17.27	159.06	17.25	9	7/14/2014	38:34.7
7104	RSPe_2	-122.445	37.938	0.04	17.32	159.46	17.35	-122.445	37.938	-0.07	17.32	159.13	17.25	9	7/14/2014	38:34.6
7105	RSPe_2	-122.445	37.938	0.09	17.27	159.62	17.35	-122.445	37.938	-0.02	17.27	159.27	17.25	9	7/14/2014	38:34.5
7106	RSPe_2	-122.445	37.938	0.04	17.28	159.72	17.31	-122.445	37.938	-0.07	17.28	159.48	17.21	9	7/14/2014	38:34.4
7107	RSPe_2	-122.445	37.938	0.09	17.19	159.84	17.28	-122.445	37.938	-0.02	17.19	159.57	17.18	9	7/14/2014	38:34.3
7108	RSPe_2	-122.445	37.938	0.04	17.17	159.98	17.21	-122.445	37.938	-0.07	17.17	159.81	17.10	9	7/14/2014	38:34.2
7109	RSPe_2	-122.445	37.938	0.09	17.16	160.13	17.25	-122.445	37.938	0.02	17.16	159.97	17.18	9	7/14/2014	38:34.1
7110	RSPe_2	-122.445	37.938	0.09	17.13	160.27	17.21	-122.445	37.938	-0.07	17.13	160.13	17.06	9	7/14/2014	38:34.0
7111	RSPe_2	-122.445	37.938	0.09	17.07	160.48	17.16	-122.445	37.938	-0.02	17.07	160.32	17.05	9	7/14/2014	38:33.9
7112	RSPe_2	-122.445	37.938	0.09	16.99	160.67	17.08	-122.445	37.938	0.02	16.99	160.53	17.01	9	7/14/2014	38:33.8
7113	RSPe_2	-122.445	37.938	0.09	17.00	160.83	17.09	-122.445	37.938	0.02	17.00	160.65	17.02	9	7/14/2014	38:33.7
7114	RSPe_2	-122.445	37.938	0.09	17.03	160.98	17.12	-122.445	37.938	0.02	17.03	160.81	17.05	9	7/14/2014	38:33.6
7115	RSPe_2	-122.445	37.938	0.09	17.05	161.14	17.14	-122.445	37.938	0.07	17.05	160.91	17.12	9	7/14/2014	38:33.5
7116	RSPe_2	-122.445	37.938	0.09	16.93	161.33	17.02	-122.445	37.938	0.07	16.93	161.02	17.00	9	7/14/2014	38:33.4
7117	RSPe_2	-122.445	37.938	0.12	16.88	161.40	17.00	-122.445	37.938	0.07	16.88	161.09	16.95	9	7/14/2014	38:33.3
7118	RSPe_2	-122.445	37.938	0.09	16.83	161.62	16.91	-122.445	37.938	0.07	16.83	161.24	16.90	9	7/14/2014	38:33.2
7119	RSPe_2	-122.445	37.938	0.12	16.72	161.79	16.84	-122.445	37.938	0.10	16.72	161.42	16.82	9	7/14/2014	38:33.1
7120	RSPe_2	-122.445	37.938	0.12	16.70	161.87	16.83	-122.445	37.938	0.07	16.70	161.47	16.77	9	7/14/2014	38:33.0
7121	RSPe_2	-122.445	37.938	0.09	16.60	161.90	16.69	-122.445	37.938	0.10	16.60	161.58	16.71	9	7/14/2014	38:32.9
7122	RSPe_2	-122.445	37.938	0.09	16.51	161.97	16.59	-122.445	37.938	0.10	16.51	161.70	16.61	9	7/14/2014	38:32.8
7123	RSPe_2	-122.445	37.938	0.12	16.54	162.04	16.67	-122.445	37.938	0.10	16.54	161.84	16.65	9	7/14/2014	38:32.7
7124	RSPe_2	-122.445	37.938	0.09	16.56	162.13	16.65	-122.445	37.938	0.07	16.56	161.98	16.63	9	7/14/2014	38:32.6
7125	RSPe_2	-122.445	37.938	0.12	16.45	162.34	16.57	-122.445	37.938	0.07	16.45	162.26	16.52	9	7/14/2014	38:32.5
7126	RSPe_2	-122.445	37.938	0.09	16.48	162.62	16.57	-122.445	37.938	0.07	16.48	162.50	16.55	9	7/14/2014	38:32.4
7127	RSPe_2	-122.445	37.938	0.12	16.46	162.67	16.58	-122.445	37.938	0.07	16.46	162.62	16.53	9	7/14/2014	38:32.3

7128	RSPe_2	-122.445	37.938	0.09	16.53	162.79	16.61	-122.445	37.938	0.07	16.53	162.76	16.60	9	7/14/2014	38:32.2
7129	RSPe_2	-122.445	37.938	0.12	16.48	162.91	16.60	-122.445	37.938	0.07	16.48	162.85	16.55	9	7/14/2014	38:32.1
7130	RSPe_2	-122.445	37.938	0.09	16.60	162.99	16.68	-122.445	37.938	0.07	16.60	162.93	16.67	9	7/14/2014	38:32.0
7131	RSPe_2	-122.445	37.938	0.12	16.48	163.12	16.60	-122.445	37.938	0.10	16.48	162.93	16.58	9	7/14/2014	38:31.9
7132	RSPe_2	-122.445	37.938	0.12	16.53	163.22	16.65	-122.445	37.938	0.10	16.53	163.04	16.63	9	7/14/2014	38:31.8
7133	RSPe_2	-122.445	37.938	0.09	16.37	163.32	16.46	-122.445	37.938	0.10	16.37	163.09	16.47	9	7/14/2014	38:31.7
7134	RSPe_2	-122.445	37.938	0.09	16.44	163.45	16.52	-122.445	37.938	0.10	16.44	163.17	16.54	9	7/14/2014	38:31.6
7135	RSPe_2	-122.445	37.938	0.12	16.41	163.54	16.53	-122.445	37.938	0.10	16.41	163.28	16.51	9	7/14/2014	38:31.5
7136	RSPe_2	-122.445	37.938	0.09	16.46	163.64	16.54	-122.445	37.938	0.02	16.46	163.38	16.48	9	7/14/2014	38:31.4
7137	RSPe_2	-122.445	37.938	0.12	16.43	163.69	16.55	-122.445	37.938	0.07	16.43	163.54	16.50	9	7/14/2014	38:31.3
7138	RSPe_2	-122.445	37.938	0.09	16.48	163.78	16.57	-122.445	37.938	0.07	16.48	163.65	16.55	9	7/14/2014	38:31.2
7139	RSPe_2	-122.445	37.938	0.12	16.54	163.91	16.67	-122.445	37.938	0.02	16.54	163.82	16.56	9	7/14/2014	38:31.1
7140	RSPe_2	-122.445	37.938	0.09	16.58	164.04	16.66	-122.445	37.938	0.07	16.58	163.97	16.64	9	7/14/2014	38:31.0
7141	RSPe_2	-122.445	37.938	0.12	16.61	164.13	16.73	-122.445	37.938	0.07	16.61	164.09	16.68	9	7/14/2014	38:30.9
7142	RSPe_2	-122.445	37.938	0.09	16.58	164.31	16.66	-122.445	37.938	0.07	16.58	164.13	16.64	9	7/14/2014	38:30.8
7143	RSPe_2	-122.445	37.938	0.12	16.59	164.34	16.71	-122.445	37.938	0.10	16.59	164.19	16.69	9	7/14/2014	38:30.7
7144	RSPe_2	-122.445	37.938	0.09	16.58	164.46	16.66	-122.445	37.938	0.07	16.58	164.22	16.64	9	7/14/2014	38:30.6
7145	RSPe_2	-122.445	37.938	0.12	16.54	164.53	16.67	-122.445	37.938	0.07	16.54	164.34	16.61	9	7/14/2014	38:30.5
7146	RSPe_2	-122.445	37.938	0.09	16.53	164.62	16.62	-122.445	37.938	0.07	16.53	164.39	16.60	9	7/14/2014	38:30.4
7147	RSPe_2	-122.445	37.938	0.12	16.53	164.68	16.66	-122.445	37.938	0.07	16.53	164.46	16.60	9	7/14/2014	38:30.3
7148	RSPe_2	-122.445	37.938	0.09	16.51	164.82	16.59	-122.445	37.938	0.07	16.51	164.58	16.57	9	7/14/2014	38:30.2
7149	RSPe_2	-122.445	37.938	0.12	16.53	164.90	16.66	-122.445	37.938	0.07	16.53	164.68	16.60	9	7/14/2014	38:30.1
7150	RSPe_2	-122.445	37.938	0.09	16.52	164.99	16.61	-122.445	37.938	0.02	16.52	164.83	16.54	9	7/14/2014	38:30.0
7151	RSPe_2	-122.445	37.938	0.12	16.52	165.13	16.64	-122.445	37.938	0.07	16.52	164.93	16.59	9	7/14/2014	38:29.9
7152	RSPe_2	-122.445	37.938	0.09	16.51	165.27	16.60	-122.445	37.938	0.07	16.51	165.07	16.58	9	7/14/2014	38:29.8
7153	RSPe_2	-122.445	37.938	0.09	16.51	165.43	16.60	-122.445	37.938	0.07	16.51	165.22	16.58	9	7/14/2014	38:29.7
7154	RSPe_2	-122.445	37.938	0.09	16.53	165.57	16.61	-122.445	37.938	0.02	16.53	165.36	16.55	9	7/14/2014	38:29.6
7155	RSPe_2	-122.445	37.938	0.09	16.53	165.73	16.62	-122.445	37.938	0.02	16.53	165.41	16.55	9	7/14/2014	38:29.5
7156	RSPe_2	-122.445	37.938	0.04	16.53	165.74	16.57	-122.445	37.938	0.02	16.53	165.45	16.55	9	7/14/2014	38:29.4
7157	RSPe_2	-122.445	37.938	0.09	16.48	165.86	16.57	-122.445	37.938	0.02	16.48	165.53	16.50	9	7/14/2014	38:29.3
7158	RSPe_2	-122.445	37.938	0.09	16.46	165.93	16.55	-122.445	37.938	0.07	16.46	165.56	16.53	9	7/14/2014	38:29.2
7159	RSPe_2	-122.445	37.938	0.09	16.46	166.03	16.55	-122.445	37.938	0.07	16.46	165.67	16.53	9	7/14/2014	38:29.1
7160	RSPe_2	-122.445	37.938	0.04	16.47	166.14	16.50	-122.445	37.938	0.02	16.47	165.80	16.48	9	7/14/2014	38:29.0

7161	RSPe_2	-122.445	37.938	0.09	16.44	166.27	16.52	-122.445	37.938	0.07	16.44	165.90	16.51	9	7/14/2014	38:28.9
7162	RSPe_2	-122.445	37.938	0.04	16.44	166.29	16.47	-122.445	37.938	-0.02	16.44	165.98	16.42	9	7/14/2014	38:28.8
7163	RSPe_2	-122.445	37.938	0.04	16.47	166.41	16.51	-122.445	37.938	0.07	16.47	166.16	16.54	9	7/14/2014	38:28.7
7164	RSPe_2	-122.445	37.938	0.04	16.44	166.47	16.47	-122.445	37.938	0.02	16.44	166.29	16.46	9	7/14/2014	38:28.6
7165	RSPe_2	-122.445	37.938	0.09	16.44	166.58	16.52	-122.445	37.938	0.02	16.44	166.45	16.46	9	7/14/2014	38:28.5
7166	RSPe_2	-122.445	37.938	0.04	16.47	166.61	16.51	-122.445	37.938	-0.07	16.47	166.61	16.41	9	7/14/2014	38:28.4
7167	RSPe_2	-122.445	37.938	0.04	16.46	166.72	16.49	-122.445	37.938	-0.02	16.46	166.71	16.44	9	7/14/2014	38:28.3
7168	RSPe_2	-122.445	37.938	0.00	16.47	166.76	16.48	-122.445	37.938	-0.02	16.47	166.82	16.46	9	7/14/2014	38:28.2
7169	RSPe_2	-122.445	37.938	0.04	16.51	166.85	16.55	-122.445	37.938	-0.07	16.51	166.95	16.45	9	7/14/2014	38:28.1
7170	RSPe_2	-122.445	37.938	0.00	16.52	167.00	16.52	-122.445	37.938	-0.07	16.52	167.09	16.46	9	7/14/2014	38:28.0
7171	RSPe_2	-122.445	37.938	0.00	16.54	167.09	16.55	-122.445	37.938	-0.02	16.54	167.20	16.53	9	7/14/2014	38:27.9
7172	RSPe_2	-122.445	37.938	-0.05	16.59	167.26	16.54	-122.445	37.938	-0.10	16.59	167.29	16.49	9	7/14/2014	38:27.8
7173	RSPe_2	-122.445	37.938	0.00	16.62	167.51	16.62	-122.445	37.938	-0.10	16.62	167.44	16.52	9	7/14/2014	38:27.7
7174	RSPe_2	-122.445	37.938	0.00	16.67	167.57	16.67	-122.445	37.938	-0.10	16.67	167.52	16.57	9	7/14/2014	38:27.6
7175	RSPe_2	-122.445	37.938	0.00	16.67	167.67	16.68	-122.445	37.938	-0.10	16.67	167.55	16.57	9	7/14/2014	38:27.5
7176	RSPe_2	-122.445	37.938	0.00	16.66	167.79	16.66	-122.445	37.938	-0.10	16.66	167.68	16.56	9	7/14/2014	38:27.4
7177	RSPe_2	-122.445	37.938	0.00	16.69	167.81	16.69	-122.445	37.938	-0.10	16.69	167.63	16.59	9	7/14/2014	38:27.3
7178	RSPe_2	-122.445	37.938	-0.05	16.75	167.87	16.70	-122.445	37.938	-0.15	16.75	167.64	16.60	9	7/14/2014	38:27.2
7179	RSPe_2	-122.445	37.938	-0.05	16.70	168.01	16.65	-122.445	37.938	-0.22	16.70	167.79	16.49	9	7/14/2014	38:27.1
7180	RSPe_2	-122.445	37.938	-0.05	16.77	168.17	16.72	-122.445	37.938	-0.18	16.77	167.87	16.58	9	7/14/2014	38:27.0
7181	RSPe_2	-122.445	37.938	-0.05	16.77	168.31	16.72	-122.445	37.938	-0.22	16.77	168.03	16.55	9	7/14/2014	38:26.9
7182	RSPe_2	-122.445	37.938	-0.08	16.81	168.47	16.72	-122.445	37.938	-0.30	16.81	168.15	16.50	9	7/14/2014	38:26.8
7183	RSPe_2	-122.445	37.938	-0.05	16.79	168.64	16.74	-122.445	37.938	-0.22	16.79	168.32	16.57	9	7/14/2014	38:26.7
7184	RSPe_2	-122.445	37.938	-0.08	16.81	168.85	16.73	-122.445	37.938	-0.22	16.81	168.51	16.59	9	7/14/2014	38:26.6
7185	RSPe_2	-122.445	37.938	-0.08	16.83	169.06	16.74	-122.445	37.938	-0.27	16.83	168.65	16.56	9	7/14/2014	38:26.5
7186	RSPe_2	-122.445	37.938	-0.08	16.84	169.21	16.75	-122.445	37.938	-0.27	16.84	168.83	16.57	9	7/14/2014	38:26.4
7187	RSPe_2	-122.445	37.938	-0.08	16.82	169.34	16.74	-122.445	37.938	-0.27	16.82	168.97	16.55	9	7/14/2014	38:26.3
7188	RSPe_2	-122.445	37.938	-0.08	16.84	169.43	16.75	-122.445	37.938	-0.22	16.84	169.09	16.62	9	7/14/2014	38:26.2
7189	RSPe_2	-122.445	37.938	-0.08	16.82	169.54	16.74	-122.445	37.938	-0.22	16.82	169.21	16.60	9	7/14/2014	38:26.1
7190	RSPe_2	-122.445	37.938	-0.08	16.84	169.58	16.75	-122.445	37.938	-0.27	16.84	169.30	16.57	9	7/14/2014	38:26.0
7191	RSPe_2	-122.445	37.938	-0.08	16.86	169.69	16.78	-122.445	37.938	-0.27	16.86	169.43	16.59	9	7/14/2014	38:25.9
7192	RSPe_2	-122.445	37.938	-0.14	16.85	169.80	16.72	-122.445	37.938	-0.30	16.85	169.58	16.55	9	7/14/2014	38:25.8
7193	RSPe_2	-122.445	37.938	-0.08	16.88	169.93	16.79	-122.445	37.938	-0.30	16.88	169.69	16.57	9	7/14/2014	38:25.7

7194	RSPe_2	-122.445	37.938	-0.14	16.94	170.00	16.81	-122.445	37.938	-0.35	16.94	169.83	16.59	9	7/14/2014	38:25.6
7195	RSPe_2	-122.445	37.938	-0.14	16.94	170.11	16.80	-122.445	37.938	-0.35	16.94	170.02	16.58	9	7/14/2014	38:25.5
7196	RSPe_2	-122.445	37.938	-0.14	16.98	170.26	16.85	-122.445	37.938	-0.39	16.98	170.18	16.59	9	7/14/2014	38:25.4
7197	RSPe_2	-122.445	37.938	-0.14	16.98	170.39	16.84	-122.445	37.938	-0.35	16.98	170.37	16.62	9	7/14/2014	38:25.3
7198	RSPe_2	-122.445	37.938	-0.14	17.01	170.58	16.88	-122.445	37.938	-0.35	17.01	170.52	16.66	9	7/14/2014	38:25.2
7199	RSPe_2	-122.445	37.938	-0.14	17.05	170.65	16.91	-122.445	37.938	-0.35	17.05	170.59	16.69	9	7/14/2014	38:25.1
7200	RSPe_2	-122.445	37.938	-0.14	17.06	170.79	16.92	-122.445	37.938	-0.39	17.06	170.67	16.67	9	7/14/2014	38:25.0
7201	RSPe_2	-122.445	37.938	-0.08	17.14	170.93	17.06	-122.445	37.938	-0.35	17.14	170.73	16.79	9	7/14/2014	38:24.9
7202	RSPe_2	-122.445	37.938	-0.14	17.14	171.05	17.01	-122.445	37.938	-0.35	17.14	170.82	16.79	9	7/14/2014	38:24.8
7203	RSPe_2	-122.445	37.938	-0.08	17.09	171.15	17.01	-122.445	37.938	-0.35	17.09	170.89	16.74	9	7/14/2014	38:24.7
7204	RSPe_2	-122.445	37.938	-0.14	17.05	171.31	16.91	-122.445	37.938	-0.39	17.05	170.95	16.66	9	7/14/2014	38:24.6
7205	RSPe_2	-122.445	37.938	-0.08	17.10	171.41	17.02	-122.445	37.938	-0.30	17.10	171.09	16.80	9	7/14/2014	38:24.5
7206	RSPe_2	-122.445	37.938	-0.14	17.02	171.54	16.89	-122.445	37.938	-0.35	17.02	171.18	16.67	9	7/14/2014	38:24.4
7207	RSPe_2	-122.445	37.938	-0.08	17.06	171.63	16.98	-122.445	37.938	-0.35	17.06	171.30	16.71	9	7/14/2014	38:24.3
7208	RSPe_2	-122.445	37.938	-0.08	17.10	171.76	17.02	-122.445	37.938	-0.39	17.10	171.47	16.71	9	7/14/2014	38:24.2
7209	RSPe_2	-122.445	37.938	-0.14	17.10	171.90	16.97	-122.445	37.938	-0.35	17.10	171.61	16.75	9	7/14/2014	38:24.1
7210	RSPe_2	-122.445	37.938	-0.14	17.05	171.95	16.92	-122.445	37.938	-0.35	17.05	171.73	16.70	9	7/14/2014	38:24.0
7211	RSPe_2	-122.445	37.938	-0.08	17.10	172.14	17.02	-122.445	37.938	-0.35	17.10	171.87	16.75	9	7/14/2014	38:23.9
7212	RSPe_2	-122.445	37.938	-0.14	17.12	172.33	16.98	-122.445	37.938	-0.39	17.12	172.10	16.73	9	7/14/2014	38:23.8
7213	RSPe_2	-122.445	37.938	-0.08	17.16	172.47	17.08	-122.445	37.938	-0.39	17.16	172.20	16.77	9	7/14/2014	38:23.7
7214	RSPe_2	-122.445	37.938	-0.08	17.15	172.62	17.07	-122.445	37.938	-0.35	17.15	172.35	16.80	9	7/14/2014	38:23.6
7215	RSPe_2	-122.445	37.938	-0.05	17.20	172.77	17.15	-122.445	37.938	-0.30	17.20	172.46	16.89	9	7/14/2014	38:23.5
7216	RSPe_2	-122.445	37.938	-0.08	17.30	172.97	17.21	-122.445	37.938	-0.35	17.30	172.59	16.94	9	7/14/2014	38:23.4
7217	RSPe_2	-122.445	37.938	-0.05	17.21	173.08	17.16	-122.445	37.938	-0.30	17.21	172.68	16.91	9	7/14/2014	38:23.3
7218	RSPe_2	-122.445	37.938	-0.08	17.12	173.21	17.03	-122.445	37.938	-0.30	17.12	172.77	16.81	9	7/14/2014	38:23.2
7219	RSPe_2	-122.445	37.938	-0.05	17.18	173.31	17.13	-122.445	37.938	-0.30	17.18	172.90	16.88	9	7/14/2014	38:23.1
7220	RSPe_2	-122.445	37.938	-0.08	17.13	173.35	17.04	-122.445	37.938	-0.30	17.13	173.01	16.83	9	7/14/2014	38:23.0
7221	RSPe_2	-122.445	37.938	-0.05	17.15	173.39	17.10	-122.445	37.938	-0.27	17.15	173.07	16.88	9	7/14/2014	38:22.9
7222	RSPe_2	-122.445	37.938	-0.05	17.10	173.41	17.05	-122.445	37.938	-0.30	17.10	173.12	16.80	9	7/14/2014	38:22.8
7223	RSPe_2	-122.445	37.938	-0.05	17.25	173.47	17.20	-122.445	37.938	-0.27	17.25	173.23	16.98	9	7/14/2014	38:22.7
7224	RSPe_2	-122.445	37.938	-0.05	17.12	173.61	17.07	-122.445	37.938	-0.27	17.12	173.41	16.85	9	7/14/2014	38:22.6
7225	RSPe_2	-122.445	37.938	-0.05	17.21	173.74	17.16	-122.445	37.938	-0.22	17.21	173.54	16.99	9	7/14/2014	38:22.5
7226	RSPe_2	-122.445	37.938	-0.05	17.23	173.88	17.18	-122.445	37.938	-0.30	17.23	173.64	16.93	9	7/14/2014	38:22.4

7227	RSPe_2	-122.445	37.938	-0.05	17.15	173.96	17.10	-122.445	37.938	-0.27	17.15	173.69	16.88	9	7/14/2014 38:22.3
7228	RSPe_2	-122.445	37.938	-0.05	17.21	174.08	17.16	-122.445	37.938	-0.27	17.21	173.77	16.95	9	7/14/2014 38:22.2
7229	RSPe_2	-122.445	37.938	0.00	17.25	174.22	17.25	-122.445	37.938	-0.27	17.25	173.88	16.98	9	7/14/2014 38:22.1
7230	RSPe_2	-122.445	37.938	-0.05	17.28	174.32	17.23	-122.445	37.938	-0.27	17.28	173.93	17.01	9	7/14/2014 38:22.0
7231	RSPe_2	-122.445	37.938	0.00	17.27	174.50	17.27	-122.445	37.938	-0.27	17.27	174.07	17.00	9	7/14/2014 38:21.9
7232	RSPe_2	-122.445	37.938	0.00	17.27	174.60	17.27	-122.445	37.938	-0.22	17.27	174.20	17.05	9	7/14/2014 38:21.8
7233	RSPe_2	-122.445	37.938	0.00	17.29	174.72	17.29	-122.445	37.938	-0.22	17.29	174.26	17.07	9	7/14/2014 38:21.7
7234	RSPe_2	-122.445	37.938	0.00	17.26	174.83	17.26	-122.445	37.938	-0.18	17.26	174.34	17.08	9	7/14/2014 38:21.6
7235	RSPe_2	-122.445	37.938	0.00	17.31	174.94	17.31	-122.445	37.938	-0.22	17.31	174.46	17.09	9	7/14/2014 38:21.5
7236	RSPe_2	-122.445	37.938	0.00	17.24	174.99	17.24	-122.445	37.938	-0.22	17.24	174.52	17.02	9	7/14/2014 38:21.4
7237	RSPe_2	-122.445	37.938	0.04	17.27	175.09	17.30	-122.445	37.938	-0.18	17.27	174.62	17.08	9	7/14/2014 38:21.3
7238	RSPe_2	-122.445	37.938	0.00	17.15	175.22	17.16	-122.445	37.938	-0.18	17.15	174.74	16.97	9	7/14/2014 38:21.2
7239	RSPe_2	-122.445	37.938	0.04	17.19	175.31	17.23	-122.445	37.938	-0.15	17.19	174.78	17.04	9	7/14/2014 38:21.1
7240	RSPe_2	-122.445	37.938	0.00	17.10	175.42	17.10	-122.445	37.938	-0.15	17.10	174.88	16.95	9	7/14/2014 38:21.0
7241	RSPe_2	-122.445	37.938	0.04	17.15	175.46	17.19	-122.445	37.938	-0.10	17.15	174.98	17.05	9	7/14/2014 38:20.9
7242	RSPe_2	-122.445	37.938	0.04	17.19	175.40	17.23	-122.445	37.938	-0.10	17.19	175.03	17.09	9	7/14/2014 38:20.8
7243	RSPe_2	-122.445	37.938	0.04	17.13	175.60	17.16	-122.445	37.938	-0.10	17.13	175.18	17.03	9	7/14/2014 38:20.7
7244	RSPe_2	-122.445	37.938	0.00	17.16	175.71	17.16	-122.445	37.938	-0.10	17.16	175.44	17.06	9	7/14/2014 38:20.6
7245	RSPe_2	-122.445	37.938	0.04	17.13	175.82	17.16	-122.445	37.938	-0.07	17.13	175.53	17.06	9	7/14/2014 38:20.5
7246	RSPe_2	-122.445	37.938	0.00	17.12	175.99	17.12	-122.445	37.938	-0.10	17.12	175.62	17.02	9	7/14/2014 38:20.4
7247	RSPe_2	-122.445	37.938	0.00	17.08	176.07	17.08	-122.445	37.938	-0.07	17.08	175.73	17.01	9	7/14/2014 38:20.3
7248	RSPe_2	-122.445	37.938	0.00	17.06	176.23	17.06	-122.445	37.938	-0.10	17.06	175.80	16.96	9	7/14/2014 38:20.2
7249	RSPe_2	-122.445	37.938	0.00	17.01	176.28	17.02	-122.445	37.938	-0.10	17.01	175.90	16.91	9	7/14/2014 38:20.1
7250	RSPe_2	-122.445	37.938	0.00	16.93	176.41	16.93	-122.445	37.938	-0.10	16.93	175.99	16.83	9	7/14/2014 38:20.0
7251	RSPe_2	-122.445	37.938	0.04	16.97	176.46	17.00	-122.445	37.938	-0.10	16.97	176.09	16.87	9	7/14/2014 38:19.9
7252	RSPe_2	-122.445	37.938	0.00	16.87	176.54	16.87	-122.445	37.938	-0.07	16.87	176.22	16.80	9	7/14/2014 38:19.8
7253	RSPe_2	-122.445	37.938	0.00	16.83	176.63	16.83	-122.445	37.938	-0.07	16.83	176.40	16.76	9	7/14/2014 38:19.7
7254	RSPe_2	-122.445	37.938	0.00	16.79	176.73	16.79	-122.445	37.938	-0.15	16.79	176.47	16.64	9	7/14/2014 38:19.6
7255	RSPe_2	-122.445	37.938	0.00	16.76	176.80	16.76	-122.445	37.938	-0.07	16.76	176.64	16.69	9	7/14/2014 38:19.5
7256	RSPe_2	-122.445	37.938	0.00	16.76	176.97	16.76	-122.445	37.938	-0.10	16.76	176.76	16.66	9	7/14/2014 38:19.4
7257	RSPe_2	-122.445	37.938	0.00	16.65	176.98	16.65	-122.445	37.938	-0.07	16.65	176.87	16.58	9	7/14/2014 38:19.3
7258	RSPe_2	-122.445	37.938	0.00	16.61	177.10	16.62	-122.445	37.938	-0.07	16.61	176.96	16.55	9	7/14/2014 38:19.2
7259	RSPe_2	-122.445	37.938	0.04	16.58	177.15	16.62	-122.445	37.938	-0.07	16.58	177.03	16.52	9	7/14/2014 38:19.1

7260	RSPe_2	-122.445	37.938	0.00	16.50	177.22	16.50	-122.445	37.938	-0.10	16.50	177.08	16.40	9	7/14/2014	38:19.0
7261	RSPe_2	-122.445	37.938	0.00	16.58	177.34	16.58	-122.445	37.938	-0.10	16.58	177.17	16.48	9	7/14/2014	38:18.9
7262	RSPe_2	-122.445	37.938	0.00	16.47	177.46	16.48	-122.445	37.938	-0.10	16.47	177.28	16.37	9	7/14/2014	38:18.8
7263	RSPe_2	-122.445	37.938	0.04	16.42	177.56	16.46	-122.445	37.938	-0.10	16.42	177.31	16.32	9	7/14/2014	38:18.7
7264	RSPe_2	-122.445	37.938	0.00	16.44	177.72	16.44	-122.445	37.938	-0.10	16.44	177.44	16.34	9	7/14/2014	38:18.6
7265	RSPe_2	-122.445	37.938	0.04	16.49	177.89	16.53	-122.445	37.938	-0.10	16.49	177.54	16.39	9	7/14/2014	38:18.5
7266	RSPe_2	-122.445	37.938	0.00	16.48	177.96	16.48	-122.445	37.938	-0.10	16.48	177.64	16.38	9	7/14/2014	38:18.4
7267	RSPe_2	-122.445	37.938	0.04	16.41	178.09	16.44	-122.445	37.938	-0.07	16.41	177.75	16.34	9	7/14/2014	38:18.3
7268	RSPe_2	-122.445	37.938	0.00	16.41	178.14	16.41	-122.445	37.938	-0.10	16.41	177.78	16.31	9	7/14/2014	38:18.2
7269	RSPe_2	-122.445	37.938	0.00	16.27	178.18	16.27	-122.445	37.938	-0.10	16.27	177.81	16.17	9	7/14/2014	38:18.1
7270	RSPe_2	-122.445	37.938	0.00	16.29	178.22	16.29	-122.445	37.938	-0.07	16.29	177.93	16.22	9	7/14/2014	38:18.0
7271	RSPe_2	-122.445	37.938	0.00	16.22	178.29	16.22	-122.445	37.938	-0.07	16.22	178.01	16.15	9	7/14/2014	38:17.9
7272	RSPe_2	-122.445	37.938	0.00	16.37	178.36	16.38	-122.445	37.938	-0.07	16.37	178.11	16.31	9	7/14/2014	38:17.8
7273	RSPe_2	-122.445	37.938	0.00	16.39	178.42	16.39	-122.445	37.938	-0.01	16.39	178.20	16.37	9	7/14/2014	38:17.7
7274	RSPe_2	-122.445	37.938	0.00	16.34	178.53	16.34	-122.445	37.938	-0.10	16.34	178.28	16.24	9	7/14/2014	38:17.6
7275	RSPe_2	-122.445	37.938	0.04	16.31	178.57	16.35	-122.445	37.938	-0.07	16.31	178.34	16.25	9	7/14/2014	38:17.5
7276	RSPe_2	-122.445	37.938	0.00	16.30	178.70	16.30	-122.445	37.938	-0.07	16.30	178.42	16.23	9	7/14/2014	38:17.4
7277	RSPe_2	-122.445	37.938	0.00	16.30	178.77	16.30	-122.445	37.938	-0.07	16.30	178.42	16.23	9	7/14/2014	38:17.3
7278	RSPe_2	-122.445	37.938	0.00	16.30	178.90	16.31	-122.445	37.938	-0.07	16.30	178.48	16.24	9	7/14/2014	38:17.2
7279	RSPe_2	-122.445	37.938	0.00	16.32	178.99	16.32	-122.445	37.938	-0.07	16.32	178.55	16.25	9	7/14/2014	38:17.1
7280	RSPe_2	-122.445	37.938	0.00	16.27	179.08	16.27	-122.445	37.938	-0.07	16.27	178.62	16.20	9	7/14/2014	38:17.0
7281	RSPe_2	-122.445	37.938	0.00	16.21	179.10	16.21	-122.445	37.938	-0.10	16.21	178.71	16.11	9	7/14/2014	38:16.9
7282	RSPe_2	-122.445	37.938	0.00	16.26	179.15	16.26	-122.445	37.938	-0.10	16.26	178.82	16.16	9	7/14/2014	38:16.8
7283	RSPe_2	-122.445	37.938	0.00	16.29	179.24	16.29	-122.445	37.938	-0.07	16.29	178.98	16.22	9	7/14/2014	38:16.7
7284	RSPe_2	-122.445	37.938	0.00	16.42	179.36	16.42	-122.445	37.938	-0.10	16.42	179.14	16.32	9	7/14/2014	38:16.6
7285	RSPe_2	-122.445	37.938	0.04	16.41	179.52	16.44	-122.445	37.938	-0.10	16.41	179.33	16.31	9	7/14/2014	38:16.5
7286	RSPe_2	-122.445	37.938	0.00	16.48	179.65	16.48	-122.445	37.938	-0.10	16.48	179.50	16.38	9	7/14/2014	38:16.4
7287	RSPe_2	-122.445	37.938	0.00	16.50	179.73	16.50	-122.445	37.938	-0.10	16.50	179.58	16.40	9	7/14/2014	38:16.3
7288	RSPe_2	-122.445	37.938	0.00	16.54	179.89	16.55	-122.445	37.938	-0.10	16.54	179.74	16.45	9	7/14/2014	38:16.2
7289	RSPe_2	-122.445	37.938	0.00	16.55	179.95	16.55	-122.445	37.938	-0.07	16.55	179.81	16.48	9	7/14/2014	38:16.1
7290	RSPe_2	-122.445	37.938	0.00	16.56	180.00	16.56	-122.445	37.938	-0.10	16.56	179.89	16.46	9	7/14/2014	38:16.0
7291	RSPe_2	-122.445	37.938	0.00	16.57	180.10	16.57	-122.445	37.938	-0.10	16.57	180.01	16.47	9	7/14/2014	38:15.9
7292	RSPe_2	-122.445	37.938	-0.05	16.67	180.23	16.62	-122.445	37.938	-0.15	16.67	180.11	16.52	9	7/14/2014	38:15.8

7293	RSPe_2	-122.445	37.938	0.00	16.64	180.29	16.64	-122.445	37.938	-0.15	16.64	180.14	16.49	9	7/14/2014	38:15.7
7294	RSPe_2	-122.445	37.938	0.00	16.73	180.41	16.73	-122.445	37.938	-0.10	16.73	180.18	16.63	9	7/14/2014	38:15.6
7295	RSPe_2	-122.445	37.938	0.00	16.67	180.45	16.68	-122.445	37.938	-0.10	16.67	180.24	16.57	9	7/14/2014	38:15.5
7296	RSPe_2	-122.445	37.938	0.00	16.74	180.56	16.74	-122.445	37.938	-0.15	16.74	180.28	16.59	9	7/14/2014	38:15.4
7297	RSPe_2	-122.445	37.938	0.00	16.66	180.69	16.66	-122.445	37.938	-0.15	16.66	180.38	16.51	9	7/14/2014	38:15.3
7298	RSPe_2	-122.445	37.938	-0.05	16.67	180.76	16.62	-122.445	37.938	-0.18	16.67	180.47	16.49	9	7/14/2014	38:15.2
7299	RSPe_2	-122.445	37.938	0.00	16.77	180.89	16.78	-122.445	37.938	-0.15	16.77	180.51	16.62	9	7/14/2014	38:15.1
7300	RSPe_2	-122.445	37.938	0.00	16.70	180.94	16.71	-122.445	37.938	-0.15	16.70	180.60	16.55	9	7/14/2014	38:15.0
7301	RSPe_2	-122.445	37.938	0.00	16.76	181.05	16.76	-122.445	37.938	-0.10	16.76	180.67	16.66	9	7/14/2014	38:14.9
7302	RSPe_2	-122.445	37.938	-0.05	16.80	181.11	16.75	-122.445	37.938	-0.18	16.80	180.78	16.61	9	7/14/2014	38:14.8
7303	RSPe_2	-122.445	37.938	0.00	16.87	181.20	16.87	-122.445	37.938	-0.18	16.87	180.87	16.68	9	7/14/2014	38:14.7
7304	RSPe_2	-122.445	37.938	-0.05	16.87	181.38	16.82	-122.445	37.938	-0.18	16.87	181.00	16.68	9	7/14/2014	38:14.6
7305	RSPe_2	-122.445	37.938	-0.05	16.82	181.51	16.77	-122.445	37.938	-0.15	16.82	181.14	16.67	9	7/14/2014	38:14.5
7306	RSPe_2	-122.445	37.938	-0.05	16.85	181.64	16.81	-122.445	37.938	-0.18	16.85	181.26	16.67	9	7/14/2014	38:14.4
7307	RSPe_2	-122.445	37.938	0.00	16.94	181.70	16.94	-122.445	37.938	-0.15	16.94	181.38	16.79	9	7/14/2014	38:14.3
7308	RSPe_2	-122.445	37.938	-0.05	16.95	181.81	16.90	-122.445	37.938	-0.18	16.95	181.50	16.77	9	7/14/2014	38:14.2
7309	RSPe_2	-122.445	37.938	-0.05	16.97	181.89	16.92	-122.445	37.938	-0.15	16.97	181.59	16.82	9	7/14/2014	38:14.1
7310	RSPe_2	-122.445	37.938	-0.05	16.92	181.95	16.87	-122.445	37.938	-0.15	16.92	181.69	16.77	9	7/14/2014	38:14.0
7311	RSPe_2	-122.445	37.938	-0.05	16.88	182.06	16.83	-122.445	37.938	-0.18	16.88	181.81	16.70	9	7/14/2014	38:13.9
7312	RSPe_2	-122.445	37.938	-0.05	16.95	182.09	16.90	-122.445	37.938	-0.18	16.95	181.86	16.77	9	7/14/2014	38:13.8
7313	RSPe_2	-122.445	37.938	-0.05	16.84	182.12	16.79	-122.445	37.938	-0.18	16.84	181.93	16.66	9	7/14/2014	38:13.7
7314	RSPe_2	-122.445	37.938	-0.05	16.81	182.32	16.76	-122.445	37.938	-0.18	16.81	182.00	16.63	9	7/14/2014	38:13.6
7315	RSPe_2	-122.445	37.938	-0.05	16.80	182.35	16.75	-122.445	37.938	-0.18	16.80	182.10	16.61	9	7/14/2014	38:13.5
7316	RSPe_2	-122.445	37.938	-0.08	16.84	182.43	16.75	-122.445	37.938	-0.22	16.84	182.09	16.62	9	7/14/2014	38:13.4
7317	RSPe_2	-122.445	37.938	-0.05	16.84	182.52	16.79	-122.445	37.938	-0.15	16.84	182.19	16.69	9	7/14/2014	38:13.3
7318	RSPe_2	-122.445	37.938	-0.08	16.77	182.57	16.69	-122.445	37.938	-0.22	16.77	182.24	16.56	9	7/14/2014	38:13.2
7319	RSPe_2	-122.445	37.938	-0.05	16.81	182.67	16.76	-122.445	37.938	-0.18	16.81	182.32	16.62	9	7/14/2014	38:13.1
7320	RSPe_2	-122.445	37.938	-0.08	16.77	182.77	16.69	-122.445	37.938	-0.18	16.77	182.47	16.59	9	7/14/2014	38:13.0
7321	RSPe_2	-122.445	37.938	-0.08	16.77	182.91	16.69	-122.445	37.938	-0.22	16.77	182.54	16.56	9	7/14/2014	38:12.9
7322	RSPe_2	-122.445	37.938	-0.08	16.77	183.03	16.69	-122.445	37.938	-0.18	16.77	182.65	16.59	9	7/14/2014	38:12.8
7323	RSPe_2	-122.445	37.938	-0.08	16.77	183.10	16.68	-122.445	37.938	-0.22	16.77	182.77	16.55	9	7/14/2014	38:12.7
7324	RSPe_2	-122.445	37.938	-0.08	16.78	183.23	16.70	-122.445	37.938	-0.18	16.78	182.93	16.60	9	7/14/2014	38:12.6
7325	RSPe_2	-122.445	37.938	-0.05	16.77	183.31	16.72	-122.445	37.938	-0.18	16.77	183.03	16.58	9	7/14/2014	38:12.5

7326	RSPe_2	-122.445	37.938	-0.13	16.77	183.32	16.63	-122.445	37.938	-0.27	16.77	183.11	16.50	9	7/14/2014	38:12.4
7327	RSPe_2	-122.445	37.938	-0.08	16.77	183.32	16.69	-122.445	37.938	-0.27	16.77	183.21	16.51	9	7/14/2014	38:12.3
7328	RSPe_2	-122.445	37.938	-0.13	16.79	183.31	16.66	-122.445	37.938	-0.30	16.79	183.26	16.49	9	7/14/2014	38:12.2
7329	RSPe_2	-122.445	37.938	-0.08	16.81	183.34	16.73	-122.445	37.938	-0.30	16.81	183.36	16.51	9	7/14/2014	38:12.1
7330	RSPe_2	-122.445	37.938	-0.13	16.83	183.50	16.69	-122.445	37.938	-0.30	16.83	183.46	16.53	9	7/14/2014	38:12.0
7331	RSPe_2	-122.445	37.938	-0.08	16.95	183.54	16.87	-122.445	37.938	-0.30	16.95	183.51	16.65	9	7/14/2014	38:11.9
7332	RSPe_2	-122.445	37.938	-0.13	16.90	183.60	16.76	-122.445	37.938	-0.30	16.90	183.50	16.60	9	7/14/2014	38:11.8
7333	RSPe_2	-122.445	37.938	-0.08	16.98	183.71	16.89	-122.445	37.938	-0.30	16.98	183.60	16.67	9	7/14/2014	38:11.7
7334	RSPe_2	-122.445	37.938	-0.13	17.00	183.78	16.87	-122.445	37.938	-0.35	17.00	183.60	16.65	9	7/14/2014	38:11.6
7335	RSPe_2	-122.445	37.938	-0.13	17.08	183.91	16.95	-122.445	37.938	-0.35	17.08	183.67	16.73	9	7/14/2014	38:11.5
7336	RSPe_2	-122.445	37.938	-0.13	17.03	184.07	16.90	-122.445	37.938	-0.35	17.03	183.75	16.68	9	7/14/2014	38:11.4
7337	RSPe_2	-122.445	37.938	-0.13	17.06	184.16	16.93	-122.445	37.938	-0.35	17.06	183.80	16.71	9	7/14/2014	38:11.3
7338	RSPe_2	-122.445	37.938	-0.17	17.12	184.31	16.95	-122.445	37.938	-0.39	17.12	183.91	16.73	9	7/14/2014	38:11.2
7339	RSPe_2	-122.445	37.938	-0.13	17.13	184.40	16.99	-122.445	37.938	-0.39	17.13	183.96	16.74	9	7/14/2014	38:11.1
7340	RSPe_2	-122.445	37.938	-0.17	17.07	184.49	16.90	-122.445	37.938	-0.39	17.07	184.08	16.68	9	7/14/2014	38:11.0
7341	RSPe_2	-122.445	37.938	-0.13	17.08	184.52	16.95	-122.445	37.938	-0.39	17.08	184.15	16.70	9	7/14/2014	38:10.9
7342	RSPe_2	-122.445	37.938	-0.17	17.11	184.49	16.94	-122.445	37.938	-0.44	17.11	184.12	16.67	9	7/14/2014	38:10.8
7343	RSPe_2	-122.445	37.938	-0.13	17.14	184.57	17.00	-122.445	37.938	-0.35	17.14	184.17	16.79	9	7/14/2014	38:10.7
7344	RSPe_2	-122.445	37.938	-0.17	17.14	184.92	16.98	-122.445	37.938	-0.39	17.14	184.44	16.76	9	7/14/2014	38:10.6
7345	RSPe_2	-122.445	37.938	-0.13	17.14	185.07	17.01	-122.445	37.938	-0.39	17.14	184.55	16.76	9	7/14/2014	38:10.5
7346	RSPe_2	-122.445	37.938	-0.17	17.12	185.06	16.95	-122.445	37.938	-0.39	17.12	184.55	16.73	9	7/14/2014	38:10.4
7347	RSPe_2	-122.445	37.938	-0.17	17.15	185.12	16.99	-122.445	37.938	-0.39	17.15	184.68	16.77	9	7/14/2014	38:10.3
7348	RSPe_2	-122.445	37.938	-0.17	17.17	185.22	17.00	-122.445	37.938	-0.39	17.17	184.84	16.78	9	7/14/2014	38:10.2
7349	RSPe_2	-122.445	37.938	-0.17	17.18	185.21	17.01	-122.445	37.938	-0.44	17.18	184.95	16.74	9	7/14/2014	38:10.1
7350	RSPe_2	-122.445	37.938	-0.17	17.19	185.34	17.02	-122.445	37.938	-0.44	17.19	185.10	16.75	9	7/14/2014	38:10.0
7351	RSPe_2	-122.445	37.938	-0.17	17.25	185.44	17.08	-122.445	37.938	-0.39	17.25	185.20	16.87	9	7/14/2014	38:09.9
7352	RSPe_2	-122.445	37.938	-0.20	17.26	185.59	17.06	-122.445	37.938	-0.52	17.26	185.42	16.74	9	7/14/2014	38:09.8
7353	RSPe_2	-122.445	37.938	-0.17	17.30	185.71	17.13	-122.445	37.938	-0.44	17.30	185.47	16.86	9	7/14/2014	38:09.7
7354	RSPe_2	-122.445	37.938	-0.20	17.31	185.75	17.11	-122.445	37.938	-0.47	17.31	185.49	16.84	9	7/14/2014	38:09.6
7355	RSPe_2	-122.445	37.938	-0.17	17.32	185.85	17.15	-122.445	37.938	-0.44	17.32	185.65	16.88	9	7/14/2014	38:09.5
7356	RSPe_2	-122.445	37.938	-0.17	17.37	185.97	17.20	-122.445	37.938	-0.52	17.37	185.71	16.85	9	7/14/2014	38:09.4
7357	RSPe_2	-122.445	37.938	-0.20	17.36	186.00	17.16	-122.445	37.938	-0.44	17.36	185.80	16.92	9	7/14/2014	38:09.3
7358	RSPe_2	-122.445	37.938	-0.20	17.44	186.03	17.23	-122.445	37.938	-0.47	17.44	185.87	16.97	9	7/14/2014	38:09.2

7359	RSPe_2	-122.445	37.938	-0.17	17.44	186.21	17.27	-122.445	37.938	-0.44	17.44	186.00	17.00	9	7/14/2014	38:09.1
7360	RSPe_2	-122.445	37.938	-0.17	17.46	186.32	17.29	-122.445	37.938	-0.44	17.46	186.04	17.02	9	7/14/2014	38:09.0
7361	RSPe_2	-122.445	37.938	-0.17	17.45	186.48	17.28	-122.445	37.938	-0.39	17.45	186.23	17.06	9	7/14/2014	38:08.9
7362	RSPe_2	-122.445	37.938	-0.20	17.49	186.70	17.29	-122.445	37.938	-0.44	17.49	186.38	17.06	9	7/14/2014	38:08.8
7363	RSPe_2	-122.445	37.938	-0.17	17.48	186.80	17.31	-122.445	37.938	-0.44	17.48	186.47	17.04	9	7/14/2014	38:08.7
7364	RSPe_2	-122.445	37.938	-0.20	17.49	186.89	17.29	-122.445	37.938	-0.44	17.49	186.47	17.06	9	7/14/2014	38:08.6
7365	RSPe_2	-122.445	37.938	-0.17	17.45	186.94	17.28	-122.445	37.938	-0.47	17.45	186.63	16.98	9	7/14/2014	38:08.5
7366	RSPe_2	-122.445	37.938	-0.17	17.52	187.07	17.36	-122.445	37.938	-0.52	17.52	186.75	17.00	9	7/14/2014	38:08.4
7367	RSPe_2	-122.445	37.938	-0.17	17.50	187.15	17.33	-122.445	37.938	-0.52	17.50	186.94	16.98	9	7/14/2014	38:08.3
7368	RSPe_2	-122.445	37.938	-0.20	17.47	187.29	17.27	-122.445	37.938	-0.52	17.47	187.03	16.95	9	7/14/2014	38:08.2
7369	RSPe_2	-122.445	37.938	-0.17	17.50	187.41	17.33	-122.445	37.938	-0.44	17.50	187.20	17.06	9	7/14/2014	38:08.1
7370	RSPe_2	-122.445	37.938	-0.20	17.52	187.51	17.32	-122.445	37.938	-0.56	17.52	187.31	16.97	9	7/14/2014	38:08.0
7371	RSPe_2	-122.445	37.938	-0.17	17.55	187.57	17.38	-122.445	37.938	-0.47	17.55	187.47	17.07	9	7/14/2014	38:07.9
7372	RSPe_2	-122.445	37.938	-0.20	17.59	187.68	17.39	-122.445	37.938	-0.44	17.59	187.56	17.16	9	7/14/2014	38:07.8
7373	RSPe_2	-122.445	37.938	-0.20	17.61	187.77	17.41	-122.445	37.938	-0.56	17.61	187.68	17.05	9	7/14/2014	38:07.7
7374	RSPe_2	-122.445	37.938	-0.20	17.67	187.87	17.47	-122.445	37.938	-0.52	17.67	187.76	17.15	9	7/14/2014	38:07.6
7375	RSPe_2	-122.445	37.938	-0.17	17.70	187.90	17.53	-122.445	37.938	-0.47	17.70	187.73	17.23	9	7/14/2014	38:07.5
7376	RSPe_2	-122.445	37.938	-0.20	17.68	188.06	17.47	-122.445	37.938	-0.47	17.68	187.80	17.21	9	7/14/2014	38:07.4
7377	RSPe_2	-122.445	37.938	-0.20	17.70	188.18	17.50	-122.445	37.938	-0.52	17.70	187.89	17.18	9	7/14/2014	38:07.3
7378	RSPe_2	-122.445	37.938	-0.20	17.77	188.32	17.57	-122.445	37.938	-0.56	17.77	188.01	17.21	9	7/14/2014	38:07.2
7379	RSPe_2	-122.445	37.938	-0.17	17.76	188.46	17.60	-122.445	37.938	-0.52	17.76	188.13	17.24	9	7/14/2014	38:07.1
7380	RSPe_2	-122.445	37.938	-0.20	17.79	188.47	17.59	-122.445	37.938	-0.52	17.79	188.26	17.27	9	7/14/2014	38:07.0
7381	RSPe_2	-122.445	37.938	-0.20	17.80	188.66	17.60	-122.445	37.938	-0.56	17.80	188.37	17.25	9	7/14/2014	38:06.9
7382	RSPe_2	-122.445	37.938	-0.20	17.82	188.77	17.62	-122.445	37.938	-0.59	17.82	188.45	17.23	9	7/14/2014	38:06.8
7383	RSPe_2	-122.445	37.938	-0.20	17.91	188.85	17.71	-122.445	37.938	-0.52	17.91	188.57	17.39	9	7/14/2014	38:06.7
7384	RSPe_2	-122.445	37.938	-0.20	17.85	189.05	17.65	-122.445	37.938	-0.59	17.85	188.62	17.26	9	7/14/2014	38:06.6
7385	RSPe_2	-122.445	37.938	-0.20	17.89	189.21	17.68	-122.445	37.938	-0.59	17.89	188.72	17.30	9	7/14/2014	38:06.5
7386	RSPe_2	-122.445	37.938	-0.20	17.90	189.22	17.70	-122.445	37.938	-0.56	17.90	188.84	17.35	9	7/14/2014	38:06.4
7387	RSPe_2	-122.445	37.938	-0.20	17.94	189.29	17.74	-122.445	37.938	-0.52	17.94	188.87	17.42	9	7/14/2014	38:06.3
7388	RSPe_2	-122.445	37.938	-0.20	17.98	189.30	17.78	-122.445	37.938	-0.56	17.98	188.94	17.42	9	7/14/2014	38:06.2
7389	RSPe_2	-122.445	37.938	-0.20	17.99	189.43	17.78	-122.445	37.938	-0.56	17.99	189.10	17.43	9	7/14/2014	38:06.1
7390	RSPe_2	-122.445	37.938	-0.25	18.02	189.60	17.76	-122.445	37.938	-0.59	18.02	189.20	17.43	9	7/14/2014	38:06.0
7391	RSPe_2	-122.445	37.938	-0.20	18.05	189.76	17.85	-122.445	37.938	-0.59	18.05	189.37	17.47	9	7/14/2014	38:05.9

7392	RSPe_2	-122.445	37.938	-0.25	18.10	189.89	17.84	-122.445	37.938	-0.59	18.10	189.50	17.51	9	7/14/2014	38:05.8
7393	RSPe_2	-122.445	37.938	-0.20	18.10	190.02	17.89	-122.445	37.938	-0.52	18.10	189.65	17.58	9	7/14/2014	38:05.7
7394	RSPe_2	-122.445	37.938	-0.25	18.10	190.09	17.84	-122.445	37.938	-0.56	18.10	189.82	17.54	9	7/14/2014	38:05.6
7395	RSPe_2	-122.445	37.938	-0.25	18.13	190.13	17.88	-122.445	37.938	-0.56	18.13	189.95	17.58	9	7/14/2014	38:05.5
7396	RSPe_2	-122.445	37.938	-0.25	18.13	190.22	17.88	-122.445	37.938	-0.64	18.13	190.00	17.49	9	7/14/2014	38:05.4
7397	RSPe_2	-122.445	37.938	-0.20	18.04	190.20	17.84	-122.445	37.938	-0.56	18.04	190.02	17.48	9	7/14/2014	38:05.3
7398	RSPe_2	-122.445	37.938	-0.20	17.99	190.23	17.79	-122.445	37.938	-0.59	17.99	190.11	17.40	9	7/14/2014	38:05.2
7399	RSPe_2	-122.445	37.938	-0.20	17.94	190.33	17.74	-122.445	37.938	-0.59	17.94	190.25	17.35	9	7/14/2014	38:05.1
7400	RSPe_2	-122.445	37.938	-0.20	17.95	190.42	17.75	-122.445	37.938	-0.56	17.95	190.32	17.39	9	7/14/2014	38:05.0
7401	RSPe_2	-122.445	37.938	-0.20	17.78	190.46	17.57	-122.445	37.938	-0.56	17.78	190.44	17.22	9	7/14/2014	38:04.9
7402	RSPe_2	-122.445	37.938	-0.20	17.68	190.60	17.48	-122.445	37.938	-0.59	17.68	190.52	17.10	9	7/14/2014	38:04.8
7403	RSPe_2	-122.445	37.938	-0.20	17.61	190.69	17.41	-122.445	37.938	-0.59	17.61	190.61	17.03	9	7/14/2014	38:04.7
7404	RSPe_2	-122.445	37.938	-0.20	17.56	190.84	17.35	-122.445	37.938	-0.59	17.56	190.61	16.97	9	7/14/2014	38:04.6
7405	RSPe_2	-122.445	37.938	-0.20	17.58	190.99	17.38	-122.445	37.938	-0.52	17.58	190.71	17.06	9	7/14/2014	38:04.5
7406	RSPe_2	-122.445	37.938	-0.25	17.53	191.13	17.28	-122.445	37.938	-0.59	17.53	190.81	16.94	9	7/14/2014	38:04.4
7407	RSPe_2	-122.445	37.938	-0.20	17.44	191.32	17.24	-122.445	37.938	-0.56	17.44	190.97	16.88	9	7/14/2014	38:04.3
7408	RSPe_2	-122.445	37.938	-0.25	17.45	191.44	17.19	-122.445	37.938	-0.56	17.45	191.05	16.89	9	7/14/2014	38:04.2
7409	RSPe_2	-122.445	37.938	-0.20	17.44	191.49	17.24	-122.445	37.938	-0.56	17.44	191.08	16.88	9	7/14/2014	38:04.1
7410	RSPe_2	-122.445	37.938	-0.20	17.28	191.44	17.08	-122.445	37.938	-0.64	17.28	191.12	16.64	9	7/14/2014	38:04.0
7411	RSPe_2	-122.445	37.938	-0.20	17.21	191.59	17.01	-122.445	37.938	-0.59	17.21	191.35	16.63	9	7/14/2014	38:03.9
7412	RSPe_2	-122.445	37.938	-0.20	17.27	191.71	17.07	-122.445	37.938	-0.56	17.27	191.56	16.71	9	7/14/2014	38:03.8
7413	RSPe_2	-122.445	37.938	-0.20	17.29	191.88	17.09	-122.445	37.938	-0.59	17.29	191.74	16.70	9	7/14/2014	38:03.7
7414	RSPe_2	-122.445	37.938	-0.20	17.20	191.96	17.00	-122.445	37.938	-0.64	17.20	191.84	16.56	9	7/14/2014	38:03.6
7415	RSPe_2	-122.445	37.938	-0.17	17.24	192.04	17.07	-122.445	37.938	-0.67	17.24	191.99	16.57	9	7/14/2014	38:03.5
7416	RSPe_2	-122.445	37.938	-0.20	17.23	192.16	17.03	-122.445	37.938	-0.64	17.23	192.06	16.59	9	7/14/2014	38:03.4
7417	RSPe_2	-122.445	37.938	-0.20	17.18	192.27	16.98	-122.445	37.938	-0.56	17.18	192.12	16.63	9	7/14/2014	38:03.3
7418	RSPe_2	-122.445	37.938	-0.20	17.20	192.41	17.00	-122.445	37.938	-0.59	17.20	192.14	16.61	9	7/14/2014	38:03.2
7419	RSPe_2	-122.445	37.938	-0.20	17.27	192.65	17.07	-122.445	37.938	-0.59	17.27	192.24	16.68	9	7/14/2014	38:03.1
7420	RSPe_2	-122.445	37.938	-0.20	17.14	192.89	16.94	-122.445	37.938	-0.59	17.14	192.38	16.55	9	7/14/2014	38:03.0
7421	RSPe_2	-122.445	37.938	-0.17	17.14	193.04	16.98	-122.445	37.938	-0.56	17.14	192.51	16.59	9	7/14/2014	38:02.9
7422	RSPe_2	-122.445	37.938	-0.20	17.09	193.04	16.89	-122.445	37.938	-0.59	17.09	192.57	16.50	9	7/14/2014	38:02.8
7423	RSPe_2	-122.445	37.938	-0.17	17.08	193.16	16.92	-122.445	37.938	-0.56	17.08	192.76	16.53	9	7/14/2014	38:02.7
7424	RSPe_2	-122.445	37.938	-0.20	17.05	193.33	16.85	-122.445	37.938	-0.56	17.05	192.94	16.50	9	7/14/2014	38:02.6

7425	RSPe_2	-122.445	37.938	-0.17	17.10	193.41	16.93	-122.445	37.938	-0.56	17.10	193.11	16.55	9	7/14/2014	38:02.5
7426	RSPe_2	-122.445	37.938	-0.17	17.01	193.54	16.85	-122.445	37.938	-0.52	17.01	193.23	16.49	9	7/14/2014	38:02.4
7427	RSPe_2	-122.445	37.938	-0.13	17.05	193.66	16.92	-122.445	37.938	-0.44	17.05	193.28	16.62	9	7/14/2014	38:02.3
7428	RSPe_2	-122.445	37.938	-0.17	17.17	193.79	17.00	-122.445	37.938	-0.47	17.17	193.40	16.70	9	7/14/2014	38:02.2
7429	RSPe_2	-122.445	37.938	-0.13	17.02	193.96	16.89	-122.445	37.938	-0.39	17.02	193.47	16.63	9	7/14/2014	38:02.1
7430	RSPe_2	-122.445	37.938	-0.17	17.07	194.04	16.90	-122.445	37.938	-0.39	17.07	193.42	16.68	9	7/14/2014	38:02.0
7431	RSPe_2	-122.445	37.938	-0.13	17.09	194.21	16.96	-122.445	37.938	-0.39	17.09	193.50	16.70	9	7/14/2014	38:01.9
7432	RSPe_2	-122.445	37.938	-0.17	17.11	194.28	16.94	-122.445	37.938	-0.39	17.11	193.62	16.72	9	7/14/2014	38:01.8
7433	RSPe_2	-122.445	37.938	-0.13	17.02	194.30	16.89	-122.445	37.938	-0.35	17.02	193.70	16.67	9	7/14/2014	38:01.7
7434	RSPe_2	-122.445	37.938	-0.13	17.08	194.23	16.94	-122.445	37.938	-0.39	17.08	193.74	16.69	9	7/14/2014	38:01.6
7435	RSPe_2	-122.445	37.938	-0.13	17.09	194.13	16.96	-122.445	37.938	-0.30	17.09	193.81	16.79	9	7/14/2014	38:01.5
7436	RSPe_2	-122.445	37.938	-0.17	17.12	194.29	16.95	-122.445	37.938	-0.35	17.12	194.16	16.76	9	7/14/2014	38:01.4
7437	RSPe_2	-122.445	37.938	-0.13	17.14	194.59	17.01	-122.445	37.938	-0.30	17.14	194.62	16.84	9	7/14/2014	38:01.3
7438	RSPe_2	-122.445	37.938	-0.13	16.53	194.69	16.40	-122.445	37.938	-0.35	16.53	194.71	16.18	9	7/14/2014	38:01.2
7439	RSPe_2	-122.445	37.938	-0.13	16.57	194.70	16.43	-122.445	37.938	-0.27	16.57	194.72	16.30	9	7/14/2014	38:01.1
7440	RSPe_2	-122.445	37.938	-0.13	16.58	194.85	16.45	-122.445	37.938	-0.30	16.58	194.87	16.28	9	7/14/2014	38:01.0
7441	RSPe_2	-122.445	37.938	-0.08	16.58	194.85	16.49	-122.445	37.938	-0.35	16.58	194.87	16.22	9	7/14/2014	38:00.9
7442	RSPe_2	-122.445	37.938	-0.13	17.18	194.98	17.05	-122.445	37.938	-0.30	17.18	194.99	16.88	9	7/14/2014	38:00.8
7443	RSPe_2	-122.445	37.938	-0.08	17.18	195.07	17.09	-122.445	37.938	-0.35	17.18	195.01	16.82	9	7/14/2014	38:00.7
7444	RSPe_2	-122.445	37.938	-0.08	17.17	195.21	17.09	-122.445	37.938	-0.30	17.17	195.08	16.87	9	7/14/2014	38:00.6
7445	RSPe_2	-122.445	37.938	-0.08	17.14	195.18	17.06	-122.445	37.938	-0.22	17.14	195.08	16.93	9	7/14/2014	38:00.5
7446	RSPe_2	-122.445	37.938	-0.08	17.16	195.21	17.08	-122.445	37.938	-0.27	17.16	195.04	16.89	9	7/14/2014	38:00.4
7447	RSPe_2	-122.445	37.938	-0.08	17.17	195.21	17.09	-122.445	37.938	-0.27	17.17	195.12	16.90	9	7/14/2014	38:00.3
7448	RSPe_2	-122.445	37.938	-0.08	17.17	195.29	17.09	-122.445	37.938	-0.35	17.17	195.23	16.82	9	7/14/2014	38:00.2
7449	RSPe_2	-122.445	37.938	-0.05	17.20	195.33	17.15	-122.445	37.938	-0.27	17.20	195.28	16.93	9	7/14/2014	38:00.1
7450	RSPe_2	-122.445	37.938	-0.08	17.21	195.43	17.13	-122.445	37.938	-0.22	17.21	195.42	16.99	9	7/14/2014	38:00.0
7451	RSPe_2	-122.445	37.938	-0.05	17.28	195.60	17.23	-122.445	37.938	-0.22	17.28	195.60	17.06	9	7/14/2014	37:59.9
7452	RSPe_2	-122.445	37.938	-0.08	17.30	195.81	17.22	-122.445	37.938	-0.22	17.30	195.65	17.08	9	7/14/2014	37:59.8
7453	RSPe_2	-122.445	37.938	-0.05	17.33	195.97	17.28	-122.445	37.938	-0.22	17.33	195.73	17.11	9	7/14/2014	37:59.7
7454	RSPe_2	-122.445	37.938	-0.08	17.36	196.11	17.28	-122.445	37.938	-0.22	17.36	195.75	17.15	9	7/14/2014	37:59.6
7455	RSPe_2	-122.445	37.938	-0.05	17.36	196.28	17.31	-122.445	37.938	-0.27	17.36	195.80	17.09	9	7/14/2014	37:59.5
7456	RSPe_2	-122.445	37.938	-0.05	17.37	196.39	17.32	-122.445	37.938	-0.27	17.37	195.89	17.10	9	7/14/2014	37:59.4
7457	RSPe_2	-122.445	37.938	-0.05	17.38	196.64	17.33	-122.445	37.938	-0.22	17.38	195.97	17.16	9	7/14/2014	37:59.3

7458	RSPe_2	-122.445	37.938	-0.08	17.37	196.77	17.29	-122.445	37.938	-0.18	17.37	196.07	17.19	9	7/14/2014	37:59.2
7459	RSPe_2	-122.445	37.938	-0.05	17.39	196.86	17.34	-122.445	37.938	-0.18	17.39	196.16	17.20	9	7/14/2014	37:59.1
7460	RSPe_2	-122.445	37.938	-0.08	17.38	197.01	17.30	-122.445	37.938	-0.22	17.38	196.26	17.16	9	7/14/2014	37:59.0
7461	RSPe_2	-122.445	37.938	-0.05	17.35	196.99	17.31	-122.445	37.938	-0.18	17.35	196.32	17.17	9	7/14/2014	37:58.9
7462	RSPe_2	-122.445	37.938	-0.05	17.35	197.10	17.30	-122.445	37.938	-0.22	17.35	196.50	17.13	9	7/14/2014	37:58.8
7463	RSPe_2	-122.445	37.938	-0.05	17.28	197.17	17.24	-122.445	37.938	-0.18	17.28	196.74	17.10	9	7/14/2014	37:58.7
7464	RSPe_2	-122.445	37.938	-0.05	17.26	197.27	17.21	-122.445	37.938	-0.22	17.26	196.97	17.04	9	7/14/2014	37:58.6
7465	RSPe_2	-122.445	37.938	-0.08	17.21	197.32	17.13	-122.445	37.938	-0.15	17.21	197.16	17.06	9	7/14/2014	37:58.5
7466	RSPe_2	-122.445	37.938	-0.05	17.19	197.39	17.14	-122.445	37.938	-0.15	17.19	197.35	17.04	9	7/14/2014	37:58.4
7467	RSPe_2	-122.445	37.938	-0.05	17.19	197.49	17.14	-122.445	37.938	-0.18	17.19	197.52	17.01	9	7/14/2014	37:58.3
7468	RSPe_2	-122.445	37.938	-0.05	17.17	197.52	17.12	-122.445	37.938	-0.18	17.17	197.56	16.99	9	7/14/2014	37:58.2
7469	RSPe_2	-122.445	37.938	0.00	17.11	197.59	17.11	-122.445	37.938	-0.10	17.11	197.62	17.01	9	7/14/2014	37:58.1
7470	RSPe_2	-122.445	37.938	-0.05	17.05	197.62	17.00	-122.445	37.938	-0.10	17.05	197.67	16.95	9	7/14/2014	37:58.0
7471	RSPe_2	-122.445	37.938	-0.05	17.05	197.63	17.00	-122.445	37.938	-0.10	17.05	197.67	16.95	9	7/14/2014	37:57.9
7472	RSPe_2	-122.445	37.938	-0.05	17.05	197.70	17.00	-122.445	37.938	-0.15	17.05	197.73	16.90	9	7/14/2014	37:57.8
7473	RSPe_2	-122.445	37.938	0.00	16.98	197.73	16.98	-122.445	37.938	-0.15	16.98	197.74	16.83	9	7/14/2014	37:57.7
7474	RSPe_2	-122.445	37.938	-0.05	16.98	197.83	16.93	-122.445	37.938	-0.15	16.98	197.84	16.83	9	7/14/2014	37:57.6
7475	RSPe_2	-122.445	37.938	0.00	16.97	197.94	16.97	-122.445	37.938	-0.10	16.97	197.96	16.87	9	7/14/2014	37:57.5
7476	RSPe_2	-122.445	37.938	-0.05	16.97	198.14	16.92	-122.445	37.938	-0.15	16.97	198.05	16.82	9	7/14/2014	37:57.4
7477	RSPe_2	-122.445	37.938	0.00	16.97	198.20	16.97	-122.445	37.938	-0.15	16.97	198.16	16.82	9	7/14/2014	37:57.3
7478	RSPe_2	-122.445	37.938	0.00	16.94	198.33	16.94	-122.445	37.938	-0.10	16.94	198.24	16.84	9	7/14/2014	37:57.2
7479	RSPe_2	-122.445	37.938	0.00	16.92	198.44	16.92	-122.445	37.938	-0.01	16.92	198.31	16.91	9	7/14/2014	37:57.1
7480	RSPe_2	-122.445	37.938	0.00	16.91	198.49	16.91	-122.445	37.938	-0.10	16.91	198.43	16.81	9	7/14/2014	37:57.0
7481	RSPe_2	-122.445	37.938	0.00	16.91	198.60	16.91	-122.445	37.938	-0.10	16.91	198.52	16.81	9	7/14/2014	37:56.9
7482	RSPe_2	-122.445	37.938	0.00	16.87	198.65	16.87	-122.445	37.938	-0.06	16.87	198.53	16.80	9	7/14/2014	37:56.8
7483	RSPe_2	-122.445	37.938	0.00	16.88	198.67	16.88	-122.445	37.938	-0.06	16.88	198.62	16.81	9	7/14/2014	37:56.7
7484	RSPe_2	-122.445	37.938	0.00	16.87	198.76	16.87	-122.445	37.938	-0.10	16.87	198.66	16.77	9	7/14/2014	37:56.6
7485	RSPe_2	-122.445	37.938	0.00	16.88	198.91	16.88	-122.445	37.938	-0.10	16.88	198.77	16.78	9	7/14/2014	37:56.5
7486	RSPe_2	-122.445	37.938	0.00	16.87	199.03	16.87	-122.445	37.938	-0.10	16.87	198.87	16.77	9	7/14/2014	37:56.4
7487	RSPe_2	-122.445	37.938	0.00	16.90	199.15	16.90	-122.445	37.938	-0.10	16.90	198.90	16.80	9	7/14/2014	37:56.3
7488	RSPe_2	-122.445	37.938	0.00	16.90	199.28	16.90	-122.445	37.938	-0.10	16.90	199.00	16.80	9	7/14/2014	37:56.2
7489	RSPe_2	-122.445	37.938	0.00	16.86	199.37	16.86	-122.445	37.938	-0.10	16.86	199.08	16.76	9	7/14/2014	37:56.1
7490	RSPe_2	-122.445	37.938	0.00	16.90	199.55	16.90	-122.445	37.938	-0.10	16.90	199.22	16.80	9	7/14/2014	37:56.0

7491	RSPe_2	-122.445	37.938	0.00	16.95	199.61	16.96	-122.445	37.938	-0.06	16.95	199.37	16.89	9	7/14/2014 37:55.9
7492	RSPe_2	-122.445	37.938	0.00	16.93	199.71	16.93	-122.445	37.938	-0.10	16.93	199.44	16.83	9	7/14/2014 37:55.8
7493	RSPe_2	-122.445	37.938	0.00	16.94	199.83	16.94	-122.445	37.938	-0.06	16.94	199.56	16.87	9	7/14/2014 37:55.7
7494	RSPe_2	-122.445	37.938	0.00	16.89	199.89	16.90	-122.445	37.938	-0.15	16.89	199.66	16.74	9	7/14/2014 37:55.6
7495	RSPe_2	-122.445	37.938	0.04	16.97	199.99	17.00	-122.445	37.938	-0.10	16.97	199.76	16.87	9	7/14/2014 37:55.5
7496	RSPe_2	-122.445	37.938	0.00	16.93	200.04	16.93	-122.445	37.938	-0.15	16.93	199.87	16.78	9	7/14/2014 37:55.4
7497	RSPe_2	-122.445	37.938	0.04	16.93	200.16	16.97	-122.445	37.938	-0.15	16.93	199.99	16.78	9	7/14/2014 37:55.3
7498	RSPe_2	-122.445	37.938	-0.05	16.92	200.28	16.87	-122.445	37.938	-0.15	16.92	200.11	16.77	9	7/14/2014 37:55.2
7499	RSPe_2	-122.445	37.938	0.00	16.90	200.37	16.90	-122.445	37.938	-0.15	16.90	200.21	16.75	9	7/14/2014 37:55.1
7500	RSPe_2	-122.445	37.938	0.00	16.96	200.45	16.97	-122.445	37.938	-0.15	16.96	200.33	16.81	9	7/14/2014 37:55.0
7501	RSPe_2	-122.445	37.938	0.00	16.91	200.61	16.92	-122.445	37.938	-0.15	16.91	200.47	16.76	9	7/14/2014 37:54.9
7502	RSPe_2	-122.445	37.938	0.00	16.98	200.65	16.98	-122.445	37.938	-0.15	16.98	200.55	16.83	9	7/14/2014 37:54.8
7503	RSPe_2	-122.445	37.938	0.00	16.93	200.76	16.93	-122.445	37.938	-0.06	16.93	200.63	16.87	9	7/14/2014 37:54.7
7504	RSPe_2	-122.445	37.938	0.00	16.97	200.85	16.97	-122.445	37.938	-0.10	16.97	200.73	16.87	9	7/14/2014 37:54.6
7505	RSPe_2	-122.445	37.938	0.00	16.98	200.93	16.98	-122.445	37.938	-0.10	16.98	200.81	16.88	9	7/14/2014 37:54.5
7506	RSPe_2	-122.445	37.938	0.00	16.98	201.02	16.99	-122.445	37.938	-0.10	16.98	200.88	16.88	9	7/14/2014 37:54.4
7507	RSPe_2	-122.445	37.938	0.04	16.97	201.09	17.00	-122.445	37.938	-0.10	16.97	200.95	16.87	9	7/14/2014 37:54.3
7508	RSPe_2	-122.445	37.938	0.00	17.00	201.21	17.01	-122.445	37.938	-0.15	17.00	201.05	16.86	9	7/14/2014 37:54.2
7509	RSPe_2	-122.445	37.938	0.04	16.99	201.40	17.03	-122.445	37.938	-0.15	16.99	201.16	16.84	9	7/14/2014 37:54.1
7510	RSPe_2	-122.445	37.938	0.00	16.98	201.58	16.99	-122.445	37.938	-0.15	16.98	201.27	16.83	9	7/14/2014 37:54.0
7511	RSPe_2	-122.445	37.938	0.01	17.02	201.69	17.03	-122.445	37.938	-0.06	17.02	201.33	16.96	9	7/14/2014 37:53.9
7512	RSPe_2	-122.445	37.938	0.01	16.99	201.85	16.99	-122.445	37.938	-0.15	16.99	201.46	16.84	9	7/14/2014 37:53.8
7513	RSPe_2	-122.445	37.938	0.04	16.97	202.02	17.01	-122.445	37.938	-0.10	16.97	201.58	16.87	9	7/14/2014 37:53.7
7514	RSPe_2	-122.445	37.938	-0.05	16.99	202.10	16.94	-122.445	37.938	-0.06	16.99	201.70	16.93	9	7/14/2014 37:53.6
7515	RSPe_2	-122.445	37.938	0.04	17.00	202.26	17.04	-122.445	37.938	-0.06	17.00	201.81	16.94	9	7/14/2014 37:53.5
7516	RSPe_2	-122.445	37.938	0.01	17.00	202.31	17.01	-122.445	37.938	-0.10	17.00	201.92	16.91	9	7/14/2014 37:53.4
7517	RSPe_2	-122.445	37.938	0.04	17.08	202.41	17.11	-122.445	37.938	-0.06	17.08	202.06	17.01	9	7/14/2014 37:53.3
7518	RSPe_2	-122.445	37.938	0.01	17.02	202.42	17.03	-122.445	37.938	-0.06	17.02	202.15	16.96	9	7/14/2014 37:53.2
7519	RSPe_2	-122.445	37.938	0.04	17.09	202.52	17.13	-122.445	37.938	-0.01	17.09	202.34	17.08	9	7/14/2014 37:53.1
7520	RSPe_2	-122.445	37.938	0.01	17.23	202.55	17.23	-122.445	37.938	-0.10	17.23	202.48	17.13	9	7/14/2014 37:53.0
7521	RSPe_2	-122.445	37.938	0.04	17.23	202.60	17.27	-122.445	37.938	-0.10	17.23	202.71	17.13	9	7/14/2014 37:52.9
7522	RSPe_2	-122.445	37.938	0.01	17.25	202.65	17.26	-122.445	37.938	-0.15	17.25	202.78	17.10	9	7/14/2014 37:52.8
7523	RSPe_2	-122.445	37.938	0.01	17.28	202.73	17.29	-122.445	37.938	-0.15	17.28	202.89	17.14	9	7/14/2014 37:52.7

7524	RSPe_2	-122.445	37.938	0.01	17.31	202.79	17.31	-122.445	37.938	-0.18	17.31	202.96	17.13	9	7/14/2014	37:52.6
7525	RSPe_2	-122.445	37.938	0.01	17.32	202.90	17.32	-122.445	37.938	-0.18	17.32	203.06	17.13	9	7/14/2014	37:52.5
7526	RSPe_2	-122.445	37.938	0.01	17.34	203.06	17.34	-122.445	37.938	-0.18	17.34	203.08	17.16	9	7/14/2014	37:52.4
7527	RSPe_2	-122.445	37.938	0.01	17.36	203.16	17.37	-122.445	37.938	-0.15	17.36	203.12	17.21	9	7/14/2014	37:52.3
7528	RSPe_2	-122.445	37.938	0.01	17.38	203.26	17.38	-122.445	37.938	-0.15	17.38	203.13	17.23	9	7/14/2014	37:52.2
7529	RSPe_2	-122.445	37.938	0.04	17.38	203.28	17.42	-122.445	37.938	-0.15	17.38	203.06	17.23	9	7/14/2014	37:52.1
7530	RSPe_2	-122.445	37.938	0.01	17.39	203.40	17.40	-122.445	37.938	-0.18	17.39	203.17	17.21	9	7/14/2014	37:52.0
7531	RSPe_2	-122.445	37.938	0.04	17.46	203.61	17.50	-122.445	37.938	-0.10	17.46	203.26	17.36	9	7/14/2014	37:51.9
7532	RSPe_2	-122.445	37.938	0.01	17.46	203.72	17.46	-122.445	37.938	-0.18	17.46	203.38	17.28	9	7/14/2014	37:51.8
7533	RSPe_2	-122.445	37.938	0.01	17.45	203.85	17.46	-122.445	37.938	-0.15	17.45	203.53	17.31	9	7/14/2014	37:51.7
7534	RSPe_2	-122.445	37.938	0.01	17.52	204.03	17.53	-122.445	37.938	-0.18	17.52	203.70	17.34	9	7/14/2014	37:51.6
7535	RSPe_2	-122.445	37.938	0.04	17.49	204.20	17.53	-122.445	37.938	-0.18	17.49	203.84	17.31	9	7/14/2014	37:51.5
7536	RSPe_2	-122.445	37.938	0.01	17.56	204.31	17.56	-122.445	37.938	-0.22	17.56	203.96	17.34	9	7/14/2014	37:51.4
7537	RSPe_2	-122.445	37.938	0.01	17.55	204.44	17.55	-122.445	37.938	-0.10	17.55	204.10	17.45	9	7/14/2014	37:51.3
7538	RSPe_2	-122.445	37.938	0.01	17.57	204.53	17.58	-122.445	37.938	-0.18	17.57	204.24	17.39	9	7/14/2014	37:51.2
7539	RSPe_2	-122.445	37.938	0.04	17.51	204.57	17.55	-122.445	37.938	-0.15	17.51	204.35	17.37	9	7/14/2014	37:51.1
7540	RSPe_2	-122.445	37.938	0.01	17.60	204.66	17.60	-122.445	37.938	-0.18	17.60	204.48	17.42	9	7/14/2014	37:51.0
7541	RSPe_2	-122.445	37.938	0.04	17.71	204.78	17.75	-122.445	37.938	-0.18	17.71	204.67	17.53	9	7/14/2014	37:50.9
7542	RSPe_2	-122.445	37.938	0.01	17.69	204.93	17.70	-122.445	37.938	-0.27	17.69	204.81	17.43	9	7/14/2014	37:50.8
7543	RSPe_2	-122.445	37.938	0.01	17.78	204.97	17.78	-122.445	37.938	-0.15	17.78	204.88	17.63	9	7/14/2014	37:50.7
7544	RSPe_2	-122.445	37.938	0.01	17.76	205.04	17.77	-122.445	37.938	-0.15	17.76	204.97	17.62	9	7/14/2014	37:50.6
7545	RSPe_2	-122.445	37.938	0.04	17.75	205.11	17.79	-122.445	37.938	-0.18	17.75	205.08	17.57	9	7/14/2014	37:50.5
7546	RSPe_2	-122.445	37.938	0.01	17.59	205.19	17.60	-122.445	37.938	-0.15	17.59	205.12	17.45	9	7/14/2014	37:50.4
7547	RSPe_2	-122.445	37.938	0.04	17.56	205.28	17.60	-122.445	37.938	-0.06	17.56	205.17	17.50	9	7/14/2014	37:50.3
7548	RSPe_2	-122.445	37.938	0.01	17.45	205.49	17.46	-122.445	37.938	-0.15	17.45	205.30	17.31	9	7/14/2014	37:50.2
7549	RSPe_2	-122.445	37.938	0.04	17.40	205.52	17.44	-122.445	37.938	-0.15	17.40	205.27	17.25	9	7/14/2014	37:50.1
7550	RSPe_2	-122.445	37.938	0.01	17.33	205.68	17.34	-122.445	37.938	-0.15	17.33	205.42	17.18	9	7/14/2014	37:50.0
7551	RSPe_2	-122.445	37.938	0.04	17.30	205.85	17.34	-122.445	37.938	-0.10	17.30	205.48	17.20	9	7/14/2014	37:49.9
7552	RSPe_2	-122.445	37.938	0.04	17.20	205.93	17.24	-122.445	37.938	-0.18	17.20	205.52	17.02	9	7/14/2014	37:49.8
7553	RSPe_2	-122.445	37.938	0.04	17.22	205.97	17.26	-122.445	37.938	-0.18	17.22	205.65	17.04	9	7/14/2014	37:49.7
7554	RSPe_2	-122.445	37.938	0.04	17.18	206.11	17.22	-122.445	37.938	-0.22	17.18	205.79	16.97	9	7/14/2014	37:49.6
7555	RSPe_2	-122.445	37.938	0.04	17.14	206.19	17.18	-122.445	37.938	-0.10	17.14	205.93	17.05	9	7/14/2014	37:49.5
7556	RSPe_2	-122.445	37.938	0.04	17.08	206.32	17.11	-122.445	37.938	-0.06	17.08	206.11	17.01	9	7/14/2014	37:49.4

7557	RSPe_2	-122.445	37.938	0.04	17.08	206.47	17.11	-122.445	37.938	-0.15	17.08	206.30	16.93	9	7/14/2014	37:49.3
7558	RSPe_2	-122.445	37.938	0.04	17.08	206.62	17.11	-122.445	37.938	-0.15	17.08	206.43	16.93	9	7/14/2014	37:49.2
7559	RSPe_2	-122.445	37.938	0.04	17.12	206.76	17.16	-122.445	37.938	-0.18	17.12	206.59	16.94	9	7/14/2014	37:49.1
7560	RSPe_2	-122.445	37.938	0.01	17.18	206.87	17.18	-122.445	37.938	-0.15	17.18	206.65	17.03	9	7/14/2014	37:49.0
7561	RSPe_2	-122.445	37.938	0.04	17.05	207.05	17.09	-122.445	37.938	-0.10	17.05	206.79	16.96	9	7/14/2014	37:48.9
7562	RSPe_2	-122.445	37.938	0.01	17.08	207.11	17.09	-122.445	37.938	-0.10	17.08	206.84	16.99	9	7/14/2014	37:48.8
7563	RSPe_2	-122.445	37.938	0.04	17.12	207.22	17.16	-122.445	37.938	-0.18	17.12	206.89	16.94	9	7/14/2014	37:48.7
7564	RSPe_2	-122.445	37.938	0.01	17.09	207.36	17.10	-122.445	37.938	-0.22	17.09	206.97	16.88	9	7/14/2014	37:48.6
7565	RSPe_2	-122.445	37.938	0.04	17.10	207.49	17.14	-122.445	37.938	-0.18	17.10	207.11	16.92	9	7/14/2014	37:48.5
7566	RSPe_2	-122.445	37.938	0.01	17.08	207.62	17.09	-122.445	37.938	-0.15	17.08	207.28	16.94	9	7/14/2014	37:48.4
7567	RSPe_2	-122.445	37.938	0.04	17.08	207.68	17.11	-122.445	37.938	-0.15	17.08	207.44	16.93	9	7/14/2014	37:48.3
7568	RSPe_2	-122.445	37.938	0.04	17.08	207.81	17.11	-122.445	37.938	-0.22	17.08	207.59	16.86	9	7/14/2014	37:48.2
7569	RSPe_2	-122.445	37.938	0.04	17.06	207.85	17.10	-122.445	37.938	-0.15	17.06	207.75	16.91	9	7/14/2014	37:48.1
7570	RSPe_2	-122.445	37.938	0.04	17.10	207.97	17.14	-122.445	37.938	-0.15	17.10	207.85	16.95	9	7/14/2014	37:48.0
7571	RSPe_2	-122.445	37.938	0.04	17.09	208.10	17.13	-122.445	37.938	-0.10	17.09	208.01	16.99	9	7/14/2014	37:47.9
7572	RSPe_2	-122.445	37.938	0.04	17.07	208.17	17.11	-122.445	37.938	-0.15	17.07	208.14	16.92	9	7/14/2014	37:47.8
7573	RSPe_2	-122.445	37.938	0.04	17.10	208.37	17.14	-122.445	37.938	-0.10	17.10	208.24	17.00	9	7/14/2014	37:47.7
7574	RSPe_2	-122.445	37.938	0.04	17.07	208.55	17.11	-122.445	37.938	-0.06	17.07	208.32	17.01	9	7/14/2014	37:47.6
7575	RSPe_2	-122.445	37.938	0.04	17.15	208.70	17.19	-122.445	37.938	-0.06	17.15	208.42	17.09	9	7/14/2014	37:47.5
7576	RSPe_2	-122.445	37.938	0.01	17.08	208.84	17.09	-122.445	37.938	-0.10	17.08	208.47	16.99	9	7/14/2014	37:47.4
7577	RSPe_2	-122.445	37.938	0.04	17.14	208.85	17.18	-122.445	37.938	-0.10	17.14	208.49	17.04	9	7/14/2014	37:47.3
7578	RSPe_2	-122.445	37.938	0.01	17.09	209.05	17.10	-122.445	37.938	-0.10	17.09	208.63	16.99	9	7/14/2014	37:47.2
7579	RSPe_2	-122.445	37.938	0.04	17.13	209.16	17.17	-122.445	37.938	-0.06	17.13	208.80	17.07	9	7/14/2014	37:47.1
7580	RSPe_2	-122.445	37.938	0.04	17.17	209.29	17.21	-122.445	37.938	-0.06	17.17	208.95	17.11	9	7/14/2014	37:47.0
7581	RSPe_2	-122.445	37.938	0.04	17.20	209.43	17.24	-122.445	37.938	-0.10	17.20	209.05	17.10	9	7/14/2014	37:46.9
7582	RSPe_2	-122.445	37.938	0.04	17.16	209.53	17.20	-122.445	37.938	-0.06	17.16	209.26	17.10	9	7/14/2014	37:46.8
7583	RSPe_2	-122.445	37.938	0.04	17.20	209.56	17.24	-122.445	37.938	-0.06	17.20	209.31	17.13	9	7/14/2014	37:46.7
7584	RSPe_2	-122.445	37.938	0.01	17.21	209.62	17.22	-122.445	37.938	-0.15	17.21	209.44	17.07	9	7/14/2014	37:46.6
7585	RSPe_2	-122.445	37.938	0.04	17.28	209.71	17.32	-122.445	37.938	-0.15	17.28	209.49	17.14	9	7/14/2014	37:46.5
7586	RSPe_2	-122.445	37.938	0.04	17.26	209.76	17.30	-122.445	37.938	-0.10	17.26	209.58	17.16	9	7/14/2014	37:46.4
7587	RSPe_2	-122.445	37.938	0.04	17.33	209.89	17.37	-122.445	37.938	-0.06	17.33	209.65	17.27	9	7/14/2014	37:46.3
7588	RSPe_2	-122.445	37.938	0.04	17.30	209.98	17.34	-122.445	37.938	-0.10	17.30	209.68	17.20	9	7/14/2014	37:46.2
7589	RSPe_2	-122.445	37.938	0.04	17.25	210.11	17.28	-122.445	37.938	-0.10	17.25	209.73	17.15	9	7/14/2014	37:46.1

7590	RSPe_2	-122.445	37.938	0.01	17.28	210.26	17.29	-122.445	37.938	-0.10	17.28	209.80	17.19	9	7/14/2014	37:46.0
7591	RSPe_2	-122.445	37.938	0.01	17.25	210.37	17.25	-122.445	37.938	-0.10	17.25	209.82	17.15	9	7/14/2014	37:45.9
7592	RSPe_2	-122.445	37.938	0.04	17.25	210.34	17.29	-122.445	37.938	-0.10	17.25	209.89	17.16	9	7/14/2014	37:45.8
7593	RSPe_2	-122.445	37.938	0.01	17.25	210.35	17.26	-122.445	37.938	-0.10	17.25	209.92	17.16	9	7/14/2014	37:45.7
7594	RSPe_2	-122.445	37.938	0.01	17.28	210.46	17.28	-122.445	37.938	-0.15	17.28	210.11	17.13	9	7/14/2014	37:45.6
7595	RSPe_2	-122.445	37.938	0.04	17.31	210.64	17.35	-122.445	37.938	-0.10	17.31	210.36	17.21	9	7/14/2014	37:45.5
7596	RSPe_2	-122.445	37.938	0.01	17.33	210.82	17.34	-122.445	37.938	-0.15	17.33	210.58	17.18	9	7/14/2014	37:45.4
7597	RSPe_2	-122.445	37.938	0.04	17.40	210.81	17.44	-122.445	37.938	-0.10	17.40	210.66	17.30	9	7/14/2014	37:45.3
7598	RSPe_2	-122.445	37.938	0.01	17.37	210.90	17.37	-122.445	37.938	-0.18	17.37	210.76	17.19	9	7/14/2014	37:45.2
7599	RSPe_2	-122.445	37.938	0.01	17.42	210.93	17.43	-122.445	37.938	-0.15	17.42	210.77	17.27	9	7/14/2014	37:45.1
7600	RSPe_2	-122.445	37.938	0.01	17.38	210.95	17.38	-122.445	37.938	-0.18	17.38	210.79	17.20	9	7/14/2014	37:45.0
7601	RSPe_2	-122.445	37.938	0.01	17.42	211.04	17.43	-122.445	37.938	-0.15	17.42	210.81	17.27	9	7/14/2014	37:44.9
7602	RSPe_2	-122.445	37.938	-0.05	17.44	211.18	17.39	-122.445	37.938	-0.15	17.44	210.95	17.29	9	7/14/2014	37:44.8
7603	RSPe_2	-122.445	37.938	0.01	17.45	211.30	17.46	-122.445	37.938	-0.18	17.45	211.01	17.27	9	7/14/2014	37:44.7
7604	RSPe_2	-122.445	37.938	-0.05	17.46	211.42	17.41	-122.445	37.938	-0.18	17.46	211.14	17.28	9	7/14/2014	37:44.6
7605	RSPe_2	-122.445	37.938	0.01	17.46	211.56	17.46	-122.445	37.938	-0.18	17.46	211.24	17.28	9	7/14/2014	37:44.5
7606	RSPe_2	-122.445	37.938	-0.05	17.45	211.66	17.41	-122.445	37.938	-0.18	17.45	211.34	17.27	9	7/14/2014	37:44.4
7607	RSPe_2	-122.445	37.938	0.01	17.45	211.66	17.45	-122.445	37.938	-0.18	17.45	211.37	17.27	9	7/14/2014	37:44.3
7608	RSPe_2	-122.445	37.938	-0.05	17.44	211.71	17.39	-122.445	37.938	-0.15	17.44	211.43	17.29	9	7/14/2014	37:44.2
7609	RSPe_2	-122.445	37.938	0.01	17.46	211.72	17.46	-122.445	37.938	-0.22	17.46	211.52	17.24	9	7/14/2014	37:44.1
7610	RSPe_2	-122.445	37.938	-0.05	17.45	211.69	17.40	-122.445	37.938	-0.18	17.45	211.54	17.27	9	7/14/2014	37:44.0
7611	RSPe_2	-122.445	37.938	0.01	17.47	211.79	17.47	-122.445	37.938	-0.18	17.47	211.70	17.29	9	7/14/2014	37:43.9
7612	RSPe_2	-122.445	37.938	-0.05	17.48	211.81	17.43	-122.445	37.938	-0.22	17.48	211.80	17.26	9	7/14/2014	37:43.8
7613	RSPe_2	-122.445	37.938	-0.05	17.50	211.87	17.45	-122.445	37.938	-0.18	17.50	211.89	17.32	9	7/14/2014	37:43.7
7614	RSPe_2	-122.445	37.938	-0.05	17.51	211.97	17.46	-122.445	37.938	-0.22	17.51	211.96	17.29	9	7/14/2014	37:43.6
7615	RSPe_2	-122.445	37.938	0.01	17.55	212.05	17.55	-122.445	37.938	-0.22	17.55	212.10	17.33	9	7/14/2014	37:43.5
7616	RSPe_2	-122.445	37.938	-0.05	17.56	212.25	17.51	-122.445	37.938	-0.22	17.56	212.21	17.35	9	7/14/2014	37:43.4
7617	RSPe_2	-122.445	37.938	-0.05	17.58	212.47	17.54	-122.445	37.938	-0.18	17.58	212.31	17.40	9	7/14/2014	37:43.3
7618	RSPe_2	-122.445	37.938	-0.05	17.61	212.51	17.57	-122.445	37.938	-0.22	17.61	212.30	17.40	9	7/14/2014	37:43.2
7619	RSPe_2	-122.445	37.938	-0.05	17.58	212.64	17.53	-122.445	37.938	-0.27	17.58	212.27	17.31	9	7/14/2014	37:43.1
7620	RSPe_2	-122.445	37.938	-0.05	17.61	212.71	17.56	-122.445	37.938	-0.18	17.61	212.30	17.43	9	7/14/2014	37:43.0
7621	RSPe_2	-122.445	37.938	-0.05	17.56	212.89	17.51	-122.445	37.938	-0.18	17.56	212.40	17.37	9	7/14/2014	37:42.9
7622	RSPe_2	-122.445	37.938	-0.08	17.59	213.04	17.51	-122.445	37.938	-0.15	17.59	212.53	17.45	9	7/14/2014	37:42.8

7623	RSPe_2	-122.445	37.938	-0.05	17.57	213.09	17.52	-122.445	37.938	-0.18	17.57	212.69	17.39	9	7/14/2014	37:42.7
7624	RSPe_2	-122.445	37.938	-0.08	17.59	213.17	17.51	-122.445	37.938	-0.22	17.59	212.86	17.38	9	7/14/2014	37:42.6
7625	RSPe_2	-122.445	37.938	-0.05	17.60	213.31	17.55	-122.445	37.938	-0.27	17.60	213.02	17.33	9	7/14/2014	37:42.5
7626	RSPe_2	-122.445	37.938	-0.05	17.61	213.33	17.57	-122.445	37.938	-0.30	17.61	213.17	17.31	9	7/14/2014	37:42.4
7627	RSPe_2	-122.445	37.938	-0.05	17.63	213.40	17.58	-122.445	37.938	-0.22	17.63	213.20	17.41	9	7/14/2014	37:42.3
7628	RSPe_2	-122.445	37.938	-0.08	17.64	213.49	17.56	-122.445	37.938	-0.22	17.64	213.31	17.42	9	7/14/2014	37:42.2
7629	RSPe_2	-122.445	37.938	-0.05	17.58	213.53	17.54	-122.445	37.938	-0.27	17.58	213.35	17.32	9	7/14/2014	37:42.1
7630	RSPe_2	-122.445	37.938	-0.08	17.57	213.62	17.49	-122.445	37.938	-0.27	17.57	213.39	17.30	9	7/14/2014	37:42.0
7631	RSPe_2	-122.445	37.938	-0.05	17.58	213.80	17.54	-122.445	37.938	-0.30	17.58	213.50	17.28	9	7/14/2014	37:41.9
7632	RSPe_2	-122.445	37.938	-0.08	17.58	213.92	17.50	-122.445	37.938	-0.26	17.58	213.57	17.32	9	7/14/2014	37:41.8
7633	RSPe_2	-122.445	37.938	-0.08	17.61	213.94	17.53	-122.445	37.938	-0.26	17.61	213.63	17.35	9	7/14/2014	37:41.7
7634	RSPe_2	-122.445	37.938	-0.08	17.65	213.93	17.57	-122.445	37.938	-0.30	17.65	213.76	17.35	9	7/14/2014	37:41.6
7635	RSPe_2	-122.445	37.938	-0.08	17.56	214.01	17.48	-122.445	37.938	-0.26	17.56	213.93	17.30	9	7/14/2014	37:41.5
7636	RSPe_2	-122.445	37.938	-0.13	17.56	214.00	17.42	-122.445	37.938	-0.35	17.56	214.07	17.21	9	7/14/2014	37:41.4
7637	RSPe_2	-122.445	37.938	-0.08	17.60	214.08	17.52	-122.445	37.938	-0.26	17.60	214.12	17.33	9	7/14/2014	37:41.3
7638	RSPe_2	-122.445	37.938	-0.08	17.66	214.10	17.58	-122.445	37.938	-0.26	17.66	214.16	17.40	9	7/14/2014	37:41.2
7639	RSPe_2	-122.445	37.938	-0.08	17.79	214.22	17.71	-122.445	37.938	-0.26	17.79	214.22	17.53	9	7/14/2014	37:41.1
7640	RSPe_2	-122.445	37.938	-0.13	17.82	214.40	17.69	-122.445	37.938	-0.30	17.82	214.32	17.52	9	7/14/2014	37:41.0
7641	RSPe_2	-122.445	37.938	-0.08	17.83	214.48	17.75	-122.445	37.938	-0.30	17.83	214.39	17.53	9	7/14/2014	37:40.9
7642	RSPe_2	-122.445	37.938	-0.13	17.72	214.57	17.59	-122.445	37.938	-0.30	17.72	214.43	17.42	9	7/14/2014	37:40.8
7643	RSPe_2	-122.445	37.938	-0.08	17.68	214.63	17.60	-122.445	37.938	-0.35	17.68	214.52	17.33	9	7/14/2014	37:40.7
7644	RSPe_2	-122.445	37.938	-0.13	17.61	214.70	17.48	-122.445	37.938	-0.35	17.61	214.58	17.26	9	7/14/2014	37:40.6
7645	RSPe_2	-122.445	37.938	-0.08	17.63	214.83	17.55	-122.445	37.938	-0.35	17.63	214.68	17.28	9	7/14/2014	37:40.5
7646	RSPe_2	-122.445	37.938	-0.13	17.63	214.87	17.50	-122.445	37.938	-0.43	17.63	214.71	17.20	9	7/14/2014	37:40.4
7647	RSPe_2	-122.445	37.938	-0.13	17.68	214.94	17.55	-122.445	37.938	-0.38	17.68	214.71	17.29	9	7/14/2014	37:40.3
7648	RSPe_2	-122.445	37.938	-0.13	17.49	215.05	17.36	-122.445	37.938	-0.43	17.49	214.87	17.06	9	7/14/2014	37:40.2
7649	RSPe_2	-122.445	37.938	-0.13	17.45	215.12	17.32	-122.445	37.938	-0.43	17.45	214.87	17.01	9	7/14/2014	37:40.1
7650	RSPe_2	-122.445	37.938	-0.17	17.52	215.16	17.36	-122.445	37.938	-0.52	17.52	215.01	17.01	9	7/14/2014	37:40.0
7651	RSPe_2	-122.445	37.938	-0.08	17.43	215.29	17.35	-122.445	37.938	-0.47	17.43	215.18	16.96	9	7/14/2014	37:39.9
7652	RSPe_2	-122.445	37.938	-0.13	17.40	215.39	17.27	-122.445	37.938	-0.52	17.40	215.34	16.88	9	7/14/2014	37:39.8
7653	RSPe_2	-122.445	37.938	-0.13	17.45	215.40	17.32	-122.445	37.939	-0.47	17.45	215.46	16.98	9	7/14/2014	37:39.7
7654	RSPe_2	-122.445	37.939	-0.13	17.45	215.49	17.32	-122.445	37.939	-0.47	17.45	215.62	16.98	9	7/14/2014	37:39.6
7655	RSPe_2	-122.445	37.939	-0.08	17.42	215.47	17.34	-122.445	37.939	-0.52	17.42	215.71	16.90	9	7/14/2014	37:39.5

7656	RSPe_2	-122.445	37.939	-0.08	17.44	215.54	17.36	-122.445	37.939	-0.52	17.44	215.78	16.92	9	7/14/2014	37:39.4
7657	RSPe_2	-122.445	37.939	-0.08	17.45	215.56	17.37	-122.445	37.939	-0.47	17.45	215.78	16.98	9	7/14/2014	37:39.3
7658	RSPe_2	-122.445	37.939	-0.13	17.47	215.60	17.34	-122.445	37.939	-0.47	17.47	215.72	17.00	9	7/14/2014	37:39.2
7659	RSPe_2	-122.445	37.939	-0.08	17.46	215.69	17.38	-122.445	37.939	-0.47	17.46	215.65	16.99	9	7/14/2014	37:39.1
7660	RSPe_2	-122.445	37.939	-0.13	17.51	215.80	17.38	-122.445	37.939	-0.52	17.51	215.65	16.99	9	7/14/2014	37:39.0
7661	RSPe_2	-122.445	37.939	-0.13	17.39	215.87	17.25	-122.445	37.939	-0.52	17.39	215.59	16.87	9	7/14/2014	37:38.9
7662	RSPe_2	-122.445	37.939	-0.13	17.38	215.96	17.25	-122.445	37.939	-0.52	17.38	215.67	16.86	9	7/14/2014	37:38.8
7663	RSPe_2	-122.445	37.939	-0.08	17.41	216.11	17.33	-122.445	37.939	-0.47	17.41	215.76	16.94	9	7/14/2014	37:38.7
7664	RSPe_2	-122.445	37.939	-0.08	17.39	216.20	17.31	-122.445	37.939	-0.55	17.39	215.87	16.83	9	7/14/2014	37:38.6
7665	RSPe_2	-122.445	37.939	-0.08	17.39	216.29	17.31	-122.445	37.939	-0.52	17.39	215.93	16.87	9	7/14/2014	37:38.5
7666	RSPe_2	-122.445	37.939	-0.08	17.37	216.38	17.29	-122.445	37.939	-0.52	17.37	216.04	16.85	9	7/14/2014	37:38.4
7667	RSPe_2	-122.445	37.939	-0.08	17.37	216.47	17.29	-122.445	37.939	-0.52	17.37	216.15	16.85	9	7/14/2014	37:38.3
7668	RSPe_2	-122.445	37.939	-0.08	17.37	216.60	17.29	-122.445	37.939	-0.52	17.37	216.26	16.85	9	7/14/2014	37:38.2
7669	RSPe_2	-122.445	37.939	-0.05	17.34	216.64	17.29	-122.445	37.939	-0.43	17.34	216.32	16.90	9	7/14/2014	37:38.1
7670	RSPe_2	-122.445	37.939	-0.08	17.34	216.67	17.26	-122.445	37.939	-0.47	17.34	216.35	16.87	9	7/14/2014	37:38.0
7671	RSPe_2	-122.445	37.939	-0.08	17.35	216.79	17.27	-122.445	37.939	-0.47	17.35	216.46	16.89	9	7/14/2014	37:37.9
7672	RSPe_2	-122.445	37.939	-0.08	17.32	216.84	17.24	-122.445	37.939	-0.47	17.32	216.57	16.85	9	7/14/2014	37:37.8
7673	RSPe_2	-122.445	37.939	-0.05	17.32	216.91	17.27	-122.445	37.939	-0.43	17.32	216.70	16.89	9	7/14/2014	37:37.7
7674	RSPe_2	-122.445	37.939	-0.08	17.33	216.95	17.25	-122.445	37.939	-0.47	17.33	216.79	16.86	9	7/14/2014	37:37.6
7675	RSPe_2	-122.445	37.939	-0.05	17.28	216.95	17.23	-122.445	37.939	-0.47	17.28	216.91	16.81	9	7/14/2014	37:37.5
7676	RSPe_2	-122.445	37.939	-0.05	17.41	217.03	17.36	-122.445	37.939	-0.43	17.41	217.01	16.97	9	7/14/2014	37:37.4
7677	RSPe_2	-122.445	37.939	0.01	17.37	217.13	17.37	-122.445	37.939	-0.38	17.37	217.08	16.98	9	7/14/2014	37:37.3
7678	RSPe_2	-122.445	37.939	-0.05	17.44	217.28	17.39	-122.445	37.939	-0.43	17.44	217.25	17.00	9	7/14/2014	37:37.2
7679	RSPe_2	-122.445	37.939	0.01	17.39	217.38	17.39	-122.445	37.939	-0.43	17.39	217.23	16.95	9	7/14/2014	37:37.1
7680	RSPe_2	-122.445	37.939	-0.05	17.47	217.49	17.42	-122.445	37.939	-0.38	17.47	217.35	17.09	9	7/14/2014	37:37.0
7681	RSPe_2	-122.445	37.939	-0.05	17.41	217.54	17.36	-122.445	37.939	-0.38	17.41	217.34	17.02	9	7/14/2014	37:36.9
7682	RSPe_2	-122.445	37.939	-0.05	17.45	217.62	17.40	-122.445	37.939	-0.43	17.45	217.36	17.01	9	7/14/2014	37:36.8
7683	RSPe_2	-122.445	37.939	0.01	17.44	217.64	17.44	-122.445	37.939	-0.38	17.44	217.37	17.05	9	7/14/2014	37:36.7
7684	RSPe_2	-122.445	37.939	0.01	17.40	217.72	17.41	-122.445	37.939	-0.38	17.40	217.39	17.02	9	7/14/2014	37:36.6
7685	RSPe_2	-122.445	37.939	0.01	17.48	217.77	17.48	-122.445	37.939	-0.35	17.48	217.39	17.13	9	7/14/2014	37:36.5
7686	RSPe_2	-122.445	37.939	0.01	17.38	217.75	17.38	-122.445	37.939	-0.35	17.38	217.47	17.03	9	7/14/2014	37:36.4
7687	RSPe_2	-122.445	37.939	0.01	17.42	217.88	17.43	-122.445	37.939	-0.38	17.42	217.61	17.04	9	7/14/2014	37:36.3
7688	RSPe_2	-122.445	37.939	-0.05	17.42	217.89	17.37	-122.445	37.939	-0.38	17.42	217.77	17.03	9	7/14/2014	37:36.2

7689	RSPe_2	-122.445	37.939	0.01	17.44	217.99	17.44	-122.445	37.939	-0.26	17.44	217.90	17.17	9	7/14/2014	37:36.1
7690	RSPe_2	-122.445	37.939	0.01	17.45	218.05	17.45	-122.445	37.939	-0.35	17.45	218.01	17.10	9	7/14/2014	37:36.0
7691	RSPe_2	-122.445	37.939	0.04	17.47	218.17	17.51	-122.445	37.939	-0.35	17.47	218.12	17.12	9	7/14/2014	37:35.9
7692	RSPe_2	-122.445	37.939	0.01	17.49	218.19	17.50	-122.445	37.939	-0.35	17.49	218.12	17.14	9	7/14/2014	37:35.8
7693	RSPe_2	-122.445	37.939	0.01	17.55	218.28	17.55	-122.445	37.939	-0.30	17.55	218.15	17.25	9	7/14/2014	37:35.7
7694	RSPe_2	-122.445	37.939	0.01	17.51	218.30	17.52	-122.445	37.939	-0.35	17.51	218.10	17.16	9	7/14/2014	37:35.6
7695	RSPe_2	-122.445	37.939	0.04	17.47	218.34	17.51	-122.445	37.939	-0.30	17.47	218.03	17.17	9	7/14/2014	37:35.5
7696	RSPe_2	-122.445	37.939	0.01	17.42	218.28	17.42	-122.445	37.939	-0.35	17.42	217.95	17.07	9	7/14/2014	37:35.4
7697	RSPe_2	-122.445	37.939	0.04	17.44	218.38	17.48	-122.445	37.939	-0.30	17.44	217.95	17.14	9	7/14/2014	37:35.3
7698	RSPe_2	-122.445	37.939	0.04	17.45	218.41	17.49	-122.445	37.939	-0.30	17.45	218.00	17.16	9	7/14/2014	37:35.2
7699	RSPe_2	-122.445	37.939	0.01	17.51	218.56	17.51	-122.445	37.939	-0.30	17.51	218.11	17.21	9	7/14/2014	37:35.1
7700	RSPe_2	-122.445	37.939	0.01	17.48	218.68	17.48	-122.445	37.939	-0.35	17.48	218.23	17.13	9	7/14/2014	37:35.0
7701	RSPe_2	-122.445	37.939	0.04	17.48	218.72	17.52	-122.445	37.939	-0.30	17.48	218.30	17.18	9	7/14/2014	37:34.9
7702	RSPe_2	-122.445	37.939	0.04	17.52	218.83	17.56	-122.445	37.939	-0.35	17.52	218.45	17.17	9	7/14/2014	37:34.8
7703	RSPe_2	-122.445	37.939	0.04	17.56	218.85	17.60	-122.445	37.939	-0.21	17.56	218.56	17.35	9	7/14/2014	37:34.7
7704	RSPe_2	-122.445	37.939	0.04	17.59	218.94	17.63	-122.445	37.939	-0.26	17.59	218.75	17.33	9	7/14/2014	37:34.6
7705	RSPe_2	-122.445	37.939	0.04	17.61	218.94	17.65	-122.445	37.939	-0.30	17.61	218.75	17.32	9	7/14/2014	37:34.5
7706	RSPe_2	-122.445	37.939	0.04	17.63	219.03	17.67	-122.445	37.939	-0.26	17.63	218.85	17.37	9	7/14/2014	37:34.4
7707	RSPe_2	-122.445	37.939	0.04	17.67	219.12	17.71	-122.445	37.939	-0.21	17.67	218.92	17.46	9	7/14/2014	37:34.3
7708	RSPe_2	-122.445	37.939	0.04	17.72	219.23	17.76	-122.445	37.939	-0.26	17.72	219.00	17.46	9	7/14/2014	37:34.2
7709	RSPe_2	-122.445	37.939	0.09	17.69	219.29	17.79	-122.445	37.939	-0.21	17.69	219.06	17.48	9	7/14/2014	37:34.1
7710	RSPe_2	-122.445	37.939	0.04	17.71	219.44	17.75	-122.445	37.939	-0.21	17.71	219.15	17.50	9	7/14/2014	37:34.0
7711	RSPe_2	-122.445	37.939	0.09	17.73	219.50	17.82	-122.445	37.939	-0.18	17.73	219.21	17.55	9	7/14/2014	37:33.9
7712	RSPe_2	-122.445	37.939	0.04	17.65	219.51	17.69	-122.445	37.939	-0.21	17.65	219.26	17.44	9	7/14/2014	37:33.8
7713	RSPe_2	-122.445	37.939	0.09	17.66	219.60	17.75	-122.445	37.939	-0.18	17.66	219.35	17.48	9	7/14/2014	37:33.7
7714	RSPe_2	-122.445	37.939	0.04	17.72	219.67	17.76	-122.445	37.939	-0.21	17.72	219.51	17.50	9	7/14/2014	37:33.6
7715	RSPe_2	-122.445	37.939	0.04	17.69	219.77	17.73	-122.445	37.939	-0.21	17.69	219.64	17.48	9	7/14/2014	37:33.5
7716	RSPe_2	-122.445	37.939	0.04	17.71	219.82	17.75	-122.445	37.939	-0.26	17.71	219.75	17.44	9	7/14/2014	37:33.4
7717	RSPe_2	-122.445	37.939	0.09	17.72	219.84	17.81	-122.445	37.939	-0.26	17.72	219.85	17.46	9	7/14/2014	37:33.3
7718	RSPe_2	-122.445	37.939	0.04	17.73	219.96	17.77	-122.445	37.939	-0.26	17.73	219.99	17.47	9	7/14/2014	37:33.2
7719	RSPe_2	-122.445	37.939	0.09	17.76	220.02	17.85	-122.445	37.939	-0.26	17.76	220.08	17.50	9	7/14/2014	37:33.1
7720	RSPe_2	-122.445	37.939	0.04	17.79	220.16	17.83	-122.445	37.939	-0.26	17.79	220.11	17.53	9	7/14/2014	37:33.0
7721	RSPe_2	-122.445	37.939	0.09	17.84	220.30	17.93	-122.445	37.939	-0.21	17.84	220.21	17.63	9	7/14/2014	37:32.9

7722	RSPe_2	-122.445	37.939	0.09	17.84	220.49	17.93	-122.445	37.939	-0.21	17.84	220.31	17.63	9	7/14/2014	37:32.8
7723	RSPe_2	-122.445	37.939	0.09	17.79	220.62	17.88	-122.445	37.939	-0.21	17.79	220.32	17.58	9	7/14/2014	37:32.7
7724	RSPe_2	-122.445	37.939	0.04	17.75	220.72	17.79	-122.445	37.939	-0.26	17.75	220.35	17.48	9	7/14/2014	37:32.6
7725	RSPe_2	-122.445	37.939	0.09	17.78	220.84	17.87	-122.445	37.939	-0.18	17.78	220.40	17.60	9	7/14/2014	37:32.5
7726	RSPe_2	-122.445	37.939	0.09	17.60	220.95	17.69	-122.445	37.939	-0.21	17.60	220.54	17.39	9	7/14/2014	37:32.4
7727	RSPe_2	-122.445	37.939	0.09	17.52	221.04	17.62	-122.445	37.939	-0.21	17.52	220.66	17.31	9	7/14/2014	37:32.3
7728	RSPe_2	-122.445	37.939	0.09	17.57	221.12	17.66	-122.445	37.939	-0.21	17.57	220.80	17.36	9	7/14/2014	37:32.2
7729	RSPe_2	-122.445	37.939	0.13	17.52	221.19	17.65	-122.445	37.939	-0.21	17.52	220.94	17.31	9	7/14/2014	37:32.1
7730	RSPe_2	-122.445	37.939	0.09	17.50	221.17	17.59	-122.445	37.939	-0.26	17.50	221.05	17.24	9	7/14/2014	37:32.0
7731	RSPe_2	-122.445	37.939	0.13	17.38	221.26	17.50	-122.445	37.939	-0.26	17.38	221.13	17.11	9	7/14/2014	37:31.9
7732	RSPe_2	-122.445	37.939	0.09	17.34	221.43	17.43	-122.445	37.939	-0.26	17.34	221.31	17.07	9	7/14/2014	37:31.8
7733	RSPe_2	-122.445	37.939	0.13	17.39	221.48	17.51	-122.445	37.939	-0.21	17.39	221.36	17.17	9	7/14/2014	37:31.7
7734	RSPe_2	-122.445	37.939	0.09	17.18	221.50	17.27	-122.445	37.939	-0.26	17.18	221.36	16.91	9	7/14/2014	37:31.6
7735	RSPe_2	-122.445	37.939	0.13	17.15	221.61	17.28	-122.445	37.939	-0.21	17.15	221.43	16.94	9	7/14/2014	37:31.5
7736	RSPe_2	-122.445	37.939	0.09	17.10	221.83	17.19	-122.445	37.939	-0.26	17.10	221.55	16.84	9	7/14/2014	37:31.4
7737	RSPe_2	-122.445	37.939	0.13	17.06	221.96	17.19	-122.445	37.939	-0.21	17.06	221.69	16.85	9	7/14/2014	37:31.3
7738	RSPe_2	-122.445	37.939	0.13	17.01	222.07	17.14	-122.445	37.939	-0.18	17.01	221.76	16.83	9	7/14/2014	37:31.2
7739	RSPe_2	-122.445	37.939	0.13	16.97	222.19	17.09	-122.445	37.939	-0.15	16.97	221.82	16.82	9	7/14/2014	37:31.1
7740	RSPe_2	-122.445	37.939	0.13	16.92	222.18	17.05	-122.445	37.939	-0.18	16.92	221.91	16.74	9	7/14/2014	37:31.0
7741	RSPe_2	-122.445	37.939	0.18	16.91	222.29	17.08	-122.445	37.939	-0.15	16.91	222.02	16.76	9	7/14/2014	37:30.9
7742	RSPe_2	-122.445	37.939	0.18	16.88	222.38	17.06	-122.445	37.939	-0.15	16.88	222.17	16.73	9	7/14/2014	37:30.8
7743	RSPe_2	-122.445	37.939	0.18	16.83	222.51	17.00	-122.445	37.939	-0.15	16.83	222.29	16.68	9	7/14/2014	37:30.7
7744	RSPe_2	-122.445	37.939	0.18	16.86	222.58	17.04	-122.445	37.939	-0.18	16.86	222.45	16.68	9	7/14/2014	37:30.6
7745	RSPe_2	-122.445	37.939	0.18	16.87	222.63	17.04	-122.445	37.939	-0.15	16.87	222.58	16.72	9	7/14/2014	37:30.5
7746	RSPe_2	-122.445	37.939	0.18	16.78	222.73	16.96	-122.445	37.939	-0.18	16.78	222.66	16.60	9	7/14/2014	37:30.4
7747	RSPe_2	-122.445	37.939	0.18	16.78	222.76	16.96	-122.445	37.939	-0.10	16.78	222.73	16.69	9	7/14/2014	37:30.3
7748	RSPe_2	-122.445	37.939	0.21	16.77	222.90	16.98	-122.445	37.939	-0.15	16.77	222.86	16.62	9	7/14/2014	37:30.2
7749	RSPe_2	-122.445	37.939	0.21	16.76	223.11	16.97	-122.445	37.939	-0.06	16.76	222.98	16.70	9	7/14/2014	37:30.1
7750	RSPe_2	-122.445	37.939	0.21	16.82	223.18	17.03	-122.445	37.939	-0.06	16.82	223.01	16.76	9	7/14/2014	37:30.0
7751	RSPe_2	-122.445	37.939	0.21	16.78	223.17	17.00	-122.445	37.939	-0.06	16.78	223.03	16.72	9	7/14/2014	37:29.9
7752	RSPe_2	-122.445	37.939	0.21	16.77	223.40	16.98	-122.445	37.939	-0.06	16.77	223.17	16.71	9	7/14/2014	37:29.8
7753	RSPe_2	-122.445	37.939	0.25	16.81	223.59	17.05	-122.445	37.939	-0.01	16.81	223.30	16.80	9	7/14/2014	37:29.7
7754	RSPe_2	-122.445	37.939	0.21	16.80	223.55	17.01	-122.445	37.939	-0.01	16.80	223.34	16.79	9	7/14/2014	37:29.6

7755	RSPe_2	-122.445	37.939	0.25	16.81	223.67	17.05	-122.445	37.939	-0.06	16.81	223.48	16.74	9	7/14/2014	37:29.5
7756	RSPe_2	-122.445	37.939	0.21	16.83	223.84	17.04	-122.445	37.939	-0.06	16.83	223.61	16.77	9	7/14/2014	37:29.4
7757	RSPe_2	-122.445	37.939	0.25	16.99	223.82	17.24	-122.445	37.939	-0.01	16.99	223.73	16.98	9	7/14/2014	37:29.3
7758	RSPe_2	-122.445	37.939	0.25	16.98	223.95	17.22	-122.445	37.939	-0.01	16.98	223.83	16.97	9	7/14/2014	37:29.2
7759	RSPe_2	-122.445	37.939	0.25	16.99	223.99	17.24	-122.445	37.939	-0.01	16.99	223.94	16.98	9	7/14/2014	37:29.1
7760	RSPe_2	-122.445	37.939	0.25	17.00	224.01	17.25	-122.445	37.939	-0.06	17.00	223.91	16.94	9	7/14/2014	37:29.0
7761	RSPe_2	-122.445	37.939	0.25	17.00	224.15	17.25	-122.445	37.939	-0.01	17.00	223.97	16.99	9	7/14/2014	37:28.9
7762	RSPe_2	-122.445	37.939	0.25	17.02	224.12	17.27	-122.445	37.939	-0.01	17.02	223.98	17.01	9	7/14/2014	37:28.8
7763	RSPe_2	-122.445	37.939	0.30	17.02	224.19	17.32	-122.445	37.939	0.02	17.02	224.01	17.04	9	7/14/2014	37:28.7
7764	RSPe_2	-122.445	37.939	0.25	17.05	224.32	17.29	-122.445	37.939	-0.01	17.05	224.09	17.04	9	7/14/2014	37:28.6
7765	RSPe_2	-122.445	37.939	0.30	17.05	224.43	17.35	-122.445	37.939	-0.01	17.05	224.19	17.04	9	7/14/2014	37:28.5
7766	RSPe_2	-122.445	37.939	0.25	17.05	224.63	17.30	-122.445	37.939	-0.01	17.05	224.33	17.04	9	7/14/2014	37:28.4
7767	RSPe_2	-122.445	37.939	0.30	17.08	224.76	17.38	-122.445	37.939	0.07	17.08	224.43	17.16	9	7/14/2014	37:28.3
7768	RSPe_2	-122.445	37.939	0.30	17.06	224.87	17.36	-122.445	37.939	0.07	17.06	224.54	17.13	9	7/14/2014	37:28.2
7769	RSPe_2	-122.445	37.939	0.33	17.05	224.99	17.38	-122.445	37.939	0.02	17.05	224.61	17.08	9	7/14/2014	37:28.1
7770	RSPe_2	-122.445	37.939	0.30	17.05	225.03	17.34	-122.445	37.939	0.07	17.05	224.69	17.12	9	7/14/2014	37:28.0
7771	RSPe_2	-122.445	37.939	0.33	17.03	225.11	17.36	-122.445	37.939	0.02	17.03	224.76	17.06	9	7/14/2014	37:27.9
7772	RSPe_2	-122.445	37.939	0.33	16.98	225.07	17.31	-122.445	37.939	0.07	16.98	224.87	17.06	9	7/14/2014	37:27.8
7773	RSPe_2	-122.445	37.939	0.38	16.98	225.06	17.36	-122.445	37.939	0.16	16.98	224.91	17.14	9	7/14/2014	37:27.7
7774	RSPe_2	-122.445	37.939	0.33	16.98	225.07	17.31	-122.445	37.939	0.07	16.98	225.03	17.05	9	7/14/2014	37:27.6
7775	RSPe_2	-122.445	37.939	0.38	17.00	225.09	17.39	-122.445	37.939	0.11	17.00	225.12	17.11	9	7/14/2014	37:27.5
7776	RSPe_2	-122.445	37.939	0.38	17.00	225.18	17.38	-122.445	37.939	0.11	17.00	225.20	17.11	9	7/14/2014	37:27.4
7777	RSPe_2	-122.445	37.939	0.38	17.04	225.30	17.42	-122.445	37.939	0.19	17.04	225.32	17.23	9	7/14/2014	37:27.3
7778	RSPe_2	-122.445	37.939	0.38	17.07	225.40	17.45	-122.445	37.939	0.16	17.07	225.33	17.23	9	7/14/2014	37:27.2
7779	RSPe_2	-122.445	37.939	0.42	17.14	225.49	17.56	-122.445	37.939	0.16	17.14	225.40	17.30	9	7/14/2014	37:27.1
7780	RSPe_2	-122.445	37.939	0.38	17.11	225.60	17.49	-122.445	37.939	0.11	17.11	225.43	17.21	9	7/14/2014	37:27.0
7781	RSPe_2	-122.445	37.939	0.42	17.08	225.69	17.50	-122.445	37.939	0.19	17.08	225.49	17.28	9	7/14/2014	37:26.9
7782	RSPe_2	-122.445	37.939	0.42	17.11	225.82	17.52	-122.445	37.939	0.28	17.11	225.59	17.38	9	7/14/2014	37:26.8
7783	RSPe_2	-122.445	37.939	0.47	17.12	225.89	17.59	-122.445	37.939	0.19	17.12	225.73	17.31	9	7/14/2014	37:26.7
7784	RSPe_2	-122.445	37.939	0.47	17.15	225.99	17.62	-122.445	37.939	0.28	17.15	225.79	17.43	9	7/14/2014	37:26.6
7785	RSPe_2	-122.445	37.939	0.47	17.09	226.05	17.56	-122.445	37.939	0.28	17.09	225.90	17.37	9	7/14/2014	37:26.5
7786	RSPe_2	-122.445	37.939	0.47	17.12	226.16	17.59	-122.445	37.939	0.23	17.12	225.99	17.35	9	7/14/2014	37:26.4
7787	RSPe_2	-122.445	37.939	0.47	17.20	226.17	17.67	-122.445	37.939	0.28	17.20	226.08	17.47	9	7/14/2014	37:26.3

7788	RSPe_2	-122.445	37.939	0.47	17.19	226.31	17.66	-122.445	37.939	0.23	17.19	226.16	17.42	9	7/14/2014	37:26.2
7789	RSPe_2	-122.445	37.939	0.50	17.16	226.35	17.66	-122.445	37.939	0.28	17.16	226.28	17.44	9	7/14/2014	37:26.1
7790	RSPe_2	-122.445	37.939	0.47	17.24	226.46	17.71	-122.445	37.939	0.23	17.24	226.39	17.47	9	7/14/2014	37:26.0
7791	RSPe_2	-122.445	37.939	0.50	17.27	226.61	17.77	-122.445	37.939	0.28	17.27	226.54	17.54	9	7/14/2014	37:25.9
7792	RSPe_2	-122.445	37.939	0.47	17.34	226.79	17.81	-122.445	37.939	0.28	17.34	226.66	17.61	9	7/14/2014	37:25.8
7793	RSPe_2	-122.445	37.939	0.50	17.40	226.84	17.90	-122.445	37.939	0.28	17.40	226.68	17.68	9	7/14/2014	37:25.7
7794	RSPe_2	-122.445	37.939	0.47	17.37	226.99	17.84	-122.445	37.939	0.36	17.37	226.83	17.73	9	7/14/2014	37:25.6
7795	RSPe_2	-122.445	37.939	0.50	17.42	227.08	17.92	-122.445	37.939	0.31	17.42	226.86	17.73	9	7/14/2014	37:25.5
7796	RSPe_2	-122.445	37.939	0.47	17.45	227.17	17.92	-122.445	37.939	0.31	17.45	226.93	17.76	9	7/14/2014	37:25.4
7797	RSPe_2	-122.445	37.939	0.50	17.44	227.24	17.94	-122.445	37.939	0.31	17.44	226.94	17.75	9	7/14/2014	37:25.3
7798	RSPe_2	-122.445	37.939	0.50	17.38	227.26	17.88	-122.445	37.939	0.36	17.38	226.99	17.74	9	7/14/2014	37:25.2
7799	RSPe_2	-122.445	37.939	0.50	17.42	227.41	17.93	-122.445	37.939	0.40	17.42	227.10	17.82	9	7/14/2014	37:25.1
7800	RSPe_2	-122.445	37.939	0.50	17.27	227.48	17.77	-122.445	37.939	0.36	17.27	227.17	17.63	9	7/14/2014	37:25.0
7801	RSPe_2	-122.445	37.939	0.50	17.21	227.51	17.72	-122.445	37.939	0.36	17.21	227.25	17.58	9	7/14/2014	37:24.9
7802	RSPe_2	-122.445	37.939	0.50	17.18	227.54	17.68	-122.445	37.939	0.36	17.18	227.23	17.54	9	7/14/2014	37:24.8
7803	RSPe_2	-122.445	37.939	0.54	17.16	227.72	17.70	-122.445	37.939	0.40	17.16	227.45	17.56	9	7/14/2014	37:24.7
7804	RSPe_2	-122.445	37.939	0.54	17.21	227.80	17.75	-122.445	37.939	0.40	17.21	227.54	17.61	9	7/14/2014	37:24.6
7805	RSPe_2	-122.445	37.939	0.54	17.07	227.85	17.61	-122.445	37.939	0.40	17.07	227.63	17.47	9	7/14/2014	37:24.5
7806	RSPe_2	-122.445	37.939	0.54	16.95	227.92	17.49	-122.445	37.939	0.40	16.95	227.69	17.35	9	7/14/2014	37:24.4
7807	RSPe_2	-122.445	37.939	0.54	16.94	227.98	17.47	-122.445	37.939	0.40	16.94	227.76	17.33	9	7/14/2014	37:24.3
7808	RSPe_2	-122.445	37.939	0.50	16.89	228.03	17.40	-122.445	37.939	0.36	16.89	227.78	17.25	9	7/14/2014	37:24.2
7809	RSPe_2	-122.445	37.939	0.54	16.73	228.10	17.26	-122.445	37.939	0.45	16.73	227.87	17.17	9	7/14/2014	37:24.1
7810	RSPe_2	-122.445	37.939	0.54	16.74	228.09	17.27	-122.445	37.939	0.45	16.74	227.89	17.18	9	7/14/2014	37:24.0
7811	RSPe_2	-122.445	37.939	0.59	16.59	228.12	17.18	-122.445	37.939	0.45	16.59	227.92	17.04	9	7/14/2014	37:23.9
7812	RSPe_2	-122.445	37.939	0.54	16.59	228.19	17.13	-122.445	37.939	0.45	16.59	228.00	17.04	9	7/14/2014	37:23.8
7813	RSPe_2	-122.445	37.939	0.59	16.53	228.23	17.12	-122.445	37.939	0.45	16.53	228.03	16.98	9	7/14/2014	37:23.7
7814	RSPe_2	-122.445	37.939	0.54	16.42	228.30	16.96	-122.445	37.939	0.45	16.42	228.08	16.87	9	7/14/2014	37:23.6
7815	RSPe_2	-122.445	37.939	0.59	16.35	228.38	16.94	-122.445	37.939	0.48	16.35	228.21	16.83	9	7/14/2014	37:23.5
7816	RSPe_2	-122.445	37.939	0.59	16.40	228.47	16.98	-122.445	37.939	0.40	16.40	228.33	16.79	9	7/14/2014	37:23.4
7817	RSPe_2	-122.445	37.939	0.59	16.30	228.52	16.89	-122.445	37.939	0.45	16.30	228.34	16.74	9	7/14/2014	37:23.3
7818	RSPe_2	-122.445	37.939	0.59	16.30	228.58	16.89	-122.445	37.939	0.45	16.30	228.43	16.75	9	7/14/2014	37:23.2
7819	RSPe_2	-122.445	37.939	0.59	16.29	228.63	16.88	-122.445	37.939	0.48	16.29	228.51	16.77	9	7/14/2014	37:23.1
7820	RSPe_2	-122.445	37.939	0.54	16.27	228.80	16.81	-122.445	37.939	0.45	16.27	228.62	16.72	9	7/14/2014	37:23.0

7821	RSPe_2	-122.445	37.939	0.59	16.34	228.82	16.93	-122.445	37.939	0.48	16.34	228.67	16.82	9	7/14/2014	37:22.9
7822	RSPe_2	-122.445	37.939	0.54	16.27	228.92	16.80	-122.445	37.939	0.48	16.27	228.77	16.75	9	7/14/2014	37:22.8
7823	RSPe_2	-122.445	37.939	0.59	16.29	228.98	16.88	-122.445	37.939	0.48	16.29	228.81	16.77	9	7/14/2014	37:22.7
7824	RSPe_2	-122.445	37.939	0.54	16.26	229.03	16.80	-122.445	37.939	0.48	16.26	228.88	16.74	9	7/14/2014	37:22.6
7825	RSPe_2	-122.445	37.939	0.59	16.26	229.09	16.85	-122.445	37.939	0.48	16.26	228.94	16.74	9	7/14/2014	37:22.5
7826	RSPe_2	-122.445	37.939	0.54	16.29	229.11	16.83	-122.445	37.939	0.40	16.29	229.06	16.68	9	7/14/2014	37:22.4
7827	RSPe_2	-122.445	37.939	0.62	16.33	229.18	16.95	-122.445	37.939	0.45	16.33	229.09	16.78	9	7/14/2014	37:22.3
7828	RSPe_2	-122.445	37.939	0.59	16.33	229.25	16.92	-122.445	37.939	0.45	16.33	229.20	16.78	9	7/14/2014	37:22.2
7829	RSPe_2	-122.445	37.939	0.59	16.35	229.29	16.94	-122.445	37.939	0.48	16.35	229.24	16.83	9	7/14/2014	37:22.1
7830	RSPe_2	-122.445	37.939	0.59	16.44	229.41	17.03	-122.445	37.939	0.48	16.44	229.34	16.92	9	7/14/2014	37:22.0
7831	RSPe_2	-122.445	37.939	0.59	16.47	229.49	17.05	-122.445	37.939	0.48	16.47	229.41	16.95	9	7/14/2014	37:21.9
7832	RSPe_2	-122.445	37.939	0.59	16.48	229.57	17.07	-122.445	37.939	0.45	16.48	229.50	16.93	9	7/14/2014	37:21.8
7833	RSPe_2	-122.445	37.939	0.62	16.53	229.64	17.16	-122.445	37.939	0.48	16.53	229.53	17.02	9	7/14/2014	37:21.7
7834	RSPe_2	-122.445	37.939	0.59	16.51	229.80	17.10	-122.445	37.939	0.45	16.51	229.60	16.96	9	7/14/2014	37:21.6
7835	RSPe_2	-122.445	37.939	0.59	16.60	229.89	17.19	-122.445	37.939	0.45	16.60	229.72	17.04	9	7/14/2014	37:21.5
7836	RSPe_2	-122.445	37.939	0.59	16.67	229.93	17.26	-122.445	37.939	0.45	16.67	229.74	17.11	9	7/14/2014	37:21.4
7837	RSPe_2	-122.445	37.939	0.62	16.65	230.02	17.27	-122.445	37.939	0.48	16.65	229.82	17.13	9	7/14/2014	37:21.3
7838	RSPe_2	-122.445	37.939	0.62	16.67	230.06	17.29	-122.445	37.939	0.48	16.67	229.94	17.15	9	7/14/2014	37:21.2
7839	RSPe_2	-122.445	37.939	0.62	16.67	230.17	17.30	-122.445	37.939	0.51	16.67	230.01	17.19	9	7/14/2014	37:21.1
7840	RSPe_2	-122.445	37.939	0.62	16.72	230.24	17.34	-122.445	37.939	0.48	16.72	230.09	17.20	9	7/14/2014	37:21.0
7841	RSPe_2	-122.445	37.939	0.62	16.72	230.30	17.34	-122.445	37.939	0.51	16.72	230.15	17.23	9	7/14/2014	37:20.9
7842	RSPe_2	-122.445	37.939	0.62	16.72	230.41	17.34	-122.445	37.939	0.48	16.72	230.20	17.20	9	7/14/2014	37:20.8
7843	RSPe_2	-122.445	37.939	0.62	16.75	230.41	17.38	-122.445	37.939	0.51	16.75	230.26	17.27	9	7/14/2014	37:20.7
7844	RSPe_2	-122.445	37.939	0.62	16.72	230.42	17.34	-122.445	37.939	0.48	16.72	230.28	17.20	9	7/14/2014	37:20.6
7845	RSPe_2	-122.445	37.939	0.62	16.76	230.50	17.38	-122.445	37.939	0.51	16.76	230.39	17.27	9	7/14/2014	37:20.5
7846	RSPe_2	-122.445	37.939	0.62	16.81	230.57	17.43	-122.445	37.939	0.48	16.81	230.41	17.29	9	7/14/2014	37:20.4
7847	RSPe_2	-122.445	37.939	0.62	16.77	230.61	17.39	-122.445	37.939	0.51	16.77	230.50	17.28	9	7/14/2014	37:20.3
7848	RSPe_2	-122.445	37.939	0.62	16.77	230.70	17.40	-122.445	37.939	0.51	16.77	230.54	17.29	9	7/14/2014	37:20.2
7849	RSPe_2	-122.445	37.939	0.67	16.74	230.75	17.42	-122.445	37.939	0.51	16.74	230.65	17.26	9	7/14/2014	37:20.1
7850	RSPe_2	-122.445	37.939	0.62	16.82	230.88	17.45	-122.445	37.939	0.57	16.82	230.76	17.39	9	7/14/2014	37:20.0
7851	RSPe_2	-122.445	37.939	0.67	16.79	230.94	17.46	-122.445	37.939	0.60	16.79	230.85	17.39	9	7/14/2014	37:19.9
7852	RSPe_2	-122.445	37.939	0.62	16.78	231.01	17.41	-122.445	37.939	0.51	16.78	230.96	17.30	9	7/14/2014	37:19.8
7853	RSPe_2	-122.445	37.939	0.67	16.81	231.05	17.48	-122.445	37.939	0.57	16.81	230.99	17.37	9	7/14/2014	37:19.7

7854	RSPe_2	-122.445	37.939	0.67	16.81	231.07	17.48	-122.445	37.939	0.57	16.81	231.05	17.37	9	7/14/2014	37:19.6
7855	RSPe_2	-122.445	37.939	0.67	16.86	231.17	17.53	-122.445	37.939	0.60	16.86	231.14	17.46	9	7/14/2014	37:19.5
7856	RSPe_2	-122.445	37.939	0.62	16.84	231.23	17.46	-122.445	37.939	0.57	16.84	231.22	17.40	9	7/14/2014	37:19.4
7857	RSPe_2	-122.445	37.939	0.67	16.84	231.30	17.52	-122.445	37.939	0.51	16.84	231.28	17.36	9	7/14/2014	37:19.3
7858	RSPe_2	-122.445	37.939	0.67	16.88	231.32	17.55	-122.445	37.939	0.48	16.88	231.33	17.36	9	7/14/2014	37:19.2
7859	RSPe_2	-122.445	37.939	0.67	16.90	231.35	17.57	-122.445	37.939	0.51	16.90	231.31	17.41	9	7/14/2014	37:19.1
7860	RSPe_2	-122.445	37.939	0.62	16.87	231.38	17.49	-122.445	37.939	0.51	16.87	231.39	17.38	9	7/14/2014	37:19.0
7861	RSPe_2	-122.445	37.939	0.67	16.89	231.52	17.57	-122.445	37.939	0.51	16.89	231.46	17.41	9	7/14/2014	37:18.9
7862	RSPe_2	-122.445	37.939	0.67	16.92	231.56	17.59	-122.445	37.939	0.51	16.92	231.51	17.43	9	7/14/2014	37:18.8
7863	RSPe_2	-122.445	37.939	0.71	16.99	231.61	17.70	-122.445	37.939	0.51	16.99	231.53	17.50	9	7/14/2014	37:18.7
7864	RSPe_2	-122.445	37.939	0.67	16.96	231.67	17.64	-122.445	37.939	0.48	16.96	231.59	17.44	9	7/14/2014	37:18.6
7865	RSPe_2	-122.445	37.939	0.67	16.98	231.81	17.66	-122.445	37.939	0.51	16.98	231.60	17.50	9	7/14/2014	37:18.5
7866	RSPe_2	-122.445	37.939	0.67	16.98	231.81	17.66	-122.445	37.939	0.51	16.98	231.63	17.50	9	7/14/2014	37:18.4
7867	RSPe_2	-122.445	37.939	0.71	17.01	231.85	17.72	-122.445	37.939	0.51	17.01	231.65	17.53	9	7/14/2014	37:18.3
7868	RSPe_2	-122.445	37.939	0.67	17.01	231.94	17.69	-122.445	37.939	0.51	17.01	231.70	17.53	9	7/14/2014	37:18.2
7869	RSPe_2	-122.445	37.939	0.67	17.05	232.07	17.73	-122.445	37.939	0.51	17.05	231.78	17.57	9	7/14/2014	37:18.1
7870	RSPe_2	-122.445	37.939	0.67	17.09	232.13	17.76	-122.445	37.939	0.51	17.09	231.78	17.60	9	7/14/2014	37:18.0
7871	RSPe_2	-122.445	37.939	0.71	17.11	232.31	17.82	-122.445	37.939	0.51	17.11	231.90	17.62	9	7/14/2014	37:17.9
7872	RSPe_2	-122.445	37.939	0.68	17.12	232.40	17.80	-122.445	37.939	0.57	17.12	232.00	17.69	9	7/14/2014	37:17.8
7873	RSPe_2	-122.445	37.939	0.71	17.15	232.49	17.86	-122.445	37.939	0.51	17.15	232.12	17.67	9	7/14/2014	37:17.7
7874	RSPe_2	-122.445	37.939	0.68	17.09	232.56	17.77	-122.445	37.939	0.60	17.09	232.21	17.69	9	7/14/2014	37:17.6
7875	RSPe_2	-122.445	37.939	0.68	17.07	232.60	17.74	-122.445	37.939	0.60	17.07	232.33	17.67	9	7/14/2014	37:17.5
7876	RSPe_2	-122.445	37.939	0.68	17.04	232.58	17.71	-122.445	37.939	0.60	17.04	232.40	17.64	9	7/14/2014	37:17.4
7877	RSPe_2	-122.445	37.939	0.71	16.94	232.56	17.65	-122.445	37.939	0.57	16.94	232.47	17.50	9	7/14/2014	37:17.3
7878	RSPe_2	-122.445	37.939	0.68	16.96	232.68	17.64	-122.445	37.939	0.57	16.96	232.60	17.53	9	7/14/2014	37:17.2
7879	RSPe_2	-122.445	37.939	0.71	16.87	232.82	17.57	-122.445	37.939	0.65	16.87	232.72	17.52	9	7/14/2014	37:17.1
7880	RSPe_2	-122.445	37.939	0.71	16.88	232.82	17.59	-122.445	37.939	0.60	16.88	232.80	17.48	9	7/14/2014	37:17.0
7881	RSPe_2	-122.445	37.939	0.71	16.78	232.91	17.49	-122.445	37.939	0.57	16.78	232.82	17.35	9	7/14/2014	37:16.9
7882	RSPe_2	-122.445	37.939	0.71	16.68	232.84	17.39	-122.445	37.939	0.60	16.68	232.80	17.28	9	7/14/2014	37:16.8
7883	RSPe_2	-122.445	37.939	0.71	16.69	232.89	17.40	-122.445	37.939	0.60	16.69	232.80	17.29	9	7/14/2014	37:16.7
7884	RSPe_2	-122.445	37.939	0.71	16.62	233.11	17.33	-122.445	37.939	0.51	16.62	232.99	17.14	9	7/14/2014	37:16.6
7885	RSPe_2	-122.445	37.939	0.71	16.72	233.15	17.43	-122.445	37.939	0.57	16.72	233.04	17.29	9	7/14/2014	37:16.5
7886	RSPe_2	-122.445	37.939	0.71	16.61	233.18	17.32	-122.445	37.939	0.60	16.61	233.02	17.21	9	7/14/2014	37:16.4

7887	RSPe_2	-122.445	37.939	0.76	16.65	233.26	17.41	-122.445	37.939	0.57	16.65	233.05	17.21	9	7/14/2014	37:16.3
7888	RSPe_2	-122.445	37.939	0.71	16.53	233.31	17.24	-122.445	37.939	0.60	16.53	233.06	17.13	9	7/14/2014	37:16.2
7889	RSPe_2	-122.445	37.939	0.76	16.53	233.35	17.30	-122.445	37.939	0.60	16.53	233.15	17.13	9	7/14/2014	37:16.1
7890	RSPe_2	-122.445	37.939	0.71	16.53	233.40	17.24	-122.445	37.939	0.57	16.53	233.19	17.09	9	7/14/2014	37:16.0
7891	RSPe_2	-122.445	37.939	0.76	16.55	233.47	17.31	-122.445	37.939	0.60	16.55	233.26	17.15	9	7/14/2014	37:15.9
7892	RSPe_2	-122.445	37.939	0.71	16.51	233.57	17.22	-122.445	37.939	0.65	16.51	233.32	17.16	9	7/14/2014	37:15.8
7893	RSPe_2	-122.445	37.939	0.76	16.49	233.61	17.25	-122.445	37.939	0.69	16.49	233.38	17.17	9	7/14/2014	37:15.7
7894	RSPe_2	-122.445	37.939	0.76	16.51	233.70	17.27	-122.445	37.939	0.65	16.51	233.39	17.16	9	7/14/2014	37:15.6
7895	RSPe_2	-122.445	37.939	0.76	16.47	233.77	17.23	-122.445	37.939	0.69	16.47	233.46	17.15	9	7/14/2014	37:15.5
7896	RSPe_2	-122.445	37.939	0.76	16.47	233.88	17.23	-122.445	37.939	0.74	16.47	233.54	17.20	9	7/14/2014	37:15.4
7897	RSPe_2	-122.445	37.939	0.76	16.42	233.90	17.18	-122.445	37.939	0.74	16.42	233.57	17.16	9	7/14/2014	37:15.3
7898	RSPe_2	-122.445	37.939	0.76	16.43	233.93	17.19	-122.445	37.939	0.74	16.43	233.61	17.16	9	7/14/2014	37:15.2
7899	RSPe_2	-122.445	37.939	0.80	16.43	233.99	17.22	-122.445	37.939	0.74	16.43	233.68	17.16	9	7/14/2014	37:15.1
7900	RSPe_2	-122.445	37.939	0.80	16.41	233.95	17.20	-122.445	37.939	0.74	16.41	233.75	17.14	9	7/14/2014	37:15.0
7901	RSPe_2	-122.445	37.939	0.80	16.39	234.10	17.18	-122.445	37.939	0.77	16.39	233.82	17.16	9	7/14/2014	37:14.9
7902	RSPe_2	-122.445	37.939	0.80	16.51	234.08	17.30	-122.445	37.939	0.74	16.51	233.88	17.24	9	7/14/2014	37:14.8
7903	RSPe_2	-122.445	37.939	0.80	16.41	234.15	17.20	-122.445	37.939	0.77	16.41	233.92	17.17	9	7/14/2014	37:14.7
7904	RSPe_2	-122.445	37.939	0.80	16.58	234.17	17.38	-122.445	37.939	0.77	16.58	234.02	17.35	9	7/14/2014	37:14.6
7905	RSPe_2	-122.445	37.939	0.85	16.51	234.26	17.35	-122.445	37.939	0.77	16.51	234.10	17.28	9	7/14/2014	37:14.5
7906	RSPe_2	-122.445	37.939	0.80	16.55	234.34	17.34	-122.445	37.939	0.85	16.55	234.21	17.40	9	7/14/2014	37:14.4
7907	RSPe_2	-122.445	37.939	0.85	16.60	234.39	17.45	-122.445	37.939	0.82	16.60	234.27	17.42	9	7/14/2014	37:14.3
7908	RSPe_2	-122.445	37.939	0.80	16.62	234.45	17.42	-122.445	37.939	0.82	16.62	234.41	17.44	9	7/14/2014	37:14.2
7909	RSPe_2	-122.445	37.939	0.85	16.58	234.52	17.43	-122.445	37.939	0.82	16.58	234.48	17.40	9	7/14/2014	37:14.1
7910	RSPe_2	-122.445	37.939	0.85	16.56	234.54	17.41	-122.445	37.939	0.77	16.56	234.52	17.33	9	7/14/2014	37:14.0
7911	RSPe_2	-122.445	37.939	0.85	16.60	234.57	17.45	-122.445	37.939	0.82	16.60	234.55	17.42	9	7/14/2014	37:13.9
7912	RSPe_2	-122.445	37.939	0.85	16.53	234.59	17.37	-122.445	37.939	0.82	16.53	234.58	17.35	9	7/14/2014	37:13.8
7913	RSPe_2	-122.445	37.939	0.85	16.53	234.74	17.37	-122.445	37.939	0.85	16.53	234.67	17.38	9	7/14/2014	37:13.7
7914	RSPe_2	-122.445	37.939	0.85	16.50	234.77	17.34	-122.445	37.939	0.82	16.50	234.71	17.32	9	7/14/2014	37:13.6
7915	RSPe_2	-122.445	37.939	0.85	16.46	234.86	17.30	-122.445	37.939	0.82	16.46	234.76	17.28	9	7/14/2014	37:13.5
7916	RSPe_2	-122.445	37.939	0.85	16.47	234.92	17.31	-122.445	37.939	0.77	16.47	234.79	17.23	9	7/14/2014	37:13.4
7917	RSPe_2	-122.445	37.939	0.85	16.47	234.97	17.31	-122.445	37.939	0.82	16.47	234.83	17.29	9	7/14/2014	37:13.3
7918	RSPe_2	-122.445	37.939	0.85	16.47	235.08	17.31	-122.445	37.939	0.82	16.47	234.90	17.29	9	7/14/2014	37:13.2
7919	RSPe_2	-122.445	37.939	0.85	16.45	235.08	17.30	-122.445	37.939	0.85	16.45	234.91	17.31	9	7/14/2014	37:13.1

7920	RSPe_2	-122.445	37.939	0.85	16.44	235.23	17.28	-122.445	37.939	0.77	16.44	235.04	17.21	9	7/14/2014	37:13.0
7921	RSPe_2	-122.445	37.939	0.85	16.48	235.27	17.33	-122.445	37.939	0.85	16.48	235.10	17.34	9	7/14/2014	37:12.9
7922	RSPe_2	-122.445	37.939	0.85	16.44	235.38	17.29	-122.445	37.939	0.82	16.44	235.16	17.26	9	7/14/2014	37:12.8
7923	RSPe_2	-122.445	37.939	0.88	16.41	235.39	17.29	-122.445	37.939	0.82	16.41	235.21	17.23	9	7/14/2014	37:12.7
7924	RSPe_2	-122.445	37.939	0.85	16.40	235.50	17.24	-122.445	37.939	0.85	16.40	235.32	17.25	9	7/14/2014	37:12.6
7925	RSPe_2	-122.445	37.939	0.88	16.43	235.50	17.31	-122.445	37.939	0.89	16.43	235.38	17.32	9	7/14/2014	37:12.5
7926	RSPe_2	-122.445	37.939	0.85	16.38	235.56	17.23	-122.445	37.939	0.85	16.38	235.43	17.24	9	7/14/2014	37:12.4
7927	RSPe_2	-122.445	37.939	0.88	16.37	235.61	17.25	-122.445	37.939	0.85	16.37	235.52	17.23	9	7/14/2014	37:12.3
7928	RSPe_2	-122.445	37.939	0.85	16.37	235.61	17.21	-122.445	37.939	0.85	16.37	235.55	17.22	9	7/14/2014	37:12.2
7929	RSPe_2	-122.445	37.939	0.85	16.35	235.65	17.20	-122.445	37.939	0.85	16.35	235.63	17.20	9	7/14/2014	37:12.1
7930	RSPe_2	-122.445	37.939	0.85	16.38	235.65	17.23	-122.445	37.939	0.82	16.38	235.65	17.20	9	7/14/2014	37:12.0
7931	RSPe_2	-122.445	37.939	0.88	16.41	235.74	17.29	-122.445	37.939	0.82	16.41	235.74	17.23	9	7/14/2014	37:11.9
7932	RSPe_2	-122.445	37.939	0.85	16.38	235.85	17.23	-122.445	37.939	0.82	16.38	235.76	17.20	9	7/14/2014	37:11.8
7933	RSPe_2	-122.445	37.939	0.85	16.36	235.89	17.20	-122.445	37.939	0.85	16.36	235.78	17.21	9	7/14/2014	37:11.7
7934	RSPe_2	-122.445	37.939	0.85	16.35	235.96	17.20	-122.445	37.939	0.82	16.35	235.82	17.17	9	7/14/2014	37:11.6
7935	RSPe_2	-122.445	37.939	0.88	16.35	235.98	17.23	-122.445	37.939	0.85	16.35	235.84	17.20	9	7/14/2014	37:11.5
7936	RSPe_2	-122.445	37.939	0.88	16.38	236.03	17.26	-122.445	37.939	0.85	16.38	235.87	17.24	9	7/14/2014	37:11.4
7937	RSPe_2	-122.445	37.939	0.88	16.33	236.05	17.21	-122.445	37.939	0.89	16.33	235.88	17.22	9	7/14/2014	37:11.3
7938	RSPe_2	-122.445	37.939	0.88	16.37	236.07	17.25	-122.445	37.939	0.89	16.37	235.84	17.26	9	7/14/2014	37:11.2
7939	RSPe_2	-122.445	37.939	0.88	16.31	236.09	17.19	-122.445	37.939	0.89	16.31	235.87	17.20	9	7/14/2014	37:11.1
7940	RSPe_2	-122.445	37.939	0.85	16.33	236.12	17.18	-122.445	37.939	0.85	16.33	235.92	17.18	9	7/14/2014	37:11.0
7941	RSPe_2	-122.445	37.939	0.88	16.31	236.18	17.19	-122.445	37.939	0.89	16.31	235.98	17.20	9	7/14/2014	37:10.9
7942	RSPe_2	-122.445	37.939	0.85	16.33	236.20	17.18	-122.445	37.939	0.89	16.33	236.02	17.22	9	7/14/2014	37:10.8
7943	RSPe_2	-122.445	37.939	0.88	16.33	236.23	17.21	-122.445	37.939	0.89	16.33	236.03	17.22	9	7/14/2014	37:10.7
7944	RSPe_2	-122.445	37.939	0.85	16.40	236.29	17.24	-122.445	37.939	0.89	16.40	236.09	17.28	9	7/14/2014	37:10.6
7945	RSPe_2	-122.445	37.939	0.88	16.37	236.34	17.25	-122.445	37.939	0.89	16.37	236.20	17.26	9	7/14/2014	37:10.5
7946	RSPe_2	-122.445	37.939	0.85	16.37	236.36	17.22	-122.445	37.939	0.89	16.37	236.21	17.26	9	7/14/2014	37:10.4
7947	RSPe_2	-122.445	37.939	0.88	16.39	236.38	17.27	-122.445	37.939	0.89	16.39	236.27	17.28	9	7/14/2014	37:10.3
7948	RSPe_2	-122.445	37.939	0.88	16.38	236.49	17.26	-122.445	37.939	0.85	16.38	236.38	17.24	9	7/14/2014	37:10.2
7949	RSPe_2	-122.445	37.939	0.88	16.38	236.54	17.26	-122.445	37.939	0.89	16.38	236.36	17.27	9	7/14/2014	37:10.1
7950	RSPe_2	-122.445	37.939	0.85	16.41	236.48	17.26	-122.445	37.939	0.85	16.41	236.38	17.26	9	7/14/2014	37:10.0
7951	RSPe_2	-122.445	37.939	0.88	16.37	236.56	17.25	-122.445	37.939	0.89	16.37	236.40	17.26	9	7/14/2014	37:09.9
7952	RSPe_2	-122.445	37.939	0.85	16.37	236.58	17.21	-122.445	37.939	0.89	16.37	236.46	17.26	9	7/14/2014	37:09.8

7953	RSPe_2	-122.445	37.939	0.88	16.37	236.63	17.25	-122.445	37.939	0.89	16.37	236.47	17.26	9	7/14/2014	37:09.7
7954	RSPe_2	-122.445	37.939	0.85	16.39	236.65	17.23	-122.445	37.939	0.85	16.39	236.51	17.24	9	7/14/2014	37:09.6
7955	RSPe_2	-122.445	37.939	0.88	16.39	236.67	17.27	-122.445	37.939	0.89	16.39	236.52	17.28	9	7/14/2014	37:09.5
7956	RSPe_2	-122.445	37.939	0.85	16.41	236.62	17.25	-122.445	37.939	0.85	16.41	236.53	17.26	9	7/14/2014	37:09.4
7957	RSPe_2	-122.445	37.939	0.85	16.42	236.72	17.27	-122.445	37.939	0.85	16.42	236.56	17.27	9	7/14/2014	37:09.3
7958	RSPe_2	-122.445	37.939	0.85	16.44	236.69	17.28	-122.445	37.939	0.85	16.44	236.64	17.29	9	7/14/2014	37:09.2
7959	RSPe_2	-122.445	37.939	0.85	16.44	236.72	17.29	-122.445	37.939	0.85	16.44	236.69	17.30	9	7/14/2014	37:09.1
7960	RSPe_2	-122.445	37.939	0.85	16.45	236.82	17.30	-122.445	37.939	0.85	16.45	236.79	17.31	9	7/14/2014	37:09.0
7961	RSPe_2	-122.445	37.939	0.85	16.48	236.80	17.33	-122.445	37.939	0.85	16.48	236.87	17.34	9	7/14/2014	37:08.9
7962	RSPe_2	-122.445	37.939	0.85	16.51	236.90	17.35	-122.445	37.939	0.85	16.51	236.87	17.36	9	7/14/2014	37:08.8
7963	RSPe_2	-122.445	37.939	0.85	16.50	236.93	17.34	-122.445	37.939	0.85	16.50	236.95	17.35	9	7/14/2014	37:08.7
7964	RSPe_2	-122.445	37.939	0.85	16.51	237.03	17.35	-122.445	37.939	0.85	16.51	237.00	17.36	9	7/14/2014	37:08.6
7965	RSPe_2	-122.445	37.939	0.88	16.51	236.98	17.39	-122.445	37.939	0.85	16.51	236.96	17.37	9	7/14/2014	37:08.5
7966	RSPe_2	-122.445	37.939	0.85	16.51	237.07	17.36	-122.445	37.939	0.82	16.51	237.02	17.33	9	7/14/2014	37:08.4
7967	RSPe_2	-122.445	37.939	0.88	16.51	237.12	17.39	-122.445	37.939	0.85	16.51	237.00	17.36	9	7/14/2014	37:08.3
7968	RSPe_2	-122.445	37.939	0.88	16.51	237.04	17.39	-122.445	37.939	0.85	16.51	237.00	17.36	9	7/14/2014	37:08.2
7969	RSPe_2	-122.445	37.939	0.88	16.54	237.07	17.42	-122.445	37.939	0.85	16.54	237.00	17.40	9	7/14/2014	37:08.1
7970	RSPe_2	-122.445	37.939	0.88	16.53	237.09	17.41	-122.445	37.939	0.85	16.53	237.00	17.39	9	7/14/2014	37:08.0
7971	RSPe_2	-122.445	37.939	0.88	16.53	237.07	17.41	-122.445	37.939	0.89	16.53	237.03	17.42	9	7/14/2014	37:07.9
7972	RSPe_2	-122.445	37.939	0.88	16.58	237.13	17.46	-122.445	37.939	0.89	16.58	237.02	17.46	9	7/14/2014	37:07.8
7973	RSPe_2	-122.445	37.939	0.88	16.57	237.23	17.45	-122.445	37.939	0.94	16.57	237.05	17.50	9	7/14/2014	37:07.7
7974	RSPe_2	-122.445	37.939	0.88	16.59	237.23	17.47	-122.445	37.939	0.89	16.59	237.08	17.48	9	7/14/2014	37:07.6
7975	RSPe_2	-122.445	37.939	0.92	16.59	237.30	17.51	-122.445	37.939	0.89	16.59	237.10	17.48	9	7/14/2014	37:07.5
7976	RSPe_2	-122.445	37.939	0.85	16.60	237.36	17.45	-122.445	37.939	0.89	16.60	237.11	17.49	9	7/14/2014	37:07.4
7977	RSPe_2	-122.445	37.939	0.88	16.60	237.38	17.48	-122.445	37.939	0.89	16.60	237.16	17.49	9	7/14/2014	37:07.3
7978	RSPe_2	-122.445	37.939	0.88	16.61	237.45	17.49	-122.445	37.939	0.85	16.61	237.24	17.47	9	7/14/2014	37:07.2
7979	RSPe_2	-122.445	37.939	0.88	16.65	237.44	17.53	-122.445	37.939	0.89	16.65	237.26	17.53	9	7/14/2014	37:07.1
7980	RSPe_2	-122.445	37.939	0.88	16.65	237.47	17.53	-122.445	37.939	0.85	16.65	237.32	17.50	9	7/14/2014	37:07.0
7981	RSPe_2	-122.445	37.939	0.88	16.67	237.55	17.55	-122.445	37.939	0.85	16.67	237.37	17.53	9	7/14/2014	37:06.9
7982	RSPe_2	-122.445	37.939	0.88	16.66	237.58	17.54	-122.445	37.939	0.82	16.66	237.38	17.48	9	7/14/2014	37:06.8
7983	RSPe_2	-122.445	37.939	0.88	16.66	237.60	17.54	-122.445	37.939	0.85	16.66	237.44	17.51	9	7/14/2014	37:06.7
7984	RSPe_2	-122.445	37.939	0.88	16.73	237.64	17.61	-122.445	37.939	0.85	16.73	237.48	17.58	9	7/14/2014	37:06.6
7985	RSPe_2	-122.445	37.939	0.92	16.72	237.67	17.63	-122.445	37.939	0.89	16.72	237.56	17.60	9	7/14/2014	37:06.5

7986	RSPe_2	-122.445	37.939	0.88	16.72	237.70	17.60	-122.445	37.939	0.94	16.72	237.55	17.66	9	7/14/2014	37:06.4
7987	RSPe_2	-122.445	37.939	0.92	16.77	237.65	17.68	-122.445	37.939	0.94	16.77	237.56	17.71	9	7/14/2014	37:06.3
7988	RSPe_2	-122.445	37.939	0.88	16.77	237.72	17.65	-122.445	37.939	0.89	16.77	237.60	17.66	9	7/14/2014	37:06.2
7989	RSPe_2	-122.445	37.939	0.92	16.72	237.72	17.64	-122.445	37.939	0.94	16.72	237.62	17.66	9	7/14/2014	37:06.1
7990	RSPe_2	-122.445	37.939	0.92	16.70	237.71	17.61	-122.445	37.939	0.94	16.70	237.60	17.64	9	7/14/2014	37:06.0
7991	RSPe_2	-122.445	37.939	0.92	16.77	237.78	17.68	-122.445	37.939	0.94	16.77	237.69	17.71	9	7/14/2014	37:05.9
7992	RSPe_2	-122.445	37.939	0.92	16.75	237.85	17.67	-122.445	37.939	0.94	16.75	237.74	17.69	9	7/14/2014	37:05.8
7993	RSPe_2	-122.445	37.939	0.92	16.77	237.91	17.68	-122.445	37.939	0.94	16.77	237.77	17.71	9	7/14/2014	37:05.7
7994	RSPe_2	-122.445	37.939	0.88	16.72	237.89	17.60	-122.445	37.939	0.89	16.72	237.76	17.61	9	7/14/2014	37:05.6
7995	RSPe_2	-122.445	37.939	0.92	16.87	237.94	17.78	-122.445	37.939	0.94	16.87	237.78	17.80	9	7/14/2014	37:05.5
7996	RSPe_2	-122.445	37.939	0.92	16.78	237.94	17.70	-122.445	37.939	0.94	16.78	237.78	17.72	9	7/14/2014	37:05.4
7997	RSPe_2	-122.445	37.939	0.92	16.77	238.02	17.68	-122.445	37.939	0.94	16.77	237.79	17.71	9	7/14/2014	37:05.3
7998	RSPe_2	-122.445	37.939	0.92	16.84	238.04	17.75	-122.445	37.939	0.94	16.84	237.79	17.78	9	7/14/2014	37:05.2
7999	RSPe_2	-122.445	37.939	0.92	16.82	238.05	17.74	-122.445	37.939	0.94	16.82	237.80	17.76	9	7/14/2014	37:05.1
8000	RSPe_2	-122.445	37.939	0.92	16.78	238.12	17.70	-122.445	37.939	0.94	16.78	237.81	17.72	9	7/14/2014	37:05.0
8001	RSPe_2	-122.445	37.939	0.92	16.76	238.10	17.68	-122.445	37.939	0.97	16.76	237.79	17.73	9	7/14/2014	37:04.9
8002	RSPe_2	-122.445	37.939	0.92	16.74	238.14	17.66	-122.445	37.939	0.94	16.74	237.83	17.68	9	7/14/2014	37:04.8
8003	RSPe_2	-122.445	37.939	0.92	16.79	238.16	17.71	-122.445	37.939	0.97	16.79	237.85	17.76	9	7/14/2014	37:04.7
8004	RSPe_2	-122.445	37.939	0.92	16.83	238.15	17.74	-122.445	37.939	0.97	16.83	237.92	17.80	9	7/14/2014	37:04.6
8005	RSPe_2	-122.445	37.939	0.92	16.77	238.23	17.68	-122.445	37.939	0.97	16.77	237.95	17.74	9	7/14/2014	37:04.5
8006	RSPe_2	-122.445	37.939	0.92	16.78	238.27	17.70	-122.445	37.939	0.97	16.78	238.03	17.76	9	7/14/2014	37:04.4
8007	RSPe_2	-122.445	37.939	0.97	16.79	238.25	17.76	-122.445	37.939	1.02	16.79	238.06	17.81	9	7/14/2014	37:04.3
8008	RSPe_2	-122.445	37.939	0.92	16.81	238.29	17.72	-122.445	37.939	1.02	16.81	238.07	17.83	9	7/14/2014	37:04.2
8009	RSPe_2	-122.445	37.939	0.92	16.79	238.36	17.71	-122.445	37.939	1.06	16.79	238.21	17.85	9	7/14/2014	37:04.1
8010	RSPe_2	-122.445	37.939	0.92	16.83	238.38	17.74	-122.445	37.939	1.06	16.83	238.20	17.89	9	7/14/2014	37:04.0
8011	RSPe_2	-122.445	37.939	0.92	16.81	238.43	17.73	-122.445	37.939	1.02	16.81	238.25	17.84	9	7/14/2014	37:03.9
8012	RSPe_2	-122.445	37.939	0.92	16.79	238.42	17.71	-122.445	37.939	0.97	16.79	238.25	17.76	9	7/14/2014	37:03.8
8013	RSPe_2	-122.445	37.939	0.92	16.84	238.48	17.76	-122.445	37.939	1.06	16.84	238.29	17.90	9	7/14/2014	37:03.7
8014	RSPe_2	-122.445	37.939	0.92	16.84	238.53	17.75	-122.445	37.939	1.02	16.84	238.29	17.86	9	7/14/2014	37:03.6
8015	RSPe_2	-122.445	37.939	0.92	16.70	238.56	17.62	-122.445	37.939	1.06	16.70	238.34	17.76	9	7/14/2014	37:03.5
8016	RSPe_2	-122.445	37.939	0.88	16.63	238.56	17.51	-122.445	37.939	1.06	16.63	238.33	17.69	9	7/14/2014	37:03.4
8017	RSPe_2	-122.445	37.939	0.92	16.61	238.56	17.53	-122.445	37.939	1.06	16.61	238.38	17.67	9	7/14/2014	37:03.3
8018	RSPe_2	-122.445	37.939	0.88	16.61	238.62	17.49	-122.445	37.939	1.06	16.61	238.38	17.67	9	7/14/2014	37:03.2

8019	RSPe_2	-122.445	37.939	0.92	16.51	238.69	17.43	-122.445	37.939	1.11	16.51	238.47	17.62	9	7/14/2014	37:03.1
8020	RSPe_2	-122.445	37.939	0.88	16.65	238.71	17.53	-122.445	37.939	1.06	16.65	238.53	17.70	9	7/14/2014	37:03.0
8021	RSPe_2	-122.445	37.939	0.92	16.41	238.79	17.32	-122.445	37.939	1.11	16.41	238.60	17.51	9	7/14/2014	37:02.9
8022	RSPe_2	-122.445	37.939	0.88	16.44	238.84	17.32	-122.445	37.939	1.06	16.44	238.69	17.50	9	7/14/2014	37:02.8
8023	RSPe_2	-122.445	37.939	0.88	16.36	238.91	17.24	-122.445	37.939	1.06	16.36	238.71	17.42	9	7/14/2014	37:02.7
8024	RSPe_2	-122.445	37.939	0.85	16.31	238.96	17.16	-122.445	37.939	1.02	16.31	238.78	17.34	9	7/14/2014	37:02.6
8025	RSPe_2	-122.445	37.939	0.88	16.36	238.91	17.24	-122.445	37.939	0.97	16.36	238.76	17.33	9	7/14/2014	37:02.5
8026	RSPe_2	-122.445	37.939	0.85	16.25	238.93	17.10	-122.445	37.939	1.02	16.25	238.75	17.27	9	7/14/2014	37:02.4
8027	RSPe_2	-122.445	37.939	0.85	16.30	239.00	17.14	-122.445	37.939	1.02	16.30	238.84	17.32	9	7/14/2014	37:02.3
8028	RSPe_2	-122.445	37.939	0.85	16.27	239.02	17.12	-122.445	37.939	1.02	16.27	238.91	17.30	9	7/14/2014	37:02.2
8029	RSPe_2	-122.445	37.939	0.85	16.18	239.09	17.03	-122.445	37.939	1.02	16.18	238.89	17.20	9	7/14/2014	37:02.1
8030	RSPe_2	-122.445	37.939	0.85	16.05	239.05	16.90	-122.445	37.939	1.02	16.05	238.87	17.08	9	7/14/2014	37:02.0
8031	RSPe_2	-122.445	37.939	0.85	16.08	239.04	16.93	-122.445	37.939	1.06	16.08	238.84	17.14	9	7/14/2014	37:01.9
8032	RSPe_2	-122.445	37.939	0.85	16.00	239.07	16.84	-122.445	37.939	1.06	16.00	238.90	17.05	9	7/14/2014	37:01.8
8033	RSPe_2	-122.445	37.939	0.85	16.09	239.18	16.94	-122.445	37.939	1.06	16.09	239.00	17.15	9	7/14/2014	37:01.7
8034	RSPe_2	-122.445	37.939	0.80	16.02	239.22	16.81	-122.445	37.939	1.06	16.02	239.04	17.08	9	7/14/2014	37:01.6
8035	RSPe_2	-122.445	37.939	0.85	15.99	239.23	16.83	-122.445	37.939	1.02	15.99	239.13	17.01	9	7/14/2014	37:01.5
8036	RSPe_2	-122.445	37.939	0.80	16.05	239.29	16.85	-122.445	37.939	0.97	16.05	239.14	17.02	9	7/14/2014	37:01.4
8037	RSPe_2	-122.445	37.939	0.80	16.01	239.29	16.81	-122.445	37.939	0.97	16.01	239.22	16.98	9	7/14/2014	37:01.3
8038	RSPe_2	-122.445	37.939	0.76	16.02	239.29	16.78	-122.445	37.939	0.97	16.02	239.25	16.99	9	7/14/2014	37:01.2
8039	RSPe_2	-122.445	37.939	0.80	16.00	239.29	16.80	-122.445	37.939	0.97	16.00	239.22	16.98	9	7/14/2014	37:01.1
8040	RSPe_2	-122.445	37.939	0.76	16.05	239.34	16.81	-122.445	37.939	0.94	16.05	239.35	16.99	9	7/14/2014	37:01.0
8041	RSPe_2	-122.445	37.939	0.76	16.00	239.40	16.76	-122.445	37.939	0.94	16.00	239.39	16.94	9	7/14/2014	37:00.9
8042	RSPe_2	-122.445	37.939	0.76	16.00	239.40	16.77	-122.445	37.939	0.89	16.00	239.40	16.89	9	7/14/2014	37:00.8
8043	RSPe_2	-122.445	37.939	0.76	16.01	239.38	16.77	-122.445	37.939	0.94	16.01	239.44	16.95	9	7/14/2014	37:00.7
8044	RSPe_2	-122.445	37.939	0.71	16.00	239.45	16.71	-122.445	37.939	0.89	16.00	239.45	16.89	9	7/14/2014	37:00.6
8045	RSPe_2	-122.445	37.939	0.76	16.05	239.40	16.81	-122.445	37.939	0.89	16.05	239.46	16.94	9	7/14/2014	37:00.5
8046	RSPe_2	-122.445	37.939	0.71	16.04	239.47	16.75	-122.445	37.939	0.89	16.04	239.49	16.93	9	7/14/2014	37:00.4
8047	RSPe_2	-122.445	37.939	0.76	16.03	239.52	16.79	-122.445	37.939	0.89	16.03	239.57	16.91	9	7/14/2014	37:00.3
8048	RSPe_2	-122.445	37.939	0.71	16.06	239.56	16.77	-122.445	37.939	0.89	16.06	239.60	16.95	9	7/14/2014	37:00.2
8049	RSPe_2	-122.445	37.939	0.71	16.06	239.58	16.77	-122.445	37.939	0.86	16.06	239.65	16.91	9	7/14/2014	37:00.1
8050	RSPe_2	-122.445	37.939	0.68	16.09	239.63	16.77	-122.445	37.939	0.86	16.09	239.65	16.94	9	7/14/2014	37:00.0
8051	RSPe_2	-122.445	37.939	0.68	16.09	239.65	16.77	-122.445	37.939	0.82	16.09	239.73	16.92	9	7/14/2014	36:59.9

8052	RSPe_2	-122.445	37.939	0.68	16.11	239.69	16.79	-122.445	37.939	0.82	16.11	239.71	16.93	9	7/14/2014	36:59.8
8053	RSPe_2	-122.445	37.939	0.68	16.17	239.67	16.85	-122.445	37.939	0.77	16.17	239.68	16.94	9	7/14/2014	36:59.7
8054	RSPe_2	-122.445	37.939	0.63	16.20	239.67	16.83	-122.445	37.939	0.77	16.20	239.74	16.97	9	7/14/2014	36:59.6
8055	RSPe_2	-122.445	37.939	0.68	16.18	239.73	16.86	-122.445	37.939	0.77	16.18	239.75	16.95	9	7/14/2014	36:59.5
8056	RSPe_2	-122.445	37.939	0.63	16.18	239.74	16.81	-122.445	37.939	0.74	16.18	239.77	16.92	9	7/14/2014	36:59.4
8057	RSPe_2	-122.445	37.939	0.63	16.23	239.73	16.86	-122.445	37.939	0.74	16.23	239.78	16.97	9	7/14/2014	36:59.3
8058	RSPe_2	-122.445	37.939	0.63	16.24	239.76	16.87	-122.445	37.939	0.69	16.24	239.79	16.93	9	7/14/2014	36:59.2
8059	RSPe_2	-122.445	37.939	0.63	16.32	239.80	16.94	-122.445	37.939	0.74	16.32	239.84	17.06	9	7/14/2014	36:59.1
8060	RSPe_2	-122.445	37.939	0.63	16.40	239.78	17.02	-122.445	37.939	0.69	16.40	239.85	17.08	9	7/14/2014	36:59.0
8061	RSPe_2	-122.445	37.939	0.63	16.33	239.81	16.95	-122.445	37.939	0.69	16.33	239.91	17.02	9	7/14/2014	36:58.9
8062	RSPe_2	-122.445	37.939	0.59	16.41	239.80	17.00	-122.445	37.939	0.69	16.41	239.93	17.09	9	7/14/2014	36:58.8
8063	RSPe_2	-122.445	37.939	0.59	16.38	239.90	16.97	-122.445	37.939	0.69	16.38	240.05	17.07	9	7/14/2014	36:58.7
8064	RSPe_2	-122.445	37.939	0.59	16.52	239.91	17.11	-122.445	37.939	0.60	16.52	240.08	17.12	9	7/14/2014	36:58.6
8065	RSPe_2	-122.445	37.939	0.59	16.48	239.99	17.07	-122.445	37.939	0.60	16.48	240.09	17.08	9	7/14/2014	36:58.5
8066	RSPe_2	-122.445	37.939	0.54	16.53	239.98	17.07	-122.445	37.939	0.60	16.53	240.08	17.13	9	7/14/2014	36:58.4
8067	RSPe_2	-122.445	37.939	0.59	16.55	240.07	17.14	-122.445	37.939	0.60	16.55	240.11	17.15	9	7/14/2014	36:58.3
8068	RSPe_2	-122.445	37.939	0.54	16.54	240.07	17.08	-122.445	37.939	0.57	16.54	240.11	17.11	9	7/14/2014	36:58.2
8069	RSPe_2	-122.445	37.939	0.54	16.57	240.12	17.10	-122.445	37.939	0.57	16.57	240.11	17.13	9	7/14/2014	36:58.1
8070	RSPe_2	-122.445	37.939	0.54	16.58	240.18	17.12	-122.445	37.939	0.57	16.58	240.13	17.15	9	7/14/2014	36:58.0
8071	RSPe_2	-122.445	37.939	0.54	16.60	240.16	17.14	-122.445	37.939	0.57	16.60	240.11	17.16	9	7/14/2014	36:57.9
8072	RSPe_2	-122.445	37.939	0.54	16.60	240.20	17.14	-122.445	37.939	0.57	16.60	240.09	17.17	9	7/14/2014	36:57.8
8073	RSPe_2	-122.445	37.939	0.54	16.61	240.21	17.15	-122.445	37.939	0.57	16.61	240.12	17.18	9	7/14/2014	36:57.7
8074	RSPe_2	-122.445	37.939	0.51	16.65	240.22	17.15	-122.445	37.939	0.52	16.65	240.11	17.16	9	7/14/2014	36:57.6
8075	RSPe_2	-122.445	37.939	0.54	16.64	240.25	17.17	-122.445	37.939	0.57	16.64	240.13	17.20	9	7/14/2014	36:57.5
8076	RSPe_2	-122.445	37.939	0.51	16.64	240.31	17.14	-122.445	37.939	0.52	16.64	240.16	17.15	9	7/14/2014	36:57.4
8077	RSPe_2	-122.445	37.939	0.54	16.65	240.36	17.18	-122.445	37.939	0.52	16.65	240.18	17.16	9	7/14/2014	36:57.3
8078	RSPe_2	-122.445	37.939	0.51	16.67	240.36	17.17	-122.445	37.939	0.52	16.67	240.25	17.18	9	7/14/2014	36:57.2
8079	RSPe_2	-122.445	37.939	0.54	16.68	240.42	17.22	-122.445	37.939	0.52	16.68	240.27	17.20	9	7/14/2014	36:57.1
8080	RSPe_2	-122.445	37.939	0.51	16.68	240.43	17.19	-122.445	37.939	0.52	16.68	240.34	17.20	9	7/14/2014	36:57.0
8081	RSPe_2	-122.445	37.939	0.51	16.72	240.49	17.23	-122.445	37.939	0.52	16.72	240.41	17.24	9	7/14/2014	36:56.9
8082	RSPe_2	-122.445	37.939	0.51	16.72	240.56	17.23	-122.445	37.939	0.48	16.72	240.53	17.20	9	7/14/2014	36:56.8
8083	RSPe_2	-122.445	37.939	0.51	16.74	240.54	17.25	-122.445	37.939	0.48	16.74	240.56	17.23	9	7/14/2014	36:56.7
8084	RSPe_2	-122.445	37.939	0.51	16.76	240.57	17.26	-122.445	37.939	0.48	16.76	240.60	17.24	9	7/14/2014	36:56.6

8085	RSPe_2	-122.445	37.939	0.51	16.76	240.65	17.26	-122.445	37.939	0.48	16.76	240.71	17.24	9	7/14/2014 36:56.5
8086	RSPe_2	-122.445	37.939	0.47	16.78	240.65	17.26	-122.445	37.939	0.45	16.78	240.71	17.23	9	7/14/2014 36:56.4
8087	RSPe_2	-122.445	37.939	0.47	16.82	240.61	17.29	-122.445	37.939	0.48	16.82	240.69	17.31	9	7/14/2014 36:56.3
8088	RSPe_2	-122.445	37.939	0.47	16.84	240.64	17.31	-122.445	37.939	0.45	16.84	240.74	17.29	9	7/14/2014 36:56.2
8089	RSPe_2	-122.445	37.939	0.47	16.89	240.65	17.36	-122.445	37.939	0.48	16.89	240.72	17.38	9	7/14/2014 36:56.1
8090	RSPe_2	-122.445	37.939	0.42	16.94	240.67	17.36	-122.445	37.939	0.45	16.94	240.78	17.39	9	7/14/2014 36:56.0
8091	RSPe_2	-122.445	37.939	0.47	16.94	240.78	17.42	-122.445	37.939	0.45	16.94	240.77	17.39	9	7/14/2014 36:55.9
8092	RSPe_2	-122.445	37.939	0.42	16.97	240.80	17.39	-122.445	37.939	0.40	16.97	240.85	17.36	9	7/14/2014 36:55.8
8093	RSPe_2	-122.445	37.939	0.47	16.98	240.89	17.45	-122.445	37.939	0.45	16.98	240.89	17.43	9	7/14/2014 36:55.7
8094	RSPe_2	-122.445	37.939	0.42	17.01	240.94	17.43	-122.445	37.939	0.40	17.01	240.97	17.41	9	7/14/2014 36:55.6
8095	RSPe_2	-122.445	37.939	0.47	17.01	241.00	17.49	-122.445	37.939	0.40	17.01	240.98	17.41	9	7/14/2014 36:55.5
8096	RSPe_2	-122.445	37.939	0.42	17.04	241.06	17.46	-122.445	37.939	0.40	17.04	241.06	17.44	9	7/14/2014 36:55.4
8097	RSPe_2	-122.445	37.939	0.47	17.13	241.12	17.60	-122.445	37.939	0.40	17.13	241.11	17.53	9	7/14/2014 36:55.3
8098	RSPe_2	-122.445	37.939	0.42	17.06	241.20	17.48	-122.445	37.939	0.40	17.06	241.21	17.46	9	7/14/2014 36:55.2
8099	RSPe_2	-122.445	37.939	0.42	17.13	241.23	17.55	-122.445	37.939	0.40	17.13	241.22	17.53	9	7/14/2014 36:55.1
8100	RSPe_2	-122.445	37.939	0.42	17.12	241.27	17.53	-122.445	37.939	0.40	17.12	241.31	17.51	9	7/14/2014 36:55.0
8101	RSPe_2	-122.445	37.939	0.47	17.14	241.31	17.61	-122.445	37.939	0.36	17.14	241.35	17.51	9	7/14/2014 36:54.9
8102	RSPe_2	-122.445	37.939	0.42	17.16	241.29	17.58	-122.445	37.939	0.36	17.16	241.41	17.52	9	7/14/2014 36:54.8
8103	RSPe_2	-122.445	37.939	0.47	17.17	241.29	17.64	-122.445	37.939	0.36	17.17	241.35	17.53	9	7/14/2014 36:54.7
8104	RSPe_2	-122.445	37.939	0.42	17.23	241.31	17.65	-122.445	37.939	0.36	17.23	241.46	17.59	9	7/14/2014 36:54.6
8105	RSPe_2	-122.445	37.939	0.47	17.23	241.40	17.70	-122.445	37.939	0.36	17.23	241.51	17.59	9	7/14/2014 36:54.5
8106	RSPe_2	-122.445	37.939	0.42	17.29	241.40	17.71	-122.445	37.939	0.36	17.29	241.51	17.66	9	7/14/2014 36:54.4
8107	RSPe_2	-122.445	37.939	0.47	17.32	241.40	17.79	-122.445	37.939	0.36	17.32	241.55	17.68	9	7/14/2014 36:54.3
8108	RSPe_2	-122.445	37.939	0.42	17.35	241.47	17.77	-122.445	37.939	0.36	17.35	241.54	17.72	9	7/14/2014 36:54.2
8109	RSPe_2	-122.445	37.939	0.42	17.36	241.51	17.78	-122.445	37.939	0.36	17.36	241.57	17.73	9	7/14/2014 36:54.1
8110	RSPe_2	-122.445	37.939	0.42	17.39	241.58	17.80	-122.445	37.939	0.31	17.39	241.62	17.70	9	7/14/2014 36:54.0
8111	RSPe_2	-122.445	37.939	0.47	17.45	241.62	17.93	-122.445	37.939	0.37	17.45	241.62	17.82	9	7/14/2014 36:53.9
8112	RSPe_2	-122.445	37.939	0.42	17.42	241.65	17.84	-122.445	37.939	0.31	17.42	241.69	17.74	9	7/14/2014 36:53.8
8113	RSPe_2	-122.445	37.939	0.42	17.45	241.67	17.87	-122.445	37.939	0.31	17.45	241.69	17.77	9	7/14/2014 36:53.7
8114	RSPe_2	-122.445	37.939	0.42	17.45	241.69	17.87	-122.445	37.939	0.31	17.45	241.70	17.77	9	7/14/2014 36:53.6
8115	RSPe_2	-122.445	37.939	0.42	17.44	241.65	17.86	-122.445	37.939	0.28	17.44	241.71	17.72	9	7/14/2014 36:53.5
8116	RSPe_2	-122.445	37.939	0.42	17.45	241.69	17.87	-122.445	37.939	0.28	17.45	241.72	17.73	9	7/14/2014 36:53.4
8117	RSPe_2	-122.445	37.939	0.42	17.49	241.71	17.91	-122.445	37.939	0.31	17.49	241.78	17.81	9	7/14/2014 36:53.3

8118	RSPe_2	-122.445	37.939	0.42	17.41	241.73	17.83	-122.445	37.939	0.28	17.41	241.78	17.69	9	7/14/2014	36:53.2
8119	RSPe_2	-122.445	37.939	0.47	17.43	241.73	17.90	-122.445	37.939	0.31	17.43	241.82	17.75	9	7/14/2014	36:53.1
8120	RSPe_2	-122.445	37.939	0.42	17.38	241.80	17.80	-122.445	37.939	0.28	17.38	241.82	17.66	9	7/14/2014	36:53.0
8121	RSPe_2	-122.445	37.939	0.47	17.33	241.85	17.80	-122.445	37.939	0.31	17.33	241.84	17.64	9	7/14/2014	36:52.9
8122	RSPe_2	-122.445	37.939	0.42	17.35	241.87	17.77	-122.445	37.939	0.28	17.35	241.87	17.63	9	7/14/2014	36:52.8
8123	RSPe_2	-122.445	37.939	0.42	17.32	241.92	17.74	-122.445	37.939	0.28	17.32	241.84	17.60	9	7/14/2014	36:52.7
8124	RSPe_2	-122.445	37.939	0.42	17.32	241.98	17.74	-122.445	37.939	0.31	17.32	241.92	17.63	9	7/14/2014	36:52.6
8125	RSPe_2	-122.445	37.939	0.47	17.38	242.04	17.85	-122.445	37.939	0.31	17.38	241.91	17.69	9	7/14/2014	36:52.5
8126	RSPe_2	-122.445	37.939	0.42	17.25	242.11	17.67	-122.445	37.939	0.31	17.25	241.98	17.57	9	7/14/2014	36:52.4
8127	RSPe_2	-122.445	37.939	0.42	17.29	242.20	17.71	-122.445	37.939	0.31	17.29	242.09	17.61	9	7/14/2014	36:52.3
8128	RSPe_2	-122.445	37.939	0.42	17.27	242.25	17.69	-122.445	37.939	0.28	17.27	242.17	17.55	9	7/14/2014	36:52.2
8129	RSPe_2	-122.445	37.939	0.47	17.28	242.29	17.76	-122.445	37.939	0.31	17.28	242.20	17.60	9	7/14/2014	36:52.1
8130	RSPe_2	-122.445	37.939	0.42	17.23	242.35	17.65	-122.445	37.939	0.31	17.23	242.27	17.54	9	7/14/2014	36:52.0
8131	RSPe_2	-122.445	37.939	0.47	17.22	242.38	17.69	-122.445	37.939	0.31	17.22	242.28	17.54	9	7/14/2014	36:51.9
8132	RSPe_2	-122.445	37.939	0.42	17.20	242.44	17.62	-122.445	37.939	0.31	17.20	242.37	17.51	9	7/14/2014	36:51.8
8133	RSPe_2	-122.445	37.939	0.42	17.25	242.51	17.67	-122.445	37.939	0.31	17.25	242.40	17.56	9	7/14/2014	36:51.7
8134	RSPe_2	-122.445	37.939	0.42	17.18	242.54	17.60	-122.445	37.939	0.31	17.18	242.40	17.49	9	7/14/2014	36:51.6
8135	RSPe_2	-122.445	37.939	0.42	17.17	242.60	17.59	-122.445	37.939	0.31	17.17	242.45	17.48	9	7/14/2014	36:51.5
8136	RSPe_2	-122.445	37.939	0.42	17.15	242.64	17.57	-122.445	37.939	0.31	17.15	242.46	17.47	9	7/14/2014	36:51.4
8137	RSPe_2	-122.445	37.939	0.47	17.11	242.67	17.58	-122.445	37.939	0.31	17.11	242.53	17.42	9	7/14/2014	36:51.3
8138	RSPe_2	-122.445	37.939	0.42	17.07	242.71	17.49	-122.445	37.939	0.31	17.07	242.56	17.38	9	7/14/2014	36:51.2
8139	RSPe_2	-122.445	37.939	0.42	17.03	242.74	17.45	-122.445	37.939	0.31	17.03	242.60	17.35	9	7/14/2014	36:51.1
8140	RSPe_2	-122.445	37.939	0.42	17.02	242.77	17.44	-122.445	37.939	0.31	17.02	242.68	17.33	9	7/14/2014	36:51.0
8141	RSPe_2	-122.445	37.939	0.42	17.03	242.82	17.45	-122.445	37.939	0.37	17.03	242.75	17.40	9	7/14/2014	36:50.9
8142	RSPe_2	-122.445	37.939	0.42	17.00	242.86	17.42	-122.445	37.939	0.31	17.00	242.86	17.31	9	7/14/2014	36:50.8
8143	RSPe_2	-122.445	37.939	0.42	17.00	242.95	17.42	-122.445	37.939	0.31	17.00	242.91	17.31	9	7/14/2014	36:50.7
8144	RSPe_2	-122.445	37.939	0.42	17.00	242.96	17.42	-122.445	37.939	0.37	17.00	242.97	17.36	9	7/14/2014	36:50.6
8145	RSPe_2	-122.445	37.939	0.42	17.00	243.05	17.42	-122.445	37.939	0.37	17.00	243.00	17.37	9	7/14/2014	36:50.5
8146	RSPe_2	-122.445	37.939	0.42	17.00	243.10	17.42	-122.445	37.939	0.31	17.00	243.01	17.32	9	7/14/2014	36:50.4
8147	RSPe_2	-122.445	37.939	0.42	17.02	243.15	17.44	-122.445	37.939	0.37	17.02	243.10	17.39	9	7/14/2014	36:50.3
8148	RSPe_2	-122.445	37.939	0.42	17.07	243.21	17.49	-122.445	37.939	0.31	17.07	243.10	17.38	9	7/14/2014	36:50.2
8149	RSPe_2	-122.445	37.939	0.42	17.05	243.29	17.47	-122.445	37.939	0.31	17.05	243.17	17.36	9	7/14/2014	36:50.1
8150	RSPe_2	-122.445	37.939	0.39	16.99	243.37	17.38	-122.445	37.939	0.31	16.99	243.30	17.30	9	7/14/2014	36:50.0

8151	RSPe_2	-122.445	37.939	0.42	17.03	243.42	17.45	-122.445	37.939	0.28	17.03	243.37	17.31	9	7/14/2014	36:49.9
8152	RSPe_2	-122.445	37.939	0.39	17.03	243.48	17.42	-122.445	37.939	0.31	17.03	243.47	17.35	9	7/14/2014	36:49.8
8153	RSPe_2	-122.445	37.939	0.42	17.03	243.57	17.45	-122.445	37.939	0.31	17.03	243.61	17.35	9	7/14/2014	36:49.7
8154	RSPe_2	-122.445	37.939	0.42	17.00	243.64	17.42	-122.445	37.939	0.28	17.00	243.71	17.28	9	7/14/2014	36:49.6
8155	RSPe_2	-122.445	37.939	0.42	17.02	243.70	17.44	-122.445	37.939	0.31	17.02	243.77	17.33	9	7/14/2014	36:49.5
8156	RSPe_2	-122.445	37.939	0.42	17.07	243.77	17.49	-122.445	37.939	0.28	17.07	243.87	17.35	9	7/14/2014	36:49.4
8157	RSPe_2	-122.445	37.939	0.42	17.11	243.80	17.53	-122.445	37.939	0.31	17.11	243.86	17.42	9	7/14/2014	36:49.3
8158	RSPe_2	-122.445	37.939	0.42	17.09	243.81	17.51	-122.445	37.939	0.28	17.09	243.90	17.37	9	7/14/2014	36:49.2
8159	RSPe_2	-122.445	37.939	0.42	17.13	243.86	17.55	-122.445	37.939	0.31	17.13	243.95	17.44	9	7/14/2014	36:49.1
8160	RSPe_2	-122.445	37.939	0.39	17.12	243.95	17.51	-122.445	37.939	0.31	17.12	244.00	17.44	9	7/14/2014	36:49.0
8161	RSPe_2	-122.445	37.939	0.42	17.13	243.97	17.55	-122.445	37.939	0.31	17.13	244.04	17.44	9	7/14/2014	36:48.9
8162	RSPe_2	-122.445	37.939	0.42	17.14	244.01	17.56	-122.445	37.939	0.28	17.14	244.04	17.42	9	7/14/2014	36:48.8
8163	RSPe_2	-122.445	37.939	0.42	17.14	244.15	17.56	-122.445	37.939	0.31	17.14	244.17	17.46	9	7/14/2014	36:48.7
8164	RSPe_2	-122.445	37.939	0.42	17.08	244.23	17.50	-122.445	37.939	0.31	17.08	244.21	17.40	9	7/14/2014	36:48.6
8165	RSPe_2	-122.445	37.939	0.47	17.14	244.30	17.61	-122.445	37.939	0.37	17.14	244.30	17.50	9	7/14/2014	36:48.5
8166	RSPe_2	-122.445	37.939	0.47	17.07	244.41	17.54	-122.445	37.939	0.37	17.07	244.30	17.43	9	7/14/2014	36:48.4
8167	RSPe_2	-122.445	37.939	0.47	17.04	244.39	17.51	-122.445	37.939	0.37	17.04	244.34	17.40	9	7/14/2014	36:48.3
8168	RSPe_2	-122.445	37.939	0.47	17.08	244.34	17.56	-122.445	37.939	0.37	17.08	244.36	17.45	9	7/14/2014	36:48.2
8169	RSPe_2	-122.445	37.939	0.51	17.01	244.41	17.52	-122.445	37.939	0.37	17.01	244.41	17.38	9	7/14/2014	36:48.1
8170	RSPe_2	-122.445	37.939	0.47	16.94	244.50	17.42	-122.445	37.939	0.31	16.94	244.51	17.26	9	7/14/2014	36:48.0
8171	RSPe_2	-122.445	37.939	0.51	16.89	244.59	17.40	-122.445	37.939	0.45	16.89	244.59	17.34	9	7/14/2014	36:47.9
8172	RSPe_2	-122.445	37.939	0.47	16.84	244.61	17.31	-122.445	37.939	0.37	16.84	244.56	17.20	9	7/14/2014	36:47.8
8173	RSPe_2	-122.445	37.939	0.51	16.82	244.63	17.33	-122.445	37.939	0.40	16.82	244.61	17.22	9	7/14/2014	36:47.7
8174	RSPe_2	-122.445	37.939	0.47	16.81	244.70	17.28	-122.445	37.939	0.45	16.81	244.67	17.26	9	7/14/2014	36:47.6
8175	RSPe_2	-122.445	37.939	0.51	16.79	244.72	17.30	-122.445	37.939	0.40	16.79	244.67	17.19	9	7/14/2014	36:47.5
8176	RSPe_2	-122.445	37.939	0.51	16.74	244.72	17.25	-122.445	37.939	0.40	16.74	244.77	17.14	9	7/14/2014	36:47.4
8177	RSPe_2	-122.445	37.939	0.51	16.76	244.72	17.27	-122.445	37.939	0.45	16.76	244.76	17.21	9	7/14/2014	36:47.3
8178	RSPe_2	-122.445	37.939	0.51	16.70	244.81	17.20	-122.445	37.939	0.40	16.70	244.82	17.10	9	7/14/2014	36:47.2
8179	RSPe_2	-122.445	37.939	0.54	16.69	244.82	17.23	-122.445	37.939	0.45	16.69	244.85	17.14	9	7/14/2014	36:47.1
8180	RSPe_2	-122.445	37.939	0.51	16.69	244.92	17.19	-122.445	37.939	0.40	16.69	244.91	17.09	9	7/14/2014	36:47.0
8181	RSPe_2	-122.445	37.939	0.54	16.67	245.03	17.21	-122.445	37.939	0.40	16.67	245.01	17.07	9	7/14/2014	36:46.9
8182	RSPe_2	-122.445	37.939	0.54	16.68	245.14	17.22	-122.445	37.939	0.40	16.68	245.12	17.08	9	7/14/2014	36:46.8
8183	RSPe_2	-122.445	37.939	0.54	16.72	245.17	17.26	-122.445	37.939	0.40	16.72	245.12	17.11	9	7/14/2014	36:46.7

8184	RSPe_2	-122.445	37.939	0.51	16.67	245.23	17.17	-122.445	37.939	0.45	16.67	245.17	17.12	9	7/14/2014	36:46.6
8185	RSPe_2	-122.445	37.939	0.54	16.70	245.23	17.24	-122.445	37.939	0.45	16.70	245.25	17.15	9	7/14/2014	36:46.5
8186	RSPe_2	-122.445	37.939	0.54	16.67	245.29	17.21	-122.445	37.939	0.45	16.67	245.29	17.12	9	7/14/2014	36:46.4
8187	RSPe_2	-122.445	37.939	0.54	16.72	245.32	17.26	-122.445	37.939	0.45	16.72	245.34	17.17	9	7/14/2014	36:46.3
8188	RSPe_2	-122.445	37.939	0.54	16.66	245.32	17.20	-122.445	37.939	0.45	16.66	245.31	17.11	9	7/14/2014	36:46.2
8189	RSPe_2	-122.445	37.939	0.59	16.70	245.36	17.29	-122.445	37.939	0.48	16.70	245.39	17.18	9	7/14/2014	36:46.1
8190	RSPe_2	-122.445	37.939	0.54	16.67	245.37	17.21	-122.445	37.939	0.48	16.67	245.40	17.16	9	7/14/2014	36:46.0
8191	RSPe_2	-122.445	37.939	0.54	16.67	245.45	17.21	-122.445	37.939	0.48	16.67	245.49	17.15	9	7/14/2014	36:45.9
8192	RSPe_2	-122.445	37.939	0.54	16.65	245.46	17.19	-122.445	37.939	0.48	16.65	245.45	17.13	9	7/14/2014	36:45.8
8193	RSPe_2	-122.445	37.939	0.59	16.60	245.52	17.20	-122.445	37.939	0.48	16.60	245.52	17.09	9	7/14/2014	36:45.7
8194	RSPe_2	-122.445	37.939	0.54	16.58	245.61	17.12	-122.445	37.939	0.45	16.58	245.60	17.03	9	7/14/2014	36:45.6
8195	RSPe_2	-122.445	37.939	0.59	16.54	245.66	17.14	-122.445	37.939	0.48	16.54	245.60	17.03	9	7/14/2014	36:45.5
8196	RSPe_2	-122.445	37.939	0.59	16.54	245.69	17.14	-122.445	37.939	0.45	16.54	245.60	16.99	9	7/14/2014	36:45.4
8197	RSPe_2	-122.445	37.939	0.59	16.43	245.77	17.02	-122.445	37.939	0.48	16.43	245.68	16.91	9	7/14/2014	36:45.3
8198	RSPe_2	-122.445	37.939	0.59	16.44	245.83	17.03	-122.445	37.939	0.52	16.44	245.71	16.95	9	7/14/2014	36:45.2
8199	RSPe_2	-122.445	37.939	0.59	16.37	245.81	16.96	-122.445	37.939	0.52	16.37	245.71	16.88	9	7/14/2014	36:45.1
8200	RSPe_2	-122.445	37.939	0.59	16.33	245.89	16.92	-122.445	37.939	0.48	16.33	245.76	16.81	9	7/14/2014	36:45.0
8201	RSPe_2	-122.445	37.939	0.59	16.32	245.90	16.91	-122.445	37.939	0.52	16.32	245.78	16.84	9	7/14/2014	36:44.9
8202	RSPe_2	-122.445	37.939	0.59	16.27	245.87	16.86	-122.445	37.939	0.57	16.27	245.84	16.84	9	7/14/2014	36:44.8
8203	RSPe_2	-122.445	37.939	0.63	16.37	245.96	16.99	-122.445	37.939	0.57	16.37	245.89	16.94	9	7/14/2014	36:44.7
8204	RSPe_2	-122.445	37.939	0.59	16.25	245.94	16.84	-122.445	37.939	0.52	16.25	245.96	16.77	9	7/14/2014	36:44.6
8205	RSPe_2	-122.445	37.939	0.63	16.28	245.99	16.91	-122.445	37.939	0.52	16.28	246.01	16.80	9	7/14/2014	36:44.5
8206	RSPe_2	-122.445	37.939	0.59	16.21	246.03	16.80	-122.445	37.939	0.60	16.21	246.03	16.81	9	7/14/2014	36:44.4
8207	RSPe_2	-122.445	37.939	0.63	16.27	246.01	16.89	-122.445	37.939	0.60	16.27	246.09	16.87	9	7/14/2014	36:44.3
8208	RSPe_2	-122.445	37.939	0.63	16.21	246.01	16.84	-122.445	37.939	0.52	16.21	246.07	16.73	9	7/14/2014	36:44.2
8209	RSPe_2	-122.445	37.939	0.63	16.23	246.07	16.85	-122.445	37.939	0.60	16.23	246.14	16.83	9	7/14/2014	36:44.1
8210	RSPe_2	-122.445	37.939	0.63	16.20	246.06	16.82	-122.445	37.939	0.60	16.20	246.13	16.80	9	7/14/2014	36:44.0
8211	RSPe_2	-122.445	37.939	0.63	16.21	246.07	16.84	-122.445	37.939	0.65	16.21	246.15	16.86	9	7/14/2014	36:43.9
8212	RSPe_2	-122.445	37.939	0.63	16.20	246.14	16.83	-122.445	37.939	0.60	16.20	246.20	16.80	9	7/14/2014	36:43.8
8213	RSPe_2	-122.445	37.939	0.63	16.23	246.14	16.85	-122.445	37.939	0.57	16.23	246.18	16.80	9	7/14/2014	36:43.7
8214	RSPe_2	-122.445	37.939	0.63	16.25	246.18	16.88	-122.445	37.939	0.60	16.25	246.18	16.85	9	7/14/2014	36:43.6
8215	RSPe_2	-122.445	37.939	0.68	16.25	246.16	16.93	-122.445	37.939	0.57	16.25	246.18	16.82	9	7/14/2014	36:43.5
8216	RSPe_2	-122.445	37.939	0.63	16.23	246.20	16.85	-122.445	37.939	0.60	16.23	246.21	16.83	9	7/14/2014	36:43.4

8217	RSPe_2	-122.445	37.939	0.68	16.22	246.19	16.90	-122.445	37.939	0.60	16.22	246.20	16.82	9	7/14/2014	36:43.3
8218	RSPe_2	-122.445	37.939	0.68	16.21	246.28	16.89	-122.445	37.939	0.57	16.21	246.29	16.78	9	7/14/2014	36:43.2
8219	RSPe_2	-122.445	37.939	0.68	16.20	246.27	16.87	-122.445	37.939	0.65	16.20	246.27	16.85	9	7/14/2014	36:43.1
8220	RSPe_2	-122.445	37.939	0.68	16.21	246.30	16.89	-122.445	37.939	0.60	16.21	246.31	16.81	9	7/14/2014	36:43.0
8221	RSPe_2	-122.445	37.939	0.71	16.17	246.32	16.88	-122.445	37.939	0.69	16.17	246.32	16.86	9	7/14/2014	36:42.9
8222	RSPe_2	-122.445	37.939	0.71	16.17	246.36	16.88	-122.445	37.939	0.69	16.17	246.34	16.86	9	7/14/2014	36:42.8
8223	RSPe_2	-122.445	37.939	0.71	16.18	246.37	16.89	-122.445	37.939	0.74	16.18	246.39	16.92	9	7/14/2014	36:42.7
8224	RSPe_2	-122.445	37.939	0.68	16.18	246.39	16.86	-122.445	37.939	0.69	16.18	246.39	16.87	9	7/14/2014	36:42.6
8225	RSPe_2	-122.445	37.939	0.71	16.19	246.36	16.90	-122.445	37.939	0.74	16.19	246.40	16.93	9	7/14/2014	36:42.5
8226	RSPe_2	-122.445	37.939	0.68	16.19	246.45	16.87	-122.445	37.939	0.69	16.19	246.45	16.88	9	7/14/2014	36:42.4
8227	RSPe_2	-122.445	37.939	0.71	16.20	246.43	16.91	-122.445	37.939	0.74	16.20	246.46	16.94	9	7/14/2014	36:42.3
8228	RSPe_2	-122.445	37.939	0.71	16.20	246.50	16.91	-122.445	37.939	0.65	16.20	246.45	16.86	9	7/14/2014	36:42.2
8229	RSPe_2	-122.445	37.939	0.71	16.19	246.47	16.90	-122.445	37.939	0.69	16.19	246.52	16.88	9	7/14/2014	36:42.1
8230	RSPe_2	-122.445	37.939	0.71	16.22	246.57	16.93	-122.445	37.939	0.69	16.22	246.56	16.91	9	7/14/2014	36:42.0
8231	RSPe_2	-122.445	37.939	0.71	16.20	246.61	16.91	-122.445	37.939	0.69	16.20	246.64	16.88	9	7/14/2014	36:41.9
8232	RSPe_2	-122.445	37.939	0.71	16.25	246.63	16.96	-122.445	37.939	0.69	16.25	246.58	16.94	9	7/14/2014	36:41.8
8233	RSPe_2	-122.445	37.939	0.76	16.23	246.63	16.99	-122.445	37.939	0.74	16.23	246.62	16.97	9	7/14/2014	36:41.7
8234	RSPe_2	-122.445	37.939	0.71	16.27	246.63	16.98	-122.445	37.939	0.74	16.27	246.61	17.00	9	7/14/2014	36:41.6
8235	RSPe_2	-122.445	37.939	0.76	16.23	246.70	17.00	-122.445	37.939	0.77	16.23	246.63	17.01	9	7/14/2014	36:41.5
8236	RSPe_2	-122.445	37.939	0.71	16.23	246.70	16.94	-122.445	37.939	0.74	16.23	246.60	16.97	9	7/14/2014	36:41.4
8237	RSPe_2	-122.445	37.939	0.76	16.25	246.68	17.01	-122.445	37.939	0.74	16.25	246.67	16.99	9	7/14/2014	36:41.3
8238	RSPe_2	-122.445	37.939	0.76	16.23	246.74	16.99	-122.445	37.939	0.74	16.23	246.71	16.97	9	7/14/2014	36:41.2
8239	RSPe_2	-122.445	37.939	0.76	16.30	246.74	17.07	-122.445	37.939	0.77	16.30	246.70	17.08	9	7/14/2014	36:41.1
8240	RSPe_2	-122.445	37.939	0.76	16.27	246.77	17.03	-122.445	37.939	0.77	16.27	246.72	17.04	9	7/14/2014	36:41.0
8241	RSPe_2	-122.445	37.939	0.76	16.28	246.79	17.05	-122.445	37.939	0.77	16.28	246.77	17.05	9	7/14/2014	36:40.9
8242	RSPe_2	-122.445	37.939	0.76	16.31	246.86	17.08	-122.445	37.939	0.82	16.31	246.85	17.14	9	7/14/2014	36:40.8
8243	RSPe_2	-122.445	37.939	0.76	16.30	246.90	17.07	-122.445	37.939	0.82	16.30	246.90	17.13	9	7/14/2014	36:40.7
8244	RSPe_2	-122.445	37.939	0.76	16.38	246.94	17.15	-122.445	37.939	0.77	16.38	246.94	17.15	9	7/14/2014	36:40.6
8245	RSPe_2	-122.445	37.939	0.80	16.34	246.92	17.14	-122.445	37.939	0.82	16.34	246.96	17.16	9	7/14/2014	36:40.5
8246	RSPe_2	-122.445	37.939	0.76	16.33	246.95	17.09	-122.445	37.939	0.82	16.33	246.98	17.15	9	7/14/2014	36:40.4
8247	RSPe_2	-122.445	37.939	0.80	16.32	246.99	17.12	-122.445	37.939	0.86	16.32	246.96	17.18	9	7/14/2014	36:40.3
8248	RSPe_2	-122.445	37.939	0.76	16.37	247.01	17.14	-122.445	37.939	0.82	16.37	247.01	17.20	9	7/14/2014	36:40.2
8249	RSPe_2	-122.445	37.939	0.80	16.32	247.01	17.12	-122.445	37.939	0.82	16.32	247.01	17.14	9	7/14/2014	36:40.1

8250	RSPe_2	-122.445	37.939	0.76	16.30	247.05	17.07	-122.445	37.939	0.82	16.30	247.06	17.13	9	7/14/2014	36:40.0
8251	RSPe_2	-122.445	37.939	0.80	16.30	247.08	17.10	-122.445	37.939	0.82	16.30	247.07	17.13	9	7/14/2014	36:39.9
8252	RSPe_2	-122.445	37.939	0.76	16.32	247.06	17.08	-122.445	37.939	0.86	16.32	247.08	17.18	9	7/14/2014	36:39.8
8253	RSPe_2	-122.445	37.939	0.80	16.35	247.08	17.15	-122.445	37.939	0.86	16.35	247.06	17.21	9	7/14/2014	36:39.7
8254	RSPe_2	-122.445	37.939	0.80	16.30	247.01	17.10	-122.445	37.939	0.86	16.30	247.01	17.15	9	7/14/2014	36:39.6
8255	RSPe_2	-122.445	37.939	0.85	16.34	247.05	17.19	-122.445	37.939	0.89	16.34	247.10	17.23	9	7/14/2014	36:39.5
8256	RSPe_2	-122.445	37.939	0.80	16.33	246.99	17.13	-122.445	37.939	0.89	16.33	247.06	17.23	9	7/14/2014	36:39.4
8257	RSPe_2	-122.445	37.939	0.85	16.39	247.04	17.24	-122.445	37.939	0.94	16.39	247.09	17.33	9	7/14/2014	36:39.3
8258	RSPe_2	-122.445	37.939	0.80	16.33	247.04	17.13	-122.445	37.939	0.89	16.33	247.14	17.23	9	7/14/2014	36:39.2
8259	RSPe_2	-122.445	37.939	0.80	16.37	247.04	17.17	-122.445	37.939	0.94	16.37	247.15	17.31	9	7/14/2014	36:39.1
8260	RSPe_2	-122.445	37.939	0.80	16.37	247.06	17.17	-122.445	37.939	0.94	16.37	247.18	17.31	9	7/14/2014	36:39.0
8261	RSPe_2	-122.445	37.939	0.80	16.41	247.06	17.21	-122.445	37.939	0.94	16.41	247.19	17.35	9	7/14/2014	36:38.9
8262	RSPe_2	-122.445	37.939	0.80	16.44	247.10	17.24	-122.445	37.939	0.89	16.44	247.17	17.33	9	7/14/2014	36:38.8
8263	RSPe_2	-122.445	37.939	0.85	16.43	247.17	17.28	-122.445	37.939	0.94	16.43	247.21	17.37	9	7/14/2014	36:38.7
8264	RSPe_2	-122.445	37.939	0.80	16.49	247.13	17.29	-122.445	37.939	0.94	16.49	247.18	17.43	9	7/14/2014	36:38.6
8265	RSPe_2	-122.445	37.939	0.85	16.50	247.13	17.35	-122.445	37.939	0.94	16.50	247.15	17.44	9	7/14/2014	36:38.5
8266	RSPe_2	-122.445	37.939	0.80	16.47	247.17	17.27	-122.445	37.939	0.94	16.47	247.17	17.42	9	7/14/2014	36:38.4
8267	RSPe_2	-122.445	37.939	0.85	16.48	247.13	17.33	-122.445	37.939	0.94	16.48	247.20	17.42	9	7/14/2014	36:38.3
8268	RSPe_2	-122.445	37.939	0.85	16.49	247.15	17.34	-122.445	37.939	0.94	16.49	247.17	17.43	9	7/14/2014	36:38.2
8269	RSPe_2	-122.445	37.939	0.85	16.50	247.17	17.35	-122.445	37.939	0.98	16.50	247.21	17.47	9	7/14/2014	36:38.1
8270	RSPe_2	-122.445	37.939	0.85	16.51	247.14	17.36	-122.445	37.939	0.98	16.51	247.28	17.49	9	7/14/2014	36:38.0
8271	RSPe_2	-122.445	37.939	0.85	16.42	247.27	17.27	-122.445	37.939	0.98	16.42	247.30	17.40	9	7/14/2014	36:37.9
8272	RSPe_2	-122.445	37.939	0.85	16.53	247.26	17.38	-122.445	37.939	0.98	16.53	247.34	17.50	9	7/14/2014	36:37.8
8273	RSPe_2	-122.445	37.939	0.85	16.51	247.33	17.36	-122.445	37.939	1.03	16.51	247.41	17.54	9	7/14/2014	36:37.7
8274	RSPe_2	-122.445	37.939	0.85	16.50	247.35	17.35	-122.445	37.939	1.03	16.50	247.43	17.52	9	7/14/2014	36:37.6
8275	RSPe_2	-122.445	37.939	0.85	16.55	247.40	17.40	-122.445	37.939	1.03	16.55	247.46	17.58	9	7/14/2014	36:37.5
8276	RSPe_2	-122.445	37.939	0.85	16.43	247.40	17.28	-122.445	37.939	1.03	16.43	247.50	17.45	9	7/14/2014	36:37.4
8277	RSPe_2	-122.445	37.939	0.85	16.51	247.46	17.36	-122.445	37.939	1.03	16.51	247.52	17.53	9	7/14/2014	36:37.3
8278	RSPe_2	-122.445	37.939	0.85	16.50	247.46	17.35	-122.445	37.939	1.03	16.50	247.54	17.52	9	7/14/2014	36:37.2
8279	RSPe_2	-122.445	37.939	0.85	16.51	247.46	17.36	-122.445	37.939	1.03	16.51	247.59	17.54	9	7/14/2014	36:37.1
8280	RSPe_2	-122.445	37.939	0.85	16.54	247.46	17.39	-122.445	37.939	1.03	16.54	247.59	17.57	9	7/14/2014	36:37.0
8281	RSPe_2	-122.445	37.939	0.85	16.57	247.51	17.41	-122.445	37.939	1.03	16.57	247.61	17.59	9	7/14/2014	36:36.9
8282	RSPe_2	-122.445	37.939	0.85	16.51	247.48	17.36	-122.445	37.939	0.98	16.51	247.63	17.49	9	7/14/2014	36:36.8

8283	RSPe_2	-122.445	37.939	0.85	16.51	247.53	17.36	-122.445	37.939	1.03	16.51	247.63	17.54	9	7/14/2014	36:36.7
8284	RSPe_2	-122.445	37.939	0.85	16.57	247.55	17.41	-122.445	37.939	1.06	16.57	247.61	17.63	9	7/14/2014	36:36.6
8285	RSPe_2	-122.445	37.939	0.85	16.56	247.57	17.41	-122.445	37.939	1.06	16.56	247.65	17.62	9	7/14/2014	36:36.5
8286	RSPe_2	-122.445	37.939	0.80	16.65	247.59	17.45	-122.445	37.939	1.03	16.65	247.68	17.68	9	7/14/2014	36:36.4
8287	RSPe_2	-122.445	37.939	0.85	16.59	247.65	17.44	-122.445	37.939	1.03	16.59	247.67	17.62	9	7/14/2014	36:36.3
8288	RSPe_2	-122.445	37.939	0.85	16.60	247.64	17.45	-122.445	37.939	1.06	16.60	247.70	17.66	9	7/14/2014	36:36.2
8289	RSPe_2	-122.445	37.939	0.88	16.57	247.66	17.45	-122.445	37.939	1.06	16.57	247.72	17.63	9	7/14/2014	36:36.1
8290	RSPe_2	-122.445	37.939	0.85	16.61	247.71	17.46	-122.445	37.939	1.03	16.61	247.82	17.64	9	7/14/2014	36:36.0
8291	RSPe_2	-122.445	37.939	0.85	16.65	247.75	17.49	-122.445	37.939	1.06	16.65	247.84	17.71	9	7/14/2014	36:35.9
8292	RSPe_2	-122.445	37.939	0.85	16.65	247.77	17.49	-122.445	37.939	1.06	16.65	247.86	17.71	9	7/14/2014	36:35.8
8293	RSPe_2	-122.445	37.939	0.85	16.67	247.86	17.52	-122.445	37.939	1.06	16.67	248.01	17.73	9	7/14/2014	36:35.7
8294	RSPe_2	-122.445	37.939	0.85	16.69	247.91	17.54	-122.445	37.939	1.06	16.69	248.06	17.75	9	7/14/2014	36:35.6
8295	RSPe_2	-122.445	37.939	0.85	16.77	247.86	17.62	-122.445	37.939	1.06	16.77	248.03	17.83	9	7/14/2014	36:35.5
8296	RSPe_2	-122.445	37.939	0.85	16.80	247.88	17.65	-122.445	37.939	1.06	16.80	248.07	17.86	9	7/14/2014	36:35.4
8297	RSPe_2	-122.445	37.939	0.85	16.77	247.86	17.62	-122.445	37.939	1.06	16.77	248.10	17.83	9	7/14/2014	36:35.3
8298	RSPe_2	-122.445	37.939	0.85	16.79	247.89	17.64	-122.445	37.939	1.11	16.79	248.10	17.90	9	7/14/2014	36:35.2
8299	RSPe_2	-122.445	37.939	0.85	16.64	247.93	17.48	-122.445	37.939	1.11	16.64	248.15	17.75	9	7/14/2014	36:35.1
8300	RSPe_2	-122.445	37.939	0.80	16.63	247.99	17.43	-122.445	37.939	1.11	16.63	248.18	17.74	9	7/14/2014	36:35.0
8301	RSPe_2	-122.445	37.939	0.85	16.65	248.04	17.49	-122.445	37.939	1.06	16.65	248.17	17.71	9	7/14/2014	36:34.9
8302	RSPe_2	-122.445	37.939	0.85	16.57	248.06	17.41	-122.445	37.939	1.06	16.57	248.17	17.63	9	7/14/2014	36:34.8
8303	RSPe_2	-122.445	37.939	0.85	16.58	248.07	17.43	-122.445	37.939	1.11	16.58	248.17	17.69	9	7/14/2014	36:34.7
8304	RSPe_2	-122.445	37.939	0.85	16.48	248.13	17.33	-122.445	37.939	1.14	16.48	248.19	17.63	9	7/14/2014	36:34.6
8305	RSPe_2	-122.445	37.939	0.85	16.51	248.15	17.36	-122.445	37.939	1.11	16.51	248.26	17.62	9	7/14/2014	36:34.5
8306	RSPe_2	-122.445	37.939	0.80	16.48	248.11	17.28	-122.445	37.939	1.11	16.48	248.25	17.59	9	7/14/2014	36:34.4
8307	RSPe_2	-122.445	37.939	0.85	16.41	248.17	17.25	-122.445	37.939	1.11	16.41	248.28	17.52	9	7/14/2014	36:34.3
8308	RSPe_2	-122.445	37.939	0.85	16.43	248.15	17.28	-122.445	37.939	1.11	16.43	248.31	17.54	9	7/14/2014	36:34.2
8309	RSPe_2	-122.445	37.939	0.85	16.32	248.16	17.17	-122.445	37.939	1.14	16.32	248.35	17.46	9	7/14/2014	36:34.1
8310	RSPe_2	-122.445	37.939	0.85	16.23	248.26	17.08	-122.445	37.939	1.11	16.23	248.44	17.34	9	7/14/2014	36:34.0
8311	RSPe_2	-122.445	37.939	0.80	16.30	248.24	17.10	-122.445	37.939	1.14	16.30	248.48	17.44	9	7/14/2014	36:33.9
8312	RSPe_2	-122.445	37.939	0.80	16.30	248.29	17.10	-122.445	37.939	1.11	16.30	248.48	17.41	9	7/14/2014	36:33.8
8313	RSPe_2	-122.445	37.939	0.80	16.18	248.31	16.98	-122.445	37.939	1.11	16.18	248.51	17.29	9	7/14/2014	36:33.7
8314	RSPe_2	-122.445	37.939	0.80	16.17	248.42	16.96	-122.445	37.939	1.11	16.17	248.57	17.28	9	7/14/2014	36:33.6
8315	RSPe_2	-122.445	37.939	0.80	16.23	248.38	17.03	-122.445	37.939	1.14	16.23	248.59	17.38	9	7/14/2014	36:33.5

8316	RSPe_2	-122.445	37.939	0.80	16.13	248.37	16.93	-122.445	37.939	1.11	16.13	248.57	17.24	9	7/14/2014	36:33.4
8317	RSPe_2	-122.445	37.939	0.80	16.03	248.37	16.83	-122.445	37.939	1.11	16.03	248.55	17.15	9	7/14/2014	36:33.3
8318	RSPe_2	-122.445	37.939	0.80	16.14	248.27	16.94	-122.445	37.939	1.11	16.14	248.52	17.25	9	7/14/2014	36:33.2
8319	RSPe_2	-122.445	37.939	0.76	16.00	248.33	16.76	-122.445	37.939	1.14	16.00	248.55	17.14	9	7/14/2014	36:33.1
8320	RSPe_2	-122.445	37.939	0.76	16.03	248.29	16.79	-122.445	37.939	1.11	16.03	248.61	17.14	9	7/14/2014	36:33.0
8321	RSPe_2	-122.445	37.939	0.80	16.00	248.27	16.79	-122.445	37.939	1.11	16.00	248.57	17.11	9	7/14/2014	36:32.9
8322	RSPe_2	-122.445	37.939	0.76	15.99	248.29	16.75	-122.445	37.939	1.11	15.99	248.61	17.10	9	7/14/2014	36:32.8
8323	RSPe_2	-122.445	37.939	0.80	16.08	248.27	16.88	-122.445	37.939	1.11	16.08	248.66	17.19	9	7/14/2014	36:32.7
8324	RSPe_2	-122.445	37.939	0.71	15.90	248.36	16.61	-122.445	37.939	1.06	15.90	248.72	16.96	9	7/14/2014	36:32.6
8325	RSPe_2	-122.445	37.939	0.76	15.95	248.31	16.71	-122.445	37.939	1.06	15.95	248.70	17.01	9	7/14/2014	36:32.5
8326	RSPe_2	-122.445	37.939	0.71	15.99	248.34	16.70	-122.445	37.939	1.03	15.99	248.64	17.01	9	7/14/2014	36:32.4
8327	RSPe_2	-122.445	37.939	0.76	15.96	248.40	16.72	-122.445	37.939	1.03	15.96	248.69	16.98	9	7/14/2014	36:32.3
8328	RSPe_2	-122.445	37.939	0.71	15.93	248.38	16.65	-122.445	37.939	1.03	15.93	248.66	16.96	9	7/14/2014	36:32.2
8329	RSPe_2	-122.445	37.939	0.71	16.05	248.38	16.76	-122.445	37.939	0.98	16.05	248.64	17.03	9	7/14/2014	36:32.1
8330	RSPe_2	-122.445	37.939	0.71	15.94	248.38	16.65	-122.445	37.939	0.98	15.94	248.57	16.91	9	7/14/2014	36:32.0
8331	RSPe_2	-122.445	37.939	0.71	15.90	248.35	16.61	-122.445	37.939	0.98	15.90	248.57	16.88	9	7/14/2014	36:31.9
8332	RSPe_2	-122.445	37.939	0.71	16.02	248.34	16.73	-122.445	37.939	0.98	16.02	248.51	16.99	9	7/14/2014	36:31.8
8333	RSPe_2	-122.445	37.939	0.71	15.93	248.31	16.65	-122.445	37.939	0.98	15.93	248.54	16.91	9	7/14/2014	36:31.7
8334	RSPe_2	-122.445	37.939	0.71	15.80	248.30	16.51	-122.445	37.939	0.94	15.80	248.51	16.74	9	7/14/2014	36:31.6
8335	RSPe_2	-122.445	37.939	0.71	15.80	248.25	16.51	-122.445	37.939	0.98	15.80	248.50	16.78	9	7/14/2014	36:31.5
8336	RSPe_2	-122.445	37.939	0.68	15.77	248.29	16.45	-122.445	37.939	0.98	15.77	248.51	16.75	9	7/14/2014	36:31.4
8337	RSPe_2	-122.445	37.939	0.71	15.79	248.27	16.50	-122.445	37.939	0.98	15.79	248.58	16.76	9	7/14/2014	36:31.3
8338	RSPe_2	-122.445	37.939	0.68	15.86	248.28	16.54	-122.445	37.939	0.98	15.86	248.58	16.84	9	7/14/2014	36:31.2
8339	RSPe_2	-122.445	37.939	0.68	15.80	248.27	16.48	-122.445	37.939	0.98	15.80	248.57	16.78	9	7/14/2014	36:31.1
8340	RSPe_2	-122.445	37.939	0.68	15.82	248.30	16.50	-122.445	37.939	0.94	15.82	248.59	16.76	9	7/14/2014	36:31.0
8341	RSPe_2	-122.445	37.939	0.68	15.82	248.34	16.50	-122.445	37.939	0.98	15.82	248.64	16.79	9	7/14/2014	36:30.9
8342	RSPe_2	-122.445	37.939	0.63	15.81	248.34	16.44	-122.445	37.939	0.94	15.81	248.66	16.75	9	7/14/2014	36:30.8
8343	RSPe_2	-122.445	37.939	0.68	15.86	248.41	16.54	-122.445	37.939	0.94	15.86	248.65	16.80	9	7/14/2014	36:30.7
8344	RSPe_2	-122.445	37.939	0.63	15.86	248.43	16.48	-122.445	37.939	0.89	15.86	248.71	16.75	9	7/14/2014	36:30.6
8345	RSPe_2	-122.445	37.939	0.63	15.92	248.43	16.55	-122.445	37.939	0.89	15.92	248.67	16.81	9	7/14/2014	36:30.5
8346	RSPe_2	-122.445	37.939	0.63	15.89	248.43	16.51	-122.445	37.939	0.86	15.89	248.67	16.74	9	7/14/2014	36:30.4
8347	RSPe_2	-122.445	37.939	0.63	15.92	248.45	16.55	-122.445	37.939	0.94	15.92	248.69	16.86	9	7/14/2014	36:30.3
8348	RSPe_2	-122.445	37.939	0.59	15.90	248.50	16.49	-122.445	37.939	0.89	15.90	248.70	16.79	9	7/14/2014	36:30.2

8349	RSPe_2	-122.445	37.939	0.63	15.88	248.54	16.51	-122.445	37.939	0.86	15.88	248.71	16.74	9	7/14/2014	36:30.1
8350	RSPe_2	-122.445	37.939	0.59	15.97	248.48	16.57	-122.445	37.939	0.86	15.97	248.69	16.83	9	7/14/2014	36:30.0
8351	RSPe_2	-122.445	37.939	0.59	15.98	248.45	16.57	-122.445	37.939	0.89	15.98	248.65	16.87	9	7/14/2014	36:29.9
8352	RSPe_2	-122.445	37.939	0.59	16.03	248.49	16.62	-122.445	37.939	0.89	16.03	248.78	16.92	9	7/14/2014	36:29.8
8353	RSPe_2	-122.445	37.939	0.59	16.00	248.52	16.59	-122.445	37.939	0.89	16.00	248.82	16.89	9	7/14/2014	36:29.7
8354	RSPe_2	-122.445	37.939	0.59	16.00	248.54	16.59	-122.445	37.939	0.86	16.00	248.91	16.85	9	7/14/2014	36:29.6
8355	RSPe_2	-122.445	37.939	0.59	16.03	248.52	16.63	-122.445	37.939	0.86	16.03	248.94	16.89	9	7/14/2014	36:29.5
8356	RSPe_2	-122.445	37.939	0.54	16.05	248.54	16.59	-122.445	37.939	0.82	16.05	248.97	16.88	9	7/14/2014	36:29.4
8357	RSPe_2	-122.445	37.939	0.54	16.09	248.50	16.63	-122.445	37.939	0.82	16.09	248.96	16.91	9	7/14/2014	36:29.3
8358	RSPe_2	-122.445	37.939	0.54	16.07	248.50	16.61	-122.445	37.939	0.77	16.07	248.98	16.85	9	7/14/2014	36:29.2
8359	RSPe_2	-122.445	37.939	0.51	16.11	248.50	16.62	-122.445	37.939	0.77	16.11	248.94	16.88	9	7/14/2014	36:29.1
8360	RSPe_2	-122.445	37.939	0.51	16.22	248.46	16.73	-122.445	37.939	0.74	16.22	248.89	16.96	9	7/14/2014	36:29.0
8361	RSPe_2	-122.445	37.939	0.54	16.20	248.45	16.74	-122.445	37.939	0.74	16.20	248.83	16.93	9	7/14/2014	36:28.9
8362	RSPe_2	-122.445	37.939	0.51	16.16	248.50	16.67	-122.445	37.939	0.74	16.16	248.89	16.90	9	7/14/2014	36:28.8
8363	RSPe_2	-122.445	37.939	0.51	16.23	248.46	16.74	-122.445	37.939	0.74	16.23	248.82	16.97	9	7/14/2014	36:28.7
8364	RSPe_2	-122.445	37.939	0.47	16.20	248.50	16.68	-122.445	37.939	0.74	16.20	248.83	16.94	9	7/14/2014	36:28.6
8365	RSPe_2	-122.445	37.939	0.51	16.18	248.55	16.69	-122.445	37.939	0.74	16.18	248.87	16.92	9	7/14/2014	36:28.5
8366	RSPe_2	-122.445	37.939	0.51	16.18	248.54	16.69	-122.445	37.939	0.74	16.18	248.91	16.92	9	7/14/2014	36:28.4
8367	RSPe_2	-122.445	37.939	0.51	16.23	248.55	16.74	-122.445	37.939	0.74	16.23	248.90	16.97	9	7/14/2014	36:28.3
8368	RSPe_2	-122.445	37.939	0.51	16.28	248.59	16.79	-122.445	37.939	0.74	16.28	248.96	17.02	9	7/14/2014	36:28.2
8369	RSPe_2	-122.445	37.939	0.51	16.25	248.62	16.76	-122.445	37.939	0.74	16.25	248.99	16.99	9	7/14/2014	36:28.1
8370	RSPe_2	-122.445	37.939	0.47	16.25	248.61	16.72	-122.445	37.939	0.69	16.25	248.98	16.94	9	7/14/2014	36:28.0
8371	RSPe_2	-122.445	37.939	0.47	16.33	248.61	16.80	-122.445	37.939	0.74	16.33	249.03	17.07	9	7/14/2014	36:27.9
8372	RSPe_2	-122.445	37.939	0.42	16.34	248.64	16.76	-122.445	37.939	0.65	16.34	249.00	17.00	9	7/14/2014	36:27.8
8373	RSPe_2	-122.445	37.939	0.47	16.32	248.70	16.79	-122.445	37.939	0.65	16.32	249.05	16.97	9	7/14/2014	36:27.7
8374	RSPe_2	-122.445	37.939	0.47	16.33	248.73	16.81	-122.445	37.939	0.65	16.33	249.05	16.99	9	7/14/2014	36:27.6
8375	RSPe_2	-122.445	37.939	0.47	16.37	248.74	16.84	-122.445	37.939	0.65	16.37	248.99	17.02	9	7/14/2014	36:27.5
8376	RSPe_2	-122.445	37.939	0.42	16.35	248.73	16.77	-122.445	37.939	0.65	16.35	249.00	17.01	9	7/14/2014	36:27.4
8377	RSPe_2	-122.445	37.939	0.42	16.37	248.77	16.79	-122.445	37.939	0.65	16.37	248.99	17.03	9	7/14/2014	36:27.3
8378	RSPe_2	-122.445	37.939	0.42	16.35	248.82	16.77	-122.445	37.939	0.60	16.35	249.01	16.95	9	7/14/2014	36:27.2
8379	RSPe_2	-122.445	37.939	0.42	16.35	248.86	16.77	-122.445	37.939	0.65	16.35	249.02	17.01	9	7/14/2014	36:27.1
8380	RSPe_2	-122.445	37.939	0.42	16.37	248.95	16.79	-122.445	37.939	0.60	16.37	249.03	16.98	9	7/14/2014	36:27.0
8381	RSPe_2	-122.445	37.939	0.42	16.38	248.95	16.80	-122.445	37.939	0.60	16.38	249.05	16.99	9	7/14/2014	36:26.9

8382	RSPe_2	-122.445	37.939	0.39	16.40	248.95	16.78	-122.445	37.939	0.60	16.40	249.10	17.00	9	7/14/2014 36:26.8
8383	RSPe_2	-122.445	37.939	0.39	16.41	248.97	16.79	-122.445	37.939	0.60	16.41	249.10	17.01	9	7/14/2014 36:26.7
8384	RSPe_2	-122.445	37.939	0.39	16.44	248.95	16.83	-122.445	37.939	0.57	16.44	249.12	17.01	9	7/14/2014 36:26.6
8385	RSPe_2	-122.445	37.939	0.39	16.48	248.97	16.87	-122.445	37.939	0.60	16.48	249.17	17.09	9	7/14/2014 36:26.5
8386	RSPe_2	-122.445	37.939	0.39	16.45	248.97	16.84	-122.445	37.939	0.57	16.45	249.17	17.02	9	7/14/2014 36:26.4
8387	RSPe_2	-122.445	37.939	0.39	16.53	249.04	16.92	-122.445	37.939	0.60	16.53	249.19	17.14	9	7/14/2014 36:26.3
8388	RSPe_2	-122.445	37.939	0.39	16.49	248.97	16.88	-122.445	37.939	0.60	16.49	249.20	17.09	9	7/14/2014 36:26.2
8389	RSPe_2	-122.445	37.939	0.39	16.52	248.99	16.91	-122.445	37.939	0.60	16.52	249.21	17.13	9	7/14/2014 36:26.1
8390	RSPe_2	-122.445	37.939	0.34	16.56	248.93	16.90	-122.445	37.939	0.57	16.56	249.19	17.13	9	7/14/2014 36:26.0
8391	RSPe_2	-122.445	37.939	0.34	16.58	248.95	16.92	-122.445	37.939	0.60	16.58	249.24	17.19	9	7/14/2014 36:25.9
8392	RSPe_2	-122.445	37.939	0.34	16.67	248.98	17.01	-122.445	37.939	0.57	16.67	249.28	17.24	9	7/14/2014 36:25.8
8393	RSPe_2	-122.445	37.939	0.34	16.67	248.99	17.01	-122.445	37.939	0.57	16.67	249.29	17.24	9	7/14/2014 36:25.7
8394	RSPe_2	-122.445	37.939	0.30	16.67	249.02	16.98	-122.445	37.939	0.52	16.67	249.35	17.19	9	7/14/2014 36:25.6
8395	RSPe_2	-122.445	37.939	0.30	16.78	249.04	17.09	-122.445	37.939	0.52	16.78	249.36	17.30	9	7/14/2014 36:25.5
8396	RSPe_2	-122.445	37.939	0.25	16.84	249.08	17.09	-122.445	37.939	0.49	16.84	249.39	17.33	9	7/14/2014 36:25.4
8397	RSPe_2	-122.445	37.939	0.30	16.81	249.11	17.11	-122.445	37.939	0.49	16.81	249.42	17.29	9	7/14/2014 36:25.3
8398	RSPe_2	-122.445	37.939	0.25	16.89	249.10	17.14	-122.445	37.939	0.49	16.89	249.43	17.38	9	7/14/2014 36:25.2
8399	RSPe_2	-122.445	37.939	0.30	16.87	249.13	17.17	-122.445	37.939	0.45	16.87	249.48	17.32	9	7/14/2014 36:25.1
8400	RSPe_2	-122.445	37.939	0.22	16.88	249.15	17.10	-122.445	37.939	0.40	16.88	249.52	17.28	9	7/14/2014 36:25.0
8401	RSPe_2	-122.445	37.939	0.25	16.92	249.21	17.17	-122.445	37.939	0.45	16.92	249.52	17.37	9	7/14/2014 36:24.9
8402	RSPe_2	-122.445	37.939	0.25	16.98	249.20	17.23	-122.445	37.939	0.40	16.98	249.50	17.38	9	7/14/2014 36:24.8
8403	RSPe_2	-122.445	37.939	0.25	16.96	249.18	17.21	-122.445	37.939	0.40	16.96	249.52	17.36	9	7/14/2014 36:24.7
8404	RSPe_2	-122.445	37.939	0.25	17.04	249.15	17.29	-122.445	37.939	0.37	17.04	249.47	17.40	9	7/14/2014 36:24.6
8405	RSPe_2	-122.445	37.939	0.25	17.05	249.11	17.30	-122.445	37.939	0.40	17.05	249.46	17.45	9	7/14/2014 36:24.5
8406	RSPe_2	-122.445	37.939	0.22	17.06	249.13	17.28	-122.445	37.939	0.37	17.06	249.50	17.43	9	7/14/2014 36:24.4
8407	RSPe_2	-122.445	37.939	0.22	17.11	249.19	17.32	-122.445	37.939	0.37	17.11	249.59	17.47	9	7/14/2014 36:24.3
8408	RSPe_2	-122.445	37.939	0.22	17.12	249.22	17.33	-122.445	37.939	0.32	17.12	249.58	17.43	9	7/14/2014 36:24.2
8409	RSPe_2	-122.445	37.939	0.22	17.15	249.26	17.37	-122.445	37.939	0.32	17.15	249.63	17.47	9	7/14/2014 36:24.1
8410	RSPe_2	-122.445	37.939	0.18	17.22	249.30	17.41	-122.445	37.939	0.32	17.22	249.63	17.54	9	7/14/2014 36:24.0
8411	RSPe_2	-122.445	37.939	0.22	17.29	249.40	17.51	-122.445	37.939	0.32	17.29	249.72	17.61	9	7/14/2014 36:23.9
8412	RSPe_2	-122.445	37.939	0.18	17.30	249.42	17.48	-122.445	37.939	0.28	17.30	249.68	17.58	9	7/14/2014 36:23.8
8413	RSPe_2	-122.445	37.939	0.22	17.28	249.40	17.49	-122.445	37.939	0.28	17.28	249.66	17.56	9	7/14/2014 36:23.7
8414	RSPe_2	-122.445	37.939	0.13	17.28	249.44	17.41	-122.445	37.939	0.23	17.28	249.63	17.51	9	7/14/2014 36:23.6

8415	RSPe_2	-122.445	37.939	0.18	17.29	249.47	17.48	-122.445	37.939	0.23	17.29	249.68	17.52	9	7/14/2014	36:23.5
8416	RSPe_2	-122.445	37.939	0.13	17.32	249.45	17.45	-122.445	37.939	0.23	17.32	249.70	17.55	9	7/14/2014	36:23.4
8417	RSPe_2	-122.445	37.939	0.18	17.31	249.47	17.49	-122.445	37.939	0.23	17.31	249.70	17.54	9	7/14/2014	36:23.3
8418	RSPe_2	-122.445	37.939	0.13	17.26	249.46	17.39	-122.445	37.939	0.20	17.26	249.75	17.46	9	7/14/2014	36:23.2
8419	RSPe_2	-122.445	37.939	0.18	17.27	249.45	17.45	-122.445	37.939	0.23	17.27	249.75	17.50	9	7/14/2014	36:23.1
8420	RSPe_2	-122.445	37.939	0.18	17.25	249.49	17.44	-122.445	37.939	0.20	17.25	249.81	17.45	9	7/14/2014	36:23.0
8421	RSPe_2	-122.445	37.939	0.13	17.28	249.51	17.41	-122.445	37.939	0.20	17.28	249.85	17.48	9	7/14/2014	36:22.9
8422	RSPe_2	-122.445	37.939	0.13	17.25	249.60	17.38	-122.445	37.939	0.20	17.25	249.93	17.44	9	7/14/2014	36:22.8
8423	RSPe_2	-122.445	37.939	0.13	17.28	249.67	17.42	-122.445	37.939	0.20	17.28	249.98	17.48	9	7/14/2014	36:22.7
8424	RSPe_2	-122.445	37.939	0.13	17.28	249.71	17.42	-122.445	37.939	0.16	17.28	250.03	17.45	9	7/14/2014	36:22.6
8425	RSPe_2	-122.445	37.939	0.13	17.28	249.78	17.42	-122.445	37.939	0.16	17.28	250.01	17.45	9	7/14/2014	36:22.5
8426	RSPe_2	-122.445	37.939	0.13	17.26	249.80	17.39	-122.445	37.939	0.16	17.26	250.09	17.42	9	7/14/2014	36:22.4
8427	RSPe_2	-122.445	37.939	0.13	17.25	249.82	17.38	-122.445	37.939	0.16	17.25	250.04	17.41	9	7/14/2014	36:22.3
8428	RSPe_2	-122.445	37.939	0.13	17.28	249.84	17.42	-122.445	37.939	0.16	17.28	250.10	17.45	9	7/14/2014	36:22.2
8429	RSPe_2	-122.445	37.939	0.13	17.28	249.84	17.41	-122.445	37.939	0.11	17.28	250.07	17.39	9	7/14/2014	36:22.1
8430	RSPe_2	-122.445	37.939	0.10	17.21	249.84	17.31	-122.445	37.939	0.11	17.21	250.10	17.32	9	7/14/2014	36:22.0
8431	RSPe_2	-122.445	37.939	0.13	17.25	249.84	17.38	-122.445	37.939	0.11	17.25	250.13	17.37	9	7/14/2014	36:21.9
8432	RSPe_2	-122.445	37.939	0.13	17.19	249.89	17.32	-122.445	37.939	0.11	17.19	250.19	17.30	9	7/14/2014	36:21.8
8433	RSPe_2	-122.445	37.939	0.13	17.18	249.89	17.31	-122.445	37.939	0.11	17.18	250.19	17.29	9	7/14/2014	36:21.7
8434	RSPe_2	-122.445	37.940	0.10	17.18	249.85	17.28	-122.445	37.940	0.11	17.18	250.18	17.29	9	7/14/2014	36:21.6
8435	RSPe_2	-122.445	37.940	0.13	17.18	249.89	17.31	-122.445	37.940	0.16	17.18	250.24	17.34	9	7/14/2014	36:21.5
8436	RSPe_2	-122.445	37.940	0.13	17.18	249.96	17.31	-122.445	37.940	0.16	17.18	250.30	17.35	9	7/14/2014	36:21.4
8437	RSPe_2	-122.445	37.940	0.13	17.19	250.06	17.32	-122.445	37.940	0.20	17.19	250.40	17.39	9	7/14/2014	36:21.3
8438	RSPe_2	-122.445	37.940	0.10	17.21	250.18	17.31	-122.445	37.940	0.11	17.21	250.46	17.32	9	7/14/2014	36:21.2
8439	RSPe_2	-122.445	37.940	0.13	17.23	250.26	17.36	-122.445	37.940	0.11	17.23	250.55	17.34	9	7/14/2014	36:21.1
8440	RSPe_2	-122.445	37.940	0.10	17.22	250.31	17.32	-122.445	37.940	0.11	17.22	250.55	17.34	9	7/14/2014	36:21.0
8441	RSPe_2	-122.445	37.940	0.10	17.24	250.40	17.34	-122.445	37.940	0.11	17.24	250.57	17.35	9	7/14/2014	36:20.9
8442	RSPe_2	-122.445	37.940	0.10	17.26	250.38	17.36	-122.445	37.940	0.08	17.26	250.59	17.34	9	7/14/2014	36:20.8
8443	RSPe_2	-122.445	37.940	0.10	17.25	250.42	17.35	-122.445	37.940	0.08	17.25	250.61	17.33	9	7/14/2014	36:20.7
8444	RSPe_2	-122.445	37.940	0.10	17.24	250.47	17.34	-122.445	37.940	0.08	17.24	250.68	17.32	9	7/14/2014	36:20.6
8445	RSPe_2	-122.445	37.940	0.10	17.22	250.47	17.32	-122.445	37.940	0.08	17.22	250.64	17.30	9	7/14/2014	36:20.5
8446	RSPe_2	-122.445	37.940	0.10	17.25	250.51	17.34	-122.445	37.940	0.08	17.25	250.72	17.32	9	7/14/2014	36:20.4
8447	RSPe_2	-122.445	37.940	0.10	17.28	250.51	17.37	-122.445	37.940	0.08	17.28	250.75	17.36	9	7/14/2014	36:20.3

8448	RSPe_2	-122.445	37.940	0.10	17.23	250.51	17.33	-122.445	37.940	0.08	17.23	250.77	17.31	9	7/14/2014	36:20.2
8449	RSPe_2	-122.445	37.940	0.10	17.24	250.55	17.34	-122.445	37.940	0.08	17.24	250.81	17.32	9	7/14/2014	36:20.1
8450	RSPe_2	-122.445	37.940	0.10	17.19	250.57	17.29	-122.445	37.940	0.11	17.19	250.83	17.30	9	7/14/2014	36:20.0
8451	RSPe_2	-122.445	37.940	0.10	17.21	250.65	17.31	-122.445	37.940	0.08	17.21	250.86	17.29	9	7/14/2014	36:19.9
8452	RSPe_2	-122.445	37.940	0.10	17.18	250.68	17.27	-122.445	37.940	0.08	17.18	250.88	17.26	9	7/14/2014	36:19.8
8453	RSPe_2	-122.445	37.940	0.10	17.18	250.71	17.27	-122.445	37.940	0.08	17.18	250.88	17.26	9	7/14/2014	36:19.7
8454	RSPe_2	-122.445	37.940	0.10	17.19	250.71	17.29	-122.445	37.940	0.08	17.19	250.90	17.27	9	7/14/2014	36:19.6
8455	RSPe_2	-122.445	37.940	0.10	17.18	250.78	17.28	-122.445	37.940	0.08	17.18	250.90	17.26	9	7/14/2014	36:19.5
8456	RSPe_2	-122.445	37.940	0.10	17.22	250.78	17.32	-122.445	37.940	0.03	17.22	250.93	17.25	9	7/14/2014	36:19.4
8457	RSPe_2	-122.445	37.940	0.10	17.18	250.76	17.27	-122.445	37.940	0.08	17.18	250.88	17.26	9	7/14/2014	36:19.3
8458	RSPe_2	-122.445	37.940	0.05	17.21	250.76	17.25	-122.445	37.940	0.03	17.21	250.90	17.24	9	7/14/2014	36:19.2
8459	RSPe_2	-122.445	37.940	0.10	17.18	250.84	17.27	-122.445	37.940	0.03	17.18	250.93	17.21	9	7/14/2014	36:19.1
8460	RSPe_2	-122.445	37.940	0.05	17.18	250.84	17.22	-122.445	37.940	-0.01	17.18	250.97	17.17	9	7/14/2014	36:19.0
8461	RSPe_2	-122.445	37.940	0.10	17.17	250.83	17.27	-122.445	37.940	-0.01	17.17	250.95	17.17	9	7/14/2014	36:18.9
8462	RSPe_2	-122.445	37.940	0.05	17.20	250.87	17.24	-122.445	37.940	-0.01	17.20	250.99	17.19	9	7/14/2014	36:18.8
8463	RSPe_2	-122.445	37.940	0.10	17.21	250.91	17.31	-122.445	37.940	0.03	17.21	251.04	17.24	9	7/14/2014	36:18.7
8464	RSPe_2	-122.445	37.940	0.05	17.18	250.95	17.23	-122.445	37.940	0.03	17.18	251.06	17.21	9	7/14/2014	36:18.6
8465	RSPe_2	-122.445	37.940	0.05	17.19	251.01	17.24	-122.445	37.940	0.08	17.19	251.13	17.27	9	7/14/2014	36:18.5
8466	RSPe_2	-122.445	37.940	0.05	17.17	251.07	17.22	-122.445	37.940	-0.01	17.17	251.13	17.17	9	7/14/2014	36:18.4
8467	RSPe_2	-122.445	37.940	0.10	17.16	251.02	17.26	-122.445	37.940	0.08	17.16	251.09	17.24	9	7/14/2014	36:18.3
8468	RSPe_2	-122.445	37.940	0.05	17.17	251.04	17.22	-122.445	37.940	0.08	17.17	251.13	17.25	9	7/14/2014	36:18.2
8469	RSPe_2	-122.445	37.940	0.10	17.16	251.09	17.26	-122.445	37.940	0.08	17.16	251.20	17.24	9	7/14/2014	36:18.1
8470	RSPe_2	-122.445	37.940	0.05	17.17	251.18	17.22	-122.445	37.940	0.03	17.17	251.29	17.20	9	7/14/2014	36:18.0
8471	RSPe_2	-122.445	37.940	0.10	17.18	251.20	17.28	-122.445	37.940	0.03	17.18	251.35	17.21	9	7/14/2014	36:17.9
8472	RSPe_2	-122.445	37.940	0.05	17.17	251.23	17.22	-122.445	37.940	0.00	17.17	251.38	17.17	9	7/14/2014	36:17.8
8473	RSPe_2	-122.445	37.940	0.10	17.19	251.26	17.29	-122.445	37.940	0.00	17.19	251.37	17.19	9	7/14/2014	36:17.7
8474	RSPe_2	-122.445	37.940	0.05	17.35	251.34	17.39	-122.445	37.940	0.03	17.35	251.51	17.38	9	7/14/2014	36:17.6
8475	RSPe_2	-122.445	37.940	0.05	17.36	251.44	17.41	-122.445	37.940	0.03	17.36	251.60	17.39	9	7/14/2014	36:17.5
8476	RSPe_2	-122.445	37.940	0.05	17.39	251.53	17.44	-122.445	37.940	0.03	17.39	251.64	17.42	9	7/14/2014	36:17.4
8477	RSPe_2	-122.445	37.940	0.05	17.39	251.54	17.44	-122.445	37.940	0.03	17.39	251.65	17.42	9	7/14/2014	36:17.3
8478	RSPe_2	-122.445	37.940	0.05	17.42	251.62	17.47	-122.445	37.940	0.03	17.42	251.68	17.45	9	7/14/2014	36:17.2
8479	RSPe_2	-122.445	37.940	0.05	17.40	251.69	17.45	-122.445	37.940	0.00	17.40	251.77	17.40	9	7/14/2014	36:17.1
8480	RSPe_2	-122.445	37.940	0.05	17.39	251.73	17.44	-122.445	37.940	0.00	17.39	251.75	17.39	9	7/14/2014	36:17.0

8481	RSPe_2	-122.445	37.940	0.05	17.41	251.78	17.45	-122.445	37.940	0.00	17.41	251.81	17.40	9	7/14/2014 36:16.9
8482	RSPe_2	-122.445	37.940	0.01	17.42	251.89	17.44	-122.445	37.940	0.03	17.42	251.91	17.45	9	7/14/2014 36:16.8
8483	RSPe_2	-122.445	37.940	0.05	17.49	251.90	17.53	-122.445	37.940	0.00	17.49	251.97	17.48	9	7/14/2014 36:16.7
8484	RSPe_2	-122.445	37.940	0.01	17.47	251.98	17.48	-122.445	37.940	0.03	17.47	252.08	17.50	9	7/14/2014 36:16.6
8485	RSPe_2	-122.445	37.940	0.05	17.49	252.04	17.53	-122.445	37.940	0.03	17.49	252.18	17.52	9	7/14/2014 36:16.5
8486	RSPe_2	-122.445	37.940	0.01	17.51	252.11	17.53	-122.445	37.940	0.00	17.51	252.27	17.51	9	7/14/2014 36:16.4
8487	RSPe_2	-122.445	37.940	0.05	17.53	252.20	17.58	-122.445	37.940	-0.06	17.53	252.35	17.48	9	7/14/2014 36:16.3
8488	RSPe_2	-122.445	37.940	0.01	17.56	252.21	17.57	-122.445	37.940	-0.06	17.56	252.39	17.50	9	7/14/2014 36:16.2
8489	RSPe_2	-122.445	37.940	0.05	17.57	252.29	17.62	-122.445	37.940	-0.09	17.57	252.42	17.48	9	7/14/2014 36:16.1
8490	RSPe_2	-122.445	37.940	0.01	17.59	252.31	17.61	-122.445	37.940	-0.09	17.59	252.41	17.50	9	7/14/2014 36:16.0
8491	RSPe_2	-122.445	37.940	0.05	17.66	252.33	17.71	-122.445	37.940	-0.06	17.66	252.46	17.61	9	7/14/2014 36:15.9
8492	RSPe_2	-122.445	37.940	0.01	17.66	252.32	17.68	-122.445	37.940	-0.09	17.66	252.50	17.57	9	7/14/2014 36:15.8
8493	RSPe_2	-122.445	37.940	0.01	17.68	252.33	17.69	-122.445	37.940	-0.14	17.68	252.52	17.54	9	7/14/2014 36:15.7
8494	RSPe_2	-122.445	37.940	0.01	17.73	252.46	17.75	-122.445	37.940	-0.14	17.73	252.59	17.59	9	7/14/2014 36:15.6
8495	RSPe_2	-122.445	37.940	0.01	17.76	252.55	17.78	-122.445	37.940	-0.06	17.76	252.72	17.71	9	7/14/2014 36:15.5
8496	RSPe_2	-122.445	37.940	0.01	17.80	252.62	17.82	-122.445	37.940	-0.14	17.80	252.69	17.66	9	7/14/2014 36:15.4
8497	RSPe_2	-122.445	37.940	0.05	17.74	252.68	17.79	-122.445	37.940	-0.09	17.74	252.79	17.65	9	7/14/2014 36:15.3
8498	RSPe_2	-122.445	37.940	0.01	17.75	252.71	17.76	-122.445	37.940	-0.09	17.75	252.79	17.66	9	7/14/2014 36:15.2
8499	RSPe_2	-122.445	37.940	0.01	17.73	252.77	17.75	-122.445	37.940	-0.14	17.73	252.86	17.59	9	7/14/2014 36:15.1
8500	RSPe_2	-122.445	37.940	0.01	17.72	252.75	17.73	-122.445	37.940	-0.09	17.72	252.90	17.63	9	7/14/2014 36:15.0
8501	RSPe_2	-122.445	37.940	0.05	17.71	252.84	17.76	-122.445	37.940	-0.06	17.71	252.95	17.65	9	7/14/2014 36:14.9
8502	RSPe_2	-122.445	37.940	-0.04	17.68	252.90	17.64	-122.445	37.940	-0.09	17.68	252.98	17.59	9	7/14/2014 36:14.8
8503	RSPe_2	-122.445	37.940	0.01	17.68	252.97	17.70	-122.445	37.940	-0.09	17.68	252.97	17.60	9	7/14/2014 36:14.7
8504	RSPe_2	-122.445	37.940	0.01	17.72	253.08	17.74	-122.445	37.940	-0.14	17.72	253.01	17.58	9	7/14/2014 36:14.6
8505	RSPe_2	-122.445	37.940	0.01	17.66	253.17	17.68	-122.445	37.940	-0.14	17.66	253.03	17.52	9	7/14/2014 36:14.5
8506	RSPe_2	-122.445	37.940	0.01	17.68	253.23	17.70	-122.445	37.940	-0.09	17.68	253.08	17.60	9	7/14/2014 36:14.4
8507	RSPe_2	-122.445	37.940	0.01	17.67	253.32	17.68	-122.445	37.940	-0.09	17.67	253.13	17.58	9	7/14/2014 36:14.3
8508	RSPe_2	-122.445	37.940	0.01	17.68	253.37	17.70	-122.445	37.940	-0.14	17.68	253.21	17.54	9	7/14/2014 36:14.2
8509	RSPe_2	-122.445	37.940	0.01	17.65	253.35	17.67	-122.445	37.940	-0.09	17.65	253.24	17.56	9	7/14/2014 36:14.1
8510	RSPe_2	-122.445	37.940	-0.04	17.64	253.30	17.60	-122.445	37.940	-0.09	17.64	253.23	17.55	9	7/14/2014 36:14.0
8511	RSPe_2	-122.445	37.940	0.01	17.72	253.32	17.73	-122.445	37.940	-0.09	17.72	253.30	17.63	9	7/14/2014 36:13.9
8512	RSPe_2	-122.445	37.940	-0.04	17.65	253.30	17.61	-122.445	37.940	-0.14	17.65	253.35	17.51	9	7/14/2014 36:13.8
8513	RSPe_2	-122.445	37.940	0.01	17.67	253.26	17.68	-122.445	37.940	-0.06	17.67	253.37	17.61	9	7/14/2014 36:13.7

8514	RSPe_2	-122.445	37.940	-0.04	17.69	253.35	17.66	-122.445	37.940	-0.09	17.69	253.45	17.61	9	7/14/2014	36:13.6
8515	RSPe_2	-122.445	37.940	0.01	17.75	253.35	17.77	-122.445	37.940	-0.09	17.75	253.48	17.66	9	7/14/2014	36:13.5
8516	RSPe_2	-122.445	37.940	0.01	17.82	253.48	17.83	-122.445	37.940	-0.14	17.82	253.61	17.68	9	7/14/2014	36:13.4
8517	RSPe_2	-122.445	37.940	0.01	17.85	253.61	17.86	-122.445	37.940	-0.09	17.85	253.72	17.76	9	7/14/2014	36:13.3
8518	RSPe_2	-122.445	37.940	-0.04	18.01	253.68	17.97	-122.445	37.940	-0.09	18.01	253.74	17.92	9	7/14/2014	36:13.2
8519	RSPe_2	-122.445	37.940	0.01	18.03	253.69	18.04	-122.445	37.940	-0.14	18.03	253.72	17.89	9	7/14/2014	36:13.1
8520	RSPe_2	-122.445	37.940	0.01	18.06	253.75	18.08	-122.445	37.940	-0.09	18.06	253.84	17.97	9	7/14/2014	36:13.0
8521	RSPe_2	-122.445	37.940	0.01	18.09	253.81	18.10	-122.445	37.940	-0.06	18.09	253.83	18.03	9	7/14/2014	36:12.9
8522	RSPe_2	-122.445	37.940	0.01	18.05	253.81	18.07	-122.445	37.940	-0.09	18.05	253.76	17.97	9	7/14/2014	36:12.8
8523	RSPe_2	-122.445	37.940	0.01	18.05	253.80	18.06	-122.445	37.940	-0.06	18.05	253.77	17.99	9	7/14/2014	36:12.7
8524	RSPe_2	-122.445	37.940	0.01	18.01	253.86	18.02	-122.445	37.940	-0.09	18.01	253.74	17.92	9	7/14/2014	36:12.6
8525	RSPe_2	-122.445	37.940	0.01	18.02	253.88	18.03	-122.445	37.940	-0.09	18.02	253.74	17.93	9	7/14/2014	36:12.5
8526	RSPe_2	-122.445	37.940	-0.04	17.98	253.86	17.94	-122.445	37.940	-0.14	17.98	253.75	17.84	9	7/14/2014	36:12.4
8527	RSPe_2	-122.445	37.940	-0.04	18.03	253.86	17.99	-122.445	37.940	-0.09	18.03	253.76	17.94	9	7/14/2014	36:12.3
8528	RSPe_2	-122.445	37.940	-0.04	17.98	253.90	17.94	-122.445	37.940	-0.14	17.98	253.90	17.84	9	7/14/2014	36:12.2
8529	RSPe_2	-122.445	37.940	-0.04	18.04	253.92	18.00	-122.445	37.940	-0.14	18.04	253.95	17.90	9	7/14/2014	36:12.1
8530	RSPe_2	-122.445	37.940	-0.04	18.03	253.93	17.99	-122.445	37.940	-0.14	18.03	254.08	17.89	9	7/14/2014	36:12.0
8531	RSPe_2	-122.445	37.940	-0.04	18.07	253.95	18.03	-122.445	37.940	-0.09	18.07	254.14	17.98	9	7/14/2014	36:11.9
8532	RSPe_2	-122.445	37.940	-0.04	18.16	253.97	18.12	-122.445	37.940	-0.14	18.16	254.21	18.02	9	7/14/2014	36:11.8
8533	RSPe_2	-122.445	37.940	-0.04	18.10	253.95	18.06	-122.445	37.940	-0.09	18.10	254.26	18.01	9	7/14/2014	36:11.7
8534	RSPe_2	-122.445	37.940	-0.04	18.15	254.00	18.11	-122.445	37.940	-0.14	18.15	254.31	18.01	9	7/14/2014	36:11.6
8535	RSPe_2	-122.445	37.940	-0.04	18.22	253.97	18.18	-122.445	37.940	-0.14	18.22	254.34	18.08	9	7/14/2014	36:11.5
8536	RSPe_2	-122.445	37.940	-0.04	18.19	254.06	18.15	-122.445	37.940	-0.14	18.19	254.40	18.05	9	7/14/2014	36:11.4
8537	RSPe_2	-122.445	37.940	-0.04	18.20	254.11	18.16	-122.445	37.940	-0.14	18.20	254.39	18.06	9	7/14/2014	36:11.3
8538	RSPe_2	-122.445	37.940	-0.04	18.18	254.12	18.14	-122.445	37.940	-0.17	18.18	254.35	18.00	9	7/14/2014	36:11.2
8539	RSPe_2	-122.445	37.940	-0.04	18.17	254.19	18.13	-122.445	37.940	-0.17	18.17	254.30	17.99	9	7/14/2014	36:11.1
8540	RSPe_2	-122.445	37.940	-0.04	18.18	254.24	18.14	-122.445	37.940	-0.21	18.18	254.28	17.97	9	7/14/2014	36:11.0
8541	RSPe_2	-122.445	37.940	-0.04	18.09	254.28	18.05	-122.445	37.940	-0.17	18.09	254.24	17.91	9	7/14/2014	36:10.9
8542	RSPe_2	-122.445	37.940	-0.07	18.07	254.33	18.00	-122.445	37.940	-0.21	18.07	254.17	17.86	9	7/14/2014	36:10.8
8543	RSPe_2	-122.445	37.940	-0.04	18.13	254.35	18.09	-122.445	37.940	-0.14	18.13	254.17	17.99	9	7/14/2014	36:10.7
8544	RSPe_2	-122.445	37.940	-0.07	17.98	254.44	17.91	-122.445	37.940	-0.21	17.98	254.28	17.77	9	7/14/2014	36:10.6
8545	RSPe_2	-122.445	37.940	-0.04	18.00	254.48	17.96	-122.445	37.940	-0.17	18.00	254.33	17.83	9	7/14/2014	36:10.5
8546	RSPe_2	-122.445	37.940	-0.07	18.00	254.44	17.93	-122.445	37.940	-0.17	18.00	254.32	17.83	9	7/14/2014	36:10.4

8547	RSPe_2	-122.445	37.940	-0.04	17.91	254.35	17.87	-122.445	37.940	-0.17	17.91	254.30	17.74	9	7/14/2014	36:10.3
8548	RSPe_2	-122.445	37.940	-0.07	17.89	254.37	17.82	-122.445	37.940	-0.17	17.89	254.41	17.72	9	7/14/2014	36:10.2
8549	RSPe_2	-122.445	37.940	-0.04	17.85	254.37	17.81	-122.445	37.940	-0.09	17.85	254.41	17.76	9	7/14/2014	36:10.1
8550	RSPe_2	-122.445	37.940	-0.07	17.79	254.52	17.71	-122.445	37.940	-0.17	17.79	254.51	17.61	9	7/14/2014	36:10.0
8551	RSPe_2	-122.445	37.940	-0.07	17.79	254.58	17.71	-122.445	37.940	-0.17	17.79	254.57	17.61	9	7/14/2014	36:09.9
8552	RSPe_2	-122.445	37.940	-0.07	17.84	254.68	17.77	-122.445	37.940	-0.21	17.84	254.66	17.63	9	7/14/2014	36:09.8
8553	RSPe_2	-122.445	37.940	-0.07	17.75	254.71	17.67	-122.445	37.940	-0.21	17.75	254.68	17.54	9	7/14/2014	36:09.7
8554	RSPe_2	-122.445	37.940	-0.07	17.80	254.73	17.73	-122.445	37.940	-0.21	17.80	254.70	17.60	9	7/14/2014	36:09.6
8555	RSPe_2	-122.445	37.940	-0.04	17.78	254.72	17.74	-122.445	37.940	-0.21	17.78	254.73	17.57	9	7/14/2014	36:09.5
8556	RSPe_2	-122.445	37.940	-0.07	17.72	254.77	17.64	-122.445	37.940	-0.21	17.72	254.76	17.51	9	7/14/2014	36:09.4
8557	RSPe_2	-122.445	37.940	-0.07	17.68	254.85	17.60	-122.445	37.940	-0.26	17.68	254.87	17.42	9	7/14/2014	36:09.3
8558	RSPe_2	-122.445	37.940	-0.07	17.53	254.82	17.46	-122.445	37.940	-0.26	17.53	254.85	17.27	9	7/14/2014	36:09.2
8559	RSPe_2	-122.445	37.940	-0.07	17.52	254.79	17.45	-122.445	37.940	-0.21	17.52	254.92	17.32	9	7/14/2014	36:09.1
8560	RSPe_2	-122.445	37.940	-0.07	17.61	254.81	17.54	-122.445	37.940	-0.21	17.61	254.97	17.41	9	7/14/2014	36:09.0
8561	RSPe_2	-122.445	37.940	-0.07	17.61	254.84	17.54	-122.445	37.940	-0.21	17.61	254.99	17.40	9	7/14/2014	36:08.9
8562	RSPe_2	-122.445	37.940	-0.07	17.67	254.90	17.60	-122.445	37.940	-0.26	17.67	255.03	17.41	9	7/14/2014	36:08.8
8563	RSPe_2	-122.445	37.940	-0.07	17.70	254.95	17.63	-122.445	37.940	-0.21	17.70	255.08	17.49	9	7/14/2014	36:08.7
8564	RSPe_2	-122.445	37.940	-0.12	17.69	255.00	17.57	-122.445	37.940	-0.21	17.69	255.10	17.49	9	7/14/2014	36:08.6
8565	RSPe_2	-122.445	37.940	-0.07	17.65	255.04	17.57	-122.445	37.940	-0.17	17.65	255.10	17.47	9	7/14/2014	36:08.5
8566	RSPe_2	-122.445	37.940	-0.07	17.70	255.04	17.63	-122.445	37.940	-0.26	17.70	255.13	17.44	9	7/14/2014	36:08.4
8567	RSPe_2	-122.445	37.940	-0.07	17.73	255.04	17.66	-122.445	37.940	-0.21	17.73	255.12	17.53	9	7/14/2014	36:08.3
8568	RSPe_2	-122.445	37.940	-0.07	17.70	255.08	17.63	-122.445	37.940	-0.21	17.70	255.10	17.49	9	7/14/2014	36:08.2
8569	RSPe_2	-122.445	37.940	-0.07	17.82	255.15	17.75	-122.445	37.940	-0.26	17.82	255.06	17.57	9	7/14/2014	36:08.1
8570	RSPe_2	-122.445	37.940	-0.07	17.78	255.19	17.70	-122.445	37.940	-0.26	17.78	255.10	17.52	9	7/14/2014	36:08.0
8571	RSPe_2	-122.445	37.940	-0.07	17.82	255.28	17.75	-122.445	37.940	-0.26	17.82	255.17	17.57	9	7/14/2014	36:07.9
8572	RSPe_2	-122.445	37.940	-0.07	17.84	255.26	17.77	-122.445	37.940	-0.26	17.84	255.13	17.58	9	7/14/2014	36:07.8
8573	RSPe_2	-122.445	37.940	-0.07	17.88	255.29	17.80	-122.445	37.940	-0.21	17.88	255.14	17.67	9	7/14/2014	36:07.7
8574	RSPe_2	-122.445	37.940	-0.07	17.89	255.35	17.81	-122.445	37.940	-0.21	17.89	255.28	17.68	9	7/14/2014	36:07.6
8575	RSPe_2	-122.445	37.940	-0.07	17.92	255.37	17.84	-122.445	37.940	-0.26	17.92	255.41	17.66	9	7/14/2014	36:07.5
8576	RSPe_2	-122.445	37.940	-0.07	17.92	255.48	17.85	-122.445	37.940	-0.21	17.92	255.45	17.72	9	7/14/2014	36:07.4
8577	RSPe_2	-122.445	37.940	-0.07	17.94	255.57	17.87	-122.445	37.940	-0.21	17.94	255.57	17.73	9	7/14/2014	36:07.3
8578	RSPe_2	-122.445	37.940	-0.07	17.96	255.62	17.88	-122.445	37.940	-0.26	17.96	255.63	17.70	9	7/14/2014	36:07.2
8579	RSPe_2	-122.445	37.940	-0.07	17.98	255.62	17.91	-122.445	37.940	-0.21	17.98	255.48	17.77	9	7/14/2014	36:07.1

8580	RSPe_2	-122.445	37.940	-0.12	17.96	255.59	17.84	-122.445	37.940	-0.26	17.96	255.47	17.70	9	7/14/2014 36:07.0
8581	RSPe_2	-122.445	37.940	-0.07	17.96	255.66	17.88	-122.445	37.940	-0.29	17.96	255.50	17.66	9	7/14/2014 36:06.9
8582	RSPe_2	-122.445	37.940	-0.07	17.99	255.68	17.91	-122.445	37.940	-0.29	17.99	255.57	17.69	9	7/14/2014 36:06.8
8583	RSPe_2	-122.445	37.940	-0.12	17.99	255.62	17.87	-122.445	37.940	-0.26	17.99	255.55	17.74	9	7/14/2014 36:06.7
8584	RSPe_2	-122.445	37.940	-0.12	17.99	255.57	17.86	-122.445	37.940	-0.29	17.99	255.48	17.69	9	7/14/2014 36:06.6
8585	RSPe_2	-122.445	37.940	-0.07	18.06	255.57	17.99	-122.445	37.940	-0.29	18.06	255.64	17.77	9	7/14/2014 36:06.5
8586	RSPe_2	-122.445	37.940	-0.12	18.12	255.66	17.99	-122.445	37.940	-0.29	18.12	255.76	17.83	9	7/14/2014 36:06.4
8587	RSPe_2	-122.445	37.940	-0.07	18.17	255.55	18.10	-122.445	37.940	-0.29	18.17	255.69	17.88	9	7/14/2014 36:06.3
8588	RSPe_2	-122.445	37.940	-0.12	18.19	255.71	18.07	-122.445	37.940	-0.26	18.19	255.85	17.94	9	7/14/2014 36:06.2
8589	RSPe_2	-122.445	37.940	-0.07	18.20	255.75	18.13	-122.445	37.940	-0.29	18.20	255.77	17.91	9	7/14/2014 36:06.1
8590	RSPe_2	-122.445	37.940	-0.12	18.18	255.79	18.05	-122.445	37.940	-0.29	18.18	255.81	17.89	9	7/14/2014 36:06.0
8591	RSPe_2	-122.445	37.940	-0.07	18.30	255.90	18.23	-122.445	37.940	-0.26	18.30	255.79	18.05	9	7/14/2014 36:05.9
8592	RSPe_2	-122.445	37.940	-0.12	18.22	255.97	18.10	-122.445	37.940	-0.29	18.22	255.79	17.93	9	7/14/2014 36:05.8
8593	RSPe_2	-122.445	37.940	-0.12	18.24	256.02	18.12	-122.445	37.940	-0.29	18.24	255.84	17.95	9	7/14/2014 36:05.7
8594	RSPe_2	-122.445	37.940	-0.12	18.38	256.00	18.26	-122.445	37.940	-0.34	18.38	255.88	18.04	9	7/14/2014 36:05.6
8595	RSPe_2	-122.445	37.940	-0.07	18.24	256.02	18.17	-122.445	37.940	-0.34	18.24	255.93	17.90	9	7/14/2014 36:05.5
8596	RSPe_2	-122.445	37.940	-0.12	18.28	256.06	18.16	-122.445	37.940	-0.29	18.28	255.99	17.99	9	7/14/2014 36:05.4
8597	RSPe_2	-122.445	37.940	-0.12	18.26	256.11	18.14	-122.445	37.940	-0.29	18.26	256.06	17.97	9	7/14/2014 36:05.3
8598	RSPe_2	-122.445	37.940	-0.12	18.19	256.15	18.07	-122.445	37.940	-0.29	18.19	256.12	17.90	9	7/14/2014 36:05.2
8599	RSPe_2	-122.445	37.940	-0.07	18.17	256.11	18.10	-122.445	37.940	-0.29	18.17	256.09	17.88	9	7/14/2014 36:05.1
8600	RSPe_2	-122.445	37.940	-0.12	18.11	256.08	17.98	-122.445	37.940	-0.29	18.11	256.12	17.82	9	7/14/2014 36:05.0
8601	RSPe_2	-122.445	37.940	-0.07	18.20	256.19	18.13	-122.445	37.940	-0.29	18.20	256.26	17.91	9	7/14/2014 36:04.9
8602	RSPe_2	-122.445	37.940	-0.12	18.15	256.17	18.03	-122.445	37.940	-0.21	18.15	256.30	17.94	9	7/14/2014 36:04.8
8603	RSPe_2	-122.445	37.940	-0.07	18.11	256.22	18.04	-122.445	37.940	-0.21	18.11	256.35	17.90	9	7/14/2014 36:04.7
8604	RSPe_2	-122.445	37.940	-0.07	18.04	256.31	17.97	-122.445	37.940	-0.29	18.04	256.44	17.75	9	7/14/2014 36:04.6
8605	RSPe_2	-122.445	37.940	-0.07	17.99	256.36	17.92	-122.445	37.940	-0.34	17.99	256.43	17.65	9	7/14/2014 36:04.5
8606	RSPe_2	-122.445	37.940	-0.12	17.96	256.29	17.83	-122.445	37.940	-0.34	17.96	256.41	17.61	9	7/14/2014 36:04.4
8607	RSPe_2	-122.445	37.940	-0.07	17.98	256.29	17.91	-122.445	37.940	-0.26	17.98	256.45	17.72	9	7/14/2014 36:04.3
8608	RSPe_2	-122.445	37.940	-0.07	17.93	256.35	17.86	-122.445	37.940	-0.26	17.93	256.46	17.67	9	7/14/2014 36:04.2
8609	RSPe_2	-122.445	37.940	-0.07	17.90	256.37	17.83	-122.445	37.940	-0.21	17.90	256.53	17.70	9	7/14/2014 36:04.1
8610	RSPe_2	-122.445	37.940	-0.07	17.90	256.35	17.83	-122.445	37.940	-0.38	17.90	256.56	17.53	9	7/14/2014 36:04.0
8611	RSPe_2	-122.445	37.940	-0.04	17.86	256.35	17.82	-122.445	37.940	-0.29	17.86	256.52	17.57	9	7/14/2014 36:03.9
8612	RSPe_2	-122.445	37.940	-0.07	17.90	256.40	17.83	-122.445	37.940	-0.29	17.90	256.57	17.61	9	7/14/2014 36:03.8

8613	RSPe_2	-122.445	37.940	-0.07	17.83	256.49	17.76	-122.445	37.940	-0.34	17.83	256.62	17.49	9	7/14/2014	36:03.7
8614	RSPe_2	-122.445	37.940	-0.07	17.84	256.58	17.77	-122.445	37.940	-0.29	17.84	256.61	17.55	9	7/14/2014	36:03.6
8615	RSPe_2	-122.445	37.940	-0.07	17.81	256.73	17.74	-122.445	37.940	-0.29	17.81	256.63	17.52	9	7/14/2014	36:03.5
8616	RSPe_2	-122.445	37.940	-0.07	17.77	256.84	17.70	-122.445	37.940	-0.29	17.77	256.64	17.48	9	7/14/2014	36:03.4
8617	RSPe_2	-122.445	37.940	-0.07	17.82	256.91	17.74	-122.445	37.940	-0.26	17.82	256.64	17.56	9	7/14/2014	36:03.3
8618	RSPe_2	-122.445	37.940	-0.07	17.72	256.97	17.65	-122.445	37.940	-0.29	17.72	256.61	17.43	9	7/14/2014	36:03.2
8619	RSPe_2	-122.445	37.940	-0.07	17.65	257.04	17.58	-122.445	37.940	-0.26	17.65	256.70	17.40	9	7/14/2014	36:03.1
8620	RSPe_2	-122.445	37.940	-0.07	17.73	257.01	17.66	-122.445	37.940	-0.29	17.73	256.61	17.44	9	7/14/2014	36:03.0
8621	RSPe_2	-122.445	37.940	-0.04	17.63	256.99	17.59	-122.445	37.940	-0.26	17.63	256.64	17.37	9	7/14/2014	36:02.9
8622	RSPe_2	-122.445	37.940	-0.07	17.67	257.06	17.60	-122.445	37.940	-0.29	17.67	256.77	17.38	9	7/14/2014	36:02.8
8623	RSPe_2	-122.445	37.940	-0.04	17.64	257.14	17.60	-122.445	37.940	-0.26	17.64	256.93	17.38	9	7/14/2014	36:02.7
8624	RSPe_2	-122.445	37.940	-0.07	17.65	257.19	17.58	-122.445	37.940	-0.21	17.65	257.01	17.45	9	7/14/2014	36:02.6
8625	RSPe_2	-122.445	37.940	-0.07	17.71	257.09	17.64	-122.445	37.940	-0.17	17.71	257.03	17.54	9	7/14/2014	36:02.5
8626	RSPe_2	-122.445	37.940	-0.07	17.67	257.08	17.60	-122.445	37.940	-0.26	17.67	257.08	17.41	9	7/14/2014	36:02.4
8627	RSPe_2	-122.445	37.940	-0.04	17.75	257.07	17.72	-122.445	37.940	-0.26	17.75	257.15	17.50	9	7/14/2014	36:02.3
8628	RSPe_2	-122.445	37.940	-0.07	17.74	257.08	17.67	-122.445	37.940	-0.26	17.74	257.28	17.48	9	7/14/2014	36:02.2
8629	RSPe_2	-122.445	37.940	-0.04	17.80	257.04	17.76	-122.445	37.940	-0.21	17.80	257.35	17.60	9	7/14/2014	36:02.1
8630	RSPe_2	-122.445	37.940	-0.04	17.79	257.02	17.75	-122.445	37.940	-0.26	17.79	257.40	17.53	9	7/14/2014	36:02.0
8631	RSPe_2	-122.445	37.940	-0.04	17.97	257.06	17.93	-122.445	37.940	-0.26	17.97	257.41	17.72	9	7/14/2014	36:01.9
8632	RSPe_2	-122.445	37.940	-0.04	17.92	257.06	17.89	-122.445	37.940	-0.26	17.92	257.43	17.67	9	7/14/2014	36:01.8
8633	RSPe_2	-122.445	37.940	-0.04	17.99	257.07	17.95	-122.445	37.940	-0.26	17.99	257.35	17.73	9	7/14/2014	36:01.7
8634	RSPe_2	-122.445	37.940	-0.04	18.00	257.06	17.96	-122.445	37.940	-0.21	18.00	257.35	17.79	9	7/14/2014	36:01.6
8635	RSPe_2	-122.445	37.940	-0.04	18.06	257.16	18.03	-122.445	37.940	-0.21	18.06	257.35	17.86	9	7/14/2014	36:01.5
8636	RSPe_2	-122.445	37.940	-0.04	18.05	257.13	18.02	-122.445	37.940	-0.29	18.05	257.26	17.76	9	7/14/2014	36:01.4
8637	RSPe_2	-122.445	37.940	-0.04	18.09	257.17	18.05	-122.445	37.940	-0.21	18.09	257.17	17.88	9	7/14/2014	36:01.3
8638	RSPe_2	-122.445	37.940	-0.04	18.08	257.20	18.04	-122.445	37.940	-0.21	18.08	257.21	17.87	9	7/14/2014	36:01.2
8639	RSPe_2	-122.445	37.940	-0.04	18.05	257.37	18.01	-122.445	37.940	-0.26	18.05	257.23	17.79	9	7/14/2014	36:01.1
8640	RSPe_2	-122.445	37.940	-0.04	18.05	257.42	18.01	-122.445	37.940	-0.21	18.05	257.29	17.84	9	7/14/2014	36:01.0
8641	RSPe_2	-122.445	37.940	-0.04	18.08	257.42	18.04	-122.445	37.940	-0.17	18.08	257.30	17.91	9	7/14/2014	36:00.9
8642	RSPe_2	-122.445	37.940	0.01	18.08	257.49	18.09	-122.445	37.940	-0.26	18.08	257.35	17.82	9	7/14/2014	36:00.8
8643	RSPe_2	-122.445	37.940	0.01	18.08	257.52	18.09	-122.445	37.940	-0.26	18.08	257.44	17.82	9	7/14/2014	36:00.7
8644	RSPe_2	-122.445	37.940	0.01	18.07	257.51	18.08	-122.445	37.940	-0.17	18.07	257.49	17.90	9	7/14/2014	36:00.6
8645	RSPe_2	-122.445	37.940	0.01	18.07	257.58	18.08	-122.445	37.940	-0.17	18.07	257.55	17.90	9	7/14/2014	36:00.5

8646	RSPe_2	-122.445	37.940	-0.04	18.08	257.62	18.04	-122.445	37.940	-0.26	18.08	257.53	17.82	9	7/14/2014	36:00.4
8647	RSPe_2	-122.445	37.940	0.01	18.08	257.62	18.09	-122.445	37.940	-0.21	18.08	257.52	17.87	9	7/14/2014	36:00.3
8648	RSPe_2	-122.445	37.940	-0.04	18.07	257.64	18.03	-122.445	37.940	-0.26	18.07	257.55	17.81	9	7/14/2014	36:00.2
8649	RSPe_2	-122.445	37.940	0.01	18.06	257.62	18.08	-122.445	37.940	-0.21	18.06	257.50	17.86	9	7/14/2014	36:00.1
8650	RSPe_2	-122.445	37.940	0.01	18.09	257.66	18.10	-122.445	37.940	-0.26	18.09	257.51	17.83	9	7/14/2014	36:00.0
8651	RSPe_2	-122.445	37.940	0.01	18.05	257.71	18.07	-122.445	37.940	-0.17	18.05	257.58	17.88	9	7/14/2014	35:59.9
8652	RSPe_2	-122.445	37.940	-0.04	18.06	257.75	18.03	-122.445	37.940	-0.17	18.06	257.60	17.89	9	7/14/2014	35:59.8
8653	RSPe_2	-122.445	37.940	0.01	17.99	257.82	18.00	-122.445	37.940	-0.17	17.99	257.64	17.81	9	7/14/2014	35:59.7
8654	RSPe_2	-122.445	37.940	0.01	17.98	257.82	17.99	-122.445	37.940	-0.17	17.98	257.66	17.81	9	7/14/2014	35:59.6
8655	RSPe_2	-122.445	37.940	0.01	17.94	257.87	17.95	-122.445	37.940	-0.14	17.94	257.69	17.80	9	7/14/2014	35:59.5
8656	RSPe_2	-122.445	37.940	-0.04	17.75	257.88	17.71	-122.445	37.940	-0.09	17.75	257.78	17.66	9	7/14/2014	35:59.4
8657	RSPe_2	-122.445	37.940	0.01	17.86	257.89	17.88	-122.445	37.940	-0.14	17.86	257.84	17.72	9	7/14/2014	35:59.3
8658	RSPe_2	-122.445	37.940	0.01	17.79	257.88	17.80	-122.445	37.940	-0.09	17.79	257.86	17.70	9	7/14/2014	35:59.2
8659	RSPe_2	-122.445	37.940	0.01	17.61	257.89	17.62	-122.445	37.940	-0.09	17.61	257.86	17.52	9	7/14/2014	35:59.1
8660	RSPe_2	-122.445	37.940	-0.04	17.63	257.91	17.59	-122.445	37.940	-0.14	17.63	257.88	17.49	9	7/14/2014	35:59.0
8661	RSPe_2	-122.445	37.940	0.01	17.57	257.88	17.58	-122.445	37.940	-0.14	17.57	257.91	17.43	9	7/14/2014	35:58.9
8662	RSPe_2	-122.445	37.940	0.01	17.58	257.93	17.60	-122.445	37.940	-0.05	17.58	257.92	17.53	9	7/14/2014	35:58.8
8663	RSPe_2	-122.445	37.940	0.01	17.44	257.93	17.45	-122.445	37.940	-0.05	17.44	257.95	17.38	9	7/14/2014	35:58.7
8664	RSPe_2	-122.445	37.940	0.01	17.34	258.00	17.35	-122.445	37.940	-0.09	17.34	258.06	17.25	9	7/14/2014	35:58.6
8665	RSPe_2	-122.445	37.940	0.01	17.34	258.00	17.35	-122.445	37.940	-0.09	17.34	258.02	17.25	9	7/14/2014	35:58.5
8666	RSPe_2	-122.445	37.940	0.01	17.25	258.05	17.27	-122.445	37.940	-0.05	17.25	258.08	17.20	9	7/14/2014	35:58.4
8667	RSPe_2	-122.445	37.940	0.05	17.23	258.07	17.28	-122.445	37.940	-0.05	17.23	258.09	17.18	9	7/14/2014	35:58.3
8668	RSPe_2	-122.445	37.940	0.01	17.31	258.04	17.32	-122.445	37.940	-0.09	17.31	258.10	17.22	9	7/14/2014	35:58.2
8669	RSPe_2	-122.445	37.940	0.05	17.21	258.09	17.26	-122.445	37.940	-0.05	17.21	258.09	17.16	9	7/14/2014	35:58.1
8670	RSPe_2	-122.445	37.940	0.01	17.19	258.09	17.21	-122.445	37.940	-0.05	17.19	258.09	17.14	9	7/14/2014	35:58.0
8671	RSPe_2	-122.445	37.940	0.05	17.22	258.05	17.27	-122.445	37.940	0.00	17.22	258.06	17.22	9	7/14/2014	35:57.9
8672	RSPe_2	-122.445	37.940	0.01	17.17	258.15	17.18	-122.445	37.940	-0.09	17.17	258.11	17.08	9	7/14/2014	35:57.8
8673	RSPe_2	-122.445	37.940	0.01	17.16	258.11	17.17	-122.445	37.940	-0.05	17.16	258.09	17.11	9	7/14/2014	35:57.7
8674	RSPe_2	-122.445	37.940	0.01	17.19	258.05	17.21	-122.445	37.940	-0.05	17.19	258.04	17.14	9	7/14/2014	35:57.6
8675	RSPe_2	-122.445	37.940	0.05	17.18	258.12	17.22	-122.445	37.940	0.00	17.18	258.13	17.17	9	7/14/2014	35:57.5
8676	RSPe_2	-122.445	37.940	0.01	17.18	258.09	17.20	-122.445	37.940	-0.09	17.18	258.11	17.09	9	7/14/2014	35:57.4
8677	RSPe_2	-122.445	37.940	0.01	17.33	258.12	17.34	-122.445	37.940	-0.05	17.33	258.14	17.28	9	7/14/2014	35:57.3
8678	RSPe_2	-122.445	37.940	0.01	17.23	258.18	17.24	-122.445	37.940	-0.05	17.23	258.20	17.18	9	7/14/2014	35:57.2

8679	RSPe_2	-122.445	37.940	0.01	17.28	258.20	17.29	-122.445	37.940	-0.09	17.28	258.25	17.19	9	7/14/2014	35:57.1
8680	RSPe_2	-122.445	37.940	0.01	17.28	258.24	17.30	-122.445	37.940	-0.14	17.28	258.27	17.14	9	7/14/2014	35:57.0
8681	RSPe_2	-122.445	37.940	0.05	17.34	258.27	17.38	-122.445	37.940	-0.05	17.34	258.33	17.28	9	7/14/2014	35:56.9
8682	RSPe_2	-122.445	37.940	0.01	17.33	258.38	17.34	-122.445	37.940	0.00	17.33	258.36	17.33	9	7/14/2014	35:56.8
8683	RSPe_2	-122.445	37.940	0.05	17.35	258.47	17.40	-122.445	37.940	-0.05	17.35	258.42	17.30	9	7/14/2014	35:56.7
8684	RSPe_2	-122.445	37.940	0.01	17.44	258.54	17.45	-122.445	37.940	-0.09	17.44	258.42	17.35	9	7/14/2014	35:56.6
8685	RSPe_2	-122.445	37.940	0.01	17.45	258.51	17.46	-122.445	37.940	-0.09	17.45	258.41	17.36	9	7/14/2014	35:56.5
8686	RSPe_2	-122.445	37.940	0.01	17.45	258.54	17.46	-122.445	37.940	-0.05	17.45	258.40	17.39	9	7/14/2014	35:56.4
8687	RSPe_2	-122.445	37.940	0.01	17.42	258.47	17.44	-122.445	37.940	0.00	17.42	258.45	17.42	9	7/14/2014	35:56.3
8688	RSPe_2	-122.445	37.940	0.01	17.45	258.49	17.46	-122.445	37.940	-0.05	17.45	258.51	17.39	9	7/14/2014	35:56.2
8689	RSPe_2	-122.445	37.940	0.01	17.45	258.43	17.46	-122.445	37.940	-0.05	17.45	258.53	17.39	9	7/14/2014	35:56.1
8690	RSPe_2	-122.445	37.940	0.01	17.44	258.43	17.45	-122.445	37.940	0.00	17.44	258.56	17.43	9	7/14/2014	35:56.0
8691	RSPe_2	-122.445	37.940	0.05	17.46	258.45	17.51	-122.445	37.940	0.00	17.46	258.62	17.46	9	7/14/2014	35:55.9
8692	RSPe_2	-122.445	37.940	0.01	17.49	258.49	17.51	-122.445	37.940	-0.05	17.49	258.68	17.44	9	7/14/2014	35:55.8
8693	RSPe_2	-122.445	37.940	0.05	17.58	258.58	17.63	-122.445	37.940	-0.05	17.58	258.72	17.53	9	7/14/2014	35:55.7
8694	RSPe_2	-122.445	37.940	0.01	17.51	258.54	17.53	-122.445	37.940	0.00	17.51	258.67	17.51	9	7/14/2014	35:55.6
8695	RSPe_2	-122.445	37.940	0.01	17.55	258.59	17.56	-122.445	37.940	-0.05	17.55	258.63	17.49	9	7/14/2014	35:55.5
8696	RSPe_2	-122.445	37.940	0.01	17.57	258.70	17.58	-122.445	37.940	-0.05	17.57	258.69	17.52	9	7/14/2014	35:55.4
8697	RSPe_2	-122.445	37.940	0.05	17.60	258.76	17.65	-122.445	37.940	0.00	17.60	258.69	17.60	9	7/14/2014	35:55.3
8698	RSPe_2	-122.445	37.940	0.01	17.70	258.76	17.72	-122.445	37.940	-0.09	17.70	258.74	17.61	9	7/14/2014	35:55.2
8699	RSPe_2	-122.445	37.940	0.01	17.65	258.81	17.67	-122.445	37.940	0.00	17.65	258.78	17.65	9	7/14/2014	35:55.1
8700	RSPe_2	-122.445	37.940	0.01	17.65	258.83	17.67	-122.445	37.940	0.00	17.65	258.80	17.65	9	7/14/2014	35:55.0
8701	RSPe_2	-122.445	37.940	0.05	17.68	258.78	17.73	-122.445	37.940	0.00	17.68	258.85	17.67	9	7/14/2014	35:54.9
8702	RSPe_2	-122.445	37.940	0.01	17.75	258.72	17.76	-122.445	37.940	0.00	17.75	258.86	17.74	9	7/14/2014	35:54.8
8703	RSPe_2	-122.445	37.940	0.05	17.78	258.72	17.82	-122.445	37.940	0.03	17.78	258.87	17.81	9	7/14/2014	35:54.7
8704	RSPe_2	-122.445	37.940	0.05	17.79	258.72	17.84	-122.445	37.940	0.00	17.79	258.96	17.79	9	7/14/2014	35:54.6
8705	RSPe_2	-122.445	37.940	0.05	17.77	258.79	17.82	-122.445	37.940	0.00	17.77	258.98	17.77	9	7/14/2014	35:54.5
8706	RSPe_2	-122.445	37.940	0.05	17.75	258.74	17.80	-122.445	37.940	-0.05	17.75	258.94	17.70	9	7/14/2014	35:54.4
8707	RSPe_2	-122.445	37.940	0.05	17.69	258.79	17.74	-122.445	37.940	0.00	17.69	258.96	17.69	9	7/14/2014	35:54.3
8708	RSPe_2	-122.445	37.940	0.05	17.73	258.83	17.78	-122.445	37.940	0.00	17.73	258.96	17.73	9	7/14/2014	35:54.2
8709	RSPe_2	-122.445	37.940	0.05	17.64	258.81	17.69	-122.445	37.940	0.00	17.64	258.92	17.64	9	7/14/2014	35:54.1
8710	RSPe_2	-122.445	37.940	0.05	17.63	258.88	17.67	-122.445	37.940	0.00	17.63	258.94	17.62	9	7/14/2014	35:54.0
8711	RSPe_2	-122.445	37.940	0.05	17.60	258.92	17.65	-122.445	37.940	0.08	17.60	259.00	17.68	9	7/14/2014	35:53.9

8712	RSPe_2	-122.445	37.940	0.05	17.56	258.98	17.61	-122.445	37.940	0.03	17.56	259.02	17.59	9	7/14/2014	35:53.8
8713	RSPe_2	-122.445	37.940	0.05	17.58	259.01	17.63	-122.445	37.940	0.00	17.58	259.01	17.58	9	7/14/2014	35:53.7
8714	RSPe_2	-122.445	37.940	0.05	17.49	259.03	17.54	-122.445	37.940	0.00	17.49	259.09	17.49	9	7/14/2014	35:53.6
8715	RSPe_2	-122.445	37.940	0.05	17.45	259.10	17.50	-122.445	37.940	0.03	17.45	259.10	17.49	9	7/14/2014	35:53.5
8716	RSPe_2	-122.445	37.940	0.05	17.45	259.05	17.50	-122.445	37.940	0.03	17.45	259.12	17.48	9	7/14/2014	35:53.4
8717	RSPe_2	-122.445	37.940	0.05	17.40	259.08	17.45	-122.445	37.940	0.03	17.40	259.16	17.43	9	7/14/2014	35:53.3
8718	RSPe_2	-122.445	37.940	0.05	17.39	259.08	17.43	-122.445	37.940	0.03	17.39	259.20	17.42	9	7/14/2014	35:53.2
8719	RSPe_2	-122.445	37.940	0.05	17.35	259.10	17.40	-122.445	37.940	0.03	17.35	259.29	17.39	9	7/14/2014	35:53.1
8720	RSPe_2	-122.445	37.940	0.05	17.34	259.03	17.39	-122.445	37.940	0.03	17.34	259.20	17.37	9	7/14/2014	35:53.0
8721	RSPe_2	-122.445	37.940	0.05	17.31	258.99	17.36	-122.445	37.940	0.03	17.31	259.16	17.34	9	7/14/2014	35:52.9
8722	RSPe_2	-122.445	37.940	0.05	17.30	258.97	17.35	-122.445	37.940	0.03	17.30	259.12	17.33	9	7/14/2014	35:52.8
8723	RSPe_2	-122.445	37.940	0.05	17.32	259.08	17.37	-122.445	37.940	0.03	17.32	259.20	17.35	9	7/14/2014	35:52.7
8724	RSPe_2	-122.445	37.940	0.05	17.30	259.10	17.35	-122.445	37.940	0.03	17.30	259.19	17.33	9	7/14/2014	35:52.6
8725	RSPe_2	-122.445	37.940	0.05	17.31	259.17	17.36	-122.445	37.940	0.03	17.31	259.23	17.34	9	7/14/2014	35:52.5
8726	RSPe_2	-122.445	37.940	0.05	17.29	259.21	17.34	-122.445	37.940	0.03	17.29	259.25	17.32	9	7/14/2014	35:52.4
8727	RSPe_2	-122.445	37.940	0.05	17.29	259.23	17.34	-122.445	37.940	0.03	17.29	259.32	17.32	9	7/14/2014	35:52.3
8728	RSPe_2	-122.445	37.940	0.02	17.28	259.21	17.30	-122.445	37.940	0.00	17.28	259.37	17.28	9	7/14/2014	35:52.2
8729	RSPe_2	-122.445	37.940	0.05	17.25	259.17	17.30	-122.445	37.940	0.08	17.25	259.34	17.33	9	7/14/2014	35:52.1
8730	RSPe_2	-122.445	37.940	0.05	17.28	259.12	17.33	-122.445	37.940	0.03	17.28	259.35	17.31	9	7/14/2014	35:52.0
8731	RSPe_2	-122.445	37.940	0.05	17.32	259.15	17.36	-122.445	37.940	0.03	17.32	259.36	17.35	9	7/14/2014	35:51.9
8732	RSPe_2	-122.445	37.940	0.05	17.35	259.15	17.40	-122.445	37.940	0.03	17.35	259.34	17.38	9	7/14/2014	35:51.8
8733	RSPe_2	-122.445	37.940	0.05	17.29	259.17	17.34	-122.445	37.940	0.03	17.29	259.37	17.32	9	7/14/2014	35:51.7
8734	RSPe_2	-122.445	37.940	0.05	17.39	259.15	17.44	-122.445	37.940	0.03	17.39	259.34	17.42	9	7/14/2014	35:51.6
8735	RSPe_2	-122.445	37.940	0.05	17.42	259.17	17.47	-122.445	37.940	0.03	17.42	259.28	17.45	9	7/14/2014	35:51.5
8736	RSPe_2	-122.445	37.940	0.05	17.41	259.28	17.46	-122.445	37.940	0.00	17.41	259.37	17.40	9	7/14/2014	35:51.4
8737	RSPe_2	-122.445	37.940	0.05	17.49	259.39	17.54	-122.445	37.940	0.03	17.49	259.43	17.52	9	7/14/2014	35:51.3
8738	RSPe_2	-122.445	37.940	0.02	17.60	259.41	17.61	-122.445	37.940	0.03	17.60	259.54	17.63	9	7/14/2014	35:51.2
8739	RSPe_2	-122.445	37.940	0.05	17.56	259.41	17.60	-122.445	37.940	0.03	17.56	259.55	17.59	9	7/14/2014	35:51.1
8740	RSPe_2	-122.445	37.940	0.02	17.55	259.53	17.56	-122.445	37.940	0.00	17.55	259.65	17.54	9	7/14/2014	35:51.0
8741	RSPe_2	-122.445	37.940	0.02	17.56	259.63	17.58	-122.445	37.940	0.03	17.56	259.77	17.59	9	7/14/2014	35:50.9
8742	RSPe_2	-122.445	37.940	0.02	17.59	259.74	17.61	-122.445	37.940	0.00	17.59	259.96	17.59	9	7/14/2014	35:50.8
8743	RSPe_2	-122.445	37.940	0.02	17.59	259.94	17.61	-122.445	37.940	0.00	17.59	260.13	17.59	9	7/14/2014	35:50.7
8744	RSPe_2	-122.445	37.940	-0.04	17.59	260.04	17.56	-122.445	37.940	-0.05	17.59	260.21	17.54	9	7/14/2014	35:50.6

8745	RSPe_2	-122.445	37.940	0.02	17.63	260.12	17.64	-122.445	37.940	0.00	17.63	260.32	17.62	9	7/14/2014	35:50.5
8746	RSPe_2	-122.445	37.940	-0.04	17.58	260.23	17.55	-122.445	37.940	0.00	17.58	260.31	17.58	9	7/14/2014	35:50.4
8747	RSPe_2	-122.445	37.940	0.02	17.56	260.34	17.58	-122.445	37.940	0.00	17.56	260.36	17.56	9	7/14/2014	35:50.3
8748	RSPe_2	-122.445	37.940	0.02	17.54	260.54	17.55	-122.445	37.940	-0.05	17.54	260.40	17.49	9	7/14/2014	35:50.2
8749	RSPe_2	-122.445	37.940	0.02	17.56	260.62	17.58	-122.445	37.940	0.03	17.56	260.44	17.59	9	7/14/2014	35:50.1
8750	RSPe_2	-122.445	37.940	-0.04	17.50	260.72	17.46	-122.445	37.940	0.00	17.50	260.58	17.50	9	7/14/2014	35:50.0
8751	RSPe_2	-122.445	37.940	0.02	17.51	260.80	17.52	-122.445	37.940	0.03	17.51	260.67	17.54	9	7/14/2014	35:49.9
8752	RSPe_2	-122.445	37.940	0.02	17.53	260.89	17.55	-122.445	37.940	0.00	17.53	260.81	17.53	9	7/14/2014	35:49.8
8753	RSPe_2	-122.445	37.940	0.02	17.56	260.96	17.57	-122.445	37.940	0.00	17.56	261.00	17.55	9	7/14/2014	35:49.7
8754	RSPe_2	-122.445	37.940	-0.04	17.56	260.96	17.52	-122.445	37.940	-0.05	17.56	261.13	17.51	9	7/14/2014	35:49.6
8755	RSPe_2	-122.445	37.940	0.02	17.63	261.01	17.65	-122.445	37.940	0.00	17.63	261.24	17.63	9	7/14/2014	35:49.5
8756	RSPe_2	-122.445	37.940	-0.04	17.60	261.07	17.56	-122.445	37.940	-0.05	17.60	261.33	17.55	9	7/14/2014	35:49.4
8757	RSPe_2	-122.445	37.940	0.02	17.65	261.09	17.66	-122.445	37.940	0.00	17.65	261.40	17.64	9	7/14/2014	35:49.3
8758	RSPe_2	-122.445	37.940	-0.04	17.70	261.18	17.66	-122.445	37.940	-0.09	17.70	261.41	17.61	9	7/14/2014	35:49.2
8759	RSPe_2	-122.445	37.940	-0.04	17.66	261.23	17.63	-122.445	37.940	-0.05	17.66	261.46	17.61	9	7/14/2014	35:49.1
8760	RSPe_2	-122.445	37.940	-0.04	17.70	261.23	17.66	-122.445	37.940	0.00	17.70	261.49	17.70	9	7/14/2014	35:49.0
8761	RSPe_2	-122.445	37.940	0.02	17.73	261.20	17.75	-122.445	37.940	-0.05	17.73	261.46	17.68	9	7/14/2014	35:48.9
8762	RSPe_2	-122.445	37.940	-0.04	17.76	261.20	17.73	-122.445	37.940	-0.05	17.76	261.49	17.71	9	7/14/2014	35:48.8
8763	RSPe_2	-122.445	37.940	0.02	17.81	261.30	17.82	-122.445	37.940	0.00	17.81	261.57	17.81	9	7/14/2014	35:48.7
8764	RSPe_2	-122.445	37.940	-0.04	17.81	261.38	17.77	-122.445	37.940	0.00	17.81	261.62	17.81	9	7/14/2014	35:48.6
8765	RSPe_2	-122.445	37.940	0.02	17.90	261.43	17.92	-122.445	37.940	0.00	17.90	261.74	17.90	9	7/14/2014	35:48.5
8766	RSPe_2	-122.445	37.940	-0.04	17.91	261.56	17.87	-122.445	37.940	-0.05	17.91	261.77	17.86	9	7/14/2014	35:48.4
8767	RSPe_2	-122.445	37.940	-0.04	17.97	261.65	17.94	-122.445	37.940	0.00	17.97	261.86	17.97	9	7/14/2014	35:48.3
8768	RSPe_2	-122.445	37.940	-0.04	17.93	261.76	17.89	-122.445	37.940	-0.05	17.93	261.94	17.88	9	7/14/2014	35:48.2
8769	RSPe_2	-122.445	37.940	0.02	17.94	261.85	17.96	-122.445	37.940	-0.05	17.94	261.93	17.89	9	7/14/2014	35:48.1
8770	RSPe_2	-122.445	37.940	-0.04	17.94	261.89	17.90	-122.445	37.940	-0.05	17.94	261.97	17.89	9	7/14/2014	35:48.0
8771	RSPe_2	-122.445	37.940	-0.04	17.98	261.98	17.94	-122.445	37.940	-0.05	17.98	262.02	17.93	9	7/14/2014	35:47.9
8772	RSPe_2	-122.445	37.940	-0.04	17.94	262.04	17.90	-122.445	37.940	-0.05	17.94	262.05	17.89	9	7/14/2014	35:47.8
8773	RSPe_2	-122.445	37.940	-0.04	17.99	262.05	17.95	-122.445	37.940	-0.05	17.99	262.11	17.93	9	7/14/2014	35:47.7
8774	RSPe_2	-122.445	37.940	-0.04	17.98	262.13	17.94	-122.445	37.940	-0.09	17.98	262.18	17.89	9	7/14/2014	35:47.6
8775	RSPe_2	-122.445	37.940	-0.04	17.95	262.11	17.91	-122.445	37.940	-0.05	17.95	262.24	17.89	9	7/14/2014	35:47.5
8776	RSPe_2	-122.445	37.940	-0.07	18.06	262.13	17.99	-122.445	37.940	-0.05	18.06	262.34	18.01	9	7/14/2014	35:47.4
8777	RSPe_2	-122.445	37.940	-0.04	18.02	262.25	17.98	-122.445	37.940	-0.09	18.02	262.48	17.93	9	7/14/2014	35:47.3

8778	RSPe_2	-122.445	37.940	-0.07	18.08	262.29	18.01	-122.445	37.940	-0.09	18.08	262.55	17.99	9	7/14/2014	35:47.2
8779	RSPe_2	-122.445	37.940	-0.04	18.07	262.35	18.03	-122.445	37.940	-0.09	18.07	262.60	17.98	9	7/14/2014	35:47.1
8780	RSPe_2	-122.445	37.940	-0.04	18.12	262.46	18.09	-122.445	37.940	-0.05	18.12	262.63	18.07	9	7/14/2014	35:47.0
8781	RSPe_2	-122.445	37.940	-0.04	18.11	262.51	18.07	-122.445	37.940	-0.05	18.11	262.70	18.05	9	7/14/2014	35:46.9
8782	RSPe_2	-122.445	37.940	-0.04	18.11	262.55	18.07	-122.445	37.940	-0.05	18.11	262.73	18.05	9	7/14/2014	35:46.8
8783	RSPe_2	-122.445	37.940	-0.04	18.01	262.67	17.97	-122.445	37.940	-0.05	18.01	262.84	17.96	9	7/14/2014	35:46.7
8784	RSPe_2	-122.445	37.940	-0.04	18.03	262.77	17.99	-122.445	37.940	-0.05	18.03	262.96	17.97	9	7/14/2014	35:46.6
8785	RSPe_2	-122.445	37.940	-0.04	17.99	262.80	17.96	-122.445	37.940	-0.05	17.99	263.12	17.94	9	7/14/2014	35:46.5
8786	RSPe_2	-122.445	37.940	-0.07	17.96	262.95	17.89	-122.445	37.940	-0.14	17.96	263.23	17.82	9	7/14/2014	35:46.4
8787	RSPe_2	-122.445	37.940	-0.04	17.99	262.98	17.95	-122.445	37.940	-0.09	17.99	263.37	17.90	9	7/14/2014	35:46.3
8788	RSPe_2	-122.445	37.940	-0.07	17.92	263.04	17.85	-122.445	37.940	-0.09	17.92	263.41	17.84	9	7/14/2014	35:46.2
8789	RSPe_2	-122.445	37.940	-0.07	17.90	263.13	17.83	-122.445	37.940	-0.09	17.90	263.56	17.82	9	7/14/2014	35:46.1
8790	RSPe_2	-122.445	37.940	-0.07	17.87	263.19	17.80	-122.445	37.940	-0.14	17.87	263.59	17.73	9	7/14/2014	35:46.0
8791	RSPe_2	-122.445	37.940	-0.04	17.85	263.28	17.82	-122.445	37.940	-0.14	17.85	263.69	17.72	9	7/14/2014	35:45.9
8792	RSPe_2	-122.445	37.940	-0.12	17.87	263.33	17.75	-122.445	37.940	-0.17	17.87	263.68	17.70	9	7/14/2014	35:45.8
8793	RSPe_2	-122.445	37.940	-0.07	17.82	263.40	17.75	-122.445	37.940	-0.14	17.82	263.68	17.69	9	7/14/2014	35:45.7
8794	RSPe_2	-122.445	37.940	-0.07	17.81	263.48	17.74	-122.445	37.940	-0.17	17.81	263.70	17.64	9	7/14/2014	35:45.6
8795	RSPe_2	-122.445	37.940	-0.07	17.79	263.53	17.71	-122.445	37.940	-0.14	17.79	263.68	17.65	9	7/14/2014	35:45.5
8796	RSPe_2	-122.445	37.940	-0.07	17.78	263.60	17.70	-122.445	37.940	-0.17	17.78	263.78	17.60	9	7/14/2014	35:45.4
8797	RSPe_2	-122.445	37.940	-0.07	17.79	263.70	17.71	-122.445	37.940	-0.14	17.79	263.83	17.65	9	7/14/2014	35:45.3
8798	RSPe_2	-122.445	37.940	-0.12	17.76	263.64	17.64	-122.445	37.940	-0.17	17.76	263.80	17.59	9	7/14/2014	35:45.2
8799	RSPe_2	-122.445	37.940	-0.07	17.77	263.70	17.70	-122.445	37.940	-0.17	17.77	263.76	17.60	9	7/14/2014	35:45.1
8800	RSPe_2	-122.445	37.940	-0.12	17.76	263.74	17.64	-122.445	37.940	-0.21	17.76	263.81	17.56	9	7/14/2014	35:45.0
8801	RSPe_2	-122.445	37.940	-0.07	17.77	263.77	17.70	-122.445	37.940	-0.21	17.77	263.79	17.57	9	7/14/2014	35:44.9
8802	RSPe_2	-122.445	37.940	-0.12	17.80	263.86	17.68	-122.445	37.940	-0.21	17.80	263.87	17.60	9	7/14/2014	35:44.8
8803	RSPe_2	-122.445	37.940	-0.07	17.78	263.90	17.70	-122.445	37.940	-0.17	17.78	263.92	17.60	9	7/14/2014	35:44.7
8804	RSPe_2	-122.445	37.940	-0.12	17.77	263.95	17.65	-122.445	37.940	-0.21	17.77	263.99	17.57	9	7/14/2014	35:44.6
8805	RSPe_2	-122.445	37.940	-0.12	17.82	264.02	17.69	-122.445	37.940	-0.21	17.82	264.08	17.61	9	7/14/2014	35:44.5
8806	RSPe_2	-122.445	37.940	-0.12	17.82	263.97	17.69	-122.445	37.940	-0.21	17.82	264.08	17.61	9	7/14/2014	35:44.4
8807	RSPe_2	-122.445	37.940	-0.12	17.81	263.92	17.69	-122.445	37.940	-0.17	17.81	264.07	17.64	9	7/14/2014	35:44.3
8808	RSPe_2	-122.445	37.940	-0.12	17.76	263.89	17.64	-122.445	37.940	-0.21	17.76	264.14	17.56	9	7/14/2014	35:44.2
8809	RSPe_2	-122.445	37.940	-0.12	17.81	263.86	17.69	-122.445	37.940	-0.21	17.81	264.09	17.60	9	7/14/2014	35:44.1
8810	RSPe_2	-122.445	37.940	-0.16	17.84	263.80	17.68	-122.445	37.940	-0.26	17.84	264.14	17.58	9	7/14/2014	35:44.0

8811	RSPe_2	-122.445	37.940	-0.12	17.83	263.80	17.71	-122.445	37.940	-0.26	17.83	264.19	17.58	9	7/14/2014 35:43.9
8812	RSPe_2	-122.445	37.940	-0.16	17.85	263.85	17.69	-122.445	37.940	-0.26	17.85	264.24	17.59	9	7/14/2014 35:43.8
8813	RSPe_2	-122.445	37.940	-0.16	17.96	263.80	17.81	-122.445	37.940	-0.26	17.96	264.23	17.71	9	7/14/2014 35:43.7
8814	RSPe_2	-122.445	37.940	-0.16	17.90	263.85	17.75	-122.445	37.940	-0.29	17.90	264.26	17.61	9	7/14/2014 35:43.6
8815	RSPe_2	-122.445	37.940	-0.16	17.99	263.80	17.83	-122.445	37.940	-0.26	17.99	264.21	17.73	9	7/14/2014 35:43.5
8816	RSPe_2	-122.445	37.940	-0.16	17.96	263.76	17.81	-122.445	37.940	-0.34	17.96	264.19	17.62	9	7/14/2014 35:43.4
8817	RSPe_2	-122.445	37.940	-0.16	18.02	263.78	17.86	-122.445	37.940	-0.29	18.02	264.11	17.73	9	7/14/2014 35:43.3
8818	RSPe_2	-122.445	37.940	-0.16	18.05	263.71	17.90	-122.445	37.940	-0.34	18.05	264.12	17.71	9	7/14/2014 35:43.2
8819	RSPe_2	-122.445	37.940	-0.16	18.07	263.71	17.91	-122.445	37.940	-0.34	18.07	264.15	17.73	9	7/14/2014 35:43.1
8820	RSPe_2	-122.445	37.940	-0.19	18.10	263.76	17.91	-122.445	37.940	-0.37	18.10	264.21	17.72	9	7/14/2014 35:43.0
8821	RSPe_2	-122.445	37.940	-0.16	18.08	263.71	17.92	-122.445	37.940	-0.37	18.08	264.19	17.71	9	7/14/2014 35:42.9
8822	RSPe_2	-122.445	37.940	-0.19	18.12	263.76	17.93	-122.445	37.940	-0.37	18.12	264.22	17.74	9	7/14/2014 35:42.8
8823	RSPe_2	-122.445	37.940	-0.19	18.13	263.74	17.94	-122.445	37.940	-0.37	18.13	264.19	17.76	9	7/14/2014 35:42.7
8824	RSPe_2	-122.445	37.940	-0.19	18.15	263.73	17.96	-122.445	37.940	-0.37	18.15	264.11	17.78	9	7/14/2014 35:42.6
8825	RSPe_2	-122.445	37.940	-0.19	18.19	263.72	18.00	-122.445	37.940	-0.37	18.19	263.98	17.81	9	7/14/2014 35:42.5
8826	RSPe_2	-122.445	37.940	-0.19	18.26	263.67	18.07	-122.445	37.940	-0.42	18.26	263.89	17.83	9	7/14/2014 35:42.4
8827	RSPe_2	-122.445	37.940	-0.19	18.26	263.71	18.07	-122.445	37.940	-0.42	18.26	263.78	17.84	9	7/14/2014 35:42.3
8828	RSPe_2	-122.445	37.940	-0.24	18.20	263.72	17.96	-122.445	37.940	-0.42	18.20	263.78	17.78	9	7/14/2014 35:42.2
8829	RSPe_2	-122.445	37.940	-0.19	18.33	263.74	18.14	-122.445	37.940	-0.42	18.33	263.80	17.90	9	7/14/2014 35:42.1
8830	RSPe_2	-122.445	37.940	-0.24	18.23	263.71	17.98	-122.445	37.940	-0.46	18.23	263.83	17.77	9	7/14/2014 35:42.0
8831	RSPe_2	-122.445	37.940	-0.24	18.24	263.72	18.00	-122.445	37.940	-0.46	18.24	263.91	17.78	9	7/14/2014 35:41.9
8832	RSPe_2	-122.445	37.940	-0.24	18.35	263.76	18.11	-122.445	37.940	-0.46	18.35	264.05	17.89	9	7/14/2014 35:41.8
8833	RSPe_2	-122.445	37.940	-0.19	18.40	263.92	18.21	-122.445	37.940	-0.46	18.40	264.18	17.94	9	7/14/2014 35:41.7
8834	RSPe_2	-122.445	37.940	-0.24	18.32	264.02	18.08	-122.445	37.940	-0.51	18.32	264.24	17.81	9	7/14/2014 35:41.6
8835	RSPe_2	-122.445	37.940	-0.24	18.34	264.03	18.10	-122.445	37.940	-0.46	18.34	264.24	17.88	9	7/14/2014 35:41.5
8836	RSPe_2	-122.445	37.940	-0.24	18.47	264.03	18.22	-122.445	37.940	-0.51	18.47	264.22	17.96	9	7/14/2014 35:41.4
8837	RSPe_2	-122.445	37.940	-0.24	18.44	264.01	18.20	-122.445	37.940	-0.51	18.44	264.22	17.93	9	7/14/2014 35:41.3
8838	RSPe_2	-122.445	37.940	-0.28	18.40	263.93	18.12	-122.445	37.940	-0.51	18.40	264.15	17.89	9	7/14/2014 35:41.2
8839	RSPe_2	-122.445	37.940	-0.24	18.43	263.79	18.19	-122.445	37.940	-0.51	18.43	264.09	17.92	9	7/14/2014 35:41.1
8840	RSPe_2	-122.445	37.940	-0.24	18.38	263.74	18.14	-122.445	37.940	-0.54	18.38	264.13	17.84	9	7/14/2014 35:41.0
8841	RSPe_2	-122.445	37.940	-0.24	18.43	263.64	18.19	-122.445	37.940	-0.51	18.43	264.07	17.92	9	7/14/2014 35:40.9
8842	RSPe_2	-122.445	37.940	-0.24	18.56	263.70	18.32	-122.445	37.940	-0.54	18.56	264.23	18.01	9	7/14/2014 35:40.8
8843	RSPe_2	-122.445	37.940	-0.24	18.71	263.75	18.47	-122.445	37.940	-0.54	18.71	264.37	18.17	9	7/14/2014 35:40.7

8844	RSPe_2	-122.445	37.940	-0.24	18.63	263.81	18.38	-122.445	37.940	-0.58	18.63	264.38	18.05	9	7/14/2014 35:40.6
8845	RSPe_2	-122.445	37.940	-0.24	18.74	263.98	18.50	-122.445	37.940	-0.54	18.74	264.58	18.20	9	7/14/2014 35:40.5
8846	RSPe_2	-122.445	37.940	-0.28	18.87	264.12	18.59	-122.445	37.940	-0.58	18.87	264.53	18.29	9	7/14/2014 35:40.4
8847	RSPe_2	-122.445	37.940	-0.24	18.81	264.21	18.57	-122.445	37.940	-0.54	18.81	264.49	18.27	9	7/14/2014 35:40.3
8848	RSPe_2	-122.445	37.940	-0.28	18.84	264.27	18.57	-122.445	37.940	-0.58	18.84	264.39	18.26	9	7/14/2014 35:40.2
8849	RSPe_2	-122.445	37.940	-0.24	18.83	264.30	18.59	-122.445	37.940	-0.58	18.83	264.32	18.25	9	7/14/2014 35:40.1
8850	RSPe_2	-122.445	37.940	-0.28	18.66	264.36	18.38	-122.445	37.940	-0.58	18.66	264.29	18.08	9	7/14/2014 35:40.0
8851	RSPe_2	-122.445	37.940	-0.28	18.63	264.29	18.35	-122.445	37.940	-0.54	18.63	264.23	18.08	9	7/14/2014 35:39.9
8852	RSPe_2	-122.445	37.940	-0.28	18.68	264.23	18.41	-122.445	37.940	-0.58	18.68	264.21	18.10	9	7/14/2014 35:39.8
8853	RSPe_2	-122.445	37.940	-0.24	18.45	264.17	18.21	-122.445	37.940	-0.58	18.45	264.27	17.87	9	7/14/2014 35:39.7
8854	RSPe_2	-122.445	37.940	-0.24	18.44	264.08	18.20	-122.445	37.940	-0.63	18.44	264.32	17.81	9	7/14/2014 35:39.6
8855	RSPe_2	-122.445	37.940	-0.24	18.38	264.10	18.14	-122.445	37.940	-0.58	18.38	264.46	17.80	9	7/14/2014 35:39.5
8856	RSPe_2	-122.445	37.940	-0.28	18.40	264.12	18.12	-122.445	37.940	-0.58	18.40	264.60	17.82	9	7/14/2014 35:39.4
8857	RSPe_2	-122.445	37.940	-0.28	18.41	264.18	18.14	-122.445	37.940	-0.58	18.41	264.72	17.84	9	7/14/2014 35:39.3
8858	RSPe_2	-122.445	37.940	-0.28	18.40	264.30	18.12	-122.445	37.940	-0.63	18.40	264.86	17.77	9	7/14/2014 35:39.2
8859	RSPe_2	-122.445	37.940	-0.24	18.43	264.41	18.19	-122.445	37.940	-0.63	18.43	264.93	17.81	9	7/14/2014 35:39.1
8860	RSPe_2	-122.445	37.940	-0.24	18.57	264.39	18.32	-122.445	37.940	-0.63	18.57	264.96	17.94	9	7/14/2014 35:39.0
8861	RSPe_2	-122.445	37.940	-0.24	18.48	264.41	18.24	-122.445	37.940	-0.63	18.48	264.93	17.85	9	7/14/2014 35:38.9
8862	RSPe_2	-122.445	37.940	-0.24	18.47	264.37	18.22	-122.445	37.940	-0.66	18.47	264.92	17.80	9	7/14/2014 35:38.8
8863	RSPe_2	-122.445	37.940	-0.24	18.44	264.35	18.20	-122.445	37.940	-0.63	18.44	264.89	17.81	9	7/14/2014 35:38.7
8864	RSPe_2	-122.445	37.940	-0.24	18.40	264.30	18.16	-122.445	37.940	-0.66	18.40	264.80	17.74	9	7/14/2014 35:38.6
8865	RSPe_2	-122.445	37.940	-0.24	18.40	264.28	18.15	-122.445	37.940	-0.66	18.40	264.72	17.73	9	7/14/2014 35:38.5
8866	RSPe_2	-122.445	37.940	-0.24	18.38	264.28	18.14	-122.445	37.940	-0.66	18.38	264.67	17.72	9	7/14/2014 35:38.4
8867	RSPe_2	-122.445	37.940	-0.24	18.41	264.41	18.17	-122.445	37.940	-0.63	18.41	264.74	17.79	9	7/14/2014 35:38.3
8868	RSPe_2	-122.445	37.940	-0.24	18.35	264.55	18.11	-122.445	37.940	-0.66	18.35	264.79	17.69	9	7/14/2014 35:38.2
8869	RSPe_2	-122.445	37.940	-0.24	18.35	264.66	18.11	-122.445	37.940	-0.66	18.35	264.85	17.69	9	7/14/2014 35:38.1
8870	RSPe_2	-122.445	37.940	-0.24	18.40	264.75	18.16	-122.445	37.940	-0.66	18.40	264.87	17.74	9	7/14/2014 35:38.0
8871	RSPe_2	-122.445	37.940	-0.19	18.34	264.79	18.15	-122.445	37.940	-0.63	18.34	264.89	17.71	9	7/14/2014 35:37.9
8872	RSPe_2	-122.445	37.940	-0.24	18.36	264.80	18.12	-122.445	37.940	-0.66	18.36	264.85	17.70	9	7/14/2014 35:37.8
8873	RSPe_2	-122.445	37.940	-0.19	18.36	264.75	18.17	-122.445	37.940	-0.63	18.36	264.86	17.74	9	7/14/2014 35:37.7
8874	RSPe_2	-122.445	37.940	-0.24	18.36	264.72	18.12	-122.445	37.940	-0.63	18.36	264.89	17.73	9	7/14/2014 35:37.6
8875	RSPe_2	-122.445	37.940	-0.24	18.30	264.75	18.06	-122.445	37.940	-0.63	18.30	264.94	17.68	9	7/14/2014 35:37.5
8876	RSPe_2	-122.445	37.940	-0.24	18.33	264.75	18.09	-122.445	37.940	-0.63	18.33	264.96	17.70	9	7/14/2014 35:37.4

8877	RSPe_2	-122.445	37.940	-0.19	18.37	264.79	18.18	-122.445	37.940	-0.66	18.37	264.99	17.71	9	7/14/2014 35:37.3
8878	RSPe_2	-122.445	37.940	-0.24	18.35	264.90	18.11	-122.445	37.940	-0.58	18.35	265.05	17.77	9	7/14/2014 35:37.2
8879	RSPe_2	-122.445	37.940	-0.19	18.43	265.01	18.24	-122.445	37.940	-0.54	18.43	265.08	17.88	9	7/14/2014 35:37.1
8880	RSPe_2	-122.445	37.940	-0.24	18.40	265.06	18.15	-122.445	37.940	-0.58	18.40	265.10	17.82	9	7/14/2014 35:37.0
8881	RSPe_2	-122.445	37.940	-0.19	18.43	265.06	18.24	-122.445	37.940	-0.54	18.43	265.16	17.88	9	7/14/2014 35:36.9
8882	RSPe_2	-122.445	37.940	-0.19	18.41	265.06	18.22	-122.445	37.940	-0.58	18.41	265.12	17.84	9	7/14/2014 35:36.8
8883	RSPe_2	-122.445	37.940	-0.19	18.50	265.01	18.31	-122.445	37.940	-0.54	18.50	265.23	17.95	9	7/14/2014 35:36.7
8884	RSPe_2	-122.445	37.940	-0.19	18.46	265.02	18.27	-122.445	37.940	-0.58	18.46	265.31	17.88	9	7/14/2014 35:36.6
8885	RSPe_2	-122.445	37.940	-0.19	18.46	265.01	18.27	-122.445	37.940	-0.58	18.46	265.32	17.88	9	7/14/2014 35:36.5
8886	RSPe_2	-122.445	37.940	-0.19	18.48	265.08	18.29	-122.445	37.940	-0.63	18.48	265.37	17.85	9	7/14/2014 35:36.4
8887	RSPe_2	-122.445	37.940	-0.19	18.47	265.11	18.28	-122.445	37.940	-0.58	18.47	265.36	17.89	9	7/14/2014 35:36.3
8888	RSPe_2	-122.445	37.940	-0.19	18.43	265.17	18.24	-122.445	37.940	-0.58	18.43	265.32	17.86	9	7/14/2014 35:36.2
8889	RSPe_2	-122.445	37.940	-0.19	18.43	265.19	18.24	-122.445	37.940	-0.54	18.43	265.30	17.89	9	7/14/2014 35:36.1
8890	RSPe_2	-122.445	37.940	-0.19	18.43	265.26	18.24	-122.445	37.940	-0.58	18.43	265.32	17.86	9	7/14/2014 35:36.0
8891	RSPe_2	-122.445	37.940	-0.19	18.41	265.37	18.22	-122.445	37.940	-0.58	18.41	265.41	17.84	9	7/14/2014 35:35.9
8892	RSPe_2	-122.445	37.940	-0.19	18.41	265.37	18.22	-122.445	37.940	-0.58	18.41	265.43	17.84	9	7/14/2014 35:35.8
8893	RSPe_2	-122.445	37.940	-0.16	18.46	265.41	18.30	-122.445	37.940	-0.51	18.46	265.50	17.95	9	7/14/2014 35:35.7
8894	RSPe_2	-122.445	37.940	-0.19	18.37	265.37	18.18	-122.445	37.940	-0.54	18.37	265.58	17.83	9	7/14/2014 35:35.6
8895	RSPe_2	-122.445	37.940	-0.16	18.36	265.37	18.20	-122.445	37.940	-0.54	18.36	265.63	17.81	9	7/14/2014 35:35.5
8896	RSPe_2	-122.445	37.940	-0.16	18.34	265.42	18.19	-122.445	37.940	-0.54	18.34	265.67	17.80	9	7/14/2014 35:35.4
8897	RSPe_2	-122.445	37.940	-0.16	18.34	265.42	18.19	-122.445	37.940	-0.51	18.34	265.68	17.83	9	7/14/2014 35:35.3
8898	RSPe_2	-122.445	37.940	-0.19	18.32	265.42	18.13	-122.445	37.940	-0.51	18.32	265.70	17.81	9	7/14/2014 35:35.2
8899	RSPe_2	-122.445	37.940	-0.16	18.33	265.37	18.17	-122.445	37.940	-0.51	18.33	265.67	17.82	9	7/14/2014 35:35.1
8900	RSPe_2	-122.445	37.940	-0.19	18.33	265.37	18.14	-122.445	37.940	-0.46	18.33	265.71	17.87	9	7/14/2014 35:35.0
8901	RSPe_2	-122.445	37.940	-0.16	18.34	265.46	18.19	-122.445	37.940	-0.51	18.34	265.79	17.83	9	7/14/2014 35:34.9
8902	RSPe_2	-122.445	37.940	-0.16	18.35	265.46	18.19	-122.445	37.940	-0.51	18.35	265.83	17.84	9	7/14/2014 35:34.8
8903	RSPe_2	-122.445	37.940	-0.16	18.35	265.46	18.19	-122.445	37.940	-0.51	18.35	265.79	17.84	9	7/14/2014 35:34.7
8904	RSPe_2	-122.445	37.940	-0.16	18.34	265.42	18.19	-122.445	37.940	-0.51	18.34	265.72	17.83	9	7/14/2014 35:34.6
8905	RSPe_2	-122.445	37.940	-0.16	18.36	265.40	18.21	-122.445	37.940	-0.46	18.36	265.73	17.91	9	7/14/2014 35:34.5
8906	RSPe_2	-122.445	37.940	-0.19	18.42	265.47	18.23	-122.445	37.940	-0.51	18.42	265.81	17.91	9	7/14/2014 35:34.4
8907	RSPe_2	-122.445	37.940	-0.16	18.46	265.62	18.30	-122.445	37.940	-0.46	18.46	265.90	18.00	9	7/14/2014 35:34.3
8908	RSPe_2	-122.445	37.940	-0.16	18.49	265.66	18.33	-122.445	37.940	-0.51	18.49	265.90	17.98	9	7/14/2014 35:34.2
8909	RSPe_2	-122.445	37.940	-0.12	18.60	265.67	18.48	-122.445	37.940	-0.46	18.60	265.94	18.15	9	7/14/2014 35:34.1

8910	RSPe_2	-122.445	37.940	-0.16	18.56	265.75	18.40	-122.445	37.940	-0.51	18.56	265.96	18.05	9	7/14/2014 35:34.0
8911	RSPe_2	-122.445	37.940	-0.12	18.51	265.78	18.39	-122.445	37.940	-0.42	18.51	266.06	18.09	9	7/14/2014 35:33.9
8912	RSPe_2	-122.445	37.940	-0.16	18.65	265.82	18.49	-122.445	37.940	-0.51	18.65	266.19	18.14	9	7/14/2014 35:33.8
8913	RSPe_2	-122.445	37.940	-0.12	18.61	265.86	18.49	-122.445	37.940	-0.46	18.61	266.24	18.15	9	7/14/2014 35:33.7
8914	RSPe_2	-122.445	37.940	-0.16	18.74	265.80	18.59	-122.445	37.940	-0.54	18.74	266.30	18.20	9	7/14/2014 35:33.6
8915	RSPe_2	-122.445	37.940	-0.12	18.85	265.80	18.73	-122.445	37.940	-0.58	18.85	266.24	18.27	9	7/14/2014 35:33.5
8916	RSPe_2	-122.445	37.940	-0.16	18.87	265.84	18.71	-122.445	37.940	-0.54	18.87	266.24	18.32	9	7/14/2014 35:33.4
8917	RSPe_2	-122.445	37.940	-0.12	18.97	265.82	18.84	-122.445	37.940	-0.51	18.97	266.25	18.46	9	7/14/2014 35:33.3
8918	RSPe_2	-122.445	37.940	-0.16	18.77	265.73	18.62	-122.445	37.940	-0.54	18.77	266.13	18.23	9	7/14/2014 35:33.2
8919	RSPe_2	-122.445	37.940	-0.12	18.78	265.87	18.66	-122.445	37.940	-0.54	18.78	266.19	18.24	9	7/14/2014 35:33.1
8920	RSPe_2	-122.445	37.940	-0.16	18.90	266.21	18.75	-122.445	37.940	-0.58	18.90	266.30	18.33	9	7/14/2014 35:33.0
8921	RSPe_2	-122.445	37.940	-0.12	18.73	266.24	18.61	-122.445	37.940	-0.54	18.73	266.26	18.19	9	7/14/2014 35:32.9
8922	RSPe_2	-122.445	37.940	-0.16	18.61	266.24	18.46	-122.445	37.940	-0.54	18.61	266.30	18.07	9	7/14/2014 35:32.8
8923	RSPe_2	-122.445	37.940	-0.16	18.70	266.33	18.54	-122.445	37.940	-0.54	18.70	266.33	18.15	9	7/14/2014 35:32.7
8924	RSPe_2	-122.445	37.940	-0.12	18.57	266.31	18.44	-122.445	37.940	-0.54	18.57	266.35	18.02	9	7/14/2014 35:32.6
8925	RSPe_2	-122.445	37.940	-0.12	18.54	266.36	18.42	-122.445	37.940	-0.54	18.54	266.46	18.00	9	7/14/2014 35:32.5
8926	RSPe_2	-122.445	37.940	-0.16	18.49	266.27	18.33	-122.445	37.940	-0.58	18.49	266.50	17.91	9	7/14/2014 35:32.4
8927	RSPe_2	-122.445	37.940	-0.12	18.55	266.13	18.43	-122.445	37.940	-0.54	18.55	266.55	18.01	9	7/14/2014 35:32.3
8928	RSPe_2	-122.445	37.940	-0.12	18.44	266.11	18.32	-122.445	37.940	-0.54	18.44	266.63	17.90	9	7/14/2014 35:32.2
8929	RSPe_2	-122.445	37.940	-0.07	18.45	265.98	18.38	-122.445	37.940	-0.58	18.45	266.64	17.87	9	7/14/2014 35:32.1
8930	RSPe_2	-122.445	37.940	-0.12	18.30	266.03	18.17	-122.445	37.940	-0.54	18.30	266.63	17.75	9	7/14/2014 35:32.0
8931	RSPe_2	-122.445	37.940	-0.07	18.35	266.11	18.28	-122.445	37.940	-0.46	18.35	266.66	17.89	9	7/14/2014 35:31.9
8932	RSPe_2	-122.445	37.940	-0.12	18.26	266.10	18.14	-122.445	37.940	-0.54	18.26	266.57	17.71	9	7/14/2014 35:31.8
8933	RSPe_2	-122.445	37.940	-0.07	18.15	266.16	18.08	-122.445	37.940	-0.46	18.15	266.53	17.69	9	7/14/2014 35:31.7
8934	RSPe_2	-122.445	37.940	-0.07	18.19	266.18	18.12	-122.445	37.940	-0.51	18.19	266.44	17.68	9	7/14/2014 35:31.6
8935	RSPe_2	-122.445	37.940	-0.04	18.03	266.23	17.99	-122.445	37.940	-0.42	18.03	266.44	17.60	9	7/14/2014 35:31.5
8936	RSPe_2	-122.445	37.940	-0.07	17.92	266.27	17.86	-122.445	37.940	-0.46	17.92	266.48	17.47	9	7/14/2014 35:31.4
8937	RSPe_2	-122.445	37.940	-0.04	17.89	266.33	17.86	-122.445	37.940	-0.51	17.89	266.44	17.38	9	7/14/2014 35:31.3
8938	RSPe_2	-122.445	37.940	-0.07	17.85	266.40	17.79	-122.445	37.940	-0.51	17.85	266.46	17.35	9	7/14/2014 35:31.2
8939	RSPe_2	-122.445	37.940	-0.04	17.84	266.40	17.81	-122.445	37.940	-0.46	17.84	266.48	17.38	9	7/14/2014 35:31.1
8940	RSPe_2	-122.445	37.940	-0.07	17.79	266.53	17.72	-122.445	37.940	-0.51	17.79	266.51	17.28	9	7/14/2014 35:31.0
8941	RSPe_2	-122.445	37.940	-0.04	17.77	266.56	17.74	-122.445	37.940	-0.46	17.77	266.55	17.31	9	7/14/2014 35:30.9
8942	RSPe_2	-122.445	37.940	-0.07	17.75	266.60	17.68	-122.445	37.940	-0.51	17.75	266.62	17.24	9	7/14/2014 35:30.8

8943	RSPe_2	-122.445	37.940	-0.04	17.72	266.58	17.68	-122.445	37.940	-0.51	17.72	266.67	17.21	9	7/14/2014	35:30.7
8944	RSPe_2	-122.445	37.940	-0.07	17.71	266.60	17.64	-122.445	37.940	-0.37	17.71	266.71	17.34	9	7/14/2014	35:30.6
8945	RSPe_2	-122.445	37.940	-0.04	17.68	266.58	17.65	-122.445	37.940	-0.42	17.68	266.68	17.26	9	7/14/2014	35:30.5
8946	RSPe_2	-122.445	37.940	-0.04	17.69	266.58	17.66	-122.445	37.940	-0.46	17.69	266.71	17.24	9	7/14/2014	35:30.4
8947	RSPe_2	-122.445	37.940	0.02	17.68	266.58	17.70	-122.445	37.940	-0.37	17.68	266.71	17.31	9	7/14/2014	35:30.3
8948	RSPe_2	-122.445	37.940	-0.04	17.67	266.58	17.63	-122.445	37.940	-0.42	17.67	266.75	17.25	9	7/14/2014	35:30.2
8949	RSPe_2	-122.445	37.940	0.02	17.72	266.49	17.74	-122.445	37.940	-0.37	17.72	266.75	17.35	9	7/14/2014	35:30.1
8950	RSPe_2	-122.445	37.940	0.02	17.75	266.52	17.76	-122.445	37.940	-0.42	17.75	266.77	17.32	9	7/14/2014	35:30.0
8951	RSPe_2	-122.445	37.940	0.02	17.78	266.62	17.79	-122.445	37.940	-0.37	17.78	266.91	17.40	9	7/14/2014	35:29.9
8952	RSPe_2	-122.445	37.940	0.02	17.93	266.59	17.95	-122.445	37.940	-0.37	17.93	266.87	17.56	9	7/14/2014	35:29.8
8953	RSPe_2	-122.445	37.940	0.02	18.06	266.63	18.08	-122.445	37.940	-0.37	18.06	266.88	17.69	9	7/14/2014	35:29.7
8954	RSPe_2	-122.445	37.940	0.02	18.07	266.69	18.09	-122.445	37.940	-0.34	18.07	266.89	17.73	9	7/14/2014	35:29.6
8955	RSPe_2	-122.445	37.940	0.02	18.07	266.74	18.09	-122.445	37.940	-0.46	18.07	266.91	17.61	9	7/14/2014	35:29.5
8956	RSPe_2	-122.445	37.940	0.02	18.10	266.72	18.12	-122.445	37.940	-0.34	18.10	266.89	17.76	9	7/14/2014	35:29.4
8957	RSPe_2	-122.445	37.940	0.05	18.11	266.74	18.16	-122.445	37.940	-0.34	18.11	266.87	17.77	9	7/14/2014	35:29.3
8958	RSPe_2	-122.445	37.940	0.02	18.12	266.76	18.14	-122.445	37.940	-0.29	18.12	266.82	17.83	9	7/14/2014	35:29.2
8959	RSPe_2	-122.445	37.940	0.05	18.16	266.83	18.21	-122.445	37.940	-0.34	18.16	266.91	17.82	9	7/14/2014	35:29.1
8960	RSPe_2	-122.445	37.940	0.02	18.12	266.90	18.14	-122.445	37.940	-0.37	18.12	266.94	17.75	9	7/14/2014	35:29.0
8961	RSPe_2	-122.445	37.940	0.05	18.40	266.96	18.45	-122.445	37.940	-0.34	18.40	267.00	18.06	9	7/14/2014	35:28.9
8962	RSPe_2	-122.445	37.940	0.02	18.30	266.89	18.32	-122.445	37.940	-0.29	18.30	267.05	18.02	9	7/14/2014	35:28.8
8963	RSPe_2	-122.445	37.940	0.05	18.35	266.94	18.40	-122.445	37.940	-0.29	18.35	267.02	18.06	9	7/14/2014	35:28.7
8964	RSPe_2	-122.445	37.940	0.02	18.42	266.85	18.43	-122.445	37.940	-0.34	18.42	267.04	18.08	9	7/14/2014	35:28.6
8965	RSPe_2	-122.445	37.940	0.05	18.43	266.88	18.49	-122.445	37.940	-0.29	18.43	267.05	18.15	9	7/14/2014	35:28.5
8966	RSPe_2	-122.445	37.940	0.05	18.39	266.83	18.44	-122.445	37.940	-0.37	18.39	266.98	18.01	9	7/14/2014	35:28.4
8967	RSPe_2	-122.445	37.940	0.05	18.56	266.85	18.61	-122.445	37.940	-0.29	18.56	267.02	18.27	9	7/14/2014	35:28.3
8968	RSPe_2	-122.445	37.940	0.05	18.59	266.83	18.64	-122.445	37.940	-0.29	18.59	266.93	18.30	9	7/14/2014	35:28.2
8969	RSPe_2	-122.445	37.940	0.10	18.60	266.86	18.71	-122.445	37.940	-0.25	18.60	267.01	18.35	9	7/14/2014	35:28.1
8970	RSPe_2	-122.445	37.940	0.05	18.72	267.03	18.77	-122.445	37.940	0.00	18.72	267.05	18.72	9	7/14/2014	35:28.0
8971	RSPe_2	-122.445	37.940	0.05	18.76	267.08	18.82	-122.445	37.940	-0.25	18.76	267.10	18.51	9	7/14/2014	35:27.9
8972	RSPe_2	-122.445	37.940	0.05	18.74	267.12	18.79	-122.445	37.940	-0.14	18.74	267.00	18.61	9	7/14/2014	35:27.8
8973	RSPe_2	-122.445	37.940	0.10	18.77	267.19	18.87	-122.445	37.940	-0.17	18.77	267.05	18.60	9	7/14/2014	35:27.7
8974	RSPe_2	-122.445	37.940	0.10	18.74	267.25	18.85	-122.445	37.940	-0.20	18.74	267.07	18.54	9	7/14/2014	35:27.6
8975	RSPe_2	-122.445	37.940	0.10	18.73	267.23	18.84	-122.445	37.940	-0.14	18.73	267.12	18.60	9	7/14/2014	35:27.5

8976	RSPe_2	-122.445	37.940	0.05	18.63	267.23	18.68	-122.445	37.940	-0.20	18.63	267.18	18.42	9	7/14/2014	35:27.4
8977	RSPe_2	-122.445	37.940	0.10	18.45	267.21	18.55	-122.445	37.940	-0.17	18.45	267.22	18.28	9	7/14/2014	35:27.3
8978	RSPe_2	-122.445	37.940	0.10	18.39	267.12	18.49	-122.445	37.940	-0.20	18.39	267.23	18.18	9	7/14/2014	35:27.2
8979	RSPe_2	-122.445	37.940	0.14	18.31	267.08	18.45	-122.445	37.940	-0.17	18.31	267.32	18.14	9	7/14/2014	35:27.1
8980	RSPe_2	-122.445	37.940	0.10	18.30	267.08	18.41	-122.445	37.940	-0.17	18.30	267.34	18.14	9	7/14/2014	35:27.0
8981	RSPe_2	-122.445	37.940	0.10	18.21	267.19	18.31	-122.445	37.940	-0.09	18.21	267.47	18.12	9	7/14/2014	35:26.9
8982	RSPe_2	-122.445	37.940	0.10	18.33	267.30	18.44	-122.445	37.940	-0.14	18.33	267.60	18.20	9	7/14/2014	35:26.8
8983	RSPe_2	-122.445	37.940	0.10	18.17	267.37	18.27	-122.445	37.940	-0.05	18.17	267.59	18.12	9	7/14/2014	35:26.7
8984	RSPe_2	-122.445	37.940	0.10	18.13	267.34	18.23	-122.445	37.940	-0.09	18.13	267.56	18.05	9	7/14/2014	35:26.6
8985	RSPe_2	-122.445	37.940	0.10	18.16	267.34	18.26	-122.445	37.940	-0.05	18.16	267.51	18.11	9	7/14/2014	35:26.5
8986	RSPe_2	-122.445	37.940	0.10	18.11	267.37	18.21	-122.445	37.940	-0.09	18.11	267.42	18.02	9	7/14/2014	35:26.4
8987	RSPe_2	-122.445	37.940	0.10	18.06	267.36	18.17	-122.445	37.940	0.00	18.06	267.39	18.06	9	7/14/2014	35:26.3
8988	RSPe_2	-122.445	37.940	0.10	17.99	267.39	18.09	-122.445	37.940	-0.14	17.99	267.40	17.85	9	7/14/2014	35:26.2
8989	RSPe_2	-122.445	37.940	0.10	17.96	267.41	18.06	-122.445	37.940	-0.09	17.96	267.39	17.87	9	7/14/2014	35:26.1
8990	RSPe_2	-122.445	37.940	0.10	17.94	267.44	18.04	-122.445	37.940	-0.14	17.94	267.45	17.80	9	7/14/2014	35:26.0
8991	RSPe_2	-122.445	37.940	0.10	17.99	267.41	18.09	-122.445	37.940	-0.09	17.99	267.50	17.90	9	7/14/2014	35:25.9
8992	RSPe_2	-122.445	37.940	0.10	17.92	267.33	18.03	-122.445	37.940	-0.14	17.92	267.48	17.79	9	7/14/2014	35:25.8
8993	RSPe_2	-122.445	37.940	0.10	17.93	267.39	18.03	-122.445	37.940	-0.09	17.93	267.52	17.85	9	7/14/2014	35:25.7
8994	RSPe_2	-122.445	37.940	0.10	17.97	267.39	18.07	-122.445	37.940	-0.14	17.97	267.53	17.84	9	7/14/2014	35:25.6
8995	RSPe_2	-122.445	37.940	0.10	17.96	267.51	18.06	-122.445	37.940	-0.09	17.96	267.56	17.87	9	7/14/2014	35:25.5
8996	RSPe_2	-122.445	37.940	0.05	18.05	267.61	18.10	-122.445	37.940	-0.17	18.05	267.61	17.88	9	7/14/2014	35:25.4
8997	RSPe_2	-122.445	37.940	0.10	18.08	267.64	18.18	-122.445	37.940	-0.17	18.08	267.59	17.91	9	7/14/2014	35:25.3
8998	RSPe_2	-122.445	37.940	0.10	17.96	267.66	18.06	-122.445	37.940	-0.09	17.96	267.63	17.87	9	7/14/2014	35:25.2
8999	RSPe_2	-122.445	37.940	0.10	17.99	267.64	18.09	-122.445	37.940	-0.05	17.99	267.65	17.93	9	7/14/2014	35:25.1
9000	RSPe_2	-122.445	37.940	0.10	17.99	267.68	18.09	-122.445	37.940	-0.09	17.99	267.63	17.90	9	7/14/2014	35:25.0
9001	RSPe_2	-122.445	37.940	0.14	18.11	267.59	18.24	-122.445	37.940	-0.09	18.11	267.61	18.02	9	7/14/2014	35:24.9
9002	RSPe_2	-122.445	37.940	0.10	18.15	267.55	18.25	-122.445	37.940	-0.14	18.15	267.67	18.01	9	7/14/2014	35:24.8
9003	RSPe_2	-122.445	37.940	0.10	18.12	267.57	18.23	-122.445	37.940	-0.09	18.12	267.67	18.04	9	7/14/2014	35:24.7
9004	RSPe_2	-122.445	37.940	0.10	18.19	267.57	18.30	-122.445	37.940	-0.09	18.19	267.73	18.11	9	7/14/2014	35:24.6
9005	RSPe_2	-122.445	37.940	0.10	18.23	267.53	18.33	-122.445	37.940	-0.05	18.23	267.74	18.17	9	7/14/2014	35:24.5
9006	RSPe_2	-122.445	37.940	0.05	18.26	267.53	18.31	-122.445	37.940	-0.14	18.26	267.81	18.13	9	7/14/2014	35:24.4
9007	RSPe_2	-122.445	37.940	0.10	18.26	267.60	18.37	-122.445	37.940	-0.14	18.26	267.86	18.13	9	7/14/2014	35:24.3
9008	RSPe_2	-122.445	37.940	0.05	18.31	267.55	18.36	-122.445	37.940	-0.05	18.31	267.81	18.26	9	7/14/2014	35:24.2

9009	RSPe_2	-122.445	37.940	0.10	18.35	267.62	18.45	-122.445	37.940	-0.14	18.35	267.83	18.21	9	7/14/2014	35:24.1
9010	RSPe_2	-122.445	37.940	0.10	18.36	267.62	18.47	-122.445	37.940	-0.14	18.36	267.86	18.23	9	7/14/2014	35:24.0
9011	RSPe_2	-122.445	37.940	0.10	18.43	267.71	18.53	-122.445	37.940	-0.14	18.43	267.94	18.29	9	7/14/2014	35:23.9
9012	RSPe_2	-122.445	37.940	0.10	18.41	267.68	18.51	-122.445	37.940	-0.14	18.41	267.92	18.28	9	7/14/2014	35:23.8
9013	RSPe_2	-122.445	37.940	0.10	18.47	267.73	18.57	-122.445	37.940	-0.14	18.47	267.97	18.34	9	7/14/2014	35:23.7
9014	RSPe_2	-122.445	37.940	0.05	18.52	267.71	18.57	-122.445	37.940	-0.17	18.52	267.97	18.35	9	7/14/2014	35:23.6
9015	RSPe_2	-122.445	37.940	0.10	18.49	267.76	18.59	-122.445	37.940	-0.17	18.49	267.99	18.32	9	7/14/2014	35:23.5
9016	RSPe_2	-122.445	37.940	0.05	18.53	267.76	18.59	-122.445	37.940	-0.17	18.53	267.99	18.37	9	7/14/2014	35:23.4
9017	RSPe_2	-122.445	37.940	0.10	18.60	267.82	18.71	-122.445	37.940	-0.20	18.60	267.99	18.40	9	7/14/2014	35:23.3
9018	RSPe_2	-122.445	37.940	0.10	18.60	267.83	18.71	-122.445	37.940	-0.25	18.60	267.95	18.35	9	7/14/2014	35:23.2
9019	RSPe_2	-122.445	37.940	0.10	18.66	267.93	18.76	-122.445	37.940	-0.14	18.66	268.06	18.52	9	7/14/2014	35:23.1
9020	RSPe_2	-122.445	37.940	0.10	18.52	268.06	18.63	-122.445	37.940	-0.17	18.52	268.10	18.36	9	7/14/2014	35:23.0
9021	RSPe_2	-122.445	37.940	0.10	18.52	268.06	18.63	-122.445	37.940	-0.20	18.52	268.08	18.32	9	7/14/2014	35:22.9
9022	RSPe_2	-122.445	37.940	0.10	18.49	268.15	18.59	-122.445	37.940	-0.17	18.49	268.17	18.32	9	7/14/2014	35:22.8
9023	RSPe_2	-122.445	37.940	0.10	18.42	268.24	18.52	-122.445	37.940	-0.09	18.42	268.20	18.33	9	7/14/2014	35:22.7
9024	RSPe_2	-122.445	37.940	0.10	18.43	268.31	18.53	-122.445	37.940	-0.17	18.43	268.30	18.26	9	7/14/2014	35:22.6
9025	RSPe_2	-122.445	37.940	0.10	18.37	268.37	18.47	-122.445	37.940	-0.20	18.37	268.31	18.17	9	7/14/2014	35:22.5
9026	RSPe_2	-122.445	37.940	0.10	18.31	268.31	18.41	-122.445	37.940	-0.17	18.31	268.37	18.14	9	7/14/2014	35:22.4
9027	RSPe_2	-122.445	37.940	0.10	18.25	268.38	18.35	-122.445	37.940	-0.17	18.25	268.43	18.08	9	7/14/2014	35:22.3
9028	RSPe_2	-122.445	37.940	0.05	18.22	268.37	18.27	-122.445	37.940	-0.20	18.22	268.48	18.02	9	7/14/2014	35:22.2
9029	RSPe_2	-122.445	37.940	0.10	18.18	268.39	18.28	-122.445	37.940	-0.20	18.18	268.51	17.97	9	7/14/2014	35:22.1
9030	RSPe_2	-122.445	37.940	0.05	18.12	268.44	18.17	-122.445	37.940	-0.20	18.12	268.52	17.92	9	7/14/2014	35:22.0
9031	RSPe_2	-122.445	37.940	0.05	18.12	268.56	18.17	-122.445	37.940	-0.17	18.12	268.59	17.95	9	7/14/2014	35:21.9
9032	RSPe_2	-122.445	37.940	0.05	18.09	268.55	18.14	-122.445	37.940	-0.17	18.09	268.60	17.92	9	7/14/2014	35:21.8
9033	RSPe_2	-122.445	37.940	0.05	18.01	268.62	18.06	-122.445	37.940	-0.17	18.01	268.64	17.84	9	7/14/2014	35:21.7
9034	RSPe_2	-122.445	37.940	0.05	18.00	268.67	18.05	-122.445	37.940	-0.17	18.00	268.70	17.83	9	7/14/2014	35:21.6
9035	RSPe_2	-122.445	37.940	0.10	17.99	268.73	18.09	-122.445	37.940	-0.20	17.99	268.71	17.78	9	7/14/2014	35:21.5
9036	RSPe_2	-122.445	37.940	0.05	17.98	268.76	18.03	-122.445	37.940	-0.17	17.98	268.84	17.81	9	7/14/2014	35:21.4
9037	RSPe_2	-122.445	37.940	0.10	17.96	268.77	18.06	-122.445	37.940	-0.17	17.96	268.79	17.79	9	7/14/2014	35:21.3
9038	RSPe_2	-122.445	37.940	0.05	17.88	268.75	17.93	-122.445	37.940	-0.17	17.88	268.79	17.71	9	7/14/2014	35:21.2
9039	RSPe_2	-122.445	37.940	0.10	17.90	268.78	18.00	-122.445	37.940	-0.17	17.90	268.84	17.73	9	7/14/2014	35:21.1
9040	RSPe_2	-122.445	37.940	0.10	17.85	268.69	17.95	-122.445	37.940	-0.20	17.85	268.76	17.64	9	7/14/2014	35:21.0
9041	RSPe_2	-122.445	37.940	0.10	17.78	268.65	17.88	-122.445	37.940	-0.17	17.78	268.73	17.61	9	7/14/2014	35:20.9

9042	RSPe_2	-122.445	37.940	0.05	17.76	268.58	17.81	-122.445	37.940	-0.20	17.76	268.73	17.56	9	7/14/2014	35:20.8
9043	RSPe_2	-122.445	37.940	0.10	17.82	268.53	17.93	-122.445	37.940	-0.20	17.82	268.74	17.62	9	7/14/2014	35:20.7
9044	RSPe_2	-122.445	37.940	0.05	17.84	268.47	17.89	-122.445	37.940	-0.20	17.84	268.82	17.64	9	7/14/2014	35:20.6
9045	RSPe_2	-122.445	37.940	0.10	17.86	268.58	17.96	-122.445	37.940	-0.17	17.86	268.80	17.69	9	7/14/2014	35:20.5
9046	RSPe_2	-122.445	37.940	0.10	18.00	268.67	18.10	-122.445	37.940	-0.17	18.00	268.90	17.83	9	7/14/2014	35:20.4
9047	RSPe_2	-122.445	37.940	0.10	17.94	268.69	18.04	-122.445	37.940	-0.17	17.94	268.90	17.77	9	7/14/2014	35:20.3
9048	RSPe_2	-122.445	37.940	0.05	18.00	268.78	18.05	-122.445	37.940	-0.17	18.00	268.98	17.83	9	7/14/2014	35:20.2
9049	RSPe_2	-122.445	37.940	0.10	18.15	268.76	18.25	-122.445	37.940	-0.17	18.15	269.02	17.98	9	7/14/2014	35:20.1
9050	RSPe_2	-122.445	37.940	0.10	18.19	268.82	18.29	-122.445	37.940	-0.17	18.19	269.04	18.02	9	7/14/2014	35:20.0
9051	RSPe_2	-122.445	37.940	0.10	18.20	268.78	18.31	-122.445	37.940	-0.17	18.20	269.00	18.03	9	7/14/2014	35:19.9
9052	RSPe_2	-122.445	37.940	0.10	18.17	268.83	18.27	-122.445	37.940	-0.20	18.17	269.04	17.97	9	7/14/2014	35:19.8
9053	RSPe_2	-122.445	37.940	0.10	18.14	268.89	18.24	-122.445	37.940	-0.20	18.14	269.00	17.94	9	7/14/2014	35:19.7
9054	RSPe_2	-122.445	37.940	0.10	18.12	268.89	18.23	-122.445	37.940	-0.20	18.12	268.97	17.92	9	7/14/2014	35:19.6
9055	RSPe_2	-122.445	37.940	0.10	18.10	268.98	18.20	-122.445	37.940	-0.20	18.10	268.97	17.89	9	7/14/2014	35:19.5
9056	RSPe_2	-122.445	37.940	0.10	18.07	268.98	18.17	-122.445	37.940	-0.17	18.07	268.95	17.90	9	7/14/2014	35:19.4
9057	RSPe_2	-122.445	37.940	0.14	18.07	269.02	18.21	-122.445	37.940	-0.17	18.07	268.98	17.90	9	7/14/2014	35:19.3
9058	RSPe_2	-122.445	37.940	0.10	18.05	269.07	18.16	-122.445	37.940	-0.20	18.05	269.02	17.85	9	7/14/2014	35:19.2
9059	RSPe_2	-122.445	37.940	0.14	18.06	269.07	18.20	-122.445	37.940	-0.17	18.06	269.02	17.90	9	7/14/2014	35:19.1
9060	RSPe_2	-122.445	37.940	0.10	18.07	269.03	18.17	-122.445	37.940	-0.17	18.07	269.07	17.90	9	7/14/2014	35:19.0
9061	RSPe_2	-122.445	37.940	0.10	18.10	269.05	18.20	-122.445	37.940	-0.14	18.10	269.09	17.96	9	7/14/2014	35:18.9
9062	RSPe_2	-122.445	37.940	0.10	18.14	269.12	18.24	-122.445	37.940	-0.14	18.14	269.15	18.00	9	7/14/2014	35:18.8
9063	RSPe_2	-122.445	37.940	0.14	18.12	269.11	18.26	-122.445	37.940	-0.17	18.12	269.18	17.96	9	7/14/2014	35:18.7
9064	RSPe_2	-122.445	37.940	0.10	18.15	269.16	18.25	-122.445	37.940	-0.17	18.15	269.20	17.98	9	7/14/2014	35:18.6
9065	RSPe_2	-122.445	37.940	0.14	18.17	269.27	18.30	-122.445	37.940	-0.20	18.17	269.29	17.96	9	7/14/2014	35:18.5
9066	RSPe_2	-122.445	37.940	0.14	18.19	269.34	18.33	-122.445	37.940	-0.14	18.19	269.31	18.05	9	7/14/2014	35:18.4
9067	RSPe_2	-122.445	37.940	0.14	18.21	269.41	18.35	-122.445	37.940	-0.17	18.21	269.34	18.04	9	7/14/2014	35:18.3
9068	RSPe_2	-122.445	37.940	0.14	18.21	269.40	18.35	-122.445	37.940	-0.20	18.21	269.35	18.01	9	7/14/2014	35:18.2
9069	RSPe_2	-122.445	37.940	0.14	18.33	269.41	18.47	-122.445	37.940	-0.17	18.33	269.36	18.17	9	7/14/2014	35:18.1
9070	RSPe_2	-122.445	37.940	0.14	18.31	269.39	18.45	-122.445	37.940	-0.14	18.31	269.36	18.17	9	7/14/2014	35:18.0
9071	RSPe_2	-122.445	37.940	0.14	18.32	269.34	18.45	-122.445	37.940	-0.17	18.32	269.34	18.15	9	7/14/2014	35:17.9
9072	RSPe_2	-122.445	37.940	0.14	18.28	269.27	18.42	-122.445	37.940	-0.17	18.28	269.21	18.11	9	7/14/2014	35:17.8
9073	RSPe_2	-122.445	37.940	0.14	18.37	269.29	18.51	-122.445	37.940	-0.17	18.37	269.35	18.20	9	7/14/2014	35:17.7
9074	RSPe_2	-122.445	37.940	0.14	18.37	269.34	18.51	-122.445	37.940	-0.17	18.37	269.38	18.20	9	7/14/2014	35:17.6

9075	RSPe_2	-122.445	37.940	0.19	18.37	269.41	18.56	-122.445	37.940	-0.14	18.37	269.46	18.24	9	7/14/2014 35:17.5
9076	RSPe_2	-122.445	37.940	0.19	18.48	269.41	18.67	-122.445	37.940	-0.08	18.48	269.45	18.40	9	7/14/2014 35:17.4
9077	RSPe_2	-122.445	37.940	0.19	18.41	269.41	18.60	-122.445	37.940	-0.14	18.41	269.45	18.28	9	7/14/2014 35:17.3
9078	RSPe_2	-122.445	37.940	0.19	18.44	269.38	18.63	-122.445	37.940	-0.08	18.44	269.47	18.36	9	7/14/2014 35:17.2
9079	RSPe_2	-122.445	37.940	0.22	18.45	269.41	18.67	-122.445	37.940	-0.05	18.45	269.45	18.40	9	7/14/2014 35:17.1
9080	RSPe_2	-122.445	37.940	0.22	18.47	269.39	18.70	-122.445	37.940	-0.05	18.47	269.41	18.42	9	7/14/2014 35:17.0
9081	RSPe_2	-122.445	37.940	0.22	18.50	269.47	18.73	-122.445	37.940	0.04	18.50	269.42	18.54	9	7/14/2014 35:16.9
9082	RSPe_2	-122.445	37.940	0.22	18.52	269.50	18.74	-122.445	37.940	-0.05	18.52	269.43	18.47	9	7/14/2014 35:16.8
9083	RSPe_2	-122.445	37.940	0.22	18.47	269.52	18.70	-122.445	37.940	0.00	18.47	269.42	18.47	9	7/14/2014 35:16.7
9084	RSPe_2	-122.445	37.940	0.22	18.52	269.50	18.75	-122.445	37.940	0.09	18.52	269.46	18.61	9	7/14/2014 35:16.6
9085	RSPe_2	-122.445	37.940	0.26	18.44	269.46	18.70	-122.445	37.940	0.04	18.44	269.41	18.48	9	7/14/2014 35:16.5
9086	RSPe_2	-122.445	37.940	0.26	18.47	269.44	18.72	-122.445	37.940	0.09	18.47	269.39	18.55	9	7/14/2014 35:16.4
9087	RSPe_2	-122.445	37.940	0.26	18.32	269.41	18.57	-122.445	37.940	0.12	18.32	269.43	18.44	9	7/14/2014 35:16.3
9088	RSPe_2	-122.445	37.940	0.22	18.31	269.46	18.53	-122.445	37.940	0.12	18.31	269.52	18.43	9	7/14/2014 35:16.2
9089	RSPe_2	-122.445	37.940	0.26	18.05	269.48	18.31	-122.445	37.940	0.12	18.05	269.57	18.17	9	7/14/2014 35:16.1
9090	RSPe_2	-122.445	37.940	0.26	18.12	269.48	18.38	-122.445	37.940	0.04	18.12	269.56	18.15	9	7/14/2014 35:16.0
9091	RSPe_2	-122.445	37.940	0.26	18.08	269.46	18.34	-122.445	37.940	0.09	18.08	269.57	18.16	9	7/14/2014 35:15.9
9092	RSPe_2	-122.445	37.940	0.26	17.94	269.46	18.20	-122.445	37.940	0.04	17.94	269.58	17.98	9	7/14/2014 35:15.8
9093	RSPe_2	-122.445	37.940	0.26	17.87	269.44	18.13	-122.445	37.940	0.09	17.87	269.52	17.96	9	7/14/2014 35:15.7
9094	RSPe_2	-122.445	37.940	0.22	17.86	269.44	18.08	-122.445	37.940	0.04	17.86	269.54	17.90	9	7/14/2014 35:15.6
9095	RSPe_2	-122.445	37.940	0.26	17.82	269.37	18.07	-122.445	37.940	0.09	17.82	269.52	17.90	9	7/14/2014 35:15.5
9096	RSPe_2	-122.445	37.940	0.22	17.76	269.46	17.99	-122.445	37.940	0.09	17.76	269.52	17.85	9	7/14/2014 35:15.4
9097	RSPe_2	-122.445	37.940	0.26	17.82	269.46	18.07	-122.445	37.940	0.12	17.82	269.48	17.94	9	7/14/2014 35:15.3
9098	RSPe_2	-122.445	37.940	0.26	17.81	269.52	18.07	-122.445	37.940	0.09	17.81	269.45	17.89	9	7/14/2014 35:15.2
9099	RSPe_2	-122.445	37.940	0.26	17.76	269.48	18.02	-122.445	37.940	0.12	17.76	269.44	17.88	9	7/14/2014 35:15.1
9100	RSPe_2	-122.445	37.940	0.26	17.64	269.51	17.90	-122.445	37.940	0.12	17.64	269.36	17.76	9	7/14/2014 35:15.0
9101	RSPe_2	-122.445	37.940	0.31	17.67	269.51	17.98	-122.445	37.940	0.12	17.67	269.40	17.79	9	7/14/2014 35:14.9
9102	RSPe_2	-122.445	37.940	0.26	17.61	269.51	17.87	-122.445	37.940	0.09	17.61	269.39	17.69	9	7/14/2014 35:14.8
9103	RSPe_2	-122.445	37.940	0.31	17.58	269.53	17.89	-122.445	37.940	0.17	17.58	269.35	17.75	9	7/14/2014 35:14.7
9104	RSPe_2	-122.445	37.941	0.26	17.57	269.46	17.83	-122.445	37.940	0.17	17.57	269.37	17.74	9	7/14/2014 35:14.6
9105	RSPe_2	-122.445	37.941	0.26	17.56	269.42	17.81	-122.445	37.941	0.17	17.56	269.29	17.73	9	7/14/2014 35:14.5
9106	RSPe_2	-122.445	37.941	0.26	17.55	269.42	17.80	-122.445	37.941	0.12	17.55	269.33	17.66	9	7/14/2014 35:14.4
9107	RSPe_2	-122.445	37.941	0.26	17.58	269.47	17.83	-122.445	37.941	0.17	17.58	269.35	17.75	9	7/14/2014 35:14.3

9108	RSPe_2	-122.445	37.941	0.26	17.58	269.47	17.83	-122.445	37.941	0.17	17.58	269.42	17.75	9	7/14/2014	35:14.2
9109	RSPe_2	-122.445	37.941	0.31	17.58	269.47	17.89	-122.445	37.941	0.17	17.58	269.42	17.75	9	7/14/2014	35:14.1
9110	RSPe_2	-122.445	37.941	0.26	17.59	269.53	17.85	-122.445	37.941	0.12	17.59	269.47	17.71	9	7/14/2014	35:14.0
9111	RSPe_2	-122.445	37.941	0.31	17.61	269.49	17.92	-122.445	37.941	0.12	17.61	269.55	17.73	9	7/14/2014	35:13.9
9112	RSPe_2	-122.445	37.941	0.26	17.63	269.54	17.89	-122.445	37.941	0.12	17.63	269.58	17.75	9	7/14/2014	35:13.8
9113	RSPe_2	-122.445	37.941	0.26	17.65	269.51	17.91	-122.445	37.941	0.20	17.65	269.66	17.86	9	7/14/2014	35:13.7
9114	RSPe_2	-122.445	37.941	0.26	17.69	269.54	17.95	-122.445	37.941	0.12	17.69	269.66	17.81	9	7/14/2014	35:13.6
9115	RSPe_2	-122.445	37.941	0.31	17.70	269.47	18.01	-122.445	37.941	0.17	17.70	269.60	17.87	9	7/14/2014	35:13.5
9116	RSPe_2	-122.445	37.941	0.26	17.76	269.45	18.02	-122.445	37.941	0.09	17.76	269.60	17.85	9	7/14/2014	35:13.4
9117	RSPe_2	-122.445	37.941	0.31	17.85	269.50	18.16	-122.445	37.941	0.09	17.85	269.60	17.94	9	7/14/2014	35:13.3
9118	RSPe_2	-122.445	37.941	0.26	17.82	269.51	18.07	-122.445	37.941	0.17	17.82	269.58	17.99	9	7/14/2014	35:13.2
9119	RSPe_2	-122.445	37.941	0.31	17.84	269.56	18.15	-122.445	37.941	0.12	17.84	269.62	17.96	9	7/14/2014	35:13.1
9120	RSPe_2	-122.445	37.941	0.31	17.85	269.58	18.16	-122.445	37.941	0.12	17.85	269.67	17.97	9	7/14/2014	35:13.0
9121	RSPe_2	-122.445	37.941	0.34	17.85	269.69	18.20	-122.445	37.941	0.17	17.85	269.73	18.02	9	7/14/2014	35:12.9
9122	RSPe_2	-122.445	37.941	0.26	17.88	269.73	18.13	-122.445	37.941	0.20	17.88	269.75	18.08	9	7/14/2014	35:12.8
9123	RSPe_2	-122.445	37.941	0.31	17.93	269.69	18.24	-122.445	37.941	0.17	17.93	269.68	18.10	9	7/14/2014	35:12.7
9124	RSPe_2	-122.445	37.941	0.31	17.89	269.71	18.20	-122.445	37.941	0.20	17.89	269.71	18.10	9	7/14/2014	35:12.6
9125	RSPe_2	-122.445	37.941	0.31	17.90	269.74	18.21	-122.445	37.941	0.20	17.90	269.67	18.11	9	7/14/2014	35:12.5
9126	RSPe_2	-122.445	37.941	0.31	17.93	269.76	18.24	-122.445	37.941	0.17	17.93	269.69	18.10	9	7/14/2014	35:12.4
9127	RSPe_2	-122.445	37.941	0.31	17.96	269.81	18.26	-122.445	37.941	0.20	17.96	269.67	18.16	9	7/14/2014	35:12.3
9128	RSPe_2	-122.445	37.941	0.31	17.97	269.76	18.28	-122.445	37.941	0.17	17.97	269.62	18.14	9	7/14/2014	35:12.2
9129	RSPe_2	-122.445	37.941	0.31	17.87	269.82	18.18	-122.445	37.941	0.17	17.87	269.68	18.04	9	7/14/2014	35:12.1
9130	RSPe_2	-122.445	37.941	0.31	17.82	269.92	18.13	-122.445	37.941	0.20	17.82	269.73	18.02	9	7/14/2014	35:12.0
9131	RSPe_2	-122.445	37.941	0.34	17.85	269.92	18.19	-122.445	37.941	0.20	17.85	269.79	18.05	9	7/14/2014	35:11.9
9132	RSPe_2	-122.445	37.941	0.31	17.77	269.94	18.08	-122.445	37.941	0.24	17.77	269.89	18.01	9	7/14/2014	35:11.8
9133	RSPe_2	-122.445	37.941	0.34	17.75	269.96	18.10	-122.445	37.941	0.20	17.75	269.93	17.96	9	7/14/2014	35:11.7
9134	RSPe_2	-122.445	37.941	0.31	17.63	269.92	17.93	-122.445	37.941	0.24	17.63	269.94	17.86	9	7/14/2014	35:11.6
9135	RSPe_2	-122.445	37.941	0.31	17.64	269.96	17.95	-122.445	37.941	0.29	17.64	269.96	17.93	9	7/14/2014	35:11.5
9136	RSPe_2	-122.445	37.941	0.31	17.61	269.99	17.92	-122.445	37.941	0.24	17.61	269.96	17.85	9	7/14/2014	35:11.4
9137	RSPe_2	-122.445	37.941	0.31	17.47	270.03	17.78	-122.445	37.941	0.24	17.47	269.98	17.71	9	7/14/2014	35:11.3
9138	RSPe_2	-122.445	37.941	0.31	17.39	270.01	17.70	-122.445	37.941	0.24	17.39	269.93	17.63	9	7/14/2014	35:11.2
9139	RSPe_2	-122.445	37.941	0.34	17.44	270.03	17.78	-122.445	37.941	0.29	17.44	269.98	17.73	9	7/14/2014	35:11.1
9140	RSPe_2	-122.445	37.941	0.31	17.42	270.10	17.73	-122.445	37.941	0.24	17.42	270.00	17.65	9	7/14/2014	35:11.0

9141	RSPe_2	-122.445	37.941	0.34	17.35	270.07	17.70	-122.445	37.941	0.24	17.35	270.03	17.59	9	7/14/2014	35:10.9
9142	RSPe_2	-122.445	37.941	0.31	17.31	270.10	17.62	-122.445	37.941	0.29	17.31	270.00	17.60	9	7/14/2014	35:10.8
9143	RSPe_2	-122.445	37.941	0.34	17.36	270.10	17.70	-122.445	37.941	0.24	17.36	270.12	17.60	9	7/14/2014	35:10.7
9144	RSPe_2	-122.445	37.941	0.31	17.32	270.14	17.63	-122.445	37.941	0.32	17.32	270.16	17.64	9	7/14/2014	35:10.6
9145	RSPe_2	-122.445	37.941	0.34	17.35	270.17	17.70	-122.445	37.941	0.32	17.35	270.21	17.68	9	7/14/2014	35:10.5
9146	RSPe_2	-122.445	37.941	0.31	17.34	270.21	17.65	-122.445	37.941	0.29	17.34	270.16	17.62	9	7/14/2014	35:10.4
9147	RSPe_2	-122.445	37.941	0.34	17.28	270.12	17.63	-122.445	37.941	0.29	17.28	270.14	17.57	9	7/14/2014	35:10.3
9148	RSPe_2	-122.445	37.941	0.31	17.35	270.14	17.66	-122.445	37.941	0.29	17.35	270.16	17.64	9	7/14/2014	35:10.2
9149	RSPe_2	-122.445	37.941	0.31	17.34	270.18	17.65	-122.445	37.941	0.37	17.34	270.12	17.71	9	7/14/2014	35:10.1
9150	RSPe_2	-122.445	37.941	0.31	17.29	270.15	17.60	-122.445	37.941	0.32	17.29	270.14	17.61	9	7/14/2014	35:10.0
9151	RSPe_2	-122.445	37.941	0.34	17.32	270.19	17.66	-122.445	37.941	0.32	17.32	270.12	17.64	9	7/14/2014	35:09.9
9152	RSPe_2	-122.445	37.941	0.31	17.35	270.19	17.66	-122.445	37.941	0.29	17.35	270.10	17.64	9	7/14/2014	35:09.8
9153	RSPe_2	-122.445	37.941	0.34	17.37	270.21	17.71	-122.445	37.941	0.32	17.37	270.13	17.69	9	7/14/2014	35:09.7
9154	RSPe_2	-122.445	37.941	0.31	17.38	270.24	17.69	-122.445	37.941	0.29	17.38	270.16	17.67	9	7/14/2014	35:09.6
9155	RSPe_2	-122.445	37.941	0.31	17.59	270.21	17.90	-122.445	37.941	0.32	17.59	270.16	17.92	9	7/14/2014	35:09.5
9156	RSPe_2	-122.445	37.941	0.34	17.49	270.28	17.83	-122.445	37.941	0.32	17.49	270.26	17.81	9	7/14/2014	35:09.4
9157	RSPe_2	-122.445	37.941	0.34	17.51	270.28	17.86	-122.445	37.941	0.37	17.51	270.34	17.89	9	7/14/2014	35:09.3
9158	RSPe_2	-122.445	37.941	0.34	17.58	270.32	17.92	-122.445	37.941	0.32	17.58	270.39	17.90	9	7/14/2014	35:09.2
9159	RSPe_2	-122.445	37.941	0.34	17.64	270.35	17.98	-122.445	37.941	0.29	17.64	270.43	17.93	9	7/14/2014	35:09.1
9160	RSPe_2	-122.445	37.941	0.31	17.67	270.35	17.98	-122.445	37.941	0.32	17.67	270.46	17.99	9	7/14/2014	35:09.0
9161	RSPe_2	-122.445	37.941	0.34	17.66	270.37	18.01	-122.445	37.941	0.37	17.66	270.55	18.04	9	7/14/2014	35:08.9
9162	RSPe_2	-122.445	37.941	0.34	17.68	270.37	18.03	-122.445	37.941	0.32	17.68	270.47	18.01	9	7/14/2014	35:08.8
9163	RSPe_2	-122.445	37.941	0.34	17.69	270.42	18.04	-122.445	37.941	0.37	17.69	270.52	18.07	9	7/14/2014	35:08.7
9164	RSPe_2	-122.445	37.941	0.34	17.72	270.44	18.06	-122.445	37.941	0.32	17.72	270.51	18.04	9	7/14/2014	35:08.6
9165	RSPe_2	-122.445	37.941	0.34	17.72	270.48	18.07	-122.445	37.941	0.37	17.72	270.54	18.10	9	7/14/2014	35:08.5
9166	RSPe_2	-122.445	37.941	0.34	17.78	270.53	18.12	-122.445	37.941	0.37	17.78	270.54	18.15	9	7/14/2014	35:08.4
9167	RSPe_2	-122.445	37.941	0.34	17.76	270.55	18.11	-122.445	37.941	0.37	17.76	270.59	18.14	9	7/14/2014	35:08.3
9168	RSPe_2	-122.445	37.941	0.34	17.77	270.57	18.11	-122.445	37.941	0.37	17.77	270.61	18.14	9	7/14/2014	35:08.2
9169	RSPe_2	-122.445	37.941	0.34	17.81	270.66	18.15	-122.445	37.941	0.37	17.81	270.68	18.18	9	7/14/2014	35:08.1
9170	RSPe_2	-122.445	37.941	0.34	17.82	270.62	18.17	-122.445	37.941	0.37	17.82	270.68	18.20	9	7/14/2014	35:08.0
9171	RSPe_2	-122.445	37.941	0.40	17.82	270.66	18.22	-122.445	37.941	0.41	17.82	270.77	18.23	9	7/14/2014	35:07.9
9172	RSPe_2	-122.445	37.941	0.34	17.84	270.64	18.18	-122.445	37.941	0.37	17.84	270.82	18.21	9	7/14/2014	35:07.8
9173	RSPe_2	-122.445	37.941	0.40	17.87	270.69	18.27	-122.445	37.941	0.46	17.87	270.86	18.33	9	7/14/2014	35:07.7

9174	RSPe_2	-122.445	37.941	0.34	17.79	270.77	18.14	-122.445	37.941	0.41	17.79	270.90	18.20	9	7/14/2014	35:07.6
9175	RSPe_2	-122.445	37.941	0.40	17.85	270.80	18.24	-122.445	37.941	0.46	17.85	270.90	18.31	9	7/14/2014	35:07.5
9176	RSPe_2	-122.445	37.941	0.34	17.72	270.75	18.07	-122.445	37.941	0.46	17.72	270.85	18.18	9	7/14/2014	35:07.4
9177	RSPe_2	-122.445	37.941	0.40	17.76	270.77	18.16	-122.445	37.941	0.46	17.76	270.82	18.22	9	7/14/2014	35:07.3
9178	RSPe_2	-122.445	37.941	0.34	17.67	270.75	18.01	-122.445	37.941	0.41	17.67	270.81	18.08	9	7/14/2014	35:07.2
9179	RSPe_2	-122.445	37.941	0.40	17.58	270.80	17.97	-122.445	37.941	0.46	17.58	270.79	18.04	9	7/14/2014	35:07.1
9180	RSPe_2	-122.445	37.941	0.34	17.56	270.86	17.91	-122.445	37.941	0.46	17.56	270.82	18.02	9	7/14/2014	35:07.0
9181	RSPe_2	-122.445	37.941	0.34	17.53	270.86	17.87	-122.445	37.941	0.46	17.53	270.80	17.99	9	7/14/2014	35:06.9
9182	RSPe_2	-122.445	37.941	0.34	17.43	270.87	17.78	-122.445	37.941	0.41	17.43	270.83	17.84	9	7/14/2014	35:06.8
9183	RSPe_2	-122.445	37.941	0.40	17.49	270.91	17.88	-122.445	37.941	0.46	17.49	270.86	17.95	9	7/14/2014	35:06.7
9184	RSPe_2	-122.445	37.941	0.34	17.56	270.93	17.91	-122.445	37.941	0.46	17.56	270.88	18.02	9	7/14/2014	35:06.6
9185	RSPe_2	-122.445	37.941	0.40	17.35	270.95	17.74	-122.445	37.941	0.49	17.35	270.89	17.84	9	7/14/2014	35:06.5
9186	RSPe_2	-122.445	37.941	0.34	17.35	270.95	17.69	-122.445	37.941	0.46	17.35	270.89	17.81	9	7/14/2014	35:06.4
9187	RSPe_2	-122.445	37.941	0.34	17.35	270.95	17.70	-122.445	37.941	0.49	17.35	270.93	17.85	9	7/14/2014	35:06.3
9188	RSPe_2	-122.445	37.941	0.34	17.35	270.98	17.69	-122.445	37.941	0.46	17.35	270.91	17.81	9	7/14/2014	35:06.2
9189	RSPe_2	-122.445	37.941	0.34	17.31	270.98	17.65	-122.445	37.941	0.49	17.31	271.00	17.80	9	7/14/2014	35:06.1
9190	RSPe_2	-122.445	37.941	0.34	17.27	271.00	17.61	-122.445	37.941	0.46	17.27	271.03	17.73	9	7/14/2014	35:06.0
9191	RSPe_2	-122.445	37.941	0.34	17.28	271.00	17.62	-122.445	37.941	0.49	17.28	271.08	17.77	9	7/14/2014	35:05.9
9192	RSPe_2	-122.445	37.941	0.34	17.32	271.00	17.66	-122.445	37.941	0.46	17.32	271.15	17.77	9	7/14/2014	35:05.8
9193	RSPe_2	-122.445	37.941	0.40	17.29	271.02	17.69	-122.445	37.941	0.46	17.29	271.18	17.75	9	7/14/2014	35:05.7
9194	RSPe_2	-122.445	37.941	0.34	17.32	271.02	17.66	-122.445	37.941	0.46	17.32	271.21	17.77	9	7/14/2014	35:05.6
9195	RSPe_2	-122.445	37.941	0.34	17.33	271.03	17.67	-122.445	37.941	0.46	17.33	271.24	17.79	9	7/14/2014	35:05.5
9196	RSPe_2	-122.445	37.941	0.34	17.32	271.07	17.66	-122.445	37.941	0.46	17.32	271.29	17.77	9	7/14/2014	35:05.4
9197	RSPe_2	-122.445	37.941	0.34	17.31	271.13	17.65	-122.445	37.941	0.49	17.31	271.28	17.80	9	7/14/2014	35:05.3
9198	RSPe_2	-122.445	37.941	0.31	17.33	271.11	17.64	-122.445	37.941	0.41	17.33	271.28	17.74	9	7/14/2014	35:05.2
9199	RSPe_2	-122.445	37.941	0.34	17.33	271.18	17.67	-122.445	37.941	0.46	17.33	271.29	17.79	9	7/14/2014	35:05.1
9200	RSPe_2	-122.445	37.941	0.31	17.37	271.21	17.68	-122.445	37.941	0.37	17.37	271.29	17.74	9	7/14/2014	35:05.0
9201	RSPe_2	-122.445	37.941	0.34	17.37	271.23	17.71	-122.445	37.941	0.46	17.37	271.27	17.83	9	7/14/2014	35:04.9
9202	RSPe_2	-122.445	37.941	0.31	17.38	271.29	17.69	-122.445	37.941	0.41	17.38	271.35	17.79	9	7/14/2014	35:04.8
9203	RSPe_2	-122.445	37.941	0.34	17.45	271.31	17.80	-122.445	37.941	0.41	17.45	271.42	17.86	9	7/14/2014	35:04.7
9204	RSPe_2	-122.445	37.941	0.31	17.42	271.34	17.73	-122.445	37.941	0.41	17.42	271.41	17.83	9	7/14/2014	35:04.6
9205	RSPe_2	-122.445	37.941	0.34	17.45	271.38	17.80	-122.445	37.941	0.46	17.45	271.49	17.91	9	7/14/2014	35:04.5
9206	RSPe_2	-122.445	37.941	0.31	17.51	271.38	17.82	-122.445	37.941	0.41	17.51	271.51	17.92	9	7/14/2014	35:04.4

9207	RSPe_2	-122.445	37.941	0.34	17.51	271.43	17.85	-122.445	37.941	0.46	17.51	271.58	17.97	9	7/14/2014	35:04.3
9208	RSPe_2	-122.445	37.941	0.34	17.53	271.40	17.87	-122.445	37.941	0.41	17.53	271.58	17.94	9	7/14/2014	35:04.2
9209	RSPe_2	-122.445	37.941	0.34	17.65	271.40	18.00	-122.445	37.941	0.46	17.65	271.63	18.11	9	7/14/2014	35:04.1
9210	RSPe_2	-122.445	37.941	0.31	17.67	271.41	17.98	-122.445	37.941	0.41	17.67	271.64	18.08	9	7/14/2014	35:04.0
9211	RSPe_2	-122.445	37.941	0.31	17.65	271.49	17.96	-122.445	37.941	0.41	17.65	271.69	18.06	9	7/14/2014	35:03.9
9212	RSPe_2	-122.445	37.941	0.31	17.68	271.51	17.99	-122.445	37.941	0.37	17.68	271.72	18.06	9	7/14/2014	35:03.8
9213	RSPe_2	-122.445	37.941	0.31	17.70	271.48	18.01	-122.445	37.941	0.41	17.70	271.69	18.11	9	7/14/2014	35:03.7
9214	RSPe_2	-122.445	37.941	0.26	17.72	271.43	17.97	-122.445	37.941	0.37	17.72	271.67	18.09	9	7/14/2014	35:03.6
9215	RSPe_2	-122.445	37.941	0.31	17.77	271.47	18.08	-122.445	37.941	0.41	17.77	271.71	18.18	9	7/14/2014	35:03.5
9216	RSPe_2	-122.445	37.941	0.26	17.76	271.57	18.02	-122.445	37.941	0.37	17.76	271.73	18.14	9	7/14/2014	35:03.4
9217	RSPe_2	-122.445	37.941	0.31	17.76	271.58	18.07	-122.445	37.941	0.41	17.76	271.76	18.17	9	7/14/2014	35:03.3
9218	RSPe_2	-122.445	37.941	0.26	17.79	271.63	18.04	-122.445	37.941	0.32	17.79	271.71	18.11	9	7/14/2014	35:03.2
9219	RSPe_2	-122.445	37.941	0.31	17.80	271.68	18.11	-122.445	37.941	0.32	17.80	271.72	18.13	9	7/14/2014	35:03.1
9220	RSPe_2	-122.445	37.941	0.26	17.82	271.72	18.07	-122.445	37.941	0.37	17.82	271.76	18.19	9	7/14/2014	35:03.0
9221	RSPe_2	-122.445	37.941	0.31	17.84	271.74	18.15	-122.445	37.941	0.32	17.84	271.78	18.16	9	7/14/2014	35:02.9
9222	RSPe_2	-122.445	37.941	0.26	17.85	271.72	18.11	-122.445	37.941	0.29	17.85	271.82	18.14	9	7/14/2014	35:02.8
9223	RSPe_2	-122.445	37.941	0.31	17.85	271.76	18.16	-122.445	37.941	0.32	17.85	271.86	18.18	9	7/14/2014	35:02.7
9224	RSPe_2	-122.445	37.941	0.31	17.90	271.74	18.21	-122.445	37.941	0.29	17.90	271.91	18.19	9	7/14/2014	35:02.6
9225	RSPe_2	-122.445	37.941	0.31	17.92	271.74	18.23	-122.445	37.941	0.32	17.92	271.96	18.25	9	7/14/2014	35:02.5
9226	RSPe_2	-122.445	37.941	0.31	17.94	271.70	18.25	-122.445	37.941	0.32	17.94	272.00	18.26	9	7/14/2014	35:02.4
9227	RSPe_2	-122.445	37.941	0.31	17.97	271.72	18.28	-122.445	37.941	0.32	17.97	271.98	18.30	9	7/14/2014	35:02.3
9228	RSPe_2	-122.445	37.941	0.26	17.97	271.70	18.23	-122.445	37.941	0.32	17.97	271.96	18.30	9	7/14/2014	35:02.2
9229	RSPe_2	-122.445	37.941	0.31	18.04	271.74	18.35	-122.445	37.941	0.37	18.04	271.98	18.41	9	7/14/2014	35:02.1
9230	RSPe_2	-122.445	37.941	0.26	18.03	271.74	18.29	-122.445	37.941	0.32	18.03	271.93	18.35	9	7/14/2014	35:02.0
9231	RSPe_2	-122.445	37.941	0.26	18.07	271.75	18.33	-122.445	37.941	0.32	18.07	271.90	18.39	9	7/14/2014	35:01.9
9232	RSPe_2	-122.445	37.941	0.22	18.07	271.79	18.29	-122.445	37.941	0.29	18.07	271.83	18.36	9	7/14/2014	35:01.8
9233	RSPe_2	-122.445	37.941	0.26	18.11	271.86	18.37	-122.445	37.941	0.32	18.11	271.83	18.43	9	7/14/2014	35:01.7
9234	RSPe_2	-122.445	37.941	0.22	18.14	271.90	18.36	-122.445	37.941	0.29	18.14	271.85	18.43	9	7/14/2014	35:01.6
9235	RSPe_2	-122.445	37.941	0.26	18.17	271.95	18.42	-122.445	37.941	0.29	18.17	271.90	18.45	9	7/14/2014	35:01.5
9236	RSPe_2	-122.445	37.941	0.26	18.21	271.96	18.47	-122.445	37.941	0.29	18.21	271.93	18.50	9	7/14/2014	35:01.4
9237	RSPe_2	-122.445	37.941	0.26	18.19	271.99	18.45	-122.445	37.941	0.29	18.19	271.97	18.48	9	7/14/2014	35:01.3
9238	RSPe_2	-122.445	37.941	0.22	18.25	271.99	18.47	-122.445	37.941	0.24	18.25	272.05	18.49	9	7/14/2014	35:01.2
9239	RSPe_2	-122.445	37.941	0.26	18.27	271.90	18.53	-122.445	37.941	0.29	18.27	271.99	18.56	9	7/14/2014	35:01.1

9240	RSPe_2	-122.445	37.941	0.26	18.20	271.88	18.46	-122.445	37.941	0.32	18.20	272.03	18.53	9	7/14/2014	35:01.0
9241	RSPe_2	-122.445	37.941	0.31	18.19	271.91	18.50	-122.445	37.941	0.29	18.19	272.08	18.48	9	7/14/2014	35:00.9
9242	RSPe_2	-122.445	37.941	0.22	18.25	271.93	18.47	-122.445	37.941	0.29	18.25	272.10	18.54	9	7/14/2014	35:00.8
9243	RSPe_2	-122.445	37.941	0.22	18.13	271.93	18.36	-122.445	37.941	0.29	18.13	272.12	18.42	9	7/14/2014	35:00.7
9244	RSPe_2	-122.445	37.941	0.26	18.13	272.01	18.39	-122.445	37.941	0.24	18.13	272.17	18.37	9	7/14/2014	35:00.6
9245	RSPe_2	-122.445	37.941	0.26	18.09	271.95	18.34	-122.445	37.941	0.24	18.09	272.14	18.33	9	7/14/2014	35:00.5
9246	RSPe_2	-122.445	37.941	0.22	18.07	271.99	18.29	-122.445	37.941	0.21	18.07	272.12	18.27	9	7/14/2014	35:00.4
9247	RSPe_2	-122.445	37.941	0.22	18.13	272.04	18.36	-122.445	37.941	0.21	18.13	272.22	18.34	9	7/14/2014	35:00.3
9248	RSPe_2	-122.445	37.941	0.22	18.05	272.10	18.28	-122.445	37.941	0.17	18.05	272.21	18.23	9	7/14/2014	35:00.2
9249	RSPe_2	-122.445	37.941	0.22	18.01	272.09	18.23	-122.445	37.941	0.21	18.01	272.24	18.22	9	7/14/2014	35:00.1
9250	RSPe_2	-122.445	37.941	0.22	17.96	272.13	18.18	-122.445	37.941	0.17	17.96	272.26	18.13	9	7/14/2014	35:00.0
9251	RSPe_2	-122.445	37.941	0.26	17.96	272.13	18.21	-122.445	37.941	0.17	17.96	272.32	18.13	9	7/14/2014	34:59.9
9252	RSPe_2	-122.445	37.941	0.22	17.92	272.13	18.15	-122.445	37.941	0.21	17.92	272.35	18.13	9	7/14/2014	34:59.8
9253	RSPe_2	-122.445	37.941	0.22	17.93	272.13	18.15	-122.445	37.941	0.17	17.93	272.37	18.10	9	7/14/2014	34:59.7
9254	RSPe_2	-122.445	37.941	0.22	17.93	272.15	18.15	-122.445	37.941	0.21	17.93	272.39	18.14	9	7/14/2014	34:59.6
9255	RSPe_2	-122.445	37.941	0.26	17.88	272.11	18.14	-122.445	37.941	0.21	17.88	272.37	18.08	9	7/14/2014	34:59.5
9256	RSPe_2	-122.445	37.941	0.22	17.89	272.18	18.11	-122.445	37.941	0.21	17.89	272.34	18.09	9	7/14/2014	34:59.4
9257	RSPe_2	-122.445	37.941	0.22	17.79	272.13	18.01	-122.445	37.941	0.17	17.79	272.35	17.96	9	7/14/2014	34:59.3
9258	RSPe_2	-122.445	37.941	0.22	17.78	272.20	18.00	-122.445	37.941	0.17	17.78	272.31	17.95	9	7/14/2014	34:59.2
9259	RSPe_2	-122.445	37.941	0.22	17.89	272.15	18.11	-122.445	37.941	0.17	17.89	272.32	18.06	9	7/14/2014	34:59.1
9260	RSPe_2	-122.445	37.941	0.22	17.69	272.18	17.92	-122.445	37.941	0.17	17.69	272.29	17.87	9	7/14/2014	34:59.0
9261	RSPe_2	-122.445	37.941	0.22	17.71	272.20	17.93	-122.445	37.941	0.17	17.71	272.26	17.88	9	7/14/2014	34:58.9
9262	RSPe_2	-122.445	37.941	0.22	17.65	272.25	17.88	-122.445	37.941	0.12	17.65	272.29	17.77	9	7/14/2014	34:58.8
9263	RSPe_2	-122.445	37.941	0.22	17.65	272.27	17.88	-122.445	37.941	0.12	17.65	272.29	17.77	9	7/14/2014	34:58.7
9264	RSPe_2	-122.445	37.941	0.22	17.68	272.29	17.90	-122.445	37.941	0.12	17.68	272.32	17.80	9	7/14/2014	34:58.6
9265	RSPe_2	-122.445	37.941	0.22	17.66	272.32	17.89	-122.445	37.941	0.17	17.66	272.38	17.83	9	7/14/2014	34:58.5
9266	RSPe_2	-122.445	37.941	0.19	17.71	272.33	17.90	-122.445	37.941	0.12	17.71	272.37	17.83	9	7/14/2014	34:58.4
9267	RSPe_2	-122.445	37.941	0.22	17.65	272.29	17.88	-122.445	37.941	0.12	17.65	272.37	17.77	9	7/14/2014	34:58.3
9268	RSPe_2	-122.445	37.941	0.19	17.67	272.36	17.86	-122.445	37.941	0.09	17.67	272.44	17.76	9	7/14/2014	34:58.2
9269	RSPe_2	-122.445	37.941	0.22	17.72	272.36	17.94	-122.445	37.941	0.09	17.72	272.39	17.80	9	7/14/2014	34:58.1
9270	RSPe_2	-122.445	37.941	0.19	17.73	272.38	17.92	-122.445	37.941	0.12	17.73	272.49	17.85	9	7/14/2014	34:58.0
9271	RSPe_2	-122.445	37.941	0.22	17.76	272.40	17.99	-122.445	37.941	0.12	17.76	272.46	17.88	9	7/14/2014	34:57.9
9272	RSPe_2	-122.445	37.941	0.19	17.81	272.44	18.00	-122.445	37.941	0.09	17.81	272.53	17.90	9	7/14/2014	34:57.8

9273	RSPe_2	-122.445	37.941	0.19	17.89	272.43	18.08	-122.445	37.941	0.09	17.89	272.60	17.97	9	7/14/2014 34:57.7
9274	RSPe_2	-122.445	37.941	0.19	17.84	272.47	18.03	-122.445	37.941	0.04	17.84	272.64	17.88	9	7/14/2014 34:57.6
9275	RSPe_2	-122.445	37.941	0.19	17.95	272.45	18.14	-122.445	37.941	0.09	17.95	272.62	18.03	9	7/14/2014 34:57.5
9276	RSPe_2	-122.445	37.941	0.19	17.94	272.43	18.13	-122.445	37.941	0.09	17.94	272.62	18.03	9	7/14/2014 34:57.4
9277	RSPe_2	-122.445	37.941	0.22	17.89	272.41	18.11	-122.445	37.941	0.09	17.89	272.56	17.97	9	7/14/2014 34:57.3
9278	RSPe_2	-122.445	37.941	0.19	17.89	272.47	18.08	-122.445	37.941	0.04	17.89	272.53	17.93	9	7/14/2014 34:57.2
9279	RSPe_2	-122.445	37.941	0.19	17.92	272.47	18.11	-122.445	37.941	0.09	17.92	272.47	18.00	9	7/14/2014 34:57.1
9280	RSPe_2	-122.445	37.941	0.14	17.94	272.52	18.08	-122.445	37.941	0.09	17.94	272.46	18.03	9	7/14/2014 34:57.0
9281	RSPe_2	-122.445	37.941	0.19	17.87	272.58	18.06	-122.445	37.941	0.09	17.87	272.49	17.96	9	7/14/2014 34:56.9
9282	RSPe_2	-122.445	37.941	0.19	17.66	272.63	17.85	-122.445	37.941	0.04	17.66	272.49	17.70	9	7/14/2014 34:56.8
9283	RSPe_2	-122.445	37.941	0.19	17.65	272.72	17.84	-122.445	37.941	0.09	17.65	272.56	17.74	9	7/14/2014 34:56.7
9284	RSPe_2	-122.445	37.941	0.19	17.66	272.70	17.85	-122.445	37.941	0.04	17.66	272.61	17.70	9	7/14/2014 34:56.6
9285	RSPe_2	-122.445	37.941	0.19	17.68	272.71	17.88	-122.445	37.941	0.09	17.68	272.67	17.77	9	7/14/2014 34:56.5
9286	RSPe_2	-122.445	37.941	0.14	17.70	272.74	17.84	-122.445	37.941	0.04	17.70	272.71	17.74	9	7/14/2014 34:56.4
9287	RSPe_2	-122.445	37.941	0.19	17.75	272.67	17.94	-122.445	37.941	0.09	17.75	272.78	17.84	9	7/14/2014 34:56.3
9288	RSPe_2	-122.445	37.941	0.19	17.72	272.70	17.91	-122.445	37.941	0.09	17.72	272.87	17.81	9	7/14/2014 34:56.2
9289	RSPe_2	-122.445	37.941	0.19	17.73	272.83	17.92	-122.445	37.941	0.04	17.73	272.96	17.77	9	7/14/2014 34:56.1
9290	RSPe_2	-122.445	37.941	0.19	17.75	272.90	17.94	-122.445	37.941	0.04	17.75	272.98	17.79	9	7/14/2014 34:56.0
9291	RSPe_2	-122.445	37.941	0.19	17.79	272.85	17.98	-122.445	37.941	0.00	17.79	273.00	17.79	9	7/14/2014 34:55.9
9292	RSPe_2	-122.445	37.941	0.19	17.76	272.83	17.95	-122.445	37.941	0.04	17.76	272.91	17.80	9	7/14/2014 34:55.8
9293	RSPe_2	-122.445	37.941	0.19	17.79	272.88	17.98	-122.445	37.941	0.04	17.79	272.97	17.82	9	7/14/2014 34:55.7
9294	RSPe_2	-122.445	37.941	0.19	17.85	272.87	18.05	-122.445	37.941	0.04	17.85	272.94	17.89	9	7/14/2014 34:55.6
9295	RSPe_2	-122.445	37.941	0.19	17.85	272.85	18.05	-122.445	37.941	0.09	17.85	272.96	17.94	9	7/14/2014 34:55.5
9296	RSPe_2	-122.445	37.941	0.14	17.95	272.90	18.09	-122.445	37.941	0.04	17.95	273.03	17.98	9	7/14/2014 34:55.4
9297	RSPe_2	-122.445	37.941	0.19	17.97	272.86	18.16	-122.445	37.941	0.09	17.97	273.05	18.06	9	7/14/2014 34:55.3
9298	RSPe_2	-122.445	37.941	0.14	17.99	272.92	18.13	-122.445	37.941	0.00	17.99	273.10	17.99	9	7/14/2014 34:55.2
9299	RSPe_2	-122.445	37.941	0.19	18.01	272.94	18.20	-122.445	37.941	0.00	18.01	273.10	18.01	9	7/14/2014 34:55.1
9300	RSPe_2	-122.445	37.941	0.14	18.04	272.99	18.18	-122.445	37.941	0.00	18.04	273.13	18.04	9	7/14/2014 34:55.0
9301	RSPe_2	-122.445	37.941	0.19	18.03	273.12	18.22	-122.445	37.941	0.04	18.03	273.17	18.06	9	7/14/2014 34:54.9
9302	RSPe_2	-122.445	37.941	0.14	18.04	273.25	18.18	-122.445	37.941	0.00	18.04	273.20	18.04	9	7/14/2014 34:54.8
9303	RSPe_2	-122.445	37.941	0.19	18.05	273.23	18.25	-122.445	37.941	0.04	18.05	273.15	18.09	9	7/14/2014 34:54.7
9304	RSPe_2	-122.445	37.941	0.19	18.05	273.25	18.25	-122.445	37.941	0.04	18.05	273.21	18.09	9	7/14/2014 34:54.6
9305	RSPe_2	-122.445	37.941	0.19	18.08	273.37	18.27	-122.445	37.941	0.09	18.08	273.19	18.17	9	7/14/2014 34:54.5

9306	RSPe_2	-122.445	37.941	0.19	18.06	273.34	18.25	-122.445	37.941	0.04	18.06	273.20	18.10	9	7/14/2014	34:54.4
9307	RSPe_2	-122.445	37.941	0.19	18.07	273.26	18.26	-122.445	37.941	0.04	18.07	273.13	18.11	9	7/14/2014	34:54.3
9308	RSPe_2	-122.445	37.941	0.19	18.09	273.21	18.28	-122.445	37.941	0.12	18.09	273.14	18.21	9	7/14/2014	34:54.2
9309	RSPe_2	-122.445	37.941	0.22	18.09	273.32	18.31	-122.445	37.941	0.12	18.09	273.21	18.21	9	7/14/2014	34:54.1
9310	RSPe_2	-122.445	37.941	0.19	18.10	273.37	18.29	-122.445	37.941	0.09	18.10	273.27	18.18	9	7/14/2014	34:54.0
9311	RSPe_2	-122.445	37.941	0.19	18.12	273.34	18.32	-122.445	37.941	0.09	18.12	273.30	18.21	9	7/14/2014	34:53.9
9312	RSPe_2	-122.445	37.941	0.19	18.15	273.37	18.34	-122.445	37.941	0.04	18.15	273.27	18.19	9	7/14/2014	34:53.8
9313	RSPe_2	-122.445	37.941	0.22	18.19	273.37	18.42	-122.445	37.941	0.09	18.19	273.28	18.28	9	7/14/2014	34:53.7
9314	RSPe_2	-122.445	37.941	0.19	18.22	273.41	18.41	-122.445	37.941	0.09	18.22	273.38	18.31	9	7/14/2014	34:53.6
9315	RSPe_2	-122.445	37.941	0.19	18.24	273.48	18.43	-122.445	37.941	0.09	18.24	273.42	18.33	9	7/14/2014	34:53.5
9316	RSPe_2	-122.445	37.941	0.19	18.26	273.57	18.45	-122.445	37.941	0.04	18.26	273.58	18.29	9	7/14/2014	34:53.4
9317	RSPe_2	-122.445	37.941	0.19	18.33	273.68	18.52	-122.445	37.941	0.09	18.33	273.63	18.41	9	7/14/2014	34:53.3
9318	RSPe_2	-122.445	37.941	0.19	18.35	273.70	18.54	-122.445	37.941	0.09	18.35	273.67	18.43	9	7/14/2014	34:53.2
9319	RSPe_2	-122.445	37.941	0.19	18.39	273.72	18.58	-122.445	37.941	0.09	18.39	273.74	18.47	9	7/14/2014	34:53.1
9320	RSPe_2	-122.445	37.941	0.19	18.46	273.72	18.65	-122.445	37.941	0.09	18.46	273.76	18.54	9	7/14/2014	34:53.0
9321	RSPe_2	-122.445	37.941	0.22	18.33	273.79	18.56	-122.445	37.941	0.12	18.33	273.83	18.45	9	7/14/2014	34:52.9
9322	RSPe_2	-122.445	37.941	0.19	18.34	273.83	18.53	-122.445	37.941	0.12	18.34	273.88	18.46	9	7/14/2014	34:52.8
9323	RSPe_2	-122.445	37.941	0.22	18.32	273.75	18.54	-122.445	37.941	0.12	18.32	273.83	18.44	9	7/14/2014	34:52.7
9324	RSPe_2	-122.445	37.941	0.19	18.24	273.75	18.43	-122.445	37.941	0.12	18.24	273.82	18.36	9	7/14/2014	34:52.6
9325	RSPe_2	-122.445	37.941	0.22	18.30	273.73	18.52	-122.445	37.941	0.17	18.30	273.82	18.47	9	7/14/2014	34:52.5
9326	RSPe_2	-122.445	37.941	0.19	18.19	273.70	18.38	-122.445	37.941	0.12	18.19	273.83	18.31	9	7/14/2014	34:52.4
9327	RSPe_2	-122.445	37.941	0.22	18.18	273.73	18.40	-122.445	37.941	0.12	18.18	273.81	18.30	9	7/14/2014	34:52.3
9328	RSPe_2	-122.445	37.941	0.19	18.18	273.84	18.37	-122.445	37.941	0.09	18.18	273.97	18.26	9	7/14/2014	34:52.2
9329	RSPe_2	-122.445	37.941	0.22	18.17	273.90	18.39	-122.445	37.941	0.12	18.17	273.97	18.29	9	7/14/2014	34:52.1
9330	RSPe_2	-122.445	37.941	0.22	18.18	273.95	18.40	-122.445	37.941	0.09	18.18	273.99	18.26	9	7/14/2014	34:52.0
9331	RSPe_2	-122.445	37.941	0.22	18.20	273.95	18.43	-122.445	37.941	0.12	18.20	274.08	18.32	9	7/14/2014	34:51.9
9332	RSPe_2	-122.445	37.941	0.22	18.17	273.97	18.40	-122.445	37.941	0.12	18.17	274.19	18.29	9	7/14/2014	34:51.8
9333	RSPe_2	-122.445	37.941	0.22	18.13	273.88	18.36	-122.445	37.941	0.17	18.13	274.15	18.30	9	7/14/2014	34:51.7
9334	RSPe_2	-122.445	37.941	0.22	18.12	273.86	18.35	-122.445	37.941	0.12	18.12	274.19	18.25	9	7/14/2014	34:51.6
9335	RSPe_2	-122.445	37.941	0.22	18.13	273.82	18.36	-122.445	37.941	0.17	18.13	274.19	18.30	9	7/14/2014	34:51.5
9336	RSPe_2	-122.445	37.941	0.22	18.12	273.71	18.34	-122.445	37.941	0.12	18.12	274.08	18.24	9	7/14/2014	34:51.4
9337	RSPe_2	-122.445	37.941	0.22	18.12	273.71	18.35	-122.445	37.941	0.12	18.12	274.04	18.25	9	7/14/2014	34:51.3
9338	RSPe_2	-122.445	37.941	0.22	18.12	273.71	18.35	-122.445	37.941	0.09	18.12	274.01	18.21	9	7/14/2014	34:51.2

9339	RSPe_2	-122.445	37.941	0.22	18.14	273.67	18.36	-122.445	37.941	0.09	18.14	273.95	18.23	9	7/14/2014 34:51.1
9340	RSPe_2	-122.445	37.941	0.22	18.17	273.74	18.39	-122.445	37.941	0.09	18.17	273.93	18.25	9	7/14/2014 34:51.0
9341	RSPe_2	-122.445	37.941	0.22	18.16	273.82	18.38	-122.445	37.941	0.12	18.16	273.98	18.28	9	7/14/2014 34:50.9
9342	RSPe_2	-122.445	37.941	0.22	18.18	273.84	18.40	-122.445	37.941	0.09	18.18	273.97	18.26	9	7/14/2014 34:50.8
9343	RSPe_2	-122.445	37.941	0.26	18.21	273.98	18.47	-122.445	37.941	0.12	18.21	274.04	18.33	9	7/14/2014 34:50.7
9344	RSPe_2	-122.445	37.941	0.22	18.24	274.00	18.46	-122.445	37.941	0.04	18.24	274.02	18.27	9	7/14/2014 34:50.6
9345	RSPe_2	-122.445	37.941	0.26	18.26	274.00	18.52	-122.445	37.941	0.04	18.26	274.06	18.30	9	7/14/2014 34:50.5
9346	RSPe_2	-122.445	37.941	0.22	18.26	274.01	18.49	-122.445	37.941	0.04	18.26	274.08	18.30	9	7/14/2014 34:50.4
9347	RSPe_2	-122.445	37.941	0.26	18.26	274.09	18.52	-122.445	37.941	0.12	18.26	274.13	18.38	9	7/14/2014 34:50.3
9348	RSPe_2	-122.445	37.941	0.22	18.19	273.98	18.42	-122.445	37.941	0.09	18.19	274.11	18.28	9	7/14/2014 34:50.2
9349	RSPe_2	-122.445	37.941	0.26	18.15	273.96	18.41	-122.445	37.941	0.09	18.15	274.07	18.24	9	7/14/2014 34:50.1
9350	RSPe_2	-122.445	37.941	0.22	18.13	273.98	18.36	-122.445	37.941	0.09	18.13	274.10	18.22	9	7/14/2014 34:50.0
9351	RSPe_2	-122.445	37.941	0.26	18.11	274.01	18.37	-122.445	37.941	0.09	18.11	274.07	18.19	9	7/14/2014 34:49.9
9352	RSPe_2	-122.445	37.941	0.22	18.11	274.02	18.33	-122.445	37.941	0.04	18.11	274.09	18.14	9	7/14/2014 34:49.8
9353	RSPe_2	-122.445	37.941	0.22	18.13	274.03	18.36	-122.445	37.941	0.04	18.13	274.07	18.17	9	7/14/2014 34:49.7
9354	RSPe_2	-122.445	37.941	0.22	18.06	274.07	18.29	-122.445	37.941	0.04	18.06	274.02	18.10	9	7/14/2014 34:49.6
9355	RSPe_2	-122.445	37.941	0.26	18.09	274.22	18.35	-122.445	37.941	0.04	18.09	274.13	18.12	9	7/14/2014 34:49.5
9356	RSPe_2	-122.445	37.941	0.22	18.04	274.34	18.26	-122.445	37.941	0.04	18.04	274.18	18.08	9	7/14/2014 34:49.4
9357	RSPe_2	-122.445	37.941	0.22	18.01	274.36	18.24	-122.445	37.941	0.04	18.01	274.24	18.05	9	7/14/2014 34:49.3
9358	RSPe_2	-122.445	37.941	0.22	18.00	274.40	18.23	-122.445	37.941	0.04	18.00	274.35	18.04	9	7/14/2014 34:49.2
9359	RSPe_2	-122.445	37.941	0.22	17.99	274.43	18.22	-122.445	37.941	0.04	17.99	274.38	18.03	9	7/14/2014 34:49.1
9360	RSPe_2	-122.445	37.941	0.22	17.96	274.40	18.18	-122.445	37.941	0.00	17.96	274.40	17.96	9	7/14/2014 34:49.0
9361	RSPe_2	-122.445	37.941	0.26	17.96	274.43	18.21	-122.445	37.941	0.09	17.96	274.40	18.04	9	7/14/2014 34:48.9
9362	RSPe_2	-122.445	37.941	0.26	17.91	274.43	18.17	-122.445	37.941	0.04	17.91	274.42	17.95	9	7/14/2014 34:48.8
9363	RSPe_2	-122.445	37.941	0.26	17.89	274.45	18.15	-122.445	37.941	0.04	17.89	274.36	17.93	9	7/14/2014 34:48.7
9364	RSPe_2	-122.445	37.941	0.22	17.85	274.49	18.08	-122.445	37.941	0.04	17.85	274.36	17.89	9	7/14/2014 34:48.6
9365	RSPe_2	-122.445	37.941	0.26	17.85	274.50	18.11	-122.445	37.941	0.17	17.85	274.34	18.02	9	7/14/2014 34:48.5
9366	RSPe_2	-122.445	37.941	0.22	17.85	274.56	18.07	-122.445	37.941	0.17	17.85	274.38	18.02	9	7/14/2014 34:48.4
9367	RSPe_2	-122.445	37.941	0.26	17.82	274.56	18.08	-122.445	37.941	0.12	17.82	274.42	17.94	9	7/14/2014 34:48.3
9368	RSPe_2	-122.445	37.941	0.22	17.82	274.54	18.05	-122.445	37.941	0.12	17.82	274.43	17.94	9	7/14/2014 34:48.2
9369	RSPe_2	-122.445	37.941	0.26	17.79	274.56	18.05	-122.445	37.941	0.12	17.79	274.38	17.91	9	7/14/2014 34:48.1
9370	RSPe_2	-122.445	37.941	0.26	17.76	274.52	18.02	-122.445	37.941	0.12	17.76	274.43	17.88	9	7/14/2014 34:48.0
9371	RSPe_2	-122.445	37.941	0.26	17.75	274.54	18.01	-122.445	37.941	0.12	17.75	274.43	17.87	9	7/14/2014 34:47.9

9372	RSPe_2	-122.445	37.941	0.26	17.77	274.58	18.03	-122.445	37.941	0.12	17.77	274.51	17.89	9	7/14/2014	34:47.8
9373	RSPe_2	-122.445	37.941	0.31	17.82	274.63	18.13	-122.445	37.941	0.17	17.82	274.55	18.00	9	7/14/2014	34:47.7
9374	RSPe_2	-122.445	37.941	0.26	17.81	274.56	18.07	-122.445	37.941	0.17	17.81	274.61	17.98	9	7/14/2014	34:47.6
9375	RSPe_2	-122.445	37.941	0.26	17.71	274.63	17.97	-122.445	37.941	0.17	17.71	274.60	17.88	9	7/14/2014	34:47.5
9376	RSPe_2	-122.445	37.941	0.26	17.69	274.54	17.95	-122.445	37.941	0.17	17.69	274.57	17.87	9	7/14/2014	34:47.4
9377	RSPe_2	-122.445	37.941	0.31	17.68	274.52	18.00	-122.445	37.941	0.21	17.68	274.49	17.89	9	7/14/2014	34:47.3
9378	RSPe_2	-122.445	37.941	0.31	17.72	274.66	18.03	-122.445	37.941	0.17	17.72	274.60	17.89	9	7/14/2014	34:47.2
9379	RSPe_2	-122.445	37.941	0.31	17.72	274.72	18.03	-122.445	37.941	0.21	17.72	274.63	17.93	9	7/14/2014	34:47.1
9380	RSPe_2	-122.445	37.941	0.31	17.67	274.72	17.98	-122.445	37.941	0.24	17.67	274.63	17.91	9	7/14/2014	34:47.0
9381	RSPe_2	-122.445	37.941	0.26	17.75	274.78	18.01	-122.445	37.941	0.29	17.75	274.67	18.04	9	7/14/2014	34:46.9
9382	RSPe_2	-122.445	37.941	0.31	17.63	274.77	17.94	-122.445	37.941	0.24	17.63	274.66	17.87	9	7/14/2014	34:46.8
9383	RSPe_2	-122.445	37.941	0.31	17.66	274.81	17.98	-122.445	37.941	0.24	17.66	274.67	17.90	9	7/14/2014	34:46.7
9384	RSPe_2	-122.445	37.941	0.31	17.61	274.84	17.92	-122.445	37.941	0.24	17.61	274.70	17.85	9	7/14/2014	34:46.6
9385	RSPe_2	-122.445	37.941	0.31	17.60	274.83	17.91	-122.445	37.941	0.29	17.60	274.72	17.89	9	7/14/2014	34:46.5
9386	RSPe_2	-122.445	37.941	0.31	17.61	274.79	17.93	-122.445	37.941	0.24	17.61	274.72	17.86	9	7/14/2014	34:46.4
9387	RSPe_2	-122.445	37.941	0.31	17.61	274.81	17.93	-122.445	37.941	0.29	17.61	274.74	17.91	9	7/14/2014	34:46.3
9388	RSPe_2	-122.445	37.941	0.31	17.64	274.85	17.95	-122.445	37.941	0.29	17.64	274.83	17.93	9	7/14/2014	34:46.2
9389	RSPe_2	-122.445	37.941	0.31	17.63	274.92	17.94	-122.445	37.941	0.29	17.63	274.87	17.92	9	7/14/2014	34:46.1
9390	RSPe_2	-122.445	37.941	0.31	17.72	274.97	18.03	-122.445	37.941	0.24	17.72	275.02	17.96	9	7/14/2014	34:46.0
9391	RSPe_2	-122.445	37.941	0.31	17.66	274.99	17.98	-122.445	37.941	0.29	17.66	275.03	17.95	9	7/14/2014	34:45.9
9392	RSPe_2	-122.445	37.941	0.31	17.72	275.08	18.03	-122.445	37.941	0.24	17.72	275.14	17.96	9	7/14/2014	34:45.8
9393	RSPe_2	-122.445	37.941	0.31	17.72	275.08	18.03	-122.445	37.941	0.24	17.72	275.14	17.96	9	7/14/2014	34:45.7
9394	RSPe_2	-122.445	37.941	0.26	17.73	275.08	17.99	-122.445	37.941	0.24	17.73	275.12	17.97	9	7/14/2014	34:45.6
9395	RSPe_2	-122.445	37.941	0.31	17.75	274.99	18.07	-122.445	37.941	0.24	17.75	275.03	17.99	9	7/14/2014	34:45.5
9396	RSPe_2	-122.445	37.941	0.31	17.76	275.04	18.08	-122.445	37.941	0.29	17.76	275.05	18.05	9	7/14/2014	34:45.4
9397	RSPe_2	-122.445	37.941	0.31	17.77	275.12	18.08	-122.445	37.941	0.32	17.77	275.12	18.10	9	7/14/2014	34:45.3
9398	RSPe_2	-122.445	37.941	0.31	17.76	275.13	18.08	-122.445	37.941	0.29	17.76	275.12	18.05	9	7/14/2014	34:45.2
9399	RSPe_2	-122.445	37.941	0.31	17.81	275.13	18.12	-122.445	37.941	0.29	17.81	275.08	18.10	9	7/14/2014	34:45.1
9400	RSPe_2	-122.445	37.941	0.26	17.79	275.10	18.05	-122.445	37.941	0.24	17.79	275.03	18.03	9	7/14/2014	34:45.0
9401	RSPe_2	-122.445	37.941	0.31	17.79	275.09	18.10	-122.445	37.941	0.29	17.79	275.08	18.08	9	7/14/2014	34:44.9
9402	RSPe_2	-122.445	37.941	0.26	17.82	275.10	18.08	-122.445	37.941	0.24	17.82	275.12	18.06	9	7/14/2014	34:44.8
9403	RSPe_2	-122.445	37.941	0.26	17.83	275.15	18.09	-122.445	37.941	0.24	17.83	275.19	18.07	9	7/14/2014	34:44.7
9404	RSPe_2	-122.445	37.941	0.26	17.90	275.13	18.16	-122.445	37.941	0.24	17.90	275.17	18.14	9	7/14/2014	34:44.6

9405	RSPe_2	-122.445	37.941	0.31	17.92	275.17	18.24	-122.445	37.941	0.24	17.92	275.19	18.17	9	7/14/2014	34:44.5
9406	RSPe_2	-122.445	37.941	0.31	17.89	275.20	18.20	-122.445	37.941	0.24	17.89	275.19	18.13	9	7/14/2014	34:44.4
9407	RSPe_2	-122.445	37.941	0.26	17.93	275.20	18.19	-122.445	37.941	0.24	17.93	275.17	18.17	9	7/14/2014	34:44.3
9408	RSPe_2	-122.445	37.941	0.26	17.93	275.20	18.19	-122.445	37.941	0.24	17.93	275.17	18.17	9	7/14/2014	34:44.2
9409	RSPe_2	-122.445	37.941	0.31	17.99	275.22	18.31	-122.445	37.941	0.24	17.99	275.19	18.23	9	7/14/2014	34:44.1
9410	RSPe_2	-122.445	37.941	0.26	17.96	275.20	18.22	-122.445	37.941	0.24	17.96	275.19	18.20	9	7/14/2014	34:44.0
9411	RSPe_2	-122.445	37.941	0.31	17.98	275.20	18.29	-122.445	37.941	0.29	17.98	275.18	18.27	9	7/14/2014	34:43.9
9412	RSPe_2	-122.445	37.941	0.26	18.00	275.17	18.26	-122.445	37.941	0.21	18.00	275.15	18.21	9	7/14/2014	34:43.8
9413	RSPe_2	-122.445	37.941	0.26	18.06	275.18	18.32	-122.445	37.941	0.21	18.06	275.16	18.27	9	7/14/2014	34:43.7
9414	RSPe_2	-122.445	37.941	0.26	18.05	275.18	18.31	-122.445	37.941	0.17	18.05	275.20	18.23	9	7/14/2014	34:43.6
9415	RSPe_2	-122.445	37.941	0.31	18.05	275.16	18.37	-122.445	37.941	0.21	18.05	275.24	18.26	9	7/14/2014	34:43.5
9416	RSPe_2	-122.445	37.941	0.23	18.10	275.25	18.33	-122.445	37.941	0.21	18.10	275.27	18.31	9	7/14/2014	34:43.4
9417	RSPe_2	-122.445	37.941	0.26	18.13	275.22	18.39	-122.445	37.941	0.21	18.13	275.29	18.34	9	7/14/2014	34:43.3
9418	RSPe_2	-122.445	37.941	0.26	18.15	275.23	18.41	-122.445	37.941	0.24	18.15	275.24	18.39	9	7/14/2014	34:43.2
9419	RSPe_2	-122.445	37.941	0.26	18.19	275.31	18.45	-122.445	37.941	0.24	18.19	275.34	18.43	9	7/14/2014	34:43.1
9420	RSPe_2	-122.445	37.941	0.23	18.29	275.40	18.51	-122.445	37.941	0.24	18.29	275.37	18.53	9	7/14/2014	34:43.0
9421	RSPe_2	-122.445	37.941	0.26	18.23	275.38	18.49	-122.445	37.941	0.29	18.23	275.38	18.52	9	7/14/2014	34:42.9
9422	RSPe_2	-122.445	37.941	0.26	18.33	275.40	18.59	-122.445	37.941	0.24	18.33	275.38	18.57	9	7/14/2014	34:42.8
9423	RSPe_2	-122.445	37.941	0.26	18.28	275.47	18.54	-122.445	37.941	0.24	18.28	275.37	18.52	9	7/14/2014	34:42.7
9424	RSPe_2	-122.445	37.941	0.23	18.34	275.56	18.57	-122.445	37.941	0.21	18.34	275.42	18.55	9	7/14/2014	34:42.6
9425	RSPe_2	-122.445	37.941	0.26	18.29	275.58	18.55	-122.445	37.941	0.24	18.29	275.36	18.53	9	7/14/2014	34:42.5
9426	RSPe_2	-122.445	37.941	0.23	18.27	275.58	18.50	-122.445	37.941	0.24	18.27	275.31	18.51	9	7/14/2014	34:42.4
9427	RSPe_2	-122.445	37.941	0.26	18.22	275.60	18.48	-122.445	37.941	0.24	18.22	275.27	18.46	9	7/14/2014	34:42.3
9428	RSPe_2	-122.445	37.941	0.23	18.12	275.58	18.35	-122.445	37.941	0.24	18.12	275.27	18.37	9	7/14/2014	34:42.2
9429	RSPe_2	-122.445	37.941	0.26	18.03	275.58	18.29	-122.445	37.941	0.29	18.03	275.32	18.32	9	7/14/2014	34:42.1
9430	RSPe_2	-122.445	37.941	0.23	17.97	275.58	18.20	-122.445	37.941	0.24	17.97	275.37	18.21	9	7/14/2014	34:42.0
9431	RSPe_2	-122.445	37.941	0.26	17.96	275.63	18.22	-122.445	37.941	0.29	17.96	275.47	18.25	9	7/14/2014	34:41.9
9432	RSPe_2	-122.445	37.941	0.23	17.87	275.61	18.10	-122.445	37.941	0.21	17.87	275.53	18.08	9	7/14/2014	34:41.8
9433	RSPe_2	-122.445	37.941	0.23	17.82	275.60	18.05	-122.445	37.941	0.21	17.82	275.58	18.03	9	7/14/2014	34:41.7
9434	RSPe_2	-122.445	37.941	0.23	17.79	275.56	18.01	-122.445	37.941	0.24	17.79	275.58	18.03	9	7/14/2014	34:41.6
9435	RSPe_2	-122.445	37.941	0.26	17.79	275.63	18.05	-122.445	37.941	0.24	17.79	275.63	18.03	9	7/14/2014	34:41.5
9436	RSPe_2	-122.445	37.941	0.23	17.77	275.57	18.00	-122.445	37.941	0.24	17.77	275.58	18.01	9	7/14/2014	34:41.4
9437	RSPe_2	-122.445	37.941	0.23	17.74	275.54	17.96	-122.445	37.941	0.24	17.74	275.52	17.98	9	7/14/2014	34:41.3

9438	RSPe_2	-122.445	37.941	0.19	17.82	275.52	18.02	-122.445	37.941	0.21	17.82	275.54	18.03	9	7/14/2014	34:41.2
9439	RSPe_2	-122.445	37.941	0.23	17.74	275.55	17.96	-122.445	37.941	0.21	17.74	275.56	17.95	9	7/14/2014	34:41.1
9440	RSPe_2	-122.445	37.941	0.19	17.73	275.61	17.92	-122.445	37.941	0.17	17.73	275.62	17.91	9	7/14/2014	34:41.0
9441	RSPe_2	-122.445	37.941	0.19	17.79	275.61	17.98	-122.445	37.941	0.24	17.79	275.63	18.03	9	7/14/2014	34:40.9
9442	RSPe_2	-122.445	37.941	0.14	17.73	275.65	17.87	-122.445	37.941	0.21	17.73	275.74	17.94	9	7/14/2014	34:40.8
9443	RSPe_2	-122.445	37.941	0.19	17.75	275.66	17.95	-122.445	37.941	0.21	17.75	275.74	17.96	9	7/14/2014	34:40.7
9444	RSPe_2	-122.445	37.941	0.14	17.79	275.66	17.93	-122.445	37.941	0.17	17.79	275.76	17.96	9	7/14/2014	34:40.6
9445	RSPe_2	-122.445	37.941	0.14	17.76	275.72	17.90	-122.445	37.941	0.17	17.76	275.81	17.94	9	7/14/2014	34:40.5
9446	RSPe_2	-122.445	37.941	0.14	17.76	275.77	17.90	-122.445	37.941	0.09	17.76	275.85	17.85	9	7/14/2014	34:40.4
9447	RSPe_2	-122.445	37.941	0.14	17.82	275.81	17.96	-122.445	37.941	0.09	17.82	275.87	17.90	9	7/14/2014	34:40.3
9448	RSPe_2	-122.445	37.941	0.14	17.76	275.83	17.90	-122.445	37.941	0.09	17.76	275.87	17.85	9	7/14/2014	34:40.2
9449	RSPe_2	-122.445	37.941	0.14	17.76	275.87	17.90	-122.445	37.941	0.09	17.76	275.83	17.85	9	7/14/2014	34:40.1
9450	RSPe_2	-122.445	37.941	0.11	17.78	275.90	17.88	-122.445	37.941	0.04	17.78	275.87	17.81	9	7/14/2014	34:40.0
9451	RSPe_2	-122.445	37.941	0.11	17.76	275.90	17.87	-122.445	37.941	0.04	17.76	275.81	17.80	9	7/14/2014	34:39.9
9452	RSPe_2	-122.445	37.941	0.11	17.87	275.88	17.98	-122.445	37.941	0.00	17.87	275.81	17.88	9	7/14/2014	34:39.8
9453	RSPe_2	-122.445	37.941	0.11	17.79	275.90	17.89	-122.445	37.941	0.00	17.79	275.81	17.79	9	7/14/2014	34:39.7
9454	RSPe_2	-122.445	37.941	0.11	17.90	275.95	18.01	-122.445	37.941	0.00	17.90	275.88	17.91	9	7/14/2014	34:39.6
9455	RSPe_2	-122.445	37.941	0.11	17.86	275.93	17.97	-122.445	37.941	0.00	17.86	275.87	17.87	9	7/14/2014	34:39.5
9456	RSPe_2	-122.445	37.941	0.06	17.95	276.01	18.00	-122.445	37.941	-0.05	17.95	275.94	17.90	9	7/14/2014	34:39.4
9457	RSPe_2	-122.445	37.941	0.11	17.92	276.04	18.03	-122.445	37.941	-0.05	17.92	276.04	17.88	9	7/14/2014	34:39.3
9458	RSPe_2	-122.445	37.941	0.06	17.93	276.08	17.99	-122.445	37.941	-0.08	17.93	276.14	17.85	9	7/14/2014	34:39.2
9459	RSPe_2	-122.445	37.941	0.11	18.03	276.06	18.13	-122.445	37.941	-0.08	18.03	276.17	17.94	9	7/14/2014	34:39.1
9460	RSPe_2	-122.445	37.941	0.02	18.08	276.06	18.10	-122.445	37.941	-0.08	18.08	276.17	18.00	9	7/14/2014	34:39.0
9461	RSPe_2	-122.445	37.941	0.06	18.05	276.08	18.11	-122.445	37.941	-0.05	18.05	276.18	18.01	9	7/14/2014	34:38.9
9462	RSPe_2	-122.445	37.941	0.06	18.13	276.11	18.19	-122.445	37.941	-0.13	18.13	276.18	18.00	9	7/14/2014	34:38.8
9463	RSPe_2	-122.445	37.941	0.06	18.16	276.19	18.21	-122.445	37.941	-0.05	18.16	276.17	18.11	9	7/14/2014	34:38.7
9464	RSPe_2	-122.445	37.941	0.02	18.17	276.26	18.19	-122.445	37.941	-0.13	18.17	276.21	18.03	9	7/14/2014	34:38.6
9465	RSPe_2	-122.445	37.941	0.06	18.20	276.28	18.26	-122.445	37.941	-0.13	18.20	276.17	18.07	9	7/14/2014	34:38.5
9466	RSPe_2	-122.445	37.941	0.02	18.24	276.26	18.26	-122.445	37.941	-0.13	18.24	276.15	18.10	9	7/14/2014	34:38.4
9467	RSPe_2	-122.445	37.941	0.06	18.26	276.22	18.31	-122.445	37.941	-0.08	18.26	276.11	18.18	9	7/14/2014	34:38.3
9468	RSPe_2	-122.445	37.941	0.02	18.32	276.26	18.34	-122.445	37.941	-0.13	18.32	276.17	18.18	9	7/14/2014	34:38.2
9469	RSPe_2	-122.445	37.941	0.06	18.32	276.24	18.37	-122.445	37.941	-0.13	18.32	276.21	18.18	9	7/14/2014	34:38.1
9470	RSPe_2	-122.445	37.941	0.06	18.34	276.24	18.40	-122.445	37.941	-0.13	18.34	276.20	18.21	9	7/14/2014	34:38.0

9471	RSPe_2	-122.445	37.941	0.06	18.33	276.28	18.39	-122.445	37.941	-0.13	18.33	276.32	18.20	9	7/14/2014	34:37.9
9472	RSPe_2	-122.445	37.941	0.06	18.37	276.31	18.43	-122.445	37.941	-0.13	18.37	276.35	18.24	9	7/14/2014	34:37.8
9473	RSPe_2	-122.445	37.941	0.06	18.37	276.40	18.43	-122.445	37.941	-0.13	18.37	276.55	18.24	9	7/14/2014	34:37.7
9474	RSPe_2	-122.445	37.941	0.02	18.36	276.46	18.39	-122.445	37.941	-0.17	18.36	276.55	18.20	9	7/14/2014	34:37.6
9475	RSPe_2	-122.445	37.941	0.06	18.36	276.45	18.41	-122.445	37.941	-0.13	18.36	276.55	18.23	9	7/14/2014	34:37.5
9476	RSPe_2	-122.445	37.941	0.06	18.35	276.49	18.40	-122.445	37.941	-0.17	18.35	276.48	18.18	9	7/14/2014	34:37.4
9477	RSPe_2	-122.445	37.941	0.06	18.35	276.46	18.40	-122.445	37.941	-0.13	18.35	276.44	18.22	9	7/14/2014	34:37.3
9478	RSPe_2	-122.445	37.941	0.06	18.36	276.38	18.41	-122.445	37.941	-0.17	18.36	276.33	18.19	9	7/14/2014	34:37.2
9479	RSPe_2	-122.445	37.941	0.11	18.36	276.26	18.47	-122.445	37.941	-0.17	18.36	276.24	18.20	9	7/14/2014	34:37.1
9480	RSPe_2	-122.445	37.941	0.06	18.37	276.18	18.43	-122.445	37.941	-0.17	18.37	276.19	18.21	9	7/14/2014	34:37.0
9481	RSPe_2	-122.445	37.941	0.11	18.47	276.18	18.57	-122.445	37.941	-0.13	18.47	276.24	18.33	9	7/14/2014	34:36.9
9482	RSPe_2	-122.445	37.941	0.06	18.44	276.16	18.50	-122.445	37.941	-0.17	18.44	276.24	18.28	9	7/14/2014	34:36.8
9483	RSPe_2	-122.445	37.941	0.11	18.45	276.20	18.55	-122.445	37.941	-0.08	18.45	276.29	18.37	9	7/14/2014	34:36.7
9484	RSPe_2	-122.445	37.941	0.06	18.53	276.38	18.59	-122.445	37.941	-0.13	18.53	276.37	18.40	9	7/14/2014	34:36.6
9485	RSPe_2	-122.445	37.941	0.11	18.52	276.38	18.63	-122.445	37.941	-0.13	18.52	276.44	18.39	9	7/14/2014	34:36.5
9486	RSPe_2	-122.445	37.941	0.06	18.52	276.51	18.57	-122.445	37.941	-0.17	18.52	276.52	18.35	9	7/14/2014	34:36.4
9487	RSPe_2	-122.445	37.941	0.11	18.59	276.71	18.69	-122.445	37.941	-0.13	18.59	276.64	18.46	9	7/14/2014	34:36.3
9488	RSPe_2	-122.445	37.941	0.06	18.66	276.76	18.72	-122.445	37.941	-0.17	18.66	276.66	18.50	9	7/14/2014	34:36.2
9489	RSPe_2	-122.445	37.941	0.11	18.63	276.82	18.74	-122.445	37.941	-0.13	18.63	276.67	18.50	9	7/14/2014	34:36.1
9490	RSPe_2	-122.445	37.941	0.06	18.61	276.71	18.67	-122.445	37.941	-0.17	18.61	276.57	18.45	9	7/14/2014	34:36.0
9491	RSPe_2	-122.445	37.941	0.11	18.54	276.63	18.65	-122.445	37.941	-0.08	18.54	276.52	18.46	9	7/14/2014	34:35.9
9492	RSPe_2	-122.445	37.941	0.11	18.57	276.58	18.67	-122.445	37.941	-0.08	18.57	276.46	18.48	9	7/14/2014	34:35.8
9493	RSPe_2	-122.445	37.941	0.11	18.60	276.58	18.71	-122.445	37.941	-0.08	18.60	276.39	18.52	9	7/14/2014	34:35.7
9494	RSPe_2	-122.445	37.941	0.11	18.57	276.58	18.68	-122.445	37.941	-0.08	18.57	276.38	18.49	9	7/14/2014	34:35.6
9495	RSPe_2	-122.445	37.941	0.14	18.50	276.50	18.65	-122.445	37.941	-0.05	18.50	276.30	18.46	9	7/14/2014	34:35.5
9496	RSPe_2	-122.445	37.941	0.14	18.43	276.48	18.58	-122.445	37.941	-0.05	18.43	276.29	18.39	9	7/14/2014	34:35.4
9497	RSPe_2	-122.445	37.941	0.14	18.48	276.48	18.62	-122.445	37.941	-0.05	18.48	276.34	18.44	9	7/14/2014	34:35.3
9498	RSPe_2	-122.445	37.941	0.14	18.43	276.54	18.58	-122.445	37.941	-0.05	18.43	276.43	18.39	9	7/14/2014	34:35.2
9499	RSPe_2	-122.445	37.941	0.19	18.39	276.59	18.58	-122.445	37.941	0.01	18.39	276.52	18.39	9	7/14/2014	34:35.1
9500	RSPe_2	-122.445	37.941	0.14	18.38	276.63	18.52	-122.445	37.941	0.01	18.38	276.59	18.38	9	7/14/2014	34:35.0
9501	RSPe_2	-122.445	37.941	0.19	18.33	276.59	18.52	-122.445	37.941	0.01	18.33	276.54	18.33	9	7/14/2014	34:34.9
9502	RSPe_2	-122.445	37.941	0.14	18.39	276.54	18.53	-122.445	37.941	-0.05	18.39	276.55	18.34	9	7/14/2014	34:34.8
9503	RSPe_2	-122.445	37.941	0.14	18.36	276.57	18.50	-122.445	37.941	0.01	18.36	276.55	18.36	9	7/14/2014	34:34.7

9504	RSPe_2	-122.445	37.941	0.14	18.30	276.61	18.45	-122.445	37.941	0.01	18.30	276.54	18.31	9	7/14/2014	34:34.6
9505	RSPe_2	-122.445	37.941	0.14	18.28	276.63	18.42	-122.445	37.941	0.01	18.28	276.55	18.28	9	7/14/2014	34:34.5
9506	RSPe_2	-122.445	37.941	0.14	18.26	276.57	18.40	-122.445	37.941	-0.05	18.26	276.47	18.21	9	7/14/2014	34:34.4
9507	RSPe_2	-122.445	37.941	0.19	18.22	276.61	18.41	-122.445	37.941	0.01	18.22	276.56	18.22	9	7/14/2014	34:34.3
9508	RSPe_2	-122.445	37.941	0.14	18.19	276.61	18.34	-122.445	37.941	0.01	18.19	276.52	18.20	9	7/14/2014	34:34.2
9509	RSPe_2	-122.445	37.941	0.19	18.11	276.55	18.30	-122.445	37.941	0.01	18.11	276.52	18.11	9	7/14/2014	34:34.1
9510	RSPe_2	-122.445	37.941	0.19	18.17	276.57	18.36	-122.445	37.941	0.04	18.17	276.55	18.20	9	7/14/2014	34:34.0
9511	RSPe_2	-122.445	37.941	0.19	18.12	276.62	18.31	-122.445	37.941	0.04	18.12	276.59	18.16	9	7/14/2014	34:33.9
9512	RSPe_2	-122.445	37.941	0.19	18.06	276.66	18.26	-122.445	37.941	0.04	18.06	276.61	18.10	9	7/14/2014	34:33.8
9513	RSPe_2	-122.445	37.941	0.23	18.03	276.59	18.26	-122.445	37.941	0.09	18.03	276.57	18.12	9	7/14/2014	34:33.7
9514	RSPe_2	-122.445	37.941	0.19	18.00	276.62	18.19	-122.445	37.941	0.09	18.00	276.57	18.09	9	7/14/2014	34:33.6
9515	RSPe_2	-122.445	37.941	0.19	18.01	276.59	18.20	-122.445	37.941	0.09	18.01	276.59	18.10	9	7/14/2014	34:33.5
9516	RSPe_2	-122.445	37.941	0.19	18.03	276.66	18.22	-122.445	37.941	0.09	18.03	276.66	18.12	9	7/14/2014	34:33.4
9517	RSPe_2	-122.445	37.941	0.19	17.98	276.75	18.17	-122.445	37.941	0.09	17.98	276.70	18.07	9	7/14/2014	34:33.3
9518	RSPe_2	-122.445	37.941	0.19	17.95	276.73	18.14	-122.445	37.941	0.09	17.95	276.73	18.04	9	7/14/2014	34:33.2
9519	RSPe_2	-122.445	37.941	0.19	17.96	276.77	18.15	-122.445	37.941	0.09	17.96	276.71	18.04	9	7/14/2014	34:33.1
9520	RSPe_2	-122.445	37.941	0.19	17.98	276.75	18.17	-122.445	37.941	0.01	17.98	276.64	17.98	9	7/14/2014	34:33.0
9521	RSPe_2	-122.445	37.941	0.19	17.99	276.71	18.18	-122.445	37.941	0.04	17.99	276.62	18.02	9	7/14/2014	34:32.9
9522	RSPe_2	-122.445	37.941	0.19	17.99	276.71	18.19	-122.445	37.941	0.09	17.99	276.61	18.08	9	7/14/2014	34:32.8
9523	RSPe_2	-122.445	37.941	0.23	17.98	276.80	18.21	-122.445	37.941	0.09	17.98	276.71	18.07	9	7/14/2014	34:32.7
9524	RSPe_2	-122.445	37.941	0.19	17.96	276.84	18.15	-122.445	37.941	0.09	17.96	276.74	18.04	9	7/14/2014	34:32.6
9525	RSPe_2	-122.445	37.941	0.19	17.99	276.89	18.18	-122.445	37.941	0.04	17.99	276.80	18.02	9	7/14/2014	34:32.5
9526	RSPe_2	-122.445	37.941	0.19	18.03	276.93	18.22	-122.445	37.941	0.04	18.03	276.77	18.06	9	7/14/2014	34:32.4
9527	RSPe_2	-122.445	37.941	0.23	17.99	276.98	18.21	-122.445	37.941	0.12	17.99	276.78	18.11	9	7/14/2014	34:32.3
9528	RSPe_2	-122.445	37.941	0.19	18.00	276.98	18.19	-122.445	37.941	0.12	18.00	276.86	18.12	9	7/14/2014	34:32.2
9529	RSPe_2	-122.445	37.941	0.19	18.04	276.95	18.23	-122.445	37.941	0.12	18.04	276.77	18.16	9	7/14/2014	34:32.1
9530	RSPe_2	-122.445	37.941	0.19	18.05	276.96	18.24	-122.445	37.941	0.12	18.05	276.80	18.17	9	7/14/2014	34:32.0
9531	RSPe_2	-122.445	37.941	0.19	18.08	276.91	18.27	-122.445	37.941	0.12	18.08	276.80	18.20	9	7/14/2014	34:31.9
9532	RSPe_2	-122.445	37.941	0.14	18.09	277.04	18.23	-122.445	37.941	0.04	18.09	276.84	18.13	9	7/14/2014	34:31.8
9533	RSPe_2	-122.445	37.941	0.19	18.33	277.09	18.53	-122.445	37.941	0.09	18.33	276.89	18.42	9	7/14/2014	34:31.7
9534	RSPe_2	-122.445	37.941	0.14	18.35	277.07	18.49	-122.445	37.941	0.09	18.35	276.84	18.44	9	7/14/2014	34:31.6
9535	RSPe_2	-122.445	37.941	0.19	18.36	276.98	18.55	-122.445	37.941	0.12	18.36	276.78	18.48	9	7/14/2014	34:31.5
9536	RSPe_2	-122.445	37.941	0.14	18.33	277.07	18.47	-122.445	37.941	0.04	18.33	276.89	18.37	9	7/14/2014	34:31.4

9537	RSPe_2	-122.445	37.941	0.19	18.32	277.05	18.51	-122.445	37.941	0.12	18.32	276.95	18.44	9	7/14/2014	34:31.3
9538	RSPe_2	-122.445	37.941	0.14	18.27	277.20	18.41	-122.445	37.941	0.09	18.27	277.04	18.36	9	7/14/2014	34:31.2
9539	RSPe_2	-122.445	37.941	0.19	18.28	277.14	18.47	-122.445	37.941	0.12	18.28	277.05	18.40	9	7/14/2014	34:31.1
9540	RSPe_2	-122.445	37.941	0.14	18.25	277.11	18.39	-122.445	37.941	0.09	18.25	277.04	18.34	9	7/14/2014	34:31.0
9541	RSPe_2	-122.445	37.941	0.19	18.21	277.09	18.40	-122.445	37.941	0.12	18.21	277.09	18.33	9	7/14/2014	34:30.9
9542	RSPe_2	-122.445	37.941	0.14	18.24	277.14	18.38	-122.445	37.941	0.09	18.24	277.18	18.32	9	7/14/2014	34:30.8
9543	RSPe_2	-122.445	37.941	0.19	18.19	277.20	18.39	-122.445	37.941	0.09	18.19	277.24	18.28	9	7/14/2014	34:30.7
9544	RSPe_2	-122.445	37.941	0.14	18.17	277.16	18.31	-122.445	37.941	0.04	18.17	277.20	18.21	9	7/14/2014	34:30.6
9545	RSPe_2	-122.445	37.941	0.19	18.26	277.11	18.45	-122.445	37.941	0.09	18.26	277.15	18.35	9	7/14/2014	34:30.5
9546	RSPe_2	-122.445	37.941	0.14	18.18	277.14	18.32	-122.445	37.941	0.04	18.18	277.18	18.22	9	7/14/2014	34:30.4
9547	RSPe_2	-122.445	37.941	0.14	18.13	277.18	18.27	-122.445	37.941	0.09	18.13	277.20	18.22	9	7/14/2014	34:30.3
9548	RSPe_2	-122.445	37.941	0.11	18.11	277.27	18.21	-122.445	37.941	0.04	18.11	277.23	18.15	9	7/14/2014	34:30.2
9549	RSPe_2	-122.445	37.941	0.14	18.12	277.29	18.26	-122.445	37.941	0.04	18.12	277.20	18.16	9	7/14/2014	34:30.1
9550	RSPe_2	-122.445	37.941	0.11	18.12	277.25	18.23	-122.445	37.941	0.04	18.12	277.10	18.16	9	7/14/2014	34:30.0
9551	RSPe_2	-122.445	37.941	0.11	18.12	277.32	18.23	-122.445	37.941	0.04	18.12	277.14	18.16	9	7/14/2014	34:29.9
9552	RSPe_2	-122.445	37.941	0.11	18.11	277.40	18.21	-122.445	37.941	0.01	18.11	277.20	18.11	9	7/14/2014	34:29.8
9553	RSPe_2	-122.445	37.941	0.11	18.12	277.45	18.23	-122.445	37.941	0.04	18.12	277.32	18.16	9	7/14/2014	34:29.7
9554	RSPe_2	-122.445	37.941	0.11	18.12	277.47	18.23	-122.445	37.941	0.01	18.12	277.38	18.13	9	7/14/2014	34:29.6
9555	RSPe_2	-122.445	37.941	0.14	18.14	277.52	18.28	-122.445	37.941	0.01	18.14	277.49	18.14	9	7/14/2014	34:29.5
9556	RSPe_2	-122.445	37.941	0.06	18.13	277.63	18.19	-122.445	37.941	-0.05	18.13	277.58	18.09	9	7/14/2014	34:29.4
9557	RSPe_2	-122.445	37.941	0.06	18.15	277.61	18.21	-122.445	37.941	0.01	18.15	277.58	18.15	9	7/14/2014	34:29.3
9558	RSPe_2	-122.445	37.941	0.06	18.11	277.63	18.16	-122.445	37.941	-0.05	18.11	277.58	18.06	9	7/14/2014	34:29.2
9559	RSPe_2	-122.445	37.941	0.06	18.12	277.63	18.18	-122.445	37.941	-0.05	18.12	277.63	18.08	9	7/14/2014	34:29.1
9560	RSPe_2	-122.445	37.941	0.06	18.08	277.63	18.14	-122.445	37.941	-0.05	18.08	277.58	18.03	9	7/14/2014	34:29.0
9561	RSPe_2	-122.445	37.941	0.11	18.08	277.56	18.19	-122.445	37.941	-0.05	18.08	277.61	18.03	9	7/14/2014	34:28.9
9562	RSPe_2	-122.445	37.941	0.06	18.12	277.57	18.18	-122.445	37.941	-0.13	18.12	277.63	17.99	9	7/14/2014	34:28.8
9563	RSPe_2	-122.445	37.941	0.06	18.07	277.50	18.13	-122.445	37.941	-0.08	18.07	277.67	17.99	9	7/14/2014	34:28.7
9564	RSPe_2	-122.445	37.941	0.06	18.08	277.50	18.14	-122.445	37.941	-0.13	18.08	277.67	17.95	9	7/14/2014	34:28.6
9565	RSPe_2	-122.445	37.941	0.06	18.08	277.55	18.14	-122.445	37.941	-0.08	18.08	277.72	18.00	9	7/14/2014	34:28.5
9566	RSPe_2	-122.445	37.941	0.06	18.12	277.59	18.17	-122.445	37.941	-0.05	18.12	277.74	18.07	9	7/14/2014	34:28.4
9567	RSPe_2	-122.445	37.941	0.11	18.12	277.57	18.23	-122.445	37.941	-0.05	18.12	277.65	18.07	9	7/14/2014	34:28.3
9568	RSPe_2	-122.445	37.941	0.11	18.10	277.57	18.21	-122.445	37.941	-0.08	18.10	277.63	18.02	9	7/14/2014	34:28.2
9569	RSPe_2	-122.445	37.941	0.06	18.12	277.74	18.18	-122.445	37.941	-0.05	18.12	277.72	18.08	9	7/14/2014	34:28.1

9570	RSPe_2	-122.445	37.941	0.06	18.14	277.82	18.20	-122.445	37.941	-0.05	18.14	277.70	18.09	9	7/14/2014	34:28.0
9571	RSPe_2	-122.445	37.941	0.11	18.13	277.81	18.24	-122.445	37.941	-0.05	18.13	277.70	18.09	9	7/14/2014	34:27.9
9572	RSPe_2	-122.445	37.941	0.06	18.14	277.88	18.20	-122.445	37.941	-0.13	18.14	277.65	18.01	9	7/14/2014	34:27.8
9573	RSPe_2	-122.445	37.941	0.06	18.16	277.92	18.21	-122.445	37.941	-0.08	18.16	277.77	18.08	9	7/14/2014	34:27.7
9574	RSPe_2	-122.445	37.941	0.06	18.16	277.84	18.21	-122.445	37.941	-0.16	18.16	277.78	17.99	9	7/14/2014	34:27.6
9575	RSPe_2	-122.445	37.941	0.06	18.16	277.83	18.21	-122.445	37.941	-0.13	18.16	277.77	18.03	9	7/14/2014	34:27.5
9576	RSPe_2	-122.445	37.941	0.02	18.33	277.67	18.35	-122.445	37.941	-0.13	18.33	277.73	18.20	9	7/14/2014	34:27.4
9577	RSPe_2	-122.445	37.941	0.06	18.21	277.55	18.26	-122.445	37.941	-0.08	18.21	277.66	18.13	9	7/14/2014	34:27.3
9578	RSPe_2	-122.445	37.941	0.06	18.28	277.62	18.33	-122.445	37.941	-0.08	18.28	277.70	18.20	9	7/14/2014	34:27.2
9579	RSPe_2	-122.445	37.941	0.06	18.47	277.75	18.52	-122.445	37.941	-0.08	18.47	277.88	18.39	9	7/14/2014	34:27.1
9580	RSPe_2	-122.445	37.941	0.06	18.39	277.95	18.44	-122.445	37.941	-0.13	18.39	278.01	18.25	9	7/14/2014	34:27.0
9581	RSPe_2	-122.445	37.941	0.06	18.45	277.91	18.50	-122.445	37.941	-0.13	18.45	277.90	18.32	9	7/14/2014	34:26.9
9582	RSPe_2	-122.445	37.941	0.06	18.47	277.88	18.52	-122.445	37.941	-0.13	18.47	277.85	18.33	9	7/14/2014	34:26.8
9583	RSPe_2	-122.445	37.941	0.06	18.47	277.89	18.53	-122.445	37.941	-0.13	18.47	277.76	18.34	9	7/14/2014	34:26.7
9584	RSPe_2	-122.445	37.941	0.06	18.50	277.86	18.56	-122.445	37.941	-0.13	18.50	277.82	18.37	9	7/14/2014	34:26.6
9585	RSPe_2	-122.445	37.941	0.06	18.50	277.91	18.56	-122.445	37.941	-0.13	18.50	277.84	18.37	9	7/14/2014	34:26.5
9586	RSPe_2	-122.445	37.941	0.06	18.54	277.91	18.60	-122.445	37.941	-0.20	18.54	277.88	18.34	9	7/14/2014	34:26.4
9587	RSPe_2	-122.445	37.941	0.06	18.56	277.85	18.61	-122.445	37.941	-0.16	18.56	277.92	18.39	9	7/14/2014	34:26.3
9588	RSPe_2	-122.445	37.941	0.06	18.61	277.88	18.67	-122.445	37.941	-0.20	18.61	277.97	18.41	9	7/14/2014	34:26.2
9589	RSPe_2	-122.445	37.941	0.06	18.65	277.91	18.71	-122.445	37.941	-0.20	18.65	278.04	18.45	9	7/14/2014	34:26.1
9590	RSPe_2	-122.445	37.941	0.06	18.69	277.98	18.75	-122.445	37.941	-0.16	18.69	278.13	18.53	9	7/14/2014	34:26.0
9591	RSPe_2	-122.445	37.941	0.06	18.74	278.02	18.80	-122.445	37.941	-0.13	18.74	278.14	18.61	9	7/14/2014	34:25.9
9592	RSPe_2	-122.445	37.941	0.06	18.75	278.11	18.81	-122.445	37.941	-0.20	18.75	278.17	18.55	9	7/14/2014	34:25.8
9593	RSPe_2	-122.445	37.941	0.11	18.76	278.04	18.87	-122.445	37.941	-0.16	18.76	278.13	18.60	9	7/14/2014	34:25.7
9594	RSPe_2	-122.445	37.941	0.06	18.76	278.09	18.82	-122.445	37.941	-0.16	18.76	278.16	18.60	9	7/14/2014	34:25.6
9595	RSPe_2	-122.445	37.941	0.11	18.72	278.04	18.83	-122.445	37.941	-0.16	18.72	278.10	18.55	9	7/14/2014	34:25.5
9596	RSPe_2	-122.445	37.941	0.06	18.72	278.01	18.77	-122.445	37.941	-0.20	18.72	278.09	18.52	9	7/14/2014	34:25.4
9597	RSPe_2	-122.445	37.941	0.06	18.66	277.96	18.71	-122.445	37.941	-0.20	18.66	278.05	18.46	9	7/14/2014	34:25.3
9598	RSPe_2	-122.445	37.941	0.06	18.63	277.94	18.68	-122.445	37.941	-0.20	18.63	278.08	18.43	9	7/14/2014	34:25.2
9599	RSPe_2	-122.445	37.941	0.11	18.67	278.01	18.78	-122.445	37.941	-0.20	18.67	278.12	18.48	9	7/14/2014	34:25.1
9600	RSPe_2	-122.445	37.941	0.06	18.62	278.16	18.68	-122.445	37.941	-0.20	18.62	278.20	18.42	9	7/14/2014	34:25.0
9601	RSPe_2	-122.445	37.941	0.06	18.63	278.18	18.68	-122.445	37.941	-0.16	18.63	278.13	18.46	9	7/14/2014	34:24.9
9602	RSPe_2	-122.445	37.941	0.06	18.58	278.20	18.64	-122.445	37.941	-0.20	18.58	278.11	18.38	9	7/14/2014	34:24.8

9603	RSPe_2	-122.445	37.941	0.11	18.65	278.29	18.76	-122.445	37.941	-0.20	18.65	278.09	18.45	9	7/14/2014	34:24.7
9604	RSPe_2	-122.445	37.941	0.11	18.65	278.32	18.76	-122.445	37.941	-0.16	18.65	278.11	18.49	9	7/14/2014	34:24.6
9605	RSPe_2	-122.445	37.941	0.11	18.57	278.31	18.68	-122.445	37.941	-0.20	18.57	278.10	18.37	9	7/14/2014	34:24.5
9606	RSPe_2	-122.445	37.941	0.11	18.52	278.43	18.63	-122.445	37.941	-0.16	18.52	278.07	18.36	9	7/14/2014	34:24.4
9607	RSPe_2	-122.445	37.941	0.11	18.52	278.52	18.63	-122.445	37.941	-0.16	18.52	278.18	18.36	9	7/14/2014	34:24.3
9608	RSPe_2	-122.445	37.941	0.11	18.52	278.60	18.63	-122.445	37.941	-0.16	18.52	278.24	18.36	9	7/14/2014	34:24.2
9609	RSPe_2	-122.445	37.941	0.11	18.52	278.60	18.63	-122.445	37.941	-0.16	18.52	278.31	18.36	9	7/14/2014	34:24.1
9610	RSPe_2	-122.445	37.941	0.06	18.50	278.54	18.56	-122.445	37.941	-0.20	18.50	278.33	18.31	9	7/14/2014	34:24.0
9611	RSPe_2	-122.445	37.941	0.14	18.50	278.58	18.65	-122.445	37.941	-0.16	18.50	278.45	18.34	9	7/14/2014	34:23.9
9612	RSPe_2	-122.445	37.941	0.11	18.50	278.56	18.61	-122.445	37.941	-0.16	18.50	278.51	18.34	9	7/14/2014	34:23.8
9613	RSPe_2	-122.445	37.941	0.11	18.52	278.56	18.63	-122.445	37.941	-0.13	18.52	278.58	18.39	9	7/14/2014	34:23.7
9614	RSPe_2	-122.445	37.941	0.06	18.50	278.54	18.56	-122.445	37.941	-0.16	18.50	278.60	18.34	9	7/14/2014	34:23.6
9615	RSPe_2	-122.445	37.941	0.11	18.50	278.54	18.60	-122.445	37.941	-0.16	18.50	278.58	18.33	9	7/14/2014	34:23.5
9616	RSPe_2	-122.445	37.941	0.11	18.51	278.56	18.62	-122.445	37.941	-0.20	18.51	278.62	18.31	9	7/14/2014	34:23.4
9617	RSPe_2	-122.445	37.941	0.14	18.47	278.61	18.62	-122.445	37.941	-0.13	18.47	278.62	18.34	9	7/14/2014	34:23.3
9618	RSPe_2	-122.445	37.941	0.11	18.48	278.57	18.59	-122.445	37.941	-0.16	18.48	278.60	18.32	9	7/14/2014	34:23.2
9619	RSPe_2	-122.445	37.941	0.11	18.45	278.74	18.56	-122.445	37.941	-0.13	18.45	278.65	18.32	9	7/14/2014	34:23.1
9620	RSPe_2	-122.445	37.941	0.11	18.44	278.80	18.55	-122.445	37.941	-0.13	18.44	278.67	18.31	9	7/14/2014	34:23.0
9621	RSPe_2	-122.445	37.941	0.11	18.43	278.79	18.54	-122.445	37.941	-0.16	18.43	278.67	18.27	9	7/14/2014	34:22.9
9622	RSPe_2	-122.445	37.941	0.11	18.41	278.79	18.52	-122.445	37.941	-0.13	18.41	278.71	18.28	9	7/14/2014	34:22.8
9623	RSPe_2	-122.445	37.941	0.14	18.43	278.79	18.57	-122.445	37.941	-0.08	18.43	278.76	18.35	9	7/14/2014	34:22.7
9624	RSPe_2	-122.445	37.941	0.11	18.39	278.72	18.49	-122.445	37.941	-0.13	18.39	278.78	18.26	9	7/14/2014	34:22.6
9625	RSPe_2	-122.445	37.941	0.14	18.42	278.72	18.56	-122.445	37.941	-0.13	18.42	278.74	18.29	9	7/14/2014	34:22.5
9626	RSPe_2	-122.445	37.941	0.11	18.30	278.70	18.41	-122.445	37.941	-0.13	18.30	278.75	18.17	9	7/14/2014	34:22.4
9627	RSPe_2	-122.445	37.941	0.14	18.32	278.66	18.46	-122.445	37.941	-0.08	18.32	278.69	18.24	9	7/14/2014	34:22.3
9628	RSPe_2	-122.445	37.941	0.14	18.28	278.66	18.42	-122.445	37.941	-0.08	18.28	278.70	18.20	9	7/14/2014	34:22.2
9629	RSPe_2	-122.445	37.941	0.14	18.29	278.61	18.43	-122.445	37.941	-0.13	18.29	278.59	18.16	9	7/14/2014	34:22.1
9630	RSPe_2	-122.445	37.941	0.11	18.30	278.59	18.40	-122.445	37.941	-0.08	18.30	278.61	18.22	9	7/14/2014	34:22.0
9631	RSPe_2	-122.445	37.941	0.19	18.29	278.72	18.48	-122.445	37.941	-0.08	18.29	278.74	18.21	9	7/14/2014	34:21.9
9632	RSPe_2	-122.445	37.941	0.14	18.33	278.75	18.47	-122.445	37.941	-0.16	18.33	278.75	18.16	9	7/14/2014	34:21.8
9633	RSPe_2	-122.445	37.941	0.19	18.34	278.75	18.54	-122.445	37.941	-0.13	18.34	278.79	18.21	9	7/14/2014	34:21.7
9634	RSPe_2	-122.445	37.941	0.14	18.40	278.83	18.54	-122.445	37.941	-0.13	18.40	278.94	18.27	9	7/14/2014	34:21.6
9635	RSPe_2	-122.445	37.941	0.14	18.33	278.79	18.47	-122.445	37.941	-0.08	18.33	278.90	18.25	9	7/14/2014	34:21.5

9636	RSPe_2	-122.445	37.941	0.14	18.40	278.82	18.55	-122.445	37.941	-0.13	18.40	278.96	18.27	9	7/14/2014	34:21.4
9637	RSPe_2	-122.445	37.941	0.19	18.37	278.90	18.56	-122.445	37.941	-0.13	18.37	279.04	18.24	9	7/14/2014	34:21.3
9638	RSPe_2	-122.445	37.941	0.19	18.55	278.88	18.75	-122.445	37.941	-0.13	18.55	279.01	18.42	9	7/14/2014	34:21.2
9639	RSPe_2	-122.445	37.941	0.19	18.53	278.91	18.73	-122.445	37.941	-0.13	18.53	278.99	18.40	9	7/14/2014	34:21.1
9640	RSPe_2	-122.445	37.941	0.14	18.66	278.86	18.80	-122.445	37.941	-0.16	18.66	278.98	18.50	9	7/14/2014	34:21.0
9641	RSPe_2	-122.445	37.941	0.19	18.60	278.77	18.80	-122.445	37.941	-0.13	18.60	278.88	18.47	9	7/14/2014	34:20.9
9642	RSPe_2	-122.445	37.941	0.14	18.59	278.82	18.74	-122.445	37.941	-0.13	18.59	278.84	18.46	9	7/14/2014	34:20.8
9643	RSPe_2	-122.445	37.941	0.19	18.66	278.64	18.85	-122.445	37.941	-0.13	18.66	278.71	18.53	9	7/14/2014	34:20.7
9644	RSPe_2	-122.445	37.941	0.19	18.63	278.58	18.83	-122.445	37.941	-0.13	18.63	278.59	18.50	9	7/14/2014	34:20.6
9645	RSPe_2	-122.445	37.941	0.23	18.68	278.91	18.91	-122.445	37.941	-0.05	18.68	278.78	18.64	9	7/14/2014	34:20.5
9646	RSPe_2	-122.445	37.941	0.19	18.66	279.17	18.86	-122.445	37.941	-0.05	18.66	279.03	18.62	9	7/14/2014	34:20.4
9647	RSPe_2	-122.445	37.941	0.23	18.70	279.33	18.93	-122.445	37.941	-0.08	18.70	279.13	18.62	9	7/14/2014	34:20.3
9648	RSPe_2	-122.445	37.941	0.14	18.69	279.37	18.83	-122.445	37.941	-0.08	18.69	279.15	18.61	9	7/14/2014	34:20.2
9649	RSPe_2	-122.445	37.941	0.19	18.70	279.31	18.90	-122.445	37.941	-0.08	18.70	279.13	18.62	9	7/14/2014	34:20.1
9650	RSPe_2	-122.445	37.941	0.19	18.64	279.35	18.84	-122.445	37.941	-0.13	18.64	279.04	18.51	9	7/14/2014	34:20.0
9651	RSPe_2	-122.445	37.941	0.19	18.60	279.24	18.80	-122.445	37.941	-0.08	18.60	279.02	18.53	9	7/14/2014	34:19.9
9652	RSPe_2	-122.445	37.941	0.19	18.59	279.17	18.78	-122.445	37.941	-0.13	18.59	278.99	18.46	9	7/14/2014	34:19.8
9653	RSPe_2	-122.445	37.941	0.23	18.56	279.16	18.78	-122.445	37.941	-0.05	18.56	279.06	18.51	9	7/14/2014	34:19.7
9654	RSPe_2	-122.445	37.941	0.19	18.58	279.16	18.77	-122.445	37.941	-0.13	18.58	279.15	18.45	9	7/14/2014	34:19.6
9655	RSPe_2	-122.445	37.941	0.19	18.58	279.14	18.77	-122.445	37.941	-0.13	18.58	279.18	18.45	9	7/14/2014	34:19.5
9656	RSPe_2	-122.445	37.941	0.19	18.52	279.07	18.71	-122.445	37.941	-0.13	18.52	279.15	18.39	9	7/14/2014	34:19.4
9657	RSPe_2	-122.445	37.941	0.19	18.47	279.05	18.67	-122.445	37.941	-0.08	18.47	279.11	18.39	9	7/14/2014	34:19.3
9658	RSPe_2	-122.445	37.941	0.19	18.41	279.07	18.61	-122.445	37.941	-0.08	18.41	279.15	18.33	9	7/14/2014	34:19.2
9659	RSPe_2	-122.445	37.941	0.23	18.41	279.14	18.64	-122.445	37.941	-0.08	18.41	279.20	18.33	9	7/14/2014	34:19.1
9660	RSPe_2	-122.445	37.941	0.19	18.40	279.25	18.60	-122.445	37.941	-0.08	18.40	279.28	18.32	9	7/14/2014	34:19.0
9661	RSPe_2	-122.445	37.941	0.23	18.37	279.25	18.60	-122.445	37.941	-0.08	18.37	279.25	18.29	9	7/14/2014	34:18.9
9662	RSPe_2	-122.445	37.941	0.19	18.36	279.20	18.55	-122.445	37.941	-0.13	18.36	279.20	18.23	9	7/14/2014	34:18.8
9663	RSPe_2	-122.445	37.941	0.23	18.34	279.34	18.57	-122.445	37.941	-0.05	18.34	279.24	18.30	9	7/14/2014	34:18.7
9664	RSPe_2	-122.445	37.941	0.19	18.41	279.36	18.61	-122.445	37.941	-0.08	18.41	279.31	18.33	9	7/14/2014	34:18.6
9665	RSPe_2	-122.445	37.941	0.23	18.28	279.34	18.51	-122.445	37.941	-0.08	18.28	279.23	18.20	9	7/14/2014	34:18.5
9666	RSPe_2	-122.445	37.941	0.23	18.27	279.34	18.50	-122.445	37.941	-0.08	18.27	279.24	18.19	9	7/14/2014	34:18.4
9667	RSPe_2	-122.445	37.941	0.23	18.24	279.32	18.47	-122.445	37.941	-0.05	18.24	279.16	18.20	9	7/14/2014	34:18.3
9668	RSPe_2	-122.445	37.941	0.23	18.21	279.36	18.44	-122.445	37.941	-0.05	18.21	279.20	18.16	9	7/14/2014	34:18.2

9669	RSPe_2	-122.445	37.941	0.23	18.22	279.45	18.45	-122.445	37.941	0.01	18.22	279.23	18.22	9	7/14/2014	34:18.1
9670	RSPe_2	-122.445	37.941	0.19	18.20	279.47	18.40	-122.445	37.941	-0.05	18.20	279.23	18.16	9	7/14/2014	34:18.0
9671	RSPe_2	-122.445	37.941	0.23	18.19	279.43	18.42	-122.445	37.941	-0.05	18.19	279.25	18.15	9	7/14/2014	34:17.9
9672	RSPe_2	-122.445	37.941	0.19	18.15	279.47	18.34	-122.445	37.941	-0.05	18.15	279.23	18.10	9	7/14/2014	34:17.8
9673	RSPe_2	-122.445	37.941	0.19	18.17	279.56	18.36	-122.445	37.941	-0.05	18.17	279.36	18.13	9	7/14/2014	34:17.7
9674	RSPe_2	-122.445	37.941	0.19	18.14	279.59	18.33	-122.445	37.941	-0.08	18.14	279.38	18.06	9	7/14/2014	34:17.6
9675	RSPe_2	-122.445	37.941	0.23	18.13	279.61	18.36	-122.445	37.941	-0.08	18.13	279.39	18.05	9	7/14/2014	34:17.5
9676	RSPe_2	-122.445	37.941	0.19	18.12	279.61	18.32	-122.445	37.941	-0.08	18.12	279.43	18.05	9	7/14/2014	34:17.4
9677	RSPe_2	-122.445	37.941	0.23	18.13	279.56	18.36	-122.445	37.941	-0.08	18.13	279.43	18.05	9	7/14/2014	34:17.3
9678	RSPe_2	-122.445	37.941	0.19	18.14	279.54	18.33	-122.445	37.941	-0.08	18.14	279.52	18.06	9	7/14/2014	34:17.2
9679	RSPe_2	-122.445	37.941	0.23	18.18	279.59	18.41	-122.445	37.941	-0.05	18.18	279.56	18.13	9	7/14/2014	34:17.1
9680	RSPe_2	-122.445	37.941	0.23	18.18	279.64	18.41	-122.445	37.941	-0.05	18.18	279.64	18.13	9	7/14/2014	34:17.0
9681	RSPe_2	-122.445	37.941	0.26	18.19	279.66	18.46	-122.445	37.941	0.04	18.19	279.65	18.23	9	7/14/2014	34:16.9
9682	RSPe_2	-122.445	37.941	0.23	18.29	279.68	18.52	-122.445	37.941	-0.05	18.29	279.70	18.24	9	7/14/2014	34:16.8
9683	RSPe_2	-122.445	37.941	0.23	18.30	279.70	18.53	-122.445	37.941	0.04	18.30	279.66	18.34	9	7/14/2014	34:16.7
9684	RSPe_2	-122.445	37.941	0.23	18.33	279.79	18.56	-122.445	37.941	-0.05	18.33	279.69	18.29	9	7/14/2014	34:16.6
9685	RSPe_2	-122.445	37.941	0.26	18.30	279.88	18.56	-122.445	37.941	-0.08	18.30	279.72	18.22	9	7/14/2014	34:16.5
9686	RSPe_2	-122.445	37.941	0.23	18.33	279.90	18.55	-122.445	37.941	-0.05	18.33	279.72	18.28	9	7/14/2014	34:16.4
9687	RSPe_2	-122.445	37.941	0.23	18.36	279.92	18.59	-122.445	37.941	0.01	18.36	279.70	18.36	9	7/14/2014	34:16.3
9688	RSPe_2	-122.445	37.941	0.23	18.29	279.92	18.52	-122.445	37.941	0.01	18.29	279.69	18.29	9	7/14/2014	34:16.2
9689	RSPe_2	-122.445	37.941	0.23	18.29	279.86	18.52	-122.445	37.941	-0.05	18.29	279.68	18.24	9	7/14/2014	34:16.1
9690	RSPe_2	-122.445	37.941	0.23	18.29	279.81	18.52	-122.445	37.941	0.01	18.29	279.59	18.29	9	7/14/2014	34:16.0
9691	RSPe_2	-122.445	37.941	0.23	18.24	279.92	18.47	-122.445	37.941	-0.05	18.24	279.70	18.20	9	7/14/2014	34:15.9
9692	RSPe_2	-122.445	37.941	0.19	18.24	280.04	18.43	-122.445	37.941	0.01	18.24	279.85	18.24	9	7/14/2014	34:15.8
9693	RSPe_2	-122.445	37.941	0.23	18.26	280.03	18.49	-122.445	37.941	0.01	18.26	279.86	18.27	9	7/14/2014	34:15.7
9694	RSPe_2	-122.445	37.941	0.19	18.30	280.12	18.50	-122.445	37.941	-0.05	18.30	279.92	18.26	9	7/14/2014	34:15.6
9695	RSPe_2	-122.445	37.941	0.23	18.26	280.19	18.48	-122.445	37.941	0.01	18.26	279.99	18.26	9	7/14/2014	34:15.5
9696	RSPe_2	-122.445	37.941	0.23	18.29	280.26	18.52	-122.445	37.941	0.01	18.29	279.99	18.29	9	7/14/2014	34:15.4
9697	RSPe_2	-122.445	37.941	0.23	18.27	280.28	18.50	-122.445	37.941	0.01	18.27	280.01	18.28	9	7/14/2014	34:15.3
9698	RSPe_2	-122.445	37.941	0.19	18.26	280.30	18.46	-122.445	37.941	-0.05	18.26	280.03	18.22	9	7/14/2014	34:15.2
9699	RSPe_2	-122.445	37.941	0.23	18.28	280.19	18.51	-122.445	37.941	0.01	18.28	280.03	18.28	9	7/14/2014	34:15.1
9700	RSPe_2	-122.445	37.941	0.23	18.26	280.26	18.49	-122.445	37.941	-0.05	18.26	280.10	18.22	9	7/14/2014	34:15.0
9701	RSPe_2	-122.445	37.941	0.23	18.32	280.24	18.54	-122.445	37.941	0.01	18.32	280.17	18.32	9	7/14/2014	34:14.9

9702	RSPe_2	-122.445	37.941	0.19	18.34	280.26	18.54	-122.445	37.941	-0.05	18.34	280.19	18.30	9	7/14/2014	34:14.8
9703	RSPe_2	-122.445	37.941	0.23	18.31	280.28	18.54	-122.445	37.941	0.04	18.31	280.22	18.35	9	7/14/2014	34:14.7
9704	RSPe_2	-122.445	37.941	0.23	18.40	280.37	18.63	-122.445	37.941	0.01	18.40	280.30	18.41	9	7/14/2014	34:14.6
9705	RSPe_2	-122.445	37.941	0.23	18.36	280.40	18.59	-122.445	37.941	0.04	18.36	280.35	18.40	9	7/14/2014	34:14.5
9706	RSPe_2	-122.445	37.941	0.19	18.61	280.37	18.81	-122.445	37.941	0.01	18.61	280.26	18.62	9	7/14/2014	34:14.4
9707	RSPe_2	-122.445	37.941	0.19	18.53	280.39	18.73	-122.445	37.941	0.01	18.53	280.28	18.54	9	7/14/2014	34:14.3
9708	RSPe_2	-122.445	37.941	0.19	18.59	280.38	18.78	-122.445	37.941	0.01	18.59	280.28	18.59	9	7/14/2014	34:14.2
9709	RSPe_2	-122.445	37.941	0.23	18.50	280.39	18.72	-122.445	37.941	0.01	18.50	280.29	18.50	9	7/14/2014	34:14.1
9710	RSPe_2	-122.445	37.941	0.19	18.63	280.40	18.83	-122.445	37.941	-0.05	18.63	280.34	18.59	9	7/14/2014	34:14.0
9711	RSPe_2	-122.445	37.941	0.23	18.65	280.48	18.88	-122.445	37.941	0.01	18.65	280.38	18.66	9	7/14/2014	34:13.9
9712	RSPe_2	-122.445	37.941	0.19	18.57	280.51	18.76	-122.445	37.941	-0.05	18.57	280.36	18.52	9	7/14/2014	34:13.8
9713	RSPe_2	-122.445	37.941	0.23	18.63	280.51	18.86	-122.445	37.941	0.01	18.63	280.41	18.64	9	7/14/2014	34:13.7
9714	RSPe_2	-122.445	37.941	0.19	18.60	280.60	18.80	-122.445	37.941	0.01	18.60	280.53	18.61	9	7/14/2014	34:13.6
9715	RSPe_2	-122.445	37.941	0.23	18.59	280.64	18.82	-122.445	37.941	0.01	18.59	280.58	18.60	9	7/14/2014	34:13.5
9716	RSPe_2	-122.445	37.941	0.19	18.76	280.62	18.96	-122.445	37.941	-0.05	18.76	280.61	18.72	9	7/14/2014	34:13.4
9717	RSPe_2	-122.445	37.941	0.23	18.69	280.62	18.92	-122.445	37.941	-0.05	18.69	280.61	18.65	9	7/14/2014	34:13.3
9718	RSPe_2	-122.445	37.941	0.19	18.66	280.58	18.86	-122.445	37.941	-0.08	18.66	280.62	18.58	9	7/14/2014	34:13.2
9719	RSPe_2	-122.445	37.941	0.19	18.73	280.53	18.93	-122.445	37.941	-0.08	18.73	280.57	18.66	9	7/14/2014	34:13.1
9720	RSPe_2	-122.445	37.941	0.19	18.92	280.56	19.12	-122.445	37.941	-0.08	18.92	280.51	18.84	9	7/14/2014	34:13.0
9721	RSPe_2	-122.445	37.941	0.19	18.81	280.56	19.01	-122.445	37.941	-0.08	18.81	280.49	18.73	9	7/14/2014	34:12.9
9722	RSPe_2	-122.445	37.941	0.19	18.84	280.51	19.04	-122.445	37.941	-0.08	18.84	280.44	18.76	9	7/14/2014	34:12.8
9723	RSPe_2	-122.445	37.941	0.19	18.73	280.52	18.92	-122.445	37.941	-0.08	18.73	280.43	18.65	9	7/14/2014	34:12.7
9724	RSPe_2	-122.445	37.941	0.14	18.76	280.58	18.90	-122.445	37.941	-0.04	18.76	280.46	18.72	9	7/14/2014	34:12.6
9725	RSPe_2	-122.445	37.941	0.19	18.75	280.67	18.94	-122.445	37.941	-0.04	18.75	280.53	18.71	9	7/14/2014	34:12.5
9726	RSPe_2	-122.445	37.941	0.19	18.75	280.78	18.94	-122.445	37.941	-0.08	18.75	280.61	18.67	9	7/14/2014	34:12.4
9727	RSPe_2	-122.445	37.941	0.19	18.84	280.83	19.04	-122.445	37.941	-0.08	18.84	280.68	18.76	9	7/14/2014	34:12.3
9728	RSPe_2	-122.445	37.941	0.19	18.74	280.91	18.94	-122.445	37.941	-0.08	18.74	280.76	18.67	9	7/14/2014	34:12.2
9729	RSPe_2	-122.445	37.941	0.19	18.67	280.89	18.87	-122.445	37.941	-0.08	18.67	280.75	18.60	9	7/14/2014	34:12.1
9730	RSPe_2	-122.445	37.941	0.19	18.73	280.85	18.93	-122.445	37.941	-0.13	18.73	280.75	18.60	9	7/14/2014	34:12.0
9731	RSPe_2	-122.445	37.941	0.19	18.67	280.83	18.87	-122.445	37.941	-0.08	18.67	280.76	18.60	9	7/14/2014	34:11.9
9732	RSPe_2	-122.445	37.941	0.14	18.53	280.83	18.68	-122.445	37.941	-0.13	18.53	280.78	18.41	9	7/14/2014	34:11.8
9733	RSPe_2	-122.445	37.941	0.19	18.50	280.76	18.70	-122.445	37.941	-0.13	18.50	280.75	18.37	9	7/14/2014	34:11.7
9734	RSPe_2	-122.445	37.941	0.14	18.52	280.78	18.66	-122.445	37.941	-0.13	18.52	280.74	18.39	9	7/14/2014	34:11.6

9735	RSPe_2	-122.445	37.941	0.19	18.47	280.81	18.66	-122.445	37.941	-0.08	18.47	280.81	18.39	9	7/14/2014	34:11.5
9736	RSPe_2	-122.445	37.941	0.14	18.40	280.87	18.54	-122.445	37.941	-0.13	18.40	280.87	18.27	9	7/14/2014	34:11.4
9737	RSPe_2	-122.445	37.941	0.19	18.43	280.92	18.62	-122.445	37.941	-0.08	18.43	280.87	18.35	9	7/14/2014	34:11.3
9738	RSPe_2	-122.445	37.941	0.14	18.38	280.92	18.52	-122.445	37.941	-0.13	18.38	280.94	18.25	9	7/14/2014	34:11.2
9739	RSPe_2	-122.445	37.941	0.19	18.46	280.98	18.65	-122.445	37.941	-0.13	18.46	280.96	18.33	9	7/14/2014	34:11.1
9740	RSPe_2	-122.445	37.941	0.14	18.39	281.05	18.53	-122.445	37.941	-0.13	18.39	280.98	18.26	9	7/14/2014	34:11.0
9741	RSPe_2	-122.445	37.941	0.14	18.37	281.09	18.51	-122.445	37.941	-0.13	18.37	281.00	18.24	9	7/14/2014	34:10.9
9742	RSPe_2	-122.445	37.941	0.14	18.36	281.14	18.50	-122.445	37.941	-0.13	18.36	281.00	18.23	9	7/14/2014	34:10.8
9743	RSPe_2	-122.445	37.941	0.19	18.27	281.16	18.47	-122.445	37.941	-0.08	18.27	281.03	18.19	9	7/14/2014	34:10.7
9744	RSPe_2	-122.445	37.941	0.14	18.22	281.19	18.36	-122.445	37.941	-0.13	18.22	281.07	18.09	9	7/14/2014	34:10.6
9745	RSPe_2	-122.445	37.941	0.19	18.17	281.25	18.36	-122.445	37.941	-0.13	18.17	281.10	18.04	9	7/14/2014	34:10.5
9746	RSPe_2	-122.445	37.941	0.14	18.17	281.25	18.31	-122.445	37.941	-0.16	18.17	281.14	18.01	9	7/14/2014	34:10.4
9747	RSPe_2	-122.445	37.941	0.19	18.27	281.34	18.47	-122.445	37.941	-0.13	18.27	281.18	18.14	9	7/14/2014	34:10.3
9748	RSPe_2	-122.445	37.941	0.14	18.26	281.37	18.40	-122.445	37.941	-0.13	18.26	281.20	18.13	9	7/14/2014	34:10.2
9749	RSPe_2	-122.445	37.941	0.19	18.26	281.30	18.45	-122.445	37.941	-0.13	18.26	281.23	18.13	9	7/14/2014	34:10.1
9750	RSPe_2	-122.445	37.941	0.14	18.24	281.32	18.38	-122.445	37.941	-0.16	18.24	281.25	18.08	9	7/14/2014	34:10.0
9751	RSPe_2	-122.445	37.941	0.14	18.23	281.28	18.37	-122.445	37.941	-0.13	18.23	281.25	18.10	9	7/14/2014	34:09.9
9752	RSPe_2	-122.445	37.941	0.14	18.22	281.30	18.36	-122.445	37.941	-0.16	18.22	281.27	18.06	9	7/14/2014	34:09.8
9753	RSPe_2	-122.445	37.941	0.19	18.20	281.30	18.40	-122.445	37.941	-0.13	18.20	281.32	18.07	9	7/14/2014	34:09.7
9754	RSPe_2	-122.445	37.941	0.19	18.26	281.39	18.45	-122.445	37.941	-0.13	18.26	281.33	18.13	9	7/14/2014	34:09.6
9755	RSPe_2	-122.445	37.941	0.14	18.34	281.39	18.49	-122.445	37.941	-0.13	18.34	281.35	18.21	9	7/14/2014	34:09.5
9756	RSPe_2	-122.445	37.941	0.14	18.33	281.43	18.48	-122.445	37.941	-0.13	18.33	281.36	18.20	9	7/14/2014	34:09.4
9757	RSPe_2	-122.445	37.941	0.19	18.26	281.48	18.46	-122.445	37.941	-0.13	18.26	281.32	18.13	9	7/14/2014	34:09.3
9758	RSPe_2	-122.445	37.941	0.14	18.33	281.44	18.47	-122.445	37.941	-0.13	18.33	281.28	18.20	9	7/14/2014	34:09.2
9759	RSPe_2	-122.445	37.941	0.14	18.34	281.48	18.49	-122.445	37.941	-0.16	18.34	281.28	18.18	9	7/14/2014	34:09.1
9760	RSPe_2	-122.445	37.941	0.14	18.33	281.48	18.48	-122.445	37.941	-0.16	18.33	281.30	18.17	9	7/14/2014	34:09.0
9761	RSPe_2	-122.445	37.941	0.19	18.40	281.64	18.59	-122.445	37.941	-0.13	18.40	281.33	18.27	9	7/14/2014	34:08.9
9762	RSPe_2	-122.445	37.941	0.14	18.30	281.53	18.45	-122.445	37.941	-0.08	18.30	281.33	18.23	9	7/14/2014	34:08.8
9763	RSPe_2	-122.445	37.941	0.19	18.53	281.59	18.73	-122.445	37.941	-0.08	18.53	281.37	18.46	9	7/14/2014	34:08.7
9764	RSPe_2	-122.445	37.941	0.19	18.40	281.57	18.59	-122.445	37.941	-0.13	18.40	281.37	18.27	9	7/14/2014	34:08.6
9765	RSPe_2	-122.445	37.941	0.19	18.48	281.59	18.68	-122.445	37.941	-0.13	18.48	281.45	18.35	9	7/14/2014	34:08.5
9766	RSPe_2	-122.445	37.941	0.14	18.43	281.57	18.57	-122.445	37.941	-0.16	18.43	281.49	18.27	9	7/14/2014	34:08.4
9767	RSPe_2	-122.445	37.941	0.19	18.58	281.55	18.77	-122.445	37.941	-0.16	18.58	281.52	18.42	9	7/14/2014	34:08.3

9768	RSPe_2	-122.445	37.941	0.19	18.70	281.55	18.90	-122.445	37.941	-0.16	18.70	281.51	18.54	9	7/14/2014	34:08.2
9769	RSPe_2	-122.445	37.941	0.19	18.70	281.64	18.90	-122.445	37.941	-0.16	18.70	281.60	18.54	9	7/14/2014	34:08.1
9770	RSPe_2	-122.445	37.941	0.19	18.78	281.62	18.98	-122.445	37.941	-0.16	18.78	281.55	18.62	9	7/14/2014	34:08.0
9771	RSPe_2	-122.445	37.941	0.19	18.73	281.69	18.92	-122.445	37.941	-0.08	18.73	281.59	18.65	9	7/14/2014	34:07.9
9772	RSPe_2	-122.445	37.941	0.19	18.73	281.70	18.93	-122.445	37.941	-0.13	18.73	281.57	18.60	9	7/14/2014	34:07.8
9773	RSPe_2	-122.445	37.941	0.19	18.77	281.75	18.97	-122.445	37.941	-0.13	18.77	281.59	18.64	9	7/14/2014	34:07.7
9774	RSPe_2	-122.445	37.941	0.19	18.79	281.80	18.98	-122.445	37.941	-0.13	18.79	281.62	18.66	9	7/14/2014	34:07.6
9775	RSPe_2	-122.445	37.941	0.23	18.84	281.89	19.07	-122.445	37.941	-0.08	18.84	281.66	18.76	9	7/14/2014	34:07.5
9776	RSPe_2	-122.445	37.941	0.14	18.81	281.89	18.96	-122.445	37.941	-0.13	18.81	281.66	18.68	9	7/14/2014	34:07.4
9777	RSPe_2	-122.445	37.941	0.19	18.86	281.89	19.05	-122.445	37.941	-0.13	18.86	281.69	18.73	9	7/14/2014	34:07.3
9778	RSPe_2	-122.445	37.941	0.19	18.87	281.89	19.06	-122.445	37.941	-0.13	18.87	281.68	18.74	9	7/14/2014	34:07.2
9779	RSPe_2	-122.445	37.941	0.19	18.80	281.86	18.99	-122.445	37.941	-0.13	18.80	281.66	18.67	9	7/14/2014	34:07.1
9780	RSPe_2	-122.445	37.941	0.19	18.85	281.82	19.05	-122.445	37.941	-0.13	18.85	281.60	18.72	9	7/14/2014	34:07.0
9781	RSPe_2	-122.445	37.941	0.19	18.91	281.82	19.11	-122.445	37.941	-0.13	18.91	281.60	18.78	9	7/14/2014	34:06.9
9782	RSPe_2	-122.445	37.941	0.19	18.92	281.78	19.12	-122.445	37.941	-0.13	18.92	281.53	18.79	9	7/14/2014	34:06.8
9783	RSPe_2	-122.445	37.941	0.19	18.97	281.78	19.17	-122.445	37.941	-0.08	18.97	281.54	18.90	9	7/14/2014	34:06.7
9784	RSPe_2	-122.445	37.941	0.19	18.95	281.87	19.15	-122.445	37.941	-0.08	18.95	281.58	18.87	9	7/14/2014	34:06.6
9785	RSPe_2	-122.445	37.941	0.19	19.11	281.89	19.31	-122.445	37.941	-0.04	19.11	281.60	19.07	9	7/14/2014	34:06.5
9786	RSPe_2	-122.445	37.941	0.19	19.01	281.87	19.21	-122.445	37.941	-0.08	19.01	281.66	18.93	9	7/14/2014	34:06.4
9787	RSPe_2	-122.445	37.941	0.23	19.03	281.83	19.26	-122.445	37.941	-0.04	19.03	281.69	18.98	9	7/14/2014	34:06.3
9788	RSPe_2	-122.445	37.941	0.19	19.10	281.81	19.29	-122.445	37.941	-0.08	19.10	281.74	19.02	9	7/14/2014	34:06.2
9789	RSPe_2	-122.445	37.941	0.23	19.02	281.79	19.25	-122.445	37.941	-0.13	19.02	281.72	18.89	9	7/14/2014	34:06.1
9790	RSPe_2	-122.445	37.941	0.19	19.10	281.81	19.29	-122.445	37.941	-0.08	19.10	281.76	19.02	9	7/14/2014	34:06.0
9791	RSPe_2	-122.445	37.941	0.23	19.08	281.80	19.31	-122.445	37.941	-0.04	19.08	281.72	19.04	9	7/14/2014	34:05.9
9792	RSPe_2	-122.445	37.941	0.19	19.17	281.90	19.36	-122.445	37.941	-0.13	19.17	281.76	19.04	9	7/14/2014	34:05.8
9793	RSPe_2	-122.445	37.941	0.23	18.96	281.94	19.19	-122.445	37.941	-0.08	18.96	281.78	18.88	9	7/14/2014	34:05.7
9794	RSPe_2	-122.445	37.941	0.23	18.97	281.94	19.20	-122.445	37.941	-0.04	18.97	281.74	18.93	9	7/14/2014	34:05.6
9795	RSPe_2	-122.445	37.941	0.23	18.87	281.99	19.10	-122.445	37.941	-0.08	18.87	281.78	18.79	9	7/14/2014	34:05.5
9796	RSPe_2	-122.445	37.941	0.23	18.87	281.94	19.10	-122.445	37.941	-0.08	18.87	281.76	18.79	9	7/14/2014	34:05.4
9797	RSPe_2	-122.445	37.941	0.23	18.69	282.01	18.92	-122.445	37.941	-0.13	18.69	281.86	18.56	9	7/14/2014	34:05.3
9798	RSPe_2	-122.445	37.941	0.23	18.71	281.99	18.94	-122.445	37.941	-0.08	18.71	281.83	18.63	9	7/14/2014	34:05.2
9799	RSPe_2	-122.445	37.941	0.23	18.47	282.01	18.70	-122.445	37.941	0.01	18.47	281.89	18.48	9	7/14/2014	34:05.1
9800	RSPe_2	-122.445	37.941	0.23	18.43	281.99	18.66	-122.445	37.941	-0.08	18.43	281.87	18.36	9	7/14/2014	34:05.0

9801	RSPe_2	-122.445	37.941	0.23	18.37	282.05	18.60	-122.445	37.941	-0.04	18.37	281.92	18.33	9	7/14/2014 34:04.9
9802	RSPe_2	-122.445	37.941	0.23	18.44	282.06	18.67	-122.445	37.941	-0.08	18.44	282.01	18.36	9	7/14/2014 34:04.8
9803	RSPe_2	-122.445	37.941	0.26	18.40	282.01	18.67	-122.445	37.941	-0.04	18.40	281.96	18.36	9	7/14/2014 34:04.7
9804	RSPe_2	-122.445	37.941	0.23	18.59	282.01	18.82	-122.445	37.941	-0.04	18.59	281.95	18.54	9	7/14/2014 34:04.6
9805	RSPe_2	-122.445	37.941	0.26	18.28	282.08	18.54	-122.445	37.941	0.01	18.28	281.99	18.29	9	7/14/2014 34:04.5
9806	RSPe_2	-122.445	37.941	0.23	18.24	282.12	18.46	-122.445	37.941	-0.04	18.24	282.03	18.19	9	7/14/2014 34:04.4
9807	RSPe_2	-122.445	37.941	0.26	18.29	282.14	18.55	-122.445	37.941	-0.04	18.29	282.03	18.24	9	7/14/2014 34:04.3
9808	RSPe_2	-122.445	37.941	0.23	18.35	282.15	18.58	-122.445	37.941	-0.08	18.35	282.06	18.27	9	7/14/2014 34:04.2
9809	RSPe_2	-122.445	37.941	0.26	18.27	282.24	18.53	-122.445	37.941	-0.04	18.27	282.04	18.23	9	7/14/2014 34:04.1
9810	RSPe_2	-122.445	37.941	0.23	18.23	282.35	18.45	-122.445	37.941	0.01	18.23	282.08	18.23	9	7/14/2014 34:04.0
9811	RSPe_2	-122.445	37.941	0.23	18.10	282.41	18.33	-122.445	37.941	0.01	18.10	282.12	18.11	9	7/14/2014 34:03.9
9812	RSPe_2	-122.445	37.941	0.23	18.15	282.46	18.38	-122.445	37.941	-0.04	18.15	282.19	18.11	9	7/14/2014 34:03.8
9813	RSPe_2	-122.445	37.941	0.26	18.12	282.46	18.38	-122.445	37.941	0.04	18.12	282.21	18.16	9	7/14/2014 34:03.7
9814	RSPe_2	-122.445	37.941	0.23	18.05	282.52	18.28	-122.445	37.941	0.04	18.05	282.30	18.10	9	7/14/2014 34:03.6
9815	RSPe_2	-122.445	37.941	0.26	18.10	282.55	18.36	-122.445	37.941	0.01	18.10	282.32	18.10	9	7/14/2014 34:03.5
9816	RSPe_2	-122.445	37.941	0.23	18.12	282.46	18.35	-122.445	37.941	-0.04	18.12	282.32	18.07	9	7/14/2014 34:03.4
9817	RSPe_2	-122.445	37.941	0.26	18.13	282.51	18.40	-122.445	37.941	0.01	18.13	282.42	18.14	9	7/14/2014 34:03.3
9818	RSPe_2	-122.445	37.941	0.23	18.09	282.57	18.32	-122.445	37.941	-0.04	18.09	282.46	18.04	9	7/14/2014 34:03.2
9819	RSPe_2	-122.445	37.941	0.26	18.10	282.57	18.36	-122.445	37.941	0.01	18.10	282.48	18.10	9	7/14/2014 34:03.1
9820	RSPe_2	-122.445	37.941	0.23	18.09	282.59	18.32	-122.445	37.941	-0.08	18.09	282.46	18.01	9	7/14/2014 34:03.0
9821	RSPe_2	-122.445	37.941	0.26	18.11	282.55	18.37	-122.445	37.941	-0.04	18.11	282.44	18.06	9	7/14/2014 34:02.9
9822	RSPe_2	-122.445	37.941	0.23	18.15	282.62	18.38	-122.445	37.941	-0.08	18.15	282.43	18.07	9	7/14/2014 34:02.8
9823	RSPe_2	-122.445	37.941	0.26	18.27	282.71	18.53	-122.445	37.941	0.01	18.27	282.46	18.28	9	7/14/2014 34:02.7
9824	RSPe_2	-122.445	37.941	0.23	18.23	282.77	18.45	-122.445	37.941	-0.04	18.23	282.54	18.18	9	7/14/2014 34:02.6
9825	RSPe_2	-122.445	37.941	0.26	18.25	282.91	18.51	-122.445	37.941	-0.04	18.25	282.64	18.20	9	7/14/2014 34:02.5
9826	RSPe_2	-122.445	37.941	0.23	18.32	282.95	18.55	-122.445	37.941	-0.04	18.32	282.75	18.27	9	7/14/2014 34:02.4
9827	RSPe_2	-122.445	37.941	0.26	18.43	282.97	18.69	-122.445	37.941	-0.04	18.43	282.72	18.38	9	7/14/2014 34:02.3
9828	RSPe_2	-122.445	37.941	0.23	18.34	282.97	18.57	-122.445	37.941	-0.04	18.34	282.81	18.30	9	7/14/2014 34:02.2
9829	RSPe_2	-122.445	37.941	0.23	18.35	282.95	18.58	-122.445	37.941	-0.04	18.35	282.84	18.30	9	7/14/2014 34:02.1
9830	RSPe_2	-122.445	37.941	0.23	18.28	282.89	18.51	-122.445	37.941	-0.04	18.28	282.81	18.23	9	7/14/2014 34:02.0
9831	RSPe_2	-122.445	37.941	0.26	18.36	282.84	18.63	-122.445	37.941	-0.04	18.36	282.86	18.32	9	7/14/2014 34:01.9
9832	RSPe_2	-122.445	37.941	0.23	18.33	282.93	18.56	-122.445	37.941	-0.04	18.33	282.92	18.28	9	7/14/2014 34:01.8
9833	RSPe_2	-122.445	37.941	0.23	18.48	282.97	18.71	-122.445	37.941	-0.04	18.48	283.04	18.44	9	7/14/2014 34:01.7

9834	RSPe_2	-122.445	37.941	0.23	18.42	283.00	18.65	-122.445	37.941	-0.13	18.42	283.06	18.29	9	7/14/2014 34:01.6
9835	RSPe_2	-122.445	37.941	0.23	18.39	283.09	18.62	-122.445	37.941	-0.08	18.39	283.10	18.31	9	7/14/2014 34:01.5
9836	RSPe_2	-122.445	37.941	0.23	18.43	283.20	18.66	-122.445	37.941	-0.08	18.43	283.15	18.35	9	7/14/2014 34:01.4
9837	RSPe_2	-122.445	37.941	0.26	18.57	283.33	18.83	-122.445	37.941	-0.08	18.57	283.19	18.49	9	7/14/2014 34:01.3
9838	RSPe_2	-122.445	37.941	0.23	18.57	283.35	18.80	-122.445	37.941	-0.04	18.57	283.17	18.53	9	7/14/2014 34:01.2
9839	RSPe_2	-122.445	37.941	0.26	18.53	283.37	18.80	-122.445	37.941	-0.04	18.53	283.10	18.49	9	7/14/2014 34:01.1
9840	RSPe_2	-122.445	37.942	0.23	18.61	283.29	18.84	-122.445	37.942	-0.08	18.61	283.10	18.53	9	7/14/2014 34:01.0
9841	RSPe_2	-122.445	37.942	0.26	18.59	283.31	18.85	-122.445	37.942	-0.08	18.59	283.14	18.51	9	7/14/2014 34:00.9
9842	RSPe_2	-122.445	37.942	0.23	18.61	283.27	18.84	-122.445	37.942	-0.08	18.61	283.11	18.53	9	7/14/2014 34:00.8
9843	RSPe_2	-122.445	37.942	0.26	18.66	283.45	18.92	-122.445	37.942	-0.13	18.66	283.26	18.53	9	7/14/2014 34:00.7
9844	RSPe_2	-122.445	37.942	0.23	18.70	283.60	18.93	-122.445	37.942	-0.08	18.70	283.44	18.62	9	7/14/2014 34:00.6
9845	RSPe_2	-122.445	37.942	0.26	18.70	283.56	18.97	-122.445	37.942	-0.13	18.70	283.52	18.57	9	7/14/2014 34:00.5
9846	RSPe_2	-122.445	37.942	0.26	18.75	283.66	19.01	-122.445	37.942	-0.19	18.75	283.62	18.56	9	7/14/2014 34:00.4
9847	RSPe_2	-122.445	37.942	0.32	18.73	283.67	19.05	-122.445	37.942	-0.08	18.73	283.62	18.66	9	7/14/2014 34:00.3
9848	RSPe_2	-122.445	37.942	0.26	18.76	283.73	19.02	-122.445	37.942	-0.16	18.76	283.75	18.60	9	7/14/2014 34:00.2
9849	RSPe_2	-122.445	37.942	0.26	18.76	283.69	19.02	-122.445	37.942	0.01	18.76	283.69	18.77	9	7/14/2014 34:00.1
9850	RSPe_2	-122.445	37.942	0.26	18.81	283.73	19.08	-122.445	37.942	-0.13	18.81	283.73	18.68	9	7/14/2014 34:00.0
9851	RSPe_2	-122.445	37.942	0.32	18.83	283.69	19.15	-122.445	37.942	-0.08	18.83	283.69	18.76	9	7/14/2014 33:59.9
9852	RSPe_2	-122.445	37.942	0.26	18.83	283.67	19.10	-122.445	37.942	-0.16	18.83	283.71	18.67	9	7/14/2014 33:59.8
9853	RSPe_2	-122.445	37.942	0.32	18.87	283.67	19.18	-122.445	37.942	-0.08	18.87	283.73	18.79	9	7/14/2014 33:59.7
9854	RSPe_2	-122.445	37.942	0.26	18.91	283.69	19.17	-122.445	37.942	-0.08	18.91	283.76	18.83	9	7/14/2014 33:59.6
9855	RSPe_2	-122.445	37.942	0.32	18.97	283.74	19.29	-122.445	37.942	-0.08	18.97	283.71	18.90	9	7/14/2014 33:59.5
9856	RSPe_2	-122.445	37.942	0.32	19.00	283.78	19.31	-122.445	37.942	-0.08	19.00	283.75	18.92	9	7/14/2014 33:59.4
9857	RSPe_2	-122.445	37.942	0.32	19.04	283.85	19.36	-122.445	37.942	-0.08	19.04	283.78	18.97	9	7/14/2014 33:59.3
9858	RSPe_2	-122.445	37.942	0.32	19.08	283.93	19.39	-122.445	37.942	-0.08	19.08	283.82	19.00	9	7/14/2014 33:59.2
9859	RSPe_2	-122.445	37.942	0.35	19.03	284.07	19.38	-122.445	37.942	-0.08	19.03	283.84	18.95	9	7/14/2014 33:59.1
9860	RSPe_2	-122.445	37.942	0.32	18.94	284.16	19.25	-122.445	37.942	-0.13	18.94	283.94	18.81	9	7/14/2014 33:59.0
9861	RSPe_2	-122.445	37.942	0.32	18.96	284.29	19.27	-122.445	37.942	0.04	18.96	284.02	19.00	9	7/14/2014 33:58.9
9862	RSPe_2	-122.445	37.942	0.32	18.92	284.31	19.24	-122.445	37.942	-0.04	18.92	284.04	18.88	9	7/14/2014 33:58.8
9863	RSPe_2	-122.445	37.942	0.32	18.89	284.29	19.20	-122.445	37.942	-0.04	18.89	284.05	18.85	9	7/14/2014 33:58.7
9864	RSPe_2	-122.445	37.942	0.35	18.80	284.27	19.15	-122.445	37.942	0.01	18.80	284.07	18.81	9	7/14/2014 33:58.6
9865	RSPe_2	-122.445	37.942	0.35	18.76	284.25	19.11	-122.445	37.942	-0.04	18.76	284.11	18.72	9	7/14/2014 33:58.5
9866	RSPe_2	-122.445	37.942	0.35	18.72	284.25	19.07	-122.445	37.942	-0.04	18.72	284.07	18.68	9	7/14/2014 33:58.4

9867	RSPe_2	-122.445	37.942	0.35	18.62	284.22	18.97	-122.445	37.942	0.01	18.62	284.13	18.63	9	7/14/2014	33:58.3
9868	RSPe_2	-122.445	37.942	0.35	18.48	284.19	18.83	-122.445	37.942	0.01	18.48	284.11	18.49	9	7/14/2014	33:58.2
9869	RSPe_2	-122.445	37.942	0.40	18.47	284.14	18.87	-122.445	37.942	0.01	18.47	284.12	18.48	9	7/14/2014	33:58.1
9870	RSPe_2	-122.445	37.942	0.35	18.47	284.14	18.82	-122.445	37.942	0.01	18.47	284.16	18.48	9	7/14/2014	33:58.0
9871	RSPe_2	-122.445	37.942	0.40	18.41	284.19	18.81	-122.445	37.942	0.04	18.41	284.12	18.45	9	7/14/2014	33:57.9
9872	RSPe_2	-122.445	37.942	0.40	18.34	284.20	18.74	-122.445	37.942	-0.04	18.34	284.09	18.30	9	7/14/2014	33:57.8
9873	RSPe_2	-122.445	37.942	0.40	18.30	284.28	18.71	-122.445	37.942	0.09	18.30	284.12	18.40	9	7/14/2014	33:57.7
9874	RSPe_2	-122.445	37.942	0.40	18.26	284.41	18.66	-122.445	37.942	0.04	18.26	284.14	18.30	9	7/14/2014	33:57.6
9875	RSPe_2	-122.445	37.942	0.40	18.24	284.26	18.64	-122.445	37.942	0.04	18.24	284.12	18.28	9	7/14/2014	33:57.5
9876	RSPe_2	-122.445	37.942	0.40	18.20	284.32	18.60	-122.445	37.942	0.04	18.20	284.20	18.24	9	7/14/2014	33:57.4
9877	RSPe_2	-122.445	37.942	0.40	18.14	284.39	18.54	-122.445	37.942	0.13	18.14	284.19	18.27	9	7/14/2014	33:57.3
9878	RSPe_2	-122.445	37.942	0.40	18.13	284.41	18.53	-122.445	37.942	0.09	18.13	284.20	18.22	9	7/14/2014	33:57.2
9879	RSPe_2	-122.445	37.942	0.40	18.16	284.49	18.56	-122.445	37.942	0.09	18.16	284.27	18.25	9	7/14/2014	33:57.1
9880	RSPe_2	-122.445	37.942	0.40	18.18	284.50	18.58	-122.445	37.942	0.09	18.18	284.30	18.27	9	7/14/2014	33:57.0
9881	RSPe_2	-122.445	37.942	0.40	18.10	284.57	18.50	-122.445	37.942	0.13	18.10	284.30	18.22	9	7/14/2014	33:56.9
9882	RSPe_2	-122.445	37.942	0.40	18.13	284.59	18.53	-122.445	37.942	0.13	18.13	284.40	18.26	9	7/14/2014	33:56.8
9883	RSPe_2	-122.445	37.942	0.44	18.10	284.61	18.53	-122.445	37.942	0.04	18.10	284.44	18.14	9	7/14/2014	33:56.7
9884	RSPe_2	-122.445	37.942	0.40	18.02	284.61	18.42	-122.445	37.942	0.01	18.02	284.48	18.03	9	7/14/2014	33:56.6
9885	RSPe_2	-122.445	37.942	0.44	18.09	284.68	18.52	-122.445	37.942	0.18	18.09	284.54	18.26	9	7/14/2014	33:56.5
9886	RSPe_2	-122.445	37.942	0.44	18.08	284.70	18.51	-122.445	37.942	0.01	18.08	284.59	18.09	9	7/14/2014	33:56.4
9887	RSPe_2	-122.445	37.942	0.44	18.13	284.73	18.57	-122.445	37.942	0.18	18.13	284.61	18.31	9	7/14/2014	33:56.3
9888	RSPe_2	-122.445	37.942	0.40	18.08	284.77	18.48	-122.445	37.942	0.18	18.08	284.61	18.26	9	7/14/2014	33:56.2
9889	RSPe_2	-122.445	37.942	0.44	18.12	284.77	18.55	-122.445	37.942	0.18	18.12	284.59	18.30	9	7/14/2014	33:56.1
9890	RSPe_2	-122.445	37.942	0.44	18.10	284.85	18.53	-122.445	37.942	0.18	18.10	284.73	18.27	9	7/14/2014	33:56.0
9891	RSPe_2	-122.445	37.942	0.44	18.24	284.95	18.68	-122.445	37.942	0.18	18.24	284.79	18.42	9	7/14/2014	33:55.9
9892	RSPe_2	-122.445	37.942	0.44	18.16	284.97	18.59	-122.445	37.942	0.13	18.16	284.86	18.28	9	7/14/2014	33:55.8
9893	RSPe_2	-122.445	37.942	0.49	18.25	284.95	18.73	-122.445	37.942	0.18	18.25	284.83	18.42	9	7/14/2014	33:55.7
9894	RSPe_2	-122.445	37.942	0.49	18.21	284.90	18.70	-122.445	37.942	0.18	18.21	284.86	18.39	9	7/14/2014	33:55.6
9895	RSPe_2	-122.445	37.942	0.49	18.21	284.93	18.70	-122.445	37.942	0.13	18.21	284.83	18.33	9	7/14/2014	33:55.5
9896	RSPe_2	-122.445	37.942	0.49	18.21	284.84	18.70	-122.445	37.942	0.21	18.21	284.83	18.42	9	7/14/2014	33:55.4
9897	RSPe_2	-122.445	37.942	0.52	18.20	284.99	18.72	-122.445	37.942	0.18	18.20	284.90	18.38	9	7/14/2014	33:55.3
9898	RSPe_2	-122.445	37.942	0.49	18.43	285.04	18.92	-122.445	37.942	0.25	18.43	284.99	18.68	9	7/14/2014	33:55.2
9899	RSPe_2	-122.445	37.942	0.52	18.33	284.99	18.85	-122.445	37.942	0.21	18.33	284.99	18.54	9	7/14/2014	33:55.1

9900	RSPe_2	-122.445	37.942	0.49	18.32	285.00	18.80	-122.445	37.942	0.21	18.32	284.97	18.53	9	7/14/2014	33:55.0
9901	RSPe_2	-122.445	37.942	0.52	18.23	284.95	18.75	-122.445	37.942	0.21	18.23	284.94	18.44	9	7/14/2014	33:54.9
9902	RSPe_2	-122.445	37.942	0.52	18.24	284.91	18.76	-122.445	37.942	0.25	18.24	284.92	18.49	9	7/14/2014	33:54.8
9903	RSPe_2	-122.445	37.942	0.52	18.50	284.97	19.02	-122.445	37.942	0.25	18.50	284.91	18.74	9	7/14/2014	33:54.7
9904	RSPe_2	-122.445	37.942	0.49	18.35	285.04	18.83	-122.445	37.942	0.25	18.35	284.94	18.59	9	7/14/2014	33:54.6
9905	RSPe_2	-122.445	37.942	0.52	18.36	285.04	18.89	-122.445	37.942	0.25	18.36	284.91	18.61	9	7/14/2014	33:54.5
9906	RSPe_2	-122.445	37.942	0.52	18.37	285.11	18.89	-122.445	37.942	0.21	18.37	284.96	18.58	9	7/14/2014	33:54.4
9907	RSPe_2	-122.445	37.942	0.52	18.40	285.13	18.92	-122.445	37.942	0.21	18.40	284.99	18.61	9	7/14/2014	33:54.3
9908	RSPe_2	-122.445	37.942	0.49	18.40	285.22	18.89	-122.445	37.942	0.21	18.40	285.06	18.61	9	7/14/2014	33:54.2
9909	RSPe_2	-122.445	37.942	0.52	18.38	285.24	18.90	-122.445	37.942	0.21	18.38	285.06	18.59	9	7/14/2014	33:54.1
9910	RSPe_2	-122.445	37.942	0.49	18.39	285.31	18.87	-122.445	37.942	0.18	18.39	285.15	18.56	9	7/14/2014	33:54.0
9911	RSPe_2	-122.445	37.942	0.52	18.39	285.37	18.91	-122.445	37.942	0.21	18.39	285.15	18.60	9	7/14/2014	33:53.9
9912	RSPe_2	-122.445	37.942	0.52	18.47	285.40	18.99	-122.445	37.942	0.18	18.47	285.19	18.64	9	7/14/2014	33:53.8
9913	RSPe_2	-122.445	37.942	0.52	18.48	285.42	19.00	-122.445	37.942	0.21	18.48	285.22	18.69	9	7/14/2014	33:53.7
9914	RSPe_2	-122.445	37.942	0.49	18.60	285.40	19.09	-122.445	37.942	0.25	18.60	285.24	18.85	9	7/14/2014	33:53.6
9915	RSPe_2	-122.445	37.942	0.52	18.52	285.36	19.04	-122.445	37.942	0.25	18.52	285.31	18.76	9	7/14/2014	33:53.5
9916	RSPe_2	-122.445	37.942	0.52	18.54	285.40	19.06	-122.445	37.942	0.25	18.54	285.37	18.79	9	7/14/2014	33:53.4
9917	RSPe_2	-122.445	37.942	0.52	18.55	285.33	19.07	-122.445	37.942	0.21	18.55	285.35	18.76	9	7/14/2014	33:53.3
9918	RSPe_2	-122.445	37.942	0.52	18.50	285.38	19.02	-122.445	37.942	0.25	18.50	285.39	18.74	9	7/14/2014	33:53.2
9919	RSPe_2	-122.445	37.942	0.52	18.70	285.54	19.22	-122.445	37.942	0.25	18.70	285.49	18.94	9	7/14/2014	33:53.1
9920	RSPe_2	-122.445	37.942	0.52	18.54	285.58	19.06	-122.445	37.942	0.25	18.54	285.55	18.79	9	7/14/2014	33:53.0
9921	RSPe_2	-122.445	37.942	0.52	18.61	285.69	19.13	-122.445	37.942	0.29	18.61	285.51	18.91	9	7/14/2014	33:52.9
9922	RSPe_2	-122.445	37.942	0.52	18.66	285.73	19.18	-122.445	37.942	0.25	18.66	285.59	18.91	9	7/14/2014	33:52.8
9923	RSPe_2	-122.445	37.942	0.56	18.63	285.80	19.18	-122.445	37.942	0.33	18.63	285.62	18.95	9	7/14/2014	33:52.7
9924	RSPe_2	-122.445	37.942	0.52	18.67	285.85	19.19	-122.445	37.942	0.25	18.67	285.64	18.92	9	7/14/2014	33:52.6
9925	RSPe_2	-122.445	37.942	0.56	18.60	285.87	19.16	-122.445	37.942	0.29	18.60	285.71	18.90	9	7/14/2014	33:52.5
9926	RSPe_2	-122.445	37.942	0.52	18.58	285.85	19.10	-122.445	37.942	0.33	18.58	285.72	18.91	9	7/14/2014	33:52.4
9927	RSPe_2	-122.445	37.942	0.56	18.55	285.85	19.11	-122.445	37.942	0.25	18.55	285.76	18.80	9	7/14/2014	33:52.3
9928	RSPe_2	-122.445	37.942	0.52	18.48	285.91	19.00	-122.445	37.942	0.25	18.48	285.80	18.73	9	7/14/2014	33:52.2
9929	RSPe_2	-122.445	37.942	0.56	18.36	285.80	18.92	-122.445	37.942	0.29	18.36	285.78	18.66	9	7/14/2014	33:52.1
9930	RSPe_2	-122.445	37.942	0.56	18.36	285.89	18.92	-122.445	37.942	0.29	18.36	285.83	18.66	9	7/14/2014	33:52.0
9931	RSPe_2	-122.445	37.942	0.56	18.31	285.94	18.86	-122.445	37.942	0.29	18.31	285.87	18.60	9	7/14/2014	33:51.9
9932	RSPe_2	-122.445	37.942	0.56	18.47	285.96	19.02	-122.445	37.942	0.29	18.47	285.93	18.76	9	7/14/2014	33:51.8

9933	RSPe_2	-122.445	37.942	0.56	18.26	286.03	18.81	-122.445	37.942	0.29	18.26	285.98	18.55	9	7/14/2014	33:51.7
9934	RSPe_2	-122.445	37.942	0.56	18.28	286.09	18.83	-122.445	37.942	0.29	18.28	286.04	18.57	9	7/14/2014	33:51.6
9935	RSPe_2	-122.445	37.942	0.56	18.24	286.16	18.80	-122.445	37.942	0.29	18.24	286.11	18.54	9	7/14/2014	33:51.5
9936	RSPe_2	-122.445	37.942	0.52	18.20	286.16	18.72	-122.445	37.942	0.25	18.20	286.13	18.45	9	7/14/2014	33:51.4
9937	RSPe_2	-122.445	37.942	0.56	18.22	286.14	18.77	-122.445	37.942	0.33	18.22	286.09	18.55	9	7/14/2014	33:51.3
9938	RSPe_2	-122.445	37.942	0.56	18.15	286.19	18.70	-122.445	37.942	0.29	18.15	286.10	18.44	9	7/14/2014	33:51.2
9939	RSPe_2	-122.445	37.942	0.56	18.08	286.10	18.63	-122.445	37.942	0.29	18.08	286.07	18.37	9	7/14/2014	33:51.1
9940	RSPe_2	-122.445	37.942	0.52	18.06	286.07	18.58	-122.445	37.942	0.29	18.06	286.11	18.36	9	7/14/2014	33:51.0
9941	RSPe_2	-122.445	37.942	0.56	18.05	286.10	18.60	-122.445	37.942	0.25	18.05	286.14	18.29	9	7/14/2014	33:50.9
9942	RSPe_2	-122.445	37.942	0.56	18.03	286.14	18.58	-122.445	37.942	0.25	18.03	286.23	18.27	9	7/14/2014	33:50.8
9943	RSPe_2	-122.445	37.942	0.56	18.03	286.14	18.59	-122.445	37.942	0.29	18.03	286.25	18.33	9	7/14/2014	33:50.7
9944	RSPe_2	-122.445	37.942	0.56	18.03	286.10	18.58	-122.445	37.942	0.30	18.03	286.21	18.32	9	7/14/2014	33:50.6
9945	RSPe_2	-122.445	37.942	0.61	18.04	286.23	18.65	-122.445	37.942	0.30	18.04	286.23	18.33	9	7/14/2014	33:50.5
9946	RSPe_2	-122.445	37.942	0.56	18.05	286.34	18.61	-122.445	37.942	0.30	18.05	286.35	18.35	9	7/14/2014	33:50.4
9947	RSPe_2	-122.445	37.942	0.56	18.09	286.41	18.64	-122.445	37.942	0.25	18.09	286.38	18.33	9	7/14/2014	33:50.3
9948	RSPe_2	-122.445	37.942	0.56	18.04	286.43	18.59	-122.445	37.942	0.30	18.04	286.36	18.33	9	7/14/2014	33:50.2
9949	RSPe_2	-122.445	37.942	0.61	18.00	286.41	18.61	-122.445	37.942	0.30	18.00	286.36	18.30	9	7/14/2014	33:50.1
9950	RSPe_2	-122.445	37.942	0.56	18.10	286.48	18.65	-122.445	37.942	0.30	18.10	286.40	18.39	9	7/14/2014	33:50.0
9951	RSPe_2	-122.445	37.942	0.56	18.04	286.57	18.59	-122.445	37.942	0.25	18.04	286.48	18.28	9	7/14/2014	33:49.9
9952	RSPe_2	-122.445	37.942	0.56	18.01	286.55	18.57	-122.445	37.942	0.25	18.01	286.49	18.26	9	7/14/2014	33:49.8
9953	RSPe_2	-122.445	37.942	0.61	18.09	286.57	18.69	-122.445	37.942	0.30	18.09	286.54	18.38	9	7/14/2014	33:49.7
9954	RSPe_2	-122.445	37.942	0.61	18.03	286.57	18.64	-122.445	37.942	0.25	18.03	286.58	18.28	9	7/14/2014	33:49.6
9955	RSPe_2	-122.445	37.942	0.61	17.99	286.54	18.59	-122.445	37.942	0.30	17.99	286.61	18.28	9	7/14/2014	33:49.5
9956	RSPe_2	-122.445	37.942	0.61	18.07	286.51	18.68	-122.445	37.942	0.30	18.07	286.58	18.37	9	7/14/2014	33:49.4
9957	RSPe_2	-122.445	37.942	0.61	18.03	286.50	18.64	-122.445	37.942	0.33	18.03	286.59	18.36	9	7/14/2014	33:49.3
9958	RSPe_2	-122.445	37.942	0.61	18.06	286.48	18.67	-122.445	37.942	0.30	18.06	286.52	18.36	9	7/14/2014	33:49.2
9959	RSPe_2	-122.445	37.942	0.61	17.97	286.48	18.58	-122.445	37.942	0.33	17.97	286.48	18.30	9	7/14/2014	33:49.1
9960	RSPe_2	-122.445	37.942	0.61	18.03	286.62	18.64	-122.445	37.942	0.33	18.03	286.61	18.36	9	7/14/2014	33:49.0
9961	RSPe_2	-122.445	37.942	0.61	18.00	286.72	18.61	-122.445	37.942	0.30	18.00	286.59	18.30	9	7/14/2014	33:48.9
9962	RSPe_2	-122.445	37.942	0.61	17.96	286.71	18.56	-122.445	37.942	0.25	17.96	286.63	18.20	9	7/14/2014	33:48.8
9963	RSPe_2	-122.445	37.942	0.64	17.95	286.73	18.59	-122.445	37.942	0.30	17.95	286.61	18.24	9	7/14/2014	33:48.7
9964	RSPe_2	-122.445	37.942	0.61	17.95	286.73	18.56	-122.445	37.942	0.25	17.95	286.61	18.19	9	7/14/2014	33:48.6
9965	RSPe_2	-122.445	37.942	0.64	17.92	286.75	18.57	-122.445	37.942	0.30	17.92	286.63	18.22	9	7/14/2014	33:48.5

9966	RSPe_2	-122.445	37.942	0.61	17.92	286.75	18.52	-122.445	37.942	0.30	17.92	286.59	18.21	9	7/14/2014 33:48.4
9967	RSPe_2	-122.445	37.942	0.61	17.92	286.77	18.53	-122.445	37.942	0.30	17.92	286.64	18.22	9	7/14/2014 33:48.3
9968	RSPe_2	-122.445	37.942	0.61	17.90	286.73	18.51	-122.445	37.942	0.30	17.90	286.61	18.20	9	7/14/2014 33:48.2
9969	RSPe_2	-122.445	37.942	0.64	17.98	286.73	18.62	-122.445	37.942	0.30	17.98	286.58	18.28	9	7/14/2014 33:48.1
9970	RSPe_2	-122.445	37.942	0.64	17.93	286.80	18.57	-122.445	37.942	0.33	17.93	286.69	18.26	9	7/14/2014 33:48.0
9971	RSPe_2	-122.445	37.942	0.64	17.92	286.82	18.56	-122.445	37.942	0.38	17.92	286.70	18.30	9	7/14/2014 33:47.9
9972	RSPe_2	-122.445	37.942	0.61	17.92	286.84	18.53	-122.445	37.942	0.38	17.92	286.73	18.31	9	7/14/2014 33:47.8
9973	RSPe_2	-122.445	37.942	0.64	17.97	286.86	18.61	-122.445	37.942	0.38	17.97	286.84	18.35	9	7/14/2014 33:47.7
9974	RSPe_2	-122.445	37.942	0.64	17.95	286.93	18.59	-122.445	37.942	0.33	17.95	286.83	18.28	9	7/14/2014 33:47.6
9975	RSPe_2	-122.445	37.942	0.64	17.95	287.00	18.59	-122.445	37.942	0.38	17.95	286.89	18.33	9	7/14/2014 33:47.5
9976	RSPe_2	-122.445	37.942	0.64	17.94	287.02	18.58	-122.445	37.942	0.30	17.94	286.89	18.24	9	7/14/2014 33:47.4
9977	RSPe_2	-122.445	37.942	0.69	17.94	287.09	18.63	-122.445	37.942	0.33	17.94	286.97	18.27	9	7/14/2014 33:47.3
9978	RSPe_2	-122.445	37.942	0.64	17.94	287.20	18.58	-122.445	37.942	0.30	17.94	287.01	18.24	9	7/14/2014 33:47.2
9979	RSPe_2	-122.445	37.942	0.69	17.96	287.17	18.66	-122.445	37.942	0.30	17.96	287.06	18.26	9	7/14/2014 33:47.1
9980	RSPe_2	-122.445	37.942	0.69	17.92	287.24	18.61	-122.445	37.942	0.33	17.92	287.06	18.25	9	7/14/2014 33:47.0
9981	RSPe_2	-122.445	37.942	0.69	17.92	287.27	18.61	-122.445	37.942	0.33	17.92	287.13	18.25	9	7/14/2014 33:46.9
9982	RSPe_2	-122.445	37.942	0.64	17.89	287.26	18.53	-122.445	37.942	0.33	17.89	287.19	18.22	9	7/14/2014 33:46.8
9983	RSPe_2	-122.445	37.942	0.73	17.88	287.25	18.60	-122.445	37.942	0.41	17.88	287.15	18.29	9	7/14/2014 33:46.7
9984	RSPe_2	-122.445	37.942	0.69	17.90	287.27	18.60	-122.445	37.942	0.33	17.90	287.26	18.23	9	7/14/2014 33:46.6
9985	RSPe_2	-122.445	37.942	0.73	17.90	287.27	18.63	-122.445	37.942	0.47	17.90	287.26	18.37	9	7/14/2014 33:46.5
9986	RSPe_2	-122.445	37.942	0.73	17.89	287.26	18.62	-122.445	37.942	0.38	17.89	287.27	18.27	9	7/14/2014 33:46.4
9987	RSPe_2	-122.445	37.942	0.78	17.97	287.25	18.75	-122.445	37.942	0.41	17.97	287.29	18.39	9	7/14/2014 33:46.3
9988	RSPe_2	-122.445	37.942	0.73	17.90	287.27	18.63	-122.445	37.942	0.47	17.90	287.31	18.37	9	7/14/2014 33:46.2
9989	RSPe_2	-122.445	37.942	0.73	17.90	287.31	18.63	-122.445	37.942	0.41	17.90	287.32	18.32	9	7/14/2014 33:46.1
9990	RSPe_2	-122.445	37.942	0.73	17.96	287.29	18.69	-122.445	37.942	0.47	17.96	287.31	18.43	9	7/14/2014 33:46.0
9991	RSPe_2	-122.445	37.942	0.78	17.93	287.29	18.71	-122.445	37.942	0.41	17.93	287.27	18.35	9	7/14/2014 33:45.9
9992	RSPe_2	-122.445	37.942	0.73	17.92	287.27	18.65	-122.445	37.942	0.41	17.92	287.18	18.34	9	7/14/2014 33:45.8
9993	RSPe_2	-122.445	37.942	0.78	17.94	287.27	18.72	-122.445	37.942	0.47	17.94	287.27	18.41	9	7/14/2014 33:45.7
9994	RSPe_2	-122.445	37.942	0.78	17.98	287.41	18.76	-122.445	37.942	0.47	17.98	287.36	18.45	9	7/14/2014 33:45.6
9995	RSPe_2	-122.445	37.942	0.78	17.99	287.45	18.77	-122.445	37.942	0.47	17.99	287.43	18.46	9	7/14/2014 33:45.5
9996	RSPe_2	-122.445	37.942	0.81	18.01	287.47	18.82	-122.445	37.942	0.47	18.01	287.40	18.48	9	7/14/2014 33:45.4
9997	RSPe_2	-122.445	37.942	0.81	18.04	287.47	18.85	-122.445	37.942	0.47	18.04	287.46	18.50	9	7/14/2014 33:45.3
9998	RSPe_2	-122.445	37.942	0.81	18.12	287.43	18.93	-122.445	37.942	0.50	18.12	287.45	18.62	9	7/14/2014 33:45.2

9999	RSPe_2	-122.445	37.942	0.81	18.02	287.45	18.83	-122.445	37.942	0.50	18.02	287.45	18.52	9	7/14/2014	33:45.1
10000	RSPe_2	-122.445	37.942	0.81	18.08	287.41	18.89	-122.445	37.942	0.53	18.08	287.45	18.61	9	7/14/2014	33:45.0
10001	RSPe_2	-122.445	37.942	0.86	18.02	287.50	18.88	-122.445	37.942	0.53	18.02	287.45	18.55	9	7/14/2014	33:44.9
10002	RSPe_2	-122.445	37.942	0.86	18.12	287.48	18.99	-122.445	37.942	0.50	18.12	287.42	18.62	9	7/14/2014	33:44.8
10003	RSPe_2	-122.445	37.942	0.86	18.08	287.52	18.94	-122.445	37.942	0.58	18.08	287.47	18.66	9	7/14/2014	33:44.7
10004	RSPe_2	-122.445	37.942	0.86	18.06	287.54	18.93	-122.445	37.942	0.53	18.06	287.45	18.60	9	7/14/2014	33:44.6
10005	RSPe_2	-122.445	37.942	0.90	18.01	287.54	18.91	-122.445	37.942	0.58	18.01	287.43	18.59	9	7/14/2014	33:44.5
10006	RSPe_2	-122.445	37.942	0.90	17.89	287.50	18.78	-122.445	37.942	0.58	17.89	287.32	18.47	9	7/14/2014	33:44.4
10007	RSPe_2	-122.445	37.942	0.90	17.81	287.50	18.71	-122.445	37.942	0.58	17.81	287.39	18.39	9	7/14/2014	33:44.3
10008	RSPe_2	-122.445	37.942	0.90	17.83	287.61	18.73	-122.445	37.942	0.62	17.83	287.43	18.45	9	7/14/2014	33:44.2
10009	RSPe_2	-122.445	37.942	0.93	17.78	287.61	18.71	-122.445	37.942	0.62	17.78	287.48	18.39	9	7/14/2014	33:44.1
10010	RSPe_2	-122.445	37.942	0.90	17.80	287.59	18.70	-122.445	37.942	0.58	17.80	287.49	18.39	9	7/14/2014	33:44.0
10011	RSPe_2	-122.445	37.942	0.93	17.75	287.61	18.68	-122.445	37.942	0.58	17.75	287.48	18.33	9	7/14/2014	33:43.9
10012	RSPe_2	-122.445	37.942	0.93	17.75	287.64	18.69	-122.445	37.942	0.62	17.75	287.51	18.37	9	7/14/2014	33:43.8
10013	RSPe_2	-122.445	37.942	0.93	17.68	287.66	18.61	-122.445	37.942	0.67	17.68	287.55	18.35	9	7/14/2014	33:43.7
10014	RSPe_2	-122.445	37.942	0.93	17.67	287.68	18.60	-122.445	37.942	0.67	17.67	287.59	18.34	9	7/14/2014	33:43.6
10015	RSPe_2	-122.445	37.942	0.93	17.75	287.70	18.69	-122.445	37.942	0.70	17.75	287.59	18.46	9	7/14/2014	33:43.5
10016	RSPe_2	-122.445	37.942	0.98	17.70	287.75	18.69	-122.445	37.942	0.75	17.70	287.62	18.45	9	7/14/2014	33:43.4
10017	RSPe_2	-122.445	37.942	0.98	17.66	287.79	18.65	-122.445	37.942	0.70	17.66	287.59	18.37	9	7/14/2014	33:43.3
10018	RSPe_2	-122.445	37.942	0.98	17.65	287.75	18.64	-122.445	37.942	0.79	17.65	287.53	18.44	9	7/14/2014	33:43.2
10019	RSPe_2	-122.445	37.942	1.02	17.59	287.67	18.61	-122.445	37.942	0.84	17.59	287.47	18.43	9	7/14/2014	33:43.1
10020	RSPe_2	-122.445	37.942	1.02	17.58	287.64	18.59	-122.445	37.942	0.84	17.58	287.40	18.41	9	7/14/2014	33:43.0
10021	RSPe_2	-122.445	37.942	1.07	17.55	287.75	18.61	-122.445	37.942	0.79	17.55	287.53	18.33	9	7/14/2014	33:42.9
10022	RSPe_2	-122.445	37.942	1.02	17.56	287.84	18.57	-122.445	37.942	0.84	17.56	287.68	18.39	9	7/14/2014	33:42.8
10023	RSPe_2	-122.445	37.942	1.02	17.58	287.84	18.60	-122.445	37.942	0.87	17.58	287.70	18.46	9	7/14/2014	33:42.7
10024	RSPe_2	-122.445	37.942	1.02	17.53	287.89	18.55	-122.445	37.942	0.87	17.53	287.73	18.40	9	7/14/2014	33:42.6
10025	RSPe_2	-122.445	37.942	1.07	17.58	287.89	18.65	-122.445	37.942	0.84	17.58	287.75	18.41	9	7/14/2014	33:42.5
10026	RSPe_2	-122.445	37.942	1.02	17.53	287.91	18.55	-122.445	37.942	0.84	17.53	287.77	18.37	9	7/14/2014	33:42.4
10027	RSPe_2	-122.445	37.942	1.07	17.49	287.86	18.56	-122.445	37.942	0.87	17.49	287.71	18.36	9	7/14/2014	33:42.3
10028	RSPe_2	-122.445	37.942	1.07	17.49	287.85	18.56	-122.445	37.942	0.87	17.49	287.71	18.36	9	7/14/2014	33:42.2
10029	RSPe_2	-122.445	37.942	1.10	17.46	287.85	18.56	-122.445	37.942	0.91	17.46	287.71	18.37	9	7/14/2014	33:42.1
10030	RSPe_2	-122.445	37.942	1.07	17.47	287.85	18.54	-122.445	37.942	0.87	17.47	287.71	18.34	9	7/14/2014	33:42.0
10031	RSPe_2	-122.445	37.942	1.10	17.36	287.80	18.47	-122.445	37.942	0.96	17.36	287.69	18.32	9	7/14/2014	33:41.9

10032	RSPe_2	-122.445	37.942	1.10	17.36	287.76	18.47	-122.445	37.942	0.91	17.36	287.65	18.27	9	7/14/2014	33:41.8
10033	RSPe_2	-122.445	37.942	1.10	17.27	287.78	18.37	-122.445	37.942	0.99	17.27	287.73	18.26	9	7/14/2014	33:41.7
10034	RSPe_2	-122.445	37.942	1.10	17.27	287.85	18.37	-122.445	37.942	0.99	17.27	287.75	18.26	9	7/14/2014	33:41.6
10035	RSPe_2	-122.445	37.942	1.15	17.24	287.78	18.39	-122.445	37.942	0.99	17.24	287.79	18.23	9	7/14/2014	33:41.5
10036	RSPe_2	-122.445	37.942	1.15	17.21	287.81	18.37	-122.445	37.942	1.04	17.21	287.80	18.26	9	7/14/2014	33:41.4
10037	RSPe_2	-122.445	37.942	1.19	17.20	287.81	18.39	-122.445	37.942	1.04	17.20	287.80	18.24	9	7/14/2014	33:41.3
10038	RSPe_2	-122.445	37.942	1.15	17.21	287.79	18.36	-122.445	37.942	1.04	17.21	287.80	18.25	9	7/14/2014	33:41.2
10039	RSPe_2	-122.445	37.942	1.15	17.18	287.81	18.33	-122.445	37.942	1.04	17.18	287.83	18.22	9	7/14/2014	33:41.1
10040	RSPe_2	-122.445	37.942	1.19	17.18	287.79	18.37	-122.445	37.942	1.04	17.18	287.83	18.22	9	7/14/2014	33:41.0
10041	RSPe_2	-122.444	37.942	1.19	17.17	287.83	18.36	-122.445	37.942	1.08	17.17	287.85	18.25	9	7/14/2014	33:40.9
10042	RSPe_2	-122.444	37.942	1.19	17.18	287.83	18.37	-122.445	37.942	1.08	17.18	287.83	18.26	9	7/14/2014	33:40.8
10043	RSPe_2	-122.444	37.942	1.19	17.18	287.86	18.37	-122.445	37.942	1.08	17.18	287.83	18.26	9	7/14/2014	33:40.7
10044	RSPe_2	-122.444	37.942	1.19	17.15	287.92	18.34	-122.445	37.942	1.08	17.15	287.85	18.23	9	7/14/2014	33:40.6
10045	RSPe_2	-122.444	37.942	1.24	17.14	287.85	18.38	-122.445	37.942	1.08	17.14	287.79	18.21	9	7/14/2014	33:40.5
10046	RSPe_2	-122.444	37.942	1.24	17.14	287.88	18.38	-122.445	37.942	1.08	17.14	287.80	18.22	9	7/14/2014	33:40.4
10047	RSPe_2	-122.444	37.942	1.24	17.12	287.90	18.36	-122.445	37.942	1.13	17.12	287.79	18.25	9	7/14/2014	33:40.3
10048	RSPe_2	-122.444	37.942	1.19	17.14	287.86	18.33	-122.445	37.942	1.13	17.14	287.83	18.27	9	7/14/2014	33:40.2
10049	RSPe_2	-122.444	37.942	1.27	17.14	287.90	18.41	-122.445	37.942	1.16	17.14	287.84	18.30	9	7/14/2014	33:40.1
10050	RSPe_2	-122.444	37.942	1.24	17.14	287.90	18.38	-122.445	37.942	1.16	17.14	287.83	18.30	9	7/14/2014	33:40.0
10051	RSPe_2	-122.444	37.942	1.24	17.15	287.95	18.39	-122.445	37.942	1.16	17.15	287.90	18.31	9	7/14/2014	33:39.9
10052	RSPe_2	-122.444	37.942	1.24	17.17	287.90	18.41	-122.445	37.942	1.16	17.17	287.90	18.33	9	7/14/2014	33:39.8
10053	RSPe_2	-122.444	37.942	1.27	17.24	288.01	18.51	-122.445	37.942	1.21	17.24	287.94	18.45	9	7/14/2014	33:39.7
10054	RSPe_2	-122.444	37.942	1.27	17.20	288.01	18.47	-122.445	37.942	1.21	17.20	287.94	18.41	9	7/14/2014	33:39.6
10055	RSPe_2	-122.444	37.942	1.27	17.22	288.04	18.50	-122.445	37.942	1.16	17.22	287.95	18.38	9	7/14/2014	33:39.5
10056	RSPe_2	-122.444	37.942	1.27	17.25	288.08	18.52	-122.445	37.942	1.13	17.25	287.97	18.37	9	7/14/2014	33:39.4
10057	RSPe_2	-122.444	37.942	1.28	17.21	288.08	18.48	-122.445	37.942	1.21	17.21	288.00	18.42	9	7/14/2014	33:39.3
10058	RSPe_2	-122.444	37.942	1.28	17.21	288.13	18.48	-122.445	37.942	1.21	17.21	288.01	18.42	9	7/14/2014	33:39.2
10059	RSPe_2	-122.444	37.942	1.31	17.24	288.21	18.55	-122.445	37.942	1.25	17.24	288.04	18.48	9	7/14/2014	33:39.1
10060	RSPe_2	-122.444	37.942	1.28	17.25	288.21	18.52	-122.445	37.942	1.21	17.25	288.10	18.46	9	7/14/2014	33:39.0
10061	RSPe_2	-122.444	37.942	1.31	17.23	288.26	18.54	-122.445	37.942	1.25	17.23	288.13	18.47	9	7/14/2014	33:38.9
10062	RSPe_2	-122.444	37.942	1.31	17.23	288.33	18.54	-122.445	37.942	1.25	17.23	288.17	18.47	9	7/14/2014	33:38.8
10063	RSPe_2	-122.444	37.942	1.31	17.27	288.37	18.58	-122.445	37.942	1.25	17.27	288.21	18.51	9	7/14/2014	33:38.7
10064	RSPe_2	-122.444	37.942	1.31	17.23	288.35	18.54	-122.445	37.942	1.25	17.23	288.21	18.47	9	7/14/2014	33:38.6

10065	RSPe_2	-122.444	37.942	1.31	17.23	288.37	18.54	-122.445	37.942	1.28	17.23	288.24	18.51	9	7/14/2014	33:38.5
10066	RSPe_2	-122.444	37.942	1.36	17.25	288.33	18.61	-122.445	37.942	1.28	17.25	288.21	18.52	9	7/14/2014	33:38.4
10067	RSPe_2	-122.444	37.942	1.36	17.23	288.33	18.59	-122.445	37.942	1.33	17.23	288.23	18.56	9	7/14/2014	33:38.3
10068	RSPe_2	-122.444	37.942	1.36	17.21	288.29	18.58	-122.445	37.942	1.33	17.21	288.22	18.54	9	7/14/2014	33:38.2
10069	RSPe_2	-122.444	37.942	1.40	17.21	288.24	18.61	-122.445	37.942	1.36	17.21	288.17	18.58	9	7/14/2014	33:38.1
10070	RSPe_2	-122.444	37.942	1.36	17.23	288.27	18.59	-122.445	37.942	1.33	17.23	288.24	18.56	9	7/14/2014	33:38.0
10071	RSPe_2	-122.444	37.942	1.40	17.22	288.24	18.62	-122.445	37.942	1.36	17.22	288.22	18.59	9	7/14/2014	33:37.9
10072	RSPe_2	-122.444	37.942	1.36	17.25	288.22	18.61	-122.445	37.942	1.36	17.25	288.27	18.62	9	7/14/2014	33:37.8
10073	RSPe_2	-122.444	37.942	1.40	17.28	288.27	18.68	-122.445	37.942	1.36	17.28	288.27	18.65	9	7/14/2014	33:37.7
10074	RSPe_2	-122.444	37.942	1.36	17.28	288.23	18.64	-122.445	37.942	1.36	17.28	288.26	18.65	9	7/14/2014	33:37.6
10075	RSPe_2	-122.444	37.942	1.40	17.25	288.25	18.65	-122.445	37.942	1.36	17.25	288.25	18.62	9	7/14/2014	33:37.5
10076	RSPe_2	-122.444	37.942	1.40	17.28	288.20	18.68	-122.445	37.942	1.36	17.28	288.22	18.65	9	7/14/2014	33:37.4
10077	RSPe_2	-122.444	37.942	1.40	17.35	288.27	18.74	-122.445	37.942	1.36	17.35	288.27	18.71	9	7/14/2014	33:37.3
10078	RSPe_2	-122.444	37.942	1.40	17.32	288.25	18.71	-122.445	37.942	1.33	17.32	288.29	18.65	9	7/14/2014	33:37.2
10079	RSPe_2	-122.444	37.942	1.40	17.31	288.23	18.71	-122.445	37.942	1.36	17.31	288.27	18.67	9	7/14/2014	33:37.1
10080	RSPe_2	-122.444	37.942	1.40	17.34	288.25	18.73	-122.445	37.942	1.36	17.34	288.27	18.70	9	7/14/2014	33:37.0
10081	RSPe_2	-122.444	37.942	1.40	17.37	288.23	18.76	-122.445	37.942	1.41	17.37	288.23	18.78	9	7/14/2014	33:36.9
10082	RSPe_2	-122.444	37.942	1.40	17.35	288.27	18.74	-122.445	37.942	1.41	17.35	288.23	18.76	9	7/14/2014	33:36.8
10083	RSPe_2	-122.444	37.942	1.45	17.35	288.30	18.80	-122.445	37.942	1.41	17.35	288.25	18.77	9	7/14/2014	33:36.7
10084	RSPe_2	-122.444	37.942	1.40	17.43	288.25	18.83	-122.445	37.942	1.41	17.43	288.22	18.85	9	7/14/2014	33:36.6
10085	RSPe_2	-122.444	37.942	1.45	17.42	288.28	18.86	-122.445	37.942	1.45	17.42	288.23	18.86	9	7/14/2014	33:36.5
10086	RSPe_2	-122.444	37.942	1.45	17.38	288.32	18.83	-122.445	37.942	1.41	17.38	288.25	18.79	9	7/14/2014	33:36.4
10087	RSPe_2	-122.444	37.942	1.45	17.40	288.35	18.85	-122.445	37.942	1.45	17.40	288.21	18.85	9	7/14/2014	33:36.3
10088	RSPe_2	-122.444	37.942	1.45	17.42	288.37	18.86	-122.445	37.942	1.41	17.42	288.25	18.83	9	7/14/2014	33:36.2
10089	RSPe_2	-122.444	37.942	1.48	17.42	288.37	18.90	-122.445	37.942	1.45	17.42	288.25	18.87	9	7/14/2014	33:36.1
10090	RSPe_2	-122.444	37.942	1.45	17.41	288.43	18.85	-122.445	37.942	1.41	17.41	288.30	18.82	9	7/14/2014	33:36.0
10091	RSPe_2	-122.444	37.942	1.48	17.41	288.44	18.89	-122.445	37.942	1.45	17.41	288.32	18.85	9	7/14/2014	33:35.9
10092	RSPe_2	-122.444	37.942	1.45	17.48	288.44	18.92	-122.445	37.942	1.45	17.48	288.36	18.92	9	7/14/2014	33:35.8
10093	RSPe_2	-122.444	37.942	1.48	17.43	288.44	18.91	-122.445	37.942	1.50	17.43	288.35	18.93	9	7/14/2014	33:35.7
10094	RSPe_2	-122.444	37.942	1.45	17.45	288.44	18.89	-122.445	37.942	1.45	17.45	288.41	18.90	9	7/14/2014	33:35.6
10095	RSPe_2	-122.444	37.942	1.48	17.45	288.46	18.94	-122.445	37.942	1.45	17.45	288.39	18.90	9	7/14/2014	33:35.5
10096	RSPe_2	-122.444	37.942	1.48	17.48	288.44	18.96	-122.445	37.942	1.50	17.48	288.43	18.97	9	7/14/2014	33:35.4
10097	RSPe_2	-122.444	37.942	1.53	17.49	288.51	19.02	-122.445	37.942	1.53	17.49	288.46	19.03	9	7/14/2014	33:35.3

10098	RSPe_2	-122.444	37.942	1.48	17.45	288.51	18.94	-122.445	37.942	1.53	17.45	288.45	18.99	9	7/14/2014	33:35.2
10099	RSPe_2	-122.444	37.942	1.53	17.50	288.51	19.03	-122.445	37.942	1.58	17.50	288.44	19.08	9	7/14/2014	33:35.1
10100	RSPe_2	-122.444	37.942	1.48	17.48	288.48	18.96	-122.445	37.942	1.58	17.48	288.46	19.06	9	7/14/2014	33:35.0
10101	RSPe_2	-122.444	37.942	1.57	17.46	288.57	19.03	-122.445	37.942	1.62	17.46	288.48	19.08	9	7/14/2014	33:34.9
10102	RSPe_2	-122.444	37.942	1.53	17.49	288.59	19.02	-122.445	37.942	1.62	17.49	288.46	19.10	9	7/14/2014	33:34.8
10103	RSPe_2	-122.444	37.942	1.57	17.46	288.58	19.03	-122.445	37.942	1.62	17.46	288.48	19.08	9	7/14/2014	33:34.7
10104	RSPe_2	-122.444	37.942	1.53	17.49	288.69	19.02	-122.445	37.942	1.62	17.49	288.51	19.11	9	7/14/2014	33:34.6
10105	RSPe_2	-122.444	37.942	1.57	17.45	288.69	19.02	-122.445	37.942	1.65	17.45	288.53	19.11	9	7/14/2014	33:34.5
10106	RSPe_2	-122.444	37.942	1.53	17.45	288.62	18.98	-122.445	37.942	1.62	17.45	288.51	19.06	9	7/14/2014	33:34.4
10107	RSPe_2	-122.444	37.942	1.57	17.50	288.69	19.07	-122.445	37.942	1.62	17.50	288.55	19.12	9	7/14/2014	33:34.3
10108	RSPe_2	-122.444	37.942	1.53	17.40	288.75	18.93	-122.445	37.942	1.62	17.40	288.66	19.02	9	7/14/2014	33:34.2
10109	RSPe_2	-122.444	37.942	1.57	17.45	288.80	19.02	-122.445	37.942	1.70	17.45	288.71	19.16	9	7/14/2014	33:34.1
10110	RSPe_2	-122.444	37.942	1.57	17.42	288.78	18.99	-122.445	37.942	1.62	17.42	288.71	19.04	9	7/14/2014	33:34.0
10111	RSPe_2	-122.444	37.942	1.57	17.45	288.71	19.01	-122.445	37.942	1.65	17.45	288.68	19.10	9	7/14/2014	33:33.9
10112	RSPe_2	-122.444	37.942	1.57	17.44	288.60	19.00	-122.445	37.942	1.74	17.44	288.62	19.17	9	7/14/2014	33:33.8
10113	RSPe_2	-122.444	37.942	1.62	17.40	288.58	19.02	-122.445	37.942	1.74	17.40	288.62	19.14	9	7/14/2014	33:33.7
10114	RSPe_2	-122.444	37.942	1.57	17.33	288.65	18.90	-122.445	37.942	1.74	17.33	288.66	19.07	9	7/14/2014	33:33.6
10115	RSPe_2	-122.444	37.942	1.57	17.31	288.70	18.88	-122.445	37.942	1.79	17.31	288.74	19.10	9	7/14/2014	33:33.5
10116	RSPe_2	-122.444	37.942	1.57	17.24	288.72	18.81	-122.445	37.942	1.74	17.24	288.76	18.98	9	7/14/2014	33:33.4
10117	RSPe_2	-122.444	37.942	1.62	17.25	288.72	18.86	-122.445	37.942	1.79	17.25	288.76	19.03	9	7/14/2014	33:33.3
10118	RSPe_2	-122.444	37.942	1.57	17.05	288.80	18.62	-122.445	37.942	1.74	17.05	288.76	18.79	9	7/14/2014	33:33.2
10119	RSPe_2	-122.444	37.942	1.62	17.11	288.83	18.72	-122.445	37.942	1.79	17.11	288.78	18.89	9	7/14/2014	33:33.1
10120	RSPe_2	-122.444	37.942	1.57	16.96	288.83	18.53	-122.445	37.942	1.74	16.96	288.71	18.70	9	7/14/2014	33:33.0
10121	RSPe_2	-122.444	37.942	1.62	16.98	288.89	18.60	-122.445	37.942	1.79	16.98	288.81	18.77	9	7/14/2014	33:32.9
10122	RSPe_2	-122.444	37.942	1.57	16.92	288.89	18.49	-122.445	37.942	1.74	16.92	288.80	18.66	9	7/14/2014	33:32.8
10123	RSPe_2	-122.444	37.942	1.62	16.80	289.01	18.42	-122.445	37.942	1.79	16.80	288.85	18.58	9	7/14/2014	33:32.7
10124	RSPe_2	-122.444	37.942	1.57	16.80	289.01	18.36	-122.445	37.942	1.79	16.80	288.85	18.58	9	7/14/2014	33:32.6
10125	RSPe_2	-122.444	37.942	1.57	16.78	289.07	18.35	-122.445	37.942	1.82	16.78	288.87	18.61	9	7/14/2014	33:32.5
10126	RSPe_2	-122.444	37.942	1.62	16.74	289.11	18.35	-122.445	37.942	1.87	16.74	288.89	18.61	9	7/14/2014	33:32.4
10127	RSPe_2	-122.444	37.942	1.62	16.74	289.14	18.36	-122.445	37.942	1.82	16.74	288.92	18.56	9	7/14/2014	33:32.3
10128	RSPe_2	-122.444	37.942	1.57	16.60	289.10	18.16	-122.445	37.942	1.82	16.60	288.92	18.42	9	7/14/2014	33:32.2
10129	RSPe_2	-122.444	37.942	1.62	16.69	289.16	18.31	-122.445	37.942	1.87	16.69	289.03	18.56	9	7/14/2014	33:32.1
10130	RSPe_2	-122.444	37.942	1.57	16.51	289.19	18.08	-122.445	37.942	1.82	16.51	289.05	18.33	9	7/14/2014	33:32.0

10131	RSPe_2	-122.444	37.942	1.57	16.61	289.21	18.18	-122.445	37.942	1.87	16.61	289.07	18.48	9	7/14/2014 33:31.9
10132	RSPe_2	-122.444	37.942	1.57	16.53	289.23	18.09	-122.445	37.942	1.87	16.53	289.11	18.40	9	7/14/2014 33:31.8
10133	RSPe_2	-122.444	37.942	1.57	16.44	289.26	18.00	-122.445	37.942	1.87	16.44	289.16	18.31	9	7/14/2014 33:31.7
10134	RSPe_2	-122.444	37.942	1.57	16.43	289.21	17.99	-122.445	37.942	1.82	16.43	289.18	18.25	9	7/14/2014 33:31.6
10135	RSPe_2	-122.444	37.942	1.57	16.43	289.19	17.99	-122.445	37.942	1.87	16.43	289.23	18.30	9	7/14/2014 33:31.5
10136	RSPe_2	-122.444	37.942	1.57	16.47	289.21	18.04	-122.445	37.942	1.87	16.47	289.29	18.35	9	7/14/2014 33:31.4
10137	RSPe_2	-122.444	37.942	1.57	16.42	289.25	17.99	-122.445	37.942	1.87	16.42	289.30	18.29	9	7/14/2014 33:31.3
10138	RSPe_2	-122.444	37.942	1.53	16.44	289.21	17.98	-122.445	37.942	1.87	16.44	289.27	18.31	9	7/14/2014 33:31.2
10139	RSPe_2	-122.444	37.942	1.62	16.41	289.17	18.02	-122.445	37.942	1.96	16.41	289.21	18.36	9	7/14/2014 33:31.1
10140	RSPe_2	-122.444	37.942	1.57	16.40	289.21	17.96	-122.445	37.942	1.96	16.40	289.23	18.35	9	7/14/2014 33:31.0
10141	RSPe_2	-122.444	37.942	1.57	16.39	289.22	17.96	-122.445	37.942	1.96	16.39	289.25	18.34	9	7/14/2014 33:30.9
10142	RSPe_2	-122.444	37.942	1.57	16.39	289.30	17.96	-122.445	37.942	1.90	16.39	289.30	18.29	9	7/14/2014 33:30.8
10143	RSPe_2	-122.444	37.942	1.57	16.38	289.26	17.95	-122.445	37.942	1.90	16.38	289.21	18.29	9	7/14/2014 33:30.7
10144	RSPe_2	-122.444	37.942	1.53	16.41	289.35	17.94	-122.445	37.942	1.87	16.41	289.34	18.28	9	7/14/2014 33:30.6
10145	RSPe_2	-122.444	37.942	1.57	16.45	289.39	18.02	-122.445	37.942	1.90	16.45	289.37	18.36	9	7/14/2014 33:30.5
10146	RSPe_2	-122.444	37.942	1.53	16.40	289.38	17.93	-122.445	37.942	1.87	16.40	289.32	18.27	9	7/14/2014 33:30.4
10147	RSPe_2	-122.444	37.942	1.53	16.43	289.39	17.96	-122.445	37.942	1.87	16.43	289.37	18.30	9	7/14/2014 33:30.3
10148	RSPe_2	-122.444	37.942	1.48	16.44	289.44	17.92	-122.445	37.942	1.87	16.44	289.38	18.31	9	7/14/2014 33:30.2
10149	RSPe_2	-122.444	37.942	1.53	16.44	289.42	17.97	-122.445	37.942	1.87	16.44	289.37	18.31	9	7/14/2014 33:30.1
10150	RSPe_2	-122.444	37.942	1.48	16.45	289.46	17.93	-122.445	37.942	1.87	16.45	289.46	18.32	9	7/14/2014 33:30.0
10151	RSPe_2	-122.444	37.942	1.53	16.47	289.51	18.00	-122.445	37.942	1.87	16.47	289.53	18.34	9	7/14/2014 33:29.9
10152	RSPe_2	-122.444	37.942	1.48	16.48	289.57	17.96	-122.445	37.942	1.87	16.48	289.57	18.35	9	7/14/2014 33:29.8
10153	RSPe_2	-122.444	37.942	1.48	16.44	289.56	17.92	-122.445	37.942	1.87	16.44	289.57	18.31	9	7/14/2014 33:29.7
10154	RSPe_2	-122.444	37.942	1.45	16.45	289.57	17.90	-122.445	37.942	1.82	16.45	289.55	18.27	9	7/14/2014 33:29.6
10155	RSPe_2	-122.444	37.942	1.45	16.51	289.53	17.96	-122.445	37.942	1.87	16.51	289.57	18.38	9	7/14/2014 33:29.5
10156	RSPe_2	-122.444	37.942	1.45	16.44	289.55	17.89	-122.445	37.942	1.82	16.44	289.62	18.26	9	7/14/2014 33:29.4
10157	RSPe_2	-122.444	37.942	1.45	16.51	289.53	17.96	-122.445	37.942	1.87	16.51	289.62	18.38	9	7/14/2014 33:29.3
10158	RSPe_2	-122.444	37.942	1.45	16.51	289.54	17.96	-122.445	37.942	1.82	16.51	289.66	18.33	9	7/14/2014 33:29.2
10159	RSPe_2	-122.444	37.942	1.45	16.54	289.47	17.99	-122.445	37.942	1.87	16.54	289.62	18.42	9	7/14/2014 33:29.1
10160	RSPe_2	-122.444	37.942	1.40	16.57	289.45	17.96	-122.445	37.942	1.82	16.57	289.64	18.39	9	7/14/2014 33:29.0
10161	RSPe_2	-122.444	37.942	1.40	16.59	289.47	17.99	-122.445	37.942	1.82	16.59	289.62	18.41	9	7/14/2014 33:28.9
10162	RSPe_2	-122.444	37.942	1.40	16.58	289.43	17.97	-122.445	37.942	1.82	16.58	289.68	18.40	9	7/14/2014 33:28.8
10163	RSPe_2	-122.444	37.942	1.40	16.58	289.45	17.98	-122.445	37.942	1.82	16.58	289.60	18.40	9	7/14/2014 33:28.7

10164	RSPe_2	-122.444	37.942	1.36	16.61	289.43	17.97	-122.445	37.942	1.82	16.61	289.61	18.43	9	7/14/2014	33:28.6
10165	RSPe_2	-122.444	37.942	1.40	16.60	289.49	18.00	-122.445	37.942	1.82	16.60	289.64	18.43	9	7/14/2014	33:28.5
10166	RSPe_2	-122.444	37.942	1.36	16.61	289.47	17.97	-122.445	37.942	1.74	16.61	289.60	18.35	9	7/14/2014	33:28.4
10167	RSPe_2	-122.444	37.942	1.36	16.70	289.50	18.06	-122.445	37.942	1.79	16.70	289.58	18.49	9	7/14/2014	33:28.3
10168	RSPe_2	-122.444	37.942	1.36	16.76	289.54	18.12	-122.445	37.942	1.74	16.76	289.58	18.50	9	7/14/2014	33:28.2
10169	RSPe_2	-122.444	37.942	1.36	16.68	289.59	18.05	-122.445	37.942	1.74	16.68	289.56	18.42	9	7/14/2014	33:28.1
10170	RSPe_2	-122.444	37.942	1.31	16.74	289.63	18.05	-122.445	37.942	1.70	16.74	289.58	18.44	9	7/14/2014	33:28.0
10171	RSPe_2	-122.444	37.942	1.31	16.72	289.68	18.03	-122.445	37.942	1.74	16.72	289.67	18.45	9	7/14/2014	33:27.9
10172	RSPe_2	-122.444	37.942	1.28	16.74	289.67	18.01	-122.445	37.942	1.70	16.74	289.62	18.44	9	7/14/2014	33:27.8
10173	RSPe_2	-122.444	37.942	1.28	16.77	289.70	18.05	-122.445	37.942	1.70	16.77	289.69	18.48	9	7/14/2014	33:27.7
10174	RSPe_2	-122.444	37.942	1.28	16.82	289.68	18.10	-122.445	37.942	1.65	16.82	289.63	18.47	9	7/14/2014	33:27.6
10175	RSPe_2	-122.444	37.942	1.28	16.79	289.68	18.07	-122.445	37.942	1.70	16.79	289.65	18.49	9	7/14/2014	33:27.5
10176	RSPe_2	-122.444	37.942	1.24	16.78	289.65	18.03	-122.445	37.942	1.65	16.78	289.67	18.44	9	7/14/2014	33:27.4
10177	RSPe_2	-122.444	37.942	1.24	16.85	289.57	18.10	-122.445	37.942	1.65	16.85	289.65	18.51	9	7/14/2014	33:27.3
10178	RSPe_2	-122.444	37.942	1.19	16.90	289.55	18.09	-122.445	37.942	1.62	16.90	289.65	18.52	9	7/14/2014	33:27.2
10179	RSPe_2	-122.444	37.942	1.24	16.96	289.55	18.20	-122.445	37.942	1.65	16.96	289.67	18.61	9	7/14/2014	33:27.1
10180	RSPe_2	-122.444	37.942	1.19	16.92	289.46	18.11	-122.445	37.942	1.62	16.92	289.65	18.54	9	7/14/2014	33:27.0
10181	RSPe_2	-122.444	37.942	1.19	17.00	289.49	18.20	-122.445	37.942	1.65	17.00	289.64	18.66	9	7/14/2014	33:26.9
10182	RSPe_2	-122.444	37.942	1.16	17.13	289.42	18.29	-122.445	37.942	1.62	17.13	289.61	18.75	9	7/14/2014	33:26.8
10183	RSPe_2	-122.444	37.942	1.19	17.13	289.39	18.32	-122.445	37.942	1.62	17.13	289.55	18.75	9	7/14/2014	33:26.7
10184	RSPe_2	-122.444	37.942	1.16	17.18	289.38	18.33	-122.445	37.942	1.62	17.18	289.57	18.79	9	7/14/2014	33:26.6
10185	RSPe_2	-122.444	37.942	1.16	17.18	289.48	18.33	-122.445	37.942	1.62	17.18	289.60	18.79	9	7/14/2014	33:26.5
10186	RSPe_2	-122.444	37.942	1.16	17.18	289.56	18.34	-122.445	37.942	1.59	17.18	289.61	18.77	9	7/14/2014	33:26.4
10187	RSPe_2	-122.444	37.942	1.16	17.29	289.55	18.45	-122.445	37.942	1.59	17.29	289.61	18.88	9	7/14/2014	33:26.3
10188	RSPe_2	-122.444	37.942	1.11	17.26	289.62	18.37	-122.445	37.942	1.53	17.26	289.67	18.79	9	7/14/2014	33:26.2
10189	RSPe_2	-122.444	37.942	1.11	17.28	289.68	18.38	-122.445	37.942	1.53	17.28	289.73	18.81	9	7/14/2014	33:26.1
10190	RSPe_2	-122.444	37.942	1.07	17.37	289.67	18.44	-122.445	37.942	1.50	17.37	289.70	18.87	9	7/14/2014	33:26.0
10191	RSPe_2	-122.444	37.942	1.07	17.34	289.66	18.41	-122.445	37.942	1.50	17.34	289.73	18.84	9	7/14/2014	33:25.9
10192	RSPe_2	-122.444	37.942	1.02	17.44	289.73	18.46	-122.445	37.942	1.41	17.44	289.73	18.85	9	7/14/2014	33:25.8
10193	RSPe_2	-122.444	37.942	1.07	17.39	289.69	18.46	-122.445	37.942	1.41	17.39	289.69	18.80	9	7/14/2014	33:25.7
10194	RSPe_2	-122.444	37.942	1.02	17.38	289.67	18.40	-122.445	37.942	1.37	17.38	289.68	18.74	9	7/14/2014	33:25.6
10195	RSPe_2	-122.444	37.942	1.02	17.39	289.73	18.41	-122.445	37.942	1.41	17.39	289.77	18.80	9	7/14/2014	33:25.5
10196	RSPe_2	-122.444	37.942	0.99	17.45	289.71	18.43	-122.445	37.942	1.37	17.45	289.82	18.81	9	7/14/2014	33:25.4

10197	RSPe_2	-122.444	37.942	0.99	17.54	289.69	18.52	-122.445	37.942	1.37	17.54	289.82	18.90	9	7/14/2014	33:25.3
10198	RSPe_2	-122.444	37.942	0.99	17.53	289.65	18.52	-122.445	37.942	1.37	17.53	289.86	18.90	9	7/14/2014	33:25.2
10199	RSPe_2	-122.444	37.942	0.99	17.58	289.61	18.57	-122.445	37.942	1.37	17.58	289.87	18.95	9	7/14/2014	33:25.1
10200	RSPe_2	-122.444	37.942	0.94	17.61	289.59	18.55	-122.445	37.942	1.37	17.61	289.87	18.98	9	7/14/2014	33:25.0
10201	RSPe_2	-122.444	37.942	0.94	17.68	289.56	18.61	-122.445	37.942	1.37	17.68	289.85	19.04	9	7/14/2014	33:24.9
10202	RSPe_2	-122.444	37.942	0.94	17.70	289.61	18.64	-122.445	37.942	1.28	17.70	289.93	18.98	9	7/14/2014	33:24.8
10203	RSPe_2	-122.444	37.942	0.94	17.67	289.72	18.60	-122.445	37.942	1.33	17.67	290.07	19.00	9	7/14/2014	33:24.7
10204	RSPe_2	-122.444	37.942	0.90	17.72	289.74	18.62	-122.445	37.942	1.28	17.72	290.14	19.00	9	7/14/2014	33:24.6
10205	RSPe_2	-122.444	37.942	0.90	17.68	289.79	18.58	-122.445	37.942	1.25	17.68	290.16	18.93	9	7/14/2014	33:24.5
10206	RSPe_2	-122.444	37.942	0.87	17.72	289.74	18.59	-122.445	37.942	1.21	17.72	290.09	18.93	9	7/14/2014	33:24.4
10207	RSPe_2	-122.444	37.942	0.87	17.73	289.74	18.60	-122.445	37.942	1.21	17.73	290.05	18.94	9	7/14/2014	33:24.3
10208	RSPe_2	-122.444	37.942	0.82	17.76	289.79	18.58	-122.445	37.942	1.16	17.76	290.03	18.93	9	7/14/2014	33:24.2
10209	RSPe_2	-122.444	37.942	0.87	17.79	289.77	18.65	-122.445	37.942	1.16	17.79	290.03	18.95	9	7/14/2014	33:24.1
10210	RSPe_2	-122.444	37.942	0.82	17.81	289.75	18.62	-122.445	37.942	1.16	17.81	289.98	18.97	9	7/14/2014	33:24.0
10211	RSPe_2	-122.444	37.942	0.82	17.89	289.68	18.71	-122.445	37.942	1.13	17.89	289.98	19.02	9	7/14/2014	33:23.9
10212	RSPe_2	-122.444	37.942	0.82	17.86	289.64	18.68	-122.445	37.942	1.13	17.86	289.85	18.99	9	7/14/2014	33:23.8
10213	RSPe_2	-122.444	37.942	0.82	17.88	289.59	18.69	-122.445	37.942	1.13	17.88	289.89	19.01	9	7/14/2014	33:23.7
10214	RSPe_2	-122.444	37.942	0.78	17.93	289.55	18.71	-122.445	37.942	1.08	17.93	289.92	19.01	9	7/14/2014	33:23.6
10215	RSPe_2	-122.444	37.942	0.78	17.99	289.57	18.77	-122.445	37.942	1.13	17.99	289.95	19.11	9	7/14/2014	33:23.5
10216	RSPe_2	-122.444	37.942	0.78	18.12	289.53	18.91	-122.445	37.942	1.08	18.12	289.94	19.20	9	7/14/2014	33:23.4
10217	RSPe_2	-122.444	37.942	0.78	18.08	289.60	18.86	-122.445	37.942	1.13	18.08	290.03	19.21	9	7/14/2014	33:23.3
10218	RSPe_2	-122.444	37.942	0.73	18.09	289.60	18.82	-122.445	37.942	1.04	18.09	290.05	19.13	9	7/14/2014	33:23.2
10219	RSPe_2	-122.444	37.942	0.73	18.15	289.66	18.88	-122.445	37.942	1.04	18.15	290.03	19.19	9	7/14/2014	33:23.1
10220	RSPe_2	-122.444	37.942	0.73	18.22	289.67	18.95	-122.445	37.942	0.99	18.22	290.05	19.21	9	7/14/2014	33:23.0
10221	RSPe_2	-122.444	37.942	0.69	18.22	289.69	18.91	-122.445	37.942	0.99	18.22	290.04	19.21	9	7/14/2014	33:22.9
10222	RSPe_2	-122.444	37.942	0.69	18.19	289.67	18.89	-122.445	37.942	0.96	18.19	289.95	19.15	9	7/14/2014	33:22.8
10223	RSPe_2	-122.444	37.942	0.69	18.23	289.66	18.92	-122.445	37.942	0.96	18.23	289.93	19.18	9	7/14/2014	33:22.7
10224	RSPe_2	-122.444	37.942	0.64	18.24	289.67	18.88	-122.445	37.942	0.91	18.24	289.90	19.15	9	7/14/2014	33:22.6
10225	RSPe_2	-122.444	37.942	0.64	18.30	289.64	18.94	-122.445	37.942	0.91	18.30	289.86	19.20	9	7/14/2014	33:22.5
10226	RSPe_2	-122.444	37.942	0.61	18.33	289.60	18.94	-122.445	37.942	0.87	18.33	289.80	19.21	9	7/14/2014	33:22.4
10227	RSPe_2	-122.444	37.942	0.64	18.32	289.58	18.96	-122.445	37.942	0.87	18.32	289.75	19.19	9	7/14/2014	33:22.3
10228	RSPe_2	-122.444	37.942	0.61	18.33	289.65	18.94	-122.445	37.942	0.87	18.33	289.81	19.20	9	7/14/2014	33:22.2
10229	RSPe_2	-122.444	37.942	0.61	18.33	289.62	18.94	-122.445	37.942	0.84	18.33	289.80	19.17	9	7/14/2014	33:22.1

10230	RSPe_2	-122.444	37.942	0.56	18.36	289.58	18.92	-122.445	37.942	0.84	18.36	289.80	19.20	9	7/14/2014	33:22.0
10231	RSPe_2	-122.444	37.942	0.61	18.35	289.54	18.96	-122.445	37.942	0.84	18.35	289.80	19.19	9	7/14/2014	33:21.9
10232	RSPe_2	-122.444	37.942	0.56	18.35	289.52	18.91	-122.445	37.942	0.79	18.35	289.82	19.14	9	7/14/2014	33:21.8
10233	RSPe_2	-122.444	37.942	0.56	18.33	289.50	18.88	-122.445	37.942	0.79	18.33	289.78	19.12	9	7/14/2014	33:21.7
10234	RSPe_2	-122.444	37.942	0.56	18.26	289.45	18.82	-122.445	37.942	0.76	18.26	289.76	19.01	9	7/14/2014	33:21.6
10235	RSPe_2	-122.444	37.942	0.56	18.26	289.41	18.82	-122.445	37.942	0.76	18.26	289.74	19.01	9	7/14/2014	33:21.5
10236	RSPe_2	-122.444	37.942	0.52	18.36	289.43	18.88	-122.445	37.942	0.70	18.36	289.81	19.06	9	7/14/2014	33:21.4
10237	RSPe_2	-122.444	37.942	0.52	18.30	289.41	18.83	-122.445	37.942	0.70	18.30	289.73	19.01	9	7/14/2014	33:21.3
10238	RSPe_2	-122.444	37.942	0.52	18.31	289.39	18.83	-122.445	37.942	0.67	18.31	289.76	18.98	9	7/14/2014	33:21.2
10239	RSPe_2	-122.444	37.942	0.52	18.31	289.37	18.83	-122.445	37.942	0.67	18.31	289.67	18.98	9	7/14/2014	33:21.1
10240	RSPe_2	-122.444	37.942	0.52	18.31	289.42	18.83	-122.445	37.942	0.67	18.31	289.71	18.98	9	7/14/2014	33:21.0
10241	RSPe_2	-122.444	37.942	0.52	18.36	289.48	18.89	-122.445	37.942	0.67	18.36	289.70	19.04	9	7/14/2014	33:20.9
10242	RSPe_2	-122.444	37.942	0.49	18.40	289.48	18.89	-122.445	37.942	0.62	18.40	289.65	19.02	9	7/14/2014	33:20.8
10243	RSPe_2	-122.444	37.942	0.49	18.30	289.53	18.79	-122.445	37.942	0.62	18.30	289.67	18.92	9	7/14/2014	33:20.7
10244	RSPe_2	-122.444	37.942	0.44	18.34	289.57	18.78	-122.445	37.942	0.59	18.34	289.71	18.93	9	7/14/2014	33:20.6
10245	RSPe_2	-122.444	37.942	0.49	18.34	289.62	18.83	-122.445	37.942	0.59	18.34	289.72	18.93	9	7/14/2014	33:20.5
10246	RSPe_2	-122.444	37.942	0.44	18.35	289.61	18.79	-122.445	37.942	0.54	18.35	289.70	18.88	9	7/14/2014	33:20.4
10247	RSPe_2	-122.444	37.942	0.44	18.34	289.53	18.78	-122.445	37.942	0.54	18.34	289.70	18.88	9	7/14/2014	33:20.3
10248	RSPe_2	-122.444	37.942	0.40	18.36	289.50	18.76	-122.445	37.942	0.50	18.36	289.67	18.86	9	7/14/2014	33:20.2
10249	RSPe_2	-122.444	37.942	0.44	18.20	289.44	18.64	-122.445	37.942	0.54	18.20	289.72	18.74	9	7/14/2014	33:20.1
10250	RSPe_2	-122.444	37.942	0.44	18.24	289.46	18.67	-122.445	37.942	0.50	18.24	289.77	18.74	9	7/14/2014	33:20.0
10251	RSPe_2	-122.444	37.942	0.44	18.17	289.42	18.60	-122.445	37.942	0.50	18.17	289.79	18.67	9	7/14/2014	33:19.9
10252	RSPe_2	-122.444	37.942	0.40	18.15	289.47	18.55	-122.445	37.942	0.50	18.15	289.81	18.65	9	7/14/2014	33:19.8
10253	RSPe_2	-122.444	37.942	0.40	18.20	289.44	18.61	-122.445	37.942	0.50	18.20	289.82	18.70	9	7/14/2014	33:19.7
10254	RSPe_2	-122.444	37.942	0.35	18.17	289.45	18.52	-122.445	37.942	0.47	18.17	289.79	18.64	9	7/14/2014	33:19.6
10255	RSPe_2	-122.444	37.942	0.40	18.18	289.51	18.58	-122.445	37.942	0.47	18.18	289.80	18.65	9	7/14/2014	33:19.5
10256	RSPe_2	-122.444	37.942	0.35	18.19	289.51	18.54	-122.445	37.942	0.42	18.19	289.75	18.60	9	7/14/2014	33:19.4
10257	RSPe_2	-122.444	37.942	0.35	18.18	289.60	18.53	-122.445	37.942	0.42	18.18	289.77	18.59	9	7/14/2014	33:19.3
10258	RSPe_2	-122.444	37.942	0.35	18.19	289.62	18.55	-122.445	37.942	0.38	18.19	289.72	18.58	9	7/14/2014	33:19.2
10259	RSPe_2	-122.444	37.942	0.35	18.23	289.65	18.58	-122.445	37.942	0.38	18.23	289.80	18.61	9	7/14/2014	33:19.1
10260	RSPe_2	-122.444	37.942	0.35	18.26	289.67	18.61	-122.445	37.942	0.33	18.26	289.77	18.59	9	7/14/2014	33:19.0
10261	RSPe_2	-122.444	37.942	0.35	18.30	289.67	18.66	-122.445	37.942	0.33	18.30	289.78	18.64	9	7/14/2014	33:18.9
10262	RSPe_2	-122.444	37.942	0.32	18.19	289.63	18.51	-122.445	37.942	0.30	18.19	289.75	18.49	9	7/14/2014	33:18.8

10263	RSPe_2	-122.444	37.942	0.35	18.21	289.61	18.56	-122.445	37.942	0.30	18.21	289.80	18.51	9	7/14/2014	33:18.7
10264	RSPe_2	-122.444	37.942	0.32	18.21	289.56	18.53	-122.445	37.942	0.25	18.21	289.77	18.46	9	7/14/2014	33:18.6
10265	RSPe_2	-122.444	37.942	0.32	18.27	289.48	18.59	-122.445	37.942	0.25	18.27	289.80	18.52	9	7/14/2014	33:18.5
10266	RSPe_2	-122.444	37.942	0.32	18.20	289.54	18.52	-122.445	37.942	0.25	18.20	289.84	18.45	9	7/14/2014	33:18.4
10267	RSPe_2	-122.444	37.942	0.32	18.29	289.50	18.61	-122.445	37.942	0.25	18.29	289.85	18.54	9	7/14/2014	33:18.3
10268	RSPe_2	-122.444	37.942	0.32	18.30	289.57	18.61	-122.445	37.942	0.25	18.30	289.85	18.54	9	7/14/2014	33:18.2
10269	RSPe_2	-122.444	37.942	0.32	18.34	289.59	18.66	-122.445	37.942	0.25	18.34	289.80	18.59	9	7/14/2014	33:18.1
10270	RSPe_2	-122.444	37.942	0.27	18.28	289.55	18.55	-122.445	37.942	0.21	18.28	289.70	18.49	9	7/14/2014	33:18.0
10271	RSPe_2	-122.444	37.942	0.32	18.28	289.59	18.60	-122.445	37.942	0.21	18.28	289.63	18.49	9	7/14/2014	33:17.9
10272	RSPe_2	-122.444	37.942	0.23	18.30	289.57	18.54	-122.445	37.942	0.21	18.30	289.58	18.52	9	7/14/2014	33:17.8
10273	RSPe_2	-122.444	37.942	0.27	18.24	289.61	18.51	-122.445	37.942	0.21	18.24	289.54	18.45	9	7/14/2014	33:17.7
10274	RSPe_2	-122.444	37.942	0.23	18.24	289.66	18.47	-122.445	37.942	0.21	18.24	289.57	18.45	9	7/14/2014	33:17.6
10275	RSPe_2	-122.444	37.942	0.27	18.25	289.61	18.51	-122.445	37.942	0.18	18.25	289.56	18.43	9	7/14/2014	33:17.5
10276	RSPe_2	-122.444	37.942	0.23	18.25	289.57	18.48	-122.445	37.942	0.13	18.25	289.57	18.38	9	7/14/2014	33:17.4
10277	RSPe_2	-122.444	37.942	0.23	18.26	289.61	18.49	-122.445	37.942	0.13	18.26	289.64	18.39	9	7/14/2014	33:17.3
10278	RSPe_2	-122.444	37.942	0.23	18.29	289.61	18.52	-122.445	37.942	0.13	18.29	289.68	18.42	9	7/14/2014	33:17.2
10279	RSPe_2	-122.444	37.942	0.23	18.32	289.64	18.55	-122.445	37.942	0.13	18.32	289.75	18.45	9	7/14/2014	33:17.1
10280	RSPe_2	-122.444	37.942	0.20	18.32	289.68	18.52	-122.445	37.942	0.10	18.32	289.79	18.41	9	7/14/2014	33:17.0
10281	RSPe_2	-122.444	37.942	0.23	18.34	289.66	18.57	-122.445	37.942	0.10	18.34	289.77	18.44	9	7/14/2014	33:16.9
10282	RSPe_2	-122.444	37.942	0.20	18.33	289.70	18.53	-122.445	37.942	0.10	18.33	289.83	18.42	9	7/14/2014	33:16.8
10283	RSPe_2	-122.444	37.942	0.23	18.34	289.69	18.58	-122.445	37.942	0.10	18.34	289.86	18.44	9	7/14/2014	33:16.7
10284	RSPe_2	-122.444	37.942	0.23	18.35	289.64	18.58	-122.445	37.942	0.10	18.35	289.86	18.44	9	7/14/2014	33:16.6
10285	RSPe_2	-122.444	37.942	0.23	18.35	289.60	18.58	-122.445	37.942	0.10	18.35	289.84	18.44	9	7/14/2014	33:16.5
10286	RSPe_2	-122.444	37.942	0.20	18.36	289.62	18.56	-122.445	37.942	0.10	18.36	289.88	18.46	9	7/14/2014	33:16.4
10287	RSPe_2	-122.444	37.942	0.23	18.36	289.60	18.59	-122.445	37.942	0.10	18.36	289.86	18.45	9	7/14/2014	33:16.3
10288	RSPe_2	-122.444	37.942	0.20	18.37	289.62	18.57	-122.445	37.942	0.10	18.37	289.86	18.47	9	7/14/2014	33:16.2
10289	RSPe_2	-122.444	37.942	0.23	18.42	289.64	18.65	-122.445	37.942	0.10	18.42	289.86	18.51	9	7/14/2014	33:16.1
10290	RSPe_2	-122.444	37.942	0.20	18.48	289.64	18.68	-122.445	37.942	0.05	18.48	289.85	18.53	9	7/14/2014	33:16.0
10291	RSPe_2	-122.444	37.942	0.23	18.42	289.64	18.65	-122.445	37.942	0.05	18.42	289.82	18.46	9	7/14/2014	33:15.9
10292	RSPe_2	-122.444	37.942	0.20	18.43	289.67	18.63	-122.445	37.942	0.01	18.43	289.82	18.45	9	7/14/2014	33:15.8
10293	RSPe_2	-122.444	37.942	0.20	18.48	289.69	18.68	-122.445	37.942	0.05	18.48	289.80	18.53	9	7/14/2014	33:15.7
10294	RSPe_2	-122.444	37.942	0.15	18.45	289.64	18.60	-122.445	37.942	0.01	18.45	289.75	18.46	9	7/14/2014	33:15.6
10295	RSPe_2	-122.444	37.942	0.20	18.47	289.65	18.67	-122.445	37.942	0.01	18.47	289.74	18.48	9	7/14/2014	33:15.5

10296	RSPe_2	-122.444	37.942	0.20	18.63	289.61	18.83	-122.445	37.942	0.01	18.63	289.69	18.64	9	7/14/2014 33:15.4
10297	RSPe_2	-122.444	37.942	0.20	18.53	289.61	18.73	-122.445	37.942	0.01	18.53	289.73	18.55	9	7/14/2014 33:15.3
10298	RSPe_2	-122.444	37.942	0.20	18.64	289.54	18.84	-122.445	37.942	0.01	18.64	289.67	18.65	9	7/14/2014 33:15.2
10299	RSPe_2	-122.444	37.942	0.20	18.52	289.58	18.72	-122.445	37.942	-0.04	18.52	289.78	18.48	9	7/14/2014 33:15.1
10300	RSPe_2	-122.444	37.942	0.20	18.56	289.59	18.76	-122.445	37.942	-0.04	18.56	289.84	18.52	9	7/14/2014 33:15.0
10301	RSPe_2	-122.444	37.942	0.23	18.67	289.63	18.91	-122.444	37.942	0.01	18.67	289.92	18.69	9	7/14/2014 33:14.9
10302	RSPe_2	-122.444	37.942	0.20	18.66	289.68	18.86	-122.444	37.942	0.01	18.66	289.93	18.67	9	7/14/2014 33:14.8
10303	RSPe_2	-122.444	37.942	0.23	18.72	289.76	18.95	-122.444	37.942	0.05	18.72	289.98	18.76	9	7/14/2014 33:14.7
10304	RSPe_2	-122.444	37.942	0.20	18.64	289.81	18.84	-122.444	37.942	0.01	18.64	289.98	18.65	9	7/14/2014 33:14.6
10305	RSPe_2	-122.444	37.942	0.23	18.69	289.81	18.92	-122.444	37.942	0.01	18.69	289.94	18.70	9	7/14/2014 33:14.5
10306	RSPe_2	-122.444	37.942	0.15	18.63	289.77	18.78	-122.444	37.942	0.01	18.63	289.82	18.64	9	7/14/2014 33:14.4
10307	RSPe_2	-122.444	37.942	0.20	18.60	289.70	18.80	-122.444	37.942	-0.04	18.60	289.76	18.57	9	7/14/2014 33:14.3
10308	RSPe_2	-122.444	37.942	0.20	18.63	289.70	18.83	-122.444	37.942	-0.04	18.63	289.70	18.59	9	7/14/2014 33:14.2
10309	RSPe_2	-122.444	37.942	0.20	18.63	289.64	18.83	-122.444	37.942	-0.04	18.63	289.64	18.59	9	7/14/2014 33:14.1
10310	RSPe_2	-122.444	37.942	0.20	18.68	289.66	18.88	-122.444	37.942	-0.04	18.68	289.67	18.64	9	7/14/2014 33:14.0
10311	RSPe_2	-122.444	37.942	0.20	18.64	289.68	18.84	-122.444	37.942	0.01	18.64	289.70	18.65	9	7/14/2014 33:13.9
10312	RSPe_2	-122.444	37.942	0.20	18.67	289.64	18.87	-122.444	37.942	-0.04	18.67	289.76	18.63	9	7/14/2014 33:13.8
10313	RSPe_2	-122.444	37.942	0.20	18.76	289.75	18.96	-122.444	37.942	-0.04	18.76	289.86	18.73	9	7/14/2014 33:13.7
10314	RSPe_2	-122.444	37.942	0.20	18.73	289.77	18.93	-122.444	37.942	-0.04	18.73	289.92	18.69	9	7/14/2014 33:13.6
10315	RSPe_2	-122.444	37.942	0.23	18.73	289.77	18.97	-122.444	37.942	0.01	18.73	289.98	18.75	9	7/14/2014 33:13.5
10316	RSPe_2	-122.444	37.942	0.20	18.84	289.82	19.04	-122.444	37.942	0.01	18.84	289.94	18.85	9	7/14/2014 33:13.4
10317	RSPe_2	-122.444	37.942	0.23	18.90	289.86	19.14	-122.444	37.942	0.05	18.90	290.02	18.95	9	7/14/2014 33:13.3
10318	RSPe_2	-122.444	37.942	0.20	18.87	289.93	19.07	-122.444	37.942	0.01	18.87	290.10	18.88	9	7/14/2014 33:13.2
10319	RSPe_2	-122.444	37.942	0.23	18.87	289.91	19.10	-122.444	37.942	0.01	18.87	290.10	18.88	9	7/14/2014 33:13.1
10320	RSPe_2	-122.444	37.942	0.20	18.90	289.95	19.10	-122.444	37.942	0.01	18.90	290.13	18.92	9	7/14/2014 33:13.0
10321	RSPe_2	-122.444	37.942	0.23	18.95	289.95	19.19	-122.444	37.942	-0.04	18.95	290.13	18.91	9	7/14/2014 33:12.9
10322	RSPe_2	-122.444	37.942	0.20	18.97	289.98	19.17	-122.444	37.942	-0.04	18.97	290.13	18.94	9	7/14/2014 33:12.8
10323	RSPe_2	-122.444	37.942	0.20	18.97	289.98	19.17	-122.444	37.942	-0.04	18.97	290.06	18.94	9	7/14/2014 33:12.7
10324	RSPe_2	-122.444	37.942	0.20	19.01	290.04	19.21	-122.444	37.942	-0.04	19.01	290.06	18.97	9	7/14/2014 33:12.6
10325	RSPe_2	-122.444	37.942	0.20	19.05	290.07	19.25	-122.444	37.942	0.01	19.05	290.07	19.06	9	7/14/2014 33:12.5
10326	RSPe_2	-122.444	37.942	0.20	19.10	290.15	19.30	-122.444	37.942	-0.04	19.10	290.04	19.07	9	7/14/2014 33:12.4
10327	RSPe_2	-122.444	37.942	0.23	18.99	290.24	19.22	-122.444	37.942	-0.04	18.99	290.17	18.95	9	7/14/2014 33:12.3
10328	RSPe_2	-122.444	37.942	0.20	19.12	290.31	19.32	-122.444	37.942	-0.07	19.12	290.17	19.05	9	7/14/2014 33:12.2

10329	RSPe_2	-122.444	37.942	0.23	19.03	290.33	19.26	-122.444	37.942	0.01	19.03	290.22	19.04	9	7/14/2014	33:12.1
10330	RSPe_2	-122.444	37.942	0.20	19.08	290.33	19.28	-122.444	37.942	0.01	19.08	290.24	19.09	9	7/14/2014	33:12.0
10331	RSPe_2	-122.444	37.942	0.23	19.10	290.38	19.34	-122.444	37.942	0.05	19.10	290.27	19.15	9	7/14/2014	33:11.9
10332	RSPe_2	-122.444	37.942	0.20	19.03	290.31	19.23	-122.444	37.942	0.01	19.03	290.28	19.04	9	7/14/2014	33:11.8
10333	RSPe_2	-122.444	37.942	0.23	18.94	290.31	19.18	-122.444	37.942	0.05	18.94	290.27	18.99	9	7/14/2014	33:11.7
10334	RSPe_2	-122.444	37.942	0.23	19.02	290.31	19.25	-122.444	37.942	0.01	19.02	290.33	19.03	9	7/14/2014	33:11.6
10335	RSPe_2	-122.444	37.942	0.23	18.81	290.32	19.05	-122.444	37.942	0.05	18.81	290.35	18.86	9	7/14/2014	33:11.5
10336	RSPe_2	-122.444	37.942	0.23	18.84	290.20	19.07	-122.444	37.942	0.05	18.84	290.30	18.89	9	7/14/2014	33:11.4
10337	RSPe_2	-122.444	37.942	0.23	18.78	290.19	19.02	-122.444	37.942	0.05	18.78	290.29	18.83	9	7/14/2014	33:11.3
10338	RSPe_2	-122.444	37.942	0.23	18.77	290.36	19.01	-122.444	37.942	0.05	18.77	290.45	18.82	9	7/14/2014	33:11.2
10339	RSPe_2	-122.444	37.942	0.23	18.76	290.34	19.00	-122.444	37.942	0.10	18.76	290.53	18.86	9	7/14/2014	33:11.1
10340	RSPe_2	-122.444	37.942	0.23	18.67	290.38	18.91	-122.444	37.942	0.05	18.67	290.53	18.72	9	7/14/2014	33:11.0
10341	RSPe_2	-122.444	37.942	0.23	18.69	290.36	18.92	-122.444	37.942	0.10	18.69	290.49	18.79	9	7/14/2014	33:10.9
10342	RSPe_2	-122.444	37.942	0.23	18.61	290.38	18.85	-122.444	37.942	0.05	18.61	290.45	18.66	9	7/14/2014	33:10.8
10343	RSPe_2	-122.444	37.942	0.27	18.59	290.41	18.86	-122.444	37.942	0.10	18.59	290.43	18.69	9	7/14/2014	33:10.7
10344	RSPe_2	-122.444	37.942	0.23	18.59	290.41	18.83	-122.444	37.942	0.10	18.59	290.36	18.69	9	7/14/2014	33:10.6
10345	RSPe_2	-122.444	37.942	0.23	18.54	290.41	18.78	-122.444	37.942	0.10	18.54	290.34	18.64	9	7/14/2014	33:10.5
10346	RSPe_2	-122.444	37.942	0.23	18.57	290.45	18.81	-122.444	37.942	0.10	18.57	290.32	18.67	9	7/14/2014	33:10.4
10347	RSPe_2	-122.444	37.942	0.27	18.43	290.41	18.70	-122.444	37.942	0.13	18.43	290.28	18.56	9	7/14/2014	33:10.3
10348	RSPe_2	-122.444	37.942	0.23	18.45	290.43	18.68	-122.444	37.942	0.10	18.45	290.32	18.54	9	7/14/2014	33:10.2
10349	RSPe_2	-122.444	37.942	0.23	18.45	290.46	18.68	-122.444	37.942	0.13	18.45	290.35	18.58	9	7/14/2014	33:10.1
10350	RSPe_2	-122.444	37.942	0.23	18.36	290.41	18.60	-122.444	37.942	0.10	18.36	290.36	18.46	9	7/14/2014	33:10.0
10351	RSPe_2	-122.444	37.942	0.27	18.33	290.39	18.60	-122.444	37.942	0.13	18.33	290.34	18.46	9	7/14/2014	33:09.9
10352	RSPe_2	-122.444	37.942	0.23	18.27	290.39	18.51	-122.444	37.942	0.10	18.27	290.39	18.37	9	7/14/2014	33:09.8
10353	RSPe_2	-122.444	37.942	0.27	18.26	290.26	18.52	-122.444	37.942	0.18	18.26	290.34	18.44	9	7/14/2014	33:09.7
10354	RSPe_2	-122.444	37.942	0.27	18.24	290.26	18.50	-122.444	37.942	0.13	18.24	290.32	18.37	9	7/14/2014	33:09.6
10355	RSPe_2	-122.444	37.942	0.27	18.21	290.26	18.48	-122.444	37.942	0.18	18.21	290.35	18.39	9	7/14/2014	33:09.5
10356	RSPe_2	-122.444	37.942	0.23	18.19	290.26	18.43	-122.444	37.942	0.18	18.19	290.35	18.37	9	7/14/2014	33:09.4
10357	RSPe_2	-122.444	37.942	0.27	18.33	290.24	18.60	-122.444	37.942	0.13	18.33	290.31	18.46	9	7/14/2014	33:09.3
10358	RSPe_2	-122.444	37.942	0.23	18.24	290.24	18.47	-122.444	37.942	0.13	18.24	290.33	18.37	9	7/14/2014	33:09.2
10359	RSPe_2	-122.444	37.942	0.27	18.23	290.24	18.49	-122.444	37.942	0.18	18.23	290.28	18.41	9	7/14/2014	33:09.1
10360	RSPe_2	-122.444	37.942	0.27	18.24	290.16	18.50	-122.444	37.942	0.22	18.24	290.24	18.45	9	7/14/2014	33:09.0
10361	RSPe_2	-122.444	37.942	0.27	18.19	290.18	18.46	-122.444	37.942	0.22	18.19	290.21	18.41	9	7/14/2014	33:08.9

10362	RSPe_2	-122.444	37.942	0.27	18.18	290.15	18.45	-122.444	37.942	0.18	18.18	290.24	18.36	9	7/14/2014 33:08.8
10363	RSPe_2	-122.444	37.942	0.27	18.29	290.20	18.56	-122.444	37.942	0.22	18.29	290.27	18.50	9	7/14/2014 33:08.7
10364	RSPe_2	-122.444	37.942	0.27	18.26	290.25	18.52	-122.444	37.942	0.18	18.26	290.31	18.44	9	7/14/2014 33:08.6
10365	RSPe_2	-122.444	37.942	0.27	18.36	290.27	18.63	-122.444	37.942	0.22	18.36	290.31	18.58	9	7/14/2014 33:08.5
10366	RSPe_2	-122.444	37.942	0.23	18.33	290.29	18.56	-122.444	37.942	0.22	18.33	290.28	18.54	9	7/14/2014 33:08.4
10367	RSPe_2	-122.444	37.942	0.27	18.43	290.32	18.70	-122.444	37.942	0.22	18.43	290.29	18.64	9	7/14/2014 33:08.3
10368	RSPe_2	-122.444	37.942	0.23	18.50	290.31	18.74	-122.444	37.942	0.22	18.50	290.31	18.72	9	7/14/2014 33:08.2
10369	RSPe_2	-122.444	37.942	0.27	18.52	290.32	18.79	-122.444	37.942	0.22	18.52	290.31	18.74	9	7/14/2014 33:08.1
10370	RSPe_2	-122.444	37.942	0.23	18.48	290.23	18.72	-122.444	37.942	0.18	18.48	290.24	18.66	9	7/14/2014 33:08.0
10371	RSPe_2	-122.444	37.942	0.27	18.68	290.23	18.95	-122.444	37.942	0.22	18.68	290.30	18.90	9	7/14/2014 33:07.9
10372	RSPe_2	-122.444	37.942	0.27	18.56	290.23	18.82	-122.444	37.942	0.22	18.56	290.33	18.77	9	7/14/2014 33:07.8
10373	RSPe_2	-122.444	37.942	0.27	18.66	290.14	18.93	-122.444	37.942	0.22	18.66	290.30	18.87	9	7/14/2014 33:07.7
10374	RSPe_2	-122.444	37.942	0.23	18.67	290.16	18.91	-122.444	37.942	0.18	18.67	290.34	18.85	9	7/14/2014 33:07.6
10375	RSPe_2	-122.444	37.942	0.27	18.70	290.12	18.96	-122.444	37.942	0.18	18.70	290.31	18.88	9	7/14/2014 33:07.5
10376	RSPe_2	-122.444	37.942	0.27	18.70	290.08	18.96	-122.444	37.942	0.18	18.70	290.34	18.88	9	7/14/2014 33:07.4
10377	RSPe_2	-122.444	37.942	0.27	18.70	290.10	18.96	-122.444	37.942	0.22	18.70	290.31	18.91	9	7/14/2014 33:07.3
10378	RSPe_2	-122.444	37.942	0.27	18.73	290.04	19.00	-122.444	37.942	0.18	18.73	290.34	18.91	9	7/14/2014 33:07.2
10379	RSPe_2	-122.444	37.942	0.27	18.73	290.05	19.00	-122.444	37.942	0.22	18.73	290.27	18.94	9	7/14/2014 33:07.1
10380	RSPe_2	-122.444	37.942	0.23	18.74	290.13	18.98	-122.444	37.942	0.18	18.74	290.30	18.92	9	7/14/2014 33:07.0
10381	RSPe_2	-122.444	37.942	0.27	18.74	290.17	19.01	-122.444	37.942	0.22	18.74	290.34	18.96	9	7/14/2014 33:06.9
10382	RSPe_2	-122.444	37.942	0.23	18.77	290.15	19.01	-122.444	37.942	0.18	18.77	290.26	18.95	9	7/14/2014 33:06.8
10383	RSPe_2	-122.444	37.942	0.23	18.81	290.24	19.05	-122.444	37.942	0.22	18.81	290.27	19.03	9	7/14/2014 33:06.7
10384	RSPe_2	-122.444	37.942	0.23	18.82	290.21	19.05	-122.444	37.942	0.18	18.82	290.23	19.00	9	7/14/2014 33:06.6
10385	RSPe_2	-122.444	37.942	0.23	18.81	290.28	19.05	-122.444	37.942	0.22	18.81	290.21	19.03	9	7/14/2014 33:06.5
10386	RSPe_2	-122.444	37.942	0.27	18.82	290.22	19.09	-122.444	37.942	0.18	18.82	290.16	19.00	9	7/14/2014 33:06.4
10387	RSPe_2	-122.444	37.942	0.27	18.89	290.22	19.16	-122.444	37.942	0.25	18.89	290.17	19.14	9	7/14/2014 33:06.3
10388	RSPe_2	-122.444	37.942	0.23	18.86	290.27	19.09	-122.444	37.942	0.22	18.86	290.17	19.07	9	7/14/2014 33:06.2
10389	RSPe_2	-122.444	37.942	0.23	18.84	290.22	19.07	-122.444	37.942	0.25	18.84	290.17	19.09	9	7/14/2014 33:06.1
10390	RSPe_2	-122.444	37.942	0.23	18.93	290.24	19.16	-122.444	37.942	0.22	18.93	290.21	19.14	9	7/14/2014 33:06.0
10391	RSPe_2	-122.444	37.942	0.27	18.93	290.22	19.20	-122.444	37.942	0.22	18.93	290.20	19.14	9	7/14/2014 33:05.9
10392	RSPe_2	-122.444	37.942	0.23	18.92	290.16	19.15	-122.444	37.942	0.22	18.92	290.23	19.14	9	7/14/2014 33:05.8
10393	RSPe_2	-122.444	37.942	0.23	18.84	290.20	19.07	-122.444	37.942	0.18	18.84	290.26	19.02	9	7/14/2014 33:05.7
10394	RSPe_2	-122.444	37.942	0.24	18.78	290.14	19.02	-122.444	37.942	0.18	18.78	290.33	18.96	9	7/14/2014 33:05.6

10395	RSPe_2	-122.444	37.942	0.24	18.82	290.09	19.05	-122.444	37.942	0.18	18.82	290.35	19.00	9	7/14/2014	33:05.5
10396	RSPe_2	-122.444	37.942	0.24	18.72	290.11	18.95	-122.444	37.942	0.18	18.72	290.37	18.90	9	7/14/2014	33:05.4
10397	RSPe_2	-122.444	37.942	0.24	18.73	290.07	18.96	-122.444	37.942	0.18	18.73	290.33	18.91	9	7/14/2014	33:05.3
10398	RSPe_2	-122.444	37.942	0.24	18.79	290.09	19.02	-122.444	37.942	0.13	18.79	290.29	18.92	9	7/14/2014	33:05.2
10399	RSPe_2	-122.444	37.942	0.24	18.59	290.03	18.83	-122.444	37.942	0.13	18.59	290.27	18.73	9	7/14/2014	33:05.1
10400	RSPe_2	-122.444	37.942	0.24	18.62	290.03	18.86	-122.444	37.942	0.13	18.62	290.21	18.75	9	7/14/2014	33:05.0
10401	RSPe_2	-122.444	37.942	0.24	18.54	289.94	18.78	-122.444	37.942	0.13	18.54	290.15	18.67	9	7/14/2014	33:04.9
10402	RSPe_2	-122.444	37.942	0.20	18.53	289.96	18.74	-122.444	37.942	0.13	18.53	290.12	18.67	9	7/14/2014	33:04.8
10403	RSPe_2	-122.444	37.942	0.24	18.49	289.86	18.72	-122.444	37.942	0.13	18.49	290.10	18.62	9	7/14/2014	33:04.7
10404	RSPe_2	-122.444	37.942	0.20	18.50	289.84	18.70	-122.444	37.942	0.13	18.50	290.09	18.63	9	7/14/2014	33:04.6
10405	RSPe_2	-122.444	37.942	0.24	18.44	289.81	18.68	-122.444	37.942	0.13	18.44	290.07	18.57	9	7/14/2014	33:04.5
10406	RSPe_2	-122.444	37.942	0.20	18.46	289.79	18.66	-122.444	37.942	0.10	18.46	290.12	18.55	9	7/14/2014	33:04.4
10407	RSPe_2	-122.444	37.942	0.24	18.43	289.84	18.67	-122.444	37.942	0.13	18.43	290.14	18.57	9	7/14/2014	33:04.3
10408	RSPe_2	-122.444	37.942	0.20	18.53	289.84	18.74	-122.444	37.942	0.05	18.53	290.17	18.58	9	7/14/2014	33:04.2
10409	RSPe_2	-122.444	37.942	0.20	18.48	289.86	18.68	-122.444	37.942	0.05	18.48	290.14	18.53	9	7/14/2014	33:04.1
10410	RSPe_2	-122.444	37.942	0.20	18.48	289.93	18.68	-122.444	37.942	0.01	18.48	290.18	18.49	9	7/14/2014	33:04.0
10411	RSPe_2	-122.444	37.942	0.20	18.49	289.99	18.69	-122.444	37.942	0.01	18.49	290.17	18.50	9	7/14/2014	33:03.9
10412	RSPe_2	-122.444	37.942	0.15	18.51	289.99	18.66	-122.444	37.942	0.01	18.51	290.16	18.52	9	7/14/2014	33:03.8
10413	RSPe_2	-122.444	37.942	0.20	18.52	290.03	18.73	-122.444	37.942	0.05	18.52	290.14	18.57	9	7/14/2014	33:03.7
10414	RSPe_2	-122.444	37.942	0.15	18.52	289.98	18.67	-122.444	37.942	0.01	18.52	290.06	18.54	9	7/14/2014	33:03.6
10415	RSPe_2	-122.444	37.942	0.15	18.48	289.93	18.63	-122.444	37.942	-0.04	18.48	290.01	18.44	9	7/14/2014	33:03.5
10416	RSPe_2	-122.444	37.942	0.15	18.44	289.91	18.59	-122.444	37.942	0.01	18.44	290.00	18.45	9	7/14/2014	33:03.4
10417	RSPe_2	-122.444	37.942	0.15	18.50	289.86	18.65	-122.444	37.942	0.01	18.50	289.95	18.51	9	7/14/2014	33:03.3
10418	RSPe_2	-122.444	37.942	0.15	18.50	289.89	18.65	-122.444	37.942	-0.04	18.50	289.99	18.47	9	7/14/2014	33:03.2
10419	RSPe_2	-122.444	37.942	0.15	18.45	289.93	18.60	-122.444	37.942	-0.04	18.45	289.98	18.41	9	7/14/2014	33:03.1
10420	RSPe_2	-122.444	37.942	0.12	18.47	289.98	18.59	-122.444	37.942	-0.04	18.47	290.02	18.43	9	7/14/2014	33:03.0
10421	RSPe_2	-122.444	37.942	0.15	18.55	290.00	18.70	-122.444	37.942	-0.04	18.55	290.06	18.51	9	7/14/2014	33:02.9
10422	RSPe_2	-122.444	37.942	0.15	18.54	290.02	18.69	-122.444	37.942	-0.04	18.54	290.11	18.50	9	7/14/2014	33:02.8
10423	RSPe_2	-122.444	37.942	0.15	18.60	290.13	18.75	-122.444	37.942	-0.04	18.60	290.22	18.57	9	7/14/2014	33:02.7
10424	RSPe_2	-122.444	37.942	0.15	18.60	290.15	18.75	-122.444	37.942	-0.04	18.60	290.28	18.57	9	7/14/2014	33:02.6
10425	RSPe_2	-122.444	37.942	0.15	18.60	290.14	18.75	-122.444	37.942	-0.07	18.60	290.31	18.53	9	7/14/2014	33:02.5
10426	RSPe_2	-122.444	37.942	0.12	18.57	290.09	18.68	-122.444	37.942	-0.12	18.57	290.28	18.44	9	7/14/2014	33:02.4
10427	RSPe_2	-122.444	37.942	0.15	18.67	290.07	18.82	-122.444	37.942	-0.12	18.67	290.33	18.55	9	7/14/2014	33:02.3

10428	RSPe_2	-122.444	37.942	0.12	18.71	290.01	18.83	-122.444	37.942	-0.16	18.71	290.30	18.56	9	7/14/2014	33:02.2
10429	RSPe_2	-122.444	37.942	0.12	18.77	290.02	18.89	-122.444	37.942	-0.12	18.77	290.33	18.65	9	7/14/2014	33:02.1
10430	RSPe_2	-122.444	37.942	0.12	18.95	290.03	19.07	-122.444	37.942	-0.16	18.95	290.31	18.80	9	7/14/2014	33:02.0
10431	RSPe_2	-122.444	37.942	0.12	18.92	289.98	19.04	-122.444	37.942	-0.12	18.92	290.23	18.80	9	7/14/2014	33:01.9
10432	RSPe_2	-122.444	37.942	0.06	18.97	290.12	19.03	-122.444	37.942	-0.12	18.97	290.24	18.84	9	7/14/2014	33:01.8
10433	RSPe_2	-122.444	37.942	0.12	19.09	290.19	19.21	-122.444	37.942	-0.12	19.09	290.23	18.97	9	7/14/2014	33:01.7
10434	RSPe_2	-122.444	37.942	0.12	19.13	290.23	19.24	-122.444	37.942	-0.19	19.13	290.20	18.94	9	7/14/2014	33:01.6
10435	RSPe_2	-122.444	37.942	0.12	19.14	290.23	19.25	-122.444	37.942	-0.12	19.14	290.16	19.01	9	7/14/2014	33:01.5
10436	RSPe_2	-122.444	37.942	0.12	19.12	290.23	19.23	-122.444	37.942	-0.16	19.12	290.20	18.96	9	7/14/2014	33:01.4
10437	RSPe_2	-122.444	37.942	0.12	19.21	290.23	19.33	-122.444	37.942	-0.12	19.21	290.23	19.09	9	7/14/2014	33:01.3
10438	RSPe_2	-122.444	37.942	0.12	19.22	290.15	19.33	-122.444	37.942	-0.16	19.22	290.31	19.06	9	7/14/2014	33:01.2
10439	RSPe_2	-122.444	37.942	0.12	19.22	290.16	19.33	-122.444	37.942	-0.16	19.22	290.38	19.06	9	7/14/2014	33:01.1
10440	RSPe_2	-122.444	37.942	0.06	19.26	290.08	19.32	-122.444	37.942	-0.19	19.26	290.36	19.07	9	7/14/2014	33:01.0
10441	RSPe_2	-122.444	37.942	0.12	19.25	290.08	19.37	-122.444	37.942	-0.19	19.25	290.32	19.06	9	7/14/2014	33:00.9
10442	RSPe_2	-122.444	37.942	0.06	19.31	289.97	19.37	-122.444	37.942	-0.19	19.31	290.13	19.12	9	7/14/2014	33:00.8
10443	RSPe_2	-122.444	37.942	0.12	19.33	290.00	19.44	-122.444	37.942	-0.19	19.33	290.12	19.14	9	7/14/2014	33:00.7
10444	RSPe_2	-122.444	37.942	0.03	19.33	290.08	19.36	-122.444	37.942	-0.19	19.33	290.10	19.14	9	7/14/2014	33:00.6
10445	RSPe_2	-122.444	37.942	0.06	19.37	290.08	19.43	-122.444	37.942	-0.24	19.37	290.10	19.12	9	7/14/2014	33:00.5
10446	RSPe_2	-122.444	37.942	0.06	19.41	289.99	19.48	-122.444	37.942	-0.24	19.41	290.04	19.17	9	7/14/2014	33:00.4
10447	RSPe_2	-122.444	37.942	0.06	19.45	289.95	19.52	-122.444	37.942	-0.28	19.45	290.10	19.18	9	7/14/2014	33:00.3
10448	RSPe_2	-122.444	37.942	0.06	19.53	290.00	19.59	-122.444	37.942	-0.33	19.53	290.18	19.20	9	7/14/2014	33:00.2
10449	RSPe_2	-122.444	37.942	0.06	19.53	290.06	19.59	-122.444	37.942	-0.24	19.53	290.23	19.29	9	7/14/2014	33:00.1
10450	RSPe_2	-122.444	37.942	0.03	19.52	290.09	19.55	-122.444	37.942	-0.24	19.52	290.26	19.28	9	7/14/2014	33:00.0
10451	RSPe_2	-122.444	37.942	0.06	19.57	290.02	19.63	-122.444	37.942	-0.24	19.57	290.17	19.33	9	7/14/2014	32:59.9
10452	RSPe_2	-122.444	37.942	0.03	19.59	289.98	19.62	-122.444	37.942	-0.28	19.59	290.11	19.32	9	7/14/2014	32:59.8
10453	RSPe_2	-122.444	37.942	0.06	19.62	289.93	19.69	-122.444	37.942	-0.24	19.62	290.07	19.38	9	7/14/2014	32:59.7
10454	RSPe_2	-122.444	37.942	0.06	19.61	289.96	19.68	-122.444	37.942	-0.28	19.61	290.05	19.34	9	7/14/2014	32:59.6
10455	RSPe_2	-122.444	37.942	0.06	19.61	290.04	19.68	-122.444	37.942	-0.24	19.61	290.06	19.37	9	7/14/2014	32:59.5
10456	RSPe_2	-122.444	37.942	0.06	19.59	290.05	19.65	-122.444	37.942	-0.28	19.59	290.04	19.32	9	7/14/2014	32:59.4
10457	RSPe_2	-122.444	37.942	0.06	19.60	290.09	19.66	-122.444	37.942	-0.28	19.60	290.02	19.33	9	7/14/2014	32:59.3
10458	RSPe_2	-122.444	37.942	0.03	19.51	290.05	19.54	-122.444	37.942	-0.33	19.51	290.02	19.19	9	7/14/2014	32:59.2
10459	RSPe_2	-122.444	37.942	0.06	19.49	290.03	19.55	-122.444	37.942	-0.28	19.49	290.03	19.21	9	7/14/2014	32:59.1
10460	RSPe_2	-122.444	37.942	0.06	19.54	289.92	19.60	-122.444	37.942	-0.33	19.54	290.02	19.21	9	7/14/2014	32:59.0

10461	RSPe_2	-122.444	37.942	0.06	19.39	289.90	19.46	-122.444	37.942	-0.28	19.39	290.04	19.12	9	7/14/2014 32:58.9
10462	RSPe_2	-122.444	37.942	0.06	19.39	289.87	19.46	-122.444	37.942	-0.28	19.39	289.99	19.12	9	7/14/2014 32:58.8
10463	RSPe_2	-122.444	37.942	0.06	19.28	289.86	19.35	-122.444	37.942	-0.28	19.28	289.99	19.01	9	7/14/2014 32:58.7
10464	RSPe_2	-122.444	37.942	0.06	19.29	289.90	19.35	-122.444	37.942	-0.28	19.29	289.98	19.02	9	7/14/2014 32:58.6
10465	RSPe_2	-122.444	37.942	0.06	19.27	289.92	19.34	-122.444	37.942	-0.28	19.27	289.94	19.00	9	7/14/2014 32:58.5
10466	RSPe_2	-122.444	37.942	0.06	19.25	289.94	19.32	-122.444	37.942	-0.28	19.25	289.88	18.98	9	7/14/2014 32:58.4
10467	RSPe_2	-122.444	37.942	0.06	19.26	289.95	19.32	-122.444	37.942	-0.28	19.26	289.94	18.98	9	7/14/2014 32:58.3
10468	RSPe_2	-122.444	37.942	0.06	19.24	289.90	19.30	-122.444	37.942	-0.24	19.24	289.87	19.00	9	7/14/2014 32:58.2
10469	RSPe_2	-122.444	37.942	0.06	19.24	289.90	19.31	-122.444	37.942	-0.33	19.24	289.88	18.92	9	7/14/2014 32:58.1
10470	RSPe_2	-122.444	37.942	0.06	19.24	289.81	19.30	-122.444	37.942	-0.28	19.24	289.88	18.96	9	7/14/2014 32:58.0
10471	RSPe_2	-122.444	37.942	0.06	19.12	289.75	19.18	-122.444	37.942	-0.28	19.12	289.81	18.84	9	7/14/2014 32:57.9
10472	RSPe_2	-122.444	37.942	0.06	19.02	289.81	19.08	-122.444	37.942	-0.19	19.02	289.92	18.83	9	7/14/2014 32:57.8
10473	RSPe_2	-122.444	37.942	0.06	19.00	289.77	19.06	-122.444	37.942	-0.24	19.00	289.88	18.76	9	7/14/2014 32:57.7
10474	RSPe_2	-122.444	37.942	0.06	19.00	289.73	19.06	-122.444	37.942	-0.28	19.00	289.86	18.72	9	7/14/2014 32:57.6
10475	RSPe_2	-122.444	37.942	0.12	18.89	289.78	19.00	-122.444	37.942	-0.24	18.89	289.86	18.65	9	7/14/2014 32:57.5
10476	RSPe_2	-122.444	37.942	0.06	18.89	289.75	18.95	-122.444	37.942	-0.28	18.89	289.83	18.61	9	7/14/2014 32:57.4
10477	RSPe_2	-122.444	37.942	0.12	18.89	289.80	19.00	-122.444	37.942	-0.24	18.89	289.84	18.65	9	7/14/2014 32:57.3
10478	RSPe_2	-122.444	37.942	0.06	18.87	289.80	18.94	-122.444	37.942	-0.24	18.87	289.83	18.63	9	7/14/2014 32:57.2
10479	RSPe_2	-122.444	37.942	0.06	18.95	289.84	19.02	-122.444	37.942	-0.28	18.95	289.81	18.68	9	7/14/2014 32:57.1
10480	RSPe_2	-122.444	37.942	0.06	18.83	289.86	18.89	-122.444	37.942	-0.24	18.83	289.82	18.59	9	7/14/2014 32:57.0
10481	RSPe_2	-122.444	37.942	0.12	18.79	289.85	18.90	-122.444	37.942	-0.24	18.79	289.78	18.55	9	7/14/2014 32:56.9
10482	RSPe_2	-122.444	37.942	0.06	18.80	289.84	18.87	-122.444	37.942	-0.24	18.80	289.77	18.56	9	7/14/2014 32:56.8
10483	RSPe_2	-122.444	37.942	0.12	18.79	289.85	18.90	-122.444	37.942	-0.19	18.79	289.69	18.60	9	7/14/2014 32:56.7
10484	RSPe_2	-122.444	37.942	0.06	18.72	289.84	18.78	-122.444	37.942	-0.24	18.72	289.71	18.48	9	7/14/2014 32:56.6
10485	RSPe_2	-122.444	37.942	0.12	18.75	289.81	18.87	-122.444	37.942	-0.16	18.75	289.71	18.59	9	7/14/2014 32:56.5
10486	RSPe_2	-122.444	37.942	0.06	18.73	289.80	18.80	-122.444	37.942	-0.19	18.73	289.73	18.54	9	7/14/2014 32:56.4
10487	RSPe_2	-122.444	37.942	0.12	18.80	289.80	18.92	-122.444	37.942	-0.24	18.80	289.78	18.56	9	7/14/2014 32:56.3
10488	RSPe_2	-122.444	37.942	0.06	18.82	289.76	18.88	-122.444	37.942	-0.24	18.82	289.78	18.58	9	7/14/2014 32:56.2
10489	RSPe_2	-122.444	37.942	0.12	18.78	289.78	18.90	-122.444	37.942	-0.19	18.78	289.85	18.59	9	7/14/2014 32:56.1
10490	RSPe_2	-122.444	37.942	0.06	18.80	289.85	18.86	-122.444	37.942	-0.24	18.80	289.98	18.56	9	7/14/2014 32:56.0
10491	RSPe_2	-122.444	37.942	0.12	18.85	289.87	18.97	-122.444	37.942	-0.19	18.85	289.96	18.66	9	7/14/2014 32:55.9
10492	RSPe_2	-122.444	37.942	0.12	18.83	289.88	18.95	-122.444	37.942	-0.19	18.83	289.98	18.64	9	7/14/2014 32:55.8
10493	RSPe_2	-122.444	37.942	0.06	18.88	289.79	18.95	-122.444	37.942	-0.16	18.88	289.98	18.73	9	7/14/2014 32:55.7

10494	RSPe_2	-122.444	37.942	0.12	18.90	289.75	19.02	-122.444	37.942	-0.19	18.90	289.92	18.71	9	7/14/2014	32:55.6
10495	RSPe_2	-122.444	37.942	0.12	18.89	289.70	19.00	-122.444	37.942	-0.19	18.89	289.92	18.70	9	7/14/2014	32:55.5
10496	RSPe_2	-122.444	37.942	0.06	18.90	289.62	18.97	-122.444	37.942	-0.19	18.90	289.89	18.71	9	7/14/2014	32:55.4
10497	RSPe_2	-122.444	37.942	0.12	18.99	289.61	19.10	-122.444	37.942	-0.16	18.99	289.81	18.83	9	7/14/2014	32:55.3
10498	RSPe_2	-122.444	37.942	0.06	18.94	289.59	19.01	-122.444	37.942	-0.19	18.94	289.81	18.75	9	7/14/2014	32:55.2
10499	RSPe_2	-122.444	37.942	0.12	18.95	289.60	19.07	-122.444	37.942	-0.12	18.95	289.76	18.83	9	7/14/2014	32:55.1
10500	RSPe_2	-122.444	37.942	0.12	18.94	289.57	19.05	-122.444	37.942	-0.19	18.94	289.81	18.75	9	7/14/2014	32:55.0
10501	RSPe_2	-122.444	37.942	0.15	18.99	289.62	19.14	-122.444	37.942	-0.16	18.99	289.76	18.83	9	7/14/2014	32:54.9
10502	RSPe_2	-122.444	37.942	0.12	18.98	289.46	19.09	-122.444	37.942	-0.16	18.98	289.69	18.82	9	7/14/2014	32:54.8
10503	RSPe_2	-122.444	37.942	0.15	19.03	289.60	19.18	-122.444	37.942	-0.16	19.03	289.77	18.88	9	7/14/2014	32:54.7
10504	RSPe_2	-122.444	37.942	0.12	19.01	289.67	19.13	-122.444	37.942	-0.16	19.01	289.83	18.86	9	7/14/2014	32:54.6
10505	RSPe_2	-122.444	37.942	0.12	19.03	289.66	19.14	-122.444	37.942	-0.12	19.03	289.82	18.90	9	7/14/2014	32:54.5
10506	RSPe_2	-122.444	37.942	0.12	19.02	289.60	19.14	-122.444	37.942	-0.16	19.02	289.75	18.86	9	7/14/2014	32:54.4
10507	RSPe_2	-122.444	37.942	0.12	19.04	289.58	19.16	-122.444	37.942	-0.16	19.04	289.71	18.89	9	7/14/2014	32:54.3
10508	RSPe_2	-122.444	37.942	0.12	19.12	289.58	19.23	-122.444	37.942	-0.19	19.12	289.69	18.93	9	7/14/2014	32:54.2
10509	RSPe_2	-122.444	37.942	0.15	19.07	289.60	19.22	-122.444	37.942	-0.16	19.07	289.62	18.91	9	7/14/2014	32:54.1
10510	RSPe_2	-122.444	37.942	0.12	19.07	289.58	19.18	-122.444	37.942	-0.19	19.07	289.57	18.88	9	7/14/2014	32:54.0
10511	RSPe_2	-122.444	37.942	0.12	19.10	289.58	19.22	-122.444	37.942	-0.16	19.10	289.56	18.95	9	7/14/2014	32:53.9
10512	RSPe_2	-122.444	37.942	0.12	19.15	289.67	19.27	-122.444	37.942	-0.24	19.15	289.57	18.91	9	7/14/2014	32:53.8
10513	RSPe_2	-122.444	37.942	0.15	19.17	289.73	19.32	-122.444	37.942	-0.19	19.17	289.60	18.98	9	7/14/2014	32:53.7
10514	RSPe_2	-122.444	37.942	0.12	19.24	289.72	19.36	-122.444	37.942	-0.24	19.24	289.62	19.00	9	7/14/2014	32:53.6
10515	RSPe_2	-122.444	37.942	0.15	19.17	289.76	19.32	-122.444	37.942	-0.19	19.17	289.71	18.98	9	7/14/2014	32:53.5
10516	RSPe_2	-122.444	37.942	0.12	19.18	289.67	19.30	-122.444	37.942	-0.16	19.18	289.71	19.03	9	7/14/2014	32:53.4
10517	RSPe_2	-122.444	37.942	0.15	19.20	289.60	19.36	-122.444	37.942	-0.16	19.20	289.80	19.05	9	7/14/2014	32:53.3
10518	RSPe_2	-122.444	37.942	0.15	19.23	289.54	19.38	-122.444	37.942	-0.16	19.23	289.78	19.07	9	7/14/2014	32:53.2
10519	RSPe_2	-122.444	37.942	0.15	19.26	289.45	19.41	-122.444	37.942	-0.12	19.26	289.76	19.14	9	7/14/2014	32:53.1
10520	RSPe_2	-122.444	37.942	0.15	19.28	289.48	19.44	-122.444	37.942	-0.12	19.28	289.80	19.16	9	7/14/2014	32:53.0
10521	RSPe_2	-122.444	37.942	0.15	19.38	289.43	19.53	-122.444	37.942	-0.12	19.38	289.70	19.25	9	7/14/2014	32:52.9
10522	RSPe_2	-122.444	37.942	0.15	19.38	289.39	19.53	-122.444	37.942	-0.16	19.38	289.69	19.22	9	7/14/2014	32:52.8
10523	RSPe_2	-122.444	37.942	0.15	19.36	289.41	19.51	-122.444	37.942	-0.16	19.36	289.58	19.21	9	7/14/2014	32:52.7
10524	RSPe_2	-122.444	37.942	0.15	19.41	289.44	19.56	-122.444	37.942	-0.16	19.41	289.52	19.25	9	7/14/2014	32:52.6
10525	RSPe_2	-122.444	37.942	0.15	19.39	289.48	19.54	-122.444	37.942	-0.12	19.39	289.52	19.27	9	7/14/2014	32:52.5
10526	RSPe_2	-122.444	37.942	0.15	19.41	289.44	19.56	-122.444	37.942	-0.16	19.41	289.43	19.25	9	7/14/2014	32:52.4

10527	RSPe_2	-122.444	37.942	0.15	19.41	289.57	19.56	-122.444	37.942	-0.12	19.41	289.65	19.28	9	7/14/2014	32:52.3
10528	RSPe_2	-122.444	37.942	0.12	19.46	289.70	19.58	-122.444	37.942	-0.16	19.46	289.82	19.31	9	7/14/2014	32:52.2
10529	RSPe_2	-122.444	37.942	0.15	19.43	289.66	19.58	-122.444	37.942	-0.16	19.43	289.90	19.27	9	7/14/2014	32:52.1
10530	RSPe_2	-122.444	37.942	0.15	19.43	289.64	19.59	-122.444	37.942	-0.16	19.43	289.92	19.28	9	7/14/2014	32:52.0
10531	RSPe_2	-122.444	37.942	0.20	19.42	289.61	19.63	-122.444	37.942	-0.16	19.42	289.90	19.27	9	7/14/2014	32:51.9
10532	RSPe_2	-122.444	37.942	0.15	19.42	289.53	19.57	-122.444	37.942	-0.16	19.42	289.90	19.27	9	7/14/2014	32:51.8
10533	RSPe_2	-122.444	37.942	0.20	19.46	289.55	19.66	-122.444	37.942	-0.12	19.46	289.77	19.34	9	7/14/2014	32:51.7
10534	RSPe_2	-122.444	37.942	0.15	19.41	289.51	19.56	-122.444	37.942	-0.16	19.41	289.71	19.26	9	7/14/2014	32:51.6
10535	RSPe_2	-122.444	37.942	0.20	19.37	289.55	19.57	-122.444	37.942	-0.12	19.37	289.71	19.24	9	7/14/2014	32:51.5
10536	RSPe_2	-122.444	37.942	0.20	19.39	289.64	19.59	-122.444	37.942	-0.16	19.39	289.73	19.24	9	7/14/2014	32:51.4
10537	RSPe_2	-122.444	37.942	0.20	19.33	289.62	19.53	-122.444	37.942	-0.12	19.33	289.71	19.21	9	7/14/2014	32:51.3
10538	RSPe_2	-122.444	37.942	0.15	19.39	289.71	19.54	-122.444	37.942	-0.12	19.39	289.73	19.27	9	7/14/2014	32:51.2
10539	RSPe_2	-122.444	37.942	0.20	19.31	289.78	19.51	-122.444	37.942	-0.12	19.31	289.86	19.19	9	7/14/2014	32:51.1
10540	RSPe_2	-122.444	37.942	0.15	19.34	289.82	19.49	-122.444	37.942	-0.12	19.34	289.86	19.21	9	7/14/2014	32:51.0
10541	RSPe_2	-122.444	37.942	0.20	19.30	289.82	19.50	-122.444	37.942	-0.12	19.30	289.93	19.17	9	7/14/2014	32:50.9
10542	RSPe_2	-122.444	37.942	0.20	19.34	289.85	19.55	-122.444	37.942	-0.12	19.34	289.93	19.22	9	7/14/2014	32:50.8
10543	RSPe_2	-122.444	37.942	0.20	19.20	289.82	19.41	-122.444	37.942	-0.12	19.20	290.00	19.08	9	7/14/2014	32:50.7
10544	RSPe_2	-122.444	37.942	0.15	19.18	289.80	19.33	-122.444	37.942	-0.12	19.18	290.02	19.06	9	7/14/2014	32:50.6
10545	RSPe_2	-122.444	37.942	0.20	19.20	289.80	19.41	-122.444	37.942	-0.12	19.20	290.02	19.08	9	7/14/2014	32:50.5
10546	RSPe_2	-122.444	37.942	0.20	19.27	289.79	19.47	-122.444	37.942	-0.16	19.27	290.06	19.11	9	7/14/2014	32:50.4
10547	RSPe_2	-122.444	37.942	0.24	19.15	289.83	19.39	-122.444	37.942	-0.12	19.15	290.04	19.03	9	7/14/2014	32:50.3
10548	RSPe_2	-122.444	37.942	0.20	19.13	289.79	19.33	-122.444	37.942	-0.12	19.13	290.07	19.01	9	7/14/2014	32:50.2
10549	RSPe_2	-122.444	37.942	0.20	19.15	289.87	19.35	-122.444	37.942	-0.16	19.15	290.13	19.00	9	7/14/2014	32:50.1
10550	RSPe_2	-122.444	37.942	0.20	19.31	289.89	19.51	-122.444	37.942	-0.16	19.31	290.11	19.15	9	7/14/2014	32:50.0
10551	RSPe_2	-122.444	37.942	0.24	19.20	289.89	19.43	-122.444	37.942	-0.12	19.20	290.12	19.08	9	7/14/2014	32:49.9
10552	RSPe_2	-122.444	37.942	0.20	19.13	289.96	19.33	-122.444	37.942	-0.12	19.13	290.18	19.01	9	7/14/2014	32:49.8
10553	RSPe_2	-122.444	37.942	0.24	18.99	289.98	19.23	-122.444	37.942	-0.07	18.99	290.22	18.92	9	7/14/2014	32:49.7
10554	RSPe_2	-122.444	37.942	0.20	19.03	290.05	19.23	-122.444	37.942	-0.12	19.03	290.22	18.91	9	7/14/2014	32:49.6
10555	RSPe_2	-122.444	37.942	0.20	18.99	290.11	19.19	-122.444	37.942	-0.12	18.99	290.24	18.87	9	7/14/2014	32:49.5
10556	RSPe_2	-122.444	37.942	0.20	18.97	290.17	19.17	-122.444	37.942	-0.16	18.97	290.23	18.81	9	7/14/2014	32:49.4
10557	RSPe_2	-122.444	37.942	0.20	18.97	290.18	19.18	-122.444	37.942	-0.12	18.97	290.22	18.85	9	7/14/2014	32:49.3
10558	RSPe_2	-122.444	37.942	0.20	18.88	290.21	19.08	-122.444	37.942	-0.16	18.88	290.14	18.73	9	7/14/2014	32:49.2
10559	RSPe_2	-122.444	37.942	0.24	18.85	290.25	19.09	-122.444	37.942	-0.12	18.85	290.16	18.73	9	7/14/2014	32:49.1

10560	RSPe_2	-122.444	37.942	0.20	18.75	290.23	18.95	-122.444	37.942	-0.16	18.75	290.11	18.60	9	7/14/2014	32:49.0
10561	RSPe_2	-122.444	37.942	0.24	18.78	290.28	19.02	-122.444	37.942	-0.12	18.78	290.10	18.66	9	7/14/2014	32:48.9
10562	RSPe_2	-122.444	37.942	0.20	18.70	290.21	18.90	-122.444	37.942	-0.12	18.70	290.10	18.57	9	7/14/2014	32:48.8
10563	RSPe_2	-122.444	37.942	0.24	18.70	290.25	18.94	-122.444	37.942	-0.12	18.70	290.12	18.58	9	7/14/2014	32:48.7
10564	RSPe_2	-122.444	37.942	0.20	18.66	290.30	18.87	-122.444	37.942	-0.12	18.66	290.23	18.54	9	7/14/2014	32:48.6
10565	RSPe_2	-122.444	37.942	0.24	18.73	290.30	18.96	-122.444	37.942	-0.12	18.73	290.28	18.61	9	7/14/2014	32:48.5
10566	RSPe_2	-122.444	37.942	0.24	18.66	290.32	18.89	-122.444	37.942	-0.12	18.66	290.38	18.54	9	7/14/2014	32:48.4
10567	RSPe_2	-122.444	37.942	0.24	18.67	290.30	18.91	-122.444	37.942	-0.12	18.67	290.43	18.55	9	7/14/2014	32:48.3
10568	RSPe_2	-122.444	37.942	0.24	18.70	290.32	18.93	-122.444	37.942	-0.12	18.70	290.54	18.57	9	7/14/2014	32:48.2
10569	RSPe_2	-122.444	37.942	0.24	18.66	290.24	18.89	-122.444	37.942	-0.12	18.66	290.54	18.54	9	7/14/2014	32:48.1
10570	RSPe_2	-122.444	37.942	0.24	18.65	290.15	18.89	-122.444	37.942	-0.16	18.65	290.54	18.49	9	7/14/2014	32:48.0
10571	RSPe_2	-122.444	37.942	0.24	18.69	290.13	18.93	-122.444	37.942	-0.12	18.69	290.55	18.57	9	7/14/2014	32:47.9
10572	RSPe_2	-122.444	37.942	0.24	18.73	290.22	18.97	-122.444	37.942	-0.12	18.73	290.59	18.61	9	7/14/2014	32:47.8
10573	RSPe_2	-122.444	37.942	0.24	18.68	290.24	18.92	-122.444	37.942	-0.16	18.68	290.61	18.53	9	7/14/2014	32:47.7
10574	RSPe_2	-122.444	37.942	0.24	18.75	290.22	18.99	-122.444	37.942	-0.19	18.75	290.59	18.56	9	7/14/2014	32:47.6
10575	RSPe_2	-122.444	37.942	0.24	18.72	290.35	18.95	-122.444	37.942	-0.16	18.72	290.53	18.56	9	7/14/2014	32:47.5
10576	RSPe_2	-122.444	37.942	0.24	18.72	290.31	18.95	-122.444	37.942	-0.12	18.72	290.48	18.60	9	7/14/2014	32:47.4
10577	RSPe_2	-122.444	37.942	0.27	18.70	290.38	18.97	-122.444	37.942	-0.07	18.70	290.44	18.62	9	7/14/2014	32:47.3
10578	RSPe_2	-122.444	37.942	0.27	18.60	290.46	18.87	-122.444	37.942	-0.12	18.60	290.48	18.48	9	7/14/2014	32:47.2
10579	RSPe_2	-122.444	37.942	0.27	18.63	290.58	18.90	-122.444	37.942	-0.04	18.63	290.52	18.59	9	7/14/2014	32:47.1
10580	RSPe_2	-122.444	37.942	0.24	18.68	290.49	18.92	-122.444	37.942	-0.07	18.68	290.53	18.61	9	7/14/2014	32:47.0
10581	RSPe_2	-122.444	37.942	0.27	18.57	290.51	18.84	-122.444	37.942	-0.04	18.57	290.52	18.53	9	7/14/2014	32:46.9
10582	RSPe_2	-122.444	37.942	0.27	18.53	290.40	18.80	-122.444	37.942	-0.12	18.53	290.44	18.41	9	7/14/2014	32:46.8
10583	RSPe_2	-122.444	37.942	0.27	18.59	290.32	18.86	-122.444	37.942	-0.07	18.59	290.38	18.52	9	7/14/2014	32:46.7
10584	RSPe_2	-122.444	37.942	0.27	18.54	290.23	18.81	-122.444	37.942	-0.12	18.54	290.35	18.42	9	7/14/2014	32:46.6
10585	RSPe_2	-122.444	37.942	0.32	18.54	290.33	18.86	-122.444	37.942	-0.12	18.54	290.40	18.42	9	7/14/2014	32:46.5
10586	RSPe_2	-122.444	37.942	0.27	18.51	290.43	18.78	-122.444	37.942	-0.07	18.51	290.44	18.44	9	7/14/2014	32:46.4
10587	RSPe_2	-122.444	37.942	0.27	18.56	290.40	18.83	-122.444	37.942	-0.07	18.56	290.42	18.49	9	7/14/2014	32:46.3
10588	RSPe_2	-122.444	37.942	0.27	18.57	290.53	18.84	-122.444	37.942	-0.07	18.57	290.49	18.49	9	7/14/2014	32:46.2
10589	RSPe_2	-122.444	37.942	0.27	18.60	290.56	18.87	-122.444	37.942	-0.07	18.60	290.56	18.53	9	7/14/2014	32:46.1
10590	RSPe_2	-122.444	37.942	0.32	18.63	290.61	18.95	-122.444	37.942	-0.04	18.63	290.53	18.60	9	7/14/2014	32:46.0
10591	RSPe_2	-122.444	37.942	0.32	18.65	290.67	18.97	-122.444	37.942	-0.04	18.65	290.63	18.61	9	7/14/2014	32:45.9
10592	RSPe_2	-122.444	37.942	0.27	18.64	290.58	18.91	-122.444	37.942	-0.07	18.64	290.55	18.57	9	7/14/2014	32:45.8

10593	RSPe_2	-122.444	37.942	0.32	18.64	290.56	18.96	-122.444	37.942	-0.04	18.64	290.56	18.61	9	7/14/2014	32:45.7
10594	RSPe_2	-122.444	37.942	0.27	18.69	290.50	18.96	-122.444	37.942	-0.12	18.69	290.52	18.57	9	7/14/2014	32:45.6
10595	RSPe_2	-122.444	37.942	0.32	18.68	290.48	19.00	-122.444	37.942	-0.04	18.68	290.56	18.64	9	7/14/2014	32:45.5
10596	RSPe_2	-122.444	37.942	0.32	18.69	290.41	19.01	-122.444	37.942	-0.04	18.69	290.54	18.65	9	7/14/2014	32:45.4
10597	RSPe_2	-122.444	37.942	0.32	18.77	290.39	19.09	-122.444	37.942	-0.04	18.77	290.58	18.73	9	7/14/2014	32:45.3
10598	RSPe_2	-122.444	37.942	0.32	18.76	290.39	19.08	-122.444	37.942	-0.04	18.76	290.62	18.72	9	7/14/2014	32:45.2
10599	RSPe_2	-122.444	37.942	0.32	18.84	290.46	19.16	-122.444	37.942	-0.04	18.84	290.72	18.80	9	7/14/2014	32:45.1
10600	RSPe_2	-122.444	37.942	0.32	18.86	290.53	19.18	-122.444	37.942	-0.04	18.86	290.72	18.82	9	7/14/2014	32:45.0
10601	RSPe_2	-122.444	37.942	0.36	18.90	290.57	19.26	-122.444	37.942	0.01	18.90	290.70	18.92	9	7/14/2014	32:44.9
10602	RSPe_2	-122.444	37.942	0.32	18.90	290.61	19.22	-122.444	37.942	-0.04	18.90	290.68	18.86	9	7/14/2014	32:44.8
10603	RSPe_2	-122.444	37.942	0.36	18.92	290.61	19.28	-122.444	37.942	0.01	18.92	290.61	18.93	9	7/14/2014	32:44.7
10604	RSPe_2	-122.444	37.942	0.32	18.89	290.55	19.21	-122.444	37.942	-0.04	18.89	290.45	18.85	9	7/14/2014	32:44.6
10605	RSPe_2	-122.444	37.942	0.36	18.93	290.60	19.28	-122.444	37.942	0.01	18.93	290.46	18.94	9	7/14/2014	32:44.5
10606	RSPe_2	-122.444	37.942	0.36	18.94	290.53	19.29	-122.444	37.942	0.01	18.94	290.39	18.95	9	7/14/2014	32:44.4
10607	RSPe_2	-122.444	37.942	0.36	18.99	290.51	19.35	-122.444	37.942	0.05	18.99	290.33	19.04	9	7/14/2014	32:44.3
10608	RSPe_2	-122.444	37.942	0.36	18.97	290.48	19.33	-122.444	37.942	0.05	18.97	290.37	19.02	9	7/14/2014	32:44.2
10609	RSPe_2	-122.444	37.942	0.41	18.96	290.55	19.37	-122.444	37.942	0.10	18.96	290.37	19.06	9	7/14/2014	32:44.1
10610	RSPe_2	-122.444	37.942	0.36	18.97	290.47	19.32	-122.444	37.942	0.05	18.97	290.42	19.01	9	7/14/2014	32:44.0
10611	RSPe_2	-122.444	37.942	0.36	19.01	290.60	19.37	-122.444	37.942	0.10	19.01	290.46	19.11	9	7/14/2014	32:43.9
10612	RSPe_2	-122.444	37.942	0.36	19.06	290.53	19.42	-122.444	37.942	0.05	19.06	290.55	19.11	9	7/14/2014	32:43.8
10613	RSPe_2	-122.444	37.942	0.41	19.10	290.62	19.51	-122.444	37.942	0.10	19.10	290.64	19.20	9	7/14/2014	32:43.7
10614	RSPe_2	-122.444	37.942	0.41	19.11	290.62	19.52	-122.444	37.942	0.13	19.11	290.69	19.25	9	7/14/2014	32:43.6
10615	RSPe_2	-122.444	37.942	0.41	19.17	290.64	19.57	-122.444	37.942	0.10	19.17	290.69	19.26	9	7/14/2014	32:43.5
10616	RSPe_2	-122.444	37.942	0.36	19.17	290.69	19.52	-122.444	37.942	0.05	19.17	290.75	19.21	9	7/14/2014	32:43.4
10617	RSPe_2	-122.444	37.942	0.41	19.15	290.71	19.55	-122.444	37.942	0.10	19.15	290.64	19.24	9	7/14/2014	32:43.3
10618	RSPe_2	-122.444	37.942	0.36	19.13	290.67	19.48	-122.444	37.942	0.13	19.13	290.67	19.26	9	7/14/2014	32:43.2
10619	RSPe_2	-122.444	37.942	0.41	19.08	290.63	19.48	-122.444	37.942	0.13	19.08	290.60	19.21	9	7/14/2014	32:43.1
10620	RSPe_2	-122.444	37.942	0.41	19.03	290.58	19.44	-122.444	37.942	0.13	19.03	290.58	19.17	9	7/14/2014	32:43.0
10621	RSPe_2	-122.444	37.942	0.41	18.80	290.48	19.21	-122.444	37.942	0.13	18.80	290.43	18.94	9	7/14/2014	32:42.9
10622	RSPe_2	-122.444	37.942	0.41	18.72	290.56	19.13	-122.444	37.942	0.18	18.72	290.49	18.90	9	7/14/2014	32:42.8
10623	RSPe_2	-122.444	37.942	0.41	18.63	290.63	19.04	-122.444	37.942	0.22	18.63	290.59	18.85	9	7/14/2014	32:42.7
10624	RSPe_2	-122.444	37.942	0.41	18.54	290.65	18.95	-122.444	37.942	0.13	18.54	290.58	18.67	9	7/14/2014	32:42.6
10625	RSPe_2	-122.444	37.942	0.41	18.54	290.67	18.95	-122.444	37.942	0.22	18.54	290.65	18.76	9	7/14/2014	32:42.5

10626	RSPe_2	-122.444	37.942	0.41	18.67	290.70	19.08	-122.444	37.942	0.18	18.67	290.70	18.86	9	7/14/2014	32:42.4
10627	RSPe_2	-122.444	37.942	0.44	18.60	290.70	19.05	-122.444	37.942	0.13	18.60	290.78	18.74	9	7/14/2014	32:42.3
10628	RSPe_2	-122.444	37.942	0.41	18.53	290.72	18.94	-122.444	37.942	0.18	18.53	290.83	18.72	9	7/14/2014	32:42.2
10629	RSPe_2	-122.444	37.942	0.44	18.41	290.77	18.85	-122.444	37.942	0.18	18.41	290.89	18.60	9	7/14/2014	32:42.1
10630	RSPe_2	-122.444	37.942	0.41	18.38	290.81	18.79	-122.444	37.942	0.13	18.38	290.89	18.51	9	7/14/2014	32:42.0
10631	RSPe_2	-122.444	37.942	0.44	18.34	290.77	18.78	-122.444	37.942	0.22	18.34	290.86	18.56	9	7/14/2014	32:41.9
10632	RSPe_2	-122.444	37.942	0.44	18.36	290.79	18.81	-122.444	37.942	0.22	18.36	290.89	18.58	9	7/14/2014	32:41.8
10633	RSPe_2	-122.444	37.942	0.44	18.32	290.79	18.76	-122.444	37.942	0.22	18.32	290.85	18.53	9	7/14/2014	32:41.7
10634	RSPe_2	-122.444	37.942	0.41	18.26	290.77	18.67	-122.444	37.942	0.22	18.26	290.83	18.48	9	7/14/2014	32:41.6
10635	RSPe_2	-122.444	37.942	0.44	18.25	290.81	18.69	-122.444	37.942	0.22	18.25	290.85	18.46	9	7/14/2014	32:41.5
10636	RSPe_2	-122.444	37.942	0.44	18.29	290.81	18.73	-122.444	37.942	0.22	18.29	290.85	18.51	9	7/14/2014	32:41.4
10637	RSPe_2	-122.444	37.942	0.44	18.31	290.86	18.75	-122.444	37.942	0.22	18.31	290.86	18.53	9	7/14/2014	32:41.3
10638	RSPe_2	-122.444	37.942	0.44	18.25	290.88	18.69	-122.444	37.942	0.22	18.25	290.92	18.46	9	7/14/2014	32:41.2
10639	RSPe_2	-122.444	37.942	0.44	18.27	290.93	18.71	-122.444	37.942	0.18	18.27	290.94	18.46	9	7/14/2014	32:41.1
10640	RSPe_2	-122.444	37.942	0.41	18.26	290.99	18.67	-122.444	37.942	0.18	18.26	291.03	18.45	9	7/14/2014	32:41.0
10641	RSPe_2	-122.444	37.943	0.44	18.34	290.99	18.78	-122.444	37.943	0.18	18.34	290.97	18.53	9	7/14/2014	32:40.9
10642	RSPe_2	-122.444	37.943	0.41	18.30	291.04	18.71	-122.444	37.943	0.18	18.30	290.99	18.49	9	7/14/2014	32:40.8
10643	RSPe_2	-122.444	37.943	0.44	18.34	291.04	18.78	-122.444	37.943	0.22	18.34	290.97	18.56	9	7/14/2014	32:40.7
10644	RSPe_2	-122.444	37.943	0.44	18.29	291.08	18.73	-122.444	37.943	0.22	18.29	290.99	18.51	9	7/14/2014	32:40.6
10645	RSPe_2	-122.444	37.943	0.44	18.34	291.06	18.78	-122.444	37.943	0.22	18.34	290.99	18.56	9	7/14/2014	32:40.5
10646	RSPe_2	-122.444	37.943	0.44	18.33	291.11	18.77	-122.444	37.943	0.25	18.33	291.08	18.58	9	7/14/2014	32:40.4
10647	RSPe_2	-122.444	37.943	0.49	18.32	291.08	18.81	-122.444	37.943	0.30	18.32	291.06	18.62	9	7/14/2014	32:40.3
10648	RSPe_2	-122.444	37.943	0.44	18.36	291.08	18.81	-122.444	37.943	0.22	18.36	291.10	18.58	9	7/14/2014	32:40.2
10649	RSPe_2	-122.444	37.943	0.49	18.36	291.05	18.85	-122.444	37.943	0.25	18.36	291.08	18.61	9	7/14/2014	32:40.1
10650	RSPe_2	-122.444	37.943	0.49	18.43	291.07	18.92	-122.444	37.943	0.25	18.43	291.10	18.68	9	7/14/2014	32:40.0
10651	RSPe_2	-122.444	37.943	0.49	18.37	291.05	18.86	-122.444	37.943	0.30	18.37	291.15	18.67	9	7/14/2014	32:39.9
10652	RSPe_2	-122.444	37.943	0.44	18.43	291.05	18.88	-122.444	37.943	0.25	18.43	291.17	18.69	9	7/14/2014	32:39.8
10653	RSPe_2	-122.444	37.943	0.49	18.45	291.07	18.94	-122.444	37.943	0.25	18.45	291.20	18.70	9	7/14/2014	32:39.7
10654	RSPe_2	-122.444	37.943	0.49	18.43	291.05	18.93	-122.444	37.943	0.25	18.43	291.22	18.69	9	7/14/2014	32:39.6
10655	RSPe_2	-122.444	37.943	0.49	18.43	291.09	18.93	-122.444	37.943	0.25	18.43	291.20	18.69	9	7/14/2014	32:39.5
10656	RSPe_2	-122.444	37.943	0.49	18.46	291.07	18.95	-122.444	37.943	0.25	18.46	291.20	18.71	9	7/14/2014	32:39.4
10657	RSPe_2	-122.444	37.943	0.49	18.46	291.05	18.95	-122.444	37.943	0.30	18.46	291.18	18.76	9	7/14/2014	32:39.3
10658	RSPe_2	-122.444	37.943	0.49	18.47	291.07	18.96	-122.444	37.943	0.30	18.47	291.18	18.77	9	7/14/2014	32:39.2

10659	RSPe_2	-122.444	37.943	0.53	18.49	291.05	19.01	-122.444	37.943	0.34	18.49	291.20	18.82	9	7/14/2014	32:39.1
10660	RSPe_2	-122.444	37.943	0.49	18.51	291.05	19.00	-122.444	37.943	0.30	18.51	291.14	18.81	9	7/14/2014	32:39.0
10661	RSPe_2	-122.444	37.943	0.53	18.53	291.01	19.06	-122.444	37.943	0.30	18.53	291.14	18.84	9	7/14/2014	32:38.9
10662	RSPe_2	-122.444	37.943	0.49	18.53	291.05	19.03	-122.444	37.943	0.30	18.53	291.15	18.84	9	7/14/2014	32:38.8
10663	RSPe_2	-122.444	37.943	0.53	18.56	291.07	19.08	-122.444	37.943	0.30	18.56	291.19	18.86	9	7/14/2014	32:38.7
10664	RSPe_2	-122.444	37.943	0.49	18.61	291.12	19.11	-122.444	37.943	0.30	18.61	291.23	18.91	9	7/14/2014	32:38.6
10665	RSPe_2	-122.444	37.943	0.53	18.63	291.21	19.15	-122.444	37.943	0.30	18.63	291.27	18.93	9	7/14/2014	32:38.5
10666	RSPe_2	-122.444	37.943	0.49	18.62	291.21	19.11	-122.444	37.943	0.25	18.62	291.25	18.87	9	7/14/2014	32:38.4
10667	RSPe_2	-122.444	37.943	0.53	18.63	291.30	19.15	-122.444	37.943	0.30	18.63	291.27	18.93	9	7/14/2014	32:38.3
10668	RSPe_2	-122.444	37.943	0.49	18.63	291.30	19.12	-122.444	37.943	0.30	18.63	291.27	18.93	9	7/14/2014	32:38.2
10669	RSPe_2	-122.444	37.943	0.53	18.59	291.32	19.12	-122.444	37.943	0.34	18.59	291.28	18.92	9	7/14/2014	32:38.1
10670	RSPe_2	-122.444	37.943	0.49	18.54	291.35	19.04	-122.444	37.943	0.30	18.54	291.28	18.84	9	7/14/2014	32:38.0
10671	RSPe_2	-122.444	37.943	0.53	18.59	291.35	19.12	-122.444	37.943	0.30	18.59	291.30	18.90	9	7/14/2014	32:37.9
10672	RSPe_2	-122.444	37.943	0.53	18.51	291.32	19.04	-122.444	37.943	0.30	18.51	291.30	18.81	9	7/14/2014	32:37.8
10673	RSPe_2	-122.444	37.943	0.53	18.33	291.32	18.86	-122.444	37.943	0.30	18.33	291.32	18.64	9	7/14/2014	32:37.7
10674	RSPe_2	-122.444	37.943	0.53	18.35	291.35	18.88	-122.444	37.943	0.34	18.35	291.35	18.68	9	7/14/2014	32:37.6
10675	RSPe_2	-122.444	37.943	0.53	18.14	291.42	18.67	-122.444	37.943	0.34	18.14	291.41	18.48	9	7/14/2014	32:37.5
10676	RSPe_2	-122.444	37.943	0.53	18.18	291.46	18.71	-122.444	37.943	0.34	18.18	291.46	18.51	9	7/14/2014	32:37.4
10677	RSPe_2	-122.444	37.943	0.53	18.10	291.50	18.63	-122.444	37.943	0.34	18.10	291.50	18.44	9	7/14/2014	32:37.3
10678	RSPe_2	-122.444	37.943	0.53	18.11	291.55	18.64	-122.444	37.943	0.34	18.11	291.50	18.44	9	7/14/2014	32:37.2
10679	RSPe_2	-122.444	37.943	0.56	18.06	291.61	18.63	-122.444	37.943	0.34	18.06	291.52	18.40	9	7/14/2014	32:37.1
10680	RSPe_2	-122.444	37.943	0.49	17.99	291.66	18.49	-122.444	37.943	0.42	17.99	291.55	18.41	9	7/14/2014	32:37.0
10681	RSPe_2	-122.444	37.943	0.56	17.92	291.70	18.49	-122.444	37.943	0.39	17.92	291.59	18.31	9	7/14/2014	32:36.9
10682	RSPe_2	-122.444	37.943	0.53	17.81	291.66	18.34	-122.444	37.943	0.39	17.81	291.55	18.20	9	7/14/2014	32:36.8
10683	RSPe_2	-122.444	37.943	0.56	17.80	291.66	18.36	-122.444	37.943	0.39	17.80	291.57	18.19	9	7/14/2014	32:36.7
10684	RSPe_2	-122.444	37.943	0.53	17.94	291.55	18.47	-122.444	37.943	0.39	17.94	291.52	18.33	9	7/14/2014	32:36.6
10685	RSPe_2	-122.444	37.943	0.56	17.81	291.54	18.37	-122.444	37.943	0.42	17.81	291.53	18.23	9	7/14/2014	32:36.5
10686	RSPe_2	-122.444	37.943	0.53	17.66	291.51	18.19	-122.444	37.943	0.39	17.66	291.57	18.05	9	7/14/2014	32:36.4
10687	RSPe_2	-122.444	37.943	0.56	17.68	291.53	18.25	-122.444	37.943	0.42	17.68	291.58	18.11	9	7/14/2014	32:36.3
10688	RSPe_2	-122.444	37.943	0.56	17.68	291.53	18.24	-122.444	37.943	0.39	17.68	291.61	18.06	9	7/14/2014	32:36.2
10689	RSPe_2	-122.444	37.943	0.56	17.70	291.60	18.26	-122.444	37.943	0.47	17.70	291.57	18.17	9	7/14/2014	32:36.1
10690	RSPe_2	-122.444	37.943	0.53	17.80	291.60	18.33	-122.444	37.943	0.47	17.80	291.66	18.27	9	7/14/2014	32:36.0
10691	RSPe_2	-122.444	37.943	0.56	17.79	291.65	18.35	-122.444	37.943	0.47	17.79	291.66	18.26	9	7/14/2014	32:35.9

10692	RSPe_2	-122.444	37.943	0.53	17.78	291.71	18.30	-122.444	37.943	0.42	17.78	291.66	18.20	9	7/14/2014	32:35.8
10693	RSPe_2	-122.444	37.943	0.53	17.75	291.76	18.28	-122.444	37.943	0.47	17.75	291.75	18.23	9	7/14/2014	32:35.7
10694	RSPe_2	-122.444	37.943	0.53	17.81	291.76	18.34	-122.444	37.943	0.47	17.81	291.68	18.28	9	7/14/2014	32:35.6
10695	RSPe_2	-122.444	37.943	0.56	17.75	291.73	18.32	-122.444	37.943	0.47	17.75	291.71	18.23	9	7/14/2014	32:35.5
10696	RSPe_2	-122.444	37.943	0.53	17.79	291.72	18.32	-122.444	37.943	0.47	17.79	291.65	18.26	9	7/14/2014	32:35.4
10697	RSPe_2	-122.444	37.943	0.56	17.80	291.69	18.36	-122.444	37.943	0.47	17.80	291.67	18.27	9	7/14/2014	32:35.3
10698	RSPe_2	-122.444	37.943	0.53	17.80	291.67	18.33	-122.444	37.943	0.47	17.80	291.65	18.27	9	7/14/2014	32:35.2
10699	RSPe_2	-122.444	37.943	0.56	17.83	291.67	18.40	-122.444	37.943	0.47	17.83	291.69	18.31	9	7/14/2014	32:35.1
10700	RSPe_2	-122.444	37.943	0.56	17.83	291.63	18.40	-122.444	37.943	0.42	17.83	291.72	18.25	9	7/14/2014	32:35.0
10701	RSPe_2	-122.444	37.943	0.56	17.93	291.66	18.49	-122.444	37.943	0.51	17.93	291.74	18.44	9	7/14/2014	32:34.9
10702	RSPe_2	-122.444	37.943	0.56	18.08	291.65	18.64	-122.444	37.943	0.47	18.08	291.80	18.55	9	7/14/2014	32:34.8
10703	RSPe_2	-122.444	37.943	0.56	18.00	291.65	18.56	-122.444	37.943	0.51	18.00	291.84	18.51	9	7/14/2014	32:34.7
10704	RSPe_2	-122.444	37.943	0.53	18.12	291.61	18.65	-122.444	37.943	0.47	18.12	291.86	18.60	9	7/14/2014	32:34.6
10705	RSPe_2	-122.444	37.943	0.56	18.12	291.59	18.68	-122.444	37.943	0.51	18.12	291.85	18.62	9	7/14/2014	32:34.5
10706	RSPe_2	-122.444	37.943	0.53	18.10	291.55	18.62	-122.444	37.943	0.42	18.10	291.81	18.52	9	7/14/2014	32:34.4
10707	RSPe_2	-122.444	37.943	0.56	18.16	291.53	18.72	-122.444	37.943	0.42	18.16	291.79	18.58	9	7/14/2014	32:34.3
10708	RSPe_2	-122.444	37.943	0.53	18.20	291.59	18.73	-122.444	37.943	0.47	18.20	291.81	18.67	9	7/14/2014	32:34.2
10709	RSPe_2	-122.444	37.943	0.53	18.19	291.61	18.72	-122.444	37.943	0.42	18.19	291.79	18.61	9	7/14/2014	32:34.1
10710	RSPe_2	-122.444	37.943	0.53	18.19	291.64	18.72	-122.444	37.943	0.39	18.19	291.74	18.57	9	7/14/2014	32:34.0
10711	RSPe_2	-122.444	37.943	0.53	18.18	291.68	18.71	-122.444	37.943	0.39	18.18	291.68	18.56	9	7/14/2014	32:33.9
10712	RSPe_2	-122.444	37.943	0.53	18.19	291.68	18.72	-122.444	37.943	0.39	18.19	291.64	18.58	9	7/14/2014	32:33.8
10713	RSPe_2	-122.444	37.943	0.56	18.18	291.68	18.74	-122.444	37.943	0.51	18.18	291.61	18.68	9	7/14/2014	32:33.7
10714	RSPe_2	-122.444	37.943	0.53	18.16	291.68	18.68	-122.444	37.943	0.47	18.16	291.64	18.63	9	7/14/2014	32:33.6
10715	RSPe_2	-122.444	37.943	0.56	18.17	291.71	18.73	-122.444	37.943	0.39	18.17	291.64	18.55	9	7/14/2014	32:33.5
10716	RSPe_2	-122.444	37.943	0.53	18.16	291.64	18.68	-122.444	37.943	0.42	18.16	291.70	18.58	9	7/14/2014	32:33.4
10717	RSPe_2	-122.444	37.943	0.56	18.16	291.67	18.72	-122.444	37.943	0.42	18.16	291.73	18.58	9	7/14/2014	32:33.3
10718	RSPe_2	-122.444	37.943	0.53	18.17	291.60	18.69	-122.444	37.943	0.39	18.17	291.71	18.55	9	7/14/2014	32:33.2
10719	RSPe_2	-122.444	37.943	0.56	18.17	291.60	18.73	-122.444	37.943	0.42	18.17	291.73	18.59	9	7/14/2014	32:33.1
10720	RSPe_2	-122.444	37.943	0.53	18.18	291.60	18.71	-122.444	37.943	0.42	18.18	291.74	18.60	9	7/14/2014	32:33.0
10721	RSPe_2	-122.444	37.943	0.56	18.19	291.67	18.76	-122.444	37.943	0.47	18.19	291.79	18.67	9	7/14/2014	32:32.9
10722	RSPe_2	-122.444	37.943	0.53	18.20	291.71	18.73	-122.444	37.943	0.39	18.20	291.86	18.59	9	7/14/2014	32:32.8
10723	RSPe_2	-122.444	37.943	0.53	18.22	291.69	18.75	-122.444	37.943	0.42	18.22	291.80	18.64	9	7/14/2014	32:32.7
10724	RSPe_2	-122.444	37.943	0.53	18.22	291.74	18.75	-122.444	37.943	0.39	18.22	291.80	18.61	9	7/14/2014	32:32.6

10725	RSPe_2	-122.444	37.943	0.53	18.22	291.71	18.75	-122.444	37.943	0.42	18.22	291.73	18.64	9	7/14/2014	32:32.5
10726	RSPe_2	-122.444	37.943	0.49	18.24	291.80	18.73	-122.444	37.943	0.42	18.24	291.78	18.66	9	7/14/2014	32:32.4
10727	RSPe_2	-122.444	37.943	0.53	18.24	291.87	18.76	-122.444	37.943	0.42	18.24	291.78	18.66	9	7/14/2014	32:32.3
10728	RSPe_2	-122.444	37.943	0.53	18.24	291.90	18.77	-122.444	37.943	0.39	18.24	291.82	18.63	9	7/14/2014	32:32.2
10729	RSPe_2	-122.444	37.943	0.53	18.24	291.87	18.77	-122.444	37.943	0.39	18.24	291.81	18.63	9	7/14/2014	32:32.1
10730	RSPe_2	-122.444	37.943	0.53	18.24	291.87	18.76	-122.444	37.943	0.47	18.24	291.84	18.71	9	7/14/2014	32:32.0
10731	RSPe_2	-122.444	37.943	0.53	18.21	291.78	18.74	-122.444	37.943	0.42	18.21	291.89	18.63	9	7/14/2014	32:31.9
10732	RSPe_2	-122.444	37.943	0.53	18.26	291.83	18.79	-122.444	37.943	0.39	18.26	291.96	18.64	9	7/14/2014	32:31.8
10733	RSPe_2	-122.444	37.943	0.53	18.30	291.77	18.83	-122.444	37.943	0.42	18.30	291.92	18.73	9	7/14/2014	32:31.7
10734	RSPe_2	-122.444	37.943	0.53	18.30	291.83	18.82	-122.444	37.943	0.39	18.30	292.02	18.68	9	7/14/2014	32:31.6
10735	RSPe_2	-122.444	37.943	0.53	18.26	291.83	18.79	-122.444	37.943	0.42	18.26	291.96	18.68	9	7/14/2014	32:31.5
10736	RSPe_2	-122.444	37.943	0.50	18.26	291.90	18.75	-122.444	37.943	0.39	18.26	292.02	18.64	9	7/14/2014	32:31.4
10737	RSPe_2	-122.444	37.943	0.53	18.26	291.96	18.79	-122.444	37.943	0.39	18.26	292.02	18.65	9	7/14/2014	32:31.3
10738	RSPe_2	-122.444	37.943	0.53	18.30	292.03	18.83	-122.444	37.943	0.34	18.30	292.05	18.64	9	7/14/2014	32:31.2
10739	RSPe_2	-122.444	37.943	0.53	18.40	292.05	18.93	-122.444	37.943	0.39	18.40	292.01	18.79	9	7/14/2014	32:31.1
10740	RSPe_2	-122.444	37.943	0.53	18.36	292.06	18.89	-122.444	37.943	0.39	18.36	292.02	18.75	9	7/14/2014	32:31.0
10741	RSPe_2	-122.444	37.943	0.53	18.41	292.06	18.94	-122.444	37.943	0.39	18.41	292.03	18.80	9	7/14/2014	32:30.9
10742	RSPe_2	-122.444	37.943	0.53	18.45	292.06	18.98	-122.444	37.943	0.39	18.45	292.05	18.84	9	7/14/2014	32:30.8
10743	RSPe_2	-122.444	37.943	0.53	18.47	292.01	18.99	-122.444	37.943	0.47	18.47	292.03	18.94	9	7/14/2014	32:30.7
10744	RSPe_2	-122.444	37.943	0.53	18.52	292.04	19.05	-122.444	37.943	0.39	18.52	292.06	18.91	9	7/14/2014	32:30.6
10745	RSPe_2	-122.444	37.943	0.53	18.62	291.97	19.15	-122.444	37.943	0.39	18.62	292.17	19.01	9	7/14/2014	32:30.5
10746	RSPe_2	-122.444	37.943	0.50	18.61	291.99	19.11	-122.444	37.943	0.34	18.61	292.17	18.95	9	7/14/2014	32:30.4
10747	RSPe_2	-122.444	37.943	0.53	18.57	291.98	19.10	-122.444	37.943	0.39	18.57	292.24	18.96	9	7/14/2014	32:30.3
10748	RSPe_2	-122.444	37.943	0.50	18.68	291.99	19.18	-122.444	37.943	0.34	18.68	292.25	19.02	9	7/14/2014	32:30.2
10749	RSPe_2	-122.444	37.943	0.53	18.76	291.93	19.29	-122.444	37.943	0.34	18.76	292.17	19.10	9	7/14/2014	32:30.1
10750	RSPe_2	-122.444	37.943	0.53	18.76	291.95	19.29	-122.444	37.943	0.34	18.76	292.15	19.10	9	7/14/2014	32:30.0
10751	RSPe_2	-122.444	37.943	0.53	18.84	292.00	19.37	-122.444	37.943	0.39	18.84	292.15	19.23	9	7/14/2014	32:29.9
10752	RSPe_2	-122.444	37.943	0.50	18.83	292.00	19.33	-122.444	37.943	0.30	18.83	292.13	19.14	9	7/14/2014	32:29.8
10753	RSPe_2	-122.444	37.943	0.50	18.88	292.07	19.38	-122.444	37.943	0.34	18.88	292.13	19.22	9	7/14/2014	32:29.7
10754	RSPe_2	-122.444	37.943	0.50	18.90	292.00	19.39	-122.444	37.943	0.30	18.90	292.08	19.20	9	7/14/2014	32:29.6
10755	RSPe_2	-122.444	37.943	0.53	18.88	292.00	19.41	-122.444	37.943	0.39	18.88	292.08	19.27	9	7/14/2014	32:29.5
10756	RSPe_2	-122.444	37.943	0.50	18.82	291.98	19.31	-122.444	37.943	0.34	18.82	292.06	19.16	9	7/14/2014	32:29.4
10757	RSPe_2	-122.444	37.943	0.53	18.76	291.93	19.29	-122.444	37.943	0.39	18.76	292.09	19.15	9	7/14/2014	32:29.3

10758	RSPe_2	-122.444	37.943	0.50	18.69	291.96	19.19	-122.444	37.943	0.34	18.69	292.08	19.03	9	7/14/2014	32:29.2
10759	RSPe_2	-122.444	37.943	0.53	18.70	291.92	19.23	-122.444	37.943	0.34	18.70	292.08	19.04	9	7/14/2014	32:29.1
10760	RSPe_2	-122.444	37.943	0.50	18.74	291.87	19.24	-122.444	37.943	0.30	18.74	292.07	19.05	9	7/14/2014	32:29.0
10761	RSPe_2	-122.444	37.943	0.50	18.65	291.83	19.14	-122.444	37.943	0.30	18.65	292.07	18.95	9	7/14/2014	32:28.9
10762	RSPe_2	-122.444	37.943	0.50	18.66	291.83	19.15	-122.444	37.943	0.34	18.66	292.02	19.00	9	7/14/2014	32:28.8
10763	RSPe_2	-122.444	37.943	0.50	18.66	291.83	19.16	-122.444	37.943	0.39	18.66	292.03	19.05	9	7/14/2014	32:28.7
10764	RSPe_2	-122.444	37.943	0.44	18.61	291.79	19.05	-122.444	37.943	0.30	18.61	291.93	18.91	9	7/14/2014	32:28.6
10765	RSPe_2	-122.444	37.943	0.50	18.62	291.77	19.12	-122.444	37.943	0.25	18.62	291.90	18.87	9	7/14/2014	32:28.5
10766	RSPe_2	-122.444	37.943	0.50	18.64	291.72	19.14	-122.444	37.943	0.25	18.64	291.89	18.90	9	7/14/2014	32:28.4
10767	RSPe_2	-122.444	37.943	0.50	18.44	291.68	18.94	-122.444	37.943	0.30	18.44	291.83	18.74	9	7/14/2014	32:28.3
10768	RSPe_2	-122.444	37.943	0.50	18.47	291.57	18.96	-122.444	37.943	0.30	18.47	291.81	18.77	9	7/14/2014	32:28.2
10769	RSPe_2	-122.444	37.943	0.53	18.47	291.50	19.00	-122.444	37.943	0.30	18.47	291.72	18.78	9	7/14/2014	32:28.1
10770	RSPe_2	-122.444	37.943	0.50	18.38	291.46	18.87	-122.444	37.943	0.30	18.38	291.71	18.68	9	7/14/2014	32:28.0
10771	RSPe_2	-122.444	37.943	0.53	18.43	291.53	18.96	-122.444	37.943	0.30	18.43	291.73	18.73	9	7/14/2014	32:27.9
10772	RSPe_2	-122.444	37.943	0.50	18.41	291.51	18.91	-122.444	37.943	0.30	18.41	291.65	18.72	9	7/14/2014	32:27.8
10773	RSPe_2	-122.444	37.943	0.53	18.33	291.49	18.86	-122.444	37.943	0.30	18.33	291.68	18.63	9	7/14/2014	32:27.7
10774	RSPe_2	-122.444	37.943	0.53	18.34	291.62	18.87	-122.444	37.943	0.30	18.34	291.64	18.64	9	7/14/2014	32:27.6
10775	RSPe_2	-122.444	37.943	0.53	18.26	291.62	18.79	-122.444	37.943	0.34	18.26	291.64	18.59	9	7/14/2014	32:27.5
10776	RSPe_2	-122.444	37.943	0.53	18.26	291.64	18.79	-122.444	37.943	0.34	18.26	291.59	18.60	9	7/14/2014	32:27.4
10777	RSPe_2	-122.444	37.943	0.53	18.21	291.62	18.74	-122.444	37.943	0.34	18.21	291.60	18.55	9	7/14/2014	32:27.3
10778	RSPe_2	-122.444	37.943	0.53	18.15	291.60	18.68	-122.444	37.943	0.34	18.15	291.57	18.49	9	7/14/2014	32:27.2
10779	RSPe_2	-122.444	37.943	0.53	18.06	291.58	18.59	-122.444	37.943	0.34	18.06	291.62	18.40	9	7/14/2014	32:27.1
10780	RSPe_2	-122.444	37.943	0.53	18.04	291.67	18.57	-122.444	37.943	0.34	18.04	291.68	18.38	9	7/14/2014	32:27.0
10781	RSPe_2	-122.444	37.943	0.56	18.04	291.63	18.60	-122.444	37.943	0.39	18.04	291.69	18.43	9	7/14/2014	32:26.9
10782	RSPe_2	-122.444	37.943	0.53	17.98	291.65	18.51	-122.444	37.943	0.39	17.98	291.73	18.37	9	7/14/2014	32:26.8
10783	RSPe_2	-122.444	37.943	0.56	17.93	291.60	18.49	-122.444	37.943	0.42	17.93	291.73	18.35	9	7/14/2014	32:26.7
10784	RSPe_2	-122.444	37.943	0.56	17.99	291.65	18.55	-122.444	37.943	0.39	17.99	291.77	18.37	9	7/14/2014	32:26.6
10785	RSPe_2	-122.444	37.943	0.56	17.88	291.65	18.44	-122.444	37.943	0.42	17.88	291.77	18.30	9	7/14/2014	32:26.5
10786	RSPe_2	-122.444	37.943	0.53	17.90	291.65	18.43	-122.444	37.943	0.42	17.90	291.77	18.32	9	7/14/2014	32:26.4
10787	RSPe_2	-122.444	37.943	0.56	17.86	291.65	18.42	-122.444	37.943	0.42	17.86	291.74	18.28	9	7/14/2014	32:26.3
10788	RSPe_2	-122.444	37.943	0.53	17.92	291.74	18.44	-122.444	37.943	0.42	17.92	291.78	18.34	9	7/14/2014	32:26.2
10789	RSPe_2	-122.444	37.943	0.56	17.80	291.74	18.37	-122.444	37.943	0.42	17.80	291.80	18.22	9	7/14/2014	32:26.1
10790	RSPe_2	-122.444	37.943	0.56	17.82	291.74	18.38	-122.444	37.943	0.47	17.82	291.76	18.29	9	7/14/2014	32:26.0

10791	RSPe_2	-122.444	37.943	0.56	17.77	291.76	18.33	-122.444	37.943	0.42	17.77	291.80	18.19	9	7/14/2014	32:25.9
10792	RSPe_2	-122.444	37.943	0.56	17.79	291.76	18.35	-122.444	37.943	0.42	17.79	291.79	18.21	9	7/14/2014	32:25.8
10793	RSPe_2	-122.444	37.943	0.56	17.77	291.73	18.33	-122.444	37.943	0.42	17.77	291.83	18.19	9	7/14/2014	32:25.7
10794	RSPe_2	-122.444	37.943	0.56	17.76	291.74	18.33	-122.444	37.943	0.39	17.76	291.85	18.15	9	7/14/2014	32:25.6
10795	RSPe_2	-122.444	37.943	0.62	17.78	291.70	18.39	-122.444	37.943	0.47	17.78	291.81	18.25	9	7/14/2014	32:25.5
10796	RSPe_2	-122.444	37.943	0.56	17.72	291.70	18.29	-122.444	37.943	0.47	17.72	291.89	18.20	9	7/14/2014	32:25.4
10797	RSPe_2	-122.444	37.943	0.56	17.75	291.72	18.31	-122.444	37.943	0.51	17.75	291.94	18.25	9	7/14/2014	32:25.3
10798	RSPe_2	-122.444	37.943	0.62	17.75	291.73	18.36	-122.444	37.943	0.47	17.75	291.90	18.22	9	7/14/2014	32:25.2
10799	RSPe_2	-122.444	37.943	0.62	17.75	291.73	18.37	-122.444	37.943	0.51	17.75	291.94	18.26	9	7/14/2014	32:25.1
10800	RSPe_2	-122.444	37.943	0.56	17.76	291.81	18.33	-122.444	37.943	0.47	17.76	291.97	18.24	9	7/14/2014	32:25.0
10801	RSPe_2	-122.444	37.943	0.62	17.76	291.83	18.38	-122.444	37.943	0.47	17.76	292.01	18.24	9	7/14/2014	32:24.9
10802	RSPe_2	-122.444	37.943	0.56	17.79	291.82	18.35	-122.444	37.943	0.47	17.79	291.97	18.26	9	7/14/2014	32:24.8
10803	RSPe_2	-122.444	37.943	0.62	17.79	291.84	18.40	-122.444	37.943	0.47	17.79	291.95	18.26	9	7/14/2014	32:24.7
10804	RSPe_2	-122.444	37.943	0.62	17.79	291.86	18.40	-122.444	37.943	0.42	17.79	291.99	18.21	9	7/14/2014	32:24.6
10805	RSPe_2	-122.444	37.943	0.62	17.84	291.91	18.46	-122.444	37.943	0.51	17.84	291.99	18.35	9	7/14/2014	32:24.5
10806	RSPe_2	-122.444	37.943	0.56	17.88	291.93	18.44	-122.444	37.943	0.47	17.88	291.97	18.35	9	7/14/2014	32:24.4
10807	RSPe_2	-122.444	37.943	0.62	17.86	291.90	18.48	-122.444	37.943	0.47	17.86	291.99	18.33	9	7/14/2014	32:24.3
10808	RSPe_2	-122.444	37.943	0.62	17.83	291.86	18.45	-122.444	37.943	0.51	17.83	292.00	18.34	9	7/14/2014	32:24.2
10809	RSPe_2	-122.444	37.943	0.62	17.88	291.89	18.49	-122.444	37.943	0.51	17.88	292.03	18.38	9	7/14/2014	32:24.1
10810	RSPe_2	-122.444	37.943	0.62	18.23	291.82	18.84	-122.444	37.943	0.51	18.23	292.04	18.73	9	7/14/2014	32:24.0
10811	RSPe_2	-122.444	37.943	0.65	17.89	291.80	18.54	-122.444	37.943	0.54	17.89	292.04	18.43	9	7/14/2014	32:23.9
10812	RSPe_2	-122.444	37.943	0.62	18.10	291.84	18.71	-122.444	37.943	0.54	18.10	292.06	18.64	9	7/14/2014	32:23.8
10813	RSPe_2	-122.444	37.943	0.65	18.05	291.85	18.70	-122.444	37.943	0.54	18.05	292.06	18.59	9	7/14/2014	32:23.7
10814	RSPe_2	-122.444	37.943	0.62	17.98	291.87	18.59	-122.444	37.943	0.51	17.98	292.01	18.49	9	7/14/2014	32:23.6
10815	RSPe_2	-122.444	37.943	0.62	18.17	291.87	18.79	-122.444	37.943	0.54	18.17	292.02	18.71	9	7/14/2014	32:23.5
10816	RSPe_2	-122.444	37.943	0.62	18.15	291.87	18.76	-122.444	37.943	0.54	18.15	292.06	18.69	9	7/14/2014	32:23.4
10817	RSPe_2	-122.444	37.943	0.62	18.30	291.89	18.91	-122.444	37.943	0.54	18.30	292.06	18.84	9	7/14/2014	32:23.3
10818	RSPe_2	-122.444	37.943	0.62	18.28	291.89	18.89	-122.444	37.943	0.54	18.28	292.07	18.82	9	7/14/2014	32:23.2
10819	RSPe_2	-122.444	37.943	0.65	18.29	291.85	18.94	-122.444	37.943	0.59	18.29	292.07	18.88	9	7/14/2014	32:23.1
10820	RSPe_2	-122.444	37.943	0.62	18.24	291.83	18.85	-122.444	37.943	0.51	18.24	292.11	18.74	9	7/14/2014	32:23.0
10821	RSPe_2	-122.444	37.943	0.65	18.39	291.81	19.03	-122.444	37.943	0.59	18.39	292.07	18.98	9	7/14/2014	32:22.9
10822	RSPe_2	-122.444	37.943	0.65	18.40	291.78	19.05	-122.444	37.943	0.59	18.40	292.08	18.99	9	7/14/2014	32:22.8
10823	RSPe_2	-122.444	37.943	0.65	18.39	291.81	19.03	-122.444	37.943	0.59	18.39	292.07	18.98	9	7/14/2014	32:22.7

10824	RSPe_2	-122.444	37.943	0.65	18.44	291.79	19.09	-122.444	37.943	0.59	18.44	292.05	19.03	9	7/14/2014	32:22.6
10825	RSPe_2	-122.444	37.943	0.65	18.40	291.79	19.04	-122.444	37.943	0.54	18.40	292.05	18.94	9	7/14/2014	32:22.5
10826	RSPe_2	-122.444	37.943	0.62	18.47	291.79	19.09	-122.444	37.943	0.54	18.47	292.00	19.01	9	7/14/2014	32:22.4
10827	RSPe_2	-122.444	37.943	0.65	18.51	291.83	19.16	-122.444	37.943	0.59	18.51	292.01	19.10	9	7/14/2014	32:22.3
10828	RSPe_2	-122.444	37.943	0.65	18.48	291.81	19.13	-122.444	37.943	0.59	18.48	292.02	19.07	9	7/14/2014	32:22.2
10829	RSPe_2	-122.444	37.943	0.65	18.46	291.83	19.10	-122.444	37.943	0.59	18.46	291.99	19.05	9	7/14/2014	32:22.1
10830	RSPe_2	-122.444	37.943	0.62	18.39	291.84	19.00	-122.444	37.943	0.59	18.39	291.99	18.98	9	7/14/2014	32:22.0
10831	RSPe_2	-122.444	37.943	0.65	18.42	291.77	19.07	-122.444	37.943	0.63	18.42	291.92	19.04	9	7/14/2014	32:21.9
10832	RSPe_2	-122.444	37.943	0.65	18.30	291.75	18.95	-122.444	37.943	0.59	18.30	291.93	18.90	9	7/14/2014	32:21.8
10833	RSPe_2	-122.444	37.943	0.70	18.27	291.81	18.97	-122.444	37.943	0.63	18.27	291.96	18.90	9	7/14/2014	32:21.7
10834	RSPe_2	-122.444	37.943	0.65	18.28	291.78	18.93	-122.444	37.943	0.63	18.28	291.95	18.90	9	7/14/2014	32:21.6
10835	RSPe_2	-122.444	37.943	0.65	18.21	291.73	18.86	-122.444	37.943	0.68	18.21	291.95	18.88	9	7/14/2014	32:21.5
10836	RSPe_2	-122.444	37.943	0.65	18.22	291.80	18.87	-122.444	37.943	0.63	18.22	291.96	18.84	9	7/14/2014	32:21.4
10837	RSPe_2	-122.444	37.943	0.65	18.20	291.75	18.85	-122.444	37.943	0.63	18.20	291.95	18.83	9	7/14/2014	32:21.3
10838	RSPe_2	-122.444	37.943	0.62	18.20	291.71	18.82	-122.444	37.943	0.59	18.20	291.88	18.79	9	7/14/2014	32:21.2
10839	RSPe_2	-122.444	37.943	0.65	18.17	291.73	18.81	-122.444	37.943	0.59	18.17	291.88	18.76	9	7/14/2014	32:21.1
10840	RSPe_2	-122.444	37.943	0.65	18.17	291.69	18.81	-122.444	37.943	0.59	18.17	291.86	18.76	9	7/14/2014	32:21.0
10841	RSPe_2	-122.444	37.943	0.65	18.18	291.69	18.83	-122.444	37.943	0.68	18.18	291.87	18.85	9	7/14/2014	32:20.9
10842	RSPe_2	-122.444	37.943	0.65	18.17	291.73	18.82	-122.444	37.943	0.63	18.17	291.88	18.80	9	7/14/2014	32:20.8
10843	RSPe_2	-122.444	37.943	0.65	18.19	291.76	18.84	-122.444	37.943	0.59	18.19	291.93	18.78	9	7/14/2014	32:20.7
10844	RSPe_2	-122.444	37.943	0.65	18.20	291.73	18.85	-122.444	37.943	0.59	18.20	291.95	18.79	9	7/14/2014	32:20.6
10845	RSPe_2	-122.444	37.943	0.65	18.19	291.69	18.84	-122.444	37.943	0.63	18.19	291.91	18.82	9	7/14/2014	32:20.5
10846	RSPe_2	-122.444	37.943	0.65	18.27	291.58	18.92	-122.444	37.943	0.63	18.27	291.89	18.90	9	7/14/2014	32:20.4
10847	RSPe_2	-122.444	37.943	0.65	18.21	291.56	18.86	-122.444	37.943	0.68	18.21	291.89	18.89	9	7/14/2014	32:20.3
10848	RSPe_2	-122.444	37.943	0.62	18.21	291.54	18.82	-122.444	37.943	0.68	18.21	292.00	18.89	9	7/14/2014	32:20.2
10849	RSPe_2	-122.444	37.943	0.65	18.24	291.54	18.89	-122.444	37.943	0.63	18.24	291.94	18.87	9	7/14/2014	32:20.1
10850	RSPe_2	-122.444	37.943	0.65	18.27	291.48	18.92	-122.444	37.943	0.63	18.27	291.98	18.90	9	7/14/2014	32:20.0
10851	RSPe_2	-122.444	37.943	0.65	18.26	291.50	18.91	-122.444	37.943	0.59	18.26	291.98	18.85	9	7/14/2014	32:19.9
10852	RSPe_2	-122.444	37.943	0.62	18.27	291.43	18.89	-122.444	37.943	0.54	18.27	291.95	18.81	9	7/14/2014	32:19.8
10853	RSPe_2	-122.444	37.943	0.65	18.32	291.41	18.97	-122.444	37.943	0.54	18.32	291.87	18.86	9	7/14/2014	32:19.7
10854	RSPe_2	-122.444	37.943	0.65	18.27	291.41	18.92	-122.444	37.943	0.54	18.27	291.87	18.81	9	7/14/2014	32:19.6
10855	RSPe_2	-122.444	37.943	0.65	18.31	291.42	18.96	-122.444	37.943	0.54	18.31	291.81	18.85	9	7/14/2014	32:19.5
10856	RSPe_2	-122.444	37.943	0.65	18.30	291.42	18.95	-122.444	37.943	0.54	18.30	291.78	18.84	9	7/14/2014	32:19.4

10857	RSPe_2	-122.444	37.943	0.65	18.32	291.42	18.97	-122.444	37.943	0.54	18.32	291.68	18.86	9	7/14/2014 32:19.3
10858	RSPe_2	-122.444	37.943	0.62	18.33	291.37	18.94	-122.444	37.943	0.54	18.33	291.63	18.87	9	7/14/2014 32:19.2
10859	RSPe_2	-122.444	37.943	0.65	18.35	291.33	19.00	-122.444	37.943	0.54	18.35	291.54	18.89	9	7/14/2014 32:19.1
10860	RSPe_2	-122.444	37.943	0.65	18.36	291.28	19.01	-122.444	37.943	0.54	18.36	291.56	18.90	9	7/14/2014 32:19.0
10861	RSPe_2	-122.444	37.943	0.65	18.37	291.27	19.02	-122.444	37.943	0.63	18.37	291.51	19.00	9	7/14/2014 32:18.9
10862	RSPe_2	-122.444	37.943	0.65	18.43	291.18	19.08	-122.444	37.943	0.59	18.43	291.48	19.03	9	7/14/2014 32:18.8
10863	RSPe_2	-122.444	37.943	0.65	18.44	291.24	19.09	-122.444	37.943	0.63	18.44	291.49	19.07	9	7/14/2014 32:18.7
10864	RSPe_2	-122.444	37.943	0.62	18.47	291.22	19.09	-122.444	37.943	0.59	18.47	291.52	19.06	9	7/14/2014 32:18.6
10865	RSPe_2	-122.444	37.943	0.65	18.68	291.29	19.33	-122.444	37.943	0.59	18.68	291.57	19.27	9	7/14/2014 32:18.5
10866	RSPe_2	-122.444	37.943	0.62	18.62	291.29	19.24	-122.444	37.943	0.54	18.62	291.55	19.16	9	7/14/2014 32:18.4
10867	RSPe_2	-122.444	37.943	0.65	18.59	291.25	19.24	-122.444	37.943	0.54	18.59	291.51	19.14	9	7/14/2014 32:18.3
10868	RSPe_2	-122.444	37.943	0.62	18.59	291.27	19.21	-122.444	37.943	0.54	18.59	291.55	19.14	9	7/14/2014 32:18.2
10869	RSPe_2	-122.444	37.943	0.65	18.63	291.29	19.28	-122.444	37.943	0.54	18.63	291.53	19.17	9	7/14/2014 32:18.1
10870	RSPe_2	-122.444	37.943	0.62	18.63	291.29	19.24	-122.444	37.943	0.54	18.63	291.60	19.17	9	7/14/2014 32:18.0
10871	RSPe_2	-122.444	37.943	0.62	18.70	291.32	19.31	-122.444	37.943	0.54	18.70	291.58	19.24	9	7/14/2014 32:17.9
10872	RSPe_2	-122.444	37.943	0.62	18.53	291.30	19.15	-122.444	37.943	0.51	18.53	291.64	19.04	9	7/14/2014 32:17.8
10873	RSPe_2	-122.444	37.943	0.62	18.65	291.34	19.27	-122.444	37.943	0.54	18.65	291.66	19.19	9	7/14/2014 32:17.7
10874	RSPe_2	-122.444	37.943	0.62	18.55	291.38	19.17	-122.444	37.943	0.54	18.55	291.75	19.09	9	7/14/2014 32:17.6
10875	RSPe_2	-122.444	37.943	0.62	18.47	291.34	19.08	-122.444	37.943	0.54	18.47	291.67	19.01	9	7/14/2014 32:17.5
10876	RSPe_2	-122.444	37.943	0.62	18.52	291.34	19.14	-122.444	37.943	0.54	18.52	291.71	19.07	9	7/14/2014 32:17.4
10877	RSPe_2	-122.444	37.943	0.62	18.48	291.39	19.10	-122.444	37.943	0.54	18.48	291.71	19.02	9	7/14/2014 32:17.3
10878	RSPe_2	-122.444	37.943	0.62	18.36	291.37	18.97	-122.444	37.943	0.51	18.36	291.76	18.87	9	7/14/2014 32:17.2
10879	RSPe_2	-122.444	37.943	0.62	18.46	291.39	19.07	-122.444	37.943	0.51	18.46	291.71	18.96	9	7/14/2014 32:17.1
10880	RSPe_2	-122.444	37.943	0.56	18.33	291.39	18.89	-122.444	37.943	0.51	18.33	291.65	18.83	9	7/14/2014 32:17.0
10881	RSPe_2	-122.444	37.943	0.62	18.25	291.34	18.86	-122.444	37.943	0.51	18.25	291.63	18.75	9	7/14/2014 32:16.9
10882	RSPe_2	-122.444	37.943	0.56	18.23	291.37	18.79	-122.444	37.943	0.47	18.23	291.63	18.70	9	7/14/2014 32:16.8
10883	RSPe_2	-122.444	37.943	0.62	18.23	291.35	18.84	-122.444	37.943	0.51	18.23	291.61	18.73	9	7/14/2014 32:16.7
10884	RSPe_2	-122.444	37.943	0.56	18.17	291.35	18.73	-122.444	37.943	0.51	18.17	291.58	18.67	9	7/14/2014 32:16.6
10885	RSPe_2	-122.444	37.943	0.62	18.13	291.26	18.75	-122.444	37.943	0.51	18.13	291.50	18.64	9	7/14/2014 32:16.5
10886	RSPe_2	-122.444	37.943	0.56	18.13	291.22	18.70	-122.444	37.943	0.47	18.13	291.50	18.61	9	7/14/2014 32:16.4
10887	RSPe_2	-122.444	37.943	0.62	18.05	291.33	18.66	-122.444	37.943	0.51	18.05	291.52	18.56	9	7/14/2014 32:16.3
10888	RSPe_2	-122.444	37.943	0.56	18.06	291.24	18.63	-122.444	37.943	0.51	18.06	291.55	18.57	9	7/14/2014 32:16.2
10889	RSPe_2	-122.444	37.943	0.62	18.05	291.15	18.66	-122.444	37.943	0.51	18.05	291.48	18.56	9	7/14/2014 32:16.1

10890	RSPe_2	-122.444	37.943	0.56	18.20	291.13	18.77	-122.444	37.943	0.47	18.20	291.44	18.68	9	7/14/2014	32:16.0
10891	RSPe_2	-122.444	37.943	0.62	18.09	291.11	18.70	-122.444	37.943	0.51	18.09	291.46	18.59	9	7/14/2014	32:15.9
10892	RSPe_2	-122.444	37.943	0.56	18.15	291.09	18.71	-122.444	37.943	0.47	18.15	291.41	18.62	9	7/14/2014	32:15.8
10893	RSPe_2	-122.444	37.943	0.62	18.03	291.09	18.65	-122.444	37.943	0.47	18.03	291.42	18.51	9	7/14/2014	32:15.7
10894	RSPe_2	-122.444	37.943	0.56	18.10	291.07	18.67	-122.444	37.943	0.47	18.10	291.40	18.58	9	7/14/2014	32:15.6
10895	RSPe_2	-122.444	37.943	0.62	17.99	291.05	18.60	-122.444	37.943	0.51	17.99	291.39	18.49	9	7/14/2014	32:15.5
10896	RSPe_2	-122.444	37.943	0.62	18.00	291.09	18.62	-122.444	37.943	0.47	18.00	291.40	18.47	9	7/14/2014	32:15.4
10897	RSPe_2	-122.444	37.943	0.62	17.96	291.07	18.57	-122.444	37.943	0.51	17.96	291.37	18.46	9	7/14/2014	32:15.3
10898	RSPe_2	-122.444	37.943	0.56	18.00	291.07	18.56	-122.444	37.943	0.47	18.00	291.33	18.47	9	7/14/2014	32:15.2
10899	RSPe_2	-122.444	37.943	0.62	18.04	291.03	18.65	-122.444	37.943	0.51	18.04	291.31	18.55	9	7/14/2014	32:15.1
10900	RSPe_2	-122.444	37.943	0.56	17.98	290.99	18.54	-122.444	37.943	0.47	17.98	291.29	18.45	9	7/14/2014	32:15.0
10901	RSPe_2	-122.444	37.943	0.62	17.99	290.94	18.61	-122.444	37.943	0.47	17.99	291.29	18.47	9	7/14/2014	32:14.9
10902	RSPe_2	-122.444	37.943	0.56	17.96	290.94	18.52	-122.444	37.943	0.51	17.96	291.29	18.46	9	7/14/2014	32:14.8
10903	RSPe_2	-122.444	37.943	0.65	18.01	290.92	18.66	-122.444	37.943	0.51	18.01	291.29	18.52	9	7/14/2014	32:14.7
10904	RSPe_2	-122.444	37.943	0.62	18.03	290.90	18.65	-122.444	37.943	0.51	18.03	291.23	18.54	9	7/14/2014	32:14.6
10905	RSPe_2	-122.444	37.943	0.62	17.94	290.86	18.56	-122.444	37.943	0.54	17.94	291.21	18.48	9	7/14/2014	32:14.5
10906	RSPe_2	-122.444	37.943	0.62	17.99	290.90	18.61	-122.444	37.943	0.51	17.99	291.20	18.50	9	7/14/2014	32:14.4
10907	RSPe_2	-122.444	37.943	0.65	17.98	290.86	18.63	-122.444	37.943	0.51	17.98	291.18	18.49	9	7/14/2014	32:14.3
10908	RSPe_2	-122.444	37.943	0.62	18.02	290.86	18.63	-122.444	37.943	0.51	18.02	291.19	18.53	9	7/14/2014	32:14.2
10909	RSPe_2	-122.444	37.943	0.65	18.07	290.84	18.72	-122.444	37.943	0.54	18.07	291.17	18.61	9	7/14/2014	32:14.1
10910	RSPe_2	-122.444	37.943	0.62	18.03	290.82	18.64	-122.444	37.943	0.54	18.03	291.10	18.57	9	7/14/2014	32:14.0
10911	RSPe_2	-122.444	37.943	0.65	18.03	290.80	18.68	-122.444	37.943	0.54	18.03	291.14	18.57	9	7/14/2014	32:13.9
10912	RSPe_2	-122.444	37.943	0.62	18.18	290.80	18.79	-122.444	37.943	0.51	18.18	291.12	18.69	9	7/14/2014	32:13.8
10913	RSPe_2	-122.444	37.943	0.65	18.11	290.73	18.76	-122.444	37.943	0.51	18.11	291.10	18.62	9	7/14/2014	32:13.7
10914	RSPe_2	-122.444	37.943	0.62	18.12	290.71	18.74	-122.444	37.943	0.51	18.12	291.10	18.63	9	7/14/2014	32:13.6
10915	RSPe_2	-122.444	37.943	0.65	18.20	290.67	18.85	-122.444	37.943	0.54	18.20	291.09	18.74	9	7/14/2014	32:13.5
10916	RSPe_2	-122.444	37.943	0.62	17.39	290.67	18.01	-122.444	37.943	0.54	17.39	291.08	17.93	9	7/14/2014	32:13.4
10917	RSPe_2	-122.444	37.943	0.65	18.26	290.67	18.91	-122.444	37.943	0.54	18.26	291.08	18.80	9	7/14/2014	32:13.3
10918	RSPe_2	-122.444	37.943	0.62	17.47	290.67	18.09	-122.444	37.943	0.54	17.47	291.06	18.01	9	7/14/2014	32:13.2
10919	RSPe_2	-122.444	37.943	0.65	18.20	290.71	18.85	-122.444	37.943	0.54	18.20	291.07	18.74	9	7/14/2014	32:13.1
10920	RSPe_2	-122.444	37.943	0.62	18.17	290.72	18.78	-122.444	37.943	0.51	18.17	291.06	18.67	9	7/14/2014	32:13.0
10921	RSPe_2	-122.444	37.943	0.65	18.13	290.74	18.78	-122.444	37.943	0.54	18.13	291.07	18.67	9	7/14/2014	32:12.9
10922	RSPe_2	-122.444	37.943	0.65	18.30	290.71	18.95	-122.444	37.943	0.51	18.30	291.06	18.80	9	7/14/2014	32:12.8

10923	RSPe_2	-122.444	37.943	0.65	18.48	290.70	19.13	-122.444	37.943	0.54	18.48	291.06	19.02	9	7/14/2014	32:12.7
10924	RSPe_2	-122.444	37.943	0.65	18.26	290.65	18.91	-122.444	37.943	0.54	18.26	291.03	18.80	9	7/14/2014	32:12.6
10925	RSPe_2	-122.444	37.943	0.65	18.40	290.63	19.05	-122.444	37.943	0.54	18.40	291.02	18.94	9	7/14/2014	32:12.5
10926	RSPe_2	-122.444	37.943	0.65	18.19	290.59	18.84	-122.444	37.943	0.51	18.19	290.98	18.70	9	7/14/2014	32:12.4
10927	RSPe_2	-122.444	37.943	0.65	18.23	290.52	18.88	-122.444	37.943	0.54	18.23	290.91	18.77	9	7/14/2014	32:12.3
10928	RSPe_2	-122.444	37.943	0.65	18.35	290.54	19.00	-122.444	37.943	0.51	18.35	290.94	18.86	9	7/14/2014	32:12.2
10929	RSPe_2	-122.444	37.943	0.65	18.14	290.52	18.79	-122.444	37.943	0.54	18.14	290.94	18.68	9	7/14/2014	32:12.1
10930	RSPe_2	-122.444	37.943	0.65	18.30	290.52	18.95	-122.444	37.943	0.51	18.30	290.92	18.80	9	7/14/2014	32:12.0
10931	RSPe_2	-122.444	37.943	0.65	18.26	290.44	18.91	-122.444	37.943	0.59	18.26	290.87	18.86	9	7/14/2014	32:11.9
10932	RSPe_2	-122.444	37.943	0.65	18.25	290.48	18.90	-122.444	37.943	0.54	18.25	290.88	18.79	9	7/14/2014	32:11.8
10933	RSPe_2	-122.444	37.943	0.65	18.24	290.46	18.89	-122.444	37.943	0.59	18.24	290.80	18.83	9	7/14/2014	32:11.7
10934	RSPe_2	-122.444	37.943	0.65	18.21	290.44	18.86	-122.444	37.943	0.54	18.21	290.79	18.75	9	7/14/2014	32:11.6
10935	RSPe_2	-122.444	37.943	0.65	18.26	290.44	18.91	-122.444	37.943	0.54	18.26	290.77	18.80	9	7/14/2014	32:11.5
10936	RSPe_2	-122.444	37.943	0.62	18.24	290.44	18.85	-122.444	37.943	0.51	18.24	290.77	18.74	9	7/14/2014	32:11.4
10937	RSPe_2	-122.444	37.943	0.65	18.24	290.42	18.89	-122.444	37.943	0.54	18.24	290.77	18.78	9	7/14/2014	32:11.3
10938	RSPe_2	-122.444	37.943	0.65	18.28	290.46	18.93	-122.444	37.943	0.51	18.28	290.81	18.79	9	7/14/2014	32:11.2
10939	RSPe_2	-122.444	37.943	0.65	18.22	290.42	18.87	-122.444	37.943	0.54	18.22	290.75	18.76	9	7/14/2014	32:11.1
10940	RSPe_2	-122.444	37.943	0.65	18.26	290.40	18.91	-122.444	37.943	0.47	18.26	290.77	18.74	9	7/14/2014	32:11.0
10941	RSPe_2	-122.444	37.943	0.65	18.25	290.36	18.90	-122.444	37.943	0.51	18.25	290.73	18.75	9	7/14/2014	32:10.9
10942	RSPe_2	-122.444	37.943	0.65	18.24	290.31	18.89	-122.444	37.943	0.51	18.24	290.68	18.75	9	7/14/2014	32:10.8
10943	RSPe_2	-122.444	37.943	0.65	18.24	290.27	18.89	-122.444	37.943	0.59	18.24	290.62	18.83	9	7/14/2014	32:10.7
10944	RSPe_2	-122.444	37.943	0.65	18.24	290.25	18.89	-122.444	37.943	0.54	18.24	290.59	18.78	9	7/14/2014	32:10.6
10945	RSPe_2	-122.444	37.943	0.65	18.22	290.23	18.87	-122.444	37.943	0.54	18.22	290.53	18.76	9	7/14/2014	32:10.5
10946	RSPe_2	-122.444	37.943	0.65	18.29	290.23	18.94	-122.444	37.943	0.54	18.29	290.53	18.83	9	7/14/2014	32:10.4
10947	RSPe_2	-122.444	37.943	0.70	18.21	290.21	18.91	-122.444	37.943	0.54	18.21	290.56	18.75	9	7/14/2014	32:10.3
10948	RSPe_2	-122.444	37.943	0.65	18.23	290.23	18.88	-122.444	37.943	0.54	18.23	290.53	18.77	9	7/14/2014	32:10.2
10949	RSPe_2	-122.444	37.943	0.65	18.21	290.23	18.86	-122.444	37.943	0.59	18.21	290.56	18.80	9	7/14/2014	32:10.1
10950	RSPe_2	-122.444	37.943	0.65	18.21	290.32	18.86	-122.444	37.943	0.59	18.21	290.60	18.80	9	7/14/2014	32:10.0
10951	RSPe_2	-122.444	37.943	0.70	18.19	290.37	18.89	-122.444	37.943	0.59	18.19	290.63	18.78	9	7/14/2014	32:09.9
10952	RSPe_2	-122.444	37.943	0.65	18.19	290.37	18.84	-122.444	37.943	0.59	18.19	290.60	18.79	9	7/14/2014	32:09.8
10953	RSPe_2	-122.444	37.943	0.70	18.17	290.30	18.87	-122.444	37.943	0.54	18.17	290.60	18.71	9	7/14/2014	32:09.7
10954	RSPe_2	-122.444	37.943	0.65	18.18	290.32	18.83	-122.444	37.943	0.59	18.18	290.56	18.77	9	7/14/2014	32:09.6
10955	RSPe_2	-122.444	37.943	0.65	18.16	290.25	18.81	-122.444	37.943	0.59	18.16	290.58	18.75	9	7/14/2014	32:09.5

10956	RSPe_2	-122.444	37.943	0.65	18.15	290.20	18.80	-122.444	37.943	0.59	18.15	290.56	18.74	9	7/14/2014	32:09.4
10957	RSPe_2	-122.444	37.943	0.70	18.12	290.19	18.83	-122.444	37.943	0.59	18.12	290.54	18.72	9	7/14/2014	32:09.3
10958	RSPe_2	-122.444	37.943	0.65	18.12	290.13	18.78	-122.444	37.943	0.54	18.12	290.55	18.67	9	7/14/2014	32:09.2
10959	RSPe_2	-122.444	37.943	0.65	18.13	290.13	18.78	-122.444	37.943	0.59	18.13	290.57	18.73	9	7/14/2014	32:09.1
10960	RSPe_2	-122.444	37.943	0.65	18.07	290.09	18.72	-122.444	37.943	0.59	18.07	290.52	18.66	9	7/14/2014	32:09.0
10961	RSPe_2	-122.444	37.943	0.70	18.12	289.97	18.83	-122.444	37.943	0.59	18.12	290.44	18.72	9	7/14/2014	32:08.9
10962	RSPe_2	-122.444	37.943	0.65	18.06	289.98	18.71	-122.444	37.943	0.59	18.06	290.46	18.66	9	7/14/2014	32:08.8
10963	RSPe_2	-122.444	37.943	0.65	18.14	289.98	18.79	-122.444	37.943	0.63	18.14	290.48	18.77	9	7/14/2014	32:08.7
10964	RSPe_2	-122.444	37.943	0.65	18.06	289.93	18.71	-122.444	37.943	0.59	18.06	290.39	18.66	9	7/14/2014	32:08.6
10965	RSPe_2	-122.444	37.943	0.70	18.10	289.91	18.80	-122.444	37.943	0.59	18.10	290.37	18.69	9	7/14/2014	32:08.5
10966	RSPe_2	-122.444	37.943	0.65	18.05	289.98	18.71	-122.444	37.943	0.59	18.05	290.37	18.65	9	7/14/2014	32:08.4
10967	RSPe_2	-122.444	37.943	0.70	18.10	289.96	18.80	-122.444	37.943	0.63	18.10	290.33	18.72	9	7/14/2014	32:08.3
10968	RSPe_2	-122.444	37.943	0.65	18.06	289.90	18.71	-122.444	37.943	0.59	18.06	290.27	18.66	9	7/14/2014	32:08.2
10969	RSPe_2	-122.444	37.943	0.70	18.06	289.94	18.77	-122.444	37.943	0.63	18.06	290.24	18.69	9	7/14/2014	32:08.1
10970	RSPe_2	-122.444	37.943	0.65	18.05	289.92	18.70	-122.444	37.943	0.59	18.05	290.27	18.64	9	7/14/2014	32:08.0
10971	RSPe_2	-122.444	37.943	0.70	18.09	289.98	18.79	-122.444	37.943	0.59	18.09	290.27	18.68	9	7/14/2014	32:07.9
10972	RSPe_2	-122.444	37.943	0.65	18.04	289.98	18.69	-122.444	37.943	0.54	18.04	290.20	18.58	9	7/14/2014	32:07.8
10973	RSPe_2	-122.444	37.943	0.65	18.03	289.97	18.68	-122.444	37.943	0.59	18.03	290.15	18.62	9	7/14/2014	32:07.7
10974	RSPe_2	-122.444	37.943	0.65	18.03	289.92	18.68	-122.444	37.943	0.54	18.03	290.15	18.57	9	7/14/2014	32:07.6
10975	RSPe_2	-122.444	37.943	0.65	18.05	289.90	18.70	-122.444	37.943	0.59	18.05	290.12	18.64	9	7/14/2014	32:07.5
10976	RSPe_2	-122.444	37.943	0.65	18.08	289.81	18.73	-122.444	37.943	0.59	18.08	290.07	18.67	9	7/14/2014	32:07.4
10977	RSPe_2	-122.444	37.943	0.65	18.00	289.79	18.65	-122.444	37.943	0.59	18.00	290.09	18.59	9	7/14/2014	32:07.3
10978	RSPe_2	-122.444	37.943	0.65	18.07	289.75	18.72	-122.444	37.943	0.54	18.07	290.12	18.61	9	7/14/2014	32:07.2
10979	RSPe_2	-122.444	37.943	0.65	18.03	289.72	18.68	-122.444	37.943	0.54	18.03	290.14	18.57	9	7/14/2014	32:07.1
10980	RSPe_2	-122.444	37.943	0.65	18.02	289.72	18.67	-122.444	37.943	0.54	18.02	290.10	18.56	9	7/14/2014	32:07.0
10981	RSPe_2	-122.444	37.943	0.65	18.05	289.68	18.70	-122.444	37.943	0.59	18.05	290.14	18.64	9	7/14/2014	32:06.9
10982	RSPe_2	-122.444	37.943	0.62	18.04	289.70	18.66	-122.444	37.943	0.59	18.04	290.11	18.63	9	7/14/2014	32:06.8
10983	RSPe_2	-122.444	37.943	0.65	18.08	289.68	18.73	-122.444	37.943	0.59	18.08	290.10	18.67	9	7/14/2014	32:06.7
10984	RSPe_2	-122.444	37.943	0.62	18.04	289.69	18.66	-122.444	37.943	0.54	18.04	290.12	18.58	9	7/14/2014	32:06.6
10985	RSPe_2	-122.444	37.943	0.65	18.10	289.68	18.75	-122.444	37.943	0.59	18.10	290.06	18.69	9	7/14/2014	32:06.5
10986	RSPe_2	-122.444	37.943	0.62	18.07	289.57	18.69	-122.444	37.943	0.54	18.07	289.92	18.61	9	7/14/2014	32:06.4
10987	RSPe_2	-122.444	37.943	0.65	18.05	289.49	18.70	-122.444	37.943	0.59	18.05	289.88	18.64	9	7/14/2014	32:06.3
10988	RSPe_2	-122.444	37.943	0.57	18.01	289.54	18.58	-122.444	37.943	0.54	18.01	289.92	18.55	9	7/14/2014	32:06.2

10989	RSPe_2	-122.444	37.943	0.62	18.03	289.58	18.64	-122.444	37.943	0.54	18.03	289.95	18.57	9	7/14/2014	32:06.1
10990	RSPe_2	-122.444	37.943	0.62	18.09	289.53	18.70	-122.444	37.943	0.54	18.09	289.90	18.63	9	7/14/2014	32:06.0
10991	RSPe_2	-122.444	37.943	0.62	18.04	289.56	18.66	-122.444	37.943	0.54	18.04	289.89	18.58	9	7/14/2014	32:05.9
10992	RSPe_2	-122.444	37.943	0.62	18.05	289.52	18.67	-122.444	37.943	0.51	18.05	289.88	18.56	9	7/14/2014	32:05.8
10993	RSPe_2	-122.444	37.943	0.62	18.09	289.43	18.70	-122.444	37.943	0.51	18.09	289.82	18.60	9	7/14/2014	32:05.7
10994	RSPe_2	-122.444	37.943	0.57	18.10	289.45	18.66	-122.444	37.943	0.51	18.10	289.82	18.61	9	7/14/2014	32:05.6
10995	RSPe_2	-122.444	37.943	0.62	18.07	289.49	18.69	-122.444	37.943	0.51	18.07	289.85	18.58	9	7/14/2014	32:05.5
10996	RSPe_2	-122.444	37.943	0.57	18.19	289.43	18.75	-122.444	37.943	0.47	18.19	289.75	18.66	9	7/14/2014	32:05.4
10997	RSPe_2	-122.444	37.943	0.62	18.12	289.36	18.74	-122.444	37.943	0.51	18.12	289.73	18.63	9	7/14/2014	32:05.3
10998	RSPe_2	-122.444	37.943	0.57	18.11	289.38	18.67	-122.444	37.943	0.47	18.11	289.77	18.58	9	7/14/2014	32:05.2
10999	RSPe_2	-122.444	37.943	0.57	18.12	289.36	18.68	-122.444	37.943	0.47	18.12	289.80	18.59	9	7/14/2014	32:05.1
11000	RSPe_2	-122.444	37.943	0.57	18.14	289.43	18.70	-122.444	37.943	0.47	18.14	289.82	18.61	9	7/14/2014	32:05.0
11001	RSPe_2	-122.444	37.943	0.57	18.15	289.39	18.71	-122.444	37.943	0.47	18.15	289.76	18.62	9	7/14/2014	32:04.9
11002	RSPe_2	-122.444	37.943	0.53	18.15	289.33	18.68	-122.444	37.943	0.47	18.15	289.74	18.62	9	7/14/2014	32:04.8
11003	RSPe_2	-122.444	37.943	0.57	18.17	289.35	18.73	-122.444	37.943	0.51	18.17	289.67	18.67	9	7/14/2014	32:04.7
11004	RSPe_2	-122.444	37.943	0.57	18.19	289.30	18.76	-122.444	37.943	0.42	18.19	289.65	18.62	9	7/14/2014	32:04.6
11005	RSPe_2	-122.444	37.943	0.57	18.19	289.28	18.75	-122.444	37.943	0.42	18.19	289.61	18.61	9	7/14/2014	32:04.5
11006	RSPe_2	-122.444	37.943	0.53	18.19	289.24	18.72	-122.444	37.943	0.39	18.19	289.59	18.58	9	7/14/2014	32:04.4
11007	RSPe_2	-122.444	37.943	0.57	18.24	289.18	18.81	-122.444	37.943	0.42	18.24	289.57	18.66	9	7/14/2014	32:04.3
11008	RSPe_2	-122.444	37.943	0.53	18.24	289.24	18.77	-122.444	37.943	0.42	18.24	289.56	18.66	9	7/14/2014	32:04.2
11009	RSPe_2	-122.444	37.943	0.53	18.26	289.26	18.79	-122.444	37.943	0.42	18.26	289.61	18.69	9	7/14/2014	32:04.1
11010	RSPe_2	-122.444	37.943	0.50	18.27	289.27	18.77	-122.444	37.943	0.39	18.27	289.62	18.66	9	7/14/2014	32:04.0
11011	RSPe_2	-122.444	37.943	0.53	18.35	289.29	18.88	-122.444	37.943	0.39	18.35	289.59	18.74	9	7/14/2014	32:03.9
11012	RSPe_2	-122.444	37.943	0.50	18.31	289.27	18.81	-122.444	37.943	0.39	18.31	289.59	18.70	9	7/14/2014	32:03.8
11013	RSPe_2	-122.444	37.943	0.53	18.35	289.24	18.88	-122.444	37.943	0.39	18.35	289.55	18.74	9	7/14/2014	32:03.7
11014	RSPe_2	-122.444	37.943	0.50	18.34	289.23	18.84	-122.444	37.943	0.39	18.34	289.59	18.73	9	7/14/2014	32:03.6
11015	RSPe_2	-122.444	37.943	0.50	18.36	289.20	18.85	-122.444	37.943	0.39	18.36	289.58	18.75	9	7/14/2014	32:03.5
11016	RSPe_2	-122.444	37.943	0.50	18.36	289.16	18.85	-122.444	37.943	0.39	18.36	289.51	18.75	9	7/14/2014	32:03.4
11017	RSPe_2	-122.444	37.943	0.53	18.34	289.09	18.87	-122.444	37.943	0.39	18.34	289.46	18.73	9	7/14/2014	32:03.3
11018	RSPe_2	-122.444	37.943	0.50	18.38	289.03	18.88	-122.444	37.943	0.34	18.38	289.43	18.72	9	7/14/2014	32:03.2
11019	RSPe_2	-122.444	37.943	0.50	18.37	289.01	18.87	-122.444	37.943	0.39	18.37	289.42	18.76	9	7/14/2014	32:03.1
11020	RSPe_2	-122.444	37.943	0.50	18.43	288.92	18.92	-122.444	37.943	0.34	18.43	289.40	18.77	9	7/14/2014	32:03.0
11021	RSPe_2	-122.444	37.943	0.53	18.50	288.92	19.03	-122.444	37.943	0.34	18.50	289.40	18.84	9	7/14/2014	32:02.9

11022	RSPe_2	-122.444	37.943	0.50	18.53	288.90	19.03	-122.444	37.943	0.34	18.53	289.44	18.87	9	7/14/2014	32:02.8
11023	RSPe_2	-122.444	37.943	0.53	18.54	288.90	19.07	-122.444	37.943	0.34	18.54	289.40	18.88	9	7/14/2014	32:02.7
11024	RSPe_2	-122.444	37.943	0.50	18.58	288.92	19.08	-122.444	37.943	0.31	18.58	289.44	18.88	9	7/14/2014	32:02.6
11025	RSPe_2	-122.444	37.943	0.50	18.56	288.92	19.05	-122.444	37.943	0.31	18.56	289.38	18.86	9	7/14/2014	32:02.5
11026	RSPe_2	-122.444	37.943	0.50	18.59	288.99	19.08	-122.444	37.943	0.34	18.59	289.33	18.93	9	7/14/2014	32:02.4
11027	RSPe_2	-122.444	37.943	0.50	18.65	288.99	19.15	-122.444	37.943	0.34	18.65	289.30	18.99	9	7/14/2014	32:02.3
11028	RSPe_2	-122.444	37.943	0.50	18.58	289.01	19.08	-122.444	37.943	0.31	18.58	289.23	18.88	9	7/14/2014	32:02.2
11029	RSPe_2	-122.444	37.943	0.50	18.57	288.99	19.07	-122.444	37.943	0.34	18.57	289.21	18.91	9	7/14/2014	32:02.1
11030	RSPe_2	-122.444	37.943	0.50	18.59	289.01	19.09	-122.444	37.943	0.31	18.59	289.20	18.90	9	7/14/2014	32:02.0
11031	RSPe_2	-122.444	37.943	0.53	18.66	289.02	19.19	-122.444	37.943	0.31	18.66	289.24	18.96	9	7/14/2014	32:01.9
11032	RSPe_2	-122.444	37.943	0.50	18.59	289.06	19.09	-122.444	37.943	0.34	18.59	289.25	18.93	9	7/14/2014	32:01.8
11033	RSPe_2	-122.444	37.943	0.50	18.57	289.06	19.07	-122.444	37.943	0.34	18.57	289.28	18.91	9	7/14/2014	32:01.7
11034	RSPe_2	-122.444	37.943	0.50	18.62	288.97	19.12	-122.444	37.943	0.34	18.62	289.25	18.96	9	7/14/2014	32:01.6
11035	RSPe_2	-122.444	37.943	0.53	18.59	288.95	19.13	-122.444	37.943	0.39	18.59	289.23	18.98	9	7/14/2014	32:01.5
11036	RSPe_2	-122.444	37.943	0.50	18.59	288.86	19.09	-122.444	37.943	0.34	18.59	289.19	18.93	9	7/14/2014	32:01.4
11037	RSPe_2	-122.444	37.943	0.50	18.59	288.87	19.09	-122.444	37.943	0.34	18.59	289.28	18.93	9	7/14/2014	32:01.3
11038	RSPe_2	-122.444	37.943	0.50	18.61	288.85	19.11	-122.444	37.943	0.34	18.61	289.25	18.95	9	7/14/2014	32:01.2
11039	RSPe_2	-122.444	37.943	0.53	18.68	288.80	19.21	-122.444	37.943	0.39	18.68	289.26	19.07	9	7/14/2014	32:01.1
11040	RSPe_2	-122.444	37.943	0.50	18.66	288.82	19.16	-122.444	37.943	0.34	18.66	289.21	19.00	9	7/14/2014	32:01.0
11041	RSPe_2	-122.444	37.943	0.50	18.71	288.76	19.21	-122.444	37.943	0.39	18.71	289.19	19.10	9	7/14/2014	32:00.9
11042	RSPe_2	-122.444	37.943	0.50	18.73	288.80	19.23	-122.444	37.943	0.34	18.73	289.22	19.07	9	7/14/2014	32:00.8
11043	RSPe_2	-122.444	37.943	0.53	18.76	288.76	19.29	-122.444	37.943	0.39	18.76	289.18	19.15	9	7/14/2014	32:00.7
11044	RSPe_2	-122.444	37.943	0.50	18.80	288.78	19.29	-122.444	37.943	0.34	18.80	289.16	19.14	9	7/14/2014	32:00.6
11045	RSPe_2	-122.444	37.943	0.53	18.79	288.70	19.32	-122.444	37.943	0.39	18.79	289.09	19.18	9	7/14/2014	32:00.5
11046	RSPe_2	-122.444	37.943	0.50	18.80	288.67	19.30	-122.444	37.943	0.34	18.80	289.11	19.14	9	7/14/2014	32:00.4
11047	RSPe_2	-122.444	37.943	0.53	18.87	288.74	19.40	-122.444	37.943	0.34	18.87	289.14	19.21	9	7/14/2014	32:00.3
11048	RSPe_2	-122.444	37.943	0.50	18.85	288.70	19.35	-122.444	37.943	0.39	18.85	289.13	19.24	9	7/14/2014	32:00.2
11049	RSPe_2	-122.444	37.943	0.53	18.87	288.77	19.40	-122.444	37.943	0.39	18.87	289.11	19.26	9	7/14/2014	32:00.1
11050	RSPe_2	-122.444	37.943	0.50	18.90	288.72	19.40	-122.444	37.943	0.34	18.90	289.16	19.24	9	7/14/2014	32:00.0
11051	RSPe_2	-122.444	37.943	0.50	18.90	288.77	19.40	-122.444	37.943	0.34	18.90	289.20	19.24	9	7/14/2014	31:59.9
11052	RSPe_2	-122.444	37.943	0.50	18.94	288.77	19.43	-122.444	37.943	0.34	18.94	289.14	19.28	9	7/14/2014	31:59.8
11053	RSPe_2	-122.444	37.943	0.53	18.98	288.70	19.51	-122.444	37.943	0.39	18.98	289.16	19.37	9	7/14/2014	31:59.7
11054	RSPe_2	-122.444	37.943	0.50	18.97	288.73	19.46	-122.444	37.943	0.39	18.97	289.16	19.36	9	7/14/2014	31:59.6

11055	RSPe_2	-122.444	37.943	0.53	18.96	288.64	19.49	-122.444	37.943	0.39	18.96	289.12	19.35	9	7/14/2014 31:59.5
11056	RSPe_2	-122.444	37.943	0.50	18.96	288.64	19.46	-122.444	37.943	0.34	18.96	289.08	19.30	9	7/14/2014 31:59.4
11057	RSPe_2	-122.444	37.943	0.53	19.00	288.57	19.53	-122.444	37.943	0.39	19.00	289.03	19.39	9	7/14/2014 31:59.3
11058	RSPe_2	-122.444	37.943	0.53	19.00	288.49	19.53	-122.444	37.943	0.39	19.00	288.97	19.39	9	7/14/2014 31:59.2
11059	RSPe_2	-122.444	37.943	0.53	19.03	288.49	19.56	-122.444	37.943	0.42	19.03	288.94	19.45	9	7/14/2014 31:59.1
11060	RSPe_2	-122.444	37.943	0.50	19.03	288.44	19.53	-122.444	37.943	0.34	19.03	288.90	19.37	9	7/14/2014 31:59.0
11061	RSPe_2	-122.444	37.943	0.53	18.98	288.48	19.51	-122.444	37.943	0.39	18.98	288.88	19.37	9	7/14/2014 31:58.9
11062	RSPe_2	-122.444	37.943	0.50	18.94	288.44	19.43	-122.444	37.943	0.34	18.94	288.86	19.28	9	7/14/2014 31:58.8
11063	RSPe_2	-122.444	37.943	0.50	18.96	288.45	19.46	-122.444	37.943	0.34	18.96	288.84	19.30	9	7/14/2014 31:58.7
11064	RSPe_2	-122.444	37.943	0.50	18.88	288.45	19.38	-122.444	37.943	0.31	18.88	288.82	19.19	9	7/14/2014 31:58.6
11065	RSPe_2	-122.444	37.943	0.50	18.81	288.45	19.31	-122.444	37.943	0.31	18.81	288.82	19.12	9	7/14/2014 31:58.5
11066	RSPe_2	-122.444	37.943	0.50	18.90	288.45	19.39	-122.444	37.943	0.31	18.90	288.79	19.20	9	7/14/2014 31:58.4
11067	RSPe_2	-122.444	37.943	0.50	18.81	288.46	19.31	-122.444	37.943	0.34	18.81	288.79	19.15	9	7/14/2014 31:58.3
11068	RSPe_2	-122.444	37.943	0.44	18.74	288.49	19.19	-122.444	37.943	0.31	18.74	288.84	19.05	9	7/14/2014 31:58.2
11069	RSPe_2	-122.444	37.943	0.50	18.70	288.49	19.20	-122.444	37.943	0.34	18.70	288.82	19.04	9	7/14/2014 31:58.1
11070	RSPe_2	-122.444	37.943	0.45	18.73	288.43	19.17	-122.444	37.943	0.31	18.73	288.80	19.03	9	7/14/2014 31:58.0
11071	RSPe_2	-122.444	37.943	0.50	18.66	288.39	19.16	-122.444	37.943	0.34	18.66	288.78	19.00	9	7/14/2014 31:57.9
11072	RSPe_2	-122.444	37.943	0.50	18.63	288.36	19.13	-122.444	37.943	0.34	18.63	288.79	18.97	9	7/14/2014 31:57.8
11073	RSPe_2	-122.444	37.943	0.50	18.62	288.32	19.12	-122.444	37.943	0.34	18.62	288.80	18.96	9	7/14/2014 31:57.7
11074	RSPe_2	-122.444	37.943	0.50	18.50	288.28	19.00	-122.444	37.943	0.34	18.50	288.76	18.84	9	7/14/2014 31:57.6
11075	RSPe_2	-122.444	37.943	0.50	18.59	288.26	19.09	-122.444	37.943	0.34	18.59	288.71	18.93	9	7/14/2014 31:57.5
11076	RSPe_2	-122.444	37.943	0.45	18.50	288.24	18.94	-122.444	37.943	0.31	18.50	288.71	18.80	9	7/14/2014 31:57.4
11077	RSPe_2	-122.444	37.943	0.50	18.46	288.24	18.95	-122.444	37.943	0.34	18.46	288.69	18.80	9	7/14/2014 31:57.3
11078	RSPe_2	-122.444	37.943	0.45	18.43	288.26	18.87	-122.444	37.943	0.31	18.43	288.69	18.73	9	7/14/2014 31:57.2
11079	RSPe_2	-122.444	37.943	0.50	18.40	288.26	18.89	-122.444	37.943	0.31	18.40	288.65	18.70	9	7/14/2014 31:57.1
11080	RSPe_2	-122.444	37.943	0.45	18.33	288.26	18.78	-122.444	37.943	0.26	18.33	288.63	18.59	9	7/14/2014 31:57.0
11081	RSPe_2	-122.444	37.943	0.45	18.36	288.28	18.81	-122.444	37.943	0.26	18.36	288.67	18.62	9	7/14/2014 31:56.9
11082	RSPe_2	-122.444	37.943	0.45	18.26	288.24	18.71	-122.444	37.943	0.26	18.26	288.61	18.52	9	7/14/2014 31:56.8
11083	RSPe_2	-122.444	37.943	0.45	18.26	288.21	18.70	-122.444	37.943	0.26	18.26	288.57	18.51	9	7/14/2014 31:56.7
11084	RSPe_2	-122.444	37.943	0.45	18.19	288.18	18.63	-122.444	37.943	0.22	18.19	288.57	18.41	9	7/14/2014 31:56.6
11085	RSPe_2	-122.444	37.943	0.45	18.17	288.18	18.61	-122.444	37.943	0.26	18.17	288.55	18.42	9	7/14/2014 31:56.5
11086	RSPe_2	-122.444	37.943	0.45	18.14	288.13	18.59	-122.444	37.943	0.26	18.14	288.50	18.40	9	7/14/2014 31:56.4
11087	RSPe_2	-122.444	37.943	0.45	18.17	288.13	18.62	-122.444	37.943	0.26	18.17	288.46	18.43	9	7/14/2014 31:56.3

11088	RSPe_2	-122.444	37.943	0.45	18.18	288.11	18.62	-122.444	37.943	0.22	18.18	288.47	18.40	9	7/14/2014	31:56.2
11089	RSPe_2	-122.444	37.943	0.45	18.09	288.09	18.53	-122.444	37.943	0.26	18.09	288.44	18.34	9	7/14/2014	31:56.1
11090	RSPe_2	-122.444	37.943	0.41	18.12	288.09	18.53	-122.444	37.943	0.22	18.12	288.42	18.34	9	7/14/2014	31:56.0
11091	RSPe_2	-122.444	37.943	0.45	18.11	288.09	18.55	-122.444	37.943	0.26	18.11	288.40	18.36	9	7/14/2014	31:55.9
11092	RSPe_2	-122.444	37.943	0.45	18.05	288.03	18.49	-122.444	37.943	0.22	18.05	288.37	18.27	9	7/14/2014	31:55.8
11093	RSPe_2	-122.444	37.943	0.45	18.10	288.01	18.54	-122.444	37.943	0.22	18.10	288.31	18.32	9	7/14/2014	31:55.7
11094	RSPe_2	-122.444	37.943	0.41	18.10	287.96	18.51	-122.444	37.943	0.22	18.10	288.33	18.32	9	7/14/2014	31:55.6
11095	RSPe_2	-122.444	37.943	0.45	18.12	287.92	18.56	-122.444	37.943	0.26	18.12	288.31	18.37	9	7/14/2014	31:55.5
11096	RSPe_2	-122.444	37.943	0.41	18.05	287.90	18.47	-122.444	37.943	0.22	18.05	288.33	18.28	9	7/14/2014	31:55.4
11097	RSPe_2	-122.444	37.943	0.45	18.06	287.85	18.51	-122.444	37.943	0.22	18.06	288.33	18.29	9	7/14/2014	31:55.3
11098	RSPe_2	-122.444	37.943	0.41	18.05	287.79	18.47	-122.444	37.943	0.22	18.05	288.27	18.28	9	7/14/2014	31:55.2
11099	RSPe_2	-122.444	37.943	0.45	18.05	287.72	18.49	-122.444	37.943	0.22	18.05	288.25	18.27	9	7/14/2014	31:55.1
11100	RSPe_2	-122.444	37.943	0.41	18.09	287.79	18.50	-122.444	37.943	0.10	18.09	288.27	18.19	9	7/14/2014	31:55.0
11101	RSPe_2	-122.444	37.943	0.41	18.10	287.84	18.51	-122.444	37.943	0.19	18.10	288.23	18.28	9	7/14/2014	31:54.9
11102	RSPe_2	-122.444	37.943	0.41	18.08	287.92	18.49	-122.444	37.943	0.19	18.08	288.20	18.27	9	7/14/2014	31:54.8
11103	RSPe_2	-122.444	37.943	0.45	18.15	287.92	18.60	-122.444	37.943	0.19	18.15	288.12	18.34	9	7/14/2014	31:54.7
11104	RSPe_2	-122.444	37.943	0.41	18.08	287.90	18.49	-122.444	37.943	0.22	18.08	288.05	18.30	9	7/14/2014	31:54.6
11105	RSPe_2	-122.444	37.943	0.45	18.09	287.95	18.53	-122.444	37.943	0.22	18.09	288.01	18.31	9	7/14/2014	31:54.5
11106	RSPe_2	-122.444	37.943	0.41	18.07	287.97	18.48	-122.444	37.943	0.19	18.07	288.03	18.26	9	7/14/2014	31:54.4
11107	RSPe_2	-122.444	37.943	0.41	18.12	287.97	18.54	-122.444	37.943	0.19	18.12	288.02	18.31	9	7/14/2014	31:54.3
11108	RSPe_2	-122.444	37.943	0.41	18.10	287.92	18.51	-122.444	37.943	0.19	18.10	287.99	18.29	9	7/14/2014	31:54.2
11109	RSPe_2	-122.444	37.943	0.41	18.15	287.91	18.56	-122.444	37.943	0.19	18.15	288.06	18.34	9	7/14/2014	31:54.1
11110	RSPe_2	-122.444	37.943	0.41	18.19	287.86	18.61	-122.444	37.943	0.22	18.19	288.10	18.42	9	7/14/2014	31:54.0
11111	RSPe_2	-122.444	37.943	0.45	18.22	287.80	18.66	-122.444	37.943	0.31	18.22	288.10	18.52	9	7/14/2014	31:53.9
11112	RSPe_2	-122.444	37.943	0.41	18.20	287.76	18.61	-122.444	37.943	0.26	18.20	288.15	18.46	9	7/14/2014	31:53.8
11113	RSPe_2	-122.444	37.943	0.41	18.24	287.78	18.65	-122.444	37.943	0.31	18.24	288.25	18.54	9	7/14/2014	31:53.7
11114	RSPe_2	-122.444	37.943	0.41	18.26	287.78	18.67	-122.444	37.943	0.26	18.26	288.21	18.51	9	7/14/2014	31:53.6
11115	RSPe_2	-122.444	37.943	0.45	18.26	287.71	18.70	-122.444	37.943	0.31	18.26	288.17	18.56	9	7/14/2014	31:53.5
11116	RSPe_2	-122.444	37.943	0.41	18.26	287.76	18.68	-122.444	37.943	0.22	18.26	288.21	18.49	9	7/14/2014	31:53.4
11117	RSPe_2	-122.444	37.943	0.41	18.35	287.85	18.76	-122.444	37.943	0.22	18.35	288.26	18.57	9	7/14/2014	31:53.3
11118	RSPe_2	-122.444	37.943	0.36	18.42	287.89	18.78	-122.444	37.943	0.22	18.42	288.22	18.64	9	7/14/2014	31:53.2
11119	RSPe_2	-122.444	37.943	0.41	18.31	287.85	18.72	-122.444	37.943	0.22	18.31	288.24	18.53	9	7/14/2014	31:53.1
11120	RSPe_2	-122.444	37.943	0.41	18.32	287.83	18.73	-122.444	37.943	0.19	18.32	288.19	18.50	9	7/14/2014	31:53.0

11121	RSPe_2	-122.444	37.943	0.36	18.36	287.82	18.72	-122.444	37.943	0.14	18.36	288.24	18.49	9	7/14/2014	31:52.9
11122	RSPe_2	-122.444	37.943	0.41	18.38	287.85	18.79	-122.444	37.943	0.14	18.38	288.20	18.52	9	7/14/2014	31:52.8
11123	RSPe_2	-122.444	37.943	0.41	18.40	287.92	18.81	-122.444	37.943	0.19	18.40	288.24	18.59	9	7/14/2014	31:52.7
11124	RSPe_2	-122.444	37.943	0.41	18.43	287.92	18.85	-122.444	37.943	0.19	18.43	288.24	18.62	9	7/14/2014	31:52.6
11125	RSPe_2	-122.444	37.943	0.41	18.45	287.96	18.86	-122.444	37.943	0.19	18.45	288.24	18.64	9	7/14/2014	31:52.5
11126	RSPe_2	-122.444	37.943	0.36	18.43	287.96	18.79	-122.444	37.943	0.22	18.43	288.26	18.65	9	7/14/2014	31:52.4
11127	RSPe_2	-122.444	37.943	0.41	18.47	287.92	18.88	-122.444	37.943	0.22	18.47	288.18	18.69	9	7/14/2014	31:52.3
11128	RSPe_2	-122.444	37.943	0.36	18.45	287.86	18.81	-122.444	37.943	0.22	18.45	288.14	18.67	9	7/14/2014	31:52.2
11129	RSPe_2	-122.444	37.943	0.41	18.58	287.77	18.99	-122.444	37.943	0.22	18.58	288.05	18.80	9	7/14/2014	31:52.1
11130	RSPe_2	-122.444	37.943	0.36	18.48	287.83	18.84	-122.444	37.943	0.19	18.48	288.14	18.67	9	7/14/2014	31:52.0
11131	RSPe_2	-122.444	37.943	0.41	18.58	287.81	18.99	-122.444	37.943	0.26	18.58	288.15	18.84	9	7/14/2014	31:51.9
11132	RSPe_2	-122.444	37.943	0.36	18.59	287.84	18.95	-122.444	37.943	0.22	18.59	288.19	18.81	9	7/14/2014	31:51.8
11133	RSPe_2	-122.444	37.943	0.41	18.56	287.90	18.97	-122.444	37.943	0.19	18.56	288.23	18.74	9	7/14/2014	31:51.7
11134	RSPe_2	-122.444	37.943	0.41	18.58	287.91	18.99	-122.444	37.943	0.22	18.58	288.27	18.80	9	7/14/2014	31:51.6
11135	RSPe_2	-122.444	37.943	0.41	18.60	287.92	19.02	-122.444	37.943	0.19	18.60	288.32	18.79	9	7/14/2014	31:51.5
11136	RSPe_2	-122.444	37.943	0.41	18.61	287.93	19.02	-122.444	37.943	0.19	18.61	288.30	18.80	9	7/14/2014	31:51.4
11137	RSPe_2	-122.444	37.943	0.41	18.72	287.93	19.13	-122.444	37.943	0.19	18.72	288.30	18.91	9	7/14/2014	31:51.3
11138	RSPe_2	-122.444	37.943	0.36	18.68	287.88	19.04	-122.444	37.943	0.14	18.68	288.30	18.82	9	7/14/2014	31:51.2
11139	RSPe_2	-122.444	37.943	0.41	18.70	287.89	19.11	-122.444	37.943	0.19	18.70	288.27	18.89	9	7/14/2014	31:51.1
11140	RSPe_2	-122.444	37.943	0.41	18.69	287.73	19.10	-122.444	37.943	0.19	18.69	288.25	18.88	9	7/14/2014	31:51.0
11141	RSPe_2	-122.444	37.943	0.41	18.78	287.71	19.19	-122.444	37.943	0.22	18.78	288.24	19.00	9	7/14/2014	31:50.9
11142	RSPe_2	-122.444	37.943	0.41	18.73	287.65	19.14	-122.444	37.943	0.14	18.73	288.17	18.87	9	7/14/2014	31:50.8
11143	RSPe_2	-122.444	37.943	0.41	18.77	287.58	19.18	-122.444	37.943	0.19	18.77	288.15	18.96	9	7/14/2014	31:50.7
11144	RSPe_2	-122.444	37.943	0.36	18.81	287.61	19.17	-122.444	37.943	0.19	18.81	288.15	19.00	9	7/14/2014	31:50.6
11145	RSPe_2	-122.444	37.943	0.41	18.84	287.67	19.25	-122.444	37.943	0.22	18.84	288.15	19.06	9	7/14/2014	31:50.5
11146	RSPe_2	-122.444	37.943	0.41	18.81	287.65	19.22	-122.444	37.943	0.19	18.81	288.10	19.00	9	7/14/2014	31:50.4
11147	RSPe_2	-122.444	37.943	0.41	18.81	287.67	19.22	-122.444	37.943	0.19	18.81	288.05	19.00	9	7/14/2014	31:50.3
11148	RSPe_2	-122.444	37.943	0.36	18.82	287.69	19.18	-122.444	37.943	0.19	18.82	288.08	19.01	9	7/14/2014	31:50.2
11149	RSPe_2	-122.444	37.943	0.41	18.80	287.70	19.21	-122.444	37.943	0.19	18.80	288.04	18.99	9	7/14/2014	31:50.1
11150	RSPe_2	-122.444	37.943	0.36	18.82	287.69	19.18	-122.444	37.943	0.19	18.82	288.02	19.01	9	7/14/2014	31:50.0
11151	RSPe_2	-122.444	37.943	0.36	18.74	287.67	19.10	-122.444	37.943	0.19	18.74	288.00	18.93	9	7/14/2014	31:49.9
11152	RSPe_2	-122.444	37.943	0.36	18.57	287.71	18.93	-122.444	37.943	0.14	18.57	287.96	18.70	9	7/14/2014	31:49.8
11153	RSPe_2	-122.444	37.943	0.41	18.53	287.65	18.95	-122.444	37.943	0.19	18.53	287.93	18.72	9	7/14/2014	31:49.7

11154	RSPe_2	-122.444	37.943	0.36	18.50	287.72	18.86	-122.444	37.943	0.14	18.50	287.89	18.63	9	7/14/2014	31:49.6
11155	RSPe_2	-122.444	37.943	0.36	18.42	287.70	18.78	-122.444	37.943	0.14	18.42	287.98	18.55	9	7/14/2014	31:49.5
11156	RSPe_2	-122.444	37.943	0.36	18.40	287.70	18.76	-122.444	37.943	0.10	18.40	287.89	18.50	9	7/14/2014	31:49.4
11157	RSPe_2	-122.444	37.943	0.36	18.38	287.72	18.74	-122.444	37.943	0.10	18.38	288.03	18.48	9	7/14/2014	31:49.3
11158	RSPe_2	-122.444	37.943	0.36	18.39	287.70	18.75	-122.444	37.943	0.10	18.39	288.02	18.49	9	7/14/2014	31:49.2
11159	RSPe_2	-122.444	37.943	0.36	18.40	287.61	18.76	-122.444	37.943	0.10	18.40	288.01	18.51	9	7/14/2014	31:49.1
11160	RSPe_2	-122.444	37.943	0.36	18.35	287.62	18.71	-122.444	37.943	0.14	18.35	288.05	18.48	9	7/14/2014	31:49.0
11161	RSPe_2	-122.444	37.943	0.36	18.35	287.61	18.71	-122.444	37.943	0.10	18.35	288.03	18.45	9	7/14/2014	31:48.9
11162	RSPe_2	-122.444	37.943	0.36	18.30	287.66	18.67	-122.444	37.943	0.10	18.30	288.11	18.41	9	7/14/2014	31:48.8
11163	RSPe_2	-122.444	37.943	0.36	18.35	287.68	18.71	-122.444	37.943	0.10	18.35	288.14	18.45	9	7/14/2014	31:48.7
11164	RSPe_2	-122.444	37.943	0.33	18.32	287.77	18.64	-122.444	37.943	0.05	18.32	288.16	18.37	9	7/14/2014	31:48.6
11165	RSPe_2	-122.444	37.943	0.36	18.33	287.69	18.69	-122.444	37.943	0.10	18.33	288.10	18.43	9	7/14/2014	31:48.5
11166	RSPe_2	-122.444	37.943	0.33	18.28	287.75	18.61	-122.444	37.943	0.10	18.28	288.14	18.38	9	7/14/2014	31:48.4
11167	RSPe_2	-122.444	37.943	0.33	18.33	287.73	18.65	-122.444	37.943	0.10	18.33	288.10	18.43	9	7/14/2014	31:48.3
11168	RSPe_2	-122.444	37.943	0.33	18.28	287.70	18.61	-122.444	37.943	0.10	18.28	288.08	18.38	9	7/14/2014	31:48.2
11169	RSPe_2	-122.444	37.943	0.36	18.28	287.71	18.64	-122.444	37.943	0.10	18.28	288.10	18.38	9	7/14/2014	31:48.1
11170	RSPe_2	-122.444	37.943	0.33	18.24	287.76	18.56	-122.444	37.943	0.10	18.24	288.10	18.34	9	7/14/2014	31:48.0
11171	RSPe_2	-122.444	37.943	0.36	18.25	287.71	18.61	-122.444	37.943	0.10	18.25	288.08	18.35	9	7/14/2014	31:47.9
11172	RSPe_2	-122.444	37.943	0.33	18.21	287.73	18.54	-122.444	37.943	0.05	18.21	288.02	18.26	9	7/14/2014	31:47.8
11173	RSPe_2	-122.444	37.943	0.36	18.26	287.75	18.62	-122.444	37.943	0.10	18.26	287.99	18.37	9	7/14/2014	31:47.7
11174	RSPe_2	-122.444	37.943	0.33	18.24	287.69	18.56	-122.444	37.943	0.05	18.24	287.92	18.29	9	7/14/2014	31:47.6
11175	RSPe_2	-122.444	37.943	0.36	18.22	287.77	18.58	-122.444	37.943	0.10	18.22	287.93	18.32	9	7/14/2014	31:47.5
11176	RSPe_2	-122.444	37.943	0.36	18.23	287.76	18.59	-122.444	37.943	0.10	18.23	288.00	18.33	9	7/14/2014	31:47.4
11177	RSPe_2	-122.444	37.943	0.36	18.24	287.76	18.60	-122.444	37.943	0.10	18.24	287.98	18.34	9	7/14/2014	31:47.3
11178	RSPe_2	-122.444	37.943	0.36	18.21	287.78	18.57	-122.444	37.943	0.10	18.21	288.06	18.31	9	7/14/2014	31:47.2
11179	RSPe_2	-122.444	37.943	0.36	18.23	287.72	18.59	-122.444	37.943	0.14	18.23	288.05	18.36	9	7/14/2014	31:47.1
11180	RSPe_2	-122.444	37.943	0.33	18.24	287.76	18.57	-122.444	37.943	0.10	18.24	288.15	18.34	9	7/14/2014	31:47.0
11181	RSPe_2	-122.444	37.943	0.36	18.26	287.76	18.62	-122.444	37.943	0.10	18.26	288.18	18.37	9	7/14/2014	31:46.9
11182	RSPe_2	-122.444	37.943	0.36	18.26	287.72	18.63	-122.444	37.943	0.05	18.26	288.18	18.32	9	7/14/2014	31:46.8
11183	RSPe_2	-122.444	37.943	0.36	18.26	287.67	18.63	-122.444	37.943	0.10	18.26	288.20	18.37	9	7/14/2014	31:46.7
11184	RSPe_2	-122.444	37.943	0.33	18.30	287.77	18.63	-122.444	37.943	0.05	18.30	288.24	18.36	9	7/14/2014	31:46.6
11185	RSPe_2	-122.444	37.943	0.36	18.32	287.70	18.68	-122.444	37.943	0.05	18.32	288.20	18.37	9	7/14/2014	31:46.5
11186	RSPe_2	-122.444	37.943	0.33	18.34	287.66	18.67	-122.444	37.943	0.05	18.34	288.18	18.40	9	7/14/2014	31:46.4

11187	RSPe_2	-122.444	37.943	0.36	18.36	287.57	18.73	-122.444	37.943	0.05	18.36	288.10	18.42	9	7/14/2014	31:46.3
11188	RSPe_2	-122.444	37.943	0.33	18.46	287.57	18.78	-122.444	37.943	0.05	18.46	288.07	18.51	9	7/14/2014	31:46.2
11189	RSPe_2	-122.444	37.943	0.33	18.47	287.61	18.79	-122.444	37.943	0.05	18.47	288.05	18.52	9	7/14/2014	31:46.1
11190	RSPe_2	-122.444	37.943	0.33	18.43	287.66	18.76	-122.444	37.943	0.05	18.43	288.03	18.48	9	7/14/2014	31:46.0
11191	RSPe_2	-122.444	37.943	0.36	18.46	287.70	18.82	-122.444	37.943	0.05	18.46	288.05	18.51	9	7/14/2014	31:45.9
11192	RSPe_2	-122.444	37.943	0.36	18.49	287.68	18.85	-122.444	37.943	0.05	18.49	288.08	18.54	9	7/14/2014	31:45.8
11193	RSPe_2	-122.444	37.943	0.36	18.52	287.73	18.88	-122.444	37.943	0.05	18.52	288.05	18.57	9	7/14/2014	31:45.7
11194	RSPe_2	-122.444	37.943	0.36	18.59	287.75	18.96	-122.444	37.943	0.02	18.59	288.05	18.61	9	7/14/2014	31:45.6
11195	RSPe_2	-122.444	37.943	0.36	18.68	287.66	19.04	-122.444	37.943	0.05	18.68	288.01	18.73	9	7/14/2014	31:45.5
11196	RSPe_2	-122.444	37.943	0.33	18.57	287.73	18.90	-122.444	37.943	0.05	18.57	288.01	18.63	9	7/14/2014	31:45.4
11197	RSPe_2	-122.444	37.943	0.36	18.66	287.62	19.02	-122.444	37.943	0.10	18.66	287.95	18.76	9	7/14/2014	31:45.3
11198	RSPe_2	-122.444	37.943	0.36	18.63	287.64	18.99	-122.444	37.943	0.05	18.63	287.97	18.68	9	7/14/2014	31:45.2
11199	RSPe_2	-122.444	37.943	0.41	18.65	287.62	19.06	-122.444	37.943	0.10	18.65	287.93	18.75	9	7/14/2014	31:45.1
11200	RSPe_2	-122.444	37.943	0.36	18.63	287.58	18.99	-122.444	37.943	0.05	18.63	287.94	18.68	9	7/14/2014	31:45.0
11201	RSPe_2	-122.444	37.943	0.36	18.63	287.56	18.99	-122.444	37.943	0.05	18.63	287.95	18.69	9	7/14/2014	31:44.9
11202	RSPe_2	-122.444	37.943	0.36	18.75	287.53	19.11	-122.444	37.943	0.05	18.75	287.97	18.80	9	7/14/2014	31:44.8
11203	RSPe_2	-122.444	37.943	0.36	18.67	287.56	19.04	-122.444	37.943	0.05	18.67	287.99	18.73	9	7/14/2014	31:44.7
11204	RSPe_2	-122.444	37.943	0.36	18.74	287.58	19.11	-122.444	37.943	0.05	18.74	288.04	18.80	9	7/14/2014	31:44.6
11205	RSPe_2	-122.444	37.943	0.36	18.83	287.61	19.19	-122.444	37.943	0.05	18.83	288.04	18.88	9	7/14/2014	31:44.5
11206	RSPe_2	-122.444	37.943	0.33	18.80	287.60	19.13	-122.444	37.943	0.05	18.80	288.04	18.85	9	7/14/2014	31:44.4
11207	RSPe_2	-122.444	37.943	0.36	18.81	287.54	19.17	-122.444	37.943	0.05	18.81	288.02	18.87	9	7/14/2014	31:44.3
11208	RSPe_2	-122.444	37.943	0.36	18.85	287.47	19.21	-122.444	37.943	0.05	18.85	287.95	18.90	9	7/14/2014	31:44.2
11209	RSPe_2	-122.444	37.943	0.36	18.90	287.41	19.27	-122.444	37.943	0.05	18.90	287.89	18.96	9	7/14/2014	31:44.1
11210	RSPe_2	-122.444	37.943	0.36	18.88	287.25	19.24	-122.444	37.943	0.05	18.88	287.75	18.94	9	7/14/2014	31:44.0
11211	RSPe_2	-122.444	37.943	0.41	18.94	287.35	19.35	-122.444	37.943	0.05	18.94	287.76	18.99	9	7/14/2014	31:43.9
11212	RSPe_2	-122.444	37.943	0.36	18.97	287.27	19.34	-122.444	37.943	0.05	18.97	287.71	19.03	9	7/14/2014	31:43.8
11213	RSPe_2	-122.444	37.943	0.41	19.03	287.30	19.45	-122.444	37.943	0.10	19.03	287.69	19.14	9	7/14/2014	31:43.7
11214	RSPe_2	-122.444	37.943	0.36	19.04	287.35	19.41	-122.444	37.943	0.10	19.04	287.65	19.15	9	7/14/2014	31:43.6
11215	RSPe_2	-122.444	37.943	0.36	19.10	287.35	19.47	-122.444	37.943	0.10	19.10	287.67	19.21	9	7/14/2014	31:43.5
11216	RSPe_2	-122.444	37.943	0.36	19.09	287.39	19.45	-122.444	37.943	0.10	19.09	287.71	19.19	9	7/14/2014	31:43.4
11217	RSPe_2	-122.444	37.943	0.41	19.20	287.37	19.62	-122.444	37.943	0.14	19.20	287.68	19.34	9	7/14/2014	31:43.3
11218	RSPe_2	-122.444	37.943	0.36	19.12	287.33	19.48	-122.444	37.943	0.10	19.12	287.63	19.22	9	7/14/2014	31:43.2
11219	RSPe_2	-122.444	37.943	0.41	19.22	287.33	19.63	-122.444	37.943	0.10	19.22	287.57	19.32	9	7/14/2014	31:43.1

11220	RSPe_2	-122.444	37.943	0.41	19.20	287.29	19.61	-122.444	37.943	0.05	19.20	287.59	19.25	9	7/14/2014	31:43.0
11221	RSPe_2	-122.444	37.943	0.41	19.20	287.30	19.62	-122.444	37.943	0.05	19.20	287.68	19.26	9	7/14/2014	31:42.9
11222	RSPe_2	-122.444	37.943	0.36	19.18	287.35	19.55	-122.444	37.943	0.02	19.18	287.72	19.20	9	7/14/2014	31:42.8
11223	RSPe_2	-122.444	37.943	0.41	19.15	287.20	19.56	-122.444	37.943	0.05	19.15	287.70	19.20	9	7/14/2014	31:42.7
11224	RSPe_2	-122.444	37.943	0.41	19.16	287.20	19.57	-122.444	37.943	0.10	19.16	287.77	19.26	9	7/14/2014	31:42.6
11225	RSPe_2	-122.444	37.943	0.41	19.13	287.13	19.54	-122.444	37.943	0.10	19.13	287.68	19.23	9	7/14/2014	31:42.5
11226	RSPe_2	-122.444	37.943	0.36	18.97	287.07	19.33	-122.444	37.943	0.05	18.97	287.61	19.02	9	7/14/2014	31:42.4
11227	RSPe_2	-122.444	37.943	0.41	19.01	287.09	19.42	-122.444	37.943	0.14	19.01	287.58	19.15	9	7/14/2014	31:42.3
11228	RSPe_2	-122.444	37.943	0.36	18.88	287.07	19.24	-122.444	37.943	0.10	18.88	287.48	18.99	9	7/14/2014	31:42.2
11229	RSPe_2	-122.444	37.943	0.41	18.80	287.03	19.22	-122.444	37.943	0.10	18.80	287.51	18.91	9	7/14/2014	31:42.1
11230	RSPe_2	-122.444	37.943	0.41	18.84	287.11	19.25	-122.444	37.943	0.10	18.84	287.53	18.94	9	7/14/2014	31:42.0
11231	RSPe_2	-122.444	37.943	0.41	18.75	287.09	19.16	-122.444	37.943	0.10	18.75	287.48	18.85	9	7/14/2014	31:41.9
11232	RSPe_2	-122.444	37.943	0.41	18.82	287.08	19.23	-122.444	37.943	0.14	18.82	287.51	18.96	9	7/14/2014	31:41.8
11233	RSPe_2	-122.444	37.943	0.41	18.77	287.11	19.18	-122.444	37.943	0.14	18.77	287.49	18.91	9	7/14/2014	31:41.7
11234	RSPe_2	-122.444	37.943	0.41	18.64	287.14	19.06	-122.444	37.943	0.05	18.64	287.51	18.70	9	7/14/2014	31:41.6
11235	RSPe_2	-122.444	37.943	0.45	18.60	287.14	19.05	-122.444	37.943	0.10	18.60	287.51	18.71	9	7/14/2014	31:41.5
11236	RSPe_2	-122.444	37.943	0.41	18.63	287.12	19.04	-122.444	37.943	0.10	18.63	287.56	18.73	9	7/14/2014	31:41.4
11237	RSPe_2	-122.444	37.943	0.45	18.61	287.12	19.06	-122.444	37.943	0.10	18.61	287.56	18.72	9	7/14/2014	31:41.3
11238	RSPe_2	-122.444	37.943	0.41	18.54	287.01	18.95	-122.444	37.943	0.10	18.54	287.47	18.65	9	7/14/2014	31:41.2
11239	RSPe_2	-122.444	37.943	0.41	18.58	287.01	18.99	-122.444	37.943	0.14	18.58	287.42	18.72	9	7/14/2014	31:41.1
11240	RSPe_2	-122.444	37.943	0.41	18.52	286.94	18.93	-122.444	37.943	0.14	18.52	287.42	18.66	9	7/14/2014	31:41.0
11241	RSPe_2	-122.444	37.943	0.45	18.62	286.95	19.07	-122.444	37.943	0.14	18.62	287.39	18.76	9	7/14/2014	31:40.9
11242	RSPe_2	-122.444	37.943	0.41	18.52	286.97	18.93	-122.444	37.943	0.14	18.52	287.38	18.66	9	7/14/2014	31:40.8
11243	RSPe_2	-122.444	37.943	0.41	18.51	286.93	18.92	-122.444	37.943	0.14	18.51	287.36	18.65	9	7/14/2014	31:40.7
11244	RSPe_2	-122.444	37.943	0.41	18.59	287.06	19.01	-122.444	37.943	0.10	18.59	287.47	18.70	9	7/14/2014	31:40.6
11245	RSPe_2	-122.444	37.943	0.41	18.50	287.14	18.92	-122.444	37.943	0.10	18.50	287.47	18.61	9	7/14/2014	31:40.5
11246	RSPe_2	-122.444	37.943	0.41	18.57	287.17	18.98	-122.444	37.943	0.10	18.57	287.52	18.67	9	7/14/2014	31:40.4
11247	RSPe_2	-122.444	37.943	0.41	18.56	287.15	18.97	-122.444	37.943	0.05	18.56	287.50	18.61	9	7/14/2014	31:40.3
11248	RSPe_2	-122.444	37.943	0.41	18.55	287.19	18.96	-122.444	37.943	0.05	18.55	287.54	18.61	9	7/14/2014	31:40.2
11249	RSPe_2	-122.444	37.943	0.41	18.57	287.11	18.99	-122.444	37.943	0.10	18.57	287.41	18.68	9	7/14/2014	31:40.1
11250	RSPe_2	-122.444	37.943	0.41	18.50	287.04	18.91	-122.444	37.943	0.05	18.50	287.43	18.55	9	7/14/2014	31:40.0
11251	RSPe_2	-122.444	37.943	0.41	18.51	287.08	18.92	-122.444	37.943	0.10	18.51	287.39	18.61	9	7/14/2014	31:39.9
11252	RSPe_2	-122.444	37.943	0.41	18.50	287.00	18.92	-122.444	37.943	0.10	18.50	287.37	18.61	9	7/14/2014	31:39.8

11253	RSPe_2	-122.444	37.943	0.45	18.50	286.89	18.95	-122.444	37.943	0.10	18.50	287.28	18.61	9	7/14/2014	31:39.7
11254	RSPe_2	-122.444	37.943	0.41	18.52	286.80	18.94	-122.444	37.943	0.14	18.52	287.17	18.66	9	7/14/2014	31:39.6
11255	RSPe_2	-122.444	37.943	0.45	18.52	286.87	18.97	-122.444	37.943	0.19	18.52	287.26	18.71	9	7/14/2014	31:39.5
11256	RSPe_2	-122.444	37.943	0.41	18.58	286.96	18.99	-122.444	37.943	0.14	18.58	287.32	18.72	9	7/14/2014	31:39.4
11257	RSPe_2	-122.444	37.943	0.41	18.63	286.92	19.04	-122.444	37.943	0.14	18.63	287.33	18.76	9	7/14/2014	31:39.3
11258	RSPe_2	-122.444	37.943	0.41	18.58	286.85	18.99	-122.444	37.943	0.10	18.58	287.29	18.68	9	7/14/2014	31:39.2
11259	RSPe_2	-122.444	37.943	0.41	18.59	286.81	19.01	-122.444	37.943	0.10	18.59	287.33	18.70	9	7/14/2014	31:39.1
11260	RSPe_2	-122.444	37.943	0.41	18.63	286.85	19.05	-122.444	37.943	0.05	18.63	287.26	18.69	9	7/14/2014	31:39.0
11261	RSPe_2	-122.444	37.943	0.41	18.71	286.81	19.12	-122.444	37.943	0.05	18.71	287.22	18.77	9	7/14/2014	31:38.9
11262	RSPe_2	-122.444	37.943	0.41	18.74	286.81	19.16	-122.444	37.943	0.02	18.74	287.18	18.76	9	7/14/2014	31:38.8
11263	RSPe_2	-122.444	37.943	0.41	18.73	286.85	19.15	-122.444	37.943	0.05	18.73	287.13	18.79	9	7/14/2014	31:38.7
11264	RSPe_2	-122.444	37.943	0.41	18.85	286.85	19.26	-122.444	37.943	0.02	18.85	287.14	18.87	9	7/14/2014	31:38.6
11265	RSPe_2	-122.444	37.943	0.41	18.80	286.92	19.22	-122.444	37.943	0.02	18.80	287.16	18.82	9	7/14/2014	31:38.5
11266	RSPe_2	-122.444	37.943	0.36	18.86	286.92	19.22	-122.444	37.943	0.02	18.86	287.11	18.88	9	7/14/2014	31:38.4
11267	RSPe_2	-122.444	37.943	0.41	18.85	287.01	19.26	-122.444	37.943	0.02	18.85	287.18	18.87	9	7/14/2014	31:38.3
11268	RSPe_2	-122.444	37.943	0.36	18.94	286.97	19.30	-122.444	37.943	0.05	18.94	287.14	18.99	9	7/14/2014	31:38.2
11269	RSPe_2	-122.444	37.943	0.41	18.96	286.95	19.37	-122.444	37.943	0.02	18.96	287.12	18.98	9	7/14/2014	31:38.1
11270	RSPe_2	-122.444	37.943	0.41	19.00	286.95	19.41	-122.444	37.943	0.05	19.00	287.12	19.05	9	7/14/2014	31:38.0
11271	RSPe_2	-122.444	37.943	0.41	18.97	286.92	19.38	-122.444	37.943	0.14	18.97	287.14	19.10	9	7/14/2014	31:37.9
11272	RSPe_2	-122.444	37.943	0.36	18.94	286.82	19.30	-122.444	37.943	0.05	18.94	287.12	18.99	9	7/14/2014	31:37.8
11273	RSPe_2	-122.444	37.943	0.41	18.98	286.77	19.39	-122.444	37.943	0.05	18.98	287.14	19.03	9	7/14/2014	31:37.7
11274	RSPe_2	-122.444	37.943	0.41	18.90	286.73	19.32	-122.444	37.943	0.05	18.90	287.17	18.96	9	7/14/2014	31:37.6
11275	RSPe_2	-122.444	37.943	0.41	18.90	286.71	19.32	-122.444	37.943	0.05	18.90	287.21	18.96	9	7/14/2014	31:37.5
11276	RSPe_2	-122.444	37.943	0.36	18.86	286.66	19.22	-122.444	37.943	0.05	18.86	287.17	18.91	9	7/14/2014	31:37.4
11277	RSPe_2	-122.444	37.943	0.41	18.86	286.60	19.27	-122.444	37.943	0.05	18.86	287.16	18.91	9	7/14/2014	31:37.3
11278	RSPe_2	-122.444	37.943	0.36	18.83	286.56	19.20	-122.444	37.943	0.02	18.83	287.15	18.85	9	7/14/2014	31:37.2
11279	RSPe_2	-122.444	37.943	0.41	18.82	286.58	19.23	-122.444	37.943	0.05	18.82	287.08	18.87	9	7/14/2014	31:37.1
11280	RSPe_2	-122.444	37.943	0.41	18.80	286.64	19.22	-122.444	37.943	0.02	18.80	287.08	18.82	9	7/14/2014	31:37.0
11281	RSPe_2	-122.444	37.943	0.41	18.80	286.73	19.21	-122.444	37.943	0.05	18.80	287.11	18.85	9	7/14/2014	31:36.9
11282	RSPe_2	-122.444	37.943	0.41	18.76	286.76	19.18	-122.444	37.943	0.05	18.76	287.06	18.82	9	7/14/2014	31:36.8
11283	RSPe_2	-122.444	37.943	0.41	18.75	286.69	19.16	-122.444	37.943	0.05	18.75	286.97	18.80	9	7/14/2014	31:36.7
11284	RSPe_2	-122.444	37.943	0.41	18.70	286.69	19.11	-122.444	37.943	0.05	18.70	286.93	18.75	9	7/14/2014	31:36.6
11285	RSPe_2	-122.444	37.943	0.41	18.68	286.67	19.09	-122.444	37.943	0.05	18.68	286.89	18.73	9	7/14/2014	31:36.5

11286	RSPe_2	-122.444	37.943	0.36	18.63	286.67	18.99	-122.444	37.943	0.05	18.63	286.89	18.69	9	7/14/2014	31:36.4
11287	RSPe_2	-122.444	37.943	0.41	18.63	286.71	19.04	-122.444	37.943	0.05	18.63	286.95	18.68	9	7/14/2014	31:36.3
11288	RSPe_2	-122.444	37.943	0.41	18.62	286.67	19.03	-122.444	37.943	0.05	18.62	286.93	18.67	9	7/14/2014	31:36.2
11289	RSPe_2	-122.444	37.943	0.41	18.64	286.63	19.06	-122.444	37.943	0.05	18.64	286.91	18.70	9	7/14/2014	31:36.1
11290	RSPe_2	-122.444	37.943	0.41	18.62	286.59	19.03	-122.444	37.943	0.05	18.62	286.96	18.67	9	7/14/2014	31:36.0
11291	RSPe_2	-122.444	37.943	0.45	18.60	286.57	19.05	-122.444	37.943	0.10	18.60	287.03	18.71	9	7/14/2014	31:35.9
11292	RSPe_2	-122.444	37.943	0.41	18.63	286.57	19.04	-122.444	37.943	0.05	18.63	287.09	18.68	9	7/14/2014	31:35.8
11293	RSPe_2	-122.444	37.943	0.45	18.63	286.52	19.07	-122.444	37.943	0.05	18.63	287.09	18.68	9	7/14/2014	31:35.7
11294	RSPe_2	-122.444	37.943	0.41	18.67	286.39	19.09	-122.444	37.943	0.06	18.67	286.98	18.73	9	7/14/2014	31:35.6
11295	RSPe_2	-122.444	37.943	0.45	18.65	286.41	19.10	-122.444	37.943	0.06	18.65	287.02	18.70	9	7/14/2014	31:35.5
11296	RSPe_2	-122.444	37.943	0.41	18.63	286.52	19.04	-122.444	37.943	0.06	18.63	287.16	18.68	9	7/14/2014	31:35.4
11297	RSPe_2	-122.444	37.943	0.45	18.64	286.59	19.09	-122.444	37.943	0.06	18.64	287.21	18.70	9	7/14/2014	31:35.3
11298	RSPe_2	-122.444	37.943	0.41	18.63	286.55	19.05	-122.444	37.943	0.02	18.63	287.13	18.65	9	7/14/2014	31:35.2
11299	RSPe_2	-122.444	37.943	0.45	18.65	286.53	19.10	-122.444	37.943	0.06	18.65	287.05	18.70	9	7/14/2014	31:35.1
11300	RSPe_2	-122.444	37.943	0.41	18.67	286.48	19.09	-122.444	37.943	0.02	18.67	286.96	18.69	9	7/14/2014	31:35.0
11301	RSPe_2	-122.444	37.943	0.45	18.67	286.48	19.12	-122.444	37.943	0.06	18.67	286.90	18.73	9	7/14/2014	31:34.9
11302	RSPe_2	-122.444	37.943	0.41	18.70	286.57	19.12	-122.444	37.943	0.02	18.70	286.88	18.72	9	7/14/2014	31:34.8
11303	RSPe_2	-122.444	37.943	0.45	18.66	286.66	19.11	-122.444	37.943	0.06	18.66	286.87	18.72	9	7/14/2014	31:34.7
11304	RSPe_2	-122.444	37.943	0.45	18.67	286.73	19.12	-122.444	37.943	0.02	18.67	286.90	18.69	9	7/14/2014	31:34.6
11305	RSPe_2	-122.444	37.943	0.45	18.69	286.75	19.14	-122.444	37.943	0.06	18.69	286.92	18.75	9	7/14/2014	31:34.5
11306	RSPe_2	-122.444	37.943	0.45	18.70	286.75	19.14	-122.444	37.943	0.06	18.70	286.92	18.75	9	7/14/2014	31:34.4
11307	RSPe_2	-122.444	37.943	0.45	18.71	286.71	19.16	-122.444	37.943	0.06	18.71	286.84	18.77	9	7/14/2014	31:34.3
11308	RSPe_2	-122.444	37.943	0.45	18.73	286.82	19.18	-122.444	37.943	0.06	18.73	286.91	18.78	9	7/14/2014	31:34.2
11309	RSPe_2	-122.444	37.943	0.45	18.74	286.82	19.19	-122.444	37.943	0.06	18.74	286.99	18.80	9	7/14/2014	31:34.1
11310	RSPe_2	-122.444	37.943	0.45	18.77	286.82	19.22	-122.444	37.943	0.06	18.77	286.99	18.83	9	7/14/2014	31:34.0
11311	RSPe_2	-122.444	37.943	0.45	18.73	286.82	19.18	-122.444	37.943	0.06	18.73	287.02	18.79	9	7/14/2014	31:33.9
11312	RSPe_2	-122.444	37.943	0.45	18.76	286.73	19.21	-122.444	37.943	0.06	18.76	287.02	18.82	9	7/14/2014	31:33.8
11313	RSPe_2	-122.444	37.943	0.50	18.79	286.71	19.29	-122.444	37.943	0.06	18.79	287.06	18.84	9	7/14/2014	31:33.7
11314	RSPe_2	-122.444	37.943	0.45	18.83	286.63	19.28	-122.444	37.943	0.06	18.83	287.09	18.88	9	7/14/2014	31:33.6
11315	RSPe_2	-122.444	37.943	0.45	18.81	286.63	19.26	-122.444	37.943	0.06	18.81	287.17	18.87	9	7/14/2014	31:33.5
11316	RSPe_2	-122.444	37.943	0.45	18.90	286.58	19.34	-122.444	37.943	0.02	18.90	287.18	18.92	9	7/14/2014	31:33.4
11317	RSPe_2	-122.444	37.943	0.45	19.08	286.56	19.52	-122.444	37.943	0.02	19.08	287.24	19.10	9	7/14/2014	31:33.3
11318	RSPe_2	-122.444	37.943	0.45	19.20	286.58	19.65	-122.444	37.943	-0.03	19.20	287.28	19.17	9	7/14/2014	31:33.2

11319	RSPe_2	-122.444	37.943	0.45	19.17	286.61	19.61	-122.444	37.943	0.02	19.17	287.29	19.19	9	7/14/2014	31:33.1
11320	RSPe_2	-122.444	37.943	0.45	19.15	286.58	19.60	-122.444	37.943	0.02	19.15	287.24	19.17	9	7/14/2014	31:33.0
11321	RSPe_2	-122.444	37.943	0.50	19.12	286.63	19.62	-122.444	37.943	0.06	19.12	287.18	19.17	9	7/14/2014	31:32.9
11322	RSPe_2	-122.444	37.943	0.45	19.17	286.67	19.62	-122.444	37.943	0.06	19.17	287.15	19.23	9	7/14/2014	31:32.8
11323	RSPe_2	-122.444	37.943	0.50	19.13	286.68	19.63	-122.444	37.943	0.06	19.13	287.05	19.18	9	7/14/2014	31:32.7
11324	RSPe_2	-122.444	37.943	0.50	19.09	286.70	19.59	-122.444	37.943	0.06	19.09	287.03	19.15	9	7/14/2014	31:32.6
11325	RSPe_2	-122.444	37.943	0.50	19.02	286.74	19.52	-122.444	37.943	0.06	19.02	287.01	19.08	9	7/14/2014	31:32.5
11326	RSPe_2	-122.444	37.943	0.45	19.01	286.72	19.46	-122.444	37.943	0.06	19.01	286.95	19.07	9	7/14/2014	31:32.4
11327	RSPe_2	-122.444	37.943	0.50	18.97	286.77	19.47	-122.444	37.943	0.06	18.97	287.05	19.02	9	7/14/2014	31:32.3
11328	RSPe_2	-122.444	37.943	0.45	18.90	286.87	19.34	-122.444	37.943	0.06	18.90	287.11	18.95	9	7/14/2014	31:32.2
11329	RSPe_2	-122.444	37.943	0.50	18.86	286.90	19.36	-122.444	37.943	0.02	18.86	287.16	18.88	9	7/14/2014	31:32.1
11330	RSPe_2	-122.444	37.943	0.50	19.01	286.88	19.51	-122.444	37.943	0.02	19.01	287.16	19.03	9	7/14/2014	31:32.0
11331	RSPe_2	-122.444	37.943	0.50	18.86	286.93	19.36	-122.444	37.943	0.02	18.86	287.20	18.88	9	7/14/2014	31:31.9
11332	RSPe_2	-122.444	37.943	0.50	18.84	286.88	19.34	-122.444	37.943	-0.03	18.84	287.27	18.81	9	7/14/2014	31:31.8
11333	RSPe_2	-122.444	37.943	0.50	18.85	286.75	19.35	-122.444	37.943	0.02	18.85	287.30	18.87	9	7/14/2014	31:31.7
11334	RSPe_2	-122.444	37.943	0.50	18.79	286.71	19.29	-122.444	37.943	0.02	18.79	287.25	18.81	9	7/14/2014	31:31.6
11335	RSPe_2	-122.444	37.943	0.50	18.78	286.66	19.28	-122.444	37.943	-0.03	18.78	287.23	18.75	9	7/14/2014	31:31.5
11336	RSPe_2	-122.444	37.943	0.50	18.80	286.69	19.30	-122.444	37.943	0.02	18.80	287.27	18.82	9	7/14/2014	31:31.4
11337	RSPe_2	-122.444	37.943	0.50	18.78	286.77	19.28	-122.444	37.943	0.02	18.78	287.27	18.80	9	7/14/2014	31:31.3
11338	RSPe_2	-122.444	37.943	0.50	18.80	286.64	19.30	-122.444	37.943	0.02	18.80	287.10	18.82	9	7/14/2014	31:31.2
11339	RSPe_2	-122.444	37.943	0.53	18.83	286.66	19.37	-122.444	37.943	0.06	18.83	287.14	18.89	9	7/14/2014	31:31.1
11340	RSPe_2	-122.444	37.943	0.50	18.87	286.75	19.37	-122.444	37.943	0.06	18.87	287.19	18.92	9	7/14/2014	31:31.0
11341	RSPe_2	-122.444	37.943	0.53	18.87	286.80	19.41	-122.444	37.943	0.02	18.87	287.21	18.89	9	7/14/2014	31:30.9
11342	RSPe_2	-122.444	37.943	0.50	19.01	286.80	19.51	-122.444	37.943	-0.03	19.01	287.21	18.98	9	7/14/2014	31:30.8
11343	RSPe_2	-122.444	37.943	0.50	18.97	286.78	19.47	-122.444	37.943	0.02	18.97	287.17	18.99	9	7/14/2014	31:30.7
11344	RSPe_2	-122.444	37.943	0.50	18.97	286.69	19.47	-122.444	37.943	-0.03	18.97	287.17	18.94	9	7/14/2014	31:30.6
11345	RSPe_2	-122.444	37.943	0.50	18.94	286.74	19.44	-122.444	37.943	-0.06	18.94	287.15	18.87	9	7/14/2014	31:30.5
11346	RSPe_2	-122.444	37.943	0.50	18.95	286.76	19.45	-122.444	37.943	-0.06	18.95	287.19	18.89	9	7/14/2014	31:30.4
11347	RSPe_2	-122.444	37.943	0.53	18.94	286.74	19.47	-122.444	37.943	-0.03	18.94	287.17	18.91	9	7/14/2014	31:30.3
11348	RSPe_2	-122.444	37.943	0.50	19.04	286.76	19.54	-122.444	37.943	-0.03	19.04	287.11	19.01	9	7/14/2014	31:30.2
11349	RSPe_2	-122.444	37.943	0.50	19.09	286.78	19.59	-122.444	37.943	-0.06	19.09	287.19	19.03	9	7/14/2014	31:30.1
11350	RSPe_2	-122.444	37.943	0.50	19.10	286.87	19.60	-122.444	37.943	-0.06	19.10	287.19	19.04	9	7/14/2014	31:30.0
11351	RSPe_2	-122.444	37.943	0.53	19.09	286.89	19.62	-122.444	37.943	-0.06	19.09	287.24	19.03	9	7/14/2014	31:29.9

11352	RSPe_2	-122.444	37.943	0.50	19.16	286.90	19.66	-122.444	37.943	-0.06	19.16	287.15	19.09	9	7/14/2014	31:29.8
11353	RSPe_2	-122.444	37.943	0.53	19.09	286.92	19.62	-122.444	37.943	0.02	19.09	287.13	19.11	9	7/14/2014	31:29.7
11354	RSPe_2	-122.444	37.943	0.53	19.13	286.81	19.66	-122.444	37.943	-0.03	19.13	287.02	19.10	9	7/14/2014	31:29.6
11355	RSPe_2	-122.444	37.943	0.53	19.09	286.85	19.62	-122.444	37.943	-0.06	19.09	287.00	19.03	9	7/14/2014	31:29.5
11356	RSPe_2	-122.444	37.943	0.53	19.12	286.77	19.65	-122.444	37.943	-0.03	19.12	287.01	19.09	9	7/14/2014	31:29.4
11357	RSPe_2	-122.444	37.943	0.57	19.10	286.70	19.67	-122.444	37.943	-0.06	19.10	286.96	19.03	9	7/14/2014	31:29.3
11358	RSPe_2	-122.444	37.943	0.53	19.12	286.66	19.65	-122.444	37.943	-0.06	19.12	286.96	19.05	9	7/14/2014	31:29.2
11359	RSPe_2	-122.444	37.943	0.53	19.15	286.59	19.68	-122.444	37.943	-0.06	19.15	286.88	19.09	9	7/14/2014	31:29.1
11360	RSPe_2	-122.444	37.943	0.53	19.17	286.66	19.71	-122.444	37.943	-0.06	19.17	286.92	19.11	9	7/14/2014	31:29.0
11361	RSPe_2	-122.444	37.943	0.57	19.20	286.81	19.77	-122.444	37.943	-0.03	19.20	287.10	19.17	9	7/14/2014	31:28.9
11362	RSPe_2	-122.444	37.943	0.57	19.24	286.86	19.80	-122.444	37.943	-0.03	19.24	287.07	19.21	9	7/14/2014	31:28.8
11363	RSPe_2	-122.444	37.943	0.57	19.24	286.91	19.81	-122.444	37.943	-0.06	19.24	287.12	19.18	9	7/14/2014	31:28.7
11364	RSPe_2	-122.444	37.943	0.57	19.23	286.86	19.79	-122.444	37.943	-0.06	19.23	287.12	19.16	9	7/14/2014	31:28.6
11365	RSPe_2	-122.444	37.943	0.57	19.22	286.84	19.79	-122.444	37.943	-0.03	19.22	287.05	19.19	9	7/14/2014	31:28.5
11366	RSPe_2	-122.444	37.943	0.57	19.22	286.81	19.79	-122.444	37.943	-0.03	19.22	287.05	19.19	9	7/14/2014	31:28.4
11367	RSPe_2	-122.444	37.943	0.57	19.24	286.73	19.80	-122.444	37.943	-0.06	19.24	286.99	19.17	9	7/14/2014	31:28.3
11368	RSPe_2	-122.444	37.943	0.57	19.27	286.75	19.84	-122.444	37.943	0.02	19.27	287.01	19.30	9	7/14/2014	31:28.2
11369	RSPe_2	-122.444	37.943	0.62	19.25	286.66	19.87	-122.444	37.943	0.02	19.25	286.95	19.27	9	7/14/2014	31:28.1
11370	RSPe_2	-122.444	37.943	0.57	19.28	286.62	19.85	-122.444	37.943	0.02	19.28	286.92	19.31	9	7/14/2014	31:28.0
11371	RSPe_2	-122.444	37.943	0.62	19.31	286.62	19.93	-122.444	37.943	0.06	19.31	286.92	19.36	9	7/14/2014	31:27.9
11372	RSPe_2	-122.444	37.943	0.62	19.30	286.64	19.92	-122.444	37.943	0.02	19.30	286.97	19.32	9	7/14/2014	31:27.8
11373	RSPe_2	-122.444	37.943	0.62	19.35	286.61	19.97	-122.444	37.943	0.02	19.35	286.99	19.37	9	7/14/2014	31:27.7
11374	RSPe_2	-122.444	37.943	0.57	19.36	286.62	19.93	-122.444	37.943	-0.03	19.36	287.06	19.33	9	7/14/2014	31:27.6
11375	RSPe_2	-122.444	37.943	0.62	19.37	286.71	19.99	-122.444	37.943	0.02	19.37	287.04	19.39	9	7/14/2014	31:27.5
11376	RSPe_2	-122.444	37.943	0.62	19.33	286.65	19.95	-122.444	37.943	0.02	19.33	287.04	19.35	9	7/14/2014	31:27.4
11377	RSPe_2	-122.444	37.943	0.65	19.32	286.82	19.98	-122.444	37.943	0.02	19.32	287.02	19.34	9	7/14/2014	31:27.3
11378	RSPe_2	-122.444	37.943	0.62	19.27	286.80	19.89	-122.444	37.943	0.06	19.27	286.95	19.32	9	7/14/2014	31:27.2
11379	RSPe_2	-122.444	37.943	0.65	19.25	286.83	19.91	-122.444	37.943	0.06	19.25	286.93	19.31	9	7/14/2014	31:27.1
11380	RSPe_2	-122.444	37.943	0.62	19.14	286.91	19.76	-122.444	37.943	0.14	19.14	286.91	19.27	9	7/14/2014	31:27.0
11381	RSPe_2	-122.444	37.943	0.65	19.10	286.96	19.76	-122.444	37.943	0.06	19.10	286.93	19.16	9	7/14/2014	31:26.9
11382	RSPe_2	-122.444	37.943	0.65	19.00	287.00	19.65	-122.444	37.943	0.06	19.00	286.93	19.05	9	7/14/2014	31:26.8
11383	RSPe_2	-122.444	37.943	0.65	18.89	287.01	19.54	-122.444	37.943	0.14	18.89	287.02	19.03	9	7/14/2014	31:26.7
11384	RSPe_2	-122.444	37.943	0.65	18.87	287.00	19.52	-122.444	37.943	0.14	18.87	287.06	19.01	9	7/14/2014	31:26.6

11385	RSPe_2	-122.444	37.943	0.65	18.78	286.98	19.44	-122.444	37.943	0.06	18.78	287.11	18.84	9	7/14/2014 31:26.5
11386	RSPe_2	-122.444	37.943	0.65	18.73	286.89	19.39	-122.444	37.943	0.06	18.73	287.09	18.79	9	7/14/2014 31:26.4
11387	RSPe_2	-122.444	37.943	0.65	18.60	286.81	19.26	-122.444	37.943	0.11	18.60	287.09	18.71	9	7/14/2014 31:26.3
11388	RSPe_2	-122.444	37.943	0.65	18.59	286.72	19.25	-122.444	37.943	0.06	18.59	287.04	18.65	9	7/14/2014 31:26.2
11389	RSPe_2	-122.444	37.943	0.70	18.61	286.81	19.32	-122.444	37.943	0.06	18.61	287.13	18.67	9	7/14/2014 31:26.1
11390	RSPe_2	-122.444	37.943	0.65	18.59	286.86	19.25	-122.444	37.943	0.06	18.59	287.16	18.65	9	7/14/2014 31:26.0
11391	RSPe_2	-122.444	37.943	0.70	18.61	286.88	19.32	-122.444	37.943	0.11	18.61	287.18	18.72	9	7/14/2014 31:25.9
11392	RSPe_2	-122.444	37.943	0.65	18.60	287.01	19.26	-122.444	37.943	0.02	18.60	287.27	18.63	9	7/14/2014 31:25.8
11393	RSPe_2	-122.444	37.943	0.70	18.66	287.03	19.36	-122.444	37.943	0.11	18.66	287.27	18.76	9	7/14/2014 31:25.7
11394	RSPe_2	-122.444	37.943	0.70	18.61	287.14	19.32	-122.444	37.943	0.14	18.61	287.34	18.75	9	7/14/2014 31:25.6
11395	RSPe_2	-122.444	37.943	0.74	18.73	287.17	19.47	-122.444	37.943	0.11	18.73	287.38	18.84	9	7/14/2014 31:25.5
11396	RSPe_2	-122.444	37.943	0.70	18.57	287.21	19.28	-122.444	37.943	0.11	18.57	287.36	18.68	9	7/14/2014 31:25.4
11397	RSPe_2	-122.444	37.943	0.74	18.58	287.21	19.32	-122.444	37.943	0.22	18.58	287.35	18.80	9	7/14/2014 31:25.3
11398	RSPe_2	-122.444	37.943	0.74	18.57	287.24	19.31	-122.444	37.943	0.19	18.57	287.38	18.76	9	7/14/2014 31:25.2
11399	RSPe_2	-122.444	37.943	0.74	18.59	287.30	19.33	-122.444	37.943	0.19	18.59	287.39	18.78	9	7/14/2014 31:25.1
11400	RSPe_2	-122.444	37.943	0.74	18.58	287.34	19.32	-122.444	37.943	0.14	18.58	287.47	18.72	9	7/14/2014 31:25.0
11401	RSPe_2	-122.444	37.943	0.74	18.64	287.33	19.38	-122.444	37.943	0.22	18.64	287.49	18.87	9	7/14/2014 31:24.9
11402	RSPe_2	-122.444	37.943	0.74	18.59	287.28	19.33	-122.444	37.943	0.22	18.59	287.50	18.81	9	7/14/2014 31:24.8
11403	RSPe_2	-122.444	37.943	0.74	18.63	287.30	19.37	-122.444	37.943	0.22	18.63	287.54	18.86	9	7/14/2014 31:24.7
11404	RSPe_2	-122.444	37.943	0.74	18.62	287.24	19.36	-122.444	37.943	0.19	18.62	287.50	18.81	9	7/14/2014 31:24.6
11405	RSPe_2	-122.444	37.943	0.79	18.59	287.28	19.38	-122.444	37.943	0.19	18.59	287.48	18.78	9	7/14/2014 31:24.5
11406	RSPe_2	-122.444	37.943	0.79	18.59	287.31	19.39	-122.444	37.943	0.14	18.59	287.54	18.73	9	7/14/2014 31:24.4
11407	RSPe_2	-122.444	37.943	0.79	18.63	287.39	19.42	-122.444	37.943	0.22	18.63	287.57	18.85	9	7/14/2014 31:24.3
11408	RSPe_2	-122.444	37.943	0.79	18.58	287.35	19.37	-122.444	37.943	0.22	18.58	287.50	18.80	9	7/14/2014 31:24.2
11409	RSPe_2	-122.444	37.943	0.83	18.65	287.42	19.48	-122.444	37.943	0.22	18.65	287.50	18.87	9	7/14/2014 31:24.1
11410	RSPe_2	-122.444	37.943	0.79	18.60	287.42	19.40	-122.444	37.943	0.19	18.60	287.46	18.80	9	7/14/2014 31:24.0
11411	RSPe_2	-122.444	37.943	0.83	18.70	287.42	19.52	-122.444	37.943	0.22	18.70	287.41	18.92	9	7/14/2014 31:23.9
11412	RSPe_2	-122.444	37.943	0.79	18.64	287.46	19.43	-122.444	37.943	0.26	18.64	287.46	18.90	9	7/14/2014 31:23.8
11413	RSPe_2	-122.444	37.943	0.83	18.61	287.46	19.44	-122.444	37.943	0.26	18.61	287.44	18.87	9	7/14/2014 31:23.7
11414	RSPe_2	-122.444	37.943	0.79	18.67	287.49	19.47	-122.444	37.943	0.31	18.67	287.50	18.98	9	7/14/2014 31:23.6
11415	RSPe_2	-122.444	37.943	0.83	18.71	287.44	19.54	-122.444	37.943	0.31	18.71	287.50	19.02	9	7/14/2014 31:23.5
11416	RSPe_2	-122.444	37.943	0.83	18.69	287.42	19.52	-122.444	37.943	0.34	18.69	287.52	19.03	9	7/14/2014 31:23.4
11417	RSPe_2	-122.444	37.943	0.83	18.70	287.49	19.53	-122.444	37.943	0.31	18.70	287.60	19.01	9	7/14/2014 31:23.3

11418	RSPe_2	-122.444	37.943	0.83	18.71	287.47	19.54	-122.444	37.943	0.31	18.71	287.62	19.02	9	7/14/2014	31:23.2
11419	RSPe_2	-122.444	37.943	0.88	18.86	287.51	19.73	-122.444	37.943	0.34	18.86	287.73	19.20	9	7/14/2014	31:23.1
11420	RSPe_2	-122.444	37.943	0.83	18.70	287.51	19.52	-122.444	37.943	0.31	18.70	287.82	19.00	9	7/14/2014	31:23.0
11421	RSPe_2	-122.444	37.943	0.88	18.87	287.54	19.75	-122.444	37.943	0.26	18.87	287.86	19.13	9	7/14/2014	31:22.9
11422	RSPe_2	-122.444	37.943	0.83	18.76	287.58	19.59	-122.444	37.943	0.26	18.76	287.89	19.02	9	7/14/2014	31:22.8
11423	RSPe_2	-122.444	37.943	0.88	18.74	287.49	19.62	-122.444	37.943	0.34	18.74	287.86	19.09	9	7/14/2014	31:22.7
11424	RSPe_2	-122.444	37.943	0.88	18.79	287.47	19.67	-122.444	37.943	0.26	18.79	287.77	19.05	9	7/14/2014	31:22.6
11425	RSPe_2	-122.444	37.943	0.91	18.71	287.41	19.62	-122.444	37.943	0.31	18.71	287.67	19.02	9	7/14/2014	31:22.5
11426	RSPe_2	-122.444	37.943	0.88	18.73	287.49	19.61	-122.444	37.943	0.34	18.73	287.69	19.07	9	7/14/2014	31:22.4
11427	RSPe_2	-122.444	37.943	0.88	18.66	287.54	19.54	-122.444	37.943	0.34	18.66	287.80	19.00	9	7/14/2014	31:22.3
11428	RSPe_2	-122.444	37.943	0.88	18.68	287.50	19.56	-122.444	37.943	0.31	18.68	287.73	18.99	9	7/14/2014	31:22.2
11429	RSPe_2	-122.444	37.943	0.88	18.62	287.47	19.50	-122.444	37.943	0.34	18.62	287.71	18.96	9	7/14/2014	31:22.1
11430	RSPe_2	-122.444	37.943	0.88	18.63	287.52	19.51	-122.444	37.943	0.34	18.63	287.71	18.98	9	7/14/2014	31:22.0
11431	RSPe_2	-122.444	37.943	0.91	18.65	287.54	19.56	-122.444	37.943	0.39	18.65	287.70	19.04	9	7/14/2014	31:21.9
11432	RSPe_2	-122.444	37.943	0.88	18.66	287.46	19.54	-122.444	37.943	0.31	18.66	287.64	18.97	9	7/14/2014	31:21.8
11433	RSPe_2	-122.444	37.943	0.91	18.66	287.52	19.57	-122.444	37.943	0.34	18.66	287.71	19.00	9	7/14/2014	31:21.7
11434	RSPe_2	-122.444	37.943	0.88	18.63	287.50	19.50	-122.444	37.943	0.31	18.63	287.72	18.93	9	7/14/2014	31:21.6
11435	RSPe_2	-122.444	37.943	0.91	18.66	287.48	19.57	-122.444	37.943	0.31	18.66	287.74	18.97	9	7/14/2014	31:21.5
11436	RSPe_2	-122.444	37.943	0.91	18.63	287.59	19.54	-122.444	37.943	0.31	18.63	287.76	18.93	9	7/14/2014	31:21.4
11437	RSPe_2	-122.444	37.943	0.91	18.63	287.62	19.54	-122.444	37.943	0.34	18.63	287.81	18.98	9	7/14/2014	31:21.3
11438	RSPe_2	-122.444	37.943	0.91	18.64	287.61	19.55	-122.444	37.943	0.31	18.64	287.79	18.95	9	7/14/2014	31:21.2
11439	RSPe_2	-122.444	37.943	0.95	18.58	287.68	19.53	-122.444	37.943	0.31	18.58	287.78	18.89	9	7/14/2014	31:21.1
11440	RSPe_2	-122.444	37.943	0.91	18.59	287.59	19.51	-122.444	37.943	0.31	18.59	287.77	18.90	9	7/14/2014	31:21.0
11441	RSPe_2	-122.444	37.943	0.95	18.58	287.62	19.53	-122.444	37.943	0.34	18.58	287.72	18.92	9	7/14/2014	31:20.9
11442	RSPe_2	-122.444	37.943	0.95	18.61	287.61	19.56	-122.444	37.943	0.31	18.61	287.75	18.92	9	7/14/2014	31:20.8
11443	RSPe_2	-122.444	37.943	0.95	18.53	287.58	19.48	-122.444	37.943	0.31	18.53	287.68	18.84	9	7/14/2014	31:20.7
11444	RSPe_2	-122.444	37.943	0.95	18.46	287.57	19.40	-122.444	37.943	0.31	18.46	287.75	18.76	9	7/14/2014	31:20.6
11445	RSPe_2	-122.444	37.943	0.95	18.47	287.59	19.41	-122.444	37.943	0.31	18.47	287.77	18.77	9	7/14/2014	31:20.5
11446	RSPe_2	-122.444	37.943	0.95	18.51	287.64	19.46	-122.444	37.943	0.31	18.51	287.79	18.82	9	7/14/2014	31:20.4
11447	RSPe_2	-122.444	37.943	1.00	18.42	287.66	19.41	-122.444	37.944	0.34	18.42	287.83	18.76	9	7/14/2014	31:20.3
11448	RSPe_2	-122.444	37.944	0.95	18.33	287.60	19.27	-122.444	37.944	0.31	18.33	287.80	18.64	9	7/14/2014	31:20.2
11449	RSPe_2	-122.444	37.944	1.00	18.34	287.60	19.34	-122.444	37.944	0.39	18.34	287.81	18.74	9	7/14/2014	31:20.1
11450	RSPe_2	-122.444	37.944	1.00	18.43	287.56	19.43	-122.444	37.944	0.34	18.43	287.79	18.78	9	7/14/2014	31:20.0

11451	RSPe_2	-122.444	37.944	1.00	18.28	287.66	19.28	-122.444	37.944	0.31	18.28	287.77	18.59	9	7/14/2014	31:19.9
11452	RSPe_2	-122.444	37.944	1.00	18.28	287.60	19.28	-122.444	37.944	0.31	18.28	287.71	18.59	9	7/14/2014	31:19.8
11453	RSPe_2	-122.444	37.944	1.03	18.19	287.69	19.22	-122.444	37.944	0.34	18.19	287.67	18.54	9	7/14/2014	31:19.7
11454	RSPe_2	-122.444	37.944	1.00	18.23	287.71	19.22	-122.444	37.944	0.26	18.23	287.75	18.48	9	7/14/2014	31:19.6
11455	RSPe_2	-122.444	37.944	1.03	18.17	287.75	19.20	-122.444	37.944	0.31	18.17	287.73	18.47	9	7/14/2014	31:19.5
11456	RSPe_2	-122.444	37.944	1.03	18.11	287.76	19.14	-122.444	37.944	0.34	18.11	287.75	18.45	9	7/14/2014	31:19.4
11457	RSPe_2	-122.444	37.944	1.03	18.11	287.73	19.14	-122.444	37.944	0.34	18.11	287.75	18.45	9	7/14/2014	31:19.3
11458	RSPe_2	-122.444	37.944	1.03	17.96	287.74	18.99	-122.444	37.944	0.34	17.96	287.75	18.30	9	7/14/2014	31:19.2
11459	RSPe_2	-122.444	37.944	1.08	18.11	287.78	19.19	-122.444	37.944	0.43	18.11	287.76	18.54	9	7/14/2014	31:19.1
11460	RSPe_2	-122.444	37.944	1.08	17.93	287.74	19.01	-122.444	37.944	0.39	17.93	287.73	18.32	9	7/14/2014	31:19.0
11461	RSPe_2	-122.444	37.944	1.08	18.04	287.76	19.12	-122.444	37.944	0.43	18.04	287.78	18.47	9	7/14/2014	31:18.9
11462	RSPe_2	-122.444	37.944	1.08	17.91	287.83	18.99	-122.444	37.944	0.43	17.91	287.82	18.34	9	7/14/2014	31:18.8
11463	RSPe_2	-122.444	37.944	1.12	17.86	287.78	18.98	-122.444	37.944	0.43	17.86	287.74	18.29	9	7/14/2014	31:18.7
11464	RSPe_2	-122.444	37.944	1.12	17.87	287.79	18.99	-122.444	37.944	0.51	17.87	287.80	18.38	9	7/14/2014	31:18.6
11465	RSPe_2	-122.444	37.944	1.12	17.85	287.83	18.97	-122.444	37.944	0.43	17.85	287.85	18.28	9	7/14/2014	31:18.5
11466	RSPe_2	-122.444	37.944	1.12	17.85	287.83	18.97	-122.444	37.944	0.43	17.85	287.93	18.28	9	7/14/2014	31:18.4
11467	RSPe_2	-122.444	37.944	1.17	17.87	287.88	19.04	-122.444	37.944	0.51	17.87	288.00	18.38	9	7/14/2014	31:18.3
11468	RSPe_2	-122.444	37.944	1.17	17.87	287.85	19.04	-122.444	37.944	0.48	17.87	287.98	18.35	9	7/14/2014	31:18.2
11469	RSPe_2	-122.444	37.944	1.20	17.89	287.81	19.09	-122.444	37.944	0.51	17.89	287.94	18.41	9	7/14/2014	31:18.1
11470	RSPe_2	-122.444	37.944	1.17	17.89	287.85	19.06	-122.444	37.944	0.39	17.89	287.94	18.29	9	7/14/2014	31:18.0
11471	RSPe_2	-122.444	37.944	1.20	17.89	287.81	19.09	-122.444	37.944	0.43	17.89	287.85	18.31	9	7/14/2014	31:17.9
11472	RSPe_2	-122.444	37.944	1.20	17.93	287.81	19.13	-122.444	37.944	0.55	17.93	287.81	18.48	9	7/14/2014	31:17.8
11473	RSPe_2	-122.444	37.944	1.25	17.86	287.73	19.12	-122.444	37.944	0.55	17.86	287.72	18.41	9	7/14/2014	31:17.7
11474	RSPe_2	-122.444	37.944	1.20	17.88	287.79	19.08	-122.444	37.944	0.55	17.88	287.76	18.42	9	7/14/2014	31:17.6
11475	RSPe_2	-122.444	37.944	1.25	17.85	287.84	19.11	-122.444	37.944	0.60	17.85	287.83	18.45	9	7/14/2014	31:17.5
11476	RSPe_2	-122.444	37.944	1.25	17.85	287.79	19.11	-122.444	37.944	0.55	17.85	287.79	18.40	9	7/14/2014	31:17.4
11477	RSPe_2	-122.444	37.944	1.29	17.85	287.84	19.14	-122.444	37.944	0.60	17.85	287.83	18.45	9	7/14/2014	31:17.3
11478	RSPe_2	-122.444	37.944	1.29	17.85	287.86	19.13	-122.444	37.944	0.68	17.85	287.83	18.53	9	7/14/2014	31:17.2
11479	RSPe_2	-122.444	37.944	1.32	17.87	287.84	19.19	-122.444	37.944	0.63	17.87	287.81	18.50	9	7/14/2014	31:17.1
11480	RSPe_2	-122.444	37.944	1.32	17.85	287.80	19.17	-122.444	37.944	0.68	17.85	287.86	18.53	9	7/14/2014	31:17.0
11481	RSPe_2	-122.444	37.944	1.37	17.95	287.86	19.32	-122.444	37.944	0.68	17.95	287.92	18.63	9	7/14/2014	31:16.9
11482	RSPe_2	-122.444	37.944	1.32	17.87	287.73	19.19	-122.444	37.944	0.63	17.87	287.86	18.50	9	7/14/2014	31:16.8
11483	RSPe_2	-122.444	37.944	1.37	17.85	287.84	19.22	-122.444	37.944	0.77	17.85	287.91	18.61	9	7/14/2014	31:16.7

11484	RSPe_2	-122.444	37.944	1.37	17.88	287.71	19.25	-122.444	37.944	0.72	17.88	287.77	18.59	9	7/14/2014	31:16.6
11485	RSPe_2	-122.444	37.944	1.37	17.88	287.77	19.25	-122.444	37.944	0.72	17.88	287.84	18.59	9	7/14/2014	31:16.5
11486	RSPe_2	-122.444	37.944	1.37	17.88	287.78	19.25	-122.444	37.944	0.68	17.88	287.81	18.56	9	7/14/2014	31:16.4
11487	RSPe_2	-122.444	37.944	1.41	17.91	287.76	19.32	-122.444	37.944	0.72	17.91	287.76	18.63	9	7/14/2014	31:16.3
11488	RSPe_2	-122.444	37.944	1.46	17.87	287.87	19.33	-122.444	37.944	0.77	17.87	287.93	18.64	9	7/14/2014	31:16.2
11489	RSPe_2	-122.444	37.944	1.46	17.89	288.00	19.35	-122.444	37.944	0.80	17.89	288.02	18.69	9	7/14/2014	31:16.1
11490	RSPe_2	-122.444	37.944	1.46	17.86	287.98	19.32	-122.444	37.944	0.85	17.86	288.00	18.71	9	7/14/2014	31:16.0
11491	RSPe_2	-122.444	37.944	1.49	17.88	287.87	19.37	-122.444	37.944	0.80	17.88	287.95	18.68	9	7/14/2014	31:15.9
11492	RSPe_2	-122.444	37.944	1.46	17.88	287.80	19.34	-122.444	37.944	0.80	17.88	287.91	18.68	9	7/14/2014	31:15.8
11493	RSPe_2	-122.444	37.944	1.54	17.88	287.83	19.42	-122.444	37.944	0.80	17.88	287.92	18.68	9	7/14/2014	31:15.7
11494	RSPe_2	-122.444	37.944	1.54	17.90	287.90	19.45	-122.444	37.944	0.88	17.90	287.91	18.79	9	7/14/2014	31:15.6
11495	RSPe_2	-122.444	37.944	1.58	17.89	287.94	19.47	-122.444	37.944	0.88	17.89	287.91	18.77	9	7/14/2014	31:15.5
11496	RSPe_2	-122.444	37.944	1.54	17.90	287.91	19.45	-122.444	37.944	0.80	17.90	287.94	18.70	9	7/14/2014	31:15.4
11497	RSPe_2	-122.444	37.944	1.58	17.89	287.94	19.47	-122.444	37.944	1.00	17.89	287.91	18.90	9	7/14/2014	31:15.3
11498	RSPe_2	-122.444	37.944	1.58	17.89	287.94	19.47	-122.444	37.944	0.97	17.89	287.93	18.86	9	7/14/2014	31:15.2
11499	RSPe_2	-122.444	37.944	1.63	17.89	287.96	19.52	-122.444	37.944	1.05	17.89	287.89	18.94	9	7/14/2014	31:15.1
11500	RSPe_2	-122.444	37.944	1.63	17.92	288.01	19.55	-122.444	37.944	1.00	17.92	287.98	18.92	9	7/14/2014	31:15.0
11501	RSPe_2	-122.444	37.944	1.66	17.92	287.92	19.58	-122.444	37.944	1.05	17.92	287.89	18.97	9	7/14/2014	31:14.9
11502	RSPe_2	-122.444	37.944	1.66	17.92	287.88	19.58	-122.444	37.944	1.05	17.92	287.94	18.97	9	7/14/2014	31:14.8
11503	RSPe_2	-122.444	37.944	1.66	17.96	287.85	19.62	-122.444	37.944	1.09	17.96	287.91	19.04	9	7/14/2014	31:14.7
11504	RSPe_2	-122.444	37.944	1.66	17.94	287.79	19.61	-122.444	37.944	1.05	17.94	287.92	19.00	9	7/14/2014	31:14.6
11505	RSPe_2	-122.444	37.944	1.75	18.02	287.83	19.77	-122.444	37.944	1.09	18.02	287.90	19.11	9	7/14/2014	31:14.5
11506	RSPe_2	-122.444	37.944	1.70	17.96	287.77	19.66	-122.444	37.944	1.14	17.96	287.87	19.10	9	7/14/2014	31:14.4
11507	RSPe_2	-122.444	37.944	1.75	18.04	287.77	19.79	-122.444	37.944	1.14	18.04	287.86	19.18	9	7/14/2014	31:14.3
11508	RSPe_2	-122.444	37.944	1.75	18.04	287.82	19.79	-122.444	37.944	1.22	18.04	287.87	19.26	9	7/14/2014	31:14.2
11509	RSPe_2	-122.444	37.944	1.78	18.03	287.72	19.81	-122.444	37.944	1.09	18.03	287.81	19.11	9	7/14/2014	31:14.1
11510	RSPe_2	-122.444	37.944	1.78	17.99	287.73	19.78	-122.444	37.944	1.29	17.99	287.81	19.28	9	7/14/2014	31:14.0
11511	RSPe_2	-122.444	37.944	1.84	18.00	287.70	19.84	-122.444	37.944	1.26	18.00	287.75	19.26	9	7/14/2014	31:13.9
11512	RSPe_2	-122.444	37.944	1.84	17.94	287.73	19.78	-122.444	37.944	1.38	17.94	287.72	19.32	9	7/14/2014	31:13.8
11513	RSPe_2	-122.444	37.944	1.87	17.74	287.73	19.61	-122.444	37.944	1.26	17.74	287.72	19.00	9	7/14/2014	31:13.7
11514	RSPe_2	-122.444	37.944	1.87	17.67	287.75	19.54	-122.444	37.944	1.29	17.67	287.70	18.96	9	7/14/2014	31:13.6
11515	RSPe_2	-122.444	37.944	1.87	17.67	287.68	19.54	-122.444	37.944	1.29	17.67	287.68	18.96	9	7/14/2014	31:13.5
11516	RSPe_2	-122.444	37.944	1.92	17.63	287.67	19.55	-122.444	37.944	1.29	17.63	287.73	18.92	9	7/14/2014	31:13.4

11517	RSPe_2	-122.444	37.944	1.92	17.67	287.71	19.59	-122.444	37.944	1.43	17.67	287.75	19.10	9	7/14/2014	31:13.3
11518	RSPe_2	-122.444	37.944	1.92	17.51	287.67	19.43	-122.444	37.944	1.38	17.51	287.74	18.88	9	7/14/2014	31:13.2
11519	RSPe_2	-122.444	37.944	1.96	17.51	287.69	19.46	-122.444	37.944	1.38	17.51	287.75	18.88	9	7/14/2014	31:13.1
11520	RSPe_2	-122.444	37.944	1.96	17.50	287.69	19.46	-122.444	37.944	1.38	17.50	287.75	18.88	9	7/14/2014	31:13.0
11521	RSPe_2	-122.444	37.944	2.01	17.48	287.67	19.48	-122.444	37.944	1.43	17.48	287.75	18.90	9	7/14/2014	31:12.9
11522	RSPe_2	-122.444	37.944	2.01	17.41	287.69	19.41	-122.444	37.944	1.38	17.41	287.71	18.78	9	7/14/2014	31:12.8
11523	RSPe_2	-122.444	37.944	2.04	17.39	287.61	19.43	-122.444	37.944	1.46	17.39	287.67	18.85	9	7/14/2014	31:12.7
11524	RSPe_2	-122.444	37.944	2.01	17.40	287.69	19.41	-122.444	37.944	1.46	17.40	287.69	18.86	9	7/14/2014	31:12.6
11525	RSPe_2	-122.444	37.944	2.08	17.38	287.59	19.45	-122.444	37.944	1.51	17.38	287.58	18.89	9	7/14/2014	31:12.5
11526	RSPe_2	-122.444	37.944	2.04	17.36	287.56	19.40	-122.444	37.944	1.46	17.36	287.56	18.82	9	7/14/2014	31:12.4
11527	RSPe_2	-122.444	37.944	2.08	17.35	287.56	19.42	-122.444	37.944	1.55	17.35	287.53	18.89	9	7/14/2014	31:12.3
11528	RSPe_2	-122.444	37.944	2.08	17.35	287.45	19.43	-122.444	37.944	1.60	17.35	287.48	18.95	9	7/14/2014	31:12.2
11529	RSPe_2	-122.444	37.944	2.13	17.35	287.52	19.47	-122.444	37.944	1.63	17.35	287.47	18.98	9	7/14/2014	31:12.1
11530	RSPe_2	-122.444	37.944	2.13	17.34	287.46	19.46	-122.444	37.944	1.63	17.34	287.48	18.97	9	7/14/2014	31:12.0
11531	RSPe_2	-122.444	37.944	2.13	17.32	287.45	19.45	-122.444	37.944	1.63	17.32	287.48	18.95	9	7/14/2014	31:11.9
11532	RSPe_2	-122.444	37.944	2.13	17.35	287.41	19.48	-122.444	37.944	1.66	17.35	287.45	19.02	9	7/14/2014	31:11.8
11533	RSPe_2	-122.444	37.944	2.16	17.35	287.48	19.51	-122.444	37.944	1.66	17.35	287.50	19.01	9	7/14/2014	31:11.7
11534	RSPe_2	-122.444	37.944	2.16	17.39	287.46	19.55	-122.444	37.944	1.63	17.39	287.48	19.02	9	7/14/2014	31:11.6
11535	RSPe_2	-122.444	37.944	2.21	17.39	287.40	19.60	-122.444	37.944	1.66	17.39	287.43	19.06	9	7/14/2014	31:11.5
11536	RSPe_2	-122.444	37.944	2.21	17.35	287.41	19.56	-122.444	37.944	1.66	17.35	287.44	19.01	9	7/14/2014	31:11.4
11537	RSPe_2	-122.444	37.944	2.21	17.36	287.35	19.57	-122.444	37.944	1.80	17.36	287.37	19.16	9	7/14/2014	31:11.3
11538	RSPe_2	-122.444	37.944	2.21	17.35	287.37	19.57	-122.444	37.944	1.80	17.35	287.44	19.15	9	7/14/2014	31:11.2
11539	RSPe_2	-122.444	37.944	2.25	17.42	287.37	19.66	-122.444	37.944	1.83	17.42	287.44	19.25	9	7/14/2014	31:11.1
11540	RSPe_2	-122.444	37.944	2.25	17.39	287.36	19.64	-122.444	37.944	1.75	17.39	287.35	19.14	9	7/14/2014	31:11.0
11541	RSPe_2	-122.444	37.944	2.30	17.39	287.37	19.69	-122.444	37.944	1.83	17.39	287.39	19.23	9	7/14/2014	31:10.9
11542	RSPe_2	-122.444	37.944	2.30	17.41	287.35	19.70	-122.444	37.944	1.83	17.41	287.39	19.24	9	7/14/2014	31:10.8
11543	RSPe_2	-122.444	37.944	2.33	17.42	287.38	19.75	-122.444	37.944	1.83	17.42	287.37	19.25	9	7/14/2014	31:10.7
11544	RSPe_2	-122.444	37.944	2.30	17.42	287.40	19.72	-122.444	37.944	1.88	17.42	287.39	19.31	9	7/14/2014	31:10.6
11545	RSPe_2	-122.444	37.944	2.33	17.44	287.36	19.77	-122.444	37.944	1.88	17.44	287.29	19.32	9	7/14/2014	31:10.5
11546	RSPe_2	-122.444	37.944	2.33	17.50	287.38	19.83	-122.444	37.944	1.88	17.50	287.35	19.38	9	7/14/2014	31:10.4
11547	RSPe_2	-122.444	37.944	2.37	17.44	287.38	19.80	-122.444	37.944	1.88	17.44	287.33	19.32	9	7/14/2014	31:10.3
11548	RSPe_2	-122.444	37.944	2.33	17.41	287.45	19.74	-122.444	37.944	1.80	17.41	287.40	19.21	9	7/14/2014	31:10.2
11549	RSPe_2	-122.444	37.944	2.37	17.31	287.44	19.68	-122.444	37.944	1.97	17.31	287.37	19.28	9	7/14/2014	31:10.1

11550	RSPe_2	-122.444	37.944	2.37	17.38	287.44	19.75	-122.444	37.944	1.88	17.38	287.35	19.26	9	7/14/2014	31:10.0
11551	RSPe_2	-122.444	37.944	2.37	17.21	287.45	19.58	-122.444	37.944	1.97	17.21	287.40	19.18	9	7/14/2014	31:09.9
11552	RSPe_2	-122.444	37.944	2.37	17.23	287.43	19.60	-122.444	37.944	2.00	17.23	287.31	19.23	9	7/14/2014	31:09.8
11553	RSPe_2	-122.444	37.944	2.42	17.08	287.36	19.49	-122.444	37.944	2.04	17.08	287.34	19.11	9	7/14/2014	31:09.7
11554	RSPe_2	-122.444	37.944	2.42	17.09	287.39	19.51	-122.444	37.944	2.00	17.09	287.27	19.09	9	7/14/2014	31:09.6
11555	RSPe_2	-122.444	37.944	2.42	16.99	287.36	19.41	-122.444	37.944	2.00	16.99	287.31	18.99	9	7/14/2014	31:09.5
11556	RSPe_2	-122.444	37.944	2.45	16.97	287.36	19.42	-122.444	37.944	2.04	16.97	287.33	19.00	9	7/14/2014	31:09.4
11557	RSPe_2	-122.444	37.944	2.45	16.86	287.43	19.31	-122.444	37.944	2.04	16.86	287.30	18.90	9	7/14/2014	31:09.3
11558	RSPe_2	-122.444	37.944	2.45	16.96	287.39	19.41	-122.444	37.944	2.04	16.96	287.31	19.00	9	7/14/2014	31:09.2
11559	RSPe_2	-122.444	37.944	2.50	16.88	287.37	19.39	-122.444	37.944	2.09	16.88	287.30	18.97	9	7/14/2014	31:09.1
11560	RSPe_2	-122.444	37.944	2.45	16.77	287.39	19.23	-122.444	37.944	2.09	16.77	287.32	18.86	9	7/14/2014	31:09.0
11561	RSPe_2	-122.444	37.944	2.50	16.79	287.36	19.29	-122.444	37.944	2.12	16.79	287.35	18.91	9	7/14/2014	31:08.9
11562	RSPe_2	-122.444	37.944	2.50	16.64	287.37	19.14	-122.444	37.944	2.12	16.64	287.32	18.76	9	7/14/2014	31:08.8
11563	RSPe_2	-122.444	37.944	2.54	16.62	287.32	19.16	-122.444	37.944	2.17	16.62	287.30	18.79	9	7/14/2014	31:08.7
11564	RSPe_2	-122.444	37.944	2.50	16.58	287.35	19.08	-122.444	37.944	2.17	16.58	287.30	18.75	9	7/14/2014	31:08.6
11565	RSPe_2	-122.444	37.944	2.59	16.51	287.31	19.10	-122.444	37.944	2.21	16.51	287.32	18.71	9	7/14/2014	31:08.5
11566	RSPe_2	-122.444	37.944	2.54	16.49	287.32	19.03	-122.444	37.944	2.21	16.49	287.33	18.70	9	7/14/2014	31:08.4
11567	RSPe_2	-122.444	37.944	2.59	16.44	287.35	19.03	-122.444	37.944	2.21	16.44	287.32	18.65	9	7/14/2014	31:08.3
11568	RSPe_2	-122.444	37.944	2.59	16.42	287.31	19.01	-122.444	37.944	2.26	16.42	287.32	18.68	9	7/14/2014	31:08.2
11569	RSPe_2	-122.444	37.944	2.62	16.41	287.28	19.03	-122.444	37.944	2.26	16.41	287.35	18.66	9	7/14/2014	31:08.1
11570	RSPe_2	-122.444	37.944	2.59	16.39	287.26	18.98	-122.444	37.944	2.26	16.39	287.28	18.65	9	7/14/2014	31:08.0
11571	RSPe_2	-122.444	37.944	2.62	16.36	287.24	18.98	-122.444	37.944	2.32	16.36	287.26	18.68	9	7/14/2014	31:07.9
11572	RSPe_2	-122.444	37.944	2.62	16.35	287.24	18.97	-122.444	37.944	2.32	16.35	287.26	18.67	9	7/14/2014	31:07.8
11573	RSPe_2	-122.444	37.944	2.67	16.33	287.22	19.01	-122.444	37.944	2.32	16.33	287.21	18.66	9	7/14/2014	31:07.7
11574	RSPe_2	-122.444	37.944	2.62	16.31	287.27	18.94	-122.444	37.944	2.38	16.31	287.27	18.69	9	7/14/2014	31:07.6
11575	RSPe_2	-122.444	37.944	2.67	16.33	287.24	19.01	-122.444	37.944	2.38	16.33	287.24	18.71	9	7/14/2014	31:07.5
11576	RSPe_2	-122.444	37.944	2.67	16.33	287.22	19.00	-122.444	37.944	2.38	16.33	287.24	18.70	9	7/14/2014	31:07.4
11577	RSPe_2	-122.444	37.944	2.71	16.29	287.27	19.00	-122.444	37.944	2.41	16.29	287.27	18.70	9	7/14/2014	31:07.3
11578	RSPe_2	-122.444	37.944	2.71	16.36	287.27	19.07	-122.444	37.944	2.38	16.36	287.33	18.73	9	7/14/2014	31:07.2
11579	RSPe_2	-122.444	37.944	2.71	16.33	287.22	19.04	-122.444	37.944	2.41	16.33	287.27	18.74	9	7/14/2014	31:07.1
11580	RSPe_2	-122.444	37.944	2.71	16.27	287.20	18.98	-122.444	37.944	2.41	16.27	287.29	18.68	9	7/14/2014	31:07.0
11581	RSPe_2	-122.444	37.944	2.71	16.30	287.21	19.01	-122.444	37.944	2.46	16.30	287.22	18.76	9	7/14/2014	31:06.9
11582	RSPe_2	-122.444	37.944	2.71	16.24	287.20	18.95	-122.444	37.944	2.46	16.24	287.27	18.70	9	7/14/2014	31:06.8

11583	RSPe_2	-122.444	37.944	2.74	16.24	287.14	18.99	-122.444	37.944	2.49	16.24	287.15	18.74	9	7/14/2014	31:06.7
11584	RSPe_2	-122.444	37.944	2.71	16.23	287.12	18.94	-122.444	37.944	2.49	16.23	287.15	18.73	9	7/14/2014	31:06.6
11585	RSPe_2	-122.444	37.944	2.74	16.24	287.16	18.99	-122.444	37.944	2.54	16.24	287.18	18.79	9	7/14/2014	31:06.5
11586	RSPe_2	-122.444	37.944	2.74	16.24	287.10	18.99	-122.444	37.944	2.54	16.24	287.20	18.79	9	7/14/2014	31:06.4
11587	RSPe_2	-122.444	37.944	2.79	16.20	287.10	19.00	-122.444	37.944	2.58	16.20	287.16	18.78	9	7/14/2014	31:06.3
11588	RSPe_2	-122.444	37.944	2.79	16.22	287.10	19.01	-122.444	37.944	2.58	16.22	287.16	18.80	9	7/14/2014	31:06.2
11589	RSPe_2	-122.444	37.944	2.79	16.20	287.05	19.00	-122.444	37.944	2.58	16.20	287.16	18.78	9	7/14/2014	31:06.1
11590	RSPe_2	-122.444	37.944	2.79	16.19	287.05	18.98	-122.444	37.944	2.58	16.19	287.11	18.77	9	7/14/2014	31:06.0
11591	RSPe_2	-122.444	37.944	2.83	16.22	286.99	19.05	-122.444	37.944	2.63	16.22	287.07	18.85	9	7/14/2014	31:05.9
11592	RSPe_2	-122.444	37.944	2.83	16.19	286.97	19.02	-122.444	37.944	2.63	16.19	287.05	18.82	9	7/14/2014	31:05.8
11593	RSPe_2	-122.444	37.944	2.83	16.17	286.97	18.99	-122.444	37.944	2.66	16.17	287.05	18.83	9	7/14/2014	31:05.7
11594	RSPe_2	-122.444	37.944	2.83	16.27	286.95	19.10	-122.444	37.944	2.66	16.27	287.05	18.93	9	7/14/2014	31:05.6
11595	RSPe_2	-122.444	37.944	2.88	16.21	286.97	19.09	-122.444	37.944	2.70	16.21	287.04	18.91	9	7/14/2014	31:05.5
11596	RSPe_2	-122.444	37.944	2.83	16.18	286.97	19.01	-122.444	37.944	2.70	16.18	287.07	18.88	9	7/14/2014	31:05.4
11597	RSPe_2	-122.444	37.944	2.88	16.20	286.95	19.08	-122.444	37.944	2.75	16.20	286.99	18.95	9	7/14/2014	31:05.3
11598	RSPe_2	-122.444	37.944	2.88	16.20	287.00	19.08	-122.444	37.944	2.75	16.20	287.08	18.95	9	7/14/2014	31:05.2
11599	RSPe_2	-122.444	37.944	2.88	16.23	287.00	19.11	-122.444	37.944	2.78	16.23	286.97	19.01	9	7/14/2014	31:05.1
11600	RSPe_2	-122.444	37.944	2.88	16.21	287.02	19.09	-122.444	37.944	2.78	16.21	287.01	18.99	9	7/14/2014	31:05.0
11601	RSPe_2	-122.444	37.944	2.88	16.22	287.04	19.10	-122.444	37.944	2.78	16.22	286.95	19.00	9	7/14/2014	31:04.9
11602	RSPe_2	-122.444	37.944	2.88	16.18	286.98	19.06	-122.444	37.944	2.78	16.18	286.97	18.96	9	7/14/2014	31:04.8
11603	RSPe_2	-122.444	37.944	2.88	16.19	286.97	19.07	-122.444	37.944	2.83	16.19	286.99	19.02	9	7/14/2014	31:04.7
11604	RSPe_2	-122.444	37.944	2.88	16.23	287.00	19.11	-122.444	37.944	2.83	16.23	286.99	19.06	9	7/14/2014	31:04.6
11605	RSPe_2	-122.444	37.944	2.91	16.20	287.02	19.12	-122.444	37.944	2.87	16.20	287.00	19.07	9	7/14/2014	31:04.5
11606	RSPe_2	-122.444	37.944	2.91	16.19	286.98	19.10	-122.444	37.944	2.92	16.19	286.96	19.11	9	7/14/2014	31:04.4
11607	RSPe_2	-122.444	37.944	2.91	16.18	287.00	19.09	-122.444	37.944	2.92	16.18	287.04	19.10	9	7/14/2014	31:04.3
11608	RSPe_2	-122.444	37.944	2.91	16.18	287.02	19.09	-122.444	37.944	2.92	16.18	287.06	19.10	9	7/14/2014	31:04.2
11609	RSPe_2	-122.444	37.944	2.97	16.17	287.00	19.13	-122.444	37.944	2.95	16.17	287.04	19.12	9	7/14/2014	31:04.1
11610	RSPe_2	-122.444	37.944	2.97	16.19	287.00	19.16	-122.444	37.944	2.95	16.19	287.06	19.14	9	7/14/2014	31:04.0
11611	RSPe_2	-122.444	37.944	2.97	16.20	286.96	19.16	-122.444	37.944	3.00	16.20	287.05	19.20	9	7/14/2014	31:03.9
11612	RSPe_2	-122.444	37.944	2.97	16.15	286.94	19.12	-122.444	37.944	3.00	16.15	287.03	19.15	9	7/14/2014	31:03.8
11613	RSPe_2	-122.444	37.944	2.97	16.17	286.94	19.13	-122.444	37.944	3.04	16.17	287.07	19.20	9	7/14/2014	31:03.7
11614	RSPe_2	-122.444	37.944	2.97	16.16	286.89	19.12	-122.444	37.944	3.04	16.16	287.03	19.19	9	7/14/2014	31:03.6
11615	RSPe_2	-122.444	37.944	3.00	16.18	286.92	19.18	-122.444	37.944	3.07	16.18	287.02	19.25	9	7/14/2014	31:03.5

11616	RSPe_2	-122.444	37.944	2.97	16.17	286.90	19.13	-122.444	37.944	3.07	16.17	287.09	19.23	9	7/14/2014 31:03.4
11617	RSPe_2	-122.444	37.944	3.00	16.19	286.95	19.19	-122.444	37.944	3.07	16.19	287.10	19.26	9	7/14/2014 31:03.3
11618	RSPe_2	-122.444	37.944	2.97	16.21	286.94	19.18	-122.444	37.944	3.07	16.21	287.16	19.28	9	7/14/2014 31:03.2
11619	RSPe_2	-122.444	37.944	3.00	16.21	286.96	19.21	-122.444	37.944	3.07	16.21	287.14	19.28	9	7/14/2014 31:03.1
11620	RSPe_2	-122.444	37.944	2.97	16.25	287.03	19.22	-122.444	37.944	3.07	16.25	287.14	19.32	9	7/14/2014 31:03.0
11621	RSPe_2	-122.444	37.944	3.00	16.24	287.05	19.24	-122.444	37.944	3.12	16.24	287.11	19.36	9	7/14/2014 31:02.9
11622	RSPe_2	-122.444	37.944	3.00	16.25	287.04	19.25	-122.444	37.944	3.07	16.25	287.10	19.32	9	7/14/2014 31:02.8
11623	RSPe_2	-122.444	37.944	2.97	16.28	287.10	19.25	-122.444	37.944	3.12	16.28	287.12	19.40	9	7/14/2014 31:02.7
11624	RSPe_2	-122.444	37.944	2.97	16.25	287.10	19.22	-122.444	37.944	3.12	16.25	287.17	19.37	9	7/14/2014 31:02.6
11625	RSPe_2	-122.444	37.944	3.00	16.25	287.14	19.25	-122.444	37.944	3.12	16.25	287.21	19.37	9	7/14/2014 31:02.5
11626	RSPe_2	-122.444	37.944	3.00	16.24	287.15	19.24	-122.444	37.944	3.12	16.24	287.24	19.36	9	7/14/2014 31:02.4
11627	RSPe_2	-122.444	37.944	3.00	16.27	287.12	19.27	-122.444	37.944	3.12	16.27	287.23	19.39	9	7/14/2014 31:02.3
11628	RSPe_2	-122.444	37.944	3.00	16.27	287.19	19.27	-122.444	37.944	3.12	16.27	287.27	19.39	9	7/14/2014 31:02.2
11629	RSPe_2	-122.444	37.944	3.00	16.26	287.15	19.26	-122.444	37.944	3.12	16.26	287.32	19.38	9	7/14/2014 31:02.1
11630	RSPe_2	-122.444	37.944	3.00	16.29	287.15	19.29	-122.444	37.944	3.15	16.29	287.27	19.44	9	7/14/2014 31:02.0
11631	RSPe_2	-122.444	37.944	3.05	16.28	287.10	19.33	-122.444	37.944	3.15	16.28	287.26	19.44	9	7/14/2014 31:01.9
11632	RSPe_2	-122.444	37.944	3.00	16.31	287.15	19.31	-122.444	37.944	3.15	16.31	287.31	19.47	9	7/14/2014 31:01.8
11633	RSPe_2	-122.444	37.944	3.05	16.30	287.19	19.35	-122.444	37.944	3.20	16.30	287.32	19.50	9	7/14/2014 31:01.7
11634	RSPe_2	-122.444	37.944	3.00	16.31	287.11	19.31	-122.444	37.944	3.15	16.31	287.30	19.47	9	7/14/2014 31:01.6
11635	RSPe_2	-122.444	37.944	3.00	16.31	287.11	19.31	-122.444	37.944	3.20	16.31	287.28	19.52	9	7/14/2014 31:01.5
11636	RSPe_2	-122.444	37.944	3.00	16.34	287.11	19.34	-122.444	37.944	3.20	16.34	287.30	19.55	9	7/14/2014 31:01.4
11637	RSPe_2	-122.444	37.944	3.05	16.28	287.13	19.33	-122.444	37.944	3.20	16.28	287.28	19.49	9	7/14/2014 31:01.3
11638	RSPe_2	-122.444	37.944	3.00	16.30	287.13	19.30	-122.444	37.944	3.20	16.30	287.30	19.50	9	7/14/2014 31:01.2
11639	RSPe_2	-122.444	37.944	3.05	16.27	287.15	19.32	-122.444	37.944	3.20	16.27	287.33	19.48	9	7/14/2014 31:01.1
11640	RSPe_2	-122.444	37.944	3.00	16.33	287.20	19.33	-122.444	37.944	3.20	16.33	287.35	19.53	9	7/14/2014 31:01.0
11641	RSPe_2	-122.444	37.944	3.00	16.28	287.20	19.28	-122.444	37.944	3.20	16.28	287.35	19.49	9	7/14/2014 31:00.9
11642	RSPe_2	-122.444	37.944	3.00	16.28	287.22	19.28	-122.444	37.944	3.20	16.28	287.33	19.49	9	7/14/2014 31:00.8
11643	RSPe_2	-122.444	37.944	3.05	16.23	287.20	19.28	-122.444	37.944	3.20	16.23	287.33	19.43	9	7/14/2014 31:00.7
11644	RSPe_2	-122.444	37.944	3.00	16.27	287.18	19.27	-122.444	37.944	3.20	16.27	287.26	19.47	9	7/14/2014 31:00.6
11645	RSPe_2	-122.444	37.944	3.00	16.25	287.18	19.25	-122.444	37.944	3.24	16.25	287.29	19.49	9	7/14/2014 31:00.5
11646	RSPe_2	-122.444	37.944	3.05	16.10	287.16	19.16	-122.444	37.944	3.24	16.10	287.33	19.34	9	7/14/2014 31:00.4
11647	RSPe_2	-122.444	37.944	3.05	16.16	287.18	19.21	-122.444	37.944	3.24	16.16	287.32	19.40	9	7/14/2014 31:00.3
11648	RSPe_2	-122.444	37.944	3.05	15.97	287.20	19.02	-122.444	37.944	3.29	15.97	287.33	19.26	9	7/14/2014 31:00.2

11649	RSPe_2	-122.444	37.944	3.05	16.09	287.21	19.14	-122.444	37.944	3.29	16.09	287.36	19.38	9	7/14/2014	31:00.1
11650	RSPe_2	-122.444	37.944	3.05	16.03	287.23	19.08	-122.444	37.944	3.29	16.03	287.37	19.32	9	7/14/2014	31:00.0
11651	RSPe_2	-122.444	37.944	3.05	15.90	287.27	18.95	-122.444	37.944	3.29	15.90	287.42	19.19	9	7/14/2014	30:59.9
11652	RSPe_2	-122.444	37.944	3.05	16.09	287.30	19.14	-122.444	37.944	3.29	16.09	287.45	19.38	9	7/14/2014	30:59.8
11653	RSPe_2	-122.444	37.944	3.05	15.90	287.29	18.95	-122.444	37.944	3.29	15.90	287.47	19.19	9	7/14/2014	30:59.7
11654	RSPe_2	-122.444	37.944	3.00	15.86	287.34	18.86	-122.444	37.944	3.29	15.86	287.49	19.15	9	7/14/2014	30:59.6
11655	RSPe_2	-122.444	37.944	3.05	15.97	287.32	19.02	-122.444	37.944	3.29	15.97	287.50	19.26	9	7/14/2014	30:59.5
11656	RSPe_2	-122.444	37.944	3.00	15.93	287.38	18.93	-122.444	37.944	3.29	15.93	287.58	19.22	9	7/14/2014	30:59.4
11657	RSPe_2	-122.444	37.944	3.05	15.83	287.40	18.89	-122.444	37.944	3.29	15.83	287.58	19.12	9	7/14/2014	30:59.3
11658	RSPe_2	-122.444	37.944	3.05	15.80	287.43	18.85	-122.444	37.944	3.29	15.80	287.60	19.09	9	7/14/2014	30:59.2
11659	RSPe_2	-122.444	37.944	3.05	15.74	287.43	18.79	-122.444	37.944	3.29	15.74	287.61	19.03	9	7/14/2014	30:59.1
11660	RSPe_2	-122.444	37.944	3.00	15.73	287.45	18.73	-122.444	37.944	3.29	15.73	287.65	19.02	9	7/14/2014	30:59.0
11661	RSPe_2	-122.444	37.944	3.05	15.61	287.50	18.66	-122.444	37.944	3.29	15.61	287.67	18.90	9	7/14/2014	30:58.9
11662	RSPe_2	-122.444	37.944	3.05	15.78	287.52	18.83	-122.444	37.944	3.29	15.78	287.67	19.07	9	7/14/2014	30:58.8
11663	RSPe_2	-122.444	37.944	3.05	15.69	287.57	18.74	-122.444	37.944	3.29	15.69	287.72	18.98	9	7/14/2014	30:58.7
11664	RSPe_2	-122.444	37.944	3.05	15.72	287.57	18.77	-122.444	37.944	3.29	15.72	287.76	19.01	9	7/14/2014	30:58.6
11665	RSPe_2	-122.444	37.944	3.05	15.52	287.61	18.57	-122.444	37.944	3.32	15.52	287.79	18.84	9	7/14/2014	30:58.5
11666	RSPe_2	-122.444	37.944	3.05	15.53	287.66	18.59	-122.444	37.944	3.32	15.53	287.87	18.86	9	7/14/2014	30:58.4
11667	RSPe_2	-122.444	37.944	3.05	15.57	287.68	18.62	-122.444	37.944	3.32	15.57	287.85	18.89	9	7/14/2014	30:58.3
11668	RSPe_2	-122.444	37.944	3.05	15.57	287.72	18.62	-122.444	37.944	3.32	15.57	287.92	18.89	9	7/14/2014	30:58.2
11669	RSPe_2	-122.444	37.944	3.05	15.48	287.72	18.54	-122.444	37.944	3.32	15.48	287.96	18.81	9	7/14/2014	30:58.1
11670	RSPe_2	-122.444	37.944	3.05	15.49	287.74	18.55	-122.444	37.944	3.32	15.49	287.93	18.82	9	7/14/2014	30:58.0
11671	RSPe_2	-122.444	37.944	3.05	15.46	287.81	18.51	-122.444	37.944	3.32	15.46	288.01	18.78	9	7/14/2014	30:57.9
11672	RSPe_2	-122.444	37.944	3.05	15.49	287.86	18.55	-122.444	37.944	3.32	15.49	288.05	18.82	9	7/14/2014	30:57.8
11673	RSPe_2	-122.444	37.944	3.05	15.50	287.88	18.55	-122.444	37.944	3.32	15.50	288.05	18.83	9	7/14/2014	30:57.7
11674	RSPe_2	-122.444	37.944	3.05	15.46	287.90	18.51	-122.444	37.944	3.29	15.46	288.07	18.75	9	7/14/2014	30:57.6
11675	RSPe_2	-122.444	37.944	3.05	15.45	287.94	18.50	-122.444	37.944	3.32	15.45	288.12	18.77	9	7/14/2014	30:57.5
11676	RSPe_2	-122.444	37.944	3.05	15.42	287.99	18.47	-122.444	37.944	3.29	15.42	288.14	18.71	9	7/14/2014	30:57.4
11677	RSPe_2	-122.444	37.944	3.05	15.43	288.01	18.48	-122.444	37.944	3.29	15.43	288.12	18.72	9	7/14/2014	30:57.3
11678	RSPe_2	-122.444	37.944	3.00	15.43	288.02	18.43	-122.444	37.944	3.29	15.43	288.14	18.72	9	7/14/2014	30:57.2
11679	RSPe_2	-122.444	37.944	3.05	15.43	287.99	18.48	-122.444	37.944	3.29	15.43	288.12	18.72	9	7/14/2014	30:57.1
11680	RSPe_2	-122.444	37.944	3.00	15.43	288.06	18.43	-122.444	37.944	3.29	15.43	288.16	18.72	9	7/14/2014	30:57.0
11681	RSPe_2	-122.444	37.944	3.00	15.43	288.04	18.43	-122.444	37.944	3.29	15.43	288.21	18.72	9	7/14/2014	30:56.9

11682	RSPe_2	-122.444	37.944	3.00	15.43	288.06	18.43	-122.444	37.944	3.29	15.43	288.21	18.72	9	7/14/2014	30:56.8
11683	RSPe_2	-122.444	37.944	3.05	15.43	288.04	18.48	-122.444	37.944	3.29	15.43	288.21	18.72	9	7/14/2014	30:56.7
11684	RSPe_2	-122.444	37.944	3.00	15.44	288.04	18.44	-122.444	37.944	3.29	15.44	288.21	18.73	9	7/14/2014	30:56.6
11685	RSPe_2	-122.444	37.944	3.00	15.43	288.07	18.43	-122.444	37.944	3.29	15.43	288.21	18.72	9	7/14/2014	30:56.5
11686	RSPe_2	-122.444	37.944	3.00	15.43	288.04	18.43	-122.444	37.944	3.29	15.43	288.24	18.72	9	7/14/2014	30:56.4
11687	RSPe_2	-122.444	37.944	3.05	15.44	288.00	18.49	-122.444	37.944	3.29	15.44	288.21	18.73	9	7/14/2014	30:56.3
11688	RSPe_2	-122.444	37.944	3.00	15.47	288.04	18.47	-122.444	37.944	3.29	15.47	288.23	18.76	9	7/14/2014	30:56.2
11689	RSPe_2	-122.444	37.944	3.00	15.46	288.00	18.46	-122.444	37.944	3.29	15.46	288.24	18.75	9	7/14/2014	30:56.1
11690	RSPe_2	-122.444	37.944	3.00	15.47	288.04	18.47	-122.444	37.944	3.24	15.47	288.26	18.71	9	7/14/2014	30:56.0
11691	RSPe_2	-122.444	37.944	3.05	15.53	288.05	18.58	-122.444	37.944	3.24	15.53	288.28	18.77	9	7/14/2014	30:55.9
11692	RSPe_2	-122.444	37.944	2.97	15.53	288.07	18.49	-122.444	37.944	3.24	15.53	288.31	18.77	9	7/14/2014	30:55.8
11693	RSPe_2	-122.444	37.944	3.00	15.53	288.09	18.53	-122.444	37.944	3.20	15.53	288.28	18.73	9	7/14/2014	30:55.7
11694	RSPe_2	-122.444	37.944	2.97	15.53	288.05	18.50	-122.444	37.944	3.20	15.53	288.26	18.74	9	7/14/2014	30:55.6
11695	RSPe_2	-122.444	37.944	3.00	15.59	288.07	18.59	-122.444	37.944	3.20	15.59	288.25	18.79	9	7/14/2014	30:55.5
11696	RSPe_2	-122.444	37.944	2.97	15.59	288.05	18.55	-122.444	37.944	3.15	15.59	288.20	18.74	9	7/14/2014	30:55.4
11697	RSPe_2	-122.444	37.944	3.00	15.61	288.00	18.61	-122.444	37.944	3.15	15.61	288.15	18.76	9	7/14/2014	30:55.3
11698	RSPe_2	-122.444	37.944	2.97	15.58	287.94	18.55	-122.444	37.944	3.12	15.58	288.11	18.70	9	7/14/2014	30:55.2
11699	RSPe_2	-122.444	37.944	3.00	15.58	287.94	18.58	-122.444	37.944	3.15	15.58	288.07	18.73	9	7/14/2014	30:55.1
11700	RSPe_2	-122.444	37.944	2.97	15.62	287.94	18.59	-122.444	37.944	3.15	15.62	288.07	18.77	9	7/14/2014	30:55.0
11701	RSPe_2	-122.444	37.944	2.97	15.57	287.87	18.54	-122.444	37.944	3.20	15.57	288.03	18.78	9	7/14/2014	30:54.9
11702	RSPe_2	-122.444	37.944	2.97	15.59	287.88	18.55	-122.444	37.944	3.15	15.59	288.00	18.74	9	7/14/2014	30:54.8
11703	RSPe_2	-122.444	37.944	3.00	15.60	287.83	18.60	-122.444	37.944	3.20	15.60	288.03	18.81	9	7/14/2014	30:54.7
11704	RSPe_2	-122.444	37.944	2.97	15.63	287.83	18.60	-122.444	37.944	3.15	15.63	288.02	18.79	9	7/14/2014	30:54.6
11705	RSPe_2	-122.444	37.944	2.97	15.66	287.79	18.63	-122.444	37.944	3.20	15.66	288.01	18.87	9	7/14/2014	30:54.5
11706	RSPe_2	-122.444	37.944	2.97	15.67	287.79	18.64	-122.444	37.944	3.15	15.67	287.98	18.83	9	7/14/2014	30:54.4
11707	RSPe_2	-122.444	37.944	3.00	15.64	287.72	18.64	-122.444	37.944	3.20	15.64	287.94	18.85	9	7/14/2014	30:54.3
11708	RSPe_2	-122.444	37.944	2.97	15.66	287.73	18.62	-122.444	37.944	3.15	15.66	287.99	18.81	9	7/14/2014	30:54.2
11709	RSPe_2	-122.444	37.944	2.97	15.69	287.75	18.65	-122.444	37.944	3.15	15.69	287.96	18.84	9	7/14/2014	30:54.1
11710	RSPe_2	-122.444	37.944	2.97	15.76	287.75	18.72	-122.444	37.944	3.12	15.76	287.90	18.88	9	7/14/2014	30:54.0
11711	RSPe_2	-122.444	37.944	2.97	15.71	287.75	18.68	-122.444	37.944	3.12	15.71	287.90	18.83	9	7/14/2014	30:53.9
11712	RSPe_2	-122.444	37.944	2.97	15.71	287.73	18.68	-122.444	37.944	3.07	15.71	287.83	18.78	9	7/14/2014	30:53.8
11713	RSPe_2	-122.444	37.944	2.97	15.76	287.70	18.73	-122.444	37.944	3.07	15.76	287.83	18.83	9	7/14/2014	30:53.7
11714	RSPe_2	-122.444	37.944	2.97	15.75	287.75	18.72	-122.444	37.944	3.07	15.75	287.82	18.82	9	7/14/2014	30:53.6

11715	RSPe_2	-122.444	37.944	2.97	15.75	287.67	18.72	-122.444	37.944	3.07	15.75	287.79	18.82	9	7/14/2014	30:53.5
11716	RSPe_2	-122.444	37.944	2.97	15.76	287.68	18.72	-122.444	37.944	3.07	15.76	287.72	18.83	9	7/14/2014	30:53.4
11717	RSPe_2	-122.444	37.944	2.97	15.77	287.67	18.74	-122.444	37.944	3.07	15.77	287.75	18.84	9	7/14/2014	30:53.3
11718	RSPe_2	-122.444	37.944	2.91	15.80	287.68	18.72	-122.444	37.944	3.07	15.80	287.72	18.87	9	7/14/2014	30:53.2
11719	RSPe_2	-122.444	37.944	2.97	15.80	287.67	18.77	-122.444	37.944	3.07	15.80	287.72	18.87	9	7/14/2014	30:53.1
11720	RSPe_2	-122.444	37.944	2.97	15.83	287.62	18.80	-122.444	37.944	3.12	15.83	287.68	18.95	9	7/14/2014	30:53.0
11721	RSPe_2	-122.444	37.944	2.97	15.99	287.67	18.95	-122.444	37.944	3.12	15.99	287.73	19.11	9	7/14/2014	30:52.9
11722	RSPe_2	-122.444	37.944	2.97	15.83	287.61	18.79	-122.444	37.944	3.15	15.83	287.71	18.98	9	7/14/2014	30:52.8
11723	RSPe_2	-122.444	37.944	3.00	15.93	287.67	18.93	-122.444	37.944	3.15	15.93	287.75	19.09	9	7/14/2014	30:52.7
11724	RSPe_2	-122.444	37.944	2.97	15.91	287.65	18.88	-122.444	37.944	3.12	15.91	287.78	19.03	9	7/14/2014	30:52.6
11725	RSPe_2	-122.444	37.944	2.97	15.92	287.64	18.89	-122.444	37.944	3.15	15.92	287.82	19.07	9	7/14/2014	30:52.5
11726	RSPe_2	-122.444	37.944	2.97	15.93	287.65	18.90	-122.444	37.944	3.12	15.93	287.82	19.06	9	7/14/2014	30:52.4
11727	RSPe_2	-122.444	37.944	2.97	15.98	287.67	18.95	-122.444	37.944	3.12	15.98	287.80	19.10	9	7/14/2014	30:52.3
11728	RSPe_2	-122.444	37.944	2.97	15.96	287.65	18.92	-122.444	37.944	3.12	15.96	287.80	19.08	9	7/14/2014	30:52.2
11729	RSPe_2	-122.444	37.944	2.97	16.00	287.65	18.96	-122.444	37.944	3.12	16.00	287.80	19.12	9	7/14/2014	30:52.1
11730	RSPe_2	-122.444	37.944	2.97	15.93	287.65	18.90	-122.444	37.944	3.07	15.93	287.80	19.00	9	7/14/2014	30:52.0
11731	RSPe_2	-122.444	37.944	2.97	16.03	287.63	19.00	-122.444	37.944	3.12	16.03	287.74	19.16	9	7/14/2014	30:51.9
11732	RSPe_2	-122.444	37.944	2.97	16.04	287.65	19.01	-122.444	37.944	3.07	16.04	287.75	19.11	9	7/14/2014	30:51.8
11733	RSPe_2	-122.444	37.944	2.97	16.07	287.67	19.04	-122.444	37.944	3.07	16.07	287.71	19.14	9	7/14/2014	30:51.7
11734	RSPe_2	-122.444	37.944	2.97	16.06	287.61	19.02	-122.444	37.944	3.07	16.06	287.68	19.13	9	7/14/2014	30:51.6
11735	RSPe_2	-122.444	37.944	2.97	16.06	287.63	19.03	-122.444	37.944	3.12	16.06	287.67	19.18	9	7/14/2014	30:51.5
11736	RSPe_2	-122.444	37.944	2.97	16.11	287.64	19.08	-122.444	37.944	3.12	16.11	287.67	19.23	9	7/14/2014	30:51.4
11737	RSPe_2	-122.444	37.944	3.00	16.09	287.63	19.09	-122.444	37.944	3.15	16.09	287.65	19.24	9	7/14/2014	30:51.3
11738	RSPe_2	-122.444	37.944	2.97	16.11	287.59	19.08	-122.444	37.944	3.15	16.11	287.61	19.27	9	7/14/2014	30:51.2
11739	RSPe_2	-122.444	37.944	3.00	16.00	287.60	19.00	-122.444	37.944	3.20	16.00	287.67	19.20	9	7/14/2014	30:51.1
11740	RSPe_2	-122.444	37.944	2.97	16.04	287.57	19.01	-122.444	37.944	3.15	16.04	287.61	19.20	9	7/14/2014	30:51.0
11741	RSPe_2	-122.444	37.944	3.00	15.99	287.60	18.99	-122.444	37.944	3.20	15.99	287.67	19.19	9	7/14/2014	30:50.9
11742	RSPe_2	-122.444	37.944	2.97	16.05	287.57	19.02	-122.444	37.944	3.15	16.05	287.61	19.21	9	7/14/2014	30:50.8
11743	RSPe_2	-122.444	37.944	2.97	16.00	287.57	18.96	-122.444	37.944	3.20	16.00	287.66	19.20	9	7/14/2014	30:50.7
11744	RSPe_2	-122.444	37.944	2.97	16.05	287.60	19.02	-122.444	37.944	3.16	16.05	287.67	19.21	9	7/14/2014	30:50.6
11745	RSPe_2	-122.444	37.944	3.00	16.01	287.57	19.01	-122.444	37.944	3.21	16.01	287.68	19.22	9	7/14/2014	30:50.5
11746	RSPe_2	-122.444	37.944	2.97	15.97	287.60	18.93	-122.444	37.944	3.16	15.97	287.68	19.12	9	7/14/2014	30:50.4
11747	RSPe_2	-122.444	37.944	3.00	15.98	287.60	18.98	-122.444	37.944	3.21	15.98	287.70	19.19	9	7/14/2014	30:50.3

11748	RSPe_2	-122.444	37.944	2.97	15.94	287.55	18.91	-122.444	37.944	3.16	15.94	287.66	19.10	9	7/14/2014	30:50.2
11749	RSPe_2	-122.444	37.944	3.00	16.01	287.55	19.01	-122.444	37.944	3.21	16.01	287.66	19.22	9	7/14/2014	30:50.1
11750	RSPe_2	-122.444	37.944	2.97	15.99	287.51	18.96	-122.444	37.944	3.16	15.99	287.66	19.14	9	7/14/2014	30:50.0
11751	RSPe_2	-122.444	37.944	3.00	15.98	287.49	18.98	-122.444	37.944	3.21	15.98	287.62	19.19	9	7/14/2014	30:49.9
11752	RSPe_2	-122.444	37.944	2.97	15.96	287.49	18.92	-122.444	37.944	3.21	15.96	287.62	19.16	9	7/14/2014	30:49.8
11753	RSPe_2	-122.444	37.944	3.00	15.97	287.47	18.97	-122.444	37.944	3.21	15.97	287.57	19.18	9	7/14/2014	30:49.7
11754	RSPe_2	-122.444	37.944	2.97	16.03	287.47	18.99	-122.444	37.944	3.21	16.03	287.58	19.23	9	7/14/2014	30:49.6
11755	RSPe_2	-122.444	37.944	3.00	15.97	287.47	18.97	-122.444	37.944	3.24	15.97	287.60	19.21	9	7/14/2014	30:49.5
11756	RSPe_2	-122.444	37.944	3.00	15.97	287.47	18.97	-122.444	37.944	3.24	15.97	287.57	19.21	9	7/14/2014	30:49.4
11757	RSPe_2	-122.444	37.944	3.00	15.95	287.45	18.95	-122.444	37.944	3.24	15.95	287.60	19.19	9	7/14/2014	30:49.3
11758	RSPe_2	-122.444	37.944	2.97	15.97	287.47	18.93	-122.444	37.944	3.24	15.97	287.58	19.21	9	7/14/2014	30:49.2
11759	RSPe_2	-122.444	37.944	2.97	15.97	287.45	18.94	-122.444	37.944	3.24	15.97	287.62	19.21	9	7/14/2014	30:49.1
11760	RSPe_2	-122.444	37.944	2.97	15.99	287.49	18.96	-122.444	37.944	3.24	15.99	287.60	19.23	9	7/14/2014	30:49.0
11761	RSPe_2	-122.444	37.944	3.00	15.94	287.45	18.94	-122.444	37.944	3.24	15.94	287.60	19.18	9	7/14/2014	30:48.9
11762	RSPe_2	-122.444	37.944	2.97	16.00	287.51	18.97	-122.444	37.944	3.24	16.00	287.62	19.24	9	7/14/2014	30:48.8
11763	RSPe_2	-122.444	37.944	3.00	15.98	287.45	18.98	-122.444	37.944	3.24	15.98	287.58	19.22	9	7/14/2014	30:48.7
11764	RSPe_2	-122.444	37.944	2.97	16.03	287.50	18.99	-122.444	37.944	3.24	16.03	287.60	19.27	9	7/14/2014	30:48.6
11765	RSPe_2	-122.444	37.944	2.97	15.99	287.52	18.96	-122.444	37.944	3.24	15.99	287.58	19.23	9	7/14/2014	30:48.5
11766	RSPe_2	-122.444	37.944	2.97	16.01	287.50	18.98	-122.444	37.944	3.24	16.01	287.60	19.25	9	7/14/2014	30:48.4
11767	RSPe_2	-122.444	37.944	3.00	16.02	287.54	19.02	-122.444	37.944	3.24	16.02	287.56	19.26	9	7/14/2014	30:48.3
11768	RSPe_2	-122.444	37.944	2.97	15.97	287.52	18.93	-122.444	37.944	3.24	15.97	287.55	19.21	9	7/14/2014	30:48.2
11769	RSPe_2	-122.444	37.944	2.97	15.97	287.52	18.93	-122.444	37.944	3.24	15.97	287.54	19.21	9	7/14/2014	30:48.1
11770	RSPe_2	-122.444	37.944	2.97	15.92	287.52	18.89	-122.444	37.944	3.24	15.92	287.54	19.16	9	7/14/2014	30:48.0
11771	RSPe_2	-122.444	37.944	2.97	15.97	287.48	18.94	-122.444	37.944	3.24	15.97	287.50	19.21	9	7/14/2014	30:47.9
11772	RSPe_2	-122.444	37.944	2.97	16.00	287.50	18.96	-122.444	37.944	3.24	16.00	287.52	19.24	9	7/14/2014	30:47.8
11773	RSPe_2	-122.444	37.944	2.97	15.93	287.52	18.90	-122.444	37.944	3.24	15.93	287.50	19.17	9	7/14/2014	30:47.7
11774	RSPe_2	-122.444	37.944	2.97	15.77	287.54	18.74	-122.444	37.944	3.24	15.77	287.52	19.01	9	7/14/2014	30:47.6
11775	RSPe_2	-122.444	37.944	3.00	15.69	287.50	18.70	-122.444	37.944	3.29	15.69	287.55	18.99	9	7/14/2014	30:47.5
11776	RSPe_2	-122.444	37.944	2.97	15.69	287.57	18.66	-122.444	37.944	3.29	15.69	287.57	18.98	9	7/14/2014	30:47.4
11777	RSPe_2	-122.444	37.944	2.97	15.68	287.55	18.65	-122.444	37.944	3.29	15.68	287.55	18.97	9	7/14/2014	30:47.3
11778	RSPe_2	-122.444	37.944	2.97	15.67	287.59	18.64	-122.444	37.944	3.24	15.67	287.61	18.91	9	7/14/2014	30:47.2
11779	RSPe_2	-122.444	37.944	2.97	15.68	287.55	18.65	-122.444	37.944	3.29	15.68	287.61	18.97	9	7/14/2014	30:47.1
11780	RSPe_2	-122.444	37.944	2.92	15.65	287.57	18.57	-122.444	37.944	3.24	15.65	287.63	18.89	9	7/14/2014	30:47.0

11781	RSPe_2	-122.444	37.944	2.97	15.62	287.57	18.59	-122.444	37.944	3.29	15.62	287.68	18.91	9	7/14/2014	30:46.9
11782	RSPe_2	-122.444	37.944	2.92	15.64	287.60	18.56	-122.444	37.944	3.29	15.64	287.63	18.93	9	7/14/2014	30:46.8
11783	RSPe_2	-122.444	37.944	2.97	15.69	287.60	18.66	-122.444	37.944	3.29	15.69	287.69	18.98	9	7/14/2014	30:46.7
11784	RSPe_2	-122.444	37.944	2.92	15.60	287.60	18.52	-122.444	37.944	3.29	15.60	287.66	18.89	9	7/14/2014	30:46.6
11785	RSPe_2	-122.444	37.944	2.97	15.56	287.60	18.52	-122.444	37.944	3.29	15.56	287.66	18.85	9	7/14/2014	30:46.5
11786	RSPe_2	-122.444	37.944	2.92	15.62	287.64	18.53	-122.444	37.944	3.24	15.62	287.66	18.86	9	7/14/2014	30:46.4
11787	RSPe_2	-122.444	37.944	2.92	15.68	287.60	18.59	-122.444	37.944	3.33	15.68	287.70	19.00	9	7/14/2014	30:46.3
11788	RSPe_2	-122.444	37.944	2.92	15.50	287.64	18.42	-122.444	37.944	3.29	15.50	287.68	18.79	9	7/14/2014	30:46.2
11789	RSPe_2	-122.444	37.944	2.97	15.54	287.62	18.51	-122.444	37.944	3.29	15.54	287.71	18.83	9	7/14/2014	30:46.1
11790	RSPe_2	-122.444	37.944	2.92	15.51	287.63	18.43	-122.444	37.944	3.24	15.51	287.73	18.75	9	7/14/2014	30:46.0
11791	RSPe_2	-122.444	37.944	2.97	15.44	287.62	18.41	-122.444	37.944	3.29	15.44	287.75	18.73	9	7/14/2014	30:45.9
11792	RSPe_2	-122.444	37.944	2.92	15.42	287.60	18.33	-122.444	37.944	3.29	15.42	287.74	18.71	9	7/14/2014	30:45.8
11793	RSPe_2	-122.444	37.944	2.92	15.38	287.58	18.29	-122.444	37.944	3.29	15.38	287.75	18.67	9	7/14/2014	30:45.7
11794	RSPe_2	-122.444	37.944	2.92	15.34	287.60	18.26	-122.444	37.944	3.29	15.34	287.77	18.63	9	7/14/2014	30:45.6
11795	RSPe_2	-122.444	37.944	2.92	15.32	287.61	18.24	-122.444	37.944	3.29	15.32	287.80	18.62	9	7/14/2014	30:45.5
11796	RSPe_2	-122.444	37.944	2.92	15.29	287.58	18.20	-122.444	37.944	3.29	15.29	287.77	18.58	9	7/14/2014	30:45.4
11797	RSPe_2	-122.444	37.944	2.92	15.28	287.58	18.19	-122.444	37.944	3.29	15.28	287.76	18.57	9	7/14/2014	30:45.3
11798	RSPe_2	-122.444	37.944	2.88	15.27	287.54	18.15	-122.444	37.944	3.29	15.27	287.73	18.56	9	7/14/2014	30:45.2
11799	RSPe_2	-122.444	37.944	2.92	15.26	287.54	18.17	-122.444	37.944	3.29	15.26	287.78	18.55	9	7/14/2014	30:45.1
11800	RSPe_2	-122.444	37.944	2.92	15.26	287.56	18.18	-122.444	37.944	3.29	15.26	287.75	18.55	9	7/14/2014	30:45.0
11801	RSPe_2	-122.444	37.944	2.92	15.27	287.59	18.19	-122.444	37.944	3.29	15.27	287.76	18.56	9	7/14/2014	30:44.9
11802	RSPe_2	-122.444	37.944	2.88	15.31	287.58	18.19	-122.444	37.944	3.24	15.31	287.76	18.55	9	7/14/2014	30:44.8
11803	RSPe_2	-122.444	37.944	2.92	15.28	287.59	18.19	-122.444	37.944	3.24	15.28	287.74	18.52	9	7/14/2014	30:44.7
11804	RSPe_2	-122.444	37.944	2.88	15.31	287.61	18.19	-122.444	37.944	3.21	15.31	287.76	18.51	9	7/14/2014	30:44.6
11805	RSPe_2	-122.444	37.944	2.88	15.29	287.61	18.18	-122.444	37.944	3.24	15.29	287.74	18.53	9	7/14/2014	30:44.5
11806	RSPe_2	-122.444	37.944	2.88	15.32	287.63	18.20	-122.444	37.944	3.21	15.32	287.76	18.52	9	7/14/2014	30:44.4
11807	RSPe_2	-122.444	37.944	2.88	15.33	287.63	18.22	-122.444	37.944	3.21	15.33	287.76	18.54	9	7/14/2014	30:44.3
11808	RSPe_2	-122.444	37.944	2.88	15.32	287.61	18.21	-122.444	37.944	3.16	15.32	287.69	18.48	9	7/14/2014	30:44.2
11809	RSPe_2	-122.444	37.944	2.88	15.33	287.59	18.22	-122.444	37.944	3.16	15.33	287.72	18.49	9	7/14/2014	30:44.1
11810	RSPe_2	-122.444	37.944	2.88	15.33	287.57	18.22	-122.444	37.944	3.16	15.33	287.70	18.49	9	7/14/2014	30:44.0
11811	RSPe_2	-122.444	37.944	2.88	15.34	287.57	18.22	-122.444	37.944	3.16	15.34	287.70	18.50	9	7/14/2014	30:43.9
11812	RSPe_2	-122.444	37.944	2.83	15.35	287.52	18.18	-122.444	37.944	3.16	15.35	287.70	18.50	9	7/14/2014	30:43.8
11813	RSPe_2	-122.444	37.944	2.88	15.35	287.50	18.23	-122.444	37.944	3.16	15.35	287.70	18.50	9	7/14/2014	30:43.7

11814	RSPe_2	-122.444	37.944	2.83	15.38	287.49	18.21	-122.444	37.944	3.16	15.38	287.68	18.53	9	7/14/2014	30:43.6
11815	RSPe_2	-122.444	37.944	2.83	15.39	287.53	18.23	-122.444	37.944	3.16	15.39	287.72	18.55	9	7/14/2014	30:43.5
11816	RSPe_2	-122.444	37.944	2.83	15.40	287.51	18.23	-122.444	37.944	3.12	15.40	287.74	18.52	9	7/14/2014	30:43.4
11817	RSPe_2	-122.444	37.944	2.83	15.41	287.51	18.24	-122.444	37.944	3.12	15.41	287.74	18.53	9	7/14/2014	30:43.3
11818	RSPe_2	-122.444	37.944	2.80	15.46	287.48	18.26	-122.444	37.944	3.07	15.46	287.75	18.53	9	7/14/2014	30:43.2
11819	RSPe_2	-122.444	37.944	2.80	15.46	287.51	18.25	-122.444	37.944	3.07	15.46	287.74	18.53	9	7/14/2014	30:43.1
11820	RSPe_2	-122.444	37.944	2.80	15.48	287.47	18.28	-122.444	37.944	3.04	15.48	287.72	18.52	9	7/14/2014	30:43.0
11821	RSPe_2	-122.444	37.944	2.80	15.50	287.51	18.30	-122.444	37.944	3.04	15.50	287.73	18.54	9	7/14/2014	30:42.9
11822	RSPe_2	-122.444	37.944	2.74	15.52	287.47	18.26	-122.444	37.944	3.00	15.52	287.69	18.52	9	7/14/2014	30:42.8
11823	RSPe_2	-122.444	37.944	2.80	15.56	287.47	18.36	-122.444	37.944	3.00	15.56	287.68	18.57	9	7/14/2014	30:42.7
11824	RSPe_2	-122.444	37.944	2.74	15.56	287.44	18.31	-122.444	37.944	3.00	15.56	287.67	18.57	9	7/14/2014	30:42.6
11825	RSPe_2	-122.444	37.944	2.80	15.61	287.42	18.41	-122.444	37.944	3.00	15.61	287.64	18.61	9	7/14/2014	30:42.5
11826	RSPe_2	-122.444	37.944	2.74	15.62	287.43	18.36	-122.444	37.944	2.95	15.62	287.62	18.57	9	7/14/2014	30:42.4
11827	RSPe_2	-122.444	37.944	2.74	15.65	287.38	18.39	-122.444	37.944	2.95	15.65	287.64	18.60	9	7/14/2014	30:42.3
11828	RSPe_2	-122.444	37.944	2.74	15.66	287.38	18.41	-122.444	37.944	2.92	15.66	287.60	18.58	9	7/14/2014	30:42.2
11829	RSPe_2	-122.444	37.944	2.74	15.70	287.39	18.45	-122.444	37.944	2.95	15.70	287.58	18.65	9	7/14/2014	30:42.1
11830	RSPe_2	-122.444	37.944	2.71	15.68	287.38	18.39	-122.444	37.944	2.92	15.68	287.60	18.60	9	7/14/2014	30:42.0
11831	RSPe_2	-122.444	37.944	2.74	15.74	287.34	18.49	-122.444	37.944	2.92	15.74	287.55	18.66	9	7/14/2014	30:41.9
11832	RSPe_2	-122.444	37.944	2.71	15.76	287.34	18.47	-122.444	37.944	2.87	15.76	287.54	18.63	9	7/14/2014	30:41.8
11833	RSPe_2	-122.444	37.944	2.71	15.77	287.34	18.48	-122.444	37.944	2.87	15.77	287.58	18.64	9	7/14/2014	30:41.7
11834	RSPe_2	-122.444	37.944	2.68	15.76	287.32	18.44	-122.444	37.944	2.83	15.76	287.56	18.60	9	7/14/2014	30:41.6
11835	RSPe_2	-122.444	37.944	2.71	15.83	287.32	18.54	-122.444	37.944	2.83	15.83	287.51	18.66	9	7/14/2014	30:41.5
11836	RSPe_2	-122.444	37.944	2.68	15.83	287.32	18.51	-122.444	37.944	2.78	15.83	287.52	18.62	9	7/14/2014	30:41.4
11837	RSPe_2	-122.444	37.944	2.68	15.87	287.34	18.55	-122.444	37.944	2.78	15.87	287.49	18.65	9	7/14/2014	30:41.3
11838	RSPe_2	-122.444	37.944	2.68	15.88	287.30	18.56	-122.444	37.944	2.75	15.88	287.47	18.63	9	7/14/2014	30:41.2
11839	RSPe_2	-122.444	37.944	2.68	15.93	287.30	18.61	-122.444	37.944	2.75	15.93	287.42	18.68	9	7/14/2014	30:41.1
11840	RSPe_2	-122.444	37.944	2.63	15.93	287.24	18.56	-122.444	37.944	2.70	15.93	287.48	18.63	9	7/14/2014	30:41.0
11841	RSPe_2	-122.444	37.944	2.63	15.93	287.21	18.55	-122.444	37.944	2.70	15.93	287.41	18.63	9	7/14/2014	30:40.9
11842	RSPe_2	-122.444	37.944	2.63	15.93	287.19	18.56	-122.444	37.944	2.70	15.93	287.41	18.63	9	7/14/2014	30:40.8
11843	RSPe_2	-122.444	37.944	2.68	15.96	287.15	18.63	-122.444	37.944	2.70	15.96	287.41	18.66	9	7/14/2014	30:40.7
11844	RSPe_2	-122.444	37.944	2.63	16.03	287.13	18.65	-122.444	37.944	2.66	16.03	287.34	18.69	9	7/14/2014	30:40.6
11845	RSPe_2	-122.444	37.944	2.68	16.03	287.06	18.70	-122.444	37.944	2.70	16.03	287.36	18.72	9	7/14/2014	30:40.5
11846	RSPe_2	-122.444	37.944	2.63	16.01	287.08	18.64	-122.444	37.944	2.66	16.01	287.32	18.68	9	7/14/2014	30:40.4

11847	RSPe_2	-122.444	37.944	2.63	16.05	287.11	18.68	-122.444	37.944	2.66	16.05	287.37	18.72	9	7/14/2014	30:40.3
11848	RSPe_2	-122.444	37.944	2.59	16.05	287.15	18.64	-122.444	37.944	2.63	16.05	287.39	18.68	9	7/14/2014	30:40.2
11849	RSPe_2	-122.444	37.944	2.63	16.06	287.16	18.69	-122.444	37.944	2.66	16.06	287.39	18.73	9	7/14/2014	30:40.1
11850	RSPe_2	-122.444	37.944	2.59	16.08	287.13	18.67	-122.444	37.944	2.63	16.08	287.37	18.71	9	7/14/2014	30:40.0
11851	RSPe_2	-122.444	37.944	2.63	16.08	287.24	18.70	-122.444	37.944	2.63	16.08	287.39	18.71	9	7/14/2014	30:39.9
11852	RSPe_2	-122.444	37.944	2.59	16.08	287.26	18.67	-122.444	37.944	2.58	16.08	287.41	18.66	9	7/14/2014	30:39.8
11853	RSPe_2	-122.444	37.944	2.59	16.16	287.29	18.75	-122.444	37.944	2.58	16.16	287.41	18.74	9	7/14/2014	30:39.7
11854	RSPe_2	-122.444	37.944	2.59	16.12	287.31	18.71	-122.444	37.944	2.55	16.12	287.44	18.67	9	7/14/2014	30:39.6
11855	RSPe_2	-122.444	37.944	2.59	16.13	287.29	18.72	-122.444	37.944	2.55	16.13	287.37	18.67	9	7/14/2014	30:39.5
11856	RSPe_2	-122.444	37.944	2.54	16.14	287.24	18.68	-122.444	37.944	2.50	16.14	287.39	18.64	9	7/14/2014	30:39.4
11857	RSPe_2	-122.444	37.944	2.59	16.17	287.27	18.76	-122.444	37.944	2.55	16.17	287.35	18.71	9	7/14/2014	30:39.3
11858	RSPe_2	-122.444	37.944	2.54	16.16	287.24	18.70	-122.444	37.944	2.50	16.16	287.39	18.66	9	7/14/2014	30:39.2
11859	RSPe_2	-122.444	37.944	2.59	16.18	287.24	18.77	-122.444	37.944	2.55	16.18	287.39	18.73	9	7/14/2014	30:39.1
11860	RSPe_2	-122.444	37.944	2.54	16.18	287.29	18.72	-122.444	37.944	2.55	16.18	287.40	18.73	9	7/14/2014	30:39.0
11861	RSPe_2	-122.444	37.944	2.59	16.19	287.25	18.78	-122.444	37.944	2.55	16.19	287.44	18.74	9	7/14/2014	30:38.9
11862	RSPe_2	-122.444	37.944	2.59	16.23	287.34	18.82	-122.444	37.944	2.55	16.23	287.46	18.78	9	7/14/2014	30:38.8
11863	RSPe_2	-122.444	37.944	2.54	16.21	287.34	18.75	-122.444	37.944	2.55	16.21	287.51	18.76	9	7/14/2014	30:38.7
11864	RSPe_2	-122.444	37.944	2.54	16.23	287.38	18.77	-122.444	37.944	2.55	16.23	287.48	18.78	9	7/14/2014	30:38.6
11865	RSPe_2	-122.444	37.944	2.59	16.23	287.38	18.83	-122.444	37.944	2.55	16.23	287.49	18.78	9	7/14/2014	30:38.5
11866	RSPe_2	-122.444	37.944	2.54	16.23	287.40	18.77	-122.444	37.944	2.50	16.23	287.55	18.73	9	7/14/2014	30:38.4
11867	RSPe_2	-122.444	37.944	2.59	16.23	287.40	18.82	-122.444	37.944	2.55	16.23	287.56	18.78	9	7/14/2014	30:38.3
11868	RSPe_2	-122.444	37.944	2.54	16.27	287.39	18.81	-122.444	37.944	2.50	16.27	287.54	18.77	9	7/14/2014	30:38.2
11869	RSPe_2	-122.444	37.944	2.59	16.26	287.45	18.85	-122.444	37.944	2.55	16.26	287.52	18.81	9	7/14/2014	30:38.1
11870	RSPe_2	-122.444	37.944	2.54	16.30	287.43	18.84	-122.444	37.944	2.50	16.30	287.55	18.79	9	7/14/2014	30:38.0
11871	RSPe_2	-122.444	37.944	2.54	16.29	287.49	18.83	-122.444	37.944	2.50	16.29	287.53	18.79	9	7/14/2014	30:37.9
11872	RSPe_2	-122.444	37.944	2.54	16.28	287.48	18.82	-122.444	37.944	2.50	16.28	287.58	18.78	9	7/14/2014	30:37.8
11873	RSPe_2	-122.444	37.944	2.59	16.27	287.52	18.86	-122.444	37.944	2.55	16.27	287.56	18.82	9	7/14/2014	30:37.7
11874	RSPe_2	-122.444	37.944	2.54	16.29	287.50	18.83	-122.444	37.944	2.50	16.29	287.56	18.79	9	7/14/2014	30:37.6
11875	RSPe_2	-122.444	37.944	2.59	16.30	287.52	18.89	-122.444	37.944	2.50	16.30	287.56	18.79	9	7/14/2014	30:37.5
11876	RSPe_2	-122.444	37.944	2.54	16.28	287.52	18.82	-122.444	37.944	2.50	16.28	287.58	18.78	9	7/14/2014	30:37.4
11877	RSPe_2	-122.444	37.944	2.59	16.33	287.48	18.92	-122.444	37.944	2.55	16.33	287.54	18.88	9	7/14/2014	30:37.3
11878	RSPe_2	-122.444	37.944	2.54	16.30	287.50	18.84	-122.444	37.944	2.55	16.30	287.56	18.84	9	7/14/2014	30:37.2
11879	RSPe_2	-122.444	37.944	2.59	16.35	287.52	18.94	-122.444	37.944	2.55	16.35	287.63	18.90	9	7/14/2014	30:37.1

11880	RSPe_2	-122.444	37.944	2.59	16.29	287.48	18.88	-122.444	37.944	2.55	16.29	287.66	18.84	9	7/14/2014	30:37.0
11881	RSPe_2	-122.444	37.944	2.63	16.32	287.55	18.95	-122.444	37.944	2.58	16.32	287.67	18.90	9	7/14/2014	30:36.9
11882	RSPe_2	-122.444	37.944	2.59	16.29	287.57	18.88	-122.444	37.944	2.55	16.29	287.70	18.84	9	7/14/2014	30:36.8
11883	RSPe_2	-122.444	37.944	2.59	16.30	287.57	18.90	-122.444	37.944	2.58	16.30	287.72	18.89	9	7/14/2014	30:36.7
11884	RSPe_2	-122.444	37.944	2.59	16.31	287.63	18.90	-122.444	37.944	2.58	16.31	287.65	18.89	9	7/14/2014	30:36.6
11885	RSPe_2	-122.444	37.944	2.59	16.33	287.65	18.93	-122.444	37.944	2.63	16.33	287.70	18.97	9	7/14/2014	30:36.5
11886	RSPe_2	-122.444	37.944	2.59	16.35	287.68	18.94	-122.444	37.944	2.58	16.35	287.72	18.93	9	7/14/2014	30:36.4
11887	RSPe_2	-122.444	37.944	2.63	16.31	287.70	18.94	-122.444	37.944	2.58	16.31	287.70	18.89	9	7/14/2014	30:36.3
11888	RSPe_2	-122.444	37.944	2.59	16.34	287.73	18.93	-122.444	37.944	2.58	16.34	287.69	18.92	9	7/14/2014	30:36.2
11889	RSPe_2	-122.444	37.944	2.59	16.32	287.66	18.91	-122.444	37.944	2.58	16.32	287.73	18.90	9	7/14/2014	30:36.1
11890	RSPe_2	-122.444	37.944	2.59	16.31	287.73	18.90	-122.444	37.944	2.58	16.31	287.70	18.89	9	7/14/2014	30:36.0
11891	RSPe_2	-122.444	37.944	2.59	16.32	287.66	18.91	-122.444	37.944	2.58	16.32	287.72	18.90	9	7/14/2014	30:35.9
11892	RSPe_2	-122.444	37.944	2.59	16.31	287.66	18.90	-122.444	37.944	2.63	16.31	287.68	18.94	9	7/14/2014	30:35.8
11893	RSPe_2	-122.444	37.944	2.63	16.33	287.68	18.96	-122.444	37.944	2.63	16.33	287.68	18.97	9	7/14/2014	30:35.7
11894	RSPe_2	-122.444	37.944	2.59	16.30	287.62	18.90	-122.444	37.944	2.63	16.30	287.68	18.94	9	7/14/2014	30:35.6
11895	RSPe_2	-122.444	37.944	2.63	16.30	287.62	18.93	-122.444	37.944	2.67	16.30	287.66	18.97	9	7/14/2014	30:35.5
11896	RSPe_2	-122.444	37.944	2.63	16.34	287.60	18.97	-122.444	37.944	2.67	16.34	287.70	19.01	9	7/14/2014	30:35.4
11897	RSPe_2	-122.444	37.944	2.63	16.31	287.58	18.94	-122.444	37.944	2.70	16.31	287.66	19.01	9	7/14/2014	30:35.3
11898	RSPe_2	-122.444	37.944	2.63	16.31	287.60	18.94	-122.444	37.944	2.67	16.31	287.75	18.98	9	7/14/2014	30:35.2
11899	RSPe_2	-122.444	37.944	2.68	16.31	287.58	18.99	-122.444	37.944	2.70	16.31	287.72	19.01	9	7/14/2014	30:35.1
11900	RSPe_2	-122.444	37.944	2.63	16.33	287.60	18.96	-122.444	37.944	2.70	16.33	287.73	19.03	9	7/14/2014	30:35.0
11901	RSPe_2	-122.444	37.944	2.63	16.33	287.58	18.96	-122.444	37.944	2.70	16.33	287.75	19.03	9	7/14/2014	30:34.9
11902	RSPe_2	-122.444	37.944	2.63	16.35	287.58	18.98	-122.444	37.944	2.67	16.35	287.73	19.02	9	7/14/2014	30:34.8
11903	RSPe_2	-122.444	37.944	2.68	16.36	287.60	19.04	-122.444	37.944	2.70	16.36	287.75	19.06	9	7/14/2014	30:34.7
11904	RSPe_2	-122.444	37.944	2.63	16.40	287.62	19.02	-122.444	37.944	2.67	16.40	287.81	19.06	9	7/14/2014	30:34.6
11905	RSPe_2	-122.444	37.944	2.68	16.37	287.60	19.05	-122.444	37.944	2.70	16.37	287.80	19.07	9	7/14/2014	30:34.5
11906	RSPe_2	-122.444	37.944	2.63	16.37	287.63	19.00	-122.444	37.944	2.67	16.37	287.80	19.04	9	7/14/2014	30:34.4
11907	RSPe_2	-122.444	37.944	2.63	16.40	287.65	19.02	-122.444	37.944	2.70	16.40	287.80	19.10	9	7/14/2014	30:34.3
11908	RSPe_2	-122.444	37.944	2.63	16.41	287.65	19.04	-122.444	37.944	2.67	16.41	287.75	19.08	9	7/14/2014	30:34.2
11909	RSPe_2	-122.444	37.944	2.68	16.39	287.65	19.07	-122.444	37.944	2.67	16.39	287.76	19.05	9	7/14/2014	30:34.1
11910	RSPe_2	-122.444	37.944	2.63	16.41	287.61	19.03	-122.444	37.944	2.67	16.41	287.74	19.07	9	7/14/2014	30:34.0
11911	RSPe_2	-122.444	37.944	2.63	16.45	287.58	19.08	-122.444	37.944	2.67	16.45	287.71	19.12	9	7/14/2014	30:33.9
11912	RSPe_2	-122.444	37.944	2.63	16.44	287.58	19.07	-122.444	37.944	2.67	16.44	287.72	19.11	9	7/14/2014	30:33.8

11913	RSPe_2	-122.444	37.944	2.68	16.46	287.61	19.14	-122.444	37.944	2.70	16.46	287.76	19.16	9	7/14/2014 30:33.7
11914	RSPe_2	-122.444	37.944	2.63	16.47	287.61	19.09	-122.444	37.944	2.67	16.47	287.78	19.13	9	7/14/2014 30:33.6
11915	RSPe_2	-122.444	37.944	2.68	16.46	287.59	19.14	-122.444	37.944	2.70	16.46	287.81	19.16	9	7/14/2014 30:33.5
11916	RSPe_2	-122.444	37.944	2.63	16.50	287.61	19.12	-122.444	37.944	2.70	16.50	287.81	19.20	9	7/14/2014 30:33.4
11917	RSPe_2	-122.444	37.944	2.68	16.51	287.63	19.18	-122.444	37.944	2.70	16.51	287.78	19.21	9	7/14/2014 30:33.3
11918	RSPe_2	-122.444	37.944	2.63	16.47	287.63	19.10	-122.444	37.944	2.70	16.47	287.74	19.17	9	7/14/2014 30:33.2
11919	RSPe_2	-122.444	37.944	2.68	16.51	287.67	19.18	-122.444	37.944	2.70	16.51	287.80	19.21	9	7/14/2014 30:33.1
11920	RSPe_2	-122.444	37.944	2.63	16.53	287.70	19.15	-122.444	37.944	2.70	16.53	287.84	19.23	9	7/14/2014 30:33.0
11921	RSPe_2	-122.444	37.944	2.68	16.50	287.73	19.17	-122.444	37.944	2.70	16.50	287.85	19.20	9	7/14/2014 30:32.9
11922	RSPe_2	-122.444	37.944	2.63	16.53	287.72	19.16	-122.444	37.944	2.67	16.53	287.88	19.20	9	7/14/2014 30:32.8
11923	RSPe_2	-122.444	37.944	2.68	16.51	287.75	19.19	-122.444	37.944	2.67	16.51	287.85	19.18	9	7/14/2014 30:32.7
11924	RSPe_2	-122.444	37.944	2.63	16.51	287.70	19.14	-122.444	37.944	2.63	16.51	287.87	19.14	9	7/14/2014 30:32.6
11925	RSPe_2	-122.444	37.944	2.68	16.51	287.70	19.19	-122.444	37.944	2.67	16.51	287.83	19.18	9	7/14/2014 30:32.5
11926	RSPe_2	-122.444	37.944	2.63	16.49	287.68	19.12	-122.444	37.944	2.67	16.49	287.81	19.16	9	7/14/2014 30:32.4
11927	RSPe_2	-122.444	37.944	2.63	16.54	287.66	19.17	-122.444	37.944	2.67	16.54	287.79	19.21	9	7/14/2014 30:32.3
11928	RSPe_2	-122.444	37.944	2.63	16.49	287.66	19.12	-122.444	37.944	2.67	16.49	287.72	19.16	9	7/14/2014 30:32.2
11929	RSPe_2	-122.444	37.944	2.68	16.39	287.64	19.07	-122.444	37.944	2.70	16.39	287.70	19.09	9	7/14/2014 30:32.1
11930	RSPe_2	-122.444	37.944	2.63	16.36	287.64	18.98	-122.444	37.944	2.67	16.36	287.74	19.02	9	7/14/2014 30:32.0
11931	RSPe_2	-122.444	37.944	2.68	16.38	287.69	19.06	-122.444	37.944	2.70	16.38	287.73	19.08	9	7/14/2014 30:31.9
11932	RSPe_2	-122.444	37.944	2.68	16.31	287.70	18.99	-122.444	37.944	2.70	16.31	287.74	19.01	9	7/14/2014 30:31.8
11933	RSPe_2	-122.444	37.944	2.63	16.35	287.77	18.98	-122.444	37.944	2.70	16.35	287.75	19.05	9	7/14/2014 30:31.7
11934	RSPe_2	-122.444	37.944	2.63	16.27	287.78	18.90	-122.444	37.944	2.70	16.27	287.77	18.97	9	7/14/2014 30:31.6
11935	RSPe_2	-122.444	37.944	2.68	16.41	287.82	19.09	-122.444	37.944	2.70	16.41	287.81	19.11	9	7/14/2014 30:31.5
11936	RSPe_2	-122.444	37.944	2.63	16.27	287.84	18.89	-122.444	37.944	2.67	16.27	287.81	18.93	9	7/14/2014 30:31.4
11937	RSPe_2	-122.444	37.944	2.68	16.37	287.82	19.05	-122.444	37.944	2.70	16.37	287.80	19.07	9	7/14/2014 30:31.3
11938	RSPe_2	-122.444	37.944	2.63	16.43	287.82	19.05	-122.444	37.944	2.67	16.43	287.83	19.09	9	7/14/2014 30:31.2
11939	RSPe_2	-122.444	37.944	2.68	16.26	287.82	18.94	-122.444	37.944	2.70	16.26	287.86	18.96	9	7/14/2014 30:31.1
11940	RSPe_2	-122.444	37.944	2.63	16.27	287.80	18.90	-122.444	37.944	2.67	16.27	287.84	18.94	9	7/14/2014 30:31.0
11941	RSPe_2	-122.444	37.944	2.63	16.24	287.78	18.87	-122.444	37.944	2.67	16.24	287.89	18.91	9	7/14/2014 30:30.9
11942	RSPe_2	-122.444	37.944	2.63	16.20	287.76	18.83	-122.444	37.944	2.63	16.20	287.84	18.83	9	7/14/2014 30:30.8
11943	RSPe_2	-122.444	37.944	2.68	16.16	287.72	18.84	-122.444	37.944	2.67	16.16	287.84	18.82	9	7/14/2014 30:30.7
11944	RSPe_2	-122.444	37.944	2.63	16.13	287.71	18.75	-122.444	37.944	2.70	16.13	287.83	18.83	9	7/14/2014 30:30.6
11945	RSPe_2	-122.444	37.944	2.68	16.13	287.71	18.81	-122.444	37.944	2.70	16.13	287.82	18.83	9	7/14/2014 30:30.5

11946	RSPe_2	-122.444	37.944	2.68	16.16	287.67	18.84	-122.444	37.944	2.70	16.16	287.81	18.86	9	7/14/2014 30:30.4
11947	RSPe_2	-122.444	37.944	2.68	16.06	287.69	18.74	-122.444	37.944	2.75	16.06	287.83	18.81	9	7/14/2014 30:30.3
11948	RSPe_2	-122.444	37.944	2.68	16.17	287.68	18.85	-122.444	37.944	2.70	16.17	287.80	18.87	9	7/14/2014 30:30.2
11949	RSPe_2	-122.444	37.944	2.68	16.04	287.67	18.72	-122.444	37.944	2.75	16.04	287.84	18.79	9	7/14/2014 30:30.1
11950	RSPe_2	-122.444	37.944	2.68	16.03	287.69	18.71	-122.444	37.944	2.70	16.03	287.80	18.73	9	7/14/2014 30:30.0
11951	RSPe_2	-122.444	37.944	2.68	16.02	287.72	18.70	-122.444	37.944	2.75	16.02	287.86	18.77	9	7/14/2014 30:29.9
11952	RSPe_2	-122.444	37.944	2.68	16.00	287.72	18.67	-122.444	37.944	2.70	16.00	287.87	18.70	9	7/14/2014 30:29.8
11953	RSPe_2	-122.444	37.944	2.68	16.00	287.78	18.68	-122.444	37.944	2.75	16.00	287.85	18.75	9	7/14/2014 30:29.7
11954	RSPe_2	-122.444	37.944	2.68	15.99	287.77	18.67	-122.444	37.944	2.70	15.99	287.87	18.69	9	7/14/2014 30:29.6
11955	RSPe_2	-122.444	37.944	2.68	16.12	287.81	18.80	-122.444	37.944	2.70	16.12	287.85	18.82	9	7/14/2014 30:29.5
11956	RSPe_2	-122.444	37.944	2.63	16.05	287.81	18.68	-122.444	37.944	2.70	16.05	287.78	18.75	9	7/14/2014 30:29.4
11957	RSPe_2	-122.444	37.944	2.68	15.99	287.81	18.67	-122.444	37.944	2.70	15.99	287.83	18.69	9	7/14/2014 30:29.3
11958	RSPe_2	-122.444	37.944	2.63	16.00	287.83	18.62	-122.444	37.944	2.70	16.00	287.79	18.70	9	7/14/2014 30:29.2
11959	RSPe_2	-122.444	37.944	2.68	15.91	287.81	18.59	-122.444	37.944	2.70	15.91	287.81	18.61	9	7/14/2014 30:29.1
11960	RSPe_2	-122.444	37.944	2.63	15.89	287.81	18.51	-122.444	37.944	2.70	15.89	287.83	18.59	9	7/14/2014 30:29.0
11961	RSPe_2	-122.444	37.944	2.68	15.86	287.79	18.54	-122.444	37.944	2.70	15.86	287.82	18.56	9	7/14/2014 30:28.9
11962	RSPe_2	-122.444	37.944	2.63	15.84	287.77	18.47	-122.444	37.944	2.70	15.84	287.85	18.54	9	7/14/2014 30:28.8
11963	RSPe_2	-122.444	37.944	2.68	15.86	287.75	18.54	-122.444	37.944	2.75	15.86	287.83	18.61	9	7/14/2014 30:28.7
11964	RSPe_2	-122.444	37.944	2.63	15.76	287.77	18.38	-122.444	37.944	2.75	15.76	287.86	18.51	9	7/14/2014 30:28.6
11965	RSPe_2	-122.444	37.944	2.68	15.82	287.79	18.50	-122.444	37.944	2.78	15.82	287.85	18.60	9	7/14/2014 30:28.5
11966	RSPe_2	-122.444	37.944	2.68	15.77	287.77	18.45	-122.444	37.944	2.75	15.77	287.90	18.52	9	7/14/2014 30:28.4
11967	RSPe_2	-122.444	37.944	2.68	15.74	287.79	18.42	-122.444	37.944	2.75	15.74	287.92	18.49	9	7/14/2014 30:28.3
11968	RSPe_2	-122.444	37.944	2.68	15.76	287.75	18.43	-122.444	37.944	2.75	15.76	287.92	18.51	9	7/14/2014 30:28.2
11969	RSPe_2	-122.444	37.944	2.68	15.74	287.82	18.42	-122.444	37.944	2.75	15.74	287.95	18.49	9	7/14/2014 30:28.1
11970	RSPe_2	-122.444	37.944	2.68	15.76	287.84	18.44	-122.444	37.944	2.75	15.76	287.96	18.51	9	7/14/2014 30:28.0
11971	RSPe_2	-122.444	37.944	2.68	15.76	287.86	18.44	-122.444	37.944	2.75	15.76	287.99	18.51	9	7/14/2014 30:27.9
11972	RSPe_2	-122.444	37.944	2.63	15.76	287.84	18.38	-122.444	37.944	2.70	15.76	287.96	18.46	9	7/14/2014 30:27.8
11973	RSPe_2	-122.444	37.944	2.68	15.76	287.86	18.44	-122.444	37.944	2.75	15.76	287.97	18.51	9	7/14/2014 30:27.7
11974	RSPe_2	-122.444	37.944	2.63	15.79	287.89	18.42	-122.444	37.944	2.70	15.79	287.97	18.49	9	7/14/2014 30:27.6
11975	RSPe_2	-122.444	37.944	2.68	15.77	287.91	18.45	-122.444	37.944	2.75	15.77	287.97	18.52	9	7/14/2014 30:27.5
11976	RSPe_2	-122.444	37.944	2.63	15.77	287.95	18.40	-122.444	37.944	2.70	15.77	288.03	18.47	9	7/14/2014 30:27.4
11977	RSPe_2	-122.444	37.944	2.63	15.78	287.95	18.41	-122.444	37.944	2.75	15.78	288.02	18.53	9	7/14/2014 30:27.3
11978	RSPe_2	-122.444	37.944	2.63	15.76	287.99	18.39	-122.444	37.944	2.70	15.76	288.06	18.46	9	7/14/2014 30:27.2

11979	RSPe_2	-122.444	37.944	2.68	15.80	288.00	18.48	-122.444	37.944	2.70	15.80	288.06	18.50	9	7/14/2014	30:27.1
11980	RSPe_2	-122.444	37.944	2.63	15.84	288.02	18.47	-122.444	37.944	2.67	15.84	288.08	18.51	9	7/14/2014	30:27.0
11981	RSPe_2	-122.444	37.944	2.68	15.83	288.00	18.51	-122.444	37.944	2.70	15.83	288.08	18.53	9	7/14/2014	30:26.9
11982	RSPe_2	-122.444	37.944	2.63	15.80	288.02	18.43	-122.444	37.944	2.70	15.80	288.15	18.50	9	7/14/2014	30:26.8
11983	RSPe_2	-122.444	37.944	2.68	15.83	287.98	18.51	-122.444	37.944	2.75	15.83	288.09	18.58	9	7/14/2014	30:26.7
11984	RSPe_2	-122.444	37.944	2.68	15.79	287.95	18.47	-122.444	37.944	2.70	15.79	288.08	18.49	9	7/14/2014	30:26.6
11985	RSPe_2	-122.444	37.944	2.63	15.81	287.98	18.44	-122.444	37.944	2.75	15.81	288.11	18.56	9	7/14/2014	30:26.5
11986	RSPe_2	-122.444	37.944	2.63	15.80	288.00	18.42	-122.444	37.944	2.75	15.80	288.08	18.55	9	7/14/2014	30:26.4
11987	RSPe_2	-122.444	37.944	2.68	15.84	288.00	18.52	-122.444	37.944	2.75	15.84	288.06	18.59	9	7/14/2014	30:26.3
11988	RSPe_2	-122.444	37.944	2.63	15.83	288.01	18.45	-122.444	37.944	2.75	15.83	288.09	18.58	9	7/14/2014	30:26.2
11989	RSPe_2	-122.444	37.944	2.68	15.84	288.02	18.52	-122.444	37.944	2.75	15.84	288.08	18.59	9	7/14/2014	30:26.1
11990	RSPe_2	-122.444	37.944	2.63	15.86	288.03	18.48	-122.444	37.944	2.75	15.86	288.07	18.61	9	7/14/2014	30:26.0
11991	RSPe_2	-122.444	37.944	2.68	15.86	288.02	18.54	-122.444	37.944	2.70	15.86	288.06	18.56	9	7/14/2014	30:25.9
11992	RSPe_2	-122.444	37.944	2.63	15.86	287.98	18.48	-122.444	37.944	2.70	15.86	288.07	18.56	9	7/14/2014	30:25.8
11993	RSPe_2	-122.444	37.944	2.63	15.89	288.05	18.51	-122.444	37.944	2.75	15.89	288.14	18.64	9	7/14/2014	30:25.7
11994	RSPe_2	-122.444	37.944	2.63	15.91	288.01	18.54	-122.444	37.944	2.70	15.91	288.13	18.61	9	7/14/2014	30:25.6
11995	RSPe_2	-122.444	37.944	2.63	15.86	288.03	18.48	-122.444	37.944	2.70	15.86	288.11	18.56	9	7/14/2014	30:25.5
11996	RSPe_2	-122.444	37.944	2.63	15.90	288.01	18.52	-122.444	37.944	2.70	15.90	288.10	18.60	9	7/14/2014	30:25.4
11997	RSPe_2	-122.444	37.944	2.63	15.90	287.98	18.53	-122.444	37.944	2.70	15.90	288.07	18.60	9	7/14/2014	30:25.3
11998	RSPe_2	-122.444	37.944	2.63	15.95	287.95	18.58	-122.444	37.944	2.70	15.95	288.05	18.65	9	7/14/2014	30:25.2
11999	RSPe_2	-122.444	37.944	2.63	15.93	287.98	18.56	-122.444	37.944	2.70	15.93	288.07	18.63	9	7/14/2014	30:25.1
12000	RSPe_2	-122.444	37.944	2.63	15.93	287.97	18.56	-122.444	37.944	2.70	15.93	288.05	18.63	9	7/14/2014	30:25.0
12001	RSPe_2	-122.444	37.944	2.63	15.96	287.98	18.58	-122.444	37.944	2.70	15.96	288.05	18.66	9	7/14/2014	30:24.9
12002	RSPe_2	-122.444	37.944	2.63	16.00	288.04	18.62	-122.444	37.944	2.67	16.00	288.09	18.66	9	7/14/2014	30:24.8
12003	RSPe_2	-122.444	37.944	2.63	15.97	288.03	18.59	-122.444	37.944	2.75	15.97	288.10	18.72	9	7/14/2014	30:24.7
12004	RSPe_2	-122.444	37.944	2.63	16.00	288.04	18.62	-122.444	37.944	2.70	16.00	288.10	18.70	9	7/14/2014	30:24.6
12005	RSPe_2	-122.444	37.944	2.63	16.00	288.08	18.63	-122.444	37.944	2.75	16.00	288.14	18.75	9	7/14/2014	30:24.5
12006	RSPe_2	-122.444	37.944	2.59	16.03	288.10	18.63	-122.444	37.944	2.70	16.03	288.16	18.74	9	7/14/2014	30:24.4
12007	RSPe_2	-122.444	37.944	2.63	16.03	288.14	18.65	-122.444	37.944	2.70	16.03	288.21	18.73	9	7/14/2014	30:24.3
12008	RSPe_2	-122.444	37.944	2.59	16.04	288.12	18.63	-122.444	37.944	2.70	16.04	288.20	18.74	9	7/14/2014	30:24.2
12009	RSPe_2	-122.444	37.944	2.63	16.05	288.19	18.68	-122.444	37.944	2.75	16.05	288.24	18.80	9	7/14/2014	30:24.1
12010	RSPe_2	-122.444	37.944	2.63	16.05	288.17	18.68	-122.444	37.944	2.70	16.05	288.21	18.75	9	7/14/2014	30:24.0
12011	RSPe_2	-122.444	37.944	2.63	16.08	288.21	18.71	-122.444	37.944	2.70	16.08	288.25	18.78	9	7/14/2014	30:23.9

12012	RSPe_2	-122.444	37.944	2.59	16.08	288.24	18.67	-122.444	37.944	2.70	16.08	288.30	18.78	9	7/14/2014	30:23.8
12013	RSPe_2	-122.444	37.944	2.63	16.18	288.26	18.81	-122.444	37.944	2.70	16.18	288.26	18.88	9	7/14/2014	30:23.7
12014	RSPe_2	-122.444	37.944	2.63	16.08	288.21	18.71	-122.444	37.944	2.70	16.08	288.30	18.78	9	7/14/2014	30:23.6
12015	RSPe_2	-122.444	37.944	2.59	16.09	288.24	18.68	-122.444	37.944	2.70	16.09	288.34	18.79	9	7/14/2014	30:23.5
12016	RSPe_2	-122.444	37.944	2.59	16.13	288.26	18.73	-122.444	37.944	2.70	16.13	288.33	18.83	9	7/14/2014	30:23.4
12017	RSPe_2	-122.444	37.944	2.59	16.18	288.26	18.77	-122.444	37.944	2.70	16.18	288.33	18.88	9	7/14/2014	30:23.3
12018	RSPe_2	-122.444	37.944	2.59	16.14	288.26	18.74	-122.444	37.944	2.67	16.14	288.39	18.81	9	7/14/2014	30:23.2
12019	RSPe_2	-122.444	37.944	2.63	16.17	288.29	18.80	-122.444	37.944	2.70	16.17	288.34	18.87	9	7/14/2014	30:23.1
12020	RSPe_2	-122.444	37.944	2.59	16.20	288.26	18.80	-122.444	37.944	2.70	16.20	288.39	18.90	9	7/14/2014	30:23.0
12021	RSPe_2	-122.444	37.944	2.63	16.23	288.29	18.86	-122.444	37.944	2.70	16.23	288.41	18.93	9	7/14/2014	30:22.9
12022	RSPe_2	-122.444	37.944	2.59	16.23	288.35	18.83	-122.444	37.944	2.67	16.23	288.48	18.90	9	7/14/2014	30:22.8
12023	RSPe_2	-122.444	37.944	2.63	16.24	288.37	18.87	-122.444	37.944	2.70	16.24	288.50	18.94	9	7/14/2014	30:22.7
12024	RSPe_2	-122.444	37.944	2.59	16.25	288.42	18.84	-122.444	37.944	2.67	16.25	288.53	18.92	9	7/14/2014	30:22.6
12025	RSPe_2	-122.444	37.944	2.59	16.26	288.44	18.85	-122.444	37.944	2.67	16.26	288.52	18.93	9	7/14/2014	30:22.5
12026	RSPe_2	-122.444	37.944	2.54	16.28	288.48	18.82	-122.444	37.944	2.67	16.28	288.57	18.95	9	7/14/2014	30:22.4
12027	RSPe_2	-122.444	37.944	2.59	16.33	288.49	18.93	-122.444	37.944	2.67	16.33	288.55	19.00	9	7/14/2014	30:22.3
12028	RSPe_2	-122.444	37.944	2.59	16.35	288.51	18.94	-122.444	37.944	2.67	16.35	288.56	19.02	9	7/14/2014	30:22.2
12029	RSPe_2	-122.444	37.944	2.59	16.35	288.55	18.94	-122.444	37.944	2.67	16.35	288.61	19.02	9	7/14/2014	30:22.1
12030	RSPe_2	-122.444	37.944	2.59	16.33	288.53	18.93	-122.444	37.944	2.63	16.33	288.57	18.97	9	7/14/2014	30:22.0
12031	RSPe_2	-122.444	37.944	2.59	16.33	288.54	18.93	-122.444	37.944	2.67	16.33	288.59	19.00	9	7/14/2014	30:21.9
12032	RSPe_2	-122.444	37.944	2.59	16.40	288.53	18.99	-122.444	37.944	2.67	16.40	288.58	19.06	9	7/14/2014	30:21.8
12033	RSPe_2	-122.444	37.944	2.59	16.40	288.51	18.99	-122.444	37.944	2.67	16.40	288.60	19.06	9	7/14/2014	30:21.7
12034	RSPe_2	-122.444	37.944	2.59	16.40	288.54	18.99	-122.444	37.944	2.67	16.40	288.64	19.06	9	7/14/2014	30:21.6
12035	RSPe_2	-122.444	37.944	2.59	16.44	288.53	19.04	-122.444	37.944	2.63	16.44	288.64	19.08	9	7/14/2014	30:21.5
12036	RSPe_2	-122.444	37.944	2.59	16.43	288.51	19.02	-122.444	37.944	2.63	16.43	288.64	19.06	9	7/14/2014	30:21.4
12037	RSPe_2	-122.444	37.944	2.59	16.47	288.54	19.06	-122.444	37.944	2.63	16.47	288.66	19.10	9	7/14/2014	30:21.3
12038	RSPe_2	-122.444	37.944	2.54	16.46	288.54	19.00	-122.444	37.944	2.63	16.46	288.69	19.09	9	7/14/2014	30:21.2
12039	RSPe_2	-122.444	37.944	2.54	16.48	288.61	19.02	-122.444	37.944	2.63	16.48	288.76	19.11	9	7/14/2014	30:21.1
12040	RSPe_2	-122.444	37.944	2.54	16.48	288.60	19.02	-122.444	37.944	2.58	16.48	288.78	19.06	9	7/14/2014	30:21.0
12041	RSPe_2	-122.444	37.944	2.54	16.49	288.61	19.03	-122.444	37.944	2.58	16.49	288.76	19.07	9	7/14/2014	30:20.9
12042	RSPe_2	-122.444	37.944	2.54	16.47	288.63	19.01	-122.444	37.944	2.55	16.47	288.80	19.01	9	7/14/2014	30:20.8
12043	RSPe_2	-122.444	37.944	2.54	16.48	288.63	19.02	-122.444	37.944	2.55	16.48	288.77	19.03	9	7/14/2014	30:20.7
12044	RSPe_2	-122.444	37.944	2.54	16.44	288.65	18.99	-122.444	37.944	2.58	16.44	288.81	19.03	9	7/14/2014	30:20.6

12045	RSPe_2	-122.444	37.944	2.51	16.48	288.59	18.99	-122.444	37.944	2.55	16.48	288.80	19.03	9	7/14/2014	30:20.5
12046	RSPe_2	-122.444	37.944	2.54	16.44	288.61	18.98	-122.444	37.944	2.50	16.44	288.78	18.94	9	7/14/2014	30:20.4
12047	RSPe_2	-122.444	37.944	2.54	16.51	288.58	19.05	-122.444	37.944	2.55	16.51	288.78	19.05	9	7/14/2014	30:20.3
12048	RSPe_2	-122.444	37.944	2.54	16.46	288.54	19.00	-122.444	37.944	2.55	16.46	288.76	19.01	9	7/14/2014	30:20.2
12049	RSPe_2	-122.444	37.944	2.54	16.39	288.59	18.93	-122.444	37.944	2.58	16.39	288.74	18.97	9	7/14/2014	30:20.1
12050	RSPe_2	-122.444	37.944	2.51	16.39	288.58	18.90	-122.444	37.944	2.55	16.39	288.80	18.94	9	7/14/2014	30:20.0
12051	RSPe_2	-122.444	37.944	2.54	16.41	288.65	18.95	-122.444	37.944	2.55	16.41	288.82	18.95	9	7/14/2014	30:19.9
12052	RSPe_2	-122.444	37.944	2.51	16.39	288.63	18.90	-122.444	37.944	2.55	16.39	288.81	18.94	9	7/14/2014	30:19.8
12053	RSPe_2	-122.444	37.944	2.51	16.41	288.74	18.92	-122.444	37.944	2.50	16.41	288.85	18.91	9	7/14/2014	30:19.7
12054	RSPe_2	-122.444	37.944	2.46	16.39	288.68	18.85	-122.444	37.944	2.50	16.39	288.81	18.89	9	7/14/2014	30:19.6
12055	RSPe_2	-122.444	37.944	2.51	16.42	288.74	18.93	-122.444	37.944	2.50	16.42	288.83	18.92	9	7/14/2014	30:19.5
12056	RSPe_2	-122.444	37.944	2.51	16.43	288.72	18.93	-122.444	37.944	2.46	16.43	288.85	18.89	9	7/14/2014	30:19.4
12057	RSPe_2	-122.444	37.944	2.46	16.44	288.74	18.89	-122.444	37.944	2.46	16.44	288.87	18.90	9	7/14/2014	30:19.3
12058	RSPe_2	-122.444	37.944	2.46	16.43	288.72	18.88	-122.444	37.944	2.41	16.43	288.89	18.84	9	7/14/2014	30:19.2
12059	RSPe_2	-122.444	37.944	2.46	16.41	288.70	18.87	-122.444	37.944	2.41	16.41	288.85	18.82	9	7/14/2014	30:19.1
12060	RSPe_2	-122.444	37.944	2.46	16.44	288.70	18.90	-122.444	37.944	2.41	16.44	288.87	18.86	9	7/14/2014	30:19.0
12061	RSPe_2	-122.444	37.944	2.46	16.41	288.68	18.86	-122.444	37.944	2.41	16.41	288.85	18.82	9	7/14/2014	30:18.9
12062	RSPe_2	-122.444	37.944	2.42	16.40	288.68	18.82	-122.444	37.944	2.38	16.40	288.85	18.77	9	7/14/2014	30:18.8
12063	RSPe_2	-122.444	37.944	2.46	16.41	288.66	18.87	-122.444	37.944	2.41	16.41	288.85	18.82	9	7/14/2014	30:18.7
12064	RSPe_2	-122.444	37.944	2.42	16.40	288.66	18.82	-122.444	37.944	2.38	16.40	288.83	18.77	9	7/14/2014	30:18.6
12065	RSPe_2	-122.444	37.944	2.46	16.39	288.62	18.85	-122.444	37.944	2.38	16.39	288.90	18.77	9	7/14/2014	30:18.5
12066	RSPe_2	-122.444	37.944	2.42	16.39	288.66	18.81	-122.444	37.944	2.38	16.39	288.85	18.77	9	7/14/2014	30:18.4
12067	RSPe_2	-122.444	37.944	2.42	16.41	288.66	18.83	-122.444	37.944	2.38	16.41	288.81	18.78	9	7/14/2014	30:18.3
12068	RSPe_2	-122.444	37.944	2.42	16.42	288.69	18.84	-122.444	37.944	2.33	16.42	288.81	18.75	9	7/14/2014	30:18.2
12069	RSPe_2	-122.444	37.944	2.42	16.44	288.66	18.86	-122.444	37.944	2.33	16.44	288.79	18.77	9	7/14/2014	30:18.1
12070	RSPe_2	-122.444	37.944	2.42	16.41	288.68	18.83	-122.444	37.944	2.29	16.41	288.77	18.70	9	7/14/2014	30:18.0
12071	RSPe_2	-122.444	37.944	2.42	16.43	288.71	18.85	-122.444	37.944	2.33	16.43	288.83	18.76	9	7/14/2014	30:17.9
12072	RSPe_2	-122.444	37.944	2.42	16.42	288.73	18.84	-122.444	37.944	2.29	16.42	288.77	18.71	9	7/14/2014	30:17.8
12073	RSPe_2	-122.444	37.944	2.42	16.41	288.71	18.83	-122.444	37.944	2.29	16.41	288.79	18.70	9	7/14/2014	30:17.7
12074	RSPe_2	-122.444	37.944	2.37	16.40	288.73	18.77	-122.444	37.944	2.29	16.40	288.75	18.69	9	7/14/2014	30:17.6
12075	RSPe_2	-122.444	37.944	2.42	16.41	288.71	18.83	-122.444	37.944	2.29	16.41	288.76	18.70	9	7/14/2014	30:17.5
12076	RSPe_2	-122.444	37.944	2.37	16.41	288.71	18.78	-122.444	37.944	2.29	16.41	288.75	18.70	9	7/14/2014	30:17.4
12077	RSPe_2	-122.444	37.944	2.42	16.44	288.71	18.86	-122.444	37.944	2.29	16.44	288.77	18.73	9	7/14/2014	30:17.3

12078	RSPe_2	-122.444	37.944	2.37	16.39	288.69	18.76	-122.444	37.944	2.26	16.39	288.77	18.65	9	7/14/2014	30:17.2
12079	RSPe_2	-122.444	37.944	2.37	16.39	288.73	18.76	-122.444	37.944	2.29	16.39	288.73	18.68	9	7/14/2014	30:17.1
12080	RSPe_2	-122.444	37.944	2.37	16.39	288.73	18.76	-122.444	37.944	2.26	16.39	288.77	18.65	9	7/14/2014	30:17.0
12081	RSPe_2	-122.444	37.944	2.42	16.41	288.71	18.83	-122.444	37.944	2.29	16.41	288.76	18.70	9	7/14/2014	30:16.9
12082	RSPe_2	-122.444	37.944	2.37	16.46	288.78	18.83	-122.444	37.944	2.26	16.46	288.78	18.72	9	7/14/2014	30:16.8
12083	RSPe_2	-122.444	37.944	2.42	16.37	288.74	18.80	-122.444	37.944	2.26	16.37	288.80	18.63	9	7/14/2014	30:16.7
12084	RSPe_2	-122.444	37.944	2.37	16.41	288.80	18.78	-122.444	37.944	2.26	16.41	288.82	18.67	9	7/14/2014	30:16.6
12085	RSPe_2	-122.444	37.944	2.37	16.37	288.82	18.74	-122.444	37.944	2.26	16.37	288.84	18.63	9	7/14/2014	30:16.5
12086	RSPe_2	-122.444	37.944	2.37	16.41	288.80	18.78	-122.444	37.944	2.26	16.41	288.84	18.67	9	7/14/2014	30:16.4
12087	RSPe_2	-122.444	37.944	2.37	16.39	288.83	18.76	-122.444	37.944	2.26	16.39	288.87	18.65	9	7/14/2014	30:16.3
12088	RSPe_2	-122.444	37.944	2.37	16.40	288.83	18.77	-122.444	37.944	2.26	16.40	288.91	18.66	9	7/14/2014	30:16.2
12089	RSPe_2	-122.444	37.944	2.42	16.37	288.78	18.79	-122.444	37.944	2.26	16.37	288.91	18.63	9	7/14/2014	30:16.1
12090	RSPe_2	-122.444	37.944	2.42	16.38	288.79	18.81	-122.444	37.944	2.21	16.38	288.90	18.59	9	7/14/2014	30:16.0
12091	RSPe_2	-122.444	37.944	2.42	16.36	288.79	18.78	-122.444	37.944	2.26	16.36	288.87	18.62	9	7/14/2014	30:15.9
12092	RSPe_2	-122.444	37.944	2.37	16.37	288.79	18.74	-122.444	37.944	2.21	16.37	288.93	18.58	9	7/14/2014	30:15.8
12093	RSPe_2	-122.444	37.944	2.42	16.37	288.78	18.80	-122.444	37.944	2.26	16.37	288.91	18.63	9	7/14/2014	30:15.7
12094	RSPe_2	-122.444	37.944	2.42	16.37	288.83	18.80	-122.444	37.944	2.21	16.37	288.95	18.58	9	7/14/2014	30:15.6
12095	RSPe_2	-122.444	37.944	2.37	16.37	288.83	18.74	-122.444	37.944	2.21	16.37	288.96	18.58	9	7/14/2014	30:15.5
12096	RSPe_2	-122.444	37.944	2.37	16.41	288.88	18.78	-122.444	37.944	2.21	16.41	289.01	18.62	9	7/14/2014	30:15.4
12097	RSPe_2	-122.444	37.944	2.42	16.44	288.87	18.86	-122.444	37.944	2.21	16.44	289.02	18.65	9	7/14/2014	30:15.3
12098	RSPe_2	-122.444	37.944	2.37	16.42	288.92	18.79	-122.444	37.944	2.18	16.42	289.03	18.60	9	7/14/2014	30:15.2
12099	RSPe_2	-122.444	37.944	2.42	16.43	288.92	18.85	-122.444	37.944	2.21	16.43	289.05	18.64	9	7/14/2014	30:15.1
12100	RSPe_2	-122.444	37.944	2.37	16.43	288.94	18.80	-122.444	37.944	2.18	16.43	289.00	18.60	9	7/14/2014	30:15.0
12101	RSPe_2	-122.444	37.944	2.42	16.44	288.92	18.86	-122.444	37.944	2.18	16.44	289.05	18.61	9	7/14/2014	30:14.9
12102	RSPe_2	-122.444	37.944	2.37	16.44	288.92	18.81	-122.444	37.944	2.18	16.44	289.01	18.61	9	7/14/2014	30:14.8
12103	RSPe_2	-122.444	37.944	2.42	16.44	288.90	18.86	-122.444	37.944	2.18	16.44	289.01	18.61	9	7/14/2014	30:14.7
12104	RSPe_2	-122.444	37.944	2.37	16.46	288.93	18.83	-122.444	37.944	2.13	16.46	289.05	18.58	9	7/14/2014	30:14.6
12105	RSPe_2	-122.444	37.944	2.42	16.46	288.86	18.88	-122.444	37.944	2.18	16.46	288.99	18.64	9	7/14/2014	30:14.5
12106	RSPe_2	-122.444	37.944	2.37	16.45	288.88	18.82	-122.444	37.944	2.13	16.45	289.01	18.58	9	7/14/2014	30:14.4
12107	RSPe_2	-122.444	37.944	2.42	16.48	288.84	18.90	-122.444	37.944	2.18	16.48	288.96	18.66	9	7/14/2014	30:14.3
12108	RSPe_2	-122.444	37.944	2.42	16.48	288.86	18.90	-122.444	37.944	2.18	16.48	288.96	18.66	9	7/14/2014	30:14.2
12109	RSPe_2	-122.444	37.944	2.42	16.48	288.88	18.90	-122.444	37.944	2.18	16.48	288.96	18.66	9	7/14/2014	30:14.1
12110	RSPe_2	-122.444	37.944	2.42	16.49	288.95	18.91	-122.444	37.944	2.13	16.49	289.03	18.62	9	7/14/2014	30:14.0

12111	RSPe_2	-122.444	37.944	2.42	16.59	288.97	19.01	-122.444	37.944	2.13	16.59	289.08	18.72	9	7/14/2014	30:13.9
12112	RSPe_2	-122.444	37.944	2.42	16.58	289.00	19.01	-122.444	37.944	2.13	16.58	289.05	18.71	9	7/14/2014	30:13.8
12113	RSPe_2	-122.444	37.944	2.46	16.64	288.97	19.09	-122.444	37.944	2.13	16.64	289.06	18.76	9	7/14/2014	30:13.7
12114	RSPe_2	-122.444	37.944	2.42	16.58	288.97	19.01	-122.444	37.944	2.13	16.58	289.01	18.71	9	7/14/2014	30:13.6
12115	RSPe_2	-122.444	37.944	2.42	16.52	288.99	18.94	-122.444	37.944	2.13	16.52	288.99	18.65	9	7/14/2014	30:13.5
12116	RSPe_2	-122.444	37.944	2.42	16.53	288.95	18.95	-122.444	37.944	2.09	16.53	288.95	18.62	9	7/14/2014	30:13.4
12117	RSPe_2	-122.444	37.944	2.46	16.54	288.91	19.00	-122.444	37.944	2.13	16.54	288.91	18.67	9	7/14/2014	30:13.3
12118	RSPe_2	-122.444	37.944	2.46	16.68	288.89	19.14	-122.444	37.944	2.18	16.68	288.86	18.86	9	7/14/2014	30:13.2
12119	RSPe_2	-122.444	37.944	2.46	16.57	288.89	19.02	-122.444	37.944	2.18	16.57	288.91	18.74	9	7/14/2014	30:13.1
12120	RSPe_2	-122.444	37.944	2.46	16.57	288.86	19.02	-122.444	37.944	2.13	16.57	288.83	18.69	9	7/14/2014	30:13.0
12121	RSPe_2	-122.444	37.944	2.51	16.54	288.78	19.05	-122.444	37.944	2.18	16.54	288.78	18.72	9	7/14/2014	30:12.9
12122	RSPe_2	-122.444	37.944	2.46	16.52	288.76	18.98	-122.444	37.944	2.18	16.52	288.78	18.70	9	7/14/2014	30:12.8
12123	RSPe_2	-122.444	37.944	2.51	16.56	288.80	19.07	-122.444	37.944	2.21	16.56	288.75	18.77	9	7/14/2014	30:12.7
12124	RSPe_2	-122.444	37.944	2.46	16.57	288.75	19.02	-122.444	37.944	2.21	16.57	288.75	18.78	9	7/14/2014	30:12.6
12125	RSPe_2	-122.444	37.944	2.51	16.56	288.83	19.07	-122.444	37.944	2.21	16.56	288.74	18.77	9	7/14/2014	30:12.5
12126	RSPe_2	-122.444	37.944	2.51	16.58	288.78	19.09	-122.444	37.944	2.26	16.58	288.78	18.84	9	7/14/2014	30:12.4
12127	RSPe_2	-122.444	37.944	2.51	16.58	288.87	19.09	-122.444	37.944	2.21	16.58	288.74	18.79	9	7/14/2014	30:12.3
12128	RSPe_2	-122.444	37.944	2.51	16.53	288.89	19.04	-122.444	37.944	2.26	16.53	288.80	18.80	9	7/14/2014	30:12.2
12129	RSPe_2	-122.444	37.944	2.51	16.60	288.89	19.11	-122.444	37.944	2.26	16.60	288.74	18.86	9	7/14/2014	30:12.1
12130	RSPe_2	-122.444	37.944	2.51	16.59	288.94	19.10	-122.444	37.944	2.26	16.59	288.78	18.85	9	7/14/2014	30:12.0
12131	RSPe_2	-122.444	37.944	2.54	16.60	289.00	19.15	-122.444	37.944	2.26	16.60	288.84	18.87	9	7/14/2014	30:11.9
12132	RSPe_2	-122.444	37.944	2.51	16.54	289.00	19.05	-122.444	37.944	2.21	16.54	288.87	18.76	9	7/14/2014	30:11.8
12133	RSPe_2	-122.444	37.944	2.54	16.59	288.98	19.13	-122.444	37.944	2.26	16.59	288.85	18.85	9	7/14/2014	30:11.7
12134	RSPe_2	-122.444	37.944	2.51	16.61	288.98	19.12	-122.444	37.944	2.26	16.61	288.89	18.88	9	7/14/2014	30:11.6
12135	RSPe_2	-122.444	37.944	2.54	16.65	288.98	19.19	-122.444	37.944	2.26	16.65	288.89	18.91	9	7/14/2014	30:11.5
12136	RSPe_2	-122.444	37.944	2.54	16.61	288.98	19.16	-122.444	37.944	2.30	16.61	288.87	18.91	9	7/14/2014	30:11.4
12137	RSPe_2	-122.444	37.944	2.59	16.59	288.92	19.18	-122.444	37.944	2.30	16.59	288.85	18.89	9	7/14/2014	30:11.3
12138	RSPe_2	-122.444	37.944	2.59	16.59	288.94	19.18	-122.444	37.944	2.33	16.59	288.85	18.92	9	7/14/2014	30:11.2
12139	RSPe_2	-122.444	37.944	2.59	16.61	289.01	19.21	-122.444	37.944	2.38	16.61	288.91	18.99	9	7/14/2014	30:11.1
12140	RSPe_2	-122.444	37.944	2.59	16.56	289.03	19.15	-122.444	37.944	2.33	16.56	288.92	18.89	9	7/14/2014	30:11.0
12141	RSPe_2	-122.444	37.944	2.63	16.60	289.03	19.23	-122.444	37.944	2.38	16.60	288.92	18.98	9	7/14/2014	30:10.9
12142	RSPe_2	-122.444	37.944	2.59	16.59	289.10	19.18	-122.444	37.944	2.38	16.59	288.98	18.97	9	7/14/2014	30:10.8
12143	RSPe_2	-122.444	37.944	2.63	16.58	289.10	19.21	-122.444	37.944	2.41	16.58	289.01	19.00	9	7/14/2014	30:10.7

12144	RSPe_2	-122.444	37.944	2.63	16.56	289.05	19.19	-122.444	37.944	2.41	16.56	288.96	18.97	9	7/14/2014	30:10.6
12145	RSPe_2	-122.444	37.944	2.68	16.58	289.10	19.26	-122.444	37.944	2.41	16.58	289.03	18.99	9	7/14/2014	30:10.5
12146	RSPe_2	-122.444	37.944	2.63	16.56	289.10	19.19	-122.444	37.944	2.41	16.56	289.01	18.97	9	7/14/2014	30:10.4
12147	RSPe_2	-122.444	37.944	2.68	16.55	289.12	19.23	-122.444	37.944	2.46	16.55	289.03	19.01	9	7/14/2014	30:10.3
12148	RSPe_2	-122.444	37.944	2.68	16.57	289.15	19.25	-122.444	37.944	2.46	16.57	289.08	19.03	9	7/14/2014	30:10.2
12149	RSPe_2	-122.444	37.944	2.68	16.60	289.16	19.28	-122.444	37.944	2.46	16.60	289.10	19.06	9	7/14/2014	30:10.1
12150	RSPe_2	-122.444	37.944	2.68	16.55	289.06	19.23	-122.444	37.944	2.46	16.55	288.96	19.01	9	7/14/2014	30:10.0
12151	RSPe_2	-122.444	37.944	2.71	16.53	289.08	19.25	-122.444	37.944	2.50	16.53	288.94	19.03	9	7/14/2014	30:09.9
12152	RSPe_2	-122.444	37.944	2.71	16.54	289.10	19.26	-122.444	37.944	2.50	16.54	289.01	19.04	9	7/14/2014	30:09.8
12153	RSPe_2	-122.444	37.944	2.71	16.58	289.17	19.29	-122.444	37.944	2.55	16.58	289.03	19.13	9	7/14/2014	30:09.7
12154	RSPe_2	-122.444	37.944	2.68	16.58	289.21	19.26	-122.444	37.944	2.55	16.58	289.03	19.13	9	7/14/2014	30:09.6
12155	RSPe_2	-122.444	37.944	2.71	16.58	289.24	19.29	-122.444	37.944	2.58	16.58	289.08	19.16	9	7/14/2014	30:09.5
12156	RSPe_2	-122.444	37.944	2.71	16.62	289.19	19.33	-122.444	37.944	2.58	16.62	289.08	19.20	9	7/14/2014	30:09.4
12157	RSPe_2	-122.444	37.944	2.75	16.57	289.22	19.31	-122.444	37.944	2.63	16.57	289.10	19.20	9	7/14/2014	30:09.3
12158	RSPe_2	-122.444	37.944	2.75	16.58	289.24	19.33	-122.444	37.944	2.67	16.58	289.19	19.25	9	7/14/2014	30:09.2
12159	RSPe_2	-122.444	37.944	2.80	16.59	289.24	19.39	-122.444	37.944	2.67	16.59	289.19	19.26	9	7/14/2014	30:09.1
12160	RSPe_2	-122.444	37.944	2.75	16.59	289.24	19.34	-122.444	37.944	2.67	16.59	289.19	19.26	9	7/14/2014	30:09.0
12161	RSPe_2	-122.444	37.944	2.80	16.57	289.28	19.37	-122.444	37.944	2.70	16.57	289.26	19.27	9	7/14/2014	30:08.9
12162	RSPe_2	-122.444	37.944	2.80	16.59	289.26	19.39	-122.444	37.944	2.70	16.59	289.27	19.29	9	7/14/2014	30:08.8
12163	RSPe_2	-122.444	37.944	2.80	16.58	289.24	19.38	-122.444	37.944	2.70	16.58	289.28	19.28	9	7/14/2014	30:08.7
12164	RSPe_2	-122.444	37.944	2.75	16.57	289.24	19.31	-122.444	37.944	2.70	16.57	289.24	19.27	9	7/14/2014	30:08.6
12165	RSPe_2	-122.444	37.944	2.83	16.59	289.22	19.42	-122.444	37.944	2.75	16.59	289.24	19.34	9	7/14/2014	30:08.5
12166	RSPe_2	-122.444	37.944	2.80	16.58	289.24	19.38	-122.444	37.944	2.75	16.58	289.26	19.33	9	7/14/2014	30:08.4
12167	RSPe_2	-122.444	37.944	2.83	16.59	289.22	19.42	-122.444	37.944	2.79	16.59	289.26	19.38	9	7/14/2014	30:08.3
12168	RSPe_2	-122.444	37.944	2.83	16.58	289.20	19.42	-122.444	37.944	2.75	16.58	289.17	19.33	9	7/14/2014	30:08.2
12169	RSPe_2	-122.444	37.944	2.83	16.60	289.18	19.43	-122.444	37.944	2.84	16.60	289.15	19.43	9	7/14/2014	30:08.1
12170	RSPe_2	-122.444	37.944	2.83	16.59	289.11	19.42	-122.444	37.944	2.84	16.59	289.07	19.43	9	7/14/2014	30:08.0
12171	RSPe_2	-122.444	37.944	2.88	16.62	289.18	19.51	-122.444	37.944	2.87	16.62	289.13	19.49	9	7/14/2014	30:07.9
12172	RSPe_2	-122.444	37.944	2.83	16.63	289.18	19.46	-122.444	37.944	2.87	16.63	289.20	19.50	9	7/14/2014	30:07.8
12173	RSPe_2	-122.444	37.944	2.88	16.68	289.14	19.57	-122.444	37.944	2.92	16.68	289.18	19.61	9	7/14/2014	30:07.7
12174	RSPe_2	-122.444	37.944	2.88	16.66	289.11	19.54	-122.444	37.944	2.92	16.66	289.17	19.58	9	7/14/2014	30:07.6
12175	RSPe_2	-122.444	37.944	2.88	16.65	289.09	19.53	-122.444	37.944	2.92	16.65	289.15	19.57	9	7/14/2014	30:07.5
12176	RSPe_2	-122.444	37.944	2.88	16.65	289.07	19.54	-122.444	37.944	2.92	16.65	289.16	19.57	9	7/14/2014	30:07.4

12177	RSPe_2	-122.444	37.944	2.88	16.65	289.07	19.54	-122.444	37.944	2.95	16.65	289.09	19.61	9	7/14/2014	30:07.3
12178	RSPe_2	-122.444	37.944	2.88	16.67	289.07	19.56	-122.444	37.944	2.95	16.67	289.14	19.63	9	7/14/2014	30:07.2
12179	RSPe_2	-122.444	37.944	2.92	16.67	289.08	19.59	-122.444	37.944	2.95	16.67	289.15	19.63	9	7/14/2014	30:07.1
12180	RSPe_2	-122.444	37.944	2.88	16.70	289.03	19.59	-122.444	37.944	2.95	16.70	289.11	19.66	9	7/14/2014	30:07.0
12181	RSPe_2	-122.444	37.944	2.92	16.67	289.08	19.59	-122.444	37.944	2.95	16.67	289.11	19.62	9	7/14/2014	30:06.9
12182	RSPe_2	-122.444	37.944	2.88	16.65	289.05	19.54	-122.444	37.944	3.01	16.65	289.11	19.66	9	7/14/2014	30:06.8
12183	RSPe_2	-122.444	37.944	2.92	16.63	289.05	19.55	-122.444	37.944	3.01	16.63	289.09	19.63	9	7/14/2014	30:06.7
12184	RSPe_2	-122.444	37.944	2.92	16.62	289.05	19.54	-122.444	37.944	3.01	16.62	289.09	19.63	9	7/14/2014	30:06.6
12185	RSPe_2	-122.444	37.944	2.92	16.60	289.03	19.52	-122.444	37.944	3.04	16.60	289.07	19.64	9	7/14/2014	30:06.5
12186	RSPe_2	-122.444	37.944	2.92	16.63	288.97	19.55	-122.444	37.944	3.01	16.63	289.05	19.63	9	7/14/2014	30:06.4
12187	RSPe_2	-122.444	37.944	2.92	16.56	289.01	19.48	-122.444	37.944	3.01	16.56	289.05	19.57	9	7/14/2014	30:06.3
12188	RSPe_2	-122.444	37.944	2.92	16.59	289.03	19.51	-122.444	37.944	3.04	16.59	289.03	19.63	9	7/14/2014	30:06.2
12189	RSPe_2	-122.444	37.944	2.92	16.46	289.04	19.38	-122.444	37.944	3.04	16.46	289.01	19.50	9	7/14/2014	30:06.1
12190	RSPe_2	-122.444	37.944	2.92	16.41	288.99	19.33	-122.444	37.944	3.04	16.41	288.96	19.45	9	7/14/2014	30:06.0
12191	RSPe_2	-122.444	37.944	2.92	16.25	289.04	19.17	-122.444	37.944	3.07	16.25	288.96	19.32	9	7/14/2014	30:05.9
12192	RSPe_2	-122.444	37.944	2.92	16.33	289.04	19.25	-122.444	37.944	3.07	16.33	288.96	19.41	9	7/14/2014	30:05.8
12193	RSPe_2	-122.444	37.944	2.92	16.21	289.06	19.13	-122.444	37.944	3.07	16.21	289.03	19.29	9	7/14/2014	30:05.7
12194	RSPe_2	-122.444	37.944	2.92	16.33	289.06	19.25	-122.444	37.944	3.08	16.33	289.03	19.41	9	7/14/2014	30:05.6
12195	RSPe_2	-122.444	37.944	2.97	16.17	289.06	19.15	-122.444	37.944	3.08	16.17	289.03	19.25	9	7/14/2014	30:05.5
12196	RSPe_2	-122.444	37.944	2.89	16.20	289.08	19.08	-122.444	37.944	3.08	16.20	289.10	19.27	9	7/14/2014	30:05.4
12197	RSPe_2	-122.444	37.944	2.92	16.28	289.08	19.20	-122.444	37.944	3.08	16.28	289.03	19.36	9	7/14/2014	30:05.3
12198	RSPe_2	-122.444	37.944	2.92	16.13	289.04	19.05	-122.444	37.944	3.08	16.13	289.03	19.21	9	7/14/2014	30:05.2
12199	RSPe_2	-122.444	37.944	2.92	16.09	289.06	19.01	-122.444	37.944	3.08	16.09	289.01	19.16	9	7/14/2014	30:05.1
12200	RSPe_2	-122.444	37.944	2.92	16.16	289.04	19.08	-122.444	37.944	3.08	16.16	289.03	19.23	9	7/14/2014	30:05.0
12201	RSPe_2	-122.444	37.944	2.92	16.02	289.06	18.94	-122.444	37.944	3.13	16.02	289.06	19.14	9	7/14/2014	30:04.9
12202	RSPe_2	-122.444	37.944	2.92	16.00	288.98	18.92	-122.444	37.944	3.13	16.00	289.01	19.13	9	7/14/2014	30:04.8
12203	RSPe_2	-122.444	37.944	2.92	15.89	288.95	18.81	-122.444	37.944	3.13	15.89	289.02	19.01	9	7/14/2014	30:04.7
12204	RSPe_2	-122.444	37.944	2.92	15.89	288.97	18.81	-122.444	37.944	3.13	15.89	289.00	19.01	9	7/14/2014	30:04.6
12205	RSPe_2	-122.444	37.944	2.92	15.95	289.02	18.87	-122.444	37.944	3.16	15.95	289.02	19.11	9	7/14/2014	30:04.5
12206	RSPe_2	-122.444	37.944	2.92	15.80	288.98	18.72	-122.444	37.944	3.13	15.80	289.01	18.92	9	7/14/2014	30:04.4
12207	RSPe_2	-122.444	37.944	2.97	15.80	289.00	18.77	-122.444	37.944	3.13	15.80	289.04	18.93	9	7/14/2014	30:04.3
12208	RSPe_2	-122.444	37.944	2.92	15.80	289.00	18.72	-122.444	37.944	3.13	15.80	289.02	18.93	9	7/14/2014	30:04.2
12209	RSPe_2	-122.444	37.944	2.97	15.77	289.00	18.74	-122.444	37.944	3.13	15.77	289.02	18.90	9	7/14/2014	30:04.1

12210	RSPe_2	-122.444	37.944	2.92	15.80	288.98	18.72	-122.444	37.944	3.13	15.80	289.01	18.92	9	7/14/2014	30:04.0
12211	RSPe_2	-122.444	37.944	2.92	15.74	288.96	18.66	-122.444	37.944	3.16	15.74	288.99	18.90	9	7/14/2014	30:03.9
12212	RSPe_2	-122.444	37.944	2.92	15.80	288.91	18.72	-122.444	37.944	3.16	15.80	288.98	18.96	9	7/14/2014	30:03.8
12213	RSPe_2	-122.444	37.944	2.92	15.77	288.95	18.69	-122.444	37.944	3.16	15.77	289.00	18.93	9	7/14/2014	30:03.7
12214	RSPe_2	-122.444	37.944	2.92	15.72	288.98	18.64	-122.444	37.944	3.16	15.72	289.02	18.88	9	7/14/2014	30:03.6
12215	RSPe_2	-122.444	37.944	2.92	15.72	288.96	18.64	-122.444	37.944	3.16	15.72	289.02	18.88	9	7/14/2014	30:03.5
12216	RSPe_2	-122.444	37.944	2.89	15.74	288.96	18.63	-122.444	37.944	3.16	15.74	289.02	18.90	9	7/14/2014	30:03.4
12217	RSPe_2	-122.444	37.944	2.92	15.73	288.96	18.65	-122.444	37.944	3.16	15.73	289.07	18.89	9	7/14/2014	30:03.3
12218	RSPe_2	-122.444	37.944	2.92	15.76	288.96	18.68	-122.444	37.944	3.16	15.76	289.05	18.92	9	7/14/2014	30:03.2
12219	RSPe_2	-122.444	37.944	2.92	15.76	288.96	18.68	-122.444	37.944	3.16	15.76	289.00	18.92	9	7/14/2014	30:03.1
12220	RSPe_2	-122.444	37.944	2.89	15.74	288.94	18.63	-122.444	37.944	3.16	15.74	289.02	18.90	9	7/14/2014	30:03.0
12221	RSPe_2	-122.444	37.944	2.89	15.74	288.92	18.63	-122.444	37.944	3.16	15.74	289.01	18.90	9	7/14/2014	30:02.9
12222	RSPe_2	-122.444	37.944	2.89	15.84	288.94	18.73	-122.444	37.944	3.13	15.84	288.98	18.97	9	7/14/2014	30:02.8
12223	RSPe_2	-122.444	37.944	2.92	15.75	288.92	18.67	-122.444	37.944	3.16	15.75	288.98	18.91	9	7/14/2014	30:02.7
12224	RSPe_2	-122.444	37.944	2.89	15.74	288.92	18.63	-122.444	37.944	3.16	15.74	289.01	18.90	9	7/14/2014	30:02.6
12225	RSPe_2	-122.444	37.944	2.89	15.76	288.92	18.64	-122.444	37.944	3.16	15.76	289.01	18.92	9	7/14/2014	30:02.5
12226	RSPe_2	-122.444	37.944	2.84	15.80	288.94	18.64	-122.444	37.944	3.13	15.80	289.03	18.93	9	7/14/2014	30:02.4
12227	RSPe_2	-122.444	37.944	2.92	15.83	288.97	18.75	-122.444	37.944	3.16	15.83	289.05	18.99	9	7/14/2014	30:02.3
12228	RSPe_2	-122.444	37.944	2.89	15.83	288.92	18.71	-122.444	37.944	3.16	15.83	289.05	18.99	9	7/14/2014	30:02.2
12229	RSPe_2	-122.444	37.944	2.89	15.83	288.92	18.72	-122.444	37.944	3.16	15.83	289.05	18.99	9	7/14/2014	30:02.1
12230	RSPe_2	-122.444	37.944	2.84	15.83	288.94	18.66	-122.444	37.944	3.16	15.83	289.03	18.99	9	7/14/2014	30:02.0
12231	RSPe_2	-122.444	37.944	2.89	15.85	288.94	18.73	-122.444	37.944	3.13	15.85	289.07	18.97	9	7/14/2014	30:01.9
12232	RSPe_2	-122.444	37.944	2.84	15.86	288.93	18.69	-122.444	37.944	3.13	15.86	289.05	18.98	9	7/14/2014	30:01.8
12233	RSPe_2	-122.444	37.944	2.84	15.86	288.88	18.70	-122.444	37.944	3.13	15.86	289.05	18.99	9	7/14/2014	30:01.7
12234	RSPe_2	-122.444	37.944	2.84	15.89	288.88	18.72	-122.444	37.944	3.13	15.89	289.05	19.01	9	7/14/2014	30:01.6
12235	RSPe_2	-122.444	37.944	2.84	15.88	288.90	18.72	-122.444	37.944	3.13	15.88	289.05	19.01	9	7/14/2014	30:01.5
12236	RSPe_2	-122.444	37.944	2.84	15.90	288.86	18.74	-122.444	37.944	3.08	15.90	289.03	18.98	9	7/14/2014	30:01.4
12237	RSPe_2	-122.444	37.944	2.84	15.89	288.86	18.72	-122.444	37.944	3.08	15.89	289.03	18.96	9	7/14/2014	30:01.3
12238	RSPe_2	-122.444	37.944	2.80	15.92	288.86	18.72	-122.444	37.944	3.04	15.92	289.05	18.96	9	7/14/2014	30:01.2
12239	RSPe_2	-122.444	37.944	2.84	15.90	288.84	18.73	-122.444	37.944	3.04	15.90	289.03	18.94	9	7/14/2014	30:01.1
12240	RSPe_2	-122.444	37.944	2.80	15.94	288.86	18.74	-122.444	37.944	3.04	15.94	289.06	18.98	9	7/14/2014	30:01.0
12241	RSPe_2	-122.444	37.944	2.84	15.95	288.82	18.78	-122.444	37.944	3.04	15.95	289.01	18.99	9	7/14/2014	30:00.9
12242	RSPe_2	-122.444	37.944	2.80	15.95	288.90	18.75	-122.444	37.944	3.04	15.95	289.05	18.99	9	7/14/2014	30:00.8

12243	RSPe_2	-122.444	37.944	2.80	15.97	288.86	18.77	-122.444	37.944	3.04	15.97	289.04	19.01	9	7/14/2014	30:00.7
12244	RSPe_2	-122.444	37.944	2.80	15.96	288.91	18.76	-122.444	37.944	3.01	15.96	289.06	18.96	9	7/14/2014	30:00.6
12245	RSPe_2	-122.444	37.944	2.80	15.99	288.91	18.79	-122.444	37.944	3.01	15.99	289.05	18.99	9	7/14/2014	30:00.5
12246	RSPe_2	-122.444	37.944	2.75	16.00	288.93	18.75	-122.444	37.944	3.01	16.00	289.08	19.00	9	7/14/2014	30:00.4
12247	RSPe_2	-122.444	37.944	2.80	16.03	288.95	18.83	-122.444	37.944	3.01	16.03	289.08	19.03	9	7/14/2014	30:00.3
12248	RSPe_2	-122.444	37.944	2.75	16.03	288.98	18.78	-122.444	37.944	2.96	16.03	289.11	18.99	9	7/14/2014	30:00.2
12249	RSPe_2	-122.444	37.944	2.75	16.03	288.96	18.78	-122.444	37.944	3.01	16.03	289.10	19.04	9	7/14/2014	30:00.1
12250	RSPe_2	-122.444	37.944	2.75	16.05	288.95	18.80	-122.444	37.944	2.96	16.05	289.11	19.01	9	7/14/2014	30:00.0
12251	RSPe_2	-122.444	37.944	2.75	16.09	288.98	18.84	-122.444	37.944	2.96	16.09	289.10	19.05	9	7/14/2014	29:59.9
12252	RSPe_2	-122.444	37.944	2.71	16.06	288.96	18.78	-122.444	37.944	2.92	16.06	289.11	18.99	9	7/14/2014	29:59.8
12253	RSPe_2	-122.444	37.944	2.75	16.07	288.98	18.82	-122.444	37.944	2.92	16.07	289.09	19.00	9	7/14/2014	29:59.7
12254	RSPe_2	-122.444	37.944	2.71	16.08	288.93	18.79	-122.444	37.944	2.92	16.08	289.10	19.00	9	7/14/2014	29:59.6
12255	RSPe_2	-122.444	37.944	2.71	16.10	288.93	18.82	-122.444	37.944	2.92	16.10	289.06	19.03	9	7/14/2014	29:59.5
12256	RSPe_2	-122.444	37.944	2.71	16.13	288.92	18.84	-122.444	37.944	2.87	16.13	289.11	19.00	9	7/14/2014	29:59.4
12257	RSPe_2	-122.444	37.944	2.71	16.14	288.92	18.86	-122.444	37.944	2.87	16.14	289.11	19.01	9	7/14/2014	29:59.3
12258	RSPe_2	-122.444	37.944	2.68	16.15	288.89	18.83	-122.444	37.944	2.87	16.15	289.17	19.02	9	7/14/2014	29:59.2
12259	RSPe_2	-122.444	37.944	2.68	16.18	288.91	18.86	-122.444	37.944	2.87	16.18	289.16	19.05	9	7/14/2014	29:59.1
12260	RSPe_2	-122.444	37.944	2.68	16.19	288.92	18.87	-122.444	37.944	2.84	16.19	289.15	19.03	9	7/14/2014	29:59.0
12261	RSPe_2	-122.444	37.944	2.68	16.20	288.96	18.88	-122.444	37.944	2.84	16.20	289.15	19.03	9	7/14/2014	29:58.9
12262	RSPe_2	-122.444	37.944	2.63	16.21	288.92	18.84	-122.444	37.944	2.79	16.21	289.17	19.00	9	7/14/2014	29:58.8
12263	RSPe_2	-122.444	37.944	2.63	16.23	288.96	18.86	-122.444	37.944	2.79	16.23	289.15	19.02	9	7/14/2014	29:58.7
12264	RSPe_2	-122.444	37.944	2.63	16.25	288.88	18.88	-122.444	37.944	2.75	16.25	289.11	19.00	9	7/14/2014	29:58.6
12265	RSPe_2	-122.444	37.944	2.63	16.26	288.90	18.89	-122.444	37.944	2.75	16.26	289.14	19.01	9	7/14/2014	29:58.5
12266	RSPe_2	-122.444	37.944	2.63	16.28	288.85	18.91	-122.444	37.944	2.75	16.28	289.11	19.03	9	7/14/2014	29:58.4
12267	RSPe_2	-122.444	37.944	2.63	16.30	288.85	18.93	-122.444	37.944	2.75	16.30	289.12	19.05	9	7/14/2014	29:58.3
12268	RSPe_2	-122.444	37.944	2.60	16.29	288.88	18.88	-122.444	37.944	2.70	16.29	289.11	18.99	9	7/14/2014	29:58.2
12269	RSPe_2	-122.444	37.944	2.60	16.32	288.85	18.91	-122.444	37.944	2.70	16.32	289.14	19.02	9	7/14/2014	29:58.1
12270	RSPe_2	-122.444	37.944	2.54	16.33	288.90	18.88	-122.444	37.944	2.67	16.33	289.20	19.00	9	7/14/2014	29:58.0
12271	RSPe_2	-122.444	37.944	2.60	16.34	288.87	18.94	-122.444	37.944	2.67	16.34	289.20	19.01	9	7/14/2014	29:57.9
12272	RSPe_2	-122.444	37.944	2.54	16.37	288.90	18.92	-122.444	37.944	2.63	16.37	289.20	19.01	9	7/14/2014	29:57.8
12273	RSPe_2	-122.444	37.944	2.60	16.38	288.90	18.98	-122.444	37.944	2.63	16.38	289.23	19.02	9	7/14/2014	29:57.7
12274	RSPe_2	-122.444	37.944	2.54	16.41	288.92	18.95	-122.444	37.944	2.58	16.41	289.23	18.99	9	7/14/2014	29:57.6
12275	RSPe_2	-122.444	37.944	2.54	16.43	288.92	18.97	-122.444	37.944	2.58	16.43	289.27	19.01	9	7/14/2014	29:57.5

12276	RSPe_2	-122.444	37.944	2.51	16.43	288.88	18.94	-122.444	37.944	2.55	16.43	289.25	18.98	9	7/14/2014	29:57.4
12277	RSPe_2	-122.444	37.944	2.54	16.43	288.88	18.97	-122.444	37.944	2.55	16.43	289.21	18.98	9	7/14/2014	29:57.3
12278	RSPe_2	-122.444	37.944	2.51	16.46	288.84	18.97	-122.444	37.944	2.50	16.46	289.20	18.96	9	7/14/2014	29:57.2
12279	RSPe_2	-122.444	37.944	2.54	16.47	288.83	19.02	-122.444	37.944	2.50	16.47	289.16	18.97	9	7/14/2014	29:57.1
12280	RSPe_2	-122.444	37.944	2.51	16.47	288.80	18.98	-122.444	37.944	2.47	16.47	289.12	18.94	9	7/14/2014	29:57.0
12281	RSPe_2	-122.444	37.944	2.51	16.49	288.86	19.00	-122.444	37.944	2.50	16.49	289.17	18.99	9	7/14/2014	29:56.9
12282	RSPe_2	-122.444	37.944	2.51	16.48	288.90	18.99	-122.444	37.944	2.47	16.48	289.19	18.95	9	7/14/2014	29:56.8
12283	RSPe_2	-122.444	37.944	2.51	16.51	288.95	19.02	-122.444	37.944	2.47	16.51	289.21	18.98	9	7/14/2014	29:56.7
12284	RSPe_2	-122.444	37.944	2.46	16.53	289.02	18.99	-122.444	37.944	2.41	16.53	289.23	18.95	9	7/14/2014	29:56.6
12285	RSPe_2	-122.444	37.944	2.46	16.56	289.04	19.02	-122.444	37.944	2.41	16.56	289.21	18.97	9	7/14/2014	29:56.5
12286	RSPe_2	-122.444	37.944	2.46	16.62	289.02	19.08	-122.444	37.944	2.38	16.62	289.23	19.00	9	7/14/2014	29:56.4
12287	RSPe_2	-122.444	37.944	2.46	16.58	289.03	19.04	-122.444	37.944	2.38	16.58	289.21	18.96	9	7/14/2014	29:56.3
12288	RSPe_2	-122.444	37.944	2.42	16.58	289.04	19.00	-122.444	37.944	2.33	16.58	289.23	18.91	9	7/14/2014	29:56.2
12289	RSPe_2	-122.444	37.944	2.46	16.63	289.04	19.09	-122.444	37.944	2.33	16.63	289.26	18.96	9	7/14/2014	29:56.1
12290	RSPe_2	-122.444	37.944	2.37	16.60	289.06	18.97	-122.444	37.944	2.30	16.60	289.26	18.89	9	7/14/2014	29:56.0
12291	RSPe_2	-122.444	37.944	2.42	16.60	289.04	19.03	-122.444	37.944	2.33	16.60	289.24	18.93	9	7/14/2014	29:55.9
12292	RSPe_2	-122.444	37.944	2.42	16.60	289.06	19.03	-122.444	37.944	2.30	16.60	289.26	18.90	9	7/14/2014	29:55.8
12293	RSPe_2	-122.444	37.944	2.42	16.63	289.02	19.05	-122.444	37.944	2.30	16.63	289.26	18.92	9	7/14/2014	29:55.7
12294	RSPe_2	-122.444	37.944	2.37	16.62	289.02	18.99	-122.444	37.944	2.26	16.62	289.26	18.88	9	7/14/2014	29:55.6
12295	RSPe_2	-122.444	37.944	2.42	16.67	289.04	19.09	-122.444	37.944	2.30	16.67	289.21	18.96	9	7/14/2014	29:55.5
12296	RSPe_2	-122.444	37.944	2.37	16.65	289.07	19.02	-122.444	37.944	2.26	16.65	289.24	18.91	9	7/14/2014	29:55.4
12297	RSPe_2	-122.444	37.944	2.42	16.67	289.11	19.09	-122.444	37.944	2.26	16.67	289.21	18.93	9	7/14/2014	29:55.3
12298	RSPe_2	-122.444	37.944	2.37	16.72	289.11	19.10	-122.444	37.944	2.21	16.72	289.26	18.93	9	7/14/2014	29:55.2
12299	RSPe_2	-122.444	37.944	2.37	16.68	289.15	19.06	-122.444	37.944	2.21	16.68	289.29	18.90	9	7/14/2014	29:55.1
12300	RSPe_2	-122.444	37.944	2.37	16.70	289.18	19.08	-122.444	37.944	2.21	16.70	289.24	18.92	9	7/14/2014	29:55.0
12301	RSPe_2	-122.444	37.944	2.37	16.79	289.20	19.16	-122.444	37.944	2.21	16.79	289.22	19.00	9	7/14/2014	29:54.9
12302	RSPe_2	-122.444	37.944	2.34	16.76	289.24	19.10	-122.444	37.944	2.18	16.76	289.26	18.94	9	7/14/2014	29:54.8
12303	RSPe_2	-122.444	37.944	2.37	16.75	289.28	19.13	-122.444	37.944	2.18	16.75	289.22	18.93	9	7/14/2014	29:54.7
12304	RSPe_2	-122.444	37.944	2.34	16.74	289.29	19.08	-122.444	37.944	2.18	16.74	289.27	18.92	9	7/14/2014	29:54.6
12305	RSPe_2	-122.444	37.944	2.37	16.80	289.29	19.17	-122.444	37.944	2.18	16.80	289.28	18.98	9	7/14/2014	29:54.5
12306	RSPe_2	-122.444	37.944	2.34	16.77	289.29	19.11	-122.444	37.944	2.13	16.77	289.26	18.90	9	7/14/2014	29:54.4
12307	RSPe_2	-122.444	37.944	2.34	16.77	289.33	19.11	-122.444	37.944	2.18	16.77	289.29	18.95	9	7/14/2014	29:54.3
12308	RSPe_2	-122.444	37.944	2.34	16.77	289.33	19.11	-122.444	37.944	2.13	16.77	289.33	18.90	9	7/14/2014	29:54.2

12309	RSPe_2	-122.444	37.944	2.34	16.78	289.36	19.12	-122.444	37.944	2.18	16.78	289.29	18.96	9	7/14/2014	29:54.1
12310	RSPe_2	-122.444	37.944	2.34	16.78	289.36	19.12	-122.444	37.944	2.18	16.78	289.31	18.96	9	7/14/2014	29:54.0
12311	RSPe_2	-122.444	37.944	2.34	16.79	289.38	19.13	-122.444	37.944	2.18	16.79	289.37	18.97	9	7/14/2014	29:53.9
12312	RSPe_2	-122.444	37.944	2.34	16.79	289.35	19.13	-122.444	37.944	2.18	16.79	289.40	18.97	9	7/14/2014	29:53.8
12313	RSPe_2	-122.444	37.944	2.34	16.80	289.36	19.14	-122.444	37.944	2.18	16.80	289.44	18.98	9	7/14/2014	29:53.7
12314	RSPe_2	-122.444	37.944	2.34	16.82	289.34	19.16	-122.444	37.944	2.13	16.82	289.44	18.95	9	7/14/2014	29:53.6
12315	RSPe_2	-122.444	37.944	2.34	16.80	289.29	19.14	-122.444	37.944	2.13	16.80	289.44	18.93	9	7/14/2014	29:53.5
12316	RSPe_2	-122.444	37.944	2.34	16.85	289.29	19.19	-122.444	37.944	2.13	16.85	289.42	18.98	9	7/14/2014	29:53.4
12317	RSPe_2	-122.444	37.944	2.34	16.83	289.23	19.17	-122.444	37.944	2.13	16.83	289.40	18.96	9	7/14/2014	29:53.3
12318	RSPe_2	-122.444	37.944	2.34	16.88	289.23	19.22	-122.444	37.944	2.13	16.88	289.40	19.01	9	7/14/2014	29:53.2
12319	RSPe_2	-122.444	37.944	2.34	16.92	289.20	19.26	-122.444	37.944	2.13	16.92	289.40	19.05	9	7/14/2014	29:53.1
12320	RSPe_2	-122.444	37.944	2.34	16.94	289.18	19.28	-122.444	37.944	2.13	16.94	289.38	19.06	9	7/14/2014	29:53.0
12321	RSPe_2	-122.444	37.944	2.34	16.94	289.14	19.28	-122.444	37.944	2.13	16.94	289.34	19.07	9	7/14/2014	29:52.9
12322	RSPe_2	-122.444	37.944	2.34	16.92	289.06	19.26	-122.444	37.944	2.13	16.92	289.25	19.05	9	7/14/2014	29:52.8
12323	RSPe_2	-122.444	37.944	2.37	16.94	289.06	19.32	-122.444	37.944	2.18	16.94	289.21	19.12	9	7/14/2014	29:52.7
12324	RSPe_2	-122.444	37.944	2.34	16.94	289.14	19.28	-122.444	37.944	2.13	16.94	289.29	19.07	9	7/14/2014	29:52.6
12325	RSPe_2	-122.444	37.944	2.37	16.93	289.10	19.30	-122.444	37.944	2.13	16.93	289.23	19.06	9	7/14/2014	29:52.5
12326	RSPe_2	-122.444	37.944	2.34	16.90	289.08	19.24	-122.444	37.944	2.13	16.90	289.20	19.03	9	7/14/2014	29:52.4
12327	RSPe_2	-122.444	37.944	2.37	16.91	289.08	19.29	-122.444	37.944	2.13	16.91	289.18	19.04	9	7/14/2014	29:52.3
12328	RSPe_2	-122.444	37.944	2.34	16.91	289.08	19.25	-122.444	37.944	2.13	16.91	289.20	19.04	9	7/14/2014	29:52.2
12329	RSPe_2	-122.444	37.944	2.34	16.94	289.11	19.28	-122.444	37.944	2.13	16.94	289.23	19.06	9	7/14/2014	29:52.1
12330	RSPe_2	-122.444	37.944	2.34	16.89	289.10	19.23	-122.444	37.944	2.09	16.89	289.21	18.99	9	7/14/2014	29:52.0
12331	RSPe_2	-122.444	37.944	2.34	16.88	289.13	19.22	-122.444	37.944	2.13	16.88	289.25	19.01	9	7/14/2014	29:51.9
12332	RSPe_2	-122.444	37.944	2.34	16.86	289.08	19.20	-122.444	37.944	2.09	16.86	289.18	18.95	9	7/14/2014	29:51.8
12333	RSPe_2	-122.444	37.944	2.37	16.85	289.11	19.23	-122.444	37.944	2.13	16.85	289.24	18.98	9	7/14/2014	29:51.7
12334	RSPe_2	-122.444	37.944	2.34	16.88	289.15	19.22	-122.444	37.944	2.09	16.88	289.24	18.98	9	7/14/2014	29:51.6
12335	RSPe_2	-122.444	37.944	2.37	16.85	289.17	19.23	-122.444	37.944	2.13	16.85	289.23	18.98	9	7/14/2014	29:51.5
12336	RSPe_2	-122.444	37.944	2.34	16.83	289.17	19.17	-122.444	37.944	2.09	16.83	289.23	18.92	9	7/14/2014	29:51.4
12337	RSPe_2	-122.444	37.944	2.37	16.86	289.15	19.23	-122.444	37.944	2.09	16.86	289.21	18.95	9	7/14/2014	29:51.3
12338	RSPe_2	-122.444	37.944	2.37	16.82	289.15	19.20	-122.444	37.944	2.13	16.82	289.21	18.95	9	7/14/2014	29:51.2
12339	RSPe_2	-122.444	37.944	2.37	16.81	289.17	19.18	-122.444	37.944	2.13	16.81	289.20	18.93	9	7/14/2014	29:51.1
12340	RSPe_2	-122.444	37.944	2.37	16.81	289.13	19.18	-122.444	37.944	2.13	16.81	289.25	18.93	9	7/14/2014	29:51.0
12341	RSPe_2	-122.444	37.944	2.37	16.80	289.17	19.17	-122.444	37.944	2.18	16.80	289.28	18.98	9	7/14/2014	29:50.9

12342	RSPe_2	-122.444	37.944	2.37	16.81	289.11	19.18	-122.444	37.944	2.13	16.81	289.26	18.93	9	7/14/2014	29:50.8
12343	RSPe_2	-122.444	37.944	2.37	16.79	289.15	19.16	-122.444	37.944	2.13	16.79	289.30	18.92	9	7/14/2014	29:50.7
12344	RSPe_2	-122.444	37.944	2.37	16.79	289.15	19.16	-122.444	37.944	2.13	16.79	289.32	18.92	9	7/14/2014	29:50.6
12345	RSPe_2	-122.444	37.944	2.37	16.78	289.09	19.16	-122.444	37.944	2.13	16.78	289.26	18.91	9	7/14/2014	29:50.5
12346	RSPe_2	-122.444	37.944	2.37	16.77	289.09	19.15	-122.444	37.944	2.09	16.77	289.28	18.87	9	7/14/2014	29:50.4
12347	RSPe_2	-122.444	37.944	2.42	16.77	289.09	19.20	-122.444	37.944	2.13	16.77	289.27	18.90	9	7/14/2014	29:50.3
12348	RSPe_2	-122.444	37.944	2.37	16.83	289.07	19.20	-122.444	37.944	2.13	16.83	289.24	18.96	9	7/14/2014	29:50.2
12349	RSPe_2	-122.444	37.944	2.42	16.77	289.02	19.20	-122.444	37.944	2.13	16.77	289.22	18.90	9	7/14/2014	29:50.1
12350	RSPe_2	-122.444	37.944	2.37	16.77	289.07	19.15	-122.444	37.944	2.09	16.77	289.24	18.87	9	7/14/2014	29:50.0
12351	RSPe_2	-122.444	37.944	2.42	16.79	289.04	19.22	-122.444	37.944	2.13	16.79	289.24	18.92	9	7/14/2014	29:49.9
12352	RSPe_2	-122.444	37.944	2.37	16.78	289.02	19.16	-122.444	37.944	2.13	16.78	289.20	18.91	9	7/14/2014	29:49.8
12353	RSPe_2	-122.444	37.944	2.42	16.77	289.02	19.20	-122.444	37.944	2.13	16.77	289.17	18.90	9	7/14/2014	29:49.7
12354	RSPe_2	-122.444	37.944	2.42	16.80	289.03	19.22	-122.444	37.944	2.09	16.80	289.24	18.89	9	7/14/2014	29:49.6
12355	RSPe_2	-122.444	37.944	2.42	16.79	289.05	19.22	-122.444	37.944	2.13	16.79	289.18	18.92	9	7/14/2014	29:49.5
12356	RSPe_2	-122.444	37.944	2.42	16.80	289.03	19.22	-122.444	37.944	2.13	16.80	289.20	18.93	9	7/14/2014	29:49.4
12357	RSPe_2	-122.444	37.944	2.46	16.79	289.07	19.25	-122.444	37.944	2.13	16.79	289.18	18.92	9	7/14/2014	29:49.3
12358	RSPe_2	-122.444	37.944	2.42	16.81	289.05	19.23	-122.444	37.944	2.09	16.81	289.17	18.90	9	7/14/2014	29:49.2
12359	RSPe_2	-122.444	37.944	2.42	16.83	289.10	19.25	-122.444	37.944	2.13	16.83	289.16	18.96	9	7/14/2014	29:49.1
12360	RSPe_2	-122.444	37.944	2.42	16.77	289.09	19.19	-122.444	37.944	2.09	16.77	289.13	18.86	9	7/14/2014	29:49.0
12361	RSPe_2	-122.444	37.944	2.46	16.77	289.10	19.23	-122.444	37.944	2.09	16.77	289.16	18.87	9	7/14/2014	29:48.9
12362	RSPe_2	-122.444	37.944	2.42	16.76	289.12	19.18	-122.444	37.944	2.09	16.76	289.13	18.85	9	7/14/2014	29:48.8
12363	RSPe_2	-122.444	37.944	2.46	16.80	289.12	19.26	-122.444	37.944	2.13	16.80	289.11	18.93	9	7/14/2014	29:48.7
12364	RSPe_2	-122.444	37.944	2.42	16.77	289.14	19.20	-122.444	37.944	2.09	16.77	289.16	18.87	9	7/14/2014	29:48.6
12365	RSPe_2	-122.444	37.944	2.46	16.79	289.14	19.25	-122.444	37.944	2.13	16.79	289.14	18.92	9	7/14/2014	29:48.5
12366	RSPe_2	-122.444	37.944	2.42	16.78	289.16	19.21	-122.444	37.944	2.13	16.78	289.22	18.91	9	7/14/2014	29:48.4
12367	RSPe_2	-122.444	37.944	2.51	16.78	289.17	19.30	-122.444	37.944	2.09	16.78	289.18	18.88	9	7/14/2014	29:48.3
12368	RSPe_2	-122.444	37.944	2.46	16.78	289.17	19.24	-122.444	37.944	2.13	16.78	289.23	18.91	9	7/14/2014	29:48.2
12369	RSPe_2	-122.444	37.944	2.46	16.78	289.19	19.24	-122.444	37.944	2.13	16.78	289.24	18.91	9	7/14/2014	29:48.1
12370	RSPe_2	-122.444	37.944	2.46	16.81	289.21	19.27	-122.444	37.944	2.09	16.81	289.29	18.91	9	7/14/2014	29:48.0
12371	RSPe_2	-122.444	37.944	2.51	16.82	289.23	19.33	-122.444	37.944	2.13	16.82	289.34	18.95	9	7/14/2014	29:47.9
12372	RSPe_2	-122.444	37.944	2.46	16.81	289.25	19.26	-122.444	37.944	2.09	16.81	289.36	18.90	9	7/14/2014	29:47.8
12373	RSPe_2	-122.444	37.944	2.51	16.80	289.21	19.31	-122.444	37.944	2.13	16.80	289.32	18.93	9	7/14/2014	29:47.7
12374	RSPe_2	-122.444	37.944	2.51	16.84	289.26	19.35	-122.444	37.944	2.09	16.84	289.34	18.94	9	7/14/2014	29:47.6

12375	RSPe_2	-122.444	37.944	2.51	16.80	289.23	19.31	-122.444	37.944	2.13	16.80	289.32	18.93	9	7/14/2014	29:47.5
12376	RSPe_2	-122.444	37.944	2.51	16.84	289.23	19.35	-122.444	37.944	2.13	16.84	289.33	18.97	9	7/14/2014	29:47.4
12377	RSPe_2	-122.444	37.944	2.55	16.86	289.21	19.41	-122.444	37.944	2.13	16.86	289.26	18.99	9	7/14/2014	29:47.3
12378	RSPe_2	-122.444	37.944	2.51	16.84	289.23	19.35	-122.444	37.944	2.13	16.84	289.31	18.97	9	7/14/2014	29:47.2
12379	RSPe_2	-122.444	37.944	2.55	16.81	289.23	19.36	-122.444	37.944	2.18	16.81	289.23	18.99	9	7/14/2014	29:47.1
12380	RSPe_2	-122.444	37.944	2.55	16.83	289.24	19.37	-122.444	37.944	2.13	16.83	289.26	18.96	9	7/14/2014	29:47.0
12381	RSPe_2	-122.444	37.944	2.55	16.84	289.24	19.39	-122.444	37.944	2.18	16.84	289.21	19.02	9	7/14/2014	29:46.9
12382	RSPe_2	-122.444	37.944	2.55	16.81	289.24	19.35	-122.444	37.944	2.13	16.81	289.21	18.93	9	7/14/2014	29:46.8
12383	RSPe_2	-122.444	37.944	2.60	16.85	289.24	19.45	-122.444	37.944	2.18	16.85	289.21	19.03	9	7/14/2014	29:46.7
12384	RSPe_2	-122.444	37.944	2.55	16.81	289.26	19.35	-122.444	37.944	2.18	16.81	289.19	18.98	9	7/14/2014	29:46.6
12385	RSPe_2	-122.444	37.944	2.63	16.83	289.23	19.46	-122.444	37.944	2.21	16.83	289.14	19.04	9	7/14/2014	29:46.5
12386	RSPe_2	-122.444	37.944	2.60	16.82	289.26	19.42	-122.444	37.944	2.18	16.82	289.19	19.00	9	7/14/2014	29:46.4
12387	RSPe_2	-122.444	37.944	2.63	16.82	289.26	19.45	-122.444	37.944	2.21	16.82	289.17	19.04	9	7/14/2014	29:46.3
12388	RSPe_2	-122.444	37.944	2.60	16.84	289.26	19.43	-122.444	37.944	2.21	16.84	289.19	19.05	9	7/14/2014	29:46.2
12389	RSPe_2	-122.444	37.944	2.63	16.82	289.26	19.45	-122.444	37.944	2.21	16.82	289.19	19.04	9	7/14/2014	29:46.1
12390	RSPe_2	-122.444	37.944	2.63	16.88	289.24	19.51	-122.444	37.944	2.21	16.88	289.15	19.10	9	7/14/2014	29:46.0
12391	RSPe_2	-122.444	37.944	2.68	16.81	289.24	19.50	-122.444	37.944	2.27	16.81	289.19	19.08	9	7/14/2014	29:45.9
12392	RSPe_2	-122.444	37.944	2.68	16.84	289.22	19.53	-122.444	37.944	2.27	16.84	289.12	19.11	9	7/14/2014	29:45.8
12393	RSPe_2	-122.444	37.944	2.68	16.82	289.20	19.51	-122.444	37.944	2.30	16.82	289.13	19.12	9	7/14/2014	29:45.7
12394	RSPe_2	-122.444	37.944	2.68	16.86	289.20	19.54	-122.444	37.944	2.27	16.86	289.15	19.13	9	7/14/2014	29:45.6
12395	RSPe_2	-122.444	37.944	2.68	16.86	289.20	19.54	-122.444	37.944	2.30	16.86	289.10	19.16	9	7/14/2014	29:45.5
12396	RSPe_2	-122.444	37.944	2.72	16.84	289.22	19.55	-122.444	37.944	2.30	16.84	289.11	19.14	9	7/14/2014	29:45.4
12397	RSPe_2	-122.444	37.944	2.72	16.84	289.24	19.56	-122.444	37.944	2.33	16.84	289.17	19.18	9	7/14/2014	29:45.3
12398	RSPe_2	-122.444	37.944	2.72	16.86	289.24	19.58	-122.444	37.944	2.30	16.86	289.14	19.16	9	7/14/2014	29:45.2
12399	RSPe_2	-122.444	37.944	2.75	16.86	289.27	19.61	-122.444	37.944	2.38	16.86	289.15	19.24	9	7/14/2014	29:45.1
12400	RSPe_2	-122.444	37.944	2.75	16.81	289.20	19.56	-122.444	37.944	2.33	16.81	289.13	19.14	9	7/14/2014	29:45.0
12401	RSPe_2	-122.444	37.944	2.75	16.81	289.27	19.56	-122.444	37.944	2.42	16.81	289.17	19.23	9	7/14/2014	29:44.9
12402	RSPe_2	-122.444	37.944	2.75	16.86	289.22	19.61	-122.444	37.944	2.38	16.86	289.15	19.24	9	7/14/2014	29:44.8
12403	RSPe_2	-122.444	37.944	2.80	16.81	289.20	19.61	-122.444	37.944	2.42	16.81	289.11	19.22	9	7/14/2014	29:44.7
12404	RSPe_2	-122.444	37.944	2.80	16.83	289.20	19.63	-122.444	37.944	2.47	16.83	289.11	19.30	9	7/14/2014	29:44.6
12405	RSPe_2	-122.444	37.944	2.84	16.80	289.22	19.63	-122.444	37.944	2.47	16.80	289.13	19.27	9	7/14/2014	29:44.5
12406	RSPe_2	-122.444	37.944	2.80	16.81	289.18	19.61	-122.444	37.944	2.47	16.81	289.09	19.28	9	7/14/2014	29:44.4
12407	RSPe_2	-122.444	37.944	2.84	16.81	289.16	19.65	-122.444	37.944	2.50	16.81	289.09	19.31	9	7/14/2014	29:44.3

12408	RSPe_2	-122.444	37.944	2.84	16.81	289.27	19.65	-122.444	37.944	2.50	16.81	289.11	19.31	9	7/14/2014	29:44.2
12409	RSPe_2	-122.444	37.944	2.84	16.79	289.25	19.63	-122.444	37.944	2.55	16.79	289.11	19.34	9	7/14/2014	29:44.1
12410	RSPe_2	-122.444	37.944	2.84	16.85	289.22	19.69	-122.444	37.944	2.59	16.85	289.09	19.44	9	7/14/2014	29:44.0
12411	RSPe_2	-122.444	37.944	2.89	16.79	289.20	19.68	-122.444	37.944	2.64	16.79	289.05	19.43	9	7/14/2014	29:43.9
12412	RSPe_2	-122.444	37.944	2.89	16.77	289.18	19.66	-122.444	37.944	2.59	16.77	289.11	19.35	9	7/14/2014	29:43.8
12413	RSPe_2	-122.444	37.944	2.89	16.84	289.23	19.73	-122.444	37.944	2.64	16.84	289.15	19.48	9	7/14/2014	29:43.7
12414	RSPe_2	-122.444	37.944	2.89	16.77	289.20	19.66	-122.444	37.944	2.64	16.77	289.16	19.41	9	7/14/2014	29:43.6
12415	RSPe_2	-122.444	37.944	2.92	16.80	289.27	19.72	-122.444	37.944	2.67	16.80	289.16	19.47	9	7/14/2014	29:43.5
12416	RSPe_2	-122.444	37.944	2.89	16.80	289.29	19.69	-122.444	37.944	2.64	16.80	289.18	19.43	9	7/14/2014	29:43.4
12417	RSPe_2	-122.444	37.944	2.92	16.77	289.25	19.69	-122.444	37.944	2.64	16.77	289.18	19.40	9	7/14/2014	29:43.3
12418	RSPe_2	-122.444	37.944	2.92	16.74	289.32	19.66	-122.444	37.944	2.67	16.74	289.18	19.41	9	7/14/2014	29:43.2
12419	RSPe_2	-122.444	37.944	2.92	16.72	289.30	19.64	-122.444	37.944	2.67	16.72	289.19	19.39	9	7/14/2014	29:43.1
12420	RSPe_2	-122.444	37.944	2.92	16.74	289.27	19.66	-122.444	37.944	2.67	16.74	289.12	19.41	9	7/14/2014	29:43.0
12421	RSPe_2	-122.444	37.944	2.97	16.73	289.27	19.70	-122.444	37.944	2.67	16.73	289.14	19.40	9	7/14/2014	29:42.9
12422	RSPe_2	-122.444	37.944	2.92	16.68	289.30	19.61	-122.444	37.944	2.76	16.68	289.16	19.44	9	7/14/2014	29:42.8
12423	RSPe_2	-122.444	37.944	2.97	16.80	289.21	19.77	-122.444	37.944	2.79	16.80	289.12	19.59	9	7/14/2014	29:42.7
12424	RSPe_2	-122.444	37.944	2.97	16.62	289.13	19.59	-122.444	37.944	2.79	16.62	289.05	19.41	9	7/14/2014	29:42.6
12425	RSPe_2	-122.444	37.944	3.01	16.51	289.10	19.51	-122.444	37.944	2.84	16.51	289.02	19.35	9	7/14/2014	29:42.5
12426	RSPe_2	-122.444	37.944	3.01	16.30	289.08	19.31	-122.444	37.944	2.84	16.30	289.03	19.14	9	7/14/2014	29:42.4
12427	RSPe_2	-122.444	37.944	3.06	16.35	289.13	19.41	-122.444	37.944	2.92	16.35	289.10	19.28	9	7/14/2014	29:42.3
12428	RSPe_2	-122.444	37.944	3.01	16.37	289.10	19.38	-122.444	37.944	2.87	16.37	289.10	19.25	9	7/14/2014	29:42.2
12429	RSPe_2	-122.444	37.944	3.01	16.21	289.06	19.22	-122.444	37.944	2.92	16.21	289.12	19.14	9	7/14/2014	29:42.1
12430	RSPe_2	-122.444	37.944	3.01	16.21	289.08	19.22	-122.444	37.944	2.87	16.21	289.14	19.09	9	7/14/2014	29:42.0
12431	RSPe_2	-122.444	37.944	3.06	16.26	289.06	19.32	-122.444	37.944	2.92	16.26	289.12	19.19	9	7/14/2014	29:41.9
12432	RSPe_2	-122.444	37.944	3.06	16.23	289.08	19.29	-122.444	37.944	2.92	16.23	289.12	19.16	9	7/14/2014	29:41.8
12433	RSPe_2	-122.444	37.944	3.06	16.20	289.08	19.26	-122.444	37.944	2.92	16.20	289.12	19.13	9	7/14/2014	29:41.7
12434	RSPe_2	-122.444	37.944	3.06	16.17	289.02	19.23	-122.444	37.944	2.96	16.17	289.08	19.13	9	7/14/2014	29:41.6
12435	RSPe_2	-122.444	37.944	3.09	16.09	288.95	19.18	-122.444	37.944	2.96	16.09	289.06	19.05	9	7/14/2014	29:41.5
12436	RSPe_2	-122.444	37.944	3.09	16.05	288.95	19.14	-122.444	37.944	2.96	16.05	289.06	19.01	9	7/14/2014	29:41.4
12437	RSPe_2	-122.444	37.944	3.13	16.10	288.95	19.23	-122.444	37.944	3.01	16.10	289.10	19.11	9	7/14/2014	29:41.3
12438	RSPe_2	-122.444	37.944	3.09	15.99	288.91	19.08	-122.444	37.944	3.04	15.99	289.06	19.03	9	7/14/2014	29:41.2
12439	RSPe_2	-122.444	37.944	3.13	16.00	288.87	19.13	-122.444	37.944	3.08	16.00	289.02	19.08	9	7/14/2014	29:41.1
12440	RSPe_2	-122.444	37.944	3.13	15.93	288.80	19.06	-122.444	37.944	3.08	15.93	288.93	19.01	9	7/14/2014	29:41.0

12441	RSPe_2	-122.444	37.944	3.18	15.86	288.71	19.04	-122.444	37.944	3.13	15.86	288.88	18.99	9	7/14/2014 29:40.9
12442	RSPe_2	-122.444	37.944	3.13	15.86	288.69	18.99	-122.444	37.944	3.13	15.86	288.84	18.99	9	7/14/2014 29:40.8
12443	RSPe_2	-122.444	37.944	3.18	15.72	288.71	18.90	-122.444	37.944	3.16	15.72	288.89	18.88	9	7/14/2014 29:40.7
12444	RSPe_2	-122.444	37.944	3.18	15.73	288.70	18.91	-122.444	37.944	3.16	15.73	288.86	18.90	9	7/14/2014 29:40.6
12445	RSPe_2	-122.444	37.944	3.18	15.84	288.72	19.02	-122.444	37.944	3.21	15.84	288.80	19.05	9	7/14/2014 29:40.5
12446	RSPe_2	-122.444	37.944	3.18	15.66	288.67	18.84	-122.444	37.944	3.21	15.66	288.75	18.87	9	7/14/2014 29:40.4
12447	RSPe_2	-122.444	37.944	3.21	15.53	288.63	18.75	-122.444	37.944	3.25	15.53	288.69	18.78	9	7/14/2014 29:40.3
12448	RSPe_2	-122.444	37.944	3.18	15.44	288.70	18.62	-122.444	37.944	3.25	15.44	288.71	18.69	9	7/14/2014 29:40.2
12449	RSPe_2	-122.444	37.944	3.21	15.42	288.67	18.63	-122.444	37.944	3.30	15.42	288.67	18.71	9	7/14/2014 29:40.1
12450	RSPe_2	-122.444	37.944	3.18	15.39	288.65	18.57	-122.444	37.944	3.30	15.39	288.64	18.68	9	7/14/2014 29:40.0
12451	RSPe_2	-122.444	37.944	3.21	15.38	288.61	18.59	-122.444	37.944	3.30	15.38	288.56	18.68	9	7/14/2014 29:39.9
12452	RSPe_2	-122.444	37.944	3.18	15.37	288.59	18.55	-122.444	37.944	3.30	15.37	288.56	18.67	9	7/14/2014 29:39.8
12453	RSPe_2	-122.444	37.944	3.21	15.36	288.68	18.57	-122.444	37.944	3.33	15.36	288.61	18.69	9	7/14/2014 29:39.7
12454	RSPe_2	-122.444	37.944	3.21	15.40	288.72	18.62	-122.444	37.944	3.33	15.40	288.60	18.73	9	7/14/2014 29:39.6
12455	RSPe_2	-122.444	37.944	3.21	15.35	288.70	18.56	-122.444	37.944	3.38	15.35	288.65	18.73	9	7/14/2014 29:39.5
12456	RSPe_2	-122.444	37.944	3.21	15.34	288.74	18.55	-122.444	37.944	3.41	15.34	288.62	18.76	9	7/14/2014 29:39.4
12457	RSPe_2	-122.444	37.944	3.26	15.35	288.74	18.61	-122.444	37.944	3.41	15.35	288.62	18.76	9	7/14/2014 29:39.3
12458	RSPe_2	-122.444	37.944	3.21	15.33	288.70	18.55	-122.444	37.944	3.41	15.33	288.56	18.75	9	7/14/2014 29:39.2
12459	RSPe_2	-122.444	37.944	3.26	15.31	288.65	18.57	-122.444	37.944	3.45	15.31	288.56	18.76	9	7/14/2014 29:39.1
12460	RSPe_2	-122.444	37.944	3.21	15.30	288.63	18.52	-122.444	37.944	3.50	15.30	288.58	18.80	9	7/14/2014 29:39.0
12461	RSPe_2	-122.444	37.944	3.26	15.29	288.66	18.56	-122.444	37.944	3.53	15.29	288.55	18.83	9	7/14/2014 29:38.9
12462	RSPe_2	-122.444	37.944	3.26	15.29	288.61	18.55	-122.444	37.944	3.50	15.29	288.56	18.79	9	7/14/2014 29:38.8
12463	RSPe_2	-122.444	37.944	3.26	15.29	288.68	18.55	-122.444	37.944	3.53	15.29	288.55	18.82	9	7/14/2014 29:38.7
12464	RSPe_2	-122.444	37.944	3.21	15.32	288.64	18.54	-122.444	37.944	3.59	15.32	288.56	18.91	9	7/14/2014 29:38.6
12465	RSPe_2	-122.444	37.944	3.26	15.29	288.63	18.56	-122.444	37.944	3.62	15.29	288.55	18.91	9	7/14/2014 29:38.5
12466	RSPe_2	-122.444	37.944	3.26	15.31	288.61	18.57	-122.444	37.944	3.62	15.31	288.58	18.93	9	7/14/2014 29:38.4
12467	RSPe_2	-122.444	37.944	3.26	15.29	288.59	18.56	-122.444	37.944	3.67	15.29	288.61	18.96	9	7/14/2014 29:38.3
12468	RSPe_2	-122.444	37.944	3.26	15.30	288.55	18.57	-122.444	37.944	3.62	15.30	288.59	18.92	9	7/14/2014 29:38.2
12469	RSPe_2	-122.444	37.944	3.30	15.30	288.51	18.60	-122.444	37.944	3.67	15.30	288.61	18.97	9	7/14/2014 29:38.1
12470	RSPe_2	-122.444	37.944	3.26	15.30	288.46	18.57	-122.444	37.944	3.67	15.30	288.55	18.97	9	7/14/2014 29:38.0
12471	RSPe_2	-122.444	37.944	3.30	15.30	288.44	18.60	-122.444	37.944	3.70	15.30	288.53	19.01	9	7/14/2014 29:37.9
12472	RSPe_2	-122.444	37.944	3.26	15.29	288.44	18.56	-122.444	37.944	3.70	15.29	288.54	19.00	9	7/14/2014 29:37.8
12473	RSPe_2	-122.444	37.944	3.30	15.28	288.44	18.58	-122.444	37.944	3.74	15.28	288.51	19.01	9	7/14/2014 29:37.7

12474	RSPe_2	-122.444	37.944	3.26	15.30	288.46	18.57	-122.444	37.944	3.70	15.30	288.53	19.01	9	7/14/2014 29:37.6
12475	RSPe_2	-122.444	37.944	3.30	15.31	288.46	18.61	-122.444	37.944	3.74	15.31	288.54	19.04	9	7/14/2014 29:37.5
12476	RSPe_2	-122.444	37.944	3.27	15.31	288.46	18.57	-122.444	37.944	3.74	15.31	288.58	19.05	9	7/14/2014 29:37.4
12477	RSPe_2	-122.444	37.944	3.30	15.28	288.48	18.58	-122.444	37.944	3.79	15.28	288.57	19.07	9	7/14/2014 29:37.3
12478	RSPe_2	-122.444	37.944	3.27	15.32	288.45	18.58	-122.444	37.944	3.74	15.32	288.52	19.06	9	7/14/2014 29:37.2
12479	RSPe_2	-122.444	37.944	3.30	15.32	288.44	18.62	-122.444	37.944	3.79	15.32	288.56	19.11	9	7/14/2014 29:37.1
12480	RSPe_2	-122.444	37.944	3.27	15.32	288.40	18.59	-122.444	37.944	3.79	15.32	288.55	19.11	9	7/14/2014 29:37.0
12481	RSPe_2	-122.444	37.944	3.27	15.32	288.42	18.58	-122.444	37.944	3.82	15.32	288.57	19.14	9	7/14/2014 29:36.9
12482	RSPe_2	-122.444	37.944	3.27	15.32	288.40	18.58	-122.444	37.944	3.79	15.32	288.56	19.11	9	7/14/2014 29:36.8
12483	RSPe_2	-122.444	37.944	3.27	15.32	288.40	18.58	-122.444	37.944	3.82	15.32	288.55	19.14	9	7/14/2014 29:36.7
12484	RSPe_2	-122.444	37.944	3.27	15.33	288.35	18.60	-122.444	37.944	3.82	15.33	288.56	19.16	9	7/14/2014 29:36.6
12485	RSPe_2	-122.444	37.944	3.27	15.35	288.40	18.61	-122.444	37.944	3.82	15.35	288.64	19.17	9	7/14/2014 29:36.5
12486	RSPe_2	-122.444	37.944	3.27	15.32	288.35	18.59	-122.444	37.944	3.79	15.32	288.64	19.11	9	7/14/2014 29:36.4
12487	RSPe_2	-122.444	37.944	3.27	15.33	288.36	18.60	-122.444	37.944	3.79	15.33	288.64	19.12	9	7/14/2014 29:36.3
12488	RSPe_2	-122.444	37.944	3.21	15.34	288.36	18.55	-122.444	37.944	3.79	15.34	288.71	19.13	9	7/14/2014 29:36.2
12489	RSPe_2	-122.444	37.944	3.27	15.36	288.38	18.62	-122.444	37.944	3.79	15.36	288.64	19.14	9	7/14/2014 29:36.1
12490	RSPe_2	-122.444	37.944	3.27	15.35	288.36	18.61	-122.444	37.944	3.79	15.35	288.69	19.13	9	7/14/2014 29:36.0
12491	RSPe_2	-122.444	37.944	3.27	15.32	288.36	18.58	-122.444	37.944	3.79	15.32	288.64	19.11	9	7/14/2014 29:35.9
12492	RSPe_2	-122.444	37.944	3.21	15.36	288.31	18.58	-122.444	37.944	3.74	15.36	288.53	19.10	9	7/14/2014 29:35.8
12493	RSPe_2	-122.444	37.944	3.21	15.32	288.30	18.53	-122.444	37.944	3.79	15.32	288.51	19.11	9	7/14/2014 29:35.7
12494	RSPe_2	-122.444	37.944	3.21	15.21	288.27	18.42	-122.444	37.944	3.79	15.21	288.51	19.00	9	7/14/2014 29:35.6
12495	RSPe_2	-122.444	37.944	3.27	15.24	288.23	18.50	-122.444	37.944	3.79	15.24	288.49	19.03	9	7/14/2014 29:35.5
12496	RSPe_2	-122.444	37.944	3.21	15.22	288.23	18.43	-122.444	37.944	3.79	15.22	288.49	19.01	9	7/14/2014 29:35.4
12497	RSPe_2	-122.444	37.944	3.21	15.16	288.21	18.38	-122.444	37.944	3.79	15.16	288.56	18.95	9	7/14/2014 29:35.3
12498	RSPe_2	-122.444	37.944	3.18	15.18	288.21	18.36	-122.444	37.944	3.74	15.18	288.60	18.92	9	7/14/2014 29:35.2
12499	RSPe_2	-122.444	37.944	3.21	15.19	288.19	18.41	-122.444	37.944	3.74	15.19	288.65	18.93	9	7/14/2014 29:35.1
12500	RSPe_2	-122.444	37.944	3.18	15.19	288.21	18.37	-122.444	37.944	3.70	15.19	288.67	18.89	9	7/14/2014 29:35.0
12501	RSPe_2	-122.444	37.944	3.18	15.21	288.19	18.39	-122.444	37.944	3.70	15.21	288.69	18.91	9	7/14/2014 29:34.9
12502	RSPe_2	-122.444	37.944	3.18	15.19	288.15	18.37	-122.444	37.944	3.67	15.19	288.71	18.86	9	7/14/2014 29:34.8
12503	RSPe_2	-122.444	37.944	3.18	15.20	288.14	18.38	-122.444	37.944	3.67	15.20	288.69	18.87	9	7/14/2014 29:34.7
12504	RSPe_2	-122.444	37.944	3.18	15.27	288.13	18.45	-122.444	37.944	3.62	15.27	288.69	18.89	9	7/14/2014 29:34.6
12505	RSPe_2	-122.444	37.944	3.21	15.24	288.12	18.45	-122.444	37.944	3.62	15.24	288.68	18.86	9	7/14/2014 29:34.5
12506	RSPe_2	-122.444	37.944	3.18	15.29	288.13	18.47	-122.444	37.944	3.59	15.29	288.65	18.87	9	7/14/2014 29:34.4

12507	RSPe_2	-122.444	37.944	3.18	15.26	288.13	18.44	-122.444	37.944	3.59	15.26	288.60	18.85	9	7/14/2014 29:34.3
12508	RSPe_2	-122.444	37.944	3.13	15.26	288.13	18.38	-122.444	37.944	3.59	15.26	288.63	18.84	9	7/14/2014 29:34.2
12509	RSPe_2	-122.444	37.944	3.13	15.29	288.13	18.42	-122.444	37.944	3.59	15.29	288.59	18.87	9	7/14/2014 29:34.1
12510	RSPe_2	-122.444	37.944	3.13	15.28	288.15	18.41	-122.444	37.944	3.54	15.28	288.58	18.81	9	7/14/2014 29:34.0
12511	RSPe_2	-122.444	37.944	3.18	15.31	288.08	18.49	-122.444	37.944	3.54	15.31	288.56	18.84	9	7/14/2014 29:33.9
12512	RSPe_2	-122.444	37.944	3.13	15.32	288.04	18.45	-122.444	37.944	3.54	15.32	288.54	18.86	9	7/14/2014 29:33.8
12513	RSPe_2	-122.444	37.944	3.13	15.29	288.02	18.42	-122.444	37.944	3.54	15.29	288.52	18.83	9	7/14/2014 29:33.7
12514	RSPe_2	-122.444	37.944	3.13	15.29	287.95	18.42	-122.444	37.944	3.50	15.29	288.50	18.79	9	7/14/2014 29:33.6
12515	RSPe_2	-122.444	37.944	3.13	15.32	287.93	18.45	-122.444	37.944	3.50	15.32	288.50	18.83	9	7/14/2014 29:33.5
12516	RSPe_2	-122.444	37.944	3.09	15.39	287.93	18.48	-122.444	37.944	3.45	15.39	288.52	18.84	9	7/14/2014 29:33.4
12517	RSPe_2	-122.444	37.944	3.09	15.36	287.93	18.46	-122.444	37.944	3.45	15.36	288.53	18.81	9	7/14/2014 29:33.3
12518	RSPe_2	-122.444	37.944	3.09	15.41	287.91	18.50	-122.444	37.944	3.42	15.41	288.46	18.83	9	7/14/2014 29:33.2
12519	RSPe_2	-122.444	37.944	3.09	15.40	287.95	18.50	-122.444	37.944	3.42	15.40	288.48	18.82	9	7/14/2014 29:33.1
12520	RSPe_2	-122.444	37.944	3.09	15.46	287.95	18.55	-122.444	37.944	3.38	15.46	288.48	18.84	9	7/14/2014 29:33.0
12521	RSPe_2	-122.444	37.944	3.09	15.46	287.98	18.56	-122.444	37.944	3.38	15.46	288.46	18.85	9	7/14/2014 29:32.9
12522	RSPe_2	-122.444	37.944	3.06	15.54	288.00	18.60	-122.444	37.944	3.33	15.54	288.41	18.87	9	7/14/2014 29:32.8
12523	RSPe_2	-122.444	37.944	3.09	15.49	288.04	18.59	-122.444	37.944	3.33	15.49	288.41	18.83	9	7/14/2014 29:32.7
12524	RSPe_2	-122.444	37.944	3.06	15.67	288.00	18.73	-122.444	37.944	3.30	15.67	288.35	18.97	9	7/14/2014 29:32.6
12525	RSPe_2	-122.444	37.944	3.09	15.53	288.02	18.63	-122.444	37.944	3.30	15.53	288.36	18.83	9	7/14/2014 29:32.5
12526	RSPe_2	-122.444	37.944	3.06	15.55	288.05	18.61	-122.444	37.944	3.25	15.55	288.33	18.80	9	7/14/2014 29:32.4
12527	RSPe_2	-122.444	37.944	3.06	15.57	288.05	18.63	-122.444	37.944	3.30	15.57	288.31	18.87	9	7/14/2014 29:32.3
12528	RSPe_2	-122.444	37.944	3.06	15.57	288.05	18.63	-122.444	37.944	3.25	15.57	288.30	18.82	9	7/14/2014 29:32.2
12529	RSPe_2	-122.444	37.944	3.06	15.60	288.05	18.66	-122.444	37.944	3.25	15.60	288.31	18.85	9	7/14/2014 29:32.1
12530	RSPe_2	-122.444	37.944	3.06	15.57	288.05	18.63	-122.444	37.944	3.25	15.57	288.28	18.82	9	7/14/2014 29:32.0
12531	RSPe_2	-122.444	37.944	3.06	15.62	288.07	18.68	-122.444	37.944	3.25	15.62	288.33	18.87	9	7/14/2014 29:31.9
12532	RSPe_2	-122.444	37.944	3.06	15.59	288.09	18.65	-122.444	37.944	3.21	15.59	288.35	18.80	9	7/14/2014 29:31.8
12533	RSPe_2	-122.444	37.944	3.06	15.57	288.14	18.63	-122.444	37.944	3.25	15.57	288.39	18.82	9	7/14/2014 29:31.7
12534	RSPe_2	-122.444	37.944	3.01	15.60	288.18	18.61	-122.444	37.944	3.21	15.60	288.42	18.82	9	7/14/2014 29:31.6
12535	RSPe_2	-122.444	37.944	3.01	15.61	288.21	18.62	-122.444	37.944	3.21	15.61	288.44	18.82	9	7/14/2014 29:31.5
12536	RSPe_2	-122.444	37.944	3.01	15.74	288.20	18.75	-122.444	37.944	3.16	15.74	288.44	18.90	9	7/14/2014 29:31.4
12537	RSPe_2	-122.444	37.944	3.01	15.73	288.27	18.74	-122.444	37.944	3.16	15.73	288.47	18.90	9	7/14/2014 29:31.3
12538	RSPe_2	-122.444	37.944	3.01	15.67	288.27	18.68	-122.444	37.944	3.13	15.67	288.46	18.80	9	7/14/2014 29:31.2
12539	RSPe_2	-122.444	37.944	3.01	15.73	288.27	18.73	-122.444	37.944	3.13	15.73	288.47	18.85	9	7/14/2014 29:31.1

12540	RSPe_2	-122.444	37.944	3.01	15.71	288.32	18.72	-122.444	37.944	3.08	15.71	288.46	18.79	9	7/14/2014	29:31.0
12541	RSPe_2	-122.444	37.944	3.01	15.78	288.31	18.79	-122.444	37.944	3.08	15.78	288.49	18.86	9	7/14/2014	29:30.9
12542	RSPe_2	-122.444	37.944	3.01	15.78	288.36	18.79	-122.444	37.944	3.08	15.78	288.48	18.86	9	7/14/2014	29:30.8
12543	RSPe_2	-122.444	37.944	3.01	15.79	288.36	18.80	-122.444	37.944	3.08	15.79	288.51	18.87	9	7/14/2014	29:30.7
12544	RSPe_2	-122.444	37.944	2.97	15.77	288.34	18.75	-122.444	37.944	3.08	15.77	288.50	18.85	9	7/14/2014	29:30.6
12545	RSPe_2	-122.444	37.944	3.01	15.71	288.38	18.72	-122.444	37.944	3.08	15.71	288.51	18.79	9	7/14/2014	29:30.5
12546	RSPe_2	-122.444	37.944	2.97	15.73	288.34	18.71	-122.444	37.944	3.08	15.73	288.51	18.81	9	7/14/2014	29:30.4
12547	RSPe_2	-122.444	37.944	2.97	15.83	288.38	18.80	-122.444	37.944	3.13	15.83	288.56	18.96	9	7/14/2014	29:30.3
12548	RSPe_2	-122.444	37.944	2.97	15.73	288.36	18.71	-122.444	37.944	3.08	15.73	288.53	18.81	9	7/14/2014	29:30.2
12549	RSPe_2	-122.444	37.944	3.01	15.75	288.36	18.76	-122.444	37.944	3.08	15.75	288.60	18.83	9	7/14/2014	29:30.1
12550	RSPe_2	-122.444	37.944	2.97	15.73	288.39	18.71	-122.444	37.944	3.08	15.73	288.60	18.81	9	7/14/2014	29:30.0
12551	RSPe_2	-122.444	37.944	2.97	15.73	288.41	18.70	-122.444	37.944	3.08	15.73	288.60	18.80	9	7/14/2014	29:29.9
12552	RSPe_2	-122.444	37.944	2.97	15.88	288.40	18.86	-122.444	37.944	3.08	15.88	288.60	18.96	9	7/14/2014	29:29.8
12553	RSPe_2	-122.444	37.944	3.01	15.73	288.43	18.74	-122.444	37.944	3.08	15.73	288.64	18.81	9	7/14/2014	29:29.7
12554	RSPe_2	-122.444	37.944	2.97	15.92	288.49	18.89	-122.444	37.944	3.04	15.92	288.70	18.96	9	7/14/2014	29:29.6
12555	RSPe_2	-122.444	37.944	3.01	15.91	288.54	18.92	-122.444	37.944	3.04	15.91	288.71	18.96	9	7/14/2014	29:29.5
12556	RSPe_2	-122.444	37.944	2.97	15.85	288.58	18.82	-122.444	37.944	3.04	15.85	288.69	18.89	9	7/14/2014	29:29.4
12557	RSPe_2	-122.444	37.944	2.97	15.80	288.61	18.78	-122.444	37.944	3.04	15.80	288.72	18.84	9	7/14/2014	29:29.3
12558	RSPe_2	-122.444	37.944	2.97	15.84	288.68	18.82	-122.444	37.944	3.04	15.84	288.73	18.89	9	7/14/2014	29:29.2
12559	RSPe_2	-122.444	37.944	2.97	15.95	288.72	18.92	-122.444	37.944	3.04	15.95	288.69	18.99	9	7/14/2014	29:29.1
12560	RSPe_2	-122.444	37.944	2.97	15.97	288.74	18.95	-122.444	37.944	3.04	15.97	288.71	19.02	9	7/14/2014	29:29.0
12561	RSPe_2	-122.444	37.944	2.97	15.83	288.74	18.81	-122.444	37.944	3.04	15.83	288.73	18.88	9	7/14/2014	29:28.9
12562	RSPe_2	-122.444	37.944	2.97	15.98	288.81	18.96	-122.444	37.944	3.04	15.98	288.76	19.02	9	7/14/2014	29:28.8
12563	RSPe_2	-122.444	37.944	2.97	15.76	288.79	18.74	-122.444	37.944	3.08	15.76	288.76	18.84	9	7/14/2014	29:28.7
12564	RSPe_2	-122.444	37.944	2.97	15.85	288.81	18.82	-122.444	37.944	3.04	15.85	288.81	18.89	9	7/14/2014	29:28.6
12565	RSPe_2	-122.444	37.944	2.97	15.93	288.79	18.91	-122.444	37.944	3.08	15.93	288.79	19.01	9	7/14/2014	29:28.5
12566	RSPe_2	-122.444	37.944	2.98	15.85	288.75	18.83	-122.444	37.944	3.08	15.85	288.80	18.93	9	7/14/2014	29:28.4
12567	RSPe_2	-122.444	37.944	3.01	15.83	288.79	18.84	-122.444	37.944	3.08	15.83	288.85	18.91	9	7/14/2014	29:28.3
12568	RSPe_2	-122.444	37.944	2.98	15.83	288.79	18.81	-122.444	37.944	3.08	15.83	288.92	18.91	9	7/14/2014	29:28.2
12569	RSPe_2	-122.444	37.944	3.01	15.74	288.81	18.75	-122.444	37.944	3.13	15.74	288.94	18.87	9	7/14/2014	29:28.1
12570	RSPe_2	-122.444	37.944	2.98	15.85	288.83	18.83	-122.444	37.944	3.13	15.85	289.05	18.98	9	7/14/2014	29:28.0
12571	RSPe_2	-122.444	37.944	3.01	15.85	288.89	18.86	-122.444	37.944	3.13	15.85	289.07	18.98	9	7/14/2014	29:27.9
12572	RSPe_2	-122.444	37.944	2.98	15.79	288.88	18.76	-122.444	37.944	3.13	15.79	289.09	18.92	9	7/14/2014	29:27.8

12573	RSPe_2	-122.444	37.944	3.01	15.80	288.95	18.81	-122.444	37.944	3.13	15.80	289.16	18.93	9	7/14/2014	29:27.7
12574	RSPe_2	-122.444	37.944	3.01	15.78	288.99	18.79	-122.444	37.944	3.08	15.78	289.22	18.86	9	7/14/2014	29:27.6
12575	RSPe_2	-122.444	37.944	3.01	15.81	289.01	18.82	-122.444	37.944	3.13	15.81	289.21	18.94	9	7/14/2014	29:27.5
12576	RSPe_2	-122.444	37.944	2.98	15.79	289.03	18.76	-122.444	37.944	3.08	15.79	289.22	18.87	9	7/14/2014	29:27.4
12577	RSPe_2	-122.444	37.944	3.01	15.79	289.10	18.80	-122.444	37.944	3.13	15.79	289.27	18.92	9	7/14/2014	29:27.3
12578	RSPe_2	-122.444	37.944	2.98	15.79	289.10	18.76	-122.444	37.944	3.08	15.79	289.27	18.87	9	7/14/2014	29:27.2
12579	RSPe_2	-122.444	37.944	3.01	15.76	289.14	18.77	-122.444	37.944	3.13	15.76	289.29	18.89	9	7/14/2014	29:27.1
12580	RSPe_2	-122.444	37.944	2.98	15.77	289.14	18.75	-122.444	37.944	3.08	15.77	289.24	18.85	9	7/14/2014	29:27.0
12581	RSPe_2	-122.444	37.944	3.01	15.73	289.19	18.74	-122.444	37.944	3.13	15.73	289.27	18.86	9	7/14/2014	29:26.9
12582	RSPe_2	-122.444	37.944	3.01	15.65	289.17	18.66	-122.444	37.944	3.08	15.65	289.27	18.73	9	7/14/2014	29:26.8
12583	RSPe_2	-122.444	37.944	3.01	15.60	289.23	18.61	-122.444	37.944	3.13	15.60	289.27	18.73	9	7/14/2014	29:26.7
12584	RSPe_2	-122.444	37.944	3.01	15.59	289.15	18.60	-122.444	37.944	3.13	15.59	289.23	18.72	9	7/14/2014	29:26.6
12585	RSPe_2	-122.444	37.944	3.01	15.56	289.17	18.57	-122.444	37.944	3.16	15.56	289.23	18.73	9	7/14/2014	29:26.5
12586	RSPe_2	-122.444	37.944	3.01	15.53	289.15	18.54	-122.444	37.944	3.13	15.53	289.23	18.66	9	7/14/2014	29:26.4
12587	RSPe_2	-122.444	37.944	3.01	15.51	289.14	18.52	-122.444	37.944	3.16	15.51	289.21	18.67	9	7/14/2014	29:26.3
12588	RSPe_2	-122.444	37.944	3.01	15.51	289.15	18.52	-122.444	37.944	3.16	15.51	289.21	18.67	9	7/14/2014	29:26.2
12589	RSPe_2	-122.444	37.944	3.06	15.46	289.13	18.52	-122.444	37.944	3.21	15.46	289.21	18.68	9	7/14/2014	29:26.1
12590	RSPe_2	-122.444	37.944	2.98	15.46	289.17	18.43	-122.444	37.944	3.21	15.46	289.21	18.67	9	7/14/2014	29:26.0
12591	RSPe_2	-122.444	37.944	3.01	15.46	289.15	18.47	-122.444	37.944	3.25	15.46	289.21	18.71	9	7/14/2014	29:25.9
12592	RSPe_2	-122.444	37.944	3.01	15.46	289.17	18.47	-122.444	37.944	3.21	15.46	289.20	18.67	9	7/14/2014	29:25.8
12593	RSPe_2	-122.444	37.944	3.06	15.46	289.17	18.52	-122.444	37.944	3.21	15.46	289.23	18.67	9	7/14/2014	29:25.7
12594	RSPe_2	-122.444	37.944	3.01	15.46	289.20	18.47	-122.444	37.944	3.21	15.46	289.26	18.67	9	7/14/2014	29:25.6
12595	RSPe_2	-122.444	37.944	3.01	15.46	289.17	18.47	-122.444	37.944	3.25	15.46	289.21	18.71	9	7/14/2014	29:25.5
12596	RSPe_2	-122.444	37.944	3.01	15.46	289.15	18.47	-122.444	37.944	3.21	15.46	289.27	18.68	9	7/14/2014	29:25.4
12597	RSPe_2	-122.444	37.944	3.06	15.47	289.13	18.53	-122.444	37.944	3.25	15.47	289.24	18.72	9	7/14/2014	29:25.3
12598	RSPe_2	-122.444	37.944	3.01	15.44	289.13	18.45	-122.444	37.944	3.25	15.44	289.25	18.69	9	7/14/2014	29:25.2
12599	RSPe_2	-122.444	37.944	3.06	15.46	289.11	18.52	-122.444	37.944	3.30	15.46	289.21	18.76	9	7/14/2014	29:25.1
12600	RSPe_2	-122.444	37.944	3.01	15.48	289.11	18.49	-122.444	37.944	3.25	15.48	289.21	18.73	9	7/14/2014	29:25.0
12601	RSPe_2	-122.444	37.944	3.06	15.46	289.10	18.52	-122.444	37.944	3.25	15.46	289.24	18.71	9	7/14/2014	29:24.9
12602	RSPe_2	-122.444	37.944	3.01	15.53	289.11	18.54	-122.444	37.944	3.25	15.53	289.19	18.78	9	7/14/2014	29:24.8
12603	RSPe_2	-122.444	37.944	3.06	15.47	289.11	18.53	-122.444	37.944	3.30	15.47	289.22	18.77	9	7/14/2014	29:24.7
12604	RSPe_2	-122.444	37.944	3.01	15.49	289.09	18.50	-122.444	37.944	3.30	15.49	289.24	18.79	9	7/14/2014	29:24.6
12605	RSPe_2	-122.444	37.944	3.01	15.53	289.13	18.54	-122.444	37.944	3.30	15.53	289.22	18.83	9	7/14/2014	29:24.5

12606	RSPe_2	-122.444	37.944	3.01	15.53	289.09	18.54	-122.444	37.944	3.30	15.53	289.26	18.83	9	7/14/2014 29:24.4
12607	RSPe_2	-122.444	37.944	3.06	15.57	289.13	18.63	-122.444	37.944	3.30	15.57	289.26	18.87	9	7/14/2014 29:24.3
12608	RSPe_2	-122.444	37.944	3.01	15.57	289.13	18.58	-122.444	37.944	3.25	15.57	289.27	18.82	9	7/14/2014 29:24.2
12609	RSPe_2	-122.444	37.944	3.01	15.60	289.09	18.61	-122.444	37.944	3.30	15.60	289.21	18.89	9	7/14/2014 29:24.1
12610	RSPe_2	-122.444	37.944	3.01	15.62	289.06	18.63	-122.444	37.944	3.30	15.62	289.24	18.92	9	7/14/2014 29:24.0
12611	RSPe_2	-122.444	37.944	3.01	15.64	289.02	18.65	-122.444	37.944	3.30	15.64	289.20	18.94	9	7/14/2014 29:23.9
12612	RSPe_2	-122.444	37.944	2.98	15.81	289.02	18.79	-122.444	37.944	3.30	15.81	289.24	19.11	9	7/14/2014 29:23.8
12613	RSPe_2	-122.444	37.944	3.01	15.71	289.01	18.72	-122.444	37.944	3.33	15.71	289.20	19.04	9	7/14/2014 29:23.7
12614	RSPe_2	-122.444	37.944	3.01	15.82	288.96	18.83	-122.444	37.944	3.30	15.82	289.18	19.12	9	7/14/2014 29:23.6
12615	RSPe_2	-122.444	37.944	3.01	15.83	288.94	18.84	-122.444	37.944	3.30	15.83	289.20	19.13	9	7/14/2014 29:23.5
12616	RSPe_2	-122.444	37.944	3.01	15.87	288.92	18.88	-122.444	37.944	3.30	15.87	289.20	19.17	9	7/14/2014 29:23.4
12617	RSPe_2	-122.444	37.944	3.01	15.85	288.91	18.86	-122.444	37.944	3.30	15.85	289.18	19.15	9	7/14/2014 29:23.3
12618	RSPe_2	-122.444	37.944	2.98	15.98	288.90	18.96	-122.444	37.944	3.30	15.98	289.15	19.28	9	7/14/2014 29:23.2
12619	RSPe_2	-122.444	37.944	2.98	15.98	288.87	18.96	-122.444	37.944	3.30	15.98	289.15	19.28	9	7/14/2014 29:23.1
12620	RSPe_2	-122.444	37.944	2.98	15.92	288.87	18.89	-122.444	37.944	3.21	15.92	289.15	19.13	9	7/14/2014 29:23.0
12621	RSPe_2	-122.444	37.944	2.98	16.01	288.87	18.99	-122.444	37.944	3.25	16.01	289.11	19.26	9	7/14/2014 29:22.9
12622	RSPe_2	-122.444	37.944	2.98	15.98	288.90	18.96	-122.444	37.944	3.25	15.98	289.11	19.23	9	7/14/2014 29:22.8
12623	RSPe_2	-122.444	37.944	2.98	15.97	288.90	18.95	-122.444	37.944	3.21	15.97	289.13	19.19	9	7/14/2014 29:22.7
12624	RSPe_2	-122.444	37.944	2.98	15.97	288.90	18.95	-122.444	37.944	3.16	15.97	289.12	19.14	9	7/14/2014 29:22.6
12625	RSPe_2	-122.444	37.944	2.98	15.98	288.86	18.96	-122.444	37.944	3.21	15.98	289.09	19.19	9	7/14/2014 29:22.5
12626	RSPe_2	-122.444	37.944	2.92	16.01	288.86	18.93	-122.444	37.944	3.16	16.01	289.09	19.17	9	7/14/2014 29:22.4
12627	RSPe_2	-122.444	37.944	2.98	15.99	288.79	18.96	-122.444	37.944	3.21	15.99	289.05	19.20	9	7/14/2014 29:22.3
12628	RSPe_2	-122.444	37.944	2.98	15.94	288.79	18.92	-122.444	37.944	3.21	15.94	289.02	19.15	9	7/14/2014 29:22.2
12629	RSPe_2	-122.444	37.944	2.98	15.95	288.73	18.93	-122.444	37.944	3.21	15.95	288.98	19.16	9	7/14/2014 29:22.1
12630	RSPe_2	-122.444	37.944	2.92	15.93	288.70	18.86	-122.444	37.944	3.21	15.93	289.03	19.15	9	7/14/2014 29:22.0
12631	RSPe_2	-122.444	37.944	2.98	15.95	288.68	18.93	-122.444	37.944	3.21	15.95	289.00	19.16	9	7/14/2014 29:21.9
12632	RSPe_2	-122.444	37.944	2.92	15.94	288.65	18.86	-122.444	37.944	3.16	15.94	288.98	19.10	9	7/14/2014 29:21.8
12633	RSPe_2	-122.444	37.944	2.92	15.95	288.68	18.87	-122.444	37.944	3.16	15.95	288.94	19.11	9	7/14/2014 29:21.7
12634	RSPe_2	-122.444	37.944	2.89	15.97	288.64	18.86	-122.444	37.944	3.13	15.97	288.95	19.10	9	7/14/2014 29:21.6
12635	RSPe_2	-122.444	37.944	2.92	15.97	288.66	18.89	-122.444	37.944	3.16	15.97	288.92	19.13	9	7/14/2014 29:21.5
12636	RSPe_2	-122.444	37.944	2.89	15.98	288.60	18.87	-122.444	37.944	3.08	15.98	288.91	19.06	9	7/14/2014 29:21.4
12637	RSPe_2	-122.444	37.944	2.92	16.00	288.59	18.92	-122.444	37.944	3.13	16.00	288.88	19.13	9	7/14/2014 29:21.3
12638	RSPe_2	-122.444	37.944	2.89	16.00	288.60	18.89	-122.444	37.944	3.08	16.00	288.85	19.08	9	7/14/2014 29:21.2

12639	RSPe_2	-122.444	37.944	2.92	16.04	288.51	18.97	-122.444	37.944	3.08	16.04	288.81	19.12	9	7/14/2014 29:21.1
12640	RSPe_2	-122.444	37.944	2.89	15.99	288.53	18.88	-122.444	37.944	3.05	15.99	288.81	19.03	9	7/14/2014 29:21.0
12641	RSPe_2	-122.444	37.944	2.89	16.00	288.51	18.89	-122.444	37.944	3.08	16.00	288.79	19.08	9	7/14/2014 29:20.9
12642	RSPe_2	-122.444	37.944	2.89	16.00	288.44	18.89	-122.444	37.944	3.05	16.00	288.72	19.05	9	7/14/2014 29:20.8
12643	RSPe_2	-122.444	37.944	2.89	16.00	288.44	18.89	-122.444	37.944	3.08	16.00	288.79	19.08	9	7/14/2014 29:20.7
12644	RSPe_2	-122.444	37.944	2.84	16.02	288.46	18.86	-122.444	37.944	3.05	16.02	288.82	19.06	9	7/14/2014 29:20.6
12645	RSPe_2	-122.444	37.944	2.89	16.05	288.51	18.94	-122.444	37.944	3.05	16.05	288.84	19.10	9	7/14/2014 29:20.5
12646	RSPe_2	-122.444	37.944	2.84	16.04	288.46	18.88	-122.444	37.944	3.05	16.04	288.86	19.09	9	7/14/2014 29:20.4
12647	RSPe_2	-122.444	37.944	2.84	16.04	288.47	18.88	-122.444	37.944	3.05	16.04	288.83	19.09	9	7/14/2014 29:20.3
12648	RSPe_2	-122.444	37.944	2.84	16.06	288.53	18.90	-122.444	37.944	3.01	16.06	288.88	19.07	9	7/14/2014 29:20.2
12649	RSPe_2	-122.444	37.944	2.84	16.07	288.54	18.91	-122.444	37.944	3.01	16.07	288.86	19.08	9	7/14/2014 29:20.1
12650	RSPe_2	-122.444	37.944	2.84	16.07	288.54	18.91	-122.444	37.944	2.96	16.07	288.88	19.03	9	7/14/2014 29:20.0
12651	RSPe_2	-122.444	37.944	2.84	16.09	288.55	18.93	-122.444	37.944	2.96	16.09	288.86	19.05	9	7/14/2014 29:19.9
12652	RSPe_2	-122.444	37.944	2.80	16.12	288.54	18.93	-122.444	37.944	2.93	16.12	288.90	19.05	9	7/14/2014 29:19.8
12653	RSPe_2	-122.444	37.944	2.80	16.12	288.55	18.93	-122.444	37.944	2.93	16.12	288.84	19.05	9	7/14/2014 29:19.7
12654	RSPe_2	-122.444	37.944	2.80	16.13	288.49	18.93	-122.444	37.944	2.88	16.13	288.84	19.00	9	7/14/2014 29:19.6
12655	RSPe_2	-122.444	37.944	2.84	16.14	288.53	18.98	-122.444	37.944	2.88	16.14	288.86	19.02	9	7/14/2014 29:19.5
12656	RSPe_2	-122.444	37.944	2.81	16.15	288.49	18.95	-122.444	37.944	2.88	16.15	288.82	19.03	9	7/14/2014 29:19.4
12657	RSPe_2	-122.444	37.944	2.81	16.15	288.51	18.95	-122.444	37.944	2.88	16.15	288.84	19.03	9	7/14/2014 29:19.3
12658	RSPe_2	-122.444	37.944	2.81	16.17	288.47	18.97	-122.444	37.944	2.88	16.17	288.82	19.04	9	7/14/2014 29:19.2
12659	RSPe_2	-122.444	37.944	2.84	16.19	288.52	19.03	-122.444	37.944	2.88	16.19	288.84	19.07	9	7/14/2014 29:19.1
12660	RSPe_2	-122.444	37.944	2.81	16.22	288.51	19.02	-122.444	37.944	2.88	16.22	288.80	19.10	9	7/14/2014 29:19.0
12661	RSPe_2	-122.444	37.944	2.84	16.23	288.54	19.07	-122.444	37.944	2.88	16.23	288.84	19.11	9	7/14/2014 29:18.9
12662	RSPe_2	-122.444	37.944	2.81	16.35	288.58	19.16	-122.444	37.944	2.84	16.35	288.89	19.19	9	7/14/2014 29:18.8
12663	RSPe_2	-122.444	37.944	2.81	16.24	288.63	19.05	-122.444	37.944	2.84	16.24	288.89	19.09	9	7/14/2014 29:18.7
12664	RSPe_2	-122.444	37.944	2.81	16.30	288.62	19.10	-122.444	37.944	2.79	16.30	288.90	19.09	9	7/14/2014 29:18.6
12665	RSPe_2	-122.444	37.944	2.81	16.30	288.67	19.10	-122.444	37.944	2.79	16.30	288.89	19.09	9	7/14/2014 29:18.5
12666	RSPe_2	-122.444	37.944	2.75	16.30	288.65	19.05	-122.444	37.944	2.76	16.30	288.89	19.06	9	7/14/2014 29:18.4
12667	RSPe_2	-122.444	37.944	2.75	16.31	288.67	19.07	-122.444	37.944	2.76	16.31	288.82	19.07	9	7/14/2014 29:18.3
12668	RSPe_2	-122.444	37.944	2.75	16.30	288.67	19.06	-122.444	37.944	2.71	16.30	288.86	19.01	9	7/14/2014 29:18.2
12669	RSPe_2	-122.444	37.944	2.75	16.32	288.67	19.07	-122.444	37.944	2.76	16.32	288.82	19.08	9	7/14/2014 29:18.1
12670	RSPe_2	-122.444	37.944	2.72	16.33	288.70	19.05	-122.444	37.944	2.71	16.33	288.80	19.04	9	7/14/2014 29:18.0
12671	RSPe_2	-122.444	37.944	2.75	16.33	288.74	19.09	-122.444	37.944	2.71	16.33	288.82	19.04	9	7/14/2014 29:17.9

12672	RSPe_2	-122.444	37.944	2.75	16.33	288.71	19.09	-122.444	37.944	2.71	16.33	288.82	19.04	9	7/14/2014	29:17.8
12673	RSPe_2	-122.444	37.944	2.75	16.33	288.67	19.09	-122.444	37.944	2.76	16.33	288.82	19.09	9	7/14/2014	29:17.7
12674	RSPe_2	-122.444	37.944	2.75	16.36	288.67	19.11	-122.444	37.944	2.71	16.36	288.82	19.07	9	7/14/2014	29:17.6
12675	RSPe_2	-122.444	37.944	2.81	16.36	288.67	19.16	-122.444	37.944	2.76	16.36	288.85	19.12	9	7/14/2014	29:17.5
12676	RSPe_2	-122.444	37.944	2.75	16.39	288.70	19.14	-122.444	37.944	2.76	16.39	288.87	19.15	9	7/14/2014	29:17.4
12677	RSPe_2	-122.444	37.944	2.81	16.37	288.74	19.18	-122.444	37.944	2.76	16.37	288.92	19.13	9	7/14/2014	29:17.3
12678	RSPe_2	-122.444	37.944	2.75	16.39	288.72	19.14	-122.444	37.944	2.76	16.39	288.86	19.15	9	7/14/2014	29:17.2
12679	RSPe_2	-122.444	37.944	2.81	16.39	288.77	19.19	-122.444	37.944	2.76	16.39	288.89	19.15	9	7/14/2014	29:17.1
12680	RSPe_2	-122.444	37.944	2.75	16.41	288.79	19.16	-122.444	37.944	2.71	16.41	288.89	19.12	9	7/14/2014	29:17.0
12681	RSPe_2	-122.444	37.944	2.75	16.43	288.79	19.18	-122.444	37.944	2.76	16.43	288.92	19.18	9	7/14/2014	29:16.9
12682	RSPe_2	-122.444	37.944	2.75	16.42	288.81	19.17	-122.444	37.944	2.71	16.42	288.96	19.13	9	7/14/2014	29:16.8
12683	RSPe_2	-122.444	37.944	2.81	16.44	288.83	19.24	-122.444	37.944	2.76	16.44	288.94	19.20	9	7/14/2014	29:16.7
12684	RSPe_2	-122.444	37.944	2.75	16.44	288.83	19.20	-122.444	37.944	2.76	16.44	288.96	19.20	9	7/14/2014	29:16.6
12685	RSPe_2	-122.444	37.944	2.75	16.44	288.88	19.20	-122.444	37.944	2.76	16.44	289.00	19.20	9	7/14/2014	29:16.5
12686	RSPe_2	-122.444	37.944	2.75	16.44	288.87	19.20	-122.444	37.944	2.71	16.44	289.01	19.15	9	7/14/2014	29:16.4
12687	RSPe_2	-122.444	37.944	2.81	16.46	288.83	19.26	-122.444	37.944	2.76	16.46	289.00	19.22	9	7/14/2014	29:16.3
12688	RSPe_2	-122.444	37.944	2.75	16.48	288.90	19.24	-122.444	37.944	2.76	16.48	289.05	19.24	9	7/14/2014	29:16.2
12689	RSPe_2	-122.444	37.944	2.81	16.56	288.90	19.36	-122.444	37.944	2.76	16.56	289.02	19.32	9	7/14/2014	29:16.1
12690	RSPe_2	-122.444	37.944	2.75	16.47	288.92	19.23	-122.444	37.944	2.76	16.47	289.10	19.23	9	7/14/2014	29:16.0
12691	RSPe_2	-122.444	37.944	2.81	16.51	288.99	19.31	-122.444	37.944	2.79	16.51	289.14	19.30	9	7/14/2014	29:15.9
12692	RSPe_2	-122.444	37.944	2.75	16.51	288.98	19.27	-122.444	37.944	2.76	16.51	289.14	19.27	9	7/14/2014	29:15.8
12693	RSPe_2	-122.444	37.944	2.81	16.54	288.99	19.35	-122.444	37.944	2.79	16.54	289.16	19.34	9	7/14/2014	29:15.7
12694	RSPe_2	-122.444	37.944	2.81	16.52	288.99	19.33	-122.444	37.944	2.79	16.52	289.17	19.31	9	7/14/2014	29:15.6
12695	RSPe_2	-122.444	37.944	2.81	16.52	289.01	19.33	-122.444	37.944	2.84	16.52	289.18	19.36	9	7/14/2014	29:15.5
12696	RSPe_2	-122.444	37.944	2.81	16.57	289.01	19.37	-122.444	37.944	2.79	16.57	289.17	19.36	9	7/14/2014	29:15.4
12697	RSPe_2	-122.444	37.944	2.84	16.53	288.99	19.37	-122.444	37.944	2.84	16.53	289.18	19.38	9	7/14/2014	29:15.3
12698	RSPe_2	-122.444	37.944	2.81	16.54	289.02	19.35	-122.444	37.944	2.79	16.54	289.20	19.34	9	7/14/2014	29:15.2
12699	RSPe_2	-122.444	37.944	2.84	16.54	289.01	19.38	-122.444	37.944	2.84	16.54	289.19	19.39	9	7/14/2014	29:15.1
12700	RSPe_2	-122.444	37.944	2.84	16.47	288.99	19.31	-122.444	37.944	2.84	16.47	289.20	19.32	9	7/14/2014	29:15.0
12701	RSPe_2	-122.444	37.944	2.84	16.54	288.97	19.38	-122.444	37.945	2.88	16.54	289.19	19.42	9	7/14/2014	29:14.9
12702	RSPe_2	-122.444	37.945	2.81	16.53	288.95	19.34	-122.444	37.945	2.84	16.53	289.20	19.38	9	7/14/2014	29:14.8
12703	RSPe_2	-122.444	37.945	2.84	16.51	288.97	19.35	-122.444	37.945	2.88	16.51	289.19	19.38	9	7/14/2014	29:14.7
12704	RSPe_2	-122.444	37.945	2.81	16.51	288.97	19.31	-122.444	37.945	2.84	16.51	289.16	19.35	9	7/14/2014	29:14.6

12705	RSPe_2	-122.444	37.945	2.84	16.53	288.93	19.37	-122.444	37.945	2.88	16.53	289.12	19.40	9	7/14/2014	29:14.5
12706	RSPe_2	-122.444	37.945	2.84	16.42	288.95	19.26	-122.444	37.945	2.88	16.42	289.12	19.30	9	7/14/2014	29:14.4
12707	RSPe_2	-122.444	37.945	2.84	16.37	288.95	19.21	-122.444	37.945	2.88	16.37	289.10	19.25	9	7/14/2014	29:14.3
12708	RSPe_2	-122.444	37.945	2.84	16.34	288.90	19.18	-122.444	37.945	2.88	16.34	289.09	19.22	9	7/14/2014	29:14.2
12709	RSPe_2	-122.444	37.945	2.84	16.33	288.90	19.17	-122.444	37.945	2.93	16.33	289.08	19.26	9	7/14/2014	29:14.1
12710	RSPe_2	-122.444	37.945	2.84	16.42	288.91	19.26	-122.444	37.945	2.88	16.42	289.03	19.30	9	7/14/2014	29:14.0
12711	RSPe_2	-122.444	37.945	2.84	16.03	288.89	18.87	-122.444	37.945	2.93	16.03	289.04	18.95	9	7/14/2014	29:13.9
12712	RSPe_2	-122.444	37.945	2.84	16.29	288.93	19.13	-122.444	37.945	2.93	16.29	289.03	19.22	9	7/14/2014	29:13.8
12713	RSPe_2	-122.444	37.945	2.84	16.02	288.93	18.86	-122.444	37.945	2.96	16.02	289.04	18.98	9	7/14/2014	29:13.7
12714	RSPe_2	-122.444	37.945	2.84	16.14	288.98	18.98	-122.444	37.945	2.96	16.14	289.03	19.10	9	7/14/2014	29:13.6
12715	RSPe_2	-122.444	37.945	2.89	16.09	289.02	18.99	-122.444	37.945	2.96	16.09	289.10	19.06	9	7/14/2014	29:13.5
12716	RSPe_2	-122.444	37.945	2.84	16.24	289.02	19.08	-122.444	37.945	2.96	16.24	289.13	19.20	9	7/14/2014	29:13.4
12717	RSPe_2	-122.444	37.945	2.89	16.18	289.02	19.07	-122.444	37.945	2.96	16.18	289.14	19.14	9	7/14/2014	29:13.3
12718	RSPe_2	-122.444	37.945	2.84	16.10	289.04	18.94	-122.444	37.945	2.93	16.10	289.17	19.03	9	7/14/2014	29:13.2
12719	RSPe_2	-122.444	37.945	2.84	16.28	289.00	19.12	-122.444	37.945	2.96	16.28	289.17	19.24	9	7/14/2014	29:13.1
12720	RSPe_2	-122.444	37.945	2.84	16.17	288.98	19.01	-122.444	37.945	2.93	16.17	289.12	19.09	9	7/14/2014	29:13.0
12721	RSPe_2	-122.444	37.945	2.84	16.09	288.96	18.93	-122.444	37.945	2.96	16.09	289.12	19.05	9	7/14/2014	29:12.9
12722	RSPe_2	-122.444	37.945	2.84	16.08	288.91	18.92	-122.444	37.945	2.93	16.08	289.08	19.01	9	7/14/2014	29:12.8
12723	RSPe_2	-122.444	37.945	2.89	16.07	288.89	18.96	-122.444	37.945	2.96	16.07	289.11	19.03	9	7/14/2014	29:12.7
12724	RSPe_2	-122.444	37.945	2.84	16.15	288.89	18.99	-122.444	37.945	2.96	16.15	289.12	19.11	9	7/14/2014	29:12.6
12725	RSPe_2	-122.444	37.945	2.89	16.09	288.87	18.98	-122.444	37.945	2.96	16.09	289.11	19.05	9	7/14/2014	29:12.5
12726	RSPe_2	-122.444	37.945	2.84	16.03	288.91	18.87	-122.444	37.945	2.96	16.03	289.11	19.00	9	7/14/2014	29:12.4
12727	RSPe_2	-122.444	37.945	2.84	15.99	288.87	18.83	-122.444	37.945	3.01	15.99	289.15	19.00	9	7/14/2014	29:12.3
12728	RSPe_2	-122.444	37.945	2.84	16.08	288.89	18.92	-122.444	37.945	3.01	16.08	289.10	19.09	9	7/14/2014	29:12.2
12729	RSPe_2	-122.444	37.945	2.89	16.12	288.86	19.01	-122.444	37.945	3.01	16.12	289.13	19.13	9	7/14/2014	29:12.1
12730	RSPe_2	-122.444	37.945	2.84	15.98	288.89	18.82	-122.444	37.945	2.96	15.98	289.15	18.94	9	7/14/2014	29:12.0
12731	RSPe_2	-122.444	37.945	2.89	16.06	288.87	18.95	-122.444	37.945	3.01	16.06	289.13	19.07	9	7/14/2014	29:11.9
12732	RSPe_2	-122.444	37.945	2.84	16.03	288.91	18.87	-122.444	37.945	2.96	16.03	289.13	18.99	9	7/14/2014	29:11.8
12733	RSPe_2	-122.444	37.945	2.84	16.06	288.87	18.90	-122.444	37.945	3.01	16.06	289.11	19.08	9	7/14/2014	29:11.7
12734	RSPe_2	-122.444	37.945	2.84	16.06	288.89	18.90	-122.444	37.945	2.96	16.06	289.12	19.02	9	7/14/2014	29:11.6
12735	RSPe_2	-122.444	37.945	2.84	16.06	288.89	18.90	-122.444	37.945	2.96	16.06	289.15	19.02	9	7/14/2014	29:11.5
12736	RSPe_2	-122.444	37.945	2.81	16.07	288.89	18.88	-122.444	37.945	2.96	16.07	289.09	19.03	9	7/14/2014	29:11.4
12737	RSPe_2	-122.444	37.945	2.84	16.08	288.92	18.92	-122.444	37.945	2.96	16.08	289.11	19.04	9	7/14/2014	29:11.3

12738	RSPe_2	-122.444	37.945	2.81	16.08	288.89	18.88	-122.444	37.945	2.93	16.08	289.02	19.01	9	7/14/2014	29:11.2
12739	RSPe_2	-122.444	37.945	2.84	16.08	288.92	18.92	-122.444	37.945	2.96	16.08	289.02	19.04	9	7/14/2014	29:11.1
12740	RSPe_2	-122.444	37.945	2.81	16.16	288.87	18.96	-122.444	37.945	2.96	16.16	288.96	19.12	9	7/14/2014	29:11.0
12741	RSPe_2	-122.444	37.945	2.84	16.08	288.96	18.92	-122.444	37.945	2.96	16.08	289.00	19.04	9	7/14/2014	29:10.9
12742	RSPe_2	-122.444	37.945	2.81	16.10	288.99	18.91	-122.444	37.945	2.96	16.10	289.01	19.07	9	7/14/2014	29:10.8
12743	RSPe_2	-122.444	37.945	2.81	16.11	289.03	18.92	-122.444	37.945	2.96	16.11	289.05	19.07	9	7/14/2014	29:10.7
12744	RSPe_2	-122.444	37.945	2.81	16.10	289.08	18.91	-122.444	37.945	2.96	16.10	289.00	19.07	9	7/14/2014	29:10.6
12745	RSPe_2	-122.444	37.945	2.81	16.19	289.14	19.00	-122.444	37.945	3.01	16.19	289.05	19.20	9	7/14/2014	29:10.5
12746	RSPe_2	-122.444	37.945	2.81	16.13	289.14	18.93	-122.444	37.945	2.96	16.13	289.00	19.09	9	7/14/2014	29:10.4
12747	RSPe_2	-122.444	37.945	2.81	16.16	289.10	18.96	-122.444	37.945	2.96	16.16	289.01	19.12	9	7/14/2014	29:10.3
12748	RSPe_2	-122.444	37.945	2.81	16.17	289.12	18.98	-122.444	37.945	2.96	16.17	289.02	19.14	9	7/14/2014	29:10.2
12749	RSPe_2	-122.444	37.945	2.81	16.16	289.11	18.96	-122.444	37.945	2.96	16.16	289.09	19.12	9	7/14/2014	29:10.1
12750	RSPe_2	-122.444	37.945	2.76	16.16	289.14	18.91	-122.444	37.945	2.96	16.16	289.13	19.12	9	7/14/2014	29:10.0
12751	RSPe_2	-122.444	37.945	2.81	16.17	289.14	18.98	-122.444	37.945	2.96	16.17	289.16	19.14	9	7/14/2014	29:09.9
12752	RSPe_2	-122.444	37.945	2.76	16.17	289.10	18.93	-122.444	37.945	2.96	16.17	289.16	19.14	9	7/14/2014	29:09.8
12753	RSPe_2	-122.444	37.945	2.76	16.20	289.14	18.95	-122.444	37.945	2.96	16.20	289.18	19.16	9	7/14/2014	29:09.7
12754	RSPe_2	-122.444	37.945	2.76	16.20	289.14	18.96	-122.444	37.945	2.96	16.20	289.27	19.16	9	7/14/2014	29:09.6
12755	RSPe_2	-122.444	37.945	2.81	16.20	289.10	19.00	-122.444	37.945	2.96	16.20	289.27	19.16	9	7/14/2014	29:09.5
12756	RSPe_2	-122.444	37.945	2.76	16.21	289.10	18.97	-122.444	37.945	2.96	16.21	289.29	19.17	9	7/14/2014	29:09.4
12757	RSPe_2	-122.444	37.945	2.76	16.25	289.05	19.01	-122.444	37.945	3.01	16.25	289.29	19.26	9	7/14/2014	29:09.3
12758	RSPe_2	-122.444	37.945	2.76	16.28	288.99	19.04	-122.444	37.945	2.96	16.28	289.29	19.24	9	7/14/2014	29:09.2
12759	RSPe_2	-122.444	37.945	2.81	16.23	288.97	19.04	-122.444	37.945	3.01	16.23	289.23	19.25	9	7/14/2014	29:09.1
12760	RSPe_2	-122.444	37.945	2.72	16.24	288.95	18.96	-122.444	37.945	2.96	16.24	289.25	19.21	9	7/14/2014	29:09.0
12761	RSPe_2	-122.444	37.945	2.76	16.27	288.94	19.03	-122.444	37.945	3.01	16.27	289.20	19.28	9	7/14/2014	29:08.9
12762	RSPe_2	-122.444	37.945	2.76	16.30	288.92	19.06	-122.444	37.945	2.96	16.30	289.21	19.27	9	7/14/2014	29:08.8
12763	RSPe_2	-122.444	37.945	2.76	16.27	288.92	19.03	-122.444	37.945	3.01	16.27	289.18	19.28	9	7/14/2014	29:08.7
12764	RSPe_2	-122.444	37.945	2.72	16.29	288.94	19.01	-122.444	37.945	2.96	16.29	289.25	19.25	9	7/14/2014	29:08.6
12765	RSPe_2	-122.444	37.945	2.76	16.33	288.95	19.08	-122.444	37.945	2.96	16.33	289.19	19.29	9	7/14/2014	29:08.5
12766	RSPe_2	-122.444	37.945	2.76	16.37	288.97	19.12	-122.444	37.945	2.96	16.37	289.23	19.33	9	7/14/2014	29:08.4
12767	RSPe_2	-122.444	37.945	2.76	16.38	288.99	19.14	-122.444	37.945	2.96	16.38	289.23	19.34	9	7/14/2014	29:08.3
12768	RSPe_2	-122.444	37.945	2.72	16.38	288.99	19.10	-122.444	37.945	2.93	16.38	289.23	19.31	9	7/14/2014	29:08.2
12769	RSPe_2	-122.444	37.945	2.76	16.41	289.02	19.17	-122.444	37.945	2.93	16.41	289.24	19.34	9	7/14/2014	29:08.1
12770	RSPe_2	-122.444	37.945	2.72	16.47	289.04	19.20	-122.444	37.945	2.88	16.47	289.23	19.35	9	7/14/2014	29:08.0

12771	RSPe_2	-122.444	37.945	2.72	16.41	289.06	19.13	-122.444	37.945	2.88	16.41	289.25	19.29	9	7/14/2014	29:07.9
12772	RSPe_2	-122.444	37.945	2.72	16.47	289.08	19.20	-122.444	37.945	2.88	16.47	289.25	19.35	9	7/14/2014	29:07.8
12773	RSPe_2	-122.444	37.945	2.72	16.45	289.11	19.17	-122.444	37.945	2.93	16.45	289.28	19.38	9	7/14/2014	29:07.7
12774	RSPe_2	-122.444	37.945	2.72	16.44	289.15	19.16	-122.444	37.945	2.88	16.44	289.28	19.32	9	7/14/2014	29:07.6
12775	RSPe_2	-122.444	37.945	2.72	16.42	289.17	19.14	-122.444	37.945	2.93	16.42	289.34	19.35	9	7/14/2014	29:07.5
12776	RSPe_2	-122.444	37.945	2.72	16.45	289.20	19.17	-122.444	37.945	2.88	16.45	289.35	19.33	9	7/14/2014	29:07.4
12777	RSPe_2	-122.444	37.945	2.72	16.38	289.24	19.10	-122.444	37.945	2.93	16.38	289.41	19.31	9	7/14/2014	29:07.3
12778	RSPe_2	-122.444	37.945	2.69	16.41	289.20	19.09	-122.444	37.945	2.88	16.41	289.39	19.28	9	7/14/2014	29:07.2
12779	RSPe_2	-122.444	37.945	2.72	16.47	289.19	19.19	-122.444	37.945	2.88	16.47	289.37	19.34	9	7/14/2014	29:07.1
12780	RSPe_2	-122.444	37.945	2.69	16.37	289.15	19.05	-122.444	37.945	2.84	16.37	289.41	19.21	9	7/14/2014	29:07.0
12781	RSPe_2	-122.444	37.945	2.72	16.34	289.15	19.06	-122.444	37.945	2.88	16.34	289.37	19.22	9	7/14/2014	29:06.9
12782	RSPe_2	-122.444	37.945	2.69	16.37	289.13	19.05	-122.444	37.945	2.84	16.37	289.35	19.21	9	7/14/2014	29:06.8
12783	RSPe_2	-122.444	37.945	2.72	16.31	289.15	19.03	-122.444	37.945	2.88	16.31	289.37	19.19	9	7/14/2014	29:06.7
12784	RSPe_2	-122.444	37.945	2.69	16.30	289.11	18.98	-122.444	37.945	2.84	16.30	289.34	19.14	9	7/14/2014	29:06.6
12785	RSPe_2	-122.444	37.945	2.69	16.36	289.11	19.04	-122.444	37.945	2.84	16.36	289.35	19.20	9	7/14/2014	29:06.5
12786	RSPe_2	-122.444	37.945	2.69	16.27	289.13	18.96	-122.444	37.945	2.84	16.27	289.39	19.12	9	7/14/2014	29:06.4
12787	RSPe_2	-122.444	37.945	2.69	16.26	289.08	18.95	-122.444	37.945	2.84	16.26	289.41	19.10	9	7/14/2014	29:06.3
12788	RSPe_2	-122.444	37.945	2.69	16.30	289.16	18.98	-122.444	37.945	2.84	16.30	289.39	19.14	9	7/14/2014	29:06.2
12789	RSPe_2	-122.444	37.945	2.69	16.34	289.14	19.03	-122.444	37.945	2.84	16.34	289.39	19.19	9	7/14/2014	29:06.1
12790	RSPe_2	-122.444	37.945	2.63	16.37	289.18	19.01	-122.444	37.945	2.79	16.37	289.43	19.17	9	7/14/2014	29:06.0
12791	RSPe_2	-122.444	37.945	2.69	16.28	289.15	18.97	-122.444	37.945	2.84	16.28	289.42	19.13	9	7/14/2014	29:05.9
12792	RSPe_2	-122.444	37.945	2.63	16.30	289.18	18.93	-122.444	37.945	2.79	16.30	289.42	19.09	9	7/14/2014	29:05.8
12793	RSPe_2	-122.444	37.945	2.63	16.30	289.20	18.93	-122.444	37.945	2.79	16.30	289.44	19.09	9	7/14/2014	29:05.7
12794	RSPe_2	-122.444	37.945	2.63	16.29	289.20	18.92	-122.444	37.945	2.79	16.29	289.43	19.08	9	7/14/2014	29:05.6
12795	RSPe_2	-122.444	37.945	2.63	16.27	289.18	18.91	-122.444	37.945	2.79	16.27	289.44	19.06	9	7/14/2014	29:05.5
12796	RSPe_2	-122.444	37.945	2.63	16.30	289.20	18.93	-122.444	37.945	2.76	16.30	289.41	19.06	9	7/14/2014	29:05.4
12797	RSPe_2	-122.444	37.945	2.63	16.28	289.18	18.92	-122.444	37.945	2.79	16.28	289.42	19.07	9	7/14/2014	29:05.3
12798	RSPe_2	-122.444	37.945	2.63	16.27	289.16	18.91	-122.444	37.945	2.76	16.27	289.41	19.03	9	7/14/2014	29:05.2
12799	RSPe_2	-122.444	37.945	2.63	16.30	289.16	18.93	-122.444	37.945	2.79	16.30	289.42	19.09	9	7/14/2014	29:05.1
12800	RSPe_2	-122.444	37.945	2.63	16.28	289.13	18.92	-122.444	37.945	2.76	16.28	289.39	19.04	9	7/14/2014	29:05.0
12801	RSPe_2	-122.444	37.945	2.63	16.30	289.18	18.94	-122.444	37.945	2.76	16.30	289.40	19.06	9	7/14/2014	29:04.9
12802	RSPe_2	-122.444	37.945	2.60	16.30	289.14	18.91	-122.444	37.945	2.71	16.30	289.40	19.01	9	7/14/2014	29:04.8
12803	RSPe_2	-122.444	37.945	2.63	16.30	289.09	18.94	-122.444	37.945	2.76	16.30	289.35	19.06	9	7/14/2014	29:04.7

12804	RSPe_2	-122.444	37.945	2.60	16.34	289.16	18.94	-122.444	37.945	2.71	16.34	289.38	19.05	9	7/14/2014	29:04.6
12805	RSPe_2	-122.444	37.945	2.63	16.32	289.12	18.95	-122.444	37.945	2.71	16.32	289.38	19.03	9	7/14/2014	29:04.5
12806	RSPe_2	-122.444	37.945	2.60	16.34	289.12	18.94	-122.444	37.945	2.67	16.34	289.35	19.02	9	7/14/2014	29:04.4
12807	RSPe_2	-122.444	37.945	2.60	16.37	289.14	18.97	-122.444	37.945	2.71	16.37	289.33	19.08	9	7/14/2014	29:04.3
12808	RSPe_2	-122.444	37.945	2.60	16.36	289.14	18.96	-122.444	37.945	2.67	16.36	289.33	19.03	9	7/14/2014	29:04.2
12809	RSPe_2	-122.444	37.945	2.60	16.36	289.14	18.96	-122.444	37.945	2.67	16.36	289.29	19.03	9	7/14/2014	29:04.1
12810	RSPe_2	-122.444	37.945	2.55	16.38	289.09	18.93	-122.444	37.945	2.67	16.38	289.31	19.06	9	7/14/2014	29:04.0
12811	RSPe_2	-122.444	37.945	2.60	16.39	289.12	18.99	-122.444	37.945	2.67	16.39	289.31	19.06	9	7/14/2014	29:03.9
12812	RSPe_2	-122.444	37.945	2.55	16.44	289.09	18.99	-122.444	37.945	2.64	16.44	289.31	19.08	9	7/14/2014	29:03.8
12813	RSPe_2	-122.444	37.945	2.60	16.41	289.08	19.01	-122.444	37.945	2.67	16.41	289.27	19.09	9	7/14/2014	29:03.7
12814	RSPe_2	-122.444	37.945	2.55	16.41	289.10	18.96	-122.444	37.945	2.64	16.41	289.31	19.05	9	7/14/2014	29:03.6
12815	RSPe_2	-122.444	37.945	2.55	16.48	289.10	19.03	-122.444	37.945	2.64	16.48	289.30	19.12	9	7/14/2014	29:03.5
12816	RSPe_2	-122.444	37.945	2.55	16.45	289.12	19.00	-122.444	37.945	2.64	16.45	289.29	19.09	9	7/14/2014	29:03.4
12817	RSPe_2	-122.444	37.945	2.55	16.52	289.05	19.07	-122.444	37.945	2.64	16.52	289.25	19.16	9	7/14/2014	29:03.3
12818	RSPe_2	-122.444	37.945	2.55	16.50	289.10	19.05	-122.444	37.945	2.59	16.50	289.29	19.09	9	7/14/2014	29:03.2
12819	RSPe_2	-122.444	37.945	2.55	16.50	289.09	19.05	-122.444	37.945	2.64	16.50	289.29	19.14	9	7/14/2014	29:03.1
12820	RSPe_2	-122.444	37.945	2.55	16.50	289.08	19.05	-122.444	37.945	2.59	16.50	289.30	19.09	9	7/14/2014	29:03.0
12821	RSPe_2	-122.444	37.945	2.55	16.50	289.12	19.05	-122.444	37.945	2.64	16.50	289.31	19.14	9	7/14/2014	29:02.9
12822	RSPe_2	-122.444	37.945	2.52	16.47	289.12	18.99	-122.444	37.945	2.59	16.47	289.33	19.06	9	7/14/2014	29:02.8
12823	RSPe_2	-122.444	37.945	2.55	16.47	289.14	19.02	-122.444	37.945	2.59	16.47	289.34	19.06	9	7/14/2014	29:02.7
12824	RSPe_2	-122.444	37.945	2.52	16.47	289.15	18.98	-122.444	37.945	2.59	16.47	289.32	19.06	9	7/14/2014	29:02.6
12825	RSPe_2	-122.444	37.945	2.55	16.47	289.19	19.02	-122.444	37.945	2.59	16.47	289.34	19.06	9	7/14/2014	29:02.5
12826	RSPe_2	-122.444	37.945	2.52	16.47	289.15	18.98	-122.444	37.945	2.56	16.47	289.34	19.02	9	7/14/2014	29:02.4
12827	RSPe_2	-122.444	37.945	2.55	16.49	289.21	19.04	-122.444	37.945	2.56	16.49	289.38	19.05	9	7/14/2014	29:02.3
12828	RSPe_2	-122.444	37.945	2.52	16.51	289.16	19.03	-122.444	37.945	2.56	16.51	289.38	19.07	9	7/14/2014	29:02.2
12829	RSPe_2	-122.444	37.945	2.55	16.50	289.21	19.05	-122.444	37.945	2.56	16.50	289.38	19.05	9	7/14/2014	29:02.1
12830	RSPe_2	-122.444	37.945	2.52	16.42	289.21	18.94	-122.444	37.945	2.51	16.42	289.45	18.93	9	7/14/2014	29:02.0
12831	RSPe_2	-122.444	37.945	2.52	16.38	289.19	18.90	-122.444	37.945	2.56	16.38	289.43	18.94	9	7/14/2014	29:01.9
12832	RSPe_2	-122.444	37.945	2.52	16.41	289.21	18.93	-122.444	37.945	2.56	16.41	289.45	18.97	9	7/14/2014	29:01.8
12833	RSPe_2	-122.444	37.945	2.55	16.37	289.21	18.92	-122.444	37.945	2.56	16.37	289.47	18.93	9	7/14/2014	29:01.7
12834	RSPe_2	-122.444	37.945	2.52	16.38	289.23	18.90	-122.444	37.945	2.51	16.38	289.49	18.89	9	7/14/2014	29:01.6
12835	RSPe_2	-122.444	37.945	2.52	16.35	289.26	18.87	-122.444	37.945	2.51	16.35	289.52	18.86	9	7/14/2014	29:01.5
12836	RSPe_2	-122.444	37.945	2.52	16.35	289.28	18.87	-122.444	37.945	2.47	16.35	289.47	18.82	9	7/14/2014	29:01.4

12837	RSPe_2	-122.444	37.945	2.52	16.35	289.32	18.87	-122.444	37.945	2.51	16.35	289.52	18.86	9	7/14/2014	29:01.3
12838	RSPe_2	-122.444	37.945	2.52	16.33	289.30	18.85	-122.444	37.945	2.47	16.33	289.49	18.80	9	7/14/2014	29:01.2
12839	RSPe_2	-122.444	37.945	2.52	16.37	289.33	18.89	-122.444	37.945	2.51	16.37	289.50	18.88	9	7/14/2014	29:01.1
12840	RSPe_2	-122.444	37.945	2.46	16.33	289.30	18.80	-122.444	37.945	2.47	16.33	289.48	18.81	9	7/14/2014	29:01.0
12841	RSPe_2	-122.444	37.945	2.52	16.38	289.30	18.90	-122.444	37.945	2.47	16.38	289.50	18.86	9	7/14/2014	29:00.9
12842	RSPe_2	-122.444	37.945	2.46	16.31	289.32	18.78	-122.444	37.945	2.47	16.31	289.49	18.79	9	7/14/2014	29:00.8
12843	RSPe_2	-122.444	37.945	2.52	16.29	289.33	18.81	-122.444	37.945	2.47	16.29	289.48	18.76	9	7/14/2014	29:00.7
12844	RSPe_2	-122.444	37.945	2.46	16.30	289.28	18.76	-122.444	37.945	2.47	16.30	289.50	18.77	9	7/14/2014	29:00.6
12845	RSPe_2	-122.444	37.945	2.52	16.30	289.28	18.82	-122.444	37.945	2.47	16.30	289.48	18.78	9	7/14/2014	29:00.5
12846	RSPe_2	-122.444	37.945	2.46	16.29	289.24	18.75	-122.444	37.945	2.42	16.29	289.49	18.71	9	7/14/2014	29:00.4
12847	RSPe_2	-122.444	37.945	2.46	16.30	289.21	18.76	-122.444	37.945	2.42	16.30	289.43	18.72	9	7/14/2014	29:00.3
12848	RSPe_2	-122.444	37.945	2.46	16.26	289.22	18.73	-122.444	37.945	2.42	16.26	289.46	18.68	9	7/14/2014	29:00.2
12849	RSPe_2	-122.444	37.945	2.52	16.27	289.22	18.79	-122.444	37.945	2.42	16.27	289.43	18.69	9	7/14/2014	29:00.1
12850	RSPe_2	-122.444	37.945	2.46	16.27	289.24	18.74	-122.444	37.945	2.42	16.27	289.46	18.69	9	7/14/2014	29:00.0
12851	RSPe_2	-122.444	37.945	2.46	16.27	289.20	18.73	-122.444	37.945	2.42	16.27	289.43	18.69	9	7/14/2014	28:59.9
12852	RSPe_2	-122.444	37.945	2.46	16.27	289.19	18.73	-122.444	37.945	2.39	16.27	289.41	18.65	9	7/14/2014	28:59.8
12853	RSPe_2	-122.444	37.945	2.46	16.27	289.18	18.74	-122.444	37.945	2.39	16.27	289.35	18.66	9	7/14/2014	28:59.7
12854	RSPe_2	-122.444	37.945	2.46	16.27	289.18	18.73	-122.444	37.945	2.39	16.27	289.36	18.65	9	7/14/2014	28:59.6
12855	RSPe_2	-122.444	37.945	2.46	16.26	289.18	18.73	-122.444	37.945	2.39	16.26	289.33	18.65	9	7/14/2014	28:59.5
12856	RSPe_2	-122.444	37.945	2.46	16.27	289.26	18.74	-122.444	37.945	2.39	16.27	289.34	18.66	9	7/14/2014	28:59.4
12857	RSPe_2	-122.444	37.945	2.46	16.31	289.26	18.78	-122.444	37.945	2.39	16.31	289.37	18.70	9	7/14/2014	28:59.3
12858	RSPe_2	-122.444	37.945	2.43	16.30	289.31	18.73	-122.444	37.945	2.39	16.30	289.35	18.69	9	7/14/2014	28:59.2
12859	RSPe_2	-122.444	37.945	2.46	16.27	289.26	18.73	-122.444	37.945	2.39	16.27	289.34	18.65	9	7/14/2014	28:59.1
12860	RSPe_2	-122.444	37.945	2.43	16.33	289.33	18.76	-122.444	37.945	2.34	16.33	289.35	18.67	9	7/14/2014	28:59.0
12861	RSPe_2	-122.444	37.945	2.46	16.27	289.31	18.74	-122.444	37.945	2.34	16.27	289.37	18.61	9	7/14/2014	28:58.9
12862	RSPe_2	-122.444	37.945	2.43	16.30	289.33	18.74	-122.444	37.945	2.34	16.30	289.33	18.64	9	7/14/2014	28:58.8
12863	RSPe_2	-122.444	37.945	2.43	16.27	289.27	18.70	-122.444	37.945	2.34	16.27	289.31	18.61	9	7/14/2014	28:58.7
12864	RSPe_2	-122.444	37.945	2.43	16.30	289.27	18.73	-122.444	37.945	2.30	16.30	289.31	18.60	9	7/14/2014	28:58.6
12865	RSPe_2	-122.444	37.945	2.43	16.30	289.27	18.73	-122.444	37.945	2.34	16.30	289.31	18.63	9	7/14/2014	28:58.5
12866	RSPe_2	-122.444	37.945	2.43	16.28	289.29	18.71	-122.444	37.945	2.34	16.28	289.31	18.62	9	7/14/2014	28:58.4
12867	RSPe_2	-122.444	37.945	2.43	16.30	289.27	18.73	-122.444	37.945	2.34	16.30	289.33	18.63	9	7/14/2014	28:58.3
12868	RSPe_2	-122.444	37.945	2.43	16.30	289.29	18.74	-122.444	37.945	2.30	16.30	289.35	18.61	9	7/14/2014	28:58.2
12869	RSPe_2	-122.444	37.945	2.43	16.32	289.31	18.75	-122.444	37.945	2.30	16.32	289.44	18.62	9	7/14/2014	28:58.1

12870	RSPe_2	-122.444	37.945	2.38	16.31	289.31	18.69	-122.444	37.945	2.30	16.31	289.46	18.62	9	7/14/2014	28:58.0
12871	RSPe_2	-122.444	37.945	2.43	16.33	289.33	18.77	-122.444	37.945	2.30	16.33	289.53	18.64	9	7/14/2014	28:57.9
12872	RSPe_2	-122.444	37.945	2.38	16.34	289.33	18.72	-122.444	37.945	2.27	16.34	289.50	18.61	9	7/14/2014	28:57.8
12873	RSPe_2	-122.444	37.945	2.43	16.37	289.38	18.80	-122.444	37.945	2.27	16.37	289.55	18.64	9	7/14/2014	28:57.7
12874	RSPe_2	-122.444	37.945	2.38	16.37	289.38	18.75	-122.444	37.945	2.27	16.37	289.55	18.64	9	7/14/2014	28:57.6
12875	RSPe_2	-122.444	37.945	2.43	16.39	289.42	18.82	-122.444	37.945	2.27	16.39	289.55	18.66	9	7/14/2014	28:57.5
12876	RSPe_2	-122.444	37.945	2.38	16.40	289.40	18.78	-122.444	37.945	2.22	16.40	289.61	18.61	9	7/14/2014	28:57.4
12877	RSPe_2	-122.444	37.945	2.38	16.38	289.40	18.76	-122.444	37.945	2.22	16.38	289.64	18.60	9	7/14/2014	28:57.3
12878	RSPe_2	-122.444	37.945	2.38	16.40	289.36	18.78	-122.444	37.945	2.22	16.40	289.58	18.61	9	7/14/2014	28:57.2
12879	RSPe_2	-122.444	37.945	2.43	16.44	289.38	18.87	-122.444	37.945	2.22	16.44	289.55	18.66	9	7/14/2014	28:57.1
12880	RSPe_2	-122.444	37.945	2.38	16.45	289.32	18.83	-122.444	37.945	2.22	16.45	289.55	18.67	9	7/14/2014	28:57.0
12881	RSPe_2	-122.444	37.945	2.43	16.44	289.32	18.87	-122.444	37.945	2.22	16.44	289.53	18.66	9	7/14/2014	28:56.9
12882	RSPe_2	-122.444	37.945	2.38	16.44	289.32	18.82	-122.444	37.945	2.22	16.44	289.55	18.66	9	7/14/2014	28:56.8
12883	RSPe_2	-122.444	37.945	2.43	16.46	289.36	18.89	-122.444	37.945	2.22	16.46	289.55	18.68	9	7/14/2014	28:56.7
12884	RSPe_2	-122.444	37.945	2.38	16.47	289.38	18.85	-122.444	37.945	2.18	16.47	289.57	18.65	9	7/14/2014	28:56.6
12885	RSPe_2	-122.444	37.945	2.38	16.47	289.45	18.85	-122.444	37.945	2.18	16.47	289.58	18.66	9	7/14/2014	28:56.5
12886	RSPe_2	-122.444	37.945	2.38	16.48	289.49	18.86	-122.444	37.945	2.13	16.48	289.64	18.62	9	7/14/2014	28:56.4
12887	RSPe_2	-122.444	37.945	2.38	16.51	289.58	18.89	-122.444	37.945	2.13	16.51	289.65	18.64	9	7/14/2014	28:56.3
12888	RSPe_2	-122.444	37.945	2.38	16.52	289.52	18.90	-122.444	37.945	2.13	16.52	289.64	18.66	9	7/14/2014	28:56.2
12889	RSPe_2	-122.444	37.945	2.38	16.53	289.56	18.91	-122.444	37.945	2.13	16.53	289.64	18.66	9	7/14/2014	28:56.1
12890	RSPe_2	-122.444	37.945	2.38	16.58	289.56	18.96	-122.444	37.945	2.10	16.58	289.68	18.68	9	7/14/2014	28:56.0
12891	RSPe_2	-122.444	37.945	2.38	16.57	289.58	18.95	-122.444	37.945	2.13	16.57	289.68	18.70	9	7/14/2014	28:55.9
12892	RSPe_2	-122.444	37.945	2.35	16.58	289.63	18.93	-122.444	37.945	2.10	16.58	289.71	18.68	9	7/14/2014	28:55.8
12893	RSPe_2	-122.444	37.945	2.38	16.57	289.61	18.95	-122.444	37.945	2.10	16.57	289.74	18.67	9	7/14/2014	28:55.7
12894	RSPe_2	-122.444	37.945	2.38	16.59	289.67	18.97	-122.444	37.945	2.05	16.59	289.75	18.64	9	7/14/2014	28:55.6
12895	RSPe_2	-122.444	37.945	2.38	16.60	289.61	18.98	-122.444	37.945	2.10	16.60	289.71	18.70	9	7/14/2014	28:55.5
12896	RSPe_2	-122.444	37.945	2.38	16.66	289.63	19.04	-122.444	37.945	2.05	16.66	289.69	18.71	9	7/14/2014	28:55.4
12897	RSPe_2	-122.444	37.945	2.38	16.63	289.58	19.01	-122.444	37.945	2.10	16.63	289.71	18.73	9	7/14/2014	28:55.3
12898	RSPe_2	-122.444	37.945	2.38	16.67	289.63	19.05	-122.444	37.945	2.05	16.67	289.73	18.72	9	7/14/2014	28:55.2
12899	RSPe_2	-122.444	37.945	2.38	16.64	289.65	19.02	-122.444	37.945	2.10	16.64	289.73	18.74	9	7/14/2014	28:55.1
12900	RSPe_2	-122.444	37.945	2.38	16.68	289.70	19.06	-122.444	37.945	2.05	16.68	289.76	18.73	9	7/14/2014	28:55.0
12901	RSPe_2	-122.444	37.945	2.38	16.67	289.72	19.05	-122.444	37.945	2.05	16.67	289.80	18.72	9	7/14/2014	28:54.9
12902	RSPe_2	-122.444	37.945	2.38	16.70	289.76	19.09	-122.444	37.945	2.05	16.70	289.82	18.75	9	7/14/2014	28:54.8

12903	RSPe_2	-122.444	37.945	2.38	16.67	289.74	19.05	-122.444	37.945	2.02	16.67	289.79	18.68	9	7/14/2014	28:54.7
12904	RSPe_2	-122.444	37.945	2.38	16.77	289.74	19.15	-122.444	37.945	2.02	16.77	289.82	18.79	9	7/14/2014	28:54.6
12905	RSPe_2	-122.444	37.945	2.38	16.72	289.76	19.10	-122.444	37.945	2.02	16.72	289.86	18.74	9	7/14/2014	28:54.5
12906	RSPe_2	-122.444	37.945	2.38	16.75	289.81	19.13	-122.444	37.945	2.02	16.75	289.87	18.77	9	7/14/2014	28:54.4
12907	RSPe_2	-122.444	37.945	2.38	16.79	289.81	19.17	-122.444	37.945	2.02	16.79	289.87	18.81	9	7/14/2014	28:54.3
12908	RSPe_2	-122.444	37.945	2.38	16.73	289.83	19.11	-122.444	37.945	2.02	16.73	289.82	18.74	9	7/14/2014	28:54.2
12909	RSPe_2	-122.444	37.945	2.38	16.84	289.83	19.22	-122.444	37.945	2.02	16.84	289.87	18.85	9	7/14/2014	28:54.1
12910	RSPe_2	-122.444	37.945	2.43	16.83	289.81	19.26	-122.444	37.945	2.02	16.83	289.87	18.84	9	7/14/2014	28:54.0
12911	RSPe_2	-122.444	37.945	2.43	16.82	289.85	19.25	-122.444	37.945	2.02	16.82	289.83	18.84	9	7/14/2014	28:53.9
12912	RSPe_2	-122.444	37.945	2.43	16.85	289.85	19.29	-122.444	37.945	2.02	16.85	289.87	18.87	9	7/14/2014	28:53.8
12913	RSPe_2	-122.444	37.945	2.43	16.84	289.83	19.27	-122.444	37.945	2.05	16.84	289.85	18.89	9	7/14/2014	28:53.7
12914	RSPe_2	-122.444	37.945	2.43	16.84	289.88	19.27	-122.444	37.945	2.02	16.84	289.88	18.85	9	7/14/2014	28:53.6
12915	RSPe_2	-122.444	37.945	2.43	16.85	289.88	19.29	-122.444	37.945	2.05	16.85	289.85	18.90	9	7/14/2014	28:53.5
12916	RSPe_2	-122.444	37.945	2.43	16.86	289.90	19.29	-122.444	37.945	2.02	16.86	289.89	18.88	9	7/14/2014	28:53.4
12917	RSPe_2	-122.444	37.945	2.46	16.88	289.92	19.34	-122.444	37.945	2.05	16.88	289.87	18.93	9	7/14/2014	28:53.3
12918	RSPe_2	-122.444	37.945	2.43	16.88	289.94	19.31	-122.444	37.945	2.05	16.88	289.89	18.93	9	7/14/2014	28:53.2
12919	RSPe_2	-122.444	37.945	2.46	16.88	289.94	19.35	-122.444	37.945	2.05	16.88	289.89	18.93	9	7/14/2014	28:53.1
12920	RSPe_2	-122.444	37.945	2.46	16.91	290.01	19.38	-122.444	37.945	2.05	16.91	289.94	18.96	9	7/14/2014	28:53.0
12921	RSPe_2	-122.444	37.945	2.52	16.93	289.94	19.45	-122.444	37.945	2.05	16.93	289.94	18.98	9	7/14/2014	28:52.9
12922	RSPe_2	-122.444	37.945	2.46	16.92	289.90	19.38	-122.444	37.945	2.05	16.92	289.89	18.97	9	7/14/2014	28:52.8
12923	RSPe_2	-122.444	37.945	2.52	16.97	289.96	19.48	-122.444	37.945	2.10	16.97	289.98	19.07	9	7/14/2014	28:52.7
12924	RSPe_2	-122.444	37.945	2.46	16.98	290.01	19.45	-122.444	37.945	2.05	16.98	290.00	19.03	9	7/14/2014	28:52.6
12925	RSPe_2	-122.444	37.945	2.52	16.96	289.99	19.48	-122.444	37.945	2.10	16.96	289.99	19.06	9	7/14/2014	28:52.5
12926	RSPe_2	-122.444	37.945	2.52	16.98	290.01	19.50	-122.444	37.945	2.05	16.98	289.96	19.03	9	7/14/2014	28:52.4
12927	RSPe_2	-122.444	37.945	2.55	16.99	290.03	19.54	-122.444	37.945	2.10	16.99	289.96	19.09	9	7/14/2014	28:52.3
12928	RSPe_2	-122.444	37.945	2.52	17.07	290.06	19.59	-122.444	37.945	2.05	17.07	290.03	19.12	9	7/14/2014	28:52.2
12929	RSPe_2	-122.444	37.945	2.55	17.01	290.10	19.57	-122.444	37.945	2.10	17.01	290.05	19.12	9	7/14/2014	28:52.1
12930	RSPe_2	-122.444	37.945	2.55	17.05	290.21	19.60	-122.444	37.945	2.10	17.05	290.11	19.15	9	7/14/2014	28:52.0
12931	RSPe_2	-122.444	37.945	2.55	17.04	290.17	19.59	-122.444	37.945	2.10	17.04	290.14	19.14	9	7/14/2014	28:51.9
12932	RSPe_2	-122.444	37.945	2.55	17.04	290.10	19.59	-122.444	37.945	2.10	17.04	290.05	19.14	9	7/14/2014	28:51.8
12933	RSPe_2	-122.444	37.945	2.55	17.09	290.12	19.64	-122.444	37.945	2.10	17.09	290.07	19.19	9	7/14/2014	28:51.7
12934	RSPe_2	-122.444	37.945	2.55	17.07	290.19	19.62	-122.444	37.945	2.10	17.07	290.13	19.17	9	7/14/2014	28:51.6
12935	RSPe_2	-122.444	37.945	2.60	17.12	290.17	19.72	-122.444	37.945	2.10	17.12	290.06	19.22	9	7/14/2014	28:51.5

12936	RSPe_2	-122.444	37.945	2.60	17.14	290.12	19.75	-122.444	37.945	2.10	17.14	290.07	19.24	9	7/14/2014	28:51.4
12937	RSPe_2	-122.444	37.945	2.60	17.12	290.24	19.72	-122.444	37.945	2.13	17.12	290.12	19.26	9	7/14/2014	28:51.3
12938	RSPe_2	-122.444	37.945	2.60	17.22	290.32	19.83	-122.444	37.945	2.10	17.22	290.18	19.32	9	7/14/2014	28:51.2
12939	RSPe_2	-122.444	37.945	2.64	17.16	290.30	19.80	-122.444	37.945	2.13	17.16	290.18	19.29	9	7/14/2014	28:51.1
12940	RSPe_2	-122.444	37.945	2.64	17.18	290.30	19.82	-122.444	37.945	2.13	17.18	290.17	19.32	9	7/14/2014	28:51.0
12941	RSPe_2	-122.444	37.945	2.69	17.20	290.39	19.89	-122.444	37.945	2.19	17.20	290.26	19.38	9	7/14/2014	28:50.9
12942	RSPe_2	-122.444	37.945	2.69	17.29	290.41	19.98	-122.444	37.945	2.13	17.29	290.32	19.43	9	7/14/2014	28:50.8
12943	RSPe_2	-122.444	37.945	2.69	17.23	290.41	19.92	-122.444	37.945	2.19	17.23	290.30	19.42	9	7/14/2014	28:50.7
12944	RSPe_2	-122.444	37.945	2.69	17.25	290.37	19.93	-122.444	37.945	2.19	17.25	290.30	19.43	9	7/14/2014	28:50.6
12945	RSPe_2	-122.444	37.945	2.72	17.14	290.37	19.86	-122.444	37.945	2.19	17.14	290.27	19.32	9	7/14/2014	28:50.5
12946	RSPe_2	-122.444	37.945	2.72	17.12	290.35	19.84	-122.444	37.945	2.22	17.12	290.32	19.34	9	7/14/2014	28:50.4
12947	RSPe_2	-122.444	37.945	2.72	17.13	290.32	19.85	-122.444	37.945	2.27	17.13	290.32	19.40	9	7/14/2014	28:50.3
12948	RSPe_2	-122.444	37.945	2.72	17.08	290.37	19.80	-122.444	37.945	2.22	17.08	290.36	19.30	9	7/14/2014	28:50.2
12949	RSPe_2	-122.444	37.945	2.72	17.12	290.37	19.84	-122.444	37.945	2.27	17.12	290.43	19.39	9	7/14/2014	28:50.1
12950	RSPe_2	-122.444	37.945	2.72	17.08	290.39	19.81	-122.444	37.945	2.22	17.08	290.43	19.30	9	7/14/2014	28:50.0
12951	RSPe_2	-122.444	37.945	2.76	17.14	290.41	19.90	-122.444	37.945	2.27	17.14	290.50	19.41	9	7/14/2014	28:49.9
12952	RSPe_2	-122.444	37.945	2.72	17.14	290.50	19.86	-122.444	37.945	2.22	17.14	290.49	19.36	9	7/14/2014	28:49.8
12953	RSPe_2	-122.444	37.945	2.76	17.13	290.50	19.89	-122.444	37.945	2.27	17.13	290.47	19.40	9	7/14/2014	28:49.7
12954	RSPe_2	-122.444	37.945	2.76	17.14	290.48	19.90	-122.444	37.945	2.22	17.14	290.48	19.36	9	7/14/2014	28:49.6
12955	RSPe_2	-122.444	37.945	2.76	17.08	290.55	19.83	-122.444	37.945	2.22	17.08	290.45	19.30	9	7/14/2014	28:49.5
12956	RSPe_2	-122.444	37.945	2.76	17.08	290.50	19.83	-122.444	37.945	2.22	17.08	290.43	19.30	9	7/14/2014	28:49.4
12957	RSPe_2	-122.444	37.945	2.76	17.12	290.57	19.87	-122.444	37.945	2.27	17.12	290.44	19.39	9	7/14/2014	28:49.3
12958	RSPe_2	-122.444	37.945	2.81	17.00	290.50	19.81	-122.444	37.945	2.22	17.00	290.43	19.22	9	7/14/2014	28:49.2
12959	RSPe_2	-122.444	37.945	2.81	16.98	290.46	19.79	-122.444	37.945	2.27	16.98	290.34	19.25	9	7/14/2014	28:49.1
12960	RSPe_2	-122.444	37.945	2.81	17.05	290.48	19.85	-122.444	37.945	2.30	17.05	290.41	19.35	9	7/14/2014	28:49.0
12961	RSPe_2	-122.444	37.945	2.81	17.01	290.50	19.82	-122.444	37.945	2.30	17.01	290.46	19.32	9	7/14/2014	28:48.9
12962	RSPe_2	-122.444	37.945	2.81	17.05	290.48	19.86	-122.444	37.945	2.30	17.05	290.45	19.36	9	7/14/2014	28:48.8
12963	RSPe_2	-122.444	37.945	2.84	17.00	290.48	19.85	-122.444	37.945	2.34	17.00	290.45	19.34	9	7/14/2014	28:48.7
12964	RSPe_2	-122.444	37.945	2.84	17.00	290.51	19.85	-122.444	37.945	2.30	17.00	290.43	19.31	9	7/14/2014	28:48.6
12965	RSPe_2	-122.444	37.945	2.84	16.97	290.50	19.81	-122.444	37.945	2.30	16.97	290.48	19.27	9	7/14/2014	28:48.5
12966	RSPe_2	-122.444	37.945	2.84	16.97	290.48	19.81	-122.444	37.945	2.34	16.97	290.41	19.30	9	7/14/2014	28:48.4
12967	RSPe_2	-122.444	37.945	2.84	16.99	290.46	19.83	-122.444	37.945	2.34	16.99	290.41	19.33	9	7/14/2014	28:48.3
12968	RSPe_2	-122.444	37.945	2.84	16.98	290.57	19.82	-122.444	37.945	2.34	16.98	290.46	19.31	9	7/14/2014	28:48.2

12969	RSPe_2	-122.444	37.945	2.89	16.94	290.53	19.83	-122.444	37.945	2.34	16.94	290.45	19.27	9	7/14/2014	28:48.1
12970	RSPe_2	-122.444	37.945	2.89	16.97	290.51	19.86	-122.444	37.945	2.34	16.97	290.41	19.30	9	7/14/2014	28:48.0
12971	RSPe_2	-122.444	37.945	2.89	17.01	290.48	19.91	-122.444	37.945	2.39	17.01	290.44	19.40	9	7/14/2014	28:47.9
12972	RSPe_2	-122.444	37.945	2.89	16.96	290.51	19.86	-122.444	37.945	2.39	16.96	290.43	19.35	9	7/14/2014	28:47.8
12973	RSPe_2	-122.444	37.945	2.93	17.03	290.51	19.96	-122.444	37.945	2.39	17.03	290.40	19.42	9	7/14/2014	28:47.7
12974	RSPe_2	-122.444	37.945	2.89	16.98	290.46	19.88	-122.444	37.945	2.39	16.98	290.39	19.37	9	7/14/2014	28:47.6
12975	RSPe_2	-122.444	37.945	2.93	16.97	290.46	19.89	-122.444	37.945	2.42	16.97	290.39	19.39	9	7/14/2014	28:47.5
12976	RSPe_2	-122.444	37.945	2.93	16.95	290.46	19.88	-122.444	37.945	2.39	16.95	290.39	19.34	9	7/14/2014	28:47.4
12977	RSPe_2	-122.444	37.945	2.98	16.96	290.40	19.94	-122.444	37.945	2.42	16.96	290.30	19.38	9	7/14/2014	28:47.3
12978	RSPe_2	-122.444	37.945	2.93	16.97	290.42	19.89	-122.444	37.945	2.39	16.97	290.30	19.36	9	7/14/2014	28:47.2
12979	RSPe_2	-122.444	37.945	2.98	16.96	290.40	19.94	-122.444	37.945	2.42	16.96	290.29	19.38	9	7/14/2014	28:47.1
12980	RSPe_2	-122.444	37.945	2.93	16.97	290.42	19.89	-122.444	37.945	2.39	16.97	290.32	19.36	9	7/14/2014	28:47.0
12981	RSPe_2	-122.444	37.945	2.98	16.98	290.42	19.96	-122.444	37.945	2.42	16.98	290.29	19.40	9	7/14/2014	28:46.9
12982	RSPe_2	-122.444	37.945	2.98	17.00	290.42	19.98	-122.444	37.945	2.47	17.00	290.26	19.47	9	7/14/2014	28:46.8
12983	RSPe_2	-122.444	37.945	3.01	16.99	290.40	20.00	-122.444	37.945	2.47	16.99	290.29	19.46	9	7/14/2014	28:46.7
12984	RSPe_2	-122.444	37.945	2.98	16.99	290.47	19.97	-122.444	37.945	2.47	16.99	290.34	19.46	9	7/14/2014	28:46.6
12985	RSPe_2	-122.444	37.945	3.01	16.98	290.49	20.00	-122.444	37.945	2.47	16.98	290.35	19.46	9	7/14/2014	28:46.5
12986	RSPe_2	-122.444	37.945	3.01	17.00	290.60	20.02	-122.444	37.945	2.47	17.00	290.42	19.48	9	7/14/2014	28:46.4
12987	RSPe_2	-122.444	37.945	3.01	17.00	290.62	20.01	-122.444	37.945	2.47	17.00	290.46	19.47	9	7/14/2014	28:46.3
12988	RSPe_2	-122.444	37.945	3.01	17.01	290.60	20.03	-122.444	37.945	2.47	17.01	290.42	19.49	9	7/14/2014	28:46.2
12989	RSPe_2	-122.444	37.945	3.07	17.04	290.55	20.10	-122.444	37.945	2.51	17.04	290.40	19.55	9	7/14/2014	28:46.1
12990	RSPe_2	-122.444	37.945	3.07	16.97	290.55	20.03	-122.444	37.945	2.51	16.97	290.44	19.47	9	7/14/2014	28:46.0
12991	RSPe_2	-122.444	37.945	3.07	16.94	290.66	20.01	-122.444	37.945	2.56	16.94	290.46	19.50	9	7/14/2014	28:45.9
12992	RSPe_2	-122.444	37.945	3.07	16.88	290.67	19.94	-122.444	37.945	2.51	16.88	290.51	19.38	9	7/14/2014	28:45.8
12993	RSPe_2	-122.444	37.945	3.10	16.93	290.71	20.03	-122.444	37.945	2.51	16.93	290.53	19.44	9	7/14/2014	28:45.7
12994	RSPe_2	-122.444	37.945	3.07	16.94	290.77	20.00	-122.444	37.945	2.56	16.94	290.62	19.49	9	7/14/2014	28:45.6
12995	RSPe_2	-122.444	37.945	3.10	16.76	290.73	19.86	-122.444	37.945	2.51	16.76	290.60	19.27	9	7/14/2014	28:45.5
12996	RSPe_2	-122.444	37.945	3.10	16.62	290.78	19.72	-122.444	37.945	2.51	16.62	290.62	19.13	9	7/14/2014	28:45.4
12997	RSPe_2	-122.444	37.945	3.13	16.53	290.80	19.67	-122.444	37.945	2.56	16.53	290.64	19.09	9	7/14/2014	28:45.3
12998	RSPe_2	-122.444	37.945	3.10	16.51	290.78	19.61	-122.444	37.945	2.56	16.51	290.66	19.06	9	7/14/2014	28:45.2
12999	RSPe_2	-122.444	37.945	3.13	16.60	290.78	19.74	-122.444	37.945	2.59	16.60	290.71	19.20	9	7/14/2014	28:45.1
13000	RSPe_2	-122.444	37.945	3.13	16.51	290.78	19.65	-122.444	37.945	2.59	16.51	290.71	19.10	9	7/14/2014	28:45.0
13001	RSPe_2	-122.444	37.945	3.13	16.35	290.78	19.48	-122.444	37.945	2.59	16.35	290.71	18.94	9	7/14/2014	28:44.9

13002	RSPe_2	-122.444	37.945	3.13	16.39	290.76	19.52	-122.444	37.945	2.59	16.39	290.69	18.98	9	7/14/2014	28:44.8
13003	RSPe_2	-122.444	37.945	3.18	16.33	290.82	19.52	-122.444	37.945	2.64	16.33	290.71	18.98	9	7/14/2014	28:44.7
13004	RSPe_2	-122.444	37.945	3.18	16.33	290.87	19.52	-122.444	37.945	2.64	16.33	290.77	18.98	9	7/14/2014	28:44.6
13005	RSPe_2	-122.444	37.945	3.22	16.40	290.93	19.61	-122.444	37.945	2.64	16.40	290.84	19.04	9	7/14/2014	28:44.5
13006	RSPe_2	-122.444	37.945	3.18	16.41	290.98	19.59	-122.444	37.945	2.64	16.41	290.88	19.05	9	7/14/2014	28:44.4
13007	RSPe_2	-122.444	37.945	3.22	16.29	291.00	19.51	-122.444	37.945	2.68	16.29	290.84	18.97	9	7/14/2014	28:44.3
13008	RSPe_2	-122.444	37.945	3.22	16.28	290.93	19.50	-122.444	37.945	2.64	16.28	290.86	18.92	9	7/14/2014	28:44.2
13009	RSPe_2	-122.444	37.945	3.27	16.32	291.02	19.59	-122.444	37.945	2.64	16.32	290.91	18.96	9	7/14/2014	28:44.1
13010	RSPe_2	-122.444	37.945	3.22	16.23	290.98	19.45	-122.444	37.945	2.64	16.23	290.90	18.87	9	7/14/2014	28:44.0
13011	RSPe_2	-122.444	37.945	3.27	16.21	291.02	19.48	-122.444	37.945	2.68	16.21	290.88	18.89	9	7/14/2014	28:43.9
13012	RSPe_2	-122.444	37.945	3.27	16.23	290.94	19.50	-122.444	37.945	2.68	16.23	290.84	18.91	9	7/14/2014	28:43.8
13013	RSPe_2	-122.444	37.945	3.31	16.00	290.96	19.31	-122.444	37.945	2.71	16.00	290.89	18.71	9	7/14/2014	28:43.7
13014	RSPe_2	-122.444	37.945	3.31	16.04	290.96	19.35	-122.444	37.945	2.76	16.04	290.89	18.80	9	7/14/2014	28:43.6
13015	RSPe_2	-122.444	37.945	3.36	15.94	290.98	19.30	-122.444	37.945	2.80	15.94	290.96	18.73	9	7/14/2014	28:43.5
13016	RSPe_2	-122.444	37.945	3.31	15.96	290.98	19.26	-122.444	37.945	2.80	15.96	290.93	18.75	9	7/14/2014	28:43.4
13017	RSPe_2	-122.444	37.945	3.36	15.93	290.98	19.29	-122.444	37.945	2.85	15.93	290.95	18.78	9	7/14/2014	28:43.3
13018	RSPe_2	-122.444	37.945	3.36	15.86	291.01	19.22	-122.444	37.945	2.85	15.86	290.93	18.71	9	7/14/2014	28:43.2
13019	RSPe_2	-122.444	37.945	3.36	15.84	291.00	19.20	-122.444	37.945	2.85	15.84	290.98	18.69	9	7/14/2014	28:43.1
13020	RSPe_2	-122.444	37.945	3.36	15.83	291.05	19.18	-122.444	37.945	2.88	15.83	290.98	18.71	9	7/14/2014	28:43.0
13021	RSPe_2	-122.444	37.945	3.44	15.91	291.05	19.35	-122.444	37.945	2.88	15.91	291.00	18.79	9	7/14/2014	28:42.9
13022	RSPe_2	-122.444	37.945	3.39	15.75	291.05	19.14	-122.444	37.945	2.88	15.75	290.95	18.63	9	7/14/2014	28:42.8
13023	RSPe_2	-122.444	37.945	3.44	15.74	291.07	19.18	-122.444	37.945	2.93	15.74	290.95	18.67	9	7/14/2014	28:42.7
13024	RSPe_2	-122.444	37.945	3.39	15.69	291.07	19.09	-122.444	37.945	2.93	15.69	290.94	18.63	9	7/14/2014	28:42.6
13025	RSPe_2	-122.444	37.945	3.44	15.69	291.07	19.14	-122.444	37.945	2.93	15.69	290.98	18.63	9	7/14/2014	28:42.5
13026	RSPe_2	-122.444	37.945	3.44	15.70	291.16	19.15	-122.444	37.945	2.93	15.70	290.98	18.63	9	7/14/2014	28:42.4
13027	RSPe_2	-122.444	37.945	3.48	15.73	291.11	19.21	-122.444	37.945	2.96	15.73	290.96	18.70	9	7/14/2014	28:42.3
13028	RSPe_2	-122.444	37.945	3.48	15.62	291.16	19.10	-122.444	37.945	3.02	15.62	290.96	18.63	9	7/14/2014	28:42.2
13029	RSPe_2	-122.444	37.945	3.48	15.57	291.20	19.05	-122.444	37.945	3.02	15.57	290.96	18.59	9	7/14/2014	28:42.1
13030	RSPe_2	-122.444	37.945	3.48	15.56	291.16	19.04	-122.444	37.945	3.05	15.56	290.96	18.61	9	7/14/2014	28:42.0
13031	RSPe_2	-122.444	37.945	3.51	15.56	291.23	19.07	-122.444	37.945	3.08	15.56	291.00	18.64	9	7/14/2014	28:41.9
13032	RSPe_2	-122.444	37.945	3.48	15.54	291.18	19.02	-122.444	37.945	3.05	15.54	290.94	18.59	9	7/14/2014	28:41.8
13033	RSPe_2	-122.444	37.945	3.51	15.53	291.21	19.04	-122.444	37.945	3.08	15.53	290.98	18.61	9	7/14/2014	28:41.7
13034	RSPe_2	-122.444	37.945	3.51	15.50	291.18	19.01	-122.444	37.945	3.08	15.50	290.98	18.59	9	7/14/2014	28:41.6

13035	RSPe_2	-122.444	37.945	3.56	15.51	291.14	19.07	-122.444	37.945	3.13	15.51	290.92	18.65	9	7/14/2014	28:41.5
13036	RSPe_2	-122.444	37.945	3.56	15.56	291.09	19.12	-122.444	37.945	3.13	15.56	290.87	18.69	9	7/14/2014	28:41.4
13037	RSPe_2	-122.444	37.945	3.60	15.45	291.08	19.04	-122.444	37.945	3.22	15.45	290.94	18.67	9	7/14/2014	28:41.3
13038	RSPe_2	-122.444	37.945	3.56	15.47	291.11	19.03	-122.444	37.945	3.25	15.47	290.96	18.73	9	7/14/2014	28:41.2
13039	RSPe_2	-122.444	37.945	3.60	15.52	291.05	19.11	-122.444	37.945	3.25	15.52	290.98	18.77	9	7/14/2014	28:41.1
13040	RSPe_2	-122.444	37.945	3.56	15.48	291.07	19.04	-122.444	37.945	3.25	15.48	291.01	18.73	9	7/14/2014	28:41.0
13041	RSPe_2	-122.444	37.945	3.60	15.47	291.05	19.07	-122.444	37.945	3.30	15.47	291.05	18.78	9	7/14/2014	28:40.9
13042	RSPe_2	-122.444	37.945	3.60	15.46	291.01	19.06	-122.444	37.945	3.30	15.46	291.00	18.77	9	7/14/2014	28:40.8
13043	RSPe_2	-122.444	37.945	3.60	15.52	291.01	19.11	-122.444	37.945	3.34	15.52	291.03	18.86	9	7/14/2014	28:40.7
13044	RSPe_2	-122.444	37.945	3.65	15.48	291.05	19.13	-122.444	37.945	3.30	15.48	291.03	18.78	9	7/14/2014	28:40.6
13045	RSPe_2	-122.444	37.945	3.65	15.50	291.06	19.15	-122.444	37.945	3.34	15.50	290.99	18.84	9	7/14/2014	28:40.5
13046	RSPe_2	-122.444	37.945	3.65	15.50	291.05	19.15	-122.444	37.945	3.34	15.50	290.96	18.84	9	7/14/2014	28:40.4
13047	RSPe_2	-122.444	37.945	3.68	15.50	291.08	19.18	-122.444	37.945	3.39	15.50	291.05	18.89	9	7/14/2014	28:40.3
13048	RSPe_2	-122.444	37.945	3.65	15.48	291.08	19.13	-122.444	37.945	3.39	15.48	291.01	18.87	9	7/14/2014	28:40.2
13049	RSPe_2	-122.444	37.945	3.68	15.55	291.03	19.23	-122.444	37.945	3.42	15.55	290.94	18.97	9	7/14/2014	28:40.1
13050	RSPe_2	-122.444	37.945	3.65	15.53	291.10	19.18	-122.444	37.945	3.42	15.53	291.03	18.95	9	7/14/2014	28:40.0
13051	RSPe_2	-122.444	37.945	3.68	15.53	291.12	19.21	-122.444	37.945	3.46	15.53	291.01	18.99	9	7/14/2014	28:39.9
13052	RSPe_2	-122.444	37.945	3.65	15.58	291.12	19.23	-122.444	37.945	3.46	15.58	291.01	19.04	9	7/14/2014	28:39.8
13053	RSPe_2	-122.444	37.945	3.68	15.59	291.12	19.27	-122.444	37.945	3.46	15.59	291.03	19.04	9	7/14/2014	28:39.7
13054	RSPe_2	-122.444	37.945	3.68	15.50	291.08	19.18	-122.444	37.945	3.46	15.50	290.96	18.96	9	7/14/2014	28:39.6
13055	RSPe_2	-122.444	37.945	3.68	15.50	291.10	19.18	-122.444	37.945	3.54	15.50	290.96	19.04	9	7/14/2014	28:39.5
13056	RSPe_2	-122.444	37.945	3.68	15.48	291.05	19.17	-122.444	37.945	3.51	15.48	290.92	18.99	9	7/14/2014	28:39.4
13057	RSPe_2	-122.444	37.945	3.73	15.50	291.08	19.23	-122.444	37.945	3.54	15.50	290.96	19.04	9	7/14/2014	28:39.3
13058	RSPe_2	-122.444	37.945	3.73	15.52	291.08	19.25	-122.444	37.945	3.54	15.52	290.99	19.06	9	7/14/2014	28:39.2
13059	RSPe_2	-122.444	37.945	3.73	15.47	291.13	19.21	-122.444	37.945	3.59	15.47	291.04	19.06	9	7/14/2014	28:39.1
13060	RSPe_2	-122.444	37.945	3.73	15.47	291.10	19.21	-122.444	37.945	3.59	15.47	291.05	19.06	9	7/14/2014	28:39.0
13061	RSPe_2	-122.444	37.945	3.73	15.48	291.17	19.22	-122.444	37.945	3.63	15.48	291.08	19.11	9	7/14/2014	28:38.9
13062	RSPe_2	-122.444	37.945	3.73	15.46	291.15	19.19	-122.444	37.945	3.63	15.46	291.10	19.08	9	7/14/2014	28:38.8
13063	RSPe_2	-122.444	37.945	3.77	15.56	291.19	19.32	-122.444	37.945	3.63	15.56	291.11	19.18	9	7/14/2014	28:38.7
13064	RSPe_2	-122.444	37.945	3.77	15.55	291.19	19.32	-122.444	37.945	3.63	15.55	291.15	19.17	9	7/14/2014	28:38.6
13065	RSPe_2	-122.444	37.945	3.77	15.47	291.23	19.24	-122.444	37.945	3.68	15.47	291.14	19.15	9	7/14/2014	28:38.5
13066	RSPe_2	-122.444	37.945	3.73	15.53	291.24	19.27	-122.444	37.945	3.68	15.53	291.14	19.21	9	7/14/2014	28:38.4
13067	RSPe_2	-122.444	37.945	3.77	15.51	291.28	19.28	-122.444	37.945	3.68	15.51	291.17	19.19	9	7/14/2014	28:38.3

13068	RSPe_2	-122.444	37.945	3.73	15.48	291.30	19.21	-122.444	37.945	3.68	15.48	291.21	19.16	9	7/14/2014 28:38.2
13069	RSPe_2	-122.444	37.945	3.77	15.53	291.32	19.30	-122.444	37.945	3.71	15.53	291.23	19.24	9	7/14/2014 28:38.1
13070	RSPe_2	-122.444	37.945	3.77	15.46	291.35	19.23	-122.444	37.945	3.71	15.46	291.23	19.17	9	7/14/2014 28:38.0
13071	RSPe_2	-122.444	37.945	3.77	15.48	291.41	19.25	-122.444	37.945	3.74	15.48	291.27	19.23	9	7/14/2014 28:37.9
13072	RSPe_2	-122.444	37.945	3.77	15.51	291.39	19.28	-122.444	37.945	3.71	15.51	291.32	19.22	9	7/14/2014 28:37.8
13073	RSPe_2	-122.444	37.945	3.80	15.48	291.41	19.28	-122.444	37.945	3.74	15.48	291.29	19.22	9	7/14/2014 28:37.7
13074	RSPe_2	-122.444	37.945	3.77	15.47	291.44	19.24	-122.444	37.945	3.74	15.47	291.37	19.22	9	7/14/2014 28:37.6
13075	RSPe_2	-122.444	37.945	3.77	15.48	291.46	19.25	-122.444	37.945	3.80	15.48	291.39	19.27	9	7/14/2014 28:37.5
13076	RSPe_2	-122.444	37.945	3.77	15.47	291.46	19.24	-122.444	37.945	3.80	15.47	291.41	19.27	9	7/14/2014 28:37.4
13077	RSPe_2	-122.444	37.945	3.80	15.48	291.48	19.29	-122.444	37.945	3.80	15.48	291.48	19.28	9	7/14/2014 28:37.3
13078	RSPe_2	-122.444	37.945	3.80	15.46	291.53	19.27	-122.444	37.945	3.80	15.46	291.48	19.26	9	7/14/2014 28:37.2
13079	RSPe_2	-122.444	37.945	3.80	15.51	291.52	19.31	-122.444	37.945	3.83	15.51	291.45	19.34	9	7/14/2014 28:37.1
13080	RSPe_2	-122.444	37.945	3.80	15.46	291.55	19.27	-122.444	37.945	3.83	15.46	291.50	19.29	9	7/14/2014 28:37.0
13081	RSPe_2	-122.444	37.945	3.80	15.46	291.57	19.26	-122.444	37.945	3.88	15.46	291.54	19.34	9	7/14/2014 28:36.9
13082	RSPe_2	-122.444	37.945	3.80	15.48	291.61	19.28	-122.444	37.945	3.83	15.48	291.58	19.31	9	7/14/2014 28:36.8
13083	RSPe_2	-122.444	37.945	3.85	15.48	291.55	19.33	-122.444	37.945	3.88	15.48	291.59	19.36	9	7/14/2014 28:36.7
13084	RSPe_2	-122.444	37.945	3.80	15.46	291.61	19.27	-122.444	37.945	3.88	15.46	291.61	19.34	9	7/14/2014 28:36.6
13085	RSPe_2	-122.444	37.945	3.80	15.47	291.61	19.27	-122.444	37.945	3.88	15.47	291.67	19.35	9	7/14/2014 28:36.5
13086	RSPe_2	-122.444	37.945	3.80	15.46	291.64	19.27	-122.444	37.945	3.91	15.46	291.68	19.38	9	7/14/2014 28:36.4
13087	RSPe_2	-122.444	37.945	3.85	15.48	291.68	19.33	-122.444	37.945	3.91	15.48	291.76	19.39	9	7/14/2014 28:36.3
13088	RSPe_2	-122.444	37.945	3.85	15.48	291.70	19.33	-122.444	37.945	3.91	15.48	291.76	19.39	9	7/14/2014 28:36.2
13089	RSPe_2	-122.444	37.945	3.85	15.47	291.70	19.33	-122.444	37.945	3.96	15.47	291.74	19.44	9	7/14/2014 28:36.1
13090	RSPe_2	-122.444	37.945	3.80	15.46	291.77	19.26	-122.444	37.945	3.96	15.46	291.78	19.42	9	7/14/2014 28:36.0
13091	RSPe_2	-122.444	37.945	3.85	15.49	291.75	19.35	-122.444	37.945	3.96	15.49	291.77	19.46	9	7/14/2014 28:35.9
13092	RSPe_2	-122.444	37.945	3.85	15.46	291.79	19.31	-122.444	37.945	3.96	15.46	291.79	19.42	9	7/14/2014 28:35.8
13093	RSPe_2	-122.444	37.945	3.85	15.43	291.79	19.28	-122.444	37.945	4.00	15.43	291.81	19.42	9	7/14/2014 28:35.7
13094	RSPe_2	-122.444	37.945	3.85	15.45	291.79	19.30	-122.444	37.945	4.00	15.45	291.79	19.44	9	7/14/2014 28:35.6
13095	RSPe_2	-122.444	37.945	3.85	15.44	291.81	19.29	-122.444	37.945	4.05	15.44	291.76	19.49	9	7/14/2014 28:35.5
13096	RSPe_2	-122.444	37.945	3.85	15.44	291.82	19.29	-122.444	37.945	4.00	15.44	291.79	19.44	9	7/14/2014 28:35.4
13097	RSPe_2	-122.444	37.945	3.85	15.43	291.84	19.28	-122.444	37.945	4.05	15.43	291.79	19.47	9	7/14/2014 28:35.3
13098	RSPe_2	-122.444	37.945	3.85	15.36	291.83	19.21	-122.444	37.945	4.05	15.36	291.83	19.40	9	7/14/2014 28:35.2
13099	RSPe_2	-122.444	37.945	3.85	15.39	291.90	19.24	-122.444	37.945	4.05	15.39	291.83	19.44	9	7/14/2014 28:35.1
13100	RSPe_2	-122.444	37.945	3.85	15.39	291.90	19.24	-122.444	37.945	4.05	15.39	291.89	19.44	9	7/14/2014 28:35.0

13101	RSPe_2	-122.444	37.945	3.89	15.34	291.97	19.23	-122.444	37.945	4.08	15.34	291.95	19.42	9	7/14/2014 28:34.9
13102	RSPe_2	-122.444	37.945	3.89	15.37	291.97	19.26	-122.444	37.945	4.08	15.37	291.98	19.45	9	7/14/2014 28:34.8
13103	RSPe_2	-122.444	37.945	3.89	15.32	292.01	19.20	-122.444	37.945	4.17	15.32	291.98	19.48	9	7/14/2014 28:34.7
13104	RSPe_2	-122.444	37.945	3.85	15.27	292.04	19.12	-122.444	37.945	4.12	15.27	291.99	19.39	9	7/14/2014 28:34.6
13105	RSPe_2	-122.444	37.945	3.89	15.33	291.99	19.22	-122.444	37.945	4.20	15.33	291.99	19.53	9	7/14/2014 28:34.5
13106	RSPe_2	-122.444	37.945	3.89	15.21	292.06	19.09	-122.444	37.945	4.20	15.21	291.99	19.41	9	7/14/2014 28:34.4
13107	RSPe_2	-122.444	37.945	3.89	15.19	292.08	19.07	-122.444	37.945	4.20	15.19	292.01	19.39	9	7/14/2014 28:34.3
13108	RSPe_2	-122.444	37.945	3.89	15.22	292.08	19.11	-122.444	37.945	4.20	15.22	291.99	19.42	9	7/14/2014 28:34.2
13109	RSPe_2	-122.444	37.945	3.94	15.14	292.12	19.08	-122.444	37.945	4.20	15.14	292.03	19.34	9	7/14/2014 28:34.1
13110	RSPe_2	-122.444	37.945	3.89	15.09	292.15	18.98	-122.444	37.945	4.20	15.09	292.08	19.30	9	7/14/2014 28:34.0
13111	RSPe_2	-122.444	37.945	3.89	15.15	292.19	19.04	-122.444	37.945	4.20	15.15	292.10	19.36	9	7/14/2014 28:33.9
13112	RSPe_2	-122.444	37.945	3.89	15.18	292.17	19.07	-122.444	37.945	4.25	15.18	292.12	19.43	9	7/14/2014 28:33.8
13113	RSPe_2	-122.444	37.945	3.89	15.13	292.21	19.01	-122.444	37.945	4.25	15.13	292.15	19.38	9	7/14/2014 28:33.7
13114	RSPe_2	-122.444	37.945	3.89	15.02	292.23	18.91	-122.444	37.945	4.25	15.02	292.17	19.28	9	7/14/2014 28:33.6
13115	RSPe_2	-122.444	37.945	3.94	15.05	292.21	18.99	-122.444	37.945	4.25	15.05	292.17	19.30	9	7/14/2014 28:33.5
13116	RSPe_2	-122.444	37.945	3.89	15.08	292.26	18.97	-122.444	37.945	4.25	15.08	292.23	19.34	9	7/14/2014 28:33.4
13117	RSPe_2	-122.444	37.945	3.94	14.98	292.28	18.92	-122.444	37.945	4.29	14.98	292.25	19.26	9	7/14/2014 28:33.3
13118	RSPe_2	-122.444	37.945	3.89	14.99	292.30	18.88	-122.444	37.945	4.29	14.99	292.27	19.28	9	7/14/2014 28:33.2
13119	RSPe_2	-122.444	37.945	3.89	15.01	292.32	18.90	-122.444	37.945	4.34	15.01	292.34	19.35	9	7/14/2014 28:33.1
13120	RSPe_2	-122.444	37.945	3.89	15.05	292.32	18.93	-122.444	37.945	4.34	15.05	292.39	19.38	9	7/14/2014 28:33.0
13121	RSPe_2	-122.444	37.945	3.94	14.92	292.37	18.86	-122.444	37.945	4.37	14.92	292.41	19.29	9	7/14/2014 28:32.9
13122	RSPe_2	-122.444	37.945	3.94	14.92	292.37	18.86	-122.444	37.945	4.34	14.92	292.47	19.26	9	7/14/2014 28:32.8
13123	RSPe_2	-122.444	37.945	3.94	14.98	292.41	18.92	-122.444	37.945	4.37	14.98	292.47	19.35	9	7/14/2014 28:32.7
13124	RSPe_2	-122.444	37.945	3.89	14.89	292.44	18.78	-122.444	37.945	4.37	14.89	292.50	19.26	9	7/14/2014 28:32.6
13125	RSPe_2	-122.444	37.945	3.94	14.82	292.44	18.76	-122.444	37.945	4.42	14.82	292.50	19.24	9	7/14/2014 28:32.5
13126	RSPe_2	-122.444	37.945	3.94	14.88	292.48	18.82	-122.444	37.945	4.42	14.88	292.48	19.30	9	7/14/2014 28:32.4
13127	RSPe_2	-122.444	37.945	3.94	14.79	292.52	18.73	-122.444	37.945	4.46	14.79	292.52	19.25	9	7/14/2014 28:32.3
13128	RSPe_2	-122.444	37.945	3.94	14.75	292.57	18.69	-122.444	37.945	4.46	14.75	292.50	19.20	9	7/14/2014 28:32.2
13129	RSPe_2	-122.444	37.945	3.94	14.82	292.59	18.76	-122.444	37.945	4.46	14.82	292.52	19.28	9	7/14/2014 28:32.1
13130	RSPe_2	-122.444	37.945	3.94	14.75	292.57	18.69	-122.444	37.945	4.46	14.75	292.45	19.21	9	7/14/2014 28:32.0
13131	RSPe_2	-122.444	37.945	3.94	14.68	292.61	18.62	-122.444	37.945	4.54	14.68	292.50	19.22	9	7/14/2014 28:31.9
13132	RSPe_2	-122.444	37.945	3.94	14.72	292.64	18.66	-122.444	37.945	4.49	14.72	292.48	19.21	9	7/14/2014 28:31.8
13133	RSPe_2	-122.444	37.945	3.94	14.70	292.64	18.64	-122.444	37.945	4.54	14.70	292.50	19.24	9	7/14/2014 28:31.7

13134	RSPe_2	-122.444	37.945	3.94	14.70	292.64	18.64	-122.444	37.945	4.54	14.70	292.52	19.24	9	7/14/2014	28:31.6
13135	RSPe_2	-122.444	37.945	3.94	14.71	292.64	18.65	-122.444	37.945	4.54	14.71	292.52	19.25	9	7/14/2014	28:31.5
13136	RSPe_2	-122.444	37.945	3.94	14.57	292.66	18.51	-122.444	37.945	4.54	14.57	292.57	19.11	9	7/14/2014	28:31.4
13137	RSPe_2	-122.444	37.945	3.94	14.56	292.61	18.50	-122.444	37.945	4.57	14.56	292.50	19.13	9	7/14/2014	28:31.3
13138	RSPe_2	-122.444	37.945	3.94	14.51	292.61	18.45	-122.444	37.945	4.57	14.51	292.52	19.08	9	7/14/2014	28:31.2
13139	RSPe_2	-122.444	37.945	3.97	14.55	292.59	18.53	-122.444	37.945	4.63	14.55	292.54	19.18	9	7/14/2014	28:31.1
13140	RSPe_2	-122.444	37.945	3.94	14.47	292.57	18.41	-122.444	37.945	4.63	14.47	292.52	19.09	9	7/14/2014	28:31.0
13141	RSPe_2	-122.444	37.945	3.97	14.50	292.57	18.47	-122.444	37.945	4.66	14.50	292.55	19.16	9	7/14/2014	28:30.9
13142	RSPe_2	-122.444	37.945	3.94	14.46	292.59	18.40	-122.444	37.945	4.66	14.46	292.55	19.12	9	7/14/2014	28:30.8
13143	RSPe_2	-122.444	37.945	3.94	14.46	292.60	18.40	-122.444	37.945	4.71	14.46	292.61	19.17	9	7/14/2014	28:30.7
13144	RSPe_2	-122.444	37.945	3.94	14.45	292.60	18.39	-122.444	37.945	4.66	14.45	292.59	19.11	9	7/14/2014	28:30.6
13145	RSPe_2	-122.444	37.945	3.94	14.45	292.62	18.39	-122.444	37.945	4.66	14.45	292.64	19.11	9	7/14/2014	28:30.5
13146	RSPe_2	-122.444	37.945	3.94	14.43	292.64	18.37	-122.444	37.945	4.66	14.43	292.65	19.09	9	7/14/2014	28:30.4
13147	RSPe_2	-122.444	37.945	3.94	14.41	292.66	18.35	-122.444	37.945	4.71	14.41	292.68	19.12	9	7/14/2014	28:30.3
13148	RSPe_2	-122.444	37.945	3.94	14.42	292.68	18.36	-122.444	37.945	4.66	14.42	292.70	19.08	9	7/14/2014	28:30.2
13149	RSPe_2	-122.444	37.945	3.94	14.43	292.70	18.37	-122.444	37.945	4.66	14.43	292.70	19.09	9	7/14/2014	28:30.1
13150	RSPe_2	-122.444	37.945	3.94	14.41	292.73	18.35	-122.444	37.945	4.66	14.41	292.76	19.07	9	7/14/2014	28:30.0
13151	RSPe_2	-122.444	37.945	3.94	14.40	292.73	18.34	-122.444	37.945	4.71	14.40	292.72	19.11	9	7/14/2014	28:29.9
13152	RSPe_2	-122.444	37.945	3.94	14.38	292.75	18.32	-122.444	37.945	4.71	14.38	292.80	19.09	9	7/14/2014	28:29.8
13153	RSPe_2	-122.444	37.945	3.94	14.41	292.75	18.35	-122.444	37.945	4.71	14.41	292.81	19.12	9	7/14/2014	28:29.7
13154	RSPe_2	-122.444	37.945	3.94	14.42	292.79	18.36	-122.444	37.945	4.71	14.42	292.81	19.13	9	7/14/2014	28:29.6
13155	RSPe_2	-122.444	37.945	3.97	14.40	292.82	18.37	-122.444	37.945	4.71	14.40	292.88	19.11	9	7/14/2014	28:29.5
13156	RSPe_2	-122.444	37.945	3.94	14.38	292.90	18.32	-122.444	37.945	4.71	14.38	292.92	19.09	9	7/14/2014	28:29.4
13157	RSPe_2	-122.444	37.945	3.94	14.39	292.90	18.33	-122.444	37.945	4.71	14.39	292.96	19.10	9	7/14/2014	28:29.3
13158	RSPe_2	-122.444	37.945	3.94	14.41	292.97	18.35	-122.444	37.945	4.71	14.41	292.97	19.12	9	7/14/2014	28:29.2
13159	RSPe_2	-122.444	37.945	3.94	14.38	293.02	18.32	-122.444	37.945	4.74	14.38	293.03	19.13	9	7/14/2014	28:29.1
13160	RSPe_2	-122.444	37.945	3.94	14.42	293.02	18.36	-122.444	37.945	4.71	14.42	293.06	19.13	9	7/14/2014	28:29.0
13161	RSPe_2	-122.444	37.945	3.94	14.36	293.04	18.30	-122.444	37.945	4.74	14.36	293.08	19.10	9	7/14/2014	28:28.9
13162	RSPe_2	-122.444	37.945	3.94	14.38	293.04	18.32	-122.444	37.945	4.71	14.38	293.08	19.09	9	7/14/2014	28:28.8
13163	RSPe_2	-122.444	37.945	3.94	14.38	293.11	18.32	-122.444	37.945	4.74	14.38	293.12	19.13	9	7/14/2014	28:28.7
13164	RSPe_2	-122.444	37.945	3.94	14.38	293.10	18.32	-122.444	37.945	4.74	14.38	293.17	19.13	9	7/14/2014	28:28.6
13165	RSPe_2	-122.444	37.945	3.94	14.42	293.15	18.36	-122.444	37.945	4.74	14.42	293.21	19.16	9	7/14/2014	28:28.5
13166	RSPe_2	-122.444	37.945	3.94	14.41	293.13	18.35	-122.444	37.945	4.71	14.41	293.19	19.12	9	7/14/2014	28:28.4

13167	RSPe_2	-122.444	37.945	3.94	14.50	293.15	18.44	-122.444	37.945	4.74	14.50	293.21	19.24	9	7/14/2014	28:28.3
13168	RSPe_2	-122.444	37.945	3.94	14.45	293.15	18.39	-122.444	37.945	4.71	14.45	293.21	19.16	9	7/14/2014	28:28.2
13169	RSPe_2	-122.444	37.945	3.94	14.42	293.19	18.36	-122.444	37.945	4.71	14.42	293.24	19.13	9	7/14/2014	28:28.1
13170	RSPe_2	-122.444	37.945	3.94	14.46	293.22	18.40	-122.444	37.945	4.71	14.46	293.25	19.17	9	7/14/2014	28:28.0
13171	RSPe_2	-122.444	37.945	3.94	14.48	293.19	18.42	-122.444	37.945	4.74	14.48	293.25	19.22	9	7/14/2014	28:27.9
13172	RSPe_2	-122.444	37.945	3.94	14.47	293.21	18.41	-122.444	37.945	4.74	14.47	293.21	19.21	9	7/14/2014	28:27.8
13173	RSPe_2	-122.444	37.945	3.94	14.46	293.19	18.40	-122.444	37.945	4.79	14.46	293.27	19.26	9	7/14/2014	28:27.7
13174	RSPe_2	-122.444	37.945	3.89	14.52	293.19	18.41	-122.444	37.945	4.74	14.52	293.27	19.27	9	7/14/2014	28:27.6
13175	RSPe_2	-122.444	37.945	3.94	14.49	293.17	18.43	-122.444	37.945	4.79	14.49	293.23	19.28	9	7/14/2014	28:27.5
[truncated: 2,495,480 more chars]
