# Supplementary material for: Chinook salmon and green sturgeon migrate through San Francisco Estuary despite large distortions in the local magnetic field produced by bridges
Source: PLoS One. 2017 Jun 2;12(6):e0169031. doi: 10.1371/journal.pone.0169031 (PMC5456031; doi:10.1371/journal.pone.0169031)
Supplement: S4 Fig — (PDF) [file pone.0169031.s004.pdf]

Record	Profile	#Longitude1	Latitude1	Z1	ALT1	T1	Alt+Depth1	Longitude2	Latitude2	Z2	ALT2	T2	Alt+Depth2	LINE	DATE	TIME
1	RWS11	-122.4510719	37.93504	-0.52	22.4	-799.2	21.8880007	-122.45106	37.93505	-0.41	22.4	-805.49	21.9980007	11	7/24/2014	17:18.0
2	RWS11	-122.4510743	37.93504	-0.49	22.4	-800.7	21.9090002	-122.45107	37.93505	-0.41	22.4	-807.2	21.9850002	11	7/24/2014	17:18.1
3	RWS11	-122.4510767	37.93504	-0.49	22.4	-802.1	21.8880003	-122.45107	37.93505	-0.41	22.4	-808.79	21.9640003	11	7/24/2014	17:18.2
4	RWS11	-122.451079	37.93504	-0.49	22.3	-803.3	21.8560007	-122.45107	37.93505	-0.41	22.3	-810.24	21.9320007	11	7/24/2014	17:18.3
5	RWS11	-122.4510814	37.93504	-0.52	22.3	-804.3	21.7780001	-122.45107	37.93505	-0.41	22.3	-811.68	21.888	11	7/24/2014	17:18.4
6	RWS11	-122.4510839	37.93504	-0.49	22.2	-805.3	21.753	-122.45108	37.93505	-0.41	22.2	-812.98	21.829	11	7/24/2014	17:18.5
7	RWS11	-122.4510863	37.93504	-0.49	22.2	-806.3	21.6800001	-122.45108	37.93506	-0.41	22.2	-814.35	21.7560001	11	7/24/2014	17:18.6
8	RWS11	-122.4510887	37.93504	-0.49	22.1	-807.3	21.5930002	-122.45108	37.93506	-0.36	22.1	-815.61	21.7190002	11	7/24/2014	17:18.7
9	RWS11	-122.4510911	37.93505	-0.49	22	-808.4	21.4920003	-122.45108	37.93506	-0.41	22	-817.05	21.5680004	11	7/24/2014	17:18.8
10	RWS11	-122.4510935	37.93505	-0.49	21.9	-809.4	21.3800008	-122.45109	37.93506	-0.36	21.9	-818.38	21.5060009	11	7/24/2014	17:18.9
11	RWS11	-122.4510971	37.93505	-0.49	21.7	-810.2	21.2579992	-122.45109	37.93506	-0.41	21.7	-819.65	21.3339992	11	7/24/2014	17:19.0
12	RWS11	-122.4510995	37.93505	-0.49	21.6	-811	21.1289995	-122.45109	37.93506	-0.41	21.6	-820.74	21.2049995	11	7/24/2014	17:19.1
13	RWS11	-122.4511019	37.93505	-0.52	21.5	-811.8	20.9599993	-122.45109	37.93506	-0.41	21.5	-821.78	21.0699993	11	7/24/2014	17:19.2
14	RWS11	-122.4511043	37.93505	-0.49	21.3	-812.4	20.8549993	-122.4511	37.93506	-0.36	21.3	-822.59	20.9809993	11	7/24/2014	17:19.3
15	RWS11	-122.4511067	37.93505	-0.52	21.2	-813	20.6800005	-122.4511	37.93506	-0.41	21.2	-823.26	20.7900005	11	7/24/2014	17:19.4
16	RWS11	-122.4511092	37.93505	-0.49	21.1	-813.6	20.5699995	-122.4511	37.93506	-0.41	21.1	-823.8	20.6459995	11	7/24/2014	17:19.5
17	RWS11	-122.4511116	37.93505	-0.52	20.9	-814.1	20.3929998	-122.4511	37.93507	-0.41	20.9	-824.18	20.5029998	11	7/24/2014	17:19.6
18	RWS11	-122.4511141	37.93505	-0.49	20.8	-814.4	20.2870004	-122.45111	37.93507	-0.36	20.8	-824.49	20.4130004	11	7/24/2014	17:19.7
19	RWS11	-122.4511165	37.93505	-0.49	20.6	-814.6	20.1539991	-122.45111	37.93507	-0.41	20.6	-824.71	20.2299991	11	7/24/2014	17:19.8
20	RWS11	-122.4511189	37.93506	-0.49	20.5	-814.8	20.0289991	-122.45111	37.93507	-0.41	20.5	-824.87	20.1049991	11	7/24/2014	17:19.9
21	RWS11	-122.4511225	37.93506	-0.52	20.4	-814.8	19.8780004	-122.45112	37.93507	-0.41	20.4	-824.96	19.9880004	11	7/24/2014	17:20.0
22	RWS11	-122.4511249	37.93506	-0.52	20.3	-814.7	19.7710001	-122.45112	37.93507	-0.36	20.3	-824.94	19.9310001	11	7/24/2014	17:20.1
23	RWS11	-122.4511274	37.93506	-0.52	20.2	-814.5	19.6730005	-122.45112	37.93507	-0.45	20.2	-824.79	19.7490005	11	7/24/2014	17:20.2
24	RWS11	-122.4511298	37.93506	-0.49	20.1	-814.3	19.6189992	-122.45112	37.93507	-0.41	20.1	-824.47	19.6949993	11	7/24/2014	17:20.3
25	RWS11	-122.4511322	37.93506	-0.52	20	-814.3	19.5049993	-122.45113	37.93507	-0.41	20	-824.25	19.6149993	11	7/24/2014	17:20.4
26	RWS11	-122.4511347	37.93506	-0.49	20	-814.2	19.4670007	-122.45113	37.93507	-0.41	20	-824.06	19.5430007	11	7/24/2014	17:20.5
27	RWS11	-122.4511372	37.93506	-0.52	19.9	-814.2	19.365	-122.45113	37.93507	-0.41	19.9	-823.98	19.4749999	11	7/24/2014	17:20.6
28	RWS11	-122.4511396	37.93506	-0.52	19.8	-814.3	19.3029997	-122.45113	37.93508	-0.41	19.8	-823.99	19.4129997	11	7/24/2014	17:20.7
29	RWS11	-122.451142	37.93506	-0.52	19.8	-814.5	19.2439997	-122.45113	37.93508	-0.45	19.8	-824.04	19.3199996	11	7/24/2014	17:20.8
30	RWS11	-122.4511444	37.93507	-0.52	19.7	-814.7	19.1879999	-122.45114	37.93508	-0.36	19.7	-824.17	19.3479999	11	7/24/2014	17:20.9
31	RWS11	-122.451148	37.93507	-0.52	19.7	-814.8	19.1369993	-122.45114	37.93508	-0.41	19.7	-824.28	19.2469993	11	7/24/2014	17:21.0
32	RWS11	-122.4511504	37.93507	-0.52	19.6	-814.7	19.0890009	-122.45114	37.93508	-0.41	19.6	-824.25	19.1990009	11	7/24/2014	17:21.1

33	RWS11	-122.4511528	37.93507	-0.52	19.6	-814.5	19.0440008	-122.45115	37.93508	-0.41	19.6	-824.11	19.1540008	11	7/24/2014	17:21.2
34	RWS11	-122.4511552	37.93507	-0.52	19.5	-814.1	19.0019991	-122.45115	37.93508	-0.41	19.5	-823.83	19.1119991	11	7/24/2014	17:21.3
35	RWS11	-122.4511576	37.93507	-0.52	19.5	-813.7	18.9640009	-122.45115	37.93508	-0.41	19.5	-823.34	19.0740009	11	7/24/2014	17:21.4
36	RWS11	-122.4511601	37.93507	-0.52	19.5	-813.1	18.9279997	-122.45115	37.93508	-0.45	19.5	-822.84	19.0039997	11	7/24/2014	17:21.5
37	RWS11	-122.4511625	37.93507	-0.57	19.4	-812.5	18.8459995	-122.45116	37.93508	-0.45	19.4	-822.22	18.9729995	11	7/24/2014	17:21.6
38	RWS11	-122.4511649	37.93507	-0.52	19.4	-812.2	18.8689997	-122.45116	37.93508	-0.41	19.4	-821.81	18.9789996	11	7/24/2014	17:21.7
39	RWS11	-122.4511673	37.93507	-0.52	19.4	-812	18.8460009	-122.45116	37.93509	-0.45	19.4	-821.47	18.9220008	11	7/24/2014	17:21.8
40	RWS11	-122.4511696	37.93507	-0.52	19.4	-811.9	18.8260004	-122.45116	37.93509	-0.45	19.4	-821.29	18.9020004	11	7/24/2014	17:21.9
41	RWS11	-122.4511732	37.93508	-0.52	19.3	-811.8	18.8090002	-122.45117	37.93509	-0.45	19.3	-821.08	18.8850002	11	7/24/2014	17:22.0
42	RWS11	-122.4511755	37.93508	-0.52	19.3	-811.4	18.7950003	-122.45117	37.93509	-0.41	19.3	-820.73	18.9050002	11	7/24/2014	17:22.1
43	RWS11	-122.4511779	37.93508	-0.52	19.3	-810.9	18.7829992	-122.45117	37.93509	-0.41	19.3	-820.18	18.8929992	11	7/24/2014	17:22.2
44	RWS11	-122.4511802	37.93508	-0.52	19.3	-810.4	18.7730009	-122.45117	37.93509	-0.41	19.3	-819.49	18.8830009	11	7/24/2014	17:22.3
45	RWS11	-122.4511826	37.93508	-0.52	19.3	-809.8	18.7660009	-122.45118	37.93509	-0.41	19.3	-818.6	18.8760009	11	7/24/2014	17:22.4
46	RWS11	-122.451185	37.93508	-0.52	19.3	-809.2	18.7609999	-122.45118	37.93509	-0.41	19.3	-817.6	18.8709998	11	7/24/2014	17:22.5
47	RWS11	-122.4511874	37.93508	-0.52	19.3	-808.5	18.7579996	-122.45118	37.93509	-0.41	19.3	-816.52	18.8679996	11	7/24/2014	17:22.6
48	RWS11	-122.4511897	37.93508	-0.52	19.3	-807.6	18.7560007	-122.45118	37.93509	-0.36	19.3	-815.32	18.9160007	11	7/24/2014	17:22.7
49	RWS11	-122.4511921	37.93508	-0.52	19.3	-806.7	18.7539999	-122.45119	37.93509	-0.41	19.3	-814.05	18.8639999	11	7/24/2014	17:22.8
50	RWS11	-122.4511944	37.93508	-0.49	19.3	-805.7	18.7819994	-122.45119	37.9351	-0.41	19.3	-812.72	18.8579994	11	7/24/2014	17:22.9
51	RWS11	-122.4511979	37.93508	-0.52	19.3	-804.8	18.7369997	-122.45119	37.9351	-0.41	19.3	-811.37	18.8469997	11	7/24/2014	17:23.0
52	RWS11	-122.4512003	37.93509	-0.49	19.2	-803.7	18.753	-122.45119	37.9351	-0.41	19.2	-810.03	18.829	11	7/24/2014	17:23.1
53	RWS11	-122.4512026	37.93509	-0.52	19.2	-802.1	18.6929991	-122.4512	37.9351	-0.36	19.2	-808.7	18.8529991	11	7/24/2014	17:23.2
54	RWS11	-122.451205	37.93509	-0.49	19.2	-800.5	18.6919992	-122.4512	37.9351	-0.41	19.2	-807.31	18.7679992	11	7/24/2014	17:23.3
55	RWS11	-122.4512074	37.93509	-0.52	19.1	-798.9	18.6170008	-122.4512	37.9351	-0.36	19.1	-805.69	18.7770008	11	7/24/2014	17:23.4
56	RWS11	-122.4512098	37.93509	-0.49	19.1	-797.1	18.6049993	-122.4512	37.9351	-0.36	19.1	-803.91	18.7309993	11	7/24/2014	17:23.5
57	RWS11	-122.4512121	37.93509	-0.52	19	-795.4	18.5200006	-122.45121	37.9351	-0.36	19	-802.01	18.6800006	11	7/24/2014	17:23.6
58	RWS11	-122.4512145	37.93509	-0.49	19	-793.7	18.4999998	-122.45121	37.9351	-0.36	19	-800.02	18.6259998	11	7/24/2014	17:23.7
59	RWS11	-122.4512169	37.93509	-0.52	18.9	-792.1	18.4090006	-122.45121	37.9351	-0.36	18.9	-798.28	18.5690006	11	7/24/2014	17:23.8
60	RWS11	-122.4512193	37.93509	-0.49	18.9	-790.3	18.385	-122.45121	37.9351	-0.36	18.9	-796.45	18.511	11	7/24/2014	17:23.9
61	RWS11	-122.4512228	37.93509	-0.49	18.8	-788.6	18.3280008	-122.45122	37.93511	-0.36	18.8	-794.54	18.4540008	11	7/24/2014	17:24.0
62	RWS11	-122.4512251	37.93509	-0.49	18.8	-786.9	18.2700002	-122.45122	37.93511	-0.41	18.8	-792.63	18.3460002	11	7/24/2014	17:24.1
63	RWS11	-122.4512275	37.93509	-0.52	18.7	-785.3	18.1800005	-122.45122	37.93511	-0.36	18.7	-790.6	18.3400005	11	7/24/2014	17:24.2
64	RWS11	-122.4512299	37.9351	-0.49	18.6	-783.6	18.1590002	-122.45122	37.93511	-0.36	18.6	-788.53	18.2850002	11	7/24/2014	17:24.3
65	RWS11	-122.4512323	37.9351	-0.52	18.6	-781.7	18.0730001	-122.45123	37.93511	-0.36	18.6	-786.4	18.2330001	11	7/24/2014	17:24.4

66	RWS11	-122.4512347	37.9351	-0.49	18.5	-779.6	18.0559995	-122.45123	37.93511	-0.36	18.5	-784.06	18.1819995	11	7/24/2014	17:24.5
67	RWS11	-122.4512371	37.9351	-0.52	18.5	-777.3	17.9720003	-122.45123	37.93511	-0.41	18.5	-781.48	18.0820003	11	7/24/2014	17:24.6
68	RWS11	-122.4512394	37.9351	-0.49	18.4	-775	17.9570005	-122.45123	37.93511	-0.36	18.4	-778.68	18.0830005	11	7/24/2014	17:24.7
69	RWS11	-122.4512418	37.9351	-0.52	18.4	-772.4	17.8729994	-122.45124	37.93511	-0.45	18.4	-775.57	17.9489994	11	7/24/2014	17:24.8
70	RWS11	-122.4512441	37.9351	-0.49	18.3	-769.5	17.8579996	-122.45124	37.93511	-0.41	18.3	-772.11	17.9339996	11	7/24/2014	17:24.9
71	RWS11	-122.4512476	37.9351	-0.52	18.3	-766.5	17.7749998	-122.45124	37.93511	-0.36	18.3	-768.41	17.9349998	11	7/24/2014	17:25.0
72	RWS11	-122.4512499	37.9351	-0.52	18.3	-763.1	17.7269995	-122.45124	37.93511	-0.41	18.3	-764.65	17.8369995	11	7/24/2014	17:25.1
73	RWS11	-122.4512522	37.9351	-0.52	18.2	-759.8	17.6779997	-122.45125	37.93512	-0.45	18.2	-760.66	17.7539997	11	7/24/2014	17:25.2
74	RWS11	-122.4512545	37.9351	-0.52	18.2	-756	17.6280004	-122.45125	37.93512	-0.41	18.2	-756.57	17.7380004	11	7/24/2014	17:25.3
75	RWS11	-122.4512569	37.9351	-0.52	18.1	-752.2	17.5779993	-122.45125	37.93512	-0.45	18.1	-752.49	17.6539993	11	7/24/2014	17:25.4
76	RWS11	-122.4512592	37.93511	-0.52	18	-748.5	17.5259993	-122.45125	37.93512	-0.41	18	-748.47	17.6359992	11	7/24/2014	17:25.5
77	RWS11	-122.4512615	37.93511	-0.52	18	-744.8	17.4729998	-122.45126	37.93512	-0.45	18	-744.69	17.5489997	11	7/24/2014	17:25.6
78	RWS11	-122.4512639	37.93511	-0.52	17.9	-741	17.4190008	-122.45126	37.93512	-0.45	17.9	-740.82	17.4950008	11	7/24/2014	17:25.7
79	RWS11	-122.4512662	37.93511	-0.57	17.9	-737.4	17.3130005	-122.45126	37.93512	-0.5	17.9	-736.76	17.3890005	11	7/24/2014	17:25.8
80	RWS11	-122.4512685	37.93511	-0.57	17.8	-733.6	17.2570007	-122.45126	37.93512	-0.45	17.8	-732.44	17.3840007	11	7/24/2014	17:25.9
81	RWS11	-122.451272	37.93511	-0.57	17.8	-729.7	17.2009991	-122.45127	37.93512	-0.5	17.8	-727.91	17.2769991	11	7/24/2014	17:26.0
82	RWS11	-122.4512744	37.93511	-0.57	17.7	-725.5	17.1460007	-122.45127	37.93512	-0.5	17.7	-723.24	17.2220007	11	7/24/2014	17:26.1
83	RWS11	-122.4512768	37.93511	-0.57	17.7	-721	17.0919998	-122.45127	37.93512	-0.5	17.7	-718.43	17.1679998	11	7/24/2014	17:26.2
84	RWS11	-122.4512792	37.93511	-0.57	17.6	-716.4	17.0390009	-122.45127	37.93512	-0.5	17.6	-713.4	17.1150009	11	7/24/2014	17:26.3
85	RWS11	-122.4512817	37.93511	-0.57	17.6	-711.7	16.9870008	-122.45128	37.93513	-0.53	17.6	-708.2	17.0290008	11	7/24/2014	17:26.4
86	RWS11	-122.4512842	37.93511	-0.57	17.5	-706.9	16.9339994	-122.45128	37.93513	-0.53	17.5	-703.1	16.9759994	11	7/24/2014	17:26.5
87	RWS11	-122.4512866	37.93511	-0.61	17.5	-702.2	16.8459999	-122.45128	37.93513	-0.53	17.5	-697.86	16.9229999	11	7/24/2014	17:26.6
88	RWS11	-122.4512891	37.93511	-0.57	17.4	-697.5	16.8280004	-122.45128	37.93513	-0.5	17.4	-692.25	16.9040004	11	7/24/2014	17:26.7
89	RWS11	-122.4512915	37.93512	-0.61	17.3	-692.6	16.7389996	-122.45129	37.93513	-0.53	17.3	-686.36	16.8159996	11	7/24/2014	17:26.8
90	RWS11	-122.451294	37.93512	-0.57	17.3	-687.7	16.7210001	-122.45129	37.93513	-0.53	17.3	-680.39	16.7630001	11	7/24/2014	17:26.9
91	RWS11	-122.4512976	37.93512	-0.61	17.2	-682.7	16.634	-122.45129	37.93513	-0.58	17.2	-674.5	16.66	11	7/24/2014	17:27.0
92	RWS11	-122.4513	37.93512	-0.61	17.2	-677.5	16.5840008	-122.45129	37.93513	-0.53	17.2	-668.82	16.6610008	11	7/24/2014	17:27.1
93	RWS11	-122.4513025	37.93512	-0.61	17.1	-672.3	16.5349991	-122.4513	37.93513	-0.58	17.1	-662.94	16.5609991	11	7/24/2014	17:27.2
94	RWS11	-122.4513049	37.93512	-0.61	17.1	-667	16.4880001	-122.4513	37.93513	-0.53	17.1	-656.73	16.5650001	11	7/24/2014	17:27.3
95	RWS11	-122.4513074	37.93512	-0.66	17.1	-661.6	16.3929995	-122.4513	37.93513	-0.58	17.1	-650.43	16.4699995	11	7/24/2014	17:27.4
96	RWS11	-122.4513099	37.93512	-0.61	17	-656.2	16.4019997	-122.4513	37.93513	-0.58	17	-643.9	16.4279997	11	7/24/2014	17:27.5
97	RWS11	-122.4513123	37.93512	-0.66	17	-650.6	16.3110007	-122.45131	37.93513	-0.58	17	-636.96	16.3880007	11	7/24/2014	17:27.6
98	RWS11	-122.4513148	37.93512	-0.61	16.9	-645.2	16.3240005	-122.45131	37.93514	-0.58	16.9	-629.77	16.3500006	11	7/24/2014	17:27.7

99	RWS11	-122.4513172	37.93512	-0.66	16.9	-639.5	16.2359999	-122.45131	37.93514	-0.62	16.9	-622.23	16.2799999	11	7/24/2014	17:27.8
100	RWS11	-122.4513196	37.93512	-0.61	16.9	-633.6	16.2499992	-122.45131	37.93514	-0.62	16.9	-614.62	16.2429993	11	7/24/2014	17:27.9
101	RWS11	-122.4513232	37.93513	-0.66	16.8	-627.8	16.1620005	-122.45132	37.93514	-0.67	16.8	-607.13	16.1550005	11	7/24/2014	17:28.0
102	RWS11	-122.4513256	37.93513	-0.66	16.8	-622	16.1240004	-122.45132	37.93514	-0.62	16.8	-599.88	16.1680004	11	7/24/2014	17:28.1
103	RWS11	-122.451328	37.93513	-0.66	16.7	-616.3	16.0860003	-122.45132	37.93514	-0.67	16.7	-592.72	16.0790003	11	7/24/2014	17:28.2
104	RWS11	-122.4513304	37.93513	-0.66	16.7	-610.5	16.0459994	-122.45132	37.93514	-0.67	16.7	-585.16	16.0389994	11	7/24/2014	17:28.3
105	RWS11	-122.4513328	37.93513	-0.66	16.7	-604.5	16.0050009	-122.45133	37.93514	-0.7	16.7	-577.47	15.9640009	11	7/24/2014	17:28.4
106	RWS11	-122.4513353	37.93513	-0.66	16.6	-598.6	15.9619997	-122.45133	37.93514	-0.62	16.6	-569.94	16.0059997	11	7/24/2014	17:28.5
107	RWS11	-122.4513377	37.93513	-0.66	16.6	-592.7	15.9179991	-122.45133	37.93514	-0.7	16.6	-562.46	15.8769991	11	7/24/2014	17:28.6
108	RWS11	-122.4513401	37.93513	-0.66	16.5	-586.8	15.8730009	-122.45133	37.93514	-0.67	16.5	-555.4	15.866001	11	7/24/2014	17:28.7
109	RWS11	-122.4513425	37.93513	-0.66	16.5	-581.1	15.8280008	-122.45134	37.93514	-0.7	16.5	-548.5	15.7870009	11	7/24/2014	17:28.8
110	RWS11	-122.451345	37.93513	-0.61	16.4	-575.1	15.8329994	-122.45134	37.93514	-0.7	16.4	-541.52	15.7409995	11	7/24/2014	17:28.9
111	RWS11	-122.4513485	37.93513	-0.66	16.4	-569.1	15.7369993	-122.45134	37.93515	-0.7	16.4	-534.52	15.6959994	11	7/24/2014	17:29.0
112	RWS11	-122.451351	37.93513	-0.61	16.4	-562.9	15.7429993	-122.45135	37.93515	-0.7	16.4	-527.43	15.6509993	11	7/24/2014	17:29.1
113	RWS11	-122.4513534	37.93513	-0.66	16.3	-556.6	15.6480005	-122.45135	37.93515	-0.74	16.3	-520.14	15.5730006	11	7/24/2014	17:29.2
114	RWS11	-122.4513558	37.93514	-0.66	16.3	-550.2	15.6039999	-122.45135	37.93515	-0.67	16.3	-512.59	15.5969999	11	7/24/2014	17:29.3
115	RWS11	-122.4513582	37.93514	-0.66	16.2	-543.5	15.5620001	-122.45135	37.93515	-0.7	16.2	-504.57	15.5210002	11	7/24/2014	17:29.4
116	RWS11	-122.4513607	37.93514	-0.66	16.2	-536.5	15.5190008	-122.45135	37.93515	-0.67	16.2	-496.35	15.5120009	11	7/24/2014	17:29.5
117	RWS11	-122.4513632	37.93514	-0.66	16.1	-529.5	15.4750002	-122.45136	37.93515	-0.7	16.1	-488.11	15.4340003	11	7/24/2014	17:29.6
118	RWS11	-122.4513656	37.93514	-0.66	16.1	-522.4	15.4290007	-122.45136	37.93515	-0.67	16.1	-479.98	15.4220007	11	7/24/2014	17:29.7
119	RWS11	-122.451368	37.93514	-0.66	16	-515.3	15.3819998	-122.45136	37.93515	-0.7	16	-472	15.3409998	11	7/24/2014	17:29.8
120	RWS11	-122.4513705	37.93514	-0.66	16	-508.1	15.3340004	-122.45136	37.93515	-0.67	16	-463.94	15.3270004	11	7/24/2014	17:29.9
121	RWS11	-122.4513741	37.93514	-0.66	15.9	-500.9	15.2849997	-122.45137	37.93515	-0.67	15.9	-455.96	15.2779997	11	7/24/2014	17:30.0
122	RWS11	-122.4513765	37.93514	-0.66	15.9	-493.6	15.2359999	-122.45137	37.93515	-0.67	15.9	-447.96	15.2289999	11	7/24/2014	17:30.1
123	RWS11	-122.4513789	37.93514	-0.66	15.8	-486.1	15.1879996	-122.45137	37.93515	-0.67	15.8	-439.76	15.1809996	11	7/24/2014	17:30.2
124	RWS11	-122.4513814	37.93514	-0.61	15.8	-478.3	15.1919996	-122.45138	37.93516	-0.67	15.8	-431.09	15.1339997	11	7/24/2014	17:30.3
125	RWS11	-122.4513839	37.93514	-0.66	15.8	-470.5	15.0950001	-122.45138	37.93516	-0.67	15.8	-421.91	15.0880001	11	7/24/2014	17:30.4
126	RWS11	-122.4513863	37.93514	-0.66	15.7	-462.4	15.0519999	-122.45138	37.93516	-0.67	15.7	-412.41	15.0449999	11	7/24/2014	17:30.5
127	RWS11	-122.4513888	37.93514	-0.66	15.7	-454.2	15.0100001	-122.45138	37.93516	-0.62	15.7	-402.4	15.0540001	11	7/24/2014	17:30.6
128	RWS11	-122.4513913	37.93515	-0.61	15.6	-445.7	15.0199997	-122.45139	37.93516	-0.67	15.6	-392.25	14.9619997	11	7/24/2014	17:30.7
129	RWS11	-122.4513938	37.93515	-0.61	15.6	-437.2	14.9799997	-122.45139	37.93516	-0.67	15.6	-382.11	14.9219998	11	7/24/2014	17:30.8
130	RWS11	-122.4513962	37.93515	-0.61	15.6	-428.6	14.9410002	-122.45139	37.93516	-0.62	15.6	-372.13	14.9340002	11	7/24/2014	17:30.9
131	RWS11	-122.4513999	37.93515	-0.61	15.5	-419.8	14.9030001	-122.45139	37.93516	-0.62	15.5	-362.3	14.8960001	11	7/24/2014	17:31.0

132	RWS11	-122.4514023	37.93515	-0.61	15.5	-411	14.8639996	-122.4514	37.93516	-0.62	15.5	-352.62	14.8569996	11	7/24/2014	17:31.1
133	RWS11	-122.4514048	37.93515	-0.61	15.4	-402.3	14.8269998	-122.4514	37.93516	-0.67	15.4	-343.03	14.7689999	11	7/24/2014	17:31.2
134	RWS11	-122.4514073	37.93515	-0.61	15.4	-393.7	14.7909996	-122.4514	37.93516	-0.58	15.4	-333.59	14.8169996	11	7/24/2014	17:31.3
135	RWS11	-122.4514098	37.93515	-0.61	15.4	-385.2	14.7559997	-122.4514	37.93516	-0.62	15.4	-324.15	14.7489998	11	7/24/2014	17:31.4
136	RWS11	-122.4514123	37.93515	-0.57	15.3	-377.1	14.7579998	-122.45141	37.93516	-0.58	15.3	-314.75	14.7489998	11	7/24/2014	17:31.5
137	RWS11	-122.4514148	37.93515	-0.61	15.3	-369.1	14.6919996	-122.45141	37.93516	-0.58	15.3	-305.42	14.7179996	11	7/24/2014	17:31.6
138	RWS11	-122.4514173	37.93515	-0.57	15.3	-361.2	14.6980003	-122.45141	37.93517	-0.53	15.3	-295.94	14.7400003	11	7/24/2014	17:31.7
139	RWS11	-122.4514198	37.93515	-0.57	15.2	-353.2	14.6709999	-122.45141	37.93517	-0.58	15.2	-286.26	14.6619999	11	7/24/2014	17:31.8
140	RWS11	-122.4514223	37.93515	-0.57	15.2	-345.1	14.6449999	-122.45142	37.93517	-0.53	15.2	-276.47	14.6869999	11	7/24/2014	17:31.9
141	RWS11	-122.451426	37.93516	-0.57	15.2	-336.7	14.6209997	-122.45142	37.93517	-0.53	15.2	-266.52	14.6629997	11	7/24/2014	17:32.0
142	RWS11	-122.4514285	37.93516	-0.52	15.2	-327.9	14.6489999	-122.45142	37.93517	-0.5	15.2	-256.23	14.6739999	11	7/24/2014	17:32.1
143	RWS11	-122.451431	37.93516	-0.57	15.1	-318.7	14.5759996	-122.45143	37.93517	-0.5	15.1	-245.74	14.6519996	11	7/24/2014	17:32.2
144	RWS11	-122.4514336	37.93516	-0.52	15.1	-309.2	14.6079996	-122.45143	37.93517	-0.5	15.1	-235.01	14.6329996	11	7/24/2014	17:32.3
145	RWS11	-122.4514361	37.93516	-0.52	15.1	-299.6	14.5919998	-122.45143	37.93517	-0.5	15.1	-224.24	14.6169998	11	7/24/2014	17:32.4
146	RWS11	-122.4514386	37.93516	-0.52	15.1	-290.1	14.5810001	-122.45143	37.93517	-0.5	15.1	-213.35	14.6060001	11	7/24/2014	17:32.5
147	RWS11	-122.4514412	37.93516	-0.52	15.1	-280.7	14.5740001	-122.45144	37.93517	-0.45	15.1	-202.56	14.6500001	11	7/24/2014	17:32.6
148	RWS11	-122.4514437	37.93516	-0.49	15.1	-271.3	14.6069997	-122.45144	37.93517	-0.45	15.1	-191.56	14.6489997	11	7/24/2014	17:32.7
149	RWS11	-122.4514462	37.93516	-0.52	15.1	-262.1	14.5779998	-122.45144	37.93517	-0.5	15.1	-180.38	14.6029998	11	7/24/2014	17:32.8
150	RWS11	-122.4514487	37.93516	-0.49	15.1	-252.8	14.6220001	-122.45144	37.93517	-0.45	15.1	-169.14	14.6640001	11	7/24/2014	17:32.9
151	RWS11	-122.4514524	37.93516	-0.52	15.1	-243.3	14.6039999	-122.45145	37.93518	-0.5	15.1	-157.74	14.6289999	11	7/24/2014	17:33.0
152	RWS11	-122.4514549	37.93516	-0.49	15.1	-233.6	14.6600002	-122.45145	37.93518	-0.45	15.1	-146.2	14.7020002	11	7/24/2014	17:33.1
153	RWS11	-122.4514573	37.93516	-0.52	15.2	-223.7	14.6520002	-122.45145	37.93518	-0.5	15.2	-134.6	14.6770002	11	7/24/2014	17:33.2
154	RWS11	-122.4514598	37.93516	-0.49	15.2	-213.6	14.7179998	-122.45145	37.93518	-0.45	15.2	-122.7	14.7599998	11	7/24/2014	17:33.3
155	RWS11	-122.4514623	37.93517	-0.49	15.2	-203.3	14.754	-122.45146	37.93518	-0.45	15.2	-110.53	14.796	11	7/24/2014	17:33.4
156	RWS11	-122.4514648	37.93517	-0.49	15.3	-193	14.7929996	-122.45146	37.93518	-0.45	15.3	-98.178	14.8349996	11	7/24/2014	17:33.5
157	RWS11	-122.4514673	37.93517	-0.49	15.3	-182.5	14.8350004	-122.45146	37.93518	-0.45	15.3	-85.799	14.8770004	11	7/24/2014	17:33.6
158	RWS11	-122.4514698	37.93517	-0.49	15.4	-171.9	14.8770002	-122.45146	37.93518	-0.41	15.4	-73.252	14.9530002	11	7/24/2014	17:33.7
159	RWS11	-122.4514723	37.93517	-0.49	15.4	-161.2	14.919	-122.45147	37.93518	-0.45	15.4	-60.871	14.961	11	7/24/2014	17:33.8
160	RWS11	-122.4514747	37.93517	-0.49	15.4	-150.5	14.9600004	-122.45147	37.93518	-0.41	15.4	-48.517	15.0360004	11	7/24/2014	17:33.9
161	RWS11	-122.4514784	37.93517	-0.49	15.5	-139.8	15.0000003	-122.45147	37.93518	-0.45	15.5	-36.367	15.0420003	11	7/24/2014	17:34.0
162	RWS11	-122.4514809	37.93517	-0.44	15.5	-129.3	15.0910003	-122.45148	37.93518	-0.45	15.5	-24.303	15.0820003	11	7/24/2014	17:34.1
163	RWS11	-122.4514834	37.93517	-0.49	15.6	-118.8	15.0789999	-122.45148	37.93518	-0.45	15.6	-12.412	15.1209998	11	7/24/2014	17:34.2
164	RWS11	-122.4514859	37.93517	-0.49	15.6	-108.4	15.1189998	-122.45148	37.93518	-0.45	15.6	-0.739	15.1609998	11	7/24/2014	17:34.3

165	RWS11	-122.4514885	37.93517	-0.49	15.6	-98.29	15.1600002	-122.45148	37.93519	-0.45	15.6	10.892	15.2020002	11	7/24/2014	17:34.4
166	RWS11	-122.4514911	37.93517	-0.44	15.7	-88.12	15.253	-122.45149	37.93519	-0.45	15.7	22.388	15.244	11	7/24/2014	17:34.5
167	RWS11	-122.4514936	37.93517	-0.49	15.7	-78.04	15.2450002	-122.45149	37.93519	-0.45	15.7	33.954	15.2870002	11	7/24/2014	17:34.6
168	RWS11	-122.4514962	37.93517	-0.44	15.8	-68.01	15.3419997	-122.45149	37.93519	-0.45	15.8	45.518	15.3329997	11	7/24/2014	17:34.7
169	RWS11	-122.4514987	37.93517	-0.49	15.8	-57.97	15.3370002	-122.45149	37.93519	-0.45	15.8	57.148	15.3790002	11	7/24/2014	17:34.8
170	RWS11	-122.4515012	37.93518	-0.44	15.9	-47.89	15.4359996	-122.4515	37.93519	-0.45	15.9	68.844	15.4269996	11	7/24/2014	17:34.9
171	RWS11	-122.451505	37.93518	-0.49	15.9	-37.7	15.4319995	-122.4515	37.93519	-0.45	15.9	80.797	15.4739995	11	7/24/2014	17:35.0
172	RWS11	-122.4515075	37.93518	-0.44	16	-27.44	15.5309999	-122.4515	37.93519	-0.41	16	92.623	15.5559999	11	7/24/2014	17:35.1
173	RWS11	-122.45151	37.93518	-0.49	16	-17.39	15.5259994	-122.4515	37.93519	-0.45	16	104.28	15.5679994	11	7/24/2014	17:35.2
174	RWS11	-122.4515126	37.93518	-0.44	16.1	-7.327	15.621	-122.45151	37.93519	-0.45	16.1	115.73	15.612	11	7/24/2014	17:35.3
175	RWS11	-122.4515151	37.93518	-0.49	16.1	2.622	15.6119998	-122.45151	37.93519	-0.45	16.1	127.13	15.6539998	11	7/24/2014	17:35.4
176	RWS11	-122.4515177	37.93518	-0.44	16.1	12.458	15.7030008	-122.45151	37.93519	-0.41	16.1	138.31	15.7280008	11	7/24/2014	17:35.5
177	RWS11	-122.4515203	37.93518	-0.49	16.2	22.126	15.6900009	-122.45151	37.93519	-0.45	16.2	149.33	15.7320009	11	7/24/2014	17:35.6
178	RWS11	-122.4515229	37.93518	-0.44	16.2	31.573	15.7779996	-122.45152	37.93519	-0.36	16.2	160.08	15.8529996	11	7/24/2014	17:35.7
179	RWS11	-122.4515254	37.93518	-0.49	16.3	40.975	15.7619995	-122.45152	37.93519	-0.45	16.3	170.66	15.8039995	11	7/24/2014	17:35.8
180	RWS11	-122.451528	37.93518	-0.44	16.3	50.358	15.8490007	-122.45152	37.9352	-0.41	16.3	181.1	15.8740007	11	7/24/2014	17:35.9
181	RWS11	-122.4515317	37.93518	-0.49	16.3	59.57	15.8330005	-122.45153	37.9352	-0.45	16.3	191.44	15.8750005	11	7/24/2014	17:36.0
182	RWS11	-122.4515343	37.93518	-0.49	16.4	68.6	15.8699993	-122.45153	37.9352	-0.41	16.4	201.52	15.9459993	11	7/24/2014	17:36.1
183	RWS11	-122.4515368	37.93519	-0.49	16.4	77.372	15.9100002	-122.45153	37.9352	-0.45	16.4	211.43	15.9520002	11	7/24/2014	17:36.2
184	RWS11	-122.4515394	37.93519	-0.49	16.4	85.941	15.9510005	-122.45153	37.9352	-0.41	16.4	221.09	16.0270006	11	7/24/2014	17:36.3
185	RWS11	-122.451542	37.93519	-0.49	16.5	94.325	15.9949993	-122.45154	37.9352	-0.45	16.5	230.56	16.0369993	11	7/24/2014	17:36.4
186	RWS11	-122.4515445	37.93519	-0.49	16.5	102.51	16.0420001	-122.45154	37.9352	-0.41	16.5	239.84	16.1180002	11	7/24/2014	17:36.5
187	RWS11	-122.4515471	37.93519	-0.49	16.6	110.54	16.0909999	-122.45154	37.9352	-0.41	16.6	248.9	16.1669999	11	7/24/2014	17:36.6
188	RWS11	-122.4515497	37.93519	-0.44	16.6	118.5	16.194	-122.45154	37.9352	-0.45	16.6	258.05	16.185	11	7/24/2014	17:36.7
189	RWS11	-122.4515522	37.93519	-0.49	16.7	126.38	16.1970008	-122.45155	37.9352	-0.45	16.7	267.09	16.2390008	11	7/24/2014	17:36.8
190	RWS11	-122.4515548	37.93519	-0.49	16.7	134.1	16.254	-122.45155	37.9352	-0.41	16.7	275.92	16.33	11	7/24/2014	17:36.9
191	RWS11	-122.4515585	37.93519	-0.49	16.8	141.56	16.3130001	-122.45155	37.9352	-0.45	16.8	284.49	16.3550001	11	7/24/2014	17:37.0
192	RWS11	-122.4515611	37.93519	-0.49	16.9	148.76	16.3759998	-122.45156	37.9352	-0.41	16.9	292.76	16.4519998	11	7/24/2014	17:37.1
193	RWS11	-122.4515636	37.93519	-0.49	16.9	155.68	16.4419998	-122.45156	37.9352	-0.45	16.9	300.67	16.4839998	11	7/24/2014	17:37.2
194	RWS11	-122.4515661	37.93519	-0.49	17	162.35	16.511	-122.45156	37.93521	-0.45	17	308.26	16.553	11	7/24/2014	17:37.3
195	RWS11	-122.4515687	37.93519	-0.52	17.1	168.64	16.55	-122.45156	37.93521	-0.45	17.1	315.52	16.626	11	7/24/2014	17:37.4
196	RWS11	-122.4515712	37.93519	-0.49	17.1	174.46	16.6609996	-122.45157	37.93521	-0.5	17.1	322.33	16.6519996	11	7/24/2014	17:37.5
197	RWS11	-122.4515738	37.93519	-0.49	17.2	179.98	16.7409996	-122.45157	37.93521	-0.5	17.2	328.79	16.7319996	11	7/24/2014	17:37.6

198	RWS11	-122.4515763	37.9352	-0.49	17.3	185.24	16.8249992	-122.45157	37.93521	-0.5	17.3	334.95	16.8159992	11	7/24/2014	17:37.7
199	RWS11	-122.4515788	37.9352	-0.49	17.4	190.3	16.9109996	-122.45157	37.93521	-0.5	17.4	340.88	16.9019996	11	7/24/2014	17:37.8
200	RWS11	-122.4515813	37.9352	-0.49	17.5	195	16.9990009	-122.45158	37.93521	-0.45	17.5	346.45	17.0410009	11	7/24/2014	17:37.9
201	RWS11	-122.451585	37.9352	-0.49	17.6	199.29	17.0870002	-122.45158	37.93521	-0.41	17.6	351.44	17.1630002	11	7/24/2014	17:38.0
202	RWS11	-122.4515875	37.9352	-0.49	17.7	203.07	17.1749996	-122.45158	37.93521	-0.41	17.7	355.86	17.2509996	11	7/24/2014	17:38.1
203	RWS11	-122.45159	37.9352	-0.49	17.8	206.63	17.2630008	-122.45158	37.93521	-0.45	17.8	359.97	17.3050008	11	7/24/2014	17:38.2
204	RWS11	-122.4515925	37.9352	-0.49	17.8	209.84	17.3489994	-122.45159	37.93521	-0.45	17.8	363.68	17.3909994	11	7/24/2014	17:38.3
205	RWS11	-122.451595	37.9352	-0.49	17.9	212.54	17.4349998	-122.45159	37.93521	-0.45	17.9	366.67	17.4769998	11	7/24/2014	17:38.4
206	RWS11	-122.4515976	37.9352	-0.49	18	214.78	17.5189994	-122.45159	37.93521	-0.45	18	369.18	17.5609994	11	7/24/2014	17:38.5
207	RWS11	-122.4516001	37.9352	-0.49	18.1	216.85	17.6029991	-122.45159	37.93521	-0.45	18.1	371.36	17.6449991	11	7/24/2014	17:38.6
208	RWS11	-122.4516026	37.9352	-0.49	18.2	218.44	17.6859992	-122.4516	37.93522	-0.45	18.2	372.83	17.7279992	11	7/24/2014	17:38.7
209	RWS11	-122.4516051	37.9352	-0.49	18.3	219.4	17.768	-122.4516	37.93522	-0.45	18.3	373.6	17.81	11	7/24/2014	17:38.8
210	RWS11	-122.4516076	37.9352	-0.49	18.3	219.86	17.8510002	-122.4516	37.93522	-0.41	18.3	373.66	17.9270002	11	7/24/2014	17:38.9
211	RWS11	-122.4516114	37.9352	-0.49	18.4	220.04	17.9330009	-122.45161	37.93522	-0.5	18.4	373.49	17.9240009	11	7/24/2014	17:39.0
212	RWS11	-122.4516139	37.93521	-0.49	18.5	220.04	18.0159992	-122.45161	37.93522	-0.45	18.5	373.16	18.0579992	11	7/24/2014	17:39.1
213	RWS11	-122.4516165	37.93521	-0.49	18.6	219.65	18.0979999	-122.45161	37.93522	-0.45	18.6	372.52	18.1399999	11	7/24/2014	17:39.2
214	RWS11	-122.4516191	37.93521	-0.49	18.7	218.79	18.1810001	-122.45161	37.93522	-0.45	18.7	371.27	18.2230001	11	7/24/2014	17:39.3
215	RWS11	-122.4516216	37.93521	-0.49	18.8	217.55	18.2630008	-122.45162	37.93522	-0.41	18.8	369.71	18.3390008	11	7/24/2014	17:39.4
216	RWS11	-122.4516243	37.93521	-0.44	18.8	216.22	18.3959996	-122.45162	37.93522	-0.41	18.8	367.82	18.4209997	11	7/24/2014	17:39.5
217	RWS11	-122.4516268	37.93521	-0.49	18.9	214.58	18.4279998	-122.45162	37.93522	-0.45	18.9	365.79	18.4699998	11	7/24/2014	17:39.6
218	RWS11	-122.4516294	37.93521	-0.49	19	212.8	18.511	-122.45162	37.93522	-0.41	19	363.39	18.587	11	7/24/2014	17:39.7
219	RWS11	-122.451632	37.93521	-0.49	19.1	210.67	18.5949996	-122.45163	37.93522	-0.41	19.1	360.84	18.6709997	11	7/24/2014	17:39.8
220	RWS11	-122.4516346	37.93521	-0.44	19.2	208.08	18.7329995	-122.45163	37.93522	-0.36	19.2	357.74	18.8079995	11	7/24/2014	17:39.9
221	RWS11	-122.4516384	37.93521	-0.49	19.3	205.11	18.7719997	-122.45163	37.93522	-0.45	19.3	354.07	18.8139997	11	7/24/2014	17:40.0
222	RWS11	-122.4516409	37.93521	-0.44	19.4	201.76	18.9150007	-122.45164	37.93523	-0.41	19.4	349.81	18.9400007	11	7/24/2014	17:40.1
223	RWS11	-122.4516435	37.93521	-0.49	19.4	198.12	18.9599994	-122.45164	37.93523	-0.45	19.4	345.25	19.0019994	11	7/24/2014	17:40.2
224	RWS11	-122.451646	37.93521	-0.44	19.5	194.16	19.1100003	-122.45164	37.93523	-0.41	19.5	340.46	19.1350004	11	7/24/2014	17:40.3
225	RWS11	-122.4516486	37.93521	-0.49	19.6	189.87	19.1579994	-122.45164	37.93523	-0.5	19.6	335.32	19.1489994	11	7/24/2014	17:40.4
226	RWS11	-122.4516512	37.93522	-0.49	19.7	185.69	19.2579998	-122.45165	37.93523	-0.41	19.7	330.28	19.3339998	11	7/24/2014	17:40.5
227	RWS11	-122.4516537	37.93522	-0.49	19.8	181.5	19.3600009	-122.45165	37.93523	-0.45	19.8	325.16	19.4020009	11	7/24/2014	17:40.6
228	RWS11	-122.4516563	37.93522	-0.44	20	177.12	19.5130002	-122.45165	37.93523	-0.41	20	319.99	19.5380002	11	7/24/2014	17:40.7
229	RWS11	-122.4516588	37.93522	-0.49	20.1	172.74	19.5630001	-122.45165	37.93523	-0.45	20.1	314.71	19.6050001	11	7/24/2014	17:40.8
230	RWS11	-122.4516614	37.93522	-0.44	20.2	168.06	19.7159993	-122.45166	37.93523	-0.41	20.2	309.07	19.7409993	11	7/24/2014	17:40.9

231	RWS11	-122.4516651	37.93522	-0.49	20.3	162.82	19.7659992	-122.45166	37.93523	-0.45	20.3	302.9	19.8079992	11	7/24/2014	17:41.0
232	RWS11	-122.4516676	37.93522	-0.44	20.4	157.28	19.9160001	-122.45166	37.93523	-0.45	20.4	296.37	19.9070001	11	7/24/2014	17:41.1
233	RWS11	-122.45167	37.93522	-0.49	20.5	151.35	19.9610008	-122.45166	37.93523	-0.5	20.5	289.43	19.9520008	11	7/24/2014	17:41.2
234	RWS11	-122.4516725	37.93522	-0.49	20.5	144.97	20.0539993	-122.45167	37.93523	-0.45	20.5	282.08	20.0959993	11	7/24/2014	17:41.3
235	RWS11	-122.451675	37.93522	-0.49	20.6	138.38	20.1420005	-122.45167	37.93523	-0.5	20.6	274.39	20.1330005	11	7/24/2014	17:41.4
236	RWS11	-122.4516775	37.93522	-0.44	20.7	131.53	20.2739999	-122.45167	37.93524	-0.45	20.7	266.54	20.2649999	11	7/24/2014	17:41.5
237	RWS11	-122.45168	37.93522	-0.44	20.8	124.51	20.3469999	-122.45167	37.93524	-0.5	20.8	258.53	20.2869999	11	7/24/2014	17:41.6
238	RWS11	-122.4516825	37.93522	-0.44	20.8	117.39	20.4090001	-122.45168	37.93524	-0.45	20.8	250.52	20.4000001	11	7/24/2014	17:41.7
239	RWS11	-122.451685	37.93522	-0.44	20.9	110.26	20.4610002	-122.45168	37.93524	-0.5	20.9	242.57	20.4010002	11	7/24/2014	17:41.8
240	RWS11	-122.4516874	37.93523	-0.44	20.9	103.04	20.5009992	-122.45168	37.93524	-0.45	20.9	234.52	20.4919992	11	7/24/2014	17:41.9
241	RWS11	-122.4516911	37.93523	-0.44	21	95.725	20.5319993	-122.45169	37.93524	-0.45	21	226.22	20.5229993	11	7/24/2014	17:42.0
242	RWS11	-122.4516935	37.93523	-0.44	21	88.183	20.5540006	-122.45169	37.93524	-0.45	21	217.71	20.5450006	11	7/24/2014	17:42.1
243	RWS11	-122.451696	37.93523	-0.49	21	80.428	20.5189994	-122.45169	37.93524	-0.5	21	208.86	20.5099994	11	7/24/2014	17:42.2
244	RWS11	-122.4516984	37.93523	-0.49	21	72.338	20.5289997	-122.45169	37.93524	-0.45	21	199.52	20.5709997	11	7/24/2014	17:42.3
245	RWS11	-122.4517009	37.93523	-0.49	21	64.154	20.5359996	-122.4517	37.93524	-0.5	21	190.01	20.5269996	11	7/24/2014	17:42.4
246	RWS11	-122.4517034	37.93523	-0.44	21	56.055	20.5899999	-122.4517	37.93524	-0.41	21	180.29	20.6149999	11	7/24/2014	17:42.5
247	RWS11	-122.4517059	37.93523	-0.44	21	47.874	20.5930001	-122.4517	37.93524	-0.5	21	170.45	20.5330001	11	7/24/2014	17:42.6
248	RWS11	-122.4517084	37.93523	-0.44	21	39.452	20.5939996	-122.4517	37.93524	-0.45	21	160.44	20.5849996	11	7/24/2014	17:42.7
249	RWS11	-122.4517109	37.93523	-0.44	21	30.593	20.595001	-122.45171	37.93524	-0.45	21	150.08	20.586001	11	7/24/2014	17:42.8
250	RWS11	-122.4517134	37.93523	-0.44	21	21.365	20.5960004	-122.45171	37.93525	-0.41	21	139.44	20.6210004	11	7/24/2014	17:42.9
251	RWS11	-122.4517171	37.93523	-0.44	21	11.96	20.5969999	-122.45171	37.93525	-0.45	21	128.69	20.5879999	11	7/24/2014	17:43.0
252	RWS11	-122.4517195	37.93523	-0.44	21	2.512	20.5990007	-122.45171	37.93525	-0.41	21	117.82	20.6240007	11	7/24/2014	17:43.1
253	RWS11	-122.451722	37.93523	-0.44	21	-7.241	20.6009996	-122.45172	37.93525	-0.45	21	106.77	20.5919996	11	7/24/2014	17:43.2
254	RWS11	-122.4517245	37.93524	-0.44	21	-16.97	20.6039998	-122.45172	37.93525	-0.41	21	95.655	20.6289998	11	7/24/2014	17:43.3
255	RWS11	-122.4517271	37.93524	-0.44	21	-26.55	20.6070001	-122.45172	37.93525	-0.45	21	84.664	20.5980001	11	7/24/2014	17:43.4
256	RWS11	-122.4517296	37.93524	-0.44	21	-36	20.6109998	-122.45172	37.93525	-0.36	21	73.943	20.6859998	11	7/24/2014	17:43.5
257	RWS11	-122.4517321	37.93524	-0.44	21.1	-45.32	20.6149995	-122.45173	37.93525	-0.41	21.1	63.431	20.6399995	11	7/24/2014	17:43.6
258	RWS11	-122.4517346	37.93524	-0.44	21.1	-54.46	20.6189992	-122.45173	37.93525	-0.41	21.1	53.171	20.6439992	11	7/24/2014	17:43.7
259	RWS11	-122.4517371	37.93524	-0.44	21.1	-63.58	20.6240003	-122.45173	37.93525	-0.41	21.1	43.004	20.6490003	11	7/24/2014	17:43.8
260	RWS11	-122.4517396	37.93524	-0.4	21.1	-72.81	20.6650008	-122.45173	37.93525	-0.36	21.1	32.77	20.7050008	11	7/24/2014	17:43.9
261	RWS11	-122.4517432	37.93524	-0.44	21.1	-82.24	20.635	-122.45174	37.93525	-0.41	21.1	22.232	20.66	11	7/24/2014	17:44.0
262	RWS11	-122.4517457	37.93524	-0.4	21.1	-91.68	20.6760005	-122.45174	37.93525	-0.36	21.1	11.54	20.7160005	11	7/24/2014	17:44.1
263	RWS11	-122.4517481	37.93524	-0.44	21.1	-100.9	20.6469991	-122.45174	37.93525	-0.36	21.1	0.849	20.7219991	11	7/24/2014	17:44.2

264	RWS11	-122.4517505	37.93524	-0.4	21.1	-110.1	20.6889991	-122.45174	37.93526	-0.36	21.1	-9.668	20.7289991	11	7/24/2014	17:44.3
265	RWS11	-122.451753	37.93524	-0.44	21.1	-119.3	20.6599996	-122.45175	37.93526	-0.33	21.1	-20.446	20.7689996	11	7/24/2014	17:44.4
266	RWS11	-122.4517554	37.93524	-0.4	21.1	-128.6	20.7010001	-122.45175	37.93526	-0.33	21.1	-31.29	20.7750001	11	7/24/2014	17:44.5
267	RWS11	-122.4517579	37.93524	-0.4	21.1	-138	20.7070006	-122.45175	37.93526	-0.33	21.1	-42.158	20.7810006	11	7/24/2014	17:44.6
268	RWS11	-122.4517603	37.93525	-0.4	21.1	-147.3	20.7119998	-122.45175	37.93526	-0.33	21.1	-52.91	20.7859998	11	7/24/2014	17:44.7
269	RWS11	-122.4517628	37.93525	-0.44	21.1	-156.8	20.68	-122.45176	37.93526	-0.33	21.1	-63.739	20.789	11	7/24/2014	17:44.8
270	RWS11	-122.4517652	37.93525	-0.35	21.1	-166.4	20.7669995	-122.45176	37.93526	-0.33	21.1	-74.724	20.7899995	11	7/24/2014	17:44.9
271	RWS11	-122.4517689	37.93525	-0.4	21.1	-176.1	20.7140006	-122.45176	37.93526	-0.29	21.1	-85.884	20.8220006	11	7/24/2014	17:45.0
272	RWS11	-122.4517713	37.93525	-0.35	21.1	-185.7	20.7590001	-122.45177	37.93526	-0.29	21.1	-96.988	20.8160001	11	7/24/2014	17:45.1
273	RWS11	-122.4517738	37.93525	-0.4	21.1	-195.3	20.6979998	-122.45177	37.93526	-0.29	21.1	-108.09	20.8059999	11	7/24/2014	17:45.2
274	RWS11	-122.4517763	37.93525	-0.35	21.1	-205	20.7319996	-122.45177	37.93526	-0.29	21.1	-119.32	20.7889997	11	7/24/2014	17:45.3
275	RWS11	-122.4517788	37.93525	-0.4	21.1	-214.4	20.6590003	-122.45177	37.93526	-0.29	21.1	-130.22	20.7670003	11	7/24/2014	17:45.4
276	RWS11	-122.4517814	37.93525	-0.35	21	-223.6	20.681001	-122.45178	37.93526	-0.29	21	-140.75	20.738001	11	7/24/2014	17:45.5
277	RWS11	-122.4517839	37.93525	-0.4	21	-232.7	20.5960006	-122.45178	37.93526	-0.29	21	-151.15	20.7040006	11	7/24/2014	17:45.6
278	RWS11	-122.4517865	37.93525	-0.35	21	-241.8	20.6069996	-122.45178	37.93527	-0.29	21	-161.28	20.6639997	11	7/24/2014	17:45.7
279	RWS11	-122.4517889	37.93525	-0.4	20.9	-250.7	20.5120009	-122.45178	37.93527	-0.29	20.9	-171.21	20.6200009	11	7/24/2014	17:45.8
280	RWS11	-122.4517915	37.93525	-0.35	20.9	-259.7	20.5150006	-122.45179	37.93527	-0.24	20.9	-181.15	20.6230006	11	7/24/2014	17:45.9
281	RWS11	-122.4517951	37.93526	-0.35	20.8	-268.4	20.464	-122.45179	37.93527	-0.29	20.8	-191	20.521	11	7/24/2014	17:46.0
282	RWS11	-122.4517976	37.93526	-0.35	20.8	-277.4	20.4089997	-122.45179	37.93527	-0.29	20.8	-200.89	20.4659997	11	7/24/2014	17:46.1
283	RWS11	-122.4518	37.93526	-0.4	20.7	-286.2	20.2999991	-122.45179	37.93527	-0.29	20.7	-210.72	20.4079991	11	7/24/2014	17:46.2
284	RWS11	-122.4518025	37.93526	-0.35	20.6	-295.2	20.2900002	-122.4518	37.93527	-0.29	20.6	-220.58	20.3470002	11	7/24/2014	17:46.3
285	RWS11	-122.451805	37.93526	-0.35	20.6	-304.3	20.2240002	-122.4518	37.93527	-0.29	20.6	-230.54	20.2810002	11	7/24/2014	17:46.4
286	RWS11	-122.4518075	37.93526	-0.35	20.5	-313.6	20.1559994	-122.4518	37.93527	-0.24	20.5	-240.52	20.2639994	11	7/24/2014	17:46.5
287	RWS11	-122.45181	37.93526	-0.35	20.4	-322.9	20.0859997	-122.4518	37.93527	-0.29	20.4	-250.52	20.1429997	11	7/24/2014	17:46.6
288	RWS11	-122.4518124	37.93526	-0.32	20.4	-332.1	20.0479992	-122.45181	37.93527	-0.24	20.4	-260.43	20.1219992	11	7/24/2014	17:46.7
289	RWS11	-122.4518149	37.93526	-0.32	20.3	-341.3	19.9739998	-122.45181	37.93527	-0.29	20.3	-270.16	19.9969998	11	7/24/2014	17:46.8
290	RWS11	-122.4518173	37.93526	-0.32	20.2	-350.2	19.8979996	-122.45181	37.93527	-0.24	20.2	-279.72	19.9719996	11	7/24/2014	17:46.9
291	RWS11	-122.4518209	37.93526	-0.35	20.1	-358.8	19.7849991	-122.45182	37.93528	-0.29	20.1	-289.13	19.8419991	11	7/24/2014	17:47.0
292	RWS11	-122.4518233	37.93526	-0.32	20.1	-367.3	19.7389992	-122.45182	37.93528	-0.24	20.1	-298.37	19.8129992	11	7/24/2014	17:47.1
293	RWS11	-122.4518258	37.93526	-0.35	20	-375.7	19.6239998	-122.45182	37.93528	-0.24	20	-307.65	19.7319998	11	7/24/2014	17:47.2
294	RWS11	-122.4518282	37.93526	-0.32	19.9	-384.1	19.5779999	-122.45182	37.93528	-0.24	19.9	-316.96	19.6519999	11	7/24/2014	17:47.3
295	RWS11	-122.4518307	37.93527	-0.32	19.8	-392.3	19.4989995	-122.45182	37.93528	-0.29	19.8	-326.21	19.5219995	11	7/24/2014	17:47.4
296	RWS11	-122.4518332	37.93527	-0.32	19.7	-400.5	19.4210003	-122.45183	37.93528	-0.24	19.7	-335.33	19.4950003	11	7/24/2014	17:47.5

297	RWS11	-122.4518356	37.93527	-0.32	19.7	-408.5	19.3459996	-122.45183	37.93528	-0.29	19.7	-344.53	19.3689996	11	7/24/2014	17:47.6
298	RWS11	-122.4518381	37.93527	-0.32	19.6	-416.4	19.2720002	-122.45183	37.93528	-0.24	19.6	-353.38	19.3460002	11	7/24/2014	17:47.7
299	RWS11	-122.4518405	37.93527	-0.32	19.5	-424.1	19.2020005	-122.45183	37.93528	-0.24	19.5	-362.16	19.2760005	11	7/24/2014	17:47.8
300	RWS11	-122.451843	37.93527	-0.32	19.5	-431.7	19.1330002	-122.45184	37.93528	-0.24	19.5	-370.67	19.2070002	11	7/24/2014	17:47.9
301	RWS11	-122.4518466	37.93527	-0.32	19.4	-439.5	19.0649994	-122.45184	37.93528	-0.29	19.4	-379.09	19.0879994	11	7/24/2014	17:48.0
302	RWS11	-122.451849	37.93527	-0.32	19.3	-446.9	18.998	-122.45184	37.93528	-0.24	19.3	-387.51	19.072	11	7/24/2014	17:48.1
303	RWS11	-122.4518515	37.93527	-0.32	19.2	-454.4	18.9310006	-122.45185	37.93528	-0.24	19.2	-395.72	19.0050006	11	7/24/2014	17:48.2
304	RWS11	-122.4518539	37.93527	-0.32	19.2	-461.8	18.8639992	-122.45185	37.93528	-0.29	19.2	-403.95	18.8869992	11	7/24/2014	17:48.3
305	RWS11	-122.4518564	37.93527	-0.35	19.1	-468.9	18.7599995	-122.45185	37.93529	-0.29	19.1	-411.85	18.8169995	11	7/24/2014	17:48.4
306	RWS11	-122.4518589	37.93527	-0.32	19	-475.9	18.7230004	-122.45185	37.93529	-0.29	19	-419.63	18.7460004	11	7/24/2014	17:48.5
307	RWS11	-122.4518614	37.93527	-0.32	19	-482.5	18.6500004	-122.45186	37.93529	-0.33	19	-427.34	18.6390004	11	7/24/2014	17:48.6
308	RWS11	-122.4518639	37.93527	-0.32	18.9	-489.1	18.5740002	-122.45186	37.93529	-0.33	18.9	-434.93	18.5630002	11	7/24/2014	17:48.7
309	RWS11	-122.4518664	37.93528	-0.35	18.8	-495.5	18.4619997	-122.45186	37.93529	-0.33	18.8	-442.54	18.4849997	11	7/24/2014	17:48.8
310	RWS11	-122.4518689	37.93528	-0.32	18.7	-501.6	18.4169993	-122.45186	37.93529	-0.33	18.7	-450.01	18.4059992	11	7/24/2014	17:48.9
311	RWS11	-122.4518725	37.93528	-0.35	18.7	-507.8	18.3019999	-122.45187	37.93529	-0.36	18.7	-457.54	18.2909999	11	7/24/2014	17:49.0
312	RWS11	-122.451875	37.93528	-0.32	18.6	-514	18.2550005	-122.45187	37.93529	-0.33	18.6	-465.16	18.2440005	11	7/24/2014	17:49.1
313	RWS11	-122.4518775	37.93528	-0.35	18.5	-520	18.1389998	-122.45187	37.93529	-0.36	18.5	-472.7	18.1279998	11	7/24/2014	17:49.2
314	RWS11	-122.45188	37.93528	-0.35	18.4	-525.9	18.0570009	-122.45187	37.93529	-0.36	18.4	-480.21	18.046001	11	7/24/2014	17:49.3
315	RWS11	-122.4518825	37.93528	-0.4	18.3	-532	17.9249997	-122.45188	37.93529	-0.36	18.3	-487.63	17.9649997	11	7/24/2014	17:49.4
316	RWS11	-122.4518851	37.93528	-0.35	18.2	-538.3	17.8950003	-122.45188	37.93529	-0.36	18.2	-494.94	17.8840003	11	7/24/2014	17:49.5
317	RWS11	-122.4518876	37.93528	-0.35	18.2	-544.6	17.8150004	-122.45188	37.93529	-0.41	18.2	-502.3	17.7540004	11	7/24/2014	17:49.6
318	RWS11	-122.4518901	37.93528	-0.35	18.1	-550.6	17.7339991	-122.45188	37.9353	-0.36	18.1	-509.52	17.7229991	11	7/24/2014	17:49.7
319	RWS11	-122.4518926	37.93528	-0.35	18	-556.4	17.6550005	-122.45189	37.9353	-0.41	18	-516.48	17.5940005	11	7/24/2014	17:49.8
320	RWS11	-122.4518951	37.93528	-0.35	17.9	-561.9	17.576	-122.45189	37.9353	-0.36	17.9	-523.2	17.5650001	11	7/24/2014	17:49.9
321	RWS11	-122.4518988	37.93529	-0.4	17.8	-567.3	17.4459996	-122.45189	37.9353	-0.36	17.8	-529.61	17.4859996	11	7/24/2014	17:50.0
322	RWS11	-122.4519012	37.93529	-0.4	17.8	-572.7	17.3669991	-122.4519	37.9353	-0.41	17.8	-536	17.3569991	11	7/24/2014	17:50.1
323	RWS11	-122.4519037	37.93529	-0.4	17.7	-578.1	17.2899994	-122.4519	37.9353	-0.41	17.7	-542.32	17.2799994	11	7/24/2014	17:50.2
324	RWS11	-122.4519061	37.93529	-0.4	17.6	-583.2	17.2139992	-122.4519	37.9353	-0.41	17.6	-548.64	17.2039992	11	7/24/2014	17:50.3
325	RWS11	-122.4519086	37.93529	-0.44	17.5	-588.1	17.1049998	-122.4519	37.9353	-0.41	17.5	-554.86	17.1299998	11	7/24/2014	17:50.4
326	RWS11	-122.4519111	37.93529	-0.4	17.5	-592.9	17.0679993	-122.45191	37.9353	-0.41	17.5	-560.88	17.0579993	11	7/24/2014	17:50.5
327	RWS11	-122.4519136	37.93529	-0.4	17.4	-597.8	16.9989991	-122.45191	37.9353	-0.41	17.4	-566.81	16.9889991	11	7/24/2014	17:50.6
328	RWS11	-122.451916	37.93529	-0.4	17.3	-602.8	16.9340004	-122.45191	37.9353	-0.41	17.3	-572.74	16.9240004	11	7/24/2014	17:50.7
329	RWS11	-122.4519185	37.93529	-0.4	17.3	-607.9	16.8710007	-122.45191	37.9353	-0.41	17.3	-578.51	16.8610007	11	7/24/2014	17:50.8

330	RWS11	-122.451921	37.93529	-0.4	17.2	-613	16.8109993	-122.45192	37.9353	-0.36	17.2	-583.99	16.8509994	11	7/24/2014	17:50.9
331	RWS11	-122.4519246	37.93529	-0.44	17.2	-618.3	16.7190001	-122.45192	37.93531	-0.45	17.2	-589.47	16.7100001	11	7/24/2014	17:51.0
332	RWS11	-122.4519271	37.93529	-0.44	17.1	-623.8	16.6620009	-122.45192	37.93531	-0.41	17.1	-595.1	16.6870009	11	7/24/2014	17:51.1
333	RWS11	-122.4519296	37.93529	-0.44	17	-629.2	16.6070006	-122.45192	37.93531	-0.45	17	-601	16.5980006	11	7/24/2014	17:51.2
334	RWS11	-122.4519322	37.93529	-0.44	17	-634.5	16.5529998	-122.45193	37.93531	-0.45	17	-606.88	16.5439998	11	7/24/2014	17:51.3
335	RWS11	-122.4519347	37.9353	-0.44	16.9	-639.5	16.4990008	-122.45193	37.93531	-0.5	16.9	-612.61	16.4390008	11	7/24/2014	17:51.4
336	RWS11	-122.4519373	37.9353	-0.44	16.9	-644.3	16.4459994	-122.45193	37.93531	-0.5	16.9	-618.31	16.3859994	11	7/24/2014	17:51.5
337	RWS11	-122.4519399	37.9353	-0.44	16.8	-649.2	16.3929999	-122.45193	37.93531	-0.45	16.8	-623.93	16.3839999	11	7/24/2014	17:51.6
338	RWS11	-122.4519424	37.9353	-0.44	16.8	-654	16.3409999	-122.45194	37.93531	-0.45	16.8	-629.46	16.3319999	11	7/24/2014	17:51.7
339	RWS11	-122.4519449	37.9353	-0.49	16.7	-658.6	16.2400006	-122.45194	37.93531	-0.45	16.7	-634.71	16.2820006	11	7/24/2014	17:51.8
340	RWS11	-122.4519475	37.9353	-0.44	16.7	-663.2	16.2409995	-122.45194	37.93531	-0.45	16.7	-639.93	16.2319995	11	7/24/2014	17:51.9
341	RWS11	-122.4519512	37.9353	-0.49	16.6	-667.5	16.1409997	-122.45195	37.93531	-0.5	16.6	-645.21	16.1319997	11	7/24/2014	17:52.0
342	RWS11	-122.4519537	37.9353	-0.49	16.6	-671.9	16.0929994	-122.45195	37.93531	-0.45	16.6	-650.61	16.1349994	11	7/24/2014	17:52.1
343	RWS11	-122.4519562	37.9353	-0.49	16.5	-676.2	16.0469998	-122.45195	37.93531	-0.5	16.5	-656.02	16.0379998	11	7/24/2014	17:52.2
344	RWS11	-122.4519587	37.9353	-0.52	16.5	-680.2	15.9679998	-122.45195	37.93532	-0.53	16.5	-661.29	15.9589998	11	7/24/2014	17:52.3
345	RWS11	-122.4519612	37.9353	-0.52	16.4	-684.1	15.9250005	-122.45196	37.93532	-0.53	16.4	-666.21	15.9160005	11	7/24/2014	17:52.4
346	RWS11	-122.4519637	37.9353	-0.49	16.4	-688.2	15.9180001	-122.45196	37.93532	-0.5	16.4	-670.88	15.9090001	11	7/24/2014	17:52.5
347	RWS11	-122.4519662	37.9353	-0.52	16.4	-691.9	15.8450006	-122.45196	37.93532	-0.5	16.4	-675.37	15.8700006	11	7/24/2014	17:52.6
348	RWS11	-122.4519687	37.93531	-0.49	16.3	-695.6	15.8429994	-122.45196	37.93532	-0.53	16.3	-679.97	15.7999994	11	7/24/2014	17:52.7
349	RWS11	-122.4519712	37.93531	-0.57	16.3	-699.1	15.7250003	-122.45197	37.93532	-0.53	16.3	-684.52	15.7670003	11	7/24/2014	17:52.8
350	RWS11	-122.4519737	37.93531	-0.52	16.3	-702.4	15.7459996	-122.45197	37.93532	-0.5	16.3	-688.99	15.7709996	11	7/24/2014	17:52.9
351	RWS11	-122.4519774	37.93531	-0.52	16.2	-705.5	15.7189992	-122.45197	37.93532	-0.53	16.2	-693.29	15.7099992	11	7/24/2014	17:53.0
352	RWS11	-122.4519798	37.93531	-0.52	16.2	-708.5	15.694999	-122.45197	37.93532	-0.53	16.2	-697.2	15.685999	11	7/24/2014	17:53.1
353	RWS11	-122.4519823	37.93531	-0.57	16.2	-711.4	15.6229991	-122.45198	37.93532	-0.53	16.2	-701.08	15.6649991	11	7/24/2014	17:53.2
354	RWS11	-122.4519848	37.93531	-0.52	16.2	-714	15.6559995	-122.45198	37.93532	-0.53	16.2	-704.77	15.6469995	11	7/24/2014	17:53.3
355	RWS11	-122.4519872	37.93531	-0.61	16.2	-716.8	15.5529993	-122.45198	37.93532	-0.58	16.2	-708.3	15.5789993	11	7/24/2014	17:53.4
356	RWS11	-122.4519897	37.93531	-0.57	16.1	-719.7	15.5729999	-122.45198	37.93532	-0.58	16.1	-711.67	15.5639999	11	7/24/2014	17:53.5
357	RWS11	-122.4519922	37.93531	-0.61	16.1	-722.6	15.5249994	-122.45199	37.93533	-0.58	16.1	-715.05	15.5509994	11	7/24/2014	17:53.6
358	RWS11	-122.4519947	37.93531	-0.57	16.1	-725.5	15.5480003	-122.45199	37.93533	-0.58	16.1	-718.31	15.5390003	11	7/24/2014	17:53.7
359	RWS11	-122.4519972	37.93531	-0.57	16.1	-728.5	15.5370006	-122.45199	37.93533	-0.58	16.1	-721.47	15.5280006	11	7/24/2014	17:53.8
360	RWS11	-122.4519996	37.93531	-0.57	16.1	-731.4	15.5260009	-122.45199	37.93533	-0.62	16.1	-724.41	15.4840009	11	7/24/2014	17:53.9
361	RWS11	-122.4520033	37.93532	-0.61	16.1	-734.3	15.4820002	-122.452	37.93533	-0.58	16.1	-727.36	15.5080001	11	7/24/2014	17:54.0
362	RWS11	-122.4520058	37.93532	-0.57	16.1	-737.3	15.5079994	-122.452	37.93533	-0.58	16.1	-730.41	15.4989994	11	7/24/2014	17:54.1

363	RWS11	-122.4520082	37.93532	-0.61	16.1	-740.3	15.465	-122.452	37.93533	-0.62	16.1	-733.44	15.4579999	11	7/24/2014	17:54.2
364	RWS11	-122.4520107	37.93532	-0.57	16.1	-743.3	15.493	-122.452	37.93533	-0.58	16.1	-736.77	15.484	11	7/24/2014	17:54.3
365	RWS11	-122.4520132	37.93532	-0.61	16.1	-746.1	15.4519995	-122.45201	37.93533	-0.58	16.1	-740.13	15.4779995	11	7/24/2014	17:54.4
366	RWS11	-122.4520157	37.93532	-0.61	16.1	-749.1	15.4470003	-122.45201	37.93533	-0.58	16.1	-743.55	15.4730003	11	7/24/2014	17:54.5
367	RWS11	-122.4520182	37.93532	-0.61	16	-752.1	15.4419993	-122.45201	37.93533	-0.62	16	-747.07	15.4349992	11	7/24/2014	17:54.6
368	RWS11	-122.4520207	37.93532	-0.61	16	-755	15.4390009	-122.45201	37.93533	-0.58	16	-750.42	15.4650009	11	7/24/2014	17:54.7
369	RWS11	-122.4520232	37.93532	-0.61	16	-757.6	15.4360007	-122.45202	37.93533	-0.62	16	-753.6	15.4290006	11	7/24/2014	17:54.8
370	RWS11	-122.4520256	37.93532	-0.61	16	-760	15.4339998	-122.45202	37.93534	-0.62	16	-756.4	15.4269998	11	7/24/2014	17:54.9
371	RWS11	-122.4520293	37.93532	-0.61	16	-762.3	15.4330004	-122.45202	37.93534	-0.67	16	-759.21	15.3750004	11	7/24/2014	17:55.0
372	RWS11	-122.4520318	37.93532	-0.61	16	-764.7	15.4320009	-122.45203	37.93534	-0.62	16	-761.83	15.4250009	11	7/24/2014	17:55.1
373	RWS11	-122.4520342	37.93533	-0.66	16	-767	15.3810009	-122.45203	37.93534	-0.67	16	-764.41	15.3740009	11	7/24/2014	17:55.2
374	RWS11	-122.4520367	37.93533	-0.61	16	-769.2	15.4330004	-122.45203	37.93534	-0.62	16	-766.74	15.4260004	11	7/24/2014	17:55.3
375	RWS11	-122.4520391	37.93533	-0.66	16	-771.3	15.3829998	-122.45203	37.93534	-0.67	16	-769.29	15.3759998	11	7/24/2014	17:55.4
376	RWS11	-122.4520416	37.93533	-0.66	16	-773.2	15.3850007	-122.45204	37.93534	-0.62	16	-771.72	15.4290006	11	7/24/2014	17:55.5
377	RWS11	-122.4520441	37.93533	-0.66	16	-775.2	15.3880009	-122.45204	37.93534	-0.67	16	-774.07	15.3810009	11	7/24/2014	17:55.6
378	RWS11	-122.4520466	37.93533	-0.61	16	-777.1	15.4419993	-122.45204	37.93534	-0.62	16	-776.1	15.4349992	11	7/24/2014	17:55.7
379	RWS11	-122.452049	37.93533	-0.66	16.1	-778.9	15.3939995	-122.45204	37.93534	-0.62	16.1	-778.04	15.4379995	11	7/24/2014	17:55.8
380	RWS11	-122.4520515	37.93533	-0.61	16.1	-780.5	15.4479998	-122.45205	37.93534	-0.62	16.1	-779.81	15.4409998	11	7/24/2014	17:55.9
381	RWS11	-122.4520551	37.93533	-0.66	16.1	-782	15.4	-122.45205	37.93534	-0.67	16.1	-781.46	15.393	11	7/24/2014	17:56.0
382	RWS11	-122.4520575	37.93533	-0.66	16.1	-783.4	15.4020008	-122.45205	37.93534	-0.67	16.1	-782.94	15.3950008	11	7/24/2014	17:56.1
383	RWS11	-122.45206	37.93533	-0.66	16.1	-784.4	15.4030003	-122.45205	37.93535	-0.67	16.1	-784.34	15.3960003	11	7/24/2014	17:56.2
384	RWS11	-122.4520624	37.93533	-0.66	16.1	-785.3	15.4039998	-122.45206	37.93535	-0.62	16.1	-785.63	15.4479997	11	7/24/2014	17:56.3
385	RWS11	-122.4520649	37.93533	-0.66	16.1	-786.1	15.4049992	-122.45206	37.93535	-0.67	16.1	-786.81	15.3979992	11	7/24/2014	17:56.4
386	RWS11	-122.4520674	37.93534	-0.66	16.1	-786.8	15.4039998	-122.45206	37.93535	-0.67	16.1	-788.19	15.3969997	11	7/24/2014	17:56.5
387	RWS11	-122.4520699	37.93534	-0.66	16.1	-787.6	15.4039998	-122.45206	37.93535	-0.7	16.1	-789.6	15.3629997	11	7/24/2014	17:56.6
388	RWS11	-122.4520723	37.93534	-0.66	16.1	-788.4	15.4020008	-122.45207	37.93535	-0.67	16.1	-790.76	15.3950008	11	7/24/2014	17:56.7
389	RWS11	-122.4520748	37.93534	-0.69	16.1	-789.1	15.3650006	-122.45207	37.93535	-0.67	16.1	-791.88	15.3920006	11	7/24/2014	17:56.8
390	RWS11	-122.4520772	37.93534	-0.66	16.1	-790.1	15.3950009	-122.45207	37.93535	-0.62	16.1	-792.85	15.4390008	11	7/24/2014	17:56.9
391	RWS11	-122.4520808	37.93534	-0.66	16	-791.1	15.3899998	-122.45207	37.93535	-0.62	16	-793.65	15.4339998	11	7/24/2014	17:57.0
392	RWS11	-122.4520833	37.93534	-0.66	16	-792.4	15.3829998	-122.45208	37.93535	-0.62	16	-794.46	15.4269998	11	7/24/2014	17:57.1
393	RWS11	-122.4520857	37.93534	-0.69	16	-793.9	15.3419998	-122.45208	37.93535	-0.62	16	-795.78	15.4199998	11	7/24/2014	17:57.2
394	RWS11	-122.4520882	37.93534	-0.66	16	-795.6	15.3680005	-122.45208	37.93535	-0.58	16	-797.45	15.4450004	11	7/24/2014	17:57.3
395	RWS11	-122.4520907	37.93534	-0.66	16	-797.2	15.3589997	-122.45208	37.93535	-0.62	16	-799.22	15.4029996	11	7/24/2014	17:57.4

396	RWS11	-122.4520932	37.93534	-0.66	16	-798.6	15.3500008	-122.45209	37.93536	-0.62	16	-800.74	15.3940008	11	7/24/2014	17:57.5
397	RWS11	-122.4520957	37.93534	-0.66	16	-799.5	15.3419995	-122.45209	37.93536	-0.62	16	-801.81	15.3859994	11	7/24/2014	17:57.6
398	RWS11	-122.4520982	37.93534	-0.66	16	-799.8	15.3329997	-122.45209	37.93536	-0.62	16	-802.42	15.3769996	11	7/24/2014	17:57.7
399	RWS11	-122.4521006	37.93535	-0.66	16	-799.9	15.3230004	-122.45209	37.93536	-0.62	16	-802.74	15.3670003	11	7/24/2014	17:57.8
400	RWS11	-122.4521031	37.93535	-0.66	16	-799.8	15.3139996	-122.4521	37.93536	-0.58	16	-802.78	15.3909996	11	7/24/2014	17:57.9
401	RWS11	-122.4521068	37.93535	-0.66	16	-799.8	15.3040003	-122.4521	37.93536	-0.62	16	-802.59	15.3480003	11	7/24/2014	17:58.0
402	RWS11	-122.4521092	37.93535	-0.61	16	-799.6	15.3439997	-122.4521	37.93536	-0.58	16	-802.11	15.3699997	11	7/24/2014	17:58.1
403	RWS11	-122.4521117	37.93535	-0.66	15.9	-799.5	15.2809996	-122.45211	37.93536	-0.62	15.9	-801.84	15.3249996	11	7/24/2014	17:58.2
404	RWS11	-122.4521142	37.93535	-0.61	15.9	-799.5	15.3209999	-122.45211	37.93536	-0.62	15.9	-801.66	15.3139999	11	7/24/2014	17:58.3
405	RWS11	-122.4521167	37.93535	-0.66	15.9	-799.6	15.2570004	-122.45211	37.93536	-0.58	15.9	-801.69	15.3340004	11	7/24/2014	17:58.4
406	RWS11	-122.4521192	37.93535	-0.66	15.9	-799.4	15.2450003	-122.45211	37.93536	-0.58	15.9	-801.71	15.3220003	11	7/24/2014	17:58.5
407	RWS11	-122.4521217	37.93535	-0.66	15.9	-799.3	15.2330002	-122.45212	37.93536	-0.58	15.9	-801.67	15.3100002	11	7/24/2014	17:58.6
408	RWS11	-122.4521243	37.93535	-0.66	15.9	-799.1	15.2210001	-122.45212	37.93537	-0.5	15.9	-801.58	15.3830001	11	7/24/2014	17:58.7
409	RWS11	-122.4521268	37.93535	-0.66	15.9	-798.8	15.2100005	-122.45212	37.93537	-0.58	15.9	-801.35	15.2870004	11	7/24/2014	17:58.8
410	RWS11	-122.4521292	37.93535	-0.66	15.9	-798.5	15.1989998	-122.45212	37.93537	-0.5	15.9	-800.91	15.3609998	11	7/24/2014	17:58.9
411	RWS11	-122.4521329	37.93535	-0.66	15.8	-797.9	15.19	-122.45213	37.93537	-0.58	15.8	-800.32	15.267	11	7/24/2014	17:59.0
412	RWS11	-122.4521354	37.93536	-0.61	15.8	-797.1	15.2320002	-122.45213	37.93537	-0.5	15.8	-799.41	15.3430001	11	7/24/2014	17:59.1
413	RWS11	-122.4521379	37.93536	-0.66	15.8	-796.1	15.1720004	-122.45213	37.93537	-0.53	15.8	-798.42	15.3000003	11	7/24/2014	17:59.2
414	RWS11	-122.4521404	37.93536	-0.61	15.8	-795.1	15.215	-122.45213	37.93537	-0.58	15.8	-797.33	15.2409999	11	7/24/2014	17:59.3
415	RWS11	-122.452143	37.93536	-0.61	15.8	-793.6	15.2060002	-122.45214	37.93537	-0.53	15.8	-796.06	15.2830001	11	7/24/2014	17:59.4
416	RWS11	-122.4521455	37.93536	-0.61	15.8	-791.8	15.1970003	-122.45214	37.93537	-0.53	15.8	-794.7	15.2740003	11	7/24/2014	17:59.5
417	RWS11	-122.452148	37.93536	-0.61	15.8	-789.9	15.1870001	-122.45214	37.93537	-0.5	15.8	-793.02	15.2980001	11	7/24/2014	17:59.6
418	RWS11	-122.4521505	37.93536	-0.61	15.8	-788	15.1769999	-122.45214	37.93537	-0.53	15.8	-791.18	15.2539998	11	7/24/2014	17:59.7
419	RWS11	-122.452153	37.93536	-0.66	15.8	-786	15.1150002	-122.45215	37.93537	-0.53	15.8	-789.36	15.2430002	11	7/24/2014	17:59.8
420	RWS11	-122.4521555	37.93536	-0.61	15.8	-783.9	15.1549996	-122.45215	37.93537	-0.53	15.8	-787.45	15.2319995	11	7/24/2014	17:59.9
421	RWS11	-122.4521592	37.93536	-0.66	15.8	-781.8	15.0929999	-122.45215	37.93538	-0.53	15.8	-785.54	15.2209998	11	7/24/2014	18:00.0
422	RWS11	-122.4521616	37.93536	-0.61	15.7	-779.7	15.1330002	-122.45216	37.93538	-0.5	15.7	-783.6	15.2440002	11	7/24/2014	18:00.1
423	RWS11	-122.4521641	37.93536	-0.61	15.7	-777.4	15.123	-122.45216	37.93538	-0.58	15.7	-781.62	15.1489999	11	7/24/2014	18:00.2
424	RWS11	-122.4521666	37.93536	-0.61	15.7	-774.9	15.1129997	-122.45216	37.93538	-0.53	15.7	-779.62	15.1899997	11	7/24/2014	18:00.3
425	RWS11	-122.4521691	37.93537	-0.66	15.7	-772.5	15.0540003	-122.45216	37.93538	-0.53	15.7	-777.65	15.1820003	11	7/24/2014	18:00.4
426	RWS11	-122.4521716	37.93537	-0.61	15.7	-769.8	15.0980004	-122.45217	37.93538	-0.5	15.7	-775.28	15.2090003	11	7/24/2014	18:00.5
427	RWS11	-122.4521741	37.93537	-0.61	15.7	-767.3	15.0930002	-122.45217	37.93538	-0.5	15.7	-772.51	15.2040002	11	7/24/2014	18:00.6
428	RWS11	-122.4521766	37.93537	-0.57	15.7	-764.7	15.1239995	-122.45217	37.93538	-0.45	15.7	-769.52	15.2509995	11	7/24/2014	18:00.7

429	RWS11	-122.4521791	37.93537	-0.61	15.7	-762.3	15.0869997	-122.45217	37.93538	-0.5	15.7	-766.21	15.1979997	11	7/24/2014	18:00.8
430	RWS11	-122.4521816	37.93537	-0.61	15.7	-759.8	15.0869997	-122.45218	37.93538	-0.45	15.7	-762.6	15.2489997	11	7/24/2014	18:00.9
431	RWS11	-122.4521853	37.93537	-0.61	15.7	-756.9	15.0880001	-122.45218	37.93538	-0.5	15.7	-758.93	15.1990001	11	7/24/2014	18:01.0
432	RWS11	-122.4521878	37.93537	-0.57	15.7	-754.1	15.1239995	-122.45218	37.93538	-0.5	15.7	-755.15	15.1999995	11	7/24/2014	18:01.1
433	RWS11	-122.4521903	37.93537	-0.61	15.7	-751.2	15.0919998	-122.45218	37.93538	-0.5	15.7	-751.39	15.2029998	11	7/24/2014	18:01.2
434	RWS11	-122.4521929	37.93537	-0.57	15.7	-748.3	15.1300001	-122.45219	37.93539	-0.5	15.7	-747.52	15.2060001	11	7/24/2014	18:01.3
435	RWS11	-122.4521955	37.93537	-0.61	15.7	-745.2	15.0989998	-122.45219	37.93539	-0.5	15.7	-743.44	15.2099998	11	7/24/2014	18:01.4
436	RWS11	-122.4521981	37.93537	-0.57	15.7	-742.1	15.1389999	-122.45219	37.93539	-0.5	15.7	-738.95	15.2149999	11	7/24/2014	18:01.5
437	RWS11	-122.4522006	37.93537	-0.57	15.7	-738.7	15.144	-122.45219	37.93539	-0.53	15.7	-734.04	15.186	11	7/24/2014	18:01.6
438	RWS11	-122.4522032	37.93538	-0.57	15.7	-735	15.1499996	-122.4522	37.93539	-0.53	15.7	-728.93	15.1919996	11	7/24/2014	18:01.7
439	RWS11	-122.4522058	37.93538	-0.61	15.7	-730.6	15.1210001	-122.4522	37.93539	-0.5	15.7	-723.6	15.2320001	11	7/24/2014	18:01.8
440	RWS11	-122.4522083	37.93538	-0.57	15.7	-726.2	15.1619996	-122.4522	37.93539	-0.5	15.7	-718.1	15.2379997	11	7/24/2014	18:01.9
441	RWS11	-122.4522121	37.93538	-0.61	15.7	-721.5	15.1330002	-122.45221	37.93539	-0.5	15.7	-712.55	15.2440002	11	7/24/2014	18:02.0
442	RWS11	-122.4522146	37.93538	-0.57	15.7	-716.8	15.1739997	-122.45221	37.93539	-0.5	15.7	-707.14	15.2499997	11	7/24/2014	18:02.1
443	RWS11	-122.4522172	37.93538	-0.61	15.8	-711.8	15.1450003	-122.45221	37.93539	-0.5	15.8	-701.68	15.2560003	11	7/24/2014	18:02.2
444	RWS11	-122.4522198	37.93538	-0.57	15.8	-706.9	15.1859998	-122.45221	37.93539	-0.53	15.8	-696.07	15.2279998	11	7/24/2014	18:02.3
445	RWS11	-122.4522223	37.93538	-0.61	15.8	-701.8	15.156	-122.45222	37.93539	-0.53	15.8	-690.35	15.2329999	11	7/24/2014	18:02.4
446	RWS11	-122.4522249	37.93538	-0.61	15.8	-696.5	15.1599997	-122.45222	37.93539	-0.5	15.8	-684.62	15.2709996	11	7/24/2014	18:02.5
447	RWS11	-122.4522275	37.93538	-0.61	15.8	-691.2	15.1640003	-122.45222	37.9354	-0.53	15.8	-678.78	15.2410003	11	7/24/2014	18:02.6
448	RWS11	-122.45223	37.93538	-0.57	15.8	-685.9	15.2010002	-122.45222	37.9354	-0.5	15.8	-672.96	15.2770002	11	7/24/2014	18:02.7
449	RWS11	-122.4522326	37.93538	-0.61	15.8	-680.5	15.168	-122.45223	37.9354	-0.53	15.8	-667.15	15.245	11	7/24/2014	18:02.8
450	RWS11	-122.4522351	37.93538	-0.57	15.8	-675.2	15.2049999	-122.45223	37.9354	-0.5	15.8	-661.43	15.2809999	11	7/24/2014	18:02.9
451	RWS11	-122.4522389	37.93539	-0.57	15.8	-669.8	15.2069997	-122.45223	37.9354	-0.5	15.8	-655.63	15.2829997	11	7/24/2014	18:03.0
452	RWS11	-122.4522414	37.93539	-0.57	15.8	-664.3	15.2110004	-122.45224	37.9354	-0.5	15.8	-649.53	15.2870004	11	7/24/2014	18:03.1
453	RWS11	-122.4522439	37.93539	-0.57	15.8	-658.5	15.2139997	-122.45224	37.9354	-0.5	15.8	-643.04	15.2899997	11	7/24/2014	18:03.2
454	RWS11	-122.4522465	37.93539	-0.57	15.8	-652.3	15.2189998	-122.45224	37.9354	-0.5	15.8	-636.16	15.2949998	11	7/24/2014	18:03.3
455	RWS11	-122.452249	37.93539	-0.57	15.8	-645.6	15.2250003	-122.45224	37.9354	-0.53	15.8	-628.96	15.2670003	11	7/24/2014	18:03.4
456	RWS11	-122.4522516	37.93539	-0.57	15.8	-638.8	15.2309999	-122.45225	37.9354	-0.5	15.8	-621.76	15.3069999	11	7/24/2014	18:03.5
457	RWS11	-122.4522541	37.93539	-0.61	15.8	-631.9	15.2029999	-122.45225	37.9354	-0.45	15.8	-614.46	15.3649999	11	7/24/2014	18:03.6
458	RWS11	-122.4522567	37.93539	-0.57	15.8	-625	15.2460002	-122.45225	37.9354	-0.45	15.8	-607.21	15.3730002	11	7/24/2014	18:03.7
459	RWS11	-122.4522592	37.93539	-0.57	15.8	-618	15.2550001	-122.45225	37.9354	-0.5	15.8	-599.84	15.3310001	11	7/24/2014	18:03.8
460	RWS11	-122.4522617	37.93539	-0.57	15.8	-610.9	15.2659997	-122.45226	37.93541	-0.45	15.8	-592.3	15.3929997	11	7/24/2014	18:03.9
461	RWS11	-122.4522655	37.93539	-0.57	15.9	-604	15.2779998	-122.45226	37.93541	-0.5	15.9	-584.75	15.3539998	11	7/24/2014	18:04.0

462	RWS11	-122.452268	37.93539	-0.57	15.9	-597.1	15.2919998	-122.45226	37.93541	-0.45	15.9	-577.2	15.4189998	11	7/24/2014	18:04.1
463	RWS11	-122.4522705	37.9354	-0.57	15.9	-590.3	15.309	-122.45226	37.93541	-0.45	15.9	-569.45	15.436	11	7/24/2014	18:04.2
464	RWS11	-122.452273	37.9354	-0.52	15.9	-583.5	15.379	-122.45227	37.93541	-0.36	15.9	-561.59	15.539	11	7/24/2014	18:04.3
465	RWS11	-122.4522756	37.9354	-0.57	15.9	-576.7	15.3500003	-122.45227	37.93541	-0.45	15.9	-553.57	15.4770003	11	7/24/2014	18:04.4
466	RWS11	-122.4522781	37.9354	-0.52	15.9	-569.8	15.4259999	-122.45227	37.93541	-0.41	15.9	-545.3	15.536	11	7/24/2014	18:04.5
467	RWS11	-122.4522807	37.9354	-0.52	16	-562.6	15.4530004	-122.45227	37.93541	-0.41	16	-536.96	15.5630004	11	7/24/2014	18:04.6
468	RWS11	-122.4522832	37.9354	-0.52	16	-555	15.4829992	-122.45228	37.93541	-0.41	16	-528.19	15.5929992	11	7/24/2014	18:04.7
469	RWS11	-122.4522857	37.9354	-0.57	16	-547	15.4650001	-122.45228	37.93541	-0.45	16	-519.14	15.5920001	11	7/24/2014	18:04.8
470	RWS11	-122.4522883	37.9354	-0.52	16.1	-538.8	15.5500005	-122.45228	37.93541	-0.41	16.1	-509.77	15.6600005	11	7/24/2014	18:04.9
471	RWS11	-122.4522921	37.9354	-0.52	16.1	-530	15.5880006	-122.45229	37.93541	-0.45	16.1	-500.08	15.6640006	11	7/24/2014	18:05.0
472	RWS11	-122.4522946	37.9354	-0.52	16.1	-520.7	15.6260007	-122.45229	37.93541	-0.36	16.1	-490.2	15.7860007	11	7/24/2014	18:05.1
473	RWS11	-122.4522971	37.9354	-0.52	16.2	-511.1	15.6669992	-122.45229	37.93542	-0.45	16.2	-480.12	15.7429992	11	7/24/2014	18:05.2
474	RWS11	-122.4522997	37.9354	-0.52	16.2	-501.8	15.7100003	-122.45229	37.93542	-0.45	16.2	-470	15.7860003	11	7/24/2014	18:05.3
475	RWS11	-122.4523023	37.9354	-0.52	16.3	-492.4	15.7559999	-122.4523	37.93542	-0.41	16.3	-459.76	15.8659999	11	7/24/2014	18:05.4
476	RWS11	-122.4523049	37.9354	-0.52	16.3	-483	15.8049997	-122.4523	37.93542	-0.45	16.3	-449.19	15.8809997	11	7/24/2014	18:05.5
477	RWS11	-122.4523075	37.93541	-0.57	16.4	-473.7	15.8059997	-122.4523	37.93542	-0.45	16.4	-438.54	15.9329997	11	7/24/2014	18:05.6
478	RWS11	-122.45231	37.93541	-0.57	16.4	-464.5	15.861	-122.4523	37.93542	-0.45	16.4	-427.62	15.988	11	7/24/2014	18:05.7
479	RWS11	-122.4523126	37.93541	-0.57	16.5	-454.9	15.9179992	-122.45231	37.93542	-0.45	16.5	-416.45	16.0449992	11	7/24/2014	18:05.8
480	RWS11	-122.4523152	37.93541	-0.57	16.5	-445.1	15.9769992	-122.45231	37.93542	-0.5	16.5	-404.91	16.0529992	11	7/24/2014	18:05.9
481	RWS11	-122.452319	37.93541	-0.57	16.6	-435.1	16.0359992	-122.45231	37.93542	-0.5	16.6	-393.13	16.1119992	11	7/24/2014	18:06.0
482	RWS11	-122.4523215	37.93541	-0.57	16.7	-424.7	16.0939998	-122.45232	37.93542	-0.45	16.7	-380.94	16.2209998	11	7/24/2014	18:06.1
483	RWS11	-122.4523241	37.93541	-0.57	16.7	-414.1	16.1499996	-122.45232	37.93542	-0.5	16.7	-368.49	16.2259996	11	7/24/2014	18:06.2
484	RWS11	-122.4523267	37.93541	-0.52	16.8	-403.3	16.2529996	-122.45232	37.93542	-0.5	16.8	-355.93	16.2779996	11	7/24/2014	18:06.3
485	RWS11	-122.4523293	37.93541	-0.57	16.8	-392.3	16.2499999	-122.45232	37.93542	-0.53	16.8	-343.37	16.2919999	11	7/24/2014	18:06.4
486	RWS11	-122.4523319	37.93541	-0.52	16.9	-381.2	16.3439992	-122.45233	37.93543	-0.5	16.9	-330.82	16.3689992	11	7/24/2014	18:06.5
487	RWS11	-122.4523345	37.93541	-0.57	16.9	-370	16.3309993	-122.45233	37.93543	-0.53	16.9	-318.34	16.3729993	11	7/24/2014	18:06.6
488	RWS11	-122.452337	37.93541	-0.57	16.9	-358.9	16.3640003	-122.45233	37.93543	-0.5	16.9	-306.15	16.4400003	11	7/24/2014	18:06.7
489	RWS11	-122.4523396	37.93541	-0.57	17	-347.7	16.3929996	-122.45233	37.93543	-0.53	17	-293.86	16.4349996	11	7/24/2014	18:06.8
490	RWS11	-122.4523422	37.93542	-0.57	17	-336.3	16.4179992	-122.45234	37.93543	-0.45	17	-281.46	16.5449992	11	7/24/2014	18:06.9
491	RWS11	-122.452346	37.93542	-0.57	17	-324.7	16.4389991	-122.45234	37.93543	-0.5	17	-268.74	16.5149991	11	7/24/2014	18:07.0
492	RWS11	-122.4523486	37.93542	-0.52	17	-312.9	16.5069993	-122.45234	37.93543	-0.45	17	-255.63	16.5829993	11	7/24/2014	18:07.1
493	RWS11	-122.4523512	37.93542	-0.57	17	-300.7	16.4720001	-122.45235	37.93543	-0.53	17	-242.1	16.5140001	11	7/24/2014	18:07.2
494	RWS11	-122.4523538	37.93542	-0.52	17.1	-288.1	16.5349992	-122.45235	37.93543	-0.5	17.1	-228.22	16.5599992	11	7/24/2014	18:07.3

495	RWS11	-122.4523564	37.93542	-0.57	17.1	-275.4	16.4960002	-122.45235	37.93543	-0.5	17.1	-213.94	16.5720002	11	7/24/2014	18:07.4
496	RWS11	-122.4523591	37.93542	-0.52	17.1	-262.5	16.5559991	-122.45235	37.93543	-0.5	17.1	-199.36	16.5809991	11	7/24/2014	18:07.5
497	RWS11	-122.4523617	37.93542	-0.57	17.1	-249.5	16.5139999	-122.45236	37.93543	-0.58	17.1	-184.45	16.5049999	11	7/24/2014	18:07.6
498	RWS11	-122.4523643	37.93542	-0.52	17.1	-236.2	16.5710004	-122.45236	37.93543	-0.53	17.1	-169.28	16.5620004	11	7/24/2014	18:07.7
499	RWS11	-122.4523669	37.93542	-0.57	17.1	-222.7	16.5240001	-122.45236	37.93544	-0.53	17.1	-154.02	16.5660001	11	7/24/2014	18:07.8
500	RWS11	-122.4523694	37.93542	-0.57	17.1	-209	16.5260009	-122.45236	37.93544	-0.5	17.1	-138.95	16.6020009	11	7/24/2014	18:07.9
501	RWS11	-122.4523732	37.93542	-0.61	17.1	-195.5	16.4899996	-122.45237	37.93544	-0.58	17.1	-123.92	16.5159996	11	7/24/2014	18:08.0
502	RWS11	-122.4523758	37.93542	-0.57	17.1	-181.9	16.5219993	-122.45237	37.93544	-0.53	17.1	-108.66	16.5639993	11	7/24/2014	18:08.1
503	RWS11	-122.4523783	37.93543	-0.61	17.1	-168.1	16.4820002	-122.45237	37.93544	-0.53	17.1	-93.173	16.5590001	11	7/24/2014	18:08.2
504	RWS11	-122.4523809	37.93543	-0.57	17.1	-154.3	16.5119991	-122.45238	37.93544	-0.53	17.1	-77.509	16.5539991	11	7/24/2014	18:08.3
505	RWS11	-122.4523834	37.93543	-0.57	17.1	-140.6	16.5069999	-122.45238	37.93544	-0.58	17.1	-61.735	16.4979999	11	7/24/2014	18:08.4
506	RWS11	-122.452386	37.93543	-0.57	17.1	-126.8	16.5030002	-122.45238	37.93544	-0.53	17.1	-45.847	16.5450002	11	7/24/2014	18:08.5
507	RWS11	-122.4523885	37.93543	-0.57	17.1	-112.9	16.4999999	-122.45238	37.93544	-0.53	17.1	-29.638	16.5419999	11	7/24/2014	18:08.6
508	RWS11	-122.4523911	37.93543	-0.57	17.1	-98.69	16.4979991	-122.45239	37.93544	-0.53	17.1	-13.382	16.5399991	11	7/24/2014	18:08.7
509	RWS11	-122.4523936	37.93543	-0.61	17.1	-84.32	16.4629992	-122.45239	37.93544	-0.58	17.1	2.895	16.4889991	11	7/24/2014	18:08.8
510	RWS11	-122.4523961	37.93543	-0.57	17.1	-69.76	16.4990005	-122.45239	37.93544	-0.53	17.1	19.127	16.5410005	11	7/24/2014	18:08.9
511	RWS11	-122.4523999	37.93543	-0.57	17.1	-55.12	16.5020008	-122.45239	37.93544	-0.53	17.1	35.466	16.5440007	11	7/24/2014	18:09.0
512	RWS11	-122.4524025	37.93543	-0.57	17.1	-40.4	16.5049991	-122.4524	37.93545	-0.53	17.1	52.248	16.5469991	11	7/24/2014	18:09.1
513	RWS11	-122.452405	37.93543	-0.57	17.1	-25.47	16.5090007	-122.4524	37.93545	-0.58	17.1	69.547	16.5000007	11	7/24/2014	18:09.2
514	RWS11	-122.4524076	37.93543	-0.57	17.1	-10.26	16.5109996	-122.4524	37.93545	-0.58	17.1	87.002	16.5019996	11	7/24/2014	18:09.3
515	RWS11	-122.4524102	37.93543	-0.57	17.1	4.922	16.5109996	-122.4524	37.93545	-0.58	17.1	104.35	16.5019996	11	7/24/2014	18:09.4
516	RWS11	-122.4524128	37.93544	-0.57	17.1	20.37	16.5079994	-122.45241	37.93545	-0.53	17.1	121.96	16.5499994	11	7/24/2014	18:09.5
517	RWS11	-122.4524154	37.93544	-0.57	17.1	35.773	16.4990005	-122.45241	37.93545	-0.58	17.1	139.72	16.4900005	11	7/24/2014	18:09.6
518	RWS11	-122.452418	37.93544	-0.57	17.1	51.025	16.4829997	-122.45241	37.93545	-0.58	17.1	157.3	16.4739997	11	7/24/2014	18:09.7
519	RWS11	-122.4524205	37.93544	-0.61	17	66.497	16.4239996	-122.45241	37.93545	-0.58	17	174.83	16.4499996	11	7/24/2014	18:09.8
520	RWS11	-122.4524231	37.93544	-0.57	17	81.942	16.427	-122.45242	37.93545	-0.58	17	192.14	16.418	11	7/24/2014	18:09.9
521	RWS11	-122.4524269	37.93544	-0.61	17	97.458	16.3530005	-122.45242	37.93545	-0.58	17	209.37	16.3790004	11	7/24/2014	18:10.0
522	RWS11	-122.4524294	37.93544	-0.57	16.9	113.01	16.3430004	-122.45242	37.93545	-0.58	16.9	226.9	16.3340004	11	7/24/2014	18:10.1
523	RWS11	-122.452432	37.93544	-0.57	16.9	128.74	16.2929992	-122.45243	37.93545	-0.5	16.9	244.82	16.3689992	11	7/24/2014	18:10.2
524	RWS11	-122.4524346	37.93544	-0.57	16.8	144.75	16.2399997	-122.45243	37.93545	-0.58	16.8	262.74	16.2309997	11	7/24/2014	18:10.3
525	RWS11	-122.4524371	37.93544	-0.57	16.8	160.91	16.1849994	-122.45243	37.93545	-0.58	16.8	280.63	16.1759994	11	7/24/2014	18:10.4
526	RWS11	-122.4524398	37.93544	-0.52	16.7	177.07	16.1809991	-122.45243	37.93546	-0.53	16.7	298.58	16.1719991	11	7/24/2014	18:10.5
527	RWS11	-122.4524423	37.93544	-0.57	16.6	193.54	16.0760002	-122.45244	37.93546	-0.53	16.6	317	16.1180002	11	7/24/2014	18:10.6

528	RWS11	-122.4524449	37.93544	-0.52	16.6	210.13	16.0759996	-122.45244	37.93546	-0.5	16.6	335.77	16.1009996	11	7/24/2014	18:10.7
529	RWS11	-122.4524475	37.93544	-0.57	16.5	226.9	15.9769992	-122.45244	37.93546	-0.58	16.5	355.13	15.9679992	11	7/24/2014	18:10.8
530	RWS11	-122.45245	37.93545	-0.52	16.5	244.22	15.9829992	-122.45244	37.93546	-0.5	16.5	374.89	16.0079992	11	7/24/2014	18:10.9
531	RWS11	-122.4524538	37.93545	-0.57	16.5	261.64	15.8929996	-122.45245	37.93546	-0.53	16.5	394.74	15.9349996	11	7/24/2014	18:11.0
532	RWS11	-122.4524564	37.93545	-0.57	16.4	279.29	15.8579997	-122.45245	37.93546	-0.53	16.4	414.77	15.8999997	11	7/24/2014	18:11.1
533	RWS11	-122.4524589	37.93545	-0.57	16.4	297.19	15.8279991	-122.45245	37.93546	-0.53	16.4	435.09	15.8699991	11	7/24/2014	18:11.2
534	RWS11	-122.4524615	37.93545	-0.57	16.4	315.13	15.8040008	-122.45246	37.93546	-0.58	16.4	455.49	15.7950008	11	7/24/2014	18:11.3
535	RWS11	-122.4524641	37.93545	-0.57	16.4	332.89	15.7849998	-122.45246	37.93546	-0.58	16.4	475.81	15.7759998	11	7/24/2014	18:11.4
536	RWS11	-122.4524667	37.93545	-0.52	16.3	350.48	15.8210004	-122.45246	37.93546	-0.53	16.3	495.82	15.8120004	11	7/24/2014	18:11.5
537	RWS11	-122.4524693	37.93545	-0.57	16.3	368.05	15.7609996	-122.45246	37.93546	-0.58	16.3	515.68	15.7519996	11	7/24/2014	18:11.6
538	RWS11	-122.4524719	37.93545	-0.52	16.3	385.42	15.8079999	-122.45247	37.93546	-0.58	16.3	535.5	15.7479999	11	7/24/2014	18:11.7
539	RWS11	-122.4524745	37.93545	-0.57	16.3	402.83	15.7569999	-122.45247	37.93546	-0.53	16.3	555.34	15.7989999	11	7/24/2014	18:11.8
540	RWS11	-122.4524771	37.93545	-0.52	16.3	420.24	15.8129991	-122.45247	37.93547	-0.58	16.3	575.23	15.7529991	11	7/24/2014	18:11.9
541	RWS11	-122.4524809	37.93545	-0.52	16.3	437.59	15.8229993	-122.45248	37.93547	-0.58	16.3	595.15	15.7629993	11	7/24/2014	18:12.0
542	RWS11	-122.4524834	37.93545	-0.52	16.4	454.93	15.8369992	-122.45248	37.93547	-0.5	16.4	614.99	15.8619992	11	7/24/2014	18:12.1
543	RWS11	-122.452486	37.93546	-0.52	16.4	472.29	15.8560002	-122.45248	37.93547	-0.5	16.4	634.87	15.8810003	11	7/24/2014	18:12.2
544	RWS11	-122.4524885	37.93546	-0.57	16.4	489.81	15.8269996	-122.45248	37.93547	-0.5	16.4	654.92	15.9029996	11	7/24/2014	18:12.3
545	RWS11	-122.4524911	37.93546	-0.52	16.4	507.51	15.9040006	-122.45249	37.93547	-0.58	16.4	674.81	15.8440006	11	7/24/2014	18:12.4
546	RWS11	-122.4524937	37.93546	-0.52	16.5	525.05	15.9329999	-122.45249	37.93547	-0.53	16.5	694.67	15.9239999	11	7/24/2014	18:12.5
547	RWS11	-122.4524963	37.93546	-0.52	16.5	542.8	15.9640001	-122.45249	37.93547	-0.5	16.5	714.74	15.9890001	11	7/24/2014	18:12.6
548	RWS11	-122.4524988	37.93546	-0.49	16.5	560.5	16.0309991	-122.45249	37.93547	-0.5	16.5	734.76	16.0219991	11	7/24/2014	18:12.7
549	RWS11	-122.4525014	37.93546	-0.52	16.6	578.27	16.03	-122.4525	37.93547	-0.5	16.6	755.01	16.055	11	7/24/2014	18:12.8
550	RWS11	-122.4525039	37.93546	-0.52	16.6	596.07	16.0640004	-122.4525	37.93547	-0.45	16.6	775.52	16.1400004	11	7/24/2014	18:12.9
551	RWS11	-122.4525077	37.93546	-0.52	16.6	613.79	16.0969995	-122.4525	37.93547	-0.5	16.6	796.15	16.1219995	11	7/24/2014	18:13.0
552	RWS11	-122.4525103	37.93546	-0.49	16.7	631.53	16.1649999	-122.4525	37.93547	-0.5	16.7	816.84	16.1559999	11	7/24/2014	18:13.1
553	RWS11	-122.4525128	37.93546	-0.52	16.7	649.3	16.1640008	-122.45251	37.93548	-0.5	16.7	837.56	16.1890008	11	7/24/2014	18:13.2
554	RWS11	-122.4525154	37.93546	-0.49	16.7	667.1	16.2309999	-122.45251	37.93548	-0.5	16.7	858.3	16.2219999	11	7/24/2014	18:13.3
555	RWS11	-122.4525179	37.93546	-0.52	16.8	685.11	16.2289994	-122.45251	37.93548	-0.5	16.8	879.19	16.2539994	11	7/24/2014	18:13.4
556	RWS11	-122.4525205	37.93546	-0.49	16.8	703.1	16.2950009	-122.45251	37.93548	-0.5	16.8	899.93	16.2860009	11	7/24/2014	18:13.5
557	RWS11	-122.4525231	37.93547	-0.52	16.8	720.96	16.294	-122.45252	37.93548	-0.5	16.8	920.68	16.319	11	7/24/2014	18:13.6
558	RWS11	-122.4525257	37.93547	-0.52	16.8	738.84	16.3259996	-122.45252	37.93548	-0.5	16.8	941.23	16.3509996	11	7/24/2014	18:13.7
559	RWS11	-122.4525282	37.93547	-0.52	16.9	756.48	16.3590005	-122.45252	37.93548	-0.5	16.9	961.49	16.3840005	11	7/24/2014	18:13.8
560	RWS11	-122.4525308	37.93547	-0.49	16.9	774.03	16.4270009	-122.45253	37.93548	-0.5	16.9	981.45	16.4180009	11	7/24/2014	18:13.9

561	RWS11	-122.4525346	37.93547	-0.49	17	791.29	16.4620008	-122.45253	37.93548	-0.53	17	1001.1	16.4190007	11	7/24/2014	18:14.0
562	RWS11	-122.4525372	37.93547	-0.49	17	808.37	16.4970006	-122.45253	37.93548	-0.5	17	1020.7	16.4880006	11	7/24/2014	18:14.1
563	RWS11	-122.4525397	37.93547	-0.52	17	825.22	16.4980004	-122.45253	37.93548	-0.53	17	1040.1	16.4890004	11	7/24/2014	18:14.2
564	RWS11	-122.4525423	37.93547	-0.49	17.1	841.85	16.5679998	-122.45254	37.93548	-0.53	17.1	1059.1	16.5249997	11	7/24/2014	18:14.3
565	RWS11	-122.4525449	37.93547	-0.49	17.1	858.11	16.6029996	-122.45254	37.93548	-0.5	17.1	1077.7	16.5939996	11	7/24/2014	18:14.4
566	RWS11	-122.4525475	37.93547	-0.49	17.1	874.11	16.6360005	-122.45254	37.93548	-0.5	17.1	1096.1	16.6270005	11	7/24/2014	18:14.5
567	RWS11	-122.4525502	37.93547	-0.49	17.2	889.79	16.6680001	-122.45254	37.93549	-0.5	17.2	1113.9	16.6590001	11	7/24/2014	18:14.6
568	RWS11	-122.4525527	37.93547	-0.49	17.2	905.01	16.6969995	-122.45255	37.93549	-0.41	17.2	1131.4	16.7729995	11	7/24/2014	18:14.7
569	RWS11	-122.4525553	37.93547	-0.49	17.2	919.84	16.7230004	-122.45255	37.93549	-0.45	17.2	1148.2	16.7650004	11	7/24/2014	18:14.8
570	RWS11	-122.4525579	37.93547	-0.49	17.2	934.47	16.7449998	-122.45255	37.93549	-0.5	17.2	1165	16.7359998	11	7/24/2014	18:14.9
571	RWS11	-122.4525616	37.93548	-0.49	17.2	948.89	16.7610005	-122.45256	37.93549	-0.45	17.2	1181.6	16.8030005	11	7/24/2014	18:15.0
572	RWS11	-122.4525642	37.93548	-0.44	17.3	962.98	16.8220008	-122.45256	37.93549	-0.5	17.3	1197.7	16.7620008	11	7/24/2014	18:15.1
573	RWS11	-122.4525667	37.93548	-0.49	17.3	976.65	16.7720002	-122.45256	37.93549	-0.5	17.3	1213.3	16.7630002	11	7/24/2014	18:15.2
574	RWS11	-122.4525692	37.93548	-0.49	17.3	990.05	16.7650003	-122.45256	37.93549	-0.41	17.3	1228.5	16.8410003	11	7/24/2014	18:15.3
575	RWS11	-122.4525718	37.93548	-0.49	17.2	1003.2	16.7489995	-122.45257	37.93549	-0.5	17.2	1243.5	16.7399995	11	7/24/2014	18:15.4
576	RWS11	-122.4525743	37.93548	-0.49	17.2	1016.2	16.7260007	-122.45257	37.93549	-0.45	17.2	1258.4	16.7680007	11	7/24/2014	18:15.5
577	RWS11	-122.4525769	37.93548	-0.49	17.2	1028.9	16.6969995	-122.45257	37.93549	-0.45	17.2	1272.9	16.7389995	11	7/24/2014	18:15.6
578	RWS11	-122.4525794	37.93548	-0.44	17.2	1041	16.7139991	-122.45257	37.93549	-0.45	17.2	1286.8	16.7049991	11	7/24/2014	18:15.7
579	RWS11	-122.4525819	37.93548	-0.49	17.1	1052.6	16.6260003	-122.45258	37.93549	-0.45	17.1	1300.2	16.6680003	11	7/24/2014	18:15.8
580	RWS11	-122.4525845	37.93548	-0.44	17.1	1063.9	16.6380008	-122.45258	37.9355	-0.41	17.1	1313.1	16.6630008	11	7/24/2014	18:15.9
581	RWS11	-122.4525882	37.93548	-0.49	17	1074.6	16.5460004	-122.45258	37.9355	-0.45	17	1325.4	16.5880004	11	7/24/2014	18:16.0
582	RWS11	-122.4525907	37.93548	-0.44	17	1084.9	16.5569995	-122.45258	37.9355	-0.45	17	1337.1	16.5479995	11	7/24/2014	18:16.1
583	RWS11	-122.4525932	37.93548	-0.49	17	1094.6	16.4649991	-122.45259	37.9355	-0.41	17	1348.2	16.5409991	11	7/24/2014	18:16.2
584	RWS11	-122.4525958	37.93549	-0.44	16.9	1103.9	16.4769996	-122.45259	37.9355	-0.36	16.9	1358.7	16.5519996	11	7/24/2014	18:16.3
585	RWS11	-122.4525984	37.93549	-0.44	16.9	1112.5	16.4389994	-122.45259	37.9355	-0.45	16.9	1368.8	16.4299994	11	7/24/2014	18:16.4
586	RWS11	-122.4526009	37.93549	-0.44	16.8	1120.9	16.4039996	-122.4526	37.9355	-0.41	16.8	1378.3	16.4289996	11	7/24/2014	18:16.5
587	RWS11	-122.4526035	37.93549	-0.49	16.8	1128.9	16.321	-122.4526	37.9355	-0.45	16.8	1387.3	16.363	11	7/24/2014	18:16.6
588	RWS11	-122.4526061	37.93549	-0.44	16.8	1136.5	16.3430007	-122.4526	37.9355	-0.41	16.8	1395.9	16.3680007	11	7/24/2014	18:16.7
589	RWS11	-122.4526086	37.93549	-0.49	16.8	1143.7	16.2680005	-122.4526	37.9355	-0.45	16.8	1404	16.3100005	11	7/24/2014	18:16.8
590	RWS11	-122.4526112	37.93549	-0.44	16.7	1150.4	16.2990001	-122.45261	37.9355	-0.41	16.7	1411.6	16.3240001	11	7/24/2014	18:16.9
591	RWS11	-122.452615	37.93549	-0.44	16.7	1157	16.2829993	-122.45261	37.9355	-0.41	16.7	1418.7	16.3079993	11	7/24/2014	18:17.0
592	RWS11	-122.4526176	37.93549	-0.44	16.7	1163.1	16.2710002	-122.45261	37.9355	-0.41	16.7	1425.5	16.2960002	11	7/24/2014	18:17.1
593	RWS11	-122.4526202	37.93549	-0.49	16.7	1168.8	16.21	-122.45261	37.93551	-0.41	16.7	1431.7	16.286	11	7/24/2014	18:17.2

594	RWS11	-122.4526229	37.93549	-0.44	16.7	1174	16.2519992	-122.45262	37.93551	-0.41	16.7	1437.3	16.2769992	11	7/24/2014	18:17.3
595	RWS11	-122.4526255	37.93549	-0.49	16.7	1178.6	16.1920003	-122.45262	37.93551	-0.41	16.7	1442.1	16.2680003	11	7/24/2014	18:17.4
596	RWS11	-122.4526282	37.93549	-0.44	16.7	1182.7	16.2330001	-122.45262	37.93551	-0.41	16.7	1446.4	16.2580001	11	7/24/2014	18:17.5
597	RWS11	-122.4526309	37.9355	-0.44	16.7	1186.1	16.2220004	-122.45263	37.93551	-0.41	16.7	1450	16.2470004	11	7/24/2014	18:17.6
598	RWS11	-122.4526335	37.9355	-0.44	16.6	1188.9	16.2080005	-122.45263	37.93551	-0.41	16.6	1452.8	16.2330005	11	7/24/2014	18:17.7
599	RWS11	-122.4526362	37.9355	-0.49	16.6	1191.3	16.1419992	-122.45263	37.93551	-0.41	16.6	1454.9	16.2179992	11	7/24/2014	18:17.8
600	RWS11	-122.4526388	37.9355	-0.44	16.6	1193	16.1740001	-122.45263	37.93551	-0.45	16.6	1456.4	16.1650001	11	7/24/2014	18:17.9
601	RWS11	-122.4526427	37.9355	-0.44	16.6	1194.1	16.1530002	-122.45264	37.93551	-0.41	16.6	1457.1	16.1780002	11	7/24/2014	18:18.0
602	RWS11	-122.4526453	37.9355	-0.44	16.6	1194.4	16.1299994	-122.45264	37.93551	-0.45	16.6	1456.9	16.1209994	11	7/24/2014	18:18.1
603	RWS11	-122.4526479	37.9355	-0.44	16.5	1193.9	16.1040004	-122.45264	37.93551	-0.45	16.5	1455.9	16.0950004	11	7/24/2014	18:18.2
604	RWS11	-122.4526505	37.9355	-0.44	16.5	1192.8	16.0760005	-122.45264	37.93551	-0.45	16.5	1454	16.0670005	11	7/24/2014	18:18.3
605	RWS11	-122.4526531	37.9355	-0.44	16.5	1190.8	16.0459998	-122.45265	37.93551	-0.45	16.5	1451.3	16.0369998	11	7/24/2014	18:18.4
606	RWS11	-122.4526557	37.9355	-0.44	16.5	1188.3	16.0140002	-122.45265	37.93552	-0.41	16.5	1448	16.0390002	11	7/24/2014	18:18.5
607	RWS11	-122.4526583	37.9355	-0.44	16.4	1185.3	15.9799998	-122.45265	37.93552	-0.45	16.4	1444.1	15.9709998	11	7/24/2014	18:18.6
608	RWS11	-122.4526609	37.9355	-0.44	16.4	1181.6	15.9440005	-122.45265	37.93552	-0.41	16.4	1439.4	15.9690005	11	7/24/2014	18:18.7
609	RWS11	-122.4526635	37.93551	-0.49	16.3	1177.4	15.8559999	-122.45266	37.93552	-0.41	16.3	1434.2	15.9319999	11	7/24/2014	18:18.8
610	RWS11	-122.452666	37.93551	-0.44	16.3	1172.5	15.8680003	-122.45266	37.93552	-0.41	16.3	1428.2	15.8930003	11	7/24/2014	18:18.9
611	RWS11	-122.4526698	37.93551	-0.44	16.3	1167.1	15.8290007	-122.45266	37.93552	-0.41	16.3	1421.6	15.8540008	11	7/24/2014	18:19.0
612	RWS11	-122.4526724	37.93551	-0.44	16.2	1161	15.7899993	-122.45267	37.93552	-0.36	16.2	1414.4	15.8649993	11	7/24/2014	18:19.1
613	RWS11	-122.4526749	37.93551	-0.44	16.2	1154.6	15.7500003	-122.45267	37.93552	-0.41	16.2	1406.5	15.7750003	11	7/24/2014	18:19.2
614	RWS11	-122.4526775	37.93551	-0.44	16.1	1147.5	15.7089999	-122.45267	37.93552	-0.41	16.1	1397.8	15.7339999	11	7/24/2014	18:19.3
615	RWS11	-122.4526801	37.93551	-0.44	16.1	1139.7	15.6700003	-122.45267	37.93552	-0.41	16.1	1388.5	15.6950004	11	7/24/2014	18:19.4
616	RWS11	-122.4526826	37.93551	-0.44	16.1	1131.2	15.6310008	-122.45268	37.93552	-0.36	16.1	1378.4	15.7060008	11	7/24/2014	18:19.5
617	RWS11	-122.4526852	37.93551	-0.44	16	1122.3	15.5940001	-122.45268	37.93552	-0.45	16	1367.6	15.5850001	11	7/24/2014	18:19.6
618	RWS11	-122.4526878	37.93551	-0.4	16	1112.8	15.5940003	-122.45268	37.93553	-0.41	16	1356.3	15.5840003	11	7/24/2014	18:19.7
619	RWS11	-122.4526903	37.93551	-0.44	16	1102.9	15.5249999	-122.45268	37.93553	-0.45	16	1344.6	15.5159999	11	7/24/2014	18:19.8
620	RWS11	-122.4526928	37.93551	-0.44	15.9	1092.6	15.4939998	-122.45269	37.93553	-0.45	15.9	1332.4	15.4849998	11	7/24/2014	18:19.9
621	RWS11	-122.4526966	37.93552	-0.44	15.9	1081.8	15.4650004	-122.45269	37.93553	-0.41	15.9	1319.7	15.4900004	11	7/24/2014	18:20.0
622	RWS11	-122.4526991	37.93552	-0.44	15.9	1070.4	15.438	-122.45269	37.93553	-0.41	15.9	1306.4	15.463	11	7/24/2014	18:20.1
623	RWS11	-122.4527016	37.93552	-0.44	15.9	1058.8	15.4139998	-122.4527	37.93553	-0.45	15.9	1292.9	15.4049998	11	7/24/2014	18:20.2
624	RWS11	-122.4527042	37.93552	-0.4	15.8	1046.9	15.4260001	-122.4527	37.93553	-0.45	15.8	1279.2	15.3820001	11	7/24/2014	18:20.3
625	RWS11	-122.4527067	37.93552	-0.44	15.8	1034.9	15.3709996	-122.4527	37.93553	-0.45	15.8	1265.1	15.3619996	11	7/24/2014	18:20.4
626	RWS11	-122.4527092	37.93552	-0.44	15.8	1022.8	15.353	-122.4527	37.93553	-0.45	15.8	1251	15.344	11	7/24/2014	18:20.5

627	RWS11	-122.4527118	37.93552	-0.44	15.8	1010.7	15.3359998	-122.45271	37.93553	-0.45	15.8	1236.9	15.3269998	11	7/24/2014	18:20.6
628	RWS11	-122.4527143	37.93552	-0.44	15.8	998.67	15.3210004	-122.45271	37.93553	-0.45	15.8	1222.7	15.3120004	11	7/24/2014	18:20.7
629	RWS11	-122.4527168	37.93552	-0.49	15.7	986.57	15.2560004	-122.45271	37.93553	-0.45	15.7	1208.5	15.2980004	11	7/24/2014	18:20.8
630	RWS11	-122.4527193	37.93552	-0.44	15.7	974.42	15.2929995	-122.45271	37.93553	-0.45	15.7	1194.1	15.2839995	11	7/24/2014	18:20.9
631	RWS11	-122.452723	37.93552	-0.49	15.7	962.09	15.2300004	-122.45272	37.93554	-0.45	15.7	1179.5	15.2720004	11	7/24/2014	18:21.0
632	RWS11	-122.4527255	37.93552	-0.44	15.7	949.73	15.2699998	-122.45272	37.93554	-0.45	15.7	1164.7	15.2609998	11	7/24/2014	18:21.1
633	RWS11	-122.4527279	37.93552	-0.49	15.7	937.24	15.2089995	-122.45272	37.93554	-0.41	15.7	1149.8	15.2849996	11	7/24/2014	18:21.2
634	RWS11	-122.4527304	37.93553	-0.44	15.7	924.62	15.2509997	-122.45272	37.93554	-0.45	15.7	1134.8	15.2419997	11	7/24/2014	18:21.3
635	RWS11	-122.4527329	37.93553	-0.49	15.7	911.49	15.1929998	-122.45273	37.93554	-0.45	15.7	1119.4	15.2349998	11	7/24/2014	18:21.4
636	RWS11	-122.4527354	37.93553	-0.44	15.7	898.02	15.2380002	-122.45273	37.93554	-0.45	15.7	1103.6	15.2290002	11	7/24/2014	18:21.5
637	RWS11	-122.4527379	37.93553	-0.44	15.7	884.11	15.2330001	-122.45273	37.93554	-0.41	15.7	1087.5	15.2580001	11	7/24/2014	18:21.6
638	RWS11	-122.4527405	37.93553	-0.44	15.7	869.82	15.2290004	-122.45273	37.93554	-0.36	15.7	1071	15.3040004	11	7/24/2014	18:21.7
639	RWS11	-122.452743	37.93553	-0.44	15.7	855.3	15.2249997	-122.45274	37.93554	-0.45	15.7	1054.4	15.2159997	11	7/24/2014	18:21.8
640	RWS11	-122.4527454	37.93553	-0.44	15.7	840.59	15.221	-122.45274	37.93554	-0.41	15.7	1037.5	15.246	11	7/24/2014	18:21.9
641	RWS11	-122.4527492	37.93553	-0.44	15.7	825.87	15.2170003	-122.45274	37.93554	-0.45	15.7	1020.4	15.2080003	11	7/24/2014	18:22.0
642	RWS11	-122.4527517	37.93553	-0.49	15.6	811.23	15.1619996	-122.45275	37.93554	-0.41	15.6	1003.3	15.2379996	11	7/24/2014	18:22.1
643	RWS11	-122.4527543	37.93553	-0.49	15.6	796.58	15.1579999	-122.45275	37.93555	-0.45	15.6	986.28	15.1999999	11	7/24/2014	18:22.2
644	RWS11	-122.4527569	37.93553	-0.44	15.6	781.74	15.2050002	-122.45275	37.93555	-0.41	15.6	968.85	15.2300002	11	7/24/2014	18:22.3
645	RWS11	-122.4527595	37.93553	-0.49	15.6	766.71	15.1490001	-122.45275	37.93555	-0.5	15.6	951.13	15.1400001	11	7/24/2014	18:22.4
646	RWS11	-122.4527621	37.93554	-0.44	15.6	751.25	15.1970004	-122.45276	37.93555	-0.44	15.6	933.14	15.1880004	11	7/24/2014	18:22.5
647	RWS11	-122.4527647	37.93554	-0.49	15.6	735.34	15.1410003	-122.45276	37.93555	-0.5	15.6	914.67	15.1320003	11	7/24/2014	18:22.6
648	RWS11	-122.4527673	37.93554	-0.44	15.6	719.01	15.1879996	-122.45276	37.93555	-0.44	15.6	895.94	15.1789996	11	7/24/2014	18:22.7
649	RWS11	-122.4527699	37.93554	-0.49	15.6	702.54	15.1320005	-122.45276	37.93555	-0.5	15.6	876.93	15.1230004	11	7/24/2014	18:22.8
650	RWS11	-122.4527725	37.93554	-0.49	15.6	685.93	15.1279998	-122.45277	37.93555	-0.41	15.6	857.97	15.2039998	11	7/24/2014	18:22.9
651	RWS11	-122.4527763	37.93554	-0.49	15.6	669	15.1229997	-122.45277	37.93555	-0.44	15.6	838.75	15.1649997	11	7/24/2014	18:23.0
652	RWS11	-122.4527788	37.93554	-0.49	15.6	652	15.119	-122.45277	37.93555	-0.5	15.6	819.36	15.11	11	7/24/2014	18:23.1
653	RWS11	-122.4527813	37.93554	-0.49	15.6	635.03	15.1150003	-122.45277	37.93555	-0.5	15.6	799.95	15.1060002	11	7/24/2014	18:23.2
654	RWS11	-122.4527839	37.93554	-0.49	15.6	618.3	15.112	-122.45278	37.93556	-0.53	15.6	780.85	15.069	11	7/24/2014	18:23.3
655	RWS11	-122.4527864	37.93554	-0.49	15.6	601.83	15.1089997	-122.45278	37.93556	-0.53	15.6	762.22	15.0659998	11	7/24/2014	18:23.4
656	RWS11	-122.4527889	37.93554	-0.49	15.6	585.57	15.1069999	-122.45278	37.93556	-0.5	15.6	743.58	15.0979999	11	7/24/2014	18:23.5
657	RWS11	-122.4527915	37.93555	-0.49	15.6	569.39	15.1069999	-122.45278	37.93556	-0.5	15.6	725.3	15.0979999	11	7/24/2014	18:23.6
658	RWS11	-122.452794	37.93555	-0.49	15.6	553.51	15.1069999	-122.45279	37.93556	-0.53	15.6	707.2	15.0639999	11	7/24/2014	18:23.7
659	RWS11	-122.4527965	37.93555	-0.52	15.6	537.69	15.0760001	-122.45279	37.93556	-0.58	15.6	689.25	15.0160002	11	7/24/2014	18:23.8

660	RWS11	-122.452799	37.93555	-0.49	15.6	522.01	15.1139998	-122.45279	37.93556	-0.5	15.6	671.4	15.1049998	11	7/24/2014	18:23.9
661	RWS11	-122.4528028	37.93555	-0.49	15.6	506.31	15.1209998	-122.4528	37.93556	-0.58	15.6	653.53	15.0269998	11	7/24/2014	18:24.0
662	RWS11	-122.4528053	37.93555	-0.49	15.6	490.59	15.1299996	-122.4528	37.93556	-0.58	15.6	635.79	15.0359997	11	7/24/2014	18:24.1
663	RWS11	-122.4528078	37.93555	-0.49	15.6	475.2	15.1410003	-122.4528	37.93556	-0.58	15.6	618.3	15.0470003	11	7/24/2014	18:24.2
664	RWS11	-122.4528103	37.93555	-0.49	15.6	459.81	15.1539998	-122.4528	37.93556	-0.53	15.6	600.9	15.1109998	11	7/24/2014	18:24.3
665	RWS11	-122.4528129	37.93555	-0.49	15.7	444.46	15.1670003	-122.45281	37.93557	-0.61	15.7	583.6	15.0400003	11	7/24/2014	18:24.4
666	RWS11	-122.4528155	37.93555	-0.49	15.7	429	15.1810002	-122.45281	37.93557	-0.53	15.7	566.15	15.1380003	11	7/24/2014	18:24.5
667	RWS11	-122.452818	37.93555	-0.49	15.7	413.65	15.1939998	-122.45281	37.93557	-0.58	15.7	548.99	15.0999998	11	7/24/2014	18:24.6
668	RWS11	-122.4528206	37.93556	-0.49	15.7	398.35	15.2059999	-122.45281	37.93557	-0.58	15.7	531.97	15.1119999	11	7/24/2014	18:24.7
669	RWS11	-122.4528231	37.93556	-0.52	15.7	383.55	15.1830005	-122.45282	37.93557	-0.61	15.7	515.49	15.0900005	11	7/24/2014	18:24.8
670	RWS11	-122.4528256	37.93556	-0.52	15.7	368.95	15.1929997	-122.45282	37.93557	-0.58	15.7	499.07	15.1329998	11	7/24/2014	18:24.9
671	RWS11	-122.4528293	37.93556	-0.52	15.7	354.27	15.2010001	-122.45282	37.93557	-0.58	15.7	482.56	15.1410002	11	7/24/2014	18:25.0
672	RWS11	-122.4528317	37.93556	-0.52	15.7	339.38	15.2069997	-122.45282	37.93557	-0.58	15.7	465.93	15.1469997	11	7/24/2014	18:25.1
673	RWS11	-122.4528342	37.93556	-0.52	15.7	323.9	15.2099999	-122.45283	37.93557	-0.61	15.7	448.87	15.1169999	11	7/24/2014	18:25.2
674	RWS11	-122.4528367	37.93556	-0.52	15.7	308.42	15.2110003	-122.45283	37.93557	-0.61	15.7	431.64	15.1180003	11	7/24/2014	18:25.3
675	RWS11	-122.4528392	37.93556	-0.52	15.7	293.21	15.2099999	-122.45283	37.93557	-0.58	15.7	414.46	15.15	11	7/24/2014	18:25.4
676	RWS11	-122.4528417	37.93556	-0.49	15.7	278.12	15.2400003	-122.45283	37.93557	-0.61	15.7	397.24	15.1130002	11	7/24/2014	18:25.5
677	RWS11	-122.4528442	37.93556	-0.52	15.7	262.93	15.1999997	-122.45284	37.93558	-0.61	15.7	379.88	15.1069997	11	7/24/2014	18:25.6
678	RWS11	-122.4528467	37.93556	-0.49	15.7	247.83	15.2249999	-122.45284	37.93558	-0.58	15.7	362.56	15.1309999	11	7/24/2014	18:25.7
679	RWS11	-122.4528491	37.93557	-0.52	15.7	232.79	15.1809996	-122.45284	37.93558	-0.61	15.7	345.27	15.0879996	11	7/24/2014	18:25.8
680	RWS11	-122.4528516	37.93557	-0.49	15.7	218.07	15.204	-122.45284	37.93558	-0.58	15.7	328.21	15.11	11	7/24/2014	18:25.9
681	RWS11	-122.4528553	37.93557	-0.52	15.7	203.25	15.1579999	-122.45285	37.93558	-0.58	15.7	311.21	15.0979999	11	7/24/2014	18:26.0
682	RWS11	-122.4528578	37.93557	-0.52	15.7	188.13	15.1459998	-122.45285	37.93558	-0.53	15.7	294.17	15.1369999	11	7/24/2014	18:26.1
683	RWS11	-122.4528603	37.93557	-0.52	15.7	173.46	15.1350001	-122.45285	37.93558	-0.58	15.7	277.55	15.0750002	11	7/24/2014	18:26.2
684	RWS11	-122.4528628	37.93557	-0.52	15.6	159.17	15.123	-122.45286	37.93558	-0.58	15.6	261.26	15.0630001	11	7/24/2014	18:26.3
685	RWS11	-122.4528654	37.93557	-0.52	15.6	145.25	15.1120004	-122.45286	37.93558	-0.58	15.6	245.34	15.0520004	11	7/24/2014	18:26.4
686	RWS11	-122.4528679	37.93557	-0.52	15.6	131.01	15.1009997	-122.45286	37.93558	-0.58	15.6	229.45	15.0409998	11	7/24/2014	18:26.5
687	RWS11	-122.4528705	37.93557	-0.52	15.6	117.07	15.0880002	-122.45286	37.93558	-0.58	15.6	213.9	15.0280002	11	7/24/2014	18:26.6
688	RWS11	-122.452873	37.93557	-0.49	15.6	103.19	15.1089997	-122.45287	37.93559	-0.53	15.6	198.55	15.0659998	11	7/24/2014	18:26.7
689	RWS11	-122.4528756	37.93557	-0.52	15.6	89.422	15.0609998	-122.45287	37.93559	-0.53	15.6	183.24	15.0519998	11	7/24/2014	18:26.8
690	RWS11	-122.4528781	37.93558	-0.52	15.6	75.715	15.0460004	-122.45287	37.93559	-0.53	15.6	167.82	15.0370004	11	7/24/2014	18:26.9
691	RWS11	-122.4528818	37.93558	-0.52	15.6	62.102	15.0290002	-122.45287	37.93559	-0.58	15.6	152.52	14.9690002	11	7/24/2014	18:27.0
692	RWS11	-122.4528843	37.93558	-0.52	15.5	48.617	15.012	-122.45288	37.93559	-0.5	15.5	137.48	15.037	11	7/24/2014	18:27.1

693	RWS11	-122.4528869	37.93558	-0.52	15.5	35.175	14.9949998	-122.45288	37.93559	-0.58	15.5	122.51	14.9349998	11	7/24/2014	18:27.2
694	RWS11	-122.4528894	37.93558	-0.52	15.5	21.994	14.9770001	-122.45288	37.93559	-0.53	15.5	107.7	14.9680002	11	7/24/2014	18:27.3
695	RWS11	-122.452892	37.93558	-0.52	15.5	8.969	14.9610003	-122.45288	37.93559	-0.5	15.5	93.145	14.9860004	11	7/24/2014	18:27.4
696	RWS11	-122.4528945	37.93558	-0.49	15.5	-3.796	14.9789996	-122.45289	37.93559	-0.44	15.5	78.832	15.0209996	11	7/24/2014	18:27.5
697	RWS11	-122.4528971	37.93558	-0.52	15.5	-16.52	14.9309996	-122.45289	37.93559	-0.5	15.5	64.387	14.9559997	11	7/24/2014	18:27.6
698	RWS11	-122.4528996	37.93558	-0.49	15.4	-29.02	14.9520001	-122.45289	37.9356	-0.44	15.4	50.118	14.9940001	11	7/24/2014	18:27.7
699	RWS11	-122.4529021	37.93558	-0.52	15.4	-41.41	14.9070004	-122.4529	37.9356	-0.5	15.4	36.127	14.9320005	11	7/24/2014	18:27.8
700	RWS11	-122.4529047	37.93558	-0.49	15.4	-53.33	14.9299998	-122.4529	37.9356	-0.41	15.4	22.212	15.0059998	11	7/24/2014	18:27.9
701	RWS11	-122.4529084	37.93559	-0.49	15.4	-65.21	14.921	-122.4529	37.9356	-0.41	15.4	8.509	14.997	11	7/24/2014	18:28.0
702	RWS11	-122.4529109	37.93559	-0.49	15.4	-77.37	14.9120002	-122.4529	37.9356	-0.41	15.4	-5.302	14.9880002	11	7/24/2014	18:28.1
703	RWS11	-122.4529134	37.93559	-0.49	15.4	-89.68	14.9030004	-122.45291	37.9356	-0.44	15.4	-18.939	14.9450004	11	7/24/2014	18:28.2
704	RWS11	-122.452916	37.93559	-0.49	15.4	-101.9	14.895	-122.45291	37.9356	-0.41	15.4	-32.467	14.971	11	7/24/2014	18:28.3
705	RWS11	-122.4529185	37.93559	-0.49	15.4	-113.9	14.8860002	-122.45291	37.9356	-0.44	15.4	-45.732	14.9280002	11	7/24/2014	18:28.4
706	RWS11	-122.4529211	37.93559	-0.49	15.4	-125.9	14.8770003	-122.45291	37.9356	-0.41	15.4	-59.005	14.9530003	11	7/24/2014	18:28.5
707	RWS11	-122.4529236	37.93559	-0.49	15.4	-137.5	14.8679996	-122.45292	37.9356	-0.44	15.4	-72.174	14.9099996	11	7/24/2014	18:28.6
708	RWS11	-122.4529262	37.93559	-0.44	15.3	-148.6	14.9090003	-122.45292	37.9356	-0.41	15.3	-85.101	14.9340003	11	7/24/2014	18:28.7
709	RWS11	-122.4529287	37.93559	-0.49	15.3	-159.8	14.8469997	-122.45292	37.93561	-0.41	15.3	-98.016	14.9229996	11	7/24/2014	18:28.8
710	RWS11	-122.4529312	37.93559	-0.44	15.3	-170.6	14.887	-122.45292	37.93561	-0.36	15.3	-110.73	14.962	11	7/24/2014	18:28.9
711	RWS11	-122.452935	37.9356	-0.49	15.3	-181.3	14.8239999	-122.45293	37.93561	-0.41	15.3	-123.28	14.8999999	11	7/24/2014	18:29.0
712	RWS11	-122.4529375	37.9356	-0.44	15.3	-192.2	14.8640002	-122.45293	37.93561	-0.36	15.3	-135.78	14.9390002	11	7/24/2014	18:29.1
713	RWS11	-122.45294	37.9356	-0.44	15.3	-203.2	14.8529996	-122.45293	37.93561	-0.33	15.3	-148.22	14.9619996	11	7/24/2014	18:29.2
714	RWS11	-122.4529425	37.9356	-0.44	15.3	-214.2	14.8430003	-122.45294	37.93561	-0.33	15.3	-160.42	14.9520003	11	7/24/2014	18:29.3
715	RWS11	-122.4529451	37.9356	-0.44	15.3	-225	14.8340005	-122.45294	37.93561	-0.33	15.3	-172.2	14.9430005	11	7/24/2014	18:29.4
716	RWS11	-122.4529476	37.9356	-0.44	15.3	-235.8	14.8249997	-122.45294	37.93561	-0.33	15.3	-183.99	14.9339997	11	7/24/2014	18:29.5
717	RWS11	-122.4529501	37.9356	-0.44	15.3	-246.5	14.8170003	-122.45294	37.93561	-0.36	15.3	-195.7	14.8920003	11	7/24/2014	18:29.6
718	RWS11	-122.4529526	37.9356	-0.44	15.2	-257.3	14.8089999	-122.45295	37.93561	-0.29	15.2	-207.38	14.9519999	11	7/24/2014	18:29.7
719	RWS11	-122.4529551	37.9356	-0.44	15.2	-267.7	14.8000001	-122.45295	37.93561	-0.33	15.2	-218.8	14.9090001	11	7/24/2014	18:29.8
720	RWS11	-122.4529576	37.9356	-0.4	15.2	-277.7	14.8269997	-122.45295	37.93562	-0.29	15.2	-229.89	14.9349997	11	7/24/2014	18:29.9
721	RWS11	-122.4529613	37.9356	-0.44	15.2	-287.6	14.7829999	-122.45295	37.93562	-0.29	15.2	-240.93	14.9259999	11	7/24/2014	18:30.0
722	RWS11	-122.4529638	37.93561	-0.4	15.2	-297.2	14.8090001	-122.45296	37.93562	-0.29	15.2	-251.85	14.917	11	7/24/2014	18:30.1
723	RWS11	-122.4529663	37.93561	-0.44	15.2	-306.9	14.7650002	-122.45296	37.93562	-0.29	15.2	-262.72	14.9080002	11	7/24/2014	18:30.2
724	RWS11	-122.4529688	37.93561	-0.4	15.2	-316.8	14.7910004	-122.45296	37.93562	-0.29	15.2	-273.66	14.8990004	11	7/24/2014	18:30.3
725	RWS11	-122.4529712	37.93561	-0.4	15.2	-326.8	14.7810002	-122.45296	37.93562	-0.29	15.2	-284.85	14.8890002	11	7/24/2014	18:30.4

726	RWS11	-122.4529737	37.93561	-0.4	15.2	-336.6	14.7720003	-122.45297	37.93562	-0.24	15.2	-295.96	14.9310003	11	7/24/2014	18:30.5
727	RWS11	-122.4529762	37.93561	-0.4	15.2	-346.5	14.7620001	-122.45297	37.93562	-0.29	15.2	-306.93	14.8700001	11	7/24/2014	18:30.6
728	RWS11	-122.4529787	37.93561	-0.4	15.2	-356.3	14.7530003	-122.45297	37.93562	-0.24	15.2	-317.96	14.9120003	11	7/24/2014	18:30.7
729	RWS11	-122.4529812	37.93561	-0.4	15.1	-366	14.7430001	-122.45297	37.93562	-0.33	15.1	-328.76	14.8170001	11	7/24/2014	18:30.8
730	RWS11	-122.4529836	37.93561	-0.4	15.1	-375.2	14.7340002	-122.45298	37.93563	-0.24	15.1	-339.16	14.8930002	11	7/24/2014	18:30.9
731	RWS11	-122.4529872	37.93561	-0.4	15.1	-384.1	14.7250004	-122.45298	37.93563	-0.29	15.1	-349.07	14.8330004	11	7/24/2014	18:31.0
732	RWS11	-122.4529897	37.93562	-0.4	15.1	-392.5	14.7159996	-122.45298	37.93563	-0.24	15.1	-358.45	14.8749996	11	7/24/2014	18:31.1
733	RWS11	-122.4529921	37.93562	-0.4	15.1	-400.9	14.7069998	-122.45298	37.93563	-0.29	15.1	-367.64	14.8149998	11	7/24/2014	18:31.2
734	RWS11	-122.4529946	37.93562	-0.4	15.1	-408.6	14.6990004	-122.45299	37.93563	-0.24	15.1	-376.45	14.8580004	11	7/24/2014	18:31.3
735	RWS11	-122.452997	37.93562	-0.4	15.1	-416.2	14.6920004	-122.45299	37.93563	-0.24	15.1	-385.05	14.8510004	11	7/24/2014	18:31.4
736	RWS11	-122.4529995	37.93562	-0.35	15.1	-423.7	14.7369999	-122.45299	37.93563	-0.24	15.1	-393.38	14.8449999	11	7/24/2014	18:31.5
737	RWS11	-122.453002	37.93562	-0.4	15.1	-430.7	14.6809998	-122.45299	37.93563	-0.21	15.1	-401.37	14.8739998	11	7/24/2014	18:31.6
738	RWS11	-122.4530044	37.93562	-0.4	15.1	-437.7	14.6770001	-122.453	37.93563	-0.24	15.1	-409.32	14.8360001	11	7/24/2014	18:31.7
739	RWS11	-122.4530069	37.93562	-0.4	15.1	-444.7	14.6750002	-122.453	37.93563	-0.24	15.1	-417.28	14.8340002	11	7/24/2014	18:31.8
740	RWS11	-122.4530093	37.93562	-0.4	15.1	-451.6	14.6739998	-122.453	37.93563	-0.24	15.1	-425.28	14.8329998	11	7/24/2014	18:31.9
741	RWS11	-122.4530129	37.93562	-0.4	15.1	-458.6	14.6739998	-122.45301	37.93564	-0.24	15.1	-433.07	14.8329998	11	7/24/2014	18:32.0
742	RWS11	-122.4530154	37.93562	-0.35	15.1	-465.4	14.7260002	-122.45301	37.93564	-0.24	15.1	-440.76	14.8340002	11	7/24/2014	18:32.1
743	RWS11	-122.4530178	37.93563	-0.4	15.1	-472	14.6759997	-122.45301	37.93564	-0.29	15.1	-448.21	14.7839997	11	7/24/2014	18:32.2
744	RWS11	-122.4530203	37.93563	-0.35	15.1	-478.5	14.7290005	-122.45301	37.93564	-0.24	15.1	-455.59	14.8370005	11	7/24/2014	18:32.3
745	RWS11	-122.4530228	37.93563	-0.4	15.1	-484.9	14.6789999	-122.45302	37.93564	-0.29	15.1	-462.83	14.7869999	11	7/24/2014	18:32.4
746	RWS11	-122.4530253	37.93563	-0.4	15.1	-491	14.6800003	-122.45302	37.93564	-0.29	15.1	-469.87	14.7880003	11	7/24/2014	18:32.5
747	RWS11	-122.4530278	37.93563	-0.4	15.1	-497	14.6800003	-122.45302	37.93564	-0.33	15.1	-476.66	14.7540003	11	7/24/2014	18:32.6
748	RWS11	-122.4530303	37.93563	-0.4	15.1	-502.8	14.6789999	-122.45302	37.93564	-0.24	15.1	-483.29	14.8379999	11	7/24/2014	18:32.7
749	RWS11	-122.4530327	37.93563	-0.4	15.1	-508.7	14.6770001	-122.45303	37.93564	-0.29	15.1	-489.84	14.7850001	11	7/24/2014	18:32.8
750	RWS11	-122.4530352	37.93563	-0.4	15.1	-514.5	14.6739998	-122.45303	37.93564	-0.29	15.1	-496.3	14.7819998	11	7/24/2014	18:32.9
751	RWS11	-122.4530388	37.93563	-0.4	15.1	-520.5	14.6700001	-122.45303	37.93565	-0.29	15.1	-502.68	14.7780001	11	7/24/2014	18:33.0
752	RWS11	-122.4530413	37.93563	-0.4	15.1	-526.2	14.665	-122.45303	37.93565	-0.24	15.1	-508.97	14.824	11	7/24/2014	18:33.1
753	RWS11	-122.4530437	37.93563	-0.4	15.1	-531.7	14.6590004	-122.45304	37.93565	-0.33	15.1	-515.09	14.7330004	11	7/24/2014	18:33.2
754	RWS11	-122.4530462	37.93564	-0.4	15.1	-536.9	14.6529999	-122.45304	37.93565	-0.33	15.1	-521.08	14.7269999	11	7/24/2014	18:33.3
755	RWS11	-122.4530487	37.93564	-0.4	15	-541.7	14.6459999	-122.45304	37.93565	-0.33	15	-526.75	14.7199999	11	7/24/2014	18:33.4
756	RWS11	-122.4530512	37.93564	-0.4	15	-545.9	14.639	-122.45304	37.93565	-0.29	15	-531.99	14.747	11	7/24/2014	18:33.5
757	RWS11	-122.4530537	37.93564	-0.4	15	-550.2	14.632	-122.45305	37.93565	-0.33	15	-537.15	14.706	11	7/24/2014	18:33.6
758	RWS11	-122.4530562	37.93564	-0.4	15	-554.4	14.625	-122.45305	37.93565	-0.36	15	-542.07	14.665	11	7/24/2014	18:33.7

759	RWS11	-122.4530586	37.93564	-0.4	15	-558.4	14.6180001	-122.45305	37.93565	-0.33	15	-546.63	14.6920001	11	7/24/2014	18:33.8
760	RWS11	-122.4530611	37.93564	-0.4	15	-562.2	14.6119995	-122.45305	37.93565	-0.29	15	-550.99	14.7199995	11	7/24/2014	18:33.9
761	RWS11	-122.4530647	37.93564	-0.44	15	-565.9	14.571	-122.45306	37.93565	-0.33	15	-555.17	14.68	11	7/24/2014	18:34.0
762	RWS11	-122.4530672	37.93564	-0.44	15	-569.6	14.5650004	-122.45306	37.93566	-0.29	15	-559.18	14.7080004	11	7/24/2014	18:34.1
763	RWS11	-122.4530697	37.93564	-0.44	15	-573.2	14.5589999	-122.45306	37.93566	-0.36	15	-563.03	14.6339999	11	7/24/2014	18:34.2
764	RWS11	-122.4530722	37.93565	-0.4	15	-576.5	14.5869999	-122.45306	37.93566	-0.29	15	-566.79	14.6949999	11	7/24/2014	18:34.3
765	RWS11	-122.4530746	37.93565	-0.44	15	-579.6	14.5460004	-122.45307	37.93566	-0.36	15	-570.28	14.6210004	11	7/24/2014	18:34.4
766	RWS11	-122.4530772	37.93565	-0.4	15	-582.5	14.5740004	-122.45307	37.93566	-0.36	15	-573.63	14.6140004	11	7/24/2014	18:34.5
767	RWS11	-122.4530796	37.93565	-0.44	15	-585.2	14.5329999	-122.45307	37.93566	-0.36	15	-576.91	14.6079999	11	7/24/2014	18:34.6
768	RWS11	-122.4530821	37.93565	-0.44	15	-587.7	14.5259999	-122.45307	37.93566	-0.33	15	-579.9	14.6349999	11	7/24/2014	18:34.7
769	RWS11	-122.4530846	37.93565	-0.44	15	-590.1	14.5189999	-122.45308	37.93566	-0.36	15	-582.74	14.5939999	11	7/24/2014	18:34.8
770	RWS11	-122.453087	37.93565	-0.44	14.9	-592.4	14.5130004	-122.45308	37.93566	-0.36	14.9	-585.33	14.5880004	11	7/24/2014	18:34.9
771	RWS11	-122.4530907	37.93565	-0.49	14.9	-594.8	14.4550004	-122.45308	37.93566	-0.36	14.9	-587.95	14.5810004	11	7/24/2014	18:35.0
772	RWS11	-122.4530931	37.93565	-0.44	14.9	-597.1	14.4990004	-122.45309	37.93567	-0.33	14.9	-590.47	14.6080004	11	7/24/2014	18:35.1
773	RWS11	-122.4530956	37.93565	-0.44	14.9	-599.3	14.4920005	-122.45309	37.93567	-0.36	14.9	-593.02	14.5670005	11	7/24/2014	18:35.2
774	RWS11	-122.453098	37.93565	-0.44	14.9	-601.7	14.4840001	-122.45309	37.93567	-0.33	14.9	-595.62	14.5930001	11	7/24/2014	18:35.3
775	RWS11	-122.4531005	37.93566	-0.44	14.9	-603.9	14.4759997	-122.45309	37.93567	-0.33	14.9	-598.3	14.5849997	11	7/24/2014	18:35.4
776	RWS11	-122.453103	37.93566	-0.44	14.9	-605.9	14.4689997	-122.4531	37.93567	-0.36	14.9	-600.86	14.5439997	11	7/24/2014	18:35.5
777	RWS11	-122.4531054	37.93566	-0.49	14.9	-607.7	14.4100003	-122.4531	37.93567	-0.36	14.9	-603.2	14.5360003	11	7/24/2014	18:35.6
778	RWS11	-122.4531079	37.93566	-0.44	14.9	-609.6	14.4549998	-122.4531	37.93567	-0.33	14.9	-605.38	14.5639998	11	7/24/2014	18:35.7
779	RWS11	-122.4531103	37.93566	-0.49	14.9	-611.3	14.3969998	-122.4531	37.93567	-0.36	14.9	-607.34	14.5229998	11	7/24/2014	18:35.8
780	RWS11	-122.4531128	37.93566	-0.44	14.9	-612.8	14.4429997	-122.45311	37.93567	-0.36	14.9	-608.92	14.5179997	11	7/24/2014	18:35.9
781	RWS11	-122.4531164	37.93566	-0.49	14.9	-614	14.3869996	-122.45311	37.93567	-0.33	14.9	-610.27	14.5469996	11	7/24/2014	18:36.0
782	RWS11	-122.4531189	37.93566	-0.44	14.9	-615	14.4339999	-122.45311	37.93567	-0.33	14.9	-611.35	14.5429999	11	7/24/2014	18:36.1
783	RWS11	-122.4531213	37.93566	-0.49	14.9	-615.6	14.3799996	-122.45311	37.93568	-0.36	14.9	-612.22	14.5059996	11	7/24/2014	18:36.2
784	RWS11	-122.4531238	37.93566	-0.44	14.9	-616	14.4289998	-122.45312	37.93568	-0.33	14.9	-612.75	14.5379998	11	7/24/2014	18:36.3
785	RWS11	-122.4531263	37.93567	-0.44	14.9	-616.3	14.4280003	-122.45312	37.93568	-0.41	14.9	-613.07	14.4530003	11	7/24/2014	18:36.4
786	RWS11	-122.4531288	37.93567	-0.44	14.9	-616.5	14.4280003	-122.45312	37.93568	-0.33	14.9	-613.42	14.5370003	11	7/24/2014	18:36.5
787	RWS11	-122.4531313	37.93567	-0.44	14.9	-616.5	14.4280003	-122.45312	37.93568	-0.36	14.9	-613.63	14.5030003	11	7/24/2014	18:36.6
788	RWS11	-122.4531338	37.93567	-0.44	14.9	-616.6	14.4300002	-122.45313	37.93568	-0.36	14.9	-613.69	14.5050002	11	7/24/2014	18:36.7
789	RWS11	-122.4531363	37.93567	-0.49	14.9	-616.6	14.381	-122.45313	37.93568	-0.36	14.9	-613.58	14.507	11	7/24/2014	18:36.8
790	RWS11	-122.4531388	37.93567	-0.49	14.9	-616.5	14.3849998	-122.45313	37.93568	-0.36	14.9	-613.24	14.5109997	11	7/24/2014	18:36.9
791	RWS11	-122.4531425	37.93567	-0.49	14.9	-616.2	14.3890004	-122.45314	37.93568	-0.41	14.9	-612.63	14.4650004	11	7/24/2014	18:37.0

792	RWS11	-122.4531449	37.93567	-0.44	14.9	-615.5	14.446	-122.45314	37.93568	-0.36	14.9	-611.76	14.521	11	7/24/2014	18:37.1
793	RWS11	-122.4531474	37.93567	-0.49	14.9	-614.6	14.4009996	-122.45314	37.93569	-0.33	14.9	-610.75	14.5609995	11	7/24/2014	18:37.2
794	RWS11	-122.4531499	37.93567	-0.44	14.9	-613.5	14.4599999	-122.45314	37.93569	-0.33	14.9	-609.59	14.5689999	11	7/24/2014	18:37.3
795	RWS11	-122.4531524	37.93567	-0.49	14.9	-612.3	14.4179997	-122.45315	37.93569	-0.41	14.9	-608.39	14.4939997	11	7/24/2014	18:37.4
796	RWS11	-122.4531549	37.93568	-0.44	14.9	-610.7	14.479	-122.45315	37.93569	-0.33	14.9	-606.87	14.588	11	7/24/2014	18:37.5
797	RWS11	-122.4531574	37.93568	-0.49	14.9	-608.7	14.4400001	-122.45315	37.93569	-0.36	14.9	-604.89	14.566	11	7/24/2014	18:37.6
798	RWS11	-122.4531599	37.93568	-0.44	14.9	-606.3	14.5039996	-122.45315	37.93569	-0.33	14.9	-602.73	14.6129996	11	7/24/2014	18:37.7
799	RWS11	-122.4531624	37.93568	-0.49	15	-604	14.4690003	-122.45316	37.93569	-0.36	15	-600.42	14.5950003	11	7/24/2014	18:37.8
800	RWS11	-122.4531649	37.93568	-0.44	15	-601.5	14.5360001	-122.45316	37.93569	-0.33	15	-597.88	14.6450001	11	7/24/2014	18:37.9
801	RWS11	-122.4531685	37.93568	-0.49	15	-599.2	14.5040002	-122.45316	37.93569	-0.36	15	-595.11	14.6300002	11	7/24/2014	18:38.0
802	RWS11	-122.453171	37.93568	-0.49	15	-596.6	14.5230002	-122.45316	37.93569	-0.33	15	-592.16	14.6830002	11	7/24/2014	18:38.1
803	RWS11	-122.4531735	37.93568	-0.49	15	-593.7	14.5420003	-122.45317	37.93569	-0.36	15	-588.9	14.6680003	11	7/24/2014	18:38.2
804	RWS11	-122.453176	37.93568	-0.49	15	-590.5	14.5599999	-122.45317	37.9357	-0.36	15	-585.3	14.6859999	11	7/24/2014	18:38.3
805	RWS11	-122.4531785	37.93568	-0.49	15.1	-586.8	14.5770001	-122.45317	37.9357	-0.36	15.1	-581.33	14.7030001	11	7/24/2014	18:38.4
806	RWS11	-122.4531811	37.93569	-0.49	15.1	-582.9	14.5929999	-122.45317	37.9357	-0.36	15.1	-577.1	14.7189999	11	7/24/2014	18:38.5
807	RWS11	-122.4531837	37.93569	-0.49	15.1	-578.7	14.6069999	-122.45318	37.9357	-0.36	15.1	-572.8	14.7329999	11	7/24/2014	18:38.6
808	RWS11	-122.4531863	37.93569	-0.49	15.1	-574.4	14.619	-122.45318	37.9357	-0.33	15.1	-568.62	14.779	11	7/24/2014	18:38.7
809	RWS11	-122.4531889	37.93569	-0.49	15.1	-570.4	14.6290002	-122.45318	37.9357	-0.36	15.1	-564.78	14.7550002	11	7/24/2014	18:38.8
810	RWS11	-122.4531915	37.93569	-0.49	15.1	-566.5	14.638	-122.45318	37.9357	-0.36	15.1	-561.11	14.764	11	7/24/2014	18:38.9
811	RWS11	-122.4531941	37.93569	-0.49	15.1	-562.6	14.6469998	-122.45319	37.9357	-0.29	15.1	-557.09	14.8409998	11	7/24/2014	18:39.0
812	RWS11	-122.4531967	37.93569	-0.49	15.1	-558.9	14.6559997	-122.45319	37.9357	-0.36	15.1	-552.93	14.7819997	11	7/24/2014	18:39.1
813	RWS11	-122.4531993	37.93569	-0.49	15.2	-555.1	14.6650004	-122.45319	37.9357	-0.33	15.2	-548.44	14.8250004	11	7/24/2014	18:39.2
814	RWS11	-122.4532018	37.93569	-0.49	15.2	-551.2	14.6760001	-122.45319	37.9357	-0.33	15.2	-543.77	14.8360001	11	7/24/2014	18:39.3
815	RWS11	-122.4532044	37.93569	-0.49	15.2	-547.1	14.6880002	-122.4532	37.93571	-0.36	15.2	-538.8	14.8140002	11	7/24/2014	18:39.4
816	RWS11	-122.4532069	37.93569	-0.49	15.2	-542.6	14.7020001	-122.4532	37.93571	-0.33	15.2	-533.52	14.8620001	11	7/24/2014	18:39.5
817	RWS11	-122.4532094	37.93569	-0.52	15.2	-537.9	14.6839999	-122.4532	37.93571	-0.36	15.2	-527.84	14.8439999	11	7/24/2014	18:39.6
818	RWS11	-122.4532119	37.9357	-0.49	15.2	-532.7	14.7350001	-122.45321	37.93571	-0.29	15.2	-521.61	14.9290001	11	7/24/2014	18:39.7
819	RWS11	-122.4532144	37.9357	-0.49	15.2	-527.3	14.7549996	-122.45321	37.93571	-0.33	15.2	-515.01	14.9149996	11	7/24/2014	18:39.8
820	RWS11	-122.4532168	37.9357	-0.49	15.3	-521.5	14.7750001	-122.45321	37.93571	-0.36	15.3	-507.91	14.9010001	11	7/24/2014	18:39.9
821	RWS11	-122.4532204	37.9357	-0.49	15.3	-515.4	14.7979999	-122.45321	37.93571	-0.36	15.3	-500.29	14.9239999	11	7/24/2014	18:40.0
822	RWS11	-122.4532229	37.9357	-0.49	15.3	-508.9	14.8209996	-122.45322	37.93571	-0.33	15.3	-492.47	14.9809996	11	7/24/2014	18:40.1
823	RWS11	-122.4532253	37.9357	-0.49	15.3	-502.2	14.8449998	-122.45322	37.93571	-0.36	15.3	-484.49	14.9709998	11	7/24/2014	18:40.2
824	RWS11	-122.4532278	37.9357	-0.44	15.4	-495.3	14.9219998	-122.45322	37.93571	-0.33	15.4	-476.25	15.0309998	11	7/24/2014	18:40.3

825	RWS11	-122.4532303	37.9357	-0.49	15.4	-488.2	14.8969998	-122.45322	37.93571	-0.41	15.4	-467.92	14.9729998	11	7/24/2014	18:40.4
826	RWS11	-122.4532328	37.9357	-0.49	15.4	-480.8	14.9249997	-122.45323	37.93572	-0.36	15.4	-459.38	15.0509997	11	7/24/2014	18:40.5
827	RWS11	-122.4532354	37.9357	-0.49	15.4	-473.5	14.954	-122.45323	37.93572	-0.41	15.4	-450.56	15.03	11	7/24/2014	18:40.6
828	RWS11	-122.453238	37.9357	-0.44	15.5	-465.5	15.0340003	-122.45323	37.93572	-0.36	15.5	-441.46	15.1090003	11	7/24/2014	18:40.7
829	RWS11	-122.4532406	37.93571	-0.49	15.5	-457.5	15.0140004	-122.45323	37.93572	-0.41	15.5	-431.92	15.0900004	11	7/24/2014	18:40.8
830	RWS11	-122.4532432	37.93571	-0.49	15.5	-449.3	15.0470004	-122.45324	37.93572	-0.41	15.5	-422.21	15.1230004	11	7/24/2014	18:40.9
831	RWS11	-122.4532458	37.93571	-0.49	15.6	-440.8	15.0809999	-122.45324	37.93572	-0.41	15.6	-412.24	15.1569998	11	7/24/2014	18:41.0
832	RWS11	-122.4532485	37.93571	-0.44	15.6	-432	15.1669997	-122.45324	37.93572	-0.41	15.6	-401.94	15.1919997	11	7/24/2014	18:41.1
833	RWS11	-122.4532511	37.93571	-0.49	15.6	-422.7	15.1530004	-122.45324	37.93572	-0.41	15.6	-391.42	15.2290003	11	7/24/2014	18:41.2
834	RWS11	-122.4532537	37.93571	-0.49	15.7	-413.1	15.1919999	-122.45325	37.93572	-0.41	15.7	-380.61	15.2679999	11	7/24/2014	18:41.3
835	RWS11	-122.4532564	37.93571	-0.49	15.7	-403.2	15.2319999	-122.45325	37.93572	-0.41	15.7	-369.46	15.3079999	11	7/24/2014	18:41.4
836	RWS11	-122.4532591	37.93571	-0.44	15.8	-393.1	15.3249997	-122.45325	37.93572	-0.41	15.8	-358.12	15.3499997	11	7/24/2014	18:41.5
837	RWS11	-122.4532617	37.93571	-0.49	15.8	-382.8	15.3180003	-122.45326	37.93573	-0.41	15.8	-346.58	15.3940003	11	7/24/2014	18:41.6
838	RWS11	-122.4532644	37.93571	-0.44	15.9	-372.3	15.4149998	-122.45326	37.93573	-0.36	15.9	-334.82	15.4899998	11	7/24/2014	18:41.7
839	RWS11	-122.453267	37.93571	-0.49	15.9	-361.7	15.4120002	-122.45326	37.93573	-0.41	15.9	-323	15.4880002	11	7/24/2014	18:41.8
840	RWS11	-122.4532697	37.93572	-0.44	15.9	-350.9	15.5139998	-122.45326	37.93573	-0.41	15.9	-310.87	15.5389998	11	7/24/2014	18:41.9
841	RWS11	-122.4532724	37.93572	-0.49	16	-340.1	15.5160003	-122.45327	37.93573	-0.41	16	-298.83	15.5920003	11	7/24/2014	18:42.0
842	RWS11	-122.4532751	37.93572	-0.44	16.1	-329.1	15.623	-122.45327	37.93573	-0.41	16.1	-286.51	15.648	11	7/24/2014	18:42.1
843	RWS11	-122.4532778	37.93572	-0.49	16.1	-317.8	15.6300006	-122.45327	37.93573	-0.41	16.1	-273.97	15.7060006	11	7/24/2014	18:42.2
844	RWS11	-122.4532805	37.93572	-0.44	16.2	-306.5	15.7419995	-122.45327	37.93573	-0.44	16.2	-261.33	15.7329995	11	7/24/2014	18:42.3
845	RWS11	-122.4532832	37.93572	-0.49	16.2	-294.9	15.7529998	-122.45328	37.93573	-0.41	16.2	-248.54	15.8289998	11	7/24/2014	18:42.4
846	RWS11	-122.453286	37.93572	-0.44	16.3	-283.2	15.8690003	-122.45328	37.93573	-0.41	16.3	-235.67	15.8940003	11	7/24/2014	18:42.5
847	RWS11	-122.4532887	37.93572	-0.49	16.4	-271.3	15.8840003	-122.45328	37.93573	-0.41	16.4	-222.38	15.9600003	11	7/24/2014	18:42.6
848	RWS11	-122.4532914	37.93572	-0.44	16.4	-259.5	16.0019997	-122.45329	37.93573	-0.41	16.4	-208.79	16.0269997	11	7/24/2014	18:42.7
849	RWS11	-122.4532941	37.93572	-0.49	16.5	-247.4	16.0179992	-122.45329	37.93574	-0.41	16.5	-194.96	16.0939992	11	7/24/2014	18:42.8
850	RWS11	-122.4532969	37.93572	-0.49	16.6	-234.8	16.086	-122.45329	37.93574	-0.41	16.6	-180.62	16.1619999	11	7/24/2014	18:42.9
851	RWS11	-122.4532996	37.93572	-0.49	16.6	-221.6	16.152	-122.45329	37.93574	-0.44	16.6	-165.92	16.194	11	7/24/2014	18:43.0
852	RWS11	-122.4533023	37.93573	-0.44	16.7	-208.2	16.2689999	-122.4533	37.93574	-0.41	16.7	-150.93	16.2939999	11	7/24/2014	18:43.1
853	RWS11	-122.453305	37.93573	-0.49	16.8	-194.7	16.2830005	-122.4533	37.93574	-0.41	16.8	-135.81	16.3590004	11	7/24/2014	18:43.2
854	RWS11	-122.4533078	37.93573	-0.44	16.8	-180.9	16.3989991	-122.4533	37.93574	-0.44	16.8	-120.4	16.3899991	11	7/24/2014	18:43.3
855	RWS11	-122.4533104	37.93573	-0.49	16.9	-167	16.4150004	-122.4533	37.93574	-0.41	16.9	-104.67	16.4910004	11	7/24/2014	18:43.4
856	RWS11	-122.4533131	37.93573	-0.49	17	-152.8	16.4829993	-122.45331	37.93574	-0.41	17	-88.607	16.5589993	11	7/24/2014	18:43.5
857	RWS11	-122.4533158	37.93573	-0.52	17	-138.3	16.5200003	-122.45331	37.93574	-0.41	17	-72.334	16.6300004	11	7/24/2014	18:43.6

858	RWS11	-122.4533184	37.93573	-0.49	17.1	-123.6	16.6289992	-122.45331	37.93574	-0.41	17.1	-55.99	16.7049992	11	7/24/2014	18:43.7
859	RWS11	-122.453321	37.93573	-0.49	17.2	-108.6	16.7070003	-122.45331	37.93574	-0.44	17.2	-39.325	16.7490003	11	7/24/2014	18:43.8
860	RWS11	-122.4533236	37.93573	-0.44	17.3	-93.13	16.8389996	-122.45332	37.93575	-0.41	17.3	-22.172	16.8639996	11	7/24/2014	18:43.9
861	RWS11	-122.4533273	37.93573	-0.49	17.4	-77.33	16.8700004	-122.45332	37.93575	-0.41	17.4	-4.524	16.9460003	11	7/24/2014	18:44.0
862	RWS11	-122.4533299	37.93573	-0.44	17.4	-61.28	17.0040005	-122.45332	37.93575	-0.41	17.4	13.455	17.0290005	11	7/24/2014	18:44.1
863	RWS11	-122.4533324	37.93574	-0.49	17.5	-44.95	17.0339999	-122.45333	37.93575	-0.44	17.5	31.871	17.0759999	11	7/24/2014	18:44.2
864	RWS11	-122.453335	37.93574	-0.49	17.6	-28.26	17.1130004	-122.45333	37.93575	-0.41	17.6	50.719	17.1890004	11	7/24/2014	18:44.3
865	RWS11	-122.4533376	37.93574	-0.49	17.7	-11.42	17.1900001	-122.45333	37.93575	-0.44	17.7	69.839	17.2320001	11	7/24/2014	18:44.4
866	RWS11	-122.4533403	37.93574	-0.44	17.7	5.754	17.3119992	-122.45333	37.93575	-0.41	17.7	89.099	17.3369992	11	7/24/2014	18:44.5
867	RWS11	-122.4533429	37.93574	-0.49	17.8	23.122	17.3250003	-122.45334	37.93575	-0.44	17.8	108.46	17.3670003	11	7/24/2014	18:44.6
868	RWS11	-122.4533455	37.93574	-0.49	17.9	40.75	17.3800006	-122.45334	37.93575	-0.41	17.9	127.92	17.4560006	11	7/24/2014	18:44.7
869	RWS11	-122.4533482	37.93574	-0.49	17.9	58.646	17.4260001	-122.45334	37.93575	-0.44	17.9	147.68	17.4680001	11	7/24/2014	18:44.8
870	RWS11	-122.4533509	37.93574	-0.49	18	76.753	17.4630008	-122.45334	37.93575	-0.44	18	167.56	17.5050008	11	7/24/2014	18:44.9
871	RWS11	-122.4533535	37.93574	-0.52	18	95.034	17.4580001	-122.45335	37.93575	-0.44	18	187.46	17.5340001	11	7/24/2014	18:45.0
872	RWS11	-122.4533562	37.93574	-0.49	18	113.39	17.513	-122.45335	37.93575	-0.41	18	207.46	17.589	11	7/24/2014	18:45.1
873	RWS11	-122.4533589	37.93574	-0.52	18	131.88	17.4939994	-122.45335	37.93576	-0.44	18	227.59	17.5699994	11	7/24/2014	18:45.2
874	RWS11	-122.4533615	37.93574	-0.49	18	150.31	17.5379996	-122.45336	37.93576	-0.44	18	247.7	17.5799996	11	7/24/2014	18:45.3
875	RWS11	-122.4533642	37.93574	-0.52	18	168.84	17.5100001	-122.45336	37.93576	-0.41	18	268.07	17.6200001	11	7/24/2014	18:45.4
876	RWS11	-122.4533669	37.93575	-0.49	18	187.49	17.546001	-122.45336	37.93576	-0.44	18	288.75	17.588001	11	7/24/2014	18:45.5
877	RWS11	-122.4533695	37.93575	-0.49	18	206.3	17.546001	-122.45336	37.93576	-0.44	18	309.66	17.588001	11	7/24/2014	18:45.6
878	RWS11	-122.4533722	37.93575	-0.49	18	225.43	17.5430007	-122.45337	37.93576	-0.41	18	330.99	17.6190007	11	7/24/2014	18:45.7
879	RWS11	-122.4533749	37.93575	-0.52	18	244.77	17.5039996	-122.45337	37.93576	-0.44	18	352.4	17.5799996	11	7/24/2014	18:45.8
880	RWS11	-122.4533776	37.93575	-0.52	18	264.43	17.4979991	-122.45337	37.93576	-0.41	18	373.97	17.6079991	11	7/24/2014	18:45.9
881	RWS11	-122.4533803	37.93575	-0.49	18	284.44	17.5260005	-122.45337	37.93576	-0.44	18	396.02	17.5680005	11	7/24/2014	18:46.0
882	RWS11	-122.4533831	37.93575	-0.49	18	304.75	17.5190005	-122.45338	37.93576	-0.44	18	418.8	17.5610005	11	7/24/2014	18:46.1
883	RWS11	-122.4533858	37.93575	-0.49	18	325.47	17.5120006	-122.45338	37.93576	-0.44	18	442.24	17.5540006	11	7/24/2014	18:46.2
884	RWS11	-122.4533886	37.93575	-0.49	18	346.46	17.5050006	-122.45338	37.93576	-0.41	18	466.05	17.5810006	11	7/24/2014	18:46.3
885	RWS11	-122.4533913	37.93575	-0.49	18	367.57	17.4969993	-122.45339	37.93577	-0.5	18	490.35	17.4879993	11	7/24/2014	18:46.4
886	RWS11	-122.4533941	37.93575	-0.49	18	389.07	17.4880004	-122.45339	37.93577	-0.44	18	514.89	17.5300004	11	7/24/2014	18:46.5
887	RWS11	-122.4533969	37.93575	-0.49	18	410.6	17.4759994	-122.45339	37.93577	-0.44	18	539.04	17.5179994	11	7/24/2014	18:46.6
888	RWS11	-122.4533997	37.93575	-0.49	17.9	432.36	17.461	-122.45339	37.93577	-0.44	17.9	563.18	17.503	11	7/24/2014	18:46.7
889	RWS11	-122.4534024	37.93576	-0.49	17.9	454.44	17.4430003	-122.4534	37.93577	-0.44	17.9	587.86	17.4850003	11	7/24/2014	18:46.8
890	RWS11	-122.4534052	37.93576	-0.49	17.9	476.78	17.421001	-122.4534	37.93577	-0.44	17.9	612.7	17.463001	11	7/24/2014	18:46.9

891	RWS11	-122.4534079	37.93576	-0.52	17.9	499.01	17.3600005	-122.4534	37.93577	-0.44	17.9	637.38	17.4360005	11	7/24/2014	18:47.0
892	RWS11	-122.4534107	37.93576	-0.49	17.9	521.63	17.3630004	-122.45341	37.93577	-0.44	17.9	662.39	17.4050004	11	7/24/2014	18:47.1
893	RWS11	-122.4534134	37.93576	-0.49	17.8	544.23	17.3280006	-122.45341	37.93577	-0.44	17.8	687.48	17.3700005	11	7/24/2014	18:47.2
894	RWS11	-122.4534161	37.93576	-0.49	17.8	566.8	17.2900004	-122.45341	37.93577	-0.44	17.8	712.45	17.3320004	11	7/24/2014	18:47.3
895	RWS11	-122.4534188	37.93576	-0.49	17.7	589.35	17.2499995	-122.45341	37.93577	-0.44	17.7	737.28	17.2919995	11	7/24/2014	18:47.4
896	RWS11	-122.4534215	37.93576	-0.49	17.7	612.03	17.2070003	-122.45342	37.93577	-0.44	17.7	762.21	17.2490003	11	7/24/2014	18:47.5
897	RWS11	-122.4534242	37.93576	-0.52	17.7	634.71	17.129999	-122.45342	37.93577	-0.5	17.7	787.21	17.1549991	11	7/24/2014	18:47.6
898	RWS11	-122.453427	37.93576	-0.49	17.6	657.42	17.1219993	-122.45342	37.93577	-0.44	17.6	812.35	17.1639993	11	7/24/2014	18:47.7
899	RWS11	-122.4534297	37.93576	-0.49	17.6	680.22	17.0799995	-122.45342	37.93578	-0.44	17.6	837.53	17.1219994	11	7/24/2014	18:47.8
900	RWS11	-122.4534325	37.93576	-0.44	17.5	703.18	17.0919999	-122.45343	37.93578	-0.44	17.5	862.6	17.0829999	11	7/24/2014	18:47.9
901	RWS11	-122.4534352	37.93576	-0.49	17.5	726.04	17.0039992	-122.45343	37.93578	-0.44	17.5	887.55	17.0459992	11	7/24/2014	18:48.0
902	RWS11	-122.453438	37.93576	-0.44	17.5	749.15	17.0210007	-122.45343	37.93578	-0.41	17.5	912.51	17.0460007	11	7/24/2014	18:48.1
903	RWS11	-122.4534408	37.93577	-0.49	17.4	772.2	16.9400001	-122.45344	37.93578	-0.41	17.4	937.47	17.016	11	7/24/2014	18:48.2
904	RWS11	-122.4534436	37.93577	-0.44	17.4	795.46	16.9639996	-122.45344	37.93578	-0.41	17.4	962.45	16.9889996	11	7/24/2014	18:48.3
905	RWS11	-122.4534464	37.93577	-0.49	17.4	818.75	16.8889995	-122.45344	37.93578	-0.44	17.4	987.52	16.9309995	11	7/24/2014	18:48.4
906	RWS11	-122.4534493	37.93577	-0.44	17.4	841.93	16.9200009	-122.45344	37.93578	-0.41	17.4	1012.6	16.9450009	11	7/24/2014	18:48.5
907	RWS11	-122.4534521	37.93577	-0.49	17.3	865.08	16.8530002	-122.45345	37.93578	-0.41	17.3	1037.6	16.9290001	11	7/24/2014	18:48.6
908	RWS11	-122.453455	37.93577	-0.44	17.3	888.34	16.8900002	-122.45345	37.93578	-0.41	17.3	1062.7	16.9150002	11	7/24/2014	18:48.7
909	RWS11	-122.4534578	37.93577	-0.49	17.3	911.61	16.8280006	-122.45345	37.93578	-0.41	17.3	1087.7	16.9040005	11	7/24/2014	18:48.8
910	RWS11	-122.4534606	37.93577	-0.44	17.3	935.08	16.8699998	-122.45346	37.93578	-0.36	17.3	1112.9	16.9449998	11	7/24/2014	18:48.9
911	RWS11	-122.4534634	37.93577	-0.49	17.3	958.71	16.8119998	-122.45346	37.93578	-0.44	17.3	1138.3	16.8539998	11	7/24/2014	18:49.0
912	RWS11	-122.4534663	37.93577	-0.44	17.3	982.44	16.8569993	-122.45346	37.93578	-0.33	17.3	1163.7	16.9659993	11	7/24/2014	18:49.1
913	RWS11	-122.453469	37.93577	-0.49	17.3	1006	16.8000007	-122.45346	37.93579	-0.44	17.3	1188.9	16.8420007	11	7/24/2014	18:49.2
914	RWS11	-122.4534718	37.93577	-0.44	17.3	1029.6	16.8459996	-122.45347	37.93579	-0.41	17.3	1214	16.8709996	11	7/24/2014	18:49.3
915	RWS11	-122.4534746	37.93577	-0.44	17.3	1053	16.8419999	-122.45347	37.93579	-0.41	17.3	1239	16.8669999	11	7/24/2014	18:49.4
916	RWS11	-122.4534773	37.93577	-0.44	17.3	1076.1	16.8389996	-122.45347	37.93579	-0.41	17.3	1263.7	16.8639996	11	7/24/2014	18:49.5
917	RWS11	-122.4534799	37.93577	-0.49	17.3	1099.3	16.7870002	-122.45348	37.93579	-0.36	17.3	1288	16.9130002	11	7/24/2014	18:49.6
918	RWS11	-122.4534826	37.93578	-0.44	17.3	1122.2	16.8380002	-122.45348	37.93579	-0.41	17.3	1312.1	16.8630002	11	7/24/2014	18:49.7
919	RWS11	-122.4534852	37.93578	-0.44	17.3	1145.4	16.8399991	-122.45348	37.93579	-0.36	17.3	1336.4	16.9149991	11	7/24/2014	18:49.8
920	RWS11	-122.4534878	37.93578	-0.44	17.3	1168.6	16.8440007	-122.45348	37.93579	-0.41	17.3	1360.6	16.8690007	11	7/24/2014	18:49.9
921	RWS11	-122.4534917	37.93578	-0.49	17.3	1191.6	16.7979999	-122.45349	37.93579	-0.41	17.3	1384.6	16.8739998	11	7/24/2014	18:50.0
922	RWS11	-122.4534943	37.93578	-0.44	17.3	1214.5	16.8540009	-122.45349	37.93579	-0.41	17.3	1408.5	16.8790009	11	7/24/2014	18:50.1
923	RWS11	-122.4534969	37.93578	-0.44	17.3	1237.3	16.8599995	-122.45349	37.93579	-0.41	17.3	1432.1	16.8849995	11	7/24/2014	18:50.2

924	RWS11	-122.4534995	37.93578	-0.44	17.3	1260	16.8660001	-122.45349	37.93579	-0.41	17.3	1455.7	16.891	11	7/24/2014	18:50.3
925	RWS11	-122.4535022	37.93578	-0.44	17.3	1282.3	16.8709992	-122.4535	37.93579	-0.41	17.3	1478.8	16.8959992	11	7/24/2014	18:50.4
926	RWS11	-122.4535049	37.93578	-0.44	17.3	1304.4	16.8750008	-122.4535	37.93579	-0.41	17.3	1501.5	16.9000008	11	7/24/2014	18:50.5
927	RWS11	-122.4535076	37.93578	-0.44	17.3	1326.2	16.8790005	-122.4535	37.93579	-0.44	17.3	1523.8	16.8700005	11	7/24/2014	18:50.6
928	RWS11	-122.4535103	37.93578	-0.44	17.3	1347.9	16.8830003	-122.45351	37.93579	-0.36	17.3	1545.9	16.9580002	11	7/24/2014	18:50.7
929	RWS11	-122.4535131	37.93578	-0.44	17.3	1369.2	16.887	-122.45351	37.9358	-0.41	17.3	1567.6	16.9119999	11	7/24/2014	18:50.8
930	RWS11	-122.4535158	37.93578	-0.44	17.3	1390.3	16.8930005	-122.45351	37.9358	-0.41	17.3	1588.7	16.9180005	11	7/24/2014	18:50.9
931	RWS11	-122.4535186	37.93578	-0.44	17.3	1411	16.9000005	-122.45351	37.9358	-0.41	17.3	1609.4	16.9250004	11	7/24/2014	18:51.0
932	RWS11	-122.4535213	37.93578	-0.44	17.3	1431.5	16.9089993	-122.45352	37.9358	-0.41	17.3	1629.8	16.9339993	11	7/24/2014	18:51.1
933	RWS11	-122.4535241	37.93579	-0.44	17.4	1451.8	16.9219998	-122.45352	37.9358	-0.41	17.4	1649.9	16.9469998	11	7/24/2014	18:51.2
934	RWS11	-122.4535269	37.93579	-0.44	17.4	1471.7	16.9380006	-122.45352	37.9358	-0.41	17.4	1669.5	16.9630005	11	7/24/2014	18:51.3
935	RWS11	-122.4535296	37.93579	-0.49	17.4	1491.3	16.9069991	-122.45353	37.9358	-0.41	17.4	1688.6	16.9829991	11	7/24/2014	18:51.4
936	RWS11	-122.4535323	37.93579	-0.44	17.4	1510.4	16.9809998	-122.45353	37.9358	-0.41	17.4	1707.2	17.0059998	11	7/24/2014	18:51.5
937	RWS11	-122.453535	37.93579	-0.44	17.4	1529.1	17.0080003	-122.45353	37.9358	-0.41	17.4	1725.4	17.0330002	11	7/24/2014	18:51.6
938	RWS11	-122.4535377	37.93579	-0.44	17.5	1547.3	17.0380009	-122.45353	37.9358	-0.44	17.5	1743.1	17.0290009	11	7/24/2014	18:51.7
939	RWS11	-122.4535403	37.93579	-0.49	17.5	1565	17.0190005	-122.45354	37.9358	-0.41	17.5	1760.1	17.0950005	11	7/24/2014	18:51.8
940	RWS11	-122.453543	37.93579	-0.44	17.5	1582.2	17.1040009	-122.45354	37.9358	-0.41	17.5	1776.6	17.1290009	11	7/24/2014	18:51.9
941	RWS11	-122.4535469	37.93579	-0.44	17.6	1598.9	17.1379994	-122.45354	37.9358	-0.44	17.6	1792.5	17.1289994	11	7/24/2014	18:52.0
942	RWS11	-122.4535495	37.93579	-0.44	17.6	1615	17.1729993	-122.45355	37.9358	-0.41	17.6	1807.7	17.1979992	11	7/24/2014	18:52.1
943	RWS11	-122.4535522	37.93579	-0.49	17.6	1630.6	17.1550002	-122.45355	37.9358	-0.41	17.6	1822.3	17.2310002	11	7/24/2014	18:52.2
944	RWS11	-122.4535549	37.93579	-0.44	17.7	1645.5	17.2389993	-122.45355	37.9358	-0.44	17.7	1836.2	17.2299992	11	7/24/2014	18:52.3
945	RWS11	-122.4535575	37.93579	-0.49	17.7	1659.8	17.2210002	-122.45355	37.93581	-0.44	17.7	1849.4	17.2630002	11	7/24/2014	18:52.4
946	RWS11	-122.4535603	37.93579	-0.49	17.7	1673.3	17.2520003	-122.45356	37.93581	-0.41	17.7	1861.7	17.3280003	11	7/24/2014	18:52.5
947	RWS11	-122.453563	37.93579	-0.49	17.8	1686	17.2819991	-122.45356	37.93581	-0.44	17.8	1873.3	17.3239991	11	7/24/2014	18:52.6
948	RWS11	-122.4535658	37.93579	-0.44	17.8	1697.8	17.3620004	-122.45356	37.93581	-0.44	17.8	1883.8	17.3530003	11	7/24/2014	18:52.7
949	RWS11	-122.4535685	37.93579	-0.49	17.8	1708.8	17.3369994	-122.45356	37.93581	-0.44	17.8	1893.4	17.3789994	11	7/24/2014	18:52.8
950	RWS11	-122.4535713	37.9358	-0.44	17.8	1719	17.4119996	-122.45357	37.93581	-0.44	17.8	1902.3	17.4029996	11	7/24/2014	18:52.9
951	RWS11	-122.453574	37.9358	-0.49	17.9	1728.3	17.381	-122.45357	37.93581	-0.44	17.9	1910.1	17.423	11	7/24/2014	18:53.0
952	RWS11	-122.4535768	37.9358	-0.44	17.9	1736.7	17.4480008	-122.45357	37.93581	-0.41	17.9	1917	17.4730008	11	7/24/2014	18:53.1
953	RWS11	-122.4535795	37.9358	-0.44	17.9	1744.3	17.4579991	-122.45358	37.93581	-0.41	17.9	1923.1	17.4829991	11	7/24/2014	18:53.2
954	RWS11	-122.4535822	37.9358	-0.44	17.9	1751	17.4609994	-122.45358	37.93581	-0.41	17.9	1928.2	17.4859994	11	7/24/2014	18:53.3
955	RWS11	-122.4535848	37.9358	-0.49	17.9	1756.8	17.4050002	-122.45358	37.93581	-0.44	17.9	1932.3	17.4470002	11	7/24/2014	18:53.4
956	RWS11	-122.4535874	37.9358	-0.44	17.9	1761.5	17.4420003	-122.45358	37.93581	-0.44	17.9	1935.5	17.4330003	11	7/24/2014	18:53.5

957	RWS11	-122.45359	37.9358	-0.49	17.9	1765.4	17.3679996	-122.45359	37.93581	-0.44	17.9	1937.8	17.4099996	11	7/24/2014	18:53.6
958	RWS11	-122.4535926	37.9358	-0.44	17.8	1768.5	17.3879994	-122.45359	37.93581	-0.41	17.8	1939.4	17.4129994	11	7/24/2014	18:53.7
959	RWS11	-122.4535951	37.9358	-0.44	17.8	1770.6	17.3499993	-122.45359	37.93581	-0.44	17.8	1940	17.3409993	11	7/24/2014	18:53.8
960	RWS11	-122.4535977	37.9358	-0.44	17.7	1771.7	17.3060006	-122.45359	37.93581	-0.41	17.7	1939.6	17.3310006	11	7/24/2014	18:53.9
961	RWS11	-122.4536014	37.9358	-0.44	17.7	1771.9	17.2580003	-122.4536	37.93581	-0.44	17.7	1938.3	17.2490003	11	7/24/2014	18:54.0
962	RWS11	-122.4536039	37.9358	-0.44	17.6	1771.2	17.2079991	-122.4536	37.93581	-0.41	17.6	1936.1	17.2329991	11	7/24/2014	18:54.1
963	RWS11	-122.4536065	37.9358	-0.44	17.6	1769.5	17.1559991	-122.4536	37.93582	-0.41	17.6	1932.8	17.180999	11	7/24/2014	18:54.2
964	RWS11	-122.453609	37.9358	-0.44	17.5	1766.6	17.1029996	-122.45361	37.93582	-0.41	17.5	1928.3	17.1279995	11	7/24/2014	18:54.3
965	RWS11	-122.4536116	37.9358	-0.44	17.5	1762.7	17.0520009	-122.45361	37.93582	-0.44	17.5	1922.7	17.0430009	11	7/24/2014	18:54.4
966	RWS11	-122.4536143	37.9358	-0.44	17.4	1757.7	17.0019997	-122.45361	37.93582	-0.44	17.4	1915.9	16.9929997	11	7/24/2014	18:54.5
967	RWS11	-122.4536169	37.9358	-0.44	17.4	1751.7	16.9539994	-122.45361	37.93582	-0.44	17.4	1908.2	16.9449994	11	7/24/2014	18:54.6
968	RWS11	-122.4536196	37.9358	-0.44	17.3	1744.7	16.9089993	-122.45362	37.93582	-0.44	17.3	1899.5	16.8999993	11	7/24/2014	18:54.7
969	RWS11	-122.4536222	37.93581	-0.44	17.3	1736.7	16.8669995	-122.45362	37.93582	-0.44	17.3	1889.6	16.8579995	11	7/24/2014	18:54.8
970	RWS11	-122.4536249	37.93581	-0.44	17.3	1727.8	16.8300008	-122.45362	37.93582	-0.44	17.3	1879	16.8210008	11	7/24/2014	18:54.9
971	RWS11	-122.4536276	37.93581	-0.44	17.2	1718.2	16.7950009	-122.45362	37.93582	-0.44	17.2	1867.7	16.7860009	11	7/24/2014	18:55.0
972	RWS11	-122.4536303	37.93581	-0.44	17.2	1707.8	16.7640008	-122.45363	37.93582	-0.44	17.2	1855.7	16.7550008	11	7/24/2014	18:55.1
973	RWS11	-122.4536329	37.93581	-0.49	17.2	1696.5	16.6860004	-122.45363	37.93582	-0.44	17.2	1843	16.7280003	11	7/24/2014	18:55.2
974	RWS11	-122.4536356	37.93581	-0.44	17.1	1684.5	16.7120007	-122.45363	37.93582	-0.44	17.1	1829.5	16.7030007	11	7/24/2014	18:55.3
975	RWS11	-122.4536382	37.93581	-0.44	17.1	1671.7	16.6910008	-122.45363	37.93582	-0.5	17.1	1815.2	16.6310008	11	7/24/2014	18:55.4
976	RWS11	-122.4536408	37.93581	-0.44	17.1	1658.2	16.6729993	-122.45364	37.93582	-0.41	17.1	1800.4	16.6979992	11	7/24/2014	18:55.5
977	RWS11	-122.4536434	37.93581	-0.49	17.1	1643.9	16.6060004	-122.45364	37.93582	-0.44	17.1	1784.7	16.6480004	11	7/24/2014	18:55.6
978	RWS11	-122.453646	37.93581	-0.44	17.1	1628.7	16.6430005	-122.45364	37.93582	-0.44	17.1	1768.2	16.6340005	11	7/24/2014	18:55.7
979	RWS11	-122.4536485	37.93581	-0.44	17.1	1612.7	16.6309995	-122.45364	37.93582	-0.44	17.1	1750.9	16.6219994	11	7/24/2014	18:55.8
980	RWS11	-122.4536511	37.93581	-0.44	17.1	1595.9	16.6209992	-122.45365	37.93582	-0.44	17.1	1732.9	16.6119992	11	7/24/2014	18:55.9
981	RWS11	-122.4536548	37.93581	-0.44	17	1578.6	16.6120004	-122.45365	37.93582	-0.44	17	1714.5	16.6030003	11	7/24/2014	18:56.0
982	RWS11	-122.4536573	37.93581	-0.44	17	1560.5	16.6050004	-122.45365	37.93582	-0.41	17	1695.4	16.6300004	11	7/24/2014	18:56.1
983	RWS11	-122.4536598	37.93581	-0.49	17	1541.9	16.5470004	-122.45366	37.93583	-0.44	17	1675.8	16.5890004	11	7/24/2014	18:56.2
984	RWS11	-122.4536624	37.93581	-0.44	17	1522.9	16.5910004	-122.45366	37.93583	-0.41	17	1655.7	16.6160004	11	7/24/2014	18:56.3
985	RWS11	-122.453665	37.93581	-0.44	17	1503.6	16.5829991	-122.45366	37.93583	-0.44	17	1635.5	16.5739991	11	7/24/2014	18:56.4
986	RWS11	-122.4536676	37.93581	-0.44	17	1483.7	16.5730008	-122.45366	37.93583	-0.44	17	1614.7	16.5640008	11	7/24/2014	18:56.5
987	RWS11	-122.4536702	37.93581	-0.44	17	1463.4	16.5609998	-122.45367	37.93583	-0.44	17	1593.5	16.5519998	11	7/24/2014	18:56.6
988	RWS11	-122.4536728	37.93581	-0.44	17	1442.6	16.5450009	-122.45367	37.93583	-0.41	17	1571.9	16.5700009	11	7/24/2014	18:56.7
989	RWS11	-122.4536754	37.93581	-0.49	17	1421.3	16.4729991	-122.45367	37.93583	-0.44	17	1549.7	16.5149991	11	7/24/2014	18:56.8

990	RWS11	-122.453678	37.93581	-0.49	16.9	1399.5	16.447	-122.45367	37.93583	-0.41	16.9	1527	16.523	11	7/24/2014	18:56.9
991	RWS11	-122.4536806	37.93582	-0.49	16.9	1377	16.4159999	-122.45368	37.93583	-0.44	16.9	1503.6	16.4579999	11	7/24/2014	18:57.0
992	RWS11	-122.4536832	37.93582	-0.44	16.9	1354.2	16.432	-122.45368	37.93583	-0.36	16.9	1479.6	16.507	11	7/24/2014	18:57.1
993	RWS11	-122.4536858	37.93582	-0.44	16.8	1330.9	16.3919991	-122.45368	37.93583	-0.41	16.8	1455.3	16.4169991	11	7/24/2014	18:57.2
994	RWS11	-122.4536883	37.93582	-0.49	16.8	1307.3	16.2970004	-122.45368	37.93583	-0.41	16.8	1430.4	16.3730004	11	7/24/2014	18:57.3
995	RWS11	-122.4536909	37.93582	-0.49	16.7	1283.5	16.2490001	-122.45369	37.93583	-0.41	16.7	1405.2	16.3250001	11	7/24/2014	18:57.4
996	RWS11	-122.4536934	37.93582	-0.44	16.7	1259.4	16.248	-122.45369	37.93583	-0.41	16.7	1379.6	16.273	11	7/24/2014	18:57.5
997	RWS11	-122.4536959	37.93582	-0.49	16.6	1235.2	16.1419997	-122.45369	37.93583	-0.41	16.6	1353.9	16.2179997	11	7/24/2014	18:57.6
998	RWS11	-122.4536983	37.93582	-0.44	16.6	1210.8	16.1339997	-122.45369	37.93583	-0.41	16.6	1327.9	16.1589997	11	7/24/2014	18:57.7
999	RWS11	-122.4537007	37.93582	-0.49	16.5	1186.3	16.0220008	-122.4537	37.93583	-0.44	16.5	1301.9	16.0640008	11	7/24/2014	18:57.8
1000	RWS11	-122.4537032	37.93582	-0.44	16.4	1161.9	16.0110005	-122.4537	37.93583	-0.41	16.4	1275.8	16.0360005	11	7/24/2014	18:57.9
1001	RWS11	-122.4537067	37.93582	-0.49	16.4	1137.4	15.8989997	-122.4537	37.93583	-0.41	16.4	1249.7	15.9749997	11	7/24/2014	18:58.0
1002	RWS11	-122.4537091	37.93582	-0.44	16.3	1112.8	15.8900002	-122.45371	37.93583	-0.41	16.3	1223.5	15.9150002	11	7/24/2014	18:58.1
1003	RWS11	-122.4537115	37.93582	-0.44	16.3	1088.1	15.8319997	-122.45371	37.93583	-0.41	16.3	1197.2	15.8569996	11	7/24/2014	18:58.2
1004	RWS11	-122.4537139	37.93582	-0.44	16.2	1063.3	15.7759999	-122.45371	37.93583	-0.41	16.2	1170.7	15.8009999	11	7/24/2014	18:58.3
1005	RWS11	-122.4537163	37.93582	-0.49	16.2	1038.3	15.6720004	-122.45371	37.93583	-0.41	16.2	1144.1	15.7480004	11	7/24/2014	18:58.4
1006	RWS11	-122.4537188	37.93582	-0.44	16.1	1013.3	15.6729993	-122.45372	37.93584	-0.41	16.1	1117.5	15.6979992	11	7/24/2014	18:58.5
1007	RWS11	-122.4537212	37.93582	-0.44	16.1	988.04	15.6269997	-122.45372	37.93584	-0.36	16.1	1090.6	15.7019997	11	7/24/2014	18:58.6
1008	RWS11	-122.4537236	37.93582	-0.44	16	962.78	15.5829991	-122.45372	37.93584	-0.41	16	1063.7	15.6079991	11	7/24/2014	18:58.7
1009	RWS11	-122.453726	37.93582	-0.44	16	937.62	15.5419997	-122.45372	37.93584	-0.36	16	1036.9	15.6169997	11	7/24/2014	18:58.8
1010	RWS11	-122.4537284	37.93582	-0.44	15.9	912.42	15.505	-122.45372	37.93584	-0.36	15.9	1010.3	15.58	11	7/24/2014	18:58.9
1011	RWS11	-122.4537319	37.93582	-0.44	15.9	887.34	15.4700002	-122.45373	37.93584	-0.41	15.9	983.7	15.4950001	11	7/24/2014	18:59.0
1012	RWS11	-122.4537343	37.93582	-0.44	15.9	862.32	15.4379996	-122.45373	37.93584	-0.36	15.9	957.35	15.5129996	11	7/24/2014	18:59.1
1013	RWS11	-122.4537367	37.93582	-0.44	15.8	837.48	15.4079999	-122.45373	37.93584	-0.41	15.8	931.37	15.4329999	11	7/24/2014	18:59.2
1014	RWS11	-122.453739	37.93583	-0.44	15.8	813.04	15.38	-122.45374	37.93584	-0.41	15.8	905.8	15.405	11	7/24/2014	18:59.3
1015	RWS11	-122.4537415	37.93583	-0.44	15.8	788.76	15.3529996	-122.45374	37.93584	-0.41	15.8	880.46	15.3779995	11	7/24/2014	18:59.4
1016	RWS11	-122.4537439	37.93583	-0.4	15.8	764.63	15.3619995	-122.45374	37.93584	-0.36	15.8	855.36	15.4019995	11	7/24/2014	18:59.5
1017	RWS11	-122.4537463	37.93583	-0.44	15.7	740.76	15.3019999	-122.45374	37.93584	-0.36	15.7	830.42	15.3769999	11	7/24/2014	18:59.6
1018	RWS11	-122.4537487	37.93583	-0.4	15.7	716.88	15.3129998	-122.45375	37.93584	-0.36	15.7	805.45	15.3529998	11	7/24/2014	18:59.7
1019	RWS11	-122.4537511	37.93583	-0.44	15.7	692.81	15.2539996	-122.45375	37.93584	-0.33	15.7	780.25	15.3629996	11	7/24/2014	18:59.8
1020	RWS11	-122.4537535	37.93583	-0.44	15.7	668.42	15.2300004	-122.45375	37.93584	-0.36	15.7	754.73	15.3050004	11	7/24/2014	18:59.9
1021	RWS11	-122.4537571	37.93583	-0.44	15.6	643.84	15.2060002	-122.45375	37.93584	-0.36	15.6	728.88	15.2810002	11	7/24/2014	19:00.0
1022	RWS11	-122.4537595	37.93583	-0.4	15.6	619.25	15.2159996	-122.45376	37.93584	-0.36	15.6	702.86	15.2559996	11	7/24/2014	19:00.1

1023	RWS11	-122.453762	37.93583	-0.44	15.6	594.54	15.156	-122.45376	37.93584	-0.36	15.6	676.6	15.231	11	7/24/2014	19:00.2
1024	RWS11	-122.4537644	37.93583	-0.4	15.6	569.87	15.165	-122.45376	37.93584	-0.36	15.6	650.3	15.205	11	7/24/2014	19:00.3
1025	RWS11	-122.4537669	37.93583	-0.44	15.5	545.45	15.1050004	-122.45376	37.93584	-0.36	15.5	624.14	15.1800004	11	7/24/2014	19:00.4
1026	RWS11	-122.4537694	37.93583	-0.4	15.5	521.18	15.1140004	-122.45377	37.93584	-0.33	15.5	598.14	15.1880004	11	7/24/2014	19:00.5
1027	RWS11	-122.4537719	37.93583	-0.44	15.5	497.21	15.0539998	-122.45377	37.93584	-0.36	15.5	572.44	15.1289998	11	7/24/2014	19:00.6
1028	RWS11	-122.4537744	37.93583	-0.4	15.5	473.7	15.0649996	-122.45377	37.93584	-0.36	15.5	547.16	15.1049996	11	7/24/2014	19:00.7
1029	RWS11	-122.4537769	37.93583	-0.44	15.4	450.79	15.0060004	-122.45377	37.93584	-0.36	15.4	522.37	15.0810004	11	7/24/2014	19:00.8
1030	RWS11	-122.4537793	37.93583	-0.4	15.4	428.32	15.0170002	-122.45378	37.93584	-0.33	15.4	498.08	15.0910002	11	7/24/2014	19:00.9
1031	RWS11	-122.453783	37.93583	-0.44	15.4	406.42	14.9590005	-122.45378	37.93585	-0.33	15.4	474.43	15.0680005	11	7/24/2014	19:01.0
1032	RWS11	-122.4537854	37.93583	-0.44	15.4	385.03	14.9359998	-122.45378	37.93585	-0.33	15.4	451.29	15.0449998	11	7/24/2014	19:01.1
1033	RWS11	-122.4537879	37.93583	-0.4	15.4	363.92	14.9490004	-122.45378	37.93585	-0.33	15.4	428.54	15.0230004	11	7/24/2014	19:01.2
1034	RWS11	-122.4537903	37.93583	-0.4	15.3	343.12	14.9270001	-122.45379	37.93585	-0.33	15.3	405.94	15.0010001	11	7/24/2014	19:01.3
1035	RWS11	-122.4537928	37.93583	-0.4	15.3	322.3	14.9060002	-122.45379	37.93585	-0.33	15.3	383.47	14.9800002	11	7/24/2014	19:01.4
1036	RWS11	-122.4537953	37.93583	-0.4	15.3	301.5	14.8850003	-122.45379	37.93585	-0.33	15.3	360.82	14.9590003	11	7/24/2014	19:01.5
1037	RWS11	-122.4537977	37.93583	-0.44	15.3	280.61	14.8299998	-122.45379	37.93585	-0.33	15.3	338.37	14.9389998	11	7/24/2014	19:01.6
1038	RWS11	-122.4538002	37.93584	-0.4	15.2	259.99	14.8450003	-122.4538	37.93585	-0.33	15.2	316.08	14.9190003	11	7/24/2014	19:01.7
1039	RWS11	-122.4538026	37.93584	-0.44	15.2	239.52	14.7899998	-122.4538	37.93585	-0.36	15.2	294.15	14.8649998	11	7/24/2014	19:01.8
1040	RWS11	-122.4538051	37.93584	-0.4	15.2	219.31	14.8059998	-122.4538	37.93585	-0.33	15.2	272.43	14.8799998	11	7/24/2014	19:01.9
1041	RWS11	-122.4538087	37.93584	-0.4	15.2	199.57	14.7869997	-122.45381	37.93585	-0.33	15.2	251.28	14.8609997	11	7/24/2014	19:02.0
1042	RWS11	-122.4538111	37.93584	-0.4	15.2	180.06	14.7690001	-122.45381	37.93585	-0.33	15.2	230.46	14.8430001	11	7/24/2014	19:02.1
1043	RWS11	-122.4538135	37.93584	-0.4	15.2	160.88	14.7510004	-122.45381	37.93585	-0.33	15.2	209.91	14.8250004	11	7/24/2014	19:02.2
1044	RWS11	-122.453816	37.93584	-0.4	15.1	141.94	14.7340002	-122.45381	37.93585	-0.33	15.1	189.68	14.8080002	11	7/24/2014	19:02.3
1045	RWS11	-122.4538184	37.93584	-0.44	15.1	123.5	14.682	-122.45381	37.93585	-0.33	15.1	169.91	14.791	11	7/24/2014	19:02.4
1046	RWS11	-122.4538209	37.93584	-0.4	15.1	105.3	14.6990004	-122.45382	37.93585	-0.29	15.1	150.45	14.8070004	11	7/24/2014	19:02.5
1047	RWS11	-122.4538233	37.93584	-0.4	15.1	87.389	14.6820002	-122.45382	37.93585	-0.33	15.1	131.35	14.7560002	11	7/24/2014	19:02.6
1048	RWS11	-122.4538258	37.93584	-0.4	15.1	69.676	14.665	-122.45382	37.93585	-0.33	15.1	112.52	14.739	11	7/24/2014	19:02.7
1049	RWS11	-122.4538282	37.93584	-0.4	15	52.194	14.6479998	-122.45382	37.93585	-0.33	15	94.024	14.7219998	11	7/24/2014	19:02.8
1050	RWS11	-122.4538306	37.93584	-0.4	15	34.851	14.632	-122.45383	37.93585	-0.29	15	75.667	14.74	11	7/24/2014	19:02.9
1051	RWS11	-122.4538343	37.93584	-0.4	15	17.678	14.6169997	-122.45383	37.93585	-0.29	15	57.433	14.7249996	11	7/24/2014	19:03.0
1052	RWS11	-122.4538367	37.93584	-0.35	15	0.573	14.6550001	-122.45383	37.93585	-0.29	15	39.32	14.7120001	11	7/24/2014	19:03.1
1053	RWS11	-122.4538391	37.93584	-0.4	15	-16.38	14.592	-122.45384	37.93585	-0.29	15	21.474	14.7	11	7/24/2014	19:03.2
1054	RWS11	-122.4538416	37.93584	-0.35	15	-33.31	14.6340002	-122.45384	37.93586	-0.24	15	3.648	14.7420002	11	7/24/2014	19:03.3
1055	RWS11	-122.453844	37.93584	-0.4	15	-50.01	14.5749998	-122.45384	37.93586	-0.29	15	-13.872	14.6829998	11	7/24/2014	19:03.4

1056	RWS11	-122.4538465	37.93584	-0.35	15	-66.68	14.6209997	-122.45384	37.93586	-0.29	15	-31.355	14.6779997	11	7/24/2014	19:03.5
1057	RWS11	-122.453849	37.93584	-0.4	15	-83.04	14.566	-122.45385	37.93586	-0.24	15	-48.658	14.725	11	7/24/2014	19:03.6
1058	RWS11	-122.4538514	37.93584	-0.35	15	-99.19	14.6150002	-122.45385	37.93586	-0.24	15	-65.804	14.7230002	11	7/24/2014	19:03.7
1059	RWS11	-122.4538539	37.93584	-0.35	15	-115.1	14.6139998	-122.45385	37.93586	-0.29	15	-82.801	14.6709997	11	7/24/2014	19:03.8
1060	RWS11	-122.4538563	37.93584	-0.35	15	-130.8	14.6139998	-122.45385	37.93586	-0.24	15	-99.607	14.7219998	11	7/24/2014	19:03.9
1061	RWS11	-122.4538599	37.93585	-0.4	15	-146.1	14.5629998	-122.45386	37.93586	-0.29	15	-116.2	14.6709997	11	7/24/2014	19:04.0
1062	RWS11	-122.4538623	37.93585	-0.35	15	-161.2	14.6139998	-122.45386	37.93586	-0.29	15	-132.52	14.6709997	11	7/24/2014	19:04.1
1063	RWS11	-122.4538647	37.93585	-0.35	15	-176	14.6139998	-122.45386	37.93586	-0.29	15	-148.59	14.6709997	11	7/24/2014	19:04.2
1064	RWS11	-122.4538671	37.93585	-0.35	15	-190.4	14.6159996	-122.45386	37.93586	-0.29	15	-164.25	14.6729996	11	7/24/2014	19:04.3
1065	RWS11	-122.4538695	37.93585	-0.35	15	-204.5	14.617	-122.45387	37.93586	-0.24	15	-179.51	14.725	11	7/24/2014	19:04.4
1066	RWS11	-122.4538719	37.93585	-0.35	15	-218.1	14.6189999	-122.45387	37.93586	-0.24	15	-194.52	14.7269999	11	7/24/2014	19:04.5
1067	RWS11	-122.4538743	37.93585	-0.35	15	-231.4	14.6220001	-122.45387	37.93586	-0.29	15	-209.03	14.6790001	11	7/24/2014	19:04.6
1068	RWS11	-122.4538767	37.93585	-0.35	15	-244.4	14.6250004	-122.45387	37.93586	-0.24	15	-223.27	14.7330004	11	7/24/2014	19:04.7
1069	RWS11	-122.4538791	37.93585	-0.35	15	-257.3	14.6290001	-122.45388	37.93586	-0.24	15	-237.35	14.7370001	11	7/24/2014	19:04.8
1070	RWS11	-122.4538815	37.93585	-0.35	15	-270.1	14.6340002	-122.45388	37.93586	-0.24	15	-251.25	14.7420002	11	7/24/2014	19:04.9
1071	RWS11	-122.453885	37.93585	-0.35	15	-282.8	14.6390003	-122.45388	37.93586	-0.24	15	-265.01	14.7470003	11	7/24/2014	19:05.0
1072	RWS11	-122.4538874	37.93585	-0.35	15	-295.4	14.6440004	-122.45388	37.93586	-0.21	15	-278.61	14.7860004	11	7/24/2014	19:05.1
1073	RWS11	-122.4538898	37.93585	-0.35	15	-307.7	14.65	-122.45389	37.93586	-0.29	15	-291.97	14.707	11	7/24/2014	19:05.2
1074	RWS11	-122.4538922	37.93585	-0.35	15	-319.8	14.6559996	-122.45389	37.93586	-0.24	15	-304.99	14.7639996	11	7/24/2014	19:05.3
1075	RWS11	-122.4538947	37.93585	-0.4	15	-331.6	14.6110001	-122.45389	37.93586	-0.29	15	-317.71	14.7190001	11	7/24/2014	19:05.4
1076	RWS11	-122.4538971	37.93585	-0.35	15	-343.2	14.6679997	-122.45389	37.93586	-0.24	15	-330.04	14.7759997	11	7/24/2014	19:05.5
1077	RWS11	-122.4538996	37.93585	-0.35	15	-354.7	14.6729998	-122.4539	37.93587	-0.29	15	-342.03	14.7299998	11	7/24/2014	19:05.6
1078	RWS11	-122.453902	37.93585	-0.35	15	-365.8	14.676	-122.4539	37.93587	-0.29	15	-353.75	14.733	11	7/24/2014	19:05.7
1079	RWS11	-122.4539044	37.93585	-0.35	15	-376.7	14.6779999	-122.4539	37.93587	-0.29	15	-365.22	14.7349999	11	7/24/2014	19:05.8
1080	RWS11	-122.4539068	37.93585	-0.35	15	-387.3	14.6779999	-122.4539	37.93587	-0.24	15	-376.35	14.7859999	11	7/24/2014	19:05.9
1081	RWS11	-122.4539104	37.93585	-0.35	15	-397.7	14.676	-122.45391	37.93587	-0.29	15	-387.36	14.733	11	7/24/2014	19:06.0
1082	RWS11	-122.4539128	37.93585	-0.35	15	-408	14.6729998	-122.45391	37.93587	-0.29	15	-398.16	14.7299998	11	7/24/2014	19:06.1
1083	RWS11	-122.4539152	37.93586	-0.4	15	-418.1	14.6180001	-122.45391	37.93587	-0.29	15	-408.77	14.726	11	7/24/2014	19:06.2
1084	RWS11	-122.4539177	37.93586	-0.35	15	-428.1	14.6639999	-122.45391	37.93587	-0.29	15	-419.17	14.7209999	11	7/24/2014	19:06.3
1085	RWS11	-122.4539201	37.93586	-0.35	15	-437.9	14.6589998	-122.45392	37.93587	-0.29	15	-429.43	14.7159998	11	7/24/2014	19:06.4
1086	RWS11	-122.4539226	37.93586	-0.35	15	-447.7	14.6550001	-122.45392	37.93587	-0.29	15	-439.46	14.7120001	11	7/24/2014	19:06.5
1087	RWS11	-122.453925	37.93586	-0.4	15	-457.4	14.6000004	-122.45392	37.93587	-0.29	15	-449.39	14.7080004	11	7/24/2014	19:06.6
1088	RWS11	-122.4539275	37.93586	-0.35	15	-466.9	14.6489996	-122.45392	37.93587	-0.29	15	-459.16	14.7059996	11	7/24/2014	19:06.7

1089	RWS11	-122.4539299	37.93586	-0.35	15	-476.4	14.6460003	-122.45393	37.93587	-0.29	15	-468.8	14.7030003	11	7/24/2014	19:06.8
1090	RWS11	-122.4539323	37.93586	-0.35	15	-485.8	14.6449999	-122.45393	37.93587	-0.24	15	-478.43	14.7529999	11	7/24/2014	19:06.9
1091	RWS11	-122.4539359	37.93586	-0.35	15	-494.9	14.6440004	-122.45393	37.93587	-0.29	15	-487.92	14.7010004	11	7/24/2014	19:07.0
1092	RWS11	-122.4539383	37.93586	-0.35	15	-503.9	14.643	-122.45393	37.93587	-0.24	15	-497.21	14.751	11	7/24/2014	19:07.1
1093	RWS11	-122.4539406	37.93586	-0.35	15	-512.5	14.6419996	-122.45394	37.93587	-0.29	15	-506.36	14.6989996	11	7/24/2014	19:07.2
1094	RWS11	-122.4539431	37.93586	-0.35	15	-521	14.6399998	-122.45394	37.93587	-0.24	15	-515.35	14.7479998	11	7/24/2014	19:07.3
1095	RWS11	-122.4539455	37.93586	-0.35	15	-529.1	14.6379999	-122.45394	37.93587	-0.29	15	-524.08	14.6949999	11	7/24/2014	19:07.4
1096	RWS11	-122.4539479	37.93586	-0.35	15	-536.9	14.6370001	-122.45394	37.93587	-0.24	15	-532.49	14.7450001	11	7/24/2014	19:07.5
1097	RWS11	-122.4539503	37.93586	-0.4	15	-544.3	14.5820003	-122.45395	37.93587	-0.29	15	-540.72	14.6900004	11	7/24/2014	19:07.6
1098	RWS11	-122.4539527	37.93586	-0.35	15	-551.4	14.6289997	-122.45395	37.93588	-0.24	15	-548.66	14.7369997	11	7/24/2014	19:07.7
1099	RWS11	-122.4539551	37.93586	-0.35	15	-558.1	14.625	-122.45395	37.93588	-0.29	15	-556.26	14.682	11	7/24/2014	19:07.8
1100	RWS11	-122.4539575	37.93586	-0.35	15	-564.5	14.6210003	-122.45395	37.93588	-0.29	15	-563.67	14.6780003	11	7/24/2014	19:07.9
1101	RWS11	-122.453961	37.93586	-0.35	15	-570.8	14.6190004	-122.45396	37.93588	-0.29	15	-570.94	14.6760004	11	7/24/2014	19:08.0
1102	RWS11	-122.4539634	37.93586	-0.35	15	-576.9	14.6160002	-122.45396	37.93588	-0.24	15	-578.2	14.7240002	11	7/24/2014	19:08.1
1103	RWS11	-122.4539658	37.93586	-0.35	15	-583.1	14.6149997	-122.45396	37.93588	-0.24	15	-585.21	14.7229998	11	7/24/2014	19:08.2
1104	RWS11	-122.4539682	37.93587	-0.35	15	-589.1	14.6160002	-122.45396	37.93588	-0.24	15	-592.11	14.7240002	11	7/24/2014	19:08.3
1105	RWS11	-122.4539706	37.93587	-0.35	15	-595.2	14.6169996	-122.45397	37.93588	-0.24	15	-599.05	14.7249996	11	7/24/2014	19:08.4
1106	RWS11	-122.453973	37.93587	-0.35	15	-601.3	14.6190004	-122.45397	37.93588	-0.24	15	-605.99	14.7270004	11	7/24/2014	19:08.5
1107	RWS11	-122.4539754	37.93587	-0.35	15	-607.4	14.6219997	-122.45397	37.93588	-0.24	15	-612.75	14.7299997	11	7/24/2014	19:08.6
1108	RWS11	-122.4539778	37.93587	-0.32	15	-613.5	14.6609998	-122.45397	37.93588	-0.29	15	-619.42	14.6839998	11	7/24/2014	19:08.7
1109	RWS11	-122.4539802	37.93587	-0.35	15	-619.5	14.6309995	-122.45398	37.93588	-0.29	15	-625.78	14.6879996	11	7/24/2014	19:08.8
1110	RWS11	-122.4539826	37.93587	-0.32	15	-625.5	14.6699997	-122.45398	37.93588	-0.29	15	-631.93	14.6929997	11	7/24/2014	19:08.9
1111	RWS11	-122.4539862	37.93587	-0.35	15	-631.3	14.6400003	-122.45398	37.93588	-0.29	15	-637.73	14.6970003	11	7/24/2014	19:09.0
1112	RWS11	-122.4539886	37.93587	-0.35	15	-636.9	14.644	-122.45398	37.93588	-0.24	15	-643.38	14.752	11	7/24/2014	19:09.1
1113	RWS11	-122.453991	37.93587	-0.35	15	-642.5	14.6470003	-122.45399	37.93588	-0.29	15	-648.95	14.7040003	11	7/24/2014	19:09.2
1114	RWS11	-122.4539934	37.93587	-0.35	15	-648	14.6499996	-122.45399	37.93588	-0.24	15	-654.15	14.7579996	11	7/24/2014	19:09.3
1115	RWS11	-122.4539959	37.93587	-0.35	15	-653.2	14.6529999	-122.45399	37.93588	-0.29	15	-659.11	14.7099999	11	7/24/2014	19:09.4
1116	RWS11	-122.4539983	37.93587	-0.35	15	-658.2	14.6549997	-122.45399	37.93588	-0.24	15	-663.99	14.7629997	11	7/24/2014	19:09.5
1117	RWS11	-122.4540008	37.93587	-0.35	15	-663.1	14.6560001	-122.454	37.93588	-0.29	15	-669.09	14.7130001	11	7/24/2014	19:09.6
1118	RWS11	-122.4540032	37.93587	-0.35	15	-668	14.658	-122.454	37.93589	-0.24	15	-674.03	14.766	11	7/24/2014	19:09.7
1119	RWS11	-122.4540057	37.93587	-0.35	15	-672.6	14.6590004	-122.454	37.93589	-0.29	15	-678.95	14.7160004	11	7/24/2014	19:09.8
1120	RWS11	-122.454008	37.93587	-0.35	15	-677.3	14.6590004	-122.454	37.93589	-0.24	15	-683.98	14.7670004	11	7/24/2014	19:09.9
1121	RWS11	-122.4540116	37.93587	-0.35	15	-681.9	14.6569996	-122.45401	37.93589	-0.29	15	-689.06	14.7139996	11	7/24/2014	19:10.0

1122	RWS11	-122.4540139	37.93587	-0.35	15	-686.4	14.6549997	-122.45401	37.93589	-0.29	15	-693.85	14.7119997	11	7/24/2014	19:10.1
1123	RWS11	-122.4540163	37.93588	-0.35	15	-690.7	14.6499996	-122.45401	37.93589	-0.29	15	-698.6	14.7069996	11	7/24/2014	19:10.2
1124	RWS11	-122.4540186	37.93588	-0.35	15	-695	14.6450004	-122.45401	37.93589	-0.29	15	-703	14.7020004	11	7/24/2014	19:10.3
1125	RWS11	-122.454021	37.93588	-0.35	15	-698.8	14.6380005	-122.45402	37.93589	-0.29	15	-707.09	14.6950005	11	7/24/2014	19:10.4
1126	RWS11	-122.4540234	37.93588	-0.35	15	-702.7	14.6309995	-122.45402	37.93589	-0.29	15	-710.91	14.6879996	11	7/24/2014	19:10.5
1127	RWS11	-122.4540257	37.93588	-0.35	15	-706	14.6230001	-122.45402	37.93589	-0.33	15	-714.46	14.6460001	11	7/24/2014	19:10.6
1128	RWS11	-122.4540281	37.93588	-0.35	15	-709.1	14.6149997	-122.45402	37.93589	-0.29	15	-717.66	14.6719998	11	7/24/2014	19:10.7
1129	RWS11	-122.4540305	37.93588	-0.35	15	-711.9	14.6070003	-122.45403	37.93589	-0.29	15	-720.5	14.6640003	11	7/24/2014	19:10.8
1130	RWS11	-122.4540328	37.93588	-0.35	14.9	-714.6	14.6000004	-122.45403	37.93589	-0.29	14.9	-723.16	14.6570004	11	7/24/2014	19:10.9
1131	RWS11	-122.4540364	37.93588	-0.35	14.9	-717.1	14.5930004	-122.45403	37.93589	-0.29	14.9	-725.59	14.6500004	11	7/24/2014	19:11.0
1132	RWS11	-122.4540388	37.93588	-0.32	14.9	-719.5	14.6220003	-122.45403	37.93589	-0.29	14.9	-728.09	14.6450003	11	7/24/2014	19:11.1
1133	RWS11	-122.4540413	37.93588	-0.35	14.9	-721.9	14.5839996	-122.45404	37.93589	-0.33	14.9	-730.49	14.6069996	11	7/24/2014	19:11.2
1134	RWS11	-122.4540438	37.93588	-0.32	14.9	-724.1	14.6159998	-122.45404	37.93589	-0.29	14.9	-732.79	14.6389998	11	7/24/2014	19:11.3
1135	RWS11	-122.4540462	37.93588	-0.35	14.9	-726.1	14.5819998	-122.45404	37.93589	-0.33	14.9	-735.22	14.6049998	11	7/24/2014	19:11.4
1136	RWS11	-122.4540488	37.93588	-0.32	14.9	-728	14.619	-122.45404	37.9359	-0.33	14.9	-737.41	14.608	11	7/24/2014	19:11.5
1137	RWS11	-122.4540513	37.93588	-0.35	14.9	-729.8	14.5909996	-122.45405	37.9359	-0.36	14.9	-739.44	14.5799996	11	7/24/2014	19:11.6
1138	RWS11	-122.4540538	37.93588	-0.32	14.9	-731.5	14.633	-122.45405	37.9359	-0.29	14.9	-741.26	14.656	11	7/24/2014	19:11.7
1139	RWS11	-122.4540563	37.93588	-0.35	15	-733.2	14.6090002	-122.45405	37.9359	-0.33	15	-742.81	14.6320002	11	7/24/2014	19:11.8
1140	RWS11	-122.4540588	37.93588	-0.32	15	-734.8	14.6550003	-122.45405	37.9359	-0.33	15	-744.16	14.6440003	11	7/24/2014	19:11.9
1141	RWS11	-122.4540624	37.93589	-0.32	15	-736.4	14.6690002	-122.45406	37.9359	-0.36	15	-745.43	14.6240002	11	7/24/2014	19:12.0
1142	RWS11	-122.4540649	37.93589	-0.32	15	-737.8	14.6830002	-122.45406	37.9359	-0.36	15	-746.48	14.6380001	11	7/24/2014	19:12.1
1143	RWS11	-122.4540673	37.93589	-0.32	15	-739	14.6959997	-122.45406	37.9359	-0.36	15	-747.3	14.6509997	11	7/24/2014	19:12.2
1144	RWS11	-122.4540697	37.93589	-0.32	15	-739.9	14.7090002	-122.45407	37.9359	-0.36	15	-747.88	14.6640002	11	7/24/2014	19:12.3
1145	RWS11	-122.4540722	37.93589	-0.32	15	-740.4	14.7199999	-122.45407	37.9359	-0.36	15	-748.29	14.6749998	11	7/24/2014	19:12.4
1146	RWS11	-122.4540746	37.93589	-0.32	15	-740.9	14.7300001	-122.45407	37.9359	-0.36	15	-748.53	14.6850001	11	7/24/2014	19:12.5
1147	RWS11	-122.4540771	37.93589	-0.35	15.1	-740.8	14.7040004	-122.45407	37.9359	-0.36	15.1	-748.65	14.6930004	11	7/24/2014	19:12.6
1148	RWS11	-122.4540795	37.93589	-0.32	15.1	-740.5	14.7429996	-122.45407	37.9359	-0.33	15.1	-748.7	14.7319996	11	7/24/2014	19:12.7
1149	RWS11	-122.4540819	37.93589	-0.35	15.1	-740	14.7110004	-122.45408	37.9359	-0.36	15.1	-748.73	14.7000004	11	7/24/2014	19:12.8
1150	RWS11	-122.4540843	37.93589	-0.35	15.1	-739.3	14.7089996	-122.45408	37.9359	-0.36	15.1	-748.83	14.6979996	11	7/24/2014	19:12.9
1151	RWS11	-122.4540879	37.93589	-0.35	15.1	-738.6	14.703	-122.45408	37.9359	-0.41	15.1	-748.78	14.6420001	11	7/24/2014	19:13.0
1152	RWS11	-122.4540903	37.93589	-0.35	15	-737.6	14.6920004	-122.45409	37.9359	-0.36	15	-748.49	14.6810004	11	7/24/2014	19:13.1
1153	RWS11	-122.4540927	37.93589	-0.35	15	-736.6	14.6759996	-122.45409	37.9359	-0.36	15	-748.25	14.6649996	11	7/24/2014	19:13.2
1154	RWS11	-122.4540952	37.93589	-0.35	15	-735.6	14.6560001	-122.45409	37.93591	-0.36	15	-747.93	14.6450001	11	7/24/2014	19:13.3

1155	RWS11	-122.4540976	37.93589	-0.4	15	-734.3	14.5820003	-122.45409	37.93591	-0.41	15	-747.56	14.5720004	11	7/24/2014	19:13.4
1156	RWS11	-122.4541001	37.93589	-0.35	15	-733	14.6059999	-122.4541	37.93591	-0.36	15	-747.18	14.5949999	11	7/24/2014	19:13.5
1157	RWS11	-122.4541025	37.93589	-0.35	14.9	-731.5	14.578	-122.4541	37.93591	-0.36	14.9	-746.66	14.567	11	7/24/2014	19:13.6
1158	RWS11	-122.4541049	37.93589	-0.35	14.9	-730	14.5489998	-122.4541	37.93591	-0.36	14.9	-746.06	14.5379998	11	7/24/2014	19:13.7
1159	RWS11	-122.4541073	37.9359	-0.35	14.9	-728.2	14.5209999	-122.4541	37.93591	-0.36	14.9	-745.16	14.5099999	11	7/24/2014	19:13.8
1160	RWS11	-122.4541097	37.9359	-0.35	14.8	-726.3	14.493	-122.4541	37.93591	-0.36	14.8	-744.25	14.482	11	7/24/2014	19:13.9
1161	RWS11	-122.4541133	37.9359	-0.4	14.8	-724.3	14.416	-122.45411	37.93591	-0.36	14.8	-743.29	14.456	11	7/24/2014	19:14.0
1162	RWS11	-122.4541157	37.9359	-0.35	14.8	-722.1	14.4420004	-122.45411	37.93591	-0.36	14.8	-742.04	14.4310004	11	7/24/2014	19:14.1
1163	RWS11	-122.4541181	37.9359	-0.4	14.8	-719.8	14.3690001	-122.45411	37.93591	-0.36	14.8	-740.58	14.409	11	7/24/2014	19:14.2
1164	RWS11	-122.4541205	37.9359	-0.35	14.7	-717.3	14.3990001	-122.45412	37.93591	-0.36	14.7	-738.93	14.3880001	11	7/24/2014	19:14.3
1165	RWS11	-122.4541229	37.9359	-0.35	14.7	-714.7	14.3800001	-122.45412	37.93591	-0.33	14.7	-737.14	14.4030001	11	7/24/2014	19:14.4
1166	RWS11	-122.4541253	37.9359	-0.35	14.7	-712.1	14.3620004	-122.45412	37.93591	-0.33	14.7	-735.23	14.3850004	11	7/24/2014	19:14.5
1167	RWS11	-122.4541278	37.9359	-0.35	14.7	-709.4	14.3439998	-122.45412	37.93591	-0.33	14.7	-733.32	14.3669998	11	7/24/2014	19:14.6
1168	RWS11	-122.4541302	37.9359	-0.35	14.7	-706.8	14.328	-122.45413	37.93591	-0.33	14.7	-731.32	14.351	11	7/24/2014	19:14.7
1169	RWS11	-122.4541326	37.9359	-0.35	14.7	-704	14.3109998	-122.45413	37.93591	-0.36	14.7	-729.15	14.2999998	11	7/24/2014	19:14.8
1170	RWS11	-122.4541349	37.9359	-0.35	14.6	-700.9	14.2939996	-122.45413	37.93591	-0.33	14.6	-726.81	14.3169996	11	7/24/2014	19:14.9
1171	RWS11	-122.4541385	37.9359	-0.4	14.6	-697.7	14.225	-122.45413	37.93592	-0.33	14.6	-724.19	14.299	11	7/24/2014	19:15.0
1172	RWS11	-122.4541409	37.9359	-0.35	14.6	-694.2	14.2569999	-122.45414	37.93592	-0.29	14.6	-721.3	14.314	11	7/24/2014	19:15.1
1173	RWS11	-122.4541433	37.9359	-0.35	14.6	-690.9	14.2379999	-122.45414	37.93592	-0.33	14.6	-718.38	14.2609999	11	7/24/2014	19:15.2
1174	RWS11	-122.4541457	37.9359	-0.35	14.6	-687.6	14.2189998	-122.45414	37.93592	-0.33	14.6	-715.36	14.2419998	11	7/24/2014	19:15.3
1175	RWS11	-122.4541481	37.93591	-0.35	14.6	-684.2	14.2010002	-122.45414	37.93592	-0.33	14.6	-712.03	14.2240002	11	7/24/2014	19:15.4
1176	RWS11	-122.4541505	37.93591	-0.35	14.5	-680.7	14.1850004	-122.45415	37.93592	-0.29	14.5	-708.65	14.2420004	11	7/24/2014	19:15.5
1177	RWS11	-122.4541529	37.93591	-0.35	14.5	-677	14.1719999	-122.45415	37.93592	-0.29	14.5	-705.17	14.2289999	11	7/24/2014	19:15.6
1178	RWS11	-122.4541553	37.93591	-0.32	14.5	-673.4	14.1970001	-122.45415	37.93592	-0.29	14.5	-701.6	14.2200001	11	7/24/2014	19:15.7
1179	RWS11	-122.4541577	37.93591	-0.35	14.5	-669.6	14.1590004	-122.45415	37.93592	-0.29	14.5	-697.82	14.2160004	11	7/24/2014	19:15.8
1180	RWS11	-122.4541601	37.93591	-0.32	14.5	-666	14.1930004	-122.45416	37.93592	-0.29	14.5	-693.99	14.2160004	11	7/24/2014	19:15.9
1181	RWS11	-122.4541637	37.93591	-0.35	14.5	-662.1	14.1639995	-122.45416	37.93592	-0.29	14.5	-690.02	14.2209995	11	7/24/2014	19:16.0
1182	RWS11	-122.454166	37.93591	-0.32	14.5	-657.9	14.2079998	-122.45416	37.93592	-0.29	14.5	-685.79	14.2309998	11	7/24/2014	19:16.1
1183	RWS11	-122.4541684	37.93591	-0.35	14.5	-653.5	14.1870002	-122.45416	37.93592	-0.33	14.5	-681.4	14.2100003	11	7/24/2014	19:16.2
1184	RWS11	-122.4541708	37.93591	-0.35	14.6	-649.2	14.2019996	-122.45417	37.93592	-0.24	14.6	-677.14	14.3099996	11	7/24/2014	19:16.3
1185	RWS11	-122.4541732	37.93591	-0.35	14.6	-644.8	14.2159996	-122.45417	37.93592	-0.29	14.6	-672.83	14.2729996	11	7/24/2014	19:16.4
1186	RWS11	-122.4541756	37.93591	-0.32	14.6	-640.3	14.2619997	-122.45417	37.93592	-0.29	14.6	-668.48	14.2849997	11	7/24/2014	19:16.5
1187	RWS11	-122.454178	37.93591	-0.35	14.6	-636.1	14.236	-122.45417	37.93593	-0.29	14.6	-664.26	14.293	11	7/24/2014	19:16.6

1188	RWS11	-122.4541804	37.93591	-0.32	14.6	-631.9	14.2719999	-122.45418	37.93593	-0.29	14.6	-660.06	14.2949999	11	7/24/2014	19:16.7
1189	RWS11	-122.4541827	37.93591	-0.35	14.6	-627.7	14.2329998	-122.45418	37.93593	-0.29	14.6	-655.85	14.2899998	11	7/24/2014	19:16.8
1190	RWS11	-122.4541851	37.93591	-0.32	14.6	-623.3	14.2569996	-122.45418	37.93593	-0.24	14.6	-651.56	14.3309996	11	7/24/2014	19:16.9
1191	RWS11	-122.4541885	37.93592	-0.35	14.6	-618.9	14.2080002	-122.45418	37.93593	-0.29	14.6	-647.23	14.2650002	11	7/24/2014	19:17.0
1192	RWS11	-122.4541909	37.93592	-0.32	14.5	-614.3	14.2219997	-122.45419	37.93593	-0.24	14.5	-642.61	14.2959997	11	7/24/2014	19:17.1
1193	RWS11	-122.4541932	37.93592	-0.35	14.5	-609.5	14.1649999	-122.45419	37.93593	-0.29	14.5	-637.78	14.2219999	11	7/24/2014	19:17.2
1194	RWS11	-122.4541955	37.93592	-0.32	14.5	-604.5	14.1740003	-122.45419	37.93593	-0.24	14.5	-632.73	14.2480003	11	7/24/2014	19:17.3
1195	RWS11	-122.4541979	37.93592	-0.35	14.5	-599.5	14.1149997	-122.45419	37.93593	-0.24	14.5	-627.53	14.2229998	11	7/24/2014	19:17.4
1196	RWS11	-122.4542002	37.93592	-0.32	14.4	-594.4	14.1249996	-122.45419	37.93593	-0.24	14.4	-622.2	14.1989996	11	7/24/2014	19:17.5
1197	RWS11	-122.4542026	37.93592	-0.35	14.4	-589.3	14.0699997	-122.4542	37.93593	-0.29	14.4	-616.71	14.1269997	11	7/24/2014	19:17.6
1198	RWS11	-122.4542049	37.93592	-0.32	14.4	-584.1	14.0849996	-122.4542	37.93593	-0.24	14.4	-611.09	14.1589996	11	7/24/2014	19:17.7
1199	RWS11	-122.4542073	37.93592	-0.32	14.4	-578.7	14.0709997	-122.4542	37.93593	-0.29	14.4	-605.32	14.0939997	11	7/24/2014	19:17.8
1200	RWS11	-122.4542097	37.93592	-0.32	14.4	-573.4	14.0619999	-122.4542	37.93593	-0.24	14.4	-599.46	14.1359999	11	7/24/2014	19:17.9
1201	RWS11	-122.4542133	37.93592	-0.32	14.4	-567.9	14.06	-122.45421	37.93593	-0.29	14.4	-593.53	14.083	11	7/24/2014	19:18.0
1202	RWS11	-122.4542157	37.93592	-0.32	14.4	-562.3	14.0650001	-122.45421	37.93593	-0.24	14.4	-587.43	14.1390001	11	7/24/2014	19:18.1
1203	RWS11	-122.4542182	37.93592	-0.35	14.4	-556.7	14.0439996	-122.45421	37.93594	-0.24	14.4	-581.29	14.1519997	11	7/24/2014	19:18.2
1204	RWS11	-122.4542207	37.93592	-0.32	14.4	-551.2	14.1	-122.45422	37.93594	-0.24	14.4	-575.01	14.174	11	7/24/2014	19:18.3
1205	RWS11	-122.4542232	37.93592	-0.35	14.4	-545.6	14.0970001	-122.45422	37.93594	-0.29	14.4	-568.66	14.1540001	11	7/24/2014	19:18.4
1206	RWS11	-122.4542258	37.93592	-0.32	14.5	-539.9	14.1699997	-122.45422	37.93594	-0.24	14.5	-562.18	14.2439997	11	7/24/2014	19:18.5
1207	RWS11	-122.4542283	37.93593	-0.32	14.5	-534	14.2169996	-122.45422	37.93594	-0.29	14.5	-555.53	14.2399996	11	7/24/2014	19:18.6
1208	RWS11	-122.4542309	37.93593	-0.32	14.6	-528.1	14.2689996	-122.45423	37.93594	-0.24	14.6	-548.86	14.3429996	11	7/24/2014	19:18.7
1209	RWS11	-122.4542334	37.93593	-0.35	14.6	-522.1	14.2930002	-122.45423	37.93594	-0.24	14.6	-542.16	14.4010002	11	7/24/2014	19:18.8
1210	RWS11	-122.4542359	37.93593	-0.32	14.7	-516.1	14.3910003	-122.45423	37.93594	-0.21	14.7	-535.5	14.4990003	11	7/24/2014	19:18.9
1211	RWS11	-122.4542395	37.93593	-0.35	14.8	-510.1	14.4250002	-122.45423	37.93594	-0.24	14.8	-528.69	14.5330002	11	7/24/2014	19:19.0
1212	RWS11	-122.454242	37.93593	-0.32	14.8	-504.1	14.534	-122.45424	37.93594	-0.21	14.8	-521.69	14.642	11	7/24/2014	19:19.1
1213	RWS11	-122.4542444	37.93593	-0.35	14.9	-498	14.5790004	-122.45424	37.93594	-0.24	14.9	-514.61	14.6870005	11	7/24/2014	19:19.2
1214	RWS11	-122.4542468	37.93593	-0.35	15	-492	14.6610002	-122.45424	37.93594	-0.24	15	-507.38	14.7690002	11	7/24/2014	19:19.3
1215	RWS11	-122.4542492	37.93593	-0.35	15.1	-485.9	14.7440004	-122.45424	37.93594	-0.24	15.1	-500.1	14.8520004	11	7/24/2014	19:19.4
1216	RWS11	-122.4542517	37.93593	-0.32	15.2	-479.8	14.8569999	-122.45425	37.93594	-0.21	15.2	-492.77	14.9649999	11	7/24/2014	19:19.5
1217	RWS11	-122.4542541	37.93593	-0.32	15.2	-473.7	14.928	-122.45425	37.93594	-0.24	15.2	-485.34	15.002	11	7/24/2014	19:19.6
1218	RWS11	-122.4542565	37.93593	-0.32	15.3	-467.5	14.9909998	-122.45425	37.93595	-0.21	15.3	-477.91	15.0989998	11	7/24/2014	19:19.7
1219	RWS11	-122.4542589	37.93593	-0.35	15.4	-461.4	15.0109997	-122.45425	37.93595	-0.21	15.4	-470.38	15.1529997	11	7/24/2014	19:19.8
1220	RWS11	-122.4542613	37.93593	-0.32	15.4	-455.2	15.0899997	-122.45426	37.93595	-0.24	15.4	-462.79	15.1639997	11	7/24/2014	19:19.9

1221	RWS11	-122.4542648	37.93593	-0.35	15.4	-449.1	15.092	-122.45426	37.93595	-0.21	15.4	-455.24	15.234	11	7/24/2014	19:20.0
1222	RWS11	-122.4542671	37.93594	-0.32	15.5	-442.7	15.1559997	-122.45426	37.93595	-0.21	15.5	-447.51	15.2639997	11	7/24/2014	19:20.1
1223	RWS11	-122.4542695	37.93594	-0.32	15.5	-436.2	15.1799999	-122.45426	37.93595	-0.21	15.5	-439.58	15.2879999	11	7/24/2014	19:20.2
1224	RWS11	-122.4542719	37.93594	-0.32	15.5	-429.6	15.1989999	-122.45427	37.93595	-0.16	15.5	-431.53	15.3569999	11	7/24/2014	19:20.3
1225	RWS11	-122.4542743	37.93594	-0.32	15.5	-422.9	15.2149997	-122.45427	37.93595	-0.24	15.5	-423.29	15.2889997	11	7/24/2014	19:20.4
1226	RWS11	-122.4542767	37.93594	-0.32	15.5	-416	15.2289997	-122.45427	37.93595	-0.16	15.5	-414.9	15.3869997	11	7/24/2014	19:20.5
1227	RWS11	-122.4542791	37.93594	-0.32	15.6	-409	15.2429996	-122.45427	37.93595	-0.21	15.6	-406.41	15.3509996	11	7/24/2014	19:20.6
1228	RWS11	-122.4542815	37.93594	-0.32	15.6	-401.7	15.258	-122.45428	37.93595	-0.16	15.6	-397.73	15.416	11	7/24/2014	19:20.7
1229	RWS11	-122.4542839	37.93594	-0.32	15.6	-394.3	15.2750002	-122.45428	37.93595	-0.21	15.6	-388.83	15.3830002	11	7/24/2014	19:20.8
1230	RWS11	-122.4542863	37.93594	-0.32	15.6	-386.7	15.2940002	-122.45428	37.93595	-0.21	15.6	-379.67	15.4020002	11	7/24/2014	19:20.9
1231	RWS11	-122.4542898	37.93594	-0.32	15.6	-379	15.317	-122.45428	37.93595	-0.21	15.6	-370.32	15.425	11	7/24/2014	19:21.0
1232	RWS11	-122.4542922	37.93594	-0.32	15.7	-371.1	15.3440004	-122.45429	37.93595	-0.21	15.7	-360.83	15.4520004	11	7/24/2014	19:21.1
1233	RWS11	-122.4542947	37.93594	-0.32	15.7	-363.2	15.376	-122.45429	37.93596	-0.16	15.7	-351.16	15.534	11	7/24/2014	19:21.2
1234	RWS11	-122.4542971	37.93594	-0.32	15.7	-354.9	15.4129997	-122.45429	37.93596	-0.21	15.7	-341.45	15.5209997	11	7/24/2014	19:21.3
1235	RWS11	-122.4542996	37.93594	-0.32	15.8	-346.8	15.4550005	-122.45429	37.93596	-0.21	15.8	-331.56	15.5630005	11	7/24/2014	19:21.4
1236	RWS11	-122.454302	37.93594	-0.32	15.8	-338.6	15.501	-122.4543	37.93596	-0.24	15.8	-321.55	15.575	11	7/24/2014	19:21.5
1237	RWS11	-122.4543045	37.93595	-0.32	15.9	-330.4	15.5499998	-122.4543	37.93596	-0.24	15.9	-311.31	15.6239998	11	7/24/2014	19:21.6
1238	RWS11	-122.454307	37.93595	-0.32	15.9	-322.2	15.6030002	-122.4543	37.93596	-0.21	15.9	-301.24	15.7110002	11	7/24/2014	19:21.7
1239	RWS11	-122.4543094	37.93595	-0.32	16	-313.8	15.6559997	-122.4543	37.93596	-0.24	16	-291.08	15.7299997	11	7/24/2014	19:21.8
1240	RWS11	-122.4543118	37.93595	-0.32	16	-305.1	15.7099996	-122.45431	37.93596	-0.21	16	-280.78	15.8179996	11	7/24/2014	19:21.9
1241	RWS11	-122.4543154	37.93595	-0.32	16.1	-296.1	15.7619997	-122.45431	37.93596	-0.24	16.1	-270.3	15.8359997	11	7/24/2014	19:22.0
1242	RWS11	-122.4543178	37.93595	-0.28	16.1	-286.8	15.8460008	-122.45431	37.93596	-0.16	16.1	-259.59	15.9700008	11	7/24/2014	19:22.1
1243	RWS11	-122.4543202	37.93595	-0.32	16.2	-277.1	15.8589998	-122.45431	37.93596	-0.21	16.2	-248.64	15.9669998	11	7/24/2014	19:22.2
1244	RWS11	-122.4543226	37.93595	-0.32	16.2	-267.1	15.9019991	-122.45432	37.93596	-0.21	16.2	-237.39	16.0099991	11	7/24/2014	19:22.3
1245	RWS11	-122.4543251	37.93595	-0.32	16.3	-256.8	15.942	-122.45432	37.93596	-0.21	16.3	-226	16.05	11	7/24/2014	19:22.4
1246	RWS11	-122.4543275	37.93595	-0.32	16.3	-246.2	15.9779993	-122.45432	37.93596	-0.21	16.3	-214.39	16.0859993	11	7/24/2014	19:22.5
1247	RWS11	-122.4543299	37.93595	-0.32	16.3	-235.6	16.0129991	-122.45432	37.93596	-0.21	16.3	-202.61	16.1209991	11	7/24/2014	19:22.6
1248	RWS11	-122.4543323	37.93595	-0.32	16.4	-224.7	16.0439993	-122.45433	37.93597	-0.21	16.4	-190.74	16.1519993	11	7/24/2014	19:22.7
1249	RWS11	-122.4543347	37.93595	-0.32	16.4	-213.9	16.0739999	-122.45433	37.93597	-0.24	16.4	-178.74	16.1479999	11	7/24/2014	19:22.8
1250	RWS11	-122.4543371	37.93595	-0.32	16.4	-203	16.1010004	-122.45433	37.93597	-0.21	16.4	-166.69	16.2090004	11	7/24/2014	19:22.9
1251	RWS11	-122.4543406	37.93595	-0.32	16.4	-192.1	16.1269994	-122.45434	37.93597	-0.24	16.4	-154.54	16.2009994	11	7/24/2014	19:23.0
1252	RWS11	-122.454343	37.93596	-0.32	16.5	-181.2	16.1500002	-122.45434	37.93597	-0.24	16.5	-142.27	16.2240002	11	7/24/2014	19:23.1
1253	RWS11	-122.4543454	37.93596	-0.32	16.5	-170.2	16.1719995	-122.45434	37.93597	-0.21	16.5	-130.02	16.2799995	11	7/24/2014	19:23.2

1254	RWS11	-122.4543478	37.93596	-0.32	16.5	-159	16.1929994	-122.45434	37.93597	-0.21	16.5	-117.57	16.3009994	11	7/24/2014	19:23.3
1255	RWS11	-122.4543502	37.93596	-0.32	16.5	-147.5	16.2129999	-122.45434	37.93597	-0.24	16.5	-104.88	16.2869999	11	7/24/2014	19:23.4
1256	RWS11	-122.4543526	37.93596	-0.32	16.5	-135.8	16.2330003	-122.45435	37.93597	-0.21	16.5	-91.919	16.3410003	11	7/24/2014	19:23.5
1257	RWS11	-122.454355	37.93596	-0.35	16.6	-124	16.2200002	-122.45435	37.93597	-0.21	16.6	-78.777	16.3620002	11	7/24/2014	19:23.6
1258	RWS11	-122.4543574	37.93596	-0.32	16.6	-112.1	16.2759996	-122.45435	37.93597	-0.21	16.6	-65.431	16.3839996	11	7/24/2014	19:23.7
1259	RWS11	-122.4543598	37.93596	-0.32	16.6	-99.99	16.3009992	-122.45435	37.93597	-0.21	16.6	-51.858	16.4089992	11	7/24/2014	19:23.8
1260	RWS11	-122.4543621	37.93596	-0.32	16.6	-87.79	16.3270002	-122.45436	37.93597	-0.21	16.6	-38.088	16.4350002	11	7/24/2014	19:23.9
1261	RWS11	-122.4543657	37.93596	-0.32	16.7	-75.34	16.3559995	-122.45436	37.93597	-0.24	16.7	-24.038	16.4299995	11	7/24/2014	19:24.0
1262	RWS11	-122.454368	37.93596	-0.32	16.7	-62.78	16.3879991	-122.45436	37.93598	-0.21	16.7	-9.747	16.4959991	11	7/24/2014	19:24.1
1263	RWS11	-122.4543704	37.93596	-0.32	16.7	-50.13	16.4210001	-122.45436	37.93598	-0.24	16.7	4.653	16.4950001	11	7/24/2014	19:24.2
1264	RWS11	-122.4543728	37.93596	-0.32	16.8	-37.22	16.4559999	-122.45437	37.93598	-0.21	16.8	19.36	16.5639999	11	7/24/2014	19:24.3
1265	RWS11	-122.4543752	37.93596	-0.35	16.8	-23.97	16.4560003	-122.45437	37.93598	-0.21	16.8	34.368	16.5980003	11	7/24/2014	19:24.4
1266	RWS11	-122.4543776	37.93596	-0.32	16.8	-10.51	16.5240007	-122.45437	37.93598	-0.24	16.8	49.603	16.5980007	11	7/24/2014	19:24.5
1267	RWS11	-122.45438	37.93597	-0.32	16.9	3.193	16.5560003	-122.45437	37.93598	-0.24	16.9	65.024	16.6300003	11	7/24/2014	19:24.6
1268	RWS11	-122.4543824	37.93597	-0.32	16.9	17.176	16.5870004	-122.45438	37.93598	-0.24	16.9	80.709	16.6610004	11	7/24/2014	19:24.7
1269	RWS11	-122.4543848	37.93597	-0.32	16.9	31.445	16.6159998	-122.45438	37.93598	-0.21	16.9	96.793	16.7239998	11	7/24/2014	19:24.8
1270	RWS11	-122.4543871	37.93597	-0.32	17	45.955	16.6449991	-122.45438	37.93598	-0.21	17	113.18	16.7529991	11	7/24/2014	19:24.9
1271	RWS11	-122.4543907	37.93597	-0.32	17	60.811	16.6719995	-122.45439	37.93598	-0.21	17	129.82	16.7799995	11	7/24/2014	19:25.0
1272	RWS11	-122.4543931	37.93597	-0.32	17	76.195	16.6989999	-122.45439	37.93598	-0.21	17	146.87	16.8069999	11	7/24/2014	19:25.1
1273	RWS11	-122.4543955	37.93597	-0.35	17	92.192	16.6910009	-122.45439	37.93598	-0.24	17	164.23	16.7990009	11	7/24/2014	19:25.2
1274	RWS11	-122.454398	37.93597	-0.32	17.1	108.49	16.751	-122.45439	37.93598	-0.24	17.1	181.78	16.825	11	7/24/2014	19:25.3
1275	RWS11	-122.4544004	37.93597	-0.35	17.1	125	16.7429991	-122.4544	37.93598	-0.24	17.1	199.5	16.8509991	11	7/24/2014	19:25.4
1276	RWS11	-122.4544029	37.93597	-0.32	17.1	141.65	16.8020006	-122.4544	37.93598	-0.24	17.1	217.25	16.8760006	11	7/24/2014	19:25.5
1277	RWS11	-122.4544053	37.93597	-0.32	17.1	158.29	16.8260008	-122.4544	37.93598	-0.29	17.1	235.07	16.8490008	11	7/24/2014	19:25.6
1278	RWS11	-122.4544078	37.93597	-0.32	17.2	175.14	16.8489996	-122.4544	37.93599	-0.24	17.2	252.95	16.9229996	11	7/24/2014	19:25.7
1279	RWS11	-122.4544102	37.93597	-0.35	17.2	192.13	16.8370008	-122.4544	37.93599	-0.29	17.2	271.18	16.8940008	11	7/24/2014	19:25.8
1280	RWS11	-122.4544127	37.93597	-0.32	17.2	209.19	16.8920007	-122.45441	37.93599	-0.29	17.2	289.51	16.9150007	11	7/24/2014	19:25.9
1281	RWS11	-122.4544163	37.93597	-0.35	17.2	226.54	16.8779993	-122.45441	37.93599	-0.24	17.2	308.13	16.9859993	11	7/24/2014	19:26.0
1282	RWS11	-122.4544187	37.93598	-0.32	17.2	244.05	16.9300008	-122.45441	37.93599	-0.24	17.2	327.06	17.0040008	11	7/24/2014	19:26.1
1283	RWS11	-122.4544211	37.93598	-0.35	17.3	261.89	16.9119997	-122.45442	37.93599	-0.24	17.3	346.26	17.0199997	11	7/24/2014	19:26.2
1284	RWS11	-122.4544235	37.93598	-0.32	17.3	279.81	16.9599996	-122.45442	37.93599	-0.24	17.3	365.67	17.0339996	11	7/24/2014	19:26.3
1285	RWS11	-122.4544259	37.93598	-0.35	17.3	297.95	16.9359998	-122.45442	37.93599	-0.29	17.3	385.22	16.9929999	11	7/24/2014	19:26.4
1286	RWS11	-122.4544284	37.93598	-0.35	17.3	316.52	16.9439993	-122.45442	37.93599	-0.29	17.3	405.14	17.0009993	11	7/24/2014	19:26.5

1287	RWS11	-122.4544308	37.93598	-0.35	17.3	335.59	16.9490003	-122.45443	37.93599	-0.29	17.3	425.41	17.0060003	11	7/24/2014	19:26.6
1288	RWS11	-122.4544333	37.93598	-0.32	17.3	354.92	16.9839998	-122.45443	37.93599	-0.29	17.3	445.99	17.0069998	11	7/24/2014	19:26.7
1289	RWS11	-122.4544356	37.93598	-0.35	17.3	374.57	16.9460001	-122.45443	37.93599	-0.29	17.3	466.86	17.0030001	11	7/24/2014	19:26.8
1290	RWS11	-122.454438	37.93598	-0.35	17.3	394.63	16.9380007	-122.45443	37.93599	-0.29	17.3	488.07	16.9950007	11	7/24/2014	19:26.9
1291	RWS11	-122.4544416	37.93598	-0.35	17.3	415.03	16.9250002	-122.45444	37.93599	-0.33	17.3	509.77	16.9480002	11	7/24/2014	19:27.0
1292	RWS11	-122.454444	37.93598	-0.35	17.3	435.59	16.908	-122.45444	37.936	-0.29	17.3	531.7	16.965	11	7/24/2014	19:27.1
1293	RWS11	-122.4544463	37.93598	-0.35	17.2	456.32	16.8849993	-122.45444	37.936	-0.29	17.2	553.88	16.9419993	11	7/24/2014	19:27.2
1294	RWS11	-122.4544487	37.93598	-0.35	17.2	476.92	16.8569994	-122.45444	37.936	-0.29	17.2	576.06	16.9139994	11	7/24/2014	19:27.3
1295	RWS11	-122.4544511	37.93598	-0.4	17.2	497.77	16.7739998	-122.45445	37.936	-0.33	17.2	598.25	16.8479998	11	7/24/2014	19:27.4
1296	RWS11	-122.4544535	37.93598	-0.35	17.1	518.78	16.7879991	-122.45445	37.936	-0.29	17.1	620.49	16.8449991	11	7/24/2014	19:27.5
1297	RWS11	-122.4544559	37.93599	-0.35	17.1	539.98	16.7470007	-122.45445	37.936	-0.33	17.1	642.66	16.7700007	11	7/24/2014	19:27.6
1298	RWS11	-122.4544583	37.93599	-0.35	17.1	561.11	16.7039995	-122.45445	37.936	-0.33	17.1	664.91	16.7269995	11	7/24/2014	19:27.7
1299	RWS11	-122.4544607	37.93599	-0.35	17	582.32	16.6600008	-122.45446	37.936	-0.33	17	687.13	16.6830008	11	7/24/2014	19:27.8
1300	RWS11	-122.4544631	37.93599	-0.35	17	603.86	16.6160002	-122.45446	37.936	-0.29	17	709.6	16.6730002	11	7/24/2014	19:27.9
1301	RWS11	-122.4544666	37.93599	-0.4	16.9	625.69	16.5209995	-122.45446	37.936	-0.33	16.9	732.31	16.5949995	11	7/24/2014	19:28.0
1302	RWS11	-122.4544689	37.93599	-0.35	16.9	647.84	16.5290003	-122.45446	37.936	-0.29	16.9	755.31	16.5860003	11	7/24/2014	19:28.1
1303	RWS11	-122.4544713	37.93599	-0.35	16.8	670.09	16.4879999	-122.45447	37.936	-0.33	16.8	778.42	16.5109999	11	7/24/2014	19:28.2
1304	RWS11	-122.4544737	37.93599	-0.4	16.8	692.62	16.3980003	-122.45447	37.936	-0.33	16.8	801.8	16.4720003	11	7/24/2014	19:28.3
1305	RWS11	-122.4544761	37.93599	-0.4	16.8	715.33	16.3600002	-122.45447	37.936	-0.33	16.8	825.44	16.4340002	11	7/24/2014	19:28.4
1306	RWS11	-122.4544785	37.93599	-0.35	16.7	738.18	16.3750009	-122.45447	37.936	-0.29	16.7	849.18	16.4320009	11	7/24/2014	19:28.5
1307	RWS11	-122.4544809	37.93599	-0.4	16.7	760.93	16.2900005	-122.45448	37.936	-0.33	16.7	872.94	16.3640005	11	7/24/2014	19:28.6
1308	RWS11	-122.4544833	37.93599	-0.35	16.7	783.65	16.3090009	-122.45448	37.93601	-0.33	16.7	896.65	16.3320009	11	7/24/2014	19:28.7
1309	RWS11	-122.4544856	37.93599	-0.4	16.6	806.33	16.2270008	-122.45448	37.93601	-0.33	16.6	920.31	16.3010008	11	7/24/2014	19:28.8
1310	RWS11	-122.454488	37.93599	-0.35	16.6	829.13	16.2489996	-122.45448	37.93601	-0.29	16.6	943.9	16.3059996	11	7/24/2014	19:28.9
1311	RWS11	-122.4544915	37.93599	-0.4	16.6	852.02	16.1690002	-122.45449	37.93601	-0.29	16.6	967.56	16.2770003	11	7/24/2014	19:29.0
1312	RWS11	-122.4544939	37.936	-0.4	16.5	875.04	16.1400009	-122.45449	37.93601	-0.29	16.5	991.38	16.2480009	11	7/24/2014	19:29.1
1313	RWS11	-122.4544962	37.936	-0.4	16.5	898.2	16.1130005	-122.45449	37.93601	-0.33	16.5	1015.3	16.1870005	11	7/24/2014	19:29.2
1314	RWS11	-122.4544987	37.936	-0.35	16.5	921.52	16.1379995	-122.45449	37.93601	-0.33	16.5	1039.4	16.1609995	11	7/24/2014	19:29.3
1315	RWS11	-122.454501	37.936	-0.4	16.5	945.12	16.0640007	-122.4545	37.93601	-0.29	16.5	1063.7	16.1720007	11	7/24/2014	19:29.4
1316	RWS11	-122.4545034	37.936	-0.4	16.4	968.99	16.0419994	-122.4545	37.93601	-0.33	16.4	1088.3	16.1159994	11	7/24/2014	19:29.5
1317	RWS11	-122.4545059	37.936	-0.4	16.4	992.93	16.0239998	-122.4545	37.93601	-0.33	16.4	1112.9	16.0979998	11	7/24/2014	19:29.6
1318	RWS11	-122.4545083	37.936	-0.4	16.4	1016.8	16.0109993	-122.4545	37.93601	-0.29	16.4	1137.5	16.1189993	11	7/24/2014	19:29.7
1319	RWS11	-122.4545106	37.936	-0.4	16.4	1040.8	16.0020004	-122.45451	37.93601	-0.33	16.4	1161.9	16.0760004	11	7/24/2014	19:29.8

1320	RWS11	-122.454513	37.936	-0.4	16.4	1064.6	15.9969994	-122.45451	37.93601	-0.29	16.4	1186.3	16.1049994	11	7/24/2014	19:29.9
1321	RWS11	-122.4545165	37.936	-0.4	16.4	1088.1	15.9980007	-122.45451	37.93601	-0.29	16.4	1210.3	16.1060007	11	7/24/2014	19:30.0
1322	RWS11	-122.4545189	37.936	-0.35	16.4	1111.5	16.0539999	-122.45451	37.93601	-0.29	16.4	1234.2	16.1109999	11	7/24/2014	19:30.1
1323	RWS11	-122.4545213	37.936	-0.4	16.4	1134.7	16.0120007	-122.45452	37.93602	-0.29	16.4	1257.8	16.1200007	11	7/24/2014	19:30.2
1324	RWS11	-122.4545236	37.936	-0.4	16.4	1157.7	16.0230003	-122.45452	37.93602	-0.29	16.4	1281.1	16.1310003	11	7/24/2014	19:30.3
1325	RWS11	-122.454526	37.936	-0.4	16.4	1180.5	16.034	-122.45452	37.93602	-0.33	16.4	1304.3	16.108	11	7/24/2014	19:30.4
1326	RWS11	-122.4545285	37.936	-0.35	16.4	1203.1	16.0950002	-122.45452	37.93602	-0.33	16.4	1327.1	16.1180002	11	7/24/2014	19:30.5
1327	RWS11	-122.4545308	37.936	-0.4	16.5	1225.4	16.0500008	-122.45453	37.93602	-0.33	16.5	1349.5	16.1240008	11	7/24/2014	19:30.6
1328	RWS11	-122.4545332	37.93601	-0.4	16.5	1247.5	16.0519997	-122.45453	37.93602	-0.33	16.5	1371.6	16.1259997	11	7/24/2014	19:30.7
1329	RWS11	-122.4545356	37.93601	-0.4	16.4	1269.1	16.0489994	-122.45453	37.93602	-0.36	16.4	1393	16.0889994	11	7/24/2014	19:30.8
1330	RWS11	-122.454538	37.93601	-0.4	16.4	1290.3	16.0430008	-122.45453	37.93602	-0.33	16.4	1413.9	16.1170008	11	7/24/2014	19:30.9
1331	RWS11	-122.4545416	37.93601	-0.4	16.4	1310.8	16.0330006	-122.45454	37.93602	-0.36	16.4	1434.1	16.0730006	11	7/24/2014	19:31.0
1332	RWS11	-122.454544	37.93601	-0.4	16.4	1330.6	16.0209995	-122.45454	37.93602	-0.33	16.4	1453.6	16.0949995	11	7/24/2014	19:31.1
1333	RWS11	-122.4545465	37.93601	-0.4	16.4	1349.6	16.0060001	-122.45454	37.93602	-0.36	16.4	1472.3	16.0460001	11	7/24/2014	19:31.2
1334	RWS11	-122.4545489	37.93601	-0.4	16.4	1367.8	15.9910007	-122.45454	37.93602	-0.36	16.4	1490.2	16.0310007	11	7/24/2014	19:31.3
1335	RWS11	-122.4545514	37.93601	-0.44	16.4	1385.2	15.9390006	-122.45455	37.93602	-0.36	16.4	1507.1	16.0140005	11	7/24/2014	19:31.4
1336	RWS11	-122.4545539	37.93601	-0.4	16.4	1401.9	15.9549995	-122.45455	37.93602	-0.36	16.4	1523.4	15.9949995	11	7/24/2014	19:31.5
1337	RWS11	-122.4545564	37.93601	-0.44	16.3	1418	15.8999991	-122.45455	37.93602	-0.36	16.3	1539	15.9749991	11	7/24/2014	19:31.6
1338	RWS11	-122.4545589	37.93601	-0.4	16.3	1433.5	15.9129997	-122.45455	37.93603	-0.36	16.3	1553.9	15.9529997	11	7/24/2014	19:31.7
1339	RWS11	-122.4545613	37.93601	-0.44	16.3	1448.2	15.8539996	-122.45456	37.93603	-0.41	16.3	1568	15.8789996	11	7/24/2014	19:31.8
1340	RWS11	-122.4545637	37.93601	-0.44	16.3	1462.2	15.8269991	-122.45456	37.93603	-0.36	16.3	1581.4	15.9019991	11	7/24/2014	19:31.9
1341	RWS11	-122.4545673	37.93601	-0.44	16.2	1475.3	15.7979998	-122.45456	37.93603	-0.41	16.2	1593.8	15.8229998	11	7/24/2014	19:32.0
1342	RWS11	-122.4545698	37.93602	-0.4	16.2	1487.6	15.8010002	-122.45456	37.93603	-0.41	16.2	1605.4	15.7910002	11	7/24/2014	19:32.1
1343	RWS11	-122.4545721	37.93602	-0.49	16.2	1498.8	15.6809998	-122.45457	37.93603	-0.41	16.2	1616	15.7569998	11	7/24/2014	19:32.2
1344	RWS11	-122.4545746	37.93602	-0.44	16.1	1509.2	15.6949992	-122.45457	37.93603	-0.41	16.1	1625.6	15.7199992	11	7/24/2014	19:32.3
1345	RWS11	-122.454577	37.93602	-0.44	16.1	1518.5	15.6569991	-122.45457	37.93603	-0.41	16.1	1634.2	15.6819991	11	7/24/2014	19:32.4
1346	RWS11	-122.4545794	37.93602	-0.44	16.1	1526.7	15.6190009	-122.45457	37.93603	-0.41	16.1	1641.8	15.6440009	11	7/24/2014	19:32.5
1347	RWS11	-122.4545818	37.93602	-0.49	16	1533.9	15.5310002	-122.45458	37.93603	-0.41	16	1648.3	15.6070002	11	7/24/2014	19:32.6
1348	RWS11	-122.4545842	37.93602	-0.49	16	1540.4	15.4939995	-122.45458	37.93603	-0.41	16	1654.3	15.5699996	11	7/24/2014	19:32.7
1349	RWS11	-122.4545866	37.93602	-0.49	15.9	1546.1	15.4589997	-122.45458	37.93603	-0.41	15.9	1659.6	15.5349997	11	7/24/2014	19:32.8
1350	RWS11	-122.454589	37.93602	-0.44	15.9	1551.4	15.4769997	-122.45458	37.93603	-0.41	15.9	1664.4	15.5019997	11	7/24/2014	19:32.9
1351	RWS11	-122.4545925	37.93602	-0.49	15.9	1556.2	15.3949996	-122.45459	37.93603	-0.41	15.9	1668.5	15.4709996	11	7/24/2014	19:33.0
1352	RWS11	-122.4545949	37.93602	-0.44	15.9	1560.5	15.4190001	-122.45459	37.93603	-0.44	15.9	1671.7	15.4100001	11	7/24/2014	19:33.1

1353	RWS11	-122.4545973	37.93602	-0.49	15.8	1563.8	15.3450003	-122.45459	37.93604	-0.44	15.8	1673.4	15.3870003	11	7/24/2014	19:33.2
1354	RWS11	-122.4545997	37.93602	-0.49	15.8	1566.3	15.3249999	-122.45459	37.93604	-0.41	15.8	1674	15.4009999	11	7/24/2014	19:33.3
1355	RWS11	-122.4546021	37.93602	-0.52	15.8	1567.5	15.2750001	-122.4546	37.93604	-0.41	15.8	1673.3	15.3850001	11	7/24/2014	19:33.4
1356	RWS11	-122.4546045	37.93602	-0.49	15.8	1567.5	15.2959996	-122.4546	37.93604	-0.41	15.8	1671.2	15.3719996	11	7/24/2014	19:33.5
1357	RWS11	-122.4546069	37.93602	-0.49	15.8	1566	15.2849999	-122.4546	37.93604	-0.44	15.8	1667.5	15.3269999	11	7/24/2014	19:33.6
1358	RWS11	-122.4546094	37.93603	-0.49	15.8	1562.8	15.2769995	-122.4546	37.93604	-0.44	15.8	1662.6	15.3189995	11	7/24/2014	19:33.7
1359	RWS11	-122.4546117	37.93603	-0.52	15.8	1558.1	15.2359996	-122.45461	37.93604	-0.44	15.8	1656.6	15.3119996	11	7/24/2014	19:33.8
1360	RWS11	-122.4546142	37.93603	-0.49	15.8	1552.2	15.2659999	-122.45461	37.93604	-0.44	15.8	1649.6	15.3079999	11	7/24/2014	19:33.9
1361	RWS11	-122.4546177	37.93603	-0.52	15.7	1545.1	15.2280002	-122.45461	37.93604	-0.44	15.7	1641.5	15.3040001	11	7/24/2014	19:34.0
1362	RWS11	-122.4546201	37.93603	-0.49	15.7	1537.2	15.2600003	-122.45461	37.93604	-0.41	15.7	1632.4	15.3360003	11	7/24/2014	19:34.1
1363	RWS11	-122.4546225	37.93603	-0.49	15.7	1528.1	15.2580004	-122.45462	37.93604	-0.5	15.7	1622.4	15.2490004	11	7/24/2014	19:34.2
1364	RWS11	-122.454625	37.93603	-0.49	15.7	1518.3	15.2559996	-122.45462	37.93604	-0.41	15.7	1611.8	15.3319996	11	7/24/2014	19:34.3
1365	RWS11	-122.4546274	37.93603	-0.52	15.7	1507.8	15.2179999	-122.45462	37.93604	-0.5	15.7	1600.2	15.2429999	11	7/24/2014	19:34.4
1366	RWS11	-122.4546299	37.93603	-0.49	15.7	1496.4	15.2469998	-122.45462	37.93604	-0.44	15.7	1587.7	15.2889998	11	7/24/2014	19:34.5
1367	RWS11	-122.4546323	37.93603	-0.52	15.7	1484.2	15.2070003	-122.45463	37.93604	-0.5	15.7	1574.5	15.2320002	11	7/24/2014	19:34.6
1368	RWS11	-122.4546347	37.93603	-0.49	15.7	1471.4	15.2340003	-122.45463	37.93604	-0.44	15.7	1560.6	15.2760003	11	7/24/2014	19:34.7
1369	RWS11	-122.4546371	37.93603	-0.52	15.7	1458.1	15.1910005	-122.45463	37.93605	-0.5	15.7	1546.1	15.2160004	11	7/24/2014	19:34.8
1370	RWS11	-122.4546395	37.93603	-0.52	15.7	1444.1	15.1830001	-122.45463	37.93605	-0.44	15.7	1530.8	15.2590001	11	7/24/2014	19:34.9
1371	RWS11	-122.4546431	37.93603	-0.52	15.7	1429.5	15.1740003	-122.45464	37.93605	-0.5	15.7	1514.9	15.1990002	11	7/24/2014	19:35.0
1372	RWS11	-122.4546454	37.93603	-0.49	15.7	1414.2	15.1999999	-122.45464	37.93605	-0.44	15.7	1498.5	15.2419999	11	7/24/2014	19:35.1
1373	RWS11	-122.4546478	37.93604	-0.52	15.7	1398.2	15.1589999	-122.45464	37.93605	-0.5	15.7	1481.4	15.1839999	11	7/24/2014	19:35.2
1374	RWS11	-122.4546502	37.93604	-0.49	15.7	1381.6	15.1879998	-122.45464	37.93605	-0.5	15.7	1463.4	15.1789998	11	7/24/2014	19:35.3
1375	RWS11	-122.4546526	37.93604	-0.52	15.7	1364.4	15.1509996	-122.45465	37.93605	-0.5	15.7	1445	15.1759995	11	7/24/2014	19:35.4
1376	RWS11	-122.454655	37.93604	-0.49	15.7	1346.5	15.1829997	-122.45465	37.93605	-0.44	15.7	1425.9	15.2249997	11	7/24/2014	19:35.5
1377	RWS11	-122.4546574	37.93604	-0.52	15.7	1327.9	15.1500001	-122.45465	37.93605	-0.53	15.7	1406.2	15.1410001	11	7/24/2014	19:35.6
1378	RWS11	-122.4546597	37.93604	-0.49	15.7	1308.8	15.1849995	-122.45465	37.93605	-0.5	15.7	1386.4	15.1759995	11	7/24/2014	19:35.7
1379	RWS11	-122.4546621	37.93604	-0.52	15.7	1289.6	15.1550002	-122.45466	37.93605	-0.44	15.7	1366.2	15.2310002	11	7/24/2014	19:35.8
1380	RWS11	-122.4546644	37.93604	-0.49	15.7	1270.2	15.1929999	-122.45466	37.93605	-0.5	15.7	1345.8	15.1839999	11	7/24/2014	19:35.9
1381	RWS11	-122.4546679	37.93604	-0.52	15.7	1250.6	15.164	-122.45466	37.93605	-0.5	15.7	1325	15.189	11	7/24/2014	19:36.0
1382	RWS11	-122.4546703	37.93604	-0.52	15.7	1230.5	15.1690002	-122.45466	37.93605	-0.5	15.7	1303.5	15.1940001	11	7/24/2014	19:36.1
1383	RWS11	-122.4546726	37.93604	-0.52	15.7	1209.9	15.1749997	-122.45467	37.93605	-0.5	15.7	1281.6	15.1999997	11	7/24/2014	19:36.2
1384	RWS11	-122.454675	37.93604	-0.49	15.7	1188.9	15.2150002	-122.45467	37.93606	-0.5	15.7	1259.2	15.2060002	11	7/24/2014	19:36.3
1385	RWS11	-122.4546773	37.93604	-0.52	15.7	1167.2	15.1860004	-122.45467	37.93606	-0.5	15.7	1236.5	15.2110003	11	7/24/2014	19:36.4

1386	RWS11	-122.4546797	37.93604	-0.49	15.7	1145.2	15.224	-122.45467	37.93606	-0.5	15.7	1213.4	15.215	11	7/24/2014	19:36.5
1387	RWS11	-122.4546821	37.93604	-0.52	15.7	1122.6	15.1919999	-122.45468	37.93606	-0.5	15.7	1189.9	15.2169999	11	7/24/2014	19:36.6
1388	RWS11	-122.4546844	37.93605	-0.49	15.7	1099.8	15.2259999	-122.45468	37.93606	-0.5	15.7	1166.4	15.2169999	11	7/24/2014	19:36.7
1389	RWS11	-122.4546868	37.93605	-0.52	15.7	1076.9	15.1900001	-122.45468	37.93606	-0.5	15.7	1142.7	15.215	11	7/24/2014	19:36.8
1390	RWS11	-122.4546892	37.93605	-0.49	15.7	1054	15.2180005	-122.45468	37.93606	-0.53	15.7	1119.2	15.1750005	11	7/24/2014	19:36.9
1391	RWS11	-122.4546927	37.93605	-0.52	15.7	1031.2	15.1740003	-122.45469	37.93606	-0.53	15.7	1095.6	15.1650003	11	7/24/2014	19:37.0
1392	RWS11	-122.4546951	37.93605	-0.49	15.7	1008.2	15.1940003	-122.45469	37.93606	-0.5	15.7	1071.9	15.1850003	11	7/24/2014	19:37.1
1393	RWS11	-122.4546974	37.93605	-0.52	15.7	985.38	15.1419997	-122.45469	37.93606	-0.5	15.7	1048.4	15.1669997	11	7/24/2014	19:37.2
1394	RWS11	-122.4546999	37.93605	-0.52	15.6	962.63	15.1209998	-122.45469	37.93606	-0.5	15.6	1025.1	15.1459998	11	7/24/2014	19:37.3
1395	RWS11	-122.4547023	37.93605	-0.52	15.6	940.05	15.0969997	-122.4547	37.93606	-0.53	15.6	1001.7	15.0879996	11	7/24/2014	19:37.4
1396	RWS11	-122.4547048	37.93605	-0.49	15.6	917.56	15.1040002	-122.4547	37.93606	-0.5	15.6	978.58	15.0950002	11	7/24/2014	19:37.5
1397	RWS11	-122.4547072	37.93605	-0.52	15.6	895.02	15.0420003	-122.4547	37.93606	-0.5	15.6	955.31	15.0670003	11	7/24/2014	19:37.6
1398	RWS11	-122.4547096	37.93605	-0.49	15.5	872.36	15.047	-122.4547	37.93606	-0.44	15.5	932.03	15.089	11	7/24/2014	19:37.7
1399	RWS11	-122.4547121	37.93605	-0.49	15.5	849.62	15.0179997	-122.45471	37.93607	-0.5	15.5	908.72	15.0089997	11	7/24/2014	19:37.8
1400	RWS11	-122.4547145	37.93605	-0.49	15.5	826.47	14.9890004	-122.45471	37.93607	-0.41	15.5	885.16	15.0650004	11	7/24/2014	19:37.9
1401	RWS11	-122.454718	37.93605	-0.52	15.4	802.9	14.9269996	-122.45471	37.93607	-0.5	15.4	861.45	14.9519996	11	7/24/2014	19:38.0
1402	RWS11	-122.4547204	37.93605	-0.49	15.4	779.27	14.9349995	-122.45471	37.93607	-0.44	15.4	837.86	14.9769995	11	7/24/2014	19:38.1
1403	RWS11	-122.4547228	37.93606	-0.49	15.4	755.66	14.9099999	-122.45472	37.93607	-0.44	15.4	814.33	14.9519999	11	7/24/2014	19:38.2
1404	RWS11	-122.4547252	37.93606	-0.44	15.4	732.1	14.9389996	-122.45472	37.93607	-0.44	15.4	790.8	14.9299996	11	7/24/2014	19:38.3
1405	RWS11	-122.4547276	37.93606	-0.49	15.4	708.97	14.8669997	-122.45472	37.93607	-0.44	15.4	767.31	14.9089997	11	7/24/2014	19:38.4
1406	RWS11	-122.45473	37.93606	-0.49	15.3	686.41	14.849	-122.45472	37.93607	-0.44	15.3	744.56	14.891	11	7/24/2014	19:38.5
1407	RWS11	-122.4547324	37.93606	-0.49	15.3	664.56	14.8319998	-122.45473	37.93607	-0.44	15.3	722.18	14.8739998	11	7/24/2014	19:38.6
1408	RWS11	-122.4547348	37.93606	-0.49	15.3	643.07	14.8170005	-122.45473	37.93607	-0.44	15.3	700.07	14.8590004	11	7/24/2014	19:38.7
1409	RWS11	-122.4547372	37.93606	-0.49	15.3	622.02	14.8020001	-122.45473	37.93607	-0.5	15.3	678.48	14.7930001	11	7/24/2014	19:38.8
1410	RWS11	-122.4547395	37.93606	-0.49	15.3	601.36	14.7880002	-122.45473	37.93607	-0.44	15.3	657.28	14.8300002	11	7/24/2014	19:38.9
1411	RWS11	-122.454743	37.93606	-0.49	15.3	580.75	14.7740002	-122.45474	37.93607	-0.44	15.3	636.23	14.8160002	11	7/24/2014	19:39.0
1412	RWS11	-122.4547453	37.93606	-0.49	15.2	560.2	14.7609997	-122.45474	37.93607	-0.44	15.2	615.25	14.8029997	11	7/24/2014	19:39.1
1413	RWS11	-122.4547477	37.93606	-0.49	15.2	539.82	14.7480002	-122.45474	37.93607	-0.44	15.2	594.52	14.7900002	11	7/24/2014	19:39.2
1414	RWS11	-122.45475	37.93606	-0.49	15.2	519.56	14.7349997	-122.45474	37.93608	-0.5	15.2	573.99	14.7259997	11	7/24/2014	19:39.3
1415	RWS11	-122.4547523	37.93606	-0.49	15.2	499.29	14.7229996	-122.45475	37.93608	-0.44	15.2	553.47	14.7649996	11	7/24/2014	19:39.4
1416	RWS11	-122.4547547	37.93606	-0.44	15.2	479.16	14.7610001	-122.45475	37.93608	-0.44	15.2	533.15	14.7520001	11	7/24/2014	19:39.5
1417	RWS11	-122.454757	37.93606	-0.49	15.2	459.45	14.6969996	-122.45475	37.93608	-0.44	15.2	513.28	14.7389996	11	7/24/2014	19:39.6
1418	RWS11	-122.4547593	37.93607	-0.49	15.2	440.21	14.6849995	-122.45475	37.93608	-0.44	15.2	493.68	14.7269995	11	7/24/2014	19:39.7

1419	RWS11	-122.4547616	37.93607	-0.49	15.2	421.36	14.672	-122.45476	37.93608	-0.44	15.2	474.54	14.714	11	7/24/2014	19:39.8
1420	RWS11	-122.4547639	37.93607	-0.49	15.1	402.99	14.6599999	-122.45476	37.93608	-0.41	15.1	455.67	14.7359999	11	7/24/2014	19:39.9
1421	RWS11	-122.4547674	37.93607	-0.49	15.1	384.82	14.6470004	-122.45476	37.93608	-0.44	15.1	437.09	14.6890004	11	7/24/2014	19:40.0
1422	RWS11	-122.4547697	37.93607	-0.44	15.1	367.1	14.6840004	-122.45476	37.93608	-0.44	15.1	418.9	14.6750004	11	7/24/2014	19:40.1
1423	RWS11	-122.4547719	37.93607	-0.49	15.1	349.49	14.6189995	-122.45477	37.93608	-0.44	15.1	400.81	14.6609995	11	7/24/2014	19:40.2
1424	RWS11	-122.4547743	37.93607	-0.44	15.1	331.86	14.6539998	-122.45477	37.93608	-0.44	15.1	382.54	14.6449997	11	7/24/2014	19:40.3
1425	RWS11	-122.4547766	37.93607	-0.49	15.1	314.38	14.5880004	-122.45477	37.93608	-0.44	15.1	364.25	14.6300004	11	7/24/2014	19:40.4
1426	RWS11	-122.4547789	37.93607	-0.44	15.1	297.1	14.6229996	-122.45477	37.93608	-0.41	15.1	346.15	14.6479996	11	7/24/2014	19:40.5
1427	RWS11	-122.4547813	37.93607	-0.49	15	280.03	14.5570002	-122.45478	37.93608	-0.44	15	328.25	14.5990002	11	7/24/2014	19:40.6
1428	RWS11	-122.4547836	37.93607	-0.44	15	263.04	14.5929999	-122.45478	37.93608	-0.41	15	310.4	14.6179999	11	7/24/2014	19:40.7
1429	RWS11	-122.4547859	37.93607	-0.49	15	246.06	14.5279999	-122.45478	37.93609	-0.41	15	292.92	14.6039999	11	7/24/2014	19:40.8
1430	RWS11	-122.4547882	37.93607	-0.44	15	229.6	14.5660004	-122.45478	37.93609	-0.41	15	275.86	14.5910004	11	7/24/2014	19:40.9
1431	RWS11	-122.4547917	37.93607	-0.49	15	213.41	14.5030003	-122.45479	37.93609	-0.44	15	259.21	14.5450003	11	7/24/2014	19:41.0
1432	RWS11	-122.454794	37.93607	-0.44	15	197.51	14.5440001	-122.45479	37.93609	-0.41	15	242.89	14.5690001	11	7/24/2014	19:41.1
1433	RWS11	-122.4547963	37.93608	-0.44	15	181.86	14.5339999	-122.45479	37.93609	-0.41	15	226.79	14.5589999	11	7/24/2014	19:41.2
1434	RWS11	-122.4547987	37.93608	-0.44	15	166.53	14.5260004	-122.45479	37.93609	-0.41	15	211.01	14.5510005	11	7/24/2014	19:41.3
1435	RWS11	-122.454801	37.93608	-0.49	15	151.49	14.4659997	-122.4548	37.93609	-0.41	15	195.59	14.5419997	11	7/24/2014	19:41.4
1436	RWS11	-122.4548034	37.93608	-0.49	14.9	136.72	14.4580002	-122.4548	37.93609	-0.41	14.9	180.39	14.5340003	11	7/24/2014	19:41.5
1437	RWS11	-122.4548058	37.93608	-0.49	14.9	122.25	14.4499999	-122.4548	37.93609	-0.41	14.9	165.43	14.5259999	11	7/24/2014	19:41.6
1438	RWS11	-122.4548081	37.93608	-0.44	14.9	108.09	14.4930005	-122.4548	37.93609	-0.41	14.9	150.61	14.5180005	11	7/24/2014	19:41.7
1439	RWS11	-122.4548105	37.93608	-0.44	14.9	93.844	14.4859995	-122.4548	37.93609	-0.44	14.9	135.65	14.4769995	11	7/24/2014	19:41.8
1440	RWS11	-122.4548128	37.93608	-0.44	14.9	79.444	14.4789996	-122.45481	37.93609	-0.41	14.9	120.64	14.5039996	11	7/24/2014	19:41.9
1441	RWS11	-122.4548163	37.93608	-0.49	14.9	65.087	14.4230004	-122.45481	37.93609	-0.44	14.9	105.62	14.4650004	11	7/24/2014	19:42.0
1442	RWS11	-122.4548187	37.93608	-0.44	14.9	50.992	14.4710001	-122.45481	37.93609	-0.41	14.9	90.767	14.4960001	11	7/24/2014	19:42.1
1443	RWS11	-122.4548211	37.93608	-0.49	14.9	37.333	14.4169999	-122.45482	37.93609	-0.44	14.9	76.235	14.4589999	11	7/24/2014	19:42.2
1444	RWS11	-122.4548235	37.93608	-0.44	14.9	23.957	14.4670004	-122.45482	37.9361	-0.41	14.9	62.001	14.4920004	11	7/24/2014	19:42.3
1445	RWS11	-122.4548259	37.93608	-0.49	14.9	10.888	14.4169999	-122.45482	37.9361	-0.44	14.9	48.264	14.4589999	11	7/24/2014	19:42.4
1446	RWS11	-122.4548283	37.93608	-0.44	14.9	-1.793	14.4679999	-122.45482	37.9361	-0.41	14.9	34.817	14.4929999	11	7/24/2014	19:42.5
1447	RWS11	-122.4548307	37.93608	-0.49	14.9	-14.34	14.4180003	-122.45483	37.9361	-0.41	14.9	21.705	14.4940003	11	7/24/2014	19:42.6
1448	RWS11	-122.4548331	37.93609	-0.44	14.9	-26.86	14.4699997	-122.45483	37.9361	-0.44	14.9	8.788	14.4609997	11	7/24/2014	19:42.7
1449	RWS11	-122.4548355	37.93609	-0.44	14.9	-39.15	14.4710001	-122.45483	37.9361	-0.41	14.9	-4.172	14.4960001	11	7/24/2014	19:42.8
1450	RWS11	-122.4548379	37.93609	-0.44	14.9	-51.43	14.4710001	-122.45483	37.9361	-0.44	14.9	-16.98	14.4620001	11	7/24/2014	19:42.9
1451	RWS11	-122.4548413	37.93609	-0.49	14.9	-63.5	14.4189997	-122.45484	37.9361	-0.44	14.9	-29.788	14.4609997	11	7/24/2014	19:43.0

1452	RWS11	-122.4548437	37.93609	-0.44	14.9	-75.43	14.4679999	-122.45484	37.9361	-0.44	14.9	-42.445	14.4589999	11	7/24/2014	19:43.1
1453	RWS11	-122.454846	37.93609	-0.49	14.9	-87.02	14.415	-122.45484	37.9361	-0.5	14.9	-54.815	14.406	11	7/24/2014	19:43.2
1454	RWS11	-122.4548484	37.93609	-0.49	14.9	-98.3	14.4130002	-122.45484	37.9361	-0.44	14.9	-66.902	14.4550002	11	7/24/2014	19:43.3
1455	RWS11	-122.4548508	37.93609	-0.49	14.9	-109.5	14.4099999	-122.45485	37.9361	-0.44	14.9	-78.575	14.4519999	11	7/24/2014	19:43.4
1456	RWS11	-122.4548532	37.93609	-0.44	14.9	-120.2	14.4579997	-122.45485	37.9361	-0.44	14.9	-89.812	14.4489996	11	7/24/2014	19:43.5
1457	RWS11	-122.4548555	37.93609	-0.49	14.9	-130.5	14.4040003	-122.45485	37.9361	-0.5	14.9	-100.68	14.3950003	11	7/24/2014	19:43.6
1458	RWS11	-122.4548579	37.93609	-0.49	14.9	-140.5	14.4010001	-122.45485	37.9361	-0.44	14.9	-111.17	14.4430001	11	7/24/2014	19:43.7
1459	RWS11	-122.4548602	37.93609	-0.49	14.9	-150.1	14.3970004	-122.45485	37.93611	-0.5	14.9	-121.35	14.3880004	11	7/24/2014	19:43.8
1460	RWS11	-122.4548625	37.93609	-0.49	14.9	-159.4	14.3920003	-122.45486	37.93611	-0.5	14.9	-131.31	14.3830003	11	7/24/2014	19:43.9
1461	RWS11	-122.454866	37.93609	-0.49	14.9	-168.6	14.3859997	-122.45486	37.93611	-0.5	14.9	-140.94	14.3769997	11	7/24/2014	19:44.0
1462	RWS11	-122.4548683	37.9361	-0.49	14.9	-177.8	14.3769999	-122.45486	37.93611	-0.5	14.9	-150.62	14.3679999	11	7/24/2014	19:44.1
1463	RWS11	-122.4548706	37.9361	-0.49	14.9	-187	14.3660002	-122.45486	37.93611	-0.5	14.9	-160.45	14.3570002	11	7/24/2014	19:44.2
1464	RWS11	-122.4548729	37.9361	-0.49	14.8	-196.3	14.3520003	-122.45487	37.93611	-0.5	14.8	-170.39	14.3430003	11	7/24/2014	19:44.3
1465	RWS11	-122.4548752	37.9361	-0.49	14.8	-205.5	14.3359996	-122.45487	37.93611	-0.53	14.8	-180.28	14.2929996	11	7/24/2014	19:44.4
1466	RWS11	-122.4548775	37.9361	-0.49	14.8	-214.7	14.3179999	-122.45487	37.93611	-0.53	14.8	-189.9	14.2749999	11	7/24/2014	19:44.5
1467	RWS11	-122.4548799	37.9361	-0.52	14.8	-223.7	14.2640004	-122.45487	37.93611	-0.53	14.8	-199.27	14.2550004	11	7/24/2014	19:44.6
1468	RWS11	-122.4548822	37.9361	-0.49	14.8	-232.4	14.2760001	-122.45488	37.93611	-0.53	14.8	-208.48	14.2330001	11	7/24/2014	19:44.7
1469	RWS11	-122.4548845	37.9361	-0.52	14.7	-241.1	14.2199998	-122.45488	37.93611	-0.58	14.7	-217.46	14.1599998	11	7/24/2014	19:44.8
1470	RWS11	-122.4548868	37.9361	-0.49	14.7	-249.6	14.2329999	-122.45488	37.93611	-0.5	14.7	-226.17	14.2239999	11	7/24/2014	19:44.9
1471	RWS11	-122.4548902	37.9361	-0.52	14.7	-257.9	14.178	-122.45488	37.93611	-0.58	14.7	-234.63	14.118	11	7/24/2014	19:45.0
1472	RWS11	-122.4548926	37.9361	-0.49	14.7	-265.9	14.1920005	-122.45489	37.93611	-0.53	14.7	-242.76	14.1490005	11	7/24/2014	19:45.1
1473	RWS11	-122.4548949	37.9361	-0.52	14.7	-273.4	14.1390004	-122.45489	37.93612	-0.58	14.7	-250.49	14.0790004	11	7/24/2014	19:45.2
1474	RWS11	-122.4548972	37.9361	-0.49	14.6	-280.8	14.1560002	-122.45489	37.93612	-0.58	14.6	-258.15	14.0620002	11	7/24/2014	19:45.3
1475	RWS11	-122.4548996	37.9361	-0.52	14.6	-288.2	14.1069999	-122.45489	37.93612	-0.61	14.6	-265.87	14.0139999	11	7/24/2014	19:45.4
1476	RWS11	-122.454902	37.9361	-0.52	14.6	-295.4	14.0949998	-122.4549	37.93612	-0.58	14.6	-273.47	14.0349998	11	7/24/2014	19:45.5
1477	RWS11	-122.4549043	37.93611	-0.52	14.6	-302.4	14.0849996	-122.4549	37.93612	-0.58	14.6	-280.87	14.0249996	11	7/24/2014	19:45.6
1478	RWS11	-122.4549067	37.93611	-0.52	14.6	-309.3	14.0779996	-122.4549	37.93612	-0.58	14.6	-288	14.0179996	11	7/24/2014	19:45.7
1479	RWS11	-122.454909	37.93611	-0.57	14.6	-315.6	14.0220004	-122.4549	37.93612	-0.61	14.6	-294.82	13.9800004	11	7/24/2014	19:45.8
1480	RWS11	-122.4549114	37.93611	-0.52	14.6	-322	14.0709996	-122.45491	37.93612	-0.58	14.6	-301.55	14.0109996	11	7/24/2014	19:45.9
1481	RWS11	-122.4549149	37.93611	-0.52	14.6	-328.2	14.0709996	-122.45491	37.93612	-0.58	14.6	-308.22	14.0109996	11	7/24/2014	19:46.0
1482	RWS11	-122.4549172	37.93611	-0.52	14.6	-334.3	14.072	-122.45491	37.93612	-0.58	14.6	-314.84	14.012	11	7/24/2014	19:46.1
1483	RWS11	-122.4549195	37.93611	-0.52	14.6	-340.3	14.0730004	-122.45491	37.93612	-0.58	14.6	-321.41	14.0130004	11	7/24/2014	19:46.2
1484	RWS11	-122.4549219	37.93611	-0.52	14.6	-346.2	14.0759997	-122.45492	37.93612	-0.58	14.6	-327.86	14.0159997	11	7/24/2014	19:46.3

1485	RWS11	-122.4549243	37.93611	-0.52	14.6	-352.1	14.0779996	-122.45492	37.93612	-0.61	14.6	-334.31	13.9849996	11	7/24/2014	19:46.4
1486	RWS11	-122.4549267	37.93611	-0.52	14.6	-357.9	14.0800004	-122.45492	37.93612	-0.58	14.6	-340.61	14.0200004	11	7/24/2014	19:46.5
1487	RWS11	-122.4549291	37.93611	-0.57	14.6	-363.3	14.0299999	-122.45492	37.93613	-0.61	14.6	-346.46	13.9879999	11	7/24/2014	19:46.6
1488	RWS11	-122.4549315	37.93611	-0.52	14.6	-368.5	14.079	-122.45493	37.93613	-0.61	14.6	-352.07	13.986	11	7/24/2014	19:46.7
1489	RWS11	-122.4549338	37.93611	-0.52	14.6	-373.5	14.0750003	-122.45493	37.93613	-0.61	14.6	-357.51	13.9820003	11	7/24/2014	19:46.8
1490	RWS11	-122.4549362	37.93611	-0.52	14.6	-378.2	14.0689998	-122.45493	37.93613	-0.61	14.6	-362.88	13.9759998	11	7/24/2014	19:46.9
1491	RWS11	-122.4549397	37.93612	-0.52	14.6	-383	14.0610004	-122.45493	37.93613	-0.61	14.6	-368.36	13.9680004	11	7/24/2014	19:47.0
1492	RWS11	-122.4549421	37.93612	-0.52	14.6	-387.7	14.0519996	-122.45494	37.93613	-0.58	14.6	-373.86	13.9919996	11	7/24/2014	19:47.1
1493	RWS11	-122.4549444	37.93612	-0.57	14.6	-392.5	13.9910003	-122.45494	37.93613	-0.61	14.6	-379.33	13.9490003	11	7/24/2014	19:47.2
1494	RWS11	-122.4549468	37.93612	-0.52	14.6	-397.6	14.0309997	-122.45494	37.93613	-0.58	14.6	-384.86	13.9709997	11	7/24/2014	19:47.3
1495	RWS11	-122.4549492	37.93612	-0.57	14.5	-402.5	13.9679996	-122.45494	37.93613	-0.61	14.5	-390.48	13.9259996	11	7/24/2014	19:47.4
1496	RWS11	-122.4549516	37.93612	-0.57	14.5	-407.5	13.9569999	-122.45495	37.93613	-0.58	14.5	-396.04	13.9479999	11	7/24/2014	19:47.5
1497	RWS11	-122.454954	37.93612	-0.57	14.5	-412.3	13.9460002	-122.45495	37.93613	-0.61	14.5	-401.59	13.9040002	11	7/24/2014	19:47.6
1498	RWS11	-122.4549564	37.93612	-0.57	14.5	-417	13.936	-122.45495	37.93613	-0.58	14.5	-406.92	13.927	11	7/24/2014	19:47.7
1499	RWS11	-122.4549588	37.93612	-0.57	14.5	-421.5	13.9270002	-122.45495	37.93613	-0.61	14.5	-411.88	13.8850002	11	7/24/2014	19:47.8
1500	RWS11	-122.4549612	37.93612	-0.52	14.5	-426.1	13.9699998	-122.45496	37.93613	-0.61	14.5	-416.64	13.8769998	11	7/24/2014	19:47.9
1501	RWS11	-122.4549647	37.93612	-0.57	14.5	-430.4	13.9139997	-122.45496	37.93614	-0.61	14.5	-421.17	13.8719997	11	7/24/2014	19:48.0
1502	RWS11	-122.4549671	37.93612	-0.57	14.5	-434.7	13.91	-122.45496	37.93614	-0.58	14.5	-425.74	13.901	11	7/24/2014	19:48.1
1503	RWS11	-122.4549694	37.93612	-0.57	14.5	-438.9	13.9080001	-122.45496	37.93614	-0.58	14.5	-430.14	13.8990001	11	7/24/2014	19:48.2
1504	RWS11	-122.4549718	37.93613	-0.57	14.5	-442.8	13.9080001	-122.45497	37.93614	-0.58	14.5	-434.23	13.8990001	11	7/24/2014	19:48.3
1505	RWS11	-122.4549742	37.93613	-0.57	14.5	-446.5	13.9089996	-122.45497	37.93614	-0.58	14.5	-438.13	13.8999996	11	7/24/2014	19:48.4
1506	RWS11	-122.4549766	37.93613	-0.57	14.5	-449.9	13.9110004	-122.45497	37.93614	-0.58	14.5	-441.79	13.9020004	11	7/24/2014	19:48.5
1507	RWS11	-122.454979	37.93613	-0.57	14.5	-453.1	13.9130002	-122.45497	37.93614	-0.58	14.5	-445.25	13.9040002	11	7/24/2014	19:48.6
1508	RWS11	-122.4549814	37.93613	-0.57	14.5	-456.2	13.9150001	-122.45498	37.93614	-0.58	14.5	-448.61	13.9060001	11	7/24/2014	19:48.7
1509	RWS11	-122.4549837	37.93613	-0.57	14.5	-459.1	13.9180003	-122.45498	37.93614	-0.58	14.5	-451.71	13.9090003	11	7/24/2014	19:48.8
1510	RWS11	-122.4549861	37.93613	-0.52	14.5	-462.1	13.9719997	-122.45498	37.93614	-0.58	14.5	-454.84	13.9119996	11	7/24/2014	19:48.9
1511	RWS11	-122.4549896	37.93613	-0.57	14.5	-465.1	13.9239999	-122.45498	37.93614	-0.58	14.5	-457.88	13.9149999	11	7/24/2014	19:49.0
1512	RWS11	-122.454992	37.93613	-0.52	14.5	-468.1	13.9780002	-122.45499	37.93614	-0.58	14.5	-460.99	13.9180002	11	7/24/2014	19:49.1
1513	RWS11	-122.4549944	37.93613	-0.57	14.5	-471.2	13.9300004	-122.45499	37.93615	-0.58	14.5	-464.03	13.9210004	11	7/24/2014	19:49.2
1514	RWS11	-122.4549968	37.93613	-0.52	14.5	-474.2	13.9839997	-122.45499	37.93615	-0.58	14.5	-466.95	13.9239997	11	7/24/2014	19:49.3
1515	RWS11	-122.4549992	37.93613	-0.57	14.5	-477	13.9349996	-122.45499	37.93615	-0.61	14.5	-469.57	13.8929996	11	7/24/2014	19:49.4
1516	RWS11	-122.4550017	37.93613	-0.52	14.5	-479.5	13.9880004	-122.455	37.93615	-0.58	14.5	-471.95	13.9280004	11	7/24/2014	19:49.5
1517	RWS11	-122.4550041	37.93614	-0.57	14.5	-482.1	13.9390003	-122.455	37.93615	-0.61	14.5	-474.23	13.8970003	11	7/24/2014	19:49.6

1518	RWS11	-122.4550065	37.93614	-0.57	14.5	-484.4	13.9410001	-122.455	37.93615	-0.58	14.5	-476.39	13.9320001	11	7/24/2014	19:49.7
1519	RWS11	-122.4550089	37.93614	-0.57	14.5	-486.5	13.943	-122.455	37.93615	-0.61	14.5	-478.34	13.901	11	7/24/2014	19:49.8
1520	RWS11	-122.4550113	37.93614	-0.52	14.5	-488.1	13.9970002	-122.45501	37.93615	-0.58	14.5	-479.96	13.9370002	11	7/24/2014	19:49.9
1521	RWS11	-122.4550148	37.93614	-0.61	14.5	-489.6	13.9130001	-122.45501	37.93615	-0.61	14.5	-481.52	13.9060001	11	7/24/2014	19:50.0
1522	RWS11	-122.4550171	37.93614	-0.52	14.5	-491	14.0009999	-122.45501	37.93615	-0.61	14.5	-483.15	13.9079999	11	7/24/2014	19:50.1
1523	RWS11	-122.4550195	37.93614	-0.57	14.5	-492.3	13.9519998	-122.45501	37.93615	-0.58	14.5	-484.74	13.9429998	11	7/24/2014	19:50.2
1524	RWS11	-122.4550218	37.93614	-0.52	14.5	-493.6	14.0029998	-122.45502	37.93615	-0.58	14.5	-486.45	13.9429998	11	7/24/2014	19:50.3
1525	RWS11	-122.4550242	37.93614	-0.57	14.5	-494.9	13.9519998	-122.45502	37.93615	-0.58	14.5	-488.26	13.9429998	11	7/24/2014	19:50.4
1526	RWS11	-122.4550266	37.93614	-0.57	14.5	-496.2	13.9499999	-122.45502	37.93615	-0.58	14.5	-489.93	13.9409999	11	7/24/2014	19:50.5
1527	RWS11	-122.4550289	37.93614	-0.57	14.5	-497.3	13.9480001	-122.45502	37.93616	-0.61	14.5	-491.33	13.9060001	11	7/24/2014	19:50.6
1528	RWS11	-122.4550313	37.93614	-0.57	14.5	-498.1	13.9440004	-122.45503	37.93616	-0.58	14.5	-492.48	13.9350004	11	7/24/2014	19:50.7
1529	RWS11	-122.4550336	37.93614	-0.57	14.5	-498.9	13.9399997	-122.45503	37.93616	-0.58	14.5	-493.5	13.9309997	11	7/24/2014	19:50.8
1530	RWS11	-122.4550359	37.93615	-0.57	14.5	-499.5	13.936	-122.45503	37.93616	-0.58	14.5	-494.3	13.927	11	7/24/2014	19:50.9
1531	RWS11	-122.4550394	37.93615	-0.57	14.5	-500.1	13.9320003	-122.45503	37.93616	-0.58	14.5	-494.91	13.9230003	11	7/24/2014	19:51.0
1532	RWS11	-122.4550417	37.93615	-0.57	14.5	-500.6	13.9279996	-122.45504	37.93616	-0.53	14.5	-495.4	13.9699996	11	7/24/2014	19:51.1
1533	RWS11	-122.455044	37.93615	-0.57	14.5	-501.2	13.9239999	-122.45504	37.93616	-0.58	14.5	-495.78	13.9149999	11	7/24/2014	19:51.2
1534	RWS11	-122.4550464	37.93615	-0.57	14.5	-501.7	13.9220001	-122.45504	37.93616	-0.53	14.5	-496.26	13.9640001	11	7/24/2014	19:51.3
1535	RWS11	-122.4550487	37.93615	-0.57	14.5	-501.9	13.9200002	-122.45504	37.93616	-0.58	14.5	-496.48	13.9110002	11	7/24/2014	19:51.4
1536	RWS11	-122.4550511	37.93615	-0.57	14.5	-502.1	13.9200002	-122.45504	37.93616	-0.58	14.5	-496.67	13.9110002	11	7/24/2014	19:51.5
1537	RWS11	-122.4550535	37.93615	-0.57	14.5	-502.3	13.9209997	-122.45505	37.93616	-0.58	14.5	-496.92	13.9119996	11	7/24/2014	19:51.6
1538	RWS11	-122.4550558	37.93615	-0.57	14.5	-502.4	13.9239999	-122.45505	37.93616	-0.58	14.5	-497.07	13.9149999	11	7/24/2014	19:51.7
1539	RWS11	-122.4550582	37.93615	-0.61	14.5	-502.3	13.8950004	-122.45505	37.93616	-0.61	14.5	-497.09	13.8880004	11	7/24/2014	19:51.8
1540	RWS11	-122.4550605	37.93615	-0.57	14.5	-502.1	13.9370004	-122.45505	37.93617	-0.58	14.5	-496.92	13.9280004	11	7/24/2014	19:51.9
1541	RWS11	-122.455064	37.93615	-0.57	14.5	-501.9	13.9460002	-122.45506	37.93617	-0.61	14.5	-496.58	13.9040002	11	7/24/2014	19:52.0
1542	RWS11	-122.4550663	37.93615	-0.57	14.5	-501.5	13.9580003	-122.45506	37.93617	-0.58	14.5	-495.86	13.9490003	11	7/24/2014	19:52.1
1543	RWS11	-122.4550686	37.93616	-0.57	14.5	-500.9	13.9709998	-122.45506	37.93617	-0.61	14.5	-494.89	13.9289998	11	7/24/2014	19:52.2
1544	RWS11	-122.455071	37.93616	-0.57	14.6	-500.2	13.9860002	-122.45506	37.93617	-0.53	14.6	-493.62	14.0280002	11	7/24/2014	19:52.3
1545	RWS11	-122.4550734	37.93616	-0.57	14.6	-499.2	14.002	-122.45507	37.93617	-0.58	14.6	-492.17	13.993	11	7/24/2014	19:52.4
1546	RWS11	-122.4550758	37.93616	-0.57	14.6	-497.7	14.0180003	-122.45507	37.93617	-0.58	14.6	-490.26	14.0090003	11	7/24/2014	19:52.5
1547	RWS11	-122.4550781	37.93616	-0.61	14.6	-496.2	13.9979997	-122.45507	37.93617	-0.61	14.6	-488.36	13.9909997	11	7/24/2014	19:52.6
1548	RWS11	-122.4550805	37.93616	-0.57	14.6	-494.5	14.0460002	-122.45507	37.93617	-0.58	14.6	-486.34	14.0370002	11	7/24/2014	19:52.7
1549	RWS11	-122.4550829	37.93616	-0.61	14.6	-492.9	14.0230002	-122.45508	37.93617	-0.58	14.6	-484.5	14.0490003	11	7/24/2014	19:52.8
1550	RWS11	-122.4550852	37.93616	-0.57	14.6	-491.4	14.0679995	-122.45508	37.93617	-0.58	14.6	-482.75	14.0589995	11	7/24/2014	19:52.9

1551	RWS11	-122.4550887	37.93616	-0.61	14.6	-489.7	14.0409999	-122.45508	37.93617	-0.61	14.6	-480.91	14.0339999	11	7/24/2014	19:53.0
1552	RWS11	-122.4550911	37.93616	-0.57	14.7	-488.1	14.0820004	-122.45508	37.93618	-0.58	14.7	-479.17	14.0730004	11	7/24/2014	19:53.1
1553	RWS11	-122.4550935	37.93616	-0.57	14.7	-486.3	14.0869996	-122.45509	37.93618	-0.58	14.7	-477.49	14.0779996	11	7/24/2014	19:53.2
1554	RWS11	-122.4550958	37.93616	-0.57	14.7	-484.7	14.0890004	-122.45509	37.93618	-0.53	14.7	-475.73	14.1310004	11	7/24/2014	19:53.3
1555	RWS11	-122.4550982	37.93617	-0.57	14.7	-483	14.0910003	-122.45509	37.93618	-0.58	14.7	-473.81	14.0820003	11	7/24/2014	19:53.4
1556	RWS11	-122.4551006	37.93617	-0.57	14.7	-481.4	14.0919997	-122.45509	37.93618	-0.53	14.7	-471.96	14.1339997	11	7/24/2014	19:53.5
1557	RWS11	-122.455103	37.93617	-0.57	14.7	-479.9	14.0930001	-122.4551	37.93618	-0.58	14.7	-470.19	14.0840001	11	7/24/2014	19:53.6
1558	RWS11	-122.4551054	37.93617	-0.57	14.7	-478.2	14.0960004	-122.4551	37.93618	-0.53	14.7	-468.31	14.1380004	11	7/24/2014	19:53.7
1559	RWS11	-122.4551077	37.93617	-0.57	14.7	-476.4	14.1009995	-122.4551	37.93618	-0.61	14.7	-466.2	14.0589995	11	7/24/2014	19:53.8
1560	RWS11	-122.4551101	37.93617	-0.57	14.7	-474.1	14.1070001	-122.4551	37.93618	-0.58	14.7	-463.63	14.0980001	11	7/24/2014	19:53.9
1561	RWS11	-122.4551136	37.93617	-0.61	14.7	-471.6	14.0800004	-122.45511	37.93618	-0.58	14.7	-460.64	14.1060004	11	7/24/2014	19:54.0
1562	RWS11	-122.4551159	37.93617	-0.57	14.7	-468.8	14.1240003	-122.45511	37.93618	-0.58	14.7	-457.33	14.1150003	11	7/24/2014	19:54.1
1563	RWS11	-122.4551182	37.93617	-0.61	14.7	-465.8	14.0980001	-122.45511	37.93618	-0.61	14.7	-453.91	14.0910001	11	7/24/2014	19:54.2
1564	RWS11	-122.4551206	37.93617	-0.57	14.7	-462.8	14.1430003	-122.45511	37.93618	-0.58	14.7	-450.4	14.1340003	11	7/24/2014	19:54.3
1565	RWS11	-122.455123	37.93617	-0.57	14.7	-459.6	14.1529996	-122.45512	37.93619	-0.61	14.7	-446.82	14.1109996	11	7/24/2014	19:54.4
1566	RWS11	-122.4551253	37.93617	-0.57	14.7	-456.5	14.1629998	-122.45512	37.93619	-0.58	14.7	-443.36	14.1539998	11	7/24/2014	19:54.5
1567	RWS11	-122.4551277	37.93617	-0.61	14.7	-453.4	14.1390004	-122.45512	37.93619	-0.58	14.7	-440.07	14.1650004	11	7/24/2014	19:54.6
1568	RWS11	-122.4551301	37.93618	-0.57	14.8	-450.3	14.1850001	-122.45512	37.93619	-0.58	14.8	-436.76	14.1760001	11	7/24/2014	19:54.7
1569	RWS11	-122.4551324	37.93618	-0.57	14.8	-447.2	14.1970002	-122.45513	37.93619	-0.58	14.8	-433.36	14.1880002	11	7/24/2014	19:54.8
1570	RWS11	-122.4551347	37.93618	-0.57	14.8	-443.8	14.2099997	-122.45513	37.93619	-0.58	14.8	-430.02	14.2009997	11	7/24/2014	19:54.9
1571	RWS11	-122.4551382	37.93618	-0.61	14.8	-440.4	14.1889997	-122.45513	37.93619	-0.58	14.8	-426.53	14.2149997	11	7/24/2014	19:55.0
1572	RWS11	-122.4551405	37.93618	-0.57	14.8	-436.8	14.2400004	-122.45513	37.93619	-0.58	14.8	-422.79	14.2310004	11	7/24/2014	19:55.1
1573	RWS11	-122.4551429	37.93618	-0.61	14.8	-432.9	14.2210002	-122.45514	37.93619	-0.58	14.8	-418.63	14.2470002	11	7/24/2014	19:55.2
1574	RWS11	-122.4551452	37.93618	-0.61	14.8	-428.8	14.2400003	-122.45514	37.93619	-0.58	14.8	-414.15	14.2660003	11	7/24/2014	19:55.3
1575	RWS11	-122.4551476	37.93618	-0.61	14.9	-424.6	14.2599998	-122.45514	37.93619	-0.61	14.9	-409.48	14.2529998	11	7/24/2014	19:55.4
1576	RWS11	-122.45515	37.93618	-0.61	14.9	-420.1	14.2809997	-122.45514	37.93619	-0.58	14.9	-404.54	14.3069997	11	7/24/2014	19:55.5
1577	RWS11	-122.4551523	37.93618	-0.61	14.9	-415.4	14.303	-122.45515	37.93619	-0.61	14.9	-399.45	14.296	11	7/24/2014	19:55.6
1578	RWS11	-122.4551547	37.93618	-0.61	14.9	-410.7	14.3270001	-122.45515	37.9362	-0.58	14.9	-394.19	14.3530002	11	7/24/2014	19:55.7
1579	RWS11	-122.455157	37.93618	-0.61	15	-405.8	14.3499999	-122.45515	37.9362	-0.61	15	-388.89	14.3429999	11	7/24/2014	19:55.8
1580	RWS11	-122.4551594	37.93618	-0.57	15	-401	14.4090001	-122.45515	37.9362	-0.58	15	-383.73	14.4000001	11	7/24/2014	19:55.9
1581	RWS11	-122.4551628	37.93619	-0.66	15	-396.2	14.3470002	-122.45516	37.9362	-0.61	15	-378.43	14.3910003	11	7/24/2014	19:56.0
1582	RWS11	-122.4551652	37.93619	-0.61	15	-391.5	14.4229999	-122.45516	37.9362	-0.58	15	-372.99	14.4489999	11	7/24/2014	19:56.1
1583	RWS11	-122.4551675	37.93619	-0.61	15.1	-386.9	14.4480004	-122.45516	37.9362	-0.61	15.1	-367.58	14.4410005	11	7/24/2014	19:56.2

1584	RWS11	-122.4551699	37.93619	-0.57	15.1	-382.2	14.5080001	-122.45516	37.9362	-0.58	15.1	-361.88	14.4990001	11	7/24/2014	19:56.3
1585	RWS11	-122.4551722	37.93619	-0.61	15.1	-377.4	14.4970002	-122.45517	37.9362	-0.61	15.1	-356.1	14.4900003	11	7/24/2014	19:56.4
1586	RWS11	-122.4551746	37.93619	-0.57	15.1	-372.4	14.5560004	-122.45517	37.9362	-0.58	15.1	-350.19	14.5470004	11	7/24/2014	19:56.5
1587	RWS11	-122.455177	37.93619	-0.61	15.1	-367.3	14.5440002	-122.45517	37.9362	-0.61	15.1	-344	14.5370002	11	7/24/2014	19:56.6
1588	RWS11	-122.4551793	37.93619	-0.57	15.2	-361.8	14.6019999	-122.45517	37.9362	-0.58	15.2	-337.81	14.5929999	11	7/24/2014	19:56.7
1589	RWS11	-122.4551817	37.93619	-0.61	15.2	-356	14.5890002	-122.45518	37.9362	-0.58	15.2	-331.29	14.6150003	11	7/24/2014	19:56.8
1590	RWS11	-122.455184	37.93619	-0.57	15.2	-349.9	14.6459996	-122.45518	37.93621	-0.53	15.2	-324.56	14.6879996	11	7/24/2014	19:56.9
1591	RWS11	-122.4551875	37.93619	-0.61	15.2	-343.6	14.6340003	-122.45518	37.93621	-0.58	15.2	-317.75	14.6600003	11	7/24/2014	19:57.0
1592	RWS11	-122.4551898	37.93619	-0.57	15.3	-337.2	14.6929995	-122.45518	37.93621	-0.53	15.3	-310.76	14.7349995	11	7/24/2014	19:57.1
1593	RWS11	-122.4551921	37.9362	-0.61	15.3	-330.8	14.6830001	-122.45519	37.93621	-0.58	15.3	-303.64	14.7090001	11	7/24/2014	19:57.2
1594	RWS11	-122.4551944	37.9362	-0.57	15.3	-324.6	14.746	-122.45519	37.93621	-0.58	15.3	-296.52	14.737	11	7/24/2014	19:57.3
1595	RWS11	-122.4551968	37.9362	-0.61	15.3	-318.3	14.7389998	-122.45519	37.93621	-0.58	15.3	-289.48	14.7649999	11	7/24/2014	19:57.4
1596	RWS11	-122.4551991	37.9362	-0.57	15.4	-312.2	14.805	-122.45519	37.93621	-0.58	15.4	-282.5	14.796	11	7/24/2014	19:57.5
1597	RWS11	-122.4552015	37.9362	-0.61	15.4	-305.8	14.8010001	-122.4552	37.93621	-0.58	15.4	-275.47	14.8270001	11	7/24/2014	19:57.6
1598	RWS11	-122.4552038	37.9362	-0.57	15.4	-299.5	14.8690001	-122.4552	37.93621	-0.58	15.4	-268.28	14.8600001	11	7/24/2014	19:57.7
1599	RWS11	-122.4552061	37.9362	-0.57	15.5	-293.1	14.9009997	-122.4552	37.93621	-0.58	15.5	-260.96	14.8919997	11	7/24/2014	19:57.8
1600	RWS11	-122.4552084	37.9362	-0.57	15.5	-286.5	14.9319999	-122.4552	37.93621	-0.58	15.5	-253.36	14.9229999	11	7/24/2014	19:57.9
1601	RWS11	-122.4552118	37.9362	-0.61	15.5	-279.5	14.9269996	-122.45521	37.93621	-0.58	15.5	-245.54	14.9529996	11	7/24/2014	19:58.0
1602	RWS11	-122.4552141	37.9362	-0.57	15.6	-272.2	14.9900004	-122.45521	37.93621	-0.53	15.6	-237.62	15.0320004	11	7/24/2014	19:58.1
1603	RWS11	-122.4552165	37.9362	-0.57	15.6	-264.7	15.0160005	-122.45521	37.93622	-0.58	15.6	-229.64	15.0070005	11	7/24/2014	19:58.2
1604	RWS11	-122.4552188	37.9362	-0.57	15.6	-257.2	15.0410001	-122.45521	37.93622	-0.53	15.6	-221.7	15.0830001	11	7/24/2014	19:58.3
1605	RWS11	-122.4552211	37.9362	-0.57	15.6	-249.6	15.0650002	-122.45521	37.93622	-0.58	15.6	-213.73	15.0560002	11	7/24/2014	19:58.4
1606	RWS11	-122.4552234	37.9362	-0.57	15.7	-242.1	15.088	-122.45522	37.93622	-0.53	15.7	-205.59	15.13	11	7/24/2014	19:58.5
1607	RWS11	-122.4552258	37.93621	-0.57	15.7	-234.6	15.1109998	-122.45522	37.93622	-0.58	15.7	-197.31	15.1019998	11	7/24/2014	19:58.6
1608	RWS11	-122.4552281	37.93621	-0.57	15.7	-227.1	15.1340005	-122.45522	37.93622	-0.53	15.7	-188.95	15.1760005	11	7/24/2014	19:58.7
1609	RWS11	-122.4552304	37.93621	-0.57	15.7	-219.5	15.1570002	-122.45522	37.93622	-0.58	15.7	-180.56	15.1480002	11	7/24/2014	19:58.8
1610	RWS11	-122.4552327	37.93621	-0.57	15.7	-211.7	15.1789996	-122.45523	37.93622	-0.53	15.7	-172.07	15.2209996	11	7/24/2014	19:58.9
1611	RWS11	-122.4552361	37.93621	-0.61	15.8	-203.7	15.1659999	-122.45523	37.93622	-0.58	15.8	-163.36	15.1919999	11	7/24/2014	19:59.0
1612	RWS11	-122.4552384	37.93621	-0.57	15.8	-195.6	15.2210004	-122.45523	37.93622	-0.53	15.8	-154.53	15.2630004	11	7/24/2014	19:59.1
1613	RWS11	-122.4552407	37.93621	-0.57	15.8	-187.5	15.2409999	-122.45523	37.93622	-0.53	15.8	-145.7	15.2829999	11	7/24/2014	19:59.2
1614	RWS11	-122.455243	37.93621	-0.57	15.8	-179.4	15.2590005	-122.45524	37.93622	-0.49	15.8	-136.92	15.3350005	11	7/24/2014	19:59.3
1615	RWS11	-122.4552453	37.93621	-0.61	15.8	-171.2	15.2409997	-122.45524	37.93622	-0.53	15.8	-128.26	15.3179997	11	7/24/2014	19:59.4
1616	RWS11	-122.4552477	37.93621	-0.52	15.9	-163	15.3439999	-122.45524	37.93623	-0.49	15.9	-119.41	15.3689999	11	7/24/2014	19:59.5

1617	RWS11	-122.45525	37.93621	-0.57	15.9	-154.7	15.3089997	-122.45524	37.93623	-0.49	15.9	-110.44	15.3849997	11	7/24/2014	19:59.6
1618	RWS11	-122.4552523	37.93621	-0.57	15.9	-146.3	15.3250005	-122.45525	37.93623	-0.49	15.9	-101.25	15.4010005	11	7/24/2014	19:59.7
1619	RWS11	-122.4552546	37.93621	-0.57	15.9	-137.8	15.3410003	-122.45525	37.93623	-0.49	15.9	-91.644	15.4170003	11	7/24/2014	19:59.8
1620	RWS11	-122.4552569	37.93622	-0.52	15.9	-129	15.4080001	-122.45525	37.93623	-0.49	15.9	-81.523	15.4330001	11	7/24/2014	19:59.9
1621	RWS11	-122.4552603	37.93622	-0.57	15.9	-119.7	15.3740003	-122.45525	37.93623	-0.44	15.9	-71.099	15.5010003	11	7/24/2014	20:00.0
1622	RWS11	-122.4552626	37.93622	-0.52	16	-110.2	15.4420005	-122.45526	37.93623	-0.49	16	-60.378	15.4670005	11	7/24/2014	20:00.1
1623	RWS11	-122.4552649	37.93622	-0.57	16	-100.3	15.4079997	-122.45526	37.93623	-0.49	16	-49.515	15.4839997	11	7/24/2014	20:00.2
1624	RWS11	-122.4552673	37.93622	-0.52	16	-90.29	15.4750004	-122.45526	37.93623	-0.44	16	-38.474	15.5510005	11	7/24/2014	20:00.3
1625	RWS11	-122.4552696	37.93622	-0.57	16	-80.13	15.4390008	-122.45526	37.93623	-0.49	16	-27.261	15.5150008	11	7/24/2014	20:00.4
1626	RWS11	-122.4552719	37.93622	-0.57	16	-69.89	15.4540002	-122.45527	37.93623	-0.44	16	-15.913	15.5810002	11	7/24/2014	20:00.5
1627	RWS11	-122.4552743	37.93622	-0.57	16	-59.62	15.4670007	-122.45527	37.93623	-0.49	16	-4.72	15.5430007	11	7/24/2014	20:00.6
1628	RWS11	-122.4552766	37.93622	-0.52	16	-49.21	15.5290003	-122.45527	37.93623	-0.49	16	6.756	15.5540004	11	7/24/2014	20:00.7
1629	RWS11	-122.4552789	37.93622	-0.57	16.1	-38.8	15.4869992	-122.45527	37.93623	-0.49	16.1	18.101	15.5629992	11	7/24/2014	20:00.8
1630	RWS11	-122.4552812	37.93622	-0.52	16.1	-28.44	15.5449992	-122.45528	37.93624	-0.44	16.1	29.533	15.6209992	11	7/24/2014	20:00.9
1631	RWS11	-122.4552846	37.93622	-0.57	16.1	-17.75	15.4990003	-122.45528	37.93624	-0.49	16.1	41.008	15.5750003	11	7/24/2014	20:01.0
1632	RWS11	-122.4552869	37.93622	-0.57	16.1	-6.97	15.5020005	-122.45528	37.93624	-0.44	16.1	52.681	15.6290005	11	7/24/2014	20:01.1
1633	RWS11	-122.4552891	37.93623	-0.57	16.1	3.94	15.5039994	-122.45528	37.93624	-0.44	16.1	64.424	15.6309994	11	7/24/2014	20:01.2
1634	RWS11	-122.4552914	37.93623	-0.52	16.1	15.001	15.5549994	-122.45529	37.93624	-0.44	16.1	76.22	15.6309994	11	7/24/2014	20:01.3
1635	RWS11	-122.4552937	37.93623	-0.57	16.1	25.977	15.503	-122.45529	37.93624	-0.44	16.1	88.03	15.63	11	7/24/2014	20:01.4
1636	RWS11	-122.455296	37.93623	-0.52	16.1	36.864	15.5530005	-122.45529	37.93624	-0.44	16.1	99.769	15.6290005	11	7/24/2014	20:01.5
1637	RWS11	-122.4552983	37.93623	-0.57	16.1	47.73	15.4999997	-122.45529	37.93624	-0.44	16.1	111.71	15.6269997	11	7/24/2014	20:01.6
1638	RWS11	-122.4553006	37.93623	-0.52	16.1	58.857	15.5490008	-122.45529	37.93624	-0.44	16.1	123.83	15.6250008	11	7/24/2014	20:01.7
1639	RWS11	-122.4553029	37.93623	-0.57	16.1	70.12	15.4969994	-122.4553	37.93624	-0.44	16.1	136.1	15.6239995	11	7/24/2014	20:01.8
1640	RWS11	-122.4553052	37.93623	-0.52	16.1	81.57	15.547	-122.4553	37.93624	-0.41	16.1	148.59	15.657	11	7/24/2014	20:01.9
1641	RWS11	-122.4553085	37.93623	-0.52	16.1	93.331	15.5460005	-122.4553	37.93624	-0.44	16.1	161.33	15.6220006	11	7/24/2014	20:02.0
1642	RWS11	-122.4553108	37.93623	-0.52	16.1	105.16	15.5449992	-122.4553	37.93624	-0.36	16.1	174.25	15.7049992	11	7/24/2014	20:02.1
1643	RWS11	-122.455313	37.93623	-0.57	16.1	117.2	15.4939992	-122.45531	37.93624	-0.41	16.1	187.42	15.6549992	11	7/24/2014	20:02.2
1644	RWS11	-122.4553153	37.93623	-0.52	16.1	129.39	15.5449992	-122.45531	37.93625	-0.41	16.1	200.85	15.6549992	11	7/24/2014	20:02.3
1645	RWS11	-122.4553176	37.93623	-0.52	16.1	141.8	15.5460005	-122.45531	37.93625	-0.41	16.1	214.47	15.6560005	11	7/24/2014	20:02.4
1646	RWS11	-122.4553199	37.93623	-0.52	16.1	154.44	15.547	-122.45531	37.93625	-0.36	16.1	228.56	15.707	11	7/24/2014	20:02.5
1647	RWS11	-122.4553222	37.93623	-0.57	16.1	167.19	15.4969994	-122.45532	37.93625	-0.36	16.1	242.81	15.7079994	11	7/24/2014	20:02.6
1648	RWS11	-122.4553245	37.93624	-0.49	16.1	180.07	15.5840002	-122.45532	37.93625	-0.41	16.1	257.18	15.6600002	11	7/24/2014	20:02.7
1649	RWS11	-122.4553268	37.93624	-0.52	16.1	193.05	15.5530005	-122.45532	37.93625	-0.41	16.1	271.79	15.6630005	11	7/24/2014	20:02.8

1650	RWS11	-122.4553291	37.93624	-0.49	16.1	206.15	15.5910002	-122.45532	37.93625	-0.36	16.1	286.53	15.7170002	11	7/24/2014	20:02.9
1651	RWS11	-122.4553324	37.93624	-0.52	16.1	219.35	15.5619994	-122.45533	37.93625	-0.41	16.1	301.24	15.6719994	11	7/24/2014	20:03.0
1652	RWS11	-122.4553347	37.93624	-0.49	16.1	232.72	15.6019999	-122.45533	37.93625	-0.41	16.1	315.99	15.6779999	11	7/24/2014	20:03.1
1653	RWS11	-122.4553369	37.93624	-0.52	16.1	246.18	15.5749999	-122.45533	37.93625	-0.36	16.1	330.96	15.7349999	11	7/24/2014	20:03.2
1654	RWS11	-122.4553392	37.93624	-0.49	16.1	259.77	15.6180006	-122.45533	37.93625	-0.36	16.1	346.22	15.7440006	11	7/24/2014	20:03.3
1655	RWS11	-122.4553415	37.93624	-0.49	16.1	273.46	15.6269995	-122.45534	37.93625	-0.36	16.1	361.52	15.7529995	11	7/24/2014	20:03.4
1656	RWS11	-122.4553438	37.93624	-0.49	16.1	287.4	15.6360003	-122.45534	37.93625	-0.36	16.1	377.05	15.7620003	11	7/24/2014	20:03.5
1657	RWS11	-122.4553461	37.93624	-0.49	16.1	301.5	15.6449992	-122.45534	37.93625	-0.36	16.1	392.72	15.7709992	11	7/24/2014	20:03.6
1658	RWS11	-122.4553484	37.93624	-0.49	16.1	315.94	15.6539999	-122.45534	37.93626	-0.36	16.1	408.49	15.7799999	11	7/24/2014	20:03.7
1659	RWS11	-122.4553507	37.93624	-0.52	16.1	330.8	15.6279994	-122.45534	37.93626	-0.36	16.1	424.46	15.7879994	11	7/24/2014	20:03.8
1660	RWS11	-122.4553529	37.93624	-0.49	16.2	345.93	15.6679999	-122.45535	37.93626	-0.36	16.2	440.76	15.7939999	11	7/24/2014	20:03.9
1661	RWS11	-122.4553563	37.93624	-0.49	16.2	361.45	15.6740004	-122.45535	37.93626	-0.36	16.2	457.25	15.8000004	11	7/24/2014	20:04.0
1662	RWS11	-122.4553586	37.93625	-0.49	16.2	377.15	15.6800009	-122.45535	37.93626	-0.36	16.2	474.1	15.8060009	11	7/24/2014	20:04.1
1663	RWS11	-122.4553609	37.93625	-0.49	16.2	393.07	15.6850001	-122.45536	37.93626	-0.36	16.2	491.27	15.8110001	11	7/24/2014	20:04.2
1664	RWS11	-122.4553632	37.93625	-0.49	16.2	409.1	15.6910006	-122.45536	37.93626	-0.36	16.2	508.57	15.8170006	11	7/24/2014	20:04.3
1665	RWS11	-122.4553655	37.93625	-0.52	16.2	425.3	15.6629992	-122.45536	37.93626	-0.36	16.2	526.18	15.8229992	11	7/24/2014	20:04.4
1666	RWS11	-122.4553678	37.93625	-0.49	16.2	441.55	15.7050005	-122.45536	37.93626	-0.36	16.2	544.01	15.8310005	11	7/24/2014	20:04.5
1667	RWS11	-122.4553701	37.93625	-0.52	16.2	457.84	15.679	-122.45536	37.93626	-0.41	16.2	561.92	15.7889999	11	7/24/2014	20:04.6
1668	RWS11	-122.4553724	37.93625	-0.49	16.2	474.16	15.7220007	-122.45537	37.93626	-0.41	16.2	579.85	15.7980007	11	7/24/2014	20:04.7
1669	RWS11	-122.4553747	37.93625	-0.52	16.2	490.49	15.6979991	-122.45537	37.93626	-0.36	16.2	597.85	15.8579991	11	7/24/2014	20:04.8
1670	RWS11	-122.4553771	37.93625	-0.49	16.2	506.9	15.7430006	-122.45537	37.93626	-0.41	16.2	616.04	15.8190006	11	7/24/2014	20:04.9
1671	RWS11	-122.4553805	37.93625	-0.49	16.2	523.46	15.7549998	-122.45537	37.93626	-0.36	16.2	634.21	15.8809998	11	7/24/2014	20:05.0
1672	RWS11	-122.4553828	37.93625	-0.52	16.3	540.15	15.7340003	-122.45538	37.93626	-0.41	16.3	652.38	15.8440003	11	7/24/2014	20:05.1
1673	RWS11	-122.4553852	37.93625	-0.49	16.3	556.93	15.7820002	-122.45538	37.93627	-0.41	16.3	670.52	15.8580002	11	7/24/2014	20:05.2
1674	RWS11	-122.4553875	37.93625	-0.49	16.3	573.75	15.7980009	-122.45538	37.93627	-0.36	16.3	688.78	15.924001	11	7/24/2014	20:05.3
1675	RWS11	-122.4553899	37.93625	-0.52	16.3	590.64	15.7830001	-122.45538	37.93627	-0.36	16.3	706.77	15.9430001	11	7/24/2014	20:05.4
1676	RWS11	-122.4553923	37.93625	-0.49	16.3	607.67	15.8400008	-122.45539	37.93627	-0.36	16.3	724.87	15.9660008	11	7/24/2014	20:05.5
1677	RWS11	-122.4553947	37.93626	-0.49	16.4	625.03	15.8669993	-122.45539	37.93627	-0.36	16.4	743.17	15.9929993	11	7/24/2014	20:05.6
1678	RWS11	-122.455397	37.93626	-0.49	16.4	642.71	15.897	-122.45539	37.93627	-0.36	16.4	761.88	16.023	11	7/24/2014	20:05.7
1679	RWS11	-122.4553994	37.93626	-0.52	16.4	660.86	15.8979998	-122.45539	37.93627	-0.41	16.4	781.04	16.0079998	11	7/24/2014	20:05.8
1680	RWS11	-122.4554017	37.93626	-0.49	16.5	679.37	15.9690005	-122.4554	37.93627	-0.32	16.5	800.67	16.1290005	11	7/24/2014	20:05.9
1681	RWS11	-122.4554052	37.93626	-0.52	16.5	698.23	15.974	-122.4554	37.93627	-0.36	16.5	820.74	16.134	11	7/24/2014	20:06.0
1682	RWS11	-122.4554076	37.93626	-0.49	16.5	717.47	16.0490004	-122.4554	37.93627	-0.36	16.5	841.11	16.1750004	11	7/24/2014	20:06.1

1683	RWS11	-122.4554099	37.93626	-0.49	16.6	736.79	16.0900008	-122.4554	37.93627	-0.41	16.6	861.52	16.1660008	11	7/24/2014	20:06.2
1684	RWS11	-122.4554123	37.93626	-0.49	16.6	756.31	16.1309992	-122.45541	37.93627	-0.41	16.6	882	16.2069992	11	7/24/2014	20:06.3
1685	RWS11	-122.4554146	37.93626	-0.49	16.7	776.12	16.1719996	-122.45541	37.93627	-0.41	16.7	902.86	16.2479996	11	7/24/2014	20:06.4
1686	RWS11	-122.455417	37.93626	-0.49	16.7	795.99	16.2129999	-122.45541	37.93627	-0.41	16.7	923.93	16.2889999	11	7/24/2014	20:06.5
1687	RWS11	-122.4554194	37.93626	-0.49	16.7	815.95	16.2510001	-122.45541	37.93627	-0.41	16.7	945.17	16.3270001	11	7/24/2014	20:06.6
1688	RWS11	-122.4554217	37.93626	-0.49	16.8	836.11	16.2890002	-122.45542	37.93628	-0.36	16.8	966.58	16.4150002	11	7/24/2014	20:06.7
1689	RWS11	-122.4554241	37.93626	-0.52	16.8	856.4	16.2920008	-122.45542	37.93628	-0.41	16.8	987.97	16.4020008	11	7/24/2014	20:06.8
1690	RWS11	-122.4554264	37.93626	-0.52	16.8	876.68	16.3289996	-122.45542	37.93628	-0.41	16.8	1009.5	16.4389996	11	7/24/2014	20:06.9
1691	RWS11	-122.4554299	37.93626	-0.49	16.9	897.03	16.4009997	-122.45542	37.93628	-0.36	16.9	1030.8	16.5269997	11	7/24/2014	20:07.0
1692	RWS11	-122.4554322	37.93627	-0.49	16.9	917.54	16.4389998	-122.45543	37.93628	-0.36	16.9	1052.2	16.5649998	11	7/24/2014	20:07.1
1693	RWS11	-122.4554345	37.93627	-0.49	17	938.22	16.4790007	-122.45543	37.93628	-0.44	17	1073.9	16.5210007	11	7/24/2014	20:07.2
1694	RWS11	-122.4554368	37.93627	-0.49	17	959.11	16.5210005	-122.45543	37.93628	-0.41	17	1095.6	16.5970005	11	7/24/2014	20:07.3
1695	RWS11	-122.4554391	37.93627	-0.52	17.1	980.16	16.5320006	-122.45543	37.93628	-0.41	17.1	1117.6	16.6420006	11	7/24/2014	20:07.4
1696	RWS11	-122.4554415	37.93627	-0.49	17.1	1001.5	16.6120001	-122.45544	37.93628	-0.41	17.1	1139.7	16.6880001	11	7/24/2014	20:07.5
1697	RWS11	-122.4554438	37.93627	-0.49	17.1	1022.9	16.6600004	-122.45544	37.93628	-0.41	17.1	1161.9	16.7360005	11	7/24/2014	20:07.6
1698	RWS11	-122.4554461	37.93627	-0.49	17.2	1044.4	16.7099997	-122.45544	37.93628	-0.36	17.2	1184.1	16.8359997	11	7/24/2014	20:07.7
1699	RWS11	-122.4554484	37.93627	-0.49	17.2	1066.1	16.7600008	-122.45544	37.93628	-0.44	17.2	1206.4	16.8020009	11	7/24/2014	20:07.8
1700	RWS11	-122.4554507	37.93627	-0.49	17.3	1087.9	16.8090006	-122.45545	37.93628	-0.41	17.3	1228.8	16.8850006	11	7/24/2014	20:07.9
1701	RWS11	-122.4554541	37.93627	-0.49	17.3	1109.7	16.856999	-122.45545	37.93628	-0.41	17.3	1251.3	16.932999	11	7/24/2014	20:08.0
1702	RWS11	-122.4554563	37.93627	-0.49	17.4	1131.9	16.9019991	-122.45545	37.93628	-0.41	17.4	1274.1	16.9779991	11	7/24/2014	20:08.1
1703	RWS11	-122.4554585	37.93627	-0.49	17.4	1154.3	16.9440008	-122.45545	37.93629	-0.44	17.4	1297	16.9860009	11	7/24/2014	20:08.2
1704	RWS11	-122.4554608	37.93627	-0.49	17.5	1176.6	16.9849993	-122.45546	37.93629	-0.41	17.5	1319.8	17.0609993	11	7/24/2014	20:08.3
1705	RWS11	-122.4554631	37.93627	-0.49	17.5	1199	17.0240008	-122.45546	37.93629	-0.44	17.5	1342.5	17.0660008	11	7/24/2014	20:08.4
1706	RWS11	-122.4554653	37.93627	-0.49	17.5	1221.5	17.0609995	-122.45546	37.93629	-0.41	17.5	1365.2	17.1369995	11	7/24/2014	20:08.5
1707	RWS11	-122.4554676	37.93627	-0.49	17.6	1243.9	17.0970007	-122.45546	37.93629	-0.44	17.6	1387.8	17.1390007	11	7/24/2014	20:08.6
1708	RWS11	-122.4554698	37.93628	-0.49	17.6	1266.2	17.1309992	-122.45546	37.93629	-0.41	17.6	1410.4	17.2069992	11	7/24/2014	20:08.7
1709	RWS11	-122.4554721	37.93628	-0.49	17.6	1288.3	17.1619993	-122.45547	37.93629	-0.49	17.6	1432.7	17.1529994	11	7/24/2014	20:08.8
1710	RWS11	-122.4554743	37.93628	-0.43	17.7	1310.3	17.2399998	-122.45547	37.93629	-0.41	17.7	1454.8	17.2649998	11	7/24/2014	20:08.9
1711	RWS11	-122.4554777	37.93628	-0.49	17.7	1332.1	17.2090002	-122.45547	37.93629	-0.41	17.7	1476.8	17.2850002	11	7/24/2014	20:09.0
1712	RWS11	-122.4554799	37.93628	-0.49	17.7	1353.8	17.2209994	-122.45547	37.93629	-0.41	17.7	1498.5	17.2969994	11	7/24/2014	20:09.1
1713	RWS11	-122.4554822	37.93628	-0.49	17.7	1375.1	17.2209994	-122.45548	37.93629	-0.41	17.7	1520	17.2969994	11	7/24/2014	20:09.2
1714	RWS11	-122.4554845	37.93628	-0.43	17.7	1396.4	17.2600003	-122.45548	37.93629	-0.44	17.7	1541.1	17.2510003	11	7/24/2014	20:09.3
1715	RWS11	-122.4554868	37.93628	-0.49	17.7	1417.3	17.1810004	-122.45548	37.93629	-0.41	17.7	1562	17.2570004	11	7/24/2014	20:09.4

1716	RWS11	-122.4554891	37.93628	-0.43	17.6	1437.9	17.1870003	-122.45548	37.93629	-0.41	17.6	1582.4	17.2120003	11	7/24/2014	20:09.5
1717	RWS11	-122.4554914	37.93628	-0.49	17.6	1458.5	17.0700003	-122.45549	37.93629	-0.44	17.6	1602.4	17.1120003	11	7/24/2014	20:09.6
1718	RWS11	-122.4554937	37.93628	-0.43	17.5	1478.7	17.0349999	-122.45549	37.93629	-0.41	17.5	1622	17.0599999	11	7/24/2014	20:09.7
1719	RWS11	-122.455496	37.93628	-0.49	17.4	1498.6	16.8769995	-122.45549	37.93629	-0.44	17.4	1641.1	16.9189995	11	7/24/2014	20:09.8
1720	RWS11	-122.4554983	37.93628	-0.43	17.2	1518.2	16.8029995	-122.45549	37.9363	-0.41	17.2	1659.6	16.8279995	11	7/24/2014	20:09.9
1721	RWS11	-122.4555017	37.93628	-0.49	17.1	1537.2	16.6150004	-122.4555	37.9363	-0.41	17.1	1677.4	16.6910004	11	7/24/2014	20:10.0
1722	RWS11	-122.455504	37.93628	-0.43	17	1555.7	16.5300007	-122.4555	37.9363	-0.36	17	1694.7	16.6050007	11	7/24/2014	20:10.1
1723	RWS11	-122.4555063	37.93628	-0.43	16.8	1573.5	16.4099999	-122.4555	37.9363	-0.41	16.8	1711.2	16.4349999	11	7/24/2014	20:10.2
1724	RWS11	-122.4555087	37.93629	-0.43	16.8	1590.6	16.3279991	-122.4555	37.9363	-0.41	16.8	1727.1	16.3529991	11	7/24/2014	20:10.3
1725	RWS11	-122.455511	37.93629	-0.43	16.7	1607.2	16.2950001	-122.45551	37.9363	-0.41	16.7	1742.4	16.3200001	11	7/24/2014	20:10.4
1726	RWS11	-122.4555133	37.93629	-0.43	16.7	1623.1	16.3120003	-122.45551	37.9363	-0.41	16.7	1757	16.3370003	11	7/24/2014	20:10.5
1727	RWS11	-122.4555157	37.93629	-0.49	16.8	1638.2	16.3209997	-122.45551	37.9363	-0.41	16.8	1770.7	16.3969998	11	7/24/2014	20:10.6
1728	RWS11	-122.455518	37.93629	-0.43	16.9	1652.6	16.4629994	-122.45551	37.9363	-0.36	16.9	1783.8	16.5379994	11	7/24/2014	20:10.7
1729	RWS11	-122.4555203	37.93629	-0.43	17	1666.1	16.5739994	-122.45552	37.9363	-0.41	17	1796.1	16.5989994	11	7/24/2014	20:10.8
1730	RWS11	-122.4555226	37.93629	-0.43	17.1	1678.8	16.6889992	-122.45552	37.9363	-0.36	17.1	1807.6	16.7639992	11	7/24/2014	20:10.9
1731	RWS11	-122.4555261	37.93629	-0.43	17.2	1690.6	16.7970009	-122.45552	37.9363	-0.36	17.2	1818.1	16.8720009	11	7/24/2014	20:11.0
1732	RWS11	-122.4555284	37.93629	-0.4	17.3	1701.2	16.9219992	-122.45552	37.9363	-0.32	17.3	1827.5	16.9959992	11	7/24/2014	20:11.1
1733	RWS11	-122.4555307	37.93629	-0.43	17.4	1710.7	16.9540005	-122.45553	37.9363	-0.36	17.4	1836.1	17.0290005	11	7/24/2014	20:11.2
1734	RWS11	-122.455533	37.93629	-0.4	17.4	1719.2	17.0329992	-122.45553	37.9363	-0.36	17.4	1843.7	17.0729992	11	7/24/2014	20:11.3
1735	RWS11	-122.4555353	37.93629	-0.43	17.5	1726.6	17.0179997	-122.45553	37.93631	-0.41	17.5	1850.2	17.0429997	11	7/24/2014	20:11.4
1736	RWS11	-122.4555376	37.93629	-0.43	17.5	1732.9	17.0160008	-122.45553	37.93631	-0.32	17.5	1855.8	17.1250008	11	7/24/2014	20:11.5
1737	RWS11	-122.45554	37.93629	-0.43	17.4	1738.2	16.9939995	-122.45553	37.93631	-0.36	17.4	1860.5	17.0689995	11	7/24/2014	20:11.6
1738	RWS11	-122.4555423	37.93629	-0.4	17.4	1742.5	16.9909994	-122.45554	37.93631	-0.32	17.4	1864.2	17.0649994	11	7/24/2014	20:11.7
1739	RWS11	-122.4555446	37.93629	-0.43	17.3	1745.9	16.9039994	-122.45554	37.93631	-0.36	17.3	1866.7	16.9789994	11	7/24/2014	20:11.8
1740	RWS11	-122.4555469	37.9363	-0.4	17.3	1748.3	16.8750002	-122.45554	37.93631	-0.32	17.3	1868.2	16.9490002	11	7/24/2014	20:11.9
1741	RWS11	-122.4555504	37.9363	-0.43	17.2	1749.8	16.7689991	-122.45555	37.93631	-0.36	17.2	1868.6	16.8439991	11	7/24/2014	20:12.0
1742	RWS11	-122.4555527	37.9363	-0.4	17.1	1750.3	16.7280008	-122.45555	37.93631	-0.32	17.1	1867.8	16.8020008	11	7/24/2014	20:12.1
1743	RWS11	-122.455555	37.9363	-0.43	17	1749.8	16.6130009	-122.45555	37.93631	-0.32	17	1865.8	16.7220009	11	7/24/2014	20:12.2
1744	RWS11	-122.4555574	37.9363	-0.4	17	1748.1	16.5679991	-122.45555	37.93631	-0.36	17	1862.4	16.6079991	11	7/24/2014	20:12.3
1745	RWS11	-122.4555598	37.9363	-0.43	16.9	1745.3	16.455	-122.45555	37.93631	-0.36	16.9	1857.7	16.5299999	11	7/24/2014	20:12.4
1746	RWS11	-122.4555622	37.9363	-0.4	16.8	1741.3	16.4149992	-122.45556	37.93631	-0.29	16.8	1851.8	16.5229992	11	7/24/2014	20:12.5
1747	RWS11	-122.4555645	37.9363	-0.43	16.7	1736	16.3090001	-122.45556	37.93631	-0.36	16.7	1844.4	16.384	11	7/24/2014	20:12.6
1748	RWS11	-122.4555669	37.9363	-0.4	16.7	1729.3	16.2770006	-122.45556	37.93631	-0.32	16.7	1835.7	16.3510006	11	7/24/2014	20:12.7

1749	RWS11	-122.4555693	37.9363	-0.43	16.6	1721.3	16.1790009	-122.45556	37.93631	-0.36	16.6	1825.8	16.2540009	11	7/24/2014	20:12.8
1750	RWS11	-122.4555716	37.9363	-0.4	16.6	1712	16.1539995	-122.45557	37.93631	-0.36	16.6	1814.8	16.1939995	11	7/24/2014	20:12.9
1751	RWS11	-122.4555751	37.9363	-0.43	16.5	1701.6	16.0620003	-122.45557	37.93632	-0.36	16.5	1803	16.1370003	11	7/24/2014	20:13.0
1752	RWS11	-122.4555775	37.9363	-0.4	16.4	1690.3	16.0429995	-122.45557	37.93632	-0.36	16.4	1790.4	16.0829994	11	7/24/2014	20:13.1
1753	RWS11	-122.4555799	37.9363	-0.43	16.4	1677.9	15.9559994	-122.45557	37.93632	-0.41	16.4	1776.9	15.9809994	11	7/24/2014	20:13.2
1754	RWS11	-122.4555823	37.9363	-0.4	16.3	1664.9	15.9400007	-122.45558	37.93632	-0.36	16.3	1762.9	15.9800007	11	7/24/2014	20:13.3
1755	RWS11	-122.4555847	37.9363	-0.43	16.3	1651	15.8549996	-122.45558	37.93632	-0.36	16.3	1748.1	15.9299996	11	7/24/2014	20:13.4
1756	RWS11	-122.4555871	37.93631	-0.4	16.2	1636.4	15.8390009	-122.45558	37.93632	-0.36	16.2	1732.5	15.8790009	11	7/24/2014	20:13.5
1757	RWS11	-122.4555895	37.93631	-0.4	16.2	1621	15.7870008	-122.45558	37.93632	-0.36	16.2	1716	15.8270008	11	7/24/2014	20:13.6
1758	RWS11	-122.455592	37.93631	-0.4	16.1	1605	15.7350008	-122.45559	37.93632	-0.36	16.1	1698.9	15.7750008	11	7/24/2014	20:13.7
1759	RWS11	-122.4555944	37.93631	-0.43	16.1	1588.2	15.6480008	-122.45559	37.93632	-0.32	16.1	1680.8	15.7570007	11	7/24/2014	20:13.8
1760	RWS11	-122.4555968	37.93631	-0.4	16	1570.6	15.6289999	-122.45559	37.93632	-0.36	16	1661.9	15.6689999	11	7/24/2014	20:13.9
1761	RWS11	-122.4556004	37.93631	-0.43	16	1552.3	15.5410004	-122.4556	37.93632	-0.36	16	1642.3	15.6160004	11	7/24/2014	20:14.0
1762	RWS11	-122.4556028	37.93631	-0.4	15.9	1533.3	15.5229999	-122.4556	37.93632	-0.32	15.9	1622.1	15.5969999	11	7/24/2014	20:14.1
1763	RWS11	-122.4556053	37.93631	-0.43	15.9	1513.5	15.4340001	-122.4556	37.93632	-0.36	15.9	1601.2	15.509	11	7/24/2014	20:14.2
1764	RWS11	-122.4556078	37.93631	-0.43	15.8	1493.2	15.3809996	-122.4556	37.93632	-0.36	15.8	1580	15.4559996	11	7/24/2014	20:14.3
1765	RWS11	-122.4556102	37.93631	-0.43	15.8	1472.5	15.3289996	-122.45561	37.93632	-0.36	15.8	1558.4	15.4039995	11	7/24/2014	20:14.4
1766	RWS11	-122.4556127	37.93631	-0.4	15.7	1451.5	15.3110001	-122.45561	37.93632	-0.36	15.7	1536.5	15.351	11	7/24/2014	20:14.5
1767	RWS11	-122.4556152	37.93631	-0.43	15.7	1430.1	15.2250004	-122.45561	37.93633	-0.36	15.7	1514.3	15.3000004	11	7/24/2014	20:14.6
1768	RWS11	-122.4556177	37.93631	-0.4	15.6	1408.4	15.2080004	-122.45561	37.93633	-0.36	15.6	1491.8	15.2480004	11	7/24/2014	20:14.7
1769	RWS11	-122.4556202	37.93631	-0.4	15.6	1386.6	15.1580002	-122.45561	37.93633	-0.41	15.6	1469.1	15.1480002	11	7/24/2014	20:14.8
1770	RWS11	-122.4556226	37.93631	-0.4	15.5	1364.5	15.1090004	-122.45562	37.93633	-0.36	15.5	1446.1	15.1490004	11	7/24/2014	20:14.9
1771	RWS11	-122.4556262	37.93632	-0.43	15.5	1342.1	15.0249997	-122.45562	37.93633	-0.36	15.5	1422.8	15.0999996	11	7/24/2014	20:15.0
1772	RWS11	-122.4556286	37.93632	-0.4	15.4	1319.5	15.0129997	-122.45562	37.93633	-0.36	15.4	1399.2	15.0529997	11	7/24/2014	20:15.1
1773	RWS11	-122.4556309	37.93632	-0.43	15.4	1296.5	14.9329996	-122.45563	37.93633	-0.36	15.4	1375.3	15.0079996	11	7/24/2014	20:15.2
1774	RWS11	-122.4556333	37.93632	-0.4	15.3	1273.1	14.9250004	-122.45563	37.93633	-0.36	15.3	1351.1	14.9650004	11	7/24/2014	20:15.3
1775	RWS11	-122.4556357	37.93632	-0.43	15.3	1249.2	14.849	-122.45563	37.93633	-0.36	15.3	1326.6	14.924	11	7/24/2014	20:15.4
1776	RWS11	-122.4556381	37.93632	-0.4	15.2	1225.1	14.8450005	-122.45563	37.93633	-0.36	15.2	1301.8	14.8850004	11	7/24/2014	20:15.5
1777	RWS11	-122.4556404	37.93632	-0.4	15.2	1200.5	14.8079998	-122.45564	37.93633	-0.36	15.2	1276.6	14.8479998	11	7/24/2014	20:15.6
1778	RWS11	-122.4556428	37.93632	-0.4	15.2	1175.7	14.7719995	-122.45564	37.93633	-0.36	15.2	1251.1	14.8119995	11	7/24/2014	20:15.7
1779	RWS11	-122.4556452	37.93632	-0.43	15.1	1150.8	14.7030001	-122.45564	37.93633	-0.36	15.1	1225.7	14.7780001	11	7/24/2014	20:15.8
1780	RWS11	-122.4556475	37.93632	-0.4	15.1	1126.1	14.7050001	-122.45564	37.93633	-0.36	15.1	1200.3	14.7450001	11	7/24/2014	20:15.9
1781	RWS11	-122.455651	37.93632	-0.4	15.1	1101.2	14.6720001	-122.45565	37.93633	-0.36	15.1	1174.9	14.7120001	11	7/24/2014	20:16.0

1782	RWS11	-122.4556534	37.93632	-0.4	15	1076.7	14.6399996	-122.45565	37.93634	-0.36	15	1149.7	14.6799996	11	7/24/2014	20:16.1
1783	RWS11	-122.4556558	37.93632	-0.43	15	1052.2	14.573	-122.45565	37.93634	-0.36	15	1124.8	14.648	11	7/24/2014	20:16.2
1784	RWS11	-122.4556582	37.93632	-0.4	15	1027.9	14.5760004	-122.45565	37.93634	-0.32	15	1100.1	14.6500004	11	7/24/2014	20:16.3
1785	RWS11	-122.4556606	37.93632	-0.43	14.9	1003.7	14.5100003	-122.45566	37.93634	-0.36	14.9	1075.5	14.5850003	11	7/24/2014	20:16.4
1786	RWS11	-122.455663	37.93632	-0.4	14.9	979.41	14.5149996	-122.45566	37.93634	-0.36	14.9	1051.1	14.5549996	11	7/24/2014	20:16.5
1787	RWS11	-122.4556654	37.93633	-0.43	14.9	955.28	14.4499998	-122.45566	37.93634	-0.36	14.9	1026.8	14.5249998	11	7/24/2014	20:16.6
1788	RWS11	-122.4556679	37.93633	-0.4	14.9	931.26	14.4550001	-122.45566	37.93634	-0.32	14.9	1002.5	14.5290001	11	7/24/2014	20:16.7
1789	RWS11	-122.4556703	37.93633	-0.4	14.8	907.3	14.4250004	-122.45567	37.93634	-0.36	14.8	978.42	14.4650004	11	7/24/2014	20:16.8
1790	RWS11	-122.4556727	37.93633	-0.4	14.8	883.71	14.3949997	-122.45567	37.93634	-0.36	14.8	954.67	14.4349997	11	7/24/2014	20:16.9
1791	RWS11	-122.4556763	37.93633	-0.4	14.8	860.23	14.365	-122.45567	37.93634	-0.36	14.8	930.99	14.4049999	11	7/24/2014	20:17.0
1792	RWS11	-122.4556787	37.93633	-0.4	14.7	837	14.3339998	-122.45567	37.93634	-0.36	14.7	907.46	14.3739998	11	7/24/2014	20:17.1
1793	RWS11	-122.4556811	37.93633	-0.4	14.7	814.17	14.3040001	-122.45568	37.93634	-0.36	14.7	884.36	14.3440001	11	7/24/2014	20:17.2
1794	RWS11	-122.4556835	37.93633	-0.4	14.7	791.71	14.2740004	-122.45568	37.93634	-0.32	14.7	861.5	14.3480004	11	7/24/2014	20:17.3
1795	RWS11	-122.4556859	37.93633	-0.4	14.6	769.44	14.2439997	-122.45568	37.93634	-0.32	14.6	838.73	14.3179997	11	7/24/2014	20:17.4
1796	RWS11	-122.4556883	37.93633	-0.4	14.6	747.28	14.2139999	-122.45568	37.93634	-0.32	14.6	816.04	14.2879999	11	7/24/2014	20:17.5
1797	RWS11	-122.4556908	37.93633	-0.4	14.6	725.28	14.1849996	-122.45569	37.93634	-0.32	14.6	793.55	14.2589997	11	7/24/2014	20:17.6
1798	RWS11	-122.4556932	37.93633	-0.4	14.6	703.47	14.1549999	-122.45569	37.93635	-0.32	14.6	771.38	14.2289999	11	7/24/2014	20:17.7
1799	RWS11	-122.4556956	37.93633	-0.4	14.5	681.74	14.1259996	-122.45569	37.93635	-0.36	14.5	749.3	14.1659996	11	7/24/2014	20:17.8
1800	RWS11	-122.455698	37.93633	-0.4	14.5	660.22	14.0979998	-122.45569	37.93635	-0.32	14.5	727.28	14.1719998	11	7/24/2014	20:17.9
1801	RWS11	-122.4557015	37.93633	-0.43	14.5	639.13	14.0360003	-122.4557	37.93635	-0.32	14.5	705.65	14.1450003	11	7/24/2014	20:18.0
1802	RWS11	-122.4557039	37.93634	-0.4	14.4	618.41	14.0470001	-122.4557	37.93635	-0.32	14.4	684.27	14.1210001	11	7/24/2014	20:18.1
1803	RWS11	-122.4557062	37.93634	-0.4	14.4	598.08	14.0249998	-122.4557	37.93635	-0.36	14.4	663.15	14.0649998	11	7/24/2014	20:18.2
1804	RWS11	-122.4557086	37.93634	-0.4	14.4	578.19	14.0050003	-122.4557	37.93635	-0.32	14.4	642.35	14.0790003	11	7/24/2014	20:18.3
1805	RWS11	-122.455711	37.93634	-0.4	14.4	558.68	13.9869997	-122.45571	37.93635	-0.36	14.4	621.9	14.0269997	11	7/24/2014	20:18.4
1806	RWS11	-122.4557134	37.93634	-0.4	14.4	539.55	13.9720003	-122.45571	37.93635	-0.32	14.4	601.62	14.0460003	11	7/24/2014	20:18.5
1807	RWS11	-122.4557159	37.93634	-0.43	14.4	520.79	13.922	-122.45571	37.93635	-0.36	14.4	581.78	13.997	11	7/24/2014	20:18.6
1808	RWS11	-122.4557183	37.93634	-0.4	14.3	502.27	13.9440004	-122.45571	37.93635	-0.32	14.3	562.26	14.0180004	11	7/24/2014	20:18.7
1809	RWS11	-122.4557208	37.93634	-0.4	14.3	483.88	13.9309999	-122.45572	37.93635	-0.36	14.3	543.05	13.9709999	11	7/24/2014	20:18.8
1810	RWS11	-122.4557233	37.93634	-0.4	14.3	465.61	13.9189999	-122.45572	37.93635	-0.32	14.3	524.34	13.9929999	11	7/24/2014	20:18.9
1811	RWS11	-122.4557258	37.93634	-0.4	14.3	447.54	13.9060003	-122.45572	37.93635	-0.36	14.3	506.03	13.9460003	11	7/24/2014	20:19.0
1812	RWS11	-122.4557282	37.93634	-0.4	14.3	429.77	13.8940002	-122.45572	37.93635	-0.36	14.3	488.08	13.9340002	11	7/24/2014	20:19.1
1813	RWS11	-122.4557307	37.93634	-0.4	14.3	412.31	13.8820002	-122.45573	37.93635	-0.36	14.3	470.47	13.9220001	11	7/24/2014	20:19.2
1814	RWS11	-122.4557332	37.93634	-0.4	14.3	395.12	13.8700001	-122.45573	37.93636	-0.36	14.3	453.19	13.9100001	11	7/24/2014	20:19.3

1815	RWS11	-122.4557356	37.93634	-0.43	14.3	378.17	13.823	-122.45573	37.93636	-0.41	14.3	436.35	13.848	11	7/24/2014	20:19.4
1816	RWS11	-122.4557381	37.93634	-0.4	14.2	361.46	13.8459999	-122.45573	37.93636	-0.36	14.2	419.77	13.8859999	11	7/24/2014	20:19.5
1817	RWS11	-122.4557405	37.93634	-0.4	14.2	345.19	13.8330004	-122.45574	37.93636	-0.41	14.2	403.57	13.8230003	11	7/24/2014	20:19.6
1818	RWS11	-122.4557429	37.93635	-0.4	14.2	329.27	13.8199999	-122.45574	37.93636	-0.36	14.2	387.51	13.8599999	11	7/24/2014	20:19.7
1819	RWS11	-122.4557452	37.93635	-0.4	14.2	313.53	13.8059999	-122.45574	37.93636	-0.41	14.2	371.44	13.7959999	11	7/24/2014	20:19.8
1820	RWS11	-122.4557476	37.93635	-0.43	14.2	298.07	13.7559996	-122.45574	37.93636	-0.36	14.2	355.54	13.8309996	11	7/24/2014	20:19.9
1821	RWS11	-122.4557511	37.93635	-0.4	14.2	282.96	13.7749998	-122.45575	37.93636	-0.41	14.2	340.02	13.7649998	11	7/24/2014	20:20.0
1822	RWS11	-122.4557534	37.93635	-0.4	14.2	268.01	13.759	-122.45575	37.93636	-0.41	14.2	324.36	13.749	11	7/24/2014	20:20.1
1823	RWS11	-122.4557558	37.93635	-0.43	14.1	253.12	13.7060004	-122.45575	37.93636	-0.36	14.1	308.66	13.7810004	11	7/24/2014	20:20.2
1824	RWS11	-122.4557582	37.93635	-0.4	14.1	238.48	13.7229998	-122.45575	37.93636	-0.36	14.1	293.06	13.7629997	11	7/24/2014	20:20.3
1825	RWS11	-122.4557606	37.93635	-0.43	14.1	224.03	13.6680003	-122.45576	37.93636	-0.41	14.1	277.73	13.6930002	11	7/24/2014	20:20.4
1826	RWS11	-122.4557631	37.93635	-0.4	14.1	209.7	13.6840002	-122.45576	37.93636	-0.36	14.1	262.28	13.7240002	11	7/24/2014	20:20.5
1827	RWS11	-122.4557655	37.93635	-0.43	14.1	195.41	13.6289997	-122.45576	37.93636	-0.36	14.1	246.97	13.7039997	11	7/24/2014	20:20.6
1828	RWS11	-122.455768	37.93635	-0.4	14	181.33	13.6440002	-122.45576	37.93636	-0.36	14	231.98	13.6840002	11	7/24/2014	20:20.7
1829	RWS11	-122.4557705	37.93635	-0.43	14	167.4	13.5889998	-122.45577	37.93637	-0.41	14	217.27	13.6139998	11	7/24/2014	20:20.8
1830	RWS11	-122.455773	37.93635	-0.4	14	153.74	13.6049997	-122.45577	37.93637	-0.36	14	202.9	13.6449997	11	7/24/2014	20:20.9
1831	RWS11	-122.4557755	37.93635	-0.43	14	140.48	13.5530005	-122.45577	37.93637	-0.36	14	188.75	13.6280005	11	7/24/2014	20:21.0
1832	RWS11	-122.4557781	37.93635	-0.4	14	127.06	13.5719997	-122.45577	37.93637	-0.36	14	174.69	13.6119997	11	7/24/2014	20:21.1
1833	RWS11	-122.4557806	37.93635	-0.43	14	113.92	13.5209999	-122.45578	37.93637	-0.36	14	160.75	13.5959999	11	7/24/2014	20:21.2
1834	RWS11	-122.4557831	37.93636	-0.4	13.9	100.81	13.5430004	-122.45578	37.93637	-0.36	13.9	146.9	13.5830004	11	7/24/2014	20:21.3
1835	RWS11	-122.4557855	37.93636	-0.43	13.9	87.722	13.4949999	-122.45578	37.93637	-0.36	13.9	133.06	13.5699999	11	7/24/2014	20:21.4
1836	RWS11	-122.455788	37.93636	-0.4	13.9	74.728	13.5179998	-122.45578	37.93637	-0.36	13.9	119.42	13.5579998	11	7/24/2014	20:21.5
1837	RWS11	-122.4557904	37.93636	-0.43	13.9	61.944	13.4720002	-122.45579	37.93637	-0.36	13.9	105.97	13.5470001	11	7/24/2014	20:21.6
1838	RWS11	-122.4557929	37.93636	-0.4	13.9	49.466	13.4969999	-122.45579	37.93637	-0.36	13.9	92.751	13.5369999	11	7/24/2014	20:21.7
1839	RWS11	-122.4557953	37.93636	-0.4	13.9	37.353	13.4869997	-122.45579	37.93637	-0.41	13.9	79.924	13.4769997	11	7/24/2014	20:21.8
1840	RWS11	-122.4557976	37.93636	-0.4	13.9	25.551	13.4779999	-122.45579	37.93637	-0.36	13.9	67.477	13.5179999	11	7/24/2014	20:21.9
1841	RWS11	-122.4558011	37.93636	-0.43	13.9	14.096	13.4340001	-122.4558	37.93637	-0.36	13.9	55.351	13.509	11	7/24/2014	20:22.0
1842	RWS11	-122.4558035	37.93636	-0.4	13.9	2.619	13.4600002	-122.4558	37.93637	-0.36	13.9	43.156	13.5000002	11	7/24/2014	20:22.1
1843	RWS11	-122.4558059	37.93636	-0.4	13.9	-8.749	13.4519998	-122.4558	37.93637	-0.41	13.9	30.917	13.4419998	11	7/24/2014	20:22.2
1844	RWS11	-122.4558083	37.93636	-0.4	13.8	-20.25	13.4440004	-122.4558	37.93637	-0.36	13.8	19.025	13.4840004	11	7/24/2014	20:22.3
1845	RWS11	-122.4558107	37.93636	-0.4	13.8	-31.5	13.4370005	-122.45581	37.93638	-0.41	13.8	7.156	13.4270004	11	7/24/2014	20:22.4
1846	RWS11	-122.4558132	37.93636	-0.4	13.8	-42.68	13.4320003	-122.45581	37.93638	-0.36	13.8	-4.43	13.4720003	11	7/24/2014	20:22.5
1847	RWS11	-122.4558156	37.93636	-0.4	13.8	-53.57	13.4290001	-122.45581	37.93638	-0.41	13.8	-15.755	13.4190001	11	7/24/2014	20:22.6

1848	RWS11	-122.4558181	37.93636	-0.4	13.8	-64.21	13.4270002	-122.45581	37.93638	-0.41	13.8	-26.663	13.4170002	11	7/24/2014	20:22.7
1849	RWS11	-122.4558206	37.93637	-0.4	13.8	-74.49	13.4270002	-122.45582	37.93638	-0.36	13.8	-37.247	13.4670002	11	7/24/2014	20:22.8
1850	RWS11	-122.4558231	37.93637	-0.4	13.8	-84.53	13.4290001	-122.45582	37.93638	-0.41	13.8	-47.588	13.4190001	11	7/24/2014	20:22.9
1851	RWS11	-122.4558256	37.93637	-0.4	13.8	-94.37	13.4320003	-122.45582	37.93638	-0.41	13.8	-57.521	13.4220003	11	7/24/2014	20:23.0
1852	RWS11	-122.4558281	37.93637	-0.4	13.8	-103.9	13.4360001	-122.45582	37.93638	-0.41	13.8	-67.207	13.426	11	7/24/2014	20:23.1
1853	RWS11	-122.4558305	37.93637	-0.43	13.8	-113.4	13.4049998	-122.45583	37.93638	-0.44	13.8	-76.702	13.3959998	11	7/24/2014	20:23.2
1854	RWS11	-122.455833	37.93637	-0.4	13.8	-122.7	13.4449999	-122.45583	37.93638	-0.44	13.8	-85.969	13.4009999	11	7/24/2014	20:23.3
1855	RWS11	-122.4558355	37.93637	-0.4	13.8	-131.8	13.45	-122.45583	37.93638	-0.44	13.8	-95.077	13.406	11	7/24/2014	20:23.4
1856	RWS11	-122.4558379	37.93637	-0.4	13.9	-140.8	13.4550001	-122.45583	37.93638	-0.41	13.9	-104.14	13.4450001	11	7/24/2014	20:23.5
1857	RWS11	-122.4558403	37.93637	-0.4	13.9	-149.8	13.4589998	-122.45583	37.93638	-0.44	13.9	-113.08	13.4149998	11	7/24/2014	20:23.6
1858	RWS11	-122.4558427	37.93637	-0.4	13.9	-158.6	13.4620001	-122.45584	37.93638	-0.44	13.9	-121.6	13.4180001	11	7/24/2014	20:23.7
1859	RWS11	-122.455845	37.93637	-0.4	13.9	-167	13.4650003	-122.45584	37.93638	-0.44	13.9	-129.81	13.4210003	11	7/24/2014	20:23.8
1860	RWS11	-122.4558473	37.93637	-0.4	13.9	-175	13.4670002	-122.45584	37.93639	-0.44	13.9	-137.66	13.4230002	11	7/24/2014	20:23.9
1861	RWS11	-122.4558508	37.93637	-0.4	13.9	-182.7	13.4679996	-122.45585	37.93639	-0.44	13.9	-145.3	13.4239996	11	7/24/2014	20:24.0
1862	RWS11	-122.4558531	37.93637	-0.4	13.9	-189.9	13.4670002	-122.45585	37.93639	-0.44	13.9	-152.73	13.4230002	11	7/24/2014	20:24.1
1863	RWS11	-122.4558554	37.93637	-0.43	13.9	-196.8	13.4300003	-122.45585	37.93639	-0.44	13.9	-160.28	13.4210003	11	7/24/2014	20:24.2
1864	RWS11	-122.4558577	37.93638	-0.4	13.9	-203.6	13.4609997	-122.45585	37.93639	-0.49	13.9	-168.06	13.3659997	11	7/24/2014	20:24.3
1865	RWS11	-122.4558601	37.93638	-0.43	13.9	-210.4	13.4200001	-122.45585	37.93639	-0.44	13.9	-175.91	13.4110001	11	7/24/2014	20:24.4
1866	RWS11	-122.4558625	37.93638	-0.4	13.8	-217	13.4460003	-122.45586	37.93639	-0.49	13.8	-183.66	13.3510003	11	7/24/2014	20:24.5
1867	RWS11	-122.4558649	37.93638	-0.43	13.8	-223.4	13.4010001	-122.45586	37.93639	-0.49	13.8	-191.4	13.3410001	11	7/24/2014	20:24.6
1868	RWS11	-122.4558673	37.93638	-0.4	13.8	-229.8	13.4250004	-122.45586	37.93639	-0.44	13.8	-198.87	13.3810004	11	7/24/2014	20:24.7
1869	RWS11	-122.4558697	37.93638	-0.43	13.8	-236.4	13.3769999	-122.45586	37.93639	-0.49	13.8	-206.27	13.3169999	11	7/24/2014	20:24.8
1870	RWS11	-122.4558722	37.93638	-0.43	13.8	-242.9	13.3640004	-122.45587	37.93639	-0.44	13.8	-213.64	13.3550004	11	7/24/2014	20:24.9
1871	RWS11	-122.4558746	37.93638	-0.43	13.8	-249.3	13.3500004	-122.45587	37.93639	-0.49	13.8	-221.02	13.2900004	11	7/24/2014	20:25.0
1872	RWS11	-122.455877	37.93638	-0.43	13.8	-255.7	13.3369999	-122.45587	37.93639	-0.49	13.8	-228.2	13.2769999	11	7/24/2014	20:25.1
1873	RWS11	-122.4558795	37.93638	-0.43	13.8	-262	13.3249998	-122.45587	37.93639	-0.49	13.8	-235.07	13.2649998	11	7/24/2014	20:25.2
1874	RWS11	-122.4558819	37.93638	-0.43	13.7	-268.1	13.3129998	-122.45588	37.93639	-0.44	13.7	-241.63	13.3039998	11	7/24/2014	20:25.3
1875	RWS11	-122.4558844	37.93638	-0.43	13.7	-274.1	13.3020001	-122.45588	37.9364	-0.49	13.7	-248.11	13.2420001	11	7/24/2014	20:25.4
1876	RWS11	-122.4558868	37.93638	-0.43	13.7	-280.1	13.2919999	-122.45588	37.9364	-0.49	13.7	-254.39	13.2319998	11	7/24/2014	20:25.5
1877	RWS11	-122.4558891	37.93638	-0.43	13.7	-286.1	13.2819996	-122.45588	37.9364	-0.49	13.7	-260.53	13.2219996	11	7/24/2014	20:25.6
1878	RWS11	-122.4558915	37.93638	-0.43	13.7	-291.9	13.2729998	-122.45589	37.9364	-0.44	13.7	-266.6	13.2639998	11	7/24/2014	20:25.7
1879	RWS11	-122.4558938	37.93638	-0.43	13.7	-297.7	13.2650004	-122.45589	37.9364	-0.49	13.7	-272.86	13.2050004	11	7/24/2014	20:25.8
1880	RWS11	-122.4558961	37.93639	-0.43	13.7	-303.4	13.257	-122.45589	37.9364	-0.44	13.7	-278.86	13.248	11	7/24/2014	20:25.9

1881	RWS11	-122.4558996	37.93639	-0.43	13.7	-309.1	13.2489996	-122.45589	37.9364	-0.49	13.7	-284.79	13.1889996	11	7/24/2014	20:26.0
1882	RWS11	-122.4559019	37.93639	-0.43	13.7	-314.6	13.2410002	-122.4559	37.9364	-0.44	13.7	-290.63	13.2320002	11	7/24/2014	20:26.1
1883	RWS11	-122.4559042	37.93639	-0.43	13.7	-320	13.2329998	-122.4559	37.9364	-0.44	13.7	-296.51	13.2239998	11	7/24/2014	20:26.2
1884	RWS11	-122.4559065	37.93639	-0.43	13.7	-325.3	13.224	-122.4559	37.9364	-0.49	13.7	-302.38	13.164	11	7/24/2014	20:26.3
1885	RWS11	-122.4559089	37.93639	-0.43	13.6	-330.6	13.2150002	-122.4559	37.9364	-0.49	13.6	-308.48	13.1550002	11	7/24/2014	20:26.4
1886	RWS11	-122.4559113	37.93639	-0.43	13.6	-335.7	13.205	-122.45591	37.9364	-0.44	13.6	-314.28	13.196	11	7/24/2014	20:26.5
1887	RWS11	-122.4559137	37.93639	-0.43	13.6	-340.6	13.1949997	-122.45591	37.9364	-0.41	13.6	-319.96	13.2199997	11	7/24/2014	20:26.6
1888	RWS11	-122.4559161	37.93639	-0.43	13.6	-345.6	13.1840001	-122.45591	37.9364	-0.49	13.6	-325.57	13.124	11	7/24/2014	20:26.7
1889	RWS11	-122.4559185	37.93639	-0.43	13.6	-350.6	13.172	-122.45591	37.9364	-0.44	13.6	-331	13.163	11	7/24/2014	20:26.8
1890	RWS11	-122.4559209	37.93639	-0.43	13.6	-355.4	13.1599999	-122.45592	37.9364	-0.44	13.6	-336.31	13.1509999	11	7/24/2014	20:26.9
1891	RWS11	-122.4559234	37.93639	-0.43	13.6	-360.2	13.1499997	-122.45592	37.93641	-0.44	13.6	-341.42	13.1409996	11	7/24/2014	20:27.0
1892	RWS11	-122.4559258	37.93639	-0.43	13.6	-364.8	13.1400004	-122.45592	37.93641	-0.41	13.6	-346.4	13.1650004	11	7/24/2014	20:27.1
1893	RWS11	-122.4559282	37.93639	-0.43	13.6	-369.4	13.132	-122.45592	37.93641	-0.44	13.6	-351.27	13.123	11	7/24/2014	20:27.2
1894	RWS11	-122.4559306	37.93639	-0.43	13.6	-373.7	13.125	-122.45593	37.93641	-0.44	13.6	-355.84	13.116	11	7/24/2014	20:27.3
1895	RWS11	-122.455933	37.9364	-0.43	13.6	-378	13.1190005	-122.45593	37.93641	-0.41	13.6	-360.21	13.1440004	11	7/24/2014	20:27.4
1896	RWS11	-122.4559354	37.9364	-0.43	13.5	-382.1	13.1149998	-122.45593	37.93641	-0.41	13.5	-364.46	13.1399998	11	7/24/2014	20:27.5
1897	RWS11	-122.4559378	37.9364	-0.43	13.5	-386	13.113	-122.45593	37.93641	-0.44	13.5	-368.23	13.1039999	11	7/24/2014	20:27.6
1898	RWS11	-122.4559401	37.9364	-0.4	13.5	-389.7	13.1469995	-122.45593	37.93641	-0.41	13.5	-372.06	13.1369995	11	7/24/2014	20:27.7
1899	RWS11	-122.4559424	37.9364	-0.43	13.5	-393.2	13.1119995	-122.45594	37.93641	-0.41	13.5	-375.74	13.1369995	11	7/24/2014	20:27.8
1900	RWS11	-122.4559447	37.9364	-0.4	13.5	-396.7	13.1490004	-122.45594	37.93641	-0.41	13.5	-379.34	13.1390003	11	7/24/2014	20:27.9
1901	RWS11	-122.4559481	37.9364	-0.43	13.6	-399.9	13.1180001	-122.45594	37.93641	-0.41	13.6	-382.76	13.143	11	7/24/2014	20:28.0
1902	RWS11	-122.4559504	37.9364	-0.43	13.6	-403	13.1219998	-122.45595	37.93641	-0.41	13.6	-386.24	13.1469998	11	7/24/2014	20:28.1
1903	RWS11	-122.4559527	37.9364	-0.4	13.6	-406.1	13.1630003	-122.45595	37.93641	-0.41	13.6	-389.45	13.1530003	11	7/24/2014	20:28.2
1904	RWS11	-122.455955	37.9364	-0.4	13.6	-408.9	13.1689999	-122.45595	37.93641	-0.41	13.6	-392.52	13.1589998	11	7/24/2014	20:28.3
1905	RWS11	-122.4559574	37.9364	-0.4	13.6	-411.7	13.1759998	-122.45595	37.93641	-0.41	13.6	-395.53	13.1659998	11	7/24/2014	20:28.4
1906	RWS11	-122.4559597	37.9364	-0.4	13.6	-414.4	13.1829998	-122.45595	37.93642	-0.36	13.6	-398.41	13.2229998	11	7/24/2014	20:28.5
1907	RWS11	-122.455962	37.9364	-0.43	13.6	-416.9	13.1529999	-122.45596	37.93642	-0.41	13.6	-401.1	13.1779999	11	7/24/2014	20:28.6
1908	RWS11	-122.4559643	37.9364	-0.4	13.6	-419.2	13.1910002	-122.45596	37.93642	-0.36	13.6	-403.67	13.2310002	11	7/24/2014	20:28.7
1909	RWS11	-122.4559666	37.9364	-0.4	13.6	-421.3	13.1919996	-122.45596	37.93642	-0.36	13.6	-406.06	13.2319996	11	7/24/2014	20:28.8
1910	RWS11	-122.4559689	37.9364	-0.4	13.6	-423.2	13.1919996	-122.45596	37.93642	-0.36	13.6	-408.37	13.2319996	11	7/24/2014	20:28.9
1911	RWS11	-122.4559723	37.93641	-0.43	13.6	-425.3	13.1540003	-122.45597	37.93642	-0.32	13.6	-410.8	13.2630003	11	7/24/2014	20:29.0
1912	RWS11	-122.4559745	37.93641	-0.4	13.6	-427.3	13.1860001	-122.45597	37.93642	-0.32	13.6	-413.47	13.2600001	11	7/24/2014	20:29.1
1913	RWS11	-122.4559768	37.93641	-0.4	13.6	-429.6	13.1820003	-122.45597	37.93642	-0.32	13.6	-416.25	13.2560003	11	7/24/2014	20:29.2

1914	RWS11	-122.4559791	37.93641	-0.35	13.6	-432.3	13.2289997	-122.45597	37.93642	-0.29	13.6	-419.36	13.2859997	11	7/24/2014	20:29.3
1915	RWS11	-122.4559814	37.93641	-0.4	13.6	-435.1	13.174	-122.45598	37.93642	-0.32	13.6	-422.49	13.248	11	7/24/2014	20:29.4
1916	RWS11	-122.4559838	37.93641	-0.35	13.6	-437.9	13.2219997	-122.45598	37.93642	-0.32	13.6	-425.79	13.2449997	11	7/24/2014	20:29.5
1917	RWS11	-122.4559861	37.93641	-0.4	13.6	-440.7	13.1689999	-122.45598	37.93642	-0.36	13.6	-428.9	13.2089998	11	7/24/2014	20:29.6
1918	RWS11	-122.4559884	37.93641	-0.35	13.6	-443.1	13.2190004	-122.45598	37.93642	-0.32	13.6	-431.46	13.2420004	11	7/24/2014	20:29.7
1919	RWS11	-122.4559907	37.93641	-0.4	13.6	-445.3	13.1689999	-122.45599	37.93642	-0.36	13.6	-433.73	13.2089998	11	7/24/2014	20:29.8
1920	RWS11	-122.4559929	37.93641	-0.4	13.6	-446.9	13.1709997	-122.45599	37.93642	-0.29	13.6	-435.36	13.2789997	11	7/24/2014	20:29.9
1921	RWS11	-122.4559964	37.93641	-0.4	13.6	-448.1	13.1759998	-122.45599	37.93642	-0.32	13.6	-436.52	13.2499998	11	7/24/2014	20:30.0
1922	RWS11	-122.4559987	37.93641	-0.4	13.6	-449.1	13.1820003	-122.45599	37.93643	-0.32	13.6	-437.37	13.2560003	11	7/24/2014	20:30.1
1923	RWS11	-122.456001	37.93641	-0.4	13.6	-450	13.1910002	-122.456	37.93643	-0.36	13.6	-438	13.2310002	11	7/24/2014	20:30.2
1924	RWS11	-122.4560033	37.93641	-0.4	13.6	-450.6	13.2010004	-122.456	37.93643	-0.32	13.6	-438.33	13.2750004	11	7/24/2014	20:30.3
1925	RWS11	-122.4560057	37.93641	-0.4	13.6	-451.2	13.2120001	-122.456	37.93643	-0.32	13.6	-438.45	13.2860001	11	7/24/2014	20:30.4
1926	RWS11	-122.456008	37.93642	-0.4	13.6	-451.6	13.2229998	-122.456	37.93643	-0.32	13.6	-438.39	13.2969998	11	7/24/2014	20:30.5
1927	RWS11	-122.4560104	37.93642	-0.4	13.6	-451.8	13.2340004	-122.45601	37.93643	-0.36	13.6	-438.08	13.2740004	11	7/24/2014	20:30.6
1928	RWS11	-122.4560127	37.93642	-0.4	13.6	-452	13.2450001	-122.45601	37.93643	-0.32	13.6	-437.78	13.3190001	11	7/24/2014	20:30.7
1929	RWS11	-122.456015	37.93642	-0.4	13.7	-452	13.2539999	-122.45601	37.93643	-0.36	13.7	-437.53	13.2939999	11	7/24/2014	20:30.8
1930	RWS11	-122.4560174	37.93642	-0.35	13.7	-451.7	13.3119999	-122.45601	37.93643	-0.36	13.7	-437.07	13.3009999	11	7/24/2014	20:30.9
1931	RWS11	-122.4560208	37.93642	-0.43	13.7	-451.3	13.2320004	-122.45602	37.93643	-0.41	13.7	-436.42	13.2570004	11	7/24/2014	20:31.0
1932	RWS11	-122.4560231	37.93642	-0.4	13.7	-450.7	13.2710001	-122.45602	37.93643	-0.36	13.7	-435.6	13.3110001	11	7/24/2014	20:31.1
1933	RWS11	-122.4560254	37.93642	-0.4	13.7	-449.8	13.2740004	-122.45602	37.93643	-0.41	13.7	-434.69	13.2640003	11	7/24/2014	20:31.2
1934	RWS11	-122.4560278	37.93642	-0.4	13.7	-448.9	13.2760002	-122.45602	37.93643	-0.36	13.7	-433.71	13.3160002	11	7/24/2014	20:31.3
1935	RWS11	-122.4560301	37.93642	-0.4	13.7	-448.1	13.2769997	-122.45602	37.93643	-0.41	13.7	-432.76	13.2669996	11	7/24/2014	20:31.4
1936	RWS11	-122.4560325	37.93642	-0.4	13.7	-447.4	13.2790005	-122.45603	37.93643	-0.41	13.7	-432.11	13.2690004	11	7/24/2014	20:31.5
1937	RWS11	-122.4560348	37.93642	-0.4	13.7	-446.9	13.2799999	-122.45603	37.93643	-0.41	13.7	-431.55	13.2699999	11	7/24/2014	20:31.6
1938	RWS11	-122.4560371	37.93642	-0.4	13.7	-446.2	13.2830002	-122.45603	37.93644	-0.36	13.7	-431.09	13.3230002	11	7/24/2014	20:31.7
1939	RWS11	-122.4560395	37.93642	-0.4	13.7	-445.6	13.285	-122.45603	37.93644	-0.41	13.7	-430.49	13.275	11	7/24/2014	20:31.8
1940	RWS11	-122.4560418	37.93642	-0.4	13.7	-444.8	13.2880003	-122.45604	37.93644	-0.36	13.7	-429.64	13.3280003	11	7/24/2014	20:31.9
1941	RWS11	-122.4560452	37.93642	-0.4	13.7	-443.9	13.2900001	-122.45604	37.93644	-0.41	13.7	-428.74	13.2800001	11	7/24/2014	20:32.0
1942	RWS11	-122.4560475	37.93643	-0.4	13.7	-442.9	13.2909996	-122.45604	37.93644	-0.36	13.7	-427.72	13.3309996	11	7/24/2014	20:32.1
1943	RWS11	-122.4560498	37.93643	-0.4	13.7	-441.8	13.2909996	-122.45604	37.93644	-0.41	13.7	-426.45	13.2809996	11	7/24/2014	20:32.2
1944	RWS11	-122.4560521	37.93643	-0.4	13.7	-440.5	13.2909996	-122.45605	37.93644	-0.36	13.7	-424.92	13.3309996	11	7/24/2014	20:32.3
1945	RWS11	-122.4560544	37.93643	-0.4	13.7	-439.1	13.2909996	-122.45605	37.93644	-0.41	13.7	-423.5	13.2809996	11	7/24/2014	20:32.4
1946	RWS11	-122.4560567	37.93643	-0.4	13.7	-437.8	13.2900001	-122.45605	37.93644	-0.32	13.7	-421.92	13.3640001	11	7/24/2014	20:32.5

1947	RWS11	-122.4560591	37.93643	-0.4	13.7	-436.6	13.2900001	-122.45605	37.93644	-0.41	13.7	-420.34	13.2800001	11	7/24/2014	20:32.6
1948	RWS11	-122.4560614	37.93643	-0.4	13.7	-435.2	13.2889997	-122.45606	37.93644	-0.36	13.7	-418.56	13.3289997	11	7/24/2014	20:32.7
1949	RWS11	-122.4560637	37.93643	-0.4	13.7	-433.8	13.2880003	-122.45606	37.93644	-0.41	13.7	-417.13	13.2780003	11	7/24/2014	20:32.8
1950	RWS11	-122.456066	37.93643	-0.4	13.7	-432.3	13.2880003	-122.45606	37.93644	-0.32	13.7	-415.51	13.3620003	11	7/24/2014	20:32.9
1951	RWS11	-122.4560694	37.93643	-0.4	13.7	-430.8	13.2880003	-122.45606	37.93644	-0.36	13.7	-413.84	13.3280003	11	7/24/2014	20:33.0
1952	RWS11	-122.4560717	37.93643	-0.4	13.7	-429.3	13.2889997	-122.45607	37.93644	-0.36	13.7	-412.28	13.3289997	11	7/24/2014	20:33.1
1953	RWS11	-122.456074	37.93643	-0.4	13.7	-427.8	13.2909996	-122.45607	37.93645	-0.36	13.7	-410.55	13.3309996	11	7/24/2014	20:33.2
1954	RWS11	-122.4560764	37.93643	-0.4	13.7	-426.1	13.2939999	-122.45607	37.93645	-0.32	13.7	-408.73	13.3679999	11	7/24/2014	20:33.3
1955	RWS11	-122.4560787	37.93643	-0.4	13.7	-424.3	13.299	-122.45607	37.93645	-0.36	13.7	-406.76	13.339	11	7/24/2014	20:33.4
1956	RWS11	-122.4560811	37.93643	-0.4	13.7	-422.5	13.3059999	-122.45608	37.93645	-0.36	13.7	-404.55	13.3459999	11	7/24/2014	20:33.5
1957	RWS11	-122.4560835	37.93643	-0.4	13.7	-420.5	13.3160002	-122.45608	37.93645	-0.32	13.7	-402.02	13.3900002	11	7/24/2014	20:33.6
1958	RWS11	-122.4560858	37.93644	-0.35	13.7	-418.4	13.3790003	-122.45608	37.93645	-0.32	13.7	-399.38	13.4020003	11	7/24/2014	20:33.7
1959	RWS11	-122.4560881	37.93644	-0.4	13.7	-416.1	13.3420002	-122.45608	37.93645	-0.36	13.7	-396.29	13.3820002	11	7/24/2014	20:33.8
1960	RWS11	-122.4560904	37.93644	-0.4	13.8	-413.6	13.3590004	-122.45609	37.93645	-0.32	13.8	-393.12	13.4330004	11	7/24/2014	20:33.9
1961	RWS11	-122.4560939	37.93644	-0.4	13.8	-410.9	13.377	-122.45609	37.93645	-0.36	13.8	-389.68	13.417	11	7/24/2014	20:34.0
1962	RWS11	-122.4560962	37.93644	-0.4	13.8	-407.7	13.3979999	-122.45609	37.93645	-0.36	13.8	-386.06	13.4379999	11	7/24/2014	20:34.1
1963	RWS11	-122.4560985	37.93644	-0.4	13.8	-404.4	13.4200003	-122.45609	37.93645	-0.36	13.8	-382.34	13.4600003	11	7/24/2014	20:34.2
1964	RWS11	-122.4561008	37.93644	-0.4	13.8	-401	13.4440004	-122.4561	37.93645	-0.32	13.8	-378.51	13.5180004	11	7/24/2014	20:34.3
1965	RWS11	-122.4561031	37.93644	-0.4	13.9	-397.3	13.4700005	-122.4561	37.93645	-0.36	13.9	-374.63	13.5100004	11	7/24/2014	20:34.4
1966	RWS11	-122.4561055	37.93644	-0.4	13.9	-393.6	13.4969999	-122.4561	37.93645	-0.36	13.9	-370.56	13.5369999	11	7/24/2014	20:34.5
1967	RWS11	-122.4561078	37.93644	-0.4	13.9	-389.8	13.5249998	-122.4561	37.93645	-0.36	13.9	-366.51	13.5649998	11	7/24/2014	20:34.6
1968	RWS11	-122.4561101	37.93644	-0.4	14	-386.2	13.5550005	-122.4561	37.93645	-0.36	14	-362.33	13.5950005	11	7/24/2014	20:34.7
1969	RWS11	-122.4561124	37.93644	-0.4	14	-382.3	13.5850002	-122.45611	37.93645	-0.41	14	-358.11	13.5750002	11	7/24/2014	20:34.8
1970	RWS11	-122.4561147	37.93644	-0.43	14	-378.5	13.5819998	-122.45611	37.93646	-0.36	14	-353.8	13.6569998	11	7/24/2014	20:34.9
1971	RWS11	-122.4561182	37.93644	-0.43	14	-374.6	13.6149998	-122.45611	37.93646	-0.36	14	-349.36	13.6899998	11	7/24/2014	20:35.0
1972	RWS11	-122.4561205	37.93644	-0.4	14.1	-370.4	13.6840002	-122.45612	37.93646	-0.41	14.1	-344.56	13.6740002	11	7/24/2014	20:35.1
1973	RWS11	-122.4561229	37.93644	-0.43	14.1	-366.2	13.6840001	-122.45612	37.93646	-0.36	14.1	-339.73	13.759	11	7/24/2014	20:35.2
1974	RWS11	-122.4561252	37.93645	-0.4	14.2	-361.8	13.7539999	-122.45612	37.93646	-0.36	14.2	-334.84	13.7939999	11	7/24/2014	20:35.3
1975	RWS11	-122.4561276	37.93645	-0.43	14.2	-357.2	13.7550001	-122.45612	37.93646	-0.41	14.2	-329.83	13.7800001	11	7/24/2014	20:35.4
1976	RWS11	-122.45613	37.93645	-0.4	14.2	-352.7	13.8269998	-122.45612	37.93646	-0.41	14.2	-324.83	13.8169998	11	7/24/2014	20:35.5
1977	RWS11	-122.4561324	37.93645	-0.43	14.3	-348	13.8310004	-122.45613	37.93646	-0.41	14.3	-319.91	13.8560003	11	7/24/2014	20:35.6
1978	RWS11	-122.4561348	37.93645	-0.43	14.3	-343.5	13.8710003	-122.45613	37.93646	-0.41	14.3	-315.07	13.8960003	11	7/24/2014	20:35.7
1979	RWS11	-122.4561372	37.93645	-0.43	14.3	-338.9	13.9130001	-122.45613	37.93646	-0.41	14.3	-309.99	13.9380001	11	7/24/2014	20:35.8

1980	RWS11	-122.4561395	37.93645	-0.43	14.4	-334.3	13.9560004	-122.45613	37.93646	-0.41	14.4	-304.83	13.9810003	11	7/24/2014	20:35.9
1981	RWS11	-122.456143	37.93645	-0.43	14.4	-329.6	13.9989996	-122.45614	37.93646	-0.41	14.4	-299.73	14.0239996	11	7/24/2014	20:36.0
1982	RWS11	-122.4561453	37.93645	-0.43	14.5	-324.8	14.0430003	-122.45614	37.93646	-0.41	14.5	-294.67	14.0680002	11	7/24/2014	20:36.1
1983	RWS11	-122.4561477	37.93645	-0.43	14.5	-320.1	14.0860005	-122.45614	37.93646	-0.41	14.5	-289.49	14.1110005	11	7/24/2014	20:36.2
1984	RWS11	-122.4561501	37.93645	-0.43	14.6	-315.5	14.1269999	-122.45614	37.93646	-0.41	14.6	-284.25	14.1519999	11	7/24/2014	20:36.3
1985	RWS11	-122.4561524	37.93645	-0.49	14.6	-310.9	14.1159998	-122.45615	37.93646	-0.41	14.6	-279.21	14.1919998	11	7/24/2014	20:36.4
1986	RWS11	-122.4561548	37.93645	-0.43	14.6	-306.2	14.205	-122.45615	37.93647	-0.41	14.6	-274.14	14.2299999	11	7/24/2014	20:36.5
1987	RWS11	-122.4561572	37.93645	-0.49	14.7	-301.5	14.1900002	-122.45615	37.93647	-0.41	14.7	-269.01	14.2660002	11	7/24/2014	20:36.6
1988	RWS11	-122.4561595	37.93645	-0.43	14.7	-296.7	14.2760001	-122.45615	37.93647	-0.41	14.7	-263.79	14.301	11	7/24/2014	20:36.7
1989	RWS11	-122.4561619	37.93645	-0.49	14.7	-291.7	14.2590004	-122.45616	37.93647	-0.41	14.7	-258.25	14.3350004	11	7/24/2014	20:36.8
1990	RWS11	-122.4561642	37.93645	-0.49	14.8	-286.5	14.291	-122.45616	37.93647	-0.41	14.8	-252.38	14.367	11	7/24/2014	20:36.9
1991	RWS11	-122.4561677	37.93646	-0.49	14.8	-281.2	14.3209997	-122.45616	37.93647	-0.41	14.8	-246.35	14.3969998	11	7/24/2014	20:37.0
1992	RWS11	-122.45617	37.93646	-0.49	14.8	-275.7	14.35	-122.45616	37.93647	-0.41	14.8	-240.1	14.426	11	7/24/2014	20:37.1
1993	RWS11	-122.4561724	37.93646	-0.49	14.9	-270.1	14.3770005	-122.45617	37.93647	-0.41	14.9	-233.58	14.4530005	11	7/24/2014	20:37.2
1994	RWS11	-122.4561747	37.93646	-0.49	14.9	-264.3	14.4039999	-122.45617	37.93647	-0.41	14.9	-226.94	14.4799999	11	7/24/2014	20:37.3
1995	RWS11	-122.4561771	37.93646	-0.49	14.9	-258.2	14.43	-122.45617	37.93647	-0.41	14.9	-220.03	14.506	11	7/24/2014	20:37.4
1996	RWS11	-122.4561795	37.93646	-0.48	14.9	-252	14.4559996	-122.45617	37.93647	-0.41	14.9	-213.07	14.5319996	11	7/24/2014	20:37.5
1997	RWS11	-122.4561819	37.93646	-0.48	15	-245.5	14.4810002	-122.45618	37.93647	-0.44	15	-205.89	14.5230002	11	7/24/2014	20:37.6
1998	RWS11	-122.4561842	37.93646	-0.43	15	-238.9	14.5580002	-122.45618	37.93647	-0.36	15	-198.46	14.6330002	11	7/24/2014	20:37.7
1999	RWS11	-122.4561866	37.93646	-0.48	15	-232.1	14.5330002	-122.45618	37.93647	-0.41	15	-190.78	14.6090002	11	7/24/2014	20:37.8
2000	RWS11	-122.4561889	37.93646	-0.48	15	-225.3	14.5590002	-122.45618	37.93647	-0.41	15	-183.25	14.6350002	11	7/24/2014	20:37.9
2001	RWS11	-122.4561924	37.93646	-0.48	15.1	-218.5	14.5870001	-122.45619	37.93647	-0.41	15.1	-175.7	14.6630001	11	7/24/2014	20:38.0
2002	RWS11	-122.4561948	37.93646	-0.43	15.1	-211.7	14.6679998	-122.45619	37.93648	-0.41	15.1	-168.14	14.6929998	11	7/24/2014	20:38.1
2003	RWS11	-122.4561971	37.93646	-0.48	15.1	-204.9	14.6469996	-122.45619	37.93648	-0.44	15.1	-160.68	14.6889996	11	7/24/2014	20:38.2
2004	RWS11	-122.4561995	37.93646	-0.43	15.2	-198.2	14.7289997	-122.45619	37.93648	-0.41	15.2	-153.2	14.7539997	11	7/24/2014	20:38.3
2005	RWS11	-122.4562019	37.93646	-0.48	15.2	-191.4	14.7100003	-122.4562	37.93648	-0.44	15.2	-145.52	14.7520002	11	7/24/2014	20:38.4
2006	RWS11	-122.4562043	37.93646	-0.43	15.2	-184.6	14.7929998	-122.4562	37.93648	-0.41	15.2	-137.38	14.8179998	11	7/24/2014	20:38.5
2007	RWS11	-122.4562067	37.93647	-0.48	15.3	-177.6	14.773	-122.4562	37.93648	-0.41	15.3	-128.99	14.849	11	7/24/2014	20:38.6
2008	RWS11	-122.4562091	37.93647	-0.43	15.3	-170.5	14.8539997	-122.4562	37.93648	-0.44	15.3	-120.59	14.8449997	11	7/24/2014	20:38.7
2009	RWS11	-122.4562114	37.93647	-0.48	15.3	-163.3	14.8309996	-122.45621	37.93648	-0.44	15.3	-112.24	14.8729996	11	7/24/2014	20:38.8
2010	RWS11	-122.4562138	37.93647	-0.48	15.3	-156	14.8549998	-122.45621	37.93648	-0.41	15.3	-103.78	14.9309998	11	7/24/2014	20:38.9
2011	RWS11	-122.4562173	37.93647	-0.48	15.4	-148.7	14.8770001	-122.45621	37.93648	-0.44	15.4	-95.465	14.9190001	11	7/24/2014	20:39.0
2012	RWS11	-122.4562197	37.93647	-0.43	15.4	-141.5	14.9459997	-122.45621	37.93648	-0.44	15.4	-87.445	14.9369997	11	7/24/2014	20:39.1

2013	RWS11	-122.456222	37.93647	-0.48	15.4	-134.3	14.9100001	-122.45622	37.93648	-0.44	15.4	-79.65	14.9520001	11	7/24/2014	20:39.2
2014	RWS11	-122.4562244	37.93647	-0.43	15.4	-127.3	14.9719997	-122.45622	37.93648	-0.44	15.4	-71.978	14.9629997	11	7/24/2014	20:39.3
2015	RWS11	-122.4562268	37.93647	-0.48	15.4	-120.4	14.9279997	-122.45622	37.93648	-0.44	15.4	-64.37	14.9699997	11	7/24/2014	20:39.4
2016	RWS11	-122.4562293	37.93647	-0.48	15.4	-113.5	14.9329998	-122.45622	37.93648	-0.41	15.4	-56.598	15.0089998	11	7/24/2014	20:39.5
2017	RWS11	-122.4562317	37.93647	-0.48	15.4	-106.5	14.9360001	-122.45623	37.93648	-0.41	15.4	-48.723	15.0120001	11	7/24/2014	20:39.6
2018	RWS11	-122.4562342	37.93647	-0.43	15.4	-99.54	14.9889999	-122.45623	37.93648	-0.41	15.4	-40.714	15.0139999	11	7/24/2014	20:39.7
2019	RWS11	-122.4562367	37.93647	-0.48	15.4	-92.43	14.9390003	-122.45623	37.93649	-0.44	15.4	-32.517	14.9810003	11	7/24/2014	20:39.8
2020	RWS11	-122.4562392	37.93647	-0.43	15.4	-85.17	14.9900003	-122.45623	37.93649	-0.41	15.4	-23.895	15.0150003	11	7/24/2014	20:39.9
2021	RWS11	-122.4562417	37.93647	-0.48	15.4	-77.83	14.9390003	-122.45624	37.93649	-0.44	15.4	-15.104	14.9810003	11	7/24/2014	20:40.0
2022	RWS11	-122.4562442	37.93647	-0.48	15.4	-70.19	14.9379999	-122.45624	37.93649	-0.41	15.4	-6.092	15.0139999	11	7/24/2014	20:40.1
2023	RWS11	-122.4562467	37.93648	-0.48	15.4	-62.41	14.9369995	-122.45624	37.93649	-0.44	15.4	3.094	14.9789995	11	7/24/2014	20:40.2
2024	RWS11	-122.4562492	37.93648	-0.43	15.4	-54.42	14.9870001	-122.45624	37.93649	-0.41	15.4	12.476	15.0120001	11	7/24/2014	20:40.3
2025	RWS11	-122.4562516	37.93648	-0.48	15.4	-46.28	14.9349997	-122.45625	37.93649	-0.44	15.4	21.749	14.9769997	11	7/24/2014	20:40.4
2026	RWS11	-122.456254	37.93648	-0.48	15.4	-37.99	14.9329998	-122.45625	37.93649	-0.44	15.4	31.176	14.9749998	11	7/24/2014	20:40.5
2027	RWS11	-122.4562565	37.93648	-0.48	15.4	-29.63	14.931	-122.45625	37.93649	-0.44	15.4	40.666	14.973	11	7/24/2014	20:40.6
2028	RWS11	-122.4562588	37.93648	-0.48	15.4	-21.25	14.9290001	-122.45625	37.93649	-0.44	15.4	50.114	14.9710001	11	7/24/2014	20:40.7
2029	RWS11	-122.4562612	37.93648	-0.48	15.4	-12.85	14.9279997	-122.45626	37.93649	-0.44	15.4	59.65	14.9699997	11	7/24/2014	20:40.8
2030	RWS11	-122.4562635	37.93648	-0.43	15.4	-4.214	14.9780003	-122.45626	37.93649	-0.44	15.4	69.401	14.9690003	11	7/24/2014	20:40.9
2031	RWS11	-122.456267	37.93648	-0.48	15.4	4.732	14.9250004	-122.45626	37.93649	-0.44	15.4	79.462	14.9670004	11	7/24/2014	20:41.0
2032	RWS11	-122.4562693	37.93648	-0.43	15.4	13.94	14.975	-122.45626	37.93649	-0.44	15.4	89.562	14.966	11	7/24/2014	20:41.1
2033	RWS11	-122.4562716	37.93648	-0.48	15.4	23.124	14.9229996	-122.45627	37.93649	-0.44	15.4	99.645	14.9649996	11	7/24/2014	20:41.2
2034	RWS11	-122.456274	37.93648	-0.48	15.4	32.483	14.9209997	-122.45627	37.93649	-0.44	15.4	109.78	14.9629997	11	7/24/2014	20:41.3
2035	RWS11	-122.4562764	37.93648	-0.48	15.4	41.907	14.9200003	-122.45627	37.9365	-0.49	15.4	120.04	14.9110003	11	7/24/2014	20:41.4
2036	RWS11	-122.4562788	37.93648	-0.43	15.4	51.443	14.9690004	-122.45627	37.9365	-0.41	15.4	130.53	14.9940004	11	7/24/2014	20:41.5
2037	RWS11	-122.4562812	37.93648	-0.48	15.4	61.041	14.917	-122.45628	37.9365	-0.44	15.4	141.14	14.959	11	7/24/2014	20:41.6
2038	RWS11	-122.4562836	37.93648	-0.48	15.4	70.621	14.9159996	-122.45628	37.9365	-0.44	15.4	151.75	14.9579996	11	7/24/2014	20:41.7
2039	RWS11	-122.456286	37.93648	-0.48	15.4	80.202	14.9159996	-122.45628	37.9365	-0.49	15.4	162.42	14.9069996	11	7/24/2014	20:41.8
2040	RWS11	-122.4562885	37.93649	-0.48	15.4	89.799	14.9159996	-122.45628	37.9365	-0.44	15.4	173.16	14.9579996	11	7/24/2014	20:41.9
2041	RWS11	-122.4562909	37.93649	-0.48	15.4	99.401	14.9159996	-122.45629	37.9365	-0.44	15.4	183.99	14.9579996	11	7/24/2014	20:42.0
2042	RWS11	-122.4562934	37.93649	-0.43	15.4	109.18	14.968	-122.45629	37.9365	-0.41	15.4	194.99	14.993	11	7/24/2014	20:42.1
2043	RWS11	-122.4562958	37.93649	-0.48	15.4	119.06	14.9200003	-122.45629	37.9365	-0.41	15.4	206.07	14.9960003	11	7/24/2014	20:42.2
2044	RWS11	-122.4562983	37.93649	-0.43	15.4	129.07	14.9739996	-122.45629	37.9365	-0.41	15.4	217.18	14.9989996	11	7/24/2014	20:42.3
2045	RWS11	-122.4563007	37.93649	-0.48	15.4	139.02	14.9290001	-122.4563	37.9365	-0.44	15.4	228.35	14.9710001	11	7/24/2014	20:42.4

2046	RWS11	-122.4563031	37.93649	-0.43	15.4	149	14.9859997	-122.4563	37.9365	-0.41	15.4	239.39	15.0109997	11	7/24/2014	20:42.5
2047	RWS11	-122.4563055	37.93649	-0.43	15.4	158.97	14.9929996	-122.4563	37.9365	-0.44	15.4	250.39	14.9839996	11	7/24/2014	20:42.6
2048	RWS11	-122.4563079	37.93649	-0.43	15.4	169.03	15.001	-122.4563	37.9365	-0.44	15.4	261.4	14.992	11	7/24/2014	20:42.7
2049	RWS11	-122.4563102	37.93649	-0.48	15.4	179	14.957	-122.45631	37.9365	-0.44	15.4	272.35	14.999	11	7/24/2014	20:42.8
2050	RWS11	-122.4563126	37.93649	-0.43	15.4	188.95	15.015	-122.45631	37.9365	-0.41	15.4	283.46	15.04	11	7/24/2014	20:42.9
2051	RWS11	-122.456316	37.93649	-0.48	15.5	198.96	14.9690001	-122.45631	37.9365	-0.41	15.5	294.71	15.0450001	11	7/24/2014	20:43.0
2052	RWS11	-122.4563184	37.93649	-0.43	15.5	209.05	15.0250002	-122.45631	37.93651	-0.41	15.5	306.12	15.0500002	11	7/24/2014	20:43.1
2053	RWS11	-122.4563207	37.93649	-0.48	15.5	219.22	14.9770005	-122.45632	37.93651	-0.44	15.5	317.82	15.0190004	11	7/24/2014	20:43.2
2054	RWS11	-122.4563231	37.93649	-0.43	15.5	229.54	15.0320002	-122.45632	37.93651	-0.41	15.5	329.56	15.0570002	11	7/24/2014	20:43.3
2055	RWS11	-122.4563255	37.93649	-0.43	15.5	240	15.0350004	-122.45632	37.93651	-0.44	15.5	341.56	15.0260004	11	7/24/2014	20:43.4
2056	RWS11	-122.4563279	37.93649	-0.43	15.5	250.62	15.0390001	-122.45632	37.93651	-0.44	15.5	353.81	15.0300001	11	7/24/2014	20:43.5
2057	RWS11	-122.4563304	37.9365	-0.48	15.5	261.44	14.9939997	-122.45633	37.93651	-0.44	15.5	366.23	15.0359997	11	7/24/2014	20:43.6
2058	RWS11	-122.4563328	37.9365	-0.43	15.5	272.45	15.0519997	-122.45633	37.93651	-0.44	15.5	378.81	15.0429997	11	7/24/2014	20:43.7
2059	RWS11	-122.4563353	37.9365	-0.48	15.5	283.72	15.0100004	-122.45633	37.93651	-0.44	15.5	391.45	15.0520004	11	7/24/2014	20:43.8
2060	RWS11	-122.4563378	37.9365	-0.48	15.5	295.08	15.0210001	-122.45633	37.93651	-0.44	15.5	404.03	15.0630001	11	7/24/2014	20:43.9
2061	RWS11	-122.4563402	37.9365	-0.48	15.5	306.58	15.0339997	-122.45634	37.93651	-0.49	15.5	416.72	15.0249997	11	7/24/2014	20:44.0
2062	RWS11	-122.4563427	37.9365	-0.43	15.5	318.14	15.1019999	-122.45634	37.93651	-0.41	15.5	429.49	15.1269999	11	7/24/2014	20:44.1
2063	RWS11	-122.4563451	37.9365	-0.48	15.6	329.93	15.0690005	-122.45634	37.93651	-0.44	15.6	442.46	15.1110005	11	7/24/2014	20:44.2
2064	RWS11	-122.4563476	37.9365	-0.43	15.6	341.94	15.1419998	-122.45634	37.93651	-0.44	15.6	455.52	15.1329998	11	7/24/2014	20:44.3
2065	RWS11	-122.45635	37.9365	-0.48	15.6	353.96	15.1160004	-122.45634	37.93651	-0.49	15.6	468.58	15.1070004	11	7/24/2014	20:44.4
2066	RWS11	-122.4563524	37.9365	-0.43	15.6	366.05	15.1959997	-122.45635	37.93651	-0.41	15.6	481.64	15.2209997	11	7/24/2014	20:44.5
2067	RWS11	-122.4563548	37.9365	-0.48	15.7	378.32	15.1759999	-122.45635	37.93651	-0.44	15.7	494.81	15.2179999	11	7/24/2014	20:44.6
2068	RWS11	-122.4563572	37.9365	-0.43	15.7	390.65	15.2630001	-122.45635	37.93651	-0.44	15.7	508.06	15.2540001	11	7/24/2014	20:44.7
2069	RWS11	-122.4563596	37.9365	-0.43	15.7	403.03	15.3010002	-122.45635	37.93652	-0.44	15.7	521.13	15.2920002	11	7/24/2014	20:44.8
2070	RWS11	-122.4563619	37.9365	-0.43	15.8	415.44	15.3410002	-122.45636	37.93652	-0.44	15.8	534.4	15.3320002	11	7/24/2014	20:44.9
2071	RWS11	-122.4563654	37.9365	-0.48	15.8	427.83	15.332	-122.45636	37.93652	-0.44	15.8	547.81	15.374	11	7/24/2014	20:45.0
2072	RWS11	-122.4563679	37.9365	-0.43	15.9	440.24	15.4240004	-122.45636	37.93652	-0.41	15.9	561.43	15.4490004	11	7/24/2014	20:45.1
2073	RWS11	-122.4563703	37.93651	-0.48	15.9	452.68	15.4139998	-122.45637	37.93652	-0.44	15.9	575.28	15.4559998	11	7/24/2014	20:45.2
2074	RWS11	-122.4563727	37.93651	-0.48	15.9	465.28	15.4510004	-122.45637	37.93652	-0.41	15.9	589.41	15.5270004	11	7/24/2014	20:45.3
2075	RWS11	-122.4563752	37.93651	-0.48	16	477.89	15.4860003	-122.45637	37.93652	-0.44	16	603.57	15.5280003	11	7/24/2014	20:45.4
2076	RWS11	-122.4563778	37.93651	-0.43	16	490.54	15.5690008	-122.45637	37.93652	-0.36	16	617.66	15.6440008	11	7/24/2014	20:45.5
2077	RWS11	-122.4563803	37.93651	-0.48	16	503.17	15.549001	-122.45638	37.93652	-0.44	16	631.67	15.5910009	11	7/24/2014	20:45.6
2078	RWS11	-122.4563829	37.93651	-0.43	16.1	515.8	15.6280008	-122.45638	37.93652	-0.41	16.1	645.62	15.6530008	11	7/24/2014	20:45.7

2079	RWS11	-122.4563855	37.93651	-0.43	16.1	528.36	15.6549994	-122.45638	37.93652	-0.41	16.1	659.54	15.6799994	11	7/24/2014	20:45.8
2080	RWS11	-122.456388	37.93651	-0.43	16.1	540.96	15.6810003	-122.45638	37.93652	-0.41	16.1	673.57	15.7060003	11	7/24/2014	20:45.9
2081	RWS11	-122.4563906	37.93651	-0.48	16.1	553.57	15.6540005	-122.45639	37.93652	-0.41	16.1	687.72	15.7300005	11	7/24/2014	20:46.0
2082	RWS11	-122.4563932	37.93651	-0.43	16.2	566.29	15.7269999	-122.45639	37.93652	-0.41	16.2	701.93	15.7519999	11	7/24/2014	20:46.1
2083	RWS11	-122.4563957	37.93651	-0.43	16.2	578.97	15.7479998	-122.45639	37.93652	-0.44	16.2	716.15	15.7389998	11	7/24/2014	20:46.2
2084	RWS11	-122.4563982	37.93651	-0.43	16.2	591.76	15.7670008	-122.45639	37.93653	-0.36	16.2	730.46	15.8420008	11	7/24/2014	20:46.3
2085	RWS11	-122.4564007	37.93651	-0.48	16.2	604.68	15.7340004	-122.4564	37.93653	-0.41	16.2	745	15.8100004	11	7/24/2014	20:46.4
2086	RWS11	-122.4564032	37.93651	-0.43	16.2	617.68	15.8020006	-122.4564	37.93653	-0.41	16.2	759.66	15.8270006	11	7/24/2014	20:46.5
2087	RWS11	-122.4564057	37.93651	-0.43	16.3	630.7	15.8179995	-122.4564	37.93653	-0.41	16.3	774.42	15.8429995	11	7/24/2014	20:46.6
2088	RWS11	-122.4564081	37.93651	-0.43	16.3	643.62	15.8340002	-122.4564	37.93653	-0.41	16.3	789.09	15.8590002	11	7/24/2014	20:46.7
2089	RWS11	-122.4564105	37.93652	-0.43	16.3	656.41	15.850001	-122.45641	37.93653	-0.44	16.3	803.51	15.8410009	11	7/24/2014	20:46.8
2090	RWS11	-122.4564129	37.93652	-0.43	16.3	669.08	15.8669992	-122.45641	37.93653	-0.44	16.3	817.94	15.8579992	11	7/24/2014	20:46.9
2091	RWS11	-122.4564164	37.93652	-0.48	16.3	681.64	15.8329994	-122.45641	37.93653	-0.41	16.3	832.02	15.9089994	11	7/24/2014	20:47.0
2092	RWS11	-122.4564188	37.93652	-0.43	16.3	694.07	15.9019991	-122.45641	37.93653	-0.41	16.3	845.99	15.9269991	11	7/24/2014	20:47.1
2093	RWS11	-122.4564212	37.93652	-0.48	16.4	706.46	15.8700001	-122.45642	37.93653	-0.44	16.4	859.77	15.9120001	11	7/24/2014	20:47.2
2094	RWS11	-122.4564236	37.93652	-0.43	16.4	718.69	15.9399992	-122.45642	37.93653	-0.41	16.4	873.35	15.9649992	11	7/24/2014	20:47.3
2095	RWS11	-122.456426	37.93652	-0.48	16.4	730.9	15.9070008	-122.45642	37.93653	-0.44	16.4	886.65	15.9490008	11	7/24/2014	20:47.4
2096	RWS11	-122.4564284	37.93652	-0.43	16.4	742.93	15.9739996	-122.45642	37.93653	-0.41	16.4	899.89	15.9989996	11	7/24/2014	20:47.5
2097	RWS11	-122.4564308	37.93652	-0.43	16.4	755.03	15.9890009	-122.45643	37.93653	-0.41	16.4	913	16.0140009	11	7/24/2014	20:47.6
2098	RWS11	-122.4564332	37.93652	-0.43	16.4	766.88	16.0000006	-122.45643	37.93653	-0.44	16.4	926.06	15.9910006	11	7/24/2014	20:47.7
2099	RWS11	-122.4564356	37.93652	-0.43	16.4	778.62	16.0070005	-122.45643	37.93653	-0.44	16.4	938.88	15.9980005	11	7/24/2014	20:47.8
2100	RWS11	-122.456438	37.93652	-0.43	16.4	790.28	16.0119997	-122.45643	37.93653	-0.44	16.4	951.52	16.0029997	11	7/24/2014	20:47.9
2101	RWS11	-122.4564416	37.93652	-0.43	16.4	801.66	16.0140005	-122.45644	37.93654	-0.41	16.4	963.92	16.0390005	11	7/24/2014	20:48.0
2102	RWS11	-122.456444	37.93652	-0.43	16.4	812.99	16.0129992	-122.45644	37.93654	-0.44	16.4	976.24	16.0039991	11	7/24/2014	20:48.1
2103	RWS11	-122.4564464	37.93652	-0.43	16.4	824.14	16.0100008	-122.45644	37.93654	-0.44	16.4	988.37	16.0010008	11	7/24/2014	20:48.2
2104	RWS11	-122.4564488	37.93652	-0.43	16.4	835.03	16.0049997	-122.45644	37.93654	-0.44	16.4	1000.2	15.9959997	11	7/24/2014	20:48.3
2105	RWS11	-122.4564512	37.93653	-0.48	16.4	845.58	15.9479992	-122.45645	37.93654	-0.44	16.4	1011.7	15.9899992	11	7/24/2014	20:48.4
2106	RWS11	-122.4564536	37.93653	-0.43	16.4	855.78	15.9900003	-122.45645	37.93654	-0.41	16.4	1022.8	16.0150003	11	7/24/2014	20:48.5
2107	RWS11	-122.4564561	37.93653	-0.43	16.4	865.66	15.9779993	-122.45645	37.93654	-0.44	16.4	1033.6	15.9689993	11	7/24/2014	20:48.6
2108	RWS11	-122.4564585	37.93653	-0.43	16.4	875.25	15.9639994	-122.45645	37.93654	-0.44	16.4	1043.9	15.9549994	11	7/24/2014	20:48.7
2109	RWS11	-122.4564609	37.93653	-0.43	16.4	884.55	15.9459997	-122.45646	37.93654	-0.41	16.4	1053.9	15.9709997	11	7/24/2014	20:48.8
2110	RWS11	-122.4564632	37.93653	-0.43	16.4	893.49	15.9259993	-122.45646	37.93654	-0.44	16.4	1063.5	15.9169993	11	7/24/2014	20:48.9
2111	RWS11	-122.4564668	37.93653	-0.48	16.3	902.15	15.8509991	-122.45646	37.93654	-0.44	16.3	1072.8	15.8929991	11	7/24/2014	20:49.0

2112	RWS11	-122.4564692	37.93653	-0.43	16.3	910.54	15.8769995	-122.45646	37.93654	-0.41	16.3	1081.7	15.9019995	11	7/24/2014	20:49.1
2113	RWS11	-122.4564716	37.93653	-0.43	16.3	918.63	15.850001	-122.45647	37.93654	-0.44	16.3	1090.3	15.8410009	11	7/24/2014	20:49.2
2114	RWS11	-122.456474	37.93653	-0.43	16.3	926.26	15.8200003	-122.45647	37.93654	-0.41	16.3	1098.4	15.8450003	11	7/24/2014	20:49.3
2115	RWS11	-122.4564764	37.93653	-0.43	16.2	933.56	15.7880007	-122.45647	37.93654	-0.49	16.2	1106.1	15.7280007	11	7/24/2014	20:49.4
2116	RWS11	-122.4564789	37.93653	-0.43	16.2	940.41	15.7549997	-122.45647	37.93654	-0.41	16.2	1113.2	15.7799997	11	7/24/2014	20:49.5
2117	RWS11	-122.4564814	37.93653	-0.43	16.2	946.92	15.7199999	-122.45648	37.93655	-0.44	16.2	1119.9	15.7109999	11	7/24/2014	20:49.6
2118	RWS11	-122.4564839	37.93653	-0.43	16.1	953.12	15.6840006	-122.45648	37.93655	-0.44	16.1	1126.3	15.6750006	11	7/24/2014	20:49.7
2119	RWS11	-122.4564864	37.93653	-0.48	16.1	958.96	15.5969994	-122.45648	37.93655	-0.49	16.1	1132.2	15.5879994	11	7/24/2014	20:49.8
2120	RWS11	-122.4564889	37.93653	-0.43	16	964.27	15.6120001	-122.45648	37.93655	-0.44	16	1137.7	15.6030001	11	7/24/2014	20:49.9
2121	RWS11	-122.4564915	37.93654	-0.43	16	969.29	15.5760008	-122.45649	37.93655	-0.44	16	1142.7	15.5670008	11	7/24/2014	20:50.0
2122	RWS11	-122.456494	37.93654	-0.43	16	973.87	15.541	-122.45649	37.93655	-0.44	16	1147.3	15.532	11	7/24/2014	20:50.1
2123	RWS11	-122.4564965	37.93654	-0.43	15.9	978.11	15.5060001	-122.45649	37.93655	-0.49	15.9	1151.4	15.4460001	11	7/24/2014	20:50.2
2124	RWS11	-122.456499	37.93654	-0.43	15.9	981.84	15.4719997	-122.45649	37.93655	-0.44	15.9	1154.9	15.4629997	11	7/24/2014	20:50.3
2125	RWS11	-122.4565015	37.93654	-0.48	15.9	985.08	15.3879997	-122.4565	37.93655	-0.44	15.9	1157.9	15.4299997	11	7/24/2014	20:50.4
2126	RWS11	-122.4565039	37.93654	-0.43	15.8	987.89	15.4070002	-122.4565	37.93655	-0.41	15.8	1160.3	15.4320002	11	7/24/2014	20:50.5
2127	RWS11	-122.4565064	37.93654	-0.48	15.8	990.33	15.3260004	-122.4565	37.93655	-0.44	15.8	1162.1	15.3680004	11	7/24/2014	20:50.6
2128	RWS11	-122.4565088	37.93654	-0.43	15.8	992.2	15.3480001	-122.4565	37.93655	-0.44	15.8	1163.4	15.3390001	11	7/24/2014	20:50.7
2129	RWS11	-122.4565112	37.93654	-0.48	15.8	993.43	15.2699997	-122.45651	37.93655	-0.49	15.8	1163.9	15.2609997	11	7/24/2014	20:50.8
2130	RWS11	-122.4565136	37.93654	-0.43	15.7	994.14	15.2929998	-122.45651	37.93655	-0.44	15.7	1164	15.2839998	11	7/24/2014	20:50.9
2131	RWS11	-122.4565171	37.93654	-0.43	15.7	994.36	15.265	-122.45651	37.93655	-0.44	15.7	1163.6	15.256	11	7/24/2014	20:51.0
2132	RWS11	-122.4565195	37.93654	-0.43	15.7	994.18	15.2370001	-122.45651	37.93656	-0.44	15.7	1162.7	15.2280001	11	7/24/2014	20:51.1
2133	RWS11	-122.4565218	37.93654	-0.48	15.6	993.65	15.1569998	-122.45652	37.93656	-0.44	15.6	1161.3	15.1989998	11	7/24/2014	20:51.2
2134	RWS11	-122.4565243	37.93654	-0.43	15.6	992.6	15.1780001	-122.45652	37.93656	-0.44	15.6	1159.3	15.1690001	11	7/24/2014	20:51.3
2135	RWS11	-122.4565267	37.93654	-0.48	15.6	990.93	15.0950005	-122.45652	37.93656	-0.44	15.6	1156.7	15.1370005	11	7/24/2014	20:51.4
2136	RWS11	-122.4565292	37.93654	-0.43	15.5	988.83	15.1129995	-122.45652	37.93656	-0.44	15.5	1153.5	15.1039995	11	7/24/2014	20:51.5
2137	RWS11	-122.4565316	37.93655	-0.48	15.5	986.37	15.0269997	-122.45653	37.93656	-0.44	15.5	1149.9	15.0689997	11	7/24/2014	20:51.6
2138	RWS11	-122.4565342	37.93655	-0.43	15.5	983.22	15.0420004	-122.45653	37.93656	-0.49	15.5	1145.4	14.9820004	11	7/24/2014	20:51.7
2139	RWS11	-122.4565367	37.93655	-0.48	15.4	979.52	14.9539997	-122.45653	37.93656	-0.44	15.4	1140.3	14.9959997	11	7/24/2014	20:51.8
2140	RWS11	-122.4565392	37.93655	-0.43	15.4	975.42	14.9669996	-122.45653	37.93656	-0.44	15.4	1134.9	14.9579996	11	7/24/2014	20:51.9
2141	RWS11	-122.4565418	37.93655	-0.43	15.4	970.76	14.9280001	-122.45654	37.93656	-0.49	15.4	1128.7	14.8680001	11	7/24/2014	20:52.0
2142	RWS11	-122.4565443	37.93655	-0.43	15.3	965.42	14.8889996	-122.45654	37.93656	-0.44	15.3	1122	14.8799996	11	7/24/2014	20:52.1
2143	RWS11	-122.4565469	37.93655	-0.43	15.3	959.64	14.85	-122.45654	37.93656	-0.49	15.3	1114.6	14.79	11	7/24/2014	20:52.2
2144	RWS11	-122.4565494	37.93655	-0.43	15.2	953.34	14.81	-122.45654	37.93656	-0.44	15.2	1106.9	14.801	11	7/24/2014	20:52.3

2145	RWS11	-122.4565519	37.93655	-0.48	15.2	946.52	14.7200005	-122.45655	37.93656	-0.49	15.2	1098.6	14.7110005	11	7/24/2014	20:52.4
2146	RWS11	-122.4565545	37.93655	-0.43	15.2	939.27	14.7330004	-122.45655	37.93656	-0.49	15.2	1089.8	14.6730004	11	7/24/2014	20:52.5
2147	RWS11	-122.4565569	37.93655	-0.43	15.1	931.5	14.6959997	-122.45655	37.93656	-0.49	15.1	1080.5	14.6359997	11	7/24/2014	20:52.6
2148	RWS11	-122.4565594	37.93655	-0.43	15.1	923.42	14.6609999	-122.45655	37.93657	-0.49	15.1	1070.7	14.6009999	11	7/24/2014	20:52.7
2149	RWS11	-122.4565618	37.93655	-0.43	15.1	914.66	14.6270004	-122.45656	37.93657	-0.44	15.1	1060.3	14.6180004	11	7/24/2014	20:52.8
2150	RWS11	-122.4565642	37.93655	-0.43	15	905.4	14.5949999	-122.45656	37.93657	-0.44	15	1049.5	14.5859999	11	7/24/2014	20:52.9
2151	RWS11	-122.4565678	37.93655	-0.43	15	895.8	14.5639997	-122.45656	37.93657	-0.44	15	1038.3	14.5549997	11	7/24/2014	20:53.0
2152	RWS11	-122.4565702	37.93656	-0.43	15	885.79	14.5359999	-122.45656	37.93657	-0.44	15	1026.7	14.5269999	11	7/24/2014	20:53.1
2153	RWS11	-122.4565726	37.93656	-0.48	14.9	875.51	14.4589998	-122.45657	37.93657	-0.49	14.9	1014.6	14.4499998	11	7/24/2014	20:53.2
2154	RWS11	-122.4565751	37.93656	-0.43	14.9	864.87	14.4859997	-122.45657	37.93657	-0.44	14.9	1002.2	14.4769997	11	7/24/2014	20:53.3
2155	RWS11	-122.4565775	37.93656	-0.48	14.9	853.92	14.4130003	-122.45657	37.93657	-0.49	14.9	989.3	14.4040003	11	7/24/2014	20:53.4
2156	RWS11	-122.45658	37.93656	-0.43	14.9	842.67	14.4430004	-122.45657	37.93657	-0.44	14.9	976.13	14.4340004	11	7/24/2014	20:53.5
2157	RWS11	-122.4565825	37.93656	-0.43	14.9	831.08	14.423	-122.45658	37.93657	-0.49	14.9	962.57	14.363	11	7/24/2014	20:53.6
2158	RWS11	-122.456585	37.93656	-0.43	14.8	819.14	14.402	-122.45658	37.93657	-0.44	14.8	948.78	14.393	11	7/24/2014	20:53.7
2159	RWS11	-122.4565876	37.93656	-0.43	14.8	807.08	14.3799997	-122.45658	37.93657	-0.49	14.8	934.81	14.3199997	11	7/24/2014	20:53.8
2160	RWS11	-122.4565901	37.93656	-0.43	14.8	794.83	14.3550001	-122.45658	37.93657	-0.44	14.8	920.63	14.3460001	11	7/24/2014	20:53.9
2161	RWS11	-122.4565927	37.93656	-0.43	14.8	782.33	14.3279997	-122.45659	37.93657	-0.49	14.8	906.26	14.2679997	11	7/24/2014	20:54.0
2162	RWS11	-122.4565953	37.93656	-0.4	14.7	769.54	14.3329999	-122.45659	37.93657	-0.49	14.7	891.63	14.238	11	7/24/2014	20:54.1
2163	RWS11	-122.4565978	37.93656	-0.43	14.7	756.41	14.2660004	-122.45659	37.93658	-0.49	14.7	876.65	14.2060004	11	7/24/2014	20:54.2
2164	RWS11	-122.4566003	37.93656	-0.43	14.7	742.96	14.232	-122.45659	37.93658	-0.49	14.7	861.39	14.172	11	7/24/2014	20:54.3
2165	RWS11	-122.4566029	37.93656	-0.43	14.6	729.42	14.1959997	-122.4566	37.93658	-0.49	14.6	846.02	14.1359997	11	7/24/2014	20:54.4
2166	RWS11	-122.4566054	37.93656	-0.43	14.6	715.57	14.1570002	-122.4566	37.93658	-0.49	14.6	830.48	14.0970002	11	7/24/2014	20:54.5
2167	RWS11	-122.4566079	37.93657	-0.48	14.6	701.81	14.0669996	-122.4566	37.93658	-0.44	14.6	815.02	14.1089996	11	7/24/2014	20:54.6
2168	RWS11	-122.4566104	37.93657	-0.43	14.5	687.78	14.0770002	-122.4566	37.93658	-0.49	14.5	799.35	14.0170002	11	7/24/2014	20:54.7
2169	RWS11	-122.4566128	37.93657	-0.43	14.5	673.8	14.0359999	-122.45661	37.93658	-0.49	14.5	783.82	13.9759999	11	7/24/2014	20:54.8
2170	RWS11	-122.4566153	37.93657	-0.43	14.4	659.91	13.9940001	-122.45661	37.93658	-0.44	14.4	768.37	13.985	11	7/24/2014	20:54.9
2171	RWS11	-122.4566189	37.93657	-0.43	14.4	646.06	13.9529997	-122.45661	37.93658	-0.49	14.4	753	13.8929997	11	7/24/2014	20:55.0
2172	RWS11	-122.4566213	37.93657	-0.43	14.3	632.3	13.9129997	-122.45662	37.93658	-0.41	14.3	737.73	13.9379997	11	7/24/2014	20:55.1
2173	RWS11	-122.4566237	37.93657	-0.43	14.3	618.72	13.8740002	-122.45662	37.93658	-0.44	14.3	722.51	13.8650002	11	7/24/2014	20:55.2
2174	RWS11	-122.4566262	37.93657	-0.43	14.3	605.16	13.8370005	-122.45662	37.93658	-0.49	14.3	707.31	13.7770005	11	7/24/2014	20:55.3
2175	RWS11	-122.4566287	37.93657	-0.43	14.2	591.62	13.8019997	-122.45662	37.93658	-0.49	14.2	692.13	13.7419997	11	7/24/2014	20:55.4
2176	RWS11	-122.4566312	37.93657	-0.43	14.2	577.83	13.7689997	-122.45663	37.93658	-0.41	14.2	676.79	13.7939997	11	7/24/2014	20:55.5
2177	RWS11	-122.4566338	37.93657	-0.43	14.2	564.03	13.7370001	-122.45663	37.93658	-0.44	14.2	661.25	13.7280001	11	7/24/2014	20:55.6

2178	RWS11	-122.4566363	37.93657	-0.43	14.1	550.2	13.7059999	-122.45663	37.93659	-0.44	14.1	645.66	13.6969999	11	7/24/2014	20:55.7
2179	RWS11	-122.4566389	37.93657	-0.43	14.1	536.36	13.6769997	-122.45663	37.93659	-0.44	14.1	630.02	13.6679997	11	7/24/2014	20:55.8
2180	RWS11	-122.4566414	37.93657	-0.43	14.1	522.46	13.6480003	-122.45664	37.93659	-0.49	14.1	614.46	13.5880003	11	7/24/2014	20:55.9
2181	RWS11	-122.456644	37.93657	-0.43	14.1	508.52	13.6190001	-122.45664	37.93659	-0.49	14.1	598.96	13.5590001	11	7/24/2014	20:56.0
2182	RWS11	-122.4566465	37.93658	-0.43	14	494.58	13.5910002	-122.45664	37.93659	-0.49	14	583.52	13.5310002	11	7/24/2014	20:56.1
2183	RWS11	-122.456649	37.93658	-0.48	14	480.56	13.5120003	-122.45664	37.93659	-0.49	14	568.13	13.5030003	11	7/24/2014	20:56.2
2184	RWS11	-122.4566516	37.93658	-0.43	14	466.83	13.5359999	-122.45665	37.93659	-0.49	14	552.87	13.4759999	11	7/24/2014	20:56.3
2185	RWS11	-122.456654	37.93658	-0.48	13.9	453.16	13.4580004	-122.45665	37.93659	-0.49	13.9	537.77	13.4490004	11	7/24/2014	20:56.4
2186	RWS11	-122.4566565	37.93658	-0.43	13.9	439.53	13.482	-122.45665	37.93659	-0.49	13.9	522.79	13.422	11	7/24/2014	20:56.5
2187	RWS11	-122.456659	37.93658	-0.48	13.9	426.03	13.4030001	-122.45665	37.93659	-0.49	13.9	507.84	13.3940001	11	7/24/2014	20:56.6
2188	RWS11	-122.4566614	37.93658	-0.43	13.9	412.62	13.4260002	-122.45666	37.93659	-0.44	13.9	493.11	13.4170002	11	7/24/2014	20:56.7
2189	RWS11	-122.4566638	37.93658	-0.48	13.8	399.25	13.3459999	-122.45666	37.93659	-0.49	13.8	478.36	13.3369999	11	7/24/2014	20:56.8
2190	RWS11	-122.4566662	37.93658	-0.43	13.8	385.86	13.3690001	-122.45666	37.93659	-0.44	13.8	463.7	13.36	11	7/24/2014	20:56.9
2191	RWS11	-122.4566697	37.93658	-0.48	13.8	372.51	13.2900002	-122.45666	37.93659	-0.49	13.8	449.02	13.2810002	11	7/24/2014	20:57.0
2192	RWS11	-122.4566721	37.93658	-0.43	13.7	359.4	13.3139997	-122.45667	37.9366	-0.49	13.7	434.62	13.2539997	11	7/24/2014	20:57.1
2193	RWS11	-122.4566745	37.93658	-0.43	13.7	346.21	13.2890001	-122.45667	37.9366	-0.49	13.7	420.29	13.2290001	11	7/24/2014	20:57.2
2194	RWS11	-122.4566769	37.93658	-0.43	13.7	333.28	13.2660004	-122.45667	37.9366	-0.49	13.7	406.3	13.2060004	11	7/24/2014	20:57.3
2195	RWS11	-122.4566793	37.93658	-0.43	13.7	320.64	13.2459999	-122.45667	37.9366	-0.53	13.7	392.67	13.1519999	11	7/24/2014	20:57.4
2196	RWS11	-122.4566818	37.93659	-0.43	13.7	308.26	13.2280003	-122.45668	37.9366	-0.44	13.7	379.39	13.2190003	11	7/24/2014	20:57.5
2197	RWS11	-122.4566843	37.93659	-0.48	13.6	296.08	13.1610005	-122.45668	37.9366	-0.49	13.6	366.09	13.1520005	11	7/24/2014	20:57.6
2198	RWS11	-122.4566868	37.93659	-0.43	13.6	284.25	13.199	-122.45668	37.9366	-0.49	13.6	353.05	13.139	11	7/24/2014	20:57.7
2199	RWS11	-122.4566893	37.93659	-0.48	13.6	272.42	13.1359999	-122.45668	37.9366	-0.53	13.6	340.01	13.0929999	11	7/24/2014	20:57.8
2200	RWS11	-122.4566918	37.93659	-0.43	13.6	260.7	13.1769997	-122.45669	37.9366	-0.49	13.6	326.97	13.1169997	11	7/24/2014	20:57.9
2201	RWS11	-122.4566943	37.93659	-0.48	13.6	249.12	13.1169998	-122.45669	37.9366	-0.49	13.6	314.18	13.1079998	11	7/24/2014	20:58.0
2202	RWS11	-122.4566969	37.93659	-0.48	13.6	237.55	13.1090004	-122.45669	37.9366	-0.49	13.6	301.47	13.1000004	11	7/24/2014	20:58.1
2203	RWS11	-122.4566994	37.93659	-0.48	13.6	226.08	13.1029999	-122.45669	37.9366	-0.49	13.6	289.14	13.0939999	11	7/24/2014	20:58.2
2204	RWS11	-122.4567019	37.93659	-0.43	13.6	214.75	13.1480003	-122.4567	37.9366	-0.49	13.6	277	13.0880003	11	7/24/2014	20:58.3
2205	RWS11	-122.4567044	37.93659	-0.48	13.6	203.51	13.0920002	-122.4567	37.9366	-0.49	13.6	264.88	13.0830002	11	7/24/2014	20:58.4
2206	RWS11	-122.4567069	37.93659	-0.43	13.6	192.5	13.1389996	-122.4567	37.9366	-0.49	13.6	253.08	13.0789996	11	7/24/2014	20:58.5
2207	RWS11	-122.4567094	37.93659	-0.48	13.6	181.57	13.0839998	-122.4567	37.93661	-0.53	13.6	241.12	13.0409998	11	7/24/2014	20:58.6
2208	RWS11	-122.4567118	37.93659	-0.48	13.6	170.75	13.0800001	-122.45671	37.93661	-0.49	13.6	229.21	13.0710001	11	7/24/2014	20:58.7
2209	RWS11	-122.4567142	37.93659	-0.48	13.6	160.02	13.075	-122.45671	37.93661	-0.53	13.6	217.26	13.032	11	7/24/2014	20:58.8
2210	RWS11	-122.4567166	37.93659	-0.48	13.6	149.35	13.0699999	-122.45671	37.93661	-0.53	13.6	205.39	13.0269999	11	7/24/2014	20:58.9

2211	RWS11	-122.4567202	37.9366	-0.48	13.5	138.83	13.0629999	-122.45671	37.93661	-0.53	13.5	193.51	13.0199999	11	7/24/2014	20:59.0
2212	RWS11	-122.4567226	37.9366	-0.48	13.5	128.34	13.056	-122.45672	37.93661	-0.49	13.5	181.71	13.047	11	7/24/2014	20:59.1
2213	RWS11	-122.456725	37.9366	-0.48	13.5	117.99	13.0479996	-122.45672	37.93661	-0.53	13.5	170.16	13.0049996	11	7/24/2014	20:59.2
2214	RWS11	-122.4567275	37.9366	-0.48	13.5	107.58	13.0400002	-122.45672	37.93661	-0.49	13.5	158.81	13.0310002	11	7/24/2014	20:59.3
2215	RWS11	-122.4567299	37.9366	-0.48	13.5	97.243	13.0310004	-122.45672	37.93661	-0.53	13.5	147.46	12.9880003	11	7/24/2014	20:59.4
2216	RWS11	-122.4567323	37.9366	-0.48	13.5	87.007	13.0219996	-122.45673	37.93661	-0.53	13.5	136.39	12.9789996	11	7/24/2014	20:59.5
2217	RWS11	-122.4567348	37.9366	-0.48	13.5	76.798	13.0140002	-122.45673	37.93661	-0.53	13.5	125.6	12.9710001	11	7/24/2014	20:59.6
2218	RWS11	-122.4567372	37.9366	-0.48	13.5	66.933	13.0050003	-122.45673	37.93661	-0.49	13.5	115.09	12.9960003	11	7/24/2014	20:59.7
2219	RWS11	-122.4567396	37.9366	-0.52	13.5	57.204	12.9629999	-122.45673	37.93661	-0.53	13.5	104.88	12.9539999	11	7/24/2014	20:59.8
2220	RWS11	-122.4567421	37.9366	-0.48	13.5	47.756	12.9889996	-122.45674	37.93661	-0.53	13.5	94.919	12.9459996	11	7/24/2014	20:59.9
2221	RWS11	-122.4567457	37.9366	-0.52	13.5	38.616	12.9470002	-122.45674	37.93662	-0.53	13.5	85.105	12.9380001	11	7/24/2014	21:00.0
2222	RWS11	-122.4567481	37.9366	-0.52	13.5	29.689	12.9389998	-122.45674	37.93662	-0.49	13.5	75.49	12.9639998	11	7/24/2014	21:00.1
2223	RWS11	-122.4567505	37.9366	-0.52	13.4	20.873	12.9319998	-122.45674	37.93662	-0.53	13.4	66.032	12.9229998	11	7/24/2014	21:00.2
2224	RWS11	-122.4567529	37.93661	-0.48	13.4	12.296	12.9589998	-122.45675	37.93662	-0.49	13.4	56.675	12.9499998	11	7/24/2014	21:00.3
2225	RWS11	-122.4567554	37.93661	-0.48	13.4	3.786	12.9519999	-122.45675	37.93662	-0.58	13.4	47.551	12.8579999	11	7/24/2014	21:00.4
2226	RWS11	-122.4567578	37.93661	-0.48	13.4	-4.483	12.9460003	-122.45675	37.93662	-0.53	13.4	38.652	12.9030003	11	7/24/2014	21:00.5
2227	RWS11	-122.4567603	37.93661	-0.48	13.4	-12.71	12.9399998	-122.45675	37.93662	-0.58	13.4	29.771	12.8459998	11	7/24/2014	21:00.6
2228	RWS11	-122.4567627	37.93661	-0.48	13.4	-20.83	12.9340002	-122.45676	37.93662	-0.49	13.4	20.911	12.9250002	11	7/24/2014	21:00.7
2229	RWS11	-122.4567651	37.93661	-0.48	13.4	-28.86	12.9279997	-122.45676	37.93662	-0.53	13.4	12.113	12.8849997	11	7/24/2014	21:00.8
2230	RWS11	-122.4567676	37.93661	-0.48	13.4	-36.97	12.9220001	-122.45676	37.93662	-0.49	13.4	3.545	12.9130001	11	7/24/2014	21:00.9
2231	RWS11	-122.4567711	37.93661	-0.52	13.4	-44.78	12.8819996	-122.45677	37.93662	-0.58	13.4	-5.052	12.8219996	11	7/24/2014	21:01.0
2232	RWS11	-122.4567735	37.93661	-0.48	13.4	-52.35	12.9100001	-122.45677	37.93662	-0.53	13.4	-13.19	12.867	11	7/24/2014	21:01.1
2233	RWS11	-122.4567759	37.93661	-0.52	13.4	-59.77	12.8690001	-122.45677	37.93662	-0.53	13.4	-21.198	12.8600001	11	7/24/2014	21:01.2
2234	RWS11	-122.4567783	37.93661	-0.48	13.4	-66.87	12.8960001	-122.45677	37.93662	-0.53	13.4	-28.901	12.8530001	11	7/24/2014	21:01.3
2235	RWS11	-122.4567808	37.93661	-0.52	13.4	-73.95	12.8550001	-122.45677	37.93663	-0.58	13.4	-36.536	12.7950001	11	7/24/2014	21:01.4
2236	RWS11	-122.4567832	37.93661	-0.48	13.4	-80.83	12.8820002	-122.45678	37.93663	-0.53	13.4	-44.086	12.8390002	11	7/24/2014	21:01.5
2237	RWS11	-122.4567856	37.93661	-0.52	13.4	-87.7	12.8399998	-122.45678	37.93663	-0.58	13.4	-51.55	12.7799998	11	7/24/2014	21:01.6
2238	RWS11	-122.456788	37.93661	-0.48	13.4	-94.55	12.8669998	-122.45678	37.93663	-0.53	13.4	-58.835	12.8239998	11	7/24/2014	21:01.7
2239	RWS11	-122.4567904	37.93662	-0.48	13.3	-101.4	12.8590004	-122.45678	37.93663	-0.58	13.3	-66.104	12.7650004	11	7/24/2014	21:01.8
2240	RWS11	-122.4567928	37.93662	-0.48	13.3	-108.5	12.8520005	-122.45679	37.93663	-0.58	13.3	-73.61	12.7580004	11	7/24/2014	21:01.9
2241	RWS11	-122.4567963	37.93662	-0.52	13.3	-115.5	12.8100001	-122.45679	37.93663	-0.58	13.3	-80.878	12.7500001	11	7/24/2014	21:02.0
2242	RWS11	-122.4567987	37.93662	-0.52	13.3	-122.3	12.7999998	-122.45679	37.93663	-0.53	13.3	-88.228	12.7909998	11	7/24/2014	21:02.1
2243	RWS11	-122.456801	37.93662	-0.52	13.3	-128.9	12.7899996	-122.4568	37.93663	-0.58	13.3	-95.67	12.7299996	11	7/24/2014	21:02.2

2244	RWS11	-122.4568034	37.93662	-0.52	13.3	-135.4	12.7779995	-122.4568	37.93663	-0.58	13.3	-103.07	12.7179995	11	7/24/2014	21:02.3
2245	RWS11	-122.4568058	37.93662	-0.52	13.3	-141.7	12.7660004	-122.4568	37.93663	-0.58	13.3	-110.35	12.7060004	11	7/24/2014	21:02.4
2246	RWS11	-122.4568082	37.93662	-0.52	13.3	-147.8	12.7540003	-122.4568	37.93663	-0.58	13.3	-117.38	12.6940003	11	7/24/2014	21:02.5
2247	RWS11	-122.4568106	37.93662	-0.52	13.3	-153.7	12.7420002	-122.4568	37.93663	-0.61	13.3	-124.14	12.6490002	11	7/24/2014	21:02.6
2248	RWS11	-122.456813	37.93662	-0.52	13.2	-159.4	12.7300001	-122.45681	37.93663	-0.58	13.2	-130.54	12.6700001	11	7/24/2014	21:02.7
2249	RWS11	-122.4568153	37.93662	-0.52	13.2	-164.7	12.7199999	-122.45681	37.93664	-0.61	13.2	-136.57	12.6269999	11	7/24/2014	21:02.8
2250	RWS11	-122.4568177	37.93662	-0.52	13.2	-169.9	12.7110001	-122.45681	37.93664	-0.58	13.2	-142.4	12.6510001	11	7/24/2014	21:02.9
2251	RWS11	-122.4568212	37.93662	-0.57	13.2	-175	12.6519997	-122.45682	37.93664	-0.61	13.2	-148.17	12.6099997	11	7/24/2014	21:03.0
2252	RWS11	-122.4568235	37.93663	-0.52	13.2	-180	12.6970002	-122.45682	37.93664	-0.58	13.2	-153.73	12.6370001	11	7/24/2014	21:03.1
2253	RWS11	-122.4568259	37.93663	-0.57	13.2	-184.9	12.641	-122.45682	37.93664	-0.58	13.2	-159.04	12.632	11	7/24/2014	21:03.2
2254	RWS11	-122.4568283	37.93663	-0.57	13.2	-189.6	12.6370003	-122.45682	37.93664	-0.58	13.2	-164.2	12.6280003	11	7/24/2014	21:03.3
2255	RWS11	-122.4568307	37.93663	-0.57	13.2	-194.2	12.6340001	-122.45682	37.93664	-0.61	13.2	-169.15	12.5920001	11	7/24/2014	21:03.4
2256	RWS11	-122.4568331	37.93663	-0.57	13.2	-198.9	12.6320002	-122.45683	37.93664	-0.58	13.2	-174.15	12.6230002	11	7/24/2014	21:03.5
2257	RWS11	-122.4568355	37.93663	-0.57	13.2	-203.4	12.6309998	-122.45683	37.93664	-0.58	13.2	-179.04	12.6219998	11	7/24/2014	21:03.6
2258	RWS11	-122.4568379	37.93663	-0.57	13.2	-207.8	12.6309998	-122.45683	37.93664	-0.58	13.2	-183.66	12.6219998	11	7/24/2014	21:03.7
2259	RWS11	-122.4568403	37.93663	-0.57	13.2	-211.9	12.6320002	-122.45683	37.93664	-0.58	13.2	-188.11	12.6230002	11	7/24/2014	21:03.8
2260	RWS11	-122.4568427	37.93663	-0.57	13.2	-216.1	12.6329997	-122.45684	37.93664	-0.58	13.2	-192.51	12.6239997	11	7/24/2014	21:03.9
2261	RWS11	-122.4568462	37.93663	-0.57	13.2	-219.9	12.6340001	-122.45684	37.93664	-0.61	13.2	-196.6	12.5920001	11	7/24/2014	21:04.0
2262	RWS11	-122.4568486	37.93663	-0.57	13.2	-223.7	12.6329997	-122.45684	37.93665	-0.61	13.2	-200.53	12.5909997	11	7/24/2014	21:04.1
2263	RWS11	-122.4568509	37.93663	-0.57	13.2	-227.3	12.6309998	-122.45685	37.93665	-0.61	13.2	-204.57	12.5889998	11	7/24/2014	21:04.2
2264	RWS11	-122.4568533	37.93663	-0.57	13.2	-231	12.6270001	-122.45685	37.93665	-0.61	13.2	-208.67	12.5850001	11	7/24/2014	21:04.3
2265	RWS11	-122.4568557	37.93663	-0.6	13.2	-234.5	12.5850002	-122.45685	37.93665	-0.61	13.2	-212.69	12.5780001	11	7/24/2014	21:04.4
2266	RWS11	-122.4568582	37.93664	-0.57	13.2	-237.9	12.6119998	-122.45685	37.93665	-0.61	13.2	-216.63	12.5699998	11	7/24/2014	21:04.5
2267	RWS11	-122.4568606	37.93664	-0.57	13.2	-241.4	12.5999997	-122.45685	37.93665	-0.61	13.2	-220.36	12.5579997	11	7/24/2014	21:04.6
2268	RWS11	-122.456863	37.93664	-0.57	13.2	-244.8	12.5859997	-122.45686	37.93665	-0.61	13.2	-223.97	12.5439997	11	7/24/2014	21:04.7
2269	RWS11	-122.4568654	37.93664	-0.6	13.1	-248.1	12.5360004	-122.45686	37.93665	-0.61	13.1	-227.58	12.5290003	11	7/24/2014	21:04.8
2270	RWS11	-122.4568677	37.93664	-0.6	13.1	-251.4	12.521	-122.45686	37.93665	-0.61	13.1	-231.14	12.514	11	7/24/2014	21:04.9
2271	RWS11	-122.4568713	37.93664	-0.6	13.1	-254.7	12.5050002	-122.45687	37.93665	-0.61	13.1	-234.88	12.4980002	11	7/24/2014	21:05.0
2272	RWS11	-122.4568736	37.93664	-0.57	13.1	-258.1	12.5249999	-122.45687	37.93665	-0.61	13.1	-238.75	12.4829999	11	7/24/2014	21:05.1
2273	RWS11	-122.456876	37.93664	-0.6	13.1	-261.5	12.476	-122.45687	37.93665	-0.61	13.1	-242.61	12.4689999	11	7/24/2014	21:05.2
2274	RWS11	-122.4568784	37.93664	-0.57	13.1	-264.9	12.497	-122.45687	37.93665	-0.61	13.1	-246.65	12.455	11	7/24/2014	21:05.3
2275	RWS11	-122.4568808	37.93664	-0.6	13.1	-268.4	12.4499999	-122.45687	37.93665	-0.66	13.1	-250.8	12.3919999	11	7/24/2014	21:05.4
2276	RWS11	-122.4568832	37.93664	-0.57	13	-271.6	12.4740002	-122.45688	37.93665	-0.61	13	-254.71	12.4320002	11	7/24/2014	21:05.5

2277	RWS11	-122.4568856	37.93664	-0.6	13	-274.8	12.429	-122.45688	37.93666	-0.61	13	-258.53	12.422	11	7/24/2014	21:05.6
2278	RWS11	-122.456888	37.93664	-0.57	13	-277.9	12.457	-122.45688	37.93666	-0.61	13	-262.15	12.415	11	7/24/2014	21:05.7
2279	RWS11	-122.4568903	37.93664	-0.6	13	-280.9	12.4160005	-122.45688	37.93666	-0.66	13	-265.55	12.3580005	11	7/24/2014	21:05.8
2280	RWS11	-122.4568927	37.93664	-0.57	13	-283.7	12.4460003	-122.45689	37.93666	-0.61	13	-268.6	12.4040003	11	7/24/2014	21:05.9
2281	RWS11	-122.4568962	37.93665	-0.6	13	-286.3	12.4080001	-122.45689	37.93666	-0.61	13	-271.35	12.4010001	11	7/24/2014	21:06.0
2282	RWS11	-122.4568986	37.93665	-0.57	13	-288.9	12.4419997	-122.45689	37.93666	-0.61	13	-273.94	12.3999997	11	7/24/2014	21:06.1
2283	RWS11	-122.4569009	37.93665	-0.6	13	-291.7	12.4069997	-122.4569	37.93666	-0.58	13	-276.79	12.4329997	11	7/24/2014	21:06.2
2284	RWS11	-122.4569033	37.93665	-0.57	13	-294.5	12.4439995	-122.4569	37.93666	-0.58	13	-279.63	12.4349995	11	7/24/2014	21:06.3
2285	RWS11	-122.4569057	37.93665	-0.6	13	-297.3	12.4139997	-122.4569	37.93666	-0.58	13	-282.2	12.4399996	11	7/24/2014	21:06.4
2286	RWS11	-122.4569081	37.93665	-0.57	13	-299.7	12.4550002	-122.4569	37.93666	-0.61	13	-284.56	12.4130002	11	7/24/2014	21:06.5
2287	RWS11	-122.4569105	37.93665	-0.6	13	-301.8	12.4279996	-122.4569	37.93666	-0.61	13	-286.55	12.4209996	11	7/24/2014	21:06.6
2288	RWS11	-122.4569128	37.93665	-0.57	13	-303.4	12.4729998	-122.45691	37.93666	-0.58	13	-288.33	12.4639998	11	7/24/2014	21:06.7
2289	RWS11	-122.4569152	37.93665	-0.6	13.1	-304.7	12.4499999	-122.45691	37.93666	-0.61	13.1	-289.76	12.4429999	11	7/24/2014	21:06.8
2290	RWS11	-122.4569175	37.93665	-0.57	13.1	-305.9	12.497	-122.45691	37.93666	-0.61	13.1	-291.09	12.455	11	7/24/2014	21:06.9
2291	RWS11	-122.456921	37.93665	-0.6	13.1	-307	12.4750005	-122.45692	37.93667	-0.61	13.1	-292.25	12.4680005	11	7/24/2014	21:07.0
2292	RWS11	-122.4569233	37.93665	-0.6	13.1	-308	12.4890004	-122.45692	37.93667	-0.61	13.1	-293.38	12.4820004	11	7/24/2014	21:07.1
2293	RWS11	-122.4569257	37.93665	-0.6	13.1	-308.9	12.5030004	-122.45692	37.93667	-0.66	13.1	-294.4	12.4450004	11	7/24/2014	21:07.2
2294	RWS11	-122.456928	37.93666	-0.6	13.1	-309.7	12.5159999	-122.45692	37.93667	-0.61	13.1	-295.11	12.5089999	11	7/24/2014	21:07.3
2295	RWS11	-122.4569304	37.93666	-0.6	13.1	-310.5	12.5310003	-122.45692	37.93667	-0.66	13.1	-295.74	12.4730002	11	7/24/2014	21:07.4
2296	RWS11	-122.4569327	37.93666	-0.6	13.1	-311.4	12.5459997	-122.45693	37.93667	-0.61	13.1	-296.42	12.5389996	11	7/24/2014	21:07.5
2297	RWS11	-122.4569351	37.93666	-0.6	13.2	-312	12.5629998	-122.45693	37.93667	-0.66	13.2	-297.1	12.5049998	11	7/24/2014	21:07.6
2298	RWS11	-122.4569374	37.93666	-0.6	13.2	-312.3	12.5789996	-122.45693	37.93667	-0.61	13.2	-297.58	12.5719996	11	7/24/2014	21:07.7
2299	RWS11	-122.4569397	37.93666	-0.6	13.2	-312.6	12.594	-122.45693	37.93667	-0.66	13.2	-298.21	12.536	11	7/24/2014	21:07.8
2300	RWS11	-122.456942	37.93666	-0.6	13.2	-312.8	12.6079999	-122.45694	37.93667	-0.61	13.2	-299.05	12.6009999	11	7/24/2014	21:07.9
2301	RWS11	-122.4569455	37.93666	-0.65	13.2	-313.2	12.569	-122.45694	37.93667	-0.66	13.2	-300.03	12.562	11	7/24/2014	21:08.0
2302	RWS11	-122.4569478	37.93666	-0.6	13.2	-313.7	12.6300002	-122.45694	37.93667	-0.66	13.2	-300.96	12.5720002	11	7/24/2014	21:08.1
2303	RWS11	-122.45695	37.93666	-0.65	13.2	-314.2	12.5849998	-122.45694	37.93667	-0.61	13.2	-301.8	12.6289998	11	7/24/2014	21:08.2
2304	RWS11	-122.4569524	37.93666	-0.6	13.2	-314.6	12.6420003	-122.45695	37.93667	-0.66	13.2	-302.67	12.5840003	11	7/24/2014	21:08.3
2305	RWS11	-122.4569547	37.93666	-0.65	13.2	-315	12.5939996	-122.45695	37.93668	-0.66	13.2	-303.5	12.5869996	11	7/24/2014	21:08.4
2306	RWS11	-122.456957	37.93666	-0.65	13.3	-315.4	12.5969999	-122.45695	37.93668	-0.61	13.3	-304.21	12.6409999	11	7/24/2014	21:08.5
2307	RWS11	-122.4569593	37.93666	-0.65	13.3	-315.6	12.5980003	-122.45695	37.93668	-0.66	13.3	-304.67	12.5910003	11	7/24/2014	21:08.6
2308	RWS11	-122.4569617	37.93666	-0.65	13.3	-315.9	12.5989997	-122.45696	37.93668	-0.61	13.3	-305.01	12.6429997	11	7/24/2014	21:08.7
2309	RWS11	-122.456964	37.93667	-0.65	13.3	-316.1	12.6009996	-122.45696	37.93668	-0.61	13.3	-305.3	12.6449996	11	7/24/2014	21:08.8

2310	RWS11	-122.4569663	37.93667	-0.6	13.3	-316.5	12.6540004	-122.45696	37.93668	-0.61	13.3	-305.36	12.6470004	11	7/24/2014	21:08.9
2311	RWS11	-122.4569697	37.93667	-0.65	13.3	-317	12.6050003	-122.45696	37.93668	-0.61	13.3	-305.36	12.6490002	11	7/24/2014	21:09.0
2312	RWS11	-122.456972	37.93667	-0.6	13.3	-317.5	12.6589996	-122.45697	37.93668	-0.61	13.3	-305.15	12.6519995	11	7/24/2014	21:09.1
2313	RWS11	-122.4569744	37.93667	-0.65	13.3	-317.9	12.6120002	-122.45697	37.93668	-0.66	13.3	-304.79	12.6050002	11	7/24/2014	21:09.2
2314	RWS11	-122.4569767	37.93667	-0.6	13.3	-318	12.6680003	-122.45697	37.93668	-0.58	13.3	-304.15	12.6940003	11	7/24/2014	21:09.3
2315	RWS11	-122.4569791	37.93667	-0.65	13.3	-317.7	12.6229999	-122.45697	37.93668	-0.61	13.3	-303.35	12.6669999	11	7/24/2014	21:09.4
2316	RWS11	-122.4569815	37.93667	-0.6	13.3	-317	12.679	-122.45698	37.93668	-0.66	13.3	-302.27	12.621	11	7/24/2014	21:09.5
2317	RWS11	-122.4569838	37.93667	-0.65	13.3	-316	12.6339996	-122.45698	37.93668	-0.66	13.3	-301	12.6269996	11	7/24/2014	21:09.6
2318	RWS11	-122.4569862	37.93667	-0.6	13.3	-314.9	12.6890003	-122.45698	37.93668	-0.61	13.3	-299.71	12.6820002	11	7/24/2014	21:09.7
2319	RWS11	-122.4569886	37.93667	-0.65	13.3	-313.8	12.6409996	-122.45698	37.93669	-0.61	13.3	-298.45	12.6849995	11	7/24/2014	21:09.8
2320	RWS11	-122.4569909	37.93667	-0.65	13.3	-312.6	12.642	-122.45699	37.93669	-0.66	13.3	-297.2	12.6349999	11	7/24/2014	21:09.9
2321	RWS11	-122.4569944	37.93667	-0.65	13.3	-311.4	12.6409996	-122.45699	37.93669	-0.66	13.3	-295.83	12.6339995	11	7/24/2014	21:10.0
2322	RWS11	-122.4569968	37.93667	-0.6	13.3	-310.4	12.6899997	-122.45699	37.93669	-0.61	13.3	-294.47	12.6829997	11	7/24/2014	21:10.1
2323	RWS11	-122.4569991	37.93668	-0.65	13.3	-309.5	12.635	-122.45699	37.93669	-0.66	13.3	-293.24	12.628	11	7/24/2014	21:10.2
2324	RWS11	-122.4570015	37.93668	-0.6	13.3	-308.8	12.6809999	-122.457	37.93669	-0.61	13.3	-292.13	12.6739999	11	7/24/2014	21:10.3
2325	RWS11	-122.4570039	37.93668	-0.6	13.3	-308.3	12.6750003	-122.457	37.93669	-0.61	13.3	-291.18	12.6680003	11	7/24/2014	21:10.4
2326	RWS11	-122.4570063	37.93668	-0.6	13.3	-307.7	12.6689998	-122.457	37.93669	-0.61	13.3	-290.25	12.6619998	11	7/24/2014	21:10.5
2327	RWS11	-122.4570087	37.93668	-0.6	13.3	-307.2	12.6630002	-122.457	37.93669	-0.61	13.3	-289.21	12.6560002	11	7/24/2014	21:10.6
2328	RWS11	-122.457011	37.93668	-0.6	13.3	-306.6	12.6580001	-122.45701	37.93669	-0.58	13.3	-288.04	12.6840001	11	7/24/2014	21:10.7
2329	RWS11	-122.4570134	37.93668	-0.6	13.3	-306	12.6549999	-122.45701	37.93669	-0.61	13.3	-286.87	12.6479998	11	7/24/2014	21:10.8
2330	RWS11	-122.4570158	37.93668	-0.6	13.3	-305	12.653	-122.45701	37.93669	-0.58	13.3	-285.55	12.679	11	7/24/2014	21:10.9
2331	RWS11	-122.4570193	37.93668	-0.6	13.3	-303.8	12.6519996	-122.45701	37.93669	-0.58	13.3	-284.08	12.6779996	11	7/24/2014	21:11.0
2332	RWS11	-122.4570216	37.93668	-0.6	13.3	-302.4	12.653	-122.45702	37.93669	-0.53	13.3	-282.52	12.73	11	7/24/2014	21:11.1
2333	RWS11	-122.457024	37.93668	-0.6	13.3	-300.8	12.6549999	-122.45702	37.9367	-0.58	13.3	-280.7	12.6809998	11	7/24/2014	21:11.2
2334	RWS11	-122.4570264	37.93668	-0.57	13.3	-299.1	12.6939995	-122.45702	37.9367	-0.53	13.3	-278.71	12.7359995	11	7/24/2014	21:11.3
2335	RWS11	-122.4570287	37.93668	-0.6	13.3	-297.2	12.6639997	-122.45702	37.9367	-0.53	13.3	-276.72	12.7409996	11	7/24/2014	21:11.4
2336	RWS11	-122.4570311	37.93668	-0.57	13.3	-295.4	12.7059996	-122.45703	37.9367	-0.58	13.3	-274.62	12.6969996	11	7/24/2014	21:11.5
2337	RWS11	-122.4570335	37.93669	-0.57	13.3	-293.3	12.7150004	-122.45703	37.9367	-0.58	13.3	-272.41	12.7060004	11	7/24/2014	21:11.6
2338	RWS11	-122.4570359	37.93669	-0.57	13.3	-291.4	12.7260001	-122.45703	37.9367	-0.53	13.3	-270.27	12.7680001	11	7/24/2014	21:11.7
2339	RWS11	-122.4570383	37.93669	-0.6	13.3	-289.3	12.7030002	-122.45703	37.9367	-0.53	13.3	-268.12	12.7800002	11	7/24/2014	21:11.8
2340	RWS11	-122.4570406	37.93669	-0.57	13.3	-287.2	12.7509997	-122.45703	37.9367	-0.53	13.3	-265.92	12.7929997	11	7/24/2014	21:11.9
2341	RWS11	-122.4570441	37.93669	-0.6	13.3	-285.1	12.7299997	-122.45704	37.9367	-0.53	13.3	-263.6	12.8069996	11	7/24/2014	21:12.0
2342	RWS11	-122.4570464	37.93669	-0.57	13.3	-282.8	12.78	-122.45704	37.9367	-0.53	13.3	-261.25	12.822	11	7/24/2014	21:12.1

2343	RWS11	-122.4570488	37.93669	-0.57	13.4	-280.6	12.7939999	-122.45704	37.9367	-0.49	13.4	-258.66	12.8699999	11	7/24/2014	21:12.2
2344	RWS11	-122.4570511	37.93669	-0.57	13.4	-278.2	12.8099997	-122.45705	37.9367	-0.49	13.4	-255.98	12.8859997	11	7/24/2014	21:12.3
2345	RWS11	-122.4570535	37.93669	-0.57	13.4	-275.7	12.8250001	-122.45705	37.9367	-0.49	13.4	-253.23	12.9010001	11	7/24/2014	21:12.4
2346	RWS11	-122.4570559	37.93669	-0.52	13.4	-273.2	12.8919998	-122.45705	37.9367	-0.49	13.4	-250.35	12.9169999	11	7/24/2014	21:12.5
2347	RWS11	-122.4570582	37.93669	-0.57	13.4	-270.5	12.858	-122.45705	37.9367	-0.49	13.4	-247.13	12.9340001	11	7/24/2014	21:12.6
2348	RWS11	-122.4570606	37.93669	-0.57	13.4	-267.7	12.8739998	-122.45705	37.93671	-0.49	13.4	-243.7	12.9499998	11	7/24/2014	21:12.7
2349	RWS11	-122.457063	37.93669	-0.57	13.5	-264.6	12.8899996	-122.45706	37.93671	-0.49	13.5	-240.16	12.9659996	11	7/24/2014	21:12.8
2350	RWS11	-122.4570653	37.93669	-0.52	13.5	-261.5	12.9579998	-122.45706	37.93671	-0.49	13.5	-236.33	12.9829998	11	7/24/2014	21:12.9
2351	RWS11	-122.4570688	37.9367	-0.57	13.5	-258.2	12.9220002	-122.45706	37.93671	-0.49	13.5	-232.48	12.9980002	11	7/24/2014	21:13.0
2352	RWS11	-122.4570711	37.9367	-0.52	13.5	-254.9	12.989	-122.45707	37.93671	-0.44	13.5	-228.5	13.065	11	7/24/2014	21:13.1
2353	RWS11	-122.4570735	37.9367	-0.57	13.5	-251.5	12.9530003	-122.45707	37.93671	-0.44	13.5	-224.58	13.0800003	11	7/24/2014	21:13.2
2354	RWS11	-122.4570758	37.9367	-0.52	13.5	-248.1	13.0189997	-122.45707	37.93671	-0.44	13.5	-220.68	13.0949997	11	7/24/2014	21:13.3
2355	RWS11	-122.4570782	37.9367	-0.57	13.6	-244.6	12.983	-122.45707	37.93671	-0.49	13.6	-216.72	13.0590001	11	7/24/2014	21:13.4
2356	RWS11	-122.4570806	37.9367	-0.52	13.6	-241	13.048	-122.45707	37.93671	-0.44	13.6	-212.69	13.124	11	7/24/2014	21:13.5
2357	RWS11	-122.457083	37.9367	-0.52	13.6	-237.3	13.0619999	-122.45708	37.93671	-0.49	13.6	-208.54	13.0869999	11	7/24/2014	21:13.6
2358	RWS11	-122.4570853	37.9367	-0.52	13.6	-233.5	13.074	-122.45708	37.93671	-0.44	13.6	-204.48	13.15	11	7/24/2014	21:13.7
2359	RWS11	-122.4570877	37.9367	-0.52	13.6	-229.8	13.0860001	-122.45708	37.93671	-0.49	13.6	-200.34	13.1110001	11	7/24/2014	21:13.8
2360	RWS11	-122.45709	37.9367	-0.52	13.6	-226	13.0969998	-122.45708	37.93671	-0.49	13.6	-196.24	13.1219998	11	7/24/2014	21:13.9
2361	RWS11	-122.4570935	37.9367	-0.52	13.6	-222.3	13.107	-122.45709	37.93671	-0.49	13.6	-192.12	13.132	11	7/24/2014	21:14.0
2362	RWS11	-122.4570958	37.9367	-0.48	13.6	-218.7	13.1499998	-122.45709	37.93672	-0.41	13.6	-187.95	13.2259998	11	7/24/2014	21:14.1
2363	RWS11	-122.4570981	37.9367	-0.52	13.6	-214.9	13.1240002	-122.45709	37.93672	-0.44	13.6	-183.5	13.2000002	11	7/24/2014	21:14.2
2364	RWS11	-122.4571005	37.9367	-0.48	13.7	-211.1	13.167	-122.45709	37.93672	-0.41	13.7	-179	13.243	11	7/24/2014	21:14.3
2365	RWS11	-122.4571028	37.9367	-0.52	13.7	-207.4	13.1419998	-122.4571	37.93672	-0.44	13.7	-174.49	13.2179999	11	7/24/2014	21:14.4
2366	RWS11	-122.4571052	37.93671	-0.52	13.7	-203.7	13.1520001	-122.4571	37.93672	-0.44	13.7	-170.01	13.2280001	11	7/24/2014	21:14.5
2367	RWS11	-122.4571075	37.93671	-0.48	13.7	-199.7	13.1980002	-122.4571	37.93672	-0.41	13.7	-165.45	13.2740002	11	7/24/2014	21:14.6
2368	RWS11	-122.4571099	37.93671	-0.48	13.7	-195.6	13.2129996	-122.4571	37.93672	-0.41	13.7	-160.61	13.2889996	11	7/24/2014	21:14.7
2369	RWS11	-122.4571122	37.93671	-0.48	13.7	-191.2	13.2299998	-122.45711	37.93672	-0.41	13.7	-155.61	13.3059998	11	7/24/2014	21:14.8
2370	RWS11	-122.4571145	37.93671	-0.48	13.7	-186.7	13.2509997	-122.45711	37.93672	-0.44	13.7	-150.55	13.2929997	11	7/24/2014	21:14.9
2371	RWS11	-122.457118	37.93671	-0.48	13.8	-181.9	13.2760002	-122.45711	37.93672	-0.44	13.8	-145.17	13.3180002	11	7/24/2014	21:15.0
2372	RWS11	-122.4571203	37.93671	-0.48	13.8	-177	13.3049996	-122.45711	37.93672	-0.44	13.8	-139.45	13.3469996	11	7/24/2014	21:15.1
2373	RWS11	-122.4571226	37.93671	-0.48	13.8	-172	13.3379996	-122.45712	37.93672	-0.41	13.8	-133.43	13.4139996	11	7/24/2014	21:15.2
2374	RWS11	-122.457125	37.93671	-0.48	13.9	-166.9	13.3750002	-122.45712	37.93672	-0.41	13.9	-127.3	13.4510002	11	7/24/2014	21:15.3
2375	RWS11	-122.4571273	37.93671	-0.52	13.9	-161.5	13.3799998	-122.45712	37.93672	-0.44	13.9	-120.81	13.4559998	11	7/24/2014	21:15.4

2376	RWS11	-122.4571297	37.93671	-0.48	13.9	-156.1	13.457	-122.45712	37.93672	-0.41	13.9	-114.45	13.533	11	7/24/2014	21:15.5
2377	RWS11	-122.4571321	37.93671	-0.48	14	-150.7	13.5020001	-122.45713	37.93673	-0.41	14	-108.2	13.5780001	11	7/24/2014	21:15.6
2378	RWS11	-122.4571345	37.93671	-0.48	14	-145.2	13.5500004	-122.45713	37.93673	-0.44	14	-101.77	13.5920004	11	7/24/2014	21:15.7
2379	RWS11	-122.4571368	37.93671	-0.48	14.1	-139.6	13.5990002	-122.45713	37.93673	-0.44	14.1	-95.222	13.6410002	11	7/24/2014	21:15.8
2380	RWS11	-122.4571391	37.93671	-0.48	14.1	-133.8	13.6499998	-122.45713	37.93673	-0.41	14.1	-88.477	13.7259998	11	7/24/2014	21:15.9
2381	RWS11	-122.4571426	37.93672	-0.52	14.2	-127.8	13.666	-122.45714	37.93673	-0.44	14.2	-81.741	13.742	11	7/24/2014	21:16.0
2382	RWS11	-122.4571449	37.93672	-0.48	14.2	-121.6	13.7500002	-122.45714	37.93673	-0.44	14.2	-74.857	13.7920002	11	7/24/2014	21:16.1
2383	RWS11	-122.4571473	37.93672	-0.48	14.3	-115.4	13.7979996	-122.45714	37.93673	-0.49	14.3	-68.012	13.7889996	11	7/24/2014	21:16.2
2384	RWS11	-122.4571496	37.93672	-0.48	14.3	-109	13.8429997	-122.45714	37.93673	-0.44	14.3	-61.097	13.8849997	11	7/24/2014	21:16.3
2385	RWS11	-122.457152	37.93672	-0.48	14.4	-102.6	13.8829996	-122.45715	37.93673	-0.41	14.4	-54.004	13.9589996	11	7/24/2014	21:16.4
2386	RWS11	-122.4571544	37.93672	-0.48	14.4	-96.2	13.9200003	-122.45715	37.93673	-0.44	14.4	-46.958	13.9620003	11	7/24/2014	21:16.5
2387	RWS11	-122.4571567	37.93672	-0.48	14.4	-89.68	13.9519999	-122.45715	37.93673	-0.44	14.4	-39.867	13.9939999	11	7/24/2014	21:16.6
2388	RWS11	-122.4571591	37.93672	-0.48	14.5	-83.2	13.9799998	-122.45715	37.93673	-0.41	14.5	-32.997	14.0559998	11	7/24/2014	21:16.7
2389	RWS11	-122.4571614	37.93672	-0.48	14.5	-76.79	14.0030005	-122.45716	37.93673	-0.49	14.5	-26.016	13.9940005	11	7/24/2014	21:16.8
2390	RWS11	-122.4571638	37.93672	-0.48	14.5	-70.33	14.0219996	-122.45716	37.93673	-0.44	14.5	-18.901	14.0639996	11	7/24/2014	21:16.9
2391	RWS11	-122.4571673	37.93672	-0.48	14.5	-63.74	14.0369999	-122.45716	37.93674	-0.49	14.5	-11.811	14.0279999	11	7/24/2014	21:17.0
2392	RWS11	-122.4571696	37.93672	-0.48	14.5	-57.17	14.049	-122.45716	37.93674	-0.44	14.5	-4.611	14.091	11	7/24/2014	21:17.1
2393	RWS11	-122.4571719	37.93672	-0.52	14.5	-50.49	14.0250002	-122.45717	37.93674	-0.49	14.5	2.677	14.0500002	11	7/24/2014	21:17.2
2394	RWS11	-122.4571743	37.93672	-0.48	14.6	-43.67	14.0660002	-122.45717	37.93674	-0.41	14.6	10.378	14.1420002	11	7/24/2014	21:17.3
2395	RWS11	-122.4571767	37.93673	-0.48	14.6	-36.88	14.0719998	-122.45717	37.93674	-0.49	14.6	17.864	14.0629998	11	7/24/2014	21:17.4
2396	RWS11	-122.4571791	37.93673	-0.48	14.6	-29.94	14.0760004	-122.45717	37.93674	-0.44	14.6	25.629	14.1180004	11	7/24/2014	21:17.5
2397	RWS11	-122.4571814	37.93673	-0.48	14.6	-22.78	14.0789997	-122.45718	37.93674	-0.41	14.6	33.724	14.1549997	11	7/24/2014	21:17.6
2398	RWS11	-122.4571838	37.93673	-0.48	14.6	-15.52	14.082	-122.45718	37.93674	-0.44	14.6	41.621	14.124	11	7/24/2014	21:17.7
2399	RWS11	-122.4571862	37.93673	-0.48	14.6	-8.166	14.0839998	-122.45718	37.93674	-0.44	14.6	49.522	14.1259998	11	7/24/2014	21:17.8
2400	RWS11	-122.4571885	37.93673	-0.48	14.6	-0.791	14.0850003	-122.45718	37.93674	-0.44	14.6	57.565	14.1270002	11	7/24/2014	21:17.9
2401	RWS11	-122.457192	37.93673	-0.48	14.6	6.845	14.0870001	-122.45719	37.93674	-0.49	14.6	65.863	14.0780001	11	7/24/2014	21:18.0
2402	RWS11	-122.4571944	37.93673	-0.48	14.6	14.372	14.0870001	-122.45719	37.93674	-0.44	14.6	74.223	14.1290001	11	7/24/2014	21:18.1
2403	RWS11	-122.4571968	37.93673	-0.48	14.6	22.137	14.0870001	-122.45719	37.93674	-0.44	14.6	83.013	14.1290001	11	7/24/2014	21:18.2
2404	RWS11	-122.4571992	37.93673	-0.48	14.6	30.078	14.0859997	-122.45719	37.93674	-0.44	14.6	92.064	14.1279997	11	7/24/2014	21:18.3
2405	RWS11	-122.4572016	37.93673	-0.48	14.6	38.042	14.0839998	-122.4572	37.93674	-0.44	14.6	101.12	14.1259998	11	7/24/2014	21:18.4
2406	RWS11	-122.457204	37.93673	-0.48	14.6	46.074	14.0809996	-122.4572	37.93675	-0.44	14.6	110.39	14.1229996	11	7/24/2014	21:18.5
2407	RWS11	-122.4572064	37.93673	-0.52	14.6	54.321	14.0429999	-122.4572	37.93675	-0.44	14.6	119.76	14.1189999	11	7/24/2014	21:18.6
2408	RWS11	-122.4572088	37.93673	-0.48	14.6	62.656	14.0719998	-122.4572	37.93675	-0.44	14.6	129.2	14.1139998	11	7/24/2014	21:18.7

2409	RWS11	-122.4572112	37.93673	-0.48	14.6	71.078	14.0669996	-122.45721	37.93675	-0.44	14.6	138.66	14.1089996	11	7/24/2014	21:18.8
2410	RWS11	-122.4572135	37.93674	-0.48	14.5	79.521	14.0610001	-122.45721	37.93675	-0.41	14.5	148.02	14.1370001	11	7/24/2014	21:18.9
2411	RWS11	-122.4572171	37.93674	-0.48	14.5	88.047	14.0540001	-122.45721	37.93675	-0.44	14.5	157.41	14.0960001	11	7/24/2014	21:19.0
2412	RWS11	-122.4572194	37.93674	-0.48	14.5	96.868	14.0479996	-122.45721	37.93675	-0.44	14.5	167.06	14.0899996	11	7/24/2014	21:19.1
2413	RWS11	-122.4572218	37.93674	-0.48	14.5	105.73	14.0409996	-122.45722	37.93675	-0.44	14.5	176.9	14.0829996	11	7/24/2014	21:19.2
2414	RWS11	-122.4572242	37.93674	-0.48	14.5	114.7	14.0350001	-122.45722	37.93675	-0.44	14.5	186.91	14.0770001	11	7/24/2014	21:19.3
2415	RWS11	-122.4572266	37.93674	-0.52	14.5	123.75	13.9949995	-122.45722	37.93675	-0.49	14.5	196.98	14.0199995	11	7/24/2014	21:19.4
2416	RWS11	-122.457229	37.93674	-0.48	14.5	132.89	14.0219996	-122.45722	37.93675	-0.44	14.5	207.2	14.0639996	11	7/24/2014	21:19.5
2417	RWS11	-122.4572314	37.93674	-0.52	14.5	142.1	13.982	-122.45723	37.93675	-0.44	14.5	217.51	14.058	11	7/24/2014	21:19.6
2418	RWS11	-122.4572337	37.93674	-0.48	14.5	151.44	14.009	-122.45723	37.93675	-0.44	14.5	227.94	14.051	11	7/24/2014	21:19.7
2419	RWS11	-122.4572361	37.93674	-0.48	14.5	160.78	14.0009997	-122.45723	37.93675	-0.49	14.5	238.46	13.9919997	11	7/24/2014	21:19.8
2420	RWS11	-122.4572385	37.93674	-0.48	14.5	170.2	13.9930002	-122.45723	37.93676	-0.44	14.5	249.02	14.0350002	11	7/24/2014	21:19.9
2421	RWS11	-122.457242	37.93674	-0.48	14.5	179.8	13.9849999	-122.45724	37.93676	-0.44	14.5	259.66	14.0269999	11	7/24/2014	21:20.0
2422	RWS11	-122.4572443	37.93674	-0.48	14.5	189.61	13.9770005	-122.45724	37.93676	-0.44	14.5	270.5	14.0190004	11	7/24/2014	21:20.1
2423	RWS11	-122.4572467	37.93674	-0.48	14.5	199.35	13.9690001	-122.45724	37.93676	-0.41	14.5	281.19	14.0450001	11	7/24/2014	21:20.2
2424	RWS11	-122.4572491	37.93675	-0.43	14.4	209.02	14.0130001	-122.45724	37.93676	-0.44	14.4	291.81	14.0040001	11	7/24/2014	21:20.3
2425	RWS11	-122.4572514	37.93675	-0.48	14.4	218.79	13.9559996	-122.45725	37.93676	-0.44	14.4	302.52	13.9979996	11	7/24/2014	21:20.4
2426	RWS11	-122.4572538	37.93675	-0.43	14.4	228.77	14.001	-122.45725	37.93676	-0.41	14.4	313.66	14.026	11	7/24/2014	21:20.5
2427	RWS11	-122.4572562	37.93675	-0.48	14.4	238.96	13.9460003	-122.45725	37.93676	-0.41	14.4	325.05	14.0220003	11	7/24/2014	21:20.6
2428	RWS11	-122.4572586	37.93675	-0.43	14.4	249.24	13.9929996	-122.45725	37.93676	-0.41	14.4	336.79	14.0179997	11	7/24/2014	21:20.7
2429	RWS11	-122.4572609	37.93675	-0.48	14.4	259.5	13.9390003	-122.45726	37.93676	-0.41	14.4	348.84	14.0150003	11	7/24/2014	21:20.8
2430	RWS11	-122.4572633	37.93675	-0.48	14.4	269.86	13.9369995	-122.45726	37.93676	-0.36	14.4	361.06	14.0629995	11	7/24/2014	21:20.9
2431	RWS11	-122.4572667	37.93675	-0.48	14.4	280.32	13.9329998	-122.45726	37.93676	-0.41	14.4	373.42	14.0089998	11	7/24/2014	21:21.0
2432	RWS11	-122.4572691	37.93675	-0.43	14.4	290.86	13.9800001	-122.45726	37.93676	-0.41	14.4	385.89	14.0050001	11	7/24/2014	21:21.1
2433	RWS11	-122.4572714	37.93675	-0.48	14.4	301.57	13.924	-122.45727	37.93676	-0.44	14.4	398.38	13.966	11	7/24/2014	21:21.2
2434	RWS11	-122.4572738	37.93675	-0.43	14.4	312.38	13.968	-122.45727	37.93676	-0.41	14.4	410.95	13.993	11	7/24/2014	21:21.3
2435	RWS11	-122.4572761	37.93675	-0.48	14.4	323.4	13.9089997	-122.45727	37.93677	-0.41	14.4	423.44	13.9849997	11	7/24/2014	21:21.4
2436	RWS11	-122.4572785	37.93675	-0.43	14.4	334.59	13.9509998	-122.45727	37.93677	-0.41	14.4	436.12	13.9759998	11	7/24/2014	21:21.5
2437	RWS11	-122.4572808	37.93675	-0.48	14.4	345.93	13.8899996	-122.45728	37.93677	-0.41	14.4	448.92	13.9659996	11	7/24/2014	21:21.6
2438	RWS11	-122.4572832	37.93675	-0.43	14.4	357.37	13.9299999	-122.45728	37.93677	-0.41	14.4	461.89	13.9549999	11	7/24/2014	21:21.7
2439	RWS11	-122.4572855	37.93676	-0.48	14.4	368.89	13.8680002	-122.45728	37.93677	-0.41	14.4	475.01	13.9440002	11	7/24/2014	21:21.8
2440	RWS11	-122.4572878	37.93676	-0.43	14.3	380.52	13.9079996	-122.45728	37.93677	-0.36	14.3	488.16	13.9829996	11	7/24/2014	21:21.9
2441	RWS11	-122.4572913	37.93676	-0.48	14.3	392.3	13.8479998	-122.45729	37.93677	-0.41	14.3	501.51	13.9239998	11	7/24/2014	21:22.0

2442	RWS11	-122.4572936	37.93676	-0.43	14.3	404.26	13.89	-122.45729	37.93677	-0.41	14.3	514.97	13.915	11	7/24/2014	21:22.1
2443	RWS11	-122.457296	37.93676	-0.48	14.3	416.36	13.832	-122.45729	37.93677	-0.44	14.3	528.32	13.874	11	7/24/2014	21:22.2
2444	RWS11	-122.4572983	37.93676	-0.43	14.3	428.53	13.8790003	-122.45729	37.93677	-0.36	14.3	541.78	13.9540003	11	7/24/2014	21:22.3
2445	RWS11	-122.4573007	37.93676	-0.48	14.3	440.7	13.8260004	-122.4573	37.93677	-0.41	14.3	555.24	13.9020004	11	7/24/2014	21:22.4
2446	RWS11	-122.4573031	37.93676	-0.43	14.3	452.96	13.8789999	-122.4573	37.93677	-0.41	14.3	568.83	13.9039999	11	7/24/2014	21:22.5
2447	RWS11	-122.4573054	37.93676	-0.48	14.3	465.34	13.8310001	-122.4573	37.93677	-0.41	14.3	582.74	13.9070001	11	7/24/2014	21:22.6
2448	RWS11	-122.4573078	37.93676	-0.43	14.3	478.01	13.8859998	-122.4573	37.93677	-0.36	14.3	596.97	13.9609999	11	7/24/2014	21:22.7
2449	RWS11	-122.4573101	37.93676	-0.43	14.3	490.92	13.8909999	-122.4573	37.93677	-0.41	14.3	611.45	13.916	11	7/24/2014	21:22.8
2450	RWS11	-122.4573124	37.93676	-0.43	14.3	504.06	13.8970005	-122.45731	37.93678	-0.36	14.3	625.91	13.9720005	11	7/24/2014	21:22.9
2451	RWS11	-122.4573159	37.93676	-0.48	14.3	517.3	13.8509996	-122.45731	37.93678	-0.36	14.3	640.37	13.9769996	11	7/24/2014	21:23.0
2452	RWS11	-122.4573182	37.93676	-0.43	14.3	530.69	13.9069997	-122.45731	37.93678	-0.36	14.3	654.95	13.9819998	11	7/24/2014	21:23.1
2453	RWS11	-122.4573205	37.93676	-0.48	14.3	544.27	13.8600004	-122.45731	37.93678	-0.41	14.3	669.79	13.9360004	11	7/24/2014	21:23.2
2454	RWS11	-122.4573229	37.93677	-0.43	14.3	557.89	13.9150001	-122.45732	37.93678	-0.36	14.3	684.86	13.9900001	11	7/24/2014	21:23.3
2455	RWS11	-122.4573252	37.93677	-0.43	14.4	571.66	13.9180004	-122.45732	37.93678	-0.41	14.4	700.1	13.9430004	11	7/24/2014	21:23.4
2456	RWS11	-122.4573275	37.93677	-0.43	14.4	585.39	13.9200002	-122.45732	37.93678	-0.36	14.4	715.48	13.9950002	11	7/24/2014	21:23.5
2457	RWS11	-122.4573299	37.93677	-0.48	14.4	599.2	13.8710001	-122.45732	37.93678	-0.36	14.4	731.01	13.9970001	11	7/24/2014	21:23.6
2458	RWS11	-122.4573322	37.93677	-0.43	14.4	613.15	13.9239999	-122.45733	37.93678	-0.36	14.4	746.9	13.999	11	7/24/2014	21:23.7
2459	RWS11	-122.4573345	37.93677	-0.43	14.4	627	13.9250003	-122.45733	37.93678	-0.36	14.4	762.86	14.0000004	11	7/24/2014	21:23.8
2460	RWS11	-122.4573368	37.93677	-0.43	14.4	640.87	13.9270002	-122.45733	37.93678	-0.36	14.4	778.76	14.0020002	11	7/24/2014	21:23.9
2461	RWS11	-122.4573403	37.93677	-0.43	14.4	654.55	13.9300005	-122.45733	37.93678	-0.36	14.4	794.78	14.0050005	11	7/24/2014	21:24.0
2462	RWS11	-122.4573426	37.93677	-0.43	14.4	668.33	13.9329998	-122.45734	37.93678	-0.36	14.4	810.83	14.0079998	11	7/24/2014	21:24.1
2463	RWS11	-122.4573449	37.93677	-0.43	14.4	682.04	13.9379999	-122.45734	37.93678	-0.36	14.4	827.02	14.0129999	11	7/24/2014	21:24.2
2464	RWS11	-122.4573473	37.93677	-0.43	14.4	695.91	13.9440004	-122.45734	37.93679	-0.36	14.4	843.28	14.0190004	11	7/24/2014	21:24.3
2465	RWS11	-122.4573496	37.93677	-0.43	14.4	709.92	13.9530002	-122.45734	37.93679	-0.36	14.4	859.71	14.0280002	11	7/24/2014	21:24.4
2466	RWS11	-122.457352	37.93677	-0.43	14.4	724.1	13.9639999	-122.45735	37.93679	-0.36	14.4	876.43	14.0389999	11	7/24/2014	21:24.5
2467	RWS11	-122.4573544	37.93677	-0.43	14.4	738.56	13.9770004	-122.45735	37.93679	-0.41	14.4	893.47	14.0020004	11	7/24/2014	21:24.6
2468	RWS11	-122.4573567	37.93677	-0.4	14.4	753.25	14.0269998	-122.45735	37.93679	-0.36	14.4	910.73	14.0669998	11	7/24/2014	21:24.7
2469	RWS11	-122.4573591	37.93678	-0.43	14.4	768.12	14.0100004	-122.45735	37.93679	-0.41	14.4	928.01	14.0350004	11	7/24/2014	21:24.8
2470	RWS11	-122.4573614	37.93678	-0.43	14.5	783.14	14.0299999	-122.45736	37.93679	-0.36	14.5	945.46	14.1049999	11	7/24/2014	21:24.9
2471	RWS11	-122.4573649	37.93678	-0.43	14.5	798.25	14.0509998	-122.45736	37.93679	-0.41	14.5	962.96	14.0759998	11	7/24/2014	21:25.0
2472	RWS11	-122.4573672	37.93678	-0.43	14.5	813.45	14.0730001	-122.45736	37.93679	-0.41	14.5	980.49	14.0980001	11	7/24/2014	21:25.1
2473	RWS11	-122.4573695	37.93678	-0.43	14.5	828.68	14.094	-122.45736	37.93679	-0.41	14.5	998.09	14.119	11	7/24/2014	21:25.2
2474	RWS11	-122.4573719	37.93678	-0.4	14.5	843.94	14.1489995	-122.45737	37.93679	-0.41	14.5	1015.7	14.1389995	11	7/24/2014	21:25.3

2475	RWS11	-122.4573742	37.93678	-0.43	14.6	859.21	14.1320001	-122.45737	37.93679	-0.41	14.6	1033.3	14.1570001	11	7/24/2014	21:25.4
2476	RWS11	-122.4573766	37.93678	-0.4	14.6	874.47	14.1829999	-122.45737	37.93679	-0.41	14.6	1050.9	14.1729999	11	7/24/2014	21:25.5
2477	RWS11	-122.457379	37.93678	-0.43	14.6	889.7	14.1610004	-122.45737	37.93679	-0.44	14.6	1068.4	14.1520004	11	7/24/2014	21:25.6
2478	RWS11	-122.4573813	37.93678	-0.4	14.6	904.9	14.2059997	-122.45738	37.93679	-0.41	14.6	1085.9	14.1959997	11	7/24/2014	21:25.7
2479	RWS11	-122.4573837	37.93678	-0.43	14.6	920.03	14.1779997	-122.45738	37.9368	-0.44	14.6	1103.3	14.1689996	11	7/24/2014	21:25.8
2480	RWS11	-122.457386	37.93678	-0.43	14.6	935.03	14.1820003	-122.45738	37.9368	-0.36	14.6	1120.5	14.2570003	11	7/24/2014	21:25.9
2481	RWS11	-122.4573894	37.93678	-0.43	14.6	949.85	14.1829998	-122.45738	37.9368	-0.44	14.6	1137.5	14.1739998	11	7/24/2014	21:26.0
2482	RWS11	-122.4573917	37.93678	-0.4	14.6	964.47	14.2159999	-122.45739	37.9368	-0.44	14.6	1154.2	14.1719999	11	7/24/2014	21:26.1
2483	RWS11	-122.4573941	37.93679	-0.43	14.6	979.1	14.1770002	-122.45739	37.9368	-0.44	14.6	1170.8	14.1680002	11	7/24/2014	21:26.2
2484	RWS11	-122.4573964	37.93679	-0.43	14.6	993.32	14.1700002	-122.45739	37.9368	-0.41	14.6	1187.1	14.1950002	11	7/24/2014	21:26.3
2485	RWS11	-122.4573987	37.93679	-0.43	14.6	1007.3	14.1619999	-122.45739	37.9368	-0.44	14.6	1203.1	14.1529999	11	7/24/2014	21:26.4
2486	RWS11	-122.4574011	37.93679	-0.43	14.6	1021.1	14.1540004	-122.4574	37.9368	-0.41	14.6	1218.9	14.1790004	11	7/24/2014	21:26.5
2487	RWS11	-122.4574034	37.93679	-0.48	14.6	1034.9	14.0930002	-122.4574	37.9368	-0.44	14.6	1234.6	14.1350002	11	7/24/2014	21:26.6
2488	RWS11	-122.4574057	37.93679	-0.43	14.6	1048.4	14.134	-122.4574	37.9368	-0.41	14.6	1250.2	14.159	11	7/24/2014	21:26.7
2489	RWS11	-122.457408	37.93679	-0.43	14.6	1061.7	14.1239997	-122.4574	37.9368	-0.44	14.6	1265.7	14.1149997	11	7/24/2014	21:26.8
2490	RWS11	-122.4574103	37.93679	-0.43	14.5	1074.8	14.1139995	-122.4574	37.9368	-0.41	14.5	1280.8	14.1389995	11	7/24/2014	21:26.9
2491	RWS11	-122.4574137	37.93679	-0.43	14.5	1087.5	14.1060001	-122.45741	37.9368	-0.41	14.5	1295.6	14.1310001	11	7/24/2014	21:27.0
2492	RWS11	-122.457416	37.93679	-0.43	14.5	1100.2	14.0990001	-122.45741	37.9368	-0.41	14.5	1310.3	14.1240001	11	7/24/2014	21:27.1
2493	RWS11	-122.4574183	37.93679	-0.43	14.5	1112.7	14.094	-122.45741	37.9368	-0.44	14.5	1324.8	14.085	11	7/24/2014	21:27.2
2494	RWS11	-122.4574206	37.93679	-0.43	14.5	1125	14.0909998	-122.45741	37.93681	-0.44	14.5	1339.3	14.0819998	11	7/24/2014	21:27.3
2495	RWS11	-122.4574229	37.93679	-0.48	14.5	1137.3	14.0399998	-122.45742	37.93681	-0.36	14.5	1353.5	14.1659998	11	7/24/2014	21:27.4
2496	RWS11	-122.4574252	37.93679	-0.43	14.5	1149.6	14.094	-122.45742	37.93681	-0.41	14.5	1367.7	14.119	11	7/24/2014	21:27.5
2497	RWS11	-122.4574275	37.93679	-0.43	14.5	1161.8	14.0990001	-122.45742	37.93681	-0.44	14.5	1381.8	14.0900001	11	7/24/2014	21:27.6
2498	RWS11	-122.4574298	37.9368	-0.43	14.5	1173.9	14.1069996	-122.45742	37.93681	-0.41	14.5	1395.6	14.1319996	11	7/24/2014	21:27.7
2499	RWS11	-122.4574321	37.9368	-0.43	14.5	1185.7	14.1160003	-122.45743	37.93681	-0.41	14.5	1409.3	14.1410003	11	7/24/2014	21:27.8
2500	RWS11	-122.4574344	37.9368	-0.43	14.6	1197.4	14.127	-122.45743	37.93681	-0.36	14.6	1422.6	14.202	11	7/24/2014	21:27.9
2501	RWS11	-122.4574379	37.9368	-0.43	14.6	1208.7	14.1379997	-122.45743	37.93681	-0.44	14.6	1435.5	14.1289997	11	7/24/2014	21:28.0
2502	RWS11	-122.4574402	37.9368	-0.43	14.6	1219.5	14.1479999	-122.45743	37.93681	-0.36	14.6	1447.7	14.2229999	11	7/24/2014	21:28.1
2503	RWS11	-122.4574425	37.9368	-0.48	14.6	1229.7	14.1059997	-122.45744	37.93681	-0.41	14.6	1459.3	14.1819997	11	7/24/2014	21:28.2
2504	RWS11	-122.4574448	37.9368	-0.43	14.6	1239.4	14.1650001	-122.45744	37.93681	-0.36	14.6	1470.3	14.2400001	11	7/24/2014	21:28.3
2505	RWS11	-122.4574472	37.9368	-0.43	14.6	1248.5	14.1709997	-122.45744	37.93681	-0.41	14.6	1480.5	14.1959997	11	7/24/2014	21:28.4
2506	RWS11	-122.4574495	37.9368	-0.43	14.6	1257	14.1750003	-122.45744	37.93681	-0.41	14.6	1489.9	14.2000004	11	7/24/2014	21:28.5
2507	RWS11	-122.4574519	37.9368	-0.43	14.6	1264.9	14.1779997	-122.45745	37.93681	-0.41	14.6	1498.5	14.2029997	11	7/24/2014	21:28.6

2508	RWS11	-122.4574543	37.9368	-0.43	14.6	1271.9	14.1790001	-122.45745	37.93681	-0.36	14.6	1506.3	14.2540001	11	7/24/2014	21:28.7
2509	RWS11	-122.4574566	37.9368	-0.43	14.6	1278.2	14.1790001	-122.45745	37.93682	-0.41	14.6	1513.1	14.2040001	11	7/24/2014	21:28.8
2510	RWS11	-122.457459	37.9368	-0.43	14.6	1284	14.1770002	-122.45745	37.93682	-0.36	14.6	1519.3	14.2520002	11	7/24/2014	21:28.9
2511	RWS11	-122.4574625	37.9368	-0.43	14.6	1289.1	14.1750003	-122.45746	37.93682	-0.36	14.6	1524.9	14.2500004	11	7/24/2014	21:29.0
2512	RWS11	-122.4574648	37.93681	-0.4	14.6	1293.5	14.2050002	-122.45746	37.93682	-0.36	14.6	1529.6	14.2450002	11	7/24/2014	21:29.1
2513	RWS11	-122.4574672	37.93681	-0.43	14.6	1297.2	14.1630003	-122.45746	37.93682	-0.41	14.6	1533.4	14.1880003	11	7/24/2014	21:29.2
2514	RWS11	-122.4574696	37.93681	-0.43	14.6	1300.2	14.1540004	-122.45746	37.93682	-0.41	14.6	1536.4	14.1790004	11	7/24/2014	21:29.3
2515	RWS11	-122.457472	37.93681	-0.43	14.6	1302.5	14.1420004	-122.45747	37.93682	-0.41	14.6	1538.7	14.1670004	11	7/24/2014	21:29.4
2516	RWS11	-122.4574744	37.93681	-0.43	14.6	1304.2	14.1259996	-122.45747	37.93682	-0.36	14.6	1540	14.2009996	11	7/24/2014	21:29.5
2517	RWS11	-122.4574768	37.93681	-0.43	14.5	1305.1	14.1069996	-122.45747	37.93682	-0.41	14.5	1540.6	14.1319996	11	7/24/2014	21:29.6
2518	RWS11	-122.4574792	37.93681	-0.43	14.5	1305.4	14.0850002	-122.45747	37.93682	-0.36	14.5	1540.3	14.1600002	11	7/24/2014	21:29.7
2519	RWS11	-122.4574815	37.93681	-0.43	14.5	1305.2	14.0599996	-122.45748	37.93682	-0.41	14.5	1539.3	14.0849996	11	7/24/2014	21:29.8
2520	RWS11	-122.4574839	37.93681	-0.43	14.5	1304.2	14.0319997	-122.45748	37.93682	-0.41	14.5	1537.6	14.0569997	11	7/24/2014	21:29.9
2521	RWS11	-122.4574874	37.93681	-0.48	14.4	1302.8	13.951	-122.45748	37.93682	-0.41	14.4	1535.1	14.027	11	7/24/2014	21:30.0
2522	RWS11	-122.4574898	37.93681	-0.43	14.4	1300.9	13.9700004	-122.45748	37.93682	-0.36	14.4	1531.9	14.0450004	11	7/24/2014	21:30.1
2523	RWS11	-122.4574921	37.93681	-0.48	14.4	1298.3	13.8869999	-122.45749	37.93683	-0.41	14.4	1527.9	13.9629999	11	7/24/2014	21:30.2
2524	RWS11	-122.4574945	37.93681	-0.43	14.3	1295.3	13.9040004	-122.45749	37.93683	-0.41	14.3	1523.2	13.9290004	11	7/24/2014	21:30.3
2525	RWS11	-122.4574969	37.93681	-0.43	14.3	1291.6	13.87	-122.45749	37.93683	-0.41	14.3	1517.9	13.895	11	7/24/2014	21:30.4
2526	RWS11	-122.4574993	37.93681	-0.43	14.3	1287.3	13.8359996	-122.45749	37.93683	-0.41	14.3	1511.7	13.8609996	11	7/24/2014	21:30.5
2527	RWS11	-122.4575016	37.93682	-0.43	14.2	1282.4	13.8009998	-122.4575	37.93683	-0.41	14.2	1505.1	13.8259998	11	7/24/2014	21:30.6
2528	RWS11	-122.457504	37.93682	-0.43	14.2	1277	13.7659999	-122.4575	37.93683	-0.41	14.2	1497.6	13.791	11	7/24/2014	21:30.7
2529	RWS11	-122.4575064	37.93682	-0.43	14.2	1271.1	13.7319996	-122.4575	37.93683	-0.36	14.2	1489.7	13.8069996	11	7/24/2014	21:30.8
2530	RWS11	-122.4575087	37.93682	-0.43	14.1	1264.8	13.6969997	-122.4575	37.93683	-0.36	14.1	1481	13.7719997	11	7/24/2014	21:30.9
2531	RWS11	-122.4575121	37.93682	-0.43	14.1	1257.8	13.6639997	-122.45751	37.93683	-0.36	14.1	1471.6	13.7389997	11	7/24/2014	21:31.0
2532	RWS11	-122.4575144	37.93682	-0.4	14.1	1250	13.6670001	-122.45751	37.93683	-0.36	14.1	1461.3	13.7070001	11	7/24/2014	21:31.1
2533	RWS11	-122.4575167	37.93682	-0.43	14	1241.6	13.6020004	-122.45751	37.93683	-0.36	14	1450.4	13.6770004	11	7/24/2014	21:31.2
2534	RWS11	-122.457519	37.93682	-0.4	14	1232.5	13.6080001	-122.45751	37.93683	-0.36	14	1438.8	13.6480001	11	7/24/2014	21:31.3
2535	RWS11	-122.4575213	37.93682	-0.43	14	1222.9	13.5450002	-122.45752	37.93683	-0.36	14	1426.4	13.6200002	11	7/24/2014	21:31.4
2536	RWS11	-122.4575236	37.93682	-0.43	13.9	1212.7	13.5170004	-122.45752	37.93683	-0.36	13.9	1413.5	13.5920004	11	7/24/2014	21:31.5
2537	RWS11	-122.4575259	37.93682	-0.43	13.9	1201.9	13.4889995	-122.45752	37.93684	-0.36	13.9	1399.8	13.5639995	11	7/24/2014	21:31.6
2538	RWS11	-122.4575282	37.93682	-0.4	13.9	1190.9	13.4959996	-122.45752	37.93684	-0.36	13.9	1385.8	13.5359997	11	7/24/2014	21:31.7
2539	RWS11	-122.4575305	37.93682	-0.43	13.9	1179.5	13.4309999	-122.45752	37.93684	-0.36	13.9	1371.2	13.5059999	11	7/24/2014	21:31.8
2540	RWS11	-122.4575328	37.93682	-0.4	13.8	1167.6	13.4340003	-122.45753	37.93684	-0.36	13.8	1356.1	13.4740003	11	7/24/2014	21:31.9

2541	RWS11	-122.4575362	37.93683	-0.43	13.8	1155.2	13.3649999	-122.45753	37.93684	-0.36	13.8	1340.5	13.4399999	11	7/24/2014	21:32.0
2542	RWS11	-122.4575385	37.93683	-0.4	13.8	1142.4	13.3630002	-122.45753	37.93684	-0.36	13.8	1324.7	13.4030002	11	7/24/2014	21:32.1
2543	RWS11	-122.4575407	37.93683	-0.43	13.7	1129.4	13.2900001	-122.45753	37.93684	-0.32	13.7	1308.3	13.3990001	11	7/24/2014	21:32.2
2544	RWS11	-122.4575431	37.93683	-0.4	13.7	1115.8	13.2839997	-122.45754	37.93684	-0.36	13.7	1291.6	13.3239998	11	7/24/2014	21:32.3
2545	RWS11	-122.4575454	37.93683	-0.43	13.6	1102	13.2069999	-122.45754	37.93684	-0.36	13.6	1274.5	13.282	11	7/24/2014	21:32.4
2546	RWS11	-122.4575477	37.93683	-0.4	13.6	1087.7	13.1980003	-122.45754	37.93684	-0.36	13.6	1257	13.2380003	11	7/24/2014	21:32.5
2547	RWS11	-122.4575501	37.93683	-0.43	13.6	1073.2	13.1189996	-122.45754	37.93684	-0.36	13.6	1239.2	13.1939997	11	7/24/2014	21:32.6
2548	RWS11	-122.4575524	37.93683	-0.4	13.5	1058.2	13.1080001	-122.45755	37.93684	-0.36	13.5	1221.1	13.1480001	11	7/24/2014	21:32.7
2549	RWS11	-122.4575547	37.93683	-0.43	13.5	1043	13.0269996	-122.45755	37.93684	-0.36	13.5	1202.7	13.1019996	11	7/24/2014	21:32.8
2550	RWS11	-122.457557	37.93683	-0.4	13.4	1027.7	13.0140002	-122.45755	37.93684	-0.36	13.4	1184.3	13.0540003	11	7/24/2014	21:32.9
2551	RWS11	-122.4575604	37.93683	-0.43	13.4	1012.3	12.9309999	-122.45755	37.93685	-0.36	13.4	1165.8	13.0059999	11	7/24/2014	21:33.0
2552	RWS11	-122.4575627	37.93683	-0.4	13.3	996.77	12.9159997	-122.45756	37.93685	-0.36	13.3	1147.4	12.9559997	11	7/24/2014	21:33.1
2553	RWS11	-122.457565	37.93683	-0.43	13.3	981.16	12.8300001	-122.45756	37.93685	-0.36	13.3	1128.9	12.9050001	11	7/24/2014	21:33.2
2554	RWS11	-122.4575673	37.93683	-0.4	13.2	965.41	12.8149999	-122.45756	37.93685	-0.36	13.2	1110.4	12.8549999	11	7/24/2014	21:33.3
2555	RWS11	-122.4575696	37.93684	-0.4	13.2	949.6	12.7649997	-122.45756	37.93685	-0.36	13.2	1091.9	12.8049997	11	7/24/2014	21:33.4
2556	RWS11	-122.457572	37.93684	-0.4	13.1	933.79	12.7170003	-122.45757	37.93685	-0.36	13.1	1073.4	12.7570003	11	7/24/2014	21:33.5
2557	RWS11	-122.4575743	37.93684	-0.4	13.1	917.85	12.669	-122.45757	37.93685	-0.41	13.1	1054.9	12.659	11	7/24/2014	21:33.6
2558	RWS11	-122.4575766	37.93684	-0.4	13	901.85	12.6230004	-122.45757	37.93685	-0.36	13	1036.5	12.6630005	11	7/24/2014	21:33.7
2559	RWS11	-122.4575789	37.93684	-0.43	13	885.77	12.5430004	-122.45757	37.93685	-0.36	13	1017.9	12.6180004	11	7/24/2014	21:33.8
2560	RWS11	-122.4575812	37.93684	-0.4	12.9	869.58	12.5350002	-122.45758	37.93685	-0.36	12.9	999.27	12.5750002	11	7/24/2014	21:33.9
2561	RWS11	-122.4575846	37.93684	-0.43	12.9	853.43	12.4589998	-122.45758	37.93685	-0.41	12.9	980.6	12.4839998	11	7/24/2014	21:34.0
2562	RWS11	-122.4575869	37.93684	-0.4	12.9	837.21	12.4550002	-122.45758	37.93685	-0.32	12.9	961.98	12.5290002	11	7/24/2014	21:34.1
2563	RWS11	-122.4575892	37.93684	-0.43	12.8	821.1	12.3850004	-122.45758	37.93685	-0.36	12.8	943.39	12.4600004	11	7/24/2014	21:34.2
2564	RWS11	-122.4575916	37.93684	-0.4	12.8	804.88	12.3870004	-122.45759	37.93685	-0.36	12.8	924.73	12.4270004	11	7/24/2014	21:34.3
2565	RWS11	-122.4575939	37.93684	-0.4	12.8	788.64	12.3560002	-122.45759	37.93685	-0.41	12.8	906.13	12.3460003	11	7/24/2014	21:34.4
2566	RWS11	-122.4575963	37.93684	-0.4	12.7	772.4	12.327	-122.45759	37.93686	-0.36	12.7	887.73	12.367	11	7/24/2014	21:34.5
2567	RWS11	-122.4575986	37.93684	-0.43	12.7	756.22	12.2659999	-122.45759	37.93686	-0.36	12.7	869.37	12.341	11	7/24/2014	21:34.6
2568	RWS11	-122.4576009	37.93684	-0.4	12.7	740.07	12.2749999	-122.4576	37.93686	-0.36	12.7	851.12	12.3149999	11	7/24/2014	21:34.7
2569	RWS11	-122.4576033	37.93684	-0.4	12.6	723.94	12.2509998	-122.4576	37.93686	-0.41	12.6	832.98	12.2409998	11	7/24/2014	21:34.8
2570	RWS11	-122.4576056	37.93685	-0.4	12.6	707.76	12.228	-122.4576	37.93686	-0.41	12.6	814.77	12.218	11	7/24/2014	21:34.9
2571	RWS11	-122.457609	37.93685	-0.4	12.6	691.54	12.2050002	-122.4576	37.93686	-0.41	12.6	796.57	12.1950002	11	7/24/2014	21:35.0
2572	RWS11	-122.4576113	37.93685	-0.4	12.6	675.39	12.1829999	-122.45761	37.93686	-0.36	12.6	778.47	12.2229999	11	7/24/2014	21:35.1
2573	RWS11	-122.4576136	37.93685	-0.43	12.6	659.32	12.127	-122.45761	37.93686	-0.36	12.6	760.52	12.202	11	7/24/2014	21:35.2

2574	RWS11	-122.457616	37.93685	-0.4	12.5	643.3	12.1410001	-122.45761	37.93686	-0.41	12.5	742.69	12.1310001	11	7/24/2014	21:35.3
2575	RWS11	-122.4576183	37.93685	-0.4	12.5	627.22	12.1209996	-122.45761	37.93686	-0.41	12.5	724.95	12.1109996	11	7/24/2014	21:35.4
2576	RWS11	-122.4576207	37.93685	-0.4	12.5	611.26	12.1010001	-122.45761	37.93686	-0.36	12.5	707.48	12.1410002	11	7/24/2014	21:35.5
2577	RWS11	-122.457623	37.93685	-0.4	12.5	595.64	12.0809997	-122.45762	37.93686	-0.41	12.5	690.37	12.0709997	11	7/24/2014	21:35.6
2578	RWS11	-122.4576254	37.93685	-0.4	12.5	580.27	12.0610002	-122.45762	37.93686	-0.36	12.5	673.46	12.1010002	11	7/24/2014	21:35.7
2579	RWS11	-122.4576277	37.93685	-0.4	12.4	565.04	12.0409997	-122.45762	37.93686	-0.41	12.4	656.78	12.0309997	11	7/24/2014	21:35.8
2580	RWS11	-122.45763	37.93685	-0.4	12.4	550.15	12.0199998	-122.45762	37.93687	-0.36	12.4	640.52	12.0599998	11	7/24/2014	21:35.9
2581	RWS11	-122.4576334	37.93685	-0.4	12.4	535.78	11.9989999	-122.45763	37.93687	-0.36	12.4	624.54	12.0389999	11	7/24/2014	21:36.0
2582	RWS11	-122.4576357	37.93685	-0.4	12.4	521.64	11.9760001	-122.45763	37.93687	-0.36	12.4	608.77	12.0160002	11	7/24/2014	21:36.1
2583	RWS11	-122.457638	37.93685	-0.4	12.4	507.57	11.9539998	-122.45763	37.93687	-0.36	12.4	592.9	11.9939998	11	7/24/2014	21:36.2
2584	RWS11	-122.4576403	37.93686	-0.4	12.3	493.7	11.9310001	-122.45763	37.93687	-0.36	12.3	577.19	11.9710001	11	7/24/2014	21:36.3
2585	RWS11	-122.4576426	37.93686	-0.4	12.3	480.09	11.9089997	-122.45764	37.93687	-0.41	12.3	561.66	11.8989998	11	7/24/2014	21:36.4
2586	RWS11	-122.4576449	37.93686	-0.4	12.3	466.45	11.886	-122.45764	37.93687	-0.32	12.3	546.24	11.96	11	7/24/2014	21:36.5
2587	RWS11	-122.4576472	37.93686	-0.43	12.3	453.24	11.8280002	-122.45764	37.93687	-0.36	12.3	531.21	11.9030002	11	7/24/2014	21:36.6
2588	RWS11	-122.4576495	37.93686	-0.4	12.2	440.3	11.8409999	-122.45764	37.93687	-0.36	12.2	516.52	11.8809999	11	7/24/2014	21:36.7
2589	RWS11	-122.4576518	37.93686	-0.4	12.2	427.56	11.8180001	-122.45765	37.93687	-0.32	12.2	502.18	11.8920001	11	7/24/2014	21:36.8
2590	RWS11	-122.457654	37.93686	-0.4	12.2	414.93	11.7970002	-122.45765	37.93687	-0.32	12.2	488.1	11.8710002	11	7/24/2014	21:36.9
2591	RWS11	-122.4576574	37.93686	-0.43	12.2	402.42	11.7410003	-122.45765	37.93687	-0.36	12.2	474.15	11.8160003	11	7/24/2014	21:37.0
2592	RWS11	-122.4576597	37.93686	-0.4	12.2	390	11.7570003	-122.45765	37.93687	-0.32	12.2	460.45	11.8310003	11	7/24/2014	21:37.1
2593	RWS11	-122.4576619	37.93686	-0.43	12.1	377.82	11.7030002	-122.45766	37.93687	-0.32	12.1	446.91	11.8120002	11	7/24/2014	21:37.2
2594	RWS11	-122.4576642	37.93686	-0.4	12.1	365.53	11.7199996	-122.45766	37.93688	-0.32	12.1	433.41	11.7939996	11	7/24/2014	21:37.3
2595	RWS11	-122.4576666	37.93686	-0.4	12.1	353.46	11.7030004	-122.45766	37.93688	-0.36	12.1	420.14	11.7430004	11	7/24/2014	21:37.4
2596	RWS11	-122.4576689	37.93686	-0.4	12.1	341.59	11.6849998	-122.45766	37.93688	-0.36	12.1	407.18	11.7249998	11	7/24/2014	21:37.5
2597	RWS11	-122.4576712	37.93686	-0.43	12.1	329.63	11.6320001	-122.45767	37.93688	-0.32	12.1	394.23	11.7410001	11	7/24/2014	21:37.6
2598	RWS11	-122.4576735	37.93687	-0.4	12	317.82	11.6480001	-122.45767	37.93688	-0.32	12	381.36	11.7220001	11	7/24/2014	21:37.7
2599	RWS11	-122.4576758	37.93687	-0.43	12	306.16	11.594	-122.45767	37.93688	-0.36	12	368.68	11.669	11	7/24/2014	21:37.8
2600	RWS11	-122.4576781	37.93687	-0.4	12	294.69	11.61	-122.45767	37.93688	-0.32	12	356.14	11.684	11	7/24/2014	21:37.9
2601	RWS11	-122.4576815	37.93687	-0.4	12	283.32	11.5909999	-122.45768	37.93688	-0.32	12	343.72	11.6649999	11	7/24/2014	21:38.0
2602	RWS11	-122.4576838	37.93687	-0.35	12	272.26	11.6229999	-122.45768	37.93688	-0.32	12	331.48	11.6459999	11	7/24/2014	21:38.1
2603	RWS11	-122.4576861	37.93687	-0.4	11.9	261.4	11.5529998	-122.45768	37.93688	-0.32	11.9	319.23	11.6269998	11	7/24/2014	21:38.2
2604	RWS11	-122.4576885	37.93687	-0.4	11.9	250.62	11.5350002	-122.45768	37.93688	-0.29	11.9	307.24	11.6430002	11	7/24/2014	21:38.3
2605	RWS11	-122.4576908	37.93687	-0.4	11.9	239.89	11.518	-122.45768	37.93688	-0.29	11.9	295.32	11.626	11	7/24/2014	21:38.4
2606	RWS11	-122.4576932	37.93687	-0.35	11.9	229.14	11.5539996	-122.45769	37.93688	-0.32	11.9	283.55	11.5769996	11	7/24/2014	21:38.5

2607	RWS11	-122.4576955	37.93687	-0.4	11.9	218.44	11.4889997	-122.45769	37.93688	-0.29	11.9	271.86	11.5969997	11	7/24/2014	21:38.6
2608	RWS11	-122.4576979	37.93687	-0.35	11.9	207.9	11.5279996	-122.45769	37.93688	-0.29	11.9	260.35	11.5849996	11	7/24/2014	21:38.7
2609	RWS11	-122.4577002	37.93687	-0.4	11.9	197.59	11.4670003	-122.45769	37.93689	-0.29	11.9	249.1	11.5750003	11	7/24/2014	21:38.8
2610	RWS11	-122.4577025	37.93687	-0.35	11.9	187.52	11.5089996	-122.4577	37.93689	-0.29	11.9	238.12	11.5659995	11	7/24/2014	21:38.9
2611	RWS11	-122.4577059	37.93687	-0.4	11.8	177.76	11.4489997	-122.4577	37.93689	-0.29	11.8	227.52	11.5569997	11	7/24/2014	21:39.0
2612	RWS11	-122.4577082	37.93688	-0.35	11.8	168.37	11.4920003	-122.4577	37.93689	-0.29	11.8	217.33	11.5490003	11	7/24/2014	21:39.1
2613	RWS11	-122.4577105	37.93688	-0.4	11.8	159.01	11.4320005	-122.4577	37.93689	-0.32	11.8	207.25	11.5060005	11	7/24/2014	21:39.2
2614	RWS11	-122.4577128	37.93688	-0.35	11.8	149.76	11.4750001	-122.45771	37.93689	-0.29	11.8	197.36	11.5320001	11	7/24/2014	21:39.3
2615	RWS11	-122.457715	37.93688	-0.4	11.8	140.72	11.4150003	-122.45771	37.93689	-0.29	11.8	187.52	11.5230003	11	7/24/2014	21:39.4
2616	RWS11	-122.4577174	37.93688	-0.35	11.8	131.76	11.4570005	-122.45771	37.93689	-0.29	11.8	177.89	11.5140005	11	7/24/2014	21:39.5
2617	RWS11	-122.4577196	37.93688	-0.35	11.8	122.91	11.4479997	-122.45771	37.93689	-0.29	11.8	168.24	11.5049997	11	7/24/2014	21:39.6
2618	RWS11	-122.4577219	37.93688	-0.35	11.8	114.12	11.4389999	-122.45772	37.93689	-0.29	11.8	158.75	11.4959998	11	7/24/2014	21:39.7
2619	RWS11	-122.4577242	37.93688	-0.35	11.8	105.44	11.43	-122.45772	37.93689	-0.32	11.8	149.45	11.453	11	7/24/2014	21:39.8
2620	RWS11	-122.4577264	37.93688	-0.4	11.8	96.665	11.3709996	-122.45772	37.93689	-0.29	11.8	140.08	11.4789996	11	7/24/2014	21:39.9
2621	RWS11	-122.4577298	37.93688	-0.35	11.8	87.85	11.4140002	-122.45772	37.93689	-0.32	11.8	130.72	11.4370002	11	7/24/2014	21:40.0
2622	RWS11	-122.457732	37.93688	-0.35	11.8	79.253	11.4059999	-122.45773	37.93689	-0.29	11.8	121.51	11.4629999	11	7/24/2014	21:40.1
2623	RWS11	-122.4577343	37.93688	-0.35	11.7	70.744	11.3989999	-122.45773	37.9369	-0.29	11.7	112.44	11.4559999	11	7/24/2014	21:40.2
2624	RWS11	-122.4577365	37.93688	-0.35	11.7	62.431	11.3930003	-122.45773	37.9369	-0.29	11.7	103.37	11.4500003	11	7/24/2014	21:40.3
2625	RWS11	-122.4577388	37.93688	-0.35	11.7	54.249	11.3869998	-122.45773	37.9369	-0.32	11.7	94.413	11.4099998	11	7/24/2014	21:40.4
2626	RWS11	-122.4577411	37.93688	-0.35	11.7	46.326	11.3819997	-122.45774	37.9369	-0.29	11.7	85.69	11.4389997	11	7/24/2014	21:40.5
2627	RWS11	-122.4577434	37.93689	-0.4	11.7	38.45	11.3250001	-122.45774	37.9369	-0.29	11.7	76.984	11.4330001	11	7/24/2014	21:40.6
2628	RWS11	-122.4577456	37.93689	-0.35	11.7	30.793	11.371	-122.45774	37.9369	-0.29	11.7	68.427	11.428	11	7/24/2014	21:40.7
2629	RWS11	-122.4577479	37.93689	-0.35	11.7	23.397	11.3650005	-122.45774	37.9369	-0.32	11.7	60.162	11.3880004	11	7/24/2014	21:40.8
2630	RWS11	-122.4577501	37.93689	-0.35	11.7	16.174	11.3600003	-122.45774	37.9369	-0.29	11.7	52.042	11.4170003	11	7/24/2014	21:40.9
2631	RWS11	-122.4577535	37.93689	-0.4	11.7	9.169	11.3029998	-122.45775	37.9369	-0.29	11.7	44.147	11.4109998	11	7/24/2014	21:41.0
2632	RWS11	-122.4577557	37.93689	-0.35	11.7	2.34	11.3469999	-122.45775	37.9369	-0.29	11.7	36.331	11.4039998	11	7/24/2014	21:41.1
2633	RWS11	-122.457758	37.93689	-0.35	11.7	-4.38	11.3410003	-122.45775	37.9369	-0.32	11.7	28.717	11.3640003	11	7/24/2014	21:41.2
2634	RWS11	-122.4577602	37.93689	-0.35	11.7	-10.9	11.3349998	-122.45775	37.9369	-0.32	11.7	21.387	11.3579997	11	7/24/2014	21:41.3
2635	RWS11	-122.4577625	37.93689	-0.35	11.7	-17.17	11.3290002	-122.45776	37.9369	-0.32	11.7	14.296	11.3520002	11	7/24/2014	21:41.4
2636	RWS11	-122.4577648	37.93689	-0.4	11.7	-23.67	11.2710002	-122.45776	37.9369	-0.29	11.7	7.422	11.3790002	11	7/24/2014	21:41.5
2637	RWS11	-122.457767	37.93689	-0.4	11.7	-29.93	11.2649997	-122.45776	37.9369	-0.32	11.7	0.568	11.3389997	11	7/24/2014	21:41.6
2638	RWS11	-122.4577693	37.93689	-0.35	11.7	-36.04	11.3100002	-122.45776	37.93691	-0.29	11.7	-6.109	11.3670001	11	7/24/2014	21:41.7
2639	RWS11	-122.4577716	37.93689	-0.4	11.6	-42.09	11.2529996	-122.45777	37.93691	-0.36	11.6	-12.676	11.2929996	11	7/24/2014	21:41.8

2640	RWS11	-122.4577738	37.93689	-0.35	11.6	-48.02	11.2969997	-122.45777	37.93691	-0.32	11.6	-19.157	11.3199996	11	7/24/2014	21:41.9
2641	RWS11	-122.4577771	37.9369	-0.4	11.6	-53.94	11.2389997	-122.45777	37.93691	-0.32	11.6	-25.591	11.3129997	11	7/24/2014	21:42.0
2642	RWS11	-122.4577793	37.9369	-0.35	11.6	-59.63	11.2829997	-122.45777	37.93691	-0.36	11.6	-31.898	11.2719997	11	7/24/2014	21:42.1
2643	RWS11	-122.4577816	37.9369	-0.4	11.6	-65.35	11.2249997	-122.45778	37.93691	-0.32	11.6	-38.074	11.2989997	11	7/24/2014	21:42.2
2644	RWS11	-122.4577838	37.9369	-0.35	11.6	-71.02	11.2689998	-122.45778	37.93691	-0.32	11.6	-44.181	11.2919998	11	7/24/2014	21:42.3
2645	RWS11	-122.457786	37.9369	-0.4	11.6	-76.67	11.2120002	-122.45778	37.93691	-0.32	11.6	-50.186	11.2860002	11	7/24/2014	21:42.4
2646	RWS11	-122.4577883	37.9369	-0.35	11.6	-82.41	11.2580001	-122.45778	37.93691	-0.29	11.6	-56.18	11.3150001	11	7/24/2014	21:42.5
2647	RWS11	-122.4577906	37.9369	-0.35	11.6	-88.23	11.2549999	-122.45778	37.93691	-0.32	11.6	-62.27	11.2779998	11	7/24/2014	21:42.6
2648	RWS11	-122.4577928	37.9369	-0.35	11.6	-93.93	11.253	-122.45779	37.93691	-0.32	11.6	-68.426	11.276	11	7/24/2014	21:42.7
2649	RWS11	-122.457795	37.9369	-0.4	11.6	-99.58	11.202	-122.45779	37.93691	-0.32	11.6	-74.353	11.276	11	7/24/2014	21:42.8
2650	RWS11	-122.4577972	37.9369	-0.35	11.6	-105.2	11.2549999	-122.45779	37.93691	-0.32	11.6	-80.139	11.2779998	11	7/24/2014	21:42.9
2651	RWS11	-122.4578005	37.9369	-0.35	11.6	-110.6	11.2569997	-122.45779	37.93691	-0.36	11.6	-85.794	11.2459997	11	7/24/2014	21:43.0
2652	RWS11	-122.4578027	37.9369	-0.4	11.6	-115.9	11.2100003	-122.4578	37.93692	-0.32	11.6	-91.31	11.2840003	11	7/24/2014	21:43.1
2653	RWS11	-122.4578049	37.9369	-0.4	11.6	-120.9	11.2150005	-122.4578	37.93692	-0.32	11.6	-96.548	11.2890005	11	7/24/2014	21:43.2
2654	RWS11	-122.4578071	37.9369	-0.4	11.6	-125.4	11.221	-122.4578	37.93692	-0.36	11.6	-101.52	11.261	11	7/24/2014	21:43.3
2655	RWS11	-122.4578093	37.9369	-0.4	11.6	-129.9	11.2269996	-122.4578	37.93692	-0.36	11.6	-106.33	11.2669996	11	7/24/2014	21:43.4
2656	RWS11	-122.4578116	37.9369	-0.35	11.6	-134.3	11.2840001	-122.45781	37.93692	-0.36	11.6	-110.95	11.2730001	11	7/24/2014	21:43.5
2657	RWS11	-122.4578137	37.93691	-0.4	11.6	-138.7	11.2380002	-122.45781	37.93692	-0.36	11.6	-115.36	11.2780002	11	7/24/2014	21:43.6
2658	RWS11	-122.457816	37.93691	-0.35	11.6	-142.7	11.293	-122.45781	37.93692	-0.36	11.6	-119.46	11.282	11	7/24/2014	21:43.7
2659	RWS11	-122.4578181	37.93691	-0.4	11.6	-146.6	11.2450002	-122.45781	37.93692	-0.41	11.6	-123.48	11.2350002	11	7/24/2014	21:43.8
2660	RWS11	-122.4578203	37.93691	-0.4	11.6	-150.5	11.2480004	-122.45781	37.93692	-0.36	11.6	-127.48	11.2880005	11	7/24/2014	21:43.9
2661	RWS11	-122.4578235	37.93691	-0.4	11.6	-154.5	11.2489999	-122.45782	37.93692	-0.36	11.6	-131.6	11.2889999	11	7/24/2014	21:44.0
2662	RWS11	-122.4578257	37.93691	-0.4	11.6	-158.4	11.2489999	-122.45782	37.93692	-0.36	11.6	-135.7	11.2889999	11	7/24/2014	21:44.1
2663	RWS11	-122.4578279	37.93691	-0.4	11.6	-162.3	11.2489999	-122.45782	37.93692	-0.36	11.6	-139.8	11.2889999	11	7/24/2014	21:44.2
2664	RWS11	-122.4578301	37.93691	-0.35	11.6	-166.2	11.2990005	-122.45782	37.93692	-0.32	11.6	-144.07	11.3220004	11	7/24/2014	21:44.3
2665	RWS11	-122.4578322	37.93691	-0.4	11.6	-170.4	11.2450002	-122.45783	37.93692	-0.41	11.6	-148.62	11.2350002	11	7/24/2014	21:44.4
2666	RWS11	-122.4578344	37.93691	-0.35	11.6	-174.5	11.293	-122.45783	37.93692	-0.36	11.6	-153.11	11.282	11	7/24/2014	21:44.5
2667	RWS11	-122.4578366	37.93691	-0.4	11.6	-178.5	11.2369998	-122.45783	37.93692	-0.36	11.6	-157.48	11.2769998	11	7/24/2014	21:44.6
2668	RWS11	-122.4578388	37.93691	-0.35	11.6	-182.5	11.2820003	-122.45783	37.93693	-0.36	11.6	-161.77	11.2710003	11	7/24/2014	21:44.7
2669	RWS11	-122.457841	37.93691	-0.4	11.6	-186.6	11.2249997	-122.45784	37.93693	-0.36	11.6	-166.16	11.2649998	11	7/24/2014	21:44.8
2670	RWS11	-122.4578432	37.93691	-0.35	11.6	-190.7	11.2700002	-122.45784	37.93693	-0.36	11.6	-170.35	11.2590002	11	7/24/2014	21:44.9
2671	RWS11	-122.4578464	37.93691	-0.4	11.6	-194.4	11.2120002	-122.45784	37.93693	-0.36	11.6	-174.34	11.2520002	11	7/24/2014	21:45.0
2672	RWS11	-122.4578486	37.93692	-0.35	11.6	-197.9	11.2560003	-122.45784	37.93693	-0.36	11.6	-177.99	11.2450002	11	7/24/2014	21:45.1

2673	RWS11	-122.4578507	37.93692	-0.43	11.6	-201.4	11.1630003	-122.45784	37.93693	-0.36	11.6	-181.52	11.2380003	11	7/24/2014	21:45.2
2674	RWS11	-122.457853	37.93692	-0.4	11.6	-204.7	11.1910003	-122.45785	37.93693	-0.36	11.6	-184.94	11.2310003	11	7/24/2014	21:45.3
2675	RWS11	-122.4578552	37.93692	-0.4	11.6	-208	11.1829999	-122.45785	37.93693	-0.36	11.6	-188.58	11.2229999	11	7/24/2014	21:45.4
2676	RWS11	-122.4578574	37.93692	-0.35	11.6	-211.2	11.227	-122.45785	37.93693	-0.36	11.6	-192.25	11.216	11	7/24/2014	21:45.5
2677	RWS11	-122.4578596	37.93692	-0.43	11.6	-214.2	11.1329996	-122.45785	37.93693	-0.36	11.6	-195.64	11.2079996	11	7/24/2014	21:45.6
2678	RWS11	-122.4578619	37.93692	-0.35	11.6	-217.1	11.2119996	-122.45786	37.93693	-0.36	11.6	-199.06	11.2009996	11	7/24/2014	21:45.7
2679	RWS11	-122.4578641	37.93692	-0.43	11.6	-219.9	11.1180002	-122.45786	37.93693	-0.36	11.6	-202.34	11.1930002	11	7/24/2014	21:45.8
2680	RWS11	-122.4578663	37.93692	-0.4	11.5	-222.6	11.1460002	-122.45786	37.93693	-0.32	11.5	-205.49	11.2200002	11	7/24/2014	21:45.9
2681	RWS11	-122.4578696	37.93692	-0.4	11.5	-225.1	11.1399997	-122.45786	37.93693	-0.36	11.5	-208.54	11.1799997	11	7/24/2014	21:46.0
2682	RWS11	-122.4578718	37.93692	-0.4	11.5	-227.8	11.1340001	-122.45787	37.93693	-0.36	11.5	-211.55	11.1740001	11	7/24/2014	21:46.1
2683	RWS11	-122.4578741	37.93692	-0.4	11.5	-230.7	11.1279996	-122.45787	37.93694	-0.36	11.5	-214.77	11.1679996	11	7/24/2014	21:46.2
2684	RWS11	-122.4578763	37.93692	-0.4	11.5	-233.5	11.122	-122.45787	37.93694	-0.36	11.5	-217.8	11.1620001	11	7/24/2014	21:46.3
2685	RWS11	-122.4578786	37.93692	-0.4	11.5	-236	11.1159995	-122.45787	37.93694	-0.36	11.5	-220.47	11.1559995	11	7/24/2014	21:46.4
2686	RWS11	-122.4578808	37.93692	-0.4	11.5	-238.4	11.1110004	-122.45788	37.93694	-0.32	11.5	-223.14	11.1850004	11	7/24/2014	21:46.5
2687	RWS11	-122.4578831	37.93693	-0.4	11.5	-241	11.1060002	-122.45788	37.93694	-0.32	11.5	-225.88	11.1800003	11	7/24/2014	21:46.6
2688	RWS11	-122.4578853	37.93693	-0.4	11.5	-243.4	11.1010001	-122.45788	37.93694	-0.36	11.5	-228.46	11.1410002	11	7/24/2014	21:46.7
2689	RWS11	-122.4578875	37.93693	-0.4	11.5	-245.5	11.0979999	-122.45788	37.93694	-0.36	11.5	-230.74	11.1379999	11	7/24/2014	21:46.8
2690	RWS11	-122.4578898	37.93693	-0.35	11.5	-247.5	11.1459996	-122.45788	37.93694	-0.32	11.5	-232.98	11.1689996	11	7/24/2014	21:46.9
2691	RWS11	-122.457893	37.93693	-0.4	11.5	-249.9	11.0940002	-122.45789	37.93694	-0.36	11.5	-235.55	11.1340002	11	7/24/2014	21:47.0
2692	RWS11	-122.4578952	37.93693	-0.4	11.5	-252.2	11.0949996	-122.45789	37.93694	-0.32	11.5	-238.18	11.1689996	11	7/24/2014	21:47.1
2693	RWS11	-122.4578974	37.93693	-0.4	11.5	-254.4	11.0970004	-122.45789	37.93694	-0.32	11.5	-240.47	11.1710004	11	7/24/2014	21:47.2
2694	RWS11	-122.4578996	37.93693	-0.4	11.5	-256.4	11.1010001	-122.45789	37.93694	-0.32	11.5	-242.5	11.1750001	11	7/24/2014	21:47.3
2695	RWS11	-122.4579018	37.93693	-0.4	11.5	-258.2	11.1069997	-122.4579	37.93694	-0.32	11.5	-244.22	11.1809997	11	7/24/2014	21:47.4
2696	RWS11	-122.457904	37.93693	-0.35	11.5	-260	11.1640002	-122.4579	37.93694	-0.32	11.5	-245.77	11.1870002	11	7/24/2014	21:47.5
2697	RWS11	-122.4579062	37.93693	-0.4	11.5	-261.6	11.1200002	-122.4579	37.93694	-0.36	11.5	-247.21	11.1600002	11	7/24/2014	21:47.6
2698	RWS11	-122.4579084	37.93693	-0.35	11.5	-263	11.1769998	-122.4579	37.93695	-0.32	11.5	-248.49	11.1999998	11	7/24/2014	21:47.7
2699	RWS11	-122.4579105	37.93693	-0.4	11.5	-264.3	11.1320003	-122.4579	37.93695	-0.32	11.5	-249.62	11.2060003	11	7/24/2014	21:47.8
2700	RWS11	-122.4579127	37.93693	-0.35	11.5	-265.5	11.1880004	-122.45791	37.93695	-0.32	11.5	-250.75	11.2110004	11	7/24/2014	21:47.9
2701	RWS11	-122.4579159	37.93693	-0.4	11.5	-266.7	11.1410001	-122.45791	37.93695	-0.32	11.5	-251.8	11.2150001	11	7/24/2014	21:48.0
2702	RWS11	-122.4579181	37.93694	-0.35	11.5	-267.9	11.1950004	-122.45791	37.93695	-0.32	11.5	-252.74	11.2180004	11	7/24/2014	21:48.1
2703	RWS11	-122.4579203	37.93694	-0.4	11.5	-269	11.1469997	-122.45791	37.93695	-0.32	11.5	-253.61	11.2209997	11	7/24/2014	21:48.2
2704	RWS11	-122.4579225	37.93694	-0.35	11.5	-270	11.1999995	-122.45792	37.93695	-0.32	11.5	-254.34	11.2229995	11	7/24/2014	21:48.3
2705	RWS11	-122.4579248	37.93694	-0.35	11.5	-271	11.2029998	-122.45792	37.93695	-0.36	11.5	-255.04	11.1919998	11	7/24/2014	21:48.4

2706	RWS11	-122.457927	37.93694	-0.35	11.6	-272.1	11.2049997	-122.45792	37.93695	-0.29	11.6	-256.02	11.2619996	11	7/24/2014	21:48.5
2707	RWS11	-122.4579292	37.93694	-0.35	11.6	-273.3	11.2079999	-122.45792	37.93695	-0.32	11.6	-257.25	11.2309999	11	7/24/2014	21:48.6
2708	RWS11	-122.4579314	37.93694	-0.35	11.6	-274.4	11.2110002	-122.45793	37.93695	-0.29	11.6	-258.38	11.2680002	11	7/24/2014	21:48.7
2709	RWS11	-122.4579337	37.93694	-0.35	11.6	-275.4	11.2140004	-122.45793	37.93695	-0.32	11.6	-259.51	11.2370004	11	7/24/2014	21:48.8
2710	RWS11	-122.4579359	37.93694	-0.35	11.6	-276.7	11.2169997	-122.45793	37.93695	-0.32	11.6	-260.71	11.2399997	11	7/24/2014	21:48.9
2711	RWS11	-122.4579392	37.93694	-0.35	11.6	-278	11.2210004	-122.45793	37.93695	-0.32	11.6	-262.06	11.2440004	11	7/24/2014	21:49.0
2712	RWS11	-122.4579414	37.93694	-0.35	11.6	-279.1	11.2250001	-122.45794	37.93695	-0.29	11.6	-263.08	11.2820001	11	7/24/2014	21:49.1
2713	RWS11	-122.4579437	37.93694	-0.35	11.6	-279.9	11.2300002	-122.45794	37.93696	-0.29	11.6	-263.76	11.2870002	11	7/24/2014	21:49.2
2714	RWS11	-122.457946	37.93694	-0.35	11.6	-280.6	11.2350003	-122.45794	37.93696	-0.29	11.6	-264.4	11.2920003	11	7/24/2014	21:49.3
2715	RWS11	-122.4579482	37.93694	-0.35	11.6	-281.5	11.2420003	-122.45794	37.93696	-0.29	11.6	-265.28	11.2990003	11	7/24/2014	21:49.4
2716	RWS11	-122.4579505	37.93694	-0.31	11.6	-282.3	11.2819999	-122.45794	37.93696	-0.29	11.6	-265.98	11.3049999	11	7/24/2014	21:49.5
2717	RWS11	-122.4579528	37.93694	-0.35	11.6	-282.9	11.2549999	-122.45795	37.93696	-0.29	11.6	-266.37	11.3119998	11	7/24/2014	21:49.6
2718	RWS11	-122.4579551	37.93695	-0.31	11.6	-283.4	11.2950003	-122.45795	37.93696	-0.29	11.6	-266.78	11.3180004	11	7/24/2014	21:49.7
2719	RWS11	-122.4579574	37.93695	-0.35	11.6	-283.9	11.2650001	-122.45795	37.93696	-0.29	11.6	-267.19	11.3220001	11	7/24/2014	21:49.8
2720	RWS11	-122.4579596	37.93695	-0.31	11.6	-284.3	11.3009999	-122.45795	37.93696	-0.24	11.6	-267.65	11.3749999	11	7/24/2014	21:49.9
2721	RWS11	-122.4579629	37.93695	-0.31	11.6	-284.5	11.3000005	-122.45796	37.93696	-0.24	11.6	-267.88	11.3740005	11	7/24/2014	21:50.0
2722	RWS11	-122.4579651	37.93695	-0.31	11.6	-284.6	11.2950003	-122.45796	37.93696	-0.2	11.6	-268.06	11.4030004	11	7/24/2014	21:50.1
2723	RWS11	-122.4579674	37.93695	-0.35	11.6	-284.8	11.253	-122.45796	37.93696	-0.24	11.6	-268.47	11.361	11	7/24/2014	21:50.2
2724	RWS11	-122.4579696	37.93695	-0.31	11.6	-285.1	11.2749999	-122.45796	37.93696	-0.2	11.6	-268.84	11.3829999	11	7/24/2014	21:50.3
2725	RWS11	-122.4579718	37.93695	-0.31	11.6	-285.4	11.261	-122.45797	37.93696	-0.2	11.6	-269.26	11.369	11	7/24/2014	21:50.4
2726	RWS11	-122.4579741	37.93695	-0.28	11.6	-285.8	11.2779998	-122.45797	37.93696	-0.15	11.6	-269.81	11.4019998	11	7/24/2014	21:50.5
2727	RWS11	-122.4579763	37.93695	-0.28	11.5	-286.4	11.2609996	-122.45797	37.93696	-0.2	11.5	-270.71	11.3349996	11	7/24/2014	21:50.6
2728	RWS11	-122.4579785	37.93695	-0.28	11.5	-287	11.2449998	-122.45797	37.93696	-0.15	11.5	-271.59	11.3689998	11	7/24/2014	21:50.7
2729	RWS11	-122.4579808	37.93695	-0.28	11.5	-287.8	11.229	-122.45798	37.93697	-0.15	11.5	-272.54	11.353	11	7/24/2014	21:50.8
2730	RWS11	-122.457983	37.93695	-0.28	11.5	-288.5	11.215	-122.45798	37.93697	-0.12	11.5	-273.42	11.373	11	7/24/2014	21:50.9
2731	RWS11	-122.4579863	37.93695	-0.28	11.5	-289.2	11.2040004	-122.45798	37.93697	-0.12	11.5	-274.25	11.3620004	11	7/24/2014	21:51.0
2732	RWS11	-122.4579885	37.93695	-0.23	11.5	-289.8	11.2469996	-122.45798	37.93697	-0.07	11.5	-274.9	11.4039996	11	7/24/2014	21:51.1
2733	RWS11	-122.4579907	37.93696	-0.23	11.5	-290.3	11.2420004	-122.45799	37.93697	-0.12	11.5	-275.36	11.3480004	11	7/24/2014	21:51.2
2734	RWS11	-122.4579929	37.93696	-0.23	11.5	-290.7	11.241	-122.45799	37.93697	-0.12	11.5	-275.55	11.347	11	7/24/2014	21:51.3
2735	RWS11	-122.4579952	37.93696	-0.23	11.5	-290.7	11.2440003	-122.45799	37.93697	-0.07	11.5	-275.28	11.4010003	11	7/24/2014	21:51.4
2736	RWS11	-122.4579974	37.93696	-0.23	11.5	-290.4	11.2510002	-122.45799	37.93697	-0.07	11.5	-274.68	11.4080002	11	7/24/2014	21:51.5
2737	RWS11	-122.4579997	37.93696	-0.23	11.5	-289.9	11.2619999	-122.45799	37.93697	-0.12	11.5	-273.87	11.3679999	11	7/24/2014	21:51.6
2738	RWS11	-122.4580019	37.93696	-0.23	11.5	-289.3	11.2759999	-122.458	37.93697	-0.07	11.5	-272.82	11.4329999	11	7/24/2014	21:51.7

2739	RWS11	-122.4580041	37.93696	-0.23	11.5	-288.4	11.2919997	-122.458	37.93697	-0.07	11.5	-271.38	11.4489997	11	7/24/2014	21:51.8
2740	RWS11	-122.4580063	37.93696	-0.23	11.5	-287.3	11.3109997	-122.458	37.93697	-0.07	11.5	-269.82	11.4679997	11	7/24/2014	21:51.9
2741	RWS11	-122.4580096	37.93696	-0.23	11.6	-286.1	11.3299998	-122.458	37.93697	-0.12	11.6	-268.27	11.4359998	11	7/24/2014	21:52.0
2742	RWS11	-122.4580117	37.93696	-0.23	11.6	-284.7	11.3500002	-122.45801	37.93697	-0.12	11.6	-266.55	11.4560002	11	7/24/2014	21:52.1
2743	RWS11	-122.4580139	37.93696	-0.23	11.6	-283.2	11.3690003	-122.45801	37.93697	-0.12	11.6	-264.88	11.4750003	11	7/24/2014	21:52.2
2744	RWS11	-122.4580161	37.93696	-0.23	11.6	-281.5	11.3880003	-122.45801	37.93698	-0.12	11.6	-263.07	11.4940003	11	7/24/2014	21:52.3
2745	RWS11	-122.4580183	37.93696	-0.23	11.6	-279.7	11.4070004	-122.45801	37.93698	-0.07	11.6	-261.2	11.5640004	11	7/24/2014	21:52.4
2746	RWS11	-122.4580205	37.93696	-0.23	11.7	-277.9	11.425	-122.45801	37.93698	-0.07	11.7	-259.13	11.582	11	7/24/2014	21:52.5
2747	RWS11	-122.4580227	37.93696	-0.23	11.7	-276	11.4440001	-122.45802	37.93698	-0.12	11.7	-257.05	11.5500001	11	7/24/2014	21:52.6
2748	RWS11	-122.458025	37.93696	-0.23	11.7	-274.2	11.4630001	-122.45802	37.93698	-0.12	11.7	-254.97	11.5690001	11	7/24/2014	21:52.7
2749	RWS11	-122.4580271	37.93697	-0.23	11.7	-272.4	11.4820002	-122.45802	37.93698	-0.12	11.7	-252.84	11.5880002	11	7/24/2014	21:52.8
2750	RWS11	-122.4580293	37.93697	-0.19	11.7	-270.6	11.5359997	-122.45802	37.93698	-0.07	11.7	-250.69	11.6589997	11	7/24/2014	21:52.9
2751	RWS11	-122.4580326	37.93697	-0.23	11.7	-269.1	11.5220002	-122.45803	37.93698	-0.12	11.7	-248.84	11.6280002	11	7/24/2014	21:53.0
2752	RWS11	-122.4580348	37.93697	-0.19	11.8	-267.5	11.5770001	-122.45803	37.93698	-0.12	11.8	-246.93	11.6490001	11	7/24/2014	21:53.1
2753	RWS11	-122.458037	37.93697	-0.23	11.8	-265.7	11.564	-122.45803	37.93698	-0.12	11.8	-244.87	11.67	11	7/24/2014	21:53.2
2754	RWS11	-122.4580393	37.93697	-0.23	11.8	-264	11.5860003	-122.45803	37.93698	-0.12	11.8	-242.67	11.6920003	11	7/24/2014	21:53.3
2755	RWS11	-122.4580415	37.93697	-0.23	11.8	-262.1	11.6070002	-122.45804	37.93698	-0.12	11.8	-240.56	11.7130002	11	7/24/2014	21:53.4
2756	RWS11	-122.4580438	37.93697	-0.19	11.9	-260.3	11.6629995	-122.45804	37.93698	-0.12	11.9	-238.49	11.7349995	11	7/24/2014	21:53.5
2757	RWS11	-122.458046	37.93697	-0.23	11.9	-258.5	11.649	-122.45804	37.93698	-0.12	11.9	-236.37	11.755	11	7/24/2014	21:53.6
2758	RWS11	-122.4580483	37.93697	-0.19	11.9	-256.7	11.7030005	-122.45804	37.93698	-0.12	11.9	-234.36	11.7750005	11	7/24/2014	21:53.7
2759	RWS11	-122.4580505	37.93697	-0.19	11.9	-254.8	11.7219996	-122.45804	37.93698	-0.12	11.9	-232.23	11.7939996	11	7/24/2014	21:53.8
2760	RWS11	-122.4580527	37.93697	-0.19	11.9	-252.9	11.7400002	-122.45805	37.93699	-0.12	11.9	-230.07	11.8120002	11	7/24/2014	21:53.9
2761	RWS11	-122.458056	37.93697	-0.19	11.9	-250.9	11.7579998	-122.45805	37.93699	-0.07	11.9	-227.92	11.8809998	11	7/24/2014	21:54.0
2762	RWS11	-122.4580582	37.93697	-0.19	12	-248.9	11.7769999	-122.45805	37.93699	-0.12	12	-225.74	11.8489999	11	7/24/2014	21:54.1
2763	RWS11	-122.4580604	37.93697	-0.23	12	-246.8	11.7619999	-122.45805	37.93699	-0.12	12	-223.46	11.8679999	11	7/24/2014	21:54.2
2764	RWS11	-122.4580627	37.93698	-0.19	12	-244.6	11.8160004	-122.45806	37.93699	-0.12	12	-221.14	11.8880004	11	7/24/2014	21:54.3
2765	RWS11	-122.4580649	37.93698	-0.23	12	-242.5	11.8030003	-122.45806	37.93699	-0.12	12	-218.79	11.9090003	11	7/24/2014	21:54.4
2766	RWS11	-122.4580671	37.93698	-0.19	12.1	-240.2	11.86	-122.45806	37.93699	-0.12	12.1	-216.21	11.9320001	11	7/24/2014	21:54.5
2767	RWS11	-122.4580694	37.93698	-0.23	12.1	-237.6	11.8489998	-122.45806	37.93699	-0.15	12.1	-213.44	11.9209998	11	7/24/2014	21:54.6
2768	RWS11	-122.4580716	37.93698	-0.23	12.1	-234.9	11.8740004	-122.45807	37.93699	-0.07	12.1	-210.36	12.0310004	11	7/24/2014	21:54.7
2769	RWS11	-122.4580738	37.93698	-0.23	12.1	-231.9	11.899	-122.45807	37.93699	-0.15	12.1	-207.11	11.971	11	7/24/2014	21:54.8
2770	RWS11	-122.458076	37.93698	-0.23	12.2	-228.7	11.925	-122.45807	37.93699	-0.15	12.2	-203.67	11.997	11	7/24/2014	21:54.9
2771	RWS11	-122.4580793	37.93698	-0.23	12.2	-225.4	11.9520005	-122.45807	37.93699	-0.15	12.2	-200.06	12.0240005	11	7/24/2014	21:55.0

2772	RWS11	-122.4580815	37.93698	-0.23	12.2	-221.9	11.9789999	-122.45808	37.93699	-0.12	12.2	-196.41	12.0849999	11	7/24/2014	21:55.1
2773	RWS11	-122.4580837	37.93698	-0.23	12.2	-218.4	12.0060004	-122.45808	37.93699	-0.15	12.2	-192.6	12.0780004	11	7/24/2014	21:55.2
2774	RWS11	-122.4580859	37.93698	-0.23	12.3	-215	12.0340002	-122.45808	37.93699	-0.12	12.3	-189.05	12.1400002	11	7/24/2014	21:55.3
2775	RWS11	-122.4580882	37.93698	-0.28	12.3	-211.4	12.0089997	-122.45808	37.93699	-0.15	12.3	-185.47	12.1329997	11	7/24/2014	21:55.4
2776	RWS11	-122.4580904	37.93698	-0.23	12.3	-207.7	12.0880001	-122.45808	37.937	-0.15	12.3	-181.56	12.1600001	11	7/24/2014	21:55.5
2777	RWS11	-122.4580926	37.93698	-0.28	12.3	-204	12.064	-122.45809	37.937	-0.15	12.3	-177.53	12.188	11	7/24/2014	21:55.6
2778	RWS11	-122.4580949	37.93698	-0.23	12.4	-200.4	12.1439999	-122.45809	37.937	-0.12	12.4	-173.6	12.2499999	11	7/24/2014	21:55.7
2779	RWS11	-122.4580971	37.93698	-0.23	12.4	-197	12.1730002	-122.45809	37.937	-0.12	12.4	-169.72	12.2790002	11	7/24/2014	21:55.8
2780	RWS11	-122.4580993	37.93699	-0.23	12.4	-193.4	12.2029999	-122.45809	37.937	-0.15	12.4	-165.77	12.2749999	11	7/24/2014	21:55.9
2781	RWS11	-122.4581026	37.93699	-0.28	12.5	-189.9	12.182	-122.4581	37.937	-0.15	12.5	-161.9	12.306	11	7/24/2014	21:56.0
2782	RWS11	-122.4581048	37.93699	-0.23	12.5	-186.5	12.2650002	-122.4581	37.937	-0.15	12.5	-158.11	12.3370002	11	7/24/2014	21:56.1
2783	RWS11	-122.4581069	37.93699	-0.28	12.5	-183	12.2449998	-122.4581	37.937	-0.15	12.5	-154.21	12.3689998	11	7/24/2014	21:56.2
2784	RWS11	-122.4581092	37.93699	-0.23	12.6	-179.4	12.3270005	-122.4581	37.937	-0.15	12.6	-150.16	12.3990005	11	7/24/2014	21:56.3
2785	RWS11	-122.4581113	37.93699	-0.28	12.6	-175.9	12.3030003	-122.45811	37.937	-0.15	12.6	-146.22	12.4270003	11	7/24/2014	21:56.4
2786	RWS11	-122.4581136	37.93699	-0.23	12.6	-172.4	12.38	-122.45811	37.937	-0.15	12.6	-142.31	12.452	11	7/24/2014	21:56.5
2787	RWS11	-122.4581158	37.93699	-0.28	12.6	-168.9	12.3509997	-122.45811	37.937	-0.15	12.6	-138.26	12.4749997	11	7/24/2014	21:56.6
2788	RWS11	-122.458118	37.93699	-0.23	12.6	-165.6	12.4230002	-122.45811	37.937	-0.12	12.6	-134.4	12.5290002	11	7/24/2014	21:56.7
2789	RWS11	-122.4581202	37.93699	-0.28	12.7	-162.2	12.3889998	-122.45811	37.937	-0.15	12.7	-130.49	12.5129998	11	7/24/2014	21:56.8
2790	RWS11	-122.4581224	37.93699	-0.23	12.7	-158.8	12.458	-122.45812	37.937	-0.15	12.7	-126.58	12.53	11	7/24/2014	21:56.9
2791	RWS11	-122.4581257	37.93699	-0.28	12.7	-155.3	12.4230002	-122.45812	37.93701	-0.2	12.7	-122.51	12.4970002	11	7/24/2014	21:57.0
2792	RWS11	-122.4581279	37.93699	-0.23	12.7	-151.6	12.4920004	-122.45812	37.93701	-0.15	12.7	-118.21	12.5640004	11	7/24/2014	21:57.1
2793	RWS11	-122.4581301	37.93699	-0.28	12.7	-147.9	12.4569997	-122.45812	37.93701	-0.15	12.7	-113.96	12.5809997	11	7/24/2014	21:57.2
2794	RWS11	-122.4581324	37.93699	-0.23	12.8	-144.1	12.5270003	-122.45813	37.93701	-0.15	12.8	-109.61	12.5990003	11	7/24/2014	21:57.3
2795	RWS11	-122.4581346	37.93699	-0.28	12.8	-140.4	12.4929999	-122.45813	37.93701	-0.2	12.8	-105.16	12.5669999	11	7/24/2014	21:57.4
2796	RWS11	-122.4581369	37.937	-0.23	12.8	-136.6	12.5650004	-122.45813	37.93701	-0.15	12.8	-100.66	12.6370004	11	7/24/2014	21:57.5
2797	RWS11	-122.4581391	37.937	-0.28	12.8	-132.9	12.5329999	-122.45813	37.93701	-0.2	12.8	-96.109	12.6069999	11	7/24/2014	21:57.6
2798	RWS11	-122.4581414	37.937	-0.23	12.8	-129.2	12.6059998	-122.45814	37.93701	-0.15	12.8	-91.563	12.6779998	11	7/24/2014	21:57.7
2799	RWS11	-122.4581436	37.937	-0.28	12.9	-125.3	12.5729998	-122.45814	37.93701	-0.15	12.9	-86.853	12.6969998	11	7/24/2014	21:57.8
2800	RWS11	-122.4581459	37.937	-0.23	12.9	-121.3	12.6430004	-122.45814	37.93701	-0.15	12.9	-81.855	12.7150004	11	7/24/2014	21:57.9
2801	RWS11	-122.4581492	37.937	-0.28	12.9	-117.2	12.6079997	-122.45814	37.93701	-0.2	12.9	-76.775	12.6819997	11	7/24/2014	21:58.0
2802	RWS11	-122.4581514	37.937	-0.23	12.9	-113.1	12.675	-122.45815	37.93701	-0.15	12.9	-71.708	12.747	11	7/24/2014	21:58.1
2803	RWS11	-122.4581536	37.937	-0.28	12.9	-108.9	12.637	-122.45815	37.93701	-0.2	12.9	-66.562	12.711	11	7/24/2014	21:58.2
2804	RWS11	-122.4581559	37.937	-0.23	12.9	-104.6	12.7029999	-122.45815	37.93701	-0.15	12.9	-61.346	12.7749999	11	7/24/2014	21:58.3

2805	RWS11	-122.4581581	37.937	-0.23	12.9	-100.3	12.7160004	-122.45815	37.93701	-0.15	12.9	-55.978	12.7880004	11	7/24/2014	21:58.4
2806	RWS11	-122.4581603	37.937	-0.23	13	-95.84	12.7280005	-122.45815	37.93701	-0.2	13	-50.721	12.7500005	11	7/24/2014	21:58.5
2807	RWS11	-122.4581626	37.937	-0.23	13	-91.2	12.7390002	-122.45816	37.93702	-0.2	13	-45.075	12.7610002	11	7/24/2014	21:58.6
2808	RWS11	-122.4581648	37.937	-0.23	13	-86.31	12.748	-122.45816	37.93702	-0.2	13	-39.291	12.77	11	7/24/2014	21:58.7
2809	RWS11	-122.458167	37.937	-0.28	13	-81.36	12.703	-122.45816	37.93702	-0.2	13	-33.358	12.777	11	7/24/2014	21:58.8
2810	RWS11	-122.4581692	37.937	-0.23	13	-76.36	12.7589997	-122.45816	37.93702	-0.2	13	-27.355	12.7809997	11	7/24/2014	21:58.9
2811	RWS11	-122.4581724	37.93701	-0.28	13	-71.32	12.7099999	-122.45817	37.93702	-0.2	13	-21.442	12.7839999	11	7/24/2014	21:59.0
2812	RWS11	-122.4581746	37.93701	-0.23	13	-66.39	12.7619999	-122.45817	37.93702	-0.2	13	-15.661	12.7839999	11	7/24/2014	21:59.1
2813	RWS11	-122.4581768	37.93701	-0.23	13	-61.41	12.7600001	-122.45817	37.93702	-0.2	13	-9.835	12.7820001	11	7/24/2014	21:59.2
2814	RWS11	-122.458179	37.93701	-0.23	13	-56.42	12.7560004	-122.45817	37.93702	-0.2	13	-4.097	12.7780004	11	7/24/2014	21:59.3
2815	RWS11	-122.4581811	37.93701	-0.28	13	-51.29	12.6979998	-122.45818	37.93702	-0.2	13	1.663	12.7719998	11	7/24/2014	21:59.4
2816	RWS11	-122.4581834	37.93701	-0.23	13	-46.16	12.7429999	-122.45818	37.93702	-0.2	13	7.379	12.7649999	11	7/24/2014	21:59.5
2817	RWS11	-122.4581855	37.93701	-0.28	13	-41.01	12.682	-122.45818	37.93702	-0.24	13	13.118	12.722	11	7/24/2014	21:59.6
2818	RWS11	-122.4581877	37.93701	-0.23	12.9	-35.73	12.7239998	-122.45818	37.93702	-0.2	12.9	18.857	12.7459998	11	7/24/2014	21:59.7
2819	RWS11	-122.4581898	37.93701	-0.28	12.9	-30.48	12.6599997	-122.45818	37.93702	-0.24	12.9	24.659	12.6999997	11	7/24/2014	21:59.8
2820	RWS11	-122.458192	37.93701	-0.23	12.9	-25.19	12.6999997	-122.45819	37.93702	-0.24	12.9	30.572	12.6879996	11	7/24/2014	21:59.9
2821	RWS11	-122.4581952	37.93701	-0.28	12.9	-19.98	12.6339997	-122.45819	37.93702	-0.24	12.9	36.334	12.6739997	11	7/24/2014	22:00.0
2822	RWS11	-122.4581973	37.93701	-0.28	12.9	-14.92	12.6190003	-122.45819	37.93702	-0.2	12.9	42.114	12.6930003	11	7/24/2014	22:00.1
2823	RWS11	-122.4581994	37.93701	-0.28	12.9	-9.704	12.6029996	-122.45819	37.93703	-0.24	12.9	47.854	12.6429996	11	7/24/2014	22:00.2
2824	RWS11	-122.4582016	37.93701	-0.23	12.9	-4.689	12.6369999	-122.4582	37.93703	-0.24	12.9	53.655	12.6249999	11	7/24/2014	22:00.3
2825	RWS11	-122.4582037	37.93701	-0.28	12.8	0.417	12.5670003	-122.4582	37.93703	-0.24	12.8	59.412	12.6070003	11	7/24/2014	22:00.4
2826	RWS11	-122.4582059	37.93701	-0.23	12.8	5.369	12.6009997	-122.4582	37.93703	-0.2	12.8	65.063	12.6229997	11	7/24/2014	22:00.5
2827	RWS11	-122.4582081	37.93701	-0.28	12.8	10.345	12.5299996	-122.4582	37.93703	-0.24	12.8	70.845	12.5699996	11	7/24/2014	22:00.6
2828	RWS11	-122.4582102	37.93702	-0.23	12.8	15.429	12.564	-122.4582	37.93703	-0.24	12.8	76.692	12.552	11	7/24/2014	22:00.7
2829	RWS11	-122.4582124	37.93702	-0.28	12.8	20.533	12.4949998	-122.45821	37.93703	-0.24	12.8	82.698	12.5349998	11	7/24/2014	22:00.8
2830	RWS11	-122.4582145	37.93702	-0.23	12.8	25.924	12.5299996	-122.45821	37.93703	-0.2	12.8	88.89	12.5519996	11	7/24/2014	22:00.9
2831	RWS11	-122.4582177	37.93702	-0.28	12.7	31.335	12.4619998	-122.45821	37.93703	-0.24	12.7	95.262	12.5019998	11	7/24/2014	22:01.0
2832	RWS11	-122.4582198	37.93702	-0.23	12.7	36.962	12.4990004	-122.45821	37.93703	-0.24	12.7	101.72	12.4870004	11	7/24/2014	22:01.1
2833	RWS11	-122.458222	37.93702	-0.28	12.7	42.878	12.4330005	-122.45822	37.93703	-0.24	12.7	108.46	12.4730004	11	7/24/2014	22:01.2
2834	RWS11	-122.4582241	37.93702	-0.23	12.7	48.791	12.472	-122.45822	37.93703	-0.24	12.7	115.28	12.46	11	7/24/2014	22:01.3
2835	RWS11	-122.4582263	37.93702	-0.28	12.7	54.766	12.4070004	-122.45822	37.93703	-0.24	12.7	122.17	12.4470004	11	7/24/2014	22:01.4
2836	RWS11	-122.4582285	37.93702	-0.28	12.7	60.815	12.3959998	-122.45822	37.93703	-0.24	12.7	129.17	12.4359998	11	7/24/2014	22:01.5
2837	RWS11	-122.4582307	37.93702	-0.28	12.7	66.877	12.3859996	-122.45823	37.93703	-0.24	12.7	136.26	12.4259996	11	7/24/2014	22:01.6

2838	RWS11	-122.4582329	37.93702	-0.23	12.7	72.877	12.4300001	-122.45823	37.93703	-0.24	12.7	143.22	12.4180001	11	7/24/2014	22:01.7
2839	RWS11	-122.458235	37.93702	-0.28	12.6	78.79	12.3719996	-122.45823	37.93703	-0.24	12.6	149.94	12.4119996	11	7/24/2014	22:01.8
2840	RWS11	-122.4582371	37.93702	-0.28	12.6	84.618	12.3679999	-122.45823	37.93704	-0.2	12.6	156.7	12.4419999	11	7/24/2014	22:01.9
2841	RWS11	-122.4582403	37.93702	-0.28	12.6	90.607	12.3649997	-122.45823	37.93704	-0.29	12.6	163.45	12.3539996	11	7/24/2014	22:02.0
2842	RWS11	-122.4582425	37.93702	-0.23	12.6	96.723	12.4169997	-122.45824	37.93704	-0.24	12.6	170.42	12.4049996	11	7/24/2014	22:02.1
2843	RWS11	-122.4582446	37.93702	-0.28	12.6	102.96	12.3679999	-122.45824	37.93704	-0.24	12.6	177.41	12.4079999	11	7/24/2014	22:02.2
2844	RWS11	-122.4582468	37.93702	-0.23	12.6	109.4	12.4239996	-122.45824	37.93704	-0.24	12.6	184.61	12.4119996	11	7/24/2014	22:02.3
2845	RWS11	-122.4582489	37.93703	-0.28	12.7	115.95	12.3780001	-122.45824	37.93704	-0.24	12.7	192.13	12.4180001	11	7/24/2014	22:02.4
2846	RWS11	-122.4582511	37.93703	-0.23	12.7	122.59	12.4379996	-122.45825	37.93704	-0.24	12.7	199.71	12.4259996	11	7/24/2014	22:02.5
2847	RWS11	-122.4582533	37.93703	-0.28	12.7	129.3	12.3959998	-122.45825	37.93704	-0.24	12.7	207.43	12.4359998	11	7/24/2014	22:02.6
2848	RWS11	-122.4582554	37.93703	-0.28	12.7	135.97	12.4070004	-122.45825	37.93704	-0.24	12.7	215.22	12.4470004	11	7/24/2014	22:02.7
2849	RWS11	-122.4582576	37.93703	-0.28	12.7	142.87	12.4189996	-122.45825	37.93704	-0.29	12.7	223.29	12.4079996	11	7/24/2014	22:02.8
2850	RWS11	-122.4582597	37.93703	-0.28	12.7	149.98	12.432	-122.45825	37.93704	-0.24	12.7	231.64	12.472	11	7/24/2014	22:02.9
2851	RWS11	-122.4582629	37.93703	-0.31	12.7	157.03	12.412	-122.45826	37.93704	-0.24	12.7	239.85	12.486	11	7/24/2014	22:03.0
2852	RWS11	-122.458265	37.93703	-0.28	12.7	164.12	12.4599999	-122.45826	37.93704	-0.24	12.7	248	12.4999999	11	7/24/2014	22:03.1
2853	RWS11	-122.4582672	37.93703	-0.31	12.8	171.37	12.4410003	-122.45826	37.93704	-0.24	12.8	256.37	12.5150003	11	7/24/2014	22:03.2
2854	RWS11	-122.4582693	37.93703	-0.28	12.8	178.61	12.4890002	-122.45826	37.93704	-0.24	12.8	264.76	12.5290002	11	7/24/2014	22:03.3
2855	RWS11	-122.4582715	37.93703	-0.28	12.8	185.77	12.5039996	-122.45827	37.93704	-0.2	12.8	272.98	12.5779996	11	7/24/2014	22:03.4
2856	RWS11	-122.4582737	37.93703	-0.28	12.8	192.72	12.5200003	-122.45827	37.93704	-0.24	12.8	281.02	12.5600003	11	7/24/2014	22:03.5
2857	RWS11	-122.4582759	37.93703	-0.28	12.8	199.64	12.5349997	-122.45827	37.93705	-0.24	12.8	289	12.5749997	11	7/24/2014	22:03.6
2858	RWS11	-122.458278	37.93703	-0.28	12.8	206.42	12.5519999	-122.45827	37.93705	-0.24	12.8	297.09	12.5919999	11	7/24/2014	22:03.7
2859	RWS11	-122.4582802	37.93703	-0.28	12.8	213.19	12.5679997	-122.45827	37.93705	-0.24	12.8	305.05	12.6079997	11	7/24/2014	22:03.8
2860	RWS11	-122.4582823	37.93703	-0.28	12.9	219.89	12.5860003	-122.45828	37.93705	-0.2	12.9	313.06	12.6600003	11	7/24/2014	22:03.9
2861	RWS11	-122.4582855	37.93703	-0.28	12.9	226.71	12.6029996	-122.45828	37.93705	-0.24	12.9	321.19	12.6429996	11	7/24/2014	22:04.0
2862	RWS11	-122.4582876	37.93704	-0.23	12.9	233.56	12.6739996	-122.45828	37.93705	-0.24	12.9	329.35	12.6619996	11	7/24/2014	22:04.1
2863	RWS11	-122.4582897	37.93704	-0.28	12.9	240.43	12.6409997	-122.45828	37.93705	-0.29	12.9	337.64	12.6299997	11	7/24/2014	22:04.2
2864	RWS11	-122.4582918	37.93704	-0.28	12.9	247.38	12.6599997	-122.45829	37.93705	-0.24	12.9	345.92	12.6999997	11	7/24/2014	22:04.3
2865	RWS11	-122.458294	37.93704	-0.28	13	254.35	12.6809996	-122.45829	37.93705	-0.24	13	354.26	12.7209996	11	7/24/2014	22:04.4
2866	RWS11	-122.4582961	37.93704	-0.28	13	261.25	12.7010001	-122.45829	37.93705	-0.24	13	362.59	12.7410001	11	7/24/2014	22:04.5
2867	RWS11	-122.4582983	37.93704	-0.28	13	268.15	12.7230004	-122.45829	37.93705	-0.29	13	370.88	12.7120004	11	7/24/2014	22:04.6
2868	RWS11	-122.4583004	37.93704	-0.28	13	275.05	12.7449998	-122.4583	37.93705	-0.24	13	379.17	12.7849998	11	7/24/2014	22:04.7
2869	RWS11	-122.4583025	37.93704	-0.31	13	282.01	12.7339995	-122.4583	37.93705	-0.29	13	387.5	12.7569995	11	7/24/2014	22:04.8
2870	RWS11	-122.4583046	37.93704	-0.28	13.1	288.89	12.7899998	-122.4583	37.93705	-0.29	13.1	395.81	12.7789998	11	7/24/2014	22:04.9

2871	RWS11	-122.4583078	37.93704	-0.28	13.1	295.79	12.8120002	-122.4583	37.93705	-0.29	13.1	404.1	12.8010002	11	7/24/2014	22:05.0
2872	RWS11	-122.4583099	37.93704	-0.28	13.1	302.67	12.8340005	-122.4583	37.93705	-0.29	13.1	412.33	12.8230005	11	7/24/2014	22:05.1
2873	RWS11	-122.4583121	37.93704	-0.31	13.1	309.46	12.8219998	-122.45831	37.93705	-0.29	13.1	420.54	12.8449998	11	7/24/2014	22:05.2
2874	RWS11	-122.4583143	37.93704	-0.28	13.2	316.24	12.8769997	-122.45831	37.93705	-0.29	13.2	428.86	12.8659997	11	7/24/2014	22:05.3
2875	RWS11	-122.4583164	37.93704	-0.28	13.2	322.96	12.8979996	-122.45831	37.93706	-0.29	13.2	437.12	12.8869996	11	7/24/2014	22:05.4
2876	RWS11	-122.4583186	37.93704	-0.28	13.2	329.63	12.9169997	-122.45831	37.93706	-0.24	13.2	445.45	12.9569997	11	7/24/2014	22:05.5
2877	RWS11	-122.4583208	37.93704	-0.28	13.2	336.16	12.9350003	-122.45832	37.93706	-0.29	13.2	453.57	12.9240003	11	7/24/2014	22:05.6
2878	RWS11	-122.458323	37.93704	-0.28	13.2	342.51	12.953	-122.45832	37.93706	-0.24	13.2	461.51	12.9929999	11	7/24/2014	22:05.7
2879	RWS11	-122.4583252	37.93704	-0.31	13.2	348.95	12.9349997	-122.45832	37.93706	-0.29	13.2	469.23	12.9579997	11	7/24/2014	22:05.8
2880	RWS11	-122.4583274	37.93705	-0.28	13.3	355.13	12.9859999	-122.45832	37.93706	-0.24	13.3	476.9	13.0259999	11	7/24/2014	22:05.9
2881	RWS11	-122.4583306	37.93705	-0.31	13.3	361.44	12.9679997	-122.45833	37.93706	-0.29	13.3	484.46	12.9909997	11	7/24/2014	22:06.0
2882	RWS11	-122.4583328	37.93705	-0.28	13.3	367.75	13.0179995	-122.45833	37.93706	-0.24	13.3	492.11	13.0579995	11	7/24/2014	22:06.1
2883	RWS11	-122.4583349	37.93705	-0.28	13.3	373.75	13.0340003	-122.45833	37.93706	-0.29	13.3	499.46	13.0230003	11	7/24/2014	22:06.2
2884	RWS11	-122.4583371	37.93705	-0.28	13.3	379.8	13.0489997	-122.45833	37.93706	-0.24	13.3	506.69	13.0889997	11	7/24/2014	22:06.3
2885	RWS11	-122.4583393	37.93705	-0.31	13.3	385.71	13.0310004	-122.45833	37.93706	-0.29	13.3	513.82	13.0540004	11	7/24/2014	22:06.4
2886	RWS11	-122.4583415	37.93705	-0.28	13.4	391.56	13.0799998	-122.45834	37.93706	-0.24	13.4	520.74	13.1199998	11	7/24/2014	22:06.5
2887	RWS11	-122.4583438	37.93705	-0.28	13.4	397.17	13.0939997	-122.45834	37.93706	-0.29	13.4	527.43	13.0829997	11	7/24/2014	22:06.6
2888	RWS11	-122.458346	37.93705	-0.28	13.4	402.6	13.1070002	-122.45834	37.93706	-0.24	13.4	533.95	13.1470002	11	7/24/2014	22:06.7
2889	RWS11	-122.4583482	37.93705	-0.28	13.4	407.94	13.1190003	-122.45834	37.93706	-0.24	13.4	540.22	13.1590003	11	7/24/2014	22:06.8
2890	RWS11	-122.4583503	37.93705	-0.28	13.4	413.15	13.1310004	-122.45835	37.93706	-0.24	13.4	546.44	13.1710004	11	7/24/2014	22:06.9
2891	RWS11	-122.4583536	37.93705	-0.31	13.4	418.25	13.1080001	-122.45835	37.93706	-0.24	13.4	552.43	13.1820001	11	7/24/2014	22:07.0
2892	RWS11	-122.4583558	37.93705	-0.28	13.4	423.18	13.1540002	-122.45835	37.93707	-0.24	13.4	558.41	13.1940002	11	7/24/2014	22:07.1
2893	RWS11	-122.458358	37.93705	-0.28	13.4	427.93	13.1649998	-122.45835	37.93707	-0.24	13.4	564.06	13.2049998	11	7/24/2014	22:07.2
2894	RWS11	-122.4583602	37.93705	-0.23	13.5	432.46	13.2289999	-122.45836	37.93707	-0.24	13.5	569.63	13.2169999	11	7/24/2014	22:07.3
2895	RWS11	-122.4583624	37.93705	-0.28	13.5	436.85	13.1879996	-122.45836	37.93707	-0.24	13.5	574.93	13.2279996	11	7/24/2014	22:07.4
2896	RWS11	-122.4583646	37.93705	-0.28	13.5	441.12	13.2000002	-122.45836	37.93707	-0.2	13.5	580.21	13.2740002	11	7/24/2014	22:07.5
2897	RWS11	-122.4583668	37.93705	-0.28	13.5	445.19	13.2090001	-122.45836	37.93707	-0.24	13.5	585.19	13.2490001	11	7/24/2014	22:07.6
2898	RWS11	-122.458369	37.93706	-0.28	13.5	449.12	13.216	-122.45836	37.93707	-0.24	13.5	590.01	13.256	11	7/24/2014	22:07.7
2899	RWS11	-122.4583712	37.93706	-0.28	13.5	452.95	13.2190003	-122.45837	37.93707	-0.2	13.5	594.68	13.2930003	11	7/24/2014	22:07.8
2900	RWS11	-122.4583734	37.93706	-0.22	13.5	456.55	13.2710003	-122.45837	37.93707	-0.2	13.5	599.07	13.2930003	11	7/24/2014	22:07.9
2901	RWS11	-122.4583766	37.93706	-0.28	13.5	460.02	13.2140002	-122.45837	37.93707	-0.2	13.5	603.31	13.2880002	11	7/24/2014	22:08.0
2902	RWS11	-122.4583788	37.93706	-0.22	13.5	463.39	13.256	-122.45837	37.93707	-0.2	13.5	607.36	13.278	11	7/24/2014	22:08.1
2903	RWS11	-122.458381	37.93706	-0.28	13.5	466.53	13.1869997	-122.45838	37.93707	-0.2	13.5	611.22	13.2609998	11	7/24/2014	22:08.2

2904	RWS11	-122.4583832	37.93706	-0.22	13.4	469.51	13.2170004	-122.45838	37.93707	-0.2	13.4	614.91	13.2390004	11	7/24/2014	22:08.3
2905	RWS11	-122.4583854	37.93706	-0.28	13.4	472.45	13.138	-122.45838	37.93707	-0.2	13.4	618.43	13.212	11	7/24/2014	22:08.4
2906	RWS11	-122.4583876	37.93706	-0.22	13.4	475.31	13.1580004	-122.45838	37.93707	-0.2	13.4	621.9	13.1800004	11	7/24/2014	22:08.5
2907	RWS11	-122.4583898	37.93706	-0.22	13.3	478.01	13.1229996	-122.45838	37.93707	-0.2	13.3	625.11	13.1449996	11	7/24/2014	22:08.6
2908	RWS11	-122.458392	37.93706	-0.22	13.3	480.36	13.0890001	-122.45839	37.93707	-0.15	13.3	628.11	13.1610001	11	7/24/2014	22:08.7
2909	RWS11	-122.4583942	37.93706	-0.28	13.3	482.74	13.006	-122.45839	37.93707	-0.2	13.3	630.91	13.08	11	7/24/2014	22:08.8
2910	RWS11	-122.4583964	37.93706	-0.22	13.3	484.76	13.0309996	-122.45839	37.93707	-0.2	13.3	633.44	13.0529996	11	7/24/2014	22:08.9
2911	RWS11	-122.4583996	37.93706	-0.22	13.2	486.36	13.0120005	-122.45839	37.93708	-0.2	13.2	635.48	13.0340005	11	7/24/2014	22:09.0
2912	RWS11	-122.4584017	37.93706	-0.19	13.2	487.57	13.0349998	-122.4584	37.93708	-0.15	13.2	637.05	13.0729998	11	7/24/2014	22:09.1
2913	RWS11	-122.4584039	37.93706	-0.22	13.2	488.59	12.9979996	-122.4584	37.93708	-0.2	13.2	638.29	13.0199996	11	7/24/2014	22:09.2
2914	RWS11	-122.458406	37.93706	-0.22	13.2	489.36	13.0040001	-122.4584	37.93708	-0.2	13.2	639.25	13.0260001	11	7/24/2014	22:09.3
2915	RWS11	-122.4584082	37.93706	-0.22	13.2	490.01	13.0169996	-122.4584	37.93708	-0.2	13.2	639.95	13.0389996	11	7/24/2014	22:09.4
2916	RWS11	-122.4584103	37.93707	-0.22	13.3	490.58	13.0339998	-122.45841	37.93708	-0.2	13.3	640.52	13.0559998	11	7/24/2014	22:09.5
2917	RWS11	-122.4584125	37.93707	-0.22	13.3	491.02	13.0540003	-122.45841	37.93708	-0.2	13.3	640.89	13.0760003	11	7/24/2014	22:09.6
2918	RWS11	-122.4584147	37.93707	-0.22	13.3	491.33	13.0750002	-122.45841	37.93708	-0.2	13.3	641.02	13.0970002	11	7/24/2014	22:09.7
2919	RWS11	-122.4584168	37.93707	-0.28	13.3	491.47	13.0409998	-122.45841	37.93708	-0.2	13.3	640.95	13.1149999	11	7/24/2014	22:09.8
2920	RWS11	-122.4584189	37.93707	-0.22	13.3	491.33	13.1060003	-122.45841	37.93708	-0.2	13.3	640.56	13.1280003	11	7/24/2014	22:09.9
2921	RWS11	-122.4584221	37.93707	-0.22	13.3	491	13.1119999	-122.45842	37.93708	-0.2	13.3	639.93	13.1339999	11	7/24/2014	22:10.0
2922	RWS11	-122.4584243	37.93707	-0.22	13.3	490.52	13.11	-122.45842	37.93708	-0.15	13.3	639	13.182	11	7/24/2014	22:10.1
2923	RWS11	-122.4584264	37.93707	-0.22	13.3	489.72	13.0990004	-122.45842	37.93708	-0.2	13.3	637.67	13.1210004	11	7/24/2014	22:10.2
2924	RWS11	-122.4584286	37.93707	-0.22	13.3	488.81	13.0809998	-122.45842	37.93708	-0.2	13.3	636.08	13.1029998	11	7/24/2014	22:10.3
2925	RWS11	-122.4584308	37.93707	-0.22	13.3	487.52	13.0540003	-122.45843	37.93708	-0.2	13.3	634.14	13.0760003	11	7/24/2014	22:10.4
2926	RWS11	-122.458433	37.93707	-0.22	13.2	486.01	13.0219998	-122.45843	37.93708	-0.2	13.2	631.84	13.0439998	11	7/24/2014	22:10.5
2927	RWS11	-122.4584352	37.93707	-0.22	13.2	484.07	12.9839996	-122.45843	37.93708	-0.2	13.2	629.2	13.0059996	11	7/24/2014	22:10.6
2928	RWS11	-122.4584374	37.93707	-0.22	13.2	481.79	12.9430002	-122.45843	37.93708	-0.2	13.2	626.08	12.9650002	11	7/24/2014	22:10.7
2929	RWS11	-122.4584396	37.93707	-0.22	13.1	479.1	12.8989996	-122.45843	37.93709	-0.15	13.1	622.5	12.9709996	11	7/24/2014	22:10.8
2930	RWS11	-122.4584418	37.93707	-0.19	13.1	476.3	12.8880005	-122.45844	37.93709	-0.2	13.1	618.73	12.8760005	11	7/24/2014	22:10.9
2931	RWS11	-122.458445	37.93707	-0.22	13	473.14	12.808	-122.45844	37.93709	-0.2	13	614.68	12.83	11	7/24/2014	22:11.0
2932	RWS11	-122.4584471	37.93707	-0.22	13	469.69	12.7610001	-122.45844	37.93709	-0.15	13	610.32	12.8330001	11	7/24/2014	22:11.1
2933	RWS11	-122.4584493	37.93707	-0.22	12.9	466.22	12.7149996	-122.45844	37.93709	-0.2	12.9	605.9	12.7369996	11	7/24/2014	22:11.2
2934	RWS11	-122.4584515	37.93708	-0.22	12.9	462.57	12.6690001	-122.45845	37.93709	-0.15	12.9	601.19	12.7410001	11	7/24/2014	22:11.3
2935	RWS11	-122.4584537	37.93708	-0.22	12.8	458.85	12.624	-122.45845	37.93709	-0.2	12.8	596.41	12.646	11	7/24/2014	22:11.4
2936	RWS11	-122.4584559	37.93708	-0.22	12.8	455.09	12.5789999	-122.45845	37.93709	-0.15	12.8	591.47	12.6509999	11	7/24/2014	22:11.5

2937	RWS11	-122.4584581	37.93708	-0.28	12.8	451.18	12.4830002	-122.45845	37.93709	-0.24	12.8	586.39	12.5230002	11	7/24/2014	22:11.6
2938	RWS11	-122.4584603	37.93708	-0.22	12.7	447.09	12.4909996	-122.45846	37.93709	-0.15	12.7	581.06	12.5629996	11	7/24/2014	22:11.7
2939	RWS11	-122.4584625	37.93708	-0.22	12.7	442.8	12.4480003	-122.45846	37.93709	-0.2	12.7	575.58	12.4700003	11	7/24/2014	22:11.8
2940	RWS11	-122.4584647	37.93708	-0.22	12.6	438.25	12.4050001	-122.45846	37.93709	-0.2	12.6	569.9	12.4270001	11	7/24/2014	22:11.9
2941	RWS11	-122.4584679	37.93708	-0.22	12.6	433.57	12.3639998	-122.45846	37.93709	-0.2	12.6	564.06	12.3859998	11	7/24/2014	22:12.0
2942	RWS11	-122.45847	37.93708	-0.22	12.6	428.84	12.3250002	-122.45847	37.93709	-0.2	12.6	558.14	12.3470002	11	7/24/2014	22:12.1
2943	RWS11	-122.4584722	37.93708	-0.22	12.5	424.02	12.2879995	-122.45847	37.93709	-0.24	12.5	552.21	12.2759995	11	7/24/2014	22:12.2
2944	RWS11	-122.4584743	37.93708	-0.22	12.5	419.14	12.2540001	-122.45847	37.93709	-0.2	12.5	546.18	12.2760001	11	7/24/2014	22:12.3
2945	RWS11	-122.4584765	37.93708	-0.28	12.4	414.26	12.1709999	-122.45847	37.93709	-0.24	12.4	540.03	12.211	11	7/24/2014	22:12.4
2946	RWS11	-122.4584787	37.93708	-0.22	12.4	409.25	12.1950001	-122.45847	37.93709	-0.2	12.4	533.82	12.2170001	11	7/24/2014	22:12.5
2947	RWS11	-122.4584809	37.93708	-0.28	12.4	404.23	12.1189999	-122.45848	37.9371	-0.2	12.4	527.45	12.1929999	11	7/24/2014	22:12.6
2948	RWS11	-122.458483	37.93708	-0.22	12.4	399.07	12.1489996	-122.45848	37.9371	-0.2	12.4	521.03	12.1709996	11	7/24/2014	22:12.7
2949	RWS11	-122.4584852	37.93708	-0.22	12.4	393.86	12.1290001	-122.45848	37.9371	-0.24	12.4	514.44	12.1170001	11	7/24/2014	22:12.8
2950	RWS11	-122.4584874	37.93708	-0.22	12.3	388.56	12.1110005	-122.45848	37.9371	-0.24	12.3	507.73	12.0990004	11	7/24/2014	22:12.9
2951	RWS11	-122.4584905	37.93708	-0.28	12.3	383.1	12.0409998	-122.45849	37.9371	-0.24	12.3	500.81	12.0809998	11	7/24/2014	22:13.0
2952	RWS11	-122.4584927	37.93708	-0.22	12.3	377.54	12.0750002	-122.45849	37.9371	-0.2	12.3	493.88	12.0970002	11	7/24/2014	22:13.1
2953	RWS11	-122.4584949	37.93709	-0.28	12.3	371.91	12.0040001	-122.45849	37.9371	-0.24	12.3	486.71	12.0440001	11	7/24/2014	22:13.2
2954	RWS11	-122.4584971	37.93709	-0.22	12.3	366.11	12.0350002	-122.45849	37.9371	-0.24	12.3	479.36	12.0230002	11	7/24/2014	22:13.3
2955	RWS11	-122.4584993	37.93709	-0.28	12.2	360.15	11.9590001	-122.45849	37.9371	-0.24	12.2	471.72	11.9990001	11	7/24/2014	22:13.4
2956	RWS11	-122.4585015	37.93709	-0.22	12.2	354.06	11.985	-122.4585	37.9371	-0.2	12.2	463.93	12.007	11	7/24/2014	22:13.5
2957	RWS11	-122.4585037	37.93709	-0.28	12.2	347.84	11.9019999	-122.4585	37.9371	-0.29	12.2	455.96	11.8909999	11	7/24/2014	22:13.6
2958	RWS11	-122.4585059	37.93709	-0.28	12.1	341.5	11.867	-122.4585	37.9371	-0.24	12.1	447.81	11.9070001	11	7/24/2014	22:13.7
2959	RWS11	-122.4585081	37.93709	-0.28	12.1	334.86	11.8270001	-122.4585	37.9371	-0.29	12.1	439.63	11.8160001	11	7/24/2014	22:13.8
2960	RWS11	-122.4585103	37.93709	-0.28	12.1	328.31	11.7830004	-122.45851	37.9371	-0.24	12.1	431.41	11.8230004	11	7/24/2014	22:13.9
2961	RWS11	-122.4585135	37.93709	-0.28	12	321.58	11.7350001	-122.45851	37.9371	-0.29	12	423.16	11.7240001	11	7/24/2014	22:14.0
2962	RWS11	-122.4585157	37.93709	-0.28	12	314.83	11.6849999	-122.45851	37.9371	-0.29	12	414.85	11.6739999	11	7/24/2014	22:14.1
2963	RWS11	-122.4585179	37.93709	-0.31	11.9	308.06	11.597	-122.45851	37.9371	-0.29	11.9	406.48	11.62	11	7/24/2014	22:14.2
2964	RWS11	-122.4585202	37.93709	-0.28	11.9	301.16	11.5759997	-122.45852	37.9371	-0.29	11.9	398.12	11.5649997	11	7/24/2014	22:14.3
2965	RWS11	-122.4585224	37.93709	-0.31	11.8	294.38	11.4859999	-122.45852	37.9371	-0.29	11.8	389.63	11.5089999	11	7/24/2014	22:14.4
2966	RWS11	-122.4585246	37.93709	-0.28	11.7	287.5	11.4620003	-122.45852	37.93711	-0.29	11.7	381.17	11.4510003	11	7/24/2014	22:14.5
2967	RWS11	-122.4585269	37.93709	-0.31	11.7	280.6	11.3699998	-122.45852	37.93711	-0.32	11.7	372.64	11.3589998	11	7/24/2014	22:14.6
2968	RWS11	-122.4585291	37.93709	-0.31	11.6	273.76	11.3100003	-122.45852	37.93711	-0.29	11.6	364.13	11.3330003	11	7/24/2014	22:14.7
2969	RWS11	-122.4585313	37.93709	-0.31	11.6	266.9	11.2499999	-122.45853	37.93711	-0.32	11.6	355.64	11.2389999	11	7/24/2014	22:14.8

2970	RWS11	-122.4585335	37.93709	-0.28	11.5	260.06	11.2240004	-122.45853	37.93711	-0.32	11.5	347.04	11.1790004	11	7/24/2014	22:14.9
2971	RWS11	-122.4585368	37.9371	-0.31	11.4	253.11	11.1310004	-122.45853	37.93711	-0.32	11.4	338.4	11.1200004	11	7/24/2014	22:15.0
2972	RWS11	-122.458539	37.9371	-0.28	11.4	246.15	11.1080002	-122.45853	37.93711	-0.29	11.4	329.68	11.0970002	11	7/24/2014	22:15.1
2973	RWS11	-122.4585412	37.9371	-0.31	11.3	239.15	11.0200003	-122.45854	37.93711	-0.32	11.3	320.92	11.0090003	11	7/24/2014	22:15.2
2974	RWS11	-122.4585434	37.9371	-0.31	11.3	232	10.9689997	-122.45854	37.93711	-0.32	11.3	312.15	10.9579997	11	7/24/2014	22:15.3
2975	RWS11	-122.4585456	37.9371	-0.31	11.2	224.82	10.9210004	-122.45854	37.93711	-0.32	11.2	303.4	10.9100004	11	7/24/2014	22:15.4
2976	RWS11	-122.4585478	37.9371	-0.31	11.2	217.56	10.8749999	-122.45854	37.93711	-0.32	11.2	294.67	10.8639999	11	7/24/2014	22:15.5
2977	RWS11	-122.4585501	37.9371	-0.31	11.1	210.32	10.8330001	-122.45855	37.93711	-0.32	11.1	285.94	10.8220001	11	7/24/2014	22:15.6
2978	RWS11	-122.4585523	37.9371	-0.31	11.1	203.07	10.7930001	-122.45855	37.93711	-0.32	11.1	277.21	10.7820001	11	7/24/2014	22:15.7
2979	RWS11	-122.4585545	37.9371	-0.31	11.1	195.75	10.7560004	-122.45855	37.93711	-0.36	11.1	268.57	10.7110004	11	7/24/2014	22:15.8
2980	RWS11	-122.4585567	37.9371	-0.31	11	188.42	10.7209996	-122.45855	37.93711	-0.32	11	259.87	10.7099996	11	7/24/2014	22:15.9
2981	RWS11	-122.4585599	37.9371	-0.31	11	181.01	10.6870002	-122.45856	37.93711	-0.32	11	251.22	10.6760002	11	7/24/2014	22:16.0
2982	RWS11	-122.4585621	37.9371	-0.31	11	173.52	10.6549996	-122.45856	37.93711	-0.32	11	242.56	10.6439996	11	7/24/2014	22:16.1
2983	RWS11	-122.4585643	37.9371	-0.31	10.9	166.16	10.6240004	-122.45856	37.93711	-0.36	10.9	233.94	10.5790004	11	7/24/2014	22:16.2
2984	RWS11	-122.4585665	37.9371	-0.31	10.9	158.79	10.5930003	-122.45856	37.93712	-0.32	10.9	225.41	10.5820003	11	7/24/2014	22:16.3
2985	RWS11	-122.4585687	37.9371	-0.31	10.9	151.5	10.5629996	-122.45856	37.93712	-0.32	10.9	216.96	10.5519996	11	7/24/2014	22:16.4
2986	RWS11	-122.4585709	37.9371	-0.31	10.8	144.3	10.5329999	-122.45857	37.93712	-0.32	10.8	208.65	10.5219999	11	7/24/2014	22:16.5
2987	RWS11	-122.4585731	37.9371	-0.35	10.8	137.19	10.4699996	-122.45857	37.93712	-0.32	10.8	200.45	10.4929996	11	7/24/2014	22:16.6
2988	RWS11	-122.4585753	37.9371	-0.31	10.8	130.05	10.4739999	-122.45857	37.93712	-0.32	10.8	192.19	10.4629999	11	7/24/2014	22:16.7
2989	RWS11	-122.4585774	37.9371	-0.35	10.8	122.99	10.4089997	-122.45857	37.93712	-0.36	10.8	184.07	10.3979997	11	7/24/2014	22:16.8
2990	RWS11	-122.4585796	37.93711	-0.31	10.7	115.87	10.4119996	-122.45857	37.93712	-0.32	10.7	175.92	10.4009996	11	7/24/2014	22:16.9
2991	RWS11	-122.4585828	37.93711	-0.35	10.7	108.95	10.3449996	-122.45858	37.93712	-0.36	10.7	167.95	10.3339996	11	7/24/2014	22:17.0
2992	RWS11	-122.458585	37.93711	-0.31	10.7	101.97	10.3439997	-122.45858	37.93712	-0.32	10.7	160	10.3329997	11	7/24/2014	22:17.1
2993	RWS11	-122.4585871	37.93711	-0.31	10.6	95.055	10.3089999	-122.45858	37.93712	-0.32	10.6	151.99	10.2979999	11	7/24/2014	22:17.2
2994	RWS11	-122.4585893	37.93711	-0.31	10.6	88.114	10.2740001	-122.45858	37.93712	-0.32	10.6	143.92	10.263	11	7/24/2014	22:17.3
2995	RWS11	-122.4585915	37.93711	-0.35	10.5	81.195	10.2039998	-122.45859	37.93712	-0.32	10.5	135.83	10.2269998	11	7/24/2014	22:17.4
2996	RWS11	-122.4585936	37.93711	-0.31	10.5	74.39	10.2019996	-122.45859	37.93712	-0.32	10.5	127.93	10.1909995	11	7/24/2014	22:17.5
2997	RWS11	-122.4585958	37.93711	-0.31	10.5	67.756	10.1669997	-122.45859	37.93712	-0.36	10.5	120.24	10.1219997	11	7/24/2014	22:17.6
2998	RWS11	-122.458598	37.93711	-0.31	10.4	61.165	10.1319999	-122.45859	37.93712	-0.32	10.4	112.66	10.1209998	11	7/24/2014	22:17.7
2999	RWS11	-122.4586001	37.93711	-0.35	10.4	54.684	10.0649999	-122.4586	37.93712	-0.32	10.4	105.09	10.0879999	11	7/24/2014	22:17.8
3000	RWS11	-122.4586023	37.93711	-0.31	10.4	48.293	10.0670003	-122.4586	37.93712	-0.36	10.4	97.644	10.0220003	11	7/24/2014	22:17.9
3001	RWS11	-122.4586055	37.93711	-0.31	10.3	41.943	10.0349997	-122.4586	37.93712	-0.36	10.3	90.316	9.98999971	11	7/24/2014	22:18.0
3002	RWS11	-122.4586077	37.93711	-0.31	10.3	35.596	10.005	-122.4586	37.93712	-0.36	10.3	83.069	9.95999998	11	7/24/2014	22:18.1

3003	RWS11	-122.4586099	37.93711	-0.31	10.3	29.2	9.97599971	-122.45861	37.93713	-0.36	10.3	75.735	9.9309997	11	7/24/2014	22:18.2
3004	RWS11	-122.458612	37.93711	-0.31	10.3	22.765	9.94799984	-122.45861	37.93713	-0.32	10.3	68.389	9.93699983	11	7/24/2014	22:18.3
3005	RWS11	-122.4586142	37.93711	-0.31	10.2	16.284	9.92100036	-122.45861	37.93713	-0.32	10.2	61.069	9.91000035	11	7/24/2014	22:18.4
3006	RWS11	-122.4586164	37.93711	-0.31	10.2	10	9.89500034	-122.45861	37.93713	-0.32	10.2	53.895	9.88400033	11	7/24/2014	22:18.5
3007	RWS11	-122.4586186	37.93711	-0.31	10.2	3.607	9.86999977	-122.45861	37.93713	-0.32	10.2	46.767	9.85899976	11	7/24/2014	22:18.6
3008	RWS11	-122.4586208	37.93711	-0.31	10.2	-2.525	9.8459996	-122.45862	37.93713	-0.32	10.2	39.692	9.83499959	11	7/24/2014	22:18.7
3009	RWS11	-122.458623	37.93712	-0.31	10.1	-8.768	9.82400024	-122.45862	37.93713	-0.36	10.1	32.667	9.77900022	11	7/24/2014	22:18.8
3010	RWS11	-122.4586252	37.93712	-0.31	10.1	-14.75	9.80199993	-122.45862	37.93713	-0.32	10.1	25.747	9.79099992	11	7/24/2014	22:18.9
3011	RWS11	-122.4586284	37.93712	-0.31	10.1	-20.73	9.78200042	-122.45862	37.93713	-0.36	10.1	18.723	9.73700041	11	7/24/2014	22:19.0
3012	RWS11	-122.4586306	37.93712	-0.31	10.1	-26.55	9.76300037	-122.45863	37.93713	-0.32	10.1	11.784	9.75200036	11	7/24/2014	22:19.1
3013	RWS11	-122.4586327	37.93712	-0.35	10.1	-32.51	9.71200016	-122.45863	37.93713	-0.32	10.1	4.89	9.73500016	11	7/24/2014	22:19.2
3014	RWS11	-122.4586349	37.93712	-0.31	10	-38.27	9.72899997	-122.45863	37.93713	-0.32	10	-1.918	9.71799996	11	7/24/2014	22:19.3
3015	RWS11	-122.4586371	37.93712	-0.35	10	-44.1	9.67999962	-122.45863	37.93713	-0.32	10	-8.595	9.70299962	11	7/24/2014	22:19.4
3016	RWS11	-122.4586393	37.93712	-0.35	10	-49.81	9.66399983	-122.45863	37.93713	-0.32	10	-15.119	9.68699983	11	7/24/2014	22:19.5
3017	RWS11	-122.4586415	37.93712	-0.31	9.99	-55.42	9.68300045	-122.45864	37.93713	-0.32	9.99	-21.599	9.67200044	11	7/24/2014	22:19.6
3018	RWS11	-122.4586437	37.93712	-0.31	9.98	-61.09	9.6669997	-122.45864	37.93713	-0.32	9.98	-28.166	9.65599969	11	7/24/2014	22:19.7
3019	RWS11	-122.4586458	37.93712	-0.35	9.96	-66.72	9.6180003	-122.45864	37.93713	-0.32	9.96	-34.473	9.6410003	11	7/24/2014	22:19.8
3020	RWS11	-122.458648	37.93712	-0.31	9.95	-72.31	9.63800037	-122.45864	37.93713	-0.32	9.95	-40.561	9.62700036	11	7/24/2014	22:19.9
3021	RWS11	-122.4586512	37.93712	-0.31	9.94	-77.93	9.62400043	-122.45865	37.93713	-0.32	9.94	-46.692	9.61300042	11	7/24/2014	22:20.0
3022	RWS11	-122.4586533	37.93712	-0.31	9.92	-83.52	9.60999954	-122.45865	37.93714	-0.32	9.92	-52.866	9.59899953	11	7/24/2014	22:20.1
3023	RWS11	-122.4586555	37.93712	-0.35	9.91	-88.98	9.5640004	-122.45865	37.93714	-0.32	9.91	-58.757	9.5870004	11	7/24/2014	22:20.2
3024	RWS11	-122.4586577	37.93712	-0.31	9.9	-94.38	9.58499992	-122.45865	37.93714	-0.32	9.9	-64.539	9.57399991	11	7/24/2014	22:20.3
3025	RWS11	-122.4586598	37.93712	-0.35	9.88	-99.69	9.53899983	-122.45866	37.93714	-0.36	9.88	-70.148	9.52799982	11	7/24/2014	22:20.4
3026	RWS11	-122.458662	37.93712	-0.31	9.87	-104.8	9.56200016	-122.45866	37.93714	-0.32	9.87	-75.606	9.55100015	11	7/24/2014	22:20.5
3027	RWS11	-122.4586642	37.93712	-0.35	9.86	-109.9	9.51799992	-122.45866	37.93714	-0.32	9.86	-80.926	9.54099992	11	7/24/2014	22:20.6
3028	RWS11	-122.4586664	37.93713	-0.31	9.85	-114.7	9.5430001	-122.45866	37.93714	-0.32	9.85	-86.102	9.53200009	11	7/24/2014	22:20.7
3029	RWS11	-122.4586685	37.93713	-0.31	9.85	-119.6	9.53499973	-122.45866	37.93714	-0.32	9.85	-91.303	9.52399972	11	7/24/2014	22:20.8
3030	RWS11	-122.4586707	37.93713	-0.31	9.84	-124.4	9.5259999	-122.45867	37.93714	-0.32	9.84	-96.447	9.5149999	11	7/24/2014	22:20.9
3031	RWS11	-122.4586738	37.93713	-0.35	9.83	-129.1	9.48300007	-122.45867	37.93714	-0.32	9.83	-101.74	9.50600007	11	7/24/2014	22:21.0
3032	RWS11	-122.458676	37.93713	-0.31	9.82	-133.8	9.5060004	-122.45867	37.93714	-0.32	9.82	-106.82	9.49500039	11	7/24/2014	22:21.1
3033	RWS11	-122.4586781	37.93713	-0.35	9.81	-138.3	9.46000031	-122.45867	37.93714	-0.32	9.81	-111.89	9.48300031	11	7/24/2014	22:21.2
3034	RWS11	-122.4586802	37.93713	-0.31	9.79	-142.6	9.48099983	-122.45868	37.93714	-0.32	9.79	-116.99	9.46999982	11	7/24/2014	22:21.3
3035	RWS11	-122.4586824	37.93713	-0.35	9.77	-146.8	9.42999962	-122.45868	37.93714	-0.32	9.77	-122.06	9.45299962	11	7/24/2014	22:21.4

3036	RWS11	-122.4586845	37.93713	-0.35	9.76	-150.9	9.41099957	-122.45868	37.93714	-0.32	9.76	-127.11	9.43399957	11	7/24/2014	22:21.5
3037	RWS11	-122.4586867	37.93713	-0.35	9.73	-155	9.38900021	-122.45868	37.93714	-0.32	9.73	-132.34	9.41200021	11	7/24/2014	22:21.6
3038	RWS11	-122.4586888	37.93713	-0.31	9.71	-159.2	9.39700019	-122.45868	37.93714	-0.32	9.71	-137.49	9.38600019	11	7/24/2014	22:21.7
3039	RWS11	-122.4586909	37.93713	-0.35	9.68	-163.6	9.3339999	-122.45869	37.93714	-0.32	9.68	-142.64	9.3569999	11	7/24/2014	22:21.8
3040	RWS11	-122.458693	37.93713	-0.35	9.65	-168.1	9.30200031	-122.45869	37.93714	-0.29	9.65	-147.95	9.35900033	11	7/24/2014	22:21.9
3041	RWS11	-122.4586961	37.93713	-0.35	9.61	-172.6	9.26700047	-122.45869	37.93715	-0.32	9.61	-153.17	9.29000047	11	7/24/2014	22:22.0
3042	RWS11	-122.4586983	37.93713	-0.31	9.58	-177.1	9.26500022	-122.45869	37.93715	-0.32	9.58	-158.21	9.25400022	11	7/24/2014	22:22.1
3043	RWS11	-122.4587003	37.93713	-0.35	9.54	-181.6	9.1919997	-122.4587	37.93715	-0.32	9.54	-163.1	9.21499971	11	7/24/2014	22:22.2
3044	RWS11	-122.4587025	37.93713	-0.31	9.5	-186	9.18700016	-122.4587	37.93715	-0.32	9.5	-167.77	9.17600015	11	7/24/2014	22:22.3
3045	RWS11	-122.4587046	37.93713	-0.35	9.46	-190.2	9.11399963	-122.4587	37.93715	-0.29	9.46	-172.31	9.17099965	11	7/24/2014	22:22.4
3046	RWS11	-122.4587067	37.93713	-0.31	9.42	-194.4	9.11099994	-122.4587	37.93715	-0.29	9.42	-176.75	9.13399994	11	7/24/2014	22:22.5
3047	RWS11	-122.4587088	37.93714	-0.35	9.39	-198.5	9.04099968	-122.4587	37.93715	-0.29	9.39	-181.17	9.09799969	11	7/24/2014	22:22.6
3048	RWS11	-122.458711	37.93714	-0.31	9.35	-202.6	9.0430001	-122.45871	37.93715	-0.29	9.35	-185.66	9.0660001	11	7/24/2014	22:22.7
3049	RWS11	-122.4587131	37.93714	-0.35	9.32	-206.6	8.97900036	-122.45871	37.93715	-0.29	9.32	-189.9	9.03600037	11	7/24/2014	22:22.8
3050	RWS11	-122.4587152	37.93714	-0.31	9.3	-210.7	8.98700035	-122.45871	37.93715	-0.29	9.3	-194.08	9.01000035	11	7/24/2014	22:22.9
3051	RWS11	-122.4587183	37.93714	-0.35	9.28	-214.6	8.93100002	-122.45871	37.93715	-0.32	9.28	-198.26	8.95400003	11	7/24/2014	22:23.0
3052	RWS11	-122.4587204	37.93714	-0.31	9.26	-218.5	8.94599998	-122.45872	37.93715	-0.29	9.26	-202.33	8.96899998	11	7/24/2014	22:23.1
3053	RWS11	-122.4587225	37.93714	-0.31	9.24	-222.1	8.93000019	-122.45872	37.93715	-0.29	9.24	-206.14	8.95300019	11	7/24/2014	22:23.2
3054	RWS11	-122.4587247	37.93714	-0.35	9.23	-225.6	8.88499954	-122.45872	37.93715	-0.32	9.23	-209.93	8.90799955	11	7/24/2014	22:23.3
3055	RWS11	-122.4587268	37.93714	-0.35	9.22	-229.1	8.87599972	-122.45872	37.93715	-0.32	9.22	-213.55	8.89899972	11	7/24/2014	22:23.4
3056	RWS11	-122.458729	37.93714	-0.31	9.22	-232.5	8.90499961	-122.45872	37.93715	-0.29	9.22	-217.1	8.92799962	11	7/24/2014	22:23.5
3057	RWS11	-122.4587311	37.93714	-0.35	9.21	-235.8	8.8680003	-122.45873	37.93715	-0.29	9.21	-220.57	8.92500031	11	7/24/2014	22:23.6
3058	RWS11	-122.4587332	37.93714	-0.35	9.21	-239	8.86699989	-122.45873	37.93715	-0.29	9.21	-224.12	8.92399991	11	7/24/2014	22:23.7
3059	RWS11	-122.4587354	37.93714	-0.35	9.21	-242.1	8.8680003	-122.45873	37.93715	-0.32	9.21	-227.57	8.8910003	11	7/24/2014	22:23.8
3060	RWS11	-122.4587375	37.93714	-0.31	9.21	-245.2	8.90299976	-122.45873	37.93715	-0.29	9.21	-230.96	8.92599976	11	7/24/2014	22:23.9
3061	RWS11	-122.4587406	37.93714	-0.35	9.22	-248.2	8.87000015	-122.45874	37.93716	-0.29	9.22	-234.27	8.92700016	11	7/24/2014	22:24.0
3062	RWS11	-122.4587427	37.93714	-0.31	9.22	-251.1	8.90499961	-122.45874	37.93716	-0.32	9.22	-237.52	8.89399961	11	7/24/2014	22:24.1
3063	RWS11	-122.4587448	37.93714	-0.35	9.22	-253.9	8.8709996	-122.45874	37.93716	-0.32	9.22	-240.64	8.89399961	11	7/24/2014	22:24.2
3064	RWS11	-122.4587469	37.93714	-0.35	9.21	-256.6	8.8680003	-122.45874	37.93716	-0.32	9.21	-243.69	8.8910003	11	7/24/2014	22:24.3
3065	RWS11	-122.4587491	37.93714	-0.35	9.21	-259.3	8.86399963	-122.45874	37.93716	-0.32	9.21	-246.73	8.88699964	11	7/24/2014	22:24.4
3066	RWS11	-122.4587512	37.93715	-0.31	9.2	-262.1	8.89200008	-122.45875	37.93716	-0.32	9.2	-249.84	8.88100007	11	7/24/2014	22:24.5
3067	RWS11	-122.4587533	37.93715	-0.35	9.19	-264.8	8.84900025	-122.45875	37.93716	-0.32	9.19	-252.85	8.87200025	11	7/24/2014	22:24.6
3068	RWS11	-122.4587555	37.93715	-0.31	9.18	-267.5	8.87199962	-122.45875	37.93716	-0.32	9.18	-255.75	8.86099961	11	7/24/2014	22:24.7

3069	RWS11	-122.4587575	37.93715	-0.35	9.17	-270.1	8.82599953	-122.45875	37.93716	-0.36	9.17	-258.52	8.81499952	11	7/24/2014	22:24.8
3070	RWS11	-122.4587596	37.93715	-0.35	9.16	-272.8	8.81100014	-122.45875	37.93716	-0.32	9.16	-261.28	8.83400014	11	7/24/2014	22:24.9
3071	RWS11	-122.4587627	37.93715	-0.35	9.14	-275.5	8.79399994	-122.45876	37.93716	-0.29	9.14	-263.85	8.85099995	11	7/24/2014	22:25.0
3072	RWS11	-122.4587648	37.93715	-0.31	9.12	-278	8.8100003	-122.45876	37.93716	-0.32	9.12	-266.39	8.79900029	11	7/24/2014	22:25.1
3073	RWS11	-122.4587668	37.93715	-0.35	9.1	-280.5	8.75700024	-122.45876	37.93716	-0.32	9.1	-269.05	8.78000024	11	7/24/2014	22:25.2
3074	RWS11	-122.4587689	37.93715	-0.31	9.08	-283	8.77000034	-122.45876	37.93716	-0.29	9.08	-271.77	8.79300034	11	7/24/2014	22:25.3
3075	RWS11	-122.458771	37.93715	-0.31	9.06	-285.6	8.74900043	-122.45877	37.93716	-0.32	9.06	-274.64	8.73800042	11	7/24/2014	22:25.4
3076	RWS11	-122.4587731	37.93715	-0.31	9.04	-288.2	8.72899997	-122.45877	37.93716	-0.32	9.04	-277.33	8.71799996	11	7/24/2014	22:25.5
3077	RWS11	-122.4587752	37.93715	-0.35	9.02	-290.8	8.67500046	-122.45877	37.93716	-0.36	9.02	-280.05	8.66400045	11	7/24/2014	22:25.6
3078	RWS11	-122.4587773	37.93715	-0.31	9	-293.3	8.69000041	-122.45877	37.93716	-0.32	9	-282.71	8.67900041	11	7/24/2014	22:25.7
3079	RWS11	-122.4587793	37.93715	-0.35	8.98	-295.9	8.63700035	-122.45877	37.93717	-0.32	8.98	-285.35	8.66000035	11	7/24/2014	22:25.8
3080	RWS11	-122.4587814	37.93715	-0.35	8.96	-298.3	8.6180003	-122.45878	37.93717	-0.32	8.96	-287.93	8.6410003	11	7/24/2014	22:25.9
3081	RWS11	-122.4587844	37.93715	-0.35	8.94	-300.6	8.5999997	-122.45878	37.93717	-0.32	8.94	-290.47	8.6229997	11	7/24/2014	22:26.0
3082	RWS11	-122.4587865	37.93715	-0.31	8.93	-302.9	8.61600006	-122.45878	37.93717	-0.32	8.93	-293.05	8.60500005	11	7/24/2014	22:26.1
3083	RWS11	-122.4587886	37.93715	-0.35	8.91	-305.3	8.56499985	-122.45878	37.93717	-0.32	8.91	-295.66	8.58799985	11	7/24/2014	22:26.2
3084	RWS11	-122.4587907	37.93715	-0.31	8.9	-307.5	8.58400047	-122.45879	37.93717	-0.32	8.9	-298.12	8.57300046	11	7/24/2014	22:26.3
3085	RWS11	-122.4587927	37.93716	-0.35	8.88	-309.7	8.53599957	-122.45879	37.93717	-0.32	8.88	-300.4	8.55899957	11	7/24/2014	22:26.4
3086	RWS11	-122.4587948	37.93716	-0.31	8.87	-311.9	8.55700004	-122.45879	37.93717	-0.32	8.87	-302.71	8.54600003	11	7/24/2014	22:26.5
3087	RWS11	-122.4587969	37.93716	-0.35	8.86	-314.1	8.51099995	-122.45879	37.93717	-0.36	8.86	-304.93	8.49999994	11	7/24/2014	22:26.6
3088	RWS11	-122.458799	37.93716	-0.31	8.85	-316.3	8.53499973	-122.45879	37.93717	-0.32	8.85	-307.08	8.52399972	11	7/24/2014	22:26.7
3089	RWS11	-122.4588011	37.93716	-0.35	8.84	-318.3	8.49100044	-122.4588	37.93717	-0.29	8.84	-309.2	8.54800045	11	7/24/2014	22:26.8
3090	RWS11	-122.4588031	37.93716	-0.31	8.83	-320.2	8.51599967	-122.4588	37.93717	-0.32	8.83	-311.3	8.50499967	11	7/24/2014	22:26.9
3091	RWS11	-122.4588062	37.93716	-0.35	8.82	-322.1	8.47400025	-122.4588	37.93717	-0.29	8.82	-313.38	8.53100026	11	7/24/2014	22:27.0
3092	RWS11	-122.4588082	37.93716	-0.31	8.81	-324.1	8.50100029	-122.4588	37.93717	-0.29	8.81	-315.57	8.52400029	11	7/24/2014	22:27.1
3093	RWS11	-122.4588103	37.93716	-0.35	8.81	-326	8.46099976	-122.45881	37.93717	-0.29	8.81	-317.61	8.51799977	11	7/24/2014	22:27.2
3094	RWS11	-122.4588124	37.93716	-0.31	8.8	-327.9	8.4890002	-122.45881	37.93717	-0.32	8.8	-319.73	8.47800019	11	7/24/2014	22:27.3
3095	RWS11	-122.4588144	37.93716	-0.31	8.8	-330	8.48400009	-122.45881	37.93717	-0.29	8.8	-321.95	8.50700009	11	7/24/2014	22:27.4
3096	RWS11	-122.4588165	37.93716	-0.31	8.79	-332	8.48000038	-122.45881	37.93717	-0.29	8.79	-324.15	8.50300038	11	7/24/2014	22:27.5
3097	RWS11	-122.4588186	37.93716	-0.31	8.79	-333.9	8.47599971	-122.45881	37.93717	-0.29	8.79	-326.31	8.49899971	11	7/24/2014	22:27.6
3098	RWS11	-122.4588206	37.93716	-0.31	8.78	-335.7	8.47300041	-122.45882	37.93718	-0.29	8.78	-328.36	8.49600041	11	7/24/2014	22:27.7
3099	RWS11	-122.4588227	37.93716	-0.31	8.78	-337.4	8.4709996	-122.45882	37.93718	-0.29	8.78	-330.32	8.4939996	11	7/24/2014	22:27.8
3100	RWS11	-122.4588247	37.93716	-0.31	8.78	-339	8.46899974	-122.45882	37.93718	-0.29	8.78	-332.22	8.49199975	11	7/24/2014	22:27.9
3101	RWS11	-122.4588278	37.93716	-0.31	8.78	-340.6	8.46899974	-122.45882	37.93718	-0.29	8.78	-333.91	8.49199975	11	7/24/2014	22:28.0

3102	RWS11	-122.4588298	37.93716	-0.31	8.78	-342.2	8.4709996	-122.45882	37.93718	-0.29	8.78	-335.47	8.4939996	11	7/24/2014	22:28.1
3103	RWS11	-122.4588319	37.93717	-0.31	8.79	-343.7	8.47599971	-122.45883	37.93718	-0.29	8.79	-336.94	8.49899971	11	7/24/2014	22:28.2
3104	RWS11	-122.458834	37.93717	-0.31	8.79	-345.2	8.48299968	-122.45883	37.93718	-0.24	8.79	-338.35	8.55699967	11	7/24/2014	22:28.3
3105	RWS11	-122.458836	37.93717	-0.35	8.8	-346.8	8.45800045	-122.45883	37.93718	-0.32	8.8	-339.82	8.48100045	11	7/24/2014	22:28.4
3106	RWS11	-122.4588381	37.93717	-0.28	8.82	-348.3	8.53899997	-122.45883	37.93718	-0.29	8.82	-341.12	8.528	11	7/24/2014	22:28.5
3107	RWS11	-122.4588402	37.93717	-0.31	8.83	-349.7	8.52000034	-122.45883	37.93718	-0.32	8.83	-342.57	8.50900033	11	7/24/2014	22:28.6
3108	RWS11	-122.4588423	37.93717	-0.31	8.85	-351.1	8.53600013	-122.45884	37.93718	-0.29	8.85	-343.92	8.55900013	11	7/24/2014	22:28.7
3109	RWS11	-122.4588443	37.93717	-0.31	8.87	-352.4	8.55500019	-122.45884	37.93718	-0.29	8.87	-345.21	8.57800019	11	7/24/2014	22:28.8
3110	RWS11	-122.4588463	37.93717	-0.31	8.89	-353.8	8.57400024	-122.45884	37.93718	-0.29	8.89	-346.69	8.59700024	11	7/24/2014	22:28.9
3111	RWS11	-122.4588493	37.93717	-0.31	8.9	-355.2	8.59399974	-122.45884	37.93718	-0.29	8.9	-348.12	8.61699975	11	7/24/2014	22:29.0
3112	RWS11	-122.4588512	37.93717	-0.31	8.93	-356.4	8.6140002	-122.45885	37.93718	-0.29	8.93	-349.44	8.6370002	11	7/24/2014	22:29.1
3113	RWS11	-122.4588532	37.93717	-0.31	8.94	-357.6	8.63199985	-122.45885	37.93718	-0.32	8.94	-350.61	8.62099984	11	7/24/2014	22:29.2
3114	RWS11	-122.4588552	37.93717	-0.28	8.96	-358.7	8.68199962	-122.45885	37.93718	-0.29	8.96	-351.76	8.67099965	11	7/24/2014	22:29.3
3115	RWS11	-122.4588571	37.93717	-0.31	8.97	-359.8	8.66199958	-122.45885	37.93718	-0.24	8.97	-353.08	8.73599957	11	7/24/2014	22:29.4
3116	RWS11	-122.4588591	37.93717	-0.31	8.98	-361	8.67399967	-122.45885	37.93719	-0.29	8.98	-354.46	8.69699967	11	7/24/2014	22:29.5
3117	RWS11	-122.458861	37.93717	-0.31	9	-362.1	8.68599975	-122.45886	37.93719	-0.29	9	-355.76	8.70899975	11	7/24/2014	22:29.6
3118	RWS11	-122.458863	37.93717	-0.28	9.01	-363.4	8.73100036	-122.45886	37.93719	-0.24	9.01	-357.19	8.77100037	11	7/24/2014	22:29.7
3119	RWS11	-122.458865	37.93717	-0.31	9.02	-364.6	8.70900047	-122.45886	37.93719	-0.24	9.02	-358.69	8.78300045	11	7/24/2014	22:29.8
3120	RWS11	-122.4588669	37.93717	-0.28	9.03	-366	8.75700039	-122.45886	37.93719	-0.24	9.03	-360.2	8.79700039	11	7/24/2014	22:29.9
3121	RWS11	-122.4588699	37.93718	-0.28	9.05	-367.2	8.77300018	-122.45886	37.93719	-0.24	9.05	-361.63	8.81300019	11	7/24/2014	22:30.0
3122	RWS11	-122.4588718	37.93718	-0.28	9.07	-368.6	8.79299968	-122.45887	37.93719	-0.24	9.07	-363.02	8.83299969	11	7/24/2014	22:30.1
3123	RWS11	-122.4588738	37.93718	-0.28	9.09	-369.7	8.815	-122.45887	37.93719	-0.24	9.09	-364.32	8.855	11	7/24/2014	22:30.2
3124	RWS11	-122.4588759	37.93718	-0.28	9.12	-370.9	8.84100002	-122.45887	37.93719	-0.2	9.12	-365.49	8.91500004	11	7/24/2014	22:30.3
3125	RWS11	-122.4588779	37.93718	-0.28	9.15	-371.9	8.87200016	-122.45887	37.93719	-0.24	9.15	-366.57	8.91200016	11	7/24/2014	22:30.4
3126	RWS11	-122.45888	37.93718	-0.28	9.18	-372.9	8.90700001	-122.45887	37.93719	-0.2	9.18	-367.52	8.98100002	11	7/24/2014	22:30.5
3127	RWS11	-122.4588821	37.93718	-0.28	9.22	-374	8.94599956	-122.45888	37.93719	-0.24	9.22	-368.54	8.98599957	11	7/24/2014	22:30.6
3128	RWS11	-122.4588841	37.93718	-0.28	9.27	-375.1	8.98800033	-122.45888	37.93719	-0.24	9.27	-369.56	9.02800034	11	7/24/2014	22:30.7
3129	RWS11	-122.4588862	37.93718	-0.28	9.31	-376.2	9.03300041	-122.45888	37.93719	-0.24	9.31	-370.58	9.07300042	11	7/24/2014	22:30.8
3130	RWS11	-122.4588882	37.93718	-0.28	9.36	-377	9.07899994	-122.45888	37.93719	-0.24	9.36	-371.31	9.11899994	11	7/24/2014	22:30.9
3131	RWS11	-122.4588913	37.93718	-0.28	9.4	-377.8	9.12599987	-122.45889	37.93719	-0.24	9.4	-372.05	9.16599987	11	7/24/2014	22:31.0
3132	RWS11	-122.4588933	37.93718	-0.22	9.45	-378.6	9.22299996	-122.45889	37.93719	-0.24	9.45	-372.74	9.21099995	11	7/24/2014	22:31.1
3133	RWS11	-122.4588954	37.93718	-0.28	9.49	-379.1	9.21400017	-122.45889	37.9372	-0.24	9.49	-373.33	9.25400017	11	7/24/2014	22:31.2
3134	RWS11	-122.4588975	37.93718	-0.28	9.53	-379.6	9.25499958	-122.45889	37.9372	-0.24	9.53	-373.83	9.29499958	11	7/24/2014	22:31.3

3135	RWS11	-122.4588996	37.93718	-0.28	9.57	-380.2	9.29200023	-122.45889	37.9372	-0.24	9.57	-374.41	9.33200024	11	7/24/2014	22:31.4
3136	RWS11	-122.4589017	37.93718	-0.28	9.6	-380.8	9.32700008	-122.4589	37.9372	-0.24	9.6	-375.04	9.36700009	11	7/24/2014	22:31.5
3137	RWS11	-122.4589037	37.93718	-0.28	9.64	-381.4	9.35899967	-122.4589	37.9372	-0.24	9.64	-375.73	9.39899968	11	7/24/2014	22:31.6
3138	RWS11	-122.4589058	37.93719	-0.28	9.66	-382	9.38799995	-122.4589	37.9372	-0.24	9.66	-376.36	9.42799996	11	7/24/2014	22:31.7
3139	RWS11	-122.4589079	37.93719	-0.28	9.69	-382.6	9.41399997	-122.4589	37.9372	-0.24	9.69	-377.01	9.45399998	11	7/24/2014	22:31.8
3140	RWS11	-122.4589099	37.93719	-0.28	9.72	-383.2	9.43800014	-122.4589	37.9372	-0.2	9.72	-377.72	9.51200016	11	7/24/2014	22:31.9
3141	RWS11	-122.4589129	37.93719	-0.28	9.74	-383.8	9.46000046	-122.45891	37.9372	-0.24	9.74	-378.33	9.50000046	11	7/24/2014	22:32.0
3142	RWS11	-122.458915	37.93719	-0.22	9.76	-384.4	9.53300038	-122.45891	37.9372	-0.2	9.76	-379	9.55500038	11	7/24/2014	22:32.1
3143	RWS11	-122.458917	37.93719	-0.28	9.78	-384.9	9.50000042	-122.45891	37.9372	-0.24	9.78	-379.61	9.54000042	11	7/24/2014	22:32.2
3144	RWS11	-122.458919	37.93719	-0.22	9.8	-385.7	9.57000008	-122.45891	37.9372	-0.2	9.8	-380.17	9.59200008	11	7/24/2014	22:32.3
3145	RWS11	-122.458921	37.93719	-0.28	9.81	-386.2	9.53599972	-122.45892	37.9372	-0.24	9.81	-380.71	9.57599972	11	7/24/2014	22:32.4
3146	RWS11	-122.4589231	37.93719	-0.22	9.83	-386.9	9.60600033	-122.45892	37.9372	-0.2	9.83	-381.32	9.62800033	11	7/24/2014	22:32.5
3147	RWS11	-122.4589251	37.93719	-0.22	9.85	-387.5	9.62399998	-122.45892	37.9372	-0.2	9.85	-381.8	9.64599998	11	7/24/2014	22:32.6
3148	RWS11	-122.4589271	37.93719	-0.22	9.87	-388.2	9.64300004	-122.45892	37.9372	-0.2	9.87	-382.43	9.66500004	11	7/24/2014	22:32.7
3149	RWS11	-122.4589291	37.93719	-0.22	9.89	-388.6	9.66399994	-122.45892	37.9372	-0.24	9.89	-382.9	9.65199994	11	7/24/2014	22:32.8
3150	RWS11	-122.4589311	37.93719	-0.22	9.91	-389.1	9.68600026	-122.45893	37.93721	-0.2	9.91	-383.46	9.70800026	11	7/24/2014	22:32.9
3151	RWS11	-122.4589341	37.93719	-0.22	9.94	-389.5	9.71000043	-122.45893	37.93721	-0.2	9.94	-383.9	9.73200043	11	7/24/2014	22:33.0
3152	RWS11	-122.4589361	37.93719	-0.22	9.96	-389.9	9.73500004	-122.45893	37.93721	-0.15	9.96	-384.4	9.80700004	11	7/24/2014	22:33.1
3153	RWS11	-122.4589381	37.93719	-0.22	9.99	-390.2	9.76200047	-122.45893	37.93721	-0.2	9.99	-384.7	9.78400047	11	7/24/2014	22:33.2
3154	RWS11	-122.4589401	37.9372	-0.22	10	-390.5	9.79000035	-122.45893	37.93721	-0.2	10	-384.94	9.81200035	11	7/24/2014	22:33.3
3155	RWS11	-122.4589421	37.9372	-0.28	10	-390.6	9.76600021	-122.45894	37.93721	-0.2	10	-385.11	9.84000023	11	7/24/2014	22:33.4
3156	RWS11	-122.4589442	37.9372	-0.22	10.1	-390.8	9.84600011	-122.45894	37.93721	-0.15	10.1	-385.39	9.9180001	11	7/24/2014	22:33.5
3157	RWS11	-122.4589462	37.9372	-0.22	10.1	-390.8	9.87399998	-122.45894	37.93721	-0.2	10.1	-385.46	9.89599998	11	7/24/2014	22:33.6
3158	RWS11	-122.4589483	37.9372	-0.22	10.1	-390.8	9.90100041	-122.45894	37.93721	-0.2	10.1	-385.37	9.92300041	11	7/24/2014	22:33.7
3159	RWS11	-122.4589503	37.9372	-0.22	10.2	-391	9.92900029	-122.45894	37.93721	-0.2	10.2	-385.42	9.95100029	11	7/24/2014	22:33.8
3160	RWS11	-122.4589524	37.9372	-0.22	10.2	-391.3	9.95700017	-122.45895	37.93721	-0.15	10.2	-385.4	10.0290002	11	7/24/2014	22:33.9
3161	RWS11	-122.4589554	37.9372	-0.22	10.2	-391.5	9.98500004	-122.45895	37.93721	-0.2	10.2	-385.31	10.007	11	7/24/2014	22:34.0
3162	RWS11	-122.4589575	37.9372	-0.19	10.2	-391.6	10.0469999	-122.45895	37.93721	-0.2	10.2	-385.09	10.0349999	11	7/24/2014	22:34.1
3163	RWS11	-122.4589596	37.9372	-0.22	10.3	-391.6	10.0409998	-122.45895	37.93721	-0.15	10.3	-384.79	10.1129998	11	7/24/2014	22:34.2
3164	RWS11	-122.4589617	37.9372	-0.19	10.3	-391.6	10.1020002	-122.45896	37.93721	-0.2	10.3	-384.46	10.0900002	11	7/24/2014	22:34.3
3165	RWS11	-122.4589638	37.9372	-0.22	10.3	-391.5	10.0949997	-122.45896	37.93722	-0.2	10.3	-384.14	10.1169997	11	7/24/2014	22:34.4
3166	RWS11	-122.4589659	37.9372	-0.22	10.3	-391.1	10.1189999	-122.45896	37.93722	-0.2	10.3	-383.62	10.1409999	11	7/24/2014	22:34.5
3167	RWS11	-122.458968	37.9372	-0.22	10.4	-390.8	10.1419996	-122.45896	37.93722	-0.2	10.4	-383.1	10.1639996	11	7/24/2014	22:34.6

3168	RWS11	-122.4589701	37.9372	-0.19	10.4	-390.4	10.1969995	-122.45896	37.93722	-0.2	10.4	-382.58	10.1849995	11	7/24/2014	22:34.7
3169	RWS11	-122.4589722	37.9372	-0.22	10.4	-389.9	10.1819996	-122.45897	37.93722	-0.2	10.4	-382.08	10.2039996	11	7/24/2014	22:34.8
3170	RWS11	-122.4589743	37.93721	-0.22	10.4	-389.3	10.2000002	-122.45897	37.93722	-0.2	10.4	-381.43	10.2220002	11	7/24/2014	22:34.9
3171	RWS11	-122.4589774	37.93721	-0.22	10.4	-388.8	10.216	-122.45897	37.93722	-0.2	10.4	-380.85	10.238	11	7/24/2014	22:35.0
3172	RWS11	-122.4589794	37.93721	-0.19	10.5	-388.3	10.2630005	-122.45897	37.93722	-0.2	10.5	-380.52	10.2510005	11	7/24/2014	22:35.1
3173	RWS11	-122.4589815	37.93721	-0.22	10.5	-388	10.2409996	-122.45898	37.93722	-0.24	10.5	-379.98	10.2289996	11	7/24/2014	22:35.2
3174	RWS11	-122.4589836	37.93721	-0.19	10.5	-387.6	10.2840004	-122.45898	37.93722	-0.2	10.5	-379.55	10.2720004	11	7/24/2014	22:35.3
3175	RWS11	-122.4589857	37.93721	-0.22	10.5	-387.2	10.2570004	-122.45898	37.93722	-0.24	10.5	-379.16	10.2450004	11	7/24/2014	22:35.4
3176	RWS11	-122.4589878	37.93721	-0.22	10.5	-386.7	10.2610001	-122.45898	37.93722	-0.2	10.5	-378.6	10.2830001	11	7/24/2014	22:35.5
3177	RWS11	-122.4589899	37.93721	-0.22	10.5	-386	10.2629999	-122.45898	37.93722	-0.24	10.5	-378.06	10.2509999	11	7/24/2014	22:35.6
3178	RWS11	-122.458992	37.93721	-0.22	10.5	-385.2	10.2640003	-122.45899	37.93722	-0.2	10.5	-377.39	10.2860003	11	7/24/2014	22:35.7
3179	RWS11	-122.4589941	37.93721	-0.28	10.5	-384.7	10.2100005	-122.45899	37.93722	-0.24	10.5	-376.82	10.2500005	11	7/24/2014	22:35.8
3180	RWS11	-122.4589962	37.93721	-0.19	10.5	-384.1	10.2930002	-122.45899	37.93722	-0.2	10.5	-376.26	10.2810002	11	7/24/2014	22:35.9
3181	RWS11	-122.4589992	37.93721	-0.22	10.5	-383.5	10.2549996	-122.45899	37.93723	-0.24	10.5	-375.7	10.2429995	11	7/24/2014	22:36.0
3182	RWS11	-122.4590013	37.93721	-0.22	10.5	-382.7	10.249	-122.459	37.93723	-0.24	10.5	-375.05	10.237	11	7/24/2014	22:36.1
3183	RWS11	-122.4590033	37.93721	-0.22	10.5	-381.9	10.2409996	-122.459	37.93723	-0.24	10.5	-374.36	10.2289996	11	7/24/2014	22:36.2
3184	RWS11	-122.4590054	37.93722	-0.22	10.5	-381.1	10.2330002	-122.459	37.93723	-0.2	10.5	-373.68	10.2550002	11	7/24/2014	22:36.3
3185	RWS11	-122.4590075	37.93722	-0.22	10.4	-380.4	10.2249998	-122.459	37.93723	-0.24	10.4	-373.08	10.2129998	11	7/24/2014	22:36.4
3186	RWS11	-122.4590095	37.93722	-0.22	10.4	-379.5	10.216	-122.459	37.93723	-0.2	10.4	-372.34	10.238	11	7/24/2014	22:36.5
3187	RWS11	-122.4590116	37.93722	-0.22	10.4	-378.7	10.2079996	-122.45901	37.93723	-0.24	10.4	-371.5	10.1959996	11	7/24/2014	22:36.6
3188	RWS11	-122.4590137	37.93722	-0.22	10.4	-377.7	10.1989998	-122.45901	37.93723	-0.2	10.4	-370.81	10.2209998	11	7/24/2014	22:36.7
3189	RWS11	-122.4590158	37.93722	-0.22	10.4	-377	10.1910004	-122.45901	37.93723	-0.2	10.4	-370.02	10.2130004	11	7/24/2014	22:36.8
3190	RWS11	-122.4590178	37.93722	-0.28	10.4	-376.1	10.1320004	-122.45901	37.93723	-0.2	10.4	-369.29	10.2060004	11	7/24/2014	22:36.9
3191	RWS11	-122.4590209	37.93722	-0.28	10.4	-375.6	10.1250004	-122.45901	37.93723	-0.2	10.4	-368.51	10.1990004	11	7/24/2014	22:37.0
3192	RWS11	-122.459023	37.93722	-0.22	10.4	-375	10.1720003	-122.45902	37.93723	-0.15	10.4	-367.82	10.2440003	11	7/24/2014	22:37.1
3193	RWS11	-122.459025	37.93722	-0.28	10.4	-374.2	10.1150002	-122.45902	37.93723	-0.24	10.4	-366.98	10.1550002	11	7/24/2014	22:37.2
3194	RWS11	-122.4590272	37.93722	-0.22	10.4	-373.3	10.1629995	-122.45902	37.93723	-0.2	10.4	-365.96	10.1849995	11	7/24/2014	22:37.3
3195	RWS11	-122.4590293	37.93722	-0.22	10.4	-372.3	10.1589998	-122.45902	37.93724	-0.24	10.4	-364.98	10.1469998	11	7/24/2014	22:37.4
3196	RWS11	-122.4590314	37.93722	-0.22	10.4	-371.4	10.1559996	-122.45903	37.93724	-0.2	10.4	-363.94	10.1779996	11	7/24/2014	22:37.5
3197	RWS11	-122.4590335	37.93722	-0.22	10.4	-370.3	10.1539997	-122.45903	37.93724	-0.24	10.4	-362.86	10.1419997	11	7/24/2014	22:37.6
3198	RWS11	-122.4590357	37.93722	-0.22	10.4	-369.3	10.1510004	-122.45903	37.93724	-0.2	10.4	-361.69	10.1730004	11	7/24/2014	22:37.7
3199	RWS11	-122.4590378	37.93723	-0.22	10.4	-368	10.1469998	-122.45903	37.93724	-0.2	10.4	-360.52	10.1689998	11	7/24/2014	22:37.8
3200	RWS11	-122.4590399	37.93723	-0.22	10.4	-366.8	10.143	-122.45903	37.93724	-0.2	10.4	-359.29	10.165	11	7/24/2014	22:37.9

3201	RWS11	-122.4590431	37.93723	-0.28	10.4	-365.5	10.0859999	-122.45904	37.93724	-0.15	10.4	-357.97	10.2099999	11	7/24/2014	22:38.0
3202	RWS11	-122.4590452	37.93723	-0.22	10.4	-364.1	10.1320004	-122.45904	37.93724	-0.2	10.4	-356.56	10.1540004	11	7/24/2014	22:38.1
3203	RWS11	-122.4590474	37.93723	-0.22	10.4	-362.9	10.1250004	-122.45904	37.93724	-0.2	10.4	-355.26	10.1470004	11	7/24/2014	22:38.2
3204	RWS11	-122.4590496	37.93723	-0.22	10.3	-361.5	10.1159996	-122.45904	37.93724	-0.2	10.3	-353.81	10.1379996	11	7/24/2014	22:38.3
3205	RWS11	-122.4590517	37.93723	-0.22	10.3	-360.3	10.1060003	-122.45905	37.93724	-0.15	10.3	-352.4	10.1780003	11	7/24/2014	22:38.4
3206	RWS11	-122.4590539	37.93723	-0.22	10.3	-359.2	10.0949997	-122.45905	37.93724	-0.2	10.3	-351.1	10.1169997	11	7/24/2014	22:38.5
3207	RWS11	-122.4590561	37.93723	-0.22	10.3	-358	10.0829996	-122.45905	37.93724	-0.15	10.3	-349.72	10.1549996	11	7/24/2014	22:38.6
3208	RWS11	-122.4590583	37.93723	-0.22	10.3	-356.6	10.0709995	-122.45905	37.93724	-0.15	10.3	-348.34	10.1429995	11	7/24/2014	22:38.7
3209	RWS11	-122.4590605	37.93723	-0.22	10.3	-355.2	10.0590004	-122.45905	37.93725	-0.2	10.3	-346.8	10.0810004	11	7/24/2014	22:38.8
3210	RWS11	-122.4590626	37.93723	-0.22	10.3	-353.7	10.0479998	-122.45906	37.93725	-0.15	10.3	-345.24	10.1199998	11	7/24/2014	22:38.9
3211	RWS11	-122.4590657	37.93723	-0.22	10.3	-352.2	10.0370001	-122.45906	37.93725	-0.15	10.3	-343.7	10.1090001	11	7/24/2014	22:39.0
3212	RWS11	-122.4590678	37.93724	-0.22	10.3	-350.9	10.0269999	-122.45906	37.93725	-0.15	10.3	-342.17	10.0989999	11	7/24/2014	22:39.1
3213	RWS11	-122.4590699	37.93724	-0.22	10.2	-349.6	10.0169996	-122.45906	37.93725	-0.15	10.2	-340.69	10.0889996	11	7/24/2014	22:39.2
3214	RWS11	-122.459072	37.93724	-0.22	10.2	-348.3	10.0090002	-122.45907	37.93725	-0.07	10.2	-339.18	10.1660002	11	7/24/2014	22:39.3
3215	RWS11	-122.4590741	37.93724	-0.22	10.2	-346.9	10.0009998	-122.45907	37.93725	-0.15	10.2	-337.64	10.0729998	11	7/24/2014	22:39.4
3216	RWS11	-122.4590762	37.93724	-0.22	10.2	-345.3	9.99399987	-122.45907	37.93725	-0.15	10.2	-335.95	10.0659999	11	7/24/2014	22:39.5
3217	RWS11	-122.4590783	37.93724	-0.22	10.2	-343.7	9.9880003	-122.45907	37.93725	-0.12	10.2	-334.26	10.0940003	11	7/24/2014	22:39.6
3218	RWS11	-122.4590804	37.93724	-0.19	10.2	-341.9	10.0159998	-122.45907	37.93725	-0.15	10.2	-332.55	10.0539998	11	7/24/2014	22:39.7
3219	RWS11	-122.4590825	37.93724	-0.22	10.2	-340.1	9.97699967	-122.45908	37.93725	-0.15	10.2	-330.8	10.0489997	11	7/24/2014	22:39.8
3220	RWS11	-122.4590846	37.93724	-0.19	10.2	-338.3	10.007	-122.45908	37.93725	-0.15	10.2	-328.89	10.045	11	7/24/2014	22:39.9
3221	RWS11	-122.4590877	37.93724	-0.22	10.2	-336.4	9.9699997	-122.45908	37.93725	-0.12	10.2	-326.96	10.0759997	11	7/24/2014	22:40.0
3222	RWS11	-122.4590898	37.93724	-0.19	10.2	-334.4	10.0010004	-122.45908	37.93725	-0.12	10.2	-324.95	10.0730004	11	7/24/2014	22:40.1
3223	RWS11	-122.4590918	37.93724	-0.22	10.2	-332.5	9.96499959	-122.45909	37.93726	-0.12	10.2	-322.85	10.0709996	11	7/24/2014	22:40.2
3224	RWS11	-122.4590939	37.93724	-0.22	10.2	-330.5	9.96400014	-122.45909	37.93726	-0.12	10.2	-320.76	10.0700001	11	7/24/2014	22:40.3
3225	RWS11	-122.4590961	37.93724	-0.22	10.2	-328.5	9.96299973	-122.45909	37.93726	-0.15	10.2	-318.54	10.0349997	11	7/24/2014	22:40.4
3226	RWS11	-122.4590982	37.93725	-0.19	10.2	-326.4	9.99699973	-122.45909	37.93726	-0.07	10.2	-316.32	10.1199997	11	7/24/2014	22:40.5
3227	RWS11	-122.4591003	37.93725	-0.22	10.2	-324.4	9.96299973	-122.45909	37.93726	-0.12	10.2	-314.1	10.0689997	11	7/24/2014	22:40.6
3228	RWS11	-122.4591025	37.93725	-0.19	10.2	-322.4	9.99800013	-122.4591	37.93726	-0.12	10.2	-311.81	10.0700001	11	7/24/2014	22:40.7
3229	RWS11	-122.4591046	37.93725	-0.22	10.2	-320.4	9.96499959	-122.4591	37.93726	-0.12	10.2	-309.53	10.0709996	11	7/24/2014	22:40.8
3230	RWS11	-122.4591067	37.93725	-0.19	10.2	-318.4	10.0010004	-122.4591	37.93726	-0.07	10.2	-307.2	10.1240004	11	7/24/2014	22:40.9
3231	RWS11	-122.4591098	37.93725	-0.22	10.2	-316.3	9.96900025	-122.4591	37.93726	-0.15	10.2	-304.86	10.0410003	11	7/24/2014	22:41.0
3232	RWS11	-122.4591118	37.93725	-0.19	10.2	-314.2	10.0059996	-122.45911	37.93726	-0.07	10.2	-302.43	10.1289996	11	7/24/2014	22:41.1
3233	RWS11	-122.4591139	37.93725	-0.22	10.2	-312	9.97600022	-122.45911	37.93726	-0.12	10.2	-300.05	10.0820002	11	7/24/2014	22:41.2

3234	RWS11	-122.459116	37.93725	-0.19	10.2	-309.8	10.0139999	-122.45911	37.93726	-0.12	10.2	-297.67	10.0859999	11	7/24/2014	22:41.3
3235	RWS11	-122.4591181	37.93725	-0.22	10.2	-307.5	9.98600045	-122.45911	37.93726	-0.12	10.2	-295.22	10.0920004	11	7/24/2014	22:41.4
3236	RWS11	-122.4591202	37.93725	-0.19	10.2	-305.3	10.0270004	-122.45911	37.93726	-0.12	10.2	-292.75	10.0990004	11	7/24/2014	22:41.5
3237	RWS11	-122.4591223	37.93725	-0.19	10.2	-303	10.0340004	-122.45912	37.93727	-0.07	10.2	-290.16	10.1570004	11	7/24/2014	22:41.6
3238	RWS11	-122.4591244	37.93725	-0.19	10.2	-300.5	10.0419998	-122.45912	37.93727	-0.12	10.2	-287.56	10.1139998	11	7/24/2014	22:41.7
3239	RWS11	-122.4591265	37.93725	-0.22	10.2	-298	10.0169996	-122.45912	37.93727	-0.12	10.2	-284.79	10.1229996	11	7/24/2014	22:41.8
3240	RWS11	-122.4591286	37.93726	-0.19	10.3	-295.5	10.0600004	-122.45912	37.93727	-0.07	10.3	-281.93	10.1830004	11	7/24/2014	22:41.9
3241	RWS11	-122.4591316	37.93726	-0.22	10.3	-292.8	10.0350002	-122.45913	37.93727	-0.12	10.3	-279.03	10.1410002	11	7/24/2014	22:42.0
3242	RWS11	-122.4591337	37.93726	-0.19	10.3	-290.2	10.0780001	-122.45913	37.93727	-0.12	10.3	-276.15	10.1500001	11	7/24/2014	22:42.1
3243	RWS11	-122.4591358	37.93726	-0.22	10.3	-287.6	10.0520004	-122.45913	37.93727	-0.12	10.3	-273.23	10.1580004	11	7/24/2014	22:42.2
3244	RWS11	-122.4591379	37.93726	-0.19	10.3	-284.9	10.0930004	-122.45913	37.93727	-0.12	10.3	-270.26	10.1650004	11	7/24/2014	22:42.3
3245	RWS11	-122.45914	37.93726	-0.19	10.3	-282	10.1000004	-122.45913	37.93727	-0.07	10.3	-267.24	10.2230004	11	7/24/2014	22:42.4
3246	RWS11	-122.4591422	37.93726	-0.19	10.3	-279	10.1059999	-122.45914	37.93727	-0.07	10.3	-264.13	10.2289999	11	7/24/2014	22:42.5
3247	RWS11	-122.4591444	37.93726	-0.19	10.3	-276	10.1110001	-122.45914	37.93727	-0.12	10.3	-260.93	10.183	11	7/24/2014	22:42.6
3248	RWS11	-122.4591465	37.93726	-0.19	10.3	-272.9	10.1169996	-122.45914	37.93727	-0.07	10.3	-257.63	10.2399996	11	7/24/2014	22:42.7
3249	RWS11	-122.4591487	37.93726	-0.19	10.3	-269.8	10.1230001	-122.45914	37.93727	-0.12	10.3	-254.35	10.1950001	11	7/24/2014	22:42.8
3250	RWS11	-122.4591509	37.93726	-0.19	10.3	-266.7	10.1289997	-122.45914	37.93728	-0.12	10.3	-251.08	10.2009997	11	7/24/2014	22:42.9
3251	RWS11	-122.4591531	37.93726	-0.19	10.3	-263.5	10.1370001	-122.45915	37.93728	-0.12	10.3	-247.7	10.2090001	11	7/24/2014	22:43.0
3252	RWS11	-122.4591553	37.93726	-0.19	10.3	-260.3	10.1450004	-122.45915	37.93728	-0.07	10.3	-244.32	10.2680004	11	7/24/2014	22:43.1
3253	RWS11	-122.4591575	37.93726	-0.22	10.3	-257	10.1209997	-122.45915	37.93728	-0.12	10.3	-240.84	10.2269997	11	7/24/2014	22:43.2
3254	RWS11	-122.4591597	37.93727	-0.19	10.4	-253.5	10.1660004	-122.45915	37.93728	-0.07	10.4	-237.24	10.2890004	11	7/24/2014	22:43.3
3255	RWS11	-122.4591619	37.93727	-0.19	10.4	-250	10.1789999	-122.45916	37.93728	-0.12	10.4	-233.47	10.2509999	11	7/24/2014	22:43.4
3256	RWS11	-122.4591641	37.93727	-0.19	10.4	-246.4	10.1929998	-122.45916	37.93728	-0.07	10.4	-229.54	10.3159998	11	7/24/2014	22:43.5
3257	RWS11	-122.4591662	37.93727	-0.19	10.4	-242.6	10.2089996	-122.45916	37.93728	-0.12	10.4	-225.49	10.2809996	11	7/24/2014	22:43.6
3258	RWS11	-122.4591683	37.93727	-0.19	10.4	-238.6	10.2250004	-122.45916	37.93728	-0.07	10.4	-221.37	10.3480004	11	7/24/2014	22:43.7
3259	RWS11	-122.4591704	37.93727	-0.19	10.4	-234.6	10.2419996	-122.45916	37.93728	-0.12	10.4	-217.15	10.3139996	11	7/24/2014	22:43.8
3260	RWS11	-122.4591725	37.93727	-0.19	10.4	-230.6	10.2589998	-122.45917	37.93728	-0.12	10.4	-212.88	10.3309998	11	7/24/2014	22:43.9
3261	RWS11	-122.4591755	37.93727	-0.19	10.5	-226.6	10.276	-122.45917	37.93728	-0.12	10.5	-208.52	10.348	11	7/24/2014	22:44.0
3262	RWS11	-122.4591776	37.93727	-0.19	10.5	-222.5	10.2930002	-122.45917	37.93728	-0.12	10.5	-204	10.3650002	11	7/24/2014	22:44.1
3263	RWS11	-122.4591796	37.93727	-0.19	10.5	-218.2	10.309	-122.45917	37.93728	-0.12	10.5	-199.31	10.381	11	7/24/2014	22:44.2
3264	RWS11	-122.4591817	37.93727	-0.19	10.5	-213.7	10.3249998	-122.45918	37.93729	-0.07	10.5	-194.45	10.4479998	11	7/24/2014	22:44.3
3265	RWS11	-122.4591838	37.93727	-0.22	10.5	-209.2	10.3049997	-122.45918	37.93729	-0.12	10.5	-189.39	10.4109997	11	7/24/2014	22:44.4
3266	RWS11	-122.4591859	37.93727	-0.19	10.5	-204.5	10.3529997	-122.45918	37.93729	-0.12	10.5	-184.18	10.4249997	11	7/24/2014	22:44.5

3267	RWS11	-122.459188	37.93727	-0.22	10.6	-199.9	10.3309998	-122.45918	37.93729	-0.15	10.6	-178.9	10.4029998	11	7/24/2014	22:44.6
3268	RWS11	-122.4591902	37.93728	-0.19	10.6	-195.3	10.3769998	-122.45918	37.93729	-0.12	10.6	-173.65	10.4489998	11	7/24/2014	22:44.7
3269	RWS11	-122.4591924	37.93728	-0.22	10.6	-190.8	10.3549999	-122.45919	37.93729	-0.15	10.6	-168.49	10.4269999	11	7/24/2014	22:44.8
3270	RWS11	-122.4591945	37.93728	-0.19	10.6	-186.2	10.401	-122.45919	37.93729	-0.12	10.6	-163.21	10.473	11	7/24/2014	22:44.9
3271	RWS11	-122.4591967	37.93728	-0.22	10.6	-181.6	10.3790001	-122.45919	37.93729	-0.15	10.6	-157.94	10.4510001	11	7/24/2014	22:45.0
3272	RWS11	-122.4591989	37.93728	-0.19	10.6	-176.9	10.4259996	-122.45919	37.93729	-0.15	10.6	-152.57	10.4639996	11	7/24/2014	22:45.1
3273	RWS11	-122.459201	37.93728	-0.22	10.6	-172.1	10.4039997	-122.45919	37.93729	-0.15	10.6	-147.12	10.4759997	11	7/24/2014	22:45.2
3274	RWS11	-122.4592032	37.93728	-0.22	10.6	-167	10.4159998	-122.4592	37.93729	-0.12	10.6	-141.52	10.5219998	11	7/24/2014	22:45.3
3275	RWS11	-122.4592053	37.93728	-0.22	10.7	-161.9	10.4279999	-122.4592	37.93729	-0.15	10.7	-135.86	10.4999999	11	7/24/2014	22:45.4
3276	RWS11	-122.4592075	37.93728	-0.19	10.7	-156.8	10.4729996	-122.4592	37.93729	-0.12	10.7	-130.28	10.5449996	11	7/24/2014	22:45.5
3277	RWS11	-122.4592096	37.93728	-0.22	10.7	-151.6	10.4509997	-122.4592	37.93729	-0.15	10.7	-124.63	10.5229996	11	7/24/2014	22:45.6
3278	RWS11	-122.4592117	37.93728	-0.22	10.7	-146.6	10.4620003	-122.45921	37.93729	-0.15	10.7	-118.97	10.5340003	11	7/24/2014	22:45.7
3279	RWS11	-122.4592137	37.93728	-0.22	10.7	-141.5	10.4719996	-122.45921	37.9373	-0.15	10.7	-113.35	10.5439996	11	7/24/2014	22:45.8
3280	RWS11	-122.4592158	37.93728	-0.22	10.7	-136.4	10.4830002	-122.45921	37.9373	-0.15	10.7	-107.69	10.5550002	11	7/24/2014	22:45.9
3281	RWS11	-122.4592188	37.93728	-0.22	10.7	-131	10.4930004	-122.45921	37.9373	-0.15	10.7	-101.9	10.5650004	11	7/24/2014	22:46.0
3282	RWS11	-122.4592208	37.93729	-0.22	10.7	-125.7	10.5029997	-122.45921	37.9373	-0.12	10.7	-96.065	10.6089997	11	7/24/2014	22:46.1
3283	RWS11	-122.4592228	37.93729	-0.22	10.7	-120.4	10.5120005	-122.45922	37.9373	-0.15	10.7	-90.174	10.5840005	11	7/24/2014	22:46.2
3284	RWS11	-122.4592249	37.93729	-0.22	10.7	-115.3	10.5210003	-122.45922	37.9373	-0.12	10.7	-84.545	10.6270003	11	7/24/2014	22:46.3
3285	RWS11	-122.4592269	37.93729	-0.22	10.8	-110.3	10.5300001	-122.45922	37.9373	-0.15	10.8	-78.915	10.6020001	11	7/24/2014	22:46.4
3286	RWS11	-122.459229	37.93729	-0.19	10.8	-105.5	10.5729999	-122.45922	37.9373	-0.12	10.8	-73.282	10.6449999	11	7/24/2014	22:46.5
3287	RWS11	-122.459231	37.93729	-0.22	10.8	-100.7	10.5490002	-122.45922	37.9373	-0.15	10.8	-67.791	10.6210002	11	7/24/2014	22:46.6
3288	RWS11	-122.459233	37.93729	-0.19	10.8	-95.95	10.5930004	-122.45923	37.9373	-0.15	10.8	-62.151	10.6310004	11	7/24/2014	22:46.7
3289	RWS11	-122.459235	37.93729	-0.22	10.8	-91.21	10.5689997	-122.45923	37.9373	-0.15	10.8	-56.557	10.6409997	11	7/24/2014	22:46.8
3290	RWS11	-122.459237	37.93729	-0.22	10.8	-86.36	10.5800003	-122.45923	37.9373	-0.15	10.8	-50.881	10.6520003	11	7/24/2014	22:46.9
3291	RWS11	-122.45924	37.93729	-0.22	10.8	-81.51	10.5920004	-122.45923	37.9373	-0.15	10.8	-45.184	10.6640004	11	7/24/2014	22:47.0
3292	RWS11	-122.459242	37.93729	-0.22	10.8	-76.58	10.6049999	-122.45924	37.9373	-0.15	10.8	-39.45	10.6769999	11	7/24/2014	22:47.1
3293	RWS11	-122.459244	37.93729	-0.22	10.8	-71.54	10.6180004	-122.45924	37.93731	-0.15	10.8	-33.601	10.6900004	11	7/24/2014	22:47.2
3294	RWS11	-122.4592461	37.93729	-0.19	10.9	-66.55	10.6660004	-122.45924	37.93731	-0.15	10.9	-27.621	10.7040004	11	7/24/2014	22:47.3
3295	RWS11	-122.4592481	37.93729	-0.22	10.9	-61.64	10.6469998	-122.45924	37.93731	-0.15	10.9	-21.644	10.7189997	11	7/24/2014	22:47.4
3296	RWS11	-122.4592501	37.93729	-0.19	10.9	-56.9	10.6969995	-122.45924	37.93731	-0.15	10.9	-15.796	10.7349995	11	7/24/2014	22:47.5
3297	RWS11	-122.4592522	37.9373	-0.22	10.9	-52.3	10.6799997	-122.45925	37.93731	-0.2	10.9	-10.211	10.7019997	11	7/24/2014	22:47.6
3298	RWS11	-122.4592542	37.9373	-0.19	10.9	-47.76	10.7309999	-122.45925	37.93731	-0.15	10.9	-4.56	10.7689999	11	7/24/2014	22:47.7
3299	RWS11	-122.4592562	37.9373	-0.22	10.9	-43.22	10.7149996	-122.45925	37.93731	-0.15	10.9	0.96	10.7869996	11	7/24/2014	22:47.8

3300	RWS11	-122.4592582	37.9373	-0.19	11	-38.86	10.7679996	-122.45925	37.93731	-0.15	11	6.394	10.8059996	11	7/24/2014	22:47.9
3301	RWS11	-122.4592612	37.9373	-0.22	11	-34.36	10.7509998	-122.45925	37.93731	-0.2	11	11.826	10.7729998	11	7/24/2014	22:48.0
3302	RWS11	-122.4592632	37.9373	-0.22	11	-29.89	10.768	-122.45926	37.93731	-0.15	11	17.28	10.84	11	7/24/2014	22:48.1
3303	RWS11	-122.4592652	37.9373	-0.22	11	-25.35	10.7839998	-122.45926	37.93731	-0.2	11	22.735	10.8059998	11	7/24/2014	22:48.2
3304	RWS11	-122.4592673	37.9373	-0.22	11	-20.79	10.7979998	-122.45926	37.93731	-0.15	11	28.187	10.8699998	11	7/24/2014	22:48.3
3305	RWS11	-122.4592693	37.9373	-0.22	11	-16.32	10.8110003	-122.45926	37.93731	-0.2	11	33.555	10.8330003	11	7/24/2014	22:48.4
3306	RWS11	-122.4592714	37.9373	-0.22	11	-11.89	10.8219999	-122.45927	37.93731	-0.15	11	38.9	10.8939999	11	7/24/2014	22:48.5
3307	RWS11	-122.4592734	37.9373	-0.28	11.1	-7.443	10.7789998	-122.45927	37.93731	-0.2	11.1	44.133	10.8529998	11	7/24/2014	22:48.6
3308	RWS11	-122.4592754	37.9373	-0.22	11.1	-3.21	10.8390001	-122.45927	37.93732	-0.15	11.1	49.282	10.9110001	11	7/24/2014	22:48.7
3309	RWS11	-122.4592775	37.9373	-0.22	11.1	1.001	10.8440003	-122.45927	37.93732	-0.2	11.1	54.328	10.8660003	11	7/24/2014	22:48.8
3310	RWS11	-122.4592795	37.9373	-0.22	11.1	5.038	10.8469996	-122.45927	37.93732	-0.15	11.1	59.28	10.9189996	11	7/24/2014	22:48.9
3311	RWS11	-122.4592825	37.9373	-0.22	11.1	9.03	10.8469996	-122.45928	37.93732	-0.15	11.1	64.144	10.9189996	11	7/24/2014	22:49.0
3312	RWS11	-122.4592845	37.93731	-0.22	11.1	12.85	10.8449997	-122.45928	37.93732	-0.15	11.1	68.877	10.9169997	11	7/24/2014	22:49.1
3313	RWS11	-122.4592866	37.93731	-0.22	11.1	16.513	10.8390001	-122.45928	37.93732	-0.2	11.1	73.462	10.8610001	11	7/24/2014	22:49.2
3314	RWS11	-122.4592886	37.93731	-0.22	11.1	20.093	10.8309998	-122.45928	37.93732	-0.15	11.1	77.911	10.9029998	11	7/24/2014	22:49.3
3315	RWS11	-122.4592907	37.93731	-0.22	11	23.476	10.8200001	-122.45928	37.93732	-0.2	11	82.19	10.8420001	11	7/24/2014	22:49.4
3316	RWS11	-122.4592927	37.93731	-0.22	11	26.725	10.8069996	-122.45929	37.93732	-0.12	11	86.267	10.9129996	11	7/24/2014	22:49.5
3317	RWS11	-122.4592948	37.93731	-0.22	11	29.671	10.7929997	-122.45929	37.93732	-0.2	11	90.022	10.8149997	11	7/24/2014	22:49.6
3318	RWS11	-122.4592969	37.93731	-0.22	11	32.442	10.7769999	-122.45929	37.93732	-0.15	11	93.667	10.8489999	11	7/24/2014	22:49.7
3319	RWS11	-122.4592989	37.93731	-0.28	11	35.106	10.7090001	-122.45929	37.93732	-0.2	11	97.198	10.7830001	11	7/24/2014	22:49.8
3320	RWS11	-122.4593009	37.93731	-0.22	11	37.874	10.7439999	-122.45929	37.93732	-0.2	11	100.76	10.7659999	11	7/24/2014	22:49.9
3321	RWS11	-122.459304	37.93731	-0.28	11	40.754	10.6760001	-122.4593	37.93732	-0.2	11	104.35	10.7500001	11	7/24/2014	22:50.0
3322	RWS11	-122.459306	37.93731	-0.22	10.9	43.588	10.7129997	-122.4593	37.93732	-0.15	10.9	107.97	10.7849997	11	7/24/2014	22:50.1
3323	RWS11	-122.4593081	37.93731	-0.28	10.9	46.446	10.6469998	-122.4593	37.93733	-0.15	10.9	111.62	10.7709998	11	7/24/2014	22:50.2
3324	RWS11	-122.4593102	37.93731	-0.22	10.9	49.308	10.6860003	-122.4593	37.93733	-0.15	10.9	115.37	10.7580003	11	7/24/2014	22:50.3
3325	RWS11	-122.4593122	37.93731	-0.28	10.9	51.988	10.6209998	-122.45931	37.93733	-0.15	10.9	119.02	10.7449998	11	7/24/2014	22:50.4
3326	RWS11	-122.4593144	37.93731	-0.22	10.9	54.566	10.6620001	-122.45931	37.93733	-0.15	10.9	122.51	10.7340001	11	7/24/2014	22:50.5
3327	RWS11	-122.4593165	37.93731	-0.28	10.9	57.096	10.5999999	-122.45931	37.93733	-0.15	10.9	125.89	10.7239999	11	7/24/2014	22:50.6
3328	RWS11	-122.4593187	37.93732	-0.22	10.9	59.579	10.6440004	-122.45931	37.93733	-0.15	10.9	129.28	10.7160004	11	7/24/2014	22:50.7
3329	RWS11	-122.4593208	37.93732	-0.22	10.9	62.114	10.6370005	-122.45931	37.93733	-0.15	10.9	132.75	10.7090005	11	7/24/2014	22:50.8
3330	RWS11	-122.459323	37.93732	-0.22	10.9	64.601	10.631	-122.45932	37.93733	-0.15	10.9	136.21	10.703	11	7/24/2014	22:50.9
3331	RWS11	-122.4593252	37.93732	-0.22	10.9	67.005	10.6270002	-122.45932	37.93733	-0.15	10.9	139.59	10.6990002	11	7/24/2014	22:51.0
3332	RWS11	-122.4593274	37.93732	-0.22	10.8	69.334	10.624	-122.45932	37.93733	-0.15	10.8	142.93	10.696	11	7/24/2014	22:51.1

3333	RWS11	-122.4593295	37.93732	-0.28	10.8	71.585	10.5700001	-122.45932	37.93733	-0.2	10.8	146.18	10.6440001	11	7/24/2014	22:51.2
3334	RWS11	-122.4593317	37.93732	-0.22	10.8	73.717	10.6189999	-122.45933	37.93733	-0.15	10.8	149.31	10.6909999	11	7/24/2014	22:51.3
3335	RWS11	-122.4593339	37.93732	-0.22	10.8	75.798	10.6159996	-122.45933	37.93733	-0.15	10.8	152.39	10.6879996	11	7/24/2014	22:51.4
3336	RWS11	-122.459336	37.93732	-0.22	10.8	77.776	10.6119999	-122.45933	37.93733	-0.2	10.8	155.32	10.6339999	11	7/24/2014	22:51.5
3337	RWS11	-122.4593381	37.93732	-0.28	10.8	79.522	10.5529999	-122.45933	37.93733	-0.2	10.8	158.14	10.6269999	11	7/24/2014	22:51.6
3338	RWS11	-122.4593402	37.93732	-0.28	10.8	81.331	10.5449995	-122.45933	37.93733	-0.2	10.8	160.85	10.6189996	11	7/24/2014	22:51.7
3339	RWS11	-122.4593424	37.93732	-0.22	10.8	82.993	10.5859999	-122.45934	37.93734	-0.2	10.8	163.57	10.6079999	11	7/24/2014	22:51.8
3340	RWS11	-122.4593444	37.93732	-0.22	10.8	84.648	10.5719999	-122.45934	37.93734	-0.15	10.8	166.13	10.6439999	11	7/24/2014	22:51.9
3341	RWS11	-122.4593475	37.93732	-0.28	10.8	86.129	10.5049996	-122.45934	37.93734	-0.2	10.8	168.35	10.5789996	11	7/24/2014	22:52.0
3342	RWS11	-122.4593496	37.93732	-0.22	10.8	87.463	10.5400004	-122.45934	37.93734	-0.2	10.8	170.43	10.5620004	11	7/24/2014	22:52.1
3343	RWS11	-122.4593517	37.93733	-0.22	10.7	88.687	10.5219998	-122.45935	37.93734	-0.24	10.7	172.51	10.5099997	11	7/24/2014	22:52.2
3344	RWS11	-122.4593539	37.93733	-0.22	10.7	89.819	10.5020002	-122.45935	37.93734	-0.15	10.7	174.44	10.5740002	11	7/24/2014	22:52.3
3345	RWS11	-122.459356	37.93733	-0.22	10.7	90.824	10.4810003	-122.45935	37.93734	-0.2	10.7	176.03	10.5030003	11	7/24/2014	22:52.4
3346	RWS11	-122.4593582	37.93733	-0.22	10.7	91.896	10.4589996	-122.45935	37.93734	-0.2	10.7	177.53	10.4809996	11	7/24/2014	22:52.5
3347	RWS11	-122.4593604	37.93733	-0.28	10.7	92.677	10.3830004	-122.45935	37.93734	-0.24	10.7	178.75	10.4230004	11	7/24/2014	22:52.6
3348	RWS11	-122.4593626	37.93733	-0.22	10.6	93.267	10.4099998	-122.45936	37.93734	-0.2	10.6	179.75	10.4319998	11	7/24/2014	22:52.7
3349	RWS11	-122.4593648	37.93733	-0.28	10.6	93.704	10.3310004	-122.45936	37.93734	-0.2	10.6	180.54	10.4050003	11	7/24/2014	22:52.8
3350	RWS11	-122.4593671	37.93733	-0.22	10.6	94.011	10.3559999	-122.45936	37.93734	-0.15	10.6	181.16	10.4279999	11	7/24/2014	22:52.9
3351	RWS11	-122.4593693	37.93733	-0.28	10.6	94.136	10.2770005	-122.45936	37.93734	-0.2	10.6	181.38	10.3510004	11	7/24/2014	22:53.0
3352	RWS11	-122.4593715	37.93733	-0.22	10.5	94.141	10.3009996	-122.45937	37.93734	-0.2	10.5	181.42	10.3229996	11	7/24/2014	22:53.1
3353	RWS11	-122.4593737	37.93733	-0.28	10.5	93.985	10.2209998	-122.45937	37.93734	-0.2	10.5	181.38	10.2949997	11	7/24/2014	22:53.2
3354	RWS11	-122.4593759	37.93733	-0.22	10.5	93.766	10.2449999	-122.45937	37.93734	-0.15	10.5	181.21	10.3169999	11	7/24/2014	22:53.3
3355	RWS11	-122.4593781	37.93733	-0.28	10.4	93.526	10.165	-122.45937	37.93735	-0.2	10.4	181.1	10.239	11	7/24/2014	22:53.4
3356	RWS11	-122.4593803	37.93733	-0.22	10.4	93.263	10.1890001	-122.45937	37.93735	-0.2	10.4	181.08	10.2110001	11	7/24/2014	22:53.5
3357	RWS11	-122.4593824	37.93733	-0.28	10.4	93.066	10.1099997	-122.45938	37.93735	-0.2	10.4	181.02	10.1839997	11	7/24/2014	22:53.6
3358	RWS11	-122.4593846	37.93733	-0.22	10.4	92.76	10.1350002	-122.45938	37.93735	-0.2	10.4	181.02	10.1570002	11	7/24/2014	22:53.7
3359	RWS11	-122.4593867	37.93734	-0.28	10.3	92.454	10.0559998	-122.45938	37.93735	-0.2	10.3	181.07	10.1299998	11	7/24/2014	22:53.8
3360	RWS11	-122.4593888	37.93734	-0.22	10.3	92.154	10.0810003	-122.45938	37.93735	-0.2	10.3	181.08	10.1030003	11	7/24/2014	22:53.9
3361	RWS11	-122.4593919	37.93734	-0.22	10.3	91.754	10.0539999	-122.45939	37.93735	-0.2	10.3	180.93	10.0759999	11	7/24/2014	22:54.0
3362	RWS11	-122.459394	37.93734	-0.22	10.3	91.236	10.0270004	-122.45939	37.93735	-0.15	10.3	180.6	10.0990004	11	7/24/2014	22:54.1
3363	RWS11	-122.4593961	37.93734	-0.28	10.2	90.755	9.94699958	-122.45939	37.93735	-0.2	10.2	180.33	10.0209996	11	7/24/2014	22:54.2
3364	RWS11	-122.4593983	37.93734	-0.22	10.2	90.23	9.97099969	-122.45939	37.93735	-0.2	10.2	179.97	9.99299969	11	7/24/2014	22:54.3
3365	RWS11	-122.4594004	37.93734	-0.28	10.2	89.443	9.89099982	-122.45939	37.93735	-0.2	10.2	179.45	9.96499981	11	7/24/2014	22:54.4

3366	RWS11	-122.4594026	37.93734	-0.22	10.1	88.683	9.91600034	-122.4594	37.93735	-0.2	10.1	178.77	9.93800034	11	7/24/2014	22:54.5
3367	RWS11	-122.4594048	37.93734	-0.22	10.1	87.721	9.88800046	-122.4594	37.93735	-0.24	10.1	177.9	9.87600046	11	7/24/2014	22:54.6
3368	RWS11	-122.459407	37.93734	-0.22	10.1	86.715	9.86100003	-122.4594	37.93735	-0.2	10.1	176.85	9.88300003	11	7/24/2014	22:54.7
3369	RWS11	-122.4594092	37.93734	-0.28	10.1	85.471	9.78300002	-122.4594	37.93735	-0.2	10.1	175.51	9.85700001	11	7/24/2014	22:54.8
3370	RWS11	-122.4594115	37.93734	-0.22	10	84.116	9.80799958	-122.45941	37.93735	-0.2	10	174	9.82999958	11	7/24/2014	22:54.9
3371	RWS11	-122.4594137	37.93734	-0.28	10	82.613	9.72999957	-122.45941	37.93736	-0.2	10	172.3	9.80399956	11	7/24/2014	22:55.0
3372	RWS11	-122.459416	37.93734	-0.22	9.98	80.952	9.75599954	-122.45941	37.93736	-0.2	9.98	170.6	9.77799954	11	7/24/2014	22:55.1
3373	RWS11	-122.4594182	37.93734	-0.22	9.95	79.295	9.73099992	-122.45941	37.93736	-0.24	9.95	168.7	9.71899992	11	7/24/2014	22:55.2
3374	RWS11	-122.4594204	37.93734	-0.22	9.93	77.377	9.70499989	-122.45941	37.93736	-0.2	9.93	166.55	9.72699989	11	7/24/2014	22:55.3
3375	RWS11	-122.4594226	37.93735	-0.28	9.9	75.388	9.62699988	-122.45942	37.93736	-0.24	9.9	164.3	9.66699988	11	7/24/2014	22:55.4
3376	RWS11	-122.4594248	37.93735	-0.22	9.88	73.295	9.65299985	-122.45942	37.93736	-0.2	9.88	161.87	9.67499985	11	7/24/2014	22:55.5
3377	RWS11	-122.4594269	37.93735	-0.22	9.85	71.048	9.62600037	-122.45942	37.93736	-0.24	9.85	159.27	9.61400038	11	7/24/2014	22:55.6
3378	RWS11	-122.4594291	37.93735	-0.22	9.82	68.671	9.59899995	-122.45942	37.93736	-0.24	9.82	156.46	9.58699995	11	7/24/2014	22:55.7
3379	RWS11	-122.4594312	37.93735	-0.28	9.8	66.158	9.51900008	-122.45943	37.93736	-0.24	9.8	153.44	9.55900007	11	7/24/2014	22:55.8
3380	RWS11	-122.4594333	37.93735	-0.22	9.77	63.562	9.54300019	-122.45943	37.93736	-0.2	9.77	150.13	9.56500019	11	7/24/2014	22:55.9
3381	RWS11	-122.4594364	37.93735	-0.22	9.74	60.729	9.51399991	-122.45943	37.93736	-0.24	9.74	146.69	9.50199991	11	7/24/2014	22:56.0
3382	RWS11	-122.4594385	37.93735	-0.22	9.71	57.954	9.48400018	-122.45943	37.93736	-0.2	9.71	143.16	9.50600018	11	7/24/2014	22:56.1
3383	RWS11	-122.4594406	37.93735	-0.28	9.68	54.947	9.40200046	-122.45944	37.93736	-0.24	9.68	139.4	9.44200045	11	7/24/2014	22:56.2
3384	RWS11	-122.4594427	37.93735	-0.22	9.65	51.822	9.42399976	-122.45944	37.93736	-0.24	9.65	135.43	9.41199976	11	7/24/2014	22:56.3
3385	RWS11	-122.4594449	37.93735	-0.22	9.62	48.549	9.39400002	-122.45944	37.93736	-0.24	9.62	131.29	9.38200003	11	7/24/2014	22:56.4
3386	RWS11	-122.459447	37.93735	-0.22	9.59	45.21	9.36400029	-122.45944	37.93736	-0.24	9.59	127.14	9.3520003	11	7/24/2014	22:56.5
3387	RWS11	-122.4594492	37.93735	-0.22	9.56	41.98	9.33500001	-122.45944	37.93737	-0.24	9.56	123.08	9.32300001	11	7/24/2014	22:56.6
3388	RWS11	-122.4594514	37.93735	-0.22	9.53	38.663	9.30599973	-122.45945	37.93737	-0.2	9.53	118.99	9.32799973	11	7/24/2014	22:56.7
3389	RWS11	-122.4594536	37.93735	-0.22	9.5	35.194	9.27799985	-122.45945	37.93737	-0.24	9.5	114.68	9.26599985	11	7/24/2014	22:56.8
3390	RWS11	-122.4594558	37.93735	-0.22	9.47	31.617	9.24999997	-122.45945	37.93737	-0.2	9.47	110.23	9.27199997	11	7/24/2014	22:56.9
3391	RWS11	-122.459458	37.93735	-0.22	9.45	28.082	9.22200009	-122.45945	37.93737	-0.24	9.45	105.7	9.2100001	11	7/24/2014	22:57.0
3392	RWS11	-122.4594602	37.93736	-0.22	9.42	24.547	9.19400021	-122.45945	37.93737	-0.2	9.42	101.28	9.21600021	11	7/24/2014	22:57.1
3393	RWS11	-122.4594623	37.93736	-0.28	9.39	20.99	9.11299995	-122.45946	37.93737	-0.2	9.39	96.89	9.18699993	11	7/24/2014	22:57.2
3394	RWS11	-122.4594645	37.93736	-0.22	9.36	17.391	9.1350002	-122.45946	37.93737	-0.2	9.36	92.424	9.1570002	11	7/24/2014	22:57.3
3395	RWS11	-122.4594666	37.93736	-0.22	9.33	13.681	9.10500047	-122.45946	37.93737	-0.2	9.33	87.866	9.12700047	11	7/24/2014	22:57.4
3396	RWS11	-122.4594687	37.93736	-0.22	9.3	10.015	9.07299992	-122.45946	37.93737	-0.2	9.3	83.303	9.09499992	11	7/24/2014	22:57.5
3397	RWS11	-122.4594708	37.93736	-0.22	9.27	6.262	9.04100034	-122.45947	37.93737	-0.24	9.27	78.745	9.02900034	11	7/24/2014	22:57.6
3398	RWS11	-122.4594729	37.93736	-0.22	9.23	2.488	9.00899979	-122.45947	37.93737	-0.2	9.23	74.033	9.03099979	11	7/24/2014	22:57.7

3399	RWS11	-122.459475	37.93736	-0.22	9.2	-1.374	8.97500035	-122.45947	37.93737	-0.2	9.2	69.252	8.99700035	11	7/24/2014	22:57.8
3400	RWS11	-122.459477	37.93736	-0.22	9.17	-5.215	8.94200036	-122.45947	37.93737	-0.2	9.17	64.386	8.96400036	11	7/24/2014	22:57.9
3401	RWS11	-122.4594801	37.93736	-0.22	9.13	-9.102	8.90900037	-122.45947	37.93737	-0.2	9.13	59.541	8.93100037	11	7/24/2014	22:58.0
3402	RWS11	-122.4594821	37.93736	-0.22	9.1	-13.05	8.87699983	-122.45948	37.93737	-0.2	9.1	54.607	8.89899983	11	7/24/2014	22:58.1
3403	RWS11	-122.4594842	37.93736	-0.22	9.07	-17	8.84500024	-122.45948	37.93737	-0.2	9.07	49.633	8.86700024	11	7/24/2014	22:58.2
3404	RWS11	-122.4594863	37.93736	-0.22	9.04	-21.13	8.81499955	-122.45948	37.93738	-0.15	9.04	44.572	8.88699956	11	7/24/2014	22:58.3
3405	RWS11	-122.4594884	37.93736	-0.22	9.01	-25.2	8.78699967	-122.45948	37.93738	-0.2	9.01	39.467	8.80899967	11	7/24/2014	22:58.4
3406	RWS11	-122.4594906	37.93736	-0.22	8.98	-29.44	8.76099965	-122.45949	37.93738	-0.2	8.98	34.294	8.78299965	11	7/24/2014	22:58.5
3407	RWS11	-122.4594928	37.93736	-0.22	8.96	-33.58	8.73600003	-122.45949	37.93738	-0.2	8.96	29.038	8.75800003	11	7/24/2014	22:58.6
3408	RWS11	-122.459495	37.93736	-0.22	8.94	-37.82	8.71300027	-122.45949	37.93738	-0.2	8.94	23.799	8.73500027	11	7/24/2014	22:58.7
3409	RWS11	-122.4594972	37.93737	-0.22	8.92	-42.09	8.69200036	-122.45949	37.93738	-0.24	8.92	18.476	8.68000036	11	7/24/2014	22:58.8
3410	RWS11	-122.4594994	37.93737	-0.22	8.9	-46.37	8.67300031	-122.45949	37.93738	-0.2	8.9	13.152	8.69500031	11	7/24/2014	22:58.9
3411	RWS11	-122.4595015	37.93737	-0.28	8.88	-50.65	8.60299972	-122.4595	37.93738	-0.24	8.88	7.762	8.64299971	11	7/24/2014	22:59.0
3412	RWS11	-122.4595038	37.93737	-0.22	8.86	-55.01	8.63800046	-122.4595	37.93738	-0.15	8.86	2.395	8.71000047	11	7/24/2014	22:59.1
3413	RWS11	-122.4595059	37.93737	-0.28	8.85	-59.33	8.56900027	-122.4595	37.93738	-0.2	8.85	-3.038	8.64300026	11	7/24/2014	22:59.2
3414	RWS11	-122.4595081	37.93737	-0.22	8.83	-63.61	8.60500047	-122.4595	37.93738	-0.2	8.83	-8.427	8.62700047	11	7/24/2014	22:59.3
3415	RWS11	-122.4595103	37.93737	-0.22	8.81	-67.84	8.58800027	-122.45951	37.93738	-0.24	8.81	-13.773	8.57600027	11	7/24/2014	22:59.4
3416	RWS11	-122.4595125	37.93737	-0.22	8.8	-72.12	8.57100007	-122.45951	37.93738	-0.2	8.8	-19.032	8.59300007	11	7/24/2014	22:59.5
3417	RWS11	-122.4595146	37.93737	-0.28	8.78	-76.42	8.50000003	-122.45951	37.93738	-0.2	8.78	-24.381	8.57400002	11	7/24/2014	22:59.6
3418	RWS11	-122.4595167	37.93737	-0.22	8.76	-80.69	8.53299996	-122.45951	37.93738	-0.2	8.76	-29.68	8.55499996	11	7/24/2014	22:59.7
3419	RWS11	-122.4595188	37.93737	-0.22	8.74	-85.06	8.51200005	-122.45951	37.93738	-0.2	8.74	-34.942	8.53400005	11	7/24/2014	22:59.8
3420	RWS11	-122.4595209	37.93737	-0.22	8.72	-89.4	8.49100015	-122.45952	37.93738	-0.2	8.72	-40.111	8.51300015	11	7/24/2014	22:59.9
3421	RWS11	-122.4595239	37.93737	-0.22	8.69	-93.78	8.46899983	-122.45952	37.93738	-0.24	8.69	-45.261	8.45699984	11	7/24/2014	23:00.0
3422	RWS11	-122.459526	37.93737	-0.22	8.67	-98.19	8.44699952	-122.45952	37.93739	-0.2	8.67	-50.345	8.46899952	11	7/24/2014	23:00.1
3423	RWS11	-122.4595281	37.93737	-0.22	8.65	-102.6	8.42599961	-122.45952	37.93739	-0.2	8.65	-55.492	8.44799961	11	7/24/2014	23:00.2
3424	RWS11	-122.4595302	37.93737	-0.22	8.63	-107	8.4049997	-122.45952	37.93739	-0.15	8.63	-60.645	8.47699971	11	7/24/2014	23:00.3
3425	RWS11	-122.4595323	37.93737	-0.22	8.61	-111.3	8.38399979	-122.45953	37.93739	-0.2	8.61	-65.857	8.40599979	11	7/24/2014	23:00.4
3426	RWS11	-122.4595344	37.93737	-0.22	8.59	-115.6	8.36299989	-122.45953	37.93739	-0.2	8.59	-71.003	8.38499989	11	7/24/2014	23:00.5
3427	RWS11	-122.4595366	37.93738	-0.22	8.57	-119.9	8.34199998	-122.45953	37.93739	-0.24	8.57	-76.201	8.32999998	11	7/24/2014	23:00.6
3428	RWS11	-122.4595387	37.93738	-0.22	8.55	-124.2	8.32199952	-122.45953	37.93739	-0.2	8.55	-81.307	8.34399952	11	7/24/2014	23:00.7
3429	RWS11	-122.4595409	37.93738	-0.22	8.52	-128.5	8.30000016	-122.45954	37.93739	-0.24	8.52	-86.562	8.28800017	11	7/24/2014	23:00.8
3430	RWS11	-122.4595431	37.93738	-0.22	8.5	-132.8	8.2770004	-122.45954	37.93739	-0.15	8.5	-91.869	8.34900041	11	7/24/2014	23:00.9
3431	RWS11	-122.4595453	37.93738	-0.22	8.48	-137.1	8.25300023	-122.45954	37.93739	-0.2	8.48	-97.123	8.27500023	11	7/24/2014	23:01.0

3432	RWS11	-122.4595475	37.93738	-0.22	8.45	-141.3	8.22799966	-122.45954	37.93739	-0.15	8.45	-102.3	8.29999967	11	7/24/2014	23:01.1
3433	RWS11	-122.4595497	37.93738	-0.28	8.43	-145.6	8.14999965	-122.45954	37.93739	-0.2	8.43	-107.49	8.22399963	11	7/24/2014	23:01.2
3434	RWS11	-122.4595519	37.93738	-0.22	8.4	-149.8	8.17500016	-122.45955	37.93739	-0.15	8.4	-112.62	8.24700017	11	7/24/2014	23:01.3
3435	RWS11	-122.4595541	37.93738	-0.22	8.37	-154	8.14900014	-122.45955	37.93739	-0.24	8.37	-117.72	8.13700014	11	7/24/2014	23:01.4
3436	RWS11	-122.4595562	37.93738	-0.22	8.35	-158.2	8.12300012	-122.45955	37.93739	-0.2	8.35	-122.76	8.14500012	11	7/24/2014	23:01.5
3437	RWS11	-122.4595583	37.93738	-0.28	8.32	-162.4	8.04500011	-122.45955	37.93739	-0.2	8.32	-127.79	8.11900009	11	7/24/2014	23:01.6
3438	RWS11	-122.4595605	37.93738	-0.22	8.3	-166.5	8.07199952	-122.45956	37.93739	-0.2	8.3	-132.63	8.09399952	11	7/24/2014	23:01.7
3439	RWS11	-122.4595626	37.93738	-0.22	8.27	-170.6	8.04800031	-122.45956	37.93739	-0.2	8.27	-137.35	8.07000031	11	7/24/2014	23:01.8
3440	RWS11	-122.4595646	37.93738	-0.22	8.25	-174.6	8.02400014	-122.45956	37.9374	-0.15	8.25	-142.07	8.09600015	11	7/24/2014	23:01.9
3441	RWS11	-122.4595677	37.93738	-0.28	8.22	-178.6	7.94799998	-122.45956	37.9374	-0.2	8.22	-146.77	8.02199997	11	7/24/2014	23:02.0
3442	RWS11	-122.4595698	37.93738	-0.22	8.2	-182.6	7.97700021	-122.45956	37.9374	-0.15	8.2	-151.57	8.04900022	11	7/24/2014	23:02.1
3443	RWS11	-122.4595718	37.93738	-0.28	8.18	-186.6	7.90100005	-122.45957	37.9374	-0.2	8.18	-156.35	7.97500004	11	7/24/2014	23:02.2
3444	RWS11	-122.4595739	37.93738	-0.22	8.15	-190.6	7.92800042	-122.45957	37.9374	-0.2	8.15	-161.21	7.95000042	11	7/24/2014	23:02.3
3445	RWS11	-122.459576	37.93739	-0.22	8.13	-194.6	7.90299985	-122.45957	37.9374	-0.2	8.13	-165.95	7.92499985	11	7/24/2014	23:02.4
3446	RWS11	-122.459578	37.93739	-0.22	8.1	-198.6	7.87699983	-122.45957	37.9374	-0.2	8.1	-170.69	7.89899983	11	7/24/2014	23:02.5
3447	RWS11	-122.4595801	37.93739	-0.22	8.07	-202.6	7.8509998	-122.45957	37.9374	-0.2	8.07	-175.3	7.8729998	11	7/24/2014	23:02.6
3448	RWS11	-122.4595822	37.93739	-0.22	8.05	-206.6	7.82400033	-122.45958	37.9374	-0.2	8.05	-179.91	7.84600033	11	7/24/2014	23:02.7
3449	RWS11	-122.4595842	37.93739	-0.22	8.02	-210.5	7.7969999	-122.45958	37.9374	-0.2	8.02	-184.35	7.8189999	11	7/24/2014	23:02.8
3450	RWS11	-122.4595863	37.93739	-0.22	7.99	-214.3	7.76999995	-122.45958	37.9374	-0.2	7.99	-188.57	7.79199995	11	7/24/2014	23:02.9
3451	RWS11	-122.4595893	37.93739	-0.22	7.97	-218.1	7.743	-122.45958	37.9374	-0.2	7.97	-192.8	7.765	11	7/24/2014	23:03.0
3452	RWS11	-122.4595913	37.93739	-0.22	7.94	-221.8	7.71600005	-122.45959	37.9374	-0.15	7.94	-196.91	7.78800006	11	7/24/2014	23:03.1
3453	RWS11	-122.4595933	37.93739	-0.22	7.91	-225.5	7.69000003	-122.45959	37.9374	-0.2	7.91	-200.89	7.71200003	11	7/24/2014	23:03.2
3454	RWS11	-122.4595954	37.93739	-0.22	7.89	-229.2	7.664	-122.45959	37.9374	-0.24	7.89	-204.96	7.65200001	11	7/24/2014	23:03.3
3455	RWS11	-122.4595974	37.93739	-0.28	7.86	-232.8	7.586	-122.45959	37.9374	-0.2	7.86	-208.94	7.65999998	11	7/24/2014	23:03.4
3456	RWS11	-122.4595995	37.93739	-0.22	7.84	-236.4	7.61100003	-122.45959	37.9374	-0.15	7.84	-213.02	7.68300004	11	7/24/2014	23:03.5
3457	RWS11	-122.4596015	37.93739	-0.28	7.81	-240	7.53000024	-122.4596	37.9374	-0.2	7.81	-217.08	7.60400023	11	7/24/2014	23:03.6
3458	RWS11	-122.4596036	37.93739	-0.22	7.78	-243.4	7.55100009	-122.4596	37.9374	-0.2	7.78	-220.85	7.57300009	11	7/24/2014	23:03.7
3459	RWS11	-122.4596056	37.93739	-0.28	7.74	-246.8	7.46600011	-122.4596	37.93741	-0.24	7.74	-224.55	7.5060001	11	7/24/2014	23:03.8
3460	RWS11	-122.4596076	37.93739	-0.22	7.71	-250.2	7.48199984	-122.4596	37.93741	-0.2	7.71	-228.41	7.50399984	11	7/24/2014	23:03.9
3461	RWS11	-122.4596107	37.93739	-0.22	7.67	-253.6	7.44199988	-122.45961	37.93741	-0.24	7.67	-232.16	7.42999989	11	7/24/2014	23:04.0
3462	RWS11	-122.4596128	37.93739	-0.22	7.62	-257	7.39900014	-122.45961	37.93741	-0.24	7.62	-235.95	7.38700014	11	7/24/2014	23:04.1
3463	RWS11	-122.4596148	37.93739	-0.22	7.58	-260.3	7.35400006	-122.45961	37.93741	-0.2	7.58	-239.67	7.37600006	11	7/24/2014	23:04.2
3464	RWS11	-122.459617	37.9374	-0.22	7.53	-263.7	7.3049998	-122.45961	37.93741	-0.2	7.53	-243.4	7.3269998	11	7/24/2014	23:04.3

3465	RWS11	-122.4596191	37.9374	-0.22	7.48	-267	7.25600001	-122.45961	37.93741	-0.24	7.48	-247.06	7.24400002	11	7/24/2014	23:04.4
3466	RWS11	-122.4596212	37.9374	-0.22	7.43	-270.1	7.20599982	-122.45962	37.93741	-0.2	7.43	-250.49	7.22799982	11	7/24/2014	23:04.5
3467	RWS11	-122.4596233	37.9374	-0.28	7.38	-273.3	7.10599998	-122.45962	37.93741	-0.24	7.38	-253.98	7.14599997	11	7/24/2014	23:04.6
3468	RWS11	-122.4596255	37.9374	-0.22	7.34	-276.5	7.11100003	-122.45962	37.93741	-0.2	7.34	-257.82	7.13300003	11	7/24/2014	23:04.7
3469	RWS11	-122.4596276	37.9374	-0.22	7.29	-279.8	7.06599995	-122.45962	37.93741	-0.2	7.29	-261.36	7.08799995	11	7/24/2014	23:04.8
3470	RWS11	-122.4596297	37.9374	-0.22	7.25	-282.8	7.02500007	-122.45962	37.93741	-0.24	7.25	-264.79	7.01300007	11	7/24/2014	23:04.9
3471	RWS11	-122.4596328	37.9374	-0.28	7.21	-285.7	6.9359999	-122.45963	37.93741	-0.24	7.21	-268.09	6.97599989	11	7/24/2014	23:05.0
3472	RWS11	-122.4596349	37.9374	-0.22	7.18	-288.6	6.95599982	-122.45963	37.93741	-0.2	7.18	-271.57	6.97799982	11	7/24/2014	23:05.1
3473	RWS11	-122.459637	37.9374	-0.28	7.15	-291.6	6.87799981	-122.45963	37.93741	-0.24	7.15	-275.02	6.9179998	11	7/24/2014	23:05.2
3474	RWS11	-122.4596391	37.9374	-0.22	7.13	-294.4	6.90899989	-122.45963	37.93741	-0.15	7.13	-278.33	6.9809999	11	7/24/2014	23:05.3
3475	RWS11	-122.4596412	37.9374	-0.28	7.12	-297.2	6.84100011	-122.45964	37.93741	-0.24	7.12	-281.66	6.8810001	11	7/24/2014	23:05.4
3476	RWS11	-122.4596433	37.9374	-0.22	7.11	-300	6.88199994	-122.45964	37.93741	-0.2	7.11	-284.91	6.90399994	11	7/24/2014	23:05.5
3477	RWS11	-122.4596455	37.9374	-0.28	7.1	-302.8	6.82299998	-122.45964	37.93742	-0.2	7.1	-288.06	6.89699997	11	7/24/2014	23:05.6
3478	RWS11	-122.4596476	37.9374	-0.22	7.1	-305.5	6.87200019	-122.45964	37.93742	-0.24	7.1	-291.14	6.86000019	11	7/24/2014	23:05.7
3479	RWS11	-122.4596497	37.9374	-0.28	7.1	-308.2	6.82100013	-122.45964	37.93742	-0.24	7.1	-294.14	6.86100012	11	7/24/2014	23:05.8
3480	RWS11	-122.4596518	37.9374	-0.22	7.1	-310.9	6.8759999	-122.45965	37.93742	-0.2	7.1	-297.13	6.8979999	11	7/24/2014	23:05.9
3481	RWS11	-122.4596549	37.93741	-0.22	7.11	-313.5	6.88100001	-122.45965	37.93742	-0.24	7.11	-299.93	6.86900002	11	7/24/2014	23:06.0
3482	RWS11	-122.459657	37.93741	-0.22	7.11	-315.9	6.88899991	-122.45965	37.93742	-0.24	7.11	-302.65	6.87699991	11	7/24/2014	23:06.1
3483	RWS11	-122.4596591	37.93741	-0.28	7.12	-318.3	6.84600022	-122.45965	37.93742	-0.24	7.12	-305.27	6.88600022	11	7/24/2014	23:06.2
3484	RWS11	-122.4596613	37.93741	-0.22	7.13	-320.6	6.90700004	-122.45966	37.93742	-0.24	7.13	-307.85	6.89500004	11	7/24/2014	23:06.3
3485	RWS11	-122.4596634	37.93741	-0.22	7.14	-323	6.91499993	-122.45966	37.93742	-0.24	7.14	-310.42	6.90299994	11	7/24/2014	23:06.4
3486	RWS11	-122.4596656	37.93741	-0.22	7.15	-325.3	6.92399976	-122.45966	37.93742	-0.2	7.15	-312.89	6.94599976	11	7/24/2014	23:06.5
3487	RWS11	-122.4596677	37.93741	-0.28	7.16	-327.6	6.88000014	-122.45966	37.93742	-0.24	7.16	-315.27	6.92000014	11	7/24/2014	23:06.6
3488	RWS11	-122.4596699	37.93741	-0.28	7.16	-329.8	6.88800004	-122.45966	37.93742	-0.24	7.16	-317.65	6.92800003	11	7/24/2014	23:06.7
3489	RWS11	-122.459672	37.93741	-0.28	7.17	-332	6.89400008	-122.45967	37.93742	-0.24	7.17	-320.1	6.93400007	11	7/24/2014	23:06.8
3490	RWS11	-122.4596741	37.93741	-0.22	7.17	-334.3	6.94999978	-122.45967	37.93742	-0.24	7.17	-322.64	6.93799978	11	7/24/2014	23:06.9
3491	RWS11	-122.4596772	37.93741	-0.28	7.18	-336.5	6.90100005	-122.45967	37.93742	-0.24	7.18	-325.21	6.94100004	11	7/24/2014	23:07.0
3492	RWS11	-122.4596793	37.93741	-0.22	7.18	-338.8	6.95300004	-122.45967	37.93742	-0.24	7.18	-327.7	6.94100004	11	7/24/2014	23:07.1
3493	RWS11	-122.4596814	37.93741	-0.28	7.18	-341.1	6.8990002	-122.45968	37.93742	-0.24	7.18	-330.15	6.93900019	11	7/24/2014	23:07.2
3494	RWS11	-122.4596835	37.93741	-0.22	7.17	-343.4	6.947	-122.45968	37.93743	-0.2	7.17	-332.53	6.969	11	7/24/2014	23:07.3
3495	RWS11	-122.4596856	37.93741	-0.28	7.17	-345.7	6.8899999	-122.45968	37.93743	-0.29	7.17	-334.89	6.87899989	11	7/24/2014	23:07.4
3496	RWS11	-122.4596877	37.93741	-0.22	7.16	-348	6.93599984	-122.45968	37.93743	-0.24	7.16	-337.34	6.92399985	11	7/24/2014	23:07.5
3497	RWS11	-122.4596898	37.93741	-0.28	7.15	-350.4	6.87599996	-122.45968	37.93743	-0.24	7.15	-340.01	6.91599995	11	7/24/2014	23:07.6

3498	RWS11	-122.4596919	37.93741	-0.22	7.14	-352.7	6.91900012	-122.45969	37.93743	-0.24	7.14	-342.52	6.90700012	11	7/24/2014	23:07.7
3499	RWS11	-122.459694	37.93742	-0.28	7.13	-354.9	6.85799983	-122.45969	37.93743	-0.24	7.13	-344.92	6.89799982	11	7/24/2014	23:07.8
3500	RWS11	-122.459696	37.93742	-0.22	7.12	-357	6.89800021	-122.45969	37.93743	-0.2	7.12	-347.24	6.92000021	11	7/24/2014	23:07.9
3501	RWS11	-122.459699	37.93742	-0.28	7.11	-359	6.83199981	-122.45969	37.93743	-0.24	7.11	-349.38	6.8719998	11	7/24/2014	23:08.0
3502	RWS11	-122.459701	37.93742	-0.22	7.09	-361	6.868	-122.4597	37.93743	-0.24	7.09	-351.24	6.85600001	11	7/24/2014	23:08.1
3503	RWS11	-122.459703	37.93742	-0.28	7.07	-362.7	6.79699996	-122.4597	37.93743	-0.24	7.07	-353.13	6.83699995	11	7/24/2014	23:08.2
3504	RWS11	-122.459705	37.93742	-0.22	7.05	-364.4	6.82800004	-122.4597	37.93743	-0.2	7.05	-355.21	6.85000004	11	7/24/2014	23:08.3
3505	RWS11	-122.459707	37.93742	-0.28	7.03	-366.2	6.75199988	-122.4597	37.93743	-0.24	7.03	-357.31	6.79199988	11	7/24/2014	23:08.4
3506	RWS11	-122.459709	37.93742	-0.28	7	-368.1	6.72499993	-122.4597	37.93743	-0.2	7	-359.49	6.79899992	11	7/24/2014	23:08.5
3507	RWS11	-122.4597109	37.93742	-0.28	6.97	-370.1	6.69700006	-122.45971	37.93743	-0.24	6.97	-361.68	6.73700005	11	7/24/2014	23:08.6
3508	RWS11	-122.4597129	37.93742	-0.22	6.94	-372	6.71899983	-122.45971	37.93743	-0.2	6.94	-363.85	6.74099983	11	7/24/2014	23:08.7
3509	RWS11	-122.4597149	37.93742	-0.28	6.91	-373.7	6.63700011	-122.45971	37.93743	-0.24	6.91	-365.88	6.67700011	11	7/24/2014	23:08.8
3510	RWS11	-122.4597169	37.93742	-0.22	6.88	-375.5	6.65899989	-122.45971	37.93743	-0.2	6.88	-367.94	6.68099989	11	7/24/2014	23:08.9
3511	RWS11	-122.4597198	37.93742	-0.22	6.85	-377.2	6.62900016	-122.45971	37.93743	-0.2	6.85	-369.87	6.65100016	11	7/24/2014	23:09.0
3512	RWS11	-122.4597218	37.93742	-0.22	6.82	-378.8	6.59999987	-122.45972	37.93744	-0.2	6.82	-371.75	6.62199987	11	7/24/2014	23:09.1
3513	RWS11	-122.4597238	37.93742	-0.22	6.79	-380.2	6.57000014	-122.45972	37.93744	-0.2	6.79	-373.53	6.59200014	11	7/24/2014	23:09.2
3514	RWS11	-122.4597258	37.93742	-0.22	6.76	-381.6	6.54099986	-122.45972	37.93744	-0.2	6.76	-375.26	6.56299986	11	7/24/2014	23:09.3
3515	RWS11	-122.4597279	37.93742	-0.22	6.74	-382.9	6.51399991	-122.45972	37.93744	-0.2	6.74	-376.91	6.53599991	11	7/24/2014	23:09.4
3516	RWS11	-122.4597299	37.93742	-0.22	6.71	-384.2	6.48699996	-122.45972	37.93744	-0.2	6.71	-378.47	6.50899996	11	7/24/2014	23:09.5
3517	RWS11	-122.459732	37.93743	-0.22	6.69	-385.6	6.4640002	-122.45973	37.93744	-0.2	6.69	-380.05	6.4860002	11	7/24/2014	23:09.6
3518	RWS11	-122.459734	37.93743	-0.22	6.67	-386.9	6.44400021	-122.45973	37.93744	-0.2	6.67	-381.69	6.46600021	11	7/24/2014	23:09.7
3519	RWS11	-122.4597361	37.93743	-0.22	6.65	-388.2	6.42700002	-122.45973	37.93744	-0.2	6.65	-383.27	6.44900002	11	7/24/2014	23:09.8
3520	RWS11	-122.4597381	37.93743	-0.22	6.64	-389.6	6.414	-122.45973	37.93744	-0.2	6.64	-384.79	6.436	11	7/24/2014	23:09.9
3521	RWS11	-122.4597411	37.93743	-0.28	6.63	-391	6.35099986	-122.45974	37.93744	-0.2	6.63	-386.15	6.42499985	11	7/24/2014	23:10.0
3522	RWS11	-122.4597432	37.93743	-0.22	6.62	-392.4	6.39499995	-122.45974	37.93744	-0.2	6.62	-387.67	6.41699995	11	7/24/2014	23:10.1
3523	RWS11	-122.4597452	37.93743	-0.28	6.61	-393.7	6.33500007	-122.45974	37.93744	-0.2	6.61	-388.88	6.40900005	11	7/24/2014	23:10.2
3524	RWS11	-122.4597473	37.93743	-0.22	6.6	-395	6.37800023	-122.45974	37.93744	-0.24	6.6	-390.11	6.36600024	11	7/24/2014	23:10.3
3525	RWS11	-122.4597494	37.93743	-0.28	6.59	-396.2	6.31699994	-122.45974	37.93744	-0.24	6.59	-391.37	6.35699993	11	7/24/2014	23:10.4
3526	RWS11	-122.4597515	37.93743	-0.22	6.58	-397.5	6.35799977	-122.45975	37.93744	-0.24	6.58	-392.65	6.34599978	11	7/24/2014	23:10.5
3527	RWS11	-122.4597536	37.93743	-0.28	6.57	-398.6	6.29299977	-122.45975	37.93744	-0.24	6.57	-393.82	6.33299977	11	7/24/2014	23:10.6
3528	RWS11	-122.4597557	37.93743	-0.22	6.55	-399.7	6.32800004	-122.45975	37.93744	-0.24	6.55	-395.1	6.31600004	11	7/24/2014	23:10.7
3529	RWS11	-122.4597578	37.93743	-0.22	6.53	-401.1	6.30899999	-122.45975	37.93745	-0.24	6.53	-396.49	6.29699999	11	7/24/2014	23:10.8
3530	RWS11	-122.4597599	37.93743	-0.22	6.51	-402.4	6.28800008	-122.45975	37.93745	-0.2	6.51	-398.02	6.31000008	11	7/24/2014	23:10.9

3531	RWS11	-122.4597629	37.93743	-0.28	6.49	-403.9	6.21299985	-122.45976	37.93745	-0.24	6.49	-399.82	6.25299984	11	7/24/2014	23:11.0
3532	RWS11	-122.459765	37.93743	-0.22	6.47	-405.3	6.24200007	-122.45976	37.93745	-0.15	6.47	-401.49	6.31400008	11	7/24/2014	23:11.1
3533	RWS11	-122.4597671	37.93743	-0.22	6.45	-406.7	6.22100016	-122.45976	37.93745	-0.24	6.45	-403.03	6.20900017	11	7/24/2014	23:11.2
3534	RWS11	-122.4597692	37.93744	-0.22	6.43	-407.9	6.20300004	-122.45976	37.93745	-0.2	6.43	-404.26	6.22500004	11	7/24/2014	23:11.3
3535	RWS11	-122.4597712	37.93744	-0.22	6.41	-408.9	6.1890001	-122.45977	37.93745	-0.24	6.41	-405.32	6.17700011	11	7/24/2014	23:11.4
3536	RWS11	-122.4597733	37.93744	-0.22	6.4	-409.9	6.17899987	-122.45977	37.93745	-0.2	6.4	-406.27	6.20099987	11	7/24/2014	23:11.5
3537	RWS11	-122.4597754	37.93744	-0.28	6.4	-410.8	6.12300017	-122.45977	37.93745	-0.24	6.4	-407.12	6.16300017	11	7/24/2014	23:11.6
3538	RWS11	-122.4597775	37.93744	-0.22	6.4	-411.6	6.17500016	-122.45977	37.93745	-0.24	6.4	-407.88	6.16300017	11	7/24/2014	23:11.7
3539	RWS11	-122.4597796	37.93744	-0.22	6.41	-412.5	6.1810002	-122.45977	37.93745	-0.24	6.41	-408.72	6.16900021	11	7/24/2014	23:11.8
3540	RWS11	-122.4597816	37.93744	-0.22	6.41	-413.4	6.19099995	-122.45978	37.93745	-0.2	6.41	-409.59	6.21299995	11	7/24/2014	23:11.9
3541	RWS11	-122.4597846	37.93744	-0.28	6.43	-414.2	6.15399984	-122.45978	37.93745	-0.24	6.43	-410.5	6.19399983	11	7/24/2014	23:12.0
3542	RWS11	-122.4597867	37.93744	-0.22	6.45	-415.1	6.22300002	-122.45978	37.93745	-0.15	6.45	-411.38	6.29500003	11	7/24/2014	23:12.1
3543	RWS11	-122.4597887	37.93744	-0.22	6.47	-415.9	6.243	-122.45978	37.93745	-0.24	6.47	-412.21	6.23100001	11	7/24/2014	23:12.2
3544	RWS11	-122.4597908	37.93744	-0.22	6.49	-416.7	6.26399991	-122.45979	37.93745	-0.2	6.49	-412.9	6.28599991	11	7/24/2014	23:12.3
3545	RWS11	-122.4597928	37.93744	-0.28	6.51	-417.4	6.23400024	-122.45979	37.93745	-0.24	6.51	-413.64	6.27400023	11	7/24/2014	23:12.4
3546	RWS11	-122.4597949	37.93744	-0.22	6.53	-418.1	6.30899999	-122.45979	37.93746	-0.2	6.53	-414.35	6.33099999	11	7/24/2014	23:12.5
3547	RWS11	-122.4597969	37.93744	-0.22	6.55	-418.8	6.33099982	-122.45979	37.93746	-0.24	6.55	-415.02	6.31899983	11	7/24/2014	23:12.6
3548	RWS11	-122.459799	37.93744	-0.22	6.58	-419.5	6.35300013	-122.45979	37.93746	-0.24	6.58	-415.76	6.34100014	11	7/24/2014	23:12.7
3549	RWS11	-122.459801	37.93744	-0.28	6.6	-420.1	6.32200006	-122.4598	37.93746	-0.2	6.6	-416.5	6.39600004	11	7/24/2014	23:12.8
3550	RWS11	-122.459803	37.93744	-0.22	6.62	-420.7	6.39499995	-122.4598	37.93746	-0.24	6.62	-417.19	6.38299996	11	7/24/2014	23:12.9
3551	RWS11	-122.459806	37.93745	-0.22	6.64	-421.2	6.41499993	-122.4598	37.93746	-0.24	6.64	-417.88	6.40299994	11	7/24/2014	23:13.0
3552	RWS11	-122.459808	37.93745	-0.22	6.66	-421.7	6.43399999	-122.4598	37.93746	-0.2	6.66	-418.58	6.45599999	11	7/24/2014	23:13.1
3553	RWS11	-122.4598101	37.93745	-0.22	6.68	-422.2	6.45399997	-122.4598	37.93746	-0.24	6.68	-419.33	6.44199997	11	7/24/2014	23:13.2
3554	RWS11	-122.4598121	37.93745	-0.22	6.7	-422.8	6.47300002	-122.45981	37.93746	-0.24	6.7	-419.98	6.46100003	11	7/24/2014	23:13.3
3555	RWS11	-122.4598141	37.93745	-0.28	6.72	-423.3	6.44100001	-122.45981	37.93746	-0.24	6.72	-420.65	6.48100001	11	7/24/2014	23:13.4
3556	RWS11	-122.4598162	37.93745	-0.22	6.74	-423.9	6.51399991	-122.45981	37.93746	-0.24	6.74	-421.39	6.50199991	11	7/24/2014	23:13.5
3557	RWS11	-122.4598182	37.93745	-0.22	6.76	-424.3	6.53499982	-122.45981	37.93746	-0.24	6.76	-421.98	6.52299982	11	7/24/2014	23:13.6
3558	RWS11	-122.4598202	37.93745	-0.22	6.78	-424.9	6.55700013	-122.45981	37.93746	-0.2	6.78	-422.46	6.57900013	11	7/24/2014	23:13.7
3559	RWS11	-122.4598223	37.93745	-0.28	6.8	-425.3	6.52699998	-122.45982	37.93746	-0.24	6.8	-423.02	6.56699997	11	7/24/2014	23:13.8
3560	RWS11	-122.4598243	37.93745	-0.22	6.82	-425.8	6.59999987	-122.45982	37.93746	-0.24	6.82	-423.5	6.58799988	11	7/24/2014	23:13.9
3561	RWS11	-122.4598273	37.93745	-0.22	6.85	-426.1	6.62200019	-122.45982	37.93746	-0.24	6.85	-423.86	6.61000019	11	7/24/2014	23:14.0
3562	RWS11	-122.4598294	37.93745	-0.22	6.87	-426.3	6.64200017	-122.45982	37.93746	-0.24	6.87	-424.15	6.63000017	11	7/24/2014	23:14.1
3563	RWS11	-122.4598314	37.93745	-0.28	6.89	-426.5	6.61000016	-122.45983	37.93747	-0.24	6.89	-424.44	6.65000015	11	7/24/2014	23:14.2

3564	RWS11	-122.4598335	37.93745	-0.22	6.91	-426.7	6.6810002	-122.45983	37.93747	-0.24	6.91	-424.76	6.66900021	11	7/24/2014	23:14.3
3565	RWS11	-122.4598356	37.93745	-0.28	6.92	-426.8	6.64699987	-122.45983	37.93747	-0.29	6.92	-424.93	6.63599986	11	7/24/2014	23:14.4
3566	RWS11	-122.4598377	37.93745	-0.22	6.94	-426.8	6.71699998	-122.45983	37.93747	-0.29	6.94	-424.98	6.65399998	11	7/24/2014	23:14.5
3567	RWS11	-122.4598398	37.93745	-0.28	6.96	-426.7	6.68099979	-122.45983	37.93747	-0.29	6.96	-425.01	6.66999978	11	7/24/2014	23:14.6
3568	RWS11	-122.459842	37.93746	-0.22	6.97	-426.7	6.74900004	-122.45984	37.93747	-0.24	6.97	-425.08	6.73700005	11	7/24/2014	23:14.7
3569	RWS11	-122.459844	37.93746	-0.28	6.99	-426.7	6.71299985	-122.45984	37.93747	-0.2	6.99	-425.19	6.78699984	11	7/24/2014	23:14.8
3570	RWS11	-122.4598462	37.93746	-0.22	7.01	-426.8	6.78100011	-122.45984	37.93747	-0.29	7.01	-425.38	6.71800011	11	7/24/2014	23:14.9
3571	RWS11	-122.4598493	37.93746	-0.28	7.02	-426.9	6.74499992	-122.45984	37.93747	-0.29	7.02	-425.47	6.73399991	11	7/24/2014	23:15.0
3572	RWS11	-122.4598515	37.93746	-0.28	7.04	-427.1	6.76100019	-122.45985	37.93747	-0.29	7.04	-425.57	6.75000018	11	7/24/2014	23:15.1
3573	RWS11	-122.4598536	37.93746	-0.28	7.05	-427.3	6.77699998	-122.45985	37.93747	-0.29	7.05	-425.69	6.76599997	11	7/24/2014	23:15.2
3574	RWS11	-122.4598558	37.93746	-0.28	7.07	-427.5	6.79299977	-122.45985	37.93747	-0.29	7.07	-425.6	6.78199977	11	7/24/2014	23:15.3
3575	RWS11	-122.459858	37.93746	-0.31	7.08	-427.6	6.77400011	-122.45985	37.93747	-0.29	7.08	-425.49	6.79700011	11	7/24/2014	23:15.4
3576	RWS11	-122.4598603	37.93746	-0.28	7.1	-427.5	6.82200006	-122.45985	37.93747	-0.29	7.1	-425.23	6.81100005	11	7/24/2014	23:15.5
3577	RWS11	-122.4598625	37.93746	-0.28	7.11	-427.2	6.83500007	-122.45986	37.93747	-0.32	7.11	-424.86	6.79000005	11	7/24/2014	23:15.6
3578	RWS11	-122.4598647	37.93746	-0.28	7.12	-426.9	6.84800008	-122.45986	37.93747	-0.32	7.12	-424.3	6.80300006	11	7/24/2014	23:15.7
3579	RWS11	-122.4598668	37.93746	-0.31	7.14	-426.5	6.82600015	-122.45986	37.93748	-0.32	7.14	-423.85	6.81500015	11	7/24/2014	23:15.8
3580	RWS11	-122.459869	37.93746	-0.31	7.15	-426.2	6.83699983	-122.45986	37.93748	-0.32	7.15	-423.54	6.82599983	11	7/24/2014	23:15.9
3581	RWS11	-122.4598722	37.93746	-0.31	7.16	-425.8	6.84600013	-122.45987	37.93748	-0.35	7.16	-423.15	6.80100015	11	7/24/2014	23:16.0
3582	RWS11	-122.4598743	37.93746	-0.31	7.16	-425.5	6.85400003	-122.45987	37.93748	-0.32	7.16	-422.81	6.84300002	11	7/24/2014	23:16.1
3583	RWS11	-122.4598764	37.93747	-0.31	7.17	-425.1	6.86000007	-122.45987	37.93748	-0.35	7.17	-422.44	6.81500009	11	7/24/2014	23:16.2
3584	RWS11	-122.4598786	37.93747	-0.31	7.17	-424.7	6.86299986	-122.45987	37.93748	-0.35	7.17	-422.14	6.81799987	11	7/24/2014	23:16.3
3585	RWS11	-122.4598807	37.93747	-0.34	7.18	-424.1	6.83200011	-122.45987	37.93748	-0.35	7.18	-421.72	6.82100013	11	7/24/2014	23:16.4
3586	RWS11	-122.4598828	37.93747	-0.31	7.18	-423.5	6.86700004	-122.45988	37.93748	-0.32	7.18	-421.34	6.85600004	11	7/24/2014	23:16.5
3587	RWS11	-122.4598849	37.93747	-0.31	7.18	-423.2	6.86799997	-122.45988	37.93748	-0.35	7.18	-421.09	6.82299998	11	7/24/2014	23:16.6
3588	RWS11	-122.459887	37.93747	-0.31	7.18	-422.9	6.8689999	-122.45988	37.93748	-0.32	7.18	-421.06	6.85799989	11	7/24/2014	23:16.7
3589	RWS11	-122.4598891	37.93747	-0.31	7.18	-422.8	6.87100023	-122.45988	37.93748	-0.35	7.18	-421.17	6.82600024	11	7/24/2014	23:16.8
3590	RWS11	-122.4598911	37.93747	-0.31	7.18	-422.7	6.87300009	-122.45989	37.93748	-0.35	7.18	-421.16	6.8280001	11	7/24/2014	23:16.9
3591	RWS11	-122.4598941	37.93747	-0.34	7.18	-422.7	6.84	-122.45989	37.93748	-0.35	7.18	-421.17	6.82900003	11	7/24/2014	23:17.0
3592	RWS11	-122.4598961	37.93747	-0.31	7.18	-422.5	6.87499994	-122.45989	37.93748	-0.35	7.18	-421.01	6.82999995	11	7/24/2014	23:17.1
3593	RWS11	-122.4598981	37.93747	-0.31	7.18	-422.2	6.87400001	-122.45989	37.93748	-0.35	7.18	-420.75	6.82900003	11	7/24/2014	23:17.2
3594	RWS11	-122.4599001	37.93747	-0.34	7.18	-421.8	6.83499989	-122.45989	37.93748	-0.35	7.18	-420.38	6.82399991	11	7/24/2014	23:17.3
3595	RWS11	-122.4599021	37.93747	-0.34	7.17	-421.4	6.82500014	-122.4599	37.93749	-0.35	7.17	-419.95	6.81400016	11	7/24/2014	23:17.4
3596	RWS11	-122.4599041	37.93747	-0.34	7.16	-420.9	6.8110002	-122.4599	37.93749	-0.35	7.16	-419.39	6.80000022	11	7/24/2014	23:17.5

3597	RWS11	-122.4599061	37.93747	-0.34	7.14	-420.4	6.79100022	-122.4599	37.93749	-0.41	7.14	-418.83	6.73000023	11	7/24/2014	23:17.6
3598	RWS11	-122.459908	37.93747	-0.34	7.11	-419.9	6.76700005	-122.4599	37.93749	-0.35	7.11	-418.26	6.75600007	11	7/24/2014	23:17.7
3599	RWS11	-122.45991	37.93747	-0.34	7.08	-419.4	6.73799977	-122.4599	37.93749	-0.35	7.08	-417.74	6.72699979	11	7/24/2014	23:17.8
3600	RWS11	-122.459912	37.93748	-0.31	7.05	-418.9	6.74100012	-122.45991	37.93749	-0.41	7.05	-417.2	6.64600012	11	7/24/2014	23:17.9
3601	RWS11	-122.4599149	37.93748	-0.34	7.02	-418.5	6.67400011	-122.45991	37.93749	-0.41	7.02	-416.68	6.61300012	11	7/24/2014	23:18.0
3602	RWS11	-122.4599169	37.93748	-0.34	6.99	-418.1	6.64299998	-122.45991	37.93749	-0.35	6.99	-416.1	6.632	11	7/24/2014	23:18.1
3603	RWS11	-122.4599189	37.93748	-0.34	6.96	-417.5	6.61400017	-122.45991	37.93749	-0.41	6.96	-415.57	6.55300018	11	7/24/2014	23:18.2
3604	RWS11	-122.459921	37.93748	-0.34	6.93	-417.1	6.58900008	-122.45992	37.93749	-0.35	6.93	-415.02	6.5780001	11	7/24/2014	23:18.3
3605	RWS11	-122.459923	37.93748	-0.4	6.91	-416.5	6.51700017	-122.45992	37.93749	-0.41	6.91	-414.41	6.50700018	11	7/24/2014	23:18.4
3606	RWS11	-122.4599251	37.93748	-0.34	6.9	-415.7	6.55299982	-122.45992	37.93749	-0.35	6.9	-413.61	6.54199985	11	7/24/2014	23:18.5
3607	RWS11	-122.4599271	37.93748	-0.4	6.89	-414.8	6.49200007	-122.45992	37.93749	-0.35	6.89	-412.73	6.53200009	11	7/24/2014	23:18.6
3608	RWS11	-122.4599292	37.93748	-0.34	6.88	-414	6.53799996	-122.45992	37.93749	-0.41	6.88	-411.82	6.47699997	11	7/24/2014	23:18.7
3609	RWS11	-122.4599312	37.93748	-0.4	6.88	-413.2	6.48699996	-122.45993	37.93749	-0.41	6.88	-411.05	6.47699997	11	7/24/2014	23:18.8
3610	RWS11	-122.4599332	37.93748	-0.4	6.89	-412.3	6.493	-122.45993	37.93749	-0.41	6.89	-410.26	6.48300001	11	7/24/2014	23:18.9
3611	RWS11	-122.4599363	37.93748	-0.4	6.9	-411.3	6.50299975	-122.45993	37.9375	-0.41	6.9	-409.37	6.49299976	11	7/24/2014	23:19.0
3612	RWS11	-122.4599384	37.93748	-0.34	6.91	-410.3	6.57000002	-122.45993	37.9375	-0.35	6.91	-408.65	6.55900005	11	7/24/2014	23:19.1
3613	RWS11	-122.4599405	37.93748	-0.4	6.94	-409.5	6.54099986	-122.45993	37.9375	-0.41	6.94	-407.94	6.53099987	11	7/24/2014	23:19.2
3614	RWS11	-122.4599426	37.93748	-0.34	6.96	-408.8	6.61799988	-122.45994	37.9375	-0.41	6.96	-407.27	6.55699989	11	7/24/2014	23:19.3
3615	RWS11	-122.4599447	37.93749	-0.4	6.99	-408.2	6.59800002	-122.45994	37.9375	-0.44	6.99	-406.62	6.55400002	11	7/24/2014	23:19.4
3616	RWS11	-122.4599468	37.93749	-0.34	7.03	-407.6	6.68499979	-122.45994	37.9375	-0.41	7.03	-405.8	6.6239998	11	7/24/2014	23:19.5
3617	RWS11	-122.4599489	37.93749	-0.4	7.07	-406.9	6.6719999	-122.45994	37.9375	-0.35	7.07	-404.91	6.71199992	11	7/24/2014	23:19.6
3618	RWS11	-122.459951	37.93749	-0.34	7.11	-406.4	6.76299986	-122.45995	37.9375	-0.41	7.11	-403.96	6.70199987	11	7/24/2014	23:19.7
3619	RWS11	-122.4599531	37.93749	-0.4	7.15	-405.6	6.75299975	-122.45995	37.9375	-0.44	7.15	-402.89	6.70899975	11	7/24/2014	23:19.8
3620	RWS11	-122.4599551	37.93749	-0.4	7.19	-404.8	6.79400012	-122.45995	37.9375	-0.44	7.19	-401.73	6.75000012	11	7/24/2014	23:19.9
3621	RWS11	-122.4599581	37.93749	-0.4	7.23	-404	6.83300015	-122.45995	37.9375	-0.44	7.23	-400.54	6.78900015	11	7/24/2014	23:20.0
3622	RWS11	-122.4599601	37.93749	-0.4	7.27	-402.9	6.87099978	-122.45995	37.9375	-0.44	7.27	-399.24	6.82699978	11	7/24/2014	23:20.1
3623	RWS11	-122.4599621	37.93749	-0.43	7.3	-401.8	6.87000018	-122.45996	37.9375	-0.44	7.3	-397.98	6.86100018	11	7/24/2014	23:20.2
3624	RWS11	-122.4599641	37.93749	-0.4	7.33	-400.6	6.93599984	-122.45996	37.9375	-0.44	7.33	-396.75	6.89199984	11	7/24/2014	23:20.3
3625	RWS11	-122.4599661	37.93749	-0.43	7.36	-399.5	6.92699987	-122.45996	37.9375	-0.49	7.36	-395.51	6.86699986	11	7/24/2014	23:20.4
3626	RWS11	-122.4599681	37.93749	-0.4	7.38	-398.5	6.98600003	-122.45996	37.9375	-0.44	7.38	-394.47	6.94200003	11	7/24/2014	23:20.5
3627	RWS11	-122.4599701	37.93749	-0.43	7.4	-397.4	6.97100002	-122.45996	37.93751	-0.49	7.4	-393.37	6.91100001	11	7/24/2014	23:20.6
3628	RWS11	-122.4599721	37.93749	-0.4	7.42	-396.4	7.02400014	-122.45997	37.93751	-0.49	7.42	-392.22	6.92900014	11	7/24/2014	23:20.7
3629	RWS11	-122.4599741	37.93749	-0.43	7.44	-395.4	7.00599986	-122.45997	37.93751	-0.49	7.44	-391.12	6.94599986	11	7/24/2014	23:20.8

3630	RWS11	-122.4599761	37.93749	-0.4	7.45	-394.4	7.0549998	-122.45997	37.93751	-0.44	7.45	-390.08	7.0109998	11	7/24/2014	23:20.9
3631	RWS11	-122.4599791	37.9375	-0.43	7.46	-393.3	7.03299981	-122.45997	37.93751	-0.49	7.46	-388.95	6.97299981	11	7/24/2014	23:21.0
3632	RWS11	-122.4599811	37.9375	-0.43	7.48	-392.3	7.04599983	-122.45998	37.93751	-0.49	7.48	-387.91	6.98599982	11	7/24/2014	23:21.1
3633	RWS11	-122.4599832	37.9375	-0.43	7.49	-391.3	7.05699998	-122.45998	37.93751	-0.49	7.49	-386.94	6.99699998	11	7/24/2014	23:21.2
3634	RWS11	-122.4599853	37.9375	-0.43	7.5	-390.4	7.06900007	-122.45998	37.93751	-0.49	7.5	-385.9	7.00900006	11	7/24/2014	23:21.3
3635	RWS11	-122.4599874	37.9375	-0.43	7.51	-389.4	7.07899982	-122.45998	37.93751	-0.49	7.51	-384.86	7.01899981	11	7/24/2014	23:21.4
3636	RWS11	-122.4599895	37.9375	-0.43	7.52	-388.4	7.08999997	-122.45998	37.93751	-0.49	7.52	-383.87	7.02999997	11	7/24/2014	23:21.5
3637	RWS11	-122.4599916	37.9375	-0.48	7.53	-387.4	7.05000013	-122.45999	37.93751	-0.49	7.53	-382.68	7.04100013	11	7/24/2014	23:21.6
3638	RWS11	-122.4599937	37.9375	-0.43	7.54	-386.6	7.11300021	-122.45999	37.93751	-0.49	7.54	-381.53	7.05300021	11	7/24/2014	23:21.7
3639	RWS11	-122.4599957	37.9375	-0.48	7.55	-385.6	7.07399982	-122.45999	37.93751	-0.52	7.55	-380.36	7.03099984	11	7/24/2014	23:21.8
3640	RWS11	-122.4599978	37.9375	-0.43	7.57	-384.6	7.13799983	-122.45999	37.93751	-0.44	7.57	-379.15	7.12899983	11	7/24/2014	23:21.9
3641	RWS11	-122.4600008	37.9375	-0.48	7.58	-383.4	7.10200018	-122.45999	37.93751	-0.52	7.58	-377.87	7.05900019	11	7/24/2014	23:22.0
3642	RWS11	-122.4600028	37.9375	-0.48	7.6	-382.3	7.1189999	-122.46	37.93751	-0.52	7.6	-376.55	7.07599992	11	7/24/2014	23:22.1
3643	RWS11	-122.4600048	37.9375	-0.48	7.62	-380.9	7.13799995	-122.46	37.93752	-0.52	7.62	-375.12	7.09499997	11	7/24/2014	23:22.2
3644	RWS11	-122.4600068	37.9375	-0.48	7.64	-379.4	7.15899986	-122.46	37.93752	-0.49	7.64	-373.56	7.14999986	11	7/24/2014	23:22.3
3645	RWS11	-122.4600088	37.9375	-0.48	7.66	-377.9	7.18300003	-122.46	37.93752	-0.52	7.66	-371.83	7.14000005	11	7/24/2014	23:22.4
3646	RWS11	-122.4600109	37.9375	-0.48	7.69	-376.3	7.20900005	-122.46	37.93752	-0.52	7.69	-370.07	7.16600007	11	7/24/2014	23:22.5
3647	RWS11	-122.4600129	37.93751	-0.48	7.72	-374.6	7.23699993	-122.46001	37.93752	-0.52	7.72	-368.41	7.19399995	11	7/24/2014	23:22.6
3648	RWS11	-122.4600149	37.93751	-0.48	7.75	-373	7.26600021	-122.46001	37.93752	-0.52	7.75	-366.8	7.22300023	11	7/24/2014	23:22.7
3649	RWS11	-122.4600168	37.93751	-0.48	7.78	-371.4	7.2979998	-122.46001	37.93752	-0.52	7.78	-365.03	7.25499982	11	7/24/2014	23:22.8
3650	RWS11	-122.4600188	37.93751	-0.48	7.81	-369.9	7.32999986	-122.46001	37.93752	-0.52	7.81	-363.45	7.28699988	11	7/24/2014	23:22.9
3651	RWS11	-122.4600218	37.93751	-0.48	7.84	-368.4	7.36299986	-122.46002	37.93752	-0.57	7.84	-361.72	7.26899987	11	7/24/2014	23:23.0
3652	RWS11	-122.4600238	37.93751	-0.48	7.88	-366.7	7.39699978	-122.46002	37.93752	-0.52	7.88	-359.97	7.35399979	11	7/24/2014	23:23.1
3653	RWS11	-122.4600257	37.93751	-0.51	7.91	-365.2	7.39599979	-122.46002	37.93752	-0.52	7.91	-358.23	7.38699979	11	7/24/2014	23:23.2
3654	RWS11	-122.4600278	37.93751	-0.48	7.95	-363.7	7.46400017	-122.46002	37.93752	-0.49	7.95	-356.72	7.45500016	11	7/24/2014	23:23.3
3655	RWS11	-122.4600298	37.93751	-0.51	7.98	-362.4	7.46300018	-122.46002	37.93752	-0.57	7.98	-355.22	7.40300018	11	7/24/2014	23:23.4
3656	RWS11	-122.4600318	37.93751	-0.48	8.01	-361.1	7.52999967	-122.46003	37.93752	-0.52	8.01	-353.64	7.48699969	11	7/24/2014	23:23.5
3657	RWS11	-122.4600339	37.93751	-0.48	8.05	-359.8	7.56400007	-122.46003	37.93752	-0.57	8.05	-351.89	7.47000009	11	7/24/2014	23:23.6
3658	RWS11	-122.4600359	37.93751	-0.48	8.08	-358.6	7.59700006	-122.46003	37.93752	-0.52	8.08	-350.14	7.55400008	11	7/24/2014	23:23.7
3659	RWS11	-122.4600379	37.93751	-0.51	8.11	-357.4	7.59600008	-122.46003	37.93753	-0.52	8.11	-348.34	7.58700007	11	7/24/2014	23:23.8
3660	RWS11	-122.4600399	37.93751	-0.51	8.14	-356	7.62799966	-122.46003	37.93753	-0.52	8.14	-346.5	7.61899966	11	7/24/2014	23:23.9
3661	RWS11	-122.460043	37.93751	-0.48	8.18	-354.8	7.69400018	-122.46004	37.93753	-0.57	8.18	-344.72	7.6000002	11	7/24/2014	23:24.0
3662	RWS11	-122.460045	37.93752	-0.48	8.21	-353.5	7.72599977	-122.46004	37.93753	-0.52	8.21	-342.9	7.68299979	11	7/24/2014	23:24.1

3663	RWS11	-122.460047	37.93752	-0.51	8.24	-352	7.72299993	-122.46004	37.93753	-0.52	8.24	-341.02	7.71399993	11	7/24/2014	23:24.2
3664	RWS11	-122.4600491	37.93752	-0.48	8.27	-350.4	7.78800005	-122.46004	37.93753	-0.57	8.27	-339.07	7.69400007	11	7/24/2014	23:24.3
3665	RWS11	-122.4600512	37.93752	-0.51	8.3	-348.7	7.78300035	-122.46005	37.93753	-0.57	8.3	-337.1	7.72300035	11	7/24/2014	23:24.4
3666	RWS11	-122.4600533	37.93752	-0.48	8.33	-346.9	7.84500021	-122.46005	37.93753	-0.57	8.33	-335	7.75100023	11	7/24/2014	23:24.5
3667	RWS11	-122.4600553	37.93752	-0.51	8.35	-345.1	7.83700025	-122.46005	37.93753	-0.57	8.35	-333.05	7.77700025	11	7/24/2014	23:24.6
3668	RWS11	-122.4600574	37.93752	-0.51	8.38	-343.4	7.86199987	-122.46005	37.93753	-0.57	8.38	-331.22	7.80199987	11	7/24/2014	23:24.7
3669	RWS11	-122.4600594	37.93752	-0.51	8.4	-341.7	7.88400018	-122.46005	37.93753	-0.57	8.4	-329.39	7.82400018	11	7/24/2014	23:24.8
3670	RWS11	-122.4600614	37.93752	-0.48	8.42	-340.1	7.93999952	-122.46006	37.93753	-0.57	8.42	-327.75	7.84599954	11	7/24/2014	23:24.9
3671	RWS11	-122.4600644	37.93752	-0.51	8.44	-338.6	7.926	-122.46006	37.93753	-0.57	8.44	-326.04	7.866	11	7/24/2014	23:25.0
3672	RWS11	-122.4600664	37.93752	-0.48	8.46	-336.9	7.97900003	-122.46006	37.93753	-0.57	8.46	-324.18	7.88500005	11	7/24/2014	23:25.1
3673	RWS11	-122.4600683	37.93752	-0.51	8.48	-335.3	7.9629997	-122.46006	37.93753	-0.57	8.48	-322.32	7.9029997	11	7/24/2014	23:25.2
3674	RWS11	-122.4600703	37.93752	-0.48	8.49	-333.8	8.01399988	-122.46006	37.93754	-0.52	8.49	-320.38	7.9709999	11	7/24/2014	23:25.3
3675	RWS11	-122.4600723	37.93752	-0.51	8.51	-332.1	7.99599969	-122.46007	37.93754	-0.57	8.51	-318.42	7.93599969	11	7/24/2014	23:25.4
3676	RWS11	-122.4600743	37.93752	-0.51	8.52	-330.5	8.00999963	-122.46007	37.93754	-0.52	8.52	-316.49	8.00099963	11	7/24/2014	23:25.5
3677	RWS11	-122.4600763	37.93752	-0.51	8.54	-328.8	8.02300012	-122.46007	37.93754	-0.61	8.54	-314.6	7.93000013	11	7/24/2014	23:25.6
3678	RWS11	-122.4600782	37.93753	-0.51	8.55	-327	8.03500021	-122.46007	37.93754	-0.57	8.55	-312.59	7.9750002	11	7/24/2014	23:25.7
3679	RWS11	-122.4600802	37.93753	-0.51	8.56	-325	8.04400003	-122.46007	37.93754	-0.57	8.56	-310.43	7.98400003	11	7/24/2014	23:25.8
3680	RWS11	-122.4600822	37.93753	-0.48	8.57	-323	8.08499998	-122.46008	37.93754	-0.61	8.57	-308.28	7.958	11	7/24/2014	23:25.9
3681	RWS11	-122.4600853	37.93753	-0.51	8.57	-321.2	8.05600011	-122.46008	37.93754	-0.61	8.57	-306.2	7.96300012	11	7/24/2014	23:26.0
3682	RWS11	-122.4600873	37.93753	-0.51	8.57	-319.4	8.05999982	-122.46008	37.93754	-0.57	8.57	-304.17	7.99999982	11	7/24/2014	23:26.1
3683	RWS11	-122.4600894	37.93753	-0.51	8.58	-317.5	8.06199968	-122.46008	37.93754	-0.57	8.58	-302.24	8.00199968	11	7/24/2014	23:26.2
3684	RWS11	-122.4600915	37.93753	-0.51	8.58	-315.8	8.06400049	-122.46009	37.93754	-0.52	8.58	-300.3	8.05500048	11	7/24/2014	23:26.3
3685	RWS11	-122.4600937	37.93753	-0.51	8.58	-314.1	8.06600034	-122.46009	37.93754	-0.57	8.58	-298.32	8.00600034	11	7/24/2014	23:26.4
3686	RWS11	-122.4600958	37.93753	-0.51	8.58	-312.3	8.06899965	-122.46009	37.93754	-0.57	8.58	-296.27	8.00899965	11	7/24/2014	23:26.5
3687	RWS11	-122.460098	37.93753	-0.51	8.59	-310.7	8.07100046	-122.46009	37.93754	-0.61	8.59	-294.3	7.97800046	11	7/24/2014	23:26.6
3688	RWS11	-122.4601002	37.93753	-0.51	8.59	-308.9	8.07300031	-122.46009	37.93754	-0.57	8.59	-292.27	8.01300031	11	7/24/2014	23:26.7
3689	RWS11	-122.4601023	37.93753	-0.51	8.59	-307.2	8.07599962	-122.4601	37.93755	-0.57	8.59	-290.16	8.01599962	11	7/24/2014	23:26.8
3690	RWS11	-122.4601044	37.93753	-0.51	8.59	-305.4	8.07899988	-122.4601	37.93755	-0.52	8.59	-288.04	8.06999987	11	7/24/2014	23:26.9
3691	RWS11	-122.4601076	37.93753	-0.51	8.6	-303.3	8.08200014	-122.4601	37.93755	-0.52	8.6	-285.75	8.07300013	11	7/24/2014	23:27.0
3692	RWS11	-122.4601097	37.93754	-0.48	8.6	-301.2	8.11799997	-122.4601	37.93755	-0.52	8.6	-283.46	8.07499999	11	7/24/2014	23:27.1
3693	RWS11	-122.4601118	37.93754	-0.57	8.6	-299	8.03499985	-122.46011	37.93755	-0.52	8.6	-281.04	8.07699984	11	7/24/2014	23:27.2
3694	RWS11	-122.4601139	37.93754	-0.48	8.6	-296.8	8.12199968	-122.46011	37.93755	-0.52	8.6	-278.54	8.0789997	11	7/24/2014	23:27.3
3695	RWS11	-122.460116	37.93754	-0.51	8.6	-294.7	8.08999956	-122.46011	37.93755	-0.52	8.6	-276.12	8.08099955	11	7/24/2014	23:27.4

3696	RWS11	-122.4601181	37.93754	-0.51	8.61	-292.6	8.09200037	-122.46011	37.93755	-0.52	8.61	-273.86	8.08300036	11	7/24/2014	23:27.5
3697	RWS11	-122.4601203	37.93754	-0.51	8.61	-290.6	8.09600008	-122.46011	37.93755	-0.52	8.61	-271.44	8.08700007	11	7/24/2014	23:27.6
3698	RWS11	-122.4601224	37.93754	-0.48	8.62	-288.5	8.13500017	-122.46012	37.93755	-0.49	8.62	-269.14	8.12600017	11	7/24/2014	23:27.7
3699	RWS11	-122.4601245	37.93754	-0.51	8.62	-286.5	8.10899961	-122.46012	37.93755	-0.52	8.62	-266.89	8.09999961	11	7/24/2014	23:27.8
3700	RWS11	-122.4601265	37.93754	-0.48	8.63	-284.5	8.15200037	-122.46012	37.93755	-0.52	8.63	-264.64	8.10900038	11	7/24/2014	23:27.9
3701	RWS11	-122.4601296	37.93754	-0.51	8.64	-282.5	8.12900007	-122.46012	37.93755	-0.52	8.64	-262.17	8.12000006	11	7/24/2014	23:28.0
3702	RWS11	-122.4601317	37.93754	-0.51	8.66	-280.4	8.1419996	-122.46013	37.93755	-0.52	8.66	-259.57	8.1329996	11	7/24/2014	23:28.1
3703	RWS11	-122.4601338	37.93754	-0.51	8.67	-278.3	8.15599954	-122.46013	37.93756	-0.57	8.67	-256.96	8.09599954	11	7/24/2014	23:28.2
3704	RWS11	-122.4601359	37.93754	-0.48	8.69	-276.1	8.20499986	-122.46013	37.93756	-0.52	8.69	-254.24	8.16199988	11	7/24/2014	23:28.3
3705	RWS11	-122.4601379	37.93754	-0.51	8.7	-273.8	8.18600023	-122.46013	37.93756	-0.57	8.7	-251.47	8.12600023	11	7/24/2014	23:28.4
3706	RWS11	-122.4601401	37.93754	-0.48	8.72	-271.5	8.23700041	-122.46013	37.93756	-0.52	8.72	-248.59	8.19400042	11	7/24/2014	23:28.5
3707	RWS11	-122.4601421	37.93755	-0.51	8.74	-269.1	8.22100008	-122.46014	37.93756	-0.49	8.74	-245.73	8.24600005	11	7/24/2014	23:28.6
3708	RWS11	-122.4601443	37.93755	-0.48	8.75	-266.8	8.2729997	-122.46014	37.93756	-0.52	8.75	-242.9	8.22999972	11	7/24/2014	23:28.7
3709	RWS11	-122.4601463	37.93755	-0.51	8.77	-264.5	8.25599992	-122.46014	37.93756	-0.52	8.77	-240.02	8.24699992	11	7/24/2014	23:28.8
3710	RWS11	-122.4601484	37.93755	-0.48	8.79	-262.2	8.30799955	-122.46014	37.93756	-0.52	8.79	-237.33	8.26499957	11	7/24/2014	23:28.9
3711	RWS11	-122.4601515	37.93755	-0.51	8.81	-259.9	8.29099977	-122.46015	37.93756	-0.57	8.81	-234.55	8.23099977	11	7/24/2014	23:29.0
3712	RWS11	-122.4601536	37.93755	-0.48	8.82	-257.5	8.34099954	-122.46015	37.93756	-0.52	8.82	-231.77	8.29799956	11	7/24/2014	23:29.1
3713	RWS11	-122.4601557	37.93755	-0.51	8.84	-255.1	8.32300031	-122.46015	37.93756	-0.52	8.84	-228.99	8.31400031	11	7/24/2014	23:29.2
3714	RWS11	-122.4601578	37.93755	-0.48	8.85	-252.8	8.37100023	-122.46015	37.93756	-0.57	8.85	-226.25	8.27700025	11	7/24/2014	23:29.3
3715	RWS11	-122.4601599	37.93755	-0.51	8.86	-250.3	8.34999979	-122.46015	37.93756	-0.52	8.86	-223.54	8.34099978	11	7/24/2014	23:29.4
3716	RWS11	-122.4601621	37.93755	-0.48	8.88	-247.9	8.3950004	-122.46016	37.93756	-0.49	8.88	-220.72	8.38600039	11	7/24/2014	23:29.5
3717	RWS11	-122.4601642	37.93755	-0.51	8.88	-245.6	8.36899984	-122.46016	37.93756	-0.52	8.88	-218.12	8.35999984	11	7/24/2014	23:29.6
3718	RWS11	-122.4601663	37.93755	-0.48	8.89	-243.3	8.40999979	-122.46016	37.93757	-0.52	8.89	-215.53	8.3669998	11	7/24/2014	23:29.7
3719	RWS11	-122.4601684	37.93755	-0.51	8.9	-241.1	8.38000047	-122.46016	37.93757	-0.52	8.9	-213.07	8.37100047	11	7/24/2014	23:29.8
3720	RWS11	-122.4601705	37.93755	-0.51	8.9	-238.8	8.38099992	-122.46016	37.93757	-0.52	8.9	-210.68	8.37199992	11	7/24/2014	23:29.9
3721	RWS11	-122.4601736	37.93756	-0.51	8.9	-236.5	8.38000047	-122.46017	37.93757	-0.52	8.9	-208.14	8.37100047	11	7/24/2014	23:30.0
3722	RWS11	-122.4601756	37.93756	-0.48	8.89	-234.2	8.41100019	-122.46017	37.93757	-0.52	8.89	-205.49	8.36800021	11	7/24/2014	23:30.1
3723	RWS11	-122.4601776	37.93756	-0.51	8.89	-231.8	8.3720001	-122.46017	37.93757	-0.57	8.89	-202.79	8.3120001	11	7/24/2014	23:30.2
3724	RWS11	-122.4601797	37.93756	-0.48	8.88	-229.3	8.39900011	-122.46017	37.93757	-0.49	8.88	-199.99	8.3900001	11	7/24/2014	23:30.3
3725	RWS11	-122.4601817	37.93756	-0.51	8.87	-226.8	8.35800016	-122.46018	37.93757	-0.49	8.87	-197.22	8.38300014	11	7/24/2014	23:30.4
3726	RWS11	-122.4601838	37.93756	-0.48	8.86	-224.4	8.38399976	-122.46018	37.93757	-0.49	8.86	-194.59	8.37499976	11	7/24/2014	23:30.5
3727	RWS11	-122.4601859	37.93756	-0.48	8.86	-222.2	8.37600034	-122.46018	37.93757	-0.49	8.86	-192.08	8.36700034	11	7/24/2014	23:30.6
3728	RWS11	-122.4601879	37.93756	-0.48	8.85	-219.9	8.36999983	-122.46018	37.93757	-0.49	8.85	-189.52	8.36099982	11	7/24/2014	23:30.7

3729	RWS11	-122.46019	37.93756	-0.48	8.85	-217.5	8.36400026	-122.46018	37.93757	-0.52	8.85	-186.88	8.32100028	11	7/24/2014	23:30.8
3730	RWS11	-122.460192	37.93756	-0.48	8.84	-215	8.35900015	-122.46019	37.93757	-0.49	8.84	-184.04	8.35000014	11	7/24/2014	23:30.9
3731	RWS11	-122.4601951	37.93756	-0.51	8.84	-212.5	8.32000005	-122.46019	37.93757	-0.49	8.84	-181.12	8.34500003	11	7/24/2014	23:31.0
3732	RWS11	-122.4601971	37.93756	-0.48	8.83	-209.8	8.35000032	-122.46019	37.93758	-0.49	8.83	-178.09	8.34100032	11	7/24/2014	23:31.1
3733	RWS11	-122.4601992	37.93756	-0.51	8.83	-207.1	8.31199968	-122.46019	37.93758	-0.52	8.83	-174.91	8.30299968	11	7/24/2014	23:31.2
3734	RWS11	-122.4602014	37.93756	-0.48	8.82	-204.3	8.34300035	-122.4602	37.93758	-0.52	8.82	-171.65	8.30000037	11	7/24/2014	23:31.3
3735	RWS11	-122.4602035	37.93756	-0.51	8.82	-201.5	8.30600011	-122.4602	37.93758	-0.49	8.82	-168.22	8.33100009	11	7/24/2014	23:31.4
3736	RWS11	-122.4602056	37.93757	-0.48	8.82	-198.7	8.33800024	-122.4602	37.93758	-0.49	8.82	-164.84	8.32900023	11	7/24/2014	23:31.5
3737	RWS11	-122.4602078	37.93757	-0.51	8.82	-195.9	8.3020004	-122.4602	37.93758	-0.52	8.82	-161.46	8.2930004	11	7/24/2014	23:31.6
3738	RWS11	-122.4602099	37.93757	-0.48	8.81	-193.1	8.33300012	-122.4602	37.93758	-0.49	8.81	-158.14	8.32400012	11	7/24/2014	23:31.7
3739	RWS11	-122.4602121	37.93757	-0.51	8.81	-190.3	8.29700029	-122.46021	37.93758	-0.52	8.81	-154.86	8.28800029	11	7/24/2014	23:31.8
3740	RWS11	-122.4602142	37.93757	-0.48	8.81	-187.6	8.32800001	-122.46021	37.93758	-0.49	8.81	-151.65	8.31900001	11	7/24/2014	23:31.9
3741	RWS11	-122.4602174	37.93757	-0.51	8.81	-184.8	8.29000032	-122.46021	37.93758	-0.52	8.81	-148.41	8.28100032	11	7/24/2014	23:32.0
3742	RWS11	-122.4602195	37.93757	-0.51	8.8	-182	8.28700006	-122.46021	37.93758	-0.52	8.8	-145.25	8.27800006	11	7/24/2014	23:32.1
3743	RWS11	-122.4602217	37.93757	-0.51	8.8	-179.3	8.28199995	-122.46022	37.93758	-0.52	8.8	-142.23	8.27299994	11	7/24/2014	23:32.2
3744	RWS11	-122.4602239	37.93757	-0.48	8.79	-176.7	8.31200022	-122.46022	37.93758	-0.52	8.79	-139.14	8.26900023	11	7/24/2014	23:32.3
3745	RWS11	-122.4602261	37.93757	-0.51	8.79	-173.8	8.27300012	-122.46022	37.93758	-0.52	8.79	-135.92	8.26400012	11	7/24/2014	23:32.4
3746	RWS11	-122.4602283	37.93757	-0.48	8.78	-170.8	8.30000013	-122.46022	37.93758	-0.49	8.78	-132.51	8.29100013	11	7/24/2014	23:32.5
3747	RWS11	-122.4602305	37.93757	-0.51	8.77	-167.8	8.25999963	-122.46022	37.93759	-0.52	8.77	-129.11	8.25099963	11	7/24/2014	23:32.6
3748	RWS11	-122.4602327	37.93757	-0.51	8.77	-164.8	8.25299966	-122.46023	37.93759	-0.52	8.77	-125.88	8.24399966	11	7/24/2014	23:32.7
3749	RWS11	-122.4602349	37.93757	-0.51	8.76	-162	8.24599969	-122.46023	37.93759	-0.57	8.76	-122.81	8.18599969	11	7/24/2014	23:32.8
3750	RWS11	-122.460237	37.93758	-0.51	8.75	-159.3	8.23899972	-122.46023	37.93759	-0.49	8.75	-119.7	8.2639997	11	7/24/2014	23:32.9
3751	RWS11	-122.4602402	37.93758	-0.51	8.75	-156.6	8.23300016	-122.46023	37.93759	-0.52	8.75	-116.54	8.22400016	11	7/24/2014	23:33.0
3752	RWS11	-122.4602424	37.93758	-0.51	8.74	-154	8.22600019	-122.46024	37.93759	-0.52	8.74	-113.07	8.21700019	11	7/24/2014	23:33.1
3753	RWS11	-122.4602445	37.93758	-0.57	8.73	-151.3	8.16800022	-122.46024	37.93759	-0.57	8.73	-109.6	8.15900022	11	7/24/2014	23:33.2
3754	RWS11	-122.4602467	37.93758	-0.51	8.73	-148.5	8.21200025	-122.46024	37.93759	-0.52	8.73	-106.16	8.20300025	11	7/24/2014	23:33.3
3755	RWS11	-122.4602489	37.93758	-0.57	8.72	-145.7	8.15400028	-122.46024	37.93759	-0.52	8.72	-102.81	8.19600028	11	7/24/2014	23:33.4
3756	RWS11	-122.4602511	37.93758	-0.51	8.71	-143.1	8.19699991	-122.46024	37.93759	-0.52	8.71	-99.606	8.1879999	11	7/24/2014	23:33.5
3757	RWS11	-122.4602532	37.93758	-0.51	8.7	-140.5	8.18800008	-122.46025	37.93759	-0.52	8.7	-96.378	8.17900008	11	7/24/2014	23:33.6
3758	RWS11	-122.4602554	37.93758	-0.51	8.69	-138	8.17900026	-122.46025	37.93759	-0.52	8.69	-93.171	8.17000026	11	7/24/2014	23:33.7
3759	RWS11	-122.4602575	37.93758	-0.57	8.68	-135.5	8.11800003	-122.46025	37.93759	-0.57	8.68	-90.012	8.10900003	11	7/24/2014	23:33.8
3760	RWS11	-122.4602596	37.93758	-0.51	8.67	-133.1	8.15800035	-122.46025	37.93759	-0.52	8.67	-86.909	8.14900035	11	7/24/2014	23:33.9
3761	RWS11	-122.4602628	37.93758	-0.51	8.66	-130.9	8.14600027	-122.46026	37.9376	-0.52	8.66	-83.86	8.13700026	11	7/24/2014	23:34.0

3762	RWS11	-122.4602649	37.93758	-0.51	8.65	-128.6	8.13400018	-122.46026	37.9376	-0.52	8.65	-80.735	8.12500018	11	7/24/2014	23:34.1
3763	RWS11	-122.460267	37.93758	-0.51	8.64	-126.4	8.12099969	-122.46026	37.9376	-0.52	8.64	-77.838	8.11199969	11	7/24/2014	23:34.2
3764	RWS11	-122.4602692	37.93759	-0.48	8.62	-124.3	8.14200014	-122.46026	37.9376	-0.52	8.62	-75.084	8.09900016	11	7/24/2014	23:34.3
3765	RWS11	-122.4602713	37.93759	-0.51	8.61	-122.3	8.09499967	-122.46027	37.9376	-0.52	8.61	-72.357	8.08599967	11	7/24/2014	23:34.4
3766	RWS11	-122.4602734	37.93759	-0.48	8.6	-120.2	8.11600012	-122.46027	37.9376	-0.49	8.6	-69.629	8.10700011	11	7/24/2014	23:34.5
3767	RWS11	-122.4602756	37.93759	-0.51	8.58	-118.2	8.06899965	-122.46027	37.9376	-0.52	8.58	-67.058	8.05999964	11	7/24/2014	23:34.6
3768	RWS11	-122.4602777	37.93759	-0.51	8.57	-116.3	8.05699956	-122.46027	37.9376	-0.52	8.57	-64.458	8.04799956	11	7/24/2014	23:34.7
3769	RWS11	-122.4602798	37.93759	-0.51	8.56	-114.2	8.04299963	-122.46027	37.9376	-0.52	8.56	-61.843	8.03399962	11	7/24/2014	23:34.8
3770	RWS11	-122.4602819	37.93759	-0.48	8.54	-112.2	8.06299967	-122.46028	37.9376	-0.52	8.54	-59.249	8.01999968	11	7/24/2014	23:34.9
3771	RWS11	-122.460285	37.93759	-0.51	8.53	-110.2	8.01299989	-122.46028	37.9376	-0.52	8.53	-56.69	8.00399989	11	7/24/2014	23:35.0
3772	RWS11	-122.4602872	37.93759	-0.51	8.51	-108.1	7.99599969	-122.46028	37.9376	-0.52	8.51	-54.143	7.98699969	11	7/24/2014	23:35.1
3773	RWS11	-122.4602893	37.93759	-0.51	8.49	-106	7.97600019	-122.46028	37.9376	-0.52	8.49	-51.693	7.96700019	11	7/24/2014	23:35.2
3774	RWS11	-122.4602914	37.93759	-0.51	8.47	-103.9	7.95599973	-122.46029	37.9376	-0.49	8.47	-49.408	7.98099971	11	7/24/2014	23:35.3
3775	RWS11	-122.4602936	37.93759	-0.51	8.45	-101.9	7.93299997	-122.46029	37.93761	-0.52	8.45	-47.316	7.92399997	11	7/24/2014	23:35.4
3776	RWS11	-122.4602957	37.93759	-0.51	8.42	-99.98	7.90800035	-122.46029	37.93761	-0.52	8.42	-45.22	7.89900035	11	7/24/2014	23:35.5
3777	RWS11	-122.4602979	37.93759	-0.51	8.4	-98.18	7.88099992	-122.46029	37.93761	-0.52	8.4	-43.321	7.87199992	11	7/24/2014	23:35.6
3778	RWS11	-122.4603	37.93759	-0.51	8.37	-96.54	7.85199964	-122.46029	37.93761	-0.52	8.37	-41.555	7.84299964	11	7/24/2014	23:35.7
3779	RWS11	-122.4603022	37.9376	-0.51	8.34	-95.1	7.82100046	-122.4603	37.93761	-0.49	8.34	-39.962	7.84600043	11	7/24/2014	23:35.8
3780	RWS11	-122.4603043	37.9376	-0.48	8.31	-93.81	7.8240003	-122.4603	37.93761	-0.49	8.31	-38.414	7.8150003	11	7/24/2014	23:35.9
3781	RWS11	-122.4603075	37.9376	-0.57	8.27	-92.59	7.70699978	-122.4603	37.93761	-0.52	8.27	-36.824	7.74899977	11	7/24/2014	23:36.0
3782	RWS11	-122.4603096	37.9376	-0.48	8.24	-91.46	7.75899976	-122.4603	37.93761	-0.49	8.24	-35.315	7.74999976	11	7/24/2014	23:36.1
3783	RWS11	-122.4603117	37.9376	-0.51	8.21	-90.45	7.69100034	-122.46031	37.93761	-0.52	8.21	-33.92	7.68200034	11	7/24/2014	23:36.2
3784	RWS11	-122.4603139	37.9376	-0.51	8.17	-89.34	7.65699995	-122.46031	37.93761	-0.49	8.17	-32.477	7.68199992	11	7/24/2014	23:36.3
3785	RWS11	-122.460316	37.9376	-0.51	8.14	-88.29	7.62299955	-122.46031	37.93761	-0.49	8.14	-31.236	7.64799953	11	7/24/2014	23:36.4
3786	RWS11	-122.4603182	37.9376	-0.48	8.1	-87.29	7.62199968	-122.46031	37.93761	-0.49	8.1	-30.057	7.61299968	11	7/24/2014	23:36.5
3787	RWS11	-122.4603204	37.9376	-0.51	8.07	-86.41	7.55400026	-122.46031	37.93761	-0.52	8.07	-28.965	7.54500026	11	7/24/2014	23:36.6
3788	RWS11	-122.4603225	37.9376	-0.48	8.03	-85.68	7.55300039	-122.46032	37.93761	-0.44	8.03	-28.181	7.59500039	11	7/24/2014	23:36.7
3789	RWS11	-122.4603246	37.9376	-0.51	8	-85.06	7.48500001	-122.46032	37.93761	-0.49	8	-27.444	7.50999999	11	7/24/2014	23:36.8
3790	RWS11	-122.4603268	37.9376	-0.48	7.97	-84.39	7.48500007	-122.46032	37.93762	-0.44	7.97	-26.678	7.52700007	11	7/24/2014	23:36.9
3791	RWS11	-122.4603299	37.9376	-0.51	7.93	-83.71	7.41700017	-122.46032	37.93762	-0.49	7.93	-25.959	7.44200015	11	7/24/2014	23:37.0
3792	RWS11	-122.460332	37.9376	-0.48	7.9	-83.1	7.41800016	-122.46033	37.93762	-0.49	7.9	-25.107	7.40900016	11	7/24/2014	23:37.1
3793	RWS11	-122.4603341	37.9376	-0.48	7.86	-82.43	7.38299984	-122.46033	37.93762	-0.44	7.86	-24.191	7.42499983	11	7/24/2014	23:37.2
3794	RWS11	-122.4603363	37.93761	-0.48	7.83	-81.73	7.34899992	-122.46033	37.93762	-0.49	7.83	-23.406	7.33999991	11	7/24/2014	23:37.3

3795	RWS11	-122.4603384	37.93761	-0.48	7.79	-81.07	7.31300014	-122.46033	37.93762	-0.44	7.79	-22.751	7.35500014	11	7/24/2014	23:37.4
3796	RWS11	-122.4603405	37.93761	-0.48	7.76	-80.53	7.27899984	-122.46033	37.93762	-0.44	7.76	-22.184	7.32099983	11	7/24/2014	23:37.5
3797	RWS11	-122.4603427	37.93761	-0.51	7.72	-79.91	7.20900005	-122.46034	37.93762	-0.49	7.72	-21.596	7.23400006	11	7/24/2014	23:37.6
3798	RWS11	-122.4603448	37.93761	-0.48	7.69	-79.39	7.20800021	-122.46034	37.93762	-0.49	7.69	-21.005	7.19900021	11	7/24/2014	23:37.7
3799	RWS11	-122.4603469	37.93761	-0.48	7.65	-78.89	7.17199996	-122.46034	37.93762	-0.49	7.65	-20.417	7.16299996	11	7/24/2014	23:37.8
3800	RWS11	-122.4603491	37.93761	-0.48	7.62	-78.41	7.13700011	-122.46034	37.93762	-0.44	7.62	-19.871	7.17900011	11	7/24/2014	23:37.9
3801	RWS11	-122.4603522	37.93761	-0.48	7.58	-78.19	7.10300019	-122.46035	37.93762	-0.44	7.58	-19.523	7.14500019	11	7/24/2014	23:38.0
3802	RWS11	-122.4603544	37.93761	-0.48	7.55	-78.06	7.0689998	-122.46035	37.93762	-0.44	7.55	-19.347	7.11099979	11	7/24/2014	23:38.1
3803	RWS11	-122.4603565	37.93761	-0.48	7.52	-77.97	7.0359998	-122.46035	37.93762	-0.44	7.52	-19.237	7.0779998	11	7/24/2014	23:38.2
3804	RWS11	-122.4603587	37.93761	-0.48	7.48	-78.11	7.00299981	-122.46035	37.93762	-0.44	7.48	-19.283	7.04499981	11	7/24/2014	23:38.3
3805	RWS11	-122.4603608	37.93761	-0.48	7.45	-78.33	6.97100022	-122.46035	37.93763	-0.44	7.45	-19.549	7.01300022	11	7/24/2014	23:38.4
3806	RWS11	-122.460363	37.93761	-0.48	7.42	-78.57	6.94000009	-122.46036	37.93763	-0.44	7.42	-19.787	6.98200008	11	7/24/2014	23:38.5
3807	RWS11	-122.4603652	37.93761	-0.48	7.39	-78.79	6.90800002	-122.46036	37.93763	-0.44	7.39	-20.093	6.95000002	11	7/24/2014	23:38.6
3808	RWS11	-122.4603674	37.93761	-0.48	7.36	-79.15	6.87699988	-122.46036	37.93763	-0.44	7.36	-20.638	6.91899988	11	7/24/2014	23:38.7
3809	RWS11	-122.4603695	37.93762	-0.48	7.33	-79.64	6.84600022	-122.46036	37.93763	-0.44	7.33	-21.293	6.88800022	11	7/24/2014	23:38.8
3810	RWS11	-122.4603717	37.93762	-0.48	7.3	-80.25	6.81500009	-122.46037	37.93763	-0.4	7.3	-22.144	6.89100006	11	7/24/2014	23:38.9
3811	RWS11	-122.4603749	37.93762	-0.48	7.26	-81.01	6.78300002	-122.46037	37.93763	-0.4	7.26	-23.016	6.859	11	7/24/2014	23:39.0
3812	RWS11	-122.460377	37.93762	-0.48	7.23	-81.89	6.75199988	-122.46037	37.93763	-0.44	7.23	-23.999	6.79399988	11	7/24/2014	23:39.1
3813	RWS11	-122.4603792	37.93762	-0.48	7.2	-82.89	6.72100022	-122.46037	37.93763	-0.44	7.2	-25.111	6.76300022	11	7/24/2014	23:39.2
3814	RWS11	-122.4603813	37.93762	-0.43	7.17	-84.02	6.74200001	-122.46038	37.93763	-0.4	7.17	-26.353	6.76699999	11	7/24/2014	23:39.3
3815	RWS11	-122.4603835	37.93762	-0.48	7.14	-85.29	6.6609998	-122.46038	37.93763	-0.44	7.14	-27.708	6.7029998	11	7/24/2014	23:39.4
3816	RWS11	-122.4603857	37.93762	-0.43	7.11	-86.57	6.68499985	-122.46038	37.93763	-0.4	7.11	-29.082	6.70999983	11	7/24/2014	23:39.5
3817	RWS11	-122.4603879	37.93762	-0.43	7.09	-87.97	6.6579999	-122.46038	37.93763	-0.44	7.09	-30.719	6.6489999	11	7/24/2014	23:39.6
3818	RWS11	-122.46039	37.93762	-0.43	7.06	-89.43	6.63299981	-122.46038	37.93763	-0.4	7.06	-32.291	6.65799978	11	7/24/2014	23:39.7
3819	RWS11	-122.4603922	37.93762	-0.43	7.04	-90.92	6.61000004	-122.46039	37.93763	-0.4	7.04	-34.011	6.63500002	11	7/24/2014	23:39.8
3820	RWS11	-122.4603943	37.93762	-0.43	7.02	-92.4	6.5869998	-122.46039	37.93763	-0.4	7.02	-35.763	6.61199978	11	7/24/2014	23:39.9
3821	RWS11	-122.4603975	37.93762	-0.43	6.99	-93.93	6.5659999	-122.46039	37.93764	-0.44	6.99	-37.64	6.55699989	11	7/24/2014	23:40.0
3822	RWS11	-122.4603997	37.93762	-0.43	6.98	-95.39	6.54699984	-122.46039	37.93764	-0.4	6.98	-39.556	6.57199982	11	7/24/2014	23:40.1
3823	RWS11	-122.4604018	37.93762	-0.43	6.96	-96.83	6.52799979	-122.4604	37.93764	-0.44	6.96	-41.414	6.51899979	11	7/24/2014	23:40.2
3824	RWS11	-122.460404	37.93762	-0.43	6.94	-98.29	6.51000014	-122.4604	37.93764	-0.4	6.94	-43.226	6.53500012	11	7/24/2014	23:40.3
3825	RWS11	-122.4604061	37.93763	-0.43	6.92	-99.72	6.49299994	-122.4604	37.93764	-0.4	6.92	-44.991	6.51799992	11	7/24/2014	23:40.4
3826	RWS11	-122.4604083	37.93763	-0.43	6.91	-101.3	6.47700015	-122.4604	37.93764	-0.4	6.91	-46.844	6.50200012	11	7/24/2014	23:40.5
3827	RWS11	-122.4604105	37.93763	-0.43	6.89	-102.9	6.4619998	-122.4604	37.93764	-0.4	6.89	-48.727	6.48699978	11	7/24/2014	23:40.6

3828	RWS11	-122.4604127	37.93763	-0.43	6.88	-104.6	6.44799986	-122.46041	37.93764	-0.4	6.88	-50.64	6.47299984	11	7/24/2014	23:40.7
3829	RWS11	-122.4604148	37.93763	-0.43	6.86	-106.3	6.43499985	-122.46041	37.93764	-0.44	6.86	-52.654	6.42599985	11	7/24/2014	23:40.8
3830	RWS11	-122.460417	37.93763	-0.43	6.85	-107.9	6.42199984	-122.46041	37.93764	-0.4	6.85	-54.612	6.44699982	11	7/24/2014	23:40.9
3831	RWS11	-122.4604202	37.93763	-0.43	6.84	-109.6	6.41000023	-122.46041	37.93764	-0.44	6.84	-56.686	6.40100023	11	7/24/2014	23:41.0
3832	RWS11	-122.4604224	37.93763	-0.43	6.83	-111.4	6.39700022	-122.46042	37.93764	-0.44	6.83	-58.694	6.38800022	11	7/24/2014	23:41.1
3833	RWS11	-122.4604245	37.93763	-0.48	6.81	-113.1	6.33199981	-122.46042	37.93764	-0.44	6.81	-60.725	6.3739998	11	7/24/2014	23:41.2
3834	RWS11	-122.4604267	37.93763	-0.43	6.8	-114.7	6.36600009	-122.46042	37.93764	-0.4	6.8	-62.643	6.39100006	11	7/24/2014	23:41.3
3835	RWS11	-122.4604289	37.93763	-0.48	6.77	-116.4	6.29400018	-122.46042	37.93764	-0.44	6.77	-64.611	6.33600017	11	7/24/2014	23:41.4
3836	RWS11	-122.4604311	37.93763	-0.43	6.75	-118.1	6.32100001	-122.46043	37.93764	-0.44	6.75	-66.685	6.31200001	11	7/24/2014	23:41.5
3837	RWS11	-122.4604333	37.93763	-0.43	6.72	-119.9	6.2909998	-122.46043	37.93765	-0.44	6.72	-68.933	6.2819998	11	7/24/2014	23:41.6
3838	RWS11	-122.4604355	37.93763	-0.43	6.68	-121.6	6.25500003	-122.46043	37.93765	-0.4	6.68	-71.199	6.28	11	7/24/2014	23:41.7
3839	RWS11	-122.4604377	37.93763	-0.43	6.64	-123.3	6.21599999	-122.46043	37.93765	-0.44	6.64	-73.513	6.20699999	11	7/24/2014	23:41.8
3840	RWS11	-122.4604399	37.93763	-0.43	6.6	-125.1	6.17199984	-122.46043	37.93765	-0.44	6.6	-75.848	6.16299984	11	7/24/2014	23:41.9
3841	RWS11	-122.4604431	37.93764	-0.43	6.56	-127	6.12700024	-122.46044	37.93765	-0.49	6.56	-78.358	6.06700024	11	7/24/2014	23:42.0
3842	RWS11	-122.4604453	37.93764	-0.43	6.51	-128.9	6.07999983	-122.46044	37.93765	-0.44	6.51	-80.759	6.07099983	11	7/24/2014	23:42.1
3843	RWS11	-122.4604474	37.93764	-0.43	6.46	-130.8	6.03399983	-122.46044	37.93765	-0.49	6.46	-83.003	5.97399983	11	7/24/2014	23:42.2
3844	RWS11	-122.4604496	37.93764	-0.43	6.42	-132.8	5.99000016	-122.46044	37.93765	-0.44	6.42	-85.36	5.98100016	11	7/24/2014	23:42.3
3845	RWS11	-122.4604518	37.93764	-0.48	6.38	-134.9	5.8990002	-122.46045	37.93765	-0.49	6.38	-88.288	5.89000019	11	7/24/2014	23:42.4
3846	RWS11	-122.460454	37.93764	-0.43	6.34	-137.1	5.91300002	-122.46045	37.93765	-0.44	6.34	-91.224	5.90400001	11	7/24/2014	23:42.5
3847	RWS11	-122.4604562	37.93764	-0.48	6.31	-139.4	5.83099988	-122.46045	37.93765	-0.49	6.31	-94.196	5.82199988	11	7/24/2014	23:42.6
3848	RWS11	-122.4604584	37.93764	-0.43	6.28	-141.8	5.85599986	-122.46045	37.93765	-0.44	6.28	-97.125	5.84699985	11	7/24/2014	23:42.7
3849	RWS11	-122.4604606	37.93764	-0.43	6.27	-144.2	5.8369998	-122.46046	37.93765	-0.44	6.27	-100.22	5.8279998	11	7/24/2014	23:42.8
3850	RWS11	-122.4604627	37.93764	-0.43	6.25	-146.6	5.82399979	-122.46046	37.93765	-0.44	6.25	-103.18	5.81499979	11	7/24/2014	23:42.9
3851	RWS11	-122.460466	37.93764	-0.48	6.25	-149.2	5.76599982	-122.46046	37.93765	-0.49	6.25	-106.26	5.75699982	11	7/24/2014	23:43.0
3852	RWS11	-122.4604682	37.93764	-0.43	6.24	-151.6	5.81400004	-122.46046	37.93765	-0.44	6.24	-109.28	5.80500004	11	7/24/2014	23:43.1
3853	RWS11	-122.4604704	37.93764	-0.43	6.24	-154.1	5.81499997	-122.46046	37.93766	-0.44	6.24	-112.33	5.80599996	11	7/24/2014	23:43.2
3854	RWS11	-122.4604726	37.93764	-0.43	6.25	-156.6	5.81900015	-122.46047	37.93766	-0.4	6.25	-115.4	5.84400013	11	7/24/2014	23:43.3
3855	RWS11	-122.4604749	37.93764	-0.43	6.25	-159.2	5.82399979	-122.46047	37.93766	-0.44	6.25	-118.68	5.81499979	11	7/24/2014	23:43.4
3856	RWS11	-122.4604772	37.93764	-0.39	6.26	-161.9	5.8639999	-122.46047	37.93766	-0.44	6.26	-121.87	5.8199999	11	7/24/2014	23:43.5
3857	RWS11	-122.4604794	37.93765	-0.43	6.26	-164.5	5.83300009	-122.46047	37.93766	-0.4	6.26	-125.13	5.85800007	11	7/24/2014	23:43.6
3858	RWS11	-122.4604817	37.93765	-0.39	6.26	-167.3	5.86900002	-122.46048	37.93766	-0.44	6.26	-128.52	5.82500002	11	7/24/2014	23:43.7
3859	RWS11	-122.4604839	37.93765	-0.43	6.26	-170	5.83400002	-122.46048	37.93766	-0.44	6.26	-131.99	5.82500002	11	7/24/2014	23:43.8
3860	RWS11	-122.4604862	37.93765	-0.43	6.26	-172.7	5.83100024	-122.46048	37.93766	-0.44	6.26	-135.42	5.82200024	11	7/24/2014	23:43.9

3861	RWS11	-122.4604895	37.93765	-0.43	6.25	-175.5	5.8250002	-122.46048	37.93766	-0.44	6.25	-138.84	5.81600019	11	7/24/2014	23:44.0
3862	RWS11	-122.4604918	37.93765	-0.43	6.25	-178.2	5.81699982	-122.46049	37.93766	-0.44	6.25	-142.28	5.80799982	11	7/24/2014	23:44.1
3863	RWS11	-122.4604941	37.93765	-0.43	6.24	-180.9	5.80700007	-122.46049	37.93766	-0.44	6.24	-145.56	5.79800007	11	7/24/2014	23:44.2
3864	RWS11	-122.4604964	37.93765	-0.39	6.22	-183.5	5.83099991	-122.46049	37.93766	-0.4	6.22	-148.78	5.82099989	11	7/24/2014	23:44.3
3865	RWS11	-122.4604987	37.93765	-0.43	6.21	-186.1	5.7829999	-122.46049	37.93766	-0.44	6.21	-152	5.7739999	11	7/24/2014	23:44.4
3866	RWS11	-122.4605011	37.93765	-0.39	6.2	-188.8	5.80399996	-122.4605	37.93766	-0.4	6.2	-155.29	5.79399994	11	7/24/2014	23:44.5
3867	RWS11	-122.4605034	37.93765	-0.43	6.18	-191.4	5.75500003	-122.4605	37.93766	-0.4	6.18	-158.63	5.78	11	7/24/2014	23:44.6
3868	RWS11	-122.4605057	37.93765	-0.43	6.17	-194.1	5.74000016	-122.4605	37.93766	-0.44	6.17	-162.04	5.73100016	11	7/24/2014	23:44.7
3869	RWS11	-122.460508	37.93765	-0.43	6.16	-196.8	5.72600022	-122.4605	37.93767	-0.44	6.16	-165.33	5.71700022	11	7/24/2014	23:44.8
3870	RWS11	-122.4605103	37.93765	-0.43	6.14	-199.5	5.71300021	-122.4605	37.93767	-0.4	6.14	-168.63	5.73800018	11	7/24/2014	23:44.9
3871	RWS11	-122.4605137	37.93765	-0.43	6.13	-202.3	5.7000002	-122.46051	37.93767	-0.4	6.13	-171.97	5.72500017	11	7/24/2014	23:45.0
3872	RWS11	-122.460516	37.93765	-0.39	6.12	-205.2	5.72200018	-122.46051	37.93767	-0.4	6.12	-175.35	5.71200016	11	7/24/2014	23:45.1
3873	RWS11	-122.4605183	37.93766	-0.43	6.11	-207.9	5.67600003	-122.46051	37.93767	-0.44	6.11	-178.73	5.66700003	11	7/24/2014	23:45.2
3874	RWS11	-122.4605206	37.93766	-0.43	6.09	-210.7	5.66499987	-122.46052	37.93767	-0.4	6.09	-182.11	5.68999985	11	7/24/2014	23:45.3
3875	RWS11	-122.4605228	37.93766	-0.43	6.08	-213.5	5.65400019	-122.46052	37.93767	-0.44	6.08	-185.33	5.64500019	11	7/24/2014	23:45.4
3876	RWS11	-122.4605251	37.93766	-0.43	6.07	-216.1	5.64399996	-122.46052	37.93767	-0.4	6.07	-188.63	5.66899994	11	7/24/2014	23:45.5
3877	RWS11	-122.4605274	37.93766	-0.43	6.06	-218.8	5.63400021	-122.46052	37.93767	-0.4	6.06	-191.94	5.65900019	11	7/24/2014	23:45.6
3878	RWS11	-122.4605297	37.93766	-0.43	6.05	-221.5	5.62499991	-122.46052	37.93767	-0.4	6.05	-195.31	5.64999989	11	7/24/2014	23:45.7
3879	RWS11	-122.460532	37.93766	-0.43	6.05	-224.2	5.61700001	-122.46053	37.93767	-0.44	6.05	-198.68	5.60800001	11	7/24/2014	23:45.8
3880	RWS11	-122.4605342	37.93766	-0.43	6.04	-226.9	5.61099997	-122.46053	37.93767	-0.4	6.04	-201.93	5.63599995	11	7/24/2014	23:45.9
3881	RWS11	-122.4605375	37.93766	-0.43	6.03	-229.5	5.60499993	-122.46053	37.93767	-0.44	6.03	-205.15	5.59599993	11	7/24/2014	23:46.0
3882	RWS11	-122.4605397	37.93766	-0.43	6.03	-232.1	5.60200015	-122.46053	37.93767	-0.4	6.03	-208.28	5.62700012	11	7/24/2014	23:46.1
3883	RWS11	-122.460542	37.93766	-0.43	6.03	-234.6	5.59799996	-122.46054	37.93767	-0.44	6.03	-211.27	5.58899996	11	7/24/2014	23:46.2
3884	RWS11	-122.4605442	37.93766	-0.43	6.03	-237.1	5.59600011	-122.46054	37.93767	-0.44	6.03	-214.22	5.5870001	11	7/24/2014	23:46.3
3885	RWS11	-122.4605464	37.93766	-0.48	6.02	-239.5	5.54199985	-122.46054	37.93768	-0.44	6.02	-217.16	5.58399984	11	7/24/2014	23:46.4
3886	RWS11	-122.4605487	37.93766	-0.43	6.02	-241.9	5.59099999	-122.46054	37.93768	-0.44	6.02	-220.1	5.58199999	11	7/24/2014	23:46.5
3887	RWS11	-122.4605509	37.93766	-0.43	6.02	-244.3	5.58900014	-122.46055	37.93768	-0.49	6.02	-222.99	5.52900013	11	7/24/2014	23:46.6
3888	RWS11	-122.4605531	37.93766	-0.43	6.02	-246.7	5.5869998	-122.46055	37.93768	-0.44	6.02	-225.77	5.5779998	11	7/24/2014	23:46.7
3889	RWS11	-122.4605553	37.93766	-0.43	6.01	-249	5.58599988	-122.46055	37.93768	-0.44	6.01	-228.65	5.57699987	11	7/24/2014	23:46.8
3890	RWS11	-122.4605575	37.93767	-0.43	6.01	-251.4	5.58599988	-122.46055	37.93768	-0.44	6.01	-231.49	5.57699987	11	7/24/2014	23:46.9
3891	RWS11	-122.4605608	37.93767	-0.43	6.01	-253.8	5.58599988	-122.46056	37.93768	-0.44	6.01	-234.23	5.57699987	11	7/24/2014	23:47.0
3892	RWS11	-122.4605629	37.93767	-0.43	6.01	-256.1	5.58599988	-122.46056	37.93768	-0.44	6.01	-236.87	5.57699987	11	7/24/2014	23:47.1
3893	RWS11	-122.4605651	37.93767	-0.43	6.02	-258.4	5.5869998	-122.46056	37.93768	-0.49	6.02	-239.37	5.5269998	11	7/24/2014	23:47.2

3894	RWS11	-122.4605673	37.93767	-0.43	6.02	-260.6	5.58800021	-122.46056	37.93768	-0.49	6.02	-241.93	5.52800021	11	7/24/2014	23:47.3
3895	RWS11	-122.4605695	37.93767	-0.48	6.02	-262.8	5.53700021	-122.46056	37.93768	-0.49	6.02	-244.39	5.52800021	11	7/24/2014	23:47.4
3896	RWS11	-122.4605717	37.93767	-0.43	6.02	-264.9	5.58800021	-122.46057	37.93768	-0.44	6.02	-246.91	5.5790002	11	7/24/2014	23:47.5
3897	RWS11	-122.4605739	37.93767	-0.48	6.02	-267	5.5359998	-122.46057	37.93768	-0.49	6.02	-249.4	5.5269998	11	7/24/2014	23:47.6
3898	RWS11	-122.4605761	37.93767	-0.48	6.01	-269	5.53300002	-122.46057	37.93768	-0.44	6.01	-252.04	5.57500002	11	7/24/2014	23:47.7
3899	RWS11	-122.4605782	37.93767	-0.51	6.01	-271	5.49600023	-122.46057	37.93768	-0.49	6.01	-254.74	5.52100024	11	7/24/2014	23:47.8
3900	RWS11	-122.4605804	37.93767	-0.48	6	-273	5.5240002	-122.46058	37.93768	-0.49	6	-257.36	5.51500019	11	7/24/2014	23:47.9
3901	RWS11	-122.4605836	37.93767	-0.48	6	-274.9	5.51800016	-122.46058	37.93769	-0.49	6	-259.79	5.50900015	11	7/24/2014	23:48.0
3902	RWS11	-122.4605858	37.93767	-0.48	5.99	-276.8	5.50999978	-122.46058	37.93769	-0.49	5.99	-262.19	5.50099978	11	7/24/2014	23:48.1
3903	RWS11	-122.4605879	37.93767	-0.48	5.98	-278.6	5.50199988	-122.46058	37.93769	-0.49	5.98	-264.45	5.49299988	11	7/24/2014	23:48.2
3904	RWS11	-122.4605901	37.93767	-0.48	5.97	-280.4	5.49300006	-122.46058	37.93769	-0.52	5.97	-266.63	5.45000005	11	7/24/2014	23:48.3
3905	RWS11	-122.4605922	37.93767	-0.48	5.96	-282.2	5.48299983	-122.46059	37.93769	-0.52	5.96	-268.72	5.43999982	11	7/24/2014	23:48.4
3906	RWS11	-122.4605944	37.93768	-0.48	5.95	-283.9	5.47400001	-122.46059	37.93769	-0.52	5.95	-270.68	5.43099999	11	7/24/2014	23:48.5
3907	RWS11	-122.4605966	37.93768	-0.51	5.94	-285.6	5.42999977	-122.46059	37.93769	-0.57	5.94	-272.63	5.36999977	11	7/24/2014	23:48.6
3908	RWS11	-122.4605988	37.93768	-0.51	5.93	-287.3	5.41900009	-122.46059	37.93769	-0.52	5.93	-274.61	5.41000009	11	7/24/2014	23:48.7
3909	RWS11	-122.460601	37.93768	-0.51	5.92	-289.1	5.40799993	-122.4606	37.93769	-0.57	5.92	-276.79	5.34799993	11	7/24/2014	23:48.8
3910	RWS11	-122.4606031	37.93768	-0.51	5.91	-290.9	5.39499992	-122.4606	37.93769	-0.52	5.91	-278.98	5.38599992	11	7/24/2014	23:48.9
3911	RWS11	-122.4606064	37.93768	-0.51	5.9	-292.7	5.38199991	-122.4606	37.93769	-0.52	5.9	-281.22	5.37299991	11	7/24/2014	23:49.0
3912	RWS11	-122.4606086	37.93768	-0.51	5.88	-294.4	5.36700004	-122.4606	37.93769	-0.52	5.88	-283.35	5.35800004	11	7/24/2014	23:49.1
3913	RWS11	-122.4606108	37.93768	-0.51	5.86	-296.2	5.35099977	-122.46061	37.93769	-0.57	5.86	-285.45	5.29099977	11	7/24/2014	23:49.2
3914	RWS11	-122.4606131	37.93768	-0.51	5.85	-297.9	5.33499998	-122.46061	37.93769	-0.52	5.85	-287.5	5.32599998	11	7/24/2014	23:49.3
3915	RWS11	-122.4606154	37.93768	-0.57	5.83	-299.6	5.26800019	-122.46061	37.93769	-0.57	5.83	-289.46	5.25900018	11	7/24/2014	23:49.4
3916	RWS11	-122.4606176	37.93768	-0.51	5.82	-301.3	5.30299991	-122.46061	37.93769	-0.52	5.82	-291.46	5.29399991	11	7/24/2014	23:49.5
3917	RWS11	-122.4606199	37.93768	-0.57	5.8	-303.1	5.23799998	-122.46061	37.93769	-0.57	5.8	-293.57	5.22899997	11	7/24/2014	23:49.6
3918	RWS11	-122.4606222	37.93768	-0.51	5.79	-304.8	5.27599996	-122.46062	37.93769	-0.52	5.79	-295.65	5.26699996	11	7/24/2014	23:49.7
3919	RWS11	-122.4606244	37.93768	-0.57	5.78	-306.6	5.21299988	-122.46062	37.9377	-0.52	5.78	-297.73	5.25499988	11	7/24/2014	23:49.8
3920	RWS11	-122.4606266	37.93768	-0.51	5.77	-308.4	5.25500005	-122.46062	37.9377	-0.57	5.77	-299.88	5.19500005	11	7/24/2014	23:49.9
3921	RWS11	-122.4606299	37.93768	-0.57	5.76	-310.2	5.19700009	-122.46062	37.9377	-0.52	5.76	-301.96	5.23900008	11	7/24/2014	23:50.0
3922	RWS11	-122.4606321	37.93768	-0.51	5.76	-312	5.24200004	-122.46063	37.9377	-0.57	5.76	-304.06	5.18200004	11	7/24/2014	23:50.1
3923	RWS11	-122.4606343	37.93769	-0.51	5.75	-313.9	5.23899978	-122.46063	37.9377	-0.52	5.75	-306.12	5.22999978	11	7/24/2014	23:50.2
3924	RWS11	-122.4606365	37.93769	-0.51	5.75	-315.8	5.23699993	-122.46063	37.9377	-0.52	5.75	-308.21	5.22799993	11	7/24/2014	23:50.3
3925	RWS11	-122.4606387	37.93769	-0.57	5.75	-317.6	5.18599993	-122.46063	37.9377	-0.57	5.75	-310.25	5.17699993	11	7/24/2014	23:50.4
3926	RWS11	-122.4606409	37.93769	-0.51	5.75	-319.4	5.23799986	-122.46064	37.9377	-0.52	5.75	-312.33	5.22899985	11	7/24/2014	23:50.5

3927	RWS11	-122.4606431	37.93769	-0.51	5.76	-321.3	5.24200004	-122.46064	37.9377	-0.52	5.76	-314.41	5.23300004	11	7/24/2014	23:50.6
3928	RWS11	-122.4606453	37.93769	-0.51	5.76	-323.1	5.24700016	-122.46064	37.9377	-0.52	5.76	-316.51	5.23800015	11	7/24/2014	23:50.7
3929	RWS11	-122.4606474	37.93769	-0.51	5.77	-324.9	5.2530002	-122.46064	37.9377	-0.52	5.77	-318.61	5.2440002	11	7/24/2014	23:50.8
3930	RWS11	-122.4606496	37.93769	-0.51	5.78	-326.7	5.2610001	-122.46064	37.9377	-0.49	5.78	-320.71	5.2860001	11	7/24/2014	23:50.9
3931	RWS11	-122.4606528	37.93769	-0.51	5.78	-328.5	5.26899999	-122.46065	37.9377	-0.49	5.78	-322.72	5.294	11	7/24/2014	23:51.0
3932	RWS11	-122.460655	37.93769	-0.51	5.79	-330.3	5.27699989	-122.46065	37.9377	-0.52	5.79	-324.72	5.26799989	11	7/24/2014	23:51.1
3933	RWS11	-122.4606572	37.93769	-0.57	5.8	-332.1	5.23299986	-122.46065	37.9377	-0.49	5.8	-326.56	5.30899987	11	7/24/2014	23:51.2
3934	RWS11	-122.4606594	37.93769	-0.51	5.8	-333.9	5.2899999	-122.46065	37.9377	-0.49	5.8	-328.46	5.31499991	11	7/24/2014	23:51.3
3935	RWS11	-122.4606616	37.93769	-0.51	5.81	-335.7	5.29400009	-122.46066	37.9377	-0.52	5.81	-330.44	5.28500009	11	7/24/2014	23:51.4
3936	RWS11	-122.4606638	37.93769	-0.51	5.81	-337.4	5.29500002	-122.46066	37.93771	-0.49	5.81	-332.36	5.32000002	11	7/24/2014	23:51.5
3937	RWS11	-122.4606661	37.93769	-0.51	5.81	-339.2	5.29400009	-122.46066	37.93771	-0.52	5.81	-334.33	5.28500009	11	7/24/2014	23:51.6
3938	RWS11	-122.4606683	37.93769	-0.48	5.8	-341	5.32399991	-122.46066	37.93771	-0.44	5.8	-336.24	5.36599991	11	7/24/2014	23:51.7
3939	RWS11	-122.4606705	37.93769	-0.51	5.8	-342.8	5.28200001	-122.46067	37.93771	-0.49	5.8	-338.04	5.30700001	11	7/24/2014	23:51.8
3940	RWS11	-122.4606726	37.93769	-0.48	5.78	-344.5	5.30499986	-122.46067	37.93771	-0.49	5.78	-339.79	5.29599985	11	7/24/2014	23:51.9
3941	RWS11	-122.4606759	37.9377	-0.51	5.77	-346.3	5.25500005	-122.46067	37.93771	-0.49	5.77	-341.5	5.28000006	11	7/24/2014	23:52.0
3942	RWS11	-122.460678	37.9377	-0.48	5.75	-348	5.26900008	-122.46067	37.93771	-0.44	5.75	-343.24	5.31100008	11	7/24/2014	23:52.1
3943	RWS11	-122.4606802	37.9377	-0.48	5.73	-349.6	5.24599984	-122.46068	37.93771	-0.49	5.73	-344.93	5.23699984	11	7/24/2014	23:52.2
3944	RWS11	-122.4606823	37.9377	-0.48	5.7	-351.1	5.21899989	-122.46068	37.93771	-0.4	5.7	-346.55	5.29499987	11	7/24/2014	23:52.3
3945	RWS11	-122.4606845	37.9377	-0.48	5.67	-352.7	5.18900016	-122.46068	37.93771	-0.49	5.67	-348.37	5.18000016	11	7/24/2014	23:52.4
3946	RWS11	-122.4606867	37.9377	-0.48	5.64	-354.3	5.15600017	-122.46068	37.93771	-0.44	5.64	-349.93	5.19800016	11	7/24/2014	23:52.5
3947	RWS11	-122.4606889	37.9377	-0.48	5.6	-355.7	5.12099984	-122.46068	37.93771	-0.44	5.6	-351.59	5.16299984	11	7/24/2014	23:52.6
3948	RWS11	-122.460691	37.9377	-0.48	5.56	-357.2	5.08400014	-122.46069	37.93771	-0.44	5.56	-353.05	5.12600014	11	7/24/2014	23:52.7
3949	RWS11	-122.4606932	37.9377	-0.48	5.53	-358.6	5.04500011	-122.46069	37.93771	-0.44	5.53	-354.67	5.0870001	11	7/24/2014	23:52.8
3950	RWS11	-122.4606953	37.9377	-0.48	5.49	-360.1	5.00500014	-122.46069	37.93771	-0.44	5.49	-356.25	5.04700014	11	7/24/2014	23:52.9
3951	RWS11	-122.4606985	37.9377	-0.48	5.45	-361.4	4.96600011	-122.46069	37.93771	-0.44	5.45	-357.72	5.00800011	11	7/24/2014	23:53.0
3952	RWS11	-122.4607007	37.9377	-0.48	5.41	-362.7	4.928	-122.4607	37.93771	-0.4	5.41	-359.17	5.00399998	11	7/24/2014	23:53.1
3953	RWS11	-122.4607029	37.9377	-0.48	5.37	-364	4.89300016	-122.4607	37.93772	-0.44	5.37	-360.67	4.93500015	11	7/24/2014	23:53.2
3954	RWS11	-122.4607051	37.9377	-0.48	5.34	-365.2	4.85900024	-122.4607	37.93772	-0.4	5.34	-361.97	4.93500021	11	7/24/2014	23:53.3
3955	RWS11	-122.4607073	37.9377	-0.48	5.31	-366.3	4.82900003	-122.4607	37.93772	-0.44	5.31	-363.25	4.87100002	11	7/24/2014	23:53.4
3956	RWS11	-122.4607095	37.9377	-0.43	5.28	-367.4	4.85200015	-122.4607	37.93772	-0.4	5.28	-364.65	4.87700012	11	7/24/2014	23:53.5
3957	RWS11	-122.4607117	37.9377	-0.43	5.26	-368.4	4.82799998	-122.46071	37.93772	-0.44	5.26	-365.87	4.81899998	11	7/24/2014	23:53.6
3958	RWS11	-122.4607139	37.93771	-0.43	5.24	-369.4	4.808	-122.46071	37.93772	-0.44	5.24	-367.04	4.79899999	11	7/24/2014	23:53.7
3959	RWS11	-122.4607161	37.93771	-0.43	5.22	-370.6	4.7920002	-122.46071	37.93772	-0.4	5.22	-368.16	4.81700018	11	7/24/2014	23:53.8

3960	RWS11	-122.4607183	37.93771	-0.43	5.21	-371.6	4.78000012	-122.46071	37.93772	-0.4	5.21	-369.25	4.8050001	11	7/24/2014	23:53.9
3961	RWS11	-122.4607216	37.93771	-0.43	5.2	-372.7	4.76999989	-122.46072	37.93772	-0.4	5.2	-370.16	4.79499987	11	7/24/2014	23:54.0
3962	RWS11	-122.4607238	37.93771	-0.43	5.19	-373.8	4.76399985	-122.46072	37.93772	-0.4	5.19	-371.2	4.78899983	11	7/24/2014	23:54.1
3963	RWS11	-122.460726	37.93771	-0.43	5.19	-374.9	4.76100007	-122.46072	37.93772	-0.44	5.19	-372.19	4.75200006	11	7/24/2014	23:54.2
3964	RWS11	-122.4607282	37.93771	-0.43	5.19	-375.8	4.76100007	-122.46072	37.93772	-0.44	5.19	-372.95	4.75200006	11	7/24/2014	23:54.3
3965	RWS11	-122.4607304	37.93771	-0.43	5.19	-376.8	4.76299992	-122.46073	37.93772	-0.44	5.19	-373.75	4.75399992	11	7/24/2014	23:54.4
3966	RWS11	-122.4607326	37.93771	-0.43	5.2	-377.7	4.76700011	-122.46073	37.93772	-0.44	5.2	-374.55	4.75800011	11	7/24/2014	23:54.5
3967	RWS11	-122.4607348	37.93771	-0.48	5.2	-378.5	4.72400001	-122.46073	37.93772	-0.44	5.2	-375.46	4.766	11	7/24/2014	23:54.6
3968	RWS11	-122.4607371	37.93771	-0.43	5.21	-379.3	4.78500023	-122.46073	37.93772	-0.44	5.21	-376.28	4.77600023	11	7/24/2014	23:54.7
3969	RWS11	-122.4607393	37.93771	-0.43	5.23	-380.1	4.79900017	-122.46073	37.93772	-0.49	5.23	-377.11	4.73900017	11	7/24/2014	23:54.8
3970	RWS11	-122.4607415	37.93771	-0.39	5.24	-381	4.84999996	-122.46074	37.93772	-0.4	5.24	-378.17	4.83999994	11	7/24/2014	23:54.9
3971	RWS11	-122.4607447	37.93771	-0.43	5.26	-381.9	4.83300009	-122.46074	37.93773	-0.44	5.26	-379.14	4.82400009	11	7/24/2014	23:55.0
3972	RWS11	-122.4607469	37.93771	-0.39	5.28	-383	4.889	-122.46074	37.93773	-0.44	5.28	-380.21	4.845	11	7/24/2014	23:55.1
3973	RWS11	-122.460749	37.93771	-0.43	5.3	-384.1	4.87499991	-122.46074	37.93773	-0.44	5.3	-381.36	4.86599991	11	7/24/2014	23:55.2
3974	RWS11	-122.4607512	37.93771	-0.39	5.33	-385	4.93200022	-122.46075	37.93773	-0.4	5.33	-382.29	4.9220002	11	7/24/2014	23:55.3
3975	RWS11	-122.4607534	37.93771	-0.43	5.35	-385.9	4.9170002	-122.46075	37.93773	-0.44	5.35	-383.09	4.9080002	11	7/24/2014	23:55.4
3976	RWS11	-122.4607556	37.93772	-0.39	5.37	-386.6	4.97200018	-122.46075	37.93773	-0.44	5.37	-383.69	4.92800018	11	7/24/2014	23:55.5
3977	RWS11	-122.4607578	37.93772	-0.43	5.38	-387.2	4.95499983	-122.46075	37.93773	-0.49	5.38	-384.37	4.89499983	11	7/24/2014	23:55.6
3978	RWS11	-122.46076	37.93772	-0.39	5.4	-387.8	5.00799996	-122.46075	37.93773	-0.49	5.4	-384.93	4.91299996	11	7/24/2014	23:55.7
3979	RWS11	-122.4607622	37.93772	-0.43	5.42	-388.5	4.98900023	-122.46076	37.93773	-0.49	5.42	-385.49	4.92900023	11	7/24/2014	23:55.8
3980	RWS11	-122.4607643	37.93772	-0.43	5.43	-388.9	5.0040001	-122.46076	37.93773	-0.44	5.43	-385.88	4.99500009	11	7/24/2014	23:55.9
3981	RWS11	-122.4607675	37.93772	-0.43	5.45	-389.4	5.01800004	-122.46076	37.93773	-0.49	5.45	-386.18	4.95800003	11	7/24/2014	23:56.0
3982	RWS11	-122.4607697	37.93772	-0.43	5.46	-389.8	5.03100005	-122.46076	37.93773	-0.49	5.46	-386.55	4.97100005	11	7/24/2014	23:56.1
3983	RWS11	-122.4607719	37.93772	-0.43	5.47	-390.1	5.0420002	-122.46077	37.93773	-0.49	5.47	-386.88	4.9820002	11	7/24/2014	23:56.2
3984	RWS11	-122.4607741	37.93772	-0.43	5.48	-390.4	5.05299988	-122.46077	37.93773	-0.49	5.48	-387.12	4.99299988	11	7/24/2014	23:56.3
3985	RWS11	-122.4607763	37.93772	-0.43	5.49	-390.8	5.06200019	-122.46077	37.93773	-0.49	5.49	-387.68	5.00200018	11	7/24/2014	23:56.4
3986	RWS11	-122.4607785	37.93772	-0.43	5.5	-391.2	5.06800023	-122.46077	37.93773	-0.44	5.5	-387.98	5.05900022	11	7/24/2014	23:56.5
3987	RWS11	-122.4607807	37.93772	-0.43	5.5	-391.6	5.07199994	-122.46078	37.93773	-0.52	5.5	-388.59	4.97799993	11	7/24/2014	23:56.6
3988	RWS11	-122.4607829	37.93772	-0.39	5.5	-392.3	5.10799986	-122.46078	37.93773	-0.49	5.5	-389.15	5.01299986	11	7/24/2014	23:56.7
3989	RWS11	-122.4607851	37.93772	-0.43	5.5	-392.8	5.07199994	-122.46078	37.93774	-0.52	5.5	-389.63	4.97799993	11	7/24/2014	23:56.8
3990	RWS11	-122.4607873	37.93772	-0.43	5.5	-393.2	5.06800023	-122.46078	37.93774	-0.49	5.5	-390.06	5.00800022	11	7/24/2014	23:56.9
3991	RWS11	-122.4607906	37.93772	-0.43	5.49	-393.5	5.06300011	-122.46079	37.93774	-0.52	5.49	-390.49	4.9690001	11	7/24/2014	23:57.0
3992	RWS11	-122.4607928	37.93772	-0.39	5.49	-393.9	5.09100014	-122.46079	37.93774	-0.49	5.49	-390.95	4.99600014	11	7/24/2014	23:57.1

3993	RWS11	-122.460795	37.93772	-0.43	5.48	-394.3	5.04699984	-122.46079	37.93774	-0.52	5.48	-391.3	4.95299983	11	7/24/2014	23:57.2
3994	RWS11	-122.4607972	37.93773	-0.43	5.47	-394.5	5.03800002	-122.46079	37.93774	-0.49	5.47	-391.6	4.97800002	11	7/24/2014	23:57.3
3995	RWS11	-122.4607995	37.93773	-0.43	5.46	-394.8	5.02900019	-122.46079	37.93774	-0.49	5.46	-391.97	4.96900019	11	7/24/2014	23:57.4
3996	RWS11	-122.4608017	37.93773	-0.43	5.45	-395.2	5.01999989	-122.4608	37.93774	-0.49	5.45	-392.34	4.95999989	11	7/24/2014	23:57.5
3997	RWS11	-122.460804	37.93773	-0.43	5.44	-395.5	5.01199999	-122.4608	37.93774	-0.52	5.44	-392.84	4.91799998	11	7/24/2014	23:57.6
3998	RWS11	-122.4608062	37.93773	-0.43	5.43	-395.9	5.0040001	-122.4608	37.93774	-0.49	5.43	-393.22	4.9440001	11	7/24/2014	23:57.7
3999	RWS11	-122.4608084	37.93773	-0.43	5.43	-396.1	4.99800006	-122.4608	37.93774	-0.49	5.43	-393.68	4.93800005	11	7/24/2014	23:57.8
4000	RWS11	-122.4608107	37.93773	-0.39	5.42	-396.4	5.02700001	-122.46081	37.93774	-0.49	5.42	-394.07	4.93200001	11	7/24/2014	23:57.9
4001	RWS11	-122.4608139	37.93773	-0.43	5.42	-396.7	4.9869999	-122.46081	37.93774	-0.49	5.42	-394.55	4.9269999	11	7/24/2014	23:58.0
4002	RWS11	-122.4608161	37.93773	-0.43	5.41	-397	4.98300019	-122.46081	37.93774	-0.44	5.41	-395	4.97400019	11	7/24/2014	23:58.1
4003	RWS11	-122.4608182	37.93773	-0.43	5.41	-397.1	4.97999993	-122.46081	37.93774	-0.49	5.41	-395.39	4.91999993	11	7/24/2014	23:58.2
4004	RWS11	-122.4608204	37.93773	-0.39	5.41	-397.3	5.01300007	-122.46082	37.93774	-0.49	5.41	-395.7	4.91800007	11	7/24/2014	23:58.3
4005	RWS11	-122.4608226	37.93773	-0.43	5.41	-397.6	4.97700015	-122.46082	37.93774	-0.49	5.41	-396	4.91700014	11	7/24/2014	23:58.4
4006	RWS11	-122.4608248	37.93773	-0.39	5.41	-397.8	5.01200014	-122.46082	37.93774	-0.44	5.41	-396.17	4.96800014	11	7/24/2014	23:58.5
4007	RWS11	-122.460827	37.93773	-0.43	5.41	-397.9	4.979	-122.46082	37.93775	-0.49	5.41	-396.39	4.919	11	7/24/2014	23:58.6
4008	RWS11	-122.4608292	37.93773	-0.43	5.41	-398	4.98099986	-122.46082	37.93775	-0.49	5.41	-396.61	4.92099985	11	7/24/2014	23:58.7
4009	RWS11	-122.4608313	37.93773	-0.43	5.41	-398.1	4.98400012	-122.46083	37.93775	-0.49	5.41	-396.67	4.92400011	11	7/24/2014	23:58.8
4010	RWS11	-122.4608335	37.93773	-0.43	5.42	-398.3	4.98799983	-122.46083	37.93775	-0.49	5.42	-396.84	4.92799982	11	7/24/2014	23:58.9
4011	RWS11	-122.4608367	37.93773	-0.43	5.42	-398.4	4.99299994	-122.46083	37.93775	-0.52	5.42	-397.04	4.89899993	11	7/24/2014	23:59.0
4012	RWS11	-122.4608389	37.93774	-0.39	5.43	-398.6	5.03399998	-122.46083	37.93775	-0.44	5.43	-397.17	4.98999998	11	7/24/2014	23:59.1
4013	RWS11	-122.4608411	37.93774	-0.43	5.43	-398.7	5.00500003	-122.46084	37.93775	-0.49	5.43	-397.24	4.94500002	11	7/24/2014	23:59.2
4014	RWS11	-122.4608433	37.93774	-0.43	5.44	-398.8	5.01100007	-122.46084	37.93775	-0.49	5.44	-397.26	4.95100006	11	7/24/2014	23:59.3
4015	RWS11	-122.4608455	37.93774	-0.43	5.45	-398.9	5.01600018	-122.46084	37.93775	-0.49	5.45	-397.26	4.95600018	11	7/24/2014	23:59.4
4016	RWS11	-122.4608477	37.93774	-0.43	5.45	-399	5.01899996	-122.46084	37.93775	-0.52	5.45	-397.43	4.92499995	11	7/24/2014	23:59.5
4017	RWS11	-122.4608499	37.93774	-0.43	5.45	-399.1	5.01899996	-122.46084	37.93775	-0.49	5.45	-397.3	4.95899996	11	7/24/2014	23:59.6
4018	RWS11	-122.4608521	37.93774	-0.43	5.45	-399.2	5.01700011	-122.46085	37.93775	-0.49	5.45	-397.41	4.95700011	11	7/24/2014	23:59.7
4019	RWS11	-122.4608543	37.93774	-0.43	5.44	-399.3	5.01399985	-122.46085	37.93775	-0.52	5.44	-397.63	4.91999984	11	7/24/2014	23:59.8
4020	RWS11	-122.4608564	37.93774	-0.43	5.44	-399.4	5.01199999	-122.46085	37.93775	-0.44	5.44	-397.67	5.00299999	11	7/24/2014	23:59.9
4021	RWS11	-122.4608597	37.93774	-0.43	5.44	-399.4	5.00900021	-122.46085	37.93775	-0.52	5.44	-397.56	4.9150002	11	7/24/2014	24:00.0
4022	RWS11	-122.4608618	37.93774	-0.43	5.43	-399.4	5.00599995	-122.46086	37.93775	-0.49	5.43	-397.5	4.94599995	11	7/24/2014	24:00.1
4023	RWS11	-122.460864	37.93774	-0.43	5.43	-399.5	5.0040001	-122.46086	37.93775	-0.52	5.43	-397.48	4.91000009	11	7/24/2014	24:00.2
4024	RWS11	-122.4608662	37.93774	-0.39	5.43	-399.4	5.03700024	-122.46086	37.93775	-0.49	5.43	-397.37	4.94200024	11	7/24/2014	24:00.3
4025	RWS11	-122.4608683	37.93774	-0.43	5.43	-399.3	4.99999991	-122.46086	37.93776	-0.52	5.43	-397.28	4.9059999	11	7/24/2014	24:00.4

4026	RWS11	-122.4608705	37.93774	-0.39	5.43	-399.1	5.03300005	-122.46087	37.93776	-0.49	5.43	-397	4.93800005	11	7/24/2014	24:00.5
4027	RWS11	-122.4608727	37.93774	-0.43	5.43	-398.8	4.9960002	-122.46087	37.93776	-0.52	5.43	-396.96	4.90200019	11	7/24/2014	24:00.6
4028	RWS11	-122.4608748	37.93774	-0.43	5.42	-398.6	4.99399987	-122.46087	37.93776	-0.49	5.42	-396.89	4.93399987	11	7/24/2014	24:00.7
4029	RWS11	-122.460877	37.93774	-0.43	5.42	-398.4	4.99200001	-122.46087	37.93776	-0.52	5.42	-396.76	4.898	11	7/24/2014	24:00.8
4030	RWS11	-122.4608791	37.93775	-0.43	5.42	-398	4.99100009	-122.46087	37.93776	-0.49	5.42	-396.7	4.93100008	11	7/24/2014	24:00.9
4031	RWS11	-122.4608823	37.93775	-0.48	5.42	-397.8	4.93900016	-122.46088	37.93776	-0.52	5.42	-396.61	4.89600015	11	7/24/2014	24:01.0
4032	RWS11	-122.4608844	37.93775	-0.43	5.42	-397.7	4.98799983	-122.46088	37.93776	-0.49	5.42	-396.59	4.92799982	11	7/24/2014	24:01.1
4033	RWS11	-122.4608866	37.93775	-0.48	5.41	-397.3	4.93499997	-122.46088	37.93776	-0.49	5.41	-396.51	4.92599997	11	7/24/2014	24:01.2
4034	RWS11	-122.4608888	37.93775	-0.43	5.41	-397.1	4.98400012	-122.46088	37.93776	-0.49	5.41	-396.51	4.92400011	11	7/24/2014	24:01.3
4035	RWS11	-122.4608909	37.93775	-0.48	5.41	-396.8	4.93099979	-122.46089	37.93776	-0.52	5.41	-396.46	4.88799977	11	7/24/2014	24:01.4
4036	RWS11	-122.4608931	37.93775	-0.43	5.41	-396.6	4.979	-122.46089	37.93776	-0.49	5.41	-396.23	4.919	11	7/24/2014	24:01.5
4037	RWS11	-122.4608953	37.93775	-0.48	5.41	-396.4	4.92500022	-122.46089	37.93776	-0.49	5.41	-396.05	4.91600022	11	7/24/2014	24:01.6
4038	RWS11	-122.4608975	37.93775	-0.43	5.4	-396.1	4.97299996	-122.46089	37.93776	-0.49	5.4	-395.86	4.91299996	11	7/24/2014	24:01.7
4039	RWS11	-122.4608996	37.93775	-0.48	5.4	-395.9	4.91900018	-122.46089	37.93776	-0.52	5.4	-395.66	4.87600017	11	7/24/2014	24:01.8
4040	RWS11	-122.4609018	37.93775	-0.43	5.39	-395.6	4.96599999	-122.4609	37.93776	-0.49	5.39	-395.4	4.90599999	11	7/24/2014	24:01.9
4041	RWS11	-122.460905	37.93775	-0.48	5.39	-395.3	4.9109998	-122.4609	37.93776	-0.52	5.39	-395.13	4.86799979	11	7/24/2014	24:02.0
4042	RWS11	-122.4609072	37.93775	-0.43	5.39	-395.1	4.95700017	-122.4609	37.93776	-0.49	5.39	-394.93	4.89700016	11	7/24/2014	24:02.1
4043	RWS11	-122.4609094	37.93775	-0.43	5.38	-395	4.95200005	-122.4609	37.93777	-0.49	5.38	-394.93	4.89200005	11	7/24/2014	24:02.2
4044	RWS11	-122.4609115	37.93775	-0.43	5.38	-394.8	4.94600001	-122.46091	37.93777	-0.44	5.38	-394.75	4.93700001	11	7/24/2014	24:02.3
4045	RWS11	-122.4609137	37.93775	-0.48	5.37	-394.6	4.88899997	-122.46091	37.93777	-0.49	5.37	-394.62	4.87999997	11	7/24/2014	24:02.4
4046	RWS11	-122.460916	37.93775	-0.48	5.36	-394.6	4.88299993	-122.46091	37.93777	-0.49	5.36	-394.58	4.87399992	11	7/24/2014	24:02.5
4047	RWS11	-122.4609182	37.93775	-0.48	5.36	-394.5	4.87699988	-122.46091	37.93777	-0.49	5.36	-394.69	4.86799988	11	7/24/2014	24:02.6
4048	RWS11	-122.4609204	37.93776	-0.43	5.35	-394.4	4.92199984	-122.46092	37.93777	-0.44	5.35	-394.49	4.91299984	11	7/24/2014	24:02.7
4049	RWS11	-122.4609226	37.93776	-0.48	5.34	-394.3	4.8649998	-122.46092	37.93777	-0.49	5.34	-394.41	4.8559998	11	7/24/2014	24:02.8
4050	RWS11	-122.4609248	37.93776	-0.43	5.34	-394.2	4.91000023	-122.46092	37.93777	-0.44	5.34	-394.35	4.90100023	11	7/24/2014	24:02.9
4051	RWS11	-122.4609281	37.93776	-0.43	5.33	-394.3	4.90400019	-122.46092	37.93777	-0.49	5.33	-394.28	4.84400019	11	7/24/2014	24:03.0
4052	RWS11	-122.4609303	37.93776	-0.43	5.33	-394.3	4.89800015	-122.46093	37.93777	-0.44	5.33	-394.09	4.88900015	11	7/24/2014	24:03.1
4053	RWS11	-122.4609325	37.93776	-0.43	5.32	-394.3	4.89100018	-122.46093	37.93777	-0.49	5.32	-393.98	4.83100018	11	7/24/2014	24:03.2
4054	RWS11	-122.4609347	37.93776	-0.43	5.31	-394.5	4.88500014	-122.46093	37.93777	-0.44	5.31	-394.09	4.87600014	11	7/24/2014	24:03.3
4055	RWS11	-122.4609369	37.93776	-0.48	5.31	-394.5	4.8280001	-122.46093	37.93777	-0.44	5.31	-394.04	4.87000009	11	7/24/2014	24:03.4
4056	RWS11	-122.4609392	37.93776	-0.43	5.3	-394.5	4.87300006	-122.46093	37.93777	-0.44	5.3	-393.78	4.86400005	11	7/24/2014	24:03.5
4057	RWS11	-122.4609414	37.93776	-0.43	5.3	-394.6	4.86899987	-122.46094	37.93777	-0.44	5.3	-393.63	4.85999987	11	7/24/2014	24:03.6
4058	RWS11	-122.4609436	37.93776	-0.39	5.29	-394.4	4.90000015	-122.46094	37.93777	-0.4	5.29	-393.46	4.89000013	11	7/24/2014	24:03.7

4059	RWS11	-122.4609458	37.93776	-0.43	5.29	-394.4	4.86299983	-122.46094	37.93777	-0.49	5.29	-393.33	4.80299982	11	7/24/2014	24:03.8
4060	RWS11	-122.4609481	37.93776	-0.39	5.29	-394.3	4.8969999	-122.46094	37.93778	-0.44	5.29	-393.11	4.8529999	11	7/24/2014	24:03.9
4061	RWS11	-122.4609513	37.93776	-0.43	5.29	-394	4.8619999	-122.46095	37.93778	-0.44	5.29	-393.07	4.8529999	11	7/24/2014	24:04.0
4062	RWS11	-122.4609535	37.93776	-0.43	5.29	-393.8	4.8619999	-122.46095	37.93778	-0.44	5.29	-392.85	4.8529999	11	7/24/2014	24:04.1
4063	RWS11	-122.4609557	37.93776	-0.43	5.29	-393.4	4.8619999	-122.46095	37.93778	-0.49	5.29	-392.55	4.8019999	11	7/24/2014	24:04.2
4064	RWS11	-122.4609579	37.93776	-0.43	5.29	-393	4.86299983	-122.46095	37.93778	-0.44	5.29	-392.35	4.85399982	11	7/24/2014	24:04.3
4065	RWS11	-122.4609601	37.93777	-0.43	5.29	-392.7	4.86500016	-122.46095	37.93778	-0.44	5.29	-392.1	4.85600016	11	7/24/2014	24:04.4
4066	RWS11	-122.4609624	37.93777	-0.39	5.3	-392.4	4.90200001	-122.46096	37.93778	-0.4	5.3	-391.82	4.89199999	11	7/24/2014	24:04.5
4067	RWS11	-122.4609646	37.93777	-0.43	5.3	-392	4.8710002	-122.46096	37.93778	-0.44	5.3	-391.56	4.8620002	11	7/24/2014	24:04.6
4068	RWS11	-122.4609668	37.93777	-0.39	5.3	-391.7	4.90999991	-122.46096	37.93778	-0.44	5.3	-391.21	4.86599991	11	7/24/2014	24:04.7
4069	RWS11	-122.460969	37.93777	-0.43	5.31	-391.2	4.88000003	-122.46096	37.93778	-0.44	5.31	-390.8	4.87100002	11	7/24/2014	24:04.8
4070	RWS11	-122.4609711	37.93777	-0.43	5.32	-390.8	4.88600007	-122.46097	37.93778	-0.44	5.32	-390.39	4.87700006	11	7/24/2014	24:04.9
4071	RWS11	-122.4609744	37.93777	-0.43	5.32	-390.3	4.89200011	-122.46097	37.93778	-0.4	5.32	-389.8	4.91700009	11	7/24/2014	24:05.0
4072	RWS11	-122.4609766	37.93777	-0.39	5.33	-389.8	4.93300015	-122.46097	37.93778	-0.4	5.33	-389.32	4.92300013	11	7/24/2014	24:05.1
4073	RWS11	-122.4609787	37.93777	-0.43	5.33	-389.4	4.90299979	-122.46097	37.93778	-0.44	5.33	-388.85	4.89399979	11	7/24/2014	24:05.2
4074	RWS11	-122.4609809	37.93777	-0.39	5.34	-389	4.9429999	-122.46098	37.93778	-0.4	5.34	-388.42	4.93299988	11	7/24/2014	24:05.3
4075	RWS11	-122.4609831	37.93777	-0.43	5.34	-388.7	4.91300002	-122.46098	37.93778	-0.4	5.34	-388.24	4.93799999	11	7/24/2014	24:05.4
4076	RWS11	-122.4609853	37.93777	-0.43	5.35	-388.4	4.91800013	-122.46098	37.93778	-0.4	5.35	-388.05	4.94300011	11	7/24/2014	24:05.5
4077	RWS11	-122.4609874	37.93777	-0.43	5.35	-388.2	4.92199984	-122.46098	37.93779	-0.4	5.35	-388.03	4.94699982	11	7/24/2014	24:05.6
4078	RWS11	-122.4609896	37.93777	-0.39	5.36	-388	4.96199995	-122.46098	37.93779	-0.4	5.36	-388.05	4.95199993	11	7/24/2014	24:05.7
4079	RWS11	-122.4609918	37.93777	-0.43	5.36	-387.9	4.93100014	-122.46099	37.93779	-0.4	5.36	-388.16	4.95600012	11	7/24/2014	24:05.8
4080	RWS11	-122.4609939	37.93777	-0.43	5.36	-387.7	4.93499985	-122.46099	37.93779	-0.35	5.36	-388.01	5.00999984	11	7/24/2014	24:05.9
4081	RWS11	-122.4609971	37.93777	-0.43	5.37	-387.5	4.93900004	-122.46099	37.93779	-0.44	5.37	-387.62	4.93000004	11	7/24/2014	24:06.0
4082	RWS11	-122.4609993	37.93778	-0.39	5.37	-387.1	4.97900015	-122.46099	37.93779	-0.4	5.37	-387.31	4.96900013	11	7/24/2014	24:06.1
4083	RWS11	-122.4610014	37.93778	-0.43	5.38	-386.8	4.9500002	-122.461	37.93779	-0.4	5.38	-387.01	4.97500017	11	7/24/2014	24:06.2
4084	RWS11	-122.4610036	37.93778	-0.43	5.38	-386.5	4.95499983	-122.461	37.93779	-0.4	5.38	-386.69	4.97999981	11	7/24/2014	24:06.3
4085	RWS11	-122.4610058	37.93778	-0.43	5.39	-386.1	4.96099988	-122.461	37.93779	-0.4	5.39	-386.25	4.98599985	11	7/24/2014	24:06.4
4086	RWS11	-122.461008	37.93778	-0.39	5.39	-385.8	5.00099999	-122.461	37.93779	-0.4	5.39	-385.87	4.99099997	11	7/24/2014	24:06.5
4087	RWS11	-122.4610101	37.93778	-0.43	5.4	-385.5	4.97100011	-122.461	37.93779	-0.35	5.4	-385.5	5.04600009	11	7/24/2014	24:06.6
4088	RWS11	-122.4610123	37.93778	-0.39	5.4	-385.3	5.00999981	-122.46101	37.93779	-0.35	5.4	-385.21	5.0499998	11	7/24/2014	24:06.7
4089	RWS11	-122.4610145	37.93778	-0.43	5.41	-385	4.97800007	-122.46101	37.93779	-0.4	5.41	-384.87	5.00300005	11	7/24/2014	24:06.8
4090	RWS11	-122.4610166	37.93778	-0.43	5.41	-384.6	4.98099986	-122.46101	37.93779	-0.35	5.41	-384.46	5.05599985	11	7/24/2014	24:06.9
4091	RWS11	-122.4610199	37.93778	-0.43	5.41	-384.2	4.98400012	-122.46101	37.93779	-0.35	5.41	-384.18	5.0590001	11	7/24/2014	24:07.0

4092	RWS11	-122.461022	37.93778	-0.39	5.42	-383.7	5.0219999	-122.46102	37.93779	-0.4	5.42	-383.85	5.01199988	11	7/24/2014	24:07.1
4093	RWS11	-122.4610242	37.93778	-0.43	5.42	-383.3	4.99000016	-122.46102	37.93779	-0.4	5.42	-383.61	5.01500013	11	7/24/2014	24:07.2
4094	RWS11	-122.4610264	37.93778	-0.39	5.42	-383.1	5.02799994	-122.46102	37.9378	-0.35	5.42	-383.57	5.06799993	11	7/24/2014	24:07.3
4095	RWS11	-122.4610286	37.93778	-0.43	5.43	-382.7	4.9960002	-122.46102	37.9378	-0.4	5.43	-383.29	5.02100018	11	7/24/2014	24:07.4
4096	RWS11	-122.4610309	37.93778	-0.43	5.43	-382.4	4.99800006	-122.46103	37.9378	-0.35	5.43	-382.97	5.07300004	11	7/24/2014	24:07.5
4097	RWS11	-122.4610331	37.93778	-0.43	5.43	-382.1	4.99999991	-122.46103	37.9378	-0.4	5.43	-382.51	5.02499989	11	7/24/2014	24:07.6
4098	RWS11	-122.4610353	37.93778	-0.39	5.43	-381.8	5.03700024	-122.46103	37.9378	-0.35	5.43	-382.06	5.07700023	11	7/24/2014	24:07.7
4099	RWS11	-122.4610375	37.93779	-0.43	5.43	-381.4	5.00300017	-122.46103	37.9378	-0.4	5.43	-381.56	5.02800015	11	7/24/2014	24:07.8
4100	RWS11	-122.4610397	37.93779	-0.39	5.43	-381.1	5.03700024	-122.46103	37.9378	-0.35	5.43	-381.06	5.07700023	11	7/24/2014	24:07.9
4101	RWS11	-122.4610429	37.93779	-0.43	5.43	-380.8	4.99999991	-122.46104	37.9378	-0.4	5.43	-380.71	5.02499989	11	7/24/2014	24:08.0
4102	RWS11	-122.4610451	37.93779	-0.39	5.43	-380.6	5.03200012	-122.46104	37.9378	-0.35	5.43	-380.35	5.07200012	11	7/24/2014	24:08.1
4103	RWS11	-122.4610472	37.93779	-0.43	5.42	-380.5	4.99299994	-122.46104	37.9378	-0.4	5.42	-379.98	5.01799992	11	7/24/2014	24:08.2
4104	RWS11	-122.4610494	37.93779	-0.39	5.42	-380.2	5.0219999	-122.46104	37.9378	-0.35	5.42	-379.61	5.06199989	11	7/24/2014	24:08.3
4105	RWS11	-122.4610516	37.93779	-0.43	5.41	-379.8	4.97999993	-122.46105	37.9378	-0.4	5.41	-379.2	5.00499991	11	7/24/2014	24:08.4
4106	RWS11	-122.4610538	37.93779	-0.39	5.4	-379.5	5.00700003	-122.46105	37.9378	-0.4	5.4	-378.81	4.99700001	11	7/24/2014	24:08.5
4107	RWS11	-122.4610559	37.93779	-0.43	5.39	-379.2	4.96400014	-122.46105	37.9378	-0.4	5.39	-378.4	4.98900011	11	7/24/2014	24:08.6
4108	RWS11	-122.4610581	37.93779	-0.39	5.39	-378.6	4.99100024	-122.46105	37.9378	-0.35	5.39	-377.96	5.03100023	11	7/24/2014	24:08.7
4109	RWS11	-122.4610603	37.93779	-0.43	5.38	-378.1	4.94699994	-122.46105	37.9378	-0.4	5.38	-377.64	4.97199991	11	7/24/2014	24:08.8
4110	RWS11	-122.4610624	37.93779	-0.39	5.37	-377.7	4.97499996	-122.46106	37.9378	-0.35	5.37	-377.15	5.01499996	11	7/24/2014	24:08.9
4111	RWS11	-122.4610656	37.93779	-0.43	5.36	-377.3	4.93200007	-122.46106	37.93781	-0.4	5.36	-376.74	4.95700005	11	7/24/2014	24:09.0
4112	RWS11	-122.4610677	37.93779	-0.39	5.36	-376.7	4.96100003	-122.46106	37.93781	-0.35	5.36	-376.37	5.00100002	11	7/24/2014	24:09.1
4113	RWS11	-122.4610698	37.93779	-0.43	5.35	-376.6	4.91999999	-122.46106	37.93781	-0.4	5.35	-376.26	4.94499996	11	7/24/2014	24:09.2
4114	RWS11	-122.461072	37.93779	-0.39	5.34	-376.4	4.94999987	-122.46107	37.93781	-0.35	5.34	-376.15	4.98999986	11	7/24/2014	24:09.3
4115	RWS11	-122.4610741	37.93779	-0.43	5.34	-376.1	4.91100016	-122.46107	37.93781	-0.35	5.34	-375.98	4.98600015	11	7/24/2014	24:09.4
4116	RWS11	-122.4610763	37.9378	-0.39	5.34	-375.8	4.94399983	-122.46107	37.93781	-0.35	5.34	-375.46	4.98399982	11	7/24/2014	24:09.5
4117	RWS11	-122.4610784	37.9378	-0.43	5.34	-375.4	4.9079999	-122.46107	37.93781	-0.4	5.34	-374.94	4.93299988	11	7/24/2014	24:09.6
4118	RWS11	-122.4610806	37.9378	-0.39	5.34	-375.1	4.9429999	-122.46108	37.93781	-0.35	5.34	-374.4	4.98299989	11	7/24/2014	24:09.7
4119	RWS11	-122.4610827	37.9378	-0.43	5.34	-374.5	4.90899983	-122.46108	37.93781	-0.4	5.34	-373.7	4.93399981	11	7/24/2014	24:09.8
4120	RWS11	-122.4610848	37.9378	-0.43	5.34	-374	4.91100016	-122.46108	37.93781	-0.35	5.34	-373.18	4.98600015	11	7/24/2014	24:09.9
4121	RWS11	-122.4610879	37.9378	-0.43	5.34	-373.7	4.91399994	-122.46108	37.93781	-0.4	5.34	-372.73	4.93899992	11	7/24/2014	24:10.0
4122	RWS11	-122.46109	37.9378	-0.39	5.35	-373.2	4.9520002	-122.46108	37.93781	-0.35	5.35	-372.17	4.99200019	11	7/24/2014	24:10.1
4123	RWS11	-122.4610921	37.9378	-0.43	5.35	-372.8	4.92199984	-122.46109	37.93781	-0.4	5.35	-371.73	4.94699982	11	7/24/2014	24:10.2
4124	RWS11	-122.4610943	37.9378	-0.39	5.36	-372.4	4.96199995	-122.46109	37.93781	-0.35	5.36	-371.24	5.00199994	11	7/24/2014	24:10.3

4125	RWS11	-122.4610964	37.9378	-0.43	5.36	-371.9	4.933	-122.46109	37.93781	-0.44	5.36	-370.78	4.92399999	11	7/24/2014	24:10.4
4126	RWS11	-122.4610985	37.9378	-0.39	5.37	-371.6	4.97499996	-122.46109	37.93781	-0.35	5.37	-370.2	5.01499996	11	7/24/2014	24:10.5
4127	RWS11	-122.4611007	37.9378	-0.43	5.38	-371.2	4.94600001	-122.4611	37.93781	-0.4	5.38	-369.68	4.97099999	11	7/24/2014	24:10.6
4128	RWS11	-122.4611028	37.9378	-0.39	5.38	-370.7	4.98700005	-122.4611	37.93782	-0.4	5.38	-368.88	4.97700003	11	7/24/2014	24:10.7
4129	RWS11	-122.461105	37.9378	-0.43	5.39	-370.2	4.95800009	-122.4611	37.93782	-0.4	5.39	-368.21	4.98300007	11	7/24/2014	24:10.8
4130	RWS11	-122.4611071	37.9378	-0.39	5.39	-369.7	4.99900013	-122.4611	37.93782	-0.4	5.39	-367.51	4.98900011	11	7/24/2014	24:10.9
4131	RWS11	-122.4611103	37.9378	-0.43	5.4	-369.3	4.97100011	-122.4611	37.93782	-0.4	5.4	-367.1	4.99600008	11	7/24/2014	24:11.0
4132	RWS11	-122.4611125	37.93781	-0.39	5.41	-368.9	5.01300007	-122.46111	37.93782	-0.4	5.41	-366.95	5.00300005	11	7/24/2014	24:11.1
4133	RWS11	-122.4611147	37.93781	-0.43	5.41	-368.6	4.98599997	-122.46111	37.93782	-0.4	5.41	-366.78	5.01099995	11	7/24/2014	24:11.2
4134	RWS11	-122.4611169	37.93781	-0.39	5.42	-368.3	5.02999979	-122.46111	37.93782	-0.4	5.42	-366.37	5.01999977	11	7/24/2014	24:11.3
4135	RWS11	-122.4611191	37.93781	-0.43	5.43	-367.9	5.00500003	-122.46111	37.93782	-0.44	5.43	-365.98	4.99600002	11	7/24/2014	24:11.4
4136	RWS11	-122.4611214	37.93781	-0.39	5.44	-367.4	5.04999977	-122.46112	37.93782	-0.4	5.44	-365.33	5.03999975	11	7/24/2014	24:11.5
4137	RWS11	-122.4611236	37.93781	-0.48	5.45	-367	4.97499993	-122.46112	37.93782	-0.4	5.45	-364.72	5.05099991	11	7/24/2014	24:11.6
4138	RWS11	-122.4611259	37.93781	-0.43	5.47	-366.4	5.03600016	-122.46112	37.93782	-0.4	5.47	-364.09	5.06100014	11	7/24/2014	24:11.7
4139	RWS11	-122.4611281	37.93781	-0.48	5.47	-365.8	4.99399999	-122.46112	37.93782	-0.4	5.47	-363.47	5.06999996	11	7/24/2014	24:11.8
4140	RWS11	-122.4611303	37.93781	-0.43	5.48	-365.3	5.05399981	-122.46112	37.93782	-0.4	5.48	-362.93	5.07899979	11	7/24/2014	24:11.9
4141	RWS11	-122.4611336	37.93781	-0.43	5.49	-364.9	5.06099978	-122.46113	37.93782	-0.44	5.49	-362.47	5.05199978	11	7/24/2014	24:12.0
4142	RWS11	-122.4611358	37.93781	-0.43	5.49	-364.4	5.0659999	-122.46113	37.93782	-0.44	5.49	-362.06	5.05699989	11	7/24/2014	24:12.1
4143	RWS11	-122.461138	37.93781	-0.43	5.5	-363.8	5.07000008	-122.46113	37.93782	-0.44	5.5	-361.58	5.06100008	11	7/24/2014	24:12.2
4144	RWS11	-122.4611403	37.93781	-0.43	5.5	-363.3	5.07299986	-122.46113	37.93783	-0.4	5.5	-361.04	5.09799984	11	7/24/2014	24:12.3
4145	RWS11	-122.4611425	37.93781	-0.43	5.5	-362.8	5.07399979	-122.46114	37.93783	-0.44	5.5	-360.57	5.06499979	11	7/24/2014	24:12.4
4146	RWS11	-122.4611447	37.93781	-0.43	5.5	-362.4	5.0750002	-122.46114	37.93783	-0.4	5.5	-360.11	5.10000017	11	7/24/2014	24:12.5
4147	RWS11	-122.4611469	37.93781	-0.48	5.51	-362	5.02500013	-122.46114	37.93783	-0.44	5.51	-359.72	5.06700012	11	7/24/2014	24:12.6
4148	RWS11	-122.4611491	37.93782	-0.43	5.51	-361.7	5.07700005	-122.46114	37.93783	-0.44	5.51	-359.51	5.06800005	11	7/24/2014	24:12.7
4149	RWS11	-122.4611513	37.93782	-0.43	5.51	-361.6	5.07700005	-122.46115	37.93783	-0.44	5.51	-359.18	5.06800005	11	7/24/2014	24:12.8
4150	RWS11	-122.4611535	37.93782	-0.43	5.51	-361.3	5.07799998	-122.46115	37.93783	-0.4	5.51	-358.94	5.10299996	11	7/24/2014	24:12.9
4151	RWS11	-122.4611567	37.93782	-0.48	5.51	-361.1	5.02799991	-122.46115	37.93783	-0.44	5.51	-358.6	5.0699999	11	7/24/2014	24:13.0
4152	RWS11	-122.4611588	37.93782	-0.43	5.51	-360.9	5.07999983	-122.46115	37.93783	-0.4	5.51	-358.19	5.10499981	11	7/24/2014	24:13.1
4153	RWS11	-122.461161	37.93782	-0.43	5.51	-360.6	5.08200017	-122.46116	37.93783	-0.44	5.51	-357.77	5.07300016	11	7/24/2014	24:13.2
4154	RWS11	-122.4611631	37.93782	-0.43	5.51	-360.3	5.08499995	-122.46116	37.93783	-0.44	5.51	-357.28	5.07599995	11	7/24/2014	24:13.3
4155	RWS11	-122.4611652	37.93782	-0.43	5.52	-360	5.08800021	-122.46116	37.93783	-0.4	5.52	-356.8	5.11300018	11	7/24/2014	24:13.4
4156	RWS11	-122.4611674	37.93782	-0.43	5.52	-359.6	5.09099999	-122.46116	37.93783	-0.44	5.52	-356.39	5.08199999	11	7/24/2014	24:13.5
4157	RWS11	-122.4611695	37.93782	-0.43	5.52	-359.3	5.09500018	-122.46116	37.93783	-0.49	5.52	-355.98	5.03500018	11	7/24/2014	24:13.6

4158	RWS11	-122.4611717	37.93782	-0.43	5.53	-358.9	5.09899989	-122.46117	37.93783	-0.49	5.53	-355.59	5.03899989	11	7/24/2014	24:13.7
4159	RWS11	-122.4611738	37.93782	-0.43	5.53	-358.7	5.104	-122.46117	37.93783	-0.49	5.53	-355.11	5.044	11	7/24/2014	24:13.8
4160	RWS11	-122.4611759	37.93782	-0.43	5.54	-358.5	5.11000004	-122.46117	37.93784	-0.44	5.54	-354.72	5.10100004	11	7/24/2014	24:13.9
4161	RWS11	-122.4611791	37.93782	-0.48	5.55	-358.2	5.06699994	-122.46117	37.93784	-0.44	5.55	-354.34	5.10899994	11	7/24/2014	24:14.0
4162	RWS11	-122.4611813	37.93782	-0.39	5.56	-358.1	5.16200024	-122.46118	37.93784	-0.44	5.56	-354.05	5.11800024	11	7/24/2014	24:14.1
4163	RWS11	-122.4611835	37.93782	-0.43	5.57	-357.9	5.13699999	-122.46118	37.93784	-0.44	5.57	-353.75	5.12799999	11	7/24/2014	24:14.2
4164	RWS11	-122.4611857	37.93782	-0.39	5.58	-357.8	5.18200022	-122.46118	37.93784	-0.4	5.58	-353.34	5.1720002	11	7/24/2014	24:14.3
4165	RWS11	-122.461188	37.93783	-0.43	5.59	-357.5	5.15899983	-122.46118	37.93784	-0.49	5.59	-352.88	5.09899983	11	7/24/2014	24:14.4
4166	RWS11	-122.4611902	37.93783	-0.43	5.6	-357.4	5.17099991	-122.46118	37.93784	-0.49	5.6	-352.45	5.11099991	11	7/24/2014	24:14.5
4167	RWS11	-122.4611924	37.93783	-0.43	5.61	-357.2	5.183	-122.46119	37.93784	-0.49	5.61	-351.95	5.123	11	7/24/2014	24:14.6
4168	RWS11	-122.4611946	37.93783	-0.39	5.63	-356.8	5.23100001	-122.46119	37.93784	-0.49	5.63	-351.39	5.13600001	11	7/24/2014	24:14.7
4169	RWS11	-122.4611968	37.93783	-0.43	5.64	-356.5	5.20800009	-122.46119	37.93784	-0.44	5.64	-350.79	5.19900009	11	7/24/2014	24:14.8
4170	RWS11	-122.461199	37.93783	-0.43	5.65	-356.1	5.22000018	-122.46119	37.93784	-0.44	5.65	-350.39	5.21100017	11	7/24/2014	24:14.9
4171	RWS11	-122.4612021	37.93783	-0.43	5.66	-356	5.23099986	-122.4612	37.93784	-0.49	5.66	-350.01	5.17099985	11	7/24/2014	24:15.0
4172	RWS11	-122.4612042	37.93783	-0.39	5.67	-355.8	5.27500015	-122.4612	37.93784	-0.49	5.67	-349.75	5.18000016	11	7/24/2014	24:15.1
4173	RWS11	-122.4612062	37.93783	-0.43	5.68	-355.5	5.24800006	-122.4612	37.93784	-0.49	5.68	-349.44	5.18800005	11	7/24/2014	24:15.2
4174	RWS11	-122.4612083	37.93783	-0.43	5.68	-355.4	5.2540001	-122.4612	37.93784	-0.49	5.68	-349.23	5.1940001	11	7/24/2014	24:15.3
4175	RWS11	-122.4612104	37.93783	-0.43	5.69	-355.3	5.25799981	-122.4612	37.93784	-0.49	5.69	-348.9	5.19799981	11	7/24/2014	24:15.4
4176	RWS11	-122.4612124	37.93783	-0.43	5.69	-355	5.26199999	-122.46121	37.93785	-0.49	5.69	-348.49	5.20199999	11	7/24/2014	24:15.5
4177	RWS11	-122.4612145	37.93783	-0.43	5.69	-354.5	5.26299992	-122.46121	37.93785	-0.49	5.69	-347.86	5.20299992	11	7/24/2014	24:15.6
4178	RWS11	-122.4612165	37.93783	-0.43	5.69	-354.1	5.26299992	-122.46121	37.93785	-0.44	5.69	-347.3	5.25399992	11	7/24/2014	24:15.7
4179	RWS11	-122.4612186	37.93783	-0.43	5.69	-353.7	5.26199999	-122.46121	37.93785	-0.52	5.69	-346.65	5.16799998	11	7/24/2014	24:15.8
4180	RWS11	-122.4612207	37.93783	-0.43	5.69	-353.1	5.26100007	-122.46122	37.93785	-0.49	5.69	-345.96	5.20100006	11	7/24/2014	24:15.9
4181	RWS11	-122.4612238	37.93784	-0.43	5.69	-352.7	5.26000014	-122.46122	37.93785	-0.57	5.69	-345.33	5.11500013	11	7/24/2014	24:16.0
4182	RWS11	-122.461226	37.93784	-0.43	5.69	-352.2	5.26100007	-122.46122	37.93785	-0.52	5.69	-344.73	5.16700006	11	7/24/2014	24:16.1
4183	RWS11	-122.4612281	37.93784	-0.43	5.69	-351.7	5.26199999	-122.46122	37.93785	-0.52	5.69	-344.2	5.16799998	11	7/24/2014	24:16.2
4184	RWS11	-122.4612303	37.93784	-0.43	5.69	-351.2	5.26299992	-122.46122	37.93785	-0.52	5.69	-343.73	5.16899991	11	7/24/2014	24:16.3
4185	RWS11	-122.4612325	37.93784	-0.48	5.69	-350.7	5.21299985	-122.46123	37.93785	-0.57	5.69	-343.14	5.11899984	11	7/24/2014	24:16.4
4186	RWS11	-122.4612348	37.93784	-0.43	5.69	-350	5.26499978	-122.46123	37.93785	-0.52	5.69	-342.54	5.17099977	11	7/24/2014	24:16.5
4187	RWS11	-122.461237	37.93784	-0.48	5.69	-349.4	5.21399978	-122.46123	37.93785	-0.52	5.69	-341.89	5.17099977	11	7/24/2014	24:16.6
4188	RWS11	-122.4612392	37.93784	-0.48	5.69	-348.8	5.21199992	-122.46123	37.93785	-0.57	5.69	-341.35	5.11799991	11	7/24/2014	24:16.7
4189	RWS11	-122.4612414	37.93784	-0.48	5.69	-348.3	5.20900014	-122.46124	37.93785	-0.57	5.69	-340.87	5.11500013	11	7/24/2014	24:16.8
4190	RWS11	-122.4612436	37.93784	-0.48	5.68	-347.6	5.20499995	-122.46124	37.93785	-0.57	5.68	-340.27	5.11099994	11	7/24/2014	24:16.9

4191	RWS11	-122.4612468	37.93784	-0.48	5.68	-347.1	5.19999984	-122.46124	37.93785	-0.61	5.68	-339.77	5.07299984	11	7/24/2014	24:17.0
4192	RWS11	-122.4612489	37.93784	-0.48	5.67	-346.6	5.1939998	-122.46124	37.93785	-0.61	5.67	-339.21	5.06699979	11	7/24/2014	24:17.1
4193	RWS11	-122.4612509	37.93784	-0.48	5.67	-346.2	5.18699983	-122.46125	37.93786	-0.61	5.67	-338.56	5.05999982	11	7/24/2014	24:17.2
4194	RWS11	-122.461253	37.93784	-0.48	5.66	-345.9	5.17999986	-122.46125	37.93786	-0.57	5.66	-338.04	5.08599985	11	7/24/2014	24:17.3
4195	RWS11	-122.4612551	37.93784	-0.51	5.65	-345.5	5.13899988	-122.46125	37.93786	-0.66	5.65	-337.54	4.99499989	11	7/24/2014	24:17.4
4196	RWS11	-122.4612572	37.93784	-0.48	5.65	-345	5.16599992	-122.46125	37.93786	-0.61	5.65	-336.98	5.03899992	11	7/24/2014	24:17.5
4197	RWS11	-122.4612592	37.93785	-0.51	5.64	-344.6	5.12599987	-122.46125	37.93786	-0.61	5.64	-336.54	5.03299987	11	7/24/2014	24:17.6
4198	RWS11	-122.4612613	37.93785	-0.48	5.63	-344	5.15199998	-122.46126	37.93786	-0.66	5.63	-336.11	4.97399998	11	7/24/2014	24:17.7
4199	RWS11	-122.4612633	37.93785	-0.51	5.62	-343.6	5.11000007	-122.46126	37.93786	-0.69	5.62	-335.65	4.9320001	11	7/24/2014	24:17.8
4200	RWS11	-122.4612654	37.93785	-0.51	5.61	-343.3	5.10099977	-122.46126	37.93786	-0.61	5.61	-335.31	5.00799978	11	7/24/2014	24:17.9
4201	RWS11	-122.4612684	37.93785	-0.51	5.61	-342.9	5.09100002	-122.46126	37.93786	-0.69	5.61	-334.99	4.91300005	11	7/24/2014	24:18.0
4202	RWS11	-122.4612705	37.93785	-0.51	5.59	-342.4	5.07999986	-122.46126	37.93786	-0.66	5.59	-334.64	4.93599987	11	7/24/2014	24:18.1
4203	RWS11	-122.4612725	37.93785	-0.57	5.58	-342	5.01599985	-122.46127	37.93786	-0.69	5.58	-334.38	4.88899988	11	7/24/2014	24:18.2
4204	RWS11	-122.4612746	37.93785	-0.51	5.57	-341.6	5.05299991	-122.46127	37.93786	-0.66	5.57	-334.1	4.90899992	11	7/24/2014	24:18.3
4205	RWS11	-122.4612767	37.93785	-0.57	5.55	-341.3	4.98600012	-122.46127	37.93786	-0.66	5.55	-333.84	4.89300013	11	7/24/2014	24:18.4
4206	RWS11	-122.4612788	37.93785	-0.51	5.53	-341	5.01999992	-122.46127	37.93786	-0.66	5.53	-333.69	4.87599993	11	7/24/2014	24:18.5
4207	RWS11	-122.4612809	37.93785	-0.57	5.51	-340.9	4.94999987	-122.46128	37.93786	-0.66	5.51	-333.58	4.85699987	11	7/24/2014	24:18.6
4208	RWS11	-122.4612831	37.93785	-0.51	5.49	-340.5	4.98099989	-122.46128	37.93786	-0.66	5.49	-333.41	4.83699989	11	7/24/2014	24:18.7
4209	RWS11	-122.4612851	37.93785	-0.57	5.47	-340.4	4.90999991	-122.46128	37.93786	-0.69	5.47	-333.56	4.78299993	11	7/24/2014	24:18.8
4210	RWS11	-122.4612872	37.93785	-0.51	5.45	-340.5	4.94099993	-122.46128	37.93787	-0.66	5.45	-333.8	4.79699993	11	7/24/2014	24:18.9
4211	RWS11	-122.4612903	37.93785	-0.57	5.44	-340.5	4.8719998	-122.46128	37.93787	-0.69	5.44	-334.06	4.74499983	11	7/24/2014	24:19.0
4212	RWS11	-122.4612924	37.93785	-0.51	5.42	-340.6	4.90700001	-122.46129	37.93787	-0.66	5.42	-334.14	4.76300001	11	7/24/2014	24:19.1
4213	RWS11	-122.4612945	37.93785	-0.57	5.41	-340.5	4.84299999	-122.46129	37.93787	-0.66	5.41	-334.07	4.75	11	7/24/2014	24:19.2
4214	RWS11	-122.4612965	37.93786	-0.57	5.4	-340.6	4.83199984	-122.46129	37.93787	-0.66	5.4	-333.99	4.73899984	11	7/24/2014	24:19.3
4215	RWS11	-122.4612986	37.93786	-0.57	5.39	-340.7	4.82399994	-122.46129	37.93787	-0.69	5.39	-333.8	4.69699997	11	7/24/2014	24:19.4
4216	RWS11	-122.4613008	37.93786	-0.57	5.38	-340.7	4.81699997	-122.4613	37.93787	-0.66	5.38	-333.6	4.72399998	11	7/24/2014	24:19.5
4217	RWS11	-122.4613029	37.93786	-0.57	5.38	-340.6	4.81199986	-122.4613	37.93787	-0.69	5.38	-333.43	4.68499988	11	7/24/2014	24:19.6
4218	RWS11	-122.461305	37.93786	-0.57	5.37	-340.6	4.80800015	-122.4613	37.93787	-0.69	5.37	-333.41	4.68100017	11	7/24/2014	24:19.7
4219	RWS11	-122.461307	37.93786	-0.6	5.37	-340.6	4.76899993	-122.4613	37.93787	-0.69	5.37	-333.5	4.67699999	11	7/24/2014	24:19.8
4220	RWS11	-122.4613091	37.93786	-0.57	5.36	-340.6	4.79899985	-122.4613	37.93787	-0.69	5.36	-333.61	4.67199987	11	7/24/2014	24:19.9
4221	RWS11	-122.4613123	37.93786	-0.6	5.36	-340.7	4.75799978	-122.46131	37.93787	-0.66	5.36	-333.73	4.69999981	11	7/24/2014	24:20.0
4222	RWS11	-122.4613144	37.93786	-0.57	5.35	-340.8	4.78599983	-122.46131	37.93787	-0.69	5.35	-333.87	4.65899986	11	7/24/2014	24:20.1
4223	RWS11	-122.4613165	37.93786	-0.6	5.34	-341	4.74199998	-122.46131	37.93787	-0.69	5.34	-334.12	4.65000004	11	7/24/2014	24:20.2

4224	RWS11	-122.4613186	37.93786	-0.57	5.33	-341.3	4.76599985	-122.46131	37.93787	-0.66	5.33	-334.49	4.67299986	11	7/24/2014	24:20.3
4225	RWS11	-122.4613208	37.93786	-0.6	5.32	-341.6	4.71799982	-122.46132	37.93787	-0.73	5.32	-334.84	4.59199983	11	7/24/2014	24:20.4
4226	RWS11	-122.461323	37.93786	-0.57	5.3	-341.9	4.73999983	-122.46132	37.93788	-0.66	5.3	-335.14	4.64699984	11	7/24/2014	24:20.5
4227	RWS11	-122.4613251	37.93786	-0.6	5.29	-342.2	4.69199979	-122.46132	37.93788	-0.69	5.29	-335.47	4.59999985	11	7/24/2014	24:20.6
4228	RWS11	-122.4613273	37.93786	-0.6	5.28	-342.4	4.67899978	-122.46132	37.93788	-0.69	5.28	-335.77	4.58699983	11	7/24/2014	24:20.7
4229	RWS11	-122.4613294	37.93786	-0.6	5.27	-342.6	4.66700017	-122.46132	37.93788	-0.69	5.27	-335.99	4.57500023	11	7/24/2014	24:20.8
4230	RWS11	-122.4613316	37.93786	-0.57	5.26	-342.8	4.69100004	-122.46133	37.93788	-0.69	5.26	-336.31	4.56400007	11	7/24/2014	24:20.9
4231	RWS11	-122.4613348	37.93787	-0.6	5.25	-343.2	4.64599979	-122.46133	37.93788	-0.69	5.25	-336.74	4.55399984	11	7/24/2014	24:21.0
4232	RWS11	-122.4613369	37.93787	-0.57	5.24	-343.6	4.67299992	-122.46133	37.93788	-0.69	5.24	-337.4	4.54599994	11	7/24/2014	24:21.1
4233	RWS11	-122.4613391	37.93787	-0.6	5.23	-344	4.63099992	-122.46133	37.93788	-0.69	5.23	-337.86	4.53899997	11	7/24/2014	24:21.2
4234	RWS11	-122.4613413	37.93787	-0.6	5.22	-344.4	4.62499988	-122.46134	37.93788	-0.69	5.22	-338.42	4.53299993	11	7/24/2014	24:21.3
4235	RWS11	-122.4613435	37.93787	-0.6	5.22	-344.8	4.6220001	-122.46134	37.93788	-0.69	5.22	-338.81	4.53000015	11	7/24/2014	24:21.4
4236	RWS11	-122.4613457	37.93787	-0.6	5.22	-345.1	4.62100017	-122.46134	37.93788	-0.69	5.22	-339.18	4.52900022	11	7/24/2014	24:21.5
4237	RWS11	-122.4613479	37.93787	-0.6	5.22	-345.4	4.62100017	-122.46134	37.93788	-0.69	5.22	-339.46	4.52900022	11	7/24/2014	24:21.6
4238	RWS11	-122.4613501	37.93787	-0.6	5.22	-345.7	4.62300003	-122.46134	37.93788	-0.66	5.22	-339.8	4.56500006	11	7/24/2014	24:21.7
4239	RWS11	-122.4613523	37.93787	-0.6	5.23	-346.1	4.62599981	-122.46135	37.93788	-0.73	5.23	-340.28	4.49999982	11	7/24/2014	24:21.8
4240	RWS11	-122.4613544	37.93787	-0.6	5.23	-346.4	4.62900007	-122.46135	37.93788	-0.69	5.23	-340.65	4.53700012	11	7/24/2014	24:21.9
4241	RWS11	-122.4613577	37.93787	-0.6	5.23	-346.8	4.63299978	-122.46135	37.93788	-0.73	5.23	-340.97	4.50699979	11	7/24/2014	24:22.0
4242	RWS11	-122.4613599	37.93787	-0.6	5.24	-347.1	4.63799989	-122.46135	37.93789	-0.73	5.24	-341.24	4.51199991	11	7/24/2014	24:22.1
4243	RWS11	-122.461362	37.93787	-0.6	5.24	-347.5	4.64200008	-122.46136	37.93789	-0.73	5.24	-341.51	4.51600009	11	7/24/2014	24:22.2
4244	RWS11	-122.4613642	37.93787	-0.6	5.25	-347.8	4.64700019	-122.46136	37.93789	-0.78	5.25	-341.93	4.47000021	11	7/24/2014	24:22.3
4245	RWS11	-122.4613664	37.93787	-0.65	5.25	-348.1	4.60099983	-122.46136	37.93789	-0.73	5.25	-342.32	4.52599984	11	7/24/2014	24:22.4
4246	RWS11	-122.4613686	37.93787	-0.65	5.26	-348.3	4.60599995	-122.46136	37.93789	-0.73	5.26	-342.64	4.53099996	11	7/24/2014	24:22.5
4247	RWS11	-122.4613708	37.93788	-0.65	5.26	-348.5	4.61100006	-122.46137	37.93789	-0.78	5.26	-342.86	4.48500007	11	7/24/2014	24:22.6
4248	RWS11	-122.4613731	37.93788	-0.6	5.27	-348.8	4.66700017	-122.46137	37.93789	-0.78	5.27	-343.16	4.49000019	11	7/24/2014	24:22.7
4249	RWS11	-122.4613752	37.93788	-0.65	5.27	-349.2	4.62099981	-122.46137	37.93789	-0.78	5.27	-343.58	4.49499983	11	7/24/2014	24:22.8
4250	RWS11	-122.4613774	37.93788	-0.6	5.28	-349.7	4.67799985	-122.46137	37.93789	-0.78	5.28	-344.05	4.50099987	11	7/24/2014	24:22.9
4251	RWS11	-122.4613806	37.93788	-0.65	5.28	-350	4.6329999	-122.46138	37.93789	-0.78	5.28	-344.38	4.50699991	11	7/24/2014	24:23.0
4252	RWS11	-122.4613828	37.93788	-0.65	5.29	-350.3	4.63899994	-122.46138	37.93789	-0.78	5.29	-344.74	4.51299995	11	7/24/2014	24:23.1
4253	RWS11	-122.4613849	37.93788	-0.65	5.3	-350.7	4.64400005	-122.46138	37.93789	-0.78	5.3	-345.2	4.51800007	11	7/24/2014	24:23.2
4254	RWS11	-122.4613871	37.93788	-0.65	5.3	-350.9	4.64699984	-122.46138	37.93789	-0.78	5.3	-345.68	4.52099985	11	7/24/2014	24:23.3
4255	RWS11	-122.4613893	37.93788	-0.65	5.3	-351.2	4.64799976	-122.46138	37.93789	-0.86	5.3	-346.26	4.43799978	11	7/24/2014	24:23.4
4256	RWS11	-122.4613915	37.93788	-0.65	5.3	-351.3	4.64699984	-122.46139	37.93789	-0.78	5.3	-346.79	4.52099985	11	7/24/2014	24:23.5

4257	RWS11	-122.4613936	37.93788	-0.69	5.3	-351.5	4.611	-122.46139	37.93789	-0.81	5.3	-347.32	4.48500001	11	7/24/2014	24:23.6
4258	RWS11	-122.4613958	37.93788	-0.65	5.29	-351.8	4.64099979	-122.46139	37.93789	-0.81	5.29	-347.93	4.48099983	11	7/24/2014	24:23.7
4259	RWS11	-122.461398	37.93788	-0.69	5.29	-352.2	4.60200018	-122.46139	37.9379	-0.86	5.29	-348.38	4.42600018	11	7/24/2014	24:23.8
4260	RWS11	-122.4614002	37.93788	-0.65	5.28	-352.8	4.63000011	-122.46139	37.9379	-0.81	5.28	-348.84	4.47000015	11	7/24/2014	24:23.9
4261	RWS11	-122.4614034	37.93788	-0.69	5.28	-353.3	4.59100002	-122.4614	37.9379	-0.81	5.28	-349.31	4.46500003	11	7/24/2014	24:24.0
4262	RWS11	-122.4614055	37.93788	-0.65	5.27	-354	4.61999989	-122.4614	37.9379	-0.81	5.27	-349.73	4.45999992	11	7/24/2014	24:24.1
4263	RWS11	-122.4614077	37.93789	-0.69	5.27	-354.2	4.58099979	-122.4614	37.9379	-0.86	5.27	-349.84	4.40499979	11	7/24/2014	24:24.2
4264	RWS11	-122.4614099	37.93789	-0.69	5.26	-354.6	4.57600015	-122.4614	37.9379	-0.81	5.26	-350.01	4.45000017	11	7/24/2014	24:24.3
4265	RWS11	-122.4614121	37.93789	-0.69	5.26	-355	4.57199997	-122.46141	37.9379	-0.89	5.26	-350.25	4.36199999	11	7/24/2014	24:24.4
4266	RWS11	-122.4614143	37.93789	-0.69	5.25	-355.3	4.56900018	-122.46141	37.9379	-0.81	5.25	-350.42	4.4430002	11	7/24/2014	24:24.5
4267	RWS11	-122.4614166	37.93789	-0.69	5.25	-355.7	4.565	-122.46141	37.9379	-0.86	5.25	-350.75	4.389	11	7/24/2014	24:24.6
4268	RWS11	-122.4614188	37.93789	-0.69	5.25	-356.1	4.56200022	-122.46141	37.9379	-0.86	5.25	-351.07	4.38600022	11	7/24/2014	24:24.7
4269	RWS11	-122.461421	37.93789	-0.69	5.24	-356.5	4.55899996	-122.46142	37.9379	-0.86	5.24	-351.42	4.38299996	11	7/24/2014	24:24.8
4270	RWS11	-122.4614231	37.93789	-0.69	5.24	-356.8	4.55499977	-122.46142	37.9379	-0.86	5.24	-351.89	4.37899977	11	7/24/2014	24:24.9
4271	RWS11	-122.4614264	37.93789	-0.72	5.24	-357.2	4.51700008	-122.46142	37.9379	-0.86	5.24	-352.35	4.37500006	11	7/24/2014	24:25.0
4272	RWS11	-122.4614285	37.93789	-0.69	5.23	-357.6	4.54699987	-122.46142	37.9379	-0.81	5.23	-352.98	4.42099988	11	7/24/2014	24:25.1
4273	RWS11	-122.4614307	37.93789	-0.72	5.23	-358.1	4.50800025	-122.46143	37.9379	-0.86	5.23	-353.49	4.36600024	11	7/24/2014	24:25.2
4274	RWS11	-122.4614329	37.93789	-0.69	5.22	-358.6	4.53700012	-122.46143	37.93791	-0.81	5.22	-353.95	4.41100013	11	7/24/2014	24:25.3
4275	RWS11	-122.4614351	37.93789	-0.72	5.22	-359.1	4.49899995	-122.46143	37.93791	-0.86	5.22	-354.43	4.35699993	11	7/24/2014	24:25.4
4276	RWS11	-122.4614373	37.93789	-0.69	5.21	-359.7	4.52900022	-122.46143	37.93791	-0.81	5.21	-354.97	4.40300024	11	7/24/2014	24:25.5
4277	RWS11	-122.4614395	37.93789	-0.69	5.21	-360.3	4.52599996	-122.46143	37.93791	-0.81	5.21	-355.73	4.39999998	11	7/24/2014	24:25.6
4278	RWS11	-122.4614416	37.93789	-0.72	5.21	-360.9	4.49100006	-122.46144	37.93791	-0.81	5.21	-356.53	4.39900005	11	7/24/2014	24:25.7
4279	RWS11	-122.4614438	37.9379	-0.69	5.21	-361.7	4.52500004	-122.46144	37.93791	-0.86	5.21	-357.51	4.34900004	11	7/24/2014	24:25.8
4280	RWS11	-122.461446	37.9379	-0.69	5.21	-362.5	4.52699989	-122.46144	37.93791	-0.78	5.21	-358.35	4.43499988	11	7/24/2014	24:25.9
4281	RWS11	-122.4614492	37.9379	-0.69	5.21	-363.3	4.52900022	-122.46144	37.93791	-0.86	5.21	-359.15	4.35300022	11	7/24/2014	24:26.0
4282	RWS11	-122.4614514	37.9379	-0.69	5.22	-364	4.53200001	-122.46145	37.93791	-0.81	5.22	-359.91	4.40600002	11	7/24/2014	24:26.1
4283	RWS11	-122.4614536	37.9379	-0.69	5.22	-364.8	4.53499979	-122.46145	37.93791	-0.81	5.22	-360.8	4.4089998	11	7/24/2014	24:26.2
4284	RWS11	-122.4614558	37.9379	-0.69	5.22	-365.5	4.53700012	-122.46145	37.93791	-0.81	5.22	-361.64	4.41100013	11	7/24/2014	24:26.3
4285	RWS11	-122.461458	37.9379	-0.69	5.22	-366.2	4.53899997	-122.46145	37.93791	-0.78	5.22	-362.49	4.44699997	11	7/24/2014	24:26.4
4286	RWS11	-122.4614602	37.9379	-0.69	5.22	-367	4.5399999	-122.46145	37.93791	-0.78	5.22	-363.18	4.44799989	11	7/24/2014	24:26.5
4287	RWS11	-122.4614625	37.9379	-0.72	5.22	-367.9	4.50599992	-122.46146	37.93791	-0.78	5.22	-364.07	4.44799989	11	7/24/2014	24:26.6
4288	RWS11	-122.4614647	37.9379	-0.69	5.22	-368.8	4.53899997	-122.46146	37.93791	-0.73	5.22	-364.98	4.49799997	11	7/24/2014	24:26.7
4289	RWS11	-122.4614669	37.9379	-0.69	5.22	-369.7	4.53700012	-122.46146	37.93791	-0.78	5.22	-365.86	4.44500011	11	7/24/2014	24:26.8

4290	RWS11	-122.4614692	37.9379	-0.65	5.22	-370.7	4.56799984	-122.46146	37.93791	-0.73	5.22	-366.69	4.49299985	11	7/24/2014	24:26.9
4291	RWS11	-122.4614725	37.9379	-0.69	5.22	-371.6	4.53000015	-122.46147	37.93792	-0.73	5.22	-367.75	4.48900014	11	7/24/2014	24:27.0
4292	RWS11	-122.4614747	37.9379	-0.65	5.21	-372.6	4.55900002	-122.46147	37.93792	-0.69	5.21	-368.72	4.51800007	11	7/24/2014	24:27.1
4293	RWS11	-122.461477	37.9379	-0.69	5.2	-373.6	4.51999992	-122.46147	37.93792	-0.73	5.2	-369.79	4.47899991	11	7/24/2014	24:27.2
4294	RWS11	-122.4614793	37.9379	-0.65	5.2	-374.5	4.54799986	-122.46147	37.93792	-0.69	5.2	-370.7	4.50699991	11	7/24/2014	24:27.3
4295	RWS11	-122.4614816	37.93791	-0.69	5.19	-375.3	4.50699991	-122.46148	37.93792	-0.69	5.19	-371.74	4.49999994	11	7/24/2014	24:27.4
4296	RWS11	-122.4614839	37.93791	-0.65	5.18	-376.3	4.53200006	-122.46148	37.93792	-0.73	5.18	-372.93	4.45700008	11	7/24/2014	24:27.5
4297	RWS11	-122.4614862	37.93791	-0.69	5.17	-377.2	4.48699993	-122.46148	37.93792	-0.69	5.17	-373.9	4.47999996	11	7/24/2014	24:27.6
4298	RWS11	-122.4614884	37.93791	-0.65	5.16	-378.1	4.50899982	-122.46148	37.93792	-0.69	5.16	-374.88	4.46799988	11	7/24/2014	24:27.7
4299	RWS11	-122.4614907	37.93791	-0.65	5.15	-378.7	4.49599981	-122.46149	37.93792	-0.69	5.15	-375.5	4.45499986	11	7/24/2014	24:27.8
4300	RWS11	-122.4614929	37.93791	-0.65	5.13	-379.4	4.48199987	-122.46149	37.93792	-0.66	5.13	-376.2	4.4749999	11	7/24/2014	24:27.9
4301	RWS11	-122.4614961	37.93791	-0.65	5.12	-380.1	4.46700001	-122.46149	37.93792	-0.66	5.12	-376.87	4.46000004	11	7/24/2014	24:28.0
4302	RWS11	-122.4614983	37.93791	-0.65	5.1	-380.8	4.45200014	-122.46149	37.93792	-0.66	5.1	-377.58	4.44500017	11	7/24/2014	24:28.1
4303	RWS11	-122.4615004	37.93791	-0.65	5.09	-381.6	4.4369998	-122.4615	37.93792	-0.61	5.09	-378.36	4.48099983	11	7/24/2014	24:28.2
4304	RWS11	-122.4615026	37.93791	-0.6	5.07	-382.3	4.472	-122.4615	37.93792	-0.66	5.07	-379.36	4.41400003	11	7/24/2014	24:28.3
4305	RWS11	-122.4615047	37.93791	-0.65	5.06	-383.1	4.40700006	-122.4615	37.93792	-0.66	5.06	-380.38	4.4000001	11	7/24/2014	24:28.4
4306	RWS11	-122.4615068	37.93791	-0.6	5.04	-384.1	4.4430002	-122.4615	37.93792	-0.61	5.04	-381.35	4.43600023	11	7/24/2014	24:28.5
4307	RWS11	-122.4615089	37.93791	-0.65	5.03	-385.1	4.37900019	-122.4615	37.93793	-0.66	5.03	-382.41	4.37200022	11	7/24/2014	24:28.6
4308	RWS11	-122.461511	37.93791	-0.6	5.02	-386.1	4.4180001	-122.46151	37.93793	-0.61	5.02	-383.37	4.41100013	11	7/24/2014	24:28.7
4309	RWS11	-122.4615131	37.93791	-0.6	5.01	-387.1	4.40799987	-122.46151	37.93793	-0.57	5.01	-384.28	4.4339999	11	7/24/2014	24:28.8
4310	RWS11	-122.4615151	37.93791	-0.6	5	-387.9	4.39999998	-122.46151	37.93793	-0.57	5	-385.25	4.426	11	7/24/2014	24:28.9
4311	RWS11	-122.4615181	37.93791	-0.65	4.99	-388.8	4.34200001	-122.46151	37.93793	-0.57	4.99	-386.14	4.41900003	11	7/24/2014	24:29.0
4312	RWS11	-122.4615201	37.93792	-0.6	4.99	-389.8	4.38699996	-122.46151	37.93793	-0.57	4.99	-387.07	4.41299999	11	7/24/2014	24:29.1
4313	RWS11	-122.4615221	37.93792	-0.6	4.98	-390.5	4.38099992	-122.46152	37.93793	-0.57	4.98	-387.85	4.40699995	11	7/24/2014	24:29.2
4314	RWS11	-122.4615241	37.93792	-0.57	4.97	-391.1	4.40899998	-122.46152	37.93793	-0.57	4.97	-388.41	4.39999998	11	7/24/2014	24:29.3
4315	RWS11	-122.4615261	37.93792	-0.57	4.97	-391.8	4.40100008	-122.46152	37.93793	-0.57	4.97	-389.15	4.39200008	11	7/24/2014	24:29.4
4316	RWS11	-122.4615281	37.93792	-0.57	4.96	-392.4	4.39199978	-122.46152	37.93793	-0.57	4.96	-389.76	4.38299978	11	7/24/2014	24:29.5
4317	RWS11	-122.46153	37.93792	-0.57	4.95	-393.1	4.3810001	-122.46152	37.93793	-0.57	4.95	-390.49	4.3720001	11	7/24/2014	24:29.6
4318	RWS11	-122.461532	37.93792	-0.57	4.93	-393.8	4.36800009	-122.46153	37.93793	-0.52	4.93	-391.25	4.41000009	11	7/24/2014	24:29.7
4319	RWS11	-122.461534	37.93792	-0.57	4.92	-394.5	4.35199982	-122.46153	37.93793	-0.57	4.92	-391.99	4.34299982	11	7/24/2014	24:29.8
4320	RWS11	-122.4615359	37.93792	-0.57	4.9	-395.2	4.33400017	-122.46153	37.93793	-0.52	4.9	-392.63	4.37600017	11	7/24/2014	24:29.9
4321	RWS11	-122.4615389	37.93792	-0.57	4.88	-395.9	4.31400019	-122.46153	37.93793	-0.52	4.88	-393.37	4.35600019	11	7/24/2014	24:30.0
4322	RWS11	-122.4615409	37.93792	-0.51	4.86	-396.7	4.34299988	-122.46154	37.93793	-0.52	4.86	-394.26	4.33399987	11	7/24/2014	24:30.1

4323	RWS11	-122.4615429	37.93792	-0.51	4.84	-397.6	4.32100004	-122.46154	37.93793	-0.49	4.84	-395.3	4.34600005	11	7/24/2014	24:30.2
4324	RWS11	-122.4615449	37.93792	-0.51	4.81	-398.3	4.2990002	-122.46154	37.93793	-0.49	4.81	-396.15	4.32400021	11	7/24/2014	24:30.3
4325	RWS11	-122.461547	37.93792	-0.51	4.79	-399.1	4.27799982	-122.46154	37.93794	-0.52	4.79	-397.05	4.26899981	11	7/24/2014	24:30.4
4326	RWS11	-122.461549	37.93792	-0.51	4.77	-399.9	4.25899976	-122.46154	37.93794	-0.49	4.77	-397.92	4.28399977	11	7/24/2014	24:30.5
4327	RWS11	-122.4615511	37.93792	-0.51	4.76	-400.6	4.24200004	-122.46155	37.93794	-0.49	4.76	-398.79	4.26700005	11	7/24/2014	24:30.6
4328	RWS11	-122.4615531	37.93792	-0.48	4.74	-401.3	4.26100019	-122.46155	37.93794	-0.49	4.74	-399.5	4.25200018	11	7/24/2014	24:30.7
4329	RWS11	-122.4615552	37.93792	-0.51	4.73	-402.1	4.21400017	-122.46155	37.93794	-0.49	4.73	-400.2	4.23900017	11	7/24/2014	24:30.8
4330	RWS11	-122.4615572	37.93792	-0.48	4.72	-402.7	4.23700002	-122.46155	37.93794	-0.44	4.72	-400.82	4.27900001	11	7/24/2014	24:30.9
4331	RWS11	-122.4615602	37.93793	-0.51	4.71	-403.1	4.19299978	-122.46155	37.93794	-0.52	4.71	-401.36	4.18399978	11	7/24/2014	24:31.0
4332	RWS11	-122.4615622	37.93793	-0.48	4.7	-403.6	4.21799996	-122.46156	37.93794	-0.44	4.7	-401.91	4.25999996	11	7/24/2014	24:31.1
4333	RWS11	-122.4615642	37.93793	-0.51	4.69	-404	4.17500013	-122.46156	37.93794	-0.49	4.69	-402.38	4.20000014	11	7/24/2014	24:31.2
4334	RWS11	-122.4615662	37.93793	-0.48	4.68	-404.4	4.20200017	-122.46156	37.93794	-0.49	4.68	-402.97	4.19300017	11	7/24/2014	24:31.3
4335	RWS11	-122.4615683	37.93793	-0.48	4.68	-404.8	4.19700006	-122.46156	37.93794	-0.49	4.68	-403.53	4.18800005	11	7/24/2014	24:31.4
4336	RWS11	-122.4615703	37.93793	-0.48	4.67	-405.2	4.1939998	-122.46157	37.93794	-0.44	4.67	-404.22	4.23599979	11	7/24/2014	24:31.5
4337	RWS11	-122.4615723	37.93793	-0.48	4.68	-405.6	4.1950002	-122.46157	37.93794	-0.44	4.68	-404.87	4.2370002	11	7/24/2014	24:31.6
4338	RWS11	-122.4615743	37.93793	-0.43	4.68	-406.1	4.24899998	-122.46157	37.93794	-0.44	4.68	-405.54	4.23999998	11	7/24/2014	24:31.7
4339	RWS11	-122.4615763	37.93793	-0.48	4.68	-406.6	4.20499995	-122.46157	37.93794	-0.44	4.68	-406.22	4.24699995	11	7/24/2014	24:31.8
4340	RWS11	-122.4615783	37.93793	-0.48	4.7	-407.1	4.21500018	-122.46157	37.93794	-0.44	4.7	-406.87	4.25700018	11	7/24/2014	24:31.9
4341	RWS11	-122.4615813	37.93793	-0.48	4.71	-407.6	4.22699979	-122.46158	37.93794	-0.4	4.71	-407.52	4.30299976	11	7/24/2014	24:32.0
4342	RWS11	-122.4615833	37.93793	-0.43	4.72	-408.1	4.2909998	-122.46158	37.93794	-0.44	4.72	-408.24	4.2819998	11	7/24/2014	24:32.1
4343	RWS11	-122.4615852	37.93793	-0.48	4.73	-408.5	4.25400022	-122.46158	37.93794	-0.4	4.73	-408.99	4.33000019	11	7/24/2014	24:32.2
4344	RWS11	-122.4615873	37.93793	-0.43	4.75	-409.1	4.32000008	-122.46158	37.93795	-0.4	4.75	-409.77	4.34500006	11	7/24/2014	24:32.3
4345	RWS11	-122.4615893	37.93793	-0.43	4.76	-409.8	4.33599988	-122.46158	37.93795	-0.44	4.76	-410.64	4.32699987	11	7/24/2014	24:32.4
4346	RWS11	-122.4615913	37.93793	-0.43	4.78	-410.5	4.35300007	-122.46159	37.93795	-0.4	4.78	-411.5	4.37800005	11	7/24/2014	24:32.5
4347	RWS11	-122.4615933	37.93793	-0.43	4.8	-411.2	4.3710002	-122.46159	37.93795	-0.4	4.8	-412.37	4.39600018	11	7/24/2014	24:32.6
4348	RWS11	-122.4615953	37.93793	-0.43	4.82	-411.9	4.38899985	-122.46159	37.93795	-0.4	4.82	-413.09	4.41399983	11	7/24/2014	24:32.7
4349	RWS11	-122.4615973	37.93794	-0.43	4.84	-412.5	4.40600005	-122.46159	37.93795	-0.44	4.84	-413.76	4.39700004	11	7/24/2014	24:32.8
4350	RWS11	-122.4615993	37.93794	-0.43	4.85	-413.2	4.42300025	-122.46159	37.93795	-0.4	4.85	-414.47	4.44800022	11	7/24/2014	24:32.9
4351	RWS11	-122.4616022	37.93794	-0.43	4.87	-413.8	4.43999997	-122.4616	37.93795	-0.44	4.87	-415.27	4.43099996	11	7/24/2014	24:33.0
4352	RWS11	-122.4616041	37.93794	-0.43	4.89	-414.4	4.45600024	-122.4616	37.93795	-0.44	4.89	-415.98	4.44700024	11	7/24/2014	24:33.1
4353	RWS11	-122.4616061	37.93794	-0.43	4.9	-414.9	4.47100011	-122.4616	37.93795	-0.4	4.9	-416.64	4.49600008	11	7/24/2014	24:33.2
4354	RWS11	-122.4616081	37.93794	-0.43	4.91	-415.4	4.48500004	-122.4616	37.93795	-0.44	4.91	-417.2	4.47600004	11	7/24/2014	24:33.3
4355	RWS11	-122.4616101	37.93794	-0.43	4.93	-415.7	4.49899998	-122.4616	37.93795	-0.4	4.93	-417.62	4.52399996	11	7/24/2014	24:33.4

4356	RWS11	-122.4616121	37.93794	-0.39	4.94	-416.1	4.54600006	-122.46161	37.93795	-0.4	4.94	-418.14	4.53600004	11	7/24/2014	24:33.5
4357	RWS11	-122.461614	37.93794	-0.43	4.95	-416.5	4.52300015	-122.46161	37.93795	-0.4	4.95	-418.68	4.54800013	11	7/24/2014	24:33.6
4358	RWS11	-122.461616	37.93794	-0.43	4.96	-416.9	4.53399983	-122.46161	37.93795	-0.4	4.96	-419.22	4.55899981	11	7/24/2014	24:33.7
4359	RWS11	-122.4616179	37.93794	-0.48	4.97	-417.2	4.49200013	-122.46161	37.93795	-0.44	4.97	-419.74	4.53400013	11	7/24/2014	24:33.8
4360	RWS11	-122.4616199	37.93794	-0.43	4.98	-417.6	4.55199996	-122.46161	37.93795	-0.44	4.98	-420.26	4.54299995	11	7/24/2014	24:33.9
4361	RWS11	-122.4616228	37.93794	-0.43	4.99	-417.9	4.55999985	-122.46162	37.93795	-0.44	4.99	-420.73	4.55099985	11	7/24/2014	24:34.0
4362	RWS11	-122.4616247	37.93794	-0.43	5	-418.2	4.56699982	-122.46162	37.93795	-0.4	5	-421.21	4.5919998	11	7/24/2014	24:34.1
4363	RWS11	-122.4616266	37.93794	-0.43	5	-418.5	4.57399979	-122.46162	37.93796	-0.44	5	-421.6	4.56499979	11	7/24/2014	24:34.2
4364	RWS11	-122.4616286	37.93794	-0.43	5.01	-418.8	4.58100024	-122.46162	37.93796	-0.4	5.01	-421.92	4.60600021	11	7/24/2014	24:34.3
4365	RWS11	-122.4616305	37.93794	-0.43	5.02	-419	4.58900014	-122.46163	37.93796	-0.44	5.02	-422.22	4.58000013	11	7/24/2014	24:34.4
4366	RWS11	-122.4616325	37.93794	-0.43	5.03	-419.4	4.59799996	-122.46163	37.93796	-0.44	5.03	-422.68	4.58899996	11	7/24/2014	24:34.5
4367	RWS11	-122.4616344	37.93794	-0.43	5.04	-419.8	4.60699978	-122.46163	37.93796	-0.44	5.04	-423.16	4.59799978	11	7/24/2014	24:34.6
4368	RWS11	-122.4616363	37.93795	-0.43	5.05	-420.1	4.61700001	-122.46163	37.93796	-0.4	5.05	-423.63	4.64199999	11	7/24/2014	24:34.7
4369	RWS11	-122.4616382	37.93795	-0.48	5.06	-420.4	4.57600024	-122.46163	37.93796	-0.44	5.06	-424.04	4.61800024	11	7/24/2014	24:34.8
4370	RWS11	-122.4616401	37.93795	-0.43	5.07	-420.7	4.63899985	-122.46163	37.93796	-0.44	5.07	-424.48	4.62999985	11	7/24/2014	24:34.9
4371	RWS11	-122.461643	37.93795	-0.43	5.08	-420.9	4.65099993	-122.46164	37.93796	-0.44	5.08	-424.85	4.64199993	11	7/24/2014	24:35.0
4372	RWS11	-122.4616448	37.93795	-0.43	5.09	-421.2	4.66399994	-122.46164	37.93796	-0.44	5.09	-425.22	4.65499994	11	7/24/2014	24:35.1
4373	RWS11	-122.4616467	37.93795	-0.43	5.11	-421.5	4.67799988	-122.46164	37.93796	-0.44	5.11	-425.63	4.66899988	11	7/24/2014	24:35.2
4374	RWS11	-122.4616486	37.93795	-0.43	5.12	-421.6	4.6909999	-122.46164	37.93796	-0.44	5.12	-425.93	4.68199989	11	7/24/2014	24:35.3
4375	RWS11	-122.4616505	37.93795	-0.48	5.13	-421.8	4.65399984	-122.46165	37.93796	-0.44	5.13	-426.19	4.69599983	11	7/24/2014	24:35.4
4376	RWS11	-122.4616524	37.93795	-0.43	5.15	-422.1	4.71899977	-122.46165	37.93796	-0.4	5.15	-426.49	4.74399975	11	7/24/2014	24:35.5
4377	RWS11	-122.4616543	37.93795	-0.48	5.16	-422.3	4.68300012	-122.46165	37.93796	-0.49	5.16	-426.8	4.67400011	11	7/24/2014	24:35.6
4378	RWS11	-122.4616562	37.93795	-0.43	5.18	-422.6	4.74800006	-122.46165	37.93796	-0.4	5.18	-427.13	4.77300003	11	7/24/2014	24:35.7
4379	RWS11	-122.4616581	37.93795	-0.48	5.19	-422.9	4.71199992	-122.46165	37.93796	-0.44	5.19	-427.46	4.75399992	11	7/24/2014	24:35.8
4380	RWS11	-122.46166	37.93795	-0.48	5.21	-423.1	4.72800019	-122.46165	37.93796	-0.44	5.21	-427.8	4.77000019	11	7/24/2014	24:35.9
4381	RWS11	-122.4616628	37.93795	-0.43	5.22	-423.5	4.79400012	-122.46166	37.93796	-0.44	5.22	-428.18	4.78500012	11	7/24/2014	24:36.0
4382	RWS11	-122.4616647	37.93795	-0.43	5.24	-423.9	4.80800006	-122.46166	37.93797	-0.4	5.24	-428.64	4.83300006	11	7/24/2014	24:36.1
4383	RWS11	-122.4616666	37.93795	-0.48	5.25	-424.3	4.76900014	-122.46166	37.93797	-0.4	5.25	-429.18	4.84500015	11	7/24/2014	24:36.2
4384	RWS11	-122.4616685	37.93795	-0.43	5.26	-424.9	4.82899997	-122.46166	37.93797	-0.4	5.26	-429.76	4.85399997	11	7/24/2014	24:36.3
4385	RWS11	-122.4616704	37.93795	-0.43	5.26	-425.4	4.83599994	-122.46167	37.93797	-0.4	5.26	-430.41	4.86099994	11	7/24/2014	24:36.4
4386	RWS11	-122.4616723	37.93795	-0.43	5.27	-426.1	4.84100005	-122.46167	37.93797	-0.35	5.27	-431.09	4.91600007	11	7/24/2014	24:36.5
4387	RWS11	-122.4616743	37.93795	-0.43	5.27	-426.8	4.84499976	-122.46167	37.93797	-0.4	5.27	-431.96	4.86999977	11	7/24/2014	24:36.6
4388	RWS11	-122.4616762	37.93796	-0.39	5.28	-427.6	4.88399994	-122.46167	37.93797	-0.35	5.28	-432.97	4.92399997	11	7/24/2014	24:36.7

4389	RWS11	-122.4616781	37.93796	-0.43	5.28	-428.5	4.85400006	-122.46167	37.93797	-0.35	5.28	-433.99	4.92900008	11	7/24/2014	24:36.8
4390	RWS11	-122.4616799	37.93796	-0.39	5.29	-429.3	4.8950001	-122.46167	37.93797	-0.35	5.29	-434.86	4.93500012	11	7/24/2014	24:36.9
4391	RWS11	-122.4616827	37.93796	-0.43	5.3	-430.1	4.86899993	-122.46168	37.93797	-0.35	5.3	-435.7	4.94399995	11	7/24/2014	24:37.0
4392	RWS11	-122.4616846	37.93796	-0.39	5.31	-431	4.91400015	-122.46168	37.93797	-0.35	5.31	-436.59	4.95400017	11	7/24/2014	24:37.1
4393	RWS11	-122.4616865	37.93796	-0.43	5.32	-431.7	4.89099976	-122.46168	37.93797	-0.35	5.32	-437.24	4.96599978	11	7/24/2014	24:37.2
4394	RWS11	-122.4616884	37.93796	-0.39	5.33	-432.2	4.94000018	-122.46168	37.93797	-0.32	5.33	-437.78	5.01400018	11	7/24/2014	24:37.3
4395	RWS11	-122.4616903	37.93796	-0.39	5.35	-432.8	4.95500004	-122.46169	37.93797	-0.35	5.35	-438.3	4.99500006	11	7/24/2014	24:37.4
4396	RWS11	-122.4616921	37.93796	-0.39	5.36	-433.4	4.97099984	-122.46169	37.93797	-0.32	5.36	-438.82	5.04499984	11	7/24/2014	24:37.5
4397	RWS11	-122.461694	37.93796	-0.39	5.38	-433.8	4.98899996	-122.46169	37.93797	-0.35	5.38	-439.13	5.02899998	11	7/24/2014	24:37.6
4398	RWS11	-122.4616959	37.93796	-0.39	5.4	-434.2	5.00700009	-122.46169	37.93797	-0.32	5.4	-439.43	5.08100009	11	7/24/2014	24:37.7
4399	RWS11	-122.4616977	37.93796	-0.43	5.42	-434.5	4.99100015	-122.46169	37.93797	-0.32	5.42	-439.75	5.10000014	11	7/24/2014	24:37.8
4400	RWS11	-122.4616996	37.93796	-0.39	5.44	-434.9	5.0450002	-122.46169	37.93797	-0.32	5.44	-440.06	5.1190002	11	7/24/2014	24:37.9
4401	RWS11	-122.4617023	37.93796	-0.39	5.46	-435.2	5.06299984	-122.4617	37.93797	-0.35	5.46	-440.41	5.10299987	11	7/24/2014	24:38.0
4402	RWS11	-122.4617041	37.93796	-0.39	5.47	-435.6	5.08099997	-122.4617	37.93798	-0.32	5.47	-440.93	5.15499997	11	7/24/2014	24:38.1
4403	RWS11	-122.4617059	37.93796	-0.39	5.49	-436	5.09800017	-122.4617	37.93798	-0.32	5.49	-441.36	5.17200017	11	7/24/2014	24:38.2
4404	RWS11	-122.4617078	37.93796	-0.39	5.51	-436.3	5.11499989	-122.4617	37.93798	-0.32	5.51	-441.84	5.18899989	11	7/24/2014	24:38.3
4405	RWS11	-122.4617096	37.93796	-0.39	5.53	-436.8	5.13200009	-122.4617	37.93798	-0.35	5.53	-442.49	5.17200011	11	7/24/2014	24:38.4
4406	RWS11	-122.4617114	37.93796	-0.39	5.54	-437.2	5.14799988	-122.46171	37.93798	-0.35	5.54	-443.04	5.1879999	11	7/24/2014	24:38.5
4407	RWS11	-122.4617132	37.93796	-0.39	5.56	-437.5	5.16400015	-122.46171	37.93798	-0.32	5.56	-443.55	5.23800015	11	7/24/2014	24:38.6
4408	RWS11	-122.461715	37.93797	-0.39	5.57	-437.8	5.17900002	-122.46171	37.93798	-0.32	5.57	-444.02	5.25300002	11	7/24/2014	24:38.7
4409	RWS11	-122.4617167	37.93797	-0.43	5.59	-438.2	5.15899989	-122.46171	37.93798	-0.35	5.59	-444.37	5.23399991	11	7/24/2014	24:38.8
4410	RWS11	-122.4617184	37.93797	-0.39	5.6	-438.5	5.20900023	-122.46171	37.93798	-0.35	5.6	-444.71	5.24900025	11	7/24/2014	24:38.9
4411	RWS11	-122.4617209	37.93797	-0.39	5.62	-438.8	5.22500002	-122.46172	37.93798	-0.35	5.62	-444.99	5.26500005	11	7/24/2014	24:39.0
4412	RWS11	-122.4617225	37.93797	-0.39	5.63	-439	5.23899996	-122.46172	37.93798	-0.35	5.63	-445.26	5.27899998	11	7/24/2014	24:39.1
4413	RWS11	-122.4617241	37.93797	-0.43	5.65	-439.1	5.21899983	-122.46172	37.93798	-0.35	5.65	-445.45	5.29399985	11	7/24/2014	24:39.2
4414	RWS11	-122.4617258	37.93797	-0.39	5.66	-439.3	5.26900017	-122.46172	37.93798	-0.35	5.66	-445.67	5.30900019	11	7/24/2014	24:39.3
4415	RWS11	-122.4617274	37.93797	-0.39	5.68	-439.4	5.28300011	-122.46172	37.93798	-0.35	5.68	-445.84	5.32300013	11	7/24/2014	24:39.4
4416	RWS11	-122.461729	37.93797	-0.39	5.69	-439.6	5.29700005	-122.46172	37.93798	-0.35	5.69	-446.06	5.33700007	11	7/24/2014	24:39.5
4417	RWS11	-122.4617306	37.93797	-0.43	5.7	-439.7	5.27400014	-122.46173	37.93798	-0.35	5.7	-446.37	5.34900016	11	7/24/2014	24:39.6
4418	RWS11	-122.4617322	37.93797	-0.39	5.71	-439.9	5.32100022	-122.46173	37.93798	-0.35	5.71	-446.64	5.36100024	11	7/24/2014	24:39.7
4419	RWS11	-122.4617338	37.93797	-0.43	5.72	-440	5.2969999	-122.46173	37.93798	-0.4	5.72	-446.94	5.32199991	11	7/24/2014	24:39.8
4420	RWS11	-122.4617354	37.93797	-0.39	5.74	-440.2	5.34300005	-122.46173	37.93798	-0.35	5.74	-447.25	5.38300008	11	7/24/2014	24:39.9
4421	RWS11	-122.4617379	37.93797	-0.43	5.75	-440.5	5.31799981	-122.46173	37.93798	-0.4	5.75	-447.62	5.34299982	11	7/24/2014	24:40.0

4422	RWS11	-122.4617396	37.93797	-0.39	5.76	-440.8	5.36399996	-122.46173	37.93798	-0.35	5.76	-447.95	5.40399998	11	7/24/2014	24:40.1
4423	RWS11	-122.4617413	37.93797	-0.43	5.77	-441.1	5.3390002	-122.46174	37.93798	-0.4	5.77	-448.25	5.3640002	11	7/24/2014	24:40.2
4424	RWS11	-122.461743	37.93797	-0.39	5.78	-441.4	5.38499987	-122.46174	37.93799	-0.35	5.78	-448.39	5.42499989	11	7/24/2014	24:40.3
4425	RWS11	-122.4617448	37.93797	-0.43	5.79	-441.7	5.3600001	-122.46174	37.93799	-0.4	5.79	-448.64	5.38500011	11	7/24/2014	24:40.4
4426	RWS11	-122.4617466	37.93797	-0.39	5.8	-442	5.40499985	-122.46174	37.93799	-0.35	5.8	-448.81	5.44499987	11	7/24/2014	24:40.5
4427	RWS11	-122.4617484	37.93797	-0.43	5.81	-442.3	5.37900016	-122.46174	37.93799	-0.35	5.81	-448.98	5.45400017	11	7/24/2014	24:40.6
4428	RWS11	-122.4617502	37.93797	-0.39	5.82	-442.7	5.42399991	-122.46174	37.93799	-0.35	5.82	-449.32	5.46399993	11	7/24/2014	24:40.7
4429	RWS11	-122.461752	37.93797	-0.43	5.83	-443	5.40000007	-122.46175	37.93799	-0.35	5.83	-449.7	5.47500008	11	7/24/2014	24:40.8
4430	RWS11	-122.4617538	37.93797	-0.39	5.84	-443.5	5.44700015	-122.46175	37.93799	-0.35	5.84	-450.18	5.48700017	11	7/24/2014	24:40.9
4431	RWS11	-122.4617565	37.93798	-0.39	5.85	-443.9	5.46000016	-122.46175	37.93799	-0.35	5.85	-450.68	5.50000018	11	7/24/2014	24:41.0
4432	RWS11	-122.4617583	37.93798	-0.39	5.87	-444.4	5.47300017	-122.46175	37.93799	-0.32	5.87	-451.28	5.54700017	11	7/24/2014	24:41.1
4433	RWS11	-122.4617601	37.93798	-0.39	5.88	-444.9	5.48499978	-122.46175	37.93799	-0.35	5.88	-451.89	5.5249998	11	7/24/2014	24:41.2
4434	RWS11	-122.461762	37.93798	-0.39	5.89	-445.4	5.49699986	-122.46176	37.93799	-0.32	5.89	-452.43	5.57099986	11	7/24/2014	24:41.3
4435	RWS11	-122.4617638	37.93798	-0.39	5.9	-445.9	5.50800002	-122.46176	37.93799	-0.32	5.9	-452.97	5.58200002	11	7/24/2014	24:41.4
4436	RWS11	-122.4617658	37.93798	-0.34	5.91	-446.3	5.57000017	-122.46176	37.93799	-0.32	5.91	-453.43	5.59300017	11	7/24/2014	24:41.5
4437	RWS11	-122.4617677	37.93798	-0.39	5.92	-446.8	5.52899992	-122.46176	37.93799	-0.32	5.92	-453.84	5.60299993	11	7/24/2014	24:41.6
4438	RWS11	-122.4617696	37.93798	-0.39	5.93	-447.3	5.53800023	-122.46176	37.93799	-0.32	5.93	-454.24	5.61200023	11	7/24/2014	24:41.7
4439	RWS11	-122.4617715	37.93798	-0.39	5.94	-447.7	5.54600012	-122.46177	37.93799	-0.35	5.94	-454.69	5.58600014	11	7/24/2014	24:41.8
4440	RWS11	-122.4617735	37.93798	-0.39	5.95	-448.3	5.55300009	-122.46177	37.93799	-0.32	5.95	-455.14	5.62700009	11	7/24/2014	24:41.9
4441	RWS11	-122.4617754	37.93798	-0.39	5.95	-448.7	5.56000006	-122.46177	37.93799	-0.32	5.95	-455.59	5.63400006	11	7/24/2014	24:42.0
4442	RWS11	-122.4617774	37.93798	-0.34	5.96	-449.3	5.61899996	-122.46177	37.93799	-0.29	5.96	-456.09	5.67599997	11	7/24/2014	24:42.1
4443	RWS11	-122.4617793	37.93798	-0.39	5.97	-449.8	5.57699978	-122.46177	37.93799	-0.32	5.97	-456.57	5.65099978	11	7/24/2014	24:42.2
4444	RWS11	-122.4617813	37.93798	-0.34	5.98	-450.3	5.63899994	-122.46178	37.93799	-0.29	5.98	-457.02	5.69599995	11	7/24/2014	24:42.3
4445	RWS11	-122.4617832	37.93798	-0.34	5.99	-450.7	5.65100002	-122.46178	37.938	-0.32	5.99	-457.42	5.67400002	11	7/24/2014	24:42.4
4446	RWS11	-122.4617852	37.93798	-0.34	6.01	-451.1	5.66499996	-122.46178	37.938	-0.32	6.01	-457.72	5.68799996	11	7/24/2014	24:42.5
4447	RWS11	-122.4617871	37.93798	-0.34	6.02	-451.5	5.6789999	-122.46178	37.938	-0.32	6.02	-457.94	5.7019999	11	7/24/2014	24:42.6
4448	RWS11	-122.461789	37.93798	-0.34	6.03	-451.7	5.69299984	-122.46178	37.938	-0.32	6.03	-458.21	5.71599984	11	7/24/2014	24:42.7
4449	RWS11	-122.4617908	37.93798	-0.34	6.05	-452.1	5.70499992	-122.46179	37.938	-0.29	6.05	-458.5	5.76199993	11	7/24/2014	24:42.8
4450	RWS11	-122.4617927	37.93798	-0.34	6.06	-452.4	5.71600008	-122.46179	37.938	-0.32	6.06	-458.8	5.73900008	11	7/24/2014	24:42.9
4451	RWS11	-122.4617954	37.93799	-0.34	6.07	-452.8	5.7249999	-122.46179	37.938	-0.32	6.07	-459.12	5.74799991	11	7/24/2014	24:43.0
4452	RWS11	-122.4617973	37.93799	-0.34	6.07	-453.1	5.73199987	-122.46179	37.938	-0.29	6.07	-459.43	5.78899989	11	7/24/2014	24:43.1
4453	RWS11	-122.4617991	37.93799	-0.34	6.08	-453.5	5.73899984	-122.46179	37.938	-0.32	6.08	-459.8	5.76199985	11	7/24/2014	24:43.2
4454	RWS11	-122.461801	37.93799	-0.31	6.09	-453.9	5.77799997	-122.4618	37.938	-0.23	6.09	-460.25	5.85199997	11	7/24/2014	24:43.3

4455	RWS11	-122.4618028	37.93799	-0.34	6.09	-454.4	5.74800014	-122.4618	37.938	-0.32	6.09	-460.79	5.77100015	11	7/24/2014	24:43.4
4456	RWS11	-122.4618047	37.93799	-0.34	6.09	-454.8	5.75199986	-122.4618	37.938	-0.29	6.09	-461.25	5.80899987	11	7/24/2014	24:43.5
4457	RWS11	-122.4618066	37.93799	-0.31	6.1	-455.3	5.78900012	-122.4618	37.938	-0.29	6.1	-461.83	5.81200013	11	7/24/2014	24:43.6
4458	RWS11	-122.4618085	37.93799	-0.31	6.1	-455.7	5.79099998	-122.4618	37.938	-0.23	6.1	-462.24	5.86499998	11	7/24/2014	24:43.7
4459	RWS11	-122.4618103	37.93799	-0.31	6.1	-456	5.79199991	-122.4618	37.938	-0.29	6.1	-462.62	5.81499991	11	7/24/2014	24:43.8
4460	RWS11	-122.4618121	37.93799	-0.31	6.1	-456.4	5.79299983	-122.46181	37.938	-0.23	6.1	-462.96	5.86699983	11	7/24/2014	24:43.9
4461	RWS11	-122.4618149	37.93799	-0.31	6.1	-456.7	5.79299983	-122.46181	37.938	-0.23	6.1	-463.39	5.86699983	11	7/24/2014	24:44.0
4462	RWS11	-122.4618167	37.93799	-0.31	6.1	-457	5.79199991	-122.46181	37.938	-0.23	6.1	-463.78	5.86599991	11	7/24/2014	24:44.1
4463	RWS11	-122.4618186	37.93799	-0.31	6.1	-457.3	5.79099998	-122.46181	37.938	-0.23	6.1	-464.2	5.86499998	11	7/24/2014	24:44.2
4464	RWS11	-122.4618204	37.93799	-0.31	6.1	-457.6	5.78900012	-122.46182	37.93801	-0.23	6.1	-464.63	5.86300012	11	7/24/2014	24:44.3
4465	RWS11	-122.4618223	37.93799	-0.31	6.09	-458	5.78499994	-122.46182	37.93801	-0.23	6.09	-465.17	5.85899994	11	7/24/2014	24:44.4
4466	RWS11	-122.4618242	37.93799	-0.31	6.09	-458.3	5.78200015	-122.46182	37.93801	-0.2	6.09	-465.6	5.89000015	11	7/24/2014	24:44.5
4467	RWS11	-122.461826	37.93799	-0.31	6.09	-458.6	5.77899989	-122.46182	37.93801	-0.23	6.09	-465.94	5.8529999	11	7/24/2014	24:44.6
4468	RWS11	-122.4618279	37.93799	-0.27	6.09	-459	5.81100005	-122.46182	37.93801	-0.15	6.09	-466.39	5.93500003	11	7/24/2014	24:44.7
4469	RWS11	-122.4618298	37.93799	-0.27	6.08	-459.3	5.81000012	-122.46182	37.93801	-0.2	6.08	-466.8	5.88400011	11	7/24/2014	24:44.8
4470	RWS11	-122.4618316	37.938	-0.27	6.08	-459.7	5.81000012	-122.46183	37.93801	-0.15	6.08	-467.25	5.9340001	11	7/24/2014	24:44.9
4471	RWS11	-122.4618344	37.938	-0.27	6.09	-460.2	5.8129999	-122.46183	37.93801	-0.15	6.09	-467.74	5.93699989	11	7/24/2014	24:45.0
4472	RWS11	-122.4618363	37.938	-0.22	6.09	-460.7	5.86800015	-122.46183	37.93801	-0.15	6.09	-468.25	5.94000015	11	7/24/2014	24:45.1
4473	RWS11	-122.4618381	37.938	-0.27	6.1	-461.2	5.82200021	-122.46183	37.93801	-0.15	6.1	-468.88	5.94600019	11	7/24/2014	24:45.2
4474	RWS11	-122.46184	37.938	-0.27	6.1	-461.6	5.82800025	-122.46183	37.93801	-0.15	6.1	-469.43	5.95200023	11	7/24/2014	24:45.3
4475	RWS11	-122.4618419	37.938	-0.27	6.11	-462	5.83600014	-122.46184	37.93801	-0.15	6.11	-469.94	5.96000013	11	7/24/2014	24:45.4
4476	RWS11	-122.4618438	37.938	-0.22	6.12	-462.6	5.89600003	-122.46184	37.93801	-0.15	6.12	-470.39	5.96800002	11	7/24/2014	24:45.5
4477	RWS11	-122.4618457	37.938	-0.22	6.13	-462.9	5.903	-122.46184	37.93801	-0.12	6.13	-470.78	6.009	11	7/24/2014	24:45.6
4478	RWS11	-122.4618476	37.938	-0.22	6.13	-463.1	5.90999997	-122.46184	37.93801	-0.12	6.13	-470.96	6.01599997	11	7/24/2014	24:45.7
4479	RWS11	-122.4618495	37.938	-0.27	6.14	-463.4	5.86599988	-122.46184	37.93801	-0.12	6.14	-471.14	6.02399987	11	7/24/2014	24:45.8
4480	RWS11	-122.4618515	37.938	-0.22	6.15	-463.5	5.92499983	-122.46185	37.93801	-0.12	6.15	-471.26	6.03099984	11	7/24/2014	24:45.9
4481	RWS11	-122.4618544	37.938	-0.22	6.16	-463.7	5.93300021	-122.46185	37.93801	-0.12	6.16	-471.32	6.03900021	11	7/24/2014	24:46.0
4482	RWS11	-122.4618563	37.938	-0.22	6.16	-463.8	5.94200003	-122.46185	37.93801	-0.12	6.16	-471.37	6.04800004	11	7/24/2014	24:46.1
4483	RWS11	-122.4618583	37.938	-0.22	6.17	-464	5.94999993	-122.46185	37.93802	-0.12	6.17	-471.52	6.05599993	11	7/24/2014	24:46.2
4484	RWS11	-122.4618603	37.938	-0.22	6.18	-464.2	5.95900023	-122.46185	37.93802	-0.12	6.18	-471.73	6.06500024	11	7/24/2014	24:46.3
4485	RWS11	-122.4618623	37.938	-0.22	6.19	-464.5	5.96800005	-122.46186	37.93802	-0.12	6.19	-471.93	6.07400006	11	7/24/2014	24:46.4
4486	RWS11	-122.4618644	37.938	-0.19	6.2	-464.7	6.00999996	-122.46186	37.93802	-0.12	6.2	-472.19	6.08199996	11	7/24/2014	24:46.5
4487	RWS11	-122.4618664	37.938	-0.22	6.21	-464.9	5.98399985	-122.46186	37.93802	-0.12	6.21	-472.37	6.08999985	11	7/24/2014	24:46.6

4488	RWS11	-122.4618684	37.93801	-0.19	6.21	-465.1	6.02499983	-122.46186	37.93802	-0.12	6.21	-472.66	6.09699982	11	7/24/2014	24:46.7
4489	RWS11	-122.4618704	37.93801	-0.22	6.22	-465.4	5.99900019	-122.46186	37.93802	-0.15	6.22	-472.93	6.07100019	11	7/24/2014	24:46.8
4490	RWS11	-122.4618723	37.93801	-0.22	6.23	-465.6	6.00600016	-122.46187	37.93802	-0.07	6.23	-473.16	6.16300017	11	7/24/2014	24:46.9
4491	RWS11	-122.4618752	37.93801	-0.22	6.24	-465.8	6.01300013	-122.46187	37.93802	-0.15	6.24	-473.44	6.08500013	11	7/24/2014	24:47.0
4492	RWS11	-122.4618771	37.93801	-0.19	6.24	-466.1	6.05300018	-122.46187	37.93802	-0.12	6.24	-473.67	6.12500018	11	7/24/2014	24:47.1
4493	RWS11	-122.461879	37.93801	-0.22	6.24	-466.3	6.02299988	-122.46187	37.93802	-0.12	6.24	-473.9	6.12899989	11	7/24/2014	24:47.2
4494	RWS11	-122.4618809	37.93801	-0.19	6.25	-466.3	6.06000015	-122.46188	37.93802	-0.12	6.25	-473.99	6.13200015	11	7/24/2014	24:47.3
4495	RWS11	-122.4618827	37.93801	-0.22	6.25	-466.4	6.02899992	-122.46188	37.93802	-0.12	6.25	-474.08	6.13499993	11	7/24/2014	24:47.4
4496	RWS11	-122.4618846	37.93801	-0.19	6.25	-466.3	6.06499979	-122.46188	37.93802	-0.12	6.25	-474.17	6.13699979	11	7/24/2014	24:47.5
4497	RWS11	-122.4618865	37.93801	-0.22	6.26	-466.3	6.03300011	-122.46188	37.93802	-0.15	6.26	-474.16	6.10500011	11	7/24/2014	24:47.6
4498	RWS11	-122.4618884	37.93801	-0.22	6.26	-466	6.03400004	-122.46188	37.93802	-0.12	6.26	-474.06	6.14000005	11	7/24/2014	24:47.7
4499	RWS11	-122.4618902	37.93801	-0.22	6.26	-466	6.03499997	-122.46188	37.93802	-0.15	6.26	-474.06	6.10699996	11	7/24/2014	24:47.8
4500	RWS11	-122.4618921	37.93801	-0.19	6.26	-465.9	6.07099983	-122.46189	37.93802	-0.12	6.26	-474.13	6.14299983	11	7/24/2014	24:47.9
4501	RWS11	-122.4618948	37.93801	-0.22	6.26	-465.8	6.03699982	-122.46189	37.93803	-0.12	6.26	-474.17	6.14299983	11	7/24/2014	24:48.0
4502	RWS11	-122.4618967	37.93801	-0.19	6.26	-465.8	6.07200024	-122.46189	37.93803	-0.12	6.26	-474.24	6.14400023	11	7/24/2014	24:48.1
4503	RWS11	-122.4618986	37.93801	-0.22	6.26	-465.8	6.03800023	-122.46189	37.93803	-0.15	6.26	-474.3	6.11000022	11	7/24/2014	24:48.2
4504	RWS11	-122.4619005	37.93801	-0.22	6.26	-465.9	6.03699982	-122.46189	37.93803	-0.12	6.26	-474.38	6.14299983	11	7/24/2014	24:48.3
4505	RWS11	-122.4619024	37.93801	-0.22	6.26	-466.1	6.03499997	-122.4619	37.93803	-0.12	6.26	-474.55	6.14099997	11	7/24/2014	24:48.4
4506	RWS11	-122.4619044	37.93802	-0.22	6.25	-466.3	6.03200018	-122.4619	37.93803	-0.12	6.25	-474.81	6.13800019	11	7/24/2014	24:48.5
4507	RWS11	-122.4619063	37.93802	-0.22	6.25	-466.6	6.028	-122.4619	37.93803	-0.12	6.25	-475.1	6.134	11	7/24/2014	24:48.6
4508	RWS11	-122.4619083	37.93802	-0.19	6.24	-466.8	6.05599996	-122.4619	37.93803	-0.12	6.24	-475.35	6.12799996	11	7/24/2014	24:48.7
4509	RWS11	-122.4619102	37.93802	-0.22	6.24	-467	6.01599991	-122.4619	37.93803	-0.12	6.24	-475.49	6.12199992	11	7/24/2014	24:48.8
4510	RWS11	-122.4619121	37.93802	-0.19	6.23	-467.1	6.04299995	-122.46191	37.93803	-0.12	6.23	-475.66	6.11499995	11	7/24/2014	24:48.9
4511	RWS11	-122.4619149	37.93802	-0.22	6.22	-467.1	6.00100005	-122.46191	37.93803	-0.12	6.22	-475.67	6.10700005	11	7/24/2014	24:49.0
4512	RWS11	-122.4619169	37.93802	-0.19	6.22	-467.2	6.02800009	-122.46191	37.93803	-0.07	6.22	-475.64	6.15100008	11	7/24/2014	24:49.1
4513	RWS11	-122.4619188	37.93802	-0.22	6.21	-467.2	5.98700011	-122.46191	37.93803	-0.07	6.21	-475.7	6.14400011	11	7/24/2014	24:49.2
4514	RWS11	-122.4619207	37.93802	-0.22	6.2	-467.3	5.98100007	-122.46191	37.93803	-0.12	6.2	-475.85	6.08700007	11	7/24/2014	24:49.3
4515	RWS11	-122.4619227	37.93802	-0.22	6.2	-467.4	5.97699988	-122.46192	37.93803	-0.12	6.2	-475.97	6.08299989	11	7/24/2014	24:49.4
4516	RWS11	-122.4619246	37.93802	-0.19	6.2	-467.6	6.00700018	-122.46192	37.93803	-0.07	6.2	-476.21	6.13000017	11	7/24/2014	24:49.5
4517	RWS11	-122.4619266	37.93802	-0.22	6.19	-467.7	5.96999991	-122.46192	37.93803	-0.12	6.19	-476.36	6.07599992	11	7/24/2014	24:49.6
4518	RWS11	-122.4619286	37.93802	-0.19	6.19	-467.8	6.00100014	-122.46192	37.93804	-0.12	6.19	-476.49	6.07300013	11	7/24/2014	24:49.7
4519	RWS11	-122.4619305	37.93802	-0.19	6.19	-467.9	5.99799988	-122.46192	37.93804	-0.07	6.19	-476.49	6.12099987	11	7/24/2014	24:49.8
4520	RWS11	-122.4619325	37.93802	-0.19	6.18	-467.9	5.99400017	-122.46193	37.93804	-0.07	6.18	-476.46	6.11700016	11	7/24/2014	24:49.9

4521	RWS11	-122.4619354	37.93802	-0.22	6.18	-468	5.95500004	-122.46193	37.93804	-0.12	6.18	-476.38	6.06100005	11	7/24/2014	24:50.0
4522	RWS11	-122.4619374	37.93803	-0.22	6.17	-468	5.94800007	-122.46193	37.93804	-0.12	6.17	-476.32	6.05400008	11	7/24/2014	24:50.1
4523	RWS11	-122.4619394	37.93803	-0.22	6.16	-467.9	5.93899977	-122.46193	37.93804	-0.12	6.16	-476.17	6.04499978	11	7/24/2014	24:50.2
4524	RWS11	-122.4619415	37.93803	-0.19	6.15	-467.8	5.96100017	-122.46194	37.93804	-0.12	6.15	-476.06	6.03300017	11	7/24/2014	24:50.3
4525	RWS11	-122.4619435	37.93803	-0.22	6.14	-467.8	5.91300023	-122.46194	37.93804	-0.12	6.14	-475.91	6.01900023	11	7/24/2014	24:50.4
4526	RWS11	-122.4619455	37.93803	-0.19	6.12	-467.8	5.93000004	-122.46194	37.93804	-0.12	6.12	-475.97	6.00200003	11	7/24/2014	24:50.5
4527	RWS11	-122.4619476	37.93803	-0.22	6.1	-467.9	5.8779999	-122.46194	37.93804	-0.12	6.1	-475.99	5.98399991	11	7/24/2014	24:50.6
4528	RWS11	-122.4619496	37.93803	-0.22	6.08	-467.8	5.85999978	-122.46194	37.93804	-0.12	6.08	-476.14	5.96599978	11	7/24/2014	24:50.7
4529	RWS11	-122.4619516	37.93803	-0.22	6.07	-468	5.84300005	-122.46195	37.93804	-0.15	6.07	-476.32	5.91500005	11	7/24/2014	24:50.8
4530	RWS11	-122.4619535	37.93803	-0.19	6.05	-468.1	5.86099979	-122.46195	37.93804	-0.07	6.05	-476.47	5.98399979	11	7/24/2014	24:50.9
4531	RWS11	-122.4619564	37.93803	-0.22	6.03	-468.2	5.81299984	-122.46195	37.93804	-0.12	6.03	-476.62	5.91899985	11	7/24/2014	24:51.0
4532	RWS11	-122.4619584	37.93803	-0.19	6.02	-468.2	5.83499977	-122.46195	37.93804	-0.12	6.02	-476.62	5.90699977	11	7/24/2014	24:51.1
4533	RWS11	-122.4619603	37.93803	-0.22	6.01	-468.2	5.79000008	-122.46195	37.93804	-0.12	6.01	-476.63	5.89600009	11	7/24/2014	24:51.2
4534	RWS11	-122.4619622	37.93803	-0.22	6	-468.3	5.77999985	-122.46196	37.93805	-0.12	6	-476.68	5.88599986	11	7/24/2014	24:51.3
4535	RWS11	-122.4619641	37.93803	-0.19	5.99	-468.3	5.80599996	-122.46196	37.93805	-0.15	5.99	-476.72	5.84399995	11	7/24/2014	24:51.4
4536	RWS11	-122.4619661	37.93803	-0.22	5.99	-468.3	5.76400006	-122.46196	37.93805	-0.12	5.99	-476.77	5.87000006	11	7/24/2014	24:51.5
4537	RWS11	-122.461968	37.93803	-0.22	5.98	-468.5	5.75600016	-122.46196	37.93805	-0.12	5.98	-476.92	5.86200017	11	7/24/2014	24:51.6
4538	RWS11	-122.46197	37.93803	-0.19	5.97	-468.5	5.7819998	-122.46196	37.93805	-0.12	5.97	-476.97	5.85399979	11	7/24/2014	24:51.7
4539	RWS11	-122.461972	37.93804	-0.22	5.96	-468.6	5.73999989	-122.46197	37.93805	-0.15	5.96	-476.99	5.81199989	11	7/24/2014	24:51.8
4540	RWS11	-122.461974	37.93804	-0.19	5.95	-468.7	5.766	-122.46197	37.93805	-0.12	5.95	-477.09	5.838	11	7/24/2014	24:51.9
4541	RWS11	-122.4619759	37.93804	-0.22	5.95	-468.8	5.7240001	-122.46197	37.93805	-0.12	5.95	-477.14	5.8300001	11	7/24/2014	24:52.0
4542	RWS11	-122.4619779	37.93804	-0.19	5.94	-468.9	5.7489998	-122.46197	37.93805	-0.07	5.94	-477.17	5.8719998	11	7/24/2014	24:52.1
4543	RWS11	-122.4619799	37.93804	-0.19	5.93	-469	5.73900005	-122.46197	37.93805	-0.12	5.93	-477.16	5.81100005	11	7/24/2014	24:52.2
4544	RWS11	-122.4619819	37.93804	-0.19	5.92	-469.1	5.7279999	-122.46198	37.93805	-0.15	5.92	-477.11	5.76599988	11	7/24/2014	24:52.3
4545	RWS11	-122.4619839	37.93804	-0.22	5.9	-469	5.6819998	-122.46198	37.93805	-0.12	5.9	-477.03	5.78799981	11	7/24/2014	24:52.4
4546	RWS11	-122.4619859	37.93804	-0.22	5.89	-468.9	5.66799986	-122.46198	37.93805	-0.12	5.89	-476.9	5.77399987	11	7/24/2014	24:52.5
4547	RWS11	-122.4619878	37.93804	-0.22	5.88	-468.7	5.65399992	-122.46198	37.93805	-0.15	5.88	-476.73	5.72599992	11	7/24/2014	24:52.6
4548	RWS11	-122.4619898	37.93804	-0.19	5.86	-468.5	5.67300007	-122.46198	37.93805	-0.12	5.86	-476.57	5.74500006	11	7/24/2014	24:52.7
4549	RWS11	-122.4619917	37.93804	-0.22	5.85	-468.3	5.62400019	-122.46199	37.93805	-0.15	5.85	-476.34	5.69600019	11	7/24/2014	24:52.8
4550	RWS11	-122.4619936	37.93804	-0.19	5.83	-468.1	5.64299986	-122.46199	37.93805	-0.15	5.83	-476.11	5.68099985	11	7/24/2014	24:52.9
4551	RWS11	-122.4619965	37.93804	-0.22	5.82	-467.8	5.59300005	-122.46199	37.93806	-0.15	5.82	-475.97	5.66500005	11	7/24/2014	24:53.0
4552	RWS11	-122.4619984	37.93804	-0.22	5.8	-467.6	5.57599986	-122.46199	37.93806	-0.12	5.8	-475.85	5.68199986	11	7/24/2014	24:53.1
4553	RWS11	-122.4620003	37.93804	-0.22	5.78	-467.5	5.56000006	-122.46199	37.93806	-0.15	5.78	-475.76	5.63200006	11	7/24/2014	24:53.2

4554	RWS11	-122.4620023	37.93804	-0.22	5.76	-467.5	5.54199994	-122.462	37.93806	-0.15	5.76	-475.63	5.61399993	11	7/24/2014	24:53.3
4555	RWS11	-122.4620043	37.93805	-0.22	5.74	-467.5	5.52299988	-122.462	37.93806	-0.15	5.74	-475.58	5.59499988	11	7/24/2014	24:53.4
4556	RWS11	-122.4620063	37.93805	-0.19	5.72	-467.4	5.53599998	-122.462	37.93806	-0.12	5.72	-475.52	5.60799998	11	7/24/2014	24:53.5
4557	RWS11	-122.4620083	37.93805	-0.22	5.7	-467.5	5.47900021	-122.462	37.93806	-0.15	5.7	-475.45	5.55100021	11	7/24/2014	24:53.6
4558	RWS11	-122.4620103	37.93805	-0.22	5.68	-467.4	5.45300019	-122.462	37.93806	-0.12	5.68	-475.48	5.55900019	11	7/24/2014	24:53.7
4559	RWS11	-122.4620124	37.93805	-0.22	5.65	-467.4	5.42499983	-122.46201	37.93806	-0.15	5.65	-475.44	5.49699983	11	7/24/2014	24:53.8
4560	RWS11	-122.4620145	37.93805	-0.22	5.62	-467.3	5.3950001	-122.46201	37.93806	-0.15	5.62	-475.35	5.4670001	11	7/24/2014	24:53.9
4561	RWS11	-122.4620165	37.93805	-0.22	5.58	-467.2	5.36200011	-122.46201	37.93806	-0.15	5.58	-475.31	5.4340001	11	7/24/2014	24:54.0
4562	RWS11	-122.4620186	37.93805	-0.22	5.55	-467.1	5.32800019	-122.46201	37.93806	-0.15	5.55	-475.24	5.40000018	11	7/24/2014	24:54.1
4563	RWS11	-122.4620207	37.93805	-0.22	5.52	-467.1	5.29399979	-122.46201	37.93806	-0.15	5.52	-475.14	5.36599979	11	7/24/2014	24:54.2
4564	RWS11	-122.4620227	37.93805	-0.22	5.48	-467	5.25999987	-122.46202	37.93806	-0.15	5.48	-475.04	5.33199987	11	7/24/2014	24:54.3
4565	RWS11	-122.4620248	37.93805	-0.22	5.45	-467	5.22699988	-122.46202	37.93806	-0.2	5.45	-474.96	5.24899988	11	7/24/2014	24:54.4
4566	RWS11	-122.4620269	37.93805	-0.22	5.42	-467.1	5.19600022	-122.46202	37.93806	-0.12	5.42	-474.96	5.30200022	11	7/24/2014	24:54.5
4567	RWS11	-122.4620289	37.93805	-0.22	5.39	-467.2	5.16500008	-122.46202	37.93807	-0.15	5.39	-474.94	5.23700008	11	7/24/2014	24:54.6
4568	RWS11	-122.4620309	37.93805	-0.22	5.36	-467.3	5.1359998	-122.46202	37.93807	-0.15	5.36	-474.92	5.2079998	11	7/24/2014	24:54.7
4569	RWS11	-122.4620329	37.93805	-0.22	5.33	-467.3	5.10799992	-122.46203	37.93807	-0.15	5.33	-474.86	5.17999992	11	7/24/2014	24:54.8
4570	RWS11	-122.4620348	37.93805	-0.22	5.3	-467.2	5.08000004	-122.46203	37.93807	-0.15	5.3	-474.79	5.15200004	11	7/24/2014	24:54.9
4571	RWS11	-122.4620378	37.93806	-0.22	5.28	-467.2	5.05300009	-122.46203	37.93807	-0.15	5.28	-474.75	5.12500009	11	7/24/2014	24:55.0
4572	RWS11	-122.4620397	37.93806	-0.19	5.25	-467.3	5.06000015	-122.46203	37.93807	-0.15	5.25	-474.79	5.09800014	11	7/24/2014	24:55.1
4573	RWS11	-122.4620417	37.93806	-0.22	5.22	-467.3	4.99900019	-122.46204	37.93807	-0.15	5.22	-474.75	5.07100019	11	7/24/2014	24:55.2
4574	RWS11	-122.4620437	37.93806	-0.22	5.2	-467.2	4.97300017	-122.46204	37.93807	-0.15	5.2	-474.72	5.04500017	11	7/24/2014	24:55.3
4575	RWS11	-122.4620457	37.93806	-0.22	5.17	-467.1	4.94600022	-122.46204	37.93807	-0.2	5.17	-474.67	4.96800022	11	7/24/2014	24:55.4
4576	RWS11	-122.4620477	37.93806	-0.22	5.14	-467.1	4.9200002	-122.46204	37.93807	-0.15	5.14	-474.57	4.99200019	11	7/24/2014	24:55.5
4577	RWS11	-122.4620498	37.93806	-0.22	5.12	-467	4.8950001	-122.46204	37.93807	-0.2	5.12	-474.53	4.9170001	11	7/24/2014	24:55.6
4578	RWS11	-122.4620518	37.93806	-0.22	5.09	-466.9	4.87	-122.46205	37.93807	-0.2	5.09	-474.4	4.892	11	7/24/2014	24:55.7
4579	RWS11	-122.4620539	37.93806	-0.22	5.07	-466.8	4.84599984	-122.46205	37.93807	-0.2	5.07	-474.34	4.86799984	11	7/24/2014	24:55.8
4580	RWS11	-122.462056	37.93806	-0.22	5.05	-466.8	4.82300007	-122.46205	37.93807	-0.2	5.05	-474.22	4.84500007	11	7/24/2014	24:55.9
4581	RWS11	-122.4620581	37.93806	-0.22	5.02	-466.7	4.79899991	-122.46205	37.93807	-0.2	5.02	-474.12	4.82099991	11	7/24/2014	24:56.0
4582	RWS11	-122.4620602	37.93806	-0.22	5	-466.6	4.77500021	-122.46205	37.93807	-0.15	5	-473.97	4.84700021	11	7/24/2014	24:56.1
4583	RWS11	-122.4620623	37.93806	-0.22	4.97	-466.5	4.75000012	-122.46206	37.93808	-0.2	4.97	-473.84	4.77200012	11	7/24/2014	24:56.2
4584	RWS11	-122.4620643	37.93806	-0.22	4.95	-466.4	4.72500002	-122.46206	37.93808	-0.15	4.95	-473.78	4.79700002	11	7/24/2014	24:56.3
4585	RWS11	-122.4620664	37.93806	-0.22	4.92	-466.5	4.69999993	-122.46206	37.93808	-0.15	4.92	-473.82	4.77199993	11	7/24/2014	24:56.4
4586	RWS11	-122.4620684	37.93806	-0.22	4.9	-466.5	4.67599976	-122.46206	37.93808	-0.15	4.9	-473.93	4.74799976	11	7/24/2014	24:56.5

4587	RWS11	-122.4620705	37.93807	-0.22	4.87	-466.6	4.65200007	-122.46206	37.93808	-0.2	4.87	-473.97	4.67400007	11	7/24/2014	24:56.6
4588	RWS11	-122.4620725	37.93807	-0.22	4.85	-466.7	4.6279999	-122.46207	37.93808	-0.15	4.85	-474.05	4.6999999	11	7/24/2014	24:56.7
4589	RWS11	-122.4620744	37.93807	-0.22	4.83	-466.7	4.60500014	-122.46207	37.93808	-0.2	4.83	-474.09	4.62700014	11	7/24/2014	24:56.8
4590	RWS11	-122.4620764	37.93807	-0.22	4.8	-466.8	4.58299983	-122.46207	37.93808	-0.15	4.8	-474.17	4.65499982	11	7/24/2014	24:56.9
4591	RWS11	-122.4620793	37.93807	-0.22	4.78	-466.9	4.56099999	-122.46207	37.93808	-0.2	4.78	-474.2	4.58299999	11	7/24/2014	24:57.0
4592	RWS11	-122.4620812	37.93807	-0.22	4.76	-467	4.53900015	-122.46207	37.93808	-0.15	4.76	-474.26	4.61100015	11	7/24/2014	24:57.1
4593	RWS11	-122.4620832	37.93807	-0.22	4.74	-467.2	4.51699984	-122.46208	37.93808	-0.2	4.74	-474.3	4.53899984	11	7/24/2014	24:57.2
4594	RWS11	-122.4620852	37.93807	-0.22	4.72	-467.4	4.49599993	-122.46208	37.93808	-0.2	4.72	-474.31	4.51799993	11	7/24/2014	24:57.3
4595	RWS11	-122.4620871	37.93807	-0.22	4.7	-467.6	4.47300017	-122.46208	37.93808	-0.2	4.7	-474.35	4.49500017	11	7/24/2014	24:57.4
4596	RWS11	-122.4620892	37.93807	-0.19	4.67	-467.8	4.48499987	-122.46208	37.93808	-0.15	4.67	-474.34	4.52299985	11	7/24/2014	24:57.5
4597	RWS11	-122.4620912	37.93807	-0.22	4.65	-468.1	4.42700016	-122.46208	37.93808	-0.2	4.65	-474.35	4.44900016	11	7/24/2014	24:57.6
4598	RWS11	-122.4620932	37.93807	-0.22	4.63	-468.3	4.40399992	-122.46209	37.93809	-0.2	4.63	-474.47	4.42599992	11	7/24/2014	24:57.7
4599	RWS11	-122.4620952	37.93807	-0.22	4.6	-468.5	4.38100016	-122.46209	37.93809	-0.2	4.6	-474.44	4.40300016	11	7/24/2014	24:57.8
4600	RWS11	-122.4620973	37.93807	-0.22	4.58	-468.5	4.35899985	-122.46209	37.93809	-0.15	4.58	-474.37	4.43099985	11	7/24/2014	24:57.9
4601	RWS11	-122.4620993	37.93807	-0.22	4.56	-468.5	4.33899987	-122.46209	37.93809	-0.2	4.56	-474.32	4.36099987	11	7/24/2014	24:58.0
4602	RWS11	-122.4621014	37.93808	-0.22	4.54	-468.6	4.31999981	-122.4621	37.93809	-0.2	4.54	-474.28	4.34199981	11	7/24/2014	24:58.1
4603	RWS11	-122.4621034	37.93808	-0.27	4.53	-468.5	4.25200003	-122.4621	37.93809	-0.23	4.53	-474.26	4.29200003	11	7/24/2014	24:58.2
4604	RWS11	-122.4621055	37.93808	-0.22	4.51	-468.4	4.28900015	-122.4621	37.93809	-0.2	4.51	-474.13	4.31100015	11	7/24/2014	24:58.3
4605	RWS11	-122.4621075	37.93808	-0.22	4.5	-468.3	4.27700007	-122.4621	37.93809	-0.2	4.5	-474.08	4.29900007	11	7/24/2014	24:58.4
4606	RWS11	-122.4621095	37.93808	-0.22	4.49	-468.3	4.26599991	-122.4621	37.93809	-0.2	4.49	-474	4.28799991	11	7/24/2014	24:58.5
4607	RWS11	-122.4621115	37.93808	-0.22	4.48	-468.1	4.25700009	-122.46211	37.93809	-0.23	4.48	-473.95	4.24500009	11	7/24/2014	24:58.6
4608	RWS11	-122.4621135	37.93808	-0.22	4.47	-468.2	4.24900019	-122.46211	37.93809	-0.2	4.47	-473.95	4.27100019	11	7/24/2014	24:58.7
4609	RWS11	-122.4621155	37.93808	-0.22	4.46	-468.1	4.24099982	-122.46211	37.93809	-0.23	4.46	-473.95	4.22899982	11	7/24/2014	24:58.8
4610	RWS11	-122.4621174	37.93808	-0.22	4.45	-468.1	4.23299992	-122.46211	37.93809	-0.2	4.45	-473.91	4.25499992	11	7/24/2014	24:58.9
4611	RWS11	-122.4621203	37.93808	-0.27	4.45	-468.2	4.17300004	-122.46211	37.93809	-0.23	4.45	-473.98	4.21300003	11	7/24/2014	24:59.0
4612	RWS11	-122.4621222	37.93808	-0.22	4.44	-468.3	4.2160002	-122.46212	37.93809	-0.2	4.44	-473.94	4.2380002	11	7/24/2014	24:59.1
4613	RWS11	-122.4621242	37.93808	-0.22	4.43	-468.4	4.2069999	-122.46212	37.9381	-0.23	4.43	-474.07	4.1949999	11	7/24/2014	24:59.2
4614	RWS11	-122.4621262	37.93808	-0.22	4.42	-468.4	4.19800007	-122.46212	37.9381	-0.15	4.42	-474	4.27000007	11	7/24/2014	24:59.3
4615	RWS11	-122.4621282	37.93808	-0.27	4.41	-468.4	4.13699979	-122.46212	37.9381	-0.23	4.41	-473.94	4.17699978	11	7/24/2014	24:59.4
4616	RWS11	-122.4621302	37.93808	-0.22	4.4	-468.4	4.17900002	-122.46212	37.9381	-0.2	4.4	-473.89	4.20100002	11	7/24/2014	24:59.5
4617	RWS11	-122.4621323	37.93809	-0.22	4.39	-468.4	4.16799986	-122.46213	37.9381	-0.23	4.39	-473.86	4.15599987	11	7/24/2014	24:59.6
4618	RWS11	-122.4621343	37.93809	-0.22	4.38	-468.3	4.15599978	-122.46213	37.9381	-0.2	4.38	-473.66	4.17799978	11	7/24/2014	24:59.7
4619	RWS11	-122.4621364	37.93809	-0.22	4.37	-468.3	4.14400017	-122.46213	37.9381	-0.2	4.37	-473.55	4.16600017	11	7/24/2014	24:59.8

4620	RWS11	-122.4621385	37.93809	-0.19	4.35	-468.5	4.16400024	-122.46213	37.9381	-0.15	4.35	-473.79	4.20200023	11	7/24/2014	24:59.9
4621	RWS11	-122.4621406	37.93809	-0.22	4.34	-468.8	4.11700022	-122.46213	37.9381	-0.2	4.34	-474.1	4.13900022	11	7/24/2014	25:00.0
4622	RWS11	-122.4621427	37.93809	-0.22	4.32	-469	4.10199988	-122.46214	37.9381	-0.2	4.32	-474.46	4.12399988	11	7/24/2014	25:00.1
4623	RWS11	-122.4621448	37.93809	-0.22	4.31	-469.2	4.08799994	-122.46214	37.9381	-0.2	4.31	-474.61	4.10999994	11	7/24/2014	25:00.2
4624	RWS11	-122.4621469	37.93809	-0.19	4.3	-469.4	4.10800001	-122.46214	37.9381	-0.15	4.3	-474.85	4.146	11	7/24/2014	25:00.3
4625	RWS11	-122.4621489	37.93809	-0.22	4.28	-469.7	4.06000006	-122.46214	37.9381	-0.2	4.28	-475.17	4.08200006	11	7/24/2014	25:00.4
4626	RWS11	-122.462151	37.93809	-0.22	4.27	-469.9	4.04700005	-122.46214	37.9381	-0.15	4.27	-475.36	4.11900005	11	7/24/2014	25:00.5
4627	RWS11	-122.462153	37.93809	-0.22	4.26	-470	4.03499997	-122.46215	37.9381	-0.2	4.26	-475.39	4.05699997	11	7/24/2014	25:00.6
4628	RWS11	-122.4621551	37.93809	-0.19	4.25	-470	4.05799982	-122.46215	37.9381	-0.2	4.25	-475.41	4.04599981	11	7/24/2014	25:00.7
4629	RWS11	-122.4621571	37.93809	-0.22	4.24	-470	4.01499999	-122.46215	37.93811	-0.2	4.24	-475.32	4.03699999	11	7/24/2014	25:00.8
4630	RWS11	-122.4621591	37.93809	-0.22	4.23	-470	4.00800002	-122.46215	37.93811	-0.2	4.23	-475.35	4.03000002	11	7/24/2014	25:00.9
4631	RWS11	-122.462162	37.93809	-0.22	4.22	-470	4.00199997	-122.46216	37.93811	-0.2	4.22	-475.3	4.02399997	11	7/24/2014	25:01.0
4632	RWS11	-122.462164	37.9381	-0.22	4.22	-470	3.99799979	-122.46216	37.93811	-0.2	4.22	-475.29	4.01999979	11	7/24/2014	25:01.1
4633	RWS11	-122.462166	37.9381	-0.22	4.22	-470	3.995	-122.46216	37.93811	-0.2	4.22	-475.41	4.017	11	7/24/2014	25:01.2
4634	RWS11	-122.462168	37.9381	-0.22	4.22	-470	3.99400008	-122.46216	37.93811	-0.15	4.22	-475.54	4.06600007	11	7/24/2014	25:01.3
4635	RWS11	-122.4621701	37.9381	-0.22	4.22	-470.1	3.99300015	-122.46216	37.93811	-0.2	4.22	-475.65	4.01500015	11	7/24/2014	25:01.4
4636	RWS11	-122.4621721	37.9381	-0.22	4.22	-470.2	3.99400008	-122.46217	37.93811	-0.15	4.22	-475.81	4.06600007	11	7/24/2014	25:01.5
4637	RWS11	-122.4621741	37.9381	-0.27	4.22	-470.3	3.94300002	-122.46217	37.93811	-0.2	4.22	-475.92	4.017	11	7/24/2014	25:01.6
4638	RWS11	-122.4621761	37.9381	-0.22	4.22	-470.5	3.99599993	-122.46217	37.93811	-0.15	4.22	-476.04	4.06799993	11	7/24/2014	25:01.7
4639	RWS11	-122.4621781	37.9381	-0.22	4.22	-470.5	3.99699986	-122.46217	37.93811	-0.2	4.22	-476.12	4.01899986	11	7/24/2014	25:01.8
4640	RWS11	-122.4621802	37.9381	-0.19	4.22	-470.6	4.0330002	-122.46217	37.93811	-0.15	4.22	-476.13	4.07100019	11	7/24/2014	25:01.9
4641	RWS11	-122.4621831	37.9381	-0.27	4.22	-470.5	3.94999999	-122.46218	37.93811	-0.2	4.22	-476.15	4.02399997	11	7/24/2014	25:02.0
4642	RWS11	-122.4621851	37.9381	-0.22	4.23	-470.5	4.00399983	-122.46218	37.93811	-0.2	4.23	-476.13	4.02599983	11	7/24/2014	25:02.1
4643	RWS11	-122.4621871	37.9381	-0.22	4.23	-470.4	4.00800002	-122.46218	37.93811	-0.2	4.23	-476.17	4.03000002	11	7/24/2014	25:02.2
4644	RWS11	-122.4621891	37.9381	-0.22	4.23	-470.4	4.0109998	-122.46218	37.93812	-0.15	4.23	-476.17	4.0829998	11	7/24/2014	25:02.3
4645	RWS11	-122.4621911	37.9381	-0.22	4.24	-470.5	4.01499999	-122.46218	37.93812	-0.2	4.24	-476.28	4.03699999	11	7/24/2014	25:02.4
4646	RWS11	-122.4621931	37.9381	-0.19	4.24	-470.8	4.05199978	-122.46219	37.93812	-0.15	4.24	-476.5	4.08999977	11	7/24/2014	25:02.5
4647	RWS11	-122.4621951	37.9381	-0.22	4.24	-471	4.02199996	-122.46219	37.93812	-0.2	4.24	-476.7	4.04399996	11	7/24/2014	25:02.6
4648	RWS11	-122.462197	37.93811	-0.22	4.25	-471.2	4.02600014	-122.46219	37.93812	-0.15	4.25	-476.89	4.09800014	11	7/24/2014	25:02.7
4649	RWS11	-122.462199	37.93811	-0.22	4.25	-471.6	4.02999985	-122.46219	37.93812	-0.15	4.25	-477.23	4.10199985	11	7/24/2014	25:02.8
4650	RWS11	-122.462201	37.93811	-0.19	4.26	-471.8	4.0699999	-122.46219	37.93812	-0.15	4.26	-477.5	4.10799989	11	7/24/2014	25:02.9
4651	RWS11	-122.4622039	37.93811	-0.22	4.26	-472	4.04199994	-122.4622	37.93812	-0.15	4.26	-477.75	4.11399993	11	7/24/2014	25:03.0
4652	RWS11	-122.4622058	37.93811	-0.22	4.27	-472.2	4.04899991	-122.4622	37.93812	-0.15	4.27	-477.87	4.1209999	11	7/24/2014	25:03.1

4653	RWS11	-122.4622077	37.93811	-0.22	4.28	-472.3	4.05499995	-122.4622	37.93812	-0.15	4.28	-477.8	4.12699994	11	7/24/2014	25:03.2
4654	RWS11	-122.4622097	37.93811	-0.22	4.28	-472.3	4.06199992	-122.4622	37.93812	-0.15	4.28	-477.77	4.13399991	11	7/24/2014	25:03.3
4655	RWS11	-122.4622116	37.93811	-0.22	4.29	-472.2	4.06799996	-122.46221	37.93812	-0.2	4.29	-477.61	4.08999996	11	7/24/2014	25:03.4
4656	RWS11	-122.4622136	37.93811	-0.19	4.3	-472.1	4.10700008	-122.46221	37.93812	-0.15	4.3	-477.43	4.14500007	11	7/24/2014	25:03.5
4657	RWS11	-122.4622155	37.93811	-0.22	4.3	-472.1	4.07900012	-122.46221	37.93812	-0.2	4.3	-477.39	4.10100012	11	7/24/2014	25:03.6
4658	RWS11	-122.4622175	37.93811	-0.22	4.31	-472.1	4.08600008	-122.46221	37.93812	-0.15	4.31	-477.41	4.15800008	11	7/24/2014	25:03.7
4659	RWS11	-122.4622194	37.93811	-0.22	4.32	-472.2	4.09300005	-122.46221	37.93813	-0.2	4.32	-477.61	4.11500005	11	7/24/2014	25:03.8
4660	RWS11	-122.4622213	37.93811	-0.22	4.32	-472.3	4.10000002	-122.46221	37.93813	-0.15	4.32	-477.82	4.17200002	11	7/24/2014	25:03.9
4661	RWS11	-122.4622242	37.93811	-0.22	4.33	-472.5	4.10699999	-122.46222	37.93813	-0.15	4.33	-478.09	4.17899999	11	7/24/2014	25:04.0
4662	RWS11	-122.4622262	37.93811	-0.19	4.34	-472.6	4.1489999	-122.46222	37.93813	-0.15	4.34	-478.22	4.18699989	11	7/24/2014	25:04.1
4663	RWS11	-122.4622281	37.93812	-0.22	4.34	-472.7	4.12199986	-122.46222	37.93813	-0.15	4.34	-478.33	4.19399986	11	7/24/2014	25:04.2
4664	RWS11	-122.4622301	37.93812	-0.19	4.35	-472.8	4.16199991	-122.46222	37.93813	-0.12	4.35	-478.38	4.23399991	11	7/24/2014	25:04.3
4665	RWS11	-122.4622321	37.93812	-0.22	4.36	-472.8	4.13399994	-122.46223	37.93813	-0.15	4.36	-478.45	4.20599994	11	7/24/2014	25:04.4
4666	RWS11	-122.462234	37.93812	-0.22	4.36	-472.9	4.13800013	-122.46223	37.93813	-0.15	4.36	-478.53	4.21000013	11	7/24/2014	25:04.5
4667	RWS11	-122.462236	37.93812	-0.22	4.36	-473	4.14299977	-122.46223	37.93813	-0.15	4.36	-478.76	4.21499977	11	7/24/2014	25:04.6
4668	RWS11	-122.462238	37.93812	-0.19	4.37	-473.1	4.18000004	-122.46223	37.93813	-0.15	4.37	-479.04	4.21800002	11	7/24/2014	25:04.7
4669	RWS11	-122.46224	37.93812	-0.22	4.37	-473.3	4.14899981	-122.46223	37.93813	-0.15	4.37	-479.4	4.22099981	11	7/24/2014	25:04.8
4670	RWS11	-122.462242	37.93812	-0.19	4.37	-473.5	4.18500015	-122.46224	37.93813	-0.12	4.37	-479.77	4.25700015	11	7/24/2014	25:04.9
4671	RWS11	-122.4622449	37.93812	-0.22	4.37	-473.7	4.15200007	-122.46224	37.93813	-0.15	4.37	-480.14	4.22400007	11	7/24/2014	25:05.0
4672	RWS11	-122.4622469	37.93812	-0.22	4.38	-473.9	4.153	-122.46224	37.93813	-0.12	4.38	-480.53	4.259	11	7/24/2014	25:05.1
4673	RWS11	-122.4622489	37.93812	-0.22	4.38	-474.1	4.15399992	-122.46224	37.93813	-0.12	4.38	-480.76	4.25999993	11	7/24/2014	25:05.2
4674	RWS11	-122.4622509	37.93812	-0.22	4.38	-474.1	4.15499985	-122.46224	37.93814	-0.12	4.38	-480.89	4.26099986	11	7/24/2014	25:05.3
4675	RWS11	-122.4622529	37.93812	-0.22	4.38	-474.1	4.15499985	-122.46225	37.93814	-0.15	4.38	-480.82	4.22699985	11	7/24/2014	25:05.4
4676	RWS11	-122.462255	37.93812	-0.22	4.38	-474.1	4.15399992	-122.46225	37.93814	-0.07	4.38	-480.86	4.31099993	11	7/24/2014	25:05.5
4677	RWS11	-122.462257	37.93812	-0.22	4.38	-474.2	4.153	-122.46225	37.93814	-0.12	4.38	-480.89	4.259	11	7/24/2014	25:05.6
4678	RWS11	-122.462259	37.93813	-0.19	4.38	-474.3	4.18700001	-122.46225	37.93814	-0.12	4.38	-481.16	4.259	11	7/24/2014	25:05.7
4679	RWS11	-122.462261	37.93813	-0.22	4.37	-474.5	4.15200007	-122.46225	37.93814	-0.12	4.37	-481.49	4.25800008	11	7/24/2014	25:05.8
4680	RWS11	-122.462263	37.93813	-0.19	4.38	-474.8	4.18700001	-122.46226	37.93814	-0.12	4.38	-481.92	4.259	11	7/24/2014	25:05.9
4681	RWS11	-122.462266	37.93813	-0.22	4.38	-475.1	4.15499985	-122.46226	37.93814	-0.15	4.38	-482.19	4.22699985	11	7/24/2014	25:06.0
4682	RWS11	-122.4622679	37.93813	-0.19	4.38	-475.3	4.19200012	-122.46226	37.93814	-0.12	4.38	-482.24	4.26400012	11	7/24/2014	25:06.1
4683	RWS11	-122.4622699	37.93813	-0.22	4.38	-475.4	4.16099989	-122.46226	37.93814	-0.15	4.38	-482.25	4.23299989	11	7/24/2014	25:06.2
4684	RWS11	-122.4622719	37.93813	-0.22	4.39	-475.5	4.16400015	-122.46227	37.93814	-0.12	4.39	-482.2	4.27000016	11	7/24/2014	25:06.3
4685	RWS11	-122.4622739	37.93813	-0.22	4.39	-475.6	4.16600001	-122.46227	37.93814	-0.15	4.39	-482.15	4.23800001	11	7/24/2014	25:06.4

4686	RWS11	-122.4622759	37.93813	-0.22	4.39	-475.7	4.16799986	-122.46227	37.93814	-0.12	4.39	-482.13	4.27399987	11	7/24/2014	25:06.5
4687	RWS11	-122.4622779	37.93813	-0.22	4.39	-475.8	4.1700002	-122.46227	37.93814	-0.12	4.39	-482.23	4.2760002	11	7/24/2014	25:06.6
4688	RWS11	-122.4622799	37.93813	-0.19	4.39	-476	4.20600006	-122.46227	37.93814	-0.12	4.39	-482.47	4.27800006	11	7/24/2014	25:06.7
4689	RWS11	-122.4622819	37.93813	-0.22	4.4	-476.2	4.17399991	-122.46228	37.93815	-0.12	4.4	-482.73	4.27999991	11	7/24/2014	25:06.8
4690	RWS11	-122.4622839	37.93813	-0.22	4.4	-476.3	4.17599976	-122.46228	37.93815	-0.07	4.4	-482.94	4.33299977	11	7/24/2014	25:06.9
4691	RWS11	-122.4622869	37.93813	-0.22	4.4	-476.5	4.17700016	-122.46228	37.93815	-0.15	4.4	-483.21	4.24900016	11	7/24/2014	25:07.0
4692	RWS11	-122.4622889	37.93813	-0.22	4.4	-476.5	4.17700016	-122.46228	37.93815	-0.12	4.4	-483.29	4.28300017	11	7/24/2014	25:07.1
4693	RWS11	-122.4622909	37.93814	-0.22	4.4	-476.6	4.17499983	-122.46228	37.93815	-0.15	4.4	-483.38	4.24699983	11	7/24/2014	25:07.2
4694	RWS11	-122.462293	37.93814	-0.19	4.39	-476.5	4.20600006	-122.46229	37.93815	-0.12	4.39	-483.33	4.27800006	11	7/24/2014	25:07.3
4695	RWS11	-122.462295	37.93814	-0.27	4.39	-476.4	4.11800021	-122.46229	37.93815	-0.15	4.39	-483.22	4.24200019	11	7/24/2014	25:07.4
4696	RWS11	-122.4622971	37.93814	-0.22	4.39	-476.2	4.16699994	-122.46229	37.93815	-0.12	4.39	-483.09	4.27299994	11	7/24/2014	25:07.5
4697	RWS11	-122.4622992	37.93814	-0.22	4.39	-476.1	4.16500008	-122.46229	37.93815	-0.12	4.39	-483.08	4.27100009	11	7/24/2014	25:07.6
4698	RWS11	-122.4623012	37.93814	-0.22	4.39	-476	4.16400015	-122.46229	37.93815	-0.07	4.39	-483.07	4.32100016	11	7/24/2014	25:07.7
4699	RWS11	-122.4623032	37.93814	-0.22	4.39	-475.9	4.16300023	-122.4623	37.93815	-0.15	4.39	-483.18	4.23500022	11	7/24/2014	25:07.8
4700	RWS11	-122.4623052	37.93814	-0.22	4.38	-475.9	4.16099989	-122.4623	37.93815	-0.12	4.38	-483.29	4.2669999	11	7/24/2014	25:07.9
4701	RWS11	-122.4623082	37.93814	-0.27	4.38	-475.8	4.10600013	-122.4623	37.93815	-0.12	4.38	-483.4	4.26400012	11	7/24/2014	25:08.0
4702	RWS11	-122.4623101	37.93814	-0.22	4.38	-475.8	4.153	-122.4623	37.93815	-0.07	4.38	-483.55	4.31	11	7/24/2014	25:08.1
4703	RWS11	-122.462312	37.93814	-0.27	4.37	-475.8	4.09400004	-122.46231	37.93815	-0.15	4.37	-483.59	4.21800002	11	7/24/2014	25:08.2
4704	RWS11	-122.462314	37.93814	-0.22	4.36	-475.7	4.1370002	-122.46231	37.93816	-0.12	4.36	-483.58	4.24300021	11	7/24/2014	25:08.3
4705	RWS11	-122.4623159	37.93814	-0.22	4.35	-475.6	4.12699997	-122.46231	37.93816	-0.12	4.35	-483.59	4.23299998	11	7/24/2014	25:08.4
4706	RWS11	-122.4623179	37.93814	-0.22	4.34	-475.6	4.11599982	-122.46231	37.93816	-0.12	4.34	-483.54	4.22199982	11	7/24/2014	25:08.5
4707	RWS11	-122.4623198	37.93815	-0.22	4.33	-475.7	4.10500014	-122.46231	37.93816	-0.15	4.33	-483.57	4.17700014	11	7/24/2014	25:08.6
4708	RWS11	-122.4623217	37.93815	-0.22	4.32	-475.7	4.09300005	-122.46232	37.93816	-0.12	4.32	-483.49	4.19900006	11	7/24/2014	25:08.7
4709	RWS11	-122.4623236	37.93815	-0.27	4.3	-475.7	4.02899998	-122.46232	37.93816	-0.15	4.3	-483.57	4.15299997	11	7/24/2014	25:08.8
4710	RWS11	-122.4623256	37.93815	-0.22	4.29	-475.8	4.06899989	-122.46232	37.93816	-0.12	4.29	-483.56	4.17499989	11	7/24/2014	25:08.9
4711	RWS11	-122.4623284	37.93815	-0.22	4.28	-476	4.0569998	-122.46232	37.93816	-0.15	4.28	-483.62	4.1289998	11	7/24/2014	25:09.0
4712	RWS11	-122.4623304	37.93815	-0.22	4.27	-476.2	4.0450002	-122.46232	37.93816	-0.12	4.27	-483.7	4.1510002	11	7/24/2014	25:09.1
4713	RWS11	-122.4623323	37.93815	-0.27	4.25	-476.4	3.9800002	-122.46233	37.93816	-0.15	4.25	-483.84	4.10400018	11	7/24/2014	25:09.2
4714	RWS11	-122.4623344	37.93815	-0.22	4.24	-476.7	4.01799977	-122.46233	37.93816	-0.15	4.24	-483.96	4.08999977	11	7/24/2014	25:09.3
4715	RWS11	-122.4623364	37.93815	-0.22	4.22	-476.9	4.0029999	-122.46233	37.93816	-0.15	4.22	-484.09	4.0749999	11	7/24/2014	25:09.4
4716	RWS11	-122.4623384	37.93815	-0.22	4.21	-477.1	3.98700011	-122.46233	37.93816	-0.15	4.21	-484.23	4.0590001	11	7/24/2014	25:09.5
4717	RWS11	-122.4623405	37.93815	-0.22	4.19	-477.4	3.97099984	-122.46233	37.93816	-0.15	4.19	-484.32	4.04299983	11	7/24/2014	25:09.6
4718	RWS11	-122.4623426	37.93815	-0.22	4.18	-477.5	3.95300019	-122.46234	37.93817	-0.12	4.18	-484.3	4.05900019	11	7/24/2014	25:09.7

4719	RWS11	-122.4623447	37.93815	-0.22	4.16	-477.7	3.93500006	-122.46234	37.93817	-0.12	4.16	-484.19	4.04100007	11	7/24/2014	25:09.8
4720	RWS11	-122.4623468	37.93815	-0.22	4.14	-477.9	3.91600001	-122.46234	37.93817	-0.12	4.14	-484.14	4.02200001	11	7/24/2014	25:09.9
4721	RWS11	-122.4623489	37.93815	-0.22	4.12	-478.1	3.89699996	-122.46234	37.93817	-0.12	4.12	-484.32	4.00299996	11	7/24/2014	25:10.0
4722	RWS11	-122.462351	37.93816	-0.22	4.1	-478.4	3.87699997	-122.46234	37.93817	-0.12	4.1	-484.64	3.98299998	11	7/24/2014	25:10.1
4723	RWS11	-122.462353	37.93816	-0.22	4.08	-478.7	3.85799992	-122.46235	37.93817	-0.12	4.08	-485.02	3.96399993	11	7/24/2014	25:10.2
4724	RWS11	-122.4623551	37.93816	-0.22	4.06	-479.1	3.83899987	-122.46235	37.93817	-0.07	4.06	-485.42	3.99599987	11	7/24/2014	25:10.3
4725	RWS11	-122.4623572	37.93816	-0.22	4.04	-479.5	3.82200015	-122.46235	37.93817	-0.07	4.04	-485.89	3.97900015	11	7/24/2014	25:10.4
4726	RWS11	-122.4623592	37.93816	-0.22	4.03	-479.9	3.80499995	-122.46235	37.93817	-0.07	4.03	-486.32	3.96199995	11	7/24/2014	25:10.5
4727	RWS11	-122.4623612	37.93816	-0.22	4.01	-480.3	3.79000008	-122.46235	37.93817	-0.12	4.01	-486.74	3.89600009	11	7/24/2014	25:10.6
4728	RWS11	-122.4623633	37.93816	-0.19	4	-480.8	3.80899999	-122.46236	37.93817	-0.07	4	-487.08	3.93199998	11	7/24/2014	25:10.7
4729	RWS11	-122.4623652	37.93816	-0.22	3.98	-481.1	3.76100004	-122.46236	37.93817	-0.07	3.98	-487.38	3.91800004	11	7/24/2014	25:10.8
4730	RWS11	-122.4623672	37.93816	-0.19	3.97	-481.5	3.78200004	-122.46236	37.93817	-0.07	3.97	-487.52	3.90500003	11	7/24/2014	25:10.9
4731	RWS11	-122.4623701	37.93816	-0.22	3.96	-481.6	3.73500001	-122.46236	37.93817	-0.07	3.96	-487.59	3.89200002	11	7/24/2014	25:11.0
4732	RWS11	-122.4623721	37.93816	-0.19	3.94	-481.8	3.75699994	-122.46237	37.93817	-0.07	3.94	-487.59	3.87999994	11	7/24/2014	25:11.1
4733	RWS11	-122.4623741	37.93816	-0.22	3.93	-482	3.70999992	-122.46237	37.93818	-0.07	3.93	-487.59	3.86699992	11	7/24/2014	25:11.2
4734	RWS11	-122.4623761	37.93816	-0.19	3.92	-482	3.73200008	-122.46237	37.93818	-0.07	3.92	-487.52	3.85500008	11	7/24/2014	25:11.3
4735	RWS11	-122.462378	37.93816	-0.22	3.91	-482	3.68500006	-122.46237	37.93818	-0.12	3.91	-487.37	3.79100007	11	7/24/2014	25:11.4
4736	RWS11	-122.46238	37.93817	-0.22	3.89	-482	3.67099988	-122.46237	37.93818	-0.07	3.89	-487.31	3.82799989	11	7/24/2014	25:11.5
4737	RWS11	-122.462382	37.93817	-0.22	3.88	-482	3.65699995	-122.46238	37.93818	-0.07	3.88	-487.29	3.81399995	11	7/24/2014	25:11.6
4738	RWS11	-122.462384	37.93817	-0.19	3.87	-482	3.67700002	-122.46238	37.93818	-0.07	3.87	-487.24	3.80000001	11	7/24/2014	25:11.7
4739	RWS11	-122.462386	37.93817	-0.22	3.85	-482	3.6279999	-122.46238	37.93818	-0.07	3.85	-487.07	3.78499991	11	7/24/2014	25:11.8
4740	RWS11	-122.462388	37.93817	-0.19	3.84	-482.1	3.64799997	-122.46238	37.93818	-0.07	3.84	-487.11	3.77099997	11	7/24/2014	25:11.9
4741	RWS11	-122.4623909	37.93817	-0.19	3.82	-482.1	3.63499996	-122.46238	37.93818	-0.07	3.82	-487.05	3.75799996	11	7/24/2014	25:12.0
4742	RWS11	-122.4623928	37.93817	-0.19	3.81	-482.2	3.62100002	-122.46239	37.93818	-0.07	3.81	-487.11	3.74400002	11	7/24/2014	25:12.1
4743	RWS11	-122.4623947	37.93817	-0.19	3.8	-482.3	3.60899994	-122.46239	37.93818	-0.07	3.8	-487.01	3.73199993	11	7/24/2014	25:12.2
4744	RWS11	-122.4623967	37.93817	-0.19	3.78	-482.4	3.59599993	-122.46239	37.93818	-0.07	3.78	-487.09	3.71899992	11	7/24/2014	25:12.3
4745	RWS11	-122.4623986	37.93817	-0.22	3.77	-482.7	3.551	-122.46239	37.93818	-0.12	3.77	-487.18	3.65700001	11	7/24/2014	25:12.4
4746	RWS11	-122.4624006	37.93817	-0.19	3.76	-482.8	3.57400009	-122.46239	37.93818	-0.07	3.76	-487.27	3.69700009	11	7/24/2014	25:12.5
4747	RWS11	-122.4624025	37.93817	-0.22	3.75	-483	3.52899992	-122.4624	37.93819	-0.07	3.75	-487.5	3.68599993	11	7/24/2014	25:12.6
4748	RWS11	-122.4624044	37.93817	-0.19	3.74	-483.2	3.55400011	-122.4624	37.93819	-0.07	3.74	-487.76	3.67700011	11	7/24/2014	25:12.7
4749	RWS11	-122.4624063	37.93817	-0.19	3.73	-483.6	3.54400012	-122.4624	37.93819	-0.12	3.73	-488.12	3.61600012	11	7/24/2014	25:12.8
4750	RWS11	-122.4624082	37.93817	-0.19	3.72	-484	3.53399989	-122.4624	37.93819	-0.07	3.72	-488.56	3.65699989	11	7/24/2014	25:12.9
4751	RWS11	-122.4624111	37.93818	-0.22	3.71	-484.3	3.48999989	-122.4624	37.93819	-0.07	3.71	-488.91	3.6469999	11	7/24/2014	25:13.0

4752	RWS11	-122.462413	37.93818	-0.19	3.7	-484.7	3.51299998	-122.46241	37.93819	-0.12	3.7	-489.28	3.58499998	11	7/24/2014	25:13.1
4753	RWS11	-122.4624148	37.93818	-0.22	3.69	-485.2	3.46599996	-122.46241	37.93819	-0.12	3.69	-489.63	3.57199997	11	7/24/2014	25:13.2
4754	RWS11	-122.4624168	37.93818	-0.19	3.67	-485.7	3.48699996	-122.46241	37.93819	-0.07	3.67	-490.21	3.60999995	11	7/24/2014	25:13.3
4755	RWS11	-122.4624187	37.93818	-0.22	3.66	-486.4	3.4410001	-122.46241	37.93819	-0.12	3.66	-490.86	3.54700011	11	7/24/2014	25:13.4
4756	RWS11	-122.4624206	37.93818	-0.19	3.65	-487	3.46300003	-122.46241	37.93819	-0.07	3.65	-491.49	3.58600003	11	7/24/2014	25:13.5
4757	RWS11	-122.4624225	37.93818	-0.22	3.64	-487.5	3.4180001	-122.46242	37.93819	-0.12	3.64	-492.06	3.52400011	11	7/24/2014	25:13.6
4758	RWS11	-122.4624245	37.93818	-0.19	3.63	-488.2	3.44300005	-122.46242	37.93819	-0.07	3.63	-492.76	3.56600004	11	7/24/2014	25:13.7
4759	RWS11	-122.4624263	37.93818	-0.22	3.62	-488.9	3.4009999	-122.46242	37.93819	-0.15	3.62	-493.46	3.4729999	11	7/24/2014	25:13.8
4760	RWS11	-122.4624282	37.93818	-0.19	3.62	-489.6	3.42799994	-122.46242	37.93819	-0.12	3.62	-494.17	3.49999994	11	7/24/2014	25:13.9
4761	RWS11	-122.462431	37.93818	-0.22	3.61	-490.4	3.38799989	-122.46242	37.93819	-0.12	3.61	-494.99	3.4939999	11	7/24/2014	25:14.0
4762	RWS11	-122.4624329	37.93818	-0.19	3.61	-491.2	3.41799995	-122.46243	37.9382	-0.15	3.61	-495.91	3.45599994	11	7/24/2014	25:14.1
4763	RWS11	-122.4624348	37.93818	-0.22	3.6	-492	3.38099992	-122.46243	37.9382	-0.12	3.6	-496.79	3.48699993	11	7/24/2014	25:14.2
4764	RWS11	-122.4624367	37.93818	-0.19	3.6	-492.8	3.41300008	-122.46243	37.9382	-0.12	3.6	-497.67	3.48500007	11	7/24/2014	25:14.3
4765	RWS11	-122.4624386	37.93818	-0.22	3.6	-493.7	3.37699997	-122.46243	37.9382	-0.12	3.6	-498.62	3.48299998	11	7/24/2014	25:14.4
4766	RWS11	-122.4624405	37.93819	-0.22	3.6	-494.7	3.37600005	-122.46243	37.9382	-0.12	3.6	-499.81	3.48200005	11	7/24/2014	25:14.5
4767	RWS11	-122.4624425	37.93819	-0.22	3.6	-495.8	3.37600005	-122.46244	37.9382	-0.15	3.6	-501.11	3.44800004	11	7/24/2014	25:14.6
4768	RWS11	-122.4624444	37.93819	-0.22	3.6	-496.8	3.37600005	-122.46244	37.9382	-0.12	3.6	-502.43	3.48200005	11	7/24/2014	25:14.7
4769	RWS11	-122.4624463	37.93819	-0.22	3.6	-497.9	3.37600005	-122.46244	37.9382	-0.15	3.6	-503.75	3.44800004	11	7/24/2014	25:14.8
4770	RWS11	-122.4624482	37.93819	-0.22	3.6	-499.1	3.3779999	-122.46244	37.9382	-0.12	3.6	-505.07	3.48399991	11	7/24/2014	25:14.9
4771	RWS11	-122.4624511	37.93819	-0.22	3.6	-500.3	3.38	-122.46244	37.9382	-0.15	3.6	-506.6	3.45199999	11	7/24/2014	25:15.0
4772	RWS11	-122.462453	37.93819	-0.22	3.6	-501.6	3.38200009	-122.46245	37.9382	-0.12	3.6	-508.25	3.48800009	11	7/24/2014	25:15.1
4773	RWS11	-122.462455	37.93819	-0.22	3.61	-502.8	3.38600004	-122.46245	37.9382	-0.15	3.61	-509.9	3.45800003	11	7/24/2014	25:15.2
4774	RWS11	-122.462457	37.93819	-0.22	3.61	-504.1	3.38999999	-122.46245	37.9382	-0.12	3.61	-511.57	3.49599999	11	7/24/2014	25:15.3
4775	RWS11	-122.462459	37.93819	-0.22	3.62	-505.4	3.39399993	-122.46245	37.9382	-0.15	3.62	-513.41	3.46599993	11	7/24/2014	25:15.4
4776	RWS11	-122.462461	37.93819	-0.22	3.62	-506.7	3.39900005	-122.46245	37.9382	-0.12	3.62	-515.1	3.50500005	11	7/24/2014	25:15.5
4777	RWS11	-122.4624631	37.93819	-0.22	3.63	-508.1	3.403	-122.46246	37.93821	-0.15	3.63	-516.83	3.47499999	11	7/24/2014	25:15.6
4778	RWS11	-122.4624651	37.93819	-0.22	3.63	-509.4	3.40699995	-122.46246	37.93821	-0.12	3.63	-518.52	3.51299995	11	7/24/2014	25:15.7
4779	RWS11	-122.4624671	37.93819	-0.22	3.63	-510.6	3.40900004	-122.46246	37.93821	-0.15	3.63	-520.17	3.48100004	11	7/24/2014	25:15.8
4780	RWS11	-122.462469	37.9382	-0.22	3.63	-511.9	3.40900004	-122.46246	37.93821	-0.15	3.63	-521.82	3.48100004	11	7/24/2014	25:15.9
4781	RWS11	-122.462472	37.9382	-0.22	3.63	-513.1	3.40600002	-122.46247	37.93821	-0.15	3.63	-523.21	3.47800002	11	7/24/2014	25:16.0
4782	RWS11	-122.4624739	37.9382	-0.22	3.62	-514	3.4009999	-122.46247	37.93821	-0.15	3.62	-524.45	3.4729999	11	7/24/2014	25:16.1
4783	RWS11	-122.4624759	37.9382	-0.27	3.61	-515.1	3.34000009	-122.46247	37.93821	-0.2	3.61	-525.65	3.41400008	11	7/24/2014	25:16.2
4784	RWS11	-122.4624779	37.9382	-0.22	3.6	-516.2	3.38099992	-122.46247	37.93821	-0.2	3.6	-526.94	3.40299992	11	7/24/2014	25:16.3

4785	RWS11	-122.4624799	37.9382	-0.27	3.59	-517.2	3.31599993	-122.46247	37.93821	-0.2	3.59	-528.19	3.38999991	11	7/24/2014	25:16.4
4786	RWS11	-122.4624819	37.9382	-0.22	3.58	-518.2	3.35600007	-122.46248	37.93821	-0.2	3.58	-529.34	3.37800007	11	7/24/2014	25:16.5
4787	RWS11	-122.4624838	37.9382	-0.27	3.57	-519.1	3.29299992	-122.46248	37.93821	-0.23	3.57	-530.43	3.33299991	11	7/24/2014	25:16.6
4788	RWS11	-122.4624858	37.9382	-0.22	3.56	-520.1	3.33600008	-122.46248	37.93821	-0.15	3.56	-531.43	3.40800008	11	7/24/2014	25:16.7
4789	RWS11	-122.4624878	37.9382	-0.27	3.55	-520.9	3.27700013	-122.46248	37.93821	-0.23	3.55	-532.34	3.31700012	11	7/24/2014	25:16.8
4790	RWS11	-122.4624897	37.9382	-0.22	3.55	-521.7	3.324	-122.46248	37.93821	-0.2	3.55	-533.12	3.346	11	7/24/2014	25:16.9
4791	RWS11	-122.4624926	37.9382	-0.27	3.54	-522.5	3.2669999	-122.46249	37.93822	-0.23	3.54	-533.98	3.30699989	11	7/24/2014	25:17.0
4792	RWS11	-122.4624946	37.9382	-0.22	3.54	-523.2	3.31499994	-122.46249	37.93822	-0.2	3.54	-534.83	3.33699994	11	7/24/2014	25:17.1
4793	RWS11	-122.4624966	37.9382	-0.27	3.53	-524.1	3.259	-122.46249	37.93822	-0.2	3.53	-535.8	3.33299999	11	7/24/2014	25:17.2
4794	RWS11	-122.4624986	37.93821	-0.22	3.53	-524.9	3.30700004	-122.46249	37.93822	-0.2	3.53	-536.74	3.32900004	11	7/24/2014	25:17.3
4795	RWS11	-122.4625005	37.93821	-0.27	3.53	-525.7	3.25200003	-122.46249	37.93822	-0.23	3.53	-537.64	3.29200003	11	7/24/2014	25:17.4
4796	RWS11	-122.4625025	37.93821	-0.27	3.52	-526.5	3.24900001	-122.4625	37.93822	-0.23	3.52	-538.35	3.289	11	7/24/2014	25:17.5
4797	RWS11	-122.4625045	37.93821	-0.27	3.52	-527.1	3.24699992	-122.4625	37.93822	-0.23	3.52	-538.88	3.28699991	11	7/24/2014	25:17.6
4798	RWS11	-122.4625065	37.93821	-0.22	3.52	-527.7	3.29700005	-122.4625	37.93822	-0.2	3.52	-539.23	3.31900005	11	7/24/2014	25:17.7
4799	RWS11	-122.4625085	37.93821	-0.27	3.52	-528.2	3.24299997	-122.4625	37.93822	-0.23	3.52	-539.46	3.28299996	11	7/24/2014	25:17.8
4800	RWS11	-122.4625104	37.93821	-0.27	3.52	-528.7	3.24100012	-122.4625	37.93822	-0.23	3.52	-539.63	3.28100011	11	7/24/2014	25:17.9
4801	RWS11	-122.4625134	37.93821	-0.27	3.51	-529.1	3.23900002	-122.46251	37.93822	-0.23	3.51	-539.73	3.27900001	11	7/24/2014	25:18.0
4802	RWS11	-122.4625153	37.93821	-0.27	3.51	-529.4	3.23800009	-122.46251	37.93822	-0.2	3.51	-539.74	3.31200008	11	7/24/2014	25:18.1
4803	RWS11	-122.4625173	37.93821	-0.27	3.51	-529.6	3.23699993	-122.46251	37.93822	-0.29	3.51	-539.61	3.22599992	11	7/24/2014	25:18.2
4804	RWS11	-122.4625193	37.93821	-0.22	3.51	-529.9	3.29000008	-122.46251	37.93822	-0.23	3.51	-539.46	3.27800009	11	7/24/2014	25:18.3
4805	RWS11	-122.4625213	37.93821	-0.27	3.51	-530	3.23900002	-122.46251	37.93823	-0.29	3.51	-539.34	3.22800002	11	7/24/2014	25:18.4
4806	RWS11	-122.4625233	37.93821	-0.22	3.52	-530	3.29499996	-122.46252	37.93823	-0.23	3.52	-539.04	3.28299996	11	7/24/2014	25:18.5
4807	RWS11	-122.4625253	37.93821	-0.27	3.52	-529.9	3.24699992	-122.46252	37.93823	-0.29	3.52	-538.58	3.23599991	11	7/24/2014	25:18.6
4808	RWS11	-122.4625273	37.93821	-0.27	3.53	-529.7	3.25299996	-122.46252	37.93823	-0.29	3.53	-538.1	3.24199995	11	7/24/2014	25:18.7
4809	RWS11	-122.4625292	37.93822	-0.27	3.54	-529.5	3.26200002	-122.46252	37.93823	-0.29	3.54	-537.65	3.25100002	11	7/24/2014	25:18.8
4810	RWS11	-122.4625312	37.93822	-0.27	3.55	-529.2	3.27200001	-122.46252	37.93823	-0.29	3.55	-537.13	3.26100001	11	7/24/2014	25:18.9
4811	RWS11	-122.4625342	37.93822	-0.27	3.56	-528.8	3.28500003	-122.46253	37.93823	-0.29	3.56	-536.44	3.27400002	11	7/24/2014	25:19.0
4812	RWS11	-122.4625361	37.93822	-0.27	3.57	-528.4	3.29899997	-122.46253	37.93823	-0.29	3.57	-535.85	3.28799996	11	7/24/2014	25:19.1
4813	RWS11	-122.4625381	37.93822	-0.27	3.59	-528.2	3.315	-122.46253	37.93823	-0.29	3.59	-535.3	3.30399999	11	7/24/2014	25:19.2
4814	RWS11	-122.4625401	37.93822	-0.27	3.6	-527.9	3.3300001	-122.46253	37.93823	-0.23	3.6	-534.91	3.37000009	11	7/24/2014	25:19.3
4815	RWS11	-122.4625421	37.93822	-0.27	3.62	-527.6	3.34499997	-122.46254	37.93823	-0.29	3.62	-534.49	3.33399996	11	7/24/2014	25:19.4
4816	RWS11	-122.4625441	37.93822	-0.27	3.63	-527.3	3.35600013	-122.46254	37.93823	-0.29	3.63	-534.03	3.34500012	11	7/24/2014	25:19.5
4817	RWS11	-122.4625461	37.93822	-0.27	3.64	-527	3.36400002	-122.46254	37.93823	-0.29	3.64	-533.58	3.35300002	11	7/24/2014	25:19.6

4818	RWS11	-122.4625481	37.93822	-0.27	3.64	-526.6	3.36700004	-122.46254	37.93823	-0.29	3.64	-533.08	3.35600004	11	7/24/2014	25:19.7
4819	RWS11	-122.4625501	37.93822	-0.27	3.64	-526.4	3.36499995	-122.46254	37.93823	-0.29	3.64	-532.57	3.35399994	11	7/24/2014	25:19.8
4820	RWS11	-122.4625521	37.93822	-0.27	3.63	-526.1	3.36000007	-122.46255	37.93824	-0.29	3.63	-532.15	3.34900007	11	7/24/2014	25:19.9
4821	RWS11	-122.4625551	37.93822	-0.27	3.63	-525.8	3.3530001	-122.46255	37.93824	-0.32	3.63	-531.68	3.30800009	11	7/24/2014	25:20.0
4822	RWS11	-122.4625571	37.93822	-0.27	3.62	-525.6	3.34400004	-122.46255	37.93824	-0.29	3.62	-531.24	3.33300003	11	7/24/2014	25:20.1
4823	RWS11	-122.4625591	37.93823	-0.27	3.61	-525.5	3.33499998	-122.46255	37.93824	-0.29	3.61	-530.94	3.32399997	11	7/24/2014	25:20.2
4824	RWS11	-122.4625611	37.93823	-0.27	3.6	-525.5	3.32499999	-122.46255	37.93824	-0.32	3.6	-530.62	3.27999997	11	7/24/2014	25:20.3
4825	RWS11	-122.4625631	37.93823	-0.27	3.59	-525.3	3.315	-122.46256	37.93824	-0.32	3.59	-530.34	3.26999998	11	7/24/2014	25:20.4
4826	RWS11	-122.4625652	37.93823	-0.27	3.58	-525.2	3.30599993	-122.46256	37.93824	-0.29	3.58	-530.18	3.29499993	11	7/24/2014	25:20.5
4827	RWS11	-122.4625672	37.93823	-0.27	3.57	-525.1	3.29700011	-122.46256	37.93824	-0.23	3.57	-530.08	3.3370001	11	7/24/2014	25:20.6
4828	RWS11	-122.4625692	37.93823	-0.27	3.56	-524.9	3.2899999	-122.46256	37.93824	-0.23	3.56	-529.92	3.32999989	11	7/24/2014	25:20.7
4829	RWS11	-122.4625712	37.93823	-0.27	3.56	-524.7	3.28299993	-122.46256	37.93824	-0.29	3.56	-529.76	3.27199993	11	7/24/2014	25:20.8
4830	RWS11	-122.4625732	37.93823	-0.27	3.55	-524.6	3.27800006	-122.46257	37.93824	-0.23	3.55	-529.72	3.31800005	11	7/24/2014	25:20.9
4831	RWS11	-122.4625762	37.93823	-0.27	3.55	-524.7	3.27299994	-122.46257	37.93824	-0.29	3.55	-529.78	3.26199993	11	7/24/2014	25:21.0
4832	RWS11	-122.4625783	37.93823	-0.22	3.54	-524.9	3.32099998	-122.46257	37.93824	-0.23	3.54	-529.79	3.30899999	11	7/24/2014	25:21.1
4833	RWS11	-122.4625803	37.93823	-0.27	3.54	-525.1	3.26500005	-122.46257	37.93825	-0.29	3.54	-529.9	3.25400004	11	7/24/2014	25:21.2
4834	RWS11	-122.4625823	37.93823	-0.27	3.54	-525.3	3.2610001	-122.46258	37.93825	-0.29	3.54	-529.88	3.25000009	11	7/24/2014	25:21.3
4835	RWS11	-122.4625844	37.93823	-0.27	3.53	-525.7	3.25800008	-122.46258	37.93825	-0.23	3.53	-530.1	3.29800007	11	7/24/2014	25:21.4
4836	RWS11	-122.4625864	37.93823	-0.27	3.53	-526	3.25699991	-122.46258	37.93825	-0.23	3.53	-530.33	3.2969999	11	7/24/2014	25:21.5
4837	RWS11	-122.4625885	37.93824	-0.27	3.53	-526.3	3.25699991	-122.46258	37.93825	-0.23	3.53	-530.58	3.2969999	11	7/24/2014	25:21.6
4838	RWS11	-122.4625905	37.93824	-0.27	3.53	-526.6	3.259	-122.46258	37.93825	-0.29	3.53	-530.97	3.248	11	7/24/2014	25:21.7
4839	RWS11	-122.4625926	37.93824	-0.27	3.54	-527	3.26299995	-122.46259	37.93825	-0.2	3.54	-531.3	3.33699994	11	7/24/2014	25:21.8
4840	RWS11	-122.4625946	37.93824	-0.22	3.55	-527.4	3.32300007	-122.46259	37.93825	-0.23	3.55	-531.74	3.31100008	11	7/24/2014	25:21.9
4841	RWS11	-122.4625976	37.93824	-0.27	3.56	-527.8	3.28200001	-122.46259	37.93825	-0.29	3.56	-532.02	3.271	11	7/24/2014	25:22.0
4842	RWS11	-122.4625996	37.93824	-0.22	3.57	-528.2	3.35000002	-122.46259	37.93825	-0.23	3.57	-532.24	3.33800003	11	7/24/2014	25:22.1
4843	RWS11	-122.4626016	37.93824	-0.27	3.59	-528.4	3.31700009	-122.4626	37.93825	-0.29	3.59	-532.47	3.30600008	11	7/24/2014	25:22.2
4844	RWS11	-122.4626037	37.93824	-0.22	3.61	-528.6	3.39200008	-122.4626	37.93825	-0.23	3.61	-532.69	3.38000008	11	7/24/2014	25:22.3
4845	RWS11	-122.4626057	37.93824	-0.27	3.64	-528.8	3.36499995	-122.4626	37.93825	-0.23	3.64	-532.88	3.40499994	11	7/24/2014	25:22.4
4846	RWS11	-122.4626078	37.93824	-0.22	3.66	-528.9	3.44200003	-122.4626	37.93825	-0.23	3.66	-533.11	3.43000004	11	7/24/2014	25:22.5
4847	RWS11	-122.4626098	37.93824	-0.27	3.69	-529	3.4149999	-122.4626	37.93825	-0.23	3.69	-533.37	3.45499989	11	7/24/2014	25:22.6
4848	RWS11	-122.4626119	37.93824	-0.22	3.71	-529.1	3.48999989	-122.46261	37.93826	-0.23	3.71	-533.82	3.4779999	11	7/24/2014	25:22.7
4849	RWS11	-122.4626139	37.93824	-0.27	3.73	-529.2	3.46099991	-122.46261	37.93826	-0.29	3.73	-534.21	3.4499999	11	7/24/2014	25:22.8
4850	RWS11	-122.4626159	37.93824	-0.27	3.76	-529.2	3.48299998	-122.46261	37.93826	-0.23	3.76	-534.56	3.52299997	11	7/24/2014	25:22.9

4851	RWS11	-122.4626189	37.93825	-0.31	3.78	-529.2	3.47000012	-122.46261	37.93826	-0.23	3.78	-534.85	3.54400012	11	7/24/2014	25:23.0
4852	RWS11	-122.4626209	37.93825	-0.27	3.8	-529.2	3.52500004	-122.46261	37.93826	-0.23	3.8	-535.09	3.56500003	11	7/24/2014	25:23.1
4853	RWS11	-122.4626229	37.93825	-0.31	3.82	-529.2	3.51100001	-122.46262	37.93826	-0.29	3.82	-535.22	3.53400001	11	7/24/2014	25:23.2
4854	RWS11	-122.462625	37.93825	-0.27	3.84	-529.1	3.56199998	-122.46262	37.93826	-0.23	3.84	-535.37	3.60199997	11	7/24/2014	25:23.3
4855	RWS11	-122.462627	37.93825	-0.31	3.85	-529	3.54300007	-122.46262	37.93826	-0.23	3.85	-535.51	3.61700007	11	7/24/2014	25:23.4
4856	RWS11	-122.462629	37.93825	-0.27	3.86	-528.8	3.58700007	-122.46262	37.93826	-0.2	3.86	-535.55	3.66100006	11	7/24/2014	25:23.5
4857	RWS11	-122.4626311	37.93825	-0.27	3.87	-528.6	3.59400004	-122.46262	37.93826	-0.23	3.87	-535.61	3.63400003	11	7/24/2014	25:23.6
4858	RWS11	-122.4626331	37.93825	-0.27	3.87	-528.4	3.5959999	-122.46263	37.93826	-0.23	3.87	-535.69	3.63599989	11	7/24/2014	25:23.7
4859	RWS11	-122.4626351	37.93825	-0.31	3.87	-528.3	3.56000003	-122.46263	37.93826	-0.29	3.87	-535.8	3.58300003	11	7/24/2014	25:23.8
4860	RWS11	-122.4626372	37.93825	-0.27	3.86	-528.2	3.588	-122.46263	37.93826	-0.23	3.86	-536.06	3.62799999	11	7/24/2014	25:23.9
4861	RWS11	-122.4626401	37.93825	-0.31	3.85	-528.3	3.544	-122.46263	37.93826	-0.23	3.85	-536.32	3.618	11	7/24/2014	25:24.0
4862	RWS11	-122.4626422	37.93825	-0.31	3.84	-528.3	3.53199992	-122.46264	37.93827	-0.23	3.84	-536.41	3.60599992	11	7/24/2014	25:24.1
4863	RWS11	-122.4626442	37.93825	-0.31	3.83	-528.2	3.51799998	-122.46264	37.93827	-0.29	3.83	-536.3	3.54099998	11	7/24/2014	25:24.2
4864	RWS11	-122.4626462	37.93825	-0.27	3.81	-528.2	3.53899997	-122.46264	37.93827	-0.23	3.81	-536.19	3.57899997	11	7/24/2014	25:24.3
4865	RWS11	-122.4626483	37.93825	-0.31	3.8	-528.1	3.49199995	-122.46264	37.93827	-0.23	3.8	-536.22	3.56599995	11	7/24/2014	25:24.4
4866	RWS11	-122.4626503	37.93826	-0.31	3.79	-528	3.48000011	-122.46264	37.93827	-0.29	3.79	-536.22	3.50300011	11	7/24/2014	25:24.5
4867	RWS11	-122.4626524	37.93826	-0.31	3.78	-528	3.47000012	-122.46265	37.93827	-0.29	3.78	-536.22	3.49300012	11	7/24/2014	25:24.6
4868	RWS11	-122.4626544	37.93826	-0.31	3.77	-528.1	3.46299991	-122.46265	37.93827	-0.29	3.77	-536.3	3.48599991	11	7/24/2014	25:24.7
4869	RWS11	-122.4626564	37.93826	-0.31	3.77	-528.2	3.45700011	-122.46265	37.93827	-0.29	3.77	-536.43	3.48000011	11	7/24/2014	25:24.8
4870	RWS11	-122.4626584	37.93826	-0.31	3.76	-528.2	3.45400009	-122.46265	37.93827	-0.29	3.76	-536.6	3.47700009	11	7/24/2014	25:24.9
4871	RWS11	-122.4626614	37.93826	-0.31	3.76	-528.5	3.45299992	-122.46266	37.93827	-0.29	3.76	-536.96	3.47599992	11	7/24/2014	25:25.0
4872	RWS11	-122.4626635	37.93826	-0.31	3.76	-528.7	3.45400009	-122.46266	37.93827	-0.29	3.76	-537.27	3.47700009	11	7/24/2014	25:25.1
4873	RWS11	-122.4626655	37.93826	-0.31	3.76	-529	3.45500001	-122.46266	37.93827	-0.23	3.76	-537.49	3.52900001	11	7/24/2014	25:25.2
4874	RWS11	-122.4626675	37.93826	-0.31	3.77	-529.2	3.45800003	-122.46266	37.93827	-0.29	3.77	-537.82	3.48100004	11	7/24/2014	25:25.3
4875	RWS11	-122.4626696	37.93826	-0.34	3.77	-529.5	3.42599988	-122.46266	37.93827	-0.29	3.77	-538.14	3.48299989	11	7/24/2014	25:25.4
4876	RWS11	-122.4626716	37.93826	-0.31	3.77	-529.8	3.46299991	-122.46267	37.93827	-0.29	3.77	-538.36	3.48599991	11	7/24/2014	25:25.5
4877	RWS11	-122.4626737	37.93826	-0.34	3.77	-530	3.43199992	-122.46267	37.93828	-0.32	3.77	-538.6	3.45499992	11	7/24/2014	25:25.6
4878	RWS11	-122.4626758	37.93826	-0.31	3.78	-530.2	3.46800002	-122.46267	37.93828	-0.32	3.78	-538.7	3.45700002	11	7/24/2014	25:25.7
4879	RWS11	-122.4626778	37.93826	-0.34	3.78	-530.4	3.43700004	-122.46267	37.93828	-0.32	3.78	-538.7	3.46000004	11	7/24/2014	25:25.8
4880	RWS11	-122.4626799	37.93826	-0.31	3.78	-530.5	3.47499999	-122.46267	37.93828	-0.29	3.78	-538.6	3.498	11	7/24/2014	25:25.9
4881	RWS11	-122.4626829	37.93827	-0.34	3.79	-530.5	3.44499993	-122.46268	37.93828	-0.32	3.79	-538.56	3.46799994	11	7/24/2014	25:26.0
4882	RWS11	-122.462685	37.93827	-0.31	3.79	-530.6	3.48499998	-122.46268	37.93828	-0.29	3.79	-538.53	3.50799999	11	7/24/2014	25:26.1
4883	RWS11	-122.4626871	37.93827	-0.34	3.8	-530.8	3.45900011	-122.46268	37.93828	-0.32	3.8	-538.73	3.48200011	11	7/24/2014	25:26.2

4884	RWS11	-122.4626892	37.93827	-0.31	3.81	-531.1	3.50300011	-122.46268	37.93828	-0.32	3.81	-539.01	3.4920001	11	7/24/2014	25:26.3
4885	RWS11	-122.4626913	37.93827	-0.34	3.82	-531.4	3.48099995	-122.46269	37.93828	-0.35	3.82	-539.41	3.46999997	11	7/24/2014	25:26.4
4886	RWS11	-122.4626935	37.93827	-0.31	3.84	-531.8	3.52899989	-122.46269	37.93828	-0.29	3.84	-539.88	3.5519999	11	7/24/2014	25:26.5
4887	RWS11	-122.4626956	37.93827	-0.34	3.85	-532.1	3.50999999	-122.46269	37.93828	-0.32	3.85	-540.38	3.53299999	11	7/24/2014	25:26.6
4888	RWS11	-122.4626977	37.93827	-0.31	3.87	-532.4	3.5590001	-122.46269	37.93828	-0.29	3.87	-540.75	3.58200011	11	7/24/2014	25:26.7
4889	RWS11	-122.4626998	37.93827	-0.34	3.88	-532.8	3.53900003	-122.46269	37.93828	-0.32	3.88	-541.24	3.56200004	11	7/24/2014	25:26.8
4890	RWS11	-122.4627018	37.93827	-0.31	3.89	-533.1	3.58699998	-122.4627	37.93828	-0.32	3.89	-541.6	3.57599998	11	7/24/2014	25:26.9
4891	RWS11	-122.4627049	37.93827	-0.39	3.91	-533.3	3.51299989	-122.4627	37.93828	-0.32	3.91	-541.89	3.58699989	11	7/24/2014	25:27.0
4892	RWS11	-122.4627069	37.93827	-0.34	3.91	-533.5	3.57299995	-122.4627	37.93829	-0.32	3.91	-542.2	3.59599996	11	7/24/2014	25:27.1
4893	RWS11	-122.4627089	37.93827	-0.34	3.92	-533.8	3.57800007	-122.4627	37.93829	-0.32	3.92	-542.48	3.60100007	11	7/24/2014	25:27.2
4894	RWS11	-122.462711	37.93827	-0.31	3.92	-534	3.61600003	-122.4627	37.93829	-0.29	3.92	-542.78	3.63900003	11	7/24/2014	25:27.3
4895	RWS11	-122.462713	37.93827	-0.34	3.92	-534.2	3.58299994	-122.46271	37.93829	-0.29	3.92	-543.01	3.63999996	11	7/24/2014	25:27.4
4896	RWS11	-122.462715	37.93828	-0.34	3.92	-534.4	3.58200002	-122.46271	37.93829	-0.29	3.92	-543.19	3.63900003	11	7/24/2014	25:27.5
4897	RWS11	-122.4627171	37.93828	-0.34	3.92	-534.7	3.58100009	-122.46271	37.93829	-0.32	3.92	-543.45	3.60400009	11	7/24/2014	25:27.6
4898	RWS11	-122.4627191	37.93828	-0.34	3.92	-534.8	3.57999992	-122.46271	37.93829	-0.29	3.92	-543.69	3.63699993	11	7/24/2014	25:27.7
4899	RWS11	-122.4627211	37.93828	-0.34	3.92	-535	3.57800007	-122.46272	37.93829	-0.29	3.92	-544	3.63500008	11	7/24/2014	25:27.8
4900	RWS11	-122.4627231	37.93828	-0.31	3.92	-535.3	3.61099991	-122.46272	37.93829	-0.29	3.92	-544.37	3.63399991	11	7/24/2014	25:27.9
4901	RWS11	-122.4627261	37.93828	-0.34	3.92	-535.5	3.57800007	-122.46272	37.93829	-0.29	3.92	-544.77	3.63500008	11	7/24/2014	25:28.0
4902	RWS11	-122.4627281	37.93828	-0.31	3.92	-535.8	3.61200008	-122.46272	37.93829	-0.29	3.92	-545.19	3.63500008	11	7/24/2014	25:28.1
4903	RWS11	-122.46273	37.93828	-0.34	3.92	-536	3.579	-122.46272	37.93829	-0.29	3.92	-545.61	3.63600001	11	7/24/2014	25:28.2
4904	RWS11	-122.462732	37.93828	-0.34	3.92	-536.3	3.57999992	-122.46273	37.93829	-0.29	3.92	-545.92	3.63699993	11	7/24/2014	25:28.3
4905	RWS11	-122.4627341	37.93828	-0.34	3.92	-536.5	3.57999992	-122.46273	37.93829	-0.23	3.92	-546.2	3.68799993	11	7/24/2014	25:28.4
4906	RWS11	-122.4627361	37.93828	-0.34	3.92	-536.7	3.5769999	-122.46273	37.93829	-0.29	3.92	-546.38	3.63399991	11	7/24/2014	25:28.5
4907	RWS11	-122.4627381	37.93828	-0.34	3.91	-536.9	3.56999993	-122.46273	37.93829	-0.32	3.91	-546.59	3.59299994	11	7/24/2014	25:28.6
4908	RWS11	-122.4627401	37.93828	-0.31	3.9	-537.1	3.59500012	-122.46273	37.9383	-0.29	3.9	-546.84	3.61800012	11	7/24/2014	25:28.7
4909	RWS11	-122.4627421	37.93828	-0.34	3.89	-537.4	3.54699993	-122.46274	37.9383	-0.32	3.89	-547.1	3.56999993	11	7/24/2014	25:28.8
4910	RWS11	-122.4627441	37.93828	-0.31	3.87	-537.7	3.56399998	-122.46274	37.9383	-0.29	3.87	-547.34	3.58699998	11	7/24/2014	25:28.9
4911	RWS11	-122.462747	37.93828	-0.34	3.85	-537.8	3.50999999	-122.46274	37.9383	-0.29	3.85	-547.42	3.567	11	7/24/2014	25:29.0
4912	RWS11	-122.462749	37.93829	-0.31	3.83	-537.9	3.52300009	-122.46274	37.9383	-0.29	3.83	-547.5	3.54600009	11	7/24/2014	25:29.1
4913	RWS11	-122.462751	37.93829	-0.34	3.81	-538.1	3.46700001	-122.46275	37.9383	-0.32	3.81	-547.66	3.49000001	11	7/24/2014	25:29.2
4914	RWS11	-122.462753	37.93829	-0.31	3.79	-538.2	3.48100004	-122.46275	37.9383	-0.29	3.79	-547.88	3.50400004	11	7/24/2014	25:29.3
4915	RWS11	-122.462755	37.93829	-0.34	3.77	-538.4	3.4289999	-122.46275	37.9383	-0.29	3.77	-547.97	3.48599991	11	7/24/2014	25:29.4
4916	RWS11	-122.462757	37.93829	-0.31	3.76	-538.6	3.44899997	-122.46275	37.9383	-0.29	3.76	-548.02	3.47199997	11	7/24/2014	25:29.5

4917	RWS11	-122.462759	37.93829	-0.34	3.74	-538.7	3.40299988	-122.46275	37.9383	-0.29	3.74	-548.04	3.45999989	11	7/24/2014	25:29.6
4918	RWS11	-122.4627611	37.93829	-0.31	3.74	-538.9	3.42800006	-122.46276	37.9383	-0.29	3.74	-548.14	3.45100006	11	7/24/2014	25:29.7
4919	RWS11	-122.462763	37.93829	-0.34	3.73	-539.1	3.38700008	-122.46276	37.9383	-0.29	3.73	-548.2	3.4440001	11	7/24/2014	25:29.8
4920	RWS11	-122.462765	37.93829	-0.34	3.72	-539.2	3.37899995	-122.46276	37.9383	-0.29	3.72	-548.24	3.43599996	11	7/24/2014	25:29.9
4921	RWS11	-122.462768	37.93829	-0.34	3.71	-539.4	3.37299991	-122.46276	37.9383	-0.32	3.71	-548.2	3.39599991	11	7/24/2014	25:30.0
4922	RWS11	-122.46277	37.93829	-0.31	3.71	-539.4	3.40200004	-122.46276	37.9383	-0.29	3.71	-548.27	3.42500004	11	7/24/2014	25:30.1
4923	RWS11	-122.462772	37.93829	-0.34	3.71	-539.5	3.36400008	-122.46277	37.9383	-0.32	3.71	-548.38	3.38700008	11	7/24/2014	25:30.2
4924	RWS11	-122.462774	37.93829	-0.31	3.7	-539.6	3.396	-122.46277	37.9383	-0.29	3.7	-548.38	3.419	11	7/24/2014	25:30.3
4925	RWS11	-122.462776	37.93829	-0.34	3.7	-539.6	3.36100006	-122.46277	37.93831	-0.32	3.7	-548.47	3.38400006	11	7/24/2014	25:30.4
4926	RWS11	-122.4627781	37.93829	-0.34	3.7	-539.7	3.36100006	-122.46277	37.93831	-0.29	3.7	-548.52	3.41800007	11	7/24/2014	25:30.5
4927	RWS11	-122.4627801	37.93829	-0.34	3.7	-539.8	3.36199999	-122.46277	37.93831	-0.32	3.7	-548.64	3.38499999	11	7/24/2014	25:30.6
4928	RWS11	-122.4627821	37.93829	-0.34	3.7	-539.9	3.36299992	-122.46278	37.93831	-0.29	3.7	-548.67	3.41999993	11	7/24/2014	25:30.7
4929	RWS11	-122.4627841	37.9383	-0.34	3.71	-540	3.36400008	-122.46278	37.93831	-0.32	3.71	-548.79	3.38700008	11	7/24/2014	25:30.8
4930	RWS11	-122.4627861	37.9383	-0.34	3.71	-540.1	3.36599994	-122.46278	37.93831	-0.29	3.71	-548.79	3.42299995	11	7/24/2014	25:30.9
4931	RWS11	-122.4627891	37.9383	-0.34	3.71	-540.1	3.36800003	-122.46278	37.93831	-0.32	3.71	-548.79	3.39100003	11	7/24/2014	25:31.0
4932	RWS11	-122.4627911	37.9383	-0.31	3.71	-540.2	3.40500006	-122.46279	37.93831	-0.29	3.71	-548.74	3.42800006	11	7/24/2014	25:31.1
4933	RWS11	-122.4627932	37.9383	-0.34	3.72	-540.2	3.375	-122.46279	37.93831	-0.32	3.72	-548.72	3.398	11	7/24/2014	25:31.2
4934	RWS11	-122.4627952	37.9383	-0.31	3.72	-540.2	3.41399989	-122.46279	37.93831	-0.29	3.72	-548.72	3.43699989	11	7/24/2014	25:31.3
4935	RWS11	-122.4627972	37.9383	-0.34	3.73	-540.3	3.38400006	-122.46279	37.93831	-0.35	3.73	-548.72	3.37300009	11	7/24/2014	25:31.4
4936	RWS11	-122.4627992	37.9383	-0.34	3.73	-540.3	3.38700008	-122.46279	37.93831	-0.32	3.73	-548.73	3.41000009	11	7/24/2014	25:31.5
4937	RWS11	-122.4628013	37.9383	-0.34	3.73	-540.3	3.38899994	-122.4628	37.93831	-0.32	3.73	-548.6	3.41199994	11	7/24/2014	25:31.6
4938	RWS11	-122.4628033	37.9383	-0.34	3.73	-540.1	3.38800001	-122.4628	37.93831	-0.29	3.73	-548.42	3.44500002	11	7/24/2014	25:31.7
4939	RWS11	-122.4628053	37.9383	-0.34	3.73	-540	3.38400006	-122.4628	37.93831	-0.32	3.73	-548.32	3.40700006	11	7/24/2014	25:31.8
4940	RWS11	-122.4628073	37.9383	-0.34	3.72	-539.9	3.37700009	-122.4628	37.93831	-0.29	3.72	-548.14	3.4340001	11	7/24/2014	25:31.9
4941	RWS11	-122.4628103	37.9383	-0.34	3.71	-539.7	3.36500001	-122.4628	37.93831	-0.32	3.71	-547.99	3.38800001	11	7/24/2014	25:32.0
4942	RWS11	-122.4628122	37.9383	-0.34	3.69	-539.7	3.34800005	-122.46281	37.93832	-0.32	3.69	-548.04	3.37100005	11	7/24/2014	25:32.1
4943	RWS11	-122.4628142	37.9383	-0.34	3.67	-539.6	3.3269999	-122.46281	37.93832	-0.35	3.67	-547.99	3.31599993	11	7/24/2014	25:32.2
4944	RWS11	-122.4628162	37.9383	-0.34	3.65	-539.5	3.3039999	-122.46281	37.93832	-0.29	3.65	-547.84	3.36099991	11	7/24/2014	25:32.3
4945	RWS11	-122.4628182	37.9383	-0.34	3.62	-539.4	3.2809999	-122.46281	37.93832	-0.32	3.62	-547.76	3.3039999	11	7/24/2014	25:32.4
4946	RWS11	-122.4628202	37.9383	-0.34	3.6	-539.4	3.25900006	-122.46281	37.93832	-0.29	3.6	-547.71	3.31600007	11	7/24/2014	25:32.5
4947	RWS11	-122.4628222	37.93831	-0.34	3.58	-539.3	3.2420001	-122.46282	37.93832	-0.32	3.58	-547.77	3.2650001	11	7/24/2014	25:32.6
4948	RWS11	-122.4628242	37.93831	-0.34	3.57	-539.2	3.23099995	-122.46282	37.93832	-0.29	3.57	-547.81	3.28799996	11	7/24/2014	25:32.7
4949	RWS11	-122.4628261	37.93831	-0.34	3.57	-539.1	3.2249999	-122.46282	37.93832	-0.32	3.57	-547.83	3.24799991	11	7/24/2014	25:32.8

4950	RWS11	-122.4628281	37.93831	-0.34	3.57	-539	3.22600007	-122.46282	37.93832	-0.32	3.57	-547.86	3.24900007	11	7/24/2014	25:32.9
4951	RWS11	-122.4628311	37.93831	-0.34	3.58	-539	3.23300004	-122.46283	37.93832	-0.32	3.58	-547.89	3.25600004	11	7/24/2014	25:33.0
4952	RWS11	-122.462833	37.93831	-0.34	3.59	-538.8	3.24600005	-122.46283	37.93832	-0.29	3.59	-547.88	3.30300006	11	7/24/2014	25:33.1
4953	RWS11	-122.462835	37.93831	-0.34	3.61	-538.8	3.2650001	-122.46283	37.93832	-0.32	3.61	-547.94	3.28800011	11	7/24/2014	25:33.2
4954	RWS11	-122.4628371	37.93831	-0.34	3.63	-538.7	3.28800011	-122.46283	37.93832	-0.29	3.63	-548.08	3.34500012	11	7/24/2014	25:33.3
4955	RWS11	-122.4628391	37.93831	-0.39	3.66	-538.6	3.26299989	-122.46283	37.93832	-0.32	3.66	-548.23	3.33699989	11	7/24/2014	25:33.4
4956	RWS11	-122.4628411	37.93831	-0.34	3.68	-538.4	3.34200001	-122.46284	37.93832	-0.29	3.68	-548.13	3.39900002	11	7/24/2014	25:33.5
4957	RWS11	-122.4628432	37.93831	-0.34	3.71	-538.3	3.36899996	-122.46284	37.93832	-0.32	3.71	-548.07	3.39199996	11	7/24/2014	25:33.6
4958	RWS11	-122.4628452	37.93831	-0.34	3.73	-538.2	3.39299989	-122.46284	37.93832	-0.29	3.73	-547.94	3.4499999	11	7/24/2014	25:33.7
4959	RWS11	-122.4628472	37.93831	-0.34	3.76	-538.1	3.41400003	-122.46284	37.93833	-0.32	3.76	-547.9	3.43700004	11	7/24/2014	25:33.8
4960	RWS11	-122.4628492	37.93831	-0.34	3.77	-538	3.4289999	-122.46284	37.93833	-0.29	3.77	-547.85	3.48599991	11	7/24/2014	25:33.9
4961	RWS11	-122.4628522	37.93831	-0.39	3.78	-538	3.38799989	-122.46285	37.93833	-0.29	3.78	-547.82	3.4959999	11	7/24/2014	25:34.0
4962	RWS11	-122.4628542	37.93831	-0.34	3.78	-538.1	3.44199991	-122.46285	37.93833	-0.29	3.78	-547.81	3.49899992	11	7/24/2014	25:34.1
4963	RWS11	-122.4628563	37.93831	-0.34	3.78	-538.2	3.43799996	-122.46285	37.93833	-0.29	3.78	-547.76	3.49499997	11	7/24/2014	25:34.2
4964	RWS11	-122.4628583	37.93832	-0.31	3.77	-538.3	3.46100006	-122.46285	37.93833	-0.29	3.77	-547.7	3.48400006	11	7/24/2014	25:34.3
4965	RWS11	-122.4628603	37.93832	-0.34	3.75	-538.4	3.41100001	-122.46285	37.93833	-0.29	3.75	-547.64	3.46800002	11	7/24/2014	25:34.4
4966	RWS11	-122.4628624	37.93832	-0.34	3.73	-538.4	3.3900001	-122.46286	37.93833	-0.29	3.73	-547.44	3.44700012	11	7/24/2014	25:34.5
4967	RWS11	-122.4628644	37.93832	-0.34	3.71	-538.4	3.36599994	-122.46286	37.93833	-0.32	3.71	-547.21	3.38899994	11	7/24/2014	25:34.6
4968	RWS11	-122.4628665	37.93832	-0.31	3.68	-538.3	3.37500009	-122.46286	37.93833	-0.23	3.68	-546.92	3.44900009	11	7/24/2014	25:34.7
4969	RWS11	-122.4628685	37.93832	-0.34	3.66	-538.2	3.31599998	-122.46286	37.93833	-0.29	3.66	-546.81	3.373	11	7/24/2014	25:34.8
4970	RWS11	-122.4628705	37.93832	-0.31	3.64	-538.1	3.32900009	-122.46287	37.93833	-0.29	3.64	-546.73	3.35200009	11	7/24/2014	25:34.9
4971	RWS11	-122.4628735	37.93832	-0.34	3.62	-538	3.27799988	-122.46287	37.93833	-0.29	3.62	-546.61	3.33499989	11	7/24/2014	25:35.0
4972	RWS11	-122.4628755	37.93832	-0.31	3.61	-538	3.3019999	-122.46287	37.93833	-0.23	3.61	-546.59	3.3759999	11	7/24/2014	25:35.1
4973	RWS11	-122.4628775	37.93832	-0.34	3.61	-537.9	3.26300001	-122.46287	37.93833	-0.29	3.61	-546.55	3.32000002	11	7/24/2014	25:35.2
4974	RWS11	-122.4628796	37.93832	-0.31	3.61	-537.9	3.29799995	-122.46287	37.93833	-0.29	3.61	-546.61	3.32099995	11	7/24/2014	25:35.3
4975	RWS11	-122.4628816	37.93832	-0.34	3.61	-537.8	3.26799989	-122.46288	37.93833	-0.29	3.61	-546.59	3.3249999	11	7/24/2014	25:35.4
4976	RWS11	-122.4628836	37.93832	-0.34	3.62	-537.7	3.27399993	-122.46288	37.93833	-0.29	3.62	-546.53	3.33099994	11	7/24/2014	25:35.5
4977	RWS11	-122.4628857	37.93832	-0.34	3.62	-537.7	3.27900004	-122.46288	37.93834	-0.29	3.62	-546.42	3.33600006	11	7/24/2014	25:35.6
4978	RWS11	-122.4628877	37.93832	-0.31	3.63	-537.5	3.3190001	-122.46288	37.93834	-0.2	3.63	-546.11	3.42700009	11	7/24/2014	25:35.7
4979	RWS11	-122.4628897	37.93832	-0.34	3.63	-537.4	3.28900003	-122.46288	37.93834	-0.32	3.63	-545.74	3.31200004	11	7/24/2014	25:35.8
4980	RWS11	-122.4628917	37.93832	-0.31	3.63	-537.2	3.32699999	-122.46289	37.93834	-0.29	3.63	-545.37	3.34999999	11	7/24/2014	25:35.9
4981	RWS11	-122.4628947	37.93832	-0.34	3.64	-537	3.296	-122.46289	37.93834	-0.29	3.64	-545.04	3.35300002	11	7/24/2014	25:36.0
4982	RWS11	-122.4628967	37.93833	-0.31	3.64	-536.9	3.33300003	-122.46289	37.93834	-0.29	3.64	-544.73	3.35600004	11	7/24/2014	25:36.1

4983	RWS11	-122.4628987	37.93833	-0.34	3.64	-536.6	3.30299997	-122.46289	37.93834	-0.23	3.64	-544.35	3.41099998	11	7/24/2014	25:36.2
4984	RWS11	-122.4629008	37.93833	-0.31	3.65	-536.4	3.3420001	-122.4629	37.93834	-0.29	3.65	-543.96	3.3650001	11	7/24/2014	25:36.3
4985	RWS11	-122.4629028	37.93833	-0.34	3.66	-536.2	3.31399989	-122.4629	37.93834	-0.29	3.66	-543.67	3.3709999	11	7/24/2014	25:36.4
4986	RWS11	-122.4629048	37.93833	-0.31	3.66	-536	3.35699996	-122.4629	37.93834	-0.29	3.66	-543.35	3.37999997	11	7/24/2014	25:36.5
4987	RWS11	-122.4629068	37.93833	-0.31	3.68	-535.7	3.36800012	-122.4629	37.93834	-0.29	3.68	-542.99	3.39100012	11	7/24/2014	25:36.6
4988	RWS11	-122.4629089	37.93833	-0.31	3.69	-535.5	3.38099989	-122.4629	37.93834	-0.23	3.69	-542.52	3.45499989	11	7/24/2014	25:36.7
4989	RWS11	-122.4629109	37.93833	-0.31	3.7	-535.1	3.39500007	-122.46291	37.93834	-0.29	3.7	-542.09	3.41800007	11	7/24/2014	25:36.8
4990	RWS11	-122.4629128	37.93833	-0.31	3.72	-534.9	3.40999994	-122.46291	37.93834	-0.29	3.72	-541.73	3.43299994	11	7/24/2014	25:36.9
4991	RWS11	-122.4629158	37.93833	-0.31	3.73	-534.6	3.42400011	-122.46291	37.93834	-0.23	3.73	-541.38	3.49800012	11	7/24/2014	25:37.0
4992	RWS11	-122.4629178	37.93833	-0.31	3.74	-534.3	3.43599996	-122.46291	37.93834	-0.29	3.74	-541.09	3.45899996	11	7/24/2014	25:37.1
4993	RWS11	-122.4629198	37.93833	-0.34	3.75	-534.1	3.41100001	-122.46291	37.93834	-0.29	3.75	-540.82	3.46800002	11	7/24/2014	25:37.2
4994	RWS11	-122.4629218	37.93833	-0.31	3.76	-533.8	3.4499999	-122.46292	37.93834	-0.29	3.76	-540.6	3.4729999	11	7/24/2014	25:37.3
4995	RWS11	-122.4629238	37.93833	-0.34	3.76	-533.6	3.41599989	-122.46292	37.93835	-0.29	3.76	-540.38	3.4729999	11	7/24/2014	25:37.4
4996	RWS11	-122.4629258	37.93833	-0.31	3.75	-533.3	3.4440001	-122.46292	37.93835	-0.29	3.75	-540.27	3.4670001	11	7/24/2014	25:37.5
4997	RWS11	-122.4629278	37.93833	-0.31	3.74	-533	3.43100008	-122.46292	37.93835	-0.29	3.74	-540.11	3.45400009	11	7/24/2014	25:37.6
4998	RWS11	-122.4629298	37.93833	-0.31	3.72	-532.7	3.41299996	-122.46292	37.93835	-0.29	3.72	-540.04	3.43599996	11	7/24/2014	25:37.7
4999	RWS11	-122.4629318	37.93833	-0.31	3.7	-532.3	3.3880001	-122.46293	37.93835	-0.29	3.7	-539.81	3.4110001	11	7/24/2014	25:37.8
5000	RWS11	-122.4629338	37.93834	-0.31	3.67	-532	3.35900006	-122.46293	37.93835	-0.23	3.67	-539.41	3.43300006	11	7/24/2014	25:37.9
5001	RWS11	-122.4629367	37.93834	-0.31	3.63	-531.6	3.3249999	-122.46293	37.93835	-0.29	3.63	-538.96	3.3479999	11	7/24/2014	25:38.0
5002	RWS11	-122.4629387	37.93834	-0.27	3.6	-531.3	3.32299989	-122.46293	37.93835	-0.29	3.6	-538.6	3.31199989	11	7/24/2014	25:38.1
5003	RWS11	-122.4629406	37.93834	-0.31	3.56	-530.9	3.25199994	-122.46294	37.93835	-0.23	3.56	-538.2	3.32599995	11	7/24/2014	25:38.2
5004	RWS11	-122.4629426	37.93834	-0.27	3.52	-530.4	3.24999994	-122.46294	37.93835	-0.23	3.52	-537.67	3.28999993	11	7/24/2014	25:38.3
5005	RWS11	-122.4629446	37.93834	-0.31	3.49	-529.9	3.18200001	-122.46294	37.93835	-0.29	3.49	-537.12	3.20500001	11	7/24/2014	25:38.4
5006	RWS11	-122.4629466	37.93834	-0.27	3.46	-529.4	3.18600005	-122.46294	37.93835	-0.23	3.46	-536.57	3.22600004	11	7/24/2014	25:38.5
5007	RWS11	-122.4629486	37.93834	-0.31	3.43	-528.9	3.12699994	-122.46294	37.93835	-0.23	3.43	-535.97	3.20099995	11	7/24/2014	25:38.6
5008	RWS11	-122.4629506	37.93834	-0.27	3.42	-528.4	3.14399999	-122.46295	37.93835	-0.23	3.42	-535.3	3.18399999	11	7/24/2014	25:38.7
5009	RWS11	-122.4629526	37.93834	-0.31	3.41	-527.9	3.09999999	-122.46295	37.93835	-0.23	3.41	-534.64	3.17399999	11	7/24/2014	25:38.8
5010	RWS11	-122.4629546	37.93834	-0.27	3.41	-527.4	3.13199991	-122.46295	37.93835	-0.23	3.41	-533.83	3.1719999	11	7/24/2014	25:38.9
5011	RWS11	-122.4629576	37.93834	-0.31	3.41	-526.9	3.10300002	-122.46295	37.93835	-0.29	3.41	-533.11	3.12600002	11	7/24/2014	25:39.0
5012	RWS11	-122.4629596	37.93834	-0.31	3.42	-526.5	3.11600003	-122.46295	37.93835	-0.23	3.42	-532.62	3.19000003	11	7/24/2014	25:39.1
5013	RWS11	-122.4629616	37.93834	-0.31	3.44	-526.1	3.13600001	-122.46296	37.93836	-0.23	3.44	-532.2	3.21000001	11	7/24/2014	25:39.2
5014	RWS11	-122.4629636	37.93834	-0.27	3.47	-525.7	3.19600004	-122.46296	37.93836	-0.23	3.47	-531.9	3.23600003	11	7/24/2014	25:39.3
5015	RWS11	-122.4629656	37.93834	-0.31	3.5	-525.4	3.19100007	-122.46296	37.93836	-0.23	3.5	-531.51	3.26500008	11	7/24/2014	25:39.4

5016	RWS11	-122.4629677	37.93834	-0.31	3.53	-525.1	3.22199997	-122.46296	37.93836	-0.23	3.53	-531.14	3.29599997	11	7/24/2014	25:39.5
5017	RWS11	-122.4629697	37.93834	-0.31	3.56	-524.8	3.25199994	-122.46296	37.93836	-0.23	3.56	-530.85	3.32599995	11	7/24/2014	25:39.6
5018	RWS11	-122.4629718	37.93834	-0.27	3.59	-524.4	3.3129999	-122.46297	37.93836	-0.23	3.59	-530.42	3.3529999	11	7/24/2014	25:39.7
5019	RWS11	-122.4629738	37.93835	-0.31	3.61	-524.1	3.30300006	-122.46297	37.93836	-0.23	3.61	-530.06	3.37700006	11	7/24/2014	25:39.8
5020	RWS11	-122.4629759	37.93835	-0.27	3.63	-523.9	3.35600013	-122.46297	37.93836	-0.23	3.63	-529.69	3.39600012	11	7/24/2014	25:39.9
5021	RWS11	-122.4629789	37.93835	-0.27	3.64	-523.5	3.37000006	-122.46297	37.93836	-0.23	3.64	-529.27	3.41000006	11	7/24/2014	25:40.0
5022	RWS11	-122.462981	37.93835	-0.27	3.65	-523.2	3.38000005	-122.46298	37.93836	-0.23	3.65	-528.8	3.42000005	11	7/24/2014	25:40.1
5023	RWS11	-122.462983	37.93835	-0.27	3.66	-522.9	3.384	-122.46298	37.93836	-0.23	3.66	-528.34	3.42399999	11	7/24/2014	25:40.2
5024	RWS11	-122.4629851	37.93835	-0.27	3.66	-522.5	3.38300008	-122.46298	37.93836	-0.23	3.66	-527.93	3.42300007	11	7/24/2014	25:40.3
5025	RWS11	-122.4629872	37.93835	-0.31	3.65	-522.1	3.34399995	-122.46298	37.93836	-0.23	3.65	-527.46	3.41799995	11	7/24/2014	25:40.4
5026	RWS11	-122.4629893	37.93835	-0.27	3.64	-521.7	3.3689999	-122.46298	37.93836	-0.2	3.64	-527.04	3.44299988	11	7/24/2014	25:40.5
5027	RWS11	-122.4629914	37.93835	-0.27	3.63	-521.2	3.35899991	-122.46299	37.93836	-0.23	3.63	-526.41	3.3989999	11	7/24/2014	25:40.6
5028	RWS11	-122.4629935	37.93835	-0.27	3.62	-520.7	3.34700006	-122.46299	37.93836	-0.23	3.62	-525.83	3.38700005	11	7/24/2014	25:40.7
5029	RWS11	-122.4629955	37.93835	-0.27	3.61	-520.2	3.33400005	-122.46299	37.93836	-0.23	3.61	-525.24	3.37400004	11	7/24/2014	25:40.8
5030	RWS11	-122.4629976	37.93835	-0.27	3.6	-519.6	3.32100004	-122.46299	37.93836	-0.23	3.6	-524.62	3.36100003	11	7/24/2014	25:40.9
5031	RWS11	-122.4630007	37.93835	-0.31	3.58	-519.1	3.27400002	-122.463	37.93836	-0.23	3.58	-524.04	3.34800002	11	7/24/2014	25:41.0
5032	RWS11	-122.4630027	37.93835	-0.27	3.57	-518.6	3.29500002	-122.463	37.93837	-0.23	3.57	-523.37	3.33500001	11	7/24/2014	25:41.1
5033	RWS11	-122.4630048	37.93835	-0.27	3.56	-518	3.28299993	-122.463	37.93837	-0.23	3.56	-522.72	3.32299992	11	7/24/2014	25:41.2
5034	RWS11	-122.4630069	37.93835	-0.22	3.54	-517.4	3.32199991	-122.463	37.93837	-0.23	3.54	-522.1	3.30999991	11	7/24/2014	25:41.3
5035	RWS11	-122.463009	37.93835	-0.27	3.53	-516.8	3.259	-122.463	37.93837	-0.2	3.53	-521.34	3.33299999	11	7/24/2014	25:41.4
5036	RWS11	-122.4630111	37.93835	-0.27	3.52	-516.2	3.24999994	-122.46301	37.93837	-0.23	3.52	-520.65	3.28999993	11	7/24/2014	25:41.5
5037	RWS11	-122.4630132	37.93836	-0.27	3.52	-515.6	3.24200004	-122.46301	37.93837	-0.23	3.52	-520.02	3.28200004	11	7/24/2014	25:41.6
5038	RWS11	-122.4630152	37.93836	-0.27	3.51	-515.1	3.23699993	-122.46301	37.93837	-0.2	3.51	-519.37	3.31099992	11	7/24/2014	25:41.7
5039	RWS11	-122.4630173	37.93836	-0.27	3.51	-514.5	3.23299998	-122.46301	37.93837	-0.23	3.51	-518.75	3.27299997	11	7/24/2014	25:41.8
5040	RWS11	-122.4630193	37.93836	-0.27	3.51	-514	3.23100013	-122.46301	37.93837	-0.2	3.51	-518.07	3.30500011	11	7/24/2014	25:41.9
5041	RWS11	-122.4630223	37.93836	-0.27	3.5	-513.4	3.22999996	-122.46302	37.93837	-0.23	3.5	-517.39	3.26999995	11	7/24/2014	25:42.0
5042	RWS11	-122.4630243	37.93836	-0.27	3.5	-512.8	3.22900003	-122.46302	37.93837	-0.2	3.5	-516.69	3.30300002	11	7/24/2014	25:42.1
5043	RWS11	-122.4630263	37.93836	-0.27	3.5	-512.3	3.22900003	-122.46302	37.93837	-0.23	3.5	-516.1	3.26900002	11	7/24/2014	25:42.2
5044	RWS11	-122.4630284	37.93836	-0.27	3.5	-511.7	3.22900003	-122.46302	37.93837	-0.2	3.5	-515.38	3.30300002	11	7/24/2014	25:42.3
5045	RWS11	-122.4630304	37.93836	-0.27	3.5	-511.1	3.2280001	-122.46303	37.93837	-0.2	3.5	-514.61	3.30200009	11	7/24/2014	25:42.4
5046	RWS11	-122.4630324	37.93836	-0.22	3.5	-510.4	3.28100002	-122.46303	37.93837	-0.23	3.5	-513.83	3.26900002	11	7/24/2014	25:42.5
5047	RWS11	-122.4630344	37.93836	-0.27	3.5	-509.9	3.22900003	-122.46303	37.93837	-0.23	3.5	-513.12	3.26900002	11	7/24/2014	25:42.6
5048	RWS11	-122.4630364	37.93836	-0.27	3.5	-509.3	3.22900003	-122.46303	37.93837	-0.2	3.5	-512.57	3.30300002	11	7/24/2014	25:42.7

5049	RWS11	-122.4630384	37.93836	-0.27	3.5	-508.6	3.22900003	-122.46303	37.93837	-0.23	3.5	-511.95	3.26900002	11	7/24/2014	25:42.8
5050	RWS11	-122.4630404	37.93836	-0.27	3.5	-508	3.22999996	-122.46304	37.93837	-0.2	3.5	-511.36	3.30399995	11	7/24/2014	25:42.9
5051	RWS11	-122.4630434	37.93836	-0.27	3.51	-507.4	3.23100013	-122.46304	37.93838	-0.23	3.51	-510.65	3.27100012	11	7/24/2014	25:43.0
5052	RWS11	-122.4630454	37.93836	-0.27	3.51	-506.8	3.23200005	-122.46304	37.93838	-0.23	3.51	-510.02	3.27200004	11	7/24/2014	25:43.1
5053	RWS11	-122.4630474	37.93836	-0.27	3.51	-506.3	3.23399991	-122.46304	37.93838	-0.2	3.51	-509.3	3.30799989	11	7/24/2014	25:43.2
5054	RWS11	-122.4630494	37.93836	-0.27	3.51	-505.7	3.23699993	-122.46304	37.93838	-0.23	3.51	-508.61	3.27699992	11	7/24/2014	25:43.3
5055	RWS11	-122.4630515	37.93836	-0.27	3.52	-505.1	3.24200004	-122.46305	37.93838	-0.23	3.52	-508.01	3.28200004	11	7/24/2014	25:43.4
5056	RWS11	-122.4630536	37.93837	-0.22	3.52	-504.6	3.301	-122.46305	37.93838	-0.2	3.52	-507.49	3.323	11	7/24/2014	25:43.5
5057	RWS11	-122.4630556	37.93837	-0.27	3.53	-504.1	3.25699991	-122.46305	37.93838	-0.23	3.53	-507.06	3.2969999	11	7/24/2014	25:43.6
5058	RWS11	-122.4630577	37.93837	-0.22	3.54	-503.6	3.31899989	-122.46305	37.93838	-0.2	3.54	-506.52	3.34099989	11	7/24/2014	25:43.7
5059	RWS11	-122.4630597	37.93837	-0.27	3.55	-503.1	3.27800006	-122.46305	37.93838	-0.23	3.55	-505.97	3.31800005	11	7/24/2014	25:43.8
5060	RWS11	-122.4630617	37.93837	-0.22	3.56	-502.5	3.34099996	-122.46306	37.93838	-0.2	3.56	-505.29	3.36299996	11	7/24/2014	25:43.9
5061	RWS11	-122.4630648	37.93837	-0.27	3.57	-501.9	3.29800004	-122.46306	37.93838	-0.23	3.57	-504.61	3.33800003	11	7/24/2014	25:44.0
5062	RWS11	-122.4630668	37.93837	-0.27	3.58	-501.4	3.30500001	-122.46306	37.93838	-0.2	3.58	-504.02	3.37899999	11	7/24/2014	25:44.1
5063	RWS11	-122.4630688	37.93837	-0.27	3.58	-500.7	3.31000012	-122.46306	37.93838	-0.2	3.58	-503.4	3.38400011	11	7/24/2014	25:44.2
5064	RWS11	-122.4630709	37.93837	-0.22	3.59	-500.2	3.36399996	-122.46307	37.93838	-0.2	3.59	-502.79	3.38599996	11	7/24/2014	25:44.3
5065	RWS11	-122.463073	37.93837	-0.27	3.59	-499.6	3.31199998	-122.46307	37.93838	-0.23	3.59	-502.24	3.35199997	11	7/24/2014	25:44.4
5066	RWS11	-122.4630751	37.93837	-0.27	3.59	-499	3.3129999	-122.46307	37.93838	-0.23	3.59	-501.69	3.3529999	11	7/24/2014	25:44.5
5067	RWS11	-122.4630771	37.93837	-0.27	3.59	-498.4	3.31400007	-122.46307	37.93838	-0.23	3.59	-501.15	3.35400006	11	7/24/2014	25:44.6
5068	RWS11	-122.4630792	37.93837	-0.27	3.59	-497.9	3.31599993	-122.46307	37.93838	-0.2	3.59	-500.68	3.38999991	11	7/24/2014	25:44.7
5069	RWS11	-122.4630813	37.93837	-0.27	3.59	-497.4	3.31800002	-122.46308	37.93839	-0.2	3.59	-500.2	3.392	11	7/24/2014	25:44.8
5070	RWS11	-122.4630833	37.93837	-0.22	3.59	-496.8	3.3720001	-122.46308	37.93839	-0.2	3.59	-499.52	3.3940001	11	7/24/2014	25:44.9
5071	RWS11	-122.4630863	37.93837	-0.27	3.59	-496.2	3.32000011	-122.46308	37.93839	-0.23	3.59	-498.95	3.3600001	11	7/24/2014	25:45.0
5072	RWS11	-122.4630883	37.93837	-0.27	3.59	-495.7	3.31800002	-122.46308	37.93839	-0.2	3.59	-498.31	3.392	11	7/24/2014	25:45.1
5073	RWS11	-122.4630904	37.93837	-0.27	3.59	-495.2	3.31199998	-122.46309	37.93839	-0.23	3.59	-497.67	3.35199997	11	7/24/2014	25:45.2
5074	RWS11	-122.4630924	37.93838	-0.22	3.58	-494.6	3.3549999	-122.46309	37.93839	-0.2	3.58	-496.95	3.3769999	11	7/24/2014	25:45.3
5075	RWS11	-122.4630945	37.93838	-0.27	3.57	-494.1	3.29100007	-122.46309	37.93839	-0.23	3.57	-496.33	3.33100006	11	7/24/2014	25:45.4
5076	RWS11	-122.4630965	37.93838	-0.27	3.55	-493.5	3.27700013	-122.46309	37.93839	-0.2	3.55	-495.77	3.35100012	11	7/24/2014	25:45.5
5077	RWS11	-122.4630986	37.93838	-0.27	3.54	-493.1	3.2610001	-122.46309	37.93839	-0.23	3.54	-495.35	3.30100009	11	7/24/2014	25:45.6
5078	RWS11	-122.4631006	37.93838	-0.27	3.52	-492.7	3.24500006	-122.4631	37.93839	-0.2	3.52	-494.96	3.31900005	11	7/24/2014	25:45.7
5079	RWS11	-122.4631026	37.93838	-0.27	3.5	-492.2	3.22900003	-122.4631	37.93839	-0.2	3.5	-494.53	3.30300002	11	7/24/2014	25:45.8
5080	RWS11	-122.4631047	37.93838	-0.22	3.49	-491.8	3.26700008	-122.4631	37.93839	-0.15	3.49	-494.16	3.33900008	11	7/24/2014	25:45.9
5081	RWS11	-122.4631077	37.93838	-0.27	3.48	-491.4	3.20300001	-122.4631	37.93839	-0.23	3.48	-493.68	3.243	11	7/24/2014	25:46.0

5082	RWS11	-122.4631097	37.93838	-0.22	3.47	-490.9	3.24599993	-122.4631	37.93839	-0.2	3.47	-493.15	3.26799993	11	7/24/2014	25:46.1
5083	RWS11	-122.4631117	37.93838	-0.27	3.46	-490.5	3.18900007	-122.46311	37.93839	-0.2	3.46	-492.65	3.26300006	11	7/24/2014	25:46.2
5084	RWS11	-122.4631138	37.93838	-0.22	3.46	-489.9	3.23800004	-122.46311	37.93839	-0.2	3.46	-492.18	3.26000004	11	7/24/2014	25:46.3
5085	RWS11	-122.4631159	37.93838	-0.27	3.46	-489.2	3.18600005	-122.46311	37.93839	-0.23	3.46	-491.61	3.22600004	11	7/24/2014	25:46.4
5086	RWS11	-122.463118	37.93838	-0.22	3.46	-488.7	3.23899996	-122.46311	37.93839	-0.15	3.46	-491.07	3.31099996	11	7/24/2014	25:46.5
5087	RWS11	-122.46312	37.93838	-0.27	3.46	-488	3.19	-122.46311	37.9384	-0.2	3.46	-490.38	3.26399998	11	7/24/2014	25:46.6
5088	RWS11	-122.4631221	37.93838	-0.22	3.47	-487.3	3.2470001	-122.46312	37.9384	-0.2	3.47	-489.71	3.2690001	11	7/24/2014	25:46.7
5089	RWS11	-122.4631241	37.93838	-0.27	3.47	-486.7	3.19999999	-122.46312	37.9384	-0.2	3.47	-489.01	3.27399997	11	7/24/2014	25:46.8
5090	RWS11	-122.4631262	37.93838	-0.22	3.48	-486	3.25599992	-122.46312	37.9384	-0.2	3.48	-488.27	3.27799992	11	7/24/2014	25:46.9
5091	RWS11	-122.4631292	37.93838	-0.27	3.48	-485.3	3.20699996	-122.46312	37.9384	-0.2	3.48	-487.58	3.28099994	11	7/24/2014	25:47.0
5092	RWS11	-122.4631312	37.93839	-0.22	3.48	-484.6	3.26100004	-122.46313	37.9384	-0.15	3.48	-486.77	3.33300003	11	7/24/2014	25:47.1
5093	RWS11	-122.4631333	37.93839	-0.27	3.48	-483.9	3.20900005	-122.46313	37.9384	-0.2	3.48	-486.05	3.28300004	11	7/24/2014	25:47.2
5094	RWS11	-122.4631353	37.93839	-0.22	3.48	-483.4	3.25800002	-122.46313	37.9384	-0.2	3.48	-485.46	3.28000002	11	7/24/2014	25:47.3
5095	RWS11	-122.4631374	37.93839	-0.27	3.48	-482.8	3.20200008	-122.46313	37.9384	-0.2	3.48	-484.86	3.27600007	11	7/24/2014	25:47.4
5096	RWS11	-122.4631395	37.93839	-0.22	3.47	-482.4	3.24899995	-122.46313	37.9384	-0.2	3.47	-484.27	3.27099995	11	7/24/2014	25:47.5
5097	RWS11	-122.4631415	37.93839	-0.27	3.47	-481.9	3.19200009	-122.46314	37.9384	-0.2	3.47	-483.8	3.26600008	11	7/24/2014	25:47.6
5098	RWS11	-122.4631436	37.93839	-0.22	3.46	-481.4	3.23999989	-122.46314	37.9384	-0.23	3.46	-483.2	3.2279999	11	7/24/2014	25:47.7
5099	RWS11	-122.4631456	37.93839	-0.27	3.46	-481	3.18399996	-122.46314	37.9384	-0.2	3.46	-482.69	3.25799994	11	7/24/2014	25:47.8
5100	RWS11	-122.4631476	37.93839	-0.27	3.46	-480.6	3.1820001	-122.46314	37.9384	-0.2	3.46	-482.18	3.25600009	11	7/24/2014	25:47.9
5101	RWS11	-122.4631507	37.93839	-0.27	3.45	-480.1	3.18199992	-122.46315	37.9384	-0.23	3.45	-481.65	3.22199993	11	7/24/2014	25:48.0
5102	RWS11	-122.4631527	37.93839	-0.22	3.46	-479.7	3.23500009	-122.46315	37.9384	-0.23	3.46	-481.1	3.22300009	11	7/24/2014	25:48.1
5103	RWS11	-122.4631547	37.93839	-0.27	3.46	-479.2	3.18400002	-122.46315	37.9384	-0.23	3.46	-480.45	3.22400002	11	7/24/2014	25:48.2
5104	RWS11	-122.4631568	37.93839	-0.22	3.46	-478.8	3.23800011	-122.46315	37.9384	-0.2	3.46	-480	3.26000011	11	7/24/2014	25:48.3
5105	RWS11	-122.4631588	37.93839	-0.27	3.46	-478.4	3.18700004	-122.46315	37.93841	-0.2	3.46	-479.53	3.26100004	11	7/24/2014	25:48.4
5106	RWS11	-122.4631608	37.93839	-0.27	3.46	-478.1	3.19000006	-122.46316	37.93841	-0.2	3.46	-479.2	3.26400006	11	7/24/2014	25:48.5
5107	RWS11	-122.4631629	37.93839	-0.27	3.46	-477.7	3.19099998	-122.46316	37.93841	-0.23	3.46	-478.99	3.23099999	11	7/24/2014	25:48.6
5108	RWS11	-122.4631649	37.93839	-0.22	3.46	-477.4	3.24399991	-122.46316	37.93841	-0.23	3.46	-478.72	3.23199992	11	7/24/2014	25:48.7
5109	RWS11	-122.4631669	37.93839	-0.27	3.46	-477	3.19099998	-122.46316	37.93841	-0.23	3.46	-478.33	3.23099999	11	7/24/2014	25:48.8
5110	RWS11	-122.4631689	37.9384	-0.27	3.46	-476.6	3.18899989	-122.46316	37.93841	-0.2	3.46	-477.82	3.26299989	11	7/24/2014	25:48.9
5111	RWS11	-122.4631719	37.9384	-0.27	3.46	-476.3	3.18499994	-122.46317	37.93841	-0.23	3.46	-477.4	3.22499995	11	7/24/2014	25:49.0
5112	RWS11	-122.4631739	37.9384	-0.27	3.45	-475.8	3.1789999	-122.46317	37.93841	-0.2	3.45	-476.96	3.2529999	11	7/24/2014	25:49.1
5113	RWS11	-122.4631759	37.9384	-0.27	3.44	-475.4	3.171	-122.46317	37.93841	-0.2	3.44	-476.56	3.245	11	7/24/2014	25:49.2
5114	RWS11	-122.463178	37.9384	-0.27	3.43	-475	3.16199994	-122.46317	37.93841	-0.2	3.43	-476.16	3.23599994	11	7/24/2014	25:49.3

5115	RWS11	-122.46318	37.9384	-0.27	3.42	-474.5	3.15199995	-122.46317	37.93841	-0.2	3.42	-475.72	3.22599995	11	7/24/2014	25:49.4
5116	RWS11	-122.463182	37.9384	-0.27	3.41	-474	3.1400001	-122.46318	37.93841	-0.2	3.41	-475.34	3.21400011	11	7/24/2014	25:49.5
5117	RWS11	-122.4631841	37.9384	-0.27	3.4	-473.5	3.12800002	-122.46318	37.93841	-0.2	3.4	-474.9	3.20200002	11	7/24/2014	25:49.6
5118	RWS11	-122.4631861	37.9384	-0.22	3.39	-473	3.16700001	-122.46318	37.93841	-0.2	3.39	-474.56	3.18900001	11	7/24/2014	25:49.7
5119	RWS11	-122.4631881	37.9384	-0.31	3.38	-472.7	3.06899992	-122.46318	37.93841	-0.23	3.38	-474.47	3.14299993	11	7/24/2014	25:49.8
5120	RWS11	-122.4631901	37.9384	-0.27	3.36	-472.4	3.08999991	-122.46318	37.93841	-0.2	3.36	-474.31	3.16399992	11	7/24/2014	25:49.9
5121	RWS11	-122.4631931	37.9384	-0.27	3.35	-472.2	3.07800007	-122.46319	37.93841	-0.23	3.35	-474.19	3.11800008	11	7/24/2014	25:50.0
5122	RWS11	-122.4631952	37.9384	-0.27	3.34	-471.9	3.06699991	-122.46319	37.93841	-0.2	3.34	-473.92	3.14099991	11	7/24/2014	25:50.1
5123	RWS11	-122.4631972	37.9384	-0.27	3.33	-471.6	3.05699992	-122.46319	37.93842	-0.23	3.33	-473.68	3.09699993	11	7/24/2014	25:50.2
5124	RWS11	-122.4631993	37.9384	-0.27	3.32	-471.4	3.0480001	-122.46319	37.93842	-0.2	3.32	-473.34	3.1220001	11	7/24/2014	25:50.3
5125	RWS11	-122.4632013	37.9384	-0.27	3.31	-471.1	3.04099989	-122.4632	37.93842	-0.23	3.31	-472.95	3.0809999	11	7/24/2014	25:50.4
5126	RWS11	-122.4632034	37.9384	-0.27	3.31	-470.7	3.03600001	-122.4632	37.93842	-0.23	3.31	-472.47	3.07600002	11	7/24/2014	25:50.5
5127	RWS11	-122.4632055	37.9384	-0.27	3.31	-470.4	3.03200006	-122.4632	37.93842	-0.23	3.31	-471.96	3.07200007	11	7/24/2014	25:50.6
5128	RWS11	-122.4632076	37.93841	-0.27	3.3	-470.1	3.02999997	-122.4632	37.93842	-0.23	3.3	-471.6	3.06999998	11	7/24/2014	25:50.7
5129	RWS11	-122.4632096	37.93841	-0.31	3.3	-469.8	2.99500003	-122.4632	37.93842	-0.28	3.3	-471.24	3.01800004	11	7/24/2014	25:50.8
5130	RWS11	-122.4632117	37.93841	-0.27	3.3	-469.4	3.02800012	-122.46321	37.93842	-0.23	3.3	-470.85	3.06800012	11	7/24/2014	25:50.9
5131	RWS11	-122.4632147	37.93841	-0.27	3.3	-469.2	3.02900004	-122.46321	37.93842	-0.23	3.3	-470.49	3.06900005	11	7/24/2014	25:51.0
5132	RWS11	-122.4632168	37.93841	-0.27	3.3	-468.8	3.02900004	-122.46321	37.93842	-0.23	3.3	-470.09	3.06900005	11	7/24/2014	25:51.1
5133	RWS11	-122.4632188	37.93841	-0.31	3.3	-468.4	2.99699989	-122.46321	37.93842	-0.28	3.3	-469.66	3.01999989	11	7/24/2014	25:51.2
5134	RWS11	-122.4632209	37.93841	-0.27	3.31	-468	3.03399992	-122.46322	37.93842	-0.28	3.31	-469.12	3.02299991	11	7/24/2014	25:51.3
5135	RWS11	-122.463223	37.93841	-0.27	3.31	-467.4	3.03600001	-122.46322	37.93842	-0.28	3.31	-468.51	3.02500001	11	7/24/2014	25:51.4
5136	RWS11	-122.4632251	37.93841	-0.27	3.31	-467.1	3.03900003	-122.46322	37.93842	-0.23	3.31	-467.99	3.07900004	11	7/24/2014	25:51.5
5137	RWS11	-122.4632271	37.93841	-0.31	3.32	-466.7	3.00899997	-122.46322	37.93842	-0.28	3.32	-467.69	3.03199998	11	7/24/2014	25:51.6
5138	RWS11	-122.4632292	37.93841	-0.27	3.32	-466.4	3.046	-122.46322	37.93842	-0.28	3.32	-467.51	3.035	11	7/24/2014	25:51.7
5139	RWS11	-122.4632313	37.93841	-0.31	3.32	-466.1	3.01500002	-122.46323	37.93842	-0.28	3.32	-467.19	3.03800002	11	7/24/2014	25:51.8
5140	RWS11	-122.4632333	37.93841	-0.27	3.33	-465.9	3.05200005	-122.46323	37.93842	-0.28	3.33	-467.12	3.04100004	11	7/24/2014	25:51.9
5141	RWS11	-122.4632364	37.93841	-0.31	3.33	-465.6	3.01899996	-122.46323	37.93843	-0.28	3.33	-466.98	3.04199997	11	7/24/2014	25:52.0
5142	RWS11	-122.4632384	37.93841	-0.27	3.33	-465.4	3.05200005	-122.46323	37.93843	-0.28	3.33	-466.78	3.04100004	11	7/24/2014	25:52.1
5143	RWS11	-122.4632405	37.93841	-0.31	3.32	-465.1	3.01500002	-122.46324	37.93843	-0.32	3.32	-466.58	3.00400004	11	7/24/2014	25:52.2
5144	RWS11	-122.4632425	37.93841	-0.31	3.32	-464.8	3.01100007	-122.46324	37.93843	-0.28	3.32	-466.37	3.03400007	11	7/24/2014	25:52.3
5145	RWS11	-122.4632446	37.93841	-0.31	3.31	-464.6	3.00599995	-122.46324	37.93843	-0.28	3.31	-466.17	3.02899995	11	7/24/2014	25:52.4
5146	RWS11	-122.4632467	37.93842	-0.27	3.31	-464.3	3.03399992	-122.46324	37.93843	-0.28	3.31	-466.08	3.02299991	11	7/24/2014	25:52.5
5147	RWS11	-122.4632487	37.93842	-0.31	3.3	-464.2	2.99599996	-122.46324	37.93843	-0.28	3.3	-466.02	3.01899996	11	7/24/2014	25:52.6

5148	RWS11	-122.4632508	37.93842	-0.27	3.3	-464	3.02699995	-122.46325	37.93843	-0.28	3.3	-465.98	3.01599994	11	7/24/2014	25:52.7
5149	RWS11	-122.4632528	37.93842	-0.31	3.3	-463.8	2.99400011	-122.46325	37.93843	-0.28	3.3	-465.98	3.01700011	11	7/24/2014	25:52.8
5150	RWS11	-122.4632549	37.93842	-0.31	3.31	-463.8	2.99800006	-122.46325	37.93843	-0.28	3.31	-466.03	3.02100006	11	7/24/2014	25:52.9
5151	RWS11	-122.4632579	37.93842	-0.31	3.31	-463.8	3.00699988	-122.46325	37.93843	-0.28	3.31	-466.26	3.02999988	11	7/24/2014	25:53.0
5152	RWS11	-122.46326	37.93842	-0.27	3.33	-463.8	3.0539999	-122.46325	37.93843	-0.28	3.33	-466.31	3.04299989	11	7/24/2014	25:53.1
5153	RWS11	-122.463262	37.93842	-0.31	3.35	-463.8	3.03800002	-122.46326	37.93843	-0.28	3.35	-466.37	3.06100002	11	7/24/2014	25:53.2
5154	RWS11	-122.4632642	37.93842	-0.27	3.37	-463.7	3.0940001	-122.46326	37.93843	-0.23	3.37	-466.36	3.13400011	11	7/24/2014	25:53.3
5155	RWS11	-122.4632662	37.93842	-0.31	3.39	-463.6	3.08700004	-122.46326	37.93843	-0.28	3.39	-466.35	3.11000004	11	7/24/2014	25:53.4
5156	RWS11	-122.4632684	37.93842	-0.27	3.42	-463.5	3.15100002	-122.46326	37.93843	-0.28	3.42	-466.33	3.14000002	11	7/24/2014	25:53.5
5157	RWS11	-122.4632705	37.93842	-0.31	3.46	-463.5	3.15000001	-122.46327	37.93843	-0.28	3.46	-466.31	3.17300001	11	7/24/2014	25:53.6
5158	RWS11	-122.4632726	37.93842	-0.27	3.49	-463.5	3.21700001	-122.46327	37.93844	-0.23	3.49	-466.33	3.25700001	11	7/24/2014	25:53.7
5159	RWS11	-122.4632747	37.93842	-0.31	3.52	-463.4	3.21500006	-122.46327	37.93844	-0.28	3.52	-466.28	3.23800007	11	7/24/2014	25:53.8
5160	RWS11	-122.4632768	37.93842	-0.27	3.55	-463.4	3.27800012	-122.46327	37.93844	-0.28	3.55	-466.16	3.26700011	11	7/24/2014	25:53.9
5161	RWS11	-122.4632799	37.93842	-0.31	3.58	-463.2	3.26800004	-122.46327	37.93844	-0.28	3.58	-466.03	3.29100004	11	7/24/2014	25:54.0
5162	RWS11	-122.463282	37.93842	-0.31	3.59	-463.1	3.285	-122.46328	37.93844	-0.28	3.59	-465.92	3.308	11	7/24/2014	25:54.1
5163	RWS11	-122.4632841	37.93843	-0.31	3.6	-462.8	3.29599991	-122.46328	37.93844	-0.28	3.6	-465.71	3.31899992	11	7/24/2014	25:54.2
5164	RWS11	-122.4632862	37.93843	-0.31	3.61	-462.7	3.3000001	-122.46328	37.93844	-0.23	3.61	-465.6	3.37400012	11	7/24/2014	25:54.3
5165	RWS11	-122.4632884	37.93843	-0.31	3.61	-462.6	3.29800001	-122.46328	37.93844	-0.28	3.61	-465.51	3.32100001	11	7/24/2014	25:54.4
5166	RWS11	-122.4632905	37.93843	-0.27	3.6	-462.5	3.32599998	-122.46329	37.93844	-0.28	3.6	-465.4	3.31499997	11	7/24/2014	25:54.5
5167	RWS11	-122.4632927	37.93843	-0.31	3.59	-462.5	3.2829999	-122.46329	37.93844	-0.32	3.59	-465.38	3.27199993	11	7/24/2014	25:54.6
5168	RWS11	-122.4632948	37.93843	-0.31	3.58	-462.4	3.27400008	-122.46329	37.93844	-0.32	3.58	-465.27	3.2630001	11	7/24/2014	25:54.7
5169	RWS11	-122.463297	37.93843	-0.31	3.57	-462.4	3.26599994	-122.46329	37.93844	-0.32	3.57	-465.34	3.25499997	11	7/24/2014	25:54.8
5170	RWS11	-122.4632991	37.93843	-0.31	3.57	-462.5	3.2599999	-122.46329	37.93844	-0.28	3.57	-465.49	3.2829999	11	7/24/2014	25:54.9
5171	RWS11	-122.4633022	37.93843	-0.31	3.56	-462.3	3.25500003	-122.4633	37.93844	-0.32	3.56	-465.45	3.24400005	11	7/24/2014	25:55.0
5172	RWS11	-122.4633044	37.93843	-0.31	3.56	-462.2	3.25299993	-122.4633	37.93844	-0.32	3.56	-465.42	3.24199995	11	7/24/2014	25:55.1
5173	RWS11	-122.4633065	37.93843	-0.31	3.56	-462.1	3.252	-122.4633	37.93844	-0.32	3.56	-465.41	3.24100003	11	7/24/2014	25:55.2
5174	RWS11	-122.4633086	37.93843	-0.27	3.56	-462	3.28500009	-122.4633	37.93844	-0.28	3.56	-465.36	3.27400008	11	7/24/2014	25:55.3
5175	RWS11	-122.4633108	37.93843	-0.31	3.56	-462	3.25100008	-122.46331	37.93845	-0.32	3.56	-465.45	3.2400001	11	7/24/2014	25:55.4
5176	RWS11	-122.4633129	37.93843	-0.31	3.56	-462	3.24999991	-122.46331	37.93845	-0.32	3.56	-465.54	3.23899993	11	7/24/2014	25:55.5
5177	RWS11	-122.4633151	37.93843	-0.34	3.56	-462.2	3.215	-122.46331	37.93845	-0.32	3.56	-465.69	3.23800001	11	7/24/2014	25:55.6
5178	RWS11	-122.4633172	37.93843	-0.31	3.56	-462.2	3.24899998	-122.46331	37.93845	-0.35	3.56	-465.73	3.204	11	7/24/2014	25:55.7
5179	RWS11	-122.4633194	37.93843	-0.34	3.56	-462.2	3.21599993	-122.46331	37.93845	-0.35	3.56	-465.75	3.20499992	11	7/24/2014	25:55.8
5180	RWS11	-122.4633215	37.93843	-0.31	3.56	-462.2	3.252	-122.46332	37.93845	-0.32	3.56	-465.73	3.24100003	11	7/24/2014	25:55.9

5181	RWS11	-122.4633246	37.93844	-0.31	3.56	-462	3.25500003	-122.46332	37.93845	-0.32	3.56	-465.71	3.24400005	11	7/24/2014	25:56.0
5182	RWS11	-122.4633268	37.93844	-0.31	3.57	-462	3.25899997	-122.46332	37.93845	-0.32	3.57	-465.64	3.248	11	7/24/2014	25:56.1
5183	RWS11	-122.4633289	37.93844	-0.31	3.57	-462	3.26299992	-122.46332	37.93845	-0.35	3.57	-465.69	3.21799994	11	7/24/2014	25:56.2
5184	RWS11	-122.463331	37.93844	-0.31	3.57	-462	3.26500002	-122.46333	37.93845	-0.32	3.57	-465.71	3.25400004	11	7/24/2014	25:56.3
5185	RWS11	-122.4633331	37.93844	-0.31	3.57	-462.1	3.26400009	-122.46333	37.93845	-0.35	3.57	-465.82	3.2190001	11	7/24/2014	25:56.4
5186	RWS11	-122.4633353	37.93844	-0.31	3.57	-462.1	3.25899997	-122.46333	37.93845	-0.32	3.57	-465.88	3.248	11	7/24/2014	25:56.5
5187	RWS11	-122.4633374	37.93844	-0.34	3.56	-462	3.21599993	-122.46333	37.93845	-0.35	3.56	-465.84	3.20499992	11	7/24/2014	25:56.6
5188	RWS11	-122.4633395	37.93844	-0.31	3.54	-461.9	3.23599997	-122.46333	37.93845	-0.32	3.54	-465.87	3.22499999	11	7/24/2014	25:56.7
5189	RWS11	-122.4633417	37.93844	-0.31	3.53	-461.9	3.21800008	-122.46334	37.93845	-0.35	3.53	-465.97	3.1730001	11	7/24/2014	25:56.8
5190	RWS11	-122.4633438	37.93844	-0.31	3.5	-461.7	3.19699994	-122.46334	37.93845	-0.32	3.5	-466	3.18599996	11	7/24/2014	25:56.9
5191	RWS11	-122.4633469	37.93844	-0.31	3.48	-461.6	3.17699996	-122.46334	37.93845	-0.32	3.48	-466.06	3.16599998	11	7/24/2014	25:57.0
5192	RWS11	-122.463349	37.93844	-0.31	3.47	-461.7	3.15900007	-122.46334	37.93845	-0.32	3.47	-466.19	3.14800009	11	7/24/2014	25:57.1
5193	RWS11	-122.4633511	37.93844	-0.31	3.45	-461.7	3.14399996	-122.46335	37.93846	-0.32	3.45	-466.47	3.13299999	11	7/24/2014	25:57.2
5194	RWS11	-122.4633532	37.93844	-0.31	3.44	-461.9	3.1349999	-122.46335	37.93846	-0.28	3.44	-466.7	3.1579999	11	7/24/2014	25:57.3
5195	RWS11	-122.4633553	37.93844	-0.31	3.44	-462	3.13000003	-122.46335	37.93846	-0.32	3.44	-466.99	3.11900005	11	7/24/2014	25:57.4
5196	RWS11	-122.4633574	37.93844	-0.31	3.43	-462.2	3.12799993	-122.46335	37.93846	-0.28	3.43	-467.36	3.15099993	11	7/24/2014	25:57.5
5197	RWS11	-122.4633595	37.93844	-0.31	3.44	-462.2	3.1290001	-122.46335	37.93846	-0.28	3.44	-467.64	3.1520001	11	7/24/2014	25:57.6
5198	RWS11	-122.4633616	37.93845	-0.31	3.44	-462.4	3.13000003	-122.46336	37.93846	-0.28	3.44	-467.92	3.15300003	11	7/24/2014	25:57.7
5199	RWS11	-122.4633637	37.93845	-0.31	3.44	-462.4	3.13099995	-122.46336	37.93846	-0.32	3.44	-468.2	3.11999997	11	7/24/2014	25:57.8
5200	RWS11	-122.4633658	37.93845	-0.31	3.44	-462.4	3.1290001	-122.46336	37.93846	-0.28	3.44	-468.42	3.1520001	11	7/24/2014	25:57.9
5201	RWS11	-122.4633689	37.93845	-0.31	3.43	-462.6	3.12399998	-122.46336	37.93846	-0.32	3.43	-468.71	3.11300001	11	7/24/2014	25:58.0
5202	RWS11	-122.463371	37.93845	-0.31	3.42	-462.6	3.11799994	-122.46337	37.93846	-0.28	3.42	-468.91	3.14099994	11	7/24/2014	25:58.1
5203	RWS11	-122.4633731	37.93845	-0.31	3.42	-462.6	3.11099997	-122.46337	37.93846	-0.28	3.42	-468.99	3.13399997	11	7/24/2014	25:58.2
5204	RWS11	-122.4633753	37.93845	-0.31	3.41	-462.6	3.10300007	-122.46337	37.93846	-0.32	3.41	-469.14	3.0920001	11	7/24/2014	25:58.3
5205	RWS11	-122.4633774	37.93845	-0.31	3.4	-462.7	3.09499994	-122.46337	37.93846	-0.28	3.4	-469.22	3.11799994	11	7/24/2014	25:58.4
5206	RWS11	-122.4633795	37.93845	-0.31	3.39	-462.6	3.08700004	-122.46337	37.93846	-0.28	3.39	-469.29	3.11000004	11	7/24/2014	25:58.5
5207	RWS11	-122.4633817	37.93845	-0.31	3.39	-462.7	3.08000007	-122.46338	37.93846	-0.28	3.39	-469.44	3.10300007	11	7/24/2014	25:58.6
5208	RWS11	-122.4633838	37.93845	-0.31	3.38	-462.8	3.07199994	-122.46338	37.93846	-0.28	3.38	-469.55	3.09499994	11	7/24/2014	25:58.7
5209	RWS11	-122.463386	37.93845	-0.31	3.37	-462.9	3.0659999	-122.46338	37.93846	-0.28	3.37	-469.73	3.0889999	11	7/24/2014	25:58.8
5210	RWS11	-122.4633881	37.93845	-0.31	3.37	-463	3.05899993	-122.46338	37.93846	-0.28	3.37	-469.87	3.08199993	11	7/24/2014	25:58.9
5211	RWS11	-122.4633912	37.93845	-0.31	3.36	-463.1	3.05299988	-122.46339	37.93847	-0.28	3.36	-470.03	3.07599989	11	7/24/2014	25:59.0
5212	RWS11	-122.4633934	37.93845	-0.31	3.36	-463.3	3.04800001	-122.46339	37.93847	-0.23	3.36	-470.22	3.12200002	11	7/24/2014	25:59.1
5213	RWS11	-122.4633955	37.93845	-0.31	3.35	-463.5	3.04400006	-122.46339	37.93847	-0.28	3.35	-470.62	3.06700006	11	7/24/2014	25:59.2

5214	RWS11	-122.4633977	37.93845	-0.27	3.35	-463.8	3.07500005	-122.46339	37.93847	-0.28	3.35	-471.11	3.06400004	11	7/24/2014	25:59.3
5215	RWS11	-122.4633998	37.93846	-0.31	3.35	-464	3.03800002	-122.46339	37.93847	-0.28	3.35	-471.52	3.06100002	11	7/24/2014	25:59.4
5216	RWS11	-122.463402	37.93846	-0.31	3.34	-464.2	3.03599992	-122.4634	37.93847	-0.28	3.34	-471.85	3.05899993	11	7/24/2014	25:59.5
5217	RWS11	-122.4634041	37.93846	-0.31	3.34	-464.1	3.03599992	-122.4634	37.93847	-0.28	3.34	-472.05	3.05899993	11	7/24/2014	25:59.6
5218	RWS11	-122.4634063	37.93846	-0.27	3.34	-464.2	3.0710001	-122.4634	37.93847	-0.23	3.34	-472.21	3.11100011	11	7/24/2014	25:59.7
5219	RWS11	-122.4634084	37.93846	-0.31	3.35	-464.3	3.03899994	-122.4634	37.93847	-0.28	3.35	-472.32	3.06199995	11	7/24/2014	25:59.8
5220	RWS11	-122.4634106	37.93846	-0.31	3.35	-464.3	3.04499999	-122.46341	37.93847	-0.32	3.35	-472.44	3.03400001	11	7/24/2014	25:59.9
5221	RWS11	-122.4634137	37.93846	-0.31	3.36	-464.3	3.05199996	-122.46341	37.93847	-0.32	3.36	-472.37	3.04099998	11	7/24/2014	26:00.0
5222	RWS11	-122.4634159	37.93846	-0.31	3.37	-464.2	3.06299987	-122.46341	37.93847	-0.32	3.37	-472.15	3.0519999	11	7/24/2014	26:00.1
5223	RWS11	-122.463418	37.93846	-0.31	3.38	-464.1	3.07499996	-122.46341	37.93847	-0.32	3.38	-471.96	3.06399998	11	7/24/2014	26:00.2
5224	RWS11	-122.4634202	37.93846	-0.31	3.4	-463.9	3.0889999	-122.46341	37.93847	-0.28	3.4	-471.71	3.1119999	11	7/24/2014	26:00.3
5225	RWS11	-122.4634223	37.93846	-0.34	3.41	-463.8	3.07000002	-122.46342	37.93847	-0.35	3.41	-471.46	3.05900002	11	7/24/2014	26:00.4
5226	RWS11	-122.4634245	37.93846	-0.31	3.43	-463.7	3.12099996	-122.46342	37.93847	-0.32	3.43	-471.4	3.10999998	11	7/24/2014	26:00.5
5227	RWS11	-122.4634266	37.93846	-0.34	3.44	-463.6	3.10399994	-122.46342	37.93847	-0.32	3.44	-471.37	3.12699994	11	7/24/2014	26:00.6
5228	RWS11	-122.4634288	37.93846	-0.31	3.46	-463.6	3.15600005	-122.46342	37.93848	-0.32	3.46	-471.35	3.14500007	11	7/24/2014	26:00.7
5229	RWS11	-122.4634308	37.93846	-0.34	3.48	-463.4	3.13900003	-122.46343	37.93848	-0.35	3.48	-471.28	3.12800002	11	7/24/2014	26:00.8
5230	RWS11	-122.4634329	37.93846	-0.31	3.5	-463.3	3.1909999	-122.46343	37.93848	-0.32	3.5	-471.27	3.17999992	11	7/24/2014	26:00.9
5231	RWS11	-122.463436	37.93846	-0.34	3.52	-463.1	3.17400011	-122.46343	37.93848	-0.35	3.52	-471.21	3.16300011	11	7/24/2014	26:01.0
5232	RWS11	-122.4634381	37.93846	-0.34	3.53	-462.9	3.19100007	-122.46343	37.93848	-0.35	3.53	-471.13	3.18000007	11	7/24/2014	26:01.1
5233	RWS11	-122.4634401	37.93847	-0.34	3.55	-462.7	3.20899996	-122.46343	37.93848	-0.4	3.55	-471.05	3.14799994	11	7/24/2014	26:01.2
5234	RWS11	-122.4634422	37.93847	-0.34	3.57	-462.5	3.22499999	-122.46344	37.93848	-0.32	3.57	-470.96	3.248	11	7/24/2014	26:01.3
5235	RWS11	-122.4634442	37.93847	-0.34	3.58	-462.2	3.24100003	-122.46344	37.93848	-0.4	3.58	-470.85	3.18000001	11	7/24/2014	26:01.4
5236	RWS11	-122.4634462	37.93847	-0.34	3.6	-462	3.25499997	-122.46344	37.93848	-0.35	3.6	-470.64	3.24399996	11	7/24/2014	26:01.5
5237	RWS11	-122.4634483	37.93847	-0.34	3.61	-461.6	3.26600012	-122.46344	37.93848	-0.35	3.61	-470.42	3.25500011	11	7/24/2014	26:01.6
5238	RWS11	-122.4634503	37.93847	-0.34	3.62	-461.3	3.27499995	-122.46345	37.93848	-0.35	3.62	-470.16	3.26399994	11	7/24/2014	26:01.7
5239	RWS11	-122.4634523	37.93847	-0.39	3.62	-460.9	3.22999999	-122.46345	37.93848	-0.4	3.62	-469.89	3.21999997	11	7/24/2014	26:01.8
5240	RWS11	-122.4634543	37.93847	-0.34	3.63	-460.6	3.28400001	-122.46345	37.93848	-0.4	3.63	-469.6	3.22299999	11	7/24/2014	26:01.9
5241	RWS11	-122.4634572	37.93847	-0.39	3.63	-460.3	3.23399994	-122.46345	37.93848	-0.4	3.63	-469.33	3.22399992	11	7/24/2014	26:02.0
5242	RWS11	-122.4634592	37.93847	-0.34	3.62	-460	3.28300008	-122.46345	37.93848	-0.4	3.62	-469.13	3.22200006	11	7/24/2014	26:02.1
5243	RWS11	-122.4634612	37.93847	-0.39	3.62	-459.7	3.2279999	-122.46346	37.93848	-0.4	3.62	-468.86	3.21799988	11	7/24/2014	26:02.2
5244	RWS11	-122.4634632	37.93847	-0.39	3.62	-459.5	3.22399995	-122.46346	37.93848	-0.44	3.62	-468.63	3.17999995	11	7/24/2014	26:02.3
5245	RWS11	-122.4634652	37.93847	-0.39	3.61	-459.2	3.21900007	-122.46346	37.93848	-0.44	3.61	-468.37	3.17500007	11	7/24/2014	26:02.4
5246	RWS11	-122.4634672	37.93847	-0.39	3.61	-459	3.21399996	-122.46346	37.93848	-0.4	3.61	-468.11	3.20399994	11	7/24/2014	26:02.5

5247	RWS11	-122.4634692	37.93847	-0.43	3.6	-458.8	3.17299992	-122.46346	37.93849	-0.44	3.6	-467.76	3.16399992	11	7/24/2014	26:02.6
5248	RWS11	-122.4634712	37.93847	-0.39	3.6	-458.6	3.20300004	-122.46347	37.93849	-0.44	3.6	-467.48	3.15900004	11	7/24/2014	26:02.7
5249	RWS11	-122.4634732	37.93847	-0.39	3.59	-458.3	3.197	-122.46347	37.93849	-0.44	3.59	-467.15	3.153	11	7/24/2014	26:02.8
5250	RWS11	-122.4634752	37.93847	-0.39	3.58	-458.2	3.19200012	-122.46347	37.93849	-0.4	3.58	-466.96	3.1820001	11	7/24/2014	26:02.9
5251	RWS11	-122.4634781	37.93847	-0.43	3.58	-458	3.15100008	-122.46347	37.93849	-0.44	3.58	-466.86	3.14200008	11	7/24/2014	26:03.0
5252	RWS11	-122.4634801	37.93848	-0.39	3.57	-457.9	3.18000004	-122.46347	37.93849	-0.44	3.57	-466.84	3.13600004	11	7/24/2014	26:03.1
5253	RWS11	-122.4634821	37.93848	-0.43	3.57	-457.8	3.13999993	-122.46348	37.93849	-0.44	3.57	-466.88	3.13099992	11	7/24/2014	26:03.2
5254	RWS11	-122.4634841	37.93848	-0.39	3.56	-457.8	3.17000005	-122.46348	37.93849	-0.44	3.56	-466.95	3.12600005	11	7/24/2014	26:03.3
5255	RWS11	-122.4634861	37.93848	-0.39	3.56	-457.8	3.1660001	-122.46348	37.93849	-0.44	3.56	-466.88	3.1220001	11	7/24/2014	26:03.4
5256	RWS11	-122.4634881	37.93848	-0.39	3.56	-457.6	3.16300008	-122.46348	37.93849	-0.44	3.56	-466.62	3.11900008	11	7/24/2014	26:03.5
5257	RWS11	-122.4634901	37.93848	-0.39	3.55	-457.5	3.16199991	-122.46348	37.93849	-0.49	3.55	-466.3	3.06699991	11	7/24/2014	26:03.6
5258	RWS11	-122.4634921	37.93848	-0.39	3.56	-457.3	3.16300008	-122.46349	37.93849	-0.44	3.56	-465.95	3.11900008	11	7/24/2014	26:03.7
5259	RWS11	-122.4634941	37.93848	-0.43	3.56	-457.2	3.1310001	-122.46349	37.93849	-0.44	3.56	-465.78	3.1220001	11	7/24/2014	26:03.8
5260	RWS11	-122.463496	37.93848	-0.39	3.56	-457.2	3.1719999	-122.46349	37.93849	-0.49	3.56	-465.73	3.0769999	11	7/24/2014	26:03.9
5261	RWS11	-122.463499	37.93848	-0.39	3.57	-457.1	3.18000004	-122.46349	37.93849	-0.44	3.57	-465.69	3.13600004	11	7/24/2014	26:04.0
5262	RWS11	-122.463501	37.93848	-0.39	3.58	-456.9	3.19099995	-122.4635	37.93849	-0.44	3.58	-465.58	3.14699996	11	7/24/2014	26:04.1
5263	RWS11	-122.4635029	37.93848	-0.43	3.6	-456.8	3.16800004	-122.4635	37.93849	-0.49	3.6	-465.47	3.10800004	11	7/24/2014	26:04.2
5264	RWS11	-122.4635049	37.93848	-0.39	3.61	-456.7	3.21600005	-122.4635	37.93849	-0.44	3.61	-465.43	3.17200005	11	7/24/2014	26:04.3
5265	RWS11	-122.4635069	37.93848	-0.43	3.62	-456.6	3.19199997	-122.4635	37.93849	-0.49	3.62	-465.35	3.13199997	11	7/24/2014	26:04.4
5266	RWS11	-122.463509	37.93848	-0.39	3.63	-456.4	3.23699996	-122.4635	37.9385	-0.44	3.63	-465.25	3.19299996	11	7/24/2014	26:04.5
5267	RWS11	-122.463511	37.93848	-0.39	3.63	-456.2	3.243	-122.46351	37.9385	-0.49	3.63	-465.12	3.148	11	7/24/2014	26:04.6
5268	RWS11	-122.463513	37.93848	-0.39	3.64	-456.1	3.24699995	-122.46351	37.9385	-0.44	3.64	-464.96	3.20299995	11	7/24/2014	26:04.7
5269	RWS11	-122.463515	37.93848	-0.43	3.64	-455.9	3.21199995	-122.46351	37.9385	-0.44	3.64	-464.8	3.20299995	11	7/24/2014	26:04.8
5270	RWS11	-122.463517	37.93848	-0.39	3.64	-455.7	3.24500009	-122.46351	37.9385	-0.49	3.64	-464.61	3.1500001	11	7/24/2014	26:04.9
5271	RWS11	-122.4635199	37.93849	-0.43	3.63	-455.5	3.20599991	-122.46351	37.9385	-0.49	3.63	-464.31	3.14599991	11	7/24/2014	26:05.0
5272	RWS11	-122.4635219	37.93849	-0.39	3.63	-455.2	3.23399994	-122.46352	37.9385	-0.49	3.63	-464.07	3.13899994	11	7/24/2014	26:05.1
5273	RWS11	-122.4635239	37.93849	-0.43	3.62	-455	3.19000012	-122.46352	37.9385	-0.44	3.62	-463.92	3.18100011	11	7/24/2014	26:05.2
5274	RWS11	-122.4635259	37.93849	-0.39	3.61	-454.9	3.21300003	-122.46352	37.9385	-0.44	3.61	-464	3.16900003	11	7/24/2014	26:05.3
5275	RWS11	-122.4635279	37.93849	-0.43	3.59	-454.7	3.16400009	-122.46352	37.9385	-0.49	3.59	-463.91	3.10400009	11	7/24/2014	26:05.4
5276	RWS11	-122.4635299	37.93849	-0.39	3.58	-454.5	3.18300006	-122.46352	37.9385	-0.44	3.58	-463.81	3.13900006	11	7/24/2014	26:05.5
5277	RWS11	-122.4635319	37.93849	-0.43	3.56	-454.4	3.12800008	-122.46353	37.9385	-0.44	3.56	-463.77	3.11900008	11	7/24/2014	26:05.6
5278	RWS11	-122.4635339	37.93849	-0.43	3.53	-454.3	3.10500008	-122.46353	37.9385	-0.44	3.53	-463.74	3.09600008	11	7/24/2014	26:05.7
5279	RWS11	-122.4635359	37.93849	-0.43	3.51	-454.2	3.07999998	-122.46353	37.9385	-0.49	3.51	-463.67	3.01999998	11	7/24/2014	26:05.8

5280	RWS11	-122.4635379	37.93849	-0.39	3.48	-454	3.08899996	-122.46353	37.9385	-0.44	3.48	-463.57	3.04499996	11	7/24/2014	26:05.9
5281	RWS11	-122.4635408	37.93849	-0.43	3.45	-453.9	3.02799994	-122.46354	37.9385	-0.44	3.45	-463.38	3.01899993	11	7/24/2014	26:06.0
5282	RWS11	-122.4635428	37.93849	-0.39	3.43	-453.8	3.03800008	-122.46354	37.9385	-0.44	3.43	-463.31	2.99400008	11	7/24/2014	26:06.1
5283	RWS11	-122.4635448	37.93849	-0.43	3.4	-453.6	2.97799999	-122.46354	37.9385	-0.44	3.4	-463.18	2.96899998	11	7/24/2014	26:06.2
5284	RWS11	-122.4635468	37.93849	-0.39	3.38	-453.6	2.98900005	-122.46354	37.9385	-0.4	3.38	-463.17	2.97900003	11	7/24/2014	26:06.3
5285	RWS11	-122.4635488	37.93849	-0.39	3.36	-453.5	2.96600005	-122.46354	37.9385	-0.4	3.36	-463.14	2.95600003	11	7/24/2014	26:06.4
5286	RWS11	-122.4635508	37.93849	-0.39	3.34	-453.5	2.94300005	-122.46355	37.93851	-0.4	3.34	-463.16	2.93300003	11	7/24/2014	26:06.5
5287	RWS11	-122.4635528	37.93849	-0.43	3.31	-453.5	2.88500005	-122.46355	37.93851	-0.4	3.31	-463.34	2.91000003	11	7/24/2014	26:06.6
5288	RWS11	-122.4635548	37.93849	-0.39	3.29	-453.5	2.89700004	-122.46355	37.93851	-0.35	3.29	-463.53	2.93700004	11	7/24/2014	26:06.7
5289	RWS11	-122.4635568	37.93849	-0.39	3.27	-453.5	2.87300012	-122.46355	37.93851	-0.4	3.27	-463.68	2.86300009	11	7/24/2014	26:06.8
5290	RWS11	-122.4635588	37.93849	-0.34	3.24	-453.5	2.89800009	-122.46355	37.93851	-0.4	3.24	-463.79	2.83700007	11	7/24/2014	26:06.9
5291	RWS11	-122.4635617	37.9385	-0.39	3.21	-453.4	2.8199999	-122.46356	37.93851	-0.4	3.21	-463.82	2.80999988	11	7/24/2014	26:07.0
5292	RWS11	-122.4635637	37.9385	-0.34	3.18	-453.4	2.84	-122.46356	37.93851	-0.35	3.18	-463.86	2.829	11	7/24/2014	26:07.1
5293	RWS11	-122.4635657	37.9385	-0.39	3.15	-453.4	2.75600001	-122.46356	37.93851	-0.4	3.15	-463.84	2.74599999	11	7/24/2014	26:07.2
5294	RWS11	-122.4635676	37.9385	-0.39	3.11	-453.3	2.72	-122.46356	37.93851	-0.35	3.11	-463.68	2.75999999	11	7/24/2014	26:07.3
5295	RWS11	-122.4635696	37.9385	-0.34	3.08	-453.2	2.73400006	-122.46356	37.93851	-0.35	3.08	-463.6	2.72300005	11	7/24/2014	26:07.4
5296	RWS11	-122.4635716	37.9385	-0.34	3.04	-453.3	2.69800004	-122.46357	37.93851	-0.32	3.04	-463.6	2.72100005	11	7/24/2014	26:07.5
5297	RWS11	-122.4635736	37.9385	-0.34	3.01	-453.3	2.66599998	-122.46357	37.93851	-0.35	3.01	-463.65	2.65499997	11	7/24/2014	26:07.6
5298	RWS11	-122.4635756	37.9385	-0.34	2.98	-453.4	2.64100012	-122.46357	37.93851	-0.32	2.98	-463.75	2.66400012	11	7/24/2014	26:07.7
5299	RWS11	-122.4635776	37.9385	-0.34	2.97	-453.4	2.62900004	-122.46357	37.93851	-0.35	2.97	-463.86	2.61800003	11	7/24/2014	26:07.8
5300	RWS11	-122.4635796	37.9385	-0.34	2.97	-453.4	2.63399991	-122.46357	37.93851	-0.32	2.97	-463.88	2.65699992	11	7/24/2014	26:07.9
5301	RWS11	-122.4635825	37.9385	-0.34	2.99	-453.5	2.65299997	-122.46358	37.93851	-0.35	2.99	-464.04	2.64199996	11	7/24/2014	26:08.0
5302	RWS11	-122.4635845	37.9385	-0.31	3.02	-453.5	2.71200004	-122.46358	37.93851	-0.32	3.02	-464.03	2.70100006	11	7/24/2014	26:08.1
5303	RWS11	-122.4635865	37.9385	-0.34	3.05	-453.4	2.70599994	-122.46358	37.93851	-0.32	3.05	-463.97	2.72899994	11	7/24/2014	26:08.2
5304	RWS11	-122.4635885	37.9385	-0.31	3.08	-453.3	2.76800004	-122.46358	37.93851	-0.28	3.08	-463.84	2.79100004	11	7/24/2014	26:08.3
5305	RWS11	-122.4635906	37.9385	-0.31	3.1	-453.2	2.79299989	-122.46359	37.93851	-0.32	3.1	-463.65	2.78199992	11	7/24/2014	26:08.4
5306	RWS11	-122.4635926	37.9385	-0.31	3.12	-453	2.81299987	-122.46359	37.93852	-0.28	3.12	-463.47	2.83599988	11	7/24/2014	26:08.5
5307	RWS11	-122.4635946	37.9385	-0.34	3.13	-452.9	2.794	-122.46359	37.93852	-0.32	3.13	-463.26	2.817	11	7/24/2014	26:08.6
5308	RWS11	-122.4635967	37.9385	-0.31	3.14	-452.7	2.83799997	-122.46359	37.93852	-0.32	3.14	-463.02	2.82699999	11	7/24/2014	26:08.7
5309	RWS11	-122.4635987	37.9385	-0.31	3.15	-452.5	2.84300008	-122.46359	37.93852	-0.32	3.15	-462.89	2.83200011	11	7/24/2014	26:08.8
5310	RWS11	-122.4636007	37.9385	-0.31	3.15	-452.4	2.84400001	-122.4636	37.93852	-0.32	3.15	-462.75	2.83300003	11	7/24/2014	26:08.9
5311	RWS11	-122.4636038	37.93851	-0.31	3.15	-452.3	2.84300008	-122.4636	37.93852	-0.32	3.15	-462.52	2.83200011	11	7/24/2014	26:09.0
5312	RWS11	-122.4636058	37.93851	-0.31	3.15	-452	2.8389999	-122.4636	37.93852	-0.28	3.15	-462.28	2.8619999	11	7/24/2014	26:09.1

5313	RWS11	-122.4636078	37.93851	-0.31	3.14	-451.8	2.83499995	-122.4636	37.93852	-0.28	3.14	-462.05	2.85799995	11	7/24/2014	26:09.2
5314	RWS11	-122.4636099	37.93851	-0.27	3.14	-451.5	2.86400008	-122.46361	37.93852	-0.28	3.14	-461.81	2.85300007	11	7/24/2014	26:09.3
5315	RWS11	-122.463612	37.93851	-0.31	3.13	-451.3	2.82599989	-122.46361	37.93852	-0.28	3.13	-461.58	2.84899989	11	7/24/2014	26:09.4
5316	RWS11	-122.463614	37.93851	-0.31	3.13	-451	2.8230001	-122.46361	37.93852	-0.28	3.13	-461.36	2.84600011	11	7/24/2014	26:09.5
5317	RWS11	-122.4636161	37.93851	-0.31	3.13	-450.8	2.82199994	-122.46361	37.93852	-0.28	3.13	-461.07	2.84499994	11	7/24/2014	26:09.6
5318	RWS11	-122.4636182	37.93851	-0.27	3.13	-450.5	2.85700011	-122.46361	37.93852	-0.28	3.13	-460.77	2.84600011	11	7/24/2014	26:09.7
5319	RWS11	-122.4636202	37.93851	-0.31	3.13	-450.2	2.82499996	-122.46362	37.93852	-0.28	3.13	-460.58	2.84799996	11	7/24/2014	26:09.8
5320	RWS11	-122.4636222	37.93851	-0.31	3.14	-449.9	2.83000007	-122.46362	37.93852	-0.28	3.14	-460.28	2.85300007	11	7/24/2014	26:09.9
5321	RWS11	-122.4636251	37.93851	-0.31	3.14	-449.6	2.83499995	-122.46362	37.93852	-0.28	3.14	-459.96	2.85799995	11	7/24/2014	26:10.0
5322	RWS11	-122.4636271	37.93851	-0.27	3.15	-449.2	2.87400007	-122.46362	37.93852	-0.28	3.15	-459.6	2.86300007	11	7/24/2014	26:10.1
5323	RWS11	-122.463629	37.93851	-0.31	3.15	-448.8	2.84499994	-122.46362	37.93852	-0.28	3.15	-459.19	2.86799994	11	7/24/2014	26:10.2
5324	RWS11	-122.463631	37.93851	-0.27	3.16	-448.5	2.88400006	-122.46363	37.93852	-0.28	3.16	-458.84	2.87300006	11	7/24/2014	26:10.3
5325	RWS11	-122.4636329	37.93851	-0.31	3.16	-448.1	2.85499993	-122.46363	37.93852	-0.28	3.16	-458.52	2.87799993	11	7/24/2014	26:10.4
5326	RWS11	-122.4636349	37.93851	-0.27	3.17	-447.8	2.89299989	-122.46363	37.93853	-0.23	3.17	-458.32	2.93299989	11	7/24/2014	26:10.5
5327	RWS11	-122.4636368	37.93851	-0.31	3.17	-447.6	2.8619999	-122.46363	37.93853	-0.28	3.17	-458.15	2.8849999	11	7/24/2014	26:10.6
5328	RWS11	-122.4636388	37.93851	-0.27	3.17	-447.3	2.89700007	-122.46363	37.93853	-0.23	3.17	-457.93	2.93700008	11	7/24/2014	26:10.7
5329	RWS11	-122.4636407	37.93851	-0.31	3.17	-447.1	2.8619999	-122.46364	37.93853	-0.28	3.17	-457.8	2.8849999	11	7/24/2014	26:10.8
5330	RWS11	-122.4636427	37.93851	-0.27	3.17	-446.9	2.89400005	-122.46364	37.93853	-0.23	3.17	-457.68	2.93400006	11	7/24/2014	26:10.9
5331	RWS11	-122.4636456	37.93851	-0.27	3.16	-446.6	2.89100003	-122.46364	37.93853	-0.28	3.16	-457.44	2.88000003	11	7/24/2014	26:11.0
5332	RWS11	-122.4636475	37.93852	-0.27	3.16	-446.3	2.88700008	-122.46364	37.93853	-0.23	3.16	-457.2	2.92700009	11	7/24/2014	26:11.1
5333	RWS11	-122.4636495	37.93852	-0.31	3.16	-446.1	2.85000005	-122.46364	37.93853	-0.23	3.16	-457.07	2.92400007	11	7/24/2014	26:11.2
5334	RWS11	-122.4636515	37.93852	-0.27	3.15	-445.8	2.88199997	-122.46365	37.93853	-0.23	3.15	-456.92	2.92199998	11	7/24/2014	26:11.3
5335	RWS11	-122.4636535	37.93852	-0.27	3.15	-445.6	2.88199997	-122.46365	37.93853	-0.23	3.15	-456.69	2.92199998	11	7/24/2014	26:11.4
5336	RWS11	-122.4636555	37.93852	-0.27	3.16	-445.2	2.8829999	-122.46365	37.93853	-0.2	3.16	-456.51	2.9569999	11	7/24/2014	26:11.5
5337	RWS11	-122.4636575	37.93852	-0.27	3.16	-445	2.88700008	-122.46365	37.93853	-0.23	3.16	-456.34	2.92700009	11	7/24/2014	26:11.6
5338	RWS11	-122.4636596	37.93852	-0.27	3.17	-444.7	2.89400005	-122.46365	37.93853	-0.23	3.17	-456.18	2.93400006	11	7/24/2014	26:11.7
5339	RWS11	-122.4636616	37.93852	-0.27	3.18	-444.5	2.90300012	-122.46366	37.93853	-0.23	3.18	-456.1	2.94300012	11	7/24/2014	26:11.8
5340	RWS11	-122.4636635	37.93852	-0.27	3.19	-444.3	2.91499996	-122.46366	37.93853	-0.2	3.19	-455.93	2.98899996	11	7/24/2014	26:11.9
5341	RWS11	-122.4636665	37.93852	-0.27	3.2	-444	2.93000007	-122.46366	37.93853	-0.23	3.2	-455.73	2.97000007	11	7/24/2014	26:12.0
5342	RWS11	-122.4636684	37.93852	-0.27	3.22	-443.8	2.94799995	-122.46366	37.93853	-0.2	3.22	-455.47	3.02199996	11	7/24/2014	26:12.1
5343	RWS11	-122.4636704	37.93852	-0.27	3.24	-443.5	2.9690001	-122.46367	37.93853	-0.23	3.24	-455.19	3.00900011	11	7/24/2014	26:12.2
5344	RWS11	-122.4636724	37.93852	-0.27	3.27	-443.3	2.9920001	-122.46367	37.93853	-0.23	3.27	-455.02	3.03200011	11	7/24/2014	26:12.3
5345	RWS11	-122.4636744	37.93852	-0.27	3.29	-443.1	3.01600003	-122.46367	37.93853	-0.23	3.29	-454.87	3.05600004	11	7/24/2014	26:12.4

5346	RWS11	-122.4636764	37.93852	-0.22	3.31	-442.9	3.09199996	-122.46367	37.93853	-0.2	3.31	-454.71	3.11399996	11	7/24/2014	26:12.5
5347	RWS11	-122.4636783	37.93852	-0.27	3.34	-442.8	3.06500006	-122.46367	37.93854	-0.2	3.34	-454.61	3.13900006	11	7/24/2014	26:12.6
5348	RWS11	-122.4636803	37.93852	-0.27	3.36	-442.7	3.08899999	-122.46368	37.93854	-0.2	3.36	-454.46	3.16299999	11	7/24/2014	26:12.7
5349	RWS11	-122.4636823	37.93852	-0.27	3.39	-442.5	3.11299992	-122.46368	37.93854	-0.2	3.39	-454.33	3.18699992	11	7/24/2014	26:12.8
5350	RWS11	-122.4636843	37.93852	-0.22	3.41	-442.4	3.18699999	-122.46368	37.93854	-0.2	3.41	-454.17	3.20899999	11	7/24/2014	26:12.9
5351	RWS11	-122.4636872	37.93852	-0.27	3.43	-442.2	3.15499997	-122.46368	37.93854	-0.15	3.43	-454.01	3.27899997	11	7/24/2014	26:13.0
5352	RWS11	-122.4636893	37.93852	-0.22	3.44	-442	3.22399993	-122.46368	37.93854	-0.15	3.44	-453.87	3.29599993	11	7/24/2014	26:13.1
5353	RWS11	-122.4636913	37.93852	-0.27	3.46	-441.9	3.18499994	-122.46369	37.93854	-0.2	3.46	-453.67	3.25899994	11	7/24/2014	26:13.2
5354	RWS11	-122.4636933	37.93853	-0.27	3.46	-441.6	3.19199991	-122.46369	37.93854	-0.15	3.46	-453.53	3.31599991	11	7/24/2014	26:13.3
5355	RWS11	-122.4636954	37.93853	-0.22	3.46	-441.4	3.24399991	-122.46369	37.93854	-0.15	3.46	-453.3	3.31599991	11	7/24/2014	26:13.4
5356	RWS11	-122.4636974	37.93853	-0.22	3.46	-441.1	3.23699994	-122.46369	37.93854	-0.15	3.46	-453.1	3.30899994	11	7/24/2014	26:13.5
5357	RWS11	-122.4636994	37.93853	-0.27	3.44	-440.9	3.17000008	-122.46369	37.93854	-0.15	3.44	-452.9	3.29400007	11	7/24/2014	26:13.6
5358	RWS11	-122.4637015	37.93853	-0.22	3.42	-440.6	3.2	-122.4637	37.93854	-0.15	3.42	-452.73	3.272	11	7/24/2014	26:13.7
5359	RWS11	-122.4637035	37.93853	-0.22	3.39	-440.3	3.17099996	-122.4637	37.93854	-0.15	3.39	-452.56	3.24299996	11	7/24/2014	26:13.8
5360	RWS11	-122.4637055	37.93853	-0.22	3.36	-440	3.13799997	-122.4637	37.93854	-0.12	3.36	-452.34	3.24399997	11	7/24/2014	26:13.9
5361	RWS11	-122.4637085	37.93853	-0.22	3.32	-439.8	3.10199995	-122.4637	37.93854	-0.15	3.32	-452.12	3.17399995	11	7/24/2014	26:14.0
5362	RWS11	-122.4637105	37.93853	-0.22	3.29	-439.5	3.06400008	-122.46371	37.93854	-0.12	3.29	-451.84	3.17000008	11	7/24/2014	26:14.1
5363	RWS11	-122.4637125	37.93853	-0.22	3.25	-439.2	3.02599998	-122.46371	37.93854	-0.12	3.25	-451.56	3.13199998	11	7/24/2014	26:14.2
5364	RWS11	-122.4637145	37.93853	-0.19	3.21	-439	3.02499989	-122.46371	37.93854	-0.15	3.21	-451.27	3.06299989	11	7/24/2014	26:14.3
5365	RWS11	-122.4637164	37.93853	-0.22	3.18	-438.7	2.95699997	-122.46371	37.93854	-0.15	3.18	-450.95	3.02899997	11	7/24/2014	26:14.4
5366	RWS11	-122.4637184	37.93853	-0.22	3.15	-438.5	2.927	-122.46371	37.93854	-0.12	3.15	-450.71	3.033	11	7/24/2014	26:14.5
5367	RWS11	-122.4637204	37.93853	-0.22	3.12	-438.3	2.89899988	-122.46372	37.93854	-0.12	3.12	-450.43	3.00499988	11	7/24/2014	26:14.6
5368	RWS11	-122.4637224	37.93853	-0.19	3.1	-438	2.90899995	-122.46372	37.93854	-0.12	3.1	-450.11	2.98099995	11	7/24/2014	26:14.7
5369	RWS11	-122.4637244	37.93853	-0.19	3.07	-437.8	2.88599995	-122.46372	37.93855	-0.12	3.07	-449.85	2.95799995	11	7/24/2014	26:14.8
5370	RWS11	-122.4637264	37.93853	-0.19	3.05	-437.5	2.86400011	-122.46372	37.93855	-0.12	3.05	-449.49	2.93600012	11	7/24/2014	26:14.9
5371	RWS11	-122.4637293	37.93853	-0.19	3.03	-437.2	2.84299996	-122.46372	37.93855	-0.15	3.03	-449.12	2.88099997	11	7/24/2014	26:15.0
5372	RWS11	-122.4637312	37.93853	-0.19	3.01	-437	2.82500008	-122.46373	37.93855	-0.12	3.01	-448.79	2.89700008	11	7/24/2014	26:15.1
5373	RWS11	-122.4637332	37.93853	-0.19	2.99	-436.6	2.80799988	-122.46373	37.93855	-0.12	2.99	-448.38	2.87999988	11	7/24/2014	26:15.2
5374	RWS11	-122.4637352	37.93853	-0.19	2.98	-436.3	2.79500011	-122.46373	37.93855	-0.12	2.98	-448.01	2.86700011	11	7/24/2014	26:15.3
5375	RWS11	-122.4637371	37.93854	-0.19	2.97	-436	2.78600004	-122.46373	37.93855	-0.15	2.97	-447.57	2.82400005	11	7/24/2014	26:15.4
5376	RWS11	-122.4637391	37.93854	-0.19	2.97	-435.7	2.78099993	-122.46373	37.93855	-0.12	2.97	-447.17	2.85299993	11	7/24/2014	26:15.5
5377	RWS11	-122.4637411	37.93854	-0.19	2.97	-435.3	2.78200009	-122.46374	37.93855	-0.12	2.97	-446.82	2.8540001	11	7/24/2014	26:15.6
5378	RWS11	-122.4637431	37.93854	-0.19	2.97	-435	2.7879999	-122.46374	37.93855	-0.12	2.97	-446.43	2.8599999	11	7/24/2014	26:15.7

5379	RWS11	-122.463745	37.93854	-0.19	2.99	-434.6	2.79999998	-122.46374	37.93855	-0.12	2.99	-445.96	2.87199999	11	7/24/2014	26:15.8
5380	RWS11	-122.463747	37.93854	-0.14	3	-434.2	2.86799994	-122.46374	37.93855	-0.12	3	-445.48	2.88899995	11	7/24/2014	26:15.9
5381	RWS11	-122.4637499	37.93854	-0.19	3.03	-433.7	2.83900002	-122.46375	37.93855	-0.15	3.03	-444.98	2.87700002	11	7/24/2014	26:16.0
5382	RWS11	-122.4637519	37.93854	-0.14	3.05	-433.2	2.91699997	-122.46375	37.93855	-0.06	3.05	-444.4	2.98899997	11	7/24/2014	26:16.1
5383	RWS11	-122.4637538	37.93854	-0.19	3.08	-432.7	2.89500001	-122.46375	37.93855	-0.12	3.08	-443.9	2.96700002	11	7/24/2014	26:16.2
5384	RWS11	-122.4637557	37.93854	-0.19	3.11	-432.2	2.92700008	-122.46375	37.93855	-0.12	3.11	-443.36	2.99900008	11	7/24/2014	26:16.3
5385	RWS11	-122.4637577	37.93854	-0.19	3.14	-431.7	2.95799997	-122.46375	37.93855	-0.12	3.14	-442.82	3.02999998	11	7/24/2014	26:16.4
5386	RWS11	-122.4637597	37.93854	-0.14	3.17	-431.2	3.03599992	-122.46376	37.93855	-0.12	3.17	-442.28	3.05699993	11	7/24/2014	26:16.5
5387	RWS11	-122.4637617	37.93854	-0.19	3.19	-430.7	3.007	-122.46376	37.93855	-0.15	3.19	-441.78	3.045	11	7/24/2014	26:16.6
5388	RWS11	-122.4637636	37.93854	-0.19	3.21	-430.3	3.02099994	-122.46376	37.93855	-0.12	3.21	-441.34	3.09299994	11	7/24/2014	26:16.7
5389	RWS11	-122.4637656	37.93854	-0.19	3.21	-429.7	3.02699998	-122.46376	37.93855	-0.15	3.21	-440.83	3.06499998	11	7/24/2014	26:16.8
5390	RWS11	-122.4637675	37.93854	-0.14	3.21	-429.2	3.07499996	-122.46376	37.93855	-0.12	3.21	-440.33	3.09599996	11	7/24/2014	26:16.9
5391	RWS11	-122.4637704	37.93854	-0.19	3.2	-428.7	3.01399997	-122.46377	37.93856	-0.12	3.2	-439.83	3.08599997	11	7/24/2014	26:17.0
5392	RWS11	-122.4637724	37.93854	-0.14	3.18	-428.2	3.04899994	-122.46377	37.93856	-0.12	3.18	-439.4	3.06999994	11	7/24/2014	26:17.1
5393	RWS11	-122.4637743	37.93854	-0.19	3.16	-427.7	2.97799996	-122.46377	37.93856	-0.12	3.16	-438.97	3.04999996	11	7/24/2014	26:17.2
5394	RWS11	-122.4637763	37.93854	-0.14	3.14	-427.2	3.00699988	-122.46377	37.93856	-0.12	3.14	-438.47	3.02799989	11	7/24/2014	26:17.3
5395	RWS11	-122.4637782	37.93854	-0.19	3.12	-426.8	2.93400005	-122.46377	37.93856	-0.15	3.12	-438.06	2.97200005	11	7/24/2014	26:17.4
5396	RWS11	-122.4637802	37.93854	-0.19	3.1	-426.3	2.91499999	-122.46378	37.93856	-0.12	3.1	-437.62	2.987	11	7/24/2014	26:17.5
5397	RWS11	-122.4637822	37.93854	-0.19	3.09	-425.8	2.89899996	-122.46378	37.93856	-0.15	3.09	-437.13	2.93699996	11	7/24/2014	26:17.6
5398	RWS11	-122.4637841	37.93855	-0.19	3.08	-425.3	2.88800004	-122.46378	37.93856	-0.15	3.08	-436.63	2.92600004	11	7/24/2014	26:17.7
5399	RWS11	-122.4637861	37.93855	-0.19	3.07	-424.8	2.88299993	-122.46378	37.93856	-0.15	3.07	-436.09	2.92099993	11	7/24/2014	26:17.8
5400	RWS11	-122.4637881	37.93855	-0.19	3.07	-424.3	2.88299993	-122.46378	37.93856	-0.12	3.07	-435.55	2.95499993	11	7/24/2014	26:17.9
5401	RWS11	-122.463791	37.93855	-0.22	3.08	-423.8	2.85400005	-122.46379	37.93856	-0.15	3.08	-435	2.92600004	11	7/24/2014	26:18.0
5402	RWS11	-122.463793	37.93855	-0.19	3.08	-423.2	2.89599994	-122.46379	37.93856	-0.12	3.08	-434.31	2.96799994	11	7/24/2014	26:18.1
5403	RWS11	-122.4637949	37.93855	-0.19	3.09	-422.7	2.90700009	-122.46379	37.93856	-0.15	3.09	-433.71	2.9450001	11	7/24/2014	26:18.2
5404	RWS11	-122.463797	37.93855	-0.19	3.11	-422.2	2.91899994	-122.46379	37.93856	-0.12	3.11	-433.16	2.99099994	11	7/24/2014	26:18.3
5405	RWS11	-122.463799	37.93855	-0.19	3.12	-421.7	2.93199995	-122.46379	37.93856	-0.2	3.12	-432.65	2.91999996	11	7/24/2014	26:18.4
5406	RWS11	-122.463801	37.93855	-0.19	3.13	-421.2	2.94700006	-122.4638	37.93856	-0.15	3.13	-432.21	2.98500006	11	7/24/2014	26:18.5
5407	RWS11	-122.463803	37.93855	-0.19	3.15	-420.8	2.96199992	-122.4638	37.93856	-0.15	3.15	-431.8	2.99999993	11	7/24/2014	26:18.6
5408	RWS11	-122.463805	37.93855	-0.19	3.16	-420.3	2.97700003	-122.4638	37.93856	-0.15	3.16	-431.33	3.01500003	11	7/24/2014	26:18.7
5409	RWS11	-122.463807	37.93855	-0.22	3.18	-419.8	2.9579999	-122.4638	37.93856	-0.15	3.18	-430.81	3.0299999	11	7/24/2014	26:18.8
5410	RWS11	-122.463809	37.93855	-0.19	3.19	-419.3	3.00600007	-122.4638	37.93856	-0.15	3.19	-430.22	3.04400007	11	7/24/2014	26:18.9
5411	RWS11	-122.463812	37.93855	-0.19	3.21	-418.8	3.02000001	-122.46381	37.93856	-0.2	3.21	-429.68	3.00800002	11	7/24/2014	26:19.0

5412	RWS11	-122.463814	37.93855	-0.19	3.22	-418.4	3.03099993	-122.46381	37.93856	-0.15	3.22	-429.2	3.06899993	11	7/24/2014	26:19.1
5413	RWS11	-122.463816	37.93855	-0.22	3.23	-417.8	3.00699992	-122.46381	37.93856	-0.15	3.23	-428.6	3.07899992	11	7/24/2014	26:19.2
5414	RWS11	-122.463818	37.93855	-0.19	3.23	-417.3	3.04799989	-122.46381	37.93857	-0.15	3.23	-428.02	3.08599989	11	7/24/2014	26:19.3
5415	RWS11	-122.46382	37.93855	-0.19	3.24	-416.8	3.05200008	-122.46382	37.93857	-0.15	3.24	-427.56	3.09000008	11	7/24/2014	26:19.4
5416	RWS11	-122.463822	37.93855	-0.19	3.24	-416.3	3.053	-122.46382	37.93857	-0.15	3.24	-427.09	3.09100001	11	7/24/2014	26:19.5
5417	RWS11	-122.463824	37.93855	-0.22	3.24	-415.9	3.01800008	-122.46382	37.93857	-0.2	3.24	-426.65	3.04000008	11	7/24/2014	26:19.6
5418	RWS11	-122.463826	37.93855	-0.19	3.24	-415.4	3.04999998	-122.46382	37.93857	-0.2	3.24	-426.23	3.03799999	11	7/24/2014	26:19.7
5419	RWS11	-122.463828	37.93855	-0.22	3.23	-414.9	3.01200004	-122.46382	37.93857	-0.2	3.23	-425.71	3.03400004	11	7/24/2014	26:19.8
5420	RWS11	-122.46383	37.93855	-0.19	3.23	-414.5	3.04200009	-122.46383	37.93857	-0.15	3.23	-425.24	3.08000009	11	7/24/2014	26:19.9
5421	RWS11	-122.4638328	37.93856	-0.22	3.22	-414	3.00299998	-122.46383	37.93857	-0.2	3.22	-424.69	3.02499998	11	7/24/2014	26:20.0
5422	RWS11	-122.4638348	37.93856	-0.19	3.22	-413.5	3.03399995	-122.46383	37.93857	-0.2	3.22	-424.18	3.02199996	11	7/24/2014	26:20.1
5423	RWS11	-122.4638367	37.93856	-0.22	3.22	-413	2.99699993	-122.46383	37.93857	-0.2	3.22	-423.6	3.01899993	11	7/24/2014	26:20.2
5424	RWS11	-122.4638387	37.93856	-0.19	3.21	-412.5	3.02799991	-122.46383	37.93857	-0.2	3.21	-423.06	3.01599991	11	7/24/2014	26:20.3
5425	RWS11	-122.4638407	37.93856	-0.22	3.21	-412.1	2.99200006	-122.46384	37.93857	-0.2	3.21	-422.5	3.01400006	11	7/24/2014	26:20.4
5426	RWS11	-122.4638426	37.93856	-0.19	3.21	-411.6	3.0220001	-122.46384	37.93857	-0.2	3.21	-421.98	3.01000011	11	7/24/2014	26:20.5
5427	RWS11	-122.4638446	37.93856	-0.22	3.2	-411.2	2.98399992	-122.46384	37.93857	-0.2	3.2	-421.5	3.00599992	11	7/24/2014	26:20.6
5428	RWS11	-122.4638466	37.93856	-0.19	3.2	-410.7	3.01300004	-122.46384	37.93857	-0.2	3.2	-420.98	3.00100005	11	7/24/2014	26:20.7
5429	RWS11	-122.4638485	37.93856	-0.22	3.19	-410.2	2.973	-122.46384	37.93857	-0.23	3.19	-420.44	2.96100001	11	7/24/2014	26:20.8
5430	RWS11	-122.4638504	37.93856	-0.19	3.19	-409.7	3.00000003	-122.46385	37.93857	-0.2	3.19	-419.86	2.98800004	11	7/24/2014	26:20.9
5431	RWS11	-122.4638533	37.93856	-0.22	3.18	-409.2	2.95900007	-122.46385	37.93857	-0.2	3.18	-419.34	2.98100007	11	7/24/2014	26:21.0
5432	RWS11	-122.4638553	37.93856	-0.19	3.17	-408.7	2.98600009	-122.46385	37.93857	-0.2	3.17	-418.74	2.9740001	11	7/24/2014	26:21.1
5433	RWS11	-122.4638572	37.93856	-0.22	3.17	-408.3	2.94499989	-122.46385	37.93857	-0.2	3.17	-418.21	2.96699989	11	7/24/2014	26:21.2
5434	RWS11	-122.4638592	37.93856	-0.19	3.16	-407.8	2.97199991	-122.46385	37.93857	-0.2	3.16	-417.68	2.95999992	11	7/24/2014	26:21.3
5435	RWS11	-122.4638612	37.93856	-0.22	3.15	-407.3	2.93200012	-122.46386	37.93857	-0.23	3.15	-417.15	2.92000012	11	7/24/2014	26:21.4
5436	RWS11	-122.4638632	37.93856	-0.22	3.15	-406.8	2.92799993	-122.46386	37.93857	-0.2	3.15	-416.65	2.94999993	11	7/24/2014	26:21.5
5437	RWS11	-122.4638652	37.93856	-0.22	3.15	-406.1	2.92600007	-122.46386	37.93858	-0.23	3.15	-416.09	2.91400008	11	7/24/2014	26:21.6
5438	RWS11	-122.4638672	37.93856	-0.22	3.15	-405.6	2.92600007	-122.46386	37.93858	-0.2	3.15	-415.55	2.94800007	11	7/24/2014	26:21.7
5439	RWS11	-122.4638691	37.93856	-0.22	3.15	-405	2.93000002	-122.46386	37.93858	-0.2	3.15	-415.05	2.95200002	11	7/24/2014	26:21.8
5440	RWS11	-122.4638711	37.93856	-0.19	3.16	-404.5	2.96899989	-122.46387	37.93858	-0.2	3.16	-414.53	2.9569999	11	7/24/2014	26:21.9
5441	RWS11	-122.463874	37.93856	-0.22	3.16	-404	2.94300003	-122.46387	37.93858	-0.2	3.16	-413.99	2.96500003	11	7/24/2014	26:22.0
5442	RWS11	-122.4638759	37.93856	-0.19	3.17	-403.3	2.98799995	-122.46387	37.93858	-0.15	3.17	-413.47	3.02599995	11	7/24/2014	26:22.1
5443	RWS11	-122.4638779	37.93856	-0.22	3.19	-402.8	2.96600004	-122.46387	37.93858	-0.23	3.19	-412.89	2.95400004	11	7/24/2014	26:22.2
5444	RWS11	-122.4638799	37.93857	-0.22	3.2	-402.1	2.97999997	-122.46388	37.93858	-0.2	3.2	-412.37	3.00199997	11	7/24/2014	26:22.3

5445	RWS11	-122.4638818	37.93857	-0.22	3.22	-401.5	2.99600001	-122.46388	37.93858	-0.2	3.22	-411.83	3.01800001	11	7/24/2014	26:22.4
5446	RWS11	-122.4638838	37.93857	-0.19	3.23	-400.8	3.04699996	-122.46388	37.93858	-0.2	3.23	-411.24	3.03499997	11	7/24/2014	26:22.5
5447	RWS11	-122.4638858	37.93857	-0.22	3.25	-400.2	3.03100009	-122.46388	37.93858	-0.2	3.25	-410.49	3.05300009	11	7/24/2014	26:22.6
5448	RWS11	-122.4638877	37.93857	-0.19	3.27	-399.4	3.08299997	-122.46388	37.93858	-0.23	3.27	-409.82	3.03699999	11	7/24/2014	26:22.7
5449	RWS11	-122.4638897	37.93857	-0.22	3.29	-398.8	3.06500001	-122.46389	37.93858	-0.2	3.29	-409.17	3.08700001	11	7/24/2014	26:22.8
5450	RWS11	-122.4638916	37.93857	-0.19	3.3	-398	3.11299995	-122.46389	37.93858	-0.2	3.3	-408.58	3.10099995	11	7/24/2014	26:22.9
5451	RWS11	-122.4638945	37.93857	-0.22	3.31	-397.4	3.09100004	-122.46389	37.93858	-0.2	3.31	-408.06	3.11300004	11	7/24/2014	26:23.0
5452	RWS11	-122.4638964	37.93857	-0.19	3.32	-396.7	3.13500002	-122.46389	37.93858	-0.2	3.32	-407.58	3.12300003	11	7/24/2014	26:23.1
5453	RWS11	-122.4638984	37.93857	-0.22	3.33	-396.1	3.11000009	-122.46389	37.93858	-0.2	3.33	-407.09	3.13200009	11	7/24/2014	26:23.2
5454	RWS11	-122.4639003	37.93857	-0.22	3.34	-395.5	3.11799999	-122.4639	37.93858	-0.2	3.34	-406.59	3.13999999	11	7/24/2014	26:23.3
5455	RWS11	-122.4639023	37.93857	-0.22	3.35	-394.9	3.12599988	-122.4639	37.93858	-0.2	3.35	-406.02	3.14799988	11	7/24/2014	26:23.4
5456	RWS11	-122.4639042	37.93857	-0.19	3.36	-394.3	3.16899994	-122.4639	37.93858	-0.2	3.36	-405.49	3.15699995	11	7/24/2014	26:23.5
5457	RWS11	-122.4639062	37.93857	-0.22	3.37	-393.7	3.1460001	-122.4639	37.93858	-0.2	3.37	-404.93	3.1680001	11	7/24/2014	26:23.6
5458	RWS11	-122.4639081	37.93857	-0.22	3.38	-393.2	3.15700002	-122.4639	37.93858	-0.2	3.38	-404.38	3.17900002	11	7/24/2014	26:23.7
5459	RWS11	-122.4639101	37.93857	-0.22	3.39	-392.7	3.1690001	-122.46391	37.93858	-0.2	3.39	-403.91	3.1910001	11	7/24/2014	26:23.8
5460	RWS11	-122.463912	37.93857	-0.22	3.4	-392.1	3.18000002	-122.46391	37.93859	-0.2	3.4	-403.24	3.20200002	11	7/24/2014	26:23.9
5461	RWS11	-122.4639148	37.93857	-0.22	3.41	-391.6	3.19099994	-122.46391	37.93859	-0.2	3.41	-402.7	3.21299994	11	7/24/2014	26:24.0
5462	RWS11	-122.4639168	37.93857	-0.19	3.42	-391.1	3.23499992	-122.46391	37.93859	-0.2	3.42	-402.11	3.22299993	11	7/24/2014	26:24.1
5463	RWS11	-122.4639187	37.93857	-0.22	3.43	-390.7	3.20900007	-122.46391	37.93859	-0.2	3.43	-401.59	3.23100007	11	7/24/2014	26:24.2
5464	RWS11	-122.4639206	37.93857	-0.22	3.44	-390	3.21500011	-122.46392	37.93859	-0.2	3.44	-400.92	3.23700011	11	7/24/2014	26:24.3
5465	RWS11	-122.4639225	37.93857	-0.22	3.44	-389.4	3.21799989	-122.46392	37.93859	-0.2	3.44	-400.32	3.23999989	11	7/24/2014	26:24.4
5466	RWS11	-122.4639244	37.93857	-0.22	3.44	-388.9	3.21799989	-122.46392	37.93859	-0.2	3.44	-399.68	3.23999989	11	7/24/2014	26:24.5
5467	RWS11	-122.4639263	37.93857	-0.22	3.44	-388.4	3.21500011	-122.46392	37.93859	-0.2	3.44	-399.13	3.23700011	11	7/24/2014	26:24.6
5468	RWS11	-122.4639282	37.93858	-0.22	3.43	-387.7	3.20999999	-122.46392	37.93859	-0.2	3.43	-398.5	3.23199999	11	7/24/2014	26:24.7
5469	RWS11	-122.4639301	37.93858	-0.22	3.42	-387.3	3.20300002	-122.46393	37.93859	-0.2	3.42	-397.89	3.22500002	11	7/24/2014	26:24.8
5470	RWS11	-122.463932	37.93858	-0.19	3.41	-386.7	3.22799996	-122.46393	37.93859	-0.2	3.41	-397.33	3.21599996	11	7/24/2014	26:24.9
5471	RWS11	-122.4639348	37.93858	-0.22	3.4	-386.1	3.18399997	-122.46393	37.93859	-0.2	3.4	-396.79	3.20599997	11	7/24/2014	26:25.0
5472	RWS11	-122.4639366	37.93858	-0.19	3.4	-385.5	3.2089999	-122.46393	37.93859	-0.15	3.4	-396.08	3.2469999	11	7/24/2014	26:25.1
5473	RWS11	-122.4639385	37.93858	-0.22	3.39	-384.9	3.16600008	-122.46393	37.93859	-0.2	3.39	-395.47	3.18800008	11	7/24/2014	26:25.2
5474	RWS11	-122.4639404	37.93858	-0.19	3.38	-384.3	3.19100001	-122.46394	37.93859	-0.2	3.38	-394.93	3.17900002	11	7/24/2014	26:25.3
5475	RWS11	-122.4639423	37.93858	-0.22	3.37	-383.8	3.15000005	-122.46394	37.93859	-0.2	3.37	-394.39	3.17200005	11	7/24/2014	26:25.4
5476	RWS11	-122.4639442	37.93858	-0.19	3.36	-383.1	3.17700008	-122.46394	37.93859	-0.2	3.36	-393.78	3.16500008	11	7/24/2014	26:25.5
5477	RWS11	-122.463946	37.93858	-0.22	3.36	-382.6	3.13799997	-122.46394	37.93859	-0.2	3.36	-393.22	3.15999997	11	7/24/2014	26:25.6

5478	RWS11	-122.4639479	37.93858	-0.19	3.36	-382	3.16800001	-122.46394	37.93859	-0.2	3.36	-392.54	3.15600002	11	7/24/2014	26:25.7
5479	RWS11	-122.4639498	37.93858	-0.22	3.35	-381.4	3.13199992	-122.46395	37.93859	-0.2	3.35	-391.96	3.15399992	11	7/24/2014	26:25.8
5480	RWS11	-122.4639517	37.93858	-0.19	3.35	-380.9	3.16599992	-122.46395	37.93859	-0.15	3.35	-391.36	3.20399992	11	7/24/2014	26:25.9
5481	RWS11	-122.4639545	37.93858	-0.22	3.35	-380.4	3.13300009	-122.46395	37.93859	-0.2	3.35	-390.9	3.15500009	11	7/24/2014	26:26.0
5482	RWS11	-122.4639563	37.93858	-0.19	3.36	-380	3.16899994	-122.46395	37.93859	-0.15	3.36	-390.3	3.20699994	11	7/24/2014	26:26.1
5483	RWS11	-122.4639582	37.93858	-0.19	3.36	-379.4	3.16899994	-122.46395	37.93859	-0.15	3.36	-389.84	3.20699994	11	7/24/2014	26:26.2
5484	RWS11	-122.4639601	37.93858	-0.19	3.36	-379.2	3.16800001	-122.46396	37.9386	-0.15	3.36	-389.43	3.20600002	11	7/24/2014	26:26.3
5485	RWS11	-122.4639621	37.93858	-0.22	3.35	-378.9	3.1289999	-122.46396	37.9386	-0.15	3.35	-389.11	3.2009999	11	7/24/2014	26:26.4
5486	RWS11	-122.463964	37.93858	-0.19	3.34	-378.4	3.15400007	-122.46396	37.9386	-0.12	3.34	-388.69	3.22600008	11	7/24/2014	26:26.5
5487	RWS11	-122.4639659	37.93858	-0.19	3.33	-378	3.13899997	-122.46396	37.9386	-0.15	3.33	-388.22	3.17699997	11	7/24/2014	26:26.6
5488	RWS11	-122.4639678	37.93858	-0.19	3.31	-377.5	3.11999992	-122.46396	37.9386	-0.15	3.31	-387.74	3.15799992	11	7/24/2014	26:26.7
5489	RWS11	-122.4639698	37.93858	-0.19	3.28	-376.9	3.09399989	-122.46397	37.9386	-0.15	3.28	-387.2	3.13199989	11	7/24/2014	26:26.8
5490	RWS11	-122.4639717	37.93858	-0.19	3.25	-376.4	3.06299999	-122.46397	37.9386	-0.15	3.25	-386.57	3.101	11	7/24/2014	26:26.9
5491	RWS11	-122.4639745	37.93859	-0.19	3.21	-376	3.02799991	-122.46397	37.9386	-0.15	3.21	-386.01	3.06599991	11	7/24/2014	26:27.0
5492	RWS11	-122.4639764	37.93859	-0.14	3.18	-375.4	3.04100004	-122.46397	37.9386	-0.15	3.18	-385.41	3.02800004	11	7/24/2014	26:27.1
5493	RWS11	-122.4639783	37.93859	-0.19	3.14	-374.9	2.95100001	-122.46397	37.9386	-0.12	3.14	-384.89	3.02300001	11	7/24/2014	26:27.2
5494	RWS11	-122.4639802	37.93859	-0.19	3.1	-374.4	2.9129999	-122.46398	37.9386	-0.12	3.1	-384.22	2.9849999	11	7/24/2014	26:27.3
5495	RWS11	-122.4639822	37.93859	-0.19	3.07	-373.9	2.87899998	-122.46398	37.9386	-0.15	3.07	-383.72	2.91699998	11	7/24/2014	26:27.4
5496	RWS11	-122.4639842	37.93859	-0.14	3.04	-373.4	2.89900008	-122.46398	37.9386	-0.12	3.04	-383.24	2.92000008	11	7/24/2014	26:27.5
5497	RWS11	-122.4639861	37.93859	-0.19	3.01	-372.9	2.82399991	-122.46398	37.9386	-0.12	3.01	-382.83	2.89599992	11	7/24/2014	26:27.6
5498	RWS11	-122.463988	37.93859	-0.14	2.99	-372.5	2.8560001	-122.46398	37.9386	-0.12	2.99	-382.38	2.8770001	11	7/24/2014	26:27.7
5499	RWS11	-122.4639899	37.93859	-0.19	2.98	-372.1	2.79200009	-122.46399	37.9386	-0.12	2.98	-381.99	2.86400009	11	7/24/2014	26:27.8
5500	RWS11	-122.4639919	37.93859	-0.14	2.97	-371.7	2.83499995	-122.46399	37.9386	-0.06	2.97	-381.53	2.90699995	11	7/24/2014	26:27.9
5501	RWS11	-122.4639947	37.93859	-0.19	2.97	-371.3	2.77900007	-122.46399	37.9386	-0.12	2.97	-381.18	2.85100008	11	7/24/2014	26:28.0
5502	RWS11	-122.4639966	37.93859	-0.19	2.96	-370.9	2.77699998	-122.46399	37.9386	-0.12	2.96	-380.73	2.84899998	11	7/24/2014	26:28.1
5503	RWS11	-122.4639985	37.93859	-0.19	2.96	-370.4	2.77600005	-122.46399	37.9386	-0.12	2.96	-380.15	2.84800006	11	7/24/2014	26:28.2
5504	RWS11	-122.4640004	37.93859	-0.14	2.96	-370	2.82499996	-122.464	37.9386	-0.06	2.96	-379.75	2.89699996	11	7/24/2014	26:28.3
5505	RWS11	-122.4640023	37.93859	-0.19	2.96	-369.5	2.77099994	-122.464	37.9386	-0.12	2.96	-379.28	2.84299994	11	7/24/2014	26:28.4
5506	RWS11	-122.4640043	37.93859	-0.14	2.95	-369.1	2.81799999	-122.464	37.9386	-0.06	2.95	-378.76	2.88999999	11	7/24/2014	26:28.5
5507	RWS11	-122.4640062	37.93859	-0.14	2.95	-368.7	2.81400004	-122.464	37.93861	-0.12	2.95	-378.37	2.83500005	11	7/24/2014	26:28.6
5508	RWS11	-122.4640081	37.93859	-0.14	2.95	-368.2	2.81000009	-122.464	37.93861	-0.12	2.95	-377.81	2.8310001	11	7/24/2014	26:28.7
5509	RWS11	-122.46401	37.93859	-0.19	2.94	-367.8	2.75499991	-122.46401	37.93861	-0.06	2.94	-377.25	2.87799991	11	7/24/2014	26:28.8
5510	RWS11	-122.4640119	37.93859	-0.14	2.94	-367.3	2.80199996	-122.46401	37.93861	-0.06	2.94	-376.79	2.87399996	11	7/24/2014	26:28.9

5511	RWS11	-122.4640147	37.93859	-0.14	2.94	-366.9	2.8000001	-122.46401	37.93861	-0.06	2.94	-376.38	2.87200011	11	7/24/2014	26:29.0
5512	RWS11	-122.4640166	37.93859	-0.14	2.93	-366.3	2.79800001	-122.46401	37.93861	-0.06	2.93	-375.8	2.87000001	11	7/24/2014	26:29.1
5513	RWS11	-122.4640185	37.9386	-0.14	2.93	-366	2.79800001	-122.46401	37.93861	-0.12	2.93	-375.43	2.81900001	11	7/24/2014	26:29.2
5514	RWS11	-122.4640204	37.9386	-0.14	2.94	-365.6	2.8000001	-122.46402	37.93861	-0.12	2.94	-375.06	2.82100011	11	7/24/2014	26:29.3
5515	RWS11	-122.4640223	37.9386	-0.14	2.94	-365.3	2.80299988	-122.46402	37.93861	-0.12	2.94	-374.69	2.82399989	11	7/24/2014	26:29.4
5516	RWS11	-122.4640242	37.9386	-0.14	2.94	-365	2.80700007	-122.46402	37.93861	-0.06	2.94	-374.41	2.87900008	11	7/24/2014	26:29.5
5517	RWS11	-122.4640261	37.9386	-0.14	2.95	-364.6	2.81400004	-122.46402	37.93861	-0.12	2.95	-373.96	2.83500005	11	7/24/2014	26:29.6
5518	RWS11	-122.464028	37.9386	-0.14	2.96	-364.3	2.82100001	-122.46402	37.93861	-0.06	2.96	-373.55	2.89300001	11	7/24/2014	26:29.7
5519	RWS11	-122.4640299	37.9386	-0.14	2.97	-363.9	2.83000007	-122.46403	37.93861	-0.06	2.97	-373.12	2.90200008	11	7/24/2014	26:29.8
5520	RWS11	-122.4640318	37.9386	-0.14	2.98	-363.6	2.84099999	-122.46403	37.93861	-0.06	2.98	-372.62	2.913	11	7/24/2014	26:29.9
5521	RWS11	-122.4640346	37.9386	-0.14	2.99	-363.2	2.85199991	-122.46403	37.93861	-0.06	2.99	-372.23	2.92399991	11	7/24/2014	26:30.0
5522	RWS11	-122.4640364	37.9386	-0.14	3	-362.8	2.86600009	-122.46403	37.93861	-0.06	3	-371.75	2.93800009	11	7/24/2014	26:30.1
5523	RWS11	-122.4640383	37.9386	-0.14	3.02	-362.5	2.88000003	-122.46403	37.93861	-0.06	3.02	-371.27	2.95200003	11	7/24/2014	26:30.2
5524	RWS11	-122.4640402	37.9386	-0.14	3.03	-362.1	2.89600006	-122.46404	37.93861	-0.06	3.03	-370.84	2.96800006	11	7/24/2014	26:30.3
5525	RWS11	-122.4640421	37.9386	-0.14	3.05	-361.7	2.91500011	-122.46404	37.93861	-0.03	3.05	-370.41	3.02100012	11	7/24/2014	26:30.4
5526	RWS11	-122.464044	37.9386	-0.14	3.07	-361.4	2.93500009	-122.46404	37.93861	-0.06	3.07	-369.93	3.0070001	11	7/24/2014	26:30.5
5527	RWS11	-122.4640459	37.9386	-0.14	3.09	-361.1	2.95699993	-122.46404	37.93861	-0.06	3.09	-369.46	3.02899993	11	7/24/2014	26:30.6
5528	RWS11	-122.4640479	37.9386	-0.1	3.12	-360.7	3.01400001	-122.46404	37.93861	-0.06	3.12	-369.03	3.05100001	11	7/24/2014	26:30.7
5529	RWS11	-122.4640497	37.9386	-0.14	3.14	-360.4	3.00100008	-122.46404	37.93862	-0.03	3.14	-368.53	3.10700008	11	7/24/2014	26:30.8
5530	RWS11	-122.4640516	37.9386	-0.1	3.16	-359.9	3.05600006	-122.46405	37.93862	-0.03	3.16	-368.01	3.12700007	11	7/24/2014	26:30.9
5531	RWS11	-122.4640544	37.9386	-0.14	3.17	-359.6	3.03800002	-122.46405	37.93862	-0.06	3.17	-367.55	3.11000002	11	7/24/2014	26:31.0
5532	RWS11	-122.4640563	37.9386	-0.1	3.19	-359.2	3.08699996	-122.46405	37.93862	-0.03	3.19	-367.21	3.15799996	11	7/24/2014	26:31.1
5533	RWS11	-122.4640582	37.9386	-0.14	3.2	-358.8	3.06499997	-122.46405	37.93862	-0.03	3.2	-366.71	3.17099998	11	7/24/2014	26:31.2
5534	RWS11	-122.4640601	37.9386	-0.1	3.21	-358.4	3.10999996	-122.46406	37.93862	-0.03	3.21	-366.25	3.18099997	11	7/24/2014	26:31.3
5535	RWS11	-122.4640621	37.93861	-0.1	3.22	-358	3.11900003	-122.46406	37.93862	-0.03	3.22	-365.8	3.19000003	11	7/24/2014	26:31.4
5536	RWS11	-122.464064	37.93861	-0.1	3.23	-357.5	3.12699992	-122.46406	37.93862	0.02	3.23	-365.17	3.24899993	11	7/24/2014	26:31.5
5537	RWS11	-122.4640659	37.93861	-0.14	3.23	-357.1	3.09899989	-122.46406	37.93862	-0.03	3.23	-364.63	3.2049999	11	7/24/2014	26:31.6
5538	RWS11	-122.4640678	37.93861	-0.14	3.24	-356.7	3.10499993	-122.46406	37.93862	-0.03	3.24	-364.15	3.21099994	11	7/24/2014	26:31.7
5539	RWS11	-122.4640697	37.93861	-0.14	3.25	-356.2	3.11099997	-122.46406	37.93862	-0.03	3.25	-363.55	3.21699998	11	7/24/2014	26:31.8
5540	RWS11	-122.4640716	37.93861	-0.1	3.25	-355.7	3.15100009	-122.46407	37.93862	-0.03	3.25	-362.97	3.22200009	11	7/24/2014	26:31.9
5541	RWS11	-122.4640744	37.93861	-0.14	3.26	-355.1	3.12099996	-122.46407	37.93862	-0.03	3.26	-362.32	3.22699997	11	7/24/2014	26:32.0
5542	RWS11	-122.4640763	37.93861	-0.14	3.26	-354.6	3.12499991	-122.46407	37.93862	-0.03	3.26	-361.67	3.23099992	11	7/24/2014	26:32.1
5543	RWS11	-122.4640782	37.93861	-0.14	3.27	-353.9	3.1290001	-122.46407	37.93862	-0.03	3.27	-361.06	3.23500011	11	7/24/2014	26:32.2

5544	RWS11	-122.4640801	37.93861	-0.1	3.27	-353.4	3.16899998	-122.46408	37.93862	-0.03	3.27	-360.3	3.23999998	11	7/24/2014	26:32.3
5545	RWS11	-122.464082	37.93861	-0.14	3.28	-352.8	3.14099994	-122.46408	37.93862	-0.03	3.28	-359.81	3.24699995	11	7/24/2014	26:32.4
5546	RWS11	-122.4640839	37.93861	-0.1	3.29	-352.3	3.18500001	-122.46408	37.93862	-0.03	3.29	-359.24	3.25600001	11	7/24/2014	26:32.5
5547	RWS11	-122.4640858	37.93861	-0.14	3.3	-351.8	3.16200009	-122.46408	37.93862	-0.03	3.3	-358.84	3.2680001	11	7/24/2014	26:32.6
5548	RWS11	-122.4640878	37.93861	-0.1	3.31	-351.3	3.21100003	-122.46408	37.93862	-0.03	3.31	-358.25	3.28200004	11	7/24/2014	26:32.7
5549	RWS11	-122.4640896	37.93861	-0.14	3.33	-350.8	3.1909999	-122.46408	37.93862	-0.03	3.33	-357.84	3.2969999	11	7/24/2014	26:32.8
5550	RWS11	-122.4640916	37.93861	-0.1	3.34	-350.3	3.24199993	-122.46409	37.93863	-0.03	3.34	-357.4	3.31299994	11	7/24/2014	26:32.9
5551	RWS11	-122.4640944	37.93861	-0.1	3.36	-349.8	3.25799996	-122.46409	37.93863	-0.03	3.36	-356.88	3.32899997	11	7/24/2014	26:33.0
5552	RWS11	-122.4640963	37.93861	-0.1	3.37	-349.3	3.2719999	-122.46409	37.93863	-0.03	3.37	-356.37	3.34299991	11	7/24/2014	26:33.1
5553	RWS11	-122.4640982	37.93861	-0.14	3.38	-348.8	3.24899998	-122.46409	37.93863	-0.03	3.38	-355.91	3.35499999	11	7/24/2014	26:33.2
5554	RWS11	-122.4641002	37.93861	-0.1	3.39	-348.4	3.29300005	-122.4641	37.93863	-0.03	3.39	-355.5	3.36400005	11	7/24/2014	26:33.3
5555	RWS11	-122.4641021	37.93861	-0.14	3.4	-347.9	3.26299992	-122.4641	37.93863	-0.03	3.4	-355.07	3.36899993	11	7/24/2014	26:33.4
5556	RWS11	-122.4641041	37.93862	-0.1	3.4	-347.5	3.29900009	-122.4641	37.93863	-0.03	3.4	-354.72	3.3700001	11	7/24/2014	26:33.5
5557	RWS11	-122.464106	37.93862	-0.14	3.4	-347	3.2599999	-122.4641	37.93863	-0.03	3.4	-354.35	3.36599991	11	7/24/2014	26:33.6
5558	RWS11	-122.464108	37.93862	-0.1	3.39	-346.5	3.28600008	-122.4641	37.93863	0.02	3.39	-354.01	3.40800008	11	7/24/2014	26:33.7
5559	RWS11	-122.4641099	37.93862	-0.14	3.38	-346.1	3.23899999	-122.4641	37.93863	-0.03	3.38	-353.46	3.345	11	7/24/2014	26:33.8
5560	RWS11	-122.4641119	37.93862	-0.1	3.36	-345.6	3.25899989	-122.46411	37.93863	-0.03	3.36	-353.12	3.3299999	11	7/24/2014	26:33.9
5561	RWS11	-122.4641147	37.93862	-0.14	3.34	-345.1	3.206	-122.46411	37.93863	-0.03	3.34	-352.73	3.31200001	11	7/24/2014	26:34.0
5562	RWS11	-122.4641167	37.93862	-0.1	3.32	-344.7	3.22100002	-122.46411	37.93863	-0.03	3.32	-352.36	3.29200003	11	7/24/2014	26:34.1
5563	RWS11	-122.4641186	37.93862	-0.14	3.3	-344.3	3.16500011	-122.46411	37.93863	-0.06	3.3	-351.97	3.23700012	11	7/24/2014	26:34.2
5564	RWS11	-122.4641205	37.93862	-0.1	3.28	-343.8	3.17899997	-122.46412	37.93863	-0.03	3.28	-351.63	3.24999997	11	7/24/2014	26:34.3
5565	RWS11	-122.4641225	37.93862	-0.1	3.26	-343.4	3.15800006	-122.46412	37.93863	-0.03	3.26	-351.24	3.22900006	11	7/24/2014	26:34.4
5566	RWS11	-122.4641245	37.93862	-0.1	3.24	-342.9	3.13800008	-122.46412	37.93863	-0.03	3.24	-350.87	3.20900008	11	7/24/2014	26:34.5
5567	RWS11	-122.4641264	37.93862	-0.14	3.22	-342.5	3.08499995	-122.46412	37.93863	-0.03	3.22	-350.5	3.19099996	11	7/24/2014	26:34.6
5568	RWS11	-122.4641284	37.93862	-0.1	3.2	-342	3.10399992	-122.46412	37.93863	-0.03	3.2	-350.11	3.17499992	11	7/24/2014	26:34.7
5569	RWS11	-122.4641303	37.93862	-0.14	3.19	-341.5	3.05499998	-122.46413	37.93863	-0.06	3.19	-349.7	3.12699998	11	7/24/2014	26:34.8
5570	RWS11	-122.4641322	37.93862	-0.1	3.18	-341.1	3.07699997	-122.46413	37.93863	-0.03	3.18	-349.38	3.14799997	11	7/24/2014	26:34.9
5571	RWS11	-122.4641351	37.93862	-0.1	3.17	-340.6	3.06499989	-122.46413	37.93864	-0.03	3.17	-348.94	3.13599989	11	7/24/2014	26:35.0
5572	RWS11	-122.464137	37.93862	-0.1	3.15	-340.1	3.05300004	-122.46413	37.93864	-0.03	3.15	-348.55	3.12400004	11	7/24/2014	26:35.1
5573	RWS11	-122.4641389	37.93862	-0.1	3.14	-339.7	3.04000003	-122.46413	37.93864	-0.06	3.14	-348.19	3.07700003	11	7/24/2014	26:35.2
5574	RWS11	-122.4641408	37.93862	-0.1	3.13	-339.2	3.02700002	-122.46414	37.93864	-0.03	3.13	-347.8	3.09800002	11	7/24/2014	26:35.3
5575	RWS11	-122.4641428	37.93862	-0.1	3.12	-338.8	3.01400001	-122.46414	37.93864	-0.06	3.12	-347.45	3.05100001	11	7/24/2014	26:35.4
5576	RWS11	-122.4641447	37.93863	-0.1	3.1	-338.3	3.00199992	-122.46414	37.93864	-0.06	3.1	-347.04	3.03899992	11	7/24/2014	26:35.5

5577	RWS11	-122.4641467	37.93863	-0.1	3.09	-337.8	2.99000008	-122.46414	37.93864	-0.06	3.09	-346.72	3.02700008	11	7/24/2014	26:35.6
5578	RWS11	-122.4641486	37.93863	-0.1	3.08	-337.4	2.97799999	-122.46414	37.93864	-0.06	3.08	-346.29	3.01499999	11	7/24/2014	26:35.7
5579	RWS11	-122.4641505	37.93863	-0.14	3.07	-337	2.933	-122.46415	37.93864	-0.06	3.07	-345.87	3.005	11	7/24/2014	26:35.8
5580	RWS11	-122.4641524	37.93863	-0.1	3.06	-336.7	2.96000011	-122.46415	37.93864	-0.06	3.06	-345.53	2.99700011	11	7/24/2014	26:35.9
5581	RWS11	-122.4641553	37.93863	-0.1	3.06	-336.3	2.95400006	-122.46415	37.93864	-0.06	3.06	-345.16	2.99100006	11	7/24/2014	26:36.0
5582	RWS11	-122.4641572	37.93863	-0.1	3.05	-336	2.9529999	-122.46415	37.93864	-0.03	3.05	-344.81	3.0239999	11	7/24/2014	26:36.1
5583	RWS11	-122.4641591	37.93863	-0.1	3.06	-335.6	2.95700008	-122.46415	37.93864	-0.06	3.06	-344.46	2.99400008	11	7/24/2014	26:36.2
5584	RWS11	-122.464161	37.93863	-0.1	3.07	-335.2	2.96599991	-122.46416	37.93864	-0.06	3.07	-344.03	3.00299991	11	7/24/2014	26:36.3
5585	RWS11	-122.4641629	37.93863	-0.1	3.08	-334.8	2.98100001	-122.46416	37.93864	-0.06	3.08	-343.64	3.01800001	11	7/24/2014	26:36.4
5586	RWS11	-122.4641648	37.93863	-0.1	3.1	-334.4	3.00000007	-122.46416	37.93864	-0.06	3.1	-343.17	3.03700007	11	7/24/2014	26:36.5
5587	RWS11	-122.4641667	37.93863	-0.1	3.13	-333.9	3.024	-122.46416	37.93864	-0.06	3.13	-342.69	3.061	11	7/24/2014	26:36.6
5588	RWS11	-122.4641687	37.93863	-0.1	3.15	-333.4	3.05099995	-122.46416	37.93864	-0.06	3.15	-342.24	3.08799995	11	7/24/2014	26:36.7
5589	RWS11	-122.4641706	37.93863	-0.1	3.18	-332.9	3.08099992	-122.46417	37.93864	-0.06	3.18	-341.74	3.11799992	11	7/24/2014	26:36.8
5590	RWS11	-122.4641725	37.93863	-0.1	3.21	-332.5	3.11200006	-122.46417	37.93865	-0.06	3.21	-341.32	3.14900006	11	7/24/2014	26:36.9
5591	RWS11	-122.4641753	37.93863	-0.14	3.24	-332.1	3.10700002	-122.46417	37.93865	-0.06	3.24	-340.92	3.17900003	11	7/24/2014	26:37.0
5592	RWS11	-122.4641772	37.93863	-0.1	3.27	-331.6	3.1699999	-122.46417	37.93865	-0.06	3.27	-340.57	3.20699991	11	7/24/2014	26:37.1
5593	RWS11	-122.4641791	37.93863	-0.14	3.29	-331.3	3.1579999	-122.46417	37.93865	-0.06	3.29	-340.18	3.22999991	11	7/24/2014	26:37.2
5594	RWS11	-122.464181	37.93863	-0.1	3.31	-330.9	3.21000011	-122.46418	37.93865	-0.06	3.31	-339.84	3.24700011	11	7/24/2014	26:37.3
5595	RWS11	-122.4641829	37.93864	-0.14	3.32	-330.5	3.18699995	-122.46418	37.93865	-0.06	3.32	-339.55	3.25899995	11	7/24/2014	26:37.4
5596	RWS11	-122.4641848	37.93864	-0.1	3.33	-330.2	3.22700007	-122.46418	37.93865	-0.03	3.33	-339.21	3.29800007	11	7/24/2014	26:37.5
5597	RWS11	-122.4641868	37.93864	-0.1	3.33	-329.9	3.2259999	-122.46418	37.93865	-0.06	3.33	-338.88	3.2629999	11	7/24/2014	26:37.6
5598	RWS11	-122.4641887	37.93864	-0.1	3.32	-329.6	3.22100002	-122.46418	37.93865	-0.06	3.32	-338.56	3.25800002	11	7/24/2014	26:37.7
5599	RWS11	-122.4641906	37.93864	-0.1	3.31	-329.4	3.21199996	-122.46419	37.93865	-0.06	3.31	-338.28	3.24899996	11	7/24/2014	26:37.8
5600	RWS11	-122.4641925	37.93864	-0.1	3.3	-329.1	3.20000011	-122.46419	37.93865	-0.06	3.3	-337.98	3.23700012	11	7/24/2014	26:37.9
5601	RWS11	-122.4641953	37.93864	-0.1	3.29	-328.8	3.18599994	-122.46419	37.93865	-0.03	3.29	-337.63	3.25699994	11	7/24/2014	26:38.0
5602	RWS11	-122.4641972	37.93864	-0.1	3.27	-328.4	3.16899998	-122.46419	37.93865	-0.03	3.27	-337.37	3.23999998	11	7/24/2014	26:38.1
5603	RWS11	-122.4641991	37.93864	-0.1	3.25	-328.1	3.14999992	-122.46419	37.93865	-0.06	3.25	-337.02	3.18699992	11	7/24/2014	26:38.2
5604	RWS11	-122.464201	37.93864	-0.1	3.23	-327.8	3.12800009	-122.4642	37.93865	-0.03	3.23	-336.76	3.19900009	11	7/24/2014	26:38.3
5605	RWS11	-122.4642029	37.93864	-0.1	3.2	-327.5	3.10399992	-122.4642	37.93865	-0.03	3.2	-336.44	3.17499992	11	7/24/2014	26:38.4
5606	RWS11	-122.4642048	37.93864	-0.1	3.18	-327.2	3.07699997	-122.4642	37.93865	-0.03	3.18	-336.09	3.14799997	11	7/24/2014	26:38.5
5607	RWS11	-122.4642067	37.93864	-0.14	3.15	-326.8	3.01299992	-122.4642	37.93865	-0.06	3.15	-335.75	3.08499993	11	7/24/2014	26:38.6
5608	RWS11	-122.4642087	37.93864	-0.1	3.12	-326.5	3.01700003	-122.4642	37.93865	-0.03	3.12	-335.42	3.08800003	11	7/24/2014	26:38.7
5609	RWS11	-122.4642106	37.93864	-0.1	3.09	-326.2	2.98599989	-122.46421	37.93866	-0.06	3.09	-335.1	3.02299989	11	7/24/2014	26:38.8

5610	RWS11	-122.4642125	37.93864	-0.1	3.06	-325.8	2.95700008	-122.46421	37.93866	-0.03	3.06	-334.75	3.02800009	11	7/24/2014	26:38.9
5611	RWS11	-122.4642153	37.93864	-0.1	3.03	-325.4	2.93100006	-122.46421	37.93866	-0.06	3.03	-334.3	2.96800006	11	7/24/2014	26:39.0
5612	RWS11	-122.4642171	37.93864	-0.1	3.01	-324.9	2.91100008	-122.46421	37.93866	-0.03	3.01	-333.93	2.98200008	11	7/24/2014	26:39.1
5613	RWS11	-122.464219	37.93864	-0.1	3	-324.5	2.8969999	-122.46421	37.93866	-0.03	3	-333.59	2.96799991	11	7/24/2014	26:39.2
5614	RWS11	-122.4642209	37.93865	-0.1	2.99	-324.1	2.88999993	-122.46422	37.93866	-0.03	2.99	-333.15	2.96099994	11	7/24/2014	26:39.3
5615	RWS11	-122.4642228	37.93865	-0.14	2.99	-323.6	2.8560001	-122.46422	37.93866	-0.03	2.99	-332.76	2.9620001	11	7/24/2014	26:39.4
5616	RWS11	-122.4642247	37.93865	-0.1	3	-323.2	2.89999992	-122.46422	37.93866	-0.03	3	-332.4	2.97099993	11	7/24/2014	26:39.5
5617	RWS11	-122.4642266	37.93865	-0.1	3.02	-322.8	2.91699988	-122.46422	37.93866	-0.03	3.02	-332.03	2.98799989	11	7/24/2014	26:39.6
5618	RWS11	-122.4642285	37.93865	-0.1	3.04	-322.5	2.93899996	-122.46422	37.93866	-0.03	3.04	-331.7	3.00999996	11	7/24/2014	26:39.7
5619	RWS11	-122.4642303	37.93865	-0.14	3.07	-322.2	2.9309999	-122.46422	37.93866	-0.03	3.07	-331.38	3.03699991	11	7/24/2014	26:39.8
5620	RWS11	-122.4642322	37.93865	-0.1	3.1	-321.8	2.99599988	-122.46423	37.93866	-0.03	3.1	-331.05	3.06699988	11	7/24/2014	26:39.9
5621	RWS11	-122.4642349	37.93865	-0.1	3.13	-321.5	3.02600009	-122.46423	37.93866	-0.06	3.13	-330.73	3.06300009	11	7/24/2014	26:40.0
5622	RWS11	-122.4642368	37.93865	-0.1	3.16	-321.2	3.05699999	-122.46423	37.93866	-0.03	3.16	-330.43	3.12799999	11	7/24/2014	26:40.1
5623	RWS11	-122.4642386	37.93865	-0.1	3.19	-320.9	3.08500011	-122.46423	37.93866	-0.03	3.19	-330.19	3.15600011	11	7/24/2014	26:40.2
5624	RWS11	-122.4642404	37.93865	-0.1	3.21	-320.7	3.11099989	-122.46423	37.93866	-0.03	3.21	-329.93	3.18199989	11	7/24/2014	26:40.3
5625	RWS11	-122.4642423	37.93865	-0.14	3.24	-320.5	3.10000005	-122.46424	37.93866	-0.03	3.24	-329.73	3.20600006	11	7/24/2014	26:40.4
5626	RWS11	-122.4642441	37.93865	-0.1	3.26	-320.2	3.15699989	-122.46424	37.93866	0.02	3.26	-329.52	3.2789999	11	7/24/2014	26:40.5
5627	RWS11	-122.464246	37.93865	-0.1	3.28	-320	3.17599995	-122.46424	37.93866	-0.06	3.28	-329.3	3.21299995	11	7/24/2014	26:40.6
5628	RWS11	-122.4642478	37.93865	-0.1	3.3	-319.8	3.19400007	-122.46424	37.93867	0.02	3.3	-329.09	3.31600008	11	7/24/2014	26:40.7
5629	RWS11	-122.4642497	37.93865	-0.1	3.31	-319.6	3.21199996	-122.46424	37.93867	-0.03	3.31	-328.89	3.28299996	11	7/24/2014	26:40.8
5630	RWS11	-122.4642515	37.93865	-0.1	3.33	-319.3	3.22899992	-122.46425	37.93867	-0.03	3.33	-328.64	3.29999992	11	7/24/2014	26:40.9
5631	RWS11	-122.4642542	37.93865	-0.14	3.35	-319.1	3.20999995	-122.46425	37.93867	-0.03	3.35	-328.43	3.31599996	11	7/24/2014	26:41.0
5632	RWS11	-122.4642561	37.93865	-0.1	3.36	-318.9	3.26099999	-122.46425	37.93867	-0.03	3.36	-328.2	3.33199999	11	7/24/2014	26:41.1
5633	RWS11	-122.4642579	37.93866	-0.14	3.38	-318.7	3.23899999	-122.46425	37.93867	-0.03	3.38	-328.05	3.345	11	7/24/2014	26:41.2
5634	RWS11	-122.4642598	37.93866	-0.1	3.39	-318.6	3.28499991	-122.46425	37.93867	-0.03	3.39	-327.85	3.35599992	11	7/24/2014	26:41.3
5635	RWS11	-122.4642617	37.93866	-0.1	3.39	-318.4	3.29300005	-122.46426	37.93867	-0.03	3.39	-327.66	3.36400005	11	7/24/2014	26:41.4
5636	RWS11	-122.4642635	37.93866	-0.1	3.4	-318.2	3.29799993	-122.46426	37.93867	-0.03	3.4	-327.51	3.36899993	11	7/24/2014	26:41.5
5637	RWS11	-122.4642654	37.93866	-0.14	3.4	-318	3.26299992	-122.46426	37.93867	-0.03	3.4	-327.31	3.36899993	11	7/24/2014	26:41.6
5638	RWS11	-122.4642673	37.93866	-0.1	3.39	-317.8	3.29399998	-122.46426	37.93867	-0.03	3.39	-327.14	3.36499998	11	7/24/2014	26:41.7
5639	RWS11	-122.4642692	37.93866	-0.1	3.39	-317.6	3.28700001	-122.46426	37.93867	-0.03	3.39	-326.94	3.35800001	11	7/24/2014	26:41.8
5640	RWS11	-122.464271	37.93866	-0.1	3.38	-317.4	3.27700002	-122.46427	37.93867	-0.03	3.38	-326.79	3.34800002	11	7/24/2014	26:41.9
5641	RWS11	-122.4642738	37.93866	-0.14	3.37	-317.2	3.2310001	-122.46427	37.93867	-0.03	3.37	-326.64	3.3370001	11	7/24/2014	26:42.0
5642	RWS11	-122.4642757	37.93866	-0.1	3.36	-317	3.25499994	-122.46427	37.93867	0.02	3.36	-326.38	3.37699995	11	7/24/2014	26:42.1

5643	RWS11	-122.4642775	37.93866	-0.1	3.35	-316.8	3.24499995	-122.46427	37.93867	-0.03	3.35	-326.16	3.31599996	11	7/24/2014	26:42.2
5644	RWS11	-122.4642794	37.93866	-0.1	3.34	-316.6	3.23700006	-122.46427	37.93867	0.02	3.34	-325.93	3.35900006	11	7/24/2014	26:42.3
5645	RWS11	-122.4642813	37.93866	-0.1	3.33	-316.4	3.22899992	-122.46428	37.93867	-0.03	3.33	-325.69	3.29999992	11	7/24/2014	26:42.4
5646	RWS11	-122.4642832	37.93866	-0.1	3.32	-316.2	3.22299988	-122.46428	37.93868	0.02	3.32	-325.48	3.34499988	11	7/24/2014	26:42.5
5647	RWS11	-122.4642851	37.93866	-0.14	3.32	-316	3.1809999	-122.46428	37.93868	-0.03	3.32	-325.3	3.28699991	11	7/24/2014	26:42.6
5648	RWS11	-122.464287	37.93866	-0.1	3.31	-315.8	3.20899994	-122.46428	37.93868	0.02	3.31	-325.11	3.33099994	11	7/24/2014	26:42.7
5649	RWS11	-122.4642889	37.93866	-0.1	3.3	-315.6	3.20100004	-122.46428	37.93868	0.02	3.3	-324.96	3.32300005	11	7/24/2014	26:42.8
5650	RWS11	-122.4642908	37.93866	-0.1	3.29	-315.5	3.19299991	-122.46428	37.93868	0.02	3.29	-324.78	3.31499991	11	7/24/2014	26:42.9
5651	RWS11	-122.4642936	37.93867	-0.1	3.29	-315.3	3.18500001	-122.46429	37.93868	0.02	3.29	-324.63	3.30700001	11	7/24/2014	26:43.0
5652	RWS11	-122.4642955	37.93867	-0.1	3.28	-315.2	3.17700011	-122.46429	37.93868	0.06	3.28	-324.5	3.33300012	11	7/24/2014	26:43.1
5653	RWS11	-122.4642974	37.93867	-0.1	3.27	-315.1	3.16800005	-122.46429	37.93868	0.02	3.27	-324.37	3.29000005	11	7/24/2014	26:43.2
5654	RWS11	-122.4642993	37.93867	-0.1	3.26	-315	3.15800006	-122.46429	37.93868	0.02	3.26	-324.31	3.28000006	11	7/24/2014	26:43.3
5655	RWS11	-122.4643012	37.93867	-0.1	3.25	-314.9	3.14599998	-122.4643	37.93868	0.02	3.25	-324.2	3.26799998	11	7/24/2014	26:43.4
5656	RWS11	-122.4643031	37.93867	-0.1	3.23	-314.8	3.13200004	-122.4643	37.93868	0.06	3.23	-324.16	3.28800004	11	7/24/2014	26:43.5
5657	RWS11	-122.464305	37.93867	-0.14	3.21	-314.8	3.07899991	-122.4643	37.93868	0.02	3.21	-324.05	3.23599991	11	7/24/2014	26:43.6
5658	RWS11	-122.464307	37.93867	-0.1	3.2	-314.6	3.0950001	-122.4643	37.93868	0.06	3.2	-323.96	3.2510001	11	7/24/2014	26:43.7
5659	RWS11	-122.4643089	37.93867	-0.1	3.17	-314.5	3.07300002	-122.4643	37.93868	0.06	3.17	-323.88	3.22900002	11	7/24/2014	26:43.8
5660	RWS11	-122.4643108	37.93867	-0.1	3.15	-314.4	3.05200011	-122.4643	37.93868	0.06	3.15	-323.71	3.20800012	11	7/24/2014	26:43.9
5661	RWS11	-122.4643136	37.93867	-0.1	3.13	-314.3	3.03099997	-122.46431	37.93868	0.02	3.13	-323.62	3.15299997	11	7/24/2014	26:44.0
5662	RWS11	-122.4643156	37.93867	-0.1	3.11	-314.2	3.01199991	-122.46431	37.93868	0.06	3.11	-323.54	3.16799992	11	7/24/2014	26:44.1
5663	RWS11	-122.4643175	37.93867	-0.1	3.1	-314	2.99599988	-122.46431	37.93869	0.06	3.1	-323.46	3.15199988	11	7/24/2014	26:44.2
5664	RWS11	-122.4643194	37.93867	-0.1	3.08	-313.9	2.98199994	-122.46431	37.93869	0.06	3.08	-323.38	3.13799994	11	7/24/2014	26:44.3
5665	RWS11	-122.4643214	37.93867	-0.1	3.07	-313.8	2.97199995	-122.46432	37.93869	0.06	3.07	-323.29	3.12799995	11	7/24/2014	26:44.4
5666	RWS11	-122.4643233	37.93867	-0.1	3.07	-313.7	2.96400005	-122.46432	37.93869	0.06	3.07	-323.23	3.12000006	11	7/24/2014	26:44.5
5667	RWS11	-122.4643253	37.93868	-0.14	3.06	-313.6	2.9250001	-122.46432	37.93869	0.02	3.06	-323.17	3.08200011	11	7/24/2014	26:44.6
5668	RWS11	-122.4643273	37.93868	-0.1	3.06	-313.5	2.95800001	-122.46432	37.93869	0.06	3.06	-323.1	3.11400001	11	7/24/2014	26:44.7
5669	RWS11	-122.4643293	37.93868	-0.1	3.06	-313.4	2.95899994	-122.46432	37.93869	0.02	3.06	-323.1	3.08099994	11	7/24/2014	26:44.8
5670	RWS11	-122.4643313	37.93868	-0.1	3.06	-313.4	2.96299989	-122.46433	37.93869	0.02	3.06	-323.06	3.08499989	11	7/24/2014	26:44.9
5671	RWS11	-122.4643343	37.93868	-0.1	3.07	-313.4	2.968	-122.46433	37.93869	0.02	3.07	-323.06	3.09000001	11	7/24/2014	26:45.0
5672	RWS11	-122.4643363	37.93868	-0.1	3.08	-313.4	2.97499997	-122.46433	37.93869	0.06	3.08	-323.1	3.13099997	11	7/24/2014	26:45.1
5673	RWS11	-122.4643384	37.93868	-0.1	3.09	-313.4	2.98400003	-122.46433	37.93869	0.06	3.09	-323.11	3.14000004	11	7/24/2014	26:45.2
5674	RWS11	-122.4643404	37.93868	-0.1	3.09	-313.4	2.9930001	-122.46433	37.93869	0.06	3.09	-323.14	3.1490001	11	7/24/2014	26:45.3
5675	RWS11	-122.4643425	37.93868	-0.1	3.1	-313.4	3.00099999	-122.46434	37.93869	0.02	3.1	-323.19	3.123	11	7/24/2014	26:45.4

5676	RWS11	-122.4643446	37.93868	-0.1	3.11	-313.5	3.00899989	-122.46434	37.93869	0.06	3.11	-323.3	3.16499989	11	7/24/2014	26:45.5
5677	RWS11	-122.4643467	37.93868	-0.1	3.12	-313.5	3.01499993	-122.46434	37.93869	0.06	3.12	-323.39	3.17099994	11	7/24/2014	26:45.6
5678	RWS11	-122.4643488	37.93868	-0.05	3.12	-313.6	3.06899996	-122.46434	37.93869	0.02	3.12	-323.54	3.13999996	11	7/24/2014	26:45.7
5679	RWS11	-122.4643508	37.93868	-0.1	3.12	-313.7	3.02000005	-122.46434	37.9387	0.06	3.12	-323.67	3.17600005	11	7/24/2014	26:45.8
5680	RWS11	-122.4643527	37.93868	-0.1	3.12	-313.8	3.02000005	-122.46435	37.9387	0.06	3.12	-323.87	3.17600005	11	7/24/2014	26:45.9
5681	RWS11	-122.4643556	37.93868	-0.1	3.12	-313.9	3.01700003	-122.46435	37.9387	0.02	3.12	-324.03	3.13900003	11	7/24/2014	26:46.0
5682	RWS11	-122.4643574	37.93868	-0.1	3.11	-314	3.01199991	-122.46435	37.9387	0.06	3.11	-324.26	3.16799992	11	7/24/2014	26:46.1
5683	RWS11	-122.4643592	37.93869	-0.1	3.1	-314	3.00199992	-122.46435	37.9387	0.02	3.1	-324.47	3.12399993	11	7/24/2014	26:46.2
5684	RWS11	-122.464361	37.93869	-0.1	3.09	-314.1	2.98799998	-122.46435	37.9387	0.06	3.09	-324.69	3.14399999	11	7/24/2014	26:46.3
5685	RWS11	-122.4643628	37.93869	-0.1	3.07	-314	2.9700001	-122.46436	37.9387	-0.03	3.07	-324.86	3.0410001	11	7/24/2014	26:46.4
5686	RWS11	-122.4643646	37.93869	-0.1	3.05	-314	2.94800002	-122.46436	37.9387	0.06	3.05	-325.01	3.10400002	11	7/24/2014	26:46.5
5687	RWS11	-122.4643663	37.93869	-0.14	3.02	-313.9	2.88699999	-122.46436	37.9387	0.02	3.02	-325.14	3.044	11	7/24/2014	26:46.6
5688	RWS11	-122.4643682	37.93869	-0.1	3	-313.7	2.89500005	-122.46436	37.9387	0.06	3	-325.25	3.05100005	11	7/24/2014	26:46.7
5689	RWS11	-122.46437	37.93869	-0.1	2.97	-313.6	2.8680001	-122.46436	37.9387	0.02	2.97	-325.34	2.9900001	11	7/24/2014	26:46.8
5690	RWS11	-122.4643718	37.93869	-0.1	2.94	-313.5	2.843	-122.46437	37.9387	0.09	2.94	-325.42	3.03200001	11	7/24/2014	26:46.9
5691	RWS11	-122.4643728	37.93869	-0.1	2.92	-313.4	2.82099993	-122.46437	37.9387	0.06	2.92	-325.43	2.97699993	11	7/24/2014	26:47.0
5692	RWS11	-122.4643756	37.93869	-0.1	2.9	-313.1	2.80399997	-122.46437	37.9387	0.06	2.9	-325.43	2.95999997	11	7/24/2014	26:47.1
5693	RWS11	-122.4643775	37.93869	-0.1	2.89	-312.9	2.79000003	-122.46437	37.9387	0.02	2.89	-325.38	2.91200003	11	7/24/2014	26:47.2
5694	RWS11	-122.4643794	37.93869	-0.1	2.88	-312.6	2.78099997	-122.46437	37.9387	0.06	2.88	-325.28	2.93699997	11	7/24/2014	26:47.3
5695	RWS11	-122.4643804	37.93869	-0.1	2.88	-312.2	2.77499992	-122.46437	37.9387	0.06	2.88	-325.04	2.93099993	11	7/24/2014	26:47.4
5696	RWS11	-122.4643832	37.93869	-0.1	2.87	-311.7	2.76899988	-122.46438	37.93871	0.09	2.87	-324.71	2.95799989	11	7/24/2014	26:47.5
5697	RWS11	-122.4643851	37.93869	-0.1	2.87	-311.1	2.76400001	-122.46438	37.93871	0.06	2.87	-324.3	2.92000001	11	7/24/2014	26:47.6
5698	RWS11	-122.464387	37.93869	-0.1	2.86	-310.5	2.75899989	-122.46438	37.93871	0.09	2.86	-323.85	2.94799989	11	7/24/2014	26:47.7
5699	RWS11	-122.464388	37.93869	-0.1	2.86	-309.8	2.75400002	-122.46438	37.93871	0.06	2.86	-323.33	2.91000002	11	7/24/2014	26:47.8
5700	RWS11	-122.4643908	37.9387	-0.1	2.85	-309.1	2.74700005	-122.46438	37.93871	0.09	2.85	-322.74	2.93600005	11	7/24/2014	26:47.9
5701	RWS11	-122.4643936	37.9387	-0.14	2.84	-308.5	2.706	-122.46439	37.93871	0.06	2.84	-322.16	2.89700001	11	7/24/2014	26:48.0
5702	RWS11	-122.4643955	37.9387	-0.1	2.84	-307.8	2.73499996	-122.46439	37.93871	0.14	2.84	-321.53	2.97499996	11	7/24/2014	26:48.1
5703	RWS11	-122.4643974	37.9387	-0.1	2.83	-307	2.73000009	-122.46439	37.93871	0.09	2.83	-320.98	2.91900009	11	7/24/2014	26:48.2
5704	RWS11	-122.4643993	37.9387	-0.1	2.83	-306.3	2.72700007	-122.46439	37.93871	0.09	2.83	-320.28	2.91600007	11	7/24/2014	26:48.3
5705	RWS11	-122.4644011	37.9387	-0.14	2.83	-305.5	2.68999997	-122.46439	37.93871	0.06	2.83	-319.59	2.88099997	11	7/24/2014	26:48.4
5706	RWS11	-122.4644031	37.9387	-0.1	2.83	-304.7	2.72499997	-122.4644	37.93871	0.09	2.83	-318.83	2.91399997	11	7/24/2014	26:48.5
5707	RWS11	-122.464405	37.9387	-0.1	2.83	-303.9	2.72700007	-122.4644	37.93871	0.09	2.83	-318.07	2.91600007	11	7/24/2014	26:48.6
5708	RWS11	-122.4644069	37.9387	-0.1	2.83	-302.9	2.73000009	-122.4644	37.93871	0.09	2.83	-317.25	2.91900009	11	7/24/2014	26:48.7

5709	RWS11	-122.4644088	37.9387	-0.1	2.84	-302.1	2.73499996	-122.4644	37.93871	0.09	2.84	-316.45	2.92399997	11	7/24/2014	26:48.8
5710	RWS11	-122.4644107	37.9387	-0.1	2.84	-301.2	2.74000008	-122.4644	37.93871	0.06	2.84	-315.58	2.89600008	11	7/24/2014	26:48.9
5711	RWS11	-122.4644135	37.9387	-0.1	2.85	-300.4	2.74599988	-122.46441	37.93871	0.02	2.85	-314.76	2.86799988	11	7/24/2014	26:49.0
5712	RWS11	-122.4644153	37.9387	-0.1	2.85	-299.6	2.75199992	-122.46441	37.93872	0.09	2.85	-313.96	2.94099993	11	7/24/2014	26:49.1
5713	RWS11	-122.4644172	37.9387	-0.1	2.86	-298.7	2.75899989	-122.46441	37.93872	0.09	2.86	-313.1	2.94799989	11	7/24/2014	26:49.2
5714	RWS11	-122.4644191	37.9387	-0.1	2.87	-297.9	2.76499993	-122.46441	37.93872	0.14	2.87	-312.25	3.00499994	11	7/24/2014	26:49.3
5715	RWS11	-122.464421	37.9387	-0.14	2.87	-297	2.73500004	-122.46441	37.93872	0.06	2.87	-311.49	2.92600005	11	7/24/2014	26:49.4
5716	RWS11	-122.4644229	37.93871	-0.1	2.87	-296.3	2.7719999	-122.46442	37.93872	0.09	2.87	-310.78	2.96099991	11	7/24/2014	26:49.5
5717	RWS11	-122.4644248	37.93871	-0.1	2.87	-295.5	2.77099998	-122.46442	37.93872	0.09	2.87	-310.06	2.95999998	11	7/24/2014	26:49.6
5718	RWS11	-122.4644266	37.93871	-0.1	2.87	-294.9	2.7660001	-122.46442	37.93872	0.09	2.87	-309.46	2.9550001	11	7/24/2014	26:49.7
5719	RWS11	-122.4644285	37.93871	-0.1	2.86	-294.3	2.75899989	-122.46442	37.93872	0.09	2.86	-308.86	2.94799989	11	7/24/2014	26:49.8
5720	RWS11	-122.4644304	37.93871	-0.05	2.85	-293.8	2.802	-122.46442	37.93872	0.09	2.85	-308.35	2.94	11	7/24/2014	26:49.9
5721	RWS11	-122.4644332	37.93871	-0.1	2.84	-293.4	2.74199993	-122.46443	37.93872	0.09	2.84	-307.87	2.93099993	11	7/24/2014	26:50.0
5722	RWS11	-122.4644351	37.93871	-0.05	2.84	-292.9	2.78699989	-122.46443	37.93872	0.14	2.84	-307.51	2.97599989	11	7/24/2014	26:50.1
5723	RWS11	-122.464436	37.93871	-0.1	2.84	-292.6	2.73400003	-122.46443	37.93872	0.09	2.84	-307.14	2.92300004	11	7/24/2014	26:50.2
5724	RWS11	-122.4644389	37.93871	-0.05	2.84	-292.3	2.78800006	-122.46443	37.93872	0.02	2.84	-306.8	2.85900006	11	7/24/2014	26:50.3
5725	RWS11	-122.4644408	37.93871	-0.1	2.85	-292	2.74499995	-122.46443	37.93872	0.09	2.85	-306.51	2.93399996	11	7/24/2014	26:50.4
5726	RWS11	-122.4644428	37.93871	-0.05	2.86	-291.8	2.81100006	-122.46444	37.93872	0.06	2.86	-306.25	2.91600006	11	7/24/2014	26:50.5
5727	RWS11	-122.4644438	37.93871	-0.1	2.88	-291.7	2.77799994	-122.46444	37.93872	0.09	2.88	-306.06	2.96699995	11	7/24/2014	26:50.6
5728	RWS11	-122.4644469	37.93871	-0.05	2.9	-291.7	2.84899993	-122.46444	37.93873	0.17	2.9	-305.8	3.07199992	11	7/24/2014	26:50.7
5729	RWS11	-122.464449	37.93871	-0.1	2.92	-291.7	2.81900007	-122.46444	37.93873	0.06	2.92	-305.64	2.97500008	11	7/24/2014	26:50.8
5730	RWS11	-122.4644511	37.93871	-0.05	2.94	-291.8	2.88899989	-122.46444	37.93873	0.09	2.94	-305.56	3.02699989	11	7/24/2014	26:50.9
5731	RWS11	-122.4644521	37.93872	-0.05	2.95	-291.9	2.904	-122.46445	37.93873	0.09	2.95	-305.43	3.042	11	7/24/2014	26:51.0
5732	RWS11	-122.4644554	37.93872	-0.05	2.96	-292.1	2.91399999	-122.46445	37.93873	0.14	2.96	-305.41	3.10299999	11	7/24/2014	26:51.1
5733	RWS11	-122.4644575	37.93872	-0.1	2.97	-292.3	2.8680001	-122.46445	37.93873	0.17	2.97	-305.41	3.14200009	11	7/24/2014	26:51.2
5734	RWS11	-122.4644597	37.93872	-0.05	2.97	-292.6	2.9190001	-122.46445	37.93873	0.14	2.97	-305.45	3.1080001	11	7/24/2014	26:51.3
5735	RWS11	-122.4644608	37.93872	-0.1	2.96	-292.9	2.86399991	-122.46445	37.93873	0.06	2.96	-305.5	3.01999991	11	7/24/2014	26:51.4
5736	RWS11	-122.4644641	37.93872	-0.05	2.96	-293.3	2.90799994	-122.46446	37.93873	0.17	2.96	-305.59	3.13099994	11	7/24/2014	26:51.5
5737	RWS11	-122.4644662	37.93872	-0.1	2.95	-293.5	2.84799988	-122.46446	37.93873	0.09	2.95	-305.65	3.03699988	11	7/24/2014	26:51.6
5738	RWS11	-122.4644683	37.93872	-0.05	2.94	-293.8	2.88700004	-122.46446	37.93873	0.17	2.94	-305.7	3.11000003	11	7/24/2014	26:51.7
5739	RWS11	-122.4644693	37.93872	-0.1	2.92	-294	2.82200009	-122.46446	37.93873	0.14	2.92	-305.67	3.0620001	11	7/24/2014	26:51.8
5740	RWS11	-122.4644724	37.93872	-0.05	2.91	-294.2	2.85799999	-122.46447	37.93873	0.17	2.91	-305.72	3.08099999	11	7/24/2014	26:51.9
5741	RWS11	-122.4644753	37.93872	-0.1	2.89	-294.3	2.79099996	-122.46447	37.93874	0.14	2.89	-305.63	3.03099996	11	7/24/2014	26:52.0

5742	RWS11	-122.4644772	37.93872	-0.05	2.88	-294.5	2.82599993	-122.46447	37.93874	0.17	2.88	-305.65	3.04899992	11	7/24/2014	26:52.1
5743	RWS11	-122.4644781	37.93872	-0.1	2.86	-294.6	2.75899989	-122.46447	37.93874	0.09	2.86	-305.57	2.94799989	11	7/24/2014	26:52.2
5744	RWS11	-122.46448	37.93872	-0.05	2.85	-294.7	2.79599996	-122.46447	37.93874	0.09	2.85	-305.57	2.93399996	11	7/24/2014	26:52.3
5745	RWS11	-122.4644818	37.93873	-0.1	2.83	-294.7	2.73100001	-122.46448	37.93874	0.17	2.83	-305.46	3.00500001	11	7/24/2014	26:52.4
5746	RWS11	-122.4644836	37.93873	-0.05	2.82	-294.7	2.76800008	-122.46448	37.93874	0.17	2.82	-305.38	2.99100007	11	7/24/2014	26:52.5
5747	RWS11	-122.4644845	37.93873	-0.05	2.8	-294.6	2.75299997	-122.46448	37.93874	0.14	2.8	-305.22	2.94199997	11	7/24/2014	26:52.6
5748	RWS11	-122.4644873	37.93873	-0.05	2.79	-294.6	2.73600001	-122.46448	37.93874	0.17	2.79	-305.1	2.95900001	11	7/24/2014	26:52.7
5749	RWS11	-122.4644891	37.93873	-0.1	2.77	-294.6	2.66599996	-122.46448	37.93874	0.09	2.77	-305.03	2.85499996	11	7/24/2014	26:52.8
5750	RWS11	-122.464491	37.93873	-0.05	2.75	-294.5	2.69799991	-122.46448	37.93874	0.17	2.75	-304.88	2.9209999	11	7/24/2014	26:52.9
5751	RWS11	-122.464492	37.93873	-0.1	2.73	-294.5	2.62999994	-122.46449	37.93874	0.14	2.73	-304.8	2.86999995	11	7/24/2014	26:53.0
5752	RWS11	-122.4644939	37.93873	-0.05	2.72	-294.4	2.66600008	-122.46449	37.93874	0.09	2.72	-304.68	2.80400008	11	7/24/2014	26:53.1
5753	RWS11	-122.4644959	37.93873	-0.1	2.71	-294.4	2.60500009	-122.46449	37.93874	0.14	2.71	-304.59	2.84500009	11	7/24/2014	26:53.2
5754	RWS11	-122.4644978	37.93873	-0.1	2.7	-294.3	2.59799988	-122.46449	37.93874	0.17	2.7	-304.42	2.87199987	11	7/24/2014	26:53.3
5755	RWS11	-122.4644988	37.93873	-0.05	2.7	-294.3	2.64700003	-122.46449	37.93874	0.14	2.7	-304.34	2.83600003	11	7/24/2014	26:53.4
5756	RWS11	-122.4645018	37.93873	-0.05	2.7	-294.4	2.64700003	-122.4645	37.93874	0.14	2.7	-304.25	2.83600003	11	7/24/2014	26:53.5
5757	RWS11	-122.4645038	37.93873	-0.1	2.7	-294.4	2.59999997	-122.4645	37.93875	0.14	2.7	-304.1	2.83999997	11	7/24/2014	26:53.6
5758	RWS11	-122.4645058	37.93873	-0.05	2.71	-294.3	2.65600009	-122.4645	37.93875	0.17	2.71	-303.95	2.87900008	11	7/24/2014	26:53.7
5759	RWS11	-122.4645068	37.93873	-0.1	2.71	-294.3	2.61099989	-122.4645	37.93875	0.14	2.71	-303.82	2.85099989	11	7/24/2014	26:53.8
5760	RWS11	-122.4645088	37.93873	-0.05	2.72	-294.2	2.6690001	-122.4645	37.93875	0.17	2.72	-303.6	2.89200009	11	7/24/2014	26:53.9
5761	RWS11	-122.4645119	37.93874	-0.1	2.72	-294.1	2.6239999	-122.46451	37.93875	0.14	2.72	-303.37	2.8639999	11	7/24/2014	26:54.0
5762	RWS11	-122.4645139	37.93874	-0.05	2.73	-294.1	2.68099995	-122.46451	37.93875	0.14	2.73	-303.17	2.86999995	11	7/24/2014	26:54.1
5763	RWS11	-122.464515	37.93874	-0.05	2.73	-293.9	2.68499989	-122.46451	37.93875	0.14	2.73	-302.89	2.87399989	11	7/24/2014	26:54.2
5764	RWS11	-122.4645182	37.93874	-0.05	2.74	-293.9	2.68900008	-122.46451	37.93875	0.17	2.74	-302.7	2.91200007	11	7/24/2014	26:54.3
5765	RWS11	-122.4645204	37.93874	-0.1	2.74	-293.8	2.64200003	-122.46451	37.93875	0.14	2.74	-302.52	2.88200003	11	7/24/2014	26:54.4
5766	RWS11	-122.4645227	37.93874	-0.05	2.75	-293.7	2.69699998	-122.46452	37.93875	0.17	2.75	-302.32	2.91999997	11	7/24/2014	26:54.5
5767	RWS11	-122.4645238	37.93874	-0.1	2.75	-293.5	2.64999992	-122.46452	37.93875	0.17	2.75	-302.09	2.92399992	11	7/24/2014	26:54.6
5768	RWS11	-122.4645262	37.93874	-0.05	2.76	-293.3	2.70600004	-122.46452	37.93875	0.14	2.76	-301.87	2.89500004	11	7/24/2014	26:54.7
5769	RWS11	-122.4645285	37.93874	-0.1	2.76	-293.2	2.66200001	-122.46452	37.93875	0.14	2.76	-301.55	2.90200001	11	7/24/2014	26:54.8
5770	RWS11	-122.4645308	37.93874	-0.05	2.77	-293	2.71999998	-122.46452	37.93875	0.14	2.77	-301.31	2.90899998	11	7/24/2014	26:54.9
5771	RWS11	-122.464532	37.93874	-0.1	2.78	-292.8	2.67800004	-122.46453	37.93875	0.14	2.78	-301.03	2.91800004	11	7/24/2014	26:55.0
5772	RWS11	-122.4645343	37.93874	-0.05	2.79	-292.6	2.73900003	-122.46453	37.93876	0.14	2.79	-300.73	2.92800003	11	7/24/2014	26:55.1
5773	RWS11	-122.4645366	37.93874	-0.1	2.8	-292.4	2.69899995	-122.46453	37.93876	0.14	2.8	-300.51	2.93899995	11	7/24/2014	26:55.2
5774	RWS11	-122.4645389	37.93874	-0.05	2.81	-292.3	2.76100011	-122.46453	37.93876	0.14	2.81	-300.27	2.95000011	11	7/24/2014	26:55.3

5775	RWS11	-122.46454	37.93875	-0.1	2.82	-292.2	2.7200001	-122.46453	37.93876	0.09	2.82	-300.08	2.9090001	11	7/24/2014	26:55.4
5776	RWS11	-122.4645423	37.93875	-0.1	2.83	-292.1	2.72899992	-122.46454	37.93876	0.14	2.83	-299.88	2.96899992	11	7/24/2014	26:55.5
5777	RWS11	-122.4645445	37.93875	-0.1	2.84	-291.9	2.73700006	-122.46454	37.93876	0.14	2.84	-299.7	2.97700006	11	7/24/2014	26:55.6
5778	RWS11	-122.4645467	37.93875	-0.05	2.85	-291.8	2.79599996	-122.46454	37.93876	0.14	2.85	-299.52	2.98499995	11	7/24/2014	26:55.7
5779	RWS11	-122.4645477	37.93875	-0.1	2.85	-291.7	2.75199992	-122.46454	37.93876	0.14	2.85	-299.3	2.99199992	11	7/24/2014	26:55.8
5780	RWS11	-122.4645498	37.93875	-0.05	2.86	-291.6	2.80999989	-122.46454	37.93876	0.09	2.86	-299.15	2.94799989	11	7/24/2014	26:55.9
5781	RWS11	-122.4645529	37.93875	-0.05	2.87	-291.5	2.8170001	-122.46455	37.93876	0.14	2.87	-298.99	3.0060001	11	7/24/2014	26:56.0
5782	RWS11	-122.464555	37.93875	-0.05	2.87	-291.4	2.82400007	-122.46455	37.93876	0.14	2.87	-298.83	3.01300007	11	7/24/2014	26:56.1
5783	RWS11	-122.464556	37.93875	-0.1	2.88	-291.3	2.77900011	-122.46455	37.93876	0.14	2.88	-298.74	3.01900011	11	7/24/2014	26:56.2
5784	RWS11	-122.4645581	37.93875	-0.05	2.88	-291.2	2.83400006	-122.46455	37.93876	0.14	2.88	-298.57	3.02300006	11	7/24/2014	26:56.3
5785	RWS11	-122.4645602	37.93875	-0.1	2.89	-291.1	2.78499991	-122.46455	37.93876	0.09	2.89	-298.5	2.97399992	11	7/24/2014	26:56.4
5786	RWS11	-122.4645623	37.93875	-0.05	2.88	-291	2.83499999	-122.46456	37.93877	0.14	2.88	-298.42	3.02399999	11	7/24/2014	26:56.5
5787	RWS11	-122.4645633	37.93875	-0.1	2.88	-290.9	2.78099997	-122.46456	37.93877	0.09	2.88	-298.32	2.96999997	11	7/24/2014	26:56.6
5788	RWS11	-122.4645655	37.93875	-0.05	2.88	-290.8	2.82700009	-122.46456	37.93877	0.14	2.88	-298.29	3.01600009	11	7/24/2014	26:56.7
5789	RWS11	-122.4645676	37.93875	-0.1	2.87	-290.7	2.77000005	-122.46456	37.93877	0.09	2.87	-298.22	2.95900005	11	7/24/2014	26:56.8
5790	RWS11	-122.4645697	37.93876	-0.1	2.86	-290.7	2.76199991	-122.46456	37.93877	0.14	2.86	-298.2	3.00199991	11	7/24/2014	26:56.9
5791	RWS11	-122.4645708	37.93876	-0.1	2.85	-290.6	2.75199992	-122.46456	37.93877	0.09	2.85	-298.18	2.94099993	11	7/24/2014	26:57.0
5792	RWS11	-122.4645729	37.93876	-0.05	2.84	-290.5	2.79299993	-122.46457	37.93877	0.14	2.84	-298.16	2.98199993	11	7/24/2014	26:57.1
5793	RWS11	-122.464575	37.93876	-0.1	2.83	-290.4	2.73199994	-122.46457	37.93877	0.14	2.83	-298.15	2.97199994	11	7/24/2014	26:57.2
5794	RWS11	-122.4645771	37.93876	-0.1	2.83	-290.3	2.72400004	-122.46457	37.93877	0.14	2.83	-298.12	2.96400005	11	7/24/2014	26:57.3
5795	RWS11	-122.4645782	37.93876	-0.1	2.82	-290.2	2.71700007	-122.46457	37.93877	0.09	2.82	-298.07	2.90600008	11	7/24/2014	26:57.4
5796	RWS11	-122.4645803	37.93876	-0.1	2.81	-290.2	2.71199996	-122.46457	37.93877	0.14	2.81	-298.09	2.95199996	11	7/24/2014	26:57.5
5797	RWS11	-122.4645823	37.93876	-0.1	2.81	-290.1	2.71000011	-122.46458	37.93877	0.09	2.81	-297.99	2.89900011	11	7/24/2014	26:57.6
5798	RWS11	-122.4645844	37.93876	-0.1	2.81	-290	2.70800001	-122.46458	37.93877	0.14	2.81	-297.92	2.94800001	11	7/24/2014	26:57.7
5799	RWS11	-122.4645854	37.93876	-0.1	2.81	-290	2.70899994	-122.46458	37.93877	0.09	2.81	-297.88	2.89799994	11	7/24/2014	26:57.8
5800	RWS11	-122.4645875	37.93876	-0.1	2.81	-289.9	2.71199996	-122.46458	37.93877	0.14	2.81	-297.82	2.95199996	11	7/24/2014	26:57.9
5801	RWS11	-122.4645905	37.93876	-0.1	2.82	-289.9	2.71700007	-122.46458	37.93878	0.14	2.82	-297.81	2.95700008	11	7/24/2014	26:58.0
5802	RWS11	-122.4645926	37.93876	-0.05	2.83	-289.9	2.77599997	-122.46459	37.93878	0.14	2.83	-297.78	2.96499997	11	7/24/2014	26:58.1
5803	RWS11	-122.4645936	37.93876	-0.1	2.84	-289.9	2.73499996	-122.46459	37.93878	0.09	2.84	-297.83	2.92399997	11	7/24/2014	26:58.2
5804	RWS11	-122.4645958	37.93876	-0.1	2.85	-290	2.74799997	-122.46459	37.93878	0.14	2.85	-297.84	2.98799998	11	7/24/2014	26:58.3
5805	RWS11	-122.4645979	37.93877	-0.1	2.86	-290	2.76199991	-122.46459	37.93878	0.09	2.86	-297.85	2.95099992	11	7/24/2014	26:58.4
5806	RWS11	-122.4646	37.93877	-0.1	2.88	-290	2.77799994	-122.46459	37.93878	0.14	2.88	-297.88	3.01799995	11	7/24/2014	26:58.5
5807	RWS11	-122.4646011	37.93877	-0.1	2.9	-290.1	2.79600007	-122.46459	37.93878	0.09	2.9	-297.96	2.98500007	11	7/24/2014	26:58.6

5808	RWS11	-122.4646032	37.93877	-0.1	2.91	-290.1	2.81399996	-122.4646	37.93878	0.14	2.91	-297.99	3.05399996	11	7/24/2014	26:58.7
5809	RWS11	-122.4646053	37.93877	-0.1	2.93	-290.2	2.83300001	-122.4646	37.93878	0.14	2.93	-298.05	3.07300001	11	7/24/2014	26:58.8
5810	RWS11	-122.4646074	37.93877	-0.1	2.95	-290.2	2.85399992	-122.4646	37.93878	0.14	2.95	-298.1	3.09399992	11	7/24/2014	26:58.9
5811	RWS11	-122.4646094	37.93877	-0.1	2.97	-290.2	2.8739999	-122.4646	37.93878	0.09	2.97	-298.23	3.0629999	11	7/24/2014	26:59.0
5812	RWS11	-122.4646115	37.93877	-0.05	3	-290.3	2.94699998	-122.4646	37.93878	0.14	3	-298.32	3.13599998	11	7/24/2014	26:59.1
5813	RWS11	-122.4646135	37.93877	-0.1	3.02	-290.3	2.91800005	-122.46461	37.93878	0.14	3.02	-298.36	3.15800005	11	7/24/2014	26:59.2
5814	RWS11	-122.4646154	37.93877	-0.1	3.04	-290.3	2.93999989	-122.46461	37.93878	0.14	3.04	-298.4	3.17999989	11	7/24/2014	26:59.3
5815	RWS11	-122.4646174	37.93877	-0.1	3.06	-290.2	2.96000011	-122.46461	37.93878	0.09	3.06	-298.31	3.14900011	11	7/24/2014	26:59.4
5816	RWS11	-122.4646193	37.93877	-0.1	3.08	-290.2	2.9759999	-122.46461	37.93879	0.14	3.08	-298.34	3.2159999	11	7/24/2014	26:59.5
5817	RWS11	-122.4646212	37.93877	-0.1	3.09	-290.2	2.98799998	-122.46461	37.93879	0.09	3.09	-298.34	3.17699999	11	7/24/2014	26:59.6
5818	RWS11	-122.4646231	37.93877	-0.1	3.1	-290.2	2.99499995	-122.46462	37.93879	0.09	3.1	-298.34	3.18399996	11	7/24/2014	26:59.7
5819	RWS11	-122.464625	37.93878	-0.1	3.1	-290.2	2.99799997	-122.46462	37.93879	0.09	3.1	-298.36	3.18699998	11	7/24/2014	26:59.8
5820	RWS11	-122.4646269	37.93878	-0.1	3.1	-290.3	2.99499995	-122.46462	37.93879	0.14	3.1	-298.36	3.23499995	11	7/24/2014	26:59.9
5821	RWS11	-122.4646298	37.93878	-0.1	3.09	-290.3	2.98800006	-122.46462	37.93879	0.09	3.09	-298.38	3.17700006	11	7/24/2014	27:00.0
5822	RWS11	-122.4646317	37.93878	-0.1	3.08	-290.3	2.9769999	-122.46462	37.93879	0.14	3.08	-298.34	3.2169999	11	7/24/2014	27:00.1
5823	RWS11	-122.4646337	37.93878	-0.1	3.06	-290.3	2.96200003	-122.46463	37.93879	0.09	3.06	-298.3	3.15100004	11	7/24/2014	27:00.2
5824	RWS11	-122.4646357	37.93878	-0.05	3.05	-290.2	2.99600008	-122.46463	37.93879	0.14	3.05	-298.28	3.18500008	11	7/24/2014	27:00.3
5825	RWS11	-122.4646377	37.93878	-0.1	3.03	-290.2	2.92800011	-122.46463	37.93879	0.14	3.03	-298.19	3.16800012	11	7/24/2014	27:00.4
5826	RWS11	-122.4646398	37.93878	-0.05	3.01	-290.2	2.96099999	-122.46463	37.93879	0.14	3.01	-298.15	3.14999999	11	7/24/2014	27:00.5
5827	RWS11	-122.4646418	37.93878	-0.1	2.99	-290.2	2.89300003	-122.46463	37.93879	0.09	2.99	-298.19	3.08200003	11	7/24/2014	27:00.6
5828	RWS11	-122.4646439	37.93878	-0.05	2.98	-290.2	2.928	-122.46464	37.93879	0.09	2.98	-298.23	3.066	11	7/24/2014	27:00.7
5829	RWS11	-122.464646	37.93878	-0.1	2.96	-290.2	2.86099996	-122.46464	37.93879	0.09	2.96	-298.24	3.04999997	11	7/24/2014	27:00.8
5830	RWS11	-122.4646481	37.93878	-0.05	2.94	-290.2	2.89599993	-122.46464	37.9388	0.09	2.94	-298.3	3.03399993	11	7/24/2014	27:00.9
5831	RWS11	-122.4646501	37.93878	-0.1	2.93	-290.1	2.8289999	-122.46464	37.9388	0.09	2.93	-298.32	3.0179999	11	7/24/2014	27:01.0
5832	RWS11	-122.4646522	37.93878	-0.05	2.91	-290.1	2.86299994	-122.46465	37.9388	0.09	2.91	-298.35	3.00099994	11	7/24/2014	27:01.1
5833	RWS11	-122.4646542	37.93879	-0.1	2.89	-290.1	2.79499998	-122.46465	37.9388	0.09	2.89	-298.37	2.98399998	11	7/24/2014	27:01.2
5834	RWS11	-122.4646562	37.93879	-0.1	2.88	-290.1	2.77800002	-122.46465	37.9388	0.09	2.88	-298.41	2.96700002	11	7/24/2014	27:01.3
5835	RWS11	-122.4646582	37.93879	-0.1	2.86	-290	2.75899997	-122.46465	37.9388	0.09	2.86	-298.46	2.94799997	11	7/24/2014	27:01.4
5836	RWS11	-122.4646602	37.93879	-0.1	2.84	-289.9	2.73999991	-122.46465	37.9388	0.14	2.84	-298.39	2.97999991	11	7/24/2014	27:01.5
5837	RWS11	-122.4646622	37.93879	-0.1	2.82	-289.9	2.7210001	-122.46466	37.9388	0.09	2.82	-298.43	2.9100001	11	7/24/2014	27:01.6
5838	RWS11	-122.4646641	37.93879	-0.1	2.8	-289.9	2.70200004	-122.46466	37.9388	0.14	2.8	-298.48	2.94200005	11	7/24/2014	27:01.7
5839	RWS11	-122.464666	37.93879	-0.1	2.79	-289.9	2.68500008	-122.46466	37.9388	0.09	2.79	-298.52	2.87400009	11	7/24/2014	27:01.8
5840	RWS11	-122.4646679	37.93879	-0.05	2.77	-289.8	2.72000005	-122.46466	37.9388	0.14	2.77	-298.55	2.90900005	11	7/24/2014	27:01.9

5841	RWS11	-122.4646707	37.93879	-0.1	2.76	-289.8	2.65500011	-122.46466	37.9388	0.09	2.76	-298.58	2.84400012	11	7/24/2014	27:02.0
5842	RWS11	-122.4646726	37.93879	-0.1	2.74	-289.8	2.64399996	-122.46467	37.9388	0.14	2.74	-298.63	2.88399996	11	7/24/2014	27:02.1
5843	RWS11	-122.4646745	37.93879	-0.14	2.74	-289.8	2.60199998	-122.46467	37.93881	0.14	2.74	-298.66	2.87699999	11	7/24/2014	27:02.2
5844	RWS11	-122.4646765	37.93879	-0.05	2.73	-289.7	2.68499997	-122.46467	37.93881	0.14	2.73	-298.61	2.87399997	11	7/24/2014	27:02.3
5845	RWS11	-122.4646784	37.93879	-0.1	2.73	-289.6	2.63499989	-122.46467	37.93881	0.09	2.73	-298.5	2.8239999	11	7/24/2014	27:02.4
5846	RWS11	-122.4646804	37.93879	-0.1	2.74	-289.5	2.64000001	-122.46467	37.93881	0.09	2.74	-298.44	2.82900001	11	7/24/2014	27:02.5
5847	RWS11	-122.4646824	37.9388	-0.1	2.75	-289.3	2.65	-122.46468	37.93881	0.09	2.75	-298.35	2.839	11	7/24/2014	27:02.6
5848	RWS11	-122.4646844	37.9388	-0.1	2.77	-289.3	2.66600003	-122.46468	37.93881	0.14	2.77	-298.34	2.90600003	11	7/24/2014	27:02.7
5849	RWS11	-122.4646864	37.9388	-0.1	2.79	-289.1	2.68800011	-122.46468	37.93881	0.09	2.79	-298.22	2.87700011	11	7/24/2014	27:02.8
5850	RWS11	-122.4646885	37.9388	-0.1	2.81	-289.1	2.71399989	-122.46468	37.93881	0.14	2.81	-298.14	2.95399989	11	7/24/2014	27:02.9
5851	RWS11	-122.4646905	37.9388	-0.1	2.85	-289.1	2.74500003	-122.46468	37.93881	0.14	2.85	-298.16	2.98500003	11	7/24/2014	27:03.0
5852	RWS11	-122.4646925	37.9388	-0.1	2.88	-289	2.77700009	-122.46469	37.93881	0.09	2.88	-298.09	2.9660001	11	7/24/2014	27:03.1
5853	RWS11	-122.4646945	37.9388	-0.1	2.91	-288.9	2.80899992	-122.46469	37.93881	0.09	2.91	-298	2.99799992	11	7/24/2014	27:03.2
5854	RWS11	-122.4646965	37.9388	-0.1	2.94	-288.9	2.83899989	-122.46469	37.93881	0.09	2.94	-297.98	3.02799989	11	7/24/2014	27:03.3
5855	RWS11	-122.4646985	37.9388	-0.1	2.97	-288.9	2.86600008	-122.46469	37.93881	0.06	2.97	-297.96	3.02200008	11	7/24/2014	27:03.4
5856	RWS11	-122.4647005	37.9388	-0.1	2.99	-288.9	2.88799991	-122.46469	37.93881	0.09	2.99	-297.9	3.07699992	11	7/24/2014	27:03.5
5857	RWS11	-122.4647025	37.9388	-0.1	3.01	-288.9	2.90600004	-122.4647	37.93881	0.06	3.01	-297.94	3.06200004	11	7/24/2014	27:03.6
5858	RWS11	-122.4647044	37.9388	-0.1	3.02	-289	2.91799989	-122.4647	37.93882	0.09	3.02	-297.92	3.10699989	11	7/24/2014	27:03.7
5859	RWS11	-122.4647063	37.9388	-0.1	3.02	-289	2.923	-122.4647	37.93882	0.02	3.02	-297.96	3.045	11	7/24/2014	27:03.8
5860	RWS11	-122.4647082	37.9388	-0.1	3.02	-289.1	2.92200007	-122.4647	37.93882	0.06	3.02	-297.92	3.07800008	11	7/24/2014	27:03.9
5861	RWS11	-122.464711	37.93881	-0.1	3.01	-289.1	2.91399994	-122.4647	37.93882	0.02	3.01	-297.94	3.03599994	11	7/24/2014	27:04.0
5862	RWS11	-122.4647129	37.93881	-0.1	3	-289.2	2.9	-122.46471	37.93882	0.06	3	-297.94	3.056	11	7/24/2014	27:04.1
5863	RWS11	-122.4647148	37.93881	-0.1	2.98	-289.2	2.88099995	-122.46471	37.93882	0.06	2.98	-297.98	3.03699995	11	7/24/2014	27:04.2
5864	RWS11	-122.4647167	37.93881	-0.1	2.96	-289.2	2.85799994	-122.46471	37.93882	0.06	2.96	-297.99	3.01399995	11	7/24/2014	27:04.3
5865	RWS11	-122.4647186	37.93881	-0.1	2.93	-289.2	2.83300009	-122.46471	37.93882	0.09	2.93	-298.07	3.02200009	11	7/24/2014	27:04.4
5866	RWS11	-122.4647206	37.93881	-0.05	2.91	-289.3	2.85899999	-122.46471	37.93882	0.06	2.91	-298.21	2.96399999	11	7/24/2014	27:04.5
5867	RWS11	-122.4647225	37.93881	-0.1	2.88	-289.4	2.78400006	-122.46472	37.93882	0.06	2.88	-298.42	2.94000006	11	7/24/2014	27:04.6
5868	RWS11	-122.4647244	37.93881	-0.1	2.86	-289.5	2.76199999	-122.46472	37.93882	0.06	2.86	-298.57	2.91799999	11	7/24/2014	27:04.7
5869	RWS11	-122.4647263	37.93881	-0.1	2.84	-289.6	2.7440001	-122.46472	37.93882	0.06	2.84	-298.66	2.9000001	11	7/24/2014	27:04.8
5870	RWS11	-122.4647282	37.93881	-0.1	2.83	-289.6	2.72800007	-122.46472	37.93882	0.06	2.83	-298.66	2.88400007	11	7/24/2014	27:04.9
5871	RWS11	-122.464731	37.93881	-0.14	2.82	-289.6	2.68000005	-122.46472	37.93882	0.02	2.82	-298.7	2.83700006	11	7/24/2014	27:05.0
5872	RWS11	-122.4647329	37.93881	-0.1	2.81	-289.6	2.70599999	-122.46473	37.93883	0.06	2.81	-298.68	2.862	11	7/24/2014	27:05.1
5873	RWS11	-122.4647348	37.93881	-0.1	2.8	-289.6	2.6980001	-122.46473	37.93883	0.02	2.8	-298.63	2.8200001	11	7/24/2014	27:05.2

5874	RWS11	-122.4647367	37.93881	-0.1	2.79	-289.6	2.69299998	-122.46473	37.93883	0.06	2.79	-298.66	2.84899998	11	7/24/2014	27:05.3
5875	RWS11	-122.4647387	37.93882	-0.14	2.79	-289.6	2.65400003	-122.46473	37.93883	0.06	2.79	-298.64	2.84500004	11	7/24/2014	27:05.4
5876	RWS11	-122.4647406	37.93882	-0.1	2.79	-289.6	2.68600001	-122.46473	37.93883	0.02	2.79	-298.62	2.80800001	11	7/24/2014	27:05.5
5877	RWS11	-122.4647425	37.93882	-0.1	2.78	-289.6	2.68399992	-122.46474	37.93883	0.02	2.78	-298.62	2.80599992	11	7/24/2014	27:05.6
5878	RWS11	-122.4647444	37.93882	-0.1	2.78	-289.5	2.68299999	-122.46474	37.93883	0.02	2.78	-298.52	2.80499999	11	7/24/2014	27:05.7
5879	RWS11	-122.4647463	37.93882	-0.14	2.78	-289.4	2.64899991	-122.46474	37.93883	0.02	2.78	-298.38	2.80599992	11	7/24/2014	27:05.8
5880	RWS11	-122.4647482	37.93882	-0.1	2.79	-289.3	2.68600001	-122.46474	37.93883	0.02	2.79	-298.36	2.80800001	11	7/24/2014	27:05.9
5881	RWS11	-122.4647509	37.93882	-0.14	2.79	-289.3	2.65599988	-122.46474	37.93883	-0.03	2.79	-298.28	2.76199989	11	7/24/2014	27:06.0
5882	RWS11	-122.4647528	37.93882	-0.1	2.8	-289.2	2.69699993	-122.46475	37.93883	0.02	2.8	-298.23	2.81899993	11	7/24/2014	27:06.1
5883	RWS11	-122.4647546	37.93882	-0.14	2.81	-289.1	2.67099999	-122.46475	37.93883	-0.03	2.81	-298.17	2.777	11	7/24/2014	27:06.2
5884	RWS11	-122.4647564	37.93882	-0.1	2.82	-289	2.71699991	-122.46475	37.93883	-0.03	2.82	-298.08	2.78799991	11	7/24/2014	27:06.3
5885	RWS11	-122.4647583	37.93882	-0.14	2.83	-288.9	2.69499992	-122.46475	37.93883	-0.06	2.83	-298.01	2.76699992	11	7/24/2014	27:06.4
5886	RWS11	-122.4647601	37.93882	-0.14	2.84	-288.9	2.70799993	-122.46475	37.93883	0.02	2.84	-297.89	2.86499994	11	7/24/2014	27:06.5
5887	RWS11	-122.4647619	37.93882	-0.14	2.86	-288.7	2.72099994	-122.46476	37.93884	-0.06	2.86	-297.76	2.79299995	11	7/24/2014	27:06.6
5888	RWS11	-122.4647638	37.93882	-0.14	2.87	-288.6	2.73399995	-122.46476	37.93884	-0.06	2.87	-297.63	2.80599996	11	7/24/2014	27:06.7
5889	RWS11	-122.4647656	37.93882	-0.14	2.88	-288.5	2.74600004	-122.46476	37.93884	-0.06	2.88	-297.57	2.81800004	11	7/24/2014	27:06.8
5890	RWS11	-122.4647675	37.93882	-0.14	2.89	-288.4	2.75600003	-122.46476	37.93884	-0.06	2.89	-297.48	2.82800003	11	7/24/2014	27:06.9
5891	RWS11	-122.4647702	37.93883	-0.19	2.9	-288.3	2.71400009	-122.46476	37.93884	-0.06	2.9	-297.37	2.83700009	11	7/24/2014	27:07.0
5892	RWS11	-122.4647721	37.93883	-0.14	2.91	-288.2	2.77200006	-122.46477	37.93884	-0.06	2.91	-297.29	2.84400006	11	7/24/2014	27:07.1
5893	RWS11	-122.464774	37.93883	-0.19	2.91	-288	2.72599994	-122.46477	37.93884	-0.06	2.91	-297.14	2.84899994	11	7/24/2014	27:07.2
5894	RWS11	-122.464776	37.93883	-0.14	2.92	-287.9	2.78099988	-122.46477	37.93884	-0.06	2.92	-296.96	2.85299989	11	7/24/2014	27:07.3
5895	RWS11	-122.4647779	37.93883	-0.19	2.92	-287.8	2.73100005	-122.46477	37.93884	-0.03	2.92	-296.85	2.88800006	11	7/24/2014	27:07.4
5896	RWS11	-122.4647799	37.93883	-0.14	2.92	-287.6	2.78299998	-122.46477	37.93884	-0.06	2.92	-296.79	2.85499998	11	7/24/2014	27:07.5
5897	RWS11	-122.4647819	37.93883	-0.14	2.92	-287.5	2.78299998	-122.46478	37.93884	-0.06	2.92	-296.7	2.85499998	11	7/24/2014	27:07.6
5898	RWS11	-122.4647839	37.93883	-0.14	2.92	-287.4	2.78299998	-122.46478	37.93884	-0.06	2.92	-296.72	2.85499998	11	7/24/2014	27:07.7
5899	RWS11	-122.4647858	37.93883	-0.19	2.92	-287.3	2.735	-122.46478	37.93884	-0.11	2.92	-296.68	2.807	11	7/24/2014	27:07.8
5900	RWS11	-122.4647878	37.93883	-0.14	2.93	-287.2	2.79100011	-122.46478	37.93884	-0.06	2.93	-296.68	2.86300012	11	7/24/2014	27:07.9
5901	RWS11	-122.4647907	37.93883	-0.19	2.93	-287.2	2.74899994	-122.46478	37.93885	-0.06	2.93	-296.68	2.87199994	11	7/24/2014	27:08.0
5902	RWS11	-122.4647927	37.93883	-0.19	2.95	-287.1	2.76299988	-122.46479	37.93885	-0.06	2.95	-296.62	2.88599988	11	7/24/2014	27:08.1
5903	RWS11	-122.4647947	37.93883	-0.19	2.97	-287.1	2.781	-122.46479	37.93885	-0.11	2.97	-296.62	2.85300001	11	7/24/2014	27:08.2
5904	RWS11	-122.4647967	37.93883	-0.14	2.99	-287	2.855	-122.46479	37.93885	-0.06	2.99	-296.46	2.92700001	11	7/24/2014	27:08.3
5905	RWS11	-122.4647986	37.93884	-0.19	3.02	-286.9	2.83000003	-122.46479	37.93885	-0.15	3.02	-296.33	2.86800003	11	7/24/2014	27:08.4
5906	RWS11	-122.4648006	37.93884	-0.19	3.04	-286.9	2.85799991	-122.46479	37.93885	-0.06	3.04	-296.23	2.98099991	11	7/24/2014	27:08.5

5907	RWS11	-122.4648026	37.93884	-0.19	3.07	-286.8	2.88699995	-122.4648	37.93885	-0.11	3.07	-296.06	2.95899995	11	7/24/2014	27:08.6
5908	RWS11	-122.4648046	37.93884	-0.19	3.1	-286.7	2.91500007	-122.4648	37.93885	-0.11	3.1	-295.93	2.98700007	11	7/24/2014	27:08.7
5909	RWS11	-122.4648066	37.93884	-0.19	3.13	-286.7	2.94200002	-122.4648	37.93885	-0.15	3.13	-295.93	2.98000002	11	7/24/2014	27:08.8
5910	RWS11	-122.4648086	37.93884	-0.14	3.15	-286.7	3.01800011	-122.4648	37.93885	-0.11	3.15	-295.91	3.03900012	11	7/24/2014	27:08.9
5911	RWS11	-122.4648115	37.93884	-0.19	3.17	-286.7	2.98899995	-122.46481	37.93885	-0.15	3.17	-295.86	3.02699995	11	7/24/2014	27:09.0
5912	RWS11	-122.4648135	37.93884	-0.19	3.19	-286.6	3.008	-122.46481	37.93885	-0.11	3.19	-295.82	3.08000001	11	7/24/2014	27:09.1
5913	RWS11	-122.4648154	37.93884	-0.19	3.21	-286.6	3.02400003	-122.46481	37.93885	-0.15	3.21	-295.8	3.06200004	11	7/24/2014	27:09.2
5914	RWS11	-122.4648174	37.93884	-0.19	3.23	-286.6	3.04000007	-122.46481	37.93885	-0.11	3.23	-295.82	3.11200007	11	7/24/2014	27:09.3
5915	RWS11	-122.4648194	37.93884	-0.22	3.24	-286.6	3.01900008	-122.46481	37.93885	-0.15	3.24	-295.77	3.09100008	11	7/24/2014	27:09.4
5916	RWS11	-122.4648214	37.93884	-0.19	3.25	-286.6	3.06499992	-122.46481	37.93886	-0.15	3.25	-295.67	3.10299993	11	7/24/2014	27:09.5
5917	RWS11	-122.4648234	37.93884	-0.19	3.26	-286.5	3.07300006	-122.46482	37.93886	-0.15	3.26	-295.52	3.11100006	11	7/24/2014	27:09.6
5918	RWS11	-122.4648254	37.93884	-0.19	3.26	-286.5	3.07799993	-122.46482	37.93886	-0.15	3.26	-295.41	3.11599994	11	7/24/2014	27:09.7
5919	RWS11	-122.4648274	37.93884	-0.19	3.26	-286.5	3.07700001	-122.46482	37.93886	-0.15	3.26	-295.3	3.11500001	11	7/24/2014	27:09.8
5920	RWS11	-122.4648293	37.93885	-0.19	3.26	-286.5	3.07099997	-122.46482	37.93886	-0.15	3.26	-295.24	3.10899997	11	7/24/2014	27:09.9
5921	RWS11	-122.4648322	37.93885	-0.19	3.24	-286.5	3.05799995	-122.46483	37.93886	-0.15	3.24	-295.2	3.09599996	11	7/24/2014	27:10.0
5922	RWS11	-122.4648342	37.93885	-0.19	3.22	-286.4	3.03799997	-122.46483	37.93886	-0.15	3.22	-295.08	3.07599998	11	7/24/2014	27:10.1
5923	RWS11	-122.4648362	37.93885	-0.19	3.2	-286.4	3.01299988	-122.46483	37.93886	-0.2	3.2	-295	3.00099988	11	7/24/2014	27:10.2
5924	RWS11	-122.4648381	37.93885	-0.19	3.17	-286.4	2.98299991	-122.46483	37.93886	-0.2	3.17	-295	2.97099991	11	7/24/2014	27:10.3
5925	RWS11	-122.4648401	37.93885	-0.19	3.14	-286.4	2.95200001	-122.46483	37.93886	-0.23	3.14	-294.94	2.90600002	11	7/24/2014	27:10.4
5926	RWS11	-122.4648422	37.93885	-0.19	3.11	-286.4	2.92200004	-122.46484	37.93886	-0.15	3.11	-294.87	2.96000004	11	7/24/2014	27:10.5
5927	RWS11	-122.4648442	37.93885	-0.22	3.08	-286.3	2.86200002	-122.46484	37.93886	-0.23	3.08	-294.79	2.85000002	11	7/24/2014	27:10.6
5928	RWS11	-122.4648462	37.93885	-0.19	3.06	-286.2	2.8750001	-122.46484	37.93886	-0.2	3.06	-294.68	2.86300011	11	7/24/2014	27:10.7
5929	RWS11	-122.4648483	37.93885	-0.19	3.05	-286.1	2.86	-122.46484	37.93886	-0.15	3.05	-294.69	2.898	11	7/24/2014	27:10.8
5930	RWS11	-122.4648503	37.93885	-0.22	3.04	-286.1	2.81600001	-122.46484	37.93886	-0.15	3.04	-294.65	2.88800001	11	7/24/2014	27:10.9
5931	RWS11	-122.4648524	37.93885	-0.22	3.03	-285.9	2.8109999	-122.46485	37.93886	-0.15	3.03	-294.61	2.8829999	11	7/24/2014	27:11.0
5932	RWS11	-122.4648544	37.93885	-0.19	3.03	-286	2.84499989	-122.46485	37.93887	-0.2	3.03	-294.68	2.8329999	11	7/24/2014	27:11.1
5933	RWS11	-122.4648565	37.93885	-0.19	3.04	-285.9	2.84900008	-122.46485	37.93887	-0.2	3.04	-294.76	2.83700009	11	7/24/2014	27:11.2
5934	RWS11	-122.4648585	37.93885	-0.19	3.04	-286.1	2.85799991	-122.46485	37.93887	-0.15	3.04	-294.98	2.89599991	11	7/24/2014	27:11.3
5935	RWS11	-122.4648605	37.93886	-0.19	3.06	-286.1	2.86999999	-122.46485	37.93887	-0.2	3.06	-295.16	2.858	11	7/24/2014	27:11.4
5936	RWS11	-122.4648626	37.93886	-0.19	3.07	-286.3	2.88699995	-122.46486	37.93887	-0.11	3.07	-295.39	2.95899995	11	7/24/2014	27:11.5
5937	RWS11	-122.4648645	37.93886	-0.22	3.09	-286.3	2.87299994	-122.46486	37.93887	-0.15	3.09	-295.48	2.94499993	11	7/24/2014	27:11.6
5938	RWS11	-122.4648665	37.93886	-0.19	3.12	-286.4	2.9310001	-122.46486	37.93887	-0.15	3.12	-295.55	2.9690001	11	7/24/2014	27:11.7
5939	RWS11	-122.4648685	37.93886	-0.19	3.14	-286.4	2.95800005	-122.46486	37.93887	-0.2	3.14	-295.57	2.94600005	11	7/24/2014	27:11.8

5940	RWS11	-122.4648705	37.93886	-0.19	3.17	-286.5	2.98800002	-122.46486	37.93887	-0.2	3.17	-295.57	2.97600003	11	7/24/2014	27:11.9
5941	RWS11	-122.4648734	37.93886	-0.22	3.21	-286.6	2.98600009	-122.46487	37.93887	-0.2	3.21	-295.52	3.00800009	11	7/24/2014	27:12.0
5942	RWS11	-122.4648753	37.93886	-0.19	3.24	-286.7	3.05300008	-122.46487	37.93887	-0.15	3.24	-295.63	3.09100008	11	7/24/2014	27:12.1
5943	RWS11	-122.4648773	37.93886	-0.22	3.27	-286.8	3.05200008	-122.46487	37.93887	-0.15	3.27	-295.68	3.12400007	11	7/24/2014	27:12.2
5944	RWS11	-122.4648793	37.93886	-0.19	3.3	-286.8	3.1179999	-122.46487	37.93887	-0.15	3.3	-295.66	3.1559999	11	7/24/2014	27:12.3
5945	RWS11	-122.4648813	37.93886	-0.22	3.33	-286.8	3.11400011	-122.46488	37.93887	-0.15	3.33	-295.59	3.18600011	11	7/24/2014	27:12.4
5946	RWS11	-122.4648833	37.93886	-0.19	3.36	-286.9	3.17500006	-122.46488	37.93887	-0.15	3.36	-295.59	3.21300006	11	7/24/2014	27:12.5
5947	RWS11	-122.4648853	37.93886	-0.19	3.38	-286.9	3.19699989	-122.46488	37.93888	-0.2	3.38	-295.5	3.1849999	11	7/24/2014	27:12.6
5948	RWS11	-122.4648873	37.93886	-0.19	3.4	-286.8	3.21299993	-122.46488	37.93888	-0.2	3.4	-295.44	3.20099993	11	7/24/2014	27:12.7
5949	RWS11	-122.4648893	37.93886	-0.19	3.41	-286.8	3.22299992	-122.46488	37.93888	-0.2	3.41	-295.36	3.21099992	11	7/24/2014	27:12.8
5950	RWS11	-122.4648913	37.93886	-0.19	3.41	-286.7	3.22500001	-122.46489	37.93888	-0.15	3.41	-295.27	3.26300001	11	7/24/2014	27:12.9
5951	RWS11	-122.4648942	37.93887	-0.19	3.4	-286.7	3.21899997	-122.46489	37.93888	-0.2	3.4	-295.27	3.20699997	11	7/24/2014	27:13.0
5952	RWS11	-122.4648962	37.93887	-0.19	3.39	-286.6	3.20299993	-122.46489	37.93888	-0.15	3.39	-295.17	3.24099994	11	7/24/2014	27:13.1
5953	RWS11	-122.4648982	37.93887	-0.19	3.37	-286.6	3.17900001	-122.46489	37.93888	-0.2	3.37	-295.12	3.16700001	11	7/24/2014	27:13.2
5954	RWS11	-122.4649002	37.93887	-0.19	3.33	-286.5	3.14699994	-122.46489	37.93888	-0.15	3.33	-294.99	3.18499994	11	7/24/2014	27:13.3
5955	RWS11	-122.4649022	37.93887	-0.22	3.29	-286.5	3.07200006	-122.4649	37.93888	-0.2	3.29	-294.96	3.09400006	11	7/24/2014	27:13.4
5956	RWS11	-122.4649043	37.93887	-0.19	3.24	-286.4	3.05899988	-122.4649	37.93888	-0.15	3.24	-294.84	3.09699988	11	7/24/2014	27:13.5
5957	RWS11	-122.4649063	37.93887	-0.19	3.19	-286.2	3.00599991	-122.4649	37.93888	-0.15	3.19	-294.68	3.04399991	11	7/24/2014	27:13.6
5958	RWS11	-122.4649083	37.93887	-0.19	3.13	-286.1	2.94800006	-122.4649	37.93888	-0.2	3.13	-294.58	2.93600006	11	7/24/2014	27:13.7
5959	RWS11	-122.4649103	37.93887	-0.22	3.07	-286	2.85299996	-122.4649	37.93888	-0.2	3.07	-294.46	2.87499996	11	7/24/2014	27:13.8
5960	RWS11	-122.4649123	37.93887	-0.19	3.01	-285.9	2.82700001	-122.46491	37.93888	-0.15	3.01	-294.34	2.86500001	11	7/24/2014	27:13.9
5961	RWS11	-122.4649152	37.93887	-0.22	2.96	-285.8	2.73799995	-122.46491	37.93888	-0.2	2.96	-294.26	2.75999995	11	7/24/2014	27:14.0
5962	RWS11	-122.4649172	37.93887	-0.19	2.92	-285.8	2.72999988	-122.46491	37.93889	-0.15	2.92	-294.22	2.76799989	11	7/24/2014	27:14.1
5963	RWS11	-122.4649192	37.93887	-0.19	2.89	-285.8	2.70899998	-122.46491	37.93889	-0.2	2.89	-294.17	2.69699998	11	7/24/2014	27:14.2
5964	RWS11	-122.4649212	37.93887	-0.19	2.9	-285.7	2.71700011	-122.46492	37.93889	-0.15	2.9	-294.08	2.75500011	11	7/24/2014	27:14.3
5965	RWS11	-122.4649231	37.93887	-0.19	2.93	-285.7	2.74499999	-122.46492	37.93889	-0.2	2.93	-294.05	2.733	11	7/24/2014	27:14.4
5966	RWS11	-122.4649251	37.93887	-0.19	2.97	-285.6	2.781	-122.46492	37.93889	-0.15	2.97	-294.04	2.81900001	11	7/24/2014	27:14.5
5967	RWS11	-122.4649271	37.93888	-0.19	3.01	-285.5	2.82000004	-122.46492	37.93889	-0.2	3.01	-294	2.80800004	11	7/24/2014	27:14.6
5968	RWS11	-122.4649291	37.93888	-0.19	3.04	-285.4	2.85399996	-122.46492	37.93889	-0.15	3.04	-294	2.89199996	11	7/24/2014	27:14.7
5969	RWS11	-122.464931	37.93888	-0.22	3.07	-285.4	2.84599999	-122.46492	37.93889	-0.2	3.07	-293.96	2.86799999	11	7/24/2014	27:14.8
5970	RWS11	-122.464933	37.93888	-0.19	3.08	-285.3	2.89800011	-122.46493	37.93889	-0.15	3.08	-293.86	2.93600011	11	7/24/2014	27:14.9
5971	RWS11	-122.4649359	37.93888	-0.22	3.09	-285.1	2.87299994	-122.46493	37.93889	-0.2	3.09	-293.76	2.89499994	11	7/24/2014	27:15.0
5972	RWS11	-122.4649378	37.93888	-0.19	3.1	-285	2.90900002	-122.46493	37.93889	-0.2	3.1	-293.65	2.89700003	11	7/24/2014	27:15.1

5973	RWS11	-122.4649398	37.93888	-0.19	3.09	-284.9	2.906	-122.46493	37.93889	-0.2	3.09	-293.46	2.89400001	11	7/24/2014	27:15.2
5974	RWS11	-122.4649417	37.93888	-0.19	3.09	-284.8	2.90099989	-122.46494	37.93889	-0.11	3.09	-293.22	2.97299989	11	7/24/2014	27:15.3
5975	RWS11	-122.4649437	37.93888	-0.22	3.08	-284.7	2.85999992	-122.46494	37.93889	-0.15	3.08	-293.1	2.93199992	11	7/24/2014	27:15.4
5976	RWS11	-122.4649457	37.93888	-0.19	3.07	-284.6	2.88799988	-122.46494	37.93889	-0.15	3.07	-293.07	2.92599988	11	7/24/2014	27:15.5
5977	RWS11	-122.4649476	37.93888	-0.19	3.07	-284.5	2.88200007	-122.46494	37.93889	-0.2	3.07	-292.94	2.87000008	11	7/24/2014	27:15.6
5978	RWS11	-122.4649496	37.93888	-0.19	3.06	-284.3	2.87799989	-122.46494	37.93889	-0.2	3.06	-292.83	2.86599989	11	7/24/2014	27:15.7
5979	RWS11	-122.4649516	37.93888	-0.22	3.06	-284.2	2.84200004	-122.46495	37.9389	-0.2	3.06	-292.75	2.86400004	11	7/24/2014	27:15.8
5980	RWS11	-122.4649535	37.93888	-0.19	3.06	-284	2.87600003	-122.46495	37.9389	-0.2	3.06	-292.6	2.86400004	11	7/24/2014	27:15.9
5981	RWS11	-122.4649564	37.93888	-0.19	3.06	-283.8	2.87799989	-122.46495	37.9389	-0.23	3.06	-292.4	2.8319999	11	7/24/2014	27:16.0
5982	RWS11	-122.4649584	37.93888	-0.19	3.07	-283.7	2.88200007	-122.46495	37.9389	-0.15	3.07	-292.27	2.92000008	11	7/24/2014	27:16.1
5983	RWS11	-122.4649603	37.93889	-0.22	3.07	-283.5	2.85299996	-122.46495	37.9389	-0.15	3.07	-292.1	2.92499995	11	7/24/2014	27:16.2
5984	RWS11	-122.4649623	37.93889	-0.19	3.08	-283.4	2.89399992	-122.46496	37.9389	-0.15	3.08	-291.9	2.93199992	11	7/24/2014	27:16.3
5985	RWS11	-122.4649643	37.93889	-0.19	3.09	-283.3	2.90099989	-122.46496	37.9389	-0.15	3.09	-291.81	2.93899989	11	7/24/2014	27:16.4
5986	RWS11	-122.4649663	37.93889	-0.19	3.1	-283.2	2.90999995	-122.46496	37.9389	-0.2	3.1	-291.69	2.89799996	11	7/24/2014	27:16.5
5987	RWS11	-122.4649683	37.93889	-0.19	3.11	-283.2	2.91999994	-122.46496	37.9389	-0.15	3.11	-291.62	2.95799994	11	7/24/2014	27:16.6
5988	RWS11	-122.4649703	37.93889	-0.19	3.12	-283.1	2.92900001	-122.46496	37.9389	-0.15	3.12	-291.58	2.96700001	11	7/24/2014	27:16.7
5989	RWS11	-122.4649722	37.93889	-0.19	3.12	-283	2.93599997	-122.46497	37.9389	-0.2	3.12	-291.51	2.92399998	11	7/24/2014	27:16.8
5990	RWS11	-122.4649742	37.93889	-0.19	3.13	-282.9	2.94100009	-122.46497	37.9389	-0.15	3.13	-291.35	2.97900009	11	7/24/2014	27:16.9
5991	RWS11	-122.4649771	37.93889	-0.19	3.13	-282.8	2.94200002	-122.46497	37.9389	-0.2	3.13	-291.21	2.93000002	11	7/24/2014	27:17.0
5992	RWS11	-122.464979	37.93889	-0.19	3.13	-282.7	2.93999992	-122.46497	37.9389	-0.11	3.13	-291.07	3.01199993	11	7/24/2014	27:17.1
5993	RWS11	-122.464981	37.93889	-0.19	3.12	-282.7	2.93299995	-122.46498	37.9389	-0.15	3.12	-291.02	2.97099996	11	7/24/2014	27:17.2
5994	RWS11	-122.4649829	37.93889	-0.19	3.11	-282.7	2.92500006	-122.46498	37.9389	-0.15	3.11	-290.96	2.96300006	11	7/24/2014	27:17.3
5995	RWS11	-122.4649849	37.93889	-0.19	3.1	-282.5	2.9139999	-122.46498	37.93891	-0.15	3.1	-290.82	2.9519999	11	7/24/2014	27:17.4
5996	RWS11	-122.4649869	37.93889	-0.19	3.09	-282.4	2.90099989	-122.46498	37.93891	-0.11	3.09	-290.7	2.97299989	11	7/24/2014	27:17.5
5997	RWS11	-122.4649888	37.93889	-0.19	3.07	-282.3	2.88600002	-122.46498	37.93891	-0.15	3.07	-290.56	2.92400002	11	7/24/2014	27:17.6
5998	RWS11	-122.4649908	37.93889	-0.14	3.06	-282.2	2.92199992	-122.46498	37.93891	-0.11	3.06	-290.43	2.94299992	11	7/24/2014	27:17.7
5999	RWS11	-122.4649928	37.93889	-0.19	3.04	-282.1	2.85499988	-122.46499	37.93891	-0.15	3.04	-290.44	2.89299989	11	7/24/2014	27:17.8
6000	RWS11	-122.4649947	37.9389	-0.19	3.03	-282	2.84099995	-122.46499	37.93891	-0.11	3.03	-290.46	2.91299995	11	7/24/2014	27:17.9
6001	RWS11	-122.4649976	37.9389	-0.19	3.01	-281.9	2.82799993	-122.46499	37.93891	-0.15	3.01	-290.5	2.86599994	11	7/24/2014	27:18.0
6002	RWS11	-122.4649996	37.9389	-0.19	3	-281.8	2.81600009	-122.46499	37.93891	-0.11	3	-290.47	2.88800009	11	7/24/2014	27:18.1
6003	RWS11	-122.4650015	37.9389	-0.19	2.99	-281.7	2.80499993	-122.465	37.93891	-0.15	2.99	-290.46	2.84299994	11	7/24/2014	27:18.2
6004	RWS11	-122.4650035	37.9389	-0.19	2.98	-281.6	2.79700004	-122.465	37.93891	-0.11	2.98	-290.4	2.86900004	11	7/24/2014	27:18.3
6005	RWS11	-122.4650056	37.9389	-0.14	2.98	-281.5	2.84100007	-122.465	37.93891	-0.06	2.98	-290.36	2.91300007	11	7/24/2014	27:18.4

6006	RWS11	-122.4650076	37.9389	-0.14	2.97	-281.4	2.83699988	-122.465	37.93891	-0.11	2.97	-290.35	2.85799988	11	7/24/2014	27:18.5
6007	RWS11	-122.4650096	37.9389	-0.14	2.97	-281.4	2.83599995	-122.465	37.93891	-0.06	2.97	-290.36	2.90799996	11	7/24/2014	27:18.6
6008	RWS11	-122.4650116	37.9389	-0.14	2.97	-281.4	2.83699988	-122.46501	37.93891	-0.03	2.97	-290.36	2.94299988	11	7/24/2014	27:18.7
6009	RWS11	-122.4650135	37.9389	-0.19	2.98	-281.3	2.79199992	-122.46501	37.93891	-0.06	2.98	-290.31	2.91499992	11	7/24/2014	27:18.8
6010	RWS11	-122.4650155	37.9389	-0.14	2.99	-281.3	2.85199998	-122.46501	37.93891	-0.06	2.99	-290.3	2.92399999	11	7/24/2014	27:18.9
6011	RWS11	-122.4650185	37.9389	-0.14	3	-281.2	2.8629999	-122.46501	37.93891	-0.11	3	-290.2	2.88399991	11	7/24/2014	27:19.0
6012	RWS11	-122.4650205	37.9389	-0.14	3.01	-281.2	2.87800001	-122.46501	37.93892	-0.06	3.01	-290.16	2.95000001	11	7/24/2014	27:19.1
6013	RWS11	-122.4650225	37.9389	-0.14	3.03	-281.1	2.89300011	-122.46502	37.93892	-0.06	3.03	-290.14	2.96500012	11	7/24/2014	27:19.2
6014	RWS11	-122.4650245	37.9389	-0.14	3.04	-281	2.90700005	-122.46502	37.93892	-0.06	3.04	-290.1	2.97900005	11	7/24/2014	27:19.3
6015	RWS11	-122.4650265	37.9389	-0.14	3.06	-280.9	2.92000006	-122.46502	37.93892	-0.11	3.06	-290.06	2.94100007	11	7/24/2014	27:19.4
6016	RWS11	-122.4650285	37.93891	-0.14	3.07	-280.9	2.93099998	-122.46502	37.93892	-0.06	3.07	-290.01	3.00299998	11	7/24/2014	27:19.5
6017	RWS11	-122.4650306	37.93891	-0.14	3.08	-280.8	2.94000004	-122.46502	37.93892	-0.06	3.08	-289.92	3.01200005	11	7/24/2014	27:19.6
6018	RWS11	-122.4650326	37.93891	-0.14	3.08	-280.7	2.94499992	-122.46503	37.93892	-0.06	3.08	-289.86	3.01699992	11	7/24/2014	27:19.7
6019	RWS11	-122.4650345	37.93891	-0.19	3.08	-280.6	2.89500009	-122.46503	37.93892	-0.06	3.08	-289.74	3.01800009	11	7/24/2014	27:19.8
6020	RWS11	-122.4650365	37.93891	-0.14	3.08	-280.5	2.9419999	-122.46503	37.93892	-0.06	3.08	-289.6	3.0139999	11	7/24/2014	27:19.9
6021	RWS11	-122.4650395	37.93891	-0.14	3.07	-280.4	2.93300007	-122.46503	37.93892	-0.06	3.07	-289.38	3.00500008	11	7/24/2014	27:20.0
6022	RWS11	-122.4650415	37.93891	-0.1	3.05	-280.3	2.9539999	-122.46504	37.93892	-0.06	3.05	-289.28	2.9909999	11	7/24/2014	27:20.1
6023	RWS11	-122.4650434	37.93891	-0.14	3.04	-280.2	2.90199994	-122.46504	37.93892	-0.06	3.04	-289.17	2.97399994	11	7/24/2014	27:20.2
6024	RWS11	-122.4650454	37.93891	-0.1	3.02	-280.2	2.91900005	-122.46504	37.93892	-0.06	3.02	-289.13	2.95600005	11	7/24/2014	27:20.3
6025	RWS11	-122.4650474	37.93891	-0.14	3	-280	2.86599992	-122.46504	37.93892	-0.06	3	-289.04	2.93799993	11	7/24/2014	27:20.4
6026	RWS11	-122.4650494	37.93891	-0.1	2.99	-279.9	2.88600006	-122.46504	37.93892	-0.06	2.99	-288.91	2.92300006	11	7/24/2014	27:20.5
6027	RWS11	-122.4650514	37.93891	-0.14	2.97	-279.8	2.83800004	-122.46505	37.93892	-0.03	2.97	-288.73	2.94400005	11	7/24/2014	27:20.6
6028	RWS11	-122.4650534	37.93891	-0.1	2.96	-279.6	2.86199989	-122.46505	37.93892	-0.03	2.96	-288.57	2.93299989	11	7/24/2014	27:20.7
6029	RWS11	-122.4650554	37.93891	-0.14	2.95	-279.5	2.81999992	-122.46505	37.93893	-0.06	2.95	-288.46	2.89199992	11	7/24/2014	27:20.8
6030	RWS11	-122.4650574	37.93891	-0.1	2.95	-279.3	2.85000005	-122.46505	37.93893	-0.03	2.95	-288.23	2.92100005	11	7/24/2014	27:20.9
6031	RWS11	-122.4650603	37.93891	-0.14	2.95	-279.2	2.81200002	-122.46505	37.93893	-0.03	2.95	-288.02	2.91800003	11	7/24/2014	27:21.0
6032	RWS11	-122.4650623	37.93891	-0.1	2.94	-279	2.84499993	-122.46506	37.93893	-0.06	2.94	-287.75	2.88199993	11	7/24/2014	27:21.1
6033	RWS11	-122.4650642	37.93892	-0.14	2.94	-278.8	2.809	-122.46506	37.93893	-0.06	2.94	-287.64	2.881	11	7/24/2014	27:21.2
6034	RWS11	-122.4650663	37.93892	-0.1	2.94	-278.7	2.84300008	-122.46506	37.93893	-0.03	2.94	-287.53	2.91400008	11	7/24/2014	27:21.3
6035	RWS11	-122.4650683	37.93892	-0.14	2.94	-278.6	2.80699991	-122.46506	37.93893	-0.06	2.94	-287.42	2.87899991	11	7/24/2014	27:21.4
6036	RWS11	-122.4650703	37.93892	-0.1	2.94	-278.4	2.84099998	-122.46506	37.93893	-0.06	2.94	-287.31	2.87799998	11	7/24/2014	27:21.5
6037	RWS11	-122.4650723	37.93892	-0.1	2.94	-278.2	2.83899989	-122.46507	37.93893	-0.06	2.94	-287.05	2.87599989	11	7/24/2014	27:21.6
6038	RWS11	-122.4650743	37.93892	-0.1	2.94	-278	2.83700003	-122.46507	37.93893	-0.03	2.94	-286.93	2.90800004	11	7/24/2014	27:21.7

6039	RWS11	-122.4650763	37.93892	-0.14	2.93	-277.9	2.79999994	-122.46507	37.93893	-0.06	2.93	-286.79	2.87199994	11	7/24/2014	27:21.8
6040	RWS11	-122.4650783	37.93892	-0.1	2.93	-277.7	2.83199992	-122.46507	37.93893	0.02	2.93	-286.69	2.95399992	11	7/24/2014	27:21.9
6041	RWS11	-122.4650812	37.93892	-0.14	2.93	-277.5	2.79299997	-122.46508	37.93893	-0.06	2.93	-286.49	2.86499997	11	7/24/2014	27:22.0
6042	RWS11	-122.4650831	37.93892	-0.1	2.92	-277.4	2.82400002	-122.46508	37.93893	-0.03	2.92	-286.34	2.89500003	11	7/24/2014	27:22.1
6043	RWS11	-122.4650851	37.93892	-0.14	2.92	-277.2	2.78500007	-122.46508	37.93893	0.02	2.92	-286.17	2.94200008	11	7/24/2014	27:22.2
6044	RWS11	-122.4650871	37.93892	-0.1	2.92	-277	2.81599989	-122.46508	37.93893	-0.06	2.92	-285.95	2.85299989	11	7/24/2014	27:22.3
6045	RWS11	-122.465089	37.93892	-0.14	2.91	-276.8	2.77699994	-122.46508	37.93894	-0.03	2.91	-285.77	2.88299994	11	7/24/2014	27:22.4
6046	RWS11	-122.465091	37.93892	-0.1	2.91	-276.6	2.80799999	-122.46509	37.93894	0.02	2.91	-285.59	2.92999999	11	7/24/2014	27:22.5
6047	RWS11	-122.465093	37.93892	-0.14	2.9	-276.5	2.76800011	-122.46509	37.93894	-0.06	2.9	-285.37	2.84000012	11	7/24/2014	27:22.6
6048	RWS11	-122.465095	37.93892	-0.1	2.9	-276.3	2.79899993	-122.46509	37.93894	-0.03	2.9	-285.2	2.86999993	11	7/24/2014	27:22.7
6049	RWS11	-122.4650969	37.93892	-0.1	2.89	-276.2	2.79400005	-122.46509	37.93894	-0.06	2.89	-285.15	2.83100005	11	7/24/2014	27:22.8
6050	RWS11	-122.4650989	37.93893	-0.1	2.89	-276.1	2.78899994	-122.46509	37.93894	0.02	2.89	-285.02	2.91099994	11	7/24/2014	27:22.9
6051	RWS11	-122.4651018	37.93893	-0.1	2.88	-275.9	2.78499999	-122.4651	37.93894	0.02	2.88	-284.95	2.90699999	11	7/24/2014	27:23.0
6052	RWS11	-122.4651037	37.93893	-0.1	2.88	-275.8	2.7829999	-122.4651	37.93894	0.02	2.88	-284.83	2.9049999	11	7/24/2014	27:23.1
6053	RWS11	-122.4651057	37.93893	-0.1	2.88	-275.7	2.78100004	-122.4651	37.93894	-0.06	2.88	-284.74	2.81800004	11	7/24/2014	27:23.2
6054	RWS11	-122.4651077	37.93893	-0.1	2.88	-275.6	2.78100004	-122.4651	37.93894	-0.03	2.88	-284.53	2.85200004	11	7/24/2014	27:23.3
6055	RWS11	-122.4651097	37.93893	-0.1	2.88	-275.4	2.78199997	-122.4651	37.93894	-0.03	2.88	-284.26	2.85299997	11	7/24/2014	27:23.4
6056	RWS11	-122.4651117	37.93893	-0.1	2.88	-275.3	2.78400006	-122.46511	37.93894	-0.03	2.88	-284.12	2.85500006	11	7/24/2014	27:23.5
6057	RWS11	-122.4651136	37.93893	-0.14	2.89	-275.1	2.75300001	-122.46511	37.93894	-0.03	2.89	-283.94	2.85900001	11	7/24/2014	27:23.6
6058	RWS11	-122.4651156	37.93893	-0.1	2.89	-275	2.79400005	-122.46511	37.93894	-0.03	2.89	-283.9	2.86500005	11	7/24/2014	27:23.7
6059	RWS11	-122.4651176	37.93893	-0.14	2.9	-274.8	2.76600002	-122.46511	37.93894	-0.06	2.9	-283.76	2.83800002	11	7/24/2014	27:23.8
6060	RWS11	-122.4651196	37.93893	-0.1	2.91	-274.7	2.81000008	-122.46511	37.93894	-0.03	2.91	-283.58	2.88100009	11	7/24/2014	27:23.9
6061	RWS11	-122.4651225	37.93893	-0.14	2.92	-274.6	2.786	-122.46512	37.93894	-0.03	2.92	-283.41	2.892	11	7/24/2014	27:24.0
6062	RWS11	-122.4651244	37.93893	-0.1	2.93	-274.5	2.83400001	-122.46512	37.93895	-0.06	2.93	-283.23	2.87100001	11	7/24/2014	27:24.1
6063	RWS11	-122.4651264	37.93893	-0.14	2.95	-274.3	2.81299995	-122.46512	37.93895	-0.06	2.95	-283.08	2.88499995	11	7/24/2014	27:24.2
6064	RWS11	-122.4651284	37.93893	-0.1	2.96	-274.2	2.86499991	-122.46512	37.93895	-0.06	2.96	-282.93	2.90199991	11	7/24/2014	27:24.3
6065	RWS11	-122.4651303	37.93893	-0.14	2.98	-274	2.84599994	-122.46512	37.93895	-0.03	2.98	-282.73	2.95199995	11	7/24/2014	27:24.4
6066	RWS11	-122.4651323	37.93894	-0.14	3	-273.8	2.8629999	-122.46513	37.93895	-0.06	3	-282.47	2.93499991	11	7/24/2014	27:24.5
6067	RWS11	-122.4651343	37.93894	-0.14	3.02	-273.7	2.8800001	-122.46513	37.93895	-0.06	3.02	-282.26	2.9520001	11	7/24/2014	27:24.6
6068	RWS11	-122.4651362	37.93894	-0.14	3.03	-273.5	2.89400004	-122.46513	37.93895	-0.06	3.03	-282.17	2.96600004	11	7/24/2014	27:24.7
6069	RWS11	-122.4651382	37.93894	-0.14	3.04	-273.4	2.90700005	-122.46513	37.93895	-0.06	3.04	-282.11	2.97900005	11	7/24/2014	27:24.8
6070	RWS11	-122.4651401	37.93894	-0.14	3.05	-273.3	2.91799997	-122.46513	37.93895	-0.06	3.05	-281.98	2.98999997	11	7/24/2014	27:24.9
6071	RWS11	-122.465143	37.93894	-0.14	3.06	-273.1	2.9260001	-122.46514	37.93895	-0.06	3.06	-281.82	2.99800011	11	7/24/2014	27:25.0

6072	RWS11	-122.465145	37.93894	-0.14	3.07	-273	2.93300007	-122.46514	37.93895	-0.06	3.07	-281.72	3.00500008	11	7/24/2014	27:25.1
6073	RWS11	-122.4651469	37.93894	-0.19	3.08	-272.8	2.88900004	-122.46514	37.93895	-0.06	3.08	-281.54	3.01200005	11	7/24/2014	27:25.2
6074	RWS11	-122.4651489	37.93894	-0.14	3.08	-272.6	2.94600008	-122.46514	37.93895	-0.06	3.08	-281.44	3.01800009	11	7/24/2014	27:25.3
6075	RWS11	-122.4651508	37.93894	-0.19	3.09	-272.5	2.90200005	-122.46515	37.93895	-0.11	3.09	-281.31	2.97400006	11	7/24/2014	27:25.4
6076	RWS11	-122.4651528	37.93894	-0.14	3.1	-272.3	2.96199988	-122.46515	37.93895	-0.06	3.1	-281.09	3.03399988	11	7/24/2014	27:25.5
6077	RWS11	-122.4651548	37.93894	-0.14	3.11	-272.1	2.97000001	-122.46515	37.93895	-0.11	3.11	-280.92	2.99100002	11	7/24/2014	27:25.6
6078	RWS11	-122.4651568	37.93894	-0.19	3.11	-271.9	2.92699991	-122.46515	37.93895	-0.03	3.11	-280.66	3.08399992	11	7/24/2014	27:25.7
6079	RWS11	-122.4651587	37.93894	-0.19	3.12	-271.7	2.93500005	-122.46515	37.93896	-0.06	3.12	-280.4	3.05800005	11	7/24/2014	27:25.8
6080	RWS11	-122.4651607	37.93894	-0.19	3.13	-271.6	2.94100009	-122.46515	37.93896	-0.11	3.13	-280.27	3.01300009	11	7/24/2014	27:25.9
6081	RWS11	-122.4651636	37.93894	-0.19	3.13	-271.5	2.94500004	-122.46516	37.93896	-0.11	3.13	-280.2	3.01700004	11	7/24/2014	27:26.0
6082	RWS11	-122.4651655	37.93894	-0.19	3.13	-271.4	2.94699989	-122.46516	37.93896	-0.11	3.13	-280.14	3.0189999	11	7/24/2014	27:26.1
6083	RWS11	-122.4651675	37.93895	-0.19	3.13	-271.3	2.94800006	-122.46516	37.93896	-0.11	3.13	-280.14	3.02000006	11	7/24/2014	27:26.2
6084	RWS11	-122.4651694	37.93895	-0.19	3.13	-271.3	2.94599997	-122.46516	37.93896	-0.11	3.13	-280.21	3.01799997	11	7/24/2014	27:26.3
6085	RWS11	-122.4651714	37.93895	-0.19	3.13	-271.3	2.94200002	-122.46517	37.93896	-0.06	3.13	-280.25	3.06500002	11	7/24/2014	27:26.4
6086	RWS11	-122.4651734	37.93895	-0.19	3.12	-271.2	2.93599997	-122.46517	37.93896	-0.06	3.12	-280.24	3.05899998	11	7/24/2014	27:26.5
6087	RWS11	-122.4651753	37.93895	-0.19	3.12	-271.1	2.92900001	-122.46517	37.93896	-0.06	3.12	-280.17	3.05200001	11	7/24/2014	27:26.6
6088	RWS11	-122.4651773	37.93895	-0.19	3.11	-271.1	2.91999994	-122.46517	37.93896	-0.06	3.11	-280.04	3.04299995	11	7/24/2014	27:26.7
6089	RWS11	-122.4651792	37.93895	-0.19	3.1	-271	2.91200005	-122.46517	37.93896	-0.06	3.1	-279.97	3.03500005	11	7/24/2014	27:26.8
6090	RWS11	-122.4651812	37.93895	-0.19	3.09	-270.8	2.90500008	-122.46518	37.93896	-0.06	3.09	-279.91	3.02800008	11	7/24/2014	27:26.9
6091	RWS11	-122.465184	37.93895	-0.19	3.09	-270.7	2.89900003	-122.46518	37.93896	-0.06	3.09	-279.83	3.02200004	11	7/24/2014	27:27.0
6092	RWS11	-122.4651859	37.93895	-0.19	3.08	-270.5	2.89500009	-122.46518	37.93896	-0.03	3.08	-279.73	3.05200009	11	7/24/2014	27:27.1
6093	RWS11	-122.4651879	37.93895	-0.19	3.08	-270.4	2.89299999	-122.46518	37.93896	-0.06	3.08	-279.69	3.016	11	7/24/2014	27:27.2
6094	RWS11	-122.4651898	37.93895	-0.14	3.08	-270.3	2.94499992	-122.46518	37.93896	-0.06	3.08	-279.65	3.01699992	11	7/24/2014	27:27.3
6095	RWS11	-122.4651917	37.93895	-0.19	3.08	-270.2	2.89600001	-122.46519	37.93896	-0.06	3.08	-279.6	3.01900002	11	7/24/2014	27:27.4
6096	RWS11	-122.4651937	37.93895	-0.19	3.09	-270.2	2.89999996	-122.46519	37.93897	-0.06	3.09	-279.58	3.02299996	11	7/24/2014	27:27.5
6097	RWS11	-122.4651956	37.93895	-0.19	3.09	-270.1	2.90299998	-122.46519	37.93897	-0.06	3.09	-279.48	3.02599999	11	7/24/2014	27:27.6
6098	RWS11	-122.4651976	37.93895	-0.14	3.09	-270	2.95600007	-122.46519	37.93897	-0.06	3.09	-279.33	3.02800008	11	7/24/2014	27:27.7
6099	RWS11	-122.4651995	37.93895	-0.19	3.09	-269.9	2.906	-122.46519	37.93897	-0.06	3.09	-279.13	3.02900001	11	7/24/2014	27:27.8
6100	RWS11	-122.4652015	37.93895	-0.14	3.09	-269.8	2.95399998	-122.4652	37.93897	-0.06	3.09	-278.95	3.02599999	11	7/24/2014	27:27.9
6101	RWS11	-122.4652043	37.93896	-0.19	3.08	-269.8	2.89699994	-122.4652	37.93897	-0.06	3.08	-278.85	3.01999994	11	7/24/2014	27:28.0
6102	RWS11	-122.4652063	37.93896	-0.14	3.07	-269.6	2.93899988	-122.4652	37.93897	-0.06	3.07	-278.77	3.01099988	11	7/24/2014	27:28.1
6103	RWS11	-122.4652082	37.93896	-0.19	3.06	-269.6	2.87600003	-122.4652	37.93897	-0.06	3.06	-278.83	2.99900004	11	7/24/2014	27:28.2
6104	RWS11	-122.4652102	37.93896	-0.14	3.05	-269.5	2.91300009	-122.4652	37.93897	-0.06	3.05	-278.83	2.9850001	11	7/24/2014	27:28.3

6105	RWS11	-122.4652122	37.93896	-0.19	3.03	-269.4	2.84799992	-122.46521	37.93897	-0.06	3.03	-278.83	2.97099992	11	7/24/2014	27:28.4
6106	RWS11	-122.4652142	37.93896	-0.14	3.02	-269.3	2.88700007	-122.46521	37.93897	-0.03	3.02	-278.81	2.99300008	11	7/24/2014	27:28.5
6107	RWS11	-122.4652161	37.93896	-0.19	3.01	-269.2	2.82600008	-122.46521	37.93897	-0.06	3.01	-278.76	2.94900008	11	7/24/2014	27:28.6
6108	RWS11	-122.4652181	37.93896	-0.14	3.01	-269.1	2.87199996	-122.46521	37.93897	-0.03	3.01	-278.63	2.97799997	11	7/24/2014	27:28.7
6109	RWS11	-122.4652201	37.93896	-0.19	3.01	-268.9	2.82099997	-122.46521	37.93897	-0.06	3.01	-278.44	2.94399997	11	7/24/2014	27:28.8
6110	RWS11	-122.4652221	37.93896	-0.14	3.01	-268.8	2.87700008	-122.46522	37.93897	-0.06	3.01	-278.2	2.94900008	11	7/24/2014	27:28.9
6111	RWS11	-122.465225	37.93896	-0.14	3.02	-268.6	2.88700007	-122.46522	37.93897	-0.11	3.02	-277.99	2.90800007	11	7/24/2014	27:29.0
6112	RWS11	-122.465227	37.93896	-0.14	3.04	-268.4	2.90100001	-122.46522	37.93897	-0.06	3.04	-277.71	2.97300001	11	7/24/2014	27:29.1
6113	RWS11	-122.465229	37.93896	-0.19	3.05	-268.2	2.86600004	-122.46522	37.93898	-0.11	3.05	-277.42	2.93800005	11	7/24/2014	27:29.2
6114	RWS11	-122.465231	37.93896	-0.19	3.07	-268	2.88399993	-122.46523	37.93898	-0.11	3.07	-277.1	2.95599993	11	7/24/2014	27:29.3
6115	RWS11	-122.465233	37.93896	-0.19	3.09	-267.8	2.90200005	-122.46523	37.93898	-0.11	3.09	-276.91	2.97400006	11	7/24/2014	27:29.4
6116	RWS11	-122.465235	37.93896	-0.14	3.11	-267.6	2.97000001	-122.46523	37.93898	-0.11	3.11	-276.6	2.99100002	11	7/24/2014	27:29.5
6117	RWS11	-122.465237	37.93896	-0.19	3.12	-267.4	2.93500005	-122.46523	37.93898	-0.11	3.12	-276.41	3.00700005	11	7/24/2014	27:29.6
6118	RWS11	-122.465239	37.93897	-0.19	3.13	-267.2	2.94800006	-122.46523	37.93898	-0.11	3.13	-276.24	3.02000006	11	7/24/2014	27:29.7
6119	RWS11	-122.4652411	37.93897	-0.19	3.14	-267.1	2.95800005	-122.46524	37.93898	-0.15	3.14	-276.04	2.99600005	11	7/24/2014	27:29.8
6120	RWS11	-122.465243	37.93897	-0.19	3.15	-266.9	2.96400009	-122.46524	37.93898	-0.15	3.15	-275.87	3.00200009	11	7/24/2014	27:29.9
6121	RWS11	-122.465246	37.93897	-0.22	3.15	-266.7	2.93300012	-122.46524	37.93898	-0.15	3.15	-275.7	3.00500011	11	7/24/2014	27:30.0
6122	RWS11	-122.465248	37.93897	-0.19	3.15	-266.5	2.96700011	-122.46524	37.93898	-0.15	3.15	-275.48	3.00500011	11	7/24/2014	27:30.1
6123	RWS11	-122.46525	37.93897	-0.19	3.15	-266.4	2.96599995	-122.46524	37.93898	-0.2	3.15	-275.2	2.95399995	11	7/24/2014	27:30.2
6124	RWS11	-122.465252	37.93897	-0.19	3.15	-266.2	2.96400009	-122.46525	37.93898	-0.2	3.15	-275	2.9520001	11	7/24/2014	27:30.3
6125	RWS11	-122.4652541	37.93897	-0.22	3.15	-266	2.928	-122.46525	37.93898	-0.15	3.15	-274.72	3	11	7/24/2014	27:30.4
6126	RWS11	-122.4652561	37.93897	-0.19	3.15	-265.9	2.9599999	-122.46525	37.93898	-0.15	3.15	-274.42	2.99799991	11	7/24/2014	27:30.5
6127	RWS11	-122.4652581	37.93897	-0.22	3.14	-265.6	2.92299989	-122.46525	37.93898	-0.23	3.14	-274.08	2.91099989	11	7/24/2014	27:30.6
6128	RWS11	-122.4652602	37.93897	-0.19	3.14	-265.5	2.95100008	-122.46525	37.93898	-0.2	3.14	-273.8	2.93900008	11	7/24/2014	27:30.7
6129	RWS11	-122.4652622	37.93897	-0.22	3.13	-265.2	2.90899995	-122.46526	37.93898	-0.23	3.13	-273.47	2.89699996	11	7/24/2014	27:30.8
6130	RWS11	-122.4652642	37.93897	-0.22	3.12	-264.8	2.89899996	-122.46526	37.93899	-0.2	3.12	-273.04	2.92099996	11	7/24/2014	27:30.9
6131	RWS11	-122.4652672	37.93897	-0.22	3.11	-264.5	2.88599995	-122.46526	37.93899	-0.2	3.11	-272.65	2.90799995	11	7/24/2014	27:31.0
6132	RWS11	-122.4652692	37.93897	-0.19	3.09	-264.3	2.90500008	-122.46526	37.93899	-0.23	3.09	-272.33	2.85900009	11	7/24/2014	27:31.1
6133	RWS11	-122.4652713	37.93897	-0.22	3.08	-264.1	2.8569999	-122.46527	37.93899	-0.23	3.08	-272.09	2.84499991	11	7/24/2014	27:31.2
6134	RWS11	-122.4652733	37.93897	-0.19	3.06	-263.9	2.87600003	-122.46527	37.93899	-0.23	3.06	-271.82	2.83000004	11	7/24/2014	27:31.3
6135	RWS11	-122.4652754	37.93898	-0.22	3.05	-263.5	2.82900003	-122.46527	37.93899	-0.23	3.05	-271.53	2.81700003	11	7/24/2014	27:31.4
6136	RWS11	-122.4652775	37.93898	-0.22	3.04	-263.2	2.81800011	-122.46527	37.93899	-0.23	3.04	-271.18	2.80600011	11	7/24/2014	27:31.5
6137	RWS11	-122.4652796	37.93898	-0.22	3.03	-262.7	2.80800012	-122.46527	37.93899	-0.28	3.03	-270.8	2.74500012	11	7/24/2014	27:31.6

6138	RWS11	-122.4652816	37.93898	-0.22	3.02	-262.3	2.80099991	-122.46528	37.93899	-0.23	3.02	-270.4	2.78899992	11	7/24/2014	27:31.7
6139	RWS11	-122.4652837	37.93898	-0.22	3.02	-261.9	2.79799989	-122.46528	37.93899	-0.28	3.02	-269.96	2.7349999	11	7/24/2014	27:31.8
6140	RWS11	-122.4652857	37.93898	-0.22	3.02	-261.4	2.79900005	-122.46528	37.93899	-0.23	3.02	-269.54	2.78700006	11	7/24/2014	27:31.9
6141	RWS11	-122.4652887	37.93898	-0.27	3.03	-261	2.75300008	-122.46528	37.93899	-0.28	3.03	-269.17	2.7420001	11	7/24/2014	27:32.0
6142	RWS11	-122.4652908	37.93898	-0.22	3.04	-260.7	2.81600001	-122.46529	37.93899	-0.23	3.04	-268.86	2.80400002	11	7/24/2014	27:32.1
6143	RWS11	-122.4652928	37.93898	-0.27	3.05	-260.4	2.78000003	-122.46529	37.93899	-0.28	3.05	-268.58	2.76900005	11	7/24/2014	27:32.2
6144	RWS11	-122.4652949	37.93898	-0.22	3.07	-260.1	2.85200003	-122.46529	37.93899	-0.23	3.07	-268.3	2.84000003	11	7/24/2014	27:32.3
6145	RWS11	-122.4652969	37.93898	-0.27	3.1	-259.9	2.82399994	-122.46529	37.93899	-0.32	3.1	-268.02	2.77899995	11	7/24/2014	27:32.4
6146	RWS11	-122.465299	37.93898	-0.27	3.12	-259.7	2.84999996	-122.46529	37.93899	-0.23	3.12	-267.83	2.88999999	11	7/24/2014	27:32.5
6147	RWS11	-122.4653011	37.93898	-0.27	3.15	-259.4	2.87900001	-122.4653	37.939	-0.28	3.15	-267.59	2.86800003	11	7/24/2014	27:32.6
6148	RWS11	-122.4653031	37.93898	-0.27	3.18	-259.2	2.90899998	-122.4653	37.939	-0.23	3.18	-267.35	2.949	11	7/24/2014	27:32.7
6149	RWS11	-122.4653051	37.93898	-0.27	3.21	-259	2.93999988	-122.4653	37.939	-0.28	3.21	-267.13	2.9289999	11	7/24/2014	27:32.8
6150	RWS11	-122.4653072	37.93898	-0.27	3.24	-258.7	2.96899992	-122.4653	37.939	-0.28	3.24	-266.86	2.95799994	11	7/24/2014	27:32.9
6151	RWS11	-122.4653102	37.93898	-0.27	3.27	-258.5	2.99599987	-122.4653	37.939	-0.32	3.27	-266.6	2.95099989	11	7/24/2014	27:33.0
6152	RWS11	-122.4653122	37.93899	-0.27	3.29	-258.3	3.01799995	-122.46531	37.939	-0.28	3.29	-266.36	3.00699997	11	7/24/2014	27:33.1
6153	RWS11	-122.4653142	37.93899	-0.31	3.31	-258.1	3.00200009	-122.46531	37.939	-0.32	3.31	-266.22	2.99100009	11	7/24/2014	27:33.2
6154	RWS11	-122.4653162	37.93899	-0.31	3.32	-257.9	3.01300001	-122.46531	37.939	-0.28	3.32	-265.97	3.03600001	11	7/24/2014	27:33.3
6155	RWS11	-122.4653183	37.93899	-0.31	3.32	-257.6	3.01600003	-122.46531	37.939	-0.32	3.32	-265.77	3.00500003	11	7/24/2014	27:33.4
6156	RWS11	-122.4653203	37.93899	-0.31	3.32	-257.4	3.01200008	-122.46531	37.939	-0.32	3.32	-265.54	3.00100008	11	7/24/2014	27:33.5
6157	RWS11	-122.4653224	37.93899	-0.31	3.31	-257.1	3	-122.46532	37.939	-0.32	3.31	-265.33	2.98899999	11	7/24/2014	27:33.6
6158	RWS11	-122.4653244	37.93899	-0.31	3.29	-256.9	2.98300004	-122.46532	37.939	-0.32	3.29	-265.13	2.97200003	11	7/24/2014	27:33.7
6159	RWS11	-122.4653264	37.93899	-0.31	3.27	-256.8	2.96300006	-122.46532	37.939	-0.32	3.27	-265.02	2.95200005	11	7/24/2014	27:33.8
6160	RWS11	-122.4653284	37.93899	-0.31	3.25	-256.7	2.94199991	-122.46532	37.939	-0.32	3.25	-264.91	2.9309999	11	7/24/2014	27:33.9
6161	RWS11	-122.4653314	37.93899	-0.34	3.23	-256.5	2.88699999	-122.46533	37.939	-0.35	3.23	-264.69	2.87599999	11	7/24/2014	27:34.0
6162	RWS11	-122.4653335	37.93899	-0.31	3.21	-256.4	2.90199995	-122.46533	37.939	-0.32	3.21	-264.49	2.89099994	11	7/24/2014	27:34.1
6163	RWS11	-122.4653355	37.93899	-0.31	3.19	-256.3	2.88400006	-122.46533	37.939	-0.32	3.19	-264.41	2.87300006	11	7/24/2014	27:34.2
6164	RWS11	-122.4653376	37.93899	-0.31	3.17	-256.2	2.8670001	-122.46533	37.939	-0.32	3.17	-264.26	2.8560001	11	7/24/2014	27:34.3
6165	RWS11	-122.4653396	37.93899	-0.34	3.16	-256	2.81799999	-122.46533	37.93901	-0.35	3.16	-264.06	2.80699998	11	7/24/2014	27:34.4
6166	RWS11	-122.4653407	37.93899	-0.31	3.14	-255.9	2.83899999	-122.46534	37.93901	-0.32	3.14	-263.96	2.82799998	11	7/24/2014	27:34.5
6167	RWS11	-122.4653438	37.93899	-0.31	3.13	-255.8	2.8269999	-122.46534	37.93901	-0.35	3.13	-263.8	2.78199989	11	7/24/2014	27:34.6
6168	RWS11	-122.4653458	37.93899	-0.31	3.12	-255.6	2.81500006	-122.46534	37.93901	-0.32	3.12	-263.62	2.80400005	11	7/24/2014	27:34.7
6169	RWS11	-122.4653479	37.93899	-0.34	3.11	-255.4	2.76899996	-122.46534	37.93901	-0.35	3.11	-263.43	2.75799996	11	7/24/2014	27:34.8
6170	RWS11	-122.46535	37.939	-0.31	3.1	-255.2	2.79099989	-122.46534	37.93901	-0.32	3.1	-263.25	2.77999988	11	7/24/2014	27:34.9

6171	RWS11	-122.465353	37.939	-0.31	3.09	-255	2.77900004	-122.46535	37.93901	-0.35	3.09	-263.07	2.73400003	11	7/24/2014	27:35.0
6172	RWS11	-122.4653551	37.939	-0.31	3.07	-254.9	2.76699996	-122.46535	37.93901	-0.35	3.07	-262.98	2.72199994	11	7/24/2014	27:35.1
6173	RWS11	-122.4653572	37.939	-0.34	3.06	-254.7	2.72100011	-122.46535	37.93901	-0.35	3.06	-262.83	2.7100001	11	7/24/2014	27:35.2
6174	RWS11	-122.4653593	37.939	-0.31	3.05	-254.5	2.74399996	-122.46535	37.93901	-0.32	3.05	-262.81	2.73299995	11	7/24/2014	27:35.3
6175	RWS11	-122.4653614	37.939	-0.34	3.04	-254.5	2.69900003	-122.46536	37.93901	-0.35	3.04	-262.76	2.68800002	11	7/24/2014	27:35.4
6176	RWS11	-122.4653636	37.939	-0.31	3.03	-254.4	2.72300005	-122.46536	37.93901	-0.32	3.03	-262.75	2.71200004	11	7/24/2014	27:35.5
6177	RWS11	-122.4653657	37.939	-0.34	3.02	-254.4	2.67999998	-122.46536	37.93901	-0.28	3.02	-262.69	2.73699999	11	7/24/2014	27:35.6
6178	RWS11	-122.4653668	37.939	-0.31	3.01	-254.3	2.70499992	-122.46536	37.93901	-0.32	3.01	-262.58	2.69399992	11	7/24/2014	27:35.7
6179	RWS11	-122.4653699	37.939	-0.31	3.01	-254.3	2.70000005	-122.46536	37.93901	-0.35	3.01	-262.51	2.65500003	11	7/24/2014	27:35.8
6180	RWS11	-122.465372	37.939	-0.31	3	-254.2	2.69700003	-122.46537	37.93901	-0.32	3	-262.34	2.68600002	11	7/24/2014	27:35.9
6181	RWS11	-122.4653751	37.939	-0.31	3.01	-254.1	2.69900012	-122.46537	37.93901	-0.28	3.01	-262.18	2.72200012	11	7/24/2014	27:36.0
6182	RWS11	-122.4653762	37.939	-0.27	3.01	-254.1	2.74000007	-122.46537	37.93901	-0.28	3.01	-262.08	2.72900009	11	7/24/2014	27:36.1
6183	RWS11	-122.4653793	37.939	-0.31	3.02	-254.1	2.71600008	-122.46537	37.93902	-0.32	3.02	-262.06	2.70500007	11	7/24/2014	27:36.2
6184	RWS11	-122.4653814	37.939	-0.27	3.04	-254.2	2.76499993	-122.46538	37.93902	-0.32	3.04	-262.1	2.71999994	11	7/24/2014	27:36.3
6185	RWS11	-122.4653835	37.939	-0.31	3.05	-254.2	2.74799991	-122.46538	37.93902	-0.32	3.05	-262.08	2.7369999	11	7/24/2014	27:36.4
6186	RWS11	-122.4653857	37.939	-0.27	3.07	-254.2	2.80000001	-122.46538	37.93902	-0.32	3.07	-262.07	2.75500003	11	7/24/2014	27:36.5
6187	RWS11	-122.4653878	37.939	-0.31	3.09	-254.2	2.78500009	-122.46538	37.93902	-0.28	3.09	-261.97	2.80800009	11	7/24/2014	27:36.6
6188	RWS11	-122.4653899	37.93901	-0.27	3.11	-254.2	2.83600003	-122.46538	37.93902	-0.23	3.11	-261.91	2.87600005	11	7/24/2014	27:36.7
6189	RWS11	-122.465392	37.93901	-0.27	3.12	-254.3	2.85200006	-122.46539	37.93902	-0.23	3.12	-261.88	2.89200008	11	7/24/2014	27:36.8
6190	RWS11	-122.4653941	37.93901	-0.27	3.14	-254.2	2.86500007	-122.46539	37.93902	-0.2	3.14	-261.76	2.93900008	11	7/24/2014	27:36.9
6191	RWS11	-122.4653972	37.93901	-0.27	3.14	-254.2	2.87200004	-122.46539	37.93902	-0.28	3.14	-261.7	2.86100006	11	7/24/2014	27:37.0
6192	RWS11	-122.4653992	37.93901	-0.27	3.15	-254.2	2.87399989	-122.46539	37.93902	-0.28	3.15	-261.72	2.86299992	11	7/24/2014	27:37.1
6193	RWS11	-122.4654013	37.93901	-0.27	3.14	-254.2	2.87099987	-122.4654	37.93902	-0.28	3.14	-261.74	2.8599999	11	7/24/2014	27:37.2
6194	RWS11	-122.4654024	37.93901	-0.27	3.13	-254.2	2.86200005	-122.4654	37.93902	-0.32	3.13	-261.83	2.81700006	11	7/24/2014	27:37.3
6195	RWS11	-122.4654055	37.93901	-0.27	3.12	-254.3	2.84799987	-122.4654	37.93902	-0.23	3.12	-261.92	2.88799989	11	7/24/2014	27:37.4
6196	RWS11	-122.4654077	37.93901	-0.27	3.1	-254.3	2.83200008	-122.4654	37.93902	-0.28	3.1	-262.02	2.8210001	11	7/24/2014	27:37.5
6197	RWS11	-122.4654098	37.93901	-0.27	3.09	-254.5	2.81499988	-122.4654	37.93902	-0.32	3.09	-262.13	2.76999989	11	7/24/2014	27:37.6
6198	RWS11	-122.4654108	37.93901	-0.27	3.07	-254.5	2.79799992	-122.46541	37.93902	-0.23	3.07	-262.09	2.83799994	11	7/24/2014	27:37.7
6199	RWS11	-122.465414	37.93901	-0.27	3.06	-254.5	2.78300005	-122.46541	37.93902	-0.32	3.06	-262.1	2.73800007	11	7/24/2014	27:37.8
6200	RWS11	-122.4654161	37.93901	-0.27	3.04	-254.5	2.77000004	-122.46541	37.93902	-0.23	3.04	-262.09	2.81000006	11	7/24/2014	27:37.9
6201	RWS11	-122.4654192	37.93901	-0.27	3.03	-254.5	2.76099998	-122.46541	37.93903	-0.32	3.03	-262.13	2.71599999	11	7/24/2014	27:38.0
6202	RWS11	-122.4654203	37.93901	-0.27	3.03	-254.6	2.75499994	-122.46542	37.93903	-0.32	3.03	-262.21	2.70999995	11	7/24/2014	27:38.1
6203	RWS11	-122.4654235	37.93901	-0.27	3.02	-254.6	2.75199991	-122.46542	37.93903	-0.23	3.02	-262.28	2.79199994	11	7/24/2014	27:38.2

6204	RWS11	-122.4654256	37.93901	-0.27	3.02	-254.7	2.75099999	-122.46542	37.93903	-0.32	3.02	-262.39	2.706	11	7/24/2014	27:38.3
6205	RWS11	-122.4654277	37.93901	-0.27	3.03	-254.7	2.75300008	-122.46542	37.93903	-0.28	3.03	-262.45	2.7420001	11	7/24/2014	27:38.4
6206	RWS11	-122.4654299	37.93902	-0.27	3.03	-254.8	2.7560001	-122.46542	37.93903	-0.32	3.03	-262.57	2.71100011	11	7/24/2014	27:38.5
6207	RWS11	-122.465432	37.93902	-0.27	3.03	-254.8	2.76000005	-122.46543	37.93903	-0.28	3.03	-262.69	2.74900007	11	7/24/2014	27:38.6
6208	RWS11	-122.4654342	37.93902	-0.22	3.04	-254.8	2.81500009	-122.46543	37.93903	-0.35	3.04	-262.76	2.68400007	11	7/24/2014	27:38.7
6209	RWS11	-122.4654363	37.93902	-0.27	3.04	-254.9	2.76600009	-122.46543	37.93903	-0.4	3.04	-262.83	2.63700011	11	7/24/2014	27:38.8
6210	RWS11	-122.4654374	37.93902	-0.27	3.04	-254.9	2.76799995	-122.46543	37.93903	-0.35	3.04	-262.89	2.68899995	11	7/24/2014	27:38.9
6211	RWS11	-122.4654416	37.93902	-0.27	3.04	-254.9	2.76899987	-122.46544	37.93903	-0.35	3.04	-262.93	2.68999988	11	7/24/2014	27:39.0
6212	RWS11	-122.4654437	37.93902	-0.22	3.04	-254.9	2.82099989	-122.46544	37.93903	-0.35	3.04	-262.99	2.68999988	11	7/24/2014	27:39.1
6213	RWS11	-122.4654458	37.93902	-0.27	3.04	-254.8	2.76700002	-122.46544	37.93903	-0.4	3.04	-263.04	2.63800004	11	7/24/2014	27:39.2
6214	RWS11	-122.4654468	37.93902	-0.27	3.04	-254.8	2.76499993	-122.46544	37.93903	-0.4	3.04	-263.14	2.63599995	11	7/24/2014	27:39.3
6215	RWS11	-122.46545	37.93902	-0.27	3.03	-254.8	2.76099998	-122.46545	37.93903	-0.35	3.03	-263.14	2.68199998	11	7/24/2014	27:39.4
6216	RWS11	-122.4654522	37.93902	-0.27	3.03	-254.6	2.75499994	-122.46545	37.93903	-0.35	3.03	-263.09	2.67599994	11	7/24/2014	27:39.5
6217	RWS11	-122.4654543	37.93902	-0.31	3.02	-254.6	2.71399999	-122.46545	37.93903	-0.35	3.02	-263.14	2.66899997	11	7/24/2014	27:39.6
6218	RWS11	-122.4654554	37.93902	-0.27	3.01	-254.5	2.741	-122.46545	37.93903	-0.4	3.01	-263.14	2.61200002	11	7/24/2014	27:39.7
6219	RWS11	-122.4654586	37.93902	-0.31	3.01	-254.3	2.69900012	-122.46545	37.93903	-0.35	3.01	-263.03	2.6540001	11	7/24/2014	27:39.8
6220	RWS11	-122.4654607	37.93902	-0.27	3	-254.1	2.72499996	-122.46546	37.93904	-0.35	3	-262.93	2.64599997	11	7/24/2014	27:39.9
6221	RWS11	-122.4654639	37.93902	-0.31	2.99	-253.9	2.68400002	-122.46546	37.93904	-0.4	2.99	-262.87	2.58900002	11	7/24/2014	27:40.0
6222	RWS11	-122.4654649	37.93902	-0.27	2.98	-253.8	2.71100003	-122.46546	37.93904	-0.4	2.98	-262.77	2.58200005	11	7/24/2014	27:40.1
6223	RWS11	-122.4654681	37.93902	-0.31	2.98	-253.6	2.671	-122.46546	37.93904	-0.4	2.98	-262.64	2.57600001	11	7/24/2014	27:40.2
6224	RWS11	-122.4654703	37.93902	-0.27	2.97	-253.5	2.69999987	-122.46547	37.93904	-0.35	2.97	-262.62	2.62099987	11	7/24/2014	27:40.3
6225	RWS11	-122.4654724	37.93902	-0.31	2.97	-253.3	2.66199994	-122.46547	37.93904	-0.4	2.97	-262.51	2.56699994	11	7/24/2014	27:40.4
6226	RWS11	-122.4654735	37.93903	-0.27	2.96	-253.2	2.6929999	-122.46547	37.93904	-0.44	2.96	-262.4	2.52999991	11	7/24/2014	27:40.5
6227	RWS11	-122.4654767	37.93903	-0.31	2.96	-253	2.65899992	-122.46547	37.93904	-0.4	2.96	-262.19	2.56399992	11	7/24/2014	27:40.6
6228	RWS11	-122.4654789	37.93903	-0.31	2.97	-252.8	2.66100001	-122.46547	37.93904	-0.32	2.97	-261.92	2.65000001	11	7/24/2014	27:40.7
6229	RWS11	-122.465481	37.93903	-0.31	2.97	-252.5	2.66499996	-122.46548	37.93904	-0.44	2.97	-261.59	2.53599995	11	7/24/2014	27:40.8
6230	RWS11	-122.4654821	37.93903	-0.31	2.98	-252.2	2.671	-122.46548	37.93904	-0.4	2.98	-261.24	2.57600001	11	7/24/2014	27:40.9
6231	RWS11	-122.4654863	37.93903	-0.31	2.99	-251.9	2.68000007	-122.46548	37.93904	-0.4	2.99	-260.93	2.58500007	11	7/24/2014	27:41.0
6232	RWS11	-122.4654884	37.93903	-0.31	3	-251.6	2.69099998	-122.46548	37.93904	-0.4	3	-260.53	2.59599999	11	7/24/2014	27:41.1
6233	RWS11	-122.4654905	37.93903	-0.31	3.01	-251.2	2.704	-122.46549	37.93904	-0.44	3.01	-260.23	2.57499999	11	7/24/2014	27:41.2
6234	RWS11	-122.4654916	37.93903	-0.31	3.02	-251	2.71799994	-122.46549	37.93904	-0.4	3.02	-259.94	2.62299994	11	7/24/2014	27:41.3
6235	RWS11	-122.4654948	37.93903	-0.34	3.04	-250.6	2.6980001	-122.46549	37.93904	-0.44	3.04	-259.66	2.6030001	11	7/24/2014	27:41.4
6236	RWS11	-122.465497	37.93903	-0.31	3.05	-250.4	2.74600005	-122.46549	37.93904	-0.4	3.05	-259.35	2.65100005	11	7/24/2014	27:41.5

6237	RWS11	-122.4654991	37.93903	-0.34	3.07	-250.1	2.72500005	-122.46549	37.93904	-0.44	3.07	-259.04	2.63000005	11	7/24/2014	27:41.6
6238	RWS11	-122.4655002	37.93903	-0.34	3.08	-249.8	2.73599997	-122.4655	37.93904	-0.44	3.08	-258.75	2.64099997	11	7/24/2014	27:41.7
6239	RWS11	-122.4655033	37.93903	-0.34	3.09	-249.5	2.74599996	-122.4655	37.93904	-0.44	3.09	-258.48	2.65099996	11	7/24/2014	27:41.8
6240	RWS11	-122.4655055	37.93903	-0.31	3.09	-249.3	2.78699994	-122.4655	37.93905	-0.44	3.09	-258.15	2.65799993	11	7/24/2014	27:41.9
6241	RWS11	-122.4655086	37.93903	-0.34	3.1	-249	2.75699988	-122.4655	37.93905	-0.49	3.1	-257.84	2.61099988	11	7/24/2014	27:42.0
6242	RWS11	-122.4655096	37.93903	-0.34	3.1	-248.8	2.7599999	-122.46551	37.93905	-0.4	3.1	-257.52	2.69899991	11	7/24/2014	27:42.1
6243	RWS11	-122.4655127	37.93903	-0.39	3.1	-248.5	2.711	-122.46551	37.93905	-0.44	3.1	-257.18	2.667	11	7/24/2014	27:42.2
6244	RWS11	-122.4655149	37.93903	-0.34	3.1	-248.2	2.76400009	-122.46551	37.93905	-0.44	3.1	-256.89	2.66900009	11	7/24/2014	27:42.3
6245	RWS11	-122.4655169	37.93903	-0.39	3.11	-248	2.71499994	-122.46551	37.93905	-0.44	3.11	-256.63	2.67099994	11	7/24/2014	27:42.4
6246	RWS11	-122.465518	37.93904	-0.34	3.11	-247.7	2.76899996	-122.46551	37.93905	-0.44	3.11	-256.37	2.67399997	11	7/24/2014	27:42.5
6247	RWS11	-122.4655212	37.93904	-0.39	3.11	-247.5	2.72199991	-122.46552	37.93905	-0.44	3.11	-256.13	2.67799991	11	7/24/2014	27:42.6
6248	RWS11	-122.4655233	37.93904	-0.39	3.12	-247.2	2.72700003	-122.46552	37.93905	-0.44	3.12	-255.78	2.68300003	11	7/24/2014	27:42.7
6249	RWS11	-122.4655253	37.93904	-0.39	3.13	-247	2.734	-122.46552	37.93905	-0.49	3.13	-255.52	2.639	11	7/24/2014	27:42.8
6250	RWS11	-122.4655264	37.93904	-0.34	3.13	-246.8	2.79400006	-122.46552	37.93905	-0.44	3.13	-255.31	2.69900006	11	7/24/2014	27:42.9
6251	RWS11	-122.4655304	37.93904	-0.39	3.14	-246.5	2.75199988	-122.46553	37.93905	-0.49	3.14	-254.98	2.65699989	11	7/24/2014	27:43.0
6252	RWS11	-122.4655325	37.93904	-0.39	3.15	-246.2	2.76099995	-122.46553	37.93905	-0.4	3.15	-254.6	2.75099996	11	7/24/2014	27:43.1
6253	RWS11	-122.4655345	37.93904	-0.39	3.16	-246	2.77000001	-122.46553	37.93905	-0.44	3.16	-254.34	2.72600001	11	7/24/2014	27:43.2
6254	RWS11	-122.4655355	37.93904	-0.39	3.17	-245.7	2.77699998	-122.46553	37.93905	-0.44	3.17	-253.97	2.73299998	11	7/24/2014	27:43.3
6255	RWS11	-122.4655386	37.93904	-0.39	3.17	-245.4	2.78300002	-122.46553	37.93905	-0.4	3.17	-253.62	2.77300003	11	7/24/2014	27:43.4
6256	RWS11	-122.4655407	37.93904	-0.39	3.18	-245.1	2.7879999	-122.46554	37.93905	-0.4	3.18	-253.23	2.77799991	11	7/24/2014	27:43.5
6257	RWS11	-122.4655427	37.93904	-0.39	3.18	-244.7	2.79099992	-122.46554	37.93905	-0.44	3.18	-252.75	2.74699992	11	7/24/2014	27:43.6
6258	RWS11	-122.4655438	37.93904	-0.39	3.18	-244.4	2.79200009	-122.46554	37.93905	-0.35	3.18	-252.35	2.83200008	11	7/24/2014	27:43.7
6259	RWS11	-122.4655469	37.93904	-0.39	3.18	-244	2.79300001	-122.46554	37.93905	-0.49	3.18	-251.9	2.69800001	11	7/24/2014	27:43.8
6260	RWS11	-122.4655489	37.93904	-0.39	3.18	-243.6	2.79200009	-122.46554	37.93905	-0.4	3.18	-251.42	2.78200009	11	7/24/2014	27:43.9
6261	RWS11	-122.4655521	37.93904	-0.39	3.18	-243.3	2.79099992	-122.46555	37.93906	-0.49	3.18	-251.07	2.69599992	11	7/24/2014	27:44.0
6262	RWS11	-122.4655531	37.93904	-0.39	3.18	-243	2.78999999	-122.46555	37.93906	-0.44	3.18	-250.65	2.74599999	11	7/24/2014	27:44.1
6263	RWS11	-122.4655563	37.93904	-0.39	3.18	-242.7	2.7879999	-122.46555	37.93906	-0.4	3.18	-250.24	2.77799991	11	7/24/2014	27:44.2
6264	RWS11	-122.4655585	37.93904	-0.39	3.18	-242.3	2.78500012	-122.46555	37.93906	-0.4	3.18	-249.82	2.77500013	11	7/24/2014	27:44.3
6265	RWS11	-122.4655607	37.93904	-0.39	3.17	-241.9	2.78200009	-122.46556	37.93906	-0.4	3.17	-249.36	2.7720001	11	7/24/2014	27:44.4
6266	RWS11	-122.4655618	37.93904	-0.39	3.17	-241.5	2.77799991	-122.46556	37.93906	-0.4	3.17	-248.97	2.76799992	11	7/24/2014	27:44.5
6267	RWS11	-122.4655651	37.93905	-0.39	3.16	-241.2	2.77300003	-122.46556	37.93906	-0.44	3.16	-248.58	2.72900003	11	7/24/2014	27:44.6
6268	RWS11	-122.4655673	37.93905	-0.34	3.16	-240.9	2.81799999	-122.46556	37.93906	-0.4	3.16	-248.28	2.757	11	7/24/2014	27:44.7
6269	RWS11	-122.4655695	37.93905	-0.39	3.15	-240.6	2.76099995	-122.46557	37.93906	-0.44	3.15	-248.06	2.71699995	11	7/24/2014	27:44.8

6270	RWS11	-122.4655706	37.93905	-0.39	3.14	-240.3	2.75300005	-122.46557	37.93906	-0.44	3.14	-247.73	2.70900005	11	7/24/2014	27:44.9
6271	RWS11	-122.4655749	37.93905	-0.39	3.14	-240	2.74499992	-122.46557	37.93906	-0.44	3.14	-247.39	2.70099992	11	7/24/2014	27:45.0
6272	RWS11	-122.465577	37.93905	-0.39	3.13	-239.6	2.73799995	-122.46557	37.93906	-0.4	3.13	-246.99	2.72799996	11	7/24/2014	27:45.1
6273	RWS11	-122.4655792	37.93905	-0.39	3.12	-239.3	2.7319999	-122.46557	37.93906	-0.49	3.12	-246.66	2.63699991	11	7/24/2014	27:45.2
6274	RWS11	-122.4655803	37.93905	-0.34	3.12	-239	2.77800003	-122.46558	37.93906	-0.49	3.12	-246.33	2.63200003	11	7/24/2014	27:45.3
6275	RWS11	-122.4655835	37.93905	-0.39	3.12	-238.6	2.72400001	-122.46558	37.93906	-0.44	3.12	-246.08	2.68000001	11	7/24/2014	27:45.4
6276	RWS11	-122.4655857	37.93905	-0.34	3.12	-238.3	2.77500001	-122.46558	37.93906	-0.4	3.12	-245.68	2.71400002	11	7/24/2014	27:45.5
6277	RWS11	-122.4655878	37.93905	-0.39	3.12	-238	2.72700003	-122.46558	37.93906	-0.44	3.12	-245.34	2.68300003	11	7/24/2014	27:45.6
6278	RWS11	-122.4655889	37.93905	-0.34	3.12	-237.6	2.7829999	-122.46558	37.93906	-0.44	3.12	-245.07	2.6879999	11	7/24/2014	27:45.7
6279	RWS11	-122.465592	37.93905	-0.39	3.13	-237.3	2.74099997	-122.46559	37.93906	-0.44	3.13	-244.7	2.69699997	11	7/24/2014	27:45.8
6280	RWS11	-122.4655942	37.93905	-0.34	3.14	-237	2.80400005	-122.46559	37.93906	-0.44	3.14	-244.34	2.70900005	11	7/24/2014	27:45.9
6281	RWS11	-122.4655973	37.93905	-0.39	3.16	-236.8	2.76699999	-122.46559	37.93907	-0.44	3.16	-244.08	2.72299999	11	7/24/2014	27:46.0
6282	RWS11	-122.4655983	37.93905	-0.34	3.17	-236.5	2.83400002	-122.46559	37.93907	-0.49	3.17	-243.78	2.68800002	11	7/24/2014	27:46.1
6283	RWS11	-122.4656014	37.93905	-0.39	3.19	-236.2	2.80099991	-122.4656	37.93907	-0.49	3.19	-243.52	2.70599991	11	7/24/2014	27:46.2
6284	RWS11	-122.4656035	37.93905	-0.34	3.21	-236	2.87199989	-122.4656	37.93907	-0.4	3.21	-243.2	2.8109999	11	7/24/2014	27:46.3
6285	RWS11	-122.4656056	37.93905	-0.39	3.23	-235.6	2.84100011	-122.4656	37.93907	-0.44	3.23	-242.86	2.79700011	11	7/24/2014	27:46.4
6286	RWS11	-122.4656067	37.93905	-0.34	3.25	-235.4	2.91300002	-122.4656	37.93907	-0.44	3.25	-242.6	2.81800002	11	7/24/2014	27:46.5
6287	RWS11	-122.4656098	37.93905	-0.39	3.27	-235.1	2.88299993	-122.46561	37.93907	-0.44	3.27	-242.33	2.83899993	11	7/24/2014	27:46.6
6288	RWS11	-122.4656119	37.93906	-0.34	3.29	-234.8	2.95399991	-122.46561	37.93907	-0.4	3.29	-242.01	2.89299992	11	7/24/2014	27:46.7
6289	RWS11	-122.4656139	37.93906	-0.39	3.31	-234.6	2.92100003	-122.46561	37.93907	-0.49	3.31	-241.73	2.82600003	11	7/24/2014	27:46.8
6290	RWS11	-122.465615	37.93906	-0.34	3.33	-234.3	2.98800007	-122.46561	37.93907	-0.44	3.33	-241.38	2.89300007	11	7/24/2014	27:46.9
6291	RWS11	-122.4656191	37.93906	-0.39	3.34	-234	2.94799998	-122.46561	37.93907	-0.44	3.34	-240.95	2.90399998	11	7/24/2014	27:47.0
6292	RWS11	-122.4656212	37.93906	-0.34	3.35	-233.6	3.00599995	-122.46562	37.93907	-0.4	3.35	-240.6	2.94499996	11	7/24/2014	27:47.1
6293	RWS11	-122.4656233	37.93906	-0.39	3.35	-233.3	2.95700005	-122.46562	37.93907	-0.49	3.35	-240.23	2.86200005	11	7/24/2014	27:47.2
6294	RWS11	-122.4656254	37.93906	-0.34	3.34	-233.1	3.0040001	-122.46562	37.93907	-0.4	3.34	-240.04	2.94300011	11	7/24/2014	27:47.3
6295	RWS11	-122.4656275	37.93906	-0.39	3.33	-232.7	2.94300011	-122.46562	37.93907	-0.49	3.33	-239.83	2.84800011	11	7/24/2014	27:47.4
6296	RWS11	-122.4656297	37.93906	-0.34	3.32	-232.4	2.979	-122.46563	37.93907	-0.44	3.32	-239.57	2.884	11	7/24/2014	27:47.5
6297	RWS11	-122.4656318	37.93906	-0.39	3.3	-232.1	2.90599993	-122.46563	37.93907	-0.49	3.3	-239.28	2.81099993	11	7/24/2014	27:47.6
6298	RWS11	-122.4656339	37.93906	-0.34	3.27	-231.7	2.9309999	-122.46563	37.93907	-0.44	3.27	-238.94	2.83599991	11	7/24/2014	27:47.7
6299	RWS11	-122.465636	37.93906	-0.39	3.24	-231.5	2.84999993	-122.46563	37.93907	-0.44	3.24	-238.64	2.80599993	11	7/24/2014	27:47.8
6300	RWS11	-122.4656381	37.93906	-0.34	3.21	-231.2	2.86900011	-122.46563	37.93907	-0.4	3.21	-238.39	2.80800012	11	7/24/2014	27:47.9
6301	RWS11	-122.4656412	37.93906	-0.39	3.18	-230.9	2.78600004	-122.46564	37.93907	-0.4	3.18	-238.18	2.77600005	11	7/24/2014	27:48.0
6302	RWS11	-122.4656433	37.93906	-0.34	3.15	-230.7	2.808	-122.46564	37.93907	-0.44	3.15	-237.93	2.713	11	7/24/2014	27:48.1

6303	RWS11	-122.4656453	37.93906	-0.39	3.12	-230.3	2.7319999	-122.46564	37.93908	-0.49	3.12	-237.68	2.63699991	11	7/24/2014 27:48.2
6304	RWS11	-122.4656475	37.93906	-0.34	3.1	-230.1	2.76199999	-122.46564	37.93908	-0.44	3.1	-237.49	2.667	11	7/24/2014 27:48.3
6305	RWS11	-122.4656495	37.93906	-0.39	3.09	-229.8	2.69499996	-122.46565	37.93908	-0.44	3.09	-237.1	2.65099996	11	7/24/2014 27:48.4
6306	RWS11	-122.4656517	37.93906	-0.34	3.07	-229.4	2.73299995	-122.46565	37.93908	-0.44	3.07	-236.58	2.63799995	11	7/24/2014 27:48.5
6307	RWS11	-122.4656537	37.93906	-0.39	3.07	-229.2	2.67400005	-122.46565	37.93908	-0.44	3.07	-236.23	2.63000005	11	7/24/2014 27:48.6
6308	RWS11	-122.4656559	37.93906	-0.34	3.06	-228.9	2.71999994	-122.46565	37.93908	-0.44	3.06	-235.94	2.62499994	11	7/24/2014 27:48.7
6309	RWS11	-122.4656579	37.93906	-0.39	3.06	-228.6	2.66499999	-122.46565	37.93908	-0.44	3.06	-235.65	2.62099999	11	7/24/2014 27:48.8
6310	RWS11	-122.46566	37.93907	-0.39	3.05	-228.3	2.6629999	-122.46566	37.93908	-0.44	3.05	-235.38	2.6189999	11	7/24/2014 27:48.9
6311	RWS11	-122.4656631	37.93907	-0.39	3.05	-228	2.66100004	-122.46566	37.93908	-0.44	3.05	-235.17	2.61700004	11	7/24/2014 27:49.0
6312	RWS11	-122.4656652	37.93907	-0.34	3.05	-227.8	2.70800009	-122.46566	37.93908	-0.4	3.05	-235.05	2.6470001	11	7/24/2014 27:49.1
6313	RWS11	-122.4656673	37.93907	-0.39	3.04	-227.5	2.65199998	-122.46566	37.93908	-0.44	3.04	-234.88	2.60799998	11	7/24/2014 27:49.2
6314	RWS11	-122.4656694	37.93907	-0.39	3.04	-227.2	2.64500001	-122.46567	37.93908	-0.44	3.04	-234.68	2.60100001	11	7/24/2014 27:49.3
6315	RWS11	-122.4656715	37.93907	-0.39	3.03	-227	2.63599995	-122.46567	37.93908	-0.44	3.03	-234.42	2.59199995	11	7/24/2014 27:49.4
6316	RWS11	-122.4656736	37.93907	-0.39	3.02	-226.7	2.62599996	-122.46567	37.93908	-0.4	3.02	-234.15	2.61599997	11	7/24/2014 27:49.5
6317	RWS11	-122.4656757	37.93907	-0.39	3.01	-226.4	2.61599997	-122.46567	37.93908	-0.44	3.01	-233.81	2.57199997	11	7/24/2014 27:49.6
6318	RWS11	-122.4656778	37.93907	-0.39	3	-226.1	2.60599998	-122.46567	37.93908	-0.4	3	-233.5	2.59599999	11	7/24/2014 27:49.7
6319	RWS11	-122.4656799	37.93907	-0.39	2.99	-225.8	2.59699991	-122.46568	37.93908	-0.44	2.99	-233.26	2.55299991	11	7/24/2014 27:49.8
6320	RWS11	-122.465682	37.93907	-0.34	2.98	-225.5	2.64099994	-122.46568	37.93908	-0.44	2.98	-232.92	2.54599994	11	7/24/2014 27:49.9
6321	RWS11	-122.4656851	37.93907	-0.39	2.97	-225.1	2.5839999	-122.46568	37.93908	-0.44	2.97	-232.58	2.5399999	11	7/24/2014 27:50.0
6322	RWS11	-122.4656872	37.93907	-0.39	2.97	-224.8	2.58200005	-122.46568	37.93908	-0.44	2.97	-232.3	2.53800005	11	7/24/2014 27:50.1
6323	RWS11	-122.4656893	37.93907	-0.39	2.97	-224.5	2.58299997	-122.46568	37.93908	-0.49	2.97	-231.95	2.48799998	11	7/24/2014 27:50.2
6324	RWS11	-122.4656914	37.93907	-0.39	2.98	-224.1	2.58699992	-122.46569	37.93908	-0.44	2.98	-231.53	2.54299992	11	7/24/2014 27:50.3
6325	RWS11	-122.4656935	37.93907	-0.43	2.99	-223.7	2.56000006	-122.46569	37.93909	-0.44	2.99	-231.13	2.55100006	11	7/24/2014 27:50.4
6326	RWS11	-122.4656957	37.93907	-0.39	3	-223.5	2.6069999	-122.46569	37.93909	-0.44	3	-230.79	2.5629999	11	7/24/2014 27:50.5
6327	RWS11	-122.4656978	37.93907	-0.39	3.02	-223.2	2.6240001	-122.46569	37.93909	-0.4	3.02	-230.48	2.61400011	11	7/24/2014 27:50.6
6328	RWS11	-122.4656999	37.93907	-0.39	3.03	-222.9	2.64299992	-122.4657	37.93909	-0.44	3.03	-230.22	2.59899992	11	7/24/2014 27:50.7
6329	RWS11	-122.465702	37.93907	-0.39	3.06	-222.6	2.66599992	-122.4657	37.93909	-0.44	3.06	-229.94	2.62199992	11	7/24/2014 27:50.8
6330	RWS11	-122.4657041	37.93907	-0.39	3.08	-222.2	2.69100001	-122.4657	37.93909	-0.44	3.08	-229.58	2.64700001	11	7/24/2014 27:50.9
6331	RWS11	-122.4657073	37.93908	-0.39	3.11	-221.9	2.71799996	-122.4657	37.93909	-0.49	3.11	-229.31	2.62299997	11	7/24/2014 27:51.0
6332	RWS11	-122.4657094	37.93908	-0.39	3.14	-221.7	2.74499992	-122.46571	37.93909	-0.44	3.14	-229.06	2.70099992	11	7/24/2014 27:51.1
6333	RWS11	-122.4657115	37.93908	-0.39	3.16	-221.3	2.7720001	-122.46571	37.93909	-0.44	3.16	-228.76	2.7280001	11	7/24/2014 27:51.2
6334	RWS11	-122.4657136	37.93908	-0.39	3.19	-221	2.79699996	-122.46571	37.93909	-0.44	3.19	-228.47	2.75299996	11	7/24/2014 27:51.3
6335	RWS11	-122.4657157	37.93908	-0.39	3.21	-220.7	2.81999996	-122.46571	37.93909	-0.44	3.21	-228.24	2.77599996	11	7/24/2014 27:51.4

6336	RWS11	-122.4657178	37.93908	-0.39	3.23	-220.4	2.83800009	-122.46571	37.93909	-0.44	3.23	-227.93	2.79400009	11	7/24/2014	27:51.5
6337	RWS11	-122.46572	37.93908	-0.43	3.24	-220	2.81499994	-122.46572	37.93909	-0.49	3.24	-227.69	2.75499994	11	7/24/2014	27:51.6
6338	RWS11	-122.4657221	37.93908	-0.39	3.25	-219.6	2.85500005	-122.46572	37.93909	-0.4	3.25	-227.35	2.84500006	11	7/24/2014	27:51.7
6339	RWS11	-122.4657242	37.93908	-0.39	3.24	-219.3	2.85299996	-122.46572	37.93909	-0.4	3.24	-227.04	2.84299996	11	7/24/2014	27:51.8
6340	RWS11	-122.4657263	37.93908	-0.39	3.24	-218.9	2.84500006	-122.46572	37.93909	-0.44	3.24	-226.75	2.80100006	11	7/24/2014	27:51.9
6341	RWS11	-122.4657294	37.93908	-0.39	3.22	-218.7	2.82999995	-122.46573	37.93909	-0.44	3.22	-226.53	2.78599995	11	7/24/2014	27:52.0
6342	RWS11	-122.4657314	37.93908	-0.39	3.2	-218.4	2.8109999	-122.46573	37.93909	-0.4	3.2	-226.3	2.80099991	11	7/24/2014	27:52.1
6343	RWS11	-122.4657335	37.93908	-0.39	3.18	-218.1	2.7879999	-122.46573	37.93909	-0.49	3.18	-226.08	2.6929999	11	7/24/2014	27:52.2
6344	RWS11	-122.4657356	37.93908	-0.39	3.15	-217.9	2.76200011	-122.46573	37.93909	-0.4	3.15	-225.79	2.75200012	11	7/24/2014	27:52.3
6345	RWS11	-122.4657377	37.93908	-0.39	3.13	-217.6	2.73600009	-122.46573	37.93909	-0.44	3.13	-225.52	2.69200009	11	7/24/2014	27:52.4
6346	RWS11	-122.4657398	37.93908	-0.39	3.1	-217.3	2.711	-122.46574	37.93909	-0.44	3.1	-225.21	2.667	11	7/24/2014	27:52.5
6347	RWS11	-122.4657419	37.93908	-0.39	3.08	-217.1	2.68799999	-122.46574	37.9391	-0.44	3.08	-224.95	2.64399999	11	7/24/2014	27:52.6
6348	RWS11	-122.465744	37.93908	-0.39	3.06	-216.8	2.66899994	-122.46574	37.9391	-0.4	3.06	-224.68	2.65899995	11	7/24/2014	27:52.7
6349	RWS11	-122.465746	37.93908	-0.39	3.05	-216.5	2.65400007	-122.46574	37.9391	-0.44	3.05	-224.34	2.61000007	11	7/24/2014	27:52.8
6350	RWS11	-122.4657481	37.93908	-0.39	3.03	-216.2	2.64299992	-122.46574	37.9391	-0.44	3.03	-223.96	2.59899992	11	7/24/2014	27:52.9
6351	RWS11	-122.4657512	37.93908	-0.39	3.03	-215.9	2.63599995	-122.46575	37.9391	-0.44	3.03	-223.64	2.59199995	11	7/24/2014	27:53.0
6352	RWS11	-122.4657532	37.93908	-0.43	3.03	-215.6	2.5990001	-122.46575	37.9391	-0.44	3.03	-223.29	2.59000009	11	7/24/2014	27:53.1
6353	RWS11	-122.4657553	37.93909	-0.43	3.03	-215.3	2.60200012	-122.46575	37.9391	-0.4	3.03	-222.87	2.62700012	11	7/24/2014	27:53.2
6354	RWS11	-122.4657574	37.93909	-0.39	3.03	-215	2.64299992	-122.46575	37.9391	-0.4	3.03	-222.57	2.63299993	11	7/24/2014	27:53.3
6355	RWS11	-122.4657595	37.93909	-0.39	3.04	-214.7	2.65299991	-122.46576	37.9391	-0.44	3.04	-222.29	2.60899991	11	7/24/2014	27:53.4
6356	RWS11	-122.4657616	37.93909	-0.39	3.06	-214.3	2.66700009	-122.46576	37.9391	-0.4	3.06	-222.08	2.65700009	11	7/24/2014	27:53.5
6357	RWS11	-122.4657637	37.93909	-0.39	3.08	-214	2.68400005	-122.46576	37.9391	-0.44	3.08	-221.82	2.64000005	11	7/24/2014	27:53.6
6358	RWS11	-122.4657658	37.93909	-0.39	3.1	-213.8	2.70400003	-122.46576	37.9391	-0.4	3.1	-221.64	2.69400004	11	7/24/2014	27:53.7
6359	RWS11	-122.4657679	37.93909	-0.39	3.12	-213.5	2.72499993	-122.46576	37.9391	-0.44	3.12	-221.38	2.68099993	11	7/24/2014	27:53.8
6360	RWS11	-122.4657699	37.93909	-0.39	3.14	-213.3	2.74499992	-122.46577	37.9391	-0.4	3.14	-221.01	2.73499992	11	7/24/2014	27:53.9
6361	RWS11	-122.465773	37.93909	-0.43	3.16	-213	2.7299999	-122.46577	37.9391	-0.44	3.16	-220.69	2.7209999	11	7/24/2014	27:54.0
6362	RWS11	-122.4657751	37.93909	-0.39	3.17	-212.6	2.78200009	-122.46577	37.9391	-0.35	3.17	-220.26	2.82200009	11	7/24/2014	27:54.1
6363	RWS11	-122.4657772	37.93909	-0.39	3.19	-212.3	2.79600003	-122.46577	37.9391	-0.44	3.19	-219.85	2.75200003	11	7/24/2014	27:54.2
6364	RWS11	-122.4657792	37.93909	-0.39	3.2	-212	2.80799988	-122.46577	37.9391	-0.4	3.2	-219.44	2.79799989	11	7/24/2014	27:54.3
6365	RWS11	-122.4657813	37.93909	-0.39	3.21	-211.7	2.81600001	-122.46578	37.9391	-0.44	3.21	-219.13	2.77200001	11	7/24/2014	27:54.4
6366	RWS11	-122.4657834	37.93909	-0.39	3.21	-211.3	2.82200006	-122.46578	37.9391	-0.4	3.21	-218.66	2.81200007	11	7/24/2014	27:54.5
6367	RWS11	-122.4657855	37.93909	-0.43	3.22	-210.8	2.79199994	-122.46578	37.9391	-0.4	3.22	-218.24	2.81699994	11	7/24/2014	27:54.6
6368	RWS11	-122.4657876	37.93909	-0.39	3.22	-210.4	2.82999995	-122.46578	37.9391	-0.4	3.22	-217.88	2.81999996	11	7/24/2014	27:54.7

6369	RWS11	-122.4657897	37.93909	-0.39	3.22	-210	2.8339999	-122.46579	37.93911	-0.44	3.22	-217.44	2.7899999	11	7/24/2014	27:54.8
6370	RWS11	-122.4657918	37.93909	-0.39	3.23	-209.6	2.83900002	-122.46579	37.93911	-0.44	3.23	-217.12	2.79500002	11	7/24/2014	27:54.9
6371	RWS11	-122.4657948	37.93909	-0.43	3.23	-209.1	2.8089999	-122.46579	37.93911	-0.44	3.23	-216.71	2.79999989	11	7/24/2014	27:55.0
6372	RWS11	-122.4657968	37.93909	-0.39	3.24	-208.7	2.8510001	-122.46579	37.93911	-0.4	3.24	-216.3	2.84100011	11	7/24/2014	27:55.1
6373	RWS11	-122.4657989	37.93909	-0.43	3.25	-208.2	2.82300007	-122.46579	37.93911	-0.35	3.25	-215.93	2.89800006	11	7/24/2014	27:55.2
6374	RWS11	-122.4658009	37.93909	-0.39	3.26	-207.8	2.86500004	-122.4658	37.93911	-0.4	3.26	-215.5	2.85500005	11	7/24/2014	27:55.3
6375	RWS11	-122.465803	37.9391	-0.43	3.26	-207.5	2.83799994	-122.4658	37.93911	-0.4	3.26	-215.14	2.86299995	11	7/24/2014	27:55.4
6376	RWS11	-122.465805	37.9391	-0.39	3.27	-207.1	2.87999991	-122.4658	37.93911	-0.4	3.27	-214.83	2.86999992	11	7/24/2014	27:55.5
6377	RWS11	-122.4658071	37.9391	-0.43	3.28	-206.8	2.85000002	-122.4658	37.93911	-0.4	3.28	-214.5	2.87500003	11	7/24/2014	27:55.6
6378	RWS11	-122.4658091	37.9391	-0.39	3.28	-206.5	2.88899997	-122.4658	37.93911	-0.35	3.28	-214.2	2.92899996	11	7/24/2014	27:55.7
6379	RWS11	-122.4658112	37.9391	-0.43	3.28	-206.1	2.85699999	-122.46581	37.93911	-0.4	3.28	-213.8	2.882	11	7/24/2014	27:55.8
6380	RWS11	-122.4658132	37.9391	-0.39	3.28	-205.8	2.89299992	-122.46581	37.93911	-0.35	3.28	-213.45	2.93299991	11	7/24/2014	27:55.9
6381	RWS11	-122.4658162	37.9391	-0.43	3.28	-205.5	2.85799992	-122.46581	37.93911	-0.35	3.28	-213.14	2.93299991	11	7/24/2014	27:56.0
6382	RWS11	-122.4658183	37.9391	-0.39	3.28	-205.1	2.89299992	-122.46581	37.93911	-0.35	3.28	-212.71	2.93299991	11	7/24/2014	27:56.1
6383	RWS11	-122.4658203	37.9391	-0.43	3.28	-204.7	2.85600007	-122.46582	37.93911	-0.4	3.28	-212.31	2.88100007	11	7/24/2014	27:56.2
6384	RWS11	-122.4658224	37.9391	-0.43	3.28	-204.4	2.85399997	-122.46582	37.93911	-0.35	3.28	-211.93	2.92899996	11	7/24/2014	27:56.3
6385	RWS11	-122.4658244	37.9391	-0.43	3.28	-203.9	2.85099995	-122.46582	37.93911	-0.44	3.28	-211.39	2.84199995	11	7/24/2014	27:56.4
6386	RWS11	-122.4658265	37.9391	-0.43	3.27	-203.4	2.84799993	-122.46582	37.93911	-0.4	3.27	-210.89	2.87299994	11	7/24/2014	27:56.5
6387	RWS11	-122.4658286	37.9391	-0.39	3.27	-203	2.87800005	-122.46582	37.93911	-0.4	3.27	-210.37	2.86800006	11	7/24/2014	27:56.6
6388	RWS11	-122.4658306	37.9391	-0.43	3.26	-202.6	2.83600008	-122.46583	37.93911	-0.4	3.26	-209.96	2.86100009	11	7/24/2014	27:56.7
6389	RWS11	-122.4658326	37.9391	-0.43	3.26	-202.1	2.82900012	-122.46583	37.93911	-0.44	3.26	-209.5	2.82000011	11	7/24/2014	27:56.8
6390	RWS11	-122.4658347	37.9391	-0.39	3.24	-201.7	2.85399988	-122.46583	37.93911	-0.44	3.24	-209.09	2.80999988	11	7/24/2014	27:56.9
6391	RWS11	-122.4658376	37.9391	-0.43	3.23	-201.3	2.8089999	-122.46583	37.93912	-0.44	3.23	-208.66	2.79999989	11	7/24/2014	27:57.0
6392	RWS11	-122.4658396	37.9391	-0.43	3.22	-200.9	2.79899991	-122.46584	37.93912	-0.44	3.22	-208.23	2.7899999	11	7/24/2014	27:57.1
6393	RWS11	-122.4658416	37.9391	-0.43	3.21	-200.5	2.78700006	-122.46584	37.93912	-0.44	3.21	-207.75	2.77800006	11	7/24/2014	27:57.2
6394	RWS11	-122.4658435	37.9391	-0.43	3.2	-200	2.7759999	-122.46584	37.93912	-0.44	3.2	-207.35	2.7669999	11	7/24/2014	27:57.3
6395	RWS11	-122.4658455	37.9391	-0.43	3.19	-199.6	2.76400006	-122.46584	37.93912	-0.49	3.19	-206.86	2.70400006	11	7/24/2014	27:57.4
6396	RWS11	-122.4658475	37.9391	-0.43	3.18	-199.1	2.7529999	-122.46584	37.93912	-0.44	3.18	-206.35	2.7439999	11	7/24/2014	27:57.5
6397	RWS11	-122.4658495	37.93911	-0.43	3.17	-198.6	2.74199998	-122.46584	37.93912	-0.44	3.17	-205.86	2.73299998	11	7/24/2014	27:57.6
6398	RWS11	-122.4658514	37.93911	-0.43	3.16	-198.1	2.73299992	-122.46585	37.93912	-0.44	3.16	-205.35	2.72399992	11	7/24/2014	27:57.7
6399	RWS11	-122.4658534	37.93911	-0.43	3.15	-197.6	2.72700012	-122.46585	37.93912	-0.49	3.15	-204.84	2.66700011	11	7/24/2014	27:57.8
6400	RWS11	-122.4658554	37.93911	-0.43	3.15	-197.2	2.722	-122.46585	37.93912	-0.44	3.15	-204.39	2.713	11	7/24/2014	27:57.9
6401	RWS11	-122.4658583	37.93911	-0.48	3.15	-196.8	2.67000008	-122.46585	37.93912	-0.44	3.15	-204.01	2.71200007	11	7/24/2014	27:58.0

6402	RWS11	-122.4658603	37.93911	-0.43	3.15	-196.4	2.72299993	-122.46586	37.93912	-0.44	3.15	-203.59	2.71399993	11	7/24/2014	27:58.1
6403	RWS11	-122.4658624	37.93911	-0.48	3.15	-196	2.67600012	-122.46586	37.93912	-0.49	3.15	-203.18	2.66700011	11	7/24/2014	27:58.2
6404	RWS11	-122.4658644	37.93911	-0.43	3.16	-195.7	2.73400009	-122.46586	37.93912	-0.44	3.16	-202.78	2.72500008	11	7/24/2014	27:58.3
6405	RWS11	-122.4658665	37.93911	-0.48	3.17	-195.3	2.69300008	-122.46586	37.93912	-0.44	3.17	-202.4	2.73500007	11	7/24/2014	27:58.4
6406	RWS11	-122.4658686	37.93911	-0.48	3.18	-195	2.70499992	-122.46586	37.93912	-0.49	3.18	-201.93	2.69599992	11	7/24/2014	27:58.5
6407	RWS11	-122.4658707	37.93911	-0.43	3.2	-194.6	2.77199996	-122.46587	37.93912	-0.44	3.2	-201.58	2.76299995	11	7/24/2014	27:58.6
6408	RWS11	-122.4658728	37.93911	-0.43	3.22	-194.3	2.79199994	-122.46587	37.93912	-0.44	3.22	-201.12	2.78299993	11	7/24/2014	27:58.7
6409	RWS11	-122.4658748	37.93911	-0.48	3.24	-193.8	2.76300001	-122.46587	37.93912	-0.44	3.24	-200.63	2.80500001	11	7/24/2014	27:58.8
6410	RWS11	-122.4658769	37.93911	-0.43	3.26	-193.4	2.83799994	-122.46587	37.93912	-0.49	3.26	-200.11	2.77799994	11	7/24/2014	27:58.9
6411	RWS11	-122.4658801	37.93911	-0.48	3.29	-192.9	2.81100011	-122.46588	37.93912	-0.49	3.29	-199.59	2.80200011	11	7/24/2014	27:59.0
6412	RWS11	-122.4658822	37.93911	-0.43	3.31	-192.3	2.88399994	-122.46588	37.93913	-0.44	3.31	-199.01	2.87499994	11	7/24/2014	27:59.1
6413	RWS11	-122.4658843	37.93911	-0.48	3.33	-191.9	2.852	-122.46588	37.93913	-0.52	3.33	-198.56	2.80900002	11	7/24/2014	27:59.2
6414	RWS11	-122.4658864	37.93911	-0.48	3.34	-191.5	2.86400008	-122.46588	37.93913	-0.44	3.34	-198.07	2.90600008	11	7/24/2014	27:59.3
6415	RWS11	-122.4658886	37.93911	-0.48	3.35	-190.9	2.86999989	-122.46588	37.93913	-0.44	3.35	-197.54	2.91199988	11	7/24/2014	27:59.4
6416	RWS11	-122.4658908	37.93911	-0.43	3.35	-190.5	2.91999996	-122.46589	37.93913	-0.49	3.35	-197	2.85999995	11	7/24/2014	27:59.5
6417	RWS11	-122.4658929	37.93911	-0.48	3.34	-189.9	2.85800004	-122.46589	37.93913	-0.44	3.34	-196.48	2.90000004	11	7/24/2014	27:59.6
6418	RWS11	-122.4658951	37.93912	-0.43	3.32	-189.5	2.89099991	-122.46589	37.93913	-0.49	3.32	-195.95	2.83099991	11	7/24/2014	27:59.7
6419	RWS11	-122.4658973	37.93912	-0.48	3.29	-189	2.81399989	-122.46589	37.93913	-0.49	3.29	-195.49	2.80499989	11	7/24/2014	27:59.8
6420	RWS11	-122.4658994	37.93912	-0.43	3.26	-188.5	2.8319999	-122.46589	37.93913	-0.52	3.26	-194.95	2.73799992	11	7/24/2014	27:59.9
6421	RWS11	-122.4659026	37.93912	-0.48	3.22	-188	2.74399996	-122.4659	37.93913	-0.52	3.22	-194.42	2.70099998	11	7/24/2014	28:00.0
6422	RWS11	-122.4659047	37.93912	-0.43	3.18	-187.5	2.755	-122.4659	37.93913	-0.49	3.18	-193.97	2.69499999	11	7/24/2014	28:00.1
6423	RWS11	-122.4659068	37.93912	-0.48	3.14	-187	2.66599989	-122.4659	37.93913	-0.49	3.14	-193.41	2.65699989	11	7/24/2014	28:00.2
6424	RWS11	-122.465909	37.93912	-0.43	3.11	-186.5	2.68100011	-122.4659	37.93913	-0.44	3.11	-192.9	2.67200011	11	7/24/2014	28:00.3
6425	RWS11	-122.4659111	37.93912	-0.48	3.08	-186	2.59899998	-122.46591	37.93913	-0.57	3.08	-192.37	2.505	11	7/24/2014	28:00.4
6426	RWS11	-122.4659133	37.93912	-0.43	3.05	-185.4	2.62500012	-122.46591	37.93913	-0.49	3.05	-191.75	2.56500012	11	7/24/2014	28:00.5
6427	RWS11	-122.4659155	37.93912	-0.48	3.03	-184.8	2.55500007	-122.46591	37.93913	-0.52	3.03	-191.18	2.51200008	11	7/24/2014	28:00.6
6428	RWS11	-122.4659176	37.93912	-0.43	3.02	-184.3	2.59300005	-122.46591	37.93913	-0.52	3.02	-190.58	2.49900007	11	7/24/2014	28:00.7
6429	RWS11	-122.4659197	37.93912	-0.48	3.01	-183.7	2.53600001	-122.46591	37.93913	-0.52	3.01	-190.04	2.49300003	11	7/24/2014	28:00.8
6430	RWS11	-122.4659218	37.93912	-0.43	3.01	-183.1	2.58600008	-122.46592	37.93913	-0.49	3.01	-189.49	2.52600008	11	7/24/2014	28:00.9
6431	RWS11	-122.465925	37.93912	-0.48	3.02	-182.8	2.53800011	-122.46592	37.93914	-0.52	3.02	-189.16	2.49500012	11	7/24/2014	28:01.0
6432	RWS11	-122.4659271	37.93912	-0.43	3.02	-182.3	2.59600008	-122.46592	37.93914	-0.49	3.02	-188.72	2.53600007	11	7/24/2014	28:01.1
6433	RWS11	-122.4659291	37.93912	-0.48	3.03	-182	2.55500007	-122.46592	37.93914	-0.49	3.03	-188.52	2.54600006	11	7/24/2014	28:01.2
6434	RWS11	-122.4659313	37.93912	-0.43	3.04	-181.6	2.61699998	-122.46593	37.93914	-0.52	3.04	-188.15	2.523	11	7/24/2014	28:01.3

6435	RWS11	-122.4659334	37.93912	-0.43	3.06	-181.2	2.63	-122.46593	37.93914	-0.49	3.06	-187.81	2.56999999	11	7/24/2014	28:01.4
6436	RWS11	-122.4659355	37.93912	-0.43	3.07	-180.9	2.64300001	-122.46593	37.93914	-0.49	3.07	-187.41	2.583	11	7/24/2014	28:01.5
6437	RWS11	-122.4659376	37.93913	-0.43	3.08	-180.4	2.65699995	-122.46593	37.93914	-0.52	3.08	-186.95	2.56299996	11	7/24/2014	28:01.6
6438	RWS11	-122.4659397	37.93913	-0.43	3.1	-179.9	2.67099988	-122.46593	37.93914	-0.52	3.1	-186.49	2.5769999	11	7/24/2014	28:01.7
6439	RWS11	-122.4659418	37.93913	-0.48	3.11	-179.4	2.6329999	-122.46594	37.93914	-0.57	3.11	-185.96	2.53899992	11	7/24/2014	28:01.8
6440	RWS11	-122.4659439	37.93913	-0.48	3.12	-179	2.64400005	-122.46594	37.93914	-0.52	3.12	-185.49	2.60100007	11	7/24/2014	28:01.9
6441	RWS11	-122.465947	37.93913	-0.48	3.13	-178.5	2.65300012	-122.46594	37.93914	-0.57	3.13	-185.02	2.55900013	11	7/24/2014	28:02.0
6442	RWS11	-122.4659491	37.93913	-0.43	3.14	-178	2.71100008	-122.46594	37.93914	-0.49	3.14	-184.53	2.65100008	11	7/24/2014	28:02.1
6443	RWS11	-122.4659512	37.93913	-0.48	3.14	-177.5	2.66499996	-122.46595	37.93914	-0.52	3.14	-184.02	2.62199998	11	7/24/2014	28:02.2
6444	RWS11	-122.4659533	37.93913	-0.48	3.14	-176.9	2.66799998	-122.46595	37.93914	-0.57	3.14	-183.47	2.574	11	7/24/2014	28:02.3
6445	RWS11	-122.4659554	37.93913	-0.48	3.15	-176.4	2.67000008	-122.46595	37.93914	-0.57	3.15	-182.96	2.57600009	11	7/24/2014	28:02.4
6446	RWS11	-122.4659576	37.93913	-0.48	3.15	-175.9	2.671	-122.46595	37.93914	-0.52	3.15	-182.44	2.62800002	11	7/24/2014	28:02.5
6447	RWS11	-122.4659597	37.93913	-0.48	3.15	-175.4	2.67199993	-122.46595	37.93914	-0.57	3.15	-181.94	2.57799995	11	7/24/2014	28:02.6
6448	RWS11	-122.4659618	37.93913	-0.48	3.15	-174.8	2.67199993	-122.46596	37.93914	-0.57	3.15	-181.42	2.57799995	11	7/24/2014	28:02.7
6449	RWS11	-122.4659639	37.93913	-0.48	3.15	-174.3	2.67199993	-122.46596	37.93914	-0.57	3.15	-180.94	2.57799995	11	7/24/2014	28:02.8
6450	RWS11	-122.465966	37.93913	-0.48	3.15	-173.8	2.671	-122.46596	37.93915	-0.49	3.15	-180.48	2.662	11	7/24/2014	28:02.9
6451	RWS11	-122.4659691	37.93913	-0.48	3.15	-173.3	2.66899991	-122.46596	37.93915	-0.52	3.15	-179.99	2.62599993	11	7/24/2014	28:03.0
6452	RWS11	-122.4659712	37.93913	-0.48	3.14	-172.9	2.66700006	-122.46597	37.93915	-0.52	3.14	-179.65	2.62400007	11	7/24/2014	28:03.1
6453	RWS11	-122.4659733	37.93913	-0.48	3.14	-172.4	2.66300011	-122.46597	37.93915	-0.52	3.14	-179.25	2.62000012	11	7/24/2014	28:03.2
6454	RWS11	-122.4659754	37.93913	-0.48	3.14	-172	2.66100001	-122.46597	37.93915	-0.49	3.14	-178.88	2.65200001	11	7/24/2014	28:03.3
6455	RWS11	-122.4659775	37.93913	-0.48	3.14	-171.7	2.65899992	-122.46597	37.93915	-0.52	3.14	-178.63	2.61599994	11	7/24/2014	28:03.4
6456	RWS11	-122.4659796	37.93914	-0.48	3.13	-171.4	2.65799999	-122.46597	37.93915	-0.49	3.13	-178.36	2.64899999	11	7/24/2014	28:03.5
6457	RWS11	-122.4659817	37.93914	-0.48	3.14	-171	2.66000009	-122.46598	37.93915	-0.52	3.14	-178.05	2.6170001	11	7/24/2014	28:03.6
6458	RWS11	-122.4659838	37.93914	-0.48	3.14	-170.6	2.66300011	-122.46598	37.93915	-0.52	3.14	-177.64	2.62000012	11	7/24/2014	28:03.7
6459	RWS11	-122.4659859	37.93914	-0.48	3.14	-170.3	2.66599989	-122.46598	37.93915	-0.52	3.14	-177.27	2.62299991	11	7/24/2014	28:03.8
6460	RWS11	-122.465988	37.93914	-0.48	3.15	-170	2.67000008	-122.46598	37.93915	-0.49	3.15	-176.97	2.66100007	11	7/24/2014	28:03.9
6461	RWS11	-122.4659911	37.93914	-0.48	3.15	-169.7	2.67400002	-122.46599	37.93915	-0.52	3.15	-176.59	2.63100004	11	7/24/2014	28:04.0
6462	RWS11	-122.4659932	37.93914	-0.43	3.15	-169.3	2.72700012	-122.46599	37.93915	-0.49	3.15	-176.23	2.66700011	11	7/24/2014	28:04.1
6463	RWS11	-122.4659953	37.93914	-0.48	3.15	-169	2.67799997	-122.46599	37.93915	-0.49	3.15	-175.87	2.66899997	11	7/24/2014	28:04.2
6464	RWS11	-122.4659974	37.93914	-0.43	3.15	-168.7	2.72899997	-122.46599	37.93915	-0.52	3.15	-175.51	2.63499999	11	7/24/2014	28:04.3
6465	RWS11	-122.4659996	37.93914	-0.48	3.15	-168.3	2.67799997	-122.46599	37.93915	-0.52	3.15	-175.13	2.63499999	11	7/24/2014	28:04.4
6466	RWS11	-122.4660017	37.93914	-0.43	3.15	-168.1	2.72800004	-122.466	37.93915	-0.52	3.15	-174.81	2.63400006	11	7/24/2014	28:04.5
6467	RWS11	-122.4660039	37.93914	-0.48	3.15	-167.8	2.67700005	-122.466	37.93915	-0.52	3.15	-174.45	2.63400006	11	7/24/2014	28:04.6

6468	RWS11	-122.466006	37.93914	-0.43	3.15	-167.5	2.72700012	-122.466	37.93916	-0.49	3.15	-174.14	2.66700011	11	7/24/2014	28:04.7
6469	RWS11	-122.4660082	37.93914	-0.43	3.15	-167.2	2.72599995	-122.466	37.93916	-0.44	3.15	-173.79	2.71699995	11	7/24/2014	28:04.8
6470	RWS11	-122.4660103	37.93914	-0.43	3.15	-166.8	2.72599995	-122.46601	37.93916	-0.49	3.15	-173.42	2.66599995	11	7/24/2014	28:04.9
6471	RWS11	-122.4660134	37.93914	-0.48	3.15	-166.6	2.67499995	-122.46601	37.93916	-0.52	3.15	-173.15	2.63199997	11	7/24/2014	28:05.0
6472	RWS11	-122.4660156	37.93914	-0.43	3.15	-166.3	2.72599995	-122.46601	37.93916	-0.49	3.15	-172.85	2.66599995	11	7/24/2014	28:05.1
6473	RWS11	-122.4660177	37.93915	-0.43	3.15	-166.1	2.72700012	-122.46601	37.93916	-0.49	3.15	-172.57	2.66700011	11	7/24/2014	28:05.2
6474	RWS11	-122.4660199	37.93915	-0.43	3.15	-165.7	2.72700012	-122.46601	37.93916	-0.49	3.15	-172.32	2.66700011	11	7/24/2014	28:05.3
6475	RWS11	-122.466022	37.93915	-0.48	3.15	-165.5	2.67700005	-122.46602	37.93916	-0.49	3.15	-172.06	2.66800004	11	7/24/2014	28:05.4
6476	RWS11	-122.4660242	37.93915	-0.43	3.15	-165.1	2.72899997	-122.46602	37.93916	-0.49	3.15	-171.76	2.66899997	11	7/24/2014	28:05.5
6477	RWS11	-122.4660264	37.93915	-0.43	3.16	-164.7	2.7299999	-122.46602	37.93916	-0.49	3.16	-171.39	2.6699999	11	7/24/2014	28:05.6
6478	RWS11	-122.4660285	37.93915	-0.43	3.16	-164.3	2.73100007	-122.46602	37.93916	-0.52	3.16	-171	2.63700008	11	7/24/2014	28:05.7
6479	RWS11	-122.4660307	37.93915	-0.48	3.16	-163.9	2.68099999	-122.46603	37.93916	-0.44	3.16	-170.6	2.72299999	11	7/24/2014	28:05.8
6480	RWS11	-122.4660328	37.93915	-0.43	3.16	-163.4	2.73299992	-122.46603	37.93916	-0.49	3.16	-170.15	2.67299992	11	7/24/2014	28:05.9
6481	RWS11	-122.466036	37.93915	-0.48	3.16	-163.1	2.68199992	-122.46603	37.93916	-0.49	3.16	-169.8	2.67299992	11	7/24/2014	28:06.0
6482	RWS11	-122.4660381	37.93915	-0.43	3.16	-162.7	2.73199999	-122.46603	37.93916	-0.49	3.16	-169.44	2.67199999	11	7/24/2014	28:06.1
6483	RWS11	-122.4660403	37.93915	-0.43	3.16	-162.3	2.73100007	-122.46603	37.93916	-0.44	3.16	-169.01	2.72200006	11	7/24/2014	28:06.2
6484	RWS11	-122.4660424	37.93915	-0.48	3.15	-161.9	2.67700005	-122.46604	37.93916	-0.52	3.15	-168.6	2.63400006	11	7/24/2014	28:06.3
6485	RWS11	-122.4660445	37.93915	-0.48	3.15	-161.5	2.67499995	-122.46604	37.93916	-0.49	3.15	-168.23	2.66599995	11	7/24/2014	28:06.4
6486	RWS11	-122.4660467	37.93915	-0.43	3.15	-161.1	2.7240001	-122.46604	37.93917	-0.52	3.15	-167.81	2.63000011	11	7/24/2014	28:06.5
6487	RWS11	-122.4660489	37.93915	-0.48	3.15	-160.7	2.6730001	-122.46604	37.93917	-0.49	3.15	-167.43	2.66400009	11	7/24/2014	28:06.6
6488	RWS11	-122.466051	37.93915	-0.48	3.15	-160.4	2.67400002	-122.46605	37.93917	-0.49	3.15	-167.04	2.66500002	11	7/24/2014	28:06.7
6489	RWS11	-122.4660531	37.93915	-0.48	3.15	-160	2.67799997	-122.46605	37.93917	-0.52	3.15	-166.66	2.63499999	11	7/24/2014	28:06.8
6490	RWS11	-122.4660552	37.93915	-0.48	3.16	-159.5	2.68600011	-122.46605	37.93917	-0.49	3.16	-166.26	2.67700011	11	7/24/2014	28:06.9
6491	RWS11	-122.4660584	37.93916	-0.48	3.17	-159.1	2.6960001	-122.46605	37.93917	-0.57	3.17	-165.89	2.60200012	11	7/24/2014	28:07.0
6492	RWS11	-122.4660605	37.93916	-0.48	3.19	-158.7	2.70900011	-122.46606	37.93917	-0.52	3.19	-165.53	2.66600013	11	7/24/2014	28:07.1
6493	RWS11	-122.4660626	37.93916	-0.48	3.2	-158.4	2.72300005	-122.46606	37.93917	-0.52	3.2	-165.2	2.68000007	11	7/24/2014	28:07.2
6494	RWS11	-122.4660647	37.93916	-0.48	3.21	-158.1	2.73799992	-122.46606	37.93917	-0.52	3.21	-164.87	2.69499993	11	7/24/2014	28:07.3
6495	RWS11	-122.4660668	37.93916	-0.48	3.23	-157.7	2.75300002	-122.46606	37.93917	-0.57	3.23	-164.54	2.65900004	11	7/24/2014	28:07.4
6496	RWS11	-122.4660689	37.93916	-0.48	3.24	-157.4	2.7650001	-122.46606	37.93917	-0.52	3.24	-164.17	2.72200012	11	7/24/2014	28:07.5
6497	RWS11	-122.466071	37.93916	-0.51	3.25	-157	2.73900002	-122.46607	37.93917	-0.52	3.25	-163.76	2.73000002	11	7/24/2014	28:07.6
6498	RWS11	-122.4660731	37.93916	-0.48	3.25	-156.5	2.7750001	-122.46607	37.93917	-0.52	3.25	-163.35	2.73200011	11	7/24/2014	28:07.7
6499	RWS11	-122.4660752	37.93916	-0.51	3.25	-156.2	2.73699993	-122.46607	37.93917	-0.52	3.25	-162.95	2.72799993	11	7/24/2014	28:07.8
6500	RWS11	-122.4660773	37.93916	-0.51	3.24	-155.8	2.72699994	-122.46607	37.93917	-0.52	3.24	-162.61	2.71799994	11	7/24/2014	28:07.9

6501	RWS11	-122.4660804	37.93916	-0.51	3.22	-155.4	2.71399993	-122.46607	37.93917	-0.52	3.22	-162.18	2.70499992	11	7/24/2014	28:08.0
6502	RWS11	-122.4660825	37.93916	-0.48	3.21	-155.1	2.73399997	-122.46608	37.93917	-0.52	3.21	-161.81	2.69099998	11	7/24/2014	28:08.1
6503	RWS11	-122.4660845	37.93916	-0.51	3.2	-154.8	2.68699998	-122.46608	37.93918	-0.57	3.2	-161.41	2.62699997	11	7/24/2014	28:08.2
6504	RWS11	-122.4660866	37.93916	-0.51	3.19	-154.3	2.67600006	-122.46608	37.93918	-0.52	3.19	-160.93	2.66700006	11	7/24/2014	28:08.3
6505	RWS11	-122.4660887	37.93916	-0.56	3.18	-153.9	2.61800009	-122.46608	37.93918	-0.57	3.18	-160.55	2.60900009	11	7/24/2014	28:08.4
6506	RWS11	-122.4660909	37.93916	-0.51	3.18	-153.5	2.66600007	-122.46609	37.93918	-0.52	3.18	-160.11	2.65700006	11	7/24/2014	28:08.5
6507	RWS11	-122.466093	37.93916	-0.56	3.18	-153.1	2.616	-122.46609	37.93918	-0.57	3.18	-159.67	2.60699999	11	7/24/2014	28:08.6
6508	RWS11	-122.4660951	37.93917	-0.51	3.18	-152.6	2.67200011	-122.46609	37.93918	-0.52	3.18	-159.28	2.66300011	11	7/24/2014	28:08.7
6509	RWS11	-122.4660972	37.93917	-0.56	3.19	-152.2	2.62999994	-122.46609	37.93918	-0.6	3.19	-158.89	2.58799994	11	7/24/2014	28:08.8
6510	RWS11	-122.4660993	37.93917	-0.51	3.2	-151.9	2.69300002	-122.46609	37.93918	-0.57	3.2	-158.55	2.63300002	11	7/24/2014	28:08.9
6511	RWS11	-122.4661024	37.93917	-0.56	3.22	-151.5	2.65599996	-122.4661	37.93918	-0.57	3.22	-158.28	2.64699996	11	7/24/2014	28:09.0
6512	RWS11	-122.4661045	37.93917	-0.51	3.23	-151.2	2.72100013	-122.4661	37.93918	-0.57	3.23	-157.97	2.66100013	11	7/24/2014	28:09.1
6513	RWS11	-122.4661065	37.93917	-0.56	3.25	-151	2.685	-122.4661	37.93918	-0.6	3.25	-157.7	2.64300001	11	7/24/2014	28:09.2
6514	RWS11	-122.4661086	37.93917	-0.56	3.26	-150.7	2.70000011	-122.4661	37.93918	-0.49	3.26	-157.42	2.77600008	11	7/24/2014	28:09.3
6515	RWS11	-122.4661108	37.93917	-0.56	3.28	-150.5	2.71700007	-122.46611	37.93918	-0.6	3.28	-157.12	2.67500007	11	7/24/2014	28:09.4
6516	RWS11	-122.4661129	37.93917	-0.56	3.3	-150.2	2.73600012	-122.46611	37.93918	-0.52	3.3	-156.86	2.77800012	11	7/24/2014	28:09.5
6517	RWS11	-122.466115	37.93917	-0.56	3.32	-150	2.7560001	-122.46611	37.93918	-0.57	3.32	-156.6	2.7470001	11	7/24/2014	28:09.6
6518	RWS11	-122.4661172	37.93917	-0.51	3.34	-149.8	2.82700008	-122.46611	37.93918	-0.57	3.34	-156.37	2.76700008	11	7/24/2014	28:09.7
6519	RWS11	-122.4661193	37.93917	-0.56	3.36	-149.5	2.79500014	-122.46611	37.93918	-0.57	3.36	-156.08	2.78600013	11	7/24/2014	28:09.8
6520	RWS11	-122.4661214	37.93917	-0.51	3.37	-149.2	2.86300009	-122.46612	37.93919	-0.6	3.37	-155.8	2.7700001	11	7/24/2014	28:09.9
6521	RWS11	-122.4661245	37.93917	-0.56	3.39	-148.9	2.82500011	-122.46612	37.93919	-0.6	3.39	-155.51	2.78300011	11	7/24/2014	28:10.0
6522	RWS11	-122.4661266	37.93917	-0.56	3.4	-148.7	2.83399993	-122.46612	37.93919	-0.57	3.4	-155.21	2.82499993	11	7/24/2014	28:10.1
6523	RWS11	-122.4661287	37.93917	-0.56	3.4	-148.5	2.83800012	-122.46612	37.93919	-0.57	3.4	-155	2.82900012	11	7/24/2014	28:10.2
6524	RWS11	-122.4661308	37.93918	-0.51	3.4	-148.3	2.88799995	-122.46613	37.93919	-0.49	3.4	-154.69	2.91299993	11	7/24/2014	28:10.3
6525	RWS11	-122.466133	37.93918	-0.56	3.39	-148	2.83099991	-122.46613	37.93919	-0.57	3.39	-154.44	2.82199991	11	7/24/2014	28:10.4
6526	RWS11	-122.4661351	37.93918	-0.51	3.38	-147.8	2.87199992	-122.46613	37.93919	-0.52	3.38	-154.19	2.86299992	11	7/24/2014	28:10.5
6527	RWS11	-122.4661372	37.93918	-0.56	3.37	-147.5	2.80699998	-122.46613	37.93919	-0.57	3.37	-153.94	2.79799998	11	7/24/2014	28:10.6
6528	RWS11	-122.4661394	37.93918	-0.51	3.35	-147.3	2.84000009	-122.46613	37.93919	-0.49	3.35	-153.7	2.86500007	11	7/24/2014	28:10.7
6529	RWS11	-122.4661415	37.93918	-0.51	3.33	-147	2.81899995	-122.46614	37.93919	-0.52	3.33	-153.47	2.80999994	11	7/24/2014	28:10.8
6530	RWS11	-122.4661436	37.93918	-0.48	3.31	-146.8	2.83100009	-122.46614	37.93919	-0.49	3.31	-153.27	2.82200009	11	7/24/2014	28:10.9
6531	RWS11	-122.4661467	37.93918	-0.51	3.28	-146.6	2.77299994	-122.46614	37.93919	-0.49	3.28	-153.02	2.79799992	11	7/24/2014	28:11.0
6532	RWS11	-122.4661487	37.93918	-0.51	3.26	-146.4	2.74999994	-122.46614	37.93919	-0.49	3.26	-152.81	2.77499992	11	7/24/2014	28:11.1
6533	RWS11	-122.4661508	37.93918	-0.51	3.24	-146.2	2.72999996	-122.46615	37.93919	-0.44	3.24	-152.54	2.80599993	11	7/24/2014	28:11.2

6534	RWS11	-122.4661529	37.93918	-0.51	3.22	-146	2.71200007	-122.46615	37.93919	-0.44	3.22	-152.35	2.78800005	11	7/24/2014	28:11.3
6535	RWS11	-122.4661551	37.93918	-0.51	3.21	-145.8	2.69699997	-122.46615	37.93919	-0.49	3.21	-152.15	2.72199994	11	7/24/2014	28:11.4
6536	RWS11	-122.4661572	37.93918	-0.48	3.2	-145.6	2.7190001	-122.46615	37.9392	-0.49	3.2	-151.92	2.7100001	11	7/24/2014	28:11.5
6537	RWS11	-122.4661593	37.93918	-0.48	3.19	-145.5	2.71000004	-122.46615	37.9392	-0.49	3.19	-151.74	2.70100003	11	7/24/2014	28:11.6
6538	RWS11	-122.4661614	37.93918	-0.48	3.18	-145.4	2.7019999	-122.46616	37.9392	-0.49	3.18	-151.64	2.6929999	11	7/24/2014	28:11.7
6539	RWS11	-122.4661635	37.93918	-0.51	3.17	-145.3	2.66300005	-122.46616	37.9392	-0.44	3.17	-151.55	2.73900002	11	7/24/2014	28:11.8
6540	RWS11	-122.4661655	37.93918	-0.48	3.17	-145.1	2.69400001	-122.46616	37.9392	-0.44	3.17	-151.38	2.736	11	7/24/2014	28:11.9
6541	RWS11	-122.4661686	37.93919	-0.48	3.17	-145	2.6929999	-122.46616	37.9392	-0.4	3.17	-151.26	2.7689999	11	7/24/2014	28:12.0
6542	RWS11	-122.4661707	37.93919	-0.48	3.17	-144.8	2.69400007	-122.46616	37.9392	-0.43	3.17	-151.07	2.73600009	11	7/24/2014	28:12.1
6543	RWS11	-122.4661728	37.93919	-0.48	3.17	-144.7	2.69700009	-122.46617	37.9392	-0.43	3.17	-150.96	2.73900011	11	7/24/2014	28:12.2
6544	RWS11	-122.4661749	37.93919	-0.43	3.18	-144.5	2.75200003	-122.46617	37.9392	-0.43	3.18	-150.81	2.74300006	11	7/24/2014	28:12.3
6545	RWS11	-122.4661769	37.93919	-0.48	3.18	-144.2	2.70700008	-122.46617	37.9392	-0.49	3.18	-150.61	2.69800007	11	7/24/2014	28:12.4
6546	RWS11	-122.466179	37.93919	-0.43	3.19	-144.1	2.76399988	-122.46617	37.9392	-0.4	3.19	-150.41	2.78899989	11	7/24/2014	28:12.5
6547	RWS11	-122.4661811	37.93919	-0.48	3.19	-143.9	2.71899992	-122.46618	37.9392	-0.4	3.19	-150.24	2.79499993	11	7/24/2014	28:12.6
6548	RWS11	-122.4661832	37.93919	-0.48	3.2	-143.6	2.72499996	-122.46618	37.9392	-0.4	3.2	-150.03	2.80099997	11	7/24/2014	28:12.7
6549	RWS11	-122.4661853	37.93919	-0.48	3.21	-143.4	2.73000008	-122.46618	37.9392	-0.4	3.21	-149.77	2.80600008	11	7/24/2014	28:12.8
6550	RWS11	-122.4661873	37.93919	-0.43	3.21	-143.2	2.78299993	-122.46618	37.9392	-0.4	3.21	-149.61	2.80799994	11	7/24/2014	28:12.9
6551	RWS11	-122.4661904	37.93919	-0.43	3.21	-143	2.78299993	-122.46618	37.9392	-0.43	3.21	-149.38	2.77399996	11	7/24/2014	28:13.0
6552	RWS11	-122.4661925	37.93919	-0.43	3.2	-142.8	2.77999991	-122.46619	37.9392	-0.35	3.2	-149.15	2.85499993	11	7/24/2014	28:13.1
6553	RWS11	-122.4661945	37.93919	-0.48	3.2	-142.5	2.72199994	-122.46619	37.93921	-0.43	3.2	-148.89	2.76399997	11	7/24/2014	28:13.2
6554	RWS11	-122.4661966	37.93919	-0.48	3.19	-142.3	2.71199995	-122.46619	37.93921	-0.4	3.19	-148.65	2.78799996	11	7/24/2014	28:13.3
6555	RWS11	-122.4661987	37.93919	-0.48	3.18	-142	2.70100003	-122.46619	37.93921	-0.4	3.18	-148.33	2.77700004	11	7/24/2014	28:13.4
6556	RWS11	-122.4662008	37.93919	-0.43	3.17	-141.8	2.74099988	-122.46619	37.93921	-0.4	3.17	-148.04	2.76599988	11	7/24/2014	28:13.5
6557	RWS11	-122.4662029	37.9392	-0.43	3.15	-141.5	2.72900003	-122.4662	37.93921	-0.35	3.15	-147.74	2.80400005	11	7/24/2014	28:13.6
6558	RWS11	-122.4662049	37.9392	-0.43	3.14	-141.3	2.71900004	-122.4662	37.93921	-0.4	3.14	-147.43	2.74400005	11	7/24/2014	28:13.7
6559	RWS11	-122.466207	37.9392	-0.43	3.13	-141	2.70999998	-122.4662	37.93921	-0.43	3.13	-147.15	2.70100001	11	7/24/2014	28:13.8
6560	RWS11	-122.4662091	37.9392	-0.43	3.13	-140.8	2.70399994	-122.4662	37.93921	-0.35	3.13	-146.8	2.77899995	11	7/24/2014	28:13.9
6561	RWS11	-122.4662121	37.9392	-0.43	3.13	-140.5	2.69999999	-122.46621	37.93921	-0.35	3.13	-146.49	2.77500001	11	7/24/2014	28:14.0
6562	RWS11	-122.4662142	37.9392	-0.43	3.12	-140.2	2.69900006	-122.46621	37.93921	-0.35	3.12	-146.13	2.77400008	11	7/24/2014	28:14.1
6563	RWS11	-122.4662162	37.9392	-0.43	3.13	-139.9	2.70099992	-122.46621	37.93921	-0.32	3.13	-145.84	2.80999991	11	7/24/2014	28:14.2
6564	RWS11	-122.4662183	37.9392	-0.43	3.13	-139.6	2.7050001	-122.46621	37.93921	-0.4	3.13	-145.51	2.73000011	11	7/24/2014	28:14.3
6565	RWS11	-122.4662204	37.9392	-0.48	3.14	-139.3	2.66100007	-122.46621	37.93921	-0.35	3.14	-145.14	2.78700009	11	7/24/2014	28:14.4
6566	RWS11	-122.4662225	37.9392	-0.43	3.15	-138.9	2.72200006	-122.46622	37.93921	-0.32	3.15	-144.77	2.83100006	11	7/24/2014	28:14.5

6567	RWS11	-122.4662246	37.9392	-0.43	3.16	-138.5	2.73299998	-122.46622	37.93921	-0.4	3.16	-144.36	2.75799999	11	7/24/2014	28:14.6
6568	RWS11	-122.4662267	37.9392	-0.43	3.17	-138.1	2.74599999	-122.46622	37.93921	-0.28	3.17	-144.05	2.889	11	7/24/2014	28:14.7
6569	RWS11	-122.4662287	37.9392	-0.48	3.18	-137.8	2.708	-122.46622	37.93922	-0.35	3.18	-143.67	2.83400002	11	7/24/2014	28:14.8
6570	RWS11	-122.4662308	37.9392	-0.43	3.2	-137.5	2.77299994	-122.46622	37.93922	-0.32	3.2	-143.31	2.88199994	11	7/24/2014	28:14.9
6571	RWS11	-122.4662339	37.9392	-0.48	3.21	-137.2	2.73499995	-122.46623	37.93922	-0.32	3.21	-143.01	2.89499995	11	7/24/2014	28:15.0
6572	RWS11	-122.466236	37.9392	-0.43	3.22	-137	2.79699987	-122.46623	37.93922	-0.35	3.22	-142.7	2.87199989	11	7/24/2014	28:15.1
6573	RWS11	-122.4662381	37.93921	-0.43	3.23	-136.7	2.80599993	-122.46623	37.93922	-0.32	3.23	-142.46	2.91499993	11	7/24/2014	28:15.2
6574	RWS11	-122.4662402	37.93921	-0.43	3.24	-136.5	2.81100005	-122.46623	37.93922	-0.28	3.24	-142.14	2.95400006	11	7/24/2014	28:15.3
6575	RWS11	-122.4662423	37.93921	-0.43	3.24	-136	2.81199998	-122.46624	37.93922	-0.4	3.24	-141.75	2.83699998	11	7/24/2014	28:15.4
6576	RWS11	-122.4662445	37.93921	-0.43	3.23	-135.9	2.80800003	-122.46624	37.93922	-0.32	3.23	-141.51	2.91700003	11	7/24/2014	28:15.5
6577	RWS11	-122.4662466	37.93921	-0.43	3.23	-135.5	2.80100006	-122.46624	37.93922	-0.28	3.23	-141.21	2.94400007	11	7/24/2014	28:15.6
6578	RWS11	-122.4662488	37.93921	-0.43	3.22	-135.2	2.792	-122.46624	37.93922	-0.28	3.22	-140.92	2.935	11	7/24/2014	28:15.7
6579	RWS11	-122.4662509	37.93921	-0.43	3.21	-135	2.78100008	-122.46625	37.93922	-0.32	3.21	-140.62	2.89000008	11	7/24/2014	28:15.8
6580	RWS11	-122.466253	37.93921	-0.39	3.19	-134.7	2.80400002	-122.46625	37.93922	-0.28	3.19	-140.34	2.912	11	7/24/2014	28:15.9
6581	RWS11	-122.4662562	37.93921	-0.43	3.18	-134.5	2.75599998	-122.46625	37.93922	-0.32	3.18	-140.07	2.86499998	11	7/24/2014	28:16.0
6582	RWS11	-122.4662583	37.93921	-0.39	3.17	-134.2	2.78000009	-122.46625	37.93922	-0.28	3.17	-139.75	2.88800007	11	7/24/2014	28:16.1
6583	RWS11	-122.4662604	37.93921	-0.43	3.16	-133.9	2.73399991	-122.46625	37.93922	-0.28	3.16	-139.43	2.87699991	11	7/24/2014	28:16.2
6584	RWS11	-122.4662626	37.93921	-0.39	3.15	-133.5	2.75700009	-122.46626	37.93923	-0.32	3.15	-139.1	2.83100006	11	7/24/2014	28:16.3
6585	RWS11	-122.4662647	37.93921	-0.43	3.13	-133.2	2.70900005	-122.46626	37.93923	-0.35	3.13	-138.67	2.78400007	11	7/24/2014	28:16.4
6586	RWS11	-122.4662669	37.93921	-0.43	3.12	-132.6	2.69300002	-122.46626	37.93923	-0.35	3.12	-138.26	2.76800004	11	7/24/2014	28:16.5
6587	RWS11	-122.466269	37.93921	-0.43	3.1	-132.2	2.67399997	-122.46626	37.93923	-0.32	3.1	-137.85	2.78299996	11	7/24/2014	28:16.6
6588	RWS11	-122.4662712	37.93921	-0.39	3.08	-131.7	2.68699992	-122.46627	37.93923	-0.32	3.08	-137.41	2.76099989	11	7/24/2014	28:16.7
6589	RWS11	-122.4662733	37.93922	-0.43	3.05	-131.4	2.62600011	-122.46627	37.93923	-0.35	3.05	-137.07	2.70100012	11	7/24/2014	28:16.8
6590	RWS11	-122.4662754	37.93922	-0.43	3.02	-130.9	2.59700006	-122.46627	37.93923	-0.32	3.02	-136.64	2.70600006	11	7/24/2014	28:16.9
6591	RWS11	-122.4662786	37.93922	-0.43	2.99	-130.5	2.56599993	-122.46627	37.93923	-0.32	2.99	-136.23	2.67499992	11	7/24/2014	28:17.0
6592	RWS11	-122.4662807	37.93922	-0.39	2.96	-130	2.56900012	-122.46627	37.93923	-0.28	2.96	-135.8	2.67700011	11	7/24/2014	28:17.1
6593	RWS11	-122.4662828	37.93922	-0.43	2.93	-129.6	2.50399989	-122.46628	37.93923	-0.35	2.93	-135.39	2.57899991	11	7/24/2014	28:17.2
6594	RWS11	-122.466285	37.93922	-0.39	2.9	-129.1	2.51400006	-122.46628	37.93923	-0.35	2.9	-134.98	2.55400005	11	7/24/2014	28:17.3
6595	RWS11	-122.4662871	37.93922	-0.43	2.88	-128.7	2.45900005	-122.46628	37.93923	-0.32	2.88	-134.61	2.56800005	11	7/24/2014	28:17.4
6596	RWS11	-122.4662893	37.93922	-0.39	2.87	-128.3	2.4799999	-122.46628	37.93923	-0.32	2.87	-134.25	2.55399987	11	7/24/2014	28:17.5
6597	RWS11	-122.4662914	37.93922	-0.39	2.86	-127.9	2.472	-122.46629	37.93923	-0.4	2.86	-133.84	2.46199998	11	7/24/2014	28:17.6
6598	RWS11	-122.4662935	37.93922	-0.39	2.86	-127.4	2.46899998	-122.46629	37.93923	-0.35	2.86	-133.47	2.50899997	11	7/24/2014	28:17.7
6599	RWS11	-122.4662956	37.93922	-0.43	2.86	-127.1	2.43499988	-122.46629	37.93923	-0.35	2.86	-133.11	2.5099999	11	7/24/2014	28:17.8

6600	RWS11	-122.4662978	37.93922	-0.39	2.87	-126.8	2.47500002	-122.46629	37.93924	-0.35	2.87	-132.79	2.51500002	11	7/24/2014	28:17.9
6601	RWS11	-122.4663009	37.93922	-0.43	2.87	-126.4	2.44799989	-122.4663	37.93924	-0.35	2.87	-132.42	2.52299991	11	7/24/2014	28:18.0
6602	RWS11	-122.466303	37.93922	-0.39	2.88	-126	2.49299991	-122.4663	37.93924	-0.35	2.88	-132.11	2.5329999	11	7/24/2014	28:18.1
6603	RWS11	-122.4663051	37.93922	-0.43	2.89	-125.8	2.46900004	-122.4663	37.93924	-0.35	2.89	-131.85	2.54400006	11	7/24/2014	28:18.2
6604	RWS11	-122.4663072	37.93923	-0.43	2.91	-125.5	2.48099989	-122.4663	37.93924	-0.4	2.91	-131.52	2.50599989	11	7/24/2014	28:18.3
6605	RWS11	-122.4663093	37.93923	-0.43	2.92	-125.1	2.4939999	-122.4663	37.93924	-0.35	2.92	-131.27	2.56899992	11	7/24/2014	28:18.4
6606	RWS11	-122.4663114	37.93923	-0.43	2.93	-124.8	2.50699991	-122.46631	37.93924	-0.32	2.93	-130.92	2.61599991	11	7/24/2014	28:18.5
6607	RWS11	-122.4663135	37.93923	-0.43	2.94	-124.5	2.51999992	-122.46631	37.93924	-0.4	2.94	-130.67	2.54499993	11	7/24/2014	28:18.6
6608	RWS11	-122.4663157	37.93923	-0.43	2.96	-124.3	2.53299993	-122.46631	37.93924	-0.35	2.96	-130.36	2.60799995	11	7/24/2014	28:18.7
6609	RWS11	-122.4663178	37.93923	-0.43	2.97	-123.9	2.54500002	-122.46631	37.93924	-0.4	2.97	-129.98	2.57000002	11	7/24/2014	28:18.8
6610	RWS11	-122.4663199	37.93923	-0.43	2.98	-123.6	2.55599993	-122.46631	37.93924	-0.35	2.98	-129.6	2.63099995	11	7/24/2014	28:18.9
6611	RWS11	-122.466323	37.93923	-0.43	2.99	-123.3	2.565	-122.46632	37.93924	-0.43	2.99	-129.3	2.55600002	11	7/24/2014	28:19.0
6612	RWS11	-122.4663251	37.93923	-0.43	3	-122.9	2.57199997	-122.46632	37.93924	-0.35	3	-128.93	2.64699998	11	7/24/2014	28:19.1
6613	RWS11	-122.4663272	37.93923	-0.48	3	-122.6	2.52600008	-122.46632	37.93924	-0.4	3	-128.63	2.60200009	11	7/24/2014	28:19.2
6614	RWS11	-122.4663293	37.93923	-0.48	3.01	-122.3	2.53000003	-122.46632	37.93924	-0.4	3.01	-128.35	2.60600004	11	7/24/2014	28:19.3
6615	RWS11	-122.4663315	37.93923	-0.48	3.01	-122	2.53499991	-122.46633	37.93925	-0.35	3.01	-128.01	2.66099992	11	7/24/2014	28:19.4
6616	RWS11	-122.4663336	37.93923	-0.48	3.02	-121.6	2.54099995	-122.46633	37.93925	-0.4	3.02	-127.67	2.61699995	11	7/24/2014	28:19.5
6617	RWS11	-122.4663358	37.93923	-0.48	3.03	-121.3	2.55000001	-122.46633	37.93925	-0.4	3.03	-127.31	2.62600002	11	7/24/2014	28:19.6
6618	RWS11	-122.4663379	37.93923	-0.48	3.04	-121	2.5620001	-122.46633	37.93925	-0.4	3.04	-126.99	2.6380001	11	7/24/2014	28:19.7
6619	RWS11	-122.4663401	37.93923	-0.51	3.05	-120.6	2.54200006	-122.46633	37.93925	-0.4	3.05	-126.67	2.65200004	11	7/24/2014	28:19.8
6620	RWS11	-122.4663422	37.93924	-0.48	3.07	-120.3	2.59200007	-122.46634	37.93925	-0.4	3.07	-126.33	2.66800007	11	7/24/2014	28:19.9
6621	RWS11	-122.4663453	37.93924	-0.51	3.09	-119.9	2.5769999	-122.46634	37.93925	-0.43	3.09	-125.95	2.65299991	11	7/24/2014	28:20.0
6622	RWS11	-122.4663474	37.93924	-0.51	3.11	-119.5	2.59800005	-122.46634	37.93925	-0.43	3.11	-125.53	2.67400005	11	7/24/2014	28:20.1
6623	RWS11	-122.4663495	37.93924	-0.51	3.13	-119.1	2.61899996	-122.46634	37.93925	-0.49	3.13	-125.18	2.64399993	11	7/24/2014	28:20.2
6624	RWS11	-122.4663517	37.93924	-0.48	3.15	-118.7	2.67500001	-122.46635	37.93925	-0.4	3.15	-124.77	2.75100002	11	7/24/2014	28:20.3
6625	RWS11	-122.4663538	37.93924	-0.51	3.17	-118.4	2.66000009	-122.46635	37.93925	-0.43	3.17	-124.33	2.73600009	11	7/24/2014	28:20.4
6626	RWS11	-122.4663559	37.93924	-0.51	3.18	-118.1	2.67499995	-122.46635	37.93925	-0.43	3.18	-124	2.75099996	11	7/24/2014	28:20.5
6627	RWS11	-122.466358	37.93924	-0.51	3.19	-117.7	2.68400002	-122.46635	37.93925	-0.4	3.19	-123.62	2.794	11	7/24/2014	28:20.6
6628	RWS11	-122.4663602	37.93924	-0.48	3.19	-117.4	2.71899992	-122.46635	37.93925	-0.4	3.19	-123.3	2.79499993	11	7/24/2014	28:20.7
6629	RWS11	-122.4663623	37.93924	-0.51	3.19	-117	2.67799997	-122.46636	37.93925	-0.49	3.19	-122.88	2.70299995	11	7/24/2014	28:20.8
6630	RWS11	-122.4663643	37.93924	-0.51	3.18	-116.8	2.66600013	-122.46636	37.93925	-0.43	3.18	-122.51	2.74200013	11	7/24/2014	28:20.9
6631	RWS11	-122.4663674	37.93924	-0.51	3.16	-116.5	2.6500001	-122.46636	37.93926	-0.43	3.16	-122.2	2.7260001	11	7/24/2014	28:21.0
6632	RWS11	-122.4663695	37.93924	-0.56	3.14	-116.1	2.58099997	-122.46636	37.93926	-0.49	3.14	-121.74	2.65699995	11	7/24/2014	28:21.1

6633	RWS11	-122.4663715	37.93924	-0.56	3.12	-115.7	2.56300008	-122.46637	37.93926	-0.43	3.12	-121.33	2.69000009	11	7/24/2014	28:21.2
6634	RWS11	-122.4663736	37.93924	-0.51	3.11	-115.4	2.59800005	-122.46637	37.93926	-0.43	3.11	-121	2.67400005	11	7/24/2014	28:21.3
6635	RWS11	-122.4663757	37.93924	-0.56	3.1	-115.2	2.53400004	-122.46637	37.93926	-0.43	3.1	-120.7	2.66100004	11	7/24/2014	28:21.4
6636	RWS11	-122.4663778	37.93925	-0.51	3.08	-115	2.57400012	-122.46637	37.93926	-0.49	3.08	-120.43	2.5990001	11	7/24/2014	28:21.5
6637	RWS11	-122.4663799	37.93925	-0.56	3.08	-114.7	2.51599991	-122.46637	37.93926	-0.43	3.08	-120.09	2.64299992	11	7/24/2014	28:21.6
6638	RWS11	-122.466382	37.93925	-0.56	3.07	-114.4	2.51100004	-122.46638	37.93926	-0.49	3.07	-119.67	2.58700001	11	7/24/2014	28:21.7
6639	RWS11	-122.466384	37.93925	-0.56	3.07	-114	2.50899994	-122.46638	37.93926	-0.49	3.07	-119.24	2.58499992	11	7/24/2014	28:21.8
6640	RWS11	-122.4663861	37.93925	-0.56	3.07	-113.7	2.51000011	-122.46638	37.93926	-0.49	3.07	-118.75	2.58600008	11	7/24/2014	28:21.9
6641	RWS11	-122.4663892	37.93925	-0.56	3.07	-113.3	2.51199996	-122.46638	37.93926	-0.49	3.07	-118.28	2.58799994	11	7/24/2014	28:22.0
6642	RWS11	-122.4663912	37.93925	-0.56	3.08	-112.8	2.51499999	-122.46639	37.93926	-0.49	3.08	-117.78	2.59099996	11	7/24/2014	28:22.1
6643	RWS11	-122.4663933	37.93925	-0.6	3.08	-112.2	2.48399991	-122.46639	37.93926	-0.52	3.08	-117.22	2.56099993	11	7/24/2014	28:22.2
6644	RWS11	-122.4663954	37.93925	-0.56	3.09	-111.7	2.52499998	-122.46639	37.93926	-0.49	3.09	-116.64	2.60099995	11	7/24/2014	28:22.3
6645	RWS11	-122.4663975	37.93925	-0.6	3.1	-111.2	2.49999994	-122.46639	37.93926	-0.57	3.1	-116.1	2.52599996	11	7/24/2014	28:22.4
6646	RWS11	-122.4663996	37.93925	-0.56	3.11	-110.7	2.54899991	-122.46639	37.93926	-0.52	3.11	-115.54	2.5909999	11	7/24/2014	28:22.5
6647	RWS11	-122.4664017	37.93925	-0.6	3.13	-110.3	2.53299993	-122.4664	37.93926	-0.6	3.13	-115.03	2.52599996	11	7/24/2014	28:22.6
6648	RWS11	-122.4664038	37.93925	-0.6	3.15	-109.9	2.55599993	-122.4664	37.93927	-0.49	3.15	-114.55	2.66699994	11	7/24/2014	28:22.7
6649	RWS11	-122.4664059	37.93925	-0.6	3.18	-109.4	2.58100003	-122.4664	37.93927	-0.52	3.18	-113.96	2.65800005	11	7/24/2014	28:22.8
6650	RWS11	-122.466408	37.93925	-0.6	3.2	-108.9	2.60599989	-122.4664	37.93927	-0.57	3.2	-113.47	2.63199991	11	7/24/2014	28:22.9
6651	RWS11	-122.466411	37.93925	-0.65	3.23	-108.5	2.57999998	-122.46641	37.93927	-0.57	3.23	-112.94	2.65700001	11	7/24/2014	28:23.0
6652	RWS11	-122.4664131	37.93925	-0.6	3.25	-108.1	2.65499991	-122.46641	37.93927	-0.52	3.25	-112.41	2.73199993	11	7/24/2014	28:23.1
6653	RWS11	-122.4664152	37.93926	-0.6	3.28	-107.6	2.68000001	-122.46641	37.93927	-0.57	3.28	-111.96	2.70600003	11	7/24/2014	28:23.2
6654	RWS11	-122.4664173	37.93926	-0.6	3.3	-107.2	2.70399994	-122.46641	37.93927	-0.57	3.3	-111.41	2.72999996	11	7/24/2014	28:23.3
6655	RWS11	-122.4664194	37.93926	-0.65	3.33	-106.7	2.67800003	-122.46641	37.93927	-0.6	3.33	-110.99	2.72200006	11	7/24/2014	28:23.4
6656	RWS11	-122.4664215	37.93926	-0.6	3.35	-106.2	2.75399989	-122.46642	37.93927	-0.57	3.35	-110.52	2.77999991	11	7/24/2014	28:23.5
6657	RWS11	-122.4664236	37.93926	-0.65	3.38	-105.8	2.72899991	-122.46642	37.93927	-0.6	3.38	-110.06	2.77299994	11	7/24/2014	28:23.6
6658	RWS11	-122.4664257	37.93926	-0.65	3.4	-105.3	2.75700003	-122.46642	37.93927	-0.6	3.4	-109.53	2.80100006	11	7/24/2014	28:23.7
6659	RWS11	-122.4664278	37.93926	-0.65	3.43	-104.9	2.787	-122.46642	37.93927	-0.65	3.43	-109.08	2.78000003	11	7/24/2014	28:23.8
6660	RWS11	-122.4664299	37.93926	-0.65	3.46	-104.6	2.81600004	-122.46642	37.93927	-0.6	3.46	-108.75	2.86000007	11	7/24/2014	28:23.9
6661	RWS11	-122.466433	37.93926	-0.68	3.49	-104.3	2.80999994	-122.46643	37.93927	-0.65	3.49	-108.44	2.83699995	11	7/24/2014	28:24.0
6662	RWS11	-122.4664351	37.93926	-0.65	3.52	-103.9	2.86800009	-122.46643	37.93927	-0.65	3.52	-108.01	2.86100012	11	7/24/2014	28:24.1
6663	RWS11	-122.4664372	37.93926	-0.68	3.53	-103.5	2.85100007	-122.46643	37.93927	-0.69	3.53	-107.66	2.84400004	11	7/24/2014	28:24.2
6664	RWS11	-122.4664393	37.93926	-0.68	3.54	-103.2	2.85899997	-122.46643	37.93927	-0.65	3.54	-107.29	2.88599998	11	7/24/2014	28:24.3
6665	RWS11	-122.4664414	37.93926	-0.68	3.54	-102.9	2.85599995	-122.46644	37.93928	-0.69	3.54	-106.92	2.84899992	11	7/24/2014	28:24.4

6666	RWS11	-122.4664435	37.93926	-0.68	3.52	-102.5	2.83999991	-122.46644	37.93928	-0.69	3.52	-106.58	2.83299989	11	7/24/2014	28:24.5
6667	RWS11	-122.4664457	37.93926	-0.68	3.49	-102.2	2.80900002	-122.46644	37.93928	-0.65	3.49	-106.21	2.83600003	11	7/24/2014	28:24.6
6668	RWS11	-122.4664478	37.93926	-0.68	3.45	-101.9	2.7650001	-122.46644	37.93928	-0.69	3.45	-105.9	2.75800008	11	7/24/2014	28:24.7
6669	RWS11	-122.4664499	37.93926	-0.68	3.39	-101.6	2.71000004	-122.46644	37.93928	-0.72	3.39	-105.65	2.66900003	11	7/24/2014	28:24.8
6670	RWS11	-122.466452	37.93927	-0.68	3.33	-101.4	2.64700007	-122.46645	37.93928	-0.69	3.33	-105.38	2.64000005	11	7/24/2014	28:24.9
6671	RWS11	-122.4664552	37.93927	-0.71	3.26	-101.1	2.54899997	-122.46645	37.93928	-0.72	3.26	-105.22	2.54199994	11	7/24/2014	28:25.0
6672	RWS11	-122.4664573	37.93927	-0.71	3.21	-100.9	2.49200004	-122.46645	37.93928	-0.72	3.21	-105.05	2.48500001	11	7/24/2014	28:25.1
6673	RWS11	-122.4664595	37.93927	-0.71	3.17	-100.7	2.45200008	-122.46645	37.93928	-0.72	3.17	-104.83	2.44500005	11	7/24/2014	28:25.2
6674	RWS11	-122.4664617	37.93927	-0.68	3.16	-100.5	2.47600007	-122.46646	37.93928	-0.72	3.16	-104.72	2.43500006	11	7/24/2014	28:25.3
6675	RWS11	-122.4664638	37.93927	-0.71	3.18	-100.4	2.463	-122.46646	37.93928	-0.72	3.18	-104.6	2.45599997	11	7/24/2014	28:25.4
6676	RWS11	-122.466466	37.93927	-0.68	3.22	-100.3	2.54200006	-122.46646	37.93928	-0.72	3.22	-104.53	2.50100005	11	7/24/2014	28:25.5
6677	RWS11	-122.4664682	37.93927	-0.71	3.28	-100.1	2.56800002	-122.46646	37.93928	-0.77	3.28	-104.53	2.50999999	11	7/24/2014	28:25.6
6678	RWS11	-122.4664704	37.93927	-0.71	3.35	-100.1	2.63099998	-122.46647	37.93928	-0.72	3.35	-104.52	2.62399995	11	7/24/2014	28:25.7
6679	RWS11	-122.4664725	37.93927	-0.71	3.4	-100.1	2.68900007	-122.46647	37.93928	-0.77	3.4	-104.53	2.63100004	11	7/24/2014	28:25.8
6680	RWS11	-122.4664747	37.93927	-0.71	3.45	-100	2.73800009	-122.46647	37.93928	-0.77	3.45	-104.55	2.68000007	11	7/24/2014	28:25.9
6681	RWS11	-122.4664778	37.93927	-0.71	3.49	-99.94	2.77500004	-122.46647	37.93929	-0.77	3.49	-104.55	2.71700001	11	7/24/2014	28:26.0
6682	RWS11	-122.46648	37.93927	-0.68	3.51	-99.9	2.83299994	-122.46647	37.93929	-0.77	3.51	-104.64	2.74099994	11	7/24/2014	28:26.1
6683	RWS11	-122.4664821	37.93927	-0.71	3.53	-99.88	2.81400007	-122.46648	37.93929	-0.77	3.53	-104.71	2.75600004	11	7/24/2014	28:26.2
6684	RWS11	-122.4664843	37.93927	-0.71	3.54	-99.74	2.82000011	-122.46648	37.93929	-0.72	3.54	-104.69	2.81300008	11	7/24/2014	28:26.3
6685	RWS11	-122.4664864	37.93927	-0.71	3.54	-99.64	2.82000011	-122.46648	37.93929	-0.77	3.54	-104.67	2.76200008	11	7/24/2014	28:26.4
6686	RWS11	-122.4664886	37.93927	-0.71	3.53	-99.6	2.815	-122.46648	37.93929	-0.72	3.53	-104.72	2.80799997	11	7/24/2014	28:26.5
6687	RWS11	-122.4664907	37.93928	-0.71	3.52	-99.56	2.80599993	-122.46649	37.93929	-0.72	3.52	-104.8	2.79899991	11	7/24/2014	28:26.6
6688	RWS11	-122.4664929	37.93928	-0.71	3.51	-99.51	2.79500002	-122.46649	37.93929	-0.77	3.51	-104.9	2.73699999	11	7/24/2014	28:26.7
6689	RWS11	-122.466495	37.93928	-0.71	3.5	-99.51	2.78100008	-122.46649	37.93929	-0.77	3.5	-105.01	2.72300005	11	7/24/2014	28:26.8
6690	RWS11	-122.4664971	37.93928	-0.68	3.48	-99.54	2.79999995	-122.46649	37.93929	-0.77	3.48	-105.16	2.70799994	11	7/24/2014	28:26.9
6691	RWS11	-122.4665003	37.93928	-0.68	3.46	-99.49	2.78399992	-122.4665	37.93929	-0.69	3.46	-105.26	2.77699989	11	7/24/2014	28:27.0
6692	RWS11	-122.4665023	37.93928	-0.71	3.45	-99.49	2.73399991	-122.4665	37.93929	-0.69	3.45	-105.39	2.76099986	11	7/24/2014	28:27.1
6693	RWS11	-122.4665044	37.93928	-0.71	3.43	-99.43	2.71699995	-122.4665	37.93929	-0.72	3.43	-105.43	2.70999992	11	7/24/2014	28:27.2
6694	RWS11	-122.4665065	37.93928	-0.68	3.42	-99.37	2.7349999	-122.4665	37.93929	-0.72	3.42	-105.45	2.69399989	11	7/24/2014	28:27.3
6695	RWS11	-122.4665087	37.93928	-0.68	3.4	-99.3	2.7190001	-122.4665	37.93929	-0.72	3.4	-105.5	2.67800009	11	7/24/2014	28:27.4
6696	RWS11	-122.4665108	37.93928	-0.68	3.39	-99.25	2.70499992	-122.46651	37.93929	-0.69	3.39	-105.53	2.69799989	11	7/24/2014	28:27.5
6697	RWS11	-122.4665129	37.93928	-0.68	3.38	-99.23	2.69400001	-122.46651	37.93929	-0.69	3.38	-105.63	2.68699998	11	7/24/2014	28:27.6
6698	RWS11	-122.466515	37.93928	-0.68	3.37	-99.21	2.68499994	-122.46651	37.93929	-0.69	3.37	-105.62	2.67799991	11	7/24/2014	28:27.7

6699	RWS11	-122.4665171	37.93928	-0.68	3.36	-99.24	2.6789999	-122.46651	37.9393	-0.69	3.36	-105.61	2.67199987	11	7/24/2014	28:27.8
6700	RWS11	-122.4665192	37.93928	-0.68	3.36	-99.26	2.67799997	-122.46651	37.9393	-0.65	3.36	-105.54	2.70499998	11	7/24/2014	28:27.9
6701	RWS11	-122.4665223	37.93928	-0.68	3.36	-99.24	2.6789999	-122.46652	37.9393	-0.69	3.36	-105.54	2.67199987	11	7/24/2014	28:28.0
6702	RWS11	-122.4665243	37.93928	-0.65	3.37	-99.24	2.71799999	-122.46652	37.9393	-0.65	3.37	-105.52	2.71100003	11	7/24/2014	28:28.1
6703	RWS11	-122.4665264	37.93928	-0.68	3.37	-99.19	2.69199991	-122.46652	37.9393	-0.65	3.37	-105.52	2.71899992	11	7/24/2014	28:28.2
6704	RWS11	-122.4665285	37.93929	-0.68	3.38	-99.15	2.70000005	-122.46652	37.9393	-0.6	3.38	-105.54	2.77800006	11	7/24/2014	28:28.3
6705	RWS11	-122.4665307	37.93929	-0.65	3.39	-99.19	2.74000007	-122.46653	37.9393	-0.6	3.39	-105.59	2.7840001	11	7/24/2014	28:28.4
6706	RWS11	-122.4665328	37.93929	-0.65	3.39	-99.13	2.74400002	-122.46653	37.9393	-0.6	3.39	-105.56	2.78800005	11	7/24/2014	28:28.5
6707	RWS11	-122.4665349	37.93929	-0.65	3.39	-99.02	2.74499995	-122.46653	37.9393	-0.65	3.39	-105.55	2.73799998	11	7/24/2014	28:28.6
6708	RWS11	-122.466537	37.93929	-0.65	3.39	-98.93	2.741	-122.46653	37.9393	-0.57	3.39	-105.44	2.81800002	11	7/24/2014	28:28.7
6709	RWS11	-122.4665391	37.93929	-0.65	3.38	-98.81	2.73499995	-122.46653	37.9393	-0.6	3.38	-105.17	2.77899998	11	7/24/2014	28:28.8
6710	RWS11	-122.4665412	37.93929	-0.65	3.37	-98.81	2.72599989	-122.46654	37.9393	-0.57	3.37	-105.05	2.80299991	11	7/24/2014	28:28.9
6711	RWS11	-122.4665444	37.93929	-0.65	3.36	-98.68	2.71499997	-122.46654	37.9393	-0.6	3.36	-104.96	2.759	11	7/24/2014	28:29.0
6712	RWS11	-122.4665465	37.93929	-0.6	3.35	-98.59	2.75399989	-122.46654	37.9393	-0.57	3.35	-104.77	2.77999991	11	7/24/2014	28:29.1
6713	RWS11	-122.4665486	37.93929	-0.6	3.34	-98.43	2.74099988	-122.46654	37.9393	-0.57	3.34	-104.67	2.7669999	11	7/24/2014	28:29.2
6714	RWS11	-122.4665507	37.93929	-0.6	3.32	-98.35	2.72600001	-122.46655	37.9393	-0.57	3.32	-104.5	2.75200003	11	7/24/2014	28:29.3
6715	RWS11	-122.4665528	37.93929	-0.6	3.31	-98.2	2.71099991	-122.46655	37.9393	-0.57	3.31	-104.27	2.73699993	11	7/24/2014	28:29.4
6716	RWS11	-122.466555	37.93929	-0.6	3.29	-97.98	2.69600004	-122.46655	37.9393	-0.57	3.29	-103.93	2.72200006	11	7/24/2014	28:29.5
6717	RWS11	-122.4665571	37.93929	-0.6	3.28	-97.89	2.68099993	-122.46655	37.93931	-0.52	3.28	-103.67	2.75799996	11	7/24/2014	28:29.6
6718	RWS11	-122.4665593	37.93929	-0.6	3.26	-97.65	2.66799992	-122.46655	37.93931	-0.52	3.26	-103.35	2.74499995	11	7/24/2014	28:29.7
6719	RWS11	-122.4665614	37.93929	-0.6	3.25	-97.5	2.65799993	-122.46656	37.93931	-0.57	3.25	-103.08	2.68399996	11	7/24/2014	28:29.8
6720	RWS11	-122.4665635	37.93929	-0.6	3.25	-97.26	2.65099996	-122.46656	37.93931	-0.52	3.25	-102.87	2.72799999	11	7/24/2014	28:29.9
6721	RWS11	-122.4665666	37.93929	-0.65	3.24	-97.06	2.59699994	-122.46656	37.93931	-0.52	3.24	-102.56	2.72499996	11	7/24/2014	28:30.0
6722	RWS11	-122.4665688	37.93929	-0.6	3.25	-96.9	2.65000004	-122.46656	37.93931	-0.49	3.25	-102.35	2.76100004	11	7/24/2014	28:30.1
6723	RWS11	-122.4665709	37.9393	-0.6	3.25	-96.7	2.65799993	-122.46657	37.93931	-0.49	3.25	-102.11	2.76899993	11	7/24/2014	28:30.2
6724	RWS11	-122.4665731	37.9393	-0.56	3.27	-96.46	2.70500004	-122.46657	37.93931	-0.49	3.27	-101.84	2.78100002	11	7/24/2014	28:30.3
6725	RWS11	-122.4665752	37.9393	-0.6	3.28	-96.17	2.68499988	-122.46657	37.93931	-0.49	3.28	-101.48	2.79599988	11	7/24/2014	28:30.4
6726	RWS11	-122.4665774	37.9393	-0.56	3.3	-95.91	2.73599994	-122.46657	37.93931	-0.49	3.3	-101.17	2.81199992	11	7/24/2014	28:30.5
6727	RWS11	-122.4665796	37.9393	-0.6	3.31	-95.65	2.71699995	-122.46657	37.93931	-0.43	3.31	-100.86	2.87899998	11	7/24/2014	28:30.6
6728	RWS11	-122.4665817	37.9393	-0.56	3.33	-95.41	2.76599991	-122.46658	37.93931	-0.49	3.33	-100.56	2.84199989	11	7/24/2014	28:30.7
6729	RWS11	-122.4665839	37.9393	-0.56	3.34	-95.17	2.77400005	-122.46658	37.93931	-0.43	3.34	-100.28	2.90100005	11	7/24/2014	28:30.8
6730	RWS11	-122.466586	37.9393	-0.56	3.34	-94.98	2.7759999	-122.46658	37.93931	-0.43	3.34	-99.974	2.90299991	11	7/24/2014	28:30.9
6731	RWS11	-122.4665892	37.9393	-0.56	3.33	-94.69	2.77100003	-122.46658	37.93931	-0.49	3.33	-99.69	2.847	11	7/24/2014	28:31.0

6732	RWS11	-122.4665913	37.9393	-0.56	3.32	-94.48	2.75899994	-122.46659	37.93931	-0.49	3.32	-99.379	2.83499992	11	7/24/2014	28:31.1
6733	RWS11	-122.4665934	37.9393	-0.56	3.3	-94.19	2.74000013	-122.46659	37.93931	-0.43	3.3	-99.118	2.86700013	11	7/24/2014	28:31.2
6734	RWS11	-122.4665956	37.9393	-0.56	3.28	-93.86	2.71500003	-122.46659	37.93931	-0.4	3.28	-98.798	2.87600002	11	7/24/2014	28:31.3
6735	RWS11	-122.4665977	37.9393	-0.56	3.25	-93.6	2.68500006	-122.46659	37.93931	-0.43	3.25	-98.514	2.81200007	11	7/24/2014	28:31.4
6736	RWS11	-122.4665999	37.9393	-0.56	3.21	-93.32	2.653	-122.4666	37.93932	-0.4	3.21	-98.177	2.81399998	11	7/24/2014	28:31.5
6737	RWS11	-122.466602	37.9393	-0.56	3.18	-93.06	2.62	-122.4666	37.93932	-0.4	3.18	-97.908	2.78099999	11	7/24/2014	28:31.6
6738	RWS11	-122.4666042	37.9393	-0.51	3.15	-92.79	2.63899994	-122.4666	37.93932	-0.4	3.15	-97.554	2.74899992	11	7/24/2014	28:31.7
6739	RWS11	-122.4666063	37.9393	-0.56	3.12	-92.52	2.55799997	-122.4666	37.93932	-0.43	3.12	-97.156	2.68499997	11	7/24/2014	28:31.8
6740	RWS11	-122.4666084	37.9393	-0.56	3.09	-92.28	2.53199995	-122.4666	37.93932	-0.4	3.09	-96.857	2.69299993	11	7/24/2014	28:31.9
6741	RWS11	-122.4666116	37.93931	-0.56	3.07	-91.94	2.50899994	-122.46661	37.93932	-0.4	3.07	-96.547	2.66999993	11	7/24/2014	28:32.0
6742	RWS11	-122.4666137	37.93931	-0.51	3.05	-91.69	2.54100013	-122.46661	37.93932	-0.4	3.05	-96.198	2.65100011	11	7/24/2014	28:32.1
6743	RWS11	-122.4666159	37.93931	-0.56	3.04	-91.31	2.4740001	-122.46661	37.93932	-0.4	3.04	-95.833	2.63500008	11	7/24/2014	28:32.2
6744	RWS11	-122.466618	37.93931	-0.51	3.02	-91.05	2.51200008	-122.46661	37.93932	-0.4	3.02	-95.568	2.62200007	11	7/24/2014	28:32.3
6745	RWS11	-122.4666202	37.93931	-0.56	3.01	-90.68	2.44999993	-122.46662	37.93932	-0.4	3.01	-95.135	2.61099991	11	7/24/2014	28:32.4
6746	RWS11	-122.4666224	37.93931	-0.51	3	-90.29	2.49300003	-122.46662	37.93932	-0.4	3	-94.656	2.60300002	11	7/24/2014	28:32.5
6747	RWS11	-122.4666245	37.93931	-0.56	3	-89.94	2.43599999	-122.46662	37.93932	-0.35	3	-94.193	2.64699998	11	7/24/2014	28:32.6
6748	RWS11	-122.4666267	37.93931	-0.51	2.99	-89.66	2.48300004	-122.46662	37.93932	-0.35	2.99	-93.763	2.64300004	11	7/24/2014	28:32.7
6749	RWS11	-122.4666288	37.93931	-0.51	2.99	-89.29	2.47900009	-122.46662	37.93932	-0.4	2.99	-93.409	2.58900008	11	7/24/2014	28:32.8
6750	RWS11	-122.4666309	37.93931	-0.48	2.99	-88.94	2.51099998	-122.46663	37.93932	-0.4	2.99	-93.001	2.58699998	11	7/24/2014	28:32.9
6751	RWS11	-122.4666341	37.93931	-0.51	2.99	-88.55	2.47600007	-122.46663	37.93932	-0.4	2.99	-92.604	2.58600006	11	7/24/2014	28:33.0
6752	RWS11	-122.4666362	37.93931	-0.48	2.99	-88.11	2.51099998	-122.46663	37.93932	-0.32	2.99	-92.077	2.67099997	11	7/24/2014	28:33.1
6753	RWS11	-122.4666383	37.93931	-0.48	2.99	-87.65	2.514	-122.46663	37.93932	-0.32	2.99	-91.742	2.67399999	11	7/24/2014	28:33.2
6754	RWS11	-122.4666405	37.93931	-0.48	2.99	-87.2	2.51899987	-122.46664	37.93932	-0.32	2.99	-91.366	2.67899987	11	7/24/2014	28:33.3
6755	RWS11	-122.4666426	37.93931	-0.51	3	-86.76	2.4920001	-122.46664	37.93933	-0.4	3	-90.942	2.60200009	11	7/24/2014	28:33.4
6756	RWS11	-122.4666448	37.93931	-0.48	3.01	-86.35	2.53399998	-122.46664	37.93933	-0.35	3.01	-90.563	2.66	11	7/24/2014	28:33.5
6757	RWS11	-122.4666469	37.93931	-0.51	3.02	-85.98	2.5079999	-122.46664	37.93933	-0.35	3.02	-90.234	2.66799989	11	7/24/2014	28:33.6
6758	RWS11	-122.466649	37.93931	-0.48	3.03	-85.58	2.55000001	-122.46664	37.93933	-0.32	3.03	-89.82	2.71000001	11	7/24/2014	28:33.7
6759	RWS11	-122.4666512	37.93931	-0.48	3.03	-85.24	2.55600005	-122.46665	37.93933	-0.35	3.03	-89.445	2.68200007	11	7/24/2014	28:33.8
6760	RWS11	-122.4666533	37.93931	-0.48	3.04	-84.87	2.56099993	-122.46665	37.93933	-0.28	3.04	-89.056	2.75499994	11	7/24/2014	28:33.9
6761	RWS11	-122.4666564	37.93932	-0.48	3.04	-84.5	2.56399995	-122.46665	37.93933	-0.35	3.04	-88.648	2.68999997	11	7/24/2014	28:34.0
6762	RWS11	-122.4666586	37.93932	-0.43	3.04	-84.13	2.61599988	-122.46665	37.93933	-0.32	3.04	-88.354	2.72499987	11	7/24/2014	28:34.1
6763	RWS11	-122.4666607	37.93932	-0.48	3.04	-83.77	2.56600004	-122.46666	37.93933	-0.35	3.04	-87.972	2.69200006	11	7/24/2014	28:34.2
6764	RWS11	-122.4666628	37.93932	-0.43	3.04	-83.45	2.61799997	-122.46666	37.93933	-0.35	3.04	-87.614	2.69299999	11	7/24/2014	28:34.3

6765	RWS11	-122.4666649	37.93932	-0.43	3.04	-83.08	2.61700004	-122.46666	37.93933	-0.28	3.04	-87.205	2.76000005	11	7/24/2014	28:34.4
6766	RWS11	-122.4666671	37.93932	-0.43	3.04	-82.73	2.61499995	-122.46666	37.93933	-0.32	3.04	-86.917	2.72399995	11	7/24/2014	28:34.5
6767	RWS11	-122.4666692	37.93932	-0.43	3.04	-82.38	2.61199993	-122.46666	37.93933	-0.32	3.04	-86.574	2.72099993	11	7/24/2014	28:34.6
6768	RWS11	-122.4666714	37.93932	-0.43	3.03	-81.99	2.60700005	-122.46667	37.93933	-0.28	3.03	-86.159	2.75000006	11	7/24/2014	28:34.7
6769	RWS11	-122.4666735	37.93932	-0.43	3.03	-81.62	2.60000008	-122.46667	37.93933	-0.35	3.03	-85.745	2.6750001	11	7/24/2014	28:34.8
6770	RWS11	-122.4666756	37.93932	-0.39	3.02	-81.16	2.62600005	-122.46667	37.93933	-0.35	3.02	-85.353	2.66600004	11	7/24/2014	28:34.9
6771	RWS11	-122.4666788	37.93932	-0.43	3.01	-80.85	2.58100003	-122.46667	37.93933	-0.35	3.01	-85.026	2.65600005	11	7/24/2014	28:35.0
6772	RWS11	-122.4666809	37.93932	-0.39	3	-80.47	2.60600007	-122.46668	37.93933	-0.35	3	-84.629	2.64600006	11	7/24/2014	28:35.1
6773	RWS11	-122.466683	37.93932	-0.43	2.99	-80.07	2.56100005	-122.46668	37.93933	-0.35	2.99	-84.312	2.63600007	11	7/24/2014	28:35.2
6774	RWS11	-122.4666852	37.93932	-0.39	2.98	-79.74	2.58600008	-122.46668	37.93934	-0.4	2.98	-83.976	2.57600006	11	7/24/2014	28:35.3
6775	RWS11	-122.4666873	37.93932	-0.43	2.97	-79.39	2.54299992	-122.46668	37.93934	-0.28	2.97	-83.606	2.68599993	11	7/24/2014	28:35.4
6776	RWS11	-122.4666895	37.93932	-0.39	2.96	-79.01	2.57199991	-122.46668	37.93934	-0.28	2.96	-83.239	2.67999989	11	7/24/2014	28:35.5
6777	RWS11	-122.4666917	37.93932	-0.43	2.96	-78.57	2.5340001	-122.46669	37.93934	-0.32	2.96	-82.782	2.6430001	11	7/24/2014	28:35.6
6778	RWS11	-122.4666938	37.93932	-0.39	2.96	-78.16	2.56799996	-122.46669	37.93934	-0.32	2.96	-82.389	2.64199993	11	7/24/2014	28:35.7
6779	RWS11	-122.466696	37.93932	-0.39	2.96	-77.67	2.57099998	-122.46669	37.93934	-0.35	2.96	-81.857	2.61099997	11	7/24/2014	28:35.8
6780	RWS11	-122.4666981	37.93933	-0.39	2.96	-77.13	2.57499993	-122.46669	37.93934	-0.32	2.96	-81.3	2.6489999	11	7/24/2014	28:35.9
6781	RWS11	-122.4667013	37.93933	-0.43	2.97	-76.63	2.54800004	-122.4667	37.93934	-0.4	2.97	-80.838	2.57300004	11	7/24/2014	28:36.0
6782	RWS11	-122.4667035	37.93933	-0.39	2.98	-76.06	2.59200013	-122.4667	37.93934	-0.4	2.98	-80.202	2.58200011	11	7/24/2014	28:36.1
6783	RWS11	-122.4667056	37.93933	-0.39	2.99	-75.45	2.60399997	-122.4667	37.93934	-0.4	2.99	-79.619	2.59399995	11	7/24/2014	28:36.2
6784	RWS11	-122.4667078	37.93933	-0.34	3.01	-74.99	2.67000008	-122.4667	37.93934	-0.4	3.01	-79.139	2.60900006	11	7/24/2014	28:36.3
6785	RWS11	-122.46671	37.93933	-0.39	3.03	-74.39	2.63600004	-122.46671	37.93934	-0.4	3.03	-78.729	2.62600002	11	7/24/2014	28:36.4
6786	RWS11	-122.4667122	37.93933	-0.39	3.04	-73.86	2.65399992	-122.46671	37.93934	-0.35	3.04	-78.224	2.69399992	11	7/24/2014	28:36.5
6787	RWS11	-122.4667144	37.93933	-0.39	3.06	-73.23	2.67399991	-122.46671	37.93934	-0.32	3.06	-77.653	2.74799988	11	7/24/2014	28:36.6
6788	RWS11	-122.4667166	37.93933	-0.34	3.08	-72.59	2.74399996	-122.46671	37.93934	-0.32	3.08	-77.025	2.76699993	11	7/24/2014	28:36.7
6789	RWS11	-122.4667188	37.93933	-0.39	3.1	-71.94	2.71200001	-122.46671	37.93934	-0.4	3.1	-76.459	2.70199999	11	7/24/2014	28:36.8
6790	RWS11	-122.466721	37.93933	-0.39	3.12	-71.29	2.72899997	-122.46672	37.93934	-0.35	3.12	-75.802	2.76899996	11	7/24/2014	28:36.9
6791	RWS11	-122.4667242	37.93933	-0.39	3.13	-70.59	2.74199998	-122.46672	37.93934	-0.4	3.13	-75.173	2.73199996	11	7/24/2014	28:37.0
6792	RWS11	-122.4667264	37.93933	-0.39	3.14	-69.94	2.7529999	-122.46672	37.93934	-0.35	3.14	-74.65	2.79299989	11	7/24/2014	28:37.1
6793	RWS11	-122.4667285	37.93933	-0.39	3.15	-69.32	2.75899994	-122.46672	37.93934	-0.4	3.15	-74.106	2.74899992	11	7/24/2014	28:37.2
6794	RWS11	-122.4667307	37.93933	-0.34	3.15	-68.58	2.81100011	-122.46673	37.93935	-0.32	3.15	-73.496	2.83400008	11	7/24/2014	28:37.3
6795	RWS11	-122.4667329	37.93933	-0.39	3.15	-67.87	2.75700009	-122.46673	37.93935	-0.4	3.15	-72.895	2.74700007	11	7/24/2014	28:37.4
6796	RWS11	-122.4667351	37.93933	-0.34	3.14	-67.12	2.79900002	-122.46673	37.93935	-0.4	3.14	-72.275	2.73800001	11	7/24/2014	28:37.5
6797	RWS11	-122.4667373	37.93933	-0.39	3.12	-66.29	2.73299992	-122.46673	37.93935	-0.4	3.12	-71.542	2.7229999	11	7/24/2014	28:37.6

6798	RWS11	-122.4667395	37.93933	-0.34	3.1	-65.53	2.76399994	-122.46673	37.93935	-0.4	3.1	-70.896	2.70299992	11	7/24/2014	28:37.7
6799	RWS11	-122.4667416	37.93933	-0.34	3.08	-64.77	2.74000001	-122.46674	37.93935	-0.4	3.08	-70.162	2.67899999	11	7/24/2014	28:37.8
6800	RWS11	-122.4667438	37.93934	-0.34	3.05	-63.96	2.71399999	-122.46674	37.93935	-0.4	3.05	-69.417	2.65299997	11	7/24/2014	28:37.9
6801	RWS11	-122.4667469	37.93934	-0.39	3.03	-63.15	2.63600004	-122.46674	37.93935	-0.4	3.03	-68.746	2.62600002	11	7/24/2014	28:38.0
6802	RWS11	-122.4667491	37.93934	-0.34	3	-62.39	2.66000009	-122.46674	37.93935	-0.4	3	-68.041	2.59900007	11	7/24/2014	28:38.1
6803	RWS11	-122.4667512	37.93934	-0.39	2.97	-61.56	2.58399999	-122.46675	37.93935	-0.32	2.97	-67.394	2.65799996	11	7/24/2014	28:38.2
6804	RWS11	-122.4667533	37.93934	-0.34	2.95	-60.65	2.61299992	-122.46675	37.93935	-0.35	2.95	-66.603	2.60199991	11	7/24/2014	28:38.3
6805	RWS11	-122.4667555	37.93934	-0.39	2.93	-59.69	2.54100001	-122.46675	37.93935	-0.43	2.93	-65.84	2.49700001	11	7/24/2014	28:38.4
6806	RWS11	-122.4667576	37.93934	-0.39	2.91	-58.79	2.52400005	-122.46675	37.93935	-0.43	2.91	-65.1	2.48000005	11	7/24/2014	28:38.5
6807	RWS11	-122.4667598	37.93934	-0.39	2.9	-57.81	2.51000011	-122.46676	37.93935	-0.35	2.9	-64.384	2.5500001	11	7/24/2014	28:38.6
6808	RWS11	-122.4667619	37.93934	-0.34	2.89	-56.81	2.55100012	-122.46676	37.93935	-0.35	2.89	-63.552	2.54000011	11	7/24/2014	28:38.7
6809	RWS11	-122.466764	37.93934	-0.39	2.88	-55.76	2.49400008	-122.46676	37.93935	-0.4	2.88	-62.746	2.48400006	11	7/24/2014	28:38.8
6810	RWS11	-122.4667661	37.93934	-0.34	2.88	-54.82	2.54200006	-122.46676	37.93935	-0.4	2.88	-61.936	2.48100004	11	7/24/2014	28:38.9
6811	RWS11	-122.4667693	37.93934	-0.39	2.88	-53.6	2.49100006	-122.46676	37.93935	-0.4	2.88	-61.09	2.48100004	11	7/24/2014	28:39.0
6812	RWS11	-122.4667714	37.93934	-0.39	2.88	-52.69	2.495	-122.46677	37.93935	-0.35	2.88	-60.193	2.535	11	7/24/2014	28:39.1
6813	RWS11	-122.4667735	37.93934	-0.39	2.89	-51.46	2.50199997	-122.46677	37.93936	-0.4	2.89	-59.299	2.49199995	11	7/24/2014	28:39.2
6814	RWS11	-122.4667756	37.93934	-0.39	2.9	-50.31	2.50899994	-122.46677	37.93936	-0.35	2.9	-58.365	2.54899994	11	7/24/2014	28:39.3
6815	RWS11	-122.4667778	37.93934	-0.39	2.91	-49.11	2.51800001	-122.46677	37.93936	-0.43	2.91	-57.425	2.47400001	11	7/24/2014	28:39.4
6816	RWS11	-122.4667799	37.93934	-0.39	2.92	-47.93	2.52700007	-122.46678	37.93936	-0.35	2.92	-56.463	2.56700006	11	7/24/2014	28:39.5
6817	RWS11	-122.4667821	37.93934	-0.39	2.93	-46.62	2.53600013	-122.46678	37.93936	-0.4	2.93	-55.398	2.52600011	11	7/24/2014	28:39.6
6818	RWS11	-122.4667842	37.93934	-0.39	2.93	-45.51	2.54400003	-122.46678	37.93936	-0.4	2.93	-54.37	2.53400001	11	7/24/2014	28:39.7
6819	RWS11	-122.4667863	37.93934	-0.39	2.94	-44.22	2.55199993	-122.46678	37.93936	-0.43	2.94	-53.386	2.50799993	11	7/24/2014	28:39.8
6820	RWS11	-122.4667884	37.93935	-0.39	2.95	-42.91	2.56000006	-122.46678	37.93936	-0.4	2.95	-52.427	2.55000004	11	7/24/2014	28:39.9
6821	RWS11	-122.4667916	37.93935	-0.39	2.96	-41.65	2.56799996	-122.46679	37.93936	-0.4	2.96	-51.317	2.55799994	11	7/24/2014	28:40.0
6822	RWS11	-122.4667937	37.93935	-0.39	2.97	-40.35	2.57700002	-122.46679	37.93936	-0.35	2.97	-50.271	2.61700001	11	7/24/2014	28:40.1
6823	RWS11	-122.4667958	37.93935	-0.39	2.98	-38.97	2.58700001	-122.46679	37.93936	-0.4	2.98	-49.14	2.57699999	11	7/24/2014	28:40.2
6824	RWS11	-122.466798	37.93935	-0.39	2.99	-37.66	2.5990001	-122.46679	37.93936	-0.32	2.99	-48.023	2.67300007	11	7/24/2014	28:40.3
6825	RWS11	-122.4668001	37.93935	-0.43	3	-36.33	2.57599992	-122.4668	37.93936	-0.4	3	-46.955	2.60099992	11	7/24/2014	28:40.4
6826	RWS11	-122.4668023	37.93935	-0.39	3.01	-35.06	2.62300003	-122.4668	37.93936	-0.4	3.01	-45.821	2.61300001	11	7/24/2014	28:40.5
6827	RWS11	-122.4668044	37.93935	-0.39	3.03	-33.75	2.63600004	-122.4668	37.93936	-0.43	3.03	-44.879	2.59200004	11	7/24/2014	28:40.6
6828	RWS11	-122.4668066	37.93935	-0.39	3.04	-32.56	2.64800012	-122.4668	37.93936	-0.4	3.04	-43.904	2.6380001	11	7/24/2014	28:40.7
6829	RWS11	-122.4668087	37.93935	-0.43	3.05	-31.29	2.62499994	-122.4668	37.93936	-0.4	3.05	-42.88	2.64999995	11	7/24/2014	28:40.8
6830	RWS11	-122.4668108	37.93935	-0.39	3.06	-30.11	2.66999996	-122.46681	37.93936	-0.4	3.06	-41.896	2.65999994	11	7/24/2014	28:40.9

6831	RWS11	-122.466814	37.93935	-0.39	3.07	-29.02	2.67900002	-122.46681	37.93936	-0.43	3.07	-40.915	2.63500002	11	7/24/2014	28:41.0
6832	RWS11	-122.4668161	37.93935	-0.39	3.08	-27.95	2.68599999	-122.46681	37.93936	-0.35	3.08	-39.958	2.72599998	11	7/24/2014	28:41.1
6833	RWS11	-122.4668183	37.93935	-0.39	3.08	-27.08	2.68999994	-122.46681	37.93937	-0.35	3.08	-39.06	2.72999993	11	7/24/2014	28:41.2
6834	RWS11	-122.4668204	37.93935	-0.39	3.08	-26.14	2.69200003	-122.46682	37.93937	-0.4	3.08	-38.253	2.68200001	11	7/24/2014	28:41.3
6835	RWS11	-122.4668226	37.93935	-0.43	3.08	-25.25	2.65600008	-122.46682	37.93937	-0.43	3.08	-37.423	2.6470001	11	7/24/2014	28:41.4
6836	RWS11	-122.4668248	37.93935	-0.39	3.08	-24.49	2.68699992	-122.46682	37.93937	-0.43	3.08	-36.727	2.64299992	11	7/24/2014	28:41.5
6837	RWS11	-122.466827	37.93935	-0.39	3.07	-23.79	2.68200004	-122.46682	37.93937	-0.35	3.07	-36.009	2.72200003	11	7/24/2014	28:41.6
6838	RWS11	-122.4668291	37.93935	-0.39	3.07	-23.25	2.67699993	-122.46682	37.93937	-0.4	3.07	-35.399	2.66699991	11	7/24/2014	28:41.7
6839	RWS11	-122.4668312	37.93936	-0.43	3.06	-22.72	2.63799995	-122.46683	37.93937	-0.4	3.06	-34.854	2.66299996	11	7/24/2014	28:41.8
6840	RWS11	-122.4668334	37.93936	-0.39	3.06	-22.31	2.66999996	-122.46683	37.93937	-0.43	3.06	-34.309	2.62599996	11	7/24/2014	28:41.9
6841	RWS11	-122.4668365	37.93936	-0.43	3.06	-22.05	2.63300008	-122.46683	37.93937	-0.43	3.06	-34.047	2.6240001	11	7/24/2014	28:42.0
6842	RWS11	-122.4668387	37.93936	-0.39	3.06	-21.85	2.6680001	-122.46683	37.93937	-0.32	3.06	-33.726	2.74200007	11	7/24/2014	28:42.1
6843	RWS11	-122.4668408	37.93936	-0.39	3.06	-21.72	2.6680001	-122.46684	37.93937	-0.4	3.06	-33.481	2.65800008	11	7/24/2014	28:42.2
6844	RWS11	-122.4668429	37.93936	-0.39	3.06	-21.7	2.66900003	-122.46684	37.93937	-0.32	3.06	-33.31	2.743	11	7/24/2014	28:42.3
6845	RWS11	-122.4668451	37.93936	-0.39	3.06	-21.83	2.66999996	-122.46684	37.93937	-0.4	3.06	-33.201	2.65999994	11	7/24/2014	28:42.4
6846	RWS11	-122.4668472	37.93936	-0.39	3.06	-22.05	2.67200005	-122.46684	37.93937	-0.32	3.06	-33.177	2.74600002	11	7/24/2014	28:42.5
6847	RWS11	-122.4668494	37.93936	-0.39	3.07	-22.29	2.67500007	-122.46684	37.93937	-0.32	3.07	-33.288	2.74900004	11	7/24/2014	28:42.6
6848	RWS11	-122.4668515	37.93936	-0.39	3.07	-22.57	2.67800009	-122.46685	37.93937	-0.35	3.07	-33.31	2.71800008	11	7/24/2014	28:42.7
6849	RWS11	-122.4668537	37.93936	-0.39	3.07	-23.05	2.68299997	-122.46685	37.93937	-0.35	3.07	-33.528	2.72299996	11	7/24/2014	28:42.8
6850	RWS11	-122.4668558	37.93936	-0.39	3.08	-23.64	2.68900001	-122.46685	37.93937	-0.32	3.08	-33.789	2.76299998	11	7/24/2014	28:42.9
6851	RWS11	-122.4668589	37.93936	-0.39	3.09	-24.19	2.69800007	-122.46685	37.93937	-0.43	3.09	-34.094	2.65400007	11	7/24/2014	28:43.0
6852	RWS11	-122.4668611	37.93936	-0.39	3.1	-24.89	2.70999992	-122.46686	37.93937	-0.4	3.1	-34.515	2.6999999	11	7/24/2014	28:43.1
6853	RWS11	-122.4668632	37.93936	-0.39	3.12	-25.63	2.72599995	-122.46686	37.93938	-0.4	3.12	-34.927	2.71599993	11	7/24/2014	28:43.2
6854	RWS11	-122.4668654	37.93936	-0.34	3.13	-26.44	2.79500008	-122.46686	37.93938	-0.4	3.13	-35.387	2.73400006	11	7/24/2014	28:43.3
6855	RWS11	-122.4668675	37.93936	-0.39	3.15	-27.25	2.76499999	-122.46686	37.93938	-0.35	3.15	-35.823	2.80499998	11	7/24/2014	28:43.4
6856	RWS11	-122.4668697	37.93936	-0.34	3.18	-28.21	2.83899999	-122.46686	37.93938	-0.32	3.18	-36.454	2.86199996	11	7/24/2014	28:43.5
6857	RWS11	-122.4668718	37.93936	-0.39	3.2	-29.17	2.81099999	-122.46687	37.93938	-0.4	3.2	-36.957	2.80099997	11	7/24/2014	28:43.6
6858	RWS11	-122.466874	37.93936	-0.34	3.22	-30.15	2.8829999	-122.46687	37.93938	-0.32	3.22	-37.589	2.90599987	11	7/24/2014	28:43.7
6859	RWS11	-122.4668762	37.93937	-0.34	3.24	-31.17	2.90100002	-122.46687	37.93938	-0.32	3.24	-38.246	2.92399999	11	7/24/2014	28:43.8
6860	RWS11	-122.4668783	37.93937	-0.34	3.25	-32.27	2.91499996	-122.46687	37.93938	-0.32	3.25	-38.836	2.93799993	11	7/24/2014	28:43.9
6861	RWS11	-122.4668815	37.93937	-0.34	3.26	-33.23	2.9230001	-122.46688	37.93938	-0.32	3.26	-39.405	2.94600007	11	7/24/2014	28:44.0
6862	RWS11	-122.4668837	37.93937	-0.34	3.27	-34.25	2.92700005	-122.46688	37.93938	-0.35	3.27	-40.037	2.91600004	11	7/24/2014	28:44.1
6863	RWS11	-122.4668858	37.93937	-0.34	3.27	-35.26	2.92600012	-122.46688	37.93938	-0.35	3.27	-40.67	2.91500011	11	7/24/2014	28:44.2

6864	RWS11	-122.466888	37.93937	-0.34	3.26	-36.2	2.921	-122.46688	37.93938	-0.32	3.26	-41.326	2.94399998	11	7/24/2014	28:44.3
6865	RWS11	-122.4668902	37.93937	-0.34	3.25	-37.11	2.91400003	-122.46689	37.93938	-0.4	3.25	-41.932	2.85300002	11	7/24/2014	28:44.4
6866	RWS11	-122.4668924	37.93937	-0.34	3.24	-37.96	2.9059999	-122.46689	37.93938	-0.35	3.24	-42.55	2.89499989	11	7/24/2014	28:44.5
6867	RWS11	-122.4668945	37.93937	-0.34	3.24	-38.75	2.898	-122.46689	37.93938	-0.4	3.24	-43.069	2.83699998	11	7/24/2014	28:44.6
6868	RWS11	-122.4668967	37.93937	-0.34	3.23	-39.47	2.8900001	-122.46689	37.93938	-0.35	3.23	-43.663	2.8790001	11	7/24/2014	28:44.7
6869	RWS11	-122.4668989	37.93937	-0.39	3.22	-40.19	2.83300006	-122.46689	37.93938	-0.43	3.22	-44.079	2.78900006	11	7/24/2014	28:44.8
6870	RWS11	-122.466901	37.93937	-0.34	3.22	-40.67	2.87899995	-122.4669	37.93938	-0.35	3.22	-44.447	2.86799994	11	7/24/2014	28:44.9
6871	RWS11	-122.4669042	37.93937	-0.34	3.22	-41.2	2.87700009	-122.4669	37.93939	-0.35	3.22	-44.799	2.86600009	11	7/24/2014	28:45.0
6872	RWS11	-122.4669063	37.93937	-0.34	3.22	-41.61	2.87800002	-122.4669	37.93939	-0.32	3.22	-45.126	2.90099999	11	7/24/2014	28:45.1
6873	RWS11	-122.4669084	37.93937	-0.39	3.22	-41.94	2.83000004	-122.4669	37.93939	-0.35	3.22	-45.385	2.87000003	11	7/24/2014	28:45.2
6874	RWS11	-122.4669105	37.93937	-0.34	3.23	-42.22	2.88700008	-122.46691	37.93939	-0.32	3.23	-45.672	2.91000006	11	7/24/2014	28:45.3
6875	RWS11	-122.4669126	37.93937	-0.34	3.23	-42.49	2.89599991	-122.46691	37.93939	-0.4	3.23	-45.931	2.83499989	11	7/24/2014	28:45.4
6876	RWS11	-122.4669148	37.93937	-0.34	3.25	-42.73	2.90700006	-122.46691	37.93939	-0.32	3.25	-46.089	2.93000004	11	7/24/2014	28:45.5
6877	RWS11	-122.4669169	37.93938	-0.34	3.26	-42.77	2.92000008	-122.46691	37.93939	-0.35	3.26	-46.266	2.90900007	11	7/24/2014	28:45.6
6878	RWS11	-122.466919	37.93938	-0.34	3.27	-42.84	2.93300009	-122.46691	37.93939	-0.35	3.27	-46.304	2.92200008	11	7/24/2014	28:45.7
6879	RWS11	-122.4669211	37.93938	-0.39	3.28	-42.9	2.89399993	-122.46692	37.93939	-0.35	3.28	-46.375	2.93399993	11	7/24/2014	28:45.8
6880	RWS11	-122.4669232	37.93938	-0.34	3.29	-42.88	2.95499992	-122.46692	37.93939	-0.32	3.29	-46.35	2.9779999	11	7/24/2014	28:45.9
6881	RWS11	-122.4669263	37.93938	-0.39	3.3	-42.77	2.91200006	-122.46692	37.93939	-0.35	3.3	-46.261	2.95200005	11	7/24/2014	28:46.0
6882	RWS11	-122.4669284	37.93938	-0.34	3.31	-42.64	2.96799994	-122.46692	37.93939	-0.32	3.31	-46.161	2.99099991	11	7/24/2014	28:46.1
6883	RWS11	-122.4669305	37.93938	-0.39	3.31	-42.47	2.91900003	-122.46693	37.93939	-0.35	3.31	-46.005	2.95900002	11	7/24/2014	28:46.2
6884	RWS11	-122.4669327	37.93938	-0.34	3.31	-42.34	2.97000003	-122.46693	37.93939	-0.49	3.31	-45.853	2.824	11	7/24/2014	28:46.3
6885	RWS11	-122.4669348	37.93938	-0.39	3.31	-42.08	2.91600001	-122.46693	37.93939	-0.35	3.31	-45.613	2.956	11	7/24/2014	28:46.4
6886	RWS11	-122.4669369	37.93938	-0.34	3.3	-41.79	2.96099997	-122.46693	37.93939	-0.32	3.3	-45.264	2.98399994	11	7/24/2014	28:46.5
6887	RWS11	-122.4669391	37.93938	-0.39	3.29	-41.6	2.90200007	-122.46693	37.93939	-0.32	3.29	-45.046	2.97600004	11	7/24/2014	28:46.6
6888	RWS11	-122.4669412	37.93938	-0.39	3.28	-41.38	2.89099991	-122.46694	37.93939	-0.32	3.28	-44.826	2.96499988	11	7/24/2014	28:46.7
6889	RWS11	-122.4669433	37.93938	-0.34	3.27	-41.01	2.9289999	-122.46694	37.93939	-0.49	3.27	-44.484	2.78299987	11	7/24/2014	28:46.8
6890	RWS11	-122.4669454	37.93938	-0.34	3.25	-40.73	2.91300011	-122.46694	37.93939	-0.32	3.25	-44.157	2.93600008	11	7/24/2014	28:46.9
6891	RWS11	-122.4669486	37.93938	-0.39	3.23	-40.33	2.84399998	-122.46694	37.9394	-0.35	3.23	-43.762	2.88399997	11	7/24/2014	28:47.0
6892	RWS11	-122.4669507	37.93938	-0.39	3.21	-39.88	2.82499993	-122.46695	37.9394	-0.32	3.21	-43.26	2.8989999	11	7/24/2014	28:47.1
6893	RWS11	-122.4669528	37.93938	-0.39	3.19	-39.44	2.80499995	-122.46695	37.9394	-0.4	3.19	-42.892	2.79499993	11	7/24/2014	28:47.2
6894	RWS11	-122.4669549	37.93938	-0.39	3.18	-39.07	2.78600013	-122.46695	37.9394	-0.32	3.18	-42.473	2.8600001	11	7/24/2014	28:47.3
6895	RWS11	-122.4669571	37.93938	-0.39	3.16	-38.59	2.76899993	-122.46695	37.9394	-0.35	3.16	-41.909	2.80899993	11	7/24/2014	28:47.4
6896	RWS11	-122.4669592	37.93939	-0.39	3.14	-38.02	2.755	-122.46695	37.9394	-0.32	3.14	-41.34	2.82899997	11	7/24/2014	28:47.5

6897	RWS11	-122.4669614	37.93939	-0.39	3.13	-37.46	2.74400008	-122.46696	37.9394	-0.32	3.13	-40.756	2.81800005	11	7/24/2014	28:47.6
6898	RWS11	-122.4669635	37.93939	-0.39	3.13	-36.98	2.73700011	-122.46696	37.9394	-0.32	3.13	-40.187	2.81100008	11	7/24/2014	28:47.7
6899	RWS11	-122.4669656	37.93939	-0.43	3.12	-36.54	2.69799989	-122.46696	37.9394	-0.35	3.12	-39.773	2.77299991	11	7/24/2014	28:47.8
6900	RWS11	-122.4669677	37.93939	-0.39	3.12	-36.06	2.73299992	-122.46696	37.9394	-0.32	3.12	-39.359	2.80699989	11	7/24/2014	28:47.9
6901	RWS11	-122.4669709	37.93939	-0.39	3.13	-35.71	2.73700011	-122.46697	37.9394	-0.4	3.13	-38.879	2.72700009	11	7/24/2014	28:48.0
6902	RWS11	-122.466973	37.93939	-0.39	3.13	-35.17	2.74199998	-122.46697	37.9394	-0.4	3.13	-38.332	2.73199996	11	7/24/2014	28:48.1
6903	RWS11	-122.4669751	37.93939	-0.43	3.14	-34.62	2.71399993	-122.46697	37.9394	-0.35	3.14	-37.746	2.78899994	11	7/24/2014	28:48.2
6904	RWS11	-122.4669772	37.93939	-0.39	3.15	-34.19	2.75599992	-122.46697	37.9394	-0.4	3.15	-37.267	2.7459999	11	7/24/2014	28:48.3
6905	RWS11	-122.4669793	37.93939	-0.43	3.15	-33.71	2.72600001	-122.46697	37.9394	-0.35	3.15	-36.742	2.80100003	11	7/24/2014	28:48.4
6906	RWS11	-122.4669815	37.93939	-0.39	3.16	-33.29	2.76700008	-122.46698	37.9394	-0.35	3.16	-36.264	2.80700007	11	7/24/2014	28:48.5
6907	RWS11	-122.4669836	37.93939	-0.43	3.16	-32.86	2.73699993	-122.46698	37.9394	-0.35	3.16	-35.783	2.81199995	11	7/24/2014	28:48.6
6908	RWS11	-122.4669857	37.93939	-0.39	3.17	-32.44	2.77899992	-122.46698	37.9394	-0.43	3.17	-35.28	2.73499992	11	7/24/2014	28:48.7
6909	RWS11	-122.4669878	37.93939	-0.43	3.18	-31.99	2.75100011	-122.46698	37.93941	-0.4	3.18	-34.847	2.77600011	11	7/24/2014	28:48.8
6910	RWS11	-122.4669899	37.93939	-0.39	3.18	-31.61	2.79400003	-122.46698	37.93941	-0.4	3.18	-34.364	2.78400001	11	7/24/2014	28:48.9
6911	RWS11	-122.4669931	37.93939	-0.39	3.19	-31.18	2.80199993	-122.46699	37.93941	-0.4	3.19	-33.887	2.79199991	11	7/24/2014	28:49.0
6912	RWS11	-122.4669952	37.93939	-0.39	3.2	-30.73	2.81000006	-122.46699	37.93941	-0.4	3.2	-33.432	2.80000004	11	7/24/2014	28:49.1
6913	RWS11	-122.4669973	37.93939	-0.43	3.2	-30.27	2.77999991	-122.46699	37.93941	-0.4	3.2	-33.014	2.80499992	11	7/24/2014	28:49.2
6914	RWS11	-122.4669994	37.9394	-0.43	3.2	-29.81	2.77999991	-122.46699	37.93941	-0.4	3.2	-32.538	2.80499992	11	7/24/2014	28:49.3
6915	RWS11	-122.4670015	37.9394	-0.43	3.2	-29.44	2.77200001	-122.467	37.93941	-0.43	3.2	-32.144	2.76300004	11	7/24/2014	28:49.4
6916	RWS11	-122.4670036	37.9394	-0.43	3.18	-28.96	2.75100011	-122.467	37.93941	-0.43	3.18	-31.706	2.74200013	11	7/24/2014	28:49.5
6917	RWS11	-122.4670058	37.9394	-0.43	3.14	-28.54	2.71600002	-122.467	37.93941	-0.52	3.14	-31.293	2.62200004	11	7/24/2014	28:49.6
6918	RWS11	-122.4670079	37.9394	-0.39	3.08	-28.11	2.69400012	-122.467	37.93941	-0.4	3.08	-30.9	2.6840001	11	7/24/2014	28:49.7
6919	RWS11	-122.46701	37.9394	-0.43	3.08	-27.7	2.65600008	-122.467	37.93941	-0.43	3.08	-30.49	2.6470001	11	7/24/2014	28:49.8
6920	RWS11	-122.4670121	37.9394	-0.43	3.08	-27.26	2.65199989	-122.46701	37.93941	-0.43	3.08	-30.074	2.64299992	11	7/24/2014	28:49.9
6921	RWS11	-122.4670152	37.9394	-0.43	3.07	-26.91	2.64799994	-122.46701	37.93941	-0.43	3.07	-29.747	2.63899997	11	7/24/2014	28:50.0
6922	RWS11	-122.4670173	37.9394	-0.39	3.07	-26.5	2.67900002	-122.46701	37.93941	-0.49	3.07	-29.333	2.58399999	11	7/24/2014	28:50.1
6923	RWS11	-122.4670194	37.9394	-0.43	3.07	-25.99	2.64099997	-122.46701	37.93941	-0.49	3.07	-28.853	2.58099997	11	7/24/2014	28:50.2
6924	RWS11	-122.4670215	37.9394	-0.39	3.06	-25.62	2.67299998	-122.46702	37.93941	-0.43	3.06	-28.447	2.62899998	11	7/24/2014	28:50.3
6925	RWS11	-122.4670237	37.9394	-0.43	3.06	-25.17	2.634	-122.46702	37.93941	-0.43	3.06	-28.015	2.62500003	11	7/24/2014	28:50.4
6926	RWS11	-122.4670258	37.9394	-0.39	3.06	-24.71	2.66500008	-122.46702	37.93942	-0.43	3.06	-27.653	2.62100008	11	7/24/2014	28:50.5
6927	RWS11	-122.467028	37.9394	-0.43	3.05	-24.45	2.62700003	-122.46702	37.93942	-0.43	3.05	-27.253	2.61800006	11	7/24/2014	28:50.6
6928	RWS11	-122.4670301	37.9394	-0.39	3.05	-24.03	2.65800011	-122.46702	37.93942	-0.43	3.05	-26.938	2.61400011	11	7/24/2014	28:50.7
6929	RWS11	-122.4670322	37.9394	-0.43	3.04	-23.68	2.6189999	-122.46703	37.93942	-0.43	3.04	-26.545	2.60999992	11	7/24/2014	28:50.8

6930	RWS11	-122.4670344	37.9394	-0.39	3.04	-23.38	2.6509999	-122.46703	37.93942	-0.52	3.04	-26.216	2.5219999	11	7/24/2014	28:50.9
6931	RWS11	-122.4670375	37.93941	-0.43	3.04	-22.99	2.61300009	-122.46703	37.93942	-0.52	3.04	-25.869	2.51900011	11	7/24/2014	28:51.0
6932	RWS11	-122.4670397	37.93941	-0.43	3.03	-22.62	2.60899991	-122.46703	37.93942	-0.52	3.03	-25.476	2.51499993	11	7/24/2014	28:51.1
6933	RWS11	-122.4670418	37.93941	-0.43	3.03	-22.25	2.60499996	-122.46704	37.93942	-0.43	3.03	-25.237	2.59599999	11	7/24/2014	28:51.2
6934	RWS11	-122.467044	37.93941	-0.39	3.03	-21.94	2.63699996	-122.46704	37.93942	-0.4	3.03	-24.889	2.62699994	11	7/24/2014	28:51.3
6935	RWS11	-122.4670462	37.93941	-0.43	3.02	-21.66	2.59799999	-122.46704	37.93942	-0.4	3.02	-24.563	2.623	11	7/24/2014	28:51.4
6936	RWS11	-122.4670484	37.93941	-0.39	3.02	-21.33	2.63	-122.46704	37.93942	-0.43	3.02	-24.215	2.586	11	7/24/2014	28:51.5
6937	RWS11	-122.4670506	37.93941	-0.43	3.02	-20.94	2.59100002	-122.46705	37.93942	-0.43	3.02	-23.909	2.58200005	11	7/24/2014	28:51.6
6938	RWS11	-122.4670527	37.93941	-0.43	3.01	-20.74	2.58700007	-122.46705	37.93942	-0.35	3.01	-23.603	2.66200009	11	7/24/2014	28:51.7
6939	RWS11	-122.4670549	37.93941	-0.43	3.01	-20.39	2.58400005	-122.46705	37.93942	-0.52	3.01	-23.297	2.49000007	11	7/24/2014	28:51.8
6940	RWS11	-122.467057	37.93941	-0.39	3.01	-20.07	2.61600006	-122.46705	37.93942	-0.43	3.01	-22.927	2.57200006	11	7/24/2014	28:51.9
6941	RWS11	-122.4670602	37.93941	-0.43	3	-19.81	2.57700008	-122.46705	37.93942	-0.43	3	-22.516	2.56800011	11	7/24/2014	28:52.0
6942	RWS11	-122.4670623	37.93941	-0.39	3.07	-19.44	2.68299997	-122.46706	37.93942	-0.4	3.07	-22.298	2.67299995	11	7/24/2014	28:52.1
6943	RWS11	-122.4670643	37.93941	-0.43	3.13	-19.22	2.70399994	-122.46706	37.93943	-0.49	3.13	-21.949	2.64399993	11	7/24/2014	28:52.2
6944	RWS11	-122.4670664	37.93941	-0.43	3.17	-18.96	2.74599999	-122.46706	37.93943	-0.4	3.17	-21.599	2.771	11	7/24/2014	28:52.3
6945	RWS11	-122.4670685	37.93941	-0.43	3.2	-18.63	2.77999991	-122.46706	37.93943	-0.4	3.2	-21.295	2.80499992	11	7/24/2014	28:52.4
6946	RWS11	-122.4670706	37.93941	-0.43	3.23	-18.43	2.80599993	-122.46707	37.93943	-0.49	3.23	-20.988	2.74599993	11	7/24/2014	28:52.5
6947	RWS11	-122.4670727	37.93941	-0.43	3.25	-18.24	2.82899994	-122.46707	37.93943	-0.43	3.25	-20.792	2.81999996	11	7/24/2014	28:52.6
6948	RWS11	-122.4670748	37.93942	-0.43	3.27	-17.93	2.84899992	-122.46707	37.93943	-0.43	3.27	-20.421	2.83999994	11	7/24/2014	28:52.7
6949	RWS11	-122.4670768	37.93942	-0.43	3.29	-17.76	2.8689999	-122.46707	37.93943	-0.4	3.29	-20.142	2.8939999	11	7/24/2014	28:52.8
6950	RWS11	-122.4670789	37.93942	-0.43	3.32	-17.54	2.89000005	-122.46707	37.93943	-0.4	3.32	-19.879	2.91500005	11	7/24/2014	28:52.9
6951	RWS11	-122.4670819	37.93942	-0.43	3.34	-17.33	2.91199988	-122.46708	37.93943	-0.49	3.34	-19.575	2.85199988	11	7/24/2014	28:53.0
6952	RWS11	-122.4670839	37.93942	-0.43	3.36	-17.02	2.93399996	-122.46708	37.93943	-0.4	3.36	-19.335	2.95899996	11	7/24/2014	28:53.1
6953	RWS11	-122.467086	37.93942	-0.43	3.38	-16.87	2.9550001	-122.46708	37.93943	-0.43	3.38	-18.985	2.94600013	11	7/24/2014	28:53.2
6954	RWS11	-122.467088	37.93942	-0.43	3.4	-16.61	2.97500008	-122.46708	37.93943	-0.52	3.4	-18.767	2.8810001	11	7/24/2014	28:53.3
6955	RWS11	-122.4670901	37.93942	-0.43	3.42	-16.43	2.99200004	-122.46708	37.93943	-0.49	3.42	-18.484	2.93200004	11	7/24/2014	28:53.4
6956	RWS11	-122.4670922	37.93942	-0.48	3.43	-16.13	2.95400006	-122.46709	37.93943	-0.43	3.43	-18.159	2.99600008	11	7/24/2014	28:53.5
6957	RWS11	-122.4670942	37.93942	-0.48	3.44	-15.8	2.96100003	-122.46709	37.93943	-0.43	3.44	-17.898	3.00300005	11	7/24/2014	28:53.6
6958	RWS11	-122.4670963	37.93942	-0.43	3.44	-15.48	3.01399988	-122.46709	37.93943	-0.49	3.44	-17.548	2.95399988	11	7/24/2014	28:53.7
6959	RWS11	-122.4670983	37.93942	-0.48	3.43	-15.13	2.95899993	-122.46709	37.93943	-0.49	3.43	-17.222	2.94999993	11	7/24/2014	28:53.8
6960	RWS11	-122.4671004	37.93942	-0.48	3.43	-14.82	2.95000011	-122.46709	37.93944	-0.49	3.43	-16.939	2.9410001	11	7/24/2014	28:53.9
6961	RWS11	-122.4671034	37.93942	-0.48	3.41	-14.58	2.93800002	-122.4671	37.93944	-0.49	3.41	-16.676	2.92900002	11	7/24/2014	28:54.0
6962	RWS11	-122.4671054	37.93942	-0.43	3.4	-14.17	2.97399992	-122.4671	37.93944	-0.49	3.4	-16.35	2.91399992	11	7/24/2014	28:54.1

6963	RWS11	-122.4671075	37.93942	-0.48	3.38	-14	2.90699989	-122.4671	37.93944	-0.49	3.38	-16.157	2.89799988	11	7/24/2014	28:54.2
6964	RWS11	-122.4671095	37.93943	-0.43	3.37	-13.69	2.94300002	-122.4671	37.93944	-0.49	3.37	-15.894	2.88300002	11	7/24/2014	28:54.3
6965	RWS11	-122.4671116	37.93943	-0.48	3.35	-13.41	2.87599999	-122.46711	37.93944	-0.49	3.35	-15.612	2.86699998	11	7/24/2014	28:54.4
6966	RWS11	-122.4671136	37.93943	-0.48	3.34	-13.08	2.86200005	-122.46711	37.93944	-0.52	3.34	-15.349	2.81900007	11	7/24/2014	28:54.5
6967	RWS11	-122.4671157	37.93943	-0.48	3.32	-12.93	2.84699994	-122.46711	37.93944	-0.43	3.32	-15.088	2.88899997	11	7/24/2014	28:54.6
6968	RWS11	-122.4671177	37.93943	-0.43	3.31	-12.56	2.884	-122.46711	37.93944	-0.43	3.31	-14.804	2.87500003	11	7/24/2014	28:54.7
6969	RWS11	-122.4671198	37.93943	-0.48	3.29	-12.31	2.8179999	-122.46711	37.93944	-0.43	3.29	-14.607	2.85999992	11	7/24/2014	28:54.8
6970	RWS11	-122.4671218	37.93943	-0.48	3.28	-12.01	2.80300003	-122.46712	37.93944	-0.43	3.28	-14.255	2.84500006	11	7/24/2014	28:54.9
6971	RWS11	-122.4671249	37.93943	-0.48	3.27	-11.73	2.78900009	-122.46712	37.93944	-0.43	3.27	-13.978	2.83100012	11	7/24/2014	28:55.0
6972	RWS11	-122.4671269	37.93943	-0.48	3.25	-11.38	2.77600008	-122.46712	37.93944	-0.49	3.25	-13.716	2.76700008	11	7/24/2014	28:55.1
6973	RWS11	-122.467129	37.93943	-0.51	3.24	-11.07	2.73099995	-122.46712	37.93944	-0.49	3.24	-13.434	2.75599992	11	7/24/2014	28:55.2
6974	RWS11	-122.4671311	37.93943	-0.48	3.23	-10.77	2.75700003	-122.46713	37.93944	-0.43	3.23	-13.127	2.79900005	11	7/24/2014	28:55.3
6975	RWS11	-122.4671332	37.93943	-0.51	3.23	-10.38	2.71700001	-122.46713	37.93944	-0.49	3.23	-12.822	2.74199998	11	7/24/2014	28:55.4
6976	RWS11	-122.4671354	37.93943	-0.48	3.22	-10.14	2.74899989	-122.46713	37.93945	-0.43	3.22	-12.495	2.79099992	11	7/24/2014	28:55.5
6977	RWS11	-122.4671376	37.93943	-0.51	3.22	-9.81	2.71499991	-122.46713	37.93945	-0.43	3.22	-12.168	2.79099992	11	7/24/2014	28:55.6
6978	RWS11	-122.4671398	37.93943	-0.48	3.23	-9.508	2.75000006	-122.46713	37.93945	-0.4	3.23	-11.975	2.82600006	11	7/24/2014	28:55.7
6979	RWS11	-122.467142	37.93943	-0.51	3.23	-9.333	2.7190001	-122.46714	37.93945	-0.4	3.23	-11.691	2.82900009	11	7/24/2014	28:55.8
6980	RWS11	-122.4671442	37.93943	-0.48	3.23	-8.962	2.75700003	-122.46714	37.93945	-0.43	3.23	-11.385	2.79900005	11	7/24/2014	28:55.9
6981	RWS11	-122.4671464	37.93944	-0.51	3.24	-8.766	2.727	-122.46714	37.93945	-0.4	3.24	-11.167	2.83699998	11	7/24/2014	28:56.0
6982	RWS11	-122.4671486	37.93944	-0.48	3.24	-8.482	2.76499993	-122.46714	37.93945	-0.43	3.24	-10.971	2.80699995	11	7/24/2014	28:56.1
6983	RWS11	-122.4671508	37.93944	-0.48	3.24	-8.264	2.76799995	-122.46715	37.93945	-0.43	3.24	-10.709	2.80999997	11	7/24/2014	28:56.2
6984	RWS11	-122.467153	37.93944	-0.51	3.24	-8.003	2.7349999	-122.46715	37.93945	-0.4	3.24	-10.47	2.84499988	11	7/24/2014	28:56.3
6985	RWS11	-122.4671553	37.93944	-0.51	3.24	-7.723	2.73399997	-122.46715	37.93945	-0.43	3.24	-10.255	2.80999997	11	7/24/2014	28:56.4
6986	RWS11	-122.4671574	37.93944	-0.48	3.24	-7.57	2.76499993	-122.46715	37.93945	-0.4	3.24	-10.015	2.84099993	11	7/24/2014	28:56.5
6987	RWS11	-122.4671596	37.93944	-0.51	3.24	-7.33	2.72600007	-122.46715	37.93945	-0.43	3.24	-9.841	2.80200008	11	7/24/2014	28:56.6
6988	RWS11	-122.4671618	37.93944	-0.51	3.23	-7.134	2.7190001	-122.46716	37.93945	-0.43	3.23	-9.731	2.79500011	11	7/24/2014	28:56.7
6989	RWS11	-122.4671639	37.93944	-0.51	3.22	-6.981	2.71000004	-122.46716	37.93945	-0.49	3.22	-9.426	2.73500001	11	7/24/2014	28:56.8
6990	RWS11	-122.467166	37.93944	-0.51	3.21	-6.675	2.70099998	-122.46716	37.93945	-0.43	3.21	-9.273	2.77699998	11	7/24/2014	28:56.9
6991	RWS11	-122.4671691	37.93944	-0.51	3.2	-6.523	2.69300008	-122.46716	37.93945	-0.49	3.2	-9.011	2.71800005	11	7/24/2014	28:57.0
6992	RWS11	-122.4671712	37.93944	-0.51	3.2	-6.305	2.68700004	-122.46717	37.93946	-0.4	3.2	-8.838	2.79700002	11	7/24/2014	28:57.1
6993	RWS11	-122.4671733	37.93944	-0.56	3.19	-6.156	2.63200009	-122.46717	37.93946	-0.43	3.19	-8.688	2.75900009	11	7/24/2014	28:57.2
6994	RWS11	-122.4671755	37.93944	-0.48	3.19	-5.872	2.71499997	-122.46717	37.93946	-0.43	3.19	-8.426	2.757	11	7/24/2014	28:57.3
6995	RWS11	-122.4671776	37.93944	-0.56	3.19	-5.763	2.63	-122.46717	37.93946	-0.43	3.19	-8.208	2.757	11	7/24/2014	28:57.4

6996	RWS11	-122.4671798	37.93945	-0.51	3.19	-5.479	2.68499994	-122.46717	37.93946	-0.43	3.19	-7.989	2.76099995	11	7/24/2014	28:57.5
6997	RWS11	-122.467182	37.93945	-0.56	3.2	-5.327	2.63900006	-122.46718	37.93946	-0.43	3.2	-7.837	2.76600006	11	7/24/2014	28:57.6
6998	RWS11	-122.4671842	37.93945	-0.51	3.21	-5.065	2.69799995	-122.46718	37.93946	-0.43	3.21	-7.575	2.77399996	11	7/24/2014	28:57.7
6999	RWS11	-122.4671864	37.93945	-0.56	3.22	-4.935	2.65600002	-122.46718	37.93946	-0.35	3.22	-7.423	2.86700001	11	7/24/2014	28:57.8
7000	RWS11	-122.4671887	37.93945	-0.51	3.23	-4.676	2.71799994	-122.46718	37.93946	-0.4	3.23	-7.143	2.82799992	11	7/24/2014	28:57.9
7001	RWS11	-122.4671909	37.93945	-0.51	3.24	-4.524	2.72799993	-122.46718	37.93946	-0.4	3.24	-6.881	2.83799991	11	7/24/2014	28:58.0
7002	RWS11	-122.4671931	37.93945	-0.51	3.25	-4.37	2.73900008	-122.46719	37.93946	-0.4	3.25	-6.794	2.84900007	11	7/24/2014	28:58.1
7003	RWS11	-122.4671954	37.93945	-0.51	3.26	-4.196	2.74799991	-122.46719	37.93946	-0.4	3.26	-6.641	2.85799989	11	7/24/2014	28:58.2
7004	RWS11	-122.4671976	37.93945	-0.48	3.27	-4.021	2.79199988	-122.46719	37.93946	-0.35	3.27	-6.444	2.91799989	11	7/24/2014	28:58.3
7005	RWS11	-122.4671998	37.93945	-0.48	3.28	-3.803	2.80000001	-122.46719	37.93946	-0.4	3.28	-6.292	2.87600002	11	7/24/2014	28:58.4
7006	RWS11	-122.467202	37.93945	-0.48	3.28	-3.695	2.80699998	-122.4672	37.93946	-0.4	3.28	-6.096	2.88299999	11	7/24/2014	28:58.5
7007	RWS11	-122.4672042	37.93945	-0.48	3.29	-3.546	2.81300002	-122.4672	37.93946	-0.35	3.29	-6.056	2.93900004	11	7/24/2014	28:58.6
7008	RWS11	-122.4672063	37.93945	-0.48	3.29	-3.414	2.8179999	-122.4672	37.93947	-0.35	3.29	-5.859	2.94399992	11	7/24/2014	28:58.7
7009	RWS11	-122.4672084	37.93945	-0.51	3.3	-3.262	2.78699994	-122.4672	37.93947	-0.35	3.3	-5.619	2.94699994	11	7/24/2014	28:58.8
7010	RWS11	-122.4672106	37.93945	-0.48	3.3	-3.153	2.82200009	-122.4672	37.93947	-0.35	3.3	-5.532	2.9480001	11	7/24/2014	28:58.9
7011	RWS11	-122.4672137	37.93946	-0.48	3.3	-3.043	2.82099992	-122.46721	37.93947	-0.4	3.3	-5.336	2.89699993	11	7/24/2014	28:59.0
7012	RWS11	-122.4672158	37.93946	-0.48	3.29	-2.825	2.8179999	-122.46721	37.93947	-0.4	3.29	-5.118	2.8939999	11	7/24/2014	28:59.1
7013	RWS11	-122.4672178	37.93946	-0.51	3.29	-2.674	2.77999997	-122.46721	37.93947	-0.4	3.29	-4.988	2.88999996	11	7/24/2014	28:59.2
7014	RWS11	-122.46722	37.93946	-0.48	3.29	-2.437	2.80900007	-122.46721	37.93947	-0.4	3.29	-4.729	2.88500008	11	7/24/2014	28:59.3
7015	RWS11	-122.4672221	37.93946	-0.51	3.28	-2.109	2.77099991	-122.46722	37.93947	-0.4	3.28	-4.511	2.88099989	11	7/24/2014	28:59.4
7016	RWS11	-122.4672243	37.93946	-0.48	3.28	-1.935	2.80099994	-122.46722	37.93947	-0.35	3.28	-4.314	2.92699996	11	7/24/2014	28:59.5
7017	RWS11	-122.4672264	37.93946	-0.48	3.27	-1.717	2.79799992	-122.46722	37.93947	-0.4	3.27	-4.096	2.87399992	11	7/24/2014	28:59.6
7018	RWS11	-122.4672286	37.93946	-0.48	3.27	-1.411	2.7949999	-122.46722	37.93947	-0.35	3.27	-3.834	2.92099991	11	7/24/2014	28:59.7
7019	RWS11	-122.4672308	37.93946	-0.51	3.27	-1.149	2.75900006	-122.46722	37.93947	-0.4	3.27	-3.572	2.86900005	11	7/24/2014	28:59.8
7020	RWS11	-122.467233	37.93946	-0.48	3.27	-0.953	2.79099995	-122.46723	37.93947	-0.4	3.27	-3.376	2.86699995	11	7/24/2014	28:59.9
7021	RWS11	-122.4672352	37.93946	-0.48	3.27	-0.692	2.79000002	-122.46723	37.93947	-0.4	3.27	-3.072	2.86600003	11	7/24/2014	29:00.0
7022	RWS11	-122.4672374	37.93946	-0.48	3.27	-0.302	2.78900009	-122.46723	37.93947	-0.35	3.27	-2.856	2.91500011	11	7/24/2014	29:00.1
7023	RWS11	-122.4672396	37.93946	-0.51	3.27	-0.172	2.75500011	-122.46723	37.93948	-0.4	3.27	-2.573	2.8650001	11	7/24/2014	29:00.2
7024	RWS11	-122.4672418	37.93946	-0.51	3.27	0.156	2.75600004	-122.46724	37.93948	-0.4	3.27	-2.442	2.86600003	11	7/24/2014	29:00.3
7025	RWS11	-122.467244	37.93946	-0.51	3.27	0.396	2.75699997	-122.46724	37.93948	-0.43	3.27	-2.115	2.83299997	11	7/24/2014	29:00.4
7026	RWS11	-122.4672461	37.93946	-0.48	3.27	0.658	2.79000002	-122.46724	37.93948	-0.4	3.27	-1.809	2.86600003	11	7/24/2014	29:00.5
7027	RWS11	-122.4672482	37.93947	-0.51	3.26	1.007	2.75399995	-122.46724	37.93948	-0.4	3.26	-1.569	2.86399993	11	7/24/2014	29:00.6
7028	RWS11	-122.4672504	37.93947	-0.48	3.26	1.181	2.78399998	-122.46724	37.93948	-0.43	3.26	-1.307	2.82600001	11	7/24/2014	29:00.7

7029	RWS11	-122.4672525	37.93947	-0.51	3.26	1.374	2.74500012	-122.46725	37.93948	-0.43	3.26	-1.093	2.82100013	11	7/24/2014	29:00.8
7030	RWS11	-122.4672545	37.93947	-0.51	3.25	1.657	2.73900008	-122.46725	37.93948	-0.43	3.25	-0.831	2.81500009	11	7/24/2014	29:00.9
7031	RWS11	-122.4672576	37.93947	-0.51	3.24	1.854	2.73300004	-122.46725	37.93948	-0.43	3.24	-0.613	2.80900005	11	7/24/2014	29:01.0
7032	RWS11	-122.4672597	37.93947	-0.48	3.24	2.072	2.764	-122.46725	37.93948	-0.43	3.24	-0.329	2.80600002	11	7/24/2014	29:01.1
7033	RWS11	-122.4672618	37.93947	-0.51	3.24	2.356	2.73000002	-122.46726	37.93948	-0.49	3.24	-0.089	2.755	11	7/24/2014	29:01.2
7034	RWS11	-122.4672639	37.93947	-0.48	3.24	2.617	2.76899987	-122.46726	37.93948	-0.43	3.24	0.107	2.8109999	11	7/24/2014	29:01.3
7035	RWS11	-122.467266	37.93947	-0.51	3.25	2.835	2.74300003	-122.46726	37.93948	-0.43	3.25	0.282	2.81900004	11	7/24/2014	29:01.4
7036	RWS11	-122.4672681	37.93947	-0.48	3.27	3.006	2.78900009	-122.46726	37.93948	-0.49	3.27	0.539	2.78000009	11	7/24/2014	29:01.5
7037	RWS11	-122.4672703	37.93947	-0.56	3.28	3.246	2.71999991	-122.46726	37.93948	-0.43	3.28	0.801	2.84699991	11	7/24/2014	29:01.6
7038	RWS11	-122.4672725	37.93947	-0.51	3.3	3.443	2.78800011	-122.46727	37.93949	-0.43	3.3	1.019	2.86400011	11	7/24/2014	29:01.7
7039	RWS11	-122.4672747	37.93947	-0.51	3.32	3.639	2.80599999	-122.46727	37.93949	-0.43	3.32	1.172	2.882	11	7/24/2014	29:01.8
7040	RWS11	-122.4672769	37.93947	-0.51	3.33	3.879	2.82200003	-122.46727	37.93949	-0.43	3.33	1.499	2.89800003	11	7/24/2014	29:01.9
7041	RWS11	-122.4672791	37.93947	-0.51	3.35	4.097	2.83500004	-122.46727	37.93949	-0.49	3.35	1.631	2.86000001	11	7/24/2014	29:02.0
7042	RWS11	-122.4672813	37.93948	-0.48	3.35	4.315	2.87699991	-122.46728	37.93949	-0.43	3.35	1.912	2.91899994	11	7/24/2014	29:02.1
7043	RWS11	-122.4672834	37.93948	-0.51	3.36	4.551	2.84599996	-122.46728	37.93949	-0.49	3.36	2.041	2.87099993	11	7/24/2014	29:02.2
7044	RWS11	-122.4672856	37.93948	-0.48	3.35	4.792	2.87599999	-122.46728	37.93949	-0.49	3.35	2.302	2.86699998	11	7/24/2014	29:02.3
7045	RWS11	-122.4672878	37.93948	-0.51	3.34	4.945	2.83400011	-122.46728	37.93949	-0.43	3.34	2.521	2.91000012	11	7/24/2014	29:02.4
7046	RWS11	-122.46729	37.93948	-0.48	3.33	5.271	2.85600001	-122.46728	37.93949	-0.43	3.33	2.696	2.89800003	11	7/24/2014	29:02.5
7047	RWS11	-122.4672921	37.93948	-0.51	3.32	5.402	2.80699992	-122.46729	37.93949	-0.43	3.32	2.935	2.88299993	11	7/24/2014	29:02.6
7048	RWS11	-122.4672942	37.93948	-0.48	3.3	5.512	2.82600003	-122.46729	37.93949	-0.43	3.3	3.088	2.86800006	11	7/24/2014	29:02.7
7049	RWS11	-122.4672963	37.93948	-0.51	3.29	5.62	2.77600002	-122.46729	37.93949	-0.49	3.29	3.197	2.801	11	7/24/2014	29:02.8
7050	RWS11	-122.4672984	37.93948	-0.48	3.27	5.857	2.79600006	-122.46729	37.93949	-0.43	3.27	3.39	2.83800009	11	7/24/2014	29:02.9
7051	RWS11	-122.4673015	37.93948	-0.51	3.26	6.032	2.74900007	-122.4673	37.93949	-0.43	3.26	3.609	2.82500008	11	7/24/2014	29:03.0
7052	RWS11	-122.4673035	37.93948	-0.51	3.25	6.141	2.73799992	-122.4673	37.93949	-0.49	3.25	3.718	2.76299989	11	7/24/2014	29:03.1
7053	RWS11	-122.4673056	37.93948	-0.56	3.24	6.381	2.67900002	-122.4673	37.9395	-0.43	3.24	4.023	2.80600002	11	7/24/2014	29:03.2
7054	RWS11	-122.4673077	37.93948	-0.51	3.23	6.665	2.72300005	-122.4673	37.9395	-0.49	3.23	4.264	2.74800003	11	7/24/2014	29:03.3
7055	RWS11	-122.4673098	37.93948	-0.56	3.23	6.927	2.6680001	-122.4673	37.9395	-0.43	3.23	4.546	2.79500011	11	7/24/2014	29:03.4
7056	RWS11	-122.4673119	37.93948	-0.48	3.23	7.145	2.75000006	-122.46731	37.9395	-0.49	3.23	4.739	2.74100006	11	7/24/2014	29:03.5
7057	RWS11	-122.467314	37.93949	-0.48	3.22	7.405	2.74899989	-122.46731	37.9395	-0.49	3.22	5.005	2.73999989	11	7/24/2014	29:03.6
7058	RWS11	-122.4673161	37.93949	-0.51	3.22	7.665	2.71399999	-122.46731	37.9395	-0.49	3.22	5.262	2.73899996	11	7/24/2014	29:03.7
7059	RWS11	-122.4673182	37.93949	-0.48	3.22	7.86	2.74899989	-122.46731	37.9395	-0.49	3.22	5.482	2.73999989	11	7/24/2014	29:03.8
7060	RWS11	-122.4673203	37.93949	-0.51	3.23	8.145	2.71600008	-122.46731	37.9395	-0.49	3.23	5.765	2.74100006	11	7/24/2014	29:03.9
7061	RWS11	-122.4673234	37.93949	-0.56	3.23	8.406	2.6680001	-122.46732	37.9395	-0.52	3.23	6.049	2.7100001	11	7/24/2014	29:04.0

7062	RWS11	-122.4673255	37.93949	-0.51	3.23	8.624	2.72300005	-122.46732	37.9395	-0.49	3.23	6.244	2.74800003	11	7/24/2014	29:04.1
7063	RWS11	-122.4673275	37.93949	-0.51	3.24	8.821	2.72799993	-122.46732	37.9395	-0.49	3.24	6.551	2.7529999	11	7/24/2014	29:04.2
7064	RWS11	-122.4673297	37.93949	-0.48	3.24	8.994	2.76799995	-122.46732	37.9395	-0.49	3.24	6.724	2.75899994	11	7/24/2014	29:04.3
7065	RWS11	-122.4673318	37.93949	-0.51	3.25	9.166	2.74099994	-122.46733	37.9395	-0.49	3.25	6.918	2.76599991	11	7/24/2014	29:04.4
7066	RWS11	-122.4673339	37.93949	-0.51	3.26	9.319	2.74699998	-122.46733	37.9395	-0.49	3.26	7.113	2.77199996	11	7/24/2014	29:04.5
7067	RWS11	-122.467336	37.93949	-0.48	3.27	9.472	2.78900009	-122.46733	37.9395	-0.43	3.27	7.289	2.83100012	11	7/24/2014	29:04.6
7068	RWS11	-122.4673381	37.93949	-0.51	3.27	9.69	2.76399994	-122.46733	37.93951	-0.49	3.27	7.463	2.78899992	11	7/24/2014	29:04.7
7069	RWS11	-122.4673402	37.93949	-0.51	3.28	9.908	2.77399993	-122.46733	37.93951	-0.49	3.28	7.66	2.79899991	11	7/24/2014	29:04.8
7070	RWS11	-122.4673423	37.93949	-0.48	3.3	10.06	2.81999999	-122.46734	37.93951	-0.49	3.3	7.834	2.81099999	11	7/24/2014	29:04.9
7071	RWS11	-122.4673453	37.9395	-0.51	3.31	10.213	2.79999995	-122.46734	37.93951	-0.43	3.31	8.007	2.87599996	11	7/24/2014	29:05.0
7072	RWS11	-122.4673474	37.9395	-0.51	3.33	10.472	2.81500006	-122.46734	37.93951	-0.43	3.33	8.288	2.89100006	11	7/24/2014	29:05.1
7073	RWS11	-122.4673495	37.9395	-0.51	3.34	10.58	2.82999992	-122.46734	37.93951	-0.49	3.34	8.419	2.8549999	11	7/24/2014	29:05.2
7074	RWS11	-122.4673515	37.9395	-0.48	3.36	10.712	2.87900001	-122.46735	37.93951	-0.43	3.36	8.529	2.92100003	11	7/24/2014	29:05.3
7075	RWS11	-122.4673536	37.9395	-0.51	3.37	10.863	2.85800004	-122.46735	37.93951	-0.43	3.37	8.703	2.93400005	11	7/24/2014	29:05.4
7076	RWS11	-122.4673557	37.9395	-0.48	3.38	10.907	2.90299994	-122.46735	37.93951	-0.43	3.38	8.79	2.94499996	11	7/24/2014	29:05.5
7077	RWS11	-122.4673577	37.9395	-0.56	3.39	10.974	2.82600009	-122.46735	37.93951	-0.49	3.39	8.876	2.90200007	11	7/24/2014	29:05.6
7078	RWS11	-122.4673598	37.9395	-0.48	3.39	11.059	2.91500002	-122.46735	37.93951	-0.43	3.39	8.941	2.95700005	11	7/24/2014	29:05.7
7079	RWS11	-122.4673619	37.9395	-0.51	3.39	11.274	2.8829999	-122.46736	37.93951	-0.49	3.39	9.134	2.90799987	11	7/24/2014	29:05.8
7080	RWS11	-122.4673639	37.9395	-0.48	3.39	11.363	2.91500002	-122.46736	37.93951	-0.49	3.39	9.288	2.90600002	11	7/24/2014	29:05.9
7081	RWS11	-122.467367	37.9395	-0.51	3.39	11.449	2.87599993	-122.46736	37.93951	-0.49	3.39	9.441	2.9009999	11	7/24/2014	29:06.0
7082	RWS11	-122.467369	37.9395	-0.43	3.38	11.558	2.95399994	-122.46736	37.93952	-0.43	3.38	9.571	2.94499996	11	7/24/2014	29:06.1
7083	RWS11	-122.467371	37.9395	-0.48	3.37	11.667	2.89599997	-122.46736	37.93952	-0.49	3.37	9.746	2.88699996	11	7/24/2014	29:06.2
7084	RWS11	-122.4673731	37.9395	-0.51	3.37	11.776	2.85500002	-122.46737	37.93952	-0.43	3.37	9.833	2.93100002	11	7/24/2014	29:06.3
7085	RWS11	-122.4673751	37.9395	-0.56	3.36	11.95	2.79700005	-122.46737	37.93952	-0.49	3.36	9.918	2.87300003	11	7/24/2014	29:06.4
7086	RWS11	-122.4673772	37.93951	-0.51	3.35	12.165	2.84299994	-122.46737	37.93952	-0.43	3.35	10.179	2.91899994	11	7/24/2014	29:06.5
7087	RWS11	-122.4673793	37.93951	-0.48	3.35	12.318	2.87200004	-122.46737	37.93952	-0.49	3.35	10.352	2.86300004	11	7/24/2014	29:06.6
7088	RWS11	-122.4673813	37.93951	-0.48	3.34	12.405	2.86800009	-122.46738	37.93952	-0.43	3.34	10.483	2.91000012	11	7/24/2014	29:06.7
7089	RWS11	-122.4673834	37.93951	-0.43	3.34	12.689	2.91600007	-122.46738	37.93952	-0.52	3.34	10.658	2.82200009	11	7/24/2014	29:06.8
7090	RWS11	-122.4673854	37.93951	-0.51	3.34	12.754	2.8269999	-122.46738	37.93952	-0.43	3.34	10.833	2.90299991	11	7/24/2014	29:06.9
7091	RWS11	-122.4673885	37.93951	-0.48	3.33	13.037	2.85600001	-122.46738	37.93952	-0.52	3.33	10.92	2.81300002	11	7/24/2014	29:07.0
7092	RWS11	-122.4673906	37.93951	-0.43	3.33	13.126	2.90199989	-122.46738	37.93952	-0.49	3.33	11.225	2.84199989	11	7/24/2014	29:07.1
7093	RWS11	-122.4673927	37.93951	-0.48	3.32	13.469	2.84399992	-122.46739	37.93952	-0.52	3.32	11.394	2.80099994	11	7/24/2014	29:07.2
7094	RWS11	-122.4673948	37.93951	-0.43	3.31	13.624	2.88700002	-122.46739	37.93952	-0.43	3.31	11.636	2.87800005	11	7/24/2014	29:07.3

7095	RWS11	-122.4673969	37.93951	-0.48	3.3	13.753	2.82799989	-122.46739	37.93952	-0.52	3.3	11.789	2.78499991	11	7/24/2014	29:07.4
7096	RWS11	-122.4673991	37.93951	-0.43	3.3	13.973	2.87099999	-122.46739	37.93952	-0.43	3.3	12.072	2.86200002	11	7/24/2014	29:07.5
7097	RWS11	-122.4674013	37.93951	-0.48	3.29	14.124	2.81399995	-122.46739	37.93953	-0.43	3.29	12.095	2.85599998	11	7/24/2014	29:07.6
7098	RWS11	-122.4674035	37.93951	-0.43	3.29	14.299	2.86000007	-122.4674	37.93953	-0.49	3.29	12.369	2.80000007	11	7/24/2014	29:07.7
7099	RWS11	-122.4674057	37.93951	-0.48	3.28	14.451	2.80699998	-122.4674	37.93953	-0.52	3.28	12.443	2.764	11	7/24/2014	29:07.8
7100	RWS11	-122.4674079	37.93951	-0.43	3.28	14.535	2.85799998	-122.4674	37.93953	-0.49	3.28	12.592	2.79799998	11	7/24/2014	29:07.9
7101	RWS11	-122.4674101	37.93952	-0.51	3.29	14.71	2.77600002	-122.4674	37.93953	-0.52	3.29	12.745	2.76700002	11	7/24/2014	29:08.0
7102	RWS11	-122.4674124	37.93952	-0.43	3.29	14.842	2.86700004	-122.46741	37.93953	-0.49	3.29	12.875	2.80700004	11	7/24/2014	29:08.1
7103	RWS11	-122.4674146	37.93952	-0.48	3.3	14.97	2.82300001	-122.46741	37.93953	-0.52	3.3	13.16	2.78000003	11	7/24/2014	29:08.2
7104	RWS11	-122.4674168	37.93952	-0.51	3.31	15.125	2.7980001	-122.46741	37.93953	-0.49	3.31	13.312	2.82300007	11	7/24/2014	29:08.3
7105	RWS11	-122.467419	37.93952	-0.56	3.32	15.321	2.75700009	-122.46741	37.93953	-0.49	3.32	13.53	2.83300006	11	7/24/2014	29:08.4
7106	RWS11	-122.4674212	37.93952	-0.48	3.33	15.496	2.85299999	-122.46741	37.93953	-0.49	3.33	13.749	2.84399998	11	7/24/2014	29:08.5
7107	RWS11	-122.4674233	37.93952	-0.51	3.34	15.692	2.82800007	-122.46742	37.93953	-0.43	3.34	13.967	2.90400007	11	7/24/2014	29:08.6
7108	RWS11	-122.4674255	37.93952	-0.43	3.35	15.905	2.92199987	-122.46742	37.93953	-0.49	3.35	14.159	2.86199987	11	7/24/2014	29:08.7
7109	RWS11	-122.4674276	37.93952	-0.48	3.35	16.125	2.87800008	-122.46742	37.93953	-0.4	3.35	14.377	2.95400009	11	7/24/2014	29:08.8
7110	RWS11	-122.4674297	37.93952	-0.48	3.36	16.343	2.88399988	-122.46742	37.93953	-0.43	3.36	14.617	2.92599991	11	7/24/2014	29:08.9
7111	RWS11	-122.4674328	37.93952	-0.56	3.36	16.582	2.80300009	-122.46743	37.93954	-0.49	3.36	14.792	2.87900007	11	7/24/2014	29:09.0
7112	RWS11	-122.467435	37.93952	-0.39	3.37	16.8	2.97700012	-122.46743	37.93954	-0.43	3.37	15.141	2.93300012	11	7/24/2014	29:09.1
7113	RWS11	-122.4674371	37.93952	-0.43	3.37	16.998	2.94499987	-122.46743	37.93954	-0.49	3.37	15.272	2.88499987	11	7/24/2014	29:09.2
7114	RWS11	-122.4674392	37.93952	-0.48	3.37	17.214	2.89599997	-122.46743	37.93954	-0.52	3.37	15.445	2.85299999	11	7/24/2014	29:09.3
7115	RWS11	-122.4674414	37.93953	-0.43	3.37	17.451	2.94900006	-122.46744	37.93954	-0.49	3.37	15.661	2.88900006	11	7/24/2014	29:09.4
7116	RWS11	-122.4674436	37.93953	-0.43	3.38	17.669	2.95099992	-122.46744	37.93954	-0.52	3.38	15.878	2.85699993	11	7/24/2014	29:09.5
7117	RWS11	-122.4674458	37.93953	-0.48	3.38	17.909	2.90200001	-122.46744	37.93954	-0.49	3.38	16.12	2.89300001	11	7/24/2014	29:09.6
7118	RWS11	-122.467448	37.93953	-0.51	3.38	18.062	2.87000012	-122.46744	37.93954	-0.52	3.38	16.249	2.86100012	11	7/24/2014	29:09.7
7119	RWS11	-122.4674503	37.93953	-0.48	3.38	18.259	2.90699989	-122.46744	37.93954	-0.52	3.38	16.446	2.8639999	11	7/24/2014	29:09.8
7120	RWS11	-122.4674525	37.93953	-0.43	3.39	18.475	2.96099991	-122.46745	37.93954	-0.49	3.39	16.664	2.9009999	11	7/24/2014	29:09.9
7121	RWS11	-122.4674547	37.93953	-0.56	3.39	18.674	2.82700002	-122.46745	37.93954	-0.49	3.39	16.817	2.903	11	7/24/2014	29:10.0
7122	RWS11	-122.467457	37.93953	-0.48	3.39	18.8	2.91400009	-122.46745	37.93954	-0.49	3.39	16.966	2.90500009	11	7/24/2014	29:10.1
7123	RWS11	-122.4674592	37.93953	-0.51	3.39	18.995	2.88000011	-122.46745	37.93954	-0.49	3.39	17.117	2.90500009	11	7/24/2014	29:10.2
7124	RWS11	-122.4674614	37.93953	-0.43	3.39	19.15	2.963	-122.46745	37.93954	-0.52	3.39	17.337	2.86900002	11	7/24/2014	29:10.3
7125	RWS11	-122.4674636	37.93953	-0.48	3.38	19.432	2.90599996	-122.46746	37.93955	-0.52	3.38	17.509	2.86299998	11	7/24/2014	29:10.4
7126	RWS11	-122.4674657	37.93953	-0.48	3.37	19.563	2.8969999	-122.46746	37.93955	-0.52	3.37	17.621	2.85399991	11	7/24/2014	29:10.5
7127	RWS11	-122.4674678	37.93953	-0.56	3.36	19.717	2.79899991	-122.46746	37.93955	-0.52	3.36	17.882	2.8409999	11	7/24/2014	29:10.6

7128	RWS11	-122.46747	37.93953	-0.48	3.36	19.956	2.88299996	-122.46746	37.93955	-0.52	3.36	18.078	2.83999997	11	7/24/2014	29:10.7
7129	RWS11	-122.467472	37.93954	-0.56	3.36	20.128	2.79600012	-122.46747	37.93955	-0.6	3.36	18.272	2.75400013	11	7/24/2014	29:10.8
7130	RWS11	-122.4674742	37.93954	-0.43	3.36	20.324	2.93000001	-122.46747	37.93955	-0.57	3.36	18.468	2.78500003	11	7/24/2014	29:10.9
7131	RWS11	-122.4674772	37.93954	-0.48	3.35	20.521	2.87699991	-122.46747	37.93955	-0.52	3.35	18.686	2.83399993	11	7/24/2014	29:11.0
7132	RWS11	-122.4674793	37.93954	-0.51	3.35	20.695	2.84100008	-122.46747	37.93955	-0.52	3.35	18.86	2.83200008	11	7/24/2014	29:11.1
7133	RWS11	-122.4674813	37.93954	-0.48	3.35	20.892	2.87299997	-122.46747	37.93955	-0.57	3.35	19.077	2.77899998	11	7/24/2014	29:11.2
7134	RWS11	-122.4674834	37.93954	-0.48	3.35	21.11	2.87200004	-122.46748	37.93955	-0.57	3.35	19.255	2.77800006	11	7/24/2014	29:11.3
7135	RWS11	-122.4674855	37.93954	-0.56	3.35	21.393	2.78499997	-122.46748	37.93955	-0.49	3.35	19.471	2.86099994	11	7/24/2014	29:11.4
7136	RWS11	-122.4674876	37.93954	-0.48	3.33	21.628	2.85500008	-122.46748	37.93955	-0.52	3.33	19.663	2.8120001	11	7/24/2014	29:11.5
7137	RWS11	-122.4674898	37.93954	-0.48	3.32	21.87	2.8409999	-122.46748	37.93955	-0.57	3.32	19.882	2.74699992	11	7/24/2014	29:11.6
7138	RWS11	-122.4674919	37.93954	-0.56	3.3	22.13	2.74299991	-122.46749	37.93955	-0.52	3.3	20.123	2.78499991	11	7/24/2014	29:11.7
7139	RWS11	-122.4674941	37.93954	-0.51	3.29	22.372	2.78299999	-122.46749	37.93956	-0.52	3.29	20.339	2.77399999	11	7/24/2014	29:11.8
7140	RWS11	-122.4674963	37.93954	-0.48	3.28	22.589	2.80699998	-122.46749	37.93956	-0.57	3.28	20.603	2.713	11	7/24/2014	29:11.9
7141	RWS11	-122.4674984	37.93954	-0.51	3.27	22.785	2.76399994	-122.46749	37.93956	-0.57	3.27	20.808	2.70399994	11	7/24/2014	29:12.0
7142	RWS11	-122.4675006	37.93954	-0.51	3.27	23.024	2.75600004	-122.46749	37.93956	-0.57	3.27	21.059	2.69600004	11	7/24/2014	29:12.1
7143	RWS11	-122.4675028	37.93955	-0.51	3.26	23.264	2.74900007	-122.4675	37.93956	-0.52	3.26	21.297	2.74000007	11	7/24/2014	29:12.2
7144	RWS11	-122.4675049	37.93955	-0.51	3.25	23.5	2.74300003	-122.4675	37.93956	-0.52	3.25	21.558	2.73400003	11	7/24/2014	29:12.3
7145	RWS11	-122.4675071	37.93955	-0.56	3.25	23.829	2.68800008	-122.4675	37.93956	-0.57	3.25	21.821	2.67900008	11	7/24/2014	29:12.4
7146	RWS11	-122.4675092	37.93955	-0.48	3.25	24.048	2.77099997	-122.4675	37.93956	-0.52	3.25	22.06	2.72799999	11	7/24/2014	29:12.5
7147	RWS11	-122.4675114	37.93955	-0.51	3.25	24.266	2.73600006	-122.4675	37.93956	-0.57	3.25	22.324	2.67600006	11	7/24/2014	29:12.6
7148	RWS11	-122.4675135	37.93955	-0.51	3.25	24.506	2.73699999	-122.46751	37.93956	-0.52	3.25	22.584	2.72799999	11	7/24/2014	29:12.7
7149	RWS11	-122.4675155	37.93955	-0.56	3.25	24.722	2.68800008	-122.46751	37.93956	-0.57	3.25	22.845	2.67900008	11	7/24/2014	29:12.8
7150	RWS11	-122.4675176	37.93955	-0.48	3.25	24.961	2.77399999	-122.46751	37.93956	-0.6	3.25	23.082	2.64700001	11	7/24/2014	29:12.9
7151	RWS11	-122.4675207	37.93955	-0.51	3.25	25.199	2.74099994	-122.46751	37.93956	-0.6	3.25	23.344	2.64799994	11	7/24/2014	29:13.0
7152	RWS11	-122.4675228	37.93955	-0.51	3.25	25.483	2.74099994	-122.46752	37.93956	-0.52	3.25	23.713	2.73199993	11	7/24/2014	29:13.1
7153	RWS11	-122.4675249	37.93955	-0.51	3.25	25.767	2.74099994	-122.46752	37.93957	-0.52	3.25	23.999	2.73199993	11	7/24/2014	29:13.2
7154	RWS11	-122.467527	37.93955	-0.48	3.25	25.963	2.77399999	-122.46752	37.93957	-0.52	3.25	24.259	2.73100001	11	7/24/2014	29:13.3
7155	RWS11	-122.4675291	37.93955	-0.51	3.25	26.096	2.74000001	-122.46752	37.93957	-0.49	3.25	24.457	2.76499999	11	7/24/2014	29:13.4
7156	RWS11	-122.4675312	37.93955	-0.48	3.25	26.334	2.77600008	-122.46752	37.93957	-0.52	3.25	24.696	2.7330001	11	7/24/2014	29:13.5
7157	RWS11	-122.4675334	37.93956	-0.48	3.25	26.549	2.77799994	-122.46753	37.93957	-0.6	3.25	24.956	2.65099996	11	7/24/2014	29:13.6
7158	RWS11	-122.4675355	37.93956	-0.51	3.26	26.699	2.74900007	-122.46753	37.93957	-0.43	3.26	25.194	2.82500008	11	7/24/2014	29:13.7
7159	RWS11	-122.4675376	37.93956	-0.56	3.26	27.051	2.70299995	-122.46753	37.93957	-0.49	3.26	25.434	2.77899992	11	7/24/2014	29:13.8
7160	RWS11	-122.4675397	37.93956	-0.56	3.27	27.291	2.70999992	-122.46753	37.93957	-0.6	3.27	25.762	2.66799992	11	7/24/2014	29:13.9

7161	RWS11	-122.4675429	37.93956	-0.56	3.28	27.464	2.71800005	-122.46754	37.93957	-0.52	3.28	25.958	2.76000005	11	7/24/2014	29:14.0
7162	RWS11	-122.467545	37.93956	-0.56	3.29	27.706	2.72599995	-122.46754	37.93957	-0.49	3.29	26.111	2.80199993	11	7/24/2014	29:14.1
7163	RWS11	-122.4675471	37.93956	-0.56	3.3	27.987	2.73599994	-122.46754	37.93957	-0.49	3.3	26.414	2.81199992	11	7/24/2014	29:14.2
7164	RWS11	-122.4675493	37.93956	-0.51	3.31	28.248	2.796	-122.46754	37.93957	-0.52	3.31	26.697	2.787	11	7/24/2014	29:14.3
7165	RWS11	-122.4675514	37.93956	-0.56	3.32	28.442	2.755	-122.46754	37.93957	-0.49	3.32	26.849	2.83099997	11	7/24/2014	29:14.4
7166	RWS11	-122.4675536	37.93956	-0.56	3.33	28.595	2.76599991	-122.46755	37.93957	-0.49	3.33	27.042	2.84199989	11	7/24/2014	29:14.5
7167	RWS11	-122.4675558	37.93956	-0.56	3.34	28.813	2.7759999	-122.46755	37.93958	-0.52	3.34	27.284	2.8179999	11	7/24/2014	29:14.6
7168	RWS11	-122.4675579	37.93956	-0.56	3.35	28.966	2.78700006	-122.46755	37.93958	-0.52	3.35	27.415	2.82900006	11	7/24/2014	29:14.7
7169	RWS11	-122.4675601	37.93956	-0.51	3.36	29.141	2.84899998	-122.46755	37.93958	-0.52	3.36	27.59	2.83999997	11	7/24/2014	29:14.8
7170	RWS11	-122.4675622	37.93956	-0.56	3.37	29.315	2.81000006	-122.46756	37.93958	-0.52	3.37	27.807	2.85200006	11	7/24/2014	29:14.9
7171	RWS11	-122.4675654	37.93957	-0.56	3.38	29.53	2.82099998	-122.46756	37.93958	-0.57	3.38	28.044	2.81199998	11	7/24/2014	29:15.0
7172	RWS11	-122.4675676	37.93957	-0.51	3.39	29.639	2.8829999	-122.46756	37.93958	-0.52	3.39	28.175	2.87399989	11	7/24/2014	29:15.1
7173	RWS11	-122.4675697	37.93957	-0.51	3.4	29.792	2.89300013	-122.46756	37.93958	-0.52	3.4	28.306	2.88400012	11	7/24/2014	29:15.2
7174	RWS11	-122.4675719	37.93957	-0.56	3.41	29.943	2.85099995	-122.46757	37.93958	-0.52	3.41	28.479	2.89299995	11	7/24/2014	29:15.3
7175	RWS11	-122.467574	37.93957	-0.56	3.42	30.076	2.85799992	-122.46757	37.93958	-0.52	3.42	28.656	2.89999992	11	7/24/2014	29:15.4
7176	RWS11	-122.4675762	37.93957	-0.51	3.42	30.12	2.91499996	-122.46757	37.93958	-0.52	3.42	28.721	2.90599996	11	7/24/2014	29:15.5
7177	RWS11	-122.4675784	37.93957	-0.51	3.43	30.291	2.92000008	-122.46757	37.93958	-0.49	3.43	28.893	2.94500005	11	7/24/2014	29:15.6
7178	RWS11	-122.4675806	37.93957	-0.51	3.43	30.421	2.9230001	-122.46757	37.93958	-0.49	3.43	28.979	2.94800007	11	7/24/2014	29:15.7
7179	RWS11	-122.4675827	37.93957	-0.56	3.43	30.55	2.87399995	-122.46758	37.93958	-0.49	3.43	29.086	2.94999993	11	7/24/2014	29:15.8
7180	RWS11	-122.4675848	37.93957	-0.48	3.43	30.705	2.95899993	-122.46758	37.93958	-0.49	3.43	29.241	2.94999993	11	7/24/2014	29:15.9
7181	RWS11	-122.467588	37.93957	-0.51	3.43	30.899	2.9230001	-122.46758	37.93959	-0.52	3.43	29.458	2.91400009	11	7/24/2014	29:16.0
7182	RWS11	-122.4675901	37.93957	-0.51	3.43	31.011	2.91899991	-122.46758	37.93959	-0.49	3.43	29.57	2.94399989	11	7/24/2014	29:16.1
7183	RWS11	-122.4675923	37.93957	-0.51	3.42	31.118	2.91400003	-122.46759	37.93959	-0.49	3.42	29.629	2.93900001	11	7/24/2014	29:16.2
7184	RWS11	-122.4675944	37.93958	-0.51	3.42	31.292	2.90799999	-122.46759	37.93959	-0.57	3.42	29.809	2.84799999	11	7/24/2014	29:16.3
7185	RWS11	-122.4675966	37.93958	-0.51	3.41	31.441	2.90100002	-122.46759	37.93959	-0.57	3.41	29.939	2.84100002	11	7/24/2014	29:16.4
7186	RWS11	-122.4675988	37.93958	-0.48	3.4	31.531	2.92800003	-122.46759	37.93959	-0.52	3.4	30.065	2.88500005	11	7/24/2014	29:16.5
7187	RWS11	-122.4676009	37.93958	-0.51	3.4	31.769	2.88599992	-122.46759	37.93959	-0.6	3.4	30.242	2.79299992	11	7/24/2014	29:16.6
7188	RWS11	-122.4676031	37.93958	-0.48	3.39	31.878	2.91400009	-122.4676	37.93959	-0.52	3.39	30.414	2.87100011	11	7/24/2014	29:16.7
7189	RWS11	-122.4676052	37.93958	-0.51	3.38	32.075	2.87299991	-122.4676	37.93959	-0.52	3.38	30.589	2.8639999	11	7/24/2014	29:16.8
7190	RWS11	-122.4676073	37.93958	-0.48	3.38	32.206	2.90100008	-122.4676	37.93959	-0.49	3.38	30.785	2.89200008	11	7/24/2014	29:16.9
7191	RWS11	-122.4676105	37.93958	-0.51	3.37	32.422	2.8599999	-122.4676	37.93959	-0.52	3.37	30.984	2.85099989	11	7/24/2014	29:17.0
7192	RWS11	-122.4676127	37.93958	-0.48	3.36	32.617	2.88699991	-122.46761	37.93959	-0.57	3.36	31.24	2.79299992	11	7/24/2014	29:17.1
7193	RWS11	-122.4676148	37.93958	-0.48	3.36	32.791	2.87999994	-122.46761	37.93959	-0.6	3.36	31.415	2.75299996	11	7/24/2014	29:17.2

7194	RWS11	-122.467617	37.93958	-0.48	3.35	33.032	2.87299997	-122.46761	37.9396	-0.52	3.35	31.655	2.82999998	11	7/24/2014	29:17.3
7195	RWS11	-122.4676192	37.93958	-0.51	3.34	33.226	2.83100009	-122.46761	37.9396	-0.49	3.34	31.83	2.85600007	11	7/24/2014	29:17.4
7196	RWS11	-122.4676214	37.93958	-0.48	3.33	33.468	2.8580001	-122.46761	37.9396	-0.52	3.33	31.98	2.81500012	11	7/24/2014	29:17.5
7197	RWS11	-122.4676235	37.93958	-0.51	3.33	33.662	2.81800008	-122.46762	37.9396	-0.52	3.33	32.179	2.80900007	11	7/24/2014	29:17.6
7198	RWS11	-122.4676257	37.93959	-0.51	3.32	33.902	2.81299996	-122.46762	37.9396	-0.49	3.32	32.332	2.83799994	11	7/24/2014	29:17.7
7199	RWS11	-122.4676279	37.93959	-0.51	3.32	34.099	2.80900002	-122.46762	37.9396	-0.52	3.32	32.524	2.80000001	11	7/24/2014	29:17.8
7200	RWS11	-122.46763	37.93959	-0.48	3.32	34.313	2.83999997	-122.46762	37.9396	-0.49	3.32	32.763	2.83099997	11	7/24/2014	29:17.9
7201	RWS11	-122.4676332	37.93959	-0.51	3.32	34.554	2.80500007	-122.46763	37.9396	-0.49	3.32	32.962	2.83000004	11	7/24/2014	29:18.0
7202	RWS11	-122.4676353	37.93959	-0.51	3.32	34.685	2.80599999	-122.46763	37.9396	-0.43	3.32	33.113	2.882	11	7/24/2014	29:18.1
7203	RWS11	-122.4676374	37.93959	-0.51	3.32	34.925	2.80999994	-122.46763	37.9396	-0.43	3.32	33.31	2.88599995	11	7/24/2014	29:18.2
7204	RWS11	-122.4676395	37.93959	-0.51	3.33	35.166	2.81500006	-122.46763	37.9396	-0.49	3.33	33.505	2.84000003	11	7/24/2014	29:18.3
7205	RWS11	-122.4676417	37.93959	-0.51	3.33	35.362	2.82400012	-122.46764	37.9396	-0.43	3.33	33.679	2.90000013	11	7/24/2014	29:18.4
7206	RWS11	-122.4676438	37.93959	-0.51	3.35	35.596	2.83500004	-122.46764	37.9396	-0.52	3.35	33.958	2.82600003	11	7/24/2014	29:18.5
7207	RWS11	-122.4676459	37.93959	-0.56	3.36	35.752	2.79700005	-122.46764	37.9396	-0.52	3.36	34.091	2.83900005	11	7/24/2014	29:18.6
7208	RWS11	-122.4676481	37.93959	-0.48	3.37	35.861	2.89800006	-122.46764	37.93961	-0.49	3.37	34.2	2.88900006	11	7/24/2014	29:18.7
7209	RWS11	-122.4676502	37.93959	-0.51	3.39	36.077	2.88000011	-122.46764	37.93961	-0.6	3.39	34.353	2.78700012	11	7/24/2014	29:18.8
7210	RWS11	-122.4676523	37.93959	-0.51	3.41	36.298	2.898	-122.46765	37.93961	-0.6	3.41	34.659	2.80500001	11	7/24/2014	29:18.9
7211	RWS11	-122.4676554	37.9396	-0.56	3.42	36.514	2.86399996	-122.46765	37.93961	-0.52	3.42	34.875	2.90599996	11	7/24/2014	29:19.0
7212	RWS11	-122.4676575	37.9396	-0.51	3.44	36.668	2.93000007	-122.46765	37.93961	-0.49	3.44	35.092	2.95500004	11	7/24/2014	29:19.1
7213	RWS11	-122.4676596	37.9396	-0.51	3.45	36.837	2.94400001	-122.46765	37.93961	-0.57	3.45	35.266	2.884	11	7/24/2014	29:19.2
7214	RWS11	-122.4676617	37.9396	-0.51	3.47	37.034	2.95600009	-122.46766	37.93961	-0.49	3.47	35.48	2.98100007	11	7/24/2014	29:19.3
7215	RWS11	-122.4676638	37.9396	-0.56	3.48	37.23	2.91500008	-122.46766	37.93961	-0.52	3.48	35.703	2.95700008	11	7/24/2014	29:19.4
7216	RWS11	-122.467666	37.9396	-0.51	3.48	37.449	2.97099996	-122.46766	37.93961	-0.57	3.48	35.941	2.91099995	11	7/24/2014	29:19.5
7217	RWS11	-122.4676681	37.9396	-0.56	3.48	37.645	2.92100012	-122.46766	37.93961	-0.57	3.48	36.159	2.91200012	11	7/24/2014	29:19.6
7218	RWS11	-122.4676702	37.9396	-0.51	3.48	37.82	2.96700001	-122.46766	37.93961	-0.6	3.48	36.378	2.87400001	11	7/24/2014	29:19.7
7219	RWS11	-122.4676723	37.9396	-0.56	3.47	38.016	2.90600002	-122.46767	37.93961	-0.52	3.47	36.487	2.94800001	11	7/24/2014	29:19.8
7220	RWS11	-122.4676744	37.9396	-0.56	3.45	38.187	2.89300001	-122.46767	37.93961	-0.52	3.45	36.743	2.935	11	7/24/2014	29:19.9
7221	RWS11	-122.4676775	37.9396	-0.56	3.44	38.406	2.8779999	-122.46767	37.93961	-0.52	3.44	36.966	2.9199999	11	7/24/2014	29:20.0
7222	RWS11	-122.4676796	37.9396	-0.56	3.42	38.58	2.86300004	-122.46767	37.93962	-0.65	3.42	37.177	2.77000004	11	7/24/2014	29:20.1
7223	RWS11	-122.4676817	37.9396	-0.56	3.41	38.709	2.85099995	-122.46768	37.93962	-0.6	3.41	37.291	2.80899996	11	7/24/2014	29:20.2
7224	RWS11	-122.4676838	37.9396	-0.56	3.4	38.842	2.84300005	-122.46768	37.93962	-0.57	3.4	37.444	2.83400005	11	7/24/2014	29:20.3
7225	RWS11	-122.4676859	37.9396	-0.56	3.4	39.014	2.83900011	-122.46768	37.93962	-0.6	3.4	37.616	2.79700011	11	7/24/2014	29:20.4
7226	RWS11	-122.467688	37.93961	-0.56	3.4	39.213	2.84000003	-122.46768	37.93962	-0.49	3.4	37.792	2.91600001	11	7/24/2014	29:20.5

7227	RWS11	-122.4676901	37.93961	-0.56	3.41	39.338	2.84499991	-122.46768	37.93962	-0.57	3.41	38.008	2.83599991	11	7/24/2014	29:20.6
7228	RWS11	-122.4676922	37.93961	-0.56	3.41	39.494	2.85399997	-122.46769	37.93962	-0.52	3.41	38.112	2.89599997	11	7/24/2014	29:20.7
7229	RWS11	-122.4676943	37.93961	-0.6	3.43	39.644	2.83199996	-122.46769	37.93962	-0.52	3.43	38.295	2.90899998	11	7/24/2014	29:20.8
7230	RWS11	-122.4676963	37.93961	-0.56	3.44	39.775	2.88200009	-122.46769	37.93962	-0.52	3.44	38.42	2.92400008	11	7/24/2014	29:20.9
7231	RWS11	-122.4676994	37.93961	-0.6	3.46	39.928	2.86499995	-122.46769	37.93962	-0.57	3.46	38.575	2.89099997	11	7/24/2014	29:21.0
7232	RWS11	-122.4677015	37.93961	-0.56	3.48	40.105	2.91999996	-122.4677	37.93962	-0.52	3.48	38.748	2.96199995	11	7/24/2014	29:21.1
7233	RWS11	-122.4677036	37.93961	-0.6	3.5	40.211	2.90600008	-122.4677	37.93962	-0.57	3.5	38.921	2.9320001	11	7/24/2014	29:21.2
7234	RWS11	-122.4677057	37.93961	-0.56	3.52	40.36	2.96100008	-122.4677	37.93962	-0.52	3.52	39.049	3.00300008	11	7/24/2014	29:21.3
7235	RWS11	-122.4677079	37.93961	-0.6	3.54	40.513	2.94300002	-122.4677	37.93962	-0.52	3.54	39.205	3.02000004	11	7/24/2014	29:21.4
7236	RWS11	-122.46771	37.93961	-0.56	3.55	40.666	2.99100006	-122.4677	37.93962	-0.49	3.55	39.333	3.06700003	11	7/24/2014	29:21.5
7237	RWS11	-122.4677121	37.93961	-0.56	3.56	40.754	2.99899995	-122.46771	37.93963	-0.57	3.56	39.445	2.98999995	11	7/24/2014	29:21.6
7238	RWS11	-122.4677142	37.93961	-0.56	3.56	40.885	3.00000012	-122.46771	37.93963	-0.43	3.56	39.549	3.12700012	11	7/24/2014	29:21.7
7239	RWS11	-122.4677163	37.93961	-0.56	3.56	40.994	2.99400008	-122.46771	37.93963	-0.57	3.56	39.62	2.98500007	11	7/24/2014	29:21.8
7240	RWS11	-122.4677184	37.93961	-0.56	3.54	41.118	2.98199999	-122.46771	37.93963	-0.57	3.54	39.744	2.97299999	11	7/24/2014	29:21.9
7241	RWS11	-122.4677215	37.93962	-0.56	3.53	41.23	2.96500003	-122.46772	37.93963	-0.52	3.53	39.831	3.00700003	11	7/24/2014	29:22.0
7242	RWS11	-122.4677236	37.93962	-0.51	3.5	41.339	2.99399996	-122.46772	37.93963	-0.49	3.5	39.897	3.01899993	11	7/24/2014	29:22.1
7243	RWS11	-122.4677256	37.93962	-0.56	3.48	41.489	2.9180001	-122.46772	37.93963	-0.52	3.48	40.006	2.9600001	11	7/24/2014	29:22.2
7244	RWS11	-122.4677277	37.93962	-0.51	3.45	41.601	2.94099998	-122.46772	37.93963	-0.4	3.45	40.115	3.05099997	11	7/24/2014	29:22.3
7245	RWS11	-122.4677298	37.93962	-0.51	3.42	41.776	2.91100001	-122.46772	37.93963	-0.49	3.42	40.246	2.93599999	11	7/24/2014	29:22.4
7246	RWS11	-122.4677319	37.93962	-0.56	3.39	41.926	2.82900012	-122.46773	37.93963	-0.43	3.39	40.443	2.95600012	11	7/24/2014	29:22.5
7247	RWS11	-122.467734	37.93962	-0.51	3.36	42.034	2.84800005	-122.46773	37.93963	-0.49	3.36	40.483	2.87300003	11	7/24/2014	29:22.6
7248	RWS11	-122.467736	37.93962	-0.51	3.33	42.206	2.81699991	-122.46773	37.93963	-0.49	3.33	40.677	2.84199989	11	7/24/2014	29:22.7
7249	RWS11	-122.4677381	37.93962	-0.56	3.3	42.403	2.73599994	-122.46773	37.93963	-0.49	3.3	40.873	2.81199992	11	7/24/2014	29:22.8
7250	RWS11	-122.4677402	37.93962	-0.51	3.27	42.537	2.75900006	-122.46773	37.93963	-0.43	3.27	41.029	2.83500007	11	7/24/2014	29:22.9
7251	RWS11	-122.4677432	37.93962	-0.56	3.24	42.752	2.68200004	-122.46774	37.93964	-0.52	3.24	41.247	2.72400004	11	7/24/2014	29:23.0
7252	RWS11	-122.4677453	37.93962	-0.51	3.22	42.905	2.71000004	-122.46774	37.93964	-0.43	3.22	41.416	2.78600004	11	7/24/2014	29:23.1
7253	RWS11	-122.4677473	37.93962	-0.56	3.2	43.057	2.63900006	-122.46774	37.93964	-0.4	3.2	41.574	2.80000004	11	7/24/2014	29:23.2
7254	RWS11	-122.4677495	37.93962	-0.51	3.18	43.272	2.6730001	-122.46774	37.93964	-0.43	3.18	41.808	2.7490001	11	7/24/2014	29:23.3
7255	RWS11	-122.4677515	37.93963	-0.56	3.17	43.469	2.60799992	-122.46775	37.93964	-0.49	3.17	42.024	2.6839999	11	7/24/2014	29:23.4
7256	RWS11	-122.4677537	37.93963	-0.48	3.16	43.644	2.68199998	-122.46775	37.93964	-0.49	3.16	42.136	2.67299998	11	7/24/2014	29:23.5
7257	RWS11	-122.4677558	37.93963	-0.51	3.15	43.797	2.64199996	-122.46775	37.93964	-0.49	3.15	42.289	2.66699994	11	7/24/2014	29:23.6
7258	RWS11	-122.4677579	37.93963	-0.48	3.15	44.015	2.67299992	-122.46775	37.93964	-0.52	3.15	42.485	2.62999994	11	7/24/2014	29:23.7
7259	RWS11	-122.46776	37.93963	-0.51	3.15	44.212	2.6400001	-122.46775	37.93964	-0.49	3.15	42.682	2.66500008	11	7/24/2014	29:23.8

7260	RWS11	-122.4677621	37.93963	-0.48	3.15	44.405	2.67899996	-122.46776	37.93964	-0.49	3.15	42.832	2.66999996	11	7/24/2014	29:23.9
7261	RWS11	-122.4677653	37.93963	-0.51	3.16	44.579	2.65299994	-122.46776	37.93964	-0.43	3.16	43.027	2.72899994	11	7/24/2014	29:24.0
7262	RWS11	-122.4677674	37.93963	-0.47	3.17	44.75	2.69699994	-122.46776	37.93964	-0.4	3.17	43.224	2.77299994	11	7/24/2014	29:24.1
7263	RWS11	-122.4677695	37.93963	-0.51	3.18	44.95	2.67400008	-122.46776	37.93964	-0.52	3.18	43.458	2.66500008	11	7/24/2014	29:24.2
7264	RWS11	-122.4677717	37.93963	-0.47	3.2	45.122	2.72100011	-122.46777	37.93964	-0.43	3.2	43.704	2.7630001	11	7/24/2014	29:24.3
7265	RWS11	-122.4677738	37.93963	-0.51	3.21	45.318	2.70000011	-122.46777	37.93964	-0.48	3.21	43.857	2.72500011	11	7/24/2014	29:24.4
7266	RWS11	-122.467776	37.93963	-0.47	3.22	45.475	2.74699989	-122.46777	37.93965	-0.48	3.22	44.07	2.73799989	11	7/24/2014	29:24.5
7267	RWS11	-122.4677782	37.93963	-0.51	3.23	45.646	2.72599989	-122.46777	37.93965	-0.48	3.23	44.25	2.7509999	11	7/24/2014	29:24.6
7268	RWS11	-122.4677804	37.93963	-0.47	3.25	45.817	2.77400008	-122.46777	37.93965	-0.52	3.25	44.459	2.73100007	11	7/24/2014	29:24.7
7269	RWS11	-122.4677825	37.93963	-0.51	3.26	45.904	2.75300008	-122.46778	37.93965	-0.4	3.26	44.618	2.86300009	11	7/24/2014	29:24.8
7270	RWS11	-122.4677846	37.93964	-0.47	3.28	46.101	2.8000001	-122.46778	37.93965	-0.35	3.28	44.833	2.92600009	11	7/24/2014	29:24.9
7271	RWS11	-122.4677878	37.93964	-0.51	3.29	46.232	2.7790001	-122.46778	37.93965	-0.43	3.29	44.939	2.85500011	11	7/24/2014	29:25.0
7272	RWS11	-122.4677899	37.93964	-0.47	3.3	46.385	2.82499996	-122.46778	37.93965	-0.43	3.3	45.093	2.86699995	11	7/24/2014	29:25.1
7273	RWS11	-122.467792	37.93964	-0.47	3.31	46.559	2.83499995	-122.46779	37.93965	-0.43	3.31	45.251	2.87699994	11	7/24/2014	29:25.2
7274	RWS11	-122.4677942	37.93964	-0.51	3.32	46.778	2.81	-122.46779	37.93965	-0.43	3.32	45.445	2.88600001	11	7/24/2014	29:25.3
7275	RWS11	-122.4677963	37.93964	-0.51	3.33	46.993	2.8179999	-122.46779	37.93965	-0.43	3.33	45.616	2.8939999	11	7/24/2014	29:25.4
7276	RWS11	-122.4677985	37.93964	-0.47	3.33	47.121	2.85700002	-122.46779	37.93965	-0.43	3.33	45.791	2.89900002	11	7/24/2014	29:25.5
7277	RWS11	-122.4678006	37.93964	-0.51	3.34	47.277	2.82600003	-122.46779	37.93965	-0.43	3.34	45.9	2.90200004	11	7/24/2014	29:25.6
7278	RWS11	-122.4678027	37.93964	-0.51	3.34	47.408	2.82799989	-122.4678	37.93965	-0.43	3.34	46.053	2.90399989	11	7/24/2014	29:25.7
7279	RWS11	-122.4678048	37.93964	-0.51	3.34	47.623	2.82699996	-122.4678	37.93965	-0.43	3.34	46.206	2.90299997	11	7/24/2014	29:25.8
7280	RWS11	-122.4678069	37.93964	-0.47	3.33	47.758	2.85799995	-122.4678	37.93965	-0.43	3.33	46.422	2.89999995	11	7/24/2014	29:25.9
7281	RWS11	-122.4678101	37.93964	-0.51	3.33	47.95	2.81999999	-122.4678	37.93966	-0.43	3.33	46.598	2.896	11	7/24/2014	29:26.0
7282	RWS11	-122.4678121	37.93964	-0.51	3.32	48.1	2.81300002	-122.46781	37.93966	-0.52	3.32	46.77	2.80400002	11	7/24/2014	29:26.1
7283	RWS11	-122.4678143	37.93964	-0.51	3.31	48.322	2.80499989	-122.46781	37.93966	-0.57	3.31	46.945	2.74499989	11	7/24/2014	29:26.2
7284	RWS11	-122.4678164	37.93964	-0.47	3.3	48.45	2.82899991	-122.46781	37.93966	-0.4	3.3	47.16	2.90499991	11	7/24/2014	29:26.3
7285	RWS11	-122.4678185	37.93965	-0.51	3.29	48.646	2.78099996	-122.46781	37.93966	-0.48	3.29	47.338	2.80599996	11	7/24/2014	29:26.4
7286	RWS11	-122.4678207	37.93965	-0.47	3.27	48.886	2.79899994	-122.46781	37.93966	-0.43	3.27	47.619	2.84099993	11	7/24/2014	29:26.5
7287	RWS11	-122.4678228	37.93965	-0.51	3.26	49.039	2.74700004	-122.46782	37.93966	-0.48	3.26	47.706	2.77200004	11	7/24/2014	29:26.6
7288	RWS11	-122.4678249	37.93965	-0.47	3.23	49.191	2.7599999	-122.46782	37.93966	-0.43	3.23	47.858	2.8019999	11	7/24/2014	29:26.7
7289	RWS11	-122.467827	37.93965	-0.51	3.21	49.385	2.70299989	-122.46782	37.93966	-0.48	3.21	48.033	2.7279999	11	7/24/2014	29:26.8
7290	RWS11	-122.4678291	37.93965	-0.51	3.19	49.582	2.67800003	-122.46782	37.93966	-0.43	3.19	48.226	2.75400004	11	7/24/2014	29:26.9
7291	RWS11	-122.4678322	37.93965	-0.51	3.16	49.8	2.65299994	-122.46783	37.93966	-0.48	3.16	48.423	2.67799994	11	7/24/2014	29:27.0
7292	RWS11	-122.4678343	37.93965	-0.51	3.14	50.015	2.62999994	-122.46783	37.93966	-0.43	3.14	48.598	2.70599994	11	7/24/2014	29:27.1

7293	RWS11	-122.4678364	37.93965	-0.51	3.12	50.193	2.6080001	-122.46783	37.93966	-0.48	3.12	48.791	2.63300011	11	7/24/2014	29:27.2
7294	RWS11	-122.4678385	37.93965	-0.51	3.1	50.429	2.58799988	-122.46783	37.93966	-0.43	3.1	48.99	2.66399989	11	7/24/2014	29:27.3
7295	RWS11	-122.4678407	37.93965	-0.51	3.08	50.517	2.57200009	-122.46783	37.93966	-0.48	3.08	49.133	2.59700009	11	7/24/2014	29:27.4
7296	RWS11	-122.4678428	37.93965	-0.47	3.07	50.776	2.59300008	-122.46784	37.93967	-0.4	3.07	49.293	2.66900009	11	7/24/2014	29:27.5
7297	RWS11	-122.4678449	37.93965	-0.47	3.06	51.019	2.58400002	-122.46784	37.93967	-0.43	3.06	49.511	2.62600002	11	7/24/2014	29:27.6
7298	RWS11	-122.467847	37.93965	-0.47	3.05	51.191	2.57700005	-122.46784	37.93967	-0.43	3.05	49.726	2.61900005	11	7/24/2014	29:27.7
7299	RWS11	-122.4678491	37.93965	-0.47	3.05	51.431	2.5730001	-122.46784	37.93967	-0.43	3.05	49.989	2.6150001	11	7/24/2014	29:27.8
7300	RWS11	-122.4678512	37.93966	-0.51	3.05	51.584	2.53600007	-122.46785	37.93967	-0.4	3.05	50.145	2.64600009	11	7/24/2014	29:27.9
7301	RWS11	-122.4678544	37.93966	-0.51	3.04	51.779	2.53300005	-122.46785	37.93967	-0.43	3.04	50.377	2.60900006	11	7/24/2014	29:28.0
7302	RWS11	-122.4678565	37.93966	-0.47	3.04	51.973	2.56400004	-122.46785	37.93967	-0.43	3.04	50.599	2.60600004	11	7/24/2014	29:28.1
7303	RWS11	-122.4678586	37.93966	-0.47	3.04	52.148	2.56199995	-122.46785	37.93967	-0.43	3.04	50.764	2.60399994	11	7/24/2014	29:28.2
7304	RWS11	-122.4678608	37.93966	-0.47	3.04	52.366	2.56000009	-122.46785	37.93967	-0.43	3.04	50.924	2.60200009	11	7/24/2014	29:28.3
7305	RWS11	-122.4678629	37.93966	-0.47	3.04	52.629	2.56000009	-122.46786	37.93967	-0.4	3.04	51.142	2.6360001	11	7/24/2014	29:28.4
7306	RWS11	-122.4678651	37.93966	-0.47	3.04	52.803	2.56199995	-122.46786	37.93967	-0.4	3.04	51.317	2.63799995	11	7/24/2014	29:28.5
7307	RWS11	-122.4678672	37.93966	-0.51	3.04	53.018	2.53199989	-122.46786	37.93967	-0.4	3.04	51.535	2.6419999	11	7/24/2014	29:28.6
7308	RWS11	-122.4678694	37.93966	-0.47	3.05	53.217	2.57199994	-122.46786	37.93967	-0.4	3.05	51.731	2.64799994	11	7/24/2014	29:28.7
7309	RWS11	-122.4678716	37.93966	-0.51	3.06	53.429	2.54699999	-122.46787	37.93967	-0.4	3.06	51.943	2.65700001	11	7/24/2014	29:28.8
7310	RWS11	-122.4678737	37.93966	-0.47	3.07	53.63	2.59099999	-122.46787	37.93967	-0.4	3.07	52.096	2.667	11	7/24/2014	29:28.9
7311	RWS11	-122.4678769	37.93966	-0.47	3.08	53.888	2.604	-122.46787	37.93968	-0.43	3.08	52.362	2.646	11	7/24/2014	29:29.0
7312	RWS11	-122.4678791	37.93966	-0.47	3.09	54.085	2.61799994	-122.46787	37.93968	-0.4	3.09	52.551	2.69399995	11	7/24/2014	29:29.1
7313	RWS11	-122.4678813	37.93966	-0.47	3.11	54.238	2.63300005	-122.46788	37.93968	-0.43	3.11	52.768	2.67500004	11	7/24/2014	29:29.2
7314	RWS11	-122.4678835	37.93966	-0.47	3.12	54.416	2.64900008	-122.46788	37.93968	-0.4	3.12	52.926	2.72500008	11	7/24/2014	29:29.3
7315	RWS11	-122.4678857	37.93967	-0.47	3.14	54.587	2.66600004	-122.46788	37.93968	-0.4	3.14	53.126	2.74200004	11	7/24/2014	29:29.4
7316	RWS11	-122.4678879	37.93967	-0.47	3.16	54.78	2.683	-122.46788	37.93968	-0.4	3.16	53.337	2.759	11	7/24/2014	29:29.5
7317	RWS11	-122.4678901	37.93967	-0.51	3.17	54.955	2.66500002	-122.46788	37.93968	-0.43	3.17	53.494	2.74100003	11	7/24/2014	29:29.6
7318	RWS11	-122.4678923	37.93967	-0.47	3.19	55.042	2.71599999	-122.46789	37.93968	-0.43	3.19	53.621	2.75799999	11	7/24/2014	29:29.7
7319	RWS11	-122.4678945	37.93967	-0.51	3.21	55.235	2.69700009	-122.46789	37.93968	-0.43	3.21	53.796	2.77300009	11	7/24/2014	29:29.8
7320	RWS11	-122.4678967	37.93967	-0.47	3.22	55.413	2.74500003	-122.46789	37.93968	-0.4	3.22	53.993	2.82100004	11	7/24/2014	29:29.9
7321	RWS11	-122.4678999	37.93967	-0.51	3.23	55.61	2.72499996	-122.46789	37.93968	-0.4	3.23	54.211	2.83499998	11	7/24/2014	29:30.0
7322	RWS11	-122.4679021	37.93967	-0.51	3.25	55.803	2.73799998	-122.4679	37.93968	-0.43	3.25	54.408	2.81399998	11	7/24/2014	29:30.1
7323	RWS11	-122.4679043	37.93967	-0.51	3.26	55.977	2.75099999	-122.4679	37.93968	-0.43	3.26	54.6	2.82699999	11	7/24/2014	29:30.2
7324	RWS11	-122.4679065	37.93967	-0.47	3.27	56.218	2.79899994	-122.4679	37.93968	-0.43	3.27	54.841	2.84099993	11	7/24/2014	29:30.3
7325	RWS11	-122.4679087	37.93967	-0.51	3.29	56.367	2.78000003	-122.4679	37.93968	-0.43	3.29	55.015	2.85600004	11	7/24/2014	29:30.4

7326	RWS11	-122.4679109	37.93967	-0.51	3.31	56.585	2.79600006	-122.4679	37.93969	-0.48	3.31	55.296	2.82100007	11	7/24/2014	29:30.5
7327	RWS11	-122.4679131	37.93967	-0.51	3.32	56.72	2.81399995	-122.46791	37.93969	-0.43	3.32	55.452	2.88999996	11	7/24/2014	29:30.6
7328	RWS11	-122.4679153	37.93967	-0.47	3.34	56.956	2.86799994	-122.46791	37.93969	-0.4	3.34	55.71	2.94399995	11	7/24/2014	29:30.7
7329	RWS11	-122.4679174	37.93967	-0.51	3.36	57.127	2.85500008	-122.46791	37.93969	-0.48	3.36	55.929	2.88000008	11	7/24/2014	29:30.8
7330	RWS11	-122.4679196	37.93968	-0.51	3.38	57.28	2.87599999	-122.46791	37.93969	-0.43	3.38	56.1	2.95199999	11	7/24/2014	29:30.9
7331	RWS11	-122.4679228	37.93968	-0.51	3.4	57.455	2.89599997	-122.46792	37.93969	-0.52	3.4	56.322	2.88699996	11	7/24/2014	29:31.0
7332	RWS11	-122.4679249	37.93968	-0.47	3.42	57.652	2.9480001	-122.46792	37.93969	-0.43	3.42	56.493	2.9900001	11	7/24/2014	29:31.1
7333	RWS11	-122.467927	37.93968	-0.51	3.44	57.805	2.92999989	-122.46792	37.93969	-0.52	3.44	56.668	2.92099988	11	7/24/2014	29:31.2
7334	RWS11	-122.4679292	37.93968	-0.47	3.45	58.023	2.97699991	-122.46792	37.93969	-0.43	3.45	56.865	3.0189999	11	7/24/2014	29:31.3
7335	RWS11	-122.4679313	37.93968	-0.51	3.46	58.176	2.95199996	-122.46793	37.93969	-0.48	3.46	57.043	2.97699997	11	7/24/2014	29:31.4
7336	RWS11	-122.4679335	37.93968	-0.51	3.47	58.325	2.95700008	-122.46793	37.93969	-0.48	3.47	57.137	2.98200008	11	7/24/2014	29:31.5
7337	RWS11	-122.4679356	37.93968	-0.51	3.47	58.54	2.958	-122.46793	37.93969	-0.48	3.47	57.385	2.98300001	11	7/24/2014	29:31.6
7338	RWS11	-122.4679378	37.93968	-0.51	3.46	58.784	2.95400006	-122.46793	37.93969	-0.48	3.46	57.6	2.97900006	11	7/24/2014	29:31.7
7339	RWS11	-122.4679399	37.93968	-0.47	3.45	59.003	2.97999993	-122.46793	37.93969	-0.48	3.45	57.8	2.97099993	11	7/24/2014	29:31.8
7340	RWS11	-122.467942	37.93968	-0.51	3.44	59.239	2.935	-122.46794	37.93969	-0.43	3.44	58.106	3.01100001	11	7/24/2014	29:31.9
7341	RWS11	-122.4679452	37.93968	-0.47	3.43	59.479	2.95500007	-122.46794	37.9397	-0.48	3.43	58.383	2.94600007	11	7/24/2014	29:32.0
7342	RWS11	-122.4679473	37.93968	-0.47	3.41	59.658	2.93900004	-122.46794	37.9397	-0.43	3.41	58.543	2.98100004	11	7/24/2014	29:32.1
7343	RWS11	-122.4679493	37.93968	-0.51	3.4	59.89	2.88999993	-122.46794	37.9397	-0.48	3.4	58.776	2.91499993	11	7/24/2014	29:32.2
7344	RWS11	-122.4679515	37.93968	-0.47	3.38	60.109	2.91	-122.46795	37.9397	-0.43	3.38	59.012	2.95199999	11	7/24/2014	29:32.3
7345	RWS11	-122.4679536	37.93969	-0.47	3.37	60.415	2.89900008	-122.46795	37.9397	-0.48	3.37	59.234	2.89000008	11	7/24/2014	29:32.4
7346	RWS11	-122.4679557	37.93969	-0.47	3.37	60.699	2.89000002	-122.46795	37.9397	-0.43	3.37	59.497	2.93200001	11	7/24/2014	29:32.5
7347	RWS11	-122.4679579	37.93969	-0.47	3.36	61.027	2.88300005	-122.46795	37.9397	-0.48	3.36	59.759	2.87400004	11	7/24/2014	29:32.6
7348	RWS11	-122.46796	37.93969	-0.47	3.35	61.35	2.8790001	-122.46795	37.9397	-0.4	3.35	60.13	2.9550001	11	7/24/2014	29:32.7
7349	RWS11	-122.4679621	37.93969	-0.47	3.35	61.726	2.87499991	-122.46796	37.9397	-0.48	3.35	60.476	2.86599991	11	7/24/2014	29:32.8
7350	RWS11	-122.4679642	37.93969	-0.47	3.35	61.985	2.87300006	-122.46796	37.9397	-0.43	3.35	60.739	2.91500005	11	7/24/2014	29:32.9
7351	RWS11	-122.4679673	37.93969	-0.51	3.35	62.396	2.83600003	-122.46796	37.9397	-0.48	3.35	61.128	2.86100003	11	7/24/2014	29:33.0
7352	RWS11	-122.4679694	37.93969	-0.47	3.34	62.724	2.86499992	-122.46796	37.9397	-0.43	3.34	61.474	2.90699992	11	7/24/2014	29:33.1
7353	RWS11	-122.4679716	37.93969	-0.47	3.33	63.073	2.85900012	-122.46797	37.9397	-0.48	3.33	61.827	2.85000011	11	7/24/2014	29:33.2
7354	RWS11	-122.4679737	37.93969	-0.47	3.33	63.379	2.85000005	-122.46797	37.9397	-0.4	3.33	62.181	2.92600006	11	7/24/2014	29:33.3
7355	RWS11	-122.4679759	37.93969	-0.47	3.31	63.795	2.8389999	-122.46797	37.9397	-0.48	3.31	62.544	2.82999989	11	7/24/2014	29:33.4
7356	RWS11	-122.467978	37.93969	-0.47	3.3	64.209	2.82700005	-122.46797	37.9397	-0.48	3.3	62.941	2.81800005	11	7/24/2014	29:33.5
7357	RWS11	-122.4679802	37.93969	-0.47	3.29	64.573	2.8159999	-122.46797	37.93971	-0.43	3.29	63.317	2.85799989	11	7/24/2014	29:33.6
7358	RWS11	-122.4679824	37.93969	-0.47	3.28	64.927	2.8059999	-122.46798	37.93971	-0.48	3.28	63.659	2.7969999	11	7/24/2014	29:33.7

7359	RWS11	-122.4679845	37.93969	-0.47	3.28	65.36	2.8000001	-122.46798	37.93971	-0.43	3.28	64.092	2.8420001	11	7/24/2014	29:33.8
7360	RWS11	-122.4679866	37.93969	-0.47	3.27	65.687	2.79599991	-122.46798	37.93971	-0.48	3.27	64.463	2.78699991	11	7/24/2014	29:33.9
7361	RWS11	-122.4679898	37.9397	-0.47	3.27	66.015	2.79599991	-122.46798	37.93971	-0.43	3.27	64.773	2.83799991	11	7/24/2014	29:34.0
7362	RWS11	-122.467992	37.9397	-0.47	3.27	66.407	2.79800001	-122.46799	37.93971	-0.48	3.27	65.118	2.789	11	7/24/2014	29:34.1
7363	RWS11	-122.4679941	37.9397	-0.47	3.28	66.754	2.80199996	-122.46799	37.93971	-0.43	3.28	65.442	2.84399995	11	7/24/2014	29:34.2
7364	RWS11	-122.4679963	37.9397	-0.47	3.28	67.016	2.80899993	-122.46799	37.93971	-0.43	3.28	65.705	2.85099992	11	7/24/2014	29:34.3
7365	RWS11	-122.4679985	37.9397	-0.51	3.29	67.318	2.78300005	-122.46799	37.93971	-0.48	3.29	65.989	2.80800006	11	7/24/2014	29:34.4
7366	RWS11	-122.4680007	37.9397	-0.42	3.3	67.65	2.87599996	-122.46799	37.93971	-0.48	3.3	66.295	2.81599995	11	7/24/2014	29:34.5
7367	RWS11	-122.4680029	37.9397	-0.42	3.31	67.908	2.88400009	-122.468	37.93971	-0.43	3.31	66.557	2.87500009	11	7/24/2014	29:34.6
7368	RWS11	-122.4680051	37.9397	-0.42	3.31	68.175	2.8899999	-122.468	37.93971	-0.43	3.31	66.859	2.88099989	11	7/24/2014	29:34.7
7369	RWS11	-122.4680073	37.9397	-0.42	3.32	68.366	2.89400008	-122.468	37.93971	-0.43	3.32	67.08	2.88500008	11	7/24/2014	29:34.8
7370	RWS11	-122.4680094	37.9397	-0.47	3.32	68.516	2.84400001	-122.468	37.93971	-0.48	3.32	67.248	2.83500001	11	7/24/2014	29:34.9
7371	RWS11	-122.4680127	37.9397	-0.42	3.32	68.647	2.89400008	-122.46801	37.93971	-0.48	3.32	67.405	2.83400008	11	7/24/2014	29:35.0
7372	RWS11	-122.4680148	37.9397	-0.39	3.32	68.8	2.92600006	-122.46801	37.93972	-0.43	3.32	67.554	2.88200006	11	7/24/2014	29:35.1
7373	RWS11	-122.468017	37.9397	-0.47	3.31	68.844	2.83600011	-122.46801	37.93972	-0.43	3.31	67.689	2.87800011	11	7/24/2014	29:35.2
7374	RWS11	-122.4680191	37.9397	-0.47	3.31	68.975	2.83199993	-122.46801	37.93972	-0.43	3.31	67.794	2.87399992	11	7/24/2014	29:35.3
7375	RWS11	-122.4680214	37.9397	-0.42	3.3	69.062	2.87999991	-122.46802	37.93972	-0.43	3.3	67.925	2.8709999	11	7/24/2014	29:35.4
7376	RWS11	-122.4680236	37.93971	-0.42	3.3	69.105	2.87700012	-122.46802	37.93972	-0.43	3.3	67.946	2.86800012	11	7/24/2014	29:35.5
7377	RWS11	-122.4680257	37.93971	-0.47	3.3	69.146	2.82499996	-122.46802	37.93972	-0.43	3.3	67.987	2.86699995	11	7/24/2014	29:35.6
7378	RWS11	-122.4680279	37.93971	-0.39	3.3	69.255	2.91200012	-122.46802	37.93972	-0.43	3.3	68.035	2.86800012	11	7/24/2014	29:35.7
7379	RWS11	-122.4680301	37.93971	-0.42	3.3	69.277	2.87800005	-122.46802	37.93972	-0.48	3.3	68.135	2.81800005	11	7/24/2014	29:35.8
7380	RWS11	-122.4680322	37.93971	-0.39	3.31	69.32	2.91600007	-122.46803	37.93972	-0.43	3.31	68.162	2.87200007	11	7/24/2014	29:35.9
7381	RWS11	-122.4680354	37.93971	-0.42	3.31	69.342	2.88299993	-122.46803	37.93972	-0.48	3.31	68.227	2.82299992	11	7/24/2014	29:36.0
7382	RWS11	-122.4680375	37.93971	-0.39	3.31	69.386	2.92000002	-122.46803	37.93972	-0.43	3.31	68.271	2.87600002	11	7/24/2014	29:36.1
7383	RWS11	-122.4680397	37.93971	-0.42	3.31	69.43	2.88599995	-122.46803	37.93972	-0.43	3.31	68.38	2.87699994	11	7/24/2014	29:36.2
7384	RWS11	-122.4680418	37.93971	-0.39	3.31	69.513	2.92300004	-122.46804	37.93972	-0.43	3.31	68.499	2.87900004	11	7/24/2014	29:36.3
7385	RWS11	-122.4680439	37.93971	-0.42	3.31	69.618	2.88800004	-122.46804	37.93972	-0.48	3.31	68.638	2.82800004	11	7/24/2014	29:36.4
7386	RWS11	-122.4680461	37.93971	-0.39	3.31	69.775	2.92300004	-122.46804	37.93972	-0.43	3.31	68.813	2.87900004	11	7/24/2014	29:36.5
7387	RWS11	-122.4680482	37.93971	-0.42	3.31	69.906	2.88800004	-122.46804	37.93973	-0.43	3.31	68.984	2.87900004	11	7/24/2014	29:36.6
7388	RWS11	-122.4680504	37.93971	-0.42	3.31	70.016	2.88700011	-122.46804	37.93973	-0.48	3.31	69.228	2.82700011	11	7/24/2014	29:36.7
7389	RWS11	-122.4680525	37.93971	-0.42	3.31	70.207	2.88599995	-122.46805	37.93973	-0.48	3.31	69.424	2.82599995	11	7/24/2014	29:36.8
7390	RWS11	-122.4680546	37.93971	-0.39	3.31	70.34	2.92000002	-122.46805	37.93973	-0.4	3.31	69.565	2.91000003	11	7/24/2014	29:36.9
7391	RWS11	-122.4680577	37.93972	-0.42	3.31	70.488	2.88500002	-122.46805	37.93973	-0.43	3.31	69.727	2.87600002	11	7/24/2014	29:37.0

7392	RWS11	-122.4680598	37.93972	-0.47	3.31	70.667	2.83499995	-122.46805	37.93973	-0.43	3.31	69.871	2.87699994	11	7/24/2014	29:37.1
7393	RWS11	-122.4680619	37.93972	-0.51	3.31	70.816	2.80300003	-122.46806	37.93973	-0.48	3.31	69.985	2.82800004	11	7/24/2014	29:37.2
7394	RWS11	-122.468064	37.93972	-0.42	3.32	70.969	2.89199999	-122.46806	37.93973	-0.43	3.32	70.12	2.88299999	11	7/24/2014	29:37.3
7395	RWS11	-122.4680661	37.93972	-0.42	3.32	71.126	2.8970001	-122.46806	37.93973	-0.48	3.32	70.247	2.8370001	11	7/24/2014	29:37.4
7396	RWS11	-122.4680682	37.93972	-0.42	3.33	71.274	2.90299991	-122.46806	37.93973	-0.43	3.33	70.465	2.8939999	11	7/24/2014	29:37.5
7397	RWS11	-122.4680703	37.93972	-0.47	3.33	71.424	2.85700002	-122.46806	37.93973	-0.48	3.33	70.615	2.84800002	11	7/24/2014	29:37.6
7398	RWS11	-122.4680724	37.93972	-0.42	3.34	71.598	2.91100004	-122.46807	37.93973	-0.43	3.34	70.789	2.90200004	11	7/24/2014	29:37.7
7399	RWS11	-122.4680745	37.93972	-0.47	3.34	71.773	2.8619999	-122.46807	37.93973	-0.52	3.34	71.008	2.81899989	11	7/24/2014	29:37.8
7400	RWS11	-122.4680766	37.93972	-0.42	3.34	71.926	2.9129999	-122.46807	37.93973	-0.43	3.34	71.183	2.90399989	11	7/24/2014	29:37.9
7401	RWS11	-122.4680797	37.93972	-0.42	3.34	72.079	2.91199997	-122.46807	37.93973	-0.48	3.34	71.414	2.85199997	11	7/24/2014	29:38.0
7402	RWS11	-122.4680818	37.93972	-0.39	3.33	72.189	2.94500011	-122.46808	37.93974	-0.43	3.33	71.584	2.90100011	11	7/24/2014	29:38.1
7403	RWS11	-122.4680838	37.93972	-0.42	3.33	72.429	2.90700009	-122.46808	37.93974	-0.48	3.33	71.777	2.84700009	11	7/24/2014	29:38.2
7404	RWS11	-122.4680859	37.93972	-0.39	3.33	72.578	2.93900007	-122.46808	37.93974	-0.48	3.33	71.939	2.84400007	11	7/24/2014	29:38.3
7405	RWS11	-122.468088	37.93972	-0.42	3.33	72.639	2.90100005	-122.46808	37.93974	-0.43	3.33	72.075	2.89200005	11	7/24/2014	29:38.4
7406	RWS11	-122.4680901	37.93972	-0.42	3.32	72.797	2.8970001	-122.46808	37.93974	-0.48	3.32	72.228	2.8370001	11	7/24/2014	29:38.5
7407	RWS11	-122.4680922	37.93973	-0.42	3.32	72.928	2.89199999	-122.46809	37.93974	-0.43	3.32	72.359	2.88299999	11	7/24/2014	29:38.6
7408	RWS11	-122.4680943	37.93973	-0.39	3.31	73.098	2.92099994	-122.46809	37.93974	-0.48	3.31	72.595	2.82599995	11	7/24/2014	29:38.7
7409	RWS11	-122.4680964	37.93973	-0.42	3.3	73.255	2.87999991	-122.46809	37.93974	-0.52	3.3	72.795	2.78599989	11	7/24/2014	29:38.8
7410	RWS11	-122.4680985	37.93973	-0.39	3.3	73.29	2.90700001	-122.46809	37.93974	-0.48	3.3	72.918	2.81200001	11	7/24/2014	29:38.9
7411	RWS11	-122.4681016	37.93973	-0.47	3.29	73.47	2.81300011	-122.4681	37.93974	-0.43	3.29	73.076	2.85500011	11	7/24/2014	29:39.0
7412	RWS11	-122.4681036	37.93973	-0.47	3.28	73.596	2.80499998	-122.4681	37.93974	-0.48	3.28	73.224	2.79599997	11	7/24/2014	29:39.1
7413	RWS11	-122.4681057	37.93973	-0.42	3.27	73.771	2.84900001	-122.4681	37.93974	-0.43	3.27	73.338	2.84	11	7/24/2014	29:39.2
7414	RWS11	-122.4681079	37.93973	-0.39	3.27	73.973	2.87899989	-122.4681	37.93974	-0.48	3.27	73.596	2.78399989	11	7/24/2014	29:39.3
7415	RWS11	-122.4681099	37.93973	-0.51	3.26	74.121	2.75400001	-122.4681	37.93974	-0.43	3.26	73.771	2.83000001	11	7/24/2014	29:39.4
7416	RWS11	-122.4681121	37.93973	-0.39	3.26	74.208	2.87000006	-122.46811	37.93974	-0.48	3.26	73.88	2.77500007	11	7/24/2014	29:39.5
7417	RWS11	-122.4681142	37.93973	-0.42	3.26	74.446	2.83299997	-122.46811	37.93974	-0.48	3.26	74.1	2.77299997	11	7/24/2014	29:39.6
7418	RWS11	-122.4681163	37.93973	-0.47	3.26	74.664	2.78199998	-122.46811	37.93975	-0.48	3.26	74.288	2.77299997	11	7/24/2014	29:39.7
7419	RWS11	-122.4681184	37.93973	-0.51	3.26	74.838	2.75000006	-122.46811	37.93975	-0.52	3.26	74.445	2.74100006	11	7/24/2014	29:39.8
7420	RWS11	-122.4681205	37.93973	-0.42	3.26	75.035	2.83900002	-122.46811	37.93975	-0.48	3.26	74.664	2.77900001	11	7/24/2014	29:39.9
7421	RWS11	-122.4681236	37.93973	-0.47	3.27	75.211	2.79299989	-122.46812	37.93975	-0.57	3.27	74.882	2.69899988	11	7/24/2014	29:40.0
7422	RWS11	-122.4681258	37.93974	-0.39	3.28	75.406	2.8860001	-122.46812	37.93975	-0.52	3.28	75.074	2.75700009	11	7/24/2014	29:40.1
7423	RWS11	-122.4681279	37.93974	-0.42	3.28	75.62	2.859	-122.46812	37.93975	-0.52	3.28	75.248	2.76499999	11	7/24/2014	29:40.2
7424	RWS11	-122.46813	37.93974	-0.39	3.29	75.797	2.90099996	-122.46812	37.93975	-0.48	3.29	75.468	2.80599996	11	7/24/2014	29:40.3

7425	RWS11	-122.4681322	37.93974	-0.42	3.3	75.95	2.8740001	-122.46813	37.93975	-0.43	3.3	75.634	2.8650001	11	7/24/2014	29:40.4
7426	RWS11	-122.4681344	37.93974	-0.39	3.31	76.076	2.91600007	-122.46813	37.93975	-0.48	3.31	75.748	2.82100007	11	7/24/2014	29:40.5
7427	RWS11	-122.4681365	37.93974	-0.39	3.31	76.256	2.92399997	-122.46813	37.93975	-0.52	3.31	75.949	2.79499996	11	7/24/2014	29:40.6
7428	RWS11	-122.4681387	37.93974	-0.39	3.32	76.404	2.93099993	-122.46813	37.93975	-0.43	3.32	76.093	2.88699993	11	7/24/2014	29:40.7
7429	RWS11	-122.4681408	37.93974	-0.42	3.33	76.54	2.90299991	-122.46813	37.93975	-0.52	3.33	76.233	2.8089999	11	7/24/2014	29:40.8
7430	RWS11	-122.4681429	37.93974	-0.39	3.33	76.753	2.94300002	-122.46814	37.93975	-0.52	3.33	76.463	2.81400001	11	7/24/2014	29:40.9
7431	RWS11	-122.4681461	37.93974	-0.47	3.33	76.946	2.85900012	-122.46814	37.93975	-0.43	3.33	76.64	2.90100011	11	7/24/2014	29:41.0
7432	RWS11	-122.4681483	37.93974	-0.42	3.33	77.034	2.91000012	-122.46814	37.93975	-0.48	3.33	76.727	2.85000011	11	7/24/2014	29:41.1
7433	RWS11	-122.4681504	37.93974	-0.42	3.33	77.165	2.90599993	-122.46814	37.93975	-0.52	3.33	76.875	2.81199992	11	7/24/2014	29:41.2
7434	RWS11	-122.4681526	37.93974	-0.39	3.32	77.405	2.93499988	-122.46815	37.93976	-0.48	3.32	76.99	2.83999988	11	7/24/2014	29:41.3
7435	RWS11	-122.4681548	37.93974	-0.39	3.32	77.515	2.92799991	-122.46815	37.93976	-0.48	3.32	77.186	2.83299991	11	7/24/2014	29:41.4
7436	RWS11	-122.4681569	37.93974	-0.39	3.31	77.688	2.91900009	-122.46815	37.93976	-0.52	3.31	77.377	2.79000008	11	7/24/2014	29:41.5
7437	RWS11	-122.4681591	37.93974	-0.39	3.3	77.904	2.9090001	-122.46815	37.93976	-0.52	3.3	77.641	2.78000009	11	7/24/2014	29:41.6
7438	RWS11	-122.4681613	37.93975	-0.39	3.29	78.101	2.89900011	-122.46816	37.93976	-0.48	3.29	77.768	2.80400011	11	7/24/2014	29:41.7
7439	RWS11	-122.4681634	37.93975	-0.42	3.28	78.254	2.85200003	-122.46816	37.93976	-0.48	3.28	77.92	2.79200003	11	7/24/2014	29:41.8
7440	RWS11	-122.4681655	37.93975	-0.47	3.26	78.516	2.78700009	-122.46816	37.93976	-0.43	3.26	78.122	2.82900009	11	7/24/2014	29:41.9
7441	RWS11	-122.4681688	37.93975	-0.39	3.25	78.686	2.85899991	-122.46816	37.93976	-0.48	3.25	78.309	2.76399991	11	7/24/2014	29:42.0
7442	RWS11	-122.4681709	37.93975	-0.39	3.23	78.866	2.84499997	-122.46817	37.93976	-0.48	3.23	78.516	2.74999997	11	7/24/2014	29:42.1
7443	RWS11	-122.468173	37.93975	-0.42	3.22	79.057	2.79699996	-122.46817	37.93976	-0.48	3.22	78.642	2.73699996	11	7/24/2014	29:42.2
7444	RWS11	-122.4681752	37.93975	-0.39	3.21	79.234	2.82000011	-122.46817	37.93976	-0.48	3.21	78.813	2.72500011	11	7/24/2014	29:42.3
7445	RWS11	-122.4681773	37.93975	-0.51	3.2	79.447	2.68899995	-122.46817	37.93976	-0.52	3.2	79.04	2.67999995	11	7/24/2014	29:42.4
7446	RWS11	-122.4681795	37.93975	-0.39	3.19	79.556	2.79999989	-122.46817	37.93976	-0.48	3.19	79.157	2.70499989	11	7/24/2014	29:42.5
7447	RWS11	-122.4681816	37.93975	-0.51	3.18	79.714	2.67299992	-122.46818	37.93976	-0.52	3.18	79.32	2.66399992	11	7/24/2014	29:42.6
7448	RWS11	-122.4681838	37.93975	-0.39	3.18	79.928	2.78899997	-122.46818	37.93976	-0.48	3.18	79.49	2.69399998	11	7/24/2014	29:42.7
7449	RWS11	-122.4681859	37.93975	-0.51	3.18	80.103	2.66800004	-122.46818	37.93976	-0.48	3.18	79.687	2.69300005	11	7/24/2014	29:42.8
7450	RWS11	-122.4681881	37.93975	-0.42	3.18	80.342	2.75499991	-122.46818	37.93977	-0.48	3.18	79.926	2.6949999	11	7/24/2014	29:42.9
7451	RWS11	-122.4681912	37.93975	-0.42	3.18	80.536	2.75900009	-122.46819	37.93977	-0.52	3.18	80.098	2.66500008	11	7/24/2014	29:43.0
7452	RWS11	-122.4681933	37.93975	-0.42	3.19	80.733	2.76600006	-122.46819	37.93977	-0.48	3.19	80.404	2.70600006	11	7/24/2014	29:43.1
7453	RWS11	-122.4681954	37.93975	-0.51	3.2	80.886	2.68999988	-122.46819	37.93977	-0.52	3.2	80.557	2.68099988	11	7/24/2014	29:43.2
7454	RWS11	-122.4681976	37.93976	-0.42	3.21	80.995	2.78600004	-122.46819	37.93977	-0.52	3.21	80.618	2.69200003	11	7/24/2014	29:43.3
7455	RWS11	-122.4681998	37.93976	-0.47	3.22	81.252	2.74699989	-122.46819	37.93977	-0.52	3.22	80.819	2.70399988	11	7/24/2014	29:43.4
7456	RWS11	-122.4682019	37.93976	-0.42	3.24	81.409	2.81200007	-122.4682	37.93977	-0.48	3.24	81.015	2.75200006	11	7/24/2014	29:43.5
7457	RWS11	-122.4682041	37.93976	-0.47	3.25	81.641	2.7770001	-122.4682	37.93977	-0.52	3.25	81.248	2.73400009	11	7/24/2014	29:43.6

7458	RWS11	-122.4682062	37.93976	-0.42	3.27	81.822	2.84500006	-122.4682	37.93977	-0.48	3.27	81.449	2.78500006	11	7/24/2014	29:43.7
7459	RWS11	-122.4682084	37.93976	-0.51	3.29	82.057	2.77700001	-122.4682	37.93977	-0.52	3.29	81.602	2.76800001	11	7/24/2014	29:43.8
7460	RWS11	-122.4682105	37.93976	-0.42	3.3	82.215	2.87899998	-122.4682	37.93977	-0.52	3.3	81.833	2.78499997	11	7/24/2014	29:43.9
7461	RWS11	-122.4682137	37.93976	-0.42	3.32	82.363	2.89299992	-122.46821	37.93977	-0.48	3.32	81.996	2.83299991	11	7/24/2014	29:44.0
7462	RWS11	-122.4682158	37.93976	-0.42	3.33	82.494	2.90299991	-122.46821	37.93977	-0.48	3.33	82.144	2.84299991	11	7/24/2014	29:44.1
7463	RWS11	-122.468218	37.93976	-0.47	3.33	82.652	2.8560001	-122.46821	37.93977	-0.52	3.33	82.258	2.81300008	11	7/24/2014	29:44.2
7464	RWS11	-122.4682202	37.93976	-0.42	3.33	82.905	2.90700009	-122.46821	37.93977	-0.48	3.33	82.484	2.84700009	11	7/24/2014	29:44.3
7465	RWS11	-122.4682223	37.93976	-0.42	3.33	83.102	2.90100005	-122.46822	37.93978	-0.52	3.33	82.642	2.80700004	11	7/24/2014	29:44.4
7466	RWS11	-122.4682245	37.93976	-0.39	3.32	83.299	2.92799991	-122.46822	37.93978	-0.48	3.32	82.839	2.83299991	11	7/24/2014	29:44.5
7467	RWS11	-122.4682267	37.93976	-0.42	3.31	83.474	2.88299993	-122.46822	37.93978	-0.48	3.31	82.992	2.82299992	11	7/24/2014	29:44.6
7468	RWS11	-122.4682289	37.93976	-0.42	3.3	83.692	2.87299994	-122.46822	37.93978	-0.48	3.3	83.266	2.81299993	11	7/24/2014	29:44.7
7469	RWS11	-122.468231	37.93976	-0.42	3.29	83.817	2.86400011	-122.46823	37.93978	-0.52	3.29	83.407	2.7700001	11	7/24/2014	29:44.8
7470	RWS11	-122.4682332	37.93977	-0.42	3.28	83.994	2.85599998	-122.46823	37.93978	-0.48	3.28	83.622	2.79599997	11	7/24/2014	29:44.9
7471	RWS11	-122.4682364	37.93977	-0.47	3.28	84.125	2.8000001	-122.46823	37.93978	-0.48	3.28	83.77	2.7910001	11	7/24/2014	29:45.0
7472	RWS11	-122.4682386	37.93977	-0.47	3.27	84.235	2.79800001	-122.46823	37.93978	-0.48	3.27	83.841	2.789	11	7/24/2014	29:45.1
7473	RWS11	-122.4682407	37.93977	-0.42	3.28	84.404	2.8510001	-122.46824	37.93978	-0.48	3.28	83.983	2.7910001	11	7/24/2014	29:45.2
7474	RWS11	-122.4682429	37.93977	-0.42	3.28	84.519	2.85400012	-122.46824	37.93978	-0.43	3.28	84.163	2.84500012	11	7/24/2014	29:45.3
7475	RWS11	-122.4682451	37.93977	-0.47	3.28	84.732	2.80899993	-122.46824	37.93978	-0.57	3.28	84.426	2.71499991	11	7/24/2014	29:45.4
7476	RWS11	-122.4682473	37.93977	-0.47	3.29	84.89	2.81700006	-122.46824	37.93978	-0.48	3.29	84.556	2.80800006	11	7/24/2014	29:45.5
7477	RWS11	-122.4682495	37.93977	-0.42	3.3	85.012	2.87700012	-122.46824	37.93978	-0.48	3.3	84.662	2.81700012	11	7/24/2014	29:45.6
7478	RWS11	-122.4682517	37.93977	-0.42	3.31	85.209	2.88700011	-122.46825	37.93978	-0.48	3.31	84.886	2.82700011	11	7/24/2014	29:45.7
7479	RWS11	-122.4682539	37.93977	-0.47	3.32	85.28	2.84700003	-122.46825	37.93978	-0.48	3.32	84.985	2.83800003	11	7/24/2014	29:45.8
7480	RWS11	-122.468256	37.93977	-0.47	3.33	85.428	2.85700002	-122.46825	37.93978	-0.48	3.33	85.099	2.84800002	11	7/24/2014	29:45.9
7481	RWS11	-122.4682593	37.93977	-0.42	3.34	85.581	2.91599992	-122.46825	37.93979	-0.48	3.34	85.236	2.85599992	11	7/24/2014	29:46.0
7482	RWS11	-122.4682615	37.93977	-0.39	3.35	85.668	2.95600003	-122.46826	37.93979	-0.48	3.35	85.373	2.86100003	11	7/24/2014	29:46.1
7483	RWS11	-122.4682636	37.93977	-0.47	3.35	85.821	2.87099996	-122.46826	37.93979	-0.48	3.35	85.471	2.86199996	11	7/24/2014	29:46.2
7484	RWS11	-122.4682658	37.93977	-0.42	3.34	85.932	2.91899994	-122.46826	37.93979	-0.43	3.34	85.636	2.90999994	11	7/24/2014	29:46.3
7485	RWS11	-122.468268	37.93978	-0.42	3.34	86.058	2.9129999	-122.46826	37.93979	-0.52	3.34	85.817	2.81899989	11	7/24/2014	29:46.4
7486	RWS11	-122.4682702	37.93978	-0.42	3.33	86.211	2.905	-122.46826	37.93979	-0.4	3.33	85.97	2.93000001	11	7/24/2014	29:46.5
7487	RWS11	-122.4682724	37.93978	-0.42	3.32	86.314	2.89500001	-122.46827	37.93979	-0.4	3.32	86.139	2.92000002	11	7/24/2014	29:46.6
7488	RWS11	-122.4682746	37.93978	-0.42	3.31	86.451	2.88400009	-122.46827	37.93979	-0.4	3.31	86.32	2.9090001	11	7/24/2014	29:46.7
7489	RWS11	-122.4682768	37.93978	-0.39	3.3	86.56	2.9090001	-122.46827	37.93979	-0.43	3.3	86.451	2.8650001	11	7/24/2014	29:46.8
7490	RWS11	-122.468279	37.93978	-0.39	3.29	86.689	2.89900011	-122.46827	37.93979	-0.43	3.29	86.53	2.85500011	11	7/24/2014	29:46.9

7491	RWS11	-122.4682822	37.93978	-0.42	3.28	86.842	2.85400012	-122.46828	37.93979	-0.4	3.28	86.644	2.87900013	11	7/24/2014	29:47.0
7492	RWS11	-122.4682843	37.93978	-0.39	3.27	87.038	2.88000005	-122.46828	37.93979	-0.43	3.27	86.858	2.83600006	11	7/24/2014	29:47.1
7493	RWS11	-122.4682865	37.93978	-0.39	3.26	87.295	2.87199992	-122.46828	37.93979	-0.35	3.26	87.06	2.91199991	11	7/24/2014	29:47.2
7494	RWS11	-122.4682887	37.93978	-0.39	3.26	87.476	2.86600012	-122.46828	37.93979	-0.35	3.26	87.278	2.90600011	11	7/24/2014	29:47.3
7495	RWS11	-122.4682909	37.93978	-0.39	3.25	87.71	2.86199993	-122.46829	37.93979	-0.35	3.25	87.47	2.90199992	11	7/24/2014	29:47.4
7496	RWS11	-122.4682931	37.93978	-0.39	3.25	87.928	2.861	-122.46829	37.93979	-0.4	3.25	87.687	2.85100001	11	7/24/2014	29:47.5
7497	RWS11	-122.4682952	37.93978	-0.39	3.25	88.193	2.86199993	-122.46829	37.9398	-0.35	3.25	87.908	2.90199992	11	7/24/2014	29:47.6
7498	RWS11	-122.4682974	37.93978	-0.39	3.26	88.406	2.86600012	-122.46829	37.9398	-0.43	3.26	88.143	2.82200012	11	7/24/2014	29:47.7
7499	RWS11	-122.4682996	37.93978	-0.39	3.26	88.603	2.87000006	-122.46829	37.9398	-0.35	3.26	88.362	2.91000006	11	7/24/2014	29:47.8
7500	RWS11	-122.4683017	37.93978	-0.34	3.27	88.821	2.92700011	-122.4683	37.9398	-0.35	3.27	88.602	2.9160001	11	7/24/2014	29:47.9
7501	RWS11	-122.4683049	37.93978	-0.39	3.27	89.062	2.88099998	-122.4683	37.9398	-0.43	3.27	88.799	2.83699998	11	7/24/2014	29:48.0
7502	RWS11	-122.4683071	37.93979	-0.34	3.28	89.259	2.9370001	-122.4683	37.9398	-0.4	3.28	89.083	2.87600011	11	7/24/2014	29:48.1
7503	RWS11	-122.4683092	37.93979	-0.39	3.28	89.476	2.89000005	-122.4683	37.9398	-0.4	3.28	89.257	2.88000005	11	7/24/2014	29:48.2
7504	RWS11	-122.4683114	37.93979	-0.39	3.28	89.714	2.8919999	-122.46831	37.9398	-0.35	3.28	89.577	2.93199989	11	7/24/2014	29:48.3
7505	RWS11	-122.4683135	37.93979	-0.39	3.28	89.948	2.89300007	-122.46831	37.9398	-0.43	3.28	89.757	2.84900007	11	7/24/2014	29:48.4
7506	RWS11	-122.4683157	37.93979	-0.34	3.28	90.173	2.94400007	-122.46831	37.9398	-0.35	3.28	90.058	2.93300006	11	7/24/2014	29:48.5
7507	RWS11	-122.4683179	37.93979	-0.39	3.28	90.342	2.89099997	-122.46831	37.9398	-0.4	3.28	90.254	2.88099998	11	7/24/2014	29:48.6
7508	RWS11	-122.4683201	37.93979	-0.34	3.28	90.567	2.93800002	-122.46831	37.9398	-0.35	3.28	90.473	2.92700002	11	7/24/2014	29:48.7
7509	RWS11	-122.4683222	37.93979	-0.39	3.27	90.778	2.88199991	-122.46832	37.9398	-0.4	3.27	90.778	2.87199992	11	7/24/2014	29:48.8
7510	RWS11	-122.4683243	37.93979	-0.39	3.27	91.044	2.87700003	-122.46832	37.9398	-0.35	3.27	91.059	2.91700003	11	7/24/2014	29:48.9
7511	RWS11	-122.4683275	37.93979	-0.39	3.26	91.191	2.87300009	-122.46832	37.9398	-0.4	3.26	91.272	2.86300009	11	7/24/2014	29:49.0
7512	RWS11	-122.4683296	37.93979	-0.39	3.26	91.387	2.8689999	-122.46832	37.9398	-0.35	3.26	91.562	2.90899989	11	7/24/2014	29:49.1
7513	RWS11	-122.4683318	37.93979	-0.39	3.26	91.628	2.86600012	-122.46833	37.9398	-0.4	3.26	91.868	2.85600013	11	7/24/2014	29:49.2
7514	RWS11	-122.4683339	37.93979	-0.39	3.25	91.825	2.86300009	-122.46833	37.93981	-0.35	3.25	92.075	2.90300009	11	7/24/2014	29:49.3
7515	RWS11	-122.4683361	37.93979	-0.39	3.25	92.043	2.861	-122.46833	37.93981	-0.4	3.25	92.371	2.85100001	11	7/24/2014	29:49.4
7516	RWS11	-122.4683382	37.93979	-0.34	3.25	92.283	2.90999991	-122.46833	37.93981	-0.4	3.25	92.561	2.84899992	11	7/24/2014	29:49.5
7517	RWS11	-122.4683404	37.93979	-0.39	3.25	92.542	2.85700005	-122.46834	37.93981	-0.35	3.25	92.908	2.89700004	11	7/24/2014	29:49.6
7518	RWS11	-122.4683426	37.93979	-0.34	3.24	92.777	2.90500003	-122.46834	37.93981	-0.4	3.24	93.148	2.84400004	11	7/24/2014	29:49.7
7519	RWS11	-122.4683447	37.9398	-0.39	3.24	92.936	2.85199994	-122.46834	37.93981	-0.35	3.24	93.323	2.89199993	11	7/24/2014	29:49.8
7520	RWS11	-122.4683468	37.9398	-0.39	3.24	93.192	2.85000008	-122.46834	37.93981	-0.35	3.24	93.629	2.89000008	11	7/24/2014	29:49.9
7521	RWS11	-122.46835	37.9398	-0.39	3.24	93.395	2.85000008	-122.46834	37.93981	-0.35	3.24	93.87	2.89000008	11	7/24/2014	29:50.0
7522	RWS11	-122.4683522	37.9398	-0.34	3.24	93.607	2.90200001	-122.46835	37.93981	-0.35	3.24	94.103	2.891	11	7/24/2014	29:50.1
7523	RWS11	-122.4683544	37.9398	-0.39	3.24	93.779	2.85400003	-122.46835	37.93981	-0.4	3.24	94.303	2.84400004	11	7/24/2014	29:50.2

7524	RWS11	-122.4683565	37.9398	-0.39	3.25	93.981	2.85700005	-122.46835	37.93981	-0.32	3.25	94.5	2.93100005	11	7/24/2014	29:50.3
7525	RWS11	-122.4683587	37.9398	-0.39	3.25	94.172	2.86199993	-122.46835	37.93981	-0.35	3.25	94.669	2.90199992	11	7/24/2014	29:50.4
7526	RWS11	-122.4683609	37.9398	-0.39	3.26	94.369	2.86700004	-122.46836	37.93981	-0.35	3.26	94.866	2.90700004	11	7/24/2014	29:50.5
7527	RWS11	-122.4683631	37.9398	-0.39	3.26	94.544	2.87300009	-122.46836	37.93981	-0.35	3.26	95.062	2.91300008	11	7/24/2014	29:50.6
7528	RWS11	-122.4683653	37.9398	-0.39	3.27	94.588	2.88000005	-122.46836	37.93981	-0.35	3.27	95.09	2.92000005	11	7/24/2014	29:50.7
7529	RWS11	-122.4683675	37.9398	-0.39	3.28	94.761	2.88700002	-122.46836	37.93981	-0.4	3.28	95.242	2.87700003	11	7/24/2014	29:50.8
7530	RWS11	-122.4683697	37.9398	-0.39	3.28	94.912	2.8919999	-122.46836	37.93981	-0.32	3.28	95.355	2.9659999	11	7/24/2014	29:50.9
7531	RWS11	-122.4683729	37.9398	-0.39	3.29	95.065	2.89700001	-122.46837	37.93982	-0.4	3.29	95.458	2.88700002	11	7/24/2014	29:51.0
7532	RWS11	-122.4683751	37.9398	-0.39	3.29	95.13	2.89900011	-122.46837	37.93982	-0.35	3.29	95.561	2.9390001	11	7/24/2014	29:51.1
7533	RWS11	-122.4683773	37.9398	-0.39	3.29	95.24	2.90000004	-122.46837	37.93982	-0.4	3.29	95.589	2.89000005	11	7/24/2014	29:51.2
7534	RWS11	-122.4683795	37.9398	-0.39	3.29	95.343	2.89799994	-122.46837	37.93982	-0.35	3.29	95.736	2.93799993	11	7/24/2014	29:51.3
7535	RWS11	-122.4683817	37.93981	-0.39	3.29	95.458	2.89600009	-122.46838	37.93982	-0.4	3.29	95.851	2.8860001	11	7/24/2014	29:51.4
7536	RWS11	-122.4683839	37.93981	-0.39	3.28	95.523	2.89300007	-122.46838	37.93982	-0.35	3.28	95.91	2.93300006	11	7/24/2014	29:51.5
7537	RWS11	-122.4683861	37.93981	-0.39	3.28	95.579	2.89000005	-122.46838	37.93982	-0.4	3.28	95.907	2.88000005	11	7/24/2014	29:51.6
7538	RWS11	-122.4683882	37.93981	-0.39	3.28	95.717	2.88700002	-122.46838	37.93982	-0.35	3.28	95.979	2.92700002	11	7/24/2014	29:51.7
7539	RWS11	-122.4683904	37.93981	-0.39	3.28	95.87	2.8860001	-122.46839	37.93982	-0.35	3.28	96.06	2.92600009	11	7/24/2014	29:51.8
7540	RWS11	-122.4683926	37.93981	-0.39	3.28	95.973	2.8860001	-122.46839	37.93982	-0.35	3.28	96.132	2.92600009	11	7/24/2014	29:51.9
7541	RWS11	-122.4683958	37.93981	-0.39	3.28	96.11	2.88700002	-122.46839	37.93982	-0.35	3.28	96.213	2.92700002	11	7/24/2014	29:52.0
7542	RWS11	-122.468398	37.93981	-0.39	3.28	96.235	2.89000005	-122.46839	37.93982	-0.32	3.28	96.322	2.96400005	11	7/24/2014	29:52.1
7543	RWS11	-122.4684001	37.93981	-0.42	3.28	96.325	2.859	-122.46839	37.93982	-0.4	3.28	96.45	2.884	11	7/24/2014	29:52.2
7544	RWS11	-122.4684023	37.93981	-0.39	3.29	96.472	2.89700001	-122.4684	37.93982	-0.32	3.29	96.537	2.97100002	11	7/24/2014	29:52.3
7545	RWS11	-122.4684045	37.93981	-0.42	3.29	96.581	2.86299995	-122.4684	37.93982	-0.35	3.29	96.696	2.93799993	11	7/24/2014	29:52.4
7546	RWS11	-122.4684067	37.93981	-0.39	3.29	96.719	2.89799994	-122.4684	37.93982	-0.4	3.29	96.865	2.88799995	11	7/24/2014	29:52.5
7547	RWS11	-122.4684089	37.93981	-0.39	3.29	96.865	2.89600009	-122.4684	37.93982	-0.43	3.29	97.084	2.85200009	11	7/24/2014	29:52.6
7548	RWS11	-122.4684111	37.93981	-0.39	3.28	96.953	2.8919999	-122.46841	37.93983	-0.35	3.28	97.149	2.93199989	11	7/24/2014	29:52.7
7549	RWS11	-122.4684132	37.93981	-0.42	3.28	97.061	2.8510001	-122.46841	37.93983	-0.32	3.28	97.258	2.9600001	11	7/24/2014	29:52.8
7550	RWS11	-122.4684154	37.93981	-0.39	3.27	97.255	2.87899989	-122.46841	37.93983	-0.35	3.27	97.364	2.91899988	11	7/24/2014	29:52.9
7551	RWS11	-122.4684186	37.93981	-0.39	3.26	97.408	2.87099999	-122.46841	37.93983	-0.35	3.26	97.473	2.91099998	11	7/24/2014	29:53.0
7552	RWS11	-122.4684208	37.93981	-0.34	3.25	97.561	2.91299993	-122.46842	37.93983	-0.4	3.25	97.577	2.85199994	11	7/24/2014	29:53.1
7553	RWS11	-122.4684229	37.93982	-0.39	3.24	97.714	2.8530001	-122.46842	37.93983	-0.4	3.24	97.773	2.84300011	11	7/24/2014	29:53.2
7554	RWS11	-122.4684251	37.93982	-0.39	3.23	97.889	2.84300011	-122.46842	37.93983	-0.35	3.23	97.905	2.88300011	11	7/24/2014	29:53.3
7555	RWS11	-122.4684273	37.93982	-0.39	3.22	97.998	2.83400005	-122.46842	37.93983	-0.4	3.22	98.101	2.82400006	11	7/24/2014	29:53.4
7556	RWS11	-122.4684295	37.93982	-0.34	3.21	98.126	2.87699991	-122.46842	37.93983	-0.32	3.21	98.213	2.89999992	11	7/24/2014	29:53.5

7557	RWS11	-122.4684317	37.93982	-0.39	3.21	98.316	2.82000011	-122.46843	37.93983	-0.35	3.21	98.419	2.8600001	11	7/24/2014	29:53.6
7558	RWS11	-122.4684339	37.93982	-0.39	3.2	98.41	2.81599993	-122.46843	37.93983	-0.35	3.2	98.584	2.85599992	11	7/24/2014	29:53.7
7559	RWS11	-122.4684361	37.93982	-0.39	3.2	98.607	2.81199998	-122.46843	37.93983	-0.35	3.2	98.775	2.85199997	11	7/24/2014	29:53.8
7560	RWS11	-122.4684382	37.93982	-0.34	3.2	98.753	2.86099988	-122.46843	37.93983	-0.35	3.2	98.95	2.84999987	11	7/24/2014	29:53.9
7561	RWS11	-122.4684414	37.93982	-0.39	3.2	98.935	2.8070001	-122.46844	37.93983	-0.35	3.2	99.125	2.84700009	11	7/24/2014	29:54.0
7562	RWS11	-122.4684436	37.93982	-0.39	3.19	99.06	2.80500001	-122.46844	37.93983	-0.35	3.19	99.3	2.845	11	7/24/2014	29:54.1
7563	RWS11	-122.4684457	37.93982	-0.39	3.19	99.216	2.80299991	-122.46844	37.93983	-0.4	3.19	99.444	2.79299992	11	7/24/2014	29:54.2
7564	RWS11	-122.4684479	37.93982	-0.34	3.19	99.406	2.85099989	-122.46844	37.93983	-0.35	3.19	99.647	2.83999988	11	7/24/2014	29:54.3
7565	RWS11	-122.46845	37.93982	-0.39	3.19	99.581	2.79800004	-122.46844	37.93983	-0.35	3.19	99.815	2.83800003	11	7/24/2014	29:54.4
7566	RWS11	-122.4684522	37.93982	-0.34	3.19	99.719	2.84800011	-122.46845	37.93984	-0.32	3.19	99.997	2.87100011	11	7/24/2014	29:54.5
7567	RWS11	-122.4684543	37.93982	-0.39	3.18	99.844	2.79500002	-122.46845	37.93984	-0.35	3.18	100.17	2.83500001	11	7/24/2014	29:54.6
7568	RWS11	-122.4684565	37.93982	-0.34	3.18	100.02	2.84399992	-122.46845	37.93984	-0.35	3.18	100.3	2.83299991	11	7/24/2014	29:54.7
7569	RWS11	-122.4684586	37.93982	-0.39	3.18	100.15	2.7899999	-122.46845	37.93984	-0.35	3.18	100.45	2.82999989	11	7/24/2014	29:54.8
7570	RWS11	-122.4684607	37.93982	-0.34	3.17	100.3	2.83600003	-122.46846	37.93984	-0.32	3.17	100.58	2.85900003	11	7/24/2014	29:54.9
7571	RWS11	-122.4684639	37.93983	-0.39	3.17	100.47	2.77800006	-122.46846	37.93984	-0.35	3.17	100.75	2.81800005	11	7/24/2014	29:55.0
7572	RWS11	-122.468466	37.93983	-0.34	3.16	100.61	2.81999999	-122.46846	37.93984	-0.32	3.16	100.93	2.84299999	11	7/24/2014	29:55.1
7573	RWS11	-122.4684681	37.93983	-0.39	3.15	100.82	2.75699991	-122.46846	37.93984	-0.32	3.15	101.19	2.83099991	11	7/24/2014	29:55.2
7574	RWS11	-122.4684703	37.93983	-0.34	3.13	100.89	2.79600006	-122.46847	37.93984	-0.35	3.13	101.32	2.78500006	11	7/24/2014	29:55.3
7575	RWS11	-122.4684724	37.93983	-0.39	3.12	100.97	2.73200005	-122.46847	37.93984	-0.32	3.12	101.41	2.80600005	11	7/24/2014	29:55.4
7576	RWS11	-122.4684746	37.93983	-0.34	3.11	101.17	2.76900011	-122.46847	37.93984	-0.28	3.11	101.61	2.82600012	11	7/24/2014	29:55.5
7577	RWS11	-122.4684767	37.93983	-0.39	3.09	101.28	2.7050001	-122.46847	37.93984	-0.28	3.09	101.75	2.81300011	11	7/24/2014	29:55.6
7578	RWS11	-122.4684789	37.93983	-0.39	3.08	101.41	2.69300002	-122.46847	37.93984	-0.28	3.08	101.86	2.80100003	11	7/24/2014	29:55.7
7579	RWS11	-122.468481	37.93983	-0.39	3.07	101.49	2.68499988	-122.46848	37.93984	-0.28	3.07	101.96	2.79299989	11	7/24/2014	29:55.8
7580	RWS11	-122.4684831	37.93983	-0.34	3.07	101.54	2.73100001	-122.46848	37.93984	-0.28	3.07	102.06	2.78800002	11	7/24/2014	29:55.9
7581	RWS11	-122.4684863	37.93983	-0.34	3.07	101.69	2.72799999	-122.46848	37.93984	-0.28	3.07	102.21	2.785	11	7/24/2014	29:56.0
7582	RWS11	-122.4684884	37.93983	-0.34	3.07	101.73	2.72700006	-122.46848	37.93984	-0.28	3.07	102.32	2.78400007	11	7/24/2014	29:56.1
7583	RWS11	-122.4684905	37.93983	-0.34	3.07	101.77	2.72700006	-122.46849	37.93984	-0.28	3.07	102.41	2.78400007	11	7/24/2014	29:56.2
7584	RWS11	-122.4684927	37.93983	-0.34	3.06	101.8	2.72599989	-122.46849	37.93985	-0.23	3.06	102.49	2.83399989	11	7/24/2014	29:56.3
7585	RWS11	-122.4684948	37.93983	-0.39	3.06	101.88	2.67300004	-122.46849	37.93985	-0.35	3.06	102.59	2.71300003	11	7/24/2014	29:56.4
7586	RWS11	-122.468497	37.93983	-0.34	3.06	101.84	2.71899992	-122.46849	37.93985	-0.23	3.06	102.69	2.82699992	11	7/24/2014	29:56.5
7587	RWS11	-122.4684991	37.93983	-0.39	3.05	101.88	2.66000003	-122.46849	37.93985	-0.35	3.05	102.82	2.70000002	11	7/24/2014	29:56.6
7588	RWS11	-122.4685013	37.93983	-0.34	3.04	101.8	2.70000011	-122.4685	37.93985	-0.28	3.04	102.89	2.75700012	11	7/24/2014	29:56.7
7589	RWS11	-122.4685034	37.93983	-0.34	3.03	101.77	2.6870001	-122.4685	37.93985	-0.32	3.03	102.99	2.7100001	11	7/24/2014	29:56.8

7590	RWS11	-122.4685056	37.93984	-0.34	3.01	101.75	2.67199999	-122.4685	37.93985	-0.23	3.01	103.12	2.77999999	11	7/24/2014	29:56.9
7591	RWS11	-122.4685087	37.93984	-0.39	2.99	101.68	2.60499996	-122.4685	37.93985	-0.23	2.99	103.21	2.76399995	11	7/24/2014	29:57.0
7592	RWS11	-122.4685109	37.93984	-0.34	2.98	101.69	2.64200002	-122.46851	37.93985	-0.28	2.98	103.28	2.69900003	11	7/24/2014	29:57.1
7593	RWS11	-122.4685131	37.93984	-0.34	2.97	101.66	2.62900001	-122.46851	37.93985	-0.28	2.97	103.39	2.68600002	11	7/24/2014	29:57.2
7594	RWS11	-122.4685153	37.93984	-0.34	2.96	101.62	2.61999995	-122.46851	37.93985	-0.28	2.96	103.49	2.67699996	11	7/24/2014	29:57.3
7595	RWS11	-122.4685175	37.93984	-0.34	2.95	101.64	2.61299998	-122.46851	37.93985	-0.28	2.95	103.61	2.66999999	11	7/24/2014	29:57.4
7596	RWS11	-122.4685197	37.93984	-0.34	2.95	101.61	2.60999995	-122.46851	37.93985	-0.28	2.95	103.69	2.66699997	11	7/24/2014	29:57.5
7597	RWS11	-122.4685219	37.93984	-0.34	2.95	101.66	2.61099988	-122.46852	37.93985	-0.28	2.95	103.84	2.66799989	11	7/24/2014	29:57.6
7598	RWS11	-122.4685241	37.93984	-0.34	2.95	101.72	2.6139999	-122.46852	37.93985	-0.28	2.95	103.96	2.67099991	11	7/24/2014	29:57.7
7599	RWS11	-122.4685262	37.93984	-0.34	2.96	101.81	2.61900002	-122.46852	37.93985	-0.23	2.96	104.13	2.72700001	11	7/24/2014	29:57.8
7600	RWS11	-122.4685284	37.93984	-0.34	2.96	101.94	2.62599999	-122.46852	37.93985	-0.28	2.96	104.25	2.683	11	7/24/2014	29:57.9
7601	RWS11	-122.4685316	37.93984	-0.34	2.97	102.16	2.63500005	-122.46853	37.93985	-0.23	2.97	104.47	2.74300005	11	7/24/2014	29:58.0
7602	RWS11	-122.4685338	37.93984	-0.3	2.98	102.42	2.67800012	-122.46853	37.93986	-0.23	2.98	104.66	2.75200011	11	7/24/2014	29:58.1
7603	RWS11	-122.468536	37.93984	-0.34	2.99	102.72	2.6540001	-122.46853	37.93986	-0.23	2.99	104.89	2.7620001	11	7/24/2014	29:58.2
7604	RWS11	-122.4685382	37.93984	-0.3	3	103.09	2.69699994	-122.46853	37.93986	-0.2	3	105.19	2.80499993	11	7/24/2014	29:58.3
7605	RWS11	-122.4685404	37.93984	-0.34	3.01	103.44	2.67299992	-122.46854	37.93986	-0.23	3.01	105.36	2.78099991	11	7/24/2014	29:58.4
7606	RWS11	-122.4685426	37.93984	-0.34	3.02	103.9	2.68400007	-122.46854	37.93986	-0.2	3.02	105.57	2.82600008	11	7/24/2014	29:58.5
7607	RWS11	-122.4685448	37.93984	-0.34	3.03	104.4	2.69499999	-122.46854	37.93986	-0.23	3.03	105.91	2.80299999	11	7/24/2014	29:58.6
7608	RWS11	-122.468547	37.93985	-0.3	3.05	104.86	2.74200001	-122.46854	37.93986	-0.2	3.05	106.09	2.85000001	11	7/24/2014	29:58.7
7609	RWS11	-122.4685492	37.93985	-0.34	3.06	105.36	2.72100002	-122.46854	37.93986	-0.23	3.06	106.34	2.82900001	11	7/24/2014	29:58.8
7610	RWS11	-122.4685514	37.93985	-0.3	3.07	105.82	2.76899996	-122.46855	37.93986	-0.2	3.07	106.54	2.87699996	11	7/24/2014	29:58.9
7611	RWS11	-122.4685546	37.93985	-0.34	3.09	106.23	2.74899989	-122.46855	37.93986	-0.28	3.09	106.74	2.8059999	11	7/24/2014	29:59.0
7612	RWS11	-122.4685568	37.93985	-0.3	3.1	106.69	2.79599991	-122.46855	37.93986	-0.23	3.1	106.93	2.8699999	11	7/24/2014	29:59.1
7613	RWS11	-122.468559	37.93985	-0.34	3.11	107.04	2.77300006	-122.46855	37.93986	-0.28	3.11	107.17	2.83000007	11	7/24/2014	29:59.2
7614	RWS11	-122.4685612	37.93985	-0.3	3.12	107.41	2.8159999	-122.46856	37.93986	-0.23	3.12	107.44	2.88999988	11	7/24/2014	29:59.3
7615	RWS11	-122.4685633	37.93985	-0.34	3.13	107.71	2.78900009	-122.46856	37.93986	-0.28	3.13	107.67	2.84600011	11	7/24/2014	29:59.4
7616	RWS11	-122.4685655	37.93985	-0.34	3.13	107.96	2.7949999	-122.46856	37.93986	-0.28	3.13	107.87	2.85199991	11	7/24/2014	29:59.5
7617	RWS11	-122.4685677	37.93985	-0.34	3.14	108.17	2.80099994	-122.46856	37.93986	-0.28	3.14	108.04	2.85799995	11	7/24/2014	29:59.6
7618	RWS11	-122.4685699	37.93985	-0.34	3.14	108.43	2.80600005	-122.46857	37.93986	-0.28	3.14	108.3	2.86300007	11	7/24/2014	29:59.7
7619	RWS11	-122.4685721	37.93985	-0.34	3.15	108.65	2.81300002	-122.46857	37.93986	-0.28	3.15	108.5	2.87000003	11	7/24/2014	29:59.8
7620	RWS11	-122.4685743	37.93985	-0.34	3.16	108.8	2.82099992	-122.46857	37.93986	-0.28	3.16	108.69	2.87799993	11	7/24/2014	29:59.9
7621	RWS11	-122.4685775	37.93985	-0.39	3.17	108.95	2.77899998	-122.46857	37.93987	-0.32	3.17	108.86	2.85299999	11	7/24/2014	30:00.0
7622	RWS11	-122.4685797	37.93985	-0.34	3.18	109.1	2.83999997	-122.46857	37.93987	-0.28	3.18	109.05	2.89699998	11	7/24/2014	30:00.1

7623	RWS11	-122.4685819	37.93985	-0.34	3.19	109.21	2.85099989	-122.46858	37.93987	-0.28	3.19	109.22	2.9079999	11	7/24/2014	30:00.2
7624	RWS11	-122.4685841	37.93985	-0.34	3.2	109.39	2.86099988	-122.46858	37.93987	-0.28	3.2	109.43	2.91799989	11	7/24/2014	30:00.3
7625	RWS11	-122.4685862	37.93985	-0.39	3.21	109.5	2.81899995	-122.46858	37.93987	-0.28	3.21	109.58	2.92699996	11	7/24/2014	30:00.4
7626	RWS11	-122.4685885	37.93985	-0.39	3.22	109.61	2.82700008	-122.46858	37.93987	-0.23	3.22	109.71	2.98600008	11	7/24/2014	30:00.5
7627	RWS11	-122.4685907	37.93986	-0.39	3.22	109.75	2.83299989	-122.46859	37.93987	-0.32	3.22	109.89	2.90699989	11	7/24/2014	30:00.6
7628	RWS11	-122.4685929	37.93986	-0.39	3.23	109.84	2.838	-122.46859	37.93987	-0.28	3.23	110.03	2.94600001	11	7/24/2014	30:00.7
7629	RWS11	-122.4685951	37.93986	-0.39	3.23	109.98	2.84100002	-122.46859	37.93987	-0.32	3.23	110.19	2.91500002	11	7/24/2014	30:00.8
7630	RWS11	-122.4685972	37.93986	-0.39	3.23	110.12	2.84300011	-122.46859	37.93987	-0.28	3.23	110.3	2.95100012	11	7/24/2014	30:00.9
7631	RWS11	-122.4686005	37.93986	-0.39	3.23	110.19	2.84400004	-122.4686	37.93987	-0.32	3.23	110.39	2.91800004	11	7/24/2014	30:01.0
7632	RWS11	-122.4686027	37.93986	-0.39	3.23	110.34	2.84300011	-122.4686	37.93987	-0.28	3.23	110.55	2.95100012	11	7/24/2014	30:01.1
7633	RWS11	-122.4686049	37.93986	-0.42	3.23	110.46	2.8050001	-122.4686	37.93987	-0.32	3.23	110.65	2.91400009	11	7/24/2014	30:01.2
7634	RWS11	-122.4686071	37.93986	-0.39	3.22	110.58	2.83599991	-122.4686	37.93987	-0.23	3.22	110.8	2.9949999	11	7/24/2014	30:01.3
7635	RWS11	-122.4686093	37.93986	-0.42	3.22	110.72	2.79600003	-122.4686	37.93987	-0.32	3.22	110.95	2.90500003	11	7/24/2014	30:01.4
7636	RWS11	-122.4686115	37.93986	-0.39	3.21	110.84	2.82400006	-122.46861	37.93987	-0.23	3.21	111.08	2.98300005	11	7/24/2014	30:01.5
7637	RWS11	-122.4686137	37.93986	-0.42	3.2	110.93	2.78	-122.46861	37.93987	-0.32	3.2	111.19	2.889	11	7/24/2014	30:01.6
7638	RWS11	-122.468616	37.93986	-0.39	3.19	111.04	2.80500001	-122.46861	37.93987	-0.28	3.19	111.34	2.91300002	11	7/24/2014	30:01.7
7639	RWS11	-122.4686182	37.93986	-0.42	3.18	111.18	2.76000002	-122.46861	37.93987	-0.32	3.18	111.5	2.86900002	11	7/24/2014	30:01.8
7640	RWS11	-122.4686204	37.93985	-0.39	3.17	111.32	2.78599995	-122.46862	37.93987	-0.28	3.17	111.67	2.89399996	11	7/24/2014	30:01.9
7641	RWS11	-122.4686237	37.93986	-0.42	3.17	111.43	2.74399999	-122.46862	37.93988	-0.35	3.17	111.77	2.81899998	11	7/24/2014	30:02.0
7642	RWS11	-122.4686259	37.93986	-0.39	3.16	111.58	2.77400011	-122.46862	37.93988	-0.28	3.16	111.95	2.88200012	11	7/24/2014	30:02.1
7643	RWS11	-122.4686281	37.93986	-0.39	3.16	111.69	2.77100009	-122.46862	37.93988	-0.32	3.16	112.03	2.84500009	11	7/24/2014	30:02.2
7644	RWS11	-122.4686303	37.93986	-0.39	3.16	111.8	2.76999992	-122.46863	37.93988	-0.28	3.16	112.21	2.87799993	11	7/24/2014	30:02.3
7645	RWS11	-122.4686326	37.93986	-0.39	3.16	111.95	2.76999992	-122.46863	37.93988	-0.28	3.16	112.39	2.87799993	11	7/24/2014	30:02.4
7646	RWS11	-122.4686348	37.93987	-0.39	3.16	112.08	2.77100009	-122.46863	37.93988	-0.28	3.16	112.48	2.8790001	11	7/24/2014	30:02.5
7647	RWS11	-122.4686371	37.93987	-0.39	3.16	112.23	2.77299994	-122.46863	37.93988	-0.32	3.16	112.65	2.84699994	11	7/24/2014	30:02.6
7648	RWS11	-122.4686393	37.93987	-0.39	3.16	112.38	2.77500004	-122.46863	37.93988	-0.28	3.16	112.75	2.88300005	11	7/24/2014	30:02.7
7649	RWS11	-122.4686415	37.93987	-0.39	3.17	112.5	2.77699989	-122.46864	37.93988	-0.28	3.17	112.9	2.8849999	11	7/24/2014	30:02.8
7650	RWS11	-122.4686437	37.93987	-0.39	3.17	112.65	2.77999991	-122.46864	37.93988	-0.32	3.17	113.1	2.85399991	11	7/24/2014	30:02.9
7651	RWS11	-122.468647	37.93987	-0.39	3.17	112.74	2.7840001	-122.46864	37.93988	-0.28	3.17	113.19	2.89200011	11	7/24/2014	30:03.0
7652	RWS11	-122.4686491	37.93987	-0.39	3.18	112.84	2.79100007	-122.46864	37.93988	-0.28	3.18	113.35	2.89900008	11	7/24/2014	30:03.1
7653	RWS11	-122.4686513	37.93987	-0.39	3.19	112.95	2.79999989	-122.46865	37.93988	-0.32	3.19	113.48	2.87399989	11	7/24/2014	30:03.2
7654	RWS11	-122.4686535	37.93987	-0.39	3.2	113.05	2.81199998	-122.46865	37.93988	-0.28	3.2	113.59	2.91999999	11	7/24/2014	30:03.3
7655	RWS11	-122.4686557	37.93987	-0.39	3.21	113.17	2.82499999	-122.46865	37.93988	-0.28	3.21	113.77	2.933	11	7/24/2014	30:03.4

7656	RWS11	-122.4686579	37.93987	-0.34	3.23	113.34	2.88999993	-122.46865	37.93988	-0.28	3.23	113.95	2.94699994	11	7/24/2014	30:03.5
7657	RWS11	-122.4686601	37.93987	-0.39	3.24	113.43	2.8530001	-122.46866	37.93988	-0.28	3.24	114.01	2.96100011	11	7/24/2014	30:03.6
7658	RWS11	-122.4686622	37.93987	-0.39	3.25	113.53	2.86499995	-122.46866	37.93988	-0.32	3.25	114.15	2.93899995	11	7/24/2014	30:03.7
7659	RWS11	-122.4686644	37.93987	-0.39	3.26	113.67	2.87300009	-122.46866	37.93988	-0.28	3.26	114.3	2.9810001	11	7/24/2014	30:03.8
7660	RWS11	-122.4686666	37.93987	-0.39	3.27	113.78	2.87600011	-122.46866	37.93989	-0.28	3.27	114.36	2.98400012	11	7/24/2014	30:03.9
7661	RWS11	-122.4686698	37.93987	-0.39	3.26	113.9	2.87400001	-122.46867	37.93989	-0.32	3.26	114.47	2.94800001	11	7/24/2014	30:04.0
7662	RWS11	-122.4686719	37.93987	-0.34	3.26	114.03	2.91700011	-122.46867	37.93989	-0.28	3.26	114.58	2.97400013	11	7/24/2014	30:04.1
7663	RWS11	-122.4686741	37.93987	-0.39	3.24	114.14	2.85199994	-122.46867	37.93989	-0.28	3.24	114.67	2.95999995	11	7/24/2014	30:04.2
7664	RWS11	-122.4686763	37.93987	-0.34	3.22	114.27	2.88500005	-122.46867	37.93989	-0.28	3.22	114.79	2.94200006	11	7/24/2014	30:04.3
7665	RWS11	-122.4686784	37.93987	-0.39	3.2	114.37	2.815	-122.46867	37.93989	-0.32	3.2	114.91	2.889	11	7/24/2014	30:04.4
7666	RWS11	-122.4686807	37.93988	-0.34	3.19	114.4	2.84800011	-122.46868	37.93989	-0.32	3.19	114.9	2.87100011	11	7/24/2014	30:04.5
7667	RWS11	-122.4686829	37.93988	-0.34	3.17	114.53	2.83200008	-122.46868	37.93989	-0.28	3.17	115.03	2.88900009	11	7/24/2014	30:04.6
7668	RWS11	-122.4686851	37.93988	-0.34	3.16	114.64	2.82099992	-122.46868	37.93989	-0.28	3.16	115.18	2.87799993	11	7/24/2014	30:04.7
7669	RWS11	-122.4686872	37.93988	-0.34	3.15	114.68	2.81399995	-122.46868	37.93989	-0.32	3.15	115.25	2.83699995	11	7/24/2014	30:04.8
7670	RWS11	-122.4686894	37.93988	-0.34	3.15	114.77	2.8120001	-122.46868	37.93989	-0.28	3.15	115.34	2.86900011	11	7/24/2014	30:04.9
7671	RWS11	-122.4686927	37.93988	-0.34	3.15	114.87	2.81500012	-122.46869	37.93989	-0.28	3.15	115.42	2.87200013	11	7/24/2014	30:05.0
7672	RWS11	-122.4686949	37.93988	-0.3	3.16	114.99	2.85499993	-122.46869	37.93989	-0.28	3.16	115.49	2.87799993	11	7/24/2014	30:05.1
7673	RWS11	-122.468697	37.93988	-0.39	3.17	115.14	2.78100008	-122.46869	37.93989	-0.28	3.17	115.59	2.88900009	11	7/24/2014	30:05.2
7674	RWS11	-122.4686993	37.93988	-0.3	3.18	115.29	2.87799993	-122.46869	37.93989	-0.28	3.18	115.8	2.90099993	11	7/24/2014	30:05.3
7675	RWS11	-122.4687015	37.93988	-0.34	3.2	115.45	2.8580001	-122.4687	37.93989	-0.28	3.2	115.94	2.91500011	11	7/24/2014	30:05.4
7676	RWS11	-122.4687038	37.93988	-0.3	3.21	115.48	2.90399995	-122.4687	37.93989	-0.28	3.21	115.94	2.92699996	11	7/24/2014	30:05.5
7677	RWS11	-122.468706	37.93988	-0.34	3.22	115.62	2.87999994	-122.4687	37.93989	-0.28	3.22	116.08	2.93699995	11	7/24/2014	30:05.6
7678	RWS11	-122.4687082	37.93988	-0.34	3.22	115.8	2.88500005	-122.4687	37.93989	-0.23	3.22	116.31	2.99300005	11	7/24/2014	30:05.7
7679	RWS11	-122.4687105	37.93988	-0.34	3.22	115.86	2.88500005	-122.46871	37.9399	-0.32	3.22	116.36	2.90800005	11	7/24/2014	30:05.8
7680	RWS11	-122.4687127	37.93988	-0.3	3.22	116.01	2.91500011	-122.46871	37.9399	-0.32	3.22	116.53	2.9040001	11	7/24/2014	30:05.9
7681	RWS11	-122.468716	37.93988	-0.34	3.21	116.16	2.87100011	-122.46871	37.9399	-0.28	3.21	116.64	2.92800012	11	7/24/2014	30:06.0
7682	RWS11	-122.4687182	37.93988	-0.3	3.2	116.27	2.89300004	-122.46871	37.9399	-0.28	3.2	116.81	2.91600004	11	7/24/2014	30:06.1
7683	RWS11	-122.4687204	37.93988	-0.34	3.18	116.4	2.84399992	-122.46872	37.9399	-0.28	3.18	116.94	2.90099993	11	7/24/2014	30:06.2
7684	RWS11	-122.4687226	37.93988	-0.3	3.16	116.51	2.86099997	-122.46872	37.9399	-0.23	3.16	117.06	2.93499996	11	7/24/2014	30:06.3
7685	RWS11	-122.4687249	37.93989	-0.34	3.15	116.66	2.81099993	-122.46872	37.9399	-0.32	3.15	117.18	2.83399993	11	7/24/2014	30:06.4
7686	RWS11	-122.4687271	37.93989	-0.3	3.13	116.75	2.82899991	-122.46872	37.9399	-0.28	3.13	117.31	2.85199991	11	7/24/2014	30:06.5
7687	RWS11	-122.4687294	37.93989	-0.34	3.12	116.94	2.7790001	-122.46872	37.9399	-0.28	3.12	117.52	2.83600011	11	7/24/2014	30:06.6
7688	RWS11	-122.4687316	37.93989	-0.3	3.1	117.07	2.79899994	-122.46873	37.9399	-0.23	3.1	117.64	2.87299992	11	7/24/2014	30:06.7

7689	RWS11	-122.4687339	37.93989	-0.3	3.09	117.2	2.78599992	-122.46873	37.9399	-0.28	3.09	117.83	2.80899993	11	7/24/2014	30:06.8
7690	RWS11	-122.4687361	37.93989	-0.3	3.08	117.4	2.77400008	-122.46873	37.9399	-0.28	3.08	117.98	2.79700008	11	7/24/2014	30:06.9
7691	RWS11	-122.4687394	37.93989	-0.3	3.07	117.51	2.76400009	-122.46873	37.9399	-0.28	3.07	118.14	2.78700009	11	7/24/2014	30:07.0
7692	RWS11	-122.4687416	37.93989	-0.3	3.06	117.73	2.75599995	-122.46874	37.9399	-0.23	3.06	118.28	2.82999994	11	7/24/2014	30:07.1
7693	RWS11	-122.4687438	37.93989	-0.34	3.06	117.88	2.71700007	-122.46874	37.9399	-0.23	3.06	118.49	2.82500006	11	7/24/2014	30:07.2
7694	RWS11	-122.4687461	37.93989	-0.3	3.05	118.07	2.74800006	-122.46874	37.9399	-0.28	3.05	118.68	2.77100006	11	7/24/2014	30:07.3
7695	RWS11	-122.4687483	37.93989	-0.34	3.05	118.24	2.71400005	-122.46874	37.9399	-0.28	3.05	118.91	2.77100006	11	7/24/2014	30:07.4
7696	RWS11	-122.4687505	37.93989	-0.34	3.05	118.34	2.7159999	-122.46875	37.9399	-0.28	3.05	118.99	2.77299991	11	7/24/2014	30:07.5
7697	RWS11	-122.4687528	37.93989	-0.34	3.06	118.49	2.72100002	-122.46875	37.9399	-0.28	3.06	119.18	2.77800003	11	7/24/2014	30:07.6
7698	RWS11	-122.4687551	37.93989	-0.3	3.07	118.64	2.76199999	-122.46875	37.93991	-0.23	3.07	119.36	2.83599998	11	7/24/2014	30:07.7
7699	RWS11	-122.4687573	37.93989	-0.34	3.08	118.77	2.73799998	-122.46875	37.93991	-0.28	3.08	119.46	2.79499999	11	7/24/2014	30:07.8
7700	RWS11	-122.4687595	37.93989	-0.34	3.09	118.89	2.75000006	-122.46875	37.93991	-0.23	3.09	119.63	2.85800005	11	7/24/2014	30:07.9
7701	RWS11	-122.4687628	37.93989	-0.34	3.1	119.03	2.76499993	-122.46876	37.93991	-0.28	3.1	119.77	2.82199994	11	7/24/2014	30:08.0
7702	RWS11	-122.468765	37.93989	-0.34	3.12	119.2	2.78099996	-122.46876	37.93991	-0.28	3.12	119.88	2.83799997	11	7/24/2014	30:08.1
7703	RWS11	-122.4687672	37.93989	-0.34	3.14	119.32	2.79899991	-122.46876	37.93991	-0.28	3.14	120	2.85599992	11	7/24/2014	30:08.2
7704	RWS11	-122.4687695	37.9399	-0.34	3.15	119.46	2.81499994	-122.46876	37.93991	-0.23	3.15	120.15	2.92299995	11	7/24/2014	30:08.3
7705	RWS11	-122.4687717	37.9399	-0.34	3.16	119.55	2.82799995	-122.46877	37.93991	-0.28	3.16	120.25	2.88499996	11	7/24/2014	30:08.4
7706	RWS11	-122.468774	37.9399	-0.34	3.18	119.68	2.83900011	-122.46877	37.93991	-0.23	3.18	120.38	2.94700012	11	7/24/2014	30:08.5
7707	RWS11	-122.4687762	37.9399	-0.34	3.18	119.82	2.84600008	-122.46877	37.93991	-0.31	3.18	120.47	2.86900008	11	7/24/2014	30:08.6
7708	RWS11	-122.4687785	37.9399	-0.34	3.19	119.85	2.8490001	-122.46877	37.93991	-0.28	3.19	120.55	2.90600011	11	7/24/2014	30:08.7
7709	RWS11	-122.4687807	37.9399	-0.34	3.18	119.99	2.847	-122.46878	37.93991	-0.28	3.18	120.64	2.90400001	11	7/24/2014	30:08.8
7710	RWS11	-122.4687829	37.9399	-0.34	3.18	120.09	2.84199989	-122.46878	37.93991	-0.28	3.18	120.82	2.8989999	11	7/24/2014	30:08.9
7711	RWS11	-122.4687861	37.9399	-0.34	3.17	120.22	2.83399999	-122.46878	37.93991	-0.31	3.17	120.88	2.85699999	11	7/24/2014	30:09.0
7712	RWS11	-122.4687883	37.9399	-0.34	3.16	120.29	2.824	-122.46878	37.93991	-0.28	3.16	120.98	2.88100001	11	7/24/2014	30:09.1
7713	RWS11	-122.4687905	37.9399	-0.34	3.15	120.44	2.81300008	-122.46879	37.93991	-0.28	3.15	121.07	2.87000009	11	7/24/2014	30:09.2
7714	RWS11	-122.4687927	37.9399	-0.34	3.14	120.53	2.80199993	-122.46879	37.93991	-0.28	3.14	121.15	2.85899994	11	7/24/2014	30:09.3
7715	RWS11	-122.4687948	37.9399	-0.34	3.13	120.68	2.79000008	-122.46879	37.93991	-0.28	3.13	121.34	2.84700009	11	7/24/2014	30:09.4
7716	RWS11	-122.468797	37.9399	-0.3	3.12	120.72	2.81200001	-122.46879	37.93991	-0.28	3.12	121.37	2.83500001	11	7/24/2014	30:09.5
7717	RWS11	-122.4687992	37.9399	-0.34	3.1	120.89	2.76599991	-122.46879	37.93991	-0.28	3.1	121.56	2.82299992	11	7/24/2014	30:09.6
7718	RWS11	-122.4688014	37.9399	-0.3	3.09	120.92	2.789	-122.4688	37.93992	-0.28	3.09	121.59	2.81200001	11	7/24/2014	30:09.7
7719	RWS11	-122.4688036	37.9399	-0.34	3.08	120.94	2.74599993	-122.4688	37.93992	-0.28	3.08	121.68	2.80299994	11	7/24/2014	30:09.8
7720	RWS11	-122.4688057	37.9399	-0.34	3.08	121.11	2.73800004	-122.4688	37.93992	-0.28	3.08	121.73	2.79500005	11	7/24/2014	30:09.9
7721	RWS11	-122.468809	37.9399	-0.34	3.07	121.17	2.73100007	-122.4688	37.93992	-0.31	3.07	121.84	2.75400007	11	7/24/2014	30:10.0

7722	RWS11	-122.4688111	37.9399	-0.3	3.06	121.2	2.75800011	-122.46881	37.93992	-0.28	3.06	121.87	2.78100011	11	7/24/2014	30:10.1
7723	RWS11	-122.4688133	37.93991	-0.34	3.06	121.31	2.71800005	-122.46881	37.93992	-0.23	3.06	121.9	2.82600006	11	7/24/2014	30:10.2
7724	RWS11	-122.4688155	37.93991	-0.3	3.05	121.46	2.74800012	-122.46881	37.93992	-0.28	3.05	122.07	2.77100012	11	7/24/2014	30:10.3
7725	RWS11	-122.4688177	37.93991	-0.34	3.05	121.48	2.71200001	-122.46881	37.93992	-0.28	3.05	122.14	2.76900002	11	7/24/2014	30:10.4
7726	RWS11	-122.4688199	37.93991	-0.3	3.05	121.52	2.74600002	-122.46882	37.93992	-0.23	3.05	122.15	2.82000002	11	7/24/2014	30:10.5
7727	RWS11	-122.4688221	37.93991	-0.34	3.05	121.66	2.71599996	-122.46882	37.93992	-0.28	3.05	122.25	2.77299997	11	7/24/2014	30:10.6
7728	RWS11	-122.4688243	37.93991	-0.34	3.06	121.74	2.72299993	-122.46882	37.93992	-0.23	3.06	122.33	2.83099994	11	7/24/2014	30:10.7
7729	RWS11	-122.4688264	37.93991	-0.34	3.07	121.77	2.73299992	-122.46882	37.93992	-0.31	3.07	122.4	2.75599992	11	7/24/2014	30:10.8
7730	RWS11	-122.4688286	37.93991	-0.3	3.08	121.87	2.77999994	-122.46882	37.93992	-0.23	3.08	122.54	2.85399994	11	7/24/2014	30:10.9
7731	RWS11	-122.4688318	37.93991	-0.34	3.1	121.89	2.76199996	-122.46883	37.93992	-0.31	3.1	122.63	2.78499997	11	7/24/2014	30:11.0
7732	RWS11	-122.468834	37.93991	-0.39	3.12	121.91	2.72900009	-122.46883	37.93992	-0.31	3.12	122.69	2.80300009	11	7/24/2014	30:11.1
7733	RWS11	-122.4688361	37.93991	-0.42	3.14	121.98	2.71299991	-122.46883	37.93992	-0.35	3.14	122.75	2.78799993	11	7/24/2014	30:11.2
7734	RWS11	-122.4688384	37.93991	-0.3	3.16	121.95	2.85499999	-122.46883	37.93992	-0.31	3.16	122.72	2.84399998	11	7/24/2014	30:11.3
7735	RWS11	-122.4688406	37.93991	-0.34	3.18	122.05	2.84499991	-122.46884	37.93992	-0.23	3.18	122.84	2.95299992	11	7/24/2014	30:11.4
7736	RWS11	-122.4688428	37.93991	-0.34	3.21	122.15	2.87	-122.46884	37.93992	-0.28	3.21	122.87	2.92700002	11	7/24/2014	30:11.5
7737	RWS11	-122.468845	37.93991	-0.34	3.23	122.14	2.8950001	-122.46884	37.93993	-0.28	3.23	122.91	2.95200011	11	7/24/2014	30:11.6
7738	RWS11	-122.4688472	37.93991	-0.34	3.26	122.2	2.91999996	-122.46884	37.93993	-0.31	3.26	122.95	2.94299996	11	7/24/2014	30:11.7
7739	RWS11	-122.4688494	37.93991	-0.34	3.28	122.19	2.94500005	-122.46884	37.93993	-0.31	3.28	123	2.96800005	11	7/24/2014	30:11.8
7740	RWS11	-122.4688516	37.93991	-0.34	3.3	122.14	2.96699989	-122.46885	37.93993	-0.28	3.3	122.95	3.0239999	11	7/24/2014	30:11.9
7741	RWS11	-122.4688548	37.93991	-0.39	3.32	122.17	2.93599987	-122.46885	37.93993	-0.31	3.32	122.94	3.00999987	11	7/24/2014	30:12.0
7742	RWS11	-122.468857	37.93992	-0.34	3.34	122.12	3.00400007	-122.46885	37.93993	-0.28	3.34	122.95	3.06100008	11	7/24/2014	30:12.1
7743	RWS11	-122.4688592	37.93992	-0.39	3.35	122.19	2.96499991	-122.46885	37.93993	-0.31	3.35	122.97	3.03899992	11	7/24/2014	30:12.2
7744	RWS11	-122.4688614	37.93992	-0.34	3.36	122.17	3.02299988	-122.46886	37.93993	-0.31	3.36	122.97	3.04599988	11	7/24/2014	30:12.3
7745	RWS11	-122.4688636	37.93992	-0.39	3.36	122.16	2.9749999	-122.46886	37.93993	-0.31	3.36	122.95	3.04899991	11	7/24/2014	30:12.4
7746	RWS11	-122.4688658	37.93992	-0.39	3.36	122.18	2.97600007	-122.46886	37.93993	-0.28	3.36	123.05	3.08400008	11	7/24/2014	30:12.5
7747	RWS11	-122.4688681	37.93992	-0.39	3.36	122.17	2.97300005	-122.46886	37.93993	-0.31	3.36	123	3.04700005	11	7/24/2014	30:12.6
7748	RWS11	-122.4688703	37.93992	-0.34	3.36	122.18	3.0200001	-122.46887	37.93993	-0.23	3.36	123.06	3.12800011	11	7/24/2014	30:12.7
7749	RWS11	-122.4688725	37.93992	-0.39	3.35	122.16	2.96300006	-122.46887	37.93993	-0.23	3.35	123.06	3.12200007	11	7/24/2014	30:12.8
7750	RWS11	-122.4688747	37.93992	-0.39	3.34	122.21	2.95499992	-122.46887	37.93993	-0.28	3.34	123.06	3.06299993	11	7/24/2014	30:12.9
7751	RWS11	-122.4688779	37.93992	-0.39	3.33	122.27	2.9460001	-122.46887	37.93993	-0.35	3.33	123.1	2.98600012	11	7/24/2014	30:13.0
7752	RWS11	-122.4688801	37.93992	-0.34	3.33	122.27	2.98800004	-122.46888	37.93993	-0.31	3.33	123.11	3.01100004	11	7/24/2014	30:13.1
7753	RWS11	-122.4688823	37.93992	-0.39	3.32	122.32	2.92799997	-122.46888	37.93993	-0.31	3.32	123.19	3.00199997	11	7/24/2014	30:13.2
7754	RWS11	-122.4688845	37.93992	-0.34	3.31	122.38	2.97100008	-122.46888	37.93993	-0.31	3.31	123.2	2.99400008	11	7/24/2014	30:13.3

7755	RWS11	-122.4688867	37.93992	-0.34	3.3	122.42	2.96500003	-122.46888	37.93993	-0.28	3.3	123.27	3.02200004	11	7/24/2014	30:13.4
7756	RWS11	-122.4688889	37.93992	-0.34	3.3	122.53	2.95999992	-122.46888	37.93994	-0.31	3.3	123.32	2.98299992	11	7/24/2014	30:13.5
7757	RWS11	-122.4688911	37.93992	-0.39	3.29	122.59	2.9059999	-122.46889	37.93994	-0.31	3.29	123.38	2.9799999	11	7/24/2014	30:13.6
7758	RWS11	-122.4688933	37.93992	-0.34	3.29	122.58	2.95500004	-122.46889	37.93994	-0.31	3.29	123.39	2.97800004	11	7/24/2014	30:13.7
7759	RWS11	-122.4688954	37.93992	-0.39	3.29	122.66	2.90299988	-122.46889	37.93994	-0.31	3.29	123.4	2.97699988	11	7/24/2014	30:13.8
7760	RWS11	-122.4688976	37.93992	-0.39	3.29	122.75	2.90199995	-122.46889	37.93994	-0.31	3.29	123.47	2.97599995	11	7/24/2014	30:13.9
7761	RWS11	-122.4689008	37.93993	-0.39	3.29	122.79	2.90199995	-122.4689	37.93994	-0.31	3.29	123.53	2.97599995	11	7/24/2014	30:14.0
7762	RWS11	-122.4689029	37.93993	-0.39	3.29	122.86	2.90199995	-122.4689	37.93994	-0.31	3.29	123.63	2.97599995	11	7/24/2014	30:14.1
7763	RWS11	-122.468905	37.93993	-0.42	3.29	122.94	2.86600003	-122.4689	37.93994	-0.31	3.29	123.66	2.97500002	11	7/24/2014	30:14.2
7764	RWS11	-122.4689072	37.93993	-0.39	3.29	122.94	2.90100002	-122.4689	37.93994	-0.31	3.29	123.74	2.97500002	11	7/24/2014	30:14.3
7765	RWS11	-122.4689093	37.93993	-0.42	3.29	123.03	2.86600003	-122.4689	37.93994	-0.28	3.29	123.86	3.00900003	11	7/24/2014	30:14.4
7766	RWS11	-122.4689115	37.93993	-0.34	3.29	123	2.95399988	-122.46891	37.93994	-0.28	3.29	123.85	3.01099989	11	7/24/2014	30:14.5
7767	RWS11	-122.4689137	37.93993	-0.34	3.29	123.07	2.9569999	-122.46891	37.93994	-0.31	3.29	123.9	2.9799999	11	7/24/2014	30:14.6
7768	RWS11	-122.4689159	37.93993	-0.39	3.3	123.14	2.91199994	-122.46891	37.93994	-0.31	3.3	123.94	2.98599994	11	7/24/2014	30:14.7
7769	RWS11	-122.468918	37.93993	-0.34	3.31	123.22	2.96999991	-122.46891	37.93994	-0.28	3.31	124	3.02699992	11	7/24/2014	30:14.8
7770	RWS11	-122.4689201	37.93993	-0.39	3.32	123.26	2.92799997	-122.46892	37.93994	-0.31	3.32	124	3.00199997	11	7/24/2014	30:14.9
7771	RWS11	-122.4689233	37.93993	-0.34	3.32	123.33	2.98699987	-122.46892	37.93994	-0.28	3.32	124.04	3.04399988	11	7/24/2014	30:15.0
7772	RWS11	-122.4689255	37.93993	-0.3	3.33	123.42	3.02800009	-122.46892	37.93994	-0.28	3.33	124.12	3.05100009	11	7/24/2014	30:15.1
7773	RWS11	-122.4689276	37.93993	-0.39	3.34	123.5	2.94700003	-122.46892	37.93994	-0.31	3.34	124.14	3.02100003	11	7/24/2014	30:15.2
7774	RWS11	-122.4689298	37.93993	-0.39	3.34	123.57	2.94899988	-122.46893	37.93994	-0.31	3.34	124.22	3.02299988	11	7/24/2014	30:15.3
7775	RWS11	-122.4689319	37.93993	-0.42	3.34	123.66	2.91500005	-122.46893	37.93995	-0.31	3.34	124.31	3.02400005	11	7/24/2014	30:15.4
7776	RWS11	-122.4689342	37.93993	-0.34	3.34	123.7	3.00199997	-122.46893	37.93995	-0.28	3.34	124.4	3.05899999	11	7/24/2014	30:15.5
7777	RWS11	-122.4689363	37.93993	-0.42	3.34	123.8	2.91699991	-122.46893	37.93995	-0.31	3.34	124.48	3.0259999	11	7/24/2014	30:15.6
7778	RWS11	-122.4689385	37.93993	-0.3	3.34	123.83	3.03699991	-122.46893	37.93995	-0.35	3.34	124.56	2.99199992	11	7/24/2014	30:15.7
7779	RWS11	-122.4689406	37.93993	-0.42	3.34	123.94	2.91599998	-122.46894	37.93995	-0.31	3.34	124.67	3.02499998	11	7/24/2014	30:15.8
7780	RWS11	-122.4689428	37.93993	-0.39	3.34	124	2.94700003	-122.46894	37.93995	-0.35	3.34	124.72	2.98700005	11	7/24/2014	30:15.9
7781	RWS11	-122.468946	37.93994	-0.39	3.33	124.07	2.94199991	-122.46894	37.93995	-0.35	3.33	124.83	2.98199993	11	7/24/2014	30:16.0
7782	RWS11	-122.4689482	37.93994	-0.39	3.32	124.17	2.93599987	-122.46894	37.93995	-0.31	3.32	124.85	3.00999987	11	7/24/2014	30:16.1
7783	RWS11	-122.4689503	37.93994	-0.42	3.32	124.25	2.89299998	-122.46895	37.93995	-0.31	3.32	124.96	3.00199997	11	7/24/2014	30:16.2
7784	RWS11	-122.4689525	37.93994	-0.39	3.31	124.35	2.91799998	-122.46895	37.93995	-0.31	3.31	125.08	2.99199998	11	7/24/2014	30:16.3
7785	RWS11	-122.4689547	37.93994	-0.42	3.29	124.45	2.8709999	-122.46895	37.93995	-0.31	3.29	125.13	2.9799999	11	7/24/2014	30:16.4
7786	RWS11	-122.468957	37.93994	-0.34	3.28	124.5	2.94399989	-122.46895	37.93995	-0.31	3.28	125.24	2.96699989	11	7/24/2014	30:16.5
7787	RWS11	-122.4689592	37.93994	-0.34	3.27	124.63	2.93099988	-122.46895	37.93995	-0.31	3.27	125.43	2.95399988	11	7/24/2014	30:16.6

7788	RWS11	-122.4689614	37.93994	-0.3	3.26	124.68	2.95300004	-122.46896	37.93995	-0.31	3.26	125.43	2.94200003	11	7/24/2014	30:16.7
7789	RWS11	-122.4689635	37.93994	-0.34	3.24	124.78	2.90699995	-122.46896	37.93995	-0.31	3.24	125.56	2.92999995	11	7/24/2014	30:16.8
7790	RWS11	-122.4689657	37.93994	-0.3	3.23	124.81	2.93000004	-122.46896	37.93995	-0.31	3.23	125.62	2.91900003	11	7/24/2014	30:16.9
7791	RWS11	-122.468969	37.93994	-0.34	3.22	124.97	2.88600004	-122.46896	37.93995	-0.31	3.22	125.77	2.90900004	11	7/24/2014	30:17.0
7792	RWS11	-122.4689712	37.93994	-0.3	3.21	125.1	2.91000006	-122.46897	37.93995	-0.23	3.21	125.86	2.98400006	11	7/24/2014	30:17.1
7793	RWS11	-122.4689733	37.93994	-0.34	3.2	125.2	2.86699998	-122.46897	37.93995	-0.31	3.2	126	2.88999999	11	7/24/2014	30:17.2
7794	RWS11	-122.4689756	37.93994	-0.3	3.19	125.37	2.89000008	-122.46897	37.93996	-0.31	3.19	126.13	2.87900007	11	7/24/2014	30:17.3
7795	RWS11	-122.4689777	37.93994	-0.39	3.18	125.43	2.79500008	-122.46897	37.93996	-0.31	3.18	126.19	2.86900008	11	7/24/2014	30:17.4
7796	RWS11	-122.46898	37.93994	-0.3	3.17	125.6	2.87000009	-122.46898	37.93996	-0.28	3.17	126.34	2.8930001	11	7/24/2014	30:17.5
7797	RWS11	-122.4689822	37.93994	-0.39	3.16	125.69	2.77600002	-122.46898	37.93996	-0.31	3.16	126.46	2.85000002	11	7/24/2014	30:17.6
7798	RWS11	-122.4689844	37.93994	-0.39	3.16	125.89	2.76799989	-122.46898	37.93996	-0.28	3.16	126.65	2.8759999	11	7/24/2014	30:17.7
7799	RWS11	-122.4689866	37.93994	-0.39	3.15	125.97	2.76300001	-122.46898	37.93996	-0.31	3.15	126.71	2.83700001	11	7/24/2014	30:17.8
7800	RWS11	-122.4689888	37.93995	-0.34	3.15	126.04	2.81099999	-122.46898	37.93996	-0.28	3.15	126.83	2.868	11	7/24/2014	30:17.9
7801	RWS11	-122.4689921	37.93995	-0.39	3.15	126.14	2.7579999	-122.46899	37.93996	-0.31	3.15	126.9	2.8319999	11	7/24/2014	30:18.0
7802	RWS11	-122.4689943	37.93995	-0.39	3.15	126.28	2.75900006	-122.46899	37.93996	-0.31	3.15	127	2.83300006	11	7/24/2014	30:18.1
7803	RWS11	-122.4689965	37.93995	-0.34	3.15	126.36	2.81199992	-122.46899	37.93996	-0.31	3.15	127.12	2.83499992	11	7/24/2014	30:18.2
7804	RWS11	-122.4689988	37.93995	-0.3	3.15	126.47	2.84899995	-122.46899	37.93996	-0.35	3.15	127.26	2.80399996	11	7/24/2014	30:18.3
7805	RWS11	-122.469001	37.93995	-0.39	3.15	126.61	2.76699996	-122.469	37.93996	-0.31	3.15	127.37	2.84099996	11	7/24/2014	30:18.4
7806	RWS11	-122.4690033	37.93995	-0.34	3.16	126.62	2.82199991	-122.469	37.93996	-0.35	3.16	127.46	2.81099993	11	7/24/2014	30:18.5
7807	RWS11	-122.4690055	37.93995	-0.34	3.16	126.67	2.82600009	-122.469	37.93996	-0.31	3.16	127.54	2.8490001	11	7/24/2014	30:18.6
7808	RWS11	-122.4690078	37.93995	-0.3	3.16	126.78	2.86199996	-122.469	37.93996	-0.23	3.16	127.62	2.93599996	11	7/24/2014	30:18.7
7809	RWS11	-122.46901	37.93995	-0.39	3.17	126.83	2.77900004	-122.46901	37.93996	-0.28	3.17	127.62	2.88700005	11	7/24/2014	30:18.8
7810	RWS11	-122.4690122	37.93995	-0.34	3.17	126.91	2.83099997	-122.46901	37.93996	-0.35	3.17	127.67	2.81999999	11	7/24/2014	30:18.9
7811	RWS11	-122.4690155	37.93995	-0.39	3.17	127	2.7809999	-122.46901	37.93996	-0.4	3.17	127.67	2.77099991	11	7/24/2014	30:19.0
7812	RWS11	-122.4690178	37.93995	-0.34	3.17	127.02	2.83099997	-122.46901	37.93996	-0.35	3.17	127.67	2.81999999	11	7/24/2014	30:19.1
7813	RWS11	-122.46902	37.93995	-0.34	3.17	127.1	2.83000004	-122.46902	37.93997	-0.31	3.17	127.72	2.85300004	11	7/24/2014	30:19.2
7814	RWS11	-122.4690223	37.93995	-0.34	3.17	127.17	2.82899988	-122.46902	37.93997	-0.35	3.17	127.79	2.8179999	11	7/24/2014	30:19.3
7815	RWS11	-122.4690246	37.93995	-0.34	3.16	127.18	2.82600009	-122.46902	37.93997	-0.43	3.16	127.79	2.7310001	11	7/24/2014	30:19.4
7816	RWS11	-122.4690268	37.93995	-0.34	3.16	127.21	2.82199991	-122.46902	37.93997	-0.35	3.16	127.86	2.81099993	11	7/24/2014	30:19.5
7817	RWS11	-122.4690291	37.93995	-0.34	3.15	127.29	2.8160001	-122.46902	37.93997	-0.4	3.15	127.94	2.75500011	11	7/24/2014	30:19.6
7818	RWS11	-122.4690314	37.93996	-0.34	3.14	127.3	2.80799997	-122.46903	37.93997	-0.4	3.14	127.97	2.74699998	11	7/24/2014	30:19.7
7819	RWS11	-122.4690336	37.93996	-0.39	3.14	127.38	2.74900007	-122.46903	37.93997	-0.4	3.14	128.02	2.73900008	11	7/24/2014	30:19.8
7820	RWS11	-122.4690358	37.93996	-0.34	3.13	127.4	2.79199994	-122.46903	37.93997	-0.4	3.13	128.03	2.73099995	11	7/24/2014	30:19.9

7821	RWS11	-122.4690392	37.93996	-0.34	3.12	127.46	2.78400004	-122.46903	37.93997	-0.4	3.12	128.1	2.72300005	11	7/24/2014	30:20.0
7822	RWS11	-122.4690414	37.93996	-0.3	3.12	127.56	2.81200001	-122.46904	37.93997	-0.4	3.12	128.17	2.71700001	11	7/24/2014	30:20.1
7823	RWS11	-122.4690436	37.93996	-0.34	3.11	127.59	2.77199996	-122.46904	37.93997	-0.4	3.11	128.2	2.71099997	11	7/24/2014	30:20.2
7824	RWS11	-122.4690458	37.93996	-0.3	3.1	127.68	2.79999992	-122.46904	37.93997	-0.4	3.1	128.26	2.70499992	11	7/24/2014	30:20.3
7825	RWS11	-122.4690481	37.93996	-0.34	3.1	127.73	2.76199996	-122.46904	37.93997	-0.4	3.1	128.33	2.70099998	11	7/24/2014	30:20.4
7826	RWS11	-122.4690503	37.93996	-0.3	3.1	127.74	2.79299995	-122.46905	37.93997	-0.4	3.1	128.36	2.69799995	11	7/24/2014	30:20.5
7827	RWS11	-122.4690526	37.93996	-0.34	3.1	127.73	2.76100004	-122.46905	37.93997	-0.35	3.1	128.37	2.75000006	11	7/24/2014	30:20.6
7828	RWS11	-122.4690548	37.93996	-0.3	3.11	127.74	2.80200002	-122.46905	37.93997	-0.4	3.11	128.35	2.70700002	11	7/24/2014	30:20.7
7829	RWS11	-122.469057	37.93996	-0.34	3.12	127.79	2.78000009	-122.46905	37.93997	-0.43	3.12	128.38	2.68500009	11	7/24/2014	30:20.8
7830	RWS11	-122.4690593	37.93996	-0.3	3.13	127.81	2.83100006	-122.46905	37.93997	-0.31	3.13	128.4	2.82000005	11	7/24/2014	30:20.9
7831	RWS11	-122.4690625	37.93996	-0.34	3.16	127.85	2.81899989	-122.46906	37.93998	-0.4	3.16	128.44	2.7579999	11	7/24/2014	30:21.0
7832	RWS11	-122.4690648	37.93996	-0.3	3.18	127.9	2.87899992	-122.46906	37.93998	-0.4	3.18	128.51	2.78399992	11	7/24/2014	30:21.1
7833	RWS11	-122.469067	37.93996	-0.34	3.21	127.89	2.8720001	-122.46906	37.93998	-0.43	3.21	128.53	2.7770001	11	7/24/2014	30:21.2
7834	RWS11	-122.4690692	37.93996	-0.34	3.23	127.98	2.89799988	-122.46906	37.93998	-0.35	3.23	128.59	2.88699991	11	7/24/2014	30:21.3
7835	RWS11	-122.4690715	37.93996	-0.34	3.26	128	2.92200005	-122.46907	37.93998	-0.4	3.26	128.68	2.86100006	11	7/24/2014	30:21.4
7836	RWS11	-122.4690737	37.93997	-0.3	3.28	128.05	2.97699997	-122.46907	37.93998	-0.4	3.28	128.7	2.88199997	11	7/24/2014	30:21.5
7837	RWS11	-122.4690759	37.93997	-0.34	3.3	128.04	2.95999992	-122.46907	37.93998	-0.43	3.3	128.76	2.86499992	11	7/24/2014	30:21.6
7838	RWS11	-122.4690782	37.93997	-0.34	3.31	128.09	2.97100008	-122.46907	37.93998	-0.4	3.31	128.76	2.91000009	11	7/24/2014	30:21.7
7839	RWS11	-122.4690804	37.93997	-0.34	3.32	128.04	2.97800004	-122.46908	37.93998	-0.43	3.32	128.75	2.88300005	11	7/24/2014	30:21.8
7840	RWS11	-122.4690826	37.93997	-0.34	3.32	128.07	2.9799999	-122.46908	37.93998	-0.4	3.32	128.8	2.91899991	11	7/24/2014	30:21.9
7841	RWS11	-122.4690858	37.93997	-0.39	3.32	128.06	2.9289999	-122.46908	37.93998	-0.4	3.32	128.89	2.91899991	11	7/24/2014	30:22.0
7842	RWS11	-122.469088	37.93997	-0.34	3.32	128.08	2.97800004	-122.46908	37.93998	-0.43	3.32	128.91	2.88300005	11	7/24/2014	30:22.1
7843	RWS11	-122.4690902	37.93997	-0.34	3.31	128.13	2.97500002	-122.46909	37.93998	-0.43	3.31	128.97	2.88000003	11	7/24/2014	30:22.2
7844	RWS11	-122.4690924	37.93997	-0.34	3.31	128.11	2.97100008	-122.46909	37.93998	-0.43	3.31	128.93	2.87600008	11	7/24/2014	30:22.3
7845	RWS11	-122.4690946	37.93997	-0.34	3.3	128.1	2.96599996	-122.46909	37.93998	-0.43	3.3	128.98	2.87099996	11	7/24/2014	30:22.4
7846	RWS11	-122.4690969	37.93997	-0.34	3.3	128.21	2.96100008	-122.46909	37.93998	-0.4	3.3	129.06	2.9000001	11	7/24/2014	30:22.5
7847	RWS11	-122.4690991	37.93997	-0.34	3.29	128.23	2.95399988	-122.46909	37.93998	-0.43	3.29	129.13	2.85899988	11	7/24/2014	30:22.6
7848	RWS11	-122.4691013	37.93997	-0.34	3.28	128.24	2.94699991	-122.4691	37.93998	-0.4	3.28	129.17	2.88599992	11	7/24/2014	30:22.7
7849	RWS11	-122.4691035	37.93997	-0.34	3.27	128.23	2.93699992	-122.4691	37.93999	-0.43	3.27	129.13	2.84199992	11	7/24/2014	30:22.8
7850	RWS11	-122.4691057	37.93997	-0.34	3.26	128.3	2.926	-122.4691	37.93999	-0.43	3.26	129.13	2.831	11	7/24/2014	30:22.9
7851	RWS11	-122.4691089	37.93997	-0.34	3.25	128.41	2.91299999	-122.4691	37.93999	-0.4	3.25	129.19	2.852	11	7/24/2014	30:23.0
7852	RWS11	-122.4691111	37.93997	-0.34	3.23	128.4	2.89699996	-122.46911	37.93999	-0.43	3.23	129.29	2.80199996	11	7/24/2014	30:23.1
7853	RWS11	-122.4691133	37.93997	-0.39	3.22	128.47	2.829	-122.46911	37.93999	-0.43	3.22	129.35	2.785	11	7/24/2014	30:23.2

7854	RWS11	-122.4691156	37.93998	-0.34	3.2	128.49	2.86099994	-122.46911	37.93999	-0.4	3.2	129.35	2.79999995	11	7/24/2014	30:23.3
7855	RWS11	-122.4691178	37.93998	-0.34	3.18	128.54	2.84099996	-122.46911	37.93999	-0.43	3.18	129.35	2.74599996	11	7/24/2014	30:23.4
7856	RWS11	-122.4691201	37.93998	-0.34	3.16	128.57	2.82199991	-122.46912	37.93999	-0.43	3.16	129.4	2.72699991	11	7/24/2014	30:23.5
7857	RWS11	-122.4691223	37.93998	-0.39	3.14	128.69	2.75399995	-122.46912	37.93999	-0.4	3.14	129.49	2.74399996	11	7/24/2014	30:23.6
7858	RWS11	-122.4691246	37.93998	-0.34	3.13	128.79	2.79000008	-122.46912	37.93999	-0.35	3.13	129.6	2.7790001	11	7/24/2014	30:23.7
7859	RWS11	-122.4691268	37.93998	-0.39	3.12	128.79	2.72799993	-122.46912	37.93999	-0.48	3.12	129.63	2.63299993	11	7/24/2014	30:23.8
7860	RWS11	-122.469129	37.93998	-0.39	3.11	128.9	2.7190001	-122.46912	37.93999	-0.35	3.11	129.73	2.75900012	11	7/24/2014	30:23.9
7861	RWS11	-122.4691323	37.93998	-0.39	3.1	129.03	2.71399999	-122.46913	37.93999	-0.4	3.1	129.84	2.704	11	7/24/2014	30:24.0
7862	RWS11	-122.4691345	37.93998	-0.34	3.1	129.03	2.76199996	-122.46913	37.93999	-0.4	3.1	129.85	2.70099998	11	7/24/2014	30:24.1
7863	RWS11	-122.4691367	37.93998	-0.39	3.1	129.12	2.71000004	-122.46913	37.93999	-0.43	3.1	129.89	2.66600004	11	7/24/2014	30:24.2
7864	RWS11	-122.469139	37.93998	-0.34	3.1	129.18	2.76199996	-122.46913	37.93999	-0.35	3.1	129.96	2.75099999	11	7/24/2014	30:24.3
7865	RWS11	-122.4691412	37.93998	-0.39	3.1	129.25	2.71399999	-122.46914	37.93999	-0.43	3.1	129.99	2.66999999	11	7/24/2014	30:24.4
7866	RWS11	-122.4691434	37.93998	-0.34	3.11	129.34	2.7700001	-122.46914	37.93999	-0.35	3.11	130.12	2.75900012	11	7/24/2014	30:24.5
7867	RWS11	-122.4691457	37.93998	-0.39	3.12	129.45	2.72800002	-122.46914	37.94	-0.43	3.12	130.2	2.68400002	11	7/24/2014	30:24.6
7868	RWS11	-122.4691479	37.93998	-0.39	3.12	129.48	2.73599991	-122.46914	37.94	-0.4	3.12	130.29	2.72599989	11	7/24/2014	30:24.7
7869	RWS11	-122.4691501	37.93998	-0.39	3.13	129.51	2.74700007	-122.46914	37.94	-0.43	3.13	130.31	2.70300007	11	7/24/2014	30:24.8
7870	RWS11	-122.4691523	37.93998	-0.39	3.14	129.6	2.75799999	-122.46915	37.94	-0.35	3.14	130.46	2.79799998	11	7/24/2014	30:24.9
7871	RWS11	-122.4691556	37.93998	-0.39	3.16	129.65	2.77199993	-122.46915	37.94	-0.43	3.16	130.53	2.72799993	11	7/24/2014	30:25.0
7872	RWS11	-122.4691578	37.93999	-0.39	3.17	129.71	2.78700003	-122.46915	37.94	-0.43	3.17	130.59	2.74300003	11	7/24/2014	30:25.1
7873	RWS11	-122.46916	37.93999	-0.39	3.19	129.71	2.80399999	-122.46915	37.94	-0.43	3.19	130.62	2.75999999	11	7/24/2014	30:25.2
7874	RWS11	-122.4691623	37.93999	-0.39	3.21	129.79	2.82099995	-122.46916	37.94	-0.35	3.21	130.7	2.86099994	11	7/24/2014	30:25.3
7875	RWS11	-122.4691645	37.93999	-0.39	3.22	129.81	2.83799991	-122.46916	37.94	-0.4	3.22	130.81	2.82799989	11	7/24/2014	30:25.4
7876	RWS11	-122.4691667	37.93999	-0.39	3.24	129.81	2.85200009	-122.46916	37.94	-0.4	3.24	130.78	2.84200007	11	7/24/2014	30:25.5
7877	RWS11	-122.469169	37.93999	-0.39	3.25	129.89	2.86399993	-122.46916	37.94	-0.43	3.25	130.9	2.81999993	11	7/24/2014	30:25.6
7878	RWS11	-122.4691713	37.93999	-0.34	3.26	129.88	2.92300007	-122.46917	37.94	-0.43	3.26	130.95	2.82800007	11	7/24/2014	30:25.7
7879	RWS11	-122.4691735	37.93999	-0.39	3.26	129.94	2.87699994	-122.46917	37.94	-0.43	3.26	130.99	2.83299994	11	7/24/2014	30:25.8
7880	RWS11	-122.4691757	37.93999	-0.39	3.27	130	2.87900004	-122.46917	37.94	-0.43	3.27	131.08	2.83500004	11	7/24/2014	30:25.9
7881	RWS11	-122.469179	37.93999	-0.39	3.27	129.99	2.87999997	-122.46917	37.94	-0.43	3.27	131.14	2.83599997	11	7/24/2014	30:26.0
7882	RWS11	-122.4691812	37.93999	-0.39	3.27	129.93	2.87999997	-122.46918	37.94	-0.43	3.27	131.11	2.83599997	11	7/24/2014	30:26.1
7883	RWS11	-122.4691833	37.93999	-0.42	3.27	129.94	2.8459999	-122.46918	37.94	-0.43	3.27	131.12	2.83699989	11	7/24/2014	30:26.2
7884	RWS11	-122.4691856	37.93999	-0.39	3.27	129.94	2.88099989	-122.46918	37.94001	-0.43	3.27	131.15	2.83699989	11	7/24/2014	30:26.3
7885	RWS11	-122.4691878	37.93999	-0.42	3.27	129.95	2.84700006	-122.46918	37.94001	-0.43	3.27	131.22	2.83800006	11	7/24/2014	30:26.4
7886	RWS11	-122.46919	37.93999	-0.39	3.27	129.96	2.88299999	-122.46918	37.94001	-0.48	3.27	131.28	2.78799999	11	7/24/2014	30:26.5

7887	RWS11	-122.4691923	37.93999	-0.39	3.27	129.93	2.88500008	-122.46919	37.94001	-0.48	3.27	131.31	2.79000008	11	7/24/2014	30:26.6
7888	RWS11	-122.4691945	37.93999	-0.34	3.27	129.81	2.93700001	-122.46919	37.94001	-0.43	3.27	131.24	2.84200001	11	7/24/2014	30:26.7
7889	RWS11	-122.4691967	37.94	-0.39	3.27	129.83	2.88500008	-122.46919	37.94001	-0.4	3.27	131.32	2.87500006	11	7/24/2014	30:26.8
7890	RWS11	-122.4691989	37.94	-0.39	3.27	129.71	2.88200006	-122.46919	37.94001	-0.4	3.27	131.23	2.87200004	11	7/24/2014	30:26.9
7891	RWS11	-122.4692021	37.94	-0.39	3.27	129.63	2.87900004	-122.4692	37.94001	-0.4	3.27	131.22	2.86900002	11	7/24/2014	30:27.0
7892	RWS11	-122.4692042	37.94	-0.39	3.26	129.54	2.87399992	-122.4692	37.94001	-0.43	3.26	131.21	2.82999992	11	7/24/2014	30:27.1
7893	RWS11	-122.4692064	37.94	-0.39	3.26	129.4	2.86900005	-122.4692	37.94001	-0.48	3.26	131.13	2.77400005	11	7/24/2014	30:27.2
7894	RWS11	-122.4692086	37.94	-0.39	3.25	129.26	2.8650001	-122.4692	37.94001	-0.43	3.25	131.11	2.8210001	11	7/24/2014	30:27.3
7895	RWS11	-122.4692107	37.94	-0.39	3.25	129.08	2.86300001	-122.46921	37.94001	-0.4	3.25	130.98	2.85299999	11	7/24/2014	30:27.4
7896	RWS11	-122.4692129	37.94	-0.39	3.25	128.86	2.86099991	-122.46921	37.94001	-0.43	3.25	130.91	2.81699991	11	7/24/2014	30:27.5
7897	RWS11	-122.4692151	37.94	-0.39	3.25	128.64	2.85900006	-122.46921	37.94001	-0.4	3.25	130.77	2.84900004	11	7/24/2014	30:27.6
7898	RWS11	-122.4692173	37.94	-0.39	3.25	128.31	2.85900006	-122.46921	37.94001	-0.4	3.25	130.62	2.84900004	11	7/24/2014	30:27.7
7899	RWS11	-122.4692194	37.94	-0.39	3.25	127.97	2.85999998	-122.46921	37.94001	-0.4	3.25	130.34	2.84999996	11	7/24/2014	30:27.8
7900	RWS11	-122.4692216	37.94	-0.34	3.25	127.57	2.914	-122.46922	37.94001	-0.4	3.25	130.05	2.85299999	11	7/24/2014	30:27.9
7901	RWS11	-122.4692248	37.94	-0.39	3.26	127.16	2.86900005	-122.46922	37.94002	-0.4	3.26	129.66	2.85900003	11	7/24/2014	30:28.0
7902	RWS11	-122.4692269	37.94	-0.34	3.27	126.62	2.92900011	-122.46922	37.94002	-0.35	3.27	129.2	2.9180001	11	7/24/2014	30:28.1
7903	RWS11	-122.4692291	37.94	-0.39	3.28	126.08	2.88999996	-122.46922	37.94002	-0.4	3.28	128.73	2.87999994	11	7/24/2014	30:28.2
7904	RWS11	-122.4692313	37.94	-0.34	3.29	125.39	2.95600006	-122.46923	37.94002	-0.4	3.29	128.07	2.89500004	11	7/24/2014	30:28.3
7905	RWS11	-122.4692334	37.94	-0.39	3.31	124.66	2.92200002	-122.46923	37.94002	-0.35	3.31	127.37	2.96200001	11	7/24/2014	30:28.4
7906	RWS11	-122.4692356	37.94001	-0.34	3.33	123.84	2.99099991	-122.46923	37.94002	-0.35	3.33	126.56	2.9799999	11	7/24/2014	30:28.5
7907	RWS11	-122.4692378	37.94001	-0.39	3.35	122.87	2.95899996	-122.46923	37.94002	-0.35	3.35	125.54	2.99899995	11	7/24/2014	30:28.6
7908	RWS11	-122.46924	37.94001	-0.34	3.36	121.89	3.02500007	-122.46923	37.94002	-0.4	3.36	124.5	2.96400005	11	7/24/2014	30:28.7
7909	RWS11	-122.4692422	37.94001	-0.34	3.36	120.76	3.02699992	-122.46924	37.94002	-0.43	3.36	123.26	2.93199992	11	7/24/2014	30:28.8
7910	RWS11	-122.4692444	37.94001	-0.34	3.37	119.51	3.02900001	-122.46924	37.94002	-0.4	3.37	121.86	2.96799999	11	7/24/2014	30:28.9
7911	RWS11	-122.4692476	37.94001	-0.34	3.37	118.03	3.03100011	-122.46924	37.94002	-0.4	3.37	120.15	2.97000009	11	7/24/2014	30:29.0
7912	RWS11	-122.4692498	37.94001	-0.34	3.37	116.48	3.03299996	-122.46924	37.94002	-0.31	3.37	118.25	3.05599996	11	7/24/2014	30:29.1
7913	RWS11	-122.469252	37.94001	-0.34	3.37	114.73	3.03500006	-122.46925	37.94002	-0.4	3.37	116.03	2.97400004	11	7/24/2014	30:29.2
7914	RWS11	-122.4692542	37.94001	-0.34	3.37	112.9	3.03699991	-122.46925	37.94002	-0.4	3.37	113.6	2.97599989	11	7/24/2014	30:29.3
7915	RWS11	-122.4692564	37.94001	-0.39	3.38	110.93	2.99300012	-122.46925	37.94002	-0.43	3.38	110.92	2.94900012	11	7/24/2014	30:29.4
7916	RWS11	-122.4692586	37.94001	-0.39	3.38	108.75	2.99499997	-122.46925	37.94002	-0.4	3.38	107.85	2.98499995	11	7/24/2014	30:29.5
7917	RWS11	-122.4692609	37.94001	-0.39	3.38	106.53	2.99300012	-122.46926	37.94002	-0.4	3.38	104.62	2.9830001	11	7/24/2014	30:29.6
7918	RWS11	-122.4692631	37.94001	-0.34	3.37	104.16	3.03800008	-122.46926	37.94003	-0.4	3.37	101.09	2.97700006	11	7/24/2014	30:29.7
7919	RWS11	-122.4692653	37.94001	-0.34	3.37	101.75	3.03100011	-122.46926	37.94003	-0.43	3.37	97.344	2.93600011	11	7/24/2014	30:29.8

7920	RWS11	-122.4692675	37.94001	-0.34	3.36	99.212	3.02100012	-122.46926	37.94003	-0.4	3.36	93.343	2.9600001	11	7/24/2014	30:29.9
7921	RWS11	-122.4692708	37.94001	-0.34	3.35	96.57	3.01200005	-122.46927	37.94003	-0.35	3.35	89.131	3.00100005	11	7/24/2014	30:30.0
7922	RWS11	-122.469273	37.94002	-0.34	3.34	93.871	3.00299999	-122.46927	37.94003	-0.4	3.34	84.765	2.94199997	11	7/24/2014	30:30.1
7923	RWS11	-122.4692752	37.94002	-0.34	3.33	91.225	2.99800012	-122.46927	37.94003	-0.35	3.33	80.321	2.98700011	11	7/24/2014	30:30.2
7924	RWS11	-122.4692774	37.94002	-0.34	3.33	88.607	2.99500009	-122.46927	37.94003	-0.35	3.33	75.857	2.98400009	11	7/24/2014	30:30.3
7925	RWS11	-122.4692796	37.94002	-0.39	3.33	85.989	2.94599995	-122.46927	37.94003	-0.43	3.33	71.522	2.90199995	11	7/24/2014	30:30.4
7926	RWS11	-122.4692818	37.94002	-0.34	3.34	83.568	3.00299999	-122.46928	37.94003	-0.35	3.34	67.379	2.99199998	11	7/24/2014	30:30.5
7927	RWS11	-122.469284	37.94002	-0.39	3.35	81.291	2.96100006	-122.46928	37.94003	-0.35	3.35	63.748	3.00100005	11	7/24/2014	30:30.6
7928	RWS11	-122.4692863	37.94002	-0.34	3.36	79.307	3.0239999	-122.46928	37.94003	-0.35	3.36	60.806	3.01299989	11	7/24/2014	30:30.7
7929	RWS11	-122.4692884	37.94002	-0.39	3.37	77.52	2.98599991	-122.46928	37.94003	-0.4	3.37	58.49	2.97599989	11	7/24/2014	30:30.8
7930	RWS11	-122.4692906	37.94002	-0.34	3.39	76.06	3.04999992	-122.46929	37.94003	-0.35	3.39	56.855	3.03899992	11	7/24/2014	30:30.9
7931	RWS11	-122.4692939	37.94002	-0.39	3.4	74.814	3.01000008	-122.46929	37.94003	-0.4	3.4	55.832	3.00000006	11	7/24/2014	30:31.0
7932	RWS11	-122.4692961	37.94002	-0.34	3.41	73.787	3.07100007	-122.46929	37.94003	-0.35	3.41	55.413	3.06000006	11	7/24/2014	30:31.1
7933	RWS11	-122.4692982	37.94002	-0.34	3.41	72.802	3.07800004	-122.46929	37.94003	-0.35	3.41	55.503	3.06700003	11	7/24/2014	30:31.2
7934	RWS11	-122.4693004	37.94002	-0.34	3.42	71.996	3.08299991	-122.46929	37.94004	-0.35	3.42	55.956	3.07199991	11	7/24/2014	30:31.3
7935	RWS11	-122.4693026	37.94002	-0.39	3.42	71.187	3.0360001	-122.4693	37.94004	-0.31	3.42	56.68	3.1100001	11	7/24/2014	30:31.4
7936	RWS11	-122.4693048	37.94002	-0.34	3.43	70.449	3.09000012	-122.4693	37.94004	-0.35	3.43	57.765	3.07900012	11	7/24/2014	30:31.5
7937	RWS11	-122.469307	37.94002	-0.34	3.43	69.662	3.09199998	-122.4693	37.94004	-0.4	3.43	58.967	3.03099996	11	7/24/2014	30:31.6
7938	RWS11	-122.4693091	37.94003	-0.34	3.43	68.657	3.095	-122.4693	37.94004	-0.31	3.43	60.059	3.118	11	7/24/2014	30:31.7
7939	RWS11	-122.4693113	37.94003	-0.34	3.43	67.432	3.09599993	-122.46931	37.94004	-0.35	3.43	61.02	3.08499992	11	7/24/2014	30:31.8
7940	RWS11	-122.4693135	37.94003	-0.34	3.43	65.969	3.095	-122.46931	37.94004	-0.31	3.43	61.817	3.118	11	7/24/2014	30:31.9
7941	RWS11	-122.4693167	37.94003	-0.39	3.43	64.221	3.04000005	-122.46931	37.94004	-0.35	3.43	62.411	3.08000004	11	7/24/2014	30:32.0
7942	RWS11	-122.4693188	37.94003	-0.39	3.42	62.022	3.03199992	-122.46931	37.94004	-0.28	3.42	62.542	3.1399999	11	7/24/2014	30:32.1
7943	RWS11	-122.469321	37.94003	-0.39	3.41	59.357	3.02000007	-122.46932	37.94004	-0.35	3.41	62.188	3.06000006	11	7/24/2014	30:32.2
7944	RWS11	-122.4693232	37.94003	-0.34	3.39	56.367	3.05200002	-122.46932	37.94004	-0.31	3.39	61.318	3.07500002	11	7/24/2014	30:32.3
7945	RWS11	-122.4693254	37.94003	-0.39	3.36	52.857	2.97700009	-122.46932	37.94004	-0.35	3.36	59.88	3.01700008	11	7/24/2014	30:32.4
7946	RWS11	-122.4693277	37.94003	-0.34	3.33	48.968	2.99600002	-122.46932	37.94004	-0.4	3.33	57.823	2.935	11	7/24/2014	30:32.5
7947	RWS11	-122.4693298	37.94003	-0.39	3.3	44.537	2.90800008	-122.46932	37.94004	-0.43	3.3	55.336	2.86400008	11	7/24/2014	30:32.6
7948	RWS11	-122.4693321	37.94003	-0.34	3.25	39.741	2.9160001	-122.46933	37.94004	-0.4	3.25	52.259	2.85500008	11	7/24/2014	30:32.7
7949	RWS11	-122.4693342	37.94003	-0.39	3.21	34.674	2.82000002	-122.46933	37.94004	-0.31	3.21	48.814	2.89400002	11	7/24/2014	30:32.8
7950	RWS11	-122.4693364	37.94003	-0.39	3.16	29.331	2.77600011	-122.46933	37.94005	-0.43	3.16	45.061	2.73200011	11	7/24/2014	30:32.9
7951	RWS11	-122.4693397	37.94003	-0.39	3.13	23.854	2.73899993	-122.46933	37.94005	-0.4	3.13	41.194	2.72899991	11	7/24/2014	30:33.0
7952	RWS11	-122.4693418	37.94003	-0.39	3.1	18.374	2.71400008	-122.46934	37.94005	-0.35	3.1	37.224	2.75400007	11	7/24/2014	30:33.1

7953	RWS11	-122.469344	37.94003	-0.39	3.09	12.983	2.70199999	-122.46934	37.94005	-0.35	3.09	33.34	2.74199998	11	7/24/2014	30:33.2
7954	RWS11	-122.4693462	37.94004	-0.34	3.09	7.791	2.74999997	-122.46934	37.94005	-0.35	3.09	29.652	2.73899996	11	7/24/2014	30:33.3
7955	RWS11	-122.4693484	37.94004	-0.39	3.09	3.1	2.6999999	-122.46934	37.94005	-0.35	3.09	26.204	2.73999989	11	7/24/2014	30:33.4
7956	RWS11	-122.4693507	37.94004	-0.34	3.09	-0.893	2.75500008	-122.46935	37.94005	-0.31	3.09	23.258	2.77800009	11	7/24/2014	30:33.5
7957	RWS11	-122.4693529	37.94004	-0.39	3.1	-4.166	2.71100006	-122.46935	37.94005	-0.43	3.1	20.904	2.66700006	11	7/24/2014	30:33.6
7958	RWS11	-122.4693551	37.94004	-0.39	3.11	-6.261	2.72100005	-122.46935	37.94005	-0.43	3.11	19.245	2.67700005	11	7/24/2014	30:33.7
7959	RWS11	-122.4693573	37.94004	-0.39	3.12	-7.464	2.73299989	-122.46935	37.94005	-0.35	3.12	18.366	2.77299988	11	7/24/2014	30:33.8
7960	RWS11	-122.4693595	37.94004	-0.39	3.13	-7.464	2.748	-122.46935	37.94005	-0.4	3.13	18.346	2.73799998	11	7/24/2014	30:33.9
7961	RWS11	-122.4693627	37.94004	-0.39	3.15	-6.505	2.76600012	-122.46936	37.94005	-0.35	3.15	18.893	2.80600011	11	7/24/2014	30:34.0
7962	RWS11	-122.4693649	37.94004	-0.39	3.17	-4.803	2.7860001	-122.46936	37.94005	-0.35	3.17	20.047	2.82600009	11	7/24/2014	30:34.1
7963	RWS11	-122.4693671	37.94004	-0.39	3.19	-2.228	2.80799994	-122.46936	37.94005	-0.35	3.19	21.76	2.84799993	11	7/24/2014	30:34.2
7964	RWS11	-122.4693693	37.94004	-0.39	3.22	1.414	2.83200011	-122.46936	37.94005	-0.31	3.22	24.299	2.90600011	11	7/24/2014	30:34.3
7965	RWS11	-122.4693715	37.94004	-0.39	3.24	5.71	2.85500011	-122.46937	37.94005	-0.35	3.24	27.486	2.8950001	11	7/24/2014	30:34.4
7966	RWS11	-122.4693738	37.94004	-0.39	3.27	10.968	2.87800011	-122.46937	37.94006	-0.31	3.27	31.259	2.95200011	11	7/24/2014	30:34.5
7967	RWS11	-122.469376	37.94004	-0.39	3.29	16.643	2.89900002	-122.46937	37.94006	-0.35	3.29	35.513	2.93900001	11	7/24/2014	30:34.6
7968	RWS11	-122.4693782	37.94004	-0.39	3.31	22.642	2.91999993	-122.46937	37.94006	-0.35	3.31	40.096	2.95999992	11	7/24/2014	30:34.7
7969	RWS11	-122.4693804	37.94004	-0.42	3.33	28.948	2.90600008	-122.46937	37.94006	-0.4	3.33	45.03	2.93100005	11	7/24/2014	30:34.8
7970	RWS11	-122.4693826	37.94004	-0.39	3.35	35.426	2.96199998	-122.46938	37.94006	-0.35	3.35	50.153	3.00199997	11	7/24/2014	30:34.9
7971	RWS11	-122.4693858	37.94005	-0.42	3.37	41.948	2.94699997	-122.46938	37.94006	-0.4	3.37	55.386	2.97199994	11	7/24/2014	30:35.0
7972	RWS11	-122.469388	37.94005	-0.39	3.39	48.454	3.00199994	-122.46938	37.94006	-0.35	3.39	60.644	3.04199994	11	7/24/2014	30:35.1
7973	RWS11	-122.4693902	37.94005	-0.39	3.41	54.775	3.02000007	-122.46938	37.94006	-0.35	3.41	65.993	3.06000006	11	7/24/2014	30:35.2
7974	RWS11	-122.4693924	37.94005	-0.39	3.42	60.78	3.03499994	-122.46939	37.94006	-0.4	3.42	71.273	3.02499992	11	7/24/2014	30:35.3
7975	RWS11	-122.4693945	37.94005	-0.42	3.43	66.562	3.00999993	-122.46939	37.94006	-0.43	3.43	76.513	3.00099993	11	7/24/2014	30:35.4
7976	RWS11	-122.4693967	37.94005	-0.39	3.44	72.125	3.05099997	-122.46939	37.94006	-0.35	3.44	81.634	3.09099996	11	7/24/2014	30:35.5
7977	RWS11	-122.4693989	37.94005	-0.42	3.44	77.467	3.01599997	-122.46939	37.94006	-0.43	3.44	86.653	3.00699997	11	7/24/2014	30:35.6
7978	RWS11	-122.4694011	37.94005	-0.42	3.43	82.462	3.00999993	-122.4694	37.94006	-0.4	3.43	91.555	3.03499991	11	7/24/2014	30:35.7
7979	RWS11	-122.4694033	37.94005	-0.42	3.42	87.391	3.00100011	-122.4694	37.94006	-0.48	3.42	96.344	2.9410001	11	7/24/2014	30:35.8
7980	RWS11	-122.4694054	37.94005	-0.42	3.41	92.043	2.98999995	-122.4694	37.94006	-0.43	3.41	101.03	2.98099995	11	7/24/2014	30:35.9
7981	RWS11	-122.4694086	37.94005	-0.42	3.4	96.447	2.98100013	-122.4694	37.94006	-0.43	3.4	105.39	2.97200012	11	7/24/2014	30:36.0
7982	RWS11	-122.4694108	37.94005	-0.42	3.4	100.62	2.97500008	-122.46941	37.94007	-0.43	3.4	109.74	2.96600008	11	7/24/2014	30:36.1
7983	RWS11	-122.4694129	37.94005	-0.42	3.4	104.5	2.97399992	-122.46941	37.94007	-0.43	3.4	113.84	2.96499991	11	7/24/2014	30:36.2
7984	RWS11	-122.4694151	37.94005	-0.42	3.4	108.27	2.9780001	-122.46941	37.94007	-0.48	3.4	117.87	2.9180001	11	7/24/2014	30:36.3
7985	RWS11	-122.4694172	37.94005	-0.47	3.41	111.76	2.93800002	-122.46941	37.94007	-0.52	3.41	121.75	2.89500004	11	7/24/2014	30:36.4

7986	RWS11	-122.4694194	37.94005	-0.42	3.42	115.06	3.00299996	-122.46941	37.94007	-0.48	3.42	125.43	2.94299996	11	7/24/2014	30:36.5
7987	RWS11	-122.4694215	37.94006	-0.47	3.44	118.11	2.97000009	-122.46942	37.94007	-0.48	3.44	128.89	2.96100008	11	7/24/2014	30:36.6
7988	RWS11	-122.4694237	37.94006	-0.47	3.46	120.93	2.98799998	-122.46942	37.94007	-0.52	3.46	132.14	2.94499999	11	7/24/2014	30:36.7
7989	RWS11	-122.4694258	37.94006	-0.47	3.48	123.5	3.00400001	-122.46942	37.94007	-0.52	3.48	135.14	2.96100003	11	7/24/2014	30:36.8
7990	RWS11	-122.469428	37.94006	-0.47	3.49	125.85	3.01799995	-122.46942	37.94007	-0.43	3.49	137.93	3.05999994	11	7/24/2014	30:36.9
7991	RWS11	-122.4694311	37.94006	-0.51	3.5	127.99	2.99500006	-122.46943	37.94007	-0.57	3.5	140.49	2.93500012	11	7/24/2014	30:37.0
7992	RWS11	-122.4694332	37.94006	-0.47	3.51	129.94	3.037	-122.46943	37.94007	-0.48	3.51	142.74	3.028	11	7/24/2014	30:37.1
7993	RWS11	-122.4694354	37.94006	-0.51	3.51	131.66	3.00699991	-122.46943	37.94007	-0.52	3.51	144.81	2.99799997	11	7/24/2014	30:37.2
7994	RWS11	-122.4694375	37.94006	-0.51	3.52	133.23	3.00800008	-122.46943	37.94007	-0.52	3.52	146.6	2.99900013	11	7/24/2014	30:37.3
7995	RWS11	-122.4694397	37.94006	-0.51	3.51	134.47	3.00699991	-122.46943	37.94007	-0.52	3.51	148.04	2.99799997	11	7/24/2014	30:37.4
7996	RWS11	-122.4694418	37.94006	-0.51	3.51	135.61	3.00299996	-122.46944	37.94007	-0.52	3.51	149.22	2.99400002	11	7/24/2014	30:37.5
7997	RWS11	-122.469444	37.94006	-0.56	3.5	136.52	2.94499999	-122.46944	37.94007	-0.52	3.5	150.19	2.98700005	11	7/24/2014	30:37.6
7998	RWS11	-122.4694461	37.94006	-0.51	3.49	137.24	2.98699993	-122.46944	37.94007	-0.48	3.49	150.77	3.01199996	11	7/24/2014	30:37.7
7999	RWS11	-122.4694483	37.94006	-0.56	3.48	137.68	2.92500001	-122.46944	37.94008	-0.57	3.48	151.08	2.91600007	11	7/24/2014	30:37.8
8000	RWS11	-122.4694504	37.94006	-0.51	3.47	138.07	2.96399993	-122.46945	37.94008	-0.48	3.47	151.17	2.98899996	11	7/24/2014	30:37.9
8001	RWS11	-122.4694536	37.94006	-0.51	3.46	138.13	2.95099992	-122.46945	37.94008	-0.48	3.46	150.92	2.97599995	11	7/24/2014	30:38.0
8002	RWS11	-122.4694557	37.94006	-0.51	3.44	138.09	2.93699998	-122.46945	37.94008	-0.52	3.44	150.47	2.92800003	11	7/24/2014	30:38.1
8003	RWS11	-122.4694578	37.94006	-0.56	3.43	137.91	2.87200004	-122.46945	37.94008	-0.52	3.43	149.8	2.91400009	11	7/24/2014	30:38.2
8004	RWS11	-122.4694599	37.94007	-0.51	3.42	137.59	2.90899986	-122.46945	37.94008	-0.48	3.42	148.98	2.9339999	11	7/24/2014	30:38.3
8005	RWS11	-122.4694621	37.94007	-0.56	3.4	137.13	2.84500009	-122.46946	37.94008	-0.48	3.4	147.95	2.92100012	11	7/24/2014	30:38.4
8006	RWS11	-122.4694642	37.94007	-0.51	3.39	136.47	2.88599986	-122.46946	37.94008	-0.52	3.39	146.76	2.87699991	11	7/24/2014	30:38.5
8007	RWS11	-122.4694664	37.94007	-0.56	3.39	135.64	2.82799989	-122.46946	37.94008	-0.52	3.39	145.34	2.86999995	11	7/24/2014	30:38.6
8008	RWS11	-122.4694685	37.94007	-0.51	3.38	134.72	2.87499994	-122.46946	37.94008	-0.52	3.38	143.75	2.866	11	7/24/2014	30:38.7
8009	RWS11	-122.4694707	37.94007	-0.56	3.38	133.48	2.82399994	-122.46947	37.94008	-0.52	3.38	141.92	2.866	11	7/24/2014	30:38.8
8010	RWS11	-122.4694728	37.94007	-0.51	3.38	132.13	2.87700003	-122.46947	37.94008	-0.48	3.38	139.94	2.90200007	11	7/24/2014	30:38.9
8011	RWS11	-122.469476	37.94007	-0.56	3.39	130.57	2.82999998	-122.46947	37.94008	-0.43	3.39	137.76	2.95700002	11	7/24/2014	30:39.0
8012	RWS11	-122.4694781	37.94007	-0.51	3.39	128.76	2.88599986	-122.46947	37.94008	-0.52	3.39	135.26	2.87699991	11	7/24/2014	30:39.1
8013	RWS11	-122.4694803	37.94007	-0.56	3.4	126.85	2.83799988	-122.46948	37.94008	-0.52	3.4	132.5	2.87999994	11	7/24/2014	30:39.2
8014	RWS11	-122.4694825	37.94007	-0.51	3.4	124.64	2.89099997	-122.46948	37.94008	-0.43	3.4	129.44	2.96700001	11	7/24/2014	30:39.3
8015	RWS11	-122.4694846	37.94007	-0.56	3.4	122.21	2.83900005	-122.46948	37.94008	-0.52	3.4	126.1	2.8810001	11	7/24/2014	30:39.4
8016	RWS11	-122.4694869	37.94007	-0.51	3.39	119.71	2.88700002	-122.46948	37.94009	-0.52	3.39	122.45	2.87800008	11	7/24/2014	30:39.5
8017	RWS11	-122.4694891	37.94007	-0.51	3.39	117	2.88199991	-122.46948	37.94009	-0.48	3.39	118.55	2.90699995	11	7/24/2014	30:39.6
8018	RWS11	-122.4694912	37.94007	-0.51	3.38	114.21	2.87700003	-122.46949	37.94009	-0.43	3.38	114.42	2.95300007	11	7/24/2014	30:39.7

8019	RWS11	-122.4694934	37.94007	-0.51	3.38	111.37	2.87300009	-122.46949	37.94009	-0.48	3.38	110.22	2.89800012	11	7/24/2014	30:39.8
8020	RWS11	-122.4694956	37.94007	-0.51	3.38	108.45	2.87099999	-122.46949	37.94009	-0.48	3.38	105.96	2.89600003	11	7/24/2014	30:39.9
8021	RWS11	-122.4694988	37.94008	-0.51	3.38	105.63	2.87199992	-122.46949	37.94009	-0.48	3.38	101.79	2.89699996	11	7/24/2014	30:40.0
8022	RWS11	-122.469501	37.94008	-0.47	3.38	102.92	2.90899998	-122.4695	37.94009	-0.48	3.38	97.812	2.89999998	11	7/24/2014	30:40.1
8023	RWS11	-122.4695032	37.94008	-0.51	3.39	100.37	2.88000005	-122.4695	37.94009	-0.48	3.39	94.129	2.90500009	11	7/24/2014	30:40.2
8024	RWS11	-122.4695054	37.94008	-0.51	3.39	98.053	2.88700002	-122.4695	37.94009	-0.48	3.39	90.834	2.91200006	11	7/24/2014	30:40.3
8025	RWS11	-122.4695076	37.94008	-0.51	3.4	96.032	2.89300007	-122.4695	37.94009	-0.43	3.4	88.041	2.9690001	11	7/24/2014	30:40.4
8026	RWS11	-122.4695098	37.94008	-0.47	3.41	94.348	2.93299991	-122.4695	37.94009	-0.43	3.41	85.795	2.9749999	11	7/24/2014	30:40.5
8027	RWS11	-122.469512	37.94008	-0.51	3.41	92.967	2.90199989	-122.46951	37.94009	-0.48	3.41	84.173	2.92699993	11	7/24/2014	30:40.6
8028	RWS11	-122.4695143	37.94008	-0.47	3.41	91.814	2.9370001	-122.46951	37.94009	-0.43	3.41	82.998	2.97900009	11	7/24/2014	30:40.7
8029	RWS11	-122.4695164	37.94008	-0.51	3.41	91.092	2.90099996	-122.46951	37.94009	-0.48	3.41	82.446	2.926	11	7/24/2014	30:40.8
8030	RWS11	-122.4695186	37.94008	-0.47	3.4	90.595	2.93200004	-122.46951	37.94009	-0.4	3.4	82.451	3.00800005	11	7/24/2014	30:40.9
8031	RWS11	-122.4695219	37.94008	-0.47	3.4	90.414	2.92399991	-122.46952	37.94009	-0.43	3.4	82.867	2.9659999	11	7/24/2014	30:41.0
8032	RWS11	-122.4695241	37.94008	-0.47	3.39	90.611	2.91399992	-122.46952	37.94009	-0.4	3.39	83.884	2.98999992	11	7/24/2014	30:41.1
8033	RWS11	-122.4695262	37.94008	-0.51	3.37	91.029	2.86699992	-122.46952	37.9401	-0.43	3.37	85.35	2.9429999	11	7/24/2014	30:41.2
8034	RWS11	-122.4695284	37.94008	-0.47	3.36	91.658	2.88699996	-122.46952	37.9401	-0.43	3.36	87.049	2.92899996	11	7/24/2014	30:41.3
8035	RWS11	-122.4695306	37.94008	-0.47	3.34	92.613	2.8720001	-122.46953	37.9401	-0.35	3.34	89.187	2.99800012	11	7/24/2014	30:41.4
8036	RWS11	-122.4695329	37.94008	-0.47	3.33	93.751	2.85799992	-122.46953	37.9401	-0.35	3.33	91.57	2.98399994	11	7/24/2014	30:41.5
8037	RWS11	-122.4695351	37.94008	-0.47	3.32	94.974	2.84600008	-122.46953	37.9401	-0.43	3.32	94.007	2.88800007	11	7/24/2014	30:41.6
8038	RWS11	-122.4695373	37.94008	-0.47	3.31	96.33	2.83700001	-122.46953	37.9401	-0.35	3.31	96.433	2.96300003	11	7/24/2014	30:41.7
8039	RWS11	-122.4695394	37.94009	-0.47	3.3	97.736	2.83099997	-122.46953	37.9401	-0.43	3.3	98.742	2.87299997	11	7/24/2014	30:41.8
8040	RWS11	-122.4695416	37.94009	-0.47	3.3	99.164	2.82900012	-122.46954	37.9401	-0.35	3.3	100.96	2.95500013	11	7/24/2014	30:41.9
8041	RWS11	-122.4695449	37.94009	-0.47	3.3	100.55	2.8319999	-122.46954	37.9401	-0.43	3.3	103	2.87399989	11	7/24/2014	30:42.0
8042	RWS11	-122.469547	37.94009	-0.47	3.31	101.85	2.83900011	-122.46954	37.9401	-0.4	3.31	104.76	2.91500011	11	7/24/2014	30:42.1
8043	RWS11	-122.4695492	37.94009	-0.47	3.32	103.18	2.85199988	-122.46954	37.9401	-0.43	3.32	106.42	2.89399987	11	7/24/2014	30:42.2
8044	RWS11	-122.4695514	37.94009	-0.47	3.34	104.37	2.87	-122.46955	37.9401	-0.43	3.34	107.73	2.912	11	7/24/2014	30:42.3
8045	RWS11	-122.4695536	37.94009	-0.47	3.36	105.42	2.89200008	-122.46955	37.9401	-0.43	3.36	108.88	2.93400007	11	7/24/2014	30:42.4
8046	RWS11	-122.4695558	37.94009	-0.47	3.39	106.42	2.9180001	-122.46955	37.9401	-0.43	3.39	109.83	2.9600001	11	7/24/2014	30:42.5
8047	RWS11	-122.469558	37.94009	-0.47	3.42	107.32	2.94500005	-122.46955	37.9401	-0.43	3.42	110.63	2.98700005	11	7/24/2014	30:42.6
8048	RWS11	-122.4695602	37.94009	-0.47	3.44	108.03	2.97299993	-122.46956	37.9401	-0.4	3.44	111.21	3.04899994	11	7/24/2014	30:42.7
8049	RWS11	-122.4695623	37.94009	-0.47	3.47	108.78	3.00100005	-122.46956	37.9401	-0.43	3.47	111.8	3.04300004	11	7/24/2014	30:42.8
8050	RWS11	-122.4695645	37.94009	-0.47	3.5	109.37	3.02700007	-122.46956	37.9401	-0.4	3.5	112.25	3.10300007	11	7/24/2014	30:42.9
8051	RWS11	-122.4695677	37.94009	-0.47	3.52	109.91	3.05000007	-122.46956	37.94011	-0.4	3.52	112.61	3.12600008	11	7/24/2014	30:43.0

8052	RWS11	-122.4695699	37.94009	-0.47	3.54	110.39	3.07000005	-122.46957	37.94011	-0.35	3.54	112.88	3.19600007	11	7/24/2014	30:43.1
8053	RWS11	-122.4695721	37.94009	-0.47	3.56	110.82	3.08700001	-122.46957	37.94011	-0.4	3.56	113.09	3.16300002	11	7/24/2014	30:43.2
8054	RWS11	-122.4695743	37.94009	-0.47	3.57	111.24	3.09799993	-122.46957	37.94011	-0.4	3.57	113.24	3.17399994	11	7/24/2014	30:43.3
8055	RWS11	-122.4695765	37.94009	-0.51	3.58	111.54	3.07200009	-122.46957	37.94011	-0.4	3.58	113.38	3.18200007	11	7/24/2014	30:43.4
8056	RWS11	-122.4695787	37.94009	-0.47	3.58	111.87	3.11000001	-122.46957	37.94011	-0.43	3.58	113.48	3.15200001	11	7/24/2014	30:43.5
8057	RWS11	-122.4695809	37.9401	-0.47	3.58	112.18	3.11099994	-122.46958	37.94011	-0.43	3.58	113.61	3.15299994	11	7/24/2014	30:43.6
8058	RWS11	-122.4695831	37.9401	-0.47	3.58	112.36	3.11000001	-122.46958	37.94011	-0.4	3.58	113.65	3.18600002	11	7/24/2014	30:43.7
8059	RWS11	-122.4695853	37.9401	-0.51	3.58	112.55	3.07399994	-122.46958	37.94011	-0.43	3.58	113.72	3.14999992	11	7/24/2014	30:43.8
8060	RWS11	-122.4695875	37.9401	-0.47	3.58	112.79	3.1049999	-122.46958	37.94011	-0.4	3.58	113.83	3.1809999	11	7/24/2014	30:43.9
8061	RWS11	-122.4695908	37.9401	-0.51	3.58	112.96	3.06900007	-122.46959	37.94011	-0.43	3.58	113.92	3.14500004	11	7/24/2014	30:44.0
8062	RWS11	-122.469593	37.9401	-0.51	3.57	113.09	3.0679999	-122.46959	37.94011	-0.43	3.57	113.87	3.14399987	11	7/24/2014	30:44.1
8063	RWS11	-122.4695952	37.9401	-0.51	3.57	113.25	3.0679999	-122.46959	37.94011	-0.43	3.57	113.92	3.14399987	11	7/24/2014	30:44.2
8064	RWS11	-122.4695974	37.9401	-0.51	3.58	113.43	3.06900007	-122.46959	37.94011	-0.4	3.58	113.93	3.17900005	11	7/24/2014	30:44.3
8065	RWS11	-122.4695997	37.9401	-0.51	3.58	113.56	3.06999999	-122.46959	37.94011	-0.48	3.58	114.02	3.09499997	11	7/24/2014	30:44.4
8066	RWS11	-122.4696019	37.9401	-0.51	3.58	113.68	3.06999999	-122.4696	37.94011	-0.43	3.58	114.07	3.14599997	11	7/24/2014	30:44.5
8067	RWS11	-122.4696042	37.9401	-0.51	3.58	113.83	3.07099992	-122.4696	37.94011	-0.48	3.58	114.2	3.0959999	11	7/24/2014	30:44.6
8068	RWS11	-122.4696064	37.9401	-0.51	3.58	113.91	3.07300001	-122.4696	37.94011	-0.43	3.58	114.28	3.14899999	11	7/24/2014	30:44.7
8069	RWS11	-122.4696087	37.9401	-0.56	3.58	114.02	3.02299994	-122.4696	37.94011	-0.43	3.58	114.35	3.14999992	11	7/24/2014	30:44.8
8070	RWS11	-122.4696109	37.9401	-0.51	3.58	114.15	3.07600003	-122.46961	37.94012	-0.48	3.58	114.41	3.10100001	11	7/24/2014	30:44.9
8071	RWS11	-122.4696142	37.9401	-0.56	3.59	114.22	3.02800006	-122.46961	37.94012	-0.48	3.59	114.45	3.10400003	11	7/24/2014	30:45.0
8072	RWS11	-122.4696164	37.9401	-0.51	3.59	114.35	3.08200008	-122.46961	37.94012	-0.48	3.59	114.54	3.10700005	11	7/24/2014	30:45.1
8073	RWS11	-122.4696186	37.9401	-0.56	3.59	114.46	3.0340001	-122.46961	37.94012	-0.48	3.59	114.62	3.11000007	11	7/24/2014	30:45.2
8074	RWS11	-122.4696208	37.9401	-0.56	3.6	114.52	3.03800005	-122.46962	37.94012	-0.48	3.6	114.68	3.11400002	11	7/24/2014	30:45.3
8075	RWS11	-122.4696231	37.9401	-0.56	3.6	114.61	3.04299992	-122.46962	37.94012	-0.48	3.6	114.75	3.1189999	11	7/24/2014	30:45.4
8076	RWS11	-122.4696253	37.94011	-0.56	3.61	114.69	3.04800004	-122.46962	37.94012	-0.51	3.61	114.76	3.09000003	11	7/24/2014	30:45.5
8077	RWS11	-122.4696276	37.94011	-0.56	3.61	114.74	3.05599993	-122.46962	37.94012	-0.48	3.61	114.79	3.13199991	11	7/24/2014	30:45.6
8078	RWS11	-122.4696298	37.94011	-0.56	3.62	114.85	3.06400007	-122.46963	37.94012	-0.48	3.62	114.82	3.14000005	11	7/24/2014	30:45.7
8079	RWS11	-122.4696321	37.94011	-0.56	3.63	114.9	3.07400006	-122.46963	37.94012	-0.51	3.63	114.89	3.11600006	11	7/24/2014	30:45.8
8080	RWS11	-122.4696343	37.94011	-0.56	3.64	114.96	3.08499998	-122.46963	37.94012	-0.48	3.64	114.93	3.16099995	11	7/24/2014	30:45.9
8081	RWS11	-122.4696376	37.94011	-0.59	3.65	115	3.06200004	-122.46963	37.94012	-0.48	3.65	114.99	3.17300004	11	7/24/2014	30:46.0
8082	RWS11	-122.4696398	37.94011	-0.56	3.66	115.07	3.10799998	-122.46964	37.94012	-0.51	3.66	114.93	3.14999998	11	7/24/2014	30:46.1
8083	RWS11	-122.469642	37.94011	-0.59	3.67	115.11	3.08200002	-122.46964	37.94012	-0.51	3.67	114.99	3.15900004	11	7/24/2014	30:46.2
8084	RWS11	-122.4696442	37.94011	-0.56	3.68	115.12	3.12199992	-122.46964	37.94012	-0.48	3.68	115.02	3.19799989	11	7/24/2014	30:46.3

8085	RWS11	-122.4696464	37.94011	-0.59	3.68	115.15	3.08699989	-122.46964	37.94012	-0.51	3.68	115.03	3.16399992	11	7/24/2014	30:46.4
8086	RWS11	-122.4696487	37.94011	-0.56	3.67	115.19	3.11700004	-122.46964	37.94012	-0.48	3.67	115.12	3.19300002	11	7/24/2014	30:46.5
8087	RWS11	-122.4696509	37.94011	-0.59	3.66	115.19	3.07200003	-122.46965	37.94012	-0.51	3.66	115.06	3.14900005	11	7/24/2014	30:46.6
8088	RWS11	-122.4696532	37.94011	-0.56	3.65	115.16	3.09400004	-122.46965	37.94012	-0.48	3.65	115.05	3.17000002	11	7/24/2014	30:46.7
8089	RWS11	-122.4696554	37.94011	-0.56	3.64	115.17	3.07899994	-122.46965	37.94013	-0.51	3.64	115.02	3.12099993	11	7/24/2014	30:46.8
8090	RWS11	-122.4696576	37.94011	-0.56	3.62	115.21	3.06400007	-122.46965	37.94013	-0.43	3.62	115.01	3.19100004	11	7/24/2014	30:46.9
8091	RWS11	-122.4696609	37.94011	-0.59	3.61	115.2	3.01600003	-122.46966	37.94013	-0.51	3.61	114.99	3.09300005	11	7/24/2014	30:47.0
8092	RWS11	-122.4696632	37.94011	-0.56	3.6	115.25	3.042	-122.46966	37.94013	-0.48	3.6	115.01	3.11799997	11	7/24/2014	30:47.1
8093	RWS11	-122.4696654	37.94011	-0.56	3.59	115.25	3.03500003	-122.46966	37.94013	-0.48	3.59	114.93	3.111	11	7/24/2014	30:47.2
8094	RWS11	-122.4696676	37.94011	-0.56	3.59	115.27	3.03100008	-122.46966	37.94013	-0.51	3.59	114.9	3.07300007	11	7/24/2014	30:47.3
8095	RWS11	-122.4696699	37.94012	-0.56	3.59	115.29	3.02999991	-122.46967	37.94013	-0.51	3.59	114.9	3.07199991	11	7/24/2014	30:47.4
8096	RWS11	-122.4696722	37.94012	-0.56	3.59	115.32	3.02899998	-122.46967	37.94013	-0.48	3.59	114.89	3.10499996	11	7/24/2014	30:47.5
8097	RWS11	-122.4696744	37.94012	-0.59	3.59	115.33	2.99300003	-122.46967	37.94013	-0.51	3.59	114.88	3.07000005	11	7/24/2014	30:47.6
8098	RWS11	-122.4696767	37.94012	-0.51	3.58	115.36	3.07600003	-122.46967	37.94013	-0.48	3.58	114.85	3.10100001	11	7/24/2014	30:47.7
8099	RWS11	-122.4696789	37.94012	-0.56	3.58	115.36	3.01899999	-122.46967	37.94013	-0.48	3.58	114.86	3.09499997	11	7/24/2014	30:47.8
8100	RWS11	-122.4696811	37.94012	-0.56	3.57	115.33	3.009	-122.46968	37.94013	-0.51	3.57	114.81	3.051	11	7/24/2014	30:47.9
8101	RWS11	-122.4696845	37.94012	-0.56	3.55	115.29	2.99500006	-122.46968	37.94013	-0.51	3.55	114.74	3.03700006	11	7/24/2014	30:48.0
8102	RWS11	-122.4696867	37.94012	-0.56	3.53	115.36	2.97600001	-122.46968	37.94013	-0.48	3.53	114.76	3.05199999	11	7/24/2014	30:48.1
8103	RWS11	-122.4696889	37.94012	-0.56	3.51	115.31	2.95099992	-122.46968	37.94013	-0.48	3.51	114.77	3.02699989	11	7/24/2014	30:48.2
8104	RWS11	-122.4696911	37.94012	-0.51	3.48	115.29	2.97300011	-122.46969	37.94013	-0.48	3.48	114.67	2.99800009	11	7/24/2014	30:48.3
8105	RWS11	-122.4696933	37.94012	-0.56	3.45	115.2	2.88900012	-122.46969	37.94013	-0.51	3.45	114.61	2.93100011	11	7/24/2014	30:48.4
8106	RWS11	-122.4696956	37.94012	-0.51	3.41	115.17	2.9040001	-122.46969	37.94013	-0.48	3.41	114.57	2.92900008	11	7/24/2014	30:48.5
8107	RWS11	-122.4696978	37.94012	-0.56	3.37	115.15	2.815	-122.46969	37.94013	-0.48	3.37	114.48	2.89099997	11	7/24/2014	30:48.6
8108	RWS11	-122.4697001	37.94012	-0.51	3.34	115.11	2.82900006	-122.4697	37.94013	-0.51	3.34	114.45	2.82000005	11	7/24/2014	30:48.7
8109	RWS11	-122.4697023	37.94012	-0.56	3.3	115.07	2.74200004	-122.4697	37.94014	-0.48	3.3	114.41	2.81800002	11	7/24/2014	30:48.8
8110	RWS11	-122.4697045	37.94012	-0.51	3.27	115	2.76099998	-122.4697	37.94014	-0.43	3.27	114.34	2.83699995	11	7/24/2014	30:48.9
8111	RWS11	-122.4697077	37.94012	-0.56	3.24	114.92	2.68399996	-122.4697	37.94014	-0.43	3.24	114.22	2.81099993	11	7/24/2014	30:49.0
8112	RWS11	-122.4697099	37.94012	-0.51	3.22	114.87	2.71499997	-122.46971	37.94014	-0.43	3.22	114.18	2.79099995	11	7/24/2014	30:49.1
8113	RWS11	-122.4697121	37.94012	-0.56	3.21	114.78	2.65000004	-122.46971	37.94014	-0.43	3.21	114.14	2.77700001	11	7/24/2014	30:49.2
8114	RWS11	-122.4697143	37.94012	-0.51	3.2	114.67	2.69499999	-122.46971	37.94014	-0.43	3.2	114.08	2.77099997	11	7/24/2014	30:49.3
8115	RWS11	-122.4697166	37.94013	-0.51	3.2	114.56	2.69400007	-122.46971	37.94014	-0.48	3.2	113.99	2.71900004	11	7/24/2014	30:49.4
8116	RWS11	-122.4697188	37.94013	-0.51	3.21	114.43	2.70000011	-122.46971	37.94014	-0.43	3.21	113.9	2.77600008	11	7/24/2014	30:49.5
8117	RWS11	-122.469721	37.94013	-0.56	3.22	114.36	2.66000003	-122.46972	37.94014	-0.48	3.22	113.84	2.736	11	7/24/2014	30:49.6

8118	RWS11	-122.4697232	37.94013	-0.47	3.23	114.23	2.76000011	-122.46972	37.94014	-0.48	3.23	113.77	2.75100011	11	7/24/2014	30:49.7
8119	RWS11	-122.4697254	37.94013	-0.51	3.25	114.12	2.74499995	-122.46972	37.94014	-0.43	3.25	113.75	2.82099992	11	7/24/2014	30:49.8
8120	RWS11	-122.4697276	37.94013	-0.47	3.27	113.99	2.801	-122.46972	37.94014	-0.4	3.27	113.7	2.877	11	7/24/2014	30:49.9
8121	RWS11	-122.4697309	37.94013	-0.51	3.3	113.83	2.79099995	-122.46973	37.94014	-0.43	3.3	113.63	2.86699992	11	7/24/2014	30:50.0
8122	RWS11	-122.4697331	37.94013	-0.47	3.32	113.7	2.85000002	-122.46973	37.94014	-0.48	3.32	113.57	2.84100002	11	7/24/2014	30:50.1
8123	RWS11	-122.4697354	37.94013	-0.47	3.35	113.58	2.87600005	-122.46973	37.94014	-0.43	3.35	113.55	2.91800004	11	7/24/2014	30:50.2
8124	RWS11	-122.4697376	37.94013	-0.47	3.37	113.51	2.9009999	-122.46973	37.94014	-0.35	3.37	113.5	3.02699992	11	7/24/2014	30:50.3
8125	RWS11	-122.4697399	37.94013	-0.47	3.4	113.42	2.926	-122.46974	37.94014	-0.43	3.4	113.4	2.96799999	11	7/24/2014	30:50.4
8126	RWS11	-122.4697421	37.94013	-0.47	3.42	113.33	2.94800007	-122.46974	37.94014	-0.4	3.42	113.37	3.02400008	11	7/24/2014	30:50.5
8127	RWS11	-122.4697444	37.94013	-0.47	3.44	113.27	2.96699989	-122.46974	37.94014	-0.48	3.44	113.26	2.95799989	11	7/24/2014	30:50.6
8128	RWS11	-122.4697466	37.94013	-0.47	3.45	113.2	2.98100007	-122.46974	37.94014	-0.4	3.45	113.22	3.05700007	11	7/24/2014	30:50.7
8129	RWS11	-122.4697489	37.94013	-0.47	3.46	113.18	2.98800004	-122.46974	37.94015	-0.4	3.46	113.17	3.06400004	11	7/24/2014	30:50.8
8130	RWS11	-122.4697511	37.94013	-0.42	3.46	113.09	3.03900003	-122.46975	37.94015	-0.4	3.46	113.13	3.06400004	11	7/24/2014	30:50.9
8131	RWS11	-122.4697545	37.94013	-0.47	3.45	113.11	2.98100007	-122.46975	37.94015	-0.43	3.45	113.06	3.02300006	11	7/24/2014	30:51.0
8132	RWS11	-122.4697567	37.94013	-0.42	3.44	113.05	3.01799989	-122.46975	37.94015	-0.35	3.44	113.1	3.09299991	11	7/24/2014	30:51.1
8133	RWS11	-122.4697589	37.94013	-0.47	3.42	113.08	2.94599998	-122.46975	37.94015	-0.35	3.42	113.15	3.072	11	7/24/2014	30:51.2
8134	RWS11	-122.4697612	37.94013	-0.42	3.39	113.11	2.97099996	-122.46976	37.94015	-0.4	3.39	113.15	2.99599996	11	7/24/2014	30:51.3
8135	RWS11	-122.4697635	37.94013	-0.47	3.36	113.11	2.89099991	-122.46976	37.94015	-0.43	3.36	113.24	2.93299991	11	7/24/2014	30:51.4
8136	RWS11	-122.4697658	37.94014	-0.42	3.33	113.18	2.91199994	-122.46976	37.94015	-0.35	3.33	113.24	2.98699996	11	7/24/2014	30:51.5
8137	RWS11	-122.4697681	37.94014	-0.42	3.3	113.24	2.8829999	-122.46976	37.94015	-0.43	3.3	113.24	2.87399989	11	7/24/2014	30:51.6
8138	RWS11	-122.4697704	37.94014	-0.39	3.28	113.32	2.89300004	-122.46977	37.94015	-0.31	3.28	113.34	2.96700004	11	7/24/2014	30:51.7
8139	RWS11	-122.4697726	37.94014	-0.42	3.26	113.42	2.83599997	-122.46977	37.94015	-0.35	3.26	113.45	2.91099998	11	7/24/2014	30:51.8
8140	RWS11	-122.4697749	37.94014	-0.39	3.24	113.41	2.85499993	-122.46977	37.94015	-0.4	3.24	113.43	2.84499994	11	7/24/2014	30:51.9
8141	RWS11	-122.4697782	37.94014	-0.42	3.23	113.5	2.80800009	-122.46977	37.94015	-0.4	3.23	113.48	2.83300009	11	7/24/2014	30:52.0
8142	RWS11	-122.4697805	37.94014	-0.39	3.22	113.55	2.83499995	-122.46978	37.94015	-0.31	3.22	113.54	2.90899995	11	7/24/2014	30:52.1
8143	RWS11	-122.4697827	37.94014	-0.42	3.22	113.58	2.796	-122.46978	37.94015	-0.35	3.22	113.58	2.87100002	11	7/24/2014	30:52.2
8144	RWS11	-122.469785	37.94014	-0.39	3.22	113.71	2.83000007	-122.46978	37.94015	-0.4	3.22	113.64	2.82000008	11	7/24/2014	30:52.3
8145	RWS11	-122.4697872	37.94014	-0.42	3.22	113.78	2.79699993	-122.46978	37.94015	-0.35	3.22	113.69	2.87199995	11	7/24/2014	30:52.4
8146	RWS11	-122.4697895	37.94014	-0.39	3.22	113.84	2.83599988	-122.46979	37.94015	-0.31	3.22	113.69	2.90999988	11	7/24/2014	30:52.5
8147	RWS11	-122.4697918	37.94014	-0.42	3.23	113.89	2.80500007	-122.46979	37.94015	-0.4	3.23	113.71	2.83000007	11	7/24/2014	30:52.6
8148	RWS11	-122.4697941	37.94014	-0.39	3.23	113.97	2.84600011	-122.46979	37.94015	-0.35	3.23	113.82	2.88600013	11	7/24/2014	30:52.7
8149	RWS11	-122.4697963	37.94014	-0.39	3.24	113.99	2.85300007	-122.46979	37.94015	-0.4	3.24	113.91	2.84300008	11	7/24/2014	30:52.8
8150	RWS11	-122.4697985	37.94014	-0.39	3.25	114.08	2.86300007	-122.46979	37.94016	-0.4	3.25	113.9	2.85300007	11	7/24/2014	30:52.9

8151	RWS11	-122.4698019	37.94014	-0.39	3.26	114.08	2.87499991	-122.4698	37.94016	-0.4	3.26	113.9	2.86499992	11	7/24/2014	30:53.0
8152	RWS11	-122.4698041	37.94014	-0.39	3.28	114.1	2.88900009	-122.4698	37.94016	-0.35	3.28	113.92	2.92900011	11	7/24/2014	30:53.1
8153	RWS11	-122.4698064	37.94014	-0.39	3.29	114.12	2.90600005	-122.4698	37.94016	-0.4	3.29	113.9	2.89600006	11	7/24/2014	30:53.2
8154	RWS11	-122.4698086	37.94014	-0.39	3.31	114.14	2.9250001	-122.4698	37.94016	-0.31	3.31	113.88	2.9990001	11	7/24/2014	30:53.3
8155	RWS11	-122.4698109	37.94014	-0.39	3.33	114.17	2.94500008	-122.46981	37.94016	-0.35	3.33	113.92	2.9850001	11	7/24/2014	30:53.4
8156	RWS11	-122.4698132	37.94015	-0.34	3.35	114.19	3.0149999	-122.46981	37.94016	-0.35	3.35	113.92	3.00399992	11	7/24/2014	30:53.5
8157	RWS11	-122.4698154	37.94015	-0.39	3.37	114.18	2.98200002	-122.46981	37.94016	-0.4	3.37	113.9	2.97200003	11	7/24/2014	30:53.6
8158	RWS11	-122.4698177	37.94015	-0.34	3.38	114.16	3.04799989	-122.46981	37.94016	-0.35	3.38	113.88	3.03699991	11	7/24/2014	30:53.7
8159	RWS11	-122.4698199	37.94015	-0.39	3.39	114.14	3.00899997	-122.46982	37.94016	-0.4	3.39	113.91	2.99899998	11	7/24/2014	30:53.8
8160	RWS11	-122.4698222	37.94015	-0.34	3.4	114.13	3.06600001	-122.46982	37.94016	-0.35	3.4	113.96	3.05500004	11	7/24/2014	30:53.9
8161	RWS11	-122.4698255	37.94015	-0.34	3.4	114.05	3.06800011	-122.46982	37.94016	-0.31	3.4	113.87	3.09100011	11	7/24/2014	30:54.0
8162	RWS11	-122.4698277	37.94015	-0.34	3.4	113.96	3.06399992	-122.46982	37.94016	-0.31	3.4	113.85	3.08699992	11	7/24/2014	30:54.1
8163	RWS11	-122.46983	37.94015	-0.39	3.39	113.98	3.00299993	-122.46983	37.94016	-0.31	3.39	113.85	3.07699993	11	7/24/2014	30:54.2
8164	RWS11	-122.4698323	37.94015	-0.34	3.37	113.89	3.03900006	-122.46983	37.94016	-0.35	3.37	113.76	3.02800009	11	7/24/2014	30:54.3
8165	RWS11	-122.4698345	37.94015	-0.34	3.35	113.83	3.01900008	-122.46983	37.94016	-0.35	3.35	113.7	3.00800011	11	7/24/2014	30:54.4
8166	RWS11	-122.4698369	37.94015	-0.3	3.33	113.76	3.03199995	-122.46983	37.94016	-0.31	3.33	113.63	3.02099994	11	7/24/2014	30:54.5
8167	RWS11	-122.4698392	37.94015	-0.34	3.31	113.71	2.97700003	-122.46983	37.94016	-0.35	3.31	113.6	2.96600005	11	7/24/2014	30:54.6
8168	RWS11	-122.4698415	37.94015	-0.34	3.29	113.54	2.9589999	-122.46984	37.94016	-0.31	3.29	113.54	2.9819999	11	7/24/2014	30:54.7
8169	RWS11	-122.4698439	37.94015	-0.34	3.28	113.43	2.94499996	-122.46984	37.94016	-0.35	3.28	113.42	2.93399999	11	7/24/2014	30:54.8
8170	RWS11	-122.4698462	37.94015	-0.34	3.27	113.32	2.93499997	-122.46984	37.94016	-0.31	3.27	113.31	2.95799997	11	7/24/2014	30:54.9
8171	RWS11	-122.4698486	37.94015	-0.34	3.27	113.22	2.9300001	-122.46984	37.94017	-0.35	3.27	113.26	2.91900012	11	7/24/2014	30:55.0
8172	RWS11	-122.469851	37.94015	-0.34	3.27	113.13	2.9300001	-122.46985	37.94017	-0.31	3.27	113.19	2.9530001	11	7/24/2014	30:55.1
8173	RWS11	-122.4698533	37.94015	-0.34	3.27	113.05	2.93400005	-122.46985	37.94017	-0.35	3.27	113.1	2.92300007	11	7/24/2014	30:55.2
8174	RWS11	-122.4698557	37.94015	-0.3	3.28	112.88	2.97599995	-122.46985	37.94017	-0.31	3.28	112.99	2.96499994	11	7/24/2014	30:55.3
8175	RWS11	-122.469858	37.94015	-0.34	3.29	112.8	2.95400003	-122.46985	37.94017	-0.31	3.29	112.86	2.97700003	11	7/24/2014	30:55.4
8176	RWS11	-122.4698604	37.94015	-0.34	3.31	112.66	2.97099999	-122.46986	37.94017	-0.31	3.31	112.79	2.99399999	11	7/24/2014	30:55.5
8177	RWS11	-122.4698627	37.94016	-0.34	3.33	112.58	2.99199989	-122.46986	37.94017	-0.35	3.33	112.78	2.98099992	11	7/24/2014	30:55.6
8178	RWS11	-122.469865	37.94016	-0.34	3.35	112.39	3.01600006	-122.46986	37.94017	-0.31	3.35	112.65	3.03900006	11	7/24/2014	30:55.7
8179	RWS11	-122.4698672	37.94016	-0.34	3.38	112.2	3.04300001	-122.46986	37.94017	-0.35	3.38	112.53	3.03200004	11	7/24/2014	30:55.8
8180	RWS11	-122.4698695	37.94016	-0.3	3.41	112.08	3.1049999	-122.46987	37.94017	-0.28	3.41	112.42	3.1279999	11	7/24/2014	30:55.9
8181	RWS11	-122.4698728	37.94016	-0.34	3.43	111.92	3.09900001	-122.46987	37.94017	-0.31	3.43	112.36	3.12200001	11	7/24/2014	30:56.0
8182	RWS11	-122.469875	37.94016	-0.34	3.46	111.77	3.1240001	-122.46987	37.94017	-0.31	3.46	112.2	3.1470001	11	7/24/2014	30:56.1
8183	RWS11	-122.4698773	37.94016	-0.34	3.48	111.68	3.14800003	-122.46987	37.94017	-0.35	3.48	112.12	3.13700005	11	7/24/2014	30:56.2

8184	RWS11	-122.4698796	37.94016	-0.34	3.5	111.48	3.16899994	-122.46988	37.94017	-0.31	3.5	112.02	3.19199994	11	7/24/2014	30:56.3
8185	RWS11	-122.4698819	37.94016	-0.34	3.52	111.35	3.18700007	-122.46988	37.94017	-0.31	3.52	111.9	3.21000007	11	7/24/2014	30:56.4
8186	RWS11	-122.4698842	37.94016	-0.34	3.54	111.2	3.20000008	-122.46988	37.94017	-0.28	3.54	111.83	3.25700009	11	7/24/2014	30:56.5
8187	RWS11	-122.4698866	37.94016	-0.34	3.55	111.02	3.21000007	-122.46988	37.94017	-0.31	3.55	111.68	3.23300007	11	7/24/2014	30:56.6
8188	RWS11	-122.469889	37.94016	-0.34	3.55	110.87	3.21700004	-122.46988	37.94017	-0.31	3.55	111.52	3.24000004	11	7/24/2014	30:56.7
8189	RWS11	-122.4698914	37.94016	-0.34	3.56	110.71	3.22099999	-122.46989	37.94017	-0.35	3.56	111.44	3.21000001	11	7/24/2014	30:56.8
8190	RWS11	-122.4698938	37.94016	-0.34	3.56	110.55	3.22400001	-122.46989	37.94017	-0.28	3.56	111.31	3.28100002	11	7/24/2014	30:56.9
8191	RWS11	-122.4698963	37.94016	-0.34	3.56	110.36	3.22499993	-122.46989	37.94018	-0.35	3.56	111.24	3.21399996	11	7/24/2014	30:57.0
8192	RWS11	-122.4698988	37.94016	-0.34	3.56	110.22	3.2260001	-122.46989	37.94018	-0.28	3.56	111.11	3.28300011	11	7/24/2014	30:57.1
8193	RWS11	-122.4699013	37.94016	-0.39	3.56	110.01	3.17600003	-122.4699	37.94018	-0.31	3.56	110.95	3.25000003	11	7/24/2014	30:57.2
8194	RWS11	-122.4699038	37.94016	-0.33	3.56	109.89	3.22899997	-122.4699	37.94018	-0.28	3.56	110.82	3.28599995	11	7/24/2014	30:57.3
8195	RWS11	-122.4699063	37.94016	-0.38	3.57	109.66	3.18000007	-122.4699	37.94018	-0.28	3.57	110.69	3.28800005	11	7/24/2014	30:57.4
8196	RWS11	-122.4699088	37.94016	-0.33	3.57	109.54	3.23199999	-122.4699	37.94018	-0.28	3.57	110.54	3.28899997	11	7/24/2014	30:57.5
8197	RWS11	-122.4699113	37.94017	-0.33	3.57	109.38	3.23199999	-122.46991	37.94018	-0.35	3.57	110.4	3.22099999	11	7/24/2014	30:57.6
8198	RWS11	-122.4699137	37.94017	-0.33	3.57	109.2	3.23100007	-122.46991	37.94018	-0.23	3.57	110.27	3.33900006	11	7/24/2014	30:57.7
8199	RWS11	-122.4699161	37.94017	-0.33	3.56	109.03	3.2299999	-122.46991	37.94018	-0.35	3.56	110.16	3.21899989	11	7/24/2014	30:57.8
8200	RWS11	-122.4699185	37.94017	-0.33	3.56	108.92	3.22800004	-122.46991	37.94018	-0.31	3.56	110.04	3.25100005	11	7/24/2014	30:57.9
8201	RWS11	-122.469922	37.94017	-0.38	3.56	108.72	3.17400002	-122.46992	37.94018	-0.31	3.56	109.9	3.24800003	11	7/24/2014	30:58.0
8202	RWS11	-122.4699243	37.94017	-0.33	3.56	108.57	3.22100008	-122.46992	37.94018	-0.23	3.56	109.78	3.32900007	11	7/24/2014	30:58.1
8203	RWS11	-122.4699267	37.94017	-0.33	3.55	108.39	3.21700013	-122.46992	37.94018	-0.31	3.55	109.7	3.24000013	11	7/24/2014	30:58.2
8204	RWS11	-122.469929	37.94017	-0.33	3.55	108.18	3.21100008	-122.46992	37.94018	-0.23	3.55	109.47	3.31900008	11	7/24/2014	30:58.3
8205	RWS11	-122.4699314	37.94017	-0.33	3.54	108.07	3.20500004	-122.46993	37.94018	-0.28	3.54	109.36	3.26200002	11	7/24/2014	30:58.4
8206	RWS11	-122.4699337	37.94017	-0.33	3.53	107.92	3.19699991	-122.46993	37.94018	-0.28	3.53	109.27	3.25399989	11	7/24/2014	30:58.5
8207	RWS11	-122.4699361	37.94017	-0.38	3.52	107.76	3.13800001	-122.46993	37.94018	-0.31	3.52	109.17	3.21200001	11	7/24/2014	30:58.6
8208	RWS11	-122.4699385	37.94017	-0.38	3.52	107.58	3.13100004	-122.46993	37.94018	-0.28	3.52	109.02	3.23900002	11	7/24/2014	30:58.7
8209	RWS11	-122.4699409	37.94017	-0.33	3.51	107.46	3.17399991	-122.46994	37.94018	-0.28	3.51	108.89	3.23099989	11	7/24/2014	30:58.8
8210	RWS11	-122.4699433	37.94017	-0.33	3.5	107.3	3.1680001	-122.46994	37.94019	-0.28	3.5	108.84	3.22500008	11	7/24/2014	30:58.9
8211	RWS11	-122.4699457	37.94017	-0.33	3.5	107.17	3.16299999	-122.46994	37.94019	-0.23	3.5	108.74	3.27099998	11	7/24/2014	30:59.0
8212	RWS11	-122.4699481	37.94017	-0.33	3.49	107.03	3.15900004	-122.46994	37.94019	-0.23	3.49	108.61	3.26700003	11	7/24/2014	30:59.1
8213	RWS11	-122.4699505	37.94017	-0.38	3.49	106.84	3.10700011	-122.46995	37.94019	-0.31	3.49	108.51	3.18100011	11	7/24/2014	30:59.2
8214	RWS11	-122.4699529	37.94017	-0.33	3.49	106.72	3.15900004	-122.46995	37.94019	-0.19	3.49	108.39	3.30100003	11	7/24/2014	30:59.3
8215	RWS11	-122.4699553	37.94018	-0.33	3.5	106.64	3.16200006	-122.46995	37.94019	-0.28	3.5	108.34	3.21900004	11	7/24/2014	30:59.4
8216	RWS11	-122.4699577	37.94018	-0.33	3.5	106.51	3.1680001	-122.46995	37.94019	-0.28	3.5	108.21	3.22500008	11	7/24/2014	30:59.5

8217	RWS11	-122.46996	37.94018	-0.33	3.51	106.38	3.17699993	-122.46996	37.94019	-0.23	3.51	108.13	3.28499992	11	7/24/2014	30:59.6
8218	RWS11	-122.4699623	37.94018	-0.33	3.52	106.31	3.18900001	-122.46996	37.94019	-0.28	3.52	108.05	3.24599999	11	7/24/2014	30:59.7
8219	RWS11	-122.4699645	37.94018	-0.38	3.54	106.18	3.15100002	-122.46996	37.94019	-0.28	3.54	108	3.259	11	7/24/2014	30:59.8
8220	RWS11	-122.4699668	37.94018	-0.33	3.55	106.07	3.21500003	-122.46996	37.94019	-0.23	3.55	107.88	3.32300003	11	7/24/2014	30:59.9
8221	RWS11	-122.4699702	37.94018	-0.38	3.56	106.01	3.17600012	-122.46997	37.94019	-0.31	3.56	107.81	3.25000012	11	7/24/2014	31:00.0
8222	RWS11	-122.4699724	37.94018	-0.33	3.57	105.98	3.23899996	-122.46997	37.94019	-0.28	3.57	107.82	3.29599994	11	7/24/2014	31:00.1
8223	RWS11	-122.4699747	37.94018	-0.38	3.58	105.83	3.19700003	-122.46997	37.94019	-0.23	3.58	107.73	3.35600002	11	7/24/2014	31:00.2
8224	RWS11	-122.469977	37.94018	-0.33	3.59	105.78	3.25599992	-122.46997	37.94019	-0.23	3.59	107.64	3.36399992	11	7/24/2014	31:00.3
8225	RWS11	-122.4699793	37.94018	-0.38	3.6	105.7	3.21099997	-122.46997	37.94019	-0.23	3.6	107.58	3.36999996	11	7/24/2014	31:00.4
8226	RWS11	-122.4699816	37.94018	-0.33	3.6	105.74	3.26599991	-122.46998	37.94019	-0.28	3.6	107.5	3.32299989	11	7/24/2014	31:00.5
8227	RWS11	-122.4699839	37.94018	-0.38	3.6	105.7	3.21600008	-122.46998	37.94019	-0.23	3.6	107.46	3.37500007	11	7/24/2014	31:00.6
8228	RWS11	-122.4699863	37.94018	-0.33	3.6	105.63	3.26599991	-122.46998	37.9402	-0.19	3.6	107.42	3.4079999	11	7/24/2014	31:00.7
8229	RWS11	-122.4699886	37.94018	-0.38	3.6	105.61	3.21099997	-122.46998	37.9402	-0.23	3.6	107.35	3.36999996	11	7/24/2014	31:00.8
8230	RWS11	-122.469991	37.94018	-0.33	3.59	105.58	3.25700009	-122.46999	37.9402	-0.28	3.59	107.31	3.31400007	11	7/24/2014	31:00.9
8231	RWS11	-122.4699934	37.94018	-0.38	3.58	105.61	3.19900012	-122.46999	37.9402	-0.23	3.58	107.27	3.35800011	11	7/24/2014	31:01.0
8232	RWS11	-122.4699958	37.94018	-0.33	3.58	105.63	3.24299991	-122.46999	37.9402	-0.23	3.58	107.19	3.35099991	11	7/24/2014	31:01.1
8233	RWS11	-122.4699981	37.94018	-0.33	3.57	105.65	3.23700011	-122.46999	37.9402	-0.31	3.57	107.22	3.26000011	11	7/24/2014	31:01.2
8234	RWS11	-122.4700005	37.94019	-0.33	3.57	105.69	3.23100007	-122.47	37.9402	-0.23	3.57	107.17	3.33900006	11	7/24/2014	31:01.3
8235	RWS11	-122.4700028	37.94019	-0.33	3.56	105.69	3.22700012	-122.47	37.9402	-0.19	3.56	107.16	3.36900011	11	7/24/2014	31:01.4
8236	RWS11	-122.4700051	37.94019	-0.33	3.56	105.73	3.222	-122.47	37.9402	-0.19	3.56	107.13	3.36399999	11	7/24/2014	31:01.5
8237	RWS11	-122.4700074	37.94019	-0.33	3.55	105.73	3.21700013	-122.47	37.9402	-0.28	3.55	107.08	3.27400011	11	7/24/2014	31:01.6
8238	RWS11	-122.4700097	37.94019	-0.33	3.55	105.82	3.21100008	-122.47	37.9402	-0.31	3.55	107.06	3.23400009	11	7/24/2014	31:01.7
8239	RWS11	-122.4700119	37.94019	-0.38	3.54	105.86	3.15300012	-122.47001	37.9402	-0.28	3.54	107.07	3.2610001	11	7/24/2014	31:01.8
8240	RWS11	-122.4700142	37.94019	-0.33	3.53	105.97	3.19699991	-122.47001	37.9402	-0.31	3.53	107.08	3.21999991	11	7/24/2014	31:01.9
8241	RWS11	-122.4700175	37.94019	-0.33	3.52	105.99	3.18900001	-122.47001	37.9402	-0.28	3.52	107.11	3.24599999	11	7/24/2014	31:02.0
8242	RWS11	-122.4700197	37.94019	-0.33	3.52	106.05	3.18100011	-122.47001	37.9402	-0.28	3.52	107.11	3.23800009	11	7/24/2014	31:02.1
8243	RWS11	-122.4700219	37.94019	-0.33	3.51	106.21	3.17200005	-122.47002	37.9402	-0.31	3.51	107.19	3.19500005	11	7/24/2014	31:02.2
8244	RWS11	-122.4700241	37.94019	-0.33	3.5	106.32	3.16299999	-122.47002	37.9402	-0.31	3.5	107.23	3.18599999	11	7/24/2014	31:02.3
8245	RWS11	-122.4700264	37.94019	-0.33	3.49	106.45	3.15399992	-122.47002	37.9402	-0.31	3.49	107.36	3.17699993	11	7/24/2014	31:02.4
8246	RWS11	-122.4700287	37.94019	-0.3	3.48	106.57	3.17600006	-122.47002	37.94021	-0.31	3.48	107.43	3.16500008	11	7/24/2014	31:02.5
8247	RWS11	-122.470031	37.94019	-0.33	3.46	106.71	3.13	-122.47003	37.94021	-0.31	3.46	107.58	3.153	11	7/24/2014	31:02.6
8248	RWS11	-122.4700333	37.94019	-0.33	3.45	106.84	3.11600006	-122.47003	37.94021	-0.31	3.45	107.59	3.13900006	11	7/24/2014	31:02.7
8249	RWS11	-122.4700356	37.94019	-0.33	3.44	106.92	3.10200012	-122.47003	37.94021	-0.28	3.44	107.56	3.1590001	11	7/24/2014	31:02.8

8250	RWS11	-122.470038	37.94019	-0.33	3.42	107.1	3.08600008	-122.47003	37.94021	-0.28	3.42	107.66	3.14300007	11	7/24/2014	31:02.9
8251	RWS11	-122.4700403	37.94019	-0.38	3.4	107.25	3.01800013	-122.47004	37.94021	-0.31	3.4	107.76	3.09200013	11	7/24/2014	31:03.0
8252	RWS11	-122.4700426	37.9402	-0.33	3.38	107.41	3.04899991	-122.47004	37.94021	-0.31	3.38	107.84	3.07199991	11	7/24/2014	31:03.1
8253	RWS11	-122.4700449	37.9402	-0.33	3.36	107.51	3.02700007	-122.47004	37.94021	-0.31	3.36	107.96	3.05000007	11	7/24/2014	31:03.2
8254	RWS11	-122.4700472	37.9402	-0.33	3.34	107.62	3.00100005	-122.47004	37.94021	-0.31	3.34	108.04	3.02400005	11	7/24/2014	31:03.3
8255	RWS11	-122.4700494	37.9402	-0.38	3.31	107.71	2.92199993	-122.47004	37.94021	-0.31	3.31	108.08	2.99599993	11	7/24/2014	31:03.4
8256	RWS11	-122.4700517	37.9402	-0.33	3.28	107.88	2.94400012	-122.47005	37.94021	-0.28	3.28	108.22	3.00100011	11	7/24/2014	31:03.5
8257	RWS11	-122.470054	37.9402	-0.33	3.25	107.99	2.91299999	-122.47005	37.94021	-0.31	3.25	108.32	2.93599999	11	7/24/2014	31:03.6
8258	RWS11	-122.4700562	37.9402	-0.33	3.22	108.1	2.88399994	-122.47005	37.94021	-0.35	3.22	108.42	2.87299994	11	7/24/2014	31:03.7
8259	RWS11	-122.4700584	37.9402	-0.33	3.19	108.27	2.85900009	-122.47005	37.94021	-0.35	3.19	108.46	2.84800008	11	7/24/2014	31:03.8
8260	RWS11	-122.4700605	37.9402	-0.33	3.17	108.34	2.83799994	-122.47006	37.94021	-0.28	3.17	108.55	2.89499992	11	7/24/2014	31:03.9
8261	RWS11	-122.4700637	37.9402	-0.38	3.16	108.49	2.77200007	-122.47006	37.94021	-0.31	3.16	108.64	2.84600008	11	7/24/2014	31:04.0
8262	RWS11	-122.4700659	37.9402	-0.33	3.15	108.57	2.81499994	-122.47006	37.94021	-0.23	3.15	108.72	2.92299993	11	7/24/2014	31:04.1
8263	RWS11	-122.4700681	37.9402	-0.38	3.15	108.66	2.76300001	-122.47006	37.94021	-0.23	3.15	108.84	2.92200001	11	7/24/2014	31:04.2
8264	RWS11	-122.4700703	37.9402	-0.33	3.16	108.73	2.82199991	-122.47007	37.94022	-0.35	3.16	108.92	2.8109999	11	7/24/2014	31:04.3
8265	RWS11	-122.4700726	37.9402	-0.33	3.17	108.77	2.83600008	-122.47007	37.94022	-0.4	3.17	108.98	2.77500007	11	7/24/2014	31:04.4
8266	RWS11	-122.4700748	37.9402	-0.33	3.19	108.79	2.8549999	-122.47007	37.94022	-0.35	3.19	108.98	2.84399989	11	7/24/2014	31:04.5
8267	RWS11	-122.4700771	37.9402	-0.38	3.21	108.86	2.82800007	-122.47007	37.94022	-0.35	3.21	109.03	2.86800006	11	7/24/2014	31:04.6
8268	RWS11	-122.4700794	37.9402	-0.33	3.24	108.86	2.903	-122.47007	37.94022	-0.35	3.24	109.02	2.89199999	11	7/24/2014	31:04.7
8269	RWS11	-122.4700817	37.94021	-0.38	3.26	108.96	2.87800002	-122.47008	37.94022	-0.31	3.26	109.03	2.95200002	11	7/24/2014	31:04.8
8270	RWS11	-122.470084	37.94021	-0.33	3.29	109.02	2.95299995	-122.47008	37.94022	-0.35	3.29	109.07	2.94199994	11	7/24/2014	31:04.9
8271	RWS11	-122.4700863	37.94021	-0.38	3.31	108.96	2.92700005	-122.47008	37.94022	-0.35	3.31	109.01	2.96700004	11	7/24/2014	31:05.0
8272	RWS11	-122.4700886	37.94021	-0.33	3.34	109.01	3.00199997	-122.47008	37.94022	-0.4	3.34	108.96	2.94099995	11	7/24/2014	31:05.1
8273	RWS11	-122.4700909	37.94021	-0.38	3.36	109	2.97600007	-122.47009	37.94022	-0.35	3.36	108.96	3.01600006	11	7/24/2014	31:05.2
8274	RWS11	-122.4700932	37.94021	-0.38	3.38	108.98	3	-122.47009	37.94022	-0.31	3.38	108.96	3.074	11	7/24/2014	31:05.3
8275	RWS11	-122.4700955	37.94021	-0.38	3.41	109	3.02399993	-122.47009	37.94022	-0.35	3.41	108.95	3.06399992	11	7/24/2014	31:05.4
8276	RWS11	-122.4700977	37.94021	-0.38	3.43	108.89	3.04500008	-122.47009	37.94022	-0.43	3.43	108.85	3.00100008	11	7/24/2014	31:05.5
8277	RWS11	-122.4701	37.94021	-0.38	3.45	108.87	3.06399989	-122.47009	37.94022	-0.4	3.45	108.81	3.05399987	11	7/24/2014	31:05.6
8278	RWS11	-122.4701022	37.94021	-0.38	3.46	108.83	3.079	-122.4701	37.94022	-0.4	3.46	108.74	3.06899998	11	7/24/2014	31:05.7
8279	RWS11	-122.4701044	37.94021	-0.38	3.48	108.83	3.09100008	-122.4701	37.94022	-0.43	3.48	108.76	3.04700008	11	7/24/2014	31:05.8
8280	RWS11	-122.4701065	37.94021	-0.38	3.48	108.76	3.09899998	-122.4701	37.94022	-0.4	3.48	108.65	3.08899996	11	7/24/2014	31:05.9
8281	RWS11	-122.4701098	37.94021	-0.38	3.49	108.69	3.10400009	-122.4701	37.94023	-0.43	3.49	108.56	3.06000009	11	7/24/2014	31:06.0
8282	RWS11	-122.4701119	37.94021	-0.38	3.49	108.67	3.10400009	-122.47011	37.94023	-0.43	3.49	108.52	3.06000009	11	7/24/2014	31:06.1

8283	RWS11	-122.4701141	37.94021	-0.38	3.48	108.65	3.0999999	-122.47011	37.94023	-0.43	3.48	108.47	3.0559999	11	7/24/2014	31:06.2
8284	RWS11	-122.4701163	37.94021	-0.38	3.47	108.65	3.08999991	-122.47011	37.94023	-0.43	3.47	108.51	3.04599991	11	7/24/2014	31:06.3
8285	RWS11	-122.4701186	37.94021	-0.42	3.46	108.63	3.04099998	-122.47011	37.94023	-0.48	3.46	108.43	2.98099998	11	7/24/2014	31:06.4
8286	RWS11	-122.4701209	37.94022	-0.38	3.44	108.56	3.05699992	-122.47012	37.94023	-0.4	3.44	108.38	3.0469999	11	7/24/2014	31:06.5
8287	RWS11	-122.4701231	37.94022	-0.42	3.42	108.56	3.00000009	-122.47012	37.94023	-0.48	3.42	108.3	2.94000009	11	7/24/2014	31:06.6
8288	RWS11	-122.4701254	37.94022	-0.38	3.4	108.56	3.01200008	-122.47012	37.94023	-0.43	3.4	108.29	2.96800008	11	7/24/2014	31:06.7
8289	RWS11	-122.4701277	37.94022	-0.38	3.37	108.56	2.98900008	-122.47012	37.94023	-0.4	3.37	108.3	2.97900006	11	7/24/2014	31:06.8
8290	RWS11	-122.47013	37.94022	-0.38	3.35	108.56	2.96700001	-122.47012	37.94023	-0.35	3.35	108.23	3.007	11	7/24/2014	31:06.9
8291	RWS11	-122.4701323	37.94022	-0.42	3.33	108.56	2.91200003	-122.47013	37.94023	-0.4	3.33	108.24	2.93700001	11	7/24/2014	31:07.0
8292	RWS11	-122.4701346	37.94022	-0.38	3.32	108.51	2.93000007	-122.47013	37.94023	-0.35	3.32	108.22	2.97000006	11	7/24/2014	31:07.1
8293	RWS11	-122.4701369	37.94022	-0.38	3.3	108.49	2.91700006	-122.47013	37.94023	-0.43	3.3	108.16	2.87300006	11	7/24/2014	31:07.2
8294	RWS11	-122.4701391	37.94022	-0.42	3.29	108.47	2.8709999	-122.47013	37.94023	-0.35	3.29	108.14	2.94599989	11	7/24/2014	31:07.3
8295	RWS11	-122.4701414	37.94022	-0.42	3.28	108.53	2.86399993	-122.47014	37.94023	-0.43	3.28	108.18	2.85499993	11	7/24/2014	31:07.4
8296	RWS11	-122.4701436	37.94022	-0.38	3.28	108.53	2.89400005	-122.47014	37.94023	-0.43	3.28	108.15	2.85000005	11	7/24/2014	31:07.5
8297	RWS11	-122.4701458	37.94022	-0.42	3.28	108.6	2.85699996	-122.47014	37.94023	-0.35	3.28	108.16	2.93199995	11	7/24/2014	31:07.6
8298	RWS11	-122.470148	37.94022	-0.38	3.28	108.57	2.89199996	-122.47014	37.94024	-0.43	3.28	108.11	2.84799996	11	7/24/2014	31:07.7
8299	RWS11	-122.4701502	37.94022	-0.42	3.28	108.6	2.85999998	-122.47014	37.94024	-0.31	3.28	108.04	2.96899998	11	7/24/2014	31:07.8
8300	RWS11	-122.4701523	37.94022	-0.42	3.29	108.53	2.8650001	-122.47015	37.94024	-0.35	3.29	107.96	2.94000009	11	7/24/2014	31:07.9
8301	RWS11	-122.4701555	37.94022	-0.42	3.29	108.44	2.87399992	-122.47015	37.94024	-0.43	3.29	107.92	2.86499992	11	7/24/2014	31:08.0
8302	RWS11	-122.4701577	37.94023	-0.38	3.31	108.46	2.92199993	-122.47015	37.94024	-0.4	3.31	107.89	2.91199991	11	7/24/2014	31:08.1
8303	RWS11	-122.4701598	37.94023	-0.42	3.32	108.42	2.90399989	-122.47015	37.94024	-0.43	3.32	107.87	2.89499989	11	7/24/2014	31:08.2
8304	RWS11	-122.4701621	37.94023	-0.38	3.35	108.33	2.96099997	-122.47016	37.94024	-0.4	3.35	107.78	2.95099995	11	7/24/2014	31:08.3
8305	RWS11	-122.4701643	37.94023	-0.42	3.37	108.24	2.95199999	-122.47016	37.94024	-0.48	3.37	107.76	2.89199999	11	7/24/2014	31:08.4
8306	RWS11	-122.4701665	37.94023	-0.42	3.4	108.18	2.98199996	-122.47016	37.94024	-0.4	3.4	107.69	3.00699994	11	7/24/2014	31:08.5
8307	RWS11	-122.4701688	37.94023	-0.42	3.43	108.13	3.01499996	-122.47016	37.94024	-0.43	3.43	107.66	3.00599995	11	7/24/2014	31:08.6
8308	RWS11	-122.4701711	37.94023	-0.42	3.47	108.07	3.04900011	-122.47017	37.94024	-0.4	3.47	107.6	3.07400009	11	7/24/2014	31:08.7
8309	RWS11	-122.4701733	37.94023	-0.47	3.5	108	3.03299996	-122.47017	37.94024	-0.4	3.5	107.54	3.10899994	11	7/24/2014	31:08.8
8310	RWS11	-122.4701756	37.94023	-0.42	3.54	107.92	3.11699995	-122.47017	37.94024	-0.31	3.54	107.49	3.22599995	11	7/24/2014	31:08.9
8311	RWS11	-122.4701779	37.94023	-0.42	3.57	107.87	3.14800009	-122.47017	37.94024	-0.4	3.57	107.41	3.17300007	11	7/24/2014	31:09.0
8312	RWS11	-122.4701802	37.94023	-0.42	3.59	107.78	3.17400011	-122.47017	37.94024	-0.35	3.59	107.39	3.2490001	11	7/24/2014	31:09.1
8313	RWS11	-122.4701825	37.94023	-0.47	3.62	107.67	3.14400002	-122.47018	37.94024	-0.48	3.62	107.3	3.13500002	11	7/24/2014	31:09.2
8314	RWS11	-122.4701847	37.94023	-0.47	3.63	107.59	3.15900013	-122.47018	37.94025	-0.4	3.63	107.2	3.2350001	11	7/24/2014	31:09.3
8315	RWS11	-122.470187	37.94023	-0.47	3.64	107.49	3.16700003	-122.47018	37.94025	-0.4	3.64	107.08	3.243	11	7/24/2014	31:09.4

8316	RWS11	-122.4701892	37.94023	-0.42	3.64	107.43	3.21800002	-122.47018	37.94025	-0.4	3.64	107.05	3.243	11	7/24/2014	31:09.5
8317	RWS11	-122.4701914	37.94023	-0.47	3.63	107.34	3.16099998	-122.47019	37.94025	-0.4	3.63	106.95	3.23699996	11	7/24/2014	31:09.6
8318	RWS11	-122.4701936	37.94024	-0.47	3.62	107.25	3.14799997	-122.47019	37.94025	-0.4	3.62	106.86	3.22399995	11	7/24/2014	31:09.7
8319	RWS11	-122.4701958	37.94024	-0.47	3.6	107.19	3.13199994	-122.47019	37.94025	-0.4	3.6	106.8	3.20799991	11	7/24/2014	31:09.8
8320	RWS11	-122.4701979	37.94024	-0.47	3.59	107.14	3.11400005	-122.47019	37.94025	-0.4	3.59	106.7	3.19000003	11	7/24/2014	31:09.9
8321	RWS11	-122.4702011	37.94024	-0.51	3.57	106.95	3.06199992	-122.4702	37.94025	-0.48	3.57	106.56	3.08699992	11	7/24/2014	31:10.0
8322	RWS11	-122.4702033	37.94024	-0.47	3.55	106.92	3.07899997	-122.4702	37.94025	-0.4	3.55	106.46	3.15499994	11	7/24/2014	31:10.1
8323	RWS11	-122.4702054	37.94024	-0.51	3.54	106.9	3.03000009	-122.4702	37.94025	-0.4	3.54	106.42	3.14000008	11	7/24/2014	31:10.2
8324	RWS11	-122.4702076	37.94024	-0.47	3.52	106.76	3.05299994	-122.4702	37.94025	-0.4	3.52	106.31	3.12899992	11	7/24/2014	31:10.3
8325	RWS11	-122.4702098	37.94024	-0.51	3.52	106.73	3.01299989	-122.4702	37.94025	-0.4	3.52	106.29	3.12299988	11	7/24/2014	31:10.4
8326	RWS11	-122.470212	37.94024	-0.51	3.52	106.66	3.01000011	-122.47021	37.94025	-0.4	3.52	106.22	3.12000009	11	7/24/2014	31:10.5
8327	RWS11	-122.4702143	37.94024	-0.51	3.52	106.61	3.01199996	-122.47021	37.94025	-0.43	3.52	106.16	3.08799997	11	7/24/2014	31:10.6
8328	RWS11	-122.4702165	37.94024	-0.51	3.52	106.55	3.01700008	-122.47021	37.94025	-0.4	3.52	106.11	3.12700006	11	7/24/2014	31:10.7
8329	RWS11	-122.4702188	37.94024	-0.51	3.53	106.46	3.0259999	-122.47021	37.94025	-0.4	3.53	105.97	3.13599989	11	7/24/2014	31:10.8
8330	RWS11	-122.4702211	37.94024	-0.51	3.54	106.35	3.03599989	-122.47022	37.94026	-0.35	3.54	105.85	3.19599989	11	7/24/2014	31:10.9
8331	RWS11	-122.4702233	37.94024	-0.51	3.55	106.3	3.04799998	-122.47022	37.94026	-0.43	3.55	105.79	3.12399998	11	7/24/2014	31:11.0
8332	RWS11	-122.4702256	37.94024	-0.51	3.57	106.2	3.06000006	-122.47022	37.94026	-0.4	3.57	105.71	3.17000005	11	7/24/2014	31:11.1
8333	RWS11	-122.4702279	37.94024	-0.56	3.58	106.18	3.02200007	-122.47022	37.94026	-0.43	3.58	105.65	3.14900008	11	7/24/2014	31:11.2
8334	RWS11	-122.4702301	37.94025	-0.51	3.59	106.08	3.08499992	-122.47022	37.94026	-0.4	3.59	105.58	3.1949999	11	7/24/2014	31:11.3
8335	RWS11	-122.4702324	37.94025	-0.51	3.6	106.02	3.09600008	-122.47023	37.94026	-0.4	3.6	105.5	3.20600006	11	7/24/2014	31:11.4
8336	RWS11	-122.4702346	37.94025	-0.51	3.61	105.9	3.10600007	-122.47023	37.94026	-0.35	3.61	105.39	3.26600006	11	7/24/2014	31:11.5
8337	RWS11	-122.4702368	37.94025	-0.51	3.62	105.87	3.11499989	-122.47023	37.94026	-0.4	3.62	105.29	3.22499987	11	7/24/2014	31:11.6
8338	RWS11	-122.4702389	37.94025	-0.51	3.63	105.78	3.12300003	-122.47023	37.94026	-0.4	3.63	105.19	3.23300001	11	7/24/2014	31:11.7
8339	RWS11	-122.4702411	37.94025	-0.51	3.64	105.6	3.13099992	-122.47024	37.94026	-0.35	3.64	105.03	3.29099992	11	7/24/2014	31:11.8
8340	RWS11	-122.4702432	37.94025	-0.51	3.64	105.54	3.13799989	-122.47024	37.94026	-0.4	3.64	104.92	3.24799988	11	7/24/2014	31:11.9
8341	RWS11	-122.4702464	37.94025	-0.51	3.65	105.43	3.14600003	-122.47024	37.94026	-0.4	3.65	104.79	3.25600001	11	7/24/2014	31:12.0
8342	RWS11	-122.4702485	37.94025	-0.51	3.66	105.32	3.15399992	-122.47024	37.94026	-0.4	3.66	104.59	3.26399991	11	7/24/2014	31:12.1
8343	RWS11	-122.4702506	37.94025	-0.56	3.67	105.23	3.11199999	-122.47024	37.94026	-0.4	3.67	104.53	3.27299997	11	7/24/2014	31:12.2
8344	RWS11	-122.4702528	37.94025	-0.51	3.68	105.18	3.17200005	-122.47025	37.94026	-0.4	3.68	104.44	3.28200004	11	7/24/2014	31:12.3
8345	RWS11	-122.470255	37.94025	-0.51	3.69	105.07	3.18100011	-122.47025	37.94027	-0.4	3.69	104.35	3.2910001	11	7/24/2014	31:12.4
8346	RWS11	-122.4702572	37.94025	-0.51	3.69	104.98	3.18900001	-122.47025	37.94027	-0.4	3.69	104.25	3.29899999	11	7/24/2014	31:12.5
8347	RWS11	-122.4702594	37.94025	-0.56	3.7	104.88	3.14499998	-122.47025	37.94027	-0.4	3.7	104.13	3.30599996	11	7/24/2014	31:12.6
8348	RWS11	-122.4702616	37.94025	-0.51	3.71	104.7	3.20299995	-122.47026	37.94027	-0.43	3.71	103.93	3.27899995	11	7/24/2014	31:12.7

8349	RWS11	-122.4702639	37.94026	-0.51	3.71	104.61	3.20800006	-122.47026	37.94027	-0.43	3.71	103.79	3.28400007	11	7/24/2014	31:12.8
8350	RWS11	-122.4702661	37.94026	-0.51	3.72	104.55	3.21299994	-122.47026	37.94027	-0.43	3.72	103.71	3.28899994	11	7/24/2014	31:12.9
8351	RWS11	-122.4702684	37.94026	-0.56	3.72	104.39	3.16599989	-122.47026	37.94027	-0.43	3.72	103.63	3.29299989	11	7/24/2014	31:13.0
8352	RWS11	-122.4702706	37.94026	-0.51	3.72	104.35	3.21999991	-122.47026	37.94027	-0.48	3.72	103.51	3.24499992	11	7/24/2014	31:13.1
8353	RWS11	-122.4702729	37.94026	-0.51	3.73	104.24	3.222	-122.47027	37.94027	-0.48	3.73	103.43	3.24700001	11	7/24/2014	31:13.2
8354	RWS11	-122.4702751	37.94026	-0.51	3.73	104.17	3.22299993	-122.47027	37.94027	-0.43	3.73	103.34	3.29899994	11	7/24/2014	31:13.3
8355	RWS11	-122.4702773	37.94026	-0.56	3.73	104.05	3.171	-122.47027	37.94027	-0.43	3.73	103.2	3.29800001	11	7/24/2014	31:13.4
8356	RWS11	-122.4702796	37.94026	-0.51	3.72	103.96	3.21999991	-122.47027	37.94027	-0.43	3.72	103.08	3.29599991	11	7/24/2014	31:13.5
8357	RWS11	-122.4702817	37.94026	-0.56	3.72	103.91	3.16400003	-122.47028	37.94027	-0.43	3.72	103	3.29100004	11	7/24/2014	31:13.6
8358	RWS11	-122.4702839	37.94026	-0.5	3.71	103.78	3.20900005	-122.47028	37.94027	-0.39	3.71	102.85	3.31900007	11	7/24/2014	31:13.7
8359	RWS11	-122.4702861	37.94026	-0.5	3.7	103.68	3.19999999	-122.47028	37.94027	-0.43	3.7	102.76	3.27599999	11	7/24/2014	31:13.8
8360	RWS11	-122.4702882	37.94026	-0.47	3.69	103.61	3.22300008	-122.47028	37.94027	-0.43	3.69	102.68	3.26500008	11	7/24/2014	31:13.9
8361	RWS11	-122.4702913	37.94026	-0.5	3.68	103.43	3.17600006	-122.47029	37.94028	-0.34	3.68	102.56	3.33600006	11	7/24/2014	31:14.0
8362	RWS11	-122.4702934	37.94026	-0.47	3.67	103.3	3.19799998	-122.47029	37.94028	-0.39	3.67	102.38	3.27399999	11	7/24/2014	31:14.1
8363	RWS11	-122.4702955	37.94026	-0.5	3.65	103.25	3.15099996	-122.47029	37.94028	-0.39	3.65	102.31	3.26099998	11	7/24/2014	31:14.2
8364	RWS11	-122.4702976	37.94027	-0.5	3.64	103.14	3.14000005	-122.47029	37.94028	-0.39	3.64	102.2	3.25000006	11	7/24/2014	31:14.3
8365	RWS11	-122.4702998	37.94027	-0.47	3.63	103.06	3.16499999	-122.47029	37.94028	-0.43	3.63	102.14	3.20699999	11	7/24/2014	31:14.4
8366	RWS11	-122.470302	37.94027	-0.47	3.63	103.03	3.15700009	-122.4703	37.94028	-0.34	3.63	102.13	3.28300008	11	7/24/2014	31:14.5
8367	RWS11	-122.4703041	37.94027	-0.5	3.62	102.92	3.11799997	-122.4703	37.94028	-0.43	3.62	102.03	3.19399998	11	7/24/2014	31:14.6
8368	RWS11	-122.4703063	37.94027	-0.47	3.62	102.99	3.14800003	-122.4703	37.94028	-0.34	3.62	102.07	3.27400002	11	7/24/2014	31:14.7
8369	RWS11	-122.4703085	37.94027	-0.5	3.62	102.97	3.11199993	-122.4703	37.94028	-0.39	3.62	102.07	3.22199994	11	7/24/2014	31:14.8
8370	RWS11	-122.4703107	37.94027	-0.47	3.61	102.94	3.14400008	-122.4703	37.94028	-0.39	3.61	102.04	3.22000009	11	7/24/2014	31:14.9
8371	RWS11	-122.4703129	37.94027	-0.5	3.61	102.92	3.11000007	-122.47031	37.94028	-0.43	3.61	101.99	3.18600008	11	7/24/2014	31:15.0
8372	RWS11	-122.4703151	37.94027	-0.47	3.62	102.94	3.14599994	-122.47031	37.94028	-0.43	3.62	101.99	3.18799993	11	7/24/2014	31:15.1
8373	RWS11	-122.4703173	37.94027	-0.5	3.62	102.88	3.11599988	-122.47031	37.94028	-0.48	3.62	101.92	3.14099988	11	7/24/2014	31:15.2
8374	RWS11	-122.4703195	37.94027	-0.47	3.63	102.88	3.15800002	-122.47031	37.94028	-0.43	3.63	101.91	3.20000002	11	7/24/2014	31:15.3
8375	RWS11	-122.4703216	37.94027	-0.5	3.64	102.83	3.13300008	-122.47032	37.94029	-0.43	3.64	101.84	3.20900008	11	7/24/2014	31:15.4
8376	RWS11	-122.4703238	37.94027	-0.47	3.65	102.79	3.17700008	-122.47032	37.94029	-0.39	3.65	101.78	3.25300008	11	7/24/2014	31:15.5
8377	RWS11	-122.4703259	37.94027	-0.5	3.66	102.68	3.15300006	-122.47032	37.94029	-0.39	3.66	101.69	3.26300007	11	7/24/2014	31:15.6
8378	RWS11	-122.470328	37.94027	-0.47	3.67	102.63	3.19599989	-122.47032	37.94029	-0.39	3.67	101.63	3.2719999	11	7/24/2014	31:15.7
8379	RWS11	-122.4703301	37.94028	-0.47	3.67	102.54	3.2030001	-122.47032	37.94029	-0.43	3.67	101.54	3.24500009	11	7/24/2014	31:15.8
8380	RWS11	-122.4703321	37.94028	-0.47	3.68	102.43	3.20700005	-122.47033	37.94029	-0.39	3.68	101.49	3.28300005	11	7/24/2014	31:15.9
8381	RWS11	-122.4703351	37.94028	-0.47	3.68	102.39	3.20700005	-122.47033	37.94029	-0.39	3.68	101.41	3.28300005	11	7/24/2014	31:16.0

8382	RWS11	-122.470337	37.94028	-0.47	3.67	102.26	3.20199993	-122.47033	37.94029	-0.39	3.67	101.32	3.27799994	11	7/24/2014	31:16.1
8383	RWS11	-122.470339	37.94028	-0.47	3.66	102.17	3.19400004	-122.47033	37.94029	-0.43	3.66	101.25	3.23600003	11	7/24/2014	31:16.2
8384	RWS11	-122.470341	37.94028	-0.47	3.65	102.04	3.18100002	-122.47033	37.94029	-0.39	3.65	101.14	3.25700003	11	7/24/2014	31:16.3
8385	RWS11	-122.470343	37.94028	-0.47	3.63	101.99	3.16499999	-122.47034	37.94029	-0.39	3.63	101.03	3.241	11	7/24/2014	31:16.4
8386	RWS11	-122.470345	37.94028	-0.47	3.62	101.93	3.14899996	-122.47034	37.94029	-0.39	3.62	100.97	3.22499996	11	7/24/2014	31:16.5
8387	RWS11	-122.4703469	37.94028	-0.47	3.6	101.82	3.13299993	-122.47034	37.94029	-0.39	3.6	100.88	3.20899993	11	7/24/2014	31:16.6
8388	RWS11	-122.4703489	37.94028	-0.47	3.59	101.69	3.11899999	-122.47034	37.94029	-0.34	3.59	100.76	3.24499997	11	7/24/2014	31:16.7
8389	RWS11	-122.4703509	37.94028	-0.47	3.58	101.62	3.109	-122.47034	37.94029	-0.34	3.58	100.71	3.23499998	11	7/24/2014	31:16.8
8390	RWS11	-122.4703529	37.94028	-0.47	3.57	101.49	3.10200003	-122.47035	37.94029	-0.34	3.57	100.59	3.22800002	11	7/24/2014	31:16.9
8391	RWS11	-122.4703559	37.94028	-0.5	3.57	101.42	3.06400007	-122.47035	37.9403	-0.39	3.57	100.55	3.17400008	11	7/24/2014	31:17.0
8392	RWS11	-122.4703579	37.94028	-0.42	3.57	101.36	3.14799991	-122.47035	37.9403	-0.39	3.57	100.46	3.17299992	11	7/24/2014	31:17.1
8393	RWS11	-122.47036	37.94028	-0.47	3.57	101.31	3.09800008	-122.47035	37.9403	-0.39	3.57	100.46	3.17400008	11	7/24/2014	31:17.2
8394	RWS11	-122.4703621	37.94028	-0.47	3.57	101.21	3.09999993	-122.47036	37.9403	-0.43	3.57	100.35	3.14199993	11	7/24/2014	31:17.3
8395	RWS11	-122.4703642	37.94029	-0.47	3.57	101.2	3.1010001	-122.47036	37.9403	-0.34	3.57	100.35	3.22700009	11	7/24/2014	31:17.4
8396	RWS11	-122.4703663	37.94029	-0.47	3.57	101.14	3.09900001	-122.47036	37.9403	-0.39	3.57	100.24	3.17500001	11	7/24/2014	31:17.5
8397	RWS11	-122.4703684	37.94029	-0.47	3.56	101.13	3.09399989	-122.47036	37.9403	-0.43	3.56	100.2	3.13599989	11	7/24/2014	31:17.6
8398	RWS11	-122.4703705	37.94029	-0.47	3.56	101.1	3.086	-122.47036	37.9403	-0.39	3.56	100.18	3.162	11	7/24/2014	31:17.7
8399	RWS11	-122.4703726	37.94029	-0.47	3.55	100.98	3.07500008	-122.47037	37.9403	-0.39	3.55	100.09	3.15100008	11	7/24/2014	31:17.8
8400	RWS11	-122.4703747	37.94029	-0.47	3.53	100.9	3.06200007	-122.47037	37.9403	-0.43	3.53	99.972	3.10400006	11	7/24/2014	31:17.9
8401	RWS11	-122.4703779	37.94029	-0.47	3.52	100.87	3.04799989	-122.47037	37.9403	-0.43	3.52	99.932	3.08999988	11	7/24/2014	31:18.0
8402	RWS11	-122.47038	37.94029	-0.47	3.5	100.82	3.03399995	-122.47037	37.9403	-0.39	3.5	99.888	3.10999995	11	7/24/2014	31:18.1
8403	RWS11	-122.4703821	37.94029	-0.5	3.49	100.79	2.98500007	-122.47038	37.9403	-0.43	3.49	99.801	3.06100008	11	7/24/2014	31:18.2
8404	RWS11	-122.4703842	37.94029	-0.47	3.47	100.74	3.00499991	-122.47038	37.9403	-0.39	3.47	99.751	3.08099991	11	7/24/2014	31:18.3
8405	RWS11	-122.4703864	37.94029	-0.5	3.46	100.69	2.95799989	-122.47038	37.9403	-0.43	3.46	99.741	3.03399989	11	7/24/2014	31:18.4
8406	RWS11	-122.4703886	37.94029	-0.47	3.45	100.61	2.98000005	-122.47038	37.94031	-0.43	3.45	99.64	3.02200004	11	7/24/2014	31:18.5
8407	RWS11	-122.4703907	37.94029	-0.5	3.44	100.56	2.93699998	-122.47038	37.94031	-0.43	3.44	99.584	3.01299998	11	7/24/2014	31:18.6
8408	RWS11	-122.4703929	37.94029	-0.47	3.43	100.56	2.96400002	-122.47039	37.94031	-0.48	3.43	99.55	2.95500001	11	7/24/2014	31:18.7
8409	RWS11	-122.4703951	37.9403	-0.5	3.43	100.47	2.92600006	-122.47039	37.94031	-0.43	3.43	99.497	3.00200006	11	7/24/2014	31:18.8
8410	RWS11	-122.4703973	37.9403	-0.5	3.43	100.43	2.92499989	-122.47039	37.94031	-0.39	3.43	99.375	3.03499991	11	7/24/2014	31:18.9
8411	RWS11	-122.4704006	37.9403	-0.5	3.43	100.34	2.92799991	-122.47039	37.94031	-0.48	3.43	99.316	2.95299992	11	7/24/2014	31:19.0
8412	RWS11	-122.4704028	37.9403	-0.47	3.44	100.23	2.96799996	-122.4704	37.94031	-0.43	3.44	99.205	3.00999996	11	7/24/2014	31:19.1
8413	RWS11	-122.470405	37.9403	-0.5	3.45	100.19	2.94300002	-122.4704	37.94031	-0.43	3.45	99.137	3.01900002	11	7/24/2014	31:19.2
8414	RWS11	-122.4704073	37.9403	-0.5	3.46	100.12	2.9550001	-122.4704	37.94031	-0.43	3.46	99.099	3.03100011	11	7/24/2014	31:19.3

8415	RWS11	-122.4704095	37.9403	-0.5	3.47	100.03	2.96900004	-122.4704	37.94031	-0.39	3.47	99.087	3.07900006	11	7/24/2014	31:19.4
8416	RWS11	-122.4704118	37.9403	-0.5	3.49	99.946	2.98299998	-122.47041	37.94031	-0.48	3.49	99.027	3.00799999	11	7/24/2014	31:19.5
8417	RWS11	-122.4704141	37.9403	-0.5	3.5	99.859	2.99800009	-122.47041	37.94031	-0.48	3.5	98.962	3.02300009	11	7/24/2014	31:19.6
8418	RWS11	-122.4704163	37.9403	-0.5	3.52	99.793	3.01299995	-122.47041	37.94031	-0.48	3.52	98.896	3.03799996	11	7/24/2014	31:19.7
8419	RWS11	-122.4704186	37.9403	-0.5	3.53	99.789	3.02699989	-122.47041	37.94031	-0.51	3.53	98.848	3.01799989	11	7/24/2014	31:19.8
8420	RWS11	-122.4704207	37.9403	-0.5	3.55	99.723	3.04100007	-122.47041	37.94032	-0.48	3.55	98.848	3.06600007	11	7/24/2014	31:19.9
8421	RWS11	-122.4704239	37.9403	-0.5	3.56	99.701	3.05400008	-122.47042	37.94032	-0.43	3.56	98.755	3.13000008	11	7/24/2014	31:20.0
8422	RWS11	-122.470426	37.9403	-0.5	3.57	99.636	3.065	-122.47042	37.94032	-0.48	3.57	98.761	3.09	11	7/24/2014	31:20.1
8423	RWS11	-122.4704281	37.94031	-0.5	3.58	99.548	3.07299989	-122.47042	37.94032	-0.51	3.58	98.667	3.06399989	11	7/24/2014	31:20.2
8424	RWS11	-122.4704302	37.94031	-0.5	3.58	99.505	3.07899994	-122.47042	37.94032	-0.48	3.58	98.614	3.10399994	11	7/24/2014	31:20.3
8425	RWS11	-122.4704323	37.94031	-0.5	3.59	99.414	3.08100003	-122.47043	37.94032	-0.48	3.59	98.517	3.10600004	11	7/24/2014	31:20.4
8426	RWS11	-122.4704344	37.94031	-0.5	3.59	99.31	3.08100003	-122.47043	37.94032	-0.48	3.59	98.467	3.10600004	11	7/24/2014	31:20.5
8427	RWS11	-122.4704365	37.94031	-0.5	3.58	99.238	3.07599992	-122.47043	37.94032	-0.48	3.58	98.369	3.10099992	11	7/24/2014	31:20.6
8428	RWS11	-122.4704386	37.94031	-0.5	3.57	99.13	3.06899995	-122.47043	37.94032	-0.43	3.57	98.32	3.14499995	11	7/24/2014	31:20.7
8429	RWS11	-122.4704407	37.94031	-0.56	3.56	99.063	3.00899988	-122.47043	37.94032	-0.48	3.56	98.27	3.08499989	11	7/24/2014	31:20.8
8430	RWS11	-122.4704428	37.94031	-0.5	3.55	98.982	3.04800004	-122.47044	37.94032	-0.48	3.55	98.21	3.07300004	11	7/24/2014	31:20.9
8431	RWS11	-122.4704459	37.94031	-0.56	3.54	98.951	2.9830001	-122.47044	37.94032	-0.43	3.54	98.142	3.1100001	11	7/24/2014	31:21.0
8432	RWS11	-122.4704481	37.94031	-0.5	3.52	98.842	3.01800007	-122.47044	37.94032	-0.43	3.52	98.076	3.09400007	11	7/24/2014	31:21.1
8433	RWS11	-122.4704503	37.94031	-0.56	3.51	98.826	2.95000011	-122.47044	37.94032	-0.48	3.51	98.017	3.02600011	11	7/24/2014	31:21.2
8434	RWS11	-122.4704525	37.94031	-0.42	3.49	98.71	3.06899992	-122.47045	37.94033	-0.43	3.49	97.967	3.05999991	11	7/24/2014	31:21.3
8435	RWS11	-122.4704547	37.94031	-0.56	3.47	98.667	2.91699988	-122.47045	37.94033	-0.48	3.47	97.901	2.99299988	11	7/24/2014	31:21.4
8436	RWS11	-122.470457	37.94031	-0.5	3.46	98.585	2.95399994	-122.47045	37.94033	-0.43	3.46	97.819	3.02999994	11	7/24/2014	31:21.5
8437	RWS11	-122.4704592	37.94031	-0.56	3.45	98.553	2.89200002	-122.47045	37.94033	-0.48	3.45	97.76	2.96800002	11	7/24/2014	31:21.6
8438	RWS11	-122.4704615	37.94032	-0.5	3.44	98.472	2.93300003	-122.47046	37.94033	-0.48	3.44	97.679	2.95800003	11	7/24/2014	31:21.7
8439	RWS11	-122.4704637	37.94032	-0.56	3.43	98.422	2.87299997	-122.47046	37.94033	-0.48	3.43	97.635	2.94899997	11	7/24/2014	31:21.8
8440	RWS11	-122.4704659	37.94032	-0.5	3.42	98.385	2.917	-122.47046	37.94033	-0.48	3.42	97.575	2.942	11	7/24/2014	31:21.9
8441	RWS11	-122.470469	37.94032	-0.56	3.41	98.356	2.85999995	-122.47046	37.94033	-0.48	3.41	97.532	2.93599996	11	7/24/2014	31:22.0
8442	RWS11	-122.4704712	37.94032	-0.5	3.41	98.34	2.90499991	-122.47046	37.94033	-0.43	3.41	97.503	2.98099992	11	7/24/2014	31:22.1
8443	RWS11	-122.4704732	37.94032	-0.56	3.4	98.287	2.84900004	-122.47047	37.94033	-0.48	3.4	97.462	2.92500004	11	7/24/2014	31:22.2
8444	RWS11	-122.4704753	37.94032	-0.56	3.4	98.271	2.84299999	-122.47047	37.94033	-0.43	3.4	97.418	2.97	11	7/24/2014	31:22.3
8445	RWS11	-122.4704774	37.94032	-0.56	3.39	98.271	2.83600003	-122.47047	37.94033	-0.48	3.39	97.412	2.91200003	11	7/24/2014	31:22.4
8446	RWS11	-122.4704795	37.94032	-0.5	3.38	98.199	2.88000005	-122.47047	37.94033	-0.48	3.38	97.331	2.90500006	11	7/24/2014	31:22.5
8447	RWS11	-122.4704816	37.94032	-0.56	3.38	98.118	2.82099992	-122.47048	37.94033	-0.48	3.38	97.287	2.89699993	11	7/24/2014	31:22.6

8448	RWS11	-122.4704837	37.94032	-0.5	3.37	98.111	2.86499995	-122.47048	37.94034	-0.43	3.37	97.214	2.94099995	11	7/24/2014	31:22.7
8449	RWS11	-122.4704858	37.94032	-0.56	3.36	97.983	2.80699998	-122.47048	37.94034	-0.48	3.36	97.136	2.88299999	11	7/24/2014	31:22.8
8450	RWS11	-122.4704878	37.94032	-0.56	3.36	97.939	2.80099994	-122.47048	37.94034	-0.43	3.36	97.036	2.92799994	11	7/24/2014	31:22.9
8451	RWS11	-122.470491	37.94032	-0.56	3.35	97.845	2.79600006	-122.47048	37.94034	-0.48	3.35	96.955	2.87200007	11	7/24/2014	31:23.0
8452	RWS11	-122.4704931	37.94033	-0.5	3.35	97.808	2.84299988	-122.47049	37.94034	-0.43	3.35	96.846	2.91899988	11	7/24/2014	31:23.1
8453	RWS11	-122.4704953	37.94033	-0.56	3.34	97.677	2.78900009	-122.47049	37.94034	-0.43	3.34	96.736	2.9160001	11	7/24/2014	31:23.2
8454	RWS11	-122.4704974	37.94033	-0.5	3.34	97.568	2.83700007	-122.47049	37.94034	-0.43	3.34	96.605	2.91300008	11	7/24/2014	31:23.3
8455	RWS11	-122.4704996	37.94033	-0.56	3.34	97.495	2.78499991	-122.47049	37.94034	-0.43	3.34	96.495	2.91199991	11	7/24/2014	31:23.4
8456	RWS11	-122.4705018	37.94033	-0.56	3.34	97.367	2.78499991	-122.4705	37.94034	-0.48	3.34	96.361	2.86099991	11	7/24/2014	31:23.5
8457	RWS11	-122.470504	37.94033	-0.56	3.34	97.345	2.78600007	-122.4705	37.94034	-0.39	3.34	96.295	2.94700009	11	7/24/2014	31:23.6
8458	RWS11	-122.4705062	37.94033	-0.56	3.34	97.236	2.78799993	-122.4705	37.94034	-0.43	3.34	96.223	2.91499993	11	7/24/2014	31:23.7
8459	RWS11	-122.4705084	37.94033	-0.56	3.35	97.126	2.79000002	-122.4705	37.94034	-0.48	3.35	96.076	2.86600003	11	7/24/2014	31:23.8
8460	RWS11	-122.4705106	37.94033	-0.5	3.35	97.039	2.84299988	-122.4705	37.94034	-0.43	3.35	95.995	2.91899988	11	7/24/2014	31:23.9
8461	RWS11	-122.4705139	37.94033	-0.56	3.35	96.973	2.7949999	-122.47051	37.94034	-0.48	3.35	95.911	2.8709999	11	7/24/2014	31:24.0
8462	RWS11	-122.4705161	37.94033	-0.5	3.35	96.904	2.84700006	-122.47051	37.94034	-0.48	3.35	95.854	2.87200007	11	7/24/2014	31:24.1
8463	RWS11	-122.4705183	37.94033	-0.56	3.35	96.838	2.79600006	-122.47051	37.94035	-0.43	3.35	95.729	2.92300007	11	7/24/2014	31:24.2
8464	RWS11	-122.4705205	37.94033	-0.5	3.35	96.663	2.8459999	-122.47051	37.94035	-0.39	3.35	95.585	2.95599991	11	7/24/2014	31:24.3
8465	RWS11	-122.4705228	37.94033	-0.56	3.35	96.597	2.79300004	-122.47052	37.94035	-0.43	3.35	95.444	2.92000005	11	7/24/2014	31:24.4
8466	RWS11	-122.470525	37.94034	-0.5	3.34	96.466	2.84000009	-122.47052	37.94035	-0.43	3.34	95.345	2.9160001	11	7/24/2014	31:24.5
8467	RWS11	-122.4705272	37.94034	-0.56	3.34	96.45	2.78499991	-122.47052	37.94035	-0.43	3.34	95.29	2.91199991	11	7/24/2014	31:24.6
8468	RWS11	-122.4705295	37.94034	-0.5	3.34	96.375	2.83100003	-122.47052	37.94035	-0.48	3.34	95.238	2.85600004	11	7/24/2014	31:24.7
8469	RWS11	-122.4705317	37.94034	-0.47	3.33	96.244	2.86200002	-122.47053	37.94035	-0.51	3.33	95.079	2.81900001	11	7/24/2014	31:24.8
8470	RWS11	-122.4705339	37.94034	-0.47	3.33	96.112	2.86200002	-122.47053	37.94035	-0.51	3.33	94.953	2.81900001	11	7/24/2014	31:24.9
8471	RWS11	-122.4705372	37.94034	-0.56	3.34	95.966	2.78099996	-122.47053	37.94035	-0.51	3.34	94.784	2.82299995	11	7/24/2014	31:25.0
8472	RWS11	-122.4705394	37.94034	-0.5	3.35	95.85	2.84100002	-122.47053	37.94035	-0.39	3.35	94.669	2.95100003	11	7/24/2014	31:25.1
8473	RWS11	-122.4705415	37.94034	-0.56	3.36	95.741	2.80300003	-122.47054	37.94035	-0.51	3.36	94.544	2.84500003	11	7/24/2014	31:25.2
8474	RWS11	-122.4705437	37.94034	-0.56	3.38	95.655	2.81999999	-122.47054	37.94035	-0.43	3.38	94.49	2.947	11	7/24/2014	31:25.3
8475	RWS11	-122.4705459	37.94034	-0.56	3.4	95.459	2.8409999	-122.47054	37.94035	-0.48	3.4	94.337	2.91699991	11	7/24/2014	31:25.4
8476	RWS11	-122.4705482	37.94034	-0.56	3.42	95.387	2.86699992	-122.47054	37.94035	-0.51	3.42	94.234	2.90899992	11	7/24/2014	31:25.5
8477	RWS11	-122.4705504	37.94034	-0.56	3.45	95.306	2.8969999	-122.47054	37.94036	-0.51	3.45	94.156	2.93899989	11	7/24/2014	31:25.6
8478	RWS11	-122.4705526	37.94034	-0.5	3.49	95.168	2.98200005	-122.47055	37.94036	-0.48	3.49	94.081	3.00700006	11	7/24/2014	31:25.7
8479	RWS11	-122.4705548	37.94034	-0.56	3.52	94.977	2.96700007	-122.47055	37.94036	-0.48	3.52	93.861	3.04300007	11	7/24/2014	31:25.8
8480	RWS11	-122.470557	37.94034	-0.5	3.56	94.864	3.05599993	-122.47055	37.94036	-0.43	3.56	93.808	3.13199994	11	7/24/2014	31:25.9

8481	RWS11	-122.4705602	37.94035	-0.56	3.6	94.749	3.04300004	-122.47055	37.94036	-0.51	3.6	93.658	3.08500004	11	7/24/2014	31:26.0
8482	RWS11	-122.4705623	37.94035	-0.56	3.63	94.646	3.07999998	-122.47056	37.94036	-0.43	3.63	93.508	3.20699999	11	7/24/2014	31:26.1
8483	RWS11	-122.4705645	37.94035	-0.56	3.67	94.471	3.116	-122.47056	37.94036	-0.48	3.67	93.317	3.192	11	7/24/2014	31:26.2
8484	RWS11	-122.4705667	37.94035	-0.56	3.7	94.274	3.14999992	-122.47056	37.94036	-0.43	3.7	93.202	3.27699992	11	7/24/2014	31:26.3
8485	RWS11	-122.4705689	37.94035	-0.56	3.74	94.143	3.18299991	-122.47056	37.94036	-0.51	3.74	93.049	3.2249999	11	7/24/2014	31:26.4
8486	RWS11	-122.4705711	37.94035	-0.56	3.77	93.986	3.21299988	-122.47057	37.94036	-0.48	3.77	92.914	3.28899989	11	7/24/2014	31:26.5
8487	RWS11	-122.4705733	37.94035	-0.59	3.8	93.898	3.20800012	-122.47057	37.94036	-0.48	3.8	92.81	3.3190001	11	7/24/2014	31:26.6
8488	RWS11	-122.4705755	37.94035	-0.56	3.83	93.745	3.2719999	-122.47057	37.94036	-0.56	3.83	92.668	3.26299989	11	7/24/2014	31:26.7
8489	RWS11	-122.4705777	37.94035	-0.56	3.86	93.57	3.30099994	-122.47057	37.94036	-0.48	3.86	92.548	3.37699994	11	7/24/2014	31:26.8
8490	RWS11	-122.4705799	37.94035	-0.56	3.88	93.445	3.32900006	-122.47057	37.94036	-0.56	3.88	92.367	3.32000005	11	7/24/2014	31:26.9
8491	RWS11	-122.4705831	37.94035	-0.59	3.91	93.264	3.32100004	-122.47058	37.94037	-0.51	3.91	92.286	3.398	11	7/24/2014	31:27.0
8492	RWS11	-122.4705852	37.94035	-0.5	3.93	93.152	3.43099993	-122.47058	37.94037	-0.56	3.93	92.108	3.37099993	11	7/24/2014	31:27.1
8493	RWS11	-122.4705874	37.94035	-0.59	3.96	93.005	3.36600012	-122.47058	37.94037	-0.56	3.96	91.993	3.39200008	11	7/24/2014	31:27.2
8494	RWS11	-122.4705895	37.94035	-0.56	3.97	92.889	3.41800004	-122.47058	37.94037	-0.6	3.97	91.845	3.37600005	11	7/24/2014	31:27.3
8495	RWS11	-122.4705917	37.94036	-0.5	3.99	92.742	3.48299998	-122.47059	37.94037	-0.51	3.99	91.67	3.47399998	11	7/24/2014	31:27.4
8496	RWS11	-122.4705939	37.94036	-0.56	4	92.605	3.44100004	-122.47059	37.94037	-0.48	4	91.577	3.51700005	11	7/24/2014	31:27.5
8497	RWS11	-122.4705961	37.94036	-0.56	4	92.479	3.44699985	-122.47059	37.94037	-0.51	4	91.407	3.48899984	11	7/24/2014	31:27.6
8498	RWS11	-122.4705983	37.94036	-0.5	4	92.339	3.50000018	-122.47059	37.94037	-0.51	4	91.382	3.49100018	11	7/24/2014	31:27.7
8499	RWS11	-122.4706004	37.94036	-0.56	4	92.214	3.44699985	-122.47059	37.94037	-0.51	4	91.207	3.48899984	11	7/24/2014	31:27.8
8500	RWS11	-122.4706026	37.94036	-0.56	4	92.06	3.44199997	-122.4706	37.94037	-0.48	4	91.092	3.51799998	11	7/24/2014	31:27.9
8501	RWS11	-122.4706058	37.94036	-0.56	3.99	91.923	3.435	-122.4706	37.94037	-0.51	3.99	90.983	3.477	11	7/24/2014	31:28.0
8502	RWS11	-122.470608	37.94036	-0.5	3.98	91.732	3.47699994	-122.4706	37.94037	-0.56	3.98	90.77	3.41699994	11	7/24/2014	31:28.1
8503	RWS11	-122.4706102	37.94036	-0.56	3.97	91.6	3.41500002	-122.4706	37.94037	-0.56	3.97	90.616	3.40600002	11	7/24/2014	31:28.2
8504	RWS11	-122.4706124	37.94036	-0.56	3.96	91.422	3.40299994	-122.47061	37.94037	-0.51	3.96	90.416	3.44499993	11	7/24/2014	31:28.3
8505	RWS11	-122.4706146	37.94036	-0.56	3.94	91.335	3.38999993	-122.47061	37.94037	-0.6	3.94	90.351	3.34799993	11	7/24/2014	31:28.4
8506	RWS11	-122.4706168	37.94036	-0.56	3.93	91.226	3.37800008	-122.47061	37.94038	-0.48	3.93	90.285	3.45400009	11	7/24/2014	31:28.5
8507	RWS11	-122.470619	37.94036	-0.56	3.92	91.029	3.366	-122.47061	37.94038	-0.51	3.92	90.17	3.40799999	11	7/24/2014	31:28.6
8508	RWS11	-122.4706213	37.94036	-0.5	3.91	90.854	3.40600008	-122.47062	37.94038	-0.56	3.91	89.963	3.34600008	11	7/24/2014	31:28.7
8509	RWS11	-122.4706235	37.94036	-0.56	3.9	90.745	3.34800011	-122.47062	37.94038	-0.51	3.9	89.804	3.3900001	11	7/24/2014	31:28.8
8510	RWS11	-122.4706257	37.94037	-0.5	3.9	90.594	3.39300007	-122.47062	37.94038	-0.48	3.9	89.663	3.41800007	11	7/24/2014	31:28.9
8511	RWS11	-122.4706289	37.94037	-0.56	3.89	90.5	3.33999997	-122.47062	37.94038	-0.51	3.89	89.494	3.38199997	11	7/24/2014	31:29.0
8512	RWS11	-122.4706312	37.94037	-0.5	3.89	90.369	3.39099997	-122.47063	37.94038	-0.51	3.89	89.385	3.38199997	11	7/24/2014	31:29.1
8513	RWS11	-122.4706334	37.94037	-0.56	3.9	90.281	3.34200007	-122.47063	37.94038	-0.48	3.9	89.232	3.41800007	11	7/24/2014	31:29.2

8514	RWS11	-122.4706356	37.94037	-0.56	3.9	90.178	3.34500009	-122.47063	37.94038	-0.48	3.9	89.128	3.42100009	11	7/24/2014	31:29.3
8515	RWS11	-122.4706378	37.94037	-0.56	3.9	89.974	3.34800011	-122.47063	37.94038	-0.51	3.9	88.968	3.3900001	11	7/24/2014	31:29.4
8516	RWS11	-122.4706401	37.94037	-0.56	3.9	89.802	3.34999996	-122.47063	37.94038	-0.48	3.9	88.769	3.42599997	11	7/24/2014	31:29.5
8517	RWS11	-122.4706423	37.94037	-0.56	3.9	89.665	3.34800011	-122.47064	37.94038	-0.51	3.9	88.599	3.3900001	11	7/24/2014	31:29.6
8518	RWS11	-122.4706446	37.94037	-0.5	3.9	89.518	3.39300007	-122.47064	37.94038	-0.43	3.9	88.435	3.46900007	11	7/24/2014	31:29.7
8519	RWS11	-122.4706468	37.94037	-0.56	3.89	89.299	3.333	-122.47064	37.94038	-0.6	3.89	88.228	3.29100001	11	7/24/2014	31:29.8
8520	RWS11	-122.470649	37.94037	-0.56	3.88	89.228	3.32099992	-122.47064	37.94039	-0.48	3.88	88.069	3.39699993	11	7/24/2014	31:29.9
8521	RWS11	-122.4706523	37.94037	-0.55	3.86	89.081	3.30700004	-122.47065	37.94039	-0.48	3.86	87.965	3.38300008	11	7/24/2014	31:30.0
8522	RWS11	-122.4706545	37.94037	-0.5	3.85	88.924	3.34299994	-122.47065	37.94039	-0.51	3.85	87.683	3.33399993	11	7/24/2014	31:30.1
8523	RWS11	-122.4706567	37.94037	-0.55	3.83	88.793	3.27700007	-122.47065	37.94039	-0.51	3.83	87.584	3.31900007	11	7/24/2014	31:30.2
8524	RWS11	-122.4706589	37.94038	-0.55	3.82	88.655	3.26499999	-122.47065	37.94039	-0.48	3.82	87.436	3.34100002	11	7/24/2014	31:30.3
8525	RWS11	-122.4706612	37.94038	-0.55	3.81	88.47	3.25400007	-122.47066	37.94039	-0.51	3.81	87.245	3.29600006	11	7/24/2014	31:30.4
8526	RWS11	-122.4706634	37.94038	-0.55	3.8	88.377	3.24400008	-122.47066	37.94039	-0.51	3.8	87.081	3.28600007	11	7/24/2014	31:30.5
8527	RWS11	-122.4706656	37.94038	-0.59	3.79	88.218	3.19900012	-122.47066	37.94039	-0.51	3.79	86.918	3.27600008	11	7/24/2014	31:30.6
8528	RWS11	-122.4706678	37.94038	-0.55	3.78	88.032	3.2240001	-122.47066	37.94039	-0.43	3.78	86.758	3.35100013	11	7/24/2014	31:30.7
8529	RWS11	-122.47067	37.94038	-0.55	3.77	87.848	3.21399987	-122.47066	37.94039	-0.48	3.77	86.629	3.2899999	11	7/24/2014	31:30.8
8530	RWS11	-122.4706722	37.94038	-0.55	3.76	87.717	3.20299995	-122.47067	37.94039	-0.48	3.76	86.427	3.27899998	11	7/24/2014	31:30.9
8531	RWS11	-122.4706754	37.94038	-0.55	3.75	87.52	3.19200003	-122.47067	37.94039	-0.48	3.75	86.28	3.26800007	11	7/24/2014	31:31.0
8532	RWS11	-122.4706776	37.94038	-0.55	3.73	87.417	3.18099988	-122.47067	37.94039	-0.48	3.73	86.165	3.25699991	11	7/24/2014	31:31.1
8533	RWS11	-122.4706798	37.94038	-0.55	3.72	87.236	3.16999996	-122.47067	37.94039	-0.48	3.72	85.995	3.24599999	11	7/24/2014	31:31.2
8534	RWS11	-122.470682	37.94038	-0.55	3.71	87.044	3.15900004	-122.47068	37.94039	-0.48	3.71	85.819	3.23500007	11	7/24/2014	31:31.3
8535	RWS11	-122.4706842	37.94038	-0.59	3.7	86.904	3.11400008	-122.47068	37.9404	-0.48	3.7	85.663	3.22500008	11	7/24/2014	31:31.4
8536	RWS11	-122.4706864	37.94038	-0.55	3.69	86.757	3.14099991	-122.47068	37.9404	-0.48	3.69	85.461	3.21699995	11	7/24/2014	31:31.5
8537	RWS11	-122.4706886	37.94038	-0.55	3.69	86.642	3.13399994	-122.47068	37.9404	-0.48	3.69	85.357	3.20999998	11	7/24/2014	31:31.6
8538	RWS11	-122.4706908	37.94038	-0.55	3.68	86.495	3.13	-122.47068	37.9404	-0.48	3.68	85.226	3.20600003	11	7/24/2014	31:31.7
8539	RWS11	-122.470693	37.94039	-0.55	3.68	86.38	3.12600005	-122.47069	37.9404	-0.48	3.68	85.117	3.20200008	11	7/24/2014	31:31.8
8540	RWS11	-122.4706951	37.94039	-0.55	3.68	86.276	3.12499988	-122.47069	37.9404	-0.43	3.68	85.008	3.25199991	11	7/24/2014	31:31.9
8541	RWS11	-122.4706984	37.94039	-0.55	3.68	86.053	3.12499988	-122.47069	37.9404	-0.48	3.68	84.807	3.20099992	11	7/24/2014	31:32.0
8542	RWS11	-122.4707005	37.94039	-0.55	3.68	85.9	3.12499988	-122.47069	37.9404	-0.48	3.68	84.741	3.20099992	11	7/24/2014	31:32.1
8543	RWS11	-122.4707027	37.94039	-0.55	3.68	85.725	3.12300003	-122.4707	37.9404	-0.43	3.68	84.545	3.25000006	11	7/24/2014	31:32.2
8544	RWS11	-122.4707049	37.94039	-0.55	3.67	85.529	3.11699998	-122.4707	37.9404	-0.43	3.67	84.419	3.24400002	11	7/24/2014	31:32.3
8545	RWS11	-122.4707071	37.94039	-0.55	3.66	85.376	3.10399997	-122.4707	37.9404	-0.43	3.66	84.277	3.23100001	11	7/24/2014	31:32.4
8546	RWS11	-122.4707093	37.94039	-0.55	3.64	85.179	3.0819999	-122.4707	37.9404	-0.43	3.64	84.064	3.20899993	11	7/24/2014	31:32.5

8547	RWS11	-122.4707115	37.94039	-0.55	3.6	84.978	3.04700005	-122.47071	37.9404	-0.48	3.6	83.907	3.12300009	11	7/24/2014	31:32.6
8548	RWS11	-122.4707137	37.94039	-0.55	3.55	84.825	2.99599993	-122.47071	37.9404	-0.39	3.55	83.732	3.15699995	11	7/24/2014	31:32.7
8549	RWS11	-122.4707159	37.94039	-0.55	3.48	84.59	2.92800009	-122.47071	37.9404	-0.39	3.48	83.606	3.08900011	11	7/24/2014	31:32.8
8550	RWS11	-122.4707181	37.94039	-0.5	3.47	84.453	2.97000003	-122.47071	37.94041	-0.39	3.47	83.464	3.08000004	11	7/24/2014	31:32.9
8551	RWS11	-122.4707214	37.94039	-0.55	3.46	84.191	2.91099989	-122.47072	37.94041	-0.43	3.46	83.295	3.03799993	11	7/24/2014	31:33.0
8552	RWS11	-122.4707236	37.94039	-0.5	3.46	84	2.95499992	-122.47072	37.94041	-0.39	3.46	83.103	3.06499994	11	7/24/2014	31:33.1
8553	RWS11	-122.4707258	37.94039	-0.55	3.45	83.799	2.89600003	-122.47072	37.94041	-0.39	3.45	82.985	3.05700004	11	7/24/2014	31:33.2
8554	RWS11	-122.4707281	37.9404	-0.5	3.44	83.575	2.93799996	-122.47072	37.94041	-0.43	3.44	82.771	3.014	11	7/24/2014	31:33.3
8555	RWS11	-122.4707303	37.9404	-0.5	3.43	83.362	2.93000007	-122.47072	37.94041	-0.34	3.43	82.569	3.09000009	11	7/24/2014	31:33.4
8556	RWS11	-122.4707326	37.9404	-0.47	3.42	83.121	2.95599994	-122.47073	37.94041	-0.39	3.42	82.29	3.03199995	11	7/24/2014	31:33.5
8557	RWS11	-122.4707349	37.9404	-0.5	3.42	82.881	2.91400003	-122.47073	37.94041	-0.39	3.42	82.115	3.02400005	11	7/24/2014	31:33.6
8558	RWS11	-122.4707372	37.9404	-0.47	3.41	82.662	2.93899998	-122.47073	37.94041	-0.34	3.41	81.887	3.065	11	7/24/2014	31:33.7
8559	RWS11	-122.4707395	37.9404	-0.47	3.4	82.353	2.93100008	-122.47073	37.94041	-0.34	3.4	81.61	3.0570001	11	7/24/2014	31:33.8
8560	RWS11	-122.4707418	37.9404	-0.42	3.39	82.025	2.97499987	-122.47074	37.94041	-0.34	3.39	81.331	3.04999989	11	7/24/2014	31:33.9
8561	RWS11	-122.4707452	37.9404	-0.47	3.38	81.719	2.91599998	-122.47074	37.94041	-0.34	3.38	81.058	3.042	11	7/24/2014	31:34.0
8562	RWS11	-122.4707476	37.9404	-0.42	3.38	81.419	2.95799991	-122.47074	37.94041	-0.34	3.38	80.828	3.03299993	11	7/24/2014	31:34.1
8563	RWS11	-122.4707499	37.9404	-0.47	3.37	81.064	2.89900002	-122.47074	37.94041	-0.34	3.37	80.561	3.02500004	11	7/24/2014	31:34.2
8564	RWS11	-122.4707524	37.9404	-0.42	3.42	80.762	3.00500008	-122.47075	37.94042	-0.31	3.42	80.319	3.11400011	11	7/24/2014	31:34.3
8565	RWS11	-122.4707548	37.9404	-0.47	3.46	80.409	2.99499997	-122.47075	37.94042	-0.34	3.46	80.059	3.12099999	11	7/24/2014	31:34.4
8566	RWS11	-122.4707572	37.9404	-0.42	3.49	80.059	3.07500002	-122.47075	37.94042	-0.31	3.49	79.786	3.18400005	11	7/24/2014	31:34.5
8567	RWS11	-122.4707596	37.9404	-0.47	3.51	79.688	3.04199991	-122.47075	37.94042	-0.34	3.51	79.521	3.16799992	11	7/24/2014	31:34.6
8568	RWS11	-122.4707621	37.94041	-0.42	3.52	79.294	3.10499999	-122.47076	37.94042	-0.31	3.52	79.18	3.21400002	11	7/24/2014	31:34.7
8569	RWS11	-122.4707644	37.94041	-0.47	3.53	78.857	3.0590001	-122.47076	37.94042	-0.34	3.53	78.818	3.18500012	11	7/24/2014	31:34.8
8570	RWS11	-122.4707668	37.94041	-0.42	3.53	78.424	3.11199996	-122.47076	37.94042	-0.31	3.53	78.55	3.22099999	11	7/24/2014	31:34.9
8571	RWS11	-122.4707703	37.94041	-0.42	3.53	77.935	3.11100003	-122.47076	37.94042	-0.34	3.53	78.18	3.18600005	11	7/24/2014	31:35.0
8572	RWS11	-122.4707727	37.94041	-0.42	3.53	77.372	3.1100001	-122.47077	37.94042	-0.31	3.53	77.716	3.21900013	11	7/24/2014	31:35.1
8573	RWS11	-122.470775	37.94041	-0.42	3.53	76.782	3.10899994	-122.47077	37.94042	-0.34	3.53	77.24	3.18399996	11	7/24/2014	31:35.2
8574	RWS11	-122.4707773	37.94041	-0.38	3.53	76.148	3.14500013	-122.47077	37.94042	-0.34	3.53	76.738	3.18500012	11	7/24/2014	31:35.3
8575	RWS11	-122.4707796	37.94041	-0.42	3.53	75.497	3.11100003	-122.47077	37.94042	-0.34	3.53	76.213	3.18600005	11	7/24/2014	31:35.4
8576	RWS11	-122.470782	37.94041	-0.38	3.53	74.797	3.15000001	-122.47078	37.94042	-0.31	3.53	75.644	3.22400001	11	7/24/2014	31:35.5
8577	RWS11	-122.4707843	37.94041	-0.42	3.54	74.051	3.12100002	-122.47078	37.94042	-0.31	3.54	75.034	3.23000005	11	7/24/2014	31:35.6
8578	RWS11	-122.4707866	37.94041	-0.38	3.55	73.154	3.16300002	-122.47078	37.94043	-0.34	3.55	74.373	3.20300001	11	7/24/2014	31:35.7
8579	RWS11	-122.4707888	37.94041	-0.42	3.55	72.258	3.13599989	-122.47078	37.94043	-0.34	3.55	73.7	3.21099991	11	7/24/2014	31:35.8

8580	RWS11	-122.4707911	37.94041	-0.38	3.56	71.17	3.17900005	-122.47079	37.94043	-0.31	3.56	72.892	3.25300005	11	7/24/2014	31:35.9
8581	RWS11	-122.4707945	37.94041	-0.42	3.57	70.082	3.1489999	-122.47079	37.94043	-0.31	3.57	72.11	3.25799993	11	7/24/2014	31:36.0
8582	RWS11	-122.4707967	37.94042	-0.38	3.57	68.857	3.18600002	-122.47079	37.94043	-0.31	3.57	71.191	3.26000002	11	7/24/2014	31:36.1
8583	RWS11	-122.470799	37.94042	-0.42	3.57	67.521	3.1489999	-122.47079	37.94043	-0.34	3.57	70.204	3.22399992	11	7/24/2014	31:36.2
8584	RWS11	-122.4708012	37.94042	-0.38	3.56	66.127	3.17699996	-122.4708	37.94043	-0.31	3.56	69.112	3.25099996	11	7/24/2014	31:36.3
8585	RWS11	-122.4708035	37.94042	-0.42	3.55	64.532	3.13100001	-122.4708	37.94043	-0.34	3.55	67.914	3.20600003	11	7/24/2014	31:36.4
8586	RWS11	-122.4708058	37.94042	-0.38	3.54	62.81	3.1520001	-122.4708	37.94043	-0.34	3.54	66.629	3.19200009	11	7/24/2014	31:36.5
8587	RWS11	-122.4708081	37.94042	-0.42	3.52	60.953	3.09999987	-122.4708	37.94043	-0.34	3.52	65.143	3.17499989	11	7/24/2014	31:36.6
8588	RWS11	-122.4708103	37.94042	-0.38	3.5	58.906	3.11499992	-122.4708	37.94043	-0.31	3.5	63.569	3.18899992	11	7/24/2014	31:36.7
8589	RWS11	-122.4708126	37.94042	-0.42	3.48	56.718	3.05899999	-122.47081	37.94043	-0.34	3.48	61.844	3.134	11	7/24/2014	31:36.8
8590	RWS11	-122.4708149	37.94042	-0.38	3.45	54.253	3.07000008	-122.47081	37.94043	-0.34	3.45	59.921	3.11000007	11	7/24/2014	31:36.9
8591	RWS11	-122.4708182	37.94042	-0.38	3.43	51.548	3.04599991	-122.47081	37.94043	-0.28	3.43	57.715	3.15399989	11	7/24/2014	31:37.0
8592	RWS11	-122.4708205	37.94042	-0.38	3.4	48.602	3.02000013	-122.47081	37.94044	-0.31	3.4	55.345	3.09400013	11	7/24/2014	31:37.1
8593	RWS11	-122.4708227	37.94042	-0.42	3.38	45.369	2.95900008	-122.47082	37.94044	-0.34	3.38	52.785	3.0340001	11	7/24/2014	31:37.2
8594	RWS11	-122.470825	37.94042	-0.38	3.35	41.84	2.96800008	-122.47082	37.94044	-0.31	3.35	49.912	3.04200009	11	7/24/2014	31:37.3
8595	RWS11	-122.4708273	37.94042	-0.42	3.33	38.06	2.90799996	-122.47082	37.94044	-0.34	3.33	46.787	2.98299998	11	7/24/2014	31:37.4
8596	RWS11	-122.4708297	37.94043	-0.38	3.3	34.131	2.91900006	-122.47082	37.94044	-0.28	3.3	43.511	3.02700004	11	7/24/2014	31:37.5
8597	RWS11	-122.470832	37.94043	-0.38	3.28	29.943	2.89699998	-122.47083	37.94044	-0.31	3.28	40.041	2.97099999	11	7/24/2014	31:37.6
8598	RWS11	-122.4708343	37.94043	-0.38	3.26	25.6	2.87799993	-122.47083	37.94044	-0.34	3.26	36.377	2.91799992	11	7/24/2014	31:37.7
8599	RWS11	-122.4708366	37.94043	-0.38	3.25	21.17	2.86399999	-122.47083	37.94044	-0.31	3.25	32.622	2.93799999	11	7/24/2014	31:37.8
8600	RWS11	-122.4708389	37.94043	-0.38	3.24	16.632	2.854	-122.47083	37.94044	-0.28	3.24	28.785	2.96199998	11	7/24/2014	31:37.9
8601	RWS11	-122.4708423	37.94043	-0.38	3.23	12.138	2.84799996	-122.47084	37.94044	-0.28	3.23	25.052	2.95599994	11	7/24/2014	31:38.0
8602	RWS11	-122.4708446	37.94043	-0.38	3.23	7.77	2.84600011	-122.47084	37.94044	-0.28	3.23	21.58	2.95400009	11	7/24/2014	31:38.1
8603	RWS11	-122.4708469	37.94043	-0.38	3.23	3.712	2.84700003	-122.47084	37.94044	-0.31	3.23	18.525	2.92100003	11	7/24/2014	31:38.2
8604	RWS11	-122.4708492	37.94043	-0.38	3.23	0.09	2.85099998	-122.47084	37.94044	-0.31	3.23	16.038	2.92499998	11	7/24/2014	31:38.3
8605	RWS11	-122.4708516	37.94043	-0.38	3.24	-2.9	2.85900012	-122.47085	37.94044	-0.28	3.24	14.161	2.9670001	11	7/24/2014	31:38.4
8606	RWS11	-122.470854	37.94043	-0.33	3.25	-5.038	2.91899994	-122.47085	37.94044	-0.28	3.25	12.962	2.97599992	11	7/24/2014	31:38.5
8607	RWS11	-122.4708563	37.94043	-0.38	3.26	-6.152	2.88000003	-122.47085	37.94045	-0.31	3.26	13.024	2.95400003	11	7/24/2014	31:38.6
8608	RWS11	-122.4708586	37.94043	-0.38	3.28	-6.111	2.89200011	-122.47085	37.94045	-0.22	3.28	13.983	3.05100009	11	7/24/2014	31:38.7
8609	RWS11	-122.470861	37.94043	-0.38	3.29	-5.02	2.90500012	-122.47085	37.94045	-0.31	3.29	16.002	2.97900012	11	7/24/2014	31:38.8
8610	RWS11	-122.4708633	37.94044	-0.33	3.3	-2.62	2.96799996	-122.47086	37.94045	-0.22	3.3	18.848	3.07599995	11	7/24/2014	31:38.9
8611	RWS11	-122.4708668	37.94044	-0.38	3.31	0.74	2.92999998	-122.47086	37.94045	-0.28	3.31	22.689	3.03799996	11	7/24/2014	31:39.0
8612	RWS11	-122.4708691	37.94044	-0.33	3.33	5.06	2.99300006	-122.47086	37.94045	-0.22	3.33	27.249	3.10100004	11	7/24/2014	31:39.1

8613	RWS11	-122.4708714	37.94044	-0.38	3.34	10.036	2.95399991	-122.47087	37.94045	-0.31	3.34	32.419	3.02799991	11	7/24/2014	31:39.2
8614	RWS11	-122.4708738	37.94044	-0.38	3.35	15.507	2.96800008	-122.47087	37.94045	-0.28	3.35	37.96	3.07600006	11	7/24/2014	31:39.3
8615	RWS11	-122.4708762	37.94044	-0.38	3.36	21.356	2.9810001	-122.47087	37.94045	-0.28	3.36	43.761	3.08900008	11	7/24/2014	31:39.4
8616	RWS11	-122.4708786	37.94044	-0.38	3.38	27.333	2.99599996	-122.47087	37.94045	-0.31	3.38	49.608	3.06999996	11	7/24/2014	31:39.5
8617	RWS11	-122.470881	37.94044	-0.38	3.39	33.355	3.0099999	-122.47087	37.94045	-0.28	3.39	55.438	3.11799988	11	7/24/2014	31:39.6
8618	RWS11	-122.4708833	37.94044	-0.38	3.41	39.268	3.02299991	-122.47088	37.94045	-0.34	3.41	60.976	3.0629999	11	7/24/2014	31:39.7
8619	RWS11	-122.4708857	37.94044	-0.38	3.42	44.901	3.035	-122.47088	37.94045	-0.31	3.42	66.15	3.109	11	7/24/2014	31:39.8
8620	RWS11	-122.470888	37.94044	-0.38	3.43	50.24	3.04599991	-122.47088	37.94046	-0.31	3.43	70.945	3.11999992	11	7/24/2014	31:39.9
8621	RWS11	-122.4708915	37.94044	-0.38	3.44	55.213	3.05400005	-122.47089	37.94046	-0.34	3.44	75.115	3.09400004	11	7/24/2014	31:40.0
8622	RWS11	-122.4708939	37.94044	-0.38	3.44	59.688	3.06100002	-122.47089	37.94046	-0.22	3.44	78.816	3.22	11	7/24/2014	31:40.1
8623	RWS11	-122.4708963	37.94045	-0.38	3.45	63.77	3.06700006	-122.47089	37.94046	-0.28	3.45	81.926	3.17500004	11	7/24/2014	31:40.2
8624	RWS11	-122.4708986	37.94045	-0.38	3.46	67.323	3.0730001	-122.47089	37.94046	-0.34	3.46	84.555	3.11300009	11	7/24/2014	31:40.3
8625	RWS11	-122.470901	37.94045	-0.38	3.46	70.426	3.07700005	-122.47089	37.94046	-0.34	3.46	86.636	3.11700004	11	7/24/2014	31:40.4
8626	RWS11	-122.4709034	37.94045	-0.38	3.46	73.014	3.08199993	-122.4709	37.94046	-0.28	3.46	88.223	3.18999991	11	7/24/2014	31:40.5
8627	RWS11	-122.4709058	37.94045	-0.38	3.47	75.392	3.08700004	-122.4709	37.94046	-0.39	3.47	89.464	3.07700002	11	7/24/2014	31:40.6
8628	RWS11	-122.4709082	37.94045	-0.38	3.47	77.315	3.09199992	-122.4709	37.94046	-0.34	3.47	90.36	3.13199991	11	7/24/2014	31:40.7
8629	RWS11	-122.4709105	37.94045	-0.38	3.48	78.867	3.09799996	-122.4709	37.94046	-0.39	3.48	90.885	3.08799994	11	7/24/2014	31:40.8
8630	RWS11	-122.4709129	37.94045	-0.38	3.49	80.168	3.10499993	-122.47091	37.94046	-0.28	3.49	91.169	3.21299991	11	7/24/2014	31:40.9
8631	RWS11	-122.4709164	37.94045	-0.42	3.5	81.112	3.07899997	-122.47091	37.94046	-0.31	3.5	91.228	3.18799999	11	7/24/2014	31:41.0
8632	RWS11	-122.4709187	37.94045	-0.38	3.51	81.847	3.12399998	-122.47091	37.94046	-0.34	3.51	90.99	3.16399997	11	7/24/2014	31:41.1
8633	RWS11	-122.4709211	37.94045	-0.42	3.52	82.328	3.09999987	-122.47091	37.94047	-0.39	3.52	90.64	3.12499988	11	7/24/2014	31:41.2
8634	RWS11	-122.4709234	37.94045	-0.38	3.53	82.656	3.14900008	-122.47092	37.94047	-0.28	3.53	90.176	3.25700006	11	7/24/2014	31:41.3
8635	RWS11	-122.4709258	37.94045	-0.42	3.55	82.853	3.12899992	-122.47092	37.94047	-0.43	3.55	89.591	3.11999995	11	7/24/2014	31:41.4
8636	RWS11	-122.4709282	37.94045	-0.42	3.56	82.875	3.14599988	-122.47092	37.94047	-0.43	3.56	88.935	3.13699991	11	7/24/2014	31:41.5
8637	RWS11	-122.4709305	37.94046	-0.42	3.58	82.787	3.16499993	-122.47092	37.94047	-0.31	3.58	88.176	3.27399996	11	7/24/2014	31:41.6
8638	RWS11	-122.4709329	37.94046	-0.42	3.6	82.608	3.18399999	-122.47093	37.94047	-0.39	3.6	87.385	3.20899999	11	7/24/2014	31:41.7
8639	RWS11	-122.4709352	37.94046	-0.42	3.62	82.368	3.20399997	-122.47093	37.94047	-0.43	3.62	86.57	3.19499999	11	7/24/2014	31:41.8
8640	RWS11	-122.4709375	37.94046	-0.42	3.64	82.149	3.22300002	-122.47093	37.94047	-0.31	3.64	85.816	3.33200005	11	7/24/2014	31:41.9
8641	RWS11	-122.470941	37.94046	-0.42	3.66	81.778	3.24099991	-122.47093	37.94047	-0.39	3.66	84.985	3.26599991	11	7/24/2014	31:42.0
8642	RWS11	-122.4709433	37.94046	-0.42	3.67	81.412	3.25600001	-122.47094	37.94047	-0.39	3.67	84.149	3.28100002	11	7/24/2014	31:42.1
8643	RWS11	-122.4709456	37.94046	-0.42	3.68	80.902	3.26699993	-122.47094	37.94047	-0.31	3.68	83.285	3.37599996	11	7/24/2014	31:42.2
8644	RWS11	-122.470948	37.94046	-0.38	3.69	80.489	3.30899993	-122.47094	37.94047	-0.34	3.69	82.489	3.34899992	11	7/24/2014	31:42.3
8645	RWS11	-122.4709503	37.94046	-0.42	3.69	80.074	3.27699992	-122.47094	37.94047	-0.43	3.69	81.751	3.26799995	11	7/24/2014	31:42.4

8646	RWS11	-122.4709527	37.94046	-0.42	3.69	79.593	3.27599999	-122.47095	37.94048	-0.39	3.69	81.008	3.301	11	7/24/2014	31:42.5
8647	RWS11	-122.4709551	37.94046	-0.42	3.69	79.09	3.27299997	-122.47095	37.94048	-0.34	3.69	80.238	3.34799999	11	7/24/2014	31:42.6
8648	RWS11	-122.4709574	37.94046	-0.42	3.69	78.593	3.26900002	-122.47095	37.94048	-0.34	3.69	79.505	3.34400004	11	7/24/2014	31:42.7
8649	RWS11	-122.4709597	37.94046	-0.42	3.68	78.085	3.26500008	-122.47095	37.94048	-0.43	3.68	78.784	3.2560001	11	7/24/2014	31:42.8
8650	RWS11	-122.4709621	37.94047	-0.42	3.68	77.627	3.26200005	-122.47096	37.94048	-0.43	3.68	78.124	3.25300008	11	7/24/2014	31:42.9
8651	RWS11	-122.4709655	37.94047	-0.42	3.68	77.08	3.25900003	-122.47096	37.94048	-0.39	3.68	77.447	3.28400004	11	7/24/2014	31:43.0
8652	RWS11	-122.4709679	37.94047	-0.38	3.68	76.643	3.29400006	-122.47096	37.94048	-0.43	3.68	76.796	3.25000006	11	7/24/2014	31:43.1
8653	RWS11	-122.4709702	37.94047	-0.42	3.68	76.25	3.25999996	-122.47096	37.94048	-0.43	3.68	76.206	3.25099999	11	7/24/2014	31:43.2
8654	RWS11	-122.4709726	37.94047	-0.42	3.68	75.9	3.26500008	-122.47097	37.94048	-0.43	3.68	75.73	3.2560001	11	7/24/2014	31:43.3
8655	RWS11	-122.4709749	37.94047	-0.42	3.69	75.467	3.27200004	-122.47097	37.94048	-0.48	3.69	75.221	3.21200007	11	7/24/2014	31:43.4
8656	RWS11	-122.4709773	37.94047	-0.42	3.7	75.092	3.28099987	-122.47097	37.94048	-0.48	3.7	74.677	3.2209999	11	7/24/2014	31:43.5
8657	RWS11	-122.4709797	37.94047	-0.42	3.71	74.738	3.2910001	-122.47097	37.94048	-0.48	3.71	74.278	3.23100013	11	7/24/2014	31:43.6
8658	RWS11	-122.4709821	37.94047	-0.42	3.72	74.415	3.30299994	-122.47098	37.94048	-0.51	3.72	73.846	3.20899993	11	7/24/2014	31:43.7
8659	RWS11	-122.4709844	37.94047	-0.47	3.73	74.087	3.26400003	-122.47098	37.94049	-0.51	3.73	73.431	3.22100002	11	7/24/2014	31:43.8
8660	RWS11	-122.4709868	37.94047	-0.42	3.75	73.781	3.32800004	-122.47098	37.94049	-0.48	3.75	73.059	3.26800007	11	7/24/2014	31:43.9
8661	RWS11	-122.4709903	37.94047	-0.42	3.76	73.519	3.34199998	-122.47098	37.94049	-0.48	3.76	72.824	3.28200001	11	7/24/2014	31:44.0
8662	RWS11	-122.4709926	37.94048	-0.42	3.78	73.217	3.35700008	-122.47099	37.94049	-0.51	3.78	72.446	3.26300007	11	7/24/2014	31:44.1
8663	RWS11	-122.4709949	37.94048	-0.42	3.79	72.881	3.37299988	-122.47099	37.94049	-0.51	3.79	72.12	3.27899987	11	7/24/2014	31:44.2
8664	RWS11	-122.4709973	37.94048	-0.42	3.81	72.597	3.38899991	-122.47099	37.94049	-0.34	3.81	71.788	3.46399993	11	7/24/2014	31:44.3
8665	RWS11	-122.4709997	37.94048	-0.42	3.82	72.296	3.40599987	-122.47099	37.94049	-0.51	3.82	71.443	3.31199986	11	7/24/2014	31:44.4
8666	RWS11	-122.471002	37.94048	-0.42	3.84	72.143	3.4219999	-122.471	37.94049	-0.48	3.84	71.18	3.36199993	11	7/24/2014	31:44.5
8667	RWS11	-122.4710044	37.94048	-0.42	3.86	71.897	3.4390001	-122.471	37.94049	-0.48	3.86	70.94	3.37900013	11	7/24/2014	31:44.6
8668	RWS11	-122.4710068	37.94048	-0.42	3.87	71.662	3.45499989	-122.471	37.94049	-0.51	3.87	70.612	3.36099988	11	7/24/2014	31:44.7
8669	RWS11	-122.4710091	37.94048	-0.42	3.89	71.396	3.47	-122.471	37.94049	-0.56	3.89	70.171	3.32499999	11	7/24/2014	31:44.8
8670	RWS11	-122.4710114	37.94048	-0.42	3.9	71.199	3.48399994	-122.471	37.94049	-0.51	3.9	69.865	3.38999993	11	7/24/2014	31:44.9
8671	RWS11	-122.4710149	37.94048	-0.47	3.91	71.024	3.4440001	-122.47101	37.9405	-0.43	3.91	69.607	3.48600012	11	7/24/2014	31:45.0
8672	RWS11	-122.4710173	37.94048	-0.42	3.92	70.762	3.50399992	-122.47101	37.9405	-0.56	3.92	69.275	3.35899991	11	7/24/2014	31:45.1
8673	RWS11	-122.4710197	37.94048	-0.42	3.93	70.456	3.50900003	-122.47101	37.9405	-0.56	3.93	68.996	3.36400002	11	7/24/2014	31:45.2
8674	RWS11	-122.471022	37.94048	-0.42	3.93	70.111	3.50999996	-122.47102	37.9405	-0.56	3.93	68.637	3.36499995	11	7/24/2014	31:45.3
8675	RWS11	-122.4710244	37.94049	-0.42	3.92	69.884	3.50600001	-122.47102	37.9405	-0.6	3.92	68.358	3.32800001	11	7/24/2014	31:45.4
8676	RWS11	-122.4710268	37.94049	-0.42	3.91	69.604	3.493	-122.47102	37.9405	-0.51	3.91	68.096	3.39899999	11	7/24/2014	31:45.5
8677	RWS11	-122.4710292	37.94049	-0.47	3.89	69.337	3.41800007	-122.47102	37.9405	-0.6	3.89	67.807	3.29100007	11	7/24/2014	31:45.6
8678	RWS11	-122.4710316	37.94049	-0.42	3.85	69.014	3.43000004	-122.47103	37.9405	-0.6	3.85	67.527	3.25200003	11	7/24/2014	31:45.7

8679	RWS11	-122.471034	37.94049	-0.47	3.79	68.774	3.32300004	-122.47103	37.9405	-0.65	3.79	67.27	3.14500004	11	7/24/2014	31:45.8
8680	RWS11	-122.4710364	37.94049	-0.42	3.72	68.621	3.29800007	-122.47103	37.9405	-0.65	3.72	67.06	3.06900007	11	7/24/2014	31:45.9
8681	RWS11	-122.4710399	37.94049	-0.47	3.62	68.354	3.15299997	-122.47103	37.9405	-0.65	3.62	66.916	2.97499996	11	7/24/2014	31:46.0
8682	RWS11	-122.4710422	37.94049	-0.42	3.62	68.18	3.20600006	-122.47104	37.9405	-0.65	3.62	66.667	2.97700006	11	7/24/2014	31:46.1
8683	RWS11	-122.4710446	37.94049	-0.47	3.63	68.005	3.15699992	-122.47104	37.9405	-0.68	3.63	66.457	2.94499993	11	7/24/2014	31:46.2
8684	RWS11	-122.4710469	37.94049	-0.47	3.63	67.83	3.15900001	-122.47104	37.94051	-0.68	3.63	66.3	2.94700003	11	7/24/2014	31:46.3
8685	RWS11	-122.4710493	37.94049	-0.47	3.63	67.699	3.16200012	-122.47104	37.94051	-0.68	3.63	66.147	2.95000011	11	7/24/2014	31:46.4
8686	RWS11	-122.4710517	37.94049	-0.47	3.63	67.546	3.16399997	-122.47105	37.94051	-0.68	3.63	65.933	2.95199996	11	7/24/2014	31:46.5
8687	RWS11	-122.4710541	37.9405	-0.47	3.63	67.351	3.1649999	-122.47105	37.94051	-0.68	3.63	65.729	2.95299989	11	7/24/2014	31:46.6
8688	RWS11	-122.4710564	37.9405	-0.47	3.63	67.193	3.167	-122.47105	37.94051	-0.71	3.63	65.615	2.921	11	7/24/2014	31:46.7
8689	RWS11	-122.4710588	37.9405	-0.5	3.64	67.132	3.13500011	-122.47105	37.94051	-0.71	3.64	65.536	2.9230001	11	7/24/2014	31:46.8
8690	RWS11	-122.4710611	37.9405	-0.47	3.64	66.975	3.17099994	-122.47105	37.94051	-0.71	3.64	65.379	2.92499995	11	7/24/2014	31:46.9
8691	RWS11	-122.4710646	37.9405	-0.5	3.64	66.822	3.13900006	-122.47106	37.94051	-0.71	3.64	65.165	2.92700005	11	7/24/2014	31:47.0
8692	RWS11	-122.4710669	37.9405	-0.47	3.64	66.716	3.17499989	-122.47106	37.94051	-0.76	3.64	65.011	2.8779999	11	7/24/2014	31:47.1
8693	RWS11	-122.4710692	37.9405	-0.47	3.64	66.604	3.17699999	-122.47106	37.94051	-0.71	3.64	64.894	2.93099999	11	7/24/2014	31:47.2
8694	RWS11	-122.4710716	37.9405	-0.47	3.7	66.468	3.23299998	-122.47107	37.94051	-0.76	3.7	64.789	2.93599999	11	7/24/2014	31:47.3
8695	RWS11	-122.4710739	37.9405	-0.5	3.74	66.363	3.24199998	-122.47107	37.94051	-0.68	3.74	64.619	3.06399995	11	7/24/2014	31:47.4
8696	RWS11	-122.4710763	37.9405	-0.47	3.78	66.298	3.30800003	-122.47107	37.94052	-0.76	3.78	64.519	3.01100004	11	7/24/2014	31:47.5
8697	RWS11	-122.4710787	37.9405	-0.5	3.8	66.206	3.29600012	-122.47107	37.94052	-0.8	3.8	64.498	2.99900007	11	7/24/2014	31:47.6
8698	RWS11	-122.471081	37.9405	-0.5	3.81	66.166	3.30900013	-122.47107	37.94052	-0.76	3.81	64.395	3.04600012	11	7/24/2014	31:47.7
8699	RWS11	-122.4710833	37.9405	-0.5	3.82	66.031	3.31499994	-122.47108	37.94052	-0.8	3.82	64.287	3.01799989	11	7/24/2014	31:47.8
8700	RWS11	-122.4710857	37.94051	-0.5	3.82	65.966	3.31499994	-122.47108	37.94052	-0.76	3.82	64.147	3.05199993	11	7/24/2014	31:47.9
8701	RWS11	-122.4710891	37.94051	-0.5	3.81	65.786	3.31099999	-122.47108	37.94052	-0.76	3.81	64.025	3.04799998	11	7/24/2014	31:48.0
8702	RWS11	-122.4710915	37.94051	-0.5	3.81	65.682	3.30400002	-122.47108	37.94052	-0.76	3.81	63.846	3.04100001	11	7/24/2014	31:48.1
8703	RWS11	-122.4710938	37.94051	-0.5	3.8	65.572	3.29600012	-122.47109	37.94052	-0.8	3.8	63.675	2.99900007	11	7/24/2014	31:48.2
8704	RWS11	-122.4710962	37.94051	-0.5	3.79	65.375	3.28899992	-122.47109	37.94052	-0.76	3.79	63.473	3.0259999	11	7/24/2014	31:48.3
8705	RWS11	-122.4710985	37.94051	-0.5	3.79	65.088	3.28499997	-122.47109	37.94052	-0.85	3.79	63.19	2.93799996	11	7/24/2014	31:48.4
8706	RWS11	-122.4711009	37.94051	-0.5	3.79	64.83	3.28700006	-122.47109	37.94052	-0.71	3.79	62.924	3.07500005	11	7/24/2014	31:48.5
8707	RWS11	-122.4711033	37.94051	-0.5	3.8	64.76	3.29600012	-122.4711	37.94052	-0.8	3.8	62.731	2.99900007	11	7/24/2014	31:48.6
8708	RWS11	-122.4711056	37.94051	-0.5	3.81	64.432	3.31000006	-122.4711	37.94052	-0.76	3.81	62.425	3.04700005	11	7/24/2014	31:48.7
8709	RWS11	-122.4711079	37.94051	-0.55	3.83	64.192	3.2750001	-122.4711	37.94053	-0.8	3.83	62.098	3.02900004	11	7/24/2014	31:48.8
8710	RWS11	-122.4711103	37.94051	-0.5	3.85	63.911	3.34399998	-122.4711	37.94053	-0.76	3.85	61.769	3.08099997	11	7/24/2014	31:48.9
8711	RWS11	-122.4711137	37.94052	-0.55	3.86	63.624	3.30999994	-122.47111	37.94053	-0.8	3.86	61.416	3.06399989	11	7/24/2014	31:49.0

8712	RWS11	-122.471116	37.94052	-0.5	3.88	63.357	3.37900007	-122.47111	37.94053	-0.71	3.88	61.071	3.16700006	11	7/24/2014	31:49.1
8713	RWS11	-122.4711183	37.94052	-0.55	3.9	63.078	3.34500003	-122.47111	37.94053	-0.8	3.9	60.717	3.09899998	11	7/24/2014	31:49.2
8714	RWS11	-122.4711207	37.94052	-0.5	3.91	62.706	3.41100013	-122.47111	37.94053	-0.76	3.91	60.328	3.14800012	11	7/24/2014	31:49.3
8715	RWS11	-122.471123	37.94052	-0.55	3.92	62.335	3.37100005	-122.47112	37.94053	-0.8	3.92	59.913	3.125	11	7/24/2014	31:49.4
8716	RWS11	-122.4711254	37.94052	-0.5	3.93	62.029	3.42800009	-122.47112	37.94053	-0.71	3.93	59.581	3.21600008	11	7/24/2014	31:49.5
8717	RWS11	-122.4711277	37.94052	-0.55	3.93	61.661	3.37300014	-122.47112	37.94053	-0.76	3.93	59.191	3.16100013	11	7/24/2014	31:49.6
8718	RWS11	-122.47113	37.94052	-0.5	3.92	61.26	3.42299998	-122.47112	37.94053	-0.8	3.92	58.751	3.12599993	11	7/24/2014	31:49.7
8719	RWS11	-122.4711323	37.94052	-0.55	3.92	60.936	3.37199998	-122.47113	37.94053	-0.76	3.92	58.336	3.15999997	11	7/24/2014	31:49.8
8720	RWS11	-122.4711347	37.94052	-0.5	3.92	60.692	3.42299998	-122.47113	37.94053	-0.76	3.92	58.07	3.15999997	11	7/24/2014	31:49.9
8721	RWS11	-122.4711381	37.94052	-0.55	3.92	60.346	3.37100005	-122.47113	37.94054	-0.76	3.92	57.68	3.15900004	11	7/24/2014	31:50.0
8722	RWS11	-122.4711404	37.94052	-0.55	3.92	60.041	3.37100005	-122.47113	37.94054	-0.76	3.92	57.313	3.15900004	11	7/24/2014	31:50.1
8723	RWS11	-122.4711428	37.94052	-0.55	3.92	59.865	3.37100005	-122.47114	37.94054	-0.8	3.92	56.98	3.125	11	7/24/2014	31:50.2
8724	RWS11	-122.4711451	37.94053	-0.55	3.92	59.665	3.37000012	-122.47114	37.94054	-0.76	3.92	56.762	3.15800011	11	7/24/2014	31:50.3
8725	RWS11	-122.4711475	37.94053	-0.55	3.92	59.468	3.37000012	-122.47114	37.94054	-0.76	3.92	56.453	3.15800011	11	7/24/2014	31:50.4
8726	RWS11	-122.4711499	37.94053	-0.55	3.92	59.228	3.37000012	-122.47114	37.94054	-0.71	3.92	56.107	3.20900011	11	7/24/2014	31:50.5
8727	RWS11	-122.4711522	37.94053	-0.55	3.92	59.075	3.36899996	-122.47115	37.94054	-0.8	3.92	55.779	3.12299991	11	7/24/2014	31:50.6
8728	RWS11	-122.4711546	37.94053	-0.55	3.92	58.9	3.36899996	-122.47115	37.94054	-0.76	3.92	55.47	3.15699995	11	7/24/2014	31:50.7
8729	RWS11	-122.471157	37.94053	-0.59	3.92	58.685	3.333	-122.47115	37.94054	-0.8	3.92	55.21	3.12199998	11	7/24/2014	31:50.8
8730	RWS11	-122.4711593	37.94053	-0.55	3.92	58.525	3.36800003	-122.47115	37.94054	-0.8	3.92	54.949	3.12199998	11	7/24/2014	31:50.9
8731	RWS11	-122.4711628	37.94053	-0.59	3.92	58.463	3.333	-122.47116	37.94054	-0.85	3.92	54.814	3.07200003	11	7/24/2014	31:51.0
8732	RWS11	-122.4711652	37.94053	-0.55	3.92	58.459	3.3670001	-122.47116	37.94054	-0.8	3.92	54.88	3.12100005	11	7/24/2014	31:51.1
8733	RWS11	-122.4711675	37.94053	-0.59	3.92	58.459	3.33200008	-122.47116	37.94055	-0.85	3.92	54.88	3.0710001	11	7/24/2014	31:51.2
8734	RWS11	-122.4711699	37.94053	-0.59	3.92	58.376	3.33200008	-122.47116	37.94055	-0.85	3.92	54.836	3.0710001	11	7/24/2014	31:51.3
8735	RWS11	-122.4711723	37.94053	-0.59	3.92	58.371	3.33099991	-122.47117	37.94055	-0.85	3.92	54.817	3.06999993	11	7/24/2014	31:51.4
8736	RWS11	-122.4711746	37.94054	-0.59	3.92	58.525	3.33099991	-122.47117	37.94055	-0.85	3.92	54.971	3.06999993	11	7/24/2014	31:51.5
8737	RWS11	-122.471177	37.94054	-0.59	3.92	58.63	3.33099991	-122.47117	37.94055	-0.85	3.92	55.148	3.06999993	11	7/24/2014	31:51.6
8738	RWS11	-122.4711794	37.94054	-0.59	3.92	58.783	3.32999998	-122.47117	37.94055	-0.85	3.92	55.334	3.06900001	11	7/24/2014	31:51.7
8739	RWS11	-122.4711818	37.94054	-0.64	3.92	58.936	3.27899998	-122.47118	37.94055	-0.85	3.92	55.567	3.06900001	11	7/24/2014	31:51.8
8740	RWS11	-122.4711841	37.94054	-0.59	3.92	59.115	3.32999998	-122.47118	37.94055	-0.8	3.92	55.859	3.11899996	11	7/24/2014	31:51.9
8741	RWS11	-122.4711876	37.94054	-0.64	3.92	59.197	3.27800006	-122.47118	37.94055	-0.85	3.92	56.054	3.06800008	11	7/24/2014	31:52.0
8742	RWS11	-122.47119	37.94054	-0.59	3.92	59.325	3.32900006	-122.47118	37.94055	-0.85	3.92	56.204	3.06800008	11	7/24/2014	31:52.1
8743	RWS11	-122.4711923	37.94054	-0.64	3.92	59.303	3.27699989	-122.47119	37.94055	-0.85	3.92	56.295	3.06699991	11	7/24/2014	31:52.2
8744	RWS11	-122.4711947	37.94054	-0.59	3.92	59.391	3.32799989	-122.47119	37.94055	-0.8	3.92	56.441	3.11699986	11	7/24/2014	31:52.3

8745	RWS11	-122.471197	37.94054	-0.59	3.92	59.369	3.32799989	-122.47119	37.94056	-0.88	3.92	56.488	3.03299987	11	7/24/2014	31:52.4
8746	RWS11	-122.4711994	37.94054	-0.59	3.91	59.238	3.32699996	-122.47119	37.94056	-0.85	3.91	56.444	3.06599998	11	7/24/2014	31:52.5
8747	RWS11	-122.4712018	37.94054	-0.64	3.91	59.171	3.27599996	-122.4712	37.94056	-0.85	3.91	56.447	3.06599998	11	7/24/2014	31:52.6
8748	RWS11	-122.4712042	37.94055	-0.59	3.91	59.107	3.32699996	-122.4712	37.94056	-0.85	3.91	56.437	3.06599998	11	7/24/2014	31:52.7
8749	RWS11	-122.4712065	37.94055	-0.59	3.91	59.037	3.32600003	-122.4712	37.94056	-0.85	3.91	56.379	3.06500006	11	7/24/2014	31:52.8
8750	RWS11	-122.4712088	37.94055	-0.59	3.91	58.819	3.32600003	-122.4712	37.94056	-0.85	3.91	56.197	3.06500006	11	7/24/2014	31:52.9
8751	RWS11	-122.4712123	37.94055	-0.59	3.91	58.648	3.32600003	-122.47121	37.94056	-0.85	3.91	56.051	3.06500006	11	7/24/2014	31:53.0
8752	RWS11	-122.4712146	37.94055	-0.59	3.91	58.513	3.32500011	-122.47121	37.94056	-0.85	3.91	55.978	3.06400013	11	7/24/2014	31:53.1
8753	RWS11	-122.4712169	37.94055	-0.59	3.91	58.293	3.32500011	-122.47121	37.94056	-0.85	3.91	55.722	3.06400013	11	7/24/2014	31:53.2
8754	RWS11	-122.4712193	37.94055	-0.59	3.91	58.077	3.32399994	-122.47121	37.94056	-0.8	3.91	55.542	3.11299992	11	7/24/2014	31:53.3
8755	RWS11	-122.4712216	37.94055	-0.64	3.91	57.815	3.27299994	-122.47122	37.94056	-0.85	3.91	55.283	3.06299996	11	7/24/2014	31:53.4
8756	RWS11	-122.471224	37.94055	-0.59	3.91	57.548	3.32399994	-122.47122	37.94056	-0.8	3.91	55.101	3.11299992	11	7/24/2014	31:53.5
8757	RWS11	-122.4712263	37.94055	-0.59	3.91	57.268	3.32300001	-122.47122	37.94056	-0.85	3.91	54.803	3.06200004	11	7/24/2014	31:53.6
8758	RWS11	-122.4712287	37.94055	-0.59	3.91	56.984	3.32300001	-122.47122	37.94057	-0.85	3.91	54.584	3.06200004	11	7/24/2014	31:53.7
8759	RWS11	-122.471231	37.94055	-0.64	3.91	56.699	3.27200001	-122.47122	37.94057	-0.8	3.91	54.408	3.11199999	11	7/24/2014	31:53.8
8760	RWS11	-122.4712333	37.94055	-0.59	3.91	56.325	3.32200009	-122.47123	37.94057	-0.8	3.91	54.078	3.11100006	11	7/24/2014	31:53.9
8761	RWS11	-122.4712367	37.94056	-0.59	3.91	55.975	3.32200009	-122.47123	37.94057	-0.8	3.91	53.743	3.11100006	11	7/24/2014	31:54.0
8762	RWS11	-122.471239	37.94056	-0.55	3.91	55.63	3.35700011	-122.47123	37.94057	-0.8	3.91	53.422	3.11100006	11	7/24/2014	31:54.1
8763	RWS11	-122.4712413	37.94056	-0.59	3.91	55.254	3.32099992	-122.47123	37.94057	-0.8	3.91	53.047	3.1099999	11	7/24/2014	31:54.2
8764	RWS11	-122.4712436	37.94056	-0.59	3.91	54.887	3.32099992	-122.47124	37.94057	-0.76	3.91	52.639	3.14399993	11	7/24/2014	31:54.3
8765	RWS11	-122.471246	37.94056	-0.59	3.91	54.471	3.31999999	-122.47124	37.94057	-0.76	3.91	52.285	3.14300001	11	7/24/2014	31:54.4
8766	RWS11	-122.4712483	37.94056	-0.55	3.91	54.096	3.35500002	-122.47124	37.94057	-0.71	3.91	51.976	3.19400001	11	7/24/2014	31:54.5
8767	RWS11	-122.4712506	37.94056	-0.59	3.91	53.659	3.31999999	-122.47124	37.94057	-0.76	3.91	51.627	3.14300001	11	7/24/2014	31:54.6
8768	RWS11	-122.4712529	37.94056	-0.55	3.91	53.31	3.35400009	-122.47125	37.94057	-0.71	3.91	51.303	3.19300008	11	7/24/2014	31:54.7
8769	RWS11	-122.4712552	37.94056	-0.59	3.91	52.96	3.31900007	-122.47125	37.94057	-0.71	3.91	50.946	3.19300008	11	7/24/2014	31:54.8
8770	RWS11	-122.4712575	37.94056	-0.59	3.91	52.614	3.31900007	-122.47125	37.94058	-0.71	3.91	50.604	3.19300008	11	7/24/2014	31:54.9
8771	RWS11	-122.4712609	37.94056	-0.59	3.87	52.283	3.27799994	-122.47125	37.94058	-0.71	3.87	50.298	3.15199995	11	7/24/2014	31:55.0
8772	RWS11	-122.4712633	37.94056	-0.55	3.8	51.98	3.24699998	-122.47126	37.94058	-0.68	3.8	49.969	3.11999995	11	7/24/2014	31:55.1
8773	RWS11	-122.4712656	37.94057	-0.59	3.71	51.693	3.11800009	-122.47126	37.94058	-0.71	3.71	49.66	2.9920001	11	7/24/2014	31:55.2
8774	RWS11	-122.4712679	37.94057	-0.55	3.59	51.299	3.03200006	-122.47126	37.94058	-0.68	3.59	49.245	2.90500003	11	7/24/2014	31:55.3
8775	RWS11	-122.4712702	37.94057	-0.59	3.58	51.015	2.99300009	-122.47126	37.94058	-0.68	3.58	48.94	2.90100008	11	7/24/2014	31:55.4
8776	RWS11	-122.4712726	37.94057	-0.55	3.58	50.731	3.02399993	-122.47127	37.94058	-0.65	3.58	48.615	2.93099988	11	7/24/2014	31:55.5
8777	RWS11	-122.4712749	37.94057	-0.55	3.57	50.426	3.01900005	-122.47127	37.94058	-0.68	3.57	48.281	2.89200002	11	7/24/2014	31:55.6

8778	RWS11	-122.4712773	37.94057	-0.55	3.57	50.013	3.01600003	-122.47127	37.94058	-0.6	3.57	47.915	2.97399998	11	7/24/2014	31:55.7
8779	RWS11	-122.4712796	37.94057	-0.55	3.57	49.701	3.01200008	-122.47127	37.94058	-0.68	3.57	47.538	2.88500005	11	7/24/2014	31:55.8
8780	RWS11	-122.4712819	37.94057	-0.55	3.56	49.333	3.00800014	-122.47128	37.94058	-0.65	3.56	47.173	2.91500008	11	7/24/2014	31:55.9
8781	RWS11	-122.4712854	37.94057	-0.55	3.56	49.023	3.00399995	-122.47128	37.94058	-0.65	3.56	46.842	2.91099989	11	7/24/2014	31:56.0
8782	RWS11	-122.4712877	37.94057	-0.5	3.55	48.721	3.051	-122.47128	37.94058	-0.6	3.55	46.467	2.95799994	11	7/24/2014	31:56.1
8783	RWS11	-122.47129	37.94057	-0.55	3.55	48.328	2.99600005	-122.47128	37.94059	-0.6	3.55	46.099	2.954	11	7/24/2014	31:56.2
8784	RWS11	-122.4712923	37.94057	-0.5	3.55	47.999	3.04400003	-122.47129	37.94059	-0.6	3.55	45.727	2.95099998	11	7/24/2014	31:56.3
8785	RWS11	-122.4712947	37.94057	-0.55	3.54	47.712	2.98799992	-122.47129	37.94059	-0.65	3.54	45.377	2.89499986	11	7/24/2014	31:56.4
8786	RWS11	-122.471297	37.94058	-0.5	3.54	47.41	3.03499997	-122.47129	37.94059	-0.56	3.54	45.05	2.97499996	11	7/24/2014	31:56.5
8787	RWS11	-122.4712994	37.94058	-0.55	3.53	47.21	2.98000002	-122.47129	37.94059	-0.56	3.53	44.834	2.97100002	11	7/24/2014	31:56.6
8788	RWS11	-122.4713017	37.94058	-0.5	3.53	46.926	3.028	-122.4713	37.94059	-0.56	3.53	44.501	2.96799999	11	7/24/2014	31:56.7
8789	RWS11	-122.471304	37.94058	-0.5	3.53	46.645	3.02300012	-122.4713	37.94059	-0.6	3.53	44.179	2.93000007	11	7/24/2014	31:56.8
8790	RWS11	-122.4713063	37.94058	-0.5	3.52	46.379	3.0200001	-122.4713	37.94059	-0.56	3.52	43.891	2.9600001	11	7/24/2014	31:56.9
8791	RWS11	-122.4713097	37.94058	-0.5	3.52	46.226	3.01599991	-122.4713	37.94059	-0.6	3.52	43.673	2.92299986	11	7/24/2014	31:57.0
8792	RWS11	-122.471312	37.94058	-0.5	3.51	46.03	3.01199996	-122.47131	37.94059	-0.56	3.51	43.451	2.95199996	11	7/24/2014	31:57.1
8793	RWS11	-122.4713143	37.94058	-0.5	3.51	45.877	3.00700009	-122.47131	37.94059	-0.56	3.51	43.255	2.94700009	11	7/24/2014	31:57.2
8794	RWS11	-122.4713166	37.94058	-0.5	3.51	45.614	3.00400007	-122.47131	37.94059	-0.56	3.51	43.017	2.94400007	11	7/24/2014	31:57.3
8795	RWS11	-122.4713189	37.94058	-0.5	3.5	45.418	3.00000012	-122.47131	37.94059	-0.56	3.5	42.777	2.94000012	11	7/24/2014	31:57.4
8796	RWS11	-122.4713212	37.94058	-0.47	3.5	45.155	3.02999991	-122.47131	37.9406	-0.51	3.5	42.536	2.98699993	11	7/24/2014	31:57.5
8797	RWS11	-122.4713234	37.94058	-0.5	3.49	44.911	2.99199998	-122.47132	37.9406	-0.56	3.49	42.227	2.93199998	11	7/24/2014	31:57.6
8798	RWS11	-122.4713257	37.94058	-0.47	3.49	44.627	3.02200001	-122.47132	37.9406	-0.51	3.49	41.987	2.97900003	11	7/24/2014	31:57.7
8799	RWS11	-122.471328	37.94059	-0.5	3.49	44.387	2.98400009	-122.47132	37.9406	-0.51	3.49	41.725	2.97500008	11	7/24/2014	31:57.8
8800	RWS11	-122.4713303	37.94059	-0.5	3.48	44.15	2.98100007	-122.47132	37.9406	-0.51	3.48	41.485	2.97200006	11	7/24/2014	31:57.9
8801	RWS11	-122.4713336	37.94059	-0.5	3.48	43.863	2.97599995	-122.47133	37.9406	-0.56	3.48	41.201	2.91599995	11	7/24/2014	31:58.0
8802	RWS11	-122.4713359	37.94059	-0.5	3.52	43.603	3.01599991	-122.47133	37.9406	-0.48	3.52	40.897	3.04099989	11	7/24/2014	31:58.1
8803	RWS11	-122.4713381	37.94059	-0.5	3.52	43.335	3.01800001	-122.47133	37.9406	-0.51	3.52	40.651	3.009	11	7/24/2014	31:58.2
8804	RWS11	-122.4713404	37.94059	-0.47	3.52	43.076	3.05500001	-122.47133	37.9406	-0.48	3.52	40.389	3.046	11	7/24/2014	31:58.3
8805	RWS11	-122.4713427	37.94059	-0.5	3.53	42.836	3.02400005	-122.47134	37.9406	-0.51	3.53	40.126	3.01500005	11	7/24/2014	31:58.4
8806	RWS11	-122.471345	37.94059	-0.47	3.53	42.552	3.06000012	-122.47134	37.9406	-0.48	3.53	39.843	3.05100012	11	7/24/2014	31:58.5
8807	RWS11	-122.4713473	37.94059	-0.5	3.53	42.311	3.028	-122.47134	37.9406	-0.48	3.53	39.583	3.05299997	11	7/24/2014	31:58.6
8808	RWS11	-122.4713496	37.94059	-0.47	3.53	42.093	3.065	-122.47134	37.9406	-0.48	3.53	39.343	3.05599999	11	7/24/2014	31:58.7
8809	RWS11	-122.4713518	37.94059	-0.5	3.54	41.849	3.03400004	-122.47135	37.94061	-0.48	3.54	39.181	3.05900002	11	7/24/2014	31:58.8
8810	RWS11	-122.4713541	37.94059	-0.47	3.54	41.59	3.07000011	-122.47135	37.94061	-0.43	3.54	38.88	3.11200011	11	7/24/2014	31:58.9

8811	RWS11	-122.4713574	37.94059	-0.47	3.54	41.281	3.07199997	-122.47135	37.94061	-0.48	3.54	38.555	3.06299996	11	7/24/2014	31:59.0
8812	RWS11	-122.4713596	37.9406	-0.47	3.54	41.087	3.07499999	-122.47135	37.94061	-0.48	3.54	38.441	3.06599998	11	7/24/2014	31:59.1
8813	RWS11	-122.4713618	37.9406	-0.47	3.55	40.866	3.07800001	-122.47136	37.94061	-0.51	3.55	38.184	3.03500003	11	7/24/2014	31:59.2
8814	RWS11	-122.4713641	37.9406	-0.47	3.55	40.607	3.0800001	-122.47136	37.94061	-0.48	3.55	37.917	3.0710001	11	7/24/2014	31:59.3
8815	RWS11	-122.4713663	37.9406	-0.47	3.55	40.363	3.08199996	-122.47136	37.94061	-0.48	3.55	37.678	3.07299995	11	7/24/2014	31:59.4
8816	RWS11	-122.4713685	37.9406	-0.47	3.55	40.12	3.08499998	-122.47136	37.94061	-0.48	3.55	37.435	3.07599998	11	7/24/2014	31:59.5
8817	RWS11	-122.4713708	37.9406	-0.47	3.56	39.882	3.088	-122.47136	37.94061	-0.48	3.56	37.193	3.079	11	7/24/2014	31:59.6
8818	RWS11	-122.471373	37.9406	-0.47	3.56	39.599	3.09000009	-122.47137	37.94061	-0.48	3.56	36.914	3.08100009	11	7/24/2014	31:59.7
8819	RWS11	-122.4713752	37.9406	-0.47	3.56	39.38	3.09199995	-122.47137	37.94061	-0.48	3.56	36.761	3.08299994	11	7/24/2014	31:59.8
8820	RWS11	-122.4713773	37.9406	-0.47	3.62	39.096	3.15099996	-122.47137	37.94061	-0.48	3.62	36.452	3.14199996	11	7/24/2014	31:59.9
8821	RWS11	-122.4713806	37.9406	-0.47	3.66	38.792	3.19399995	-122.47137	37.94061	-0.48	3.66	36.148	3.18499994	11	7/24/2014	32:00.0
8822	RWS11	-122.4713827	37.9406	-0.47	3.69	38.546	3.22399992	-122.47138	37.94061	-0.43	3.69	35.906	3.26599991	11	7/24/2014	32:00.1
8823	RWS11	-122.4713849	37.9406	-0.47	3.71	38.309	3.24200004	-122.47138	37.94062	-0.48	3.71	35.666	3.23300004	11	7/24/2014	32:00.2
8824	RWS11	-122.471387	37.9406	-0.47	3.72	38.066	3.25299996	-122.47138	37.94062	-0.43	3.72	35.425	3.29499996	11	7/24/2014	32:00.3
8825	RWS11	-122.4713892	37.9406	-0.47	3.72	37.829	3.25699991	-122.47138	37.94062	-0.48	3.72	35.183	3.24799991	11	7/24/2014	32:00.4
8826	RWS11	-122.4713914	37.9406	-0.47	3.73	37.52	3.25800008	-122.47139	37.94062	-0.43	3.73	34.882	3.30000007	11	7/24/2014	32:00.5
8827	RWS11	-122.4713936	37.94061	-0.47	3.72	37.325	3.25500005	-122.47139	37.94062	-0.48	3.72	34.621	3.24600005	11	7/24/2014	32:00.6
8828	RWS11	-122.4713957	37.94061	-0.47	3.72	37.082	3.25100011	-122.47139	37.94062	-0.43	3.72	34.395	3.2930001	11	7/24/2014	32:00.7
8829	RWS11	-122.4713979	37.94061	-0.47	3.71	36.907	3.24599999	-122.47139	37.94062	-0.48	3.71	34.201	3.23699999	11	7/24/2014	32:00.8
8830	RWS11	-122.4714	37.94061	-0.47	3.71	36.689	3.23900002	-122.47139	37.94062	-0.48	3.71	34.024	3.23000002	11	7/24/2014	32:00.9
8831	RWS11	-122.4714032	37.94061	-0.47	3.7	36.449	3.23099989	-122.4714	37.94062	-0.48	3.7	33.83	3.22199988	11	7/24/2014	32:01.0
8832	RWS11	-122.4714054	37.94061	-0.47	3.69	36.143	3.22200006	-122.4714	37.94062	-0.43	3.69	33.589	3.26400006	11	7/24/2014	32:01.1
8833	RWS11	-122.4714075	37.94061	-0.5	3.68	35.992	3.17999995	-122.4714	37.94062	-0.48	3.68	33.329	3.20499992	11	7/24/2014	32:01.2
8834	RWS11	-122.4714097	37.94061	-0.47	3.67	35.68	3.20399994	-122.4714	37.94062	-0.43	3.67	33.059	3.24599993	11	7/24/2014	32:01.3
8835	RWS11	-122.4714119	37.94061	-0.47	3.66	35.462	3.19399995	-122.47141	37.94062	-0.48	3.66	32.845	3.18499994	11	7/24/2014	32:01.4
8836	RWS11	-122.4714141	37.94061	-0.47	3.65	35.246	3.18300003	-122.47141	37.94062	-0.43	3.65	32.603	3.22500002	11	7/24/2014	32:01.5
8837	RWS11	-122.4714163	37.94061	-0.47	3.64	35.025	3.17200011	-122.47141	37.94063	-0.48	3.64	32.428	3.16300011	11	7/24/2014	32:01.6
8838	RWS11	-122.4714185	37.94061	-0.47	3.63	34.831	3.16000003	-122.47141	37.94063	-0.48	3.63	32.232	3.15100002	11	7/24/2014	32:01.7
8839	RWS11	-122.4714207	37.94061	-0.47	3.62	34.546	3.14900011	-122.47141	37.94063	-0.43	3.62	31.927	3.1910001	11	7/24/2014	32:01.8
8840	RWS11	-122.4714228	37.94061	-0.47	3.61	34.369	3.14000005	-122.47142	37.94063	-0.43	3.61	31.704	3.18200004	11	7/24/2014	32:01.9
8841	RWS11	-122.471426	37.94062	-0.47	3.6	34.238	3.13199991	-122.47142	37.94063	-0.43	3.6	31.555	3.17399991	11	7/24/2014	32:02.0
8842	RWS11	-122.4714282	37.94062	-0.47	3.6	34.038	3.12700003	-122.47142	37.94063	-0.43	3.6	31.398	3.16900003	11	7/24/2014	32:02.1
8843	RWS11	-122.4714303	37.94062	-0.47	3.59	33.866	3.12400001	-122.47142	37.94063	-0.48	3.59	31.182	3.11500001	11	7/24/2014	32:02.2

8844	RWS11	-122.4714325	37.94062	-0.47	3.59	33.672	3.12199992	-122.47143	37.94063	-0.43	3.59	30.983	3.16399992	11	7/24/2014	32:02.3
8845	RWS11	-122.4714347	37.94062	-0.5	3.59	33.494	3.08799994	-122.47143	37.94063	-0.43	3.59	30.834	3.16399992	11	7/24/2014	32:02.4
8846	RWS11	-122.4714369	37.94062	-0.47	3.59	33.36	3.12400001	-122.47143	37.94063	-0.43	3.59	30.673	3.16600001	11	7/24/2014	32:02.5
8847	RWS11	-122.471439	37.94062	-0.47	3.6	33.163	3.12700003	-122.47143	37.94063	-0.43	3.6	30.457	3.16900003	11	7/24/2014	32:02.6
8848	RWS11	-122.4714412	37.94062	-0.47	3.6	33.011	3.13099998	-122.47144	37.94063	-0.43	3.6	30.326	3.17299998	11	7/24/2014	32:02.7
8849	RWS11	-122.4714433	37.94062	-0.47	3.6	32.882	3.13599992	-122.47144	37.94063	-0.43	3.6	30.195	3.17799991	11	7/24/2014	32:02.8
8850	RWS11	-122.4714455	37.94062	-0.5	3.61	32.683	3.10700005	-122.47144	37.94063	-0.48	3.61	30.06	3.13200003	11	7/24/2014	32:02.9
8851	RWS11	-122.4714487	37.94062	-0.5	3.61	32.529	3.11199993	-122.47144	37.94064	-0.43	3.61	29.891	3.1879999	11	7/24/2014	32:03.0
8852	RWS11	-122.4714508	37.94062	-0.47	3.62	32.376	3.15100002	-122.47144	37.94064	-0.43	3.62	29.796	3.19300002	11	7/24/2014	32:03.1
8853	RWS11	-122.4714529	37.94062	-0.5	3.62	32.223	3.12099999	-122.47145	37.94064	-0.48	3.62	29.689	3.14599997	11	7/24/2014	32:03.2
8854	RWS11	-122.4714551	37.94062	-0.5	3.63	32.002	3.12600011	-122.47145	37.94064	-0.43	3.63	29.429	3.20200008	11	7/24/2014	32:03.3
8855	RWS11	-122.4714572	37.94063	-0.5	3.63	31.786	3.12900013	-122.47145	37.94064	-0.48	3.63	29.206	3.1540001	11	7/24/2014	32:03.4
8856	RWS11	-122.4714594	37.94063	-0.47	3.63	31.611	3.16599989	-122.47145	37.94064	-0.43	3.63	29.036	3.20799989	11	7/24/2014	32:03.5
8857	RWS11	-122.4714615	37.94063	-0.5	3.63	31.39	3.13199991	-122.47146	37.94064	-0.48	3.63	28.858	3.15699989	11	7/24/2014	32:03.6
8858	RWS11	-122.4714637	37.94063	-0.47	3.63	31.217	3.16400003	-122.47146	37.94064	-0.43	3.63	28.659	3.20600003	11	7/24/2014	32:03.7
8859	RWS11	-122.4714658	37.94063	-0.5	3.63	30.952	3.12400001	-122.47146	37.94064	-0.43	3.63	28.44	3.19999999	11	7/24/2014	32:03.8
8860	RWS11	-122.471468	37.94063	-0.47	3.61	30.819	3.14700007	-122.47146	37.94064	-0.39	3.61	28.2	3.22300008	11	7/24/2014	32:03.9
8861	RWS11	-122.4714711	37.94063	-0.5	3.6	30.605	3.09400004	-122.47146	37.94064	-0.43	3.6	27.984	3.17000002	11	7/24/2014	32:04.0
8862	RWS11	-122.4714733	37.94063	-0.5	3.57	30.406	3.06599993	-122.47147	37.94064	-0.43	3.57	27.744	3.1419999	11	7/24/2014	32:04.1
8863	RWS11	-122.4714754	37.94063	-0.5	3.53	30.253	3.02599996	-122.47147	37.94064	-0.43	3.53	27.567	3.10199994	11	7/24/2014	32:04.2
8864	RWS11	-122.4714776	37.94063	-0.47	3.47	30.057	3.00600004	-122.47147	37.94064	-0.43	3.47	27.331	3.04800004	11	7/24/2014	32:04.3
8865	RWS11	-122.4714797	37.94063	-0.5	3.4	29.859	2.90399998	-122.47147	37.94064	-0.48	3.4	27.106	2.92899996	11	7/24/2014	32:04.4
8866	RWS11	-122.4714819	37.94063	-0.47	3.32	29.704	2.85400009	-122.47148	37.94065	-0.39	3.32	26.862	2.9300001	11	7/24/2014	32:04.5
8867	RWS11	-122.471484	37.94063	-0.5	3.22	29.575	2.71999997	-122.47148	37.94065	-0.43	3.22	26.715	2.79599994	11	7/24/2014	32:04.6
8868	RWS11	-122.4714862	37.94063	-0.47	3.22	29.398	2.75	-122.47148	37.94065	-0.39	3.22	26.48	2.82600001	11	7/24/2014	32:04.7
8869	RWS11	-122.4714883	37.94063	-0.5	3.21	29.225	2.71200007	-122.47148	37.94065	-0.43	3.21	26.255	2.78800005	11	7/24/2014	32:04.8
8870	RWS11	-122.4714904	37.94064	-0.47	3.21	29.05	2.7420001	-122.47148	37.94065	-0.39	3.21	26.103	2.81800011	11	7/24/2014	32:04.9
8871	RWS11	-122.4714936	37.94064	-0.47	3.2	28.848	2.73699999	-122.47149	37.94065	-0.43	3.2	25.88	2.77899998	11	7/24/2014	32:05.0
8872	RWS11	-122.4714957	37.94064	-0.47	3.2	28.676	2.73399997	-122.47149	37.94065	-0.39	3.2	25.661	2.80999997	11	7/24/2014	32:05.1
8873	RWS11	-122.4714978	37.94064	-0.47	3.2	28.523	2.72900009	-122.47149	37.94065	-0.43	3.2	25.445	2.77100009	11	7/24/2014	32:05.2
8874	RWS11	-122.4714999	37.94064	-0.47	3.19	28.282	2.7249999	-122.47149	37.94065	-0.39	3.19	25.183	2.80099991	11	7/24/2014	32:05.3
8875	RWS11	-122.471502	37.94064	-0.5	3.19	28.108	2.68699998	-122.4715	37.94065	-0.39	3.19	24.945	2.79699996	11	7/24/2014	32:05.4
8876	RWS11	-122.4715042	37.94064	-0.47	3.18	27.933	2.71700001	-122.4715	37.94065	-0.39	3.18	24.679	2.79300001	11	7/24/2014	32:05.5

8877	RWS11	-122.4715063	37.94064	-0.47	3.18	27.754	2.71300006	-122.4715	37.94065	-0.39	3.18	24.546	2.78900006	11	7/24/2014	32:05.6
8878	RWS11	-122.4715084	37.94064	-0.47	3.17	27.602	2.70799994	-122.4715	37.94065	-0.39	3.17	24.307	2.78399995	11	7/24/2014	32:05.7
8879	RWS11	-122.4715105	37.94064	-0.47	3.17	27.407	2.704	-122.4715	37.94065	-0.39	3.17	24.109	2.78	11	7/24/2014	32:05.8
8880	RWS11	-122.4715126	37.94064	-0.47	3.17	27.23	2.70099998	-122.47151	37.94065	-0.39	3.17	23.978	2.77699998	11	7/24/2014	32:05.9
8881	RWS11	-122.4715157	37.94064	-0.47	3.16	27.077	2.6960001	-122.47151	37.94066	-0.39	3.16	23.847	2.7720001	11	7/24/2014	32:06.0
8882	RWS11	-122.4715178	37.94064	-0.42	3.16	26.883	2.74299991	-122.47151	37.94066	-0.39	3.16	23.696	2.76799992	11	7/24/2014	32:06.1
8883	RWS11	-122.4715199	37.94064	-0.47	3.15	26.856	2.68799996	-122.47151	37.94066	-0.34	3.15	23.54	2.81399998	11	7/24/2014	32:06.2
8884	RWS11	-122.471522	37.94064	-0.47	3.15	26.662	2.68400002	-122.47152	37.94066	-0.34	3.15	23.408	2.81000003	11	7/24/2014	32:06.3
8885	RWS11	-122.4715242	37.94065	-0.47	3.15	26.529	2.6789999	-122.47152	37.94066	-0.39	3.15	23.3	2.75499991	11	7/24/2014	32:06.4
8886	RWS11	-122.4715263	37.94065	-0.47	3.14	26.356	2.67499995	-122.47152	37.94066	-0.34	3.14	23.146	2.80099997	11	7/24/2014	32:06.5
8887	RWS11	-122.4715285	37.94065	-0.42	3.15	26.201	2.73899996	-122.47152	37.94066	-0.39	3.15	22.907	2.76399997	11	7/24/2014	32:06.6
8888	RWS11	-122.4715306	37.94065	-0.42	3.16	26.093	2.74899995	-122.47152	37.94066	-0.34	3.16	22.817	2.82399997	11	7/24/2014	32:06.7
8889	RWS11	-122.4715327	37.94065	-0.47	3.17	25.872	2.70600009	-122.47153	37.94066	-0.34	3.17	22.556	2.83200011	11	7/24/2014	32:06.8
8890	RWS11	-122.4715348	37.94065	-0.42	3.18	25.719	2.76499999	-122.47153	37.94066	-0.34	3.18	22.441	2.84	11	7/24/2014	32:06.9
8891	RWS11	-122.471538	37.94065	-0.47	3.19	25.588	2.72099996	-122.47153	37.94066	-0.39	3.19	22.226	2.79699996	11	7/24/2014	32:07.0
8892	RWS11	-122.4715401	37.94065	-0.42	3.19	25.369	2.77899992	-122.47153	37.94066	-0.34	3.19	22.052	2.85399994	11	7/24/2014	32:07.1
8893	RWS11	-122.4715423	37.94065	-0.42	3.2	25.214	2.78499997	-122.47154	37.94066	-0.34	3.2	21.875	2.85999998	11	7/24/2014	32:07.2
8894	RWS11	-122.4715444	37.94065	-0.42	3.21	24.934	2.79199994	-122.47154	37.94066	-0.34	3.21	21.636	2.86699995	11	7/24/2014	32:07.3
8895	RWS11	-122.4715466	37.94065	-0.42	3.22	24.754	2.80000007	-122.47154	37.94066	-0.34	3.22	21.391	2.87500009	11	7/24/2014	32:07.4
8896	RWS11	-122.4715488	37.94065	-0.42	3.23	24.514	2.81000006	-122.47154	37.94067	-0.34	3.23	21.22	2.88500008	11	7/24/2014	32:07.5
8897	RWS11	-122.4715509	37.94065	-0.47	3.24	24.317	2.77099991	-122.47154	37.94067	-0.31	3.24	21	2.9309999	11	7/24/2014	32:07.6
8898	RWS11	-122.4715531	37.94065	-0.42	3.25	24.057	2.83799994	-122.47155	37.94067	-0.34	3.25	20.758	2.91299996	11	7/24/2014	32:07.7
8899	RWS11	-122.4715553	37.94065	-0.42	3.27	23.924	2.85699999	-122.47155	37.94067	-0.34	3.27	20.607	2.93200001	11	7/24/2014	32:07.8
8900	RWS11	-122.4715574	37.94066	-0.42	3.3	23.708	2.87900007	-122.47155	37.94067	-0.34	3.3	20.434	2.95400009	11	7/24/2014	32:07.9
8901	RWS11	-122.4715606	37.94066	-0.42	3.32	23.552	2.903	-122.47155	37.94067	-0.31	3.32	20.321	3.01199999	11	7/24/2014	32:08.0
8902	RWS11	-122.4715627	37.94066	-0.38	3.35	23.398	2.96499994	-122.47156	37.94067	-0.34	3.35	20.211	3.00499997	11	7/24/2014	32:08.1
8903	RWS11	-122.4715648	37.94066	-0.42	3.37	23.267	2.9569999	-122.47156	37.94067	-0.34	3.37	20.144	3.03199992	11	7/24/2014	32:08.2
8904	RWS11	-122.4715669	37.94066	-0.42	3.4	23.134	2.98500001	-122.47156	37.94067	-0.31	3.4	20.013	3.09400001	11	7/24/2014	32:08.3
8905	RWS11	-122.4715691	37.94066	-0.42	3.43	23.049	3.01400006	-122.47156	37.94067	-0.34	3.43	19.971	3.08900008	11	7/24/2014	32:08.4
8906	RWS11	-122.4715712	37.94066	-0.42	3.46	22.984	3.0430001	-122.47157	37.94067	-0.31	3.46	19.968	3.1520001	11	7/24/2014	32:08.5
8907	RWS11	-122.4715734	37.94066	-0.42	3.49	22.917	3.07199991	-122.47157	37.94067	-0.31	3.49	19.97	3.1809999	11	7/24/2014	32:08.6
8908	RWS11	-122.4715755	37.94066	-0.42	3.52	22.849	3.10099995	-122.47157	37.94067	-0.31	3.52	19.922	3.20999995	11	7/24/2014	32:08.7
8909	RWS11	-122.4715776	37.94066	-0.42	3.55	22.805	3.12900007	-122.47157	37.94067	-0.31	3.55	19.857	3.23800007	11	7/24/2014	32:08.8

8910	RWS11	-122.4715798	37.94066	-0.38	3.57	22.759	3.19000009	-122.47157	37.94067	-0.27	3.57	19.924	3.2980001	11	7/24/2014	32:08.9
8911	RWS11	-122.4715829	37.94066	-0.42	3.6	22.87	3.17999995	-122.47158	37.94068	-0.31	3.6	19.945	3.28899994	11	7/24/2014	32:09.0
8912	RWS11	-122.471585	37.94066	-0.42	3.62	22.87	3.20299995	-122.47158	37.94068	-0.31	3.62	20.009	3.31199995	11	7/24/2014	32:09.1
8913	RWS11	-122.4715872	37.94066	-0.42	3.64	22.869	3.2240001	-122.47158	37.94068	-0.31	3.64	20.129	3.33300009	11	7/24/2014	32:09.2
8914	RWS11	-122.4715893	37.94066	-0.42	3.66	22.932	3.24299991	-122.47158	37.94068	-0.27	3.66	20.333	3.38599992	11	7/24/2014	32:09.3
8915	RWS11	-122.4715915	37.94067	-0.42	3.68	23.017	3.26000011	-122.47159	37.94068	-0.31	3.68	20.529	3.36900011	11	7/24/2014	32:09.4
8916	RWS11	-122.4715937	37.94067	-0.38	3.69	23.041	3.3109999	-122.47159	37.94068	-0.31	3.69	20.684	3.3849999	11	7/24/2014	32:09.5
8917	RWS11	-122.4715958	37.94067	-0.42	3.71	23.085	3.29100001	-122.47159	37.94068	-0.31	3.71	20.879	3.40000001	11	7/24/2014	32:09.6
8918	RWS11	-122.471598	37.94067	-0.42	3.72	23.238	3.30400002	-122.47159	37.94068	-0.31	3.72	21.184	3.41300002	11	7/24/2014	32:09.7
8919	RWS11	-122.4716001	37.94067	-0.42	3.73	23.3	3.31499994	-122.47159	37.94068	-0.31	3.73	21.425	3.42399994	11	7/24/2014	32:09.8
8920	RWS11	-122.4716023	37.94067	-0.38	3.74	23.255	3.35800007	-122.4716	37.94068	-0.31	3.74	21.573	3.43200007	11	7/24/2014	32:09.9
8921	RWS11	-122.4716054	37.94067	-0.42	3.74	23.255	3.32700002	-122.4716	37.94068	-0.34	3.74	21.748	3.40200004	11	7/24/2014	32:10.0
8922	RWS11	-122.4716075	37.94067	-0.42	3.74	23.253	3.32700002	-122.4716	37.94068	-0.31	3.74	21.923	3.43600002	11	7/24/2014	32:10.1
8923	RWS11	-122.4716096	37.94067	-0.42	3.74	23.19	3.32199991	-122.4716	37.94068	-0.31	3.74	22.075	3.4309999	11	7/24/2014	32:10.2
8924	RWS11	-122.4716118	37.94067	-0.42	3.73	23.124	3.31300008	-122.47161	37.94068	-0.27	3.73	22.141	3.45600009	11	7/24/2014	32:10.3
8925	RWS11	-122.4716139	37.94067	-0.42	3.72	22.969	3.301	-122.47161	37.94068	-0.34	3.72	22.205	3.37600002	11	7/24/2014	32:10.4
8926	RWS11	-122.471616	37.94067	-0.42	3.7	22.902	3.28599989	-122.47161	37.94069	-0.31	3.7	22.266	3.39499989	11	7/24/2014	32:10.5
8927	RWS11	-122.4716181	37.94067	-0.47	3.69	22.662	3.2190001	-122.47161	37.94069	-0.31	3.69	22.268	3.3790001	11	7/24/2014	32:10.6
8928	RWS11	-122.4716203	37.94067	-0.42	3.67	22.356	3.25400007	-122.47161	37.94069	-0.31	3.67	22.202	3.36300007	11	7/24/2014	32:10.7
8929	RWS11	-122.4716224	37.94067	-0.42	3.65	22.05	3.23899996	-122.47162	37.94069	-0.31	3.65	22.071	3.34799996	11	7/24/2014	32:10.8
8930	RWS11	-122.4716245	37.94068	-0.42	3.64	21.616	3.22599995	-122.47162	37.94069	-0.27	3.64	21.853	3.36899996	11	7/24/2014	32:10.9
8931	RWS11	-122.4716276	37.94068	-0.42	3.63	21.132	3.21500003	-122.47162	37.94069	-0.31	3.63	21.546	3.32400003	11	7/24/2014	32:11.0
8932	RWS11	-122.4716297	37.94068	-0.38	3.63	20.586	3.24399999	-122.47162	37.94069	-0.31	3.63	21.085	3.31799999	11	7/24/2014	32:11.1
8933	RWS11	-122.4716318	37.94068	-0.42	3.62	20.018	3.20800006	-122.47163	37.94069	-0.27	3.62	20.539	3.35100007	11	7/24/2014	32:11.2
8934	RWS11	-122.4716339	37.94068	-0.38	3.63	19.341	3.24600008	-122.47163	37.94069	-0.27	3.63	19.82	3.35400009	11	7/24/2014	32:11.3
8935	RWS11	-122.4716361	37.94068	-0.42	3.63	18.708	3.21899998	-122.47163	37.94069	-0.31	3.63	18.991	3.32799998	11	7/24/2014	32:11.4
8936	RWS11	-122.4716382	37.94068	-0.38	3.65	17.9	3.2649999	-122.47163	37.94069	-0.27	3.65	17.963	3.37299991	11	7/24/2014	32:11.5
8937	RWS11	-122.4716404	37.94068	-0.42	3.66	17.094	3.24299991	-122.47163	37.94069	-0.31	3.66	16.853	3.35199991	11	7/24/2014	32:11.6
8938	RWS11	-122.4716425	37.94068	-0.38	3.67	16.351	3.28900006	-122.47164	37.94069	-0.31	3.67	15.715	3.36300007	11	7/24/2014	32:11.7
8939	RWS11	-122.4716446	37.94068	-0.42	3.68	15.498	3.26000011	-122.47164	37.94069	-0.31	3.68	14.513	3.36900011	11	7/24/2014	32:11.8
8940	RWS11	-122.4716468	37.94068	-0.38	3.67	14.754	3.29200009	-122.47164	37.94069	-0.31	3.67	13.401	3.36600009	11	7/24/2014	32:11.9
8941	RWS11	-122.47165	37.94068	-0.42	3.66	14.013	3.24299991	-122.47164	37.9407	-0.34	3.66	12.353	3.31799993	11	7/24/2014	32:12.0
8942	RWS11	-122.4716521	37.94068	-0.42	3.63	13.207	3.21200001	-122.47165	37.9407	-0.31	3.63	11.218	3.32100001	11	7/24/2014	32:12.1

8943	RWS11	-122.4716543	37.94068	-0.42	3.58	12.529	3.16299999	-122.47165	37.9407	-0.31	3.58	10.303	3.27199998	11	7/24/2014	32:12.2
8944	RWS11	-122.4716565	37.94068	-0.38	3.51	11.875	3.12800005	-122.47165	37.9407	-0.31	3.51	9.43	3.20200005	11	7/24/2014	32:12.3
8945	RWS11	-122.4716587	37.94068	-0.38	3.5	11.259	3.11999992	-122.47165	37.9407	-0.39	3.5	8.575	3.10999992	11	7/24/2014	32:12.4
8946	RWS11	-122.471661	37.94069	-0.42	3.49	10.672	3.07799995	-122.47166	37.9407	-0.31	3.49	7.942	3.18699995	11	7/24/2014	32:12.5
8947	RWS11	-122.4716632	37.94069	-0.42	3.49	10.125	3.07099998	-122.47166	37.9407	-0.31	3.49	7.264	3.17999998	11	7/24/2014	32:12.6
8948	RWS11	-122.4716654	37.94069	-0.42	3.48	9.798	3.06400001	-122.47166	37.9407	-0.31	3.48	6.742	3.17300001	11	7/24/2014	32:12.7
8949	RWS11	-122.4716676	37.94069	-0.42	3.47	9.341	3.05700004	-122.47166	37.9407	-0.34	3.47	6.306	3.13200006	11	7/24/2014	32:12.8
8950	RWS11	-122.4716698	37.94069	-0.38	3.47	8.946	3.08500007	-122.47166	37.9407	-0.31	3.47	5.848	3.15900007	11	7/24/2014	32:12.9
8951	RWS11	-122.471673	37.94069	-0.42	3.46	8.617	3.0430001	-122.47167	37.9407	-0.34	3.46	5.582	3.11800012	11	7/24/2014	32:13.0
8952	RWS11	-122.4716752	37.94069	-0.38	3.45	8.29	3.07099989	-122.47167	37.9407	-0.31	3.45	5.211	3.14499989	11	7/24/2014	32:13.1
8953	RWS11	-122.4716773	37.94069	-0.42	3.44	8.028	3.028	-122.47167	37.9407	-0.31	3.44	4.928	3.13699999	11	7/24/2014	32:13.2
8954	RWS11	-122.4716795	37.94069	-0.38	3.44	7.831	3.05600002	-122.47167	37.9407	-0.31	3.44	4.732	3.13000003	11	7/24/2014	32:13.3
8955	RWS11	-122.4716817	37.94069	-0.42	3.43	7.635	3.01400006	-122.47168	37.9407	-0.34	3.43	4.486	3.08900008	11	7/24/2014	32:13.4
8956	RWS11	-122.4716839	37.94069	-0.38	3.42	7.395	3.04300001	-122.47168	37.9407	-0.31	3.42	4.294	3.11700001	11	7/24/2014	32:13.5
8957	RWS11	-122.4716861	37.94069	-0.42	3.42	7.238	2.99999988	-122.47168	37.94071	-0.34	3.42	4.074	3.0749999	11	7/24/2014	32:13.6
8958	RWS11	-122.4716883	37.94069	-0.38	3.41	7.107	3.02799991	-122.47168	37.94071	-0.31	3.41	3.942	3.10199991	11	7/24/2014	32:13.7
8959	RWS11	-122.4716905	37.94069	-0.42	3.4	7.043	2.98599994	-122.47168	37.94071	-0.31	3.4	3.833	3.09499994	11	7/24/2014	32:13.8
8960	RWS11	-122.4716926	37.94069	-0.42	3.4	7.02	2.9799999	-122.47169	37.94071	-0.31	3.4	3.833	3.0889999	11	7/24/2014	32:13.9
8961	RWS11	-122.4716959	37.9407	-0.42	3.39	6.954	2.972	-122.47169	37.94071	-0.31	3.39	3.79	3.081	11	7/24/2014	32:14.0
8962	RWS11	-122.4716981	37.9407	-0.42	3.38	6.954	2.96500003	-122.47169	37.94071	-0.31	3.38	3.658	3.07400003	11	7/24/2014	32:14.1
8963	RWS11	-122.4717003	37.9407	-0.42	3.37	6.907	2.95800006	-122.47169	37.94071	-0.31	3.37	3.546	3.06700006	11	7/24/2014	32:14.2
8964	RWS11	-122.4717026	37.9407	-0.38	3.37	6.842	2.98600009	-122.4717	37.94071	-0.34	3.37	3.436	3.02600011	11	7/24/2014	32:14.3
8965	RWS11	-122.4717048	37.9407	-0.42	3.36	6.819	2.94399989	-122.4717	37.94071	-0.34	3.36	3.284	3.0189999	11	7/24/2014	32:14.4
8966	RWS11	-122.4717071	37.9407	-0.38	3.35	6.819	2.97199991	-122.4717	37.94071	-0.31	3.35	3.196	3.04599991	11	7/24/2014	32:14.5
8967	RWS11	-122.4717094	37.9407	-0.42	3.35	6.799	2.92900002	-122.4717	37.94071	-0.39	3.35	3.088	2.95400003	11	7/24/2014	32:14.6
8968	RWS11	-122.4717116	37.9407	-0.42	3.39	6.732	2.97699988	-122.47171	37.94071	-0.27	3.39	2.956	3.11999989	11	7/24/2014	32:14.7
8969	RWS11	-122.4717139	37.9407	-0.47	3.43	6.86	2.96199989	-122.47171	37.94071	-0.34	3.43	2.909	3.08799991	11	7/24/2014	32:14.8
8970	RWS11	-122.4717161	37.9407	-0.42	3.46	6.815	3.04000008	-122.47171	37.94071	-0.34	3.46	2.865	3.1150001	11	7/24/2014	32:14.9
8971	RWS11	-122.4717194	37.9407	-0.42	3.48	6.816	3.06000006	-122.47171	37.94071	-0.34	3.48	2.778	3.13500008	11	7/24/2014	32:15.0
8972	RWS11	-122.4717216	37.9407	-0.42	3.49	6.859	3.07199991	-122.47172	37.94072	-0.31	3.49	2.713	3.1809999	11	7/24/2014	32:15.1
8973	RWS11	-122.4717238	37.9407	-0.42	3.5	6.793	3.08000004	-122.47172	37.94072	-0.34	3.5	2.625	3.15500006	11	7/24/2014	32:15.2
8974	RWS11	-122.471726	37.9407	-0.42	3.5	6.729	3.08300006	-122.47172	37.94072	-0.31	3.5	2.516	3.19200006	11	7/24/2014	32:15.3
8975	RWS11	-122.4717283	37.9407	-0.42	3.5	6.771	3.0819999	-122.47172	37.94072	-0.31	3.5	2.275	3.1909999	11	7/24/2014	32:15.4

8976	RWS11	-122.4717305	37.9407	-0.42	3.49	6.638	3.07700002	-122.47172	37.94072	-0.31	3.49	2.228	3.18600002	11	7/24/2014	32:15.5
8977	RWS11	-122.4717327	37.94071	-0.42	3.48	6.571	3.06899989	-122.47173	37.94072	-0.31	3.48	2.032	3.17799988	11	7/24/2014	32:15.6
8978	RWS11	-122.4717349	37.94071	-0.42	3.47	6.398	3.0589999	-122.47173	37.94072	-0.31	3.47	2.01	3.16799989	11	7/24/2014	32:15.7
8979	RWS11	-122.4717371	37.94071	-0.42	3.46	6.353	3.04599988	-122.47173	37.94072	-0.31	3.46	1.769	3.15499988	11	7/24/2014	32:15.8
8980	RWS11	-122.4717393	37.94071	-0.42	3.45	6.07	3.03199995	-122.47173	37.94072	-0.31	3.45	1.574	3.14099994	11	7/24/2014	32:15.9
8981	RWS11	-122.4717425	37.94071	-0.47	3.43	5.829	2.96499991	-122.47174	37.94072	-0.34	3.43	1.289	3.09099993	11	7/24/2014	32:16.0
8982	RWS11	-122.4717447	37.94071	-0.42	3.42	5.542	2.99999988	-122.47174	37.94072	-0.34	3.42	1.002	3.0749999	11	7/24/2014	32:16.1
8983	RWS11	-122.4717468	37.94071	-0.42	3.4	5.215	2.98299992	-122.47174	37.94072	-0.34	3.4	0.762	3.05799994	11	7/24/2014	32:16.2
8984	RWS11	-122.471749	37.94071	-0.42	3.38	4.844	2.96400011	-122.47174	37.94072	-0.31	3.38	0.413	3.0730001	11	7/24/2014	32:16.3
8985	RWS11	-122.4717512	37.94071	-0.42	3.36	4.386	2.94500005	-122.47175	37.94072	-0.34	3.36	0.108	3.02000007	11	7/24/2014	32:16.4
8986	RWS11	-122.4717534	37.94071	-0.42	3.34	4.059	2.92500007	-122.47175	37.94072	-0.31	3.34	-0.328	3.03400007	11	7/24/2014	32:16.5
8987	RWS11	-122.4717555	37.94071	-0.42	3.32	3.577	2.90399992	-122.47175	37.94072	-0.31	3.32	-0.548	3.01299992	11	7/24/2014	32:16.6
8988	RWS11	-122.4717577	37.94071	-0.42	3.3	3.073	2.88300002	-122.47175	37.94072	-0.31	3.3	-0.856	2.99200001	11	7/24/2014	32:16.7
8989	RWS11	-122.4717599	37.94071	-0.42	3.28	2.549	2.86200011	-122.47175	37.94073	-0.31	3.28	-1.14	2.97100011	11	7/24/2014	32:16.8
8990	RWS11	-122.471762	37.94071	-0.42	3.26	2.178	2.84199989	-122.47176	37.94073	-0.31	3.26	-1.336	2.95099989	11	7/24/2014	32:16.9
8991	RWS11	-122.4717651	37.94071	-0.42	3.24	1.676	2.82499993	-122.47176	37.94073	-0.31	3.24	-1.598	2.93399993	11	7/24/2014	32:17.0
8992	RWS11	-122.4717673	37.94071	-0.42	3.23	1.24	2.81099999	-122.47176	37.94073	-0.31	3.23	-1.86	2.91999999	11	7/24/2014	32:17.1
8993	RWS11	-122.4717694	37.94072	-0.42	3.22	0.825	2.80000007	-122.47176	37.94073	-0.31	3.22	-2.144	2.90900007	11	7/24/2014	32:17.2
8994	RWS11	-122.4717715	37.94072	-0.38	3.21	0.494	2.82699993	-122.47177	37.94073	-0.31	3.21	-2.431	2.90099993	11	7/24/2014	32:17.3
8995	RWS11	-122.4717737	37.94072	-0.42	3.2	0.167	2.78599989	-122.47177	37.94073	-0.31	3.2	-2.671	2.89499989	11	7/24/2014	32:17.4
8996	RWS11	-122.4717758	37.94072	-0.42	3.2	-0.16	2.78299987	-122.47177	37.94073	-0.31	3.2	-2.955	2.89199987	11	7/24/2014	32:17.5
8997	RWS11	-122.4717779	37.94072	-0.42	3.2	-0.401	2.78000009	-122.47177	37.94073	-0.31	3.2	-3.064	2.88900009	11	7/24/2014	32:17.6
8998	RWS11	-122.4717801	37.94072	-0.38	3.19	-0.684	2.81299999	-122.47177	37.94073	-0.27	3.19	-3.391	2.921	11	7/24/2014	32:17.7
8999	RWS11	-122.4717822	37.94072	-0.42	3.19	-0.882	2.77499998	-122.47178	37.94073	-0.31	3.19	-3.786	2.88399997	11	7/24/2014	32:17.8
9000	RWS11	-122.4717843	37.94072	-0.38	3.19	-1.125	2.80699995	-122.47178	37.94073	-0.27	3.19	-4.115	2.91499996	11	7/24/2014	32:17.9
9001	RWS11	-122.4717874	37.94072	-0.42	3.18	-1.277	2.76800001	-122.47178	37.94073	-0.31	3.18	-4.421	2.877	11	7/24/2014	32:18.0
9002	RWS11	-122.4717895	37.94072	-0.38	3.18	-1.43	2.79699996	-122.47178	37.94073	-0.31	3.18	-4.704	2.87099996	11	7/24/2014	32:18.1
9003	RWS11	-122.4717916	37.94072	-0.42	3.17	-1.605	2.755	-122.47179	37.94073	-0.27	3.17	-5.031	2.898	11	7/24/2014	32:18.2
9004	RWS11	-122.4717938	37.94072	-0.38	3.16	-1.604	2.78200009	-122.47179	37.94073	-0.27	3.16	-5.293	2.8900001	11	7/24/2014	32:18.3
9005	RWS11	-122.4717959	37.94072	-0.42	3.15	-1.779	2.73899996	-122.47179	37.94073	-0.27	3.15	-5.555	2.88199997	11	7/24/2014	32:18.4
9006	RWS11	-122.471798	37.94072	-0.38	3.15	-1.867	2.76699999	-122.47179	37.94074	-0.27	3.15	-5.752	2.875	11	7/24/2014	32:18.5
9007	RWS11	-122.4718002	37.94072	-0.38	3.14	-1.827	2.76000002	-122.47179	37.94074	-0.31	3.14	-6.039	2.83400002	11	7/24/2014	32:18.6
9008	RWS11	-122.4718023	37.94072	-0.38	3.13	-2.001	2.75300005	-122.4718	37.94074	-0.31	3.13	-6.301	2.82700005	11	7/24/2014	32:18.7

9009	RWS11	-122.4718044	37.94072	-0.42	3.13	-2.067	2.71100008	-122.4718	37.94074	-0.27	3.13	-6.476	2.85400009	11	7/24/2014	32:18.8
9010	RWS11	-122.4718065	37.94072	-0.38	3.12	-2.067	2.73899987	-122.4718	37.94074	-0.27	3.12	-6.694	2.84699988	11	7/24/2014	32:18.9
9011	RWS11	-122.4718097	37.94073	-0.38	3.11	-2.133	2.7319999	-122.4718	37.94074	-0.27	3.11	-6.89	2.83999991	11	7/24/2014	32:19.0
9012	RWS11	-122.4718118	37.94073	-0.38	3.11	-2.154	2.72599995	-122.47181	37.94074	-0.27	3.11	-7.108	2.83399993	11	7/24/2014	32:19.1
9013	RWS11	-122.471814	37.94073	-0.38	3.1	-2.179	2.72100008	-122.47181	37.94074	-0.27	3.1	-7.351	2.82900006	11	7/24/2014	32:19.2
9014	RWS11	-122.4718161	37.94073	-0.38	3.1	-2.158	2.71800005	-122.47181	37.94074	-0.27	3.1	-7.504	2.82600003	11	7/24/2014	32:19.3
9015	RWS11	-122.4718183	37.94073	-0.38	3.1	-2.179	2.71699989	-122.47181	37.94074	-0.22	3.1	-7.548	2.87599988	11	7/24/2014	32:19.4
9016	RWS11	-122.4718205	37.94073	-0.38	3.1	-2.092	2.71800005	-122.47182	37.94074	-0.22	3.1	-7.591	2.87700005	11	7/24/2014	32:19.5
9017	RWS11	-122.4718227	37.94073	-0.41	3.1	-1.984	2.68499991	-122.47182	37.94074	-0.27	3.1	-7.591	2.82799989	11	7/24/2014	32:19.6
9018	RWS11	-122.4718249	37.94073	-0.38	3.11	-1.961	2.72500002	-122.47182	37.94074	-0.22	3.11	-7.592	2.88400002	11	7/24/2014	32:19.7
9019	RWS11	-122.471827	37.94073	-0.38	3.11	-1.943	2.73100007	-122.47182	37.94074	-0.27	3.11	-7.595	2.83900005	11	7/24/2014	32:19.8
9020	RWS11	-122.4718292	37.94073	-0.38	3.12	-1.877	2.73700011	-122.47182	37.94074	-0.27	3.12	-7.464	2.84500009	11	7/24/2014	32:19.9
9021	RWS11	-122.4718324	37.94073	-0.38	3.13	-1.79	2.745	-122.47183	37.94074	-0.27	3.13	-7.224	2.85299999	11	7/24/2014	32:20.0
9022	RWS11	-122.4718346	37.94073	-0.38	3.13	-1.79	2.75199997	-122.47183	37.94074	-0.22	3.13	-7.137	2.91099997	11	7/24/2014	32:20.1
9023	RWS11	-122.4718368	37.94073	-0.38	3.14	-1.725	2.76000011	-122.47183	37.94075	-0.31	3.14	-6.897	2.83400011	11	7/24/2014	32:20.2
9024	RWS11	-122.471839	37.94073	-0.38	3.15	-1.682	2.76899993	-122.47183	37.94075	-0.22	3.15	-6.527	2.92799993	11	7/24/2014	32:20.3
9025	RWS11	-122.4718412	37.94073	-0.38	3.16	-1.576	2.778	-122.47184	37.94075	-0.22	3.16	-6.137	2.93699999	11	7/24/2014	32:20.4
9026	RWS11	-122.4718435	37.94073	-0.38	3.17	-1.467	2.78799999	-122.47184	37.94075	-0.27	3.17	-5.788	2.89599997	11	7/24/2014	32:20.5
9027	RWS11	-122.4718457	37.94073	-0.38	3.18	-1.423	2.79799998	-122.47184	37.94075	-0.27	3.18	-5.417	2.90599996	11	7/24/2014	32:20.6
9028	RWS11	-122.4718479	37.94074	-0.33	3.19	-1.358	2.85700011	-122.47184	37.94075	-0.27	3.19	-5.046	2.91400009	11	7/24/2014	32:20.7
9029	RWS11	-122.4718501	37.94074	-0.41	3.19	-1.183	2.77800009	-122.47185	37.94075	-0.27	3.19	-4.632	2.92100006	11	7/24/2014	32:20.8
9030	RWS11	-122.4718523	37.94074	-0.38	3.2	-1.097	2.81700003	-122.47185	37.94075	-0.27	3.2	-4.24	2.92500001	11	7/24/2014	32:20.9
9031	RWS11	-122.4718556	37.94074	-0.38	3.2	-0.991	2.81899989	-122.47185	37.94075	-0.27	3.2	-3.763	2.92699987	11	7/24/2014	32:21.0
9032	RWS11	-122.4718578	37.94074	-0.38	3.2	-0.86	2.81899989	-122.47185	37.94075	-0.27	3.2	-3.414	2.92699987	11	7/24/2014	32:21.1
9033	RWS11	-122.47186	37.94074	-0.38	3.2	-0.773	2.8160001	-122.47185	37.94075	-0.31	3.2	-3.131	2.8900001	11	7/24/2014	32:21.2
9034	RWS11	-122.4718622	37.94074	-0.38	3.19	-0.728	2.81199992	-122.47186	37.94075	-0.27	3.19	-2.672	2.9199999	11	7/24/2014	32:21.3
9035	RWS11	-122.4718644	37.94074	-0.38	3.19	-0.554	2.80600011	-122.47186	37.94075	-0.31	3.19	-2.455	2.88000011	11	7/24/2014	32:21.4
9036	RWS11	-122.4718666	37.94074	-0.38	3.18	-0.576	2.79799998	-122.47186	37.94075	-0.27	3.18	-2.236	2.90599996	11	7/24/2014	32:21.5
9037	RWS11	-122.4718689	37.94074	-0.38	3.17	-0.534	2.79000008	-122.47186	37.94075	-0.31	3.17	-2.171	2.86400008	11	7/24/2014	32:21.6
9038	RWS11	-122.4718711	37.94074	-0.38	3.16	-0.537	2.78199995	-122.47187	37.94075	-0.27	3.16	-2.109	2.88999993	11	7/24/2014	32:21.7
9039	RWS11	-122.4718733	37.94074	-0.38	3.15	-0.493	2.77400005	-122.47187	37.94075	-0.27	3.15	-2.153	2.88200003	11	7/24/2014	32:21.8
9040	RWS11	-122.4718755	37.94074	-0.38	3.15	-0.406	2.76800001	-122.47187	37.94075	-0.31	3.15	-2.043	2.84200001	11	7/24/2014	32:21.9
9041	RWS11	-122.4718788	37.94074	-0.38	3.14	-0.428	2.76299989	-122.47187	37.94076	-0.27	3.14	-2.196	2.87099987	11	7/24/2014	32:22.0

9042	RWS11	-122.4718809	37.94074	-0.38	3.14	-0.449	2.76100004	-122.47188	37.94076	-0.27	3.14	-2.307	2.86900002	11	7/24/2014	32:22.1
9043	RWS11	-122.4718831	37.94074	-0.41	3.14	-0.494	2.7279999	-122.47188	37.94076	-0.31	3.14	-2.459	2.83699989	11	7/24/2014	32:22.2
9044	RWS11	-122.4718854	37.94074	-0.38	3.15	-0.519	2.76800001	-122.47188	37.94076	-0.31	3.15	-2.702	2.84200001	11	7/24/2014	32:22.3
9045	RWS11	-122.4718876	37.94074	-0.41	3.16	-0.519	2.74200007	-122.47188	37.94076	-0.31	3.16	-2.855	2.85100007	11	7/24/2014	32:22.4
9046	RWS11	-122.4718898	37.94075	-0.38	3.17	-0.541	2.78799999	-122.47188	37.94076	-0.31	3.17	-2.964	2.86199999	11	7/24/2014	32:22.5
9047	RWS11	-122.471892	37.94075	-0.41	3.18	-0.562	2.766	-122.47189	37.94076	-0.31	3.18	-3.117	2.875	11	7/24/2014	32:22.6
9048	RWS11	-122.4718942	37.94075	-0.38	3.19	-0.584	2.81499994	-122.47189	37.94076	-0.31	3.19	-3.247	2.88899994	11	7/24/2014	32:22.7
9049	RWS11	-122.4718964	37.94075	-0.41	3.21	-0.738	2.7969999	-122.47189	37.94076	-0.34	3.21	-3.401	2.87199989	11	7/24/2014	32:22.8
9050	RWS11	-122.4718986	37.94075	-0.38	3.23	-0.807	2.85000002	-122.47189	37.94076	-0.31	3.23	-3.601	2.92400002	11	7/24/2014	32:22.9
9051	RWS11	-122.4719019	37.94075	-0.38	3.25	-0.828	2.86900008	-122.4719	37.94076	-0.31	3.25	-3.819	2.94300008	11	7/24/2014	32:23.0
9052	RWS11	-122.4719041	37.94075	-0.38	3.27	-1.003	2.88999999	-122.4719	37.94076	-0.31	3.27	-3.906	2.96399999	11	7/24/2014	32:23.1
9053	RWS11	-122.4719062	37.94075	-0.38	3.29	-1.024	2.91200006	-122.4719	37.94076	-0.34	3.29	-4.19	2.95200005	11	7/24/2014	32:23.2
9054	RWS11	-122.4719085	37.94075	-0.38	3.32	-1.178	2.93500006	-122.4719	37.94076	-0.31	3.32	-4.408	3.00900006	11	7/24/2014	32:23.3
9055	RWS11	-122.4719107	37.94075	-0.38	3.34	-1.309	2.95899999	-122.47191	37.94076	-0.27	3.34	-4.671	3.06699997	11	7/24/2014	32:23.4
9056	RWS11	-122.4719129	37.94075	-0.38	3.36	-1.443	2.98299992	-122.47191	37.94076	-0.31	3.36	-4.826	3.05699992	11	7/24/2014	32:23.5
9057	RWS11	-122.4719151	37.94075	-0.41	3.39	-1.574	2.97099993	-122.47191	37.94076	-0.31	3.39	-5.11	3.07999992	11	7/24/2014	32:23.6
9058	RWS11	-122.4719173	37.94075	-0.38	3.41	-1.705	3.03000009	-122.47191	37.94076	-0.31	3.41	-5.285	3.10400009	11	7/24/2014	32:23.7
9059	RWS11	-122.4719195	37.94075	-0.41	3.43	-1.77	3.0180001	-122.47191	37.94077	-0.31	3.43	-5.459	3.12700009	11	7/24/2014	32:23.8
9060	RWS11	-122.4719217	37.94075	-0.38	3.45	-1.902	3.074	-122.47192	37.94077	-0.31	3.45	-5.656	3.148	11	7/24/2014	32:23.9
9061	RWS11	-122.4719249	37.94075	-0.41	3.47	-2.054	3.05899999	-122.47192	37.94077	-0.31	3.47	-5.852	3.16799998	11	7/24/2014	32:24.0
9062	RWS11	-122.4719271	37.94075	-0.38	3.49	-2.186	3.11200011	-122.47192	37.94077	-0.31	3.49	-5.984	3.18600011	11	7/24/2014	32:24.1
9063	RWS11	-122.4719293	37.94075	-0.41	3.51	-2.32	3.09400007	-122.47192	37.94077	-0.31	3.51	-6.205	3.20300007	11	7/24/2014	32:24.2
9064	RWS11	-122.4719315	37.94075	-0.38	3.53	-2.495	3.1450001	-122.47193	37.94077	-0.31	3.53	-6.336	3.2190001	11	7/24/2014	32:24.3
9065	RWS11	-122.4719337	37.94076	-0.41	3.54	-2.494	3.12499997	-122.47193	37.94077	-0.31	3.54	-6.51	3.23399997	11	7/24/2014	32:24.4
9066	RWS11	-122.4719359	37.94076	-0.38	3.56	-2.647	3.17500007	-122.47193	37.94077	-0.27	3.56	-6.62	3.28300005	11	7/24/2014	32:24.5
9067	RWS11	-122.4719381	37.94076	-0.41	3.57	-2.734	3.15400001	-122.47193	37.94077	-0.34	3.57	-6.816	3.229	11	7/24/2014	32:24.6
9068	RWS11	-122.4719404	37.94076	-0.41	3.58	-2.8	3.1660001	-122.47194	37.94077	-0.31	3.58	-6.925	3.2750001	11	7/24/2014	32:24.7
9069	RWS11	-122.4719426	37.94076	-0.41	3.59	-2.935	3.17799994	-122.47194	37.94077	-0.31	3.59	-6.929	3.28699994	11	7/24/2014	32:24.8
9070	RWS11	-122.4719448	37.94076	-0.38	3.6	-2.891	3.2240001	-122.47194	37.94077	-0.31	3.6	-6.929	3.2980001	11	7/24/2014	32:24.9
9071	RWS11	-122.471948	37.94076	-0.41	3.61	-2.891	3.197	-122.47194	37.94077	-0.34	3.61	-6.929	3.27199998	11	7/24/2014	32:25.0
9072	RWS11	-122.4719502	37.94076	-0.41	3.62	-2.891	3.20399997	-122.47195	37.94077	-0.31	3.62	-6.864	3.31299996	11	7/24/2014	32:25.1
9073	RWS11	-122.4719524	37.94076	-0.41	3.62	-3.023	3.20699999	-122.47195	37.94077	-0.34	3.62	-6.951	3.28199998	11	7/24/2014	32:25.2
9074	RWS11	-122.4719547	37.94076	-0.38	3.62	-3.089	3.24199998	-122.47195	37.94077	-0.31	3.62	-7.039	3.31599998	11	7/24/2014	32:25.3

9075	RWS11	-122.4719569	37.94076	-0.41	3.62	-3.092	3.20300004	-122.47195	37.94077	-0.34	3.62	-6.977	3.27800003	11	7/24/2014	32:25.4
9076	RWS11	-122.4719591	37.94076	-0.38	3.61	-3.157	3.23199999	-122.47195	37.94077	-0.31	3.61	-7.042	3.30599999	11	7/24/2014	32:25.5
9077	RWS11	-122.4719614	37.94076	-0.41	3.6	-3.157	3.18799993	-122.47196	37.94077	-0.31	3.6	-7.042	3.29699993	11	7/24/2014	32:25.6
9078	RWS11	-122.4719636	37.94076	-0.38	3.59	-3.157	3.21299994	-122.47196	37.94078	-0.31	3.59	-7.02	3.28699994	11	7/24/2014	32:25.7
9079	RWS11	-122.4719658	37.94076	-0.41	3.58	-3.026	3.16799995	-122.47196	37.94078	-0.34	3.58	-6.868	3.24299994	11	7/24/2014	32:25.8
9080	RWS11	-122.471968	37.94076	-0.41	3.57	-3.026	3.15700004	-122.47196	37.94078	-0.34	3.57	-6.78	3.23200002	11	7/24/2014	32:25.9
9081	RWS11	-122.4719713	37.94076	-0.41	3.56	-3.051	3.14700004	-122.47197	37.94078	-0.34	3.56	-6.696	3.22200003	11	7/24/2014	32:26.0
9082	RWS11	-122.4719735	37.94076	-0.41	3.55	-3.029	3.13700005	-122.47197	37.94078	-0.34	3.55	-6.543	3.21200004	11	7/24/2014	32:26.1
9083	RWS11	-122.4719757	37.94076	-0.47	3.54	-2.876	3.07799992	-122.47197	37.94078	-0.34	3.54	-6.478	3.20399991	11	7/24/2014	32:26.2
9084	RWS11	-122.4719779	37.94077	-0.41	3.54	-2.876	3.12199995	-122.47197	37.94078	-0.31	3.54	-6.172	3.23099995	11	7/24/2014	32:26.3
9085	RWS11	-122.4719801	37.94077	-0.41	3.53	-2.811	3.11599991	-122.47198	37.94078	-0.34	3.53	-6.042	3.1909999	11	7/24/2014	32:26.4
9086	RWS11	-122.4719824	37.94077	-0.41	3.53	-2.747	3.11100003	-122.47198	37.94078	-0.34	3.53	-5.933	3.18600002	11	7/24/2014	32:26.5
9087	RWS11	-122.4719846	37.94077	-0.41	3.52	-2.749	3.10700008	-122.47198	37.94078	-0.34	3.52	-5.675	3.18200007	11	7/24/2014	32:26.6
9088	RWS11	-122.4719868	37.94077	-0.41	3.52	-2.728	3.10199997	-122.47198	37.94078	-0.34	3.52	-5.609	3.17699996	11	7/24/2014	32:26.7
9089	RWS11	-122.471989	37.94077	-0.47	3.51	-2.575	3.04799995	-122.47198	37.94078	-0.34	3.51	-5.347	3.17399994	11	7/24/2014	32:26.8
9090	RWS11	-122.4719912	37.94077	-0.41	3.51	-2.466	3.09599993	-122.47199	37.94078	-0.34	3.51	-5.151	3.17099991	11	7/24/2014	32:26.9
9091	RWS11	-122.4719945	37.94077	-0.41	3.51	-2.444	3.09400007	-122.47199	37.94078	-0.34	3.51	-5.042	3.16900006	11	7/24/2014	32:27.0
9092	RWS11	-122.4719967	37.94077	-0.41	3.51	-2.466	3.09400007	-122.47199	37.94078	-0.39	3.51	-5.02	3.11900008	11	7/24/2014	32:27.1
9093	RWS11	-122.4719989	37.94077	-0.47	3.51	-2.554	3.04300007	-122.47199	37.94078	-0.34	3.51	-5.108	3.16900006	11	7/24/2014	32:27.2
9094	RWS11	-122.4720012	37.94077	-0.41	3.51	-2.513	3.09599993	-122.472	37.94078	-0.34	3.51	-5.177	3.17099991	11	7/24/2014	32:27.3
9095	RWS11	-122.4720034	37.94077	-0.41	3.51	-2.645	3.09800002	-122.472	37.94078	-0.39	3.51	-5.439	3.12300003	11	7/24/2014	32:27.4
9096	RWS11	-122.4720057	37.94077	-0.41	3.51	-2.775	3.09899995	-122.472	37.94078	-0.39	3.51	-5.657	3.12399995	11	7/24/2014	32:27.5
9097	RWS11	-122.4720079	37.94077	-0.41	3.52	-2.906	3.10000011	-122.472	37.94079	-0.39	3.52	-6.028	3.12500012	11	7/24/2014	32:27.6
9098	RWS11	-122.4720102	37.94077	-0.41	3.51	-3.146	3.09899995	-122.47201	37.94079	-0.34	3.51	-6.42	3.17399994	11	7/24/2014	32:27.7
9099	RWS11	-122.4720124	37.94077	-0.41	3.51	-3.344	3.09800002	-122.47201	37.94079	-0.39	3.51	-6.901	3.12300003	11	7/24/2014	32:27.8
9100	RWS11	-122.4720147	37.94077	-0.41	3.51	-3.565	3.09700009	-122.47201	37.94079	-0.34	3.51	-7.428	3.17200008	11	7/24/2014	32:27.9
9101	RWS11	-122.472018	37.94077	-0.47	3.51	-3.805	3.044	-122.47201	37.94079	-0.39	3.51	-7.865	3.12	11	7/24/2014	32:28.0
9102	RWS11	-122.4720202	37.94077	-0.47	3.51	-3.87	3.04199991	-122.47202	37.94079	-0.39	3.51	-8.366	3.11799991	11	7/24/2014	32:28.1
9103	RWS11	-122.4720225	37.94078	-0.47	3.51	-4.089	3.04000005	-122.47202	37.94079	-0.39	3.51	-8.715	3.11600006	11	7/24/2014	32:28.2
9104	RWS11	-122.4720248	37.94078	-0.41	3.5	-4.22	3.08800003	-122.47202	37.94079	-0.34	3.5	-9.174	3.16300002	11	7/24/2014	32:28.3
9105	RWS11	-122.4720271	37.94078	-0.41	3.5	-4.395	3.08500001	-122.47202	37.94079	-0.39	3.5	-9.436	3.11000001	11	7/24/2014	32:28.4
9106	RWS11	-122.4720293	37.94078	-0.41	3.49	-4.529	3.07999989	-122.47202	37.94079	-0.39	3.49	-9.854	3.1049999	11	7/24/2014	32:28.5
9107	RWS11	-122.4720316	37.94078	-0.47	3.49	-4.681	3.02300009	-122.47203	37.94079	-0.34	3.49	-10.247	3.14900008	11	7/24/2014	32:28.6

9108	RWS11	-122.4720339	37.94078	-0.41	3.48	-4.856	3.06599995	-122.47203	37.94079	-0.39	3.48	-10.421	3.09099996	11	7/24/2014	32:28.7
9109	RWS11	-122.4720362	37.94078	-0.47	3.47	-4.834	3.00700006	-122.47203	37.94079	-0.39	3.47	-10.618	3.08300006	11	7/24/2014	32:28.8
9110	RWS11	-122.4720384	37.94078	-0.41	3.46	-4.966	3.04800007	-122.47203	37.94079	-0.39	3.46	-10.683	3.07300007	11	7/24/2014	32:28.9
9111	RWS11	-122.4720418	37.94078	-0.47	3.45	-5.075	2.98499998	-122.47204	37.94079	-0.34	3.45	-10.902	3.11099997	11	7/24/2014	32:29.0
9112	RWS11	-122.472044	37.94078	-0.47	3.44	-5.079	2.97100005	-122.47204	37.94079	-0.39	3.44	-11.08	3.04700005	11	7/24/2014	32:29.1
9113	RWS11	-122.4720463	37.94078	-0.47	3.42	-5.144	2.95500001	-122.47204	37.94079	-0.34	3.42	-11.058	3.081	11	7/24/2014	32:29.2
9114	RWS11	-122.4720486	37.94078	-0.41	3.4	-5.187	2.98800012	-122.47204	37.94079	-0.34	3.4	-11.102	3.06300011	11	7/24/2014	32:29.3
9115	RWS11	-122.4720508	37.94078	-0.47	3.38	-5.253	2.91800007	-122.47205	37.94079	-0.39	3.38	-11.167	2.99400008	11	7/24/2014	32:29.4
9116	RWS11	-122.4720531	37.94078	-0.41	3.37	-5.318	2.95000002	-122.47205	37.94079	-0.39	3.37	-11.21	2.97500002	11	7/24/2014	32:29.5
9117	RWS11	-122.4720554	37.94078	-0.47	3.35	-5.34	2.87999997	-122.47205	37.9408	-0.34	3.35	-11.211	3.00599995	11	7/24/2014	32:29.6
9118	RWS11	-122.4720577	37.94078	-0.41	3.33	-5.407	2.914	-122.47205	37.9408	-0.34	3.33	-11.255	2.98899999	11	7/24/2014	32:29.7
9119	RWS11	-122.47206	37.94078	-0.47	3.31	-5.453	2.84699997	-122.47206	37.9408	-0.39	3.31	-11.28	2.92299998	11	7/24/2014	32:29.8
9120	RWS11	-122.4720622	37.94078	-0.41	3.3	-5.366	2.88499996	-122.47206	37.9408	-0.34	3.3	-11.062	2.95999995	11	7/24/2014	32:29.9
9121	RWS11	-122.4720656	37.94078	-0.47	3.29	-5.431	2.82399997	-122.47206	37.9408	-0.39	3.29	-11.062	2.89999998	11	7/24/2014	32:30.0
9122	RWS11	-122.4720679	37.94078	-0.41	3.28	-5.279	2.86599991	-122.47206	37.9408	-0.34	3.28	-10.931	2.9409999	11	7/24/2014	32:30.1
9123	RWS11	-122.4720702	37.94079	-0.47	3.27	-5.301	2.80799994	-122.47207	37.9408	-0.39	3.27	-10.909	2.88399994	11	7/24/2014	32:30.2
9124	RWS11	-122.4720726	37.94079	-0.41	3.27	-5.257	2.85499999	-122.47207	37.9408	-0.34	3.27	-10.756	2.92999998	11	7/24/2014	32:30.3
9125	RWS11	-122.4720749	37.94079	-0.47	3.27	-5.239	2.8019999	-122.47207	37.9408	-0.39	3.27	-10.717	2.8779999	11	7/24/2014	32:30.4
9126	RWS11	-122.4720772	37.94079	-0.41	3.27	-5.218	2.8529999	-122.47207	37.9408	-0.34	3.27	-10.649	2.92799988	11	7/24/2014	32:30.5
9127	RWS11	-122.4720796	37.94079	-0.47	3.27	-5.195	2.8019999	-122.47208	37.9408	-0.39	3.27	-10.543	2.8779999	11	7/24/2014	32:30.6
9128	RWS11	-122.4720819	37.94079	-0.41	3.27	-5.108	2.8529999	-122.47208	37.9408	-0.31	3.27	-10.411	2.96199989	11	7/24/2014	32:30.7
9129	RWS11	-122.4720843	37.94079	-0.47	3.27	-5.021	2.80300006	-122.47208	37.9408	-0.34	3.27	-10.455	2.92900005	11	7/24/2014	32:30.8
9130	RWS11	-122.4720866	37.94079	-0.41	3.27	-5.088	2.85400006	-122.47208	37.9408	-0.34	3.27	-10.391	2.92900005	11	7/24/2014	32:30.9
9131	RWS11	-122.4720901	37.94079	-0.41	3.27	-5.047	2.8529999	-122.47209	37.9408	-0.34	3.27	-10.35	2.92799988	11	7/24/2014	32:31.0
9132	RWS11	-122.4720924	37.94079	-0.41	3.27	-4.959	2.8529999	-122.47209	37.9408	-0.31	3.27	-10.306	2.96199989	11	7/24/2014	32:31.1
9133	RWS11	-122.4720947	37.94079	-0.47	3.27	-5.003	2.80099997	-122.47209	37.9408	-0.31	3.27	-10.241	2.96099997	11	7/24/2014	32:31.2
9134	RWS11	-122.4720971	37.94079	-0.41	3.27	-4.981	2.85000011	-122.47209	37.9408	-0.34	3.27	-10.175	2.9250001	11	7/24/2014	32:31.3
9135	RWS11	-122.4720994	37.94079	-0.47	3.26	-5.003	2.79799995	-122.4721	37.9408	-0.34	3.26	-10.197	2.92399994	11	7/24/2014	32:31.4
9136	RWS11	-122.4721018	37.94079	-0.41	3.26	-5.069	2.84700009	-122.4721	37.94081	-0.34	3.26	-10.176	2.92200008	11	7/24/2014	32:31.5
9137	RWS11	-122.4721042	37.94079	-0.47	3.26	-5.051	2.794	-122.4721	37.94081	-0.34	3.26	-10.179	2.91999999	11	7/24/2014	32:31.6
9138	RWS11	-122.4721066	37.94079	-0.41	3.26	-5.051	2.845	-122.4721	37.94081	-0.27	3.26	-10.223	2.98799998	11	7/24/2014	32:31.7
9139	RWS11	-122.4721089	37.94079	-0.41	3.26	-5.051	2.845	-122.4721	37.94081	-0.34	3.26	-10.092	2.91999999	11	7/24/2014	32:31.8
9140	RWS11	-122.4721113	37.94079	-0.41	3.26	-5.051	2.84599993	-122.47211	37.94081	-0.31	3.26	-10.049	2.95499992	11	7/24/2014	32:31.9

9141	RWS11	-122.4721148	37.94079	-0.41	3.26	-4.964	2.84700009	-122.47211	37.94081	-0.39	3.26	-9.961	2.8720001	11	7/24/2014	32:32.0
9142	RWS11	-122.4721172	37.9408	-0.41	3.27	-4.92	2.85000011	-122.47211	37.94081	-0.34	3.27	-9.743	2.9250001	11	7/24/2014	32:32.1
9143	RWS11	-122.4721195	37.9408	-0.47	3.27	-4.745	2.80099997	-122.47212	37.94081	-0.39	3.27	-9.59	2.87699997	11	7/24/2014	32:32.2
9144	RWS11	-122.472122	37.9408	-0.41	3.27	-4.705	2.8529999	-122.47212	37.94081	-0.34	3.27	-9.42	2.92799988	11	7/24/2014	32:32.3
9145	RWS11	-122.4721244	37.9408	-0.47	3.27	-4.488	2.8019999	-122.47212	37.94081	-0.39	3.27	-9.245	2.8779999	11	7/24/2014	32:32.4
9146	RWS11	-122.4721268	37.9408	-0.41	3.26	-4.422	2.84800002	-122.47212	37.94081	-0.39	3.26	-9.071	2.87300003	11	7/24/2014	32:32.5
9147	RWS11	-122.4721292	37.9408	-0.47	3.26	-4.225	2.78900012	-122.47212	37.94081	-0.34	3.26	-8.853	2.91500011	11	7/24/2014	32:32.6
9148	RWS11	-122.4721317	37.9408	-0.41	3.24	-4.182	2.82500002	-122.47213	37.94081	-0.39	3.24	-8.721	2.85000002	11	7/24/2014	32:32.7
9149	RWS11	-122.4721341	37.9408	-0.47	3.22	-4.182	2.75300011	-122.47213	37.94081	-0.39	3.22	-8.809	2.82900012	11	7/24/2014	32:32.8
9150	RWS11	-122.4721365	37.9408	-0.41	3.19	-4.141	2.77699992	-122.47213	37.94081	-0.34	3.19	-8.703	2.85199991	11	7/24/2014	32:32.9
9151	RWS11	-122.4721401	37.9408	-0.47	3.16	-4.011	2.69500002	-122.47214	37.94081	-0.39	3.16	-8.746	2.77100003	11	7/24/2014	32:33.0
9152	RWS11	-122.4721425	37.9408	-0.41	3.13	-3.945	2.71099994	-122.47214	37.94081	-0.39	3.13	-8.616	2.73599994	11	7/24/2014	32:33.1
9153	RWS11	-122.4721449	37.9408	-0.41	3.09	-3.967	2.67600009	-122.47214	37.94081	-0.42	3.09	-8.637	2.66700009	11	7/24/2014	32:33.2
9154	RWS11	-122.4721474	37.9408	-0.47	3.06	-3.814	2.59	-122.47214	37.94081	-0.39	3.06	-8.528	2.66600001	11	7/24/2014	32:33.3
9155	RWS11	-122.4721498	37.9408	-0.47	3.02	-3.837	2.55600008	-122.47215	37.94082	-0.42	3.02	-8.442	2.59800008	11	7/24/2014	32:33.4
9156	RWS11	-122.4721523	37.9408	-0.47	2.99	-3.709	2.52600011	-122.47215	37.94082	-0.34	2.99	-8.292	2.6520001	11	7/24/2014	32:33.5
9157	RWS11	-122.4721548	37.9408	-0.47	2.96	-3.578	2.498	-122.47215	37.94082	-0.34	2.96	-8.205	2.62399998	11	7/24/2014	32:33.6
9158	RWS11	-122.4721573	37.9408	-0.47	2.94	-3.491	2.47499999	-122.47215	37.94082	-0.39	2.94	-8.14	2.551	11	7/24/2014	32:33.7
9159	RWS11	-122.4721597	37.9408	-0.47	2.92	-3.338	2.45599994	-122.47216	37.94082	-0.34	2.92	-8.074	2.58199993	11	7/24/2014	32:33.8
9160	RWS11	-122.4721622	37.9408	-0.41	2.91	-3.185	2.49099991	-122.47216	37.94082	-0.39	2.91	-7.987	2.51599991	11	7/24/2014	32:33.9
9161	RWS11	-122.4721658	37.94081	-0.47	2.89	-3.034	2.4269999	-122.47216	37.94082	-0.42	2.89	-7.813	2.46899989	11	7/24/2014	32:34.0
9162	RWS11	-122.4721682	37.94081	-0.47	2.88	-2.819	2.41599998	-122.47216	37.94082	-0.39	2.88	-7.62	2.49199998	11	7/24/2014	32:34.1
9163	RWS11	-122.4721707	37.94081	-0.47	2.87	-2.687	2.40699992	-122.47217	37.94082	-0.42	2.87	-7.489	2.44899991	11	7/24/2014	32:34.2
9164	RWS11	-122.4721732	37.94081	-0.47	2.86	-2.513	2.39800009	-122.47217	37.94082	-0.42	2.86	-7.401	2.44000009	11	7/24/2014	32:34.3
9165	RWS11	-122.4721757	37.94081	-0.47	2.86	-2.361	2.38999996	-122.47217	37.94082	-0.42	2.86	-7.183	2.43199995	11	7/24/2014	32:34.4
9166	RWS11	-122.4721781	37.94081	-0.47	2.85	-2.12	2.38299999	-122.47217	37.94082	-0.39	2.85	-6.943	2.45899999	11	7/24/2014	32:34.5
9167	RWS11	-122.4721806	37.94081	-0.5	2.84	-1.902	2.34299994	-122.47218	37.94082	-0.42	2.84	-6.878	2.41899994	11	7/24/2014	32:34.6
9168	RWS11	-122.4721831	37.94081	-0.47	2.84	-1.75	2.37200007	-122.47218	37.94082	-0.39	2.84	-6.552	2.44800007	11	7/24/2014	32:34.7
9169	RWS11	-122.4721855	37.94081	-0.47	2.83	-1.579	2.36699995	-122.47218	37.94082	-0.47	2.83	-6.337	2.35799995	11	7/24/2014	32:34.8
9170	RWS11	-122.472188	37.94081	-0.47	2.83	-1.492	2.36200008	-122.47218	37.94082	-0.42	2.83	-6.315	2.40400007	11	7/24/2014	32:34.9
9171	RWS11	-122.4721916	37.94081	-0.5	2.82	-1.295	2.32299995	-122.47219	37.94082	-0.47	2.82	-6.249	2.34799996	11	7/24/2014	32:35.0
9172	RWS11	-122.472194	37.94081	-0.47	2.82	-1.208	2.35200009	-122.47219	37.94082	-0.47	2.82	-6.053	2.34300008	11	7/24/2014	32:35.1
9173	RWS11	-122.4721964	37.94081	-0.5	2.81	-1.033	2.31200004	-122.47219	37.94082	-0.47	2.81	-5.966	2.33700004	11	7/24/2014	32:35.2

9174	RWS11	-122.4721989	37.94081	-0.5	2.81	-0.925	2.30699992	-122.47219	37.94083	-0.47	2.81	-5.945	2.33199993	11	7/24/2014	32:35.3
9175	RWS11	-122.4722013	37.94081	-0.5	2.8	-0.95	2.3039999	-122.4722	37.94083	-0.47	2.8	-5.948	2.32899991	11	7/24/2014	32:35.4
9176	RWS11	-122.4722038	37.94081	-0.5	2.8	-0.885	2.30399996	-122.4722	37.94083	-0.47	2.8	-5.86	2.32899997	11	7/24/2014	32:35.5
9177	RWS11	-122.4722062	37.94081	-0.5	2.81	-0.775	2.30600005	-122.4722	37.94083	-0.47	2.81	-5.773	2.33100006	11	7/24/2014	32:35.6
9178	RWS11	-122.4722087	37.94081	-0.5	2.81	-0.797	2.31099993	-122.4722	37.94083	-0.42	2.81	-5.926	2.38699993	11	7/24/2014	32:35.7
9179	RWS11	-122.4722111	37.94081	-0.5	2.82	-0.797	2.31999999	-122.47221	37.94083	-0.47	2.82	-6.013	2.345	11	7/24/2014	32:35.8
9180	RWS11	-122.4722135	37.94082	-0.5	2.83	-0.689	2.33399993	-122.47221	37.94083	-0.47	2.83	-5.774	2.35899994	11	7/24/2014	32:35.9
9181	RWS11	-122.4722171	37.94082	-0.55	2.85	-0.67	2.30100006	-122.47221	37.94083	-0.51	2.85	-5.581	2.34300005	11	7/24/2014	32:36.0
9182	RWS11	-122.4722196	37.94082	-0.5	2.87	-0.561	2.37500006	-122.47222	37.94083	-0.47	2.87	-5.537	2.40000007	11	7/24/2014	32:36.1
9183	RWS11	-122.472222	37.94082	-0.5	2.9	-0.583	2.40299994	-122.47222	37.94083	-0.47	2.9	-5.45	2.42799994	11	7/24/2014	32:36.2
9184	RWS11	-122.4722245	37.94082	-0.5	2.93	-0.539	2.435	-122.47222	37.94083	-0.47	2.93	-5.363	2.46000001	11	7/24/2014	32:36.3
9185	RWS11	-122.472227	37.94082	-0.55	2.97	-0.561	2.42099994	-122.47222	37.94083	-0.47	2.97	-5.384	2.49699995	11	7/24/2014	32:36.4
9186	RWS11	-122.4722295	37.94082	-0.5	3.01	-0.605	2.514	-122.47223	37.94083	-0.47	3.01	-5.363	2.539	11	7/24/2014	32:36.5
9187	RWS11	-122.472232	37.94082	-0.55	3.06	-0.671	2.509	-122.47223	37.94083	-0.47	3.06	-5.473	2.58500001	11	7/24/2014	32:36.6
9188	RWS11	-122.4722344	37.94082	-0.5	3.11	-0.74	2.61099988	-122.47223	37.94083	-0.47	3.11	-5.498	2.63599989	11	7/24/2014	32:36.7
9189	RWS11	-122.4722369	37.94082	-0.55	3.16	-0.914	2.61400002	-122.47223	37.94083	-0.47	3.16	-5.781	2.69000003	11	7/24/2014	32:36.8
9190	RWS11	-122.4722394	37.94082	-0.5	3.22	-1.067	2.72100002	-122.47223	37.94083	-0.47	3.22	-5.999	2.74600002	11	7/24/2014	32:36.9
9191	RWS11	-122.472243	37.94082	-0.5	3.27	-1.219	2.77499992	-122.47224	37.94083	-0.47	3.27	-6.13	2.79999992	11	7/24/2014	32:37.0
9192	RWS11	-122.4722455	37.94082	-0.5	3.33	-1.504	2.82699996	-122.47224	37.94084	-0.47	3.33	-6.327	2.85199997	11	7/24/2014	32:37.1
9193	RWS11	-122.4722479	37.94082	-0.55	3.37	-1.636	2.82100004	-122.47224	37.94084	-0.47	3.37	-6.502	2.89700004	11	7/24/2014	32:37.2
9194	RWS11	-122.4722504	37.94082	-0.5	3.41	-1.856	2.90800005	-122.47225	37.94084	-0.51	3.41	-6.527	2.89900005	11	7/24/2014	32:37.3
9195	RWS11	-122.4722529	37.94082	-0.5	3.43	-2.097	2.93199998	-122.47225	37.94084	-0.47	3.43	-6.593	2.95699999	11	7/24/2014	32:37.4
9196	RWS11	-122.4722554	37.94082	-0.5	3.44	-2.249	2.93999988	-122.47225	37.94084	-0.47	3.44	-6.658	2.96499988	11	7/24/2014	32:37.5
9197	RWS11	-122.4722579	37.94082	-0.5	3.43	-2.424	2.92800003	-122.47225	37.94084	-0.47	3.43	-6.745	2.95300004	11	7/24/2014	32:37.6
9198	RWS11	-122.4722604	37.94083	-0.5	3.4	-2.576	2.8969999	-122.47226	37.94084	-0.47	3.4	-6.811	2.9219999	11	7/24/2014	32:37.7
9199	RWS11	-122.4722628	37.94083	-0.55	3.34	-2.795	2.79400009	-122.47226	37.94084	-0.51	3.34	-6.963	2.83600008	11	7/24/2014	32:37.8
9200	RWS11	-122.4722653	37.94083	-0.5	3.27	-2.886	2.77499992	-122.47226	37.94084	-0.47	3.27	-7.011	2.79999992	11	7/24/2014	32:37.9
9201	RWS11	-122.4722689	37.94083	-0.5	3.19	-2.886	2.69100004	-122.47226	37.94084	-0.47	3.19	-6.946	2.71600005	11	7/24/2014	32:38.0
9202	RWS11	-122.4722713	37.94083	-0.5	3.1	-2.973	2.60500008	-122.47227	37.94084	-0.47	3.1	-6.946	2.63000008	11	7/24/2014	32:38.1
9203	RWS11	-122.4722738	37.94083	-0.55	3.03	-3.126	2.47699994	-122.47227	37.94084	-0.51	3.03	-6.967	2.51899993	11	7/24/2014	32:38.2
9204	RWS11	-122.4722762	37.94083	-0.5	2.98	-3.257	2.48100001	-122.47227	37.94084	-0.47	2.98	-7.055	2.50600001	11	7/24/2014	32:38.3
9205	RWS11	-122.4722787	37.94083	-0.5	2.97	-3.344	2.47499996	-122.47227	37.94084	-0.56	2.97	-7.317	2.41499996	11	7/24/2014	32:38.4
9206	RWS11	-122.4722812	37.94083	-0.5	3.02	-3.414	2.51600009	-122.47228	37.94084	-0.51	3.02	-7.211	2.50700009	11	7/24/2014	32:38.5

9207	RWS11	-122.4722836	37.94083	-0.55	3.09	-3.479	2.54400009	-122.47228	37.94084	-0.47	3.09	-7.168	2.62000009	11	7/24/2014	32:38.6
9208	RWS11	-122.4722861	37.94083	-0.5	3.19	-3.61	2.69599992	-122.47228	37.94084	-0.47	3.19	-7.364	2.72099993	11	7/24/2014	32:38.7
9209	RWS11	-122.4722886	37.94083	-0.5	3.3	-3.806	2.80200011	-122.47228	37.94084	-0.51	3.3	-7.386	2.7930001	11	7/24/2014	32:38.8
9210	RWS11	-122.4722911	37.94083	-0.47	3.4	-3.872	2.93900004	-122.47229	37.94084	-0.47	3.4	-7.648	2.93000004	11	7/24/2014	32:38.9
9211	RWS11	-122.4722947	37.94083	-0.5	3.49	-4.134	2.99599987	-122.47229	37.94085	-0.51	3.49	-7.822	2.98699987	11	7/24/2014	32:39.0
9212	RWS11	-122.4722972	37.94083	-0.5	3.57	-4.309	3.07300001	-122.47229	37.94085	-0.42	3.57	-8.085	3.14900002	11	7/24/2014	32:39.1
9213	RWS11	-122.4722997	37.94083	-0.5	3.63	-4.486	3.13500005	-122.4723	37.94085	-0.51	3.63	-8.415	3.12600005	11	7/24/2014	32:39.2
9214	RWS11	-122.4723022	37.94083	-0.5	3.68	-4.639	3.18199998	-122.4723	37.94085	-0.47	3.68	-8.523	3.20699999	11	7/24/2014	32:39.3
9215	RWS11	-122.4723047	37.94083	-0.5	3.71	-4.596	3.2159999	-122.4723	37.94085	-0.47	3.71	-8.525	3.24099991	11	7/24/2014	32:39.4
9216	RWS11	-122.4723073	37.94084	-0.5	3.74	-4.878	3.23799998	-122.4723	37.94085	-0.47	3.74	-8.72	3.26299998	11	7/24/2014	32:39.5
9217	RWS11	-122.4723098	37.94084	-0.5	3.75	-4.967	3.25000006	-122.47231	37.94085	-0.47	3.75	-8.807	3.27500007	11	7/24/2014	32:39.6
9218	RWS11	-122.4723124	37.94084	-0.47	3.75	-5.141	3.28800002	-122.47231	37.94085	-0.47	3.75	-8.786	3.27900001	11	7/24/2014	32:39.7
9219	RWS11	-122.4723149	37.94084	-0.5	3.75	-5.057	3.25000006	-122.47231	37.94085	-0.47	3.75	-8.789	3.27500007	11	7/24/2014	32:39.8
9220	RWS11	-122.4723173	37.94084	-0.5	3.74	-5.122	3.2389999	-122.47231	37.94085	-0.47	3.74	-8.702	3.26399991	11	7/24/2014	32:39.9
9221	RWS11	-122.4723211	37.94084	-0.5	3.72	-5.144	3.22100002	-122.47232	37.94085	-0.51	3.72	-8.68	3.21200001	11	7/24/2014	32:40.0
9222	RWS11	-122.4723235	37.94084	-0.5	3.69	-5.145	3.19599992	-122.47232	37.94085	-0.51	3.69	-8.637	3.18699992	11	7/24/2014	32:40.1
9223	RWS11	-122.472326	37.94084	-0.55	3.66	-4.97	3.11199993	-122.47232	37.94085	-0.51	3.66	-8.418	3.15399992	11	7/24/2014	32:40.2
9224	RWS11	-122.4723286	37.94084	-0.47	3.62	-4.884	3.1579999	-122.47232	37.94085	-0.47	3.62	-8.31	3.1489999	11	7/24/2014	32:40.3
9225	RWS11	-122.4723311	37.94084	-0.5	3.58	-4.995	3.07999998	-122.47233	37.94085	-0.51	3.58	-8.335	3.07099998	11	7/24/2014	32:40.4
9226	RWS11	-122.4723336	37.94084	-0.5	3.53	-5.04	3.03399998	-122.47233	37.94085	-0.51	3.53	-8.401	3.02499998	11	7/24/2014	32:40.5
9227	RWS11	-122.4723361	37.94084	-0.5	3.49	-5.148	2.991	-122.47233	37.94085	-0.51	3.49	-8.575	2.98199999	11	7/24/2014	32:40.6
9228	RWS11	-122.4723386	37.94084	-0.5	3.46	-5.127	2.95899993	-122.47233	37.94085	-0.56	3.46	-8.51	2.89899993	11	7/24/2014	32:40.7
9229	RWS11	-122.4723411	37.94084	-0.55	3.45	-5.017	2.89700001	-122.47234	37.94086	-0.59	3.45	-8.401	2.85500002	11	7/24/2014	32:40.8
9230	RWS11	-122.4723435	37.94084	-0.5	3.46	-5.192	2.95700008	-122.47234	37.94086	-0.56	3.46	-8.444	2.89700007	11	7/24/2014	32:40.9
9231	RWS11	-122.4723472	37.94084	-0.55	3.47	-5.215	2.92300004	-122.47234	37.94086	-0.59	3.47	-8.445	2.88100004	11	7/24/2014	32:41.0
9232	RWS11	-122.4723497	37.94084	-0.5	3.48	-5.262	2.98599988	-122.47235	37.94086	-0.59	3.48	-8.514	2.89299989	11	7/24/2014	32:41.1
9233	RWS11	-122.4723521	37.94084	-0.55	3.48	-5.37	2.93000001	-122.47235	37.94086	-0.59	3.48	-8.492	2.88800001	11	7/24/2014	32:41.2
9234	RWS11	-122.4723546	37.94084	-0.5	3.45	-5.283	2.95499998	-122.47235	37.94086	-0.56	3.45	-8.492	2.89499998	11	7/24/2014	32:41.3
9235	RWS11	-122.4723571	37.94085	-0.55	3.45	-5.414	2.90199989	-122.47235	37.94086	-0.59	3.45	-8.426	2.8599999	11	7/24/2014	32:41.4
9236	RWS11	-122.4723596	37.94085	-0.5	3.45	-5.327	2.95100003	-122.47236	37.94086	-0.64	3.45	-8.317	2.80700004	11	7/24/2014	32:41.5
9237	RWS11	-122.472362	37.94085	-0.55	3.45	-5.241	2.89799994	-122.47236	37.94086	-0.64	3.45	-8.166	2.80499995	11	7/24/2014	32:41.6
9238	RWS11	-122.4723645	37.94085	-0.55	3.45	-5.287	2.89600009	-122.47236	37.94086	-0.64	3.45	-8.212	2.80300009	11	7/24/2014	32:41.7
9239	RWS11	-122.472367	37.94085	-0.55	3.44	-5.309	2.89499992	-122.47236	37.94086	-0.64	3.44	-8.147	2.80199993	11	7/24/2014	32:41.8

9240	RWS11	-122.4723694	37.94085	-0.55	3.44	-5.266	2.8919999	-122.47237	37.94086	-0.64	3.44	-8.125	2.79899991	11	7/24/2014	32:41.9
9241	RWS11	-122.4723731	37.94085	-0.58	3.44	-5.178	2.85500008	-122.47237	37.94086	-0.64	3.44	-7.994	2.79700005	11	7/24/2014	32:42.0
9242	RWS11	-122.4723755	37.94085	-0.58	3.44	-5.156	2.85299999	-122.47237	37.94086	-0.64	3.44	-7.907	2.79499996	11	7/24/2014	32:42.1
9243	RWS11	-122.472378	37.94085	-0.58	3.44	-5.201	2.85200006	-122.47237	37.94086	-0.68	3.44	-7.864	2.76000005	11	7/24/2014	32:42.2
9244	RWS11	-122.4723805	37.94085	-0.55	3.43	-5.138	2.884	-122.47238	37.94086	-0.64	3.43	-7.78	2.79100001	11	7/24/2014	32:42.3
9245	RWS11	-122.472383	37.94085	-0.58	3.43	-5.27	2.84800011	-122.47238	37.94086	-0.68	3.43	-7.78	2.7560001	11	7/24/2014	32:42.4
9246	RWS11	-122.4723855	37.94085	-0.55	3.43	-5.291	2.88099998	-122.47238	37.94086	-0.64	3.43	-7.78	2.78799999	11	7/24/2014	32:42.5
9247	RWS11	-122.472388	37.94085	-0.58	3.43	-5.488	2.84399992	-122.47238	37.94086	-0.71	3.43	-7.889	2.71799988	11	7/24/2014	32:42.6
9248	RWS11	-122.4723905	37.94085	-0.58	3.43	-5.684	2.84100014	-122.47239	37.94087	-0.68	3.43	-8.042	2.74900013	11	7/24/2014	32:42.7
9249	RWS11	-122.472393	37.94085	-0.58	3.42	-5.969	2.83999997	-122.47239	37.94087	-0.71	3.42	-8.37	2.71399993	11	7/24/2014	32:42.8
9250	RWS11	-122.4723955	37.94085	-0.58	3.42	-6.386	2.83800012	-122.47239	37.94087	-0.71	3.42	-8.635	2.71200007	11	7/24/2014	32:42.9
9251	RWS11	-122.4723991	37.94085	-0.64	3.42	-6.67	2.78500003	-122.47239	37.94087	-0.71	3.42	-9.006	2.70999998	11	7/24/2014	32:43.0
9252	RWS11	-122.4724016	37.94085	-0.58	3.42	-7.041	2.83399993	-122.4724	37.94087	-0.68	3.42	-9.137	2.74199992	11	7/24/2014	32:43.1
9253	RWS11	-122.4724041	37.94086	-0.64	3.42	-7.39	2.78100008	-122.4724	37.94087	-0.68	3.42	-9.245	2.74000007	11	7/24/2014	32:43.2
9254	RWS11	-122.4724066	37.94086	-0.58	3.41	-7.979	2.82999998	-122.4724	37.94087	-0.68	3.41	-9.726	2.73799998	11	7/24/2014	32:43.3
9255	RWS11	-122.4724091	37.94086	-0.58	3.41	-8.765	2.82800013	-122.4724	37.94087	-0.71	3.41	-10.204	2.70200008	11	7/24/2014	32:43.4
9256	RWS11	-122.4724117	37.94086	-0.58	3.41	-9.355	2.82600003	-122.47241	37.94087	-0.68	3.41	-10.665	2.73400003	11	7/24/2014	32:43.5
9257	RWS11	-122.4724142	37.94086	-0.64	3.41	-10.23	2.77299994	-122.47241	37.94087	-0.71	3.41	-11.148	2.69799989	11	7/24/2014	32:43.6
9258	RWS11	-122.4724167	37.94086	-0.58	3.41	-11.06	2.82200009	-122.47241	37.94087	-0.76	3.41	-11.585	2.64500004	11	7/24/2014	32:43.7
9259	RWS11	-122.4724192	37.94086	-0.64	3.4	-11.82	2.76899999	-122.47241	37.94087	-0.8	3.4	-11.955	2.60899997	11	7/24/2014	32:43.8
9260	RWS11	-122.4724217	37.94086	-0.64	3.4	-12.65	2.76700014	-122.47242	37.94087	-0.76	3.4	-12.348	2.64100009	11	7/24/2014	32:43.9
9261	RWS11	-122.4724255	37.94086	-0.64	3.33	-13.83	2.69099993	-122.47242	37.94087	-0.76	3.33	-12.807	2.56499988	11	7/24/2014	32:44.0
9262	RWS11	-122.472428	37.94086	-0.58	3.32	-14.94	2.73499995	-122.47242	37.94087	-0.8	3.32	-13.287	2.52399993	11	7/24/2014	32:44.1
9263	RWS11	-122.4724305	37.94086	-0.64	3.31	-16.24	2.67699999	-122.47243	37.94087	-0.76	3.31	-13.967	2.55099994	11	7/24/2014	32:44.2
9264	RWS11	-122.472433	37.94086	-0.58	3.3	-17.7	2.71899992	-122.47243	37.94087	-0.8	3.3	-14.6	2.5079999	11	7/24/2014	32:44.3
9265	RWS11	-122.4724356	37.94086	-0.64	3.3	-19.22	2.66099995	-122.47243	37.94087	-0.85	3.3	-15.254	2.45099992	11	7/24/2014	32:44.4
9266	RWS11	-122.4724381	37.94086	-0.64	3.29	-20.86	2.65399998	-122.47243	37.94088	-0.76	3.29	-15.996	2.52799994	11	7/24/2014	32:44.5
9267	RWS11	-122.4724407	37.94086	-0.64	3.28	-22.72	2.64700001	-122.47244	37.94088	-0.76	3.28	-17.044	2.52099997	11	7/24/2014	32:44.6
9268	RWS11	-122.4724432	37.94086	-0.58	3.27	-24.59	2.68899995	-122.47244	37.94088	-0.76	3.27	-17.829	2.51199991	11	7/24/2014	32:44.7
9269	RWS11	-122.4724457	37.94086	-0.64	3.27	-26.45	2.63199991	-122.47244	37.94088	-0.76	3.27	-18.663	2.50599986	11	7/24/2014	32:44.8
9270	RWS11	-122.4724483	37.94086	-0.64	3.26	-28.33	2.62400001	-122.47244	37.94088	-0.76	3.26	-19.491	2.49799997	11	7/24/2014	32:44.9
9271	RWS11	-122.472452	37.94086	-0.64	3.25	-30.07	2.61799997	-122.47245	37.94088	-0.71	3.25	-20.299	2.54299992	11	7/24/2014	32:45.0
9272	RWS11	-122.4724545	37.94087	-0.64	3.24	-31.67	2.60899991	-122.47245	37.94088	-0.76	3.24	-21.04	2.48299986	11	7/24/2014	32:45.1

9273	RWS11	-122.472457	37.94087	-0.64	3.24	-32.97	2.60199994	-122.47245	37.94088	-0.76	3.24	-21.782	2.47599989	11	7/24/2014	32:45.2
9274	RWS11	-122.4724596	37.94087	-0.64	3.23	-34.04	2.59499997	-122.47246	37.94088	-0.71	3.23	-22.371	2.51999992	11	7/24/2014	32:45.3
9275	RWS11	-122.4724621	37.94087	-0.64	3.12	-34.94	2.48399991	-122.47246	37.94088	-0.76	3.12	-22.982	2.35799986	11	7/24/2014	32:45.4
9276	RWS11	-122.4724647	37.94087	-0.64	3.11	-35.27	2.47399992	-122.47246	37.94088	-0.71	3.11	-23.335	2.39899987	11	7/24/2014	32:45.5
9277	RWS11	-122.4724673	37.94087	-0.64	3.1	-35.22	2.46600002	-122.47246	37.94088	-0.68	3.1	-23.573	2.42500001	11	7/24/2014	32:45.6
9278	RWS11	-122.4724698	37.94087	-0.58	3.09	-34.66	2.50799996	-122.47247	37.94088	-0.68	3.09	-23.532	2.41599995	11	7/24/2014	32:45.7
9279	RWS11	-122.4724724	37.94087	-0.58	3.09	-33.57	2.50000006	-122.47247	37.94088	-0.71	3.09	-23.314	2.37400001	11	7/24/2014	32:45.8
9280	RWS11	-122.4724749	37.94087	-0.58	3.08	-32.13	2.49000007	-122.47247	37.94088	-0.68	3.08	-23.094	2.39800006	11	7/24/2014	32:45.9
9281	RWS11	-122.4724786	37.94087	-0.58	3.07	-30.25	2.48199993	-122.47247	37.94088	-0.68	3.07	-22.484	2.38999993	11	7/24/2014	32:46.0
9282	RWS11	-122.4724812	37.94087	-0.55	3.06	-27.77	2.50800008	-122.47248	37.94088	-0.71	3.06	-21.767	2.34700006	11	7/24/2014	32:46.1
9283	RWS11	-122.4724837	37.94087	-0.58	3.05	-25.04	2.46499997	-122.47248	37.94088	-0.64	3.05	-20.873	2.40699995	11	7/24/2014	32:46.2
9284	RWS11	-122.4724863	37.94087	-0.55	3.04	-22.11	2.48999995	-122.47248	37.94088	-0.64	3.04	-19.912	2.39699996	11	7/24/2014	32:46.3
9285	RWS11	-122.4724888	37.94087	-0.58	3.03	-18.97	2.44700009	-122.47248	37.94089	-0.64	3.03	-18.777	2.38900006	11	7/24/2014	32:46.4
9286	RWS11	-122.4724914	37.94087	-0.55	3.02	-15.74	2.47299999	-122.47249	37.94089	-0.59	3.02	-17.534	2.43099999	11	7/24/2014	32:46.5
9287	RWS11	-122.472494	37.94087	-0.55	3.02	-12.78	2.46600002	-122.47249	37.94089	-0.64	3.02	-16.336	2.37300003	11	7/24/2014	32:46.6
9288	RWS11	-122.4724965	37.94087	-0.55	3.01	-9.922	2.4550001	-122.47249	37.94089	-0.59	3.01	-15.029	2.41300011	11	7/24/2014	32:46.7
9289	RWS11	-122.4724991	37.94087	-0.55	2.9	-7.282	2.3459999	-122.47249	37.94089	-0.64	2.9	-13.763	2.2529999	11	7/24/2014	32:46.8
9290	RWS11	-122.4725016	37.94087	-0.5	2.81	-4.969	2.30699998	-122.4725	37.94089	-0.56	2.81	-12.629	2.24699998	11	7/24/2014	32:46.9
9291	RWS11	-122.4725053	37.94088	-0.55	2.75	-2.94	2.19600004	-122.4725	37.94089	-0.59	2.75	-11.538	2.15400004	11	7/24/2014	32:47.0
9292	RWS11	-122.4725078	37.94088	-0.5	2.72	-0.976	2.22599989	-122.4725	37.94089	-0.56	2.72	-10.403	2.16599989	11	7/24/2014	32:47.1
9293	RWS11	-122.4725103	37.94088	-0.55	2.74	0.595	2.19200009	-122.47251	37.94089	-0.59	2.74	-9.312	2.1500001	11	7/24/2014	32:47.2
9294	RWS11	-122.4725128	37.94088	-0.5	2.78	1.881	2.28300005	-122.47251	37.94089	-0.56	2.78	-8.353	2.22300005	11	7/24/2014	32:47.3
9295	RWS11	-122.4725154	37.94088	-0.5	2.83	3.077	2.33200008	-122.47251	37.94089	-0.56	2.83	-7.396	2.27200007	11	7/24/2014	32:47.4
9296	RWS11	-122.4725179	37.94088	-0.5	2.88	3.886	2.37800008	-122.47251	37.94089	-0.51	2.88	-6.567	2.36900008	11	7/24/2014	32:47.5
9297	RWS11	-122.4725204	37.94088	-0.5	2.91	4.452	2.41500002	-122.47252	37.94089	-0.56	2.91	-5.913	2.35500002	11	7/24/2014	32:47.6
9298	RWS11	-122.4725229	37.94088	-0.5	2.93	4.736	2.435	-122.47252	37.94089	-0.56	2.93	-5.411	2.375	11	7/24/2014	32:47.7
9299	RWS11	-122.4725254	37.94088	-0.5	2.93	4.911	2.43400007	-122.47252	37.94089	-0.59	2.93	-4.953	2.34100008	11	7/24/2014	32:47.8
9300	RWS11	-122.4725279	37.94088	-0.47	2.91	4.997	2.44299999	-122.47252	37.94089	-0.51	2.91	-4.583	2.39999998	11	7/24/2014	32:47.9
9301	RWS11	-122.4725316	37.94088	-0.5	2.86	4.798	2.36200005	-122.47253	37.94089	-0.56	2.86	-4.171	2.30200005	11	7/24/2014	32:48.0
9302	RWS11	-122.4725341	37.94088	-0.47	2.79	4.558	2.32700005	-122.47253	37.94089	-0.56	2.79	-3.866	2.23300004	11	7/24/2014	32:48.1
9303	RWS11	-122.4725367	37.94088	-0.5	2.71	4.274	2.21100003	-122.47253	37.9409	-0.56	2.71	-3.669	2.15100002	11	7/24/2014	32:48.2
9304	RWS11	-122.4725392	37.94088	-0.47	2.63	3.773	2.16099992	-122.47253	37.9409	-0.51	2.63	-3.626	2.11799991	11	7/24/2014	32:48.3
9305	RWS11	-122.4725418	37.94088	-0.5	2.56	3.293	2.05699998	-122.47254	37.9409	-0.56	2.56	-3.713	1.99699998	11	7/24/2014	32:48.4

9306	RWS11	-122.4725443	37.94088	-0.5	2.52	2.898	2.02000004	-122.47254	37.9409	-0.51	2.52	-3.605	2.01100004	11	7/24/2014	32:48.5
9307	RWS11	-122.4725469	37.94088	-0.5	2.53	2.459	2.02700001	-122.47254	37.9409	-0.51	2.53	-3.739	2.01800001	11	7/24/2014	32:48.6
9308	RWS11	-122.4725495	37.94088	-0.5	2.58	2.088	2.083	-122.47255	37.9409	-0.47	2.58	-3.586	2.10800001	11	7/24/2014	32:48.7
9309	RWS11	-122.472552	37.94089	-0.5	2.68	1.674	2.17800003	-122.47255	37.9409	-0.47	2.68	-3.739	2.20300004	11	7/24/2014	32:48.8
9310	RWS11	-122.4725546	37.94089	-0.47	2.79	1.325	2.32700005	-122.47255	37.9409	-0.47	2.79	-3.848	2.31800005	11	7/24/2014	32:48.9
9311	RWS11	-122.4725583	37.94089	-0.47	2.91	0.997	2.44699994	-122.47255	37.9409	-0.42	2.91	-3.87	2.48899993	11	7/24/2014	32:49.0
9312	RWS11	-122.4725608	37.94089	-0.47	3.03	0.648	2.56100002	-122.47256	37.9409	-0.47	3.03	-3.957	2.55200002	11	7/24/2014	32:49.1
9313	RWS11	-122.4725634	37.94089	-0.5	3.13	0.45	2.62999994	-122.47256	37.9409	-0.42	3.13	-4.023	2.70599994	11	7/24/2014	32:49.2
9314	RWS11	-122.4725659	37.94089	-0.47	3.22	0.121	2.75299993	-122.47256	37.9409	-0.47	3.22	-4.245	2.74399993	11	7/24/2014	32:49.3
9315	RWS11	-122.4725685	37.94089	-0.47	3.29	-0.141	2.82599989	-122.47256	37.9409	-0.42	3.29	-4.266	2.86799988	11	7/24/2014	32:49.4
9316	RWS11	-122.472571	37.94089	-0.47	3.35	-0.469	2.88600007	-122.47257	37.9409	-0.42	3.35	-4.354	2.92800006	11	7/24/2014	32:49.5
9317	RWS11	-122.4725735	37.94089	-0.47	3.4	-0.578	2.93399993	-122.47257	37.9409	-0.42	3.4	-4.441	2.97599992	11	7/24/2014	32:49.6
9318	RWS11	-122.4725761	37.94089	-0.47	3.44	-0.883	2.97100011	-122.47257	37.9409	-0.42	3.44	-4.528	3.0130001	11	7/24/2014	32:49.7
9319	RWS11	-122.4725786	37.94089	-0.5	3.46	-1.036	2.96499997	-122.47257	37.9409	-0.42	3.46	-4.66	3.04099998	11	7/24/2014	32:49.8
9320	RWS11	-122.4725811	37.94089	-0.47	3.48	-1.127	3.01999989	-122.47258	37.9409	-0.47	3.48	-4.62	3.01099989	11	7/24/2014	32:49.9
9321	RWS11	-122.4725848	37.94089	-0.47	3.5	-1.28	3.03700009	-122.47258	37.9409	-0.42	3.5	-4.75	3.07900009	11	7/24/2014	32:50.0
9322	RWS11	-122.4725873	37.94089	-0.47	3.52	-1.433	3.05100003	-122.47258	37.94091	-0.39	3.52	-4.816	3.12700003	11	7/24/2014	32:50.1
9323	RWS11	-122.4725898	37.94089	-0.47	3.53	-1.564	3.06100002	-122.47259	37.94091	-0.47	3.53	-4.794	3.05200002	11	7/24/2014	32:50.2
9324	RWS11	-122.4725923	37.94089	-0.47	3.54	-1.52	3.07000008	-122.47259	37.94091	-0.42	3.54	-4.772	3.11200008	11	7/24/2014	32:50.3
9325	RWS11	-122.4725949	37.94089	-0.5	3.54	-1.739	3.04199988	-122.47259	37.94091	-0.42	3.54	-4.948	3.11799988	11	7/24/2014	32:50.4
9326	RWS11	-122.4725974	37.94089	-0.47	3.54	-1.633	3.07899991	-122.47259	37.94091	-0.39	3.54	-4.842	3.15499991	11	7/24/2014	32:50.5
9327	RWS11	-122.4725999	37.94089	-0.47	3.55	-1.764	3.08000007	-122.4726	37.94091	-0.47	3.55	-4.951	3.07100007	11	7/24/2014	32:50.6
9328	RWS11	-122.4726025	37.9409	-0.47	3.55	-1.786	3.08000007	-122.4726	37.94091	-0.42	3.55	-4.994	3.12200007	11	7/24/2014	32:50.7
9329	RWS11	-122.472605	37.9409	-0.47	3.54	-1.83	3.07700005	-122.4726	37.94091	-0.47	3.54	-5.016	3.06800005	11	7/24/2014	32:50.8
9330	RWS11	-122.4726075	37.9409	-0.47	3.54	-1.829	3.07400003	-122.4726	37.94091	-0.47	3.54	-4.973	3.06500003	11	7/24/2014	32:50.9
9331	RWS11	-122.4726112	37.9409	-0.47	3.54	-1.917	3.07000008	-122.47261	37.94091	-0.47	3.54	-4.907	3.06100008	11	7/24/2014	32:51.0
9332	RWS11	-122.4726137	37.9409	-0.47	3.53	-1.917	3.06499997	-122.47261	37.94091	-0.42	3.53	-4.886	3.10699996	11	7/24/2014	32:51.1
9333	RWS11	-122.4726162	37.9409	-0.47	3.52	-1.965	3.058	-122.47261	37.94091	-0.47	3.52	-4.999	3.04899999	11	7/24/2014	32:51.2
9334	RWS11	-122.4726188	37.9409	-0.47	3.51	-2.074	3.04899994	-122.47261	37.94091	-0.42	3.51	-5.108	3.09099993	11	7/24/2014	32:51.3
9335	RWS11	-122.4726213	37.9409	-0.5	3.5	-2.073	3.00400001	-122.47262	37.94091	-0.47	3.5	-5.064	3.02900001	11	7/24/2014	32:51.4
9336	RWS11	-122.4726238	37.9409	-0.5	3.49	-2.139	2.99000007	-122.47262	37.94091	-0.42	3.49	-5.042	3.06600007	11	7/24/2014	32:51.5
9337	RWS11	-122.4726264	37.9409	-0.5	3.47	-2.073	2.97599989	-122.47262	37.94091	-0.47	3.47	-5.064	3.0009999	11	7/24/2014	32:51.6
9338	RWS11	-122.4726289	37.9409	-0.47	3.46	-2.118	2.99500003	-122.47262	37.94091	-0.47	3.46	-5.043	2.98600003	11	7/24/2014	32:51.7

9339	RWS11	-122.4726314	37.9409	-0.5	3.45	-2.099	2.94800001	-122.47263	37.94091	-0.47	3.45	-5.024	2.97300002	11	7/24/2014	32:51.8
9340	RWS11	-122.4726339	37.9409	-0.46	3.44	-2.1	2.97300005	-122.47263	37.94091	-0.47	3.44	-5.09	2.96400005	11	7/24/2014	32:51.9
9341	RWS11	-122.4726376	37.9409	-0.5	3.43	-2.099	2.933	-122.47263	37.94092	-0.47	3.43	-4.981	2.958	11	7/24/2014	32:52.0
9342	RWS11	-122.4726401	37.9409	-0.46	3.43	-2.209	2.96300006	-122.47264	37.94092	-0.47	3.43	-5.024	2.95400006	11	7/24/2014	32:52.1
9343	RWS11	-122.4726426	37.9409	-0.5	3.42	-2.099	2.92600003	-122.47264	37.94092	-0.51	3.42	-4.981	2.917	11	7/24/2014	32:52.2
9344	RWS11	-122.4726451	37.9409	-0.46	3.42	-2.079	2.95600009	-122.47264	37.94092	-0.42	3.42	-4.938	2.99800009	11	7/24/2014	32:52.3
9345	RWS11	-122.4726476	37.9409	-0.5	3.41	-2.038	2.91399994	-122.47264	37.94092	-0.47	3.41	-4.832	2.93899995	11	7/24/2014	32:52.4
9346	RWS11	-122.4726501	37.94091	-0.46	3.4	-1.907	2.93700004	-122.47265	37.94092	-0.51	3.4	-4.788	2.89399999	11	7/24/2014	32:52.5
9347	RWS11	-122.4726526	37.94091	-0.5	3.39	-1.907	2.89000002	-122.47265	37.94092	-0.47	3.39	-4.701	2.91500002	11	7/24/2014	32:52.6
9348	RWS11	-122.4726552	37.94091	-0.46	3.37	-1.819	2.90899992	-122.47265	37.94092	-0.47	3.37	-4.745	2.89999992	11	7/24/2014	32:52.7
9349	RWS11	-122.4726576	37.94091	-0.5	3.36	-1.82	2.86099997	-122.47265	37.94092	-0.51	3.36	-4.614	2.85199994	11	7/24/2014	32:52.8
9350	RWS11	-122.4726601	37.94091	-0.46	3.35	-1.711	2.8829999	-122.47266	37.94092	-0.51	3.35	-4.636	2.83999985	11	7/24/2014	32:52.9
9351	RWS11	-122.4726637	37.94091	-0.5	3.34	-1.557	2.84099999	-122.47266	37.94092	-0.51	3.34	-4.505	2.83199996	11	7/24/2014	32:53.0
9352	RWS11	-122.4726662	37.94091	-0.46	3.34	-1.561	2.87599993	-122.47266	37.94092	-0.51	3.34	-4.464	2.83299989	11	7/24/2014	32:53.1
9353	RWS11	-122.4726686	37.94091	-0.5	3.35	-1.605	2.85199991	-122.47266	37.94092	-0.51	3.35	-4.682	2.84299988	11	7/24/2014	32:53.2
9354	RWS11	-122.4726711	37.94091	-0.5	3.37	-1.648	2.86900011	-122.47267	37.94092	-0.47	3.37	-4.835	2.89400011	11	7/24/2014	32:53.3
9355	RWS11	-122.4726736	37.94091	-0.5	3.39	-1.67	2.89200011	-122.47267	37.94092	-0.51	3.39	-4.922	2.88300008	11	7/24/2014	32:53.4
9356	RWS11	-122.4726761	37.94091	-0.5	3.42	-1.757	2.91900006	-122.47267	37.94092	-0.51	3.42	-4.966	2.91000003	11	7/24/2014	32:53.5
9357	RWS11	-122.4726786	37.94091	-0.5	3.44	-1.78	2.94600001	-122.47267	37.94092	-0.56	3.44	-5.054	2.88599998	11	7/24/2014	32:53.6
9358	RWS11	-122.472681	37.94091	-0.5	3.47	-1.805	2.97299996	-122.47268	37.94092	-0.56	3.47	-5.057	2.91299993	11	7/24/2014	32:53.7
9359	RWS11	-122.4726835	37.94091	-0.55	3.5	-1.827	2.94899988	-122.47268	37.94092	-0.51	3.5	-5.013	2.99099988	11	7/24/2014	32:53.8
9360	RWS11	-122.4726859	37.94091	-0.5	3.52	-1.783	3.02500001	-122.47268	37.94093	-0.59	3.52	-5.079	2.93199998	11	7/24/2014	32:53.9
9361	RWS11	-122.4726896	37.94091	-0.55	3.55	-1.892	2.99600005	-122.47269	37.94093	-0.59	3.55	-5.101	2.95400006	11	7/24/2014	32:54.0
9362	RWS11	-122.472692	37.94091	-0.5	3.56	-1.892	3.06400004	-122.47269	37.94093	-0.59	3.56	-5.166	2.97100002	11	7/24/2014	32:54.1
9363	RWS11	-122.4726945	37.94091	-0.55	3.57	-2.024	3.02499986	-122.47269	37.94093	-0.64	3.57	-5.276	2.93199986	11	7/24/2014	32:54.2
9364	RWS11	-122.472697	37.94091	-0.5	3.58	-1.918	3.08400002	-122.47269	37.94093	-0.56	3.58	-5.192	3.02399999	11	7/24/2014	32:54.3
9365	RWS11	-122.4726995	37.94091	-0.55	3.59	-1.918	3.03600001	-122.4727	37.94093	-0.59	3.59	-5.127	2.99400002	11	7/24/2014	32:54.4
9366	RWS11	-122.472702	37.94092	-0.55	3.59	-1.874	3.03600001	-122.4727	37.94093	-0.59	3.59	-4.952	2.99400002	11	7/24/2014	32:54.5
9367	RWS11	-122.4727045	37.94092	-0.58	3.58	-1.853	2.99599993	-122.4727	37.94093	-0.64	3.58	-4.908	2.9379999	11	7/24/2014	32:54.6
9368	RWS11	-122.472707	37.94092	-0.55	3.57	-1.896	3.023	-122.4727	37.94093	-0.64	3.57	-5.018	2.93000001	11	7/24/2014	32:54.7
9369	RWS11	-122.4727094	37.94092	-0.55	3.56	-1.918	3.01200008	-122.47271	37.94093	-0.64	3.56	-4.799	2.91900009	11	7/24/2014	32:54.8
9370	RWS11	-122.4727119	37.94092	-0.55	3.55	-1.766	3	-122.47271	37.94093	-0.64	3.55	-4.757	2.90700001	11	7/24/2014	32:54.9
9371	RWS11	-122.4727156	37.94092	-0.58	3.54	-1.726	2.95299995	-122.47271	37.94093	-0.68	3.54	-4.738	2.86099994	11	7/24/2014	32:55.0

9372	RWS11	-122.4727181	37.94092	-0.55	3.52	-1.835	2.9749999	-122.47271	37.94093	-0.68	3.52	-4.76	2.84799993	11	7/24/2014	32:55.1
9373	RWS11	-122.4727206	37.94092	-0.58	3.51	-1.922	2.92699993	-122.47272	37.94093	-0.68	3.51	-4.891	2.83499992	11	7/24/2014	32:55.2
9374	RWS11	-122.4727231	37.94092	-0.58	3.5	-1.966	2.91299999	-122.47272	37.94093	-0.68	3.5	-4.934	2.82099998	11	7/24/2014	32:55.3
9375	RWS11	-122.4727256	37.94092	-0.58	3.48	-2.009	2.89699996	-122.47272	37.94093	-0.71	3.48	-5.021	2.77099997	11	7/24/2014	32:55.4
9376	RWS11	-122.4727281	37.94092	-0.58	3.46	-1.967	2.88099992	-122.47272	37.94093	-0.68	3.46	-5.11	2.78899992	11	7/24/2014	32:55.5
9377	RWS11	-122.4727306	37.94092	-0.63	3.45	-2.035	2.81599998	-122.47273	37.94093	-0.68	3.45	-5.265	2.77499998	11	7/24/2014	32:55.6
9378	RWS11	-122.4727332	37.94092	-0.63	3.44	-1.926	2.80599999	-122.47273	37.94093	-0.71	3.44	-5.309	2.73100001	11	7/24/2014	32:55.7
9379	RWS11	-122.4727357	37.94092	-0.63	3.43	-2.013	2.79999995	-122.47273	37.94094	-0.71	3.43	-5.331	2.72499996	11	7/24/2014	32:55.8
9380	RWS11	-122.4727381	37.94092	-0.63	3.43	-2.1	2.79999995	-122.47273	37.94094	-0.71	3.43	-5.44	2.72499996	11	7/24/2014	32:55.9
9381	RWS11	-122.4727418	37.94092	-0.63	3.44	-2.144	2.8039999	-122.47274	37.94094	-0.71	3.44	-5.462	2.72899991	11	7/24/2014	32:56.0
9382	RWS11	-122.4727443	37.94092	-0.63	3.45	-2.276	2.81200004	-122.47274	37.94094	-0.68	3.45	-5.66	2.77100003	11	7/24/2014	32:56.1
9383	RWS11	-122.4727468	37.94092	-0.63	3.46	-2.432	2.82500005	-122.47274	37.94094	-0.71	3.46	-5.837	2.75000006	11	7/24/2014	32:56.2
9384	RWS11	-122.4727493	37.94092	-0.63	3.47	-2.715	2.83999991	-122.47275	37.94094	-0.71	3.47	-6.099	2.76499993	11	7/24/2014	32:56.3
9385	RWS11	-122.4727517	37.94093	-0.67	3.49	-2.803	2.824	-122.47275	37.94094	-0.71	3.49	-6.295	2.78300005	11	7/24/2014	32:56.4
9386	RWS11	-122.4727542	37.94093	-0.63	3.51	-3.043	2.87800002	-122.47275	37.94094	-0.68	3.51	-6.491	2.83700001	11	7/24/2014	32:56.5
9387	RWS11	-122.4727567	37.94093	-0.63	3.53	-3.13	2.89899993	-122.47275	37.94094	-0.68	3.53	-6.622	2.85799992	11	7/24/2014	32:56.6
9388	RWS11	-122.4727592	37.94093	-0.63	3.56	-3.261	2.92000008	-122.47276	37.94094	-0.68	3.56	-6.884	2.87900007	11	7/24/2014	32:56.7
9389	RWS11	-122.4727617	37.94093	-0.67	3.58	-3.349	2.90699995	-122.47276	37.94094	-0.71	3.58	-6.907	2.866	11	7/24/2014	32:56.8
9390	RWS11	-122.4727641	37.94093	-0.63	3.6	-3.33	2.96000004	-122.47276	37.94094	-0.71	3.6	-6.932	2.88500005	11	7/24/2014	32:56.9
9391	RWS11	-122.4727678	37.94093	-0.67	3.61	-3.505	2.94399989	-122.47276	37.94094	-0.76	3.61	-7.215	2.85199994	11	7/24/2014	32:57.0
9392	RWS11	-122.4727702	37.94093	-0.63	3.63	-3.57	2.99399996	-122.47277	37.94094	-0.71	3.63	-7.303	2.91899997	11	7/24/2014	32:57.1
9393	RWS11	-122.4727727	37.94093	-0.67	3.64	-3.723	2.972	-122.47277	37.94094	-0.76	3.64	-7.39	2.88000005	11	7/24/2014	32:57.2
9394	RWS11	-122.4727752	37.94093	-0.63	3.65	-3.701	3.0150001	-122.47277	37.94094	-0.68	3.65	-7.521	2.9740001	11	7/24/2014	32:57.3
9395	RWS11	-122.4727777	37.94093	-0.67	3.66	-3.855	2.98699987	-122.47277	37.94094	-0.71	3.66	-7.675	2.94599992	11	7/24/2014	32:57.4
9396	RWS11	-122.4727803	37.94093	-0.67	3.66	-3.902	2.98999989	-122.47278	37.94094	-0.71	3.66	-7.808	2.94899994	11	7/24/2014	32:57.5
9397	RWS11	-122.4727828	37.94093	-0.67	3.66	-4.098	2.98999989	-122.47278	37.94094	-0.71	3.66	-8.005	2.94899994	11	7/24/2014	32:57.6
9398	RWS11	-122.4727854	37.94093	-0.67	3.66	-4.12	2.98999989	-122.47278	37.94095	-0.71	3.66	-8.136	2.94899994	11	7/24/2014	32:57.7
9399	RWS11	-122.472788	37.94093	-0.67	3.66	-4.207	2.98999989	-122.47278	37.94095	-0.76	3.66	-8.114	2.89799994	11	7/24/2014	32:57.8
9400	RWS11	-122.4727906	37.94093	-0.67	3.66	-4.36	2.98800004	-122.47279	37.94095	-0.71	3.66	-8.179	2.94700009	11	7/24/2014	32:57.9
9401	RWS11	-122.4727932	37.94093	-0.67	3.65	-4.426	2.98299992	-122.47279	37.94095	-0.76	3.65	-8.268	2.89099997	11	7/24/2014	32:58.0
9402	RWS11	-122.4727958	37.94093	-0.67	3.64	-4.517	2.97299993	-122.47279	37.94095	-0.76	3.64	-8.227	2.88099998	11	7/24/2014	32:58.1
1	RWD11	-122.451	37.935	-0.41	15.80	-430.77	15.39	-122.451	37.935	-0.21	15.80	-445.84	15.58	11	8/1/2014	06:45.1
2	RWD11	-122.451	37.935	-0.41	15.84	-432.70	15.43	-122.451	37.935	-0.18	15.84	-447.58	15.66	11	8/1/2014	06:45.2

3	RWD11	-122.451	37.935	-0.37	15.83	-435.07	15.46	-122.451	37.935	-0.21	15.83	-449.47	15.62	11	8/1/2014	06:45.3
4	RWD11	-122.451	37.935	-0.37	15.85	-437.59	15.47	-122.451	37.935	-0.18	15.85	-451.63	15.67	11	8/1/2014	06:45.4
5	RWD11	-122.451	37.935	-0.37	15.84	-440.37	15.47	-122.451	37.935	-0.18	15.84	-454.08	15.66	11	8/1/2014	06:45.5
6	RWD11	-122.451	37.935	-0.41	15.86	-443.16	15.45	-122.451	37.935	-0.13	15.86	-456.80	15.73	11	8/1/2014	06:45.6
7	RWD11	-122.451	37.935	-0.37	15.84	-445.96	15.47	-122.451	37.935	-0.04	15.84	-459.68	15.80	11	8/1/2014	06:45.7
8	RWD11	-122.451	37.935	-0.41	15.84	-448.63	15.43	-122.451	37.935	-0.09	15.84	-462.68	15.75	11	8/1/2014	06:45.8
9	RWD11	-122.451	37.935	-0.37	15.86	-451.02	15.48	-122.451	37.935	-0.04	15.86	-465.59	15.81	11	8/1/2014	06:45.9
10	RWD11	-122.451	37.935	-0.37	15.88	-453.26	15.51	-122.451	37.935	-0.01	15.88	-468.48	15.87	11	8/1/2014	06:46.0
11	RWD11	-122.451	37.935	-0.34	15.90	-455.36	15.56	-122.451	37.935	0.08	15.90	-471.25	15.98	11	8/1/2014	06:46.1
12	RWD11	-122.451	37.935	-0.37	15.93	-457.48	15.55	-122.451	37.935	0.03	15.93	-473.94	15.95	11	8/1/2014	06:46.2
13	RWD11	-122.451	37.935	-0.34	15.93	-459.77	15.59	-122.451	37.935	0.08	15.93	-476.69	16.01	11	8/1/2014	06:46.3
14	RWD11	-122.451	37.935	-0.34	15.97	-462.41	15.63	-122.451	37.935	0.16	15.97	-479.52	16.13	11	8/1/2014	06:46.4
15	RWD11	-122.451	37.935	-0.29	16.02	-465.33	15.73	-122.451	37.935	0.16	16.02	-482.53	16.18	11	8/1/2014	06:46.5
16	RWD11	-122.451	37.935	-0.34	16.00	-468.49	15.66	-122.451	37.935	0.19	16.00	-485.73	16.20	11	8/1/2014	06:46.6
17	RWD11	-122.451	37.935	-0.34	16.03	-471.99	15.69	-122.451	37.935	0.28	16.03	-489.19	16.31	11	8/1/2014	06:46.7
18	RWD11	-122.451	37.935	-0.29	16.03	-475.56	15.75	-122.451	37.935	0.28	16.03	-492.78	16.31	11	8/1/2014	06:46.8
19	RWD11	-122.451	37.935	-0.29	16.05	-479.19	15.76	-122.451	37.935	0.33	16.05	-496.46	16.38	11	8/1/2014	06:46.9
20	RWD11	-122.451	37.935	-0.29	16.05	-482.85	15.76	-122.451	37.935	0.36	16.05	-500.28	16.42	11	8/1/2014	06:47.0
21	RWD11	-122.451	37.935	-0.25	16.06	-486.49	15.80	-122.451	37.935	0.36	16.06	-504.16	16.42	11	8/1/2014	06:47.1
22	RWD11	-122.451	37.935	-0.25	16.05	-490.14	15.80	-122.451	37.935	0.36	16.05	-508.11	16.42	11	8/1/2014	06:47.2
23	RWD11	-122.451	37.935	-0.25	16.13	-493.70	15.87	-122.451	37.935	0.40	16.13	-512.01	16.52	11	8/1/2014	06:47.3
24	RWD11	-122.451	37.935	-0.25	16.11	-497.07	15.86	-122.451	37.935	0.45	16.11	-515.73	16.56	11	8/1/2014	06:47.4
25	RWD11	-122.451	37.935	-0.20	16.10	-500.27	15.90	-122.451	37.935	0.45	16.10	-519.28	16.55	11	8/1/2014	06:47.5
26	RWD11	-122.451	37.935	-0.20	16.15	-503.31	15.95	-122.451	37.935	0.48	16.15	-522.63	16.63	11	8/1/2014	06:47.6
27	RWD11	-122.451	37.935	-0.20	16.20	-506.16	15.99	-122.451	37.935	0.53	16.20	-525.81	16.73	11	8/1/2014	06:47.7
28	RWD11	-122.451	37.935	-0.20	16.32	-508.95	16.12	-122.451	37.935	0.48	16.32	-528.91	16.80	11	8/1/2014	06:47.8
29	RWD11	-122.451	37.935	-0.17	16.30	-511.73	16.13	-122.451	37.935	0.57	16.30	-531.88	16.86	11	8/1/2014	06:47.9
30	RWD11	-122.451	37.935	-0.17	16.39	-514.56	16.22	-122.451	37.935	0.62	16.39	-535.00	17.01	11	8/1/2014	06:48.0
31	RWD11	-122.451	37.935	-0.17	16.39	-517.46	16.22	-122.451	37.935	0.62	16.39	-538.02	17.01	11	8/1/2014	06:48.1
32	RWD11	-122.451	37.935	-0.17	16.48	-520.54	16.31	-122.451	37.935	0.65	16.48	-541.24	17.13	11	8/1/2014	06:48.2
33	RWD11	-122.451	37.935	-0.12	16.51	-523.64	16.39	-122.451	37.935	0.70	16.51	-544.50	17.21	11	8/1/2014	06:48.3
34	RWD11	-122.451	37.935	-0.12	16.53	-526.98	16.42	-122.451	37.935	0.70	16.53	-547.90	17.24	11	8/1/2014	06:48.4
35	RWD11	-122.451	37.935	-0.08	16.58	-530.32	16.49	-122.451	37.935	0.77	16.58	-551.46	17.35	11	8/1/2014	06:48.5

36	RWD11	-122.451	37.935	-0.08	16.65	-533.74	16.57	-122.451	37.935	0.74	16.65	-555.05	17.39	11	8/1/2014	06:48.6
37	RWD11	-122.451	37.935	-0.08	16.67	-537.18	16.59	-122.451	37.935	0.77	16.67	-558.64	17.44	11	8/1/2014	06:48.7
38	RWD11	-122.451	37.935	-0.08	16.77	-540.60	16.69	-122.451	37.935	0.82	16.77	-562.27	17.59	11	8/1/2014	06:48.8
39	RWD11	-122.451	37.935	0.00	16.82	-544.03	16.82	-122.451	37.935	0.82	16.82	-565.94	17.64	11	8/1/2014	06:48.9
40	RWD11	-122.451	37.935	-0.03	16.85	-547.49	16.82	-122.451	37.935	0.85	16.85	-569.59	17.71	11	8/1/2014	06:49.0
41	RWD11	-122.451	37.935	0.00	16.90	-550.94	16.90	-122.451	37.935	0.85	16.90	-573.24	17.75	11	8/1/2014	06:49.1
42	RWD11	-122.451	37.935	0.00	17.05	-554.40	17.05	-122.451	37.935	0.90	17.05	-576.87	17.96	11	8/1/2014	06:49.2
43	RWD11	-122.451	37.935	0.04	17.00	-557.83	17.04	-122.451	37.935	0.94	17.00	-580.45	17.94	11	8/1/2014	06:49.3
44	RWD11	-122.451	37.935	0.04	17.11	-561.20	17.14	-122.451	37.935	0.94	17.11	-584.05	18.05	11	8/1/2014	06:49.4
45	RWD11	-122.451	37.935	0.09	17.15	-564.46	17.24	-122.451	37.935	0.99	17.15	-587.53	18.14	11	8/1/2014	06:49.5
46	RWD11	-122.451	37.935	0.09	17.19	-567.72	17.28	-122.451	37.935	0.99	17.19	-591.08	18.18	11	8/1/2014	06:49.6
47	RWD11	-122.451	37.935	0.12	17.20	-570.96	17.32	-122.451	37.935	1.02	17.20	-594.56	18.22	11	8/1/2014	06:49.7
48	RWD11	-122.451	37.935	0.12	17.25	-574.14	17.37	-122.451	37.935	1.06	17.25	-598.15	18.31	11	8/1/2014	06:49.8
49	RWD11	-122.451	37.935	0.17	17.33	-577.31	17.50	-122.451	37.935	1.06	17.33	-601.65	18.39	11	8/1/2014	06:49.9
50	RWD11	-122.451	37.935	0.12	17.32	-580.40	17.44	-122.451	37.935	1.02	17.32	-605.10	18.34	11	8/1/2014	06:50.0
51	RWD11	-122.451	37.935	0.17	17.41	-583.51	17.58	-122.451	37.935	1.11	17.41	-608.51	18.51	11	8/1/2014	06:50.1
52	RWD11	-122.451	37.935	0.17	17.43	-586.46	17.60	-122.451	37.935	1.06	17.43	-611.70	18.49	11	8/1/2014	06:50.2
53	RWD11	-122.451	37.935	0.21	17.48	-589.49	17.68	-122.451	37.935	1.11	17.48	-614.95	18.58	11	8/1/2014	06:50.3
54	RWD11	-122.451	37.935	0.17	17.49	-592.57	17.67	-122.451	37.935	1.14	17.49	-618.13	18.63	11	8/1/2014	06:50.4
55	RWD11	-122.451	37.935	0.21	17.58	-595.52	17.79	-122.451	37.935	1.14	17.58	-621.21	18.73	11	8/1/2014	06:50.5
56	RWD11	-122.451	37.935	0.21	17.65	-598.51	17.85	-122.451	37.935	1.19	17.65	-624.27	18.84	11	8/1/2014	06:50.6
57	RWD11	-122.451	37.935	0.26	17.67	-601.50	17.93	-122.451	37.935	1.19	17.67	-627.35	18.86	11	8/1/2014	06:50.7
58	RWD11	-122.451	37.935	0.26	17.75	-604.54	18.01	-122.451	37.935	1.23	17.75	-630.43	18.97	11	8/1/2014	06:50.8
59	RWD11	-122.451	37.935	0.29	17.79	-607.60	18.08	-122.451	37.935	1.23	17.79	-633.61	19.02	11	8/1/2014	06:50.9
60	RWD11	-122.451	37.935	0.26	17.79	-610.52	18.05	-122.451	37.935	1.23	17.79	-636.73	19.02	11	8/1/2014	06:51.0
61	RWD11	-122.451	37.935	0.34	17.82	-613.41	18.17	-122.451	37.935	1.28	17.82	-639.81	19.10	11	8/1/2014	06:51.1
62	RWD11	-122.451	37.935	0.29	17.93	-616.20	18.22	-122.451	37.935	1.28	17.93	-642.86	19.21	11	8/1/2014	06:51.2
63	RWD11	-122.451	37.935	0.34	17.94	-618.94	18.29	-122.451	37.935	1.31	17.94	-645.88	19.25	11	8/1/2014	06:51.3
64	RWD11	-122.451	37.935	0.34	17.99	-621.63	18.34	-122.451	37.935	1.28	17.99	-648.83	19.27	11	8/1/2014	06:51.4
65	RWD11	-122.451	37.935	0.38	18.03	-624.27	18.41	-122.451	37.935	1.28	18.03	-651.75	19.31	11	8/1/2014	06:51.5
66	RWD11	-122.451	37.935	0.38	18.02	-626.94	18.40	-122.451	37.935	1.31	18.02	-654.68	19.33	11	8/1/2014	06:51.6
67	RWD11	-122.451	37.935	0.41	18.10	-629.57	18.51	-122.451	37.935	1.31	18.10	-657.57	19.41	11	8/1/2014	06:51.7
68	RWD11	-122.451	37.935	0.41	18.12	-632.26	18.54	-122.451	37.935	1.36	18.12	-660.48	19.49	11	8/1/2014	06:51.8

69	RWD11	-122.451	37.935	0.41	18.14	-634.96	18.55	-122.451	37.935	1.36	18.14	-663.40	19.50	11	8/1/2014	06:51.9
70	RWD11	-122.451	37.935	0.41	18.22	-637.58	18.63	-122.451	37.935	1.36	18.22	-666.22	19.58	11	8/1/2014	06:52.0
71	RWD11	-122.451	37.935	0.46	18.25	-640.20	18.71	-122.451	37.935	1.40	18.25	-669.04	19.64	11	8/1/2014	06:52.1
72	RWD11	-122.451	37.935	0.46	18.27	-642.72	18.74	-122.451	37.935	1.40	18.27	-671.70	19.67	11	8/1/2014	06:52.2
73	RWD11	-122.451	37.935	0.50	18.34	-645.24	18.84	-122.451	37.935	1.40	18.34	-674.40	19.74	11	8/1/2014	06:52.3
74	RWD11	-122.451	37.935	0.46	18.36	-647.71	18.82	-122.451	37.935	1.40	18.36	-676.97	19.75	11	8/1/2014	06:52.4
75	RWD11	-122.451	37.935	0.55	18.42	-650.17	18.97	-122.451	37.935	1.48	18.42	-679.56	19.90	11	8/1/2014	06:52.5
76	RWD11	-122.451	37.935	0.50	18.43	-652.58	18.93	-122.451	37.935	1.48	18.43	-682.12	19.91	11	8/1/2014	06:52.6
77	RWD11	-122.451	37.935	0.55	18.55	-655.01	19.10	-122.451	37.935	1.48	18.55	-684.64	20.03	11	8/1/2014	06:52.7
78	RWD11	-122.451	37.935	0.55	18.66	-657.53	19.21	-122.451	37.935	1.48	18.66	-687.22	20.14	11	8/1/2014	06:52.8
79	RWD11	-122.451	37.935	0.58	18.66	-660.07	19.25	-122.451	37.935	1.51	18.66	-689.80	20.18	11	8/1/2014	06:52.9
80	RWD11	-122.451	37.935	0.58	18.71	-662.61	19.30	-122.451	37.935	1.51	18.71	-692.39	20.23	11	8/1/2014	06:53.0
81	RWD11	-122.451	37.935	0.58	18.80	-665.15	19.39	-122.451	37.935	1.57	18.80	-694.97	20.37	11	8/1/2014	06:53.1
82	RWD11	-122.451	37.935	0.63	18.91	-667.73	19.55	-122.451	37.935	1.57	18.91	-697.55	20.48	11	8/1/2014	06:53.2
83	RWD11	-122.451	37.935	0.63	18.93	-670.25	19.56	-122.451	37.935	1.57	18.93	-700.15	20.49	11	8/1/2014	06:53.3
84	RWD11	-122.451	37.935	0.63	18.97	-672.81	19.61	-122.451	37.935	1.57	18.97	-702.71	20.54	11	8/1/2014	06:53.4
85	RWD11	-122.451	37.935	0.67	19.05	-675.26	19.72	-122.451	37.935	1.60	19.05	-705.31	20.65	11	8/1/2014	06:53.5
86	RWD11	-122.451	37.935	0.67	19.06	-677.69	19.73	-122.451	37.935	1.57	19.06	-707.85	20.62	11	8/1/2014	06:53.6
87	RWD11	-122.451	37.935	0.72	19.14	-680.14	19.86	-122.451	37.935	1.60	19.14	-710.46	20.73	11	8/1/2014	06:53.7
88	RWD11	-122.451	37.935	0.67	19.17	-682.58	19.84	-122.451	37.935	1.65	19.17	-713.13	20.82	11	8/1/2014	06:53.8
89	RWD11	-122.451	37.935	0.72	19.20	-685.05	19.93	-122.451	37.935	1.65	19.20	-715.77	20.85	11	8/1/2014	06:53.9
90	RWD11	-122.451	37.935	0.72	19.25	-687.52	19.97	-122.451	37.935	1.65	19.25	-718.46	20.90	11	8/1/2014	06:54.0
91	RWD11	-122.451	37.935	0.76	19.37	-689.98	20.12	-122.451	37.935	1.73	19.37	-721.13	21.10	11	8/1/2014	06:54.1
92	RWD11	-122.451	37.935	0.76	19.48	-692.43	20.23	-122.451	37.935	1.73	19.48	-723.73	21.21	11	8/1/2014	06:54.2
93	RWD11	-122.451	37.935	0.79	19.57	-694.97	20.36	-122.451	37.935	1.73	19.57	-726.35	21.30	11	8/1/2014	06:54.3
94	RWD11	-122.451	37.935	0.79	19.59	-697.44	20.38	-122.451	37.935	1.77	19.59	-729.02	21.36	11	8/1/2014	06:54.4
95	RWD11	-122.451	37.935	0.84	19.68	-699.91	20.52	-122.451	37.935	1.80	19.68	-731.56	21.48	11	8/1/2014	06:54.5
96	RWD11	-122.451	37.935	0.84	19.76	-702.39	20.60	-122.451	37.935	1.77	19.76	-734.06	21.53	11	8/1/2014	06:54.6
97	RWD11	-122.451	37.935	0.88	19.75	-704.88	20.63	-122.451	37.935	1.77	19.75	-736.55	21.52	11	8/1/2014	06:54.7
98	RWD11	-122.451	37.935	0.84	19.83	-707.29	20.67	-122.451	37.935	1.77	19.83	-739.05	21.60	11	8/1/2014	06:54.8
99	RWD11	-122.451	37.935	0.88	19.92	-709.66	20.79	-122.451	37.935	1.77	19.92	-741.48	21.68	11	8/1/2014	06:54.9
100	RWD11	-122.451	37.935	0.88	19.92	-711.96	20.79	-122.451	37.935	1.80	19.92	-743.80	21.72	11	8/1/2014	06:55.0
101	RWD11	-122.451	37.935	0.93	19.97	-714.20	20.89	-122.451	37.935	1.80	19.97	-746.10	21.77	11	8/1/2014	06:55.1

102	RWD11	-122.451	37.935	0.93	20.00	-716.37	20.93	-122.451	37.935	1.85	20.00	-748.47	21.85	11	8/1/2014	06:55.2
103	RWD11	-122.451	37.935	0.96	20.08	-718.56	21.04	-122.451	37.935	1.80	20.08	-750.81	21.88	11	8/1/2014	06:55.3
104	RWD11	-122.451	37.935	0.96	20.05	-720.61	21.01	-122.451	37.935	1.85	20.05	-753.01	21.90	11	8/1/2014	06:55.4
105	RWD11	-122.451	37.935	0.96	20.10	-722.63	21.06	-122.451	37.935	1.89	20.10	-755.27	21.99	11	8/1/2014	06:55.5
106	RWD11	-122.451	37.935	0.96	20.15	-724.65	21.11	-122.451	37.935	1.85	20.15	-757.52	22.01	11	8/1/2014	06:55.6
107	RWD11	-122.451	37.935	1.01	20.19	-726.78	21.21	-122.451	37.935	1.89	20.19	-759.84	22.08	11	8/1/2014	06:55.7
108	RWD11	-122.451	37.935	1.01	20.17	-728.89	21.18	-122.451	37.935	1.89	20.17	-762.08	22.05	11	8/1/2014	06:55.8
109	RWD11	-122.451	37.935	1.01	20.17	-730.83	21.18	-122.451	37.935	1.94	20.17	-764.25	22.10	11	8/1/2014	06:55.9
110	RWD11	-122.451	37.935	1.01	20.17	-732.85	21.18	-122.451	37.935	1.89	20.17	-766.38	22.05	11	8/1/2014	06:56.0
111	RWD11	-122.451	37.935	1.05	20.14	-734.78	21.19	-122.451	37.935	1.94	20.14	-768.45	22.08	11	8/1/2014	06:56.1
112	RWD11	-122.451	37.935	1.05	20.18	-736.80	21.23	-122.451	37.935	1.94	20.18	-770.51	22.12	11	8/1/2014	06:56.2
113	RWD11	-122.451	37.935	1.08	20.13	-738.74	21.21	-122.451	37.935	1.97	20.13	-772.53	22.10	11	8/1/2014	06:56.3
114	RWD11	-122.451	37.935	1.05	20.14	-740.66	21.19	-122.451	37.935	1.94	20.14	-774.56	22.08	11	8/1/2014	06:56.4
115	RWD11	-122.451	37.935	1.08	20.17	-742.61	21.25	-122.451	37.935	1.94	20.17	-776.60	22.10	11	8/1/2014	06:56.5
116	RWD11	-122.451	37.935	1.08	20.08	-744.53	21.16	-122.451	37.935	1.94	20.08	-778.64	22.02	11	8/1/2014	06:56.6
117	RWD11	-122.451	37.935	1.13	20.08	-746.42	21.22	-122.451	37.935	1.97	20.08	-780.67	22.05	11	8/1/2014	06:56.7
118	RWD11	-122.451	37.935	1.13	20.06	-748.33	21.19	-122.451	37.935	1.97	20.06	-782.71	22.03	11	8/1/2014	06:56.8
119	RWD11	-122.451	37.935	1.17	20.05	-750.27	21.21	-122.451	37.935	2.02	20.05	-784.77	22.07	11	8/1/2014	06:56.9
120	RWD11	-122.451	37.935	1.13	20.07	-752.14	21.20	-122.451	37.935	1.97	20.07	-786.77	22.04	11	8/1/2014	06:57.0
121	RWD11	-122.451	37.935	1.17	20.06	-753.99	21.22	-122.451	37.935	2.02	20.06	-788.73	22.08	11	8/1/2014	06:57.1
122	RWD11	-122.451	37.935	1.17	20.03	-755.81	21.19	-122.451	37.935	2.06	20.03	-790.68	22.08	11	8/1/2014	06:57.2
123	RWD11	-122.451	37.935	1.22	20.06	-757.71	21.27	-122.451	37.935	2.06	20.06	-792.60	22.11	11	8/1/2014	06:57.3
124	RWD11	-122.451	37.935	1.22	20.02	-759.60	21.24	-122.451	37.935	2.06	20.02	-794.56	22.08	11	8/1/2014	06:57.4
125	RWD11	-122.451	37.935	1.22	20.02	-761.53	21.24	-122.451	37.935	2.06	20.02	-796.55	22.08	11	8/1/2014	06:57.5
126	RWD11	-122.451	37.935	1.22	19.99	-763.49	21.20	-122.451	37.935	2.06	19.99	-798.51	22.04	11	8/1/2014	06:57.6
127	RWD11	-122.451	37.935	1.25	19.97	-765.49	21.22	-122.451	37.935	2.11	19.97	-800.51	22.08	11	8/1/2014	06:57.7
128	RWD11	-122.451	37.935	1.25	19.94	-767.38	21.19	-122.451	37.935	2.11	19.94	-802.49	22.04	11	8/1/2014	06:57.8
129	RWD11	-122.451	37.935	1.30	19.95	-769.36	21.25	-122.451	37.935	2.14	19.95	-804.51	22.09	11	8/1/2014	06:57.9
130	RWD11	-122.451	37.935	1.25	19.93	-771.34	21.18	-122.451	37.935	2.14	19.93	-806.55	22.07	11	8/1/2014	06:58.0
131	RWD11	-122.451	37.935	1.30	19.90	-773.29	21.20	-122.451	37.935	2.14	19.90	-808.62	22.04	11	8/1/2014	06:58.1
132	RWD11	-122.451	37.935	1.25	19.91	-775.19	21.16	-122.451	37.935	2.14	19.91	-810.64	22.05	11	8/1/2014	06:58.2
133	RWD11	-122.451	37.935	1.34	19.92	-776.97	21.25	-122.451	37.935	2.14	19.92	-812.55	22.06	11	8/1/2014	06:58.3
134	RWD11	-122.451	37.935	1.30	19.87	-778.84	21.17	-122.451	37.935	2.17	19.87	-814.49	22.04	11	8/1/2014	06:58.4

135	RWD11	-122.451	37.935	1.34	19.86	-780.63	21.20	-122.451	37.935	2.22	19.86	-816.40	22.09	11	8/1/2014 06:58.5
136	RWD11	-122.451	37.935	1.34	19.93	-782.39	21.27	-122.451	37.935	2.22	19.93	-818.27	22.15	11	8/1/2014 06:58.6
137	RWD11	-122.451	37.935	1.39	19.79	-784.13	21.18	-122.451	37.935	2.26	19.79	-820.10	22.05	11	8/1/2014 06:58.7
138	RWD11	-122.451	37.935	1.34	19.78	-785.98	21.12	-122.451	37.935	2.26	19.78	-822.04	22.04	11	8/1/2014 06:58.8
139	RWD11	-122.451	37.935	1.42	19.71	-787.79	21.13	-122.451	37.935	2.31	19.71	-823.91	22.02	11	8/1/2014 06:58.9
140	RWD11	-122.451	37.935	1.39	19.78	-789.68	21.17	-122.451	37.935	2.31	19.78	-825.90	22.09	11	8/1/2014 06:59.0
141	RWD11	-122.451	37.935	1.42	19.56	-791.54	20.98	-122.451	37.935	2.31	19.56	-827.83	21.87	11	8/1/2014 06:59.1
142	RWD11	-122.451	37.935	1.42	19.43	-793.44	20.85	-122.451	37.935	2.31	19.43	-829.77	21.74	11	8/1/2014 06:59.2
143	RWD11	-122.451	37.935	1.46	19.34	-795.29	20.79	-122.451	37.935	2.31	19.34	-831.67	21.65	11	8/1/2014 06:59.3
144	RWD11	-122.451	37.935	1.42	19.31	-797.12	20.73	-122.451	37.935	2.31	19.31	-833.51	21.62	11	8/1/2014 06:59.4
145	RWD11	-122.451	37.935	1.46	19.25	-798.88	20.71	-122.451	37.935	2.31	19.25	-835.28	21.56	11	8/1/2014 06:59.5
146	RWD11	-122.451	37.935	1.46	19.24	-800.60	20.69	-122.451	37.935	2.31	19.24	-837.08	21.55	11	8/1/2014 06:59.6
147	RWD11	-122.451	37.935	1.51	19.23	-802.32	20.73	-122.451	37.935	2.34	19.23	-838.81	21.57	11	8/1/2014 06:59.7
148	RWD11	-122.451	37.935	1.51	19.07	-804.08	20.57	-122.451	37.935	2.31	19.07	-840.65	21.38	11	8/1/2014 06:59.8
149	RWD11	-122.451	37.935	1.51	19.08	-805.78	20.58	-122.451	37.935	2.34	19.08	-842.37	21.42	11	8/1/2014 06:59.9
150	RWD11	-122.451	37.935	1.51	19.05	-807.52	20.56	-122.451	37.935	2.34	19.05	-844.18	21.39	11	8/1/2014 07:00.0
151	RWD11	-122.451	37.935	1.54	18.90	-809.20	20.44	-122.451	37.935	2.39	18.90	-845.94	21.30	11	8/1/2014 07:00.1
152	RWD11	-122.451	37.935	1.54	18.95	-810.90	20.49	-122.451	37.935	2.39	18.95	-847.69	21.34	11	8/1/2014 07:00.2
153	RWD11	-122.451	37.935	1.54	18.70	-812.55	20.24	-122.451	37.935	2.43	18.70	-849.53	21.12	11	8/1/2014 07:00.3
154	RWD11	-122.451	37.935	1.54	18.59	-814.15	20.14	-122.451	37.935	2.39	18.59	-851.17	20.99	11	8/1/2014 07:00.4
155	RWD11	-122.451	37.935	1.59	18.55	-815.69	20.14	-122.451	37.935	2.43	18.55	-852.78	20.98	11	8/1/2014 07:00.5
156	RWD11	-122.451	37.935	1.59	18.45	-817.16	20.04	-122.451	37.935	2.39	18.45	-854.34	20.84	11	8/1/2014 07:00.6
157	RWD11	-122.451	37.935	1.63	18.38	-818.66	20.01	-122.451	37.935	2.43	18.38	-855.93	20.81	11	8/1/2014 07:00.7
158	RWD11	-122.451	37.935	1.59	18.36	-820.14	19.96	-122.451	37.935	2.43	18.36	-857.40	20.79	11	8/1/2014 07:00.8
159	RWD11	-122.451	37.935	1.63	18.27	-821.52	19.90	-122.451	37.935	2.43	18.27	-858.87	20.70	11	8/1/2014 07:00.9
160	RWD11	-122.451	37.935	1.63	18.23	-822.83	19.85	-122.451	37.935	2.43	18.23	-860.24	20.65	11	8/1/2014 07:01.0
161	RWD11	-122.451	37.935	1.68	18.05	-824.19	19.73	-122.451	37.935	2.48	18.05	-861.60	20.53	11	8/1/2014 07:01.1
162	RWD11	-122.451	37.935	1.68	18.08	-825.46	19.76	-122.451	37.935	2.48	18.08	-862.93	20.56	11	8/1/2014 07:01.2
163	RWD11	-122.451	37.935	1.71	17.99	-826.77	19.71	-122.451	37.935	2.48	17.99	-864.21	20.47	11	8/1/2014 07:01.3
164	RWD11	-122.451	37.935	1.68	17.91	-828.01	19.59	-122.451	37.935	2.48	17.91	-865.51	20.39	11	8/1/2014 07:01.4
165	RWD11	-122.451	37.935	1.71	17.74	-829.32	19.45	-122.451	37.935	2.51	17.74	-866.84	20.25	11	8/1/2014 07:01.5
166	RWD11	-122.451	37.935	1.71	17.72	-830.69	19.43	-122.451	37.935	2.51	17.72	-868.18	20.23	11	8/1/2014 07:01.6
167	RWD11	-122.451	37.935	1.76	17.66	-832.03	19.43	-122.451	37.935	2.55	17.66	-869.54	20.21	11	8/1/2014 07:01.7

168	RWD11	-122.451	37.935	1.76	17.56	-833.43	19.32	-122.451	37.935	2.55	17.56	-870.93	20.10	11	8/1/2014	07:01.8
169	RWD11	-122.451	37.935	1.76	17.45	-834.85	19.22	-122.451	37.935	2.55	17.45	-872.39	20.00	11	8/1/2014	07:01.9
170	RWD11	-122.451	37.935	1.76	17.43	-836.35	19.20	-122.451	37.935	2.55	17.43	-873.83	19.98	11	8/1/2014	07:02.0
171	RWD11	-122.451	37.935	1.80	17.29	-837.84	19.09	-122.451	37.935	2.55	17.29	-875.37	19.84	11	8/1/2014	07:02.1
172	RWD11	-122.451	37.935	1.83	17.18	-839.45	19.01	-122.451	37.935	2.55	17.18	-876.93	19.72	11	8/1/2014	07:02.2
173	RWD11	-122.451	37.935	1.88	17.07	-840.94	18.95	-122.451	37.935	2.55	17.07	-878.43	19.62	11	8/1/2014	07:02.3
174	RWD11	-122.451	37.935	2.00	17.04	-842.46	19.04	-122.451	37.935	2.55	17.04	-880.02	19.58	11	8/1/2014	07:02.4
175	RWD11	-122.451	37.935	2.05	16.93	-843.99	18.98	-122.451	37.935	2.63	16.93	-881.57	19.56	11	8/1/2014	07:02.5
176	RWD11	-122.451	37.935	2.05	16.87	-845.43	18.92	-122.451	37.935	2.60	16.87	-883.09	19.46	11	8/1/2014	07:02.6
177	RWD11	-122.451	37.935	2.05	16.78	-846.89	18.84	-122.451	37.935	2.60	16.78	-884.62	19.38	11	8/1/2014	07:02.7
178	RWD11	-122.451	37.935	2.05	16.81	-848.22	18.86	-122.451	37.935	2.63	16.81	-886.11	19.44	11	8/1/2014	07:02.8
179	RWD11	-122.451	37.935	2.05	16.56	-849.67	18.61	-122.451	37.935	2.68	16.56	-887.58	19.24	11	8/1/2014	07:02.9
180	RWD11	-122.451	37.935	2.21	16.53	-851.09	18.74	-122.451	37.935	2.68	16.53	-889.12	19.21	11	8/1/2014	07:03.0
181	RWD11	-122.451	37.935	2.26	16.47	-852.53	18.73	-122.451	37.935	2.71	16.47	-890.66	19.18	11	8/1/2014	07:03.1
182	RWD11	-122.451	37.935	2.21	16.29	-853.97	18.50	-122.451	37.935	2.71	16.29	-892.20	19.00	11	8/1/2014	07:03.2
183	RWD11	-122.451	37.935	2.26	16.34	-855.43	18.60	-122.451	37.935	2.71	16.34	-893.73	19.06	11	8/1/2014	07:03.3
184	RWD11	-122.451	37.935	2.21	16.17	-856.89	18.38	-122.451	37.935	2.71	16.17	-895.28	18.89	11	8/1/2014	07:03.4
185	RWD11	-122.451	37.935	2.26	16.03	-858.29	18.29	-122.451	37.935	2.77	16.03	-896.70	18.80	11	8/1/2014	07:03.5
186	RWD11	-122.451	37.935	2.26	15.94	-859.69	18.20	-122.451	37.935	2.77	15.94	-898.17	18.71	11	8/1/2014	07:03.6
187	RWD11	-122.451	37.935	2.26	15.81	-861.07	18.07	-122.451	37.935	2.80	15.81	-899.57	18.61	11	8/1/2014	07:03.7
188	RWD11	-122.451	37.935	2.21	15.81	-862.46	18.02	-122.451	37.935	2.77	15.81	-900.94	18.58	11	8/1/2014	07:03.8
189	RWD11	-122.451	37.935	2.26	15.69	-863.82	17.95	-122.451	37.935	2.80	15.69	-902.32	18.49	11	8/1/2014	07:03.9
190	RWD11	-122.451	37.935	2.26	15.45	-865.20	17.71	-122.451	37.935	2.80	15.45	-903.64	18.25	11	8/1/2014	07:04.0
191	RWD11	-122.451	37.935	2.29	15.39	-866.49	17.69	-122.451	37.935	2.83	15.39	-904.94	18.23	11	8/1/2014	07:04.1
192	RWD11	-122.451	37.935	2.26	15.45	-867.80	17.71	-122.451	37.935	2.83	15.45	-906.16	18.28	11	8/1/2014	07:04.2
193	RWD11	-122.451	37.935	2.29	15.22	-869.03	17.51	-122.451	37.935	2.83	15.22	-907.42	18.05	11	8/1/2014	07:04.3
194	RWD11	-122.451	37.935	2.26	15.11	-870.25	17.37	-122.451	37.935	2.83	15.11	-908.59	17.94	11	8/1/2014	07:04.4
195	RWD11	-122.451	37.935	2.43	15.08	-871.39	17.51	-122.451	37.935	2.88	15.08	-909.76	17.97	11	8/1/2014	07:04.5
196	RWD11	-122.451	37.935	2.38	14.92	-872.58	17.30	-122.451	37.935	2.88	14.92	-910.90	17.80	11	8/1/2014	07:04.6
197	RWD11	-122.451	37.935	2.43	14.77	-873.67	17.20	-122.451	37.935	2.88	14.77	-911.98	17.65	11	8/1/2014	07:04.7
198	RWD11	-122.451	37.935	2.38	14.82	-874.78	17.20	-122.451	37.935	2.88	14.82	-913.09	17.71	11	8/1/2014	07:04.8
199	RWD11	-122.451	37.935	2.43	14.68	-875.94	17.11	-122.451	37.935	2.92	14.68	-914.21	17.59	11	8/1/2014	07:04.9
200	RWD11	-122.451	37.935	2.43	14.54	-877.10	16.97	-122.451	37.935	2.92	14.54	-915.28	17.45	11	8/1/2014	07:05.0

201	RWD11	-122.451	37.935	2.43	14.49	-878.32	16.92	-122.451	37.935	2.92	14.49	-916.45	17.41	11	8/1/2014	07:05.1
202	RWD11	-122.451	37.935	2.38	14.45	-879.55	16.83	-122.451	37.935	2.92	14.45	-917.58	17.36	11	8/1/2014	07:05.2
203	RWD11	-122.451	37.935	2.43	14.28	-880.72	16.72	-122.451	37.935	2.97	14.28	-918.72	17.25	11	8/1/2014	07:05.3
204	RWD11	-122.451	37.935	2.43	14.22	-881.85	16.65	-122.451	37.935	2.92	14.22	-919.78	17.14	11	8/1/2014	07:05.4
205	RWD11	-122.451	37.935	2.46	14.22	-882.97	16.68	-122.451	37.935	2.97	14.22	-920.89	17.18	11	8/1/2014	07:05.5
206	RWD11	-122.451	37.935	2.43	14.18	-883.98	16.61	-122.451	37.935	2.97	14.18	-921.92	17.15	11	8/1/2014	07:05.6
207	RWD11	-122.451	37.935	2.46	14.04	-885.08	16.51	-122.451	37.935	3.00	14.04	-922.91	17.05	11	8/1/2014	07:05.7
208	RWD11	-122.451	37.935	2.55	14.06	-886.13	16.61	-122.451	37.935	3.00	14.06	-923.96	17.06	11	8/1/2014	07:05.8
209	RWD11	-122.451	37.935	2.59	13.98	-887.19	16.56	-122.451	37.935	3.05	13.98	-924.89	17.03	11	8/1/2014	07:05.9
210	RWD11	-122.451	37.935	2.59	13.87	-888.17	16.46	-122.451	37.935	3.00	13.87	-925.81	16.88	11	8/1/2014	07:06.0
211	RWD11	-122.451	37.935	2.59	13.90	-889.16	16.49	-122.451	37.935	3.05	13.90	-926.69	16.96	11	8/1/2014	07:06.1
212	RWD11	-122.451	37.935	2.59	13.80	-890.16	16.38	-122.451	37.935	3.05	13.80	-927.56	16.85	11	8/1/2014	07:06.2
213	RWD11	-122.451	37.935	2.64	13.79	-891.10	16.42	-122.451	37.935	3.09	13.79	-928.42	16.88	11	8/1/2014	07:06.3
214	RWD11	-122.451	37.935	2.59	13.66	-892.08	16.25	-122.451	37.935	3.05	13.66	-929.30	16.72	11	8/1/2014	07:06.4
215	RWD11	-122.451	37.935	2.59	13.64	-893.09	16.23	-122.451	37.935	3.09	13.64	-930.22	16.73	11	8/1/2014	07:06.5
216	RWD11	-122.451	37.935	2.59	13.61	-894.05	16.20	-122.451	37.935	3.09	13.61	-931.21	16.70	11	8/1/2014	07:06.6
217	RWD11	-122.451	37.935	2.64	13.56	-895.09	16.20	-122.451	37.935	3.09	13.56	-932.18	16.65	11	8/1/2014	07:06.7
218	RWD11	-122.451	37.935	2.59	13.54	-896.02	16.12	-122.451	37.935	3.09	13.54	-933.06	16.62	11	8/1/2014	07:06.8
219	RWD11	-122.451	37.935	2.59	13.53	-897.01	16.11	-122.451	37.935	3.14	13.53	-933.99	16.66	11	8/1/2014	07:06.9
220	RWD11	-122.451	37.935	2.59	13.44	-898.00	16.02	-122.451	37.935	3.17	13.44	-934.98	16.61	11	8/1/2014	07:07.0
221	RWD11	-122.451	37.935	2.64	13.39	-899.05	16.02	-122.451	37.935	3.17	13.39	-935.94	16.56	11	8/1/2014	07:07.1
222	RWD11	-122.451	37.935	2.59	13.27	-900.06	15.86	-122.451	37.935	3.17	13.27	-936.93	16.44	11	8/1/2014	07:07.2
223	RWD11	-122.451	37.935	2.64	13.27	-900.97	15.91	-122.451	37.935	3.21	13.27	-937.80	16.48	11	8/1/2014	07:07.3
224	RWD11	-122.451	37.935	2.76	13.28	-901.83	16.04	-122.451	37.935	3.21	13.28	-938.66	16.49	11	8/1/2014	07:07.4
225	RWD11	-122.451	37.935	2.81	13.17	-902.71	15.98	-122.451	37.935	3.21	13.17	-939.45	16.38	11	8/1/2014	07:07.5
226	RWD11	-122.451	37.935	2.76	13.06	-903.58	15.81	-122.451	37.935	3.21	13.06	-940.20	16.26	11	8/1/2014	07:07.6
227	RWD11	-122.451	37.935	2.81	12.99	-904.31	15.79	-122.451	37.935	3.21	12.99	-940.91	16.19	11	8/1/2014	07:07.7
228	RWD11	-122.451	37.935	2.81	12.95	-905.04	15.75	-122.451	37.935	3.21	12.95	-941.54	16.15	11	8/1/2014	07:07.8
229	RWD11	-122.451	37.935	2.81	12.90	-905.75	15.71	-122.451	37.935	3.26	12.90	-942.19	16.16	11	8/1/2014	07:07.9
230	RWD11	-122.451	37.935	2.81	12.85	-906.38	15.65	-122.451	37.935	3.26	12.85	-942.79	16.10	11	8/1/2014	07:08.0
231	RWD11	-122.451	37.935	2.81	12.85	-907.03	15.65	-122.451	37.935	3.34	12.85	-943.34	16.19	11	8/1/2014	07:08.1
232	RWD11	-122.451	37.935	2.81	12.75	-907.61	15.56	-122.451	37.935	3.29	12.75	-943.94	16.04	11	8/1/2014	07:08.2
233	RWD11	-122.451	37.935	2.81	12.67	-908.21	15.48	-122.451	37.935	3.29	12.67	-944.40	15.96	11	8/1/2014	07:08.3

234	RWD11	-122.451	37.935	2.81	12.60	-908.72	15.41	-122.451	37.935	3.29	12.60	-944.84	15.89	11	8/1/2014	07:08.4
235	RWD11	-122.451	37.935	2.84	12.56	-909.14	15.40	-122.451	37.935	3.34	12.56	-945.19	15.90	11	8/1/2014	07:08.5
236	RWD11	-122.451	37.935	2.81	12.49	-909.50	15.30	-122.451	37.935	3.29	12.49	-945.45	15.78	11	8/1/2014	07:08.6
237	RWD11	-122.451	37.935	2.84	12.51	-909.83	15.35	-122.451	37.935	3.29	12.51	-945.70	15.80	11	8/1/2014	07:08.7
238	RWD11	-122.451	37.935	2.81	12.35	-910.13	15.16	-122.451	37.935	3.34	12.35	-945.92	15.70	11	8/1/2014	07:08.8
239	RWD11	-122.451	37.935	2.84	12.35	-910.41	15.20	-122.451	37.935	3.34	12.35	-946.02	15.70	11	8/1/2014	07:08.9
240	RWD11	-122.451	37.935	2.81	12.31	-910.65	15.12	-122.451	37.935	3.34	12.31	-946.15	15.65	11	8/1/2014	07:09.0
241	RWD11	-122.451	37.935	2.84	12.16	-910.99	15.00	-122.451	37.935	3.38	12.16	-946.35	15.54	11	8/1/2014	07:09.1
242	RWD11	-122.451	37.935	2.93	12.16	-911.33	15.09	-122.451	37.935	3.34	12.16	-946.49	15.50	11	8/1/2014	07:09.2
243	RWD11	-122.451	37.935	2.96	12.07	-911.62	15.03	-122.451	37.935	3.38	12.07	-946.60	15.44	11	8/1/2014	07:09.3
244	RWD11	-122.451	37.935	2.96	12.12	-911.87	15.08	-122.451	37.935	3.38	12.12	-946.71	15.50	11	8/1/2014	07:09.4
245	RWD11	-122.451	37.935	2.96	12.01	-912.11	14.97	-122.451	37.935	3.43	12.01	-946.76	15.43	11	8/1/2014	07:09.5
246	RWD11	-122.451	37.935	2.96	11.87	-912.23	14.83	-122.451	37.935	3.43	11.87	-946.71	15.29	11	8/1/2014	07:09.6
247	RWD11	-122.451	37.935	2.96	11.84	-912.26	14.80	-122.451	37.935	3.43	11.84	-946.53	15.26	11	8/1/2014	07:09.7
248	RWD11	-122.451	37.935	2.96	11.77	-912.23	14.73	-122.451	37.935	3.43	11.77	-946.31	15.20	11	8/1/2014	07:09.8
249	RWD11	-122.451	37.935	3.01	11.69	-912.15	14.70	-122.451	37.935	3.43	11.69	-945.99	15.12	11	8/1/2014	07:09.9
250	RWD11	-122.451	37.935	2.96	11.63	-911.91	14.59	-122.451	37.935	3.43	11.63	-945.55	15.06	11	8/1/2014	07:10.0
251	RWD11	-122.451	37.935	3.01	11.58	-911.52	14.60	-122.451	37.935	3.46	11.58	-944.99	15.04	11	8/1/2014	07:10.1
252	RWD11	-122.451	37.935	2.96	11.53	-911.12	14.50	-122.451	37.935	3.43	11.53	-944.36	14.96	11	8/1/2014	07:10.2
253	RWD11	-122.451	37.935	3.01	11.47	-910.65	14.48	-122.451	37.935	3.46	11.47	-943.68	14.92	11	8/1/2014	07:10.3
254	RWD11	-122.451	37.935	2.96	11.40	-910.12	14.36	-122.451	37.935	3.46	11.40	-942.93	14.86	11	8/1/2014	07:10.4
255	RWD11	-122.451	37.935	3.01	11.36	-909.41	14.37	-122.451	37.935	3.46	11.36	-941.97	14.82	11	8/1/2014	07:10.5
256	RWD11	-122.451	37.935	3.01	11.28	-908.72	14.30	-122.451	37.935	3.46	11.28	-941.08	14.74	11	8/1/2014	07:10.6
257	RWD11	-122.451	37.935	3.01	11.26	-907.93	14.27	-122.451	37.935	3.46	11.26	-940.08	14.72	11	8/1/2014	07:10.7
258	RWD11	-122.451	37.935	2.96	11.20	-907.07	14.16	-122.451	37.935	3.46	11.20	-939.05	14.65	11	8/1/2014	07:10.8
259	RWD11	-122.451	37.935	3.01	11.13	-906.18	14.15	-122.451	37.935	3.51	11.13	-937.95	14.64	11	8/1/2014	07:10.9
260	RWD11	-122.451	37.935	3.01	11.06	-905.17	14.08	-122.451	37.935	3.51	11.06	-936.75	14.57	11	8/1/2014	07:11.0
261	RWD11	-122.451	37.935	3.01	11.09	-904.14	14.10	-122.451	37.935	3.46	11.09	-935.41	14.55	11	8/1/2014	07:11.1
262	RWD11	-122.451	37.935	3.01	10.96	-903.04	13.97	-122.451	37.935	3.51	10.96	-934.10	14.47	11	8/1/2014	07:11.2
263	RWD11	-122.451	37.935	3.01	10.94	-901.90	13.95	-122.451	37.935	3.51	10.94	-932.64	14.45	11	8/1/2014	07:11.3
264	RWD11	-122.451	37.935	3.01	10.87	-900.65	13.88	-122.451	37.935	3.51	10.87	-931.06	14.38	11	8/1/2014	07:11.4
265	RWD11	-122.451	37.935	3.01	10.89	-899.17	13.90	-122.451	37.935	3.51	10.89	-929.28	14.40	11	8/1/2014	07:11.5
266	RWD11	-122.451	37.935	3.01	10.78	-897.50	13.79	-122.451	37.935	3.46	10.78	-927.31	14.24	11	8/1/2014	07:11.6

267	RWD11	-122.451	37.935	3.01	10.73	-895.76	13.74	-122.451	37.935	3.46	10.73	-925.19	14.19	11	8/1/2014	07:11.7
268	RWD11	-122.451	37.935	3.01	10.76	-893.78	13.77	-122.451	37.935	3.46	10.76	-922.83	14.22	11	8/1/2014	07:11.8
269	RWD11	-122.451	37.935	3.01	10.66	-891.63	13.67	-122.451	37.935	3.51	10.66	-920.25	14.17	11	8/1/2014	07:11.9
270	RWD11	-122.451	37.935	3.01	10.64	-889.34	13.65	-122.451	37.935	3.46	10.64	-917.45	14.09	11	8/1/2014	07:12.0
271	RWD11	-122.451	37.935	3.01	10.59	-886.97	13.60	-122.451	37.935	3.46	10.59	-914.50	14.04	11	8/1/2014	07:12.1
272	RWD11	-122.451	37.935	2.96	10.54	-884.52	13.50	-122.451	37.935	3.42	10.54	-911.35	13.96	11	8/1/2014	07:12.2
273	RWD11	-122.451	37.935	3.01	10.50	-882.09	13.51	-122.451	37.935	3.42	10.50	-908.19	13.92	11	8/1/2014	07:12.3
274	RWD11	-122.451	37.935	3.01	10.46	-879.66	13.47	-122.451	37.935	3.42	10.46	-905.10	13.88	11	8/1/2014	07:12.4
275	RWD11	-122.451	37.935	3.01	10.43	-877.28	13.44	-122.451	37.935	3.42	10.43	-902.03	13.85	11	8/1/2014	07:12.5
276	RWD11	-122.451	37.935	3.01	10.42	-874.90	13.43	-122.451	37.935	3.37	10.42	-899.03	13.79	11	8/1/2014	07:12.6
277	RWD11	-122.451	37.935	3.01	10.41	-872.64	13.42	-122.451	37.935	3.42	10.41	-896.06	13.83	11	8/1/2014	07:12.7
278	RWD11	-122.451	37.935	3.01	10.40	-870.28	13.41	-122.451	37.935	3.37	10.40	-893.00	13.77	11	8/1/2014	07:12.8
279	RWD11	-122.451	37.935	3.01	10.36	-867.87	13.38	-122.451	37.935	3.46	10.36	-889.93	13.82	11	8/1/2014	07:12.9
280	RWD11	-122.451	37.935	3.01	10.36	-865.40	13.37	-122.451	37.935	3.37	10.36	-886.82	13.73	11	8/1/2014	07:13.0
281	RWD11	-122.451	37.935	3.01	10.32	-862.86	13.34	-122.451	37.935	3.42	10.32	-883.67	13.75	11	8/1/2014	07:13.1
282	RWD11	-122.451	37.935	3.01	10.30	-860.34	13.32	-122.451	37.935	3.42	10.30	-880.52	13.73	11	8/1/2014	07:13.2
283	RWD11	-122.451	37.935	3.01	10.30	-857.70	13.32	-122.451	37.935	3.42	10.30	-877.19	13.73	11	8/1/2014	07:13.3
284	RWD11	-122.451	37.935	3.01	10.26	-854.94	13.27	-122.451	37.935	3.42	10.26	-873.72	13.68	11	8/1/2014	07:13.4
285	RWD11	-122.451	37.935	3.01	10.25	-852.14	13.26	-122.451	37.935	3.37	10.25	-870.10	13.62	11	8/1/2014	07:13.5
286	RWD11	-122.451	37.935	3.01	10.21	-849.18	13.22	-122.451	37.935	3.42	10.21	-866.47	13.63	11	8/1/2014	07:13.6
287	RWD11	-122.451	37.935	3.01	10.21	-846.07	13.22	-122.451	37.935	3.37	10.21	-862.56	13.58	11	8/1/2014	07:13.7
288	RWD11	-122.451	37.935	3.01	10.19	-842.75	13.20	-122.451	37.935	3.34	10.19	-858.60	13.53	11	8/1/2014	07:13.8
289	RWD11	-122.451	37.935	3.01	10.16	-839.34	13.18	-122.451	37.935	3.37	10.16	-854.40	13.54	11	8/1/2014	07:13.9
290	RWD11	-122.451	37.935	3.01	10.14	-835.79	13.15	-122.451	37.935	3.34	10.14	-850.14	13.48	11	8/1/2014	07:14.0
291	RWD11	-122.451	37.935	3.01	10.13	-832.16	13.14	-122.451	37.935	3.34	10.13	-845.81	13.47	11	8/1/2014	07:14.1
292	RWD11	-122.451	37.935	3.01	10.12	-828.51	13.13	-122.451	37.935	3.34	10.12	-841.33	13.46	11	8/1/2014	07:14.2
293	RWD11	-122.451	37.935	3.01	10.09	-824.79	13.10	-122.451	37.935	3.37	10.09	-836.96	13.46	11	8/1/2014	07:14.3
294	RWD11	-122.451	37.935	3.01	10.06	-820.98	13.07	-122.451	37.935	3.34	10.06	-832.45	13.40	11	8/1/2014	07:14.4
295	RWD11	-122.451	37.935	3.05	10.06	-817.17	13.10	-122.451	37.935	3.34	10.06	-827.92	13.40	11	8/1/2014	07:14.5
296	RWD11	-122.451	37.935	3.01	10.06	-813.38	13.07	-122.451	37.935	3.34	10.06	-823.32	13.40	11	8/1/2014	07:14.6
297	RWD11	-122.451	37.935	3.05	10.03	-809.58	13.08	-122.451	37.935	3.37	10.03	-818.68	13.41	11	8/1/2014	07:14.7
298	RWD11	-122.451	37.935	2.96	10.07	-805.75	13.03	-122.451	37.935	3.29	10.07	-814.02	13.36	11	8/1/2014	07:14.8
299	RWD11	-122.451	37.935	3.01	10.06	-801.92	13.07	-122.451	37.935	3.34	10.06	-809.40	13.40	11	8/1/2014	07:14.9

300	RWD11	-122.451	37.935	3.01	10.06	-798.17	13.07	-122.451	37.935	3.29	10.06	-804.79	13.34	11	8/1/2014	07:15.0
301	RWD11	-122.451	37.935	3.01	10.12	-794.36	13.13	-122.451	37.935	3.34	10.12	-800.13	13.46	11	8/1/2014	07:15.1
302	RWD11	-122.451	37.935	3.01	10.13	-790.58	13.14	-122.451	37.935	3.29	10.13	-795.49	13.42	11	8/1/2014	07:15.2
303	RWD11	-122.451	37.935	3.05	10.13	-786.75	13.17	-122.451	37.935	3.34	10.13	-790.82	13.47	11	8/1/2014	07:15.3
304	RWD11	-122.451	37.935	3.01	10.10	-782.99	13.11	-122.451	37.935	3.34	10.10	-786.21	13.44	11	8/1/2014	07:15.4
305	RWD11	-122.451	37.935	3.05	10.13	-779.10	13.18	-122.451	37.935	3.34	10.13	-781.41	13.47	11	8/1/2014	07:15.5
306	RWD11	-122.451	37.935	3.01	10.15	-775.04	13.17	-122.451	37.935	3.34	10.15	-776.47	13.49	11	8/1/2014	07:15.6
307	RWD11	-122.451	37.935	3.01	10.16	-770.70	13.18	-122.451	37.935	3.34	10.16	-771.33	13.50	11	8/1/2014	07:15.7
308	RWD11	-122.451	37.935	3.01	10.17	-766.16	13.18	-122.451	37.935	3.29	10.17	-765.98	13.46	11	8/1/2014	07:15.8
309	RWD11	-122.451	37.935	3.01	10.22	-761.48	13.23	-122.451	37.935	3.29	10.22	-760.48	13.51	11	8/1/2014	07:15.9
310	RWD11	-122.451	37.935	3.01	10.27	-756.60	13.28	-122.451	37.935	3.29	10.27	-754.74	13.55	11	8/1/2014	07:16.0
311	RWD11	-122.451	37.935	3.01	10.29	-751.42	13.30	-122.451	37.935	3.29	10.29	-748.75	13.58	11	8/1/2014	07:16.1
312	RWD11	-122.451	37.935	3.01	10.29	-746.20	13.31	-122.451	37.935	3.26	10.29	-742.56	13.55	11	8/1/2014	07:16.2
313	RWD11	-122.451	37.935	3.01	10.36	-741.02	13.37	-122.451	37.935	3.29	10.36	-736.35	13.65	11	8/1/2014	07:16.3
314	RWD11	-122.451	37.935	3.01	10.37	-735.93	13.38	-122.451	37.935	3.26	10.37	-730.27	13.63	11	8/1/2014	07:16.4
315	RWD11	-122.451	37.935	3.05	10.43	-731.11	13.47	-122.451	37.935	3.26	10.43	-724.27	13.68	11	8/1/2014	07:16.5
316	RWD11	-122.451	37.935	3.01	10.44	-726.43	13.45	-122.451	37.935	3.26	10.44	-718.34	13.70	11	8/1/2014	07:16.6
317	RWD11	-122.451	37.935	3.01	10.48	-721.98	13.49	-122.451	37.935	3.26	10.48	-712.64	13.73	11	8/1/2014	07:16.7
318	RWD11	-122.451	37.935	3.01	10.50	-717.85	13.51	-122.451	37.935	3.26	10.50	-707.16	13.76	11	8/1/2014	07:16.8
319	RWD11	-122.451	37.935	3.05	10.53	-714.02	13.58	-122.451	37.935	3.29	10.53	-701.98	13.82	11	8/1/2014	07:16.9
320	RWD11	-122.452	37.935	3.01	10.56	-710.24	13.58	-122.451	37.935	3.26	10.56	-696.97	13.82	11	8/1/2014	07:17.0
321	RWD11	-122.452	37.935	3.05	10.60	-706.52	13.64	-122.451	37.935	3.29	10.60	-691.92	13.89	11	8/1/2014	07:17.1
322	RWD11	-122.452	37.935	3.01	10.63	-702.72	13.64	-122.451	37.935	3.29	10.63	-687.00	13.91	11	8/1/2014	07:17.2
323	RWD11	-122.452	37.935	3.05	10.63	-698.85	13.67	-122.451	37.935	3.37	10.63	-682.10	14.00	11	8/1/2014	07:17.3
324	RWD11	-122.452	37.935	3.01	10.69	-694.85	13.70	-122.452	37.935	3.34	10.69	-676.90	14.03	11	8/1/2014	07:17.4
325	RWD11	-122.452	37.935	3.05	10.71	-690.51	13.76	-122.452	37.935	3.34	10.71	-671.55	14.05	11	8/1/2014	07:17.5
326	RWD11	-122.452	37.935	3.01	10.73	-686.02	13.74	-122.452	37.935	3.29	10.73	-665.94	14.01	11	8/1/2014	07:17.6
327	RWD11	-122.452	37.935	3.05	10.76	-681.34	13.80	-122.452	37.935	3.34	10.76	-660.20	14.10	11	8/1/2014	07:17.7
328	RWD11	-122.452	37.935	3.01	10.84	-676.46	13.86	-122.452	37.935	3.29	10.84	-654.21	14.13	11	8/1/2014	07:17.8
329	RWD11	-122.452	37.935	3.01	10.90	-671.36	13.91	-122.452	37.935	3.34	10.90	-648.01	14.24	11	8/1/2014	07:17.9
330	RWD11	-122.452	37.935	3.01	11.01	-666.18	14.02	-122.452	37.935	3.29	11.01	-641.65	14.30	11	8/1/2014	07:18.0
331	RWD11	-122.452	37.935	3.05	11.10	-661.02	14.14	-122.452	37.935	3.29	11.10	-635.29	14.38	11	8/1/2014	07:18.1
332	RWD11	-122.452	37.935	3.01	11.13	-655.89	14.14	-122.452	37.935	3.29	11.13	-628.95	14.42	11	8/1/2014	07:18.2

333	RWD11	-122.452	37.935	3.01	11.20	-650.92	14.22	-122.452	37.935	3.29	11.20	-622.78	14.49	11	8/1/2014 07:18.3
334	RWD11	-122.452	37.935	3.01	11.30	-645.93	14.31	-122.452	37.935	3.29	11.30	-616.52	14.59	11	8/1/2014 07:18.4
335	RWD11	-122.452	37.935	3.05	11.31	-641.05	14.35	-122.452	37.935	3.29	11.31	-610.47	14.59	11	8/1/2014 07:18.5
336	RWD11	-122.452	37.935	3.01	11.33	-636.41	14.34	-122.452	37.935	3.29	11.33	-604.63	14.62	11	8/1/2014 07:18.6
337	RWD11	-122.452	37.935	3.05	11.38	-631.88	14.43	-122.452	37.935	3.29	11.38	-598.94	14.67	11	8/1/2014 07:18.7
338	RWD11	-122.452	37.935	3.01	11.41	-627.41	14.42	-122.452	37.935	3.29	11.41	-593.35	14.70	11	8/1/2014 07:18.8
339	RWD11	-122.452	37.935	3.05	11.52	-622.95	14.57	-122.452	37.935	3.29	11.52	-587.73	14.81	11	8/1/2014 07:18.9
340	RWD11	-122.452	37.935	3.01	11.51	-618.47	14.52	-122.452	37.935	3.29	11.51	-582.18	14.80	11	8/1/2014 07:19.0
341	RWD11	-122.452	37.935	3.05	11.60	-614.02	14.64	-122.452	37.935	3.34	11.60	-576.68	14.94	11	8/1/2014 07:19.1
342	RWD11	-122.452	37.935	3.01	11.67	-609.49	14.69	-122.452	37.935	3.37	11.67	-571.13	15.05	11	8/1/2014 07:19.2
343	RWD11	-122.452	37.935	3.05	11.75	-604.75	14.80	-122.452	37.935	3.29	11.75	-565.41	15.04	11	8/1/2014 07:19.3
344	RWD11	-122.452	37.935	3.01	11.80	-599.83	14.81	-122.452	37.935	3.29	11.80	-559.41	15.09	11	8/1/2014 07:19.4
345	RWD11	-122.452	37.935	3.05	11.81	-594.76	14.86	-122.452	37.935	3.29	11.81	-553.39	15.10	11	8/1/2014 07:19.5
346	RWD11	-122.452	37.935	3.01	11.94	-589.64	14.95	-122.452	37.935	3.34	11.94	-547.11	15.28	11	8/1/2014 07:19.6
347	RWD11	-122.452	37.935	3.05	12.03	-584.38	15.08	-122.452	37.935	3.26	12.03	-540.85	15.29	11	8/1/2014 07:19.7
348	RWD11	-122.452	37.935	3.05	12.06	-579.11	15.11	-122.452	37.935	3.29	12.06	-534.42	15.35	11	8/1/2014 07:19.8
349	RWD11	-122.452	37.935	3.05	12.18	-573.87	15.22	-122.452	37.935	3.29	12.18	-527.95	15.47	11	8/1/2014 07:19.9
350	RWD11	-122.452	37.935	3.01	12.20	-568.55	15.21	-122.452	37.935	3.26	12.20	-521.49	15.45	11	8/1/2014 07:20.0
351	RWD11	-122.452	37.935	3.05	12.35	-563.25	15.40	-122.452	37.935	3.29	12.35	-515.00	15.64	11	8/1/2014 07:20.1
352	RWD11	-122.452	37.935	3.01	12.44	-558.02	15.45	-122.452	37.935	3.26	12.44	-508.57	15.69	11	8/1/2014 07:20.2
353	RWD11	-122.452	37.935	3.01	12.53	-552.89	15.54	-122.452	37.935	3.26	12.53	-502.23	15.79	11	8/1/2014 07:20.3
354	RWD11	-122.452	37.935	3.01	12.58	-547.82	15.60	-122.452	37.935	3.20	12.58	-495.85	15.79	11	8/1/2014 07:20.4
355	RWD11	-122.452	37.935	3.01	12.67	-542.79	15.68	-122.452	37.935	3.20	12.67	-489.63	15.88	11	8/1/2014 07:20.5
356	RWD11	-122.452	37.935	3.01	12.82	-537.97	15.84	-122.452	37.935	3.20	12.82	-483.49	16.03	11	8/1/2014 07:20.6
357	RWD11	-122.452	37.935	3.01	12.91	-533.16	15.92	-122.452	37.935	3.20	12.91	-477.48	16.11	11	8/1/2014 07:20.7
358	RWD11	-122.452	37.935	3.01	12.92	-528.50	15.93	-122.452	37.935	3.20	12.92	-471.70	16.12	11	8/1/2014 07:20.8
359	RWD11	-122.452	37.935	3.01	13.03	-523.96	16.04	-122.452	37.935	3.20	13.03	-466.13	16.23	11	8/1/2014 07:20.9
360	RWD11	-122.452	37.935	3.01	13.19	-519.61	16.20	-122.452	37.935	3.17	13.19	-460.76	16.36	11	8/1/2014 07:21.0
361	RWD11	-122.452	37.935	3.01	13.33	-515.37	16.34	-122.452	37.935	3.17	13.33	-455.57	16.50	11	8/1/2014 07:21.1
362	RWD11	-122.452	37.935	3.01	13.44	-511.25	16.45	-122.452	37.935	3.17	13.44	-450.41	16.61	11	8/1/2014 07:21.2
363	RWD11	-122.452	37.935	3.01	13.46	-507.28	16.47	-122.452	37.935	3.14	13.46	-445.47	16.59	11	8/1/2014 07:21.3
364	RWD11	-122.452	37.935	3.01	13.62	-503.53	16.63	-122.452	37.935	3.14	13.62	-440.72	16.76	11	8/1/2014 07:21.4
365	RWD11	-122.452	37.935	3.01	13.69	-499.81	16.70	-122.452	37.935	3.14	13.69	-436.12	16.83	11	8/1/2014 07:21.5

366	RWD11	-122.452	37.935	2.96	13.73	-496.21	16.69	-122.452	37.935	3.09	13.73	-431.65	16.82	11	8/1/2014	07:21.6
367	RWD11	-122.452	37.935	3.01	13.84	-492.63	16.85	-122.452	37.935	3.14	13.84	-427.35	16.98	11	8/1/2014	07:21.7
368	RWD11	-122.452	37.935	2.96	13.90	-489.20	16.86	-122.452	37.935	3.09	13.90	-423.21	16.98	11	8/1/2014	07:21.8
369	RWD11	-122.452	37.935	3.01	14.14	-485.75	17.15	-122.452	37.935	3.09	14.14	-419.12	17.22	11	8/1/2014	07:21.9
370	RWD11	-122.452	37.935	2.96	14.22	-482.53	17.18	-122.452	37.935	3.05	14.22	-415.30	17.27	11	8/1/2014	07:22.0
371	RWD11	-122.452	37.935	3.01	14.32	-479.39	17.33	-122.452	37.935	3.09	14.32	-411.63	17.41	11	8/1/2014	07:22.1
372	RWD11	-122.452	37.935	2.96	14.46	-476.33	17.42	-122.452	37.935	3.00	14.46	-408.10	17.46	11	8/1/2014	07:22.2
373	RWD11	-122.452	37.935	3.01	14.55	-473.37	17.56	-122.452	37.935	3.05	14.55	-404.74	17.60	11	8/1/2014	07:22.3
374	RWD11	-122.452	37.935	2.96	14.68	-470.55	17.64	-122.452	37.935	3.00	14.68	-401.56	17.68	11	8/1/2014	07:22.4
375	RWD11	-122.452	37.935	2.96	14.90	-467.91	17.86	-122.452	37.935	3.05	14.90	-398.59	17.95	11	8/1/2014	07:22.5
376	RWD11	-122.452	37.935	2.96	14.99	-465.42	17.95	-122.452	37.935	3.00	14.99	-395.82	17.99	11	8/1/2014	07:22.6
377	RWD11	-122.452	37.935	2.96	14.99	-463.23	17.95	-122.452	37.935	2.97	14.99	-393.31	17.96	11	8/1/2014	07:22.7
378	RWD11	-122.452	37.935	2.96	15.07	-461.28	18.03	-122.452	37.935	3.00	15.07	-391.05	18.07	11	8/1/2014	07:22.8
379	RWD11	-122.452	37.935	2.96	15.21	-459.50	18.17	-122.452	37.935	2.97	15.21	-389.06	18.17	11	8/1/2014	07:22.9
380	RWD11	-122.452	37.935	2.96	15.27	-457.88	18.23	-122.452	37.935	2.97	15.27	-387.22	18.24	11	8/1/2014	07:23.0
381	RWD11	-122.452	37.935	2.96	15.48	-456.47	18.44	-122.452	37.935	2.97	15.48	-385.62	18.45	11	8/1/2014	07:23.1
382	RWD11	-122.452	37.935	2.96	15.43	-455.28	18.39	-122.452	37.935	2.97	15.43	-384.21	18.40	11	8/1/2014	07:23.2
383	RWD11	-122.452	37.935	2.96	15.57	-454.26	18.53	-122.452	37.935	2.97	15.57	-383.06	18.54	11	8/1/2014	07:23.3
384	RWD11	-122.452	37.935	2.93	15.70	-453.48	18.63	-122.452	37.935	2.97	15.70	-382.20	18.67	11	8/1/2014	07:23.4
385	RWD11	-122.452	37.935	2.96	15.77	-452.92	18.73	-122.452	37.935	2.97	15.77	-381.72	18.74	11	8/1/2014	07:23.5
386	RWD11	-122.452	37.935	2.92	15.87	-452.64	18.80	-122.452	37.935	2.92	15.87	-381.52	18.79	11	8/1/2014	07:23.6
387	RWD11	-122.452	37.935	2.96	16.06	-452.49	19.02	-122.452	37.935	2.92	16.06	-381.57	18.97	11	8/1/2014	07:23.7
388	RWD11	-122.452	37.935	2.92	16.17	-452.55	19.09	-122.452	37.935	2.88	16.17	-381.72	19.05	11	8/1/2014	07:23.8
389	RWD11	-122.452	37.935	2.92	16.30	-452.79	19.23	-122.452	37.935	2.88	16.30	-382.15	19.19	11	8/1/2014	07:23.9
390	RWD11	-122.452	37.935	2.92	16.55	-453.25	19.47	-122.452	37.935	2.88	16.55	-382.82	19.43	11	8/1/2014	07:24.0
391	RWD11	-122.452	37.935	2.92	16.65	-453.92	19.57	-122.452	37.935	2.88	16.65	-383.73	19.53	11	8/1/2014	07:24.1
392	RWD11	-122.452	37.935	2.92	16.60	-454.72	19.53	-122.452	37.935	2.88	16.60	-384.90	19.49	11	8/1/2014	07:24.2
393	RWD11	-122.452	37.935	2.92	16.84	-455.71	19.77	-122.452	37.935	2.88	16.84	-386.24	19.73	11	8/1/2014	07:24.3
394	RWD11	-122.452	37.935	2.87	17.03	-456.77	19.91	-122.452	37.935	2.83	17.03	-387.78	19.86	11	8/1/2014	07:24.4
395	RWD11	-122.452	37.935	2.92	17.11	-458.01	20.03	-122.452	37.935	2.83	17.11	-389.56	19.94	11	8/1/2014	07:24.5
396	RWD11	-122.452	37.935	2.87	17.23	-459.38	20.10	-122.452	37.935	2.83	17.23	-391.44	20.06	11	8/1/2014	07:24.6
397	RWD11	-122.452	37.935	2.92	17.29	-460.91	20.22	-122.452	37.935	2.80	17.29	-393.59	20.09	11	8/1/2014	07:24.7
398	RWD11	-122.452	37.935	2.87	17.26	-462.61	20.13	-122.452	37.935	2.76	17.26	-395.80	20.02	11	8/1/2014	07:24.8

399	RWD11	-122.452	37.935	2.87	17.46	-464.42	20.33	-122.452	37.935	2.76	17.46	-398.18	20.22	11	8/1/2014	07:24.9
400	RWD11	-122.452	37.935	2.87	17.42	-466.48	20.29	-122.452	37.935	2.76	17.42	-400.89	20.18	11	8/1/2014	07:25.0
401	RWD11	-122.452	37.935	2.87	17.47	-468.69	20.34	-122.452	37.935	2.80	17.47	-403.81	20.27	11	8/1/2014	07:25.1
402	RWD11	-122.452	37.935	2.84	17.59	-471.33	20.43	-122.452	37.935	2.76	17.59	-407.10	20.36	11	8/1/2014	07:25.2
403	RWD11	-122.452	37.935	2.87	17.59	-474.11	20.47	-122.452	37.935	2.80	17.59	-410.68	20.39	11	8/1/2014	07:25.3
404	RWD11	-122.452	37.935	2.84	17.63	-476.96	20.47	-122.452	37.935	2.76	17.63	-414.35	20.40	11	8/1/2014	07:25.4
405	RWD11	-122.452	37.935	2.87	17.63	-479.99	20.50	-122.452	37.935	2.80	17.63	-418.25	20.42	11	8/1/2014	07:25.5
406	RWD11	-122.452	37.935	2.84	17.65	-483.29	20.49	-122.452	37.935	2.76	17.65	-422.45	20.42	11	8/1/2014	07:25.6
407	RWD11	-122.452	37.935	2.84	17.74	-486.82	20.58	-122.452	37.935	2.76	17.74	-426.93	20.50	11	8/1/2014	07:25.7
408	RWD11	-122.452	37.935	2.84	17.76	-490.02	20.60	-122.452	37.935	2.71	17.76	-431.16	20.48	11	8/1/2014	07:25.8
409	RWD11	-122.452	37.935	2.87	17.85	-493.35	20.73	-122.452	37.935	2.71	17.85	-435.39	20.57	11	8/1/2014	07:25.9
410	RWD11	-122.452	37.935	2.84	17.88	-496.91	20.72	-122.452	37.935	2.68	17.88	-439.91	20.56	11	8/1/2014	07:26.0
411	RWD11	-122.452	37.935	2.84	17.90	-500.48	20.74	-122.452	37.935	2.68	17.90	-444.50	20.58	11	8/1/2014	07:26.1
412	RWD11	-122.452	37.935	2.84	17.89	-504.10	20.73	-122.452	37.935	2.68	17.89	-449.18	20.57	11	8/1/2014	07:26.2
413	RWD11	-122.452	37.935	2.84	17.92	-507.82	20.76	-122.452	37.935	2.68	17.92	-453.85	20.59	11	8/1/2014	07:26.3
414	RWD11	-122.452	37.935	2.81	17.96	-511.44	20.76	-122.452	37.935	2.68	17.96	-458.52	20.63	11	8/1/2014	07:26.4
415	RWD11	-122.452	37.935	2.81	17.95	-515.16	20.75	-122.452	37.935	2.68	17.95	-463.25	20.63	11	8/1/2014	07:26.5
416	RWD11	-122.452	37.935	2.81	17.97	-518.95	20.78	-122.452	37.935	2.63	17.97	-468.09	20.60	11	8/1/2014	07:26.6
417	RWD11	-122.452	37.935	2.84	17.96	-522.87	20.80	-122.452	37.935	2.63	17.96	-472.96	20.59	11	8/1/2014	07:26.7
418	RWD11	-122.452	37.935	2.81	17.96	-526.95	20.77	-122.452	37.935	2.63	17.96	-478.05	20.59	11	8/1/2014	07:26.8
419	RWD11	-122.452	37.935	2.81	18.02	-531.14	20.82	-122.452	37.935	2.63	18.02	-483.25	20.65	11	8/1/2014	07:26.9
420	RWD11	-122.452	37.935	2.81	18.01	-535.44	20.82	-122.452	37.935	2.59	18.01	-488.63	20.60	11	8/1/2014	07:27.0
421	RWD11	-122.452	37.935	2.75	18.02	-539.87	20.77	-122.452	37.935	2.63	18.02	-494.10	20.65	11	8/1/2014	07:27.1
422	RWD11	-122.452	37.935	2.75	17.99	-544.57	20.74	-122.452	37.935	2.59	17.99	-499.94	20.58	11	8/1/2014	07:27.2
423	RWD11	-122.452	37.935	2.81	18.05	-549.41	20.86	-122.452	37.935	2.59	18.05	-505.96	20.65	11	8/1/2014	07:27.3
424	RWD11	-122.452	37.935	2.75	18.03	-554.29	20.78	-122.452	37.935	2.59	18.03	-512.09	20.62	11	8/1/2014	07:27.4
425	RWD11	-122.452	37.935	2.75	18.10	-559.21	20.86	-122.452	37.935	2.54	18.10	-518.26	20.65	11	8/1/2014	07:27.5
426	RWD11	-122.452	37.935	2.75	18.07	-564.07	20.82	-122.452	37.935	2.51	18.07	-524.27	20.58	11	8/1/2014	07:27.6
427	RWD11	-122.452	37.935	2.75	18.05	-568.86	20.81	-122.452	37.935	2.54	18.05	-530.16	20.60	11	8/1/2014	07:27.7
428	RWD11	-122.452	37.935	2.72	18.00	-573.61	20.72	-122.452	37.935	2.51	18.00	-536.03	20.51	11	8/1/2014	07:27.8
429	RWD11	-122.452	37.935	2.75	17.97	-578.23	20.73	-122.452	37.935	2.51	17.97	-541.78	20.48	11	8/1/2014	07:27.9
430	RWD11	-122.452	37.935	2.72	17.95	-582.88	20.67	-122.452	37.935	2.54	17.95	-547.45	20.49	11	8/1/2014	07:28.0
431	RWD11	-122.452	37.935	2.72	17.91	-587.58	20.63	-122.452	37.935	2.48	17.91	-553.10	20.39	11	8/1/2014	07:28.1

432	RWD11	-122.452	37.935	2.72	17.89	-592.29	20.61	-122.452	37.935	2.48	17.89	-558.86	20.36	11	8/1/2014	07:28.2
433	RWD11	-122.452	37.935	2.72	17.85	-597.05	20.57	-122.452	37.935	2.51	17.85	-564.54	20.36	11	8/1/2014	07:28.3
434	RWD11	-122.452	37.935	2.72	17.76	-601.92	20.48	-122.452	37.935	2.48	17.76	-570.34	20.24	11	8/1/2014	07:28.4
435	RWD11	-122.452	37.935	2.72	17.85	-606.84	20.57	-122.452	37.935	2.51	17.85	-576.13	20.36	11	8/1/2014	07:28.5
436	RWD11	-122.452	37.935	2.67	17.72	-611.90	20.39	-122.452	37.935	2.48	17.72	-582.15	20.20	11	8/1/2014	07:28.6
437	RWD11	-122.452	37.935	2.67	17.67	-617.05	20.34	-122.452	37.935	2.48	17.67	-588.17	20.15	11	8/1/2014	07:28.7
438	RWD11	-122.452	37.935	2.67	17.58	-622.26	20.24	-122.452	37.935	2.51	17.58	-594.31	20.09	11	8/1/2014	07:28.8
439	RWD11	-122.452	37.935	2.67	17.56	-627.52	20.22	-122.452	37.935	2.48	17.56	-600.53	20.03	11	8/1/2014	07:28.9
440	RWD11	-122.452	37.935	2.67	17.44	-632.81	20.11	-122.452	37.935	2.42	17.44	-606.73	19.86	11	8/1/2014	07:29.0
441	RWD11	-122.452	37.935	2.67	17.34	-638.14	20.00	-122.452	37.935	2.48	17.34	-612.97	19.81	11	8/1/2014	07:29.1
442	RWD11	-122.452	37.935	2.63	17.26	-643.47	19.89	-122.452	37.935	2.42	17.26	-619.22	19.69	11	8/1/2014	07:29.2
443	RWD11	-122.452	37.935	2.67	17.21	-648.73	19.88	-122.452	37.935	2.42	17.21	-625.50	19.64	11	8/1/2014	07:29.3
444	RWD11	-122.452	37.935	2.63	17.15	-653.90	19.79	-122.452	37.935	2.42	17.15	-631.75	19.58	11	8/1/2014	07:29.4
445	RWD11	-122.452	37.935	2.67	17.01	-659.06	19.68	-122.452	37.935	2.48	17.01	-638.02	19.49	11	8/1/2014	07:29.5
446	RWD11	-122.452	37.935	2.63	17.08	-664.19	19.72	-122.452	37.935	2.42	17.08	-644.24	19.51	11	8/1/2014	07:29.6
447	RWD11	-122.452	37.935	2.63	16.97	-669.40	19.60	-122.452	37.935	2.42	16.97	-650.56	19.39	11	8/1/2014	07:29.7
448	RWD11	-122.452	37.935	2.58	16.83	-674.55	19.41	-122.452	37.935	2.39	16.83	-656.75	19.22	11	8/1/2014	07:29.8
449	RWD11	-122.452	37.935	2.63	16.70	-679.71	19.33	-122.452	37.935	2.39	16.70	-662.79	19.09	11	8/1/2014	07:29.9
450	RWD11	-122.452	37.935	2.58	16.65	-684.72	19.23	-122.452	37.935	2.39	16.65	-668.75	19.04	11	8/1/2014	07:30.0
451	RWD11	-122.452	37.935	2.58	16.66	-689.60	19.24	-122.452	37.935	2.34	16.66	-674.44	19.00	11	8/1/2014	07:30.1
452	RWD11	-122.452	37.935	2.58	16.57	-694.29	19.15	-122.452	37.935	2.39	16.57	-679.81	18.96	11	8/1/2014	07:30.2
453	RWD11	-122.452	37.935	2.58	16.49	-698.89	19.07	-122.452	37.935	2.34	16.49	-685.08	18.83	11	8/1/2014	07:30.3
454	RWD11	-122.452	37.935	2.58	16.48	-703.49	19.06	-122.452	37.935	2.34	16.48	-690.37	18.82	11	8/1/2014	07:30.4
455	RWD11	-122.452	37.935	2.58	16.44	-708.28	19.02	-122.452	37.935	2.34	16.44	-695.65	18.78	11	8/1/2014	07:30.5
456	RWD11	-122.452	37.935	2.55	16.37	-712.93	18.92	-122.452	37.935	2.31	16.37	-700.99	18.68	11	8/1/2014	07:30.6
457	RWD11	-122.452	37.935	2.58	16.37	-717.78	18.96	-122.452	37.935	2.34	16.37	-706.47	18.71	11	8/1/2014	07:30.7
458	RWD11	-122.452	37.935	2.55	16.38	-722.77	18.93	-122.452	37.935	2.31	16.38	-711.99	18.69	11	8/1/2014	07:30.8
459	RWD11	-122.452	37.935	2.55	16.22	-727.87	18.77	-122.452	37.935	2.31	16.22	-717.71	18.53	11	8/1/2014	07:30.9
460	RWD11	-122.452	37.935	2.55	16.14	-732.74	18.69	-122.452	37.935	2.22	16.14	-723.45	18.36	11	8/1/2014	07:31.0
461	RWD11	-122.452	37.935	2.55	16.12	-737.71	18.67	-122.452	37.935	2.26	16.12	-728.93	18.38	11	8/1/2014	07:31.1
462	RWD11	-122.452	37.935	2.51	16.00	-742.46	18.51	-122.452	37.935	2.22	16.00	-734.52	18.22	11	8/1/2014	07:31.2
463	RWD11	-122.452	37.935	2.55	15.94	-747.10	18.49	-122.452	37.935	2.22	15.94	-739.94	18.16	11	8/1/2014	07:31.3
464	RWD11	-122.452	37.935	2.51	15.86	-751.77	18.37	-122.452	37.935	2.22	15.86	-745.46	18.08	11	8/1/2014	07:31.4

465	RWD11	-122.452	37.935	2.55	15.88	-756.28	18.43	-122.452	37.935	2.22	15.88	-750.69	18.10	11	8/1/2014	07:31.5
466	RWD11	-122.452	37.935	2.51	15.79	-760.67	18.30	-122.452	37.935	2.17	15.79	-755.84	17.96	11	8/1/2014	07:31.6
467	RWD11	-122.452	37.935	2.55	15.74	-764.84	18.29	-122.452	37.935	2.22	15.74	-760.87	17.96	11	8/1/2014	07:31.7
468	RWD11	-122.452	37.935	2.51	15.64	-768.99	18.15	-122.452	37.935	2.17	15.64	-765.69	17.81	11	8/1/2014	07:31.8
469	RWD11	-122.452	37.935	2.51	15.67	-773.01	18.19	-122.452	37.935	2.17	15.67	-770.39	17.84	11	8/1/2014	07:31.9
470	RWD11	-122.452	37.935	2.46	15.51	-776.90	17.97	-122.452	37.935	2.17	15.51	-774.94	17.68	11	8/1/2014	07:32.0
471	RWD11	-122.452	37.935	2.51	15.47	-780.81	17.99	-122.452	37.935	2.17	15.47	-779.40	17.64	11	8/1/2014	07:32.1
472	RWD11	-122.452	37.935	2.46	15.39	-784.76	17.86	-122.452	37.935	2.17	15.39	-783.89	17.57	11	8/1/2014	07:32.2
473	RWD11	-122.452	37.935	2.51	15.36	-788.78	17.88	-122.452	37.935	2.22	15.36	-788.41	17.58	11	8/1/2014	07:32.3
474	RWD11	-122.452	37.935	2.46	15.32	-792.83	17.78	-122.452	37.935	2.17	15.32	-793.01	17.49	11	8/1/2014	07:32.4
475	RWD11	-122.452	37.935	2.51	15.28	-797.02	17.79	-122.452	37.935	2.22	15.28	-797.71	17.50	11	8/1/2014	07:32.5
476	RWD11	-122.452	37.935	2.46	15.20	-801.38	17.66	-122.452	37.935	2.17	15.20	-802.62	17.37	11	8/1/2014	07:32.6
477	RWD11	-122.452	37.935	2.46	15.13	-805.87	17.59	-122.452	37.935	2.22	15.13	-807.66	17.35	11	8/1/2014	07:32.7
478	RWD11	-122.452	37.935	2.43	15.09	-810.45	17.52	-122.452	37.935	2.17	15.09	-812.79	17.26	11	8/1/2014	07:32.8
479	RWD11	-122.452	37.935	2.46	15.09	-814.99	17.56	-122.452	37.935	2.26	15.09	-817.90	17.35	11	8/1/2014	07:32.9
480	RWD11	-122.452	37.935	2.46	14.88	-819.52	17.34	-122.452	37.935	2.22	14.88	-823.06	17.10	11	8/1/2014	07:33.0
481	RWD11	-122.452	37.935	2.46	14.85	-824.08	17.32	-122.452	37.935	2.22	14.85	-828.16	17.07	11	8/1/2014	07:33.1
482	RWD11	-122.452	37.935	2.43	14.73	-828.53	17.16	-122.452	37.935	2.22	14.73	-833.28	16.95	11	8/1/2014	07:33.2
483	RWD11	-122.452	37.935	2.46	14.70	-832.81	17.16	-122.452	37.935	2.22	14.70	-838.21	16.92	11	8/1/2014	07:33.3
484	RWD11	-122.452	37.935	2.43	14.52	-836.97	16.94	-122.452	37.935	2.22	14.52	-843.03	16.74	11	8/1/2014	07:33.4
485	RWD11	-122.452	37.935	2.43	14.47	-841.02	16.90	-122.452	37.935	2.26	14.47	-847.77	16.72	11	8/1/2014	07:33.5
486	RWD11	-122.452	37.935	2.43	14.42	-844.86	16.85	-122.452	37.935	2.22	14.42	-852.31	16.64	11	8/1/2014	07:33.6
487	RWD11	-122.452	37.935	2.46	14.27	-848.54	16.74	-122.452	37.935	2.26	14.27	-856.67	16.53	11	8/1/2014	07:33.7
488	RWD11	-122.452	37.935	2.43	14.30	-852.22	16.73	-122.452	37.935	2.26	14.30	-861.08	16.55	11	8/1/2014	07:33.8
489	RWD11	-122.452	37.935	2.46	14.22	-855.98	16.68	-122.452	37.935	2.26	14.22	-865.38	16.48	11	8/1/2014	07:33.9
490	RWD11	-122.452	37.935	2.43	14.14	-859.57	16.56	-122.452	37.935	2.26	14.14	-869.53	16.39	11	8/1/2014	07:34.0
491	RWD11	-122.452	37.935	2.43	14.15	-863.17	16.58	-122.452	37.935	2.26	14.15	-873.59	16.41	11	8/1/2014	07:34.1
492	RWD11	-122.452	37.935	2.43	14.08	-866.79	16.51	-122.452	37.935	2.26	14.08	-877.66	16.33	11	8/1/2014	07:34.2
493	RWD11	-122.452	37.935	2.46	13.99	-870.51	16.45	-122.452	37.935	2.34	13.99	-881.78	16.33	11	8/1/2014	07:34.3
494	RWD11	-122.452	37.935	2.43	14.03	-874.34	16.46	-122.452	37.935	2.31	14.03	-886.02	16.34	11	8/1/2014	07:34.4
495	RWD11	-122.452	37.935	2.46	13.93	-878.19	16.39	-122.452	37.935	2.31	13.93	-890.26	16.23	11	8/1/2014	07:34.5
496	RWD11	-122.452	37.935	2.43	13.90	-882.28	16.33	-122.452	37.935	2.34	13.90	-894.66	16.25	11	8/1/2014	07:34.6
497	RWD11	-122.452	37.935	2.43	13.83	-886.44	16.26	-122.452	37.935	2.39	13.83	-899.25	16.22	11	8/1/2014	07:34.7

498	RWD11	-122.452	37.935	2.43	13.75	-890.80	16.18	-122.452	37.935	2.39	13.75	-904.04	16.14	11	8/1/2014	07:34.8
499	RWD11	-122.452	37.935	2.43	13.75	-895.23	16.18	-122.452	37.935	2.39	13.75	-908.99	16.14	11	8/1/2014	07:34.9
500	RWD11	-122.452	37.935	2.43	13.69	-899.69	16.12	-122.452	37.935	2.34	13.69	-914.05	16.03	11	8/1/2014	07:35.0
501	RWD11	-122.452	37.935	2.46	13.56	-904.02	16.03	-122.452	37.935	2.39	13.56	-918.94	15.96	11	8/1/2014	07:35.1
502	RWD11	-122.452	37.935	2.43	13.54	-908.05	15.97	-122.452	37.935	2.42	13.54	-923.63	15.96	11	8/1/2014	07:35.2
503	RWD11	-122.452	37.935	2.46	13.47	-911.92	15.93	-122.452	37.935	2.42	13.47	-928.20	15.89	11	8/1/2014	07:35.3
504	RWD11	-122.452	37.935	2.43	13.42	-915.73	15.85	-122.452	37.935	2.42	13.42	-932.59	15.84	11	8/1/2014	07:35.4
505	RWD11	-122.452	37.935	2.46	13.36	-919.38	15.82	-122.452	37.935	2.42	13.36	-936.89	15.78	11	8/1/2014	07:35.5
506	RWD11	-122.452	37.935	2.43	13.29	-922.90	15.72	-122.452	37.935	2.42	13.29	-940.97	15.71	11	8/1/2014	07:35.6
507	RWD11	-122.452	37.935	2.46	13.26	-926.46	15.72	-122.452	37.935	2.48	13.26	-944.90	15.73	11	8/1/2014	07:35.7
508	RWD11	-122.452	37.935	2.43	13.26	-929.89	15.69	-122.452	37.935	2.51	13.26	-948.78	15.77	11	8/1/2014	07:35.8
509	RWD11	-122.452	37.935	2.46	13.18	-933.50	15.64	-122.452	37.935	2.51	13.18	-952.65	15.69	11	8/1/2014	07:35.9
510	RWD11	-122.452	37.935	2.46	13.17	-937.28	15.63	-122.452	37.935	2.51	13.17	-956.60	15.68	11	8/1/2014	07:36.0
511	RWD11	-122.452	37.935	2.51	13.15	-941.22	15.66	-122.452	37.935	2.54	13.15	-960.68	15.69	11	8/1/2014	07:36.1
512	RWD11	-122.452	37.935	2.46	13.09	-945.41	15.55	-122.452	37.935	2.54	13.09	-964.95	15.63	11	8/1/2014	07:36.2
513	RWD11	-122.452	37.935	2.51	13.12	-949.60	15.63	-122.452	37.935	2.59	13.12	-969.31	15.71	11	8/1/2014	07:36.3
514	RWD11	-122.452	37.935	2.46	13.08	-953.93	15.54	-122.452	37.935	2.59	13.08	-973.86	15.68	11	8/1/2014	07:36.4
515	RWD11	-122.452	37.935	2.51	12.96	-958.28	15.47	-122.452	37.935	2.63	12.96	-978.45	15.58	11	8/1/2014	07:36.5
516	RWD11	-122.452	37.935	2.51	12.91	-962.70	15.42	-122.452	37.935	2.63	12.91	-983.12	15.54	11	8/1/2014	07:36.6
517	RWD11	-122.452	37.935	2.51	12.88	-967.03	15.39	-122.452	37.935	2.68	12.88	-987.83	15.56	11	8/1/2014	07:36.7
518	RWD11	-122.452	37.935	2.46	12.85	-971.32	15.31	-122.452	37.935	2.68	12.85	-992.59	15.53	11	8/1/2014	07:36.8
519	RWD11	-122.452	37.935	2.51	12.80	-975.67	15.32	-122.452	37.935	2.71	12.80	-997.39	15.52	11	8/1/2014	07:36.9
520	RWD11	-122.452	37.935	2.51	12.82	-979.91	15.34	-122.452	37.935	2.71	12.82	######	15.54	11	8/1/2014	07:37.0
521	RWD11	-122.452	37.935	2.51	12.72	-984.02	15.23	-122.452	37.935	2.76	12.72	######	15.48	11	8/1/2014	07:37.1
522	RWD11	-122.452	37.935	2.51	12.66	-987.97	15.18	-122.452	37.935	2.76	12.66	######	15.43	11	8/1/2014	07:37.2
523	RWD11	-122.452	37.935	2.55	12.65	-991.81	15.20	-122.452	37.935	2.80	12.65	######	15.45	11	8/1/2014	07:37.3
524	RWD11	-122.452	37.935	2.51	12.58	-995.50	15.09	-122.452	37.935	2.76	12.58	######	15.34	11	8/1/2014	07:37.4
525	RWD11	-122.452	37.935	2.55	12.55	-999.06	15.10	-122.452	37.935	2.80	12.55	######	15.34	11	8/1/2014	07:37.5
526	RWD11	-122.452	37.935	2.51	12.51	######	15.02	-122.452	37.935	2.83	12.51	######	15.34	11	8/1/2014	07:37.6
527	RWD11	-122.452	37.935	2.58	12.46	######	15.04	-122.452	37.935	2.88	12.46	######	15.34	11	8/1/2014	07:37.7
528	RWD11	-122.452	37.935	2.55	12.44	######	14.99	-122.452	37.935	2.83	12.44	######	15.27	11	8/1/2014	07:37.8
529	RWD11	-122.452	37.935	2.58	12.40	######	14.98	-122.452	37.935	2.88	12.40	######	15.28	11	8/1/2014	07:37.9
530	RWD11	-122.452	37.935	2.55	12.38	######	14.93	-122.452	37.935	2.88	12.38	######	15.26	11	8/1/2014	07:38.0

531	RWD11	-122.452	37.935	2.58	12.34	######	14.92	-122.452	37.935	2.91	12.34	######	15.25	11	8/1/2014	07:38.1
532	RWD11	-122.452	37.935	2.58	12.32	######	14.90	-122.452	37.935	2.97	12.32	######	15.28	11	8/1/2014	07:38.2
533	RWD11	-122.452	37.935	2.63	12.28	######	14.91	-122.452	37.935	3.00	12.28	######	15.28	11	8/1/2014	07:38.3
534	RWD11	-122.452	37.935	2.58	12.31	######	14.89	-122.452	37.935	3.00	12.31	######	15.31	11	8/1/2014	07:38.4
535	RWD11	-122.452	37.935	2.63	12.25	######	14.88	-122.452	37.935	3.05	12.25	######	15.30	11	8/1/2014	07:38.5
536	RWD11	-122.452	37.935	2.63	12.22	######	14.86	-122.452	37.935	3.00	12.22	######	15.22	11	8/1/2014	07:38.6
537	RWD11	-122.452	37.935	2.63	12.19	######	14.83	-122.452	37.935	3.05	12.19	######	15.24	11	8/1/2014	07:38.7
538	RWD11	-122.452	37.935	2.63	12.16	######	14.79	-122.452	37.935	3.05	12.16	######	15.21	11	8/1/2014	07:38.8
539	RWD11	-122.452	37.935	2.67	12.15	######	14.81	-122.452	37.935	3.05	12.15	######	15.20	11	8/1/2014	07:38.9
540	RWD11	-122.452	37.935	2.67	12.11	######	14.78	-122.452	37.935	3.05	12.11	######	15.16	11	8/1/2014	07:39.0
541	RWD11	-122.452	37.935	2.67	12.11	######	14.77	-122.452	37.935	3.08	12.11	######	15.19	11	8/1/2014	07:39.1
542	RWD11	-122.452	37.935	2.67	12.12	######	14.79	-122.452	37.935	3.08	12.12	######	15.21	11	8/1/2014	07:39.2
543	RWD11	-122.452	37.935	2.72	12.10	######	14.82	-122.452	37.935	3.08	12.10	######	15.18	11	8/1/2014	07:39.3
544	RWD11	-122.452	37.935	2.72	12.07	######	14.79	-122.452	37.935	3.08	12.07	######	15.15	11	8/1/2014	07:39.4
545	RWD11	-122.452	37.935	2.72	12.04	######	14.76	-122.452	37.935	3.13	12.04	######	15.17	11	8/1/2014	07:39.5
546	RWD11	-122.452	37.935	2.72	12.05	######	14.77	-122.452	37.935	3.08	12.05	######	15.13	11	8/1/2014	07:39.6
547	RWD11	-122.452	37.935	2.75	12.03	######	14.78	-122.452	37.935	3.13	12.03	######	15.17	11	8/1/2014	07:39.7
548	RWD11	-122.452	37.935	2.72	12.02	######	14.73	-122.452	37.935	3.13	12.02	######	15.15	11	8/1/2014	07:39.8
549	RWD11	-122.452	37.935	2.75	12.02	######	14.77	-122.452	37.935	3.13	12.02	######	15.15	11	8/1/2014	07:39.9
550	RWD11	-122.452	37.935	2.75	11.99	######	14.74	-122.452	37.935	3.13	11.99	######	15.12	11	8/1/2014	07:40.0
551	RWD11	-122.452	37.935	2.75	11.98	######	14.74	-122.452	37.935	3.13	11.98	######	15.12	11	8/1/2014	07:40.1
552	RWD11	-122.452	37.935	2.72	12.03	######	14.75	-122.452	37.935	3.17	12.03	######	15.20	11	8/1/2014	07:40.2
553	RWD11	-122.452	37.935	2.75	11.97	######	14.72	-122.452	37.935	3.17	11.97	######	15.14	11	8/1/2014	07:40.3
554	RWD11	-122.452	37.935	2.75	11.98	######	14.73	-122.452	37.935	3.13	11.98	######	15.11	11	8/1/2014	07:40.4
555	RWD11	-122.452	37.935	2.80	11.98	######	14.79	-122.452	37.935	3.17	11.98	######	15.15	11	8/1/2014	07:40.5
556	RWD11	-122.452	37.935	2.75	11.96	######	14.71	-122.452	37.935	3.17	11.96	######	15.13	11	8/1/2014	07:40.6
557	RWD11	-122.452	37.935	2.80	11.96	######	14.77	-122.452	37.935	3.20	11.96	######	15.16	11	8/1/2014	07:40.7
558	RWD11	-122.452	37.935	2.80	11.98	######	14.79	-122.452	37.935	3.20	11.98	######	15.19	11	8/1/2014	07:40.8
559	RWD11	-122.452	37.935	2.84	11.96	######	14.80	-122.452	37.935	3.20	11.96	######	15.16	11	8/1/2014	07:40.9
560	RWD11	-122.452	37.935	2.80	11.96	######	14.77	-122.452	37.935	3.25	11.96	######	15.21	11	8/1/2014	07:41.0
561	RWD11	-122.452	37.935	2.84	11.96	######	14.80	-122.452	37.935	3.25	11.96	######	15.21	11	8/1/2014	07:41.1
562	RWD11	-122.452	37.935	2.80	11.97	######	14.77	-122.452	37.935	3.20	11.97	######	15.17	11	8/1/2014	07:41.2
563	RWD11	-122.452	37.935	2.84	11.97	######	14.81	-122.452	37.935	3.25	11.97	######	15.22	11	8/1/2014	07:41.3

564	RWD11	-122.452	37.935	2.84	11.97	######	14.81	-122.452	37.935	3.20	11.97	######	15.17	11	8/1/2014	07:41.4
565	RWD11	-122.452	37.935	2.84	11.98	######	14.82	-122.452	37.935	3.20	11.98	######	15.19	11	8/1/2014	07:41.5
566	RWD11	-122.452	37.935	2.84	11.96	######	14.80	-122.452	37.935	3.20	11.96	######	15.16	11	8/1/2014	07:41.6
567	RWD11	-122.452	37.935	2.84	11.98	######	14.82	-122.452	37.935	3.20	11.98	######	15.18	11	8/1/2014	07:41.7
568	RWD11	-122.452	37.935	2.84	11.99	######	14.83	-122.452	37.935	3.20	11.99	######	15.19	11	8/1/2014	07:41.8
569	RWD11	-122.452	37.935	2.87	12.00	######	14.87	-122.452	37.935	3.20	12.00	######	15.20	11	8/1/2014	07:41.9
570	RWD11	-122.452	37.935	2.84	11.99	######	14.83	-122.452	37.935	3.20	11.99	######	15.19	11	8/1/2014	07:42.0
571	RWD11	-122.452	37.935	2.87	11.99	######	14.86	-122.452	37.935	3.25	11.99	######	15.24	11	8/1/2014	07:42.1
572	RWD11	-122.452	37.935	2.87	11.98	######	14.85	-122.452	37.935	3.25	11.98	######	15.23	11	8/1/2014	07:42.2
573	RWD11	-122.452	37.935	2.87	11.96	######	14.83	-122.452	37.935	3.25	11.96	######	15.21	11	8/1/2014	07:42.3
574	RWD11	-122.452	37.935	2.87	11.95	######	14.82	-122.452	37.935	3.25	11.95	######	15.20	11	8/1/2014	07:42.4
575	RWD11	-122.452	37.935	2.87	11.94	######	14.81	-122.452	37.935	3.34	11.94	######	15.28	11	8/1/2014	07:42.5
576	RWD11	-122.452	37.935	2.87	11.92	######	14.79	-122.452	37.935	3.25	11.92	######	15.17	11	8/1/2014	07:42.6
577	RWD11	-122.452	37.935	2.87	11.92	######	14.79	-122.452	37.935	3.34	11.92	######	15.26	11	8/1/2014	07:42.7
578	RWD11	-122.452	37.935	2.87	11.89	######	14.77	-122.452	37.935	3.29	11.89	######	15.18	11	8/1/2014	07:42.8
579	RWD11	-122.452	37.935	2.92	11.89	######	14.82	-122.452	37.935	3.29	11.89	######	15.18	11	8/1/2014	07:42.9
580	RWD11	-122.452	37.935	2.87	11.88	######	14.76	-122.452	37.935	3.25	11.88	######	15.14	11	8/1/2014	07:43.0
581	RWD11	-122.452	37.935	2.92	11.91	######	14.83	-122.452	37.935	3.25	11.91	######	15.16	11	8/1/2014	07:43.1
582	RWD11	-122.452	37.935	2.87	11.88	######	14.76	-122.452	37.935	3.29	11.88	######	15.17	11	8/1/2014	07:43.2
583	RWD11	-122.452	37.935	2.92	11.87	######	14.79	-122.452	37.935	3.29	11.87	######	15.15	11	8/1/2014	07:43.3
584	RWD11	-122.452	37.935	2.92	11.86	######	14.79	-122.452	37.935	3.25	11.86	######	15.11	11	8/1/2014	07:43.4
585	RWD11	-122.452	37.935	2.92	11.88	######	14.80	-122.452	37.935	3.25	11.88	######	15.13	11	8/1/2014	07:43.5
586	RWD11	-122.452	37.935	2.92	11.86	######	14.79	-122.452	37.935	3.29	11.86	######	15.15	11	8/1/2014	07:43.6
587	RWD11	-122.452	37.935	2.92	11.85	######	14.77	-122.452	37.935	3.25	11.85	######	15.10	11	8/1/2014	07:43.7
588	RWD11	-122.452	37.935	2.92	11.81	######	14.74	-122.452	37.935	3.25	11.81	######	15.07	11	8/1/2014	07:43.8
589	RWD11	-122.452	37.935	2.92	11.81	######	14.74	-122.452	37.935	3.29	11.81	######	15.10	11	8/1/2014	07:43.9
590	RWD11	-122.452	37.935	2.92	11.80	######	14.72	-122.452	37.935	3.29	11.80	######	15.09	11	8/1/2014	07:44.0
591	RWD11	-122.452	37.935	2.92	11.77	######	14.69	-122.452	37.935	3.34	11.77	######	15.11	11	8/1/2014	07:44.1
592	RWD11	-122.452	37.935	2.92	11.76	######	14.68	-122.452	37.935	3.29	11.76	######	15.05	11	8/1/2014	07:44.2
593	RWD11	-122.452	37.935	2.92	11.78	######	14.70	-122.452	37.935	3.34	11.78	######	15.11	11	8/1/2014	07:44.3
594	RWD11	-122.452	37.935	2.92	11.75	######	14.68	-122.452	37.935	3.34	11.75	######	15.09	11	8/1/2014	07:44.4
595	RWD11	-122.452	37.935	3.10	11.74	######	14.84	-122.452	37.935	3.37	11.74	######	15.12	11	8/1/2014	07:44.5
596	RWD11	-122.452	37.935	3.04	11.73	######	14.77	-122.452	37.935	3.34	11.73	######	15.07	11	8/1/2014	07:44.6

597	RWD11	-122.452	37.935	3.10	11.72	######	14.81	-122.452	37.935	3.37	11.72	######	15.09	11	8/1/2014 07:44.7
598	RWD11	-122.452	37.935	3.04	11.71	######	14.75	-122.452	37.935	3.34	11.71	######	15.04	11	8/1/2014 07:44.8
599	RWD11	-122.452	37.935	3.10	11.72	######	14.81	-122.452	37.935	3.37	11.72	######	15.09	11	8/1/2014 07:44.9
600	RWD11	-122.452	37.935	3.10	11.72	######	14.81	-122.452	37.935	3.34	11.72	######	15.05	11	8/1/2014 07:45.0
601	RWD11	-122.452	37.935	3.04	11.70	######	14.74	-122.452	37.935	3.34	11.70	######	15.04	11	8/1/2014 07:45.1
602	RWD11	-122.452	37.935	3.04	11.69	######	14.73	-122.452	37.935	3.34	11.69	######	15.03	11	8/1/2014 07:45.2
603	RWD11	-122.452	37.935	3.10	11.69	######	14.78	-122.452	37.935	3.37	11.69	######	15.06	11	8/1/2014 07:45.3
604	RWD11	-122.452	37.935	3.04	11.70	######	14.74	-122.452	37.935	3.37	11.70	######	15.07	11	8/1/2014 07:45.4
605	RWD11	-122.452	37.935	3.10	11.67	######	14.76	-122.452	37.935	3.42	11.67	######	15.09	11	8/1/2014 07:45.5
606	RWD11	-122.452	37.935	3.10	11.67	######	14.77	-122.452	37.935	3.37	11.67	######	15.05	11	8/1/2014 07:45.6
607	RWD11	-122.452	37.935	3.10	11.66	######	14.75	-122.452	37.935	3.42	11.66	######	15.08	11	8/1/2014 07:45.7
608	RWD11	-122.452	37.935	3.04	11.68	######	14.73	-122.452	37.935	3.37	11.68	######	15.06	11	8/1/2014 07:45.8
609	RWD11	-122.452	37.935	3.10	11.68	######	14.78	-122.452	37.935	3.37	11.68	######	15.06	11	8/1/2014 07:45.9
610	RWD11	-122.452	37.935	3.10	11.67	######	14.76	-122.452	37.935	3.37	11.67	######	15.04	11	8/1/2014 07:46.0
611	RWD11	-122.452	37.935	3.10	11.66	######	14.75	-122.452	37.935	3.37	11.66	######	15.03	11	8/1/2014 07:46.1
612	RWD11	-122.452	37.935	3.04	11.66	######	14.70	-122.452	37.935	3.37	11.66	######	15.03	11	8/1/2014 07:46.2
613	RWD11	-122.452	37.935	3.10	11.66	######	14.75	-122.452	37.935	3.37	11.66	######	15.03	11	8/1/2014 07:46.3
614	RWD11	-122.452	37.935	3.10	11.65	######	14.74	-122.452	37.935	3.37	11.65	######	15.02	11	8/1/2014 07:46.4
615	RWD11	-122.452	37.935	3.10	11.64	######	14.73	-122.452	37.935	3.42	11.64	######	15.06	11	8/1/2014 07:46.5
616	RWD11	-122.452	37.935	3.10	11.60	######	14.70	-122.452	37.935	3.42	11.60	######	15.03	11	8/1/2014 07:46.6
617	RWD11	-122.452	37.935	3.10	11.62	######	14.72	-122.452	37.935	3.42	11.62	######	15.04	11	8/1/2014 07:46.7
618	RWD11	-122.452	37.935	3.10	11.60	######	14.70	-122.452	37.935	3.42	11.60	######	15.03	11	8/1/2014 07:46.8
619	RWD11	-122.452	37.935	3.13	11.60	######	14.73	-122.452	37.935	3.46	11.60	######	15.06	11	8/1/2014 07:46.9
620	RWD11	-122.452	37.935	3.10	11.60	######	14.69	-122.452	37.935	3.46	11.60	######	15.06	11	8/1/2014 07:47.0
621	RWD11	-122.452	37.935	3.13	11.62	######	14.75	-122.452	37.935	3.46	11.62	######	15.08	11	8/1/2014 07:47.1
622	RWD11	-122.452	37.935	3.13	11.61	######	14.74	-122.452	37.935	3.46	11.61	######	15.07	11	8/1/2014 07:47.2
623	RWD11	-122.452	37.935	3.13	11.59	######	14.72	-122.452	37.935	3.46	11.59	######	15.04	11	8/1/2014 07:47.3
624	RWD11	-122.452	37.935	3.13	11.61	######	14.74	-122.452	37.935	3.46	11.61	######	15.07	11	8/1/2014 07:47.4
625	RWD11	-122.452	37.935	3.13	11.58	######	14.71	-122.452	37.935	3.46	11.58	######	15.04	11	8/1/2014 07:47.5
626	RWD11	-122.452	37.935	3.13	11.59	######	14.72	-122.452	37.935	3.46	11.59	######	15.04	11	8/1/2014 07:47.6
627	RWD11	-122.452	37.935	3.13	11.61	######	14.74	-122.452	37.935	3.54	11.61	######	15.16	11	8/1/2014 07:47.7
628	RWD11	-122.452	37.935	3.13	11.57	######	14.70	-122.452	37.935	3.51	11.57	######	15.07	11	8/1/2014 07:47.8
629	RWD11	-122.452	37.935	3.13	11.56	######	14.69	-122.452	37.935	3.54	11.56	######	15.10	11	8/1/2014 07:47.9

630	RWD11	-122.452	37.935	3.13	11.55	######	14.67	-122.452	37.935	3.54	11.55	######	15.09	11	8/1/2014	07:48.0
631	RWD11	-122.452	37.935	3.18	11.53	######	14.71	-122.452	37.935	3.58	11.53	######	15.10	11	8/1/2014	07:48.1
632	RWD11	-122.452	37.935	3.13	11.52	######	14.65	-122.452	37.935	3.58	11.52	######	15.10	11	8/1/2014	07:48.2
633	RWD11	-122.452	37.935	3.34	11.48	######	14.82	-122.452	37.935	3.63	11.48	######	15.11	11	8/1/2014	07:48.3
634	RWD11	-122.452	37.935	3.30	11.48	######	14.78	-122.452	37.935	3.63	11.48	######	15.10	11	8/1/2014	07:48.4
635	RWD11	-122.452	37.935	3.34	11.46	######	14.80	-122.452	37.935	3.66	11.46	######	15.12	11	8/1/2014	07:48.5
636	RWD11	-122.452	37.935	3.30	11.44	######	14.74	-122.452	37.935	3.66	11.44	######	15.10	11	8/1/2014	07:48.6
637	RWD11	-122.452	37.935	3.34	11.43	######	14.76	-122.452	37.935	3.66	11.43	######	15.09	11	8/1/2014	07:48.7
638	RWD11	-122.452	37.935	3.34	11.41	######	14.75	-122.452	37.935	3.71	11.41	######	15.12	11	8/1/2014	07:48.8
639	RWD11	-122.452	37.935	3.34	11.41	######	14.74	-122.452	37.935	3.71	11.41	######	15.12	11	8/1/2014	07:48.9
640	RWD11	-122.452	37.935	3.34	11.39	######	14.73	-122.452	37.935	3.74	11.39	######	15.13	11	8/1/2014	07:49.0
641	RWD11	-122.452	37.935	3.34	11.38	######	14.72	-122.452	37.935	3.74	11.38	######	15.13	11	8/1/2014	07:49.1
642	RWD11	-122.452	37.935	3.34	11.38	######	14.72	-122.452	37.935	3.74	11.38	######	15.13	11	8/1/2014	07:49.2
643	RWD11	-122.452	37.935	3.38	11.37	######	14.76	-122.452	37.935	3.74	11.37	######	15.12	11	8/1/2014	07:49.3
644	RWD11	-122.452	37.935	3.47	11.40	######	14.87	-122.452	37.935	3.79	11.40	######	15.19	11	8/1/2014	07:49.4
645	RWD11	-122.452	37.935	3.51	11.37	######	14.87	-122.452	37.935	3.79	11.37	######	15.16	11	8/1/2014	07:49.5
646	RWD11	-122.452	37.935	3.47	11.37	######	14.84	-122.452	37.935	3.79	11.37	######	15.17	11	8/1/2014	07:49.6
647	RWD11	-122.452	37.935	3.47	11.41	######	14.88	-122.452	37.935	3.83	11.41	######	15.24	11	8/1/2014	07:49.7
648	RWD11	-122.452	37.935	3.47	11.40	######	14.87	-122.452	37.935	3.83	11.40	######	15.22	11	8/1/2014	07:49.8
649	RWD11	-122.452	37.935	3.51	11.44	-997.32	14.95	-122.452	37.935	3.79	11.44	######	15.24	11	8/1/2014	07:49.9
650	RWD11	-122.452	37.935	3.47	11.44	-992.90	14.92	-122.452	37.935	3.79	11.44	######	15.24	11	8/1/2014	07:50.0
651	RWD11	-122.452	37.935	3.51	11.47	-988.13	14.97	-122.452	37.935	3.79	11.47	######	15.26	11	8/1/2014	07:50.1
652	RWD11	-122.452	37.935	3.47	11.51	-983.24	14.98	-122.452	37.935	3.79	11.51	######	15.30	11	8/1/2014	07:50.2
653	RWD11	-122.452	37.935	3.51	11.51	-978.18	15.01	-122.452	37.935	3.79	11.51	-998.85	15.30	11	8/1/2014	07:50.3
654	RWD11	-122.452	37.935	3.47	11.55	-973.07	15.02	-122.452	37.935	3.79	11.55	-992.87	15.34	11	8/1/2014	07:50.4
655	RWD11	-122.452	37.935	3.51	11.57	-967.67	15.07	-122.452	37.935	3.79	11.57	-986.73	15.36	11	8/1/2014	07:50.5
656	RWD11	-122.452	37.935	3.47	11.61	-962.19	15.09	-122.452	37.935	3.79	11.61	-980.44	15.41	11	8/1/2014	07:50.6
657	RWD11	-122.452	37.935	3.51	11.66	-956.43	15.16	-122.452	37.935	3.79	11.66	-973.98	15.45	11	8/1/2014	07:50.7
658	RWD11	-122.452	37.935	3.47	11.72	-950.57	15.19	-122.452	37.935	3.79	11.72	-967.25	15.51	11	8/1/2014	07:50.8
659	RWD11	-122.452	37.935	3.51	11.74	-944.40	15.24	-122.452	37.935	3.74	11.74	-960.39	15.48	11	8/1/2014	07:50.9
660	RWD11	-122.452	37.935	3.47	11.81	-938.03	15.28	-122.452	37.935	3.74	11.81	-953.31	15.56	11	8/1/2014	07:51.0
661	RWD11	-122.452	37.935	3.51	11.80	-931.53	15.30	-122.452	37.935	3.79	11.80	-946.09	15.59	11	8/1/2014	07:51.1
662	RWD11	-122.452	37.935	3.47	11.83	-925.00	15.30	-122.452	37.935	3.71	11.83	-938.99	15.54	11	8/1/2014	07:51.2

663	RWD11	-122.452	37.935	3.51	11.88	-918.64	15.38	-122.452	37.935	3.74	11.88	-931.90	15.62	11	8/1/2014	07:51.3
664	RWD11	-122.452	37.935	3.47	11.95	-912.44	15.42	-122.452	37.935	3.74	11.95	-925.03	15.69	11	8/1/2014	07:51.4
665	RWD11	-122.452	37.935	3.51	11.98	-906.39	15.48	-122.452	37.935	3.74	11.98	-918.33	15.72	11	8/1/2014	07:51.5
666	RWD11	-122.452	37.935	3.47	11.95	-900.53	15.42	-122.452	37.935	3.74	11.95	-911.84	15.69	11	8/1/2014	07:51.6
667	RWD11	-122.452	37.935	3.51	11.97	-894.95	15.47	-122.452	37.935	3.74	11.97	-905.59	15.71	11	8/1/2014	07:51.7
668	RWD11	-122.452	37.935	3.47	11.97	-889.44	15.44	-122.452	37.935	3.79	11.97	-899.47	15.76	11	8/1/2014	07:51.8
669	RWD11	-122.452	37.935	3.51	12.01	-883.88	15.51	-122.452	37.935	3.79	12.01	-893.33	15.80	11	8/1/2014	07:51.9
670	RWD11	-122.452	37.935	3.51	12.04	-878.22	15.54	-122.452	37.935	3.83	12.04	-887.10	15.87	11	8/1/2014	07:52.0
671	RWD11	-122.452	37.935	3.51	12.03	-872.23	15.54	-122.452	37.935	3.83	12.03	-880.52	15.86	11	8/1/2014	07:52.1
672	RWD11	-122.452	37.935	3.51	12.09	-865.90	15.59	-122.452	37.935	3.83	12.09	-873.64	15.91	11	8/1/2014	07:52.2
673	RWD11	-122.452	37.935	3.56	12.13	-859.26	15.68	-122.452	37.935	3.83	12.13	-866.28	15.96	11	8/1/2014	07:52.3
674	RWD11	-122.452	37.935	3.51	12.09	-852.16	15.60	-122.452	37.935	3.83	12.09	-858.44	15.92	11	8/1/2014	07:52.4
675	RWD11	-122.452	37.935	3.51	12.15	-844.85	15.65	-122.452	37.935	3.83	12.15	-850.25	15.97	11	8/1/2014	07:52.5
676	RWD11	-122.452	37.935	3.47	12.28	-837.28	15.75	-122.452	37.935	3.83	12.28	-841.83	16.11	11	8/1/2014	07:52.6
677	RWD11	-122.452	37.935	3.51	12.26	-829.46	15.77	-122.452	37.935	3.79	12.26	-833.04	16.06	11	8/1/2014	07:52.7
678	RWD11	-122.452	37.935	3.51	12.35	-821.47	15.86	-122.452	37.935	3.83	12.35	-824.08	16.18	11	8/1/2014	07:52.8
679	RWD11	-122.452	37.935	3.51	12.39	-813.34	15.89	-122.452	37.935	3.83	12.39	-814.99	16.21	11	8/1/2014	07:52.9
680	RWD11	-122.452	37.935	3.51	12.43	-805.22	15.94	-122.452	37.935	3.79	12.43	-805.82	16.23	11	8/1/2014	07:53.0
681	RWD11	-122.452	37.935	3.51	12.46	-797.05	15.97	-122.452	37.935	3.79	12.46	-796.65	16.26	11	8/1/2014	07:53.1
682	RWD11	-122.452	37.935	3.51	12.48	-788.87	15.98	-122.452	37.935	3.79	12.48	-787.53	16.27	11	8/1/2014	07:53.2
683	RWD11	-122.452	37.935	3.51	12.52	-780.62	16.02	-122.452	37.935	3.79	12.52	-778.27	16.31	11	8/1/2014	07:53.3
684	RWD11	-122.452	37.935	3.51	12.55	-772.30	16.05	-122.452	37.935	3.79	12.55	-768.92	16.34	11	8/1/2014	07:53.4
685	RWD11	-122.452	37.935	3.51	12.56	-763.77	16.07	-122.452	37.935	3.83	12.56	-759.25	16.39	11	8/1/2014	07:53.5
686	RWD11	-122.452	37.935	3.51	12.56	-754.98	16.07	-122.452	37.935	3.79	12.56	-749.21	16.36	11	8/1/2014	07:53.6
687	RWD11	-122.452	37.935	3.56	12.56	-745.83	16.12	-122.452	37.935	3.79	12.56	-738.75	16.36	11	8/1/2014	07:53.7
688	RWD11	-122.452	37.935	3.51	12.58	-736.24	16.08	-122.452	37.935	3.79	12.58	-727.79	16.37	11	8/1/2014	07:53.8
689	RWD11	-122.452	37.935	3.51	12.58	-726.30	16.08	-122.452	37.935	3.79	12.58	-716.62	16.37	11	8/1/2014	07:53.9
690	RWD11	-122.452	37.935	3.47	12.59	-716.22	16.06	-122.452	37.935	3.74	12.59	-705.16	16.34	11	8/1/2014	07:54.0
691	RWD11	-122.452	37.935	3.51	12.62	-706.01	16.12	-122.452	37.935	3.79	12.62	-693.73	16.41	11	8/1/2014	07:54.1
692	RWD11	-122.452	37.935	3.51	12.62	-695.83	16.12	-122.452	37.935	3.74	12.62	-682.23	16.36	11	8/1/2014	07:54.2
693	RWD11	-122.452	37.935	3.51	12.63	-685.38	16.14	-122.452	37.935	3.74	12.63	-670.48	16.38	11	8/1/2014	07:54.3
694	RWD11	-122.452	37.935	3.51	12.69	-674.94	16.19	-122.452	37.935	3.71	12.69	-658.79	16.39	11	8/1/2014	07:54.4
695	RWD11	-122.452	37.935	3.51	12.69	-664.67	16.19	-122.452	37.935	3.74	12.69	-647.32	16.43	11	8/1/2014	07:54.5

696	RWD11	-122.452	37.935	3.51	12.70	-654.65	16.21	-122.452	37.935	3.71	12.70	-636.13	16.41	11	8/1/2014 07:54.6
697	RWD11	-122.452	37.935	3.51	12.72	-644.94	16.23	-122.452	37.935	3.74	12.72	-625.18	16.47	11	8/1/2014 07:54.7
698	RWD11	-122.452	37.935	3.51	12.69	-635.51	16.19	-122.452	37.935	3.74	12.69	-614.55	16.43	11	8/1/2014 07:54.8
699	RWD11	-122.452	37.935	3.51	12.71	-626.37	16.21	-122.452	37.935	3.74	12.71	-604.17	16.45	11	8/1/2014 07:54.9
700	RWD11	-122.452	37.935	3.51	12.69	-617.55	16.20	-122.452	37.935	3.74	12.69	-594.18	16.44	11	8/1/2014 07:55.0
701	RWD11	-122.452	37.935	3.51	12.68	-608.99	16.18	-122.452	37.935	3.79	12.68	-584.38	16.47	11	8/1/2014 07:55.1
702	RWD11	-122.452	37.935	3.51	12.65	-600.42	16.16	-122.452	37.935	3.79	12.65	-574.70	16.45	11	8/1/2014 07:55.2
703	RWD11	-122.452	37.935	3.56	12.63	-591.71	16.19	-122.452	37.935	3.83	12.63	-564.88	16.46	11	8/1/2014 07:55.3
704	RWD11	-122.452	37.935	3.51	12.61	-582.65	16.11	-122.452	37.935	3.79	12.61	-554.67	16.40	11	8/1/2014 07:55.4
705	RWD11	-122.452	37.935	3.56	12.60	-573.24	16.16	-122.452	37.935	3.83	12.60	-544.11	16.43	11	8/1/2014 07:55.5
706	RWD11	-122.452	37.935	3.51	12.56	-563.65	16.06	-122.452	37.935	3.83	12.56	-533.20	16.38	11	8/1/2014 07:55.6
707	RWD11	-122.452	37.935	3.56	12.54	-553.73	16.10	-122.452	37.935	3.83	12.54	-521.93	16.37	11	8/1/2014 07:55.7
708	RWD11	-122.452	37.935	3.51	12.54	-543.69	16.05	-122.452	37.935	3.83	12.54	-510.36	16.37	11	8/1/2014 07:55.8
709	RWD11	-122.452	37.935	3.56	12.50	-533.49	16.06	-122.452	37.935	3.83	12.50	-498.56	16.33	11	8/1/2014 07:55.9
710	RWD11	-122.452	37.935	3.51	12.52	-523.14	16.03	-122.452	37.935	3.83	12.52	-486.53	16.35	11	8/1/2014 07:56.0
711	RWD11	-122.452	37.935	3.56	12.49	-512.66	16.05	-122.452	37.935	3.88	12.49	-474.36	16.37	11	8/1/2014 07:56.1
712	RWD11	-122.452	37.935	3.56	12.49	-502.08	16.04	-122.452	37.935	3.88	12.49	-462.03	16.37	11	8/1/2014 07:56.2
713	RWD11	-122.452	37.935	3.56	12.45	-491.27	16.01	-122.452	37.935	3.91	12.45	-449.54	16.37	11	8/1/2014 07:56.3
714	RWD11	-122.452	37.935	3.56	12.47	-480.17	16.03	-122.452	37.935	3.88	12.47	-436.83	16.35	11	8/1/2014 07:56.4
715	RWD11	-122.452	37.935	3.56	12.45	-468.78	16.01	-122.452	37.935	3.88	12.45	-423.84	16.33	11	8/1/2014 07:56.5
716	RWD11	-122.452	37.935	3.56	12.41	-457.09	15.96	-122.452	37.935	3.88	12.41	-410.66	16.29	11	8/1/2014 07:56.6
717	RWD11	-122.452	37.935	3.56	12.47	-445.14	16.03	-122.452	37.935	3.88	12.47	-397.13	16.35	11	8/1/2014 07:56.7
718	RWD11	-122.452	37.935	3.56	12.44	-433.06	16.00	-122.452	37.935	3.88	12.44	-383.45	16.32	11	8/1/2014 07:56.8
719	RWD11	-122.452	37.935	3.56	12.50	-420.80	16.06	-122.452	37.935	3.88	12.50	-369.59	16.38	11	8/1/2014 07:56.9
720	RWD11	-122.452	37.935	3.56	12.39	-408.44	15.95	-122.452	37.935	3.88	12.39	-355.64	16.27	11	8/1/2014 07:57.0
721	RWD11	-122.452	37.935	3.59	12.37	-395.93	15.96	-122.452	37.935	3.88	12.37	-341.57	16.25	11	8/1/2014 07:57.1
722	RWD11	-122.452	37.935	3.56	12.40	-383.20	15.96	-122.452	37.935	3.88	12.40	-327.44	16.28	11	8/1/2014 07:57.2
723	RWD11	-122.452	37.935	3.59	12.39	-370.10	15.98	-122.452	37.935	3.88	12.39	-312.93	16.27	11	8/1/2014 07:57.3
724	RWD11	-122.452	37.935	3.56	12.32	-356.74	15.88	-122.452	37.935	3.88	12.32	-298.03	16.20	11	8/1/2014 07:57.4
725	RWD11	-122.452	37.935	3.56	12.37	-342.69	15.93	-122.452	37.935	3.91	12.37	-282.58	16.28	11	8/1/2014 07:57.5
726	RWD11	-122.452	37.935	3.56	12.30	-328.14	15.86	-122.452	37.935	3.88	12.30	-266.43	16.18	11	8/1/2014 07:57.6
727	RWD11	-122.452	37.935	3.59	12.29	-312.96	15.88	-122.452	37.935	3.91	12.29	-249.59	16.21	11	8/1/2014 07:57.7
728	RWD11	-122.452	37.935	3.56	12.32	-297.38	15.88	-122.452	37.935	3.88	12.32	-232.28	16.20	11	8/1/2014 07:57.8

729	RWD11	-122.452	37.935	3.56	12.30	-281.51	15.86	-122.452	37.935	3.88	12.30	-214.55	16.18	11	8/1/2014	07:57.9
730	RWD11	-122.452	37.935	3.56	12.25	-265.42	15.81	-122.452	37.935	3.88	12.25	-196.42	16.13	11	8/1/2014	07:58.0
731	RWD11	-122.452	37.935	3.59	12.25	-249.40	15.84	-122.452	37.935	3.88	12.25	-178.14	16.13	11	8/1/2014	07:58.1
732	RWD11	-122.452	37.935	3.56	12.22	-233.40	15.78	-122.452	37.935	3.83	12.22	-159.72	16.05	11	8/1/2014	07:58.2
733	RWD11	-122.452	37.935	3.56	12.23	-217.62	15.79	-122.452	37.935	3.83	12.23	-141.43	16.06	11	8/1/2014	07:58.3
734	RWD11	-122.452	37.935	3.56	12.25	-202.26	15.80	-122.452	37.935	3.88	12.25	-123.45	16.13	11	8/1/2014	07:58.4
735	RWD11	-122.452	37.935	3.56	12.16	-187.12	15.72	-122.452	37.935	3.88	12.16	-105.76	16.04	11	8/1/2014	07:58.5
736	RWD11	-122.452	37.935	3.56	12.13	-172.17	15.68	-122.452	37.935	3.83	12.13	-88.14	15.96	11	8/1/2014	07:58.6
737	RWD11	-122.452	37.935	3.59	12.12	-157.22	15.71	-122.452	37.935	3.88	12.12	-70.66	16.00	11	8/1/2014	07:58.7
738	RWD11	-122.452	37.935	3.59	12.02	-142.25	15.61	-122.452	37.935	3.88	12.02	-53.02	15.90	11	8/1/2014	07:58.8
739	RWD11	-122.452	37.935	3.59	11.98	-126.99	15.57	-122.452	37.935	3.91	11.98	-35.06	15.90	11	8/1/2014	07:58.9
740	RWD11	-122.452	37.935	3.59	11.93	-111.27	15.52	-122.452	37.935	3.88	11.93	-16.54	15.81	11	8/1/2014	07:59.0
741	RWD11	-122.452	37.935	3.59	11.91	-95.00	15.50	-122.452	37.935	3.91	11.91	2.62	15.82	11	8/1/2014	07:59.1
742	RWD11	-122.452	37.935	3.56	11.86	-78.14	15.42	-122.452	37.935	3.88	11.86	22.23	15.74	11	8/1/2014	07:59.2
743	RWD11	-122.452	37.935	3.59	11.84	-60.64	15.43	-122.452	37.935	3.95	11.84	42.61	15.78	11	8/1/2014	07:59.3
744	RWD11	-122.452	37.935	3.59	11.81	-42.36	15.40	-122.452	37.935	3.88	11.81	63.79	15.69	11	8/1/2014	07:59.4
745	RWD11	-122.452	37.935	3.59	11.78	-23.56	15.37	-122.452	37.935	3.91	11.78	85.86	15.70	11	8/1/2014	07:59.5
746	RWD11	-122.452	37.935	3.56	11.77	-4.18	15.33	-122.452	37.935	3.88	11.77	108.54	15.65	11	8/1/2014	07:59.6
747	RWD11	-122.452	37.935	3.59	11.77	15.41	15.36	-122.452	37.935	3.88	11.77	131.57	15.65	11	8/1/2014	07:59.7
748	RWD11	-122.452	37.935	3.56	11.75	35.02	15.31	-122.452	37.935	3.88	11.75	154.63	15.63	11	8/1/2014	07:59.8
749	RWD11	-122.452	37.935	3.59	11.74	54.28	15.33	-122.452	37.935	3.88	11.74	177.41	15.62	11	8/1/2014	07:59.9
750	RWD11	-122.452	37.935	3.59	11.75	73.16	15.34	-122.452	37.935	3.83	11.75	199.75	15.58	11	8/1/2014	08:00.0
751	RWD11	-122.452	37.935	3.59	11.73	91.54	15.32	-122.452	37.935	3.88	11.73	221.61	15.61	11	8/1/2014	08:00.1
752	RWD11	-122.452	37.935	3.59	11.74	109.54	15.33	-122.452	37.935	3.88	11.74	243.08	15.62	11	8/1/2014	08:00.2
753	RWD11	-122.452	37.935	3.59	11.78	127.50	15.37	-122.452	37.935	3.88	11.78	264.27	15.66	11	8/1/2014	08:00.3
754	RWD11	-122.452	37.935	3.59	11.82	145.19	15.41	-122.452	37.935	3.88	11.82	285.23	15.70	11	8/1/2014	08:00.4
755	RWD11	-122.452	37.935	3.59	11.81	163.14	15.40	-122.452	37.935	3.88	11.81	306.19	15.69	11	8/1/2014	08:00.5
756	RWD11	-122.452	37.935	3.59	11.85	181.23	15.44	-122.452	37.935	3.88	11.85	327.31	15.73	11	8/1/2014	08:00.6
757	RWD11	-122.452	37.935	3.59	11.90	199.54	15.49	-122.452	37.935	3.88	11.90	348.57	15.78	11	8/1/2014	08:00.7
758	RWD11	-122.452	37.935	3.59	11.94	218.10	15.53	-122.452	37.935	3.91	11.94	370.07	15.85	11	8/1/2014	08:00.8
759	RWD11	-122.452	37.935	3.59	11.98	236.97	15.57	-122.452	37.935	3.91	11.98	391.83	15.90	11	8/1/2014	08:00.9
760	RWD11	-122.452	37.935	3.59	12.00	256.09	15.59	-122.452	37.935	3.91	12.00	413.99	15.91	11	8/1/2014	08:01.0
761	RWD11	-122.453	37.935	3.59	12.02	275.68	15.61	-122.452	37.935	3.95	12.02	436.68	15.97	11	8/1/2014	08:01.1

762	RWD11	-122.453	37.935	3.59	12.06	295.81	15.65	-122.452	37.935	3.91	12.06	460.23	15.97	11	8/1/2014 08:01.2
763	RWD11	-122.453	37.935	3.63	12.09	316.57	15.71	-122.452	37.935	3.95	12.09	484.52	16.03	11	8/1/2014 08:01.3
764	RWD11	-122.453	37.935	3.59	12.11	337.72	15.70	-122.453	37.935	3.95	12.11	509.52	16.06	11	8/1/2014 08:01.4
765	RWD11	-122.453	37.935	3.71	12.17	359.35	15.88	-122.453	37.935	3.95	12.17	535.10	16.12	11	8/1/2014 08:01.5
766	RWD11	-122.453	37.935	3.71	12.18	381.22	15.89	-122.453	37.935	3.91	12.18	561.30	16.09	11	8/1/2014 08:01.6
767	RWD11	-122.453	37.935	3.71	12.21	403.54	15.92	-122.453	37.935	3.91	12.21	587.95	16.12	11	8/1/2014 08:01.7
768	RWD11	-122.453	37.935	3.71	12.22	426.14	15.93	-122.453	37.935	3.91	12.22	615.01	16.14	11	8/1/2014 08:01.8
769	RWD11	-122.453	37.935	3.71	12.27	448.87	15.98	-122.453	37.935	3.91	12.27	642.13	16.18	11	8/1/2014 08:01.9
770	RWD11	-122.453	37.935	3.68	12.34	471.36	16.01	-122.453	37.935	3.88	12.34	669.02	16.22	11	8/1/2014 08:02.0
771	RWD11	-122.453	37.935	3.71	12.33	493.43	16.04	-122.453	37.935	3.91	12.33	695.28	16.24	11	8/1/2014 08:02.1
772	RWD11	-122.453	37.935	3.67	12.34	514.85	16.01	-122.453	37.935	3.88	12.34	720.94	16.22	11	8/1/2014 08:02.2
773	RWD11	-122.453	37.935	3.71	12.38	535.73	16.09	-122.453	37.935	3.91	12.38	745.99	16.29	11	8/1/2014 08:02.3
774	RWD11	-122.453	37.935	3.71	12.38	556.19	16.09	-122.453	37.935	3.91	12.38	770.52	16.29	11	8/1/2014 08:02.4
775	RWD11	-122.453	37.935	3.71	12.39	576.56	16.10	-122.453	37.935	3.91	12.39	794.91	16.30	11	8/1/2014 08:02.5
776	RWD11	-122.453	37.935	3.71	12.39	597.05	16.10	-122.453	37.935	3.91	12.39	819.26	16.30	11	8/1/2014 08:02.6
777	RWD11	-122.453	37.935	3.71	12.47	617.91	16.18	-122.453	37.935	3.94	12.47	844.07	16.42	11	8/1/2014 08:02.7
778	RWD11	-122.453	37.935	3.67	12.44	639.49	16.11	-122.453	37.935	3.94	12.44	869.73	16.38	11	8/1/2014 08:02.8
779	RWD11	-122.453	37.935	3.71	12.49	661.88	16.20	-122.453	37.935	3.94	12.49	896.38	16.43	11	8/1/2014 08:02.9
780	RWD11	-122.453	37.935	3.71	12.47	684.92	16.18	-122.453	37.935	3.91	12.47	924.03	16.38	11	8/1/2014 08:03.0
781	RWD11	-122.453	37.935	3.71	12.49	708.36	16.20	-122.453	37.935	3.94	12.49	952.38	16.44	11	8/1/2014 08:03.1
782	RWD11	-122.453	37.935	3.71	12.55	732.03	16.26	-122.453	37.935	3.94	12.55	981.13	16.49	11	8/1/2014 08:03.2
783	RWD11	-122.453	37.935	3.71	12.58	755.73	16.29	-122.453	37.935	3.94	12.58	1010.19	16.53	11	8/1/2014 08:03.3
784	RWD11	-122.453	37.935	3.71	12.60	779.17	16.31	-122.453	37.935	3.91	12.60	1038.91	16.51	11	8/1/2014 08:03.4
785	RWD11	-122.453	37.935	3.71	12.66	802.01	16.37	-122.453	37.936	3.91	12.66	1067.07	16.57	11	8/1/2014 08:03.5
786	RWD11	-122.453	37.935	3.71	12.75	824.06	16.46	-122.453	37.936	3.88	12.75	1094.33	16.63	11	8/1/2014 08:03.6
787	RWD11	-122.453	37.935	3.71	12.69	845.22	16.39	-122.453	37.936	3.91	12.69	1120.43	16.60	11	8/1/2014 08:03.7
788	RWD11	-122.453	37.935	3.71	12.75	865.41	16.46	-122.453	37.936	3.91	12.75	1145.57	16.66	11	8/1/2014 08:03.8
789	RWD11	-122.453	37.935	3.71	12.75	884.63	16.46	-122.453	37.936	3.91	12.75	1169.40	16.66	11	8/1/2014 08:03.9
790	RWD11	-122.453	37.935	3.67	12.77	903.15	16.44	-122.453	37.936	3.91	12.77	1192.39	16.68	11	8/1/2014 08:04.0
791	RWD11	-122.453	37.935	3.71	12.69	921.14	16.40	-122.453	37.936	3.94	12.69	1214.75	16.64	11	8/1/2014 08:04.1
792	RWD11	-122.453	37.935	3.71	12.70	938.69	16.41	-122.453	37.936	3.91	12.70	1236.57	16.61	11	8/1/2014 08:04.2
793	RWD11	-122.453	37.935	3.71	12.65	955.75	16.36	-122.453	37.936	4.00	12.65	1257.83	16.65	11	8/1/2014 08:04.3
794	RWD11	-122.453	37.935	3.71	12.69	972.34	16.39	-122.453	37.936	3.94	12.69	1278.57	16.63	11	8/1/2014 08:04.4

795	RWD11	-122.453	37.935	3.71	12.61	988.59	16.32	-122.453	37.936	4.00	12.61	1298.91	16.61	11	8/1/2014	08:04.5
796	RWD11	-122.453	37.935	3.71	12.57	######	16.28	-122.453	37.936	4.00	12.57	1318.65	16.57	11	8/1/2014	08:04.6
797	RWD11	-122.453	37.935	3.76	12.54	######	16.30	-122.453	37.936	4.03	12.54	1338.15	16.57	11	8/1/2014	08:04.7
798	RWD11	-122.453	37.935	3.71	12.52	######	16.23	-122.453	37.936	4.03	12.52	1357.57	16.55	11	8/1/2014	08:04.8
799	RWD11	-122.453	37.935	3.76	12.47	######	16.23	-122.453	37.936	4.08	12.47	1377.17	16.55	11	8/1/2014	08:04.9
800	RWD11	-122.453	37.935	3.71	12.47	######	16.18	-122.453	37.936	4.08	12.47	1397.16	16.55	11	8/1/2014	08:05.0
801	RWD11	-122.453	37.935	3.76	12.42	######	16.18	-122.453	37.936	4.08	12.42	1417.37	16.50	11	8/1/2014	08:05.1
802	RWD11	-122.453	37.935	3.71	12.37	######	16.08	-122.453	37.936	4.03	12.37	1437.71	16.40	11	8/1/2014	08:05.2
803	RWD11	-122.453	37.935	3.76	12.37	######	16.13	-122.453	37.936	4.08	12.37	1457.85	16.45	11	8/1/2014	08:05.3
804	RWD11	-122.453	37.935	3.76	12.30	######	16.06	-122.453	37.936	4.08	12.30	1477.39	16.38	11	8/1/2014	08:05.4
805	RWD11	-122.453	37.935	3.76	12.27	######	16.03	-122.453	37.936	4.08	12.27	1496.07	16.35	11	8/1/2014	08:05.5
806	RWD11	-122.453	37.935	3.71	12.24	######	15.95	-122.453	37.936	4.08	12.24	1514.04	16.32	11	8/1/2014	08:05.6
807	RWD11	-122.453	37.936	3.76	12.26	######	16.02	-122.453	37.936	4.11	12.26	1531.26	16.38	11	8/1/2014	08:05.7
808	RWD11	-122.453	37.936	3.76	12.23	######	15.99	-122.453	37.936	4.11	12.23	1547.92	16.34	11	8/1/2014	08:05.8
809	RWD11	-122.453	37.936	3.76	12.16	######	15.92	-122.453	37.936	4.16	12.16	1563.87	16.33	11	8/1/2014	08:05.9
810	RWD11	-122.453	37.936	3.76	12.13	######	15.89	-122.453	37.936	4.16	12.13	1579.13	16.29	11	8/1/2014	08:06.0
811	RWD11	-122.453	37.936	3.93	12.09	######	16.02	-122.453	37.936	4.20	12.09	1594.03	16.28	11	8/1/2014	08:06.1
812	RWD11	-122.453	37.936	3.93	12.08	######	16.01	-122.453	37.936	4.23	12.08	1608.41	16.31	11	8/1/2014	08:06.2
813	RWD11	-122.453	37.936	3.93	12.01	######	15.94	-122.453	37.936	4.23	12.01	1622.25	16.24	11	8/1/2014	08:06.3
814	RWD11	-122.453	37.936	3.93	11.95	######	15.88	-122.453	37.936	4.23	11.95	1635.73	16.18	11	8/1/2014	08:06.4
815	RWD11	-122.453	37.936	3.93	11.94	######	15.87	-122.453	37.936	4.28	11.94	1648.72	16.22	11	8/1/2014	08:06.5
816	RWD11	-122.453	37.936	3.93	11.92	######	15.85	-122.453	37.936	4.28	11.92	1661.38	16.20	11	8/1/2014	08:06.6
817	RWD11	-122.453	37.936	3.97	11.88	######	15.85	-122.453	37.936	4.37	11.88	1673.74	16.25	11	8/1/2014	08:06.7
818	RWD11	-122.453	37.936	3.93	11.81	######	15.74	-122.453	37.936	4.37	11.81	1685.80	16.18	11	8/1/2014	08:06.8
819	RWD11	-122.453	37.936	3.97	11.85	######	15.82	-122.453	37.936	4.37	11.85	1697.57	16.22	11	8/1/2014	08:06.9
820	RWD11	-122.453	37.936	3.97	11.75	######	15.72	-122.453	37.936	4.37	11.75	1708.88	16.12	11	8/1/2014	08:07.0
821	RWD11	-122.453	37.936	4.09	11.72	######	15.81	-122.453	37.936	4.40	11.72	1719.58	16.12	11	8/1/2014	08:07.1
822	RWD11	-122.453	37.936	4.09	11.70	######	15.79	-122.453	37.936	4.40	11.70	1729.57	16.10	11	8/1/2014	08:07.2
823	RWD11	-122.453	37.936	4.09	11.68	######	15.77	-122.453	37.936	4.45	11.68	1738.74	16.14	11	8/1/2014	08:07.3
824	RWD11	-122.453	37.936	4.09	11.67	######	15.76	-122.453	37.936	4.45	11.67	1747.05	16.13	11	8/1/2014	08:07.4
825	RWD11	-122.453	37.936	4.14	11.65	######	15.79	-122.453	37.936	4.45	11.65	1754.56	16.11	11	8/1/2014	08:07.5
826	RWD11	-122.453	37.936	4.09	11.60	######	15.68	-122.453	37.936	4.49	11.60	1761.32	16.09	11	8/1/2014	08:07.6
827	RWD11	-122.453	37.936	4.09	11.61	######	15.70	-122.453	37.936	4.54	11.61	1767.50	16.15	11	8/1/2014	08:07.7

828	RWD11	-122.453	37.936	4.14	11.56	######	15.70	-122.453	37.936	4.54	11.56	1772.99	16.10	11	8/1/2014	08:07.8
829	RWD11	-122.453	37.936	4.22	11.53	######	15.76	-122.453	37.936	4.57	11.53	1777.83	16.11	11	8/1/2014	08:07.9
830	RWD11	-122.453	37.936	4.22	11.57	######	15.79	-122.453	37.936	4.57	11.57	1782.00	16.14	11	8/1/2014	08:08.0
831	RWD11	-122.453	37.936	4.26	11.48	######	15.74	-122.453	37.936	4.61	11.48	1785.34	16.09	11	8/1/2014	08:08.1
832	RWD11	-122.453	37.936	4.26	11.44	######	15.69	-122.453	37.936	4.66	11.44	1787.83	16.09	11	8/1/2014	08:08.2
833	RWD11	-122.453	37.936	4.26	11.39	######	15.65	-122.453	37.936	4.69	11.39	1789.45	16.08	11	8/1/2014	08:08.3
834	RWD11	-122.453	37.936	4.26	11.37	######	15.63	-122.453	37.936	4.69	11.37	1789.76	16.06	11	8/1/2014	08:08.4
835	RWD11	-122.453	37.936	4.34	11.31	######	15.65	-122.453	37.936	4.74	11.31	1788.72	16.05	11	8/1/2014	08:08.5
836	RWD11	-122.453	37.936	4.34	11.28	######	15.63	-122.453	37.936	4.69	11.28	1786.09	15.97	11	8/1/2014	08:08.6
837	RWD11	-122.453	37.936	4.34	11.25	######	15.59	-122.453	37.936	4.74	11.25	1781.89	15.99	11	8/1/2014	08:08.7
838	RWD11	-122.453	37.936	4.34	11.25	######	15.59	-122.453	37.936	4.69	11.25	1776.24	15.94	11	8/1/2014	08:08.8
839	RWD11	-122.453	37.936	4.38	11.20	######	15.58	-122.453	37.936	4.74	11.20	1769.24	15.95	11	8/1/2014	08:08.9
840	RWD11	-122.453	37.936	4.34	11.14	######	15.49	-122.453	37.936	4.69	11.14	1760.98	15.83	11	8/1/2014	08:09.0
841	RWD11	-122.453	37.936	4.38	11.09	######	15.47	-122.453	37.936	4.74	11.09	1751.58	15.83	11	8/1/2014	08:09.1
842	RWD11	-122.453	37.936	4.34	11.06	######	15.41	-122.453	37.936	4.69	11.06	1741.10	15.75	11	8/1/2014	08:09.2
843	RWD11	-122.453	37.936	4.34	11.09	######	15.43	-122.453	37.936	4.69	11.09	1729.71	15.78	11	8/1/2014	08:09.3
844	RWD11	-122.453	37.936	4.34	11.02	######	15.36	-122.453	37.936	4.66	11.02	1717.73	15.68	11	8/1/2014	08:09.4
845	RWD11	-122.453	37.936	4.34	10.99	######	15.34	-122.453	37.936	4.74	10.99	1705.05	15.74	11	8/1/2014	08:09.5
846	RWD11	-122.453	37.936	4.34	10.97	######	15.31	-122.453	37.936	4.69	10.97	1691.62	15.66	11	8/1/2014	08:09.6
847	RWD11	-122.453	37.936	4.38	10.94	######	15.31	-122.453	37.936	4.74	10.94	1677.21	15.68	11	8/1/2014	08:09.7
848	RWD11	-122.453	37.936	4.34	10.90	######	15.24	-122.453	37.936	4.69	10.90	1661.70	15.59	11	8/1/2014	08:09.8
849	RWD11	-122.453	37.936	4.38	10.91	######	15.29	-122.453	37.936	4.69	10.91	1644.85	15.60	11	8/1/2014	08:09.9
850	RWD11	-122.453	37.936	4.38	10.86	######	15.24	-122.453	37.936	4.69	10.86	1626.60	15.55	11	8/1/2014	08:10.0
851	RWD11	-122.453	37.936	4.38	10.84	######	15.22	-122.453	37.936	4.69	10.84	1606.70	15.53	11	8/1/2014	08:10.1
852	RWD11	-122.453	37.936	4.34	10.81	######	15.15	-122.453	37.936	4.69	10.81	1585.14	15.50	11	8/1/2014	08:10.2
853	RWD11	-122.453	37.936	4.34	10.81	######	15.15	-122.453	37.936	4.69	10.81	1562.18	15.50	11	8/1/2014	08:10.3
854	RWD11	-122.453	37.936	4.34	10.80	######	15.14	-122.453	37.936	4.61	10.80	1537.90	15.40	11	8/1/2014	08:10.4
855	RWD11	-122.453	37.936	4.34	10.77	######	15.12	-122.453	37.936	4.57	10.77	1512.44	15.35	11	8/1/2014	08:10.5
856	RWD11	-122.453	37.936	4.29	10.78	######	15.07	-122.453	37.936	4.57	10.78	1486.31	15.35	11	8/1/2014	08:10.6
857	RWD11	-122.453	37.936	4.34	10.79	######	15.13	-122.453	37.936	4.57	10.79	1460.41	15.36	11	8/1/2014	08:10.7
858	RWD11	-122.453	37.936	4.29	10.81	######	15.10	-122.453	37.936	4.54	10.81	1435.11	15.35	11	8/1/2014	08:10.8
859	RWD11	-122.453	37.936	4.34	10.79	######	15.13	-122.453	37.936	4.49	10.79	1410.06	15.28	11	8/1/2014	08:10.9
860	RWD11	-122.453	37.936	4.29	10.79	######	15.08	-122.453	37.936	4.49	10.79	1384.70	15.28	11	8/1/2014	08:11.0

861	RWD11	-122.453	37.936	4.34	10.76	######	15.11	-122.453	37.936	4.49	10.76	1359.15	15.25	11	8/1/2014	08:11.1
862	RWD11	-122.453	37.936	4.29	10.76	######	15.05	-122.453	37.936	4.49	10.76	1333.33	15.25	11	8/1/2014	08:11.2
863	RWD11	-122.453	37.936	4.34	10.78	######	15.12	-122.453	37.936	4.45	10.78	1307.24	15.23	11	8/1/2014	08:11.3
864	RWD11	-122.453	37.936	4.29	10.76	999.97	15.05	-122.453	37.936	4.45	10.76	1280.46	15.21	11	8/1/2014	08:11.4
865	RWD11	-122.453	37.936	4.29	10.74	978.44	15.03	-122.453	37.936	4.45	10.74	1252.39	15.19	11	8/1/2014	08:11.5
866	RWD11	-122.453	37.936	4.29	10.73	955.41	15.02	-122.453	37.936	4.40	10.73	1222.71	15.13	11	8/1/2014	08:11.6
867	RWD11	-122.453	37.936	4.29	10.75	931.11	15.04	-122.453	37.936	4.40	10.75	1191.63	15.15	11	8/1/2014	08:11.7
868	RWD11	-122.453	37.936	4.26	10.80	905.89	15.05	-122.453	37.936	4.37	10.80	1159.67	15.16	11	8/1/2014	08:11.8
869	RWD11	-122.453	37.936	4.29	10.84	880.20	15.13	-122.453	37.936	4.37	10.84	1127.32	15.21	11	8/1/2014	08:11.9
870	RWD11	-122.453	37.936	4.26	10.89	854.51	15.14	-122.453	37.936	4.28	10.89	1095.23	15.17	11	8/1/2014	08:12.0
871	RWD11	-122.453	37.936	4.26	10.87	829.41	15.13	-122.453	37.936	4.28	10.87	1063.73	15.16	11	8/1/2014	08:12.1
872	RWD11	-122.453	37.936	4.22	10.90	805.04	15.12	-122.453	37.936	4.20	10.90	1033.24	15.10	11	8/1/2014	08:12.2
873	RWD11	-122.453	37.936	4.26	10.92	781.75	15.18	-122.453	37.936	4.20	10.92	1004.00	15.12	11	8/1/2014	08:12.3
874	RWD11	-122.453	37.936	4.22	10.94	759.64	15.16	-122.453	37.936	4.16	10.94	976.23	15.10	11	8/1/2014	08:12.4
875	RWD11	-122.453	37.936	4.22	10.94	738.48	15.16	-122.453	37.936	4.16	10.94	949.65	15.10	11	8/1/2014	08:12.5
876	RWD11	-122.453	37.936	4.17	10.94	717.91	15.11	-122.453	37.936	4.11	10.94	924.02	15.05	11	8/1/2014	08:12.6
877	RWD11	-122.453	37.936	4.22	10.95	697.86	15.17	-122.453	37.936	4.11	10.95	899.10	15.07	11	8/1/2014	08:12.7
878	RWD11	-122.453	37.936	4.17	11.01	678.23	15.18	-122.453	37.936	4.08	11.01	874.82	15.09	11	8/1/2014	08:12.8
879	RWD11	-122.453	37.936	4.22	10.96	658.75	15.18	-122.453	37.936	4.11	10.96	850.75	15.07	11	8/1/2014	08:12.9
880	RWD11	-122.453	37.936	4.17	10.97	639.15	15.14	-122.453	37.936	4.08	10.97	826.63	15.05	11	8/1/2014	08:13.0
881	RWD11	-122.453	37.936	4.17	10.95	619.13	15.12	-122.453	37.936	4.11	10.95	802.25	15.07	11	8/1/2014	08:13.1
882	RWD11	-122.453	37.936	4.17	10.94	598.82	15.11	-122.453	37.936	4.08	10.94	777.55	15.02	11	8/1/2014	08:13.2
883	RWD11	-122.453	37.936	4.17	10.94	577.84	15.11	-122.453	37.936	4.08	10.94	752.24	15.02	11	8/1/2014	08:13.3
884	RWD11	-122.453	37.936	4.17	10.93	555.98	15.10	-122.453	37.936	4.08	10.93	726.23	15.01	11	8/1/2014	08:13.4
885	RWD11	-122.453	37.936	4.14	10.96	533.38	15.09	-122.453	37.936	4.03	10.96	699.47	14.99	11	8/1/2014	08:13.5
886	RWD11	-122.453	37.936	4.14	10.97	510.29	15.10	-122.453	37.936	4.00	10.97	672.39	14.96	11	8/1/2014	08:13.6
887	RWD11	-122.453	37.936	4.14	10.98	487.58	15.12	-122.453	37.936	4.00	10.98	645.81	14.98	11	8/1/2014	08:13.7
888	RWD11	-122.453	37.936	4.09	11.00	466.23	15.09	-122.453	37.936	3.94	11.00	620.69	14.95	11	8/1/2014	08:13.8
889	RWD11	-122.453	37.936	4.14	10.99	446.51	15.13	-122.453	37.936	3.94	10.99	597.53	14.94	11	8/1/2014	08:13.9
890	RWD11	-122.453	37.936	4.05	11.03	428.26	15.08	-122.453	37.936	3.91	11.03	576.11	14.94	11	8/1/2014	08:14.0
891	RWD11	-122.453	37.936	4.09	11.01	411.09	15.10	-122.453	37.936	3.94	11.01	556.01	14.96	11	8/1/2014	08:14.1
892	RWD11	-122.453	37.936	4.05	10.99	394.80	15.05	-122.453	37.936	3.94	10.99	537.10	14.94	11	8/1/2014	08:14.2
893	RWD11	-122.453	37.936	4.09	10.98	379.19	15.07	-122.453	37.936	3.94	10.98	518.80	14.93	11	8/1/2014	08:14.3

894	RWD11	-122.453	37.936	4.05	10.97	363.60	15.02	-122.453	37.936	3.94	10.97	500.60	14.92	11	8/1/2014 08:14.4
895	RWD11	-122.453	37.936	4.09	10.97	347.75	15.06	-122.453	37.936	4.00	10.97	482.14	14.97	11	8/1/2014 08:14.5
896	RWD11	-122.453	37.936	4.05	10.95	331.70	15.00	-122.453	37.936	3.94	10.95	463.44	14.90	11	8/1/2014 08:14.6
897	RWD11	-122.453	37.936	4.09	10.94	315.36	15.02	-122.453	37.936	4.00	10.94	444.55	14.93	11	8/1/2014 08:14.7
898	RWD11	-122.453	37.936	4.05	10.93	298.55	14.98	-122.453	37.936	4.00	10.93	425.18	14.92	11	8/1/2014 08:14.8
899	RWD11	-122.453	37.936	4.09	10.94	280.95	15.02	-122.453	37.936	4.00	10.94	405.13	14.93	11	8/1/2014 08:14.9
900	RWD11	-122.453	37.936	4.05	10.90	262.93	14.95	-122.453	37.936	3.99	10.90	384.46	14.90	11	8/1/2014 08:15.0
901	RWD11	-122.453	37.936	4.05	10.91	244.89	14.96	-122.453	37.936	3.99	10.91	363.77	14.91	11	8/1/2014 08:15.1
902	RWD11	-122.453	37.936	4.00	10.90	226.95	14.90	-122.453	37.936	3.99	10.90	343.08	14.89	11	8/1/2014 08:15.2
903	RWD11	-122.453	37.936	4.05	10.89	209.47	14.94	-122.453	37.936	3.99	10.89	322.76	14.88	11	8/1/2014 08:15.3
904	RWD11	-122.453	37.936	4.05	10.89	192.60	14.94	-122.453	37.936	3.94	10.89	303.00	14.83	11	8/1/2014 08:15.4
905	RWD11	-122.453	37.936	4.00	10.90	176.57	14.90	-122.453	37.936	3.94	10.90	284.02	14.85	11	8/1/2014 08:15.5
906	RWD11	-122.453	37.936	4.00	10.89	161.45	14.89	-122.453	37.936	3.94	10.89	266.00	14.83	11	8/1/2014 08:15.6
907	RWD11	-122.453	37.936	4.05	10.91	146.79	14.96	-122.453	37.936	3.99	10.91	248.56	14.91	11	8/1/2014 08:15.7
908	RWD11	-122.453	37.936	4.00	10.88	132.56	14.88	-122.453	37.936	3.99	10.88	231.57	14.88	11	8/1/2014 08:15.8
909	RWD11	-122.453	37.936	4.05	10.88	118.43	14.93	-122.453	37.936	4.03	10.88	214.86	14.91	11	8/1/2014 08:15.9
910	RWD11	-122.453	37.936	4.00	10.87	104.33	14.87	-122.453	37.936	4.03	10.87	198.35	14.90	11	8/1/2014 08:16.0
911	RWD11	-122.453	37.936	4.05	10.84	90.04	14.89	-122.453	37.936	4.08	10.84	181.69	14.92	11	8/1/2014 08:16.1
912	RWD11	-122.453	37.936	4.00	10.83	75.66	14.83	-122.453	37.936	4.03	10.83	165.21	14.86	11	8/1/2014 08:16.2
913	RWD11	-122.453	37.936	4.05	10.82	61.26	14.87	-122.453	37.936	4.08	10.82	148.71	14.90	11	8/1/2014 08:16.3
914	RWD11	-122.453	37.936	4.05	10.80	46.75	14.85	-122.453	37.936	4.08	10.80	132.30	14.88	11	8/1/2014 08:16.4
915	RWD11	-122.453	37.936	4.05	10.79	32.20	14.84	-122.453	37.936	4.08	10.79	115.92	14.87	11	8/1/2014 08:16.5
916	RWD11	-122.453	37.936	4.00	10.74	17.93	14.74	-122.453	37.936	4.11	10.74	99.77	14.85	11	8/1/2014 08:16.6
917	RWD11	-122.453	37.936	4.05	10.74	3.55	14.79	-122.453	37.936	4.16	10.74	83.80	14.91	11	8/1/2014 08:16.7
918	RWD11	-122.453	37.936	4.05	10.71	-10.70	14.76	-122.453	37.936	4.16	10.71	67.81	14.88	11	8/1/2014 08:16.8
919	RWD11	-122.453	37.936	4.05	10.71	-25.06	14.76	-122.453	37.936	4.16	10.71	51.79	14.88	11	8/1/2014 08:16.9
920	RWD11	-122.453	37.936	4.05	10.70	-39.48	14.75	-122.453	37.936	4.20	10.70	35.71	14.90	11	8/1/2014 08:17.0
921	RWD11	-122.453	37.936	4.09	10.68	-53.81	14.76	-122.453	37.936	4.20	10.68	19.72	14.88	11	8/1/2014 08:17.1
922	RWD11	-122.453	37.936	4.05	10.68	-68.10	14.73	-122.453	37.936	4.20	10.68	3.74	14.88	11	8/1/2014 08:17.2
923	RWD11	-122.453	37.936	4.09	10.67	-82.22	14.75	-122.453	37.936	4.28	10.67	-11.99	14.95	11	8/1/2014 08:17.3
924	RWD11	-122.453	37.936	4.05	10.64	-96.27	14.69	-122.453	37.936	4.23	10.64	-27.52	14.87	11	8/1/2014 08:17.4
925	RWD11	-122.453	37.936	4.09	10.65	-110.17	14.74	-122.453	37.936	4.28	10.65	-42.89	14.93	11	8/1/2014 08:17.5
926	RWD11	-122.453	37.936	4.09	10.60	-123.83	14.68	-122.453	37.936	4.28	10.60	-57.98	14.88	11	8/1/2014 08:17.6

927	RWD11	-122.453	37.936	4.09	10.59	-137.33	14.67	-122.453	37.936	4.32	10.59	-72.92	14.90	11	8/1/2014	08:17.7
928	RWD11	-122.453	37.936	4.09	10.56	-150.59	14.64	-122.453	37.936	4.32	10.56	-87.55	14.87	11	8/1/2014	08:17.8
929	RWD11	-122.453	37.936	4.14	10.55	-163.73	14.68	-122.453	37.936	4.37	10.55	-102.10	14.92	11	8/1/2014	08:17.9
930	RWD11	-122.453	37.936	4.09	10.52	-176.81	14.60	-122.453	37.936	4.37	10.52	-116.56	14.88	11	8/1/2014	08:18.0
931	RWD11	-122.453	37.936	4.14	10.50	-189.88	14.64	-122.453	37.936	4.45	10.50	-131.04	14.95	11	8/1/2014	08:18.1
932	RWD11	-122.453	37.936	4.14	10.47	-203.03	14.61	-122.453	37.936	4.45	10.47	-145.59	14.92	11	8/1/2014	08:18.2
933	RWD11	-122.453	37.936	4.17	10.43	-216.32	14.60	-122.453	37.936	4.49	10.43	-160.31	14.92	11	8/1/2014	08:18.3
934	RWD11	-122.453	37.936	4.14	10.40	-229.83	14.54	-122.453	37.936	4.49	10.40	-175.24	14.89	11	8/1/2014	08:18.4
935	RWD11	-122.453	37.936	4.17	10.39	-243.52	14.56	-122.453	37.936	4.54	10.39	-190.36	14.92	11	8/1/2014	08:18.5
936	RWD11	-122.453	37.936	4.17	10.35	-257.36	14.52	-122.453	37.936	4.54	10.35	-205.64	14.88	11	8/1/2014	08:18.6
937	RWD11	-122.453	37.936	4.22	10.36	-271.41	14.58	-122.453	37.936	4.60	10.36	-221.24	14.96	11	8/1/2014	08:18.7
938	RWD11	-122.453	37.936	4.22	10.31	-285.79	14.53	-122.453	37.936	4.60	10.31	-237.31	14.91	11	8/1/2014	08:18.8
939	RWD11	-122.453	37.936	4.26	10.29	-300.54	14.55	-122.453	37.936	4.60	10.29	-253.98	14.90	11	8/1/2014	08:18.9
940	RWD11	-122.453	37.936	4.22	10.27	-315.56	14.49	-122.453	37.936	4.60	10.27	-270.98	14.88	11	8/1/2014	08:19.0
941	RWD11	-122.453	37.936	4.26	10.31	-330.39	14.57	-122.453	37.936	4.66	10.31	-287.95	14.96	11	8/1/2014	08:19.1
942	RWD11	-122.453	37.936	4.22	10.28	-344.82	14.50	-122.453	37.936	4.60	10.28	-304.45	14.88	11	8/1/2014	08:19.2
943	RWD11	-122.453	37.936	4.26	10.27	-358.59	14.53	-122.453	37.936	4.60	10.27	-320.11	14.88	11	8/1/2014	08:19.3
944	RWD11	-122.453	37.936	4.26	10.27	-371.56	14.53	-122.453	37.936	4.60	10.27	-334.89	14.88	11	8/1/2014	08:19.4
945	RWD11	-122.453	37.936	4.26	10.27	-383.86	14.53	-122.453	37.936	4.60	10.27	-348.86	14.88	11	8/1/2014	08:19.5
946	RWD11	-122.453	37.936	4.26	10.25	-395.72	14.50	-122.453	37.936	4.66	10.25	-362.17	14.90	11	8/1/2014	08:19.6
947	RWD11	-122.453	37.936	4.26	10.22	-407.35	14.48	-122.453	37.936	4.66	10.22	-375.12	14.88	11	8/1/2014	08:19.7
948	RWD11	-122.453	37.936	4.26	10.21	-418.89	14.46	-122.453	37.936	4.66	10.21	-387.92	14.86	11	8/1/2014	08:19.8
949	RWD11	-122.453	37.936	4.26	10.20	-430.32	14.45	-122.453	37.936	4.66	10.20	-400.65	14.85	11	8/1/2014	08:19.9
950	RWD11	-122.453	37.936	4.26	10.20	-441.95	14.45	-122.453	37.936	4.66	10.20	-413.45	14.85	11	8/1/2014	08:20.0
951	RWD11	-122.453	37.936	4.29	10.18	-453.77	14.47	-122.453	37.936	4.69	10.18	-426.52	14.87	11	8/1/2014	08:20.1
952	RWD11	-122.453	37.936	4.29	10.16	-465.73	14.45	-122.453	37.936	4.66	10.16	-439.74	14.82	11	8/1/2014	08:20.2
953	RWD11	-122.453	37.936	4.29	10.15	-477.65	14.44	-122.453	37.936	4.69	10.15	-452.84	14.84	11	8/1/2014	08:20.3
954	RWD11	-122.453	37.936	4.29	10.17	-489.38	14.46	-122.453	37.936	4.66	10.17	-465.83	14.83	11	8/1/2014	08:20.4
955	RWD11	-122.453	37.936	4.29	10.20	-501.00	14.49	-122.453	37.936	4.66	10.20	-478.58	14.86	11	8/1/2014	08:20.5
956	RWD11	-122.453	37.936	4.29	10.22	-512.33	14.51	-122.453	37.936	4.66	10.22	-491.14	14.87	11	8/1/2014	08:20.6
957	RWD11	-122.453	37.936	4.29	10.19	-523.43	14.48	-122.453	37.936	4.66	10.19	-503.48	14.84	11	8/1/2014	08:20.7
958	RWD11	-122.453	37.936	4.29	10.21	-533.93	14.50	-122.453	37.936	4.60	10.21	-515.22	14.81	11	8/1/2014	08:20.8
959	RWD11	-122.453	37.936	4.29	10.22	-543.82	14.51	-122.453	37.936	4.60	10.22	-526.45	14.82	11	8/1/2014	08:20.9

960	RWD11	-122.453	37.936	4.29	10.25	-553.21	14.54	-122.453	37.936	4.60	10.25	-537.12	14.85	11	8/1/2014	08:21.0
961	RWD11	-122.453	37.936	4.29	10.27	-562.30	14.56	-122.453	37.936	4.60	10.27	-547.46	14.87	11	8/1/2014	08:21.1
962	RWD11	-122.453	37.936	4.26	10.24	-571.01	14.50	-122.453	37.936	4.57	10.24	-557.40	14.81	11	8/1/2014	08:21.2
963	RWD11	-122.453	37.936	4.29	10.23	-579.65	14.52	-122.453	37.936	4.60	10.23	-567.24	14.84	11	8/1/2014	08:21.3
964	RWD11	-122.453	37.936	4.26	10.27	-588.24	14.53	-122.453	37.936	4.57	10.27	-577.02	14.84	11	8/1/2014	08:21.4
965	RWD11	-122.453	37.936	4.29	10.26	-596.98	14.55	-122.453	37.936	4.60	10.26	-586.78	14.86	11	8/1/2014	08:21.5
966	RWD11	-122.453	37.936	4.29	10.26	-605.74	14.55	-122.453	37.936	4.57	10.26	-596.72	14.83	11	8/1/2014	08:21.6
967	RWD11	-122.453	37.936	4.29	10.28	-614.70	14.57	-122.453	37.936	4.57	10.28	-606.66	14.85	11	8/1/2014	08:21.7
968	RWD11	-122.453	37.936	4.26	10.28	-623.70	14.53	-122.453	37.936	4.57	10.28	-616.95	14.85	11	8/1/2014	08:21.8
969	RWD11	-122.453	37.936	4.29	10.27	-632.75	14.56	-122.453	37.936	4.54	10.27	-627.07	14.81	11	8/1/2014	08:21.9
970	RWD11	-122.453	37.936	4.26	10.27	-641.59	14.52	-122.453	37.936	4.57	10.27	-637.08	14.84	11	8/1/2014	08:22.0
971	RWD11	-122.453	37.936	4.29	10.27	-650.26	14.56	-122.453	37.936	4.57	10.27	-646.98	14.84	11	8/1/2014	08:22.1
972	RWD11	-122.453	37.936	4.29	10.32	-658.71	14.61	-122.453	37.936	4.49	10.32	-656.70	14.81	11	8/1/2014	08:22.2
973	RWD11	-122.453	37.936	4.26	10.32	-666.90	14.58	-122.453	37.936	4.49	10.32	-666.07	14.81	11	8/1/2014	08:22.3
974	RWD11	-122.453	37.936	4.26	10.37	-674.89	14.63	-122.453	37.936	4.40	10.37	-675.12	14.77	11	8/1/2014	08:22.4
975	RWD11	-122.453	37.936	4.26	10.44	-682.50	14.70	-122.453	37.936	4.40	10.44	-683.78	14.84	11	8/1/2014	08:22.5
976	RWD11	-122.453	37.936	4.26	10.44	-689.87	14.70	-122.453	37.936	4.40	10.44	-692.06	14.84	11	8/1/2014	08:22.6
977	RWD11	-122.453	37.936	4.26	10.46	-697.01	14.72	-122.453	37.936	4.37	10.46	-699.99	14.83	11	8/1/2014	08:22.7
978	RWD11	-122.453	37.936	4.22	10.48	-703.60	14.70	-122.453	37.936	4.32	10.48	-707.51	14.80	11	8/1/2014	08:22.8
979	RWD11	-122.453	37.936	4.22	10.48	-710.03	14.70	-122.453	37.936	4.28	10.48	-714.71	14.76	11	8/1/2014	08:22.9
980	RWD11	-122.453	37.936	4.22	10.54	-716.22	14.76	-122.453	37.936	4.23	10.54	-721.58	14.77	11	8/1/2014	08:23.0
981	RWD11	-122.453	37.936	4.22	10.53	-722.17	14.76	-122.453	37.936	4.23	10.53	-728.33	14.77	11	8/1/2014	08:23.1
982	RWD11	-122.453	37.936	4.17	10.56	-728.06	14.73	-122.453	37.936	4.20	10.56	-734.78	14.76	11	8/1/2014	08:23.2
983	RWD11	-122.453	37.936	4.17	10.58	-733.89	14.75	-122.453	37.936	4.20	10.58	-741.27	14.78	11	8/1/2014	08:23.3
984	RWD11	-122.453	37.936	4.17	10.64	-739.73	14.81	-122.453	37.936	4.16	10.64	-747.61	14.80	11	8/1/2014	08:23.4
985	RWD11	-122.453	37.936	4.17	10.65	-745.43	14.82	-122.453	37.936	4.16	10.65	-753.95	14.82	11	8/1/2014	08:23.5
986	RWD11	-122.453	37.936	4.17	10.68	-751.19	14.85	-122.453	37.936	4.11	10.68	-760.16	14.79	11	8/1/2014	08:23.6
987	RWD11	-122.453	37.936	4.17	10.68	-756.86	14.85	-122.453	37.936	4.11	10.68	-766.29	14.79	11	8/1/2014	08:23.7
988	RWD11	-122.453	37.936	4.14	10.69	-762.41	14.82	-122.453	37.936	4.08	10.69	-772.39	14.77	11	8/1/2014	08:23.8
989	RWD11	-122.453	37.936	4.14	10.74	-767.73	14.88	-122.453	37.936	4.03	10.74	-778.21	14.77	11	8/1/2014	08:23.9
990	RWD11	-122.453	37.936	4.09	10.78	-772.98	14.86	-122.453	37.936	3.99	10.78	-784.13	14.77	11	8/1/2014	08:24.0
991	RWD11	-122.453	37.936	4.14	10.77	-778.03	14.91	-122.453	37.936	3.99	10.77	-789.72	14.77	11	8/1/2014	08:24.1
992	RWD11	-122.453	37.936	4.09	10.79	-782.95	14.88	-122.453	37.936	3.94	10.79	-795.23	14.73	11	8/1/2014	08:24.2

993	RWD11	-122.453	37.936	4.09	10.83	-787.65	14.92	-122.453	37.936	3.91	10.83	-800.56	14.74	11	8/1/2014 08:24.3
994	RWD11	-122.453	37.936	4.05	10.89	-792.25	14.94	-122.453	37.936	3.88	10.89	-805.73	14.76	11	8/1/2014 08:24.4
995	RWD11	-122.453	37.936	4.09	10.83	-796.64	14.92	-122.453	37.936	3.88	10.83	-810.72	14.71	11	8/1/2014 08:24.5
996	RWD11	-122.453	37.936	4.05	10.85	-800.96	14.90	-122.453	37.936	3.83	10.85	-815.56	14.68	11	8/1/2014 08:24.6
997	RWD11	-122.453	37.936	4.05	10.86	-805.17	14.91	-122.453	37.936	3.83	10.86	-820.24	14.69	11	8/1/2014 08:24.7
998	RWD11	-122.453	37.936	4.00	10.93	-809.23	14.93	-122.453	37.936	3.79	10.93	-824.76	14.72	11	8/1/2014 08:24.8
999	RWD11	-122.453	37.936	4.00	10.95	-813.32	14.95	-122.453	37.936	3.79	10.95	-829.22	14.74	11	8/1/2014 08:24.9
1000	RWD11	-122.453	37.936	4.00	10.94	-817.34	14.94	-122.453	37.936	3.79	10.94	-833.70	14.73	11	8/1/2014 08:25.0
1001	RWD11	-122.453	37.936	4.00	10.94	-821.37	14.93	-122.453	37.936	3.79	10.94	-838.19	14.73	11	8/1/2014 08:25.1
1002	RWD11	-122.453	37.936	3.96	10.98	-825.40	14.95	-122.453	37.936	3.71	10.98	-842.78	14.69	11	8/1/2014 08:25.2
1003	RWD11	-122.453	37.936	3.96	10.97	-829.37	14.94	-122.453	37.936	3.71	10.97	-847.18	14.68	11	8/1/2014 08:25.3
1004	RWD11	-122.453	37.936	3.93	11.01	-833.18	14.94	-122.453	37.936	3.62	11.01	-851.35	14.63	11	8/1/2014 08:25.4
1005	RWD11	-122.453	37.936	3.93	11.11	-836.83	15.04	-122.453	37.936	3.62	11.11	-855.36	14.74	11	8/1/2014 08:25.5
1006	RWD11	-122.453	37.936	3.93	11.07	-840.14	15.01	-122.453	37.936	3.57	11.07	-859.08	14.65	11	8/1/2014 08:25.6
1007	RWD11	-122.453	37.936	3.93	11.09	-843.45	15.02	-122.453	37.936	3.57	11.09	-862.64	14.66	11	8/1/2014 08:25.7
1008	RWD11	-122.453	37.936	3.88	11.09	-846.58	14.97	-122.453	37.936	3.54	11.09	-866.13	14.63	11	8/1/2014 08:25.8
1009	RWD11	-122.453	37.936	3.88	11.12	-849.73	15.00	-122.453	37.936	3.54	11.12	-869.55	14.66	11	8/1/2014 08:25.9
1010	RWD11	-122.453	37.936	3.85	11.10	-852.71	14.94	-122.453	37.936	3.54	11.10	-872.79	14.63	11	8/1/2014 08:26.0
1011	RWD11	-122.453	37.936	3.88	11.11	-855.73	14.98	-122.453	37.936	3.54	11.11	-876.06	14.64	11	8/1/2014 08:26.1
1012	RWD11	-122.453	37.936	3.85	11.11	-858.67	14.95	-122.453	37.936	3.54	11.11	-879.24	14.64	11	8/1/2014 08:26.2
1013	RWD11	-122.453	37.936	3.85	11.11	-861.67	14.96	-122.453	37.936	3.54	11.11	-882.46	14.65	11	8/1/2014 08:26.3
1014	RWD11	-122.453	37.936	3.85	11.16	-864.51	15.00	-122.453	37.936	3.50	11.16	-885.67	14.66	11	8/1/2014 08:26.4
1015	RWD11	-122.453	37.936	3.85	11.13	-867.29	14.97	-122.453	37.936	3.50	11.13	-888.70	14.63	11	8/1/2014 08:26.5
1016	RWD11	-122.453	37.936	3.79	11.14	-869.99	14.94	-122.453	37.936	3.45	11.14	-891.78	14.60	11	8/1/2014 08:26.6
1017	RWD11	-122.453	37.936	3.79	11.15	-872.66	14.94	-122.453	37.936	3.45	11.15	-894.75	14.60	11	8/1/2014 08:26.7
1018	RWD11	-122.453	37.936	3.76	11.15	-875.25	14.91	-122.453	37.936	3.42	11.15	-897.58	14.57	11	8/1/2014 08:26.8
1019	RWD11	-122.453	37.936	3.79	11.13	-877.63	14.93	-122.453	37.936	3.42	11.13	-900.28	14.55	11	8/1/2014 08:26.9
1020	RWD11	-122.453	37.936	3.76	11.18	-879.81	14.94	-122.453	37.936	3.37	11.18	-902.72	14.55	11	8/1/2014 08:27.0
1021	RWD11	-122.453	37.936	3.76	11.20	-881.90	14.96	-122.453	37.936	3.37	11.20	-905.07	14.57	11	8/1/2014 08:27.1
1022	RWD11	-122.453	37.936	3.71	11.14	-883.89	14.85	-122.453	37.936	3.34	11.14	-907.40	14.48	11	8/1/2014 08:27.2
1023	RWD11	-122.453	37.936	3.76	11.18	-885.96	14.93	-122.453	37.936	3.37	11.18	-909.65	14.54	11	8/1/2014 08:27.3
1024	RWD11	-122.453	37.936	3.71	11.17	-888.02	14.87	-122.453	37.936	3.37	11.17	-911.95	14.54	11	8/1/2014 08:27.4
1025	RWD11	-122.453	37.936	3.71	11.14	-890.12	14.85	-122.453	37.936	3.37	11.14	-914.16	14.51	11	8/1/2014 08:27.5

1026	RWD11	-122.453	37.936	3.71	11.13	-892.18	14.84	-122.453	37.936	3.34	11.13	-916.39	14.47	11	8/1/2014	08:27.6
1027	RWD11	-122.453	37.936	3.71	11.14	-894.28	14.85	-122.453	37.936	3.37	11.14	-918.63	14.51	11	8/1/2014	08:27.7
1028	RWD11	-122.453	37.936	3.67	11.13	-896.32	14.80	-122.453	37.936	3.37	11.13	-920.88	14.50	11	8/1/2014	08:27.8
1029	RWD11	-122.453	37.936	3.71	11.12	-898.24	14.83	-122.453	37.936	3.37	11.12	-923.10	14.49	11	8/1/2014	08:27.9
1030	RWD11	-122.453	37.936	3.67	11.11	-900.16	14.78	-122.453	37.936	3.37	11.11	-925.24	14.47	11	8/1/2014	08:28.0
1031	RWD11	-122.453	37.936	3.67	11.10	-902.08	14.77	-122.453	37.936	3.42	11.10	-927.45	14.51	11	8/1/2014	08:28.1
1032	RWD11	-122.453	37.936	3.62	11.12	-903.88	14.74	-122.453	37.936	3.33	11.12	-929.52	14.46	11	8/1/2014	08:28.2
1033	RWD11	-122.453	37.936	3.67	11.08	-905.61	14.75	-122.453	37.936	3.37	11.08	-931.42	14.45	11	8/1/2014	08:28.3
1034	RWD11	-122.453	37.936	3.67	11.08	-907.18	14.75	-122.453	37.936	3.33	11.08	-933.30	14.41	11	8/1/2014	08:28.4
1035	RWD11	-122.453	37.936	3.67	11.10	-908.62	14.77	-122.453	37.936	3.33	11.10	-934.99	14.43	11	8/1/2014	08:28.5
1036	RWD11	-122.453	37.936	3.62	11.08	-910.01	14.70	-122.453	37.936	3.28	11.08	-936.60	14.36	11	8/1/2014	08:28.6
1037	RWD11	-122.453	37.936	3.67	11.06	-911.31	14.74	-122.453	37.936	3.28	11.06	-938.03	14.35	11	8/1/2014	08:28.7
1038	RWD11	-122.453	37.936	3.62	11.06	-912.61	14.69	-122.453	37.936	3.28	11.06	-939.46	14.35	11	8/1/2014	08:28.8
1039	RWD11	-122.453	37.936	3.62	11.06	-913.92	14.68	-122.453	37.936	3.33	11.06	-940.85	14.39	11	8/1/2014	08:28.9
1040	RWD11	-122.453	37.936	3.62	11.04	-915.22	14.66	-122.453	37.936	3.33	11.04	-942.19	14.37	11	8/1/2014	08:29.0
1041	RWD11	-122.453	37.936	3.62	11.02	-916.57	14.64	-122.453	37.936	3.37	11.02	-943.58	14.39	11	8/1/2014	08:29.1
1042	RWD11	-122.453	37.936	3.59	11.07	-917.92	14.66	-122.453	37.936	3.37	11.07	-944.97	14.44	11	8/1/2014	08:29.2
1043	RWD11	-122.453	37.936	3.62	11.00	-919.28	14.63	-122.453	37.936	3.42	11.00	-946.31	14.42	11	8/1/2014	08:29.3
1044	RWD11	-122.453	37.936	3.62	11.02	-920.60	14.64	-122.453	37.936	3.42	11.02	-947.59	14.44	11	8/1/2014	08:29.4
1045	RWD11	-122.453	37.936	3.62	11.01	-921.88	14.63	-122.453	37.936	3.42	11.01	-948.94	14.43	11	8/1/2014	08:29.5
1046	RWD11	-122.453	37.936	3.62	10.97	-923.04	14.59	-122.453	37.936	3.42	10.97	-950.13	14.39	11	8/1/2014	08:29.6
1047	RWD11	-122.453	37.936	3.62	10.97	-924.11	14.59	-122.453	37.936	3.42	10.97	-951.24	14.39	11	8/1/2014	08:29.7
1048	RWD11	-122.453	37.936	3.59	10.98	-924.95	14.57	-122.453	37.936	3.37	10.98	-952.15	14.35	11	8/1/2014	08:29.8
1049	RWD11	-122.453	37.936	3.59	11.02	-925.63	14.61	-122.453	37.936	3.37	11.02	-952.80	14.39	11	8/1/2014	08:29.9
1050	RWD11	-122.453	37.936	3.59	10.98	-926.17	14.57	-122.453	37.936	3.33	10.98	-953.38	14.32	11	8/1/2014	08:30.0
1051	RWD11	-122.453	37.936	3.59	11.00	-926.58	14.59	-122.453	37.936	3.37	11.00	-953.74	14.37	11	8/1/2014	08:30.1
1052	RWD11	-122.453	37.936	3.59	10.99	-927.05	14.58	-122.453	37.936	3.33	10.99	-954.19	14.32	11	8/1/2014	08:30.2
1053	RWD11	-122.453	37.936	3.62	10.99	-927.45	14.62	-122.453	37.936	3.37	10.99	-954.53	14.36	11	8/1/2014	08:30.3
1054	RWD11	-122.453	37.936	3.59	11.02	-927.86	14.61	-122.453	37.936	3.37	11.02	-954.95	14.39	11	8/1/2014	08:30.4
1055	RWD11	-122.453	37.936	3.59	10.99	-928.27	14.58	-122.453	37.936	3.37	10.99	-955.30	14.36	11	8/1/2014	08:30.5
1056	RWD11	-122.453	37.936	3.59	10.99	-928.74	14.58	-122.453	37.936	3.37	10.99	-955.71	14.36	11	8/1/2014	08:30.6
1057	RWD11	-122.453	37.936	3.59	10.97	-929.11	14.56	-122.453	37.936	3.42	10.97	-956.07	14.39	11	8/1/2014	08:30.7
1058	RWD11	-122.453	37.936	3.59	10.97	-929.57	14.55	-122.453	37.936	3.42	10.97	-956.44	14.39	11	8/1/2014	08:30.8

1059	RWD11	-122.453	37.936	3.59	10.96	-930.04	14.54	-122.453	37.936	3.42	10.96	-956.86	14.38	11	8/1/2014	08:30.9
1060	RWD11	-122.453	37.936	3.59	11.00	-930.40	14.59	-122.453	37.936	3.45	11.00	-957.07	14.46	11	8/1/2014	08:31.0
1061	RWD11	-122.453	37.936	3.59	10.97	-930.61	14.56	-122.453	37.936	3.45	10.97	-957.18	14.42	11	8/1/2014	08:31.1
1062	RWD11	-122.453	37.936	3.59	10.97	-930.70	14.56	-122.453	37.936	3.45	10.97	-957.13	14.42	11	8/1/2014	08:31.2
1063	RWD11	-122.453	37.936	3.59	10.99	-930.61	14.58	-122.453	37.936	3.45	10.99	-956.86	14.45	11	8/1/2014	08:31.3
1064	RWD11	-122.453	37.936	3.59	11.00	-930.35	14.59	-122.453	37.936	3.42	11.00	-956.33	14.42	11	8/1/2014	08:31.4
1065	RWD11	-122.453	37.936	3.59	11.00	-929.83	14.59	-122.453	37.936	3.42	11.00	-955.63	14.42	11	8/1/2014	08:31.5
1066	RWD11	-122.453	37.936	3.59	10.99	-929.34	14.58	-122.453	37.936	3.42	10.99	-954.83	14.41	11	8/1/2014	08:31.6
1067	RWD11	-122.453	37.936	3.59	10.99	-928.87	14.58	-122.453	37.936	3.42	10.99	-954.16	14.41	11	8/1/2014	08:31.7
1068	RWD11	-122.453	37.936	3.59	11.04	-928.45	14.63	-122.453	37.936	3.42	11.04	-953.54	14.46	11	8/1/2014	08:31.8
1069	RWD11	-122.453	37.936	3.59	11.02	-927.97	14.61	-122.453	37.936	3.45	11.02	-952.83	14.47	11	8/1/2014	08:31.9
1070	RWD11	-122.453	37.936	3.59	11.02	-927.55	14.61	-122.453	37.936	3.45	11.02	-952.25	14.47	11	8/1/2014	08:32.0
1071	RWD11	-122.453	37.936	3.59	11.04	-927.12	14.63	-122.453	37.936	3.50	11.04	-951.54	14.54	11	8/1/2014	08:32.1
1072	RWD11	-122.453	37.936	3.55	11.00	-926.58	14.56	-122.453	37.936	3.50	11.00	-950.89	14.51	11	8/1/2014	08:32.2
1073	RWD11	-122.453	37.936	3.59	10.99	-926.01	14.58	-122.453	37.936	3.50	10.99	-950.23	14.50	11	8/1/2014	08:32.3
1074	RWD11	-122.453	37.936	3.59	10.99	-925.42	14.58	-122.453	37.936	3.50	10.99	-949.55	14.50	11	8/1/2014	08:32.4
1075	RWD11	-122.453	37.936	3.59	10.99	-924.73	14.58	-122.453	37.936	3.57	10.99	-948.81	14.57	11	8/1/2014	08:32.5
1076	RWD11	-122.453	37.936	3.59	11.00	-923.99	14.59	-122.453	37.936	3.54	11.00	-947.93	14.54	11	8/1/2014	08:32.6
1077	RWD11	-122.453	37.936	3.59	11.00	-923.05	14.59	-122.453	37.936	3.57	11.00	-946.96	14.57	11	8/1/2014	08:32.7
1078	RWD11	-122.453	37.936	3.59	11.01	-922.01	14.60	-122.453	37.936	3.54	11.01	-945.80	14.55	11	8/1/2014	08:32.8
1079	RWD11	-122.453	37.936	3.59	11.11	-920.83	14.70	-122.453	37.936	3.57	11.11	-944.39	14.68	11	8/1/2014	08:32.9
1080	RWD11	-122.453	37.936	3.59	11.07	-919.47	14.66	-122.453	37.936	3.57	11.07	-942.78	14.65	11	8/1/2014	08:33.0
1081	RWD11	-122.453	37.936	3.59	11.06	-918.03	14.64	-122.453	37.936	3.62	11.06	-941.06	14.68	11	8/1/2014	08:33.1
1082	RWD11	-122.453	37.936	3.59	11.08	-916.44	14.67	-122.453	37.936	3.54	11.08	-939.25	14.62	11	8/1/2014	08:33.2
1083	RWD11	-122.453	37.936	3.62	11.07	-914.97	14.70	-122.453	37.936	3.57	11.07	-937.39	14.65	11	8/1/2014	08:33.3
1084	RWD11	-122.453	37.936	3.59	11.07	-913.57	14.66	-122.453	37.936	3.57	11.07	-935.61	14.65	11	8/1/2014	08:33.4
1085	RWD11	-122.453	37.936	3.62	11.07	-912.21	14.70	-122.453	37.936	3.57	11.07	-933.84	14.65	11	8/1/2014	08:33.5
1086	RWD11	-122.453	37.936	3.59	11.06	-910.84	14.65	-122.453	37.936	3.57	11.06	-932.13	14.64	11	8/1/2014	08:33.6
1087	RWD11	-122.453	37.936	3.62	11.08	-909.53	14.70	-122.453	37.936	3.66	11.08	-930.40	14.74	11	8/1/2014	08:33.7
1088	RWD11	-122.453	37.936	3.59	11.10	-908.28	14.68	-122.453	37.936	3.62	11.10	-928.75	14.72	11	8/1/2014	08:33.8
1089	RWD11	-122.453	37.936	3.62	11.07	-906.90	14.70	-122.453	37.936	3.66	11.07	-926.95	14.73	11	8/1/2014	08:33.9
1090	RWD11	-122.453	37.936	3.59	11.07	-905.46	14.66	-122.453	37.936	3.66	11.07	-925.03	14.73	11	8/1/2014	08:34.0
1091	RWD11	-122.453	37.936	3.62	11.07	-904.05	14.70	-122.453	37.936	3.71	11.07	-923.08	14.78	11	8/1/2014	08:34.1

1092	RWD11	-122.453	37.936	3.59	11.06	-902.44	14.65	-122.453	37.936	3.66	11.06	-920.98	14.72	11	8/1/2014	08:34.2
1093	RWD11	-122.453	37.936	3.62	11.07	-900.66	14.70	-122.453	37.936	3.74	11.07	-918.71	14.81	11	8/1/2014	08:34.3
1094	RWD11	-122.453	37.936	3.59	11.13	-898.65	14.72	-122.453	37.936	3.71	11.13	-916.26	14.83	11	8/1/2014	08:34.4
1095	RWD11	-122.453	37.936	3.62	11.14	-896.33	14.77	-122.453	37.936	3.71	11.14	-913.43	14.85	11	8/1/2014	08:34.5
1096	RWD11	-122.453	37.936	3.59	11.14	-893.67	14.73	-122.453	37.936	3.71	11.14	-910.36	14.85	11	8/1/2014	08:34.6
1097	RWD11	-122.453	37.936	3.62	11.18	-890.94	14.80	-122.453	37.936	3.71	11.18	-907.09	14.88	11	8/1/2014	08:34.7
1098	RWD11	-122.453	37.936	3.59	11.21	-888.15	14.80	-122.453	37.936	3.66	11.21	-903.87	14.87	11	8/1/2014	08:34.8
1099	RWD11	-122.453	37.936	3.62	11.20	-885.45	14.83	-122.453	37.936	3.71	11.20	-900.66	14.91	11	8/1/2014	08:34.9
1100	RWD11	-122.453	37.936	3.62	11.23	-882.92	14.85	-122.453	37.936	3.66	11.23	-897.73	14.88	11	8/1/2014	08:35.0
1101	RWD11	-122.453	37.936	3.62	11.25	-880.60	14.87	-122.453	37.936	3.71	11.25	-894.86	14.96	11	8/1/2014	08:35.1
1102	RWD11	-122.453	37.936	3.59	11.25	-878.31	14.84	-122.453	37.936	3.74	11.25	-892.18	14.99	11	8/1/2014	08:35.2
1103	RWD11	-122.453	37.936	3.62	11.26	-876.12	14.88	-122.453	37.936	3.74	11.26	-889.45	15.00	11	8/1/2014	08:35.3
1104	RWD11	-122.453	37.936	3.62	11.25	-873.88	14.87	-122.453	37.936	3.74	11.25	-886.70	14.99	11	8/1/2014	08:35.4
1105	RWD11	-122.453	37.936	3.62	11.25	-871.56	14.87	-122.453	37.936	3.79	11.25	-883.88	15.04	11	8/1/2014	08:35.5
1106	RWD11	-122.453	37.936	3.62	11.30	-869.15	14.92	-122.453	37.936	3.79	11.30	-880.97	15.09	11	8/1/2014	08:35.6
1107	RWD11	-122.453	37.936	3.67	11.30	-866.62	14.97	-122.453	37.936	3.83	11.30	-877.82	15.12	11	8/1/2014	08:35.7
1108	RWD11	-122.453	37.936	3.67	11.29	-863.84	14.96	-122.453	37.936	3.83	11.29	-874.50	15.11	11	8/1/2014	08:35.8
1109	RWD11	-122.453	37.936	3.67	11.37	-860.82	15.04	-122.453	37.936	3.83	11.37	-870.88	15.19	11	8/1/2014	08:35.9
1110	RWD11	-122.453	37.936	3.67	11.34	-857.42	15.01	-122.453	37.936	3.83	11.34	-866.98	15.16	11	8/1/2014	08:36.0
1111	RWD11	-122.453	37.936	3.67	11.39	-853.68	15.06	-122.453	37.936	3.83	11.39	-862.76	15.22	11	8/1/2014	08:36.1
1112	RWD11	-122.453	37.936	3.67	11.47	-849.90	15.14	-122.453	37.936	3.79	11.47	-858.40	15.26	11	8/1/2014	08:36.2
1113	RWD11	-122.453	37.936	3.67	11.56	-846.07	15.23	-122.453	37.936	3.83	11.56	-854.08	15.39	11	8/1/2014	08:36.3
1114	RWD11	-122.453	37.936	3.67	11.51	-842.33	15.19	-122.453	37.936	3.83	11.51	-849.80	15.34	11	8/1/2014	08:36.4
1115	RWD11	-122.453	37.936	3.67	11.52	-838.64	15.20	-122.453	37.936	3.83	11.52	-845.64	15.35	11	8/1/2014	08:36.5
1116	RWD11	-122.453	37.936	3.67	11.56	-835.25	15.23	-122.453	37.936	3.83	11.56	-841.72	15.39	11	8/1/2014	08:36.6
1117	RWD11	-122.453	37.936	3.67	11.58	-831.93	15.26	-122.453	37.936	3.88	11.58	-837.92	15.46	11	8/1/2014	08:36.7
1118	RWD11	-122.453	37.936	3.67	11.58	-828.90	15.26	-122.453	37.936	3.88	11.58	-834.35	15.46	11	8/1/2014	08:36.8
1119	RWD11	-122.453	37.936	3.71	11.62	-825.81	15.33	-122.453	37.936	3.91	11.62	-830.82	15.53	11	8/1/2014	08:36.9
1120	RWD11	-122.453	37.936	3.67	11.70	-822.93	15.37	-122.453	37.936	3.91	11.70	-827.32	15.61	11	8/1/2014	08:37.0
1121	RWD11	-122.453	37.936	3.71	11.68	-820.03	15.39	-122.453	37.936	3.94	11.68	-823.86	15.63	11	8/1/2014	08:37.1
1122	RWD11	-122.453	37.936	3.71	11.66	-817.06	15.37	-122.453	37.936	3.94	11.66	-820.43	15.60	11	8/1/2014	08:37.2
1123	RWD11	-122.453	37.936	3.71	11.68	-814.05	15.39	-122.453	37.936	3.99	11.68	-816.89	15.68	11	8/1/2014	08:37.3
1124	RWD11	-122.453	37.936	3.71	11.70	-810.91	15.41	-122.453	37.936	4.03	11.70	-813.20	15.73	11	8/1/2014	08:37.4

1125	RWD11	-122.453	37.936	3.76	11.74	-807.48	15.50	-122.453	37.936	4.03	11.74	-809.25	15.77	11	8/1/2014	08:37.5
1126	RWD11	-122.453	37.936	3.71	11.85	-803.57	15.55	-122.453	37.936	4.03	11.85	-804.75	15.87	11	8/1/2014	08:37.6
1127	RWD11	-122.453	37.936	3.76	11.81	-799.01	15.57	-122.453	37.936	4.03	11.81	-799.69	15.84	11	8/1/2014	08:37.7
1128	RWD11	-122.453	37.936	3.71	11.98	-794.04	15.68	-122.453	37.936	3.99	11.98	-794.09	15.97	11	8/1/2014	08:37.8
1129	RWD11	-122.453	37.936	3.76	12.05	-789.01	15.81	-122.453	37.936	3.99	12.05	-788.47	16.05	11	8/1/2014	08:37.9
1130	RWD11	-122.453	37.936	3.76	12.16	-784.09	15.92	-122.453	37.936	3.99	12.16	-782.83	16.16	11	8/1/2014	08:38.0
1131	RWD11	-122.453	37.936	3.76	12.09	-779.34	15.85	-122.453	37.936	3.99	12.09	-777.39	16.09	11	8/1/2014	08:38.1
1132	RWD11	-122.453	37.936	3.76	12.23	-774.95	15.99	-122.453	37.936	4.03	12.23	-772.24	16.26	11	8/1/2014	08:38.2
1133	RWD11	-122.453	37.936	3.76	12.16	-770.74	15.92	-122.453	37.936	4.03	12.16	-767.27	16.19	11	8/1/2014	08:38.3
1134	RWD11	-122.453	37.936	3.76	12.17	-766.75	15.93	-122.453	37.936	4.03	12.17	-762.52	16.20	11	8/1/2014	08:38.4
1135	RWD11	-122.453	37.936	3.76	12.18	-762.98	15.94	-122.453	37.936	4.08	12.18	-757.95	16.26	11	8/1/2014	08:38.5
1136	RWD11	-122.453	37.936	3.76	12.18	-759.31	15.94	-122.453	37.936	4.08	12.18	-753.45	16.26	11	8/1/2014	08:38.6
1137	RWD11	-122.453	37.936	3.79	12.19	-755.74	15.99	-122.453	37.936	4.11	12.19	-749.07	16.30	11	8/1/2014	08:38.7
1138	RWD11	-122.453	37.936	3.79	12.19	-752.21	15.99	-122.453	37.936	4.16	12.19	-744.75	16.36	11	8/1/2014	08:38.8
1139	RWD11	-122.453	37.936	3.84	12.20	-748.54	16.04	-122.453	37.936	4.16	12.20	-740.38	16.36	11	8/1/2014	08:38.9
1140	RWD11	-122.453	37.936	3.79	12.18	-744.82	15.97	-122.453	37.936	4.16	12.18	-736.00	16.34	11	8/1/2014	08:39.0
1141	RWD11	-122.453	37.936	3.84	12.18	-740.89	16.03	-122.453	37.936	4.23	12.18	-731.42	16.41	11	8/1/2014	08:39.1
1142	RWD11	-122.453	37.936	3.84	12.21	-736.61	16.05	-122.453	37.936	4.23	12.21	-726.54	16.44	11	8/1/2014	08:39.2
1143	RWD11	-122.453	37.936	3.84	12.26	-731.73	16.11	-122.453	37.936	4.23	12.26	-721.00	16.49	11	8/1/2014	08:39.3
1144	RWD11	-122.453	37.936	3.84	12.31	-726.06	16.15	-122.453	37.936	4.23	12.31	-714.68	16.54	11	8/1/2014	08:39.4
1145	RWD11	-122.453	37.936	3.88	12.35	-719.91	16.23	-122.453	37.936	4.23	12.35	-707.80	16.59	11	8/1/2014	08:39.5
1146	RWD11	-122.453	37.936	3.84	12.41	-713.53	16.25	-122.453	37.936	4.20	12.41	-700.60	16.61	11	8/1/2014	08:39.6
1147	RWD11	-122.453	37.936	3.88	12.50	-707.16	16.38	-122.453	37.936	4.23	12.50	-693.38	16.73	11	8/1/2014	08:39.7
1148	RWD11	-122.453	37.936	3.88	12.51	-701.15	16.39	-122.453	37.936	4.20	12.51	-686.33	16.71	11	8/1/2014	08:39.8
1149	RWD11	-122.453	37.936	3.88	12.56	-695.36	16.43	-122.453	37.936	4.23	12.56	-679.56	16.79	11	8/1/2014	08:39.9
1150	RWD11	-122.453	37.936	3.88	12.67	-689.78	16.55	-122.453	37.936	4.23	12.67	-672.98	16.90	11	8/1/2014	08:40.0
1151	RWD11	-122.453	37.936	3.93	12.63	-684.36	16.56	-122.453	37.936	4.28	12.63	-666.71	16.91	11	8/1/2014	08:40.1
1152	RWD11	-122.453	37.936	3.93	12.71	-678.92	16.64	-122.453	37.936	4.28	12.71	-660.49	16.99	11	8/1/2014	08:40.2
1153	RWD11	-122.453	37.936	3.93	12.71	-673.65	16.64	-122.453	37.936	4.32	12.71	-654.33	17.02	11	8/1/2014	08:40.3
1154	RWD11	-122.453	37.936	3.93	12.67	-668.24	16.60	-122.453	37.936	4.32	12.67	-648.03	16.99	11	8/1/2014	08:40.4
1155	RWD11	-122.453	37.936	3.96	12.71	-662.69	16.67	-122.453	37.936	4.32	12.71	-641.71	17.02	11	8/1/2014	08:40.5
1156	RWD11	-122.453	37.936	3.93	12.74	-656.93	16.67	-122.453	37.936	4.32	12.74	-635.21	17.06	11	8/1/2014	08:40.6
1157	RWD11	-122.453	37.936	3.96	12.71	-650.96	16.67	-122.453	37.936	4.37	12.71	-628.49	17.07	11	8/1/2014	08:40.7

1158	RWD11	-122.453	37.936	3.96	12.73	-644.76	16.70	-122.453	37.936	4.36	12.73	-621.65	17.10	11	8/1/2014	08:40.8
1159	RWD11	-122.453	37.936	4.00	12.72	-638.36	16.71	-122.453	37.936	4.40	12.72	-614.66	17.12	11	8/1/2014	08:40.9
1160	RWD11	-122.453	37.936	3.96	12.72	-631.48	16.68	-122.453	37.936	4.40	12.72	-607.19	17.12	11	8/1/2014	08:41.0
1161	RWD11	-122.453	37.936	4.00	12.77	-623.96	16.76	-122.453	37.936	4.40	12.77	-599.11	17.16	11	8/1/2014	08:41.1
1162	RWD11	-122.453	37.936	4.00	12.78	-615.81	16.77	-122.453	37.936	4.36	12.78	-590.38	17.14	11	8/1/2014	08:41.2
1163	RWD11	-122.453	37.936	4.00	12.78	-607.15	16.77	-122.453	37.936	4.36	12.78	-581.19	17.14	11	8/1/2014	08:41.3
1164	RWD11	-122.453	37.936	4.00	12.80	-598.30	16.80	-122.453	37.936	4.36	12.80	-571.61	17.17	11	8/1/2014	08:41.4
1165	RWD11	-122.453	37.936	4.05	12.84	-589.33	16.89	-122.453	37.936	4.36	12.84	-561.96	17.20	11	8/1/2014	08:41.5
1166	RWD11	-122.453	37.936	4.00	12.81	-580.73	16.81	-122.453	37.936	4.32	12.81	-552.56	17.12	11	8/1/2014	08:41.6
1167	RWD11	-122.453	37.936	4.05	12.91	-572.40	16.96	-122.453	37.936	4.36	12.91	-543.45	17.27	11	8/1/2014	08:41.7
1168	RWD11	-122.453	37.936	4.05	12.83	-564.46	16.88	-122.453	37.936	4.40	12.83	-534.69	17.23	11	8/1/2014	08:41.8
1169	RWD11	-122.453	37.936	4.05	12.82	-556.70	16.87	-122.453	37.936	4.40	12.82	-526.17	17.22	11	8/1/2014	08:41.9
1170	RWD11	-122.453	37.936	4.05	12.83	-549.21	16.88	-122.453	37.936	4.40	12.83	-517.82	17.23	11	8/1/2014	08:42.0
1171	RWD11	-122.453	37.936	4.08	12.82	-541.78	16.91	-122.453	37.936	4.45	12.82	-509.65	17.27	11	8/1/2014	08:42.1
1172	RWD11	-122.453	37.936	4.05	12.85	-534.45	16.89	-122.453	37.936	4.45	12.85	-501.45	17.30	11	8/1/2014	08:42.2
1173	RWD11	-122.453	37.936	4.08	12.81	-527.05	16.89	-122.453	37.936	4.45	12.81	-493.21	17.26	11	8/1/2014	08:42.3
1174	RWD11	-122.453	37.936	4.08	12.82	-519.53	16.90	-122.453	37.936	4.48	12.82	-484.92	17.30	11	8/1/2014	08:42.4
1175	RWD11	-122.453	37.936	4.13	12.83	-511.89	16.97	-122.453	37.936	4.48	12.83	-476.49	17.32	11	8/1/2014	08:42.5
1176	RWD11	-122.453	37.936	4.08	12.82	-503.84	16.90	-122.453	37.936	4.53	12.82	-467.63	17.35	11	8/1/2014	08:42.6
1177	RWD11	-122.453	37.936	4.13	12.81	-495.13	16.94	-122.453	37.936	4.57	12.81	-458.26	17.38	11	8/1/2014	08:42.7
1178	RWD11	-122.453	37.936	4.13	12.80	-485.61	16.94	-122.453	37.936	4.53	12.80	-448.07	17.34	11	8/1/2014	08:42.8
1179	RWD11	-122.453	37.936	4.13	12.81	-475.34	16.94	-122.453	37.936	4.53	12.81	-437.05	17.34	11	8/1/2014	08:42.9
1180	RWD11	-122.453	37.936	4.13	12.81	-464.49	16.94	-122.453	37.936	4.48	12.81	-425.42	17.29	11	8/1/2014	08:43.0
1181	RWD11	-122.453	37.936	4.13	12.82	-453.40	16.95	-122.453	37.936	4.48	12.82	-413.49	17.30	11	8/1/2014	08:43.1
1182	RWD11	-122.453	37.936	4.13	12.82	-442.21	16.96	-122.453	37.936	4.48	12.82	-401.54	17.31	11	8/1/2014	08:43.2
1183	RWD11	-122.453	37.936	4.13	12.83	-431.08	16.97	-122.453	37.936	4.53	12.83	-389.52	17.37	11	8/1/2014	08:43.3
1184	RWD11	-122.453	37.936	4.13	12.85	-420.01	16.98	-122.453	37.936	4.53	12.85	-377.48	17.38	11	8/1/2014	08:43.4
1185	RWD11	-122.453	37.936	4.17	12.79	-408.94	16.96	-122.453	37.936	4.53	12.79	-365.46	17.33	11	8/1/2014	08:43.5
1186	RWD11	-122.453	37.936	4.17	12.81	-397.79	16.98	-122.453	37.936	4.53	12.81	-353.29	17.34	11	8/1/2014	08:43.6
1187	RWD11	-122.453	37.936	4.17	12.81	-386.47	16.98	-122.453	37.936	4.53	12.81	-340.92	17.34	11	8/1/2014	08:43.7
1188	RWD11	-122.453	37.936	4.17	12.84	-374.93	17.01	-122.453	37.936	4.57	12.84	-328.30	17.41	11	8/1/2014	08:43.8
1189	RWD11	-122.453	37.936	4.17	12.83	-362.97	17.00	-122.453	37.936	4.53	12.83	-315.39	17.37	11	8/1/2014	08:43.9
1190	RWD11	-122.453	37.936	4.17	12.77	-350.80	16.93	-122.453	37.936	4.53	12.77	-302.23	17.30	11	8/1/2014	08:44.0

1191	RWD11	-122.453	37.936	4.17	12.75	-338.24	16.92	-122.453	37.936	4.57	12.75	-288.74	17.32	11	8/1/2014	08:44.1
1192	RWD11	-122.453	37.936	4.17	12.77	-325.42	16.94	-122.453	37.936	4.53	12.77	-274.88	17.31	11	8/1/2014	08:44.2
1193	RWD11	-122.453	37.936	4.22	12.76	-311.85	16.97	-122.453	37.936	4.57	12.76	-260.38	17.32	11	8/1/2014	08:44.3
1194	RWD11	-122.453	37.936	4.17	12.77	-297.27	16.94	-122.453	37.936	4.57	12.77	-244.97	17.34	11	8/1/2014	08:44.4
1195	RWD11	-122.453	37.936	4.22	12.77	-281.60	16.98	-122.453	37.936	4.57	12.77	-228.63	17.33	11	8/1/2014	08:44.5
1196	RWD11	-122.453	37.936	4.17	12.78	-264.84	16.95	-122.453	37.936	4.48	12.78	-211.34	17.26	11	8/1/2014	08:44.6
1197	RWD11	-122.453	37.936	4.22	12.76	-247.32	16.97	-122.453	37.936	4.53	12.76	-193.32	17.29	11	8/1/2014	08:44.7
1198	RWD11	-122.453	37.936	4.17	12.78	-229.24	16.95	-122.453	37.936	4.48	12.78	-174.71	17.26	11	8/1/2014	08:44.8
1199	RWD11	-122.453	37.936	4.17	12.79	-210.96	16.95	-122.453	37.936	4.48	12.79	-155.83	17.27	11	8/1/2014	08:44.9
1200	RWD11	-122.453	37.936	4.17	12.79	-192.73	16.95	-122.453	37.936	4.45	12.79	-136.89	17.24	11	8/1/2014	08:45.0
1201	RWD11	-122.453	37.936	4.22	12.82	-174.61	17.04	-122.453	37.936	4.45	12.82	-118.02	17.27	11	8/1/2014	08:45.1
1202	RWD11	-122.453	37.936	4.17	12.86	-156.50	17.02	-122.453	37.936	4.45	12.86	-99.19	17.31	11	8/1/2014	08:45.2
1203	RWD11	-122.453	37.936	4.22	12.82	-138.35	17.04	-122.453	37.936	4.45	12.82	-80.29	17.27	11	8/1/2014	08:45.3
1204	RWD11	-122.453	37.936	4.17	12.82	-119.96	16.99	-122.453	37.936	4.40	12.82	-61.14	17.22	11	8/1/2014	08:45.4
1205	RWD11	-122.453	37.936	4.22	12.80	-101.37	17.02	-122.453	37.936	4.45	12.80	-41.74	17.25	11	8/1/2014	08:45.5
1206	RWD11	-122.453	37.936	4.17	12.82	-82.42	16.99	-122.453	37.936	4.40	12.82	-21.93	17.22	11	8/1/2014	08:45.6
1207	RWD11	-122.453	37.936	4.17	12.81	-62.82	16.98	-122.453	37.936	4.45	12.81	-1.40	17.26	11	8/1/2014	08:45.7
1208	RWD11	-122.453	37.936	4.17	12.82	-42.35	16.99	-122.453	37.936	4.40	12.82	20.03	17.22	11	8/1/2014	08:45.8
1209	RWD11	-122.453	37.936	4.22	12.86	-20.71	17.07	-122.453	37.936	4.40	12.86	42.74	17.26	11	8/1/2014	08:45.9
1210	RWD11	-122.453	37.936	4.17	12.84	2.29	17.01	-122.453	37.936	4.36	12.84	66.69	17.20	11	8/1/2014	08:46.0
1211	RWD11	-122.453	37.936	4.17	12.91	26.53	17.08	-122.453	37.936	4.36	12.91	91.83	17.27	11	8/1/2014	08:46.1
1212	RWD11	-122.453	37.936	4.17	12.95	51.81	17.12	-122.453	37.936	4.28	12.95	118.03	17.23	11	8/1/2014	08:46.2
1213	RWD11	-122.453	37.936	4.17	13.00	77.52	17.17	-122.453	37.936	4.28	13.00	144.61	17.28	11	8/1/2014	08:46.3
1214	RWD11	-122.453	37.936	4.13	13.03	102.91	17.17	-122.453	37.936	4.23	13.03	171.00	17.26	11	8/1/2014	08:46.4
1215	RWD11	-122.453	37.936	4.13	13.04	127.98	17.17	-122.453	37.936	4.23	13.04	197.17	17.27	11	8/1/2014	08:46.5
1216	RWD11	-122.453	37.936	4.08	13.13	152.93	17.22	-122.453	37.936	4.20	13.13	223.32	17.33	11	8/1/2014	08:46.6
1217	RWD11	-122.453	37.936	4.13	13.11	178.04	17.24	-122.453	37.936	4.20	13.11	249.62	17.31	11	8/1/2014	08:46.7
1218	RWD11	-122.453	37.936	4.08	13.14	203.16	17.22	-122.453	37.936	4.16	13.14	275.95	17.30	11	8/1/2014	08:46.8
1219	RWD11	-122.453	37.936	4.13	13.18	228.68	17.31	-122.453	37.936	4.16	13.18	302.73	17.34	11	8/1/2014	08:46.9
1220	RWD11	-122.453	37.936	4.13	13.23	254.93	17.36	-122.453	37.936	4.16	13.23	330.23	17.39	11	8/1/2014	08:47.0
1221	RWD11	-122.453	37.936	4.13	13.28	282.23	17.41	-122.453	37.936	4.11	13.28	358.65	17.39	11	8/1/2014	08:47.1
1222	RWD11	-122.454	37.936	4.08	13.34	310.91	17.43	-122.453	37.936	4.08	13.34	388.27	17.42	11	8/1/2014	08:47.2
1223	RWD11	-122.454	37.936	4.08	13.40	341.04	17.48	-122.453	37.936	4.08	13.40	419.24	17.48	11	8/1/2014	08:47.3

1224	RWD11	-122.454	37.936	4.05	13.44	372.68	17.48	-122.454	37.936	4.03	13.44	451.60	17.46	11	8/1/2014	08:47.4
1225	RWD11	-122.454	37.936	4.08	13.47	405.50	17.56	-122.454	37.936	4.03	13.47	485.19	17.50	11	8/1/2014	08:47.5
1226	RWD11	-122.454	37.936	4.05	13.50	439.38	17.55	-122.454	37.936	3.91	13.50	519.82	17.41	11	8/1/2014	08:47.6
1227	RWD11	-122.454	37.936	4.05	13.59	473.92	17.64	-122.454	37.936	3.94	13.59	555.15	17.53	11	8/1/2014	08:47.7
1228	RWD11	-122.454	37.936	4.00	13.63	509.05	17.62	-122.454	37.936	3.91	13.63	590.99	17.53	11	8/1/2014	08:47.8
1229	RWD11	-122.454	37.936	4.00	13.68	544.32	17.68	-122.454	37.936	3.88	13.68	626.87	17.56	11	8/1/2014	08:47.9
1230	RWD11	-122.454	37.936	3.96	13.73	579.45	17.69	-122.454	37.936	3.79	13.73	662.53	17.52	11	8/1/2014	08:48.0
1231	RWD11	-122.454	37.936	4.00	13.79	614.60	17.79	-122.454	37.936	3.79	13.79	698.20	17.58	11	8/1/2014	08:48.1
1232	RWD11	-122.454	37.936	3.93	13.87	649.73	17.80	-122.454	37.936	3.79	13.87	734.01	17.66	11	8/1/2014	08:48.2
1233	RWD11	-122.454	37.936	3.93	13.90	685.07	17.82	-122.454	37.936	3.74	13.90	770.09	17.63	11	8/1/2014	08:48.3
1234	RWD11	-122.454	37.936	3.93	13.90	720.82	17.83	-122.454	37.936	3.74	13.90	806.56	17.64	11	8/1/2014	08:48.4
1235	RWD11	-122.454	37.936	3.93	13.90	757.29	17.83	-122.454	37.936	3.70	13.90	843.58	17.61	11	8/1/2014	08:48.5
1236	RWD11	-122.454	37.936	3.88	13.93	794.69	17.80	-122.454	37.936	3.70	13.93	881.49	17.63	11	8/1/2014	08:48.6
1237	RWD11	-122.454	37.936	3.88	13.92	833.34	17.80	-122.454	37.936	3.65	13.92	920.42	17.58	11	8/1/2014	08:48.7
1238	RWD11	-122.454	37.936	3.84	13.96	873.23	17.80	-122.454	37.936	3.62	13.96	960.48	17.58	11	8/1/2014	08:48.8
1239	RWD11	-122.454	37.936	3.88	13.99	914.11	17.87	-122.454	37.936	3.62	13.99	1001.62	17.61	11	8/1/2014	08:48.9
1240	RWD11	-122.454	37.936	3.79	14.04	955.80	17.84	-122.454	37.936	3.57	14.04	1043.56	17.61	11	8/1/2014	08:49.0
1241	RWD11	-122.454	37.936	3.84	14.08	997.98	17.92	-122.454	37.936	3.54	14.08	1085.99	17.62	11	8/1/2014	08:49.1
1242	RWD11	-122.454	37.936	3.79	14.16	######	17.95	-122.454	37.936	3.45	14.16	1128.52	17.61	11	8/1/2014	08:49.2
1243	RWD11	-122.454	37.936	3.79	14.16	######	17.95	-122.454	37.936	3.45	14.16	1170.50	17.61	11	8/1/2014	08:49.3
1244	RWD11	-122.454	37.936	3.76	14.22	######	17.98	-122.454	37.936	3.33	14.22	1211.47	17.55	11	8/1/2014	08:49.4
1245	RWD11	-122.454	37.936	3.76	14.21	######	17.96	-122.454	37.936	3.37	14.21	1251.10	17.57	11	8/1/2014	08:49.5
1246	RWD11	-122.454	37.936	3.71	14.27	######	17.98	-122.454	37.936	3.33	14.27	1289.16	17.60	11	8/1/2014	08:49.6
1247	RWD11	-122.454	37.936	3.71	14.24	######	17.95	-122.454	37.936	3.33	14.24	1325.83	17.58	11	8/1/2014	08:49.7
1248	RWD11	-122.454	37.936	3.67	14.27	######	17.94	-122.454	37.936	3.25	14.27	1360.89	17.52	11	8/1/2014	08:49.8
1249	RWD11	-122.454	37.936	3.67	14.27	######	17.94	-122.454	37.936	3.28	14.27	1394.97	17.55	11	8/1/2014	08:49.9
1250	RWD11	-122.454	37.936	3.67	14.27	######	17.94	-122.454	37.936	3.20	14.27	1428.15	17.47	11	8/1/2014	08:50.0
1251	RWD11	-122.454	37.936	3.67	14.28	######	17.96	-122.454	37.936	3.20	14.28	1460.60	17.48	11	8/1/2014	08:50.1
1252	RWD11	-122.454	37.936	3.62	14.35	######	17.98	-122.454	37.936	3.16	14.35	1492.21	17.52	11	8/1/2014	08:50.2
1253	RWD11	-122.454	37.936	3.59	14.29	######	17.88	-122.454	37.936	3.16	14.29	1522.54	17.46	11	8/1/2014	08:50.3
1254	RWD11	-122.454	37.936	3.59	14.36	######	17.95	-122.454	37.936	3.16	14.36	1551.64	17.52	11	8/1/2014	08:50.4
1255	RWD11	-122.454	37.936	3.59	14.34	######	17.92	-122.454	37.936	3.13	14.34	1579.16	17.47	11	8/1/2014	08:50.5
1256	RWD11	-122.454	37.936	3.55	14.34	######	17.89	-122.454	37.936	3.08	14.34	1604.96	17.42	11	8/1/2014	08:50.6

1257	RWD11	-122.454	37.936	3.59	14.35	######	17.93	-122.454	37.936	3.08	14.35	1628.71	17.42	11	8/1/2014	08:50.7
1258	RWD11	-122.454	37.936	3.55	14.35	######	17.90	-122.454	37.936	3.05	14.35	1650.32	17.39	11	8/1/2014	08:50.8
1259	RWD11	-122.454	37.936	3.55	14.38	######	17.94	-122.454	37.936	3.05	14.38	1670.07	17.43	11	8/1/2014	08:50.9
1260	RWD11	-122.454	37.936	3.50	14.37	######	17.87	-122.454	37.936	3.05	14.37	1687.91	17.41	11	8/1/2014	08:51.0
1261	RWD11	-122.454	37.936	3.50	14.43	######	17.93	-122.454	37.936	2.99	14.43	1703.87	17.43	11	8/1/2014	08:51.1
1262	RWD11	-122.454	37.936	3.47	14.41	######	17.88	-122.454	37.936	2.99	14.41	1718.11	17.40	11	8/1/2014	08:51.2
1263	RWD11	-122.454	37.936	3.50	14.39	######	17.89	-122.454	37.936	3.05	14.39	1730.59	17.44	11	8/1/2014	08:51.3
1264	RWD11	-122.454	37.936	3.47	14.65	######	18.11	-122.454	37.936	2.99	14.65	1741.14	17.64	11	8/1/2014	08:51.4
1265	RWD11	-122.454	37.936	3.50	14.55	######	18.05	-122.454	37.936	2.99	14.55	1749.55	17.54	11	8/1/2014	08:51.5
1266	RWD11	-122.454	37.936	3.41	14.48	######	17.90	-122.454	37.936	2.96	14.48	1755.99	17.45	11	8/1/2014	08:51.6
1267	RWD11	-122.454	37.936	3.47	14.47	######	17.94	-122.454	37.936	2.99	14.47	1760.27	17.46	11	8/1/2014	08:51.7
1268	RWD11	-122.454	37.936	3.41	14.41	######	17.82	-122.454	37.936	2.96	14.41	1762.31	17.37	11	8/1/2014	08:51.8
1269	RWD11	-122.454	37.936	3.41	14.42	######	17.84	-122.454	37.936	2.96	14.42	1762.47	17.38	11	8/1/2014	08:51.9
1270	RWD11	-122.454	37.936	3.38	14.28	######	17.67	-122.454	37.936	2.91	14.28	1760.50	17.19	11	8/1/2014	08:52.0
1271	RWD11	-122.454	37.936	3.41	14.25	######	17.67	-122.454	37.936	2.91	14.25	1756.64	17.16	11	8/1/2014	08:52.1
1272	RWD11	-122.454	37.936	3.38	14.21	######	17.59	-122.454	37.936	2.96	14.21	1750.94	17.17	11	8/1/2014	08:52.2
1273	RWD11	-122.454	37.936	3.38	14.10	######	17.48	-122.454	37.936	2.91	14.10	1743.39	17.01	11	8/1/2014	08:52.3
1274	RWD11	-122.454	37.936	3.38	14.11	######	17.50	-122.454	37.936	2.91	14.11	1733.80	17.02	11	8/1/2014	08:52.4
1275	RWD11	-122.454	37.936	3.38	13.99	######	17.37	-122.454	37.936	2.91	13.99	1722.18	16.90	11	8/1/2014	08:52.5
1276	RWD11	-122.454	37.936	3.33	13.96	######	17.29	-122.454	37.936	2.91	13.96	1708.39	16.87	11	8/1/2014	08:52.6
1277	RWD11	-122.454	37.936	3.38	13.94	######	17.32	-122.454	37.936	2.91	13.94	1692.35	16.85	11	8/1/2014	08:52.7
1278	RWD11	-122.454	37.936	3.33	13.86	######	17.19	-122.454	37.936	2.88	13.86	1674.29	16.73	11	8/1/2014	08:52.8
1279	RWD11	-122.454	37.936	3.33	13.77	######	17.10	-122.454	37.936	2.88	13.77	1654.53	16.65	11	8/1/2014	08:52.9
1280	RWD11	-122.454	37.936	3.33	13.69	######	17.02	-122.454	37.936	2.88	13.69	1633.00	16.57	11	8/1/2014	08:53.0
1281	RWD11	-122.454	37.936	3.33	13.73	######	17.06	-122.454	37.936	2.88	13.73	1609.73	16.61	11	8/1/2014	08:53.1
1282	RWD11	-122.454	37.936	3.30	13.63	######	16.93	-122.454	37.936	2.88	13.63	1584.70	16.51	11	8/1/2014	08:53.2
1283	RWD11	-122.454	37.936	3.30	13.54	######	16.83	-122.454	37.936	2.91	13.54	1558.00	16.45	11	8/1/2014	08:53.3
1284	RWD11	-122.454	37.936	3.30	13.52	######	16.82	-122.454	37.936	2.88	13.52	1529.50	16.40	11	8/1/2014	08:53.4
1285	RWD11	-122.454	37.936	3.30	13.45	######	16.74	-122.454	37.936	2.83	13.45	1499.40	16.27	11	8/1/2014	08:53.5
1286	RWD11	-122.454	37.936	3.24	13.39	######	16.63	-122.454	37.936	2.88	13.39	1467.94	16.26	11	8/1/2014	08:53.6
1287	RWD11	-122.454	37.936	3.24	13.36	######	16.60	-122.454	37.936	2.82	13.36	1435.46	16.18	11	8/1/2014	08:53.7
1288	RWD11	-122.454	37.936	3.24	13.32	######	16.56	-122.454	37.936	2.82	13.32	1402.21	16.14	11	8/1/2014	08:53.8
1289	RWD11	-122.454	37.936	3.24	13.29	######	16.53	-122.454	37.936	2.82	13.29	1368.48	16.11	11	8/1/2014	08:53.9

1290	RWD11	-122.454	37.936	3.24	13.23	######	16.47	-122.454	37.936	2.79	13.23	1334.47	16.02	11	8/1/2014	08:54.0
1291	RWD11	-122.454	37.936	3.24	13.19	######	16.43	-122.454	37.936	2.82	13.19	1300.49	16.01	11	8/1/2014	08:54.1
1292	RWD11	-122.454	37.936	3.21	13.12	######	16.33	-122.454	37.936	2.82	13.12	1266.37	15.94	11	8/1/2014	08:54.2
1293	RWD11	-122.454	37.936	3.21	13.08	######	16.29	-122.454	37.936	2.82	13.08	1232.03	15.91	11	8/1/2014	08:54.3
1294	RWD11	-122.454	37.936	3.21	13.06	######	16.26	-122.454	37.936	2.82	13.06	1197.51	15.88	11	8/1/2014	08:54.4
1295	RWD11	-122.454	37.936	3.21	13.02	######	16.23	-122.454	37.936	2.82	13.02	1162.85	15.84	11	8/1/2014	08:54.5
1296	RWD11	-122.454	37.936	3.17	12.96	######	16.14	-122.454	37.936	2.88	12.96	1127.76	15.84	11	8/1/2014	08:54.6
1297	RWD11	-122.454	37.936	3.21	12.93	######	16.13	-122.454	37.936	2.82	12.93	1092.20	15.75	11	8/1/2014	08:54.7
1298	RWD11	-122.454	37.936	3.17	12.87	######	16.05	-122.454	37.936	2.79	12.87	1056.26	15.66	11	8/1/2014	08:54.8
1299	RWD11	-122.454	37.936	3.21	12.86	995.51	16.07	-122.454	37.936	2.79	12.86	1020.23	15.65	11	8/1/2014	08:54.9
1300	RWD11	-122.454	37.936	3.17	12.82	960.27	15.99	-122.454	37.936	2.79	12.82	984.59	15.61	11	8/1/2014	08:55.0
1301	RWD11	-122.454	37.936	3.21	12.80	925.70	16.01	-122.454	37.936	2.82	12.80	949.63	15.63	11	8/1/2014	08:55.1
1302	RWD11	-122.454	37.936	3.17	12.77	891.78	15.95	-122.454	37.936	2.79	12.77	915.24	15.56	11	8/1/2014	08:55.2
1303	RWD11	-122.454	37.936	3.17	12.70	858.36	15.88	-122.454	37.936	2.79	12.70	881.50	15.49	11	8/1/2014	08:55.3
1304	RWD11	-122.454	37.936	3.17	12.71	825.52	15.88	-122.454	37.936	2.79	12.71	848.27	15.50	11	8/1/2014	08:55.4
1305	RWD11	-122.454	37.936	3.17	12.60	793.20	15.78	-122.454	37.936	2.79	12.60	815.56	15.39	11	8/1/2014	08:55.5
1306	RWD11	-122.454	37.936	3.17	12.61	761.13	15.78	-122.454	37.936	2.79	12.61	783.08	15.40	11	8/1/2014	08:55.6
1307	RWD11	-122.454	37.936	3.17	12.57	729.37	15.75	-122.454	37.936	2.82	12.57	750.92	15.40	11	8/1/2014	08:55.7
1308	RWD11	-122.454	37.936	3.12	12.52	697.51	15.64	-122.454	37.936	2.82	12.52	718.50	15.34	11	8/1/2014	08:55.8
1309	RWD11	-122.454	37.936	3.17	12.48	665.30	15.65	-122.454	37.936	2.82	12.48	685.89	15.30	11	8/1/2014	08:55.9
1310	RWD11	-122.454	37.936	3.12	12.49	632.72	15.61	-122.454	37.936	2.82	12.49	653.00	15.31	11	8/1/2014	08:56.0
1311	RWD11	-122.454	37.936	3.12	12.42	599.95	15.54	-122.454	37.936	2.82	12.42	619.93	15.24	11	8/1/2014	08:56.1
1312	RWD11	-122.454	37.936	3.12	12.41	567.46	15.53	-122.454	37.936	2.79	12.41	587.14	15.20	11	8/1/2014	08:56.2
1313	RWD11	-122.454	37.936	3.17	12.39	535.84	15.56	-122.454	37.936	2.79	12.39	555.13	15.18	11	8/1/2014	08:56.3
1314	RWD11	-122.454	37.936	3.12	12.34	505.51	15.46	-122.454	37.936	2.82	12.34	524.22	15.16	11	8/1/2014	08:56.4
1315	RWD11	-122.454	37.936	3.12	12.34	476.40	15.46	-122.454	37.936	2.79	12.34	494.58	15.13	11	8/1/2014	08:56.5
1316	RWD11	-122.454	37.936	3.12	12.30	448.49	15.42	-122.454	37.936	2.82	12.30	466.03	15.12	11	8/1/2014	08:56.6
1317	RWD11	-122.454	37.936	3.12	12.28	421.55	15.40	-122.454	37.936	2.82	12.28	438.36	15.10	11	8/1/2014	08:56.7
1318	RWD11	-122.454	37.936	3.12	12.25	395.07	15.38	-122.454	37.936	2.79	12.25	411.33	15.04	11	8/1/2014	08:56.8
1319	RWD11	-122.454	37.936	3.12	12.24	368.89	15.36	-122.454	37.936	2.82	12.24	384.67	15.06	11	8/1/2014	08:56.9
1320	RWD11	-122.454	37.936	3.09	12.22	342.77	15.30	-122.454	37.936	2.82	12.22	357.98	15.04	11	8/1/2014	08:57.0
1321	RWD11	-122.454	37.936	3.09	12.19	316.51	15.28	-122.454	37.936	2.82	12.19	331.39	15.02	11	8/1/2014	08:57.1
1322	RWD11	-122.454	37.936	3.09	12.14	290.16	15.23	-122.454	37.936	2.82	12.14	304.51	14.96	11	8/1/2014	08:57.2

1323	RWD11	-122.454	37.936	3.12	12.15	263.42	15.28	-122.454	37.936	2.82	12.15	277.40	14.98	11	8/1/2014	08:57.3
1324	RWD11	-122.454	37.936	3.09	12.13	236.63	15.22	-122.454	37.936	2.82	12.13	250.10	14.95	11	8/1/2014	08:57.4
1325	RWD11	-122.454	37.936	3.12	12.10	209.98	15.22	-122.454	37.936	2.82	12.10	222.99	14.92	11	8/1/2014	08:57.5
1326	RWD11	-122.454	37.936	3.09	12.09	184.22	15.17	-122.454	37.936	2.79	12.09	196.66	14.88	11	8/1/2014	08:57.6
1327	RWD11	-122.454	37.936	3.12	12.08	159.75	15.20	-122.454	37.936	2.82	12.08	171.57	14.90	11	8/1/2014	08:57.7
1328	RWD11	-122.454	37.936	3.09	12.02	136.53	15.10	-122.454	37.936	2.79	12.02	147.79	14.81	11	8/1/2014	08:57.8
1329	RWD11	-122.454	37.936	3.12	12.01	114.18	15.13	-122.454	37.936	2.82	12.01	124.84	14.83	11	8/1/2014	08:57.9
1330	RWD11	-122.454	37.936	3.09	11.99	92.55	15.08	-122.454	37.936	2.82	11.99	102.59	14.81	11	8/1/2014	08:58.0
1331	RWD11	-122.454	37.936	3.09	11.98	71.36	15.07	-122.454	37.936	2.82	11.98	80.82	14.80	11	8/1/2014	08:58.1
1332	RWD11	-122.454	37.936	3.09	11.96	50.59	15.05	-122.454	37.936	2.82	11.96	59.42	14.79	11	8/1/2014	08:58.2
1333	RWD11	-122.454	37.936	3.09	11.95	30.02	15.04	-122.454	37.936	2.88	11.95	38.27	14.82	11	8/1/2014	08:58.3
1334	RWD11	-122.454	37.936	3.04	11.92	9.64	14.96	-122.454	37.936	2.82	11.92	17.43	14.74	11	8/1/2014	08:58.4
1335	RWD11	-122.454	37.936	3.09	11.90	-10.37	14.99	-122.454	37.936	2.88	11.90	-3.24	14.77	11	8/1/2014	08:58.5
1336	RWD11	-122.454	37.936	3.09	11.90	-30.08	14.99	-122.454	37.936	2.82	11.90	-23.47	14.72	11	8/1/2014	08:58.6
1337	RWD11	-122.454	37.936	3.09	11.86	-49.29	14.95	-122.454	37.936	2.88	11.86	-43.28	14.74	11	8/1/2014	08:58.7
1338	RWD11	-122.454	37.936	3.09	11.85	-68.19	14.93	-122.454	37.936	2.88	11.85	-62.91	14.72	11	8/1/2014	08:58.8
1339	RWD11	-122.454	37.936	3.09	11.85	-86.86	14.93	-122.454	37.936	2.88	11.85	-82.15	14.72	11	8/1/2014	08:58.9
1340	RWD11	-122.454	37.936	3.09	11.81	-105.29	14.90	-122.454	37.936	2.88	11.81	-101.15	14.69	11	8/1/2014	08:59.0
1341	RWD11	-122.454	37.936	3.09	11.80	-123.71	14.89	-122.454	37.936	2.88	11.80	-120.18	14.67	11	8/1/2014	08:59.1
1342	RWD11	-122.454	37.936	3.04	11.80	-142.22	14.84	-122.454	37.936	2.88	11.80	-139.25	14.67	11	8/1/2014	08:59.2
1343	RWD11	-122.454	37.936	3.04	11.83	-160.52	14.87	-122.454	37.936	2.91	11.83	-158.14	14.74	11	8/1/2014	08:59.3
1344	RWD11	-122.454	37.936	3.09	11.79	-178.45	14.88	-122.454	37.936	2.88	11.79	-176.61	14.67	11	8/1/2014	08:59.4
1345	RWD11	-122.454	37.936	3.09	11.79	-195.76	14.88	-122.454	37.936	2.88	11.79	-194.57	14.67	11	8/1/2014	08:59.5
1346	RWD11	-122.454	37.936	3.04	11.77	-212.63	14.81	-122.454	37.936	2.82	11.77	-212.08	14.59	11	8/1/2014	08:59.6
1347	RWD11	-122.454	37.936	3.04	11.76	-228.90	14.80	-122.454	37.936	2.88	11.76	-229.11	14.64	11	8/1/2014	08:59.7
1348	RWD11	-122.454	37.936	3.09	11.75	-244.60	14.84	-122.454	37.936	2.82	11.75	-245.52	14.58	11	8/1/2014	08:59.8
1349	RWD11	-122.454	37.936	3.09	11.74	-260.00	14.83	-122.454	37.936	2.91	11.74	-261.59	14.65	11	8/1/2014	08:59.9
1350	RWD11	-122.454	37.936	3.04	11.74	-275.10	14.78	-122.454	37.936	2.88	11.74	-277.39	14.61	11	8/1/2014	09:00.0
1351	RWD11	-122.454	37.936	3.09	11.74	-290.06	14.83	-122.454	37.936	2.88	11.74	-292.94	14.61	11	8/1/2014	09:00.1
1352	RWD11	-122.454	37.936	3.04	11.73	-304.59	14.77	-122.454	37.936	2.88	11.73	-308.11	14.60	11	8/1/2014	09:00.2
1353	RWD11	-122.454	37.936	3.09	11.74	-318.93	14.83	-122.454	37.936	2.91	11.74	-323.04	14.65	11	8/1/2014	09:00.3
1354	RWD11	-122.454	37.936	3.04	11.74	-333.01	14.78	-122.454	37.936	2.88	11.74	-337.81	14.61	11	8/1/2014	09:00.4
1355	RWD11	-122.454	37.936	3.04	11.73	-347.06	14.77	-122.454	37.936	2.91	11.73	-352.52	14.64	11	8/1/2014	09:00.5

1356	RWD11	-122.454	37.936	3.04	11.76	-360.88	14.80	-122.454	37.936	2.91	11.76	-367.00	14.67	11	8/1/2014	09:00.6
1357	RWD11	-122.454	37.936	3.04	11.72	-374.61	14.76	-122.454	37.936	2.91	11.72	-381.34	14.63	11	8/1/2014	09:00.7
1358	RWD11	-122.454	37.936	3.04	11.73	-387.97	14.77	-122.454	37.936	2.88	11.73	-395.35	14.60	11	8/1/2014	09:00.8
1359	RWD11	-122.454	37.936	3.04	11.72	-400.90	14.75	-122.454	37.936	2.91	11.72	-408.98	14.62	11	8/1/2014	09:00.9
1360	RWD11	-122.454	37.936	3.04	11.72	-413.23	14.76	-122.454	37.936	2.82	11.72	-421.90	14.55	11	8/1/2014	09:01.0
1361	RWD11	-122.454	37.936	3.09	11.72	-425.18	14.80	-122.454	37.936	2.88	11.72	-434.47	14.59	11	8/1/2014	09:01.1
1362	RWD11	-122.454	37.936	3.04	11.68	-436.72	14.72	-122.454	37.936	2.82	11.68	-446.65	14.51	11	8/1/2014	09:01.2
1363	RWD11	-122.454	37.936	3.04	11.69	-447.92	14.73	-122.454	37.936	2.91	11.69	-458.44	14.60	11	8/1/2014	09:01.3
1364	RWD11	-122.454	37.936	3.04	11.67	-458.84	14.71	-122.454	37.936	2.88	11.67	-469.98	14.55	11	8/1/2014	09:01.4
1365	RWD11	-122.454	37.936	3.09	11.67	-469.60	14.76	-122.454	37.936	2.91	11.67	-481.35	14.58	11	8/1/2014	09:01.5
1366	RWD11	-122.454	37.936	3.04	11.66	-480.34	14.70	-122.454	37.936	2.91	11.66	-492.68	14.57	11	8/1/2014	09:01.6
1367	RWD11	-122.454	37.936	3.04	11.65	-490.91	14.69	-122.454	37.936	2.91	11.65	-503.80	14.56	11	8/1/2014	09:01.7
1368	RWD11	-122.454	37.936	3.04	11.65	-501.43	14.68	-122.454	37.936	2.88	11.65	-514.82	14.52	11	8/1/2014	09:01.8
1369	RWD11	-122.454	37.936	3.04	11.65	-511.80	14.69	-122.454	37.936	2.91	11.65	-525.79	14.56	11	8/1/2014	09:01.9
1370	RWD11	-122.454	37.936	3.04	11.64	-521.94	14.67	-122.454	37.936	2.91	11.64	-536.42	14.54	11	8/1/2014	09:02.0
1371	RWD11	-122.454	37.936	3.04	11.62	-531.77	14.66	-122.454	37.936	2.91	11.62	-546.71	14.53	11	8/1/2014	09:02.1
1372	RWD11	-122.454	37.936	3.04	11.64	-541.08	14.67	-122.454	37.936	2.91	11.64	-556.54	14.54	11	8/1/2014	09:02.2
1373	RWD11	-122.454	37.936	3.04	11.64	-550.05	14.67	-122.454	37.936	2.91	11.64	-566.02	14.54	11	8/1/2014	09:02.3
1374	RWD11	-122.454	37.936	3.04	11.64	-558.79	14.67	-122.454	37.936	2.91	11.64	-575.18	14.54	11	8/1/2014	09:02.4
1375	RWD11	-122.454	37.936	3.09	11.62	-567.29	14.71	-122.454	37.936	2.91	11.62	-584.13	14.53	11	8/1/2014	09:02.5
1376	RWD11	-122.454	37.936	3.04	11.60	-575.59	14.64	-122.454	37.936	2.91	11.60	-592.90	14.51	11	8/1/2014	09:02.6
1377	RWD11	-122.454	37.936	3.09	11.59	-583.80	14.68	-122.454	37.936	2.99	11.59	-601.54	14.58	11	8/1/2014	09:02.7
1378	RWD11	-122.454	37.936	3.04	11.58	-591.97	14.62	-122.454	37.936	2.96	11.58	-610.17	14.54	11	8/1/2014	09:02.8
1379	RWD11	-122.454	37.936	3.09	11.57	-600.26	14.66	-122.454	37.936	3.04	11.57	-618.97	14.61	11	8/1/2014	09:02.9
1380	RWD11	-122.454	37.936	3.09	11.56	-608.60	14.65	-122.454	37.936	2.99	11.56	-627.79	14.55	11	8/1/2014	09:03.0
1381	RWD11	-122.454	37.936	3.09	11.56	-616.98	14.65	-122.454	37.936	3.04	11.56	-636.66	14.60	11	8/1/2014	09:03.1
1382	RWD11	-122.454	37.936	3.04	11.55	-625.45	14.58	-122.454	37.936	3.04	11.55	-645.64	14.59	11	8/1/2014	09:03.2
1383	RWD11	-122.454	37.936	3.09	11.53	-634.00	14.62	-122.454	37.936	3.08	11.53	-654.69	14.61	11	8/1/2014	09:03.3
1384	RWD11	-122.454	37.936	3.04	11.53	-642.38	14.57	-122.454	37.936	2.99	11.53	-663.67	14.53	11	8/1/2014	09:03.4
1385	RWD11	-122.454	37.936	3.09	11.52	-650.66	14.61	-122.454	37.936	3.04	11.52	-672.48	14.57	11	8/1/2014	09:03.5
1386	RWD11	-122.454	37.936	3.09	11.55	-658.54	14.63	-122.454	37.936	3.04	11.55	-680.97	14.59	11	8/1/2014	09:03.6
1387	RWD11	-122.454	37.936	3.09	11.51	-666.22	14.60	-122.454	37.936	3.04	11.51	-689.22	14.55	11	8/1/2014	09:03.7
1388	RWD11	-122.454	37.936	3.04	11.51	-673.60	14.55	-122.454	37.936	3.04	11.51	-697.08	14.56	11	8/1/2014	09:03.8

1389	RWD11	-122.454	37.936	3.09	11.49	-680.78	14.58	-122.454	37.936	3.04	11.49	-704.71	14.54	11	8/1/2014	09:03.9
1390	RWD11	-122.454	37.936	3.04	11.47	-687.85	14.50	-122.454	37.936	3.08	11.47	-712.23	14.54	11	8/1/2014	09:04.0
1391	RWD11	-122.454	37.936	3.09	11.52	-694.84	14.61	-122.454	37.936	3.08	11.52	-719.50	14.60	11	8/1/2014	09:04.1
1392	RWD11	-122.454	37.936	3.09	11.45	-701.80	14.54	-122.454	37.936	3.08	11.45	-726.76	14.53	11	8/1/2014	09:04.2
1393	RWD11	-122.454	37.936	3.09	11.44	-708.61	14.53	-122.454	37.936	3.13	11.44	-734.15	14.57	11	8/1/2014	09:04.3
1394	RWD11	-122.454	37.936	3.09	11.45	-715.61	14.54	-122.454	37.936	3.13	11.45	-741.41	14.58	11	8/1/2014	09:04.4
1395	RWD11	-122.454	37.936	3.09	11.43	-722.32	14.52	-122.454	37.936	3.16	11.43	-748.70	14.59	11	8/1/2014	09:04.5
1396	RWD11	-122.454	37.936	3.09	11.44	-729.11	14.53	-122.454	37.936	3.13	11.44	-755.96	14.57	11	8/1/2014	09:04.6
1397	RWD11	-122.454	37.936	3.12	11.42	-735.64	14.54	-122.454	37.936	3.16	11.42	-763.09	14.59	11	8/1/2014	09:04.7
1398	RWD11	-122.454	37.936	3.09	11.41	-742.04	14.50	-122.454	37.936	3.13	11.41	-770.14	14.54	11	8/1/2014	09:04.8
1399	RWD11	-122.454	37.936	3.12	11.40	-748.32	14.52	-122.454	37.936	3.16	11.40	-777.04	14.56	11	8/1/2014	09:04.9
1400	RWD11	-122.454	37.936	3.09	11.40	-754.35	14.48	-122.454	37.936	3.16	11.40	-783.65	14.56	11	8/1/2014	09:05.0
1401	RWD11	-122.454	37.936	3.12	11.37	-760.19	14.49	-122.454	37.936	3.16	11.37	-790.01	14.53	11	8/1/2014	09:05.1
1402	RWD11	-122.454	37.936	3.09	11.36	-765.82	14.45	-122.454	37.936	3.16	11.36	-796.21	14.52	11	8/1/2014	09:05.2
1403	RWD11	-122.454	37.936	3.12	11.35	-771.33	14.48	-122.454	37.936	3.25	11.35	-802.22	14.60	11	8/1/2014	09:05.3
1404	RWD11	-122.454	37.936	3.09	11.31	-776.74	14.40	-122.454	37.936	3.20	11.31	-808.09	14.51	11	8/1/2014	09:05.4
1405	RWD11	-122.454	37.936	3.12	11.31	-782.00	14.43	-122.454	37.936	3.25	11.31	-813.80	14.55	11	8/1/2014	09:05.5
1406	RWD11	-122.454	37.936	3.09	11.29	-787.18	14.38	-122.454	37.936	3.20	11.29	-819.47	14.49	11	8/1/2014	09:05.6
1407	RWD11	-122.454	37.936	3.12	11.27	-792.38	14.40	-122.454	37.936	3.25	11.27	-825.06	14.52	11	8/1/2014	09:05.7
1408	RWD11	-122.454	37.936	3.09	11.25	-797.42	14.34	-122.454	37.936	3.25	11.25	-830.63	14.50	11	8/1/2014	09:05.8
1409	RWD11	-122.454	37.936	3.12	11.25	-802.46	14.37	-122.454	37.936	3.28	11.25	-836.12	14.53	11	8/1/2014	09:05.9
1410	RWD11	-122.454	37.936	3.12	11.21	-807.42	14.34	-122.454	37.936	3.28	11.21	-841.56	14.49	11	8/1/2014	09:06.0
1411	RWD11	-122.454	37.936	3.12	11.22	-812.42	14.34	-122.454	37.936	3.28	11.22	-847.03	14.50	11	8/1/2014	09:06.1
1412	RWD11	-122.454	37.936	3.12	11.24	-817.28	14.37	-122.454	37.936	3.33	11.24	-852.35	14.58	11	8/1/2014	09:06.2
1413	RWD11	-122.454	37.936	3.17	11.18	-822.03	14.36	-122.454	37.936	3.33	11.18	-857.47	14.51	11	8/1/2014	09:06.3
1414	RWD11	-122.454	37.936	3.12	11.18	-826.62	14.30	-122.454	37.936	3.33	11.18	-862.50	14.51	11	8/1/2014	09:06.4
1415	RWD11	-122.454	37.936	3.12	11.16	-831.09	14.28	-122.454	37.936	3.37	11.16	-867.30	14.52	11	8/1/2014	09:06.5
1416	RWD11	-122.454	37.936	3.12	11.16	-835.41	14.28	-122.454	37.936	3.33	11.16	-872.00	14.49	11	8/1/2014	09:06.6
1417	RWD11	-122.454	37.936	3.12	11.13	-839.53	14.25	-122.454	37.936	3.37	11.13	-876.44	14.49	11	8/1/2014	09:06.7
1418	RWD11	-122.454	37.936	3.12	11.12	-843.55	14.24	-122.454	37.936	3.37	11.12	-880.80	14.49	11	8/1/2014	09:06.8
1419	RWD11	-122.454	37.936	3.17	11.11	-847.40	14.28	-122.454	37.936	3.37	11.11	-885.04	14.47	11	8/1/2014	09:06.9
1420	RWD11	-122.454	37.936	3.17	11.09	-851.17	14.26	-122.454	37.936	3.42	11.09	-889.14	14.51	11	8/1/2014	09:07.0
1421	RWD11	-122.454	37.936	3.17	11.06	-854.94	14.23	-122.454	37.936	3.42	11.06	-893.30	14.47	11	8/1/2014	09:07.1

1422	RWD11	-122.454	37.936	3.17	11.04	-858.75	14.22	-122.454	37.936	3.45	11.04	-897.49	14.49	11	8/1/2014	09:07.2
1423	RWD11	-122.454	37.936	3.17	11.00	-862.56	14.18	-122.454	37.936	3.45	11.00	-901.68	14.45	11	8/1/2014	09:07.3
1424	RWD11	-122.454	37.936	3.17	10.99	-866.34	14.16	-122.454	37.936	3.50	10.99	-905.83	14.49	11	8/1/2014	09:07.4
1425	RWD11	-122.454	37.936	3.21	10.99	-870.08	14.20	-122.454	37.936	3.50	10.99	-910.00	14.49	11	8/1/2014	09:07.5
1426	RWD11	-122.454	37.936	3.17	10.97	-873.72	14.15	-122.454	37.936	3.50	10.97	-914.07	14.47	11	8/1/2014	09:07.6
1427	RWD11	-122.454	37.936	3.24	10.96	-877.27	14.20	-122.454	37.936	3.50	10.96	-918.08	14.46	11	8/1/2014	09:07.7
1428	RWD11	-122.454	37.936	3.21	10.95	-880.73	14.16	-122.454	37.936	3.50	10.95	-921.92	14.45	11	8/1/2014	09:07.8
1429	RWD11	-122.454	37.936	3.21	10.93	-884.09	14.14	-122.454	37.936	3.53	10.93	-925.71	14.46	11	8/1/2014	09:07.9
1430	RWD11	-122.454	37.936	3.24	10.92	-887.28	14.16	-122.454	37.936	3.53	10.92	-929.25	14.45	11	8/1/2014	09:08.0
1431	RWD11	-122.454	37.936	3.24	10.89	-890.27	14.13	-122.454	37.936	3.53	10.89	-932.62	14.42	11	8/1/2014	09:08.1
1432	RWD11	-122.454	37.936	3.24	10.87	-893.05	14.11	-122.454	37.936	3.53	10.87	-935.84	14.41	11	8/1/2014	09:08.2
1433	RWD11	-122.454	37.936	3.24	10.90	-895.83	14.14	-122.454	37.936	3.57	10.90	-939.02	14.47	11	8/1/2014	09:08.3
1434	RWD11	-122.454	37.936	3.24	10.84	-898.51	14.09	-122.454	37.936	3.57	10.84	-942.10	14.41	11	8/1/2014	09:08.4
1435	RWD11	-122.454	37.936	3.29	10.86	-901.05	14.16	-122.454	37.936	3.62	10.86	-945.07	14.48	11	8/1/2014	09:08.5
1436	RWD11	-122.454	37.936	3.24	10.83	-903.60	14.07	-122.454	37.936	3.57	10.83	-948.06	14.40	11	8/1/2014	09:08.6
1437	RWD11	-122.454	37.936	3.29	10.80	-906.11	14.10	-122.454	37.936	3.65	10.80	-950.94	14.46	11	8/1/2014	09:08.7
1438	RWD11	-122.454	37.936	3.29	10.80	-908.52	14.09	-122.454	37.936	3.65	10.80	-953.79	14.45	11	8/1/2014	09:08.8
1439	RWD11	-122.454	37.936	3.33	10.80	-910.91	14.12	-122.454	37.936	3.65	10.80	-956.56	14.45	11	8/1/2014	09:08.9
1440	RWD11	-122.454	37.936	3.29	10.76	-913.16	14.06	-122.454	37.936	3.65	10.76	-959.28	14.42	11	8/1/2014	09:09.0
1441	RWD11	-122.454	37.936	3.33	10.76	-915.28	14.09	-122.454	37.936	3.65	10.76	-961.82	14.41	11	8/1/2014	09:09.1
1442	RWD11	-122.454	37.936	3.29	10.76	-917.30	14.06	-122.454	37.936	3.70	10.76	-964.22	14.47	11	8/1/2014	09:09.2
1443	RWD11	-122.454	37.936	3.33	10.73	-919.19	14.06	-122.454	37.936	3.70	10.73	-966.59	14.44	11	8/1/2014	09:09.3
1444	RWD11	-122.454	37.936	3.33	10.71	-920.97	14.04	-122.454	37.936	3.70	10.71	-968.72	14.41	11	8/1/2014	09:09.4
1445	RWD11	-122.454	37.936	3.33	10.69	-922.58	14.02	-122.454	37.936	3.70	10.69	-970.80	14.40	11	8/1/2014	09:09.5
1446	RWD11	-122.454	37.936	3.33	10.72	-924.08	14.05	-122.454	37.936	3.70	10.72	-972.71	14.42	11	8/1/2014	09:09.6
1447	RWD11	-122.454	37.936	3.38	10.69	-925.41	14.07	-122.454	37.936	3.70	10.69	-974.54	14.40	11	8/1/2014	09:09.7
1448	RWD11	-122.454	37.936	3.33	10.68	-926.69	14.01	-122.454	37.936	3.70	10.68	-976.25	14.38	11	8/1/2014	09:09.8
1449	RWD11	-122.454	37.936	3.38	10.68	-927.74	14.06	-122.454	37.936	3.79	10.68	-977.84	14.47	11	8/1/2014	09:09.9
1450	RWD11	-122.454	37.936	3.38	10.69	-928.65	14.07	-122.454	37.936	3.74	10.69	-979.21	14.43	11	8/1/2014	09:10.0
1451	RWD11	-122.454	37.936	3.41	10.66	-929.40	14.07	-122.454	37.936	3.79	10.66	-980.56	14.44	11	8/1/2014	09:10.1
1452	RWD11	-122.454	37.936	3.38	10.70	-930.08	14.08	-122.454	37.936	3.79	10.70	-981.73	14.49	11	8/1/2014	09:10.2
1453	RWD11	-122.454	37.936	3.41	10.66	-930.56	14.07	-122.454	37.936	3.79	10.66	-982.75	14.44	11	8/1/2014	09:10.3
1454	RWD11	-122.454	37.936	3.41	10.67	-930.93	14.08	-122.454	37.936	3.79	10.67	-983.66	14.45	11	8/1/2014	09:10.4

1455	RWD11	-122.454	37.936	3.41	10.60	-931.24	14.02	-122.454	37.936	3.79	10.60	-984.47	14.39	11	8/1/2014	09:10.5
1456	RWD11	-122.454	37.936	3.41	10.63	-931.43	14.04	-122.454	37.936	3.79	10.63	-985.17	14.41	11	8/1/2014	09:10.6
1457	RWD11	-122.454	37.936	3.46	10.60	-931.43	14.06	-122.454	37.936	3.82	10.60	-985.68	14.42	11	8/1/2014	09:10.7
1458	RWD11	-122.454	37.936	3.41	10.64	-931.43	14.05	-122.454	37.936	3.79	10.64	-986.08	14.43	11	8/1/2014	09:10.8
1459	RWD11	-122.454	37.936	3.46	10.58	-931.28	14.05	-122.454	37.936	3.82	10.58	-986.45	14.40	11	8/1/2014	09:10.9
1460	RWD11	-122.454	37.936	3.46	10.57	-931.15	14.04	-122.454	37.936	3.82	10.57	-986.74	14.39	11	8/1/2014	09:11.0
1461	RWD11	-122.454	37.936	3.50	10.59	-930.81	14.09	-122.454	37.936	3.87	10.59	-986.91	14.46	11	8/1/2014	09:11.1
1462	RWD11	-122.454	37.936	3.50	10.56	-930.33	14.05	-122.454	37.936	3.87	10.56	-986.91	14.43	11	8/1/2014	09:11.2
1463	RWD11	-122.454	37.936	3.50	10.52	-929.74	14.02	-122.454	37.936	3.91	10.52	-986.80	14.42	11	8/1/2014	09:11.3
1464	RWD11	-122.454	37.936	3.50	10.53	-929.00	14.03	-122.454	37.936	3.87	10.53	-986.57	14.40	11	8/1/2014	09:11.4
1465	RWD11	-122.454	37.936	3.50	10.48	-928.09	13.98	-122.454	37.936	3.91	10.48	-986.22	14.39	11	8/1/2014	09:11.5
1466	RWD11	-122.454	37.936	3.50	10.50	-927.14	14.00	-122.454	37.936	3.91	10.50	-985.76	14.41	11	8/1/2014	09:11.6
1467	RWD11	-122.454	37.936	3.55	10.46	-925.93	14.01	-122.454	37.936	3.91	10.46	-985.15	14.37	11	8/1/2014	09:11.7
1468	RWD11	-122.454	37.936	3.55	10.49	-924.74	14.04	-122.454	37.936	3.94	10.49	-984.46	14.43	11	8/1/2014	09:11.8
1469	RWD11	-122.454	37.936	3.58	10.46	-923.49	14.04	-122.454	37.936	3.94	10.46	-983.78	14.40	11	8/1/2014	09:11.9
1470	RWD11	-122.454	37.936	3.55	10.46	-922.00	14.01	-122.454	37.936	3.94	10.46	-982.86	14.40	11	8/1/2014	09:12.0
1471	RWD11	-122.454	37.936	3.58	10.42	-920.35	14.00	-122.454	37.936	3.99	10.42	-981.77	14.41	11	8/1/2014	09:12.1
1472	RWD11	-122.454	37.936	3.58	10.42	-918.43	14.00	-122.454	37.936	3.99	10.42	-980.45	14.41	11	8/1/2014	09:12.2
1473	RWD11	-122.454	37.936	3.62	10.40	-916.34	14.01	-122.454	37.936	4.03	10.40	-978.93	14.42	11	8/1/2014	09:12.3
1474	RWD11	-122.454	37.936	3.58	10.39	-913.90	13.97	-122.454	37.936	4.03	10.39	-977.08	14.41	11	8/1/2014	09:12.4
1475	RWD11	-122.454	37.936	3.67	10.42	-911.04	14.09	-122.454	37.936	4.03	10.42	-974.85	14.44	11	8/1/2014	09:12.5
1476	RWD11	-122.454	37.936	3.62	10.36	-907.95	13.98	-122.454	37.936	4.03	10.36	-972.26	14.39	11	8/1/2014	09:12.6
1477	RWD11	-122.454	37.936	3.67	10.36	-904.50	14.03	-122.454	37.936	4.03	10.36	-969.33	14.38	11	8/1/2014	09:12.7
1478	RWD11	-122.454	37.936	3.62	10.36	-900.74	13.98	-122.454	37.936	4.08	10.36	-966.09	14.44	11	8/1/2014	09:12.8
1479	RWD11	-122.454	37.936	3.67	10.34	-896.76	14.01	-122.454	37.936	4.08	10.34	-962.62	14.41	11	8/1/2014	09:12.9
1480	RWD11	-122.454	37.936	3.67	10.32	-892.62	13.99	-122.454	37.936	4.08	10.32	-958.98	14.40	11	8/1/2014	09:13.0
1481	RWD11	-122.454	37.936	3.70	10.32	-888.29	14.02	-122.454	37.936	4.11	10.32	-955.09	14.43	11	8/1/2014	09:13.1
1482	RWD11	-122.454	37.936	3.67	10.32	-883.90	13.99	-122.454	37.936	4.11	10.32	-950.99	14.43	11	8/1/2014	09:13.2
1483	RWD11	-122.454	37.936	3.70	10.30	-879.37	14.01	-122.454	37.936	4.11	10.30	-946.85	14.41	11	8/1/2014	09:13.3
1484	RWD11	-122.454	37.936	3.70	10.27	-874.66	13.97	-122.454	37.936	4.11	10.27	-942.43	14.38	11	8/1/2014	09:13.4
1485	RWD11	-122.454	37.936	3.70	10.29	-869.56	13.99	-122.454	37.936	4.11	10.29	-937.69	14.40	11	8/1/2014	09:13.5
1486	RWD11	-122.454	37.936	3.70	10.27	-864.11	13.97	-122.454	37.936	4.11	10.27	-932.59	14.38	11	8/1/2014	09:13.6
1487	RWD11	-122.454	37.936	3.76	10.28	-858.36	14.03	-122.454	37.936	4.16	10.28	-927.20	14.44	11	8/1/2014	09:13.7

1488	RWD11	-122.454	37.936	3.70	10.29	-852.15	13.99	-122.454	37.936	4.16	10.29	-921.36	14.45	11	8/1/2014	09:13.8
1489	RWD11	-122.454	37.936	3.79	10.28	-845.42	14.07	-122.454	37.936	4.16	10.28	-915.05	14.44	11	8/1/2014	09:13.9
1490	RWD11	-122.454	37.936	3.76	10.29	-838.30	14.05	-122.454	37.936	4.16	10.29	-908.31	14.45	11	8/1/2014	09:14.0
1491	RWD11	-122.454	37.936	3.79	10.32	-830.93	14.11	-122.454	37.936	4.16	10.32	-901.26	14.48	11	8/1/2014	09:14.1
1492	RWD11	-122.454	37.936	3.79	10.31	-823.36	14.10	-122.454	37.936	4.16	10.31	-893.99	14.47	11	8/1/2014	09:14.2
1493	RWD11	-122.454	37.936	3.79	10.29	-815.78	14.08	-122.454	37.936	4.19	10.29	-886.62	14.49	11	8/1/2014	09:14.3
1494	RWD11	-122.454	37.936	3.79	10.29	-808.21	14.08	-122.454	37.936	4.16	10.29	-879.24	14.45	11	8/1/2014	09:14.4
1495	RWD11	-122.454	37.936	3.84	10.29	-800.74	14.13	-122.454	37.936	4.19	10.29	-872.06	14.48	11	8/1/2014	09:14.5
1496	RWD11	-122.454	37.936	3.79	10.29	-793.40	14.08	-122.454	37.936	4.19	10.29	-864.91	14.48	11	8/1/2014	09:14.6
1497	RWD11	-122.454	37.936	3.84	10.27	-786.21	14.11	-122.454	37.936	4.19	10.27	-857.89	14.46	11	8/1/2014	09:14.7
1498	RWD11	-122.454	37.936	3.79	10.28	-779.16	14.07	-122.454	37.936	4.19	10.28	-851.02	14.47	11	8/1/2014	09:14.8
1499	RWD11	-122.454	37.936	3.84	10.24	-772.15	14.08	-122.454	37.936	4.23	10.24	-844.25	14.47	11	8/1/2014	09:14.9
1500	RWD11	-122.454	37.936	3.84	10.22	-765.15	14.07	-122.454	37.936	4.23	10.22	-837.39	14.45	11	8/1/2014	09:15.0
1501	RWD11	-122.454	37.936	3.88	10.21	-758.03	14.08	-122.454	37.936	4.28	10.21	-830.50	14.49	11	8/1/2014	09:15.1
1502	RWD11	-122.454	37.936	3.84	10.20	-750.83	14.04	-122.454	37.936	4.28	10.20	-823.57	14.48	11	8/1/2014	09:15.2
1503	RWD11	-122.454	37.936	3.88	10.20	-743.43	14.07	-122.454	37.936	4.28	10.20	-816.43	14.47	11	8/1/2014	09:15.3
1504	RWD11	-122.454	37.936	3.88	10.21	-735.63	14.08	-122.454	37.936	4.28	10.21	-809.01	14.49	11	8/1/2014	09:15.4
1505	RWD11	-122.454	37.936	3.93	10.16	-727.57	14.09	-122.454	37.936	4.31	10.16	-801.33	14.48	11	8/1/2014	09:15.5
1506	RWD11	-122.454	37.936	3.88	10.14	-719.31	14.01	-122.454	37.936	4.31	10.14	-793.47	14.45	11	8/1/2014	09:15.6
1507	RWD11	-122.454	37.936	3.93	10.10	-710.97	14.03	-122.454	37.936	4.31	10.10	-785.47	14.41	11	8/1/2014	09:15.7
1508	RWD11	-122.454	37.936	3.93	10.08	-702.57	14.01	-122.454	37.936	4.31	10.08	-777.49	14.39	11	8/1/2014	09:15.8
1509	RWD11	-122.454	37.936	3.93	9.99	-694.35	13.91	-122.454	37.936	4.31	9.99	-769.52	14.30	11	8/1/2014	09:15.9
1510	RWD11	-122.454	37.936	3.93	10.02	-686.25	13.94	-122.454	37.936	4.31	10.02	-761.65	14.33	11	8/1/2014	09:16.0
1511	RWD11	-122.454	37.936	3.96	9.95	-678.47	13.91	-122.454	37.936	4.36	9.95	-754.13	14.31	11	8/1/2014	09:16.1
1512	RWD11	-122.454	37.936	3.93	9.94	-671.01	13.87	-122.454	37.936	4.31	9.94	-746.88	14.25	11	8/1/2014	09:16.2
1513	RWD11	-122.454	37.936	3.96	9.94	-663.89	13.90	-122.454	37.936	4.36	9.94	-740.03	14.30	11	8/1/2014	09:16.3
1514	RWD11	-122.454	37.936	3.96	9.97	-657.01	13.93	-122.454	37.936	4.36	9.97	-733.34	14.33	11	8/1/2014	09:16.4
1515	RWD11	-122.454	37.936	3.99	9.96	-650.28	13.96	-122.454	37.936	4.40	9.96	-726.97	14.36	11	8/1/2014	09:16.5
1516	RWD11	-122.454	37.936	3.96	9.91	-643.66	13.87	-122.454	37.936	4.36	9.91	-720.57	14.27	11	8/1/2014	09:16.6
1517	RWD11	-122.454	37.936	3.99	9.95	-637.15	13.94	-122.454	37.936	4.40	9.95	-714.44	14.34	11	8/1/2014	09:16.7
1518	RWD11	-122.454	37.936	3.99	10.01	-630.78	14.00	-122.454	37.936	4.40	10.01	-708.34	14.41	11	8/1/2014	09:16.8
1519	RWD11	-122.454	37.936	4.05	10.01	-624.64	14.05	-122.454	37.936	4.40	10.01	-702.42	14.41	11	8/1/2014	09:16.9
1520	RWD11	-122.454	37.936	3.99	10.13	-618.68	14.13	-122.454	37.936	4.40	10.13	-696.70	14.53	11	8/1/2014	09:17.0

1521	RWD11	-122.454	37.936	3.99	10.12	-612.99	14.11	-122.454	37.936	4.45	10.12	-691.22	14.56	11	8/1/2014	09:17.1
1522	RWD11	-122.454	37.936	3.99	10.14	-607.71	14.13	-122.454	37.936	4.40	10.14	-686.12	14.54	11	8/1/2014	09:17.2
1523	RWD11	-122.454	37.936	4.05	9.80	-602.92	13.85	-122.454	37.936	4.40	9.80	-681.49	14.20	11	8/1/2014	09:17.3
1524	RWD11	-122.454	37.936	3.99	10.15	-598.55	14.15	-122.454	37.936	4.40	10.15	-677.31	14.55	11	8/1/2014	09:17.4
1525	RWD11	-122.454	37.936	4.05	10.18	-594.71	14.23	-122.454	37.936	4.40	10.18	-673.53	14.58	11	8/1/2014	09:17.5
1526	RWD11	-122.454	37.936	4.05	10.25	-591.46	14.29	-122.454	37.936	4.40	10.25	-670.45	14.65	11	8/1/2014	09:17.6
1527	RWD11	-122.454	37.936	4.08	10.27	-588.77	14.35	-122.454	37.936	4.45	10.27	-667.83	14.71	11	8/1/2014	09:17.7
1528	RWD11	-122.454	37.936	4.05	10.30	-586.34	14.35	-122.454	37.936	4.40	10.30	-665.55	14.70	11	8/1/2014	09:17.8
1529	RWD11	-122.454	37.936	4.08	10.29	-584.31	14.37	-122.454	37.936	4.40	10.29	-663.59	14.68	11	8/1/2014	09:17.9
1530	RWD11	-122.454	37.936	4.05	10.34	-582.77	14.38	-122.454	37.936	4.40	10.34	-662.12	14.73	11	8/1/2014	09:18.0
1531	RWD11	-122.454	37.936	4.08	10.36	-581.52	14.44	-122.454	37.936	4.45	10.36	-660.92	14.81	11	8/1/2014	09:18.1
1532	RWD11	-122.454	37.936	4.08	10.35	-580.61	14.43	-122.454	37.936	4.45	10.35	-660.03	14.80	11	8/1/2014	09:18.2
1533	RWD11	-122.454	37.936	4.08	10.34	-580.01	14.42	-122.454	37.936	4.48	10.34	-659.43	14.82	11	8/1/2014	09:18.3
1534	RWD11	-122.454	37.936	4.08	10.37	-579.71	14.45	-122.454	37.936	4.45	10.37	-659.06	14.82	11	8/1/2014	09:18.4
1535	RWD11	-122.454	37.936	4.13	10.37	-579.70	14.50	-122.454	37.936	4.48	10.37	-659.02	14.85	11	8/1/2014	09:18.5
1536	RWD11	-122.454	37.936	4.08	10.40	-580.15	14.49	-122.454	37.936	4.48	10.40	-659.29	14.89	11	8/1/2014	09:18.6
1537	RWD11	-122.454	37.936	4.13	10.44	-580.82	14.57	-122.454	37.936	4.48	10.44	-659.81	14.92	11	8/1/2014	09:18.7
1538	RWD11	-122.454	37.936	4.22	10.43	-581.78	14.64	-122.454	37.936	4.45	10.43	-660.56	14.87	11	8/1/2014	09:18.8
1539	RWD11	-122.454	37.936	4.22	10.44	-583.03	14.66	-122.454	37.936	4.48	10.44	-661.48	14.92	11	8/1/2014	09:18.9
1540	RWD11	-122.454	37.936	4.22	10.45	-584.48	14.66	-122.454	37.936	4.45	10.45	-662.66	14.90	11	8/1/2014	09:19.0
1541	RWD11	-122.454	37.936	4.22	10.49	-586.31	14.70	-122.454	37.936	4.45	10.49	-664.09	14.93	11	8/1/2014	09:19.1
1542	RWD11	-122.454	37.936	4.17	10.48	-588.17	14.65	-122.454	37.936	4.40	10.48	-665.71	14.88	11	8/1/2014	09:19.2
1543	RWD11	-122.454	37.936	4.22	10.39	-590.26	14.60	-122.454	37.936	4.45	10.39	-667.42	14.83	11	8/1/2014	09:19.3
1544	RWD11	-122.454	37.936	4.16	10.34	-592.41	14.51	-122.454	37.936	4.45	10.34	-669.25	14.79	11	8/1/2014	09:19.4
1545	RWD11	-122.454	37.936	4.22	10.30	-594.51	14.52	-122.454	37.936	4.45	10.30	-670.99	14.75	11	8/1/2014	09:19.5
1546	RWD11	-122.454	37.936	4.22	10.28	-596.64	14.49	-122.454	37.936	4.48	10.28	-672.70	14.76	11	8/1/2014	09:19.6
1547	RWD11	-122.454	37.936	4.22	10.26	-598.80	14.47	-122.454	37.936	4.48	10.26	-674.42	14.74	11	8/1/2014	09:19.7
1548	RWD11	-122.454	37.936	4.22	10.23	-601.06	14.45	-122.454	37.936	4.48	10.23	-676.18	14.71	11	8/1/2014	09:19.8
1549	RWD11	-122.454	37.936	4.22	10.25	-603.25	14.46	-122.454	37.936	4.53	10.25	-677.77	14.78	11	8/1/2014	09:19.9
1550	RWD11	-122.454	37.936	4.22	10.27	-605.46	14.49	-122.454	37.936	4.53	10.27	-679.31	14.80	11	8/1/2014	09:20.0
1551	RWD11	-122.454	37.936	4.22	10.26	-607.80	14.47	-122.454	37.936	4.53	10.26	-680.86	14.79	11	8/1/2014	09:20.1
1552	RWD11	-122.454	37.936	4.22	10.27	-610.16	14.49	-122.454	37.936	4.53	10.27	-682.39	14.80	11	8/1/2014	09:20.2
1553	RWD11	-122.454	37.936	4.25	10.25	-612.53	14.50	-122.454	37.936	4.53	10.25	-683.78	14.78	11	8/1/2014	09:20.3

1554	RWD11	-122.454	37.936	4.22	10.26	-614.74	14.47	-122.454	37.936	4.48	10.26	-685.02	14.74	11	8/1/2014 09:20.4
1555	RWD11	-122.454	37.936	4.25	10.30	-616.88	14.55	-122.454	37.936	4.53	10.30	-686.18	14.84	11	8/1/2014 09:20.5
1556	RWD11	-122.454	37.936	4.22	10.76	-618.92	14.98	-122.454	37.936	4.48	10.76	-687.23	15.24	11	8/1/2014 09:20.6
1557	RWD11	-122.454	37.936	4.22	10.73	-620.92	14.95	-122.454	37.936	4.53	10.73	-688.18	15.26	11	8/1/2014 09:20.7
1558	RWD11	-122.454	37.936	4.22	10.73	-622.79	14.94	-122.454	37.936	4.53	10.73	-689.04	15.26	11	8/1/2014 09:20.8
1559	RWD11	-122.454	37.936	4.22	10.73	-624.56	14.94	-122.454	37.936	4.53	10.73	-689.81	15.26	11	8/1/2014 09:20.9
1560	RWD11	-122.454	37.936	4.22	10.71	-626.39	14.93	-122.454	37.936	4.53	10.71	-690.49	15.24	11	8/1/2014 09:21.0
1561	RWD11	-122.454	37.936	4.25	10.72	-628.09	14.97	-122.454	37.936	4.53	10.72	-691.05	15.25	11	8/1/2014 09:21.1
1562	RWD11	-122.454	37.936	4.22	10.72	-629.69	14.94	-122.454	37.936	4.53	10.72	-691.45	15.25	11	8/1/2014 09:21.2
1563	RWD11	-122.454	37.936	4.25	10.72	-631.24	14.97	-122.454	37.936	4.53	10.72	-691.76	15.25	11	8/1/2014 09:21.3
1564	RWD11	-122.454	37.936	4.22	10.74	-632.60	14.96	-122.454	37.936	4.53	10.74	-691.90	15.27	11	8/1/2014 09:21.4
1565	RWD11	-122.454	37.936	4.25	10.77	-633.73	15.03	-122.454	37.936	4.53	10.77	-691.79	15.31	11	8/1/2014 09:21.5
1566	RWD11	-122.454	37.936	4.22	10.83	-634.70	15.05	-122.454	37.936	4.48	10.83	-691.49	15.31	11	8/1/2014 09:21.6
1567	RWD11	-122.454	37.936	4.25	10.85	-635.53	15.10	-122.454	37.936	4.53	10.85	-691.03	15.38	11	8/1/2014 09:21.7
1568	RWD11	-122.454	37.936	4.22	10.88	-636.12	15.10	-122.454	37.936	4.48	10.88	-690.33	15.36	11	8/1/2014 09:21.8
1569	RWD11	-122.454	37.936	4.25	10.96	-636.37	15.21	-122.454	37.936	4.48	10.96	-689.40	15.44	11	8/1/2014 09:21.9
1570	RWD11	-122.454	37.936	4.22	10.98	-636.47	15.20	-122.454	37.936	4.48	10.98	-688.26	15.46	11	8/1/2014 09:22.0
1571	RWD11	-122.454	37.936	4.25	11.03	-636.45	15.28	-122.454	37.936	4.48	11.03	-687.08	15.51	11	8/1/2014 09:22.1
1572	RWD11	-122.454	37.936	4.22	11.08	-636.26	15.30	-122.454	37.936	4.48	11.08	-685.69	15.56	11	8/1/2014 09:22.2
1573	RWD11	-122.454	37.936	4.25	11.20	-635.91	15.45	-122.454	37.936	4.48	11.20	-684.21	15.68	11	8/1/2014 09:22.3
1574	RWD11	-122.454	37.936	4.25	11.23	-635.41	15.49	-122.454	37.936	4.48	11.23	-682.53	15.72	11	8/1/2014 09:22.4
1575	RWD11	-122.454	37.936	4.25	11.18	-634.57	15.43	-122.454	37.936	4.53	11.18	-680.62	15.71	11	8/1/2014 09:22.5
1576	RWD11	-122.454	37.936	4.25	11.20	-633.73	15.45	-122.454	37.936	4.48	11.20	-678.68	15.68	11	8/1/2014 09:22.6
1577	RWD11	-122.454	37.936	4.25	11.26	-632.63	15.51	-122.454	37.936	4.53	11.26	-676.51	15.79	11	8/1/2014 09:22.7
1578	RWD11	-122.454	37.936	4.25	11.26	-631.26	15.51	-122.454	37.936	4.48	11.26	-674.12	15.74	11	8/1/2014 09:22.8
1579	RWD11	-122.454	37.936	4.25	11.31	-629.80	15.56	-122.454	37.936	4.53	11.31	-671.55	15.84	11	8/1/2014 09:22.9
1580	RWD11	-122.454	37.936	4.22	11.37	-628.14	15.59	-122.454	37.936	4.48	11.37	-668.86	15.85	11	8/1/2014 09:23.0
1581	RWD11	-122.454	37.936	4.25	11.34	-626.21	15.59	-122.454	37.936	4.53	11.34	-665.91	15.88	11	8/1/2014 09:23.1
1582	RWD11	-122.454	37.936	4.25	11.35	-624.03	15.60	-122.454	37.936	4.53	11.35	-662.75	15.88	11	8/1/2014 09:23.2
1583	RWD11	-122.454	37.936	4.25	11.42	-621.71	15.67	-122.454	37.936	4.53	11.42	-659.40	15.95	11	8/1/2014 09:23.3
1584	RWD11	-122.454	37.936	4.25	11.41	-619.23	15.66	-122.454	37.936	4.48	11.41	-655.82	15.89	11	8/1/2014 09:23.4
1585	RWD11	-122.454	37.936	4.25	11.41	-616.53	15.66	-122.454	37.936	4.53	11.41	-652.02	15.94	11	8/1/2014 09:23.5
1586	RWD11	-122.454	37.936	4.25	11.42	-613.60	15.67	-122.454	37.936	4.48	11.42	-647.93	15.90	11	8/1/2014 09:23.6

1587	RWD11	-122.454	37.936	4.25	11.43	-610.35	15.68	-122.454	37.936	4.48	11.43	-643.64	15.91	11	8/1/2014	09:23.7
1588	RWD11	-122.454	37.936	4.25	11.43	-606.94	15.68	-122.454	37.936	4.48	11.43	-639.09	15.91	11	8/1/2014	09:23.8
1589	RWD11	-122.454	37.936	4.25	11.45	-603.41	15.70	-122.454	37.936	4.48	11.45	-634.44	15.93	11	8/1/2014	09:23.9
1590	RWD11	-122.454	37.936	4.25	11.48	-599.73	15.73	-122.454	37.936	4.48	11.48	-629.62	15.96	11	8/1/2014	09:24.0
1591	RWD11	-122.454	37.936	4.25	11.48	-595.88	15.73	-122.454	37.936	4.48	11.48	-624.67	15.96	11	8/1/2014	09:24.1
1592	RWD11	-122.454	37.936	4.25	11.53	-591.95	15.79	-122.454	37.936	4.53	11.53	-619.60	16.07	11	8/1/2014	09:24.2
1593	RWD11	-122.454	37.936	4.25	11.65	-587.86	15.90	-122.454	37.936	4.48	11.65	-614.31	16.13	11	8/1/2014	09:24.3
1594	RWD11	-122.454	37.936	4.25	11.61	-583.47	15.87	-122.454	37.936	4.48	11.61	-608.81	16.10	11	8/1/2014	09:24.4
1595	RWD11	-122.454	37.936	4.25	11.63	-578.91	15.88	-122.454	37.936	4.53	11.63	-603.08	16.16	11	8/1/2014	09:24.5
1596	RWD11	-122.454	37.936	4.25	11.73	-574.08	15.98	-122.454	37.936	4.48	11.73	-597.15	16.21	11	8/1/2014	09:24.6
1597	RWD11	-122.454	37.936	4.25	11.73	-569.07	15.98	-122.454	37.936	4.48	11.73	-590.97	16.21	11	8/1/2014	09:24.7
1598	RWD11	-122.454	37.936	4.25	11.74	-563.79	16.00	-122.454	37.936	4.48	11.74	-584.63	16.23	11	8/1/2014	09:24.8
1599	RWD11	-122.454	37.936	4.28	11.78	-558.39	16.06	-122.454	37.936	4.53	11.78	-578.21	16.31	11	8/1/2014	09:24.9
1600	RWD11	-122.454	37.936	4.25	11.84	-552.76	16.09	-122.454	37.936	4.48	11.84	-571.54	16.32	11	8/1/2014	09:25.0
1601	RWD11	-122.454	37.936	4.28	11.83	-546.93	16.11	-122.454	37.936	4.53	11.83	-564.64	16.36	11	8/1/2014	09:25.1
1602	RWD11	-122.454	37.936	4.25	11.81	-540.85	16.06	-122.454	37.936	4.48	11.81	-557.47	16.29	11	8/1/2014	09:25.2
1603	RWD11	-122.454	37.936	4.28	11.88	-534.51	16.16	-122.454	37.936	4.53	11.88	-549.99	16.41	11	8/1/2014	09:25.3
1604	RWD11	-122.454	37.936	4.25	11.85	-527.76	16.10	-122.454	37.936	4.53	11.85	-542.10	16.38	11	8/1/2014	09:25.4
1605	RWD11	-122.454	37.936	4.25	11.89	-520.73	16.14	-122.454	37.936	4.53	11.89	-533.76	16.42	11	8/1/2014	09:25.5
1606	RWD11	-122.454	37.936	4.25	11.91	-513.31	16.17	-122.454	37.936	4.48	11.91	-525.10	16.40	11	8/1/2014	09:25.6
1607	RWD11	-122.454	37.936	4.25	11.92	-505.64	16.17	-122.454	37.936	4.53	11.92	-516.00	16.45	11	8/1/2014	09:25.7
1608	RWD11	-122.454	37.936	4.25	11.95	-497.85	16.20	-122.454	37.936	4.45	11.95	-506.72	16.39	11	8/1/2014	09:25.8
1609	RWD11	-122.454	37.936	4.25	11.96	-489.97	16.21	-122.454	37.936	4.48	11.96	-497.38	16.44	11	8/1/2014	09:25.9
1610	RWD11	-122.454	37.936	4.25	12.01	-482.05	16.26	-122.454	37.936	4.53	12.01	-487.96	16.54	11	8/1/2014	09:26.0
1611	RWD11	-122.454	37.936	4.25	12.04	-474.08	16.29	-122.454	37.936	4.53	12.04	-478.60	16.57	11	8/1/2014	09:26.1
1612	RWD11	-122.454	37.936	4.25	12.02	-465.98	16.27	-122.454	37.936	4.45	12.02	-469.09	16.46	11	8/1/2014	09:26.2
1613	RWD11	-122.454	37.936	4.28	12.02	-457.80	16.31	-122.454	37.936	4.48	12.02	-459.51	16.50	11	8/1/2014	09:26.3
1614	RWD11	-122.454	37.936	4.25	12.02	-449.48	16.27	-122.454	37.936	4.48	12.02	-449.69	16.50	11	8/1/2014	09:26.4
1615	RWD11	-122.454	37.936	4.25	12.07	-440.97	16.32	-122.454	37.936	4.53	12.07	-439.74	16.60	11	8/1/2014	09:26.5
1616	RWD11	-122.454	37.936	4.25	12.01	-432.12	16.26	-122.454	37.936	4.48	12.01	-429.37	16.49	11	8/1/2014	09:26.6
1617	RWD11	-122.454	37.936	4.28	12.00	-423.03	16.28	-122.454	37.936	4.53	12.00	-418.71	16.53	11	8/1/2014	09:26.7
1618	RWD11	-122.454	37.936	4.25	11.98	-413.52	16.24	-122.454	37.936	4.53	11.98	-407.69	16.52	11	8/1/2014	09:26.8
1619	RWD11	-122.454	37.936	4.28	11.95	-403.58	16.24	-122.454	37.936	4.53	11.95	-396.08	16.48	11	8/1/2014	09:26.9

1620	RWD11	-122.454	37.936	4.25	11.94	-393.09	16.19	-122.454	37.936	4.48	11.94	-383.80	16.42	11	8/1/2014	09:27.0
1621	RWD11	-122.454	37.936	4.25	11.94	-382.14	16.19	-122.454	37.936	4.53	11.94	-371.05	16.47	11	8/1/2014	09:27.1
1622	RWD11	-122.454	37.936	4.25	11.94	-370.81	16.19	-122.454	37.936	4.48	11.94	-357.77	16.42	11	8/1/2014	09:27.2
1623	RWD11	-122.454	37.936	4.28	11.88	-359.31	16.17	-122.454	37.936	4.53	11.88	-344.28	16.42	11	8/1/2014	09:27.3
1624	RWD11	-122.454	37.936	4.25	11.87	-347.87	16.12	-122.454	37.936	4.48	11.87	-330.89	16.35	11	8/1/2014	09:27.4
1625	RWD11	-122.454	37.936	4.28	11.84	-336.68	16.12	-122.454	37.936	4.53	11.84	-317.61	16.37	11	8/1/2014	09:27.5
1626	RWD11	-122.454	37.936	4.25	11.82	-325.82	16.07	-122.454	37.936	4.53	11.82	-304.64	16.36	11	8/1/2014	09:27.6
1627	RWD11	-122.454	37.936	4.28	11.81	-314.97	16.10	-122.454	37.936	4.53	11.81	-291.71	16.34	11	8/1/2014	09:27.7
1628	RWD11	-122.454	37.936	4.28	11.84	-304.06	16.12	-122.454	37.936	4.57	11.84	-278.73	16.40	11	8/1/2014	09:27.8
1629	RWD11	-122.454	37.936	4.28	11.79	-292.79	16.08	-122.454	37.936	4.57	11.79	-265.47	16.36	11	8/1/2014	09:27.9
1630	RWD11	-122.454	37.936	4.28	11.79	-280.95	16.08	-122.454	37.936	4.57	11.79	-251.60	16.36	11	8/1/2014	09:28.0
1631	RWD11	-122.454	37.936	4.28	11.80	-268.58	16.08	-122.454	37.936	4.57	11.80	-237.20	16.36	11	8/1/2014	09:28.1
1632	RWD11	-122.454	37.936	4.28	11.81	-255.34	16.09	-122.454	37.936	4.57	11.81	-221.92	16.37	11	8/1/2014	09:28.2
1633	RWD11	-122.454	37.936	4.34	11.85	-241.29	16.18	-122.454	37.936	4.60	11.85	-205.73	16.44	11	8/1/2014	09:28.3
1634	RWD11	-122.454	37.936	4.28	11.84	-226.43	16.12	-122.454	37.936	4.60	11.84	-188.74	16.44	11	8/1/2014	09:28.4
1635	RWD11	-122.454	37.936	4.34	11.81	-211.01	16.14	-122.454	37.936	4.60	11.81	-171.04	16.41	11	8/1/2014	09:28.5
1636	RWD11	-122.454	37.936	4.28	11.81	-195.16	16.09	-122.454	37.936	4.57	11.81	-152.95	16.37	11	8/1/2014	09:28.6
1637	RWD11	-122.454	37.936	4.28	11.81	-179.40	16.10	-122.454	37.936	4.60	11.81	-134.71	16.41	11	8/1/2014	09:28.7
1638	RWD11	-122.454	37.936	4.28	11.81	-163.76	16.09	-122.454	37.936	4.60	11.81	-116.65	16.41	11	8/1/2014	09:28.8
1639	RWD11	-122.454	37.936	4.34	11.79	-148.38	16.13	-122.454	37.936	4.65	11.79	-98.81	16.44	11	8/1/2014	09:28.9
1640	RWD11	-122.454	37.936	4.34	11.78	-133.12	16.12	-122.454	37.936	4.65	11.78	-81.08	16.43	11	8/1/2014	09:29.0
1641	RWD11	-122.454	37.936	4.34	11.85	-117.91	16.19	-122.454	37.936	4.68	11.85	-63.43	16.53	11	8/1/2014	09:29.1
1642	RWD11	-122.454	37.936	4.28	11.78	-102.57	16.07	-122.454	37.936	4.65	11.78	-45.65	16.43	11	8/1/2014	09:29.2
1643	RWD11	-122.454	37.936	4.37	11.81	-87.08	16.18	-122.454	37.936	4.68	11.81	-27.66	16.50	11	8/1/2014	09:29.3
1644	RWD11	-122.454	37.936	4.34	11.78	-71.23	16.11	-122.454	37.936	4.68	11.78	-9.40	16.46	11	8/1/2014	09:29.4
1645	RWD11	-122.454	37.936	4.37	11.81	-55.04	16.18	-122.454	37.936	4.77	11.81	9.18	16.58	11	8/1/2014	09:29.5
1646	RWD11	-122.454	37.936	4.37	11.81	-38.19	16.18	-122.454	37.936	4.77	11.81	28.33	16.58	11	8/1/2014	09:29.6
1647	RWD11	-122.454	37.936	4.46	11.74	-20.62	16.20	-122.454	37.936	4.82	11.74	48.20	16.56	11	8/1/2014	09:29.7
1648	RWD11	-122.454	37.936	4.51	11.75	-2.03	16.26	-122.454	37.936	4.82	11.75	69.15	16.57	11	8/1/2014	09:29.8
1649	RWD11	-122.454	37.936	4.54	11.75	17.88	16.30	-122.454	37.936	4.82	11.75	91.51	16.57	11	8/1/2014	09:29.9
1650	RWD11	-122.454	37.936	4.51	11.76	39.02	16.27	-122.454	37.936	4.82	11.76	115.13	16.58	11	8/1/2014	09:30.0
1651	RWD11	-122.454	37.936	4.51	11.72	60.98	16.23	-122.454	37.936	4.82	11.72	139.67	16.54	11	8/1/2014	09:30.1
1652	RWD11	-122.454	37.936	4.51	11.68	83.52	16.19	-122.454	37.936	4.82	11.68	164.79	16.50	11	8/1/2014	09:30.2

1653	RWD11	-122.454	37.936	4.54	11.67	106.17	16.22	-122.454	37.936	4.85	11.67	190.15	16.53	11	8/1/2014 09:30.3
1654	RWD11	-122.454	37.936	4.51	11.63	128.93	16.14	-122.454	37.936	4.85	11.63	215.69	16.48	11	8/1/2014 09:30.4
1655	RWD11	-122.454	37.936	4.54	11.60	151.67	16.15	-122.454	37.936	4.90	11.60	241.23	16.51	11	8/1/2014 09:30.5
1656	RWD11	-122.454	37.936	4.51	11.59	174.43	16.10	-122.454	37.936	4.90	11.59	266.98	16.49	11	8/1/2014 09:30.6
1657	RWD11	-122.454	37.936	4.54	11.55	197.50	16.09	-122.454	37.936	4.94	11.55	293.05	16.49	11	8/1/2014 09:30.7
1658	RWD11	-122.454	37.936	4.54	11.52	220.98	16.06	-122.454	37.936	4.97	11.52	319.70	16.49	11	8/1/2014 09:30.8
1659	RWD11	-122.454	37.936	4.54	11.51	245.24	16.05	-122.454	37.936	4.97	11.51	347.14	16.48	11	8/1/2014 09:30.9
1660	RWD11	-122.454	37.936	4.54	11.47	270.54	16.01	-122.454	37.936	4.97	11.47	375.68	16.44	11	8/1/2014 09:31.0
1661	RWD11	-122.454	37.936	4.59	11.44	296.89	16.03	-122.454	37.936	5.02	11.44	405.34	16.46	11	8/1/2014 09:31.1
1662	RWD11	-122.454	37.936	4.63	11.41	324.40	16.04	-122.454	37.936	5.02	11.41	436.33	16.43	11	8/1/2014 09:31.2
1663	RWD11	-122.454	37.936	4.71	11.38	353.08	16.10	-122.454	37.936	5.02	11.38	468.59	16.41	11	8/1/2014 09:31.3
1664	RWD11	-122.454	37.936	4.71	11.37	382.63	16.08	-122.454	37.936	5.02	11.37	501.98	16.39	11	8/1/2014 09:31.4
1665	RWD11	-122.454	37.936	4.71	11.33	413.26	16.04	-122.454	37.936	5.06	11.33	536.49	16.38	11	8/1/2014 09:31.5
1666	RWD11	-122.454	37.936	4.71	11.35	444.80	16.07	-122.454	37.936	5.02	11.35	571.90	16.37	11	8/1/2014 09:31.6
1667	RWD11	-122.454	37.936	4.71	11.30	476.79	16.01	-122.454	37.936	5.06	11.30	607.85	16.35	11	8/1/2014 09:31.7
1668	RWD11	-122.454	37.936	4.71	11.28	508.98	16.00	-122.454	37.936	5.06	11.28	644.11	16.34	11	8/1/2014 09:31.8
1669	RWD11	-122.454	37.936	4.71	11.26	541.00	15.97	-122.454	37.936	5.11	11.26	680.29	16.36	11	8/1/2014 09:31.9
1670	RWD11	-122.454	37.936	4.71	11.23	572.75	15.95	-122.454	37.936	5.06	11.23	716.34	16.29	11	8/1/2014 09:32.0
1671	RWD11	-122.454	37.936	4.71	11.22	604.49	15.93	-122.454	37.936	5.11	11.22	752.51	16.33	11	8/1/2014 09:32.1
1672	RWD11	-122.454	37.936	4.71	11.19	636.36	15.90	-122.454	37.936	5.11	11.19	788.74	16.30	11	8/1/2014 09:32.2
1673	RWD11	-122.454	37.936	4.75	11.18	668.59	15.92	-122.454	37.936	5.14	11.18	825.37	16.32	11	8/1/2014 09:32.3
1674	RWD11	-122.454	37.936	4.75	11.19	701.46	15.94	-122.454	37.936	5.14	11.19	862.67	16.33	11	8/1/2014 09:32.4
1675	RWD11	-122.455	37.936	4.75	11.13	735.16	15.88	-122.454	37.936	5.19	11.13	900.73	16.32	11	8/1/2014 09:32.5
1676	RWD11	-122.455	37.936	4.75	11.13	770.04	15.88	-122.454	37.936	5.19	11.13	939.79	16.32	11	8/1/2014 09:32.6
1677	RWD11	-122.455	37.936	4.75	11.12	806.07	15.87	-122.454	37.936	5.19	11.12	979.96	16.31	11	8/1/2014 09:32.7
1678	RWD11	-122.455	37.936	4.75	11.09	843.21	15.84	-122.454	37.936	5.19	11.09	1021.28	16.28	11	8/1/2014 09:32.8
1679	RWD11	-122.455	37.936	4.80	11.08	881.37	15.88	-122.455	37.936	5.22	11.08	1063.62	16.30	11	8/1/2014 09:32.9
1680	RWD11	-122.455	37.936	4.75	11.12	920.37	15.87	-122.455	37.936	5.22	11.12	1106.83	16.35	11	8/1/2014 09:33.0
1681	RWD11	-122.455	37.936	4.92	11.03	960.27	15.94	-122.455	37.936	5.28	11.03	1150.89	16.30	11	8/1/2014 09:33.1
1682	RWD11	-122.455	37.936	4.88	10.98	######	15.87	-122.455	37.936	5.28	10.98	1195.91	16.26	11	8/1/2014 09:33.2
1683	RWD11	-122.455	37.936	4.92	10.93	######	15.85	-122.455	37.936	5.28	10.93	1241.76	16.20	11	8/1/2014 09:33.3
1684	RWD11	-122.455	37.936	4.88	10.91	######	15.80	-122.455	37.936	5.28	10.91	1288.69	16.19	11	8/1/2014 09:33.4
1685	RWD11	-122.455	37.936	4.88	10.89	######	15.77	-122.455	37.936	5.28	10.89	1336.61	16.16	11	8/1/2014 09:33.5

1686	RWD11	-122.455	37.936	4.88	10.86	######	15.75	-122.455	37.936	5.31	10.86	1385.61	16.17	11	8/1/2014	09:33.6
1687	RWD11	-122.455	37.936	4.92	10.83	######	15.74	-122.455	37.936	5.31	10.83	1435.55	16.14	11	8/1/2014	09:33.7
1688	RWD11	-122.455	37.936	4.88	10.83	######	15.71	-122.455	37.936	5.31	10.83	1486.31	16.14	11	8/1/2014	09:33.8
1689	RWD11	-122.455	37.936	4.92	10.80	######	15.72	-122.455	37.936	5.31	10.80	1537.42	16.11	11	8/1/2014	09:33.9
1690	RWD11	-122.455	37.936	4.88	10.80	######	15.68	-122.455	37.936	5.31	10.80	1589.17	16.11	11	8/1/2014	09:34.0
1691	RWD11	-122.455	37.936	4.92	10.81	######	15.73	-122.455	37.936	5.34	10.81	1641.68	16.16	11	8/1/2014	09:34.1
1692	RWD11	-122.455	37.936	4.92	10.75	######	15.67	-122.455	37.936	5.31	10.75	1695.31	16.06	11	8/1/2014	09:34.2
1693	RWD11	-122.455	37.936	4.92	10.76	######	15.68	-122.455	37.936	5.34	10.76	1749.75	16.11	11	8/1/2014	09:34.3
1694	RWD11	-122.455	37.936	4.92	10.76	######	15.68	-122.455	37.936	5.31	10.76	1805.09	16.07	11	8/1/2014	09:34.4
1695	RWD11	-122.455	37.936	4.97	10.78	######	15.75	-122.455	37.936	5.34	10.78	1861.14	16.12	11	8/1/2014	09:34.5
1696	RWD11	-122.455	37.936	4.92	10.77	######	15.69	-122.455	37.936	5.34	10.77	1918.15	16.12	11	8/1/2014	09:34.6
1697	RWD11	-122.455	37.936	4.97	10.79	######	15.76	-122.455	37.936	5.34	10.79	1975.54	16.13	11	8/1/2014	09:34.7
1698	RWD11	-122.455	37.936	4.92	10.79	######	15.71	-122.455	37.936	5.31	10.79	2032.65	16.10	11	8/1/2014	09:34.8
1699	RWD11	-122.455	37.936	4.92	10.83	######	15.74	-122.455	37.936	5.31	10.83	2088.99	16.14	11	8/1/2014	09:34.9
1700	RWD11	-122.455	37.936	4.92	10.81	######	15.73	-122.455	37.936	5.31	10.81	2144.56	16.12	11	8/1/2014	09:35.0
1701	RWD11	-122.455	37.936	4.97	10.78	######	15.75	-122.455	37.936	5.34	10.78	2198.96	16.12	11	8/1/2014	09:35.1
1702	RWD11	-122.455	37.936	4.92	10.76	######	15.68	-122.455	37.936	5.31	10.76	2252.48	16.07	11	8/1/2014	09:35.2
1703	RWD11	-122.455	37.936	4.97	10.73	######	15.70	-122.455	37.936	5.34	10.73	2304.89	16.08	11	8/1/2014	09:35.3
1704	RWD11	-122.455	37.936	4.97	10.70	######	15.67	-122.455	37.936	5.34	10.70	2356.44	16.05	11	8/1/2014	09:35.4
1705	RWD11	-122.455	37.936	4.97	10.68	######	15.65	-122.455	37.936	5.39	10.68	2407.61	16.07	11	8/1/2014	09:35.5
1706	RWD11	-122.455	37.936	4.97	10.64	######	15.61	-122.455	37.936	5.34	10.64	2458.98	15.98	11	8/1/2014	09:35.6
1707	RWD11	-122.455	37.936	4.97	10.61	######	15.58	-122.455	37.936	5.39	10.61	2511.11	16.00	11	8/1/2014	09:35.7
1708	RWD11	-122.455	37.936	4.97	10.58	######	15.55	-122.455	37.936	5.39	10.58	2563.76	15.98	11	8/1/2014	09:35.8
1709	RWD11	-122.455	37.936	4.97	10.54	######	15.51	-122.455	37.936	5.39	10.54	2615.86	15.93	11	8/1/2014	09:35.9
1710	RWD11	-122.455	37.936	4.97	10.51	######	15.48	-122.455	37.936	5.34	10.51	2666.70	15.86	11	8/1/2014	09:36.0
1711	RWD11	-122.455	37.936	4.97	10.47	######	15.44	-122.455	37.936	5.34	10.47	2715.81	15.81	11	8/1/2014	09:36.1
1712	RWD11	-122.455	37.936	4.97	10.41	######	15.38	-122.455	37.936	5.34	10.41	2762.60	15.75	11	8/1/2014	09:36.2
1713	RWD11	-122.455	37.936	5.00	10.38	######	15.38	-122.455	37.936	5.34	10.38	2806.90	15.72	11	8/1/2014	09:36.3
1714	RWD11	-122.455	37.936	4.97	10.35	######	15.32	-122.455	37.936	5.34	10.35	2848.87	15.69	11	8/1/2014	09:36.4
1715	RWD11	-122.455	37.936	5.00	10.34	######	15.34	-122.455	37.936	5.34	10.34	2888.43	15.68	11	8/1/2014	09:36.5
1716	RWD11	-122.455	37.936	4.97	10.27	######	15.23	-122.455	37.936	5.34	10.27	2926.55	15.61	11	8/1/2014	09:36.6
1717	RWD11	-122.455	37.936	5.00	10.23	######	15.23	-122.455	37.936	5.39	10.23	2963.55	15.63	11	8/1/2014	09:36.7
1718	RWD11	-122.455	37.936	4.97	10.19	######	15.15	-122.455	37.936	5.39	10.19	2999.21	15.58	11	8/1/2014	09:36.8

1719	RWD11	-122.455	37.936	5.00	10.16	######	15.17	-122.455	37.936	5.43	10.16	3033.80	15.59	11	8/1/2014	09:36.9
1720	RWD11	-122.455	37.936	5.09	10.16	######	15.25	-122.455	37.936	5.39	10.16	3067.07	15.56	11	8/1/2014	09:37.0
1721	RWD11	-122.455	37.936	5.09	10.12	######	15.20	-122.455	37.936	5.43	10.12	3098.31	15.54	11	8/1/2014	09:37.1
1722	RWD11	-122.455	37.936	5.09	10.06	######	15.15	-122.455	37.936	5.39	10.06	3127.31	15.46	11	8/1/2014	09:37.2
1723	RWD11	-122.455	37.936	5.09	10.03	######	15.12	-122.455	37.936	5.39	10.03	3153.64	15.43	11	8/1/2014	09:37.3
1724	RWD11	-122.455	37.936	5.09	10.00	######	15.09	-122.455	37.936	5.39	10.00	3176.98	15.40	11	8/1/2014	09:37.4
1725	RWD11	-122.455	37.936	5.09	10.00	######	15.09	-122.455	37.936	5.43	10.00	3196.60	15.43	11	8/1/2014	09:37.5
1726	RWD11	-122.455	37.936	5.09	9.98	######	15.07	-122.455	37.936	5.39	9.98	3212.43	15.37	11	8/1/2014	09:37.6
1727	RWD11	-122.455	37.936	5.09	9.94	######	15.03	-122.455	37.936	5.39	9.94	3224.66	15.33	11	8/1/2014	09:37.7
1728	RWD11	-122.455	37.936	5.04	9.92	######	14.95	-122.455	37.936	5.34	9.92	3233.16	15.26	11	8/1/2014	09:37.8
1729	RWD11	-122.455	37.936	5.09	9.90	######	14.99	-122.455	37.936	5.39	9.90	3238.70	15.30	11	8/1/2014	09:37.9
1730	RWD11	-122.455	37.936	5.09	9.88	######	14.96	-122.455	37.936	5.39	9.88	3241.17	15.27	11	8/1/2014	09:38.0
1731	RWD11	-122.455	37.936	5.09	9.85	######	14.94	-122.455	37.936	5.39	9.85	3240.63	15.25	11	8/1/2014	09:38.1
1732	RWD11	-122.455	37.936	5.04	9.83	######	14.87	-122.455	37.936	5.39	9.83	3236.64	15.23	11	8/1/2014	09:38.2
1733	RWD11	-122.455	37.936	5.09	9.82	######	14.90	-122.455	37.936	5.43	9.82	3229.18	15.24	11	8/1/2014	09:38.3
1734	RWD11	-122.455	37.936	5.09	9.80	######	14.89	-122.455	37.936	5.39	9.80	3217.83	15.19	11	8/1/2014	09:38.4
1735	RWD11	-122.455	37.936	5.09	9.78	######	14.87	-122.455	37.936	5.39	9.78	3202.65	15.17	11	8/1/2014	09:38.5
1736	RWD11	-122.455	37.936	5.04	9.76	######	14.80	-122.455	37.936	5.39	9.76	3183.51	15.16	11	8/1/2014	09:38.6
1737	RWD11	-122.455	37.936	5.09	9.75	######	14.84	-122.455	37.936	5.39	9.75	3160.31	15.15	11	8/1/2014	09:38.7
1738	RWD11	-122.455	37.936	5.09	9.69	######	14.78	-122.455	37.936	5.34	9.69	3132.56	15.04	11	8/1/2014	09:38.8
1739	RWD11	-122.455	37.936	5.09	9.72	######	14.80	-122.455	37.936	5.34	9.72	3100.63	15.06	11	8/1/2014	09:38.9
1740	RWD11	-122.455	37.936	5.09	9.65	######	14.74	-122.455	37.936	5.34	9.65	3065.26	15.00	11	8/1/2014	09:39.0
1741	RWD11	-122.455	37.936	5.09	9.72	######	14.80	-122.455	37.936	5.34	9.72	3027.07	15.06	11	8/1/2014	09:39.1
1742	RWD11	-122.455	37.936	5.04	9.61	######	14.65	-122.455	37.936	5.34	9.61	2986.74	14.96	11	8/1/2014	09:39.2
1743	RWD11	-122.455	37.936	5.09	9.61	######	14.70	-122.455	37.936	5.34	9.61	2944.39	14.96	11	8/1/2014	09:39.3
1744	RWD11	-122.455	37.936	5.04	9.63	######	14.67	-122.455	37.936	5.31	9.63	2900.03	14.94	11	8/1/2014	09:39.4
1745	RWD11	-122.455	37.936	5.09	9.63	######	14.72	-122.455	37.936	5.34	9.63	2853.51	14.97	11	8/1/2014	09:39.5
1746	RWD11	-122.455	37.936	5.09	9.72	######	14.80	-122.455	37.936	5.34	9.72	2804.51	15.06	11	8/1/2014	09:39.6
1747	RWD11	-122.455	37.936	5.09	9.71	######	14.80	-122.455	37.936	5.34	9.71	2752.71	15.05	11	8/1/2014	09:39.7
1748	RWD11	-122.455	37.936	5.09	9.70	######	14.79	-122.455	37.936	5.34	9.70	2698.23	15.04	11	8/1/2014	09:39.8
1749	RWD11	-122.455	37.936	5.09	9.68	######	14.77	-122.455	37.936	5.31	9.68	2641.16	14.99	11	8/1/2014	09:39.9
1750	RWD11	-122.455	37.936	5.04	9.69	######	14.73	-122.455	37.936	5.31	9.69	2582.03	15.00	11	8/1/2014	09:40.0
1751	RWD11	-122.455	37.936	5.09	9.71	######	14.80	-122.455	37.936	5.31	9.71	2521.59	15.02	11	8/1/2014	09:40.1

1752	RWD11	-122.455	37.936	5.04	9.70	######	14.74	-122.455	37.936	5.28	9.70	2460.47	14.97	11	8/1/2014	09:40.2
1753	RWD11	-122.455	37.936	5.09	9.73	######	14.81	-122.455	37.936	5.28	9.73	2399.76	15.00	11	8/1/2014	09:40.3
1754	RWD11	-122.455	37.936	5.04	9.73	######	14.77	-122.455	37.936	5.28	9.73	2339.34	15.01	11	8/1/2014	09:40.4
1755	RWD11	-122.455	37.936	5.09	9.73	######	14.82	-122.455	37.936	5.31	9.73	2279.63	15.04	11	8/1/2014	09:40.5
1756	RWD11	-122.455	37.936	5.04	9.72	######	14.75	-122.455	37.936	5.28	9.72	2220.18	14.99	11	8/1/2014	09:40.6
1757	RWD11	-122.455	37.936	5.09	9.74	######	14.83	-122.455	37.936	5.28	9.74	2160.66	15.01	11	8/1/2014	09:40.7
1758	RWD11	-122.455	37.936	5.04	9.72	######	14.75	-122.455	37.936	5.28	9.72	2101.08	14.99	11	8/1/2014	09:40.8
1759	RWD11	-122.455	37.936	5.09	9.75	######	14.83	-122.455	37.936	5.28	9.75	2041.52	15.02	11	8/1/2014	09:40.9
1760	RWD11	-122.455	37.936	5.09	9.73	######	14.82	-122.455	37.936	5.28	9.73	1981.84	15.01	11	8/1/2014	09:41.0
1761	RWD11	-122.455	37.936	5.04	9.73	######	14.77	-122.455	37.936	5.28	9.73	1921.62	15.01	11	8/1/2014	09:41.1
1762	RWD11	-122.455	37.936	5.04	9.74	######	14.78	-122.455	37.936	5.28	9.74	1860.31	15.01	11	8/1/2014	09:41.2
1763	RWD11	-122.455	37.936	5.09	9.75	######	14.84	-122.455	37.936	5.28	9.75	1797.83	15.03	11	8/1/2014	09:41.3
1764	RWD11	-122.455	37.936	5.04	9.75	######	14.78	-122.455	37.936	5.22	9.75	1734.50	14.97	11	8/1/2014	09:41.4
1765	RWD11	-122.455	37.936	5.04	9.78	######	14.82	-122.455	37.936	5.22	9.78	1671.10	15.00	11	8/1/2014	09:41.5
1766	RWD11	-122.455	37.936	5.00	9.82	######	14.82	-122.455	37.936	5.19	9.82	1608.85	15.01	11	8/1/2014	09:41.6
1767	RWD11	-122.455	37.936	5.04	9.79	######	14.83	-122.455	37.936	5.22	9.79	1548.82	15.02	11	8/1/2014	09:41.7
1768	RWD11	-122.455	37.936	5.00	9.82	######	14.82	-122.455	37.936	5.19	9.82	1491.29	15.01	11	8/1/2014	09:41.8
1769	RWD11	-122.455	37.936	5.04	9.80	######	14.84	-122.455	37.936	5.19	9.80	1436.19	14.99	11	8/1/2014	09:41.9
1770	RWD11	-122.455	37.936	5.04	9.83	######	14.87	-122.455	37.936	5.22	9.83	1382.79	15.06	11	8/1/2014	09:42.0
1771	RWD11	-122.455	37.936	5.04	9.82	######	14.86	-122.455	37.936	5.19	9.82	1330.54	15.01	11	8/1/2014	09:42.1
1772	RWD11	-122.455	37.936	5.00	9.82	######	14.82	-122.455	37.936	5.14	9.82	1279.38	14.96	11	8/1/2014	09:42.2
1773	RWD11	-122.455	37.936	5.00	9.85	######	14.86	-122.455	37.936	5.19	9.85	1228.84	15.05	11	8/1/2014	09:42.3
1774	RWD11	-122.455	37.936	5.04	9.84	######	14.88	-122.455	37.936	5.19	9.84	1178.80	15.03	11	8/1/2014	09:42.4
1775	RWD11	-122.455	37.936	5.04	9.86	######	14.90	-122.455	37.936	5.19	9.86	1128.75	15.06	11	8/1/2014	09:42.5
1776	RWD11	-122.455	37.936	5.00	9.83	######	14.83	-122.455	37.936	5.14	9.83	1078.75	14.97	11	8/1/2014	09:42.6
1777	RWD11	-122.455	37.936	5.04	9.84	######	14.88	-122.455	37.936	5.14	9.84	1029.17	14.98	11	8/1/2014	09:42.7
1778	RWD11	-122.455	37.936	5.00	9.84	987.61	14.84	-122.455	37.936	5.11	9.84	980.26	14.94	11	8/1/2014	09:42.8
1779	RWD11	-122.455	37.936	5.00	9.84	940.67	14.84	-122.455	37.936	5.14	9.84	932.72	14.98	11	8/1/2014	09:42.9
1780	RWD11	-122.455	37.936	5.00	9.82	895.44	14.82	-122.455	37.936	5.11	9.82	886.73	14.93	11	8/1/2014	09:43.0
1781	RWD11	-122.455	37.936	5.00	9.82	852.17	14.82	-122.455	37.936	5.11	9.82	842.63	14.92	11	8/1/2014	09:43.1
1782	RWD11	-122.455	37.936	4.97	9.85	810.40	14.82	-122.455	37.936	5.11	9.85	800.10	14.95	11	8/1/2014	09:43.2
1783	RWD11	-122.455	37.936	5.00	9.80	769.80	14.80	-122.455	37.936	5.11	9.80	758.95	14.91	11	8/1/2014	09:43.3
1784	RWD11	-122.455	37.936	5.00	9.78	730.23	14.78	-122.455	37.936	5.11	9.78	719.11	14.88	11	8/1/2014	09:43.4

1785	RWD11	-122.455	37.936	5.00	9.80	691.25	14.80	-122.455	37.936	5.11	9.80	679.98	14.91	11	8/1/2014	09:43.5
1786	RWD11	-122.455	37.936	5.00	9.77	652.80	14.77	-122.455	37.936	5.05	9.77	641.47	14.82	11	8/1/2014	09:43.6
1787	RWD11	-122.455	37.936	5.00	9.75	615.09	14.76	-122.455	37.936	5.05	9.75	603.67	14.81	11	8/1/2014	09:43.7
1788	RWD11	-122.455	37.936	4.97	9.78	578.15	14.75	-122.455	37.936	5.02	9.78	566.72	14.80	11	8/1/2014	09:43.8
1789	RWD11	-122.455	37.936	5.00	9.78	542.14	14.79	-122.455	37.936	5.05	9.78	530.62	14.84	11	8/1/2014	09:43.9
1790	RWD11	-122.455	37.936	4.97	9.79	507.13	14.76	-122.455	37.936	5.02	9.79	495.58	14.81	11	8/1/2014	09:44.0
1791	RWD11	-122.455	37.936	4.97	9.75	473.15	14.71	-122.455	37.936	5.02	9.75	461.58	14.77	11	8/1/2014	09:44.1
1792	RWD11	-122.455	37.936	4.97	9.76	440.15	14.73	-122.455	37.936	5.02	9.76	428.63	14.78	11	8/1/2014	09:44.2
1793	RWD11	-122.455	37.936	4.97	9.77	408.29	14.74	-122.455	37.936	5.02	9.77	396.81	14.79	11	8/1/2014	09:44.3
1794	RWD11	-122.455	37.936	4.92	9.75	377.73	14.66	-122.455	37.936	4.97	9.75	366.20	14.72	11	8/1/2014	09:44.4
1795	RWD11	-122.455	37.936	4.97	9.74	348.28	14.71	-122.455	37.936	4.97	9.74	336.93	14.71	11	8/1/2014	09:44.5
1796	RWD11	-122.455	37.936	4.92	9.73	319.66	14.65	-122.455	37.936	4.97	9.73	308.37	14.70	11	8/1/2014	09:44.6
1797	RWD11	-122.455	37.936	4.97	9.72	291.69	14.68	-122.455	37.936	4.97	9.72	280.64	14.69	11	8/1/2014	09:44.7
1798	RWD11	-122.455	37.936	4.92	9.73	264.46	14.64	-122.455	37.936	4.94	9.73	253.61	14.66	11	8/1/2014	09:44.8
1799	RWD11	-122.455	37.936	4.97	9.69	237.79	14.66	-122.455	37.936	4.97	9.69	227.17	14.66	11	8/1/2014	09:44.9
1800	RWD11	-122.455	37.936	4.92	9.69	211.60	14.61	-122.455	37.936	4.94	9.69	201.23	14.63	11	8/1/2014	09:45.0
1801	RWD11	-122.455	37.936	4.92	9.69	185.75	14.61	-122.455	37.936	4.97	9.69	175.72	14.66	11	8/1/2014	09:45.1
1802	RWD11	-122.455	37.936	4.92	9.69	160.31	14.61	-122.455	37.936	4.94	9.69	150.77	14.63	11	8/1/2014	09:45.2
1803	RWD11	-122.455	37.936	4.92	9.68	135.41	14.59	-122.455	37.936	4.94	9.68	126.34	14.61	11	8/1/2014	09:45.3
1804	RWD11	-122.455	37.936	4.92	9.68	111.35	14.60	-122.455	37.936	4.90	9.68	102.68	14.59	11	8/1/2014	09:45.4
1805	RWD11	-122.455	37.936	4.92	9.65	88.10	14.57	-122.455	37.936	4.94	9.65	79.88	14.59	11	8/1/2014	09:45.5
1806	RWD11	-122.455	37.936	4.88	9.65	65.97	14.53	-122.455	37.936	4.90	9.65	58.03	14.55	11	8/1/2014	09:45.6
1807	RWD11	-122.455	37.936	4.88	9.65	44.79	14.54	-122.455	37.936	4.94	9.65	37.02	14.59	11	8/1/2014	09:45.7
1808	RWD11	-122.455	37.936	4.88	9.65	24.21	14.54	-122.455	37.936	4.85	9.65	16.66	14.51	11	8/1/2014	09:45.8
1809	RWD11	-122.455	37.936	4.92	9.64	4.38	14.55	-122.455	37.936	4.90	9.64	-3.11	14.54	11	8/1/2014	09:45.9
1810	RWD11	-122.455	37.936	4.88	9.67	-15.11	14.55	-122.455	37.936	4.90	9.67	-22.37	14.57	11	8/1/2014	09:46.0
1811	RWD11	-122.455	37.936	4.88	9.61	-34.09	14.49	-122.455	37.936	4.90	9.61	-41.23	14.51	11	8/1/2014	09:46.1
1812	RWD11	-122.455	37.936	4.83	9.62	-52.75	14.45	-122.455	37.936	4.85	9.62	-59.71	14.47	11	8/1/2014	09:46.2
1813	RWD11	-122.455	37.936	4.88	9.64	-71.16	14.52	-122.455	37.936	4.90	9.64	-78.01	14.54	11	8/1/2014	09:46.3
1814	RWD11	-122.455	37.936	4.88	9.59	-89.58	14.47	-122.455	37.936	4.85	9.59	-96.19	14.44	11	8/1/2014	09:46.4
1815	RWD11	-122.455	37.936	4.88	9.58	-107.95	14.46	-122.455	37.936	4.90	9.58	-114.34	14.48	11	8/1/2014	09:46.5
1816	RWD11	-122.455	37.936	4.88	9.63	-126.16	14.51	-122.455	37.936	4.85	9.63	-132.31	14.48	11	8/1/2014	09:46.6
1817	RWD11	-122.455	37.936	4.88	9.58	-144.02	14.46	-122.455	37.936	4.85	9.58	-149.83	14.43	11	8/1/2014	09:46.7

1818	RWD11	-122.455	37.936	4.83	9.59	-161.44	14.42	-122.455	37.936	4.82	9.59	-167.10	14.40	11	8/1/2014	09:46.8
1819	RWD11	-122.455	37.936	4.83	9.60	-178.46	14.43	-122.455	37.936	4.82	9.60	-183.96	14.42	11	8/1/2014	09:46.9
1820	RWD11	-122.455	37.936	4.83	9.62	-194.86	14.45	-122.455	37.936	4.82	9.62	-200.35	14.44	11	8/1/2014	09:47.0
1821	RWD11	-122.455	37.936	4.88	9.59	-210.60	14.47	-122.455	37.936	4.82	9.59	-216.09	14.41	11	8/1/2014	09:47.1
1822	RWD11	-122.455	37.936	4.80	9.61	-225.78	14.40	-122.455	37.936	4.77	9.61	-231.32	14.37	11	8/1/2014	09:47.2
1823	RWD11	-122.455	37.936	4.83	9.60	-240.52	14.43	-122.455	37.936	4.82	9.60	-246.09	14.42	11	8/1/2014	09:47.3
1824	RWD11	-122.455	37.936	4.80	9.61	-254.84	14.41	-122.455	37.936	4.77	9.61	-260.57	14.38	11	8/1/2014	09:47.4
1825	RWD11	-122.455	37.936	4.83	9.59	-268.89	14.42	-122.455	37.936	4.77	9.59	-274.74	14.36	11	8/1/2014	09:47.5
1826	RWD11	-122.455	37.936	4.80	9.59	-282.73	14.39	-122.455	37.936	4.77	9.59	-288.68	14.36	11	8/1/2014	09:47.6
1827	RWD11	-122.455	37.936	4.83	9.65	-296.48	14.48	-122.455	37.936	4.77	9.65	-302.54	14.41	11	8/1/2014	09:47.7
1828	RWD11	-122.455	37.936	4.80	9.60	-310.06	14.40	-122.455	37.936	4.77	9.60	-316.27	14.37	11	8/1/2014	09:47.8
1829	RWD11	-122.455	37.936	4.83	9.61	-323.46	14.44	-122.455	37.936	4.77	9.61	-329.78	14.37	11	8/1/2014	09:47.9
1830	RWD11	-122.455	37.936	4.80	9.59	-336.61	14.39	-122.455	37.936	4.73	9.59	-343.00	14.32	11	8/1/2014	09:48.0
1831	RWD11	-122.455	37.936	4.80	9.59	-349.43	14.39	-122.455	37.936	4.73	9.59	-356.00	14.32	11	8/1/2014	09:48.1
1832	RWD11	-122.455	37.936	4.80	9.61	-361.90	14.41	-122.455	37.936	4.73	9.61	-368.61	14.35	11	8/1/2014	09:48.2
1833	RWD11	-122.455	37.936	4.80	9.61	-374.07	14.40	-122.455	37.936	4.68	9.61	-380.99	14.29	11	8/1/2014	09:48.3
1834	RWD11	-122.455	37.936	4.74	9.61	-385.87	14.36	-122.455	37.936	4.68	9.61	-392.88	14.30	11	8/1/2014	09:48.4
1835	RWD11	-122.455	37.936	4.80	9.60	-397.45	14.40	-122.455	37.936	4.73	9.60	-404.73	14.33	11	8/1/2014	09:48.5
1836	RWD11	-122.455	37.936	4.74	9.62	-408.91	14.37	-122.455	37.936	4.68	9.62	-416.36	14.31	11	8/1/2014	09:48.6
1837	RWD11	-122.455	37.936	4.74	9.64	-420.04	14.38	-122.455	37.936	4.68	9.64	-427.63	14.32	11	8/1/2014	09:48.7
1838	RWD11	-122.455	37.936	4.71	9.68	-430.87	14.39	-122.455	37.936	4.65	9.68	-438.61	14.33	11	8/1/2014	09:48.8
1839	RWD11	-122.455	37.936	4.74	9.67	-441.28	14.42	-122.455	37.936	4.65	9.67	-449.20	14.32	11	8/1/2014	09:48.9
1840	RWD11	-122.455	37.936	4.74	9.72	-451.38	14.46	-122.455	37.936	4.60	9.72	-459.58	14.31	11	8/1/2014	09:49.0
1841	RWD11	-122.455	37.936	4.74	9.76	-461.26	14.51	-122.455	37.936	4.65	9.76	-469.70	14.41	11	8/1/2014	09:49.1
1842	RWD11	-122.455	37.936	4.71	9.74	-470.86	14.45	-122.455	37.936	4.56	9.74	-479.60	14.30	11	8/1/2014	09:49.2
1843	RWD11	-122.455	37.936	4.71	9.74	-480.32	14.45	-122.455	37.936	4.60	9.74	-489.29	14.34	11	8/1/2014	09:49.3
1844	RWD11	-122.455	37.936	4.71	9.73	-489.51	14.44	-122.455	37.936	4.56	9.73	-498.79	14.29	11	8/1/2014	09:49.4
1845	RWD11	-122.455	37.936	4.71	9.74	-498.58	14.45	-122.455	37.936	4.60	9.74	-508.18	14.34	11	8/1/2014	09:49.5
1846	RWD11	-122.455	37.936	4.71	9.75	-507.45	14.46	-122.455	37.936	4.56	9.75	-517.35	14.31	11	8/1/2014	09:49.6
1847	RWD11	-122.455	37.936	4.71	9.75	-516.05	14.46	-122.455	37.936	4.56	9.75	-526.29	14.32	11	8/1/2014	09:49.7
1848	RWD11	-122.455	37.936	4.66	9.75	-524.41	14.41	-122.455	37.936	4.53	9.75	-534.98	14.28	11	8/1/2014	09:49.8
1849	RWD11	-122.455	37.936	4.66	9.75	-532.57	14.41	-122.455	37.936	4.56	9.75	-543.50	14.32	11	8/1/2014	09:49.9
1850	RWD11	-122.455	37.936	4.63	9.73	-540.51	14.36	-122.455	37.936	4.53	9.73	-551.77	14.26	11	8/1/2014	09:50.0

1851	RWD11	-122.455	37.936	4.66	9.73	-548.19	14.39	-122.455	37.936	4.53	9.73	-559.74	14.26	11	8/1/2014	09:50.1
1852	RWD11	-122.455	37.936	4.66	9.75	-555.82	14.41	-122.455	37.936	4.53	9.75	-567.64	14.28	11	8/1/2014	09:50.2
1853	RWD11	-122.455	37.936	4.66	9.78	-563.50	14.44	-122.455	37.936	4.53	9.78	-575.59	14.31	11	8/1/2014	09:50.3
1854	RWD11	-122.455	37.936	4.63	9.72	-571.09	14.34	-122.455	37.936	4.53	9.72	-583.49	14.25	11	8/1/2014	09:50.4
1855	RWD11	-122.455	37.936	4.66	9.72	-578.73	14.37	-122.455	37.936	4.56	9.72	-591.39	14.28	11	8/1/2014	09:50.5
1856	RWD11	-122.455	37.936	4.63	9.74	-586.39	14.36	-122.455	37.936	4.56	9.74	-599.41	14.30	11	8/1/2014	09:50.6
1857	RWD11	-122.455	37.936	4.66	9.76	-594.07	14.42	-122.455	37.936	4.53	9.76	-607.47	14.29	11	8/1/2014	09:50.7
1858	RWD11	-122.455	37.936	4.63	9.73	-601.76	14.35	-122.455	37.936	4.53	9.73	-615.44	14.26	11	8/1/2014	09:50.8
1859	RWD11	-122.455	37.936	4.63	9.73	-609.22	14.36	-122.455	37.936	4.53	9.73	-623.35	14.26	11	8/1/2014	09:50.9
1860	RWD11	-122.455	37.936	4.63	9.71	-616.37	14.33	-122.455	37.936	4.48	9.71	-630.87	14.19	11	8/1/2014	09:51.0
1861	RWD11	-122.455	37.936	4.63	9.70	-623.29	14.32	-122.455	37.936	4.53	9.70	-638.19	14.23	11	8/1/2014	09:51.1
1862	RWD11	-122.455	37.936	4.59	9.69	-629.93	14.28	-122.455	37.936	4.48	9.69	-645.20	14.17	11	8/1/2014	09:51.2
1863	RWD11	-122.455	37.936	4.63	9.66	-636.46	14.29	-122.455	37.936	4.48	9.66	-652.06	14.14	11	8/1/2014	09:51.3
1864	RWD11	-122.455	37.936	4.59	9.65	-642.70	14.24	-122.455	37.936	4.48	9.65	-658.72	14.13	11	8/1/2014	09:51.4
1865	RWD11	-122.455	37.936	4.59	9.61	-649.08	14.20	-122.455	37.936	4.53	9.61	-665.34	14.14	11	8/1/2014	09:51.5
1866	RWD11	-122.455	37.936	4.59	9.59	-655.37	14.18	-122.455	37.936	4.48	9.59	-672.07	14.07	11	8/1/2014	09:51.6
1867	RWD11	-122.455	37.936	4.59	9.58	-661.82	14.17	-122.455	37.936	4.48	9.58	-678.81	14.06	11	8/1/2014	09:51.7
1868	RWD11	-122.455	37.936	4.59	9.61	-668.18	14.21	-122.455	37.936	4.48	9.61	-685.58	14.09	11	8/1/2014	09:51.8
1869	RWD11	-122.455	37.936	4.59	9.53	-674.58	14.12	-122.455	37.936	4.48	9.53	-692.31	14.01	11	8/1/2014	09:51.9
1870	RWD11	-122.455	37.936	4.59	9.52	-680.99	14.11	-122.455	37.936	4.48	9.52	-699.05	13.99	11	8/1/2014	09:52.0
1871	RWD11	-122.455	37.936	4.59	9.56	-687.28	14.15	-122.455	37.936	4.48	9.56	-705.71	14.04	11	8/1/2014	09:52.1
1872	RWD11	-122.455	37.936	4.54	9.49	-693.36	14.03	-122.455	37.936	4.45	9.49	-712.22	13.94	11	8/1/2014	09:52.2
1873	RWD11	-122.455	37.936	4.54	9.49	-699.19	14.03	-122.455	37.936	4.48	9.49	-718.46	13.97	11	8/1/2014	09:52.3
1874	RWD11	-122.455	37.936	4.54	9.45	-704.86	13.99	-122.455	37.936	4.45	9.45	-724.62	13.89	11	8/1/2014	09:52.4
1875	RWD11	-122.455	37.936	4.59	9.48	-710.43	14.08	-122.455	37.936	4.48	9.48	-730.49	13.96	11	8/1/2014	09:52.5
1876	RWD11	-122.455	37.936	4.54	9.45	-715.65	13.99	-122.455	37.936	4.45	9.45	-736.16	13.90	11	8/1/2014	09:52.6
1877	RWD11	-122.455	37.936	4.54	9.47	-720.89	14.01	-122.455	37.936	4.48	9.47	-741.75	13.95	11	8/1/2014	09:52.7
1878	RWD11	-122.455	37.936	4.54	9.42	-725.94	13.96	-122.455	37.936	4.45	9.42	-747.19	13.87	11	8/1/2014	09:52.8
1879	RWD11	-122.455	37.936	4.54	9.48	-731.03	14.02	-122.455	37.936	4.45	9.48	-752.62	13.92	11	8/1/2014	09:52.9
1880	RWD11	-122.455	37.936	4.54	9.43	-736.06	13.97	-122.455	37.936	4.45	9.43	-757.98	13.88	11	8/1/2014	09:53.0
1881	RWD11	-122.455	37.936	4.54	9.42	-741.05	13.96	-122.455	37.936	4.45	9.42	-763.28	13.87	11	8/1/2014	09:53.1
1882	RWD11	-122.455	37.936	4.51	9.41	-745.91	13.91	-122.455	37.936	4.45	9.41	-768.55	13.85	11	8/1/2014	09:53.2
1883	RWD11	-122.455	37.936	4.54	9.41	-750.74	13.95	-122.455	37.936	4.45	9.41	-773.72	13.86	11	8/1/2014	09:53.3

1884	RWD11	-122.455	37.936	4.54	9.42	-755.57	13.96	-122.455	37.936	4.45	9.42	-778.86	13.87	11	8/1/2014	09:53.4
1885	RWD11	-122.455	37.936	4.54	9.41	-760.30	13.95	-122.455	37.936	4.45	9.41	-783.93	13.86	11	8/1/2014	09:53.5
1886	RWD11	-122.455	37.936	4.51	9.44	-764.94	13.94	-122.455	37.936	4.45	9.44	-788.88	13.88	11	8/1/2014	09:53.6
1887	RWD11	-122.455	37.936	4.54	9.41	-769.52	13.95	-122.455	37.936	4.45	9.41	-793.77	13.86	11	8/1/2014	09:53.7
1888	RWD11	-122.455	37.936	4.51	9.48	-773.97	13.99	-122.455	37.936	4.45	9.48	-798.57	13.93	11	8/1/2014	09:53.8
1889	RWD11	-122.455	37.936	4.51	9.43	-778.35	13.94	-122.455	37.936	4.45	9.43	-803.31	13.88	11	8/1/2014	09:53.9
1890	RWD11	-122.455	37.936	4.51	9.43	-782.54	13.94	-122.455	37.936	4.45	9.43	-807.87	13.88	11	8/1/2014	09:54.0
1891	RWD11	-122.455	37.936	4.54	9.40	-786.73	13.94	-122.455	37.936	4.48	9.40	-812.38	13.88	11	8/1/2014	09:54.1
1892	RWD11	-122.455	37.936	4.51	9.47	-790.90	13.97	-122.455	37.936	4.48	9.47	-816.89	13.95	11	8/1/2014	09:54.2
1893	RWD11	-122.455	37.936	4.51	9.39	-794.98	13.90	-122.455	37.936	4.48	9.39	-821.32	13.87	11	8/1/2014	09:54.3
1894	RWD11	-122.455	37.936	4.51	9.41	-799.05	13.92	-122.455	37.936	4.48	9.41	-825.68	13.89	11	8/1/2014	09:54.4
1895	RWD11	-122.455	37.936	4.54	9.31	-803.13	13.85	-122.455	37.936	4.53	9.31	-830.05	13.84	11	8/1/2014	09:54.5
1896	RWD11	-122.455	37.936	4.54	9.35	-807.30	13.89	-122.455	37.936	4.53	9.35	-834.47	13.88	11	8/1/2014	09:54.6
1897	RWD11	-122.455	37.936	4.54	9.30	-811.55	13.84	-122.455	37.936	4.56	9.30	-839.03	13.86	11	8/1/2014	09:54.7
1898	RWD11	-122.455	37.936	4.54	9.27	-815.92	13.81	-122.455	37.936	4.56	9.27	-843.60	13.83	11	8/1/2014	09:54.8
1899	RWD11	-122.455	37.936	4.54	9.26	-820.17	13.80	-122.455	37.936	4.53	9.26	-848.14	13.79	11	8/1/2014	09:54.9
1900	RWD11	-122.455	37.936	4.51	9.27	-824.36	13.78	-122.455	37.936	4.53	9.27	-852.63	13.80	11	8/1/2014	09:55.0
1901	RWD11	-122.455	37.936	4.54	9.23	-828.42	13.77	-122.455	37.936	4.56	9.23	-857.02	13.79	11	8/1/2014	09:55.1
1902	RWD11	-122.455	37.936	4.51	9.24	-832.31	13.75	-122.455	37.936	4.53	9.24	-861.19	13.77	11	8/1/2014	09:55.2
1903	RWD11	-122.455	37.936	4.54	9.21	-835.99	13.75	-122.455	37.936	4.56	9.21	-865.27	13.77	11	8/1/2014	09:55.3
1904	RWD11	-122.455	37.936	4.54	9.22	-839.57	13.76	-122.455	37.936	4.53	9.22	-869.11	13.75	11	8/1/2014	09:55.4
1905	RWD11	-122.455	37.936	4.54	9.18	-843.20	13.72	-122.455	37.936	4.60	9.18	-872.94	13.78	11	8/1/2014	09:55.5
1906	RWD11	-122.455	37.936	4.54	9.17	-846.77	13.71	-122.455	37.936	4.60	9.17	-876.78	13.77	11	8/1/2014	09:55.6
1907	RWD11	-122.455	37.936	4.54	9.17	-850.43	13.71	-122.455	37.936	4.60	9.17	-880.62	13.77	11	8/1/2014	09:55.7
1908	RWD11	-122.455	37.936	4.54	9.15	-854.15	13.69	-122.455	37.936	4.60	9.15	-884.46	13.74	11	8/1/2014	09:55.8
1909	RWD11	-122.455	37.936	4.54	9.24	-857.79	13.78	-122.455	37.936	4.60	9.24	-888.30	13.84	11	8/1/2014	09:55.9
1910	RWD11	-122.455	37.936	4.54	9.18	-861.39	13.72	-122.455	37.936	4.60	9.18	-892.00	13.77	11	8/1/2014	09:56.0
1911	RWD11	-122.455	37.936	4.54	9.19	-864.94	13.73	-122.455	37.936	4.60	9.19	-895.75	13.79	11	8/1/2014	09:56.1
1912	RWD11	-122.455	37.936	4.54	9.21	-868.27	13.75	-122.455	37.936	4.60	9.21	-899.19	13.80	11	8/1/2014	09:56.2
1913	RWD11	-122.455	37.936	4.54	9.22	-871.41	13.76	-122.455	37.936	4.60	9.22	-902.51	13.82	11	8/1/2014	09:56.3
1914	RWD11	-122.455	37.936	4.54	9.18	-874.39	13.72	-122.455	37.936	4.56	9.18	-905.69	13.75	11	8/1/2014	09:56.4
1915	RWD11	-122.455	37.936	4.54	9.21	-877.26	13.75	-122.455	37.936	4.60	9.21	-908.68	13.80	11	8/1/2014	09:56.5
1916	RWD11	-122.455	37.936	4.54	9.18	-879.85	13.72	-122.455	37.936	4.56	9.18	-911.46	13.74	11	8/1/2014	09:56.6

1917	RWD11	-122.455	37.936	4.59	9.20	-882.39	13.79	-122.455	37.936	4.60	9.20	-914.23	13.80	11	8/1/2014	09:56.7
1918	RWD11	-122.455	37.936	4.54	9.18	-884.94	13.72	-122.455	37.936	4.60	9.18	-916.97	13.77	11	8/1/2014	09:56.8
1919	RWD11	-122.455	37.936	4.59	9.17	-887.32	13.76	-122.455	37.936	4.60	9.17	-919.49	13.77	11	8/1/2014	09:56.9
1920	RWD11	-122.455	37.936	4.54	9.18	-889.61	13.72	-122.455	37.936	4.60	9.18	-922.01	13.78	11	8/1/2014	09:57.0
1921	RWD11	-122.455	37.936	4.54	9.18	-891.99	13.72	-122.455	37.936	4.65	9.18	-924.52	13.83	11	8/1/2014	09:57.1
1922	RWD11	-122.455	37.936	4.54	9.15	-894.15	13.69	-122.455	37.936	4.60	9.15	-926.84	13.75	11	8/1/2014	09:57.2
1923	RWD11	-122.455	37.936	4.59	9.18	-896.11	13.78	-122.455	37.936	4.65	9.18	-928.91	13.83	11	8/1/2014	09:57.3
1924	RWD11	-122.455	37.936	4.54	9.15	-897.89	13.69	-122.455	37.936	4.60	9.15	-930.95	13.74	11	8/1/2014	09:57.4
1925	RWD11	-122.455	37.936	4.54	9.15	-899.61	13.69	-122.455	37.936	4.60	9.15	-932.85	13.75	11	8/1/2014	09:57.5
1926	RWD11	-122.455	37.936	4.54	9.15	-901.11	13.69	-122.455	37.936	4.56	9.15	-934.60	13.71	11	8/1/2014	09:57.6
1927	RWD11	-122.455	37.936	4.54	9.14	-902.54	13.68	-122.455	37.936	4.60	9.14	-936.18	13.73	11	8/1/2014	09:57.7
1928	RWD11	-122.455	37.936	4.54	9.15	-903.77	13.69	-122.455	37.936	4.56	9.15	-937.56	13.72	11	8/1/2014	09:57.8
1929	RWD11	-122.455	37.936	4.54	9.14	-905.14	13.68	-122.455	37.936	4.60	9.14	-939.00	13.73	11	8/1/2014	09:57.9
1930	RWD11	-122.455	37.936	4.54	9.14	-906.45	13.68	-122.455	37.936	4.60	9.14	-940.35	13.73	11	8/1/2014	09:58.0
1931	RWD11	-122.455	37.936	4.54	9.17	-907.77	13.71	-122.455	37.936	4.65	9.17	-941.75	13.82	11	8/1/2014	09:58.1
1932	RWD11	-122.455	37.936	4.54	9.15	-909.11	13.68	-122.455	37.936	4.60	9.15	-943.07	13.74	11	8/1/2014	09:58.2
1933	RWD11	-122.455	37.936	4.59	9.13	-910.41	13.72	-122.455	37.936	4.65	9.13	-944.42	13.78	11	8/1/2014	09:58.3
1934	RWD11	-122.455	37.936	4.54	9.11	-911.72	13.65	-122.455	37.936	4.60	9.11	-945.68	13.71	11	8/1/2014	09:58.4
1935	RWD11	-122.455	37.936	4.59	9.12	-912.89	13.71	-122.455	37.936	4.68	9.12	-946.94	13.80	11	8/1/2014	09:58.5
1936	RWD11	-122.455	37.936	4.54	9.12	-914.02	13.66	-122.455	37.936	4.65	9.12	-948.06	13.77	11	8/1/2014	09:58.6
1937	RWD11	-122.455	37.936	4.59	9.13	-914.88	13.72	-122.455	37.936	4.65	9.13	-949.03	13.78	11	8/1/2014	09:58.7
1938	RWD11	-122.455	37.936	4.54	9.15	-915.66	13.69	-122.455	37.936	4.65	9.15	-949.81	13.80	11	8/1/2014	09:58.8
1939	RWD11	-122.455	37.936	4.59	9.15	-916.16	13.74	-122.455	37.936	4.65	9.15	-950.46	13.79	11	8/1/2014	09:58.9
1940	RWD11	-122.455	37.936	4.54	9.25	-916.50	13.79	-122.455	37.936	4.60	9.25	-950.85	13.85	11	8/1/2014	09:59.0
1941	RWD11	-122.455	37.936	4.59	9.17	-916.73	13.76	-122.455	37.936	4.60	9.17	-951.14	13.76	11	8/1/2014	09:59.1
1942	RWD11	-122.455	37.936	4.54	9.18	-916.82	13.72	-122.455	37.936	4.56	9.18	-951.10	13.75	11	8/1/2014	09:59.2
1943	RWD11	-122.455	37.936	4.54	9.22	-916.93	13.75	-122.455	37.936	4.60	9.22	-951.11	13.81	11	8/1/2014	09:59.3
1944	RWD11	-122.455	37.936	4.54	9.24	-916.99	13.78	-122.455	37.936	4.60	9.24	-950.99	13.84	11	8/1/2014	09:59.4
1945	RWD11	-122.455	37.936	4.54	9.22	-917.04	13.76	-122.455	37.936	4.60	9.22	-950.85	13.82	11	8/1/2014	09:59.5
1946	RWD11	-122.455	37.936	4.54	9.22	-917.04	13.76	-122.455	37.936	4.60	9.22	-950.60	13.82	11	8/1/2014	09:59.6
1947	RWD11	-122.455	37.936	4.54	9.24	-916.93	13.78	-122.455	37.936	4.60	9.24	-950.29	13.84	11	8/1/2014	09:59.7
1948	RWD11	-122.455	37.936	4.54	9.24	-916.61	13.78	-122.455	37.936	4.56	9.24	-949.79	13.81	11	8/1/2014	09:59.8
1949	RWD11	-122.455	37.936	4.54	9.28	-916.12	13.82	-122.455	37.936	4.60	9.28	-949.10	13.87	11	8/1/2014	09:59.9

1950	RWD11	-122.455	37.936	4.54	9.30	-915.37	13.84	-122.455	37.936	4.56	9.30	-948.28	13.86	11	8/1/2014	10:00.0
1951	RWD11	-122.455	37.936	4.54	9.28	-914.42	13.82	-122.455	37.936	4.56	9.28	-947.21	13.84	11	8/1/2014	10:00.1
1952	RWD11	-122.455	37.936	4.54	9.27	-913.29	13.81	-122.455	37.936	4.53	9.27	-946.00	13.80	11	8/1/2014	10:00.2
1953	RWD11	-122.455	37.936	4.54	9.31	-911.92	13.85	-122.455	37.936	4.53	9.31	-944.48	13.84	11	8/1/2014	10:00.3
1954	RWD11	-122.455	37.936	4.50	9.35	-910.35	13.85	-122.455	37.936	4.48	9.35	-942.89	13.82	11	8/1/2014	10:00.4
1955	RWD11	-122.455	37.936	4.54	9.31	-908.84	13.85	-122.455	37.936	4.53	9.31	-941.31	13.84	11	8/1/2014	10:00.5
1956	RWD11	-122.455	37.936	4.50	9.35	-907.37	13.86	-122.455	37.936	4.48	9.35	-939.71	13.83	11	8/1/2014	10:00.6
1957	RWD11	-122.455	37.936	4.50	9.41	-905.89	13.91	-122.455	37.936	4.53	9.41	-938.18	13.94	11	8/1/2014	10:00.7
1958	RWD11	-122.455	37.936	4.50	9.39	-904.37	13.90	-122.455	37.936	4.48	9.39	-936.55	13.87	11	8/1/2014	10:00.8
1959	RWD11	-122.455	37.936	4.54	9.37	-902.88	13.91	-122.455	37.936	4.48	9.37	-934.93	13.85	11	8/1/2014	10:00.9
1960	RWD11	-122.455	37.936	4.50	9.40	-901.36	13.90	-122.455	37.936	4.48	9.40	-933.25	13.88	11	8/1/2014	10:01.0
1961	RWD11	-122.455	37.936	4.54	9.38	-899.74	13.92	-122.455	37.936	4.48	9.38	-931.49	13.86	11	8/1/2014	10:01.1
1962	RWD11	-122.455	37.936	4.50	9.40	-897.81	13.90	-122.455	37.936	4.48	9.40	-929.48	13.88	11	8/1/2014	10:01.2
1963	RWD11	-122.455	37.936	4.54	9.38	-895.77	13.92	-122.455	37.936	4.48	9.38	-927.28	13.86	11	8/1/2014	10:01.3
1964	RWD11	-122.455	37.936	4.45	9.40	-893.66	13.85	-122.455	37.936	4.44	9.40	-925.04	13.84	11	8/1/2014	10:01.4
1965	RWD11	-122.455	37.936	4.50	9.41	-891.36	13.92	-122.455	37.936	4.48	9.41	-922.71	13.89	11	8/1/2014	10:01.5
1966	RWD11	-122.455	37.936	4.45	9.42	-889.00	13.87	-122.455	37.936	4.44	9.42	-920.23	13.86	11	8/1/2014	10:01.6
1967	RWD11	-122.455	37.936	4.45	9.43	-886.52	13.88	-122.455	37.936	4.44	9.43	-917.64	13.87	11	8/1/2014	10:01.7
1968	RWD11	-122.455	37.936	4.45	9.43	-884.19	13.88	-122.455	37.936	4.44	9.43	-915.17	13.87	11	8/1/2014	10:01.8
1969	RWD11	-122.455	37.936	4.50	9.41	-881.91	13.92	-122.455	37.936	4.44	9.41	-912.73	13.86	11	8/1/2014	10:01.9
1970	RWD11	-122.455	37.936	4.45	9.42	-879.67	13.87	-122.455	37.936	4.39	9.42	-910.31	13.81	11	8/1/2014	10:02.0
1971	RWD11	-122.455	37.936	4.50	9.43	-877.45	13.93	-122.455	37.936	4.44	9.43	-907.83	13.87	11	8/1/2014	10:02.1
1972	RWD11	-122.455	37.936	4.45	9.43	-875.30	13.88	-122.455	37.936	4.44	9.43	-905.41	13.87	11	8/1/2014	10:02.2
1973	RWD11	-122.455	37.936	4.45	9.45	-873.24	13.90	-122.455	37.936	4.44	9.45	-902.99	13.89	11	8/1/2014	10:02.3
1974	RWD11	-122.455	37.936	4.45	9.47	-871.18	13.92	-122.455	37.936	4.44	9.47	-900.55	13.91	11	8/1/2014	10:02.4
1975	RWD11	-122.455	37.936	4.45	9.45	-869.07	13.91	-122.455	37.936	4.44	9.45	-898.09	13.90	11	8/1/2014	10:02.5
1976	RWD11	-122.455	37.936	4.45	9.43	-866.95	13.88	-122.455	37.936	4.44	9.43	-895.56	13.87	11	8/1/2014	10:02.6
1977	RWD11	-122.455	37.936	4.50	9.45	-864.66	13.96	-122.455	37.936	4.48	9.45	-892.87	13.93	11	8/1/2014	10:02.7
1978	RWD11	-122.455	37.936	4.45	9.44	-862.25	13.89	-122.455	37.936	4.44	9.44	-890.12	13.88	11	8/1/2014	10:02.8
1979	RWD11	-122.455	37.936	4.45	9.45	-859.77	13.90	-122.455	37.936	4.44	9.45	-887.21	13.89	11	8/1/2014	10:02.9
1980	RWD11	-122.455	37.936	4.45	9.45	-857.27	13.91	-122.455	37.936	4.44	9.45	-884.33	13.90	11	8/1/2014	10:03.0
1981	RWD11	-122.455	37.936	4.50	9.48	-854.60	13.98	-122.455	37.936	4.44	9.48	-881.24	13.92	11	8/1/2014	10:03.1
1982	RWD11	-122.455	37.936	4.45	9.46	-851.82	13.91	-122.455	37.936	4.44	9.46	-878.06	13.91	11	8/1/2014	10:03.2

1983	RWD11	-122.455	37.936	4.45	9.48	-848.88	13.93	-122.455	37.936	4.44	9.48	-874.72	13.92	11	8/1/2014	10:03.3
1984	RWD11	-122.455	37.936	4.45	9.48	-845.83	13.93	-122.455	37.936	4.44	9.48	-871.26	13.92	11	8/1/2014	10:03.4
1985	RWD11	-122.455	37.936	4.45	9.50	-842.68	13.95	-122.455	37.936	4.44	9.50	-867.67	13.94	11	8/1/2014	10:03.5
1986	RWD11	-122.455	37.936	4.42	9.53	-839.39	13.95	-122.455	37.936	4.39	9.53	-864.02	13.92	11	8/1/2014	10:03.6
1987	RWD11	-122.455	37.936	4.45	9.52	-836.15	13.97	-122.455	37.936	4.44	9.52	-860.30	13.96	11	8/1/2014	10:03.7
1988	RWD11	-122.455	37.936	4.45	9.55	-833.05	14.00	-122.455	37.936	4.44	9.55	-856.83	13.99	11	8/1/2014	10:03.8
1989	RWD11	-122.455	37.936	4.45	9.55	-830.02	14.01	-122.455	37.936	4.44	9.55	-853.31	14.00	11	8/1/2014	10:03.9
1990	RWD11	-122.455	37.936	4.45	9.56	-826.98	14.01	-122.455	37.936	4.44	9.56	-849.74	14.00	11	8/1/2014	10:04.0
1991	RWD11	-122.455	37.936	4.45	9.59	-823.68	14.04	-122.455	37.936	4.44	9.59	-846.01	14.03	11	8/1/2014	10:04.1
1992	RWD11	-122.455	37.936	4.45	9.57	-820.29	14.02	-122.455	37.936	4.44	9.57	-842.18	14.01	11	8/1/2014	10:04.2
1993	RWD11	-122.455	37.936	4.45	9.60	-816.72	14.05	-122.455	37.936	4.48	9.60	-838.16	14.08	11	8/1/2014	10:04.3
1994	RWD11	-122.455	37.936	4.45	9.65	-813.04	14.11	-122.455	37.936	4.44	9.65	-834.02	14.10	11	8/1/2014	10:04.4
1995	RWD11	-122.455	37.936	4.45	9.71	-809.14	14.16	-122.455	37.936	4.48	9.71	-829.62	14.19	11	8/1/2014	10:04.5
1996	RWD11	-122.455	37.936	4.42	9.69	-804.98	14.11	-122.455	37.936	4.44	9.69	-824.91	14.14	11	8/1/2014	10:04.6
1997	RWD11	-122.455	37.936	4.45	9.75	-800.72	14.20	-122.455	37.936	4.44	9.75	-820.04	14.19	11	8/1/2014	10:04.7
1998	RWD11	-122.455	37.936	4.45	9.73	-796.36	14.18	-122.455	37.936	4.44	9.73	-815.10	14.17	11	8/1/2014	10:04.8
1999	RWD11	-122.455	37.936	4.45	9.82	-791.92	14.27	-122.455	37.936	4.44	9.82	-810.03	14.26	11	8/1/2014	10:04.9
2000	RWD11	-122.455	37.936	4.42	9.78	-787.47	14.20	-122.455	37.936	4.44	9.78	-804.89	14.22	11	8/1/2014	10:05.0
2001	RWD11	-122.455	37.936	4.45	9.81	-783.09	14.26	-122.455	37.936	4.44	9.81	-799.85	14.25	11	8/1/2014	10:05.1
2002	RWD11	-122.455	37.936	4.42	9.85	-778.79	14.27	-122.455	37.936	4.44	9.85	-794.85	14.29	11	8/1/2014	10:05.2
2003	RWD11	-122.455	37.936	4.45	9.89	-774.53	14.34	-122.455	37.936	4.48	9.89	-789.95	14.36	11	8/1/2014	10:05.3
2004	RWD11	-122.455	37.936	4.45	9.90	-770.20	14.36	-122.455	37.936	4.44	9.90	-784.94	14.35	11	8/1/2014	10:05.4
2005	RWD11	-122.455	37.936	4.45	9.89	-765.99	14.34	-122.455	37.936	4.48	9.89	-780.07	14.36	11	8/1/2014	10:05.5
2006	RWD11	-122.455	37.936	4.45	9.89	-761.69	14.35	-122.455	37.936	4.48	9.89	-775.13	14.37	11	8/1/2014	10:05.6
2007	RWD11	-122.455	37.936	4.45	9.91	-757.28	14.36	-122.455	37.936	4.48	9.91	-770.07	14.39	11	8/1/2014	10:05.7
2008	RWD11	-122.455	37.936	4.45	9.95	-752.67	14.41	-122.455	37.936	4.48	9.95	-765.04	14.43	11	8/1/2014	10:05.8
2009	RWD11	-122.455	37.936	4.45	9.95	-748.11	14.40	-122.455	37.936	4.48	9.95	-759.75	14.42	11	8/1/2014	10:05.9
2010	RWD11	-122.455	37.936	4.42	9.93	-743.18	14.35	-122.455	37.936	4.48	9.93	-754.35	14.41	11	8/1/2014	10:06.0
2011	RWD11	-122.455	37.936	4.45	9.95	-738.13	14.40	-122.455	37.936	4.48	9.95	-748.71	14.42	11	8/1/2014	10:06.1
2012	RWD11	-122.455	37.936	4.45	9.95	-732.97	14.40	-122.455	37.936	4.48	9.95	-743.02	14.42	11	8/1/2014	10:06.2
2013	RWD11	-122.455	37.936	4.45	10.01	-727.85	14.46	-122.455	37.936	4.53	10.01	-737.25	14.54	11	8/1/2014	10:06.3
2014	RWD11	-122.455	37.936	4.45	9.99	-722.56	14.45	-122.455	37.936	4.53	9.99	-731.40	14.52	11	8/1/2014	10:06.4
2015	RWD11	-122.455	37.936	4.45	10.02	-717.16	14.48	-122.455	37.936	4.53	10.02	-725.47	14.55	11	8/1/2014	10:06.5

2016	RWD11	-122.455	37.936	4.45	10.06	-711.82	14.52	-122.455	37.936	4.53	10.06	-719.46	14.59	11	8/1/2014	10:06.6
2017	RWD11	-122.455	37.936	4.45	10.10	-706.34	14.55	-122.455	37.936	4.53	10.10	-713.29	14.63	11	8/1/2014	10:06.7
2018	RWD11	-122.455	37.936	4.45	10.06	-700.64	14.52	-122.455	37.936	4.53	10.06	-706.99	14.59	11	8/1/2014	10:06.8
2019	RWD11	-122.455	37.936	4.50	10.13	-694.77	14.64	-122.455	37.936	4.53	10.13	-700.41	14.66	11	8/1/2014	10:06.9
2020	RWD11	-122.455	37.936	4.45	10.19	-688.83	14.64	-122.455	37.936	4.53	10.19	-693.83	14.71	11	8/1/2014	10:07.0
2021	RWD11	-122.455	37.936	4.45	10.22	-682.83	14.67	-122.455	37.936	4.53	10.22	-687.12	14.75	11	8/1/2014	10:07.1
2022	RWD11	-122.455	37.936	4.45	10.20	-676.89	14.65	-122.455	37.936	4.53	10.20	-680.45	14.72	11	8/1/2014	10:07.2
2023	RWD11	-122.455	37.936	4.50	10.23	-671.07	14.74	-122.455	37.936	4.53	10.23	-673.89	14.76	11	8/1/2014	10:07.3
2024	RWD11	-122.455	37.936	4.45	10.26	-665.25	14.71	-122.455	37.936	4.56	10.26	-667.38	14.82	11	8/1/2014	10:07.4
2025	RWD11	-122.455	37.936	4.50	10.22	-659.60	14.73	-122.455	37.936	4.56	10.22	-661.05	14.79	11	8/1/2014	10:07.5
2026	RWD11	-122.455	37.936	4.45	10.26	-653.90	14.71	-122.455	37.936	4.56	10.26	-654.68	14.82	11	8/1/2014	10:07.6
2027	RWD11	-122.455	37.936	4.50	10.23	-648.15	14.74	-122.455	37.936	4.60	10.23	-648.21	14.83	11	8/1/2014	10:07.7
2028	RWD11	-122.455	37.936	4.50	10.24	-642.37	14.74	-122.455	37.936	4.56	10.24	-641.69	14.80	11	8/1/2014	10:07.8
2029	RWD11	-122.455	37.936	4.50	10.23	-636.28	14.74	-122.455	37.936	4.60	10.23	-634.83	14.83	11	8/1/2014	10:07.9
2030	RWD11	-122.455	37.936	4.50	10.26	-629.85	14.76	-122.455	37.936	4.60	10.26	-627.62	14.85	11	8/1/2014	10:08.0
2031	RWD11	-122.455	37.936	4.50	10.25	-622.95	14.75	-122.455	37.936	4.60	10.25	-619.99	14.84	11	8/1/2014	10:08.1
2032	RWD11	-122.455	37.936	4.45	10.25	-615.70	14.70	-122.455	37.936	4.60	10.25	-611.95	14.84	11	8/1/2014	10:08.2
2033	RWD11	-122.455	37.936	4.50	10.25	-608.15	14.75	-122.455	37.936	4.60	10.25	-603.57	14.84	11	8/1/2014	10:08.3
2034	RWD11	-122.455	37.936	4.50	10.26	-600.56	14.76	-122.455	37.936	4.65	10.26	-595.02	14.90	11	8/1/2014	10:08.4
2035	RWD11	-122.455	37.936	4.50	10.30	-593.05	14.81	-122.455	37.936	4.60	10.30	-586.47	14.90	11	8/1/2014	10:08.5
2036	RWD11	-122.455	37.936	4.50	10.29	-585.89	14.80	-122.455	37.936	4.60	10.29	-578.19	14.89	11	8/1/2014	10:08.6
2037	RWD11	-122.455	37.936	4.50	10.32	-579.03	14.83	-122.455	37.936	4.65	10.32	-570.21	14.97	11	8/1/2014	10:08.7
2038	RWD11	-122.455	37.936	4.50	10.43	-572.38	14.93	-122.455	37.936	4.65	10.43	-562.44	15.07	11	8/1/2014	10:08.8
2039	RWD11	-122.455	37.936	4.50	10.38	-565.91	14.88	-122.455	37.936	4.65	10.38	-554.78	15.03	11	8/1/2014	10:08.9
2040	RWD11	-122.455	37.936	4.50	10.37	-559.35	14.88	-122.455	37.936	4.65	10.37	-547.13	15.02	11	8/1/2014	10:09.0
2041	RWD11	-122.455	37.936	4.54	10.38	-552.77	14.92	-122.455	37.936	4.68	10.38	-539.46	15.06	11	8/1/2014	10:09.1
2042	RWD11	-122.455	37.936	4.50	10.42	-545.93	14.92	-122.455	37.936	4.65	10.42	-531.57	15.06	11	8/1/2014	10:09.2
2043	RWD11	-122.455	37.936	4.54	10.46	-538.83	15.00	-122.455	37.936	4.68	10.46	-523.51	15.14	11	8/1/2014	10:09.3
2044	RWD11	-122.455	37.936	4.54	10.44	-531.37	14.98	-122.455	37.936	4.68	10.44	-515.04	15.12	11	8/1/2014	10:09.4
2045	RWD11	-122.455	37.936	4.54	10.47	-523.56	15.01	-122.455	37.936	4.68	10.47	-506.33	15.15	11	8/1/2014	10:09.5
2046	RWD11	-122.455	37.936	4.54	10.46	-515.40	15.00	-122.455	37.936	4.68	10.46	-497.26	15.14	11	8/1/2014	10:09.6
2047	RWD11	-122.455	37.936	4.54	10.50	-507.00	15.04	-122.455	37.936	4.68	10.50	-487.89	15.18	11	8/1/2014	10:09.7
2048	RWD11	-122.455	37.936	4.54	10.53	-498.48	15.07	-122.455	37.936	4.68	10.53	-478.19	15.21	11	8/1/2014	10:09.8

2049	RWD11	-122.455	37.936	4.54	10.53	-489.95	15.07	-122.455	37.936	4.68	10.53	-468.55	15.22	11	8/1/2014	10:09.9
2050	RWD11	-122.455	37.936	4.54	10.56	-481.76	15.10	-122.455	37.936	4.68	10.56	-459.12	15.25	11	8/1/2014	10:10.0
2051	RWD11	-122.455	37.936	4.54	10.58	-473.65	15.12	-122.455	37.936	4.68	10.58	-449.87	15.26	11	8/1/2014	10:10.1
2052	RWD11	-122.455	37.936	4.54	10.60	-465.79	15.14	-122.455	37.936	4.68	10.60	-440.90	15.28	11	8/1/2014	10:10.2
2053	RWD11	-122.455	37.936	4.54	10.67	-458.13	15.21	-122.455	37.936	4.73	10.67	-432.15	15.41	11	8/1/2014	10:10.3
2054	RWD11	-122.455	37.936	4.59	10.64	-450.60	15.23	-122.455	37.936	4.73	10.64	-423.48	15.37	11	8/1/2014	10:10.4
2055	RWD11	-122.455	37.936	4.59	10.65	-443.08	15.24	-122.455	37.936	4.73	10.65	-414.88	15.38	11	8/1/2014	10:10.5
2056	RWD11	-122.455	37.936	4.59	10.66	-435.42	15.25	-122.455	37.936	4.73	10.66	-406.15	15.39	11	8/1/2014	10:10.6
2057	RWD11	-122.455	37.936	4.59	10.65	-427.69	15.24	-122.455	37.936	4.76	10.65	-397.29	15.42	11	8/1/2014	10:10.7
2058	RWD11	-122.455	37.936	4.54	10.67	-419.77	15.20	-122.455	37.936	4.73	10.67	-388.27	15.40	11	8/1/2014	10:10.8
2059	RWD11	-122.455	37.936	4.59	10.69	-411.68	15.28	-122.455	37.936	4.76	10.69	-379.07	15.45	11	8/1/2014	10:10.9
2060	RWD11	-122.455	37.936	4.59	10.72	-403.62	15.31	-122.455	37.936	4.76	10.72	-369.88	15.48	11	8/1/2014	10:11.0
2061	RWD11	-122.455	37.936	4.62	10.73	-395.56	15.36	-122.455	37.936	4.76	10.73	-360.67	15.50	11	8/1/2014	10:11.1
2062	RWD11	-122.455	37.936	4.59	10.76	-387.41	15.35	-122.455	37.936	4.76	10.76	-351.48	15.53	11	8/1/2014	10:11.2
2063	RWD11	-122.455	37.936	4.62	10.77	-379.57	15.40	-122.455	37.936	4.76	10.77	-342.25	15.54	11	8/1/2014	10:11.3
2064	RWD11	-122.455	37.936	4.59	10.73	-371.73	15.32	-122.455	37.936	4.82	10.73	-333.29	15.55	11	8/1/2014	10:11.4
2065	RWD11	-122.455	37.936	4.62	10.75	-364.10	15.37	-122.455	37.936	4.82	10.75	-324.40	15.56	11	8/1/2014	10:11.5
2066	RWD11	-122.455	37.936	4.62	10.75	-356.47	15.37	-122.455	37.936	4.82	10.75	-315.60	15.56	11	8/1/2014	10:11.6
2067	RWD11	-122.455	37.936	4.62	10.73	-348.86	15.35	-122.455	37.936	4.85	10.73	-306.81	15.57	11	8/1/2014	10:11.7
2068	RWD11	-122.455	37.936	4.62	10.73	-341.35	15.36	-122.455	37.936	4.85	10.73	-298.12	15.58	11	8/1/2014	10:11.8
2069	RWD11	-122.455	37.936	4.66	10.71	-333.79	15.37	-122.455	37.936	4.90	10.71	-289.43	15.61	11	8/1/2014	10:11.9
2070	RWD11	-122.455	37.936	4.62	10.73	-326.11	15.36	-122.455	37.936	4.90	10.73	-280.69	15.63	11	8/1/2014	10:12.0
2071	RWD11	-122.455	37.936	4.66	10.72	-318.25	15.38	-122.455	37.936	4.90	10.72	-271.66	15.62	11	8/1/2014	10:12.1
2072	RWD11	-122.455	37.936	4.66	10.76	-310.02	15.42	-122.455	37.936	4.90	10.76	-262.40	15.66	11	8/1/2014	10:12.2
2073	RWD11	-122.455	37.936	4.66	10.74	-301.61	15.40	-122.455	37.936	4.93	10.74	-252.84	15.68	11	8/1/2014	10:12.3
2074	RWD11	-122.455	37.936	4.66	10.73	-292.88	15.38	-122.455	37.936	4.93	10.73	-243.01	15.66	11	8/1/2014	10:12.4
2075	RWD11	-122.455	37.936	4.71	10.73	-283.93	15.43	-122.455	37.936	4.93	10.73	-232.91	15.66	11	8/1/2014	10:12.5
2076	RWD11	-122.455	37.936	4.66	10.73	-274.83	15.38	-122.455	37.936	4.93	10.73	-222.54	15.66	11	8/1/2014	10:12.6
2077	RWD11	-122.455	37.936	4.71	10.72	-265.80	15.43	-122.455	37.936	4.93	10.72	-212.22	15.65	11	8/1/2014	10:12.7
2078	RWD11	-122.455	37.936	4.71	10.71	-256.89	15.42	-122.455	37.936	4.93	10.71	-201.94	15.65	11	8/1/2014	10:12.8
2079	RWD11	-122.455	37.936	4.71	10.69	-247.99	15.40	-122.455	37.936	4.97	10.69	-191.65	15.66	11	8/1/2014	10:12.9
2080	RWD11	-122.455	37.936	4.71	10.71	-238.97	15.42	-122.455	37.936	5.02	10.71	-181.24	15.73	11	8/1/2014	10:13.0
2081	RWD11	-122.455	37.936	4.71	10.70	-229.79	15.41	-122.455	37.936	5.02	10.70	-170.69	15.72	11	8/1/2014	10:13.1

2082	RWD11	-122.455	37.936	4.71	10.70	-220.28	15.41	-122.455	37.936	5.02	10.70	-159.71	15.72	11	8/1/2014	10:13.2
2083	RWD11	-122.455	37.936	4.74	10.71	-210.29	15.45	-122.455	37.936	5.02	10.71	-148.34	15.73	11	8/1/2014	10:13.3
2084	RWD11	-122.455	37.936	4.74	10.69	-199.87	15.44	-122.455	37.936	5.02	10.69	-136.54	15.71	11	8/1/2014	10:13.4
2085	RWD11	-122.455	37.936	4.74	10.68	-188.77	15.42	-122.455	37.936	5.05	10.68	-124.14	15.73	11	8/1/2014	10:13.5
2086	RWD11	-122.455	37.936	4.71	10.69	-177.19	15.40	-122.455	37.936	5.02	10.69	-111.11	15.71	11	8/1/2014	10:13.6
2087	RWD11	-122.455	37.936	4.74	10.71	-165.08	15.45	-122.455	37.936	5.02	10.71	-97.45	15.73	11	8/1/2014	10:13.7
2088	RWD11	-122.455	37.936	4.74	10.70	-152.58	15.45	-122.455	37.936	4.97	10.70	-83.52	15.67	11	8/1/2014	10:13.8
2089	RWD11	-122.455	37.936	4.74	10.73	-139.87	15.47	-122.455	37.936	5.02	10.73	-69.26	15.74	11	8/1/2014	10:13.9
2090	RWD11	-122.455	37.936	4.74	10.77	-127.28	15.52	-122.455	37.936	4.97	10.77	-55.19	15.74	11	8/1/2014	10:14.0
2091	RWD11	-122.455	37.936	4.79	10.68	-114.93	15.47	-122.455	37.936	4.97	10.68	-41.37	15.65	11	8/1/2014	10:14.1
2092	RWD11	-122.455	37.936	4.74	10.69	-102.68	15.44	-122.455	37.936	4.97	10.69	-27.71	15.66	11	8/1/2014	10:14.2
2093	RWD11	-122.455	37.936	4.74	10.67	-90.61	15.41	-122.455	37.936	5.02	10.67	-14.24	15.68	11	8/1/2014	10:14.3
2094	RWD11	-122.455	37.936	4.74	10.64	-78.56	15.38	-122.455	37.936	4.97	10.64	-0.84	15.61	11	8/1/2014	10:14.4
2095	RWD11	-122.455	37.936	4.79	10.64	-66.45	15.43	-122.455	37.936	5.02	10.64	12.52	15.65	11	8/1/2014	10:14.5
2096	RWD11	-122.455	37.936	4.79	10.64	-54.20	15.43	-122.455	37.936	5.02	10.64	25.85	15.66	11	8/1/2014	10:14.6
2097	RWD11	-122.455	37.936	4.79	10.60	-41.80	15.40	-122.455	37.936	5.02	10.60	39.41	15.62	11	8/1/2014	10:14.7
2098	RWD11	-122.455	37.936	4.74	10.62	-29.21	15.36	-122.455	37.936	5.02	10.62	53.16	15.64	11	8/1/2014	10:14.8
2099	RWD11	-122.455	37.936	4.79	10.60	-16.31	15.39	-122.455	37.936	5.02	10.60	67.10	15.62	11	8/1/2014	10:14.9
2100	RWD11	-122.455	37.936	4.79	10.60	-2.95	15.39	-122.455	37.936	5.02	10.60	81.57	15.62	11	8/1/2014	10:15.0
2101	RWD11	-122.455	37.936	4.79	10.59	10.86	15.38	-122.455	37.936	5.02	10.59	96.39	15.61	11	8/1/2014	10:15.1
2102	RWD11	-122.455	37.936	4.74	10.60	24.94	15.34	-122.455	37.936	5.02	10.60	111.52	15.62	11	8/1/2014	10:15.2
2103	RWD11	-122.455	37.936	4.79	10.59	39.28	15.38	-122.455	37.936	5.05	10.59	126.84	15.64	11	8/1/2014	10:15.3
2104	RWD11	-122.455	37.936	4.79	10.58	53.64	15.38	-122.455	37.936	5.02	10.58	142.16	15.60	11	8/1/2014	10:15.4
2105	RWD11	-122.455	37.936	4.79	10.57	67.78	15.37	-122.455	37.936	5.02	10.57	157.47	15.59	11	8/1/2014	10:15.5
2106	RWD11	-122.455	37.936	4.79	10.59	81.77	15.38	-122.455	37.936	5.02	10.59	172.35	15.61	11	8/1/2014	10:15.6
2107	RWD11	-122.455	37.936	4.79	10.61	95.43	15.40	-122.455	37.936	5.05	10.61	187.01	15.66	11	8/1/2014	10:15.7
2108	RWD11	-122.455	37.936	4.79	10.60	108.96	15.39	-122.455	37.936	5.02	10.60	201.44	15.62	11	8/1/2014	10:15.8
2109	RWD11	-122.455	37.936	4.83	10.56	122.39	15.39	-122.455	37.936	5.05	10.56	215.88	15.62	11	8/1/2014	10:15.9
2110	RWD11	-122.455	37.936	4.79	10.56	136.22	15.36	-122.455	37.936	5.05	10.56	230.43	15.62	11	8/1/2014	10:16.0
2111	RWD11	-122.455	37.936	4.83	10.55	150.48	15.38	-122.455	37.936	5.05	10.55	245.41	15.60	11	8/1/2014	10:16.1
2112	RWD11	-122.455	37.936	4.79	10.58	165.10	15.38	-122.455	37.936	5.10	10.58	260.73	15.68	11	8/1/2014	10:16.2
2113	RWD11	-122.455	37.936	4.83	10.55	180.20	15.38	-122.455	37.936	5.10	10.55	276.41	15.65	11	8/1/2014	10:16.3
2114	RWD11	-122.455	37.936	4.79	10.53	195.68	15.33	-122.455	37.936	5.10	10.53	292.39	15.64	11	8/1/2014	10:16.4

2115	RWD11	-122.455	37.936	4.83	10.56	211.49	15.38	-122.455	37.936	5.10	10.56	308.76	15.66	11	8/1/2014	10:16.5
2116	RWD11	-122.455	37.936	4.83	10.58	227.52	15.41	-122.455	37.936	5.10	10.58	325.34	15.68	11	8/1/2014	10:16.6
2117	RWD11	-122.455	37.936	4.83	10.60	243.69	15.43	-122.455	37.936	5.10	10.60	342.03	15.70	11	8/1/2014	10:16.7
2118	RWD11	-122.455	37.936	4.83	10.58	259.90	15.41	-122.455	37.936	5.10	10.58	358.89	15.68	11	8/1/2014	10:16.8
2119	RWD11	-122.455	37.936	4.83	10.60	276.14	15.43	-122.455	37.936	5.10	10.60	375.76	15.70	11	8/1/2014	10:16.9
2120	RWD11	-122.455	37.936	4.83	10.60	292.40	15.43	-122.455	37.936	5.10	10.60	392.65	15.71	11	8/1/2014	10:17.0
2121	RWD11	-122.455	37.936	4.88	10.64	308.69	15.52	-122.455	37.936	5.14	10.64	409.54	15.77	11	8/1/2014	10:17.1
2122	RWD11	-122.455	37.936	4.83	10.61	325.14	15.44	-122.455	37.936	5.14	10.61	426.54	15.75	11	8/1/2014	10:17.2
2123	RWD11	-122.455	37.936	4.88	10.63	341.94	15.51	-122.455	37.936	5.14	10.63	443.85	15.76	11	8/1/2014	10:17.3
2124	RWD11	-122.455	37.936	4.83	10.66	359.29	15.48	-122.455	37.936	5.14	10.66	461.52	15.79	11	8/1/2014	10:17.4
2125	RWD11	-122.455	37.936	4.88	10.69	377.19	15.57	-122.455	37.936	5.14	10.69	479.65	15.82	11	8/1/2014	10:17.5
2126	RWD11	-122.455	37.936	4.88	10.67	395.73	15.55	-122.455	37.936	5.14	10.67	498.26	15.80	11	8/1/2014	10:17.6
2127	RWD11	-122.455	37.936	4.88	10.71	414.69	15.59	-122.455	37.936	5.14	10.71	517.25	15.85	11	8/1/2014	10:17.7
2128	RWD11	-122.455	37.936	4.88	10.70	434.21	15.58	-122.455	37.936	5.14	10.70	536.74	15.84	11	8/1/2014	10:17.8
2129	RWD11	-122.455	37.936	4.88	10.73	454.29	15.61	-122.455	37.936	5.19	10.73	556.66	15.92	11	8/1/2014	10:17.9
2130	RWD11	-122.455	37.936	4.88	10.82	474.81	15.70	-122.455	37.936	5.14	10.82	576.96	15.95	11	8/1/2014	10:18.0
2131	RWD11	-122.455	37.936	4.88	10.80	495.83	15.68	-122.455	37.936	5.14	10.80	597.48	15.93	11	8/1/2014	10:18.1
2132	RWD11	-122.455	37.936	4.88	10.89	517.19	15.77	-122.455	37.936	5.19	10.89	618.30	16.08	11	8/1/2014	10:18.2
2133	RWD11	-122.455	37.936	4.91	10.83	539.02	15.75	-122.455	37.936	5.19	10.83	639.43	16.02	11	8/1/2014	10:18.3
2134	RWD11	-122.455	37.936	4.88	10.87	561.25	15.75	-122.455	37.936	5.14	10.87	660.88	16.01	11	8/1/2014	10:18.4
2135	RWD11	-122.455	37.936	4.91	10.90	583.83	15.82	-122.455	37.936	5.14	10.90	682.49	16.04	11	8/1/2014	10:18.5
2136	RWD11	-122.455	37.936	4.91	10.97	606.72	15.89	-122.455	37.936	5.19	10.97	704.29	16.16	11	8/1/2014	10:18.6
2137	RWD11	-122.455	37.936	4.91	10.94	629.79	15.86	-122.455	37.936	5.19	10.94	726.21	16.13	11	8/1/2014	10:18.7
2138	RWD11	-122.455	37.936	4.91	10.97	653.23	15.88	-122.455	37.936	5.14	10.97	748.39	16.10	11	8/1/2014	10:18.8
2139	RWD11	-122.455	37.936	4.91	11.03	677.08	15.94	-122.455	37.936	5.19	11.03	770.72	16.22	11	8/1/2014	10:18.9
2140	RWD11	-122.455	37.936	4.88	11.07	701.39	15.95	-122.455	37.936	5.19	11.07	793.31	16.26	11	8/1/2014	10:19.0
2141	RWD11	-122.455	37.936	4.91	11.10	726.41	16.01	-122.455	37.936	5.19	11.10	816.44	16.28	11	8/1/2014	10:19.1
2142	RWD11	-122.455	37.936	4.91	11.17	752.20	16.08	-122.455	37.936	5.14	11.17	839.95	16.30	11	8/1/2014	10:19.2
2143	RWD11	-122.455	37.936	4.91	11.28	778.46	16.20	-122.455	37.936	5.19	11.28	863.93	16.47	11	8/1/2014	10:19.3
2144	RWD11	-122.455	37.936	4.88	11.34	805.01	16.22	-122.455	37.936	5.19	11.34	888.04	16.52	11	8/1/2014	10:19.4
2145	RWD11	-122.455	37.936	4.91	11.33	831.86	16.24	-122.455	37.936	5.19	11.33	912.27	16.51	11	8/1/2014	10:19.5
2146	RWD11	-122.455	37.936	4.88	11.37	859.07	16.25	-122.455	37.936	5.19	11.37	936.79	16.56	11	8/1/2014	10:19.6
2147	RWD11	-122.455	37.936	4.91	11.44	886.61	16.36	-122.455	37.936	5.19	11.44	961.53	16.63	11	8/1/2014	10:19.7

2148	RWD11	-122.455	37.936	4.91	11.45	914.55	16.37	-122.455	37.936	5.19	11.45	986.41	16.64	11	8/1/2014	10:19.8
2149	RWD11	-122.455	37.936	4.91	11.45	943.01	16.37	-122.455	37.936	5.19	11.45	1011.61	16.64	11	8/1/2014	10:19.9
2150	RWD11	-122.455	37.936	4.91	11.48	971.88	16.40	-122.455	37.936	5.19	11.48	1037.10	16.67	11	8/1/2014	10:20.0
2151	RWD11	-122.455	37.936	4.91	11.52	######	16.44	-122.455	37.936	5.22	11.52	1062.84	16.74	11	8/1/2014	10:20.1
2152	RWD11	-122.455	37.936	4.91	11.60	######	16.52	-122.455	37.936	5.22	11.60	1088.78	16.83	11	8/1/2014	10:20.2
2153	RWD11	-122.455	37.936	4.97	11.58	######	16.55	-122.455	37.936	5.19	11.58	1114.84	16.77	11	8/1/2014	10:20.3
2154	RWD11	-122.455	37.936	4.91	11.58	######	16.49	-122.455	37.936	5.19	11.58	1141.22	16.76	11	8/1/2014	10:20.4
2155	RWD11	-122.455	37.936	4.97	11.58	######	16.54	-122.455	37.936	5.22	11.58	1167.88	16.80	11	8/1/2014	10:20.5
2156	RWD11	-122.455	37.936	4.91	11.62	######	16.54	-122.455	37.936	5.22	11.62	1195.01	16.84	11	8/1/2014	10:20.6
2157	RWD11	-122.455	37.936	4.97	11.62	######	16.59	-122.455	37.936	5.22	11.62	1222.39	16.84	11	8/1/2014	10:20.7
2158	RWD11	-122.455	37.936	4.91	11.60	######	16.52	-122.455	37.936	5.22	11.60	1249.95	16.83	11	8/1/2014	10:20.8
2159	RWD11	-122.455	37.936	4.97	11.65	######	16.61	-122.455	37.936	5.27	11.65	1277.72	16.92	11	8/1/2014	10:20.9
2160	RWD11	-122.455	37.936	4.91	11.72	######	16.63	-122.455	37.936	5.22	11.72	1305.60	16.94	11	8/1/2014	10:21.0
2161	RWD11	-122.455	37.936	4.97	11.69	######	16.65	-122.455	37.936	5.27	11.69	1333.48	16.96	11	8/1/2014	10:21.1
2162	RWD11	-122.455	37.936	4.97	11.69	######	16.65	-122.455	37.936	5.22	11.69	1361.33	16.91	11	8/1/2014	10:21.2
2163	RWD11	-122.455	37.936	4.97	11.67	######	16.63	-122.455	37.936	5.27	11.67	1389.27	16.94	11	8/1/2014	10:21.3
2164	RWD11	-122.455	37.936	4.97	11.67	######	16.63	-122.455	37.936	5.27	11.67	1417.45	16.94	11	8/1/2014	10:21.4
2165	RWD11	-122.455	37.936	5.00	11.66	######	16.66	-122.455	37.936	5.31	11.66	1445.87	16.97	11	8/1/2014	10:21.5
2166	RWD11	-122.455	37.936	4.97	11.67	######	16.63	-122.455	37.936	5.27	11.67	1474.70	16.94	11	8/1/2014	10:21.6
2167	RWD11	-122.455	37.936	5.00	11.65	######	16.65	-122.455	37.936	5.31	11.65	1503.74	16.96	11	8/1/2014	10:21.7
2168	RWD11	-122.455	37.936	4.97	11.66	######	16.62	-122.455	37.936	5.27	11.66	1533.15	16.93	11	8/1/2014	10:21.8
2169	RWD11	-122.455	37.936	4.97	11.67	######	16.63	-122.455	37.936	5.31	11.67	1563.24	16.97	11	8/1/2014	10:21.9
2170	RWD11	-122.455	37.936	4.97	11.73	######	16.69	-122.455	37.936	5.27	11.73	1593.76	17.00	11	8/1/2014	10:22.0
2171	RWD11	-122.455	37.936	5.00	11.70	######	16.70	-122.455	37.936	5.27	11.70	1624.69	16.97	11	8/1/2014	10:22.1
2172	RWD11	-122.455	37.936	5.00	11.74	######	16.74	-122.455	37.936	5.27	11.74	1655.76	17.01	11	8/1/2014	10:22.2
2173	RWD11	-122.455	37.936	5.00	11.72	######	16.72	-122.455	37.936	5.31	11.72	1686.96	17.03	11	8/1/2014	10:22.3
2174	RWD11	-122.455	37.936	4.97	11.75	######	16.72	-122.455	37.936	5.27	11.75	1718.05	17.03	11	8/1/2014	10:22.4
2175	RWD11	-122.455	37.936	5.00	11.77	######	16.77	-122.455	37.936	5.31	11.77	1748.98	17.08	11	8/1/2014	10:22.5
2176	RWD11	-122.455	37.936	5.00	11.79	######	16.79	-122.455	37.936	5.31	11.79	1779.75	17.10	11	8/1/2014	10:22.6
2177	RWD11	-122.455	37.936	5.00	11.79	######	16.79	-122.455	37.936	5.31	11.79	1810.55	17.10	11	8/1/2014	10:22.7
2178	RWD11	-122.455	37.936	5.00	11.84	######	16.84	-122.455	37.936	5.27	11.84	1841.19	17.11	11	8/1/2014	10:22.8
2179	RWD11	-122.455	37.936	5.00	11.85	######	16.85	-122.455	37.936	5.27	11.85	1871.61	17.12	11	8/1/2014	10:22.9
2180	RWD11	-122.455	37.936	5.00	11.87	######	16.87	-122.455	37.936	5.31	11.87	1901.78	17.17	11	8/1/2014	10:23.0

2181	RWD11	-122.455	37.936	5.00	11.90	######	16.90	-122.455	37.936	5.31	11.90	1931.66	17.21	11	8/1/2014	10:23.1
2182	RWD11	-122.455	37.936	5.00	11.90	######	16.90	-122.455	37.936	5.27	11.90	1961.04	17.17	11	8/1/2014	10:23.2
2183	RWD11	-122.455	37.936	5.03	11.91	######	16.95	-122.455	37.936	5.27	11.91	1989.37	17.19	11	8/1/2014	10:23.3
2184	RWD11	-122.455	37.936	5.00	11.88	######	16.88	-122.455	37.936	5.27	11.88	2016.28	17.15	11	8/1/2014	10:23.4
2185	RWD11	-122.455	37.936	5.00	11.87	######	16.87	-122.455	37.936	5.27	11.87	2041.82	17.14	11	8/1/2014	10:23.5
2186	RWD11	-122.455	37.936	5.00	11.85	######	16.85	-122.455	37.936	5.27	11.85	2065.99	17.12	11	8/1/2014	10:23.6
2187	RWD11	-122.455	37.936	5.00	11.83	######	16.83	-122.455	37.936	5.30	11.83	2088.98	17.13	11	8/1/2014	10:23.7
2188	RWD11	-122.456	37.936	5.00	11.82	######	16.82	-122.455	37.936	5.27	11.82	2110.96	17.09	11	8/1/2014	10:23.8
2189	RWD11	-122.456	37.936	5.00	11.80	######	16.80	-122.455	37.936	5.30	11.80	2132.18	17.10	11	8/1/2014	10:23.9
2190	RWD11	-122.456	37.936	5.00	11.81	######	16.81	-122.455	37.936	5.27	11.81	2152.67	17.08	11	8/1/2014	10:24.0
2191	RWD11	-122.456	37.936	5.03	11.99	######	17.02	-122.456	37.936	5.30	11.99	2172.20	17.29	11	8/1/2014	10:24.1
2192	RWD11	-122.456	37.936	5.00	11.81	######	16.81	-122.456	37.936	5.22	11.81	2190.60	17.03	11	8/1/2014	10:24.2
2193	RWD11	-122.456	37.936	5.00	11.82	######	16.82	-122.456	37.936	5.27	11.82	2207.56	17.09	11	8/1/2014	10:24.3
2194	RWD11	-122.456	37.936	5.00	11.99	######	16.99	-122.456	37.936	5.22	11.99	2222.82	17.21	11	8/1/2014	10:24.4
2195	RWD11	-122.456	37.936	5.03	11.95	######	16.98	-122.456	37.936	5.22	11.95	2236.38	17.17	11	8/1/2014	10:24.5
2196	RWD11	-122.456	37.936	5.00	11.96	######	16.96	-122.456	37.936	5.22	11.96	2248.16	17.18	11	8/1/2014	10:24.6
2197	RWD11	-122.456	37.936	5.03	11.98	######	17.01	-122.456	37.936	5.22	11.98	2258.10	17.20	11	8/1/2014	10:24.7
2198	RWD11	-122.456	37.936	5.00	11.95	######	16.95	-122.456	37.936	5.22	11.95	2266.38	17.17	11	8/1/2014	10:24.8
2199	RWD11	-122.456	37.936	5.03	11.98	######	17.02	-122.456	37.936	5.22	11.98	2273.34	17.20	11	8/1/2014	10:24.9
2200	RWD11	-122.456	37.936	5.00	11.95	######	16.95	-122.456	37.936	5.22	11.95	2279.05	17.17	11	8/1/2014	10:25.0
2201	RWD11	-122.456	37.936	5.00	11.95	######	16.94	-122.456	37.936	5.22	11.95	2283.35	17.17	11	8/1/2014	10:25.1
2202	RWD11	-122.456	37.936	5.00	11.91	######	16.91	-122.456	37.936	5.22	11.91	2286.34	17.13	11	8/1/2014	10:25.2
2203	RWD11	-122.456	37.936	5.03	11.85	######	16.88	-122.456	37.936	5.22	11.85	2287.94	17.07	11	8/1/2014	10:25.3
2204	RWD11	-122.456	37.936	5.00	11.85	######	16.84	-122.456	37.936	5.22	11.85	2288.17	17.07	11	8/1/2014	10:25.4
2205	RWD11	-122.456	37.936	5.00	11.79	######	16.79	-122.456	37.936	5.22	11.79	2287.02	17.01	11	8/1/2014	10:25.5
2206	RWD11	-122.456	37.936	5.00	11.78	######	16.78	-122.456	37.936	5.22	11.78	2284.53	17.00	11	8/1/2014	10:25.6
2207	RWD11	-122.456	37.936	5.03	11.78	######	16.81	-122.456	37.936	5.22	11.78	2280.51	17.00	11	8/1/2014	10:25.7
2208	RWD11	-122.456	37.936	5.00	11.74	######	16.74	-122.456	37.936	5.19	11.74	2274.97	16.92	11	8/1/2014	10:25.8
2209	RWD11	-122.456	37.936	5.03	11.75	######	16.79	-122.456	37.936	5.22	11.75	2267.91	16.97	11	8/1/2014	10:25.9
2210	RWD11	-122.456	37.936	5.00	11.73	######	16.73	-122.456	37.936	5.19	11.73	2259.44	16.91	11	8/1/2014	10:26.0
2211	RWD11	-122.456	37.936	5.00	11.65	######	16.65	-122.456	37.936	5.22	11.65	2249.54	16.87	11	8/1/2014	10:26.1
2212	RWD11	-122.456	37.936	5.00	11.62	######	16.62	-122.456	37.936	5.19	11.62	2238.29	16.81	11	8/1/2014	10:26.2
2213	RWD11	-122.456	37.936	5.03	11.57	######	16.60	-122.456	37.936	5.19	11.57	2225.85	16.75	11	8/1/2014	10:26.3

2214	RWD11	-122.456	37.936	5.00	11.56	######	16.56	-122.456	37.936	5.19	11.56	2212.17	16.75	11	8/1/2014	10:26.4
2215	RWD11	-122.456	37.936	5.03	11.47	######	16.50	-122.456	37.936	5.19	11.47	2197.33	16.65	11	8/1/2014	10:26.5
2216	RWD11	-122.456	37.936	5.00	11.44	######	16.44	-122.456	37.936	5.14	11.44	2181.32	16.58	11	8/1/2014	10:26.6
2217	RWD11	-122.456	37.936	5.00	11.34	######	16.34	-122.456	37.936	5.19	11.34	2164.24	16.53	11	8/1/2014	10:26.7
2218	RWD11	-122.456	37.936	5.00	11.31	######	16.30	-122.456	37.936	5.19	11.31	2146.14	16.49	11	8/1/2014	10:26.8
2219	RWD11	-122.456	37.936	5.00	11.24	######	16.24	-122.456	37.936	5.19	11.24	2127.04	16.43	11	8/1/2014	10:26.9
2220	RWD11	-122.456	37.936	5.00	11.24	######	16.24	-122.456	37.936	5.14	11.24	2107.00	16.38	11	8/1/2014	10:27.0
2221	RWD11	-122.456	37.936	5.00	11.15	######	16.15	-122.456	37.936	5.19	11.15	2086.09	16.34	11	8/1/2014	10:27.1
2222	RWD11	-122.456	37.936	5.00	11.11	######	16.11	-122.456	37.936	5.14	11.11	2064.39	16.25	11	8/1/2014	10:27.2
2223	RWD11	-122.456	37.936	5.00	11.07	######	16.07	-122.456	37.936	5.14	11.07	2041.92	16.21	11	8/1/2014	10:27.3
2224	RWD11	-122.456	37.936	5.00	11.02	######	16.02	-122.456	37.936	5.14	11.02	2018.74	16.16	11	8/1/2014	10:27.4
2225	RWD11	-122.456	37.936	5.00	10.97	######	15.97	-122.456	37.936	5.19	10.97	1994.86	16.16	11	8/1/2014	10:27.5
2226	RWD11	-122.456	37.936	5.00	10.94	######	15.93	-122.456	37.936	5.14	10.94	1970.30	16.07	11	8/1/2014	10:27.6
2227	RWD11	-122.456	37.936	5.00	10.88	######	15.88	-122.456	37.936	5.19	10.88	1945.18	16.07	11	8/1/2014	10:27.7
2228	RWD11	-122.456	37.936	5.00	10.89	######	15.89	-122.456	37.936	5.19	10.89	1919.30	16.07	11	8/1/2014	10:27.8
2229	RWD11	-122.456	37.936	5.00	10.83	######	15.83	-122.456	37.936	5.19	10.83	1892.86	16.02	11	8/1/2014	10:27.9
2230	RWD11	-122.456	37.936	5.00	10.80	######	15.79	-122.456	37.936	5.19	10.80	1865.82	15.98	11	8/1/2014	10:28.0
2231	RWD11	-122.456	37.936	5.03	10.75	######	15.78	-122.456	37.936	5.19	10.75	1838.35	15.94	11	8/1/2014	10:28.1
2232	RWD11	-122.456	37.936	5.00	10.71	######	15.71	-122.456	37.936	5.19	10.71	1810.57	15.90	11	8/1/2014	10:28.2
2233	RWD11	-122.456	37.936	5.03	10.66	######	15.69	-122.456	37.936	5.22	10.66	1782.49	15.88	11	8/1/2014	10:28.3
2234	RWD11	-122.456	37.936	5.00	10.67	######	15.66	-122.456	37.936	5.19	10.67	1754.11	15.85	11	8/1/2014	10:28.4
2235	RWD11	-122.456	37.936	5.03	10.64	######	15.67	-122.456	37.936	5.22	10.64	1725.56	15.86	11	8/1/2014	10:28.5
2236	RWD11	-122.456	37.936	5.00	10.56	######	15.56	-122.456	37.936	5.19	10.56	1696.94	15.75	11	8/1/2014	10:28.6
2237	RWD11	-122.456	37.936	5.03	10.52	######	15.55	-122.456	37.936	5.22	10.52	1668.27	15.74	11	8/1/2014	10:28.7
2238	RWD11	-122.456	37.936	5.00	10.46	######	15.46	-122.456	37.936	5.22	10.46	1639.54	15.68	11	8/1/2014	10:28.8
2239	RWD11	-122.456	37.936	5.03	10.46	######	15.49	-122.456	37.936	5.27	10.46	1610.79	15.73	11	8/1/2014	10:28.9
2240	RWD11	-122.456	37.936	5.03	10.36	######	15.40	-122.456	37.936	5.27	10.36	1581.99	15.63	11	8/1/2014	10:29.0
2241	RWD11	-122.456	37.936	5.03	10.38	######	15.41	-122.456	37.936	5.30	10.38	1553.02	15.68	11	8/1/2014	10:29.1
2242	RWD11	-122.456	37.936	5.00	10.36	######	15.36	-122.456	37.936	5.30	10.36	1523.89	15.67	11	8/1/2014	10:29.2
2243	RWD11	-122.456	37.936	5.08	10.36	######	15.44	-122.456	37.936	5.34	10.36	1494.52	15.70	11	8/1/2014	10:29.3
2244	RWD11	-122.456	37.936	5.03	10.22	######	15.26	-122.456	37.936	5.30	10.22	1464.93	15.53	11	8/1/2014	10:29.4
2245	RWD11	-122.456	37.936	5.03	10.15	######	15.18	-122.456	37.936	5.34	10.15	1435.30	15.49	11	8/1/2014	10:29.5
2246	RWD11	-122.456	37.936	5.03	10.09	######	15.13	-122.456	37.936	5.34	10.09	1405.41	15.43	11	8/1/2014	10:29.6

2247	RWD11	-122.456	37.936	5.03	10.09	######	15.12	-122.456	37.936	5.34	10.09	1375.26	15.42	11	8/1/2014 10:29.7
2248	RWD11	-122.456	37.936	5.03	9.94	######	14.97	-122.456	37.936	5.34	9.94	1345.08	15.28	11	8/1/2014 10:29.8
2249	RWD11	-122.456	37.936	5.08	9.89	######	14.98	-122.456	37.936	5.39	9.89	1315.02	15.28	11	8/1/2014 10:29.9
2250	RWD11	-122.456	37.936	5.08	9.82	######	14.91	-122.456	37.936	5.39	9.82	1285.27	15.21	11	8/1/2014 10:30.0
2251	RWD11	-122.456	37.936	5.08	9.82	######	14.91	-122.456	37.936	5.39	9.82	1255.85	15.21	11	8/1/2014 10:30.1
2252	RWD11	-122.456	37.936	5.08	9.68	######	14.77	-122.456	37.936	5.39	9.68	1226.76	15.07	11	8/1/2014 10:30.2
2253	RWD11	-122.456	37.936	5.12	9.63	######	14.75	-122.456	37.936	5.42	9.63	1198.14	15.05	11	8/1/2014 10:30.3
2254	RWD11	-122.456	37.936	5.08	9.66	######	14.75	-122.456	37.936	5.42	9.66	1169.84	15.08	11	8/1/2014 10:30.4
2255	RWD11	-122.456	37.936	5.12	9.58	######	14.69	-122.456	37.936	5.47	9.58	1141.65	15.05	11	8/1/2014 10:30.5
2256	RWD11	-122.456	37.936	5.08	9.57	######	14.65	-122.456	37.936	5.42	9.57	1113.62	14.99	11	8/1/2014 10:30.6
2257	RWD11	-122.456	37.936	5.12	9.47	######	14.59	-122.456	37.936	5.47	9.47	1085.57	14.94	11	8/1/2014 10:30.7
2258	RWD11	-122.456	37.936	5.08	9.50	######	14.58	-122.456	37.936	5.47	9.50	1057.45	14.97	11	8/1/2014 10:30.8
2259	RWD11	-122.456	37.936	5.12	9.40	######	14.52	-122.456	37.936	5.51	9.40	1029.39	14.91	11	8/1/2014 10:30.9
2260	RWD11	-122.456	37.936	5.08	9.34	######	14.42	-122.456	37.936	5.51	9.34	1001.46	14.85	11	8/1/2014 10:31.0
2261	RWD11	-122.456	37.936	5.17	9.30	######	14.47	-122.456	37.936	5.51	9.30	973.80	14.81	11	8/1/2014 10:31.1
2262	RWD11	-122.456	37.936	5.12	9.26	990.09	14.38	-122.456	37.936	5.51	9.26	946.58	14.77	11	8/1/2014 10:31.2
2263	RWD11	-122.456	37.936	5.12	9.27	957.45	14.39	-122.456	37.936	5.51	9.27	919.92	14.78	11	8/1/2014 10:31.3
2264	RWD11	-122.456	37.936	5.12	9.24	925.71	14.36	-122.456	37.936	5.51	9.24	893.93	14.75	11	8/1/2014 10:31.4
2265	RWD11	-122.456	37.936	5.17	9.14	894.85	14.31	-122.456	37.936	5.56	9.14	868.58	14.70	11	8/1/2014 10:31.5
2266	RWD11	-122.456	37.936	5.17	9.12	864.80	14.29	-122.456	37.936	5.56	9.12	843.66	14.68	11	8/1/2014 10:31.6
2267	RWD11	-122.456	37.936	5.26	9.10	835.28	14.35	-122.456	37.936	5.56	9.10	819.09	14.66	11	8/1/2014 10:31.7
2268	RWD11	-122.456	37.936	5.26	9.05	806.02	14.30	-122.456	37.936	5.56	9.05	794.58	14.60	11	8/1/2014 10:31.8
2269	RWD11	-122.456	37.936	5.26	9.01	776.93	14.27	-122.456	37.936	5.59	9.01	770.14	14.61	11	8/1/2014 10:31.9
2270	RWD11	-122.456	37.936	5.26	9.05	747.46	14.31	-122.456	37.936	5.59	9.05	745.43	14.64	11	8/1/2014 10:32.0
2271	RWD11	-122.456	37.936	5.29	8.96	717.47	14.25	-122.456	37.936	5.59	8.96	720.23	14.55	11	8/1/2014 10:32.1
2272	RWD11	-122.456	37.936	5.26	8.92	686.78	14.18	-122.456	37.936	5.59	8.92	694.32	14.51	11	8/1/2014 10:32.2
2273	RWD11	-122.456	37.936	5.29	8.91	655.65	14.20	-122.456	37.936	5.59	8.91	667.85	14.50	11	8/1/2014 10:32.3
2274	RWD11	-122.456	37.936	5.26	8.88	624.66	14.13	-122.456	37.936	5.59	8.88	641.27	14.47	11	8/1/2014 10:32.4
2275	RWD11	-122.456	37.936	5.29	8.89	594.21	14.18	-122.456	37.936	5.56	8.89	614.86	14.45	11	8/1/2014 10:32.5
2276	RWD11	-122.456	37.936	5.26	8.88	565.13	14.13	-122.456	37.936	5.51	8.88	589.41	14.38	11	8/1/2014 10:32.6
2277	RWD11	-122.456	37.936	5.26	8.87	537.46	14.12	-122.456	37.936	5.56	8.87	565.16	14.43	11	8/1/2014 10:32.7
2278	RWD11	-122.456	37.936	5.26	8.86	511.06	14.11	-122.456	37.936	5.47	8.86	541.78	14.33	11	8/1/2014 10:32.8
2279	RWD11	-122.456	37.936	5.26	8.87	485.49	14.12	-122.456	37.936	5.47	8.87	519.13	14.34	11	8/1/2014 10:32.9

2280	RWD11	-122.456	37.936	5.20	8.85	460.51	14.06	-122.456	37.936	5.47	8.85	496.78	14.33	11	8/1/2014	10:33.0
2281	RWD11	-122.456	37.936	5.26	8.86	435.88	14.11	-122.456	37.936	5.47	8.86	474.74	14.33	11	8/1/2014	10:33.1
2282	RWD11	-122.456	37.936	5.20	8.87	411.67	14.07	-122.456	37.936	5.47	8.87	452.71	14.34	11	8/1/2014	10:33.2
2283	RWD11	-122.456	37.936	5.26	8.84	387.34	14.09	-122.456	37.936	5.42	8.84	430.49	14.26	11	8/1/2014	10:33.3
2284	RWD11	-122.456	37.936	5.20	8.83	363.15	14.03	-122.456	37.936	5.39	8.83	408.26	14.22	11	8/1/2014	10:33.4
2285	RWD11	-122.456	37.936	5.20	8.84	339.09	14.04	-122.456	37.936	5.39	8.84	385.96	14.23	11	8/1/2014	10:33.5
2286	RWD11	-122.456	37.936	5.17	8.86	315.41	14.03	-122.456	37.936	5.30	8.86	364.06	14.16	11	8/1/2014	10:33.6
2287	RWD11	-122.456	37.936	5.20	8.84	292.45	14.04	-122.456	37.936	5.30	8.84	342.67	14.14	11	8/1/2014	10:33.7
2288	RWD11	-122.456	37.936	5.17	8.84	270.51	14.01	-122.456	37.936	5.22	8.84	321.99	14.06	11	8/1/2014	10:33.8
2289	RWD11	-122.456	37.936	5.17	8.84	249.88	14.01	-122.456	37.936	5.19	8.84	302.40	14.03	11	8/1/2014	10:33.9
2290	RWD11	-122.456	37.936	5.12	8.86	230.21	13.98	-122.456	37.936	5.14	8.86	283.51	14.00	11	8/1/2014	10:34.0
2291	RWD11	-122.456	37.936	5.17	8.87	211.21	14.04	-122.456	37.936	5.10	8.87	265.18	13.97	11	8/1/2014	10:34.1
2292	RWD11	-122.456	37.936	5.12	8.92	192.65	14.04	-122.456	37.936	5.05	8.92	247.20	13.97	11	8/1/2014	10:34.2
2293	RWD11	-122.456	37.936	5.12	8.88	174.36	14.00	-122.456	37.936	5.05	8.88	229.34	13.93	11	8/1/2014	10:34.3
2294	RWD11	-122.456	37.936	5.08	8.94	156.25	14.02	-122.456	37.936	5.02	8.94	211.64	13.95	11	8/1/2014	10:34.4
2295	RWD11	-122.456	37.936	5.08	8.94	138.33	14.02	-122.456	37.936	4.97	8.94	193.88	13.90	11	8/1/2014	10:34.5
2296	RWD11	-122.456	37.936	5.03	8.98	120.70	14.02	-122.456	37.936	4.90	8.98	176.29	13.88	11	8/1/2014	10:34.6
2297	RWD11	-122.456	37.936	5.03	9.01	103.35	14.05	-122.456	37.936	4.90	9.01	158.96	13.91	11	8/1/2014	10:34.7
2298	RWD11	-122.456	37.936	5.00	8.98	86.48	13.98	-122.456	37.936	4.81	8.98	142.06	13.80	11	8/1/2014	10:34.8
2299	RWD11	-122.456	37.936	5.00	9.03	70.31	14.03	-122.456	37.936	4.81	9.03	125.64	13.84	11	8/1/2014	10:34.9
2300	RWD11	-122.456	37.936	4.96	9.07	54.55	14.03	-122.456	37.936	4.73	9.07	109.57	13.80	11	8/1/2014	10:35.0
2301	RWD11	-122.456	37.936	4.96	9.05	38.92	14.02	-122.456	37.936	4.68	9.05	93.75	13.73	11	8/1/2014	10:35.1
2302	RWD11	-122.456	37.936	4.91	9.12	23.49	14.03	-122.456	37.936	4.64	9.12	77.96	13.77	11	8/1/2014	10:35.2
2303	RWD11	-122.456	37.936	4.91	9.09	8.23	14.00	-122.456	37.936	4.59	9.09	62.12	13.68	11	8/1/2014	10:35.3
2304	RWD11	-122.456	37.936	4.88	9.15	-6.93	14.03	-122.456	37.936	4.56	9.15	46.33	13.71	11	8/1/2014	10:35.4
2305	RWD11	-122.456	37.936	4.88	9.15	-21.86	14.03	-122.456	37.936	4.56	9.15	30.72	13.71	11	8/1/2014	10:35.5
2306	RWD11	-122.456	37.936	4.83	9.15	-36.62	13.98	-122.456	37.936	4.44	9.15	15.27	13.59	11	8/1/2014	10:35.6
2307	RWD11	-122.456	37.936	4.83	9.19	-51.13	14.02	-122.456	37.936	4.48	9.19	-0.21	13.67	11	8/1/2014	10:35.7
2308	RWD11	-122.456	37.936	4.79	9.21	-65.69	14.00	-122.456	37.936	4.39	9.21	-15.73	13.60	11	8/1/2014	10:35.8
2309	RWD11	-122.456	37.936	4.74	9.22	-80.12	13.96	-122.456	37.936	4.36	9.22	-31.24	13.58	11	8/1/2014	10:35.9
2310	RWD11	-122.456	37.936	4.71	9.28	-94.36	13.99	-122.456	37.936	4.27	9.28	-46.52	13.55	11	8/1/2014	10:36.0
2311	RWD11	-122.456	37.936	4.71	9.29	-108.33	14.00	-122.456	37.936	4.27	9.29	-61.77	13.56	11	8/1/2014	10:36.1
2312	RWD11	-122.456	37.936	4.66	9.39	-122.28	14.05	-122.456	37.936	4.22	9.39	-77.03	13.61	11	8/1/2014	10:36.2

2313	RWD11	-122.456	37.936	4.62	9.43	-135.90	14.05	-122.456	37.936	4.22	9.43	-92.07	13.65	11	8/1/2014	10:36.3
2314	RWD11	-122.456	37.936	4.59	9.41	-149.26	14.00	-122.456	37.936	4.10	9.41	-106.74	13.51	11	8/1/2014	10:36.4
2315	RWD11	-122.456	37.936	4.59	9.44	-162.40	14.02	-122.456	37.936	4.10	9.44	-121.14	13.54	11	8/1/2014	10:36.5
2316	RWD11	-122.456	37.936	4.54	9.48	-175.45	14.01	-122.456	37.936	4.07	9.48	-135.39	13.55	11	8/1/2014	10:36.6
2317	RWD11	-122.456	37.936	4.54	9.49	-188.35	14.03	-122.456	37.936	4.02	9.49	-149.56	13.51	11	8/1/2014	10:36.7
2318	RWD11	-122.456	37.936	4.50	9.49	-201.03	13.99	-122.456	37.936	3.98	9.49	-163.55	13.48	11	8/1/2014	10:36.8
2319	RWD11	-122.456	37.936	4.45	9.60	-213.62	14.05	-122.456	37.936	3.93	9.60	-177.44	13.53	11	8/1/2014	10:36.9
2320	RWD11	-122.456	37.936	4.42	9.59	-225.99	14.00	-122.456	37.936	3.87	9.59	-191.12	13.45	11	8/1/2014	10:37.0
2321	RWD11	-122.456	37.936	4.42	9.65	-238.14	14.07	-122.456	37.936	3.87	9.65	-204.62	13.52	11	8/1/2014	10:37.1
2322	RWD11	-122.456	37.936	4.36	9.61	-249.95	13.98	-122.456	37.936	3.82	9.61	-217.77	13.43	11	8/1/2014	10:37.2
2323	RWD11	-122.456	37.936	4.33	9.69	-261.50	14.02	-122.456	37.936	3.82	9.69	-230.57	13.51	11	8/1/2014	10:37.3
2324	RWD11	-122.456	37.936	4.28	9.71	-272.92	13.99	-122.456	37.936	3.73	9.71	-243.22	13.44	11	8/1/2014	10:37.4
2325	RWD11	-122.456	37.936	4.33	9.73	-284.00	14.06	-122.456	37.936	3.78	9.73	-255.55	13.51	11	8/1/2014	10:37.5
2326	RWD11	-122.456	37.936	4.28	9.77	-294.92	14.05	-122.456	37.936	3.70	9.77	-267.57	13.47	11	8/1/2014	10:37.6
2327	RWD11	-122.456	37.936	4.28	9.78	-305.69	14.06	-122.456	37.936	3.70	9.78	-279.57	13.48	11	8/1/2014	10:37.7
2328	RWD11	-122.456	37.936	4.24	9.78	-316.39	14.03	-122.456	37.936	3.65	9.78	-291.40	13.43	11	8/1/2014	10:37.8
2329	RWD11	-122.456	37.936	4.24	9.78	-327.00	14.03	-122.456	37.936	3.65	9.78	-302.98	13.43	11	8/1/2014	10:37.9
2330	RWD11	-122.456	37.936	4.16	9.84	-337.38	14.00	-122.456	37.936	3.61	9.84	-314.55	13.45	11	8/1/2014	10:38.0
2331	RWD11	-122.456	37.936	4.21	9.89	-347.64	14.10	-122.456	37.936	3.61	9.89	-325.84	13.50	11	8/1/2014	10:38.1
2332	RWD11	-122.456	37.936	4.13	9.84	-357.83	13.96	-122.456	37.936	3.56	9.84	-337.25	13.40	11	8/1/2014	10:38.2
2333	RWD11	-122.456	37.936	4.16	9.86	-367.68	14.02	-122.456	37.936	3.53	9.86	-348.36	13.39	11	8/1/2014	10:38.3
2334	RWD11	-122.456	37.936	4.13	9.89	-377.54	14.02	-122.456	37.936	3.53	9.89	-359.17	13.42	11	8/1/2014	10:38.4
2335	RWD11	-122.456	37.936	4.13	9.90	-387.18	14.03	-122.456	37.936	3.53	9.90	-369.96	13.43	11	8/1/2014	10:38.5
2336	RWD11	-122.456	37.936	4.07	9.89	-396.71	13.97	-122.456	37.936	3.49	9.89	-380.66	13.39	11	8/1/2014	10:38.6
2337	RWD11	-122.456	37.936	4.07	9.91	-406.22	13.98	-122.456	37.936	3.49	9.91	-391.21	13.40	11	8/1/2014	10:38.7
2338	RWD11	-122.456	37.936	4.04	9.93	-415.56	13.97	-122.456	37.936	3.44	9.93	-401.58	13.37	11	8/1/2014	10:38.8
2339	RWD11	-122.456	37.936	4.04	9.88	-424.76	13.92	-122.456	37.936	3.44	9.88	-411.87	13.32	11	8/1/2014	10:38.9
2340	RWD11	-122.456	37.936	3.99	9.91	-433.86	13.90	-122.456	37.936	3.41	9.91	-421.99	13.32	11	8/1/2014	10:39.0
2341	RWD11	-122.456	37.936	3.99	9.89	-442.94	13.87	-122.456	37.936	3.44	9.89	-432.04	13.33	11	8/1/2014	10:39.1
2342	RWD11	-122.456	37.936	3.95	9.89	-451.78	13.85	-122.456	37.936	3.36	9.89	-441.88	13.25	11	8/1/2014	10:39.2
2343	RWD11	-122.456	37.936	3.95	9.93	-460.63	13.89	-122.456	37.936	3.36	9.93	-451.58	13.29	11	8/1/2014	10:39.3
2344	RWD11	-122.456	37.936	3.95	9.89	-469.26	13.85	-122.456	37.936	3.36	9.89	-461.17	13.25	11	8/1/2014	10:39.4
2345	RWD11	-122.456	37.936	3.95	9.91	-477.83	13.86	-122.456	37.936	3.36	9.91	-470.60	13.27	11	8/1/2014	10:39.5

2346	RWD11	-122.456	37.936	3.92	9.92	-486.24	13.84	-122.456	37.936	3.36	9.92	-479.85	13.27	11	8/1/2014	10:39.6
2347	RWD11	-122.456	37.936	3.95	9.91	-494.65	13.86	-122.456	37.936	3.32	9.91	-489.18	13.23	11	8/1/2014	10:39.7
2348	RWD11	-122.456	37.936	3.87	9.91	-503.01	13.78	-122.456	37.936	3.32	9.91	-498.28	13.23	11	8/1/2014	10:39.8
2349	RWD11	-122.456	37.936	3.92	9.90	-511.26	13.82	-122.456	37.936	3.32	9.90	-507.45	13.23	11	8/1/2014	10:39.9
2350	RWD11	-122.456	37.936	3.87	9.90	-519.45	13.77	-122.456	37.936	3.27	9.90	-516.59	13.18	11	8/1/2014	10:40.0
2351	RWD11	-122.456	37.936	3.87	9.89	-527.62	13.76	-122.456	37.936	3.32	9.89	-525.64	13.22	11	8/1/2014	10:40.1
2352	RWD11	-122.456	37.936	3.87	9.92	-535.68	13.79	-122.456	37.936	3.27	9.92	-534.56	13.20	11	8/1/2014	10:40.2
2353	RWD11	-122.456	37.936	3.83	9.92	-543.60	13.76	-122.456	37.936	3.27	9.92	-543.30	13.20	11	8/1/2014	10:40.3
2354	RWD11	-122.456	37.936	3.83	9.94	-551.38	13.77	-122.456	37.936	3.27	9.94	-551.82	13.21	11	8/1/2014	10:40.4
2355	RWD11	-122.456	37.936	3.83	9.95	-558.84	13.78	-122.456	37.936	3.27	9.95	-560.13	13.22	11	8/1/2014	10:40.5
2356	RWD11	-122.456	37.936	3.83	9.92	-566.13	13.75	-122.456	37.936	3.27	9.92	-568.22	13.19	11	8/1/2014	10:40.6
2357	RWD11	-122.456	37.936	3.83	9.95	-573.23	13.79	-122.456	37.936	3.24	9.95	-576.13	13.19	11	8/1/2014	10:40.7
2358	RWD11	-122.456	37.936	3.75	9.93	-580.24	13.68	-122.456	37.936	3.24	9.93	-583.94	13.17	11	8/1/2014	10:40.8
2359	RWD11	-122.456	37.936	3.78	9.89	-587.16	13.67	-122.456	37.936	3.24	9.89	-591.60	13.12	11	8/1/2014	10:40.9
2360	RWD11	-122.456	37.936	3.78	9.95	-593.93	13.73	-122.456	37.936	3.19	9.95	-599.12	13.13	11	8/1/2014	10:41.0
2361	RWD11	-122.456	37.936	3.78	9.93	-600.62	13.72	-122.456	37.936	3.24	9.93	-606.49	13.17	11	8/1/2014	10:41.1
2362	RWD11	-122.456	37.936	3.75	9.90	-607.17	13.65	-122.456	37.936	3.19	9.90	-613.80	13.09	11	8/1/2014	10:41.2
2363	RWD11	-122.456	37.936	3.75	9.88	-613.65	13.62	-122.456	37.936	3.19	9.88	-620.99	13.07	11	8/1/2014	10:41.3
2364	RWD11	-122.456	37.936	3.75	9.93	-620.04	13.68	-122.456	37.936	3.19	9.93	-628.07	13.12	11	8/1/2014	10:41.4
2365	RWD11	-122.456	37.936	3.75	9.89	-626.17	13.63	-122.456	37.936	3.19	9.89	-635.01	13.07	11	8/1/2014	10:41.5
2366	RWD11	-122.456	37.936	3.70	9.86	-632.21	13.56	-122.456	37.936	3.19	9.86	-641.65	13.05	11	8/1/2014	10:41.6
2367	RWD11	-122.456	37.936	3.75	9.89	-638.06	13.63	-122.456	37.936	3.19	9.89	-648.15	13.07	11	8/1/2014	10:41.7
2368	RWD11	-122.456	37.936	3.70	9.87	-643.69	13.57	-122.456	37.936	3.15	9.87	-654.35	13.02	11	8/1/2014	10:41.8
2369	RWD11	-122.456	37.936	3.70	9.95	-649.20	13.64	-122.456	37.936	3.19	9.95	-660.47	13.13	11	8/1/2014	10:41.9
2370	RWD11	-122.456	37.936	3.66	9.89	-654.55	13.56	-122.456	37.936	3.15	9.89	-666.38	13.05	11	8/1/2014	10:42.0
2371	RWD11	-122.456	37.936	3.70	9.87	-659.88	13.57	-122.456	37.936	3.19	9.87	-672.27	13.06	11	8/1/2014	10:42.1
2372	RWD11	-122.456	37.936	3.66	9.88	-665.07	13.54	-122.456	37.936	3.15	9.88	-677.95	13.03	11	8/1/2014	10:42.2
2373	RWD11	-122.456	37.936	3.66	9.89	-670.21	13.56	-122.456	37.936	3.15	9.89	-683.54	13.05	11	8/1/2014	10:42.3
2374	RWD11	-122.456	37.936	3.66	9.90	-675.28	13.57	-122.456	37.936	3.15	9.90	-689.11	13.06	11	8/1/2014	10:42.4
2375	RWD11	-122.456	37.936	3.66	9.91	-680.18	13.57	-122.456	37.936	3.19	9.91	-694.47	13.10	11	8/1/2014	10:42.5
2376	RWD11	-122.456	37.936	3.61	9.88	-684.98	13.49	-122.456	37.936	3.15	9.88	-699.78	13.03	11	8/1/2014	10:42.6
2377	RWD11	-122.456	37.936	3.66	9.91	-689.71	13.57	-122.456	37.936	3.15	9.91	-704.90	13.06	11	8/1/2014	10:42.7
2378	RWD11	-122.456	37.936	3.61	9.87	-694.25	13.48	-122.456	37.936	3.15	9.87	-710.01	13.02	11	8/1/2014	10:42.8

2379	RWD11	-122.456	37.936	3.61	9.92	-698.79	13.53	-122.456	37.936	3.15	9.92	-714.94	13.07	11	8/1/2014	10:42.9
2380	RWD11	-122.456	37.936	3.61	9.88	-703.17	13.49	-122.456	37.936	3.15	9.88	-719.80	13.03	11	8/1/2014	10:43.0
2381	RWD11	-122.456	37.936	3.61	9.84	-707.26	13.45	-122.456	37.936	3.19	9.84	-724.53	13.03	11	8/1/2014	10:43.1
2382	RWD11	-122.456	37.936	3.61	9.85	-711.39	13.46	-122.456	37.936	3.15	9.85	-729.05	13.00	11	8/1/2014	10:43.2
2383	RWD11	-122.456	37.936	3.61	9.87	-715.24	13.48	-122.456	37.936	3.19	9.87	-733.48	13.06	11	8/1/2014	10:43.3
2384	RWD11	-122.456	37.936	3.61	9.85	-719.22	13.46	-122.456	37.936	3.15	9.85	-737.86	13.00	11	8/1/2014	10:43.4
2385	RWD11	-122.456	37.936	3.61	9.85	-722.85	13.47	-122.456	37.936	3.15	9.85	-741.97	13.01	11	8/1/2014	10:43.5
2386	RWD11	-122.456	37.936	3.58	9.87	-726.53	13.45	-122.456	37.936	3.15	9.87	-745.92	13.02	11	8/1/2014	10:43.6
2387	RWD11	-122.456	37.936	3.61	9.85	-729.86	13.46	-122.456	37.936	3.24	9.85	-749.60	13.09	11	8/1/2014	10:43.7
2388	RWD11	-122.456	37.936	3.58	9.86	-733.09	13.44	-122.456	37.936	3.15	9.86	-753.15	13.02	11	8/1/2014	10:43.8
2389	RWD11	-122.456	37.936	3.61	9.82	-736.19	13.43	-122.456	37.936	3.19	9.82	-756.49	13.00	11	8/1/2014	10:43.9
2390	RWD11	-122.456	37.936	3.58	9.86	-739.11	13.44	-122.456	37.936	3.15	9.86	-759.63	13.02	11	8/1/2014	10:44.0
2391	RWD11	-122.456	37.936	3.58	9.85	-741.82	13.43	-122.456	37.936	3.15	9.85	-762.57	13.01	11	8/1/2014	10:44.1
2392	RWD11	-122.456	37.936	3.58	9.86	-744.51	13.44	-122.456	37.936	3.15	9.86	-765.46	13.02	11	8/1/2014	10:44.2
2393	RWD11	-122.456	37.936	3.58	9.89	-746.99	13.47	-122.456	37.936	3.15	9.89	-768.08	13.05	11	8/1/2014	10:44.3
2394	RWD11	-122.456	37.936	3.58	9.83	-749.27	13.41	-122.456	37.936	3.15	9.83	-770.60	12.99	11	8/1/2014	10:44.4
2395	RWD11	-122.456	37.936	3.58	9.84	-751.42	13.42	-122.456	37.936	3.15	9.84	-772.83	12.99	11	8/1/2014	10:44.5
2396	RWD11	-122.456	37.936	3.54	9.84	-753.48	13.38	-122.456	37.936	3.15	9.84	-775.03	12.99	11	8/1/2014	10:44.6
2397	RWD11	-122.456	37.936	3.58	9.87	-755.33	13.45	-122.456	37.936	3.15	9.87	-777.07	13.02	11	8/1/2014	10:44.7
2398	RWD11	-122.456	37.936	3.54	9.87	-757.03	13.41	-122.456	37.936	3.15	9.87	-778.92	13.02	11	8/1/2014	10:44.8
2399	RWD11	-122.456	37.936	3.58	9.87	-758.52	13.45	-122.456	37.936	3.15	9.87	-780.60	13.02	11	8/1/2014	10:44.9
2400	RWD11	-122.456	37.936	3.54	9.87	-759.89	13.41	-122.456	37.936	3.19	9.87	-782.09	13.06	11	8/1/2014	10:45.0
2401	RWD11	-122.456	37.936	3.58	9.91	-761.10	13.48	-122.456	37.936	3.19	9.91	-783.42	13.10	11	8/1/2014	10:45.1
2402	RWD11	-122.456	37.936	3.54	9.93	-762.09	13.48	-122.456	37.936	3.15	9.93	-784.47	13.09	11	8/1/2014	10:45.2
2403	RWD11	-122.456	37.936	3.58	9.95	-762.93	13.52	-122.456	37.936	3.15	9.95	-785.33	13.10	11	8/1/2014	10:45.3
2404	RWD11	-122.456	37.936	3.54	10.01	-763.49	13.55	-122.456	37.936	3.12	10.01	-785.94	13.13	11	8/1/2014	10:45.4
2405	RWD11	-122.456	37.936	3.58	9.99	-763.90	13.57	-122.456	37.936	3.12	9.99	-786.29	13.11	11	8/1/2014	10:45.5
2406	RWD11	-122.456	37.936	3.49	9.95	-764.31	13.45	-122.456	37.936	3.12	9.95	-786.52	13.08	11	8/1/2014	10:45.6
2407	RWD11	-122.456	37.936	3.54	9.99	-764.35	13.53	-122.456	37.936	3.12	9.99	-786.56	13.11	11	8/1/2014	10:45.7
2408	RWD11	-122.456	37.936	3.49	9.99	-764.36	13.49	-122.456	37.936	3.12	9.99	-786.37	13.11	11	8/1/2014	10:45.8
2409	RWD11	-122.456	37.936	3.54	9.99	-764.14	13.54	-122.456	37.936	3.12	9.99	-785.99	13.11	11	8/1/2014	10:45.9
2410	RWD11	-122.456	37.936	3.49	9.99	-763.78	13.49	-122.456	37.936	3.07	9.99	-785.52	13.06	11	8/1/2014	10:46.0
2411	RWD11	-122.456	37.936	3.49	10.01	-763.30	13.50	-122.456	37.936	3.12	10.01	-784.79	13.13	11	8/1/2014	10:46.1

2412	RWD11	-122.456	37.936	3.49	9.99	-762.66	13.49	-122.456	37.936	3.07	9.99	-783.82	13.06	11	8/1/2014	10:46.2
2413	RWD11	-122.456	37.936	3.49	10.04	-761.87	13.53	-122.456	37.936	3.07	10.04	-782.75	13.11	11	8/1/2014	10:46.3
2414	RWD11	-122.456	37.936	3.46	10.03	-760.97	13.49	-122.456	37.937	3.07	10.03	-781.57	13.10	11	8/1/2014	10:46.4
2415	RWD11	-122.456	37.936	3.49	10.05	-759.91	13.54	-122.456	37.937	3.04	10.05	-780.30	13.08	11	8/1/2014	10:46.5
2416	RWD11	-122.456	37.936	3.46	10.06	-758.75	13.51	-122.456	37.937	3.04	10.06	-778.79	13.09	11	8/1/2014	10:46.6
2417	RWD11	-122.456	37.936	3.46	10.09	-757.45	13.54	-122.456	37.937	3.07	10.09	-777.16	13.15	11	8/1/2014	10:46.7
2418	RWD11	-122.456	37.936	3.46	10.09	-756.13	13.54	-122.456	37.937	3.04	10.09	-775.44	13.12	11	8/1/2014	10:46.8
2419	RWD11	-122.456	37.936	3.49	10.06	-754.78	13.55	-122.456	37.937	3.04	10.06	-773.72	13.09	11	8/1/2014	10:46.9
2420	RWD11	-122.456	37.936	3.41	10.04	-753.34	13.45	-122.456	37.937	3.04	10.04	-771.94	13.08	11	8/1/2014	10:47.0
2421	RWD11	-122.456	37.936	3.46	10.05	-751.88	13.50	-122.456	37.937	3.04	10.05	-770.11	13.08	11	8/1/2014	10:47.1
2422	RWD11	-122.456	37.936	3.41	10.06	-750.46	13.47	-122.456	37.937	3.04	10.06	-768.35	13.10	11	8/1/2014	10:47.2
2423	RWD11	-122.456	37.936	3.46	10.03	-749.24	13.49	-122.456	37.937	3.07	10.03	-766.64	13.10	11	8/1/2014	10:47.3
2424	RWD11	-122.456	37.936	3.41	10.03	-747.93	13.44	-122.456	37.937	3.04	10.03	-765.04	13.07	11	8/1/2014	10:47.4
2425	RWD11	-122.456	37.936	3.41	10.02	-746.70	13.42	-122.456	37.937	3.07	10.02	-763.46	13.09	11	8/1/2014	10:47.5
2426	RWD11	-122.456	37.936	3.41	10.03	-745.43	13.44	-122.456	37.937	3.07	10.03	-761.95	13.10	11	8/1/2014	10:47.6
2427	RWD11	-122.456	37.936	3.46	10.00	-744.17	13.46	-122.456	37.937	3.07	10.00	-760.34	13.07	11	8/1/2014	10:47.7
2428	RWD11	-122.456	37.936	3.41	10.02	-742.75	13.42	-122.456	37.937	3.07	10.02	-758.64	13.09	11	8/1/2014	10:47.8
2429	RWD11	-122.456	37.936	3.41	10.02	-741.20	13.43	-122.456	37.937	3.12	10.02	-756.78	13.15	11	8/1/2014	10:47.9
2430	RWD11	-122.456	37.936	3.41	10.01	-739.68	13.41	-122.456	37.937	3.07	10.01	-754.78	13.08	11	8/1/2014	10:48.0
2431	RWD11	-122.456	37.936	3.41	10.01	-737.80	13.41	-122.456	37.937	3.12	10.01	-752.56	13.13	11	8/1/2014	10:48.1
2432	RWD11	-122.456	37.936	3.41	10.01	-735.91	13.41	-122.456	37.937	3.07	10.01	-750.33	13.08	11	8/1/2014	10:48.2
2433	RWD11	-122.456	37.936	3.41	10.00	-733.94	13.41	-122.456	37.937	3.12	10.00	-747.85	13.12	11	8/1/2014	10:48.3
2434	RWD11	-122.456	37.936	3.41	9.99	-731.77	13.40	-122.456	37.937	3.12	9.99	-745.21	13.11	11	8/1/2014	10:48.4
2435	RWD11	-122.456	37.936	3.46	10.02	-729.51	13.47	-122.456	37.937	3.12	10.02	-742.48	13.14	11	8/1/2014	10:48.5
2436	RWD11	-122.456	37.936	3.41	10.01	-727.28	13.41	-122.456	37.937	3.12	10.01	-739.58	13.13	11	8/1/2014	10:48.6
2437	RWD11	-122.456	37.936	3.41	9.98	-724.76	13.38	-122.456	37.937	3.12	9.98	-736.42	13.10	11	8/1/2014	10:48.7
2438	RWD11	-122.456	37.936	3.41	10.00	-722.24	13.41	-122.456	37.937	3.12	10.00	-733.26	13.12	11	8/1/2014	10:48.8
2439	RWD11	-122.456	37.936	3.41	9.99	-719.58	13.40	-122.456	37.937	3.15	9.99	-729.78	13.15	11	8/1/2014	10:48.9
2440	RWD11	-122.456	37.936	3.41	10.02	-716.83	13.43	-122.456	37.937	3.12	10.02	-726.23	13.15	11	8/1/2014	10:49.0
2441	RWD11	-122.456	37.937	3.41	9.99	-714.05	13.40	-122.456	37.937	3.15	9.99	-722.60	13.15	11	8/1/2014	10:49.1
2442	RWD11	-122.456	37.937	3.41	9.99	-711.24	13.40	-122.456	37.937	3.15	9.99	-718.99	13.15	11	8/1/2014	10:49.2
2443	RWD11	-122.456	37.937	3.40	10.00	-708.38	13.41	-122.456	37.937	3.15	10.00	-715.44	13.16	11	8/1/2014	10:49.3
2444	RWD11	-122.456	37.937	3.40	9.95	-705.37	13.36	-122.456	37.937	3.15	9.95	-711.62	13.11	11	8/1/2014	10:49.4

2445	RWD11	-122.456	37.937	3.40	9.95	-702.41	13.35	-122.456	37.937	3.15	9.95	-707.81	13.10	11	8/1/2014	10:49.5
2446	RWD11	-122.456	37.937	3.40	9.93	-699.25	13.34	-122.456	37.937	3.15	9.93	-704.03	13.09	11	8/1/2014	10:49.6
2447	RWD11	-122.456	37.937	3.40	9.94	-696.21	13.34	-122.456	37.937	3.19	9.94	-700.24	13.13	11	8/1/2014	10:49.7
2448	RWD11	-122.456	37.937	3.40	9.97	-693.25	13.38	-122.456	37.937	3.19	9.97	-696.50	13.16	11	8/1/2014	10:49.8
2449	RWD11	-122.456	37.937	3.40	9.91	-690.35	13.31	-122.456	37.937	3.24	9.91	-692.92	13.15	11	8/1/2014	10:49.9
2450	RWD11	-122.456	37.937	3.40	9.91	-687.36	13.31	-122.456	37.937	3.24	9.91	-689.37	13.15	11	8/1/2014	10:50.0
2451	RWD11	-122.456	37.937	3.40	9.90	-684.48	13.31	-122.456	37.937	3.24	9.90	-685.93	13.14	11	8/1/2014	10:50.1
2452	RWD11	-122.456	37.937	3.40	9.90	-681.64	13.31	-122.456	37.937	3.24	9.90	-682.58	13.14	11	8/1/2014	10:50.2
2453	RWD11	-122.456	37.937	3.40	9.91	-678.76	13.31	-122.456	37.937	3.27	9.91	-679.10	13.18	11	8/1/2014	10:50.3
2454	RWD11	-122.456	37.937	3.40	9.87	-675.84	13.27	-122.456	37.937	3.27	9.87	-675.59	13.14	11	8/1/2014	10:50.4
2455	RWD11	-122.456	37.937	3.46	9.88	-672.89	13.33	-122.456	37.937	3.32	9.88	-671.98	13.20	11	8/1/2014	10:50.5
2456	RWD11	-122.456	37.937	3.40	9.85	-669.76	13.26	-122.456	37.937	3.32	9.85	-668.20	13.18	11	8/1/2014	10:50.6
2457	RWD11	-122.456	37.937	3.46	9.84	-666.57	13.30	-122.456	37.937	3.36	9.84	-664.22	13.20	11	8/1/2014	10:50.7
2458	RWD11	-122.456	37.937	3.40	9.86	-663.18	13.27	-122.456	37.937	3.36	9.86	-660.16	13.22	11	8/1/2014	10:50.8
2459	RWD11	-122.456	37.937	3.40	9.83	-659.61	13.24	-122.456	37.937	3.36	9.83	-655.86	13.19	11	8/1/2014	10:50.9
2460	RWD11	-122.456	37.937	3.40	9.82	-655.96	13.23	-122.456	37.937	3.36	9.82	-651.54	13.18	11	8/1/2014	10:51.0
2461	RWD11	-122.456	37.937	3.46	9.82	-652.26	13.27	-122.456	37.937	3.41	9.82	-647.14	13.22	11	8/1/2014	10:51.1
2462	RWD11	-122.456	37.937	3.40	9.87	-648.54	13.27	-122.456	37.937	3.36	9.87	-642.77	13.23	11	8/1/2014	10:51.2
2463	RWD11	-122.456	37.937	3.46	9.85	-644.80	13.31	-122.456	37.937	3.41	9.85	-638.32	13.26	11	8/1/2014	10:51.3
2464	RWD11	-122.456	37.937	3.40	9.82	-640.94	13.23	-122.456	37.937	3.41	9.82	-633.82	13.23	11	8/1/2014	10:51.4
2465	RWD11	-122.456	37.937	3.46	9.90	-637.15	13.36	-122.456	37.937	3.44	9.90	-629.39	13.34	11	8/1/2014	10:51.5
2466	RWD11	-122.456	37.937	3.40	9.83	-633.22	13.24	-122.456	37.937	3.49	9.83	-624.83	13.32	11	8/1/2014	10:51.6
2467	RWD11	-122.456	37.937	3.46	9.85	-629.28	13.31	-122.456	37.937	3.49	9.85	-620.15	13.35	11	8/1/2014	10:51.7
2468	RWD11	-122.456	37.937	3.46	9.83	-625.22	13.29	-122.456	37.937	3.49	9.83	-615.28	13.32	11	8/1/2014	10:51.8
2469	RWD11	-122.456	37.937	3.46	9.83	-621.01	13.29	-122.456	37.937	3.53	9.83	-610.31	13.36	11	8/1/2014	10:51.9
2470	RWD11	-122.456	37.937	3.46	9.83	-616.78	13.29	-122.456	37.937	3.53	9.83	-605.38	13.36	11	8/1/2014	10:52.0
2471	RWD11	-122.456	37.937	3.49	9.84	-612.46	13.33	-122.456	37.937	3.56	9.84	-600.34	13.40	11	8/1/2014	10:52.1
2472	RWD11	-122.456	37.937	3.46	9.82	-608.14	13.27	-122.456	37.937	3.56	9.82	-595.32	13.38	11	8/1/2014	10:52.2
2473	RWD11	-122.456	37.937	3.49	9.85	-603.69	13.34	-122.456	37.937	3.56	9.85	-590.30	13.41	11	8/1/2014	10:52.3
2474	RWD11	-122.456	37.937	3.46	9.78	-599.26	13.24	-122.456	37.937	3.56	9.78	-585.10	13.34	11	8/1/2014	10:52.4
2475	RWD11	-122.456	37.937	3.49	9.80	-594.79	13.29	-122.456	37.937	3.61	9.80	-579.94	13.41	11	8/1/2014	10:52.5
2476	RWD11	-122.456	37.937	3.49	9.84	-590.31	13.33	-122.456	37.937	3.61	9.84	-574.64	13.45	11	8/1/2014	10:52.6
2477	RWD11	-122.456	37.937	3.49	9.87	-585.91	13.36	-122.456	37.937	3.64	9.87	-569.36	13.51	11	8/1/2014	10:52.7

2478	RWD11	-122.456	37.937	3.46	9.82	-581.49	13.27	-122.456	37.937	3.64	9.82	-563.95	13.46	11	8/1/2014	10:52.8
2479	RWD11	-122.456	37.937	3.49	9.82	-577.11	13.31	-122.456	37.937	3.69	9.82	-558.59	13.52	11	8/1/2014	10:52.9
2480	RWD11	-122.456	37.937	3.49	9.82	-572.74	13.31	-122.456	37.937	3.69	9.82	-553.16	13.52	11	8/1/2014	10:53.0
2481	RWD11	-122.456	37.937	3.49	9.83	-568.23	13.32	-122.456	37.937	3.73	9.83	-547.68	13.56	11	8/1/2014	10:53.1
2482	RWD11	-122.456	37.937	3.49	9.87	-563.57	13.36	-122.456	37.937	3.73	9.87	-542.10	13.60	11	8/1/2014	10:53.2
2483	RWD11	-122.456	37.937	3.49	9.89	-558.81	13.38	-122.456	37.937	3.78	9.89	-536.40	13.67	11	8/1/2014	10:53.3
2484	RWD11	-122.456	37.937	3.54	9.87	-553.81	13.41	-122.456	37.937	3.78	9.87	-530.44	13.65	11	8/1/2014	10:53.4
2485	RWD11	-122.456	37.937	3.54	9.92	-548.59	13.46	-122.456	37.937	3.78	9.92	-524.21	13.70	11	8/1/2014	10:53.5
2486	RWD11	-122.456	37.937	3.54	9.94	-542.98	13.48	-122.456	37.937	3.78	9.94	-517.49	13.72	11	8/1/2014	10:53.6
2487	RWD11	-122.456	37.937	3.58	9.95	-537.05	13.52	-122.456	37.937	3.81	9.95	-510.38	13.76	11	8/1/2014	10:53.7
2488	RWD11	-122.456	37.937	3.54	10.02	-530.82	13.56	-122.456	37.937	3.78	10.02	-502.99	13.80	11	8/1/2014	10:53.8
2489	RWD11	-122.456	37.937	3.58	10.02	-524.25	13.60	-122.456	37.937	3.81	10.02	-495.19	13.84	11	8/1/2014	10:53.9
2490	RWD11	-122.456	37.937	3.54	10.07	-517.21	13.61	-122.456	37.937	3.81	10.07	-486.97	13.89	11	8/1/2014	10:54.0
2491	RWD11	-122.456	37.937	3.58	10.18	-509.79	13.76	-122.456	37.937	3.81	10.18	-478.29	13.99	11	8/1/2014	10:54.1
2492	RWD11	-122.456	37.937	3.54	10.20	-501.86	13.74	-122.456	37.937	3.81	10.20	-469.15	14.01	11	8/1/2014	10:54.2
2493	RWD11	-122.456	37.937	3.58	10.22	-493.50	13.79	-122.456	37.937	3.81	10.22	-459.58	14.03	11	8/1/2014	10:54.3
2494	RWD11	-122.456	37.937	3.58	10.29	-484.54	13.86	-122.456	37.937	3.78	10.29	-449.53	14.07	11	8/1/2014	10:54.4
2495	RWD11	-122.456	37.937	3.61	10.42	-475.24	14.03	-122.456	37.937	3.78	10.42	-439.08	14.20	11	8/1/2014	10:54.5
2496	RWD11	-122.456	37.937	3.58	10.42	-465.87	13.99	-122.456	37.937	3.78	10.42	-428.55	14.20	11	8/1/2014	10:54.6
2497	RWD11	-122.456	37.937	3.61	10.50	-456.45	14.11	-122.456	37.937	3.78	10.50	-417.93	14.28	11	8/1/2014	10:54.7
2498	RWD11	-122.456	37.937	3.58	10.52	-446.99	14.09	-122.456	37.937	3.73	10.52	-407.08	14.25	11	8/1/2014	10:54.8
2499	RWD11	-122.456	37.937	3.58	10.60	-437.53	14.17	-122.456	37.937	3.78	10.60	-396.06	14.38	11	8/1/2014	10:54.9
2500	RWD11	-122.456	37.937	3.58	10.63	-428.13	14.20	-122.456	37.937	3.78	10.63	-385.06	14.40	11	8/1/2014	10:55.0
2501	RWD11	-122.456	37.937	3.61	10.68	-418.63	14.29	-122.456	37.937	3.78	10.68	-373.95	14.46	11	8/1/2014	10:55.1
2502	RWD11	-122.456	37.937	3.58	10.73	-408.93	14.30	-122.456	37.937	3.73	10.73	-362.72	14.45	11	8/1/2014	10:55.2
2503	RWD11	-122.456	37.937	3.58	10.72	-399.04	14.30	-122.456	37.937	3.73	10.72	-351.24	14.45	11	8/1/2014	10:55.3
2504	RWD11	-122.456	37.937	3.58	10.75	-389.10	14.32	-122.456	37.937	3.73	10.75	-339.70	14.48	11	8/1/2014	10:55.4
2505	RWD11	-122.456	37.937	3.61	10.80	-379.17	14.41	-122.456	37.937	3.73	10.80	-328.10	14.53	11	8/1/2014	10:55.5
2506	RWD11	-122.456	37.937	3.58	10.90	-369.14	14.47	-122.456	37.937	3.69	10.90	-316.37	14.59	11	8/1/2014	10:55.6
2507	RWD11	-122.456	37.937	3.61	10.90	-359.10	14.51	-122.456	37.937	3.73	10.90	-304.61	14.63	11	8/1/2014	10:55.7
2508	RWD11	-122.456	37.937	3.58	10.92	-349.01	14.49	-122.456	37.937	3.69	10.92	-292.90	14.61	11	8/1/2014	10:55.8
2509	RWD11	-122.456	37.937	3.61	10.91	-338.89	14.52	-122.456	37.937	3.69	10.91	-281.19	14.61	11	8/1/2014	10:55.9
2510	RWD11	-122.456	37.937	3.58	10.94	-328.87	14.51	-122.456	37.937	3.64	10.94	-269.56	14.58	11	8/1/2014	10:56.0

2511	RWD11	-122.456	37.937	3.58	10.98	-318.74	14.56	-122.456	37.937	3.69	10.98	-257.95	14.68	11	8/1/2014	10:56.1
2512	RWD11	-122.456	37.937	3.58	10.99	-308.83	14.57	-122.456	37.937	3.69	10.99	-246.51	14.69	11	8/1/2014	10:56.2
2513	RWD11	-122.456	37.937	3.58	11.06	-298.98	14.64	-122.456	37.937	3.69	11.06	-235.27	14.76	11	8/1/2014	10:56.3
2514	RWD11	-122.456	37.937	3.58	11.08	-289.41	14.66	-122.456	37.937	3.69	11.08	-224.29	14.77	11	8/1/2014	10:56.4
2515	RWD11	-122.456	37.937	3.61	11.09	-280.01	14.70	-122.456	37.937	3.69	11.09	-213.43	14.79	11	8/1/2014	10:56.5
2516	RWD11	-122.456	37.937	3.58	11.11	-270.64	14.69	-122.456	37.937	3.69	11.11	-202.80	14.81	11	8/1/2014	10:56.6
2517	RWD11	-122.456	37.937	3.61	11.20	-261.24	14.82	-122.456	37.937	3.69	11.20	-192.12	14.90	11	8/1/2014	10:56.7
2518	RWD11	-122.456	37.937	3.58	11.16	-251.72	14.74	-122.456	37.937	3.69	11.16	-181.32	14.85	11	8/1/2014	10:56.8
2519	RWD11	-122.456	37.937	3.61	11.15	-241.98	14.76	-122.456	37.937	3.69	11.15	-170.38	14.84	11	8/1/2014	10:56.9
2520	RWD11	-122.456	37.937	3.58	11.23	-232.04	14.81	-122.456	37.937	3.69	11.23	-159.16	14.92	11	8/1/2014	10:57.0
2521	RWD11	-122.456	37.937	3.61	11.20	-222.00	14.81	-122.456	37.937	3.69	11.20	-147.86	14.89	11	8/1/2014	10:57.1
2522	RWD11	-122.456	37.937	3.58	11.25	-211.79	14.83	-122.456	37.937	3.69	11.25	-136.50	14.95	11	8/1/2014	10:57.2
2523	RWD11	-122.456	37.937	3.58	11.26	-201.46	14.83	-122.456	37.937	3.69	11.26	-124.93	14.95	11	8/1/2014	10:57.3
2524	RWD11	-122.456	37.937	3.58	11.25	-190.98	14.83	-122.456	37.937	3.64	11.25	-113.20	14.90	11	8/1/2014	10:57.4
2525	RWD11	-122.456	37.937	3.61	11.26	-180.35	14.87	-122.456	37.937	3.69	11.26	-101.31	14.95	11	8/1/2014	10:57.5
2526	RWD11	-122.456	37.937	3.58	11.28	-169.68	14.86	-122.456	37.937	3.69	11.28	-89.29	14.98	11	8/1/2014	10:57.6
2527	RWD11	-122.456	37.937	3.61	11.33	-158.92	14.94	-122.456	37.937	3.73	11.33	-77.27	15.06	11	8/1/2014	10:57.7
2528	RWD11	-122.456	37.937	3.58	11.30	-148.01	14.87	-122.456	37.937	3.69	11.30	-65.10	14.99	11	8/1/2014	10:57.8
2529	RWD11	-122.456	37.937	3.61	11.33	-137.06	14.94	-122.456	37.937	3.69	11.33	-52.77	15.02	11	8/1/2014	10:57.9
2530	RWD11	-122.456	37.937	3.58	11.34	-125.88	14.91	-122.456	37.937	3.69	11.34	-40.28	15.03	11	8/1/2014	10:58.0
2531	RWD11	-122.456	37.937	3.61	11.34	-114.61	14.95	-122.456	37.937	3.69	11.34	-27.63	15.04	11	8/1/2014	10:58.1
2532	RWD11	-122.456	37.937	3.58	11.36	-103.36	14.93	-122.456	37.937	3.64	11.36	-14.82	15.00	11	8/1/2014	10:58.2
2533	RWD11	-122.456	37.937	3.61	11.34	-92.02	14.95	-122.456	37.937	3.69	11.34	-2.26	15.04	11	8/1/2014	10:58.3
2534	RWD11	-122.456	37.937	3.58	11.34	-80.74	14.92	-122.456	37.937	3.64	11.34	10.50	14.99	11	8/1/2014	10:58.4
2535	RWD11	-122.456	37.937	3.61	11.34	-69.40	14.95	-122.456	37.937	3.69	11.34	23.15	15.04	11	8/1/2014	10:58.5
2536	RWD11	-122.456	37.937	3.61	11.31	-58.15	14.93	-122.456	37.937	3.64	11.31	35.72	14.96	11	8/1/2014	10:58.6
2537	RWD11	-122.456	37.937	3.61	11.34	-46.87	14.95	-122.456	37.937	3.69	11.34	48.20	15.03	11	8/1/2014	10:58.7
2538	RWD11	-122.456	37.937	3.58	11.33	-35.85	14.90	-122.456	37.937	3.69	11.33	60.52	15.02	11	8/1/2014	10:58.8
2539	RWD11	-122.456	37.937	3.61	11.32	-25.04	14.93	-122.456	37.937	3.73	11.32	72.60	15.05	11	8/1/2014	10:58.9
2540	RWD11	-122.456	37.937	3.61	11.31	-14.28	14.92	-122.456	37.937	3.69	11.31	84.56	15.00	11	8/1/2014	10:59.0
2541	RWD11	-122.456	37.937	3.61	11.30	-3.64	14.91	-122.456	37.937	3.73	11.30	96.42	15.03	11	8/1/2014	10:59.1
2542	RWD11	-122.456	37.937	3.58	11.29	6.98	14.86	-122.456	37.937	3.73	11.29	108.28	15.02	11	8/1/2014	10:59.2
2543	RWD11	-122.456	37.937	3.61	11.27	17.60	14.88	-122.456	37.937	3.73	11.27	120.08	15.00	11	8/1/2014	10:59.3

2544	RWD11	-122.456	37.937	3.61	11.28	28.24	14.89	-122.456	37.937	3.73	11.28	131.99	15.01	11	8/1/2014	10:59.4
2545	RWD11	-122.456	37.937	3.61	11.26	39.04	14.87	-122.456	37.937	3.78	11.26	143.91	15.04	11	8/1/2014	10:59.5
2546	RWD11	-122.456	37.937	3.61	11.24	49.92	14.86	-122.456	37.937	3.73	11.24	155.88	14.97	11	8/1/2014	10:59.6
2547	RWD11	-122.456	37.937	3.61	11.23	60.85	14.85	-122.456	37.937	3.73	11.23	167.94	14.96	11	8/1/2014	10:59.7
2548	RWD11	-122.456	37.937	3.61	11.25	71.89	14.86	-122.456	37.937	3.73	11.25	179.92	14.98	11	8/1/2014	10:59.8
2549	RWD11	-122.456	37.937	3.61	11.22	82.82	14.83	-122.456	37.937	3.78	11.22	191.93	15.00	11	8/1/2014	10:59.9
2550	RWD11	-122.456	37.937	3.61	11.20	93.80	14.81	-122.456	37.937	3.78	11.20	203.82	14.98	11	8/1/2014	11:00.0
2551	RWD11	-122.456	37.937	3.66	11.20	104.67	14.86	-122.456	37.937	3.78	11.20	215.75	14.98	11	8/1/2014	11:00.1
2552	RWD11	-122.456	37.937	3.61	11.18	115.44	14.79	-122.456	37.937	3.78	11.18	227.44	14.96	11	8/1/2014	11:00.2
2553	RWD11	-122.456	37.937	3.66	11.18	126.15	14.84	-122.456	37.937	3.81	11.18	239.13	14.99	11	8/1/2014	11:00.3
2554	RWD11	-122.456	37.937	3.61	11.16	136.82	14.77	-122.456	37.937	3.81	11.16	250.69	14.97	11	8/1/2014	11:00.4
2555	RWD11	-122.456	37.937	3.66	11.15	147.26	14.81	-122.456	37.937	3.87	11.15	262.19	15.01	11	8/1/2014	11:00.5
2556	RWD11	-122.456	37.937	3.61	11.10	157.77	14.71	-122.456	37.937	3.87	11.10	273.75	14.96	11	8/1/2014	11:00.6
2557	RWD11	-122.456	37.937	3.66	11.09	168.42	14.75	-122.456	37.937	3.87	11.09	285.39	14.96	11	8/1/2014	11:00.7
2558	RWD11	-122.456	37.937	3.61	11.07	179.30	14.69	-122.456	37.937	3.87	11.07	297.22	14.94	11	8/1/2014	11:00.8
2559	RWD11	-122.456	37.937	3.66	11.06	190.25	14.73	-122.456	37.937	3.90	11.06	309.25	14.96	11	8/1/2014	11:00.9
2560	RWD11	-122.456	37.937	3.66	11.06	201.47	14.72	-122.456	37.937	3.90	11.06	321.33	14.96	11	8/1/2014	11:01.0
2561	RWD11	-122.456	37.937	3.66	11.10	212.60	14.76	-122.456	37.937	3.93	11.10	333.48	15.03	11	8/1/2014	11:01.1
2562	RWD11	-122.456	37.937	3.66	11.10	223.82	14.76	-122.456	37.937	3.93	11.10	345.71	15.03	11	8/1/2014	11:01.2
2563	RWD11	-122.456	37.937	3.70	11.03	235.00	14.72	-122.456	37.937	3.93	11.03	357.93	14.96	11	8/1/2014	11:01.3
2564	RWD11	-122.456	37.937	3.66	11.06	246.17	14.72	-122.456	37.937	3.93	11.06	370.10	14.99	11	8/1/2014	11:01.4
2565	RWD11	-122.456	37.937	3.70	11.01	257.38	14.71	-122.456	37.937	3.98	11.01	382.30	14.99	11	8/1/2014	11:01.5
2566	RWD11	-122.456	37.937	3.66	11.01	268.63	14.67	-122.456	37.937	3.98	11.01	394.44	14.99	11	8/1/2014	11:01.6
2567	RWD11	-122.456	37.937	3.70	11.01	279.89	14.71	-122.456	37.937	4.02	11.01	406.54	15.03	11	8/1/2014	11:01.7
2568	RWD11	-122.456	37.937	3.70	10.99	291.20	14.69	-122.456	37.937	4.02	10.99	418.85	15.01	11	8/1/2014	11:01.8
2569	RWD11	-122.456	37.937	3.75	10.97	302.52	14.71	-122.456	37.937	4.07	10.97	431.05	15.03	11	8/1/2014	11:01.9
2570	RWD11	-122.456	37.937	3.70	10.97	314.04	14.67	-122.456	37.937	4.07	10.97	443.51	15.04	11	8/1/2014	11:02.0
2571	RWD11	-122.456	37.937	3.75	10.95	325.73	14.70	-122.456	37.937	4.10	10.95	456.15	15.05	11	8/1/2014	11:02.1
2572	RWD11	-122.456	37.937	3.75	10.96	337.69	14.70	-122.456	37.937	4.10	10.96	469.07	15.06	11	8/1/2014	11:02.2
2573	RWD11	-122.456	37.937	3.75	10.96	349.98	14.70	-122.456	37.937	4.15	10.96	482.27	15.11	11	8/1/2014	11:02.3
2574	RWD11	-122.456	37.937	3.75	10.93	362.51	14.67	-122.456	37.937	4.15	10.93	495.81	15.08	11	8/1/2014	11:02.4
2575	RWD11	-122.456	37.937	3.78	10.92	375.34	14.70	-122.456	37.937	4.15	10.92	509.54	15.07	11	8/1/2014	11:02.5
2576	RWD11	-122.456	37.937	3.78	10.90	388.33	14.68	-122.456	37.937	4.15	10.90	523.54	15.05	11	8/1/2014	11:02.6

2577	RWD11	-122.456	37.937	3.78	10.88	401.45	14.66	-122.456	37.937	4.18	10.88	537.63	15.07	11	8/1/2014	11:02.7
2578	RWD11	-122.456	37.937	3.78	10.84	414.55	14.62	-122.456	37.937	4.18	10.84	551.73	15.03	11	8/1/2014	11:02.8
2579	RWD11	-122.456	37.937	3.83	10.86	427.61	14.70	-122.456	37.937	4.22	10.86	565.77	15.08	11	8/1/2014	11:02.9
2580	RWD11	-122.456	37.937	3.83	10.82	440.75	14.65	-122.456	37.937	4.22	10.82	579.86	15.04	11	8/1/2014	11:03.0
2581	RWD11	-122.456	37.937	3.87	10.81	454.07	14.68	-122.456	37.937	4.27	10.81	594.00	15.08	11	8/1/2014	11:03.1
2582	RWD11	-122.456	37.937	3.87	10.78	467.66	14.65	-122.456	37.937	4.27	10.78	608.38	15.05	11	8/1/2014	11:03.2
2583	RWD11	-122.456	37.937	3.87	10.79	481.63	14.66	-122.456	37.937	4.27	10.79	623.15	15.06	11	8/1/2014	11:03.3
2584	RWD11	-122.456	37.937	3.87	10.78	496.12	14.65	-122.456	37.937	4.27	10.78	638.35	15.05	11	8/1/2014	11:03.4
2585	RWD11	-122.456	37.937	3.92	10.77	511.07	14.69	-122.456	37.937	4.30	10.77	654.02	15.08	11	8/1/2014	11:03.5
2586	RWD11	-122.456	37.937	3.92	10.84	526.46	14.76	-122.456	37.937	4.30	10.84	670.13	15.15	11	8/1/2014	11:03.6
2587	RWD11	-122.456	37.937	3.92	10.83	541.99	14.75	-122.456	37.937	4.30	10.83	686.51	15.13	11	8/1/2014	11:03.7
2588	RWD11	-122.456	37.937	3.92	10.86	557.71	14.78	-122.456	37.937	4.35	10.86	703.06	15.21	11	8/1/2014	11:03.8
2589	RWD11	-122.456	37.937	3.95	10.89	573.44	14.84	-122.456	37.937	4.35	10.89	719.69	15.24	11	8/1/2014	11:03.9
2590	RWD11	-122.456	37.937	3.92	10.94	589.35	14.85	-122.456	37.937	4.35	10.94	736.44	15.29	11	8/1/2014	11:04.0
2591	RWD11	-122.456	37.937	3.95	10.89	605.26	14.84	-122.456	37.937	4.35	10.89	753.28	15.24	11	8/1/2014	11:04.1
2592	RWD11	-122.456	37.937	3.95	10.90	621.40	14.85	-122.456	37.937	4.39	10.90	770.22	15.29	11	8/1/2014	11:04.2
2593	RWD11	-122.456	37.937	3.99	10.92	637.75	14.90	-122.456	37.937	4.44	10.92	787.41	15.36	11	8/1/2014	11:04.3
2594	RWD11	-122.456	37.937	3.99	10.94	654.48	14.92	-122.456	37.937	4.44	10.94	804.85	15.37	11	8/1/2014	11:04.4
2595	RWD11	-122.456	37.937	3.99	10.97	671.56	14.95	-122.456	37.937	4.47	10.97	822.70	15.44	11	8/1/2014	11:04.5
2596	RWD11	-122.456	37.937	3.99	10.94	689.15	14.93	-122.456	37.937	4.44	10.94	841.00	15.38	11	8/1/2014	11:04.6
2597	RWD11	-122.456	37.937	4.04	10.95	707.03	14.99	-122.456	37.937	4.47	10.95	859.66	15.42	11	8/1/2014	11:04.7
2598	RWD11	-122.456	37.937	4.04	11.00	725.37	15.04	-122.456	37.937	4.47	11.00	878.74	15.48	11	8/1/2014	11:04.8
2599	RWD11	-122.456	37.937	4.07	10.99	744.16	15.06	-122.456	37.937	4.52	10.99	898.31	15.51	11	8/1/2014	11:04.9
2600	RWD11	-122.456	37.937	4.04	11.05	763.54	15.09	-122.456	37.937	4.52	11.05	918.36	15.58	11	8/1/2014	11:05.0
2601	RWD11	-122.456	37.937	4.07	11.07	783.35	15.15	-122.456	37.937	4.52	11.07	939.04	15.60	11	8/1/2014	11:05.1
2602	RWD11	-122.456	37.937	4.07	11.07	803.53	15.15	-122.456	37.937	4.52	11.07	960.01	15.60	11	8/1/2014	11:05.2
2603	RWD11	-122.456	37.937	4.07	11.10	824.05	15.17	-122.456	37.937	4.56	11.10	981.31	15.65	11	8/1/2014	11:05.3
2604	RWD11	-122.456	37.937	4.07	11.10	844.78	15.17	-122.456	37.937	4.56	11.10	1002.88	15.65	11	8/1/2014	11:05.4
2605	RWD11	-122.456	37.937	4.12	11.11	865.96	15.24	-122.456	37.937	4.56	11.11	1024.87	15.67	11	8/1/2014	11:05.5
2606	RWD11	-122.456	37.937	4.12	11.16	887.49	15.28	-122.456	37.937	4.59	11.16	1047.28	15.75	11	8/1/2014	11:05.6
2607	RWD11	-122.456	37.937	4.16	11.19	909.50	15.35	-122.456	37.937	4.64	11.19	1069.95	15.83	11	8/1/2014	11:05.7
2608	RWD11	-122.456	37.937	4.16	11.18	932.07	15.33	-122.456	37.937	4.59	11.18	1093.19	15.77	11	8/1/2014	11:05.8
2609	RWD11	-122.456	37.937	4.16	11.23	955.40	15.39	-122.456	37.937	4.64	11.23	1117.11	15.88	11	8/1/2014	11:05.9

2610	RWD11	-122.456	37.937	4.16	11.23	979.50	15.39	-122.456	37.937	4.64	11.23	1141.89	15.88	11	8/1/2014	11:06.0
2611	RWD11	-122.456	37.937	4.21	11.28	######	15.49	-122.456	37.937	4.64	11.28	1167.34	15.93	11	8/1/2014	11:06.1
2612	RWD11	-122.456	37.937	4.16	11.29	######	15.45	-122.456	37.937	4.68	11.29	1193.47	15.96	11	8/1/2014	11:06.2
2613	RWD11	-122.456	37.937	4.21	11.35	######	15.56	-122.456	37.937	4.68	11.35	1219.99	16.03	11	8/1/2014	11:06.3
2614	RWD11	-122.456	37.937	4.21	11.37	######	15.58	-122.456	37.937	4.68	11.37	1247.06	16.04	11	8/1/2014	11:06.4
2615	RWD11	-122.456	37.937	4.21	11.40	######	15.60	-122.456	37.937	4.68	11.40	1274.63	16.07	11	8/1/2014	11:06.5
2616	RWD11	-122.456	37.937	4.21	11.37	######	15.58	-122.456	37.937	4.68	11.37	1302.57	16.04	11	8/1/2014	11:06.6
2617	RWD11	-122.456	37.937	4.24	11.39	######	15.63	-122.456	37.937	4.73	11.39	1331.07	16.12	11	8/1/2014	11:06.7
2618	RWD11	-122.456	37.937	4.21	11.40	######	15.60	-122.456	37.937	4.73	11.40	1360.23	16.12	11	8/1/2014	11:06.8
2619	RWD11	-122.456	37.937	4.24	11.44	######	15.68	-122.456	37.937	4.73	11.44	1390.04	16.16	11	8/1/2014	11:06.9
2620	RWD11	-122.456	37.937	4.28	11.44	######	15.71	-122.456	37.937	4.73	11.44	1420.48	16.16	11	8/1/2014	11:07.0
2621	RWD11	-122.456	37.937	4.28	11.41	######	15.68	-122.456	37.937	4.76	11.41	1451.42	16.17	11	8/1/2014	11:07.1
2622	RWD11	-122.456	37.937	4.28	11.41	######	15.68	-122.456	37.937	4.73	11.41	1482.82	16.13	11	8/1/2014	11:07.2
2623	RWD11	-122.456	37.937	4.33	11.42	######	15.75	-122.456	37.937	4.76	11.42	1514.71	16.18	11	8/1/2014	11:07.3
2624	RWD11	-122.456	37.937	4.28	11.48	######	15.75	-122.456	37.937	4.73	11.48	1547.01	16.20	11	8/1/2014	11:07.4
2625	RWD11	-122.456	37.937	4.33	11.46	######	15.79	-122.456	37.937	4.76	11.46	1579.50	16.22	11	8/1/2014	11:07.5
2626	RWD11	-122.456	37.937	4.33	11.45	######	15.78	-122.456	37.937	4.81	11.45	1612.07	16.26	11	8/1/2014	11:07.6
2627	RWD11	-122.456	37.937	4.33	11.51	######	15.84	-122.456	37.937	4.76	11.51	1644.69	16.27	11	8/1/2014	11:07.7
2628	RWD11	-122.456	37.937	4.33	11.47	######	15.79	-122.456	37.937	4.76	11.47	1677.29	16.23	11	8/1/2014	11:07.8
2629	RWD11	-122.456	37.937	4.33	11.48	######	15.80	-122.456	37.937	4.81	11.48	1710.01	16.29	11	8/1/2014	11:07.9
2630	RWD11	-122.456	37.937	4.33	11.51	######	15.84	-122.456	37.937	4.81	11.51	1743.07	16.32	11	8/1/2014	11:08.0
2631	RWD11	-122.456	37.937	4.36	11.53	######	15.90	-122.456	37.937	4.81	11.53	1776.48	16.35	11	8/1/2014	11:08.1
2632	RWD11	-122.456	37.937	4.36	11.55	######	15.91	-122.456	37.937	4.81	11.55	1810.16	16.36	11	8/1/2014	11:08.2
2633	RWD11	-122.456	37.937	4.36	11.47	######	15.83	-122.456	37.937	4.84	11.47	1843.95	16.31	11	8/1/2014	11:08.3
2634	RWD11	-122.456	37.937	4.36	11.46	######	15.82	-122.456	37.937	4.81	11.46	1877.95	16.27	11	8/1/2014	11:08.4
2635	RWD11	-122.456	37.937	4.41	11.47	######	15.88	-122.456	37.937	4.84	11.47	1912.20	16.31	11	8/1/2014	11:08.5
2636	RWD11	-122.456	37.937	4.36	11.43	######	15.79	-122.456	37.937	4.84	11.43	1946.79	16.27	11	8/1/2014	11:08.6
2637	RWD11	-122.456	37.937	4.41	11.39	######	15.80	-122.456	37.937	4.84	11.39	1981.43	16.24	11	8/1/2014	11:08.7
2638	RWD11	-122.456	37.937	4.41	11.35	######	15.77	-122.456	37.937	4.84	11.35	2016.05	16.20	11	8/1/2014	11:08.8
2639	RWD11	-122.456	37.937	4.45	11.34	######	15.78	-122.456	37.937	4.84	11.34	2050.44	16.18	11	8/1/2014	11:08.9
2640	RWD11	-122.456	37.937	4.41	11.34	######	15.75	-122.456	37.937	4.84	11.34	2084.42	16.18	11	8/1/2014	11:09.0
2641	RWD11	-122.456	37.937	4.45	11.31	######	15.76	-122.456	37.937	4.90	11.31	2117.86	16.21	11	8/1/2014	11:09.1
2642	RWD11	-122.456	37.937	4.50	11.29	######	15.79	-122.456	37.937	4.90	11.29	2150.62	16.18	11	8/1/2014	11:09.2

2643	RWD11	-122.456	37.937	4.50	11.27	######	15.77	-122.456	37.937	4.90	11.27	2183.08	16.17	11	8/1/2014	11:09.3
2644	RWD11	-122.456	37.937	4.50	11.31	######	15.81	-122.456	37.937	4.93	11.31	2215.24	16.24	11	8/1/2014	11:09.4
2645	RWD11	-122.456	37.937	4.50	11.31	######	15.81	-122.456	37.937	4.93	11.31	2247.09	16.24	11	8/1/2014	11:09.5
2646	RWD11	-122.456	37.937	4.50	11.34	######	15.84	-122.456	37.937	4.93	11.34	2278.67	16.27	11	8/1/2014	11:09.6
2647	RWD11	-122.456	37.937	4.53	11.27	######	15.81	-122.456	37.937	4.96	11.27	2310.00	16.24	11	8/1/2014	11:09.7
2648	RWD11	-122.456	37.937	4.50	11.23	######	15.73	-122.456	37.937	4.96	11.23	2340.98	16.19	11	8/1/2014	11:09.8
2649	RWD11	-122.456	37.937	4.53	11.23	######	15.76	-122.456	37.937	4.96	11.23	2371.55	16.19	11	8/1/2014	11:09.9
2650	RWD11	-122.456	37.937	4.50	11.18	######	15.68	-122.456	37.937	4.96	11.18	2401.62	16.14	11	8/1/2014	11:10.0
2651	RWD11	-122.456	37.937	4.53	11.16	######	15.69	-122.456	37.937	4.96	11.16	2431.16	16.12	11	8/1/2014	11:10.1
2652	RWD11	-122.456	37.937	4.53	11.15	######	15.68	-122.456	37.937	4.96	11.15	2459.91	16.11	11	8/1/2014	11:10.2
2653	RWD11	-122.456	37.937	4.53	11.09	######	15.62	-122.456	37.937	4.96	11.09	2487.46	16.05	11	8/1/2014	11:10.3
2654	RWD11	-122.456	37.937	4.53	11.10	######	15.63	-122.456	37.937	4.96	11.10	2513.64	16.06	11	8/1/2014	11:10.4
2655	RWD11	-122.456	37.937	4.59	11.04	######	15.62	-122.456	37.937	5.01	11.04	2538.17	16.05	11	8/1/2014	11:10.5
2656	RWD11	-122.456	37.937	4.59	11.00	######	15.59	-122.456	37.937	4.96	11.00	2561.01	15.97	11	8/1/2014	11:10.6
2657	RWD11	-122.456	37.937	4.59	10.97	######	15.55	-122.456	37.937	5.01	10.97	2582.31	15.98	11	8/1/2014	11:10.7
2658	RWD11	-122.456	37.937	4.59	11.01	######	15.60	-122.456	37.937	5.01	11.01	2602.50	16.02	11	8/1/2014	11:10.8
2659	RWD11	-122.456	37.937	4.59	10.94	######	15.52	-122.456	37.937	5.01	10.94	2621.31	15.95	11	8/1/2014	11:10.9
2660	RWD11	-122.456	37.937	4.59	10.88	######	15.47	-122.456	37.937	5.01	10.88	2638.85	15.89	11	8/1/2014	11:11.0
2661	RWD11	-122.456	37.937	4.59	10.85	######	15.44	-122.456	37.937	5.05	10.85	2655.11	15.90	11	8/1/2014	11:11.1
2662	RWD11	-122.456	37.937	4.59	10.81	######	15.40	-122.456	37.937	5.01	10.81	2669.99	15.83	11	8/1/2014	11:11.2
2663	RWD11	-122.456	37.937	4.62	10.78	######	15.40	-122.456	37.937	5.05	10.78	2683.44	15.83	11	8/1/2014	11:11.3
2664	RWD11	-122.456	37.937	4.62	10.76	######	15.38	-122.456	37.937	5.05	10.76	2694.89	15.81	11	8/1/2014	11:11.4
2665	RWD11	-122.456	37.937	4.62	10.75	######	15.37	-122.456	37.937	5.05	10.75	2704.74	15.80	11	8/1/2014	11:11.5
2666	RWD11	-122.456	37.937	4.62	10.73	######	15.34	-122.456	37.937	5.05	10.73	2712.45	15.77	11	8/1/2014	11:11.6
2667	RWD11	-122.456	37.937	4.65	10.73	######	15.38	-122.456	37.937	5.10	10.73	2718.24	15.82	11	8/1/2014	11:11.7
2668	RWD11	-122.456	37.937	4.62	10.72	######	15.34	-122.456	37.937	5.05	10.72	2721.97	15.77	11	8/1/2014	11:11.8
2669	RWD11	-122.456	37.937	4.62	10.70	######	15.32	-122.456	37.937	5.05	10.70	2723.75	15.75	11	8/1/2014	11:11.9
2670	RWD11	-122.456	37.937	4.62	10.70	######	15.32	-122.456	37.937	5.05	10.70	2723.91	15.75	11	8/1/2014	11:12.0
2671	RWD11	-122.456	37.937	4.65	10.65	######	15.30	-122.456	37.937	5.10	10.65	2722.62	15.75	11	8/1/2014	11:12.1
2672	RWD11	-122.457	37.937	4.65	10.59	######	15.24	-122.456	37.937	5.10	10.59	2720.10	15.69	11	8/1/2014	11:12.2
2673	RWD11	-122.457	37.937	4.65	10.56	######	15.21	-122.456	37.937	5.10	10.56	2716.30	15.65	11	8/1/2014	11:12.3
2674	RWD11	-122.457	37.937	4.65	10.52	######	15.17	-122.456	37.937	5.10	10.52	2710.88	15.62	11	8/1/2014	11:12.4
2675	RWD11	-122.457	37.937	4.70	10.49	######	15.19	-122.457	37.937	5.13	10.49	2703.83	15.62	11	8/1/2014	11:12.5

2676	RWD11	-122.457	37.937	4.65	10.41	######	15.06	-122.457	37.937	5.13	10.41	2694.68	15.54	11	8/1/2014	11:12.6
2677	RWD11	-122.457	37.937	4.70	10.39	######	15.09	-122.457	37.937	5.13	10.39	2683.36	15.52	11	8/1/2014	11:12.7
2678	RWD11	-122.457	37.937	4.65	10.36	######	15.02	-122.457	37.937	5.10	10.36	2669.69	15.46	11	8/1/2014	11:12.8
2679	RWD11	-122.457	37.937	4.74	10.23	######	14.97	-122.457	37.937	5.13	10.23	2653.81	15.37	11	8/1/2014	11:12.9
[truncated: 222,155 more chars]
