# Supplementary material for: Chinook salmon and green sturgeon migrate through San Francisco Estuary despite large distortions in the local magnetic field produced by bridges
Source: PLoS One. 2017 Jun 2;12(6):e0169031. doi: 10.1371/journal.pone.0169031 (PMC5456031; doi:10.1371/journal.pone.0169031)
Supplement: S1 Fig — (PDF) [file pone.0169031.s001.pdf]

| #X | Y | Z | ALT | GRAD | DATE | TIME | LINE |
|---|---|---|---|---|---|---|---|
| -1409.495 | 19716.261 | 0.471 | 15.978 | 0.126664 | 7/10/2014 | 35:11.3 | 2 |
| -1409.822 | 19716.279 | 0.468 | 15.961 | 0.111243 | 7/10/2014 | 35:11.4 | 2 |
| -1410.04 | 19716.292 | 0.471 | 15.936 | 0.149326 | 7/10/2014 | 35:11.5 | 2 |
| -1410.204 | 19716.302 | 0.479 | 15.912 | 0.140856 | 7/10/2014 | 35:11.6 | 2 |
| -1410.477 | 19716.318 | 0.481 | 15.891 | 0.138407 | 7/10/2014 | 35:11.7 | 2 |
| -1410.754 | 19716.333 | 0.476 | 15.87 | 0.133765 | 7/10/2014 | 35:11.8 | 2 |
| -1410.975 | 19716.346 | 0.476 | 15.848 | 0.14141 | 7/10/2014 | 35:11.9 | 2 |
| -1411.03 | 19716.35 | 0.47 | 15.837 | 0.143371 | 7/10/2014 | 35:11.9 | 2 |
| -1411.365 | 19716.369 | 0.479 | 15.811 | 0.148153 | 7/10/2014 | 35:12.1 | 2 |
| -1411.645 | 19716.385 | 0.482 | 15.799 | 0.132365 | 7/10/2014 | 35:12.2 | 2 |
| -1411.869 | 19716.398 | 0.484 | 15.788 | 0.127513 | 7/10/2014 | 35:12.3 | 2 |
| -1412.034 | 19716.409 | 0.485 | 15.783 | 0.136908 | 7/10/2014 | 35:12.4 | 2 |
| -1412.484 | 19716.435 | 0.491 | 15.781 | 0.138055 | 7/10/2014 | 35:12.5 | 2 |
| -1412.595 | 19716.441 | 0.493 | 15.78 | 0.120913 | 7/10/2014 | 35:12.6 | 2 |
| -1412.876 | 19716.458 | 0.498 | 15.776 | 0.109918 | 7/10/2014 | 35:12.7 | 2 |
| -1412.932 | 19716.461 | 0.495 | 15.778 | 0.111013 | 7/10/2014 | 35:12.7 | 2 |
| -1413.272 | 19716.481 | 0.487 | 15.768 | 0.100162 | 7/10/2014 | 35:12.9 | 2 |
| -1413.557 | 19716.499 | 0.495 | 15.777 | 0.115136 | 7/10/2014 | 35:13.0 | 2 |
| -1413.614 | 19716.503 | 0.491 | 15.78 | 0.112404 | 7/10/2014 | 35:13.0 | 2 |
| -1413.841 | 19716.517 | 0.485 | 15.773 | 0.13435 | 7/10/2014 | 35:13.1 | 2 |
| -1414.182 | 19716.537 | 0.491 | 15.765 | 0.152962 | 7/10/2014 | 35:13.3 | 2 |
| -1414.521 | 19716.558 | 0.481 | 15.763 | 0.146533 | 7/10/2014 | 35:13.4 | 2 |
| -1414.747 | 19716.572 | 0.486 | 15.766 | 0.133982 | 7/10/2014 | 35:13.5 | 2 |
| -1414.86 | 19716.579 | 0.484 | 15.762 | 0.115507 | 7/10/2014 | 35:13.6 | 2 |
| -1415.2 | 19716.6 | 0.479 | 15.77 | 0.100606 | 7/10/2014 | 35:13.7 | 2 |
| -1415.485 | 19716.617 | 0.479 | 15.767 | 0.0825602 | 7/10/2014 | 35:13.8 | 2 |
| -1415.542 | 19716.62 | 0.482 | 15.77 | 0.0725908 | 7/10/2014 | 35:13.8 | 2 |
| -1415.769 | 19716.635 | 0.461 | 15.776 | 0.0696079 | 7/10/2014 | 35:13.9 | 2 |
| -1416.116 | 19716.656 | 0.468 | 15.798 | 0.0663038 | 7/10/2014 | 35:14.1 | 2 |
| -1416.402 | 19716.674 | 0.455 | 15.812 | 0.0657897 | 7/10/2014 | 35:14.2 | 2 |
| -1416.458 | 19716.678 | 0.447 | 15.816 | 0.0705931 | 7/10/2014 | 35:14.2 | 2 |
| -1416.8 | 19716.698 | 0.455 | 15.861 | 0.0675412 | 7/10/2014 | 35:14.4 | 2 |
| -1417.256 | 19716.727 | 0.453 | 15.879 | 0.0761844 | 7/10/2014 | 35:14.5 | 2 |
| -1417.368 | 19716.733 | 0.445 | 15.886 | 0.0827525 | 7/10/2014 | 35:14.6 | 2 |
| -1417.596 | 19716.748 | 0.448 | 15.901 | 0.066233 | 7/10/2014 | 35:14.7 | 2 |
| -1417.712 | 19716.755 | 0.444 | 15.912 | 0.0688723 | 7/10/2014 | 35:14.7 | 2 |
| -1418.055 | 19716.777 | 0.441 | 15.918 | 0.075033 | 7/10/2014 | 35:14.9 | 2 |
| -1418.342 | 19716.796 | 0.439 | 15.924 | 0.065173 | 7/10/2014 | 35:15.0 | 2 |
| -1418.399 | 19716.799 | 0.43 | 15.914 | 0.0735294 | 7/10/2014 | 35:15.0 | 2 |
| -1418.628 | 19716.813 | 0.43 | 15.914 | 0.057131 | 7/10/2014 | 35:15.1 | 2 |
| -1418.97 | 19716.834 | 0.433 | 15.915 | 0.0562753 | 7/10/2014 | 35:15.3 | 2 |
| -1419.31 | 19716.855 | 0.426 | 15.911 | 0.0735521 | 7/10/2014 | 35:15.4 | 2 |
| -1419.479 | 19716.867 | 0.426 | 15.912 | 0.0608331 | 7/10/2014 | 35:15.5 | 2 |
| -1419.648 | 19716.879 | 0.421 | 15.91 | 0.0653406 | 7/10/2014 | 35:15.6 | 2 |
| -1419.987 | 19716.899 | 0.421 | 15.906 | 0.0751599 | 7/10/2014 | 35:15.7 | 2 |
| -1420.268 | 19716.917 | 0.426 | 15.902 | 0.117224 | 7/10/2014 | 35:15.8 | 2 |

| | | | | | | | |
|---|---|---|---|---|---|---|---|
| -1723.718 | 19601.832 | 2.178 | 13.629 | 0.513682 | 7/15/2014 | 14:20.3 | 5 |
| -1723.96 | 19601.834 | 2.17 | 13.612 | 0.511601 | 7/15/2014 | 14:20.4 | 5 |
| -1724.198 | 19601.835 | 2.162 | 13.601 | 0.485551 | 7/15/2014 | 14:20.5 | 5 |
| -1724.437 | 19601.838 | 2.162 | 13.592 | 0.481337 | 7/15/2014 | 14:20.6 | 5 |
| -1724.73 | 19601.839 | 2.162 | 13.589 | 0.46327 | 7/15/2014 | 14:20.7 | 5 |
| -1724.85 | 19601.84 | 2.161 | 13.584 | 0.460047 | 7/15/2014 | 14:20.8 | 5 |
| -1725.085 | 19601.841 | 2.165 | 13.577 | 0.443659 | 7/15/2014 | 14:20.9 | 5 |
| -1725.433 | 19601.842 | 2.158 | 13.571 | 0.422569 | 7/15/2014 | 14:21.0 | 5 |
| -1725.664 | 19601.842 | 2.155 | 13.568 | 0.406623 | 7/15/2014 | 14:21.1 | 5 |
| -1725.895 | 19601.843 | 2.157 | 13.564 | 0.394944 | 7/15/2014 | 14:21.2 | 5 |
| -1726.124 | 19601.844 | 2.157 | 13.562 | 0.388253 | 7/15/2014 | 14:21.3 | 5 |
| -1726.41 | 19601.844 | 2.154 | 13.555 | 0.377039 | 7/15/2014 | 14:21.4 | 5 |
| -1726.637 | 19601.843 | 2.154 | 13.555 | 0.364203 | 7/15/2014 | 14:21.5 | 5 |
| -1726.863 | 19601.843 | 2.154 | 13.556 | 0.350992 | 7/15/2014 | 14:21.6 | 5 |
| -1727.148 | 19601.842 | 2.157 | 13.56 | 0.339792 | 7/15/2014 | 14:21.7 | 5 |
| -1727.375 | 19601.842 | 2.157 | 13.564 | 0.330292 | 7/15/2014 | 14:21.8 | 5 |
| -1727.604 | 19601.84 | 2.157 | 13.576 | 0.319619 | 7/15/2014 | 14:21.9 | 5 |
| -1727.833 | 19601.84 | 2.155 | 13.59 | 0.311076 | 7/15/2014 | 14:22.0 | 5 |
| -1728.062 | 19601.838 | 2.155 | 13.599 | 0.303276 | 7/15/2014 | 14:22.1 | 5 |
| -1728.291 | 19601.837 | 2.15 | 13.62 | 0.297119 | 7/15/2014 | 14:22.2 | 5 |
| -1728.519 | 19601.835 | 2.147 | 13.626 | 0.288418 | 7/15/2014 | 14:22.3 | 5 |
| -1728.804 | 19601.833 | 2.147 | 13.64 | 0.276961 | 7/15/2014 | 14:22.4 | 5 |
| -1729.03 | 19601.832 | 2.141 | 13.642 | 0.272315 | 7/15/2014 | 14:22.5 | 5 |
| -1729.258 | 19601.829 | 2.141 | 13.644 | 0.266739 | 7/15/2014 | 14:22.6 | 5 |
| -1729.544 | 19601.827 | 2.139 | 13.64 | 0.263556 | 7/15/2014 | 14:22.7 | 5 |
| -1729.774 | 19601.823 | 2.141 | 13.638 | 0.262777 | 7/15/2014 | 14:22.8 | 5 |
| -1730.004 | 19601.821 | 2.141 | 13.632 | 0.266809 | 7/15/2014 | 14:22.9 | 5 |
| -1730.239 | 19601.818 | 2.147 | 13.623 | 0.271434 | 7/15/2014 | 14:23.0 | 5 |
| -1730.355 | 19601.817 | 2.148 | 13.621 | 0.267542 | 7/15/2014 | 14:23.1 | 5 |
| -1730.585 | 19601.814 | 2.144 | 13.616 | 0.272782 | 7/15/2014 | 14:23.2 | 5 |
| -1730.821 | 19601.812 | 2.148 | 13.62 | 0.274575 | 7/15/2014 | 14:23.3 | 5 |
| -1731.227 | 19601.806 | 2.139 | 13.624 | 0.272622 | 7/15/2014 | 14:23.4 | 5 |
| -1731.462 | 19601.802 | 2.139 | 13.628 | 0.269078 | 7/15/2014 | 14:23.5 | 5 |
| -1731.696 | 19601.798 | 2.134 | 13.627 | 0.271294 | 7/15/2014 | 14:23.6 | 5 |
| -1731.99 | 19601.793 | 2.131 | 13.631 | 0.25347 | 7/15/2014 | 14:23.7 | 5 |
| -1732.108 | 19601.791 | 2.126 | 13.632 | 0.240922 | 7/15/2014 | 14:23.8 | 5 |
| -1732.346 | 19601.787 | 2.12 | 13.635 | 0.23143 | 7/15/2014 | 14:23.9 | 5 |
| -1732.583 | 19601.783 | 2.112 | 13.632 | 0.249238 | 7/15/2014 | 14:24.0 | 5 |
| -1732.822 | 19601.778 | 2.116 | 13.638 | 0.235645 | 7/15/2014 | 14:24.1 | 5 |
| -1733.06 | 19601.773 | 2.112 | 13.634 | 0.235741 | 7/15/2014 | 14:24.2 | 5 |
| -1733.299 | 19601.77 | 2.112 | 13.634 | 0.229734 | 7/15/2014 | 14:24.3 | 5 |
| -1733.715 | 19601.76 | 2.114 | 13.628 | 0.229875 | 7/15/2014 | 14:24.4 | 5 |
| -1733.951 | 19601.755 | 2.117 | 13.615 | 0.249694 | 7/15/2014 | 14:24.5 | 5 |
| -1734.189 | 19601.75 | 2.12 | 13.599 | 0.22072 | 7/15/2014 | 14:24.6 | 5 |
| -1734.486 | 19601.743 | 2.123 | 13.594 | 0.210868 | 7/15/2014 | 14:24.7 | 5 |
| -1734.605 | 19601.74 | 2.12 | 13.582 | 0.206133 | 7/15/2014 | 14:24.8 | 5 |
| -1734.844 | 19601.734 | 2.127 | 13.578 | 0.2003 | 7/15/2014 | 14:24.9 | 5 |

| | | | | | | | |
|---|---|---|---|---|---|---|---|
| -1735.082 | 19601.729 | 2.123 | 13.569 | 0.206959 | 7/15/2014 | 14:25.0 | 5 |
| -1735.322 | 19601.723 | 2.123 | 13.572 | 0.19631 | 7/15/2014 | 14:25.1 | 5 |
| -1735.558 | 19601.718 | 2.117 | 13.562 | 0.175976 | 7/15/2014 | 14:25.2 | 5 |
| -1735.796 | 19601.711 | 2.117 | 13.569 | 0.169324 | 7/15/2014 | 14:25.3 | 5 |
| -1736.207 | 19601.701 | 2.109 | 13.577 | 0.17404 | 7/15/2014 | 14:25.4 | 5 |
| -1736.443 | 19601.693 | 2.103 | 13.582 | 0.168337 | 7/15/2014 | 14:25.5 | 5 |
| -1736.676 | 19601.688 | 2.095 | 13.586 | 0.156785 | 7/15/2014 | 14:25.6 | 5 |
| -1736.969 | 19601.68 | 2.093 | 13.592 | 0.157983 | 7/15/2014 | 14:25.7 | 5 |
| -1737.086 | 19601.678 | 2.088 | 13.596 | 0.165303 | 7/15/2014 | 14:25.8 | 5 |
| -1737.321 | 19601.67 | 2.091 | 13.602 | 0.211568 | 7/15/2014 | 14:25.9 | 5 |
| -1737.555 | 19601.665 | 2.085 | 13.612 | 0.173462 | 7/15/2014 | 14:26.0 | 5 |
| -1737.791 | 19601.658 | 2.087 | 13.618 | 0.204248 | 7/15/2014 | 14:26.1 | 5 |
| -1738.026 | 19601.65 | 2.085 | 13.628 | 0.236434 | 7/15/2014 | 14:26.2 | 5 |
| -1738.26 | 19601.644 | 2.085 | 13.631 | 0.264795 | 7/15/2014 | 14:26.3 | 5 |
| -1738.668 | 19601.631 | 2.084 | 13.627 | 0.256004 | 7/15/2014 | 14:26.4 | 5 |
| -1738.9 | 19601.623 | 2.082 | 13.624 | 0.236207 | 7/15/2014 | 14:26.5 | 5 |
| -1739.133 | 19601.616 | 2.08 | 13.626 | 0.250894 | 7/15/2014 | 14:26.6 | 5 |
| -1739.423 | 19601.608 | 2.078 | 13.62 | 0.228204 | 7/15/2014 | 14:26.7 | 5 |
| -1739.541 | 19601.604 | 2.075 | 13.619 | 0.216894 | 7/15/2014 | 14:26.8 | 5 |
| -1739.777 | 19601.596 | 2.075 | 13.609 | 0.211772 | 7/15/2014 | 14:26.9 | 5 |
| -1740.011 | 19601.588 | 2.07 | 13.608 | 0.247052 | 7/15/2014 | 14:27.0 | 5 |
| -1740.248 | 19601.58 | 2.068 | 13.615 | 0.239634 | 7/15/2014 | 14:27.1 | 5 |
| -1740.482 | 19601.572 | 2.065 | 13.616 | 0.264836 | 7/15/2014 | 14:27.2 | 5 |
| -1740.718 | 19601.564 | 2.065 | 13.626 | 0.256336 | 7/15/2014 | 14:27.3 | 5 |
| -1741.13 | 19601.552 | 2.061 | 13.637 | 0.273332 | 7/15/2014 | 14:27.4 | 5 |
| -1741.365 | 19601.541 | 2.059 | 13.645 | 0.255916 | 7/15/2014 | 14:27.5 | 5 |
| -1741.6 | 19601.533 | 2.057 | 13.654 | 0.253597 | 7/15/2014 | 14:27.6 | 5 |
| -1741.895 | 19601.523 | 2.057 | 13.656 | 0.244319 | 7/15/2014 | 14:27.7 | 5 |
| -1742.014 | 19601.52 | 2.056 | 13.657 | 0.219426 | 7/15/2014 | 14:27.8 | 5 |
| -1742.251 | 19601.512 | 2.056 | 13.661 | 0.234516 | 7/15/2014 | 14:27.9 | 5 |
| -1742.488 | 19601.502 | 2.053 | 13.661 | 0.250984 | 7/15/2014 | 14:28.0 | 5 |
| -1742.728 | 19601.494 | 2.056 | 13.661 | 0.235914 | 7/15/2014 | 14:28.1 | 5 |
| -1742.965 | 19601.484 | 2.056 | 13.651 | 0.240902 | 7/15/2014 | 14:28.2 | 5 |
| -1743.203 | 19601.476 | 2.058 | 13.644 | 0.23075 | 7/15/2014 | 14:28.3 | 5 |
| -1743.618 | 19601.461 | 2.054 | 13.635 | 0.260746 | 7/15/2014 | 14:28.4 | 5 |
| -1743.854 | 19601.451 | 2.054 | 13.632 | 0.254861 | 7/15/2014 | 14:28.5 | 5 |
| -1744.091 | 19601.441 | 2.052 | 13.621 | 0.242607 | 7/15/2014 | 14:28.6 | 5 |
| -1744.387 | 19601.431 | 2.05 | 13.607 | 0.263231 | 7/15/2014 | 14:28.7 | 5 |
| -1744.507 | 19601.427 | 2.048 | 13.6 | 0.265016 | 7/15/2014 | 14:28.8 | 5 |
| -1744.746 | 19601.417 | 2.046 | 13.592 | 0.260561 | 7/15/2014 | 14:28.9 | 5 |
| -1744.984 | 19601.407 | 2.044 | 13.587 | 0.254615 | 7/15/2014 | 14:29.0 | 5 |
| -1745.224 | 19601.398 | 2.044 | 13.59 | 0.240405 | 7/15/2014 | 14:29.1 | 5 |
| -1745.462 | 19601.389 | 2.044 | 13.593 | 0.245677 | 7/15/2014 | 14:29.2 | 5 |
| -1745.701 | 19601.379 | 2.044 | 13.602 | 0.248133 | 7/15/2014 | 14:29.3 | 5 |
| -1746.116 | 19601.362 | 2.048 | 13.623 | 0.211383 | 7/15/2014 | 14:29.4 | 5 |
| -1746.352 | 19601.353 | 2.048 | 13.636 | 0.238632 | 7/15/2014 | 14:29.5 | 5 |
| -1746.589 | 19601.343 | 2.05 | 13.641 | 0.224267 | 7/15/2014 | 14:29.6 | 5 |

| | | | | | | | |
|---|---|---|---|---|---|---|---|
| -1746.884 | 19601.33 | 2.05 | 13.639 | 0.208874 | 7/15/2014 | 14:29.7 | 5 |
| -1747.004 | 19601.326 | 2.051 | 13.639 | 0.202743 | 7/15/2014 | 14:29.8 | 5 |
| -1747.241 | 19601.316 | 2.053 | 13.635 | 0.201226 | 7/15/2014 | 14:29.9 | 5 |
| -1747.478 | 19601.306 | 2.053 | 13.618 | 0.212769 | 7/15/2014 | 14:30.0 | 5 |
| -1747.715 | 19601.296 | 2.048 | 13.603 | 0.22168 | 7/15/2014 | 14:30.1 | 5 |
| -1747.951 | 19601.286 | 2.046 | 13.583 | 0.208104 | 7/15/2014 | 14:30.2 | 5 |
| -1748.188 | 19601.275 | 2.051 | 13.575 | 0.197516 | 7/15/2014 | 14:30.3 | 5 |
| -1748.599 | 19601.258 | 2.05 | 13.573 | 0.20554 | 7/15/2014 | 14:30.4 | 5 |
| -1748.832 | 19601.25 | 2.047 | 13.578 | 0.201105 | 7/15/2014 | 14:30.5 | 5 |
| -1749.066 | 19601.238 | 2.045 | 13.576 | 0.200488 | 7/15/2014 | 14:30.6 | 5 |
| -1749.357 | 19601.226 | 2.047 | 13.582 | 0.191621 | 7/15/2014 | 14:30.7 | 5 |
| -1749.476 | 19601.221 | 2.05 | 13.587 | 0.189857 | 7/15/2014 | 14:30.8 | 5 |
| -1749.711 | 19601.211 | 2.052 | 13.595 | 0.199993 | 7/15/2014 | 14:30.9 | 5 |
| -1749.946 | 19601.201 | 2.052 | 13.597 | 0.208485 | 7/15/2014 | 14:31.0 | 5 |
| -1750.241 | 19601.188 | 2.054 | 13.602 | 0.201656 | 7/15/2014 | 14:31.1 | 5 |
| -1750.416 | 19601.182 | 2.05 | 13.61 | 0.200325 | 7/15/2014 | 14:31.2 | 5 |
| -1750.651 | 19601.17 | 2.053 | 13.613 | 0.202046 | 7/15/2014 | 14:31.3 | 5 |
| -1751.059 | 19601.152 | 2.054 | 13.625 | 0.21203 | 7/15/2014 | 14:31.4 | 5 |
| -1751.291 | 19601.143 | 2.054 | 13.63 | 0.198702 | 7/15/2014 | 14:31.5 | 5 |
| -1751.523 | 19601.132 | 2.052 | 13.641 | 0.196414 | 7/15/2014 | 14:31.6 | 5 |
| -1751.87 | 19601.117 | 2.048 | 13.649 | 0.199576 | 7/15/2014 | 14:31.7 | 5 |
| -1751.928 | 19601.115 | 2.046 | 13.654 | 0.198328 | 7/15/2014 | 14:31.8 | 5 |
| -1752.162 | 19601.104 | 2.051 | 13.658 | 0.186445 | 7/15/2014 | 14:31.9 | 5 |
| -1752.51 | 19601.089 | 2.054 | 13.664 | 0.190402 | 7/15/2014 | 14:32.0 | 5 |
| -1752.742 | 19601.078 | 2.054 | 13.668 | 0.183501 | 7/15/2014 | 14:32.1 | 5 |
| -1753.031 | 19601.064 | 2.052 | 13.674 | 0.182984 | 7/15/2014 | 14:32.2 | 5 |
| -1753.146 | 19601.06 | 2.051 | 13.679 | 0.18425 | 7/15/2014 | 14:32.3 | 5 |
| -1753.49 | 19601.045 | 2.048 | 13.693 | 0.190393 | 7/15/2014 | 14:32.4 | 5 |
| -1753.72 | 19601.035 | 2.05 | 13.699 | 0.202083 | 7/15/2014 | 14:32.5 | 5 |
| -1753.949 | 19601.024 | 2.048 | 13.705 | 0.183266 | 7/15/2014 | 14:32.6 | 5 |
| -1754.237 | 19601.013 | 2.046 | 13.709 | 0.180082 | 7/15/2014 | 14:32.7 | 5 |
| -1754.468 | 19601.002 | 2.04 | 13.715 | 0.17966 | 7/15/2014 | 14:32.8 | 5 |
| -1754.758 | 19600.988 | 2.037 | 13.711 | 0.181052 | 7/15/2014 | 14:32.9 | 5 |
| -1754.816 | 19600.986 | 2.036 | 13.715 | 0.17562 | 7/15/2014 | 14:33.0 | 5 |
| -1755.05 | 19600.977 | 2.039 | 13.7 | 0.177248 | 7/15/2014 | 14:33.1 | 5 |
| -1755.283 | 19600.967 | 2.036 | 13.689 | 0.169039 | 7/15/2014 | 14:33.2 | 5 |
| -1755.517 | 19600.955 | 2.034 | 13.674 | 0.173715 | 7/15/2014 | 14:33.3 | 5 |
| -1755.927 | 19600.938 | 2.028 | 13.662 | 0.163226 | 7/15/2014 | 14:33.4 | 5 |
| -1756.16 | 19600.929 | 2.028 | 13.662 | 0.162078 | 7/15/2014 | 14:33.5 | 5 |
| -1756.394 | 19600.918 | 2.028 | 13.657 | 0.155931 | 7/15/2014 | 14:33.6 | 5 |
| -1756.687 | 19600.906 | 2.025 | 13.66 | 0.145848 | 7/15/2014 | 14:33.7 | 5 |
| -1756.806 | 19600.9 | 2.022 | 13.662 | 0.141159 | 7/15/2014 | 14:33.8 | 5 |
| -1757.042 | 19600.89 | 2.022 | 13.672 | 0.133291 | 7/15/2014 | 14:33.9 | 5 |
| -1757.279 | 19600.881 | 2.022 | 13.686 | 0.143793 | 7/15/2014 | 14:34.0 | 5 |
| -1757.516 | 19600.869 | 2.028 | 13.703 | 0.155591 | 7/15/2014 | 14:34.1 | 5 |
| -1757.752 | 19600.86 | 2.03 | 13.712 | 0.156167 | 7/15/2014 | 14:34.2 | 5 |
| -1757.988 | 19600.85 | 2.027 | 13.722 | 0.172795 | 7/15/2014 | 14:34.3 | 5 |

| | | | | | | | |
|---|---|---|---|---|---|---|---|
| -1758.4 | 19600.832 | 2.02 | 13.741 | 0.219957 | 7/15/2014 | 14:34.4 | 5 |
| -1758.634 | 19600.822 | 2.02 | 13.754 | 0.201498 | 7/15/2014 | 14:34.5 | 5 |
| -1758.87 | 19600.813 | 2.02 | 13.763 | 0.18997 | 7/15/2014 | 14:34.6 | 5 |
| -1759.162 | 19600.799 | 2.017 | 13.776 | 0.169037 | 7/15/2014 | 14:34.7 | 5 |
| -1759.281 | 19600.795 | 2.013 | 13.779 | 0.173097 | 7/15/2014 | 14:34.8 | 5 |
| -1759.517 | 19600.785 | 2.011 | 13.788 | 0.181364 | 7/15/2014 | 14:34.9 | 5 |
| -1759.751 | 19600.774 | 2.007 | 13.785 | 0.190431 | 7/15/2014 | 14:35.0 | 5 |
| -1760.049 | 19600.762 | 2.011 | 13.782 | 0.148547 | 7/15/2014 | 14:35.1 | 5 |
| -1760.225 | 19600.755 | 2.007 | 13.783 | 0.161057 | 7/15/2014 | 14:35.2 | 5 |
| -1760.462 | 19600.745 | 2.007 | 13.789 | 0.15615 | 7/15/2014 | 14:35.3 | 5 |
| -1760.873 | 19600.728 | 2.001 | 13.785 | 0.160557 | 7/15/2014 | 14:35.4 | 5 |
| -1761.108 | 19600.719 | 2.001 | 13.779 | 0.148346 | 7/15/2014 | 14:35.5 | 5 |
| -1761.343 | 19600.708 | 2.001 | 13.777 | 0.150145 | 7/15/2014 | 14:35.6 | 5 |
| -1761.698 | 19600.692 | 2.008 | 13.782 | 0.15139 | 7/15/2014 | 14:35.7 | 5 |
| -1761.758 | 19600.69 | 2.008 | 13.783 | 0.147113 | 7/15/2014 | 14:35.8 | 5 |
| -1761.996 | 19600.681 | 2.008 | 13.788 | 0.149557 | 7/15/2014 | 14:35.9 | 5 |
| -1762.235 | 19600.67 | 2.006 | 13.784 | 0.160277 | 7/15/2014 | 14:36.0 | 5 |
| -1762.477 | 19600.661 | 2.016 | 13.792 | 0.145225 | 7/15/2014 | 14:36.1 | 5 |
| -1762.716 | 19600.65 | 2.008 | 13.802 | 0.152773 | 7/15/2014 | 14:36.2 | 5 |
| -1762.959 | 19600.64 | 2.008 | 13.81 | 0.15093 | 7/15/2014 | 14:36.3 | 5 |
| -1763.382 | 19600.622 | 2.001 | 13.812 | 0.157814 | 7/15/2014 | 14:36.4 | 5 |
| -1763.625 | 19600.611 | 2.003 | 13.81 | 0.161327 | 7/15/2014 | 14:36.5 | 5 |
| -1763.867 | 19600.601 | 2.006 | 13.807 | 0.160013 | 7/15/2014 | 14:36.6 | 5 |
| -1764.171 | 19600.589 | 2.009 | 13.803 | 0.167664 | 7/15/2014 | 14:36.7 | 5 |
| -1764.292 | 19600.583 | 2.006 | 13.8 | 0.160713 | 7/15/2014 | 14:36.8 | 5 |
| -1764.538 | 19600.572 | 2.015 | 13.789 | 0.15253 | 7/15/2014 | 14:36.9 | 5 |
| -1764.782 | 19600.563 | 2.015 | 13.78 | 0.151861 | 7/15/2014 | 14:37.0 | 5 |
| -1765.028 | 19600.552 | 2.023 | 13.774 | 0.143173 | 7/15/2014 | 14:37.1 | 5 |
| -1765.274 | 19600.541 | 2.027 | 13.761 | 0.14051 | 7/15/2014 | 14:37.2 | 5 |
| -1765.52 | 19600.53 | 2.023 | 13.759 | 0.137305 | 7/15/2014 | 14:37.3 | 5 |
| -1765.945 | 19600.513 | 2.023 | 13.766 | 0.13423 | 7/15/2014 | 14:37.4 | 5 |
| -1766.188 | 19600.502 | 2.023 | 13.769 | 0.138599 | 7/15/2014 | 14:37.5 | 5 |
| -1766.432 | 19600.491 | 2.019 | 13.775 | 0.13509 | 7/15/2014 | 14:37.6 | 5 |
| -1766.735 | 19600.479 | 2.019 | 13.787 | 0.137664 | 7/15/2014 | 14:37.7 | 5 |
| -1766.858 | 19600.474 | 2.018 | 13.794 | 0.137416 | 7/15/2014 | 14:37.8 | 5 |
| -1767.104 | 19600.463 | 2.018 | 13.811 | 0.135115 | 7/15/2014 | 14:37.9 | 5 |
| -1767.348 | 19600.451 | 2.016 | 13.82 | 0.132123 | 7/15/2014 | 14:38.0 | 5 |
| -1767.594 | 19600.443 | 2.013 | 13.825 | 0.129213 | 7/15/2014 | 14:38.1 | 5 |
| -1767.84 | 19600.432 | 2.017 | 13.834 | 0.124917 | 7/15/2014 | 14:38.2 | 5 |
| -1768.086 | 19600.421 | 2.023 | 13.843 | 0.119919 | 7/15/2014 | 14:38.3 | 5 |
| -1768.513 | 19600.403 | 2.025 | 13.848 | 0.117328 | 7/15/2014 | 14:38.4 | 5 |
| -1768.815 | 19600.391 | 2.025 | 13.849 | 0.116479 | 7/15/2014 | 14:38.5 | 5 |
| -1768.938 | 19600.385 | 2.024 | 13.849 | 0.114155 | 7/15/2014 | 14:38.6 | 5 |
| -1769.299 | 19600.371 | 2.032 | 13.857 | 0.110597 | 7/15/2014 | 14:38.7 | 5 |
| -1769.422 | 19600.365 | 2.033 | 13.86 | 0.108964 | 7/15/2014 | 14:38.8 | 5 |
| -1769.664 | 19600.354 | 2.033 | 13.868 | 0.118343 | 7/15/2014 | 14:38.9 | 5 |
| -1769.905 | 19600.344 | 2.03 | 13.873 | 0.103822 | 7/15/2014 | 14:39.0 | 5 |

| | | | | | | | |
|---|---|---|---|---|---|---|---|
| -1770.146 | 19600.334 | 2.03 | 13.877 | 0.112545 | 7/15/2014 | 14:39.1 | 5 |
| -1770.563 | 19600.316 | 2.034 | 13.871 | 0.107572 | 7/15/2014 | 14:39.2 | 5 |
| -1770.623 | 19600.314 | 2.038 | 13.873 | 0.110219 | 7/15/2014 | 14:39.3 | 5 |
| -1771.034 | 19600.297 | 2.035 | 13.866 | 0.118075 | 7/15/2014 | 14:39.4 | 5 |
| -1771.267 | 19600.285 | 2.031 | 13.87 | 0.107083 | 7/15/2014 | 14:39.5 | 5 |
| -1771.5 | 19600.276 | 2.035 | 13.872 | 0.132965 | 7/15/2014 | 14:39.6 | 5 |
| -1771.789 | 19600.264 | 2.038 | 13.867 | 0.111335 | 7/15/2014 | 14:39.7 | 5 |
| -1771.907 | 19600.259 | 2.039 | 13.864 | 0.10343 | 7/15/2014 | 14:39.8 | 5 |
| -1772.139 | 19600.25 | 2.041 | 13.868 | 0.114483 | 7/15/2014 | 14:39.9 | 5 |
| -1772.487 | 19600.234 | 2.042 | 13.87 | 0.112729 | 7/15/2014 | 14:40.0 | 5 |
| -1772.722 | 19600.225 | 2.044 | 13.874 | 0.104502 | 7/15/2014 | 14:40.1 | 5 |
| -1772.953 | 19600.215 | 2.044 | 13.87 | 0.0955496 | 7/15/2014 | 14:40.2 | 5 |
| -1773.185 | 19600.205 | 2.047 | 13.866 | 0.0969926 | 7/15/2014 | 14:40.3 | 5 |
| -1773.471 | 19600.193 | 2.043 | 13.863 | 0.0950116 | 7/15/2014 | 14:40.4 | 5 |
| -1773.702 | 19600.184 | 2.038 | 13.867 | 0.0984014 | 7/15/2014 | 14:40.5 | 5 |
| -1773.932 | 19600.174 | 2.033 | 13.873 | 0.108768 | 7/15/2014 | 14:40.6 | 5 |
| -1774.222 | 19600.162 | 2.031 | 13.886 | 0.0999093 | 7/15/2014 | 14:40.7 | 5 |
| -1774.339 | 19600.156 | 2.029 | 13.883 | 0.101834 | 7/15/2014 | 14:40.8 | 5 |
| -1774.573 | 19600.148 | 2.029 | 13.897 | 0.125219 | 7/15/2014 | 14:40.9 | 5 |
| -1774.807 | 19600.137 | 2.029 | 13.918 | 0.114232 | 7/15/2014 | 14:41.0 | 5 |
| -1775.044 | 19600.129 | 2.032 | 13.932 | 0.121747 | 7/15/2014 | 14:41.1 | 5 |
| -1775.279 | 19600.117 | 2.028 | 13.948 | 0.12122 | 7/15/2014 | 14:41.2 | 5 |
| -1775.515 | 19600.109 | 2.032 | 13.971 | 0.111944 | 7/15/2014 | 14:41.3 | 5 |
| -1775.93 | 19600.091 | 2.038 | 13.992 | 0.113055 | 7/15/2014 | 14:41.4 | 5 |
| -1776.165 | 19600.082 | 2.04 | 14.006 | 0.115906 | 7/15/2014 | 14:41.5 | 5 |
| -1776.401 | 19600.072 | 2.038 | 14.016 | 0.102841 | 7/15/2014 | 14:41.6 | 5 |
| -1776.695 | 19600.061 | 2.036 | 14.023 | 0.100936 | 7/15/2014 | 14:41.7 | 5 |
| -1776.815 | 19600.055 | 2.035 | 14.032 | 0.0974295 | 7/15/2014 | 14:41.8 | 5 |
| -1777.053 | 19600.047 | 2.039 | 14.044 | 0.108858 | 7/15/2014 | 14:41.9 | 5 |
| -1777.29 | 19600.035 | 2.041 | 14.054 | 0.0952635 | 7/15/2014 | 14:42.0 | 5 |
| -1777.528 | 19600.026 | 2.038 | 14.073 | 0.10285 | 7/15/2014 | 14:42.1 | 5 |
| -1777.763 | 19600.017 | 2.027 | 14.087 | 0.100438 | 7/15/2014 | 14:42.2 | 5 |
| -1777.998 | 19600.008 | 2.021 | 14.103 | 0.111916 | 7/15/2014 | 14:42.3 | 5 |
| -1778.406 | 19599.992 | 2.018 | 14.121 | 0.120131 | 7/15/2014 | 14:42.4 | 5 |
| -1778.641 | 19599.983 | 2.017 | 14.14 | 0.11063 | 7/15/2014 | 14:42.5 | 5 |
| -1778.873 | 19599.973 | 2.017 | 14.155 | 0.123055 | 7/15/2014 | 14:42.6 | 5 |
| -1779.165 | 19599.963 | 2.015 | 14.159 | 0.131706 | 7/15/2014 | 14:42.7 | 5 |
| -1779.283 | 19599.958 | 2.017 | 14.159 | 0.12562 | 7/15/2014 | 14:42.8 | 5 |
| -1779.517 | 19599.949 | 2.023 | 14.162 | 0.138578 | 7/15/2014 | 14:42.9 | 5 |
| -1779.753 | 19599.941 | 2.029 | 14.162 | 0.130011 | 7/15/2014 | 14:43.0 | 5 |
| -1779.988 | 19599.932 | 2.038 | 14.158 | 0.11489 | 7/15/2014 | 14:43.1 | 5 |
| -1780.224 | 19599.923 | 2.04 | 14.146 | 0.118304 | 7/15/2014 | 14:43.2 | 5 |
| -1780.46 | 19599.914 | 2.04 | 14.133 | 0.109572 | 7/15/2014 | 14:43.3 | 5 |
| -1780.872 | 19599.9 | 2.041 | 14.102 | 0.100741 | 7/15/2014 | 14:43.4 | 5 |
| -1781.107 | 19599.892 | 2.038 | 14.087 | 0.0942437 | 7/15/2014 | 14:43.5 | 5 |
| -1781.342 | 19599.883 | 2.038 | 14.068 | 0.0878841 | 7/15/2014 | 14:43.6 | 5 |
| -1781.636 | 19599.871 | 2.032 | 14.055 | 0.0864686 | 7/15/2014 | 14:43.7 | 5 |

| | | | | | | | |
|---|---|---|---|---|---|---|---|
| -1781.756 | 19599.867 | 2.028 | 14.046 | 0.0834572 | 7/15/2014 | 14:43.8 | 5 |
| -1781.993 | 19599.859 | 2.028 | 14.035 | 0.0814708 | 7/15/2014 | 14:43.9 | 5 |
| -1782.23 | 19599.852 | 2.028 | 14.022 | 0.0801276 | 7/15/2014 | 14:44.0 | 5 |
| -1782.467 | 19599.844 | 2.028 | 14.016 | 0.0737044 | 7/15/2014 | 14:44.1 | 5 |
| -1782.704 | 19599.835 | 2.026 | 14.013 | 0.0733363 | 7/15/2014 | 14:44.2 | 5 |
| -1782.94 | 19599.826 | 2.029 | 14.013 | 0.0714789 | 7/15/2014 | 14:44.3 | 5 |
| -1783.351 | 19599.813 | 2.031 | 14.015 | 0.0767085 | 7/15/2014 | 14:44.4 | 5 |
| -1783.585 | 19599.805 | 2.034 | 14.016 | 0.102048 | 7/15/2014 | 14:44.5 | 5 |
| -1783.819 | 19599.797 | 2.034 | 14.017 | 0.0973023 | 7/15/2014 | 14:44.6 | 5 |
| -1784.109 | 19599.787 | 2.037 | 14.019 | 0.110782 | 7/15/2014 | 14:44.7 | 5 |
| -1784.228 | 19599.783 | 2.038 | 14.018 | 0.13468 | 7/15/2014 | 14:44.8 | 5 |
| -1784.462 | 19599.775 | 2.043 | 14.018 | 0.155394 | 7/15/2014 | 14:44.9 | 5 |
| -1784.695 | 19599.769 | 2.047 | 14.016 | 0.148308 | 7/15/2014 | 14:45.0 | 5 |
| -1784.929 | 19599.762 | 2.05 | 14.016 | 0.151099 | 7/15/2014 | 14:45.1 | 5 |
| -1785.277 | 19599.75 | 2.049 | 14.017 | 0.142125 | 7/15/2014 | 14:45.2 | 5 |
| -1785.509 | 19599.742 | 2.049 | 14.025 | 0.135339 | 7/15/2014 | 14:45.3 | 5 |
| -1785.797 | 19599.733 | 2.049 | 14.023 | 0.11682 | 7/15/2014 | 14:45.4 | 5 |
| -1786.027 | 19599.727 | 2.047 | 14.024 | 0.101485 | 7/15/2014 | 14:45.5 | 5 |
| -1786.257 | 19599.72 | 2.047 | 14.021 | 0.105834 | 7/15/2014 | 14:45.6 | 5 |
| -1786.545 | 19599.712 | 2.045 | 14.026 | 0.105342 | 7/15/2014 | 14:45.7 | 5 |
| -1786.661 | 19599.708 | 2.043 | 14.027 | 0.0966209 | 7/15/2014 | 14:45.8 | 5 |
| -1786.894 | 19599.701 | 2.047 | 14.031 | 0.0955055 | 7/15/2014 | 14:45.9 | 5 |
| -1787.126 | 19599.693 | 2.045 | 14.024 | 0.101655 | 7/15/2014 | 14:46.0 | 5 |
| -1787.361 | 19599.688 | 2.05 | 14.015 | 0.111477 | 7/15/2014 | 14:46.1 | 5 |
| -1787.594 | 19599.682 | 2.047 | 14.022 | 0.107859 | 7/15/2014 | 14:46.2 | 5 |
| -1787.83 | 19599.674 | 2.052 | 14.03 | 0.110932 | 7/15/2014 | 14:46.3 | 5 |
| -1788.238 | 19599.663 | 2.051 | 14.046 | 0.1399 | 7/15/2014 | 14:46.4 | 5 |
| -1788.471 | 19599.657 | 2.053 | 14.045 | 0.137686 | 7/15/2014 | 14:46.5 | 5 |
| -1788.705 | 19599.65 | 2.051 | 14.048 | 0.158468 | 7/15/2014 | 14:46.6 | 5 |
| -1788.994 | 19599.643 | 2.049 | 14.051 | 0.128103 | 7/15/2014 | 14:46.7 | 5 |
| -1789.113 | 19599.64 | 2.047 | 14.056 | 0.129937 | 7/15/2014 | 14:46.8 | 5 |
| -1789.349 | 19599.633 | 2.05 | 14.049 | 0.13365 | 7/15/2014 | 14:46.9 | 5 |
| -1789.582 | 19599.628 | 2.045 | 14.048 | 0.115303 | 7/15/2014 | 14:47.0 | 5 |
| -1789.816 | 19599.621 | 2.043 | 14.034 | 0.10559 | 7/15/2014 | 14:47.1 | 5 |
| -1790.048 | 19599.615 | 2.038 | 14.019 | 0.107788 | 7/15/2014 | 14:47.2 | 5 |
| -1790.281 | 19599.609 | 2.033 | 14.011 | 0.101627 | 7/15/2014 | 14:47.3 | 5 |
| -1790.684 | 19599.599 | 2.032 | 13.989 | 0.102398 | 7/15/2014 | 14:47.4 | 5 |
| -1790.914 | 19599.594 | 2.032 | 13.973 | 0.0928286 | 7/15/2014 | 14:47.5 | 5 |
| -1791.143 | 19599.589 | 2.03 | 13.96 | 0.101566 | 7/15/2014 | 14:47.6 | 5 |
| -1791.43 | 19599.582 | 2.03 | 13.941 | 0.106312 | 7/15/2014 | 14:47.7 | 5 |
| -1791.661 | 19599.576 | 2.032 | 13.937 | 0.0872939 | 7/15/2014 | 14:47.8 | 5 |
| -1791.893 | 19599.57 | 2.032 | 13.923 | 0.106813 | 7/15/2014 | 14:47.9 | 5 |
| -1792.125 | 19599.565 | 2.029 | 13.909 | 0.0965585 | 7/15/2014 | 14:48.0 | 5 |
| -1792.416 | 19599.559 | 2.029 | 13.898 | 0.0858748 | 7/15/2014 | 14:48.1 | 5 |
| -1792.53 | 19599.557 | 2.027 | 13.896 | 0.0878711 | 7/15/2014 | 14:48.2 | 5 |
| -1792.762 | 19599.553 | 2.029 | 13.887 | 0.0808949 | 7/15/2014 | 14:48.3 | 5 |
| -1793.106 | 19599.546 | 2.029 | 13.89 | 0.0779013 | 7/15/2014 | 14:48.4 | 5 |

| | | | | | | | |
|---|---|---|---|---|---|---|---|
| -1793.335 | 19599.541 | 2.031 | 13.887 | 0.0799082 | 7/15/2014 | 14:48.5 | 5 |
| -1793.564 | 19599.536 | 2.029 | 13.885 | 0.0709493 | 7/15/2014 | 14:48.6 | 5 |
| -1793.848 | 19599.531 | 2.026 | 13.888 | 0.0722353 | 7/15/2014 | 14:48.7 | 5 |
| -1794.137 | 19599.525 | 2.031 | 13.893 | 0.100252 | 7/15/2014 | 14:48.8 | 5 |
| -1794.366 | 19599.521 | 2.031 | 13.901 | 0.0960994 | 7/15/2014 | 14:48.9 | 5 |
| -1794.537 | 19599.52 | 2.031 | 13.915 | 0.0895176 | 7/15/2014 | 14:49.0 | 5 |
| -1794.764 | 19599.516 | 2.028 | 13.931 | 0.0984292 | 7/15/2014 | 14:49.1 | 5 |
| -1794.992 | 19599.513 | 2.03 | 13.931 | 0.104048 | 7/15/2014 | 14:49.2 | 5 |
| -1795.218 | 19599.509 | 2.028 | 13.946 | 0.128546 | 7/15/2014 | 14:49.3 | 5 |
| -1795.5 | 19599.505 | 2.03 | 13.963 | 0.122463 | 7/15/2014 | 14:49.4 | 5 |
| -1795.724 | 19599.502 | 2.03 | 13.979 | 0.0979224 | 7/15/2014 | 14:49.5 | 5 |
| -1795.948 | 19599.499 | 2.03 | 13.992 | 0.101132 | 7/15/2014 | 14:49.6 | 5 |
| -1796.23 | 19599.496 | 2.03 | 14.005 | 0.101517 | 7/15/2014 | 14:49.7 | 5 |
| -1796.456 | 19599.493 | 2.027 | 14.009 | 0.120942 | 7/15/2014 | 14:49.8 | 5 |
| -1796.682 | 19599.491 | 2.027 | 14.012 | 0.0978134 | 7/15/2014 | 14:49.9 | 5 |
| -1796.911 | 19599.488 | 2.019 | 14.02 | 0.0813522 | 7/15/2014 | 14:50.0 | 5 |
| -1797.14 | 19599.487 | 2.016 | 14.017 | 0.0770444 | 7/15/2014 | 14:50.1 | 5 |
| -1797.368 | 19599.484 | 2.016 | 14.013 | 0.0948655 | 7/15/2014 | 14:50.2 | 5 |
| -1797.597 | 19599.483 | 2.02 | 14.017 | 0.105974 | 7/15/2014 | 14:50.3 | 5 |
| -1797.88 | 19599.48 | 2.017 | 14.009 | 0.11153 | 7/15/2014 | 14:50.4 | 5 |
| -1798.109 | 19599.479 | 2.014 | 14.001 | 0.0994756 | 7/15/2014 | 14:50.5 | 5 |
| -1798.338 | 19599.478 | 2.015 | 13.992 | 0.123101 | 7/15/2014 | 14:50.6 | 5 |
| -1798.624 | 19599.476 | 2.012 | 13.983 | 0.121046 | 7/15/2014 | 14:50.7 | 5 |
| -1798.74 | 19599.476 | 2.016 | 13.977 | 0.128363 | 7/15/2014 | 14:50.8 | 5 |
| -1798.974 | 19599.473 | 2.016 | 13.963 | 0.12231 | 7/15/2014 | 14:50.9 | 5 |
| -1799.207 | 19599.473 | 2.01 | 13.941 | 0.132263 | 7/15/2014 | 14:51.0 | 5 |
| -1799.44 | 19599.472 | 2.006 | 13.921 | 0.108581 | 7/15/2014 | 14:51.1 | 5 |
| -1799.673 | 19599.471 | 2.002 | 13.918 | 0.0974371 | 7/15/2014 | 14:51.2 | 5 |
| -1799.908 | 19599.471 | 2.008 | 13.913 | 0.0975409 | 7/15/2014 | 14:51.3 | 5 |
| -1800.316 | 19599.469 | 2.004 | 13.904 | 0.124809 | 7/15/2014 | 14:51.4 | 5 |
| -1800.55 | 19599.468 | 2.004 | 13.905 | 0.128541 | 7/15/2014 | 14:51.5 | 5 |
| -1800.784 | 19599.468 | 2.01 | 13.914 | 0.128156 | 7/15/2014 | 14:51.6 | 5 |
| -1801.076 | 19599.468 | 2.01 | 13.922 | 0.142712 | 7/15/2014 | 14:51.7 | 5 |
| -1801.193 | 19599.468 | 2.011 | 13.923 | 0.147976 | 7/15/2014 | 14:51.8 | 5 |
| -1801.431 | 19599.469 | 2.014 | 13.92 | 0.132477 | 7/15/2014 | 14:51.9 | 5 |
| -1801.667 | 19599.469 | 2.008 | 13.914 | 0.142983 | 7/15/2014 | 14:52.0 | 5 |
| -1801.903 | 19599.468 | 2.008 | 13.912 | 0.120738 | 7/15/2014 | 14:52.1 | 5 |
| -1802.137 | 19599.468 | 2.008 | 13.916 | 0.136603 | 7/15/2014 | 14:52.2 | 5 |
| -1802.374 | 19599.468 | 2.012 | 13.913 | 0.127017 | 7/15/2014 | 14:52.3 | 5 |
| -1802.783 | 19599.47 | 2.009 | 13.901 | 0.123166 | 7/15/2014 | 14:52.4 | 5 |
| -1803.018 | 19599.471 | 2.012 | 13.902 | 0.144613 | 7/15/2014 | 14:52.5 | 5 |
| -1803.251 | 19599.472 | 2.012 | 13.899 | 0.128102 | 7/15/2014 | 14:52.6 | 5 |
| -1803.542 | 19599.473 | 2.015 | 13.915 | 0.121986 | 7/15/2014 | 14:52.7 | 5 |
| -1803.659 | 19599.473 | 2.017 | 13.921 | 0.110735 | 7/15/2014 | 14:52.8 | 5 |
| -1803.895 | 19599.474 | 2.017 | 13.927 | 0.121955 | 7/15/2014 | 14:52.9 | 5 |
| -1804.127 | 19599.476 | 2.007 | 13.933 | 0.127412 | 7/15/2014 | 14:53.0 | 5 |
| -1804.363 | 19599.477 | 2.007 | 13.95 | 0.115366 | 7/15/2014 | 14:53.1 | 5 |

| | | | | | | | |
|---|---|---|---|---|---|---|---|
| -1804.597 | 19599.479 | 2.001 | 13.975 | 0.115903 | 7/15/2014 | 14:53.2 | 5 |
| -1804.83 | 19599.479 | 1.994 | 13.997 | 0.1262 | 7/15/2014 | 14:53.3 | 5 |
| -1805.236 | 19599.482 | 1.988 | 14.033 | 0.151759 | 7/15/2014 | 14:53.4 | 5 |
| -1805.467 | 19599.484 | 1.981 | 14.044 | 0.137566 | 7/15/2014 | 14:53.5 | 5 |
| -1805.699 | 19599.486 | 1.984 | 14.078 | 0.139708 | 7/15/2014 | 14:53.6 | 5 |
| -1805.99 | 19599.488 | 1.991 | 14.102 | 0.139662 | 7/15/2014 | 14:53.7 | 5 |
| -1806.107 | 19599.49 | 1.989 | 14.114 | 0.135783 | 7/15/2014 | 14:53.8 | 5 |
| -1806.341 | 19599.492 | 1.989 | 14.128 | 0.135085 | 7/15/2014 | 14:53.9 | 5 |
| -1806.575 | 19599.493 | 1.986 | 14.144 | 0.140731 | 7/15/2014 | 14:54.0 | 5 |
| -1806.811 | 19599.496 | 1.994 | 14.156 | 0.126733 | 7/15/2014 | 14:54.1 | 5 |
| -1807.044 | 19599.499 | 1.993 | 14.156 | 0.118721 | 7/15/2014 | 14:54.2 | 5 |
| -1807.282 | 19599.501 | 1.994 | 14.162 | 0.108302 | 7/15/2014 | 14:54.3 | 5 |
| -1807.694 | 19599.506 | 1.986 | 14.137 | 0.111916 | 7/15/2014 | 14:54.4 | 5 |
| -1807.928 | 19599.51 | 1.98 | 14.133 | 0.10293 | 7/15/2014 | 14:54.5 | 5 |
| -1808.163 | 19599.511 | 1.974 | 14.134 | 0.090077 | 7/15/2014 | 14:54.6 | 5 |
| -1808.459 | 19599.516 | 1.97 | 14.121 | 0.0937055 | 7/15/2014 | 14:54.7 | 5 |
| -1808.577 | 19599.518 | 1.96 | 14.119 | 0.0972121 | 7/15/2014 | 14:54.8 | 5 |
| -1808.818 | 19599.521 | 1.961 | 14.115 | 0.0891369 | 7/15/2014 | 14:54.9 | 5 |
| -1809.054 | 19599.523 | 1.957 | 14.118 | 0.097567 | 7/15/2014 | 14:55.0 | 5 |
| -1809.296 | 19599.527 | 1.955 | 14.113 | 0.0834339 | 7/15/2014 | 14:55.1 | 5 |
| -1809.533 | 19599.531 | 1.945 | 14.125 | 0.0857161 | 7/15/2014 | 14:55.2 | 5 |
| -1809.772 | 19599.534 | 1.945 | 14.138 | 0.111939 | 7/15/2014 | 14:55.3 | 5 |
| -1810.19 | 19599.541 | 1.947 | 14.13 | 0.105696 | 7/15/2014 | 14:55.4 | 5 |
| -1810.428 | 19599.545 | 1.943 | 14.137 | 0.123339 | 7/15/2014 | 14:55.5 | 5 |
| -1810.666 | 19599.549 | 1.934 | 14.143 | 0.101762 | 7/15/2014 | 14:55.6 | 5 |
| -1810.964 | 19599.555 | 1.927 | 14.146 | 0.119279 | 7/15/2014 | 14:55.7 | 5 |
| -1811.084 | 19599.557 | 1.922 | 14.155 | 0.124029 | 7/15/2014 | 14:55.8 | 5 |
| -1811.324 | 19599.561 | 1.922 | 14.166 | 0.121485 | 7/15/2014 | 14:55.9 | 5 |
| -1811.563 | 19599.564 | 1.917 | 14.161 | 0.137564 | 7/15/2014 | 14:56.0 | 5 |
| -1811.804 | 19599.568 | 1.914 | 14.17 | 0.138904 | 7/15/2014 | 14:56.1 | 5 |
| -1812.102 | 19599.574 | 1.909 | 14.183 | 0.132945 | 7/15/2014 | 14:56.2 | 5 |
| -1812.281 | 19599.578 | 1.909 | 14.193 | 0.149394 | 7/15/2014 | 14:56.3 | 5 |
| -1812.696 | 19599.587 | 1.907 | 14.204 | 0.152119 | 7/15/2014 | 14:56.4 | 5 |
| -1812.931 | 19599.592 | 1.904 | 14.207 | 0.140535 | 7/15/2014 | 14:56.5 | 5 |
| -1813.166 | 19599.596 | 1.904 | 14.217 | 0.140097 | 7/15/2014 | 14:56.6 | 5 |
| -1813.456 | 19599.603 | 1.904 | 14.23 | 0.136352 | 7/15/2014 | 14:56.7 | 5 |
| -1813.748 | 19599.609 | 1.9 | 14.231 | 0.127777 | 7/15/2014 | 14:56.8 | 5 |
| -1813.808 | 19599.611 | 1.9 | 14.235 | 0.132047 | 7/15/2014 | 14:56.9 | 5 |
| -1814.155 | 19599.619 | 1.896 | 14.228 | 0.127485 | 7/15/2014 | 14:57.0 | 5 |
| -1814.387 | 19599.624 | 1.893 | 14.222 | 0.131721 | 7/15/2014 | 14:57.1 | 5 |
| -1814.617 | 19599.631 | 1.89 | 14.21 | 0.143236 | 7/15/2014 | 14:57.2 | 5 |
| -1814.845 | 19599.635 | 1.895 | 14.207 | 0.146459 | 7/15/2014 | 14:57.3 | 5 |
| -1815.129 | 19599.644 | 1.897 | 14.19 | 0.12525 | 7/15/2014 | 14:57.4 | 5 |
| -1815.354 | 19599.649 | 1.897 | 14.169 | 0.165349 | 7/15/2014 | 14:57.5 | 5 |
| -1815.58 | 19599.654 | 1.899 | 14.164 | 0.15236 | 7/15/2014 | 14:57.6 | 5 |
| -1815.861 | 19599.663 | 1.902 | 14.151 | 0.151259 | 7/15/2014 | 14:57.7 | 5 |
| -1816.088 | 19599.668 | 1.908 | 14.15 | 0.168751 | 7/15/2014 | 14:57.8 | 5 |

| | | | | | | | |
|---|---|---|---|---|---|---|---|
| -1816.315 | 19599.676 | 1.911 | 14.155 | 0.145742 | 7/15/2014 | 14:57.9 | 5 |
| -1816.543 | 19599.681 | 1.916 | 14.147 | 0.164043 | 7/15/2014 | 14:58.0 | 5 |
| -1816.772 | 19599.688 | 1.914 | 14.136 | 0.180606 | 7/15/2014 | 14:58.1 | 5 |
| -1817 | 19599.694 | 1.912 | 14.129 | 0.148036 | 7/15/2014 | 14:58.2 | 5 |
| -1817.228 | 19599.7 | 1.912 | 14.13 | 0.146028 | 7/15/2014 | 14:58.3 | 5 |
| -1817.511 | 19599.708 | 1.917 | 14.115 | 0.153559 | 7/15/2014 | 14:58.4 | 5 |
| -1817.738 | 19599.715 | 1.919 | 14.097 | 0.129328 | 7/15/2014 | 14:58.5 | 5 |
| -1817.966 | 19599.723 | 1.917 | 14.084 | 0.138805 | 7/15/2014 | 14:58.6 | 5 |
| -1818.252 | 19599.73 | 1.917 | 14.065 | 0.122936 | 7/15/2014 | 14:58.7 | 5 |
| -1818.482 | 19599.736 | 1.917 | 14.056 | 0.120647 | 7/15/2014 | 14:58.8 | 5 |
| -1818.771 | 19599.746 | 1.919 | 14.049 | 0.120119 | 7/15/2014 | 14:58.9 | 5 |
| -1818.885 | 19599.749 | 1.92 | 14.039 | 0.120718 | 7/15/2014 | 14:59.0 | 5 |
| -1819.176 | 19599.758 | 1.921 | 14.033 | 0.114342 | 7/15/2014 | 14:59.1 | 5 |
| -1819.406 | 19599.766 | 1.918 | 14.038 | 0.118778 | 7/15/2014 | 14:59.2 | 5 |
| -1819.694 | 19599.773 | 1.92 | 14.042 | 0.112796 | 7/15/2014 | 14:59.3 | 5 |
| -1819.864 | 19599.778 | 1.921 | 14.046 | 0.111137 | 7/15/2014 | 14:59.4 | 5 |
| -1820.15 | 19599.787 | 1.933 | 14.054 | 0.114273 | 7/15/2014 | 14:59.5 | 5 |
| -1820.601 | 19599.803 | 1.931 | 14.063 | 0.127638 | 7/15/2014 | 14:59.7 | 5 |
| -1820.66 | 19599.805 | 1.933 | 14.065 | 0.135376 | 7/15/2014 | 14:59.7 | 5 |
| -1820.887 | 19599.813 | 1.938 | 14.067 | 0.140842 | 7/15/2014 | 14:59.8 | 5 |
| -1821.114 | 19599.819 | 1.944 | 14.074 | 0.139191 | 7/15/2014 | 14:59.9 | 5 |
| -1821.399 | 19599.827 | 1.944 | 14.078 | 0.16074 | 7/15/2014 | 15:00.0 | 5 |
| -1821.622 | 19599.834 | 1.941 | 14.099 | 0.166108 | 7/15/2014 | 15:00.1 | 5 |
| -1821.735 | 19599.838 | 1.939 | 14.1 | 0.183793 | 7/15/2014 | 15:00.2 | 5 |
| -1821.959 | 19599.846 | 1.935 | 14.109 | 0.195328 | 7/15/2014 | 15:00.3 | 5 |
| -1822.289 | 19599.857 | 1.936 | 14.116 | 0.238934 | 7/15/2014 | 15:00.4 | 5 |
| -1822.509 | 19599.865 | 1.941 | 14.125 | 0.245593 | 7/15/2014 | 15:00.5 | 5 |
| -1822.726 | 19599.872 | 1.936 | 14.129 | 0.267628 | 7/15/2014 | 15:00.6 | 5 |
| -1822.996 | 19599.88 | 1.936 | 14.134 | 0.287769 | 7/15/2014 | 15:00.7 | 5 |
| -1823.269 | 19599.889 | 1.941 | 14.145 | 0.320315 | 7/15/2014 | 15:00.8 | 5 |
| -1823.487 | 19599.896 | 1.947 | 14.131 | 0.335098 | 7/15/2014 | 15:00.9 | 5 |
| -1823.648 | 19599.902 | 1.952 | 14.125 | 0.350103 | 7/15/2014 | 15:01.0 | 5 |
| -1823.866 | 19599.909 | 1.957 | 14.118 | 0.342689 | 7/15/2014 | 15:01.1 | 5 |
| -1824.082 | 19599.916 | 1.96 | 14.116 | 0.317571 | 7/15/2014 | 15:01.2 | 5 |
| -1824.296 | 19599.924 | 1.962 | 14.11 | 0.298069 | 7/15/2014 | 15:01.3 | 5 |
| -1824.563 | 19599.932 | 1.97 | 14.093 | 0.26257 | 7/15/2014 | 15:01.4 | 5 |
| -1824.881 | 19599.941 | 1.975 | 14.067 | 0.245449 | 7/15/2014 | 15:01.6 | 5 |
| -1825.146 | 19599.951 | 1.975 | 14.042 | 0.248857 | 7/15/2014 | 15:01.7 | 5 |
| -1825.254 | 19599.955 | 1.977 | 14.035 | 0.264455 | 7/15/2014 | 15:01.7 | 5 |
| -1825.468 | 19599.96 | 1.977 | 14.022 | 0.293391 | 7/15/2014 | 15:01.8 | 5 |
| -1825.682 | 19599.967 | 1.979 | 14.009 | 0.291776 | 7/15/2014 | 15:01.9 | 5 |
| -1825.897 | 19599.975 | 1.979 | 13.995 | 0.297964 | 7/15/2014 | 15:02.0 | 5 |
| -1826.114 | 19599.981 | 1.982 | 13.986 | 0.27945 | 7/15/2014 | 15:02.1 | 5 |
| -1826.328 | 19599.987 | 1.982 | 13.985 | 0.285001 | 7/15/2014 | 15:02.2 | 5 |
| -1826.542 | 19599.994 | 1.982 | 13.978 | 0.254194 | 7/15/2014 | 15:02.3 | 5 |
| -1826.809 | 19600.003 | 1.985 | 13.976 | 0.246877 | 7/15/2014 | 15:02.4 | 5 |
| -1827.022 | 19600.008 | 1.985 | 13.982 | 0.224378 | 7/15/2014 | 15:02.5 | 5 |

| | | | | | | | |
|---|---|---|---|---|---|---|---|
| -1827.237 | 19600.016 | 1.988 | 13.986 | 0.213721 | 7/15/2014 | 15:02.6 | 5 |
| -1827.502 | 19600.023 | 1.988 | 13.985 | 0.208354 | 7/15/2014 | 15:02.7 | 5 |
| -1827.717 | 19600.03 | 1.991 | 13.985 | 0.200353 | 7/15/2014 | 15:02.8 | 5 |
| -1827.933 | 19600.035 | 1.988 | 13.98 | 0.196935 | 7/15/2014 | 15:02.9 | 5 |
| -1828.15 | 19600.043 | 1.994 | 13.977 | 0.220557 | 7/15/2014 | 15:03.0 | 5 |
| -1828.365 | 19600.048 | 1.997 | 13.982 | 0.22164 | 7/15/2014 | 15:03.1 | 5 |
| -1828.58 | 19600.055 | 1.997 | 13.983 | 0.228797 | 7/15/2014 | 15:03.2 | 5 |
| -1828.795 | 19600.062 | 2 | 13.979 | 0.242943 | 7/15/2014 | 15:03.3 | 5 |
| -1829.06 | 19600.068 | 2.003 | 13.979 | 0.245865 | 7/15/2014 | 15:03.4 | 5 |
| -1829.271 | 19600.075 | 2.006 | 13.979 | 0.260046 | 7/15/2014 | 15:03.5 | 5 |
| -1829.639 | 19600.085 | 2.015 | 13.97 | 0.239085 | 7/15/2014 | 15:03.7 | 5 |
| -1829.748 | 19600.089 | 2.02 | 13.967 | 0.233788 | 7/15/2014 | 15:03.7 | 5 |
| -1829.958 | 19600.094 | 2.018 | 13.96 | 0.200605 | 7/15/2014 | 15:03.8 | 5 |
| -1830.168 | 19600.1 | 2.018 | 13.95 | 0.195711 | 7/15/2014 | 15:03.9 | 5 |
| -1830.38 | 19600.106 | 2.024 | 13.952 | 0.172159 | 7/15/2014 | 15:04.0 | 5 |
| -1830.59 | 19600.111 | 2.026 | 13.948 | 0.177806 | 7/15/2014 | 15:04.1 | 5 |
| -1830.8 | 19600.117 | 2.028 | 13.944 | 0.168744 | 7/15/2014 | 15:04.2 | 5 |
| -1831.008 | 19600.123 | 2.028 | 13.942 | 0.154818 | 7/15/2014 | 15:04.3 | 5 |
| -1831.369 | 19600.134 | 2.022 | 13.949 | 0.17682 | 7/15/2014 | 15:04.5 | 5 |
| -1831.573 | 19600.139 | 2.017 | 13.955 | 0.162039 | 7/15/2014 | 15:04.6 | 5 |
| -1831.828 | 19600.145 | 2.014 | 13.961 | 0.170025 | 7/15/2014 | 15:04.7 | 5 |
| -1831.933 | 19600.148 | 2.016 | 13.967 | 0.17073 | 7/15/2014 | 15:04.7 | 5 |
| -1832.138 | 19600.154 | 2.011 | 13.967 | 0.155196 | 7/15/2014 | 15:04.8 | 5 |
| -1832.345 | 19600.159 | 2.009 | 13.978 | 0.159446 | 7/15/2014 | 15:04.9 | 5 |
| -1832.553 | 19600.164 | 2.006 | 13.988 | 0.162508 | 7/15/2014 | 15:05.0 | 5 |
| -1832.759 | 19600.17 | 2.006 | 13.998 | 0.133857 | 7/15/2014 | 15:05.1 | 5 |
| -1832.966 | 19600.176 | 2.006 | 14.006 | 0.123438 | 7/15/2014 | 15:05.2 | 5 |
| -1833.169 | 19600.18 | 2.01 | 14.008 | 0.125218 | 7/15/2014 | 15:05.3 | 5 |
| -1833.528 | 19600.189 | 2.016 | 14.021 | 0.117256 | 7/15/2014 | 15:05.5 | 5 |
| -1833.731 | 19600.195 | 2.021 | 14.036 | 0.119122 | 7/15/2014 | 15:05.6 | 5 |
| -1833.983 | 19600.201 | 2.021 | 14.047 | 0.0784255 | 7/15/2014 | 15:05.7 | 5 |
| -1834.088 | 19600.203 | 2.024 | 14.05 | 0.0695681 | 7/15/2014 | 15:05.7 | 5 |
| -1834.292 | 19600.208 | 2.02 | 14.059 | 0.0754097 | 7/15/2014 | 15:05.8 | 5 |
| -1834.497 | 19600.213 | 2.02 | 14.07 | 0.0744706 | 7/15/2014 | 15:05.9 | 5 |
| -1834.7 | 19600.218 | 2.015 | 14.082 | 0.0726427 | 7/15/2014 | 15:06.0 | 5 |
| -1834.906 | 19600.223 | 2.015 | 14.094 | 0.0553615 | 7/15/2014 | 15:06.1 | 5 |
| -1835.11 | 19600.229 | 2.013 | 14.104 | 0.037334 | 7/15/2014 | 15:06.2 | 5 |
| -1835.311 | 19600.232 | 2.01 | 14.111 | 0.0412189 | 7/15/2014 | 15:06.3 | 5 |
| -1835.712 | 19600.242 | 2.009 | 14.119 | 0.0406761 | 7/15/2014 | 15:06.5 | 5 |
| -1835.813 | 19600.244 | 2.01 | 14.122 | 0.0405099 | 7/15/2014 | 15:06.5 | 5 |
| -1836.113 | 19600.25 | 2.013 | 14.123 | 0.0669842 | 7/15/2014 | 15:06.7 | 5 |
| -1836.214 | 19600.253 | 2.015 | 14.125 | 0.0727927 | 7/15/2014 | 15:06.7 | 5 |
| -1836.418 | 19600.259 | 2.02 | 14.127 | 0.087217 | 7/15/2014 | 15:06.8 | 5 |
| -1836.622 | 19600.264 | 2.025 | 14.126 | 0.0813952 | 7/15/2014 | 15:06.9 | 5 |
| -1836.825 | 19600.268 | 2.027 | 14.12 | 0.0998241 | 7/15/2014 | 15:07.0 | 5 |
| -1837.029 | 19600.271 | 2.027 | 14.114 | 0.114108 | 7/15/2014 | 15:07.1 | 5 |
| -1837.386 | 19600.28 | 2.029 | 14.105 | 0.114952 | 7/15/2014 | 15:07.3 | 5 |

| | | | | | | | |
|---|---|---|---|---|---|---|---|
| -1837.437 | 19600.282 | 2.032 | 14.106 | 0.106713 | 7/15/2014 | 15:07.3 | 5 |
| -1837.789 | 19600.289 | 2.032 | 14.102 | 0.108574 | 7/15/2014 | 15:07.5 | 5 |
| -1837.994 | 19600.295 | 2.037 | 14.092 | 0.11233 | 7/15/2014 | 15:07.6 | 5 |
| -1838.246 | 19600.301 | 2.037 | 14.084 | 0.102 | 7/15/2014 | 15:07.7 | 5 |
| -1838.349 | 19600.303 | 2.036 | 14.081 | 0.101801 | 7/15/2014 | 15:07.7 | 5 |
| -1838.552 | 19600.307 | 2.036 | 14.08 | 0.115236 | 7/15/2014 | 15:07.8 | 5 |
| -1838.758 | 19600.312 | 2.044 | 14.08 | 0.0965459 | 7/15/2014 | 15:07.9 | 5 |
| -1838.962 | 19600.315 | 2.044 | 14.078 | 0.109036 | 7/15/2014 | 15:08.0 | 5 |
| -1839.166 | 19600.319 | 2.041 | 14.064 | 0.113891 | 7/15/2014 | 15:08.1 | 5 |
| -1839.37 | 19600.324 | 2.045 | 14.056 | 0.118399 | 7/15/2014 | 15:08.2 | 5 |
| -1839.572 | 19600.328 | 2.043 | 14.051 | 0.123352 | 7/15/2014 | 15:08.3 | 5 |
| -1839.975 | 19600.338 | 2.046 | 14.05 | 0.123864 | 7/15/2014 | 15:08.5 | 5 |
| -1840.076 | 19600.34 | 2.047 | 14.049 | 0.127594 | 7/15/2014 | 15:08.5 | 5 |
| -1840.376 | 19600.346 | 2.05 | 14.053 | 0.120046 | 7/15/2014 | 15:08.7 | 5 |
| -1840.477 | 19600.348 | 2.049 | 14.051 | 0.118409 | 7/15/2014 | 15:08.7 | 5 |
| -1840.678 | 19600.352 | 2.056 | 14.053 | 0.116734 | 7/15/2014 | 15:08.8 | 5 |
| -1840.882 | 19600.355 | 2.062 | 14.063 | 0.105065 | 7/15/2014 | 15:08.9 | 5 |
| -1841.082 | 19600.359 | 2.063 | 14.073 | 0.107996 | 7/15/2014 | 15:09.0 | 5 |
| -1841.286 | 19600.363 | 2.068 | 14.083 | 0.0889571 | 7/15/2014 | 15:09.1 | 5 |
| -1841.64 | 19600.371 | 2.072 | 14.095 | 0.0863014 | 7/15/2014 | 15:09.3 | 5 |
| -1841.689 | 19600.372 | 2.073 | 14.1 | 0.0946603 | 7/15/2014 | 15:09.3 | 5 |
| -1842.042 | 19600.379 | 2.078 | 14.11 | 0.0777651 | 7/15/2014 | 15:09.5 | 5 |
| -1842.243 | 19600.383 | 2.083 | 14.118 | 0.0783033 | 7/15/2014 | 15:09.6 | 5 |
| -1842.494 | 19600.387 | 2.081 | 14.124 | 0.095917 | 7/15/2014 | 15:09.7 | 5 |
| -1842.595 | 19600.389 | 2.084 | 14.127 | 0.0852733 | 7/15/2014 | 15:09.7 | 5 |
| -1842.799 | 19600.393 | 2.086 | 14.132 | 0.0808717 | 7/15/2014 | 15:09.8 | 5 |
| -1843.003 | 19600.396 | 2.086 | 14.141 | 0.0938103 | 7/15/2014 | 15:09.9 | 5 |
| -1843.21 | 19600.4 | 2.086 | 14.147 | 0.0999697 | 7/15/2014 | 15:10.0 | 5 |
| -1843.469 | 19600.405 | 2.088 | 14.15 | 0.161341 | 7/15/2014 | 15:10.1 | 5 |
| -1843.622 | 19600.408 | 2.09 | 14.153 | 0.158415 | 7/15/2014 | 15:10.2 | 5 |
| -1843.827 | 19600.411 | 2.094 | 14.159 | 0.185182 | 7/15/2014 | 15:10.3 | 5 |
| -1844.184 | 19600.417 | 2.093 | 14.156 | 0.207469 | 7/15/2014 | 15:10.5 | 5 |
| -1844.389 | 19600.421 | 2.096 | 14.169 | 0.248695 | 7/15/2014 | 15:10.6 | 5 |
| -1844.643 | 19600.425 | 2.094 | 14.167 | 0.238547 | 7/15/2014 | 15:10.7 | 5 |
| -1844.747 | 19600.427 | 2.099 | 14.164 | 0.24205 | 7/15/2014 | 15:10.7 | 5 |
| -1845.109 | 19600.433 | 2.096 | 14.144 | 0.20081 | 7/15/2014 | 15:10.9 | 5 |
| -1845.161 | 19600.434 | 2.099 | 14.145 | 0.203978 | 7/15/2014 | 15:10.9 | 5 |
| -1845.368 | 19600.437 | 2.097 | 14.133 | 0.167246 | 7/15/2014 | 15:11.0 | 5 |
| -1845.574 | 19600.441 | 2.091 | 14.111 | 0.144106 | 7/15/2014 | 15:11.1 | 5 |
| -1845.78 | 19600.443 | 2.091 | 14.105 | 0.14345 | 7/15/2014 | 15:11.2 | 5 |
| -1845.985 | 19600.446 | 2.09 | 14.098 | 0.110806 | 7/15/2014 | 15:11.3 | 5 |
| -1846.341 | 19600.452 | 2.085 | 14.074 | 0.0970745 | 7/15/2014 | 15:11.5 | 5 |
| -1846.545 | 19600.455 | 2.087 | 14.063 | 0.103004 | 7/15/2014 | 15:11.6 | 5 |
| -1846.799 | 19600.458 | 2.084 | 14.062 | 0.0990421 | 7/15/2014 | 15:11.7 | 5 |
| -1846.902 | 19600.46 | 2.085 | 14.058 | 0.100716 | 7/15/2014 | 15:11.7 | 5 |
| -1847.107 | 19600.463 | 2.085 | 14.061 | 0.0890538 | 7/15/2014 | 15:11.8 | 5 |
| -1847.313 | 19600.466 | 2.087 | 14.072 | 0.0921386 | 7/15/2014 | 15:11.9 | 5 |

| | | | | | | | |
|---|---|---|---|---|---|---|---|
| -1847.52 | 19600.468 | 2.084 | 14.064 | 0.111068 | 7/15/2014 | 15:12.0 | 5 |
| -1847.725 | 19600.471 | 2.084 | 14.063 | 0.143017 | 7/15/2014 | 15:12.1 | 5 |
| -1847.931 | 19600.475 | 2.081 | 14.061 | 0.133264 | 7/15/2014 | 15:12.2 | 5 |
| -1848.136 | 19600.477 | 2.084 | 14.065 | 0.138033 | 7/15/2014 | 15:12.3 | 5 |
| -1848.496 | 19600.481 | 2.086 | 14.068 | 0.129336 | 7/15/2014 | 15:12.5 | 5 |
| -1848.702 | 19600.482 | 2.091 | 14.067 | 0.162694 | 7/15/2014 | 15:12.6 | 5 |
| -1848.959 | 19600.486 | 2.091 | 14.067 | 0.141533 | 7/15/2014 | 15:12.7 | 5 |
| -1849.062 | 19600.487 | 2.092 | 14.061 | 0.140664 | 7/15/2014 | 15:12.7 | 5 |
| -1849.27 | 19600.49 | 2.096 | 14.059 | 0.119865 | 7/15/2014 | 15:12.8 | 5 |
| -1849.48 | 19600.492 | 2.099 | 14.062 | 0.158308 | 7/15/2014 | 15:12.9 | 5 |
| -1849.691 | 19600.495 | 2.104 | 14.061 | 0.150867 | 7/15/2014 | 15:13.0 | 5 |
| -1849.902 | 19600.496 | 2.106 | 14.057 | 0.159785 | 7/15/2014 | 15:13.1 | 5 |
| -1850.113 | 19600.5 | 2.106 | 14.056 | 0.182462 | 7/15/2014 | 15:13.2 | 5 |
| -1850.379 | 19600.501 | 2.106 | 14.05 | 0.143259 | 7/15/2014 | 15:13.3 | 5 |
| -1850.695 | 19600.505 | 2.106 | 14.049 | 0.163654 | 7/15/2014 | 15:13.5 | 5 |
| -1850.8 | 19600.507 | 2.106 | 14.05 | 0.16498 | 7/15/2014 | 15:13.5 | 5 |
| -1851.014 | 19600.508 | 2.106 | 14.045 | 0.183475 | 7/15/2014 | 15:13.6 | 5 |
| -1851.285 | 19600.511 | 2.108 | 14.047 | 0.154055 | 7/15/2014 | 15:13.7 | 5 |
| -1851.502 | 19600.512 | 2.114 | 14.038 | 0.151359 | 7/15/2014 | 15:13.8 | 5 |
| -1851.721 | 19600.515 | 2.114 | 14.036 | 0.148246 | 7/15/2014 | 15:13.9 | 5 |
| -1851.997 | 19600.517 | 2.118 | 14.029 | 0.153532 | 7/15/2014 | 15:14.0 | 5 |
| -1852.165 | 19600.519 | 2.122 | 14.018 | 0.153033 | 7/15/2014 | 15:14.1 | 5 |
| -1852.388 | 19600.521 | 2.124 | 14.017 | 0.138098 | 7/15/2014 | 15:14.2 | 5 |
| -1852.609 | 19600.521 | 2.126 | 14.002 | 0.111843 | 7/15/2014 | 15:14.3 | 5 |
| -1852.888 | 19600.524 | 2.131 | 13.994 | 0.107317 | 7/15/2014 | 15:14.4 | 5 |
| -1853.112 | 19600.525 | 2.131 | 13.974 | 0.11124 | 7/15/2014 | 15:14.5 | 5 |
| -1853.337 | 19600.528 | 2.128 | 13.968 | 0.100018 | 7/15/2014 | 15:14.6 | 5 |
| -1853.619 | 19600.53 | 2.13 | 13.962 | 0.0972266 | 7/15/2014 | 15:14.7 | 5 |
| -1853.846 | 19600.532 | 2.13 | 13.96 | 0.0890155 | 7/15/2014 | 15:14.8 | 5 |
| -1854.132 | 19600.534 | 2.128 | 13.96 | 0.0985145 | 7/15/2014 | 15:14.9 | 5 |
| -1854.246 | 19600.534 | 2.126 | 13.953 | 0.0979004 | 7/15/2014 | 15:15.0 | 5 |
| -1854.532 | 19600.537 | 2.127 | 13.951 | 0.101307 | 7/15/2014 | 15:15.1 | 5 |
| -1854.762 | 19600.538 | 2.131 | 13.951 | 0.11912 | 7/15/2014 | 15:15.2 | 5 |
| -1855.046 | 19600.542 | 2.136 | 13.949 | 0.121157 | 7/15/2014 | 15:15.3 | 5 |
| -1855.272 | 19600.543 | 2.139 | 13.957 | 0.131675 | 7/15/2014 | 15:15.4 | 5 |
| -1855.498 | 19600.545 | 2.141 | 13.956 | 0.147941 | 7/15/2014 | 15:15.5 | 5 |
| -1855.951 | 19600.548 | 2.144 | 13.959 | 0.130656 | 7/15/2014 | 15:15.7 | 5 |
| -1856.008 | 19600.549 | 2.147 | 13.958 | 0.146878 | 7/15/2014 | 15:15.7 | 5 |
| -1856.237 | 19600.55 | 2.15 | 13.957 | 0.147082 | 7/15/2014 | 15:15.8 | 5 |
| -1856.467 | 19600.552 | 2.152 | 13.964 | 0.129902 | 7/15/2014 | 15:15.9 | 5 |
| -1856.753 | 19600.555 | 2.149 | 13.971 | 0.131827 | 7/15/2014 | 15:16.0 | 5 |
| -1856.927 | 19600.556 | 2.144 | 13.98 | 0.154361 | 7/15/2014 | 15:16.1 | 5 |
| -1857.157 | 19600.558 | 2.144 | 13.98 | 0.156252 | 7/15/2014 | 15:16.2 | 5 |
| -1857.386 | 19600.56 | 2.144 | 13.985 | 0.171708 | 7/15/2014 | 15:16.3 | 5 |
| -1857.671 | 19600.561 | 2.147 | 14.002 | 0.168654 | 7/15/2014 | 15:16.4 | 5 |
| -1857.9 | 19600.563 | 2.147 | 14 | 0.189598 | 7/15/2014 | 15:16.5 | 5 |
| -1858.13 | 19600.564 | 2.147 | 14.005 | 0.186499 | 7/15/2014 | 15:16.6 | 5 |

| | | | | | | | |
|---|---|---|---|---|---|---|---|
| -1858.418 | 19600.567 | 2.145 | 14.015 | 0.178354 | 7/15/2014 | 15:16.7 | 5 |
| -1858.534 | 19600.568 | 2.146 | 14.017 | 0.171643 | 7/15/2014 | 15:16.8 | 5 |
| -1858.769 | 19600.569 | 2.152 | 14.02 | 0.179379 | 7/15/2014 | 15:16.9 | 5 |
| -1859.061 | 19600.572 | 2.152 | 14.029 | 0.165388 | 7/15/2014 | 15:17.0 | 5 |
| -1859.238 | 19600.572 | 2.156 | 14.032 | 0.163985 | 7/15/2014 | 15:17.1 | 5 |
| -1859.47 | 19600.574 | 2.148 | 14.03 | 0.150426 | 7/15/2014 | 15:17.2 | 5 |
| -1859.707 | 19600.576 | 2.148 | 14.041 | 0.144121 | 7/15/2014 | 15:17.3 | 5 |
| -1860.119 | 19600.58 | 2.147 | 14.047 | 0.149045 | 7/15/2014 | 15:17.4 | 5 |
| -1860.354 | 19600.581 | 2.149 | 14.046 | 0.147136 | 7/15/2014 | 15:17.5 | 5 |
| -1860.649 | 19600.584 | 2.152 | 14.049 | 0.160657 | 7/15/2014 | 15:17.6 | 5 |
| -1860.885 | 19600.585 | 2.152 | 14.045 | 0.17081 | 7/15/2014 | 15:17.7 | 5 |
| -1861.003 | 19600.586 | 2.151 | 14.049 | 0.174632 | 7/15/2014 | 15:17.8 | 5 |
| -1861.241 | 19600.588 | 2.158 | 14.05 | 0.179176 | 7/15/2014 | 15:17.9 | 5 |
| -1861.48 | 19600.59 | 2.166 | 14.052 | 0.146172 | 7/15/2014 | 15:18.0 | 5 |
| -1861.721 | 19600.592 | 2.175 | 14.055 | 0.172871 | 7/15/2014 | 15:18.1 | 5 |
| -1861.958 | 19600.593 | 2.175 | 14.067 | 0.174133 | 7/15/2014 | 15:18.2 | 5 |
| -1862.2 | 19600.596 | 2.183 | 14.073 | 0.159067 | 7/15/2014 | 15:18.3 | 5 |
| -1862.618 | 19600.599 | 2.18 | 14.079 | 0.162961 | 7/15/2014 | 15:18.4 | 5 |
| -1862.857 | 19600.6 | 2.18 | 14.084 | 0.158845 | 7/15/2014 | 15:18.5 | 5 |
| -1863.096 | 19600.603 | 2.18 | 14.084 | 0.176653 | 7/15/2014 | 15:18.6 | 5 |
| -1863.395 | 19600.604 | 2.175 | 14.083 | 0.168593 | 7/15/2014 | 15:18.7 | 5 |
| -1863.515 | 19600.604 | 2.17 | 14.083 | 0.166063 | 7/15/2014 | 15:18.8 | 5 |
| -1863.817 | 19600.608 | 2.174 | 14.081 | 0.180257 | 7/15/2014 | 15:18.9 | 5 |
| -1864.057 | 19600.609 | 2.164 | 14.078 | 0.175871 | 7/15/2014 | 15:19.0 | 5 |
| -1864.239 | 19600.611 | 2.168 | 14.081 | 0.169875 | 7/15/2014 | 15:19.1 | 5 |
| -1864.479 | 19600.613 | 2.167 | 14.081 | 0.184174 | 7/15/2014 | 15:19.2 | 5 |
| -1864.782 | 19600.616 | 2.17 | 14.077 | 0.179009 | 7/15/2014 | 15:19.3 | 5 |
| -1865.141 | 19600.619 | 2.169 | 14.085 | 0.197392 | 7/15/2014 | 15:19.4 | 5 |
| -1865.44 | 19600.622 | 2.171 | 14.089 | 0.192605 | 7/15/2014 | 15:19.5 | 5 |
| -1865.68 | 19600.624 | 2.171 | 14.093 | 0.194935 | 7/15/2014 | 15:19.6 | 5 |
| -1865.921 | 19600.625 | 2.177 | 14.104 | 0.203163 | 7/15/2014 | 15:19.7 | 5 |
| -1866.041 | 19600.627 | 2.175 | 14.104 | 0.201559 | 7/15/2014 | 15:19.8 | 5 |
| -1866.468 | 19600.631 | 2.171 | 14.112 | 0.200809 | 7/15/2014 | 15:19.9 | 5 |
| -1866.527 | 19600.632 | 2.169 | 14.12 | 0.204269 | 7/15/2014 | 15:20.0 | 5 |
| -1866.774 | 19600.634 | 2.173 | 14.127 | 0.198308 | 7/15/2014 | 15:20.1 | 5 |
| -1867.017 | 19600.636 | 2.165 | 14.133 | 0.192227 | 7/15/2014 | 15:20.2 | 5 |
| -1867.264 | 19600.638 | 2.163 | 14.145 | 0.193092 | 7/15/2014 | 15:20.3 | 5 |
| -1867.693 | 19600.642 | 2.156 | 14.151 | 0.191564 | 7/15/2014 | 15:20.4 | 5 |
| -1867.938 | 19600.645 | 2.156 | 14.152 | 0.192096 | 7/15/2014 | 15:20.5 | 5 |
| -1868.184 | 19600.647 | 2.156 | 14.156 | 0.192127 | 7/15/2014 | 15:20.6 | 5 |
| -1868.492 | 19600.65 | 2.156 | 14.162 | 0.193288 | 7/15/2014 | 15:20.7 | 5 |
| -1868.616 | 19600.651 | 2.151 | 14.165 | 0.190297 | 7/15/2014 | 15:20.8 | 5 |
| -1868.865 | 19600.652 | 2.153 | 14.172 | 0.191614 | 7/15/2014 | 15:20.9 | 5 |
| -1869.111 | 19600.656 | 2.151 | 14.168 | 0.189557 | 7/15/2014 | 15:21.0 | 5 |
| -1869.361 | 19600.658 | 2.155 | 14.166 | 0.189866 | 7/15/2014 | 15:21.1 | 5 |
| -1869.607 | 19600.66 | 2.149 | 14.17 | 0.191638 | 7/15/2014 | 15:21.2 | 5 |
| -1869.855 | 19600.664 | 2.145 | 14.178 | 0.197152 | 7/15/2014 | 15:21.3 | 5 |

| | | | | | | | |
|---|---|---|---|---|---|---|---|
| -1870.286 | 19600.668 | 2.138 | 14.187 | 0.188382 | 7/15/2014 | 15:21.4 | 5 |
| -1870.532 | 19600.67 | 2.133 | 14.201 | 0.190024 | 7/15/2014 | 15:21.5 | 5 |
| -1870.776 | 19600.673 | 2.13 | 14.196 | 0.197464 | 7/15/2014 | 15:21.6 | 5 |
| -1871.083 | 19600.675 | 2.124 | 14.195 | 0.190676 | 7/15/2014 | 15:21.7 | 5 |
| -1871.207 | 19600.676 | 2.119 | 14.199 | 0.196403 | 7/15/2014 | 15:21.8 | 5 |
| -1871.454 | 19600.68 | 2.123 | 14.201 | 0.177447 | 7/15/2014 | 15:21.9 | 5 |
| -1871.7 | 19600.682 | 2.123 | 14.197 | 0.188186 | 7/15/2014 | 15:22.0 | 5 |
| -1871.947 | 19600.684 | 2.125 | 14.188 | 0.183101 | 7/15/2014 | 15:22.1 | 5 |
| -1872.193 | 19600.688 | 2.123 | 14.185 | 0.183658 | 7/15/2014 | 15:22.2 | 5 |
| -1872.439 | 19600.689 | 2.125 | 14.174 | 0.180314 | 7/15/2014 | 15:22.3 | 5 |
| -1872.867 | 19600.693 | 2.124 | 14.188 | 0.179453 | 7/15/2014 | 15:22.4 | 5 |
| -1873.111 | 19600.696 | 2.124 | 14.192 | 0.17942 | 7/15/2014 | 15:22.5 | 5 |
| -1873.351 | 19600.698 | 2.116 | 14.199 | 0.170489 | 7/15/2014 | 15:22.6 | 5 |
| -1873.651 | 19600.701 | 2.111 | 14.202 | 0.159065 | 7/15/2014 | 15:22.7 | 5 |
| -1873.775 | 19600.703 | 2.107 | 14.208 | 0.144892 | 7/15/2014 | 15:22.8 | 5 |
| -1874.073 | 19600.706 | 2.104 | 14.212 | 0.14024 | 7/15/2014 | 15:22.9 | 5 |
| -1874.255 | 19600.708 | 2.101 | 14.216 | 0.157325 | 7/15/2014 | 15:23.0 | 5 |
| -1889.74 | 19806.051 | 1.677 | 14.871 | 0.143143 | 7/15/2014 | 20:15.9 | 6 |
| -1889.492 | 19806.054 | 1.68 | 14.868 | 0.134084 | 7/15/2014 | 20:16.0 | 6 |
| -1889.242 | 19806.059 | 1.686 | 14.87 | 0.130853 | 7/15/2014 | 20:16.1 | 6 |
| -1888.996 | 19806.063 | 1.683 | 14.87 | 0.143651 | 7/15/2014 | 20:16.2 | 6 |
| -1888.747 | 19806.067 | 1.693 | 14.872 | 0.134505 | 7/15/2014 | 20:16.3 | 6 |
| -1888.311 | 19806.076 | 1.689 | 14.879 | 0.149343 | 7/15/2014 | 20:16.4 | 6 |
| -1888.189 | 19806.078 | 1.693 | 14.88 | 0.139759 | 7/15/2014 | 20:16.5 | 6 |
| -1887.938 | 19806.082 | 1.687 | 14.872 | 0.15828 | 7/15/2014 | 20:16.6 | 6 |
| -1887.682 | 19806.088 | 1.688 | 14.865 | 0.153729 | 7/15/2014 | 20:16.7 | 6 |
| -1887.427 | 19806.094 | 1.685 | 14.864 | 0.126103 | 7/15/2014 | 20:16.8 | 6 |
| -1887.167 | 19806.099 | 1.687 | 14.857 | 0.133202 | 7/15/2014 | 20:16.9 | 6 |
| -1886.909 | 19806.104 | 1.677 | 14.853 | 0.134501 | 7/15/2014 | 20:17.0 | 6 |
| -1886.648 | 19806.111 | 1.677 | 14.834 | 0.146545 | 7/15/2014 | 20:17.1 | 6 |
| -1886.387 | 19806.116 | 1.68 | 14.828 | 0.11838 | 7/15/2014 | 20:17.2 | 6 |
| -1886.123 | 19806.122 | 1.686 | 14.829 | 0.157453 | 7/15/2014 | 20:17.3 | 6 |
| -1885.861 | 19806.129 | 1.683 | 14.835 | 0.143888 | 7/15/2014 | 20:17.4 | 6 |
| -1885.599 | 19806.135 | 1.687 | 14.832 | 0.152785 | 7/15/2014 | 20:17.5 | 6 |
| -1885.34 | 19806.141 | 1.685 | 14.833 | 0.106856 | 7/15/2014 | 20:17.6 | 6 |
| -1885.08 | 19806.148 | 1.686 | 14.841 | 0.134771 | 7/15/2014 | 20:17.7 | 6 |
| -1884.76 | 19806.155 | 1.686 | 14.843 | 0.142899 | 7/15/2014 | 20:17.8 | 6 |
| -1884.501 | 19806.162 | 1.693 | 14.84 | 0.0866839 | 7/15/2014 | 20:17.9 | 6 |
| -1884.244 | 19806.169 | 1.693 | 14.829 | 0.12219 | 7/15/2014 | 20:18.0 | 6 |
| -1883.986 | 19806.175 | 1.693 | 14.815 | 0.0914195 | 7/15/2014 | 20:18.1 | 6 |
| -1883.728 | 19806.182 | 1.691 | 14.805 | 0.105617 | 7/15/2014 | 20:18.2 | 6 |
| -1883.471 | 19806.189 | 1.693 | 14.797 | 0.0740662 | 7/15/2014 | 20:18.3 | 6 |
| -1883.151 | 19806.197 | 1.698 | 14.78 | 0.0839095 | 7/15/2014 | 20:18.4 | 6 |
| -1882.892 | 19806.205 | 1.7 | 14.778 | 0.0607986 | 7/15/2014 | 20:18.5 | 6 |
| -1882.632 | 19806.212 | 1.692 | 14.766 | 0.0595867 | 7/15/2014 | 20:18.6 | 6 |
| -1882.37 | 19806.219 | 1.692 | 14.755 | 0.0760664 | 7/15/2014 | 20:18.7 | 6 |
| -1882.042 | 19806.229 | 1.697 | 14.743 | 0.051421 | 7/15/2014 | 20:18.8 | 6 |

| | | | | | | | |
|---|---|---|---|---|---|---|---|
| -1881.845 | 19806.234 | 1.702 | 14.735 | 0.0578877 | 7/15/2014 | 20:18.9 | 6 |
| -1881.582 | 19806.242 | 1.697 | 14.723 | 0.0587623 | 7/15/2014 | 20:19.0 | 6 |
| -1881.318 | 19806.25 | 1.697 | 14.713 | 0.0521612 | 7/15/2014 | 20:19.1 | 6 |
| -1881.055 | 19806.258 | 1.697 | 14.701 | 0.0693026 | 7/15/2014 | 20:19.2 | 6 |
| -1880.793 | 19806.266 | 1.702 | 14.694 | 0.0411612 | 7/15/2014 | 20:19.3 | 6 |
| -1880.34 | 19806.279 | 1.698 | 14.699 | 0.0575497 | 7/15/2014 | 20:19.4 | 6 |
| -1880.275 | 19806.281 | 1.697 | 14.701 | 0.0521934 | 7/15/2014 | 20:19.5 | 6 |
| -1880.017 | 19806.289 | 1.697 | 14.699 | 0.0500497 | 7/15/2014 | 20:19.6 | 6 |
| -1879.759 | 19806.297 | 1.704 | 14.699 | 0.0887651 | 7/15/2014 | 20:19.7 | 6 |
| -1879.441 | 19806.308 | 1.702 | 14.709 | 0.0595273 | 7/15/2014 | 20:19.8 | 6 |
| -1879.184 | 19806.316 | 1.702 | 14.729 | 0.0864782 | 7/15/2014 | 20:19.9 | 6 |
| -1878.927 | 19806.324 | 1.699 | 14.742 | 0.0775859 | 7/15/2014 | 20:20.0 | 6 |
| -1878.668 | 19806.332 | 1.699 | 14.759 | 0.0820261 | 7/15/2014 | 20:20.1 | 6 |
| -1878.408 | 19806.342 | 1.702 | 14.764 | 0.056178 | 7/15/2014 | 20:20.2 | 6 |
| -1878.147 | 19806.35 | 1.702 | 14.777 | 0.0545542 | 7/15/2014 | 20:20.3 | 6 |
| -1877.823 | 19806.361 | 1.699 | 14.793 | 0.0528775 | 7/15/2014 | 20:20.4 | 6 |
| -1877.557 | 19806.371 | 1.702 | 14.803 | 0.0365668 | 7/15/2014 | 20:20.5 | 6 |
| -1877.291 | 19806.381 | 1.697 | 14.8 | 0.0646429 | 7/15/2014 | 20:20.6 | 6 |
| -1877.022 | 19806.389 | 1.697 | 14.804 | 0.0639032 | 7/15/2014 | 20:20.7 | 6 |
| -1876.752 | 19806.4 | 1.694 | 14.805 | 0.0611909 | 7/15/2014 | 20:20.8 | 6 |
| -1876.479 | 19806.408 | 1.695 | 14.813 | 0.076158 | 7/15/2014 | 20:20.9 | 6 |
| -1876.209 | 19806.419 | 1.69 | 14.813 | 0.0494252 | 7/15/2014 | 20:21.0 | 6 |
| -1875.937 | 19806.428 | 1.685 | 14.815 | 0.0411903 | 7/15/2014 | 20:21.1 | 6 |
| -1875.667 | 19806.439 | 1.673 | 14.817 | 0.0354892 | 7/15/2014 | 20:21.2 | 6 |
| -1875.396 | 19806.447 | 1.673 | 14.825 | 0.0419627 | 7/15/2014 | 20:21.3 | 6 |
| -1875.129 | 19806.459 | 1.663 | 14.833 | 0.0307577 | 7/15/2014 | 20:21.4 | 6 |
| -1874.861 | 19806.468 | 1.663 | 14.842 | 0.0972505 | 7/15/2014 | 20:21.5 | 6 |
| -1874.599 | 19806.479 | 1.657 | 14.845 | 0.0801913 | 7/15/2014 | 20:21.6 | 6 |
| -1874.336 | 19806.488 | 1.657 | 14.857 | 0.120203 | 7/15/2014 | 20:21.7 | 6 |
| -1874.014 | 19806.5 | 1.647 | 14.866 | 0.122635 | 7/15/2014 | 20:21.8 | 6 |
| -1873.753 | 19806.51 | 1.651 | 14.873 | 0.17169 | 7/15/2014 | 20:21.9 | 6 |
| -1873.496 | 19806.52 | 1.647 | 14.872 | 0.196928 | 7/15/2014 | 20:22.0 | 6 |
| -1873.236 | 19806.53 | 1.651 | 14.873 | 0.219591 | 7/15/2014 | 20:22.1 | 6 |
| -1872.98 | 19806.541 | 1.639 | 14.882 | 0.25551 | 7/15/2014 | 20:22.2 | 6 |
| -1872.722 | 19806.551 | 1.642 | 14.88 | 0.284889 | 7/15/2014 | 20:22.3 | 6 |
| -1872.405 | 19806.563 | 1.639 | 14.877 | 0.341399 | 7/15/2014 | 20:22.4 | 6 |
| -1872.148 | 19806.574 | 1.635 | 14.872 | 0.275463 | 7/15/2014 | 20:22.5 | 6 |
| -1871.892 | 19806.582 | 1.629 | 14.861 | 0.298501 | 7/15/2014 | 20:22.6 | 6 |
| -1871.633 | 19806.594 | 1.626 | 14.854 | 0.259774 | 7/15/2014 | 20:22.7 | 6 |
| -1871.312 | 19806.607 | 1.617 | 14.853 | 0.245044 | 7/15/2014 | 20:22.8 | 6 |
| -1871.05 | 19806.617 | 1.623 | 14.849 | 0.211676 | 7/15/2014 | 20:22.9 | 6 |
| -1870.789 | 19806.629 | 1.613 | 14.847 | 0.205299 | 7/15/2014 | 20:23.0 | 6 |
| -1870.525 | 19806.64 | 1.611 | 14.842 | 0.201236 | 7/15/2014 | 20:23.1 | 6 |
| -1870.261 | 19806.649 | 1.618 | 14.851 | 0.186214 | 7/15/2014 | 20:23.2 | 6 |
| -1869.996 | 19806.661 | 1.618 | 14.851 | 0.190774 | 7/15/2014 | 20:23.3 | 6 |
| -1869.666 | 19806.675 | 1.62 | 14.851 | 0.185535 | 7/15/2014 | 20:23.4 | 6 |
| -1869.4 | 19806.686 | 1.634 | 14.861 | 0.182317 | 7/15/2014 | 20:23.5 | 6 |

| | | | | | | | |
|---|---|---|---|---|---|---|---|
| -1869.134 | 19806.697 | 1.631 | 14.867 | 0.183323 | 7/15/2014 | 20:23.6 | 6 |
| -1868.866 | 19806.708 | 1.637 | 14.879 | 0.186239 | 7/15/2014 | 20:23.7 | 6 |
| -1868.533 | 19806.724 | 1.64 | 14.887 | 0.183575 | 7/15/2014 | 20:23.8 | 6 |
| -1868.266 | 19806.734 | 1.642 | 14.878 | 0.18304 | 7/15/2014 | 20:23.9 | 6 |
| -1867.998 | 19806.746 | 1.648 | 14.888 | 0.180894 | 7/15/2014 | 20:24.0 | 6 |
| -1867.729 | 19806.758 | 1.648 | 14.894 | 0.182957 | 7/15/2014 | 20:24.1 | 6 |
| -1867.462 | 19806.77 | 1.647 | 14.884 | 0.194287 | 7/15/2014 | 20:24.2 | 6 |
| -1867.197 | 19806.781 | 1.653 | 14.873 | 0.204983 | 7/15/2014 | 20:24.3 | 6 |
| -1866.867 | 19806.797 | 1.653 | 14.86 | 0.185478 | 7/15/2014 | 20:24.4 | 6 |
| -1866.602 | 19806.808 | 1.656 | 14.857 | 0.200783 | 7/15/2014 | 20:24.5 | 6 |
| -1866.339 | 19806.819 | 1.658 | 14.864 | 0.217751 | 7/15/2014 | 20:24.6 | 6 |
| -1866.076 | 19806.832 | 1.661 | 14.874 | 0.203981 | 7/15/2014 | 20:24.7 | 6 |
| -1865.749 | 19806.848 | 1.661 | 14.877 | 0.166399 | 7/15/2014 | 20:24.8 | 6 |
| -1865.487 | 19806.859 | 1.661 | 14.891 | 0.188094 | 7/15/2014 | 20:24.9 | 6 |
| -1865.225 | 19806.871 | 1.66 | 14.896 | 0.206023 | 7/15/2014 | 20:25.0 | 6 |
| -1864.961 | 19806.884 | 1.669 | 14.903 | 0.170963 | 7/15/2014 | 20:25.1 | 6 |
| -1864.701 | 19806.896 | 1.669 | 14.907 | 0.156937 | 7/15/2014 | 20:25.2 | 6 |
| -1864.44 | 19806.908 | 1.669 | 14.919 | 0.133502 | 7/15/2014 | 20:25.3 | 6 |
| -1864.117 | 19806.924 | 1.667 | 14.926 | 0.141992 | 7/15/2014 | 20:25.4 | 6 |
| -1863.857 | 19806.936 | 1.677 | 14.92 | 0.121097 | 7/15/2014 | 20:25.5 | 6 |
| -1863.6 | 19806.949 | 1.672 | 14.916 | 0.111287 | 7/15/2014 | 20:25.6 | 6 |
| -1863.34 | 19806.962 | 1.675 | 14.911 | 0.112291 | 7/15/2014 | 20:25.7 | 6 |
| -1863.021 | 19806.978 | 1.668 | 14.907 | 0.11783 | 7/15/2014 | 20:25.8 | 6 |
| -1862.763 | 19806.99 | 1.666 | 14.904 | 0.114531 | 7/15/2014 | 20:25.9 | 6 |
| -1862.505 | 19807.002 | 1.666 | 14.894 | 0.105899 | 7/15/2014 | 20:26.0 | 6 |
| -1862.245 | 19807.014 | 1.668 | 14.877 | 0.107754 | 7/15/2014 | 20:26.1 | 6 |
| -1861.99 | 19807.028 | 1.661 | 14.87 | 0.10298 | 7/15/2014 | 20:26.2 | 6 |
| -1861.731 | 19807.041 | 1.666 | 14.86 | 0.10225 | 7/15/2014 | 20:26.3 | 6 |
| -1861.413 | 19807.056 | 1.662 | 14.869 | 0.113075 | 7/15/2014 | 20:26.4 | 6 |
| -1861.157 | 19807.069 | 1.66 | 14.869 | 0.105406 | 7/15/2014 | 20:26.5 | 6 |
| -1860.902 | 19807.082 | 1.662 | 14.859 | 0.100537 | 7/15/2014 | 20:26.6 | 6 |
| -1860.647 | 19807.094 | 1.663 | 14.854 | 0.0986055 | 7/15/2014 | 20:26.7 | 6 |
| -1860.328 | 19807.11 | 1.661 | 14.836 | 0.104307 | 7/15/2014 | 20:26.8 | 6 |
| -1860.071 | 19807.123 | 1.661 | 14.844 | 0.114961 | 7/15/2014 | 20:26.9 | 6 |
| -1859.815 | 19807.136 | 1.656 | 14.833 | 0.13809 | 7/15/2014 | 20:27.0 | 6 |
| -1859.557 | 19807.149 | 1.659 | 14.812 | 0.113864 | 7/15/2014 | 20:27.1 | 6 |
| -1859.3 | 19807.162 | 1.659 | 14.807 | 0.111283 | 7/15/2014 | 20:27.2 | 6 |
| -1859.043 | 19807.175 | 1.659 | 14.795 | 0.133727 | 7/15/2014 | 20:27.3 | 6 |
| -1858.724 | 19807.191 | 1.658 | 14.779 | 0.145286 | 7/15/2014 | 20:27.4 | 6 |
| -1858.466 | 19807.203 | 1.667 | 14.774 | 0.127584 | 7/15/2014 | 20:27.5 | 6 |
| -1858.21 | 19807.216 | 1.664 | 14.753 | 0.105777 | 7/15/2014 | 20:27.6 | 6 |
| -1857.952 | 19807.229 | 1.663 | 14.745 | 0.115572 | 7/15/2014 | 20:27.7 | 6 |
| -1857.631 | 19807.244 | 1.657 | 14.749 | 0.109378 | 7/15/2014 | 20:27.8 | 6 |
| -1857.371 | 19807.257 | 1.657 | 14.742 | 0.0825425 | 7/15/2014 | 20:27.9 | 6 |
| -1857.112 | 19807.271 | 1.651 | 14.757 | 0.0921456 | 7/15/2014 | 20:28.0 | 6 |
| -1856.852 | 19807.283 | 1.649 | 14.756 | 0.0822407 | 7/15/2014 | 20:28.1 | 6 |
| -1856.592 | 19807.295 | 1.643 | 14.765 | 0.098058 | 7/15/2014 | 20:28.2 | 6 |

| | | | | | | | |
|---|---|---|---|---|---|---|---|
| -1856.331 | 19807.308 | 1.653 | 14.769 | 0.0917839 | 7/15/2014 | 20:28.3 | 6 |
| -1856.009 | 19807.322 | 1.654 | 14.78 | 0.104765 | 7/15/2014 | 20:28.4 | 6 |
| -1855.749 | 19807.336 | 1.656 | 14.774 | 0.119213 | 7/15/2014 | 20:28.5 | 6 |
| -1855.49 | 19807.35 | 1.662 | 14.764 | 0.104971 | 7/15/2014 | 20:28.6 | 6 |
| -1855.23 | 19807.361 | 1.675 | 14.755 | 0.106613 | 7/15/2014 | 20:28.7 | 6 |
| -1854.908 | 19807.377 | 1.677 | 14.75 | 0.105847 | 7/15/2014 | 20:28.8 | 6 |
| -1854.647 | 19807.389 | 1.678 | 14.749 | 0.107017 | 7/15/2014 | 20:28.9 | 6 |
| -1854.39 | 19807.4 | 1.675 | 14.744 | 0.105224 | 7/15/2014 | 20:29.0 | 6 |
| -1854.129 | 19807.414 | 1.683 | 14.731 | 0.101887 | 7/15/2014 | 20:29.1 | 6 |
| -1853.872 | 19807.426 | 1.687 | 14.74 | 0.120072 | 7/15/2014 | 20:29.2 | 6 |
| -1853.613 | 19807.438 | 1.684 | 14.738 | 0.112116 | 7/15/2014 | 20:29.3 | 6 |
| -1853.294 | 19807.454 | 1.681 | 14.725 | 0.125064 | 7/15/2014 | 20:29.4 | 6 |
| -1853.037 | 19807.466 | 1.693 | 14.728 | 0.123448 | 7/15/2014 | 20:29.5 | 6 |
| -1852.782 | 19807.479 | 1.699 | 14.711 | 0.112724 | 7/15/2014 | 20:29.6 | 6 |
| -1852.525 | 19807.49 | 1.699 | 14.703 | 0.0994363 | 7/15/2014 | 20:29.7 | 6 |
| -1852.207 | 19807.504 | 1.699 | 14.724 | 0.0926148 | 7/15/2014 | 20:29.8 | 6 |
| -1851.948 | 19807.516 | 1.702 | 14.705 | 0.0809239 | 7/15/2014 | 20:29.9 | 6 |
| -1851.69 | 19807.529 | 1.708 | 14.719 | 0.0772267 | 7/15/2014 | 20:30.0 | 6 |
| -1851.431 | 19807.541 | 1.712 | 14.741 | 0.0725281 | 7/15/2014 | 20:30.1 | 6 |
| -1851.172 | 19807.555 | 1.704 | 14.741 | 0.066567 | 7/15/2014 | 20:30.2 | 6 |
| -1850.912 | 19807.566 | 1.704 | 14.754 | 0.0763595 | 7/15/2014 | 20:30.3 | 6 |
| -1850.59 | 19807.58 | 1.71 | 14.766 | 0.0717459 | 7/15/2014 | 20:30.4 | 6 |
| -1850.329 | 19807.592 | 1.71 | 14.748 | 0.0808791 | 7/15/2014 | 20:30.5 | 6 |
| -1850.068 | 19807.604 | 1.706 | 14.754 | 0.0873787 | 7/15/2014 | 20:30.6 | 6 |
| -1849.806 | 19807.616 | 1.706 | 14.746 | 0.102481 | 7/15/2014 | 20:30.7 | 6 |
| -1849.479 | 19807.631 | 1.699 | 14.735 | 0.106362 | 7/15/2014 | 20:30.8 | 6 |
| -1849.212 | 19807.643 | 1.699 | 14.735 | 0.107324 | 7/15/2014 | 20:30.9 | 6 |
| -1848.947 | 19807.654 | 1.689 | 14.74 | 0.106016 | 7/15/2014 | 20:31.0 | 6 |
| -1848.678 | 19807.666 | 1.687 | 14.742 | 0.125096 | 7/15/2014 | 20:31.1 | 6 |
| -1848.412 | 19807.678 | 1.687 | 14.747 | 0.106509 | 7/15/2014 | 20:31.2 | 6 |
| -1848.143 | 19807.689 | 1.693 | 14.755 | 0.0896026 | 7/15/2014 | 20:31.3 | 6 |
| -1847.811 | 19807.703 | 1.693 | 14.767 | 0.0869269 | 7/15/2014 | 20:31.4 | 6 |
| -1847.542 | 19807.715 | 1.7 | 14.77 | 0.0761737 | 7/15/2014 | 20:31.5 | 6 |
| -1847.277 | 19807.727 | 1.7 | 14.774 | 0.0732664 | 7/15/2014 | 20:31.6 | 6 |
| -1847.008 | 19807.738 | 1.71 | 14.771 | 0.0635235 | 7/15/2014 | 20:31.7 | 6 |
| -1846.679 | 19807.753 | 1.7 | 14.779 | 0.0645278 | 7/15/2014 | 20:31.8 | 6 |
| -1846.411 | 19807.764 | 1.706 | 14.785 | 0.0663986 | 7/15/2014 | 20:31.9 | 6 |
| -1846.147 | 19807.775 | 1.694 | 14.795 | 0.068949 | 7/15/2014 | 20:32.0 | 6 |
| -1845.884 | 19807.787 | 1.69 | 14.797 | 0.0732917 | 7/15/2014 | 20:32.1 | 6 |
| -1845.623 | 19807.799 | 1.687 | 14.804 | 0.0774828 | 7/15/2014 | 20:32.2 | 6 |
| -1845.36 | 19807.811 | 1.69 | 14.811 | 0.075614 | 7/15/2014 | 20:32.3 | 6 |
| -1845.039 | 19807.823 | 1.68 | 14.824 | 0.0785355 | 7/15/2014 | 20:32.4 | 6 |
| -1844.781 | 19807.834 | 1.683 | 14.832 | 0.0822021 | 7/15/2014 | 20:32.5 | 6 |
| -1844.526 | 19807.846 | 1.673 | 14.849 | 0.089936 | 7/15/2014 | 20:32.6 | 6 |
| -1844.271 | 19807.857 | 1.679 | 14.846 | 0.0973572 | 7/15/2014 | 20:32.7 | 6 |
| -1843.952 | 19807.871 | 1.679 | 14.85 | 0.104624 | 7/15/2014 | 20:32.8 | 6 |
| -1843.695 | 19807.882 | 1.673 | 14.861 | 0.11436 | 7/15/2014 | 20:32.9 | 6 |

| | | | | | | | |
|---|---|---|---|---|---|---|---|
| -1843.439 | 19807.891 | 1.67 | 14.867 | 0.102703 | 7/15/2014 | 20:33.0 | 6 |
| -1843.182 | 19807.902 | 1.676 | 14.872 | 0.102005 | 7/15/2014 | 20:33.1 | 6 |
| -1842.925 | 19807.914 | 1.673 | 14.879 | 0.0924413 | 7/15/2014 | 20:33.2 | 6 |
| -1842.669 | 19807.926 | 1.671 | 14.878 | 0.100441 | 7/15/2014 | 20:33.3 | 6 |
| -1842.349 | 19807.938 | 1.665 | 14.902 | 0.101325 | 7/15/2014 | 20:33.4 | 6 |
| -1842.093 | 19807.948 | 1.659 | 14.922 | 0.100695 | 7/15/2014 | 20:33.5 | 6 |
| -1841.838 | 19807.959 | 1.659 | 14.942 | 0.113159 | 7/15/2014 | 20:33.6 | 6 |
| -1841.582 | 19807.969 | 1.649 | 14.969 | 0.109627 | 7/15/2014 | 20:33.7 | 6 |
| -1841.265 | 19807.982 | 1.637 | 14.999 | 0.133656 | 7/15/2014 | 20:33.8 | 6 |
| -1841.01 | 19807.994 | 1.637 | 15.031 | 0.106651 | 7/15/2014 | 20:33.9 | 6 |
| -1840.755 | 19808.003 | 1.631 | 15.068 | 0.118455 | 7/15/2014 | 20:34.0 | 6 |
| -1840.501 | 19808.014 | 1.628 | 15.088 | 0.10807 | 7/15/2014 | 20:34.1 | 6 |
| -1840.248 | 19808.025 | 1.622 | 15.116 | 0.0997547 | 7/15/2014 | 20:34.2 | 6 |
| -1839.995 | 19808.033 | 1.619 | 15.133 | 0.102897 | 7/15/2014 | 20:34.3 | 6 |
| -1839.684 | 19808.047 | 1.616 | 15.144 | 0.105928 | 7/15/2014 | 20:34.4 | 6 |
| -1839.433 | 19808.057 | 1.622 | 15.143 | 0.105976 | 7/15/2014 | 20:34.5 | 6 |
| -1839.184 | 19808.067 | 1.614 | 15.143 | 0.127378 | 7/15/2014 | 20:34.6 | 6 |
| -1838.934 | 19808.076 | 1.611 | 15.129 | 0.133141 | 7/15/2014 | 20:34.7 | 6 |
| -1838.622 | 19808.09 | 1.608 | 15.126 | 0.126632 | 7/15/2014 | 20:34.8 | 6 |
| -1838.37 | 19808.098 | 1.605 | 15.11 | 0.126872 | 7/15/2014 | 20:34.9 | 6 |
| -1838.117 | 19808.109 | 1.6 | 15.101 | 0.122289 | 7/15/2014 | 20:35.0 | 6 |
| -1837.864 | 19808.117 | 1.604 | 15.097 | 0.122971 | 7/15/2014 | 20:35.1 | 6 |
| -1837.611 | 19808.127 | 1.598 | 15.088 | 0.0989632 | 7/15/2014 | 20:35.2 | 6 |
| -1837.357 | 19808.137 | 1.598 | 15.08 | 0.0881788 | 7/15/2014 | 20:35.3 | 6 |
| -1837.04 | 19808.148 | 1.598 | 15.076 | 0.104851 | 7/15/2014 | 20:35.4 | 6 |
| -1836.785 | 19808.156 | 1.595 | 15.071 | 0.0898374 | 7/15/2014 | 20:35.5 | 6 |
| -1836.53 | 19808.167 | 1.592 | 15.064 | 0.0829418 | 7/15/2014 | 20:35.6 | 6 |
| -1836.273 | 19808.176 | 1.588 | 15.066 | 0.102424 | 7/15/2014 | 20:35.7 | 6 |
| -1835.95 | 19808.186 | 1.575 | 15.055 | 0.0888777 | 7/15/2014 | 20:35.8 | 6 |
| -1835.689 | 19808.195 | 1.575 | 15.057 | 0.111214 | 7/15/2014 | 20:35.9 | 6 |
| -1835.428 | 19808.203 | 1.571 | 15.043 | 0.119131 | 7/15/2014 | 20:36.0 | 6 |
| -1835.165 | 19808.211 | 1.568 | 15.033 | 0.108917 | 7/15/2014 | 20:36.1 | 6 |
| -1834.904 | 19808.22 | 1.555 | 15.018 | 0.106471 | 7/15/2014 | 20:36.2 | 6 |
| -1834.64 | 19808.229 | 1.555 | 15.019 | 0.11446 | 7/15/2014 | 20:36.3 | 6 |
| -1834.312 | 19808.238 | 1.553 | 15.003 | 0.133608 | 7/15/2014 | 20:36.4 | 6 |
| -1834.046 | 19808.246 | 1.557 | 14.995 | 0.122536 | 7/15/2014 | 20:36.5 | 6 |
| -1833.781 | 19808.254 | 1.545 | 14.988 | 0.0890583 | 7/15/2014 | 20:36.6 | 6 |
| -1833.513 | 19808.262 | 1.541 | 14.985 | 0.102902 | 7/15/2014 | 20:36.7 | 6 |
| -1833.182 | 19808.271 | 1.538 | 14.981 | 0.106988 | 7/15/2014 | 20:36.8 | 6 |
| -1832.912 | 19808.279 | 1.543 | 14.984 | 0.0717028 | 7/15/2014 | 20:36.9 | 6 |
| -1832.643 | 19808.285 | 1.537 | 14.975 | 0.0681312 | 7/15/2014 | 20:37.0 | 6 |
| -1832.372 | 19808.293 | 1.536 | 14.979 | 0.0561367 | 7/15/2014 | 20:37.1 | 6 |
| -1832.103 | 19808.3 | 1.53 | 14.981 | 0.0577422 | 7/15/2014 | 20:37.2 | 6 |
| -1831.832 | 19808.308 | 1.532 | 14.976 | 0.0597896 | 7/15/2014 | 20:37.3 | 6 |
| -1831.497 | 19808.316 | 1.524 | 14.977 | 0.064363 | 7/15/2014 | 20:37.4 | 6 |
| -1831.227 | 19808.323 | 1.514 | 14.997 | 0.065875 | 7/15/2014 | 20:37.5 | 6 |
| -1830.958 | 19808.328 | 1.512 | 15 | 0.0661603 | 7/15/2014 | 20:37.6 | 6 |

| | | | | | | | |
|---|---|---|---|---|---|---|---|
| -1830.689 | 19808.335 | 1.512 | 15.008 | 0.0755651 | 7/15/2014 | 20:37.7 | 6 |
| -1830.356 | 19808.343 | 1.497 | 15.021 | 0.0786259 | 7/15/2014 | 20:37.8 | 6 |
| -1830.087 | 19808.349 | 1.495 | 15.037 | 0.0725555 | 7/15/2014 | 20:37.9 | 6 |
| -1829.82 | 19808.354 | 1.478 | 15.062 | 0.0735052 | 7/15/2014 | 20:38.0 | 6 |
| -1829.552 | 19808.36 | 1.475 | 15.072 | 0.0749818 | 7/15/2014 | 20:38.1 | 6 |
| -1829.287 | 19808.365 | 1.469 | 15.062 | 0.0801939 | 7/15/2014 | 20:38.2 | 6 |
| -1829.021 | 19808.371 | 1.463 | 15.068 | 0.0928826 | 7/15/2014 | 20:38.3 | 6 |
| -1828.695 | 19808.378 | 1.451 | 15.074 | 0.103871 | 7/15/2014 | 20:38.4 | 6 |
| -1828.432 | 19808.383 | 1.457 | 15.067 | 0.107077 | 7/15/2014 | 20:38.5 | 6 |
| -1828.173 | 19808.386 | 1.45 | 15.057 | 0.112761 | 7/15/2014 | 20:38.6 | 6 |
| -1827.912 | 19808.393 | 1.462 | 15.049 | 0.131175 | 7/15/2014 | 20:38.7 | 6 |
| -1827.59 | 19808.397 | 1.461 | 15.05 | 0.125065 | 7/15/2014 | 20:38.8 | 6 |
| -1827.329 | 19808.402 | 1.47 | 15.05 | 0.126695 | 7/15/2014 | 20:38.9 | 6 |
| -1827.071 | 19808.406 | 1.47 | 15.056 | 0.129277 | 7/15/2014 | 20:39.0 | 6 |
| -1826.81 | 19808.41 | 1.476 | 15.052 | 0.120288 | 7/15/2014 | 20:39.1 | 6 |
| -1826.551 | 19808.413 | 1.473 | 15.062 | 0.134667 | 7/15/2014 | 20:39.2 | 6 |
| -1826.291 | 19808.418 | 1.473 | 15.067 | 0.100587 | 7/15/2014 | 20:39.3 | 6 |
| -1825.969 | 19808.422 | 1.465 | 15.05 | 0.115639 | 7/15/2014 | 20:39.4 | 6 |
| -1825.709 | 19808.425 | 1.469 | 15.042 | 0.112108 | 7/15/2014 | 20:39.5 | 6 |
| -1825.45 | 19808.429 | 1.459 | 15.032 | 0.0971744 | 7/15/2014 | 20:39.6 | 6 |
| -1825.19 | 19808.431 | 1.451 | 15.013 | 0.100745 | 7/15/2014 | 20:39.7 | 6 |
| -1824.862 | 19808.435 | 1.445 | 15.004 | 0.091455 | 7/15/2014 | 20:39.8 | 6 |
| -1824.598 | 19808.438 | 1.442 | 14.985 | 0.122566 | 7/15/2014 | 20:39.9 | 6 |
| -1824.333 | 19808.439 | 1.435 | 14.982 | 0.0947688 | 7/15/2014 | 20:40.0 | 6 |
| -1824.066 | 19808.441 | 1.43 | 14.986 | 0.0921978 | 7/15/2014 | 20:40.1 | 6 |
| -1823.8 | 19808.443 | 1.416 | 14.99 | 0.0902538 | 7/15/2014 | 20:40.2 | 6 |
| -1823.533 | 19808.446 | 1.407 | 14.995 | 0.106425 | 7/15/2014 | 20:40.3 | 6 |
| -1823.201 | 19808.448 | 1.401 | 15.014 | 0.0949507 | 7/15/2014 | 20:40.4 | 6 |
| -1822.932 | 19808.45 | 1.396 | 15.027 | 0.0825586 | 7/15/2014 | 20:40.5 | 6 |
| -1822.665 | 19808.451 | 1.382 | 15.045 | 0.0865417 | 7/15/2014 | 20:40.6 | 6 |
| -1822.395 | 19808.453 | 1.374 | 15.052 | 0.0909123 | 7/15/2014 | 20:40.7 | 6 |
| -1822.059 | 19808.453 | 1.365 | 15.075 | 0.10344 | 7/15/2014 | 20:40.8 | 6 |
| -1821.788 | 19808.455 | 1.359 | 15.094 | 0.100149 | 7/15/2014 | 20:40.9 | 6 |
| -1821.518 | 19808.455 | 1.35 | 15.125 | 0.0914223 | 7/15/2014 | 20:41.0 | 6 |
| -1821.245 | 19808.456 | 1.344 | 15.142 | 0.107138 | 7/15/2014 | 20:41.1 | 6 |
| -1820.974 | 19808.456 | 1.332 | 15.161 | 0.111914 | 7/15/2014 | 20:41.2 | 6 |
| -1820.703 | 19808.458 | 1.326 | 15.173 | 0.107313 | 7/15/2014 | 20:41.3 | 6 |
| -1820.365 | 19808.457 | 1.311 | 15.195 | 0.110671 | 7/15/2014 | 20:41.4 | 6 |
| -1820.093 | 19808.457 | 1.301 | 15.208 | 0.124283 | 7/15/2014 | 20:41.5 | 6 |
| -1819.822 | 19808.457 | 1.289 | 15.218 | 0.145761 | 7/15/2014 | 20:41.6 | 6 |
| -1819.548 | 19808.457 | 1.283 | 15.227 | 0.127277 | 7/15/2014 | 20:41.7 | 6 |
| -1819.206 | 19808.456 | 1.269 | 15.237 | 0.128312 | 7/15/2014 | 20:41.8 | 6 |
| -1819.069 | 19808.457 | 1.261 | 15.242 | 0.130997 | 7/15/2014 | 20:41.8 | 6 |
| -1818.793 | 19808.456 | 1.248 | 15.243 | 0.111132 | 7/15/2014 | 20:41.9 | 6 |
| -1818.518 | 19808.455 | 1.238 | 15.248 | 0.102795 | 7/15/2014 | 20:42.0 | 6 |
| -1818.241 | 19808.455 | 1.227 | 15.238 | 0.0837151 | 7/15/2014 | 20:42.1 | 6 |
| -1817.964 | 19808.455 | 1.212 | 15.23 | 0.0809431 | 7/15/2014 | 20:42.2 | 6 |

| | | | | | | | |
|---|---|---|---|---|---|---|---|
| -1817.479 | 19808.452 | 1.198 | 15.227 | 0.0973231 | 7/15/2014 | 20:42.4 | 6 |
| -1817.201 | 19808.452 | 1.195 | 15.225 | 0.111625 | 7/15/2014 | 20:42.5 | 6 |
| -1816.925 | 19808.45 | 1.191 | 15.231 | 0.135213 | 7/15/2014 | 20:42.6 | 6 |
| -1816.648 | 19808.449 | 1.188 | 15.234 | 0.132381 | 7/15/2014 | 20:42.7 | 6 |
| -1816.304 | 19808.448 | 1.179 | 15.25 | 0.149265 | 7/15/2014 | 20:42.8 | 6 |
| -1816.163 | 19808.447 | 1.182 | 15.261 | 0.15429 | 7/15/2014 | 20:42.8 | 6 |
| -1815.887 | 19808.445 | 1.179 | 15.273 | 0.179664 | 7/15/2014 | 20:42.9 | 6 |
| -1815.61 | 19808.445 | 1.166 | 15.299 | 0.202616 | 7/15/2014 | 20:43.0 | 6 |
| -1815.334 | 19808.442 | 1.151 | 15.324 | 0.214045 | 7/15/2014 | 20:43.1 | 6 |
| -1815.06 | 19808.441 | 1.136 | 15.366 | 0.210554 | 7/15/2014 | 20:43.2 | 6 |
| -1814.581 | 19808.438 | 1.109 | 15.396 | 0.183468 | 7/15/2014 | 20:43.4 | 6 |
| -1814.307 | 19808.437 | 1.097 | 15.413 | 0.184789 | 7/15/2014 | 20:43.5 | 6 |
| -1814.034 | 19808.436 | 1.076 | 15.427 | 0.185709 | 7/15/2014 | 20:43.6 | 6 |
| -1813.761 | 19808.432 | 1.06 | 15.431 | 0.200351 | 7/15/2014 | 20:43.7 | 6 |
| -1813.422 | 19808.43 | 1.048 | 15.433 | 0.177945 | 7/15/2014 | 20:43.8 | 6 |
| -1813.148 | 19808.427 | 1.046 | 15.418 | 0.214706 | 7/15/2014 | 20:43.9 | 6 |
| -1812.875 | 19808.426 | 1.043 | 15.42 | 0.202597 | 7/15/2014 | 20:44.0 | 6 |
| -1812.602 | 19808.423 | 1.043 | 15.411 | 0.168037 | 7/15/2014 | 20:44.1 | 6 |
| -1812.33 | 19808.421 | 1.038 | 15.399 | 0.191023 | 7/15/2014 | 20:44.2 | 6 |
| -1812.057 | 19808.418 | 1.038 | 15.382 | 0.158781 | 7/15/2014 | 20:44.3 | 6 |
| -1811.72 | 19808.414 | 1.035 | 15.376 | 0.172515 | 7/15/2014 | 20:44.4 | 6 |
| -1811.449 | 19808.412 | 1.031 | 15.362 | 0.178836 | 7/15/2014 | 20:44.5 | 6 |
| -1811.181 | 19808.41 | 1.019 | 15.353 | 0.179944 | 7/15/2014 | 20:44.6 | 6 |
| -1810.912 | 19808.406 | 1.015 | 15.341 | 0.188708 | 7/15/2014 | 20:44.7 | 6 |
| -1810.58 | 19808.402 | 1.007 | 15.339 | 0.137079 | 7/15/2014 | 20:44.8 | 6 |
| -1810.313 | 19808.4 | 0.999 | 15.356 | 0.141664 | 7/15/2014 | 20:44.9 | 6 |
| -1810.046 | 19808.396 | 0.987 | 15.369 | 0.161673 | 7/15/2014 | 20:45.0 | 6 |
| -1809.779 | 19808.394 | 0.981 | 15.386 | 0.133037 | 7/15/2014 | 20:45.1 | 6 |
| -1809.514 | 19808.391 | 0.973 | 15.409 | 0.148379 | 7/15/2014 | 20:45.2 | 6 |
| -1809.25 | 19808.388 | 0.963 | 15.436 | 0.136253 | 7/15/2014 | 20:45.3 | 6 |
| -1808.923 | 19808.383 | 0.951 | 15.472 | 0.156077 | 7/15/2014 | 20:45.4 | 6 |
| -1808.661 | 19808.381 | 0.942 | 15.509 | 0.143331 | 7/15/2014 | 20:45.5 | 6 |
| -1808.4 | 19808.377 | 0.934 | 15.546 | 0.11411 | 7/15/2014 | 20:45.6 | 6 |
| -1808.138 | 19808.373 | 0.928 | 15.576 | 0.108749 | 7/15/2014 | 20:45.7 | 6 |
| -1807.812 | 19808.369 | 0.918 | 15.59 | 0.0874087 | 7/15/2014 | 20:45.8 | 6 |
| -1807.549 | 19808.366 | 0.908 | 15.606 | 0.0952963 | 7/15/2014 | 20:45.9 | 6 |
| -1807.286 | 19808.362 | 0.9 | 15.605 | 0.106126 | 7/15/2014 | 20:46.0 | 6 |
| -1807.023 | 19808.359 | 0.9 | 15.597 | 0.141573 | 7/15/2014 | 20:46.1 | 6 |
| -1806.76 | 19808.355 | 0.893 | 15.582 | 0.137996 | 7/15/2014 | 20:46.2 | 6 |
| -1806.497 | 19808.352 | 0.889 | 15.55 | 0.155823 | 7/15/2014 | 20:46.3 | 6 |
| -1806.17 | 19808.349 | 0.885 | 15.538 | 0.178614 | 7/15/2014 | 20:46.4 | 6 |
| -1805.906 | 19808.345 | 0.885 | 15.535 | 0.154338 | 7/15/2014 | 20:46.5 | 6 |
| -1805.643 | 19808.341 | 0.883 | 15.527 | 0.16305 | 7/15/2014 | 20:46.6 | 6 |
| -1805.377 | 19808.338 | 0.885 | 15.528 | 0.15929 | 7/15/2014 | 20:46.7 | 6 |
| -1805.046 | 19808.333 | 0.877 | 15.524 | 0.148187 | 7/15/2014 | 20:46.8 | 6 |
| -1804.778 | 19808.329 | 0.872 | 15.523 | 0.110342 | 7/15/2014 | 20:46.9 | 6 |
| -1804.509 | 19808.326 | 0.867 | 15.52 | 0.073691 | 7/15/2014 | 20:47.0 | 6 |

| | | | | | | | |
|---|---|---|---|---|---|---|---|
| -1804.239 | 19808.323 | 0.864 | 15.522 | 0.0841553 | 7/15/2014 | 20:47.1 | 6 |
| -1803.969 | 19808.319 | 0.86 | 15.511 | 0.0582136 | 7/15/2014 | 20:47.2 | 6 |
| -1803.697 | 19808.316 | 0.856 | 15.502 | 0.0758362 | 7/15/2014 | 20:47.3 | 6 |
| -1803.361 | 19808.312 | 0.851 | 15.493 | 0.0685727 | 7/15/2014 | 20:47.4 | 6 |
| -1803.089 | 19808.308 | 0.853 | 15.488 | 0.084272 | 7/15/2014 | 20:47.5 | 6 |
| -1802.819 | 19808.304 | 0.853 | 15.484 | 0.0649421 | 7/15/2014 | 20:47.6 | 6 |
| -1802.547 | 19808.301 | 0.853 | 15.502 | 0.0817354 | 7/15/2014 | 20:47.7 | 6 |
| -1802.209 | 19808.297 | 0.847 | 15.512 | 0.107869 | 7/15/2014 | 20:47.8 | 6 |
| -1801.938 | 19808.293 | 0.845 | 15.53 | 0.128322 | 7/15/2014 | 20:47.9 | 6 |
| -1801.667 | 19808.289 | 0.845 | 15.557 | 0.0979893 | 7/15/2014 | 20:48.0 | 6 |
| -1801.396 | 19808.285 | 0.843 | 15.576 | 0.084309 | 7/15/2014 | 20:48.1 | 6 |
| -1801.127 | 19808.281 | 0.84 | 15.594 | 0.115979 | 7/15/2014 | 20:48.2 | 6 |
| -1800.859 | 19808.278 | 0.838 | 15.604 | 0.0969968 | 7/15/2014 | 20:48.3 | 6 |
| -1800.526 | 19808.275 | 0.832 | 15.599 | 0.110436 | 7/15/2014 | 20:48.4 | 6 |
| -1800.259 | 19808.271 | 0.838 | 15.58 | 0.108289 | 7/15/2014 | 20:48.5 | 6 |
| -1799.995 | 19808.269 | 0.834 | 15.561 | 0.147533 | 7/15/2014 | 20:48.6 | 6 |
| -1799.729 | 19808.266 | 0.835 | 15.532 | 0.157191 | 7/15/2014 | 20:48.7 | 6 |
| -1799.399 | 19808.261 | 0.838 | 15.508 | 0.128895 | 7/15/2014 | 20:48.8 | 6 |
| -1799.132 | 19808.259 | 0.832 | 15.481 | 0.127129 | 7/15/2014 | 20:48.9 | 6 |
| -1798.867 | 19808.256 | 0.831 | 15.458 | 0.122108 | 7/15/2014 | 20:49.0 | 6 |
| -1798.599 | 19808.254 | 0.837 | 15.439 | 0.154352 | 7/15/2014 | 20:49.1 | 6 |
| -1798.333 | 19808.252 | 0.831 | 15.424 | 0.182531 | 7/15/2014 | 20:49.2 | 6 |
| -1798.066 | 19808.25 | 0.837 | 15.402 | 0.172857 | 7/15/2014 | 20:49.3 | 6 |
| -1797.736 | 19808.245 | 0.835 | 15.38 | 0.205243 | 7/15/2014 | 20:49.4 | 6 |
| -1797.469 | 19808.244 | 0.841 | 15.365 | 0.208177 | 7/15/2014 | 20:49.5 | 6 |
| -1797.205 | 19808.241 | 0.844 | 15.348 | 0.207252 | 7/15/2014 | 20:49.6 | 6 |
| -1796.939 | 19808.24 | 0.841 | 15.324 | 0.155056 | 7/15/2014 | 20:49.7 | 6 |
| -1796.61 | 19808.239 | 0.838 | 15.297 | 0.0856487 | 7/15/2014 | 20:49.8 | 6 |
| -1796.344 | 19808.237 | 0.847 | 15.275 | 0.115266 | 7/15/2014 | 20:49.9 | 6 |
| -1796.08 | 19808.234 | 0.841 | 15.261 | 0.11778 | 7/15/2014 | 20:50.0 | 6 |
| -1795.814 | 19808.234 | 0.845 | 15.249 | 0.132755 | 7/15/2014 | 20:50.1 | 6 |
| -1795.551 | 19808.233 | 0.833 | 15.239 | 0.157478 | 7/15/2014 | 20:50.2 | 6 |
| -1795.287 | 19808.231 | 0.833 | 15.237 | 0.149519 | 7/15/2014 | 20:50.3 | 6 |
| -1794.961 | 19808.231 | 0.839 | 15.256 | 0.189008 | 7/15/2014 | 20:50.4 | 6 |
| -1794.699 | 19808.23 | 0.843 | 15.283 | 0.147488 | 7/15/2014 | 20:50.5 | 6 |
| -1794.438 | 19808.229 | 0.833 | 15.309 | 0.154176 | 7/15/2014 | 20:50.6 | 6 |
| -1794.175 | 19808.229 | 0.839 | 15.336 | 0.17293 | 7/15/2014 | 20:50.7 | 6 |
| -1793.849 | 19808.229 | 0.833 | 15.36 | 0.142952 | 7/15/2014 | 20:50.8 | 6 |
| -1793.585 | 19808.229 | 0.833 | 15.378 | 0.224176 | 7/15/2014 | 20:50.9 | 6 |
| -1793.32 | 19808.229 | 0.831 | 15.397 | 0.204082 | 7/15/2014 | 20:51.0 | 6 |
| -1793.054 | 19808.229 | 0.827 | 15.403 | 0.250433 | 7/15/2014 | 20:51.1 | 6 |
| -1792.788 | 19808.23 | 0.821 | 15.404 | 0.225363 | 7/15/2014 | 20:51.2 | 6 |
| -1792.522 | 19808.229 | 0.817 | 15.404 | 0.214153 | 7/15/2014 | 20:51.3 | 6 |
| -1792.191 | 19808.231 | 0.805 | 15.402 | 0.180809 | 7/15/2014 | 20:51.4 | 6 |
| -1791.924 | 19808.231 | 0.805 | 15.402 | 0.181039 | 7/15/2014 | 20:51.5 | 6 |
| -1791.657 | 19808.234 | 0.799 | 15.4 | 0.156157 | 7/15/2014 | 20:51.6 | 6 |
| -1791.39 | 19808.235 | 0.796 | 15.395 | 0.150533 | 7/15/2014 | 20:51.7 | 6 |

| | | | | | | | |
|---|---|---|---|---|---|---|---|
| -1791.057 | 19808.237 | 0.788 | 15.4 | 0.151446 | 7/15/2014 | 20:51.8 | 6 |
| -1790.789 | 19808.239 | 0.788 | 15.401 | 0.15183 | 7/15/2014 | 20:51.9 | 6 |
| -1790.522 | 19808.241 | 0.779 | 15.406 | 0.203133 | 7/15/2014 | 20:52.0 | 6 |
| -1790.253 | 19808.242 | 0.779 | 15.412 | 0.155584 | 7/15/2014 | 20:52.1 | 6 |
| -1789.987 | 19808.246 | 0.767 | 15.413 | 0.206445 | 7/15/2014 | 20:52.2 | 6 |
| -1789.722 | 19808.249 | 0.767 | 15.417 | 0.205512 | 7/15/2014 | 20:52.3 | 6 |
| -1789.393 | 19808.252 | 0.76 | 15.424 | 0.224717 | 7/15/2014 | 20:52.4 | 6 |
| -1789.129 | 19808.256 | 0.756 | 15.425 | 0.234167 | 7/15/2014 | 20:52.5 | 6 |
| -1788.869 | 19808.259 | 0.742 | 15.434 | 0.203263 | 7/15/2014 | 20:52.6 | 6 |
| -1788.608 | 19808.261 | 0.744 | 15.442 | 0.230986 | 7/15/2014 | 20:52.7 | 6 |
| -1788.288 | 19808.266 | 0.742 | 15.456 | 0.190794 | 7/15/2014 | 20:52.8 | 6 |
| -1788.03 | 19808.271 | 0.744 | 15.473 | 0.196992 | 7/15/2014 | 20:52.9 | 6 |
| -1787.773 | 19808.274 | 0.733 | 15.494 | 0.21125 | 7/15/2014 | 20:53.0 | 6 |
| -1787.517 | 19808.279 | 0.728 | 15.517 | 0.166912 | 7/15/2014 | 20:53.1 | 6 |
| -1787.263 | 19808.283 | 0.726 | 15.537 | 0.165991 | 7/15/2014 | 20:53.2 | 6 |
| -1787.011 | 19808.287 | 0.728 | 15.553 | 0.154574 | 7/15/2014 | 20:53.3 | 6 |
| -1786.698 | 19808.293 | 0.726 | 15.57 | 0.147527 | 7/15/2014 | 20:53.4 | 6 |
| -1786.327 | 19808.302 | 0.721 | 15.581 | 0.143142 | 7/15/2014 | 20:53.5 | 6 |
| -1786.081 | 19808.306 | 0.719 | 15.59 | 0.11227 | 7/15/2014 | 20:53.6 | 6 |
| -1785.775 | 19808.313 | 0.719 | 15.594 | 0.0802065 | 7/15/2014 | 20:53.7 | 6 |
| -1785.649 | 19808.315 | 0.72 | 15.594 | 0.0721463 | 7/15/2014 | 20:53.8 | 6 |
| -1785.404 | 19808.321 | 0.717 | 15.59 | 0.0677836 | 7/15/2014 | 20:53.9 | 6 |
| -1785.159 | 19808.327 | 0.711 | 15.58 | 0.0694157 | 7/15/2014 | 20:54.0 | 6 |
| -1784.913 | 19808.332 | 0.711 | 15.565 | 0.0769807 | 7/15/2014 | 20:54.1 | 6 |
| -1784.67 | 19808.34 | 0.699 | 15.548 | 0.0792083 | 7/15/2014 | 20:54.2 | 6 |
| -1784.426 | 19808.346 | 0.699 | 15.539 | 0.0760293 | 7/15/2014 | 20:54.3 | 6 |
| -1784.003 | 19808.356 | 0.69 | 15.531 | 0.0812355 | 7/15/2014 | 20:54.4 | 6 |
| -1783.761 | 19808.363 | 0.687 | 15.529 | 0.0859223 | 7/15/2014 | 20:54.5 | 6 |
| -1783.52 | 19808.37 | 0.683 | 15.538 | 0.096805 | 7/15/2014 | 20:54.6 | 6 |
| -1783.215 | 19808.379 | 0.679 | 15.549 | 0.0671399 | 7/15/2014 | 20:54.7 | 6 |
| -1783.094 | 19808.383 | 0.672 | 15.555 | 0.0775011 | 7/15/2014 | 20:54.8 | 6 |
| -1782.848 | 19808.391 | 0.676 | 15.573 | 0.0483496 | 7/15/2014 | 20:54.9 | 6 |
| -1782.602 | 19808.398 | 0.667 | 15.588 | 0.0350214 | 7/15/2014 | 20:55.0 | 6 |
| -1782.354 | 19808.406 | 0.667 | 15.606 | 0.017923 | 7/15/2014 | 20:55.1 | 6 |
| -1782.105 | 19808.414 | 0.665 | 15.619 | 0.0385501 | 7/15/2014 | 20:55.2 | 6 |
| -1781.855 | 19808.424 | 0.662 | 15.628 | 0.0772742 | 7/15/2014 | 20:55.3 | 6 |
| -1781.544 | 19808.434 | 0.654 | 15.632 | 0.144034 | 7/15/2014 | 20:55.4 | 6 |
| -1781.291 | 19808.442 | 0.654 | 15.655 | 0.1658 | 7/15/2014 | 20:55.5 | 6 |
| -1781.038 | 19808.452 | 0.651 | 15.669 | 0.223938 | 7/15/2014 | 20:55.6 | 6 |
| -1780.782 | 19808.463 | 0.648 | 15.679 | 0.257862 | 7/15/2014 | 20:55.7 | 6 |
| -1780.46 | 19808.475 | 0.642 | 15.692 | 0.180545 | 7/15/2014 | 20:55.8 | 6 |
| -1780.2 | 19808.485 | 0.639 | 15.704 | 0.137937 | 7/15/2014 | 20:55.9 | 6 |
| -1779.938 | 19808.495 | 0.639 | 15.709 | 0.11061 | 7/15/2014 | 20:56.0 | 6 |
| -1779.675 | 19808.505 | 0.639 | 15.71 | 0.110891 | 7/15/2014 | 20:56.1 | 6 |
| -1779.411 | 19808.516 | 0.629 | 15.692 | 0.148314 | 7/15/2014 | 20:56.2 | 6 |
| -1779.146 | 19808.527 | 0.626 | 15.681 | 0.196467 | 7/15/2014 | 20:56.3 | 6 |
| -1778.817 | 19808.542 | 0.623 | 15.68 | 0.233296 | 7/15/2014 | 20:56.4 | 6 |

| | | | | | | | |
|---|---|---|---|---|---|---|---|
| -1778.551 | 19808.553 | 0.617 | 15.679 | 0.243327 | 7/15/2014 | 20:56.5 | 6 |
| -1778.286 | 19808.564 | 0.604 | 15.683 | 0.240872 | 7/15/2014 | 20:56.6 | 6 |
| -1778.021 | 19808.576 | 0.592 | 15.698 | 0.219875 | 7/15/2014 | 20:56.7 | 6 |
| -1777.693 | 19808.59 | 0.588 | 15.713 | 0.213176 | 7/15/2014 | 20:56.8 | 6 |
| -1777.43 | 19808.602 | 0.588 | 15.73 | 0.219024 | 7/15/2014 | 20:56.9 | 6 |
| -1777.167 | 19808.615 | 0.579 | 15.75 | 0.145552 | 7/15/2014 | 20:57.0 | 6 |
| -1776.906 | 19808.627 | 0.569 | 15.768 | 0.111838 | 7/15/2014 | 20:57.1 | 6 |
| -1776.646 | 19808.64 | 0.563 | 15.785 | 0.0476907 | 7/15/2014 | 20:57.2 | 6 |
| -1776.387 | 19808.652 | 0.561 | 15.791 | 0.139461 | 7/15/2014 | 20:57.3 | 6 |
| -1776.068 | 19808.666 | 0.553 | 15.797 | 0.140176 | 7/15/2014 | 20:57.4 | 6 |
| -1775.815 | 19808.678 | 0.551 | 15.803 | 0.114339 | 7/15/2014 | 20:57.5 | 6 |
| -1775.563 | 19808.691 | 0.542 | 15.807 | 0.130773 | 7/15/2014 | 20:57.6 | 6 |
| -1775.312 | 19808.704 | 0.54 | 15.82 | 0.0968852 | 7/15/2014 | 20:57.7 | 6 |
| -1775.001 | 19808.721 | 0.534 | 15.821 | 0.110048 | 7/15/2014 | 20:57.8 | 6 |
| -1774.75 | 19808.732 | 0.53 | 15.821 | 0.107975 | 7/15/2014 | 20:57.9 | 6 |
| -1774.501 | 19808.744 | 0.524 | 15.827 | 0.137917 | 7/15/2014 | 20:58.0 | 6 |
| -1774.252 | 19808.758 | 0.522 | 15.826 | 0.154199 | 7/15/2014 | 20:58.1 | 6 |
| -1774.005 | 19808.771 | 0.505 | 15.846 | 0.156724 | 7/15/2014 | 20:58.2 | 6 |
| -1773.758 | 19808.784 | 0.501 | 15.855 | 0.157696 | 7/15/2014 | 20:58.3 | 6 |
| -1773.327 | 19808.807 | 0.493 | 15.859 | 0.193218 | 7/15/2014 | 20:58.4 | 6 |
| -1773.081 | 19808.82 | 0.491 | 15.864 | 0.159178 | 7/15/2014 | 20:58.5 | 6 |
| -1772.836 | 19808.832 | 0.489 | 15.869 | 0.144339 | 7/15/2014 | 20:58.6 | 6 |
| -1772.528 | 19808.85 | 0.487 | 15.873 | 0.144437 | 7/15/2014 | 20:58.7 | 6 |
| -1772.404 | 19808.855 | 0.485 | 15.882 | 0.129147 | 7/15/2014 | 20:58.8 | 6 |
| -1772.156 | 19808.87 | 0.49 | 15.871 | 0.0868803 | 7/15/2014 | 20:58.9 | 6 |
| -1771.906 | 19808.883 | 0.49 | 15.871 | 0.0945368 | 7/15/2014 | 20:59.0 | 6 |
| -1771.656 | 19808.896 | 0.493 | 15.871 | 0.103761 | 7/15/2014 | 20:59.1 | 6 |
| -1771.405 | 19808.91 | 0.483 | 15.862 | 0.187703 | 7/15/2014 | 20:59.2 | 6 |
| -1771.154 | 19808.924 | 0.485 | 15.856 | 0.202432 | 7/15/2014 | 20:59.3 | 6 |
| -1770.841 | 19808.941 | 0.475 | 15.847 | 0.174298 | 7/15/2014 | 20:59.4 | 6 |
| -1770.589 | 19808.955 | 0.475 | 15.834 | 0.232376 | 7/15/2014 | 20:59.5 | 6 |
| -1770.336 | 19808.969 | 0.476 | 15.82 | 0.228672 | 7/15/2014 | 20:59.6 | 6 |
| -1770.082 | 19808.982 | 0.476 | 15.815 | 0.224744 | 7/15/2014 | 20:59.7 | 6 |
| -1769.767 | 19809 | 0.476 | 15.803 | 0.148928 | 7/15/2014 | 20:59.8 | 6 |
| -1769.513 | 19809.014 | 0.48 | 15.8 | 0.169786 | 7/15/2014 | 20:59.9 | 6 |
| -1769.196 | 19809.033 | 0.475 | 15.798 | 0.155515 | 7/15/2014 | 21:00.0 | 6 |
| -1769.068 | 19809.039 | 0.484 | 15.795 | 0.156342 | 7/15/2014 | 21:00.0 | 6 |
| -1768.751 | 19809.057 | 0.486 | 15.804 | 0.0954239 | 7/15/2014 | 21:00.2 | 6 |
| -1768.498 | 19809.07 | 0.486 | 15.807 | 0.0383694 | 7/15/2014 | 21:00.3 | 6 |
| -1768.183 | 19809.088 | 0.478 | 15.8 | 0.0386082 | 7/15/2014 | 21:00.4 | 6 |
| -1767.931 | 19809.102 | 0.481 | 15.8 | 0.0461934 | 7/15/2014 | 21:00.5 | 6 |
| -1767.489 | 19809.127 | 0.474 | 15.781 | 0.124538 | 7/15/2014 | 21:00.6 | 6 |
| -1767.426 | 19809.13 | 0.476 | 15.779 | 0.1824 | 7/15/2014 | 21:00.7 | 6 |
| -1767.108 | 19809.148 | 0.473 | 15.764 | 0.127062 | 7/15/2014 | 21:00.8 | 6 |
| -1766.853 | 19809.162 | 0.471 | 15.754 | 0.136511 | 7/15/2014 | 21:00.9 | 6 |
| -1766.596 | 19809.176 | 0.467 | 15.739 | 0.172796 | 7/15/2014 | 21:01.0 | 6 |
| -1766.336 | 19809.191 | 0.472 | 15.724 | 0.141479 | 7/15/2014 | 21:01.1 | 6 |

| | | | | | | | |
|---|---|---|---|---|---|---|---|
| -1766.077 | 19809.205 | 0.47 | 15.705 | 0.122504 | 7/15/2014 | 21:01.2 | 6 |
| -1765.818 | 19809.219 | 0.472 | 15.694 | 0.154871 | 7/15/2014 | 21:01.3 | 6 |
| -1765.494 | 19809.238 | 0.475 | 15.689 | 0.193835 | 7/15/2014 | 21:01.4 | 6 |
| -1765.233 | 19809.252 | 0.475 | 15.682 | 0.225461 | 7/15/2014 | 21:01.5 | 6 |
| -1764.971 | 19809.268 | 0.477 | 15.681 | 0.216183 | 7/15/2014 | 21:01.6 | 6 |
| -1764.708 | 19809.281 | 0.477 | 15.693 | 0.242023 | 7/15/2014 | 21:01.7 | 6 |
| -1764.382 | 19809.3 | 0.471 | 15.714 | 0.229683 | 7/15/2014 | 21:01.8 | 6 |
| -1764.118 | 19809.313 | 0.468 | 15.741 | 0.220335 | 7/15/2014 | 21:01.9 | 6 |
| -1763.855 | 19809.328 | 0.466 | 15.768 | 0.181358 | 7/15/2014 | 21:02.0 | 6 |
| -1763.592 | 19809.343 | 0.466 | 15.796 | 0.163617 | 7/15/2014 | 21:02.1 | 6 |
| -1763.33 | 19809.357 | 0.458 | 15.82 | 0.13115 | 7/15/2014 | 21:02.2 | 6 |
| -1763.069 | 19809.371 | 0.455 | 15.836 | 0.112655 | 7/15/2014 | 21:02.3 | 6 |
| -1762.745 | 19809.389 | 0.447 | 15.839 | 0.130664 | 7/15/2014 | 21:02.4 | 6 |
| -1762.486 | 19809.403 | 0.45 | 15.831 | 0.11585 | 7/15/2014 | 21:02.5 | 6 |
| -1762.228 | 19809.416 | 0.45 | 15.811 | 0.18593 | 7/15/2014 | 21:02.6 | 6 |
| -1761.97 | 19809.431 | 0.45 | 15.789 | 0.297532 | 7/15/2014 | 21:02.7 | 6 |
| -1761.649 | 19809.447 | 0.449 | 15.764 | 0.31106 | 7/15/2014 | 21:02.8 | 6 |
| -1761.391 | 19809.461 | 0.457 | 15.743 | 0.26037 | 7/15/2014 | 21:02.9 | 6 |
| -1761.133 | 19809.474 | 0.466 | 15.729 | 0.265906 | 7/15/2014 | 21:03.0 | 6 |
| -1760.874 | 19809.488 | 0.475 | 15.713 | 0.21891 | 7/15/2014 | 21:03.1 | 6 |
| -1760.616 | 19809.5 | 0.471 | 15.708 | 0.229491 | 7/15/2014 | 21:03.2 | 6 |
| -1760.357 | 19809.516 | 0.477 | 15.702 | 0.147311 | 7/15/2014 | 21:03.3 | 6 |
| -1760.036 | 19809.531 | 0.479 | 15.698 | 0.0557051 | 7/15/2014 | 21:03.4 | 6 |
| -1759.777 | 19809.544 | 0.489 | 15.689 | 0.123583 | 7/15/2014 | 21:03.5 | 6 |
| -1759.517 | 19809.558 | 0.489 | 15.672 | 0.0937336 | 7/15/2014 | 21:03.6 | 6 |
| -1759.256 | 19809.57 | 0.487 | 15.649 | 0.1572 | 7/15/2014 | 21:03.7 | 6 |
| -1758.926 | 19809.586 | 0.49 | 15.631 | 0.104496 | 7/15/2014 | 21:03.8 | 6 |
| -1758.66 | 19809.602 | 0.503 | 15.609 | 0.140025 | 7/15/2014 | 21:03.9 | 6 |
| -1758.391 | 19809.613 | 0.503 | 15.593 | 0.18676 | 7/15/2014 | 21:04.0 | 6 |
| -1758.122 | 19809.628 | 0.506 | 15.579 | 0.156409 | 7/15/2014 | 21:04.1 | 6 |
| -1757.851 | 19809.641 | 0.5 | 15.568 | 0.152113 | 7/15/2014 | 21:04.2 | 6 |
| -1757.58 | 19809.653 | 0.51 | 15.556 | 0.157126 | 7/15/2014 | 21:04.3 | 6 |
| -1757.241 | 19809.67 | 0.512 | 15.545 | 0.141396 | 7/15/2014 | 21:04.4 | 6 |
| -1756.968 | 19809.684 | 0.521 | 15.528 | 0.119646 | 7/15/2014 | 21:04.5 | 6 |
| -1756.695 | 19809.697 | 0.521 | 15.516 | 0.0833421 | 7/15/2014 | 21:04.6 | 6 |
| -1756.423 | 19809.711 | 0.527 | 15.498 | 0.0831684 | 7/15/2014 | 21:04.7 | 6 |
| -1756.086 | 19809.727 | 0.533 | 15.48 | 0.120597 | 7/15/2014 | 21:04.8 | 6 |
| -1755.815 | 19809.739 | 0.545 | 15.463 | 0.0845059 | 7/15/2014 | 21:04.9 | 6 |
| -1755.545 | 19809.752 | 0.547 | 15.447 | 0.118534 | 7/15/2014 | 21:05.0 | 6 |
| -1755.276 | 19809.764 | 0.551 | 15.43 | 0.106029 | 7/15/2014 | 21:05.1 | 6 |
| -1755.009 | 19809.777 | 0.545 | 15.414 | 0.0908134 | 7/15/2014 | 21:05.2 | 6 |
| -1754.745 | 19809.79 | 0.553 | 15.393 | 0.114409 | 7/15/2014 | 21:05.3 | 6 |
| -1754.416 | 19809.806 | 0.553 | 15.378 | 0.0881672 | 7/15/2014 | 21:05.4 | 6 |
| -1754.155 | 19809.817 | 0.556 | 15.36 | 0.0925687 | 7/15/2014 | 21:05.5 | 6 |
| -1753.896 | 19809.828 | 0.556 | 15.342 | 0.0976885 | 7/15/2014 | 21:05.6 | 6 |
| -1753.636 | 19809.84 | 0.56 | 15.325 | 0.111545 | 7/15/2014 | 21:05.7 | 6 |
| -1753.314 | 19809.855 | 0.569 | 15.317 | 0.144504 | 7/15/2014 | 21:05.8 | 6 |

| | | | | | | | |
|---|---|---|---|---|---|---|---|
| -1753.055 | 19809.868 | 0.58 | 15.312 | 0.114493 | 7/15/2014 | 21:05.9 | 6 |
| -1752.796 | 19809.88 | 0.58 | 15.312 | 0.118617 | 7/15/2014 | 21:06.0 | 6 |
| -1752.537 | 19809.891 | 0.591 | 15.312 | 0.11507 | 7/15/2014 | 21:06.1 | 6 |
| -1752.278 | 19809.9 | 0.599 | 15.316 | 0.118749 | 7/15/2014 | 21:06.2 | 6 |
| -1752.019 | 19809.912 | 0.608 | 15.313 | 0.108523 | 7/15/2014 | 21:06.3 | 6 |
| -1751.698 | 19809.928 | 0.614 | 15.313 | 0.0923913 | 7/15/2014 | 21:06.4 | 6 |
| -1751.439 | 19809.939 | 0.614 | 15.316 | 0.119832 | 7/15/2014 | 21:06.5 | 6 |
| -1751.182 | 19809.951 | 0.617 | 15.318 | 0.106342 | 7/15/2014 | 21:06.6 | 6 |
| -1750.924 | 19809.96 | 0.62 | 15.316 | 0.147035 | 7/15/2014 | 21:06.7 | 6 |
| -1750.6 | 19809.973 | 0.62 | 15.306 | 0.136315 | 7/15/2014 | 21:06.8 | 6 |
| -1750.34 | 19809.984 | 0.626 | 15.288 | 0.109819 | 7/15/2014 | 21:06.9 | 6 |
| -1750.079 | 19809.996 | 0.628 | 15.283 | 0.148891 | 7/15/2014 | 21:07.0 | 6 |
| -1749.817 | 19810.006 | 0.632 | 15.269 | 0.140225 | 7/15/2014 | 21:07.1 | 6 |
| -1749.556 | 19810.018 | 0.641 | 15.256 | 0.174732 | 7/15/2014 | 21:07.2 | 6 |
| -1749.294 | 19810.026 | 0.655 | 15.245 | 0.160483 | 7/15/2014 | 21:07.3 | 6 |
| -1748.969 | 19810.039 | 0.667 | 15.238 | 0.157721 | 7/15/2014 | 21:07.4 | 6 |
| -1748.706 | 19810.05 | 0.682 | 15.249 | 0.21476 | 7/15/2014 | 21:07.5 | 6 |
| -1748.443 | 19810.061 | 0.69 | 15.261 | 0.174146 | 7/15/2014 | 21:07.6 | 6 |
| -1748.18 | 19810.068 | 0.703 | 15.275 | 0.170988 | 7/15/2014 | 21:07.7 | 6 |
| -1747.851 | 19810.082 | 0.709 | 15.295 | 0.162047 | 7/15/2014 | 21:07.8 | 6 |
| -1747.586 | 19810.091 | 0.726 | 15.292 | 0.158905 | 7/15/2014 | 21:07.9 | 6 |
| -1747.321 | 19810.101 | 0.728 | 15.298 | 0.186837 | 7/15/2014 | 21:08.0 | 6 |
| -1747.055 | 19810.11 | 0.74 | 15.286 | 0.170603 | 7/15/2014 | 21:08.1 | 6 |
| -1746.79 | 19810.119 | 0.74 | 15.264 | 0.153971 | 7/15/2014 | 21:08.2 | 6 |
| -1746.524 | 19810.128 | 0.74 | 15.242 | 0.1753 | 7/15/2014 | 21:08.3 | 6 |
| -1746.195 | 19810.14 | 0.736 | 15.221 | 0.180075 | 7/15/2014 | 21:08.4 | 6 |
| -1745.93 | 19810.148 | 0.745 | 15.194 | 0.223103 | 7/15/2014 | 21:08.5 | 6 |
| -1745.664 | 19810.157 | 0.739 | 15.187 | 0.215947 | 7/15/2014 | 21:08.6 | 6 |
| -1745.399 | 19810.165 | 0.739 | 15.181 | 0.221716 | 7/15/2014 | 21:08.7 | 6 |
| -1745.068 | 19810.175 | 0.73 | 15.187 | 0.236079 | 7/15/2014 | 21:08.8 | 6 |
| -1744.8 | 19810.184 | 0.731 | 15.192 | 0.238078 | 7/15/2014 | 21:08.9 | 6 |
| -1744.532 | 19810.19 | 0.728 | 15.205 | 0.260109 | 7/15/2014 | 21:09.0 | 6 |
| -1744.263 | 19810.199 | 0.738 | 15.218 | 0.205004 | 7/15/2014 | 21:09.1 | 6 |
| -1743.995 | 19810.207 | 0.729 | 15.23 | 0.196744 | 7/15/2014 | 21:09.2 | 6 |
| -1743.726 | 19810.213 | 0.723 | 15.238 | 0.189302 | 7/15/2014 | 21:09.3 | 6 |
| -1743.393 | 19810.222 | 0.715 | 15.235 | 0.19029 | 7/15/2014 | 21:09.4 | 6 |
| -1743.124 | 19810.229 | 0.717 | 15.23 | 0.203805 | 7/15/2014 | 21:09.5 | 6 |
| -1742.856 | 19810.234 | 0.715 | 15.231 | 0.176728 | 7/15/2014 | 21:09.6 | 6 |
| -1742.588 | 19810.241 | 0.714 | 15.226 | 0.224857 | 7/15/2014 | 21:09.7 | 6 |
| -1742.255 | 19810.249 | 0.701 | 15.219 | 0.249706 | 7/15/2014 | 21:09.8 | 6 |
| -1741.987 | 19810.255 | 0.7 | 15.222 | 0.265859 | 7/15/2014 | 21:09.9 | 6 |
| -1741.719 | 19810.26 | 0.695 | 15.234 | 0.30792 | 7/15/2014 | 21:10.0 | 6 |
| -1741.451 | 19810.266 | 0.69 | 15.251 | 0.28967 | 7/15/2014 | 21:10.1 | 6 |
| -1741.185 | 19810.27 | 0.68 | 15.274 | 0.291112 | 7/15/2014 | 21:10.2 | 6 |
| -1740.919 | 19810.274 | 0.671 | 15.298 | 0.269796 | 7/15/2014 | 21:10.3 | 6 |
| -1740.59 | 19810.28 | 0.66 | 15.32 | 0.270714 | 7/15/2014 | 21:10.4 | 6 |
| -1740.326 | 19810.285 | 0.66 | 15.335 | 0.294214 | 7/15/2014 | 21:10.5 | 6 |

| | | | | | | | |
|---|---|---|---|---|---|---|---|
| -1740.063 | 19810.288 | 0.653 | 15.356 | 0.276261 | 7/15/2014 | 21:10.6 | 6 |
| -1739.799 | 19810.291 | 0.653 | 15.369 | 0.285146 | 7/15/2014 | 21:10.7 | 6 |
| -1739.47 | 19810.295 | 0.647 | 15.38 | 0.269346 | 7/15/2014 | 21:10.8 | 6 |
| -1739.204 | 19810.298 | 0.649 | 15.397 | 0.223085 | 7/15/2014 | 21:10.9 | 6 |
| -1738.938 | 19810.301 | 0.649 | 15.422 | 0.246357 | 7/15/2014 | 21:11.0 | 6 |
| -1738.671 | 19810.304 | 0.655 | 15.438 | 0.215531 | 7/15/2014 | 21:11.1 | 6 |
| -1738.405 | 19810.306 | 0.65 | 15.444 | 0.228906 | 7/15/2014 | 21:11.2 | 6 |
| -1738.138 | 19810.307 | 0.653 | 15.446 | 0.255022 | 7/15/2014 | 21:11.3 | 6 |
| -1737.806 | 19810.309 | 0.653 | 15.453 | 0.2748 | 7/15/2014 | 21:11.4 | 6 |
| -1737.538 | 19810.31 | 0.667 | 15.451 | 0.381848 | 7/15/2014 | 21:11.5 | 6 |
| -1737.271 | 19810.311 | 0.67 | 15.44 | 0.367841 | 7/15/2014 | 21:11.6 | 6 |
| -1737.003 | 19810.312 | 0.675 | 15.41 | 0.401959 | 7/15/2014 | 21:11.7 | 6 |
| -1736.67 | 19810.313 | 0.672 | 15.393 | 0.340476 | 7/15/2014 | 21:11.8 | 6 |
| -1736.401 | 19810.312 | 0.683 | 15.39 | 0.336289 | 7/15/2014 | 21:11.9 | 6 |
| -1736.133 | 19810.312 | 0.686 | 15.388 | 0.350475 | 7/15/2014 | 21:12.0 | 6 |
| -1735.864 | 19810.311 | 0.694 | 15.38 | 0.321197 | 7/15/2014 | 21:12.1 | 6 |
| -1735.597 | 19810.31 | 0.69 | 15.372 | 0.285515 | 7/15/2014 | 21:12.2 | 6 |
| -1735.33 | 19810.309 | 0.69 | 15.358 | 0.31221 | 7/15/2014 | 21:12.3 | 6 |
| -1734.999 | 19810.308 | 0.69 | 15.355 | 0.360619 | 7/15/2014 | 21:12.4 | 6 |
| -1734.734 | 19810.306 | 0.701 | 15.349 | 0.39127 | 7/15/2014 | 21:12.5 | 6 |
| -1734.471 | 19810.305 | 0.699 | 15.342 | 0.382665 | 7/15/2014 | 21:12.6 | 6 |
| -1734.207 | 19810.303 | 0.701 | 15.33 | 0.422992 | 7/15/2014 | 21:12.7 | 6 |
| -1733.88 | 19810.299 | 0.69 | 15.321 | 0.352493 | 7/15/2014 | 21:12.8 | 6 |
| -1733.617 | 19810.297 | 0.69 | 15.326 | 0.381298 | 7/15/2014 | 21:12.9 | 6 |
| -1733.354 | 19810.294 | 0.69 | 15.325 | 0.352254 | 7/15/2014 | 21:13.0 | 6 |
| -1733.091 | 19810.291 | 0.69 | 15.322 | 0.336084 | 7/15/2014 | 21:13.1 | 6 |
| -1732.829 | 19810.288 | 0.682 | 15.316 | 0.304003 | 7/15/2014 | 21:13.2 | 6 |
| -1732.568 | 19810.285 | 0.682 | 15.306 | 0.320137 | 7/15/2014 | 21:13.3 | 6 |
| -1732.244 | 19810.28 | 0.677 | 15.293 | 0.383339 | 7/15/2014 | 21:13.4 | 6 |
| -1731.984 | 19810.277 | 0.682 | 15.285 | 0.355668 | 7/15/2014 | 21:13.5 | 6 |
| -1731.723 | 19810.273 | 0.677 | 15.275 | 0.406975 | 7/15/2014 | 21:13.6 | 6 |
| -1731.462 | 19810.269 | 0.672 | 15.273 | 0.369158 | 7/15/2014 | 21:13.7 | 6 |
| -1731.135 | 19810.264 | 0.667 | 15.289 | 0.384679 | 7/15/2014 | 21:13.8 | 6 |
| -1730.872 | 19810.259 | 0.667 | 15.306 | 0.355667 | 7/15/2014 | 21:13.9 | 6 |
| -1730.607 | 19810.254 | 0.661 | 15.336 | 0.401315 | 7/15/2014 | 21:14.0 | 6 |
| -1730.341 | 19810.25 | 0.659 | 15.365 | 0.386963 | 7/15/2014 | 21:14.1 | 6 |
| -1730.076 | 19810.244 | 0.65 | 15.397 | 0.342577 | 7/15/2014 | 21:14.2 | 6 |
| -1729.809 | 19810.239 | 0.647 | 15.427 | 0.397472 | 7/15/2014 | 21:14.3 | 6 |
| -1729.478 | 19810.232 | 0.643 | 15.465 | 0.391281 | 7/15/2014 | 21:14.4 | 6 |
| -1729.212 | 19810.228 | 0.639 | 15.504 | 0.441496 | 7/15/2014 | 21:14.5 | 6 |
| -1728.946 | 19810.221 | 0.627 | 15.555 | 0.451304 | 7/15/2014 | 21:14.6 | 6 |
| -1728.679 | 19810.215 | 0.615 | 15.582 | 0.454124 | 7/15/2014 | 21:14.7 | 6 |
| -1728.349 | 19810.207 | 0.608 | 15.612 | 0.488781 | 7/15/2014 | 21:14.8 | 6 |
| -1728.082 | 19810.201 | 0.598 | 15.635 | 0.455438 | 7/15/2014 | 21:14.9 | 6 |
| -1727.817 | 19810.193 | 0.586 | 15.653 | 0.536049 | 7/15/2014 | 21:15.0 | 6 |
| -1727.551 | 19810.188 | 0.574 | 15.663 | 0.487128 | 7/15/2014 | 21:15.1 | 6 |
| -1727.286 | 19810.18 | 0.562 | 15.651 | 0.486829 | 7/15/2014 | 21:15.2 | 6 |

| | | | | | | | |
|---|---|---|---|---|---|---|---|
| -1727.023 | 19810.172 | 0.553 | 15.633 | 0.505438 | 7/15/2014 | 21:15.3 | 6 |
| -1726.695 | 19810.164 | 0.545 | 15.636 | 0.560247 | 7/15/2014 | 21:15.4 | 6 |
| -1726.432 | 19810.156 | 0.536 | 15.634 | 0.613735 | 7/15/2014 | 21:15.5 | 6 |
| -1726.169 | 19810.146 | 0.534 | 15.633 | 0.562032 | 7/15/2014 | 21:15.6 | 6 |
| -1725.906 | 19810.139 | 0.531 | 15.636 | 0.635214 | 7/15/2014 | 21:15.7 | 6 |
| -1725.575 | 19810.127 | 0.526 | 15.641 | 0.532964 | 7/15/2014 | 21:15.8 | 6 |
| -1725.308 | 19810.119 | 0.515 | 15.65 | 0.567736 | 7/15/2014 | 21:15.9 | 6 |
| -1725.041 | 19810.109 | 0.512 | 15.676 | 0.480744 | 7/15/2014 | 21:16.0 | 6 |
| -1724.772 | 19810.1 | 0.504 | 15.692 | 0.457169 | 7/15/2014 | 21:16.1 | 6 |
| -1724.502 | 19810.088 | 0.493 | 15.716 | 0.468378 | 7/15/2014 | 21:16.2 | 6 |
| -1724.232 | 19810.078 | 0.485 | 15.733 | 0.431508 | 7/15/2014 | 21:16.3 | 6 |
| -1723.896 | 19810.064 | 0.476 | 15.745 | 0.518378 | 7/15/2014 | 21:16.4 | 6 |
| -1723.625 | 19810.055 | 0.469 | 15.763 | 0.464137 | 7/15/2014 | 21:16.5 | 6 |
| -1723.354 | 19810.043 | 0.465 | 15.772 | 0.58182 | 7/15/2014 | 21:16.6 | 6 |
| -1723.082 | 19810.03 | 0.459 | 15.765 | 0.601314 | 7/15/2014 | 21:16.7 | 6 |
| -1722.745 | 19810.016 | 0.449 | 15.761 | 0.59404 | 7/15/2014 | 21:16.8 | 6 |
| -1722.473 | 19810.002 | 0.448 | 15.754 | 0.554922 | 7/15/2014 | 21:16.9 | 6 |
| -1722.202 | 19809.99 | 0.442 | 15.748 | 0.526602 | 7/15/2014 | 21:17.0 | 6 |
| -1721.931 | 19809.977 | 0.436 | 15.758 | 0.636587 | 7/15/2014 | 21:17.1 | 6 |
| -1721.661 | 19809.965 | 0.431 | 15.776 | 0.545254 | 7/15/2014 | 21:17.2 | 6 |
| -1721.393 | 19809.951 | 0.431 | 15.795 | 0.545148 | 7/15/2014 | 21:17.3 | 6 |
| -1721.06 | 19809.936 | 0.422 | 15.808 | 0.528706 | 7/15/2014 | 21:17.4 | 6 |
| -1720.794 | 19809.92 | 0.426 | 15.819 | 0.583691 | 7/15/2014 | 21:17.5 | 6 |
| -1720.531 | 19809.906 | 0.418 | 15.832 | 0.659459 | 7/15/2014 | 21:17.6 | 6 |
| -1720.268 | 19809.893 | 0.416 | 15.842 | 0.56713 | 7/15/2014 | 21:17.7 | 6 |
| -1719.944 | 19809.876 | 0.41 | 15.843 | 0.609211 | 7/15/2014 | 21:17.8 | 6 |
| -1719.684 | 19809.861 | 0.41 | 15.826 | 0.62813 | 7/15/2014 | 21:17.9 | 6 |
| -1719.425 | 19809.848 | 0.408 | 15.813 | 0.656739 | 7/15/2014 | 21:18.0 | 6 |
| -1719.167 | 19809.832 | 0.41 | 15.807 | 0.657104 | 7/15/2014 | 21:18.1 | 6 |
| -1718.911 | 19809.816 | 0.406 | 15.804 | 0.672592 | 7/15/2014 | 21:18.2 | 6 |
| -1718.656 | 19809.803 | 0.408 | 15.802 | 0.599805 | 7/15/2014 | 21:18.3 | 6 |
| -1718.34 | 19809.784 | 0.401 | 15.804 | 0.648236 | 7/15/2014 | 21:18.4 | 6 |
| -1718.088 | 19809.769 | 0.405 | 15.81 | 0.687569 | 7/15/2014 | 21:18.5 | 6 |
| -1717.837 | 19809.753 | 0.399 | 15.818 | 0.718583 | 7/15/2014 | 21:18.6 | 6 |
| -1717.585 | 19809.736 | 0.399 | 15.825 | 0.72788 | 7/15/2014 | 21:18.7 | 6 |
| -1717.27 | 19809.716 | 0.389 | 15.829 | 0.65257 | 7/15/2014 | 21:18.8 | 6 |
| -1717.016 | 19809.699 | 0.387 | 15.834 | 0.734908 | 7/15/2014 | 21:18.9 | 6 |
| -1716.76 | 19809.683 | 0.382 | 15.839 | 0.696564 | 7/15/2014 | 21:19.0 | 6 |
| -1716.503 | 19809.666 | 0.382 | 15.843 | 0.684873 | 7/15/2014 | 21:19.1 | 6 |
| -1716.246 | 19809.647 | 0.375 | 15.846 | 0.671472 | 7/15/2014 | 21:19.2 | 6 |
| -1715.987 | 19809.629 | 0.375 | 15.861 | 0.652223 | 7/15/2014 | 21:19.3 | 6 |
| -1715.665 | 19809.606 | 0.363 | 15.874 | 0.770146 | 7/15/2014 | 21:19.4 | 6 |
| -1715.404 | 19809.588 | 0.369 | 15.888 | 0.83799 | 7/15/2014 | 21:19.5 | 6 |
| -1715.142 | 19809.569 | 0.369 | 15.907 | 0.865941 | 7/15/2014 | 21:19.6 | 6 |
| -1714.879 | 19809.551 | 0.363 | 15.926 | 0.806644 | 7/15/2014 | 21:19.7 | 6 |
| -1714.55 | 19809.525 | 0.357 | 15.947 | 0.802342 | 7/15/2014 | 21:19.8 | 6 |
| -1714.284 | 19809.505 | 0.357 | 15.968 | 0.847323 | 7/15/2014 | 21:19.9 | 6 |

| | | | | | | | |
|---|---|---|---|---|---|---|---|
| -1714.017 | 19809.484 | 0.353 | 15.984 | 0.880741 | 7/15/2014 | 21:20.0 | 6 |
| -1713.75 | 19809.465 | 0.359 | 16.009 | 0.861699 | 7/15/2014 | 21:20.1 | 6 |
| -1713.483 | 19809.443 | 0.351 | 16.032 | 0.898363 | 7/15/2014 | 21:20.2 | 6 |
| -1713.216 | 19809.424 | 0.351 | 16.042 | 0.957732 | 7/15/2014 | 21:20.3 | 6 |
| -1712.885 | 19809.396 | 0.357 | 16.052 | 1.07401 | 7/15/2014 | 21:20.4 | 6 |
| -1712.618 | 19809.375 | 0.365 | 16.056 | 1.09401 | 7/15/2014 | 21:20.5 | 6 |
| -1712.352 | 19809.354 | 0.369 | 16.054 | 1.09882 | 7/15/2014 | 21:20.6 | 6 |
| -1712.086 | 19809.332 | 0.375 | 16.046 | 1.15888 | 7/15/2014 | 21:20.7 | 6 |
| -1711.753 | 19809.305 | 0.377 | 16.011 | 1.15235 | 7/15/2014 | 21:20.8 | 6 |
| -1711.485 | 19809.283 | 0.392 | 15.975 | 1.14877 | 7/15/2014 | 21:20.9 | 6 |
| -1711.217 | 19809.26 | 0.4 | 15.945 | 1.16531 | 7/15/2014 | 21:21.0 | 6 |
| -1710.949 | 19809.237 | 0.412 | 15.913 | 1.14812 | 7/15/2014 | 21:21.1 | 6 |
| -1710.681 | 19809.214 | 0.412 | 15.884 | 1.09096 | 7/15/2014 | 21:21.2 | 6 |
| -1710.413 | 19809.19 | 0.421 | 15.856 | 1.08351 | 7/15/2014 | 21:21.3 | 6 |
| -1710.08 | 19809.162 | 0.423 | 15.829 | 1.08133 | 7/15/2014 | 21:21.4 | 6 |
| -1709.812 | 19809.139 | 0.433 | 15.822 | 1.11068 | 7/15/2014 | 21:21.5 | 6 |
| -1709.544 | 19809.114 | 0.429 | 15.815 | 1.1697 | 7/15/2014 | 21:21.6 | 6 |
| -1709.275 | 19809.09 | 0.433 | 15.809 | 1.24702 | 7/15/2014 | 21:21.7 | 6 |
| -1708.94 | 19809.059 | 0.433 | 15.801 | 1.23625 | 7/15/2014 | 21:21.8 | 6 |
| -1708.67 | 19809.034 | 0.443 | 15.788 | 1.34081 | 7/15/2014 | 21:21.9 | 6 |
| -1708.4 | 19809.009 | 0.44 | 15.776 | 1.41947 | 7/15/2014 | 21:22.0 | 6 |
| -1708.129 | 19808.984 | 0.454 | 15.759 | 1.47709 | 7/15/2014 | 21:22.1 | 6 |
| -1707.859 | 19808.958 | 0.456 | 15.744 | 1.47859 | 7/15/2014 | 21:22.2 | 6 |
| -1707.589 | 19808.933 | 0.466 | 15.727 | 1.57043 | 7/15/2014 | 21:22.3 | 6 |
| -1707.254 | 19808.901 | 0.472 | 15.708 | 1.70431 | 7/15/2014 | 21:22.4 | 6 |
| -1706.984 | 19808.876 | 0.48 | 15.708 | 1.67855 | 7/15/2014 | 21:22.5 | 6 |
| -1706.716 | 19808.85 | 0.486 | 15.695 | 1.65556 | 7/15/2014 | 21:22.6 | 6 |
| -1706.448 | 19808.824 | 0.501 | 15.687 | 1.74162 | 7/15/2014 | 21:22.7 | 6 |
| -1706.113 | 19808.791 | 0.503 | 15.692 | 1.67015 | 7/15/2014 | 21:22.8 | 6 |
| -1705.845 | 19808.766 | 0.512 | 15.7 | 1.68596 | 7/15/2014 | 21:22.9 | 6 |
| -1705.576 | 19808.739 | 0.515 | 15.704 | 1.7298 | 7/15/2014 | 21:23.0 | 6 |
| -1705.307 | 19808.713 | 0.518 | 15.708 | 1.76118 | 7/15/2014 | 21:23.1 | 6 |
| -1705.039 | 19808.687 | 0.518 | 15.698 | 1.84538 | 7/15/2014 | 21:23.2 | 6 |
| -1704.771 | 19808.66 | 0.526 | 15.71 | 1.85378 | 7/15/2014 | 21:23.3 | 6 |
| -1704.439 | 19808.627 | 0.529 | 15.723 | 1.96725 | 7/15/2014 | 21:23.4 | 6 |
| -1704.172 | 19808.601 | 0.538 | 15.731 | 1.98556 | 7/15/2014 | 21:23.5 | 6 |
| -1703.906 | 19808.573 | 0.538 | 15.739 | 2.00257 | 7/15/2014 | 21:23.6 | 6 |
| -1703.639 | 19808.546 | 0.547 | 15.752 | 2.03547 | 7/15/2014 | 21:23.7 | 6 |
| -1703.307 | 19808.513 | 0.549 | 15.772 | 2.02469 | 7/15/2014 | 21:23.8 | 6 |
| -1702.972 | 19808.479 | 0.559 | 15.78 | 2.00784 | 7/15/2014 | 21:23.9 | 6 |
| -1702.838 | 19808.465 | 0.558 | 15.788 | 2.01954 | 7/15/2014 | 21:23.9 | 6 |
| -1702.503 | 19808.43 | 0.564 | 15.771 | 2.15067 | 7/15/2014 | 21:24.1 | 6 |
| -1702.236 | 19808.402 | 0.56 | 15.766 | 2.22684 | 7/15/2014 | 21:24.2 | 6 |
| -1701.968 | 19808.375 | 0.568 | 15.757 | 2.29267 | 7/15/2014 | 21:24.3 | 6 |
| -1701.636 | 19808.339 | 0.568 | 15.75 | 2.38614 | 7/15/2014 | 21:24.4 | 6 |
| -1701.171 | 19808.291 | 0.574 | 15.737 | 2.52575 | 7/15/2014 | 21:24.5 | 6 |
| -1701.104 | 19808.284 | 0.571 | 15.733 | 2.49586 | 7/15/2014 | 21:24.6 | 6 |

| | | | | | | | |
|---|---|---|---|---|---|---|---|
| -1700.839 | 19808.256 | 0.576 | 15.736 | 2.54137 | 7/15/2014 | 21:24.7 | 6 |
| -1700.51 | 19808.221 | 0.573 | 15.733 | 2.55182 | 7/15/2014 | 21:24.8 | 6 |
| -1700.245 | 19808.193 | 0.581 | 15.726 | 2.57922 | 7/15/2014 | 21:24.9 | 6 |
| -1699.981 | 19808.164 | 0.58 | 15.722 | 2.63929 | 7/15/2014 | 21:25.0 | 6 |
| -1699.716 | 19808.137 | 0.584 | 15.714 | 2.68324 | 7/15/2014 | 21:25.1 | 6 |
| -1699.451 | 19808.109 | 0.588 | 15.709 | 2.72969 | 7/15/2014 | 21:25.2 | 6 |
| -1699.188 | 19808.081 | 0.597 | 15.701 | 2.77451 | 7/15/2014 | 21:25.3 | 6 |
| -1698.861 | 19808.046 | 0.598 | 15.69 | 2.82868 | 7/15/2014 | 21:25.4 | 6 |
| -1698.6 | 19808.018 | 0.604 | 15.681 | 2.89524 | 7/15/2014 | 21:25.5 | 6 |
| -1698.339 | 19807.99 | 0.604 | 15.672 | 2.91866 | 7/15/2014 | 21:25.6 | 6 |
| -1698.078 | 19807.963 | 0.613 | 15.662 | 2.97614 | 7/15/2014 | 21:25.7 | 6 |
| -1697.753 | 19807.93 | 0.618 | 15.644 | 2.99844 | 7/15/2014 | 21:25.8 | 6 |
| -1697.491 | 19807.9 | 0.621 | 15.622 | 3.05644 | 7/15/2014 | 21:25.9 | 6 |
| -1697.23 | 19807.873 | 0.625 | 15.601 | 3.11593 | 7/15/2014 | 21:26.0 | 6 |
| -1696.968 | 19807.844 | 0.633 | 15.581 | 3.1717 | 7/15/2014 | 21:26.1 | 6 |
| -1696.707 | 19807.816 | 0.638 | 15.562 | 3.23036 | 7/15/2014 | 21:26.2 | 6 |
| -1696.447 | 19807.789 | 0.641 | 15.545 | 3.27943 | 7/15/2014 | 21:26.3 | 6 |
| -1696.124 | 19807.756 | 0.647 | 15.528 | 3.32962 | 7/15/2014 | 21:26.4 | 6 |
| -1695.865 | 19807.728 | 0.655 | 15.516 | 3.38537 | 7/15/2014 | 21:26.5 | 6 |
| -1695.606 | 19807.699 | 0.66 | 15.504 | 3.43555 | 7/15/2014 | 21:26.6 | 6 |
| -1695.348 | 19807.671 | 0.667 | 15.489 | 3.49377 | 7/15/2014 | 21:26.7 | 6 |
| -1695.026 | 19807.637 | 0.672 | 15.47 | 3.55651 | 7/15/2014 | 21:26.8 | 6 |
| -1694.767 | 19807.609 | 0.684 | 15.45 | 3.62956 | 7/15/2014 | 21:26.9 | 6 |
| -1694.508 | 19807.582 | 0.692 | 15.428 | 3.70659 | 7/15/2014 | 21:27.0 | 6 |
| -1694.248 | 19807.554 | 0.705 | 15.402 | 3.79164 | 7/15/2014 | 21:27.1 | 6 |
| -1693.99 | 19807.524 | 0.717 | 15.379 | 3.87934 | 7/15/2014 | 21:27.2 | 6 |
| -1693.731 | 19807.497 | 0.729 | 15.36 | 3.98018 | 7/15/2014 | 21:27.3 | 6 |
| -1693.41 | 19807.463 | 0.736 | 15.349 | 4.10085 | 7/15/2014 | 21:27.4 | 6 |
| -1693.152 | 19807.435 | 0.752 | 15.347 | 4.21978 | 7/15/2014 | 21:27.5 | 6 |
| -1692.895 | 19807.406 | 0.762 | 15.341 | 4.34222 | 7/15/2014 | 21:27.6 | 6 |
| -1692.637 | 19807.378 | 0.778 | 15.345 | 4.45939 | 7/15/2014 | 21:27.7 | 6 |
| -1692.317 | 19807.344 | 0.778 | 15.348 | 4.54304 | 7/15/2014 | 21:27.8 | 6 |
| -1692.058 | 19807.316 | 0.78 | 15.344 | 4.62715 | 7/15/2014 | 21:27.9 | 6 |
| -1691.799 | 19807.288 | 0.786 | 15.335 | 4.70052 | 7/15/2014 | 21:28.0 | 6 |
| -1691.541 | 19807.26 | 0.807 | 15.321 | 4.77446 | 7/15/2014 | 21:28.1 | 6 |
| -1691.283 | 19807.23 | 0.819 | 15.309 | 4.82343 | 7/15/2014 | 21:28.2 | 6 |
| -1691.025 | 19807.203 | 0.834 | 15.295 | 4.91442 | 7/15/2014 | 21:28.3 | 6 |
| -1690.705 | 19807.17 | 0.848 | 15.278 | 5.06403 | 7/15/2014 | 21:28.4 | 6 |
| -1690.447 | 19807.143 | 0.875 | 15.269 | 5.18854 | 7/15/2014 | 21:28.5 | 6 |
| -1690.19 | 19807.114 | 0.895 | 15.272 | 5.32102 | 7/15/2014 | 21:28.6 | 6 |
| -1689.932 | 19807.087 | 0.913 | 15.267 | 5.45368 | 7/15/2014 | 21:28.7 | 6 |
| -1689.609 | 19807.052 | 0.926 | 15.259 | 5.48306 | 7/15/2014 | 21:28.8 | 6 |
| -1689.349 | 19807.023 | 0.943 | 15.24 | 5.58868 | 7/15/2014 | 21:28.9 | 6 |
| -1689.088 | 19806.996 | 0.961 | 15.227 | 5.69049 | 7/15/2014 | 21:29.0 | 6 |
| -1688.826 | 19806.969 | 0.976 | 15.206 | 5.8077 | 7/15/2014 | 21:29.1 | 6 |
| -1688.564 | 19806.941 | 0.985 | 15.176 | 5.85921 | 7/15/2014 | 21:29.2 | 6 |
| -1688.3 | 19806.914 | 1.004 | 15.146 | 5.95631 | 7/15/2014 | 21:29.3 | 6 |

| | | | | | | | |
|---|---|---|---|---|---|---|---|
| -1687.974 | 19806.879 | 1.019 | 15.119 | 6.30198 | 7/15/2014 | 21:29.4 | 6 |
| -1687.711 | 19806.851 | 1.031 | 15.101 | 6.4103 | 7/15/2014 | 21:29.5 | 6 |
| -1687.448 | 19806.823 | 1.043 | 15.084 | 6.6059 | 7/15/2014 | 21:29.6 | 6 |
| -1687.185 | 19806.796 | 1.055 | 15.061 | 6.78487 | 7/15/2014 | 21:29.7 | 6 |
| -1686.856 | 19806.762 | 1.068 | 15.041 | 6.69095 | 7/15/2014 | 21:29.8 | 6 |
| -1686.592 | 19806.734 | 1.083 | 15.021 | 6.90466 | 7/15/2014 | 21:29.9 | 6 |
| -1686.329 | 19806.707 | 1.088 | 15.011 | 7.11843 | 7/15/2014 | 21:30.0 | 6 |
| -1686.065 | 19806.68 | 1.105 | 15 | 7.38229 | 7/15/2014 | 21:30.1 | 6 |
| -1685.802 | 19806.653 | 1.12 | 14.986 | 7.53544 | 7/15/2014 | 21:30.2 | 6 |
| -1685.541 | 19806.626 | 1.128 | 14.975 | 7.65351 | 7/15/2014 | 21:30.3 | 6 |
| -1685.215 | 19806.592 | 1.13 | 14.968 | 8.27599 | 7/15/2014 | 21:30.4 | 6 |
| -1684.955 | 19806.565 | 1.137 | 14.961 | 8.34794 | 7/15/2014 | 21:30.5 | 6 |
| -1684.695 | 19806.539 | 1.148 | 14.955 | 8.4374 | 7/15/2014 | 21:30.6 | 6 |
| -1684.435 | 19806.513 | 1.166 | 14.938 | 8.48524 | 7/15/2014 | 21:30.7 | 6 |
| -1684.111 | 19806.48 | 1.166 | 14.929 | 8.01225 | 7/15/2014 | 21:30.8 | 6 |
| -1683.849 | 19806.454 | 1.169 | 14.921 | 8.05229 | 7/15/2014 | 21:30.9 | 6 |
| -1683.587 | 19806.427 | 1.181 | 14.914 | 8.08935 | 7/15/2014 | 21:31.0 | 6 |
| -1683.325 | 19806.401 | 1.197 | 14.919 | 8.10658 | 7/15/2014 | 21:31.1 | 6 |
| -1683.062 | 19806.374 | 1.204 | 14.917 | 8.10447 | 7/15/2014 | 21:31.2 | 6 |
| -1682.8 | 19806.35 | 1.207 | 14.918 | 8.05991 | 7/15/2014 | 21:31.3 | 6 |
| -1682.474 | 19806.316 | 1.207 | 14.91 | 8.58163 | 7/15/2014 | 21:31.4 | 6 |
| -1682.211 | 19806.291 | 1.222 | 14.9 | 8.56094 | 7/15/2014 | 21:31.5 | 6 |
| -1681.95 | 19806.265 | 1.232 | 14.887 | 8.53097 | 7/15/2014 | 21:31.6 | 6 |
| -1681.688 | 19806.24 | 1.235 | 14.88 | 8.53572 | 7/15/2014 | 21:31.7 | 6 |
| -1681.363 | 19806.207 | 1.237 | 14.859 | 7.98596 | 7/15/2014 | 21:31.8 | 6 |
| -1681.1 | 19806.184 | 1.248 | 14.853 | 7.95072 | 7/15/2014 | 21:31.9 | 6 |
| -1680.839 | 19806.158 | 1.256 | 14.846 | 7.90776 | 7/15/2014 | 21:32.0 | 6 |
| -1680.577 | 19806.133 | 1.262 | 14.842 | 7.81402 | 7/15/2014 | 21:32.1 | 6 |
| -1680.318 | 19806.107 | 1.262 | 14.839 | 7.71139 | 7/15/2014 | 21:32.2 | 6 |
| -1680.058 | 19806.084 | 1.27 | 14.837 | 7.5748 | 7/15/2014 | 21:32.3 | 6 |
| -1679.736 | 19806.053 | 1.273 | 14.83 | 7.81751 | 7/15/2014 | 21:32.4 | 6 |
| -1679.478 | 19806.029 | 1.285 | 14.83 | 7.64884 | 7/15/2014 | 21:32.5 | 6 |
| -1679.221 | 19806.006 | 1.289 | 14.833 | 7.47846 | 7/15/2014 | 21:32.6 | 6 |
| -1678.965 | 19805.981 | 1.298 | 14.834 | 7.31479 | 7/15/2014 | 21:32.7 | 6 |
| -1678.647 | 19805.951 | 1.306 | 14.832 | 6.90429 | 7/15/2014 | 21:32.8 | 6 |
| -1678.39 | 19805.928 | 1.316 | 14.834 | 6.77703 | 7/15/2014 | 21:32.9 | 6 |
| -1678.134 | 19805.906 | 1.315 | 14.832 | 6.64742 | 7/15/2014 | 21:33.0 | 6 |
| -1677.876 | 19805.883 | 1.329 | 14.835 | 6.53075 | 7/15/2014 | 21:33.1 | 6 |
| -1677.621 | 19805.859 | 1.332 | 14.826 | 6.39156 | 7/15/2014 | 21:33.2 | 6 |
| -1677.365 | 19805.837 | 1.338 | 14.809 | 6.26881 | 7/15/2014 | 21:33.3 | 6 |
| -1677.048 | 19805.809 | 1.336 | 14.796 | 6.22948 | 7/15/2014 | 21:33.4 | 6 |
| -1676.795 | 19805.785 | 1.342 | 14.787 | 6.08724 | 7/15/2014 | 21:33.5 | 6 |
| -1676.542 | 19805.766 | 1.351 | 14.775 | 5.96431 | 7/15/2014 | 21:33.6 | 6 |
| -1676.288 | 19805.742 | 1.361 | 14.766 | 5.83924 | 7/15/2014 | 21:33.7 | 6 |
| -1675.974 | 19805.715 | 1.361 | 14.748 | 5.70935 | 7/15/2014 | 21:33.8 | 6 |
| -1675.72 | 19805.694 | 1.371 | 14.735 | 5.6291 | 7/15/2014 | 21:33.9 | 6 |
| -1675.467 | 19805.674 | 1.377 | 14.723 | 5.55649 | 7/15/2014 | 21:34.0 | 6 |

| | | | | | | | |
|---|---|---|---|---|---|---|---|
| -1675.214 | 19805.65 | 1.385 | 14.712 | 5.49048 | 7/15/2014 | 21:34.1 | 6 |
| -1674.962 | 19805.631 | 1.383 | 14.703 | 5.43396 | 7/15/2014 | 21:34.2 | 6 |
| -1674.71 | 19805.609 | 1.382 | 14.686 | 5.38175 | 7/15/2014 | 21:34.3 | 6 |
| -1674.397 | 19805.584 | 1.39 | 14.677 | 5.33299 | 7/15/2014 | 21:34.4 | 6 |
| -1674.145 | 19805.564 | 1.393 | 14.664 | 5.29324 | 7/15/2014 | 21:34.5 | 6 |
| -1673.894 | 19805.545 | 1.389 | 14.665 | 5.25584 | 7/15/2014 | 21:34.6 | 6 |
| -1673.641 | 19805.523 | 1.391 | 14.662 | 5.20498 | 7/15/2014 | 21:34.7 | 6 |
| -1673.325 | 19805.499 | 1.391 | 14.651 | 5.1449 | 7/15/2014 | 21:34.8 | 6 |
| -1673.068 | 19805.479 | 1.406 | 14.647 | 5.10367 | 7/15/2014 | 21:34.9 | 6 |
| -1672.812 | 19805.46 | 1.41 | 14.648 | 5.06086 | 7/15/2014 | 21:35.0 | 6 |
| -1672.554 | 19805.44 | 1.413 | 14.637 | 5.01675 | 7/15/2014 | 21:35.1 | 6 |
| -1672.295 | 19805.421 | 1.421 | 14.633 | 4.95057 | 7/15/2014 | 21:35.2 | 6 |
| -1672.036 | 19805.402 | 1.434 | 14.615 | 4.91956 | 7/15/2014 | 21:35.3 | 6 |
| -1671.714 | 19805.378 | 1.44 | 14.596 | 4.95618 | 7/15/2014 | 21:35.4 | 6 |
| -1671.453 | 19805.358 | 1.45 | 14.581 | 4.90323 | 7/15/2014 | 21:35.5 | 6 |
| -1671.194 | 19805.341 | 1.447 | 14.561 | 4.86545 | 7/15/2014 | 21:35.6 | 6 |
| -1670.934 | 19805.321 | 1.457 | 14.546 | 4.8318 | 7/15/2014 | 21:35.7 | 6 |
| -1670.611 | 19805.299 | 1.457 | 14.534 | 4.66249 | 7/15/2014 | 21:35.8 | 6 |
| -1670.352 | 19805.282 | 1.462 | 14.527 | 4.62216 | 7/15/2014 | 21:35.9 | 6 |
| -1670.094 | 19805.264 | 1.456 | 14.525 | 4.58117 | 7/15/2014 | 21:36.0 | 6 |
| -1669.835 | 19805.246 | 1.458 | 14.53 | 4.48969 | 7/15/2014 | 21:36.1 | 6 |
| -1669.579 | 19805.229 | 1.458 | 14.524 | 4.456 | 7/15/2014 | 21:36.2 | 6 |
| -1669.323 | 19805.212 | 1.463 | 14.519 | 4.36578 | 7/15/2014 | 21:36.3 | 6 |
| -1669.007 | 19805.191 | 1.46 | 14.507 | 4.47378 | 7/15/2014 | 21:36.4 | 6 |
| -1668.754 | 19805.176 | 1.466 | 14.493 | 4.38329 | 7/15/2014 | 21:36.5 | 6 |
| -1668.503 | 19805.16 | 1.469 | 14.481 | 4.29301 | 7/15/2014 | 21:36.6 | 6 |
| -1668.251 | 19805.145 | 1.478 | 14.461 | 4.24336 | 7/15/2014 | 21:36.7 | 6 |
| -1667.939 | 19805.124 | 1.475 | 14.446 | 3.93096 | 7/15/2014 | 21:36.8 | 6 |
| -1667.687 | 19805.11 | 1.48 | 14.445 | 3.85967 | 7/15/2014 | 21:36.9 | 6 |
| -1667.437 | 19805.095 | 1.484 | 14.448 | 3.79526 | 7/15/2014 | 21:37.0 | 6 |
| -1667.186 | 19805.081 | 1.49 | 14.458 | 3.74494 | 7/15/2014 | 21:37.1 | 6 |
| -1666.934 | 19805.066 | 1.486 | 14.475 | 3.69783 | 7/15/2014 | 21:37.2 | 6 |
| -1666.684 | 19805.053 | 1.485 | 14.488 | 3.642 | 7/15/2014 | 21:37.3 | 6 |
| -1666.373 | 19805.034 | 1.485 | 14.497 | 3.83311 | 7/15/2014 | 21:37.4 | 6 |
| -1666.122 | 19805.021 | 1.491 | 14.5 | 3.77546 | 7/15/2014 | 21:37.5 | 6 |
| -1665.872 | 19805.008 | 1.492 | 14.507 | 3.74717 | 7/15/2014 | 21:37.6 | 6 |
| -1665.621 | 19804.996 | 1.495 | 14.508 | 3.64114 | 7/15/2014 | 21:37.7 | 6 |
| -1665.305 | 19804.979 | 1.495 | 14.504 | 3.32199 | 7/15/2014 | 21:37.8 | 6 |
| -1665.049 | 19804.967 | 1.497 | 14.499 | 3.22688 | 7/15/2014 | 21:37.9 | 6 |
| -1664.793 | 19804.954 | 1.501 | 14.495 | 3.13825 | 7/15/2014 | 21:38.0 | 6 |
| -1664.535 | 19804.941 | 1.509 | 14.495 | 3.0429 | 7/15/2014 | 21:38.1 | 6 |
| -1664.277 | 19804.93 | 1.512 | 14.494 | 2.87264 | 7/15/2014 | 21:38.2 | 6 |
| -1664.018 | 19804.918 | 1.52 | 14.485 | 2.81396 | 7/15/2014 | 21:38.3 | 6 |
| -1663.695 | 19804.902 | 1.514 | 14.477 | 2.9125 | 7/15/2014 | 21:38.4 | 6 |
| -1663.434 | 19804.892 | 1.519 | 14.473 | 2.80929 | 7/15/2014 | 21:38.5 | 6 |
| -1663.172 | 19804.881 | 1.525 | 14.464 | 2.71104 | 7/15/2014 | 21:38.6 | 6 |
| -1662.91 | 19804.869 | 1.527 | 14.455 | 2.62273 | 7/15/2014 | 21:38.7 | 6 |

| | | | | | | | |
|---|---|---|---|---|---|---|---|
| -1662.583 | 19804.855 | 1.521 | 14.45 | 2.41499 | 7/15/2014 | 21:38.8 | 6 |
| -1662.321 | 19804.846 | 1.521 | 14.453 | 2.32388 | 7/15/2014 | 21:38.9 | 6 |
| -1662.058 | 19804.835 | 1.515 | 14.485 | 2.24682 | 7/15/2014 | 21:39.0 | 6 |
| -1661.794 | 19804.825 | 1.521 | 14.515 | 2.2008 | 7/15/2014 | 21:39.1 | 6 |
| -1661.531 | 19804.816 | 1.51 | 14.543 | 2.12072 | 7/15/2014 | 21:39.2 | 6 |
| -1661.269 | 19804.807 | 1.51 | 14.589 | 2.04488 | 7/15/2014 | 21:39.3 | 6 |
| -1660.944 | 19804.795 | 1.506 | 14.636 | 2.12903 | 7/15/2014 | 21:39.4 | 6 |
| -1660.684 | 19804.786 | 1.5 | 14.681 | 2.09024 | 7/15/2014 | 21:39.5 | 6 |
| -1660.424 | 19804.778 | 1.496 | 14.719 | 2.02164 | 7/15/2014 | 21:39.6 | 6 |
| -1660.165 | 19804.77 | 1.492 | 14.729 | 1.99509 | 7/15/2014 | 21:39.7 | 6 |
| -1659.842 | 19804.76 | 1.486 | 14.749 | 1.80128 | 7/15/2014 | 21:39.8 | 6 |
| -1659.582 | 19804.752 | 1.491 | 14.767 | 1.80013 | 7/15/2014 | 21:39.9 | 6 |
| -1659.323 | 19804.744 | 1.483 | 14.769 | 1.76941 | 7/15/2014 | 21:40.0 | 6 |
| -1659.064 | 19804.736 | 1.482 | 14.773 | 1.69185 | 7/15/2014 | 21:40.1 | 6 |
| -1658.806 | 19804.73 | 1.482 | 14.775 | 1.65787 | 7/15/2014 | 21:40.2 | 6 |
| -1658.548 | 19804.724 | 1.485 | 14.777 | 1.60939 | 7/15/2014 | 21:40.3 | 6 |
| -1658.229 | 19804.715 | 1.479 | 14.78 | 1.67994 | 7/15/2014 | 21:40.4 | 6 |
| -1657.974 | 19804.708 | 1.479 | 14.779 | 1.62406 | 7/15/2014 | 21:40.5 | 6 |
| -1657.721 | 19804.701 | 1.474 | 14.79 | 1.55658 | 7/15/2014 | 21:40.6 | 6 |
| -1657.467 | 19804.695 | 1.474 | 14.797 | 1.51717 | 7/15/2014 | 21:40.7 | 6 |
| -1657.153 | 19804.689 | 1.472 | 14.804 | 1.39847 | 7/15/2014 | 21:40.8 | 6 |
| -1656.901 | 19804.684 | 1.472 | 14.812 | 1.35073 | 7/15/2014 | 21:40.9 | 6 |
| -1656.651 | 19804.679 | 1.46 | 14.823 | 1.34946 | 7/15/2014 | 21:41.0 | 6 |
| -1656.401 | 19804.674 | 1.463 | 14.838 | 1.31899 | 7/15/2014 | 21:41.1 | 6 |
| -1656.153 | 19804.668 | 1.463 | 14.85 | 1.32688 | 7/15/2014 | 21:41.2 | 6 |
| -1655.905 | 19804.663 | 1.462 | 14.853 | 1.33091 | 7/15/2014 | 21:41.3 | 6 |
| -1655.477 | 19804.657 | 1.46 | 14.87 | 1.386 | 7/15/2014 | 21:41.4 | 6 |
| -1655.234 | 19804.652 | 1.457 | 14.875 | 1.3683 | 7/15/2014 | 21:41.5 | 6 |
| -1654.993 | 19804.649 | 1.454 | 14.886 | 1.35467 | 7/15/2014 | 21:41.6 | 6 |
| -1654.691 | 19804.646 | 1.456 | 14.896 | 1.23691 | 7/15/2014 | 21:41.7 | 6 |
| -1654.568 | 19804.643 | 1.454 | 14.899 | 1.21617 | 7/15/2014 | 21:41.8 | 6 |
| -1654.326 | 19804.639 | 1.45 | 14.901 | 1.15908 | 7/15/2014 | 21:41.9 | 6 |
| -1654.085 | 19804.636 | 1.448 | 14.903 | 1.14041 | 7/15/2014 | 21:42.0 | 6 |
| -1653.843 | 19804.634 | 1.451 | 14.899 | 1.08607 | 7/15/2014 | 21:42.1 | 6 |
| -1653.602 | 19804.631 | 1.445 | 14.896 | 1.03716 | 7/15/2014 | 21:42.2 | 6 |
| -1653.362 | 19804.629 | 1.445 | 14.896 | 1.00453 | 7/15/2014 | 21:42.3 | 6 |
| -1652.942 | 19804.625 | 1.449 | 14.892 | 1.05742 | 7/15/2014 | 21:42.4 | 6 |
| -1652.703 | 19804.623 | 1.45 | 14.889 | 1.02855 | 7/15/2014 | 21:42.5 | 6 |
| -1652.464 | 19804.622 | 1.45 | 14.888 | 1.00134 | 7/15/2014 | 21:42.6 | 6 |
| -1652.164 | 19804.62 | 1.45 | 14.888 | 0.959356 | 7/15/2014 | 21:42.7 | 6 |
| -1652.043 | 19804.619 | 1.446 | 14.892 | 0.961117 | 7/15/2014 | 21:42.8 | 6 |
| -1651.801 | 19804.619 | 1.453 | 14.886 | 0.890189 | 7/15/2014 | 21:42.9 | 6 |
| -1651.558 | 19804.617 | 1.454 | 14.889 | 0.881186 | 7/15/2014 | 21:43.0 | 6 |
| -1651.315 | 19804.616 | 1.457 | 14.887 | 0.833592 | 7/15/2014 | 21:43.1 | 6 |
| -1651.072 | 19804.617 | 1.451 | 14.888 | 0.837453 | 7/15/2014 | 21:43.2 | 6 |
| -1650.828 | 19804.617 | 1.45 | 14.888 | 0.816189 | 7/15/2014 | 21:43.3 | 6 |
| -1650.405 | 19804.618 | 1.456 | 14.885 | 0.852826 | 7/15/2014 | 21:43.4 | 6 |

| | | | | | | | |
|---|---|---|---|---|---|---|---|
| -1650.162 | 19804.62 | 1.456 | 14.885 | 0.875945 | 7/15/2014 | 21:43.5 | 6 |
| -1649.922 | 19804.62 | 1.456 | 14.884 | 0.853427 | 7/15/2014 | 21:43.6 | 6 |
| -1649.62 | 19804.622 | 1.455 | 14.88 | 0.836485 | 7/15/2014 | 21:43.7 | 6 |
| -1649.497 | 19804.623 | 1.453 | 14.881 | 0.819987 | 7/15/2014 | 21:43.8 | 6 |
| -1649.255 | 19804.623 | 1.461 | 14.88 | 0.819374 | 7/15/2014 | 21:43.9 | 6 |
| -1649.013 | 19804.626 | 1.456 | 14.878 | 0.828969 | 7/15/2014 | 21:44.0 | 6 |
| -1648.771 | 19804.629 | 1.456 | 14.879 | 0.802611 | 7/15/2014 | 21:44.1 | 6 |
| -1648.53 | 19804.632 | 1.456 | 14.882 | 0.809404 | 7/15/2014 | 21:44.2 | 6 |
| -1648.29 | 19804.634 | 1.462 | 14.882 | 0.814726 | 7/15/2014 | 21:44.3 | 6 |
| -1647.871 | 19804.64 | 1.46 | 14.872 | 0.851213 | 7/15/2014 | 21:44.4 | 6 |
| -1647.632 | 19804.643 | 1.458 | 14.862 | 0.812732 | 7/15/2014 | 21:44.5 | 6 |
| -1647.394 | 19804.646 | 1.453 | 14.844 | 0.811999 | 7/15/2014 | 21:44.6 | 6 |
| -1647.094 | 19804.652 | 1.457 | 14.833 | 0.761256 | 7/15/2014 | 21:44.7 | 6 |
| -1646.974 | 19804.654 | 1.455 | 14.825 | 0.743659 | 7/15/2014 | 21:44.8 | 6 |
| -1646.733 | 19804.657 | 1.455 | 14.802 | 0.720033 | 7/15/2014 | 21:44.9 | 6 |
| -1646.491 | 19804.662 | 1.457 | 14.774 | 0.703242 | 7/15/2014 | 21:45.0 | 6 |
| -1646.247 | 19804.667 | 1.461 | 14.749 | 0.65847 | 7/15/2014 | 21:45.1 | 6 |
| -1646.004 | 19804.673 | 1.457 | 14.727 | 0.664316 | 7/15/2014 | 21:45.2 | 6 |
| -1645.761 | 19804.678 | 1.466 | 14.714 | 0.632864 | 7/15/2014 | 21:45.3 | 6 |
| -1645.334 | 19804.688 | 1.472 | 14.696 | 0.626706 | 7/15/2014 | 21:45.4 | 6 |
| -1645.092 | 19804.693 | 1.472 | 14.678 | 0.627069 | 7/15/2014 | 21:45.5 | 6 |
| -1644.849 | 19804.699 | 1.474 | 14.674 | 0.604226 | 7/15/2014 | 21:45.6 | 6 |
| -1644.543 | 19804.709 | 1.474 | 14.671 | 0.578611 | 7/15/2014 | 21:45.7 | 6 |
| -1644.42 | 19804.712 | 1.474 | 14.671 | 0.573108 | 7/15/2014 | 21:45.8 | 6 |
| -1644.175 | 19804.719 | 1.482 | 14.672 | 0.552494 | 7/15/2014 | 21:45.9 | 6 |
| -1643.929 | 19804.727 | 1.485 | 14.67 | 0.541376 | 7/15/2014 | 21:46.0 | 6 |
| -1643.682 | 19804.733 | 1.487 | 14.669 | 0.540683 | 7/15/2014 | 21:46.1 | 6 |
| -1643.437 | 19804.74 | 1.49 | 14.667 | 0.521151 | 7/15/2014 | 21:46.2 | 6 |
| -1643.191 | 19804.749 | 1.5 | 14.671 | 0.496923 | 7/15/2014 | 21:46.3 | 6 |
| -1642.763 | 19804.764 | 1.498 | 14.671 | 0.502148 | 7/15/2014 | 21:46.4 | 6 |
| -1642.518 | 19804.773 | 1.501 | 14.673 | 0.492415 | 7/15/2014 | 21:46.5 | 6 |
| -1642.273 | 19804.781 | 1.503 | 14.671 | 0.487724 | 7/15/2014 | 21:46.6 | 6 |
| -1641.967 | 19804.793 | 1.503 | 14.671 | 0.470623 | 7/15/2014 | 21:46.7 | 6 |
| -1641.843 | 19804.797 | 1.503 | 14.673 | 0.459553 | 7/15/2014 | 21:46.8 | 6 |
| -1641.596 | 19804.808 | 1.5 | 14.673 | 0.475872 | 7/15/2014 | 21:46.9 | 6 |
| -1641.349 | 19804.816 | 1.497 | 14.666 | 0.461624 | 7/15/2014 | 21:47.0 | 6 |
| -1641.1 | 19804.826 | 1.503 | 14.651 | 0.459023 | 7/15/2014 | 21:47.1 | 6 |
| -1640.852 | 19804.838 | 1.503 | 14.649 | 0.466041 | 7/15/2014 | 21:47.2 | 6 |
| -1640.603 | 19804.848 | 1.503 | 14.635 | 0.457856 | 7/15/2014 | 21:47.3 | 6 |
| -1640.294 | 19804.861 | 1.503 | 14.625 | 0.470024 | 7/15/2014 | 21:47.4 | 6 |
| -1640.045 | 19804.873 | 1.5 | 14.611 | 0.468815 | 7/15/2014 | 21:47.5 | 6 |
| -1639.796 | 19804.883 | 1.504 | 14.593 | 0.46421 | 7/15/2014 | 21:47.6 | 6 |
| -1639.546 | 19804.895 | 1.506 | 14.581 | 0.457958 | 7/15/2014 | 21:47.7 | 6 |
| -1639.234 | 19804.909 | 1.504 | 14.573 | 0.451225 | 7/15/2014 | 21:47.8 | 6 |
| -1638.982 | 19804.922 | 1.504 | 14.559 | 0.449531 | 7/15/2014 | 21:47.9 | 6 |
| -1638.73 | 19804.934 | 1.501 | 14.555 | 0.406919 | 7/15/2014 | 21:48.0 | 6 |
| -1638.477 | 19804.945 | 1.501 | 14.546 | 0.418211 | 7/15/2014 | 21:48.1 | 6 |

| | | | | | | | |
|---|---|---|---|---|---|---|---|
| -1638.162 | 19804.961 | 1.504 | 14.543 | 0.388081 | 7/15/2014 | 21:48.2 | 6 |
| -1638.037 | 19804.967 | 1.505 | 14.544 | 0.386297 | 7/15/2014 | 21:48.2 | 6 |
| -1637.66 | 19804.985 | 1.498 | 14.542 | 0.381391 | 7/15/2014 | 21:48.4 | 6 |
| -1637.409 | 19804.999 | 1.5 | 14.539 | 0.363854 | 7/15/2014 | 21:48.5 | 6 |
| -1637.158 | 19805.012 | 1.503 | 14.537 | 0.392554 | 7/15/2014 | 21:48.6 | 6 |
| -1636.907 | 19805.023 | 1.495 | 14.531 | 0.357239 | 7/15/2014 | 21:48.7 | 6 |
| -1636.531 | 19805.044 | 1.486 | 14.53 | 0.372481 | 7/15/2014 | 21:48.8 | 6 |
| -1636.154 | 19805.064 | 1.475 | 14.531 | 0.343947 | 7/15/2014 | 21:48.9 | 6 |
| -1636.091 | 19805.067 | 1.472 | 14.532 | 0.335764 | 7/15/2014 | 21:49.0 | 6 |
| -1635.84 | 19805.081 | 1.472 | 14.539 | 0.340084 | 7/15/2014 | 21:49.1 | 6 |
| -1635.588 | 19805.095 | 1.46 | 14.545 | 0.328823 | 7/15/2014 | 21:49.2 | 6 |
| -1635.338 | 19805.107 | 1.454 | 14.555 | 0.317648 | 7/15/2014 | 21:49.3 | 6 |
| -1635.027 | 19805.125 | 1.454 | 14.562 | 0.309464 | 7/15/2014 | 21:49.4 | 6 |
| -1634.774 | 19805.139 | 1.455 | 14.57 | 0.300608 | 7/15/2014 | 21:49.5 | 6 |
| -1634.525 | 19805.152 | 1.446 | 14.585 | 0.305102 | 7/15/2014 | 21:49.6 | 6 |
| -1634.274 | 19805.166 | 1.453 | 14.591 | 0.291896 | 7/15/2014 | 21:49.7 | 6 |
| -1633.96 | 19805.185 | 1.456 | 14.593 | 0.28817 | 7/15/2014 | 21:49.8 | 6 |
| -1633.708 | 19805.197 | 1.456 | 14.599 | 0.292137 | 7/15/2014 | 21:49.9 | 6 |
| -1633.456 | 19805.212 | 1.456 | 14.601 | 0.294227 | 7/15/2014 | 21:50.0 | 6 |
| -1633.201 | 19805.226 | 1.466 | 14.614 | 0.294396 | 7/15/2014 | 21:50.1 | 6 |
| -1632.949 | 19805.239 | 1.469 | 14.642 | 0.302344 | 7/15/2014 | 21:50.2 | 6 |
| -1632.695 | 19805.253 | 1.484 | 14.645 | 0.31535 | 7/15/2014 | 21:50.3 | 6 |
| -1632.381 | 19805.271 | 1.481 | 14.661 | 0.316424 | 7/15/2014 | 21:50.4 | 6 |
| -1632.128 | 19805.285 | 1.483 | 14.67 | 0.316114 | 7/15/2014 | 21:50.5 | 6 |
| -1631.814 | 19805.303 | 1.493 | 14.68 | 0.297815 | 7/15/2014 | 21:50.6 | 6 |
| -1631.624 | 19805.313 | 1.501 | 14.684 | 0.295721 | 7/15/2014 | 21:50.7 | 6 |
| -1631.31 | 19805.33 | 1.498 | 14.684 | 0.288269 | 7/15/2014 | 21:50.8 | 6 |
| -1631.057 | 19805.344 | 1.504 | 14.673 | 0.279441 | 7/15/2014 | 21:50.9 | 6 |
| -1630.805 | 19805.357 | 1.506 | 14.664 | 0.272087 | 7/15/2014 | 21:51.0 | 6 |
| -1630.551 | 19805.371 | 1.515 | 14.653 | 0.27334 | 7/15/2014 | 21:51.1 | 6 |
| -1630.236 | 19805.389 | 1.517 | 14.639 | 0.289896 | 7/15/2014 | 21:51.2 | 6 |
| -1630.045 | 19805.398 | 1.523 | 14.627 | 0.277089 | 7/15/2014 | 21:51.3 | 6 |
| -1629.733 | 19805.416 | 1.527 | 14.617 | 0.284741 | 7/15/2014 | 21:51.4 | 6 |
| -1629.48 | 19805.429 | 1.54 | 14.599 | 0.299107 | 7/15/2014 | 21:51.5 | 6 |
| -1629.229 | 19805.441 | 1.54 | 14.575 | 0.320531 | 7/15/2014 | 21:51.6 | 6 |
| -1628.976 | 19805.455 | 1.544 | 14.556 | 0.307895 | 7/15/2014 | 21:51.7 | 6 |
| -1628.663 | 19805.473 | 1.548 | 14.538 | 0.303899 | 7/15/2014 | 21:51.8 | 6 |
| -1628.409 | 19805.484 | 1.557 | 14.517 | 0.310463 | 7/15/2014 | 21:51.9 | 6 |
| -1628.157 | 19805.498 | 1.562 | 14.493 | 0.30983 | 7/15/2014 | 21:52.0 | 6 |
| -1627.903 | 19805.51 | 1.568 | 14.474 | 0.290817 | 7/15/2014 | 21:52.1 | 6 |
| -1627.651 | 19805.523 | 1.571 | 14.451 | 0.29186 | 7/15/2014 | 21:52.2 | 6 |
| -1627.397 | 19805.536 | 1.58 | 14.439 | 0.283587 | 7/15/2014 | 21:52.3 | 6 |
| -1627.085 | 19805.553 | 1.586 | 14.434 | 0.282502 | 7/15/2014 | 21:52.4 | 6 |
| -1626.833 | 19805.564 | 1.592 | 14.421 | 0.279505 | 7/15/2014 | 21:52.5 | 6 |
| -1626.583 | 19805.576 | 1.586 | 14.419 | 0.270193 | 7/15/2014 | 21:52.6 | 6 |
| -1626.33 | 19805.588 | 1.59 | 14.414 | 0.269317 | 7/15/2014 | 21:52.7 | 6 |
| -1626.015 | 19805.604 | 1.588 | 14.416 | 0.262372 | 7/15/2014 | 21:52.8 | 6 |

| | | | | | | | |
|---|---|---|---|---|---|---|---|
| -1625.762 | 19805.614 | 1.588 | 14.423 | 0.268201 | 7/15/2014 | 21:52.9 | 6 |
| -1625.446 | 19805.629 | 1.588 | 14.423 | 0.262633 | 7/15/2014 | 21:53.0 | 6 |
| -1625.32 | 19805.635 | 1.586 | 14.422 | 0.265642 | 7/15/2014 | 21:53.0 | 6 |
| -1625.067 | 19805.646 | 1.59 | 14.429 | 0.261105 | 7/15/2014 | 21:53.1 | 6 |
| -1624.749 | 19805.66 | 1.592 | 14.456 | 0.270232 | 7/15/2014 | 21:53.3 | 6 |
| -1624.434 | 19805.674 | 1.59 | 14.47 | 0.265803 | 7/15/2014 | 21:53.4 | 6 |
| -1624.181 | 19805.686 | 1.596 | 14.474 | 0.256318 | 7/15/2014 | 21:53.5 | 6 |
| -1623.74 | 19805.703 | 1.586 | 14.479 | 0.255596 | 7/15/2014 | 21:53.6 | 6 |
| -1623.424 | 19805.717 | 1.586 | 14.485 | 0.261783 | 7/15/2014 | 21:53.7 | 6 |
| -1623.36 | 19805.72 | 1.58 | 14.49 | 0.254537 | 7/15/2014 | 21:53.8 | 6 |
| -1623.106 | 19805.73 | 1.583 | 14.491 | 0.251912 | 7/15/2014 | 21:53.9 | 6 |
| -1622.851 | 19805.74 | 1.585 | 14.481 | 0.249061 | 7/15/2014 | 21:54.0 | 6 |
| -1622.597 | 19805.752 | 1.591 | 14.483 | 0.251909 | 7/15/2014 | 21:54.1 | 6 |
| -1622.342 | 19805.761 | 1.582 | 14.487 | 0.246152 | 7/15/2014 | 21:54.2 | 6 |
| -1622.088 | 19805.771 | 1.585 | 14.488 | 0.244116 | 7/15/2014 | 21:54.3 | 6 |
| -1621.773 | 19805.783 | 1.588 | 14.489 | 0.238771 | 7/15/2014 | 21:54.4 | 6 |
| -1621.519 | 19805.795 | 1.59 | 14.489 | 0.241667 | 7/15/2014 | 21:54.5 | 6 |
| -1621.266 | 19805.804 | 1.58 | 14.489 | 0.229183 | 7/15/2014 | 21:54.6 | 6 |
| -1621.011 | 19805.814 | 1.58 | 14.484 | 0.222592 | 7/15/2014 | 21:54.7 | 6 |
| -1620.694 | 19805.825 | 1.574 | 14.482 | 0.217465 | 7/15/2014 | 21:54.8 | 6 |
| -1620.44 | 19805.836 | 1.578 | 14.473 | 0.217078 | 7/15/2014 | 21:54.9 | 6 |
| -1620.187 | 19805.844 | 1.571 | 14.467 | 0.208751 | 7/15/2014 | 21:55.0 | 6 |
| -1619.93 | 19805.855 | 1.572 | 14.453 | 0.211166 | 7/15/2014 | 21:55.1 | 6 |
| -1619.677 | 19805.864 | 1.566 | 14.437 | 0.20269 | 7/15/2014 | 21:55.2 | 6 |
| -1619.423 | 19805.873 | 1.573 | 14.423 | 0.199873 | 7/15/2014 | 21:55.3 | 6 |
| -1619.107 | 19805.885 | 1.571 | 14.41 | 0.195877 | 7/15/2014 | 21:55.4 | 6 |
| -1618.854 | 19805.895 | 1.571 | 14.392 | 0.192704 | 7/15/2014 | 21:55.5 | 6 |
| -1618.603 | 19805.902 | 1.571 | 14.382 | 0.190274 | 7/15/2014 | 21:55.6 | 6 |
| -1618.349 | 19805.912 | 1.58 | 14.373 | 0.190393 | 7/15/2014 | 21:55.7 | 6 |
| -1618.036 | 19805.922 | 1.58 | 14.379 | 0.187485 | 7/15/2014 | 21:55.8 | 6 |
| -1617.783 | 19805.93 | 1.589 | 14.38 | 0.189627 | 7/15/2014 | 21:55.9 | 6 |
| -1617.531 | 19805.938 | 1.591 | 14.386 | 0.189819 | 7/15/2014 | 21:56.0 | 6 |
| -1617.278 | 19805.947 | 1.594 | 14.391 | 0.190088 | 7/15/2014 | 21:56.1 | 6 |
| -1617.027 | 19805.955 | 1.594 | 14.389 | 0.192239 | 7/15/2014 | 21:56.2 | 6 |
| -1616.774 | 19805.963 | 1.602 | 14.386 | 0.1992 | 7/15/2014 | 21:56.3 | 6 |
| -1616.463 | 19805.973 | 1.606 | 14.382 | 0.194427 | 7/15/2014 | 21:56.4 | 6 |
| -1616.211 | 19805.98 | 1.611 | 14.366 | 0.20614 | 7/15/2014 | 21:56.5 | 6 |
| -1615.961 | 19805.988 | 1.606 | 14.35 | 0.208318 | 7/15/2014 | 21:56.6 | 6 |
| -1615.709 | 19805.995 | 1.608 | 14.333 | 0.219817 | 7/15/2014 | 21:56.7 | 6 |
| -1615.394 | 19806.004 | 1.611 | 14.313 | 0.220683 | 7/15/2014 | 21:56.8 | 6 |
| -1615.139 | 19806.011 | 1.625 | 14.3 | 0.216507 | 7/15/2014 | 21:56.9 | 6 |
| -1614.886 | 19806.018 | 1.622 | 14.286 | 0.231393 | 7/15/2014 | 21:57.0 | 6 |
| -1614.63 | 19806.024 | 1.624 | 14.271 | 0.219281 | 7/15/2014 | 21:57.1 | 6 |
| -1614.374 | 19806.031 | 1.624 | 14.261 | 0.220847 | 7/15/2014 | 21:57.2 | 6 |
| -1614.116 | 19806.038 | 1.639 | 14.253 | 0.213546 | 7/15/2014 | 21:57.3 | 6 |
| -1613.796 | 19806.045 | 1.651 | 14.249 | 0.21615 | 7/15/2014 | 21:57.4 | 6 |
| -1613.537 | 19806.051 | 1.658 | 14.244 | 0.215904 | 7/15/2014 | 21:57.5 | 6 |

| | | | | | | | |
|---|---|---|---|---|---|---|---|
| -1613.279 | 19806.057 | 1.652 | 14.236 | 0.215814 | 7/15/2014 | 21:57.6 | 6 |
| -1613.019 | 19806.062 | 1.668 | 14.227 | 0.21968 | 7/15/2014 | 21:57.7 | 6 |
| -1612.692 | 19806.068 | 1.674 | 14.225 | 0.217675 | 7/15/2014 | 21:57.8 | 6 |
| -1612.428 | 19806.074 | 1.68 | 14.22 | 0.22092 | 7/15/2014 | 21:57.9 | 6 |
| -1612.164 | 19806.077 | 1.677 | 14.22 | 0.220711 | 7/15/2014 | 21:58.0 | 6 |
| -1611.899 | 19806.082 | 1.675 | 14.21 | 0.223548 | 7/15/2014 | 21:58.1 | 6 |
| -1611.634 | 19806.087 | 1.677 | 14.204 | 0.226711 | 7/15/2014 | 21:58.2 | 6 |
| -1611.368 | 19806.091 | 1.681 | 14.205 | 0.22904 | 7/15/2014 | 21:58.3 | 6 |
| -1611.036 | 19806.097 | 1.675 | 14.215 | 0.236948 | 7/15/2014 | 21:58.4 | 6 |
| -1610.769 | 19806.1 | 1.68 | 14.215 | 0.241432 | 7/15/2014 | 21:58.5 | 6 |
| -1610.503 | 19806.103 | 1.681 | 14.217 | 0.245836 | 7/15/2014 | 21:58.6 | 6 |
| -1610.236 | 19806.105 | 1.684 | 14.214 | 0.255339 | 7/15/2014 | 21:58.7 | 6 |
| -1609.903 | 19806.11 | 1.678 | 14.215 | 0.251915 | 7/15/2014 | 21:58.8 | 6 |
| -1609.634 | 19806.113 | 1.68 | 14.219 | 0.259747 | 7/15/2014 | 21:58.9 | 6 |
| -1609.365 | 19806.116 | 1.682 | 14.216 | 0.260633 | 7/15/2014 | 21:59.0 | 6 |
| -1609.095 | 19806.117 | 1.682 | 14.204 | 0.261174 | 7/15/2014 | 21:59.1 | 6 |
| -1608.828 | 19806.121 | 1.675 | 14.198 | 0.262388 | 7/15/2014 | 21:59.2 | 6 |
| -1608.561 | 19806.122 | 1.677 | 14.192 | 0.265705 | 7/15/2014 | 21:59.3 | 6 |
| -1608.229 | 19806.126 | 1.675 | 14.19 | 0.264403 | 7/15/2014 | 21:59.4 | 6 |
| -1607.962 | 19806.127 | 1.686 | 14.187 | 0.285926 | 7/15/2014 | 21:59.5 | 6 |
| -1607.698 | 19806.129 | 1.684 | 14.178 | 0.300681 | 7/15/2014 | 21:59.6 | 6 |
| -1607.433 | 19806.129 | 1.684 | 14.177 | 0.338529 | 7/15/2014 | 21:59.7 | 6 |
| -1607.105 | 19806.131 | 1.681 | 14.182 | 0.348755 | 7/15/2014 | 21:59.8 | 6 |
| -1606.842 | 19806.131 | 1.684 | 14.185 | 0.35453 | 7/15/2014 | 21:59.9 | 6 |
| -1606.579 | 19806.132 | 1.684 | 14.186 | 0.354128 | 7/15/2014 | 22:00.0 | 6 |
| -1606.315 | 19806.132 | 1.684 | 14.192 | 0.33719 | 7/15/2014 | 22:00.1 | 6 |
| -1606.054 | 19806.133 | 1.676 | 14.192 | 0.325058 | 7/15/2014 | 22:00.2 | 6 |
| -1605.793 | 19806.132 | 1.679 | 14.204 | 0.283344 | 7/15/2014 | 22:00.3 | 6 |
| -1605.47 | 19806.131 | 1.676 | 14.209 | 0.268279 | 7/15/2014 | 22:00.4 | 6 |
| -1605.211 | 19806.131 | 1.676 | 14.209 | 0.228893 | 7/15/2014 | 22:00.5 | 6 |
| -1604.954 | 19806.131 | 1.67 | 14.213 | 0.218185 | 7/15/2014 | 22:00.6 | 6 |
| -1604.695 | 19806.129 | 1.668 | 14.22 | 0.192435 | 7/15/2014 | 22:00.7 | 6 |
| -1604.373 | 19806.128 | 1.664 | 14.22 | 0.170576 | 7/15/2014 | 22:00.8 | 6 |
| -1604.112 | 19806.126 | 1.66 | 14.226 | 0.165328 | 7/15/2014 | 22:00.9 | 6 |
| -1603.851 | 19806.124 | 1.65 | 14.224 | 0.148931 | 7/15/2014 | 22:01.0 | 6 |
| -1603.589 | 19806.123 | 1.65 | 14.225 | 0.151254 | 7/15/2014 | 22:01.1 | 6 |
| -1603.326 | 19806.121 | 1.652 | 14.229 | 0.135707 | 7/15/2014 | 22:01.2 | 6 |
| -1603.062 | 19806.117 | 1.658 | 14.228 | 0.138398 | 7/15/2014 | 22:01.3 | 6 |
| -1602.734 | 19806.114 | 1.654 | 14.226 | 0.155367 | 7/15/2014 | 22:01.4 | 6 |
| -1602.465 | 19806.111 | 1.654 | 14.234 | 0.144976 | 7/15/2014 | 22:01.5 | 6 |
| -1602.196 | 19806.108 | 1.658 | 14.239 | 0.169137 | 7/15/2014 | 22:01.6 | 6 |
| -1601.925 | 19806.105 | 1.667 | 14.246 | 0.15735 | 7/15/2014 | 22:01.7 | 6 |
| -1601.654 | 19806.102 | 1.663 | 14.262 | 0.16479 | 7/15/2014 | 22:01.8 | 6 |
| -1601.38 | 19806.098 | 1.657 | 14.277 | 0.167915 | 7/15/2014 | 22:01.9 | 6 |
| -1601.107 | 19806.093 | 1.647 | 14.291 | 0.141607 | 7/15/2014 | 22:02.0 | 6 |
| -1600.831 | 19806.088 | 1.651 | 14.313 | 0.140732 | 7/15/2014 | 22:02.1 | 6 |
| -1600.559 | 19806.084 | 1.651 | 14.323 | 0.115369 | 7/15/2014 | 22:02.2 | 6 |

| | | | | | | | |
|---|---|---|---|---|---|---|---|
| -1600.286 | 19806.079 | 1.647 | 14.34 | 0.128548 | 7/15/2014 | 22:02.3 | 6 |
| -1600.015 | 19806.074 | 1.631 | 14.356 | 0.116969 | 7/15/2014 | 22:02.4 | 6 |
| -1599.744 | 19806.069 | 1.627 | 14.367 | 0.150874 | 7/15/2014 | 22:02.5 | 6 |
| -1599.477 | 19806.063 | 1.619 | 14.377 | 0.120428 | 7/15/2014 | 22:02.6 | 6 |
| -1599.208 | 19806.058 | 1.616 | 14.389 | 0.14072 | 7/15/2014 | 22:02.7 | 6 |
| -1598.878 | 19806.051 | 1.603 | 14.389 | 0.132984 | 7/15/2014 | 22:02.8 | 6 |
| -1598.612 | 19806.044 | 1.597 | 14.396 | 0.109285 | 7/15/2014 | 22:02.9 | 6 |
| -1598.344 | 19806.039 | 1.584 | 14.399 | 0.125649 | 7/15/2014 | 22:03.0 | 6 |
| -1598.078 | 19806.032 | 1.582 | 14.402 | 0.0930677 | 7/15/2014 | 22:03.1 | 6 |
| -1597.811 | 19806.025 | 1.577 | 14.407 | 0.112404 | 7/15/2014 | 22:03.2 | 6 |
| -1597.542 | 19806.019 | 1.579 | 14.413 | 0.112227 | 7/15/2014 | 22:03.3 | 6 |
| -1597.208 | 19806.01 | 1.571 | 14.417 | 0.136709 | 7/15/2014 | 22:03.4 | 6 |
| -1596.937 | 19806.002 | 1.577 | 14.432 | 0.154485 | 7/15/2014 | 22:03.5 | 6 |
| -1596.664 | 19805.996 | 1.569 | 14.444 | 0.139976 | 7/15/2014 | 22:03.6 | 6 |
| -1596.389 | 19805.988 | 1.571 | 14.456 | 0.157022 | 7/15/2014 | 22:03.7 | 6 |
| -1596.115 | 19805.98 | 1.569 | 14.467 | 0.142089 | 7/15/2014 | 22:03.8 | 6 |
| -1595.837 | 19805.973 | 1.563 | 14.477 | 0.153091 | 7/15/2014 | 22:03.9 | 6 |
| -1595.562 | 19805.965 | 1.553 | 14.487 | 0.121473 | 7/15/2014 | 22:04.0 | 6 |
| -1595.284 | 19805.956 | 1.553 | 14.502 | 0.137487 | 7/15/2014 | 22:04.1 | 6 |
| -1595.008 | 19805.947 | 1.542 | 14.512 | 0.123398 | 7/15/2014 | 22:04.2 | 6 |
| -1594.732 | 19805.939 | 1.538 | 14.529 | 0.124309 | 7/15/2014 | 22:04.3 | 6 |
| -1594.46 | 19805.931 | 1.526 | 14.548 | 0.132448 | 7/15/2014 | 22:04.4 | 6 |
| -1594.187 | 19805.922 | 1.529 | 14.561 | 0.122182 | 7/15/2014 | 22:04.5 | 6 |
| -1593.918 | 19805.914 | 1.519 | 14.574 | 0.142121 | 7/15/2014 | 22:04.6 | 6 |
| -1593.647 | 19805.903 | 1.51 | 14.586 | 0.136346 | 7/15/2014 | 22:04.7 | 6 |
| -1593.313 | 19805.891 | 1.501 | 14.599 | 0.167657 | 7/15/2014 | 22:04.8 | 6 |
| -1593.043 | 19805.882 | 1.497 | 14.614 | 0.159522 | 7/15/2014 | 22:04.9 | 6 |
| -1592.776 | 19805.872 | 1.491 | 14.629 | 0.190356 | 7/15/2014 | 22:05.0 | 6 |
| -1592.505 | 19805.861 | 1.485 | 14.647 | 0.173665 | 7/15/2014 | 22:05.1 | 6 |
| -1592.235 | 19805.852 | 1.473 | 14.674 | 0.189976 | 7/15/2014 | 22:05.2 | 6 |
| -1591.963 | 19805.84 | 1.473 | 14.697 | 0.166131 | 7/15/2014 | 22:05.3 | 6 |
| -1591.626 | 19805.826 | 1.466 | 14.714 | 0.157962 | 7/15/2014 | 22:05.4 | 6 |
| -1591.351 | 19805.815 | 1.463 | 14.731 | 0.168875 | 7/15/2014 | 22:05.5 | 6 |
| -1591.077 | 19805.805 | 1.457 | 14.752 | 0.1399 | 7/15/2014 | 22:05.6 | 6 |
| -1590.801 | 19805.793 | 1.451 | 14.763 | 0.165885 | 7/15/2014 | 22:05.7 | 6 |
| -1590.524 | 19805.781 | 1.452 | 14.775 | 0.129564 | 7/15/2014 | 22:05.8 | 6 |
| -1590.247 | 19805.768 | 1.446 | 14.782 | 0.141507 | 7/15/2014 | 22:05.9 | 6 |
| -1589.972 | 19805.756 | 1.437 | 14.787 | 0.126267 | 7/15/2014 | 22:06.0 | 6 |
| -1589.695 | 19805.744 | 1.44 | 14.81 | 0.114628 | 7/15/2014 | 22:06.1 | 6 |
| -1589.422 | 19805.732 | 1.435 | 14.823 | 0.0951807 | 7/15/2014 | 22:06.2 | 6 |
| -1589.149 | 19805.72 | 1.431 | 14.834 | 0.0736224 | 7/15/2014 | 22:06.3 | 6 |
| -1588.881 | 19805.707 | 1.429 | 14.841 | 0.109493 | 7/15/2014 | 22:06.4 | 6 |
| -1588.613 | 19805.693 | 1.425 | 14.842 | 0.0802762 | 7/15/2014 | 22:06.5 | 6 |
| -1588.35 | 19805.682 | 1.423 | 14.843 | 0.0493771 | 7/15/2014 | 22:06.6 | 6 |
| -1588.087 | 19805.668 | 1.425 | 14.85 | 0.0605621 | 7/15/2014 | 22:06.7 | 6 |
| -1587.763 | 19805.652 | 1.418 | 14.838 | 0.0721203 | 7/15/2014 | 22:06.8 | 6 |
| -1587.502 | 19805.64 | 1.418 | 14.835 | 0.0528125 | 7/15/2014 | 22:06.9 | 6 |

| | | | | | | | |
|---|---|---|---|---|---|---|---|
| -1587.242 | 19805.625 | 1.413 | 14.827 | 0.0575068 | 7/15/2014 | 22:07.0 | 6 |
| -1586.981 | 19805.612 | 1.413 | 14.825 | 0.064018 | 7/15/2014 | 22:07.1 | 6 |
| -1586.722 | 19805.6 | 1.405 | 14.823 | 0.0886976 | 7/15/2014 | 22:07.2 | 6 |
| -1586.46 | 19805.584 | 1.405 | 14.82 | 0.102775 | 7/15/2014 | 22:07.3 | 6 |
| -1586.135 | 19805.566 | 1.396 | 14.82 | 0.0767564 | 7/15/2014 | 22:07.4 | 6 |
| -1585.87 | 19805.553 | 1.396 | 14.829 | 0.138185 | 7/15/2014 | 22:07.5 | 6 |
| -1585.604 | 19805.537 | 1.393 | 14.842 | 0.100886 | 7/15/2014 | 22:07.6 | 6 |
| -1585.336 | 19805.522 | 1.389 | 14.866 | 0.0941929 | 7/15/2014 | 22:07.7 | 6 |
| -1585.068 | 19805.508 | 1.379 | 14.884 | 0.128036 | 7/15/2014 | 22:07.8 | 6 |
| -1584.796 | 19805.492 | 1.379 | 14.908 | 0.0961763 | 7/15/2014 | 22:07.9 | 6 |
| -1584.526 | 19805.477 | 1.373 | 14.931 | 0.100505 | 7/15/2014 | 22:08.0 | 6 |
| -1584.254 | 19805.461 | 1.376 | 14.948 | 0.0719708 | 7/15/2014 | 22:08.1 | 6 |
| -1583.984 | 19805.445 | 1.372 | 14.966 | 0.0627581 | 7/15/2014 | 22:08.2 | 6 |
| -1583.714 | 19805.43 | 1.372 | 14.977 | 0.0552386 | 7/15/2014 | 22:08.3 | 6 |
| -1583.447 | 19805.414 | 1.367 | 14.971 | 0.0340262 | 7/15/2014 | 22:08.4 | 6 |
| -1583.18 | 19805.398 | 1.373 | 14.973 | 0.0288324 | 7/15/2014 | 22:08.5 | 6 |
| -1582.918 | 19805.383 | 1.373 | 14.965 | 0.0159698 | 7/15/2014 | 22:08.6 | 6 |
| -1582.656 | 19805.368 | 1.376 | 14.952 | 0.0136944 | 7/15/2014 | 22:08.7 | 6 |
| -1582.338 | 19805.35 | 1.373 | 14.944 | 0.0116596 | 7/15/2014 | 22:08.8 | 6 |
| -1582.081 | 19805.335 | 1.371 | 14.93 | 0.0222515 | 7/15/2014 | 22:08.9 | 6 |
| -1581.826 | 19805.319 | 1.371 | 14.92 | 0.0380965 | 7/15/2014 | 22:09.0 | 6 |
| -1581.571 | 19805.305 | 1.373 | 14.918 | 0.0366754 | 7/15/2014 | 22:09.1 | 6 |
| -1581.319 | 19805.289 | 1.362 | 14.911 | 0.0602526 | 7/15/2014 | 22:09.2 | 6 |
| -1581.066 | 19805.275 | 1.36 | 14.905 | 0.0673762 | 7/15/2014 | 22:09.3 | 6 |
| -1580.753 | 19805.258 | 1.358 | 14.901 | 0.0914371 | 7/15/2014 | 22:09.4 | 6 |
| -1580.501 | 19805.243 | 1.355 | 14.896 | 0.0868757 | 7/15/2014 | 22:09.5 | 6 |
| -1580.248 | 19805.229 | 1.355 | 14.895 | 0.0821859 | 7/15/2014 | 22:09.6 | 6 |
| -1579.993 | 19805.215 | 1.35 | 14.889 | 0.0882609 | 7/15/2014 | 22:09.7 | 6 |
| -1579.739 | 19805.199 | 1.345 | 14.881 | 0.0824037 | 7/15/2014 | 22:09.8 | 6 |
| -1579.483 | 19805.185 | 1.354 | 14.889 | 0.0864401 | 7/15/2014 | 22:09.9 | 6 |
| -1579.225 | 19805.171 | 1.354 | 14.89 | 0.0646286 | 7/15/2014 | 22:10.0 | 6 |
| -1578.968 | 19805.156 | 1.351 | 14.894 | 0.0599978 | 7/15/2014 | 22:10.1 | 6 |
| -1578.712 | 19805.142 | 1.354 | 14.9 | 0.0638337 | 7/15/2014 | 22:10.2 | 6 |
| -1578.455 | 19805.129 | 1.354 | 14.903 | 0.0465753 | 7/15/2014 | 22:10.3 | 6 |
| -1578.2 | 19805.113 | 1.348 | 14.906 | 0.0394971 | 7/15/2014 | 22:10.4 | 6 |
| -1577.945 | 19805.099 | 1.35 | 14.912 | 0.048437 | 7/15/2014 | 22:10.5 | 6 |
| -1577.691 | 19805.085 | 1.35 | 14.906 | 0.0647027 | 7/15/2014 | 22:10.6 | 6 |
| -1577.438 | 19805.072 | 1.353 | 14.906 | 0.059306 | 7/15/2014 | 22:10.7 | 6 |
| -1577.124 | 19805.054 | 1.346 | 14.908 | 0.0922414 | 7/15/2014 | 22:10.8 | 6 |
| -1576.871 | 19805.041 | 1.344 | 14.904 | 0.0967597 | 7/15/2014 | 22:10.9 | 6 |
| -1576.618 | 19805.025 | 1.349 | 14.907 | 0.112174 | 7/15/2014 | 22:11.0 | 6 |
| -1576.365 | 19805.013 | 1.36 | 14.911 | 0.120298 | 7/15/2014 | 22:11.1 | 6 |
| -1576.112 | 19804.999 | 1.362 | 14.913 | 0.108879 | 7/15/2014 | 22:11.2 | 6 |
| -1575.858 | 19804.986 | 1.36 | 14.91 | 0.118496 | 7/15/2014 | 22:11.3 | 6 |
| -1575.544 | 19804.969 | 1.36 | 14.914 | 0.0787885 | 7/15/2014 | 22:11.4 | 6 |
| -1575.289 | 19804.955 | 1.368 | 14.906 | 0.0800146 | 7/15/2014 | 22:11.5 | 6 |
| -1575.034 | 19804.941 | 1.368 | 14.902 | 0.0671183 | 7/15/2014 | 22:11.6 | 6 |

| | | | | | | | |
|---|---|---|---|---|---|---|---|
| -1574.778 | 19804.928 | 1.374 | 14.896 | 0.0798524 | 7/15/2014 | 22:11.7 | 6 |
| -1574.458 | 19804.911 | 1.378 | 14.887 | 0.0662382 | 7/15/2014 | 22:11.8 | 6 |
| -1574.2 | 19804.898 | 1.381 | 14.88 | 0.0843855 | 7/15/2014 | 22:11.9 | 6 |
| -1573.942 | 19804.884 | 1.385 | 14.866 | 0.0578626 | 7/15/2014 | 22:12.0 | 6 |
| -1573.683 | 19804.871 | 1.39 | 14.854 | 0.0946928 | 7/15/2014 | 22:12.1 | 6 |
| -1573.425 | 19804.859 | 1.392 | 14.853 | 0.0879643 | 7/15/2014 | 22:12.2 | 6 |
| -1573.165 | 19804.845 | 1.4 | 14.843 | 0.0741692 | 7/15/2014 | 22:12.3 | 6 |
| -1572.845 | 19804.828 | 1.397 | 14.83 | 0.0900712 | 7/15/2014 | 22:12.4 | 6 |
| -1572.587 | 19804.816 | 1.4 | 14.824 | 0.0682615 | 7/15/2014 | 22:12.5 | 6 |
| -1572.331 | 19804.803 | 1.403 | 14.814 | 0.129809 | 7/15/2014 | 22:12.6 | 6 |
| -1572.073 | 19804.791 | 1.406 | 14.823 | 0.100786 | 7/15/2014 | 22:12.7 | 6 |
| -1571.757 | 19804.776 | 1.401 | 14.821 | 0.0890478 | 7/15/2014 | 22:12.8 | 6 |
| -1571.5 | 19804.765 | 1.407 | 14.819 | 0.0680255 | 7/15/2014 | 22:12.9 | 6 |
| -1571.246 | 19804.752 | 1.408 | 14.828 | 0.0637173 | 7/15/2014 | 22:13.0 | 6 |
| -1570.991 | 19804.74 | 1.405 | 14.833 | 0.0574349 | 7/15/2014 | 22:13.1 | 6 |
| -1570.737 | 19804.729 | 1.396 | 14.839 | 0.0391592 | 7/15/2014 | 22:13.2 | 6 |
| -1570.483 | 19804.717 | 1.395 | 14.842 | 0.0431209 | 7/15/2014 | 22:13.3 | 6 |
| -1570.168 | 19804.702 | 1.396 | 14.837 | 0.0549514 | 7/15/2014 | 22:13.4 | 6 |
| -1569.914 | 19804.693 | 1.402 | 14.841 | 0.0572007 | 7/15/2014 | 22:13.5 | 6 |
| -1569.661 | 19804.682 | 1.396 | 14.844 | 0.0571115 | 7/15/2014 | 22:13.6 | 6 |
| -1569.407 | 19804.671 | 1.392 | 14.843 | 0.0608949 | 7/15/2014 | 22:13.7 | 6 |
| -1569.152 | 19804.66 | 1.396 | 14.846 | 0.0490965 | 7/15/2014 | 22:13.8 | 6 |
| -1568.897 | 19804.648 | 1.404 | 14.846 | 0.0349146 | 7/15/2014 | 22:13.9 | 6 |
| -1568.643 | 19804.639 | 1.402 | 14.849 | 0.0348535 | 7/15/2014 | 22:14.0 | 6 |
| -1568.389 | 19804.629 | 1.398 | 14.847 | 0.0323994 | 7/15/2014 | 22:14.1 | 6 |
| -1568.136 | 19804.619 | 1.395 | 14.844 | 0.0835955 | 7/15/2014 | 22:14.2 | 6 |
| -1567.885 | 19804.609 | 1.401 | 14.838 | 0.0388397 | 7/15/2014 | 22:14.3 | 6 |
| -1567.515 | 19804.596 | 1.407 | 14.824 | 0.0675791 | 7/15/2014 | 22:14.4 | 6 |
| -1567.269 | 19804.586 | 1.404 | 14.812 | 0.0933094 | 7/15/2014 | 22:14.5 | 6 |
| -1567.025 | 19804.578 | 1.401 | 14.804 | 0.110348 | 7/15/2014 | 22:14.6 | 6 |
| -1566.725 | 19804.566 | 1.401 | 14.792 | 0.0904903 | 7/15/2014 | 22:14.7 | 6 |
| -1566.601 | 19804.563 | 1.403 | 14.787 | 0.0784927 | 7/15/2014 | 22:14.8 | 6 |
| -1566.361 | 19804.554 | 1.403 | 14.773 | 0.141361 | 7/15/2014 | 22:14.9 | 6 |
| -1566.121 | 19804.545 | 1.397 | 14.766 | 0.0974304 | 7/15/2014 | 22:15.0 | 6 |
| -1565.881 | 19804.537 | 1.394 | 14.766 | 0.148418 | 7/15/2014 | 22:15.1 | 6 |
| -1565.642 | 19804.529 | 1.391 | 14.757 | 0.116859 | 7/15/2014 | 22:15.2 | 6 |
| -1565.403 | 19804.521 | 1.391 | 14.749 | 0.125828 | 7/15/2014 | 22:15.3 | 6 |
| -1564.98 | 19804.508 | 1.382 | 14.739 | 0.132267 | 7/15/2014 | 22:15.4 | 6 |
| -1564.739 | 19804.502 | 1.383 | 14.739 | 0.12277 | 7/15/2014 | 22:15.5 | 6 |
| -1564.494 | 19804.494 | 1.383 | 14.74 | 0.165579 | 7/15/2014 | 22:15.6 | 6 |
| -1564.248 | 19804.487 | 1.381 | 14.738 | 0.136169 | 7/15/2014 | 22:15.7 | 6 |
| -1564 | 19804.48 | 1.379 | 14.734 | 0.148962 | 7/15/2014 | 22:15.8 | 6 |
| -1563.878 | 19804.477 | 1.374 | 14.734 | 0.133458 | 7/15/2014 | 22:15.9 | 6 |
| -1563.628 | 19804.469 | 1.369 | 14.74 | 0.153181 | 7/15/2014 | 22:16.0 | 6 |
| -1563.375 | 19804.463 | 1.374 | 14.743 | 0.155287 | 7/15/2014 | 22:16.1 | 6 |
| -1563.125 | 19804.458 | 1.369 | 14.747 | 0.135897 | 7/15/2014 | 22:16.2 | 6 |
| -1562.872 | 19804.451 | 1.361 | 14.756 | 0.104518 | 7/15/2014 | 22:16.3 | 6 |

| | | | | | | | |
|---|---|---|---|---|---|---|---|
| -1562.622 | 19804.446 | 1.358 | 14.761 | 0.0989427 | 7/15/2014 | 22:16.4 | 6 |
| -1562.37 | 19804.441 | 1.358 | 14.765 | 0.121377 | 7/15/2014 | 22:16.5 | 6 |
| -1562 | 19804.434 | 1.355 | 14.775 | 0.113925 | 7/15/2014 | 22:16.6 | 6 |
| -1561.692 | 19804.43 | 1.35 | 14.786 | 0.110646 | 7/15/2014 | 22:16.7 | 6 |
| -1561.566 | 19804.427 | 1.345 | 14.791 | 0.111596 | 7/15/2014 | 22:16.8 | 6 |
| -1561.319 | 19804.422 | 1.344 | 14.802 | 0.119348 | 7/15/2014 | 22:16.9 | 6 |
| -1561.071 | 19804.418 | 1.34 | 14.803 | 0.121598 | 7/15/2014 | 22:17.0 | 6 |
| -1560.823 | 19804.415 | 1.335 | 14.813 | 0.116707 | 7/15/2014 | 22:17.1 | 6 |
| -1560.577 | 19804.411 | 1.327 | 14.822 | 0.113096 | 7/15/2014 | 22:17.2 | 6 |
| -1560.329 | 19804.408 | 1.321 | 14.823 | 0.101613 | 7/15/2014 | 22:17.3 | 6 |
| -1559.892 | 19804.404 | 1.314 | 14.829 | 0.0919926 | 7/15/2014 | 22:17.4 | 6 |
| -1559.771 | 19804.402 | 1.315 | 14.828 | 0.0960265 | 7/15/2014 | 22:17.5 | 6 |
| -1559.518 | 19804.4 | 1.308 | 14.826 | 0.101344 | 7/15/2014 | 22:17.6 | 6 |
| -1559.266 | 19804.398 | 1.311 | 14.828 | 0.0802358 | 7/15/2014 | 22:17.7 | 6 |
| -1559.013 | 19804.396 | 1.299 | 14.836 | 0.100658 | 7/15/2014 | 22:17.8 | 6 |
| -1558.758 | 19804.395 | 1.293 | 14.839 | 0.119163 | 7/15/2014 | 22:17.9 | 6 |
| -1558.502 | 19804.395 | 1.289 | 14.849 | 0.0822792 | 7/15/2014 | 22:18.0 | 6 |
| -1558.246 | 19804.393 | 1.289 | 14.856 | 0.093714 | 7/15/2014 | 22:18.1 | 6 |
| -1557.991 | 19804.393 | 1.283 | 14.86 | 0.0885186 | 7/15/2014 | 22:18.2 | 6 |
| -1557.736 | 19804.392 | 1.28 | 14.871 | 0.0826441 | 7/15/2014 | 22:18.3 | 6 |
| -1557.484 | 19804.393 | 1.271 | 14.88 | 0.0613701 | 7/15/2014 | 22:18.4 | 6 |
| -1557.233 | 19804.393 | 1.271 | 14.879 | 0.0669702 | 7/15/2014 | 22:18.5 | 6 |
| -1556.861 | 19804.394 | 1.26 | 14.885 | 0.0970529 | 7/15/2014 | 22:18.6 | 6 |
| -1556.551 | 19804.395 | 1.255 | 14.886 | 0.10013 | 7/15/2014 | 22:18.7 | 6 |
| -1556.425 | 19804.395 | 1.248 | 14.888 | 0.109254 | 7/15/2014 | 22:18.8 | 6 |
| -1556.177 | 19804.396 | 1.246 | 14.897 | 0.131576 | 7/15/2014 | 22:18.9 | 6 |
| -1555.929 | 19804.398 | 1.237 | 14.897 | 0.118946 | 7/15/2014 | 22:19.0 | 6 |
| -1555.68 | 19804.4 | 1.231 | 14.895 | 0.137611 | 7/15/2014 | 22:19.1 | 6 |
| -1555.431 | 19804.402 | 1.227 | 14.893 | 0.163793 | 7/15/2014 | 22:19.2 | 6 |
| -1555.181 | 19804.404 | 1.231 | 14.894 | 0.179614 | 7/15/2014 | 22:19.3 | 6 |
| -1554.871 | 19804.406 | 1.225 | 14.898 | 0.194529 | 7/15/2014 | 22:19.4 | 6 |
| -1554.618 | 19804.41 | 1.221 | 14.898 | 0.210409 | 7/15/2014 | 22:19.5 | 6 |
| -1554.365 | 19804.413 | 1.214 | 14.894 | 0.211221 | 7/15/2014 | 22:19.6 | 6 |
| -1554.109 | 19804.416 | 1.214 | 14.905 | 0.222188 | 7/15/2014 | 22:19.7 | 6 |
| -1553.854 | 19804.421 | 1.208 | 14.918 | 0.226048 | 7/15/2014 | 22:19.8 | 6 |
| -1553.596 | 19804.424 | 1.208 | 14.928 | 0.229673 | 7/15/2014 | 22:19.9 | 6 |
| -1553.341 | 19804.428 | 1.198 | 14.953 | 0.219356 | 7/15/2014 | 22:20.0 | 6 |
| -1553.083 | 19804.432 | 1.202 | 14.981 | 0.210811 | 7/15/2014 | 22:20.1 | 6 |
| -1552.829 | 19804.437 | 1.192 | 15.015 | 0.244108 | 7/15/2014 | 22:20.2 | 6 |
| -1552.574 | 19804.441 | 1.186 | 15.051 | 0.196933 | 7/15/2014 | 22:20.3 | 6 |
| -1552.321 | 19804.446 | 1.183 | 15.085 | 0.190802 | 7/15/2014 | 22:20.4 | 6 |
| -1552.07 | 19804.452 | 1.186 | 15.114 | 0.154195 | 7/15/2014 | 22:20.5 | 6 |
| -1551.705 | 19804.461 | 1.172 | 15.155 | 0.185921 | 7/15/2014 | 22:20.6 | 6 |
| -1551.4 | 19804.467 | 1.166 | 15.173 | 0.171727 | 7/15/2014 | 22:20.7 | 6 |
| -1551.275 | 19804.47 | 1.161 | 15.181 | 0.177041 | 7/15/2014 | 22:20.8 | 6 |
| -1551.032 | 19804.477 | 1.161 | 15.192 | 0.194433 | 7/15/2014 | 22:20.9 | 6 |
| -1550.791 | 19804.482 | 1.156 | 15.194 | 0.176225 | 7/15/2014 | 22:21.0 | 6 |

| | | | | | | | |
|---|---|---|---|---|---|---|---|
| -1550.549 | 19804.489 | 1.148 | 15.194 | 0.221242 | 7/15/2014 | 22:21.1 | 6 |
| -1550.311 | 19804.496 | 1.135 | 15.19 | 0.179599 | 7/15/2014 | 22:21.2 | 6 |
| -1550.071 | 19804.502 | 1.138 | 15.185 | 0.202705 | 7/15/2014 | 22:21.3 | 6 |
| -1549.656 | 19804.515 | 1.131 | 15.17 | 0.17989 | 7/15/2014 | 22:21.4 | 6 |
| -1549.418 | 19804.523 | 1.126 | 15.166 | 0.179086 | 7/15/2014 | 22:21.5 | 6 |
| -1549.181 | 19804.531 | 1.122 | 15.157 | 0.19078 | 7/15/2014 | 22:21.6 | 6 |
| -1548.884 | 19804.54 | 1.115 | 15.151 | 0.162183 | 7/15/2014 | 22:21.7 | 6 |
| -1548.764 | 19804.545 | 1.115 | 15.145 | 0.164159 | 7/15/2014 | 22:21.8 | 6 |
| -1548.523 | 19804.553 | 1.107 | 15.144 | 0.160916 | 7/15/2014 | 22:21.9 | 6 |
| -1548.282 | 19804.562 | 1.093 | 15.144 | 0.161846 | 7/15/2014 | 22:22.0 | 6 |
| -1548.04 | 19804.571 | 1.089 | 15.147 | 0.168018 | 7/15/2014 | 22:22.1 | 6 |
| -1547.796 | 19804.58 | 1.082 | 15.149 | 0.160266 | 7/15/2014 | 22:22.2 | 6 |
| -1547.553 | 19804.59 | 1.078 | 15.151 | 0.161346 | 7/15/2014 | 22:22.3 | 6 |
| -1547.125 | 19804.605 | 1.064 | 15.162 | 0.159183 | 7/15/2014 | 22:22.4 | 6 |
| -1546.88 | 19804.617 | 1.058 | 15.176 | 0.166622 | 7/15/2014 | 22:22.5 | 6 |
| -1546.634 | 19804.627 | 1.051 | 15.187 | 0.156862 | 7/15/2014 | 22:22.6 | 6 |
| -1546.324 | 19804.643 | 1.046 | 15.189 | 0.166584 | 7/15/2014 | 22:22.7 | 6 |
| -1546.2 | 19804.646 | 1.039 | 15.195 | 0.159504 | 7/15/2014 | 22:22.8 | 6 |
| -1545.949 | 19804.658 | 1.028 | 15.201 | 0.194391 | 7/15/2014 | 22:22.9 | 6 |
| -1545.696 | 19804.67 | 1.017 | 15.201 | 0.166013 | 7/15/2014 | 22:23.0 | 6 |
| -1545.441 | 19804.681 | 1.011 | 15.202 | 0.19687 | 7/15/2014 | 22:23.1 | 6 |
| -1545.187 | 19804.693 | 1.003 | 15.207 | 0.184389 | 7/15/2014 | 22:23.2 | 6 |
| -1544.932 | 19804.706 | 0.999 | 15.204 | 0.227087 | 7/15/2014 | 22:23.3 | 6 |
| -1544.614 | 19804.723 | 0.989 | 15.208 | 0.237049 | 7/15/2014 | 22:23.4 | 6 |
| -1544.356 | 19804.736 | 0.987 | 15.221 | 0.226195 | 7/15/2014 | 22:23.5 | 6 |
| -1544.096 | 19804.748 | 0.981 | 15.234 | 0.272527 | 7/15/2014 | 22:23.6 | 6 |
| -1543.835 | 19804.763 | 0.977 | 15.25 | 0.232622 | 7/15/2014 | 22:23.7 | 6 |
| -1543.509 | 19804.781 | 0.971 | 15.267 | 0.241638 | 7/15/2014 | 22:23.8 | 6 |
| -1543.245 | 19804.796 | 0.961 | 15.295 | 0.222924 | 7/15/2014 | 22:23.9 | 6 |
| -1542.979 | 19804.811 | 0.949 | 15.317 | 0.204254 | 7/15/2014 | 22:24.0 | 6 |
| -1542.712 | 19804.827 | 0.955 | 15.338 | 0.18535 | 7/15/2014 | 22:24.1 | 6 |
| -1542.446 | 19804.842 | 0.947 | 15.358 | 0.168338 | 7/15/2014 | 22:24.2 | 6 |
| -1542.177 | 19804.857 | 0.949 | 15.385 | 0.157777 | 7/15/2014 | 22:24.3 | 6 |
| -1541.845 | 19804.878 | 0.938 | 15.411 | 0.142186 | 7/15/2014 | 22:24.4 | 6 |
| -1541.576 | 19804.895 | 0.935 | 15.439 | 0.131551 | 7/15/2014 | 22:24.5 | 6 |
| -1541.307 | 19804.911 | 0.93 | 15.455 | 0.121897 | 7/15/2014 | 22:24.6 | 6 |
| -1541.038 | 19804.929 | 0.933 | 15.475 | 0.129026 | 7/15/2014 | 22:24.7 | 6 |
| -1540.703 | 19804.951 | 0.923 | 15.49 | 0.119175 | 7/15/2014 | 22:24.8 | 6 |
| -1540.432 | 19804.969 | 0.919 | 15.504 | 0.117491 | 7/15/2014 | 22:24.9 | 6 |
| -1540.162 | 19804.986 | 0.908 | 15.509 | 0.106012 | 7/15/2014 | 22:25.0 | 6 |
| -1539.891 | 19805.005 | 0.91 | 15.517 | 0.106997 | 7/15/2014 | 22:25.1 | 6 |
| -1539.623 | 19805.023 | 0.9 | 15.521 | 0.110528 | 7/15/2014 | 22:25.2 | 6 |
| -1539.354 | 19805.042 | 0.906 | 15.528 | 0.111268 | 7/15/2014 | 22:25.3 | 6 |
| -1539.02 | 19805.065 | 0.891 | 15.532 | 0.120189 | 7/15/2014 | 22:25.4 | 6 |
| -1538.752 | 19805.086 | 0.885 | 15.536 | 0.132313 | 7/15/2014 | 22:25.5 | 6 |
| -1538.485 | 19805.104 | 0.876 | 15.542 | 0.199243 | 7/15/2014 | 22:25.6 | 6 |
| -1538.218 | 19805.124 | 0.87 | 15.558 | 0.184955 | 7/15/2014 | 22:25.7 | 6 |

| | | | | | | | |
|---|---|---|---|---|---|---|---|
| -1537.887 | 19805.149 | 0.864 | 15.567 | 0.211884 | 7/15/2014 | 22:25.8 | 6 |
| -1537.62 | 19805.169 | 0.856 | 15.579 | 0.238028 | 7/15/2014 | 22:25.9 | 6 |
| -1537.353 | 19805.189 | 0.838 | 15.592 | 0.244777 | 7/15/2014 | 22:26.0 | 6 |
| -1537.086 | 19805.211 | 0.834 | 15.612 | 0.24218 | 7/15/2014 | 22:26.1 | 6 |
| -1536.82 | 19805.23 | 0.821 | 15.633 | 0.200211 | 7/15/2014 | 22:26.2 | 6 |
| -1536.555 | 19805.252 | 0.809 | 15.654 | 0.21038 | 7/15/2014 | 22:26.3 | 6 |
| -1536.226 | 19805.278 | 0.794 | 15.666 | 0.168215 | 7/15/2014 | 22:26.4 | 6 |
| -1535.962 | 19805.299 | 0.786 | 15.686 | 0.117129 | 7/15/2014 | 22:26.5 | 6 |
| -1535.699 | 19805.321 | 0.782 | 15.704 | 0.0900761 | 7/15/2014 | 22:26.6 | 6 |
| -1535.437 | 19805.344 | 0.776 | 15.718 | 0.0790498 | 7/15/2014 | 22:26.7 | 6 |
| -1535.11 | 19805.369 | 0.761 | 15.737 | 0.085844 | 7/15/2014 | 22:26.8 | 6 |
| -1534.847 | 19805.393 | 0.75 | 15.75 | 0.0763528 | 7/15/2014 | 22:26.9 | 6 |
| -1534.518 | 19805.421 | 0.746 | 15.767 | 0.079359 | 7/15/2014 | 22:27.0 | 6 |
| -1534.386 | 19805.432 | 0.746 | 15.777 | 0.0823683 | 7/15/2014 | 22:27.0 | 6 |
| -1534.059 | 19805.46 | 0.726 | 15.803 | 0.0875443 | 7/15/2014 | 22:27.2 | 6 |
| -1533.797 | 19805.484 | 0.72 | 15.819 | 0.0921367 | 7/15/2014 | 22:27.3 | 6 |
| -1533.473 | 19805.512 | 0.711 | 15.834 | 0.0974584 | 7/15/2014 | 22:27.4 | 6 |
| -1533.214 | 19805.535 | 0.709 | 15.845 | 0.0992611 | 7/15/2014 | 22:27.5 | 6 |
| -1532.761 | 19805.576 | 0.703 | 15.86 | 0.10332 | 7/15/2014 | 22:27.6 | 6 |
| -1532.695 | 19805.582 | 0.698 | 15.866 | 0.109313 | 7/15/2014 | 22:27.7 | 6 |
| -1532.373 | 19805.61 | 0.689 | 15.871 | 0.114914 | 7/15/2014 | 22:27.8 | 6 |
| -1532.113 | 19805.635 | 0.683 | 15.873 | 0.113139 | 7/15/2014 | 22:27.9 | 6 |
| -1531.854 | 19805.659 | 0.675 | 15.873 | 0.124841 | 7/15/2014 | 22:28.0 | 6 |
| -1531.594 | 19805.684 | 0.675 | 15.873 | 0.146333 | 7/15/2014 | 22:28.1 | 6 |
| -1531.336 | 19805.707 | 0.665 | 15.868 | 0.157317 | 7/15/2014 | 22:28.2 | 6 |
| -1531.077 | 19805.731 | 0.663 | 15.861 | 0.176798 | 7/15/2014 | 22:28.3 | 6 |
| -1530.757 | 19805.762 | 0.657 | 15.853 | 0.184621 | 7/15/2014 | 22:28.4 | 6 |
| -1530.499 | 19805.787 | 0.657 | 15.841 | 0.235705 | 7/15/2014 | 22:28.5 | 6 |
| -1530.243 | 19805.812 | 0.653 | 15.831 | 0.228092 | 7/15/2014 | 22:28.6 | 6 |
| -1529.986 | 19805.838 | 0.657 | 15.826 | 0.197601 | 7/15/2014 | 22:28.7 | 6 |
| -1529.667 | 19805.868 | 0.651 | 15.818 | 0.201722 | 7/15/2014 | 22:28.8 | 6 |
| -1529.411 | 19805.894 | 0.66 | 15.817 | 0.24206 | 7/15/2014 | 22:28.9 | 6 |
| -1529.155 | 19805.92 | 0.666 | 15.815 | 0.198642 | 7/15/2014 | 22:29.0 | 6 |
| -1528.898 | 19805.946 | 0.67 | 15.815 | 0.172621 | 7/15/2014 | 22:29.1 | 6 |
| -1528.643 | 19805.971 | 0.67 | 15.817 | 0.162154 | 7/15/2014 | 22:29.2 | 6 |
| -1528.388 | 19805.998 | 0.674 | 15.824 | 0.180123 | 7/15/2014 | 22:29.3 | 6 |
| -1528.072 | 19806.03 | 0.674 | 15.828 | 0.193772 | 7/15/2014 | 22:29.4 | 6 |
| -1527.818 | 19806.057 | 0.674 | 15.834 | 0.128204 | 7/15/2014 | 22:29.5 | 6 |
| -1527.565 | 19806.084 | 0.668 | 15.856 | 0.132512 | 7/15/2014 | 22:29.6 | 6 |
| -1527.312 | 19806.11 | 0.668 | 15.868 | 0.0973996 | 7/15/2014 | 22:29.7 | 6 |
| -1526.995 | 19806.143 | 0.668 | 15.881 | 0.0876379 | 7/15/2014 | 22:29.8 | 6 |
| -1526.74 | 19806.17 | 0.668 | 15.886 | 0.0524734 | 7/15/2014 | 22:29.9 | 6 |
| -1526.485 | 19806.198 | 0.67 | 15.878 | 0.0397671 | 7/15/2014 | 22:30.0 | 6 |
| -1526.229 | 19806.227 | 0.666 | 15.868 | 0.0638282 | 7/15/2014 | 22:30.1 | 6 |
| -1525.974 | 19806.254 | 0.671 | 15.86 | 0.0852255 | 7/15/2014 | 22:30.2 | 6 |
| -1525.717 | 19806.281 | 0.685 | 15.826 | 0.104604 | 7/15/2014 | 22:30.3 | 6 |
| -1525.399 | 19806.314 | 0.687 | 15.816 | 0.111823 | 7/15/2014 | 22:30.4 | 6 |

| | | | | | | | |
|---|---|---|---|---|---|---|---|
| -1525.143 | 19806.344 | 0.698 | 15.806 | 0.111536 | 7/15/2014 | 22:30.5 | 6 |
| -1524.887 | 19806.372 | 0.706 | 15.807 | 0.107062 | 7/15/2014 | 22:30.6 | 6 |
| -1524.631 | 19806.4 | 0.721 | 15.818 | 0.0928459 | 7/15/2014 | 22:30.7 | 6 |
| -1524.313 | 19806.436 | 0.727 | 15.825 | 0.10293 | 7/15/2014 | 22:30.8 | 6 |
| -1524.056 | 19806.463 | 0.733 | 15.831 | 0.0888122 | 7/15/2014 | 22:30.9 | 6 |
| -1523.801 | 19806.49 | 0.731 | 15.836 | 0.108399 | 7/15/2014 | 22:31.0 | 6 |
| -1523.544 | 19806.52 | 0.739 | 15.828 | 0.120682 | 7/15/2014 | 22:31.1 | 6 |
| -1523.289 | 19806.549 | 0.736 | 15.822 | 0.152326 | 7/15/2014 | 22:31.2 | 6 |
| -1523.035 | 19806.576 | 0.742 | 15.805 | 0.171189 | 7/15/2014 | 22:31.3 | 6 |
| -1522.72 | 19806.611 | 0.739 | 15.785 | 0.161227 | 7/15/2014 | 22:31.4 | 6 |
| -1522.468 | 19806.641 | 0.75 | 15.766 | 0.183441 | 7/15/2014 | 22:31.5 | 6 |
| -1522.217 | 19806.668 | 0.754 | 15.748 | 0.155635 | 7/15/2014 | 22:31.6 | 6 |
| -1521.966 | 19806.695 | 0.756 | 15.735 | 0.147084 | 7/15/2014 | 22:31.7 | 6 |
| -1521.655 | 19806.732 | 0.753 | 15.721 | 0.108705 | 7/15/2014 | 22:31.8 | 6 |
| -1521.405 | 19806.76 | 0.759 | 15.711 | 0.114118 | 7/15/2014 | 22:31.9 | 6 |
| -1521.155 | 19806.787 | 0.761 | 15.712 | 0.115182 | 7/15/2014 | 22:32.0 | 6 |
| -1520.906 | 19806.817 | 0.766 | 15.712 | 0.103292 | 7/15/2014 | 22:32.1 | 6 |
| -1520.657 | 19806.846 | 0.766 | 15.706 | 0.129894 | 7/15/2014 | 22:32.2 | 6 |
| -1520.408 | 19806.873 | 0.769 | 15.702 | 0.109772 | 7/15/2014 | 22:32.3 | 6 |
| -1519.977 | 19806.923 | 0.779 | 15.684 | 0.118728 | 7/15/2014 | 22:32.4 | 6 |
| -1519.731 | 19806.951 | 0.782 | 15.681 | 0.0678903 | 7/15/2014 | 22:32.5 | 6 |
| -1519.485 | 19806.979 | 0.783 | 15.665 | 0.0907613 | 7/15/2014 | 22:32.6 | 6 |
| -1519.178 | 19807.015 | 0.785 | 15.644 | 0.087107 | 7/15/2014 | 22:32.7 | 6 |
| -1519.054 | 19807.029 | 0.785 | 15.634 | 0.0867514 | 7/15/2014 | 22:32.8 | 6 |
| -1518.806 | 19807.057 | 0.795 | 15.625 | 0.116886 | 7/15/2014 | 22:32.9 | 6 |
| -1518.56 | 19807.084 | 0.797 | 15.6 | 0.163215 | 7/15/2014 | 22:33.0 | 6 |
| -1518.311 | 19807.112 | 0.807 | 15.582 | 0.187617 | 7/15/2014 | 22:33.1 | 6 |
| -1518.065 | 19807.142 | 0.809 | 15.555 | 0.19827 | 7/15/2014 | 22:33.2 | 6 |
| -1517.816 | 19807.17 | 0.825 | 15.527 | 0.196754 | 7/15/2014 | 22:33.3 | 6 |
| -1517.384 | 19807.219 | 0.844 | 15.501 | 0.160222 | 7/15/2014 | 22:33.4 | 6 |
| -1517.262 | 19807.232 | 0.852 | 15.489 | 0.15527 | 7/15/2014 | 22:33.5 | 6 |
| -1517.016 | 19807.26 | 0.852 | 15.474 | 0.121965 | 7/15/2014 | 22:33.6 | 6 |
| -1516.586 | 19807.307 | 0.867 | 15.484 | 0.0899195 | 7/15/2014 | 22:33.7 | 6 |
| -1516.462 | 19807.321 | 0.87 | 15.48 | 0.0835955 | 7/15/2014 | 22:33.8 | 6 |
| -1516.214 | 19807.35 | 0.882 | 15.502 | 0.0573843 | 7/15/2014 | 22:33.9 | 6 |
| -1515.965 | 19807.379 | 0.887 | 15.525 | 0.0805609 | 7/15/2014 | 22:34.0 | 6 |
| -1515.716 | 19807.405 | 0.891 | 15.551 | 0.0615821 | 7/15/2014 | 22:34.1 | 6 |
| -1515.469 | 19807.433 | 0.891 | 15.571 | 0.0954992 | 7/15/2014 | 22:34.2 | 6 |
| -1515.22 | 19807.461 | 0.903 | 15.592 | 0.0859763 | 7/15/2014 | 22:34.3 | 6 |
| -1514.912 | 19807.494 | 0.903 | 15.616 | 0.0940831 | 7/15/2014 | 22:34.4 | 6 |
| -1514.664 | 19807.521 | 0.906 | 15.619 | 0.103526 | 7/15/2014 | 22:34.5 | 6 |
| -1514.417 | 19807.551 | 0.906 | 15.619 | 0.100953 | 7/15/2014 | 22:34.6 | 6 |
| -1514.169 | 19807.577 | 0.909 | 15.611 | 0.111097 | 7/15/2014 | 22:34.7 | 6 |
| -1513.86 | 19807.61 | 0.913 | 15.599 | 0.101092 | 7/15/2014 | 22:34.8 | 6 |
| -1513.611 | 19807.639 | 0.922 | 15.588 | 0.119558 | 7/15/2014 | 22:34.9 | 6 |
| -1513.362 | 19807.665 | 0.926 | 15.571 | 0.130638 | 7/15/2014 | 22:35.0 | 6 |
| -1513.113 | 19807.691 | 0.938 | 15.545 | 0.154491 | 7/15/2014 | 22:35.1 | 6 |

| | | | | | | | |
|---|---|---|---|---|---|---|---|
| -1512.865 | 19807.719 | 0.945 | 15.53 | 0.160214 | 7/15/2014 | 22:35.2 | 6 |
| -1512.617 | 19807.746 | 0.958 | 15.504 | 0.183788 | 7/15/2014 | 22:35.3 | 6 |
| -1512.186 | 19807.793 | 0.971 | 15.475 | 0.186524 | 7/15/2014 | 22:35.4 | 6 |
| -1511.939 | 19807.818 | 0.977 | 15.449 | 0.18979 | 7/15/2014 | 22:35.5 | 6 |
| -1511.694 | 19807.845 | 0.983 | 15.426 | 0.187946 | 7/15/2014 | 22:35.6 | 6 |
| -1511.387 | 19807.876 | 0.995 | 15.4 | 0.183768 | 7/15/2014 | 22:35.7 | 6 |
| -1511.263 | 19807.89 | 0.998 | 15.389 | 0.191255 | 7/15/2014 | 22:35.8 | 6 |
| -1511.016 | 19807.916 | 1.013 | 15.367 | 0.161173 | 7/15/2014 | 22:35.9 | 6 |
| -1510.769 | 19807.943 | 1.022 | 15.361 | 0.146204 | 7/15/2014 | 22:36.0 | 6 |
| -1510.52 | 19807.97 | 1.038 | 15.342 | 0.149247 | 7/15/2014 | 22:36.1 | 6 |
| -1510.274 | 19807.994 | 1.052 | 15.327 | 0.124476 | 7/15/2014 | 22:36.2 | 6 |
| -1510.026 | 19808.021 | 1.069 | 15.333 | 0.108091 | 7/15/2014 | 22:36.3 | 6 |
| -1509.594 | 19808.065 | 1.082 | 15.341 | 0.10245 | 7/15/2014 | 22:36.4 | 6 |
| -1509.472 | 19808.078 | 1.086 | 15.345 | 0.101382 | 7/15/2014 | 22:36.5 | 6 |
| -1509.226 | 19808.104 | 1.096 | 15.354 | 0.112313 | 7/15/2014 | 22:36.6 | 6 |
| -1508.795 | 19808.147 | 1.114 | 15.361 | 0.130926 | 7/15/2014 | 22:36.7 | 6 |
| -1508.671 | 19808.16 | 1.113 | 15.371 | 0.135606 | 7/15/2014 | 22:36.8 | 6 |
| -1508.423 | 19808.186 | 1.119 | 15.376 | 0.148687 | 7/15/2014 | 22:36.9 | 6 |
| -1508.176 | 19808.209 | 1.125 | 15.369 | 0.152238 | 7/15/2014 | 22:37.0 | 6 |
| -1507.927 | 19808.235 | 1.141 | 15.367 | 0.173819 | 7/15/2014 | 22:37.1 | 6 |
| -1507.68 | 19808.259 | 1.151 | 15.351 | 0.189497 | 7/15/2014 | 22:37.2 | 6 |
| -1507.433 | 19808.283 | 1.163 | 15.335 | 0.18979 | 7/15/2014 | 22:37.3 | 6 |
| -1507.003 | 19808.326 | 1.178 | 15.313 | 0.194797 | 7/15/2014 | 22:37.4 | 6 |
| -1506.759 | 19808.35 | 1.189 | 15.295 | 0.195865 | 7/15/2014 | 22:37.5 | 6 |
| -1506.514 | 19808.373 | 1.205 | 15.284 | 0.199661 | 7/15/2014 | 22:37.6 | 6 |
| -1506.21 | 19808.402 | 1.221 | 15.27 | 0.191077 | 7/15/2014 | 22:37.7 | 6 |
| -1506.086 | 19808.414 | 1.227 | 15.259 | 0.188844 | 7/15/2014 | 22:37.8 | 6 |
| -1505.84 | 19808.438 | 1.242 | 15.248 | 0.167568 | 7/15/2014 | 22:37.9 | 6 |
| -1505.594 | 19808.461 | 1.248 | 15.234 | 0.198151 | 7/15/2014 | 22:38.0 | 6 |
| -1505.348 | 19808.484 | 1.254 | 15.218 | 0.163564 | 7/15/2014 | 22:38.1 | 6 |
| -1505.103 | 19808.508 | 1.262 | 15.209 | 0.157481 | 7/15/2014 | 22:38.2 | 6 |
| -1504.857 | 19808.53 | 1.271 | 15.192 | 0.139058 | 7/15/2014 | 22:38.3 | 6 |
| -1504.428 | 19808.569 | 1.27 | 15.174 | 0.140984 | 7/15/2014 | 22:38.4 | 6 |
| -1504.184 | 19808.591 | 1.272 | 15.167 | 0.140409 | 7/15/2014 | 22:38.5 | 6 |
| -1503.939 | 19808.613 | 1.274 | 15.162 | 0.132182 | 7/15/2014 | 22:38.6 | 6 |
| -1503.633 | 19808.641 | 1.274 | 15.156 | 0.120913 | 7/15/2014 | 22:38.7 | 6 |
| -1503.509 | 19808.652 | 1.276 | 15.153 | 0.118342 | 7/15/2014 | 22:38.8 | 6 |
| -1503.262 | 19808.675 | 1.28 | 15.139 | 0.130859 | 7/15/2014 | 22:38.9 | 6 |
| -1503.015 | 19808.695 | 1.277 | 15.117 | 0.127672 | 7/15/2014 | 22:39.0 | 6 |
| -1502.766 | 19808.717 | 1.289 | 15.1 | 0.121656 | 7/15/2014 | 22:39.1 | 6 |
| -1502.519 | 19808.737 | 1.288 | 15.098 | 0.121454 | 7/15/2014 | 22:39.2 | 6 |
| -1502.272 | 19808.76 | 1.292 | 15.085 | 0.114951 | 7/15/2014 | 22:39.3 | 6 |
| -1501.843 | 19808.795 | 1.297 | 15.094 | 0.124604 | 7/15/2014 | 22:39.4 | 6 |
| -1501.6 | 19808.816 | 1.302 | 15.096 | 0.117998 | 7/15/2014 | 22:39.5 | 6 |
| -1501.359 | 19808.836 | 1.304 | 15.107 | 0.113761 | 7/15/2014 | 22:39.6 | 6 |
| -1501.06 | 19808.859 | 1.309 | 15.127 | 0.112641 | 7/15/2014 | 22:39.7 | 6 |
| -1500.936 | 19808.87 | 1.308 | 15.135 | 0.112189 | 7/15/2014 | 22:39.8 | 6 |

| | | | | | | | |
|---|---|---|---|---|---|---|---|
| -1500.696 | 19808.89 | 1.311 | 15.139 | 0.121918 | 7/15/2014 | 22:39.9 | 6 |
| -1500.459 | 19808.908 | 1.311 | 15.148 | 0.13179 | 7/15/2014 | 22:40.0 | 6 |
| -1500.222 | 19808.927 | 1.316 | 15.148 | 0.138785 | 7/15/2014 | 22:40.1 | 6 |
| -1499.989 | 19808.945 | 1.314 | 15.137 | 0.151854 | 7/15/2014 | 22:40.2 | 6 |
| -1499.644 | 19808.971 | 1.322 | 15.132 | 0.164082 | 7/15/2014 | 22:40.3 | 6 |
| -1499.357 | 19808.993 | 1.327 | 15.125 | 0.172247 | 7/15/2014 | 22:40.4 | 6 |
| -1499.132 | 19809.01 | 1.335 | 15.115 | 0.179818 | 7/15/2014 | 22:40.5 | 6 |
| -1498.908 | 19809.026 | 1.344 | 15.109 | 0.197176 | 7/15/2014 | 22:40.6 | 6 |
| -1498.631 | 19809.046 | 1.352 | 15.107 | 0.182307 | 7/15/2014 | 22:40.7 | 6 |
| -1498.41 | 19809.063 | 1.355 | 15.099 | 0.189408 | 7/15/2014 | 22:40.8 | 6 |
| -1498.188 | 19809.077 | 1.364 | 15.088 | 0.197312 | 7/15/2014 | 22:40.9 | 6 |
| -1497.967 | 19809.093 | 1.373 | 15.078 | 0.187811 | 7/15/2014 | 22:41.0 | 6 |
| -1497.747 | 19809.108 | 1.38 | 15.061 | 0.183438 | 7/15/2014 | 22:41.1 | 6 |
| -1497.528 | 19809.123 | 1.382 | 15.048 | 0.18662 | 7/15/2014 | 22:41.2 | 6 |
| -1497.31 | 19809.138 | 1.388 | 15.041 | 0.175362 | 7/15/2014 | 22:41.3 | 6 |
| -1497.04 | 19809.155 | 1.391 | 15.035 | 0.176892 | 7/15/2014 | 22:41.4 | 6 |
| -1496.825 | 19809.17 | 1.396 | 15.02 | 0.17632 | 7/15/2014 | 22:41.5 | 6 |
| -1496.61 | 19809.183 | 1.402 | 15.02 | 0.159431 | 7/15/2014 | 22:41.6 | 6 |
| -1496.34 | 19809.2 | 1.405 | 15.015 | 0.155241 | 7/15/2014 | 22:41.7 | 6 |
| -1496.123 | 19809.214 | 1.407 | 15.012 | 0.156523 | 7/15/2014 | 22:41.8 | 6 |
| -1495.905 | 19809.228 | 1.413 | 15.008 | 0.156954 | 7/15/2014 | 22:41.9 | 6 |
| -1495.685 | 19809.24 | 1.422 | 15.004 | 0.152543 | 7/15/2014 | 22:42.0 | 6 |
| -1495.465 | 19809.253 | 1.425 | 14.998 | 0.14971 | 7/15/2014 | 22:42.1 | 6 |
| -1495.246 | 19809.266 | 1.43 | 14.984 | 0.151153 | 7/15/2014 | 22:42.2 | 6 |
| -1495.027 | 19809.277 | 1.436 | 14.971 | 0.157481 | 7/15/2014 | 22:42.3 | 6 |
| -1494.754 | 19809.294 | 1.443 | 14.947 | 0.168199 | 7/15/2014 | 22:42.4 | 6 |
| -1494.535 | 19809.306 | 1.451 | 14.927 | 0.17261 | 7/15/2014 | 22:42.5 | 6 |
| -1494.317 | 19809.318 | 1.453 | 14.907 | 0.175118 | 7/15/2014 | 22:42.6 | 6 |
| -1494.97 | 19806.603 | 1.938 | 14.272 | 0.0842427 | 7/15/2014 | 33:57.1 | 0 |
| -1494.753 | 19806.582 | 1.938 | 14.27 | 0.112011 | 7/15/2014 | 33:57.2 | 0 |
| -1494.536 | 19806.563 | 1.938 | 14.27 | 0.108051 | 7/15/2014 | 33:57.3 | 0 |
| -1494.268 | 19806.538 | 1.94 | 14.27 | 0.0972831 | 7/15/2014 | 33:57.4 | 0 |
| -1494.051 | 19806.518 | 1.943 | 14.272 | 0.105301 | 7/15/2014 | 33:57.5 | 0 |
| -1493.836 | 19806.497 | 1.945 | 14.274 | 0.0943102 | 7/15/2014 | 33:57.6 | 0 |
| -1493.565 | 19806.471 | 1.946 | 14.276 | 0.101043 | 7/15/2014 | 33:57.7 | 0 |
| -1493.349 | 19806.45 | 1.944 | 14.278 | 0.101687 | 7/15/2014 | 33:57.8 | 0 |
| -1493.131 | 19806.429 | 1.946 | 14.281 | 0.0892174 | 7/15/2014 | 33:57.9 | 0 |
| -1492.913 | 19806.407 | 1.948 | 14.284 | 0.0868808 | 7/15/2014 | 33:58.0 | 0 |
| -1492.696 | 19806.387 | 1.948 | 14.288 | 0.0940964 | 7/15/2014 | 33:58.1 | 0 |
| -1492.479 | 19806.365 | 1.951 | 14.292 | 0.0736649 | 7/15/2014 | 33:58.2 | 0 |
| -1492.263 | 19806.343 | 1.953 | 14.296 | 0.0781646 | 7/15/2014 | 33:58.3 | 0 |
| -1491.995 | 19806.316 | 1.953 | 14.302 | 0.0634716 | 7/15/2014 | 33:58.4 | 0 |
| -1491.78 | 19806.294 | 1.959 | 14.309 | 0.0662199 | 7/15/2014 | 33:58.5 | 0 |
| -1491.565 | 19806.272 | 1.963 | 14.317 | 0.0648282 | 7/15/2014 | 33:58.6 | 0 |
| -1491.298 | 19806.246 | 1.963 | 14.326 | 0.0675645 | 7/15/2014 | 33:58.7 | 0 |
| -1491.082 | 19806.224 | 1.966 | 14.335 | 0.0675254 | 7/15/2014 | 33:58.8 | 0 |
| -1490.866 | 19806.201 | 1.968 | 14.345 | 0.072708 | 7/15/2014 | 33:58.9 | 0 |

| | | | | | | | |
|---|---|---|---|---|---|---|---|
| -1490.65 | 19806.178 | 1.968 | 14.354 | 0.075251 | 7/15/2014 | 33:59.0 | 0 |
| -1490.434 | 19806.155 | 1.971 | 14.362 | 0.0856887 | 7/15/2014 | 33:59.1 | 0 |
| -1490.219 | 19806.132 | 1.975 | 14.369 | 0.0840633 | 7/15/2014 | 33:59.2 | 0 |
| -1490.004 | 19806.109 | 1.975 | 14.374 | 0.0765345 | 7/15/2014 | 33:59.3 | 0 |
| -1489.738 | 19806.083 | 1.975 | 14.379 | 0.0839134 | 7/15/2014 | 33:59.4 | 0 |
| -1489.526 | 19806.06 | 1.975 | 14.382 | 0.0765396 | 7/15/2014 | 33:59.5 | 0 |
| -1489.314 | 19806.037 | 1.977 | 14.384 | 0.0798186 | 7/15/2014 | 33:59.6 | 0 |
| -1489.051 | 19806.008 | 1.98 | 14.385 | 0.0748757 | 7/15/2014 | 33:59.7 | 0 |
| -1488.84 | 19805.984 | 1.98 | 14.386 | 0.0666789 | 7/15/2014 | 33:59.8 | 0 |
| -1488.628 | 19805.961 | 1.98 | 14.387 | 0.0690803 | 7/15/2014 | 33:59.9 | 0 |
| -1488.416 | 19805.938 | 1.982 | 14.387 | 0.0648229 | 7/15/2014 | 34:00.0 | 0 |
| -1488.204 | 19805.915 | 1.984 | 14.389 | 0.0811291 | 7/15/2014 | 34:00.1 | 0 |
| -1487.994 | 19805.892 | 1.987 | 14.39 | 0.0737431 | 7/15/2014 | 34:00.2 | 0 |
| -1487.785 | 19805.869 | 1.991 | 14.392 | 0.0703852 | 7/15/2014 | 34:00.3 | 0 |
| -1487.422 | 19805.827 | 1.991 | 14.394 | 0.110238 | 7/15/2014 | 34:00.5 | 0 |
| -1487.214 | 19805.803 | 1.993 | 14.396 | 0.109713 | 7/15/2014 | 34:00.6 | 0 |
| -1486.954 | 19805.773 | 1.993 | 14.397 | 0.122551 | 7/15/2014 | 34:00.7 | 0 |
| -1486.849 | 19805.762 | 1.995 | 14.398 | 0.143305 | 7/15/2014 | 34:00.7 | 0 |
| -1486.638 | 19805.738 | 1.995 | 14.398 | 0.107341 | 7/15/2014 | 34:00.8 | 0 |
| -1486.427 | 19805.713 | 1.989 | 14.398 | 0.127267 | 7/15/2014 | 34:00.9 | 0 |
| -1486.215 | 19805.689 | 1.987 | 14.397 | 0.135074 | 7/15/2014 | 34:01.0 | 0 |
| -1486.002 | 19805.664 | 1.987 | 14.396 | 0.109635 | 7/15/2014 | 34:01.1 | 0 |
| -1485.789 | 19805.64 | 1.985 | 14.396 | 0.110053 | 7/15/2014 | 34:01.2 | 0 |
| -1485.576 | 19805.614 | 1.982 | 14.396 | 0.121982 | 7/15/2014 | 34:01.3 | 0 |
| -1485.311 | 19805.584 | 1.982 | 14.396 | 0.11508 | 7/15/2014 | 34:01.4 | 0 |
| -1485.097 | 19805.56 | 1.982 | 14.397 | 0.113932 | 7/15/2014 | 34:01.5 | 0 |
| -1484.882 | 19805.533 | 1.98 | 14.399 | 0.106294 | 7/15/2014 | 34:01.6 | 0 |
| -1484.613 | 19805.503 | 1.983 | 14.402 | 0.106819 | 7/15/2014 | 34:01.7 | 0 |
| -1484.395 | 19805.478 | 1.985 | 14.407 | 0.10974 | 7/15/2014 | 34:01.8 | 0 |
| -1484.175 | 19805.451 | 1.989 | 14.413 | 0.110376 | 7/15/2014 | 34:01.9 | 0 |
| -1483.957 | 19805.426 | 1.987 | 14.42 | 0.111519 | 7/15/2014 | 34:02.0 | 0 |
| -1483.736 | 19805.4 | 1.987 | 14.427 | 0.0970426 | 7/15/2014 | 34:02.1 | 0 |
| -1483.517 | 19805.374 | 1.987 | 14.435 | 0.102375 | 7/15/2014 | 34:02.2 | 0 |
| -1483.299 | 19805.349 | 1.984 | 14.443 | 0.0965431 | 7/15/2014 | 34:02.3 | 0 |
| -1483.027 | 19805.316 | 1.986 | 14.451 | 0.102583 | 7/15/2014 | 34:02.4 | 0 |
| -1482.808 | 19805.291 | 1.983 | 14.459 | 0.0905437 | 7/15/2014 | 34:02.5 | 0 |
| -1482.59 | 19805.264 | 1.981 | 14.466 | 0.0845721 | 7/15/2014 | 34:02.6 | 0 |
| -1482.319 | 19805.232 | 1.973 | 14.472 | 0.0830243 | 7/15/2014 | 34:02.7 | 0 |
| -1482.1 | 19805.207 | 1.976 | 14.478 | 0.0822301 | 7/15/2014 | 34:02.8 | 0 |
| -1481.881 | 19805.18 | 1.976 | 14.483 | 0.0712253 | 7/15/2014 | 34:02.9 | 0 |
| -1481.663 | 19805.154 | 1.973 | 14.488 | 0.0743928 | 7/15/2014 | 34:03.0 | 0 |
| -1481.445 | 19805.128 | 1.973 | 14.493 | 0.0790291 | 7/15/2014 | 34:03.1 | 0 |
| -1481.228 | 19805.102 | 1.97 | 14.497 | 0.0775129 | 7/15/2014 | 34:03.2 | 0 |
| -1481.013 | 19805.076 | 1.97 | 14.5 | 0.0743179 | 7/15/2014 | 34:03.3 | 0 |
| -1480.746 | 19805.044 | 1.967 | 14.502 | 0.0787755 | 7/15/2014 | 34:03.4 | 0 |
| -1480.533 | 19805.02 | 1.967 | 14.503 | 0.0825035 | 7/15/2014 | 34:03.5 | 0 |
| -1480.322 | 19804.992 | 1.962 | 14.501 | 0.0859728 | 7/15/2014 | 34:03.6 | 0 |

| | | | | | | | |
|---|---|---|---|---|---|---|---|
| -1480.061 | 19804.962 | 1.959 | 14.498 | 0.0938846 | 7/15/2014 | 34:03.7 | 0 |
| -1479.849 | 19804.937 | 1.959 | 14.492 | 0.104373 | 7/15/2014 | 34:03.8 | 0 |
| -1479.638 | 19804.91 | 1.958 | 14.483 | 0.103408 | 7/15/2014 | 34:03.9 | 0 |
| -1479.428 | 19804.887 | 1.956 | 14.471 | 0.111039 | 7/15/2014 | 34:04.0 | 0 |
| -1479.217 | 19804.861 | 1.956 | 14.457 | 0.109792 | 7/15/2014 | 34:04.1 | 0 |
| -1479.008 | 19804.836 | 1.959 | 14.44 | 0.113609 | 7/15/2014 | 34:04.2 | 0 |
| -1478.801 | 19804.811 | 1.959 | 14.421 | 0.100328 | 7/15/2014 | 34:04.3 | 0 |
| -1478.441 | 19804.769 | 1.964 | 14.391 | 0.104599 | 7/15/2014 | 34:04.5 | 0 |
| -1478.234 | 19804.743 | 1.97 | 14.371 | 0.0830049 | 7/15/2014 | 34:04.6 | 0 |
| -1477.978 | 19804.713 | 1.97 | 14.351 | 0.0761004 | 7/15/2014 | 34:04.7 | 0 |
| -1477.874 | 19804.701 | 1.971 | 14.34 | 0.0654643 | 7/15/2014 | 34:04.7 | 0 |
| -1477.668 | 19804.678 | 1.975 | 14.321 | 0.0658029 | 7/15/2014 | 34:04.8 | 0 |
| -1477.461 | 19804.651 | 1.978 | 14.303 | 0.0667163 | 7/15/2014 | 34:04.9 | 0 |
| -1477.254 | 19804.628 | 1.975 | 14.287 | 0.0699977 | 7/15/2014 | 34:05.0 | 0 |
| -1477.046 | 19804.604 | 1.975 | 14.273 | 0.0722453 | 7/15/2014 | 34:05.1 | 0 |
| -1476.84 | 19804.579 | 1.975 | 14.261 | 0.0648489 | 7/15/2014 | 34:05.2 | 0 |
| -1476.633 | 19804.555 | 1.975 | 14.251 | 0.0768664 | 7/15/2014 | 34:05.3 | 0 |
| -1476.274 | 19804.512 | 1.977 | 14.245 | 0.0770607 | 7/15/2014 | 34:05.5 | 0 |
| -1476.069 | 19804.488 | 1.979 | 14.243 | 0.0859354 | 7/15/2014 | 34:05.6 | 0 |
| -1475.813 | 19804.458 | 1.976 | 14.245 | 0.0706052 | 7/15/2014 | 34:05.7 | 0 |
| -1475.71 | 19804.446 | 1.976 | 14.245 | 0.0724968 | 7/15/2014 | 34:05.7 | 0 |
| -1475.502 | 19804.422 | 1.981 | 14.251 | 0.0750565 | 7/15/2014 | 34:05.8 | 0 |
| -1475.294 | 19804.398 | 1.986 | 14.26 | 0.0712803 | 7/15/2014 | 34:05.9 | 0 |
| -1475.086 | 19804.375 | 1.991 | 14.271 | 0.0760104 | 7/15/2014 | 34:06.0 | 0 |
| -1474.876 | 19804.35 | 1.993 | 14.285 | 0.0767613 | 7/15/2014 | 34:06.1 | 0 |
| -1474.668 | 19804.324 | 1.995 | 14.3 | 0.0863761 | 7/15/2014 | 34:06.2 | 0 |
| -1474.461 | 19804.301 | 1.995 | 14.317 | 0.082373 | 7/15/2014 | 34:06.3 | 0 |
| -1474.096 | 19804.26 | 1.995 | 14.342 | 0.0680767 | 7/15/2014 | 34:06.5 | 0 |
| -1473.886 | 19804.235 | 2 | 14.359 | 0.0592748 | 7/15/2014 | 34:06.6 | 0 |
| -1473.622 | 19804.205 | 1.998 | 14.376 | 0.0656676 | 7/15/2014 | 34:06.7 | 0 |
| -1473.518 | 19804.193 | 1.998 | 14.384 | 0.0571895 | 7/15/2014 | 34:06.7 | 0 |
| -1473.303 | 19804.169 | 1.998 | 14.4 | 0.0771131 | 7/15/2014 | 34:06.8 | 0 |
| -1473.088 | 19804.145 | 1.996 | 14.414 | 0.055459 | 7/15/2014 | 34:06.9 | 0 |
| -1472.872 | 19804.119 | 1.996 | 14.427 | 0.0666173 | 7/15/2014 | 34:07.0 | 0 |
| -1472.654 | 19804.096 | 1.996 | 14.437 | 0.0880995 | 7/15/2014 | 34:07.1 | 0 |
| -1472.436 | 19804.07 | 1.996 | 14.445 | 0.0839874 | 7/15/2014 | 34:07.2 | 0 |
| -1472.218 | 19804.046 | 1.991 | 14.45 | 0.113904 | 7/15/2014 | 34:07.3 | 0 |
| -1471.946 | 19804.016 | 1.991 | 14.452 | 0.112122 | 7/15/2014 | 34:07.4 | 0 |
| -1471.726 | 19803.992 | 1.993 | 14.451 | 0.117199 | 7/15/2014 | 34:07.5 | 0 |
| -1471.507 | 19803.967 | 1.991 | 14.447 | 0.133834 | 7/15/2014 | 34:07.6 | 0 |
| -1471.232 | 19803.936 | 1.993 | 14.439 | 0.120798 | 7/15/2014 | 34:07.7 | 0 |
| -1471.012 | 19803.912 | 1.988 | 14.428 | 0.120611 | 7/15/2014 | 34:07.8 | 0 |
| -1470.789 | 19803.887 | 1.988 | 14.416 | 0.129946 | 7/15/2014 | 34:07.9 | 0 |
| -1470.567 | 19803.863 | 1.991 | 14.401 | 0.121306 | 7/15/2014 | 34:08.0 | 0 |
| -1470.345 | 19803.84 | 1.991 | 14.386 | 0.128315 | 7/15/2014 | 34:08.1 | 0 |
| -1470.123 | 19803.816 | 1.991 | 14.372 | 0.121513 | 7/15/2014 | 34:08.2 | 0 |
| -1469.904 | 19803.791 | 1.985 | 14.358 | 0.128961 | 7/15/2014 | 34:08.3 | 0 |

| | | | | | | | |
|---|---|---|---|---|---|---|---|
| -1469.631 | 19803.762 | 1.985 | 14.347 | 0.126909 | 7/15/2014 | 34:08.4 | 0 |
| -1469.415 | 19803.738 | 1.98 | 14.339 | 0.105111 | 7/15/2014 | 34:08.5 | 0 |
| -1469.198 | 19803.716 | 1.977 | 14.333 | 0.104437 | 7/15/2014 | 34:08.6 | 0 |
| -1468.929 | 19803.688 | 1.977 | 14.331 | 0.0987904 | 7/15/2014 | 34:08.7 | 0 |
| -1468.713 | 19803.664 | 1.973 | 14.332 | 0.10052 | 7/15/2014 | 34:08.8 | 0 |
| -1468.496 | 19803.642 | 1.976 | 14.337 | 0.0968015 | 7/15/2014 | 34:08.9 | 0 |
| -1468.28 | 19803.619 | 1.97 | 14.344 | 0.113714 | 7/15/2014 | 34:09.0 | 0 |
| -1468.064 | 19803.596 | 1.97 | 14.353 | 0.112952 | 7/15/2014 | 34:09.1 | 0 |
| -1467.85 | 19803.574 | 1.968 | 14.364 | 0.115485 | 7/15/2014 | 34:09.2 | 0 |
| -1467.636 | 19803.552 | 1.971 | 14.374 | 0.127716 | 7/15/2014 | 34:09.3 | 0 |
| -1467.369 | 19803.524 | 1.974 | 14.385 | 0.118081 | 7/15/2014 | 34:09.4 | 0 |
| -1467.157 | 19803.502 | 1.97 | 14.394 | 0.122572 | 7/15/2014 | 34:09.5 | 0 |
| -1466.944 | 19803.48 | 1.973 | 14.4 | 0.122463 | 7/15/2014 | 34:09.6 | 0 |
| -1466.675 | 19803.453 | 1.969 | 14.404 | 0.112904 | 7/15/2014 | 34:09.7 | 0 |
| -1466.458 | 19803.43 | 1.975 | 14.404 | 0.106667 | 7/15/2014 | 34:09.8 | 0 |
| -1466.24 | 19803.409 | 1.975 | 14.401 | 0.102373 | 7/15/2014 | 34:09.9 | 0 |
| -1466.02 | 19803.387 | 1.974 | 14.395 | 0.108384 | 7/15/2014 | 34:10.0 | 0 |
| -1465.799 | 19803.364 | 1.974 | 14.388 | 0.122752 | 7/15/2014 | 34:10.1 | 0 |
| -1465.579 | 19803.343 | 1.971 | 14.379 | 0.112371 | 7/15/2014 | 34:10.2 | 0 |
| -1465.357 | 19803.321 | 1.971 | 14.37 | 0.117152 | 7/15/2014 | 34:10.3 | 0 |
| -1465.08 | 19803.293 | 1.968 | 14.361 | 0.115389 | 7/15/2014 | 34:10.4 | 0 |
| -1464.858 | 19803.271 | 1.965 | 14.355 | 0.120521 | 7/15/2014 | 34:10.5 | 0 |
| -1464.633 | 19803.249 | 1.96 | 14.35 | 0.126541 | 7/15/2014 | 34:10.6 | 0 |
| -1464.349 | 19803.223 | 1.96 | 14.348 | 0.115326 | 7/15/2014 | 34:10.7 | 0 |
| -1464.122 | 19803.201 | 1.958 | 14.347 | 0.121274 | 7/15/2014 | 34:10.8 | 0 |
| -1463.893 | 19803.18 | 1.949 | 14.348 | 0.119478 | 7/15/2014 | 34:10.9 | 0 |
| -1463.661 | 19803.158 | 1.952 | 14.351 | 0.117332 | 7/15/2014 | 34:11.0 | 0 |
| -1463.43 | 19803.137 | 1.949 | 14.354 | 0.117441 | 7/15/2014 | 34:11.1 | 0 |
| -1463.2 | 19803.115 | 1.947 | 14.358 | 0.120661 | 7/15/2014 | 34:11.2 | 0 |
| -1463.085 | 19803.104 | 1.949 | 14.36 | 0.121946 | 7/15/2014 | 34:11.3 | 0 |
| -1462.68 | 19803.068 | 1.943 | 14.367 | 0.14845 | 7/15/2014 | 34:11.4 | 0 |
| -1462.45 | 19803.049 | 1.935 | 14.371 | 0.113393 | 7/15/2014 | 34:11.5 | 0 |
| -1462.219 | 19803.028 | 1.928 | 14.377 | 0.128203 | 7/15/2014 | 34:11.6 | 0 |
| -1461.926 | 19803.002 | 1.928 | 14.383 | 0.125662 | 7/15/2014 | 34:11.7 | 0 |
| -1461.81 | 19802.992 | 1.92 | 14.386 | 0.128153 | 7/15/2014 | 34:11.8 | 0 |
| -1461.574 | 19802.971 | 1.916 | 14.394 | 0.123715 | 7/15/2014 | 34:11.9 | 0 |
| -1461.338 | 19802.951 | 1.907 | 14.404 | 0.110932 | 7/15/2014 | 34:12.0 | 0 |
| -1461.1 | 19802.932 | 1.905 | 14.417 | 0.104808 | 7/15/2014 | 34:12.1 | 0 |
| -1460.863 | 19802.912 | 1.901 | 14.431 | 0.117838 | 7/15/2014 | 34:12.2 | 0 |
| -1460.624 | 19802.893 | 1.905 | 14.447 | 0.1001 | 7/15/2014 | 34:12.3 | 0 |
| -1460.21 | 19802.857 | 1.904 | 14.47 | 0.094937 | 7/15/2014 | 34:12.4 | 0 |
| -1459.973 | 19802.839 | 1.898 | 14.484 | 0.0930252 | 7/15/2014 | 34:12.5 | 0 |
| -1459.737 | 19802.82 | 1.895 | 14.496 | 0.108956 | 7/15/2014 | 34:12.6 | 0 |
| -1459.442 | 19802.797 | 1.891 | 14.506 | 0.109537 | 7/15/2014 | 34:12.7 | 0 |
| -1459.321 | 19802.787 | 1.891 | 14.512 | 0.0986679 | 7/15/2014 | 34:12.8 | 0 |
| -1459.083 | 19802.768 | 1.888 | 14.517 | 0.108616 | 7/15/2014 | 34:12.9 | 0 |
| -1458.848 | 19802.75 | 1.878 | 14.521 | 0.0869692 | 7/15/2014 | 34:13.0 | 0 |

| | | | | | | | |
|---|---|---|---|---|---|---|---|
| -1458.612 | 19802.73 | 1.878 | 14.523 | 0.107376 | 7/15/2014 | 34:13.1 | 0 |
| -1458.377 | 19802.714 | 1.878 | 14.525 | 0.108245 | 7/15/2014 | 34:13.2 | 0 |
| -1458.143 | 19802.696 | 1.873 | 14.528 | 0.0928833 | 7/15/2014 | 34:13.3 | 0 |
| -1457.736 | 19802.666 | 1.872 | 14.539 | 0.0930507 | 7/15/2014 | 34:13.4 | 0 |
| -1457.505 | 19802.647 | 1.873 | 14.549 | 0.0934986 | 7/15/2014 | 34:13.5 | 0 |
| -1457.274 | 19802.631 | 1.869 | 14.56 | 0.117941 | 7/15/2014 | 34:13.6 | 0 |
| -1456.985 | 19802.609 | 1.864 | 14.574 | 0.10918 | 7/15/2014 | 34:13.7 | 0 |
| -1456.869 | 19802.602 | 1.86 | 14.581 | 0.105715 | 7/15/2014 | 34:13.8 | 0 |
| -1456.635 | 19802.585 | 1.851 | 14.598 | 0.115928 | 7/15/2014 | 34:13.9 | 0 |
| -1456.402 | 19802.568 | 1.844 | 14.616 | 0.125064 | 7/15/2014 | 34:14.0 | 0 |
| -1456.165 | 19802.552 | 1.846 | 14.634 | 0.140924 | 7/15/2014 | 34:14.1 | 0 |
| -1455.932 | 19802.535 | 1.834 | 14.651 | 0.139259 | 7/15/2014 | 34:14.2 | 0 |
| -1455.695 | 19802.519 | 1.834 | 14.669 | 0.142689 | 7/15/2014 | 34:14.3 | 0 |
| -1455.283 | 19802.491 | 1.826 | 14.693 | 0.148023 | 7/15/2014 | 34:14.4 | 0 |
| -1455.047 | 19802.476 | 1.821 | 14.708 | 0.138987 | 7/15/2014 | 34:14.5 | 0 |
| -1454.81 | 19802.46 | 1.814 | 14.723 | 0.134283 | 7/15/2014 | 34:14.6 | 0 |
| -1454.51 | 19802.44 | 1.817 | 14.736 | 0.127296 | 7/15/2014 | 34:14.7 | 0 |
| -1454.392 | 19802.433 | 1.812 | 14.743 | 0.127234 | 7/15/2014 | 34:14.8 | 0 |
| -1454.149 | 19802.417 | 1.806 | 14.755 | 0.113077 | 7/15/2014 | 34:14.9 | 0 |
| -1453.907 | 19802.401 | 1.791 | 14.765 | 0.113588 | 7/15/2014 | 34:15.0 | 0 |
| -1453.662 | 19802.387 | 1.782 | 14.773 | 0.0968935 | 7/15/2014 | 34:15.1 | 0 |
| -1453.42 | 19802.372 | 1.77 | 14.78 | 0.114862 | 7/15/2014 | 34:15.2 | 0 |
| -1453.175 | 19802.356 | 1.77 | 14.785 | 0.103757 | 7/15/2014 | 34:15.3 | 0 |
| -1452.749 | 19802.332 | 1.768 | 14.788 | 0.103848 | 7/15/2014 | 34:15.4 | 0 |
| -1452.507 | 19802.317 | 1.76 | 14.789 | 0.119596 | 7/15/2014 | 34:15.5 | 0 |
| -1452.265 | 19802.303 | 1.762 | 14.788 | 0.103299 | 7/15/2014 | 34:15.6 | 0 |
| -1451.967 | 19802.287 | 1.76 | 14.785 | 0.105945 | 7/15/2014 | 34:15.7 | 0 |
| -1451.844 | 19802.28 | 1.759 | 14.784 | 0.0989169 | 7/15/2014 | 34:15.8 | 0 |
| -1451.603 | 19802.266 | 1.771 | 14.78 | 0.093037 | 7/15/2014 | 34:15.9 | 0 |
| -1451.366 | 19802.253 | 1.769 | 14.775 | 0.0757495 | 7/15/2014 | 34:16.0 | 0 |
| -1451.128 | 19802.24 | 1.769 | 14.768 | 0.0839771 | 7/15/2014 | 34:16.1 | 0 |
| -1450.894 | 19802.228 | 1.76 | 14.76 | 0.0880789 | 7/15/2014 | 34:16.2 | 0 |
| -1450.548 | 19802.21 | 1.761 | 14.746 | 0.102781 | 7/15/2014 | 34:16.3 | 0 |
| -1450.261 | 19802.196 | 1.759 | 14.735 | 0.0957826 | 7/15/2014 | 34:16.4 | 0 |
| -1450.032 | 19802.184 | 1.761 | 14.724 | 0.109676 | 7/15/2014 | 34:16.5 | 0 |
| -1449.804 | 19802.172 | 1.758 | 14.712 | 0.11944 | 7/15/2014 | 34:16.6 | 0 |
| -1449.52 | 19802.158 | 1.758 | 14.698 | 0.0991322 | 7/15/2014 | 34:16.7 | 0 |
| -1449.291 | 19802.146 | 1.758 | 14.684 | 0.109903 | 7/15/2014 | 34:16.8 | 0 |
| -1449.06 | 19802.135 | 1.758 | 14.67 | 0.0895259 | 7/15/2014 | 34:16.9 | 0 |
| -1448.829 | 19802.126 | 1.753 | 14.655 | 0.0848031 | 7/15/2014 | 34:17.0 | 0 |
| -1448.597 | 19802.115 | 1.75 | 14.64 | 0.105006 | 7/15/2014 | 34:17.1 | 0 |
| -1448.484 | 19802.109 | 1.747 | 14.633 | 0.091292 | 7/15/2014 | 34:17.2 | 0 |
| -1448.252 | 19802.098 | 1.745 | 14.618 | 0.0778776 | 7/15/2014 | 34:17.3 | 0 |
| -1447.844 | 19802.08 | 1.745 | 14.596 | 0.0847707 | 7/15/2014 | 34:17.4 | 0 |
| -1447.611 | 19802.068 | 1.748 | 14.582 | 0.0756802 | 7/15/2014 | 34:17.5 | 0 |
| -1447.377 | 19802.059 | 1.75 | 14.569 | 0.0888017 | 7/15/2014 | 34:17.6 | 0 |
| -1447.084 | 19802.047 | 1.755 | 14.557 | 0.0878644 | 7/15/2014 | 34:17.7 | 0 |

| | | | | | | | |
|---|---|---|---|---|---|---|---|
| -1446.965 | 19802.041 | 1.759 | 14.551 | 0.0779072 | 7/15/2014 | 34:17.8 | 0 |
| -1446.728 | 19802.03 | 1.768 | 14.54 | 0.0942217 | 7/15/2014 | 34:17.9 | 0 |
| -1446.492 | 19802.021 | 1.77 | 14.53 | 0.0814259 | 7/15/2014 | 34:18.0 | 0 |
| -1446.253 | 19802.012 | 1.775 | 14.52 | 0.0973189 | 7/15/2014 | 34:18.1 | 0 |
| -1446.018 | 19802 | 1.772 | 14.511 | 0.087349 | 7/15/2014 | 34:18.2 | 0 |
| -1445.781 | 19801.992 | 1.775 | 14.502 | 0.0974529 | 7/15/2014 | 34:18.3 | 0 |
| -1445.371 | 19801.975 | 1.769 | 14.49 | 0.108894 | 7/15/2014 | 34:18.4 | 0 |
| -1445.136 | 19801.966 | 1.763 | 14.484 | 0.101956 | 7/15/2014 | 34:18.5 | 0 |
| -1444.902 | 19801.955 | 1.761 | 14.479 | 0.0931007 | 7/15/2014 | 34:18.6 | 0 |
| -1444.611 | 19801.945 | 1.759 | 14.477 | 0.111273 | 7/15/2014 | 34:18.7 | 0 |
| -1444.493 | 19801.939 | 1.756 | 14.474 | 0.106018 | 7/15/2014 | 34:18.8 | 0 |
| -1444.258 | 19801.932 | 1.753 | 14.476 | 0.115462 | 7/15/2014 | 34:18.9 | 0 |
| -1444.024 | 19801.922 | 1.748 | 14.481 | 0.104895 | 7/15/2014 | 34:19.0 | 0 |
| -1443.79 | 19801.912 | 1.752 | 14.491 | 0.0865726 | 7/15/2014 | 34:19.1 | 0 |
| -1443.557 | 19801.904 | 1.746 | 14.505 | 0.09826 | 7/15/2014 | 34:19.2 | 0 |
| -1443.325 | 19801.895 | 1.749 | 14.523 | 0.100087 | 7/15/2014 | 34:19.3 | 0 |
| -1442.921 | 19801.88 | 1.746 | 14.557 | 0.0877385 | 7/15/2014 | 34:19.4 | 0 |
| -1442.692 | 19801.873 | 1.743 | 14.581 | 0.0928432 | 7/15/2014 | 34:19.5 | 0 |
| -1442.463 | 19801.865 | 1.745 | 14.605 | 0.0940941 | 7/15/2014 | 34:19.6 | 0 |
| -1442.18 | 19801.853 | 1.742 | 14.628 | 0.0904562 | 7/15/2014 | 34:19.7 | 0 |
| -1441.952 | 19801.846 | 1.739 | 14.649 | 0.0865484 | 7/15/2014 | 34:19.8 | 0 |
| -1441.725 | 19801.838 | 1.739 | 14.667 | 0.0991204 | 7/15/2014 | 34:19.9 | 0 |
| -1441.497 | 19801.83 | 1.739 | 14.682 | 0.108359 | 7/15/2014 | 34:20.0 | 0 |
| -1441.27 | 19801.821 | 1.742 | 14.693 | 0.119277 | 7/15/2014 | 34:20.1 | 0 |
| -1441.045 | 19801.813 | 1.735 | 14.701 | 0.109009 | 7/15/2014 | 34:20.2 | 0 |
| -1440.822 | 19801.807 | 1.732 | 14.704 | 0.13412 | 7/15/2014 | 34:20.3 | 0 |
| -1440.543 | 19801.796 | 1.724 | 14.705 | 0.126637 | 7/15/2014 | 34:20.4 | 0 |
| -1440.321 | 19801.789 | 1.724 | 14.703 | 0.136742 | 7/15/2014 | 34:20.5 | 0 |
| -1440.1 | 19801.781 | 1.724 | 14.699 | 0.0871582 | 7/15/2014 | 34:20.6 | 0 |
| -1439.823 | 19801.771 | 1.724 | 14.692 | 0.101521 | 7/15/2014 | 34:20.7 | 0 |
| -1439.599 | 19801.764 | 1.721 | 14.685 | 0.0856873 | 7/15/2014 | 34:20.8 | 0 |
| -1439.375 | 19801.755 | 1.718 | 14.675 | 0.0808846 | 7/15/2014 | 34:20.9 | 0 |
| -1439.151 | 19801.747 | 1.72 | 14.665 | 0.0745484 | 7/15/2014 | 34:21.0 | 0 |
| -1438.925 | 19801.74 | 1.723 | 14.655 | 0.0813984 | 7/15/2014 | 34:21.1 | 0 |
| -1438.7 | 19801.73 | 1.731 | 14.644 | 0.0809315 | 7/15/2014 | 34:21.2 | 0 |
| -1438.475 | 19801.723 | 1.73 | 14.633 | 0.0846812 | 7/15/2014 | 34:21.3 | 0 |
| -1438.195 | 19801.713 | 1.733 | 14.622 | 0.0864769 | 7/15/2014 | 34:21.4 | 0 |
| -1437.97 | 19801.705 | 1.736 | 14.611 | 0.0871514 | 7/15/2014 | 34:21.5 | 0 |
| -1437.744 | 19801.697 | 1.736 | 14.6 | 0.0853934 | 7/15/2014 | 34:21.6 | 0 |
| -1437.46 | 19801.687 | 1.739 | 14.589 | 0.0968234 | 7/15/2014 | 34:21.7 | 0 |
| -1437.232 | 19801.68 | 1.739 | 14.579 | 0.0979715 | 7/15/2014 | 34:21.8 | 0 |
| -1437.002 | 19801.669 | 1.742 | 14.568 | 0.0976241 | 7/15/2014 | 34:21.9 | 0 |
| -1436.772 | 19801.66 | 1.74 | 14.558 | 0.138794 | 7/15/2014 | 34:22.0 | 0 |
| -1436.541 | 19801.652 | 1.743 | 14.548 | 0.123465 | 7/15/2014 | 34:22.1 | 0 |
| -1436.311 | 19801.645 | 1.74 | 14.539 | 0.118515 | 7/15/2014 | 34:22.2 | 0 |
| -1436.081 | 19801.637 | 1.737 | 14.53 | 0.135944 | 7/15/2014 | 34:22.3 | 0 |
| -1435.796 | 19801.626 | 1.737 | 14.521 | 0.137069 | 7/15/2014 | 34:22.4 | 0 |

| | | | | | | | |
|---|---|---|---|---|---|---|---|
| -1435.568 | 19801.617 | 1.731 | 14.514 | 0.145608 | 7/15/2014 | 34:22.5 | 0 |
| -1435.337 | 19801.608 | 1.731 | 14.508 | 0.167846 | 7/15/2014 | 34:22.6 | 0 |
| -1435.048 | 19801.598 | 1.727 | 14.503 | 0.118154 | 7/15/2014 | 34:22.7 | 0 |
| -1434.932 | 19801.594 | 1.724 | 14.5 | 0.117911 | 7/15/2014 | 34:22.8 | 0 |
| -1434.7 | 19801.584 | 1.724 | 14.498 | 0.160172 | 7/15/2014 | 34:22.9 | 0 |
| -1434.466 | 19801.576 | 1.724 | 14.497 | 0.123742 | 7/15/2014 | 34:23.0 | 0 |
| -1434.232 | 19801.565 | 1.724 | 14.498 | 0.169805 | 7/15/2014 | 34:23.1 | 0 |
| -1433.999 | 19801.557 | 1.728 | 14.501 | 0.108822 | 7/15/2014 | 34:23.2 | 0 |
| -1433.764 | 19801.548 | 1.735 | 14.505 | 0.111099 | 7/15/2014 | 34:23.3 | 0 |
| -1433.353 | 19801.533 | 1.737 | 14.513 | 0.151292 | 7/15/2014 | 34:23.4 | 0 |
| -1433.119 | 19801.521 | 1.734 | 14.519 | 0.143984 | 7/15/2014 | 34:23.5 | 0 |
| -1432.883 | 19801.514 | 1.734 | 14.525 | 0.168948 | 7/15/2014 | 34:23.6 | 0 |
| -1432.589 | 19801.5 | 1.734 | 14.531 | 0.118164 | 7/15/2014 | 34:23.7 | 0 |
| -1432.47 | 19801.496 | 1.735 | 14.534 | 0.173182 | 7/15/2014 | 34:23.8 | 0 |
| -1432.231 | 19801.485 | 1.735 | 14.538 | 0.131755 | 7/15/2014 | 34:23.9 | 0 |
| -1431.992 | 19801.477 | 1.728 | 14.541 | 0.141587 | 7/15/2014 | 34:24.0 | 0 |
| -1431.752 | 19801.467 | 1.724 | 14.541 | 0.147771 | 7/15/2014 | 34:24.1 | 0 |
| -1431.514 | 19801.455 | 1.72 | 14.54 | 0.140492 | 7/15/2014 | 34:24.2 | 0 |
| -1431.273 | 19801.447 | 1.73 | 14.536 | 0.152827 | 7/15/2014 | 34:24.3 | 0 |
| -1430.855 | 19801.428 | 1.731 | 14.526 | 0.158973 | 7/15/2014 | 34:24.4 | 0 |
| -1430.615 | 19801.418 | 1.731 | 14.518 | 0.166231 | 7/15/2014 | 34:24.5 | 0 |
| -1430.378 | 19801.408 | 1.731 | 14.51 | 0.167433 | 7/15/2014 | 34:24.6 | 0 |
| -1430.079 | 19801.395 | 1.735 | 14.502 | 0.152529 | 7/15/2014 | 34:24.7 | 0 |
| -1429.959 | 19801.389 | 1.736 | 14.497 | 0.145054 | 7/15/2014 | 34:24.8 | 0 |
| -1429.717 | 19801.379 | 1.743 | 14.489 | 0.152188 | 7/15/2014 | 34:24.9 | 0 |
| -1429.478 | 19801.369 | 1.743 | 14.482 | 0.154566 | 7/15/2014 | 34:25.0 | 0 |
| -1429.236 | 19801.357 | 1.753 | 14.478 | 0.13761 | 7/15/2014 | 34:25.1 | 0 |
| -1428.998 | 19801.348 | 1.747 | 14.476 | 0.132086 | 7/15/2014 | 34:25.2 | 0 |
| -1428.758 | 19801.336 | 1.751 | 14.477 | 0.131762 | 7/15/2014 | 34:25.3 | 0 |
| -1428.344 | 19801.318 | 1.75 | 14.488 | 0.138588 | 7/15/2014 | 34:25.4 | 0 |
| -1428.109 | 19801.307 | 1.75 | 14.499 | 0.126999 | 7/15/2014 | 34:25.5 | 0 |
| -1427.873 | 19801.297 | 1.75 | 14.512 | 0.132547 | 7/15/2014 | 34:25.6 | 0 |
| -1427.581 | 19801.283 | 1.745 | 14.529 | 0.122537 | 7/15/2014 | 34:25.7 | 0 |
| -1427.462 | 19801.277 | 1.738 | 14.537 | 0.124295 | 7/15/2014 | 34:25.8 | 0 |
| -1427.227 | 19801.267 | 1.745 | 14.557 | 0.1283 | 7/15/2014 | 34:25.9 | 0 |
| -1426.993 | 19801.258 | 1.741 | 14.578 | 0.149194 | 7/15/2014 | 34:26.0 | 0 |
| -1426.645 | 19801.24 | 1.742 | 14.608 | 0.130497 | 7/15/2014 | 34:26.1 | 0 |
| -1426.414 | 19801.229 | 1.744 | 14.628 | 0.149835 | 7/15/2014 | 34:26.2 | 0 |
| -1426.183 | 19801.218 | 1.747 | 14.646 | 0.158932 | 7/15/2014 | 34:26.3 | 0 |
| -1425.897 | 19801.204 | 1.747 | 14.662 | 0.133035 | 7/15/2014 | 34:26.4 | 0 |
| -1425.669 | 19801.194 | 1.751 | 14.676 | 0.165906 | 7/15/2014 | 34:26.5 | 0 |
| -1425.441 | 19801.184 | 1.748 | 14.688 | 0.144957 | 7/15/2014 | 34:26.6 | 0 |
| -1425.156 | 19801.169 | 1.752 | 14.698 | 0.140533 | 7/15/2014 | 34:26.7 | 0 |
| -1424.926 | 19801.157 | 1.752 | 14.705 | 0.147436 | 7/15/2014 | 34:26.8 | 0 |
| -1424.695 | 19801.146 | 1.754 | 14.711 | 0.127293 | 7/15/2014 | 34:26.9 | 0 |
| -1424.464 | 19801.134 | 1.752 | 14.714 | 0.108854 | 7/15/2014 | 34:27.0 | 0 |
| -1424.232 | 19801.122 | 1.754 | 14.715 | 0.138234 | 7/15/2014 | 34:27.1 | 0 |

| | | | | | | | |
|---|---|---|---|---|---|---|---|
| -1424.001 | 19801.111 | 1.754 | 14.715 | 0.122663 | 7/15/2014 | 34:27.2 | 0 |
| -1423.888 | 19801.105 | 1.754 | 14.715 | 0.139923 | 7/15/2014 | 34:27.3 | 0 |
| -1423.482 | 19801.085 | 1.754 | 14.708 | 0.137873 | 7/15/2014 | 34:27.4 | 0 |
| -1423.25 | 19801.073 | 1.751 | 14.701 | 0.137303 | 7/15/2014 | 34:27.5 | 0 |
| -1423.018 | 19801.062 | 1.753 | 14.692 | 0.119761 | 7/15/2014 | 34:27.6 | 0 |
| -1422.727 | 19801.045 | 1.747 | 14.682 | 0.12043 | 7/15/2014 | 34:27.7 | 0 |
| -1422.609 | 19801.039 | 1.747 | 14.677 | 0.13096 | 7/15/2014 | 34:27.8 | 0 |
| -1422.372 | 19801.027 | 1.745 | 14.665 | 0.108838 | 7/15/2014 | 34:27.9 | 0 |
| -1422.136 | 19801.016 | 1.743 | 14.652 | 0.103448 | 7/15/2014 | 34:28.0 | 0 |
| -1421.898 | 19801.003 | 1.746 | 14.639 | 0.0890754 | 7/15/2014 | 34:28.1 | 0 |
| -1421.661 | 19800.99 | 1.742 | 14.626 | 0.0929327 | 7/15/2014 | 34:28.2 | 0 |
| -1421.422 | 19800.979 | 1.746 | 14.616 | 0.102984 | 7/15/2014 | 34:28.3 | 0 |
| -1421.007 | 19800.955 | 1.738 | 14.605 | 0.101766 | 7/15/2014 | 34:28.4 | 0 |
| -1420.77 | 19800.942 | 1.734 | 14.601 | 0.106828 | 7/15/2014 | 34:28.5 | 0 |
| -1420.534 | 19800.93 | 1.728 | 14.599 | 0.141602 | 7/15/2014 | 34:28.6 | 0 |
| -1420.237 | 19800.913 | 1.723 | 14.601 | 0.114501 | 7/15/2014 | 34:28.7 | 0 |
| -1420.118 | 19800.906 | 1.717 | 14.6 | 0.104682 | 7/15/2014 | 34:28.8 | 0 |
| -1419.879 | 19800.894 | 1.713 | 14.607 | 0.122106 | 7/15/2014 | 34:28.9 | 0 |
| -1419.641 | 19800.879 | 1.706 | 14.616 | 0.130265 | 7/15/2014 | 34:29.0 | 0 |
| -1419.402 | 19800.866 | 1.702 | 14.628 | 0.110077 | 7/15/2014 | 34:29.1 | 0 |
| -1419.166 | 19800.853 | 1.696 | 14.642 | 0.0866803 | 7/15/2014 | 34:29.2 | 0 |
| -1418.93 | 19800.839 | 1.696 | 14.656 | 0.0621098 | 7/15/2014 | 34:29.3 | 0 |
| -1418.519 | 19800.815 | 1.699 | 14.674 | 0.0690628 | 7/15/2014 | 34:29.4 | 0 |
| -1418.285 | 19800.802 | 1.699 | 14.684 | 0.0618831 | 7/15/2014 | 34:29.5 | 0 |
| -1418.052 | 19800.789 | 1.699 | 14.691 | 0.0636469 | 7/15/2014 | 34:29.6 | 0 |
| -1417.761 | 19800.771 | 1.697 | 14.695 | 0.0732972 | 7/15/2014 | 34:29.7 | 0 |
| -1417.643 | 19800.765 | 1.697 | 14.698 | 0.0776214 | 7/15/2014 | 34:29.8 | 0 |
| -1417.408 | 19800.751 | 1.699 | 14.696 | 0.0745259 | 7/15/2014 | 34:29.9 | 0 |
| -1417.173 | 19800.737 | 1.694 | 14.692 | 0.0804506 | 7/15/2014 | 34:30.0 | 0 |
| -1416.938 | 19800.724 | 1.694 | 14.685 | 0.074311 | 7/15/2014 | 34:30.1 | 0 |
| -1416.703 | 19800.709 | 1.686 | 14.677 | 0.0776601 | 7/15/2014 | 34:30.2 | 0 |
| -1416.469 | 19800.696 | 1.683 | 14.668 | 0.0808181 | 7/15/2014 | 34:30.3 | 0 |
| -1416.062 | 19800.671 | 1.674 | 14.653 | 0.0624791 | 7/15/2014 | 34:30.4 | 0 |
| -1415.83 | 19800.658 | 1.67 | 14.644 | 0.084446 | 7/15/2014 | 34:30.5 | 0 |
| -1415.599 | 19800.644 | 1.666 | 14.635 | 0.0820114 | 7/15/2014 | 34:30.6 | 0 |
| -1415.311 | 19800.627 | 1.666 | 14.627 | 0.0822026 | 7/15/2014 | 34:30.7 | 0 |
| -1415.193 | 19800.619 | 1.661 | 14.623 | 0.0865552 | 7/15/2014 | 34:30.8 | 0 |
| -1414.849 | 19800.598 | 1.663 | 14.615 | 0.0973336 | 7/15/2014 | 34:30.9 | 0 |
| -1414.618 | 19800.584 | 1.661 | 14.611 | 0.0716458 | 7/15/2014 | 34:31.0 | 0 |
| -1414.387 | 19800.569 | 1.661 | 14.609 | 0.0746958 | 7/15/2014 | 34:31.1 | 0 |
| -1414.158 | 19800.556 | 1.669 | 14.608 | 0.0615123 | 7/15/2014 | 34:31.2 | 0 |
| -1413.93 | 19800.541 | 1.673 | 14.609 | 0.0508374 | 7/15/2014 | 34:31.3 | 0 |
| -1413.647 | 19800.523 | 1.678 | 14.612 | 0.0450127 | 7/15/2014 | 34:31.4 | 0 |
| -1413.422 | 19800.508 | 1.68 | 14.616 | 0.0367853 | 7/15/2014 | 34:31.5 | 0 |
| -1413.197 | 19800.494 | 1.686 | 14.621 | 0.0361958 | 7/15/2014 | 34:31.6 | 0 |
| -1412.916 | 19800.476 | 1.686 | 14.627 | 0.0269398 | 7/15/2014 | 34:31.7 | 0 |
| -1412.69 | 19800.461 | 1.691 | 14.634 | 0.04197 | 7/15/2014 | 34:31.8 | 0 |

| | | | | | | | |
|---|---|---|---|---|---|---|---|
| -1412.462 | 19800.445 | 1.698 | 14.641 | 0.0545425 | 7/15/2014 | 34:31.9 | 0 |
| -1412.234 | 19800.431 | 1.7 | 14.649 | 0.0616518 | 7/15/2014 | 34:32.0 | 0 |
| -1412.007 | 19800.415 | 1.7 | 14.656 | 0.101416 | 7/15/2014 | 34:32.1 | 0 |
| -1411.78 | 19800.398 | 1.697 | 14.663 | 0.0996871 | 7/15/2014 | 34:32.2 | 0 |
| -1411.554 | 19800.384 | 1.695 | 14.668 | 0.125668 | 7/15/2014 | 34:32.3 | 0 |
| -1411.271 | 19800.364 | 1.697 | 14.672 | 0.142166 | 7/15/2014 | 34:32.4 | 0 |
| -1411.045 | 19800.349 | 1.7 | 14.675 | 0.138815 | 7/15/2014 | 34:32.5 | 0 |
| -1410.816 | 19800.332 | 1.703 | 14.676 | 0.135376 | 7/15/2014 | 34:32.6 | 0 |
| -1410.531 | 19800.312 | 1.697 | 14.675 | 0.158041 | 7/15/2014 | 34:32.7 | 0 |
| -1410.301 | 19800.296 | 1.694 | 14.672 | 0.104233 | 7/15/2014 | 34:32.8 | 0 |
| -1410.069 | 19800.277 | 1.694 | 14.667 | 0.142572 | 7/15/2014 | 34:32.9 | 0 |
| -1409.955 | 19800.27 | 1.696 | 14.665 | 0.126807 | 7/15/2014 | 34:33.0 | 0 |
| -1409.721 | 19800.254 | 1.698 | 14.657 | 0.0988227 | 7/15/2014 | 34:33.1 | 0 |
| -1409.489 | 19800.236 | 1.692 | 14.647 | 0.09484 | 7/15/2014 | 34:33.2 | 0 |
| -1409.254 | 19800.218 | 1.695 | 14.635 | 0.0960597 | 7/15/2014 | 34:33.3 | 0 |
| -1408.846 | 19800.188 | 1.686 | 14.612 | 0.0654144 | 7/15/2014 | 34:33.4 | 0 |
| -1408.612 | 19800.17 | 1.686 | 14.594 | 0.112951 | 7/15/2014 | 34:33.5 | 0 |
| -1408.379 | 19800.15 | 1.686 | 14.576 | 0.0851102 | 7/15/2014 | 34:33.6 | 0 |
| -1408.086 | 19800.129 | 1.688 | 14.555 | 0.103609 | 7/15/2014 | 34:33.7 | 0 |
| -1407.968 | 19800.119 | 1.689 | 14.545 | 0.0983373 | 7/15/2014 | 34:33.8 | 0 |
| -1407.732 | 19800.1 | 1.691 | 14.524 | 0.086104 | 7/15/2014 | 34:33.9 | 0 |
| -1407.497 | 19800.082 | 1.683 | 14.503 | 0.0787765 | 7/15/2014 | 34:34.0 | 0 |
| -1407.262 | 19800.063 | 1.691 | 14.483 | 0.0920207 | 7/15/2014 | 34:34.1 | 0 |
| -1407.027 | 19800.043 | 1.689 | 14.465 | 0.067844 | 7/15/2014 | 34:34.2 | 0 |
| -1406.793 | 19800.023 | 1.691 | 14.45 | 0.0666695 | 7/15/2014 | 34:34.3 | 0 |
| -1406.385 | 19799.99 | 1.693 | 14.439 | 0.0511281 | 7/15/2014 | 34:34.4 | 0 |
| -1406.155 | 19799.971 | 1.691 | 14.434 | 0.0461723 | 7/15/2014 | 34:34.5 | 0 |
| -1405.924 | 19799.951 | 1.691 | 14.434 | 0.0355677 | 7/15/2014 | 34:34.6 | 0 |
| -1405.635 | 19799.926 | 1.693 | 14.439 | 0.0475699 | 7/15/2014 | 34:34.7 | 0 |
| -1405.519 | 19799.916 | 1.689 | 14.44 | 0.0515959 | 7/15/2014 | 34:34.8 | 0 |
| -1405.286 | 19799.896 | 1.695 | 14.45 | 0.0480239 | 7/15/2014 | 34:34.9 | 0 |
| -1405.055 | 19799.875 | 1.692 | 14.464 | 0.0486735 | 7/15/2014 | 34:35.0 | 0 |
| -1404.706 | 19799.846 | 1.693 | 14.487 | 0.0766043 | 7/15/2014 | 34:35.1 | 0 |
| -1404.591 | 19799.836 | 1.689 | 14.495 | 0.0909153 | 7/15/2014 | 34:35.2 | 0 |
| -1404.359 | 19799.816 | 1.691 | 14.511 | 0.0882688 | 7/15/2014 | 34:35.3 | 0 |
| -1403.956 | 19799.779 | 1.693 | 14.531 | 0.0794275 | 7/15/2014 | 34:35.4 | 0 |
| -1403.727 | 19799.76 | 1.693 | 14.543 | 0.0944506 | 7/15/2014 | 34:35.5 | 0 |
| -1403.497 | 19799.736 | 1.695 | 14.552 | 0.0864726 | 7/15/2014 | 34:35.6 | 0 |
| -1403.21 | 19799.712 | 1.695 | 14.558 | 0.0903401 | 7/15/2014 | 34:35.7 | 0 |
| -1403.095 | 19799.702 | 1.695 | 14.562 | 0.0752086 | 7/15/2014 | 34:35.8 | 0 |
| -1402.748 | 19799.672 | 1.698 | 14.563 | 0.0970421 | 7/15/2014 | 34:35.9 | 0 |
| -1402.517 | 19799.649 | 1.701 | 14.562 | 0.0777637 | 7/15/2014 | 34:36.0 | 0 |
| -1402.285 | 19799.629 | 1.698 | 14.559 | 0.0940612 | 7/15/2014 | 34:36.1 | 0 |
| -1402.055 | 19799.609 | 1.701 | 14.555 | 0.0933302 | 7/15/2014 | 34:36.2 | 0 |
| -1401.826 | 19799.587 | 1.705 | 14.55 | 0.0830886 | 7/15/2014 | 34:36.3 | 0 |
| -1401.541 | 19799.562 | 1.705 | 14.545 | 0.0998505 | 7/15/2014 | 34:36.4 | 0 |
| -1401.313 | 19799.541 | 1.705 | 14.541 | 0.115598 | 7/15/2014 | 34:36.5 | 0 |

| | | | | | | | |
|---|---|---|---|---|---|---|---|
| -1401.087 | 19799.521 | 1.703 | 14.537 | 0.12711 | 7/15/2014 | 34:36.6 | 0 |
| -1400.803 | 19799.495 | 1.699 | 14.535 | 0.138754 | 7/15/2014 | 34:36.7 | 0 |
| -1400.574 | 19799.475 | 1.699 | 14.534 | 0.135637 | 7/15/2014 | 34:36.8 | 0 |
| -1400.346 | 19799.454 | 1.699 | 14.533 | 0.125775 | 7/15/2014 | 34:36.9 | 0 |
| -1400.117 | 19799.433 | 1.701 | 14.534 | 0.154835 | 7/15/2014 | 34:37.0 | 0 |
| -1399.888 | 19799.411 | 1.704 | 14.536 | 0.12949 | 7/15/2014 | 34:37.1 | 0 |
| -1399.66 | 19799.391 | 1.706 | 14.538 | 0.141179 | 7/15/2014 | 34:37.2 | 0 |
| -1399.434 | 19799.371 | 1.711 | 14.541 | 0.119775 | 7/15/2014 | 34:37.3 | 0 |
| -1399.151 | 19799.345 | 1.716 | 14.543 | 0.13963 | 7/15/2014 | 34:37.4 | 0 |
| -1398.924 | 19799.324 | 1.718 | 14.545 | 0.146461 | 7/15/2014 | 34:37.5 | 0 |
| -1398.698 | 19799.303 | 1.715 | 14.546 | 0.15776 | 7/15/2014 | 34:37.6 | 0 |
| -1398.413 | 19799.276 | 1.715 | 14.546 | 0.151205 | 7/15/2014 | 34:37.7 | 0 |
| -1398.183 | 19799.255 | 1.71 | 14.545 | 0.162526 | 7/15/2014 | 34:37.8 | 0 |
| -1397.954 | 19799.234 | 1.705 | 14.543 | 0.152452 | 7/15/2014 | 34:37.9 | 0 |
| -1397.722 | 19799.213 | 1.699 | 14.541 | 0.155818 | 7/15/2014 | 34:38.0 | 0 |
| -1397.491 | 19799.192 | 1.694 | 14.538 | 0.167479 | 7/15/2014 | 34:38.1 | 0 |
| -1397.26 | 19799.17 | 1.694 | 14.535 | 0.142415 | 7/15/2014 | 34:38.2 | 0 |
| -1397.146 | 19799.159 | 1.694 | 14.534 | 0.14305 | 7/15/2014 | 34:38.3 | 0 |
| -1396.743 | 19799.122 | 1.695 | 14.534 | 0.142275 | 7/15/2014 | 34:38.4 | 0 |
| -1396.513 | 19799.101 | 1.697 | 14.537 | 0.14021 | 7/15/2014 | 34:38.5 | 0 |
| -1396.284 | 19799.08 | 1.698 | 14.542 | 0.141189 | 7/15/2014 | 34:38.6 | 0 |
| -1395.994 | 19799.053 | 1.7 | 14.551 | 0.138074 | 7/15/2014 | 34:38.7 | 0 |
| -1395.878 | 19799.043 | 1.7 | 14.554 | 0.128197 | 7/15/2014 | 34:38.8 | 0 |
| -1395.645 | 19799.02 | 1.702 | 14.567 | 0.130496 | 7/15/2014 | 34:38.9 | 0 |
| -1395.414 | 19798.999 | 1.696 | 14.582 | 0.106314 | 7/15/2014 | 34:39.0 | 0 |
| -1395.18 | 19798.977 | 1.699 | 14.599 | 0.096462 | 7/15/2014 | 34:39.1 | 0 |
| -1394.948 | 19798.955 | 1.693 | 14.617 | 0.106702 | 7/15/2014 | 34:39.2 | 0 |
| -1394.715 | 19798.933 | 1.693 | 14.635 | 0.0929931 | 7/15/2014 | 34:39.3 | 0 |
| -1394.309 | 19798.896 | 1.688 | 14.66 | 0.0882939 | 7/15/2014 | 34:39.4 | 0 |
| -1394.079 | 19798.873 | 1.686 | 14.676 | 0.0988574 | 7/15/2014 | 34:39.5 | 0 |
| -1393.848 | 19798.853 | 1.689 | 14.689 | 0.0989711 | 7/15/2014 | 34:39.6 | 0 |
| -1393.559 | 19798.824 | 1.689 | 14.701 | 0.0995541 | 7/15/2014 | 34:39.7 | 0 |
| -1393.443 | 19798.813 | 1.688 | 14.707 | 0.0900128 | 7/15/2014 | 34:39.8 | 0 |
| -1393.21 | 19798.791 | 1.688 | 14.715 | 0.0967369 | 7/15/2014 | 34:39.9 | 0 |
| -1392.977 | 19798.77 | 1.683 | 14.721 | 0.0906083 | 7/15/2014 | 34:40.0 | 0 |
| -1392.744 | 19798.747 | 1.681 | 14.725 | 0.118156 | 7/15/2014 | 34:40.1 | 0 |
| -1392.511 | 19798.726 | 1.681 | 14.726 | 0.0927007 | 7/15/2014 | 34:40.2 | 0 |
| -1392.279 | 19798.704 | 1.681 | 14.725 | 0.0925981 | 7/15/2014 | 34:40.3 | 0 |
| -1391.874 | 19798.665 | 1.675 | 14.719 | 0.0952827 | 7/15/2014 | 34:40.4 | 0 |
| -1391.644 | 19798.644 | 1.675 | 14.713 | 0.0974101 | 7/15/2014 | 34:40.5 | 0 |
| -1391.412 | 19798.621 | 1.675 | 14.706 | 0.0890481 | 7/15/2014 | 34:40.6 | 0 |
| -1391.123 | 19798.594 | 1.677 | 14.697 | 0.0825119 | 7/15/2014 | 34:40.7 | 0 |
| -1391.006 | 19798.583 | 1.68 | 14.693 | 0.0826046 | 7/15/2014 | 34:40.8 | 0 |
| -1390.773 | 19798.562 | 1.677 | 14.681 | 0.0818208 | 7/15/2014 | 34:40.9 | 0 |
| -1390.539 | 19798.538 | 1.675 | 14.67 | 0.0803142 | 7/15/2014 | 34:41.0 | 0 |
| -1390.304 | 19798.517 | 1.675 | 14.658 | 0.0784725 | 7/15/2014 | 34:41.1 | 0 |
| -1390.07 | 19798.494 | 1.675 | 14.647 | 0.0765796 | 7/15/2014 | 34:41.2 | 0 |

| | | | | | | | |
|---|---|---|---|---|---|---|---|
| -1389.837 | 19798.472 | 1.675 | 14.637 | 0.0908722 | 7/15/2014 | 34:41.3 | 0 |
| -1389.429 | 19798.434 | 1.673 | 14.628 | 0.077715 | 7/15/2014 | 34:41.4 | 0 |
| -1389.197 | 19798.412 | 1.671 | 14.625 | 0.0712633 | 7/15/2014 | 34:41.5 | 0 |
| -1388.966 | 19798.39 | 1.671 | 14.625 | 0.0696571 | 7/15/2014 | 34:41.6 | 0 |
| -1388.677 | 19798.363 | 1.669 | 14.629 | 0.0680773 | 7/15/2014 | 34:41.7 | 0 |
| -1388.559 | 19798.352 | 1.667 | 14.63 | 0.06787 | 7/15/2014 | 34:41.8 | 0 |
| -1388.327 | 19798.329 | 1.665 | 14.638 | 0.0740996 | 7/15/2014 | 34:41.9 | 0 |
| -1387.981 | 19798.296 | 1.66 | 14.658 | 0.082802 | 7/15/2014 | 34:42.0 | 0 |
| -1387.75 | 19798.274 | 1.658 | 14.672 | 0.0719328 | 7/15/2014 | 34:42.1 | 0 |
| -1387.521 | 19798.253 | 1.656 | 14.687 | 0.0913529 | 7/15/2014 | 34:42.2 | 0 |
| -1387.292 | 19798.231 | 1.652 | 14.701 | 0.0711894 | 7/15/2014 | 34:42.3 | 0 |
| -1387.008 | 19798.205 | 1.654 | 14.715 | 0.0796986 | 7/15/2014 | 34:42.4 | 0 |
| -1386.783 | 19798.182 | 1.656 | 14.726 | 0.070033 | 7/15/2014 | 34:42.5 | 0 |
| -1386.557 | 19798.162 | 1.658 | 14.735 | 0.0721336 | 7/15/2014 | 34:42.6 | 0 |
| -1386.275 | 19798.134 | 1.656 | 14.741 | 0.0626791 | 7/15/2014 | 34:42.7 | 0 |
| -1386.05 | 19798.112 | 1.654 | 14.743 | 0.0626847 | 7/15/2014 | 34:42.8 | 0 |
| -1385.822 | 19798.091 | 1.652 | 14.744 | 0.0605769 | 7/15/2014 | 34:42.9 | 0 |
| -1385.595 | 19798.068 | 1.65 | 14.742 | 0.0696415 | 7/15/2014 | 34:43.0 | 0 |
| -1385.366 | 19798.047 | 1.65 | 14.739 | 0.0673004 | 7/15/2014 | 34:43.1 | 0 |
| -1385.139 | 19798.023 | 1.646 | 14.735 | 0.0876635 | 7/15/2014 | 34:43.2 | 0 |
| -1384.913 | 19798.003 | 1.638 | 14.732 | 0.0674229 | 7/15/2014 | 34:43.3 | 0 |
| -1384.63 | 19797.975 | 1.632 | 14.729 | 0.0834605 | 7/15/2014 | 34:43.4 | 0 |
| -1384.404 | 19797.952 | 1.628 | 14.728 | 0.0878788 | 7/15/2014 | 34:43.5 | 0 |
| -1384.175 | 19797.93 | 1.628 | 14.729 | 0.0743135 | 7/15/2014 | 34:43.6 | 0 |
| -1383.888 | 19797.9 | 1.625 | 14.733 | 0.08412 | 7/15/2014 | 34:43.7 | 0 |
| -1383.774 | 19797.89 | 1.622 | 14.734 | 0.0770762 | 7/15/2014 | 34:43.8 | 0 |
| -1383.541 | 19797.866 | 1.625 | 14.741 | 0.0762957 | 7/15/2014 | 34:43.9 | 0 |
| -1383.309 | 19797.844 | 1.621 | 14.751 | 0.0817502 | 7/15/2014 | 34:44.0 | 0 |
| -1383.073 | 19797.819 | 1.627 | 14.763 | 0.081548 | 7/15/2014 | 34:44.1 | 0 |
| -1382.838 | 19797.796 | 1.63 | 14.777 | 0.0902395 | 7/15/2014 | 34:44.2 | 0 |
| -1382.602 | 19797.772 | 1.636 | 14.793 | 0.0790214 | 7/15/2014 | 34:44.3 | 0 |
| -1382.188 | 19797.73 | 1.637 | 14.817 | 0.0692941 | 7/15/2014 | 34:44.4 | 0 |
| -1381.951 | 19797.706 | 1.634 | 14.832 | 0.0658566 | 7/15/2014 | 34:44.5 | 0 |
| -1381.713 | 19797.682 | 1.632 | 14.846 | 0.0684609 | 7/15/2014 | 34:44.6 | 0 |
| -1381.415 | 19797.65 | 1.63 | 14.859 | 0.0623519 | 7/15/2014 | 34:44.7 | 0 |
| -1381.294 | 19797.639 | 1.627 | 14.866 | 0.0666105 | 7/15/2014 | 34:44.8 | 0 |
| -1381.052 | 19797.615 | 1.625 | 14.876 | 0.0626035 | 7/15/2014 | 34:44.9 | 0 |
| -1380.811 | 19797.59 | 1.615 | 14.884 | 0.0624509 | 7/15/2014 | 34:45.0 | 0 |
| -1380.568 | 19797.565 | 1.605 | 14.889 | 0.0609762 | 7/15/2014 | 34:45.1 | 0 |
| -1380.327 | 19797.54 | 1.596 | 14.891 | 0.060831 | 7/15/2014 | 34:45.2 | 0 |
| -1380.084 | 19797.516 | 1.599 | 14.892 | 0.0594412 | 7/15/2014 | 34:45.3 | 0 |
| -1379.661 | 19797.471 | 1.593 | 14.888 | 0.0633495 | 7/15/2014 | 34:45.4 | 0 |
| -1379.418 | 19797.447 | 1.59 | 14.885 | 0.0726774 | 7/15/2014 | 34:45.5 | 0 |
| -1379.177 | 19797.421 | 1.584 | 14.88 | 0.0765403 | 7/15/2014 | 34:45.6 | 0 |
| -1378.873 | 19797.391 | 1.585 | 14.874 | 0.080483 | 7/15/2014 | 34:45.7 | 0 |
| -1378.751 | 19797.378 | 1.586 | 14.871 | 0.0904808 | 7/15/2014 | 34:45.8 | 0 |
| -1378.506 | 19797.353 | 1.59 | 14.865 | 0.0959569 | 7/15/2014 | 34:45.9 | 0 |

| | | | | | | | |
|---|---|---|---|---|---|---|---|
| -1378.26 | 19797.328 | 1.59 | 14.858 | 0.0927357 | 7/15/2014 | 34:46.0 | 0 |
| -1378.014 | 19797.301 | 1.59 | 14.851 | 0.0870665 | 7/15/2014 | 34:46.1 | 0 |
| -1377.769 | 19797.277 | 1.593 | 14.845 | 0.0815916 | 7/15/2014 | 34:46.2 | 0 |
| -1377.524 | 19797.25 | 1.597 | 14.839 | 0.0726444 | 7/15/2014 | 34:46.3 | 0 |
| -1377.097 | 19797.207 | 1.594 | 14.832 | 0.0590426 | 7/15/2014 | 34:46.4 | 0 |
| -1376.853 | 19797.181 | 1.593 | 14.829 | 0.0562995 | 7/15/2014 | 34:46.5 | 0 |
| -1376.609 | 19797.157 | 1.59 | 14.827 | 0.0527306 | 7/15/2014 | 34:46.6 | 0 |
| -1376.306 | 19797.125 | 1.585 | 14.827 | 0.0533584 | 7/15/2014 | 34:46.7 | 0 |
| -1376.182 | 19797.113 | 1.582 | 14.826 | 0.0583034 | 7/15/2014 | 34:46.8 | 0 |
| -1375.938 | 19797.087 | 1.578 | 14.828 | 0.0677174 | 7/15/2014 | 34:46.9 | 0 |
| -1375.693 | 19797.062 | 1.575 | 14.831 | 0.0612754 | 7/15/2014 | 34:47.0 | 0 |
| -1375.449 | 19797.037 | 1.575 | 14.836 | 0.0460426 | 7/15/2014 | 34:47.1 | 0 |
| -1375.207 | 19797.011 | 1.566 | 14.842 | 0.0543096 | 7/15/2014 | 34:47.2 | 0 |
| -1374.964 | 19796.987 | 1.566 | 14.848 | 0.0345915 | 7/15/2014 | 34:47.3 | 0 |
| -1374.542 | 19796.943 | 1.57 | 14.859 | 0.0715148 | 7/15/2014 | 34:47.4 | 0 |
| -1374.302 | 19796.919 | 1.575 | 14.866 | 0.0566567 | 7/15/2014 | 34:47.5 | 0 |
| -1374.062 | 19796.894 | 1.575 | 14.873 | 0.0756105 | 7/15/2014 | 34:47.6 | 0 |
| -1373.763 | 19796.863 | 1.573 | 14.88 | 0.0915053 | 7/15/2014 | 34:47.7 | 0 |
| -1373.642 | 19796.85 | 1.573 | 14.883 | 0.081528 | 7/15/2014 | 34:47.8 | 0 |
| -1373.4 | 19796.826 | 1.581 | 14.889 | 0.114112 | 7/15/2014 | 34:47.9 | 0 |
| -1373.159 | 19796.8 | 1.579 | 14.895 | 0.0759055 | 7/15/2014 | 34:48.0 | 0 |
| -1372.917 | 19796.775 | 1.579 | 14.901 | 0.0898542 | 7/15/2014 | 34:48.1 | 0 |
| -1372.676 | 19796.752 | 1.575 | 14.905 | 0.10085 | 7/15/2014 | 34:48.2 | 0 |
| -1372.436 | 19796.727 | 1.563 | 14.908 | 0.120716 | 7/15/2014 | 34:48.3 | 0 |
| -1372.017 | 19796.683 | 1.556 | 14.91 | 0.124317 | 7/15/2014 | 34:48.4 | 0 |
| -1371.776 | 19796.658 | 1.549 | 14.909 | 0.133391 | 7/15/2014 | 34:48.5 | 0 |
| -1371.537 | 19796.634 | 1.543 | 14.907 | 0.14249 | 7/15/2014 | 34:48.6 | 0 |
| -1371.237 | 19796.604 | 1.538 | 14.903 | 0.144657 | 7/15/2014 | 34:48.7 | 0 |
| -1371.115 | 19796.592 | 1.533 | 14.902 | 0.149171 | 7/15/2014 | 34:48.8 | 0 |
| -1370.874 | 19796.567 | 1.524 | 14.895 | 0.145117 | 7/15/2014 | 34:48.9 | 0 |
| -1370.63 | 19796.542 | 1.528 | 14.888 | 0.126752 | 7/15/2014 | 34:49.0 | 0 |
| -1370.387 | 19796.517 | 1.53 | 14.882 | 0.0995558 | 7/15/2014 | 34:49.1 | 0 |
| -1370.144 | 19796.492 | 1.527 | 14.876 | 0.0775721 | 7/15/2014 | 34:49.2 | 0 |
| -1369.899 | 19796.469 | 1.532 | 14.871 | 0.0502819 | 7/15/2014 | 34:49.3 | 0 |
| -1369.475 | 19796.427 | 1.533 | 14.873 | 0.0251109 | 7/15/2014 | 34:49.4 | 0 |
| -1369.234 | 19796.403 | 1.533 | 14.877 | 0.0288908 | 7/15/2014 | 34:49.5 | 0 |
| -1368.992 | 19796.38 | 1.531 | 14.885 | 0.0353331 | 7/15/2014 | 34:49.6 | 0 |
| -1368.691 | 19796.35 | 1.526 | 14.897 | 0.0389356 | 7/15/2014 | 34:49.7 | 0 |
| -1368.568 | 19796.338 | 1.521 | 14.902 | 0.0363584 | 7/15/2014 | 34:49.8 | 0 |
| -1368.325 | 19796.315 | 1.524 | 14.92 | 0.032797 | 7/15/2014 | 34:49.9 | 0 |
| -1368.085 | 19796.292 | 1.516 | 14.94 | 0.041773 | 7/15/2014 | 34:50.0 | 0 |
| -1367.843 | 19796.269 | 1.506 | 14.961 | 0.0791433 | 7/15/2014 | 34:50.1 | 0 |
| -1367.604 | 19796.247 | 1.498 | 14.983 | 0.0653386 | 7/15/2014 | 34:50.2 | 0 |
| -1367.366 | 19796.225 | 1.498 | 15.005 | 0.0784768 | 7/15/2014 | 34:50.3 | 0 |
| -1366.953 | 19796.187 | 1.491 | 15.033 | 0.124128 | 7/15/2014 | 34:50.4 | 0 |
| -1366.72 | 19796.165 | 1.485 | 15.049 | 0.137 | 7/15/2014 | 34:50.5 | 0 |
| -1366.487 | 19796.145 | 1.477 | 15.063 | 0.126239 | 7/15/2014 | 34:50.6 | 0 |

| | | | | | | | |
|---|---|---|---|---|---|---|---|
| -1366.197 | 19796.117 | 1.477 | 15.075 | 0.124996 | 7/15/2014 | 34:50.7 | 0 |
| -1366.079 | 19796.107 | 1.477 | 15.081 | 0.15077 | 7/15/2014 | 34:50.8 | 0 |
| -1365.844 | 19796.086 | 1.477 | 15.088 | 0.147974 | 7/15/2014 | 34:50.9 | 0 |
| -1365.612 | 19796.066 | 1.471 | 15.093 | 0.124573 | 7/15/2014 | 34:51.0 | 0 |
| -1365.379 | 19796.046 | 1.478 | 15.096 | 0.129399 | 7/15/2014 | 34:51.1 | 0 |
| -1365.148 | 19796.025 | 1.475 | 15.098 | 0.110663 | 7/15/2014 | 34:51.2 | 0 |
| -1364.914 | 19796.006 | 1.482 | 15.097 | 0.11567 | 7/15/2014 | 34:51.3 | 0 |
| -1364.509 | 19795.971 | 1.487 | 15.094 | 0.101642 | 7/15/2014 | 34:51.4 | 0 |
| -1364.276 | 19795.951 | 1.49 | 15.09 | 0.113765 | 7/15/2014 | 34:51.5 | 0 |
| -1364.044 | 19795.932 | 1.49 | 15.085 | 0.134554 | 7/15/2014 | 34:51.6 | 0 |
| -1363.753 | 19795.906 | 1.487 | 15.079 | 0.129009 | 7/15/2014 | 34:51.7 | 0 |
| -1363.636 | 19795.896 | 1.485 | 15.076 | 0.141079 | 7/15/2014 | 34:51.8 | 0 |
| -1363.399 | 19795.877 | 1.485 | 15.067 | 0.133167 | 7/15/2014 | 34:51.9 | 0 |
| -1363.162 | 19795.857 | 1.478 | 15.057 | 0.136973 | 7/15/2014 | 34:52.0 | 0 |
| -1362.923 | 19795.838 | 1.475 | 15.046 | 0.149185 | 7/15/2014 | 34:52.1 | 0 |
| -1362.684 | 19795.818 | 1.467 | 15.034 | 0.155513 | 7/15/2014 | 34:52.2 | 0 |
| -1362.444 | 19795.799 | 1.46 | 15.021 | 0.134749 | 7/15/2014 | 34:52.3 | 0 |
| -1362.025 | 19795.764 | 1.462 | 15.001 | 0.159564 | 7/15/2014 | 34:52.4 | 0 |
| -1361.783 | 19795.744 | 1.462 | 14.989 | 0.182004 | 7/15/2014 | 34:52.5 | 0 |
| -1361.542 | 19795.726 | 1.462 | 14.977 | 0.197762 | 7/15/2014 | 34:52.6 | 0 |
| -1361.239 | 19795.702 | 1.462 | 14.968 | 0.210872 | 7/15/2014 | 34:52.7 | 0 |
| -1361.118 | 19795.692 | 1.46 | 14.962 | 0.209036 | 7/15/2014 | 34:52.8 | 0 |
| -1360.872 | 19795.674 | 1.464 | 14.955 | 0.224762 | 7/15/2014 | 34:52.9 | 0 |
| -1360.627 | 19795.654 | 1.464 | 14.951 | 0.23685 | 7/15/2014 | 34:53.0 | 0 |
| -1360.38 | 19795.636 | 1.464 | 14.951 | 0.215652 | 7/15/2014 | 34:53.1 | 0 |
| -1360.135 | 19795.617 | 1.457 | 14.956 | 0.229724 | 7/15/2014 | 34:53.2 | 0 |
| -1359.888 | 19795.6 | 1.461 | 14.965 | 0.192708 | 7/15/2014 | 34:53.3 | 0 |
| -1359.458 | 19795.568 | 1.457 | 14.99 | 0.199081 | 7/15/2014 | 34:53.4 | 0 |
| -1359.213 | 19795.551 | 1.452 | 15.01 | 0.178665 | 7/15/2014 | 34:53.5 | 0 |
| -1358.968 | 19795.532 | 1.452 | 15.033 | 0.163884 | 7/15/2014 | 34:53.6 | 0 |
| -1358.662 | 19795.511 | 1.452 | 15.056 | 0.194248 | 7/15/2014 | 34:53.7 | 0 |
| -1358.538 | 19795.502 | 1.449 | 15.069 | 0.169947 | 7/15/2014 | 34:53.8 | 0 |
| -1358.292 | 19795.485 | 1.451 | 15.093 | 0.173953 | 7/15/2014 | 34:53.9 | 0 |
| -1358.045 | 19795.469 | 1.442 | 15.115 | 0.187781 | 7/15/2014 | 34:54.0 | 0 |
| -1357.797 | 19795.451 | 1.445 | 15.133 | 0.15996 | 7/15/2014 | 34:54.1 | 0 |
| -1357.553 | 19795.435 | 1.442 | 15.146 | 0.219614 | 7/15/2014 | 34:54.2 | 0 |
| -1357.308 | 19795.418 | 1.448 | 15.154 | 0.171376 | 7/15/2014 | 34:54.3 | 0 |
| -1356.883 | 19795.39 | 1.443 | 15.153 | 0.189969 | 7/15/2014 | 34:54.4 | 0 |
| -1356.642 | 19795.375 | 1.443 | 15.149 | 0.206088 | 7/15/2014 | 34:54.5 | 0 |
| -1356.402 | 19795.359 | 1.446 | 15.142 | 0.186703 | 7/15/2014 | 34:54.6 | 0 |
| -1356.104 | 19795.34 | 1.451 | 15.132 | 0.194918 | 7/15/2014 | 34:54.7 | 0 |
| -1355.982 | 19795.332 | 1.448 | 15.128 | 0.17769 | 7/15/2014 | 34:54.8 | 0 |
| -1355.743 | 19795.317 | 1.45 | 15.117 | 0.198421 | 7/15/2014 | 34:54.9 | 0 |
| -1355.504 | 19795.303 | 1.444 | 15.106 | 0.148861 | 7/15/2014 | 34:55.0 | 0 |
| -1355.267 | 19795.288 | 1.444 | 15.097 | 0.142078 | 7/15/2014 | 34:55.1 | 0 |
| -1355.031 | 19795.274 | 1.442 | 15.09 | 0.130084 | 7/15/2014 | 34:55.2 | 0 |
| -1354.796 | 19795.26 | 1.439 | 15.086 | 0.126282 | 7/15/2014 | 34:55.3 | 0 |

| | | | | | | | |
|---|---|---|---|---|---|---|---|
| -1354.39 | 19795.236 | 1.433 | 15.087 | 0.12984 | 7/15/2014 | 34:55.4 | 0 |
| -1354.16 | 19795.223 | 1.433 | 15.091 | 0.13534 | 7/15/2014 | 34:55.5 | 0 |
| -1353.931 | 19795.21 | 1.431 | 15.098 | 0.161352 | 7/15/2014 | 34:55.6 | 0 |
| -1353.647 | 19795.194 | 1.431 | 15.109 | 0.175391 | 7/15/2014 | 34:55.7 | 0 |
| -1353.419 | 19795.181 | 1.431 | 15.123 | 0.174014 | 7/15/2014 | 34:55.8 | 0 |
| -1353.192 | 19795.168 | 1.426 | 15.14 | 0.201481 | 7/15/2014 | 34:55.9 | 0 |
| -1352.963 | 19795.155 | 1.426 | 15.161 | 0.228252 | 7/15/2014 | 34:56.0 | 0 |
| -1352.737 | 19795.144 | 1.424 | 15.184 | 0.217008 | 7/15/2014 | 34:56.1 | 0 |
| -1352.51 | 19795.131 | 1.424 | 15.21 | 0.217507 | 7/15/2014 | 34:56.2 | 0 |
| -1352.286 | 19795.119 | 1.421 | 15.237 | 0.214525 | 7/15/2014 | 34:56.3 | 0 |
| -1352.007 | 19795.104 | 1.421 | 15.266 | 0.217858 | 7/15/2014 | 34:56.4 | 0 |
| -1351.783 | 19795.093 | 1.424 | 15.295 | 0.199213 | 7/15/2014 | 34:56.5 | 0 |
| -1351.559 | 19795.081 | 1.426 | 15.323 | 0.205894 | 7/15/2014 | 34:56.6 | 0 |
| -1351.276 | 19795.066 | 1.429 | 15.351 | 0.189073 | 7/15/2014 | 34:56.7 | 0 |
| -1351.049 | 19795.055 | 1.431 | 15.376 | 0.196437 | 7/15/2014 | 34:56.8 | 0 |
| -1350.819 | 19795.044 | 1.433 | 15.4 | 0.215444 | 7/15/2014 | 34:56.9 | 0 |
| -1350.588 | 19795.033 | 1.433 | 15.421 | 0.194665 | 7/15/2014 | 34:57.0 | 0 |
| -1350.356 | 19795.021 | 1.433 | 15.439 | 0.223486 | 7/15/2014 | 34:57.1 | 0 |
| -1350.244 | 19795.017 | 1.433 | 15.449 | 0.24239 | 7/15/2014 | 34:57.2 | 0 |
| -1350.011 | 19795.005 | 1.433 | 15.463 | 0.239627 | 7/15/2014 | 34:57.3 | 0 |
| -1349.6 | 19794.986 | 1.444 | 15.476 | 0.258973 | 7/15/2014 | 34:57.4 | 0 |
| -1349.364 | 19794.975 | 1.453 | 15.482 | 0.301251 | 7/15/2014 | 34:57.5 | 0 |
| -1349.128 | 19794.964 | 1.456 | 15.486 | 0.305183 | 7/15/2014 | 34:57.6 | 0 |
| -1348.832 | 19794.951 | 1.46 | 15.488 | 0.31786 | 7/15/2014 | 34:57.7 | 0 |
| -1348.713 | 19794.945 | 1.464 | 15.489 | 0.304863 | 7/15/2014 | 34:57.8 | 0 |
| -1348.473 | 19794.936 | 1.47 | 15.487 | 0.330079 | 7/15/2014 | 34:57.9 | 0 |
| -1348.233 | 19794.924 | 1.473 | 15.483 | 0.298818 | 7/15/2014 | 34:58.0 | 0 |
| -1347.993 | 19794.916 | 1.476 | 15.478 | 0.333599 | 7/15/2014 | 34:58.1 | 0 |
| -1347.753 | 19794.904 | 1.476 | 15.471 | 0.298764 | 7/15/2014 | 34:58.2 | 0 |
| -1347.514 | 19794.895 | 1.473 | 15.463 | 0.292881 | 7/15/2014 | 34:58.3 | 0 |
| -1347.1 | 19794.877 | 1.476 | 15.446 | 0.279289 | 7/15/2014 | 34:58.4 | 0 |
| -1346.865 | 19794.869 | 1.48 | 15.434 | 0.274355 | 7/15/2014 | 34:58.5 | 0 |
| -1346.633 | 19794.857 | 1.485 | 15.42 | 0.260997 | 7/15/2014 | 34:58.6 | 0 |
| -1346.345 | 19794.848 | 1.487 | 15.405 | 0.249357 | 7/15/2014 | 34:58.7 | 0 |
| -1346.225 | 19794.842 | 1.487 | 15.398 | 0.245169 | 7/15/2014 | 34:58.8 | 0 |
| -1345.885 | 19794.83 | 1.483 | 15.374 | 0.228129 | 7/15/2014 | 34:58.9 | 0 |
| -1345.656 | 19794.819 | 1.488 | 15.358 | 0.230178 | 7/15/2014 | 34:59.0 | 0 |
| -1345.43 | 19794.812 | 1.49 | 15.342 | 0.218663 | 7/15/2014 | 34:59.1 | 0 |
| -1345.204 | 19794.803 | 1.497 | 15.327 | 0.197884 | 7/15/2014 | 34:59.2 | 0 |
| -1344.984 | 19794.793 | 1.497 | 15.313 | 0.20535 | 7/15/2014 | 34:59.3 | 0 |
| -1344.708 | 19794.783 | 1.497 | 15.301 | 0.216958 | 7/15/2014 | 34:59.4 | 0 |
| -1344.492 | 19794.775 | 1.499 | 15.291 | 0.199533 | 7/15/2014 | 34:59.5 | 0 |
| -1344.276 | 19794.768 | 1.504 | 15.282 | 0.235402 | 7/15/2014 | 34:59.6 | 0 |
| -1344.007 | 19794.756 | 1.509 | 15.275 | 0.235084 | 7/15/2014 | 34:59.7 | 0 |
| -1343.792 | 19794.749 | 1.513 | 15.27 | 0.256633 | 7/15/2014 | 34:59.8 | 0 |
| -1343.576 | 19794.74 | 1.516 | 15.265 | 0.293559 | 7/15/2014 | 34:59.9 | 0 |
| -1343.361 | 19794.732 | 1.516 | 15.262 | 0.304797 | 7/15/2014 | 35:00.0 | 0 |

| | | | | | | | |
|---|---|---|---|---|---|---|---|
| -1343.146 | 19794.725 | 1.519 | 15.26 | 0.302516 | 7/15/2014 | 35:00.1 | 0 |
| -1342.933 | 19794.717 | 1.519 | 15.257 | 0.345294 | 7/15/2014 | 35:00.2 | 0 |
| -1342.721 | 19794.709 | 1.522 | 15.253 | 0.335522 | 7/15/2014 | 35:00.3 | 0 |
| -1342.352 | 19794.696 | 1.527 | 15.245 | 0.346055 | 7/15/2014 | 35:00.5 | 0 |
| -1342.142 | 19794.689 | 1.53 | 15.238 | 0.353374 | 7/15/2014 | 35:00.6 | 0 |
| -1341.884 | 19794.68 | 1.53 | 15.229 | 0.339453 | 7/15/2014 | 35:00.7 | 0 |
| -1341.776 | 19794.676 | 1.531 | 15.226 | 0.339048 | 7/15/2014 | 35:00.7 | 0 |
| -1341.568 | 19794.668 | 1.534 | 15.214 | 0.348681 | 7/15/2014 | 35:00.8 | 0 |
| -1341.36 | 19794.66 | 1.539 | 15.201 | 0.326335 | 7/15/2014 | 35:00.9 | 0 |
| -1341.152 | 19794.653 | 1.542 | 15.186 | 0.31627 | 7/15/2014 | 35:01.0 | 0 |
| -1340.944 | 19794.646 | 1.545 | 15.17 | 0.307003 | 7/15/2014 | 35:01.1 | 0 |
| -1340.738 | 19794.64 | 1.55 | 15.154 | 0.291672 | 7/15/2014 | 35:01.2 | 0 |
| -1340.534 | 19794.633 | 1.55 | 15.138 | 0.280506 | 7/15/2014 | 35:01.3 | 0 |
| -1340.181 | 19794.621 | 1.554 | 15.114 | 0.270199 | 7/15/2014 | 35:01.5 | 0 |
| -1339.98 | 19794.613 | 1.558 | 15.1 | 0.265363 | 7/15/2014 | 35:01.6 | 0 |
| -1339.73 | 19794.604 | 1.56 | 15.086 | 0.26269 | 7/15/2014 | 35:01.7 | 0 |
| -1339.629 | 19794.602 | 1.56 | 15.079 | 0.259802 | 7/15/2014 | 35:01.7 | 0 |
| -1339.428 | 19794.596 | 1.565 | 15.068 | 0.259182 | 7/15/2014 | 35:01.8 | 0 |
| -1339.125 | 19794.584 | 1.573 | 15.056 | 0.257327 | 7/15/2014 | 35:02.0 | 0 |
| -1338.924 | 19794.578 | 1.573 | 15.049 | 0.256135 | 7/15/2014 | 35:02.1 | 0 |
| -1338.721 | 19794.571 | 1.58 | 15.043 | 0.258236 | 7/15/2014 | 35:02.2 | 0 |
| -1338.622 | 19794.568 | 1.581 | 15.04 | 0.256935 | 7/15/2014 | 35:02.2 | 0 |
| -1338.421 | 19794.561 | 1.586 | 15.037 | 0.254878 | 7/15/2014 | 35:02.3 | 0 |
| -1338.073 | 19794.55 | 1.593 | 15.034 | 0.262667 | 7/15/2014 | 35:02.5 | 0 |
| -1337.874 | 19794.543 | 1.601 | 15.034 | 0.260858 | 7/15/2014 | 35:02.6 | 0 |
| -1337.625 | 19794.534 | 1.609 | 15.034 | 0.267737 | 7/15/2014 | 35:02.7 | 0 |
| -1337.525 | 19794.53 | 1.613 | 15.034 | 0.270039 | 7/15/2014 | 35:02.7 | 0 |
| -1337.325 | 19794.525 | 1.618 | 15.035 | 0.270601 | 7/15/2014 | 35:02.8 | 0 |
| -1337.023 | 19794.514 | 1.627 | 15.042 | 0.270504 | 7/15/2014 | 35:03.0 | 0 |
| -1336.822 | 19794.508 | 1.627 | 15.048 | 0.264946 | 7/15/2014 | 35:03.1 | 0 |
| -1336.62 | 19794.501 | 1.632 | 15.055 | 0.262794 | 7/15/2014 | 35:03.2 | 0 |
| -1336.422 | 19794.494 | 1.631 | 15.065 | 0.261503 | 7/15/2014 | 35:03.3 | 0 |
| -1336.221 | 19794.486 | 1.636 | 15.078 | 0.257425 | 7/15/2014 | 35:03.4 | 0 |
| -1335.975 | 19794.479 | 1.636 | 15.092 | 0.257665 | 7/15/2014 | 35:03.5 | 0 |
| -1335.777 | 19794.473 | 1.638 | 15.11 | 0.249506 | 7/15/2014 | 35:03.6 | 0 |
| -1335.533 | 19794.463 | 1.638 | 15.13 | 0.24184 | 7/15/2014 | 35:03.7 | 0 |
| -1335.336 | 19794.455 | 1.64 | 15.153 | 0.238026 | 7/15/2014 | 35:03.8 | 0 |
| -1335.14 | 19794.45 | 1.64 | 15.177 | 0.248899 | 7/15/2014 | 35:03.9 | 0 |
| -1334.941 | 19794.443 | 1.648 | 15.203 | 0.233872 | 7/15/2014 | 35:04.0 | 0 |
| -1334.744 | 19794.436 | 1.652 | 15.23 | 0.237929 | 7/15/2014 | 35:04.1 | 0 |
| -1334.547 | 19794.428 | 1.658 | 15.256 | 0.244832 | 7/15/2014 | 35:04.2 | 0 |
| -1334.351 | 19794.421 | 1.66 | 15.282 | 0.236748 | 7/15/2014 | 35:04.3 | 0 |
| -1334.157 | 19794.415 | 1.664 | 15.305 | 0.238864 | 7/15/2014 | 35:04.4 | 0 |
| -1333.917 | 19794.406 | 1.668 | 15.327 | 0.237998 | 7/15/2014 | 35:04.5 | 0 |
| -1333.724 | 19794.398 | 1.672 | 15.345 | 0.233873 | 7/15/2014 | 35:04.6 | 0 |
| -1333.485 | 19794.39 | 1.674 | 15.361 | 0.245735 | 7/15/2014 | 35:04.7 | 0 |
| -1333.291 | 19794.384 | 1.677 | 15.373 | 0.241888 | 7/15/2014 | 35:04.8 | 0 |

| | | | | | | | |
|---|---|---|---|---|---|---|---|
| -1333.097 | 19794.377 | 1.677 | 15.382 | 0.239621 | 7/15/2014 | 35:04.9 | 0 |
| -1332.902 | 19794.369 | 1.677 | 15.389 | 0.21867 | 7/15/2014 | 35:05.0 | 0 |
| -1332.707 | 19794.361 | 1.677 | 15.393 | 0.222004 | 7/15/2014 | 35:05.1 | 0 |
| -1332.512 | 19794.354 | 1.682 | 15.396 | 0.225072 | 7/15/2014 | 35:05.2 | 0 |
| -1332.318 | 19794.348 | 1.685 | 15.397 | 0.216286 | 7/15/2014 | 35:05.3 | 0 |
| -1332.124 | 19794.342 | 1.689 | 15.396 | 0.232128 | 7/15/2014 | 35:05.4 | 0 |
| -1331.884 | 19794.332 | 1.693 | 15.395 | 0.225932 | 7/15/2014 | 35:05.5 | 0 |
| -1331.692 | 19794.325 | 1.7 | 15.392 | 0.247668 | 7/15/2014 | 35:05.6 | 0 |
| -1331.451 | 19794.316 | 1.704 | 15.389 | 0.240379 | 7/15/2014 | 35:05.7 | 0 |
| -1331.258 | 19794.311 | 1.711 | 15.384 | 0.252426 | 7/15/2014 | 35:05.8 | 0 |
| -1331.063 | 19794.303 | 1.716 | 15.379 | 0.25662 | 7/15/2014 | 35:05.9 | 0 |
| -1330.867 | 19794.295 | 1.722 | 15.374 | 0.253342 | 7/15/2014 | 35:06.0 | 0 |
| -1330.672 | 19794.287 | 1.726 | 15.368 | 0.259406 | 7/15/2014 | 35:06.1 | 0 |
| -1330.475 | 19794.281 | 1.736 | 15.362 | 0.250916 | 7/15/2014 | 35:06.2 | 0 |
| -1330.282 | 19794.275 | 1.738 | 15.355 | 0.244487 | 7/15/2014 | 35:06.3 | 0 |
| -1330.088 | 19794.268 | 1.748 | 15.348 | 0.241986 | 7/15/2014 | 35:06.4 | 0 |
| -1329.848 | 19794.258 | 1.751 | 15.341 | 0.242099 | 7/15/2014 | 35:06.5 | 0 |
| -1329.654 | 19794.252 | 1.76 | 15.333 | 0.232126 | 7/15/2014 | 35:06.6 | 0 |
| -1329.414 | 19794.242 | 1.765 | 15.325 | 0.230278 | 7/15/2014 | 35:06.7 | 0 |
| -1329.217 | 19794.234 | 1.774 | 15.315 | 0.224806 | 7/15/2014 | 35:06.8 | 0 |
| -1329.02 | 19794.227 | 1.78 | 15.304 | 0.22783 | 7/15/2014 | 35:06.9 | 0 |
| -1328.82 | 19794.219 | 1.793 | 15.292 | 0.223174 | 7/15/2014 | 35:07.0 | 0 |
| -1328.624 | 19794.213 | 1.794 | 15.278 | 0.248062 | 7/15/2014 | 35:07.1 | 0 |
| -1328.424 | 19794.205 | 1.804 | 15.261 | 0.220423 | 7/15/2014 | 35:07.2 | 0 |
| -1328.227 | 19794.198 | 1.807 | 15.243 | 0.234723 | 7/15/2014 | 35:07.3 | 0 |
| -1328.029 | 19794.19 | 1.814 | 15.223 | 0.236253 | 7/15/2014 | 35:07.4 | 0 |
| -1327.784 | 19794.182 | 1.817 | 15.201 | 0.235516 | 7/15/2014 | 35:07.5 | 0 |
| -1327.585 | 19794.174 | 1.825 | 15.179 | 0.233633 | 7/15/2014 | 35:07.6 | 0 |
| -1327.337 | 19794.164 | 1.83 | 15.155 | 0.229293 | 7/15/2014 | 35:07.7 | 0 |
| -1327.236 | 19794.16 | 1.835 | 15.143 | 0.229782 | 7/15/2014 | 35:07.7 | 0 |
| -1327.035 | 19794.152 | 1.84 | 15.119 | 0.215494 | 7/15/2014 | 35:07.8 | 0 |
| -1326.732 | 19794.141 | 1.854 | 15.084 | 0.235519 | 7/15/2014 | 35:08.0 | 0 |
| -1326.632 | 19794.138 | 1.855 | 15.072 | 0.226106 | 7/15/2014 | 35:08.0 | 0 |
| -1326.331 | 19794.126 | 1.864 | 15.041 | 0.219261 | 7/15/2014 | 35:08.2 | 0 |
| -1326.23 | 19794.123 | 1.869 | 15.03 | 0.21887 | 7/15/2014 | 35:08.2 | 0 |
| -1325.932 | 19794.111 | 1.878 | 15.004 | 0.224885 | 7/15/2014 | 35:08.4 | 0 |
| -1325.685 | 19794.102 | 1.885 | 14.988 | 0.228677 | 7/15/2014 | 35:08.5 | 0 |
| -1325.487 | 19794.094 | 1.896 | 14.974 | 0.232302 | 7/15/2014 | 35:08.6 | 0 |
| -1325.246 | 19794.084 | 1.898 | 14.962 | 0.240339 | 7/15/2014 | 35:08.7 | 0 |
| -1325.048 | 19794.076 | 1.907 | 14.951 | 0.225545 | 7/15/2014 | 35:08.8 | 0 |
| -1324.854 | 19794.068 | 1.91 | 14.941 | 0.250444 | 7/15/2014 | 35:08.9 | 0 |
| -1324.656 | 19794.061 | 1.92 | 14.933 | 0.249795 | 7/15/2014 | 35:09.0 | 0 |
| -1324.461 | 19794.055 | 1.924 | 14.926 | 0.266667 | 7/15/2014 | 35:09.1 | 0 |
| -1324.265 | 19794.047 | 1.934 | 14.921 | 0.262445 | 7/15/2014 | 35:09.2 | 0 |
| -1324.071 | 19794.039 | 1.94 | 14.917 | 0.268394 | 7/15/2014 | 35:09.3 | 0 |
| -1323.877 | 19794.031 | 1.953 | 14.914 | 0.286767 | 7/15/2014 | 35:09.4 | 0 |
| -1323.639 | 19794.021 | 1.955 | 14.912 | 0.281609 | 7/15/2014 | 35:09.5 | 0 |

| | | | | | | | |
|---|---|---|---|---|---|---|---|
| -1323.446 | 19794.014 | 1.967 | 14.911 | 0.286903 | 7/15/2014 | 35:09.6 | 0 |
| -1323.208 | 19794.003 | 1.972 | 14.912 | 0.286973 | 7/15/2014 | 35:09.7 | 0 |
| -1323.013 | 19793.994 | 1.981 | 14.914 | 0.271244 | 7/15/2014 | 35:09.8 | 0 |
| -1322.818 | 19793.989 | 1.989 | 14.917 | 0.27887 | 7/15/2014 | 35:09.9 | 0 |
| -1322.621 | 19793.98 | 1.998 | 14.923 | 0.272816 | 7/15/2014 | 35:10.0 | 0 |
| -1322.425 | 19793.973 | 2.004 | 14.93 | 0.281029 | 7/15/2014 | 35:10.1 | 0 |
| -1322.226 | 19793.965 | 2.014 | 14.939 | 0.250934 | 7/15/2014 | 35:10.2 | 0 |
| -1322.031 | 19793.957 | 2.014 | 14.95 | 0.257565 | 7/15/2014 | 35:10.3 | 0 |
| -1321.833 | 19793.949 | 2.023 | 14.962 | 0.251266 | 7/15/2014 | 35:10.4 | 0 |
| -1321.591 | 19793.938 | 2.025 | 14.975 | 0.24875 | 7/15/2014 | 35:10.5 | 0 |
| -1321.393 | 19793.93 | 2.032 | 14.989 | 0.259546 | 7/15/2014 | 35:10.6 | 0 |
| -1321.147 | 19793.919 | 2.034 | 15.003 | 0.235571 | 7/15/2014 | 35:10.7 | 0 |
| -1321.046 | 19793.914 | 2.036 | 15.01 | 0.248684 | 7/15/2014 | 35:10.7 | 0 |
| -1320.747 | 19793.902 | 2.039 | 15.028 | 0.246503 | 7/15/2014 | 35:10.9 | 0 |
| -1320.545 | 19793.895 | 2.046 | 15.038 | 0.244945 | 7/15/2014 | 35:11.0 | 0 |
| -1320.345 | 19793.887 | 2.049 | 15.047 | 0.244922 | 7/15/2014 | 35:11.1 | 0 |
| -1320.142 | 19793.879 | 2.057 | 15.053 | 0.258516 | 7/15/2014 | 35:11.2 | 0 |
| -1319.943 | 19793.869 | 2.06 | 15.056 | 0.2532 | 7/15/2014 | 35:11.3 | 0 |
| -1319.743 | 19793.861 | 2.068 | 15.057 | 0.261276 | 7/15/2014 | 35:11.4 | 0 |
| -1319.497 | 19793.85 | 2.071 | 15.055 | 0.269974 | 7/15/2014 | 35:11.5 | 0 |
| -1319.296 | 19793.842 | 2.079 | 15.052 | 0.272137 | 7/15/2014 | 35:11.6 | 0 |
| -1319.048 | 19793.83 | 2.082 | 15.046 | 0.25735 | 7/15/2014 | 35:11.7 | 0 |
| -1318.948 | 19793.826 | 2.086 | 15.043 | 0.269872 | 7/15/2014 | 35:11.7 | 0 |
| -1318.746 | 19793.818 | 2.09 | 15.035 | 0.24248 | 7/15/2014 | 35:11.8 | 0 |
| -1318.543 | 19793.811 | 2.09 | 15.024 | 0.254558 | 7/15/2014 | 35:11.9 | 0 |
| -1318.34 | 19793.801 | 2.096 | 15.012 | 0.253628 | 7/15/2014 | 35:12.0 | 0 |
| -1318.135 | 19793.791 | 2.1 | 14.999 | 0.247139 | 7/15/2014 | 35:12.1 | 0 |
| -1317.932 | 19793.783 | 2.103 | 14.984 | 0.240335 | 7/15/2014 | 35:12.2 | 0 |
| -1317.728 | 19793.775 | 2.107 | 14.967 | 0.237144 | 7/15/2014 | 35:12.3 | 0 |
| -1317.373 | 19793.758 | 2.113 | 14.941 | 0.243888 | 7/15/2014 | 35:12.5 | 0 |
| -1317.168 | 19793.75 | 2.123 | 14.923 | 0.252622 | 7/15/2014 | 35:12.6 | 0 |
| -1316.915 | 19793.738 | 2.122 | 14.905 | 0.226841 | 7/15/2014 | 35:12.7 | 0 |
| -1316.811 | 19793.734 | 2.128 | 14.896 | 0.216009 | 7/15/2014 | 35:12.7 | 0 |
| -1316.604 | 19793.725 | 2.131 | 14.879 | 0.219615 | 7/15/2014 | 35:12.8 | 0 |
| -1316.398 | 19793.717 | 2.135 | 14.864 | 0.217746 | 7/15/2014 | 35:12.9 | 0 |
| -1316.189 | 19793.707 | 2.138 | 14.851 | 0.215166 | 7/15/2014 | 35:13.0 | 0 |
| -1315.982 | 19793.697 | 2.141 | 14.841 | 0.195992 | 7/15/2014 | 35:13.1 | 0 |
| -1315.773 | 19793.689 | 2.144 | 14.835 | 0.190677 | 7/15/2014 | 35:13.2 | 0 |
| -1315.566 | 19793.68 | 2.144 | 14.832 | 0.1841 | 7/15/2014 | 35:13.3 | 0 |
| -1315.205 | 19793.664 | 2.143 | 14.838 | 0.19739 | 7/15/2014 | 35:13.5 | 0 |
| -1314.996 | 19793.656 | 2.148 | 14.845 | 0.177821 | 7/15/2014 | 35:13.6 | 0 |
| -1314.738 | 19793.646 | 2.146 | 14.855 | 0.191702 | 7/15/2014 | 35:13.7 | 0 |
| -1314.633 | 19793.64 | 2.147 | 14.86 | 0.189926 | 7/15/2014 | 35:13.7 | 0 |
| -1314.425 | 19793.631 | 2.147 | 14.874 | 0.179941 | 7/15/2014 | 35:13.8 | 0 |
| -1314.213 | 19793.623 | 2.15 | 14.891 | 0.196755 | 7/15/2014 | 35:13.9 | 0 |
| -1314.002 | 19793.612 | 2.155 | 14.909 | 0.188236 | 7/15/2014 | 35:14.0 | 0 |
| -1313.791 | 19793.604 | 2.158 | 14.928 | 0.18322 | 7/15/2014 | 35:14.1 | 0 |

| | | | | | | | |
|---|---|---|---|---|---|---|---|
| -1313.58 | 19793.596 | 2.162 | 14.947 | 0.182039 | 7/15/2014 | 35:14.2 | 0 |
| -1313.37 | 19793.588 | 2.165 | 14.966 | 0.196426 | 7/15/2014 | 35:14.3 | 0 |
| -1313.004 | 19793.571 | 2.17 | 14.992 | 0.196274 | 7/15/2014 | 35:14.5 | 0 |
| -1312.798 | 19793.563 | 2.175 | 15.009 | 0.206295 | 7/15/2014 | 35:14.6 | 0 |
| -1312.539 | 19793.553 | 2.175 | 15.024 | 0.189469 | 7/15/2014 | 35:14.7 | 0 |
| -1312.432 | 19793.548 | 2.177 | 15.032 | 0.197923 | 7/15/2014 | 35:14.7 | 0 |
| -1312.224 | 19793.539 | 2.175 | 15.044 | 0.197921 | 7/15/2014 | 35:14.8 | 0 |
| -1312.015 | 19793.529 | 2.175 | 15.055 | 0.201522 | 7/15/2014 | 35:14.9 | 0 |
| -1311.807 | 19793.521 | 2.175 | 15.063 | 0.210661 | 7/15/2014 | 35:15.0 | 0 |
| -1311.599 | 19793.514 | 2.177 | 15.07 | 0.200211 | 7/15/2014 | 35:15.1 | 0 |
| -1311.391 | 19793.504 | 2.175 | 15.075 | 0.194604 | 7/15/2014 | 35:15.2 | 0 |
| -1311.187 | 19793.496 | 2.177 | 15.078 | 0.189507 | 7/15/2014 | 35:15.3 | 0 |
| -1310.829 | 19793.482 | 2.181 | 15.08 | 0.195406 | 7/15/2014 | 35:15.5 | 0 |
| -1310.624 | 19793.474 | 2.187 | 15.08 | 0.189996 | 7/15/2014 | 35:15.6 | 0 |
| -1310.37 | 19793.463 | 2.189 | 15.079 | 0.182634 | 7/15/2014 | 35:15.7 | 0 |
| -1310.267 | 19793.459 | 2.193 | 15.079 | 0.177694 | 7/15/2014 | 35:15.7 | 0 |
| -1310.06 | 19793.449 | 2.198 | 15.077 | 0.17548 | 7/15/2014 | 35:15.8 | 0 |
| -1309.853 | 19793.441 | 2.2 | 15.076 | 0.176022 | 7/15/2014 | 35:15.9 | 0 |
| -1309.645 | 19793.434 | 2.203 | 15.073 | 0.162381 | 7/15/2014 | 35:16.0 | 0 |
| -1309.436 | 19793.426 | 2.203 | 15.07 | 0.161778 | 7/15/2014 | 35:16.1 | 0 |
| -1309.227 | 19793.418 | 2.203 | 15.067 | 0.147007 | 7/15/2014 | 35:16.2 | 0 |
| -1309.018 | 19793.409 | 2.203 | 15.063 | 0.15968 | 7/15/2014 | 35:16.3 | 0 |
| -1308.654 | 19793.396 | 2.199 | 15.056 | 0.148992 | 7/15/2014 | 35:16.5 | 0 |
| -1308.444 | 19793.387 | 2.199 | 15.051 | 0.153972 | 7/15/2014 | 35:16.6 | 0 |
| -1308.183 | 19793.377 | 2.194 | 15.046 | 0.139391 | 7/15/2014 | 35:16.7 | 0 |
| -1308.077 | 19793.373 | 2.194 | 15.043 | 0.148547 | 7/15/2014 | 35:16.7 | 0 |
| -1307.865 | 19793.365 | 2.197 | 15.038 | 0.150172 | 7/15/2014 | 35:16.8 | 0 |
| -1307.653 | 19793.357 | 2.197 | 15.033 | 0.166292 | 7/15/2014 | 35:16.9 | 0 |
| -1307.438 | 19793.349 | 2.2 | 15.029 | 0.152056 | 7/15/2014 | 35:17.0 | 0 |
| -1307.226 | 19793.342 | 2.2 | 15.025 | 0.155345 | 7/15/2014 | 35:17.1 | 0 |
| -1307.012 | 19793.334 | 2.206 | 15.023 | 0.179302 | 7/15/2014 | 35:17.2 | 0 |
| -1306.802 | 19793.326 | 2.206 | 15.023 | 0.151955 | 7/15/2014 | 35:17.3 | 0 |
| -1306.434 | 19793.313 | 2.212 | 15.025 | 0.178456 | 7/15/2014 | 35:17.5 | 0 |
| -1306.224 | 19793.305 | 2.215 | 15.028 | 0.167451 | 7/15/2014 | 35:17.6 | 0 |
| -1305.967 | 19793.295 | 2.214 | 15.032 | 0.165775 | 7/15/2014 | 35:17.7 | 0 |
| -1305.859 | 19793.29 | 2.216 | 15.033 | 0.167835 | 7/15/2014 | 35:17.7 | 0 |
| -1305.651 | 19793.283 | 2.218 | 15.039 | 0.165215 | 7/15/2014 | 35:17.8 | 0 |
| -1305.443 | 19793.276 | 2.221 | 15.047 | 0.170158 | 7/15/2014 | 35:17.9 | 0 |
| -1305.235 | 19793.27 | 2.221 | 15.055 | 0.163635 | 7/15/2014 | 35:18.0 | 0 |
| -1305.027 | 19793.262 | 2.226 | 15.065 | 0.166122 | 7/15/2014 | 35:18.1 | 0 |
| -1304.822 | 19793.254 | 2.226 | 15.076 | 0.165376 | 7/15/2014 | 35:18.2 | 0 |
| -1304.619 | 19793.248 | 2.228 | 15.087 | 0.164355 | 7/15/2014 | 35:18.3 | 0 |
| -1304.268 | 19793.234 | 2.233 | 15.105 | 0.165418 | 7/15/2014 | 35:18.5 | 0 |
| -1304.067 | 19793.229 | 2.236 | 15.116 | 0.154882 | 7/15/2014 | 35:18.6 | 0 |
| -1303.818 | 19793.221 | 2.236 | 15.127 | 0.150356 | 7/15/2014 | 35:18.7 | 0 |
| -1303.616 | 19793.215 | 2.241 | 15.136 | 0.148166 | 7/15/2014 | 35:18.8 | 0 |
| -1303.414 | 19793.207 | 2.244 | 15.145 | 0.142259 | 7/15/2014 | 35:18.9 | 0 |

| | | | | | | | |
|---|---|---|---|---|---|---|---|
| -1303.21 | 19793.201 | 2.249 | 15.152 | 0.137786 | 7/15/2014 | 35:19.0 | 0 |
| -1303.11 | 19793.199 | 2.25 | 15.156 | 0.136802 | 7/15/2014 | 35:19.0 | 0 |
| -1302.907 | 19793.191 | 2.25 | 15.161 | 0.128186 | 7/15/2014 | 35:19.1 | 0 |
| -1302.705 | 19793.185 | 2.25 | 15.164 | 0.13745 | 7/15/2014 | 35:19.2 | 0 |
| -1302.504 | 19793.18 | 2.25 | 15.167 | 0.125801 | 7/15/2014 | 35:19.3 | 0 |
| -1302.148 | 19793.168 | 2.256 | 15.172 | 0.130924 | 7/15/2014 | 35:19.5 | 0 |
| -1301.943 | 19793.162 | 2.261 | 15.175 | 0.152856 | 7/15/2014 | 35:19.6 | 0 |
| -1301.686 | 19793.153 | 2.26 | 15.18 | 0.152725 | 7/15/2014 | 35:19.7 | 0 |
| -1301.583 | 19793.151 | 2.26 | 15.181 | 0.170547 | 7/15/2014 | 35:19.7 | 0 |
| -1301.375 | 19793.145 | 2.262 | 15.187 | 0.157561 | 7/15/2014 | 35:19.8 | 0 |
| -1301.165 | 19793.138 | 2.262 | 15.194 | 0.165984 | 7/15/2014 | 35:19.9 | 0 |
| -1300.954 | 19793.133 | 2.265 | 15.203 | 0.175655 | 7/15/2014 | 35:20.0 | 0 |
| -1300.74 | 19793.125 | 2.27 | 15.212 | 0.180374 | 7/15/2014 | 35:20.1 | 0 |
| -1300.528 | 19793.12 | 2.27 | 15.222 | 0.176981 | 7/15/2014 | 35:20.2 | 0 |
| -1300.315 | 19793.113 | 2.272 | 15.231 | 0.155989 | 7/15/2014 | 35:20.3 | 0 |
| -1300.05 | 19793.106 | 2.272 | 15.238 | 0.162181 | 7/15/2014 | 35:20.4 | 0 |
| -1299.838 | 19793.101 | 2.272 | 15.243 | 0.161321 | 7/15/2014 | 35:20.5 | 0 |
| -1299.626 | 19793.094 | 2.27 | 15.245 | 0.151555 | 7/15/2014 | 35:20.6 | 0 |
| -1299.362 | 19793.088 | 2.275 | 15.244 | 0.154714 | 7/15/2014 | 35:20.7 | 0 |
| -1299.148 | 19793.081 | 2.279 | 15.237 | 0.157558 | 7/15/2014 | 35:20.8 | 0 |
| -1298.937 | 19793.077 | 2.275 | 15.227 | 0.161239 | 7/15/2014 | 35:20.9 | 0 |
| -1298.725 | 19793.07 | 2.275 | 15.211 | 0.16676 | 7/15/2014 | 35:21.0 | 0 |
| -1298.514 | 19793.066 | 2.275 | 15.192 | 0.168616 | 7/15/2014 | 35:21.1 | 0 |
| -1298.305 | 19793.061 | 2.275 | 15.17 | 0.171093 | 7/15/2014 | 35:21.2 | 0 |
| -1298.099 | 19793.056 | 2.278 | 15.145 | 0.183334 | 7/15/2014 | 35:21.3 | 0 |
| -1297.745 | 19793.047 | 2.281 | 15.108 | 0.190438 | 7/15/2014 | 35:21.5 | 0 |
| -1297.544 | 19793.042 | 2.283 | 15.084 | 0.197975 | 7/15/2014 | 35:21.6 | 0 |
| -1297.3 | 19793.037 | 2.282 | 15.062 | 0.202213 | 7/15/2014 | 35:21.7 | 0 |
| -1297.103 | 19793.032 | 2.289 | 15.043 | 0.200267 | 7/15/2014 | 35:21.8 | 0 |
| -1296.907 | 19793.027 | 2.294 | 15.027 | 0.201838 | 7/15/2014 | 35:21.9 | 0 |
| -1296.712 | 19793.023 | 2.299 | 15.016 | 0.208883 | 7/15/2014 | 35:22.0 | 0 |
| -1296.518 | 19793.019 | 2.303 | 15.01 | 0.224354 | 7/15/2014 | 35:22.1 | 0 |
| -1296.324 | 19793.016 | 2.308 | 15.008 | 0.217039 | 7/15/2014 | 35:22.2 | 0 |
| -1296.134 | 19793.01 | 2.31 | 15.01 | 0.230201 | 7/15/2014 | 35:22.3 | 0 |
| -1295.945 | 19793.007 | 2.315 | 15.018 | 0.232687 | 7/15/2014 | 35:22.4 | 0 |
| -1295.616 | 19793 | 2.32 | 15.039 | 0.243307 | 7/15/2014 | 35:22.5 | 0 |
| -1295.379 | 19792.995 | 2.322 | 15.054 | 0.262973 | 7/15/2014 | 35:22.6 | 0 |
| -1295.286 | 19792.992 | 2.322 | 15.062 | 0.268653 | 7/15/2014 | 35:22.7 | 0 |
| -1295.095 | 19792.989 | 2.324 | 15.082 | 0.262535 | 7/15/2014 | 35:22.8 | 0 |
| -1294.904 | 19792.984 | 2.327 | 15.104 | 0.26544 | 7/15/2014 | 35:22.9 | 0 |
| -1294.711 | 19792.981 | 2.33 | 15.127 | 0.272577 | 7/15/2014 | 35:23.0 | 0 |
| -1294.516 | 19792.977 | 2.333 | 15.15 | 0.279445 | 7/15/2014 | 35:23.1 | 0 |
| -1294.321 | 19792.974 | 2.336 | 15.173 | 0.286674 | 7/15/2014 | 35:23.2 | 0 |
| -1294.125 | 19792.969 | 2.342 | 15.193 | 0.290781 | 7/15/2014 | 35:23.3 | 0 |
| -1293.929 | 19792.966 | 2.349 | 15.212 | 0.300914 | 7/15/2014 | 35:23.4 | 0 |
| -1293.687 | 19792.961 | 2.352 | 15.227 | 0.310788 | 7/15/2014 | 35:23.5 | 0 |
| -1293.49 | 19792.957 | 2.356 | 15.239 | 0.304108 | 7/15/2014 | 35:23.6 | 0 |

| | | | | | | | |
|---|---|---|---|---|---|---|---|
| -1293.246 | 19792.953 | 2.359 | 15.247 | 0.304012 | 7/15/2014 | 35:23.7 | 0 |
| -1293.145 | 19792.951 | 2.361 | 15.252 | 0.307237 | 7/15/2014 | 35:23.7 | 0 |
| -1292.851 | 19792.945 | 2.365 | 15.252 | 0.313461 | 7/15/2014 | 35:23.9 | 0 |
| -1292.652 | 19792.941 | 2.369 | 15.249 | 0.347218 | 7/15/2014 | 35:24.0 | 0 |
| -1292.454 | 19792.938 | 2.371 | 15.244 | 0.339793 | 7/15/2014 | 35:24.1 | 0 |
| -1292.257 | 19792.935 | 2.371 | 15.236 | 0.34844 | 7/15/2014 | 35:24.2 | 0 |
| -1292.062 | 19792.932 | 2.371 | 15.226 | 0.354531 | 7/15/2014 | 35:24.3 | 0 |
| -1291.868 | 19792.928 | 2.375 | 15.215 | 0.382697 | 7/15/2014 | 35:24.4 | 0 |
| -1291.631 | 19792.924 | 2.377 | 15.203 | 0.394794 | 7/15/2014 | 35:24.5 | 0 |
| -1291.441 | 19792.921 | 2.382 | 15.19 | 0.382648 | 7/15/2014 | 35:24.6 | 0 |
| -1291.209 | 19792.917 | 2.382 | 15.178 | 0.382794 | 7/15/2014 | 35:24.7 | 0 |
| -1291.021 | 19792.913 | 2.389 | 15.166 | 0.354115 | 7/15/2014 | 35:24.8 | 0 |
| -1290.834 | 19792.911 | 2.392 | 15.154 | 0.380166 | 7/15/2014 | 35:24.9 | 0 |
| -1290.646 | 19792.907 | 2.4 | 15.144 | 0.349376 | 7/15/2014 | 35:25.0 | 0 |
| -1290.459 | 19792.904 | 2.405 | 15.135 | 0.365375 | 7/15/2014 | 35:25.1 | 0 |
| -1290.273 | 19792.901 | 2.411 | 15.127 | 0.329355 | 7/15/2014 | 35:25.2 | 0 |
| -1290.089 | 19792.898 | 2.411 | 15.121 | 0.358349 | 7/15/2014 | 35:25.3 | 0 |
| -1289.905 | 19792.896 | 2.417 | 15.117 | 0.349309 | 7/15/2014 | 35:25.4 | 0 |
| -1289.587 | 19792.891 | 2.422 | 15.115 | 0.371929 | 7/15/2014 | 35:25.5 | 0 |
| -1289.36 | 19792.886 | 2.428 | 15.115 | 0.384382 | 7/15/2014 | 35:25.6 | 0 |
| -1289.268 | 19792.885 | 2.427 | 15.114 | 0.388379 | 7/15/2014 | 35:25.7 | 0 |
| -1289.085 | 19792.883 | 2.432 | 15.116 | 0.38115 | 7/15/2014 | 35:25.8 | 0 |
| -1288.902 | 19792.879 | 2.432 | 15.12 | 0.381229 | 7/15/2014 | 35:25.9 | 0 |
| -1288.718 | 19792.876 | 2.436 | 15.124 | 0.405292 | 7/15/2014 | 35:26.0 | 0 |
| -1288.534 | 19792.873 | 2.436 | 15.129 | 0.382154 | 7/15/2014 | 35:26.1 | 0 |
| -1288.35 | 19792.87 | 2.442 | 15.133 | 0.380108 | 7/15/2014 | 35:26.2 | 0 |
| -1288.168 | 19792.867 | 2.446 | 15.138 | 0.392156 | 7/15/2014 | 35:26.3 | 0 |
| -1287.986 | 19792.864 | 2.453 | 15.142 | 0.368946 | 7/15/2014 | 35:26.4 | 0 |
| -1287.673 | 19792.857 | 2.461 | 15.146 | 0.378924 | 7/15/2014 | 35:26.5 | 0 |
| -1287.45 | 19792.855 | 2.467 | 15.147 | 0.364504 | 7/15/2014 | 35:26.6 | 0 |
| -1287.36 | 19792.854 | 2.469 | 15.148 | 0.359827 | 7/15/2014 | 35:26.7 | 0 |
| -1287.179 | 19792.85 | 2.477 | 15.148 | 0.35042 | 7/15/2014 | 35:26.8 | 0 |
| -1287.001 | 19792.846 | 2.48 | 15.145 | 0.354408 | 7/15/2014 | 35:26.9 | 0 |
| -1286.82 | 19792.844 | 2.486 | 15.141 | 0.346273 | 7/15/2014 | 35:27.0 | 0 |
| -1286.641 | 19792.841 | 2.489 | 15.136 | 0.356184 | 7/15/2014 | 35:27.1 | 0 |
| -1286.375 | 19792.836 | 2.493 | 15.126 | 0.314399 | 7/15/2014 | 35:27.2 | 0 |
| -1286.197 | 19792.832 | 2.497 | 15.119 | 0.331053 | 7/15/2014 | 35:27.3 | 0 |
| -1286.022 | 19792.828 | 2.499 | 15.112 | 0.323571 | 7/15/2014 | 35:27.4 | 0 |
| -1285.805 | 19792.824 | 2.502 | 15.106 | 0.317746 | 7/15/2014 | 35:27.5 | 0 |
| -1285.588 | 19792.82 | 2.504 | 15.1 | 0.29287 | 7/15/2014 | 35:27.6 | 0 |
| -1285.413 | 19792.816 | 2.508 | 15.096 | 0.296367 | 7/15/2014 | 35:27.7 | 0 |
| -1285.24 | 19792.813 | 2.51 | 15.094 | 0.303763 | 7/15/2014 | 35:27.8 | 0 |
| -1285.063 | 19792.81 | 2.518 | 15.094 | 0.262397 | 7/15/2014 | 35:27.9 | 0 |
| -1284.887 | 19792.807 | 2.521 | 15.096 | 0.288445 | 7/15/2014 | 35:28.0 | 0 |
| -1284.71 | 19792.803 | 2.527 | 15.101 | 0.304243 | 7/15/2014 | 35:28.1 | 0 |
| -1284.534 | 19792.799 | 2.535 | 15.108 | 0.271578 | 7/15/2014 | 35:28.2 | 0 |
| -1284.358 | 19792.795 | 2.539 | 15.117 | 0.255267 | 7/15/2014 | 35:28.3 | 0 |

| | | | | | | | |
|---|---|---|---|---|---|---|---|
| -1284.184 | 19792.791 | 2.545 | 15.127 | 0.263993 | 7/15/2014 | 35:28.4 | 0 |
| -1283.967 | 19792.787 | 2.549 | 15.139 | 0.260496 | 7/15/2014 | 35:28.5 | 0 |
| -1283.745 | 19792.783 | 2.555 | 15.152 | 0.263765 | 7/15/2014 | 35:28.6 | 0 |
| -1283.659 | 19792.781 | 2.556 | 15.159 | 0.263496 | 7/15/2014 | 35:28.7 | 0 |
| -1283.48 | 19792.776 | 2.562 | 15.172 | 0.253263 | 7/15/2014 | 35:28.8 | 0 |
| -1283.302 | 19792.772 | 2.562 | 15.185 | 0.232085 | 7/15/2014 | 35:28.9 | 0 |
| -1283.12 | 19792.768 | 2.57 | 15.197 | 0.226934 | 7/15/2014 | 35:29.0 | 0 |
| -1282.938 | 19792.764 | 2.573 | 15.208 | 0.232844 | 7/15/2014 | 35:29.1 | 0 |
| -1282.756 | 19792.76 | 2.577 | 15.218 | 0.214011 | 7/15/2014 | 35:29.2 | 0 |
| -1282.575 | 19792.755 | 2.58 | 15.226 | 0.21358 | 7/15/2014 | 35:29.3 | 0 |
| -1282.394 | 19792.75 | 2.585 | 15.232 | 0.214581 | 7/15/2014 | 35:29.4 | 0 |
| -1282.078 | 19792.744 | 2.595 | 15.24 | 0.196148 | 7/15/2014 | 35:29.5 | 0 |
| -1281.853 | 19792.737 | 2.599 | 15.244 | 0.199648 | 7/15/2014 | 35:29.6 | 0 |
| -1281.762 | 19792.735 | 2.603 | 15.247 | 0.203053 | 7/15/2014 | 35:29.7 | 0 |
| -1281.579 | 19792.729 | 2.609 | 15.25 | 0.202224 | 7/15/2014 | 35:29.8 | 0 |
| -1281.396 | 19792.725 | 2.613 | 15.254 | 0.205432 | 7/15/2014 | 35:29.9 | 0 |
| -1281.211 | 19792.721 | 2.617 | 15.257 | 0.194428 | 7/15/2014 | 35:30.0 | 0 |
| -1281.028 | 19792.714 | 2.617 | 15.261 | 0.19809 | 7/15/2014 | 35:30.1 | 0 |
| -1280.843 | 19792.709 | 2.623 | 15.264 | 0.204433 | 7/15/2014 | 35:30.2 | 0 |
| -1280.663 | 19792.705 | 2.625 | 15.267 | 0.1853 | 7/15/2014 | 35:30.3 | 0 |
| -1280.482 | 19792.699 | 2.625 | 15.269 | 0.200396 | 7/15/2014 | 35:30.4 | 0 |
| -1280.169 | 19792.689 | 2.626 | 15.271 | 0.183505 | 7/15/2014 | 35:30.5 | 0 |
| -1279.947 | 19792.683 | 2.627 | 15.271 | 0.170536 | 7/15/2014 | 35:30.6 | 0 |
| -1279.857 | 19792.68 | 2.627 | 15.272 | 0.16068 | 7/15/2014 | 35:30.7 | 0 |
| -1279.675 | 19792.674 | 2.63 | 15.27 | 0.185154 | 7/15/2014 | 35:30.8 | 0 |
| -1279.495 | 19792.668 | 2.633 | 15.268 | 0.172084 | 7/15/2014 | 35:30.9 | 0 |
| -1279.313 | 19792.662 | 2.638 | 15.263 | 0.171951 | 7/15/2014 | 35:31.0 | 0 |
| -1279.134 | 19792.656 | 2.638 | 15.258 | 0.182794 | 7/15/2014 | 35:31.1 | 0 |
| -1278.954 | 19792.65 | 2.643 | 15.251 | 0.174224 | 7/15/2014 | 35:31.2 | 0 |
| -1278.775 | 19792.644 | 2.649 | 15.243 | 0.211094 | 7/15/2014 | 35:31.3 | 0 |
| -1278.594 | 19792.637 | 2.658 | 15.234 | 0.226747 | 7/15/2014 | 35:31.4 | 0 |
| -1278.284 | 19792.625 | 2.664 | 15.217 | 0.242013 | 7/15/2014 | 35:31.5 | 0 |
| -1278.061 | 19792.615 | 2.67 | 15.206 | 0.239572 | 7/15/2014 | 35:31.6 | 0 |
| -1277.972 | 19792.613 | 2.672 | 15.2 | 0.253957 | 7/15/2014 | 35:31.7 | 0 |
| -1277.791 | 19792.606 | 2.678 | 15.188 | 0.255584 | 7/15/2014 | 35:31.8 | 0 |
| -1277.611 | 19792.599 | 2.683 | 15.174 | 0.275883 | 7/15/2014 | 35:31.9 | 0 |
| -1277.429 | 19792.592 | 2.689 | 15.16 | 0.276351 | 7/15/2014 | 35:32.0 | 0 |
| -1277.247 | 19792.584 | 2.693 | 15.145 | 0.261901 | 7/15/2014 | 35:32.1 | 0 |
| -1277.063 | 19792.576 | 2.7 | 15.13 | 0.243854 | 7/15/2014 | 35:32.2 | 0 |
| -1276.884 | 19792.568 | 2.702 | 15.115 | 0.274383 | 7/15/2014 | 35:32.3 | 0 |
| -1276.701 | 19792.561 | 2.708 | 15.099 | 0.255915 | 7/15/2014 | 35:32.4 | 0 |
| -1276.39 | 19792.546 | 2.71 | 15.077 | 0.266941 | 7/15/2014 | 35:32.5 | 0 |
| -1276.164 | 19792.536 | 2.714 | 15.063 | 0.277504 | 7/15/2014 | 35:32.6 | 0 |
| -1276.074 | 19792.532 | 2.714 | 15.056 | 0.260548 | 7/15/2014 | 35:32.7 | 0 |
| -1275.89 | 19792.523 | 2.722 | 15.043 | 0.251676 | 7/15/2014 | 35:32.8 | 0 |
| -1275.708 | 19792.514 | 2.722 | 15.032 | 0.246832 | 7/15/2014 | 35:32.9 | 0 |
| -1275.521 | 19792.506 | 2.725 | 15.023 | 0.238887 | 7/15/2014 | 35:33.0 | 0 |

| | | | | | | | |
|---|---|---|---|---|---|---|---|
| -1275.34 | 19792.496 | 2.722 | 15.015 | 0.224127 | 7/15/2014 | 35:33.1 | 0 |
| -1275.153 | 19792.488 | 2.725 | 15.009 | 0.228463 | 7/15/2014 | 35:33.2 | 0 |
| -1274.971 | 19792.479 | 2.725 | 15.006 | 0.212575 | 7/15/2014 | 35:33.3 | 0 |
| -1274.789 | 19792.469 | 2.73 | 15.005 | 0.206622 | 7/15/2014 | 35:33.4 | 0 |
| -1274.468 | 19792.452 | 2.74 | 15.009 | 0.198901 | 7/15/2014 | 35:33.5 | 0 |
| -1274.239 | 19792.439 | 2.745 | 15.013 | 0.21217 | 7/15/2014 | 35:33.6 | 0 |
| -1274.149 | 19792.434 | 2.744 | 15.014 | 0.208728 | 7/15/2014 | 35:33.7 | 0 |
| -1273.963 | 19792.425 | 2.749 | 15.02 | 0.213859 | 7/15/2014 | 35:33.8 | 0 |
| -1273.778 | 19792.414 | 2.752 | 15.028 | 0.226261 | 7/15/2014 | 35:33.9 | 0 |
| -1273.589 | 19792.403 | 2.762 | 15.037 | 0.203142 | 7/15/2014 | 35:34.0 | 0 |
| -1273.402 | 19792.393 | 2.766 | 15.046 | 0.237749 | 7/15/2014 | 35:34.1 | 0 |
| -1273.215 | 19792.381 | 2.773 | 15.056 | 0.235012 | 7/15/2014 | 35:34.2 | 0 |
| -1273.029 | 19792.371 | 2.777 | 15.064 | 0.225658 | 7/15/2014 | 35:34.3 | 0 |
| -1272.843 | 19792.36 | 2.78 | 15.072 | 0.244957 | 7/15/2014 | 35:34.4 | 0 |
| -1272.612 | 19792.345 | 2.782 | 15.08 | 0.248804 | 7/15/2014 | 35:34.5 | 0 |
| -1272.427 | 19792.334 | 2.789 | 15.086 | 0.240122 | 7/15/2014 | 35:34.6 | 0 |
| -1272.195 | 19792.319 | 2.793 | 15.09 | 0.234578 | 7/15/2014 | 35:34.7 | 0 |
| -1272.005 | 19792.306 | 2.799 | 15.094 | 0.248022 | 7/15/2014 | 35:34.8 | 0 |
| -1271.819 | 19792.295 | 2.799 | 15.096 | 0.235421 | 7/15/2014 | 35:34.9 | 0 |
| -1271.628 | 19792.281 | 2.807 | 15.097 | 0.240603 | 7/15/2014 | 35:35.0 | 0 |
| -1271.439 | 19792.27 | 2.807 | 15.097 | 0.255912 | 7/15/2014 | 35:35.1 | 0 |
| -1271.249 | 19792.258 | 2.813 | 15.097 | 0.234929 | 7/15/2014 | 35:35.2 | 0 |
| -1271.062 | 19792.243 | 2.816 | 15.096 | 0.250539 | 7/15/2014 | 35:35.3 | 0 |
| -1270.873 | 19792.23 | 2.821 | 15.095 | 0.26877 | 7/15/2014 | 35:35.4 | 0 |
| -1270.64 | 19792.214 | 2.821 | 15.094 | 0.262784 | 7/15/2014 | 35:35.5 | 0 |
| -1270.452 | 19792.2 | 2.827 | 15.093 | 0.278695 | 7/15/2014 | 35:35.6 | 0 |
| -1270.222 | 19792.184 | 2.824 | 15.092 | 0.270064 | 7/15/2014 | 35:35.7 | 0 |
| -1270.031 | 19792.169 | 2.827 | 15.091 | 0.27518 | 7/15/2014 | 35:35.8 | 0 |
| -1269.844 | 19792.156 | 2.824 | 15.091 | 0.246418 | 7/15/2014 | 35:35.9 | 0 |
| -1269.654 | 19792.143 | 2.83 | 15.09 | 0.28488 | 7/15/2014 | 35:36.0 | 0 |
| -1269.467 | 19792.127 | 2.83 | 15.09 | 0.224787 | 7/15/2014 | 35:36.1 | 0 |
| -1269.278 | 19792.112 | 2.833 | 15.091 | 0.261793 | 7/15/2014 | 35:36.2 | 0 |
| -1269.091 | 19792.098 | 2.83 | 15.092 | 0.254833 | 7/15/2014 | 35:36.3 | 0 |
| -1268.905 | 19792.083 | 2.833 | 15.093 | 0.246435 | 7/15/2014 | 35:36.4 | 0 |
| -1268.585 | 19792.057 | 2.829 | 15.098 | 0.256402 | 7/15/2014 | 35:36.5 | 0 |
| -1268.354 | 19792.037 | 2.834 | 15.101 | 0.26585 | 7/15/2014 | 35:36.6 | 0 |
| -1268.261 | 19792.031 | 2.836 | 15.103 | 0.285836 | 7/15/2014 | 35:36.7 | 0 |
| -1268.075 | 19792.016 | 2.842 | 15.107 | 0.265775 | 7/15/2014 | 35:36.8 | 0 |
| -1267.89 | 19792 | 2.842 | 15.113 | 0.285379 | 7/15/2014 | 35:36.9 | 0 |
| -1267.703 | 19791.984 | 2.844 | 15.12 | 0.292573 | 7/15/2014 | 35:37.0 | 0 |
| -1267.518 | 19791.967 | 2.846 | 15.127 | 0.30279 | 7/15/2014 | 35:37.1 | 0 |
| -1267.33 | 19791.951 | 2.853 | 15.135 | 0.314157 | 7/15/2014 | 35:37.2 | 0 |
| -1267.146 | 19791.934 | 2.853 | 15.143 | 0.319468 | 7/15/2014 | 35:37.3 | 0 |
| -1266.96 | 19791.918 | 2.859 | 15.152 | 0.320169 | 7/15/2014 | 35:37.4 | 0 |
| -1266.638 | 19791.889 | 2.864 | 15.164 | 0.299071 | 7/15/2014 | 35:37.5 | 0 |
| -1266.407 | 19791.867 | 2.865 | 15.172 | 0.311782 | 7/15/2014 | 35:37.6 | 0 |
| -1266.316 | 19791.859 | 2.864 | 15.177 | 0.32985 | 7/15/2014 | 35:37.7 | 0 |

| | | | | | | | |
|---|---|---|---|---|---|---|---|
| -1266.128 | 19791.842 | 2.867 | 15.184 | 0.268704 | 7/15/2014 | 35:37.8 | 0 |
| -1265.941 | 19791.822 | 2.867 | 15.191 | 0.293233 | 7/15/2014 | 35:37.9 | 0 |
| -1265.752 | 19791.806 | 2.869 | 15.198 | 0.279039 | 7/15/2014 | 35:38.0 | 0 |
| -1265.565 | 19791.786 | 2.866 | 15.204 | 0.279367 | 7/15/2014 | 35:38.1 | 0 |
| -1265.375 | 19791.769 | 2.867 | 15.209 | 0.26068 | 7/15/2014 | 35:38.2 | 0 |
| -1265.188 | 19791.75 | 2.865 | 15.214 | 0.260059 | 7/15/2014 | 35:38.3 | 0 |
| -1264.999 | 19791.73 | 2.867 | 15.218 | 0.263465 | 7/15/2014 | 35:38.4 | 0 |
| -1264.767 | 19791.707 | 2.867 | 15.223 | 0.246742 | 7/15/2014 | 35:38.5 | 0 |
| -1264.579 | 19791.688 | 2.867 | 15.227 | 0.285664 | 7/15/2014 | 35:38.6 | 0 |
| -1264.344 | 19791.662 | 2.863 | 15.231 | 0.244243 | 7/15/2014 | 35:38.7 | 0 |
| -1264.152 | 19791.644 | 2.863 | 15.235 | 0.25482 | 7/15/2014 | 35:38.8 | 0 |
| -1263.962 | 19791.623 | 2.861 | 15.239 | 0.235062 | 7/15/2014 | 35:38.9 | 0 |
| -1263.768 | 19791.603 | 2.865 | 15.243 | 0.258952 | 7/15/2014 | 35:39.0 | 0 |
| -1263.574 | 19791.581 | 2.865 | 15.247 | 0.248846 | 7/15/2014 | 35:39.1 | 0 |
| -1263.381 | 19791.56 | 2.865 | 15.251 | 0.255594 | 7/15/2014 | 35:39.2 | 0 |
| -1263.187 | 19791.538 | 2.865 | 15.254 | 0.266913 | 7/15/2014 | 35:39.3 | 0 |
| -1262.993 | 19791.517 | 2.865 | 15.257 | 0.26138 | 7/15/2014 | 35:39.4 | 0 |
| -1262.752 | 19791.49 | 2.865 | 15.259 | 0.281152 | 7/15/2014 | 35:39.5 | 0 |
| -1262.556 | 19791.468 | 2.867 | 15.26 | 0.275621 | 7/15/2014 | 35:39.6 | 0 |
| -1262.309 | 19791.438 | 2.865 | 15.26 | 0.264221 | 7/15/2014 | 35:39.7 | 0 |
| -1262.211 | 19791.428 | 2.865 | 15.261 | 0.25201 | 7/15/2014 | 35:39.7 | 0 |
| -1262.009 | 19791.404 | 2.865 | 15.26 | 0.265735 | 7/15/2014 | 35:39.8 | 0 |
| -1261.807 | 19791.381 | 2.863 | 15.259 | 0.230252 | 7/15/2014 | 35:39.9 | 0 |
| -1261.603 | 19791.356 | 2.865 | 15.258 | 0.249051 | 7/15/2014 | 35:40.0 | 0 |
| -1261.398 | 19791.331 | 2.865 | 15.257 | 0.228777 | 7/15/2014 | 35:40.1 | 0 |
| -1261.191 | 19791.308 | 2.867 | 15.256 | 0.215911 | 7/15/2014 | 35:40.2 | 0 |
| -1260.987 | 19791.281 | 2.865 | 15.257 | 0.206845 | 7/15/2014 | 35:40.3 | 0 |
| -1260.627 | 19791.236 | 2.868 | 15.261 | 0.240861 | 7/15/2014 | 35:40.5 | 0 |
| -1260.421 | 19791.211 | 2.872 | 15.265 | 0.209978 | 7/15/2014 | 35:40.6 | 0 |
| -1260.164 | 19791.179 | 2.87 | 15.272 | 0.231083 | 7/15/2014 | 35:40.7 | 0 |
| -1260.058 | 19791.166 | 2.873 | 15.274 | 0.200711 | 7/15/2014 | 35:40.7 | 0 |
| -1259.849 | 19791.139 | 2.876 | 15.284 | 0.226146 | 7/15/2014 | 35:40.8 | 0 |
| -1259.64 | 19791.112 | 2.876 | 15.296 | 0.219193 | 7/15/2014 | 35:40.9 | 0 |
| -1259.431 | 19791.085 | 2.876 | 15.311 | 0.245164 | 7/15/2014 | 35:41.0 | 0 |
| -1259.221 | 19791.059 | 2.878 | 15.327 | 0.238049 | 7/15/2014 | 35:41.1 | 0 |
| -1259.012 | 19791.03 | 2.878 | 15.345 | 0.238503 | 7/15/2014 | 35:41.2 | 0 |
| -1258.805 | 19791.002 | 2.878 | 15.365 | 0.264561 | 7/15/2014 | 35:41.3 | 0 |
| -1258.443 | 19790.955 | 2.876 | 15.395 | 0.249015 | 7/15/2014 | 35:41.5 | 0 |
| -1258.235 | 19790.927 | 2.88 | 15.416 | 0.250902 | 7/15/2014 | 35:41.6 | 0 |
| -1257.978 | 19790.892 | 2.876 | 15.436 | 0.270141 | 7/15/2014 | 35:41.7 | 0 |
| -1257.873 | 19790.878 | 2.878 | 15.447 | 0.263563 | 7/15/2014 | 35:41.7 | 0 |
| -1257.665 | 19790.85 | 2.876 | 15.467 | 0.240003 | 7/15/2014 | 35:41.8 | 0 |
| -1257.455 | 19790.82 | 2.876 | 15.487 | 0.234588 | 7/15/2014 | 35:41.9 | 0 |
| -1257.245 | 19790.79 | 2.874 | 15.507 | 0.247425 | 7/15/2014 | 35:42.0 | 0 |
| -1257.034 | 19790.762 | 2.874 | 15.526 | 0.257547 | 7/15/2014 | 35:42.1 | 0 |
| -1256.824 | 19790.732 | 2.874 | 15.544 | 0.261047 | 7/15/2014 | 35:42.2 | 0 |
| -1256.614 | 19790.702 | 2.874 | 15.562 | 0.239272 | 7/15/2014 | 35:42.3 | 0 |

| | | | | | | | |
|---|---|---|---|---|---|---|---|
| -1256.25 | 19790.65 | 2.871 | 15.588 | 0.225112 | 7/15/2014 | 35:42.5 | 0 |
| -1256.04 | 19790.621 | 2.872 | 15.605 | 0.246283 | 7/15/2014 | 35:42.6 | 0 |
| -1255.78 | 19790.583 | 2.871 | 15.622 | 0.231032 | 7/15/2014 | 35:42.7 | 0 |
| -1255.673 | 19790.567 | 2.872 | 15.63 | 0.219284 | 7/15/2014 | 35:42.7 | 0 |
| -1255.463 | 19790.537 | 2.872 | 15.647 | 0.209974 | 7/15/2014 | 35:42.8 | 0 |
| -1255.252 | 19790.506 | 2.868 | 15.664 | 0.201376 | 7/15/2014 | 35:42.9 | 0 |
| -1255.04 | 19790.475 | 2.868 | 15.681 | 0.213466 | 7/15/2014 | 35:43.0 | 0 |
| -1254.828 | 19790.443 | 2.868 | 15.699 | 0.184554 | 7/15/2014 | 35:43.1 | 0 |
| -1254.617 | 19790.413 | 2.865 | 15.717 | 0.192026 | 7/15/2014 | 35:43.2 | 0 |
| -1254.407 | 19790.383 | 2.862 | 15.736 | 0.168194 | 7/15/2014 | 35:43.3 | 0 |
| -1254.044 | 19790.328 | 2.859 | 15.763 | 0.179967 | 7/15/2014 | 35:43.5 | 0 |
| -1253.834 | 19790.297 | 2.858 | 15.781 | 0.161696 | 7/15/2014 | 35:43.6 | 0 |
| -1253.578 | 19790.258 | 2.854 | 15.798 | 0.168448 | 7/15/2014 | 35:43.7 | 0 |
| -1253.471 | 19790.242 | 2.856 | 15.807 | 0.15594 | 7/15/2014 | 35:43.7 | 0 |
| -1253.263 | 19790.211 | 2.85 | 15.822 | 0.163581 | 7/15/2014 | 35:43.8 | 0 |
| -1253.054 | 19790.18 | 2.847 | 15.836 | 0.147068 | 7/15/2014 | 35:43.9 | 0 |
| -1252.845 | 19790.147 | 2.847 | 15.849 | 0.156337 | 7/15/2014 | 35:44.0 | 0 |
| -1252.637 | 19790.115 | 2.847 | 15.86 | 0.150096 | 7/15/2014 | 35:44.1 | 0 |
| -1252.43 | 19790.084 | 2.847 | 15.87 | 0.146956 | 7/15/2014 | 35:44.2 | 0 |
| -1252.223 | 19790.053 | 2.842 | 15.879 | 0.142284 | 7/15/2014 | 35:44.3 | 0 |
| -1251.863 | 19789.997 | 2.833 | 15.892 | 0.161906 | 7/15/2014 | 35:44.5 | 0 |
| -1251.657 | 19789.965 | 2.828 | 15.901 | 0.162526 | 7/15/2014 | 35:44.6 | 0 |
| -1251.399 | 19789.926 | 2.821 | 15.91 | 0.159017 | 7/15/2014 | 35:44.7 | 0 |
| -1251.295 | 19789.909 | 2.819 | 15.914 | 0.15843 | 7/15/2014 | 35:44.7 | 0 |
| -1251.086 | 19789.875 | 2.815 | 15.924 | 0.161292 | 7/15/2014 | 35:44.8 | 0 |
| -1250.875 | 19789.844 | 2.808 | 15.934 | 0.137725 | 7/15/2014 | 35:44.9 | 0 |
| -1250.663 | 19789.811 | 2.8 | 15.945 | 0.171466 | 7/15/2014 | 35:45.0 | 0 |
| -1250.451 | 19789.777 | 2.795 | 15.957 | 0.125222 | 7/15/2014 | 35:45.1 | 0 |
| -1250.238 | 19789.744 | 2.79 | 15.968 | 0.1413 | 7/15/2014 | 35:45.2 | 0 |
| -1250.025 | 19789.71 | 2.785 | 15.98 | 0.145776 | 7/15/2014 | 35:45.3 | 0 |
| -1249.76 | 19789.669 | 2.785 | 15.991 | 0.139284 | 7/15/2014 | 35:45.4 | 0 |
| -1249.546 | 19789.635 | 2.783 | 16.003 | 0.145563 | 7/15/2014 | 35:45.5 | 0 |
| -1249.332 | 19789.602 | 2.776 | 16.013 | 0.150207 | 7/15/2014 | 35:45.6 | 0 |
| -1249.063 | 19789.559 | 2.77 | 16.024 | 0.138842 | 7/15/2014 | 35:45.7 | 0 |
| -1248.846 | 19789.523 | 2.768 | 16.033 | 0.16012 | 7/15/2014 | 35:45.8 | 0 |
| -1248.629 | 19789.49 | 2.763 | 16.041 | 0.138123 | 7/15/2014 | 35:45.9 | 0 |
| -1248.41 | 19789.455 | 2.761 | 16.049 | 0.125261 | 7/15/2014 | 35:46.0 | 0 |
| -1248.193 | 19789.421 | 2.755 | 16.055 | 0.14667 | 7/15/2014 | 35:46.1 | 0 |
| -1247.977 | 19789.387 | 2.744 | 16.06 | 0.135827 | 7/15/2014 | 35:46.2 | 0 |
| -1247.76 | 19789.351 | 2.735 | 16.064 | 0.142696 | 7/15/2014 | 35:46.3 | 0 |
| -1247.493 | 19789.309 | 2.727 | 16.066 | 0.153519 | 7/15/2014 | 35:46.4 | 0 |
| -1247.277 | 19789.273 | 2.72 | 16.066 | 0.139657 | 7/15/2014 | 35:46.5 | 0 |
| -1247.061 | 19789.239 | 2.709 | 16.065 | 0.14676 | 7/15/2014 | 35:46.6 | 0 |
| -1246.793 | 19789.196 | 2.701 | 16.063 | 0.150422 | 7/15/2014 | 35:46.7 | 0 |
| -1246.576 | 19789.162 | 2.693 | 16.06 | 0.150874 | 7/15/2014 | 35:46.8 | 0 |
| -1246.359 | 19789.127 | 2.687 | 16.055 | 0.140497 | 7/15/2014 | 35:46.9 | 0 |
| -1246.141 | 19789.091 | 2.685 | 16.05 | 0.162178 | 7/15/2014 | 35:47.0 | 0 |

| | | | | | | | |
|---|---|---|---|---|---|---|---|
| -1245.924 | 19789.057 | 2.68 | 16.044 | 0.152006 | 7/15/2014 | 35:47.1 | 0 |
| -1245.706 | 19789.021 | 2.676 | 16.038 | 0.165192 | 7/15/2014 | 35:47.2 | 0 |
| -1245.489 | 19788.986 | 2.673 | 16.033 | 0.159292 | 7/15/2014 | 35:47.3 | 0 |
| -1245.218 | 19788.942 | 2.67 | 16.029 | 0.183525 | 7/15/2014 | 35:47.4 | 0 |
| -1245.001 | 19788.907 | 2.664 | 16.027 | 0.190563 | 7/15/2014 | 35:47.5 | 0 |
| -1244.783 | 19788.872 | 2.659 | 16.027 | 0.189011 | 7/15/2014 | 35:47.6 | 0 |
| -1244.511 | 19788.829 | 2.653 | 16.03 | 0.175571 | 7/15/2014 | 35:47.7 | 0 |
| -1244.293 | 19788.794 | 2.645 | 16.036 | 0.162874 | 7/15/2014 | 35:47.8 | 0 |
| -1244.071 | 19788.759 | 2.638 | 16.047 | 0.18009 | 7/15/2014 | 35:47.9 | 0 |
| -1243.851 | 19788.723 | 2.634 | 16.063 | 0.196371 | 7/15/2014 | 35:48.0 | 0 |
| -1243.631 | 19788.687 | 2.626 | 16.083 | 0.183134 | 7/15/2014 | 35:48.1 | 0 |
| -1243.411 | 19788.651 | 2.619 | 16.108 | 0.174595 | 7/15/2014 | 35:48.2 | 0 |
| -1243.19 | 19788.615 | 2.613 | 16.138 | 0.203725 | 7/15/2014 | 35:48.3 | 0 |
| -1242.917 | 19788.572 | 2.603 | 16.172 | 0.18531 | 7/15/2014 | 35:48.4 | 0 |
| -1242.697 | 19788.537 | 2.597 | 16.208 | 0.235452 | 7/15/2014 | 35:48.5 | 0 |
| -1242.477 | 19788.502 | 2.588 | 16.247 | 0.246499 | 7/15/2014 | 35:48.6 | 0 |
| -1242.202 | 19788.457 | 2.585 | 16.287 | 0.219438 | 7/15/2014 | 35:48.7 | 0 |
| -1241.982 | 19788.422 | 2.58 | 16.326 | 0.240809 | 7/15/2014 | 35:48.8 | 0 |
| -1241.76 | 19788.387 | 2.576 | 16.364 | 0.250106 | 7/15/2014 | 35:48.9 | 0 |
| -1241.538 | 19788.351 | 2.571 | 16.398 | 0.254721 | 7/15/2014 | 35:49.0 | 0 |
| -1241.316 | 19788.315 | 2.569 | 16.429 | 0.287367 | 7/15/2014 | 35:49.1 | 0 |
| -1241.093 | 19788.279 | 2.569 | 16.455 | 0.250674 | 7/15/2014 | 35:49.2 | 0 |
| -1240.873 | 19788.244 | 2.565 | 16.475 | 0.244063 | 7/15/2014 | 35:49.3 | 0 |
| -1240.597 | 19788.199 | 2.563 | 16.491 | 0.247742 | 7/15/2014 | 35:49.4 | 0 |
| -1240.377 | 19788.165 | 2.56 | 16.502 | 0.252176 | 7/15/2014 | 35:49.5 | 0 |
| -1240.156 | 19788.129 | 2.555 | 16.508 | 0.235764 | 7/15/2014 | 35:49.6 | 0 |
| -1239.88 | 19788.085 | 2.548 | 16.511 | 0.20822 | 7/15/2014 | 35:49.7 | 0 |
| -1239.658 | 19788.05 | 2.543 | 16.512 | 0.195543 | 7/15/2014 | 35:49.8 | 0 |
| -1239.434 | 19788.014 | 2.535 | 16.511 | 0.194971 | 7/15/2014 | 35:49.9 | 0 |
| -1239.211 | 19787.978 | 2.531 | 16.51 | 0.179531 | 7/15/2014 | 35:50.0 | 0 |
| -1238.986 | 19787.941 | 2.531 | 16.51 | 0.190374 | 7/15/2014 | 35:50.1 | 0 |
| -1238.764 | 19787.907 | 2.528 | 16.512 | 0.190012 | 7/15/2014 | 35:50.2 | 0 |
| -1238.542 | 19787.871 | 2.523 | 16.515 | 0.195964 | 7/15/2014 | 35:50.3 | 0 |
| -1238.265 | 19787.827 | 2.518 | 16.52 | 0.195766 | 7/15/2014 | 35:50.4 | 0 |
| -1238.045 | 19787.792 | 2.515 | 16.526 | 0.188349 | 7/15/2014 | 35:50.5 | 0 |
| -1237.823 | 19787.757 | 2.511 | 16.533 | 0.185967 | 7/15/2014 | 35:50.6 | 0 |
| -1237.548 | 19787.713 | 2.508 | 16.541 | 0.193812 | 7/15/2014 | 35:50.7 | 0 |
| -1237.326 | 19787.678 | 2.502 | 16.547 | 0.18082 | 7/15/2014 | 35:50.8 | 0 |
| -1237.104 | 19787.643 | 2.491 | 16.552 | 0.165173 | 7/15/2014 | 35:50.9 | 0 |
| -1236.879 | 19787.607 | 2.485 | 16.554 | 0.155433 | 7/15/2014 | 35:51.0 | 0 |
| -1236.656 | 19787.572 | 2.482 | 16.554 | 0.161721 | 7/15/2014 | 35:51.1 | 0 |
| -1236.435 | 19787.537 | 2.478 | 16.551 | 0.165374 | 7/15/2014 | 35:51.2 | 0 |
| -1236.213 | 19787.502 | 2.471 | 16.547 | 0.172491 | 7/15/2014 | 35:51.3 | 0 |
| -1235.938 | 19787.459 | 2.464 | 16.541 | 0.17394 | 7/15/2014 | 35:51.4 | 0 |
| -1235.716 | 19787.424 | 2.459 | 16.534 | 0.192323 | 7/15/2014 | 35:51.5 | 0 |
| -1235.494 | 19787.39 | 2.457 | 16.528 | 0.197489 | 7/15/2014 | 35:51.6 | 0 |
| -1235.214 | 19787.346 | 2.454 | 16.523 | 0.201276 | 7/15/2014 | 35:51.7 | 0 |

| | | | | | | | |
|---|---|---|---|---|---|---|---|
| -1234.987 | 19787.311 | 2.452 | 16.519 | 0.19747 | 7/15/2014 | 35:51.8 | 0 |
| -1234.761 | 19787.275 | 2.444 | 16.517 | 0.210448 | 7/15/2014 | 35:51.9 | 0 |
| -1234.533 | 19787.24 | 2.44 | 16.517 | 0.183673 | 7/15/2014 | 35:52.0 | 0 |
| -1234.306 | 19787.205 | 2.437 | 16.518 | 0.193923 | 7/15/2014 | 35:52.1 | 0 |
| -1234.194 | 19787.188 | 2.436 | 16.519 | 0.18778 | 7/15/2014 | 35:52.2 | 0 |
| -1233.963 | 19787.153 | 2.432 | 16.523 | 0.186126 | 7/15/2014 | 35:52.3 | 0 |
| -1233.563 | 19787.092 | 2.423 | 16.534 | 0.194749 | 7/15/2014 | 35:52.4 | 0 |
| -1233.333 | 19787.057 | 2.421 | 16.542 | 0.193872 | 7/15/2014 | 35:52.5 | 0 |
| -1233.106 | 19787.023 | 2.416 | 16.551 | 0.187308 | 7/15/2014 | 35:52.6 | 0 |
| -1232.824 | 19786.98 | 2.41 | 16.561 | 0.196731 | 7/15/2014 | 35:52.7 | 0 |
| -1232.708 | 19786.963 | 2.405 | 16.566 | 0.189164 | 7/15/2014 | 35:52.8 | 0 |
| -1232.366 | 19786.913 | 2.406 | 16.583 | 0.202611 | 7/15/2014 | 35:52.9 | 0 |
| -1232.14 | 19786.88 | 2.403 | 16.593 | 0.214718 | 7/15/2014 | 35:53.0 | 0 |
| -1231.913 | 19786.846 | 2.4 | 16.604 | 0.206757 | 7/15/2014 | 35:53.1 | 0 |
| -1231.686 | 19786.813 | 2.398 | 16.614 | 0.208903 | 7/15/2014 | 35:53.2 | 0 |
| -1231.463 | 19786.781 | 2.394 | 16.624 | 0.215272 | 7/15/2014 | 35:53.3 | 0 |
| -1231.186 | 19786.74 | 2.392 | 16.634 | 0.212688 | 7/15/2014 | 35:53.4 | 0 |
| -1230.964 | 19786.709 | 2.392 | 16.642 | 0.211625 | 7/15/2014 | 35:53.5 | 0 |
| -1230.742 | 19786.677 | 2.392 | 16.65 | 0.206642 | 7/15/2014 | 35:53.6 | 0 |
| -1230.468 | 19786.639 | 2.389 | 16.657 | 0.185501 | 7/15/2014 | 35:53.7 | 0 |
| -1230.246 | 19786.607 | 2.384 | 16.663 | 0.182716 | 7/15/2014 | 35:53.8 | 0 |
| -1230.021 | 19786.576 | 2.381 | 16.667 | 0.179425 | 7/15/2014 | 35:53.9 | 0 |
| -1229.799 | 19786.545 | 2.378 | 16.671 | 0.182421 | 7/15/2014 | 35:54.0 | 0 |
| -1229.575 | 19786.514 | 2.374 | 16.673 | 0.178295 | 7/15/2014 | 35:54.1 | 0 |
| -1229.353 | 19786.483 | 2.368 | 16.674 | 0.177056 | 7/15/2014 | 35:54.2 | 0 |
| -1229.13 | 19786.453 | 2.365 | 16.675 | 0.17887 | 7/15/2014 | 35:54.3 | 0 |
| -1228.855 | 19786.415 | 2.362 | 16.676 | 0.176665 | 7/15/2014 | 35:54.4 | 0 |
| -1228.636 | 19786.387 | 2.357 | 16.676 | 0.192608 | 7/15/2014 | 35:54.5 | 0 |
| -1228.416 | 19786.356 | 2.357 | 16.676 | 0.189902 | 7/15/2014 | 35:54.6 | 0 |
| -1228.144 | 19786.32 | 2.354 | 16.677 | 0.16951 | 7/15/2014 | 35:54.7 | 0 |
| -1227.925 | 19786.291 | 2.351 | 16.678 | 0.177068 | 7/15/2014 | 35:54.8 | 0 |
| -1227.708 | 19786.263 | 2.352 | 16.679 | 0.178337 | 7/15/2014 | 35:54.9 | 0 |
| -1227.49 | 19786.234 | 2.355 | 16.68 | 0.169621 | 7/15/2014 | 35:55.0 | 0 |
| -1227.275 | 19786.206 | 2.353 | 16.681 | 0.172642 | 7/15/2014 | 35:55.1 | 0 |
| -1227.061 | 19786.179 | 2.35 | 16.682 | 0.165566 | 7/15/2014 | 35:55.2 | 0 |
| -1226.848 | 19786.151 | 2.35 | 16.683 | 0.164577 | 7/15/2014 | 35:55.3 | 0 |
| -1226.482 | 19786.104 | 2.349 | 16.684 | 0.175233 | 7/15/2014 | 35:55.5 | 0 |
| -1226.271 | 19786.078 | 2.351 | 16.685 | 0.166731 | 7/15/2014 | 35:55.6 | 0 |
| -1226.012 | 19786.046 | 2.349 | 16.686 | 0.158476 | 7/15/2014 | 35:55.7 | 0 |
| -1225.906 | 19786.032 | 2.347 | 16.686 | 0.157744 | 7/15/2014 | 35:55.7 | 0 |
| -1225.697 | 19786.007 | 2.345 | 16.688 | 0.159117 | 7/15/2014 | 35:55.8 | 0 |
| -1225.486 | 19785.98 | 2.348 | 16.691 | 0.154094 | 7/15/2014 | 35:55.9 | 0 |
| -1225.275 | 19785.955 | 2.351 | 16.695 | 0.150756 | 7/15/2014 | 35:56.0 | 0 |
| -1225.065 | 19785.929 | 2.351 | 16.701 | 0.149889 | 7/15/2014 | 35:56.1 | 0 |
| -1224.854 | 19785.903 | 2.35 | 16.708 | 0.145141 | 7/15/2014 | 35:56.2 | 0 |
| -1224.645 | 19785.879 | 2.353 | 16.716 | 0.142607 | 7/15/2014 | 35:56.3 | 0 |
| -1224.282 | 19785.835 | 2.349 | 16.73 | 0.137906 | 7/15/2014 | 35:56.5 | 0 |

| | | | | | | | |
|---|---|---|---|---|---|---|---|
| -1224.074 | 19785.812 | 2.354 | 16.739 | 0.136925 | 7/15/2014 | 35:56.6 | 0 |
| -1223.819 | 19785.782 | 2.351 | 16.747 | 0.133249 | 7/15/2014 | 35:56.7 | 0 |
| -1223.712 | 19785.77 | 2.35 | 16.752 | 0.132112 | 7/15/2014 | 35:56.7 | 0 |
| -1223.507 | 19785.745 | 2.348 | 16.759 | 0.128422 | 7/15/2014 | 35:56.8 | 0 |
| -1223.299 | 19785.722 | 2.348 | 16.763 | 0.127141 | 7/15/2014 | 35:56.9 | 0 |
| -1223.095 | 19785.699 | 2.349 | 16.766 | 0.124448 | 7/15/2014 | 35:57.0 | 0 |
| -1222.891 | 19785.675 | 2.346 | 16.766 | 0.120931 | 7/15/2014 | 35:57.1 | 0 |
| -1222.688 | 19785.652 | 2.346 | 16.763 | 0.116919 | 7/15/2014 | 35:57.2 | 0 |
| -1222.485 | 19785.63 | 2.346 | 16.759 | 0.112239 | 7/15/2014 | 35:57.3 | 0 |
| -1222.137 | 19785.593 | 2.338 | 16.751 | 0.107584 | 7/15/2014 | 35:57.5 | 0 |
| -1221.938 | 19785.57 | 2.338 | 16.745 | 0.110251 | 7/15/2014 | 35:57.6 | 0 |
| -1221.689 | 19785.544 | 2.335 | 16.74 | 0.102617 | 7/15/2014 | 35:57.7 | 0 |
| -1221.488 | 19785.523 | 2.335 | 16.736 | 0.100579 | 7/15/2014 | 35:57.8 | 0 |
| -1221.389 | 19785.512 | 2.332 | 16.734 | 0.0964904 | 7/15/2014 | 35:57.8 | 0 |
| -1221.187 | 19785.491 | 2.332 | 16.733 | 0.0945821 | 7/15/2014 | 35:57.9 | 0 |
| -1220.982 | 19785.469 | 2.334 | 16.734 | 0.0960616 | 7/15/2014 | 35:58.0 | 0 |
| -1220.779 | 19785.449 | 2.334 | 16.739 | 0.0960144 | 7/15/2014 | 35:58.1 | 0 |
| -1220.575 | 19785.427 | 2.334 | 16.749 | 0.100377 | 7/15/2014 | 35:58.2 | 0 |
| -1220.37 | 19785.406 | 2.334 | 16.762 | 0.110702 | 7/15/2014 | 35:58.3 | 0 |
| -1220.008 | 19785.369 | 2.332 | 16.793 | 0.116911 | 7/15/2014 | 35:58.5 | 0 |
| -1219.8 | 19785.35 | 2.332 | 16.816 | 0.134088 | 7/15/2014 | 35:58.6 | 0 |
| -1219.538 | 19785.323 | 2.328 | 16.842 | 0.130451 | 7/15/2014 | 35:58.7 | 0 |
| -1219.433 | 19785.313 | 2.328 | 16.854 | 0.130732 | 7/15/2014 | 35:58.7 | 0 |
| -1219.219 | 19785.293 | 2.323 | 16.884 | 0.12896 | 7/15/2014 | 35:58.8 | 0 |
| -1219.004 | 19785.271 | 2.319 | 16.913 | 0.134995 | 7/15/2014 | 35:58.9 | 0 |
| -1218.786 | 19785.25 | 2.319 | 16.942 | 0.135089 | 7/15/2014 | 35:59.0 | 0 |
| -1218.569 | 19785.229 | 2.317 | 16.969 | 0.127984 | 7/15/2014 | 35:59.1 | 0 |
| -1218.35 | 19785.208 | 2.315 | 16.993 | 0.131517 | 7/15/2014 | 35:59.2 | 0 |
| -1218.132 | 19785.188 | 2.313 | 17.015 | 0.137408 | 7/15/2014 | 35:59.3 | 0 |
| -1217.86 | 19785.162 | 2.311 | 17.033 | 0.141017 | 7/15/2014 | 35:59.4 | 0 |
| -1217.641 | 19785.143 | 2.311 | 17.048 | 0.132687 | 7/15/2014 | 35:59.5 | 0 |
| -1217.423 | 19785.122 | 2.308 | 17.059 | 0.140616 | 7/15/2014 | 35:59.6 | 0 |
| -1217.15 | 19785.098 | 2.308 | 17.068 | 0.132207 | 7/15/2014 | 35:59.7 | 0 |
| -1216.932 | 19785.078 | 2.304 | 17.074 | 0.139339 | 7/15/2014 | 35:59.8 | 0 |
| -1216.713 | 19785.058 | 2.302 | 17.079 | 0.132864 | 7/15/2014 | 35:59.9 | 0 |
| -1216.495 | 19785.038 | 2.298 | 17.082 | 0.120464 | 7/15/2014 | 36:00.0 | 0 |
| -1216.278 | 19785.02 | 2.293 | 17.084 | 0.135366 | 7/15/2014 | 36:00.1 | 0 |
| -1216.062 | 19785 | 2.288 | 17.085 | 0.145209 | 7/15/2014 | 36:00.2 | 0 |
| -1215.848 | 19784.982 | 2.283 | 17.086 | 0.131704 | 7/15/2014 | 36:00.3 | 0 |
| -1215.479 | 19784.951 | 2.281 | 17.088 | 0.1376 | 7/15/2014 | 36:00.5 | 0 |
| -1215.27 | 19784.934 | 2.279 | 17.089 | 0.139403 | 7/15/2014 | 36:00.6 | 0 |
| -1215.013 | 19784.911 | 2.272 | 17.09 | 0.138505 | 7/15/2014 | 36:00.7 | 0 |
| -1214.906 | 19784.902 | 2.27 | 17.091 | 0.139614 | 7/15/2014 | 36:00.7 | 0 |
| -1214.699 | 19784.885 | 2.27 | 17.093 | 0.132518 | 7/15/2014 | 36:00.8 | 0 |
| -1214.491 | 19784.867 | 2.267 | 17.095 | 0.137773 | 7/15/2014 | 36:00.9 | 0 |
| -1214.283 | 19784.852 | 2.267 | 17.098 | 0.137315 | 7/15/2014 | 36:01.0 | 0 |
| -1214.078 | 19784.834 | 2.264 | 17.101 | 0.13608 | 7/15/2014 | 36:01.1 | 0 |

| | | | | | | | |
|---|---|---|---|---|---|---|---|
| -1213.872 | 19784.818 | 2.261 | 17.104 | 0.133589 | 7/15/2014 | 36:01.2 | 0 |
| -1213.67 | 19784.803 | 2.254 | 17.107 | 0.13143 | 7/15/2014 | 36:01.3 | 0 |
| -1213.318 | 19784.775 | 2.244 | 17.112 | 0.135066 | 7/15/2014 | 36:01.5 | 0 |
| -1213.114 | 19784.76 | 2.244 | 17.116 | 0.129172 | 7/15/2014 | 36:01.6 | 0 |
| -1212.863 | 19784.74 | 2.239 | 17.118 | 0.126207 | 7/15/2014 | 36:01.7 | 0 |
| -1212.76 | 19784.732 | 2.239 | 17.12 | 0.125633 | 7/15/2014 | 36:01.7 | 0 |
| -1212.557 | 19784.717 | 2.231 | 17.122 | 0.125793 | 7/15/2014 | 36:01.8 | 0 |
| -1212.351 | 19784.701 | 2.229 | 17.123 | 0.125844 | 7/15/2014 | 36:01.9 | 0 |
| -1212.147 | 19784.687 | 2.223 | 17.123 | 0.121235 | 7/15/2014 | 36:02.0 | 0 |
| -1211.942 | 19784.672 | 2.219 | 17.121 | 0.120873 | 7/15/2014 | 36:02.1 | 0 |
| -1211.737 | 19784.657 | 2.215 | 17.117 | 0.11577 | 7/15/2014 | 36:02.2 | 0 |
| -1211.532 | 19784.643 | 2.213 | 17.111 | 0.119114 | 7/15/2014 | 36:02.3 | 0 |
| -1211.175 | 19784.617 | 2.207 | 17.094 | 0.114607 | 7/15/2014 | 36:02.5 | 0 |
| -1210.971 | 19784.603 | 2.204 | 17.081 | 0.113402 | 7/15/2014 | 36:02.6 | 0 |
| -1210.719 | 19784.584 | 2.199 | 17.066 | 0.109212 | 7/15/2014 | 36:02.7 | 0 |
| -1210.615 | 19784.577 | 2.197 | 17.059 | 0.108456 | 7/15/2014 | 36:02.7 | 0 |
| -1210.412 | 19784.564 | 2.195 | 17.041 | 0.106111 | 7/15/2014 | 36:02.8 | 0 |
| -1210.208 | 19784.55 | 2.192 | 17.021 | 0.104594 | 7/15/2014 | 36:02.9 | 0 |
| -1210.005 | 19784.535 | 2.189 | 17 | 0.104726 | 7/15/2014 | 36:03.0 | 0 |
| -1209.704 | 19784.516 | 2.181 | 16.97 | 0.102013 | 7/15/2014 | 36:03.2 | 0 |
| -1209.505 | 19784.502 | 2.174 | 16.951 | 0.0990675 | 7/15/2014 | 36:03.3 | 0 |
| -1209.305 | 19784.489 | 2.168 | 16.933 | 0.107569 | 7/15/2014 | 36:03.4 | 0 |
| -1209.058 | 19784.473 | 2.162 | 16.917 | 0.0925285 | 7/15/2014 | 36:03.5 | 0 |
| -1208.861 | 19784.461 | 2.159 | 16.902 | 0.0920024 | 7/15/2014 | 36:03.6 | 0 |
| -1208.615 | 19784.444 | 2.152 | 16.89 | 0.0913989 | 7/15/2014 | 36:03.7 | 0 |
| -1208.417 | 19784.432 | 2.147 | 16.88 | 0.0835077 | 7/15/2014 | 36:03.8 | 0 |
| -1208.219 | 19784.42 | 2.142 | 16.873 | 0.0715573 | 7/15/2014 | 36:03.9 | 0 |
| -1208.018 | 19784.407 | 2.14 | 16.868 | 0.0744634 | 7/15/2014 | 36:04.0 | 0 |
| -1207.82 | 19784.395 | 2.133 | 16.865 | 0.073028 | 7/15/2014 | 36:04.1 | 0 |
| -1207.62 | 19784.382 | 2.133 | 16.864 | 0.0862588 | 7/15/2014 | 36:04.2 | 0 |
| -1207.421 | 19784.371 | 2.128 | 16.865 | 0.0721937 | 7/15/2014 | 36:04.3 | 0 |
| -1207.323 | 19784.365 | 2.126 | 16.865 | 0.0707951 | 7/15/2014 | 36:04.3 | 0 |
| -1206.977 | 19784.343 | 2.119 | 16.87 | 0.100946 | 7/15/2014 | 36:04.5 | 0 |
| -1206.779 | 19784.332 | 2.116 | 16.874 | 0.112056 | 7/15/2014 | 36:04.6 | 0 |
| -1206.534 | 19784.317 | 2.11 | 16.879 | 0.0904785 | 7/15/2014 | 36:04.7 | 0 |
| -1206.336 | 19784.306 | 2.107 | 16.884 | 0.0803419 | 7/15/2014 | 36:04.8 | 0 |
| -1206.139 | 19784.295 | 2.102 | 16.889 | 0.0964583 | 7/15/2014 | 36:04.9 | 0 |
| -1205.94 | 19784.282 | 2.1 | 16.894 | 0.0864646 | 7/15/2014 | 36:05.0 | 0 |
| -1205.743 | 19784.271 | 2.09 | 16.898 | 0.143115 | 7/15/2014 | 36:05.1 | 0 |
| -1205.545 | 19784.261 | 2.088 | 16.901 | 0.129292 | 7/15/2014 | 36:05.2 | 0 |
| -1205.35 | 19784.249 | 2.084 | 16.903 | 0.130374 | 7/15/2014 | 36:05.3 | 0 |
| -1205.155 | 19784.238 | 2.084 | 16.903 | 0.16678 | 7/15/2014 | 36:05.4 | 0 |
| -1204.916 | 19784.224 | 2.082 | 16.902 | 0.178554 | 7/15/2014 | 36:05.5 | 0 |
| -1204.724 | 19784.213 | 2.084 | 16.899 | 0.183603 | 7/15/2014 | 36:05.6 | 0 |
| -1204.487 | 19784.2 | 2.082 | 16.893 | 0.188101 | 7/15/2014 | 36:05.7 | 0 |
| -1204.296 | 19784.189 | 2.085 | 16.886 | 0.208154 | 7/15/2014 | 36:05.8 | 0 |
| -1204.106 | 19784.178 | 2.085 | 16.876 | 0.183771 | 7/15/2014 | 36:05.9 | 0 |

| | | | | | | | |
|---|---|---|---|---|---|---|---|
| -1203.914 | 19784.169 | 2.092 | 16.865 | 0.184802 | 7/15/2014 | 36:06.0 | 0 |
| -1203.723 | 19784.158 | 2.092 | 16.853 | 0.201115 | 7/15/2014 | 36:06.1 | 0 |
| -1203.532 | 19784.147 | 2.095 | 16.84 | 0.162346 | 7/15/2014 | 36:06.2 | 0 |
| -1203.343 | 19784.137 | 2.098 | 16.826 | 0.186783 | 7/15/2014 | 36:06.3 | 0 |
| -1203.155 | 19784.127 | 2.101 | 16.813 | 0.192676 | 7/15/2014 | 36:06.4 | 0 |
| -1202.921 | 19784.114 | 2.101 | 16.799 | 0.204939 | 7/15/2014 | 36:06.5 | 0 |
| -1202.734 | 19784.104 | 2.104 | 16.787 | 0.194377 | 7/15/2014 | 36:06.6 | 0 |
| -1202.501 | 19784.092 | 2.104 | 16.775 | 0.190617 | 7/15/2014 | 36:06.7 | 0 |
| -1202.313 | 19784.082 | 2.106 | 16.765 | 0.210272 | 7/15/2014 | 36:06.8 | 0 |
| -1202.125 | 19784.072 | 2.106 | 16.757 | 0.218996 | 7/15/2014 | 36:06.9 | 0 |
| -1201.935 | 19784.062 | 2.109 | 16.75 | 0.238048 | 7/15/2014 | 36:07.0 | 0 |
| -1201.747 | 19784.052 | 2.106 | 16.744 | 0.230261 | 7/15/2014 | 36:07.1 | 0 |
| -1201.557 | 19784.042 | 2.108 | 16.74 | 0.251135 | 7/15/2014 | 36:07.2 | 0 |
| -1201.369 | 19784.033 | 2.108 | 16.738 | 0.222193 | 7/15/2014 | 36:07.3 | 0 |
| -1201.183 | 19784.021 | 2.111 | 16.738 | 0.251242 | 7/15/2014 | 36:07.4 | 0 |
| -1200.95 | 19784.012 | 2.111 | 16.739 | 0.253689 | 7/15/2014 | 36:07.5 | 0 |
| -1200.765 | 19784 | 2.111 | 16.742 | 0.247519 | 7/15/2014 | 36:07.6 | 0 |
| -1200.533 | 19783.989 | 2.113 | 16.747 | 0.23326 | 7/15/2014 | 36:07.7 | 0 |
| -1200.347 | 19783.979 | 2.115 | 16.754 | 0.241435 | 7/15/2014 | 36:07.8 | 0 |
| -1200.161 | 19783.97 | 2.115 | 16.763 | 0.224319 | 7/15/2014 | 36:07.9 | 0 |
| -1199.972 | 19783.959 | 2.118 | 16.775 | 0.215868 | 7/15/2014 | 36:08.0 | 0 |
| -1199.784 | 19783.951 | 2.118 | 16.788 | 0.235336 | 7/15/2014 | 36:08.1 | 0 |
| -1199.596 | 19783.939 | 2.121 | 16.804 | 0.208584 | 7/15/2014 | 36:08.2 | 0 |
| -1199.41 | 19783.932 | 2.121 | 16.821 | 0.222995 | 7/15/2014 | 36:08.3 | 0 |
| -1199.224 | 19783.921 | 2.123 | 16.841 | 0.21726 | 7/15/2014 | 36:08.4 | 0 |
| -1198.897 | 19783.905 | 2.127 | 16.874 | 0.221946 | 7/15/2014 | 36:08.5 | 0 |
| -1198.805 | 19783.9 | 2.127 | 16.885 | 0.218892 | 7/15/2014 | 36:08.6 | 0 |
| -1198.57 | 19783.889 | 2.125 | 16.909 | 0.220544 | 7/15/2014 | 36:08.7 | 0 |
| -1198.379 | 19783.878 | 2.129 | 16.933 | 0.224038 | 7/15/2014 | 36:08.8 | 0 |
| -1198.188 | 19783.869 | 2.129 | 16.958 | 0.211931 | 7/15/2014 | 36:08.9 | 0 |
| -1197.993 | 19783.858 | 2.129 | 16.983 | 0.230037 | 7/15/2014 | 36:09.0 | 0 |
| -1197.8 | 19783.85 | 2.125 | 17.008 | 0.220082 | 7/15/2014 | 36:09.1 | 0 |
| -1197.604 | 19783.839 | 2.129 | 17.032 | 0.221574 | 7/15/2014 | 36:09.2 | 0 |
| -1197.41 | 19783.83 | 2.129 | 17.055 | 0.231502 | 7/15/2014 | 36:09.3 | 0 |
| -1197.214 | 19783.82 | 2.134 | 17.077 | 0.225635 | 7/15/2014 | 36:09.4 | 0 |
| -1196.972 | 19783.809 | 2.134 | 17.099 | 0.232143 | 7/15/2014 | 36:09.5 | 0 |
| -1196.775 | 19783.798 | 2.14 | 17.119 | 0.232434 | 7/15/2014 | 36:09.6 | 0 |
| -1196.532 | 19783.786 | 2.138 | 17.139 | 0.230623 | 7/15/2014 | 36:09.7 | 0 |
| -1196.333 | 19783.776 | 2.147 | 17.159 | 0.232925 | 7/15/2014 | 36:09.8 | 0 |
| -1196.136 | 19783.768 | 2.149 | 17.177 | 0.238834 | 7/15/2014 | 36:09.9 | 0 |
| -1195.936 | 19783.756 | 2.155 | 17.194 | 0.238575 | 7/15/2014 | 36:10.0 | 0 |
| -1195.738 | 19783.748 | 2.155 | 17.21 | 0.234585 | 7/15/2014 | 36:10.1 | 0 |
| -1195.539 | 19783.736 | 2.16 | 17.223 | 0.235039 | 7/15/2014 | 36:10.2 | 0 |
| -1195.343 | 19783.729 | 2.155 | 17.234 | 0.235897 | 7/15/2014 | 36:10.3 | 0 |
| -1195.147 | 19783.719 | 2.158 | 17.242 | 0.235944 | 7/15/2014 | 36:10.4 | 0 |
| -1194.906 | 19783.706 | 2.153 | 17.247 | 0.238415 | 7/15/2014 | 36:10.5 | 0 |
| -1194.71 | 19783.697 | 2.158 | 17.248 | 0.23706 | 7/15/2014 | 36:10.6 | 0 |

| | | | | | | | |
|---|---|---|---|---|---|---|---|
| -1194.468 | 19783.688 | 2.156 | 17.245 | 0.238739 | 7/15/2014 | 36:10.7 | 0 |
| -1194.274 | 19783.676 | 2.159 | 17.24 | 0.238072 | 7/15/2014 | 36:10.8 | 0 |
| -1194.078 | 19783.668 | 2.159 | 17.232 | 0.239624 | 7/15/2014 | 36:10.9 | 0 |
| -1193.882 | 19783.658 | 2.162 | 17.221 | 0.241675 | 7/15/2014 | 36:11.0 | 0 |
| -1193.688 | 19783.65 | 2.16 | 17.209 | 0.237464 | 7/15/2014 | 36:11.1 | 0 |
| -1193.491 | 19783.641 | 2.164 | 17.196 | 0.240961 | 7/15/2014 | 36:11.2 | 0 |
| -1193.299 | 19783.631 | 2.164 | 17.183 | 0.241655 | 7/15/2014 | 36:11.3 | 0 |
| -1193.106 | 19783.623 | 2.171 | 17.17 | 0.243219 | 7/15/2014 | 36:11.4 | 0 |
| -1192.867 | 19783.613 | 2.171 | 17.158 | 0.243373 | 7/15/2014 | 36:11.5 | 0 |
| -1192.676 | 19783.603 | 2.176 | 17.148 | 0.249133 | 7/15/2014 | 36:11.6 | 0 |
| -1192.442 | 19783.593 | 2.178 | 17.139 | 0.243126 | 7/15/2014 | 36:11.7 | 0 |
| -1192.253 | 19783.585 | 2.182 | 17.132 | 0.236332 | 7/15/2014 | 36:11.8 | 0 |
| -1192.066 | 19783.577 | 2.182 | 17.127 | 0.2539 | 7/15/2014 | 36:11.9 | 0 |
| -1191.876 | 19783.567 | 2.189 | 17.124 | 0.248071 | 7/15/2014 | 36:12.0 | 0 |
| -1191.689 | 19783.56 | 2.191 | 17.123 | 0.252268 | 7/15/2014 | 36:12.1 | 0 |
| -1191.502 | 19783.551 | 2.196 | 17.124 | 0.243452 | 7/15/2014 | 36:12.2 | 0 |
| -1191.317 | 19783.544 | 2.196 | 17.126 | 0.258897 | 7/15/2014 | 36:12.3 | 0 |
| -1191.131 | 19783.534 | 2.203 | 17.129 | 0.252599 | 7/15/2014 | 36:12.4 | 0 |
| -1190.81 | 19783.521 | 2.204 | 17.133 | 0.263432 | 7/15/2014 | 36:12.5 | 0 |
| -1190.578 | 19783.511 | 2.207 | 17.135 | 0.259302 | 7/15/2014 | 36:12.6 | 0 |
| -1190.489 | 19783.507 | 2.206 | 17.136 | 0.251586 | 7/15/2014 | 36:12.7 | 0 |
| -1190.299 | 19783.499 | 2.216 | 17.137 | 0.249538 | 7/15/2014 | 36:12.8 | 0 |
| -1190.111 | 19783.491 | 2.219 | 17.135 | 0.246675 | 7/15/2014 | 36:12.9 | 0 |
| -1189.918 | 19783.482 | 2.228 | 17.131 | 0.258933 | 7/15/2014 | 36:13.0 | 0 |
| -1189.729 | 19783.476 | 2.231 | 17.125 | 0.232796 | 7/15/2014 | 36:13.1 | 0 |
| -1189.534 | 19783.465 | 2.24 | 17.116 | 0.241475 | 7/15/2014 | 36:13.2 | 0 |
| -1189.342 | 19783.457 | 2.24 | 17.106 | 0.237124 | 7/15/2014 | 36:13.3 | 0 |
| -1189.146 | 19783.449 | 2.25 | 17.094 | 0.223722 | 7/15/2014 | 36:13.4 | 0 |
| -1188.906 | 19783.439 | 2.25 | 17.08 | 0.238445 | 7/15/2014 | 36:13.5 | 0 |
| -1188.708 | 19783.432 | 2.256 | 17.065 | 0.228428 | 7/15/2014 | 36:13.6 | 0 |
| -1188.461 | 19783.42 | 2.256 | 17.049 | 0.206736 | 7/15/2014 | 36:13.7 | 0 |
| -1188.36 | 19783.416 | 2.258 | 17.041 | 0.201411 | 7/15/2014 | 36:13.7 | 0 |
| -1188.16 | 19783.408 | 2.258 | 17.025 | 0.22876 | 7/15/2014 | 36:13.8 | 0 |
| -1187.958 | 19783.398 | 2.26 | 17.009 | 0.200334 | 7/15/2014 | 36:13.9 | 0 |
| -1187.755 | 19783.391 | 2.26 | 16.994 | 0.189419 | 7/15/2014 | 36:14.0 | 0 |
| -1187.552 | 19783.381 | 2.26 | 16.979 | 0.214215 | 7/15/2014 | 36:14.1 | 0 |
| -1187.35 | 19783.373 | 2.26 | 16.966 | 0.180612 | 7/15/2014 | 36:14.2 | 0 |
| -1187.149 | 19783.365 | 2.262 | 16.953 | 0.187393 | 7/15/2014 | 36:14.3 | 0 |
| -1186.796 | 19783.348 | 2.263 | 16.937 | 0.183589 | 7/15/2014 | 36:14.5 | 0 |
| -1186.593 | 19783.34 | 2.267 | 16.926 | 0.157457 | 7/15/2014 | 36:14.6 | 0 |
| -1186.34 | 19783.328 | 2.265 | 16.915 | 0.143999 | 7/15/2014 | 36:14.7 | 0 |
| -1186.238 | 19783.324 | 2.266 | 16.91 | 0.142968 | 7/15/2014 | 36:14.7 | 0 |
| -1186.032 | 19783.314 | 2.266 | 16.897 | 0.127508 | 7/15/2014 | 36:14.8 | 0 |
| -1185.824 | 19783.305 | 2.268 | 16.884 | 0.126998 | 7/15/2014 | 36:14.9 | 0 |
| -1185.615 | 19783.297 | 2.27 | 16.868 | 0.118878 | 7/15/2014 | 36:15.0 | 0 |
| -1185.405 | 19783.285 | 2.27 | 16.851 | 0.0933969 | 7/15/2014 | 36:15.1 | 0 |
| -1185.196 | 19783.277 | 2.27 | 16.832 | 0.110224 | 7/15/2014 | 36:15.2 | 0 |

| | | | | | | | |
|---|---|---|---|---|---|---|---|
| -1184.986 | 19783.268 | 2.27 | 16.813 | 0.104961 | 7/15/2014 | 36:15.3 | 0 |
| -1184.623 | 19783.25 | 2.269 | 16.793 | 0.115923 | 7/15/2014 | 36:15.5 | 0 |
| -1184.413 | 19783.242 | 2.271 | 16.783 | 0.106863 | 7/15/2014 | 36:15.6 | 0 |
| -1184.155 | 19783.229 | 2.269 | 16.779 | 0.110954 | 7/15/2014 | 36:15.7 | 0 |
| -1184.048 | 19783.225 | 2.27 | 16.774 | 0.110268 | 7/15/2014 | 36:15.7 | 0 |
| -1183.838 | 19783.215 | 2.268 | 16.779 | 0.116761 | 7/15/2014 | 36:15.8 | 0 |
| -1183.63 | 19783.205 | 2.268 | 16.792 | 0.111505 | 7/15/2014 | 36:15.9 | 0 |
| -1183.421 | 19783.195 | 2.268 | 16.812 | 0.12113 | 7/15/2014 | 36:16.0 | 0 |
| -1183.214 | 19783.187 | 2.264 | 16.84 | 0.120443 | 7/15/2014 | 36:16.1 | 0 |
| -1183.009 | 19783.176 | 2.262 | 16.874 | 0.108939 | 7/15/2014 | 36:16.2 | 0 |
| -1182.804 | 19783.168 | 2.262 | 16.912 | 0.112681 | 7/15/2014 | 36:16.3 | 0 |
| -1182.454 | 19783.15 | 2.261 | 16.979 | 0.109157 | 7/15/2014 | 36:16.5 | 0 |
| -1182.253 | 19783.143 | 2.263 | 17.023 | 0.119204 | 7/15/2014 | 36:16.6 | 0 |
| -1182.006 | 19783.129 | 2.263 | 17.065 | 0.103375 | 7/15/2014 | 36:16.7 | 0 |
| -1181.807 | 19783.121 | 2.265 | 17.105 | 0.0914375 | 7/15/2014 | 36:16.8 | 0 |
| -1181.609 | 19783.11 | 2.263 | 17.14 | 0.0887357 | 7/15/2014 | 36:16.9 | 0 |
| -1181.408 | 19783.102 | 2.267 | 17.168 | 0.0896951 | 7/15/2014 | 36:17.0 | 0 |
| -1181.209 | 19783.093 | 2.267 | 17.187 | 0.0849966 | 7/15/2014 | 36:17.1 | 0 |
| -1181.01 | 19783.082 | 2.267 | 17.198 | 0.0826038 | 7/15/2014 | 36:17.2 | 0 |
| -1180.814 | 19783.074 | 2.265 | 17.199 | 0.0824675 | 7/15/2014 | 36:17.3 | 0 |
| -1180.616 | 19783.064 | 2.265 | 17.191 | 0.0841229 | 7/15/2014 | 36:17.4 | 0 |
| -1180.372 | 19783.053 | 2.261 | 17.175 | 0.0868399 | 7/15/2014 | 36:17.5 | 0 |
| -1180.176 | 19783.044 | 2.259 | 17.152 | 0.0938159 | 7/15/2014 | 36:17.6 | 0 |
| -1179.931 | 19783.031 | 2.255 | 17.125 | 0.102347 | 7/15/2014 | 36:17.7 | 0 |
| -1179.731 | 19783.021 | 2.257 | 17.094 | 0.101145 | 7/15/2014 | 36:17.8 | 0 |
| -1179.531 | 19783.013 | 2.254 | 17.061 | 0.11238 | 7/15/2014 | 36:17.9 | 0 |
| -1179.329 | 19783.002 | 2.255 | 17.028 | 0.12175 | 7/15/2014 | 36:18.0 | 0 |
| -1179.129 | 19782.994 | 2.254 | 16.995 | 0.137838 | 7/15/2014 | 36:18.1 | 0 |
| -1179.03 | 19782.988 | 2.253 | 16.978 | 0.12955 | 7/15/2014 | 36:18.1 | 0 |
| -1178.828 | 19782.979 | 2.256 | 16.947 | 0.136279 | 7/15/2014 | 36:18.2 | 0 |
| -1178.626 | 19782.969 | 2.26 | 16.918 | 0.143352 | 7/15/2014 | 36:18.3 | 0 |
| -1178.274 | 19782.952 | 2.263 | 16.878 | 0.141288 | 7/15/2014 | 36:18.5 | 0 |
| -1178.072 | 19782.941 | 2.269 | 16.853 | 0.146773 | 7/15/2014 | 36:18.6 | 0 |
| -1177.82 | 19782.93 | 2.269 | 16.829 | 0.144905 | 7/15/2014 | 36:18.7 | 0 |
| -1177.717 | 19782.924 | 2.273 | 16.817 | 0.143032 | 7/15/2014 | 36:18.7 | 0 |
| -1177.514 | 19782.915 | 2.275 | 16.794 | 0.138758 | 7/15/2014 | 36:18.8 | 0 |
| -1177.308 | 19782.904 | 2.277 | 16.772 | 0.139809 | 7/15/2014 | 36:18.9 | 0 |
| -1177.102 | 19782.894 | 2.28 | 16.75 | 0.137412 | 7/15/2014 | 36:19.0 | 0 |
| -1176.898 | 19782.885 | 2.28 | 16.73 | 0.133568 | 7/15/2014 | 36:19.1 | 0 |
| -1176.693 | 19782.874 | 2.283 | 16.711 | 0.137681 | 7/15/2014 | 36:19.2 | 0 |
| -1176.491 | 19782.863 | 2.283 | 16.693 | 0.138552 | 7/15/2014 | 36:19.3 | 0 |
| -1176.138 | 19782.846 | 2.284 | 16.673 | 0.148706 | 7/15/2014 | 36:19.5 | 0 |
| -1175.935 | 19782.836 | 2.289 | 16.661 | 0.142641 | 7/15/2014 | 36:19.6 | 0 |
| -1175.685 | 19782.822 | 2.289 | 16.653 | 0.150991 | 7/15/2014 | 36:19.7 | 0 |
| -1175.582 | 19782.816 | 2.291 | 16.648 | 0.148571 | 7/15/2014 | 36:19.7 | 0 |
| -1175.379 | 19782.809 | 2.293 | 16.645 | 0.149781 | 7/15/2014 | 36:19.8 | 0 |
| -1175.176 | 19782.797 | 2.295 | 16.647 | 0.149769 | 7/15/2014 | 36:19.9 | 0 |

| | | | | | | | |
|---|---|---|---|---|---|---|---|
| -1174.972 | 19782.785 | 2.297 | 16.652 | 0.139888 | 7/15/2014 | 36:20.0 | 0 |
| -1174.77 | 19782.777 | 2.297 | 16.662 | 0.136113 | 7/15/2014 | 36:20.1 | 0 |
| -1174.566 | 19782.766 | 2.303 | 16.675 | 0.129971 | 7/15/2014 | 36:20.2 | 0 |
| -1174.364 | 19782.755 | 2.303 | 16.691 | 0.127968 | 7/15/2014 | 36:20.3 | 0 |
| -1174.013 | 19782.735 | 2.304 | 16.721 | 0.118408 | 7/15/2014 | 36:20.5 | 0 |
| -1173.81 | 19782.727 | 2.307 | 16.741 | 0.112298 | 7/15/2014 | 36:20.6 | 0 |
| -1173.563 | 19782.713 | 2.304 | 16.762 | 0.115548 | 7/15/2014 | 36:20.7 | 0 |
| -1173.46 | 19782.707 | 2.306 | 16.773 | 0.112284 | 7/15/2014 | 36:20.7 | 0 |
| -1173.258 | 19782.697 | 2.306 | 16.793 | 0.111562 | 7/15/2014 | 36:20.8 | 0 |
| -1173.056 | 19782.686 | 2.308 | 16.812 | 0.124338 | 7/15/2014 | 36:20.9 | 0 |
| -1172.854 | 19782.674 | 2.305 | 16.828 | 0.133214 | 7/15/2014 | 36:21.0 | 0 |
| -1172.55 | 19782.658 | 2.309 | 16.844 | 0.12762 | 7/15/2014 | 36:21.2 | 0 |
| -1172.451 | 19782.653 | 2.305 | 16.851 | 0.130136 | 7/15/2014 | 36:21.2 | 0 |
| -1172.251 | 19782.643 | 2.303 | 16.857 | 0.141034 | 7/15/2014 | 36:21.3 | 0 |
| -1171.905 | 19782.624 | 2.3 | 16.857 | 0.139457 | 7/15/2014 | 36:21.5 | 0 |
| -1171.707 | 19782.613 | 2.3 | 16.854 | 0.138819 | 7/15/2014 | 36:21.6 | 0 |
| -1171.462 | 19782.6 | 2.3 | 16.849 | 0.141908 | 7/15/2014 | 36:21.7 | 0 |
| -1171.263 | 19782.588 | 2.303 | 16.841 | 0.144294 | 7/15/2014 | 36:21.8 | 0 |
| -1171.067 | 19782.578 | 2.3 | 16.831 | 0.149348 | 7/15/2014 | 36:21.9 | 0 |
| -1170.866 | 19782.568 | 2.303 | 16.82 | 0.156887 | 7/15/2014 | 36:22.0 | 0 |
| -1170.67 | 19782.557 | 2.3 | 16.808 | 0.152493 | 7/15/2014 | 36:22.1 | 0 |
| -1170.47 | 19782.545 | 2.305 | 16.796 | 0.149265 | 7/15/2014 | 36:22.2 | 0 |
| -1170.275 | 19782.534 | 2.302 | 16.785 | 0.152754 | 7/15/2014 | 36:22.3 | 0 |
| -1170.077 | 19782.523 | 2.309 | 16.776 | 0.141938 | 7/15/2014 | 36:22.4 | 0 |
| -1169.832 | 19782.511 | 2.312 | 16.769 | 0.135308 | 7/15/2014 | 36:22.5 | 0 |
| -1169.636 | 19782.5 | 2.312 | 16.765 | 0.133443 | 7/15/2014 | 36:22.6 | 0 |
| -1169.391 | 19782.487 | 2.309 | 16.764 | 0.130974 | 7/15/2014 | 36:22.7 | 0 |
| -1169.19 | 19782.476 | 2.312 | 16.767 | 0.118902 | 7/15/2014 | 36:22.8 | 0 |
| -1168.991 | 19782.465 | 2.309 | 16.774 | 0.121721 | 7/15/2014 | 36:22.9 | 0 |
| -1168.787 | 19782.453 | 2.309 | 16.784 | 0.121087 | 7/15/2014 | 36:23.0 | 0 |
| -1168.69 | 19782.447 | 2.308 | 16.788 | 0.117537 | 7/15/2014 | 36:23.0 | 0 |
| -1168.486 | 19782.436 | 2.311 | 16.803 | 0.11152 | 7/15/2014 | 36:23.1 | 0 |
| -1168.283 | 19782.425 | 2.308 | 16.821 | 0.117341 | 7/15/2014 | 36:23.2 | 0 |
| -1168.081 | 19782.414 | 2.311 | 16.84 | 0.120189 | 7/15/2014 | 36:23.3 | 0 |
| -1167.729 | 19782.395 | 2.311 | 16.868 | 0.124305 | 7/15/2014 | 36:23.5 | 0 |
| -1167.526 | 19782.383 | 2.317 | 16.886 | 0.130245 | 7/15/2014 | 36:23.6 | 0 |
| -1167.277 | 19782.369 | 2.317 | 16.903 | 0.137185 | 7/15/2014 | 36:23.7 | 0 |
| -1167.172 | 19782.363 | 2.32 | 16.912 | 0.140558 | 7/15/2014 | 36:23.7 | 0 |
| -1166.969 | 19782.352 | 2.325 | 16.926 | 0.144792 | 7/15/2014 | 36:23.8 | 0 |
| -1166.767 | 19782.341 | 2.325 | 16.937 | 0.149466 | 7/15/2014 | 36:23.9 | 0 |
| -1166.468 | 19782.324 | 2.324 | 16.95 | 0.160271 | 7/15/2014 | 36:24.1 | 0 |
| -1166.266 | 19782.313 | 2.324 | 16.956 | 0.166246 | 7/15/2014 | 36:24.2 | 0 |
| -1166.067 | 19782.301 | 2.324 | 16.961 | 0.172323 | 7/15/2014 | 36:24.3 | 0 |
| -1165.87 | 19782.291 | 2.329 | 16.964 | 0.17637 | 7/15/2014 | 36:24.4 | 0 |
| -1165.626 | 19782.276 | 2.329 | 16.964 | 0.187401 | 7/15/2014 | 36:24.5 | 0 |
| -1165.432 | 19782.266 | 2.332 | 16.963 | 0.197601 | 7/15/2014 | 36:24.6 | 0 |
| -1165.192 | 19782.252 | 2.33 | 16.958 | 0.20288 | 7/15/2014 | 36:24.7 | 0 |

| | | | | | | | |
|---|---|---|---|---|---|---|---|
| -1164.999 | 19782.242 | 2.334 | 16.951 | 0.217158 | 7/15/2014 | 36:24.8 | 0 |
| -1164.807 | 19782.23 | 2.334 | 16.941 | 0.216496 | 7/15/2014 | 36:24.9 | 0 |
| -1164.612 | 19782.219 | 2.344 | 16.927 | 0.218133 | 7/15/2014 | 36:25.0 | 0 |
| -1164.419 | 19782.208 | 2.348 | 16.91 | 0.220299 | 7/15/2014 | 36:25.1 | 0 |
| -1164.226 | 19782.198 | 2.354 | 16.891 | 0.216696 | 7/15/2014 | 36:25.2 | 0 |
| -1164.035 | 19782.187 | 2.354 | 16.869 | 0.224636 | 7/15/2014 | 36:25.3 | 0 |
| -1163.842 | 19782.176 | 2.361 | 16.845 | 0.200537 | 7/15/2014 | 36:25.4 | 0 |
| -1163.605 | 19782.161 | 2.363 | 16.82 | 0.188144 | 7/15/2014 | 36:25.5 | 0 |
| -1163.412 | 19782.15 | 2.366 | 16.795 | 0.181402 | 7/15/2014 | 36:25.6 | 0 |
| -1163.168 | 19782.137 | 2.368 | 16.77 | 0.16428 | 7/15/2014 | 36:25.7 | 0 |
| -1162.97 | 19782.125 | 2.372 | 16.746 | 0.160978 | 7/15/2014 | 36:25.8 | 0 |
| -1162.771 | 19782.113 | 2.372 | 16.724 | 0.151252 | 7/15/2014 | 36:25.9 | 0 |
| -1162.57 | 19782.103 | 2.372 | 16.703 | 0.139617 | 7/15/2014 | 36:26.0 | 0 |
| -1162.475 | 19782.097 | 2.372 | 16.692 | 0.139788 | 7/15/2014 | 36:26.0 | 0 |
| -1162.272 | 19782.084 | 2.372 | 16.674 | 0.134252 | 7/15/2014 | 36:26.1 | 0 |
| -1162.068 | 19782.072 | 2.369 | 16.659 | 0.140594 | 7/15/2014 | 36:26.2 | 0 |
| -1161.863 | 19782.061 | 2.371 | 16.645 | 0.153171 | 7/15/2014 | 36:26.3 | 0 |
| -1161.505 | 19782.04 | 2.371 | 16.63 | 0.154937 | 7/15/2014 | 36:26.5 | 0 |
| -1161.3 | 19782.027 | 2.371 | 16.621 | 0.147867 | 7/15/2014 | 36:26.6 | 0 |
| -1161.045 | 19782.012 | 2.367 | 16.613 | 0.139261 | 7/15/2014 | 36:26.7 | 0 |
| -1160.94 | 19782.006 | 2.367 | 16.609 | 0.141414 | 7/15/2014 | 36:26.7 | 0 |
| -1160.733 | 19781.993 | 2.367 | 16.604 | 0.151643 | 7/15/2014 | 36:26.8 | 0 |
| -1160.526 | 19781.981 | 2.367 | 16.6 | 0.13388 | 7/15/2014 | 36:26.9 | 0 |
| -1160.318 | 19781.969 | 2.367 | 16.597 | 0.126867 | 7/15/2014 | 36:27.0 | 0 |
| -1160.112 | 19781.956 | 2.367 | 16.595 | 0.137142 | 7/15/2014 | 36:27.1 | 0 |
| -1159.908 | 19781.944 | 2.367 | 16.595 | 0.133614 | 7/15/2014 | 36:27.2 | 0 |
| -1159.706 | 19781.931 | 2.367 | 16.595 | 0.130815 | 7/15/2014 | 36:27.3 | 0 |
| -1159.359 | 19781.91 | 2.37 | 16.595 | 0.144544 | 7/15/2014 | 36:27.5 | 0 |
| -1159.161 | 19781.898 | 2.379 | 16.596 | 0.145476 | 7/15/2014 | 36:27.6 | 0 |
| -1158.919 | 19781.884 | 2.379 | 16.596 | 0.146978 | 7/15/2014 | 36:27.7 | 0 |
| -1158.722 | 19781.871 | 2.387 | 16.596 | 0.151874 | 7/15/2014 | 36:27.8 | 0 |
| -1158.528 | 19781.858 | 2.387 | 16.595 | 0.156604 | 7/15/2014 | 36:27.9 | 0 |
| -1158.332 | 19781.847 | 2.392 | 16.593 | 0.161911 | 7/15/2014 | 36:28.0 | 0 |
| -1158.138 | 19781.835 | 2.395 | 16.59 | 0.166976 | 7/15/2014 | 36:28.1 | 0 |
| -1157.943 | 19781.822 | 2.397 | 16.586 | 0.167225 | 7/15/2014 | 36:28.2 | 0 |
| -1157.752 | 19781.811 | 2.394 | 16.581 | 0.175125 | 7/15/2014 | 36:28.3 | 0 |
| -1157.561 | 19781.799 | 2.397 | 16.574 | 0.180019 | 7/15/2014 | 36:28.4 | 0 |
| -1157.325 | 19781.784 | 2.395 | 16.566 | 0.184853 | 7/15/2014 | 36:28.5 | 0 |
| -1157.135 | 19781.772 | 2.4 | 16.557 | 0.200967 | 7/15/2014 | 36:28.6 | 0 |
| -1156.902 | 19781.757 | 2.396 | 16.546 | 0.184882 | 7/15/2014 | 36:28.7 | 0 |
| -1156.712 | 19781.745 | 2.402 | 16.534 | 0.191663 | 7/15/2014 | 36:28.8 | 0 |
| -1156.523 | 19781.734 | 2.402 | 16.521 | 0.189749 | 7/15/2014 | 36:28.9 | 0 |
| -1156.333 | 19781.722 | 2.406 | 16.506 | 0.179168 | 7/15/2014 | 36:29.0 | 0 |
| -1156.144 | 19781.71 | 2.409 | 16.491 | 0.18171 | 7/15/2014 | 36:29.1 | 0 |
| -1155.953 | 19781.698 | 2.419 | 16.474 | 0.171355 | 7/15/2014 | 36:29.2 | 0 |
| -1155.766 | 19781.687 | 2.421 | 16.456 | 0.170857 | 7/15/2014 | 36:29.3 | 0 |
| -1155.577 | 19781.675 | 2.424 | 16.438 | 0.159389 | 7/15/2014 | 36:29.4 | 0 |

| | | | | | | | |
|---|---|---|---|---|---|---|---|
| -1155.344 | 19781.66 | 2.426 | 16.418 | 0.164973 | 7/15/2014 | 36:29.5 | 0 |
| -1155.155 | 19781.648 | 2.436 | 16.399 | 0.152635 | 7/15/2014 | 36:29.6 | 0 |
| -1154.921 | 19781.634 | 2.436 | 16.379 | 0.147622 | 7/15/2014 | 36:29.7 | 0 |
| -1154.732 | 19781.621 | 2.438 | 16.359 | 0.150217 | 7/15/2014 | 36:29.8 | 0 |
| -1154.541 | 19781.609 | 2.438 | 16.339 | 0.143355 | 7/15/2014 | 36:29.9 | 0 |
| -1154.347 | 19781.598 | 2.445 | 16.32 | 0.144609 | 7/15/2014 | 36:30.0 | 0 |
| -1154.159 | 19781.586 | 2.442 | 16.302 | 0.144928 | 7/15/2014 | 36:30.1 | 0 |
| -1153.964 | 19781.573 | 2.45 | 16.284 | 0.14842 | 7/15/2014 | 36:30.2 | 0 |
| -1153.774 | 19781.562 | 2.45 | 16.267 | 0.150198 | 7/15/2014 | 36:30.3 | 0 |
| -1153.58 | 19781.55 | 2.453 | 16.25 | 0.149641 | 7/15/2014 | 36:30.4 | 0 |
| -1153.345 | 19781.534 | 2.447 | 16.234 | 0.148388 | 7/15/2014 | 36:30.5 | 0 |
| -1153.151 | 19781.521 | 2.453 | 16.218 | 0.152138 | 7/15/2014 | 36:30.6 | 0 |
| -1152.91 | 19781.506 | 2.459 | 16.203 | 0.155726 | 7/15/2014 | 36:30.7 | 0 |
| -1152.714 | 19781.494 | 2.461 | 16.187 | 0.158909 | 7/15/2014 | 36:30.8 | 0 |
| -1152.521 | 19781.482 | 2.456 | 16.171 | 0.15904 | 7/15/2014 | 36:30.9 | 0 |
| -1152.322 | 19781.469 | 2.459 | 16.155 | 0.156017 | 7/15/2014 | 36:31.0 | 0 |
| -1152.125 | 19781.456 | 2.459 | 16.139 | 0.164134 | 7/15/2014 | 36:31.1 | 0 |
| -1151.926 | 19781.444 | 2.464 | 16.123 | 0.159433 | 7/15/2014 | 36:31.2 | 0 |
| -1151.73 | 19781.432 | 2.467 | 16.108 | 0.158252 | 7/15/2014 | 36:31.3 | 0 |
| -1151.532 | 19781.419 | 2.473 | 16.094 | 0.161361 | 7/15/2014 | 36:31.4 | 0 |
| -1151.287 | 19781.403 | 2.473 | 16.081 | 0.151397 | 7/15/2014 | 36:31.5 | 0 |
| -1151.09 | 19781.392 | 2.473 | 16.07 | 0.14964 | 7/15/2014 | 36:31.6 | 0 |
| -1150.847 | 19781.376 | 2.473 | 16.061 | 0.139988 | 7/15/2014 | 36:31.7 | 0 |
| -1150.646 | 19781.363 | 2.478 | 16.053 | 0.142954 | 7/15/2014 | 36:31.8 | 0 |
| -1150.445 | 19781.351 | 2.481 | 16.048 | 0.138898 | 7/15/2014 | 36:31.9 | 0 |
| -1150.244 | 19781.339 | 2.481 | 16.045 | 0.132846 | 7/15/2014 | 36:32.0 | 0 |
| -1150.044 | 19781.327 | 2.478 | 16.043 | 0.13506 | 7/15/2014 | 36:32.1 | 0 |
| -1149.842 | 19781.313 | 2.478 | 16.043 | 0.122861 | 7/15/2014 | 36:32.2 | 0 |
| -1149.643 | 19781.302 | 2.475 | 16.044 | 0.130485 | 7/15/2014 | 36:32.3 | 0 |
| -1149.545 | 19781.296 | 2.474 | 16.045 | 0.121632 | 7/15/2014 | 36:32.3 | 0 |
| -1149.199 | 19781.274 | 2.466 | 16.048 | 0.148225 | 7/15/2014 | 36:32.5 | 0 |
| -1148.999 | 19781.263 | 2.469 | 16.05 | 0.122784 | 7/15/2014 | 36:32.6 | 0 |
| -1148.753 | 19781.247 | 2.466 | 16.052 | 0.123892 | 7/15/2014 | 36:32.7 | 0 |
| -1148.554 | 19781.235 | 2.463 | 16.055 | 0.123129 | 7/15/2014 | 36:32.8 | 0 |
| -1148.358 | 19781.224 | 2.457 | 16.058 | 0.121725 | 7/15/2014 | 36:32.9 | 0 |
| -1148.156 | 19781.212 | 2.46 | 16.063 | 0.136876 | 7/15/2014 | 36:33.0 | 0 |
| -1147.96 | 19781.199 | 2.455 | 16.069 | 0.130007 | 7/15/2014 | 36:33.1 | 0 |
| -1147.759 | 19781.188 | 2.457 | 16.077 | 0.134217 | 7/15/2014 | 36:33.2 | 0 |
| -1147.563 | 19781.177 | 2.457 | 16.087 | 0.128503 | 7/15/2014 | 36:33.3 | 0 |
| -1147.367 | 19781.166 | 2.463 | 16.099 | 0.140634 | 7/15/2014 | 36:33.4 | 0 |
| -1147.125 | 19781.15 | 2.463 | 16.112 | 0.137209 | 7/15/2014 | 36:33.5 | 0 |
| -1146.932 | 19781.139 | 2.466 | 16.126 | 0.139901 | 7/15/2014 | 36:33.6 | 0 |
| -1146.695 | 19781.125 | 2.466 | 16.14 | 0.143597 | 7/15/2014 | 36:33.7 | 0 |
| -1146.5 | 19781.114 | 2.472 | 16.152 | 0.135501 | 7/15/2014 | 36:33.8 | 0 |
| -1146.308 | 19781.102 | 2.472 | 16.163 | 0.13173 | 7/15/2014 | 36:33.9 | 0 |
| -1146.113 | 19781.091 | 2.479 | 16.17 | 0.121235 | 7/15/2014 | 36:34.0 | 0 |
| -1145.919 | 19781.08 | 2.479 | 16.174 | 0.117407 | 7/15/2014 | 36:34.1 | 0 |

| | | | | | | | |
|---|---|---|---|---|---|---|---|
| -1145.727 | 19781.07 | 2.482 | 16.173 | 0.11071 | 7/15/2014 | 36:34.2 | 0 |
| -1145.535 | 19781.059 | 2.479 | 16.167 | 0.106316 | 7/15/2014 | 36:34.3 | 0 |
| -1145.344 | 19781.048 | 2.479 | 16.158 | 0.100772 | 7/15/2014 | 36:34.4 | 0 |
| -1145.11 | 19781.035 | 2.476 | 16.144 | 0.0955417 | 7/15/2014 | 36:34.5 | 0 |
| -1144.918 | 19781.023 | 2.479 | 16.127 | 0.0956555 | 7/15/2014 | 36:34.6 | 0 |
| -1144.681 | 19781.01 | 2.479 | 16.107 | 0.0933469 | 7/15/2014 | 36:34.7 | 0 |
| -1144.489 | 19780.999 | 2.483 | 16.085 | 0.0957553 | 7/15/2014 | 36:34.8 | 0 |
| -1144.298 | 19780.989 | 2.481 | 16.063 | 0.0995339 | 7/15/2014 | 36:34.9 | 0 |
| -1144.104 | 19780.979 | 2.483 | 16.039 | 0.10211 | 7/15/2014 | 36:35.0 | 0 |
| -1143.911 | 19780.967 | 2.483 | 16.017 | 0.102551 | 7/15/2014 | 36:35.1 | 0 |
| -1143.717 | 19780.958 | 2.488 | 15.995 | 0.11175 | 7/15/2014 | 36:35.2 | 0 |
| -1143.526 | 19780.947 | 2.488 | 15.974 | 0.11292 | 7/15/2014 | 36:35.3 | 0 |
| -1143.332 | 19780.937 | 2.492 | 15.955 | 0.117952 | 7/15/2014 | 36:35.4 | 0 |
| -1143.092 | 19780.923 | 2.49 | 15.939 | 0.119608 | 7/15/2014 | 36:35.5 | 0 |
| -1142.899 | 19780.913 | 2.491 | 15.924 | 0.125555 | 7/15/2014 | 36:35.6 | 0 |
| -1142.658 | 19780.9 | 2.488 | 15.913 | 0.120525 | 7/15/2014 | 36:35.7 | 0 |
| -1142.462 | 19780.889 | 2.488 | 15.904 | 0.127984 | 7/15/2014 | 36:35.8 | 0 |
| -1142.266 | 19780.879 | 2.486 | 15.898 | 0.137344 | 7/15/2014 | 36:35.9 | 0 |
| -1142.068 | 19780.869 | 2.488 | 15.896 | 0.121991 | 7/15/2014 | 36:36.0 | 0 |
| -1141.871 | 19780.858 | 2.486 | 15.897 | 0.124088 | 7/15/2014 | 36:36.1 | 0 |
| -1141.672 | 19780.849 | 2.49 | 15.901 | 0.128702 | 7/15/2014 | 36:36.2 | 0 |
| -1141.476 | 19780.838 | 2.488 | 15.908 | 0.127956 | 7/15/2014 | 36:36.3 | 0 |
| -1141.281 | 19780.828 | 2.491 | 15.917 | 0.121033 | 7/15/2014 | 36:36.4 | 0 |
| -1141.037 | 19780.814 | 2.489 | 15.929 | 0.130479 | 7/15/2014 | 36:36.5 | 0 |
| -1140.841 | 19780.805 | 2.492 | 15.941 | 0.121669 | 7/15/2014 | 36:36.6 | 0 |
| -1140.598 | 19780.793 | 2.492 | 15.954 | 0.116132 | 7/15/2014 | 36:36.7 | 0 |
| -1140.4 | 19780.783 | 2.494 | 15.967 | 0.119525 | 7/15/2014 | 36:36.8 | 0 |
| -1140.204 | 19780.772 | 2.492 | 15.979 | 0.130022 | 7/15/2014 | 36:36.9 | 0 |
| -1140.005 | 19780.764 | 2.496 | 15.989 | 0.108415 | 7/15/2014 | 36:37.0 | 0 |
| -1139.809 | 19780.754 | 2.494 | 15.997 | 0.107086 | 7/15/2014 | 36:37.1 | 0 |
| -1139.612 | 19780.744 | 2.496 | 16.002 | 0.11547 | 7/15/2014 | 36:37.2 | 0 |
| -1139.416 | 19780.734 | 2.496 | 16.006 | 0.107616 | 7/15/2014 | 36:37.3 | 0 |
| -1139.22 | 19780.727 | 2.5 | 16.007 | 0.101595 | 7/15/2014 | 36:37.4 | 0 |
| -1138.978 | 19780.713 | 2.5 | 16.006 | 0.101924 | 7/15/2014 | 36:37.5 | 0 |
| -1138.785 | 19780.705 | 2.504 | 16.003 | 0.0961418 | 7/15/2014 | 36:37.6 | 0 |
| -1138.543 | 19780.693 | 2.504 | 16 | 0.0860525 | 7/15/2014 | 36:37.7 | 0 |
| -1138.345 | 19780.685 | 2.51 | 15.995 | 0.0867881 | 7/15/2014 | 36:37.8 | 0 |
| -1138.15 | 19780.676 | 2.508 | 15.991 | 0.0855011 | 7/15/2014 | 36:37.9 | 0 |
| -1137.952 | 19780.666 | 2.51 | 15.987 | 0.0831893 | 7/15/2014 | 36:38.0 | 0 |
| -1137.754 | 19780.658 | 2.51 | 15.982 | 0.0781563 | 7/15/2014 | 36:38.1 | 0 |
| -1137.555 | 19780.65 | 2.517 | 15.979 | 0.0700197 | 7/15/2014 | 36:38.2 | 0 |
| -1137.357 | 19780.64 | 2.517 | 15.976 | 0.0673003 | 7/15/2014 | 36:38.3 | 0 |
| -1137.16 | 19780.631 | 2.517 | 15.974 | 0.0617355 | 7/15/2014 | 36:38.4 | 0 |
| -1136.918 | 19780.621 | 2.515 | 15.972 | 0.0604474 | 7/15/2014 | 36:38.5 | 0 |
| -1136.719 | 19780.612 | 2.52 | 15.971 | 0.0600969 | 7/15/2014 | 36:38.6 | 0 |
| -1136.476 | 19780.603 | 2.515 | 15.971 | 0.0579387 | 7/15/2014 | 36:38.7 | 0 |
| -1136.277 | 19780.594 | 2.517 | 15.971 | 0.0648938 | 7/15/2014 | 36:38.8 | 0 |

| | | | | | | | |
|---|---|---|---|---|---|---|---|
| -1136.079 | 19780.586 | 2.515 | 15.972 | 0.0683583 | 7/15/2014 | 36:38.9 | 0 |
| -1135.88 | 19780.578 | 2.517 | 15.972 | 0.0595757 | 7/15/2014 | 36:39.0 | 0 |
| -1135.681 | 19780.569 | 2.514 | 15.973 | 0.0640514 | 7/15/2014 | 36:39.1 | 0 |
| -1135.483 | 19780.561 | 2.514 | 15.973 | 0.0666415 | 7/15/2014 | 36:39.2 | 0 |
| -1135.288 | 19780.553 | 2.512 | 15.972 | 0.0651232 | 7/15/2014 | 36:39.3 | 0 |
| -1135.093 | 19780.545 | 2.514 | 15.971 | 0.0683828 | 7/15/2014 | 36:39.4 | 0 |
| -1134.853 | 19780.536 | 2.509 | 15.969 | 0.0688491 | 7/15/2014 | 36:39.5 | 0 |
| -1134.661 | 19780.529 | 2.511 | 15.967 | 0.0740592 | 7/15/2014 | 36:39.6 | 0 |
| -1134.426 | 19780.52 | 2.508 | 15.964 | 0.0835189 | 7/15/2014 | 36:39.7 | 0 |
| -1134.235 | 19780.512 | 2.508 | 15.96 | 0.0724746 | 7/15/2014 | 36:39.8 | 0 |
| -1134.046 | 19780.506 | 2.506 | 15.955 | 0.0810164 | 7/15/2014 | 36:39.9 | 0 |
| -1133.857 | 19780.498 | 2.506 | 15.949 | 0.0850045 | 7/15/2014 | 36:40.0 | 0 |
| -1133.668 | 19780.492 | 2.506 | 15.943 | 0.0720456 | 7/15/2014 | 36:40.1 | 0 |
| -1133.48 | 19780.484 | 2.508 | 15.936 | 0.0816619 | 7/15/2014 | 36:40.2 | 0 |
| -1133.293 | 19780.478 | 2.506 | 15.929 | 0.0758365 | 7/15/2014 | 36:40.3 | 0 |
| -1133.108 | 19780.473 | 2.508 | 15.921 | 0.0727039 | 7/15/2014 | 36:40.4 | 0 |
| -1132.788 | 19780.461 | 2.505 | 15.908 | 0.0757268 | 7/15/2014 | 36:40.5 | 0 |
| -1132.556 | 19780.452 | 2.507 | 15.899 | 0.0788772 | 7/15/2014 | 36:40.6 | 0 |
| -1132.465 | 19780.45 | 2.508 | 15.894 | 0.0789612 | 7/15/2014 | 36:40.7 | 0 |
| -1132.279 | 19780.443 | 2.512 | 15.885 | 0.0819117 | 7/15/2014 | 36:40.8 | 0 |
| -1132.092 | 19780.439 | 2.512 | 15.875 | 0.0847352 | 7/15/2014 | 36:40.9 | 0 |
| -1131.904 | 19780.432 | 2.512 | 15.865 | 0.0907755 | 7/15/2014 | 36:41.0 | 0 |
| -1131.716 | 19780.426 | 2.512 | 15.855 | 0.0909848 | 7/15/2014 | 36:41.1 | 0 |
| -1131.526 | 19780.42 | 2.517 | 15.845 | 0.0993058 | 7/15/2014 | 36:41.2 | 0 |
| -1131.338 | 19780.414 | 2.515 | 15.836 | 0.0964477 | 7/15/2014 | 36:41.3 | 0 |
| -1131.147 | 19780.408 | 2.518 | 15.826 | 0.100323 | 7/15/2014 | 36:41.4 | 0 |
| -1130.91 | 19780.4 | 2.518 | 15.816 | 0.100079 | 7/15/2014 | 36:41.5 | 0 |
| -1130.719 | 19780.395 | 2.523 | 15.807 | 0.0945227 | 7/15/2014 | 36:41.6 | 0 |
| -1130.475 | 19780.389 | 2.518 | 15.798 | 0.0906045 | 7/15/2014 | 36:41.7 | 0 |
| -1130.279 | 19780.381 | 2.521 | 15.79 | 0.104908 | 7/15/2014 | 36:41.8 | 0 |
| -1130.08 | 19780.375 | 2.521 | 15.782 | 0.092083 | 7/15/2014 | 36:41.9 | 0 |
| -1129.879 | 19780.369 | 2.521 | 15.774 | 0.0926942 | 7/15/2014 | 36:42.0 | 0 |
| -1129.678 | 19780.362 | 2.518 | 15.768 | 0.0915507 | 7/15/2014 | 36:42.1 | 0 |
| -1129.58 | 19780.359 | 2.52 | 15.764 | 0.085431 | 7/15/2014 | 36:42.1 | 0 |
| -1129.378 | 19780.354 | 2.52 | 15.758 | 0.0770943 | 7/15/2014 | 36:42.2 | 0 |
| -1129.176 | 19780.348 | 2.518 | 15.754 | 0.090784 | 7/15/2014 | 36:42.3 | 0 |
| -1128.824 | 19780.339 | 2.511 | 15.749 | 0.107229 | 7/15/2014 | 36:42.5 | 0 |
| -1128.621 | 19780.332 | 2.511 | 15.747 | 0.0820637 | 7/15/2014 | 36:42.6 | 0 |
| -1128.371 | 19780.324 | 2.502 | 15.746 | 0.0789187 | 7/15/2014 | 36:42.7 | 0 |
| -1128.268 | 19780.322 | 2.5 | 15.745 | 0.0743096 | 7/15/2014 | 36:42.7 | 0 |
| -1128.065 | 19780.316 | 2.496 | 15.745 | 0.0719026 | 7/15/2014 | 36:42.8 | 0 |
| -1127.862 | 19780.312 | 2.488 | 15.747 | 0.0851834 | 7/15/2014 | 36:42.9 | 0 |
| -1127.659 | 19780.305 | 2.483 | 15.749 | 0.0647611 | 7/15/2014 | 36:43.0 | 0 |
| -1127.361 | 19780.298 | 2.475 | 15.756 | 0.073628 | 7/15/2014 | 36:43.2 | 0 |
| -1127.163 | 19780.292 | 2.471 | 15.76 | 0.0647844 | 7/15/2014 | 36:43.3 | 0 |
| -1126.964 | 19780.287 | 2.471 | 15.766 | 0.0688294 | 7/15/2014 | 36:43.4 | 0 |
| -1126.722 | 19780.281 | 2.466 | 15.772 | 0.0730145 | 7/15/2014 | 36:43.5 | 0 |

| | | | | | | | |
|---|---|---|---|---|---|---|---|
| -1126.529 | 19780.277 | 2.468 | 15.779 | 0.0782556 | 7/15/2014 | 36:43.6 | 0 |
| -1126.293 | 19780.271 | 2.465 | 15.787 | 0.0907068 | 7/15/2014 | 36:43.7 | 0 |
| -1126.102 | 19780.266 | 2.468 | 15.795 | 0.0932806 | 7/15/2014 | 36:43.8 | 0 |
| -1125.912 | 19780.261 | 2.466 | 15.804 | 0.0923734 | 7/15/2014 | 36:43.9 | 0 |
| -1125.722 | 19780.256 | 2.466 | 15.814 | 0.0984354 | 7/15/2014 | 36:44.0 | 0 |
| -1125.532 | 19780.25 | 2.466 | 15.824 | 0.116566 | 7/15/2014 | 36:44.1 | 0 |
| -1125.342 | 19780.246 | 2.466 | 15.836 | 0.113093 | 7/15/2014 | 36:44.2 | 0 |
| -1125.156 | 19780.242 | 2.459 | 15.849 | 0.0985331 | 7/15/2014 | 36:44.3 | 0 |
| -1124.968 | 19780.237 | 2.459 | 15.862 | 0.123699 | 7/15/2014 | 36:44.4 | 0 |
| -1124.645 | 19780.229 | 2.451 | 15.883 | 0.107497 | 7/15/2014 | 36:44.5 | 0 |
| -1124.412 | 19780.223 | 2.447 | 15.897 | 0.104948 | 7/15/2014 | 36:44.6 | 0 |
| -1124.319 | 19780.22 | 2.443 | 15.904 | 0.103006 | 7/15/2014 | 36:44.7 | 0 |
| -1124.131 | 19780.216 | 2.441 | 15.916 | 0.108765 | 7/15/2014 | 36:44.8 | 0 |
| -1123.942 | 19780.211 | 2.44 | 15.927 | 0.106488 | 7/15/2014 | 36:44.9 | 0 |
| -1123.75 | 19780.205 | 2.438 | 15.936 | 0.109504 | 7/15/2014 | 36:45.0 | 0 |
| -1123.559 | 19780.201 | 2.436 | 15.943 | 0.113571 | 7/15/2014 | 36:45.1 | 0 |
| -1123.367 | 19780.195 | 2.438 | 15.948 | 0.118028 | 7/15/2014 | 36:45.2 | 0 |
| -1123.176 | 19780.19 | 2.433 | 15.95 | 0.135478 | 7/15/2014 | 36:45.3 | 0 |
| -1122.984 | 19780.186 | 2.433 | 15.949 | 0.133638 | 7/15/2014 | 36:45.4 | 0 |
| -1122.744 | 19780.178 | 2.43 | 15.947 | 0.133878 | 7/15/2014 | 36:45.5 | 0 |
| -1122.55 | 19780.174 | 2.43 | 15.942 | 0.129242 | 7/15/2014 | 36:45.6 | 0 |
| -1122.306 | 19780.168 | 2.427 | 15.936 | 0.128387 | 7/15/2014 | 36:45.7 | 0 |
| -1122.111 | 19780.16 | 2.424 | 15.929 | 0.130048 | 7/15/2014 | 36:45.8 | 0 |
| -1121.913 | 19780.156 | 2.419 | 15.921 | 0.124884 | 7/15/2014 | 36:45.9 | 0 |
| -1121.715 | 19780.15 | 2.416 | 15.913 | 0.132981 | 7/15/2014 | 36:46.0 | 0 |
| -1121.515 | 19780.145 | 2.406 | 15.904 | 0.122633 | 7/15/2014 | 36:46.1 | 0 |
| -1121.316 | 19780.141 | 2.406 | 15.895 | 0.122471 | 7/15/2014 | 36:46.2 | 0 |
| -1121.118 | 19780.133 | 2.404 | 15.887 | 0.122079 | 7/15/2014 | 36:46.3 | 0 |
| -1120.918 | 19780.128 | 2.404 | 15.879 | 0.12969 | 7/15/2014 | 36:46.4 | 0 |
| -1120.673 | 19780.121 | 2.401 | 15.872 | 0.143731 | 7/15/2014 | 36:46.5 | 0 |
| -1120.475 | 19780.114 | 2.404 | 15.865 | 0.13458 | 7/15/2014 | 36:46.6 | 0 |
| -1120.227 | 19780.107 | 2.401 | 15.86 | 0.132956 | 7/15/2014 | 36:46.7 | 0 |
| -1120.128 | 19780.104 | 2.402 | 15.856 | 0.130957 | 7/15/2014 | 36:46.7 | 0 |
| -1119.925 | 19780.1 | 2.405 | 15.852 | 0.135158 | 7/15/2014 | 36:46.8 | 0 |
| -1119.723 | 19780.094 | 2.408 | 15.849 | 0.134072 | 7/15/2014 | 36:46.9 | 0 |
| -1119.519 | 19780.087 | 2.411 | 15.847 | 0.140664 | 7/15/2014 | 36:47.0 | 0 |
| -1119.316 | 19780.081 | 2.414 | 15.846 | 0.134851 | 7/15/2014 | 36:47.1 | 0 |
| -1119.113 | 19780.074 | 2.417 | 15.846 | 0.138488 | 7/15/2014 | 36:47.2 | 0 |
| -1118.909 | 19780.069 | 2.42 | 15.847 | 0.133867 | 7/15/2014 | 36:47.3 | 0 |
| -1118.555 | 19780.057 | 2.42 | 15.85 | 0.143926 | 7/15/2014 | 36:47.5 | 0 |
| -1118.351 | 19780.053 | 2.422 | 15.852 | 0.14597 | 7/15/2014 | 36:47.6 | 0 |
| -1118.098 | 19780.043 | 2.42 | 15.855 | 0.139367 | 7/15/2014 | 36:47.7 | 0 |
| -1117.994 | 19780.04 | 2.424 | 15.856 | 0.135879 | 7/15/2014 | 36:47.7 | 0 |
| -1117.788 | 19780.033 | 2.424 | 15.86 | 0.142827 | 7/15/2014 | 36:47.8 | 0 |
| -1117.581 | 19780.027 | 2.42 | 15.864 | 0.135348 | 7/15/2014 | 36:47.9 | 0 |
| -1117.373 | 19780.021 | 2.417 | 15.867 | 0.13823 | 7/15/2014 | 36:48.0 | 0 |
| -1117.167 | 19780.014 | 2.414 | 15.871 | 0.142424 | 7/15/2014 | 36:48.1 | 0 |

| | | | | | | | |
|---|---|---|---|---|---|---|---|
| -1116.961 | 19780.006 | 2.408 | 15.873 | 0.136532 | 7/15/2014 | 36:48.2 | 0 |
| -1116.755 | 19780.002 | 2.404 | 15.875 | 0.124135 | 7/15/2014 | 36:48.3 | 0 |
| -1116.398 | 19779.988 | 2.4 | 15.875 | 0.123547 | 7/15/2014 | 36:48.5 | 0 |
| -1116.194 | 19779.98 | 2.397 | 15.874 | 0.125661 | 7/15/2014 | 36:48.6 | 0 |
| -1115.945 | 19779.975 | 2.391 | 15.871 | 0.114756 | 7/15/2014 | 36:48.7 | 0 |
| -1115.839 | 19779.971 | 2.392 | 15.87 | 0.116034 | 7/15/2014 | 36:48.7 | 0 |
| -1115.638 | 19779.963 | 2.388 | 15.866 | 0.106588 | 7/15/2014 | 36:48.8 | 0 |
| -1115.437 | 19779.955 | 2.388 | 15.861 | 0.103839 | 7/15/2014 | 36:48.9 | 0 |
| -1115.139 | 19779.947 | 2.385 | 15.853 | 0.107182 | 7/15/2014 | 36:49.1 | 0 |
| -1114.936 | 19779.939 | 2.387 | 15.847 | 0.104965 | 7/15/2014 | 36:49.2 | 0 |
| -1114.74 | 19779.932 | 2.384 | 15.843 | 0.115852 | 7/15/2014 | 36:49.3 | 0 |
| -1114.542 | 19779.924 | 2.384 | 15.839 | 0.11958 | 7/15/2014 | 36:49.4 | 0 |
| -1114.298 | 19779.918 | 2.382 | 15.836 | 0.120715 | 7/15/2014 | 36:49.5 | 0 |
| -1114.103 | 19779.91 | 2.38 | 15.834 | 0.126534 | 7/15/2014 | 36:49.6 | 0 |
| -1113.861 | 19779.9 | 2.376 | 15.834 | 0.129788 | 7/15/2014 | 36:49.7 | 0 |
| -1113.664 | 19779.894 | 2.376 | 15.834 | 0.129052 | 7/15/2014 | 36:49.8 | 0 |
| -1113.468 | 19779.887 | 2.376 | 15.834 | 0.143391 | 7/15/2014 | 36:49.9 | 0 |
| -1113.267 | 19779.879 | 2.378 | 15.835 | 0.140848 | 7/15/2014 | 36:50.0 | 0 |
| -1113.071 | 19779.871 | 2.375 | 15.836 | 0.13592 | 7/15/2014 | 36:50.1 | 0 |
| -1112.871 | 19779.863 | 2.379 | 15.838 | 0.136078 | 7/15/2014 | 36:50.2 | 0 |
| -1112.674 | 19779.857 | 2.377 | 15.838 | 0.143338 | 7/15/2014 | 36:50.3 | 0 |
| -1112.475 | 19779.85 | 2.382 | 15.839 | 0.140346 | 7/15/2014 | 36:50.4 | 0 |
| -1112.23 | 19779.84 | 2.383 | 15.839 | 0.144493 | 7/15/2014 | 36:50.5 | 0 |
| -1112.031 | 19779.832 | 2.387 | 15.838 | 0.141331 | 7/15/2014 | 36:50.6 | 0 |
| -1111.781 | 19779.822 | 2.387 | 15.837 | 0.139609 | 7/15/2014 | 36:50.7 | 0 |
| -1111.681 | 19779.818 | 2.387 | 15.836 | 0.146846 | 7/15/2014 | 36:50.7 | 0 |
| -1111.479 | 19779.811 | 2.389 | 15.834 | 0.150946 | 7/15/2014 | 36:50.8 | 0 |
| -1111.276 | 19779.803 | 2.389 | 15.832 | 0.150217 | 7/15/2014 | 36:50.9 | 0 |
| -1111.073 | 19779.795 | 2.389 | 15.83 | 0.133948 | 7/15/2014 | 36:51.0 | 0 |
| -1110.869 | 19779.786 | 2.391 | 15.827 | 0.136415 | 7/15/2014 | 36:51.1 | 0 |
| -1110.666 | 19779.778 | 2.391 | 15.824 | 0.126705 | 7/15/2014 | 36:51.2 | 0 |
| -1110.464 | 19779.77 | 2.389 | 15.821 | 0.130967 | 7/15/2014 | 36:51.3 | 0 |
| -1110.109 | 19779.756 | 2.391 | 15.815 | 0.1271 | 7/15/2014 | 36:51.5 | 0 |
| -1109.907 | 19779.748 | 2.394 | 15.812 | 0.118804 | 7/15/2014 | 36:51.6 | 0 |
| -1109.653 | 19779.736 | 2.394 | 15.807 | 0.123137 | 7/15/2014 | 36:51.7 | 0 |
| -1109.551 | 19779.732 | 2.395 | 15.805 | 0.12797 | 7/15/2014 | 36:51.7 | 0 |
| -1109.346 | 19779.724 | 2.395 | 15.8 | 0.127931 | 7/15/2014 | 36:51.8 | 0 |
| -1109.139 | 19779.716 | 2.395 | 15.795 | 0.129501 | 7/15/2014 | 36:51.9 | 0 |
| -1108.93 | 19779.705 | 2.398 | 15.788 | 0.134838 | 7/15/2014 | 36:52.0 | 0 |
| -1108.723 | 19779.697 | 2.4 | 15.781 | 0.136863 | 7/15/2014 | 36:52.1 | 0 |
| -1108.513 | 19779.688 | 2.403 | 15.773 | 0.142135 | 7/15/2014 | 36:52.2 | 0 |
| -1108.307 | 19779.68 | 2.407 | 15.765 | 0.151024 | 7/15/2014 | 36:52.3 | 0 |
| -1107.944 | 19779.662 | 2.407 | 15.752 | 0.144059 | 7/15/2014 | 36:52.5 | 0 |
| -1107.738 | 19779.652 | 2.41 | 15.743 | 0.146516 | 7/15/2014 | 36:52.6 | 0 |
| -1107.479 | 19779.641 | 2.41 | 15.734 | 0.14817 | 7/15/2014 | 36:52.7 | 0 |
| -1107.372 | 19779.635 | 2.413 | 15.73 | 0.14648 | 7/15/2014 | 36:52.7 | 0 |
| -1107.163 | 19779.627 | 2.413 | 15.721 | 0.148999 | 7/15/2014 | 36:52.8 | 0 |

| | | | | | | | |
|---|---|---|---|---|---|---|---|
| -1106.954 | 19779.615 | 2.413 | 15.712 | 0.148932 | 7/15/2014 | 36:52.9 | 0 |
| -1106.745 | 19779.606 | 2.413 | 15.704 | 0.148786 | 7/15/2014 | 36:53.0 | 0 |
| -1106.537 | 19779.596 | 2.41 | 15.696 | 0.154645 | 7/15/2014 | 36:53.1 | 0 |
| -1106.327 | 19779.586 | 2.413 | 15.688 | 0.160757 | 7/15/2014 | 36:53.2 | 0 |
| -1106.122 | 19779.574 | 2.413 | 15.679 | 0.169652 | 7/15/2014 | 36:53.3 | 0 |
| -1105.763 | 19779.557 | 2.416 | 15.666 | 0.17639 | 7/15/2014 | 36:53.5 | 0 |
| -1105.556 | 19779.547 | 2.422 | 15.658 | 0.183696 | 7/15/2014 | 36:53.6 | 0 |
| -1105.305 | 19779.533 | 2.419 | 15.649 | 0.185007 | 7/15/2014 | 36:53.7 | 0 |
| -1105.2 | 19779.528 | 2.423 | 15.645 | 0.185227 | 7/15/2014 | 36:53.7 | 0 |
| -1104.996 | 19779.519 | 2.425 | 15.637 | 0.187858 | 7/15/2014 | 36:53.8 | 0 |
| -1104.791 | 19779.508 | 2.428 | 15.629 | 0.213017 | 7/15/2014 | 36:53.9 | 0 |
| -1104.587 | 19779.496 | 2.428 | 15.622 | 0.196252 | 7/15/2014 | 36:54.0 | 0 |
| -1104.382 | 19779.486 | 2.425 | 15.617 | 0.194357 | 7/15/2014 | 36:54.1 | 0 |
| -1104.179 | 19779.475 | 2.427 | 15.613 | 0.205178 | 7/15/2014 | 36:54.2 | 0 |
| -1103.977 | 19779.463 | 2.424 | 15.611 | 0.20401 | 7/15/2014 | 36:54.3 | 0 |
| -1103.627 | 19779.443 | 2.424 | 15.613 | 0.222863 | 7/15/2014 | 36:54.5 | 0 |
| -1103.425 | 19779.434 | 2.427 | 15.615 | 0.237295 | 7/15/2014 | 36:54.6 | 0 |
| -1103.179 | 19779.42 | 2.42 | 15.618 | 0.221834 | 7/15/2014 | 36:54.7 | 0 |
| -1103.077 | 19779.414 | 2.42 | 15.62 | 0.240295 | 7/15/2014 | 36:54.7 | 0 |
| -1102.877 | 19779.402 | 2.42 | 15.624 | 0.270545 | 7/15/2014 | 36:54.8 | 0 |
| -1102.576 | 19779.385 | 2.428 | 15.63 | 0.288331 | 7/15/2014 | 36:55.0 | 0 |
| -1102.378 | 19779.373 | 2.423 | 15.633 | 0.308085 | 7/15/2014 | 36:55.1 | 0 |
| -1102.176 | 19779.361 | 2.429 | 15.635 | 0.325192 | 7/15/2014 | 36:55.2 | 0 |
| -1101.979 | 19779.35 | 2.427 | 15.636 | 0.330601 | 7/15/2014 | 36:55.3 | 0 |
| -1101.781 | 19779.338 | 2.432 | 15.635 | 0.319544 | 7/15/2014 | 36:55.4 | 0 |
| -1101.54 | 19779.324 | 2.431 | 15.634 | 0.361759 | 7/15/2014 | 36:55.5 | 0 |
| -1101.344 | 19779.313 | 2.439 | 15.632 | 0.339697 | 7/15/2014 | 36:55.6 | 0 |
| -1101.103 | 19779.297 | 2.44 | 15.629 | 0.321804 | 7/15/2014 | 36:55.7 | 0 |
| -1100.909 | 19779.285 | 2.445 | 15.627 | 0.3123 | 7/15/2014 | 36:55.8 | 0 |
| -1100.717 | 19779.273 | 2.444 | 15.625 | 0.288749 | 7/15/2014 | 36:55.9 | 0 |
| -1100.519 | 19779.262 | 2.452 | 15.623 | 0.283383 | 7/15/2014 | 36:56.0 | 0 |
| -1100.324 | 19779.249 | 2.454 | 15.623 | 0.274289 | 7/15/2014 | 36:56.1 | 0 |
| -1100.13 | 19779.235 | 2.456 | 15.623 | 0.274751 | 7/15/2014 | 36:56.2 | 0 |
| -1099.938 | 19779.225 | 2.454 | 15.625 | 0.248086 | 7/15/2014 | 36:56.3 | 0 |
| -1099.744 | 19779.213 | 2.456 | 15.627 | 0.262572 | 7/15/2014 | 36:56.4 | 0 |
| -1099.506 | 19779.196 | 2.454 | 15.631 | 0.269193 | 7/15/2014 | 36:56.5 | 0 |
| -1099.312 | 19779.184 | 2.454 | 15.634 | 0.222045 | 7/15/2014 | 36:56.6 | 0 |
| -1099.069 | 19779.167 | 2.451 | 15.638 | 0.227554 | 7/15/2014 | 36:56.7 | 0 |
| -1098.872 | 19779.155 | 2.456 | 15.641 | 0.207781 | 7/15/2014 | 36:56.8 | 0 |
| -1098.674 | 19779.141 | 2.454 | 15.644 | 0.165693 | 7/15/2014 | 36:56.9 | 0 |
| -1098.473 | 19779.127 | 2.458 | 15.647 | 0.175782 | 7/15/2014 | 36:57.0 | 0 |
| -1098.273 | 19779.113 | 2.458 | 15.649 | 0.156784 | 7/15/2014 | 36:57.1 | 0 |
| -1098.176 | 19779.107 | 2.461 | 15.65 | 0.144444 | 7/15/2014 | 36:57.1 | 0 |
| -1097.976 | 19779.094 | 2.464 | 15.651 | 0.126007 | 7/15/2014 | 36:57.2 | 0 |
| -1097.772 | 19779.079 | 2.47 | 15.652 | 0.130868 | 7/15/2014 | 36:57.3 | 0 |
| -1097.416 | 19779.054 | 2.474 | 15.651 | 0.133121 | 7/15/2014 | 36:57.5 | 0 |
| -1097.213 | 19779.04 | 2.477 | 15.65 | 0.14045 | 7/15/2014 | 36:57.6 | 0 |

| | | | | | | | |
|---|---|---|---|---|---|---|---|
| -1096.957 | 19779.021 | 2.474 | 15.648 | 0.139947 | 7/15/2014 | 36:57.7 | 0 |
| -1096.853 | 19779.014 | 2.477 | 15.648 | 0.116541 | 7/15/2014 | 36:57.7 | 0 |
| -1096.646 | 19778.998 | 2.471 | 15.645 | 0.145446 | 7/15/2014 | 36:57.8 | 0 |
| -1096.437 | 19778.983 | 2.471 | 15.643 | 0.136502 | 7/15/2014 | 36:57.9 | 0 |
| -1096.226 | 19778.968 | 2.474 | 15.64 | 0.142455 | 7/15/2014 | 36:58.0 | 0 |
| -1096.017 | 19778.953 | 2.47 | 15.636 | 0.149584 | 7/15/2014 | 36:58.1 | 0 |
| -1095.807 | 19778.937 | 2.47 | 15.632 | 0.138784 | 7/15/2014 | 36:58.2 | 0 |
| -1095.598 | 19778.92 | 2.467 | 15.626 | 0.105939 | 7/15/2014 | 36:58.3 | 0 |
| -1095.235 | 19778.893 | 2.466 | 15.615 | 0.137036 | 7/15/2014 | 36:58.5 | 0 |
| -1095.027 | 19778.876 | 2.471 | 15.607 | 0.101135 | 7/15/2014 | 36:58.6 | 0 |
| -1094.77 | 19778.855 | 2.473 | 15.598 | 0.104198 | 7/15/2014 | 36:58.7 | 0 |
| -1094.663 | 19778.848 | 2.476 | 15.594 | 0.106161 | 7/15/2014 | 36:58.7 | 0 |
| -1094.456 | 19778.832 | 2.474 | 15.583 | 0.0899099 | 7/15/2014 | 36:58.8 | 0 |
| -1094.248 | 19778.815 | 2.474 | 15.572 | 0.0877021 | 7/15/2014 | 36:58.9 | 0 |
| -1094.039 | 19778.797 | 2.474 | 15.56 | 0.103027 | 7/15/2014 | 36:59.0 | 0 |
| -1093.831 | 19778.781 | 2.474 | 15.548 | 0.107772 | 7/15/2014 | 36:59.1 | 0 |
| -1093.624 | 19778.766 | 2.474 | 15.537 | 0.118512 | 7/15/2014 | 36:59.2 | 0 |
| -1093.417 | 19778.747 | 2.47 | 15.526 | 0.119544 | 7/15/2014 | 36:59.3 | 0 |
| -1093.061 | 19778.719 | 2.47 | 15.515 | 0.133963 | 7/15/2014 | 36:59.5 | 0 |
| -1092.858 | 19778.701 | 2.47 | 15.508 | 0.138578 | 7/15/2014 | 36:59.6 | 0 |
| -1092.605 | 19778.68 | 2.467 | 15.504 | 0.142125 | 7/15/2014 | 36:59.7 | 0 |
| -1092.502 | 19778.67 | 2.468 | 15.501 | 0.143748 | 7/15/2014 | 36:59.7 | 0 |
| -1092.298 | 19778.654 | 2.468 | 15.498 | 0.139199 | 7/15/2014 | 36:59.8 | 0 |
| -1092.093 | 19778.637 | 2.471 | 15.497 | 0.130323 | 7/15/2014 | 36:59.9 | 0 |
| -1091.887 | 19778.619 | 2.471 | 15.497 | 0.137501 | 7/15/2014 | 37:00.0 | 0 |
| -1091.681 | 19778.602 | 2.471 | 15.498 | 0.121371 | 7/15/2014 | 37:00.1 | 0 |
| -1091.475 | 19778.585 | 2.473 | 15.499 | 0.145385 | 7/15/2014 | 37:00.2 | 0 |
| -1091.271 | 19778.566 | 2.47 | 15.499 | 0.128736 | 7/15/2014 | 37:00.3 | 0 |
| -1090.914 | 19778.533 | 2.47 | 15.497 | 0.141998 | 7/15/2014 | 37:00.5 | 0 |
| -1090.71 | 19778.517 | 2.472 | 15.494 | 0.141828 | 7/15/2014 | 37:00.6 | 0 |
| -1090.454 | 19778.492 | 2.472 | 15.49 | 0.152612 | 7/15/2014 | 37:00.7 | 0 |
| -1090.351 | 19778.484 | 2.472 | 15.489 | 0.162175 | 7/15/2014 | 37:00.7 | 0 |
| -1090.142 | 19778.466 | 2.477 | 15.483 | 0.155489 | 7/15/2014 | 37:00.8 | 0 |
| -1089.934 | 19778.447 | 2.477 | 15.476 | 0.162665 | 7/15/2014 | 37:00.9 | 0 |
| -1089.725 | 19778.43 | 2.474 | 15.468 | 0.161233 | 7/15/2014 | 37:01.0 | 0 |
| -1089.516 | 19778.41 | 2.474 | 15.459 | 0.160362 | 7/15/2014 | 37:01.1 | 0 |
| -1089.307 | 19778.39 | 2.474 | 15.45 | 0.171442 | 7/15/2014 | 37:01.2 | 0 |
| -1089.099 | 19778.371 | 2.474 | 15.441 | 0.177051 | 7/15/2014 | 37:01.3 | 0 |
| -1088.734 | 19778.336 | 2.469 | 15.428 | 0.163268 | 7/15/2014 | 37:01.5 | 0 |
| -1088.523 | 19778.317 | 2.468 | 15.419 | 0.168409 | 7/15/2014 | 37:01.6 | 0 |
| -1088.263 | 19778.293 | 2.463 | 15.411 | 0.176537 | 7/15/2014 | 37:01.7 | 0 |
| -1088.157 | 19778.284 | 2.464 | 15.407 | 0.185652 | 7/15/2014 | 37:01.7 | 0 |
| -1087.946 | 19778.264 | 2.462 | 15.4 | 0.165903 | 7/15/2014 | 37:01.8 | 0 |
| -1087.732 | 19778.242 | 2.462 | 15.393 | 0.154901 | 7/15/2014 | 37:01.9 | 0 |
| -1087.518 | 19778.223 | 2.462 | 15.387 | 0.15498 | 7/15/2014 | 37:02.0 | 0 |
| -1087.305 | 19778.202 | 2.462 | 15.381 | 0.147238 | 7/15/2014 | 37:02.1 | 0 |
| -1087.09 | 19778.181 | 2.467 | 15.375 | 0.149642 | 7/15/2014 | 37:02.2 | 0 |

| | | | | | | | |
|---|---|---|---|---|---|---|---|
| -1086.877 | 19778.159 | 2.467 | 15.368 | 0.151171 | 7/15/2014 | 37:02.3 | 0 |
| -1086.612 | 19778.135 | 2.468 | 15.362 | 0.116718 | 7/15/2014 | 37:02.4 | 0 |
| -1086.399 | 19778.112 | 2.472 | 15.356 | 0.13734 | 7/15/2014 | 37:02.5 | 0 |
| -1086.185 | 19778.091 | 2.475 | 15.349 | 0.150442 | 7/15/2014 | 37:02.6 | 0 |
| -1085.917 | 19778.065 | 2.475 | 15.343 | 0.132665 | 7/15/2014 | 37:02.7 | 0 |
| -1085.702 | 19778.043 | 2.475 | 15.337 | 0.122813 | 7/15/2014 | 37:02.8 | 0 |
| -1085.487 | 19778.022 | 2.475 | 15.331 | 0.114422 | 7/15/2014 | 37:02.9 | 0 |
| -1085.271 | 19777.999 | 2.473 | 15.327 | 0.122052 | 7/15/2014 | 37:03.0 | 0 |
| -1085.055 | 19777.978 | 2.476 | 15.323 | 0.101621 | 7/15/2014 | 37:03.1 | 0 |
| -1084.84 | 19777.955 | 2.473 | 15.32 | 0.0787018 | 7/15/2014 | 37:03.2 | 0 |
| -1084.626 | 19777.933 | 2.469 | 15.319 | 0.0637663 | 7/15/2014 | 37:03.3 | 0 |
| -1084.358 | 19777.905 | 2.472 | 15.319 | 0.0520135 | 7/15/2014 | 37:03.4 | 0 |
| -1084.145 | 19777.883 | 2.472 | 15.32 | 0.0438117 | 7/15/2014 | 37:03.5 | 0 |
| -1083.932 | 19777.861 | 2.468 | 15.324 | 0.0433012 | 7/15/2014 | 37:03.6 | 0 |
| -1083.667 | 19777.833 | 2.468 | 15.328 | 0.038004 | 7/15/2014 | 37:03.7 | 0 |
| -1083.453 | 19777.81 | 2.47 | 15.334 | 0.0354392 | 7/15/2014 | 37:03.8 | 0 |
| -1083.239 | 19777.787 | 2.467 | 15.34 | 0.0380342 | 7/15/2014 | 37:03.9 | 0 |
| -1083.023 | 19777.764 | 2.469 | 15.346 | 0.0326382 | 7/15/2014 | 37:04.0 | 0 |
| -1082.808 | 19777.74 | 2.469 | 15.352 | 0.0311457 | 7/15/2014 | 37:04.1 | 0 |
| -1082.595 | 19777.717 | 2.464 | 15.358 | 0.027578 | 7/15/2014 | 37:04.2 | 0 |
| -1082.38 | 19777.693 | 2.464 | 15.362 | 0.0198692 | 7/15/2014 | 37:04.3 | 0 |
| -1082.115 | 19777.665 | 2.467 | 15.365 | 0.0237465 | 7/15/2014 | 37:04.4 | 0 |
| -1081.902 | 19777.642 | 2.467 | 15.367 | 0.0126894 | 7/15/2014 | 37:04.5 | 0 |
| -1081.688 | 19777.618 | 2.467 | 15.367 | 0.0220609 | 7/15/2014 | 37:04.6 | 0 |
| -1081.42 | 19777.587 | 2.467 | 15.364 | 0.0256055 | 7/15/2014 | 37:04.7 | 0 |
| -1081.204 | 19777.563 | 2.465 | 15.36 | 0.0124999 | 7/15/2014 | 37:04.8 | 0 |
| -1080.987 | 19777.537 | 2.465 | 15.354 | 0.0234909 | 7/15/2014 | 37:04.9 | 0 |
| -1080.767 | 19777.514 | 2.467 | 15.346 | 0.0272723 | 7/15/2014 | 37:05.0 | 0 |
| -1080.548 | 19777.488 | 2.467 | 15.336 | 0.02603 | 7/15/2014 | 37:05.1 | 0 |
| -1080.331 | 19777.463 | 2.462 | 15.326 | 0.0281613 | 7/15/2014 | 37:05.2 | 0 |
| -1080.112 | 19777.439 | 2.462 | 15.314 | 0.0296828 | 7/15/2014 | 37:05.3 | 0 |
| -1079.841 | 19777.406 | 2.459 | 15.303 | 0.0426899 | 7/15/2014 | 37:05.4 | 0 |
| -1079.624 | 19777.382 | 2.459 | 15.291 | 0.0342651 | 7/15/2014 | 37:05.5 | 0 |
| -1079.407 | 19777.355 | 2.459 | 15.28 | 0.0624105 | 7/15/2014 | 37:05.6 | 0 |
| -1079.137 | 19777.324 | 2.459 | 15.27 | 0.057201 | 7/15/2014 | 37:05.7 | 0 |
| -1078.92 | 19777.298 | 2.459 | 15.262 | 0.0713123 | 7/15/2014 | 37:05.8 | 0 |
| -1078.703 | 19777.271 | 2.459 | 15.255 | 0.0971697 | 7/15/2014 | 37:05.9 | 0 |
| -1078.487 | 19777.247 | 2.459 | 15.25 | 0.110811 | 7/15/2014 | 37:06.0 | 0 |
| -1078.27 | 19777.221 | 2.462 | 15.248 | 0.141939 | 7/15/2014 | 37:06.1 | 0 |
| -1078.055 | 19777.194 | 2.462 | 15.248 | 0.143586 | 7/15/2014 | 37:06.2 | 0 |
| -1077.843 | 19777.169 | 2.462 | 15.252 | 0.164735 | 7/15/2014 | 37:06.3 | 0 |
| -1077.475 | 19777.124 | 2.459 | 15.265 | 0.185625 | 7/15/2014 | 37:06.5 | 0 |
| -1077.265 | 19777.099 | 2.461 | 15.277 | 0.17634 | 7/15/2014 | 37:06.6 | 0 |
| -1077.007 | 19777.066 | 2.459 | 15.292 | 0.146506 | 7/15/2014 | 37:06.7 | 0 |
| -1076.901 | 19777.054 | 2.46 | 15.299 | 0.144756 | 7/15/2014 | 37:06.7 | 0 |
| -1076.691 | 19777.028 | 2.462 | 15.319 | 0.0958214 | 7/15/2014 | 37:06.8 | 0 |
| -1076.482 | 19777.002 | 2.459 | 15.341 | 0.119934 | 7/15/2014 | 37:06.9 | 0 |

| | | | | | | | |
|---|---|---|---|---|---|---|---|
| -1076.273 | 19776.976 | 2.459 | 15.367 | 0.0855497 | 7/15/2014 | 37:07.0 | 0 |
| -1076.065 | 19776.95 | 2.459 | 15.393 | 0.0566501 | 7/15/2014 | 37:07.1 | 0 |
| -1075.856 | 19776.925 | 2.461 | 15.42 | 0.0445287 | 7/15/2014 | 37:07.2 | 0 |
| -1075.648 | 19776.898 | 2.461 | 15.447 | 0.0465547 | 7/15/2014 | 37:07.3 | 0 |
| -1075.288 | 19776.852 | 2.46 | 15.481 | 0.0479667 | 7/15/2014 | 37:07.5 | 0 |
| -1075.08 | 19776.826 | 2.462 | 15.501 | 0.0471615 | 7/15/2014 | 37:07.6 | 0 |
| -1074.823 | 19776.793 | 2.46 | 15.518 | 0.0526618 | 7/15/2014 | 37:07.7 | 0 |
| -1074.718 | 19776.779 | 2.461 | 15.528 | 0.0555528 | 7/15/2014 | 37:07.7 | 0 |
| -1074.509 | 19776.753 | 2.459 | 15.539 | 0.0397103 | 7/15/2014 | 37:07.8 | 0 |
| -1074.3 | 19776.727 | 2.459 | 15.546 | 0.0412383 | 7/15/2014 | 37:07.9 | 0 |
| -1074.088 | 19776.699 | 2.461 | 15.549 | 0.0356542 | 7/15/2014 | 37:08.0 | 0 |
| -1073.876 | 19776.672 | 2.461 | 15.55 | 0.0231033 | 7/15/2014 | 37:08.1 | 0 |
| -1073.665 | 19776.645 | 2.464 | 15.547 | 0.0241908 | 7/15/2014 | 37:08.2 | 0 |
| -1073.454 | 19776.616 | 2.464 | 15.542 | 0.0359435 | 7/15/2014 | 37:08.3 | 0 |
| -1073.191 | 19776.582 | 2.464 | 15.536 | 0.050648 | 7/15/2014 | 37:08.4 | 0 |
| -1072.979 | 19776.555 | 2.467 | 15.528 | 0.0363869 | 7/15/2014 | 37:08.5 | 0 |
| -1072.767 | 19776.527 | 2.469 | 15.519 | 0.0631067 | 7/15/2014 | 37:08.6 | 0 |
| -1072.5 | 19776.492 | 2.472 | 15.509 | 0.0436451 | 7/15/2014 | 37:08.7 | 0 |
| -1072.288 | 19776.465 | 2.473 | 15.5 | 0.048819 | 7/15/2014 | 37:08.8 | 0 |
| -1072.072 | 19776.438 | 2.472 | 15.49 | 0.0489905 | 7/15/2014 | 37:08.9 | 0 |
| -1071.858 | 19776.407 | 2.47 | 15.481 | 0.0602452 | 7/15/2014 | 37:09.0 | 0 |
| -1071.641 | 19776.379 | 2.47 | 15.473 | 0.0751237 | 7/15/2014 | 37:09.1 | 0 |
| -1071.427 | 19776.352 | 2.468 | 15.464 | 0.0647141 | 7/15/2014 | 37:09.2 | 0 |
| -1071.212 | 19776.324 | 2.465 | 15.456 | 0.089198 | 7/15/2014 | 37:09.3 | 0 |
| -1070.944 | 19776.287 | 2.464 | 15.449 | 0.0909047 | 7/15/2014 | 37:09.4 | 0 |
| -1070.732 | 19776.26 | 2.46 | 15.441 | 0.101311 | 7/15/2014 | 37:09.5 | 0 |
| -1070.516 | 19776.232 | 2.457 | 15.435 | 0.0959337 | 7/15/2014 | 37:09.6 | 0 |
| -1070.248 | 19776.196 | 2.455 | 15.429 | 0.0989161 | 7/15/2014 | 37:09.7 | 0 |
| -1070.031 | 19776.166 | 2.451 | 15.423 | 0.107158 | 7/15/2014 | 37:09.8 | 0 |
| -1069.814 | 19776.139 | 2.451 | 15.418 | 0.100967 | 7/15/2014 | 37:09.9 | 0 |
| -1069.594 | 19776.109 | 2.453 | 15.413 | 0.106421 | 7/15/2014 | 37:10.0 | 0 |
| -1069.375 | 19776.08 | 2.453 | 15.41 | 0.109934 | 7/15/2014 | 37:10.1 | 0 |
| -1069.157 | 19776.051 | 2.451 | 15.407 | 0.114497 | 7/15/2014 | 37:10.2 | 0 |
| -1068.938 | 19776.023 | 2.454 | 15.405 | 0.119471 | 7/15/2014 | 37:10.3 | 0 |
| -1068.667 | 19775.986 | 2.456 | 15.404 | 0.115953 | 7/15/2014 | 37:10.4 | 0 |
| -1068.449 | 19775.955 | 2.458 | 15.403 | 0.121195 | 7/15/2014 | 37:10.5 | 0 |
| -1068.23 | 19775.928 | 2.46 | 15.403 | 0.145529 | 7/15/2014 | 37:10.6 | 0 |
| -1067.957 | 19775.889 | 2.458 | 15.404 | 0.141374 | 7/15/2014 | 37:10.7 | 0 |
| -1067.737 | 19775.861 | 2.458 | 15.404 | 0.125763 | 7/15/2014 | 37:10.8 | 0 |
| -1067.517 | 19775.83 | 2.456 | 15.404 | 0.13523 | 7/15/2014 | 37:10.9 | 0 |
| -1067.295 | 19775.801 | 2.456 | 15.403 | 0.142206 | 7/15/2014 | 37:11.0 | 0 |
| -1067.074 | 19775.771 | 2.453 | 15.402 | 0.147729 | 7/15/2014 | 37:11.1 | 0 |
| -1066.855 | 19775.74 | 2.449 | 15.4 | 0.133325 | 7/15/2014 | 37:11.2 | 0 |
| -1066.635 | 19775.711 | 2.447 | 15.396 | 0.134327 | 7/15/2014 | 37:11.3 | 0 |
| -1066.363 | 19775.672 | 2.447 | 15.392 | 0.133653 | 7/15/2014 | 37:11.4 | 0 |
| -1066.145 | 19775.645 | 2.449 | 15.385 | 0.151222 | 7/15/2014 | 37:11.5 | 0 |
| -1065.928 | 19775.613 | 2.447 | 15.378 | 0.136916 | 7/15/2014 | 37:11.6 | 0 |

| | | | | | | | |
|---|---|---|---|---|---|---|---|
| -1065.657 | 19775.576 | 2.447 | 15.368 | 0.130879 | 7/15/2014 | 37:11.7 | 0 |
| -1065.439 | 19775.546 | 2.449 | 15.358 | 0.126531 | 7/15/2014 | 37:11.8 | 0 |
| -1065.221 | 19775.517 | 2.449 | 15.346 | 0.151602 | 7/15/2014 | 37:11.9 | 0 |
| -1065.002 | 19775.486 | 2.451 | 15.333 | 0.152461 | 7/15/2014 | 37:12.0 | 0 |
| -1064.782 | 19775.455 | 2.451 | 15.32 | 0.172912 | 7/15/2014 | 37:12.1 | 0 |
| -1064.564 | 19775.426 | 2.449 | 15.307 | 0.165443 | 7/15/2014 | 37:12.2 | 0 |
| -1064.346 | 19775.396 | 2.447 | 15.294 | 0.165447 | 7/15/2014 | 37:12.3 | 0 |
| -1064.077 | 19775.358 | 2.445 | 15.283 | 0.185015 | 7/15/2014 | 37:12.4 | 0 |
| -1063.859 | 19775.328 | 2.445 | 15.273 | 0.184426 | 7/15/2014 | 37:12.5 | 0 |
| -1063.642 | 19775.298 | 2.443 | 15.264 | 0.164191 | 7/15/2014 | 37:12.6 | 0 |
| -1063.372 | 19775.262 | 2.436 | 15.258 | 0.168206 | 7/15/2014 | 37:12.7 | 0 |
| -1063.151 | 19775.23 | 2.434 | 15.253 | 0.162089 | 7/15/2014 | 37:12.8 | 0 |
| -1062.93 | 19775.199 | 2.431 | 15.25 | 0.171513 | 7/15/2014 | 37:12.9 | 0 |
| -1062.709 | 19775.169 | 2.429 | 15.25 | 0.188533 | 7/15/2014 | 37:13.0 | 0 |
| -1062.49 | 19775.138 | 2.423 | 15.25 | 0.185863 | 7/15/2014 | 37:13.1 | 0 |
| -1062.269 | 19775.107 | 2.416 | 15.252 | 0.179922 | 7/15/2014 | 37:13.2 | 0 |
| -1062.049 | 19775.076 | 2.41 | 15.255 | 0.200069 | 7/15/2014 | 37:13.3 | 0 |
| -1061.777 | 19775.039 | 2.401 | 15.26 | 0.207138 | 7/15/2014 | 37:13.4 | 0 |
| -1061.557 | 19775.008 | 2.401 | 15.264 | 0.194136 | 7/15/2014 | 37:13.5 | 0 |
| -1061.339 | 19774.978 | 2.395 | 15.269 | 0.201912 | 7/15/2014 | 37:13.6 | 0 |
| -1061.066 | 19774.939 | 2.392 | 15.274 | 0.184743 | 7/15/2014 | 37:13.7 | 0 |
| -1060.849 | 19774.908 | 2.386 | 15.278 | 0.164 | 7/15/2014 | 37:13.8 | 0 |
| -1060.629 | 19774.878 | 2.386 | 15.282 | 0.198185 | 7/15/2014 | 37:13.9 | 0 |
| -1060.409 | 19774.848 | 2.383 | 15.285 | 0.194402 | 7/15/2014 | 37:14.0 | 0 |
| -1060.189 | 19774.816 | 2.383 | 15.287 | 0.175281 | 7/15/2014 | 37:14.1 | 0 |
| -1059.97 | 19774.785 | 2.383 | 15.288 | 0.204197 | 7/15/2014 | 37:14.2 | 0 |
| -1059.753 | 19774.755 | 2.376 | 15.288 | 0.195171 | 7/15/2014 | 37:14.3 | 0 |
| -1059.48 | 19774.717 | 2.374 | 15.287 | 0.205695 | 7/15/2014 | 37:14.4 | 0 |
| -1059.262 | 19774.686 | 2.366 | 15.285 | 0.194299 | 7/15/2014 | 37:14.5 | 0 |
| -1059.041 | 19774.656 | 2.361 | 15.284 | 0.201559 | 7/15/2014 | 37:14.6 | 0 |
| -1058.762 | 19774.617 | 2.352 | 15.282 | 0.192753 | 7/15/2014 | 37:14.7 | 0 |
| -1058.537 | 19774.586 | 2.346 | 15.281 | 0.197906 | 7/15/2014 | 37:14.8 | 0 |
| -1058.309 | 19774.555 | 2.339 | 15.281 | 0.202718 | 7/15/2014 | 37:14.9 | 0 |
| -1058.08 | 19774.522 | 2.331 | 15.282 | 0.200242 | 7/15/2014 | 37:15.0 | 0 |
| -1057.968 | 19774.507 | 2.332 | 15.283 | 0.208631 | 7/15/2014 | 37:15.1 | 0 |
| -1057.737 | 19774.475 | 2.33 | 15.286 | 0.206799 | 7/15/2014 | 37:15.2 | 0 |
| -1057.503 | 19774.442 | 2.33 | 15.292 | 0.185687 | 7/15/2014 | 37:15.3 | 0 |
| -1057.092 | 19774.386 | 2.33 | 15.304 | 0.194189 | 7/15/2014 | 37:15.4 | 0 |
| -1056.856 | 19774.354 | 2.332 | 15.312 | 0.190851 | 7/15/2014 | 37:15.5 | 0 |
| -1056.619 | 19774.321 | 2.335 | 15.321 | 0.181278 | 7/15/2014 | 37:15.6 | 0 |
| -1056.321 | 19774.28 | 2.333 | 15.33 | 0.162383 | 7/15/2014 | 37:15.7 | 0 |
| -1056.201 | 19774.264 | 2.334 | 15.334 | 0.177227 | 7/15/2014 | 37:15.8 | 0 |
| -1055.96 | 19774.23 | 2.331 | 15.343 | 0.166947 | 7/15/2014 | 37:15.9 | 0 |
| -1055.721 | 19774.197 | 2.326 | 15.35 | 0.179583 | 7/15/2014 | 37:16.0 | 0 |
| -1055.479 | 19774.165 | 2.322 | 15.355 | 0.165383 | 7/15/2014 | 37:16.1 | 0 |
| -1055.24 | 19774.131 | 2.315 | 15.359 | 0.195929 | 7/15/2014 | 37:16.2 | 0 |
| -1054.999 | 19774.099 | 2.315 | 15.359 | 0.16844 | 7/15/2014 | 37:16.3 | 0 |

| | | | | | | | |
|---|---|---|---|---|---|---|---|
| -1054.583 | 19774.041 | 2.309 | 15.352 | 0.173887 | 7/15/2014 | 37:16.4 | 0 |
| -1054.347 | 19774.01 | 2.309 | 15.345 | 0.170166 | 7/15/2014 | 37:16.5 | 0 |
| -1054.112 | 19773.977 | 2.309 | 15.334 | 0.132457 | 7/15/2014 | 37:16.6 | 0 |
| -1053.82 | 19773.938 | 2.311 | 15.319 | 0.117512 | 7/15/2014 | 37:16.7 | 0 |
| -1053.7 | 19773.92 | 2.309 | 15.313 | 0.0905746 | 7/15/2014 | 37:16.8 | 0 |
| -1053.463 | 19773.889 | 2.316 | 15.294 | 0.0963967 | 7/15/2014 | 37:16.9 | 0 |
| -1053.232 | 19773.855 | 2.311 | 15.271 | 0.119139 | 7/15/2014 | 37:17.0 | 0 |
| -1052.998 | 19773.824 | 2.314 | 15.247 | 0.111293 | 7/15/2014 | 37:17.1 | 0 |
| -1052.766 | 19773.793 | 2.307 | 15.221 | 0.12277 | 7/15/2014 | 37:17.2 | 0 |
| -1052.421 | 19773.748 | 2.303 | 15.18 | 0.144601 | 7/15/2014 | 37:17.3 | 0 |
| -1052.136 | 19773.709 | 2.301 | 15.154 | 0.158503 | 7/15/2014 | 37:17.4 | 0 |
| -1051.907 | 19773.678 | 2.298 | 15.13 | 0.162344 | 7/15/2014 | 37:17.5 | 0 |
| -1051.68 | 19773.646 | 2.294 | 15.109 | 0.166978 | 7/15/2014 | 37:17.6 | 0 |
| -1051.393 | 19773.607 | 2.292 | 15.092 | 0.147175 | 7/15/2014 | 37:17.7 | 0 |
| -1051.162 | 19773.577 | 2.29 | 15.081 | 0.145382 | 7/15/2014 | 37:17.8 | 0 |
| -1051.048 | 19773.563 | 2.292 | 15.073 | 0.141106 | 7/15/2014 | 37:17.9 | 0 |
| -1050.816 | 19773.532 | 2.292 | 15.07 | 0.128237 | 7/15/2014 | 37:18.0 | 0 |
| -1050.582 | 19773.5 | 2.292 | 15.073 | 0.13199 | 7/15/2014 | 37:18.1 | 0 |
| -1050.35 | 19773.469 | 2.286 | 15.082 | 0.127242 | 7/15/2014 | 37:18.2 | 0 |
| -1050.114 | 19773.438 | 2.286 | 15.096 | 0.126523 | 7/15/2014 | 37:18.3 | 0 |
| -1049.702 | 19773.383 | 2.281 | 15.128 | 0.133174 | 7/15/2014 | 37:18.4 | 0 |
| -1049.468 | 19773.353 | 2.278 | 15.15 | 0.13304 | 7/15/2014 | 37:18.5 | 0 |
| -1049.23 | 19773.319 | 2.275 | 15.174 | 0.150939 | 7/15/2014 | 37:18.6 | 0 |
| -1048.936 | 19773.281 | 2.27 | 15.197 | 0.152919 | 7/15/2014 | 37:18.7 | 0 |
| -1048.818 | 19773.266 | 2.267 | 15.21 | 0.163519 | 7/15/2014 | 37:18.8 | 0 |
| -1048.578 | 19773.234 | 2.267 | 15.232 | 0.141797 | 7/15/2014 | 37:18.9 | 0 |
| -1048.341 | 19773.203 | 2.261 | 15.25 | 0.142987 | 7/15/2014 | 37:19.0 | 0 |
| -1048.102 | 19773.172 | 2.261 | 15.265 | 0.137573 | 7/15/2014 | 37:19.1 | 0 |
| -1047.865 | 19773.141 | 2.255 | 15.274 | 0.140367 | 7/15/2014 | 37:19.2 | 0 |
| -1047.628 | 19773.107 | 2.251 | 15.279 | 0.138267 | 7/15/2014 | 37:19.3 | 0 |
| -1047.218 | 19773.056 | 2.246 | 15.276 | 0.123515 | 7/15/2014 | 37:19.4 | 0 |
| -1046.985 | 19773.025 | 2.243 | 15.27 | 0.141632 | 7/15/2014 | 37:19.5 | 0 |
| -1046.752 | 19772.994 | 2.241 | 15.262 | 0.132269 | 7/15/2014 | 37:19.6 | 0 |
| -1046.463 | 19772.957 | 2.238 | 15.252 | 0.136391 | 7/15/2014 | 37:19.7 | 0 |
| -1046.344 | 19772.941 | 2.236 | 15.246 | 0.127555 | 7/15/2014 | 37:19.8 | 0 |
| -1046.111 | 19772.911 | 2.236 | 15.234 | 0.136501 | 7/15/2014 | 37:19.9 | 0 |
| -1045.88 | 19772.882 | 2.234 | 15.22 | 0.13032 | 7/15/2014 | 37:20.0 | 0 |
| -1045.647 | 19772.853 | 2.238 | 15.206 | 0.156842 | 7/15/2014 | 37:20.1 | 0 |
| -1045.415 | 19772.823 | 2.235 | 15.192 | 0.144345 | 7/15/2014 | 37:20.2 | 0 |
| -1045.185 | 19772.793 | 2.233 | 15.178 | 0.16122 | 7/15/2014 | 37:20.3 | 0 |
| -1044.784 | 19772.743 | 2.226 | 15.156 | 0.163558 | 7/15/2014 | 37:20.4 | 0 |
| -1044.557 | 19772.714 | 2.227 | 15.142 | 0.177156 | 7/15/2014 | 37:20.5 | 0 |
| -1044.329 | 19772.687 | 2.224 | 15.127 | 0.163189 | 7/15/2014 | 37:20.6 | 0 |
| -1044.045 | 19772.651 | 2.224 | 15.113 | 0.170916 | 7/15/2014 | 37:20.7 | 0 |
| -1043.815 | 19772.622 | 2.221 | 15.099 | 0.159428 | 7/15/2014 | 37:20.8 | 0 |
| -1043.585 | 19772.594 | 2.218 | 15.086 | 0.164086 | 7/15/2014 | 37:20.9 | 0 |
| -1043.356 | 19772.566 | 2.223 | 15.073 | 0.161049 | 7/15/2014 | 37:21.0 | 0 |

| | | | | | | | |
|---|---|---|---|---|---|---|---|
| -1043.127 | 19772.538 | 2.223 | 15.062 | 0.147765 | 7/15/2014 | 37:21.1 | 0 |
| -1042.898 | 19772.509 | 2.226 | 15.053 | 0.137826 | 7/15/2014 | 37:21.2 | 0 |
| -1042.671 | 19772.481 | 2.223 | 15.045 | 0.135735 | 7/15/2014 | 37:21.3 | 0 |
| -1042.388 | 19772.447 | 2.226 | 15.039 | 0.125303 | 7/15/2014 | 37:21.4 | 0 |
| -1042.162 | 19772.419 | 2.231 | 15.035 | 0.153711 | 7/15/2014 | 37:21.5 | 0 |
| -1041.937 | 19772.393 | 2.231 | 15.033 | 0.11154 | 7/15/2014 | 37:21.6 | 0 |
| -1041.658 | 19772.359 | 2.233 | 15.034 | 0.145399 | 7/15/2014 | 37:21.7 | 0 |
| -1041.434 | 19772.332 | 2.233 | 15.036 | 0.16861 | 7/15/2014 | 37:21.8 | 0 |
| -1041.209 | 19772.306 | 2.233 | 15.041 | 0.144299 | 7/15/2014 | 37:21.9 | 0 |
| -1040.985 | 19772.278 | 2.23 | 15.047 | 0.188188 | 7/15/2014 | 37:22.0 | 0 |
| -1040.76 | 19772.252 | 2.23 | 15.054 | 0.191935 | 7/15/2014 | 37:22.1 | 0 |
| -1040.538 | 19772.226 | 2.225 | 15.063 | 0.20363 | 7/15/2014 | 37:22.2 | 0 |
| -1040.317 | 19772.2 | 2.223 | 15.072 | 0.232385 | 7/15/2014 | 37:22.3 | 0 |
| -1040.043 | 19772.168 | 2.221 | 15.082 | 0.206517 | 7/15/2014 | 37:22.4 | 0 |
| -1039.824 | 19772.143 | 2.218 | 15.091 | 0.20864 | 7/15/2014 | 37:22.5 | 0 |
| -1039.606 | 19772.119 | 2.218 | 15.099 | 0.251193 | 7/15/2014 | 37:22.6 | 0 |
| -1039.333 | 19772.086 | 2.218 | 15.107 | 0.201556 | 7/15/2014 | 37:22.7 | 0 |
| -1039.113 | 19772.062 | 2.217 | 15.114 | 0.201271 | 7/15/2014 | 37:22.8 | 0 |
| -1038.894 | 19772.037 | 2.217 | 15.119 | 0.192655 | 7/15/2014 | 37:22.9 | 0 |
| -1038.672 | 19772.011 | 2.222 | 15.122 | 0.173532 | 7/15/2014 | 37:23.0 | 0 |
| -1038.452 | 19771.986 | 2.219 | 15.124 | 0.194021 | 7/15/2014 | 37:23.1 | 0 |
| -1038.231 | 19771.963 | 2.219 | 15.123 | 0.190121 | 7/15/2014 | 37:23.2 | 0 |
| -1038.011 | 19771.938 | 2.217 | 15.12 | 0.191263 | 7/15/2014 | 37:23.3 | 0 |
| -1037.738 | 19771.907 | 2.211 | 15.115 | 0.199587 | 7/15/2014 | 37:23.4 | 0 |
| -1037.517 | 19771.883 | 2.211 | 15.108 | 0.194294 | 7/15/2014 | 37:23.5 | 0 |
| -1037.298 | 19771.859 | 2.209 | 15.099 | 0.20711 | 7/15/2014 | 37:23.6 | 0 |
| -1037.023 | 19771.828 | 2.204 | 15.088 | 0.222436 | 7/15/2014 | 37:23.7 | 0 |
| -1036.8 | 19771.805 | 2.198 | 15.076 | 0.2009 | 7/15/2014 | 37:23.8 | 0 |
| -1036.577 | 19771.781 | 2.193 | 15.064 | 0.208956 | 7/15/2014 | 37:23.9 | 0 |
| -1036.353 | 19771.757 | 2.191 | 15.051 | 0.208517 | 7/15/2014 | 37:24.0 | 0 |
| -1036.13 | 19771.733 | 2.188 | 15.038 | 0.210961 | 7/15/2014 | 37:24.1 | 0 |
| -1035.906 | 19771.708 | 2.188 | 15.026 | 0.208153 | 7/15/2014 | 37:24.2 | 0 |
| -1035.683 | 19771.685 | 2.186 | 15.015 | 0.210474 | 7/15/2014 | 37:24.3 | 0 |
| -1035.408 | 19771.655 | 2.184 | 15.004 | 0.207174 | 7/15/2014 | 37:24.4 | 0 |
| -1035.187 | 19771.633 | 2.181 | 14.994 | 0.213037 | 7/15/2014 | 37:24.5 | 0 |
| -1034.964 | 19771.609 | 2.175 | 14.986 | 0.208194 | 7/15/2014 | 37:24.6 | 0 |
| -1034.691 | 19771.58 | 2.172 | 14.978 | 0.20637 | 7/15/2014 | 37:24.7 | 0 |
| -1034.47 | 19771.559 | 2.172 | 14.971 | 0.20114 | 7/15/2014 | 37:24.8 | 0 |
| -1034.25 | 19771.534 | 2.169 | 14.965 | 0.204595 | 7/15/2014 | 37:24.9 | 0 |
| -1034.03 | 19771.511 | 2.164 | 14.959 | 0.205 | 7/15/2014 | 37:25.0 | 0 |
| -1033.81 | 19771.489 | 2.157 | 14.953 | 0.197103 | 7/15/2014 | 37:25.1 | 0 |
| -1033.593 | 19771.467 | 2.157 | 14.947 | 0.187339 | 7/15/2014 | 37:25.2 | 0 |
| -1033.376 | 19771.444 | 2.157 | 14.942 | 0.187014 | 7/15/2014 | 37:25.3 | 0 |
| -1033.109 | 19771.416 | 2.157 | 14.936 | 0.181854 | 7/15/2014 | 37:25.4 | 0 |
| -1032.896 | 19771.396 | 2.157 | 14.931 | 0.179932 | 7/15/2014 | 37:25.5 | 0 |
| -1032.53 | 19771.357 | 2.152 | 14.922 | 0.180707 | 7/15/2014 | 37:25.7 | 0 |
| -1032.422 | 19771.346 | 2.154 | 14.92 | 0.181962 | 7/15/2014 | 37:25.7 | 0 |

| | | | | | | | |
|---|---|---|---|---|---|---|---|
| -1032.211 | 19771.326 | 2.151 | 14.914 | 0.182224 | 7/15/2014 | 37:25.8 | 0 |
| -1031.999 | 19771.304 | 2.149 | 14.909 | 0.182849 | 7/15/2014 | 37:25.9 | 0 |
| -1031.79 | 19771.282 | 2.142 | 14.903 | 0.181216 | 7/15/2014 | 37:26.0 | 0 |
| -1031.579 | 19771.261 | 2.14 | 14.898 | 0.177311 | 7/15/2014 | 37:26.1 | 0 |
| -1031.37 | 19771.24 | 2.137 | 14.894 | 0.173917 | 7/15/2014 | 37:26.2 | 0 |
| -1031.163 | 19771.219 | 2.135 | 14.89 | 0.173783 | 7/15/2014 | 37:26.3 | 0 |
| -1030.801 | 19771.183 | 2.132 | 14.887 | 0.167558 | 7/15/2014 | 37:26.5 | 0 |
| -1030.595 | 19771.162 | 2.132 | 14.885 | 0.16799 | 7/15/2014 | 37:26.6 | 0 |
| -1030.339 | 19771.137 | 2.13 | 14.883 | 0.165348 | 7/15/2014 | 37:26.7 | 0 |
| -1030.235 | 19771.126 | 2.132 | 14.882 | 0.165428 | 7/15/2014 | 37:26.7 | 0 |
| -1030.027 | 19771.105 | 2.132 | 14.882 | 0.166373 | 7/15/2014 | 37:26.8 | 0 |
| -1029.819 | 19771.085 | 2.129 | 14.882 | 0.167665 | 7/15/2014 | 37:26.9 | 0 |
| -1029.611 | 19771.064 | 2.127 | 14.883 | 0.170233 | 7/15/2014 | 37:27.0 | 0 |
| -1029.401 | 19771.044 | 2.127 | 14.884 | 0.171229 | 7/15/2014 | 37:27.1 | 0 |
| -1029.192 | 19771.022 | 2.127 | 14.885 | 0.175324 | 7/15/2014 | 37:27.2 | 0 |
| -1028.985 | 19771.002 | 2.123 | 14.886 | 0.178666 | 7/15/2014 | 37:27.3 | 0 |
| -1028.622 | 19770.967 | 2.119 | 14.889 | 0.188169 | 7/15/2014 | 37:27.5 | 0 |
| -1028.414 | 19770.946 | 2.119 | 14.891 | 0.19073 | 7/15/2014 | 37:27.6 | 0 |
| -1028.154 | 19770.921 | 2.115 | 14.894 | 0.181749 | 7/15/2014 | 37:27.7 | 0 |
| -1028.049 | 19770.911 | 2.116 | 14.895 | 0.183802 | 7/15/2014 | 37:27.7 | 0 |
| -1027.839 | 19770.89 | 2.114 | 14.899 | 0.191102 | 7/15/2014 | 37:27.8 | 0 |
| -1027.626 | 19770.869 | 2.112 | 14.905 | 0.183494 | 7/15/2014 | 37:27.9 | 0 |
| -1027.414 | 19770.849 | 2.11 | 14.912 | 0.191779 | 7/15/2014 | 37:28.0 | 0 |
| -1027.202 | 19770.827 | 2.109 | 14.921 | 0.190578 | 7/15/2014 | 37:28.1 | 0 |
| -1026.99 | 19770.808 | 2.104 | 14.931 | 0.180143 | 7/15/2014 | 37:28.2 | 0 |
| -1026.779 | 19770.786 | 2.104 | 14.942 | 0.186775 | 7/15/2014 | 37:28.3 | 0 |
| -1026.515 | 19770.762 | 2.099 | 14.955 | 0.207265 | 7/15/2014 | 37:28.4 | 0 |
| -1026.305 | 19770.74 | 2.092 | 14.969 | 0.211244 | 7/15/2014 | 37:28.5 | 0 |
| -1026.093 | 19770.721 | 2.089 | 14.983 | 0.21443 | 7/15/2014 | 37:28.6 | 0 |
| -1025.828 | 19770.694 | 2.083 | 14.998 | 0.207068 | 7/15/2014 | 37:28.7 | 0 |
| -1025.616 | 19770.674 | 2.081 | 15.013 | 0.193983 | 7/15/2014 | 37:28.8 | 0 |
| -1025.403 | 19770.653 | 2.074 | 15.028 | 0.20043 | 7/15/2014 | 37:28.9 | 0 |
| -1025.188 | 19770.633 | 2.074 | 15.043 | 0.196013 | 7/15/2014 | 37:29.0 | 0 |
| -1024.976 | 19770.613 | 2.068 | 15.057 | 0.159617 | 7/15/2014 | 37:29.1 | 0 |
| -1024.764 | 19770.592 | 2.068 | 15.071 | 0.152022 | 7/15/2014 | 37:29.2 | 0 |
| -1024.552 | 19770.572 | 2.074 | 15.085 | 0.150008 | 7/15/2014 | 37:29.3 | 0 |
| -1024.187 | 19770.537 | 2.075 | 15.105 | 0.130436 | 7/15/2014 | 37:29.5 | 0 |
| -1023.977 | 19770.518 | 2.077 | 15.118 | 0.138709 | 7/15/2014 | 37:29.6 | 0 |
| -1023.721 | 19770.492 | 2.075 | 15.131 | 0.131841 | 7/15/2014 | 37:29.7 | 0 |
| -1023.613 | 19770.482 | 2.077 | 15.138 | 0.127434 | 7/15/2014 | 37:29.7 | 0 |
| -1023.406 | 19770.464 | 2.081 | 15.152 | 0.126634 | 7/15/2014 | 37:29.8 | 0 |
| -1023.198 | 19770.443 | 2.081 | 15.166 | 0.138343 | 7/15/2014 | 37:29.9 | 0 |
| -1022.989 | 19770.424 | 2.084 | 15.182 | 0.128528 | 7/15/2014 | 37:30.0 | 0 |
| -1022.783 | 19770.404 | 2.077 | 15.199 | 0.114195 | 7/15/2014 | 37:30.1 | 0 |
| -1022.576 | 19770.385 | 2.077 | 15.217 | 0.127561 | 7/15/2014 | 37:30.2 | 0 |
| -1022.373 | 19770.367 | 2.074 | 15.238 | 0.12488 | 7/15/2014 | 37:30.3 | 0 |
| -1022.02 | 19770.333 | 2.069 | 15.272 | 0.106451 | 7/15/2014 | 37:30.5 | 0 |

| | | | | | | | |
|---|---|---|---|---|---|---|---|
| -1021.819 | 19770.316 | 2.069 | 15.296 | 0.105223 | 7/15/2014 | 37:30.6 | 0 |
| -1021.572 | 19770.293 | 2.063 | 15.321 | 0.0823527 | 7/15/2014 | 37:30.7 | 0 |
| -1021.469 | 19770.283 | 2.065 | 15.333 | 0.0773897 | 7/15/2014 | 37:30.7 | 0 |
| -1021.268 | 19770.264 | 2.062 | 15.358 | 0.0835536 | 7/15/2014 | 37:30.8 | 0 |
| -1020.968 | 19770.236 | 2.066 | 15.391 | 0.081187 | 7/15/2014 | 37:31.0 | 0 |
| -1020.77 | 19770.219 | 2.063 | 15.411 | 0.0733227 | 7/15/2014 | 37:31.1 | 0 |
| -1020.57 | 19770.201 | 2.066 | 15.427 | 0.122754 | 7/15/2014 | 37:31.2 | 0 |
| -1020.373 | 19770.182 | 2.066 | 15.441 | 0.05563 | 7/15/2014 | 37:31.3 | 0 |
| -1020.175 | 19770.166 | 2.069 | 15.451 | 0.0685534 | 7/15/2014 | 37:31.4 | 0 |
| -1019.931 | 19770.143 | 2.066 | 15.458 | 0.0800852 | 7/15/2014 | 37:31.5 | 0 |
| -1019.735 | 19770.125 | 2.066 | 15.461 | 0.0590101 | 7/15/2014 | 37:31.6 | 0 |
| -1019.492 | 19770.104 | 2.063 | 15.461 | 0.0623736 | 7/15/2014 | 37:31.7 | 0 |
| -1019.293 | 19770.084 | 2.069 | 15.459 | 0.0434954 | 7/15/2014 | 37:31.8 | 0 |
| -1019.098 | 19770.066 | 2.063 | 15.455 | 0.0613021 | 7/15/2014 | 37:31.9 | 0 |
| -1018.899 | 19770.049 | 2.066 | 15.449 | 0.0549376 | 7/15/2014 | 37:32.0 | 0 |
| -1018.702 | 19770.029 | 2.066 | 15.441 | 0.080485 | 7/15/2014 | 37:32.1 | 0 |
| -1018.502 | 19770.013 | 2.072 | 15.432 | 0.0637617 | 7/15/2014 | 37:32.2 | 0 |
| -1018.306 | 19769.994 | 2.069 | 15.422 | 0.089463 | 7/15/2014 | 37:32.3 | 0 |
| -1018.11 | 19769.977 | 2.072 | 15.411 | 0.068678 | 7/15/2014 | 37:32.4 | 0 |
| -1017.866 | 19769.955 | 2.072 | 15.399 | 0.0394769 | 7/15/2014 | 37:32.5 | 0 |
| -1017.669 | 19769.936 | 2.075 | 15.385 | 0.0543893 | 7/15/2014 | 37:32.6 | 0 |
| -1017.426 | 19769.915 | 2.069 | 15.371 | 0.0240327 | 7/15/2014 | 37:32.7 | 0 |
| -1017.23 | 19769.896 | 2.069 | 15.356 | 0.035334 | 7/15/2014 | 37:32.8 | 0 |
| -1017.033 | 19769.879 | 2.066 | 15.341 | 0.0210449 | 7/15/2014 | 37:32.9 | 0 |
| -1016.834 | 19769.86 | 2.066 | 15.326 | 0.0185848 | 7/15/2014 | 37:33.0 | 0 |
| -1016.637 | 19769.844 | 2.063 | 15.312 | 0.0474757 | 7/15/2014 | 37:33.1 | 0 |
| -1016.439 | 19769.824 | 2.066 | 15.299 | 0.0211328 | 7/15/2014 | 37:33.2 | 0 |
| -1016.244 | 19769.809 | 2.063 | 15.288 | 0.0263548 | 7/15/2014 | 37:33.3 | 0 |
| -1016.05 | 19769.789 | 2.063 | 15.278 | 0.0347243 | 7/15/2014 | 37:33.4 | 0 |
| -1015.807 | 19769.769 | 2.06 | 15.27 | 0.0416919 | 7/15/2014 | 37:33.5 | 0 |
| -1015.614 | 19769.752 | 2.063 | 15.264 | 0.043297 | 7/15/2014 | 37:33.6 | 0 |
| -1015.372 | 19769.73 | 2.063 | 15.26 | 0.0443395 | 7/15/2014 | 37:33.7 | 0 |
| -1015.176 | 19769.713 | 2.069 | 15.257 | 0.045119 | 7/15/2014 | 37:33.8 | 0 |
| -1014.981 | 19769.697 | 2.069 | 15.255 | 0.0395394 | 7/15/2014 | 37:33.9 | 0 |
| -1014.783 | 19769.678 | 2.069 | 15.252 | 0.0219906 | 7/15/2014 | 37:34.0 | 0 |
| -1014.588 | 19769.662 | 2.066 | 15.249 | 0.0341763 | 7/15/2014 | 37:34.1 | 0 |
| -1014.391 | 19769.643 | 2.069 | 15.245 | 0.0295138 | 7/15/2014 | 37:34.2 | 0 |
| -1014.196 | 19769.627 | 2.072 | 15.239 | 0.0396291 | 7/15/2014 | 37:34.3 | 0 |
| -1014 | 19769.61 | 2.077 | 15.231 | 0.0593176 | 7/15/2014 | 37:34.4 | 0 |
| -1013.759 | 19769.588 | 2.075 | 15.22 | 0.047006 | 7/15/2014 | 37:34.5 | 0 |
| -1013.563 | 19769.572 | 2.08 | 15.207 | 0.0529346 | 7/15/2014 | 37:34.6 | 0 |
| -1013.324 | 19769.553 | 2.077 | 15.191 | 0.0566142 | 7/15/2014 | 37:34.7 | 0 |
| -1013.128 | 19769.533 | 2.08 | 15.174 | 0.0591611 | 7/15/2014 | 37:34.8 | 0 |
| -1012.934 | 19769.518 | 2.082 | 15.156 | 0.0582497 | 7/15/2014 | 37:34.9 | 0 |
| -1012.737 | 19769.5 | 2.088 | 15.138 | 0.0508874 | 7/15/2014 | 37:35.0 | 0 |
| -1012.543 | 19769.484 | 2.088 | 15.119 | 0.0534718 | 7/15/2014 | 37:35.1 | 0 |
| -1012.345 | 19769.467 | 2.093 | 15.102 | 0.038138 | 7/15/2014 | 37:35.2 | 0 |

| | | | | | | | |
|---|---|---|---|---|---|---|---|
| -1012.153 | 19769.449 | 2.09 | 15.087 | 0.030159 | 7/15/2014 | 37:35.3 | 0 |
| -1011.958 | 19769.434 | 2.095 | 15.073 | 0.01998 | 7/15/2014 | 37:35.4 | 0 |
| -1011.719 | 19769.414 | 2.095 | 15.062 | 0.0115323 | 7/15/2014 | 37:35.5 | 0 |
| -1011.526 | 19769.396 | 2.1 | 15.053 | 0.0128925 | 7/15/2014 | 37:35.6 | 0 |
| -1011.285 | 19769.377 | 2.1 | 15.047 | 0.0259392 | 7/15/2014 | 37:35.7 | 0 |
| -1011.089 | 19769.36 | 2.103 | 15.043 | 0.0153454 | 7/15/2014 | 37:35.8 | 0 |
| -1010.896 | 19769.344 | 2.1 | 15.041 | 0.0258438 | 7/15/2014 | 37:35.9 | 0 |
| -1010.696 | 19769.327 | 2.105 | 15.042 | 0.0205049 | 7/15/2014 | 37:36.0 | 0 |
| -1010.5 | 19769.311 | 2.103 | 15.043 | 0.0315647 | 7/15/2014 | 37:36.1 | 0 |
| -1010.301 | 19769.295 | 2.111 | 15.046 | 0.0420074 | 7/15/2014 | 37:36.2 | 0 |
| -1010.104 | 19769.277 | 2.111 | 15.05 | 0.0549298 | 7/15/2014 | 37:36.3 | 0 |
| -1009.907 | 19769.262 | 2.112 | 15.053 | 0.0570364 | 7/15/2014 | 37:36.4 | 0 |
| -1009.662 | 19769.242 | 2.112 | 15.056 | 0.0630434 | 7/15/2014 | 37:36.5 | 0 |
| -1009.465 | 19769.226 | 2.114 | 15.059 | 0.0692713 | 7/15/2014 | 37:36.6 | 0 |
| -1009.219 | 19769.205 | 2.112 | 15.061 | 0.076931 | 7/15/2014 | 37:36.7 | 0 |
| -1009.117 | 19769.197 | 2.117 | 15.062 | 0.0793962 | 7/15/2014 | 37:36.7 | 0 |
| -1008.818 | 19769.174 | 2.117 | 15.063 | 0.0752505 | 7/15/2014 | 37:36.9 | 0 |
| -1008.618 | 19769.158 | 2.118 | 15.062 | 0.0693716 | 7/15/2014 | 37:37.0 | 0 |
| -1008.417 | 19769.141 | 2.116 | 15.061 | 0.0651833 | 7/15/2014 | 37:37.1 | 0 |
| -1008.217 | 19769.125 | 2.117 | 15.06 | 0.0653113 | 7/15/2014 | 37:37.2 | 0 |
| -1008.018 | 19769.109 | 2.117 | 15.058 | 0.050081 | 7/15/2014 | 37:37.3 | 0 |
| -1007.82 | 19769.094 | 2.117 | 15.056 | 0.0491876 | 7/15/2014 | 37:37.4 | 0 |
| -1007.576 | 19769.074 | 2.114 | 15.053 | 0.0462246 | 7/15/2014 | 37:37.5 | 0 |
| -1007.379 | 19769.059 | 2.116 | 15.05 | 0.0359705 | 7/15/2014 | 37:37.6 | 0 |
| -1007.136 | 19769.039 | 2.116 | 15.045 | 0.0305977 | 7/15/2014 | 37:37.7 | 0 |
| -1006.94 | 19769.023 | 2.118 | 15.04 | 0.0373911 | 7/15/2014 | 37:37.8 | 0 |
| -1006.746 | 19769.009 | 2.118 | 15.034 | 0.0317429 | 7/15/2014 | 37:37.9 | 0 |
| -1006.55 | 19768.994 | 2.118 | 15.026 | 0.0393769 | 7/15/2014 | 37:38.0 | 0 |
| -1006.355 | 19768.979 | 2.121 | 15.018 | 0.0453548 | 7/15/2014 | 37:38.1 | 0 |
| -1006.161 | 19768.963 | 2.124 | 15.009 | 0.0531924 | 7/15/2014 | 37:38.2 | 0 |
| -1005.97 | 19768.948 | 2.124 | 15 | 0.0665944 | 7/15/2014 | 37:38.3 | 0 |
| -1005.776 | 19768.934 | 2.128 | 14.99 | 0.0739748 | 7/15/2014 | 37:38.4 | 0 |
| -1005.54 | 19768.914 | 2.128 | 14.981 | 0.0866371 | 7/15/2014 | 37:38.5 | 0 |
| -1005.349 | 19768.898 | 2.133 | 14.973 | 0.0872703 | 7/15/2014 | 37:38.6 | 0 |
| -1005.114 | 19768.882 | 2.131 | 14.965 | 0.0842098 | 7/15/2014 | 37:38.7 | 0 |
| -1004.923 | 19768.868 | 2.136 | 14.959 | 0.0888121 | 7/15/2014 | 37:38.8 | 0 |
| -1004.733 | 19768.854 | 2.139 | 14.954 | 0.0823821 | 7/15/2014 | 37:38.9 | 0 |
| -1004.541 | 19768.838 | 2.138 | 14.95 | 0.0921649 | 7/15/2014 | 37:39.0 | 0 |
| -1004.349 | 19768.823 | 2.14 | 14.947 | 0.0901442 | 7/15/2014 | 37:39.1 | 0 |
| -1004.156 | 19768.811 | 2.146 | 14.946 | 0.0935301 | 7/15/2014 | 37:39.2 | 0 |
| -1003.965 | 19768.795 | 2.149 | 14.945 | 0.101491 | 7/15/2014 | 37:39.3 | 0 |
| -1003.773 | 19768.779 | 2.155 | 14.945 | 0.108843 | 7/15/2014 | 37:39.4 | 0 |
| -1003.536 | 19768.763 | 2.157 | 14.945 | 0.114332 | 7/15/2014 | 37:39.5 | 0 |
| -1003.343 | 19768.749 | 2.167 | 14.946 | 0.114703 | 7/15/2014 | 37:39.6 | 0 |
| -1003.103 | 19768.731 | 2.169 | 14.946 | 0.121112 | 7/15/2014 | 37:39.7 | 0 |
| -1002.906 | 19768.717 | 2.178 | 14.946 | 0.124365 | 7/15/2014 | 37:39.8 | 0 |
| -1002.711 | 19768.701 | 2.183 | 14.946 | 0.121987 | 7/15/2014 | 37:39.9 | 0 |

| | | | | | | | |
|---|---|---|---|---|---|---|---|
| -1002.512 | 19768.688 | 2.19 | 14.945 | 0.128299 | 7/15/2014 | 37:40.0 | 0 |
| -1002.314 | 19768.673 | 2.196 | 14.944 | 0.125419 | 7/15/2014 | 37:40.1 | 0 |
| -1002.115 | 19768.658 | 2.204 | 14.944 | 0.12925 | 7/15/2014 | 37:40.2 | 0 |
| -1001.917 | 19768.644 | 2.21 | 14.943 | 0.12503 | 7/15/2014 | 37:40.3 | 0 |
| -1001.818 | 19768.637 | 2.213 | 14.942 | 0.12802 | 7/15/2014 | 37:40.3 | 0 |
| -1001.472 | 19768.611 | 2.218 | 14.941 | 0.124499 | 7/15/2014 | 37:40.5 | 0 |
| -1001.272 | 19768.597 | 2.226 | 14.94 | 0.12102 | 7/15/2014 | 37:40.6 | 0 |
| -1001.024 | 19768.578 | 2.226 | 14.938 | 0.122443 | 7/15/2014 | 37:40.7 | 0 |
| -1000.922 | 19768.571 | 2.232 | 14.938 | 0.126674 | 7/15/2014 | 37:40.7 | 0 |
| -1000.721 | 19768.557 | 2.232 | 14.935 | 0.119034 | 7/15/2014 | 37:40.8 | 0 |
| -1000.518 | 19768.543 | 2.232 | 14.932 | 0.120493 | 7/15/2014 | 37:40.9 | 0 |
| -1000.315 | 19768.527 | 2.236 | 14.927 | 0.110084 | 7/15/2014 | 37:41.0 | 0 |
| -1000.113 | 19768.512 | 2.236 | 14.922 | 0.106683 | 7/15/2014 | 37:41.1 | 0 |
| -999.911 | 19768.498 | 2.238 | 14.916 | 0.112868 | 7/15/2014 | 37:41.2 | 0 |
| -999.709 | 19768.483 | 2.24 | 14.909 | 0.107218 | 7/15/2014 | 37:41.3 | 0 |
| -999.36 | 19768.458 | 2.241 | 14.9 | 0.109225 | 7/15/2014 | 37:41.5 | 0 |
| -999.16 | 19768.444 | 2.245 | 14.894 | 0.114408 | 7/15/2014 | 37:41.6 | 0 |
| -998.913 | 19768.427 | 2.247 | 14.89 | 0.105793 | 7/15/2014 | 37:41.7 | 0 |
| -998.811 | 19768.419 | 2.249 | 14.886 | 0.109431 | 7/15/2014 | 37:41.7 | 0 |
| -998.513 | 19768.397 | 2.252 | 14.885 | 0.10637 | 7/15/2014 | 37:41.9 | 0 |
| -998.313 | 19768.384 | 2.256 | 14.885 | 0.104589 | 7/15/2014 | 37:42.0 | 0 |
| -998.114 | 19768.371 | 2.256 | 14.887 | 0.111961 | 7/15/2014 | 37:42.1 | 0 |
| -997.914 | 19768.355 | 2.258 | 14.891 | 0.113137 | 7/15/2014 | 37:42.2 | 0 |
| -997.716 | 19768.342 | 2.256 | 14.897 | 0.099013 | 7/15/2014 | 37:42.3 | 0 |
| -997.518 | 19768.328 | 2.258 | 14.904 | 0.103452 | 7/15/2014 | 37:42.4 | 0 |
| -997.276 | 19768.311 | 2.256 | 14.911 | 0.131676 | 7/15/2014 | 37:42.5 | 0 |
| -997.078 | 19768.298 | 2.258 | 14.919 | 0.10588 | 7/15/2014 | 37:42.6 | 0 |
| -996.835 | 19768.28 | 2.258 | 14.927 | 0.112906 | 7/15/2014 | 37:42.7 | 0 |
| -996.637 | 19768.267 | 2.259 | 14.933 | 0.120328 | 7/15/2014 | 37:42.8 | 0 |
| -996.441 | 19768.254 | 2.261 | 14.937 | 0.126114 | 7/15/2014 | 37:42.9 | 0 |
| -996.242 | 19768.239 | 2.264 | 14.939 | 0.130097 | 7/15/2014 | 37:43.0 | 0 |
| -996.044 | 19768.226 | 2.266 | 14.938 | 0.146543 | 7/15/2014 | 37:43.1 | 0 |
| -995.844 | 19768.212 | 2.275 | 14.935 | 0.142237 | 7/15/2014 | 37:43.2 | 0 |
| -995.647 | 19768.198 | 2.277 | 14.928 | 0.154971 | 7/15/2014 | 37:43.3 | 0 |
| -995.446 | 19768.185 | 2.286 | 14.92 | 0.157599 | 7/15/2014 | 37:43.4 | 0 |
| -995.201 | 19768.168 | 2.291 | 14.909 | 0.157245 | 7/15/2014 | 37:43.5 | 0 |
| -995 | 19768.153 | 2.298 | 14.896 | 0.160532 | 7/15/2014 | 37:43.6 | 0 |
| -994.753 | 19768.137 | 2.3 | 14.881 | 0.162957 | 7/15/2014 | 37:43.7 | 0 |
| -994.653 | 19768.13 | 2.303 | 14.874 | 0.162359 | 7/15/2014 | 37:43.7 | 0 |
| -994.449 | 19768.116 | 2.309 | 14.859 | 0.162222 | 7/15/2014 | 37:43.8 | 0 |
| -994.246 | 19768.102 | 2.315 | 14.843 | 0.165541 | 7/15/2014 | 37:43.9 | 0 |
| -994.042 | 19768.088 | 2.32 | 14.827 | 0.165099 | 7/15/2014 | 37:44.0 | 0 |
| -993.836 | 19768.073 | 2.327 | 14.811 | 0.16002 | 7/15/2014 | 37:44.1 | 0 |
| -993.632 | 19768.06 | 2.329 | 14.796 | 0.157305 | 7/15/2014 | 37:44.2 | 0 |
| -993.426 | 19768.045 | 2.332 | 14.781 | 0.14935 | 7/15/2014 | 37:44.3 | 0 |
| -993.068 | 19768.021 | 2.334 | 14.762 | 0.138907 | 7/15/2014 | 37:44.5 | 0 |
| -992.86 | 19768.007 | 2.337 | 14.75 | 0.135183 | 7/15/2014 | 37:44.6 | 0 |

| | | | | | | | |
|---|---|---|---|---|---|---|---|
| -992.601 | 19767.988 | 2.337 | 14.74 | 0.131077 | 7/15/2014 | 37:44.7 | 0 |
| -992.498 | 19767.981 | 2.337 | 14.734 | 0.124543 | 7/15/2014 | 37:44.7 | 0 |
| -992.287 | 19767.967 | 2.337 | 14.725 | 0.117402 | 7/15/2014 | 37:44.8 | 0 |
| -992.077 | 19767.952 | 2.337 | 14.717 | 0.116409 | 7/15/2014 | 37:44.9 | 0 |
| -991.863 | 19767.938 | 2.337 | 14.709 | 0.124002 | 7/15/2014 | 37:45.0 | 0 |
| -991.651 | 19767.923 | 2.337 | 14.702 | 0.120867 | 7/15/2014 | 37:45.1 | 0 |
| -991.438 | 19767.907 | 2.342 | 14.696 | 0.126848 | 7/15/2014 | 37:45.2 | 0 |
| -991.227 | 19767.894 | 2.34 | 14.689 | 0.129959 | 7/15/2014 | 37:45.3 | 0 |
| -990.961 | 19767.875 | 2.344 | 14.683 | 0.139396 | 7/15/2014 | 37:45.4 | 0 |
| -990.749 | 19767.86 | 2.346 | 14.677 | 0.14472 | 7/15/2014 | 37:45.5 | 0 |
| -990.537 | 19767.846 | 2.348 | 14.671 | 0.15412 | 7/15/2014 | 37:45.6 | 0 |
| -990.272 | 19767.826 | 2.352 | 14.665 | 0.157298 | 7/15/2014 | 37:45.7 | 0 |
| -990.061 | 19767.812 | 2.354 | 14.661 | 0.164083 | 7/15/2014 | 37:45.8 | 0 |
| -989.848 | 19767.797 | 2.356 | 14.657 | 0.166894 | 7/15/2014 | 37:45.9 | 0 |
| -989.636 | 19767.782 | 2.356 | 14.655 | 0.170124 | 7/15/2014 | 37:46.0 | 0 |
| -989.423 | 19767.767 | 2.36 | 14.654 | 0.169067 | 7/15/2014 | 37:46.1 | 0 |
| -989.214 | 19767.752 | 2.363 | 14.656 | 0.171076 | 7/15/2014 | 37:46.2 | 0 |
| -989.005 | 19767.737 | 2.363 | 14.659 | 0.17088 | 7/15/2014 | 37:46.3 | 0 |
| -988.644 | 19767.711 | 2.367 | 14.669 | 0.164781 | 7/15/2014 | 37:46.5 | 0 |
| -988.439 | 19767.696 | 2.37 | 14.677 | 0.165429 | 7/15/2014 | 37:46.6 | 0 |
| -988.185 | 19767.68 | 2.37 | 14.686 | 0.170355 | 7/15/2014 | 37:46.7 | 0 |
| -988.08 | 19767.672 | 2.373 | 14.691 | 0.167318 | 7/15/2014 | 37:46.7 | 0 |
| -987.874 | 19767.656 | 2.378 | 14.702 | 0.16044 | 7/15/2014 | 37:46.8 | 0 |
| -987.671 | 19767.642 | 2.378 | 14.713 | 0.165534 | 7/15/2014 | 37:46.9 | 0 |
| -987.467 | 19767.628 | 2.378 | 14.724 | 0.169724 | 7/15/2014 | 37:47.0 | 0 |
| -987.262 | 19767.613 | 2.38 | 14.734 | 0.176617 | 7/15/2014 | 37:47.1 | 0 |
| -987.06 | 19767.599 | 2.377 | 14.743 | 0.182928 | 7/15/2014 | 37:47.2 | 0 |
| -986.858 | 19767.584 | 2.376 | 14.75 | 0.186722 | 7/15/2014 | 37:47.3 | 0 |
| -986.51 | 19767.561 | 2.377 | 14.756 | 0.186885 | 7/15/2014 | 37:47.5 | 0 |
| -986.312 | 19767.545 | 2.38 | 14.759 | 0.204327 | 7/15/2014 | 37:47.6 | 0 |
| -986.067 | 19767.528 | 2.378 | 14.759 | 0.191618 | 7/15/2014 | 37:47.7 | 0 |
| -985.869 | 19767.514 | 2.383 | 14.757 | 0.195801 | 7/15/2014 | 37:47.8 | 0 |
| -985.673 | 19767.5 | 2.383 | 14.754 | 0.182801 | 7/15/2014 | 37:47.9 | 0 |
| -985.475 | 19767.485 | 2.39 | 14.748 | 0.17889 | 7/15/2014 | 37:48.0 | 0 |
| -985.278 | 19767.473 | 2.392 | 14.74 | 0.16955 | 7/15/2014 | 37:48.1 | 0 |
| -985.081 | 19767.457 | 2.399 | 14.73 | 0.186461 | 7/15/2014 | 37:48.2 | 0 |
| -984.887 | 19767.444 | 2.399 | 14.719 | 0.171434 | 7/15/2014 | 37:48.3 | 0 |
| -984.693 | 19767.43 | 2.403 | 14.705 | 0.170506 | 7/15/2014 | 37:48.4 | 0 |
| -984.453 | 19767.412 | 2.403 | 14.69 | 0.18664 | 7/15/2014 | 37:48.5 | 0 |
| -984.258 | 19767.397 | 2.407 | 14.674 | 0.185867 | 7/15/2014 | 37:48.6 | 0 |
| -984.017 | 19767.381 | 2.409 | 14.657 | 0.195919 | 7/15/2014 | 37:48.7 | 0 |
| -983.821 | 19767.366 | 2.415 | 14.64 | 0.195693 | 7/15/2014 | 37:48.8 | 0 |
| -983.626 | 19767.354 | 2.415 | 14.623 | 0.209146 | 7/15/2014 | 37:48.9 | 0 |
| -983.428 | 19767.338 | 2.422 | 14.606 | 0.197922 | 7/15/2014 | 37:49.0 | 0 |
| -983.23 | 19767.323 | 2.425 | 14.591 | 0.205147 | 7/15/2014 | 37:49.1 | 0 |
| -983.031 | 19767.31 | 2.431 | 14.576 | 0.208389 | 7/15/2014 | 37:49.2 | 0 |
| -982.833 | 19767.295 | 2.431 | 14.563 | 0.196178 | 7/15/2014 | 37:49.3 | 0 |

| | | | | | | | |
|---|---|---|---|---|---|---|---|
| -982.736 | 19767.288 | 2.437 | 14.556 | 0.193848 | 7/15/2014 | 37:49.3 | 0 |
| -982.388 | 19767.262 | 2.438 | 14.538 | 0.2125 | 7/15/2014 | 37:49.5 | 0 |
| -982.188 | 19767.248 | 2.444 | 14.526 | 0.222395 | 7/15/2014 | 37:49.6 | 0 |
| -981.939 | 19767.23 | 2.441 | 14.513 | 0.220804 | 7/15/2014 | 37:49.7 | 0 |
| -981.838 | 19767.223 | 2.443 | 14.507 | 0.239182 | 7/15/2014 | 37:49.7 | 0 |
| -981.634 | 19767.207 | 2.443 | 14.494 | 0.2493 | 7/15/2014 | 37:49.8 | 0 |
| -981.432 | 19767.193 | 2.443 | 14.48 | 0.268205 | 7/15/2014 | 37:49.9 | 0 |
| -981.227 | 19767.18 | 2.446 | 14.464 | 0.288896 | 7/15/2014 | 37:50.0 | 0 |
| -981.024 | 19767.164 | 2.446 | 14.448 | 0.287678 | 7/15/2014 | 37:50.1 | 0 |
| -980.819 | 19767.148 | 2.449 | 14.432 | 0.290847 | 7/15/2014 | 37:50.2 | 0 |
| -980.616 | 19767.135 | 2.453 | 14.417 | 0.290123 | 7/15/2014 | 37:50.3 | 0 |
| -980.265 | 19767.109 | 2.452 | 14.397 | 0.28454 | 7/15/2014 | 37:50.5 | 0 |
| -980.061 | 19767.095 | 2.458 | 14.385 | 0.269703 | 7/15/2014 | 37:50.6 | 0 |
| -979.812 | 19767.077 | 2.458 | 14.377 | 0.248598 | 7/15/2014 | 37:50.7 | 0 |
| -979.709 | 19767.07 | 2.459 | 14.371 | 0.23887 | 7/15/2014 | 37:50.7 | 0 |
| -979.507 | 19767.056 | 2.459 | 14.366 | 0.220211 | 7/15/2014 | 37:50.8 | 0 |
| -979.304 | 19767.041 | 2.462 | 14.365 | 0.207614 | 7/15/2014 | 37:50.9 | 0 |
| -979.1 | 19767.026 | 2.462 | 14.367 | 0.185095 | 7/15/2014 | 37:51.0 | 0 |
| -978.896 | 19767.013 | 2.465 | 14.371 | 0.191249 | 7/15/2014 | 37:51.1 | 0 |
| -978.695 | 19766.997 | 2.465 | 14.377 | 0.1708 | 7/15/2014 | 37:51.2 | 0 |
| -978.493 | 19766.983 | 2.469 | 14.385 | 0.170165 | 7/15/2014 | 37:51.3 | 0 |
| -978.144 | 19766.958 | 2.469 | 14.396 | 0.174615 | 7/15/2014 | 37:51.5 | 0 |
| -977.943 | 19766.944 | 2.474 | 14.403 | 0.184934 | 7/15/2014 | 37:51.6 | 0 |
| -977.695 | 19766.928 | 2.474 | 14.408 | 0.170534 | 7/15/2014 | 37:51.7 | 0 |
| -977.593 | 19766.92 | 2.477 | 14.412 | 0.178542 | 7/15/2014 | 37:51.7 | 0 |
| -977.392 | 19766.905 | 2.477 | 14.415 | 0.183691 | 7/15/2014 | 37:51.8 | 0 |
| -977.191 | 19766.891 | 2.477 | 14.415 | 0.197825 | 7/15/2014 | 37:51.9 | 0 |
| -976.989 | 19766.877 | 2.479 | 14.412 | 0.193993 | 7/15/2014 | 37:52.0 | 0 |
| -976.787 | 19766.862 | 2.481 | 14.408 | 0.202961 | 7/15/2014 | 37:52.1 | 0 |
| -976.586 | 19766.848 | 2.484 | 14.401 | 0.201303 | 7/15/2014 | 37:52.2 | 0 |
| -976.386 | 19766.834 | 2.486 | 14.393 | 0.199927 | 7/15/2014 | 37:52.3 | 0 |
| -976.036 | 19766.81 | 2.488 | 14.381 | 0.216712 | 7/15/2014 | 37:52.5 | 0 |
| -975.836 | 19766.795 | 2.492 | 14.372 | 0.221721 | 7/15/2014 | 37:52.6 | 0 |
| -975.586 | 19766.777 | 2.494 | 14.364 | 0.205011 | 7/15/2014 | 37:52.7 | 0 |
| -975.485 | 19766.771 | 2.496 | 14.36 | 0.208833 | 7/15/2014 | 37:52.7 | 0 |
| -975.282 | 19766.756 | 2.501 | 14.353 | 0.198259 | 7/15/2014 | 37:52.8 | 0 |
| -975.079 | 19766.741 | 2.505 | 14.347 | 0.206696 | 7/15/2014 | 37:52.9 | 0 |
| -974.874 | 19766.727 | 2.507 | 14.343 | 0.189274 | 7/15/2014 | 37:53.0 | 0 |
| -974.669 | 19766.712 | 2.507 | 14.339 | 0.171744 | 7/15/2014 | 37:53.1 | 0 |
| -974.464 | 19766.698 | 2.507 | 14.337 | 0.168743 | 7/15/2014 | 37:53.2 | 0 |
| -974.259 | 19766.684 | 2.509 | 14.336 | 0.151794 | 7/15/2014 | 37:53.3 | 0 |
| -973.901 | 19766.658 | 2.514 | 14.336 | 0.149376 | 7/15/2014 | 37:53.5 | 0 |
| -973.695 | 19766.644 | 2.516 | 14.337 | 0.160362 | 7/15/2014 | 37:53.6 | 0 |
| -973.437 | 19766.625 | 2.512 | 14.339 | 0.140599 | 7/15/2014 | 37:53.7 | 0 |
| -973.333 | 19766.617 | 2.511 | 14.34 | 0.146434 | 7/15/2014 | 37:53.7 | 0 |
| -973.125 | 19766.604 | 2.514 | 14.342 | 0.155144 | 7/15/2014 | 37:53.8 | 0 |
| -972.915 | 19766.589 | 2.514 | 14.346 | 0.17149 | 7/15/2014 | 37:53.9 | 0 |

| | | | | | | | |
|---|---|---|---|---|---|---|---|
| -972.704 | 19766.574 | 2.514 | 14.349 | 0.158482 | 7/15/2014 | 37:54.0 | 0 |
| -972.493 | 19766.559 | 2.514 | 14.351 | 0.163914 | 7/15/2014 | 37:54.1 | 0 |
| -972.283 | 19766.544 | 2.511 | 14.354 | 0.180812 | 7/15/2014 | 37:54.2 | 0 |
| -972.073 | 19766.528 | 2.508 | 14.355 | 0.17656 | 7/15/2014 | 37:54.3 | 0 |
| -971.709 | 19766.504 | 2.511 | 14.352 | 0.186181 | 7/15/2014 | 37:54.5 | 0 |
| -971.501 | 19766.489 | 2.515 | 14.349 | 0.18411 | 7/15/2014 | 37:54.6 | 0 |
| -971.244 | 19766.472 | 2.513 | 14.344 | 0.172019 | 7/15/2014 | 37:54.7 | 0 |
| -971.137 | 19766.465 | 2.515 | 14.342 | 0.180027 | 7/15/2014 | 37:54.7 | 0 |
| -970.929 | 19766.45 | 2.515 | 14.335 | 0.18258 | 7/15/2014 | 37:54.8 | 0 |
| -970.721 | 19766.435 | 2.515 | 14.326 | 0.165885 | 7/15/2014 | 37:54.9 | 0 |
| -970.513 | 19766.42 | 2.515 | 14.316 | 0.16719 | 7/15/2014 | 37:55.0 | 0 |
| -970.305 | 19766.406 | 2.515 | 14.306 | 0.167225 | 7/15/2014 | 37:55.1 | 0 |
| -970.098 | 19766.393 | 2.512 | 14.294 | 0.148768 | 7/15/2014 | 37:55.2 | 0 |
| -969.893 | 19766.378 | 2.509 | 14.283 | 0.149074 | 7/15/2014 | 37:55.3 | 0 |
| -969.534 | 19766.354 | 2.506 | 14.267 | 0.165698 | 7/15/2014 | 37:55.5 | 0 |
| -969.33 | 19766.34 | 2.509 | 14.258 | 0.139406 | 7/15/2014 | 37:55.6 | 0 |
| -969.073 | 19766.321 | 2.509 | 14.249 | 0.147557 | 7/15/2014 | 37:55.7 | 0 |
| -968.971 | 19766.314 | 2.509 | 14.244 | 0.142902 | 7/15/2014 | 37:55.7 | 0 |
| -968.763 | 19766.301 | 2.512 | 14.237 | 0.132546 | 7/15/2014 | 37:55.8 | 0 |
| -968.555 | 19766.287 | 2.512 | 14.232 | 0.133759 | 7/15/2014 | 37:55.9 | 0 |
| -968.346 | 19766.272 | 2.512 | 14.228 | 0.126779 | 7/15/2014 | 37:56.0 | 0 |
| -968.135 | 19766.258 | 2.512 | 14.225 | 0.129525 | 7/15/2014 | 37:56.1 | 0 |
| -967.924 | 19766.244 | 2.512 | 14.222 | 0.109173 | 7/15/2014 | 37:56.2 | 0 |
| -967.715 | 19766.229 | 2.51 | 14.221 | 0.13343 | 7/15/2014 | 37:56.3 | 0 |
| -967.347 | 19766.205 | 2.501 | 14.218 | 0.138246 | 7/15/2014 | 37:56.5 | 0 |
| -967.137 | 19766.191 | 2.501 | 14.216 | 0.152539 | 7/15/2014 | 37:56.6 | 0 |
| -966.874 | 19766.174 | 2.492 | 14.213 | 0.139366 | 7/15/2014 | 37:56.7 | 0 |
| -966.768 | 19766.166 | 2.49 | 14.212 | 0.147353 | 7/15/2014 | 37:56.7 | 0 |
| -966.554 | 19766.152 | 2.49 | 14.208 | 0.141076 | 7/15/2014 | 37:56.8 | 0 |
| -966.342 | 19766.139 | 2.488 | 14.203 | 0.152959 | 7/15/2014 | 37:56.9 | 0 |
| -966.127 | 19766.124 | 2.488 | 14.198 | 0.137065 | 7/15/2014 | 37:57.0 | 0 |
| -965.914 | 19766.11 | 2.49 | 14.193 | 0.139931 | 7/15/2014 | 37:57.1 | 0 |
| -965.701 | 19766.096 | 2.492 | 14.187 | 0.132755 | 7/15/2014 | 37:57.2 | 0 |
| -965.489 | 19766.083 | 2.494 | 14.181 | 0.124672 | 7/15/2014 | 37:57.3 | 0 |
| -965.122 | 19766.059 | 2.494 | 14.171 | 0.150571 | 7/15/2014 | 37:57.5 | 0 |
| -964.909 | 19766.045 | 2.5 | 14.163 | 0.142028 | 7/15/2014 | 37:57.6 | 0 |
| -964.648 | 19766.028 | 2.499 | 14.154 | 0.163522 | 7/15/2014 | 37:57.7 | 0 |
| -964.541 | 19766.021 | 2.502 | 14.151 | 0.164284 | 7/15/2014 | 37:57.7 | 0 |
| -964.329 | 19766.008 | 2.5 | 14.141 | 0.155866 | 7/15/2014 | 37:57.8 | 0 |
| -964.118 | 19765.993 | 2.497 | 14.129 | 0.170381 | 7/15/2014 | 37:57.9 | 0 |
| -963.904 | 19765.981 | 2.495 | 14.117 | 0.179759 | 7/15/2014 | 37:58.0 | 0 |
| -963.692 | 19765.968 | 2.495 | 14.103 | 0.192861 | 7/15/2014 | 37:58.1 | 0 |
| -963.482 | 19765.954 | 2.493 | 14.089 | 0.180673 | 7/15/2014 | 37:58.2 | 0 |
| -963.271 | 19765.94 | 2.491 | 14.076 | 0.193208 | 7/15/2014 | 37:58.3 | 0 |
| -962.904 | 19765.919 | 2.489 | 14.056 | 0.174348 | 7/15/2014 | 37:58.5 | 0 |
| -962.693 | 19765.905 | 2.491 | 14.045 | 0.222938 | 7/15/2014 | 37:58.6 | 0 |
| -962.431 | 19765.89 | 2.491 | 14.035 | 0.174326 | 7/15/2014 | 37:58.7 | 0 |

| | | | | | | | |
|---|---|---|---|---|---|---|---|
| -962.324 | 19765.884 | 2.491 | 14.029 | 0.174262 | 7/15/2014 | 37:58.7 | 0 |
| -962.112 | 19765.87 | 2.493 | 14.021 | 0.167315 | 7/15/2014 | 37:58.8 | 0 |
| -961.9 | 19765.856 | 2.491 | 14.015 | 0.178357 | 7/15/2014 | 37:58.9 | 0 |
| -961.687 | 19765.844 | 2.491 | 14.01 | 0.155464 | 7/15/2014 | 37:59.0 | 0 |
| -961.473 | 19765.831 | 2.491 | 14.007 | 0.139914 | 7/15/2014 | 37:59.1 | 0 |
| -961.26 | 19765.818 | 2.489 | 14.004 | 0.132479 | 7/15/2014 | 37:59.2 | 0 |
| -961.045 | 19765.806 | 2.491 | 14.002 | 0.102119 | 7/15/2014 | 37:59.3 | 0 |
| -960.779 | 19765.79 | 2.491 | 13.999 | 0.134052 | 7/15/2014 | 37:59.4 | 0 |
| -960.566 | 19765.776 | 2.486 | 13.996 | 0.10274 | 7/15/2014 | 37:59.5 | 0 |
| -960.351 | 19765.764 | 2.481 | 13.992 | 0.113145 | 7/15/2014 | 37:59.6 | 0 |
| -960.078 | 19765.748 | 2.478 | 13.987 | 0.104506 | 7/15/2014 | 37:59.7 | 0 |
| -959.86 | 19765.734 | 2.475 | 13.98 | 0.110183 | 7/15/2014 | 37:59.8 | 0 |
| -959.639 | 19765.721 | 2.472 | 13.973 | 0.134226 | 7/15/2014 | 37:59.9 | 0 |
| -959.416 | 19765.709 | 2.472 | 13.964 | 0.13492 | 7/15/2014 | 38:00.0 | 0 |
| -959.194 | 19765.694 | 2.467 | 13.955 | 0.125625 | 7/15/2014 | 38:00.1 | 0 |
| -958.971 | 19765.682 | 2.461 | 13.946 | 0.131817 | 7/15/2014 | 38:00.2 | 0 |
| -958.749 | 19765.669 | 2.461 | 13.938 | 0.124755 | 7/15/2014 | 38:00.3 | 0 |
| -958.472 | 19765.652 | 2.461 | 13.929 | 0.12778 | 7/15/2014 | 38:00.4 | 0 |
| -958.249 | 19765.64 | 2.461 | 13.922 | 0.136883 | 7/15/2014 | 38:00.5 | 0 |
| -958.026 | 19765.626 | 2.461 | 13.916 | 0.120168 | 7/15/2014 | 38:00.6 | 0 |
| -957.748 | 19765.61 | 2.461 | 13.911 | 0.119713 | 7/15/2014 | 38:00.7 | 0 |
| -957.526 | 19765.596 | 2.459 | 13.907 | 0.120146 | 7/15/2014 | 38:00.8 | 0 |
| -957.302 | 19765.584 | 2.459 | 13.903 | 0.117248 | 7/15/2014 | 38:00.9 | 0 |
| -957.08 | 19765.571 | 2.46 | 13.901 | 0.111225 | 7/15/2014 | 38:01.0 | 0 |
| -956.857 | 19765.559 | 2.458 | 13.9 | 0.105109 | 7/15/2014 | 38:01.1 | 0 |
| -956.637 | 19765.546 | 2.455 | 13.901 | 0.108128 | 7/15/2014 | 38:01.2 | 0 |
| -956.417 | 19765.533 | 2.453 | 13.902 | 0.110434 | 7/15/2014 | 38:01.3 | 0 |
| -956.145 | 19765.517 | 2.451 | 13.905 | 0.118901 | 7/15/2014 | 38:01.4 | 0 |
| -955.929 | 19765.504 | 2.445 | 13.91 | 0.11907 | 7/15/2014 | 38:01.5 | 0 |
| -955.714 | 19765.492 | 2.44 | 13.917 | 0.146946 | 7/15/2014 | 38:01.6 | 0 |
| -955.446 | 19765.479 | 2.438 | 13.926 | 0.132011 | 7/15/2014 | 38:01.7 | 0 |
| -955.23 | 19765.466 | 2.433 | 13.937 | 0.147606 | 7/15/2014 | 38:01.8 | 0 |
| -955.015 | 19765.453 | 2.433 | 13.95 | 0.136899 | 7/15/2014 | 38:01.9 | 0 |
| -954.799 | 19765.44 | 2.43 | 13.966 | 0.148886 | 7/15/2014 | 38:02.0 | 0 |
| -954.585 | 19765.428 | 2.427 | 13.984 | 0.134781 | 7/15/2014 | 38:02.1 | 0 |
| -954.369 | 19765.416 | 2.427 | 14.004 | 0.137518 | 7/15/2014 | 38:02.2 | 0 |
| -954.156 | 19765.404 | 2.427 | 14.026 | 0.126544 | 7/15/2014 | 38:02.3 | 0 |
| -953.888 | 19765.391 | 2.427 | 14.05 | 0.129551 | 7/15/2014 | 38:02.4 | 0 |
| -953.675 | 19765.378 | 2.427 | 14.075 | 0.118494 | 7/15/2014 | 38:02.5 | 0 |
| -953.461 | 19765.365 | 2.427 | 14.101 | 0.122391 | 7/15/2014 | 38:02.6 | 0 |
| -953.192 | 19765.35 | 2.424 | 14.127 | 0.114039 | 7/15/2014 | 38:02.7 | 0 |
| -952.975 | 19765.336 | 2.424 | 14.152 | 0.111211 | 7/15/2014 | 38:02.8 | 0 |
| -952.756 | 19765.324 | 2.421 | 14.174 | 0.10817 | 7/15/2014 | 38:02.9 | 0 |
| -952.537 | 19765.313 | 2.412 | 14.193 | 0.10187 | 7/15/2014 | 38:03.0 | 0 |
| -952.315 | 19765.299 | 2.407 | 14.207 | 0.10272 | 7/15/2014 | 38:03.1 | 0 |
| -952.094 | 19765.287 | 2.404 | 14.217 | 0.114301 | 7/15/2014 | 38:03.2 | 0 |
| -951.872 | 19765.274 | 2.401 | 14.222 | 0.102739 | 7/15/2014 | 38:03.3 | 0 |

| | | | | | | | |
|---|---|---|---|---|---|---|---|
| -951.598 | 19765.258 | 2.401 | 14.222 | 0.105911 | 7/15/2014 | 38:03.4 | 0 |
| -951.376 | 19765.244 | 2.401 | 14.219 | 0.107866 | 7/15/2014 | 38:03.5 | 0 |
| -951.153 | 19765.231 | 2.398 | 14.212 | 0.103668 | 7/15/2014 | 38:03.6 | 0 |
| -950.876 | 19765.215 | 2.398 | 14.202 | 0.112883 | 7/15/2014 | 38:03.7 | 0 |
| -950.652 | 19765.201 | 2.398 | 14.191 | 0.104838 | 7/15/2014 | 38:03.8 | 0 |
| -950.427 | 19765.188 | 2.4 | 14.179 | 0.10015 | 7/15/2014 | 38:03.9 | 0 |
| -950.201 | 19765.176 | 2.394 | 14.166 | 0.0998633 | 7/15/2014 | 38:04.0 | 0 |
| -949.976 | 19765.162 | 2.389 | 14.153 | 0.0897773 | 7/15/2014 | 38:04.1 | 0 |
| -949.753 | 19765.148 | 2.38 | 14.142 | 0.0967327 | 7/15/2014 | 38:04.2 | 0 |
| -949.53 | 19765.136 | 2.373 | 14.131 | 0.100273 | 7/15/2014 | 38:04.3 | 0 |
| -949.253 | 19765.117 | 2.37 | 14.121 | 0.102116 | 7/15/2014 | 38:04.4 | 0 |
| -949.033 | 19765.105 | 2.365 | 14.112 | 0.0928487 | 7/15/2014 | 38:04.5 | 0 |
| -948.811 | 19765.092 | 2.365 | 14.105 | 0.105499 | 7/15/2014 | 38:04.6 | 0 |
| -948.536 | 19765.075 | 2.363 | 14.099 | 0.0983546 | 7/15/2014 | 38:04.7 | 0 |
| -948.315 | 19765.063 | 2.363 | 14.094 | 0.112766 | 7/15/2014 | 38:04.8 | 0 |
| -948.094 | 19765.048 | 2.357 | 14.09 | 0.104189 | 7/15/2014 | 38:04.9 | 0 |
| -947.872 | 19765.034 | 2.357 | 14.086 | 0.104738 | 7/15/2014 | 38:05.0 | 0 |
| -947.649 | 19765.02 | 2.359 | 14.083 | 0.108272 | 7/15/2014 | 38:05.1 | 0 |
| -947.43 | 19765.007 | 2.359 | 14.079 | 0.108058 | 7/15/2014 | 38:05.2 | 0 |
| -947.209 | 19764.993 | 2.361 | 14.075 | 0.104707 | 7/15/2014 | 38:05.3 | 0 |
| -946.936 | 19764.976 | 2.359 | 14.07 | 0.101145 | 7/15/2014 | 38:05.4 | 0 |
| -946.72 | 19764.962 | 2.357 | 14.065 | 0.0984817 | 7/15/2014 | 38:05.5 | 0 |
| -946.503 | 19764.948 | 2.355 | 14.06 | 0.0945768 | 7/15/2014 | 38:05.6 | 0 |
| -946.231 | 19764.93 | 2.358 | 14.055 | 0.0932197 | 7/15/2014 | 38:05.7 | 0 |
| -946.012 | 19764.916 | 2.355 | 14.049 | 0.0902719 | 7/15/2014 | 38:05.8 | 0 |
| -945.795 | 19764.901 | 2.35 | 14.044 | 0.0928822 | 7/15/2014 | 38:05.9 | 0 |
| -945.576 | 19764.887 | 2.348 | 14.041 | 0.0932304 | 7/15/2014 | 38:06.0 | 0 |
| -945.359 | 19764.872 | 2.345 | 14.038 | 0.0952857 | 7/15/2014 | 38:06.1 | 0 |
| -945.142 | 19764.857 | 2.345 | 14.038 | 0.0967683 | 7/15/2014 | 38:06.2 | 0 |
| -944.927 | 19764.843 | 2.342 | 14.039 | 0.102365 | 7/15/2014 | 38:06.3 | 0 |
| -944.657 | 19764.825 | 2.341 | 14.043 | 0.107536 | 7/15/2014 | 38:06.4 | 0 |
| -944.442 | 19764.811 | 2.338 | 14.049 | 0.107856 | 7/15/2014 | 38:06.5 | 0 |
| -944.227 | 19764.795 | 2.338 | 14.058 | 0.106219 | 7/15/2014 | 38:06.6 | 0 |
| -943.957 | 19764.775 | 2.338 | 14.07 | 0.103757 | 7/15/2014 | 38:06.7 | 0 |
| -943.74 | 19764.761 | 2.333 | 14.084 | 0.101511 | 7/15/2014 | 38:06.8 | 0 |
| -943.519 | 19764.745 | 2.33 | 14.1 | 0.0991031 | 7/15/2014 | 38:06.9 | 0 |
| -943.3 | 19764.729 | 2.324 | 14.118 | 0.0908174 | 7/15/2014 | 38:07.0 | 0 |
| -943.079 | 19764.715 | 2.322 | 14.137 | 0.0842101 | 7/15/2014 | 38:07.1 | 0 |
| -942.859 | 19764.698 | 2.32 | 14.158 | 0.0851525 | 7/15/2014 | 38:07.2 | 0 |
| -942.638 | 19764.682 | 2.318 | 14.179 | 0.0735283 | 7/15/2014 | 38:07.3 | 0 |
| -942.364 | 19764.662 | 2.314 | 14.201 | 0.0714546 | 7/15/2014 | 38:07.4 | 0 |
| -942.144 | 19764.646 | 2.311 | 14.221 | 0.0681712 | 7/15/2014 | 38:07.5 | 0 |
| -941.922 | 19764.631 | 2.311 | 14.24 | 0.076476 | 7/15/2014 | 38:07.6 | 0 |
| -941.645 | 19764.611 | 2.307 | 14.256 | 0.0879798 | 7/15/2014 | 38:07.7 | 0 |
| -941.423 | 19764.595 | 2.305 | 14.27 | 0.0820729 | 7/15/2014 | 38:07.8 | 0 |
| -941.198 | 19764.577 | 2.305 | 14.281 | 0.098672 | 7/15/2014 | 38:07.9 | 0 |
| -940.975 | 19764.561 | 2.305 | 14.288 | 0.0920854 | 7/15/2014 | 38:08.0 | 0 |

| | | | | | | | |
|---|---|---|---|---|---|---|---|
| -940.752 | 19764.545 | 2.303 | 14.291 | 0.102025 | 7/15/2014 | 38:08.1 | 0 |
| -940.53 | 19764.528 | 2.305 | 14.291 | 0.105997 | 7/15/2014 | 38:08.2 | 0 |
| -940.308 | 19764.512 | 2.305 | 14.287 | 0.109178 | 7/15/2014 | 38:08.3 | 0 |
| -940.035 | 19764.492 | 2.308 | 14.28 | 0.0975238 | 7/15/2014 | 38:08.4 | 0 |
| -939.816 | 19764.476 | 2.308 | 14.27 | 0.105305 | 7/15/2014 | 38:08.5 | 0 |
| -939.598 | 19764.458 | 2.31 | 14.258 | 0.096591 | 7/15/2014 | 38:08.6 | 0 |
| -939.328 | 19764.438 | 2.31 | 14.245 | 0.0936031 | 7/15/2014 | 38:08.7 | 0 |
| -939.11 | 19764.422 | 2.31 | 14.23 | 0.0848571 | 7/15/2014 | 38:08.8 | 0 |
| -938.892 | 19764.405 | 2.312 | 14.215 | 0.0829218 | 7/15/2014 | 38:08.9 | 0 |
| -938.674 | 19764.387 | 2.31 | 14.201 | 0.0820866 | 7/15/2014 | 38:09.0 | 0 |
| -938.458 | 19764.371 | 2.308 | 14.187 | 0.0795295 | 7/15/2014 | 38:09.1 | 0 |
| -938.243 | 19764.355 | 2.301 | 14.175 | 0.0953461 | 7/15/2014 | 38:09.2 | 0 |
| -938.028 | 19764.337 | 2.301 | 14.165 | 0.0907343 | 7/15/2014 | 38:09.3 | 0 |
| -937.762 | 19764.316 | 2.299 | 14.157 | 0.0957937 | 7/15/2014 | 38:09.4 | 0 |
| -937.548 | 19764.301 | 2.297 | 14.15 | 0.128506 | 7/15/2014 | 38:09.5 | 0 |
| -937.335 | 19764.283 | 2.294 | 14.147 | 0.121042 | 7/15/2014 | 38:09.6 | 0 |
| -937.068 | 19764.262 | 2.292 | 14.145 | 0.128711 | 7/15/2014 | 38:09.7 | 0 |
| -936.853 | 19764.244 | 2.29 | 14.145 | 0.139035 | 7/15/2014 | 38:09.8 | 0 |
| -936.637 | 19764.229 | 2.29 | 14.148 | 0.133227 | 7/15/2014 | 38:09.9 | 0 |
| -936.419 | 19764.209 | 2.294 | 14.151 | 0.13004 | 7/15/2014 | 38:10.0 | 0 |
| -936.201 | 19764.191 | 2.294 | 14.156 | 0.134498 | 7/15/2014 | 38:10.1 | 0 |
| -935.984 | 19764.176 | 2.297 | 14.162 | 0.110434 | 7/15/2014 | 38:10.2 | 0 |
| -935.767 | 19764.156 | 2.297 | 14.168 | 0.118 | 7/15/2014 | 38:10.3 | 0 |
| -935.498 | 19764.135 | 2.297 | 14.173 | 0.0982156 | 7/15/2014 | 38:10.4 | 0 |
| -935.28 | 19764.117 | 2.297 | 14.176 | 0.0970141 | 7/15/2014 | 38:10.5 | 0 |
| -935.064 | 19764.1 | 2.299 | 14.179 | 0.0851945 | 7/15/2014 | 38:10.6 | 0 |
| -934.791 | 19764.076 | 2.299 | 14.178 | 0.0899983 | 7/15/2014 | 38:10.7 | 0 |
| -934.573 | 19764.057 | 2.299 | 14.174 | 0.0812792 | 7/15/2014 | 38:10.8 | 0 |
| -934.354 | 19764.04 | 2.301 | 14.168 | 0.0849249 | 7/15/2014 | 38:10.9 | 0 |
| -934.135 | 19764.021 | 2.297 | 14.158 | 0.0808415 | 7/15/2014 | 38:11.0 | 0 |
| -933.917 | 19764.002 | 2.297 | 14.145 | 0.0810084 | 7/15/2014 | 38:11.1 | 0 |
| -933.699 | 19763.984 | 2.297 | 14.129 | 0.0809866 | 7/15/2014 | 38:11.2 | 0 |
| -933.482 | 19763.965 | 2.297 | 14.111 | 0.0810015 | 7/15/2014 | 38:11.3 | 0 |
| -933.211 | 19763.941 | 2.297 | 14.091 | 0.0815208 | 7/15/2014 | 38:11.4 | 0 |
| -932.996 | 19763.923 | 2.297 | 14.071 | 0.082036 | 7/15/2014 | 38:11.5 | 0 |
| -932.782 | 19763.905 | 2.292 | 14.05 | 0.0825858 | 7/15/2014 | 38:11.6 | 0 |
| -932.512 | 19763.881 | 2.29 | 14.029 | 0.0830887 | 7/15/2014 | 38:11.7 | 0 |
| -932.295 | 19763.862 | 2.292 | 14.009 | 0.084243 | 7/15/2014 | 38:11.8 | 0 |
| -932.077 | 19763.843 | 2.292 | 13.989 | 0.0863189 | 7/15/2014 | 38:11.9 | 0 |
| -931.858 | 19763.823 | 2.294 | 13.97 | 0.102959 | 7/15/2014 | 38:12.0 | 0 |
| -931.639 | 19763.803 | 2.294 | 13.952 | 0.08586 | 7/15/2014 | 38:12.1 | 0 |
| -931.421 | 19763.784 | 2.294 | 13.935 | 0.0945045 | 7/15/2014 | 38:12.2 | 0 |
| -931.203 | 19763.766 | 2.294 | 13.92 | 0.0912302 | 7/15/2014 | 38:12.3 | 0 |
| -930.932 | 19763.74 | 2.297 | 13.906 | 0.0867648 | 7/15/2014 | 38:12.4 | 0 |
| -930.714 | 19763.722 | 2.297 | 13.894 | 0.0874031 | 7/15/2014 | 38:12.5 | 0 |
| -930.495 | 19763.702 | 2.299 | 13.884 | 0.103778 | 7/15/2014 | 38:12.6 | 0 |
| -930.221 | 19763.678 | 2.299 | 13.877 | 0.0879641 | 7/15/2014 | 38:12.7 | 0 |

| | | | | | | | |
|---|---|---|---|---|---|---|---|
| -930.001 | 19763.657 | 2.297 | 13.871 | 0.11021 | 7/15/2014 | 38:12.8 | 0 |
| -929.78 | 19763.637 | 2.297 | 13.869 | 0.11008 | 7/15/2014 | 38:12.9 | 0 |
| -929.558 | 19763.617 | 2.297 | 13.869 | 0.115714 | 7/15/2014 | 38:13.0 | 0 |
| -929.336 | 19763.598 | 2.297 | 13.873 | 0.136016 | 7/15/2014 | 38:13.1 | 0 |
| -929.114 | 19763.577 | 2.297 | 13.881 | 0.128915 | 7/15/2014 | 38:13.2 | 0 |
| -928.892 | 19763.557 | 2.297 | 13.892 | 0.126402 | 7/15/2014 | 38:13.3 | 0 |
| -928.618 | 19763.532 | 2.294 | 13.907 | 0.134289 | 7/15/2014 | 38:13.4 | 0 |
| -928.399 | 19763.512 | 2.296 | 13.925 | 0.126783 | 7/15/2014 | 38:13.5 | 0 |
| -928.179 | 19763.49 | 2.298 | 13.947 | 0.11769 | 7/15/2014 | 38:13.6 | 0 |
| -927.907 | 19763.466 | 2.3 | 13.971 | 0.128415 | 7/15/2014 | 38:13.7 | 0 |
| -927.687 | 19763.445 | 2.302 | 13.997 | 0.103466 | 7/15/2014 | 38:13.8 | 0 |
| -927.469 | 19763.425 | 2.299 | 14.024 | 0.110722 | 7/15/2014 | 38:13.9 | 0 |
| -927.248 | 19763.403 | 2.297 | 14.052 | 0.108961 | 7/15/2014 | 38:14.0 | 0 |
| -927.028 | 19763.383 | 2.297 | 14.078 | 0.110166 | 7/15/2014 | 38:14.1 | 0 |
| -926.811 | 19763.361 | 2.295 | 14.104 | 0.106271 | 7/15/2014 | 38:14.2 | 0 |
| -926.594 | 19763.341 | 2.292 | 14.126 | 0.106263 | 7/15/2014 | 38:14.3 | 0 |
| -926.326 | 19763.315 | 2.29 | 14.147 | 0.103991 | 7/15/2014 | 38:14.4 | 0 |
| -926.111 | 19763.294 | 2.288 | 14.164 | 0.102239 | 7/15/2014 | 38:14.5 | 0 |
| -925.899 | 19763.273 | 2.284 | 14.179 | 0.103371 | 7/15/2014 | 38:14.6 | 0 |
| -925.63 | 19763.247 | 2.287 | 14.19 | 0.101806 | 7/15/2014 | 38:14.7 | 0 |
| -925.414 | 19763.226 | 2.291 | 14.199 | 0.102509 | 7/15/2014 | 38:14.8 | 0 |
| -925.198 | 19763.204 | 2.291 | 14.205 | 0.101499 | 7/15/2014 | 38:14.9 | 0 |
| -924.981 | 19763.183 | 2.296 | 14.209 | 0.102143 | 7/15/2014 | 38:15.0 | 0 |
| -924.766 | 19763.162 | 2.298 | 14.211 | 0.114806 | 7/15/2014 | 38:15.1 | 0 |
| -924.55 | 19763.14 | 2.298 | 14.211 | 0.106528 | 7/15/2014 | 38:15.2 | 0 |
| -924.335 | 19763.117 | 2.3 | 14.209 | 0.105082 | 7/15/2014 | 38:15.3 | 0 |
| -924.067 | 19763.091 | 2.305 | 14.206 | 0.101891 | 7/15/2014 | 38:15.4 | 0 |
| -923.851 | 19763.069 | 2.307 | 14.203 | 0.0994793 | 7/15/2014 | 38:15.5 | 0 |
| -923.636 | 19763.047 | 2.31 | 14.198 | 0.0970455 | 7/15/2014 | 38:15.6 | 0 |
| -923.367 | 19763.019 | 2.312 | 14.192 | 0.0978067 | 7/15/2014 | 38:15.7 | 0 |
| -923.15 | 19762.998 | 2.316 | 14.186 | 0.0986128 | 7/15/2014 | 38:15.8 | 0 |
| -922.933 | 19762.975 | 2.316 | 14.179 | 0.0973081 | 7/15/2014 | 38:15.9 | 0 |
| -922.713 | 19762.951 | 2.318 | 14.171 | 0.101224 | 7/15/2014 | 38:16.0 | 0 |
| -922.496 | 19762.929 | 2.318 | 14.162 | 0.107851 | 7/15/2014 | 38:16.1 | 0 |
| -922.277 | 19762.906 | 2.317 | 14.152 | 0.107555 | 7/15/2014 | 38:16.2 | 0 |
| -922.061 | 19762.883 | 2.317 | 14.142 | 0.111037 | 7/15/2014 | 38:16.3 | 0 |
| -921.792 | 19762.854 | 2.317 | 14.131 | 0.115074 | 7/15/2014 | 38:16.4 | 0 |
| -921.576 | 19762.832 | 2.322 | 14.119 | 0.115081 | 7/15/2014 | 38:16.5 | 0 |
| -921.363 | 19762.809 | 2.322 | 14.108 | 0.116053 | 7/15/2014 | 38:16.6 | 0 |
| -921.096 | 19762.779 | 2.327 | 14.098 | 0.117439 | 7/15/2014 | 38:16.7 | 0 |
| -920.883 | 19762.756 | 2.33 | 14.089 | 0.111084 | 7/15/2014 | 38:16.8 | 0 |
| -920.669 | 19762.732 | 2.336 | 14.082 | 0.120098 | 7/15/2014 | 38:16.9 | 0 |
| -920.457 | 19762.71 | 2.339 | 14.079 | 0.106795 | 7/15/2014 | 38:17.0 | 0 |
| -920.245 | 19762.688 | 2.342 | 14.079 | 0.109516 | 7/15/2014 | 38:17.1 | 0 |
| -920.033 | 19762.664 | 2.342 | 14.083 | 0.106978 | 7/15/2014 | 38:17.2 | 0 |
| -919.824 | 19762.641 | 2.342 | 14.092 | 0.105682 | 7/15/2014 | 38:17.3 | 0 |
| -919.463 | 19762.6 | 2.342 | 14.113 | 0.112305 | 7/15/2014 | 38:17.5 | 0 |

| | | | | | | | |
|---|---|---|---|---|---|---|---|
| -919.256 | 19762.576 | 2.342 | 14.13 | 0.114661 | 7/15/2014 | 38:17.6 | 0 |
| -918.999 | 19762.547 | 2.342 | 14.149 | 0.0987506 | 7/15/2014 | 38:17.7 | 0 |
| -918.895 | 19762.536 | 2.342 | 14.158 | 0.112804 | 7/15/2014 | 38:17.7 | 0 |
| -918.689 | 19762.514 | 2.344 | 14.18 | 0.111849 | 7/15/2014 | 38:17.8 | 0 |
| -918.48 | 19762.49 | 2.346 | 14.204 | 0.115735 | 7/15/2014 | 38:17.9 | 0 |
| -918.27 | 19762.467 | 2.35 | 14.227 | 0.122558 | 7/15/2014 | 38:18.0 | 0 |
| -918.061 | 19762.443 | 2.35 | 14.25 | 0.138145 | 7/15/2014 | 38:18.1 | 0 |
| -917.852 | 19762.42 | 2.358 | 14.271 | 0.111028 | 7/15/2014 | 38:18.2 | 0 |
| -917.644 | 19762.396 | 2.358 | 14.291 | 0.125219 | 7/15/2014 | 38:18.3 | 0 |
| -917.278 | 19762.354 | 2.362 | 14.312 | 0.126766 | 7/15/2014 | 38:18.5 | 0 |
| -917.068 | 19762.33 | 2.366 | 14.324 | 0.118454 | 7/15/2014 | 38:18.6 | 0 |
| -916.804 | 19762.301 | 2.368 | 14.332 | 0.106809 | 7/15/2014 | 38:18.7 | 0 |
| -916.698 | 19762.289 | 2.371 | 14.337 | 0.102581 | 7/15/2014 | 38:18.7 | 0 |
| -916.485 | 19762.266 | 2.371 | 14.34 | 0.109728 | 7/15/2014 | 38:18.8 | 0 |
| -916.27 | 19762.24 | 2.373 | 14.339 | 0.103453 | 7/15/2014 | 38:18.9 | 0 |
| -916.054 | 19762.217 | 2.37 | 14.336 | 0.0876526 | 7/15/2014 | 38:19.0 | 0 |
| -915.836 | 19762.189 | 2.372 | 14.331 | 0.0794326 | 7/15/2014 | 38:19.1 | 0 |
| -915.621 | 19762.166 | 2.375 | 14.324 | 0.0881721 | 7/15/2014 | 38:19.2 | 0 |
| -915.404 | 19762.142 | 2.377 | 14.317 | 0.0718766 | 7/15/2014 | 38:19.3 | 0 |
| -915.135 | 19762.111 | 2.381 | 14.309 | 0.0885776 | 7/15/2014 | 38:19.4 | 0 |
| -914.917 | 19762.088 | 2.384 | 14.302 | 0.0785677 | 7/15/2014 | 38:19.5 | 0 |
| -914.702 | 19762.061 | 2.384 | 14.295 | 0.0961564 | 7/15/2014 | 38:19.6 | 0 |
| -914.429 | 19762.031 | 2.389 | 14.289 | 0.0923835 | 7/15/2014 | 38:19.7 | 0 |
| -914.211 | 19762.005 | 2.392 | 14.284 | 0.0786108 | 7/15/2014 | 38:19.8 | 0 |
| -913.991 | 19761.98 | 2.394 | 14.281 | 0.0896166 | 7/15/2014 | 38:19.9 | 0 |
| -913.772 | 19761.956 | 2.394 | 14.278 | 0.0799238 | 7/15/2014 | 38:20.0 | 0 |
| -913.553 | 19761.931 | 2.394 | 14.277 | 0.0735171 | 7/15/2014 | 38:20.1 | 0 |
| -913.334 | 19761.906 | 2.394 | 14.277 | 0.0614716 | 7/15/2014 | 38:20.2 | 0 |
| -913.119 | 19761.881 | 2.389 | 14.278 | 0.0583772 | 7/15/2014 | 38:20.3 | 0 |
| -912.848 | 19761.85 | 2.392 | 14.281 | 0.0569651 | 7/15/2014 | 38:20.4 | 0 |
| -912.634 | 19761.825 | 2.392 | 14.285 | 0.055478 | 7/15/2014 | 38:20.5 | 0 |
| -912.419 | 19761.801 | 2.395 | 14.291 | 0.0617504 | 7/15/2014 | 38:20.6 | 0 |
| -912.155 | 19761.771 | 2.399 | 14.298 | 0.060588 | 7/15/2014 | 38:20.7 | 0 |
| -911.942 | 19761.747 | 2.399 | 14.307 | 0.0639112 | 7/15/2014 | 38:20.8 | 0 |
| -911.728 | 19761.723 | 2.404 | 14.317 | 0.0759697 | 7/15/2014 | 38:20.9 | 0 |
| -911.515 | 19761.697 | 2.404 | 14.328 | 0.0769171 | 7/15/2014 | 38:21.0 | 0 |
| -911.303 | 19761.672 | 2.409 | 14.339 | 0.0988819 | 7/15/2014 | 38:21.1 | 0 |
| -911.091 | 19761.648 | 2.412 | 14.351 | 0.0973026 | 7/15/2014 | 38:21.2 | 0 |
| -910.881 | 19761.625 | 2.412 | 14.362 | 0.0943518 | 7/15/2014 | 38:21.3 | 0 |
| -910.521 | 19761.584 | 2.409 | 14.377 | 0.0910586 | 7/15/2014 | 38:21.5 | 0 |
| -910.314 | 19761.561 | 2.413 | 14.387 | 0.0939289 | 7/15/2014 | 38:21.6 | 0 |
| -910.06 | 19761.531 | 2.413 | 14.395 | 0.0820538 | 7/15/2014 | 38:21.7 | 0 |
| -909.954 | 19761.518 | 2.412 | 14.399 | 0.0805401 | 7/15/2014 | 38:21.7 | 0 |
| -909.75 | 19761.494 | 2.412 | 14.405 | 0.0799409 | 7/15/2014 | 38:21.8 | 0 |
| -909.544 | 19761.471 | 2.412 | 14.409 | 0.0742232 | 7/15/2014 | 38:21.9 | 0 |
| -909.339 | 19761.448 | 2.412 | 14.412 | 0.076517 | 7/15/2014 | 38:22.0 | 0 |
| -909.135 | 19761.426 | 2.409 | 14.414 | 0.073547 | 7/15/2014 | 38:22.1 | 0 |

| | | | | | | | |
|---|---|---|---|---|---|---|---|
| -908.932 | 19761.402 | 2.409 | 14.414 | 0.0738415 | 7/15/2014 | 38:22.2 | 0 |
| -908.73 | 19761.379 | 2.412 | 14.413 | 0.0742733 | 7/15/2014 | 38:22.3 | 0 |
| -908.381 | 19761.338 | 2.411 | 14.407 | 0.0798264 | 7/15/2014 | 38:22.5 | 0 |
| -908.181 | 19761.316 | 2.408 | 14.402 | 0.0845322 | 7/15/2014 | 38:22.6 | 0 |
| -907.93 | 19761.287 | 2.408 | 14.395 | 0.0859138 | 7/15/2014 | 38:22.7 | 0 |
| -907.828 | 19761.276 | 2.408 | 14.393 | 0.0896433 | 7/15/2014 | 38:22.7 | 0 |
| -907.628 | 19761.254 | 2.405 | 14.384 | 0.0890493 | 7/15/2014 | 38:22.8 | 0 |
| -907.423 | 19761.23 | 2.408 | 14.374 | 0.093526 | 7/15/2014 | 38:22.9 | 0 |
| -907.219 | 19761.207 | 2.408 | 14.362 | 0.0930617 | 7/15/2014 | 38:23.0 | 0 |
| -907.016 | 19761.184 | 2.408 | 14.35 | 0.0980116 | 7/15/2014 | 38:23.1 | 0 |
| -906.811 | 19761.16 | 2.411 | 14.337 | 0.101298 | 7/15/2014 | 38:23.2 | 0 |
| -906.608 | 19761.139 | 2.408 | 14.325 | 0.105152 | 7/15/2014 | 38:23.3 | 0 |
| -906.25 | 19761.098 | 2.415 | 14.307 | 0.136052 | 7/15/2014 | 38:23.5 | 0 |
| -906.043 | 19761.074 | 2.42 | 14.297 | 0.117604 | 7/15/2014 | 38:23.6 | 0 |
| -905.787 | 19761.045 | 2.42 | 14.289 | 0.103003 | 7/15/2014 | 38:23.7 | 0 |
| -905.683 | 19761.033 | 2.422 | 14.284 | 0.104532 | 7/15/2014 | 38:23.7 | 0 |
| -905.475 | 19761.01 | 2.419 | 14.278 | 0.124387 | 7/15/2014 | 38:23.8 | 0 |
| -905.264 | 19760.986 | 2.416 | 14.276 | 0.117776 | 7/15/2014 | 38:23.9 | 0 |
| -905.054 | 19760.963 | 2.413 | 14.276 | 0.101212 | 7/15/2014 | 38:24.0 | 0 |
| -904.843 | 19760.939 | 2.409 | 14.279 | 0.144686 | 7/15/2014 | 38:24.1 | 0 |
| -904.632 | 19760.915 | 2.407 | 14.284 | 0.0926358 | 7/15/2014 | 38:24.2 | 0 |
| -904.421 | 19760.891 | 2.404 | 14.292 | 0.101534 | 7/15/2014 | 38:24.3 | 0 |
| -904.055 | 19760.85 | 2.399 | 14.311 | 0.117674 | 7/15/2014 | 38:24.5 | 0 |
| -903.844 | 19760.826 | 2.404 | 14.325 | 0.0903284 | 7/15/2014 | 38:24.6 | 0 |
| -903.583 | 19760.797 | 2.404 | 14.341 | 0.10552 | 7/15/2014 | 38:24.7 | 0 |
| -903.476 | 19760.785 | 2.407 | 14.348 | 0.117413 | 7/15/2014 | 38:24.7 | 0 |
| -903.264 | 19760.762 | 2.407 | 14.368 | 0.0703179 | 7/15/2014 | 38:24.8 | 0 |
| -903.049 | 19760.737 | 2.407 | 14.39 | 0.121665 | 7/15/2014 | 38:24.9 | 0 |
| -902.836 | 19760.713 | 2.404 | 14.416 | 0.118554 | 7/15/2014 | 38:25.0 | 0 |
| -902.622 | 19760.689 | 2.404 | 14.444 | 0.103336 | 7/15/2014 | 38:25.1 | 0 |
| -902.408 | 19760.666 | 2.404 | 14.474 | 0.102102 | 7/15/2014 | 38:25.2 | 0 |
| -902.194 | 19760.643 | 2.404 | 14.507 | 0.116246 | 7/15/2014 | 38:25.3 | 0 |
| -901.932 | 19760.613 | 2.396 | 14.54 | 0.0921298 | 7/15/2014 | 38:25.4 | 0 |
| -901.722 | 19760.59 | 2.39 | 14.574 | 0.124322 | 7/15/2014 | 38:25.5 | 0 |
| -901.51 | 19760.566 | 2.388 | 14.606 | 0.0873615 | 7/15/2014 | 38:25.6 | 0 |
| -901.248 | 19760.539 | 2.389 | 14.637 | 0.102472 | 7/15/2014 | 38:25.7 | 0 |
| -901.037 | 19760.516 | 2.391 | 14.664 | 0.0841421 | 7/15/2014 | 38:25.8 | 0 |
| -900.826 | 19760.492 | 2.391 | 14.687 | 0.0993191 | 7/15/2014 | 38:25.9 | 0 |
| -900.615 | 19760.469 | 2.391 | 14.705 | 0.0913297 | 7/15/2014 | 38:26.0 | 0 |
| -900.406 | 19760.447 | 2.388 | 14.718 | 0.122735 | 7/15/2014 | 38:26.1 | 0 |
| -900.2 | 19760.424 | 2.391 | 14.726 | 0.0998361 | 7/15/2014 | 38:26.2 | 0 |
| -899.994 | 19760.401 | 2.391 | 14.73 | 0.0861249 | 7/15/2014 | 38:26.3 | 0 |
| -899.64 | 19760.364 | 2.388 | 14.724 | 0.0793985 | 7/15/2014 | 38:26.5 | 0 |
| -899.438 | 19760.342 | 2.387 | 14.717 | 0.091266 | 7/15/2014 | 38:26.6 | 0 |
| -899.19 | 19760.314 | 2.38 | 14.708 | 0.0864548 | 7/15/2014 | 38:26.7 | 0 |
| -899.087 | 19760.303 | 2.38 | 14.704 | 0.0843946 | 7/15/2014 | 38:26.7 | 0 |
| -898.791 | 19760.273 | 2.373 | 14.683 | 0.0758154 | 7/15/2014 | 38:26.9 | 0 |

| | | | | | | | |
|---|---|---|---|---|---|---|---|
| -898.592 | 19760.251 | 2.374 | 14.668 | 0.0746817 | 7/15/2014 | 38:27.0 | 0 |
| -898.395 | 19760.229 | 2.368 | 14.652 | 0.100039 | 7/15/2014 | 38:27.1 | 0 |
| -898.196 | 19760.209 | 2.368 | 14.635 | 0.0725461 | 7/15/2014 | 38:27.2 | 0 |
| -898.001 | 19760.188 | 2.363 | 14.618 | 0.0903153 | 7/15/2014 | 38:27.3 | 0 |
| -897.806 | 19760.168 | 2.365 | 14.601 | 0.0960321 | 7/15/2014 | 38:27.4 | 0 |
| -897.567 | 19760.143 | 2.363 | 14.584 | 0.0935131 | 7/15/2014 | 38:27.5 | 0 |
| -897.373 | 19760.123 | 2.365 | 14.568 | 0.076728 | 7/15/2014 | 38:27.6 | 0 |
| -897.135 | 19760.099 | 2.366 | 14.552 | 0.0843779 | 7/15/2014 | 38:27.7 | 0 |
| -896.942 | 19760.078 | 2.372 | 14.537 | 0.076945 | 7/15/2014 | 38:27.8 | 0 |
| -896.749 | 19760.058 | 2.37 | 14.524 | 0.0580081 | 7/15/2014 | 38:27.9 | 0 |
| -896.555 | 19760.038 | 2.373 | 14.512 | 0.0727656 | 7/15/2014 | 38:28.0 | 0 |
| -896.361 | 19760.019 | 2.373 | 14.502 | 0.0701676 | 7/15/2014 | 38:28.1 | 0 |
| -896.165 | 19759.999 | 2.375 | 14.493 | 0.0634133 | 7/15/2014 | 38:28.2 | 0 |
| -895.974 | 19759.979 | 2.373 | 14.487 | 0.0563111 | 7/15/2014 | 38:28.3 | 0 |
| -895.779 | 19759.96 | 2.375 | 14.483 | 0.0577333 | 7/15/2014 | 38:28.4 | 0 |
| -895.541 | 19759.936 | 2.375 | 14.482 | 0.0502916 | 7/15/2014 | 38:28.5 | 0 |
| -895.348 | 19759.916 | 2.377 | 14.482 | 0.0842626 | 7/15/2014 | 38:28.6 | 0 |
| -895.109 | 19759.892 | 2.373 | 14.485 | 0.0624403 | 7/15/2014 | 38:28.7 | 0 |
| -894.913 | 19759.873 | 2.377 | 14.489 | 0.0519918 | 7/15/2014 | 38:28.8 | 0 |
| -894.719 | 19759.854 | 2.38 | 14.495 | 0.0621028 | 7/15/2014 | 38:28.9 | 0 |
| -894.523 | 19759.835 | 2.385 | 14.503 | 0.0676764 | 7/15/2014 | 38:29.0 | 0 |
| -894.328 | 19759.815 | 2.383 | 14.512 | 0.0616899 | 7/15/2014 | 38:29.1 | 0 |
| -894.131 | 19759.796 | 2.388 | 14.522 | 0.0858456 | 7/15/2014 | 38:29.2 | 0 |
| -893.938 | 19759.776 | 2.386 | 14.534 | 0.0859936 | 7/15/2014 | 38:29.3 | 0 |
| -893.744 | 19759.757 | 2.391 | 14.546 | 0.0598626 | 7/15/2014 | 38:29.4 | 0 |
| -893.505 | 19759.733 | 2.391 | 14.557 | 0.0809345 | 7/15/2014 | 38:29.5 | 0 |
| -893.313 | 19759.716 | 2.393 | 14.568 | 0.0878041 | 7/15/2014 | 38:29.6 | 0 |
| -893.074 | 19759.692 | 2.391 | 14.577 | 0.0777633 | 7/15/2014 | 38:29.7 | 0 |
| -892.882 | 19759.675 | 2.391 | 14.584 | 0.0846642 | 7/15/2014 | 38:29.8 | 0 |
| -892.69 | 19759.656 | 2.391 | 14.588 | 0.0649586 | 7/15/2014 | 38:29.9 | 0 |
| -892.496 | 19759.638 | 2.395 | 14.589 | 0.0374997 | 7/15/2014 | 38:30.0 | 0 |
| -892.306 | 19759.619 | 2.391 | 14.585 | 0.0583261 | 7/15/2014 | 38:30.1 | 0 |
| -892.113 | 19759.6 | 2.395 | 14.578 | 0.0377807 | 7/15/2014 | 38:30.2 | 0 |
| -891.923 | 19759.583 | 2.391 | 14.567 | 0.0500543 | 7/15/2014 | 38:30.3 | 0 |
| -891.734 | 19759.565 | 2.391 | 14.554 | 0.0631233 | 7/15/2014 | 38:30.4 | 0 |
| -891.498 | 19759.543 | 2.393 | 14.537 | 0.0615948 | 7/15/2014 | 38:30.5 | 0 |
| -891.311 | 19759.526 | 2.396 | 14.52 | 0.071174 | 7/15/2014 | 38:30.6 | 0 |
| -891.076 | 19759.505 | 2.396 | 14.501 | 0.0884555 | 7/15/2014 | 38:30.7 | 0 |
| -890.888 | 19759.487 | 2.393 | 14.482 | 0.0832762 | 7/15/2014 | 38:30.8 | 0 |
| -890.699 | 19759.47 | 2.391 | 14.463 | 0.081347 | 7/15/2014 | 38:30.9 | 0 |
| -890.508 | 19759.451 | 2.395 | 14.445 | 0.0948108 | 7/15/2014 | 38:31.0 | 0 |
| -890.318 | 19759.436 | 2.395 | 14.429 | 0.0859407 | 7/15/2014 | 38:31.1 | 0 |
| -890.126 | 19759.418 | 2.397 | 14.413 | 0.0757338 | 7/15/2014 | 38:31.2 | 0 |
| -889.936 | 19759.4 | 2.394 | 14.4 | 0.0717319 | 7/15/2014 | 38:31.3 | 0 |
| -889.747 | 19759.385 | 2.396 | 14.388 | 0.0589213 | 7/15/2014 | 38:31.4 | 0 |
| -889.512 | 19759.364 | 2.396 | 14.379 | 0.0579199 | 7/15/2014 | 38:31.5 | 0 |
| -889.323 | 19759.346 | 2.396 | 14.372 | 0.0840843 | 7/15/2014 | 38:31.6 | 0 |

| | | | | | | | |
|---|---|---|---|---|---|---|---|
| -889.085 | 19759.325 | 2.399 | 14.367 | 0.0447065 | 7/15/2014 | 38:31.7 | 0 |
| -888.894 | 19759.309 | 2.402 | 14.364 | 0.0641197 | 7/15/2014 | 38:31.8 | 0 |
| -888.702 | 19759.293 | 2.4 | 14.364 | 0.0645431 | 7/15/2014 | 38:31.9 | 0 |
| -888.508 | 19759.276 | 2.4 | 14.367 | 0.0603256 | 7/15/2014 | 38:32.0 | 0 |
| -888.315 | 19759.26 | 2.398 | 14.373 | 0.0638617 | 7/15/2014 | 38:32.1 | 0 |
| -888.12 | 19759.244 | 2.404 | 14.381 | 0.056092 | 7/15/2014 | 38:32.2 | 0 |
| -887.929 | 19759.229 | 2.399 | 14.391 | 0.081898 | 7/15/2014 | 38:32.3 | 0 |
| -887.738 | 19759.211 | 2.4 | 14.403 | 0.0642198 | 7/15/2014 | 38:32.4 | 0 |
| -887.5 | 19759.191 | 2.4 | 14.416 | 0.0827366 | 7/15/2014 | 38:32.5 | 0 |
| -887.311 | 19759.176 | 2.4 | 14.428 | 0.0631657 | 7/15/2014 | 38:32.6 | 0 |
| -887.076 | 19759.158 | 2.4 | 14.44 | 0.112788 | 7/15/2014 | 38:32.7 | 0 |
| -886.884 | 19759.143 | 2.405 | 14.45 | 0.0640893 | 7/15/2014 | 38:32.8 | 0 |
| -886.696 | 19759.127 | 2.405 | 14.456 | 0.107873 | 7/15/2014 | 38:32.9 | 0 |
| -886.501 | 19759.111 | 2.41 | 14.46 | 0.102779 | 7/15/2014 | 38:33.0 | 0 |
| -886.314 | 19759.098 | 2.407 | 14.46 | 0.0836323 | 7/15/2014 | 38:33.1 | 0 |
| -886.124 | 19759.082 | 2.41 | 14.455 | 0.0844046 | 7/15/2014 | 38:33.2 | 0 |
| -885.935 | 19759.067 | 2.408 | 14.447 | 0.0642275 | 7/15/2014 | 38:33.3 | 0 |
| -885.748 | 19759.053 | 2.408 | 14.434 | 0.0846634 | 7/15/2014 | 38:33.4 | 0 |
| -885.514 | 19759.036 | 2.41 | 14.418 | 0.0382224 | 7/15/2014 | 38:33.5 | 0 |
| -885.327 | 19759.021 | 2.41 | 14.4 | 0.0679036 | 7/15/2014 | 38:33.6 | 0 |
| -885.093 | 19759.004 | 2.409 | 14.379 | 0.0437821 | 7/15/2014 | 38:33.7 | 0 |
| -884.902 | 19758.988 | 2.413 | 14.357 | 0.0533426 | 7/15/2014 | 38:33.8 | 0 |
| -884.715 | 19758.977 | 2.411 | 14.335 | 0.0473852 | 7/15/2014 | 38:33.9 | 0 |
| -884.524 | 19758.961 | 2.412 | 14.312 | 0.0480677 | 7/15/2014 | 38:34.0 | 0 |
| -884.333 | 19758.947 | 2.414 | 14.29 | 0.0660178 | 7/15/2014 | 38:34.1 | 0 |
| -884.142 | 19758.934 | 2.416 | 14.27 | 0.0591708 | 7/15/2014 | 38:34.2 | 0 |
| -883.953 | 19758.92 | 2.418 | 14.251 | 0.0552049 | 7/15/2014 | 38:34.3 | 0 |
| -883.762 | 19758.907 | 2.421 | 14.235 | 0.0600785 | 7/15/2014 | 38:34.4 | 0 |
| -883.529 | 19758.891 | 2.423 | 14.221 | 0.0594812 | 7/15/2014 | 38:34.5 | 0 |
| -883.34 | 19758.877 | 2.423 | 14.21 | 0.0706073 | 7/15/2014 | 38:34.6 | 0 |
| -883.108 | 19758.861 | 2.42 | 14.202 | 0.100611 | 7/15/2014 | 38:34.7 | 0 |
| -882.921 | 19758.848 | 2.425 | 14.197 | 0.0620577 | 7/15/2014 | 38:34.8 | 0 |
| -882.734 | 19758.835 | 2.425 | 14.196 | 0.070135 | 7/15/2014 | 38:34.9 | 0 |
| -882.547 | 19758.821 | 2.428 | 14.198 | 0.0533225 | 7/15/2014 | 38:35.0 | 0 |
| -882.36 | 19758.809 | 2.428 | 14.202 | 0.0625591 | 7/15/2014 | 38:35.1 | 0 |
| -882.172 | 19758.797 | 2.43 | 14.209 | 0.0438269 | 7/15/2014 | 38:35.2 | 0 |
| -881.991 | 19758.784 | 2.428 | 14.219 | 0.0605074 | 7/15/2014 | 38:35.3 | 0 |
| -881.807 | 19758.772 | 2.433 | 14.231 | 0.0472343 | 7/15/2014 | 38:35.4 | 0 |
| -881.491 | 19758.752 | 2.432 | 14.25 | 0.0578658 | 7/15/2014 | 38:35.5 | 0 |
| -881.264 | 19758.737 | 2.432 | 14.263 | 0.0452867 | 7/15/2014 | 38:35.6 | 0 |
| -881.174 | 19758.731 | 2.43 | 14.27 | 0.0503359 | 7/15/2014 | 38:35.7 | 0 |
| -880.992 | 19758.718 | 2.43 | 14.282 | 0.0467334 | 7/15/2014 | 38:35.8 | 0 |
| -880.808 | 19758.707 | 2.428 | 14.293 | 0.044379 | 7/15/2014 | 38:35.9 | 0 |
| -880.621 | 19758.694 | 2.428 | 14.301 | 0.0455149 | 7/15/2014 | 38:36.0 | 0 |
| -880.437 | 19758.683 | 2.425 | 14.307 | 0.0517776 | 7/15/2014 | 38:36.1 | 0 |
| -880.249 | 19758.672 | 2.43 | 14.31 | 0.0442726 | 7/15/2014 | 38:36.2 | 0 |
| -880.065 | 19758.659 | 2.425 | 14.309 | 0.038912 | 7/15/2014 | 38:36.3 | 0 |

| | | | | | | | |
|---|---|---|---|---|---|---|---|
| -879.879 | 19758.649 | 2.428 | 14.306 | 0.0454976 | 7/15/2014 | 38:36.4 | 0 |
| -879.648 | 19758.635 | 2.428 | 14.301 | 0.0371538 | 7/15/2014 | 38:36.5 | 0 |
| -879.461 | 19758.622 | 2.43 | 14.293 | 0.0707697 | 7/15/2014 | 38:36.6 | 0 |
| -879.227 | 19758.609 | 2.43 | 14.284 | 0.0503589 | 7/15/2014 | 38:36.7 | 0 |
| -879.036 | 19758.598 | 2.433 | 14.274 | 0.0445442 | 7/15/2014 | 38:36.8 | 0 |
| -878.846 | 19758.586 | 2.433 | 14.263 | 0.0558678 | 7/15/2014 | 38:36.9 | 0 |
| -878.654 | 19758.575 | 2.433 | 14.252 | 0.0564903 | 7/15/2014 | 38:37.0 | 0 |
| -878.463 | 19758.563 | 2.428 | 14.24 | 0.0537091 | 7/15/2014 | 38:37.1 | 0 |
| -878.27 | 19758.553 | 2.428 | 14.229 | 0.0475407 | 7/15/2014 | 38:37.2 | 0 |
| -878.08 | 19758.542 | 2.425 | 14.219 | 0.0495626 | 7/15/2014 | 38:37.3 | 0 |
| -877.889 | 19758.53 | 2.428 | 14.208 | 0.0743999 | 7/15/2014 | 38:37.4 | 0 |
| -877.655 | 19758.519 | 2.423 | 14.198 | 0.0699103 | 7/15/2014 | 38:37.5 | 0 |
| -877.464 | 19758.508 | 2.426 | 14.188 | 0.0678327 | 7/15/2014 | 38:37.6 | 0 |
| -877.232 | 19758.494 | 2.421 | 14.179 | 0.0840206 | 7/15/2014 | 38:37.7 | 0 |
| -877.044 | 19758.484 | 2.421 | 14.17 | 0.100433 | 7/15/2014 | 38:37.8 | 0 |
| -876.855 | 19758.474 | 2.419 | 14.161 | 0.104922 | 7/15/2014 | 38:37.9 | 0 |
| -876.664 | 19758.464 | 2.422 | 14.152 | 0.0797342 | 7/15/2014 | 38:38.0 | 0 |
| -876.476 | 19758.455 | 2.42 | 14.144 | 0.0929625 | 7/15/2014 | 38:38.1 | 0 |
| -876.287 | 19758.443 | 2.42 | 14.135 | 0.0832785 | 7/15/2014 | 38:38.2 | 0 |
| -876.099 | 19758.436 | 2.415 | 14.126 | 0.0807041 | 7/15/2014 | 38:38.3 | 0 |
| -875.911 | 19758.425 | 2.417 | 14.117 | 0.0611815 | 7/15/2014 | 38:38.4 | 0 |
| -875.587 | 19758.408 | 2.411 | 14.105 | 0.0582804 | 7/15/2014 | 38:38.5 | 0 |
| -875.353 | 19758.398 | 2.411 | 14.098 | 0.0610334 | 7/15/2014 | 38:38.6 | 0 |
| -875.261 | 19758.394 | 2.411 | 14.094 | 0.0467274 | 7/15/2014 | 38:38.7 | 0 |
| -875.071 | 19758.384 | 2.412 | 14.088 | 0.0686103 | 7/15/2014 | 38:38.8 | 0 |
| -874.883 | 19758.375 | 2.408 | 14.083 | 0.0466575 | 7/15/2014 | 38:38.9 | 0 |
| -874.692 | 19758.365 | 2.41 | 14.079 | 0.0555216 | 7/15/2014 | 38:39.0 | 0 |
| -874.501 | 19758.357 | 2.408 | 14.078 | 0.0755713 | 7/15/2014 | 38:39.1 | 0 |
| -874.308 | 19758.348 | 2.411 | 14.078 | 0.0710932 | 7/15/2014 | 38:39.2 | 0 |
| -874.117 | 19758.338 | 2.409 | 14.079 | 0.0774844 | 7/15/2014 | 38:39.3 | 0 |
| -873.926 | 19758.33 | 2.411 | 14.083 | 0.0773538 | 7/15/2014 | 38:39.4 | 0 |
| -873.689 | 19758.32 | 2.411 | 14.087 | 0.0624763 | 7/15/2014 | 38:39.5 | 0 |
| -873.497 | 19758.312 | 2.416 | 14.093 | 0.0737744 | 7/15/2014 | 38:39.6 | 0 |
| -873.258 | 19758.301 | 2.411 | 14.098 | 0.0505897 | 7/15/2014 | 38:39.7 | 0 |
| -873.065 | 19758.293 | 2.411 | 14.104 | 0.0534722 | 7/15/2014 | 38:39.8 | 0 |
| -872.87 | 19758.285 | 2.41 | 14.108 | 0.037148 | 7/15/2014 | 38:39.9 | 0 |
| -872.673 | 19758.276 | 2.41 | 14.112 | 0.0450885 | 7/15/2014 | 38:40.0 | 0 |
| -872.479 | 19758.268 | 2.407 | 14.114 | 0.0299283 | 7/15/2014 | 38:40.1 | 0 |
| -872.283 | 19758.262 | 2.409 | 14.114 | 0.0263873 | 7/15/2014 | 38:40.2 | 0 |
| -872.091 | 19758.254 | 2.406 | 14.112 | 0.0277427 | 7/15/2014 | 38:40.3 | 0 |
| -871.898 | 19758.246 | 2.406 | 14.108 | 0.0230787 | 7/15/2014 | 38:40.4 | 0 |
| -871.662 | 19758.236 | 2.401 | 14.103 | 0.0316706 | 7/15/2014 | 38:40.5 | 0 |
| -871.473 | 19758.23 | 2.406 | 14.096 | 0.031794 | 7/15/2014 | 38:40.6 | 0 |
| -871.239 | 19758.221 | 2.406 | 14.088 | 0.0352764 | 7/15/2014 | 38:40.7 | 0 |
| -871.05 | 19758.215 | 2.411 | 14.079 | 0.042315 | 7/15/2014 | 38:40.8 | 0 |
| -870.863 | 19758.208 | 2.411 | 14.069 | 0.0426505 | 7/15/2014 | 38:40.9 | 0 |
| -870.674 | 19758.203 | 2.413 | 14.059 | 0.0493908 | 7/15/2014 | 38:41.0 | 0 |

| | | | | | | | |
|---|---|---|---|---|---|---|---|
| -870.489 | 19758.196 | 2.413 | 14.049 | 0.0565826 | 7/15/2014 | 38:41.1 | 0 |
| -870.302 | 19758.189 | 2.421 | 14.039 | 0.0591086 | 7/15/2014 | 38:41.2 | 0 |
| -870.118 | 19758.184 | 2.421 | 14.029 | 0.109448 | 7/15/2014 | 38:41.3 | 0 |
| -869.935 | 19758.179 | 2.428 | 14.019 | 0.0797975 | 7/15/2014 | 38:41.4 | 0 |
| -869.62 | 19758.168 | 2.432 | 14.005 | 0.0964975 | 7/15/2014 | 38:41.5 | 0 |
| -869.397 | 19758.162 | 2.434 | 13.995 | 0.104027 | 7/15/2014 | 38:41.6 | 0 |
| -869.307 | 19758.159 | 2.432 | 13.991 | 0.0970047 | 7/15/2014 | 38:41.7 | 0 |
| -869.125 | 19758.154 | 2.44 | 13.981 | 0.0975135 | 7/15/2014 | 38:41.8 | 0 |
| -868.947 | 19758.149 | 2.439 | 13.971 | 0.109076 | 7/15/2014 | 38:41.9 | 0 |
| -868.764 | 19758.145 | 2.444 | 13.961 | 0.0973159 | 7/15/2014 | 38:42.0 | 0 |
| -868.583 | 19758.141 | 2.444 | 13.95 | 0.0840552 | 7/15/2014 | 38:42.1 | 0 |
| -868.402 | 19758.136 | 2.452 | 13.938 | 0.103372 | 7/15/2014 | 38:42.2 | 0 |
| -868.223 | 19758.131 | 2.452 | 13.925 | 0.0984691 | 7/15/2014 | 38:42.3 | 0 |
| -868.042 | 19758.127 | 2.455 | 13.912 | 0.092807 | 7/15/2014 | 38:42.4 | 0 |
| -867.731 | 19758.119 | 2.456 | 13.89 | 0.101326 | 7/15/2014 | 38:42.5 | 0 |
| -867.507 | 19758.115 | 2.461 | 13.875 | 0.0982066 | 7/15/2014 | 38:42.6 | 0 |
| -867.418 | 19758.113 | 2.46 | 13.868 | 0.0956799 | 7/15/2014 | 38:42.7 | 0 |
| -867.236 | 19758.109 | 2.466 | 13.853 | 0.105578 | 7/15/2014 | 38:42.8 | 0 |
| -867.055 | 19758.105 | 2.466 | 13.838 | 0.122248 | 7/15/2014 | 38:42.9 | 0 |
| -866.873 | 19758.102 | 2.466 | 13.823 | 0.123584 | 7/15/2014 | 38:43.0 | 0 |
| -866.69 | 19758.1 | 2.466 | 13.808 | 0.123233 | 7/15/2014 | 38:43.1 | 0 |
| -866.507 | 19758.096 | 2.47 | 13.794 | 0.131695 | 7/15/2014 | 38:43.2 | 0 |
| -866.326 | 19758.092 | 2.473 | 13.782 | 0.13029 | 7/15/2014 | 38:43.3 | 0 |
| -866.144 | 19758.089 | 2.477 | 13.77 | 0.129688 | 7/15/2014 | 38:43.4 | 0 |
| -865.828 | 19758.084 | 2.479 | 13.758 | 0.151586 | 7/15/2014 | 38:43.5 | 0 |
| -865.602 | 19758.081 | 2.479 | 13.751 | 0.141128 | 7/15/2014 | 38:43.6 | 0 |
| -865.512 | 19758.079 | 2.48 | 13.746 | 0.127893 | 7/15/2014 | 38:43.7 | 0 |
| -865.328 | 19758.076 | 2.484 | 13.743 | 0.128658 | 7/15/2014 | 38:43.8 | 0 |
| -865.143 | 19758.075 | 2.484 | 13.742 | 0.124922 | 7/15/2014 | 38:43.9 | 0 |
| -864.957 | 19758.072 | 2.484 | 13.744 | 0.118593 | 7/15/2014 | 38:44.0 | 0 |
| -864.771 | 19758.069 | 2.482 | 13.749 | 0.119542 | 7/15/2014 | 38:44.1 | 0 |
| -864.583 | 19758.067 | 2.484 | 13.756 | 0.121552 | 7/15/2014 | 38:44.2 | 0 |
| -864.398 | 19758.065 | 2.482 | 13.766 | 0.111834 | 7/15/2014 | 38:44.3 | 0 |
| -864.212 | 19758.063 | 2.486 | 13.777 | 0.108633 | 7/15/2014 | 38:44.4 | 0 |
| -863.983 | 19758.062 | 2.484 | 13.79 | 0.111465 | 7/15/2014 | 38:44.5 | 0 |
| -863.796 | 19758.061 | 2.488 | 13.804 | 0.116683 | 7/15/2014 | 38:44.6 | 0 |
| -863.567 | 19758.059 | 2.488 | 13.819 | 0.112593 | 7/15/2014 | 38:44.7 | 0 |
| -863.381 | 19758.057 | 2.492 | 13.832 | 0.111628 | 7/15/2014 | 38:44.8 | 0 |
| -863.195 | 19758.056 | 2.495 | 13.842 | 0.113383 | 7/15/2014 | 38:44.9 | 0 |
| -863.008 | 19758.055 | 2.497 | 13.85 | 0.112214 | 7/15/2014 | 38:45.0 | 0 |
| -862.821 | 19758.054 | 2.497 | 13.854 | 0.114123 | 7/15/2014 | 38:45.1 | 0 |
| -862.635 | 19758.054 | 2.499 | 13.854 | 0.113679 | 7/15/2014 | 38:45.2 | 0 |
| -862.452 | 19758.053 | 2.497 | 13.849 | 0.128863 | 7/15/2014 | 38:45.3 | 0 |
| -862.267 | 19758.053 | 2.499 | 13.84 | 0.113874 | 7/15/2014 | 38:45.4 | 0 |
| -861.949 | 19758.052 | 2.5 | 13.817 | 0.125803 | 7/15/2014 | 38:45.5 | 0 |
| -861.723 | 19758.051 | 2.498 | 13.801 | 0.123194 | 7/15/2014 | 38:45.6 | 0 |
| -861.632 | 19758.051 | 2.497 | 13.793 | 0.119976 | 7/15/2014 | 38:45.7 | 0 |

| | | | | | | | |
|---|---|---|---|---|---|---|---|
| -861.449 | 19758.051 | 2.497 | 13.775 | 0.130939 | 7/15/2014 | 38:45.8 | 0 |
| -861.267 | 19758.052 | 2.495 | 13.757 | 0.126374 | 7/15/2014 | 38:45.9 | 0 |
| -861.082 | 19758.053 | 2.497 | 13.741 | 0.151798 | 7/15/2014 | 38:46.0 | 0 |
| -860.899 | 19758.053 | 2.497 | 13.727 | 0.123858 | 7/15/2014 | 38:46.1 | 0 |
| -860.714 | 19758.054 | 2.502 | 13.717 | 0.119581 | 7/15/2014 | 38:46.2 | 0 |
| -860.531 | 19758.054 | 2.502 | 13.71 | 0.13381 | 7/15/2014 | 38:46.3 | 0 |
| -860.347 | 19758.055 | 2.508 | 13.706 | 0.123439 | 7/15/2014 | 38:46.4 | 0 |
| -860.029 | 19758.057 | 2.508 | 13.707 | 0.12448 | 7/15/2014 | 38:46.5 | 0 |
| -859.801 | 19758.057 | 2.51 | 13.708 | 0.132934 | 7/15/2014 | 38:46.6 | 0 |
| -859.71 | 19758.058 | 2.51 | 13.708 | 0.141849 | 7/15/2014 | 38:46.7 | 0 |
| -859.522 | 19758.06 | 2.518 | 13.711 | 0.162187 | 7/15/2014 | 38:46.8 | 0 |
| -859.337 | 19758.061 | 2.518 | 13.713 | 0.145856 | 7/15/2014 | 38:46.9 | 0 |
| -859.149 | 19758.063 | 2.518 | 13.713 | 0.160746 | 7/15/2014 | 38:47.0 | 0 |
| -858.962 | 19758.063 | 2.513 | 13.711 | 0.163539 | 7/15/2014 | 38:47.1 | 0 |
| -858.771 | 19758.066 | 2.516 | 13.705 | 0.150609 | 7/15/2014 | 38:47.2 | 0 |
| -858.586 | 19758.067 | 2.508 | 13.696 | 0.151861 | 7/15/2014 | 38:47.3 | 0 |
| -858.395 | 19758.07 | 2.516 | 13.684 | 0.139022 | 7/15/2014 | 38:47.4 | 0 |
| -858.163 | 19758.071 | 2.513 | 13.67 | 0.146719 | 7/15/2014 | 38:47.5 | 0 |
| -857.974 | 19758.073 | 2.513 | 13.654 | 0.130555 | 7/15/2014 | 38:47.6 | 0 |
| -857.739 | 19758.076 | 2.508 | 13.638 | 0.131852 | 7/15/2014 | 38:47.7 | 0 |
| -857.548 | 19758.08 | 2.51 | 13.624 | 0.0998407 | 7/15/2014 | 38:47.8 | 0 |
| -857.357 | 19758.081 | 2.51 | 13.611 | 0.101864 | 7/15/2014 | 38:47.9 | 0 |
| -857.163 | 19758.084 | 2.512 | 13.601 | 0.106853 | 7/15/2014 | 38:48.0 | 0 |
| -856.97 | 19758.087 | 2.509 | 13.595 | 0.0913816 | 7/15/2014 | 38:48.1 | 0 |
| -856.775 | 19758.09 | 2.511 | 13.593 | 0.0923734 | 7/15/2014 | 38:48.2 | 0 |
| -856.584 | 19758.092 | 2.509 | 13.596 | 0.0831483 | 7/15/2014 | 38:48.3 | 0 |
| -856.391 | 19758.095 | 2.511 | 13.602 | 0.0856396 | 7/15/2014 | 38:48.4 | 0 |
| -856.154 | 19758.098 | 2.514 | 13.613 | 0.0891024 | 7/15/2014 | 38:48.5 | 0 |
| -855.963 | 19758.102 | 2.514 | 13.627 | 0.0935632 | 7/15/2014 | 38:48.6 | 0 |
| -855.723 | 19758.105 | 2.511 | 13.644 | 0.0814583 | 7/15/2014 | 38:48.7 | 0 |
| -855.53 | 19758.108 | 2.511 | 13.663 | 0.0781786 | 7/15/2014 | 38:48.8 | 0 |
| -855.335 | 19758.112 | 2.511 | 13.684 | 0.0887999 | 7/15/2014 | 38:48.9 | 0 |
| -855.14 | 19758.115 | 2.511 | 13.704 | 0.0871985 | 7/15/2014 | 38:49.0 | 0 |
| -854.945 | 19758.118 | 2.509 | 13.724 | 0.10846 | 7/15/2014 | 38:49.1 | 0 |
| -854.748 | 19758.122 | 2.507 | 13.742 | 0.0957893 | 7/15/2014 | 38:49.2 | 0 |
| -854.555 | 19758.125 | 2.504 | 13.757 | 0.107301 | 7/15/2014 | 38:49.3 | 0 |
| -854.361 | 19758.129 | 2.504 | 13.769 | 0.123926 | 7/15/2014 | 38:49.4 | 0 |
| -854.119 | 19758.134 | 2.502 | 13.777 | 0.12519 | 7/15/2014 | 38:49.5 | 0 |
| -853.925 | 19758.138 | 2.502 | 13.781 | 0.124958 | 7/15/2014 | 38:49.6 | 0 |
| -853.682 | 19758.143 | 2.5 | 13.782 | 0.131564 | 7/15/2014 | 38:49.7 | 0 |
| -853.485 | 19758.145 | 2.5 | 13.779 | 0.134877 | 7/15/2014 | 38:49.8 | 0 |
| -853.287 | 19758.148 | 2.5 | 13.773 | 0.111207 | 7/15/2014 | 38:49.9 | 0 |
| -853.088 | 19758.152 | 2.502 | 13.764 | 0.129447 | 7/15/2014 | 38:50.0 | 0 |
| -852.889 | 19758.156 | 2.5 | 13.754 | 0.108955 | 7/15/2014 | 38:50.1 | 0 |
| -852.689 | 19758.16 | 2.498 | 13.743 | 0.0747013 | 7/15/2014 | 38:50.2 | 0 |
| -852.491 | 19758.164 | 2.494 | 13.732 | 0.108138 | 7/15/2014 | 38:50.3 | 0 |
| -852.292 | 19758.168 | 2.493 | 13.72 | 0.114016 | 7/15/2014 | 38:50.4 | 0 |

| | | | | | | | |
|---|---|---|---|---|---|---|---|
| -852.047 | 19758.173 | 2.487 | 13.709 | 0.0852784 | 7/15/2014 | 38:50.5 | 0 |
| -851.847 | 19758.176 | 2.487 | 13.698 | 0.0913697 | 7/15/2014 | 38:50.6 | 0 |
| -851.599 | 19758.181 | 2.48 | 13.688 | 0.10948 | 7/15/2014 | 38:50.7 | 0 |
| -851.499 | 19758.183 | 2.477 | 13.683 | 0.110789 | 7/15/2014 | 38:50.7 | 0 |
| -851.296 | 19758.188 | 2.472 | 13.675 | 0.0957803 | 7/15/2014 | 38:50.8 | 0 |
| -851.095 | 19758.19 | 2.469 | 13.667 | 0.106168 | 7/15/2014 | 38:50.9 | 0 |
| -850.891 | 19758.194 | 2.469 | 13.66 | 0.109862 | 7/15/2014 | 38:51.0 | 0 |
| -850.688 | 19758.198 | 2.466 | 13.653 | 0.105105 | 7/15/2014 | 38:51.1 | 0 |
| -850.486 | 19758.202 | 2.466 | 13.646 | 0.119036 | 7/15/2014 | 38:51.2 | 0 |
| -850.284 | 19758.205 | 2.469 | 13.64 | 0.130477 | 7/15/2014 | 38:51.3 | 0 |
| -849.933 | 19758.212 | 2.469 | 13.631 | 0.130075 | 7/15/2014 | 38:51.5 | 0 |
| -849.73 | 19758.217 | 2.474 | 13.626 | 0.140687 | 7/15/2014 | 38:51.6 | 0 |
| -849.481 | 19758.221 | 2.475 | 13.623 | 0.142557 | 7/15/2014 | 38:51.7 | 0 |
| -849.378 | 19758.223 | 2.475 | 13.62 | 0.135894 | 7/15/2014 | 38:51.7 | 0 |
| -849.175 | 19758.227 | 2.478 | 13.618 | 0.144496 | 7/15/2014 | 38:51.8 | 0 |
| -848.972 | 19758.229 | 2.475 | 13.618 | 0.138156 | 7/15/2014 | 38:51.9 | 0 |
| -848.768 | 19758.233 | 2.475 | 13.619 | 0.135209 | 7/15/2014 | 38:52.0 | 0 |
| -848.565 | 19758.237 | 2.472 | 13.622 | 0.111824 | 7/15/2014 | 38:52.1 | 0 |
| -848.363 | 19758.24 | 2.467 | 13.627 | 0.11814 | 7/15/2014 | 38:52.2 | 0 |
| -848.159 | 19758.244 | 2.464 | 13.633 | 0.0972258 | 7/15/2014 | 38:52.3 | 0 |
| -847.804 | 19758.25 | 2.459 | 13.645 | 0.076849 | 7/15/2014 | 38:52.5 | 0 |
| -847.598 | 19758.254 | 2.458 | 13.654 | 0.0924144 | 7/15/2014 | 38:52.6 | 0 |
| -847.342 | 19758.258 | 2.449 | 13.663 | 0.0800581 | 7/15/2014 | 38:52.7 | 0 |
| -847.24 | 19758.261 | 2.45 | 13.668 | 0.0791083 | 7/15/2014 | 38:52.7 | 0 |
| -847.033 | 19758.264 | 2.445 | 13.678 | 0.081623 | 7/15/2014 | 38:52.8 | 0 |
| -846.822 | 19758.267 | 2.442 | 13.687 | 0.0862084 | 7/15/2014 | 38:52.9 | 0 |
| -846.61 | 19758.27 | 2.442 | 13.696 | 0.0878241 | 7/15/2014 | 38:53.0 | 0 |
| -846.396 | 19758.273 | 2.442 | 13.705 | 0.0890057 | 7/15/2014 | 38:53.1 | 0 |
| -846.182 | 19758.276 | 2.44 | 13.712 | 0.0944365 | 7/15/2014 | 38:53.2 | 0 |
| -845.967 | 19758.279 | 2.44 | 13.719 | 0.0972934 | 7/15/2014 | 38:53.3 | 0 |
| -845.701 | 19758.283 | 2.438 | 13.726 | 0.0912922 | 7/15/2014 | 38:53.4 | 0 |
| -845.484 | 19758.287 | 2.44 | 13.732 | 0.0928129 | 7/15/2014 | 38:53.5 | 0 |
| -845.267 | 19758.289 | 2.438 | 13.737 | 0.0913496 | 7/15/2014 | 38:53.6 | 0 |
| -844.994 | 19758.293 | 2.44 | 13.743 | 0.0880587 | 7/15/2014 | 38:53.7 | 0 |
| -844.774 | 19758.295 | 2.438 | 13.748 | 0.0938492 | 7/15/2014 | 38:53.8 | 0 |
| -844.553 | 19758.299 | 2.436 | 13.752 | 0.0978763 | 7/15/2014 | 38:53.9 | 0 |
| -844.332 | 19758.301 | 2.434 | 13.757 | 0.10233 | 7/15/2014 | 38:54.0 | 0 |
| -844.11 | 19758.303 | 2.432 | 13.76 | 0.106909 | 7/15/2014 | 38:54.1 | 0 |
| -843.89 | 19758.307 | 2.43 | 13.763 | 0.122234 | 7/15/2014 | 38:54.2 | 0 |
| -843.67 | 19758.308 | 2.424 | 13.766 | 0.134711 | 7/15/2014 | 38:54.3 | 0 |
| -843.398 | 19758.313 | 2.424 | 13.767 | 0.125561 | 7/15/2014 | 38:54.4 | 0 |
| -843.18 | 19758.313 | 2.422 | 13.769 | 0.127251 | 7/15/2014 | 38:54.5 | 0 |
| -842.964 | 19758.316 | 2.424 | 13.769 | 0.120675 | 7/15/2014 | 38:54.6 | 0 |
| -842.695 | 19758.319 | 2.421 | 13.77 | 0.121102 | 7/15/2014 | 38:54.7 | 0 |
| -842.478 | 19758.32 | 2.421 | 13.771 | 0.108692 | 7/15/2014 | 38:54.8 | 0 |
| -842.263 | 19758.324 | 2.418 | 13.772 | 0.107646 | 7/15/2014 | 38:54.9 | 0 |
| -842.045 | 19758.324 | 2.42 | 13.775 | 0.115113 | 7/15/2014 | 38:55.0 | 0 |

| | | | | | | | |
|---|---|---|---|---|---|---|---|
| -841.83 | 19758.327 | 2.423 | 13.779 | 0.118722 | 7/15/2014 | 38:55.1 | 0 |
| -841.616 | 19758.328 | 2.423 | 13.784 | 0.126424 | 7/15/2014 | 38:55.2 | 0 |
| -841.405 | 19758.33 | 2.42 | 13.791 | 0.117807 | 7/15/2014 | 38:55.3 | 0 |
| -841.038 | 19758.334 | 2.413 | 13.806 | 0.135385 | 7/15/2014 | 38:55.5 | 0 |
| -840.826 | 19758.334 | 2.416 | 13.815 | 0.115894 | 7/15/2014 | 38:55.6 | 0 |
| -840.568 | 19758.338 | 2.41 | 13.826 | 0.133877 | 7/15/2014 | 38:55.7 | 0 |
| -840.461 | 19758.338 | 2.412 | 13.831 | 0.131095 | 7/15/2014 | 38:55.7 | 0 |
| -840.251 | 19758.339 | 2.406 | 13.841 | 0.128722 | 7/15/2014 | 38:55.8 | 0 |
| -840.04 | 19758.34 | 2.403 | 13.851 | 0.124277 | 7/15/2014 | 38:55.9 | 0 |
| -839.829 | 19758.34 | 2.405 | 13.859 | 0.14272 | 7/15/2014 | 38:56.0 | 0 |
| -839.617 | 19758.342 | 2.409 | 13.865 | 0.145284 | 7/15/2014 | 38:56.1 | 0 |
| -839.408 | 19758.344 | 2.409 | 13.869 | 0.12411 | 7/15/2014 | 38:56.2 | 0 |
| -839.199 | 19758.344 | 2.412 | 13.87 | 0.145085 | 7/15/2014 | 38:56.3 | 0 |
| -838.837 | 19758.346 | 2.41 | 13.869 | 0.134923 | 7/15/2014 | 38:56.5 | 0 |
| -838.628 | 19758.346 | 2.416 | 13.868 | 0.128297 | 7/15/2014 | 38:56.6 | 0 |
| -838.371 | 19758.346 | 2.416 | 13.866 | 0.134296 | 7/15/2014 | 38:56.7 | 0 |
| -838.264 | 19758.346 | 2.418 | 13.865 | 0.127078 | 7/15/2014 | 38:56.7 | 0 |
| -838.054 | 19758.347 | 2.416 | 13.862 | 0.119347 | 7/15/2014 | 38:56.8 | 0 |
| -837.845 | 19758.348 | 2.413 | 13.86 | 0.134854 | 7/15/2014 | 38:56.9 | 0 |
| -837.635 | 19758.348 | 2.413 | 13.858 | 0.120791 | 7/15/2014 | 38:57.0 | 0 |
| -837.425 | 19758.348 | 2.41 | 13.856 | 0.117856 | 7/15/2014 | 38:57.1 | 0 |
| -837.216 | 19758.348 | 2.41 | 13.855 | 0.112292 | 7/15/2014 | 38:57.2 | 0 |
| -837.007 | 19758.347 | 2.406 | 13.853 | 0.108611 | 7/15/2014 | 38:57.3 | 0 |
| -836.643 | 19758.346 | 2.4 | 13.844 | 0.105579 | 7/15/2014 | 38:57.5 | 0 |
| -836.433 | 19758.346 | 2.4 | 13.838 | 0.102916 | 7/15/2014 | 38:57.6 | 0 |
| -836.172 | 19758.344 | 2.394 | 13.829 | 0.100028 | 7/15/2014 | 38:57.7 | 0 |
| -836.066 | 19758.344 | 2.394 | 13.826 | 0.0998161 | 7/15/2014 | 38:57.7 | 0 |
| -835.854 | 19758.343 | 2.394 | 13.815 | 0.0930131 | 7/15/2014 | 38:57.8 | 0 |
| -835.641 | 19758.342 | 2.397 | 13.804 | 0.0961645 | 7/15/2014 | 38:57.9 | 0 |
| -835.426 | 19758.341 | 2.397 | 13.791 | 0.100479 | 7/15/2014 | 38:58.0 | 0 |
| -835.213 | 19758.338 | 2.4 | 13.778 | 0.0934344 | 7/15/2014 | 38:58.1 | 0 |
| -834.996 | 19758.338 | 2.403 | 13.767 | 0.095505 | 7/15/2014 | 38:58.2 | 0 |
| -834.782 | 19758.336 | 2.406 | 13.756 | 0.0912101 | 7/15/2014 | 38:58.3 | 0 |
| -834.513 | 19758.333 | 2.41 | 13.747 | 0.0901359 | 7/15/2014 | 38:58.4 | 0 |
| -834.297 | 19758.33 | 2.41 | 13.738 | 0.0931402 | 7/15/2014 | 38:58.5 | 0 |
| -834.079 | 19758.328 | 2.41 | 13.73 | 0.0876388 | 7/15/2014 | 38:58.6 | 0 |
| -833.807 | 19758.326 | 2.41 | 13.723 | 0.0895874 | 7/15/2014 | 38:58.7 | 0 |
| -833.588 | 19758.322 | 2.406 | 13.717 | 0.0908121 | 7/15/2014 | 38:58.8 | 0 |
| -833.366 | 19758.32 | 2.403 | 13.712 | 0.0921079 | 7/15/2014 | 38:58.9 | 0 |
| -833.145 | 19758.317 | 2.397 | 13.706 | 0.0939251 | 7/15/2014 | 38:59.0 | 0 |
| -832.922 | 19758.314 | 2.397 | 13.701 | 0.103746 | 7/15/2014 | 38:59.1 | 0 |
| -832.702 | 19758.311 | 2.394 | 13.696 | 0.109201 | 7/15/2014 | 38:59.2 | 0 |
| -832.479 | 19758.308 | 2.397 | 13.691 | 0.119991 | 7/15/2014 | 38:59.3 | 0 |
| -832.204 | 19758.305 | 2.397 | 13.686 | 0.132937 | 7/15/2014 | 38:59.4 | 0 |
| -831.986 | 19758.301 | 2.397 | 13.681 | 0.150197 | 7/15/2014 | 38:59.5 | 0 |
| -831.765 | 19758.296 | 2.4 | 13.676 | 0.172674 | 7/15/2014 | 38:59.6 | 0 |
| -831.493 | 19758.292 | 2.403 | 13.671 | 0.156547 | 7/15/2014 | 38:59.7 | 0 |

| | | | | | | | |
|---|---|---|---|---|---|---|---|
| -831.273 | 19758.288 | 2.408 | 13.667 | 0.151157 | 7/15/2014 | 38:59.8 | 0 |
| -831.053 | 19758.284 | 2.408 | 13.663 | 0.151288 | 7/15/2014 | 38:59.9 | 0 |
| -830.832 | 19758.28 | 2.411 | 13.66 | 0.144854 | 7/15/2014 | 39:00.0 | 0 |
| -830.613 | 19758.277 | 2.411 | 13.657 | 0.140967 | 7/15/2014 | 39:00.1 | 0 |
| -830.397 | 19758.273 | 2.408 | 13.655 | 0.127925 | 7/15/2014 | 39:00.2 | 0 |
| -830.179 | 19758.268 | 2.408 | 13.654 | 0.119613 | 7/15/2014 | 39:00.3 | 0 |
| -829.91 | 19758.262 | 2.408 | 13.653 | 0.118266 | 7/15/2014 | 39:00.4 | 0 |
| -829.697 | 19758.258 | 2.404 | 13.654 | 0.119078 | 7/15/2014 | 39:00.5 | 0 |
| -829.481 | 19758.254 | 2.408 | 13.655 | 0.133907 | 7/15/2014 | 39:00.6 | 0 |
| -829.211 | 19758.248 | 2.408 | 13.657 | 0.123385 | 7/15/2014 | 39:00.7 | 0 |
| -828.995 | 19758.242 | 2.41 | 13.659 | 0.12883 | 7/15/2014 | 39:00.8 | 0 |
| -828.778 | 19758.238 | 2.412 | 13.661 | 0.132784 | 7/15/2014 | 39:00.9 | 0 |
| -828.557 | 19758.231 | 2.415 | 13.663 | 0.175997 | 7/15/2014 | 39:01.0 | 0 |
| -828.337 | 19758.226 | 2.417 | 13.664 | 0.173655 | 7/15/2014 | 39:01.1 | 0 |
| -828.117 | 19758.221 | 2.419 | 13.664 | 0.203913 | 7/15/2014 | 39:01.2 | 0 |
| -827.898 | 19758.216 | 2.424 | 13.663 | 0.162114 | 7/15/2014 | 39:01.3 | 0 |
| -827.624 | 19758.209 | 2.424 | 13.661 | 0.198721 | 7/15/2014 | 39:01.4 | 0 |
| -827.403 | 19758.201 | 2.427 | 13.656 | 0.211128 | 7/15/2014 | 39:01.5 | 0 |
| -827.181 | 19758.195 | 2.427 | 13.65 | 0.222778 | 7/15/2014 | 39:01.6 | 0 |
| -826.902 | 19758.187 | 2.424 | 13.642 | 0.154601 | 7/15/2014 | 39:01.7 | 0 |
| -826.678 | 19758.18 | 2.427 | 13.633 | 0.156802 | 7/15/2014 | 39:01.8 | 0 |
| -826.452 | 19758.174 | 2.424 | 13.622 | 0.150984 | 7/15/2014 | 39:01.9 | 0 |
| -826.226 | 19758.167 | 2.424 | 13.612 | 0.129851 | 7/15/2014 | 39:02.0 | 0 |
| -825.999 | 19758.16 | 2.422 | 13.601 | 0.10919 | 7/15/2014 | 39:02.1 | 0 |
| -825.773 | 19758.152 | 2.42 | 13.59 | 0.104233 | 7/15/2014 | 39:02.2 | 0 |
| -825.548 | 19758.145 | 2.418 | 13.58 | 0.0940796 | 7/15/2014 | 39:02.3 | 0 |
| -825.268 | 19758.136 | 2.416 | 13.57 | 0.0962987 | 7/15/2014 | 39:02.4 | 0 |
| -825.044 | 19758.128 | 2.416 | 13.562 | 0.101591 | 7/15/2014 | 39:02.5 | 0 |
| -824.82 | 19758.121 | 2.414 | 13.555 | 0.108539 | 7/15/2014 | 39:02.6 | 0 |
| -824.54 | 19758.111 | 2.414 | 13.549 | 0.110643 | 7/15/2014 | 39:02.7 | 0 |
| -824.316 | 19758.104 | 2.409 | 13.544 | 0.109074 | 7/15/2014 | 39:02.8 | 0 |
| -824.089 | 19758.094 | 2.409 | 13.541 | 0.114636 | 7/15/2014 | 39:02.9 | 0 |
| -823.863 | 19758.086 | 2.411 | 13.54 | 0.104209 | 7/15/2014 | 39:03.0 | 0 |
| -823.637 | 19758.078 | 2.411 | 13.54 | 0.104909 | 7/15/2014 | 39:03.1 | 0 |
| -823.412 | 19758.07 | 2.411 | 13.542 | 0.0983169 | 7/15/2014 | 39:03.2 | 0 |
| -823.187 | 19758.061 | 2.411 | 13.546 | 0.0934294 | 7/15/2014 | 39:03.3 | 0 |
| -822.91 | 19758.051 | 2.408 | 13.552 | 0.0953755 | 7/15/2014 | 39:03.4 | 0 |
| -822.687 | 19758.043 | 2.408 | 13.56 | 0.0936332 | 7/15/2014 | 39:03.5 | 0 |
| -822.463 | 19758.031 | 2.413 | 13.57 | 0.0923347 | 7/15/2014 | 39:03.6 | 0 |
| -822.184 | 19758.021 | 2.41 | 13.581 | 0.0956063 | 7/15/2014 | 39:03.7 | 0 |
| -821.961 | 19758.012 | 2.406 | 13.592 | 0.106552 | 7/15/2014 | 39:03.8 | 0 |
| -821.734 | 19758.002 | 2.403 | 13.603 | 0.103908 | 7/15/2014 | 39:03.9 | 0 |
| -821.509 | 19757.992 | 2.4 | 13.614 | 0.107616 | 7/15/2014 | 39:04.0 | 0 |
| -821.284 | 19757.982 | 2.4 | 13.622 | 0.114678 | 7/15/2014 | 39:04.1 | 0 |
| -821.059 | 19757.973 | 2.4 | 13.629 | 0.11609 | 7/15/2014 | 39:04.2 | 0 |
| -820.834 | 19757.963 | 2.4 | 13.634 | 0.11595 | 7/15/2014 | 39:04.3 | 0 |
| -820.555 | 19757.95 | 2.4 | 13.637 | 0.122863 | 7/15/2014 | 39:04.4 | 0 |

| | | | | | | | |
|---|---|---|---|---|---|---|---|
| -820.335 | 19757.939 | 2.405 | 13.637 | 0.12738 | 7/15/2014 | 39:04.5 | 0 |
| -820.112 | 19757.928 | 2.409 | 13.634 | 0.124747 | 7/15/2014 | 39:04.6 | 0 |
| -819.838 | 19757.916 | 2.414 | 13.631 | 0.122495 | 7/15/2014 | 39:04.7 | 0 |
| -819.615 | 19757.905 | 2.419 | 13.625 | 0.120318 | 7/15/2014 | 39:04.8 | 0 |
| -819.393 | 19757.895 | 2.422 | 13.619 | 0.109265 | 7/15/2014 | 39:04.9 | 0 |
| -819.172 | 19757.885 | 2.428 | 13.613 | 0.103725 | 7/15/2014 | 39:05.0 | 0 |
| -818.949 | 19757.873 | 2.433 | 13.606 | 0.101926 | 7/15/2014 | 39:05.1 | 0 |
| -818.731 | 19757.861 | 2.431 | 13.599 | 0.0946511 | 7/15/2014 | 39:05.2 | 0 |
| -818.511 | 19757.85 | 2.434 | 13.592 | 0.0906959 | 7/15/2014 | 39:05.3 | 0 |
| -818.241 | 19757.837 | 2.434 | 13.584 | 0.105624 | 7/15/2014 | 39:05.4 | 0 |
| -818.023 | 19757.826 | 2.437 | 13.577 | 0.094088 | 7/15/2014 | 39:05.5 | 0 |
| -817.806 | 19757.816 | 2.437 | 13.57 | 0.085177 | 7/15/2014 | 39:05.6 | 0 |
| -817.534 | 19757.801 | 2.434 | 13.563 | 0.0854649 | 7/15/2014 | 39:05.7 | 0 |
| -817.313 | 19757.789 | 2.434 | 13.556 | 0.0915268 | 7/15/2014 | 39:05.8 | 0 |
| -817.093 | 19757.777 | 2.431 | 13.549 | 0.0857866 | 7/15/2014 | 39:05.9 | 0 |
| -816.87 | 19757.766 | 2.433 | 13.543 | 0.0947659 | 7/15/2014 | 39:06.0 | 0 |
| -816.649 | 19757.754 | 2.43 | 13.537 | 0.0780788 | 7/15/2014 | 39:06.1 | 0 |
| -816.425 | 19757.742 | 2.433 | 13.532 | 0.0903549 | 7/15/2014 | 39:06.2 | 0 |
| -816.205 | 19757.73 | 2.428 | 13.528 | 0.105835 | 7/15/2014 | 39:06.3 | 0 |
| -815.927 | 19757.715 | 2.428 | 13.524 | 0.0819123 | 7/15/2014 | 39:06.4 | 0 |
| -815.704 | 19757.701 | 2.43 | 13.522 | 0.0896234 | 7/15/2014 | 39:06.5 | 0 |
| -815.482 | 19757.689 | 2.425 | 13.521 | 0.0800248 | 7/15/2014 | 39:06.6 | 0 |
| -815.2 | 19757.674 | 2.425 | 13.521 | 0.0694482 | 7/15/2014 | 39:06.7 | 0 |
| -814.974 | 19757.661 | 2.422 | 13.522 | 0.0864685 | 7/15/2014 | 39:06.8 | 0 |
| -814.746 | 19757.648 | 2.42 | 13.525 | 0.0544453 | 7/15/2014 | 39:06.9 | 0 |
| -814.519 | 19757.635 | 2.414 | 13.53 | 0.0707212 | 7/15/2014 | 39:07.0 | 0 |
| -814.29 | 19757.622 | 2.416 | 13.536 | 0.0549503 | 7/15/2014 | 39:07.1 | 0 |
| -814.064 | 19757.607 | 2.413 | 13.543 | 0.0469957 | 7/15/2014 | 39:07.2 | 0 |
| -813.837 | 19757.595 | 2.411 | 13.552 | 0.0455265 | 7/15/2014 | 39:07.3 | 0 |
| -813.556 | 19757.578 | 2.411 | 13.563 | 0.0472709 | 7/15/2014 | 39:07.4 | 0 |
| -813.33 | 19757.565 | 2.409 | 13.574 | 0.0502521 | 7/15/2014 | 39:07.5 | 0 |
| -813.105 | 19757.552 | 2.409 | 13.586 | 0.0426542 | 7/15/2014 | 39:07.6 | 0 |
| -812.829 | 19757.535 | 2.409 | 13.598 | 0.0779921 | 7/15/2014 | 39:07.7 | 0 |
| -812.606 | 19757.522 | 2.406 | 13.61 | 0.047705 | 7/15/2014 | 39:07.8 | 0 |
| -812.383 | 19757.508 | 2.401 | 13.621 | 0.050111 | 7/15/2014 | 39:07.9 | 0 |
| -812.16 | 19757.495 | 2.399 | 13.63 | 0.0796011 | 7/15/2014 | 39:08.0 | 0 |
| -811.939 | 19757.481 | 2.399 | 13.637 | 0.0633753 | 7/15/2014 | 39:08.1 | 0 |
| -811.72 | 19757.468 | 2.399 | 13.643 | 0.0586336 | 7/15/2014 | 39:08.2 | 0 |
| -811.5 | 19757.454 | 2.396 | 13.646 | 0.0700019 | 7/15/2014 | 39:08.3 | 0 |
| -811.231 | 19757.438 | 2.393 | 13.648 | 0.0589233 | 7/15/2014 | 39:08.4 | 0 |
| -811.017 | 19757.425 | 2.387 | 13.648 | 0.05591 | 7/15/2014 | 39:08.5 | 0 |
| -810.802 | 19757.41 | 2.384 | 13.647 | 0.0686577 | 7/15/2014 | 39:08.6 | 0 |
| -810.534 | 19757.394 | 2.387 | 13.645 | 0.0604744 | 7/15/2014 | 39:08.7 | 0 |
| -810.319 | 19757.38 | 2.387 | 13.644 | 0.0666064 | 7/15/2014 | 39:08.8 | 0 |
| -810.102 | 19757.366 | 2.387 | 13.642 | 0.0788898 | 7/15/2014 | 39:08.9 | 0 |
| -809.883 | 19757.352 | 2.392 | 13.641 | 0.0736392 | 7/15/2014 | 39:09.0 | 0 |
| -809.667 | 19757.338 | 2.395 | 13.641 | 0.0711173 | 7/15/2014 | 39:09.1 | 0 |

| | | | | | | | |
|---|---|---|---|---|---|---|---|
| -809.449 | 19757.323 | 2.395 | 13.641 | 0.0820818 | 7/15/2014 | 39:09.2 | 0 |
| -809.23 | 19757.309 | 2.401 | 13.643 | 0.0613739 | 7/15/2014 | 39:09.3 | 0 |
| -808.958 | 19757.29 | 2.404 | 13.645 | 0.0584609 | 7/15/2014 | 39:09.4 | 0 |
| -808.738 | 19757.275 | 2.4 | 13.647 | 0.0537309 | 7/15/2014 | 39:09.5 | 0 |
| -808.516 | 19757.261 | 2.4 | 13.649 | 0.0490727 | 7/15/2014 | 39:09.6 | 0 |
| -808.234 | 19757.241 | 2.397 | 13.652 | 0.038536 | 7/15/2014 | 39:09.7 | 0 |
| -808.009 | 19757.226 | 2.388 | 13.654 | 0.0440429 | 7/15/2014 | 39:09.8 | 0 |
| -807.78 | 19757.21 | 2.385 | 13.655 | 0.0547758 | 7/15/2014 | 39:09.9 | 0 |
| -807.549 | 19757.193 | 2.385 | 13.656 | 0.0468757 | 7/15/2014 | 39:10.0 | 0 |
| -807.318 | 19757.178 | 2.382 | 13.655 | 0.0499647 | 7/15/2014 | 39:10.1 | 0 |
| -807.205 | 19757.17 | 2.38 | 13.655 | 0.0435557 | 7/15/2014 | 39:10.2 | 0 |
| -806.973 | 19757.153 | 2.383 | 13.653 | 0.0460963 | 7/15/2014 | 39:10.3 | 0 |
| -806.562 | 19757.125 | 2.382 | 13.646 | 0.042096 | 7/15/2014 | 39:10.4 | 0 |
| -806.328 | 19757.106 | 2.385 | 13.641 | 0.0405275 | 7/15/2014 | 39:10.5 | 0 |
| -806.093 | 19757.091 | 2.385 | 13.636 | 0.0493249 | 7/15/2014 | 39:10.6 | 0 |
| -805.797 | 19757.069 | 2.385 | 13.63 | 0.0467963 | 7/15/2014 | 39:10.7 | 0 |
| -805.677 | 19757.062 | 2.383 | 13.627 | 0.0476853 | 7/15/2014 | 39:10.8 | 0 |
| -805.439 | 19757.044 | 2.381 | 13.622 | 0.0520357 | 7/15/2014 | 39:10.9 | 0 |
| -805.199 | 19757.026 | 2.375 | 13.619 | 0.043601 | 7/15/2014 | 39:11.0 | 0 |
| -804.96 | 19757.01 | 2.373 | 13.618 | 0.0466565 | 7/15/2014 | 39:11.1 | 0 |
| -804.721 | 19756.991 | 2.364 | 13.62 | 0.0480309 | 7/15/2014 | 39:11.2 | 0 |
| -804.481 | 19756.975 | 2.36 | 13.625 | 0.080693 | 7/15/2014 | 39:11.3 | 0 |
| -804.064 | 19756.944 | 2.358 | 13.64 | 0.0581831 | 7/15/2014 | 39:11.4 | 0 |
| -803.828 | 19756.927 | 2.353 | 13.653 | 0.0473494 | 7/15/2014 | 39:11.5 | 0 |
| -803.59 | 19756.909 | 2.355 | 13.669 | 0.0720346 | 7/15/2014 | 39:11.6 | 0 |
| -803.297 | 19756.889 | 2.355 | 13.687 | 0.0570423 | 7/15/2014 | 39:11.7 | 0 |
| -803.177 | 19756.88 | 2.355 | 13.695 | 0.0479528 | 7/15/2014 | 39:11.8 | 0 |
| -802.938 | 19756.861 | 2.36 | 13.716 | 0.0415277 | 7/15/2014 | 39:11.9 | 0 |
| -802.703 | 19756.845 | 2.358 | 13.737 | 0.0366039 | 7/15/2014 | 39:12.0 | 0 |
| -802.466 | 19756.827 | 2.36 | 13.758 | 0.030965 | 7/15/2014 | 39:12.1 | 0 |
| -802.233 | 19756.81 | 2.355 | 13.777 | 0.0663164 | 7/15/2014 | 39:12.2 | 0 |
| -801.998 | 19756.793 | 2.358 | 13.795 | 0.0258447 | 7/15/2014 | 39:12.3 | 0 |
| -801.593 | 19756.764 | 2.351 | 13.814 | 0.0337305 | 7/15/2014 | 39:12.4 | 0 |
| -801.36 | 19756.746 | 2.349 | 13.823 | 0.0697832 | 7/15/2014 | 39:12.5 | 0 |
| -801.129 | 19756.729 | 2.345 | 13.829 | 0.0553656 | 7/15/2014 | 39:12.6 | 0 |
| -800.839 | 19756.708 | 2.342 | 13.832 | 0.0689726 | 7/15/2014 | 39:12.7 | 0 |
| -800.723 | 19756.699 | 2.34 | 13.834 | 0.0395077 | 7/15/2014 | 39:12.8 | 0 |
| -800.49 | 19756.683 | 2.34 | 13.831 | 0.0667063 | 7/15/2014 | 39:12.9 | 0 |
| -800.255 | 19756.665 | 2.338 | 13.824 | 0.0415697 | 7/15/2014 | 39:13.0 | 0 |
| -800.02 | 19756.648 | 2.341 | 13.815 | 0.0552468 | 7/15/2014 | 39:13.1 | 0 |
| -799.785 | 19756.631 | 2.341 | 13.804 | 0.0366618 | 7/15/2014 | 39:13.2 | 0 |
| -799.549 | 19756.613 | 2.343 | 13.79 | 0.0394465 | 7/15/2014 | 39:13.3 | 0 |
| -799.137 | 19756.583 | 2.337 | 13.767 | 0.0386761 | 7/15/2014 | 39:13.4 | 0 |
| -798.901 | 19756.565 | 2.337 | 13.752 | 0.0349065 | 7/15/2014 | 39:13.5 | 0 |
| -798.663 | 19756.547 | 2.341 | 13.736 | 0.0421989 | 7/15/2014 | 39:13.6 | 0 |
| -798.366 | 19756.526 | 2.341 | 13.72 | 0.0339341 | 7/15/2014 | 39:13.7 | 0 |
| -798.247 | 19756.517 | 2.339 | 13.712 | 0.0357042 | 7/15/2014 | 39:13.8 | 0 |

| | | | | | | | |
|---|---|---|---|---|---|---|---|
| -798.006 | 19756.499 | 2.339 | 13.697 | 0.0497593 | 7/15/2014 | 39:13.9 | 0 |
| -797.767 | 19756.48 | 2.337 | 13.683 | 0.0395778 | 7/15/2014 | 39:14.0 | 0 |
| -797.525 | 19756.464 | 2.342 | 13.671 | 0.0150757 | 7/15/2014 | 39:14.1 | 0 |
| -797.284 | 19756.445 | 2.342 | 13.66 | 0.0525931 | 7/15/2014 | 39:14.2 | 0 |
| -797.041 | 19756.426 | 2.342 | 13.649 | 0.0493455 | 7/15/2014 | 39:14.3 | 0 |
| -796.62 | 19756.396 | 2.334 | 13.636 | 0.0822072 | 7/15/2014 | 39:14.4 | 0 |
| -796.38 | 19756.377 | 2.334 | 13.628 | 0.0582692 | 7/15/2014 | 39:14.5 | 0 |
| -796.138 | 19756.359 | 2.334 | 13.62 | 0.0712424 | 7/15/2014 | 39:14.6 | 0 |
| -795.837 | 19756.337 | 2.332 | 13.613 | 0.109693 | 7/15/2014 | 39:14.7 | 0 |
| -795.715 | 19756.327 | 2.328 | 13.609 | 0.0881113 | 7/15/2014 | 39:14.8 | 0 |
| -795.471 | 19756.309 | 2.332 | 13.602 | 0.0469024 | 7/15/2014 | 39:14.9 | 0 |
| -795.227 | 19756.29 | 2.328 | 13.594 | 0.0793226 | 7/15/2014 | 39:15.0 | 0 |
| -794.982 | 19756.271 | 2.332 | 13.586 | 0.0488827 | 7/15/2014 | 39:15.1 | 0 |
| -794.739 | 19756.253 | 2.326 | 13.578 | 0.0747264 | 7/15/2014 | 39:15.2 | 0 |
| -794.494 | 19756.236 | 2.326 | 13.569 | 0.0555192 | 7/15/2014 | 39:15.3 | 0 |
| -794.068 | 19756.203 | 2.328 | 13.555 | 0.0646191 | 7/15/2014 | 39:15.4 | 0 |
| -793.825 | 19756.186 | 2.33 | 13.545 | 0.103771 | 7/15/2014 | 39:15.5 | 0 |
| -793.581 | 19756.166 | 2.33 | 13.536 | 0.0926091 | 7/15/2014 | 39:15.6 | 0 |
| -793.278 | 19756.143 | 2.328 | 13.526 | 0.090999 | 7/15/2014 | 39:15.7 | 0 |
| -793.155 | 19756.134 | 2.326 | 13.521 | 0.0780291 | 7/15/2014 | 39:15.8 | 0 |
| -792.908 | 19756.116 | 2.332 | 13.512 | 0.10241 | 7/15/2014 | 39:15.9 | 0 |
| -792.663 | 19756.098 | 2.33 | 13.504 | 0.105468 | 7/15/2014 | 39:16.0 | 0 |
| -792.417 | 19756.079 | 2.324 | 13.497 | 0.098782 | 7/15/2014 | 39:16.1 | 0 |
| -792.172 | 19756.062 | 2.318 | 13.492 | 0.0821798 | 7/15/2014 | 39:16.2 | 0 |
| -791.926 | 19756.043 | 2.314 | 13.489 | 0.070464 | 7/15/2014 | 39:16.3 | 0 |
| -791.5 | 19756.01 | 2.31 | 13.491 | 0.0852893 | 7/15/2014 | 39:16.4 | 0 |
| -791.255 | 19755.993 | 2.308 | 13.494 | 0.0770293 | 7/15/2014 | 39:16.5 | 0 |
| -791.012 | 19755.975 | 2.304 | 13.499 | 0.0752776 | 7/15/2014 | 39:16.6 | 0 |
| -790.709 | 19755.951 | 2.301 | 13.507 | 0.0743589 | 7/15/2014 | 39:16.7 | 0 |
| -790.585 | 19755.942 | 2.302 | 13.511 | 0.0791863 | 7/15/2014 | 39:16.8 | 0 |
| -790.339 | 19755.926 | 2.302 | 13.523 | 0.0784955 | 7/15/2014 | 39:16.9 | 0 |
| -790.095 | 19755.906 | 2.3 | 13.536 | 0.0816455 | 7/15/2014 | 39:17.0 | 0 |
| -789.849 | 19755.888 | 2.302 | 13.552 | 0.078378 | 7/15/2014 | 39:17.1 | 0 |
| -789.605 | 19755.871 | 2.296 | 13.568 | 0.0814963 | 7/15/2014 | 39:17.2 | 0 |
| -789.36 | 19755.852 | 2.298 | 13.584 | 0.0824458 | 7/15/2014 | 39:17.3 | 0 |
| -788.934 | 19755.82 | 2.298 | 13.604 | 0.0842447 | 7/15/2014 | 39:17.4 | 0 |
| -788.691 | 19755.804 | 2.296 | 13.616 | 0.0845634 | 7/15/2014 | 39:17.5 | 0 |
| -788.447 | 19755.785 | 2.298 | 13.625 | 0.0917576 | 7/15/2014 | 39:17.6 | 0 |
| -788.142 | 19755.763 | 2.298 | 13.632 | 0.0875366 | 7/15/2014 | 39:17.7 | 0 |
| -788.02 | 19755.754 | 2.296 | 13.636 | 0.0888977 | 7/15/2014 | 39:17.8 | 0 |
| -787.775 | 19755.735 | 2.298 | 13.637 | 0.0900382 | 7/15/2014 | 39:17.9 | 0 |
| -787.529 | 19755.719 | 2.296 | 13.635 | 0.0952104 | 7/15/2014 | 39:18.0 | 0 |
| -787.281 | 19755.699 | 2.294 | 13.631 | 0.0960563 | 7/15/2014 | 39:18.1 | 0 |
| -787.036 | 19755.682 | 2.286 | 13.624 | 0.0972414 | 7/15/2014 | 39:18.2 | 0 |
| -786.789 | 19755.664 | 2.284 | 13.616 | 0.107072 | 7/15/2014 | 39:18.3 | 0 |
| -786.361 | 19755.633 | 2.271 | 13.602 | 0.106413 | 7/15/2014 | 39:18.4 | 0 |
| -786.117 | 19755.614 | 2.263 | 13.592 | 0.106955 | 7/15/2014 | 39:18.5 | 0 |

| | | | | | | | |
|---|---|---|---|---|---|---|---|
| -785.87 | 19755.598 | 2.259 | 13.582 | 0.108464 | 7/15/2014 | 39:18.6 | 0 |
| -785.563 | 19755.574 | 2.254 | 13.572 | 0.103969 | 7/15/2014 | 39:18.7 | 0 |
| -785.439 | 19755.565 | 2.25 | 13.567 | 0.105274 | 7/15/2014 | 39:18.8 | 0 |
| -785.19 | 19755.547 | 2.25 | 13.558 | 0.100145 | 7/15/2014 | 39:18.9 | 0 |
| -784.944 | 19755.53 | 2.246 | 13.548 | 0.0950782 | 7/15/2014 | 39:19.0 | 0 |
| -784.693 | 19755.512 | 2.249 | 13.539 | 0.0921063 | 7/15/2014 | 39:19.1 | 0 |
| -784.445 | 19755.492 | 2.242 | 13.531 | 0.0889002 | 7/15/2014 | 39:19.2 | 0 |
| -784.195 | 19755.476 | 2.242 | 13.522 | 0.085867 | 7/15/2014 | 39:19.3 | 0 |
| -783.886 | 19755.453 | 2.234 | 13.514 | 0.08547 | 7/15/2014 | 39:19.4 | 0 |
| -783.635 | 19755.434 | 2.231 | 13.505 | 0.0863527 | 7/15/2014 | 39:19.5 | 0 |
| -783.386 | 19755.418 | 2.223 | 13.496 | 0.0858462 | 7/15/2014 | 39:19.6 | 0 |
| -783.137 | 19755.398 | 2.22 | 13.487 | 0.0901969 | 7/15/2014 | 39:19.7 | 0 |
| -782.826 | 19755.375 | 2.211 | 13.478 | 0.088184 | 7/15/2014 | 39:19.8 | 0 |
| -782.574 | 19755.357 | 2.209 | 13.468 | 0.0924979 | 7/15/2014 | 39:19.9 | 0 |
| -782.324 | 19755.34 | 2.203 | 13.459 | 0.0880053 | 7/15/2014 | 39:20.0 | 0 |
| -782.073 | 19755.32 | 2.203 | 13.45 | 0.107699 | 7/15/2014 | 39:20.1 | 0 |
| -781.824 | 19755.304 | 2.198 | 13.441 | 0.0895139 | 7/15/2014 | 39:20.2 | 0 |
| -781.575 | 19755.285 | 2.198 | 13.432 | 0.0938515 | 7/15/2014 | 39:20.3 | 0 |
| -781.145 | 19755.253 | 2.197 | 13.422 | 0.100863 | 7/15/2014 | 39:20.4 | 0 |
| -780.901 | 19755.235 | 2.197 | 13.416 | 0.102568 | 7/15/2014 | 39:20.5 | 0 |
| -780.657 | 19755.219 | 2.199 | 13.412 | 0.138643 | 7/15/2014 | 39:20.6 | 0 |
| -780.357 | 19755.195 | 2.197 | 13.41 | 0.134419 | 7/15/2014 | 39:20.7 | 0 |
| -780.233 | 19755.187 | 2.195 | 13.409 | 0.15806 | 7/15/2014 | 39:20.8 | 0 |
| -779.992 | 19755.17 | 2.195 | 13.41 | 0.124155 | 7/15/2014 | 39:20.9 | 0 |
| -779.754 | 19755.151 | 2.187 | 13.416 | 0.16178 | 7/15/2014 | 39:21.0 | 0 |
| -779.514 | 19755.134 | 2.184 | 13.425 | 0.119053 | 7/15/2014 | 39:21.1 | 0 |
| -779.279 | 19755.117 | 2.172 | 13.438 | 0.151891 | 7/15/2014 | 39:21.2 | 0 |
| -779.042 | 19755.101 | 2.166 | 13.457 | 0.128819 | 7/15/2014 | 39:21.3 | 0 |
| -778.637 | 19755.07 | 2.15 | 13.498 | 0.101347 | 7/15/2014 | 39:21.4 | 0 |
| -778.406 | 19755.055 | 2.142 | 13.528 | 0.145374 | 7/15/2014 | 39:21.5 | 0 |
| -778.175 | 19755.037 | 2.139 | 13.563 | 0.0873981 | 7/15/2014 | 39:21.6 | 0 |
| -777.885 | 19755.017 | 2.134 | 13.599 | 0.139216 | 7/15/2014 | 39:21.7 | 0 |
| -777.77 | 19755.009 | 2.128 | 13.618 | 0.109908 | 7/15/2014 | 39:21.8 | 0 |
| -777.535 | 19754.991 | 2.128 | 13.657 | 0.08246 | 7/15/2014 | 39:21.9 | 0 |
| -777.302 | 19754.976 | 2.124 | 13.695 | 0.0870405 | 7/15/2014 | 39:22.0 | 0 |
| -777.068 | 19754.959 | 2.124 | 13.728 | 0.0920123 | 7/15/2014 | 39:22.1 | 0 |
| -776.832 | 19754.942 | 2.124 | 13.757 | 0.0615268 | 7/15/2014 | 39:22.2 | 0 |
| -776.596 | 19754.925 | 2.126 | 13.78 | 0.103356 | 7/15/2014 | 39:22.3 | 0 |
| -776.184 | 19754.896 | 2.115 | 13.798 | 0.0646681 | 7/15/2014 | 39:22.4 | 0 |
| -775.949 | 19754.88 | 2.113 | 13.806 | 0.0887927 | 7/15/2014 | 39:22.5 | 0 |
| -775.712 | 19754.863 | 2.111 | 13.809 | 0.0969483 | 7/15/2014 | 39:22.6 | 0 |
| -775.418 | 19754.844 | 2.106 | 13.807 | 0.0719341 | 7/15/2014 | 39:22.7 | 0 |
| -775.297 | 19754.835 | 2.103 | 13.806 | 0.0739622 | 7/15/2014 | 39:22.8 | 0 |
| -775.06 | 19754.818 | 2.098 | 13.799 | 0.062791 | 7/15/2014 | 39:22.9 | 0 |
| -774.823 | 19754.803 | 2.086 | 13.789 | 0.0605934 | 7/15/2014 | 39:23.0 | 0 |
| -774.584 | 19754.785 | 2.09 | 13.778 | 0.0467908 | 7/15/2014 | 39:23.1 | 0 |
| -774.349 | 19754.769 | 2.084 | 13.766 | 0.035928 | 7/15/2014 | 39:23.2 | 0 |

| | | | | | | | |
|---|---|---|---|---|---|---|---|
| -774.112 | 19754.753 | 2.084 | 13.754 | 0.0368105 | 7/15/2014 | 39:23.3 | 0 |
| -773.702 | 19754.725 | 2.083 | 13.736 | 0.0318621 | 7/15/2014 | 39:23.4 | 0 |
| -773.47 | 19754.71 | 2.084 | 13.725 | 0.0573995 | 7/15/2014 | 39:23.5 | 0 |
| -773.24 | 19754.694 | 2.084 | 13.714 | 0.0663379 | 7/15/2014 | 39:23.6 | 0 |
| -772.95 | 19754.674 | 2.09 | 13.704 | 0.0697982 | 7/15/2014 | 39:23.7 | 0 |
| -772.833 | 19754.665 | 2.087 | 13.699 | 0.0593811 | 7/15/2014 | 39:23.8 | 0 |
| -772.489 | 19754.644 | 2.088 | 13.688 | 0.0736191 | 7/15/2014 | 39:23.9 | 0 |
| -772.258 | 19754.627 | 2.088 | 13.682 | 0.075354 | 7/15/2014 | 39:24.0 | 0 |
| -772.028 | 19754.612 | 2.081 | 13.678 | 0.0719557 | 7/15/2014 | 39:24.1 | 0 |
| -771.798 | 19754.598 | 2.078 | 13.675 | 0.0830113 | 7/15/2014 | 39:24.2 | 0 |
| -771.571 | 19754.583 | 2.072 | 13.674 | 0.0980024 | 7/15/2014 | 39:24.3 | 0 |
| -771.287 | 19754.563 | 2.066 | 13.674 | 0.0981931 | 7/15/2014 | 39:24.4 | 0 |
| -771.06 | 19754.55 | 2.059 | 13.677 | 0.106915 | 7/15/2014 | 39:24.5 | 0 |
| -770.832 | 19754.534 | 2.056 | 13.681 | 0.104869 | 7/15/2014 | 39:24.6 | 0 |
| -770.546 | 19754.516 | 2.053 | 13.688 | 0.101045 | 7/15/2014 | 39:24.7 | 0 |
| -770.315 | 19754.502 | 2.05 | 13.696 | 0.0994169 | 7/15/2014 | 39:24.8 | 0 |
| -770.082 | 19754.486 | 2.05 | 13.706 | 0.0909349 | 7/15/2014 | 39:24.9 | 0 |
| -769.969 | 19754.479 | 2.047 | 13.711 | 0.0923542 | 7/15/2014 | 39:25.0 | 0 |
| -769.733 | 19754.464 | 2.055 | 13.723 | 0.0935313 | 7/15/2014 | 39:25.1 | 0 |
| -769.5 | 19754.449 | 2.058 | 13.736 | 0.0948968 | 7/15/2014 | 39:25.2 | 0 |
| -769.264 | 19754.434 | 2.062 | 13.749 | 0.0991801 | 7/15/2014 | 39:25.3 | 0 |
| -768.851 | 19754.407 | 2.059 | 13.765 | 0.0899809 | 7/15/2014 | 39:25.4 | 0 |
| -768.616 | 19754.393 | 2.059 | 13.774 | 0.0893145 | 7/15/2014 | 39:25.5 | 0 |
| -768.383 | 19754.378 | 2.056 | 13.781 | 0.0879388 | 7/15/2014 | 39:25.6 | 0 |
| -768.089 | 19754.358 | 2.056 | 13.786 | 0.0987362 | 7/15/2014 | 39:25.7 | 0 |
| -767.97 | 19754.351 | 2.051 | 13.789 | 0.0980853 | 7/15/2014 | 39:25.8 | 0 |
| -767.735 | 19754.335 | 2.047 | 13.789 | 0.0861753 | 7/15/2014 | 39:25.9 | 0 |
| -767.5 | 19754.32 | 2.044 | 13.786 | 0.087096 | 7/15/2014 | 39:26.0 | 0 |
| -767.264 | 19754.305 | 2.047 | 13.78 | 0.103549 | 7/15/2014 | 39:26.1 | 0 |
| -766.918 | 19754.282 | 2.047 | 13.766 | 0.102841 | 7/15/2014 | 39:26.2 | 0 |
| -766.69 | 19754.268 | 2.047 | 13.755 | 0.118408 | 7/15/2014 | 39:26.3 | 0 |
| -766.404 | 19754.249 | 2.047 | 13.744 | 0.103358 | 7/15/2014 | 39:26.4 | 0 |
| -766.177 | 19754.234 | 2.047 | 13.731 | 0.119625 | 7/15/2014 | 39:26.5 | 0 |
| -765.951 | 19754.221 | 2.047 | 13.719 | 0.118375 | 7/15/2014 | 39:26.6 | 0 |
| -765.668 | 19754.202 | 2.047 | 13.706 | 0.107734 | 7/15/2014 | 39:26.7 | 0 |
| -765.444 | 19754.188 | 2.044 | 13.694 | 0.079932 | 7/15/2014 | 39:26.8 | 0 |
| -765.216 | 19754.174 | 2.046 | 13.683 | 0.0651103 | 7/15/2014 | 39:26.9 | 0 |
| -764.99 | 19754.159 | 2.043 | 13.674 | 0.0720132 | 7/15/2014 | 39:27.0 | 0 |
| -764.765 | 19754.146 | 2.043 | 13.666 | 0.0593318 | 7/15/2014 | 39:27.1 | 0 |
| -764.538 | 19754.131 | 2.046 | 13.66 | 0.0603572 | 7/15/2014 | 39:27.2 | 0 |
| -764.316 | 19754.117 | 2.046 | 13.656 | 0.0632308 | 7/15/2014 | 39:27.3 | 0 |
| -764.036 | 19754.1 | 2.053 | 13.656 | 0.0741933 | 7/15/2014 | 39:27.4 | 0 |
| -763.816 | 19754.086 | 2.053 | 13.659 | 0.0698225 | 7/15/2014 | 39:27.5 | 0 |
| -763.592 | 19754.072 | 2.059 | 13.667 | 0.0763819 | 7/15/2014 | 39:27.6 | 0 |
| -763.316 | 19754.056 | 2.059 | 13.678 | 0.0816181 | 7/15/2014 | 39:27.7 | 0 |
| -763.094 | 19754.041 | 2.062 | 13.694 | 0.0874005 | 7/15/2014 | 39:27.8 | 0 |
| -762.871 | 19754.027 | 2.065 | 13.714 | 0.0931735 | 7/15/2014 | 39:27.9 | 0 |

| | | | | | | | |
|---|---|---|---|---|---|---|---|
| -762.649 | 19754.014 | 2.068 | 13.737 | 0.0996173 | 7/15/2014 | 39:28.0 | 0 |
| -762.426 | 19754 | 2.071 | 13.762 | 0.104449 | 7/15/2014 | 39:28.1 | 0 |
| -762.205 | 19753.986 | 2.071 | 13.787 | 0.0989021 | 7/15/2014 | 39:28.2 | 0 |
| -761.984 | 19753.973 | 2.071 | 13.812 | 0.102164 | 7/15/2014 | 39:28.3 | 0 |
| -761.711 | 19753.956 | 2.068 | 13.835 | 0.0948581 | 7/15/2014 | 39:28.4 | 0 |
| -761.49 | 19753.94 | 2.071 | 13.855 | 0.100335 | 7/15/2014 | 39:28.5 | 0 |
| -761.271 | 19753.928 | 2.073 | 13.87 | 0.0989179 | 7/15/2014 | 39:28.6 | 0 |
| -760.993 | 19753.911 | 2.076 | 13.879 | 0.10333 | 7/15/2014 | 39:28.7 | 0 |
| -760.773 | 19753.896 | 2.076 | 13.884 | 0.104414 | 7/15/2014 | 39:28.8 | 0 |
| -760.548 | 19753.883 | 2.079 | 13.883 | 0.120027 | 7/15/2014 | 39:28.9 | 0 |
| -760.324 | 19753.868 | 2.079 | 13.878 | 0.120974 | 7/15/2014 | 39:29.0 | 0 |
| -760.101 | 19753.854 | 2.083 | 13.868 | 0.114795 | 7/15/2014 | 39:29.1 | 0 |
| -759.875 | 19753.839 | 2.083 | 13.855 | 0.116109 | 7/15/2014 | 39:29.2 | 0 |
| -759.65 | 19753.825 | 2.079 | 13.839 | 0.113627 | 7/15/2014 | 39:29.3 | 0 |
| -759.368 | 19753.808 | 2.074 | 13.82 | 0.122018 | 7/15/2014 | 39:29.4 | 0 |
| -759.142 | 19753.792 | 2.071 | 13.8 | 0.107914 | 7/15/2014 | 39:29.5 | 0 |
| -758.916 | 19753.778 | 2.065 | 13.779 | 0.0949429 | 7/15/2014 | 39:29.6 | 0 |
| -758.631 | 19753.76 | 2.058 | 13.758 | 0.107798 | 7/15/2014 | 39:29.7 | 0 |
| -758.401 | 19753.745 | 2.055 | 13.737 | 0.11227 | 7/15/2014 | 39:29.8 | 0 |
| -758.169 | 19753.729 | 2.052 | 13.717 | 0.0999974 | 7/15/2014 | 39:29.9 | 0 |
| -757.936 | 19753.715 | 2.055 | 13.698 | 0.118689 | 7/15/2014 | 39:30.0 | 0 |
| -757.82 | 19753.707 | 2.057 | 13.688 | 0.107866 | 7/15/2014 | 39:30.1 | 0 |
| -757.589 | 19753.691 | 2.054 | 13.671 | 0.103133 | 7/15/2014 | 39:30.2 | 0 |
| -757.356 | 19753.677 | 2.057 | 13.656 | 0.123008 | 7/15/2014 | 39:30.3 | 0 |
| -756.948 | 19753.65 | 2.061 | 13.638 | 0.122355 | 7/15/2014 | 39:30.4 | 0 |
| -756.714 | 19753.635 | 2.061 | 13.627 | 0.113629 | 7/15/2014 | 39:30.5 | 0 |
| -756.48 | 19753.619 | 2.061 | 13.618 | 0.122571 | 7/15/2014 | 39:30.6 | 0 |
| -756.187 | 19753.6 | 2.059 | 13.611 | 0.114848 | 7/15/2014 | 39:30.7 | 0 |
| -756.069 | 19753.592 | 2.055 | 13.607 | 0.134953 | 7/15/2014 | 39:30.8 | 0 |
| -755.832 | 19753.575 | 2.055 | 13.602 | 0.126467 | 7/15/2014 | 39:30.9 | 0 |
| -755.596 | 19753.56 | 2.045 | 13.598 | 0.12977 | 7/15/2014 | 39:31.0 | 0 |
| -755.358 | 19753.544 | 2.045 | 13.597 | 0.117067 | 7/15/2014 | 39:31.1 | 0 |
| -755.123 | 19753.528 | 2.039 | 13.597 | 0.146095 | 7/15/2014 | 39:31.2 | 0 |
| -754.886 | 19753.513 | 2.039 | 13.598 | 0.165096 | 7/15/2014 | 39:31.3 | 0 |
| -754.476 | 19753.485 | 2.039 | 13.603 | 0.17935 | 7/15/2014 | 39:31.4 | 0 |
| -754.244 | 19753.469 | 2.041 | 13.608 | 0.171611 | 7/15/2014 | 39:31.5 | 0 |
| -754.011 | 19753.453 | 2.048 | 13.613 | 0.222889 | 7/15/2014 | 39:31.6 | 0 |
| -753.725 | 19753.435 | 2.05 | 13.619 | 0.225922 | 7/15/2014 | 39:31.7 | 0 |
| -753.607 | 19753.427 | 2.048 | 13.622 | 0.222503 | 7/15/2014 | 39:31.8 | 0 |
| -753.268 | 19753.404 | 2.052 | 13.632 | 0.217256 | 7/15/2014 | 39:31.9 | 0 |
| -753.038 | 19753.389 | 2.055 | 13.639 | 0.217296 | 7/15/2014 | 39:32.0 | 0 |
| -752.811 | 19753.374 | 2.053 | 13.646 | 0.193509 | 7/15/2014 | 39:32.1 | 0 |
| -752.586 | 19753.358 | 2.051 | 13.652 | 0.218179 | 7/15/2014 | 39:32.2 | 0 |
| -752.362 | 19753.344 | 2.049 | 13.658 | 0.179452 | 7/15/2014 | 39:32.3 | 0 |
| -752.087 | 19753.325 | 2.042 | 13.663 | 0.175914 | 7/15/2014 | 39:32.4 | 0 |
| -751.866 | 19753.311 | 2.04 | 13.667 | 0.172595 | 7/15/2014 | 39:32.5 | 0 |
| -751.647 | 19753.296 | 2.037 | 13.67 | 0.144715 | 7/15/2014 | 39:32.6 | 0 |

| | | | | | | | |
|---|---|---|---|---|---|---|---|
| -751.37 | 19753.277 | 2.035 | 13.673 | 0.131828 | 7/15/2014 | 39:32.7 | 0 |
| -751.15 | 19753.263 | 2.032 | 13.674 | 0.149896 | 7/15/2014 | 39:32.8 | 0 |
| -750.926 | 19753.248 | 2.034 | 13.675 | 0.120477 | 7/15/2014 | 39:32.9 | 0 |
| -750.702 | 19753.232 | 2.036 | 13.674 | 0.118801 | 7/15/2014 | 39:33.0 | 0 |
| -750.479 | 19753.217 | 2.036 | 13.673 | 0.133885 | 7/15/2014 | 39:33.1 | 0 |
| -750.254 | 19753.203 | 2.036 | 13.672 | 0.133511 | 7/15/2014 | 39:33.2 | 0 |
| -750.029 | 19753.187 | 2.036 | 13.669 | 0.13789 | 7/15/2014 | 39:33.3 | 0 |
| -749.749 | 19753.168 | 2.034 | 13.666 | 0.134607 | 7/15/2014 | 39:33.4 | 0 |
| -749.523 | 19753.152 | 2.034 | 13.662 | 0.121498 | 7/15/2014 | 39:33.5 | 0 |
| -749.296 | 19753.138 | 2.032 | 13.658 | 0.119654 | 7/15/2014 | 39:33.6 | 0 |
| -749.009 | 19753.117 | 2.029 | 13.655 | 0.105906 | 7/15/2014 | 39:33.7 | 0 |
| -748.78 | 19753.101 | 2.024 | 13.651 | 0.075291 | 7/15/2014 | 39:33.8 | 0 |
| -748.549 | 19753.086 | 2.022 | 13.647 | 0.0579132 | 7/15/2014 | 39:33.9 | 0 |
| -748.315 | 19753.068 | 2.022 | 13.645 | 0.0670347 | 7/15/2014 | 39:34.0 | 0 |
| -748.2 | 19753.061 | 2.022 | 13.643 | 0.0674482 | 7/15/2014 | 39:34.1 | 0 |
| -747.968 | 19753.045 | 2.02 | 13.642 | 0.0461586 | 7/15/2014 | 39:34.2 | 0 |
| -747.734 | 19753.028 | 2.022 | 13.643 | 0.0701698 | 7/15/2014 | 39:34.3 | 0 |
| -747.327 | 19753 | 2.024 | 13.648 | 0.0797312 | 7/15/2014 | 39:34.4 | 0 |
| -747.095 | 19752.982 | 2.024 | 13.653 | 0.0822167 | 7/15/2014 | 39:34.5 | 0 |
| -746.863 | 19752.967 | 2.026 | 13.658 | 0.102227 | 7/15/2014 | 39:34.6 | 0 |
| -746.573 | 19752.945 | 2.023 | 13.665 | 0.0730947 | 7/15/2014 | 39:34.7 | 0 |
| -746.455 | 19752.938 | 2.021 | 13.669 | 0.0968817 | 7/15/2014 | 39:34.8 | 0 |
| -746.22 | 19752.921 | 2.023 | 13.676 | 0.0865311 | 7/15/2014 | 39:34.9 | 0 |
| -745.987 | 19752.903 | 2.018 | 13.684 | 0.11796 | 7/15/2014 | 39:35.0 | 0 |
| -745.752 | 19752.887 | 2.016 | 13.692 | 0.0768843 | 7/15/2014 | 39:35.1 | 0 |
| -745.519 | 19752.87 | 2.01 | 13.7 | 0.0838324 | 7/15/2014 | 39:35.2 | 0 |
| -745.287 | 19752.854 | 2.004 | 13.708 | 0.0814568 | 7/15/2014 | 39:35.3 | 0 |
| -744.881 | 19752.824 | 1.999 | 13.718 | 0.0771643 | 7/15/2014 | 39:35.4 | 0 |
| -744.65 | 19752.808 | 2.002 | 13.725 | 0.0802213 | 7/15/2014 | 39:35.5 | 0 |
| -744.42 | 19752.79 | 1.997 | 13.731 | 0.072464 | 7/15/2014 | 39:35.6 | 0 |
| -744.131 | 19752.769 | 1.993 | 13.736 | 0.0790348 | 7/15/2014 | 39:35.7 | 0 |
| -744.015 | 19752.761 | 1.989 | 13.739 | 0.0781751 | 7/15/2014 | 39:35.8 | 0 |
| -743.783 | 19752.743 | 1.989 | 13.743 | 0.0741675 | 7/15/2014 | 39:35.9 | 0 |
| -743.55 | 19752.727 | 1.989 | 13.746 | 0.0881122 | 7/15/2014 | 39:36.0 | 0 |
| -743.316 | 19752.709 | 1.992 | 13.746 | 0.104197 | 7/15/2014 | 39:36.1 | 0 |
| -743.085 | 19752.692 | 1.986 | 13.744 | 0.0894224 | 7/15/2014 | 39:36.2 | 0 |
| -742.851 | 19752.675 | 1.984 | 13.74 | 0.112014 | 7/15/2014 | 39:36.3 | 0 |
| -742.446 | 19752.644 | 1.985 | 13.726 | 0.125775 | 7/15/2014 | 39:36.4 | 0 |
| -742.214 | 19752.627 | 1.985 | 13.715 | 0.131681 | 7/15/2014 | 39:36.5 | 0 |
| -741.981 | 19752.609 | 1.985 | 13.704 | 0.13563 | 7/15/2014 | 39:36.6 | 0 |
| -741.689 | 19752.587 | 1.985 | 13.692 | 0.122619 | 7/15/2014 | 39:36.7 | 0 |
| -741.572 | 19752.579 | 1.983 | 13.686 | 0.135971 | 7/15/2014 | 39:36.8 | 0 |
| -741.336 | 19752.562 | 1.986 | 13.675 | 0.125826 | 7/15/2014 | 39:36.9 | 0 |
| -741.101 | 19752.543 | 1.984 | 13.668 | 0.126438 | 7/15/2014 | 39:37.0 | 0 |
| -740.863 | 19752.524 | 1.986 | 13.666 | 0.145166 | 7/15/2014 | 39:37.1 | 0 |
| -740.626 | 19752.508 | 1.982 | 13.671 | 0.150618 | 7/15/2014 | 39:37.2 | 0 |
| -740.389 | 19752.488 | 1.986 | 13.682 | 0.163866 | 7/15/2014 | 39:37.3 | 0 |

| | | | | | | | |
|---|---|---|---|---|---|---|---|
| -739.974 | 19752.456 | 1.988 | 13.717 | 0.164742 | 7/15/2014 | 39:37.4 | 0 |
| -739.736 | 19752.438 | 1.991 | 13.746 | 0.174534 | 7/15/2014 | 39:37.5 | 0 |
| -739.499 | 19752.42 | 1.991 | 13.781 | 0.179571 | 7/15/2014 | 39:37.6 | 0 |
| -739.201 | 19752.396 | 1.991 | 13.819 | 0.180389 | 7/15/2014 | 39:37.7 | 0 |
| -739.081 | 19752.387 | 1.993 | 13.838 | 0.18566 | 7/15/2014 | 39:37.8 | 0 |
| -738.841 | 19752.368 | 1.997 | 13.88 | 0.163379 | 7/15/2014 | 39:37.9 | 0 |
| -738.601 | 19752.35 | 1.993 | 13.92 | 0.194229 | 7/15/2014 | 39:38.0 | 0 |
| -738.361 | 19752.33 | 1.993 | 13.957 | 0.160084 | 7/15/2014 | 39:38.1 | 0 |
| -738.121 | 19752.311 | 1.989 | 13.988 | 0.168843 | 7/15/2014 | 39:38.2 | 0 |
| -737.882 | 19752.293 | 1.986 | 14.012 | 0.162526 | 7/15/2014 | 39:38.3 | 0 |
| -737.465 | 19752.258 | 1.976 | 14.029 | 0.14746 | 7/15/2014 | 39:38.4 | 0 |
| -737.23 | 19752.239 | 1.971 | 14.035 | 0.141772 | 7/15/2014 | 39:38.5 | 0 |
| -736.993 | 19752.221 | 1.968 | 14.035 | 0.138089 | 7/15/2014 | 39:38.6 | 0 |
| -736.701 | 19752.197 | 1.963 | 14.029 | 0.152733 | 7/15/2014 | 39:38.7 | 0 |
| -736.58 | 19752.188 | 1.96 | 14.027 | 0.126252 | 7/15/2014 | 39:38.8 | 0 |
| -736.343 | 19752.168 | 1.958 | 14.015 | 0.121186 | 7/15/2014 | 39:38.9 | 0 |
| -736.111 | 19752.148 | 1.952 | 14 | 0.157063 | 7/15/2014 | 39:39.0 | 0 |
| -735.874 | 19752.129 | 1.956 | 13.984 | 0.143484 | 7/15/2014 | 39:39.1 | 0 |
| -735.644 | 19752.111 | 1.947 | 13.967 | 0.139202 | 7/15/2014 | 39:39.2 | 0 |
| -735.297 | 19752.082 | 1.946 | 13.944 | 0.149538 | 7/15/2014 | 39:39.3 | 0 |
| -735.012 | 19752.059 | 1.94 | 13.929 | 0.174787 | 7/15/2014 | 39:39.4 | 0 |
| -734.782 | 19752.039 | 1.938 | 13.915 | 0.167779 | 7/15/2014 | 39:39.5 | 0 |
| -734.556 | 19752.02 | 1.933 | 13.902 | 0.143758 | 7/15/2014 | 39:39.6 | 0 |
| -734.271 | 19751.996 | 1.931 | 13.889 | 0.153383 | 7/15/2014 | 39:39.7 | 0 |
| -734.044 | 19751.979 | 1.927 | 13.877 | 0.151325 | 7/15/2014 | 39:39.8 | 0 |
| -733.815 | 19751.959 | 1.927 | 13.866 | 0.158119 | 7/15/2014 | 39:39.9 | 0 |
| -733.586 | 19751.939 | 1.927 | 13.855 | 0.179713 | 7/15/2014 | 39:40.0 | 0 |
| -733.358 | 19751.92 | 1.923 | 13.845 | 0.166714 | 7/15/2014 | 39:40.1 | 0 |
| -733.128 | 19751.9 | 1.921 | 13.835 | 0.177686 | 7/15/2014 | 39:40.2 | 0 |
| -732.9 | 19751.881 | 1.921 | 13.825 | 0.214189 | 7/15/2014 | 39:40.3 | 0 |
| -732.617 | 19751.855 | 1.923 | 13.816 | 0.18791 | 7/15/2014 | 39:40.4 | 0 |
| -732.391 | 19751.836 | 1.921 | 13.808 | 0.236204 | 7/15/2014 | 39:40.5 | 0 |
| -732.162 | 19751.816 | 1.919 | 13.8 | 0.223689 | 7/15/2014 | 39:40.6 | 0 |
| -731.878 | 19751.793 | 1.914 | 13.794 | 0.215426 | 7/15/2014 | 39:40.7 | 0 |
| -731.65 | 19751.773 | 1.909 | 13.79 | 0.200271 | 7/15/2014 | 39:40.8 | 0 |
| -731.419 | 19751.754 | 1.909 | 13.788 | 0.214718 | 7/15/2014 | 39:40.9 | 0 |
| -731.188 | 19751.732 | 1.909 | 13.788 | 0.215857 | 7/15/2014 | 39:41.0 | 0 |
| -730.959 | 19751.711 | 1.907 | 13.79 | 0.235669 | 7/15/2014 | 39:41.1 | 0 |
| -730.729 | 19751.691 | 1.907 | 13.795 | 0.230526 | 7/15/2014 | 39:41.2 | 0 |
| -730.5 | 19751.672 | 1.907 | 13.801 | 0.242433 | 7/15/2014 | 39:41.3 | 0 |
| -730.215 | 19751.647 | 1.907 | 13.81 | 0.243281 | 7/15/2014 | 39:41.4 | 0 |
| -729.988 | 19751.627 | 1.91 | 13.819 | 0.25465 | 7/15/2014 | 39:41.5 | 0 |
| -729.758 | 19751.607 | 1.913 | 13.829 | 0.247581 | 7/15/2014 | 39:41.6 | 0 |
| -729.47 | 19751.58 | 1.913 | 13.839 | 0.210433 | 7/15/2014 | 39:41.7 | 0 |
| -729.356 | 19751.57 | 1.912 | 13.844 | 0.220577 | 7/15/2014 | 39:41.8 | 0 |
| -729.122 | 19751.551 | 1.914 | 13.853 | 0.216654 | 7/15/2014 | 39:41.9 | 0 |
| -728.891 | 19751.531 | 1.906 | 13.858 | 0.183731 | 7/15/2014 | 39:42.0 | 0 |

| | | | | | | | |
|---|---|---|---|---|---|---|---|
| -728.656 | 19751.509 | 1.91 | 13.861 | 0.159177 | 7/15/2014 | 39:42.1 | 0 |
| -728.424 | 19751.488 | 1.908 | 13.86 | 0.171559 | 7/15/2014 | 39:42.2 | 0 |
| -728.188 | 19751.468 | 1.913 | 13.855 | 0.165317 | 7/15/2014 | 39:42.3 | 0 |
| -727.78 | 19751.431 | 1.909 | 13.837 | 0.156685 | 7/15/2014 | 39:42.4 | 0 |
| -727.546 | 19751.41 | 1.906 | 13.823 | 0.11697 | 7/15/2014 | 39:42.5 | 0 |
| -727.312 | 19751.391 | 1.904 | 13.807 | 0.111284 | 7/15/2014 | 39:42.6 | 0 |
| -727.018 | 19751.363 | 1.904 | 13.788 | 0.124854 | 7/15/2014 | 39:42.7 | 0 |
| -726.9 | 19751.354 | 1.9 | 13.779 | 0.113485 | 7/15/2014 | 39:42.8 | 0 |
| -726.663 | 19751.331 | 1.9 | 13.758 | 0.106711 | 7/15/2014 | 39:42.9 | 0 |
| -726.427 | 19751.311 | 1.891 | 13.737 | 0.118786 | 7/15/2014 | 39:43.0 | 0 |
| -726.192 | 19751.29 | 1.891 | 13.717 | 0.100431 | 7/15/2014 | 39:43.1 | 0 |
| -725.956 | 19751.268 | 1.889 | 13.698 | 0.132992 | 7/15/2014 | 39:43.2 | 0 |
| -725.721 | 19751.248 | 1.892 | 13.681 | 0.137121 | 7/15/2014 | 39:43.3 | 0 |
| -725.313 | 19751.211 | 1.893 | 13.661 | 0.16472 | 7/15/2014 | 39:43.4 | 0 |
| -725.08 | 19751.188 | 1.897 | 13.651 | 0.173292 | 7/15/2014 | 39:43.5 | 0 |
| -724.848 | 19751.168 | 1.897 | 13.643 | 0.187621 | 7/15/2014 | 39:43.6 | 0 |
| -724.559 | 19751.143 | 1.897 | 13.638 | 0.206383 | 7/15/2014 | 39:43.7 | 0 |
| -724.442 | 19751.131 | 1.896 | 13.635 | 0.201771 | 7/15/2014 | 39:43.8 | 0 |
| -724.209 | 19751.111 | 1.898 | 13.636 | 0.230883 | 7/15/2014 | 39:43.9 | 0 |
| -723.977 | 19751.089 | 1.896 | 13.639 | 0.184279 | 7/15/2014 | 39:44.0 | 0 |
| -723.744 | 19751.068 | 1.896 | 13.644 | 0.213607 | 7/15/2014 | 39:44.1 | 0 |
| -723.512 | 19751.048 | 1.89 | 13.652 | 0.241353 | 7/15/2014 | 39:44.2 | 0 |
| -723.281 | 19751.025 | 1.887 | 13.663 | 0.216148 | 7/15/2014 | 39:44.3 | 0 |
| -722.877 | 19750.988 | 1.891 | 13.681 | 0.218452 | 7/15/2014 | 39:44.4 | 0 |
| -722.647 | 19750.969 | 1.892 | 13.694 | 0.22211 | 7/15/2014 | 39:44.5 | 0 |
| -722.417 | 19750.948 | 1.892 | 13.706 | 0.253979 | 7/15/2014 | 39:44.6 | 0 |
| -722.126 | 19750.922 | 1.892 | 13.718 | 0.214325 | 7/15/2014 | 39:44.7 | 0 |
| -722.011 | 19750.912 | 1.891 | 13.725 | 0.196659 | 7/15/2014 | 39:44.8 | 0 |
| -721.777 | 19750.889 | 1.897 | 13.736 | 0.221487 | 7/15/2014 | 39:44.9 | 0 |
| -721.542 | 19750.869 | 1.893 | 13.745 | 0.220529 | 7/15/2014 | 39:45.0 | 0 |
| -721.306 | 19750.846 | 1.89 | 13.754 | 0.222228 | 7/15/2014 | 39:45.1 | 0 |
| -721.071 | 19750.826 | 1.885 | 13.761 | 0.209322 | 7/15/2014 | 39:45.2 | 0 |
| -720.835 | 19750.805 | 1.885 | 13.767 | 0.205775 | 7/15/2014 | 39:45.3 | 0 |
| -720.421 | 19750.768 | 1.882 | 13.773 | 0.224318 | 7/15/2014 | 39:45.4 | 0 |
| -720.185 | 19750.745 | 1.879 | 13.775 | 0.204562 | 7/15/2014 | 39:45.5 | 0 |
| -719.95 | 19750.725 | 1.873 | 13.774 | 0.210944 | 7/15/2014 | 39:45.6 | 0 |
| -719.654 | 19750.697 | 1.873 | 13.771 | 0.196703 | 7/15/2014 | 39:45.7 | 0 |
| -719.535 | 19750.688 | 1.873 | 13.771 | 0.199587 | 7/15/2014 | 39:45.8 | 0 |
| -719.297 | 19750.665 | 1.873 | 13.764 | 0.199235 | 7/15/2014 | 39:45.9 | 0 |
| -719.059 | 19750.645 | 1.869 | 13.754 | 0.205811 | 7/15/2014 | 39:46.0 | 0 |
| -718.82 | 19750.622 | 1.872 | 13.742 | 0.209239 | 7/15/2014 | 39:46.1 | 0 |
| -718.583 | 19750.601 | 1.87 | 13.729 | 0.222856 | 7/15/2014 | 39:46.2 | 0 |
| -718.347 | 19750.579 | 1.873 | 13.715 | 0.228378 | 7/15/2014 | 39:46.3 | 0 |
| -717.935 | 19750.542 | 1.88 | 13.695 | 0.258108 | 7/15/2014 | 39:46.4 | 0 |
| -717.702 | 19750.521 | 1.88 | 13.682 | 0.238575 | 7/15/2014 | 39:46.5 | 0 |
| -717.468 | 19750.499 | 1.877 | 13.671 | 0.249136 | 7/15/2014 | 39:46.6 | 0 |
| -717.177 | 19750.473 | 1.877 | 13.662 | 0.235746 | 7/15/2014 | 39:46.7 | 0 |

| | | | | | | | |
|---|---|---|---|---|---|---|---|
| -717.058 | 19750.462 | 1.876 | 13.657 | 0.230547 | 7/15/2014 | 39:46.8 | 0 |
| -716.825 | 19750.44 | 1.876 | 13.651 | 0.239407 | 7/15/2014 | 39:46.9 | 0 |
| -716.592 | 19750.42 | 1.872 | 13.646 | 0.245856 | 7/15/2014 | 39:47.0 | 0 |
| -716.357 | 19750.397 | 1.874 | 13.643 | 0.232931 | 7/15/2014 | 39:47.1 | 0 |
| -716.126 | 19750.377 | 1.872 | 13.643 | 0.248818 | 7/15/2014 | 39:47.2 | 0 |
| -715.895 | 19750.355 | 1.872 | 13.644 | 0.236092 | 7/15/2014 | 39:47.3 | 0 |
| -715.492 | 19750.318 | 1.87 | 13.652 | 0.255448 | 7/15/2014 | 39:47.4 | 0 |
| -715.262 | 19750.297 | 1.87 | 13.66 | 0.254319 | 7/15/2014 | 39:47.5 | 0 |
| -715.032 | 19750.276 | 1.867 | 13.671 | 0.266152 | 7/15/2014 | 39:47.6 | 0 |
| -714.744 | 19750.248 | 1.865 | 13.684 | 0.247749 | 7/15/2014 | 39:47.7 | 0 |
| -714.628 | 19750.238 | 1.86 | 13.69 | 0.245156 | 7/15/2014 | 39:47.8 | 0 |
| -714.397 | 19750.218 | 1.856 | 13.709 | 0.257284 | 7/15/2014 | 39:47.9 | 0 |
| -714.164 | 19750.195 | 1.856 | 13.733 | 0.244536 | 7/15/2014 | 39:48.0 | 0 |
| -713.931 | 19750.174 | 1.856 | 13.76 | 0.240241 | 7/15/2014 | 39:48.1 | 0 |
| -713.699 | 19750.152 | 1.85 | 13.792 | 0.24934 | 7/15/2014 | 39:48.2 | 0 |
| -713.464 | 19750.13 | 1.85 | 13.828 | 0.231687 | 7/15/2014 | 39:48.3 | 0 |
| -713.055 | 19750.092 | 1.852 | 13.89 | 0.237678 | 7/15/2014 | 39:48.4 | 0 |
| -712.821 | 19750.07 | 1.854 | 13.934 | 0.235505 | 7/15/2014 | 39:48.5 | 0 |
| -712.586 | 19750.048 | 1.854 | 13.98 | 0.244203 | 7/15/2014 | 39:48.6 | 0 |
| -712.293 | 19750.021 | 1.849 | 14.028 | 0.236435 | 7/15/2014 | 39:48.7 | 0 |
| -712.176 | 19750.01 | 1.847 | 14.052 | 0.222503 | 7/15/2014 | 39:48.8 | 0 |
| -711.936 | 19749.986 | 1.854 | 14.099 | 0.220133 | 7/15/2014 | 39:48.9 | 0 |
| -711.7 | 19749.965 | 1.847 | 14.144 | 0.212472 | 7/15/2014 | 39:49.0 | 0 |
| -711.46 | 19749.943 | 1.85 | 14.186 | 0.214352 | 7/15/2014 | 39:49.1 | 0 |
| -711.223 | 19749.92 | 1.84 | 14.223 | 0.215316 | 7/15/2014 | 39:49.2 | 0 |
| -710.984 | 19749.897 | 1.84 | 14.253 | 0.202638 | 7/15/2014 | 39:49.3 | 0 |
| -710.57 | 19749.858 | 1.837 | 14.28 | 0.201132 | 7/15/2014 | 39:49.4 | 0 |
| -710.334 | 19749.835 | 1.837 | 14.29 | 0.215932 | 7/15/2014 | 39:49.5 | 0 |
| -710.098 | 19749.813 | 1.835 | 14.292 | 0.219597 | 7/15/2014 | 39:49.6 | 0 |
| -709.803 | 19749.785 | 1.835 | 14.287 | 0.219064 | 7/15/2014 | 39:49.7 | 0 |
| -709.683 | 19749.773 | 1.832 | 14.286 | 0.213786 | 7/15/2014 | 39:49.8 | 0 |
| -709.443 | 19749.751 | 1.835 | 14.268 | 0.237362 | 7/15/2014 | 39:49.9 | 0 |
| -709.208 | 19749.729 | 1.825 | 14.245 | 0.231299 | 7/15/2014 | 39:50.0 | 0 |
| -708.969 | 19749.706 | 1.825 | 14.218 | 0.260342 | 7/15/2014 | 39:50.1 | 0 |
| -708.733 | 19749.684 | 1.819 | 14.187 | 0.254498 | 7/15/2014 | 39:50.2 | 0 |
| -708.495 | 19749.661 | 1.819 | 14.155 | 0.252143 | 7/15/2014 | 39:50.3 | 0 |
| -708.083 | 19749.621 | 1.816 | 14.108 | 0.26019 | 7/15/2014 | 39:50.4 | 0 |
| -707.848 | 19749.599 | 1.812 | 14.077 | 0.254269 | 7/15/2014 | 39:50.5 | 0 |
| -707.614 | 19749.577 | 1.809 | 14.048 | 0.259592 | 7/15/2014 | 39:50.6 | 0 |
| -707.319 | 19749.549 | 1.809 | 14.021 | 0.253436 | 7/15/2014 | 39:50.7 | 0 |
| -707.2 | 19749.538 | 1.808 | 14.007 | 0.252389 | 7/15/2014 | 39:50.8 | 0 |
| -706.963 | 19749.515 | 1.805 | 13.983 | 0.249222 | 7/15/2014 | 39:50.9 | 0 |
| -706.724 | 19749.492 | 1.801 | 13.961 | 0.255602 | 7/15/2014 | 39:51.0 | 0 |
| -706.485 | 19749.469 | 1.801 | 13.942 | 0.252935 | 7/15/2014 | 39:51.1 | 0 |
| -706.248 | 19749.446 | 1.795 | 13.924 | 0.25539 | 7/15/2014 | 39:51.2 | 0 |
| -706.009 | 19749.424 | 1.799 | 13.907 | 0.249342 | 7/15/2014 | 39:51.3 | 0 |
| -705.592 | 19749.385 | 1.797 | 13.885 | 0.252249 | 7/15/2014 | 39:51.4 | 0 |

| | | | | | | | |
|---|---|---|---|---|---|---|---|
| -705.355 | 19749.361 | 1.797 | 13.871 | 0.258295 | 7/15/2014 | 39:51.5 | 0 |
| -705.118 | 19749.338 | 1.801 | 13.858 | 0.244364 | 7/15/2014 | 39:51.6 | 0 |
| -704.821 | 19749.31 | 1.801 | 13.845 | 0.244736 | 7/15/2014 | 39:51.7 | 0 |
| -704.701 | 19749.299 | 1.799 | 13.838 | 0.241315 | 7/15/2014 | 39:51.8 | 0 |
| -704.462 | 19749.276 | 1.801 | 13.826 | 0.24161 | 7/15/2014 | 39:51.9 | 0 |
| -704.223 | 19749.254 | 1.797 | 13.814 | 0.240585 | 7/15/2014 | 39:52.0 | 0 |
| -703.985 | 19749.231 | 1.797 | 13.802 | 0.24087 | 7/15/2014 | 39:52.1 | 0 |
| -703.748 | 19749.208 | 1.791 | 13.791 | 0.237301 | 7/15/2014 | 39:52.2 | 0 |
| -703.511 | 19749.186 | 1.791 | 13.78 | 0.238578 | 7/15/2014 | 39:52.3 | 0 |
| -703.099 | 19749.146 | 1.782 | 13.767 | 0.240188 | 7/15/2014 | 39:52.4 | 0 |
| -702.865 | 19749.125 | 1.782 | 13.76 | 0.24218 | 7/15/2014 | 39:52.5 | 0 |
| -702.632 | 19749.103 | 1.785 | 13.754 | 0.242895 | 7/15/2014 | 39:52.6 | 0 |
| -702.343 | 19749.075 | 1.783 | 13.749 | 0.244584 | 7/15/2014 | 39:52.7 | 0 |
| -702.224 | 19749.063 | 1.782 | 13.746 | 0.246732 | 7/15/2014 | 39:52.8 | 0 |
| -701.991 | 19749.043 | 1.788 | 13.744 | 0.248793 | 7/15/2014 | 39:52.9 | 0 |
| -701.76 | 19749.02 | 1.789 | 13.744 | 0.254849 | 7/15/2014 | 39:53.0 | 0 |
| -701.414 | 19748.987 | 1.797 | 13.749 | 0.257886 | 7/15/2014 | 39:53.1 | 0 |
| -701.183 | 19748.967 | 1.8 | 13.754 | 0.259875 | 7/15/2014 | 39:53.2 | 0 |
| -700.955 | 19748.945 | 1.797 | 13.761 | 0.261943 | 7/15/2014 | 39:53.3 | 0 |
| -700.672 | 19748.918 | 1.794 | 13.772 | 0.262614 | 7/15/2014 | 39:53.4 | 0 |
| -700.447 | 19748.898 | 1.794 | 13.785 | 0.263284 | 7/15/2014 | 39:53.5 | 0 |
| -700.223 | 19748.876 | 1.791 | 13.801 | 0.258736 | 7/15/2014 | 39:53.6 | 0 |
| -699.944 | 19748.852 | 1.788 | 13.821 | 0.257034 | 7/15/2014 | 39:53.7 | 0 |
| -699.719 | 19748.828 | 1.785 | 13.845 | 0.250788 | 7/15/2014 | 39:53.8 | 0 |
| -699.495 | 19748.809 | 1.782 | 13.871 | 0.248236 | 7/15/2014 | 39:53.9 | 0 |
| -699.271 | 19748.788 | 1.785 | 13.901 | 0.246131 | 7/15/2014 | 39:54.0 | 0 |
| -699.046 | 19748.767 | 1.788 | 13.931 | 0.245289 | 7/15/2014 | 39:54.1 | 0 |
| -698.823 | 19748.746 | 1.791 | 13.963 | 0.245399 | 7/15/2014 | 39:54.2 | 0 |
| -698.6 | 19748.726 | 1.791 | 13.993 | 0.247581 | 7/15/2014 | 39:54.3 | 0 |
| -698.322 | 19748.7 | 1.794 | 14.022 | 0.246524 | 7/15/2014 | 39:54.4 | 0 |
| -698.099 | 19748.678 | 1.797 | 14.049 | 0.249612 | 7/15/2014 | 39:54.5 | 0 |
| -697.874 | 19748.658 | 1.801 | 14.072 | 0.25564 | 7/15/2014 | 39:54.6 | 0 |
| -697.59 | 19748.633 | 1.801 | 14.092 | 0.255148 | 7/15/2014 | 39:54.7 | 0 |
| -697.36 | 19748.61 | 1.799 | 14.11 | 0.262382 | 7/15/2014 | 39:54.8 | 0 |
| -697.127 | 19748.59 | 1.795 | 14.125 | 0.262959 | 7/15/2014 | 39:54.9 | 0 |
| -697.015 | 19748.58 | 1.792 | 14.133 | 0.254355 | 7/15/2014 | 39:55.0 | 0 |
| -696.779 | 19748.558 | 1.799 | 14.146 | 0.26402 | 7/15/2014 | 39:55.1 | 0 |
| -696.544 | 19748.535 | 1.796 | 14.16 | 0.268215 | 7/15/2014 | 39:55.2 | 0 |
| -696.305 | 19748.515 | 1.798 | 14.175 | 0.257001 | 7/15/2014 | 39:55.3 | 0 |
| -695.887 | 19748.477 | 1.799 | 14.2 | 0.254887 | 7/15/2014 | 39:55.4 | 0 |
| -695.649 | 19748.454 | 1.802 | 14.216 | 0.244933 | 7/15/2014 | 39:55.5 | 0 |
| -695.409 | 19748.434 | 1.804 | 14.233 | 0.242756 | 7/15/2014 | 39:55.6 | 0 |
| -695.11 | 19748.405 | 1.804 | 14.25 | 0.228353 | 7/15/2014 | 39:55.7 | 0 |
| -694.988 | 19748.394 | 1.802 | 14.259 | 0.21685 | 7/15/2014 | 39:55.8 | 0 |
| -694.746 | 19748.372 | 1.807 | 14.275 | 0.23187 | 7/15/2014 | 39:55.9 | 0 |
| -694.506 | 19748.35 | 1.801 | 14.288 | 0.21525 | 7/15/2014 | 39:56.0 | 0 |
| -694.267 | 19748.328 | 1.802 | 14.299 | 0.210074 | 7/15/2014 | 39:56.1 | 0 |

| | | | | | | | |
|---|---|---|---|---|---|---|---|
| -694.029 | 19748.308 | 1.797 | 14.307 | 0.220043 | 7/15/2014 | 39:56.2 | 0 |
| -693.792 | 19748.285 | 1.797 | 14.311 | 0.195386 | 7/15/2014 | 39:56.3 | 0 |
| -693.382 | 19748.249 | 1.795 | 14.308 | 0.204049 | 7/15/2014 | 39:56.4 | 0 |
| -693.148 | 19748.228 | 1.799 | 14.302 | 0.187775 | 7/15/2014 | 39:56.5 | 0 |
| -692.915 | 19748.206 | 1.796 | 14.293 | 0.195873 | 7/15/2014 | 39:56.6 | 0 |
| -692.624 | 19748.18 | 1.8 | 14.281 | 0.187479 | 7/15/2014 | 39:56.7 | 0 |
| -692.506 | 19748.17 | 1.8 | 14.276 | 0.182489 | 7/15/2014 | 39:56.8 | 0 |
| -692.271 | 19748.148 | 1.802 | 14.26 | 0.194286 | 7/15/2014 | 39:56.9 | 0 |
| -692.036 | 19748.128 | 1.799 | 14.242 | 0.197661 | 7/15/2014 | 39:57.0 | 0 |
| -691.799 | 19748.107 | 1.799 | 14.224 | 0.20678 | 7/15/2014 | 39:57.1 | 0 |
| -691.563 | 19748.086 | 1.795 | 14.205 | 0.216913 | 7/15/2014 | 39:57.2 | 0 |
| -691.325 | 19748.064 | 1.794 | 14.187 | 0.223369 | 7/15/2014 | 39:57.3 | 0 |
| -690.913 | 19748.027 | 1.792 | 14.163 | 0.238948 | 7/15/2014 | 39:57.4 | 0 |
| -690.677 | 19748.008 | 1.789 | 14.149 | 0.246703 | 7/15/2014 | 39:57.5 | 0 |
| -690.442 | 19747.987 | 1.787 | 14.137 | 0.245184 | 7/15/2014 | 39:57.6 | 0 |
| -690.148 | 19747.961 | 1.787 | 14.127 | 0.23805 | 7/15/2014 | 39:57.7 | 0 |
| -690.028 | 19747.95 | 1.785 | 14.122 | 0.239374 | 7/15/2014 | 39:57.8 | 0 |
| -689.791 | 19747.93 | 1.791 | 14.115 | 0.233394 | 7/15/2014 | 39:57.9 | 0 |
| -689.556 | 19747.909 | 1.789 | 14.111 | 0.219238 | 7/15/2014 | 39:58.0 | 0 |
| -689.321 | 19747.887 | 1.789 | 14.11 | 0.218321 | 7/15/2014 | 39:58.1 | 0 |
| -689.086 | 19747.867 | 1.785 | 14.111 | 0.202551 | 7/15/2014 | 39:58.2 | 0 |
| -688.853 | 19747.848 | 1.789 | 14.117 | 0.195602 | 7/15/2014 | 39:58.3 | 0 |
| -688.448 | 19747.813 | 1.787 | 14.136 | 0.193116 | 7/15/2014 | 39:58.4 | 0 |
| -688.217 | 19747.793 | 1.787 | 14.155 | 0.191748 | 7/15/2014 | 39:58.5 | 0 |
| -687.988 | 19747.772 | 1.787 | 14.179 | 0.192493 | 7/15/2014 | 39:58.6 | 0 |
| -687.702 | 19747.747 | 1.787 | 14.209 | 0.190096 | 7/15/2014 | 39:58.7 | 0 |
| -687.471 | 19747.727 | 1.787 | 14.245 | 0.191668 | 7/15/2014 | 39:58.8 | 0 |
| -687.239 | 19747.707 | 1.79 | 14.285 | 0.191517 | 7/15/2014 | 39:58.9 | 0 |
| -687.008 | 19747.689 | 1.79 | 14.327 | 0.197965 | 7/15/2014 | 39:59.0 | 0 |
| -686.776 | 19747.67 | 1.793 | 14.368 | 0.1958 | 7/15/2014 | 39:59.1 | 0 |
| -686.545 | 19747.65 | 1.79 | 14.406 | 0.197821 | 7/15/2014 | 39:59.2 | 0 |
| -686.431 | 19747.641 | 1.793 | 14.428 | 0.196051 | 7/15/2014 | 39:59.3 | 0 |
| -686.029 | 19747.606 | 1.79 | 14.46 | 0.210977 | 7/15/2014 | 39:59.4 | 0 |
| -685.799 | 19747.586 | 1.793 | 14.472 | 0.209148 | 7/15/2014 | 39:59.5 | 0 |
| -685.569 | 19747.566 | 1.793 | 14.473 | 0.216065 | 7/15/2014 | 39:59.6 | 0 |
| -685.28 | 19747.545 | 1.793 | 14.463 | 0.22633 | 7/15/2014 | 39:59.7 | 0 |
| -685.164 | 19747.535 | 1.793 | 14.46 | 0.233781 | 7/15/2014 | 39:59.8 | 0 |
| -684.932 | 19747.516 | 1.795 | 14.434 | 0.227419 | 7/15/2014 | 39:59.9 | 0 |
| -684.698 | 19747.498 | 1.799 | 14.399 | 0.23897 | 7/15/2014 | 40:00.0 | 0 |
| -684.464 | 19747.479 | 1.808 | 14.36 | 0.232186 | 7/15/2014 | 40:00.1 | 0 |
| -684.23 | 19747.459 | 1.806 | 14.316 | 0.23839 | 7/15/2014 | 40:00.2 | 0 |
| -683.996 | 19747.439 | 1.812 | 14.271 | 0.230519 | 7/15/2014 | 40:00.3 | 0 |
| -683.586 | 19747.407 | 1.812 | 14.203 | 0.232243 | 7/15/2014 | 40:00.4 | 0 |
| -683.351 | 19747.389 | 1.812 | 14.16 | 0.239497 | 7/15/2014 | 40:00.5 | 0 |
| -683.116 | 19747.371 | 1.81 | 14.119 | 0.233194 | 7/15/2014 | 40:00.6 | 0 |
| -682.821 | 19747.348 | 1.807 | 14.079 | 0.235666 | 7/15/2014 | 40:00.7 | 0 |
| -682.702 | 19747.338 | 1.802 | 14.059 | 0.237586 | 7/15/2014 | 40:00.8 | 0 |

| | | | | | | | |
|---|---|---|---|---|---|---|---|
| -682.463 | 19747.318 | 1.802 | 14.024 | 0.243341 | 7/15/2014 | 40:00.9 | 0 |
| -682.224 | 19747.3 | 1.793 | 13.991 | 0.242633 | 7/15/2014 | 40:01.0 | 0 |
| -681.982 | 19747.281 | 1.793 | 13.96 | 0.24265 | 7/15/2014 | 40:01.1 | 0 |
| -681.741 | 19747.264 | 1.79 | 13.932 | 0.247337 | 7/15/2014 | 40:01.2 | 0 |
| -681.501 | 19747.244 | 1.793 | 13.905 | 0.247879 | 7/15/2014 | 40:01.3 | 0 |
| -681.08 | 19747.213 | 1.794 | 13.87 | 0.247344 | 7/15/2014 | 40:01.4 | 0 |
| -680.839 | 19747.193 | 1.796 | 13.849 | 0.250507 | 7/15/2014 | 40:01.5 | 0 |
| -680.599 | 19747.176 | 1.799 | 13.831 | 0.254026 | 7/15/2014 | 40:01.6 | 0 |
| -680.296 | 19747.153 | 1.802 | 13.818 | 0.260927 | 7/15/2014 | 40:01.7 | 0 |
| -680.174 | 19747.145 | 1.802 | 13.809 | 0.266556 | 7/15/2014 | 40:01.8 | 0 |
| -679.931 | 19747.126 | 1.802 | 13.803 | 0.285209 | 7/15/2014 | 40:01.9 | 0 |
| -679.689 | 19747.108 | 1.796 | 13.802 | 0.309853 | 7/15/2014 | 40:02.0 | 0 |
| -679.442 | 19747.091 | 1.797 | 13.81 | 0.347359 | 7/15/2014 | 40:02.1 | 0 |
| -679.2 | 19747.072 | 1.794 | 13.825 | 0.376052 | 7/15/2014 | 40:02.2 | 0 |
| -678.955 | 19747.055 | 1.801 | 13.849 | 0.392035 | 7/15/2014 | 40:02.3 | 0 |
| -678.531 | 19747.025 | 1.797 | 13.907 | 0.439206 | 7/15/2014 | 40:02.4 | 0 |
| -678.289 | 19747.008 | 1.797 | 13.95 | 0.408725 | 7/15/2014 | 40:02.5 | 0 |
| -678.049 | 19746.991 | 1.8 | 13.998 | 0.428768 | 7/15/2014 | 40:02.6 | 0 |
| -677.747 | 19746.97 | 1.805 | 14.049 | 0.399098 | 7/15/2014 | 40:02.7 | 0 |
| -677.625 | 19746.961 | 1.806 | 14.075 | 0.388401 | 7/15/2014 | 40:02.8 | 0 |
| -677.382 | 19746.944 | 1.81 | 14.128 | 0.382336 | 7/15/2014 | 40:02.9 | 0 |
| -677.141 | 19746.928 | 1.8 | 14.176 | 0.35228 | 7/15/2014 | 40:03.0 | 0 |
| -676.898 | 19746.912 | 1.804 | 14.219 | 0.319901 | 7/15/2014 | 40:03.1 | 0 |
| -676.658 | 19746.895 | 1.8 | 14.254 | 0.325098 | 7/15/2014 | 40:03.2 | 0 |
| -676.417 | 19746.879 | 1.802 | 14.281 | 0.309113 | 7/15/2014 | 40:03.3 | 0 |
| -676.001 | 19746.851 | 1.802 | 14.301 | 0.313987 | 7/15/2014 | 40:03.4 | 0 |
| -675.764 | 19746.835 | 1.805 | 14.309 | 0.306358 | 7/15/2014 | 40:03.5 | 0 |
| -675.528 | 19746.82 | 1.805 | 14.31 | 0.292177 | 7/15/2014 | 40:03.6 | 0 |
| -675.234 | 19746.801 | 1.81 | 14.306 | 0.298294 | 7/15/2014 | 40:03.7 | 0 |
| -675.115 | 19746.792 | 1.81 | 14.304 | 0.302577 | 7/15/2014 | 40:03.8 | 0 |
| -674.878 | 19746.776 | 1.816 | 14.293 | 0.288576 | 7/15/2014 | 40:03.9 | 0 |
| -674.641 | 19746.762 | 1.816 | 14.279 | 0.31171 | 7/15/2014 | 40:04.0 | 0 |
| -674.404 | 19746.745 | 1.827 | 14.264 | 0.314766 | 7/15/2014 | 40:04.1 | 0 |
| -674.169 | 19746.73 | 1.827 | 14.248 | 0.308805 | 7/15/2014 | 40:04.2 | 0 |
| -673.933 | 19746.716 | 1.829 | 14.231 | 0.346393 | 7/15/2014 | 40:04.3 | 0 |
| -673.522 | 19746.689 | 1.83 | 14.207 | 0.357968 | 7/15/2014 | 40:04.4 | 0 |
| -673.287 | 19746.674 | 1.83 | 14.191 | 0.364802 | 7/15/2014 | 40:04.5 | 0 |
| -673.05 | 19746.659 | 1.835 | 14.175 | 0.346076 | 7/15/2014 | 40:04.6 | 0 |
| -672.754 | 19746.64 | 1.838 | 14.159 | 0.311174 | 7/15/2014 | 40:04.7 | 0 |
| -672.636 | 19746.633 | 1.833 | 14.151 | 0.321368 | 7/15/2014 | 40:04.8 | 0 |
| -672.396 | 19746.616 | 1.839 | 14.135 | 0.302394 | 7/15/2014 | 40:04.9 | 0 |
| -672.157 | 19746.603 | 1.837 | 14.119 | 0.271843 | 7/15/2014 | 40:05.0 | 0 |
| -671.915 | 19746.587 | 1.839 | 14.102 | 0.244549 | 7/15/2014 | 40:05.1 | 0 |
| -671.673 | 19746.57 | 1.839 | 14.084 | 0.225068 | 7/15/2014 | 40:05.2 | 0 |
| -671.43 | 19746.556 | 1.843 | 14.066 | 0.220083 | 7/15/2014 | 40:05.3 | 0 |
| -671.005 | 19746.529 | 1.834 | 14.039 | 0.18821 | 7/15/2014 | 40:05.4 | 0 |
| -670.762 | 19746.512 | 1.831 | 14.021 | 0.152053 | 7/15/2014 | 40:05.5 | 0 |

| | | | | | | | |
|---|---|---|---|---|---|---|---|
| -670.518 | 19746.498 | 1.828 | 14.003 | 0.141921 | 7/15/2014 | 40:05.6 | 0 |
| -670.212 | 19746.479 | 1.823 | 13.986 | 0.143954 | 7/15/2014 | 40:05.7 | 0 |
| -670.089 | 19746.47 | 1.818 | 13.977 | 0.141816 | 7/15/2014 | 40:05.8 | 0 |
| -669.842 | 19746.455 | 1.818 | 13.963 | 0.137134 | 7/15/2014 | 40:05.9 | 0 |
| -669.594 | 19746.438 | 1.815 | 13.951 | 0.134211 | 7/15/2014 | 40:06.0 | 0 |
| -669.346 | 19746.424 | 1.815 | 13.942 | 0.134024 | 7/15/2014 | 40:06.1 | 0 |
| -669.098 | 19746.406 | 1.815 | 13.937 | 0.12008 | 7/15/2014 | 40:06.2 | 0 |
| -668.85 | 19746.392 | 1.815 | 13.938 | 0.107159 | 7/15/2014 | 40:06.3 | 0 |
| -668.418 | 19746.364 | 1.809 | 13.956 | 0.088612 | 7/15/2014 | 40:06.4 | 0 |
| -668.295 | 19746.356 | 1.811 | 13.959 | 0.0739492 | 7/15/2014 | 40:06.5 | 0 |
| -667.928 | 19746.334 | 1.811 | 13.999 | 0.0595148 | 7/15/2014 | 40:06.6 | 0 |
| -667.624 | 19746.314 | 1.809 | 14.033 | 0.0518121 | 7/15/2014 | 40:06.7 | 0 |
| -667.498 | 19746.306 | 1.809 | 14.047 | 0.0453933 | 7/15/2014 | 40:06.8 | 0 |
| -667.252 | 19746.29 | 1.811 | 14.092 | 0.0415819 | 7/15/2014 | 40:06.9 | 0 |
| -667.008 | 19746.275 | 1.809 | 14.144 | 0.0508387 | 7/15/2014 | 40:07.0 | 0 |
| -666.763 | 19746.26 | 1.814 | 14.201 | 0.0619715 | 7/15/2014 | 40:07.1 | 0 |
| -666.522 | 19746.244 | 1.814 | 14.261 | 0.0652975 | 7/15/2014 | 40:07.2 | 0 |
| -666.28 | 19746.229 | 1.814 | 14.324 | 0.076522 | 7/15/2014 | 40:07.3 | 0 |
| -665.863 | 19746.203 | 1.814 | 14.413 | 0.089016 | 7/15/2014 | 40:07.4 | 0 |
| -665.625 | 19746.188 | 1.816 | 14.47 | 0.0950558 | 7/15/2014 | 40:07.5 | 0 |
| -665.388 | 19746.174 | 1.816 | 14.521 | 0.108191 | 7/15/2014 | 40:07.6 | 0 |
| -665.093 | 19746.154 | 1.816 | 14.567 | 0.0928123 | 7/15/2014 | 40:07.7 | 0 |
| -664.974 | 19746.146 | 1.814 | 14.592 | 0.101371 | 7/15/2014 | 40:07.8 | 0 |
| -664.736 | 19746.131 | 1.816 | 14.626 | 0.0926855 | 7/15/2014 | 40:07.9 | 0 |
| -664.5 | 19746.116 | 1.814 | 14.653 | 0.0890839 | 7/15/2014 | 40:08.0 | 0 |
| -664.262 | 19746.102 | 1.82 | 14.671 | 0.0952566 | 7/15/2014 | 40:08.1 | 0 |
| -664.026 | 19746.087 | 1.82 | 14.682 | 0.107312 | 7/15/2014 | 40:08.2 | 0 |
| -663.789 | 19746.071 | 1.822 | 14.685 | 0.0652523 | 7/15/2014 | 40:08.3 | 0 |
| -663.378 | 19746.044 | 1.814 | 14.675 | 0.111076 | 7/15/2014 | 40:08.4 | 0 |
| -663.142 | 19746.028 | 1.816 | 14.664 | 0.0955912 | 7/15/2014 | 40:08.5 | 0 |
| -662.907 | 19746.013 | 1.811 | 14.649 | 0.101501 | 7/15/2014 | 40:08.6 | 0 |
| -662.611 | 19745.993 | 1.811 | 14.63 | 0.0948922 | 7/15/2014 | 40:08.7 | 0 |
| -662.492 | 19745.985 | 1.805 | 14.621 | 0.0752775 | 7/15/2014 | 40:08.8 | 0 |
| -662.253 | 19745.97 | 1.803 | 14.598 | 0.114087 | 7/15/2014 | 40:08.9 | 0 |
| -662.016 | 19745.954 | 1.794 | 14.575 | 0.109751 | 7/15/2014 | 40:09.0 | 0 |
| -661.773 | 19745.938 | 1.798 | 14.551 | 0.0949509 | 7/15/2014 | 40:09.1 | 0 |
| -661.533 | 19745.921 | 1.792 | 14.527 | 0.111468 | 7/15/2014 | 40:09.2 | 0 |
| -661.292 | 19745.904 | 1.789 | 14.504 | 0.108492 | 7/15/2014 | 40:09.3 | 0 |
| -660.872 | 19745.876 | 1.79 | 14.472 | 0.114697 | 7/15/2014 | 40:09.4 | 0 |
| -660.632 | 19745.859 | 1.79 | 14.452 | 0.0944968 | 7/15/2014 | 40:09.5 | 0 |
| -660.393 | 19745.844 | 1.792 | 14.434 | 0.0838495 | 7/15/2014 | 40:09.6 | 0 |
| -660.095 | 19745.822 | 1.794 | 14.418 | 0.0843041 | 7/15/2014 | 40:09.7 | 0 |
| -659.973 | 19745.813 | 1.794 | 14.409 | 0.0785964 | 7/15/2014 | 40:09.8 | 0 |
| -659.731 | 19745.797 | 1.798 | 14.395 | 0.0855414 | 7/15/2014 | 40:09.9 | 0 |
| -659.491 | 19745.78 | 1.798 | 14.382 | 0.0686528 | 7/15/2014 | 40:10.0 | 0 |
| -659.251 | 19745.764 | 1.8 | 14.371 | 0.0660802 | 7/15/2014 | 40:10.1 | 0 |
| -659.012 | 19745.746 | 1.8 | 14.36 | 0.0649919 | 7/15/2014 | 40:10.2 | 0 |

| | | | | | | | |
|---|---|---|---|---|---|---|---|
| -658.774 | 19745.73 | 1.8 | 14.35 | 0.0657724 | 7/15/2014 | 40:10.3 | 0 |
| -658.362 | 19745.7 | 1.8 | 14.333 | 0.0554761 | 7/15/2014 | 40:10.4 | 0 |
| -658.126 | 19745.683 | 1.803 | 14.322 | 0.0445562 | 7/15/2014 | 40:10.5 | 0 |
| -657.891 | 19745.666 | 1.805 | 14.31 | 0.0530047 | 7/15/2014 | 40:10.6 | 0 |
| -657.598 | 19745.644 | 1.807 | 14.297 | 0.0592136 | 7/15/2014 | 40:10.7 | 0 |
| -657.478 | 19745.635 | 1.806 | 14.291 | 0.0635456 | 7/15/2014 | 40:10.8 | 0 |
| -657.243 | 19745.619 | 1.809 | 14.277 | 0.0673526 | 7/15/2014 | 40:10.9 | 0 |
| -657.007 | 19745.601 | 1.811 | 14.263 | 0.0715791 | 7/15/2014 | 40:11.0 | 0 |
| -656.771 | 19745.584 | 1.817 | 14.248 | 0.0632729 | 7/15/2014 | 40:11.1 | 0 |
| -656.536 | 19745.565 | 1.817 | 14.234 | 0.0790586 | 7/15/2014 | 40:11.2 | 0 |
| -656.301 | 19745.547 | 1.821 | 14.219 | 0.104652 | 7/15/2014 | 40:11.3 | 0 |
| -655.891 | 19745.516 | 1.82 | 14.199 | 0.10282 | 7/15/2014 | 40:11.4 | 0 |
| -655.658 | 19745.5 | 1.82 | 14.185 | 0.0985087 | 7/15/2014 | 40:11.5 | 0 |
| -655.424 | 19745.48 | 1.825 | 14.173 | 0.115353 | 7/15/2014 | 40:11.6 | 0 |
| -655.133 | 19745.459 | 1.825 | 14.161 | 0.11246 | 7/15/2014 | 40:11.7 | 0 |
| -655.014 | 19745.449 | 1.823 | 14.155 | 0.106532 | 7/15/2014 | 40:11.8 | 0 |
| -654.779 | 19745.43 | 1.826 | 14.145 | 0.104638 | 7/15/2014 | 40:11.9 | 0 |
| -654.543 | 19745.412 | 1.822 | 14.137 | 0.110731 | 7/15/2014 | 40:12.0 | 0 |
| -654.307 | 19745.395 | 1.828 | 14.13 | 0.10266 | 7/15/2014 | 40:12.1 | 0 |
| -654.072 | 19745.377 | 1.828 | 14.125 | 0.10338 | 7/15/2014 | 40:12.2 | 0 |
| -653.835 | 19745.357 | 1.834 | 14.124 | 0.0928774 | 7/15/2014 | 40:12.3 | 0 |
| -653.426 | 19745.324 | 1.832 | 14.13 | 0.113644 | 7/15/2014 | 40:12.4 | 0 |
| -653.192 | 19745.305 | 1.832 | 14.138 | 0.113002 | 7/15/2014 | 40:12.5 | 0 |
| -652.96 | 19745.287 | 1.835 | 14.151 | 0.0849742 | 7/15/2014 | 40:12.6 | 0 |
| -652.67 | 19745.264 | 1.84 | 14.169 | 0.0946515 | 7/15/2014 | 40:12.7 | 0 |
| -652.551 | 19745.254 | 1.838 | 14.177 | 0.0924617 | 7/15/2014 | 40:12.8 | 0 |
| -652.318 | 19745.236 | 1.846 | 14.203 | 0.0915473 | 7/15/2014 | 40:12.9 | 0 |
| -651.974 | 19745.207 | 1.845 | 14.248 | 0.0959442 | 7/15/2014 | 40:13.0 | 0 |
| -651.742 | 19745.188 | 1.853 | 14.276 | 0.101311 | 7/15/2014 | 40:13.1 | 0 |
| -651.513 | 19745.168 | 1.855 | 14.304 | 0.0990655 | 7/15/2014 | 40:13.2 | 0 |
| -651.286 | 19745.15 | 1.858 | 14.327 | 0.110727 | 7/15/2014 | 40:13.3 | 0 |
| -651.002 | 19745.127 | 1.858 | 14.345 | 0.11126 | 7/15/2014 | 40:13.4 | 0 |
| -650.777 | 19745.109 | 1.858 | 14.356 | 0.120919 | 7/15/2014 | 40:13.5 | 0 |
| -650.553 | 19745.09 | 1.859 | 14.36 | 0.0919872 | 7/15/2014 | 40:13.6 | 0 |
| -650.274 | 19745.066 | 1.861 | 14.357 | 0.11422 | 7/15/2014 | 40:13.7 | 0 |
| -650.049 | 19745.046 | 1.863 | 14.348 | 0.109665 | 7/15/2014 | 40:13.8 | 0 |
| -649.824 | 19745.026 | 1.861 | 14.334 | 0.112827 | 7/15/2014 | 40:13.9 | 0 |
| -649.598 | 19745.007 | 1.864 | 14.318 | 0.118884 | 7/15/2014 | 40:14.0 | 0 |
| -649.373 | 19744.989 | 1.868 | 14.299 | 0.119342 | 7/15/2014 | 40:14.1 | 0 |
| -649.147 | 19744.969 | 1.875 | 14.281 | 0.0999716 | 7/15/2014 | 40:14.2 | 0 |
| -648.922 | 19744.949 | 1.877 | 14.263 | 0.125586 | 7/15/2014 | 40:14.3 | 0 |
| -648.643 | 19744.926 | 1.882 | 14.246 | 0.121917 | 7/15/2014 | 40:14.4 | 0 |
| -648.419 | 19744.905 | 1.884 | 14.232 | 0.13919 | 7/15/2014 | 40:14.5 | 0 |
| -648.194 | 19744.886 | 1.886 | 14.219 | 0.14414 | 7/15/2014 | 40:14.6 | 0 |
| -647.91 | 19744.86 | 1.888 | 14.208 | 0.133403 | 7/15/2014 | 40:14.7 | 0 |
| -647.682 | 19744.841 | 1.89 | 14.198 | 0.129663 | 7/15/2014 | 40:14.8 | 0 |
| -647.451 | 19744.818 | 1.893 | 14.188 | 0.148101 | 7/15/2014 | 40:14.9 | 0 |

| | | | | | | | |
|---|---|---|---|---|---|---|---|
| -647.221 | 19744.799 | 1.893 | 14.178 | 0.142701 | 7/15/2014 | 40:15.0 | 0 |
| -646.989 | 19744.777 | 1.897 | 14.165 | 0.144696 | 7/15/2014 | 40:15.1 | 0 |
| -646.876 | 19744.768 | 1.894 | 14.16 | 0.135071 | 7/15/2014 | 40:15.2 | 0 |
| -646.646 | 19744.748 | 1.9 | 14.144 | 0.14145 | 7/15/2014 | 40:15.3 | 0 |
| -646.241 | 19744.711 | 1.907 | 14.111 | 0.14104 | 7/15/2014 | 40:15.4 | 0 |
| -646.011 | 19744.689 | 1.913 | 14.088 | 0.164566 | 7/15/2014 | 40:15.5 | 0 |
| -645.78 | 19744.668 | 1.918 | 14.063 | 0.15513 | 7/15/2014 | 40:15.6 | 0 |
| -645.494 | 19744.643 | 1.92 | 14.037 | 0.126546 | 7/15/2014 | 40:15.7 | 0 |
| -645.377 | 19744.631 | 1.921 | 14.023 | 0.119695 | 7/15/2014 | 40:15.8 | 0 |
| -645.034 | 19744.6 | 1.927 | 13.986 | 0.113652 | 7/15/2014 | 40:15.9 | 0 |
| -644.803 | 19744.578 | 1.929 | 13.964 | 0.110309 | 7/15/2014 | 40:16.0 | 0 |
| -644.574 | 19744.559 | 1.929 | 13.944 | 0.107409 | 7/15/2014 | 40:16.1 | 0 |
| -644.345 | 19744.535 | 1.927 | 13.928 | 0.106023 | 7/15/2014 | 40:16.2 | 0 |
| -644.119 | 19744.516 | 1.925 | 13.915 | 0.107861 | 7/15/2014 | 40:16.3 | 0 |
| -643.838 | 19744.488 | 1.92 | 13.904 | 0.113508 | 7/15/2014 | 40:16.4 | 0 |
| -643.614 | 19744.469 | 1.918 | 13.897 | 0.113665 | 7/15/2014 | 40:16.5 | 0 |
| -643.39 | 19744.447 | 1.918 | 13.891 | 0.120201 | 7/15/2014 | 40:16.6 | 0 |
| -643.111 | 19744.422 | 1.916 | 13.886 | 0.125616 | 7/15/2014 | 40:16.7 | 0 |
| -642.886 | 19744.4 | 1.916 | 13.882 | 0.130105 | 7/15/2014 | 40:16.8 | 0 |
| -642.66 | 19744.379 | 1.916 | 13.877 | 0.127039 | 7/15/2014 | 40:16.9 | 0 |
| -642.434 | 19744.357 | 1.921 | 13.872 | 0.124292 | 7/15/2014 | 40:17.0 | 0 |
| -642.207 | 19744.336 | 1.925 | 13.865 | 0.122647 | 7/15/2014 | 40:17.1 | 0 |
| -641.981 | 19744.315 | 1.927 | 13.856 | 0.121882 | 7/15/2014 | 40:17.2 | 0 |
| -641.755 | 19744.294 | 1.929 | 13.846 | 0.104069 | 7/15/2014 | 40:17.3 | 0 |
| -641.474 | 19744.268 | 1.926 | 13.833 | 0.0945731 | 7/15/2014 | 40:17.4 | 0 |
| -641.248 | 19744.245 | 1.93 | 13.819 | 0.0891219 | 7/15/2014 | 40:17.5 | 0 |
| -641.022 | 19744.225 | 1.93 | 13.805 | 0.0883034 | 7/15/2014 | 40:17.6 | 0 |
| -640.738 | 19744.196 | 1.928 | 13.79 | 0.0746463 | 7/15/2014 | 40:17.7 | 0 |
| -640.508 | 19744.176 | 1.925 | 13.776 | 0.0765315 | 7/15/2014 | 40:17.8 | 0 |
| -640.278 | 19744.153 | 1.923 | 13.763 | 0.0773035 | 7/15/2014 | 40:17.9 | 0 |
| -640.047 | 19744.131 | 1.923 | 13.754 | 0.0840743 | 7/15/2014 | 40:18.0 | 0 |
| -639.816 | 19744.108 | 1.923 | 13.75 | 0.110527 | 7/15/2014 | 40:18.1 | 0 |
| -639.702 | 19744.098 | 1.922 | 13.746 | 0.0891327 | 7/15/2014 | 40:18.2 | 0 |
| -639.47 | 19744.075 | 1.926 | 13.75 | 0.114283 | 7/15/2014 | 40:18.3 | 0 |
| -639.068 | 19744.036 | 1.927 | 13.776 | 0.138255 | 7/15/2014 | 40:18.4 | 0 |
| -638.838 | 19744.015 | 1.932 | 13.8 | 0.145859 | 7/15/2014 | 40:18.5 | 0 |
| -638.61 | 19743.991 | 1.935 | 13.833 | 0.156727 | 7/15/2014 | 40:18.6 | 0 |
| -638.325 | 19743.964 | 1.935 | 13.872 | 0.142396 | 7/15/2014 | 40:18.7 | 0 |
| -638.095 | 19743.941 | 1.935 | 13.917 | 0.139109 | 7/15/2014 | 40:18.8 | 0 |
| -637.865 | 19743.919 | 1.935 | 13.965 | 0.119072 | 7/15/2014 | 40:18.9 | 0 |
| -637.635 | 19743.897 | 1.935 | 14.014 | 0.0991749 | 7/15/2014 | 40:19.0 | 0 |
| -637.404 | 19743.874 | 1.937 | 14.063 | 0.0801238 | 7/15/2014 | 40:19.1 | 0 |
| -637.176 | 19743.853 | 1.937 | 14.11 | 0.107034 | 7/15/2014 | 40:19.2 | 0 |
| -636.948 | 19743.829 | 1.937 | 14.153 | 0.0590824 | 7/15/2014 | 40:19.3 | 0 |
| -636.667 | 19743.801 | 1.937 | 14.19 | 0.0619604 | 7/15/2014 | 40:19.4 | 0 |
| -636.441 | 19743.779 | 1.939 | 14.221 | 0.0696086 | 7/15/2014 | 40:19.5 | 0 |
| -636.217 | 19743.758 | 1.939 | 14.246 | 0.0685846 | 7/15/2014 | 40:19.6 | 0 |

| | | | | | | | |
|---|---|---|---|---|---|---|---|
| -635.935 | 19743.73 | 1.941 | 14.266 | 0.0736089 | 7/15/2014 | 40:19.7 | 0 |
| -635.709 | 19743.708 | 1.941 | 14.28 | 0.0761619 | 7/15/2014 | 40:19.8 | 0 |
| -635.483 | 19743.687 | 1.941 | 14.29 | 0.0640591 | 7/15/2014 | 40:19.9 | 0 |
| -635.256 | 19743.664 | 1.938 | 14.295 | 0.0889987 | 7/15/2014 | 40:20.0 | 0 |
| -635.029 | 19743.641 | 1.938 | 14.297 | 0.10565 | 7/15/2014 | 40:20.1 | 0 |
| -634.803 | 19743.619 | 1.938 | 14.296 | 0.0897773 | 7/15/2014 | 40:20.2 | 0 |
| -634.578 | 19743.597 | 1.941 | 14.292 | 0.0968429 | 7/15/2014 | 40:20.3 | 0 |
| -634.298 | 19743.569 | 1.942 | 14.287 | 0.128478 | 7/15/2014 | 40:20.4 | 0 |
| -634.072 | 19743.548 | 1.942 | 14.279 | 0.115662 | 7/15/2014 | 40:20.5 | 0 |
| -633.848 | 19743.526 | 1.942 | 14.27 | 0.116012 | 7/15/2014 | 40:20.6 | 0 |
| -633.566 | 19743.499 | 1.94 | 14.26 | 0.10197 | 7/15/2014 | 40:20.7 | 0 |
| -633.339 | 19743.477 | 1.943 | 14.249 | 0.101892 | 7/15/2014 | 40:20.8 | 0 |
| -633.11 | 19743.454 | 1.943 | 14.236 | 0.0893926 | 7/15/2014 | 40:20.9 | 0 |
| -632.88 | 19743.432 | 1.943 | 14.222 | 0.0857101 | 7/15/2014 | 40:21.0 | 0 |
| -632.652 | 19743.41 | 1.943 | 14.207 | 0.0709806 | 7/15/2014 | 40:21.1 | 0 |
| -632.422 | 19743.388 | 1.945 | 14.191 | 0.100211 | 7/15/2014 | 40:21.2 | 0 |
| -632.193 | 19743.365 | 1.949 | 14.174 | 0.104905 | 7/15/2014 | 40:21.3 | 0 |
| -631.909 | 19743.338 | 1.949 | 14.156 | 0.0933847 | 7/15/2014 | 40:21.4 | 0 |
| -631.68 | 19743.316 | 1.954 | 14.137 | 0.0929539 | 7/15/2014 | 40:21.5 | 0 |
| -631.451 | 19743.294 | 1.954 | 14.118 | 0.0902027 | 7/15/2014 | 40:21.6 | 0 |
| -631.165 | 19743.267 | 1.957 | 14.099 | 0.11342 | 7/15/2014 | 40:21.7 | 0 |
| -631.048 | 19743.256 | 1.957 | 14.089 | 0.114092 | 7/15/2014 | 40:21.8 | 0 |
| -630.703 | 19743.223 | 1.959 | 14.061 | 0.0894454 | 7/15/2014 | 40:21.9 | 0 |
| -630.471 | 19743.199 | 1.961 | 14.043 | 0.0987941 | 7/15/2014 | 40:22.0 | 0 |
| -630.239 | 19743.178 | 1.963 | 14.025 | 0.0992248 | 7/15/2014 | 40:22.1 | 0 |
| -630.008 | 19743.156 | 1.966 | 14.009 | 0.105482 | 7/15/2014 | 40:22.2 | 0 |
| -629.894 | 19743.146 | 1.967 | 14.001 | 0.119882 | 7/15/2014 | 40:22.3 | 0 |
| -629.491 | 19743.106 | 1.972 | 13.98 | 0.102819 | 7/15/2014 | 40:22.4 | 0 |
| -629.262 | 19743.085 | 1.972 | 13.968 | 0.109624 | 7/15/2014 | 40:22.5 | 0 |
| -629.033 | 19743.063 | 1.972 | 13.958 | 0.124654 | 7/15/2014 | 40:22.6 | 0 |
| -628.745 | 19743.035 | 1.972 | 13.95 | 0.113339 | 7/15/2014 | 40:22.7 | 0 |
| -628.629 | 19743.024 | 1.971 | 13.945 | 0.120367 | 7/15/2014 | 40:22.8 | 0 |
| -628.396 | 19743.003 | 1.971 | 13.941 | 0.127153 | 7/15/2014 | 40:22.9 | 0 |
| -628.164 | 19742.98 | 1.969 | 13.941 | 0.121843 | 7/15/2014 | 40:23.0 | 0 |
| -627.93 | 19742.958 | 1.969 | 13.943 | 0.138785 | 7/15/2014 | 40:23.1 | 0 |
| -627.697 | 19742.938 | 1.966 | 13.949 | 0.148766 | 7/15/2014 | 40:23.2 | 0 |
| -627.464 | 19742.914 | 1.971 | 13.959 | 0.143604 | 7/15/2014 | 40:23.3 | 0 |
| -627.057 | 19742.877 | 1.973 | 13.978 | 0.156808 | 7/15/2014 | 40:23.4 | 0 |
| -626.824 | 19742.854 | 1.977 | 13.992 | 0.148296 | 7/15/2014 | 40:23.5 | 0 |
| -626.593 | 19742.834 | 1.979 | 14.004 | 0.145921 | 7/15/2014 | 40:23.6 | 0 |
| -626.303 | 19742.807 | 1.979 | 14.015 | 0.137098 | 7/15/2014 | 40:23.7 | 0 |
| -626.185 | 19742.797 | 1.98 | 14.023 | 0.136294 | 7/15/2014 | 40:23.8 | 0 |
| -625.95 | 19742.773 | 1.983 | 14.031 | 0.137558 | 7/15/2014 | 40:23.9 | 0 |
| -625.717 | 19742.753 | 1.978 | 14.039 | 0.128229 | 7/15/2014 | 40:24.0 | 0 |
| -625.481 | 19742.73 | 1.98 | 14.047 | 0.131757 | 7/15/2014 | 40:24.1 | 0 |
| -625.247 | 19742.708 | 1.981 | 14.059 | 0.128598 | 7/15/2014 | 40:24.2 | 0 |
| -625.013 | 19742.688 | 1.981 | 14.075 | 0.123397 | 7/15/2014 | 40:24.3 | 0 |

| | | | | | | | |
|---|---|---|---|---|---|---|---|
| -624.607 | 19742.65 | 1.978 | 14.114 | 0.107996 | 7/15/2014 | 40:24.4 | 0 |
| -624.373 | 19742.629 | 1.98 | 14.143 | 0.0984576 | 7/15/2014 | 40:24.5 | 0 |
| -624.141 | 19742.607 | 1.98 | 14.175 | 0.108687 | 7/15/2014 | 40:24.6 | 0 |
| -623.851 | 19742.581 | 1.983 | 14.209 | 0.0712814 | 7/15/2014 | 40:24.7 | 0 |
| -623.734 | 19742.57 | 1.981 | 14.228 | 0.0642212 | 7/15/2014 | 40:24.8 | 0 |
| -623.499 | 19742.549 | 1.98 | 14.26 | 0.0672351 | 7/15/2014 | 40:24.9 | 0 |
| -623.265 | 19742.528 | 1.978 | 14.288 | 0.0754861 | 7/15/2014 | 40:25.0 | 0 |
| -623.03 | 19742.506 | 1.98 | 14.31 | 0.0747193 | 7/15/2014 | 40:25.1 | 0 |
| -622.796 | 19742.486 | 1.98 | 14.326 | 0.0726288 | 7/15/2014 | 40:25.2 | 0 |
| -622.562 | 19742.464 | 1.983 | 14.336 | 0.0837568 | 7/15/2014 | 40:25.3 | 0 |
| -622.156 | 19742.429 | 1.987 | 14.339 | 0.104568 | 7/15/2014 | 40:25.4 | 0 |
| -621.925 | 19742.408 | 1.99 | 14.337 | 0.116263 | 7/15/2014 | 40:25.5 | 0 |
| -621.694 | 19742.389 | 1.99 | 14.332 | 0.127988 | 7/15/2014 | 40:25.6 | 0 |
| -621.405 | 19742.361 | 1.992 | 14.325 | 0.14452 | 7/15/2014 | 40:25.7 | 0 |
| -621.288 | 19742.352 | 1.992 | 14.321 | 0.153393 | 7/15/2014 | 40:25.8 | 0 |
| -621.056 | 19742.331 | 1.995 | 14.311 | 0.156731 | 7/15/2014 | 40:25.9 | 0 |
| -620.708 | 19742.301 | 1.992 | 14.295 | 0.157187 | 7/15/2014 | 40:26.0 | 0 |
| -620.477 | 19742.281 | 1.988 | 14.285 | 0.152219 | 7/15/2014 | 40:26.1 | 0 |
| -620.246 | 19742.262 | 1.983 | 14.276 | 0.147187 | 7/15/2014 | 40:26.2 | 0 |
| -620.014 | 19742.242 | 1.979 | 14.269 | 0.138545 | 7/15/2014 | 40:26.3 | 0 |
| -619.728 | 19742.217 | 1.975 | 14.265 | 0.130754 | 7/15/2014 | 40:26.4 | 0 |
| -619.499 | 19742.197 | 1.971 | 14.264 | 0.113811 | 7/15/2014 | 40:26.5 | 0 |
| -619.269 | 19742.178 | 1.969 | 14.267 | 0.106581 | 7/15/2014 | 40:26.6 | 0 |
| -618.982 | 19742.154 | 1.967 | 14.274 | 0.0950819 | 7/15/2014 | 40:26.7 | 0 |
| -618.867 | 19742.145 | 1.963 | 14.276 | 0.0923138 | 7/15/2014 | 40:26.8 | 0 |
| -618.634 | 19742.125 | 1.968 | 14.29 | 0.0857509 | 7/15/2014 | 40:26.9 | 0 |
| -618.401 | 19742.105 | 1.968 | 14.308 | 0.0924603 | 7/15/2014 | 40:27.0 | 0 |
| -618.168 | 19742.086 | 1.971 | 14.331 | 0.092828 | 7/15/2014 | 40:27.1 | 0 |
| -617.936 | 19742.068 | 1.971 | 14.358 | 0.099251 | 7/15/2014 | 40:27.2 | 0 |
| -617.702 | 19742.049 | 1.973 | 14.386 | 0.111521 | 7/15/2014 | 40:27.3 | 0 |
| -617.295 | 19742.016 | 1.971 | 14.426 | 0.120327 | 7/15/2014 | 40:27.4 | 0 |
| -617.063 | 19741.996 | 1.971 | 14.449 | 0.122828 | 7/15/2014 | 40:27.5 | 0 |
| -616.832 | 19741.979 | 1.967 | 14.469 | 0.127934 | 7/15/2014 | 40:27.6 | 0 |
| -616.541 | 19741.955 | 1.961 | 14.482 | 0.12717 | 7/15/2014 | 40:27.7 | 0 |
| -616.423 | 19741.947 | 1.957 | 14.492 | 0.139405 | 7/15/2014 | 40:27.8 | 0 |
| -616.189 | 19741.928 | 1.962 | 14.495 | 0.132229 | 7/15/2014 | 40:27.9 | 0 |
| -615.956 | 19741.91 | 1.96 | 14.492 | 0.126286 | 7/15/2014 | 40:28.0 | 0 |
| -615.722 | 19741.891 | 1.96 | 14.48 | 0.142663 | 7/15/2014 | 40:28.1 | 0 |
| -615.489 | 19741.871 | 1.957 | 14.462 | 0.13876 | 7/15/2014 | 40:28.2 | 0 |
| -615.256 | 19741.855 | 1.957 | 14.438 | 0.139086 | 7/15/2014 | 40:28.3 | 0 |
| -614.85 | 19741.822 | 1.954 | 14.388 | 0.134839 | 7/15/2014 | 40:28.4 | 0 |
| -614.618 | 19741.805 | 1.957 | 14.352 | 0.140479 | 7/15/2014 | 40:28.5 | 0 |
| -614.388 | 19741.787 | 1.95 | 14.313 | 0.151275 | 7/15/2014 | 40:28.6 | 0 |
| -614.098 | 19741.766 | 1.946 | 14.272 | 0.144362 | 7/15/2014 | 40:28.7 | 0 |
| -613.981 | 19741.756 | 1.945 | 14.251 | 0.153174 | 7/15/2014 | 40:28.8 | 0 |
| -613.748 | 19741.738 | 1.941 | 14.21 | 0.155176 | 7/15/2014 | 40:28.9 | 0 |
| -613.514 | 19741.721 | 1.938 | 14.169 | 0.165501 | 7/15/2014 | 40:29.0 | 0 |

| | | | | | | | |
|---|---|---|---|---|---|---|---|
| -613.281 | 19741.701 | 1.934 | 14.13 | 0.156973 | 7/15/2014 | 40:29.1 | 0 |
| -613.047 | 19741.686 | 1.932 | 14.095 | 0.157416 | 7/15/2014 | 40:29.2 | 0 |
| -612.814 | 19741.667 | 1.928 | 14.063 | 0.156008 | 7/15/2014 | 40:29.3 | 0 |
| -612.407 | 19741.638 | 1.924 | 14.029 | 0.15054 | 7/15/2014 | 40:29.4 | 0 |
| -612.176 | 19741.619 | 1.92 | 14.012 | 0.156887 | 7/15/2014 | 40:29.5 | 0 |
| -611.944 | 19741.604 | 1.92 | 14.002 | 0.161472 | 7/15/2014 | 40:29.6 | 0 |
| -611.654 | 19741.582 | 1.916 | 13.998 | 0.159775 | 7/15/2014 | 40:29.7 | 0 |
| -611.537 | 19741.574 | 1.914 | 13.995 | 0.164742 | 7/15/2014 | 40:29.8 | 0 |
| -611.302 | 19741.557 | 1.916 | 14.003 | 0.164239 | 7/15/2014 | 40:29.9 | 0 |
| -611.069 | 19741.54 | 1.914 | 14.018 | 0.179239 | 7/15/2014 | 40:30.0 | 0 |
| -610.834 | 19741.522 | 1.914 | 14.039 | 0.158862 | 7/15/2014 | 40:30.1 | 0 |
| -610.601 | 19741.506 | 1.911 | 14.065 | 0.159125 | 7/15/2014 | 40:30.2 | 0 |
| -610.367 | 19741.489 | 1.914 | 14.095 | 0.169777 | 7/15/2014 | 40:30.3 | 0 |
| -609.959 | 19741.461 | 1.907 | 14.14 | 0.146628 | 7/15/2014 | 40:30.4 | 0 |
| -609.725 | 19741.444 | 1.907 | 14.169 | 0.131052 | 7/15/2014 | 40:30.5 | 0 |
| -609.493 | 19741.429 | 1.903 | 14.197 | 0.133497 | 7/15/2014 | 40:30.6 | 0 |
| -609.199 | 19741.408 | 1.901 | 14.223 | 0.121721 | 7/15/2014 | 40:30.7 | 0 |
| -609.082 | 19741.4 | 1.899 | 14.236 | 0.115055 | 7/15/2014 | 40:30.8 | 0 |
| -608.845 | 19741.383 | 1.902 | 14.258 | 0.0875015 | 7/15/2014 | 40:30.9 | 0 |
| -608.61 | 19741.366 | 1.896 | 14.277 | 0.0791343 | 7/15/2014 | 40:31.0 | 0 |
| -608.371 | 19741.35 | 1.896 | 14.294 | 0.0805933 | 7/15/2014 | 40:31.1 | 0 |
| -608.135 | 19741.334 | 1.894 | 14.309 | 0.086917 | 7/15/2014 | 40:31.2 | 0 |
| -607.896 | 19741.318 | 1.897 | 14.322 | 0.0887685 | 7/15/2014 | 40:31.3 | 0 |
| -607.482 | 19741.29 | 1.901 | 14.34 | 0.103139 | 7/15/2014 | 40:31.4 | 0 |
| -607.244 | 19741.273 | 1.901 | 14.35 | 0.108763 | 7/15/2014 | 40:31.5 | 0 |
| -607.009 | 19741.258 | 1.901 | 14.359 | 0.114587 | 7/15/2014 | 40:31.6 | 0 |
| -606.713 | 19741.237 | 1.904 | 14.367 | 0.113515 | 7/15/2014 | 40:31.7 | 0 |
| -606.593 | 19741.229 | 1.905 | 14.371 | 0.111138 | 7/15/2014 | 40:31.8 | 0 |
| -606.354 | 19741.213 | 1.909 | 14.377 | 0.109382 | 7/15/2014 | 40:31.9 | 0 |
| -606.115 | 19741.197 | 1.909 | 14.381 | 0.103939 | 7/15/2014 | 40:32.0 | 0 |
| -605.877 | 19741.181 | 1.909 | 14.383 | 0.096435 | 7/15/2014 | 40:32.1 | 0 |
| -605.638 | 19741.166 | 1.906 | 14.384 | 0.10261 | 7/15/2014 | 40:32.2 | 0 |
| -605.4 | 19741.149 | 1.908 | 14.382 | 0.0862671 | 7/15/2014 | 40:32.3 | 0 |
| -604.987 | 19741.122 | 1.901 | 14.374 | 0.106365 | 7/15/2014 | 40:32.4 | 0 |
| -604.753 | 19741.105 | 1.898 | 14.367 | 0.110849 | 7/15/2014 | 40:32.5 | 0 |
| -604.516 | 19741.091 | 1.896 | 14.358 | 0.10847 | 7/15/2014 | 40:32.6 | 0 |
| -604.224 | 19741.07 | 1.89 | 14.348 | 0.109918 | 7/15/2014 | 40:32.7 | 0 |
| -604.104 | 19741.063 | 1.887 | 14.343 | 0.102061 | 7/15/2014 | 40:32.8 | 0 |
| -603.867 | 19741.048 | 1.89 | 14.33 | 0.107475 | 7/15/2014 | 40:32.9 | 0 |
| -603.631 | 19741.032 | 1.887 | 14.316 | 0.0938822 | 7/15/2014 | 40:33.0 | 0 |
| -603.394 | 19741.017 | 1.891 | 14.3 | 0.0925675 | 7/15/2014 | 40:33.1 | 0 |
| -603.159 | 19741.001 | 1.891 | 14.284 | 0.0865994 | 7/15/2014 | 40:33.2 | 0 |
| -602.923 | 19740.984 | 1.895 | 14.266 | 0.0966718 | 7/15/2014 | 40:33.3 | 0 |
| -602.513 | 19740.958 | 1.899 | 14.239 | 0.0940943 | 7/15/2014 | 40:33.4 | 0 |
| -602.28 | 19740.943 | 1.899 | 14.22 | 0.115757 | 7/15/2014 | 40:33.5 | 0 |
| -602.046 | 19740.928 | 1.899 | 14.202 | 0.106684 | 7/15/2014 | 40:33.6 | 0 |
| -601.756 | 19740.908 | 1.896 | 14.184 | 0.105168 | 7/15/2014 | 40:33.7 | 0 |

| | | | | | | | |
|---|---|---|---|---|---|---|---|
| -601.637 | 19740.9 | 1.896 | 14.175 | 0.118307 | 7/15/2014 | 40:33.8 | 0 |
| -601.402 | 19740.884 | 1.896 | 14.159 | 0.0993573 | 7/15/2014 | 40:33.9 | 0 |
| -601.166 | 19740.87 | 1.898 | 14.147 | 0.121661 | 7/15/2014 | 40:34.0 | 0 |
| -600.931 | 19740.854 | 1.896 | 14.138 | 0.114509 | 7/15/2014 | 40:34.1 | 0 |
| -600.698 | 19740.839 | 1.89 | 14.134 | 0.105445 | 7/15/2014 | 40:34.2 | 0 |
| -600.461 | 19740.823 | 1.896 | 14.136 | 0.117103 | 7/15/2014 | 40:34.3 | 0 |
| -600.053 | 19740.796 | 1.89 | 14.158 | 0.121537 | 7/15/2014 | 40:34.4 | 0 |
| -599.818 | 19740.78 | 1.891 | 14.179 | 0.13374 | 7/15/2014 | 40:34.5 | 0 |
| -599.584 | 19740.766 | 1.891 | 14.208 | 0.129179 | 7/15/2014 | 40:34.6 | 0 |
| -599.292 | 19740.745 | 1.884 | 14.243 | 0.124824 | 7/15/2014 | 40:34.7 | 0 |
| -599.175 | 19740.738 | 1.882 | 14.26 | 0.132787 | 7/15/2014 | 40:34.8 | 0 |
| -598.936 | 19740.723 | 1.886 | 14.304 | 0.145125 | 7/15/2014 | 40:34.9 | 0 |
| -598.701 | 19740.706 | 1.88 | 14.352 | 0.144573 | 7/15/2014 | 40:35.0 | 0 |
| -598.463 | 19740.691 | 1.88 | 14.401 | 0.166942 | 7/15/2014 | 40:35.1 | 0 |
| -598.225 | 19740.676 | 1.876 | 14.448 | 0.175742 | 7/15/2014 | 40:35.2 | 0 |
| -597.987 | 19740.66 | 1.879 | 14.492 | 0.173026 | 7/15/2014 | 40:35.3 | 0 |
| -597.573 | 19740.633 | 1.882 | 14.546 | 0.197668 | 7/15/2014 | 40:35.4 | 0 |
| -597.336 | 19740.617 | 1.886 | 14.578 | 0.180255 | 7/15/2014 | 40:35.5 | 0 |
| -597.099 | 19740.602 | 1.888 | 14.603 | 0.192134 | 7/15/2014 | 40:35.6 | 0 |
| -596.803 | 19740.582 | 1.89 | 14.624 | 0.178786 | 7/15/2014 | 40:35.7 | 0 |
| -596.683 | 19740.574 | 1.893 | 14.635 | 0.170863 | 7/15/2014 | 40:35.8 | 0 |
| -596.441 | 19740.559 | 1.899 | 14.648 | 0.194588 | 7/15/2014 | 40:35.9 | 0 |
| -596.202 | 19740.544 | 1.899 | 14.656 | 0.196419 | 7/15/2014 | 40:36.0 | 0 |
| -595.96 | 19740.527 | 1.9 | 14.659 | 0.184031 | 7/15/2014 | 40:36.1 | 0 |
| -595.721 | 19740.513 | 1.896 | 14.659 | 0.202157 | 7/15/2014 | 40:36.2 | 0 |
| -595.481 | 19740.496 | 1.899 | 14.655 | 0.175255 | 7/15/2014 | 40:36.3 | 0 |
| -595.064 | 19740.469 | 1.897 | 14.642 | 0.223715 | 7/15/2014 | 40:36.4 | 0 |
| -594.825 | 19740.453 | 1.898 | 14.631 | 0.209849 | 7/15/2014 | 40:36.5 | 0 |
| -594.588 | 19740.438 | 1.898 | 14.618 | 0.168482 | 7/15/2014 | 40:36.6 | 0 |
| -594.292 | 19740.419 | 1.9 | 14.604 | 0.167606 | 7/15/2014 | 40:36.7 | 0 |
| -594.171 | 19740.412 | 1.901 | 14.597 | 0.150206 | 7/15/2014 | 40:36.8 | 0 |
| -593.934 | 19740.396 | 1.905 | 14.58 | 0.19012 | 7/15/2014 | 40:36.9 | 0 |
| -593.697 | 19740.381 | 1.905 | 14.564 | 0.147536 | 7/15/2014 | 40:37.0 | 0 |
| -593.459 | 19740.365 | 1.913 | 14.547 | 0.127274 | 7/15/2014 | 40:37.1 | 0 |
| -593.225 | 19740.35 | 1.913 | 14.531 | 0.126577 | 7/15/2014 | 40:37.2 | 0 |
| -592.99 | 19740.334 | 1.915 | 14.515 | 0.163745 | 7/15/2014 | 40:37.3 | 0 |
| -592.582 | 19740.308 | 1.92 | 14.493 | 0.194338 | 7/15/2014 | 40:37.4 | 0 |
| -592.35 | 19740.293 | 1.92 | 14.479 | 0.147089 | 7/15/2014 | 40:37.5 | 0 |
| -592.117 | 19740.278 | 1.922 | 14.466 | 0.187856 | 7/15/2014 | 40:37.6 | 0 |
| -591.825 | 19740.26 | 1.92 | 14.454 | 0.167266 | 7/15/2014 | 40:37.7 | 0 |
| -591.709 | 19740.252 | 1.919 | 14.447 | 0.170543 | 7/15/2014 | 40:37.8 | 0 |
| -591.473 | 19740.235 | 1.921 | 14.435 | 0.170504 | 7/15/2014 | 40:37.9 | 0 |
| -591.238 | 19740.22 | 1.919 | 14.424 | 0.159272 | 7/15/2014 | 40:38.0 | 0 |
| -590.999 | 19740.204 | 1.921 | 14.413 | 0.129802 | 7/15/2014 | 40:38.1 | 0 |
| -590.763 | 19740.189 | 1.915 | 14.401 | 0.159391 | 7/15/2014 | 40:38.2 | 0 |
| -590.525 | 19740.174 | 1.915 | 14.389 | 0.134052 | 7/15/2014 | 40:38.3 | 0 |
| -590.107 | 19740.146 | 1.915 | 14.37 | 0.158238 | 7/15/2014 | 40:38.4 | 0 |

| | | | | | | | |
|---|---|---|---|---|---|---|---|
| -589.868 | 19740.13 | 1.919 | 14.357 | 0.155432 | 7/15/2014 | 40:38.5 | 0 |
| -589.629 | 19740.114 | 1.919 | 14.342 | 0.147912 | 7/15/2014 | 40:38.6 | 0 |
| -589.329 | 19740.094 | 1.921 | 14.328 | 0.163281 | 7/15/2014 | 40:38.7 | 0 |
| -589.208 | 19740.086 | 1.92 | 14.321 | 0.159422 | 7/15/2014 | 40:38.8 | 0 |
| -588.965 | 19740.07 | 1.922 | 14.305 | 0.172017 | 7/15/2014 | 40:38.9 | 0 |
| -588.722 | 19740.053 | 1.925 | 14.289 | 0.157733 | 7/15/2014 | 40:39.0 | 0 |
| -588.478 | 19740.037 | 1.927 | 14.274 | 0.162014 | 7/15/2014 | 40:39.1 | 0 |
| -588.235 | 19740.021 | 1.927 | 14.259 | 0.181011 | 7/15/2014 | 40:39.2 | 0 |
| -587.991 | 19740.005 | 1.931 | 14.245 | 0.169607 | 7/15/2014 | 40:39.3 | 0 |
| -587.568 | 19739.976 | 1.937 | 14.228 | 0.186855 | 7/15/2014 | 40:39.4 | 0 |
| -587.325 | 19739.959 | 1.942 | 14.22 | 0.186287 | 7/15/2014 | 40:39.5 | 0 |
| -587.083 | 19739.942 | 1.945 | 14.214 | 0.187669 | 7/15/2014 | 40:39.6 | 0 |
| -586.78 | 19739.922 | 1.949 | 14.213 | 0.171953 | 7/15/2014 | 40:39.7 | 0 |
| -586.658 | 19739.914 | 1.951 | 14.211 | 0.158061 | 7/15/2014 | 40:39.8 | 0 |
| -586.413 | 19739.896 | 1.955 | 14.217 | 0.174302 | 7/15/2014 | 40:39.9 | 0 |
| -586.168 | 19739.88 | 1.951 | 14.229 | 0.173362 | 7/15/2014 | 40:40.0 | 0 |
| -585.923 | 19739.863 | 1.951 | 14.248 | 0.149048 | 7/15/2014 | 40:40.1 | 0 |
| -585.679 | 19739.846 | 1.948 | 14.275 | 0.160497 | 7/15/2014 | 40:40.2 | 0 |
| -585.434 | 19739.829 | 1.948 | 14.309 | 0.139414 | 7/15/2014 | 40:40.3 | 0 |
| -585.007 | 19739.8 | 1.946 | 14.377 | 0.158254 | 7/15/2014 | 40:40.4 | 0 |
| -584.762 | 19739.782 | 1.944 | 14.425 | 0.178192 | 7/15/2014 | 40:40.5 | 0 |
| -584.517 | 19739.766 | 1.941 | 14.477 | 0.154942 | 7/15/2014 | 40:40.6 | 0 |
| -584.211 | 19739.744 | 1.941 | 14.531 | 0.162127 | 7/15/2014 | 40:40.7 | 0 |
| -584.087 | 19739.735 | 1.939 | 14.559 | 0.146482 | 7/15/2014 | 40:40.8 | 0 |
| -583.84 | 19739.718 | 1.943 | 14.613 | 0.147349 | 7/15/2014 | 40:40.9 | 0 |
| -583.593 | 19739.701 | 1.943 | 14.664 | 0.156595 | 7/15/2014 | 40:41.0 | 0 |
| -583.346 | 19739.684 | 1.943 | 14.709 | 0.152259 | 7/15/2014 | 40:41.1 | 0 |
| -583.098 | 19739.666 | 1.945 | 14.748 | 0.11692 | 7/15/2014 | 40:41.2 | 0 |
| -582.851 | 19739.648 | 1.948 | 14.78 | 0.129631 | 7/15/2014 | 40:41.3 | 0 |
| -582.42 | 19739.617 | 1.948 | 14.808 | 0.140446 | 7/15/2014 | 40:41.4 | 0 |
| -582.176 | 19739.6 | 1.951 | 14.821 | 0.128266 | 7/15/2014 | 40:41.5 | 0 |
| -581.93 | 19739.582 | 1.953 | 14.828 | 0.127729 | 7/15/2014 | 40:41.6 | 0 |
| -581.628 | 19739.561 | 1.951 | 14.827 | 0.124405 | 7/15/2014 | 40:41.7 | 0 |
| -581.504 | 19739.552 | 1.951 | 14.828 | 0.125533 | 7/15/2014 | 40:41.8 | 0 |
| -581.259 | 19739.534 | 1.947 | 14.82 | 0.129435 | 7/15/2014 | 40:41.9 | 0 |
| -581.016 | 19739.517 | 1.941 | 14.808 | 0.12318 | 7/15/2014 | 40:42.0 | 0 |
| -580.772 | 19739.498 | 1.944 | 14.792 | 0.13302 | 7/15/2014 | 40:42.1 | 0 |
| -580.529 | 19739.48 | 1.941 | 14.775 | 0.167251 | 7/15/2014 | 40:42.2 | 0 |
| -580.288 | 19739.463 | 1.941 | 14.757 | 0.166642 | 7/15/2014 | 40:42.3 | 0 |
| -579.871 | 19739.432 | 1.94 | 14.729 | 0.177433 | 7/15/2014 | 40:42.4 | 0 |
| -579.633 | 19739.414 | 1.942 | 14.711 | 0.190818 | 7/15/2014 | 40:42.5 | 0 |
| -579.396 | 19739.396 | 1.942 | 14.693 | 0.203095 | 7/15/2014 | 40:42.6 | 0 |
| -579.1 | 19739.375 | 1.948 | 14.675 | 0.170361 | 7/15/2014 | 40:42.7 | 0 |
| -578.979 | 19739.366 | 1.947 | 14.666 | 0.172778 | 7/15/2014 | 40:42.8 | 0 |
| -578.742 | 19739.348 | 1.953 | 14.65 | 0.154754 | 7/15/2014 | 40:42.9 | 0 |
| -578.504 | 19739.329 | 1.953 | 14.633 | 0.161894 | 7/15/2014 | 40:43.0 | 0 |
| -578.266 | 19739.312 | 1.955 | 14.617 | 0.152087 | 7/15/2014 | 40:43.1 | 0 |

| | | | | | | | |
|---|---|---|---|---|---|---|---|
| -578.03 | 19739.293 | 1.955 | 14.601 | 0.151112 | 7/15/2014 | 40:43.2 | 0 |
| -577.794 | 19739.274 | 1.958 | 14.585 | 0.149305 | 7/15/2014 | 40:43.3 | 0 |
| -577.382 | 19739.244 | 1.957 | 14.561 | 0.146553 | 7/15/2014 | 40:43.4 | 0 |
| -577.147 | 19739.225 | 1.957 | 14.546 | 0.138821 | 7/15/2014 | 40:43.5 | 0 |
| -576.911 | 19739.206 | 1.957 | 14.531 | 0.11917 | 7/15/2014 | 40:43.6 | 0 |
| -576.614 | 19739.183 | 1.957 | 14.518 | 0.128331 | 7/15/2014 | 40:43.7 | 0 |
| -576.497 | 19739.173 | 1.955 | 14.511 | 0.124268 | 7/15/2014 | 40:43.8 | 0 |
| -576.258 | 19739.153 | 1.958 | 14.499 | 0.108359 | 7/15/2014 | 40:43.9 | 0 |
| -576.018 | 19739.134 | 1.958 | 14.488 | 0.103799 | 7/15/2014 | 40:44.0 | 0 |
| -575.776 | 19739.115 | 1.964 | 14.479 | 0.102283 | 7/15/2014 | 40:44.1 | 0 |
| -575.535 | 19739.096 | 1.964 | 14.471 | 0.0959285 | 7/15/2014 | 40:44.2 | 0 |
| -575.293 | 19739.076 | 1.964 | 14.465 | 0.0943533 | 7/15/2014 | 40:44.3 | 0 |
| -574.868 | 19739.04 | 1.963 | 14.46 | 0.0908811 | 7/15/2014 | 40:44.4 | 0 |
| -574.626 | 19739.021 | 1.959 | 14.458 | 0.0873458 | 7/15/2014 | 40:44.5 | 0 |
| -574.383 | 19738.998 | 1.959 | 14.458 | 0.0838094 | 7/15/2014 | 40:44.6 | 0 |
| -574.079 | 19738.974 | 1.959 | 14.46 | 0.0817955 | 7/15/2014 | 40:44.7 | 0 |
| -573.956 | 19738.964 | 1.958 | 14.46 | 0.0909658 | 7/15/2014 | 40:44.8 | 0 |
| -573.711 | 19738.943 | 1.96 | 14.466 | 0.0810886 | 7/15/2014 | 40:44.9 | 0 |
| -573.467 | 19738.923 | 1.96 | 14.474 | 0.0750599 | 7/15/2014 | 40:45.0 | 0 |
| -573.221 | 19738.9 | 1.963 | 14.484 | 0.083864 | 7/15/2014 | 40:45.1 | 0 |
| -572.978 | 19738.881 | 1.96 | 14.498 | 0.0930552 | 7/15/2014 | 40:45.2 | 0 |
| -572.735 | 19738.857 | 1.966 | 14.516 | 0.0853922 | 7/15/2014 | 40:45.3 | 0 |
| -572.313 | 19738.821 | 1.967 | 14.555 | 0.0920404 | 7/15/2014 | 40:45.4 | 0 |
| -572.073 | 19738.801 | 1.967 | 14.584 | 0.109904 | 7/15/2014 | 40:45.5 | 0 |
| -571.836 | 19738.779 | 1.967 | 14.618 | 0.108193 | 7/15/2014 | 40:45.6 | 0 |
| -571.539 | 19738.752 | 1.969 | 14.656 | 0.113932 | 7/15/2014 | 40:45.7 | 0 |
| -571.418 | 19738.742 | 1.969 | 14.674 | 0.111988 | 7/15/2014 | 40:45.8 | 0 |
| -571.179 | 19738.721 | 1.973 | 14.717 | 0.0964494 | 7/15/2014 | 40:45.9 | 0 |
| -570.941 | 19738.699 | 1.973 | 14.76 | 0.0851464 | 7/15/2014 | 40:46.0 | 0 |
| -570.702 | 19738.677 | 1.976 | 14.803 | 0.0856024 | 7/15/2014 | 40:46.1 | 0 |
| -570.466 | 19738.656 | 1.976 | 14.844 | 0.0996849 | 7/15/2014 | 40:46.2 | 0 |
| -570.23 | 19738.633 | 1.978 | 14.88 | 0.0751616 | 7/15/2014 | 40:46.3 | 0 |
| -569.819 | 19738.596 | 1.982 | 14.921 | 0.0724524 | 7/15/2014 | 40:46.4 | 0 |
| -569.584 | 19738.574 | 1.984 | 14.943 | 0.0784921 | 7/15/2014 | 40:46.5 | 0 |
| -569.348 | 19738.551 | 1.99 | 14.959 | 0.0747701 | 7/15/2014 | 40:46.6 | 0 |
| -569.055 | 19738.523 | 1.992 | 14.968 | 0.0762104 | 7/15/2014 | 40:46.7 | 0 |
| -568.937 | 19738.513 | 1.993 | 14.975 | 0.0677964 | 7/15/2014 | 40:46.8 | 0 |
| -568.7 | 19738.49 | 1.999 | 14.975 | 0.0798617 | 7/15/2014 | 40:46.9 | 0 |
| -568.462 | 19738.467 | 1.999 | 14.969 | 0.0659371 | 7/15/2014 | 40:47.0 | 0 |
| -568.222 | 19738.444 | 2.006 | 14.957 | 0.0661004 | 7/15/2014 | 40:47.1 | 0 |
| -567.984 | 19738.421 | 2.006 | 14.94 | 0.0746279 | 7/15/2014 | 40:47.2 | 0 |
| -567.744 | 19738.396 | 2.006 | 14.918 | 0.0698013 | 7/15/2014 | 40:47.3 | 0 |
| -567.327 | 19738.356 | 2.007 | 14.877 | 0.0712764 | 7/15/2014 | 40:47.4 | 0 |
| -567.088 | 19738.333 | 2.014 | 14.848 | 0.0688397 | 7/15/2014 | 40:47.5 | 0 |
| -566.849 | 19738.309 | 2.017 | 14.817 | 0.064867 | 7/15/2014 | 40:47.6 | 0 |
| -566.55 | 19738.278 | 2.02 | 14.787 | 0.0614238 | 7/15/2014 | 40:47.7 | 0 |
| -566.43 | 19738.267 | 2.017 | 14.771 | 0.0580833 | 7/15/2014 | 40:47.8 | 0 |

| | | | | | | | |
|---|---|---|---|---|---|---|---|
| -566.189 | 19738.242 | 2.021 | 14.741 | 0.0572171 | 7/15/2014 | 40:47.9 | 0 |
| -565.948 | 19738.219 | 2.025 | 14.714 | 0.053641 | 7/15/2014 | 40:48.0 | 0 |
| -565.707 | 19738.194 | 2.025 | 14.69 | 0.0578243 | 7/15/2014 | 40:48.1 | 0 |
| -565.467 | 19738.169 | 2.025 | 14.669 | 0.0497819 | 7/15/2014 | 40:48.2 | 0 |
| -565.226 | 19738.145 | 2.021 | 14.653 | 0.0665615 | 7/15/2014 | 40:48.3 | 0 |
| -564.81 | 19738.102 | 2.017 | 14.637 | 0.0616318 | 7/15/2014 | 40:48.4 | 0 |
| -564.572 | 19738.077 | 2.02 | 14.629 | 0.0541511 | 7/15/2014 | 40:48.5 | 0 |
| -564.337 | 19738.053 | 2.026 | 14.624 | 0.0745515 | 7/15/2014 | 40:48.6 | 0 |
| -564.042 | 19738.022 | 2.029 | 14.62 | 0.0523708 | 7/15/2014 | 40:48.7 | 0 |
| -563.922 | 19738.01 | 2.027 | 14.619 | 0.0570466 | 7/15/2014 | 40:48.8 | 0 |
| -563.685 | 19737.984 | 2.033 | 14.619 | 0.067155 | 7/15/2014 | 40:48.9 | 0 |
| -563.451 | 19737.959 | 2.039 | 14.619 | 0.0591747 | 7/15/2014 | 40:49.0 | 0 |
| -563.215 | 19737.936 | 2.049 | 14.62 | 0.0812863 | 7/15/2014 | 40:49.1 | 0 |
| -562.982 | 19737.911 | 2.049 | 14.62 | 0.0557773 | 7/15/2014 | 40:49.2 | 0 |
| -562.75 | 19737.885 | 2.052 | 14.62 | 0.0708617 | 7/15/2014 | 40:49.3 | 0 |
| -562.346 | 19737.844 | 2.052 | 14.618 | 0.0913132 | 7/15/2014 | 40:49.4 | 0 |
| -562.118 | 19737.817 | 2.052 | 14.617 | 0.0781729 | 7/15/2014 | 40:49.5 | 0 |
| -561.89 | 19737.793 | 2.052 | 14.615 | 0.0989244 | 7/15/2014 | 40:49.6 | 0 |
| -561.604 | 19737.764 | 2.05 | 14.615 | 0.072134 | 7/15/2014 | 40:49.7 | 0 |
| -561.373 | 19737.738 | 2.052 | 14.615 | 0.0997161 | 7/15/2014 | 40:49.8 | 0 |
| -561.143 | 19737.713 | 2.052 | 14.617 | 0.0731883 | 7/15/2014 | 40:49.9 | 0 |
| -560.912 | 19737.689 | 2.052 | 14.621 | 0.0751758 | 7/15/2014 | 40:50.0 | 0 |
| -560.68 | 19737.664 | 2.052 | 14.627 | 0.0638506 | 7/15/2014 | 40:50.1 | 0 |
| -560.567 | 19737.65 | 2.049 | 14.629 | 0.0648025 | 7/15/2014 | 40:50.2 | 0 |
| -560.336 | 19737.626 | 2.052 | 14.639 | 0.0658185 | 7/15/2014 | 40:50.3 | 0 |
| -559.933 | 19737.582 | 2.05 | 14.658 | 0.0667365 | 7/15/2014 | 40:50.4 | 0 |
| -559.703 | 19737.557 | 2.05 | 14.669 | 0.069821 | 7/15/2014 | 40:50.5 | 0 |
| -559.475 | 19737.533 | 2.045 | 14.678 | 0.0712572 | 7/15/2014 | 40:50.6 | 0 |
| -559.188 | 19737.502 | 2.047 | 14.684 | 0.0778127 | 7/15/2014 | 40:50.7 | 0 |
| -559.071 | 19737.488 | 2.046 | 14.689 | 0.0771993 | 7/15/2014 | 40:50.8 | 0 |
| -558.84 | 19737.464 | 2.048 | 14.689 | 0.0756304 | 7/15/2014 | 40:50.9 | 0 |
| -558.497 | 19737.426 | 2.045 | 14.673 | 0.086769 | 7/15/2014 | 40:51.0 | 0 |
| -558.266 | 19737.398 | 2.042 | 14.66 | 0.089527 | 7/15/2014 | 40:51.1 | 0 |
| -558.037 | 19737.375 | 2.04 | 14.642 | 0.0778175 | 7/15/2014 | 40:51.2 | 0 |
| -557.809 | 19737.351 | 2.036 | 14.621 | 0.0831943 | 7/15/2014 | 40:51.3 | 0 |
| -557.526 | 19737.318 | 2.033 | 14.599 | 0.0853732 | 7/15/2014 | 40:51.4 | 0 |
| -557.3 | 19737.295 | 2.028 | 14.579 | 0.0969543 | 7/15/2014 | 40:51.5 | 0 |
| -557.074 | 19737.268 | 2.028 | 14.561 | 0.0763176 | 7/15/2014 | 40:51.6 | 0 |
| -556.793 | 19737.236 | 2.028 | 14.548 | 0.0725269 | 7/15/2014 | 40:51.7 | 0 |
| -556.567 | 19737.211 | 2.028 | 14.541 | 0.0748022 | 7/15/2014 | 40:51.8 | 0 |
| -556.34 | 19737.187 | 2.025 | 14.542 | 0.0757829 | 7/15/2014 | 40:51.9 | 0 |
| -556.111 | 19737.161 | 2.027 | 14.55 | 0.0713558 | 7/15/2014 | 40:52.0 | 0 |
| -555.883 | 19737.135 | 2.027 | 14.567 | 0.0712775 | 7/15/2014 | 40:52.1 | 0 |
| -555.656 | 19737.111 | 2.032 | 14.592 | 0.0695693 | 7/15/2014 | 40:52.2 | 0 |
| -555.43 | 19737.084 | 2.032 | 14.625 | 0.0797253 | 7/15/2014 | 40:52.3 | 0 |
| -555.149 | 19737.051 | 2.032 | 14.663 | 0.0713467 | 7/15/2014 | 40:52.4 | 0 |
| -554.925 | 19737.027 | 2.029 | 14.705 | 0.0715221 | 7/15/2014 | 40:52.5 | 0 |

| | | | | | | | |
|---|---|---|---|---|---|---|---|
| -554.701 | 19737.001 | 2.029 | 14.748 | 0.0711902 | 7/15/2014 | 40:52.6 | 0 |
| -554.419 | 19736.97 | 2.032 | 14.79 | 0.0748939 | 7/15/2014 | 40:52.7 | 0 |
| -554.194 | 19736.943 | 2.03 | 14.83 | 0.0749718 | 7/15/2014 | 40:52.8 | 0 |
| -553.967 | 19736.918 | 2.03 | 14.865 | 0.0756873 | 7/15/2014 | 40:52.9 | 0 |
| -553.742 | 19736.893 | 2.025 | 14.894 | 0.0774535 | 7/15/2014 | 40:53.0 | 0 |
| -553.515 | 19736.865 | 2.025 | 14.918 | 0.0728124 | 7/15/2014 | 40:53.1 | 0 |
| -553.291 | 19736.841 | 2.019 | 14.935 | 0.0715616 | 7/15/2014 | 40:53.2 | 0 |
| -553.066 | 19736.814 | 2.019 | 14.947 | 0.070293 | 7/15/2014 | 40:53.3 | 0 |
| -552.787 | 19736.782 | 2.016 | 14.954 | 0.0653743 | 7/15/2014 | 40:53.4 | 0 |
| -552.564 | 19736.757 | 2.016 | 14.957 | 0.0648227 | 7/15/2014 | 40:53.5 | 0 |
| -552.339 | 19736.73 | 2.016 | 14.956 | 0.0715685 | 7/15/2014 | 40:53.6 | 0 |
| -552.057 | 19736.699 | 2.016 | 14.952 | 0.06496 | 7/15/2014 | 40:53.7 | 0 |
| -551.83 | 19736.672 | 2.021 | 14.945 | 0.0538016 | 7/15/2014 | 40:53.8 | 0 |
| -551.604 | 19736.646 | 2.018 | 14.935 | 0.0573717 | 7/15/2014 | 40:53.9 | 0 |
| -551.372 | 19736.619 | 2.024 | 14.923 | 0.050376 | 7/15/2014 | 40:54.0 | 0 |
| -551.143 | 19736.593 | 2.024 | 14.909 | 0.0605781 | 7/15/2014 | 40:54.1 | 0 |
| -551.031 | 19736.58 | 2.025 | 14.903 | 0.0521107 | 7/15/2014 | 40:54.2 | 0 |
| -550.801 | 19736.554 | 2.029 | 14.886 | 0.0635497 | 7/15/2014 | 40:54.3 | 0 |
| -550.397 | 19736.506 | 2.026 | 14.857 | 0.0589285 | 7/15/2014 | 40:54.4 | 0 |
| -550.167 | 19736.479 | 2.026 | 14.836 | 0.0639607 | 7/15/2014 | 40:54.5 | 0 |
| -549.938 | 19736.452 | 2.024 | 14.815 | 0.0703442 | 7/15/2014 | 40:54.6 | 0 |
| -549.647 | 19736.418 | 2.027 | 14.792 | 0.0723068 | 7/15/2014 | 40:54.7 | 0 |
| -549.53 | 19736.405 | 2.023 | 14.781 | 0.0917323 | 7/15/2014 | 40:54.8 | 0 |
| -549.297 | 19736.378 | 2.023 | 14.758 | 0.0732933 | 7/15/2014 | 40:54.9 | 0 |
| -549.064 | 19736.35 | 2.019 | 14.735 | 0.0947328 | 7/15/2014 | 40:55.0 | 0 |
| -548.829 | 19736.322 | 2.023 | 14.713 | 0.0848944 | 7/15/2014 | 40:55.1 | 0 |
| -548.596 | 19736.295 | 2.02 | 14.692 | 0.0843813 | 7/15/2014 | 40:55.2 | 0 |
| -548.363 | 19736.269 | 2.02 | 14.673 | 0.0844998 | 7/15/2014 | 40:55.3 | 0 |
| -547.956 | 19736.221 | 2.019 | 14.65 | 0.0811068 | 7/15/2014 | 40:55.4 | 0 |
| -547.726 | 19736.194 | 2.019 | 14.638 | 0.0830126 | 7/15/2014 | 40:55.5 | 0 |
| -547.493 | 19736.167 | 2.019 | 14.629 | 0.0891601 | 7/15/2014 | 40:55.6 | 0 |
| -547.205 | 19736.133 | 2.016 | 14.623 | 0.0899192 | 7/15/2014 | 40:55.7 | 0 |
| -547.087 | 19736.119 | 2.013 | 14.62 | 0.0932623 | 7/15/2014 | 40:55.8 | 0 |
| -546.853 | 19736.092 | 2.013 | 14.621 | 0.0873411 | 7/15/2014 | 40:55.9 | 0 |
| -546.62 | 19736.064 | 2.013 | 14.626 | 0.0871363 | 7/15/2014 | 40:56.0 | 0 |
| -546.386 | 19736.038 | 2.013 | 14.636 | 0.0887304 | 7/15/2014 | 40:56.1 | 0 |
| -546.154 | 19736.013 | 2.009 | 14.651 | 0.0767611 | 7/15/2014 | 40:56.2 | 0 |
| -545.92 | 19735.985 | 2.009 | 14.669 | 0.071265 | 7/15/2014 | 40:56.3 | 0 |
| -545.514 | 19735.938 | 2.004 | 14.702 | 0.06824 | 7/15/2014 | 40:56.4 | 0 |
| -545.281 | 19735.91 | 2.008 | 14.724 | 0.0721882 | 7/15/2014 | 40:56.5 | 0 |
| -545.049 | 19735.885 | 2.008 | 14.745 | 0.0761263 | 7/15/2014 | 40:56.6 | 0 |
| -544.759 | 19735.851 | 2.004 | 14.766 | 0.0590325 | 7/15/2014 | 40:56.7 | 0 |
| -544.641 | 19735.838 | 2.003 | 14.777 | 0.0647659 | 7/15/2014 | 40:56.8 | 0 |
| -544.406 | 19735.813 | 1.999 | 14.795 | 0.0791664 | 7/15/2014 | 40:56.9 | 0 |
| -544.17 | 19735.785 | 1.997 | 14.81 | 0.0625969 | 7/15/2014 | 40:57.0 | 0 |
| -543.933 | 19735.758 | 2.003 | 14.821 | 0.0775653 | 7/15/2014 | 40:57.1 | 0 |
| -543.697 | 19735.73 | 1.997 | 14.829 | 0.0815083 | 7/15/2014 | 40:57.2 | 0 |

| | | | | | | | |
|---|---|---|---|---|---|---|---|
| -543.46 | 19735.705 | 1.997 | 14.834 | 0.0720278 | 7/15/2014 | 40:57.3 | 0 |
| -543.047 | 19735.658 | 1.991 | 14.834 | 0.086611 | 7/15/2014 | 40:57.4 | 0 |
| -542.812 | 19735.633 | 1.989 | 14.832 | 0.0907319 | 7/15/2014 | 40:57.5 | 0 |
| -542.573 | 19735.605 | 1.99 | 14.828 | 0.085151 | 7/15/2014 | 40:57.6 | 0 |
| -542.278 | 19735.572 | 1.987 | 14.821 | 0.107488 | 7/15/2014 | 40:57.7 | 0 |
| -542.16 | 19735.559 | 1.984 | 14.818 | 0.101751 | 7/15/2014 | 40:57.8 | 0 |
| -541.918 | 19735.531 | 1.987 | 14.808 | 0.0970159 | 7/15/2014 | 40:57.9 | 0 |
| -541.681 | 19735.507 | 1.984 | 14.797 | 0.0902315 | 7/15/2014 | 40:58.0 | 0 |
| -541.44 | 19735.48 | 1.986 | 14.786 | 0.0887854 | 7/15/2014 | 40:58.1 | 0 |
| -541.201 | 19735.453 | 1.982 | 14.776 | 0.0905924 | 7/15/2014 | 40:58.2 | 0 |
| -540.96 | 19735.426 | 1.988 | 14.766 | 0.102788 | 7/15/2014 | 40:58.3 | 0 |
| -540.541 | 19735.381 | 1.986 | 14.756 | 0.0871407 | 7/15/2014 | 40:58.4 | 0 |
| -540.302 | 19735.354 | 1.986 | 14.751 | 0.0960173 | 7/15/2014 | 40:58.5 | 0 |
| -540.063 | 19735.328 | 1.988 | 14.748 | 0.0903187 | 7/15/2014 | 40:58.6 | 0 |
| -539.764 | 19735.296 | 1.986 | 14.747 | 0.0874687 | 7/15/2014 | 40:58.7 | 0 |
| -539.643 | 19735.282 | 1.986 | 14.746 | 0.0839474 | 7/15/2014 | 40:58.8 | 0 |
| -539.399 | 19735.256 | 1.988 | 14.749 | 0.0670735 | 7/15/2014 | 40:58.9 | 0 |
| -539.157 | 19735.229 | 1.982 | 14.754 | 0.0607226 | 7/15/2014 | 40:59.0 | 0 |
| -538.913 | 19735.204 | 1.982 | 14.76 | 0.0612321 | 7/15/2014 | 40:59.1 | 0 |
| -538.67 | 19735.178 | 1.979 | 14.768 | 0.0501451 | 7/15/2014 | 40:59.2 | 0 |
| -538.427 | 19735.15 | 1.979 | 14.776 | 0.0459307 | 7/15/2014 | 40:59.3 | 0 |
| -538.003 | 19735.106 | 1.977 | 14.79 | 0.0381415 | 7/15/2014 | 40:59.4 | 0 |
| -537.761 | 19735.081 | 1.98 | 14.8 | 0.0497972 | 7/15/2014 | 40:59.5 | 0 |
| -537.517 | 19735.055 | 1.983 | 14.809 | 0.0533114 | 7/15/2014 | 40:59.6 | 0 |
| -537.215 | 19735.022 | 1.985 | 14.818 | 0.0379137 | 7/15/2014 | 40:59.7 | 0 |
| -537.092 | 19735.01 | 1.987 | 14.823 | 0.0654964 | 7/15/2014 | 40:59.8 | 0 |
| -536.847 | 19734.984 | 1.993 | 14.831 | 0.0442908 | 7/15/2014 | 40:59.9 | 0 |
| -536.604 | 19734.96 | 1.993 | 14.839 | 0.0696185 | 7/15/2014 | 41:00.0 | 0 |
| -536.358 | 19734.934 | 1.999 | 14.845 | 0.0703926 | 7/15/2014 | 41:00.1 | 0 |
| -536.116 | 19734.908 | 1.995 | 14.851 | 0.0536642 | 7/15/2014 | 41:00.2 | 0 |
| -535.87 | 19734.883 | 1.999 | 14.857 | 0.0777366 | 7/15/2014 | 41:00.3 | 0 |
| -535.448 | 19734.838 | 1.991 | 14.862 | 0.0685892 | 7/15/2014 | 41:00.4 | 0 |
| -535.205 | 19734.813 | 1.989 | 14.865 | 0.066489 | 7/15/2014 | 41:00.5 | 0 |
| -534.965 | 19734.788 | 1.986 | 14.867 | 0.0728488 | 7/15/2014 | 41:00.6 | 0 |
| -534.663 | 19734.758 | 1.986 | 14.869 | 0.0722051 | 7/15/2014 | 41:00.7 | 0 |
| -534.542 | 19734.745 | 1.982 | 14.87 | 0.0856682 | 7/15/2014 | 41:00.8 | 0 |
| -534.298 | 19734.721 | 1.984 | 14.871 | 0.079028 | 7/15/2014 | 41:00.9 | 0 |
| -534.059 | 19734.696 | 1.978 | 14.871 | 0.0982659 | 7/15/2014 | 41:01.0 | 0 |
| -533.817 | 19734.671 | 1.98 | 14.873 | 0.0905836 | 7/15/2014 | 41:01.1 | 0 |
| -533.576 | 19734.646 | 1.978 | 14.875 | 0.0964206 | 7/15/2014 | 41:01.2 | 0 |
| -533.337 | 19734.621 | 1.978 | 14.878 | 0.0914211 | 7/15/2014 | 41:01.3 | 0 |
| -532.92 | 19734.579 | 1.972 | 14.891 | 0.0909671 | 7/15/2014 | 41:01.4 | 0 |
| -532.682 | 19734.557 | 1.969 | 14.903 | 0.0894943 | 7/15/2014 | 41:01.5 | 0 |
| -532.447 | 19734.532 | 1.968 | 14.919 | 0.0821466 | 7/15/2014 | 41:01.6 | 0 |
| -532.152 | 19734.503 | 1.965 | 14.94 | 0.0981575 | 7/15/2014 | 41:01.7 | 0 |
| -532.032 | 19734.49 | 1.962 | 14.949 | 0.0880742 | 7/15/2014 | 41:01.8 | 0 |
| -531.795 | 19734.467 | 1.962 | 14.978 | 0.103547 | 7/15/2014 | 41:01.9 | 0 |

| | | | | | | | |
|---|---|---|---|---|---|---|---|
| -531.559 | 19734.443 | 1.958 | 15.014 | 0.0841971 | 7/15/2014 | 41:02.0 | 0 |
| -531.321 | 19734.419 | 1.961 | 15.054 | 0.0970095 | 7/15/2014 | 41:02.1 | 0 |
| -531.084 | 19734.396 | 1.961 | 15.098 | 0.104085 | 7/15/2014 | 41:02.2 | 0 |
| -530.848 | 19734.372 | 1.961 | 15.145 | 0.113313 | 7/15/2014 | 41:02.3 | 0 |
| -530.437 | 19734.333 | 1.959 | 15.21 | 0.10573 | 7/15/2014 | 41:02.4 | 0 |
| -530.202 | 19734.31 | 1.963 | 15.249 | 0.111579 | 7/15/2014 | 41:02.5 | 0 |
| -529.966 | 19734.287 | 1.963 | 15.284 | 0.111976 | 7/15/2014 | 41:02.6 | 0 |
| -529.671 | 19734.258 | 1.963 | 15.312 | 0.105094 | 7/15/2014 | 41:02.7 | 0 |
| -529.552 | 19734.246 | 1.962 | 15.329 | 0.0948178 | 7/15/2014 | 41:02.8 | 0 |
| -529.314 | 19734.223 | 1.962 | 15.345 | 0.101156 | 7/15/2014 | 41:02.9 | 0 |
| -529.076 | 19734.2 | 1.963 | 15.354 | 0.0959093 | 7/15/2014 | 41:03.0 | 0 |
| -528.835 | 19734.177 | 1.965 | 15.357 | 0.100066 | 7/15/2014 | 41:03.1 | 0 |
| -528.594 | 19734.154 | 1.965 | 15.353 | 0.096174 | 7/15/2014 | 41:03.2 | 0 |
| -528.354 | 19734.132 | 1.968 | 15.344 | 0.0916757 | 7/15/2014 | 41:03.3 | 0 |
| -527.932 | 19734.091 | 1.968 | 15.321 | 0.0944611 | 7/15/2014 | 41:03.4 | 0 |
| -527.69 | 19734.068 | 1.971 | 15.304 | 0.0929899 | 7/15/2014 | 41:03.5 | 0 |
| -527.448 | 19734.046 | 1.971 | 15.286 | 0.090958 | 7/15/2014 | 41:03.6 | 0 |
| -527.143 | 19734.016 | 1.973 | 15.267 | 0.0933789 | 7/15/2014 | 41:03.7 | 0 |
| -527.021 | 19734.005 | 1.971 | 15.257 | 0.093214 | 7/15/2014 | 41:03.8 | 0 |
| -526.775 | 19733.981 | 1.971 | 15.239 | 0.0918079 | 7/15/2014 | 41:03.9 | 0 |
| -526.529 | 19733.957 | 1.967 | 15.223 | 0.0969876 | 7/15/2014 | 41:04.0 | 0 |
| -526.279 | 19733.935 | 1.97 | 15.21 | 0.0984498 | 7/15/2014 | 41:04.1 | 0 |
| -526.033 | 19733.912 | 1.97 | 15.2 | 0.102489 | 7/15/2014 | 41:04.2 | 0 |
| -525.785 | 19733.889 | 1.97 | 15.196 | 0.110424 | 7/15/2014 | 41:04.3 | 0 |
| -525.477 | 19733.86 | 1.97 | 15.196 | 0.108819 | 7/15/2014 | 41:04.4 | 0 |
| -525.229 | 19733.837 | 1.97 | 15.202 | 0.114728 | 7/15/2014 | 41:04.5 | 0 |
| -524.981 | 19733.814 | 1.97 | 15.214 | 0.119249 | 7/15/2014 | 41:04.6 | 0 |
| -524.731 | 19733.791 | 1.973 | 15.232 | 0.11428 | 7/15/2014 | 41:04.7 | 0 |
| -524.424 | 19733.762 | 1.967 | 15.254 | 0.114409 | 7/15/2014 | 41:04.8 | 0 |
| -524.174 | 19733.74 | 1.967 | 15.281 | 0.117514 | 7/15/2014 | 41:04.9 | 0 |
| -523.925 | 19733.718 | 1.967 | 15.311 | 0.112787 | 7/15/2014 | 41:05.0 | 0 |
| -523.676 | 19733.694 | 1.967 | 15.342 | 0.11525 | 7/15/2014 | 41:05.1 | 0 |
| -523.428 | 19733.673 | 1.964 | 15.373 | 0.122215 | 7/15/2014 | 41:05.2 | 0 |
| -523.18 | 19733.649 | 1.964 | 15.402 | 0.116658 | 7/15/2014 | 41:05.3 | 0 |
| -522.75 | 19733.609 | 1.959 | 15.436 | 0.110151 | 7/15/2014 | 41:05.4 | 0 |
| -522.504 | 19733.59 | 1.959 | 15.456 | 0.111586 | 7/15/2014 | 41:05.5 | 0 |
| -522.258 | 19733.566 | 1.959 | 15.47 | 0.108569 | 7/15/2014 | 41:05.6 | 0 |
| -521.953 | 19733.539 | 1.954 | 15.479 | 0.101518 | 7/15/2014 | 41:05.7 | 0 |
| -521.83 | 19733.528 | 1.952 | 15.485 | 0.0997331 | 7/15/2014 | 41:05.8 | 0 |
| -521.582 | 19733.506 | 1.954 | 15.487 | 0.0979328 | 7/15/2014 | 41:05.9 | 0 |
| -521.338 | 19733.484 | 1.948 | 15.483 | 0.10501 | 7/15/2014 | 41:06.0 | 0 |
| -521.092 | 19733.461 | 1.951 | 15.473 | 0.100272 | 7/15/2014 | 41:06.1 | 0 |
| -520.85 | 19733.441 | 1.945 | 15.458 | 0.100556 | 7/15/2014 | 41:06.2 | 0 |
| -520.605 | 19733.418 | 1.944 | 15.439 | 0.09943 | 7/15/2014 | 41:06.3 | 0 |
| -520.185 | 19733.383 | 1.941 | 15.399 | 0.111298 | 7/15/2014 | 41:06.4 | 0 |
| -519.945 | 19733.361 | 1.941 | 15.369 | 0.11781 | 7/15/2014 | 41:06.5 | 0 |
| -519.707 | 19733.34 | 1.941 | 15.337 | 0.133517 | 7/15/2014 | 41:06.6 | 0 |

| | | | | | | | |
|---|---|---|---|---|---|---|---|
| -519.41 | 19733.313 | 1.941 | 15.303 | 0.138978 | 7/15/2014 | 41:06.7 | 0 |
| -519.29 | 19733.304 | 1.941 | 15.286 | 0.138803 | 7/15/2014 | 41:06.8 | 0 |
| -519.05 | 19733.281 | 1.947 | 15.25 | 0.141143 | 7/15/2014 | 41:06.9 | 0 |
| -518.814 | 19733.262 | 1.945 | 15.214 | 0.121871 | 7/15/2014 | 41:07.0 | 0 |
| -518.577 | 19733.242 | 1.948 | 15.179 | 0.150757 | 7/15/2014 | 41:07.1 | 0 |
| -518.341 | 19733.222 | 1.942 | 15.145 | 0.131397 | 7/15/2014 | 41:07.2 | 0 |
| -518.105 | 19733.201 | 1.946 | 15.113 | 0.118326 | 7/15/2014 | 41:07.3 | 0 |
| -517.694 | 19733.165 | 1.94 | 15.072 | 0.119089 | 7/15/2014 | 41:07.4 | 0 |
| -517.46 | 19733.145 | 1.938 | 15.049 | 0.126641 | 7/15/2014 | 41:07.5 | 0 |
| -517.226 | 19733.125 | 1.938 | 15.03 | 0.11747 | 7/15/2014 | 41:07.6 | 0 |
| -516.933 | 19733.102 | 1.941 | 15.017 | 0.120106 | 7/15/2014 | 41:07.7 | 0 |
| -516.815 | 19733.092 | 1.94 | 15.008 | 0.12861 | 7/15/2014 | 41:07.8 | 0 |
| -516.577 | 19733.072 | 1.942 | 15.004 | 0.113511 | 7/15/2014 | 41:07.9 | 0 |
| -516.341 | 19733.05 | 1.939 | 15.006 | 0.113269 | 7/15/2014 | 41:08.0 | 0 |
| -516.1 | 19733.029 | 1.941 | 15.017 | 0.115892 | 7/15/2014 | 41:08.1 | 0 |
| -515.864 | 19733.01 | 1.942 | 15.036 | 0.101629 | 7/15/2014 | 41:08.2 | 0 |
| -515.624 | 19732.99 | 1.945 | 15.063 | 0.117168 | 7/15/2014 | 41:08.3 | 0 |
| -515.206 | 19732.957 | 1.94 | 15.121 | 0.0894239 | 7/15/2014 | 41:08.4 | 0 |
| -514.965 | 19732.936 | 1.94 | 15.162 | 0.103729 | 7/15/2014 | 41:08.5 | 0 |
| -514.726 | 19732.914 | 1.94 | 15.206 | 0.0790339 | 7/15/2014 | 41:08.6 | 0 |
| -514.424 | 19732.891 | 1.944 | 15.251 | 0.06788 | 7/15/2014 | 41:08.7 | 0 |
| -514.303 | 19732.881 | 1.944 | 15.275 | 0.0600044 | 7/15/2014 | 41:08.8 | 0 |
| -514.059 | 19732.859 | 1.948 | 15.317 | 0.0592525 | 7/15/2014 | 41:08.9 | 0 |
| -513.816 | 19732.84 | 1.948 | 15.354 | 0.054227 | 7/15/2014 | 41:09.0 | 0 |
| -513.571 | 19732.82 | 1.954 | 15.383 | 0.0491574 | 7/15/2014 | 41:09.1 | 0 |
| -513.326 | 19732.799 | 1.954 | 15.403 | 0.0448497 | 7/15/2014 | 41:09.2 | 0 |
| -513.08 | 19732.777 | 1.956 | 15.415 | 0.0410984 | 7/15/2014 | 41:09.3 | 0 |
| -512.652 | 19732.744 | 1.957 | 15.415 | 0.0335539 | 7/15/2014 | 41:09.4 | 0 |
| -512.408 | 19732.723 | 1.951 | 15.41 | 0.0249724 | 7/15/2014 | 41:09.5 | 0 |
| -512.162 | 19732.703 | 1.947 | 15.4 | 0.0342308 | 7/15/2014 | 41:09.6 | 0 |
| -511.855 | 19732.678 | 1.95 | 15.386 | 0.0317652 | 7/15/2014 | 41:09.7 | 0 |
| -511.731 | 19732.668 | 1.947 | 15.379 | 0.0242621 | 7/15/2014 | 41:09.8 | 0 |
| -511.484 | 19732.646 | 1.947 | 15.36 | 0.0304694 | 7/15/2014 | 41:09.9 | 0 |
| -511.236 | 19732.625 | 1.945 | 15.342 | 0.0346295 | 7/15/2014 | 41:10.0 | 0 |
| -510.989 | 19732.605 | 1.941 | 15.323 | 0.0520354 | 7/15/2014 | 41:10.1 | 0 |
| -510.742 | 19732.586 | 1.941 | 15.306 | 0.05633 | 7/15/2014 | 41:10.2 | 0 |
| -510.495 | 19732.564 | 1.947 | 15.29 | 0.0591673 | 7/15/2014 | 41:10.3 | 0 |
| -510.065 | 19732.529 | 1.948 | 15.271 | 0.0690247 | 7/15/2014 | 41:10.4 | 0 |
| -509.821 | 19732.509 | 1.948 | 15.259 | 0.0663731 | 7/15/2014 | 41:10.5 | 0 |
| -509.577 | 19732.488 | 1.948 | 15.249 | 0.0756215 | 7/15/2014 | 41:10.6 | 0 |
| -509.275 | 19732.463 | 1.948 | 15.241 | 0.0725193 | 7/15/2014 | 41:10.7 | 0 |
| -509.15 | 19732.453 | 1.951 | 15.236 | 0.0713979 | 7/15/2014 | 41:10.8 | 0 |
| -508.907 | 19732.432 | 1.947 | 15.23 | 0.0842649 | 7/15/2014 | 41:10.9 | 0 |
| -508.665 | 19732.412 | 1.944 | 15.224 | 0.079019 | 7/15/2014 | 41:11.0 | 0 |
| -508.423 | 19732.393 | 1.94 | 15.219 | 0.0801056 | 7/15/2014 | 41:11.1 | 0 |
| -508.182 | 19732.373 | 1.936 | 15.215 | 0.08153 | 7/15/2014 | 41:11.2 | 0 |
| -507.942 | 19732.354 | 1.933 | 15.212 | 0.0794841 | 7/15/2014 | 41:11.3 | 0 |

| | | | | | | | |
|---|---|---|---|---|---|---|---|
| -507.526 | 19732.318 | 1.932 | 15.209 | 0.0819094 | 7/15/2014 | 41:11.4 | 0 |
| -507.29 | 19732.299 | 1.929 | 15.209 | 0.0786812 | 7/15/2014 | 41:11.5 | 0 |
| -507.053 | 19732.279 | 1.934 | 15.21 | 0.0710003 | 7/15/2014 | 41:11.6 | 0 |
| -506.76 | 19732.256 | 1.937 | 15.213 | 0.0810019 | 7/15/2014 | 41:11.7 | 0 |
| -506.64 | 19732.246 | 1.939 | 15.214 | 0.0742352 | 7/15/2014 | 41:11.8 | 0 |
| -506.404 | 19732.227 | 1.942 | 15.221 | 0.0662398 | 7/15/2014 | 41:11.9 | 0 |
| -506.167 | 19732.207 | 1.946 | 15.231 | 0.0688706 | 7/15/2014 | 41:12.0 | 0 |
| -505.93 | 19732.188 | 1.952 | 15.244 | 0.0665434 | 7/15/2014 | 41:12.1 | 0 |
| -505.693 | 19732.168 | 1.949 | 15.261 | 0.069489 | 7/15/2014 | 41:12.2 | 0 |
| -505.456 | 19732.148 | 1.952 | 15.279 | 0.0745665 | 7/15/2014 | 41:12.3 | 0 |
| -505.043 | 19732.115 | 1.945 | 15.312 | 0.0917847 | 7/15/2014 | 41:12.4 | 0 |
| -504.806 | 19732.097 | 1.945 | 15.334 | 0.0854475 | 7/15/2014 | 41:12.5 | 0 |
| -504.569 | 19732.078 | 1.945 | 15.356 | 0.105647 | 7/15/2014 | 41:12.6 | 0 |
| -504.269 | 19732.053 | 1.941 | 15.377 | 0.11814 | 7/15/2014 | 41:12.7 | 0 |
| -504.15 | 19732.043 | 1.938 | 15.388 | 0.123826 | 7/15/2014 | 41:12.8 | 0 |
| -503.908 | 19732.023 | 1.938 | 15.407 | 0.104476 | 7/15/2014 | 41:12.9 | 0 |
| -503.666 | 19732.004 | 1.934 | 15.424 | 0.117397 | 7/15/2014 | 41:13.0 | 0 |
| -503.421 | 19731.984 | 1.936 | 15.438 | 0.0841956 | 7/15/2014 | 41:13.1 | 0 |
| -503.176 | 19731.966 | 1.936 | 15.45 | 0.108476 | 7/15/2014 | 41:13.2 | 0 |
| -502.931 | 19731.946 | 1.936 | 15.459 | 0.0931932 | 7/15/2014 | 41:13.3 | 0 |
| -502.501 | 19731.91 | 1.935 | 15.464 | 0.0749084 | 7/15/2014 | 41:13.4 | 0 |
| -502.38 | 19731.9 | 1.936 | 15.467 | 0.0789878 | 7/15/2014 | 41:13.5 | 0 |
| -502.133 | 19731.881 | 1.936 | 15.467 | 0.0637529 | 7/15/2014 | 41:13.6 | 0 |
| -501.704 | 19731.847 | 1.935 | 15.458 | 0.0912714 | 7/15/2014 | 41:13.7 | 0 |
| -501.578 | 19731.837 | 1.936 | 15.456 | 0.0791307 | 7/15/2014 | 41:13.8 | 0 |
| -501.331 | 19731.815 | 1.933 | 15.445 | 0.0876171 | 7/15/2014 | 41:13.9 | 0 |
| -501.084 | 19731.795 | 1.929 | 15.43 | 0.109615 | 7/15/2014 | 41:14.0 | 0 |
| -500.837 | 19731.775 | 1.927 | 15.412 | 0.103881 | 7/15/2014 | 41:14.1 | 0 |
| -500.591 | 19731.756 | 1.923 | 15.391 | 0.103718 | 7/15/2014 | 41:14.2 | 0 |
| -500.346 | 19731.736 | 1.923 | 15.368 | 0.106381 | 7/15/2014 | 41:14.3 | 0 |
| -499.919 | 19731.702 | 1.921 | 15.33 | 0.148583 | 7/15/2014 | 41:14.4 | 0 |
| -499.677 | 19731.682 | 1.925 | 15.304 | 0.104254 | 7/15/2014 | 41:14.5 | 0 |
| -499.434 | 19731.662 | 1.93 | 15.279 | 0.0948098 | 7/15/2014 | 41:14.6 | 0 |
| -499.134 | 19731.639 | 1.93 | 15.255 | 0.133769 | 7/15/2014 | 41:14.7 | 0 |
| -499.011 | 19731.629 | 1.933 | 15.243 | 0.137106 | 7/15/2014 | 41:14.8 | 0 |
| -498.768 | 19731.609 | 1.939 | 15.222 | 0.129445 | 7/15/2014 | 41:14.9 | 0 |
| -498.526 | 19731.59 | 1.939 | 15.205 | 0.131832 | 7/15/2014 | 41:15.0 | 0 |
| -498.284 | 19731.57 | 1.941 | 15.193 | 0.123337 | 7/15/2014 | 41:15.1 | 0 |
| -498.042 | 19731.551 | 1.941 | 15.187 | 0.160024 | 7/15/2014 | 41:15.2 | 0 |
| -497.8 | 19731.531 | 1.944 | 15.187 | 0.175971 | 7/15/2014 | 41:15.3 | 0 |
| -497.381 | 19731.496 | 1.942 | 15.205 | 0.141724 | 7/15/2014 | 41:15.4 | 0 |
| -497.141 | 19731.477 | 1.946 | 15.224 | 0.149889 | 7/15/2014 | 41:15.5 | 0 |
| -496.902 | 19731.457 | 1.946 | 15.249 | 0.142164 | 7/15/2014 | 41:15.6 | 0 |
| -496.603 | 19731.434 | 1.948 | 15.28 | 0.0980831 | 7/15/2014 | 41:15.7 | 0 |
| -496.482 | 19731.424 | 1.951 | 15.294 | 0.103463 | 7/15/2014 | 41:15.8 | 0 |
| -496.24 | 19731.404 | 1.957 | 15.332 | 0.0943725 | 7/15/2014 | 41:15.9 | 0 |
| -496 | 19731.384 | 1.953 | 15.371 | 0.122114 | 7/15/2014 | 41:16.0 | 0 |

| | | | | | | | |
|---|---|---|---|---|---|---|---|
| -495.759 | 19731.363 | 1.956 | 15.409 | 0.104196 | 7/15/2014 | 41:16.1 | 0 |
| -495.519 | 19731.342 | 1.956 | 15.444 | 0.130043 | 7/15/2014 | 41:16.2 | 0 |
| -495.277 | 19731.322 | 1.961 | 15.474 | 0.109137 | 7/15/2014 | 41:16.3 | 0 |
| -494.857 | 19731.289 | 1.959 | 15.507 | 0.150721 | 7/15/2014 | 41:16.4 | 0 |
| -494.618 | 19731.27 | 1.963 | 15.525 | 0.161312 | 7/15/2014 | 41:16.5 | 0 |
| -494.379 | 19731.25 | 1.963 | 15.538 | 0.15686 | 7/15/2014 | 41:16.6 | 0 |
| -494.08 | 19731.225 | 1.966 | 15.546 | 0.159517 | 7/15/2014 | 41:16.7 | 0 |
| -493.959 | 19731.213 | 1.966 | 15.551 | 0.143098 | 7/15/2014 | 41:16.8 | 0 |
| -493.718 | 19731.193 | 1.97 | 15.553 | 0.193708 | 7/15/2014 | 41:16.9 | 0 |
| -493.478 | 19731.174 | 1.97 | 15.552 | 0.195418 | 7/15/2014 | 41:17.0 | 0 |
| -493.236 | 19731.154 | 1.975 | 15.549 | 0.178346 | 7/15/2014 | 41:17.1 | 0 |
| -492.995 | 19731.133 | 1.978 | 15.543 | 0.169046 | 7/15/2014 | 41:17.2 | 0 |
| -492.755 | 19731.113 | 1.98 | 15.534 | 0.182253 | 7/15/2014 | 41:17.3 | 0 |
| -492.335 | 19731.078 | 1.986 | 15.515 | 0.180935 | 7/15/2014 | 41:17.4 | 0 |
| -492.096 | 19731.057 | 1.988 | 15.499 | 0.176761 | 7/15/2014 | 41:17.5 | 0 |
| -491.857 | 19731.037 | 1.991 | 15.482 | 0.164001 | 7/15/2014 | 41:17.6 | 0 |
| -491.557 | 19731.012 | 1.991 | 15.462 | 0.163909 | 7/15/2014 | 41:17.7 | 0 |
| -491.436 | 19731 | 1.992 | 15.452 | 0.161104 | 7/15/2014 | 41:17.8 | 0 |
| -491.194 | 19730.98 | 1.992 | 15.429 | 0.187296 | 7/15/2014 | 41:17.9 | 0 |
| -490.952 | 19730.961 | 1.996 | 15.406 | 0.144436 | 7/15/2014 | 41:18.0 | 0 |
| -490.709 | 19730.939 | 2 | 15.382 | 0.152718 | 7/15/2014 | 41:18.1 | 0 |
| -490.467 | 19730.918 | 1.999 | 15.358 | 0.138546 | 7/15/2014 | 41:18.2 | 0 |
| -490.224 | 19730.898 | 2.003 | 15.337 | 0.145161 | 7/15/2014 | 41:18.3 | 0 |
| -489.801 | 19730.861 | 2.004 | 15.311 | 0.146356 | 7/15/2014 | 41:18.4 | 0 |
| -489.562 | 19730.841 | 2.007 | 15.298 | 0.125912 | 7/15/2014 | 41:18.5 | 0 |
| -489.32 | 19730.82 | 2.013 | 15.289 | 0.146472 | 7/15/2014 | 41:18.6 | 0 |
| -489.02 | 19730.793 | 2.01 | 15.285 | 0.125753 | 7/15/2014 | 41:18.7 | 0 |
| -488.898 | 19730.783 | 2.01 | 15.282 | 0.122284 | 7/15/2014 | 41:18.8 | 0 |
| -488.654 | 19730.764 | 2.013 | 15.286 | 0.140649 | 7/15/2014 | 41:18.9 | 0 |
| -488.414 | 19730.74 | 2.013 | 15.296 | 0.127841 | 7/15/2014 | 41:19.0 | 0 |
| -488.169 | 19730.721 | 2.021 | 15.313 | 0.114036 | 7/15/2014 | 41:19.1 | 0 |
| -487.928 | 19730.7 | 2.013 | 15.334 | 0.117726 | 7/15/2014 | 41:19.2 | 0 |
| -487.685 | 19730.678 | 2.017 | 15.36 | 0.114087 | 7/15/2014 | 41:19.3 | 0 |
| -487.264 | 19730.641 | 2.016 | 15.403 | 0.135494 | 7/15/2014 | 41:19.4 | 0 |
| -487.023 | 19730.621 | 2.016 | 15.43 | 0.12461 | 7/15/2014 | 41:19.5 | 0 |
| -486.782 | 19730.598 | 2.016 | 15.456 | 0.126002 | 7/15/2014 | 41:19.6 | 0 |
| -486.48 | 19730.572 | 2.016 | 15.48 | 0.136324 | 7/15/2014 | 41:19.7 | 0 |
| -486.359 | 19730.563 | 2.013 | 15.493 | 0.12375 | 7/15/2014 | 41:19.8 | 0 |
| -486.113 | 19730.541 | 2.023 | 15.51 | 0.140423 | 7/15/2014 | 41:19.9 | 0 |
| -485.872 | 19730.519 | 2.016 | 15.523 | 0.156526 | 7/15/2014 | 41:20.0 | 0 |
| -485.625 | 19730.498 | 2.02 | 15.531 | 0.141384 | 7/15/2014 | 41:20.1 | 0 |
| -485.383 | 19730.477 | 2.012 | 15.535 | 0.142982 | 7/15/2014 | 41:20.2 | 0 |
| -485.136 | 19730.455 | 2.02 | 15.536 | 0.160728 | 7/15/2014 | 41:20.3 | 0 |
| -484.71 | 19730.417 | 2.015 | 15.532 | 0.165989 | 7/15/2014 | 41:20.4 | 0 |
| -484.467 | 19730.396 | 2.012 | 15.528 | 0.195947 | 7/15/2014 | 41:20.5 | 0 |
| -484.223 | 19730.375 | 2.01 | 15.523 | 0.176758 | 7/15/2014 | 41:20.6 | 0 |
| -483.918 | 19730.349 | 2.01 | 15.519 | 0.179863 | 7/15/2014 | 41:20.7 | 0 |

| | | | | | | | |
|---|---|---|---|---|---|---|---|
| -483.795 | 19730.337 | 2.006 | 15.516 | 0.176658 | 7/15/2014 | 41:20.8 | 0 |
| -483.547 | 19730.316 | 2.016 | 15.512 | 0.190486 | 7/15/2014 | 41:20.9 | 0 |
| -483.303 | 19730.293 | 2.012 | 15.509 | 0.1922 | 7/15/2014 | 41:21.0 | 0 |
| -483.054 | 19730.272 | 2.016 | 15.508 | 0.190881 | 7/15/2014 | 41:21.1 | 0 |
| -482.811 | 19730.25 | 2.009 | 15.51 | 0.168427 | 7/15/2014 | 41:21.2 | 0 |
| -482.563 | 19730.229 | 2.012 | 15.514 | 0.184749 | 7/15/2014 | 41:21.3 | 0 |
| -482.136 | 19730.19 | 2.014 | 15.529 | 0.186 | 7/15/2014 | 41:21.4 | 0 |
| -481.891 | 19730.169 | 2.014 | 15.54 | 0.173525 | 7/15/2014 | 41:21.5 | 0 |
| -481.647 | 19730.146 | 2.009 | 15.554 | 0.174491 | 7/15/2014 | 41:21.6 | 0 |
| -481.341 | 19730.121 | 2.006 | 15.57 | 0.167655 | 7/15/2014 | 41:21.7 | 0 |
| -481.219 | 19730.109 | 2.005 | 15.578 | 0.169021 | 7/15/2014 | 41:21.8 | 0 |
| -480.97 | 19730.086 | 2.009 | 15.596 | 0.162149 | 7/15/2014 | 41:21.9 | 0 |
| -480.726 | 19730.064 | 2.005 | 15.615 | 0.157991 | 7/15/2014 | 41:22.0 | 0 |
| -480.478 | 19730.043 | 2.006 | 15.633 | 0.156312 | 7/15/2014 | 41:22.1 | 0 |
| -480.234 | 19730.021 | 1.999 | 15.649 | 0.157263 | 7/15/2014 | 41:22.2 | 0 |
| -479.987 | 19729.998 | 2.003 | 15.663 | 0.155689 | 7/15/2014 | 41:22.3 | 0 |
| -479.562 | 19729.961 | 2.007 | 15.677 | 0.146234 | 7/15/2014 | 41:22.4 | 0 |
| -479.319 | 19729.938 | 2.007 | 15.683 | 0.141787 | 7/15/2014 | 41:22.5 | 0 |
| -479.079 | 19729.916 | 2.007 | 15.685 | 0.139268 | 7/15/2014 | 41:22.6 | 0 |
| -478.777 | 19729.89 | 2.01 | 15.683 | 0.136191 | 7/15/2014 | 41:22.7 | 0 |
| -478.654 | 19729.878 | 2.006 | 15.683 | 0.138287 | 7/15/2014 | 41:22.8 | 0 |
| -478.411 | 19729.855 | 2.01 | 15.676 | 0.133126 | 7/15/2014 | 41:22.9 | 0 |
| -478.171 | 19729.833 | 2.006 | 15.665 | 0.132105 | 7/15/2014 | 41:23.0 | 0 |
| -477.929 | 19729.813 | 2.006 | 15.651 | 0.133388 | 7/15/2014 | 41:23.1 | 0 |
| -477.691 | 19729.791 | 1.999 | 15.635 | 0.136153 | 7/15/2014 | 41:23.2 | 0 |
| -477.453 | 19729.77 | 2.002 | 15.616 | 0.141073 | 7/15/2014 | 41:23.3 | 0 |
| -477.037 | 19729.731 | 2.003 | 15.584 | 0.139942 | 7/15/2014 | 41:23.4 | 0 |
| -476.802 | 19729.709 | 2 | 15.561 | 0.138889 | 7/15/2014 | 41:23.5 | 0 |
| -476.566 | 19729.688 | 2.003 | 15.539 | 0.135789 | 7/15/2014 | 41:23.6 | 0 |
| -476.274 | 19729.662 | 2.007 | 15.516 | 0.141048 | 7/15/2014 | 41:23.7 | 0 |
| -476.156 | 19729.651 | 2.006 | 15.504 | 0.138118 | 7/15/2014 | 41:23.8 | 0 |
| -475.919 | 19729.63 | 2.013 | 15.482 | 0.13655 | 7/15/2014 | 41:23.9 | 0 |
| -475.685 | 19729.607 | 2.013 | 15.462 | 0.138492 | 7/15/2014 | 41:24.0 | 0 |
| -475.448 | 19729.586 | 2.021 | 15.443 | 0.139333 | 7/15/2014 | 41:24.1 | 0 |
| -475.216 | 19729.564 | 2.017 | 15.428 | 0.143603 | 7/15/2014 | 41:24.2 | 0 |
| -474.98 | 19729.543 | 2.024 | 15.417 | 0.143642 | 7/15/2014 | 41:24.3 | 0 |
| -474.574 | 19729.507 | 2.019 | 15.413 | 0.146652 | 7/15/2014 | 41:24.4 | 0 |
| -474.341 | 19729.485 | 2.023 | 15.416 | 0.15029 | 7/15/2014 | 41:24.5 | 0 |
| -474.111 | 19729.464 | 2.025 | 15.425 | 0.152646 | 7/15/2014 | 41:24.6 | 0 |
| -473.821 | 19729.436 | 2.025 | 15.44 | 0.150572 | 7/15/2014 | 41:24.7 | 0 |
| -473.705 | 19729.426 | 2.023 | 15.446 | 0.149869 | 7/15/2014 | 41:24.8 | 0 |
| -473.47 | 19729.403 | 2.027 | 15.471 | 0.15123 | 7/15/2014 | 41:24.9 | 0 |
| -473.24 | 19729.383 | 2.02 | 15.5 | 0.156336 | 7/15/2014 | 41:25.0 | 0 |
| -473.004 | 19729.361 | 2.027 | 15.532 | 0.153037 | 7/15/2014 | 41:25.1 | 0 |
| -472.771 | 19729.34 | 2.027 | 15.565 | 0.152045 | 7/15/2014 | 41:25.2 | 0 |
| -472.538 | 19729.317 | 2.027 | 15.598 | 0.154169 | 7/15/2014 | 41:25.3 | 0 |
| -472.131 | 19729.279 | 2.023 | 15.64 | 0.155921 | 7/15/2014 | 41:25.4 | 0 |

| | | | | | | | |
|---|---|---|---|---|---|---|---|
| -471.898 | 19729.257 | 2.026 | 15.665 | 0.163682 | 7/15/2014 | 41:25.5 | 0 |
| -471.667 | 19729.236 | 2.026 | 15.686 | 0.166777 | 7/15/2014 | 41:25.6 | 0 |
| -471.375 | 19729.209 | 2.032 | 15.702 | 0.169704 | 7/15/2014 | 41:25.7 | 0 |
| -471.257 | 19729.198 | 2.032 | 15.712 | 0.167975 | 7/15/2014 | 41:25.8 | 0 |
| -471.022 | 19729.176 | 2.032 | 15.722 | 0.165194 | 7/15/2014 | 41:25.9 | 0 |
| -470.787 | 19729.153 | 2.032 | 15.728 | 0.177753 | 7/15/2014 | 41:26.0 | 0 |
| -470.551 | 19729.132 | 2.034 | 15.732 | 0.176989 | 7/15/2014 | 41:26.1 | 0 |
| -470.316 | 19729.108 | 2.034 | 15.733 | 0.170228 | 7/15/2014 | 41:26.2 | 0 |
| -470.079 | 19729.088 | 2.037 | 15.732 | 0.170626 | 7/15/2014 | 41:26.3 | 0 |
| -469.666 | 19729.048 | 2.037 | 15.73 | 0.154477 | 7/15/2014 | 41:26.4 | 0 |
| -469.429 | 19729.025 | 2.037 | 15.728 | 0.156936 | 7/15/2014 | 41:26.5 | 0 |
| -469.193 | 19729.003 | 2.037 | 15.727 | 0.147999 | 7/15/2014 | 41:26.6 | 0 |
| -468.895 | 19728.975 | 2.037 | 15.726 | 0.135433 | 7/15/2014 | 41:26.7 | 0 |
| -468.777 | 19728.963 | 2.034 | 15.726 | 0.130886 | 7/15/2014 | 41:26.8 | 0 |
| -468.536 | 19728.94 | 2.038 | 15.726 | 0.133887 | 7/15/2014 | 41:26.9 | 0 |
| -468.296 | 19728.917 | 2.032 | 15.728 | 0.144719 | 7/15/2014 | 41:27.0 | 0 |
| -468.053 | 19728.894 | 2.034 | 15.731 | 0.137425 | 7/15/2014 | 41:27.1 | 0 |
| -467.81 | 19728.869 | 2.034 | 15.734 | 0.149354 | 7/15/2014 | 41:27.2 | 0 |
| -467.568 | 19728.847 | 2.034 | 15.738 | 0.142475 | 7/15/2014 | 41:27.3 | 0 |
| -467.142 | 19728.806 | 2.03 | 15.744 | 0.160708 | 7/15/2014 | 41:27.4 | 0 |
| -466.9 | 19728.782 | 2.029 | 15.747 | 0.161519 | 7/15/2014 | 41:27.5 | 0 |
| -466.656 | 19728.758 | 2.031 | 15.75 | 0.161846 | 7/15/2014 | 41:27.6 | 0 |
| -466.349 | 19728.729 | 2.03 | 15.751 | 0.170792 | 7/15/2014 | 41:27.7 | 0 |
| -466.226 | 19728.716 | 2.027 | 15.753 | 0.161743 | 7/15/2014 | 41:27.8 | 0 |
| -465.977 | 19728.691 | 2.027 | 15.752 | 0.173766 | 7/15/2014 | 41:27.9 | 0 |
| -465.732 | 19728.668 | 2.023 | 15.749 | 0.17165 | 7/15/2014 | 41:28.0 | 0 |
| -465.481 | 19728.644 | 2.027 | 15.744 | 0.165022 | 7/15/2014 | 41:28.1 | 0 |
| -465.234 | 19728.62 | 2.021 | 15.736 | 0.169603 | 7/15/2014 | 41:28.2 | 0 |
| -464.986 | 19728.595 | 2.022 | 15.726 | 0.156922 | 7/15/2014 | 41:28.3 | 0 |
| -464.679 | 19728.565 | 2.018 | 15.712 | 0.190607 | 7/15/2014 | 41:28.4 | 0 |
| -464.43 | 19728.54 | 2.022 | 15.696 | 0.170818 | 7/15/2014 | 41:28.5 | 0 |
| -464.184 | 19728.516 | 2.022 | 15.678 | 0.17542 | 7/15/2014 | 41:28.6 | 0 |
| -463.756 | 19728.474 | 2.02 | 15.646 | 0.181852 | 7/15/2014 | 41:28.7 | 0 |
| -463.631 | 19728.462 | 2.022 | 15.636 | 0.176969 | 7/15/2014 | 41:28.8 | 0 |
| -463.383 | 19728.438 | 2.022 | 15.613 | 0.201816 | 7/15/2014 | 41:28.9 | 0 |
| -463.138 | 19728.413 | 2.022 | 15.59 | 0.178914 | 7/15/2014 | 41:29.0 | 0 |
| -462.892 | 19728.389 | 2.023 | 15.566 | 0.193598 | 7/15/2014 | 41:29.1 | 0 |
| -462.647 | 19728.364 | 2.02 | 15.543 | 0.169919 | 7/15/2014 | 41:29.2 | 0 |
| -462.405 | 19728.34 | 2.02 | 15.521 | 0.16919 | 7/15/2014 | 41:29.3 | 0 |
| -461.983 | 19728.299 | 2.022 | 15.493 | 0.165135 | 7/15/2014 | 41:29.4 | 0 |
| -461.742 | 19728.274 | 2.025 | 15.476 | 0.164959 | 7/15/2014 | 41:29.5 | 0 |
| -461.504 | 19728.251 | 2.028 | 15.463 | 0.149802 | 7/15/2014 | 41:29.6 | 0 |
| -461.207 | 19728.222 | 2.028 | 15.453 | 0.155741 | 7/15/2014 | 41:29.7 | 0 |
| -461.086 | 19728.21 | 2.027 | 15.447 | 0.156029 | 7/15/2014 | 41:29.8 | 0 |
| -460.848 | 19728.186 | 2.031 | 15.444 | 0.153451 | 7/15/2014 | 41:29.9 | 0 |
| -460.611 | 19728.162 | 2.031 | 15.446 | 0.146122 | 7/15/2014 | 41:30.0 | 0 |
| -460.374 | 19728.139 | 2.035 | 15.453 | 0.160654 | 7/15/2014 | 41:30.1 | 0 |

| | | | | | | | |
|---|---|---|---|---|---|---|---|
| -460.139 | 19728.114 | 2.031 | 15.466 | 0.139974 | 7/15/2014 | 41:30.2 | 0 |
| -459.904 | 19728.092 | 2.031 | 15.485 | 0.14602 | 7/15/2014 | 41:30.3 | 0 |
| -459.495 | 19728.051 | 2.025 | 15.526 | 0.152565 | 7/15/2014 | 41:30.4 | 0 |
| -459.264 | 19728.027 | 2.018 | 15.556 | 0.15173 | 7/15/2014 | 41:30.5 | 0 |
| -459.03 | 19728.004 | 2.018 | 15.587 | 0.134024 | 7/15/2014 | 41:30.6 | 0 |
| -458.738 | 19727.975 | 2.012 | 15.619 | 0.13132 | 7/15/2014 | 41:30.7 | 0 |
| -458.621 | 19727.963 | 2.009 | 15.636 | 0.129186 | 7/15/2014 | 41:30.8 | 0 |
| -458.383 | 19727.939 | 2.012 | 15.666 | 0.11869 | 7/15/2014 | 41:30.9 | 0 |
| -458.146 | 19727.915 | 2.009 | 15.692 | 0.116962 | 7/15/2014 | 41:31.0 | 0 |
| -457.907 | 19727.892 | 2.012 | 15.712 | 0.111833 | 7/15/2014 | 41:31.1 | 0 |
| -457.669 | 19727.867 | 2.012 | 15.727 | 0.111871 | 7/15/2014 | 41:31.2 | 0 |
| -457.428 | 19727.843 | 2.02 | 15.736 | 0.125033 | 7/15/2014 | 41:31.3 | 0 |
| -457.007 | 19727.801 | 2.018 | 15.738 | 0.126823 | 7/15/2014 | 41:31.4 | 0 |
| -456.764 | 19727.776 | 2.02 | 15.737 | 0.140875 | 7/15/2014 | 41:31.5 | 0 |
| -456.522 | 19727.751 | 2.016 | 15.734 | 0.139576 | 7/15/2014 | 41:31.6 | 0 |
| -456.216 | 19727.721 | 2.016 | 15.73 | 0.135818 | 7/15/2014 | 41:31.7 | 0 |
| -456.093 | 19727.708 | 2.014 | 15.728 | 0.135909 | 7/15/2014 | 41:31.8 | 0 |
| -455.847 | 19727.683 | 2.014 | 15.724 | 0.12135 | 7/15/2014 | 41:31.9 | 0 |
| -455.599 | 19727.656 | 2.008 | 15.721 | 0.12297 | 7/15/2014 | 41:32.0 | 0 |
| -455.351 | 19727.632 | 2.004 | 15.72 | 0.133859 | 7/15/2014 | 41:32.1 | 0 |
| -455.102 | 19727.606 | 2.004 | 15.72 | 0.12661 | 7/15/2014 | 41:32.2 | 0 |
| -454.853 | 19727.58 | 2.01 | 15.722 | 0.125509 | 7/15/2014 | 41:32.3 | 0 |
| -454.543 | 19727.549 | 2.01 | 15.723 | 0.136429 | 7/15/2014 | 41:32.4 | 0 |
| -454.293 | 19727.523 | 2.01 | 15.723 | 0.138915 | 7/15/2014 | 41:32.5 | 0 |
| -454.043 | 19727.496 | 2.014 | 15.722 | 0.160286 | 7/15/2014 | 41:32.6 | 0 |
| -453.792 | 19727.471 | 2.02 | 15.717 | 0.192476 | 7/15/2014 | 41:32.7 | 0 |
| -453.479 | 19727.438 | 2.024 | 15.709 | 0.181218 | 7/15/2014 | 41:32.8 | 0 |
| -453.226 | 19727.412 | 2.024 | 15.697 | 0.176089 | 7/15/2014 | 41:32.9 | 0 |
| -452.973 | 19727.386 | 2.016 | 15.681 | 0.183139 | 7/15/2014 | 41:33.0 | 0 |
| -452.718 | 19727.359 | 2.016 | 15.662 | 0.211444 | 7/15/2014 | 41:33.1 | 0 |
| -452.465 | 19727.332 | 2.012 | 15.641 | 0.175538 | 7/15/2014 | 41:33.2 | 0 |
| -452.211 | 19727.306 | 2.009 | 15.619 | 0.170723 | 7/15/2014 | 41:33.3 | 0 |
| -451.893 | 19727.271 | 2.005 | 15.597 | 0.14755 | 7/15/2014 | 41:33.4 | 0 |
| -451.639 | 19727.246 | 2.005 | 15.575 | 0.150164 | 7/15/2014 | 41:33.5 | 0 |
| -451.385 | 19727.219 | 1.999 | 15.556 | 0.174335 | 7/15/2014 | 41:33.6 | 0 |
| -451.127 | 19727.191 | 2.003 | 15.54 | 0.1178 | 7/15/2014 | 41:33.7 | 0 |
| -450.809 | 19727.156 | 1.999 | 15.527 | 0.119447 | 7/15/2014 | 41:33.8 | 0 |
| -450.55 | 19727.129 | 2.003 | 15.519 | 0.097388 | 7/15/2014 | 41:33.9 | 0 |
| -450.292 | 19727.102 | 1.997 | 15.516 | 0.124688 | 7/15/2014 | 41:34.0 | 0 |
| -450.032 | 19727.074 | 1.994 | 15.517 | 0.121381 | 7/15/2014 | 41:34.1 | 0 |
| -449.775 | 19727.045 | 1.984 | 15.524 | 0.0855166 | 7/15/2014 | 41:34.2 | 0 |
| -449.514 | 19727.018 | 1.991 | 15.537 | 0.114299 | 7/15/2014 | 41:34.3 | 0 |
| -449.195 | 19726.982 | 1.987 | 15.554 | 0.120861 | 7/15/2014 | 41:34.4 | 0 |
| -448.935 | 19726.953 | 1.991 | 15.577 | 0.111998 | 7/15/2014 | 41:34.5 | 0 |
| -448.68 | 19726.926 | 1.984 | 15.603 | 0.123527 | 7/15/2014 | 41:34.6 | 0 |
| -448.422 | 19726.898 | 1.987 | 15.631 | 0.132511 | 7/15/2014 | 41:34.7 | 0 |
| -448.103 | 19726.861 | 1.983 | 15.66 | 0.139165 | 7/15/2014 | 41:34.8 | 0 |

| | | | | | | | |
|---|---|---|---|---|---|---|---|
| -447.845 | 19726.834 | 1.983 | 15.69 | 0.139578 | 7/15/2014 | 41:34.9 | 0 |
| -447.59 | 19726.805 | 1.975 | 15.718 | 0.122869 | 7/15/2014 | 41:35.0 | 0 |
| -447.331 | 19726.775 | 1.969 | 15.743 | 0.14645 | 7/15/2014 | 41:35.1 | 0 |
| -447.077 | 19726.748 | 1.962 | 15.766 | 0.128563 | 7/15/2014 | 41:35.2 | 0 |
| -446.822 | 19726.719 | 1.965 | 15.785 | 0.126488 | 7/15/2014 | 41:35.3 | 0 |
| -446.506 | 19726.684 | 1.956 | 15.8 | 0.122743 | 7/15/2014 | 41:35.4 | 0 |
| -446.252 | 19726.654 | 1.953 | 15.812 | 0.100739 | 7/15/2014 | 41:35.5 | 0 |
| -446 | 19726.625 | 1.949 | 15.822 | 0.116682 | 7/15/2014 | 41:35.6 | 0 |
| -445.746 | 19726.598 | 1.951 | 15.828 | 0.10545 | 7/15/2014 | 41:35.7 | 0 |
| -445.43 | 19726.563 | 1.951 | 15.832 | 0.096523 | 7/15/2014 | 41:35.8 | 0 |
| -445.176 | 19726.531 | 1.954 | 15.834 | 0.103306 | 7/15/2014 | 41:35.9 | 0 |
| -444.921 | 19726.502 | 1.951 | 15.832 | 0.0978393 | 7/15/2014 | 41:36.0 | 0 |
| -444.665 | 19726.473 | 1.958 | 15.828 | 0.112728 | 7/15/2014 | 41:36.1 | 0 |
| -444.41 | 19726.443 | 1.961 | 15.821 | 0.10416 | 7/15/2014 | 41:36.2 | 0 |
| -444.154 | 19726.414 | 1.961 | 15.812 | 0.0948149 | 7/15/2014 | 41:36.3 | 0 |
| -443.835 | 19726.378 | 1.958 | 15.8 | 0.0942733 | 7/15/2014 | 41:36.4 | 0 |
| -443.577 | 19726.348 | 1.955 | 15.786 | 0.0936804 | 7/15/2014 | 41:36.5 | 0 |
| -443.319 | 19726.316 | 1.949 | 15.772 | 0.0930023 | 7/15/2014 | 41:36.6 | 0 |
| -443.06 | 19726.287 | 1.949 | 15.757 | 0.100263 | 7/15/2014 | 41:36.7 | 0 |
| -442.734 | 19726.248 | 1.946 | 15.742 | 0.0983573 | 7/15/2014 | 41:36.8 | 0 |
| -442.469 | 19726.217 | 1.946 | 15.73 | 0.100192 | 7/15/2014 | 41:36.9 | 0 |
| -442.203 | 19726.186 | 1.946 | 15.721 | 0.105138 | 7/15/2014 | 41:37.0 | 0 |
| -441.936 | 19726.154 | 1.946 | 15.714 | 0.106659 | 7/15/2014 | 41:37.1 | 0 |
| -441.668 | 19726.123 | 1.949 | 15.712 | 0.113864 | 7/15/2014 | 41:37.2 | 0 |
| -441.398 | 19726.091 | 1.947 | 15.715 | 0.112326 | 7/15/2014 | 41:37.3 | 0 |
| -441.062 | 19726.051 | 1.938 | 15.722 | 0.122687 | 7/15/2014 | 41:37.4 | 0 |
| -440.789 | 19726.02 | 1.938 | 15.733 | 0.112676 | 7/15/2014 | 41:37.5 | 0 |
| -440.515 | 19725.985 | 1.93 | 15.748 | 0.116504 | 7/15/2014 | 41:37.6 | 0 |
| -440.24 | 19725.953 | 1.923 | 15.767 | 0.112003 | 7/15/2014 | 41:37.7 | 0 |
| -439.895 | 19725.912 | 1.916 | 15.788 | 0.112495 | 7/15/2014 | 41:37.8 | 0 |
| -439.755 | 19725.896 | 1.91 | 15.799 | 0.111418 | 7/15/2014 | 41:37.8 | 0 |
| -439.477 | 19725.862 | 1.91 | 15.825 | 0.103298 | 7/15/2014 | 41:37.9 | 0 |
| -439.197 | 19725.83 | 1.91 | 15.851 | 0.104261 | 7/15/2014 | 41:38.0 | 0 |
| -438.916 | 19725.795 | 1.903 | 15.878 | 0.106038 | 7/15/2014 | 41:38.1 | 0 |
| -438.634 | 19725.762 | 1.9 | 15.906 | 0.107784 | 7/15/2014 | 41:38.2 | 0 |
| -438.139 | 19725.702 | 1.903 | 15.948 | 0.127157 | 7/15/2014 | 41:38.4 | 0 |
| -437.857 | 19725.669 | 1.906 | 15.975 | 0.130804 | 7/15/2014 | 41:38.5 | 0 |
| -437.576 | 19725.635 | 1.899 | 16.002 | 0.152905 | 7/15/2014 | 41:38.6 | 0 |
| -437.292 | 19725.6 | 1.899 | 16.027 | 0.155479 | 7/15/2014 | 41:38.7 | 0 |
| -436.94 | 19725.557 | 1.89 | 16.052 | 0.155278 | 7/15/2014 | 41:38.8 | 0 |
| -436.796 | 19725.54 | 1.89 | 16.065 | 0.162166 | 7/15/2014 | 41:38.8 | 0 |
| -436.513 | 19725.507 | 1.878 | 16.087 | 0.165515 | 7/15/2014 | 41:38.9 | 0 |
| -436.231 | 19725.473 | 1.871 | 16.107 | 0.16322 | 7/15/2014 | 41:39.0 | 0 |
| -435.95 | 19725.438 | 1.868 | 16.124 | 0.146757 | 7/15/2014 | 41:39.1 | 0 |
| -435.668 | 19725.402 | 1.868 | 16.138 | 0.150881 | 7/15/2014 | 41:39.2 | 0 |
| -435.179 | 19725.343 | 1.862 | 16.151 | 0.140382 | 7/15/2014 | 41:39.4 | 0 |
| -434.9 | 19725.311 | 1.866 | 16.155 | 0.132024 | 7/15/2014 | 41:39.5 | 0 |

| | | | | | | | |
|---|---|---|---|---|---|---|---|
| -434.624 | 19725.276 | 1.87 | 16.156 | 0.121676 | 7/15/2014 | 41:39.6 | 0 |
| -434.347 | 19725.242 | 1.867 | 16.152 | 0.120536 | 7/15/2014 | 41:39.7 | 0 |
| -434.005 | 19725.2 | 1.859 | 16.144 | 0.117088 | 7/15/2014 | 41:39.8 | 0 |
| -433.864 | 19725.184 | 1.856 | 16.14 | 0.115007 | 7/15/2014 | 41:39.8 | 0 |
| -433.592 | 19725.149 | 1.854 | 16.126 | 0.115124 | 7/15/2014 | 41:39.9 | 0 |
| -433.186 | 19725.101 | 1.85 | 16.099 | 0.121793 | 7/15/2014 | 41:40.1 | 0 |
| -432.916 | 19725.067 | 1.844 | 16.081 | 0.123086 | 7/15/2014 | 41:40.2 | 0 |
| -432.647 | 19725.035 | 1.844 | 16.062 | 0.13011 | 7/15/2014 | 41:40.3 | 0 |
| -432.314 | 19724.994 | 1.844 | 16.044 | 0.137989 | 7/15/2014 | 41:40.4 | 0 |
| -432.049 | 19724.962 | 1.846 | 16.029 | 0.143656 | 7/15/2014 | 41:40.5 | 0 |
| -431.786 | 19724.931 | 1.837 | 16.017 | 0.131927 | 7/15/2014 | 41:40.6 | 0 |
| -431.522 | 19724.897 | 1.839 | 16.009 | 0.131604 | 7/15/2014 | 41:40.7 | 0 |
| -431.197 | 19724.857 | 1.839 | 16.006 | 0.135701 | 7/15/2014 | 41:40.8 | 0 |
| -430.934 | 19724.826 | 1.845 | 16.009 | 0.136371 | 7/15/2014 | 41:40.9 | 0 |
| -430.673 | 19724.794 | 1.835 | 16.017 | 0.143965 | 7/15/2014 | 41:41.0 | 0 |
| -430.41 | 19724.763 | 1.835 | 16.029 | 0.141615 | 7/15/2014 | 41:41.1 | 0 |
| -430.151 | 19724.731 | 1.829 | 16.045 | 0.147128 | 7/15/2014 | 41:41.2 | 0 |
| -429.889 | 19724.699 | 1.829 | 16.062 | 0.158744 | 7/15/2014 | 41:41.3 | 0 |
| -429.569 | 19724.66 | 1.819 | 16.08 | 0.125007 | 7/15/2014 | 41:41.4 | 0 |
| -429.308 | 19724.629 | 1.817 | 16.096 | 0.129947 | 7/15/2014 | 41:41.5 | 0 |
| -429.052 | 19724.598 | 1.804 | 16.11 | 0.123183 | 7/15/2014 | 41:41.6 | 0 |
| -428.792 | 19724.566 | 1.807 | 16.12 | 0.12351 | 7/15/2014 | 41:41.7 | 0 |
| -428.472 | 19724.528 | 1.806 | 16.127 | 0.1087 | 7/15/2014 | 41:41.8 | 0 |
| -428.211 | 19724.497 | 1.816 | 16.13 | 0.105983 | 7/15/2014 | 41:41.9 | 0 |
| -427.954 | 19724.467 | 1.812 | 16.13 | 0.122817 | 7/15/2014 | 41:42.0 | 0 |
| -427.692 | 19724.436 | 1.816 | 16.13 | 0.115216 | 7/15/2014 | 41:42.1 | 0 |
| -427.436 | 19724.404 | 1.812 | 16.129 | 0.115029 | 7/15/2014 | 41:42.2 | 0 |
| -427.175 | 19724.373 | 1.82 | 16.128 | 0.114542 | 7/15/2014 | 41:42.3 | 0 |
| -426.856 | 19724.335 | 1.812 | 16.129 | 0.135318 | 7/15/2014 | 41:42.4 | 0 |
| -426.596 | 19724.304 | 1.81 | 16.132 | 0.119337 | 7/15/2014 | 41:42.5 | 0 |
| -426.341 | 19724.274 | 1.803 | 16.137 | 0.125764 | 7/15/2014 | 41:42.6 | 0 |
| -426.081 | 19724.244 | 1.81 | 16.143 | 0.114177 | 7/15/2014 | 41:42.7 | 0 |
| -425.762 | 19724.205 | 1.806 | 16.152 | 0.119843 | 7/15/2014 | 41:42.8 | 0 |
| -425.503 | 19724.176 | 1.803 | 16.162 | 0.120942 | 7/15/2014 | 41:42.9 | 0 |
| -425.245 | 19724.145 | 1.799 | 16.173 | 0.109161 | 7/15/2014 | 41:43.0 | 0 |
| -424.986 | 19724.115 | 1.805 | 16.185 | 0.11346 | 7/15/2014 | 41:43.1 | 0 |
| -424.728 | 19724.085 | 1.805 | 16.197 | 0.0976689 | 7/15/2014 | 41:43.2 | 0 |
| -424.47 | 19724.055 | 1.802 | 16.208 | 0.0970187 | 7/15/2014 | 41:43.3 | 0 |
| -424.151 | 19724.018 | 1.793 | 16.22 | 0.100549 | 7/15/2014 | 41:43.4 | 0 |
| -423.894 | 19723.988 | 1.796 | 16.231 | 0.104838 | 7/15/2014 | 41:43.5 | 0 |
| -423.638 | 19723.959 | 1.797 | 16.242 | 0.0992245 | 7/15/2014 | 41:43.6 | 0 |
| -423.381 | 19723.93 | 1.797 | 16.253 | 0.1029 | 7/15/2014 | 41:43.7 | 0 |
| -423.06 | 19723.893 | 1.795 | 16.264 | 0.120616 | 7/15/2014 | 41:43.8 | 0 |
| -422.801 | 19723.863 | 1.795 | 16.276 | 0.12382 | 7/15/2014 | 41:43.9 | 0 |
| -422.543 | 19723.834 | 1.793 | 16.289 | 0.123626 | 7/15/2014 | 41:44.0 | 0 |
| -422.283 | 19723.805 | 1.799 | 16.303 | 0.128657 | 7/15/2014 | 41:44.1 | 0 |
| -422.025 | 19723.775 | 1.79 | 16.318 | 0.129957 | 7/15/2014 | 41:44.2 | 0 |

| | | | | | | | |
|---|---|---|---|---|---|---|---|
| -421.765 | 19723.746 | 1.789 | 16.334 | 0.123307 | 7/15/2014 | 41:44.3 | 0 |
| -421.444 | 19723.709 | 1.78 | 16.351 | 0.101138 | 7/15/2014 | 41:44.4 | 0 |
| -421.185 | 19723.682 | 1.777 | 16.366 | 0.102385 | 7/15/2014 | 41:44.5 | 0 |
| -420.928 | 19723.652 | 1.774 | 16.38 | 0.0952215 | 7/15/2014 | 41:44.6 | 0 |
| -420.669 | 19723.625 | 1.773 | 16.391 | 0.0978444 | 7/15/2014 | 41:44.7 | 0 |
| -420.349 | 19723.588 | 1.765 | 16.398 | 0.09619 | 7/15/2014 | 41:44.8 | 0 |
| -420.09 | 19723.561 | 1.77 | 16.4 | 0.105165 | 7/15/2014 | 41:44.9 | 0 |
| -419.834 | 19723.531 | 1.767 | 16.396 | 0.127719 | 7/15/2014 | 41:45.0 | 0 |
| -419.576 | 19723.504 | 1.77 | 16.388 | 0.143549 | 7/15/2014 | 41:45.1 | 0 |
| -419.32 | 19723.477 | 1.761 | 16.374 | 0.141089 | 7/15/2014 | 41:45.2 | 0 |
| -419.064 | 19723.449 | 1.757 | 16.356 | 0.137692 | 7/15/2014 | 41:45.3 | 0 |
| -418.748 | 19723.415 | 1.753 | 16.335 | 0.147444 | 7/15/2014 | 41:45.4 | 0 |
| -418.494 | 19723.389 | 1.755 | 16.312 | 0.141303 | 7/15/2014 | 41:45.5 | 0 |
| -418.242 | 19723.361 | 1.747 | 16.289 | 0.130092 | 7/15/2014 | 41:45.6 | 0 |
| -417.988 | 19723.335 | 1.744 | 16.266 | 0.121124 | 7/15/2014 | 41:45.7 | 0 |
| -417.671 | 19723.302 | 1.744 | 16.246 | 0.112555 | 7/15/2014 | 41:45.8 | 0 |
| -417.416 | 19723.275 | 1.754 | 16.229 | 0.108951 | 7/15/2014 | 41:45.9 | 0 |
| -417.161 | 19723.25 | 1.745 | 16.216 | 0.102435 | 7/15/2014 | 41:46.0 | 0 |
| -416.906 | 19723.225 | 1.741 | 16.209 | 0.103431 | 7/15/2014 | 41:46.1 | 0 |
| -416.651 | 19723.197 | 1.739 | 16.208 | 0.122772 | 7/15/2014 | 41:46.2 | 0 |
| -416.395 | 19723.173 | 1.742 | 16.213 | 0.112828 | 7/15/2014 | 41:46.3 | 0 |
| -416.076 | 19723.141 | 1.736 | 16.226 | 0.131588 | 7/15/2014 | 41:46.4 | 0 |
| -415.821 | 19723.115 | 1.734 | 16.244 | 0.128301 | 7/15/2014 | 41:46.5 | 0 |
| -415.565 | 19723.091 | 1.727 | 16.268 | 0.123034 | 7/15/2014 | 41:46.6 | 0 |
| -415.309 | 19723.065 | 1.736 | 16.295 | 0.123126 | 7/15/2014 | 41:46.7 | 0 |
| -414.988 | 19723.034 | 1.736 | 16.324 | 0.107433 | 7/15/2014 | 41:46.8 | 0 |
| -414.73 | 19723.01 | 1.732 | 16.352 | 0.111839 | 7/15/2014 | 41:46.9 | 0 |
| -414.473 | 19722.985 | 1.723 | 16.377 | 0.0994439 | 7/15/2014 | 41:47.0 | 0 |
| -414.213 | 19722.961 | 1.725 | 16.397 | 0.110356 | 7/15/2014 | 41:47.1 | 0 |
| -413.954 | 19722.938 | 1.725 | 16.41 | 0.0913851 | 7/15/2014 | 41:47.2 | 0 |
| -413.696 | 19722.913 | 1.721 | 16.417 | 0.0747525 | 7/15/2014 | 41:47.3 | 0 |
| -413.376 | 19722.884 | 1.715 | 16.416 | 0.101495 | 7/15/2014 | 41:47.4 | 0 |
| -413.119 | 19722.86 | 1.718 | 16.409 | 0.105692 | 7/15/2014 | 41:47.5 | 0 |
| -412.863 | 19722.838 | 1.718 | 16.396 | 0.0679048 | 7/15/2014 | 41:47.6 | 0 |
| -412.605 | 19722.814 | 1.727 | 16.378 | 0.0677147 | 7/15/2014 | 41:47.7 | 0 |
| -412.289 | 19722.787 | 1.724 | 16.356 | 0.111165 | 7/15/2014 | 41:47.8 | 0 |
| -412.034 | 19722.766 | 1.72 | 16.333 | 0.104393 | 7/15/2014 | 41:47.9 | 0 |
| -411.782 | 19722.744 | 1.72 | 16.309 | 0.0935272 | 7/15/2014 | 41:48.0 | 0 |
| -411.529 | 19722.724 | 1.723 | 16.286 | 0.110178 | 7/15/2014 | 41:48.1 | 0 |
| -411.278 | 19722.703 | 1.717 | 16.265 | 0.112032 | 7/15/2014 | 41:48.2 | 0 |
| -411.029 | 19722.682 | 1.713 | 16.248 | 0.111644 | 7/15/2014 | 41:48.3 | 0 |
| -410.599 | 19722.646 | 1.7 | 16.235 | 0.115629 | 7/15/2014 | 41:48.4 | 0 |
| -410.353 | 19722.628 | 1.7 | 16.231 | 0.129223 | 7/15/2014 | 41:48.5 | 0 |
| -410.111 | 19722.609 | 1.694 | 16.234 | 0.130454 | 7/15/2014 | 41:48.6 | 0 |
| -409.808 | 19722.586 | 1.687 | 16.244 | 0.121008 | 7/15/2014 | 41:48.7 | 0 |
| -409.684 | 19722.576 | 1.68 | 16.247 | 0.135452 | 7/15/2014 | 41:48.8 | 0 |
| -409.44 | 19722.558 | 1.68 | 16.267 | 0.125092 | 7/15/2014 | 41:48.9 | 0 |

| | | | | | | | |
|---|---|---|---|---|---|---|---|
| -409.197 | 19722.539 | 1.678 | 16.292 | 0.142306 | 7/15/2014 | 41:49.0 | 0 |
| -408.952 | 19722.521 | 1.686 | 16.322 | 0.114495 | 7/15/2014 | 41:49.1 | 0 |
| -408.709 | 19722.503 | 1.684 | 16.353 | 0.116604 | 7/15/2014 | 41:49.2 | 0 |
| -408.465 | 19722.485 | 1.684 | 16.386 | 0.13193 | 7/15/2014 | 41:49.3 | 0 |
| -408.039 | 19722.455 | 1.683 | 16.43 | 0.119386 | 7/15/2014 | 41:49.4 | 0 |
| -407.796 | 19722.438 | 1.683 | 16.457 | 0.121432 | 7/15/2014 | 41:49.5 | 0 |
| -407.551 | 19722.422 | 1.683 | 16.481 | 0.110122 | 7/15/2014 | 41:49.6 | 0 |
| -407.244 | 19722.4 | 1.681 | 16.501 | 0.106979 | 7/15/2014 | 41:49.7 | 0 |
| -407.122 | 19722.392 | 1.675 | 16.512 | 0.102836 | 7/15/2014 | 41:49.8 | 0 |
| -406.873 | 19722.375 | 1.671 | 16.527 | 0.0990278 | 7/15/2014 | 41:49.9 | 0 |
| -406.625 | 19722.359 | 1.663 | 16.539 | 0.0930333 | 7/15/2014 | 41:50.0 | 0 |
| -406.374 | 19722.343 | 1.661 | 16.549 | 0.0995654 | 7/15/2014 | 41:50.1 | 0 |
| -406.125 | 19722.328 | 1.65 | 16.558 | 0.111274 | 7/15/2014 | 41:50.2 | 0 |
| -405.873 | 19722.313 | 1.65 | 16.566 | 0.108343 | 7/15/2014 | 41:50.3 | 0 |
| -405.562 | 19722.292 | 1.639 | 16.575 | 0.114079 | 7/15/2014 | 41:50.4 | 0 |
| -405.31 | 19722.276 | 1.637 | 16.584 | 0.110394 | 7/15/2014 | 41:50.5 | 0 |
| -405.06 | 19722.262 | 1.629 | 16.593 | 0.116893 | 7/15/2014 | 41:50.6 | 0 |
| -404.807 | 19722.247 | 1.629 | 16.603 | 0.121633 | 7/15/2014 | 41:50.7 | 0 |
| -404.493 | 19722.229 | 1.619 | 16.612 | 0.0981599 | 7/15/2014 | 41:50.8 | 0 |
| -404.238 | 19722.215 | 1.617 | 16.621 | 0.100203 | 7/15/2014 | 41:50.9 | 0 |
| -403.985 | 19722.201 | 1.605 | 16.628 | 0.0750193 | 7/15/2014 | 41:51.0 | 0 |
| -403.73 | 19722.186 | 1.605 | 16.633 | 0.0704432 | 7/15/2014 | 41:51.1 | 0 |
| -403.478 | 19722.173 | 1.593 | 16.635 | 0.0579669 | 7/15/2014 | 41:51.2 | 0 |
| -403.222 | 19722.158 | 1.587 | 16.635 | 0.0522383 | 7/15/2014 | 41:51.3 | 0 |
| -402.908 | 19722.142 | 1.568 | 16.631 | 0.05215 | 7/15/2014 | 41:51.4 | 0 |
| -402.653 | 19722.129 | 1.566 | 16.624 | 0.051901 | 7/15/2014 | 41:51.5 | 0 |
| -402.4 | 19722.116 | 1.56 | 16.615 | 0.0573294 | 7/15/2014 | 41:51.6 | 0 |
| -402.146 | 19722.102 | 1.56 | 16.603 | 0.0613711 | 7/15/2014 | 41:51.7 | 0 |
| -401.829 | 19722.088 | 1.551 | 16.588 | 0.0599013 | 7/15/2014 | 41:51.8 | 0 |
| -401.573 | 19722.074 | 1.551 | 16.573 | 0.0607581 | 7/15/2014 | 41:51.9 | 0 |
| -401.318 | 19722.063 | 1.552 | 16.556 | 0.0570178 | 7/15/2014 | 41:52.0 | 0 |
| -401.062 | 19722.051 | 1.552 | 16.538 | 0.0596415 | 7/15/2014 | 41:52.1 | 0 |
| -400.807 | 19722.038 | 1.546 | 16.521 | 0.0503273 | 7/15/2014 | 41:52.2 | 0 |
| -400.552 | 19722.025 | 1.543 | 16.504 | 0.0481256 | 7/15/2014 | 41:52.3 | 0 |
| -400.236 | 19722.012 | 1.538 | 16.488 | 0.0533041 | 7/15/2014 | 41:52.4 | 0 |
| -399.982 | 19722.002 | 1.532 | 16.474 | 0.0589075 | 7/15/2014 | 41:52.5 | 0 |
| -399.73 | 19721.99 | 1.516 | 16.462 | 0.0656143 | 7/15/2014 | 41:52.6 | 0 |
| -399.476 | 19721.979 | 1.508 | 16.454 | 0.0756724 | 7/15/2014 | 41:52.7 | 0 |
| -399.162 | 19721.967 | 1.498 | 16.449 | 0.0869301 | 7/15/2014 | 41:52.8 | 0 |
| -398.908 | 19721.955 | 1.494 | 16.449 | 0.100884 | 7/15/2014 | 41:52.9 | 0 |
| -398.656 | 19721.945 | 1.486 | 16.454 | 0.111027 | 7/15/2014 | 41:53.0 | 0 |
| -398.402 | 19721.938 | 1.476 | 16.464 | 0.111947 | 7/15/2014 | 41:53.1 | 0 |
| -398.15 | 19721.927 | 1.467 | 16.48 | 0.109858 | 7/15/2014 | 41:53.2 | 0 |
| -397.897 | 19721.916 | 1.47 | 16.501 | 0.102615 | 7/15/2014 | 41:53.3 | 0 |
| -397.584 | 19721.906 | 1.463 | 16.527 | 0.0871706 | 7/15/2014 | 41:53.4 | 0 |
| -397.333 | 19721.896 | 1.463 | 16.557 | 0.0775848 | 7/15/2014 | 41:53.5 | 0 |
| -397.083 | 19721.889 | 1.454 | 16.589 | 0.078507 | 7/15/2014 | 41:53.6 | 0 |

| | | | | | | | |
|---|---|---|---|---|---|---|---|
| -396.831 | 19721.881 | 1.451 | 16.621 | 0.0687988 | 7/15/2014 | 41:53.7 | 0 |
| -396.519 | 19721.87 | 1.451 | 16.652 | 0.0621931 | 7/15/2014 | 41:53.8 | 0 |
| -396.267 | 19721.861 | 1.454 | 16.681 | 0.062923 | 7/15/2014 | 41:53.9 | 0 |
| -396.015 | 19721.854 | 1.454 | 16.708 | 0.0651209 | 7/15/2014 | 41:54.0 | 0 |
| -395.764 | 19721.846 | 1.458 | 16.73 | 0.0667422 | 7/15/2014 | 41:54.1 | 0 |
| -395.514 | 19721.838 | 1.458 | 16.749 | 0.0816454 | 7/15/2014 | 41:54.2 | 0 |
| -395.265 | 19721.83 | 1.467 | 16.764 | 0.0823516 | 7/15/2014 | 41:54.3 | 0 |
| -394.832 | 19721.818 | 1.47 | 16.779 | 0.0991099 | 7/15/2014 | 41:54.4 | 0 |
| -394.586 | 19721.811 | 1.47 | 16.787 | 0.105677 | 7/15/2014 | 41:54.5 | 0 |
| -394.34 | 19721.803 | 1.467 | 16.791 | 0.126225 | 7/15/2014 | 41:54.6 | 0 |
| -394.036 | 19721.795 | 1.467 | 16.791 | 0.100611 | 7/15/2014 | 41:54.7 | 0 |
| -393.91 | 19721.791 | 1.467 | 16.792 | 0.0954616 | 7/15/2014 | 41:54.8 | 0 |
| -393.666 | 19721.783 | 1.467 | 16.788 | 0.10757 | 7/15/2014 | 41:54.9 | 0 |
| -393.421 | 19721.778 | 1.461 | 16.779 | 0.0926673 | 7/15/2014 | 41:55.0 | 0 |
| -393.176 | 19721.771 | 1.454 | 16.766 | 0.0717678 | 7/15/2014 | 41:55.1 | 0 |
| -392.932 | 19721.765 | 1.451 | 16.75 | 0.0963191 | 7/15/2014 | 41:55.2 | 0 |
| -392.688 | 19721.758 | 1.444 | 16.73 | 0.0702051 | 7/15/2014 | 41:55.3 | 0 |
| -392.264 | 19721.748 | 1.442 | 16.695 | 0.0888609 | 7/15/2014 | 41:55.4 | 0 |
| -392.022 | 19721.741 | 1.437 | 16.672 | 0.0945887 | 7/15/2014 | 41:55.5 | 0 |
| -391.779 | 19721.736 | 1.435 | 16.648 | 0.103534 | 7/15/2014 | 41:55.6 | 0 |
| -391.475 | 19721.729 | 1.43 | 16.625 | 0.103356 | 7/15/2014 | 41:55.7 | 0 |
| -391.353 | 19721.726 | 1.43 | 16.613 | 0.0816227 | 7/15/2014 | 41:55.8 | 0 |
| -391.107 | 19721.72 | 1.428 | 16.592 | 0.112532 | 7/15/2014 | 41:55.9 | 0 |
| -390.861 | 19721.714 | 1.43 | 16.575 | 0.0996601 | 7/15/2014 | 41:56.0 | 0 |
| -390.614 | 19721.708 | 1.434 | 16.562 | 0.137251 | 7/15/2014 | 41:56.1 | 0 |
| -390.368 | 19721.702 | 1.425 | 16.554 | 0.112821 | 7/15/2014 | 41:56.2 | 0 |
| -390.12 | 19721.697 | 1.428 | 16.55 | 0.117852 | 7/15/2014 | 41:56.3 | 0 |
| -389.688 | 19721.688 | 1.426 | 16.557 | 0.141276 | 7/15/2014 | 41:56.4 | 0 |
| -389.442 | 19721.683 | 1.426 | 16.566 | 0.101281 | 7/15/2014 | 41:56.5 | 0 |
| -389.195 | 19721.676 | 1.423 | 16.579 | 0.114144 | 7/15/2014 | 41:56.6 | 0 |
| -388.889 | 19721.669 | 1.416 | 16.596 | 0.0851701 | 7/15/2014 | 41:56.7 | 0 |
| -388.763 | 19721.666 | 1.411 | 16.604 | 0.0918989 | 7/15/2014 | 41:56.8 | 0 |
| -388.515 | 19721.662 | 1.414 | 16.625 | 0.100197 | 7/15/2014 | 41:56.9 | 0 |
| -388.268 | 19721.656 | 1.409 | 16.647 | 0.059761 | 7/15/2014 | 41:57.0 | 0 |
| -388.02 | 19721.65 | 1.412 | 16.668 | 0.0812051 | 7/15/2014 | 41:57.1 | 0 |
| -387.773 | 19721.646 | 1.409 | 16.687 | 0.0970626 | 7/15/2014 | 41:57.2 | 0 |
| -387.526 | 19721.639 | 1.409 | 16.703 | 0.0744491 | 7/15/2014 | 41:57.3 | 0 |
| -387.098 | 19721.63 | 1.409 | 16.716 | 0.0820549 | 7/15/2014 | 41:57.4 | 0 |
| -386.855 | 19721.625 | 1.409 | 16.721 | 0.0796151 | 7/15/2014 | 41:57.5 | 0 |
| -386.612 | 19721.619 | 1.409 | 16.723 | 0.096021 | 7/15/2014 | 41:57.6 | 0 |
| -386.311 | 19721.612 | 1.412 | 16.72 | 0.0714085 | 7/15/2014 | 41:57.7 | 0 |
| -386.187 | 19721.609 | 1.409 | 16.72 | 0.0767862 | 7/15/2014 | 41:57.8 | 0 |
| -385.944 | 19721.604 | 1.412 | 16.711 | 0.0673352 | 7/15/2014 | 41:57.9 | 0 |
| -385.702 | 19721.6 | 1.418 | 16.699 | 0.0824831 | 7/15/2014 | 41:58.0 | 0 |
| -385.46 | 19721.594 | 1.42 | 16.684 | 0.0752867 | 7/15/2014 | 41:58.1 | 0 |
| -385.221 | 19721.588 | 1.417 | 16.667 | 0.068472 | 7/15/2014 | 41:58.2 | 0 |
| -384.98 | 19721.583 | 1.426 | 16.649 | 0.0728988 | 7/15/2014 | 41:58.3 | 0 |

| | | | | | | | |
|---|---|---|---|---|---|---|---|
| -384.564 | 19721.573 | 1.429 | 16.618 | 0.085393 | 7/15/2014 | 41:58.4 | 0 |
| -384.327 | 19721.568 | 1.426 | 16.597 | 0.0971762 | 7/15/2014 | 41:58.5 | 0 |
| -384.09 | 19721.563 | 1.426 | 16.576 | 0.0938594 | 7/15/2014 | 41:58.6 | 0 |
| -383.796 | 19721.557 | 1.42 | 16.556 | 0.0829773 | 7/15/2014 | 41:58.7 | 0 |
| -383.677 | 19721.554 | 1.419 | 16.545 | 0.0828735 | 7/15/2014 | 41:58.8 | 0 |
| -383.437 | 19721.549 | 1.429 | 16.527 | 0.107625 | 7/15/2014 | 41:58.9 | 0 |
| -383.199 | 19721.544 | 1.421 | 16.511 | 0.0854482 | 7/15/2014 | 41:59.0 | 0 |
| -382.959 | 19721.537 | 1.421 | 16.499 | 0.083535 | 7/15/2014 | 41:59.1 | 0 |
| -382.721 | 19721.533 | 1.415 | 16.492 | 0.0949364 | 7/15/2014 | 41:59.2 | 0 |
| -382.481 | 19721.527 | 1.418 | 16.489 | 0.0886651 | 7/15/2014 | 41:59.3 | 0 |
| -382.063 | 19721.518 | 1.422 | 16.502 | 0.0907715 | 7/15/2014 | 41:59.4 | 0 |
| -381.825 | 19721.512 | 1.425 | 16.515 | 0.0859085 | 7/15/2014 | 41:59.5 | 0 |
| -381.585 | 19721.507 | 1.422 | 16.534 | 0.08382 | 7/15/2014 | 41:59.6 | 0 |
| -381.284 | 19721.498 | 1.425 | 16.557 | 0.0822164 | 7/15/2014 | 41:59.7 | 0 |
| -381.164 | 19721.496 | 1.425 | 16.568 | 0.0917463 | 7/15/2014 | 41:59.8 | 0 |
| -380.921 | 19721.49 | 1.435 | 16.596 | 0.0885027 | 7/15/2014 | 41:59.9 | 0 |
| -380.678 | 19721.484 | 1.435 | 16.624 | 0.0835355 | 7/15/2014 | 42:00.0 | 0 |
| -380.434 | 19721.479 | 1.441 | 16.65 | 0.100127 | 7/15/2014 | 42:00.1 | 0 |
| -380.192 | 19721.473 | 1.441 | 16.672 | 0.084718 | 7/15/2014 | 42:00.2 | 0 |
| -379.948 | 19721.466 | 1.444 | 16.688 | 0.112338 | 7/15/2014 | 42:00.3 | 0 |
| -379.521 | 19721.455 | 1.453 | 16.698 | 0.111835 | 7/15/2014 | 42:00.4 | 0 |
| -379.279 | 19721.448 | 1.458 | 16.7 | 0.100568 | 7/15/2014 | 42:00.5 | 0 |
| -379.035 | 19721.442 | 1.463 | 16.696 | 0.116608 | 7/15/2014 | 42:00.6 | 0 |
| -378.731 | 19721.435 | 1.466 | 16.687 | 0.0837407 | 7/15/2014 | 42:00.7 | 0 |
| -378.608 | 19721.432 | 1.465 | 16.684 | 0.107677 | 7/15/2014 | 42:00.8 | 0 |
| -378.363 | 19721.424 | 1.468 | 16.669 | 0.0949338 | 7/15/2014 | 42:00.9 | 0 |
| -378.115 | 19721.417 | 1.473 | 16.65 | 0.114907 | 7/15/2014 | 42:01.0 | 0 |
| -377.868 | 19721.412 | 1.478 | 16.629 | 0.0837613 | 7/15/2014 | 42:01.1 | 0 |
| -377.623 | 19721.404 | 1.475 | 16.606 | 0.0930571 | 7/15/2014 | 42:01.2 | 0 |
| -377.375 | 19721.397 | 1.478 | 16.582 | 0.0699563 | 7/15/2014 | 42:01.3 | 0 |
| -376.945 | 19721.386 | 1.48 | 16.546 | 0.0740186 | 7/15/2014 | 42:01.4 | 0 |
| -376.7 | 19721.379 | 1.487 | 16.522 | 0.0537186 | 7/15/2014 | 42:01.5 | 0 |
| -376.453 | 19721.372 | 1.493 | 16.499 | 0.0545352 | 7/15/2014 | 42:01.6 | 0 |
| -376.146 | 19721.363 | 1.493 | 16.476 | 0.0440136 | 7/15/2014 | 42:01.7 | 0 |
| -376.022 | 19721.359 | 1.494 | 16.465 | 0.0501242 | 7/15/2014 | 42:01.8 | 0 |
| -375.772 | 19721.352 | 1.5 | 16.445 | 0.0544145 | 7/15/2014 | 42:01.9 | 0 |
| -375.524 | 19721.347 | 1.504 | 16.426 | 0.0730332 | 7/15/2014 | 42:02.0 | 0 |
| -375.274 | 19721.338 | 1.516 | 16.408 | 0.0427196 | 7/15/2014 | 42:02.1 | 0 |
| -375.027 | 19721.331 | 1.518 | 16.392 | 0.0734015 | 7/15/2014 | 42:02.2 | 0 |
| -374.779 | 19721.324 | 1.518 | 16.378 | 0.0996046 | 7/15/2014 | 42:02.3 | 0 |
| -374.345 | 19721.311 | 1.521 | 16.362 | 0.0847245 | 7/15/2014 | 42:02.4 | 0 |
| -374.099 | 19721.303 | 1.523 | 16.353 | 0.071642 | 7/15/2014 | 42:02.5 | 0 |
| -373.851 | 19721.296 | 1.528 | 16.347 | 0.0526413 | 7/15/2014 | 42:02.6 | 0 |
| -373.542 | 19721.285 | 1.528 | 16.345 | 0.0875308 | 7/15/2014 | 42:02.7 | 0 |
| -373.418 | 19721.281 | 1.525 | 16.342 | 0.060684 | 7/15/2014 | 42:02.8 | 0 |
| -373.169 | 19721.274 | 1.526 | 16.345 | 0.0434818 | 7/15/2014 | 42:02.9 | 0 |
| -372.919 | 19721.265 | 1.526 | 16.351 | 0.045909 | 7/15/2014 | 42:03.0 | 0 |

| | | | | | | | |
|---|---|---|---|---|---|---|---|
| -372.668 | 19721.258 | 1.532 | 16.36 | 0.0411024 | 7/15/2014 | 42:03.1 | 0 |
| -372.42 | 19721.25 | 1.534 | 16.373 | 0.0408299 | 7/15/2014 | 42:03.2 | 0 |
| -372.168 | 19721.24 | 1.54 | 16.389 | 0.036723 | 7/15/2014 | 42:03.3 | 0 |
| -371.859 | 19721.229 | 1.537 | 16.406 | 0.0306368 | 7/15/2014 | 42:03.4 | 0 |
| -371.608 | 19721.222 | 1.539 | 16.424 | 0.0509328 | 7/15/2014 | 42:03.5 | 0 |
| -371.358 | 19721.212 | 1.538 | 16.443 | 0.0396041 | 7/15/2014 | 42:03.6 | 0 |
| -371.105 | 19721.203 | 1.544 | 16.46 | 0.0380867 | 7/15/2014 | 42:03.7 | 0 |
| -370.793 | 19721.191 | 1.544 | 16.475 | 0.0386306 | 7/15/2014 | 42:03.8 | 0 |
| -370.538 | 19721.183 | 1.548 | 16.487 | 0.035564 | 7/15/2014 | 42:03.9 | 0 |
| -370.286 | 19721.172 | 1.544 | 16.496 | 0.0305519 | 7/15/2014 | 42:04.0 | 0 |
| -370.031 | 19721.164 | 1.551 | 16.502 | 0.0296162 | 7/15/2014 | 42:04.1 | 0 |
| -369.777 | 19721.153 | 1.555 | 16.504 | 0.0298879 | 7/15/2014 | 42:04.2 | 0 |
| -369.522 | 19721.145 | 1.562 | 16.504 | 0.0381881 | 7/15/2014 | 42:04.3 | 0 |
| -369.208 | 19721.131 | 1.562 | 16.5 | 0.0324377 | 7/15/2014 | 42:04.4 | 0 |
| -368.952 | 19721.121 | 1.566 | 16.494 | 0.0341881 | 7/15/2014 | 42:04.5 | 0 |
| -368.7 | 19721.111 | 1.562 | 16.487 | 0.0402342 | 7/15/2014 | 42:04.6 | 0 |
| -368.444 | 19721.102 | 1.572 | 16.477 | 0.0458911 | 7/15/2014 | 42:04.7 | 0 |
| -368.128 | 19721.088 | 1.572 | 16.466 | 0.0589122 | 7/15/2014 | 42:04.8 | 0 |
| -367.872 | 19721.079 | 1.578 | 16.454 | 0.0408647 | 7/15/2014 | 42:04.9 | 0 |
| -367.618 | 19721.068 | 1.581 | 16.441 | 0.0549751 | 7/15/2014 | 42:05.0 | 0 |
| -367.362 | 19721.057 | 1.584 | 16.426 | 0.0510967 | 7/15/2014 | 42:05.1 | 0 |
| -367.108 | 19721.047 | 1.581 | 16.41 | 0.0446762 | 7/15/2014 | 42:05.2 | 0 |
| -366.852 | 19721.035 | 1.589 | 16.394 | 0.0496957 | 7/15/2014 | 42:05.3 | 0 |
| -366.535 | 19721.022 | 1.587 | 16.377 | 0.0628782 | 7/15/2014 | 42:05.4 | 0 |
| -366.281 | 19721.013 | 1.587 | 16.361 | 0.0707106 | 7/15/2014 | 42:05.5 | 0 |
| -366.027 | 19721.002 | 1.584 | 16.345 | 0.0709706 | 7/15/2014 | 42:05.6 | 0 |
| -365.774 | 19720.99 | 1.582 | 16.33 | 0.0897974 | 7/15/2014 | 42:05.7 | 0 |
| -365.459 | 19720.977 | 1.582 | 16.316 | 0.0711795 | 7/15/2014 | 42:05.8 | 0 |
| -365.204 | 19720.965 | 1.585 | 16.304 | 0.0677725 | 7/15/2014 | 42:05.9 | 0 |
| -364.95 | 19720.954 | 1.581 | 16.294 | 0.0538617 | 7/15/2014 | 42:06.0 | 0 |
| -364.697 | 19720.942 | 1.581 | 16.285 | 0.0465308 | 7/15/2014 | 42:06.1 | 0 |
| -364.444 | 19720.931 | 1.581 | 16.278 | 0.0456471 | 7/15/2014 | 42:06.2 | 0 |
| -364.192 | 19720.918 | 1.581 | 16.273 | 0.0402697 | 7/15/2014 | 42:06.3 | 0 |
| -363.878 | 19720.904 | 1.58 | 16.27 | 0.0382236 | 7/15/2014 | 42:06.4 | 0 |
| -363.628 | 19720.893 | 1.585 | 16.268 | 0.0355951 | 7/15/2014 | 42:06.5 | 0 |
| -363.377 | 19720.881 | 1.587 | 16.268 | 0.0348163 | 7/15/2014 | 42:06.6 | 0 |
| -363.125 | 19720.868 | 1.592 | 16.269 | 0.0476306 | 7/15/2014 | 42:06.7 | 0 |
| -362.812 | 19720.853 | 1.594 | 16.272 | 0.0491214 | 7/15/2014 | 42:06.8 | 0 |
| -362.559 | 19720.84 | 1.599 | 16.276 | 0.0392847 | 7/15/2014 | 42:06.9 | 0 |
| -362.307 | 19720.826 | 1.598 | 16.282 | 0.0466274 | 7/15/2014 | 42:07.0 | 0 |
| -362.052 | 19720.814 | 1.6 | 16.29 | 0.0703259 | 7/15/2014 | 42:07.1 | 0 |
| -361.797 | 19720.803 | 1.598 | 16.3 | 0.0811371 | 7/15/2014 | 42:07.2 | 0 |
| -361.542 | 19720.789 | 1.595 | 16.311 | 0.0659849 | 7/15/2014 | 42:07.3 | 0 |
| -361.226 | 19720.772 | 1.593 | 16.325 | 0.0727604 | 7/15/2014 | 42:07.4 | 0 |
| -360.969 | 19720.76 | 1.593 | 16.339 | 0.0727062 | 7/15/2014 | 42:07.5 | 0 |
| -360.715 | 19720.746 | 1.593 | 16.354 | 0.0807284 | 7/15/2014 | 42:07.6 | 0 |
| -360.458 | 19720.732 | 1.599 | 16.367 | 0.0527433 | 7/15/2014 | 42:07.7 | 0 |

| | | | | | | | |
|---|---|---|---|---|---|---|---|
| -360.137 | 19720.717 | 1.599 | 16.379 | 0.0457299 | 7/15/2014 | 42:07.8 | 0 |
| -359.879 | 19720.702 | 1.601 | 16.391 | 0.0630384 | 7/15/2014 | 42:07.9 | 0 |
| -359.621 | 19720.688 | 1.606 | 16.401 | 0.0492223 | 7/15/2014 | 42:08.0 | 0 |
| -359.361 | 19720.674 | 1.611 | 16.409 | 0.0730051 | 7/15/2014 | 42:08.1 | 0 |
| -359.103 | 19720.661 | 1.611 | 16.415 | 0.0638019 | 7/15/2014 | 42:08.2 | 0 |
| -358.845 | 19720.646 | 1.611 | 16.42 | 0.0712765 | 7/15/2014 | 42:08.3 | 0 |
| -358.525 | 19720.629 | 1.611 | 16.422 | 0.0700116 | 7/15/2014 | 42:08.4 | 0 |
| -358.267 | 19720.615 | 1.616 | 16.423 | 0.0577327 | 7/15/2014 | 42:08.5 | 0 |
| -358.01 | 19720.6 | 1.618 | 16.419 | 0.0495937 | 7/15/2014 | 42:08.6 | 0 |
| -357.752 | 19720.586 | 1.621 | 16.413 | 0.0553777 | 7/15/2014 | 42:08.7 | 0 |
| -357.431 | 19720.567 | 1.618 | 16.404 | 0.0346654 | 7/15/2014 | 42:08.8 | 0 |
| -357.172 | 19720.554 | 1.621 | 16.393 | 0.0653812 | 7/15/2014 | 42:08.9 | 0 |
| -356.914 | 19720.539 | 1.627 | 16.382 | 0.0493551 | 7/15/2014 | 42:09.0 | 0 |
| -356.655 | 19720.524 | 1.633 | 16.37 | 0.0597309 | 7/15/2014 | 42:09.1 | 0 |
| -356.396 | 19720.51 | 1.633 | 16.36 | 0.0781144 | 7/15/2014 | 42:09.2 | 0 |
| -356.138 | 19720.496 | 1.636 | 16.35 | 0.0688486 | 7/15/2014 | 42:09.3 | 0 |
| -355.818 | 19720.477 | 1.636 | 16.341 | 0.085098 | 7/15/2014 | 42:09.4 | 0 |
| -355.561 | 19720.463 | 1.642 | 16.334 | 0.0720196 | 7/15/2014 | 42:09.5 | 0 |
| -355.304 | 19720.447 | 1.642 | 16.327 | 0.112588 | 7/15/2014 | 42:09.6 | 0 |
| -355.047 | 19720.433 | 1.639 | 16.32 | 0.107604 | 7/15/2014 | 42:09.7 | 0 |
| -354.728 | 19720.414 | 1.633 | 16.313 | 0.104965 | 7/15/2014 | 42:09.8 | 0 |
| -354.47 | 19720.4 | 1.641 | 16.305 | 0.105204 | 7/15/2014 | 42:09.9 | 0 |
| -354.214 | 19720.385 | 1.641 | 16.298 | 0.121978 | 7/15/2014 | 42:10.0 | 0 |
| -353.956 | 19720.369 | 1.647 | 16.289 | 0.102433 | 7/15/2014 | 42:10.1 | 0 |
| -353.7 | 19720.354 | 1.644 | 16.281 | 0.111947 | 7/15/2014 | 42:10.2 | 0 |
| -353.444 | 19720.34 | 1.652 | 16.273 | 0.0972002 | 7/15/2014 | 42:10.3 | 0 |
| -353.128 | 19720.32 | 1.652 | 16.266 | 0.106243 | 7/15/2014 | 42:10.4 | 0 |
| -352.873 | 19720.306 | 1.658 | 16.26 | 0.13526 | 7/15/2014 | 42:10.5 | 0 |
| -352.619 | 19720.29 | 1.656 | 16.257 | 0.110136 | 7/15/2014 | 42:10.6 | 0 |
| -352.365 | 19720.275 | 1.652 | 16.256 | 0.142004 | 7/15/2014 | 42:10.7 | 0 |
| -352.051 | 19720.256 | 1.648 | 16.259 | 0.108941 | 7/15/2014 | 42:10.8 | 0 |
| -351.796 | 19720.241 | 1.654 | 16.266 | 0.143002 | 7/15/2014 | 42:10.9 | 0 |
| -351.544 | 19720.226 | 1.654 | 16.276 | 0.151132 | 7/15/2014 | 42:11.0 | 0 |
| -351.289 | 19720.21 | 1.663 | 16.291 | 0.118988 | 7/15/2014 | 42:11.1 | 0 |
| -351.035 | 19720.194 | 1.665 | 16.31 | 0.149587 | 7/15/2014 | 42:11.2 | 0 |
| -350.782 | 19720.18 | 1.662 | 16.331 | 0.139177 | 7/15/2014 | 42:11.3 | 0 |
| -350.468 | 19720.16 | 1.662 | 16.355 | 0.16403 | 7/15/2014 | 42:11.4 | 0 |
| -350.215 | 19720.145 | 1.665 | 16.379 | 0.147698 | 7/15/2014 | 42:11.5 | 0 |
| -349.963 | 19720.129 | 1.659 | 16.402 | 0.141869 | 7/15/2014 | 42:11.6 | 0 |
| -349.709 | 19720.113 | 1.659 | 16.422 | 0.133051 | 7/15/2014 | 42:11.7 | 0 |
| -349.392 | 19720.094 | 1.651 | 16.44 | 0.122946 | 7/15/2014 | 42:11.8 | 0 |
| -349.137 | 19720.078 | 1.647 | 16.452 | 0.105843 | 7/15/2014 | 42:11.9 | 0 |
| -348.882 | 19720.062 | 1.645 | 16.46 | 0.118793 | 7/15/2014 | 42:12.0 | 0 |
| -348.624 | 19720.046 | 1.647 | 16.463 | 0.0995441 | 7/15/2014 | 42:12.1 | 0 |
| -348.368 | 19720.03 | 1.641 | 16.461 | 0.101046 | 7/15/2014 | 42:12.2 | 0 |
| -348.111 | 19720.015 | 1.645 | 16.455 | 0.102414 | 7/15/2014 | 42:12.3 | 0 |
| -347.791 | 19719.994 | 1.639 | 16.445 | 0.105764 | 7/15/2014 | 42:12.4 | 0 |

| | | | | | | | |
|---|---|---|---|---|---|---|---|
| -347.532 | 19719.979 | 1.645 | 16.433 | 0.118205 | 7/15/2014 | 42:12.5 | 0 |
| -347.275 | 19719.961 | 1.643 | 16.419 | 0.111771 | 7/15/2014 | 42:12.6 | 0 |
| -347.017 | 19719.945 | 1.64 | 16.404 | 0.12806 | 7/15/2014 | 42:12.7 | 0 |
| -346.694 | 19719.925 | 1.638 | 16.389 | 0.138997 | 7/15/2014 | 42:12.8 | 0 |
| -346.434 | 19719.909 | 1.635 | 16.376 | 0.147509 | 7/15/2014 | 42:12.9 | 0 |
| -346.173 | 19719.893 | 1.629 | 16.365 | 0.152157 | 7/15/2014 | 42:13.0 | 0 |
| -345.912 | 19719.877 | 1.624 | 16.356 | 0.146069 | 7/15/2014 | 42:13.1 | 0 |
| -345.651 | 19719.859 | 1.614 | 16.351 | 0.137262 | 7/15/2014 | 42:13.2 | 0 |
| -345.391 | 19719.843 | 1.609 | 16.35 | 0.127523 | 7/15/2014 | 42:13.3 | 0 |
| -345.068 | 19719.822 | 1.604 | 16.354 | 0.121506 | 7/15/2014 | 42:13.4 | 0 |
| -344.808 | 19719.807 | 1.599 | 16.362 | 0.116172 | 7/15/2014 | 42:13.5 | 0 |
| -344.548 | 19719.791 | 1.597 | 16.375 | 0.128183 | 7/15/2014 | 42:13.6 | 0 |
| -344.288 | 19719.774 | 1.594 | 16.393 | 0.128326 | 7/15/2014 | 42:13.7 | 0 |
| -343.964 | 19719.754 | 1.594 | 16.415 | 0.136252 | 7/15/2014 | 42:13.8 | 0 |
| -343.704 | 19719.737 | 1.597 | 16.44 | 0.1417 | 7/15/2014 | 42:13.9 | 0 |
| -343.444 | 19719.721 | 1.597 | 16.468 | 0.158976 | 7/15/2014 | 42:14.0 | 0 |
| -343.183 | 19719.705 | 1.599 | 16.497 | 0.157486 | 7/15/2014 | 42:14.1 | 0 |
| -342.924 | 19719.689 | 1.593 | 16.526 | 0.160628 | 7/15/2014 | 42:14.2 | 0 |
| -342.665 | 19719.673 | 1.593 | 16.553 | 0.165412 | 7/15/2014 | 42:14.3 | 0 |
| -342.343 | 19719.653 | 1.59 | 16.578 | 0.154643 | 7/15/2014 | 42:14.4 | 0 |
| -342.085 | 19719.638 | 1.588 | 16.6 | 0.159173 | 7/15/2014 | 42:14.5 | 0 |
| -341.827 | 19719.62 | 1.58 | 16.618 | 0.148118 | 7/15/2014 | 42:14.6 | 0 |
| -341.57 | 19719.605 | 1.571 | 16.632 | 0.145462 | 7/15/2014 | 42:14.7 | 0 |
| -341.249 | 19719.586 | 1.565 | 16.642 | 0.145435 | 7/15/2014 | 42:14.8 | 0 |
| -340.99 | 19719.569 | 1.567 | 16.648 | 0.144121 | 7/15/2014 | 42:14.9 | 0 |
| -340.732 | 19719.555 | 1.565 | 16.649 | 0.147898 | 7/15/2014 | 42:15.0 | 0 |
| -340.473 | 19719.538 | 1.565 | 16.646 | 0.151058 | 7/15/2014 | 42:15.1 | 0 |
| -340.215 | 19719.522 | 1.559 | 16.64 | 0.14776 | 7/15/2014 | 42:15.2 | 0 |
| -339.956 | 19719.507 | 1.561 | 16.629 | 0.147678 | 7/15/2014 | 42:15.3 | 0 |
| -339.635 | 19719.488 | 1.564 | 16.615 | 0.149382 | 7/15/2014 | 42:15.4 | 0 |
| -339.377 | 19719.471 | 1.57 | 16.598 | 0.150709 | 7/15/2014 | 42:15.5 | 0 |
| -339.12 | 19719.457 | 1.565 | 16.579 | 0.153653 | 7/15/2014 | 42:15.6 | 0 |
| -338.863 | 19719.44 | 1.561 | 16.557 | 0.15861 | 7/15/2014 | 42:15.7 | 0 |
| -338.542 | 19719.422 | 1.555 | 16.534 | 0.157252 | 7/15/2014 | 42:15.8 | 0 |
| -338.283 | 19719.405 | 1.561 | 16.51 | 0.157488 | 7/15/2014 | 42:15.9 | 0 |
| -338.025 | 19719.391 | 1.563 | 16.486 | 0.158265 | 7/15/2014 | 42:16.0 | 0 |
| -337.766 | 19719.374 | 1.566 | 16.464 | 0.162342 | 7/15/2014 | 42:16.1 | 0 |
| -337.51 | 19719.359 | 1.556 | 16.444 | 0.166885 | 7/15/2014 | 42:16.2 | 0 |
| -337.253 | 19719.345 | 1.563 | 16.427 | 0.165783 | 7/15/2014 | 42:16.3 | 0 |
| -336.934 | 19719.326 | 1.563 | 16.414 | 0.157806 | 7/15/2014 | 42:16.4 | 0 |
| -336.679 | 19719.311 | 1.569 | 16.406 | 0.154042 | 7/15/2014 | 42:16.5 | 0 |
| -336.424 | 19719.295 | 1.565 | 16.403 | 0.155612 | 7/15/2014 | 42:16.6 | 0 |
| -336.171 | 19719.279 | 1.561 | 16.405 | 0.151587 | 7/15/2014 | 42:16.7 | 0 |
| -335.859 | 19719.262 | 1.554 | 16.413 | 0.15634 | 7/15/2014 | 42:16.8 | 0 |
| -335.605 | 19719.247 | 1.558 | 16.425 | 0.149849 | 7/15/2014 | 42:16.9 | 0 |
| -335.353 | 19719.234 | 1.554 | 16.442 | 0.176873 | 7/15/2014 | 42:17.0 | 0 |
| -335.101 | 19719.219 | 1.558 | 16.461 | 0.187195 | 7/15/2014 | 42:17.1 | 0 |

| | | | | | | | |
|---|---|---|---|---|---|---|---|
| -334.852 | 19719.204 | 1.548 | 16.482 | 0.165724 | 7/15/2014 | 42:17.2 | 0 |
| -334.603 | 19719.189 | 1.546 | 16.503 | 0.178793 | 7/15/2014 | 42:17.3 | 0 |
| -334.171 | 19719.165 | 1.547 | 16.529 | 0.184145 | 7/15/2014 | 42:17.4 | 0 |
| -333.925 | 19719.15 | 1.547 | 16.543 | 0.185511 | 7/15/2014 | 42:17.5 | 0 |
| -333.679 | 19719.139 | 1.55 | 16.554 | 0.166104 | 7/15/2014 | 42:17.6 | 0 |
| -333.371 | 19719.12 | 1.547 | 16.561 | 0.160793 | 7/15/2014 | 42:17.7 | 0 |
| -333.248 | 19719.113 | 1.542 | 16.565 | 0.175308 | 7/15/2014 | 42:17.8 | 0 |
| -332.998 | 19719.1 | 1.552 | 16.566 | 0.144131 | 7/15/2014 | 42:17.9 | 0 |
| -332.751 | 19719.085 | 1.554 | 16.564 | 0.134323 | 7/15/2014 | 42:18.0 | 0 |
| -332.5 | 19719.071 | 1.561 | 16.561 | 0.115581 | 7/15/2014 | 42:18.1 | 0 |
| -332.253 | 19719.057 | 1.554 | 16.557 | 0.0993609 | 7/15/2014 | 42:18.2 | 0 |
| -332.003 | 19719.044 | 1.554 | 16.554 | 0.114289 | 7/15/2014 | 42:18.3 | 0 |
| -331.693 | 19719.026 | 1.547 | 16.553 | 0.0927831 | 7/15/2014 | 42:18.4 | 0 |
| -331.441 | 19719.014 | 1.55 | 16.555 | 0.10155 | 7/15/2014 | 42:18.5 | 0 |
| -331.193 | 19718.998 | 1.541 | 16.56 | 0.0951349 | 7/15/2014 | 42:18.6 | 0 |
| -330.942 | 19718.986 | 1.537 | 16.569 | 0.0927428 | 7/15/2014 | 42:18.7 | 0 |
| -330.629 | 19718.968 | 1.527 | 16.582 | 0.0945515 | 7/15/2014 | 42:18.8 | 0 |
| -330.376 | 19718.954 | 1.527 | 16.598 | 0.088094 | 7/15/2014 | 42:18.9 | 0 |
| -330.123 | 19718.94 | 1.525 | 16.617 | 0.0865211 | 7/15/2014 | 42:19.0 | 0 |
| -329.867 | 19718.928 | 1.525 | 16.636 | 0.0775686 | 7/15/2014 | 42:19.1 | 0 |
| -329.615 | 19718.914 | 1.521 | 16.656 | 0.085494 | 7/15/2014 | 42:19.2 | 0 |
| -329.361 | 19718.9 | 1.521 | 16.674 | 0.0810523 | 7/15/2014 | 42:19.3 | 0 |
| -329.045 | 19718.884 | 1.519 | 16.688 | 0.0875174 | 7/15/2014 | 42:19.4 | 0 |
| -328.791 | 19718.87 | 1.522 | 16.698 | 0.118783 | 7/15/2014 | 42:19.5 | 0 |
| -328.538 | 19718.856 | 1.522 | 16.703 | 0.086481 | 7/15/2014 | 42:19.6 | 0 |
| -328.284 | 19718.844 | 1.525 | 16.702 | 0.097222 | 7/15/2014 | 42:19.7 | 0 |
| -327.968 | 19718.828 | 1.522 | 16.696 | 0.0727813 | 7/15/2014 | 42:19.8 | 0 |
| -327.713 | 19718.814 | 1.526 | 16.684 | 0.0732035 | 7/15/2014 | 42:19.9 | 0 |
| -327.46 | 19718.802 | 1.526 | 16.669 | 0.0857392 | 7/15/2014 | 42:20.0 | 0 |
| -327.205 | 19718.789 | 1.532 | 16.65 | 0.0436651 | 7/15/2014 | 42:20.1 | 0 |
| -326.952 | 19718.775 | 1.53 | 16.63 | 0.0924229 | 7/15/2014 | 42:20.2 | 0 |
| -326.7 | 19718.764 | 1.53 | 16.609 | 0.0900409 | 7/15/2014 | 42:20.3 | 0 |
| -326.386 | 19718.748 | 1.527 | 16.588 | 0.125551 | 7/15/2014 | 42:20.4 | 0 |
| -326.135 | 19718.736 | 1.53 | 16.568 | 0.14637 | 7/15/2014 | 42:20.5 | 0 |
| -325.886 | 19718.724 | 1.521 | 16.551 | 0.127329 | 7/15/2014 | 42:20.6 | 0 |
| -325.636 | 19718.712 | 1.521 | 16.536 | 0.190728 | 7/15/2014 | 42:20.7 | 0 |
| -325.325 | 19718.697 | 1.515 | 16.524 | 0.141322 | 7/15/2014 | 42:20.8 | 0 |
| -325.075 | 19718.686 | 1.513 | 16.515 | 0.127566 | 7/15/2014 | 42:20.9 | 0 |
| -324.825 | 19718.674 | 1.51 | 16.511 | 0.118824 | 7/15/2014 | 42:21.0 | 0 |
| -324.575 | 19718.662 | 1.513 | 16.51 | 0.130311 | 7/15/2014 | 42:21.1 | 0 |
| -324.328 | 19718.65 | 1.508 | 16.513 | 0.166435 | 7/15/2014 | 42:21.2 | 0 |
| -324.078 | 19718.639 | 1.51 | 16.52 | 0.0989145 | 7/15/2014 | 42:21.3 | 0 |
| -323.646 | 19718.62 | 1.51 | 16.539 | 0.131933 | 7/15/2014 | 42:21.4 | 0 |
| -323.401 | 19718.609 | 1.51 | 16.555 | 0.142029 | 7/15/2014 | 42:21.5 | 0 |
| -323.156 | 19718.598 | 1.512 | 16.573 | 0.137135 | 7/15/2014 | 42:21.6 | 0 |
| -322.851 | 19718.584 | 1.512 | 16.593 | 0.142244 | 7/15/2014 | 42:21.7 | 0 |
| -322.727 | 19718.578 | 1.51 | 16.603 | 0.120256 | 7/15/2014 | 42:21.8 | 0 |

| | | | | | | | |
|---|---|---|---|---|---|---|---|
| -322.481 | 19718.566 | 1.515 | 16.624 | 0.0937573 | 7/15/2014 | 42:21.9 | 0 |
| -322.236 | 19718.556 | 1.518 | 16.645 | 0.101104 | 7/15/2014 | 42:22.0 | 0 |
| -321.99 | 19718.546 | 1.522 | 16.663 | 0.107337 | 7/15/2014 | 42:22.1 | 0 |
| -321.747 | 19718.535 | 1.522 | 16.678 | 0.067972 | 7/15/2014 | 42:22.2 | 0 |
| -321.503 | 19718.523 | 1.529 | 16.689 | 0.0633046 | 7/15/2014 | 42:22.3 | 0 |
| -321.082 | 19718.506 | 1.526 | 16.693 | 0.0595326 | 7/15/2014 | 42:22.4 | 0 |
| -320.84 | 19718.496 | 1.529 | 16.692 | 0.0604387 | 7/15/2014 | 42:22.5 | 0 |
| -320.6 | 19718.485 | 1.532 | 16.686 | 0.0730299 | 7/15/2014 | 42:22.6 | 0 |
| -320.298 | 19718.473 | 1.53 | 16.677 | 0.0667443 | 7/15/2014 | 42:22.7 | 0 |
| -320.177 | 19718.467 | 1.53 | 16.673 | 0.0469707 | 7/15/2014 | 42:22.8 | 0 |
| -319.934 | 19718.457 | 1.53 | 16.658 | 0.0451599 | 7/15/2014 | 42:22.9 | 0 |
| -319.691 | 19718.447 | 1.524 | 16.64 | 0.0426651 | 7/15/2014 | 42:23.0 | 0 |
| -319.447 | 19718.438 | 1.526 | 16.621 | 0.0432919 | 7/15/2014 | 42:23.1 | 0 |
| -319.203 | 19718.428 | 1.524 | 16.602 | 0.047529 | 7/15/2014 | 42:23.2 | 0 |
| -318.958 | 19718.418 | 1.524 | 16.582 | 0.0337529 | 7/15/2014 | 42:23.3 | 0 |
| -318.529 | 19718.399 | 1.524 | 16.555 | 0.02611 | 7/15/2014 | 42:23.4 | 0 |
| -318.285 | 19718.391 | 1.526 | 16.538 | 0.0181335 | 7/15/2014 | 42:23.5 | 0 |
| -318.04 | 19718.381 | 1.526 | 16.523 | 0.0223409 | 7/15/2014 | 42:23.6 | 0 |
| -317.733 | 19718.369 | 1.528 | 16.51 | 0.0309965 | 7/15/2014 | 42:23.7 | 0 |
| -317.609 | 19718.363 | 1.529 | 16.503 | 0.0450208 | 7/15/2014 | 42:23.8 | 0 |
| -317.362 | 19718.355 | 1.535 | 16.493 | 0.0640937 | 7/15/2014 | 42:23.9 | 0 |
| -317.113 | 19718.344 | 1.538 | 16.485 | 0.0330646 | 7/15/2014 | 42:24.0 | 0 |
| -316.865 | 19718.335 | 1.542 | 16.478 | 0.0912815 | 7/15/2014 | 42:24.1 | 0 |
| -316.617 | 19718.324 | 1.538 | 16.473 | 0.0556266 | 7/15/2014 | 42:24.2 | 0 |
| -316.371 | 19718.316 | 1.538 | 16.469 | 0.0541111 | 7/15/2014 | 42:24.3 | 0 |
| -315.94 | 19718.299 | 1.543 | 16.467 | 0.0795585 | 7/15/2014 | 42:24.4 | 0 |
| -315.696 | 19718.291 | 1.547 | 16.467 | 0.0554115 | 7/15/2014 | 42:24.5 | 0 |
| -315.452 | 19718.281 | 1.547 | 16.468 | 0.0999815 | 7/15/2014 | 42:24.6 | 0 |
| -315.146 | 19718.27 | 1.543 | 16.47 | 0.100106 | 7/15/2014 | 42:24.7 | 0 |
| -315.022 | 19718.264 | 1.542 | 16.471 | 0.116325 | 7/15/2014 | 42:24.8 | 0 |
| -314.777 | 19718.256 | 1.542 | 16.475 | 0.0815713 | 7/15/2014 | 42:24.9 | 0 |
| -314.531 | 19718.248 | 1.542 | 16.479 | 0.104677 | 7/15/2014 | 42:25.0 | 0 |
| -314.284 | 19718.236 | 1.546 | 16.484 | 0.0863485 | 7/15/2014 | 42:25.1 | 0 |
| -314.041 | 19718.229 | 1.54 | 16.489 | 0.106849 | 7/15/2014 | 42:25.2 | 0 |
| -313.795 | 19718.221 | 1.54 | 16.493 | 0.0954489 | 7/15/2014 | 42:25.3 | 0 |
| -313.369 | 19718.204 | 1.538 | 16.495 | 0.0835875 | 7/15/2014 | 42:25.4 | 0 |
| -313.127 | 19718.197 | 1.541 | 16.496 | 0.104514 | 7/15/2014 | 42:25.5 | 0 |
| -312.885 | 19718.188 | 1.544 | 16.495 | 0.0801495 | 7/15/2014 | 42:25.6 | 0 |
| -312.584 | 19718.177 | 1.544 | 16.493 | 0.0841011 | 7/15/2014 | 42:25.7 | 0 |
| -312.461 | 19718.173 | 1.543 | 16.492 | 0.0692852 | 7/15/2014 | 42:25.8 | 0 |
| -312.219 | 19718.164 | 1.542 | 16.488 | 0.0717975 | 7/15/2014 | 42:25.9 | 0 |
| -311.978 | 19718.156 | 1.538 | 16.483 | 0.0797863 | 7/15/2014 | 42:26.0 | 0 |
| -311.737 | 19718.147 | 1.538 | 16.478 | 0.0704576 | 7/15/2014 | 42:26.1 | 0 |
| -311.497 | 19718.141 | 1.538 | 16.473 | 0.0723434 | 7/15/2014 | 42:26.2 | 0 |
| -311.259 | 19718.131 | 1.535 | 16.467 | 0.0878096 | 7/15/2014 | 42:26.3 | 0 |
| -310.846 | 19718.119 | 1.531 | 16.459 | 0.105942 | 7/15/2014 | 42:26.4 | 0 |
| -310.612 | 19718.109 | 1.531 | 16.454 | 0.0996175 | 7/15/2014 | 42:26.5 | 0 |

| | | | | | | | |
|---|---|---|---|---|---|---|---|
| -310.379 | 19718.103 | 1.531 | 16.449 | 0.0733429 | 7/15/2014 | 42:26.6 | 0 |
| -310.089 | 19718.094 | 1.534 | 16.444 | 0.0755449 | 7/15/2014 | 42:26.7 | 0 |
| -309.97 | 19718.09 | 1.535 | 16.441 | 0.0849158 | 7/15/2014 | 42:26.8 | 0 |
| -309.736 | 19718.082 | 1.535 | 16.437 | 0.0759806 | 7/15/2014 | 42:26.9 | 0 |
| -309.389 | 19718.071 | 1.537 | 16.431 | 0.107478 | 7/15/2014 | 42:27.0 | 0 |
| -309.156 | 19718.063 | 1.537 | 16.427 | 0.0757405 | 7/15/2014 | 42:27.1 | 0 |
| -308.923 | 19718.055 | 1.543 | 16.424 | 0.0687507 | 7/15/2014 | 42:27.2 | 0 |
| -308.694 | 19718.048 | 1.547 | 16.42 | 0.127655 | 7/15/2014 | 42:27.3 | 0 |
| -308.407 | 19718.039 | 1.553 | 16.417 | 0.0774986 | 7/15/2014 | 42:27.4 | 0 |
| -308.178 | 19718.031 | 1.553 | 16.414 | 0.0859855 | 7/15/2014 | 42:27.5 | 0 |
| -307.95 | 19718.023 | 1.558 | 16.41 | 0.0929801 | 7/15/2014 | 42:27.6 | 0 |
| -307.666 | 19718.015 | 1.564 | 16.407 | 0.0863137 | 7/15/2014 | 42:27.7 | 0 |
| -307.436 | 19718.008 | 1.569 | 16.404 | 0.0988363 | 7/15/2014 | 42:27.8 | 0 |
| -307.205 | 19718 | 1.572 | 16.401 | 0.113713 | 7/15/2014 | 42:27.9 | 0 |
| -306.975 | 19717.992 | 1.574 | 16.398 | 0.0846526 | 7/15/2014 | 42:28.0 | 0 |
| -306.745 | 19717.984 | 1.574 | 16.397 | 0.129905 | 7/15/2014 | 42:28.1 | 0 |
| -306.515 | 19717.977 | 1.579 | 16.396 | 0.104199 | 7/15/2014 | 42:28.2 | 0 |
| -306.287 | 19717.969 | 1.579 | 16.396 | 0.11294 | 7/15/2014 | 42:28.3 | 0 |
| -306.002 | 19717.96 | 1.579 | 16.397 | 0.108536 | 7/15/2014 | 42:28.4 | 0 |
| -305.773 | 19717.953 | 1.582 | 16.4 | 0.0879331 | 7/15/2014 | 42:28.5 | 0 |
| -305.547 | 19717.947 | 1.584 | 16.404 | 0.0849279 | 7/15/2014 | 42:28.6 | 0 |
| -305.262 | 19717.938 | 1.586 | 16.409 | 0.0856901 | 7/15/2014 | 42:28.7 | 0 |
| -305.033 | 19717.93 | 1.586 | 16.415 | 0.0859274 | 7/15/2014 | 42:28.8 | 0 |
| -304.802 | 19717.922 | 1.591 | 16.423 | 0.0891235 | 7/15/2014 | 42:28.9 | 0 |
| -304.573 | 19717.914 | 1.588 | 16.431 | 0.0850694 | 7/15/2014 | 42:29.0 | 0 |
| -304.341 | 19717.906 | 1.593 | 16.44 | 0.0855679 | 7/15/2014 | 42:29.1 | 0 |
| -304.111 | 19717.898 | 1.596 | 16.449 | 0.0855072 | 7/15/2014 | 42:29.2 | 0 |
| -303.883 | 19717.894 | 1.592 | 16.459 | 0.100035 | 7/15/2014 | 42:29.3 | 0 |
| -303.598 | 19717.885 | 1.592 | 16.47 | 0.0880656 | 7/15/2014 | 42:29.4 | 0 |
| -303.369 | 19717.877 | 1.596 | 16.48 | 0.0849405 | 7/15/2014 | 42:29.5 | 0 |
| -303.141 | 19717.869 | 1.598 | 16.489 | 0.0848159 | 7/15/2014 | 42:29.6 | 0 |
| -302.857 | 19717.859 | 1.598 | 16.498 | 0.0853908 | 7/15/2014 | 42:29.7 | 0 |
| -302.627 | 19717.852 | 1.604 | 16.506 | 0.0867601 | 7/15/2014 | 42:29.8 | 0 |
| -302.397 | 19717.845 | 1.606 | 16.513 | 0.0877617 | 7/15/2014 | 42:29.9 | 0 |
| -302.168 | 19717.839 | 1.606 | 16.519 | 0.0890385 | 7/15/2014 | 42:30.0 | 0 |
| -301.938 | 19717.832 | 1.611 | 16.524 | 0.0897622 | 7/15/2014 | 42:30.1 | 0 |
| -301.709 | 19717.824 | 1.611 | 16.528 | 0.0922613 | 7/15/2014 | 42:30.2 | 0 |
| -301.482 | 19717.816 | 1.617 | 16.53 | 0.0916243 | 7/15/2014 | 42:30.3 | 0 |
| -301.198 | 19717.807 | 1.622 | 16.532 | 0.0905616 | 7/15/2014 | 42:30.4 | 0 |
| -300.973 | 19717.799 | 1.629 | 16.533 | 0.0902473 | 7/15/2014 | 42:30.5 | 0 |
| -300.747 | 19717.792 | 1.631 | 16.532 | 0.104287 | 7/15/2014 | 42:30.6 | 0 |
| -300.466 | 19717.784 | 1.637 | 16.531 | 0.101147 | 7/15/2014 | 42:30.7 | 0 |
| -300.24 | 19717.777 | 1.644 | 16.53 | 0.116965 | 7/15/2014 | 42:30.8 | 0 |
| -300.014 | 19717.77 | 1.651 | 16.527 | 0.115752 | 7/15/2014 | 42:30.9 | 0 |
| -299.787 | 19717.762 | 1.664 | 16.524 | 0.109736 | 7/15/2014 | 42:31.0 | 0 |
| -299.56 | 19717.754 | 1.668 | 16.52 | 0.141465 | 7/15/2014 | 42:31.1 | 0 |
| -299.335 | 19717.746 | 1.674 | 16.516 | 0.134652 | 7/15/2014 | 42:31.2 | 0 |

| | | | | | | | |
|---|---|---|---|---|---|---|---|
| -299.109 | 19717.738 | 1.678 | 16.511 | 0.147029 | 7/15/2014 | 42:31.3 | 0 |
| -298.83 | 19717.729 | 1.685 | 16.506 | 0.145526 | 7/15/2014 | 42:31.4 | 0 |
| -298.606 | 19717.723 | 1.687 | 16.5 | 0.116697 | 7/15/2014 | 42:31.5 | 0 |
| -298.381 | 19717.715 | 1.695 | 16.494 | 0.138824 | 7/15/2014 | 42:31.6 | 0 |
| -298.097 | 19717.705 | 1.7 | 16.487 | 0.129395 | 7/15/2014 | 42:31.7 | 0 |
| -297.869 | 19717.697 | 1.703 | 16.48 | 0.0960077 | 7/15/2014 | 42:31.8 | 0 |
| -297.641 | 19717.689 | 1.709 | 16.471 | 0.096075 | 7/15/2014 | 42:31.9 | 0 |
| -297.409 | 19717.682 | 1.716 | 16.462 | 0.0814061 | 7/15/2014 | 42:32.0 | 0 |
| -297.179 | 19717.674 | 1.725 | 16.453 | 0.0887837 | 7/15/2014 | 42:32.1 | 0 |
| -296.949 | 19717.666 | 1.73 | 16.442 | 0.122931 | 7/15/2014 | 42:32.2 | 0 |
| -296.835 | 19717.662 | 1.739 | 16.437 | 0.110709 | 7/15/2014 | 42:32.3 | 0 |
| -296.431 | 19717.648 | 1.749 | 16.42 | 0.115433 | 7/15/2014 | 42:32.4 | 0 |
| -296.199 | 19717.641 | 1.758 | 16.408 | 0.137125 | 7/15/2014 | 42:32.5 | 0 |
| -295.968 | 19717.633 | 1.765 | 16.397 | 0.140949 | 7/15/2014 | 42:32.6 | 0 |
| -295.678 | 19717.623 | 1.774 | 16.387 | 0.151331 | 7/15/2014 | 42:32.7 | 0 |
| -295.561 | 19717.619 | 1.776 | 16.381 | 0.15889 | 7/15/2014 | 42:32.8 | 0 |
| -295.326 | 19717.611 | 1.782 | 16.372 | 0.117648 | 7/15/2014 | 42:32.9 | 0 |
| -295.093 | 19717.602 | 1.789 | 16.363 | 0.158756 | 7/15/2014 | 42:33.0 | 0 |
| -294.859 | 19717.594 | 1.794 | 16.356 | 0.151354 | 7/15/2014 | 42:33.1 | 0 |
| -294.625 | 19717.586 | 1.797 | 16.35 | 0.166758 | 7/15/2014 | 42:33.2 | 0 |
| -294.391 | 19717.578 | 1.801 | 16.345 | 0.153117 | 7/15/2014 | 42:33.3 | 0 |
| -293.983 | 19717.563 | 1.813 | 16.342 | 0.18038 | 7/15/2014 | 42:33.4 | 0 |
| -293.75 | 19717.555 | 1.817 | 16.34 | 0.201691 | 7/15/2014 | 42:33.5 | 0 |
| -293.517 | 19717.545 | 1.824 | 16.34 | 0.181506 | 7/15/2014 | 42:33.6 | 0 |
| -293.222 | 19717.533 | 1.829 | 16.34 | 0.177827 | 7/15/2014 | 42:33.7 | 0 |
| -293.105 | 19717.529 | 1.834 | 16.34 | 0.148428 | 7/15/2014 | 42:33.8 | 0 |
| -292.869 | 19717.521 | 1.84 | 16.342 | 0.167385 | 7/15/2014 | 42:33.9 | 0 |
| -292.631 | 19717.514 | 1.846 | 16.344 | 0.127039 | 7/15/2014 | 42:34.0 | 0 |
| -292.392 | 19717.503 | 1.849 | 16.346 | 0.140726 | 7/15/2014 | 42:34.1 | 0 |
| -292.156 | 19717.494 | 1.846 | 16.348 | 0.121044 | 7/15/2014 | 42:34.2 | 0 |
| -291.915 | 19717.484 | 1.854 | 16.351 | 0.122579 | 7/15/2014 | 42:34.3 | 0 |
| -291.498 | 19717.467 | 1.858 | 16.355 | 0.108835 | 7/15/2014 | 42:34.4 | 0 |
| -291.26 | 19717.459 | 1.86 | 16.356 | 0.119061 | 7/15/2014 | 42:34.5 | 0 |
| -291.02 | 19717.449 | 1.865 | 16.358 | 0.128214 | 7/15/2014 | 42:34.6 | 0 |
| -290.722 | 19717.436 | 1.865 | 16.359 | 0.11473 | 7/15/2014 | 42:34.7 | 0 |
| -290.602 | 19717.432 | 1.866 | 16.36 | 0.124561 | 7/15/2014 | 42:34.8 | 0 |
| -290.359 | 19717.422 | 1.876 | 16.36 | 0.115821 | 7/15/2014 | 42:34.9 | 0 |
| -290.119 | 19717.412 | 1.873 | 16.359 | 0.140245 | 7/15/2014 | 42:35.0 | 0 |
| -289.876 | 19717.4 | 1.876 | 16.357 | 0.137439 | 7/15/2014 | 42:35.1 | 0 |
| -289.636 | 19717.392 | 1.872 | 16.353 | 0.111894 | 7/15/2014 | 42:35.2 | 0 |
| -289.393 | 19717.381 | 1.874 | 16.347 | 0.119073 | 7/15/2014 | 42:35.3 | 0 |
| -288.97 | 19717.361 | 1.872 | 16.334 | 0.111118 | 7/15/2014 | 42:35.4 | 0 |
| -288.729 | 19717.352 | 1.872 | 16.324 | 0.131017 | 7/15/2014 | 42:35.5 | 0 |
| -288.487 | 19717.341 | 1.869 | 16.313 | 0.109744 | 7/15/2014 | 42:35.6 | 0 |
| -288.182 | 19717.326 | 1.874 | 16.301 | 0.110458 | 7/15/2014 | 42:35.7 | 0 |
| -288.061 | 19717.321 | 1.874 | 16.295 | 0.126918 | 7/15/2014 | 42:35.8 | 0 |
| -287.815 | 19717.311 | 1.881 | 16.282 | 0.121737 | 7/15/2014 | 42:35.9 | 0 |

| | | | | | | | |
|---|---|---|---|---|---|---|---|
| -287.57 | 19717.299 | 1.884 | 16.269 | 0.123216 | 7/15/2014 | 42:36.0 | 0 |
| -287.323 | 19717.287 | 1.89 | 16.257 | 0.123067 | 7/15/2014 | 42:36.1 | 0 |
| -287.077 | 19717.275 | 1.89 | 16.245 | 0.10895 | 7/15/2014 | 42:36.2 | 0 |
| -286.83 | 19717.265 | 1.896 | 16.235 | 0.104383 | 7/15/2014 | 42:36.3 | 0 |
| -286.399 | 19717.244 | 1.9 | 16.224 | 0.110029 | 7/15/2014 | 42:36.4 | 0 |
| -286.154 | 19717.23 | 1.897 | 16.218 | 0.111647 | 7/15/2014 | 42:36.5 | 0 |
| -285.909 | 19717.219 | 1.897 | 16.213 | 0.113499 | 7/15/2014 | 42:36.6 | 0 |
| -285.604 | 19717.204 | 1.894 | 16.21 | 0.121041 | 7/15/2014 | 42:36.7 | 0 |
| -285.479 | 19717.198 | 1.893 | 16.208 | 0.120675 | 7/15/2014 | 42:36.8 | 0 |
| -285.233 | 19717.186 | 1.893 | 16.208 | 0.122389 | 7/15/2014 | 42:36.9 | 0 |
| -284.987 | 19717.174 | 1.889 | 16.208 | 0.124916 | 7/15/2014 | 42:37.0 | 0 |
| -284.741 | 19717.162 | 1.889 | 16.209 | 0.134982 | 7/15/2014 | 42:37.1 | 0 |
| -284.496 | 19717.146 | 1.889 | 16.211 | 0.134641 | 7/15/2014 | 42:37.2 | 0 |
| -284.252 | 19717.135 | 1.889 | 16.213 | 0.129885 | 7/15/2014 | 42:37.3 | 0 |
| -283.827 | 19717.112 | 1.887 | 16.215 | 0.126598 | 7/15/2014 | 42:37.4 | 0 |
| -283.585 | 19717.1 | 1.887 | 16.216 | 0.122663 | 7/15/2014 | 42:37.5 | 0 |
| -283.341 | 19717.086 | 1.887 | 16.217 | 0.120429 | 7/15/2014 | 42:37.6 | 0 |
| -283.038 | 19717.07 | 1.887 | 16.217 | 0.122245 | 7/15/2014 | 42:37.7 | 0 |
| -282.916 | 19717.063 | 1.885 | 16.217 | 0.119047 | 7/15/2014 | 42:37.8 | 0 |
| -282.671 | 19717.051 | 1.882 | 16.216 | 0.120102 | 7/15/2014 | 42:37.9 | 0 |
| -282.426 | 19717.036 | 1.882 | 16.214 | 0.122114 | 7/15/2014 | 42:38.0 | 0 |
| -282.179 | 19717.022 | 1.878 | 16.211 | 0.113688 | 7/15/2014 | 42:38.1 | 0 |
| -281.934 | 19717.009 | 1.879 | 16.207 | 0.11421 | 7/15/2014 | 42:38.2 | 0 |
| -281.685 | 19716.994 | 1.887 | 16.204 | 0.112895 | 7/15/2014 | 42:38.3 | 0 |
| -281.256 | 19716.969 | 1.888 | 16.198 | 0.117824 | 7/15/2014 | 42:38.4 | 0 |
| -281.012 | 19716.956 | 1.892 | 16.194 | 0.127315 | 7/15/2014 | 42:38.5 | 0 |
| -280.766 | 19716.94 | 1.895 | 16.191 | 0.130325 | 7/15/2014 | 42:38.6 | 0 |
| -280.462 | 19716.923 | 1.895 | 16.188 | 0.130321 | 7/15/2014 | 42:38.7 | 0 |
| -280.338 | 19716.915 | 1.898 | 16.186 | 0.137968 | 7/15/2014 | 42:38.8 | 0 |
| -280.09 | 19716.9 | 1.9 | 16.184 | 0.126778 | 7/15/2014 | 42:38.9 | 0 |
| -279.845 | 19716.885 | 1.893 | 16.183 | 0.118528 | 7/15/2014 | 42:39.0 | 0 |
| -279.598 | 19716.869 | 1.893 | 16.182 | 0.114005 | 7/15/2014 | 42:39.1 | 0 |
| -279.354 | 19716.854 | 1.889 | 16.182 | 0.102564 | 7/15/2014 | 42:39.2 | 0 |
| -279.108 | 19716.838 | 1.893 | 16.182 | 0.0992941 | 7/15/2014 | 42:39.3 | 0 |
| -278.685 | 19716.811 | 1.9 | 16.182 | 0.0869042 | 7/15/2014 | 42:39.4 | 0 |
| -278.443 | 19716.795 | 1.907 | 16.181 | 0.0885784 | 7/15/2014 | 42:39.5 | 0 |
| -278.202 | 19716.779 | 1.912 | 16.18 | 0.091417 | 7/15/2014 | 42:39.6 | 0 |
| -277.902 | 19716.758 | 1.912 | 16.178 | 0.0949372 | 7/15/2014 | 42:39.7 | 0 |
| -277.78 | 19716.75 | 1.914 | 16.177 | 0.0973904 | 7/15/2014 | 42:39.8 | 0 |
| -277.536 | 19716.733 | 1.921 | 16.173 | 0.104788 | 7/15/2014 | 42:39.9 | 0 |
| -277.295 | 19716.718 | 1.914 | 16.168 | 0.109333 | 7/15/2014 | 42:40.0 | 0 |
| -277.052 | 19716.699 | 1.914 | 16.162 | 0.112961 | 7/15/2014 | 42:40.1 | 0 |
| -276.812 | 19716.684 | 1.91 | 16.155 | 0.117567 | 7/15/2014 | 42:40.2 | 0 |
| -276.571 | 19716.666 | 1.91 | 16.146 | 0.121225 | 7/15/2014 | 42:40.3 | 0 |
| -276.154 | 19716.635 | 1.903 | 16.132 | 0.132405 | 7/15/2014 | 42:40.4 | 0 |
| -275.917 | 19716.619 | 1.907 | 16.122 | 0.13833 | 7/15/2014 | 42:40.5 | 0 |
| -275.68 | 19716.602 | 1.907 | 16.112 | 0.139745 | 7/15/2014 | 42:40.6 | 0 |

| | | | | | | | |
|---|---|---|---|---|---|---|---|
| -275.383 | 19716.579 | 1.91 | 16.101 | 0.124756 | 7/15/2014 | 42:40.7 | 0 |
| -275.264 | 19716.571 | 1.908 | 16.096 | 0.127782 | 7/15/2014 | 42:40.8 | 0 |
| -275.025 | 19716.553 | 1.911 | 16.085 | 0.130159 | 7/15/2014 | 42:40.9 | 0 |
| -274.788 | 19716.535 | 1.914 | 16.074 | 0.103854 | 7/15/2014 | 42:41.0 | 0 |
| -274.551 | 19716.518 | 1.917 | 16.063 | 0.106134 | 7/15/2014 | 42:41.1 | 0 |
| -274.315 | 19716.5 | 1.917 | 16.051 | 0.0978185 | 7/15/2014 | 42:41.2 | 0 |
| -274.079 | 19716.48 | 1.917 | 16.039 | 0.0919366 | 7/15/2014 | 42:41.3 | 0 |
| -273.669 | 19716.448 | 1.912 | 16.02 | 0.0935401 | 7/15/2014 | 42:41.4 | 0 |
| -273.436 | 19716.432 | 1.909 | 16.006 | 0.101537 | 7/15/2014 | 42:41.5 | 0 |
| -273.201 | 19716.411 | 1.909 | 15.991 | 0.108657 | 7/15/2014 | 42:41.6 | 0 |
| -272.909 | 19716.389 | 1.9 | 15.976 | 0.0913427 | 7/15/2014 | 42:41.7 | 0 |
| -272.791 | 19716.379 | 1.897 | 15.968 | 0.104125 | 7/15/2014 | 42:41.8 | 0 |
| -272.553 | 19716.359 | 1.9 | 15.952 | 0.115521 | 7/15/2014 | 42:41.9 | 0 |
| -272.316 | 19716.34 | 1.9 | 15.935 | 0.0891186 | 7/15/2014 | 42:42.0 | 0 |
| -272.079 | 19716.32 | 1.904 | 15.918 | 0.0884384 | 7/15/2014 | 42:42.1 | 0 |
| -271.842 | 19716.301 | 1.908 | 15.901 | 0.0634854 | 7/15/2014 | 42:42.2 | 0 |
| -271.603 | 19716.28 | 1.915 | 15.884 | 0.0772657 | 7/15/2014 | 42:42.3 | 0 |
| -271.186 | 19716.244 | 1.916 | 15.862 | 0.0573123 | 7/15/2014 | 42:42.4 | 0 |
| -270.946 | 19716.225 | 1.92 | 15.848 | 0.0518901 | 7/15/2014 | 42:42.5 | 0 |
| -270.706 | 19716.205 | 1.92 | 15.835 | 0.0590746 | 7/15/2014 | 42:42.6 | 0 |
| -270.406 | 19716.178 | 1.916 | 15.824 | 0.0720541 | 7/15/2014 | 42:42.7 | 0 |
| -270.286 | 19716.166 | 1.915 | 15.818 | 0.0689333 | 7/15/2014 | 42:42.8 | 0 |
| -270.041 | 19716.146 | 1.911 | 15.808 | 0.0508492 | 7/15/2014 | 42:42.9 | 0 |
| -269.797 | 19716.125 | 1.908 | 15.8 | 0.060927 | 7/15/2014 | 42:43.0 | 0 |
| -269.552 | 19716.102 | 1.908 | 15.793 | 0.0582632 | 7/15/2014 | 42:43.1 | 0 |
| -269.309 | 19716.081 | 1.9 | 15.786 | 0.0505596 | 7/15/2014 | 42:43.2 | 0 |
| -269.064 | 19716.06 | 1.904 | 15.78 | 0.0473077 | 7/15/2014 | 42:43.3 | 0 |
| -268.639 | 19716.02 | 1.909 | 15.773 | 0.0502681 | 7/15/2014 | 42:43.4 | 0 |
| -268.396 | 19715.998 | 1.912 | 15.768 | 0.0539195 | 7/15/2014 | 42:43.5 | 0 |
| -268.155 | 19715.976 | 1.92 | 15.765 | 0.0568468 | 7/15/2014 | 42:43.6 | 0 |
| -267.858 | 19715.947 | 1.923 | 15.762 | 0.0599792 | 7/15/2014 | 42:43.7 | 0 |
| -267.735 | 19715.937 | 1.922 | 15.76 | 0.0578975 | 7/15/2014 | 42:43.8 | 0 |
| -267.495 | 19715.913 | 1.93 | 15.758 | 0.069601 | 7/15/2014 | 42:43.9 | 0 |
| -267.257 | 19715.892 | 1.93 | 15.757 | 0.0758624 | 7/15/2014 | 42:44.0 | 0 |
| -267.021 | 19715.868 | 1.93 | 15.758 | 0.08114 | 7/15/2014 | 42:44.1 | 0 |
| -266.787 | 19715.846 | 1.927 | 15.759 | 0.092444 | 7/15/2014 | 42:44.2 | 0 |
| -266.441 | 19715.813 | 1.93 | 15.764 | 0.0856737 | 7/15/2014 | 42:44.3 | 0 |
| -266.154 | 19715.784 | 1.928 | 15.768 | 0.0876336 | 7/15/2014 | 42:44.4 | 0 |
| -265.927 | 19715.762 | 1.93 | 15.772 | 0.0970657 | 7/15/2014 | 42:44.5 | 0 |
| -265.7 | 19715.739 | 1.932 | 15.776 | 0.0818644 | 7/15/2014 | 42:44.6 | 0 |
| -265.418 | 19715.71 | 1.932 | 15.781 | 0.0863789 | 7/15/2014 | 42:44.7 | 0 |
| -265.189 | 19715.688 | 1.934 | 15.786 | 0.0999911 | 7/15/2014 | 42:44.8 | 0 |
| -264.961 | 19715.664 | 1.934 | 15.791 | 0.0918674 | 7/15/2014 | 42:44.9 | 0 |
| -264.73 | 19715.642 | 1.939 | 15.795 | 0.0922623 | 7/15/2014 | 42:45.0 | 0 |
| -264.5 | 19715.617 | 1.939 | 15.799 | 0.0868343 | 7/15/2014 | 42:45.1 | 0 |
| -264.27 | 19715.594 | 1.941 | 15.802 | 0.0589467 | 7/15/2014 | 42:45.2 | 0 |
| -264.157 | 19715.582 | 1.941 | 15.803 | 0.0647552 | 7/15/2014 | 42:45.3 | 0 |

| | | | | | | | |
|---|---|---|---|---|---|---|---|
| -263.754 | 19715.541 | 1.941 | 15.806 | 0.0678714 | 7/15/2014 | 42:45.4 | 0 |
| -263.523 | 19715.517 | 1.941 | 15.807 | 0.0580954 | 7/15/2014 | 42:45.5 | 0 |
| -263.291 | 19715.493 | 1.941 | 15.807 | 0.0692961 | 7/15/2014 | 42:45.6 | 0 |
| -262.997 | 19715.463 | 1.943 | 15.807 | 0.055266 | 7/15/2014 | 42:45.7 | 0 |
| -262.881 | 19715.45 | 1.941 | 15.807 | 0.0612699 | 7/15/2014 | 42:45.8 | 0 |
| -262.644 | 19715.424 | 1.943 | 15.806 | 0.0794169 | 7/15/2014 | 42:45.9 | 0 |
| -262.407 | 19715.4 | 1.938 | 15.804 | 0.072694 | 7/15/2014 | 42:46.0 | 0 |
| -262.168 | 19715.375 | 1.938 | 15.803 | 0.0618998 | 7/15/2014 | 42:46.1 | 0 |
| -261.93 | 19715.35 | 1.932 | 15.802 | 0.0810099 | 7/15/2014 | 42:46.2 | 0 |
| -261.689 | 19715.325 | 1.934 | 15.801 | 0.0683181 | 7/15/2014 | 42:46.3 | 0 |
| -261.269 | 19715.279 | 1.937 | 15.801 | 0.0925294 | 7/15/2014 | 42:46.4 | 0 |
| -261.029 | 19715.252 | 1.937 | 15.802 | 0.0912465 | 7/15/2014 | 42:46.5 | 0 |
| -260.79 | 19715.227 | 1.937 | 15.803 | 0.0951305 | 7/15/2014 | 42:46.6 | 0 |
| -260.489 | 19715.193 | 1.937 | 15.804 | 0.0968508 | 7/15/2014 | 42:46.7 | 0 |
| -260.368 | 19715.181 | 1.936 | 15.805 | 0.0855125 | 7/15/2014 | 42:46.8 | 0 |
| -260.124 | 19715.154 | 1.942 | 15.807 | 0.0959577 | 7/15/2014 | 42:46.9 | 0 |
| -259.883 | 19715.129 | 1.936 | 15.809 | 0.0930357 | 7/15/2014 | 42:47.0 | 0 |
| -259.639 | 19715.102 | 1.936 | 15.811 | 0.0864844 | 7/15/2014 | 42:47.1 | 0 |
| -259.397 | 19715.074 | 1.934 | 15.813 | 0.0849656 | 7/15/2014 | 42:47.2 | 0 |
| -259.157 | 19715.047 | 1.931 | 15.816 | 0.0853557 | 7/15/2014 | 42:47.3 | 0 |
| -258.737 | 19715 | 1.935 | 15.818 | 0.0862908 | 7/15/2014 | 42:47.4 | 0 |
| -258.498 | 19714.973 | 1.935 | 15.82 | 0.0846259 | 7/15/2014 | 42:47.5 | 0 |
| -258.259 | 19714.947 | 1.935 | 15.821 | 0.0811127 | 7/15/2014 | 42:47.6 | 0 |
| -257.961 | 19714.913 | 1.938 | 15.822 | 0.0798703 | 7/15/2014 | 42:47.7 | 0 |
| -257.84 | 19714.899 | 1.937 | 15.823 | 0.0779829 | 7/15/2014 | 42:47.8 | 0 |
| -257.599 | 19714.872 | 1.937 | 15.823 | 0.0832879 | 7/15/2014 | 42:47.9 | 0 |
| -257.358 | 19714.845 | 1.936 | 15.823 | 0.0846309 | 7/15/2014 | 42:48.0 | 0 |
| -257.117 | 19714.816 | 1.938 | 15.822 | 0.0926218 | 7/15/2014 | 42:48.1 | 0 |
| -256.877 | 19714.789 | 1.932 | 15.821 | 0.100986 | 7/15/2014 | 42:48.2 | 0 |
| -256.637 | 19714.762 | 1.935 | 15.82 | 0.104089 | 7/15/2014 | 42:48.3 | 0 |
| -256.217 | 19714.712 | 1.934 | 15.817 | 0.123985 | 7/15/2014 | 42:48.4 | 0 |
| -255.977 | 19714.685 | 1.936 | 15.816 | 0.107864 | 7/15/2014 | 42:48.5 | 0 |
| -255.737 | 19714.657 | 1.938 | 15.815 | 0.121594 | 7/15/2014 | 42:48.6 | 0 |
| -255.435 | 19714.621 | 1.941 | 15.815 | 0.103094 | 7/15/2014 | 42:48.7 | 0 |
| -255.314 | 19714.607 | 1.938 | 15.814 | 0.107307 | 7/15/2014 | 42:48.8 | 0 |
| -255.071 | 19714.58 | 1.942 | 15.815 | 0.121973 | 7/15/2014 | 42:48.9 | 0 |
| -254.828 | 19714.551 | 1.94 | 15.817 | 0.11437 | 7/15/2014 | 42:49.0 | 0 |
| -254.582 | 19714.521 | 1.944 | 15.819 | 0.123599 | 7/15/2014 | 42:49.1 | 0 |
| -254.337 | 19714.493 | 1.944 | 15.823 | 0.13884 | 7/15/2014 | 42:49.2 | 0 |
| -254.093 | 19714.464 | 1.944 | 15.828 | 0.118353 | 7/15/2014 | 42:49.3 | 0 |
| -253.664 | 19714.413 | 1.947 | 15.838 | 0.135224 | 7/15/2014 | 42:49.4 | 0 |
| -253.42 | 19714.385 | 1.949 | 15.844 | 0.115543 | 7/15/2014 | 42:49.5 | 0 |
| -253.176 | 19714.354 | 1.952 | 15.851 | 0.110305 | 7/15/2014 | 42:49.6 | 0 |
| -252.87 | 19714.318 | 1.954 | 15.858 | 0.106205 | 7/15/2014 | 42:49.7 | 0 |
| -252.746 | 19714.304 | 1.953 | 15.861 | 0.103129 | 7/15/2014 | 42:49.8 | 0 |
| -252.499 | 19714.273 | 1.955 | 15.867 | 0.0901759 | 7/15/2014 | 42:49.9 | 0 |
| -252.253 | 19714.244 | 1.958 | 15.872 | 0.114024 | 7/15/2014 | 42:50.0 | 0 |

| | | | | | | | |
|---|---|---|---|---|---|---|---|
| -252.007 | 19714.215 | 1.96 | 15.875 | 0.110773 | 7/15/2014 | 42:50.1 | 0 |
| -251.761 | 19714.185 | 1.957 | 15.876 | 0.131233 | 7/15/2014 | 42:50.2 | 0 |
| -251.517 | 19714.155 | 1.96 | 15.877 | 0.128836 | 7/15/2014 | 42:50.3 | 0 |
| -251.091 | 19714.102 | 1.96 | 15.876 | 0.13025 | 7/15/2014 | 42:50.4 | 0 |
| -250.848 | 19714.072 | 1.965 | 15.874 | 0.151653 | 7/15/2014 | 42:50.5 | 0 |
| -250.606 | 19714.043 | 1.968 | 15.873 | 0.128049 | 7/15/2014 | 42:50.6 | 0 |
| -250.306 | 19714.006 | 1.971 | 15.871 | 0.148872 | 7/15/2014 | 42:50.7 | 0 |
| -250.183 | 19713.99 | 1.972 | 15.87 | 0.133179 | 7/15/2014 | 42:50.8 | 0 |
| -249.941 | 19713.961 | 1.975 | 15.868 | 0.132652 | 7/15/2014 | 42:50.9 | 0 |
| -249.7 | 19713.93 | 1.971 | 15.866 | 0.108957 | 7/15/2014 | 42:51.0 | 0 |
| -249.458 | 19713.899 | 1.977 | 15.865 | 0.132029 | 7/15/2014 | 42:51.1 | 0 |
| -249.218 | 19713.87 | 1.977 | 15.864 | 0.130371 | 7/15/2014 | 42:51.2 | 0 |
| -248.977 | 19713.84 | 1.98 | 15.864 | 0.124948 | 7/15/2014 | 42:51.3 | 0 |
| -248.56 | 19713.786 | 1.98 | 15.863 | 0.121547 | 7/15/2014 | 42:51.4 | 0 |
| -248.322 | 19713.757 | 1.977 | 15.863 | 0.13671 | 7/15/2014 | 42:51.5 | 0 |
| -248.083 | 19713.727 | 1.977 | 15.863 | 0.115112 | 7/15/2014 | 42:51.6 | 0 |
| -247.782 | 19713.688 | 1.977 | 15.863 | 0.112742 | 7/15/2014 | 42:51.7 | 0 |
| -247.663 | 19713.674 | 1.974 | 15.863 | 0.107779 | 7/15/2014 | 42:51.8 | 0 |
| -247.419 | 19713.642 | 1.976 | 15.862 | 0.106705 | 7/15/2014 | 42:51.9 | 0 |
| -247.177 | 19713.609 | 1.97 | 15.862 | 0.11552 | 7/15/2014 | 42:52.0 | 0 |
| -246.931 | 19713.578 | 1.972 | 15.862 | 0.112623 | 7/15/2014 | 42:52.1 | 0 |
| -246.687 | 19713.548 | 1.97 | 15.862 | 0.111478 | 7/15/2014 | 42:52.2 | 0 |
| -246.439 | 19713.516 | 1.978 | 15.862 | 0.128243 | 7/15/2014 | 42:52.3 | 0 |
| -246.133 | 19713.477 | 1.982 | 15.862 | 0.120936 | 7/15/2014 | 42:52.4 | 0 |
| -245.885 | 19713.442 | 1.988 | 15.862 | 0.11831 | 7/15/2014 | 42:52.5 | 0 |
| -245.638 | 19713.41 | 1.988 | 15.862 | 0.120781 | 7/15/2014 | 42:52.6 | 0 |
| -245.389 | 19713.379 | 1.994 | 15.863 | 0.125274 | 7/15/2014 | 42:52.7 | 0 |
| -245.08 | 19713.338 | 1.988 | 15.864 | 0.127712 | 7/15/2014 | 42:52.8 | 0 |
| -244.829 | 19713.305 | 1.994 | 15.865 | 0.138673 | 7/15/2014 | 42:52.9 | 0 |
| -244.579 | 19713.271 | 1.986 | 15.866 | 0.132887 | 7/15/2014 | 42:53.0 | 0 |
| -244.328 | 19713.238 | 1.986 | 15.868 | 0.132505 | 7/15/2014 | 42:53.1 | 0 |
| -244.079 | 19713.205 | 1.982 | 15.87 | 0.134567 | 7/15/2014 | 42:53.2 | 0 |
| -243.829 | 19713.174 | 1.982 | 15.874 | 0.135844 | 7/15/2014 | 42:53.3 | 0 |
| -243.52 | 19713.131 | 1.982 | 15.877 | 0.139881 | 7/15/2014 | 42:53.4 | 0 |
| -243.271 | 19713.099 | 1.985 | 15.882 | 0.137522 | 7/15/2014 | 42:53.5 | 0 |
| -243.023 | 19713.064 | 1.981 | 15.886 | 0.139882 | 7/15/2014 | 42:53.6 | 0 |
| -242.773 | 19713.032 | 1.987 | 15.892 | 0.137838 | 7/15/2014 | 42:53.7 | 0 |
| -242.464 | 19712.99 | 1.981 | 15.897 | 0.138747 | 7/15/2014 | 42:53.8 | 0 |
| -242.213 | 19712.957 | 1.981 | 15.903 | 0.128957 | 7/15/2014 | 42:53.9 | 0 |
| -241.963 | 19712.924 | 1.978 | 15.908 | 0.118971 | 7/15/2014 | 42:54.0 | 0 |
| -241.712 | 19712.89 | 1.975 | 15.914 | 0.112759 | 7/15/2014 | 42:54.1 | 0 |
| -241.462 | 19712.855 | 1.975 | 15.919 | 0.103763 | 7/15/2014 | 42:54.2 | 0 |
| -241.212 | 19712.822 | 1.975 | 15.924 | 0.0965366 | 7/15/2014 | 42:54.3 | 0 |
| -240.902 | 19712.781 | 1.971 | 15.929 | 0.0896615 | 7/15/2014 | 42:54.4 | 0 |
| -240.652 | 19712.746 | 1.974 | 15.933 | 0.0914085 | 7/15/2014 | 42:54.5 | 0 |
| -240.403 | 19712.715 | 1.974 | 15.937 | 0.0920301 | 7/15/2014 | 42:54.6 | 0 |
| -240.153 | 19712.68 | 1.974 | 15.941 | 0.0900333 | 7/15/2014 | 42:54.7 | 0 |

| | | | | | | | |
|---|---|---|---|---|---|---|---|
| -239.842 | 19712.637 | 1.971 | 15.944 | 0.0969304 | 7/15/2014 | 42:54.8 | 0 |
| -239.592 | 19712.604 | 1.971 | 15.947 | 0.102043 | 7/15/2014 | 42:54.9 | 0 |
| -239.342 | 19712.57 | 1.966 | 15.95 | 0.108458 | 7/15/2014 | 42:55.0 | 0 |
| -239.091 | 19712.535 | 1.966 | 15.952 | 0.0994301 | 7/15/2014 | 42:55.1 | 0 |
| -238.841 | 19712.503 | 1.966 | 15.953 | 0.101778 | 7/15/2014 | 42:55.2 | 0 |
| -238.592 | 19712.468 | 1.97 | 15.954 | 0.0963471 | 7/15/2014 | 42:55.3 | 0 |
| -238.282 | 19712.426 | 1.972 | 15.955 | 0.0947225 | 7/15/2014 | 42:55.4 | 0 |
| -238.035 | 19712.392 | 1.972 | 15.955 | 0.0911084 | 7/15/2014 | 42:55.5 | 0 |
| -237.789 | 19712.357 | 1.97 | 15.955 | 0.091808 | 7/15/2014 | 42:55.6 | 0 |
| -237.54 | 19712.324 | 1.978 | 15.955 | 0.0929057 | 7/15/2014 | 42:55.7 | 0 |
| -237.233 | 19712.282 | 1.978 | 15.954 | 0.0977272 | 7/15/2014 | 42:55.8 | 0 |
| -236.987 | 19712.247 | 1.982 | 15.954 | 0.0971942 | 7/15/2014 | 42:55.9 | 0 |
| -236.74 | 19712.214 | 1.978 | 15.953 | 0.108871 | 7/15/2014 | 42:56.0 | 0 |
| -236.494 | 19712.18 | 1.982 | 15.952 | 0.108505 | 7/15/2014 | 42:56.1 | 0 |
| -236.249 | 19712.145 | 1.985 | 15.952 | 0.110628 | 7/15/2014 | 42:56.2 | 0 |
| -236.004 | 19712.113 | 1.991 | 15.951 | 0.111445 | 7/15/2014 | 42:56.3 | 0 |
| -235.579 | 19712.054 | 1.986 | 15.951 | 0.117727 | 7/15/2014 | 42:56.4 | 0 |
| -235.338 | 19712.02 | 1.986 | 15.951 | 0.13189 | 7/15/2014 | 42:56.5 | 0 |
| -235.096 | 19711.985 | 1.986 | 15.95 | 0.119751 | 7/15/2014 | 42:56.6 | 0 |
| -234.795 | 19711.944 | 1.986 | 15.95 | 0.130024 | 7/15/2014 | 42:56.7 | 0 |
| -234.673 | 19711.927 | 1.983 | 15.95 | 0.129065 | 7/15/2014 | 42:56.8 | 0 |
| -234.43 | 19711.895 | 1.987 | 15.95 | 0.121247 | 7/15/2014 | 42:56.9 | 0 |
| -234.188 | 19711.859 | 1.987 | 15.95 | 0.125091 | 7/15/2014 | 42:57.0 | 0 |
| -233.945 | 19711.827 | 1.987 | 15.95 | 0.116006 | 7/15/2014 | 42:57.1 | 0 |
| -233.703 | 19711.793 | 1.991 | 15.95 | 0.118097 | 7/15/2014 | 42:57.2 | 0 |
| -233.462 | 19711.758 | 1.994 | 15.95 | 0.127731 | 7/15/2014 | 42:57.3 | 0 |
| -233.042 | 19711.699 | 1.992 | 15.95 | 0.111433 | 7/15/2014 | 42:57.4 | 0 |
| -232.802 | 19711.666 | 1.995 | 15.95 | 0.119598 | 7/15/2014 | 42:57.5 | 0 |
| -232.562 | 19711.632 | 1.995 | 15.95 | 0.120738 | 7/15/2014 | 42:57.6 | 0 |
| -232.262 | 19711.591 | 1.995 | 15.95 | 0.120279 | 7/15/2014 | 42:57.7 | 0 |
| -232.141 | 19711.573 | 1.998 | 15.95 | 0.106898 | 7/15/2014 | 42:57.8 | 0 |
| -231.898 | 19711.539 | 2.004 | 15.949 | 0.0850045 | 7/15/2014 | 42:57.9 | 0 |
| -231.657 | 19711.505 | 2.004 | 15.949 | 0.0999115 | 7/15/2014 | 42:58.0 | 0 |
| -231.413 | 19711.47 | 2.011 | 15.948 | 0.0948245 | 7/15/2014 | 42:58.1 | 0 |
| -231.172 | 19711.436 | 2.011 | 15.947 | 0.0888086 | 7/15/2014 | 42:58.2 | 0 |
| -230.929 | 19711.401 | 2.011 | 15.946 | 0.0910901 | 7/15/2014 | 42:58.3 | 0 |
| -230.503 | 19711.341 | 2.016 | 15.944 | 0.0951579 | 7/15/2014 | 42:58.4 | 0 |
| -230.26 | 19711.307 | 2.018 | 15.944 | 0.120527 | 7/15/2014 | 42:58.5 | 0 |
| -230.016 | 19711.271 | 2.018 | 15.944 | 0.109174 | 7/15/2014 | 42:58.6 | 0 |
| -229.707 | 19711.228 | 2.018 | 15.944 | 0.119569 | 7/15/2014 | 42:58.7 | 0 |
| -229.586 | 19711.21 | 2.016 | 15.944 | 0.127983 | 7/15/2014 | 42:58.8 | 0 |
| -229.336 | 19711.174 | 2.022 | 15.945 | 0.131211 | 7/15/2014 | 42:58.9 | 0 |
| -229.087 | 19711.139 | 2.022 | 15.946 | 0.13855 | 7/15/2014 | 42:59.0 | 0 |
| -228.834 | 19711.103 | 2.029 | 15.947 | 0.120378 | 7/15/2014 | 42:59.1 | 0 |
| -228.585 | 19711.066 | 2.025 | 15.948 | 0.11658 | 7/15/2014 | 42:59.2 | 0 |
| -228.33 | 19711.031 | 2.029 | 15.95 | 0.127194 | 7/15/2014 | 42:59.3 | 0 |
| -228.019 | 19710.986 | 2.025 | 15.952 | 0.11392 | 7/15/2014 | 42:59.4 | 0 |

| | | | | | | | |
|---|---|---|---|---|---|---|---|
| -227.764 | 19710.949 | 2.035 | 15.954 | 0.11704 | 7/15/2014 | 42:59.5 | 0 |
| -227.513 | 19710.913 | 2.035 | 15.957 | 0.122957 | 7/15/2014 | 42:59.6 | 0 |
| -227.259 | 19710.877 | 2.041 | 15.961 | 0.132692 | 7/15/2014 | 42:59.7 | 0 |
| -226.948 | 19710.832 | 2.037 | 15.965 | 0.129603 | 7/15/2014 | 42:59.8 | 0 |
| -226.695 | 19710.797 | 2.038 | 15.969 | 0.13436 | 7/15/2014 | 42:59.9 | 0 |
| -226.446 | 19710.761 | 2.028 | 15.974 | 0.145403 | 7/15/2014 | 43:00.0 | 0 |
| -226.194 | 19710.724 | 2.029 | 15.98 | 0.145639 | 7/15/2014 | 43:00.1 | 0 |
| -225.946 | 19710.687 | 2.016 | 15.986 | 0.143606 | 7/15/2014 | 43:00.2 | 0 |
| -225.698 | 19710.651 | 2.013 | 15.992 | 0.13538 | 7/15/2014 | 43:00.3 | 0 |
| -225.271 | 19710.59 | 2.009 | 16 | 0.137449 | 7/15/2014 | 43:00.4 | 0 |
| -225.026 | 19710.555 | 2.01 | 16.006 | 0.132923 | 7/15/2014 | 43:00.5 | 0 |
| -224.786 | 19710.52 | 2.013 | 16.01 | 0.120488 | 7/15/2014 | 43:00.6 | 0 |
| -224.485 | 19710.474 | 2.018 | 16.014 | 0.119221 | 7/15/2014 | 43:00.7 | 0 |
| -224.363 | 19710.455 | 2.017 | 16.016 | 0.119976 | 7/15/2014 | 43:00.8 | 0 |
| -224.12 | 19710.42 | 2.026 | 16.018 | 0.0873731 | 7/15/2014 | 43:00.9 | 0 |
| -223.88 | 19710.385 | 2.029 | 16.02 | 0.101776 | 7/15/2014 | 43:01.0 | 0 |
| -223.638 | 19710.35 | 2.039 | 16.021 | 0.0858461 | 7/15/2014 | 43:01.1 | 0 |
| -223.399 | 19710.314 | 2.041 | 16.022 | 0.0920252 | 7/15/2014 | 43:01.2 | 0 |
| -223.16 | 19710.279 | 2.047 | 16.023 | 0.0921346 | 7/15/2014 | 43:01.3 | 0 |
| -222.747 | 19710.217 | 2.046 | 16.024 | 0.105429 | 7/15/2014 | 43:01.4 | 0 |
| -222.51 | 19710.182 | 2.046 | 16.024 | 0.13636 | 7/15/2014 | 43:01.5 | 0 |
| -222.272 | 19710.146 | 2.044 | 16.025 | 0.121272 | 7/15/2014 | 43:01.6 | 0 |
| -221.977 | 19710.104 | 2.039 | 16.025 | 0.138675 | 7/15/2014 | 43:01.7 | 0 |
| -221.858 | 19710.086 | 2.035 | 16.025 | 0.133522 | 7/15/2014 | 43:01.8 | 0 |
| -221.619 | 19710.051 | 2.035 | 16.025 | 0.143253 | 7/15/2014 | 43:01.9 | 0 |
| -221.381 | 19710.016 | 2.025 | 16.024 | 0.130535 | 7/15/2014 | 43:02.0 | 0 |
| -221.142 | 19709.979 | 2.021 | 16.023 | 0.129736 | 7/15/2014 | 43:02.1 | 0 |
| -220.903 | 19709.943 | 2.023 | 16.021 | 0.100357 | 7/15/2014 | 43:02.2 | 0 |
| -220.664 | 19709.908 | 2.026 | 16.019 | 0.100026 | 7/15/2014 | 43:02.3 | 0 |
| -220.25 | 19709.846 | 2.034 | 16.015 | 0.0835887 | 7/15/2014 | 43:02.4 | 0 |
| -220.014 | 19709.811 | 2.042 | 16.012 | 0.0796428 | 7/15/2014 | 43:02.5 | 0 |
| -219.781 | 19709.775 | 2.045 | 16.009 | 0.089152 | 7/15/2014 | 43:02.6 | 0 |
| -219.491 | 19709.73 | 2.056 | 16.006 | 0.0798498 | 7/15/2014 | 43:02.7 | 0 |
| -219.37 | 19709.713 | 2.058 | 16.005 | 0.0864589 | 7/15/2014 | 43:02.8 | 0 |
| -219.137 | 19709.678 | 2.066 | 16.002 | 0.092541 | 7/15/2014 | 43:02.9 | 0 |
| -218.798 | 19709.624 | 2.067 | 15.999 | 0.112482 | 7/15/2014 | 43:03.0 | 0 |
| -218.569 | 19709.589 | 2.067 | 15.997 | 0.125015 | 7/15/2014 | 43:03.1 | 0 |
| -218.343 | 19709.554 | 2.067 | 15.994 | 0.133274 | 7/15/2014 | 43:03.2 | 0 |
| -218.12 | 19709.52 | 2.063 | 15.991 | 0.132896 | 7/15/2014 | 43:03.3 | 0 |
| -217.843 | 19709.477 | 2.06 | 15.988 | 0.142831 | 7/15/2014 | 43:03.4 | 0 |
| -217.623 | 19709.441 | 2.055 | 15.984 | 0.142925 | 7/15/2014 | 43:03.5 | 0 |
| -217.405 | 19709.406 | 2.05 | 15.979 | 0.150009 | 7/15/2014 | 43:03.6 | 0 |
| -217.132 | 19709.362 | 2.05 | 15.974 | 0.140567 | 7/15/2014 | 43:03.7 | 0 |
| -216.911 | 19709.328 | 2.055 | 15.969 | 0.11771 | 7/15/2014 | 43:03.8 | 0 |
| -216.691 | 19709.292 | 2.058 | 15.964 | 0.101742 | 7/15/2014 | 43:03.9 | 0 |
| -216.468 | 19709.257 | 2.062 | 15.958 | 0.110741 | 7/15/2014 | 43:04.0 | 0 |
| -216.246 | 19709.221 | 2.07 | 15.953 | 0.101164 | 7/15/2014 | 43:04.1 | 0 |

| | | | | | | | |
|---|---|---|---|---|---|---|---|
| -216.024 | 19709.186 | 2.077 | 15.948 | 0.0966402 | 7/15/2014 | 43:04.2 | 0 |
| -215.802 | 19709.148 | 2.084 | 15.943 | 0.0896316 | 7/15/2014 | 43:04.3 | 0 |
| -215.525 | 19709.103 | 2.09 | 15.939 | 0.0980638 | 7/15/2014 | 43:04.4 | 0 |
| -215.301 | 19709.066 | 2.092 | 15.935 | 0.130931 | 7/15/2014 | 43:04.5 | 0 |
| -215.076 | 19709.029 | 2.094 | 15.932 | 0.142143 | 7/15/2014 | 43:04.6 | 0 |
| -214.793 | 19708.981 | 2.094 | 15.929 | 0.112849 | 7/15/2014 | 43:04.7 | 0 |
| -214.564 | 19708.944 | 2.094 | 15.927 | 0.121696 | 7/15/2014 | 43:04.8 | 0 |
| -214.335 | 19708.908 | 2.092 | 15.925 | 0.145667 | 7/15/2014 | 43:04.9 | 0 |
| -214.221 | 19708.889 | 2.09 | 15.924 | 0.149955 | 7/15/2014 | 43:05.0 | 0 |
| -213.991 | 19708.848 | 2.09 | 15.923 | 0.129572 | 7/15/2014 | 43:05.1 | 0 |
| -213.762 | 19708.81 | 2.093 | 15.922 | 0.132526 | 7/15/2014 | 43:05.2 | 0 |
| -213.533 | 19708.771 | 2.095 | 15.921 | 0.125474 | 7/15/2014 | 43:05.3 | 0 |
| -213.133 | 19708.703 | 2.099 | 15.918 | 0.12094 | 7/15/2014 | 43:05.4 | 0 |
| -212.907 | 19708.665 | 2.104 | 15.917 | 0.137221 | 7/15/2014 | 43:05.5 | 0 |
| -212.681 | 19708.627 | 2.11 | 15.915 | 0.121224 | 7/15/2014 | 43:05.6 | 0 |
| -212.403 | 19708.578 | 2.112 | 15.913 | 0.130929 | 7/15/2014 | 43:05.7 | 0 |
| -212.179 | 19708.539 | 2.115 | 15.912 | 0.122996 | 7/15/2014 | 43:05.8 | 0 |
| -211.955 | 19708.501 | 2.118 | 15.91 | 0.119681 | 7/15/2014 | 43:05.9 | 0 |
| -211.732 | 19708.462 | 2.127 | 15.909 | 0.120601 | 7/15/2014 | 43:06.0 | 0 |
| -211.51 | 19708.424 | 2.13 | 15.908 | 0.159996 | 7/15/2014 | 43:06.1 | 0 |
| -211.29 | 19708.385 | 2.133 | 15.908 | 0.150618 | 7/15/2014 | 43:06.2 | 0 |
| -211.072 | 19708.346 | 2.133 | 15.908 | 0.167919 | 7/15/2014 | 43:06.3 | 0 |
| -210.801 | 19708.299 | 2.13 | 15.908 | 0.196231 | 7/15/2014 | 43:06.4 | 0 |
| -210.586 | 19708.261 | 2.13 | 15.908 | 0.201489 | 7/15/2014 | 43:06.5 | 0 |
| -210.37 | 19708.222 | 2.13 | 15.909 | 0.232655 | 7/15/2014 | 43:06.6 | 0 |
| -210.096 | 19708.173 | 2.127 | 15.909 | 0.191392 | 7/15/2014 | 43:06.7 | 0 |
| -209.877 | 19708.133 | 2.122 | 15.909 | 0.201435 | 7/15/2014 | 43:06.8 | 0 |
| -209.656 | 19708.094 | 2.118 | 15.909 | 0.195538 | 7/15/2014 | 43:06.9 | 0 |
| -209.433 | 19708.053 | 2.121 | 15.908 | 0.193777 | 7/15/2014 | 43:07.0 | 0 |
| -209.208 | 19708.012 | 2.129 | 15.906 | 0.155108 | 7/15/2014 | 43:07.1 | 0 |
| -208.983 | 19707.972 | 2.139 | 15.903 | 0.15095 | 7/15/2014 | 43:07.2 | 0 |
| -208.874 | 19707.951 | 2.144 | 15.902 | 0.136471 | 7/15/2014 | 43:07.3 | 0 |
| -208.476 | 19707.879 | 2.152 | 15.894 | 0.145417 | 7/15/2014 | 43:07.4 | 0 |
| -208.246 | 19707.836 | 2.161 | 15.889 | 0.150948 | 7/15/2014 | 43:07.5 | 0 |
| -208.016 | 19707.795 | 2.168 | 15.884 | 0.136399 | 7/15/2014 | 43:07.6 | 0 |
| -207.723 | 19707.74 | 2.175 | 15.878 | 0.124088 | 7/15/2014 | 43:07.7 | 0 |
| -207.607 | 19707.719 | 2.175 | 15.876 | 0.136998 | 7/15/2014 | 43:07.8 | 0 |
| -207.37 | 19707.676 | 2.175 | 15.871 | 0.154682 | 7/15/2014 | 43:07.9 | 0 |
| -207.132 | 19707.631 | 2.164 | 15.867 | 0.222516 | 7/15/2014 | 43:08.0 | 0 |
| -206.892 | 19707.588 | 2.162 | 15.863 | 0.228232 | 7/15/2014 | 43:08.1 | 0 |
| -206.651 | 19707.543 | 2.16 | 15.861 | 0.209091 | 7/15/2014 | 43:08.2 | 0 |
| -206.409 | 19707.498 | 2.157 | 15.86 | 0.259106 | 7/15/2014 | 43:08.3 | 0 |
| -205.986 | 19707.418 | 2.152 | 15.859 | 0.242989 | 7/15/2014 | 43:08.4 | 0 |
| -205.866 | 19707.396 | 2.152 | 15.858 | 0.239817 | 7/15/2014 | 43:08.5 | 0 |
| -205.622 | 19707.351 | 2.152 | 15.859 | 0.206658 | 7/15/2014 | 43:08.6 | 0 |
| -205.378 | 19707.307 | 2.161 | 15.859 | 0.200813 | 7/15/2014 | 43:08.7 | 0 |
| -205.073 | 19707.25 | 2.161 | 15.859 | 0.196049 | 7/15/2014 | 43:08.8 | 0 |

| | | | | | | | |
|---|---|---|---|---|---|---|---|
| -204.827 | 19707.203 | 2.161 | 15.859 | 0.17926 | 7/15/2014 | 43:08.9 | 0 |
| -204.581 | 19707.156 | 2.161 | 15.86 | 0.184707 | 7/15/2014 | 43:09.0 | 0 |
| -204.335 | 19707.11 | 2.161 | 15.86 | 0.190366 | 7/15/2014 | 43:09.1 | 0 |
| -204.089 | 19707.065 | 2.161 | 15.861 | 0.19112 | 7/15/2014 | 43:09.2 | 0 |
| -203.843 | 19707.02 | 2.158 | 15.862 | 0.203796 | 7/15/2014 | 43:09.3 | 0 |
| -203.538 | 19706.963 | 2.15 | 15.864 | 0.216593 | 7/15/2014 | 43:09.4 | 0 |
| -203.292 | 19706.916 | 2.15 | 15.866 | 0.234495 | 7/15/2014 | 43:09.5 | 0 |
| -203.047 | 19706.869 | 2.146 | 15.869 | 0.236607 | 7/15/2014 | 43:09.6 | 0 |
| -202.8 | 19706.825 | 2.143 | 15.874 | 0.239285 | 7/15/2014 | 43:09.7 | 0 |
| -202.491 | 19706.768 | 2.139 | 15.879 | 0.24021 | 7/15/2014 | 43:09.8 | 0 |
| -202.241 | 19706.721 | 2.135 | 15.885 | 0.239075 | 7/15/2014 | 43:09.9 | 0 |
| -201.992 | 19706.674 | 2.128 | 15.893 | 0.241519 | 7/15/2014 | 43:10.0 | 0 |
| -201.738 | 19706.627 | 2.128 | 15.9 | 0.240521 | 7/15/2014 | 43:10.1 | 0 |
| -201.486 | 19706.58 | 2.122 | 15.908 | 0.220437 | 7/15/2014 | 43:10.2 | 0 |
| -201.233 | 19706.535 | 2.122 | 15.916 | 0.229475 | 7/15/2014 | 43:10.3 | 0 |
| -200.919 | 19706.477 | 2.116 | 15.924 | 0.229624 | 7/15/2014 | 43:10.4 | 0 |
| -200.664 | 19706.43 | 2.116 | 15.932 | 0.226914 | 7/15/2014 | 43:10.5 | 0 |
| -200.409 | 19706.382 | 2.11 | 15.939 | 0.230982 | 7/15/2014 | 43:10.6 | 0 |
| -200.15 | 19706.335 | 2.113 | 15.946 | 0.227237 | 7/15/2014 | 43:10.7 | 0 |
| -199.829 | 19706.277 | 2.107 | 15.953 | 0.228415 | 7/15/2014 | 43:10.8 | 0 |
| -199.568 | 19706.229 | 2.107 | 15.959 | 0.231167 | 7/15/2014 | 43:10.9 | 0 |
| -199.31 | 19706.184 | 2.098 | 15.965 | 0.232963 | 7/15/2014 | 43:11.0 | 0 |
| -199.048 | 19706.136 | 2.098 | 15.972 | 0.215996 | 7/15/2014 | 43:11.1 | 0 |
| -198.788 | 19706.088 | 2.088 | 15.978 | 0.211996 | 7/15/2014 | 43:11.2 | 0 |
| -198.526 | 19706.04 | 2.088 | 15.985 | 0.213457 | 7/15/2014 | 43:11.3 | 0 |
| -198.202 | 19705.982 | 2.082 | 15.993 | 0.210612 | 7/15/2014 | 43:11.4 | 0 |
| -197.94 | 19705.935 | 2.082 | 16.001 | 0.209086 | 7/15/2014 | 43:11.5 | 0 |
| -197.679 | 19705.889 | 2.075 | 16.01 | 0.206756 | 7/15/2014 | 43:11.6 | 0 |
| -197.415 | 19705.841 | 2.078 | 16.019 | 0.217999 | 7/15/2014 | 43:11.7 | 0 |
| -197.089 | 19705.783 | 2.078 | 16.028 | 0.215494 | 7/15/2014 | 43:11.8 | 0 |
| -196.824 | 19705.736 | 2.078 | 16.037 | 0.227267 | 7/15/2014 | 43:11.9 | 0 |
| -196.56 | 19705.689 | 2.066 | 16.046 | 0.234027 | 7/15/2014 | 43:12.0 | 0 |
| -196.293 | 19705.642 | 2.063 | 16.055 | 0.241236 | 7/15/2014 | 43:12.1 | 0 |
| -196.028 | 19705.596 | 2.057 | 16.063 | 0.252623 | 7/15/2014 | 43:12.2 | 0 |
| -195.762 | 19705.548 | 2.052 | 16.07 | 0.255031 | 7/15/2014 | 43:12.3 | 0 |
| -195.431 | 19705.491 | 2.037 | 16.077 | 0.259842 | 7/15/2014 | 43:12.4 | 0 |
| -195.164 | 19705.445 | 2.025 | 16.083 | 0.2607 | 7/15/2014 | 43:12.5 | 0 |
| -194.9 | 19705.398 | 2.013 | 16.088 | 0.251193 | 7/15/2014 | 43:12.6 | 0 |
| -194.631 | 19705.353 | 2.017 | 16.094 | 0.250933 | 7/15/2014 | 43:12.7 | 0 |
| -194.298 | 19705.295 | 2.008 | 16.098 | 0.240663 | 7/15/2014 | 43:12.8 | 0 |
| -194.03 | 19705.249 | 2 | 16.103 | 0.237193 | 7/15/2014 | 43:12.9 | 0 |
| -193.763 | 19705.204 | 1.99 | 16.107 | 0.235477 | 7/15/2014 | 43:13.0 | 0 |
| -193.494 | 19705.158 | 1.986 | 16.112 | 0.227924 | 7/15/2014 | 43:13.1 | 0 |
| -193.227 | 19705.112 | 1.982 | 16.117 | 0.230052 | 7/15/2014 | 43:13.2 | 0 |
| -192.958 | 19705.068 | 1.982 | 16.122 | 0.239655 | 7/15/2014 | 43:13.3 | 0 |
| -192.625 | 19705.011 | 1.972 | 16.127 | 0.24831 | 7/15/2014 | 43:13.4 | 0 |
| -192.357 | 19704.967 | 1.966 | 16.131 | 0.260095 | 7/15/2014 | 43:13.5 | 0 |

| | | | | | | | |
|---|---|---|---|---|---|---|---|
| -192.091 | 19704.923 | 1.962 | 16.135 | 0.273664 | 7/15/2014 | 43:13.6 | 0 |
| -191.823 | 19704.879 | 1.964 | 16.139 | 0.277395 | 7/15/2014 | 43:13.7 | 0 |
| -191.488 | 19704.823 | 1.958 | 16.142 | 0.279045 | 7/15/2014 | 43:13.8 | 0 |
| -191.216 | 19704.778 | 1.952 | 16.144 | 0.265053 | 7/15/2014 | 43:13.9 | 0 |
| -190.945 | 19704.734 | 1.941 | 16.146 | 0.251075 | 7/15/2014 | 43:14.0 | 0 |
| -190.811 | 19704.712 | 1.939 | 16.147 | 0.241991 | 7/15/2014 | 43:14.0 | 0 |
| -190.538 | 19704.668 | 1.934 | 16.148 | 0.221157 | 7/15/2014 | 43:14.1 | 0 |
| -190.266 | 19704.624 | 1.932 | 16.149 | 0.206342 | 7/15/2014 | 43:14.2 | 0 |
| -189.787 | 19704.547 | 1.924 | 16.152 | 0.189443 | 7/15/2014 | 43:14.4 | 0 |
| -189.511 | 19704.503 | 1.927 | 16.155 | 0.189192 | 7/15/2014 | 43:14.5 | 0 |
| -189.238 | 19704.46 | 1.924 | 16.159 | 0.191728 | 7/15/2014 | 43:14.6 | 0 |
| -188.962 | 19704.416 | 1.93 | 16.164 | 0.20244 | 7/15/2014 | 43:14.7 | 0 |
| -188.617 | 19704.362 | 1.925 | 16.171 | 0.212787 | 7/15/2014 | 43:14.8 | 0 |
| -188.478 | 19704.341 | 1.923 | 16.174 | 0.219834 | 7/15/2014 | 43:14.8 | 0 |
| -188.2 | 19704.297 | 1.916 | 16.183 | 0.233106 | 7/15/2014 | 43:14.9 | 0 |
| -187.922 | 19704.254 | 1.913 | 16.193 | 0.254252 | 7/15/2014 | 43:15.0 | 0 |
| -187.645 | 19704.211 | 1.905 | 16.204 | 0.262739 | 7/15/2014 | 43:15.1 | 0 |
| -187.367 | 19704.168 | 1.896 | 16.217 | 0.271621 | 7/15/2014 | 43:15.2 | 0 |
| -186.879 | 19704.095 | 1.883 | 16.237 | 0.26598 | 7/15/2014 | 43:15.4 | 0 |
| -186.6 | 19704.053 | 1.881 | 16.252 | 0.258888 | 7/15/2014 | 43:15.5 | 0 |
| -186.323 | 19704.01 | 1.876 | 16.267 | 0.255212 | 7/15/2014 | 43:15.6 | 0 |
| -186.043 | 19703.969 | 1.868 | 16.282 | 0.233637 | 7/15/2014 | 43:15.7 | 0 |
| -185.695 | 19703.916 | 1.865 | 16.297 | 0.233712 | 7/15/2014 | 43:15.8 | 0 |
| -185.553 | 19703.895 | 1.865 | 16.305 | 0.229865 | 7/15/2014 | 43:15.8 | 0 |
| -185.273 | 19703.854 | 1.858 | 16.32 | 0.222609 | 7/15/2014 | 43:15.9 | 0 |
| -184.99 | 19703.813 | 1.858 | 16.336 | 0.228359 | 7/15/2014 | 43:16.0 | 0 |
| -184.709 | 19703.771 | 1.858 | 16.35 | 0.227932 | 7/15/2014 | 43:16.1 | 0 |
| -184.427 | 19703.731 | 1.847 | 16.365 | 0.242558 | 7/15/2014 | 43:16.2 | 0 |
| -183.931 | 19703.659 | 1.846 | 16.384 | 0.225309 | 7/15/2014 | 43:16.4 | 0 |
| -183.647 | 19703.62 | 1.84 | 16.395 | 0.210215 | 7/15/2014 | 43:16.5 | 0 |
| -183.365 | 19703.578 | 1.833 | 16.406 | 0.217241 | 7/15/2014 | 43:16.6 | 0 |
| -183.081 | 19703.539 | 1.826 | 16.415 | 0.202417 | 7/15/2014 | 43:16.7 | 0 |
| -182.727 | 19703.489 | 1.808 | 16.424 | 0.202768 | 7/15/2014 | 43:16.8 | 0 |
| -182.582 | 19703.469 | 1.81 | 16.428 | 0.194513 | 7/15/2014 | 43:16.8 | 0 |
| -182.296 | 19703.431 | 1.796 | 16.436 | 0.195406 | 7/15/2014 | 43:16.9 | 0 |
| -182.01 | 19703.391 | 1.798 | 16.444 | 0.188176 | 7/15/2014 | 43:17.0 | 0 |
| -181.725 | 19703.352 | 1.794 | 16.451 | 0.193028 | 7/15/2014 | 43:17.1 | 0 |
| -181.439 | 19703.313 | 1.79 | 16.458 | 0.196598 | 7/15/2014 | 43:17.2 | 0 |
| -180.939 | 19703.245 | 1.788 | 16.47 | 0.19817 | 7/15/2014 | 43:17.4 | 0 |
| -180.653 | 19703.209 | 1.794 | 16.478 | 0.198299 | 7/15/2014 | 43:17.5 | 0 |
| -180.368 | 19703.17 | 1.792 | 16.487 | 0.196468 | 7/15/2014 | 43:17.6 | 0 |
| -180.082 | 19703.133 | 1.789 | 16.495 | 0.205151 | 7/15/2014 | 43:17.7 | 0 |
| -179.728 | 19703.088 | 1.783 | 16.504 | 0.213008 | 7/15/2014 | 43:17.8 | 0 |
| -179.584 | 19703.068 | 1.786 | 16.508 | 0.213195 | 7/15/2014 | 43:17.8 | 0 |
| -179.299 | 19703.032 | 1.776 | 16.517 | 0.216055 | 7/15/2014 | 43:17.9 | 0 |
| -179.014 | 19702.996 | 1.776 | 16.527 | 0.2235 | 7/15/2014 | 43:18.0 | 0 |
| -178.731 | 19702.96 | 1.769 | 16.536 | 0.213251 | 7/15/2014 | 43:18.1 | 0 |

| | | | | | | | |
|---|---|---|---|---|---|---|---|
| -178.446 | 19702.924 | 1.762 | 16.544 | 0.212217 | 7/15/2014 | 43:18.2 | 0 |
| -177.954 | 19702.864 | 1.739 | 16.557 | 0.205363 | 7/15/2014 | 43:18.4 | 0 |
| -177.671 | 19702.83 | 1.736 | 16.566 | 0.198472 | 7/15/2014 | 43:18.5 | 0 |
| -177.391 | 19702.796 | 1.72 | 16.574 | 0.190949 | 7/15/2014 | 43:18.6 | 0 |
| -177.11 | 19702.762 | 1.711 | 16.582 | 0.187858 | 7/15/2014 | 43:18.7 | 0 |
| -176.76 | 19702.721 | 1.701 | 16.589 | 0.186753 | 7/15/2014 | 43:18.8 | 0 |
| -176.618 | 19702.703 | 1.695 | 16.593 | 0.186986 | 7/15/2014 | 43:18.8 | 0 |
| -176.337 | 19702.669 | 1.7 | 16.599 | 0.19103 | 7/15/2014 | 43:18.9 | 0 |
| -176.056 | 19702.637 | 1.697 | 16.604 | 0.19003 | 7/15/2014 | 43:19.0 | 0 |
| -175.777 | 19702.604 | 1.685 | 16.608 | 0.190063 | 7/15/2014 | 43:19.1 | 0 |
| -175.497 | 19702.572 | 1.685 | 16.611 | 0.18888 | 7/15/2014 | 43:19.2 | 0 |
| -175.007 | 19702.516 | 1.678 | 16.614 | 0.195188 | 7/15/2014 | 43:19.4 | 0 |
| -174.725 | 19702.482 | 1.676 | 16.616 | 0.207106 | 7/15/2014 | 43:19.5 | 0 |
| -174.447 | 19702.453 | 1.666 | 16.618 | 0.204729 | 7/15/2014 | 43:19.6 | 0 |
| -174.167 | 19702.422 | 1.664 | 16.62 | 0.2113 | 7/15/2014 | 43:19.7 | 0 |
| -173.818 | 19702.383 | 1.659 | 16.622 | 0.212684 | 7/15/2014 | 43:19.8 | 0 |
| -173.676 | 19702.367 | 1.657 | 16.623 | 0.214964 | 7/15/2014 | 43:19.8 | 0 |
| -173.394 | 19702.334 | 1.654 | 16.627 | 0.219389 | 7/15/2014 | 43:19.9 | 0 |
| -173.112 | 19702.303 | 1.65 | 16.631 | 0.211058 | 7/15/2014 | 43:20.0 | 0 |
| -172.831 | 19702.273 | 1.644 | 16.636 | 0.203774 | 7/15/2014 | 43:20.1 | 0 |
| -172.548 | 19702.243 | 1.635 | 16.642 | 0.19925 | 7/15/2014 | 43:20.2 | 0 |
| -172.055 | 19702.189 | 1.625 | 16.653 | 0.179045 | 7/15/2014 | 43:20.4 | 0 |
| -171.773 | 19702.16 | 1.617 | 16.66 | 0.170979 | 7/15/2014 | 43:20.5 | 0 |
| -171.491 | 19702.129 | 1.609 | 16.667 | 0.168523 | 7/15/2014 | 43:20.6 | 0 |
| -171.209 | 19702.1 | 1.605 | 16.673 | 0.165477 | 7/15/2014 | 43:20.7 | 0 |
| -170.858 | 19702.063 | 1.595 | 16.68 | 0.166383 | 7/15/2014 | 43:20.8 | 0 |
| -170.715 | 19702.049 | 1.593 | 16.683 | 0.164914 | 7/15/2014 | 43:20.8 | 0 |
| -170.433 | 19702.019 | 1.589 | 16.689 | 0.180292 | 7/15/2014 | 43:20.9 | 0 |
| -170.152 | 19701.991 | 1.586 | 16.694 | 0.188593 | 7/15/2014 | 43:21.0 | 0 |
| -169.87 | 19701.961 | 1.583 | 16.699 | 0.176781 | 7/15/2014 | 43:21.1 | 0 |
| -169.589 | 19701.934 | 1.576 | 16.703 | 0.195135 | 7/15/2014 | 43:21.2 | 0 |
| -169.097 | 19701.884 | 1.574 | 16.71 | 0.181904 | 7/15/2014 | 43:21.4 | 0 |
| -168.817 | 19701.855 | 1.574 | 16.714 | 0.164509 | 7/15/2014 | 43:21.5 | 0 |
| -168.537 | 19701.828 | 1.576 | 16.718 | 0.175579 | 7/15/2014 | 43:21.6 | 0 |
| -168.255 | 19701.8 | 1.572 | 16.722 | 0.169825 | 7/15/2014 | 43:21.7 | 0 |
| -167.903 | 19701.766 | 1.564 | 16.726 | 0.164803 | 7/15/2014 | 43:21.8 | 0 |
| -167.762 | 19701.751 | 1.563 | 16.728 | 0.173925 | 7/15/2014 | 43:21.8 | 0 |
| -167.478 | 19701.725 | 1.563 | 16.731 | 0.163201 | 7/15/2014 | 43:21.9 | 0 |
| -167.194 | 19701.697 | 1.553 | 16.734 | 0.159166 | 7/15/2014 | 43:22.0 | 0 |
| -166.909 | 19701.67 | 1.553 | 16.737 | 0.151177 | 7/15/2014 | 43:22.1 | 0 |
| -166.623 | 19701.644 | 1.546 | 16.74 | 0.14985 | 7/15/2014 | 43:22.2 | 0 |
| -166.121 | 19701.596 | 1.547 | 16.743 | 0.149399 | 7/15/2014 | 43:22.4 | 0 |
| -165.833 | 19701.569 | 1.553 | 16.745 | 0.149508 | 7/15/2014 | 43:22.5 | 0 |
| -165.546 | 19701.542 | 1.544 | 16.748 | 0.15717 | 7/15/2014 | 43:22.6 | 0 |
| -165.257 | 19701.516 | 1.541 | 16.752 | 0.162931 | 7/15/2014 | 43:22.7 | 0 |
| -164.898 | 19701.484 | 1.532 | 16.756 | 0.171051 | 7/15/2014 | 43:22.8 | 0 |
| -164.752 | 19701.471 | 1.53 | 16.757 | 0.174585 | 7/15/2014 | 43:22.8 | 0 |

| | | | | | | | |
|---|---|---|---|---|---|---|---|
| -164.461 | 19701.443 | 1.527 | 16.763 | 0.18393 | 7/15/2014 | 43:22.9 | 0 |
| -164.172 | 19701.417 | 1.501 | 16.769 | 0.190933 | 7/15/2014 | 43:23.0 | 0 |
| -163.88 | 19701.391 | 1.494 | 16.776 | 0.196339 | 7/15/2014 | 43:23.1 | 0 |
| -163.59 | 19701.364 | 1.48 | 16.784 | 0.183123 | 7/15/2014 | 43:23.2 | 0 |
| -163.08 | 19701.32 | 1.477 | 16.794 | 0.160629 | 7/15/2014 | 43:23.4 | 0 |
| -162.79 | 19701.294 | 1.474 | 16.8 | 0.149772 | 7/15/2014 | 43:23.5 | 0 |
| -162.501 | 19701.269 | 1.468 | 16.804 | 0.140098 | 7/15/2014 | 43:23.6 | 0 |
| -162.209 | 19701.244 | 1.466 | 16.808 | 0.135243 | 7/15/2014 | 43:23.7 | 0 |
| -161.846 | 19701.211 | 1.454 | 16.811 | 0.133721 | 7/15/2014 | 43:23.8 | 0 |
| -161.698 | 19701.198 | 1.456 | 16.812 | 0.130144 | 7/15/2014 | 43:23.8 | 0 |
| -161.405 | 19701.174 | 1.452 | 16.813 | 0.139316 | 7/15/2014 | 43:23.9 | 0 |
| -161.113 | 19701.149 | 1.448 | 16.813 | 0.150746 | 7/15/2014 | 43:24.0 | 0 |
| -160.821 | 19701.125 | 1.439 | 16.812 | 0.157982 | 7/15/2014 | 43:24.1 | 0 |
| -160.529 | 19701.1 | 1.435 | 16.811 | 0.176555 | 7/15/2014 | 43:24.2 | 0 |
| -160.018 | 19701.057 | 1.435 | 16.809 | 0.168001 | 7/15/2014 | 43:24.4 | 0 |
| -159.728 | 19701.033 | 1.438 | 16.807 | 0.153169 | 7/15/2014 | 43:24.5 | 0 |
| -159.437 | 19701.01 | 1.432 | 16.806 | 0.145632 | 7/15/2014 | 43:24.6 | 0 |
| -159.147 | 19700.987 | 1.432 | 16.804 | 0.137412 | 7/15/2014 | 43:24.7 | 0 |
| -158.788 | 19700.957 | 1.429 | 16.802 | 0.133189 | 7/15/2014 | 43:24.8 | 0 |
| -158.641 | 19700.945 | 1.428 | 16.801 | 0.119188 | 7/15/2014 | 43:24.8 | 0 |
| -158.352 | 19700.922 | 1.421 | 16.799 | 0.129357 | 7/15/2014 | 43:24.9 | 0 |
| -158.065 | 19700.9 | 1.423 | 16.797 | 0.124351 | 7/15/2014 | 43:25.0 | 0 |
| -157.779 | 19700.877 | 1.42 | 16.795 | 0.136723 | 7/15/2014 | 43:25.1 | 0 |
| -157.494 | 19700.856 | 1.411 | 16.794 | 0.143639 | 7/15/2014 | 43:25.2 | 0 |
| -156.999 | 19700.818 | 1.415 | 16.794 | 0.149127 | 7/15/2014 | 43:25.4 | 0 |
| -156.715 | 19700.796 | 1.415 | 16.795 | 0.154704 | 7/15/2014 | 43:25.5 | 0 |
| -156.435 | 19700.775 | 1.418 | 16.797 | 0.150734 | 7/15/2014 | 43:25.6 | 0 |
| -156.153 | 19700.754 | 1.416 | 16.799 | 0.15624 | 7/15/2014 | 43:25.7 | 0 |
| -155.804 | 19700.729 | 1.413 | 16.803 | 0.159112 | 7/15/2014 | 43:25.8 | 0 |
| -155.661 | 19700.717 | 1.422 | 16.804 | 0.166693 | 7/15/2014 | 43:25.8 | 0 |
| -155.381 | 19700.697 | 1.422 | 16.808 | 0.169405 | 7/15/2014 | 43:25.9 | 0 |
| -155.1 | 19700.677 | 1.419 | 16.813 | 0.149479 | 7/15/2014 | 43:26.0 | 0 |
| -154.819 | 19700.656 | 1.422 | 16.817 | 0.140275 | 7/15/2014 | 43:26.1 | 0 |
| -154.539 | 19700.637 | 1.422 | 16.821 | 0.142918 | 7/15/2014 | 43:26.2 | 0 |
| -154.049 | 19700.602 | 1.415 | 16.826 | 0.134671 | 7/15/2014 | 43:26.4 | 0 |
| -153.769 | 19700.582 | 1.424 | 16.828 | 0.130859 | 7/15/2014 | 43:26.5 | 0 |
| -153.489 | 19700.563 | 1.419 | 16.83 | 0.131532 | 7/15/2014 | 43:26.6 | 0 |
| -153.209 | 19700.544 | 1.419 | 16.83 | 0.128353 | 7/15/2014 | 43:26.7 | 0 |
| -152.859 | 19700.521 | 1.416 | 16.83 | 0.119228 | 7/15/2014 | 43:26.8 | 0 |
| -152.718 | 19700.511 | 1.415 | 16.83 | 0.114175 | 7/15/2014 | 43:26.8 | 0 |
| -152.436 | 19700.492 | 1.415 | 16.829 | 0.101668 | 7/15/2014 | 43:26.9 | 0 |
| -152.155 | 19700.473 | 1.415 | 16.827 | 0.112157 | 7/15/2014 | 43:27.0 | 0 |
| -151.872 | 19700.454 | 1.408 | 16.825 | 0.111671 | 7/15/2014 | 43:27.1 | 0 |
| -151.59 | 19700.437 | 1.408 | 16.823 | 0.12405 | 7/15/2014 | 43:27.2 | 0 |
| -151.095 | 19700.405 | 1.41 | 16.822 | 0.126005 | 7/15/2014 | 43:27.4 | 0 |
| -150.812 | 19700.387 | 1.401 | 16.821 | 0.143561 | 7/15/2014 | 43:27.5 | 0 |
| -150.529 | 19700.369 | 1.399 | 16.822 | 0.170418 | 7/15/2014 | 43:27.6 | 0 |

| | | | | | | | |
|---|---|---|---|---|---|---|---|
| -150.244 | 19700.352 | 1.402 | 16.823 | 0.154685 | 7/15/2014 | 43:27.7 | 0 |
| -149.889 | 19700.33 | 1.388 | 16.824 | 0.171452 | 7/15/2014 | 43:27.8 | 0 |
| -149.746 | 19700.32 | 1.385 | 16.825 | 0.188286 | 7/15/2014 | 43:27.8 | 0 |
| -149.458 | 19700.305 | 1.381 | 16.828 | 0.169142 | 7/15/2014 | 43:27.9 | 0 |
| -149.171 | 19700.287 | 1.377 | 16.83 | 0.166103 | 7/15/2014 | 43:28.0 | 0 |
| -148.882 | 19700.271 | 1.384 | 16.832 | 0.151819 | 7/15/2014 | 43:28.1 | 0 |
| -148.594 | 19700.255 | 1.37 | 16.834 | 0.173534 | 7/15/2014 | 43:28.2 | 0 |
| -148.087 | 19700.227 | 1.364 | 16.839 | 0.163445 | 7/15/2014 | 43:28.4 | 0 |
| -147.794 | 19700.211 | 1.376 | 16.842 | 0.170843 | 7/15/2014 | 43:28.5 | 0 |
| -147.504 | 19700.195 | 1.376 | 16.845 | 0.179443 | 7/15/2014 | 43:28.6 | 0 |
| -147.212 | 19700.181 | 1.37 | 16.849 | 0.182382 | 7/15/2014 | 43:28.7 | 0 |
| -146.847 | 19700.162 | 1.362 | 16.854 | 0.191442 | 7/15/2014 | 43:28.8 | 0 |
| -146.698 | 19700.154 | 1.369 | 16.856 | 0.202689 | 7/15/2014 | 43:28.8 | 0 |
| -146.405 | 19700.141 | 1.365 | 16.861 | 0.202723 | 7/15/2014 | 43:28.9 | 0 |
| -146.11 | 19700.125 | 1.372 | 16.868 | 0.209656 | 7/15/2014 | 43:29.0 | 0 |
| -145.816 | 19700.111 | 1.368 | 16.875 | 0.211953 | 7/15/2014 | 43:29.1 | 0 |
| -145.521 | 19700.099 | 1.358 | 16.883 | 0.208987 | 7/15/2014 | 43:29.2 | 0 |
| -145.006 | 19700.076 | 1.367 | 16.897 | 0.202842 | 7/15/2014 | 43:29.4 | 0 |
| -144.712 | 19700.064 | 1.367 | 16.907 | 0.178967 | 7/15/2014 | 43:29.5 | 0 |
| -144.42 | 19700.051 | 1.371 | 16.918 | 0.169926 | 7/15/2014 | 43:29.6 | 0 |
| -144.128 | 19700.039 | 1.365 | 16.93 | 0.183172 | 7/15/2014 | 43:29.7 | 0 |
| -143.766 | 19700.025 | 1.367 | 16.942 | 0.156958 | 7/15/2014 | 43:29.8 | 0 |
| -143.617 | 19700.019 | 1.375 | 16.947 | 0.159708 | 7/15/2014 | 43:29.8 | 0 |
| -143.327 | 19700.01 | 1.364 | 16.96 | 0.166052 | 7/15/2014 | 43:29.9 | 0 |
| -143.038 | 19699.999 | 1.361 | 16.973 | 0.176376 | 7/15/2014 | 43:30.0 | 0 |
| -142.75 | 19699.988 | 1.365 | 16.986 | 0.187354 | 7/15/2014 | 43:30.1 | 0 |
| -142.464 | 19699.979 | 1.358 | 16.999 | 0.183823 | 7/15/2014 | 43:30.2 | 0 |
| -141.966 | 19699.963 | 1.362 | 17.017 | 0.214946 | 7/15/2014 | 43:30.4 | 0 |
| -141.682 | 19699.955 | 1.36 | 17.029 | 0.203481 | 7/15/2014 | 43:30.5 | 0 |
| -141.401 | 19699.946 | 1.355 | 17.038 | 0.206243 | 7/15/2014 | 43:30.6 | 0 |
| -141.119 | 19699.939 | 1.361 | 17.047 | 0.203984 | 7/15/2014 | 43:30.7 | 0 |
| -140.771 | 19699.93 | 1.355 | 17.054 | 0.201838 | 7/15/2014 | 43:30.8 | 0 |
| -140.628 | 19699.926 | 1.358 | 17.057 | 0.202101 | 7/15/2014 | 43:30.8 | 0 |
| -140.348 | 19699.92 | 1.351 | 17.061 | 0.196758 | 7/15/2014 | 43:30.9 | 0 |
| -140.069 | 19699.913 | 1.344 | 17.063 | 0.197722 | 7/15/2014 | 43:31.0 | 0 |
| -139.79 | 19699.906 | 1.344 | 17.064 | 0.198135 | 7/15/2014 | 43:31.1 | 0 |
| -139.513 | 19699.902 | 1.351 | 17.063 | 0.205573 | 7/15/2014 | 43:31.2 | 0 |
| -139.03 | 19699.892 | 1.343 | 17.059 | 0.20913 | 7/15/2014 | 43:31.4 | 0 |
| -138.753 | 19699.888 | 1.351 | 17.056 | 0.212219 | 7/15/2014 | 43:31.5 | 0 |
| -138.479 | 19699.883 | 1.357 | 17.053 | 0.211285 | 7/15/2014 | 43:31.6 | 0 |
| -138.203 | 19699.878 | 1.36 | 17.049 | 0.20999 | 7/15/2014 | 43:31.7 | 0 |
| -137.86 | 19699.873 | 1.354 | 17.044 | 0.213749 | 7/15/2014 | 43:31.8 | 0 |
| -137.72 | 19699.87 | 1.353 | 17.042 | 0.21864 | 7/15/2014 | 43:31.8 | 0 |
| -137.445 | 19699.867 | 1.357 | 17.037 | 0.218091 | 7/15/2014 | 43:31.9 | 0 |
| -137.168 | 19699.864 | 1.364 | 17.032 | 0.210642 | 7/15/2014 | 43:32.0 | 0 |
| -136.891 | 19699.86 | 1.354 | 17.027 | 0.215433 | 7/15/2014 | 43:32.1 | 0 |
| -136.614 | 19699.858 | 1.344 | 17.021 | 0.237854 | 7/15/2014 | 43:32.2 | 0 |

| | | | | | | | |
|---|---|---|---|---|---|---|---|
| -136.127 | 19699.854 | 1.357 | 17.011 | 0.235157 | 7/15/2014 | 43:32.4 | 0 |
| -135.849 | 19699.852 | 1.357 | 17.003 | 0.220395 | 7/15/2014 | 43:32.5 | 0 |
| -135.572 | 19699.851 | 1.349 | 16.994 | 0.267346 | 7/15/2014 | 43:32.6 | 0 |
| -135.294 | 19699.848 | 1.351 | 16.985 | 0.261185 | 7/15/2014 | 43:32.7 | 0 |
| -134.95 | 19699.848 | 1.351 | 16.974 | 0.245723 | 7/15/2014 | 43:32.8 | 0 |
| -134.808 | 19699.846 | 1.357 | 16.969 | 0.262772 | 7/15/2014 | 43:32.8 | 0 |
| -134.531 | 19699.847 | 1.35 | 16.958 | 0.22256 | 7/15/2014 | 43:32.9 | 0 |
| -134.255 | 19699.846 | 1.347 | 16.946 | 0.272898 | 7/15/2014 | 43:33.0 | 0 |
| -133.979 | 19699.846 | 1.35 | 16.934 | 0.236888 | 7/15/2014 | 43:33.1 | 0 |
| -133.704 | 19699.846 | 1.357 | 16.922 | 0.253112 | 7/15/2014 | 43:33.2 | 0 |
| -133.226 | 19699.847 | 1.354 | 16.906 | 0.273372 | 7/15/2014 | 43:33.4 | 0 |
| -132.952 | 19699.849 | 1.354 | 16.896 | 0.315935 | 7/15/2014 | 43:33.5 | 0 |
| -132.68 | 19699.851 | 1.351 | 16.888 | 0.25523 | 7/15/2014 | 43:33.6 | 0 |
| -132.406 | 19699.851 | 1.351 | 16.88 | 0.2633 | 7/15/2014 | 43:33.7 | 0 |
| -132.064 | 19699.854 | 1.349 | 16.873 | 0.244234 | 7/15/2014 | 43:33.8 | 0 |
| -131.927 | 19699.854 | 1.347 | 16.869 | 0.196824 | 7/15/2014 | 43:33.8 | 0 |
| -131.652 | 19699.856 | 1.34 | 16.863 | 0.208014 | 7/15/2014 | 43:33.9 | 0 |
| -131.375 | 19699.858 | 1.344 | 16.858 | 0.163206 | 7/15/2014 | 43:34.0 | 0 |
| -131.098 | 19699.862 | 1.348 | 16.852 | 0.166809 | 7/15/2014 | 43:34.1 | 0 |
| -130.821 | 19699.863 | 1.355 | 16.848 | 0.163247 | 7/15/2014 | 43:34.2 | 0 |
| -130.333 | 19699.869 | 1.349 | 16.841 | 0.131323 | 7/15/2014 | 43:34.4 | 0 |
| -130.054 | 19699.873 | 1.355 | 16.837 | 0.141295 | 7/15/2014 | 43:34.5 | 0 |
| -129.775 | 19699.876 | 1.359 | 16.834 | 0.131986 | 7/15/2014 | 43:34.6 | 0 |
| -129.495 | 19699.88 | 1.361 | 16.832 | 0.150362 | 7/15/2014 | 43:34.7 | 0 |
| -129.146 | 19699.884 | 1.357 | 16.831 | 0.12087 | 7/15/2014 | 43:34.8 | 0 |
| -129.004 | 19699.886 | 1.354 | 16.83 | 0.128436 | 7/15/2014 | 43:34.8 | 0 |
| -128.724 | 19699.89 | 1.351 | 16.832 | 0.141531 | 7/15/2014 | 43:34.9 | 0 |
| -128.443 | 19699.894 | 1.35 | 16.835 | 0.122303 | 7/15/2014 | 43:35.0 | 0 |
| -128.162 | 19699.898 | 1.35 | 16.84 | 0.118928 | 7/15/2014 | 43:35.1 | 0 |
| -127.881 | 19699.902 | 1.345 | 16.846 | 0.115354 | 7/15/2014 | 43:35.2 | 0 |
| -127.393 | 19699.91 | 1.338 | 16.858 | 0.150871 | 7/15/2014 | 43:35.4 | 0 |
| -127.114 | 19699.915 | 1.341 | 16.867 | 0.143082 | 7/15/2014 | 43:35.5 | 0 |
| -126.838 | 19699.919 | 1.338 | 16.876 | 0.174412 | 7/15/2014 | 43:35.6 | 0 |
| -126.559 | 19699.925 | 1.341 | 16.884 | 0.196583 | 7/15/2014 | 43:35.7 | 0 |
| -126.216 | 19699.931 | 1.333 | 16.893 | 0.166801 | 7/15/2014 | 43:35.8 | 0 |
| -126.075 | 19699.933 | 1.333 | 16.897 | 0.196014 | 7/15/2014 | 43:35.8 | 0 |
| -125.8 | 19699.938 | 1.333 | 16.905 | 0.192044 | 7/15/2014 | 43:35.9 | 0 |
| -125.39 | 19699.947 | 1.329 | 16.917 | 0.193094 | 7/15/2014 | 43:36.1 | 0 |
| -125.118 | 19699.952 | 1.326 | 16.925 | 0.198138 | 7/15/2014 | 43:36.2 | 0 |
| -124.845 | 19699.958 | 1.329 | 16.933 | 0.195084 | 7/15/2014 | 43:36.3 | 0 |
| -124.509 | 19699.966 | 1.326 | 16.941 | 0.229762 | 7/15/2014 | 43:36.4 | 0 |
| -124.24 | 19699.973 | 1.328 | 16.95 | 0.230501 | 7/15/2014 | 43:36.5 | 0 |
| -123.973 | 19699.978 | 1.328 | 16.958 | 0.207782 | 7/15/2014 | 43:36.6 | 0 |
| -123.706 | 19699.984 | 1.332 | 16.968 | 0.244204 | 7/15/2014 | 43:36.7 | 0 |
| -123.379 | 19699.993 | 1.328 | 16.977 | 0.18893 | 7/15/2014 | 43:36.8 | 0 |
| -123.113 | 19699.999 | 1.328 | 16.986 | 0.1746 | 7/15/2014 | 43:36.9 | 0 |
| -122.852 | 19700.006 | 1.324 | 16.995 | 0.179063 | 7/15/2014 | 43:37.0 | 0 |

| | | | | | | | |
|---|---|---|---|---|---|---|---|
| -122.589 | 19700.014 | 1.324 | 17.004 | 0.13763 | 7/15/2014 | 43:37.1 | 0 |
| -122.328 | 19700.02 | 1.321 | 17.012 | 0.138724 | 7/15/2014 | 43:37.2 | 0 |
| -122.069 | 19700.027 | 1.321 | 17.02 | 0.113655 | 7/15/2014 | 43:37.3 | 0 |
| -121.75 | 19700.036 | 1.315 | 17.027 | 0.152068 | 7/15/2014 | 43:37.4 | 0 |
| -121.494 | 19700.042 | 1.322 | 17.032 | 0.137824 | 7/15/2014 | 43:37.5 | 0 |
| -121.243 | 19700.049 | 1.318 | 17.036 | 0.117539 | 7/15/2014 | 43:37.6 | 0 |
| -120.991 | 19700.057 | 1.326 | 17.038 | 0.110029 | 7/15/2014 | 43:37.7 | 0 |
| -120.682 | 19700.066 | 1.329 | 17.038 | 0.120088 | 7/15/2014 | 43:37.8 | 0 |
| -120.432 | 19700.074 | 1.333 | 17.037 | 0.124787 | 7/15/2014 | 43:37.9 | 0 |
| -120.187 | 19700.081 | 1.329 | 17.034 | 0.126793 | 7/15/2014 | 43:38.0 | 0 |
| -119.939 | 19700.087 | 1.339 | 17.029 | 0.13975 | 7/15/2014 | 43:38.1 | 0 |
| -119.696 | 19700.094 | 1.335 | 17.023 | 0.139941 | 7/15/2014 | 43:38.2 | 0 |
| -119.453 | 19700.102 | 1.339 | 17.016 | 0.127097 | 7/15/2014 | 43:38.3 | 0 |
| -119.034 | 19700.115 | 1.338 | 17.003 | 0.0985871 | 7/15/2014 | 43:38.4 | 0 |
| -118.797 | 19700.123 | 1.338 | 16.994 | 0.082654 | 7/15/2014 | 43:38.5 | 0 |
| -118.561 | 19700.131 | 1.342 | 16.984 | 0.0877739 | 7/15/2014 | 43:38.6 | 0 |
| -118.267 | 19700.14 | 1.345 | 16.974 | 0.067375 | 7/15/2014 | 43:38.7 | 0 |
| -118.148 | 19700.143 | 1.342 | 16.97 | 0.0526476 | 7/15/2014 | 43:38.8 | 0 |
| -117.912 | 19700.15 | 1.349 | 16.959 | 0.0918559 | 7/15/2014 | 43:38.9 | 0 |
| -117.678 | 19700.158 | 1.349 | 16.949 | 0.0938199 | 7/15/2014 | 43:39.0 | 0 |
| -117.442 | 19700.166 | 1.349 | 16.938 | 0.127696 | 7/15/2014 | 43:39.1 | 0 |
| -117.21 | 19700.174 | 1.345 | 16.927 | 0.157 | 7/15/2014 | 43:39.2 | 0 |
| -116.976 | 19700.182 | 1.349 | 16.916 | 0.134855 | 7/15/2014 | 43:39.3 | 0 |
| -116.574 | 19700.195 | 1.354 | 16.899 | 0.237081 | 7/15/2014 | 43:39.4 | 0 |
| -116.345 | 19700.202 | 1.354 | 16.887 | 0.259921 | 7/15/2014 | 43:39.5 | 0 |
| -116.117 | 19700.209 | 1.354 | 16.874 | 0.2871 | 7/15/2014 | 43:39.6 | 0 |
| -115.833 | 19700.219 | 1.36 | 16.862 | 0.293805 | 7/15/2014 | 43:39.7 | 0 |
| -115.605 | 19700.227 | 1.365 | 16.849 | 0.291401 | 7/15/2014 | 43:39.8 | 0 |
| -115.375 | 19700.234 | 1.37 | 16.836 | 0.309055 | 7/15/2014 | 43:39.9 | 0 |
| -115.146 | 19700.242 | 1.377 | 16.823 | 0.305671 | 7/15/2014 | 43:40.0 | 0 |
| -114.919 | 19700.249 | 1.381 | 16.81 | 0.311095 | 7/15/2014 | 43:40.1 | 0 |
| -114.692 | 19700.255 | 1.386 | 16.798 | 0.277521 | 7/15/2014 | 43:40.2 | 0 |
| -114.466 | 19700.262 | 1.396 | 16.787 | 0.286546 | 7/15/2014 | 43:40.3 | 0 |
| -114.184 | 19700.271 | 1.404 | 16.776 | 0.283445 | 7/15/2014 | 43:40.4 | 0 |
| -113.96 | 19700.279 | 1.409 | 16.767 | 0.264251 | 7/15/2014 | 43:40.5 | 0 |
| -113.735 | 19700.287 | 1.416 | 16.759 | 0.260057 | 7/15/2014 | 43:40.6 | 0 |
| -113.454 | 19700.295 | 1.422 | 16.752 | 0.227724 | 7/15/2014 | 43:40.7 | 0 |
| -113.228 | 19700.302 | 1.424 | 16.747 | 0.225259 | 7/15/2014 | 43:40.8 | 0 |
| -113 | 19700.309 | 1.428 | 16.743 | 0.230038 | 7/15/2014 | 43:40.9 | 0 |
| -112.773 | 19700.315 | 1.43 | 16.741 | 0.207257 | 7/15/2014 | 43:41.0 | 0 |
| -112.544 | 19700.322 | 1.43 | 16.74 | 0.20604 | 7/15/2014 | 43:41.1 | 0 |
| -112.316 | 19700.33 | 1.433 | 16.739 | 0.21614 | 7/15/2014 | 43:41.2 | 0 |
| -112.089 | 19700.338 | 1.439 | 16.739 | 0.19929 | 7/15/2014 | 43:41.3 | 0 |
| -111.807 | 19700.345 | 1.445 | 16.739 | 0.204732 | 7/15/2014 | 43:41.4 | 0 |
| -111.581 | 19700.352 | 1.452 | 16.739 | 0.203196 | 7/15/2014 | 43:41.5 | 0 |
| -111.354 | 19700.359 | 1.461 | 16.739 | 0.214218 | 7/15/2014 | 43:41.6 | 0 |
| -111.072 | 19700.368 | 1.464 | 16.738 | 0.19973 | 7/15/2014 | 43:41.7 | 0 |

| | | | | | | | |
|---|---|---|---|---|---|---|---|
| -110.845 | 19700.375 | 1.472 | 16.737 | 0.188189 | 7/15/2014 | 43:41.8 | 0 |
| -110.616 | 19700.382 | 1.483 | 16.735 | 0.176255 | 7/15/2014 | 43:41.9 | 0 |
| -110.388 | 19700.389 | 1.488 | 16.734 | 0.176721 | 7/15/2014 | 43:42.0 | 0 |
| -110.16 | 19700.396 | 1.493 | 16.731 | 0.17724 | 7/15/2014 | 43:42.1 | 0 |
| -109.935 | 19700.402 | 1.499 | 16.729 | 0.197079 | 7/15/2014 | 43:42.2 | 0 |
| -109.709 | 19700.409 | 1.502 | 16.726 | 0.210341 | 7/15/2014 | 43:42.3 | 0 |
| -109.43 | 19700.418 | 1.505 | 16.723 | 0.244073 | 7/15/2014 | 43:42.4 | 0 |
| -109.207 | 19700.426 | 1.51 | 16.719 | 0.280255 | 7/15/2014 | 43:42.5 | 0 |
| -108.984 | 19700.433 | 1.516 | 16.714 | 0.302239 | 7/15/2014 | 43:42.6 | 0 |
| -108.709 | 19700.439 | 1.519 | 16.709 | 0.336644 | 7/15/2014 | 43:42.7 | 0 |
| -108.487 | 19700.447 | 1.524 | 16.702 | 0.367651 | 7/15/2014 | 43:42.8 | 0 |
| -108.265 | 19700.455 | 1.528 | 16.695 | 0.382312 | 7/15/2014 | 43:42.9 | 0 |
| -108.043 | 19700.462 | 1.53 | 16.686 | 0.365276 | 7/15/2014 | 43:43.0 | 0 |
| -107.822 | 19700.467 | 1.535 | 16.677 | 0.457167 | 7/15/2014 | 43:43.1 | 0 |
| -107.603 | 19700.475 | 1.539 | 16.668 | 0.441522 | 7/15/2014 | 43:43.2 | 0 |
| -107.384 | 19700.482 | 1.545 | 16.658 | 0.48773 | 7/15/2014 | 43:43.3 | 0 |
| -107.114 | 19700.49 | 1.549 | 16.647 | 0.540418 | 7/15/2014 | 43:43.4 | 0 |
| -106.897 | 19700.497 | 1.553 | 16.637 | 0.525611 | 7/15/2014 | 43:43.5 | 0 |
| -106.683 | 19700.504 | 1.555 | 16.626 | 0.515606 | 7/15/2014 | 43:43.6 | 0 |
| -106.416 | 19700.514 | 1.562 | 16.616 | 0.504847 | 7/15/2014 | 43:43.7 | 0 |
| -106.201 | 19700.518 | 1.569 | 16.605 | 0.439232 | 7/15/2014 | 43:43.8 | 0 |
| -105.986 | 19700.525 | 1.577 | 16.595 | 0.447666 | 7/15/2014 | 43:43.9 | 0 |
| -105.771 | 19700.533 | 1.585 | 16.586 | 0.398443 | 7/15/2014 | 43:44.0 | 0 |
| -105.558 | 19700.539 | 1.589 | 16.577 | 0.361648 | 7/15/2014 | 43:44.1 | 0 |
| -105.344 | 19700.546 | 1.596 | 16.568 | 0.357719 | 7/15/2014 | 43:44.2 | 0 |
| -105.133 | 19700.551 | 1.604 | 16.56 | 0.331731 | 7/15/2014 | 43:44.3 | 0 |
| -104.766 | 19700.563 | 1.619 | 16.548 | 0.318005 | 7/15/2014 | 43:44.5 | 0 |
| -104.556 | 19700.568 | 1.626 | 16.54 | 0.30951 | 7/15/2014 | 43:44.6 | 0 |
| -104.296 | 19700.577 | 1.631 | 16.533 | 0.298331 | 7/15/2014 | 43:44.7 | 0 |
| -104.19 | 19700.581 | 1.634 | 16.529 | 0.295017 | 7/15/2014 | 43:44.7 | 0 |
| -103.982 | 19700.586 | 1.638 | 16.521 | 0.294092 | 7/15/2014 | 43:44.8 | 0 |
| -103.771 | 19700.592 | 1.649 | 16.513 | 0.291699 | 7/15/2014 | 43:44.9 | 0 |
| -103.561 | 19700.598 | 1.658 | 16.504 | 0.291215 | 7/15/2014 | 43:45.0 | 0 |
| -103.351 | 19700.604 | 1.666 | 16.494 | 0.290744 | 7/15/2014 | 43:45.1 | 0 |
| -103.142 | 19700.61 | 1.671 | 16.484 | 0.291249 | 7/15/2014 | 43:45.2 | 0 |
| -102.934 | 19700.616 | 1.678 | 16.473 | 0.291245 | 7/15/2014 | 43:45.3 | 0 |
| -102.572 | 19700.627 | 1.692 | 16.454 | 0.294824 | 7/15/2014 | 43:45.5 | 0 |
| -102.365 | 19700.633 | 1.703 | 16.442 | 0.286179 | 7/15/2014 | 43:45.6 | 0 |
| -102.108 | 19700.639 | 1.71 | 16.429 | 0.279489 | 7/15/2014 | 43:45.7 | 0 |
| -102.003 | 19700.643 | 1.715 | 16.422 | 0.276444 | 7/15/2014 | 43:45.7 | 0 |
| -101.794 | 19700.647 | 1.724 | 16.409 | 0.270775 | 7/15/2014 | 43:45.8 | 0 |
| -101.586 | 19700.654 | 1.728 | 16.396 | 0.267155 | 7/15/2014 | 43:45.9 | 0 |
| -101.376 | 19700.659 | 1.737 | 16.382 | 0.263619 | 7/15/2014 | 43:46.0 | 0 |
| -101.167 | 19700.666 | 1.744 | 16.37 | 0.26001 | 7/15/2014 | 43:46.1 | 0 |
| -100.958 | 19700.671 | 1.753 | 16.357 | 0.265889 | 7/15/2014 | 43:46.2 | 0 |
| -100.749 | 19700.677 | 1.759 | 16.346 | 0.257653 | 7/15/2014 | 43:46.3 | 0 |
| -100.389 | 19700.687 | 1.77 | 16.33 | 0.250727 | 7/15/2014 | 43:46.5 | 0 |

| | | | | | | | |
|---|---|---|---|---|---|---|---|
| -100.181 | 19700.692 | 1.781 | 16.32 | 0.252502 | 7/15/2014 | 43:46.6 | 0 |
| -99.926 | 19700.699 | 1.787 | 16.311 | 0.25016 | 7/15/2014 | 43:46.7 | 0 |
| -99.82 | 19700.701 | 1.794 | 16.306 | 0.249157 | 7/15/2014 | 43:46.7 | 0 |
| -99.613 | 19700.707 | 1.801 | 16.297 | 0.24867 | 7/15/2014 | 43:46.8 | 0 |
| -99.405 | 19700.712 | 1.808 | 16.289 | 0.249258 | 7/15/2014 | 43:46.9 | 0 |
| -99.196 | 19700.718 | 1.816 | 16.282 | 0.250985 | 7/15/2014 | 43:47.0 | 0 |
| -98.988 | 19700.723 | 1.825 | 16.274 | 0.253746 | 7/15/2014 | 43:47.1 | 0 |
| -98.782 | 19700.728 | 1.833 | 16.266 | 0.261325 | 7/15/2014 | 43:47.2 | 0 |
| -98.577 | 19700.734 | 1.844 | 16.258 | 0.2591 | 7/15/2014 | 43:47.3 | 0 |
| -98.221 | 19700.742 | 1.855 | 16.245 | 0.263593 | 7/15/2014 | 43:47.5 | 0 |
| -98.019 | 19700.747 | 1.865 | 16.236 | 0.261783 | 7/15/2014 | 43:47.6 | 0 |
| -97.768 | 19700.753 | 1.872 | 16.227 | 0.263117 | 7/15/2014 | 43:47.7 | 0 |
| -97.664 | 19700.756 | 1.88 | 16.223 | 0.259637 | 7/15/2014 | 43:47.7 | 0 |
| -97.462 | 19700.76 | 1.885 | 16.214 | 0.259257 | 7/15/2014 | 43:47.8 | 0 |
| -97.26 | 19700.765 | 1.891 | 16.205 | 0.26128 | 7/15/2014 | 43:47.9 | 0 |
| -97.057 | 19700.771 | 1.898 | 16.197 | 0.266341 | 7/15/2014 | 43:48.0 | 0 |
| -96.755 | 19700.777 | 1.909 | 16.188 | 0.275565 | 7/15/2014 | 43:48.2 | 0 |
| -96.555 | 19700.781 | 1.914 | 16.182 | 0.274863 | 7/15/2014 | 43:48.3 | 0 |
| -96.354 | 19700.786 | 1.923 | 16.176 | 0.284514 | 7/15/2014 | 43:48.4 | 0 |
| -96.106 | 19700.791 | 1.929 | 16.171 | 0.30297 | 7/15/2014 | 43:48.5 | 0 |
| -95.909 | 19700.795 | 1.939 | 16.167 | 0.31015 | 7/15/2014 | 43:48.6 | 0 |
| -95.661 | 19700.802 | 1.948 | 16.163 | 0.297328 | 7/15/2014 | 43:48.7 | 0 |
| -95.46 | 19700.806 | 1.958 | 16.158 | 0.297905 | 7/15/2014 | 43:48.8 | 0 |
| -95.259 | 19700.811 | 1.962 | 16.154 | 0.284188 | 7/15/2014 | 43:48.9 | 0 |
| -95.057 | 19700.814 | 1.97 | 16.15 | 0.279367 | 7/15/2014 | 43:49.0 | 0 |
| -94.957 | 19700.816 | 1.974 | 16.147 | 0.288789 | 7/15/2014 | 43:49.0 | 0 |
| -94.754 | 19700.82 | 1.979 | 16.142 | 0.252774 | 7/15/2014 | 43:49.1 | 0 |
| -94.551 | 19700.824 | 1.987 | 16.137 | 0.241505 | 7/15/2014 | 43:49.2 | 0 |
| -94.35 | 19700.828 | 1.994 | 16.131 | 0.218666 | 7/15/2014 | 43:49.3 | 0 |
| -93.998 | 19700.836 | 2.006 | 16.119 | 0.199583 | 7/15/2014 | 43:49.5 | 0 |
| -93.798 | 19700.84 | 2.012 | 16.111 | 0.210292 | 7/15/2014 | 43:49.6 | 0 |
| -93.548 | 19700.844 | 2.019 | 16.102 | 0.17499 | 7/15/2014 | 43:49.7 | 0 |
| -93.445 | 19700.846 | 2.024 | 16.098 | 0.167363 | 7/15/2014 | 43:49.7 | 0 |
| -93.242 | 19700.85 | 2.03 | 16.087 | 0.174483 | 7/15/2014 | 43:49.8 | 0 |
| -93.04 | 19700.854 | 2.037 | 16.076 | 0.178228 | 7/15/2014 | 43:49.9 | 0 |
| -92.836 | 19700.857 | 2.045 | 16.063 | 0.192062 | 7/15/2014 | 43:50.0 | 0 |
| -92.634 | 19700.86 | 2.052 | 16.049 | 0.20596 | 7/15/2014 | 43:50.1 | 0 |
| -92.431 | 19700.863 | 2.063 | 16.035 | 0.197654 | 7/15/2014 | 43:50.2 | 0 |
| -92.232 | 19700.867 | 2.068 | 16.02 | 0.194141 | 7/15/2014 | 43:50.3 | 0 |
| -91.883 | 19700.872 | 2.084 | 15.997 | 0.235535 | 7/15/2014 | 43:50.5 | 0 |
| -91.683 | 19700.875 | 2.097 | 15.981 | 0.209482 | 7/15/2014 | 43:50.6 | 0 |
| -91.437 | 19700.879 | 2.105 | 15.966 | 0.186398 | 7/15/2014 | 43:50.7 | 0 |
| -91.235 | 19700.883 | 2.121 | 15.95 | 0.182972 | 7/15/2014 | 43:50.8 | 0 |
| -91.034 | 19700.885 | 2.13 | 15.935 | 0.160669 | 7/15/2014 | 43:50.9 | 0 |
| -90.831 | 19700.887 | 2.141 | 15.92 | 0.166494 | 7/15/2014 | 43:51.0 | 0 |
| -90.63 | 19700.89 | 2.145 | 15.906 | 0.164979 | 7/15/2014 | 43:51.1 | 0 |
| -90.529 | 19700.892 | 2.152 | 15.899 | 0.167597 | 7/15/2014 | 43:51.1 | 0 |

| | | | | | | | |
|---|---|---|---|---|---|---|---|
| -90.327 | 19700.895 | 2.163 | 15.885 | 0.170033 | 7/15/2014 | 43:51.2 | 0 |
| -90.127 | 19700.896 | 2.17 | 15.87 | 0.150732 | 7/15/2014 | 43:51.3 | 0 |
| -89.777 | 19700.9 | 2.178 | 15.849 | 0.191141 | 7/15/2014 | 43:51.5 | 0 |
| -89.575 | 19700.903 | 2.189 | 15.834 | 0.191732 | 7/15/2014 | 43:51.6 | 0 |
| -89.324 | 19700.905 | 2.194 | 15.82 | 0.197522 | 7/15/2014 | 43:51.7 | 0 |
| -89.223 | 19700.906 | 2.198 | 15.812 | 0.19611 | 7/15/2014 | 43:51.7 | 0 |
| -89.019 | 19700.909 | 2.209 | 15.797 | 0.199833 | 7/15/2014 | 43:51.8 | 0 |
| -88.815 | 19700.912 | 2.22 | 15.782 | 0.189832 | 7/15/2014 | 43:51.9 | 0 |
| -88.611 | 19700.914 | 2.228 | 15.766 | 0.225925 | 7/15/2014 | 43:52.0 | 0 |
| -88.406 | 19700.916 | 2.236 | 15.75 | 0.208612 | 7/15/2014 | 43:52.1 | 0 |
| -88.203 | 19700.918 | 2.244 | 15.734 | 0.180192 | 7/15/2014 | 43:52.2 | 0 |
| -87.998 | 19700.919 | 2.256 | 15.719 | 0.199198 | 7/15/2014 | 43:52.3 | 0 |
| -87.643 | 19700.922 | 2.266 | 15.697 | 0.199589 | 7/15/2014 | 43:52.5 | 0 |
| -87.439 | 19700.924 | 2.276 | 15.683 | 0.184845 | 7/15/2014 | 43:52.6 | 0 |
| -87.185 | 19700.927 | 2.283 | 15.67 | 0.172693 | 7/15/2014 | 43:52.7 | 0 |
| -87.082 | 19700.928 | 2.287 | 15.663 | 0.171699 | 7/15/2014 | 43:52.7 | 0 |
| -86.877 | 19700.929 | 2.298 | 15.65 | 0.163574 | 7/15/2014 | 43:52.8 | 0 |
| -86.67 | 19700.931 | 2.305 | 15.639 | 0.179258 | 7/15/2014 | 43:52.9 | 0 |
| -86.462 | 19700.932 | 2.318 | 15.629 | 0.146666 | 7/15/2014 | 43:53.0 | 0 |
| -86.255 | 19700.933 | 2.326 | 15.619 | 0.146956 | 7/15/2014 | 43:53.1 | 0 |
| -86.047 | 19700.935 | 2.337 | 15.61 | 0.143614 | 7/15/2014 | 43:53.2 | 0 |
| -85.841 | 19700.936 | 2.345 | 15.603 | 0.138199 | 7/15/2014 | 43:53.3 | 0 |
| -85.478 | 19700.938 | 2.353 | 15.594 | 0.124351 | 7/15/2014 | 43:53.5 | 0 |
| -85.271 | 19700.939 | 2.357 | 15.588 | 0.122432 | 7/15/2014 | 43:53.6 | 0 |
| -85.013 | 19700.94 | 2.358 | 15.583 | 0.112335 | 7/15/2014 | 43:53.7 | 0 |
| -84.908 | 19700.941 | 2.364 | 15.581 | 0.100912 | 7/15/2014 | 43:53.7 | 0 |
| -84.697 | 19700.942 | 2.367 | 15.577 | 0.11261 | 7/15/2014 | 43:53.8 | 0 |
| -84.487 | 19700.944 | 2.369 | 15.574 | 0.0927237 | 7/15/2014 | 43:53.9 | 0 |
| -84.275 | 19700.945 | 2.373 | 15.57 | 0.0925779 | 7/15/2014 | 43:54.0 | 0 |
| -84.063 | 19700.946 | 2.378 | 15.567 | 0.0992756 | 7/15/2014 | 43:54.1 | 0 |
| -83.852 | 19700.946 | 2.384 | 15.564 | 0.118669 | 7/15/2014 | 43:54.2 | 0 |
| -83.641 | 19700.949 | 2.39 | 15.561 | 0.0979097 | 7/15/2014 | 43:54.3 | 0 |
| -83.275 | 19700.95 | 2.401 | 15.555 | 0.111278 | 7/15/2014 | 43:54.5 | 0 |
| -83.063 | 19700.951 | 2.408 | 15.552 | 0.104263 | 7/15/2014 | 43:54.6 | 0 |
| -82.803 | 19700.953 | 2.413 | 15.548 | 0.111052 | 7/15/2014 | 43:54.7 | 0 |
| -82.695 | 19700.953 | 2.418 | 15.546 | 0.112094 | 7/15/2014 | 43:54.7 | 0 |
| -82.483 | 19700.955 | 2.423 | 15.542 | 0.139006 | 7/15/2014 | 43:54.8 | 0 |
| -82.271 | 19700.955 | 2.429 | 15.538 | 0.106201 | 7/15/2014 | 43:54.9 | 0 |
| -82.057 | 19700.956 | 2.433 | 15.533 | 0.127818 | 7/15/2014 | 43:55.0 | 0 |
| -81.845 | 19700.957 | 2.44 | 15.528 | 0.133387 | 7/15/2014 | 43:55.1 | 0 |
| -81.633 | 19700.958 | 2.445 | 15.523 | 0.123154 | 7/15/2014 | 43:55.2 | 0 |
| -81.422 | 19700.958 | 2.449 | 15.517 | 0.124112 | 7/15/2014 | 43:55.3 | 0 |
| -81.056 | 19700.96 | 2.457 | 15.506 | 0.116918 | 7/15/2014 | 43:55.5 | 0 |
| -80.846 | 19700.962 | 2.463 | 15.499 | 0.110503 | 7/15/2014 | 43:55.6 | 0 |
| -80.585 | 19700.961 | 2.467 | 15.491 | 0.123311 | 7/15/2014 | 43:55.7 | 0 |
| -80.478 | 19700.961 | 2.47 | 15.487 | 0.109301 | 7/15/2014 | 43:55.7 | 0 |
| -80.269 | 19700.963 | 2.474 | 15.478 | 0.107122 | 7/15/2014 | 43:55.8 | 0 |

| | | | | | | | |
|---|---|---|---|---|---|---|---|
| -80.057 | 19700.963 | 2.477 | 15.469 | 0.10667 | 7/15/2014 | 43:55.9 | 0 |
| -79.846 | 19700.963 | 2.479 | 15.459 | 0.115802 | 7/15/2014 | 43:56.0 | 0 |
| -79.634 | 19700.963 | 2.481 | 15.45 | 0.103196 | 7/15/2014 | 43:56.1 | 0 |
| -79.422 | 19700.964 | 2.484 | 15.441 | 0.130398 | 7/15/2014 | 43:56.2 | 0 |
| -79.214 | 19700.965 | 2.486 | 15.431 | 0.119751 | 7/15/2014 | 43:56.3 | 0 |
| -78.851 | 19700.965 | 2.489 | 15.418 | 0.117742 | 7/15/2014 | 43:56.5 | 0 |
| -78.643 | 19700.966 | 2.497 | 15.409 | 0.127785 | 7/15/2014 | 43:56.6 | 0 |
| -78.386 | 19700.966 | 2.503 | 15.4 | 0.109717 | 7/15/2014 | 43:56.7 | 0 |
| -78.28 | 19700.966 | 2.506 | 15.395 | 0.101545 | 7/15/2014 | 43:56.7 | 0 |
| -78.074 | 19700.966 | 2.513 | 15.386 | 0.0919228 | 7/15/2014 | 43:56.8 | 0 |
| -77.865 | 19700.966 | 2.521 | 15.377 | 0.096557 | 7/15/2014 | 43:56.9 | 0 |
| -77.66 | 19700.966 | 2.525 | 15.368 | 0.0845035 | 7/15/2014 | 43:57.0 | 0 |
| -77.453 | 19700.967 | 2.531 | 15.36 | 0.0913179 | 7/15/2014 | 43:57.1 | 0 |
| -77.249 | 19700.967 | 2.536 | 15.352 | 0.0900378 | 7/15/2014 | 43:57.2 | 0 |
| -77.045 | 19700.967 | 2.539 | 15.344 | 0.0882171 | 7/15/2014 | 43:57.3 | 0 |
| -76.691 | 19700.967 | 2.536 | 15.335 | 0.112034 | 7/15/2014 | 43:57.5 | 0 |
| -76.489 | 19700.967 | 2.538 | 15.329 | 0.129933 | 7/15/2014 | 43:57.6 | 0 |
| -76.236 | 19700.966 | 2.54 | 15.323 | 0.10048 | 7/15/2014 | 43:57.7 | 0 |
| -76.135 | 19700.966 | 2.539 | 15.321 | 0.114086 | 7/15/2014 | 43:57.7 | 0 |
| -75.932 | 19700.966 | 2.542 | 15.316 | 0.0860166 | 7/15/2014 | 43:57.8 | 0 |
| -75.725 | 19700.966 | 2.544 | 15.312 | 0.0887584 | 7/15/2014 | 43:57.9 | 0 |
| -75.519 | 19700.965 | 2.546 | 15.309 | 0.0698896 | 7/15/2014 | 43:58.0 | 0 |
| -75.312 | 19700.965 | 2.551 | 15.306 | 0.0670467 | 7/15/2014 | 43:58.1 | 0 |
| -75.102 | 19700.965 | 2.562 | 15.304 | 0.0645608 | 7/15/2014 | 43:58.2 | 0 |
| -74.896 | 19700.964 | 2.567 | 15.302 | 0.0658248 | 7/15/2014 | 43:58.3 | 0 |
| -74.53 | 19700.963 | 2.571 | 15.299 | 0.0643034 | 7/15/2014 | 43:58.5 | 0 |
| -74.319 | 19700.962 | 2.574 | 15.297 | 0.0625729 | 7/15/2014 | 43:58.6 | 0 |
| -74.055 | 19700.961 | 2.574 | 15.295 | 0.0593473 | 7/15/2014 | 43:58.7 | 0 |
| -73.95 | 19700.96 | 2.576 | 15.294 | 0.0575869 | 7/15/2014 | 43:58.7 | 0 |
| -73.735 | 19700.961 | 2.573 | 15.292 | 0.0579338 | 7/15/2014 | 43:58.8 | 0 |
| -73.516 | 19700.96 | 2.577 | 15.29 | 0.060652 | 7/15/2014 | 43:58.9 | 0 |
| -73.299 | 19700.958 | 2.577 | 15.288 | 0.0702207 | 7/15/2014 | 43:59.0 | 0 |
| -73.081 | 19700.958 | 2.575 | 15.286 | 0.0751499 | 7/15/2014 | 43:59.1 | 0 |
| -72.863 | 19700.957 | 2.575 | 15.283 | 0.0658874 | 7/15/2014 | 43:59.2 | 0 |
| -72.643 | 19700.956 | 2.578 | 15.281 | 0.0735141 | 7/15/2014 | 43:59.3 | 0 |
| -72.374 | 19700.954 | 2.58 | 15.279 | 0.0822112 | 7/15/2014 | 43:59.4 | 0 |
| -72.156 | 19700.954 | 2.585 | 15.277 | 0.0802301 | 7/15/2014 | 43:59.5 | 0 |
| -71.937 | 19700.954 | 2.59 | 15.275 | 0.0808447 | 7/15/2014 | 43:59.6 | 0 |
| -71.668 | 19700.952 | 2.589 | 15.273 | 0.0889994 | 7/15/2014 | 43:59.7 | 0 |
| -71.451 | 19700.952 | 2.587 | 15.272 | 0.0848271 | 7/15/2014 | 43:59.8 | 0 |
| -71.232 | 19700.95 | 2.59 | 15.271 | 0.0828037 | 7/15/2014 | 43:59.9 | 0 |
| -71.014 | 19700.95 | 2.588 | 15.269 | 0.0746825 | 7/15/2014 | 44:00.0 | 0 |
| -70.797 | 19700.948 | 2.588 | 15.268 | 0.0851282 | 7/15/2014 | 44:00.1 | 0 |
| -70.582 | 19700.949 | 2.586 | 15.267 | 0.0851205 | 7/15/2014 | 44:00.2 | 0 |
| -70.369 | 19700.948 | 2.584 | 15.265 | 0.0855362 | 7/15/2014 | 44:00.3 | 0 |
| -70.002 | 19700.947 | 2.582 | 15.261 | 0.0977358 | 7/15/2014 | 44:00.5 | 0 |
| -69.791 | 19700.947 | 2.587 | 15.258 | 0.0959932 | 7/15/2014 | 44:00.6 | 0 |

| | | | | | | | |
|---|---|---|---|---|---|---|---|
| -69.534 | 19700.945 | 2.587 | 15.255 | 0.113089 | 7/15/2014 | 44:00.7 | 0 |
| -69.427 | 19700.945 | 2.587 | 15.253 | 0.106563 | 7/15/2014 | 44:00.7 | 0 |
| -69.219 | 19700.944 | 2.592 | 15.249 | 0.109201 | 7/15/2014 | 44:00.8 | 0 |
| -69.013 | 19700.945 | 2.594 | 15.245 | 0.123018 | 7/15/2014 | 44:00.9 | 0 |
| -68.805 | 19700.943 | 2.598 | 15.241 | 0.112971 | 7/15/2014 | 44:01.0 | 0 |
| -68.599 | 19700.943 | 2.602 | 15.236 | 0.112145 | 7/15/2014 | 44:01.1 | 0 |
| -68.394 | 19700.943 | 2.607 | 15.232 | 0.114713 | 7/15/2014 | 44:01.2 | 0 |
| -68.191 | 19700.943 | 2.611 | 15.227 | 0.109353 | 7/15/2014 | 44:01.3 | 0 |
| -67.84 | 19700.941 | 2.61 | 15.218 | 0.113637 | 7/15/2014 | 44:01.5 | 0 |
| -67.638 | 19700.941 | 2.614 | 15.213 | 0.112324 | 7/15/2014 | 44:01.6 | 0 |
| -67.388 | 19700.941 | 2.614 | 15.207 | 0.114109 | 7/15/2014 | 44:01.7 | 0 |
| -67.286 | 19700.94 | 2.615 | 15.204 | 0.115002 | 7/15/2014 | 44:01.7 | 0 |
| -67.082 | 19700.939 | 2.622 | 15.197 | 0.118411 | 7/15/2014 | 44:01.8 | 0 |
| -66.881 | 19700.939 | 2.622 | 15.19 | 0.122437 | 7/15/2014 | 44:01.9 | 0 |
| -66.677 | 19700.939 | 2.625 | 15.182 | 0.12739 | 7/15/2014 | 44:02.0 | 0 |
| -66.473 | 19700.939 | 2.625 | 15.174 | 0.130338 | 7/15/2014 | 44:02.1 | 0 |
| -66.269 | 19700.938 | 2.631 | 15.166 | 0.133679 | 7/15/2014 | 44:02.2 | 0 |
| -66.065 | 19700.937 | 2.636 | 15.158 | 0.139033 | 7/15/2014 | 44:02.3 | 0 |
| -65.707 | 19700.937 | 2.649 | 15.147 | 0.137586 | 7/15/2014 | 44:02.5 | 0 |
| -65.501 | 19700.935 | 2.655 | 15.139 | 0.143748 | 7/15/2014 | 44:02.6 | 0 |
| -65.246 | 19700.934 | 2.654 | 15.131 | 0.133555 | 7/15/2014 | 44:02.7 | 0 |
| -65.144 | 19700.934 | 2.658 | 15.127 | 0.139145 | 7/15/2014 | 44:02.7 | 0 |
| -64.934 | 19700.933 | 2.664 | 15.12 | 0.132885 | 7/15/2014 | 44:02.8 | 0 |
| -64.723 | 19700.931 | 2.672 | 15.113 | 0.124954 | 7/15/2014 | 44:02.9 | 0 |
| -64.512 | 19700.93 | 2.677 | 15.107 | 0.118614 | 7/15/2014 | 44:03.0 | 0 |
| -64.301 | 19700.929 | 2.682 | 15.101 | 0.126504 | 7/15/2014 | 44:03.1 | 0 |
| -64.089 | 19700.929 | 2.687 | 15.095 | 0.121556 | 7/15/2014 | 44:03.2 | 0 |
| -63.879 | 19700.927 | 2.687 | 15.09 | 0.119941 | 7/15/2014 | 44:03.3 | 0 |
| -63.512 | 19700.926 | 2.691 | 15.083 | 0.121955 | 7/15/2014 | 44:03.5 | 0 |
| -63.3 | 19700.923 | 2.695 | 15.079 | 0.124736 | 7/15/2014 | 44:03.6 | 0 |
| -63.038 | 19700.922 | 2.695 | 15.076 | 0.127022 | 7/15/2014 | 44:03.7 | 0 |
| -62.93 | 19700.922 | 2.697 | 15.074 | 0.13211 | 7/15/2014 | 44:03.7 | 0 |
| -62.718 | 19700.92 | 2.697 | 15.071 | 0.129135 | 7/15/2014 | 44:03.8 | 0 |
| -62.504 | 19700.919 | 2.702 | 15.069 | 0.128857 | 7/15/2014 | 44:03.9 | 0 |
| -62.292 | 19700.918 | 2.699 | 15.067 | 0.132307 | 7/15/2014 | 44:04.0 | 0 |
| -62.078 | 19700.918 | 2.701 | 15.067 | 0.133876 | 7/15/2014 | 44:04.1 | 0 |
| -61.866 | 19700.917 | 2.701 | 15.066 | 0.139153 | 7/15/2014 | 44:04.2 | 0 |
| -61.655 | 19700.914 | 2.703 | 15.067 | 0.13926 | 7/15/2014 | 44:04.3 | 0 |
| -61.29 | 19700.913 | 2.703 | 15.068 | 0.141762 | 7/15/2014 | 44:04.5 | 0 |
| -61.081 | 19700.912 | 2.708 | 15.069 | 0.144477 | 7/15/2014 | 44:04.6 | 0 |
| -60.823 | 19700.91 | 2.706 | 15.07 | 0.142859 | 7/15/2014 | 44:04.7 | 0 |
| -60.717 | 19700.91 | 2.703 | 15.07 | 0.153002 | 7/15/2014 | 44:04.7 | 0 |
| -60.507 | 19700.91 | 2.703 | 15.071 | 0.162243 | 7/15/2014 | 44:04.8 | 0 |
| -60.299 | 19700.908 | 2.704 | 15.072 | 0.174866 | 7/15/2014 | 44:04.9 | 0 |
| -60.089 | 19700.907 | 2.707 | 15.072 | 0.186393 | 7/15/2014 | 44:05.0 | 0 |
| -59.881 | 19700.906 | 2.711 | 15.072 | 0.173462 | 7/15/2014 | 44:05.1 | 0 |
| -59.672 | 19700.905 | 2.717 | 15.071 | 0.192194 | 7/15/2014 | 44:05.2 | 0 |

| | | | | | | | |
|---|---|---|---|---|---|---|---|
| -59.466 | 19700.904 | 2.72 | 15.07 | 0.197783 | 7/15/2014 | 44:05.3 | 0 |
| -59.108 | 19700.902 | 2.724 | 15.067 | 0.220045 | 7/15/2014 | 44:05.5 | 0 |
| -58.901 | 19700.901 | 2.731 | 15.065 | 0.206368 | 7/15/2014 | 44:05.6 | 0 |
| -58.647 | 19700.899 | 2.734 | 15.062 | 0.196384 | 7/15/2014 | 44:05.7 | 0 |
| -58.543 | 19700.899 | 2.737 | 15.061 | 0.188633 | 7/15/2014 | 44:05.7 | 0 |
| -58.335 | 19700.897 | 2.74 | 15.058 | 0.216086 | 7/15/2014 | 44:05.8 | 0 |
| -58.126 | 19700.896 | 2.744 | 15.055 | 0.202374 | 7/15/2014 | 44:05.9 | 0 |
| -57.919 | 19700.896 | 2.744 | 15.052 | 0.194863 | 7/15/2014 | 44:06.0 | 0 |
| -57.71 | 19700.894 | 2.746 | 15.048 | 0.164697 | 7/15/2014 | 44:06.1 | 0 |
| -57.502 | 19700.893 | 2.748 | 15.044 | 0.12661 | 7/15/2014 | 44:06.2 | 0 |
| -57.293 | 19700.891 | 2.751 | 15.04 | 0.129699 | 7/15/2014 | 44:06.3 | 0 |
| -56.932 | 19700.888 | 2.755 | 15.033 | 0.121776 | 7/15/2014 | 44:06.5 | 0 |
| -56.724 | 19700.886 | 2.762 | 15.028 | 0.116573 | 7/15/2014 | 44:06.6 | 0 |
| -56.464 | 19700.883 | 2.764 | 15.024 | 0.119265 | 7/15/2014 | 44:06.7 | 0 |
| -56.36 | 19700.883 | 2.766 | 15.021 | 0.127266 | 7/15/2014 | 44:06.7 | 0 |
| -56.152 | 19700.882 | 2.771 | 15.016 | 0.1271 | 7/15/2014 | 44:06.8 | 0 |
| -55.94 | 19700.879 | 2.777 | 15.011 | 0.127055 | 7/15/2014 | 44:06.9 | 0 |
| -55.73 | 19700.878 | 2.78 | 15.006 | 0.128139 | 7/15/2014 | 44:07.0 | 0 |
| -55.519 | 19700.875 | 2.782 | 15.001 | 0.145624 | 7/15/2014 | 44:07.1 | 0 |
| -55.309 | 19700.873 | 2.79 | 14.997 | 0.130664 | 7/15/2014 | 44:07.2 | 0 |
| -55.1 | 19700.871 | 2.793 | 14.992 | 0.143509 | 7/15/2014 | 44:07.3 | 0 |
| -54.735 | 19700.866 | 2.792 | 14.984 | 0.133902 | 7/15/2014 | 44:07.5 | 0 |
| -54.526 | 19700.865 | 2.797 | 14.979 | 0.134272 | 7/15/2014 | 44:07.6 | 0 |
| -54.264 | 19700.861 | 2.799 | 14.973 | 0.140077 | 7/15/2014 | 44:07.7 | 0 |
| -54.159 | 19700.861 | 2.799 | 14.97 | 0.138858 | 7/15/2014 | 44:07.7 | 0 |
| -53.948 | 19700.858 | 2.801 | 14.964 | 0.146119 | 7/15/2014 | 44:07.8 | 0 |
| -53.734 | 19700.856 | 2.807 | 14.958 | 0.144835 | 7/15/2014 | 44:07.9 | 0 |
| -53.522 | 19700.854 | 2.805 | 14.951 | 0.167283 | 7/15/2014 | 44:08.0 | 0 |
| -53.308 | 19700.85 | 2.807 | 14.943 | 0.162816 | 7/15/2014 | 44:08.1 | 0 |
| -53.095 | 19700.848 | 2.811 | 14.935 | 0.156329 | 7/15/2014 | 44:08.2 | 0 |
| -52.882 | 19700.846 | 2.816 | 14.926 | 0.163436 | 7/15/2014 | 44:08.3 | 0 |
| -52.617 | 19700.842 | 2.82 | 14.918 | 0.174208 | 7/15/2014 | 44:08.4 | 0 |
| -52.406 | 19700.839 | 2.822 | 14.908 | 0.173363 | 7/15/2014 | 44:08.5 | 0 |
| -52.193 | 19700.837 | 2.827 | 14.899 | 0.170455 | 7/15/2014 | 44:08.6 | 0 |
| -51.93 | 19700.832 | 2.827 | 14.89 | 0.177124 | 7/15/2014 | 44:08.7 | 0 |
| -51.717 | 19700.829 | 2.831 | 14.88 | 0.166746 | 7/15/2014 | 44:08.8 | 0 |
| -51.503 | 19700.826 | 2.833 | 14.871 | 0.170125 | 7/15/2014 | 44:08.9 | 0 |
| -51.292 | 19700.824 | 2.833 | 14.862 | 0.173111 | 7/15/2014 | 44:09.0 | 0 |
| -51.079 | 19700.82 | 2.837 | 14.853 | 0.162706 | 7/15/2014 | 44:09.1 | 0 |
| -50.869 | 19700.816 | 2.837 | 14.845 | 0.171316 | 7/15/2014 | 44:09.2 | 0 |
| -50.659 | 19700.813 | 2.839 | 14.837 | 0.179374 | 7/15/2014 | 44:09.3 | 0 |
| -50.297 | 19700.807 | 2.842 | 14.826 | 0.152481 | 7/15/2014 | 44:09.5 | 0 |
| -50.089 | 19700.804 | 2.847 | 14.82 | 0.156453 | 7/15/2014 | 44:09.6 | 0 |
| -49.831 | 19700.8 | 2.849 | 14.813 | 0.157233 | 7/15/2014 | 44:09.7 | 0 |
| -49.726 | 19700.799 | 2.852 | 14.81 | 0.151464 | 7/15/2014 | 44:09.7 | 0 |
| -49.518 | 19700.795 | 2.852 | 14.804 | 0.144902 | 7/15/2014 | 44:09.8 | 0 |
| -49.311 | 19700.79 | 2.852 | 14.799 | 0.135637 | 7/15/2014 | 44:09.9 | 0 |

| | | | | | | | |
|---|---|---|---|---|---|---|---|
| -49.102 | 19700.787 | 2.854 | 14.794 | 0.139314 | 7/15/2014 | 44:10.0 | 0 |
| -48.893 | 19700.783 | 2.854 | 14.789 | 0.152187 | 7/15/2014 | 44:10.1 | 0 |
| -48.686 | 19700.777 | 2.854 | 14.785 | 0.154672 | 7/15/2014 | 44:10.2 | 0 |
| -48.478 | 19700.773 | 2.854 | 14.782 | 0.147467 | 7/15/2014 | 44:10.3 | 0 |
| -48.117 | 19700.768 | 2.856 | 14.778 | 0.160385 | 7/15/2014 | 44:10.5 | 0 |
| -47.91 | 19700.764 | 2.859 | 14.775 | 0.163371 | 7/15/2014 | 44:10.6 | 0 |
| -47.652 | 19700.757 | 2.863 | 14.773 | 0.167423 | 7/15/2014 | 44:10.7 | 0 |
| -47.548 | 19700.756 | 2.866 | 14.772 | 0.165749 | 7/15/2014 | 44:10.7 | 0 |
| -47.34 | 19700.752 | 2.869 | 14.771 | 0.173133 | 7/15/2014 | 44:10.8 | 0 |
| -47.132 | 19700.745 | 2.874 | 14.77 | 0.16712 | 7/15/2014 | 44:10.9 | 0 |
| -46.922 | 19700.74 | 2.877 | 14.769 | 0.192888 | 7/15/2014 | 44:11.0 | 0 |
| -46.712 | 19700.735 | 2.883 | 14.768 | 0.191504 | 7/15/2014 | 44:11.1 | 0 |
| -46.504 | 19700.73 | 2.885 | 14.767 | 0.202799 | 7/15/2014 | 44:11.2 | 0 |
| -46.296 | 19700.727 | 2.89 | 14.767 | 0.203379 | 7/15/2014 | 44:11.3 | 0 |
| -45.934 | 19700.717 | 2.89 | 14.766 | 0.216034 | 7/15/2014 | 44:11.5 | 0 |
| -45.727 | 19700.711 | 2.893 | 14.765 | 0.209041 | 7/15/2014 | 44:11.6 | 0 |
| -45.468 | 19700.704 | 2.893 | 14.765 | 0.219863 | 7/15/2014 | 44:11.7 | 0 |
| -45.364 | 19700.701 | 2.893 | 14.765 | 0.20932 | 7/15/2014 | 44:11.7 | 0 |
| -45.154 | 19700.694 | 2.899 | 14.764 | 0.227078 | 7/15/2014 | 44:11.8 | 0 |
| -44.943 | 19700.689 | 2.899 | 14.764 | 0.201732 | 7/15/2014 | 44:11.9 | 0 |
| -44.733 | 19700.684 | 2.904 | 14.763 | 0.226867 | 7/15/2014 | 44:12.0 | 0 |
| -44.523 | 19700.676 | 2.907 | 14.763 | 0.251735 | 7/15/2014 | 44:12.1 | 0 |
| -44.311 | 19700.67 | 2.913 | 14.763 | 0.217613 | 7/15/2014 | 44:12.2 | 0 |
| -44.102 | 19700.663 | 2.916 | 14.763 | 0.232969 | 7/15/2014 | 44:12.3 | 0 |
| -43.734 | 19700.652 | 2.915 | 14.763 | 0.251685 | 7/15/2014 | 44:12.5 | 0 |
| -43.522 | 19700.645 | 2.919 | 14.762 | 0.249331 | 7/15/2014 | 44:12.6 | 0 |
| -43.261 | 19700.637 | 2.913 | 14.761 | 0.242557 | 7/15/2014 | 44:12.7 | 0 |
| -43.154 | 19700.633 | 2.916 | 14.761 | 0.229914 | 7/15/2014 | 44:12.7 | 0 |
| -42.941 | 19700.625 | 2.914 | 14.76 | 0.240099 | 7/15/2014 | 44:12.8 | 0 |
| -42.727 | 19700.617 | 2.911 | 14.758 | 0.251123 | 7/15/2014 | 44:12.9 | 0 |
| -42.513 | 19700.61 | 2.905 | 14.755 | 0.243304 | 7/15/2014 | 44:13.0 | 0 |
| -42.297 | 19700.602 | 2.902 | 14.751 | 0.240778 | 7/15/2014 | 44:13.1 | 0 |
| -42.082 | 19700.594 | 2.902 | 14.747 | 0.245266 | 7/15/2014 | 44:13.2 | 0 |
| -41.869 | 19700.586 | 2.902 | 14.743 | 0.248601 | 7/15/2014 | 44:13.3 | 0 |
| -41.602 | 19700.574 | 2.902 | 14.737 | 0.300302 | 7/15/2014 | 44:13.4 | 0 |
| -41.388 | 19700.566 | 2.91 | 14.731 | 0.264093 | 7/15/2014 | 44:13.5 | 0 |
| -41.176 | 19700.557 | 2.91 | 14.725 | 0.288626 | 7/15/2014 | 44:13.6 | 0 |
| -40.909 | 19700.545 | 2.915 | 14.719 | 0.277658 | 7/15/2014 | 44:13.7 | 0 |
| -40.694 | 19700.535 | 2.917 | 14.712 | 0.291315 | 7/15/2014 | 44:13.8 | 0 |
| -40.479 | 19700.525 | 2.92 | 14.705 | 0.281969 | 7/15/2014 | 44:13.9 | 0 |
| -40.263 | 19700.517 | 2.92 | 14.697 | 0.279657 | 7/15/2014 | 44:14.0 | 0 |
| -40.047 | 19700.505 | 2.923 | 14.689 | 0.271817 | 7/15/2014 | 44:14.1 | 0 |
| -39.831 | 19700.496 | 2.92 | 14.682 | 0.29733 | 7/15/2014 | 44:14.2 | 0 |
| -39.617 | 19700.484 | 2.92 | 14.674 | 0.307101 | 7/15/2014 | 44:14.3 | 0 |
| -39.351 | 19700.471 | 2.92 | 14.666 | 0.30336 | 7/15/2014 | 44:14.4 | 0 |
| -39.137 | 19700.461 | 2.92 | 14.658 | 0.310279 | 7/15/2014 | 44:14.5 | 0 |
| -38.925 | 19700.449 | 2.923 | 14.651 | 0.316321 | 7/15/2014 | 44:14.6 | 0 |

| | | | | | | | |
|---|---|---|---|---|---|---|---|
| -38.657 | 19700.435 | 2.926 | 14.644 | 0.288905 | 7/15/2014 | 44:14.7 | 0 |
| -38.444 | 19700.423 | 2.926 | 14.637 | 0.275127 | 7/15/2014 | 44:14.8 | 0 |
| -38.227 | 19700.411 | 2.928 | 14.631 | 0.247429 | 7/15/2014 | 44:14.9 | 0 |
| -38.01 | 19700.398 | 2.934 | 14.624 | 0.233601 | 7/15/2014 | 44:15.0 | 0 |
| -37.795 | 19700.387 | 2.931 | 14.619 | 0.218886 | 7/15/2014 | 44:15.1 | 0 |
| -37.58 | 19700.374 | 2.931 | 14.613 | 0.205442 | 7/15/2014 | 44:15.2 | 0 |
| -37.366 | 19700.361 | 2.931 | 14.609 | 0.185801 | 7/15/2014 | 44:15.3 | 0 |
| -37.1 | 19700.345 | 2.928 | 14.604 | 0.197816 | 7/15/2014 | 44:15.4 | 0 |
| -36.887 | 19700.332 | 2.926 | 14.6 | 0.20258 | 7/15/2014 | 44:15.5 | 0 |
| -36.672 | 19700.318 | 2.926 | 14.597 | 0.219605 | 7/15/2014 | 44:15.6 | 0 |
| -36.403 | 19700.3 | 2.922 | 14.595 | 0.219027 | 7/15/2014 | 44:15.7 | 0 |
| -36.188 | 19700.287 | 2.919 | 14.593 | 0.186397 | 7/15/2014 | 44:15.8 | 0 |
| -35.973 | 19700.272 | 2.919 | 14.593 | 0.212643 | 7/15/2014 | 44:15.9 | 0 |
| -35.755 | 19700.258 | 2.919 | 14.594 | 0.218536 | 7/15/2014 | 44:16.0 | 0 |
| -35.539 | 19700.243 | 2.914 | 14.595 | 0.206435 | 7/15/2014 | 44:16.1 | 0 |
| -35.321 | 19700.228 | 2.917 | 14.599 | 0.171437 | 7/15/2014 | 44:16.2 | 0 |
| -35.103 | 19700.213 | 2.916 | 14.603 | 0.20414 | 7/15/2014 | 44:16.3 | 0 |
| -34.833 | 19700.192 | 2.913 | 14.609 | 0.15999 | 7/15/2014 | 44:16.4 | 0 |
| -34.614 | 19700.178 | 2.913 | 14.615 | 0.236725 | 7/15/2014 | 44:16.5 | 0 |
| -34.397 | 19700.16 | 2.91 | 14.622 | 0.177567 | 7/15/2014 | 44:16.6 | 0 |
| -34.122 | 19700.139 | 2.91 | 14.629 | 0.179364 | 7/15/2014 | 44:16.7 | 0 |
| -33.902 | 19700.123 | 2.907 | 14.636 | 0.191643 | 7/15/2014 | 44:16.8 | 0 |
| -33.678 | 19700.104 | 2.907 | 14.644 | 0.199396 | 7/15/2014 | 44:16.9 | 0 |
| -33.452 | 19700.087 | 2.907 | 14.651 | 0.181424 | 7/15/2014 | 44:17.0 | 0 |
| -33.231 | 19700.068 | 2.902 | 14.658 | 0.216227 | 7/15/2014 | 44:17.1 | 0 |
| -33.005 | 19700.051 | 2.902 | 14.664 | 0.182196 | 7/15/2014 | 44:17.2 | 0 |
| -32.781 | 19700.031 | 2.902 | 14.671 | 0.187175 | 7/15/2014 | 44:17.3 | 0 |
| -32.502 | 19700.008 | 2.9 | 14.676 | 0.206428 | 7/15/2014 | 44:17.4 | 0 |
| -32.278 | 19699.989 | 2.898 | 14.682 | 0.197708 | 7/15/2014 | 44:17.5 | 0 |
| -32.051 | 19699.969 | 2.901 | 14.688 | 0.217693 | 7/15/2014 | 44:17.6 | 0 |
| -31.769 | 19699.943 | 2.901 | 14.693 | 0.172824 | 7/15/2014 | 44:17.7 | 0 |
| -31.545 | 19699.924 | 2.898 | 14.699 | 0.191563 | 7/15/2014 | 44:17.8 | 0 |
| -31.315 | 19699.902 | 2.9 | 14.703 | 0.22409 | 7/15/2014 | 44:17.9 | 0 |
| -31.086 | 19699.882 | 2.898 | 14.707 | 0.23193 | 7/15/2014 | 44:18.0 | 0 |
| -30.859 | 19699.86 | 2.894 | 14.711 | 0.210753 | 7/15/2014 | 44:18.1 | 0 |
| -30.629 | 19699.839 | 2.894 | 14.714 | 0.225886 | 7/15/2014 | 44:18.2 | 0 |
| -30.4 | 19699.816 | 2.892 | 14.716 | 0.219989 | 7/15/2014 | 44:18.3 | 0 |
| -30.118 | 19699.789 | 2.889 | 14.717 | 0.259976 | 7/15/2014 | 44:18.4 | 0 |
| -29.892 | 19699.768 | 2.884 | 14.718 | 0.22547 | 7/15/2014 | 44:18.5 | 0 |
| -29.664 | 19699.744 | 2.88 | 14.717 | 0.22029 | 7/15/2014 | 44:18.6 | 0 |
| -29.381 | 19699.716 | 2.873 | 14.716 | 0.23087 | 7/15/2014 | 44:18.7 | 0 |
| -29.153 | 19699.692 | 2.87 | 14.714 | 0.232194 | 7/15/2014 | 44:18.8 | 0 |
| -28.924 | 19699.669 | 2.865 | 14.711 | 0.232464 | 7/15/2014 | 44:18.9 | 0 |
| -28.694 | 19699.645 | 2.863 | 14.708 | 0.232615 | 7/15/2014 | 44:19.0 | 0 |
| -28.465 | 19699.621 | 2.861 | 14.704 | 0.226809 | 7/15/2014 | 44:19.1 | 0 |
| -28.238 | 19699.596 | 2.857 | 14.7 | 0.226267 | 7/15/2014 | 44:19.2 | 0 |
| -28.01 | 19699.572 | 2.853 | 14.696 | 0.225348 | 7/15/2014 | 44:19.3 | 0 |

| | | | | | | | |
|---|---|---|---|---|---|---|---|
| -27.726 | 19699.541 | 2.853 | 14.691 | 0.220584 | 7/15/2014 | 44:19.4 | 0 |
| -27.5 | 19699.516 | 2.857 | 14.686 | 0.221652 | 7/15/2014 | 44:19.5 | 0 |
| -27.274 | 19699.491 | 2.857 | 14.682 | 0.186876 | 7/15/2014 | 44:19.6 | 0 |
| -26.991 | 19699.458 | 2.86 | 14.677 | 0.183627 | 7/15/2014 | 44:19.7 | 0 |
| -26.762 | 19699.434 | 2.86 | 14.672 | 0.179001 | 7/15/2014 | 44:19.8 | 0 |
| -26.534 | 19699.406 | 2.854 | 14.668 | 0.186818 | 7/15/2014 | 44:19.9 | 0 |
| -26.304 | 19699.381 | 2.852 | 14.664 | 0.174182 | 7/15/2014 | 44:20.0 | 0 |
| -26.074 | 19699.354 | 2.852 | 14.661 | 0.161628 | 7/15/2014 | 44:20.1 | 0 |
| -25.845 | 19699.325 | 2.85 | 14.658 | 0.18053 | 7/15/2014 | 44:20.2 | 0 |
| -25.617 | 19699.3 | 2.843 | 14.655 | 0.206492 | 7/15/2014 | 44:20.3 | 0 |
| -25.331 | 19699.265 | 2.836 | 14.652 | 0.219774 | 7/15/2014 | 44:20.4 | 0 |
| -25.104 | 19699.238 | 2.827 | 14.65 | 0.231466 | 7/15/2014 | 44:20.5 | 0 |
| -24.874 | 19699.21 | 2.822 | 14.647 | 0.258004 | 7/15/2014 | 44:20.6 | 0 |
| -24.587 | 19699.175 | 2.822 | 14.644 | 0.22611 | 7/15/2014 | 44:20.7 | 0 |
| -24.471 | 19699.161 | 2.817 | 14.643 | 0.23904 | 7/15/2014 | 44:20.8 | 0 |
| -24.24 | 19699.132 | 2.817 | 14.64 | 0.248084 | 7/15/2014 | 44:20.9 | 0 |
| -24.011 | 19699.104 | 2.807 | 14.636 | 0.250769 | 7/15/2014 | 44:21.0 | 0 |
| -23.775 | 19699.074 | 2.809 | 14.633 | 0.212198 | 7/15/2014 | 44:21.1 | 0 |
| -23.546 | 19699.046 | 2.805 | 14.629 | 0.246452 | 7/15/2014 | 44:21.2 | 0 |
| -23.311 | 19699.015 | 2.811 | 14.626 | 0.216142 | 7/15/2014 | 44:21.3 | 0 |
| -22.904 | 19698.964 | 2.806 | 14.623 | 0.266292 | 7/15/2014 | 44:21.4 | 0 |
| -22.673 | 19698.934 | 2.803 | 14.622 | 0.250236 | 7/15/2014 | 44:21.5 | 0 |
| -22.44 | 19698.904 | 2.8 | 14.622 | 0.2381 | 7/15/2014 | 44:21.6 | 0 |
| -22.148 | 19698.865 | 2.797 | 14.623 | 0.245792 | 7/15/2014 | 44:21.7 | 0 |
| -22.032 | 19698.851 | 2.795 | 14.623 | 0.214643 | 7/15/2014 | 44:21.8 | 0 |
| -21.793 | 19698.818 | 2.795 | 14.626 | 0.21758 | 7/15/2014 | 44:21.9 | 0 |
| -21.559 | 19698.789 | 2.786 | 14.631 | 0.197 | 7/15/2014 | 44:22.0 | 0 |
| -21.32 | 19698.756 | 2.782 | 14.637 | 0.181475 | 7/15/2014 | 44:22.1 | 0 |
| -21.084 | 19698.725 | 2.775 | 14.644 | 0.177337 | 7/15/2014 | 44:22.2 | 0 |
| -20.843 | 19698.691 | 2.775 | 14.652 | 0.172957 | 7/15/2014 | 44:22.3 | 0 |
| -20.43 | 19698.635 | 2.764 | 14.667 | 0.184381 | 7/15/2014 | 44:22.4 | 0 |
| -20.191 | 19698.602 | 2.762 | 14.676 | 0.185866 | 7/15/2014 | 44:22.5 | 0 |
| -19.954 | 19698.57 | 2.759 | 14.686 | 0.202682 | 7/15/2014 | 44:22.6 | 0 |
| -19.658 | 19698.526 | 2.752 | 14.694 | 0.206121 | 7/15/2014 | 44:22.7 | 0 |
| -19.537 | 19698.511 | 2.75 | 14.699 | 0.214714 | 7/15/2014 | 44:22.8 | 0 |
| -19.295 | 19698.477 | 2.748 | 14.708 | 0.250795 | 7/15/2014 | 44:22.9 | 0 |
| -19.056 | 19698.442 | 2.738 | 14.715 | 0.261961 | 7/15/2014 | 44:23.0 | 0 |
| -18.816 | 19698.408 | 2.738 | 14.72 | 0.278959 | 7/15/2014 | 44:23.1 | 0 |
| -18.576 | 19698.373 | 2.732 | 14.724 | 0.310152 | 7/15/2014 | 44:23.2 | 0 |
| -18.336 | 19698.34 | 2.732 | 14.727 | 0.33819 | 7/15/2014 | 44:23.3 | 0 |
| -17.92 | 19698.277 | 2.726 | 14.729 | 0.346562 | 7/15/2014 | 44:23.4 | 0 |
| -17.683 | 19698.243 | 2.726 | 14.73 | 0.313936 | 7/15/2014 | 44:23.5 | 0 |
| -17.445 | 19698.207 | 2.726 | 14.731 | 0.314305 | 7/15/2014 | 44:23.6 | 0 |
| -17.15 | 19698.164 | 2.724 | 14.732 | 0.27613 | 7/15/2014 | 44:23.7 | 0 |
| -17.03 | 19698.146 | 2.721 | 14.732 | 0.25742 | 7/15/2014 | 44:23.8 | 0 |
| -16.791 | 19698.11 | 2.723 | 14.733 | 0.218926 | 7/15/2014 | 44:23.9 | 0 |
| -16.555 | 19698.074 | 2.712 | 14.735 | 0.238411 | 7/15/2014 | 44:24.0 | 0 |

| | | | | | | | |
|---|---|---|---|---|---|---|---|
| -16.315 | 19698.038 | 2.709 | 14.738 | 0.216978 | 7/15/2014 | 44:24.1 | 0 |
| -16.079 | 19698.002 | 2.697 | 14.743 | 0.22822 | 7/15/2014 | 44:24.2 | 0 |
| -15.841 | 19697.965 | 2.691 | 14.749 | 0.210887 | 7/15/2014 | 44:24.3 | 0 |
| -15.43 | 19697.902 | 2.67 | 14.761 | 0.234575 | 7/15/2014 | 44:24.4 | 0 |
| -15.195 | 19697.864 | 2.661 | 14.77 | 0.250413 | 7/15/2014 | 44:24.5 | 0 |
| -14.961 | 19697.827 | 2.65 | 14.779 | 0.235295 | 7/15/2014 | 44:24.6 | 0 |
| -14.665 | 19697.781 | 2.647 | 14.788 | 0.235993 | 7/15/2014 | 44:24.7 | 0 |
| -14.546 | 19697.763 | 2.641 | 14.793 | 0.224601 | 7/15/2014 | 44:24.8 | 0 |
| -14.31 | 19697.726 | 2.637 | 14.802 | 0.254817 | 7/15/2014 | 44:24.9 | 0 |
| -14.075 | 19697.688 | 2.626 | 14.811 | 0.222449 | 7/15/2014 | 44:25.0 | 0 |
| -13.836 | 19697.649 | 2.626 | 14.819 | 0.243574 | 7/15/2014 | 44:25.1 | 0 |
| -13.601 | 19697.611 | 2.617 | 14.827 | 0.227122 | 7/15/2014 | 44:25.2 | 0 |
| -13.363 | 19697.572 | 2.611 | 14.833 | 0.247146 | 7/15/2014 | 44:25.3 | 0 |
| -12.951 | 19697.504 | 2.602 | 14.841 | 0.270722 | 7/15/2014 | 44:25.4 | 0 |
| -12.716 | 19697.467 | 2.595 | 14.846 | 0.241452 | 7/15/2014 | 44:25.5 | 0 |
| -12.482 | 19697.428 | 2.589 | 14.85 | 0.247732 | 7/15/2014 | 44:25.6 | 0 |
| -12.186 | 19697.378 | 2.586 | 14.852 | 0.233981 | 7/15/2014 | 44:25.7 | 0 |
| -12.068 | 19697.359 | 2.581 | 14.854 | 0.232847 | 7/15/2014 | 44:25.8 | 0 |
| -11.829 | 19697.319 | 2.577 | 14.856 | 0.218131 | 7/15/2014 | 44:25.9 | 0 |
| -11.594 | 19697.28 | 2.568 | 14.856 | 0.228813 | 7/15/2014 | 44:26.0 | 0 |
| -11.355 | 19697.238 | 2.565 | 14.856 | 0.209469 | 7/15/2014 | 44:26.1 | 0 |
| -11.119 | 19697.2 | 2.556 | 14.855 | 0.213587 | 7/15/2014 | 44:26.2 | 0 |
| -10.882 | 19697.159 | 2.55 | 14.854 | 0.199691 | 7/15/2014 | 44:26.3 | 0 |
| -10.467 | 19697.089 | 2.542 | 14.852 | 0.211376 | 7/15/2014 | 44:26.4 | 0 |
| -10.231 | 19697.049 | 2.537 | 14.852 | 0.207932 | 7/15/2014 | 44:26.5 | 0 |
| -9.995 | 19697.01 | 2.535 | 14.851 | 0.203218 | 7/15/2014 | 44:26.6 | 0 |
| -9.7 | 19696.957 | 2.532 | 14.851 | 0.20405 | 7/15/2014 | 44:26.7 | 0 |
| -9.581 | 19696.938 | 2.529 | 14.851 | 0.207567 | 7/15/2014 | 44:26.8 | 0 |
| -9.341 | 19696.896 | 2.526 | 14.852 | 0.211011 | 7/15/2014 | 44:26.9 | 0 |
| -9.103 | 19696.855 | 2.516 | 14.854 | 0.198213 | 7/15/2014 | 44:27.0 | 0 |
| -8.864 | 19696.813 | 2.513 | 14.856 | 0.193262 | 7/15/2014 | 44:27.1 | 0 |
| -8.625 | 19696.771 | 2.507 | 14.858 | 0.191131 | 7/15/2014 | 44:27.2 | 0 |
| -8.387 | 19696.73 | 2.503 | 14.861 | 0.194425 | 7/15/2014 | 44:27.3 | 0 |
| -7.971 | 19696.657 | 2.491 | 14.865 | 0.194945 | 7/15/2014 | 44:27.4 | 0 |
| -7.732 | 19696.614 | 2.487 | 14.868 | 0.203276 | 7/15/2014 | 44:27.5 | 0 |
| -7.496 | 19696.573 | 2.481 | 14.87 | 0.217856 | 7/15/2014 | 44:27.6 | 0 |
| -7.197 | 19696.52 | 2.481 | 14.871 | 0.203708 | 7/15/2014 | 44:27.7 | 0 |
| -7.076 | 19696.498 | 2.479 | 14.873 | 0.20863 | 7/15/2014 | 44:27.8 | 0 |
| -6.836 | 19696.455 | 2.475 | 14.873 | 0.212372 | 7/15/2014 | 44:27.9 | 0 |
| -6.595 | 19696.413 | 2.469 | 14.873 | 0.2025 | 7/15/2014 | 44:28.0 | 0 |
| -6.352 | 19696.37 | 2.469 | 14.872 | 0.209264 | 7/15/2014 | 44:28.1 | 0 |
| -6.11 | 19696.326 | 2.467 | 14.87 | 0.194525 | 7/15/2014 | 44:28.2 | 0 |
| -5.867 | 19696.282 | 2.467 | 14.867 | 0.181438 | 7/15/2014 | 44:28.3 | 0 |
| -5.442 | 19696.205 | 2.468 | 14.859 | 0.204938 | 7/15/2014 | 44:28.4 | 0 |
| -5.201 | 19696.162 | 2.464 | 14.853 | 0.200145 | 7/15/2014 | 44:28.5 | 0 |
| -4.959 | 19696.118 | 2.462 | 14.847 | 0.228246 | 7/15/2014 | 44:28.6 | 0 |
| -4.655 | 19696.064 | 2.46 | 14.84 | 0.204901 | 7/15/2014 | 44:28.7 | 0 |

| | | | | | | | |
|---|---|---|---|---|---|---|---|
| -4.533 | 19696.042 | 2.458 | 14.836 | 0.216826 | 7/15/2014 | 44:28.8 | 0 |
| -4.287 | 19695.996 | 2.455 | 14.829 | 0.224329 | 7/15/2014 | 44:28.9 | 0 |
| -4.043 | 19695.952 | 2.45 | 14.823 | 0.2267 | 7/15/2014 | 44:29.0 | 0 |
| -3.797 | 19695.906 | 2.448 | 14.817 | 0.227353 | 7/15/2014 | 44:29.1 | 0 |
| -3.553 | 19695.863 | 2.439 | 14.813 | 0.238156 | 7/15/2014 | 44:29.2 | 0 |
| -3.31 | 19695.818 | 2.442 | 14.81 | 0.215795 | 7/15/2014 | 44:29.3 | 0 |
| -2.885 | 19695.741 | 2.439 | 14.811 | 0.245458 | 7/15/2014 | 44:29.4 | 0 |
| -2.645 | 19695.695 | 2.436 | 14.814 | 0.223334 | 7/15/2014 | 44:29.5 | 0 |
| -2.403 | 19695.652 | 2.436 | 14.818 | 0.209542 | 7/15/2014 | 44:29.6 | 0 |
| -2.105 | 19695.598 | 2.433 | 14.824 | 0.218355 | 7/15/2014 | 44:29.7 | 0 |
| -1.983 | 19695.576 | 2.432 | 14.827 | 0.213949 | 7/15/2014 | 44:29.8 | 0 |
| -1.741 | 19695.53 | 2.432 | 14.835 | 0.201805 | 7/15/2014 | 44:29.9 | 0 |
| -1.502 | 19695.486 | 2.426 | 14.844 | 0.20054 | 7/15/2014 | 44:30.0 | 0 |
| -1.26 | 19695.442 | 2.426 | 14.853 | 0.193776 | 7/15/2014 | 44:30.1 | 0 |
| -1.023 | 19695.398 | 2.419 | 14.862 | 0.200844 | 7/15/2014 | 44:30.2 | 0 |
| -0.782 | 19695.354 | 2.419 | 14.869 | 0.211263 | 7/15/2014 | 44:30.3 | 0 |
| -0.369 | 19695.279 | 2.415 | 14.877 | 0.204826 | 7/15/2014 | 44:30.4 | 0 |
| -0.133 | 19695.234 | 2.412 | 14.881 | 0.208867 | 7/15/2014 | 44:30.5 | 0 |
| 0.102 | 19695.191 | 2.408 | 14.883 | 0.20501 | 7/15/2014 | 44:30.6 | 0 |
| 0.4 | 19695.138 | 2.405 | 14.885 | 0.202793 | 7/15/2014 | 44:30.7 | 0 |
| 0.518 | 19695.116 | 2.401 | 14.886 | 0.205238 | 7/15/2014 | 44:30.8 | 0 |
| 0.757 | 19695.07 | 2.401 | 14.885 | 0.22493 | 7/15/2014 | 44:30.9 | 0 |
| 0.994 | 19695.027 | 2.395 | 14.885 | 0.204141 | 7/15/2014 | 44:31.0 | 0 |
| 1.234 | 19694.984 | 2.392 | 14.883 | 0.205562 | 7/15/2014 | 44:31.1 | 0 |
| 1.471 | 19694.941 | 2.382 | 14.882 | 0.219557 | 7/15/2014 | 44:31.2 | 0 |
| 1.711 | 19694.896 | 2.382 | 14.881 | 0.210695 | 7/15/2014 | 44:31.3 | 0 |
| 2.128 | 19694.821 | 2.375 | 14.878 | 0.216459 | 7/15/2014 | 44:31.4 | 0 |
| 2.365 | 19694.777 | 2.371 | 14.875 | 0.204429 | 7/15/2014 | 44:31.5 | 0 |
| 2.603 | 19694.734 | 2.369 | 14.872 | 0.218004 | 7/15/2014 | 44:31.6 | 0 |
| 2.898 | 19694.68 | 2.367 | 14.868 | 0.209726 | 7/15/2014 | 44:31.7 | 0 |
| 3.019 | 19694.658 | 2.367 | 14.867 | 0.206178 | 7/15/2014 | 44:31.8 | 0 |
| 3.259 | 19694.613 | 2.367 | 14.862 | 0.212482 | 7/15/2014 | 44:31.9 | 0 |
| 3.496 | 19694.571 | 2.358 | 14.855 | 0.220312 | 7/15/2014 | 44:32.0 | 0 |
| 3.735 | 19694.528 | 2.356 | 14.848 | 0.209179 | 7/15/2014 | 44:32.1 | 0 |
| 3.972 | 19694.485 | 2.35 | 14.84 | 0.214892 | 7/15/2014 | 44:32.2 | 0 |
| 4.211 | 19694.442 | 2.35 | 14.832 | 0.21369 | 7/15/2014 | 44:32.3 | 0 |
| 4.623 | 19694.366 | 2.342 | 14.82 | 0.207692 | 7/15/2014 | 44:32.4 | 0 |
| 4.859 | 19694.324 | 2.338 | 14.813 | 0.204816 | 7/15/2014 | 44:32.5 | 0 |
| 5.093 | 19694.282 | 2.336 | 14.806 | 0.203931 | 7/15/2014 | 44:32.6 | 0 |
| 5.387 | 19694.229 | 2.336 | 14.8 | 0.200797 | 7/15/2014 | 44:32.7 | 0 |
| 5.506 | 19694.208 | 2.334 | 14.797 | 0.201478 | 7/15/2014 | 44:32.8 | 0 |
| 5.743 | 19694.165 | 2.334 | 14.792 | 0.202758 | 7/15/2014 | 44:32.9 | 0 |
| 5.977 | 19694.122 | 2.324 | 14.789 | 0.198955 | 7/15/2014 | 44:33.0 | 0 |
| 6.217 | 19694.079 | 2.328 | 14.786 | 0.196973 | 7/15/2014 | 44:33.1 | 0 |
| 6.453 | 19694.037 | 2.318 | 14.785 | 0.192134 | 7/15/2014 | 44:33.2 | 0 |
| 6.692 | 19693.994 | 2.318 | 14.784 | 0.187542 | 7/15/2014 | 44:33.3 | 0 |
| 7.107 | 19693.92 | 2.31 | 14.786 | 0.182997 | 7/15/2014 | 44:33.4 | 0 |

| | | | | | | | |
|---|---|---|---|---|---|---|---|
| 7.343 | 19693.877 | 2.305 | 14.787 | 0.175363 | 7/15/2014 | 44:33.5 | 0 |
| 7.579 | 19693.834 | 2.3 | 14.788 | 0.171106 | 7/15/2014 | 44:33.6 | 0 |
| 7.878 | 19693.781 | 2.297 | 14.79 | 0.16913 | 7/15/2014 | 44:33.7 | 0 |
| 7.996 | 19693.76 | 2.293 | 14.791 | 0.166188 | 7/15/2014 | 44:33.8 | 0 |
| 8.239 | 19693.716 | 2.293 | 14.794 | 0.163431 | 7/15/2014 | 44:33.9 | 0 |
| 8.48 | 19693.673 | 2.285 | 14.797 | 0.161348 | 7/15/2014 | 44:34.0 | 0 |
| 8.723 | 19693.63 | 2.285 | 14.8 | 0.160883 | 7/15/2014 | 44:34.1 | 0 |
| 8.964 | 19693.586 | 2.276 | 14.804 | 0.15795 | 7/15/2014 | 44:34.2 | 0 |
| 9.207 | 19693.543 | 2.276 | 14.809 | 0.159214 | 7/15/2014 | 44:34.3 | 0 |
| 9.63 | 19693.468 | 2.273 | 14.819 | 0.162971 | 7/15/2014 | 44:34.4 | 0 |
| 9.872 | 19693.424 | 2.271 | 14.827 | 0.159463 | 7/15/2014 | 44:34.5 | 0 |
| 10.114 | 19693.381 | 2.265 | 14.835 | 0.172873 | 7/15/2014 | 44:34.6 | 0 |
| 10.416 | 19693.328 | 2.263 | 14.844 | 0.168785 | 7/15/2014 | 44:34.7 | 0 |
| 10.538 | 19693.306 | 2.26 | 14.848 | 0.17092 | 7/15/2014 | 44:34.8 | 0 |
| 10.782 | 19693.263 | 2.26 | 14.858 | 0.161538 | 7/15/2014 | 44:34.9 | 0 |
| 11.024 | 19693.22 | 2.252 | 14.867 | 0.152238 | 7/15/2014 | 44:35.0 | 0 |
| 11.268 | 19693.177 | 2.252 | 14.875 | 0.14809 | 7/15/2014 | 44:35.1 | 0 |
| 11.51 | 19693.134 | 2.243 | 14.882 | 0.139802 | 7/15/2014 | 44:35.2 | 0 |
| 11.752 | 19693.091 | 2.243 | 14.887 | 0.139337 | 7/15/2014 | 44:35.3 | 0 |
| 12.172 | 19693.017 | 2.245 | 14.89 | 0.13148 | 7/15/2014 | 44:35.4 | 0 |
| 12.41 | 19692.975 | 2.243 | 14.891 | 0.13113 | 7/15/2014 | 44:35.5 | 0 |
| 12.65 | 19692.933 | 2.241 | 14.89 | 0.128499 | 7/15/2014 | 44:35.6 | 0 |
| 12.947 | 19692.881 | 2.238 | 14.888 | 0.130456 | 7/15/2014 | 44:35.7 | 0 |
| 13.068 | 19692.859 | 2.237 | 14.887 | 0.131217 | 7/15/2014 | 44:35.8 | 0 |
| 13.309 | 19692.817 | 2.241 | 14.883 | 0.121131 | 7/15/2014 | 44:35.9 | 0 |
| 13.548 | 19692.775 | 2.238 | 14.879 | 0.130178 | 7/15/2014 | 44:36.0 | 0 |
| 13.789 | 19692.732 | 2.234 | 14.874 | 0.129493 | 7/15/2014 | 44:36.1 | 0 |
| 14.027 | 19692.692 | 2.232 | 14.868 | 0.135453 | 7/15/2014 | 44:36.2 | 0 |
| 14.266 | 19692.65 | 2.232 | 14.862 | 0.133003 | 7/15/2014 | 44:36.3 | 0 |
| 14.681 | 19692.578 | 2.227 | 14.853 | 0.135991 | 7/15/2014 | 44:36.4 | 0 |
| 14.919 | 19692.538 | 2.227 | 14.847 | 0.145061 | 7/15/2014 | 44:36.5 | 0 |
| 15.155 | 19692.498 | 2.225 | 14.841 | 0.130633 | 7/15/2014 | 44:36.6 | 0 |
| 15.451 | 19692.445 | 2.221 | 14.836 | 0.132367 | 7/15/2014 | 44:36.7 | 0 |
| 15.57 | 19692.426 | 2.221 | 14.832 | 0.123559 | 7/15/2014 | 44:36.8 | 0 |
| 15.808 | 19692.385 | 2.223 | 14.827 | 0.126949 | 7/15/2014 | 44:36.9 | 0 |
| 16.045 | 19692.345 | 2.22 | 14.823 | 0.115752 | 7/15/2014 | 44:37.0 | 0 |
| 16.283 | 19692.305 | 2.22 | 14.819 | 0.124172 | 7/15/2014 | 44:37.1 | 0 |
| 16.519 | 19692.266 | 2.216 | 14.815 | 0.110536 | 7/15/2014 | 44:37.2 | 0 |
| 16.755 | 19692.227 | 2.216 | 14.812 | 0.108644 | 7/15/2014 | 44:37.3 | 0 |
| 17.167 | 19692.156 | 2.209 | 14.808 | 0.100785 | 7/15/2014 | 44:37.4 | 0 |
| 17.401 | 19692.118 | 2.206 | 14.805 | 0.0993753 | 7/15/2014 | 44:37.5 | 0 |
| 17.635 | 19692.08 | 2.201 | 14.802 | 0.0836017 | 7/15/2014 | 44:37.6 | 0 |
| 17.928 | 19692.032 | 2.204 | 14.799 | 0.0655275 | 7/15/2014 | 44:37.7 | 0 |
| 18.046 | 19692.013 | 2.204 | 14.798 | 0.0586577 | 7/15/2014 | 44:37.8 | 0 |
| 18.281 | 19691.974 | 2.204 | 14.795 | 0.0717517 | 7/15/2014 | 44:37.9 | 0 |
| 18.516 | 19691.936 | 2.204 | 14.792 | 0.0578156 | 7/15/2014 | 44:38.0 | 0 |
| 18.753 | 19691.897 | 2.208 | 14.789 | 0.0617804 | 7/15/2014 | 44:38.1 | 0 |

| | | | | | | | |
|---|---|---|---|---|---|---|---|
| 18.989 | 19691.859 | 2.208 | 14.785 | 0.06005 | 7/15/2014 | 44:38.2 | 0 |
| 19.224 | 19691.822 | 2.21 | 14.782 | 0.0792787 | 7/15/2014 | 44:38.3 | 0 |
| 19.635 | 19691.757 | 2.206 | 14.775 | 0.105145 | 7/15/2014 | 44:38.4 | 0 |
| 19.869 | 19691.72 | 2.203 | 14.77 | 0.105205 | 7/15/2014 | 44:38.5 | 0 |
| 20.103 | 19691.683 | 2.203 | 14.764 | 0.108536 | 7/15/2014 | 44:38.6 | 0 |
| 20.396 | 19691.638 | 2.206 | 14.757 | 0.113435 | 7/15/2014 | 44:38.7 | 0 |
| 20.514 | 19691.618 | 2.206 | 14.754 | 0.116027 | 7/15/2014 | 44:38.8 | 0 |
| 20.75 | 19691.582 | 2.21 | 14.746 | 0.115754 | 7/15/2014 | 44:38.9 | 0 |
| 20.984 | 19691.547 | 2.21 | 14.738 | 0.114404 | 7/15/2014 | 44:39.0 | 0 |
| 21.221 | 19691.508 | 2.214 | 14.728 | 0.114525 | 7/15/2014 | 44:39.1 | 0 |
| 21.456 | 19691.473 | 2.21 | 14.718 | 0.102196 | 7/15/2014 | 44:39.2 | 0 |
| 21.692 | 19691.437 | 2.21 | 14.707 | 0.121995 | 7/15/2014 | 44:39.3 | 0 |
| 22.102 | 19691.375 | 2.206 | 14.689 | 0.108819 | 7/15/2014 | 44:39.4 | 0 |
| 22.336 | 19691.34 | 2.203 | 14.677 | 0.106881 | 7/15/2014 | 44:39.5 | 0 |
| 22.569 | 19691.307 | 2.203 | 14.665 | 0.110486 | 7/15/2014 | 44:39.6 | 0 |
| 22.86 | 19691.262 | 2.2 | 14.653 | 0.108224 | 7/15/2014 | 44:39.7 | 0 |
| 22.977 | 19691.244 | 2.199 | 14.646 | 0.114155 | 7/15/2014 | 44:39.8 | 0 |
| 23.215 | 19691.209 | 2.206 | 14.635 | 0.103318 | 7/15/2014 | 44:39.9 | 0 |
| 23.448 | 19691.174 | 2.203 | 14.625 | 0.104223 | 7/15/2014 | 44:40.0 | 0 |
| 23.686 | 19691.14 | 2.21 | 14.616 | 0.121382 | 7/15/2014 | 44:40.1 | 0 |
| 23.922 | 19691.105 | 2.21 | 14.609 | 0.0975955 | 7/15/2014 | 44:40.2 | 0 |
| 24.157 | 19691.072 | 2.214 | 14.604 | 0.0935605 | 7/15/2014 | 44:40.3 | 0 |
| 24.567 | 19691.014 | 2.21 | 14.605 | 0.0888061 | 7/15/2014 | 44:40.4 | 0 |
| 24.802 | 19690.98 | 2.213 | 14.607 | 0.0849151 | 7/15/2014 | 44:40.5 | 0 |
| 25.035 | 19690.946 | 2.206 | 14.611 | 0.101585 | 7/15/2014 | 44:40.6 | 0 |
| 25.33 | 19690.905 | 2.206 | 14.618 | 0.072465 | 7/15/2014 | 44:40.7 | 0 |
| 25.449 | 19690.889 | 2.203 | 14.621 | 0.0858484 | 7/15/2014 | 44:40.8 | 0 |
| 25.687 | 19690.855 | 2.207 | 14.631 | 0.0790502 | 7/15/2014 | 44:40.9 | 0 |
| 25.922 | 19690.822 | 2.199 | 14.642 | 0.0782584 | 7/15/2014 | 44:41.0 | 0 |
| 26.161 | 19690.789 | 2.203 | 14.653 | 0.0972565 | 7/15/2014 | 44:41.1 | 0 |
| 26.398 | 19690.758 | 2.193 | 14.664 | 0.0879956 | 7/15/2014 | 44:41.2 | 0 |
| 26.635 | 19690.724 | 2.19 | 14.674 | 0.074558 | 7/15/2014 | 44:41.3 | 0 |
| 27.05 | 19690.668 | 2.19 | 14.685 | 0.0889566 | 7/15/2014 | 44:41.4 | 0 |
| 27.288 | 19690.637 | 2.19 | 14.691 | 0.0873049 | 7/15/2014 | 44:41.5 | 0 |
| 27.524 | 19690.604 | 2.183 | 14.694 | 0.0766068 | 7/15/2014 | 44:41.6 | 0 |
| 27.819 | 19690.564 | 2.181 | 14.696 | 0.0770888 | 7/15/2014 | 44:41.7 | 0 |
| 27.939 | 19690.549 | 2.177 | 14.697 | 0.0776996 | 7/15/2014 | 44:41.8 | 0 |
| 28.178 | 19690.516 | 2.18 | 14.695 | 0.0814685 | 7/15/2014 | 44:41.9 | 0 |
| 28.416 | 19690.484 | 2.18 | 14.69 | 0.0900171 | 7/15/2014 | 44:42.0 | 0 |
| 28.654 | 19690.453 | 2.182 | 14.682 | 0.0884878 | 7/15/2014 | 44:42.1 | 0 |
| 28.891 | 19690.422 | 2.176 | 14.672 | 0.0879301 | 7/15/2014 | 44:42.2 | 0 |
| 29.128 | 19690.392 | 2.176 | 14.659 | 0.0877832 | 7/15/2014 | 44:42.3 | 0 |
| 29.54 | 19690.338 | 2.175 | 14.634 | 0.0966545 | 7/15/2014 | 44:42.4 | 0 |
| 29.774 | 19690.308 | 2.175 | 14.616 | 0.096716 | 7/15/2014 | 44:42.5 | 0 |
| 30.009 | 19690.277 | 2.175 | 14.597 | 0.0925121 | 7/15/2014 | 44:42.6 | 0 |
| 30.301 | 19690.239 | 2.175 | 14.577 | 0.113134 | 7/15/2014 | 44:42.7 | 0 |
| 30.42 | 19690.224 | 2.174 | 14.567 | 0.115644 | 7/15/2014 | 44:42.8 | 0 |

| | | | | | | | |
|---|---|---|---|---|---|---|---|
| 30.655 | 19690.195 | 2.176 | 14.547 | 0.134603 | 7/15/2014 | 44:42.9 | 0 |
| 30.89 | 19690.166 | 2.174 | 14.528 | 0.138051 | 7/15/2014 | 44:43.0 | 0 |
| 31.126 | 19690.136 | 2.174 | 14.51 | 0.12143 | 7/15/2014 | 44:43.1 | 0 |
| 31.36 | 19690.106 | 2.169 | 14.496 | 0.164767 | 7/15/2014 | 44:43.2 | 0 |
| 31.596 | 19690.078 | 2.171 | 14.485 | 0.138378 | 7/15/2014 | 44:43.3 | 0 |
| 32.006 | 19690.026 | 2.17 | 14.48 | 0.141539 | 7/15/2014 | 44:43.4 | 0 |
| 32.24 | 19689.999 | 2.17 | 14.48 | 0.142 | 7/15/2014 | 44:43.5 | 0 |
| 32.473 | 19689.971 | 2.168 | 14.486 | 0.144371 | 7/15/2014 | 44:43.6 | 0 |
| 32.768 | 19689.935 | 2.166 | 14.497 | 0.161913 | 7/15/2014 | 44:43.7 | 0 |
| 32.886 | 19689.92 | 2.166 | 14.501 | 0.132096 | 7/15/2014 | 44:43.8 | 0 |
| 33.125 | 19689.893 | 2.169 | 14.519 | 0.124081 | 7/15/2014 | 44:43.9 | 0 |
| 33.361 | 19689.864 | 2.164 | 14.541 | 0.153028 | 7/15/2014 | 44:44.0 | 0 |
| 33.601 | 19689.835 | 2.162 | 14.564 | 0.150923 | 7/15/2014 | 44:44.1 | 0 |
| 33.841 | 19689.807 | 2.157 | 14.589 | 0.12577 | 7/15/2014 | 44:44.2 | 0 |
| 34.08 | 19689.779 | 2.157 | 14.613 | 0.138377 | 7/15/2014 | 44:44.3 | 0 |
| 34.5 | 19689.729 | 2.158 | 14.645 | 0.123201 | 7/15/2014 | 44:44.4 | 0 |
| 34.74 | 19689.701 | 2.16 | 14.664 | 0.132904 | 7/15/2014 | 44:44.5 | 0 |
| 34.979 | 19689.675 | 2.155 | 14.681 | 0.135727 | 7/15/2014 | 44:44.6 | 0 |
| 35.279 | 19689.64 | 2.155 | 14.695 | 0.131346 | 7/15/2014 | 44:44.7 | 0 |
| 35.401 | 19689.625 | 2.156 | 14.703 | 0.125761 | 7/15/2014 | 44:44.8 | 0 |
| 35.643 | 19689.598 | 2.156 | 14.712 | 0.135295 | 7/15/2014 | 44:44.9 | 0 |
| 35.885 | 19689.57 | 2.151 | 14.72 | 0.128163 | 7/15/2014 | 44:45.0 | 0 |
| 36.128 | 19689.541 | 2.151 | 14.725 | 0.128477 | 7/15/2014 | 44:45.1 | 0 |
| 36.369 | 19689.515 | 2.142 | 14.728 | 0.132276 | 7/15/2014 | 44:45.2 | 0 |
| 36.61 | 19689.489 | 2.14 | 14.729 | 0.129364 | 7/15/2014 | 44:45.3 | 0 |
| 37.031 | 19689.441 | 2.141 | 14.725 | 0.13148 | 7/15/2014 | 44:45.4 | 0 |
| 37.27 | 19689.416 | 2.138 | 14.721 | 0.135906 | 7/15/2014 | 44:45.5 | 0 |
| 37.509 | 19689.389 | 2.138 | 14.714 | 0.135297 | 7/15/2014 | 44:45.6 | 0 |
| 37.807 | 19689.355 | 2.131 | 14.705 | 0.140006 | 7/15/2014 | 44:45.7 | 0 |
| 37.929 | 19689.343 | 2.129 | 14.701 | 0.139935 | 7/15/2014 | 44:45.8 | 0 |
| 38.171 | 19689.318 | 2.129 | 14.689 | 0.140752 | 7/15/2014 | 44:45.9 | 0 |
| 38.41 | 19689.291 | 2.122 | 14.675 | 0.148901 | 7/15/2014 | 44:46.0 | 0 |
| 38.654 | 19689.266 | 2.125 | 14.66 | 0.139331 | 7/15/2014 | 44:46.1 | 0 |
| 38.894 | 19689.241 | 2.116 | 14.645 | 0.138049 | 7/15/2014 | 44:46.2 | 0 |
| 39.136 | 19689.215 | 2.116 | 14.63 | 0.138699 | 7/15/2014 | 44:46.3 | 0 |
| 39.555 | 19689.17 | 2.113 | 14.612 | 0.138498 | 7/15/2014 | 44:46.4 | 0 |
| 39.795 | 19689.146 | 2.113 | 14.602 | 0.140987 | 7/15/2014 | 44:46.5 | 0 |
| 40.034 | 19689.12 | 2.113 | 14.595 | 0.13586 | 7/15/2014 | 44:46.6 | 0 |
| 40.338 | 19689.091 | 2.116 | 14.591 | 0.139949 | 7/15/2014 | 44:46.7 | 0 |
| 40.458 | 19689.078 | 2.111 | 14.588 | 0.138834 | 7/15/2014 | 44:46.8 | 0 |
| 40.702 | 19689.053 | 2.121 | 14.588 | 0.134886 | 7/15/2014 | 44:46.9 | 0 |
| 40.943 | 19689.028 | 2.12 | 14.591 | 0.144527 | 7/15/2014 | 44:47.0 | 0 |
| 41.187 | 19689.003 | 2.12 | 14.596 | 0.152047 | 7/15/2014 | 44:47.1 | 0 |
| 41.43 | 19688.979 | 2.113 | 14.602 | 0.153049 | 7/15/2014 | 44:47.2 | 0 |
| 41.673 | 19688.955 | 2.113 | 14.61 | 0.15565 | 7/15/2014 | 44:47.3 | 0 |
| 42.1 | 19688.913 | 2.108 | 14.62 | 0.196245 | 7/15/2014 | 44:47.4 | 0 |
| 42.342 | 19688.889 | 2.105 | 14.627 | 0.209161 | 7/15/2014 | 44:47.5 | 0 |

| | | | | | | | |
|---|---|---|---|---|---|---|---|
| 42.583 | 19688.865 | 2.098 | 14.633 | 0.221148 | 7/15/2014 | 44:47.6 | 0 |
| 42.885 | 19688.836 | 2.095 | 14.637 | 0.197406 | 7/15/2014 | 44:47.7 | 0 |
| 43.008 | 19688.824 | 2.095 | 14.64 | 0.209301 | 7/15/2014 | 44:47.8 | 0 |
| 43.252 | 19688.801 | 2.102 | 14.642 | 0.235037 | 7/15/2014 | 44:47.9 | 0 |
| 43.493 | 19688.777 | 2.1 | 14.643 | 0.244559 | 7/15/2014 | 44:48.0 | 0 |
| 43.735 | 19688.754 | 2.1 | 14.643 | 0.214855 | 7/15/2014 | 44:48.1 | 0 |
| 43.976 | 19688.73 | 2.097 | 14.643 | 0.241201 | 7/15/2014 | 44:48.2 | 0 |
| 44.215 | 19688.709 | 2.095 | 14.641 | 0.220343 | 7/15/2014 | 44:48.3 | 0 |
| 44.63 | 19688.67 | 2.093 | 14.638 | 0.235501 | 7/15/2014 | 44:48.4 | 0 |
| 44.867 | 19688.647 | 2.091 | 14.636 | 0.23088 | 7/15/2014 | 44:48.5 | 0 |
| 45.101 | 19688.627 | 2.084 | 14.634 | 0.243202 | 7/15/2014 | 44:48.6 | 0 |
| 45.393 | 19688.6 | 2.084 | 14.632 | 0.22236 | 7/15/2014 | 44:48.7 | 0 |
| 45.512 | 19688.588 | 2.083 | 14.631 | 0.205542 | 7/15/2014 | 44:48.8 | 0 |
| 45.746 | 19688.568 | 2.085 | 14.63 | 0.232491 | 7/15/2014 | 44:48.9 | 0 |
| 45.98 | 19688.545 | 2.091 | 14.63 | 0.203599 | 7/15/2014 | 44:49.0 | 0 |
| 46.215 | 19688.524 | 2.094 | 14.63 | 0.214777 | 7/15/2014 | 44:49.1 | 0 |
| 46.447 | 19688.504 | 2.089 | 14.63 | 0.206781 | 7/15/2014 | 44:49.2 | 0 |
| 46.681 | 19688.484 | 2.091 | 14.631 | 0.18109 | 7/15/2014 | 44:49.3 | 0 |
| 47.088 | 19688.447 | 2.088 | 14.632 | 0.188774 | 7/15/2014 | 44:49.4 | 0 |
| 47.32 | 19688.428 | 2.085 | 14.633 | 0.158818 | 7/15/2014 | 44:49.5 | 0 |
| 47.554 | 19688.406 | 2.085 | 14.634 | 0.177728 | 7/15/2014 | 44:49.6 | 0 |
| 47.847 | 19688.383 | 2.078 | 14.635 | 0.166079 | 7/15/2014 | 44:49.7 | 0 |
| 47.963 | 19688.373 | 2.076 | 14.636 | 0.154031 | 7/15/2014 | 44:49.8 | 0 |
| 48.202 | 19688.352 | 2.08 | 14.637 | 0.15737 | 7/15/2014 | 44:49.9 | 0 |
| 48.44 | 19688.332 | 2.076 | 14.638 | 0.131615 | 7/15/2014 | 44:50.0 | 0 |
| 48.681 | 19688.311 | 2.078 | 14.639 | 0.138476 | 7/15/2014 | 44:50.1 | 0 |
| 48.921 | 19688.291 | 2.078 | 14.64 | 0.133754 | 7/15/2014 | 44:50.2 | 0 |
| 49.162 | 19688.271 | 2.083 | 14.64 | 0.148313 | 7/15/2014 | 44:50.3 | 0 |
| 49.585 | 19688.236 | 2.082 | 14.64 | 0.142699 | 7/15/2014 | 44:50.4 | 0 |
| 49.827 | 19688.215 | 2.084 | 14.64 | 0.151713 | 7/15/2014 | 44:50.5 | 0 |
| 50.069 | 19688.195 | 2.084 | 14.639 | 0.173736 | 7/15/2014 | 44:50.6 | 0 |
| 50.373 | 19688.172 | 2.087 | 14.638 | 0.169338 | 7/15/2014 | 44:50.7 | 0 |
| 50.496 | 19688.162 | 2.085 | 14.638 | 0.18721 | 7/15/2014 | 44:50.8 | 0 |
| 50.741 | 19688.143 | 2.083 | 14.636 | 0.185556 | 7/15/2014 | 44:50.9 | 0 |
| 50.985 | 19688.122 | 2.078 | 14.633 | 0.190333 | 7/15/2014 | 44:51.0 | 0 |
| 51.232 | 19688.102 | 2.078 | 14.63 | 0.227518 | 7/15/2014 | 44:51.1 | 0 |
| 51.476 | 19688.082 | 2.07 | 14.626 | 0.221765 | 7/15/2014 | 44:51.2 | 0 |
| 51.721 | 19688.063 | 2.07 | 14.621 | 0.228023 | 7/15/2014 | 44:51.3 | 0 |
| 52.144 | 19688.031 | 2.068 | 14.613 | 0.2211 | 7/15/2014 | 44:51.4 | 0 |
| 52.384 | 19688.012 | 2.066 | 14.607 | 0.207051 | 7/15/2014 | 44:51.5 | 0 |
| 52.625 | 19687.993 | 2.071 | 14.6 | 0.211487 | 7/15/2014 | 44:51.6 | 0 |
| 52.922 | 19687.971 | 2.075 | 14.592 | 0.186456 | 7/15/2014 | 44:51.7 | 0 |
| 53.044 | 19687.961 | 2.074 | 14.589 | 0.185551 | 7/15/2014 | 44:51.8 | 0 |
| 53.282 | 19687.943 | 2.078 | 14.58 | 0.187107 | 7/15/2014 | 44:51.9 | 0 |
| 53.519 | 19687.926 | 2.076 | 14.57 | 0.177832 | 7/15/2014 | 44:52.0 | 0 |
| 53.757 | 19687.908 | 2.078 | 14.56 | 0.183038 | 7/15/2014 | 44:52.1 | 0 |
| 53.989 | 19687.891 | 2.07 | 14.549 | 0.196994 | 7/15/2014 | 44:52.2 | 0 |

| | | | | | | | |
|---|---|---|---|---|---|---|---|
| 54.222 | 19687.874 | 2.07 | 14.537 | 0.225837 | 7/15/2014 | 44:52.3 | 0 |
| 54.625 | 19687.846 | 2.066 | 14.518 | 0.232744 | 7/15/2014 | 44:52.4 | 0 |
| 54.851 | 19687.83 | 2.062 | 14.506 | 0.244352 | 7/15/2014 | 44:52.5 | 0 |
| 55.078 | 19687.813 | 2.059 | 14.492 | 0.249457 | 7/15/2014 | 44:52.6 | 0 |
| 55.363 | 19687.793 | 2.059 | 14.479 | 0.25439 | 7/15/2014 | 44:52.7 | 0 |
| 55.59 | 19687.778 | 2.061 | 14.465 | 0.247214 | 7/15/2014 | 44:52.8 | 0 |
| 55.818 | 19687.763 | 2.064 | 14.451 | 0.248048 | 7/15/2014 | 44:52.9 | 0 |
| 56.047 | 19687.748 | 2.064 | 14.437 | 0.287083 | 7/15/2014 | 44:53.0 | 0 |
| 56.276 | 19687.732 | 2.064 | 14.423 | 0.272761 | 7/15/2014 | 44:53.1 | 0 |
| 56.505 | 19687.717 | 2.064 | 14.409 | 0.240744 | 7/15/2014 | 44:53.2 | 0 |
| 56.733 | 19687.701 | 2.064 | 14.395 | 0.275168 | 7/15/2014 | 44:53.3 | 0 |
| 57.018 | 19687.683 | 2.072 | 14.381 | 0.27724 | 7/15/2014 | 44:53.4 | 0 |
| 57.247 | 19687.668 | 2.074 | 14.368 | 0.306823 | 7/15/2014 | 44:53.5 | 0 |
| 57.476 | 19687.653 | 2.072 | 14.355 | 0.309118 | 7/15/2014 | 44:53.6 | 0 |
| 57.766 | 19687.635 | 2.072 | 14.343 | 0.259629 | 7/15/2014 | 44:53.7 | 0 |
| 57.881 | 19687.627 | 2.073 | 14.336 | 0.25745 | 7/15/2014 | 44:53.8 | 0 |
| 58.117 | 19687.612 | 2.078 | 14.325 | 0.309161 | 7/15/2014 | 44:53.9 | 0 |
| 58.351 | 19687.598 | 2.076 | 14.314 | 0.313492 | 7/15/2014 | 44:54.0 | 0 |
| 58.59 | 19687.583 | 2.08 | 14.305 | 0.334171 | 7/15/2014 | 44:54.1 | 0 |
| 58.825 | 19687.568 | 2.076 | 14.297 | 0.319059 | 7/15/2014 | 44:54.2 | 0 |
| 59.063 | 19687.555 | 2.076 | 14.29 | 0.317715 | 7/15/2014 | 44:54.3 | 0 |
| 59.477 | 19687.529 | 2.076 | 14.284 | 0.389956 | 7/15/2014 | 44:54.4 | 0 |
| 59.712 | 19687.515 | 2.076 | 14.281 | 0.367282 | 7/15/2014 | 44:54.5 | 0 |
| 59.947 | 19687.502 | 2.079 | 14.281 | 0.363282 | 7/15/2014 | 44:54.6 | 0 |
| 60.239 | 19687.484 | 2.081 | 14.282 | 0.351497 | 7/15/2014 | 44:54.7 | 0 |
| 60.359 | 19687.478 | 2.079 | 14.282 | 0.355087 | 7/15/2014 | 44:54.8 | 0 |
| 60.593 | 19687.465 | 2.082 | 14.287 | 0.330543 | 7/15/2014 | 44:54.9 | 0 |
| 60.826 | 19687.451 | 2.082 | 14.294 | 0.344995 | 7/15/2014 | 44:55.0 | 0 |
| 61.172 | 19687.432 | 2.081 | 14.312 | 0.373376 | 7/15/2014 | 44:55.1 | 0 |
| 61.402 | 19687.418 | 2.081 | 14.325 | 0.379995 | 7/15/2014 | 44:55.2 | 0 |
| 61.63 | 19687.406 | 2.078 | 14.341 | 0.365593 | 7/15/2014 | 44:55.3 | 0 |
| 61.915 | 19687.391 | 2.073 | 14.358 | 0.416749 | 7/15/2014 | 44:55.4 | 0 |
| 62.14 | 19687.379 | 2.071 | 14.376 | 0.424496 | 7/15/2014 | 44:55.5 | 0 |
| 62.366 | 19687.367 | 2.068 | 14.393 | 0.416458 | 7/15/2014 | 44:55.6 | 0 |
| 62.65 | 19687.353 | 2.066 | 14.41 | 0.430086 | 7/15/2014 | 44:55.7 | 0 |
| 62.88 | 19687.339 | 2.064 | 14.425 | 0.394707 | 7/15/2014 | 44:55.8 | 0 |
| 63.11 | 19687.326 | 2.062 | 14.437 | 0.404434 | 7/15/2014 | 44:55.9 | 0 |
| 63.341 | 19687.314 | 2.062 | 14.448 | 0.439277 | 7/15/2014 | 44:56.0 | 0 |
| 63.576 | 19687.303 | 2.065 | 14.455 | 0.397494 | 7/15/2014 | 44:56.1 | 0 |
| 63.688 | 19687.297 | 2.063 | 14.46 | 0.408591 | 7/15/2014 | 44:56.2 | 0 |
| 63.921 | 19687.285 | 2.063 | 14.463 | 0.426322 | 7/15/2014 | 44:56.3 | 0 |
| 64.332 | 19687.265 | 2.063 | 14.464 | 0.438369 | 7/15/2014 | 44:56.4 | 0 |
| 64.567 | 19687.253 | 2.06 | 14.464 | 0.458323 | 7/15/2014 | 44:56.5 | 0 |
| 64.804 | 19687.24 | 2.06 | 14.463 | 0.468733 | 7/15/2014 | 44:56.6 | 0 |
| 65.101 | 19687.225 | 2.06 | 14.461 | 0.43694 | 7/15/2014 | 44:56.7 | 0 |
| 65.221 | 19687.219 | 2.057 | 14.459 | 0.460555 | 7/15/2014 | 44:56.8 | 0 |
| 65.46 | 19687.207 | 2.057 | 14.456 | 0.421001 | 7/15/2014 | 44:56.9 | 0 |

| | | | | | | | |
|---|---|---|---|---|---|---|---|
| 65.701 | 19687.195 | 2.054 | 14.453 | 0.434225 | 7/15/2014 | 44:57.0 | 0 |
| 65.942 | 19687.184 | 2.054 | 14.45 | 0.438849 | 7/15/2014 | 44:57.1 | 0 |
| 66.18 | 19687.172 | 2.046 | 14.447 | 0.454009 | 7/15/2014 | 44:57.2 | 0 |
| 66.422 | 19687.16 | 2.05 | 14.445 | 0.413193 | 7/15/2014 | 44:57.3 | 0 |
| 66.841 | 19687.139 | 2.045 | 14.443 | 0.440186 | 7/15/2014 | 44:57.4 | 0 |
| 67.079 | 19687.127 | 2.039 | 14.442 | 0.442533 | 7/15/2014 | 44:57.5 | 0 |
| 67.318 | 19687.115 | 2.036 | 14.441 | 0.446252 | 7/15/2014 | 44:57.6 | 0 |
| 67.614 | 19687.102 | 2.032 | 14.441 | 0.416093 | 7/15/2014 | 44:57.7 | 0 |
| 67.734 | 19687.096 | 2.028 | 14.441 | 0.390877 | 7/15/2014 | 44:57.8 | 0 |
| 67.975 | 19687.084 | 2.032 | 14.441 | 0.420581 | 7/15/2014 | 44:57.9 | 0 |
| 68.212 | 19687.073 | 2.025 | 14.44 | 0.42258 | 7/15/2014 | 44:58.0 | 0 |
| 68.453 | 19687.063 | 2.028 | 14.44 | 0.413677 | 7/15/2014 | 44:58.1 | 0 |
| 68.689 | 19687.051 | 2.021 | 14.438 | 0.418817 | 7/15/2014 | 44:58.2 | 0 |
| 68.927 | 19687.039 | 2.025 | 14.436 | 0.424029 | 7/15/2014 | 44:58.3 | 0 |
| 69.338 | 19687.021 | 2.019 | 14.431 | 0.435845 | 7/15/2014 | 44:58.4 | 0 |
| 69.571 | 19687.01 | 2.014 | 14.427 | 0.457952 | 7/15/2014 | 44:58.5 | 0 |
| 69.803 | 19686.999 | 2.011 | 14.423 | 0.418153 | 7/15/2014 | 44:58.6 | 0 |
| 70.094 | 19686.986 | 2.011 | 14.418 | 0.404202 | 7/15/2014 | 44:58.7 | 0 |
| 70.211 | 19686.98 | 2.008 | 14.416 | 0.413556 | 7/15/2014 | 44:58.8 | 0 |
| 70.445 | 19686.97 | 2.008 | 14.41 | 0.39747 | 7/15/2014 | 44:58.9 | 0 |
| 70.791 | 19686.953 | 2.003 | 14.403 | 0.415825 | 7/15/2014 | 44:59.0 | 0 |
| 71.024 | 19686.943 | 2.003 | 14.398 | 0.418004 | 7/15/2014 | 44:59.1 | 0 |
| 71.253 | 19686.934 | 1.999 | 14.393 | 0.42094 | 7/15/2014 | 44:59.2 | 0 |
| 71.484 | 19686.922 | 2.002 | 14.388 | 0.434265 | 7/15/2014 | 44:59.3 | 0 |
| 71.769 | 19686.91 | 1.999 | 14.384 | 0.44056 | 7/15/2014 | 44:59.4 | 0 |
| 71.997 | 19686.899 | 1.997 | 14.38 | 0.463522 | 7/15/2014 | 44:59.5 | 0 |
| 72.225 | 19686.889 | 1.997 | 14.376 | 0.43527 | 7/15/2014 | 44:59.6 | 0 |
| 72.511 | 19686.875 | 1.997 | 14.372 | 0.44422 | 7/15/2014 | 44:59.7 | 0 |
| 72.742 | 19686.866 | 1.995 | 14.368 | 0.444353 | 7/15/2014 | 44:59.8 | 0 |
| 72.973 | 19686.855 | 1.995 | 14.363 | 0.435046 | 7/15/2014 | 44:59.9 | 0 |
| 73.206 | 19686.844 | 1.995 | 14.358 | 0.457736 | 7/15/2014 | 45:00.0 | 0 |
| 73.439 | 19686.836 | 1.995 | 14.352 | 0.468015 | 7/15/2014 | 45:00.1 | 0 |
| 73.553 | 19686.83 | 1.995 | 14.35 | 0.449949 | 7/15/2014 | 45:00.2 | 0 |
| 73.786 | 19686.818 | 1.997 | 14.344 | 0.476752 | 7/15/2014 | 45:00.3 | 0 |
| 74.196 | 19686.801 | 1.994 | 14.334 | 0.504547 | 7/15/2014 | 45:00.4 | 0 |
| 74.431 | 19686.791 | 1.991 | 14.328 | 0.526047 | 7/15/2014 | 45:00.5 | 0 |
| 74.667 | 19686.779 | 1.986 | 14.321 | 0.517295 | 7/15/2014 | 45:00.6 | 0 |
| 74.966 | 19686.766 | 1.98 | 14.315 | 0.516014 | 7/15/2014 | 45:00.7 | 0 |
| 75.085 | 19686.762 | 1.979 | 14.312 | 0.515093 | 7/15/2014 | 45:00.8 | 0 |
| 75.327 | 19686.75 | 1.973 | 14.306 | 0.509027 | 7/15/2014 | 45:00.9 | 0 |
| 75.57 | 19686.74 | 1.968 | 14.3 | 0.509608 | 7/15/2014 | 45:01.0 | 0 |
| 75.816 | 19686.73 | 1.968 | 14.294 | 0.497057 | 7/15/2014 | 45:01.1 | 0 |
| 76.062 | 19686.719 | 1.963 | 14.288 | 0.483501 | 7/15/2014 | 45:01.2 | 0 |
| 76.309 | 19686.707 | 1.966 | 14.282 | 0.481173 | 7/15/2014 | 45:01.3 | 0 |
| 76.617 | 19686.693 | 1.966 | 14.275 | 0.480101 | 7/15/2014 | 45:01.4 | 0 |
| 76.867 | 19686.682 | 1.966 | 14.267 | 0.485917 | 7/15/2014 | 45:01.5 | 0 |
| 77.118 | 19686.674 | 1.966 | 14.258 | 0.490964 | 7/15/2014 | 45:01.6 | 0 |

| | | | | | | | |
|---|---|---|---|---|---|---|---|
| 77.37 | 19686.662 | 1.963 | 14.248 | 0.498404 | 7/15/2014 | 45:01.7 | 0 |
| 77.684 | 19686.648 | 1.955 | 14.237 | 0.450488 | 7/15/2014 | 45:01.8 | 0 |
| 77.94 | 19686.637 | 1.952 | 14.225 | 0.460037 | 7/15/2014 | 45:01.9 | 0 |
| 78.194 | 19686.625 | 1.946 | 14.211 | 0.445291 | 7/15/2014 | 45:02.0 | 0 |
| 78.449 | 19686.613 | 1.949 | 14.197 | 0.441376 | 7/15/2014 | 45:02.1 | 0 |
| 78.704 | 19686.604 | 1.949 | 14.182 | 0.449858 | 7/15/2014 | 45:02.2 | 0 |
| 78.96 | 19686.592 | 1.953 | 14.167 | 0.422206 | 7/15/2014 | 45:02.3 | 0 |
| 79.276 | 19686.578 | 1.957 | 14.151 | 0.455396 | 7/15/2014 | 45:02.4 | 0 |
| 79.532 | 19686.566 | 1.96 | 14.135 | 0.432966 | 7/15/2014 | 45:02.5 | 0 |
| 79.785 | 19686.556 | 1.957 | 14.119 | 0.440641 | 7/15/2014 | 45:02.6 | 0 |
| 80.041 | 19686.545 | 1.954 | 14.105 | 0.43242 | 7/15/2014 | 45:02.7 | 0 |
| 80.358 | 19686.529 | 1.945 | 14.091 | 0.422126 | 7/15/2014 | 45:02.8 | 0 |
| 80.616 | 19686.52 | 1.942 | 14.08 | 0.429606 | 7/15/2014 | 45:02.9 | 0 |
| 80.873 | 19686.508 | 1.936 | 14.07 | 0.420081 | 7/15/2014 | 45:03.0 | 0 |
| 81.129 | 19686.496 | 1.929 | 14.063 | 0.422795 | 7/15/2014 | 45:03.1 | 0 |
| 81.384 | 19686.485 | 1.923 | 14.058 | 0.416196 | 7/15/2014 | 45:03.2 | 0 |
| 81.642 | 19686.475 | 1.927 | 14.056 | 0.416852 | 7/15/2014 | 45:03.3 | 0 |
| 81.958 | 19686.459 | 1.925 | 14.057 | 0.411895 | 7/15/2014 | 45:03.4 | 0 |
| 82.214 | 19686.449 | 1.928 | 14.06 | 0.408322 | 7/15/2014 | 45:03.5 | 0 |
| 82.469 | 19686.438 | 1.924 | 14.065 | 0.400798 | 7/15/2014 | 45:03.6 | 0 |
| 82.722 | 19686.428 | 1.926 | 14.072 | 0.397827 | 7/15/2014 | 45:03.7 | 0 |
| 83.035 | 19686.414 | 1.924 | 14.081 | 0.399988 | 7/15/2014 | 45:03.8 | 0 |
| 83.289 | 19686.402 | 1.93 | 14.091 | 0.393498 | 7/15/2014 | 45:03.9 | 0 |
| 83.538 | 19686.391 | 1.924 | 14.102 | 0.395006 | 7/15/2014 | 45:04.0 | 0 |
| 83.789 | 19686.379 | 1.925 | 14.113 | 0.394901 | 7/15/2014 | 45:04.1 | 0 |
| 84.037 | 19686.369 | 1.923 | 14.124 | 0.397765 | 7/15/2014 | 45:04.2 | 0 |
| 84.285 | 19686.359 | 1.923 | 14.135 | 0.392461 | 7/15/2014 | 45:04.3 | 0 |
| 84.71 | 19686.342 | 1.918 | 14.149 | 0.389606 | 7/15/2014 | 45:04.4 | 0 |
| 84.952 | 19686.331 | 1.92 | 14.157 | 0.397545 | 7/15/2014 | 45:04.5 | 0 |
| 85.192 | 19686.32 | 1.92 | 14.163 | 0.389161 | 7/15/2014 | 45:04.6 | 0 |
| 85.488 | 19686.307 | 1.922 | 14.169 | 0.390262 | 7/15/2014 | 45:04.7 | 0 |
| 85.61 | 19686.303 | 1.92 | 14.172 | 0.38856 | 7/15/2014 | 45:04.8 | 0 |
| 85.848 | 19686.293 | 1.93 | 14.175 | 0.39219 | 7/15/2014 | 45:04.9 | 0 |
| 86.084 | 19686.282 | 1.932 | 14.177 | 0.387249 | 7/15/2014 | 45:05.0 | 0 |
| 86.321 | 19686.273 | 1.938 | 14.177 | 0.389541 | 7/15/2014 | 45:05.1 | 0 |
| 86.554 | 19686.263 | 1.938 | 14.177 | 0.388241 | 7/15/2014 | 45:05.2 | 0 |
| 86.789 | 19686.254 | 1.942 | 14.176 | 0.389664 | 7/15/2014 | 45:05.3 | 0 |
| 87.192 | 19686.236 | 1.936 | 14.173 | 0.3936 | 7/15/2014 | 45:05.4 | 0 |
| 87.422 | 19686.229 | 1.933 | 14.17 | 0.395135 | 7/15/2014 | 45:05.5 | 0 |
| 87.65 | 19686.219 | 1.927 | 14.167 | 0.400165 | 7/15/2014 | 45:05.6 | 0 |
| 87.938 | 19686.207 | 1.927 | 14.164 | 0.404947 | 7/15/2014 | 45:05.7 | 0 |
| 88.169 | 19686.197 | 1.93 | 14.16 | 0.405059 | 7/15/2014 | 45:05.8 | 0 |
| 88.401 | 19686.188 | 1.93 | 14.156 | 0.410957 | 7/15/2014 | 45:05.9 | 0 |
| 88.632 | 19686.18 | 1.93 | 14.151 | 0.412304 | 7/15/2014 | 45:06.0 | 0 |
| 88.867 | 19686.17 | 1.935 | 14.146 | 0.415373 | 7/15/2014 | 45:06.1 | 0 |
| 88.98 | 19686.166 | 1.936 | 14.144 | 0.41766 | 7/15/2014 | 45:06.2 | 0 |
| 89.213 | 19686.157 | 1.948 | 14.138 | 0.419618 | 7/15/2014 | 45:06.3 | 0 |

| | | | | | | | |
|---|---|---|---|---|---|---|---|
| 89.62 | 19686.141 | 1.954 | 14.128 | 0.420653 | 7/15/2014 | 45:06.4 | 0 |
| 89.853 | 19686.132 | 1.958 | 14.121 | 0.423547 | 7/15/2014 | 45:06.5 | 0 |
| 90.087 | 19686.122 | 1.96 | 14.113 | 0.422204 | 7/15/2014 | 45:06.6 | 0 |
| 90.381 | 19686.111 | 1.963 | 14.105 | 0.415742 | 7/15/2014 | 45:06.7 | 0 |
| 90.499 | 19686.107 | 1.965 | 14.101 | 0.410545 | 7/15/2014 | 45:06.8 | 0 |
| 90.739 | 19686.097 | 1.969 | 14.092 | 0.410417 | 7/15/2014 | 45:06.9 | 0 |
| 90.977 | 19686.088 | 1.963 | 14.082 | 0.405651 | 7/15/2014 | 45:07.0 | 0 |
| 91.219 | 19686.08 | 1.963 | 14.072 | 0.405785 | 7/15/2014 | 45:07.1 | 0 |
| 91.46 | 19686.069 | 1.959 | 14.062 | 0.403509 | 7/15/2014 | 45:07.2 | 0 |
| 91.704 | 19686.061 | 1.965 | 14.05 | 0.394347 | 7/15/2014 | 45:07.3 | 0 |
| 92.125 | 19686.045 | 1.963 | 14.032 | 0.415086 | 7/15/2014 | 45:07.4 | 0 |
| 92.367 | 19686.035 | 1.968 | 14.019 | 0.398297 | 7/15/2014 | 45:07.5 | 0 |
| 92.606 | 19686.027 | 1.97 | 14.007 | 0.403516 | 7/15/2014 | 45:07.6 | 0 |
| 92.905 | 19686.017 | 1.976 | 13.994 | 0.40249 | 7/15/2014 | 45:07.7 | 0 |
| 93.028 | 19686.011 | 1.98 | 13.987 | 0.402646 | 7/15/2014 | 45:07.8 | 0 |
| 93.269 | 19686.002 | 1.989 | 13.975 | 0.400074 | 7/15/2014 | 45:07.9 | 0 |
| 93.507 | 19685.992 | 1.987 | 13.963 | 0.391629 | 7/15/2014 | 45:08.0 | 0 |
| 93.745 | 19685.984 | 1.993 | 13.952 | 0.382855 | 7/15/2014 | 45:08.1 | 0 |
| 93.98 | 19685.977 | 1.989 | 13.941 | 0.383833 | 7/15/2014 | 45:08.2 | 0 |
| 94.215 | 19685.969 | 1.993 | 13.931 | 0.380366 | 7/15/2014 | 45:08.3 | 0 |
| 94.62 | 19685.953 | 1.998 | 13.917 | 0.372686 | 7/15/2014 | 45:08.4 | 0 |
| 94.849 | 19685.944 | 1.998 | 13.909 | 0.372527 | 7/15/2014 | 45:08.5 | 0 |
| 95.08 | 19685.936 | 2 | 13.9 | 0.377873 | 7/15/2014 | 45:08.6 | 0 |
| 95.365 | 19685.926 | 2.003 | 13.891 | 0.368847 | 7/15/2014 | 45:08.7 | 0 |
| 95.596 | 19685.918 | 2.01 | 13.883 | 0.379006 | 7/15/2014 | 45:08.8 | 0 |
| 95.828 | 19685.91 | 2.013 | 13.875 | 0.376246 | 7/15/2014 | 45:08.9 | 0 |
| 96.06 | 19685.902 | 2.015 | 13.866 | 0.370799 | 7/15/2014 | 45:09.0 | 0 |
| 96.293 | 19685.894 | 2.019 | 13.858 | 0.374122 | 7/15/2014 | 45:09.1 | 0 |
| 96.406 | 19685.89 | 2.016 | 13.854 | 0.379849 | 7/15/2014 | 45:09.2 | 0 |
| 96.64 | 19685.88 | 2.022 | 13.846 | 0.376476 | 7/15/2014 | 45:09.3 | 0 |
| 97.045 | 19685.865 | 2.024 | 13.835 | 0.387327 | 7/15/2014 | 45:09.4 | 0 |
| 97.278 | 19685.857 | 2.029 | 13.828 | 0.384063 | 7/15/2014 | 45:09.5 | 0 |
| 97.51 | 19685.85 | 2.027 | 13.822 | 0.377772 | 7/15/2014 | 45:09.6 | 0 |
| 97.801 | 19685.84 | 2.032 | 13.815 | 0.351851 | 7/15/2014 | 45:09.7 | 0 |
| 97.918 | 19685.836 | 2.032 | 13.812 | 0.359204 | 7/15/2014 | 45:09.8 | 0 |
| 98.153 | 19685.827 | 2.037 | 13.805 | 0.333541 | 7/15/2014 | 45:09.9 | 0 |
| 98.386 | 19685.816 | 2.037 | 13.798 | 0.316601 | 7/15/2014 | 45:10.0 | 0 |
| 98.622 | 19685.81 | 2.039 | 13.79 | 0.314163 | 7/15/2014 | 45:10.1 | 0 |
| 98.855 | 19685.801 | 2.039 | 13.781 | 0.309447 | 7/15/2014 | 45:10.2 | 0 |
| 99.088 | 19685.793 | 2.042 | 13.771 | 0.311868 | 7/15/2014 | 45:10.3 | 0 |
| 99.493 | 19685.779 | 2.048 | 13.752 | 0.311126 | 7/15/2014 | 45:10.4 | 0 |
| 99.721 | 19685.771 | 2.05 | 13.738 | 0.328356 | 7/15/2014 | 45:10.5 | 0 |
| 99.95 | 19685.762 | 2.057 | 13.723 | 0.332557 | 7/15/2014 | 45:10.6 | 0 |
| 100.232 | 19685.753 | 2.059 | 13.707 | 0.32489 | 7/15/2014 | 45:10.7 | 0 |
| 100.46 | 19685.744 | 2.065 | 13.688 | 0.329584 | 7/15/2014 | 45:10.8 | 0 |
| 100.688 | 19685.736 | 2.074 | 13.669 | 0.33646 | 7/15/2014 | 45:10.9 | 0 |
| 100.914 | 19685.729 | 2.082 | 13.648 | 0.32392 | 7/15/2014 | 45:11.0 | 0 |

| | | | | | | | |
|---|---|---|---|---|---|---|---|
| 101.142 | 19685.721 | 2.085 | 13.625 | 0.341045 | 7/15/2014 | 45:11.1 | 0 |
| 101.365 | 19685.713 | 2.088 | 13.601 | 0.320471 | 7/15/2014 | 45:11.2 | 0 |
| 101.588 | 19685.705 | 2.091 | 13.576 | 0.321476 | 7/15/2014 | 45:11.3 | 0 |
| 101.866 | 19685.695 | 2.091 | 13.55 | 0.338922 | 7/15/2014 | 45:11.4 | 0 |
| 102.087 | 19685.688 | 2.091 | 13.523 | 0.351125 | 7/15/2014 | 45:11.5 | 0 |
| 102.31 | 19685.68 | 2.091 | 13.496 | 0.312051 | 7/15/2014 | 45:11.6 | 0 |
| 102.59 | 19685.67 | 2.091 | 13.469 | 0.322219 | 7/15/2014 | 45:11.7 | 0 |
| 102.815 | 19685.662 | 2.089 | 13.443 | 0.302761 | 7/15/2014 | 45:11.8 | 0 |
| 103.042 | 19685.654 | 2.092 | 13.419 | 0.347631 | 7/15/2014 | 45:11.9 | 0 |
| 103.269 | 19685.645 | 2.095 | 13.396 | 0.330269 | 7/15/2014 | 45:12.0 | 0 |
| 103.499 | 19685.638 | 2.1 | 13.376 | 0.322823 | 7/15/2014 | 45:12.1 | 0 |
| 103.728 | 19685.629 | 2.102 | 13.359 | 0.344529 | 7/15/2014 | 45:12.2 | 0 |
| 103.84 | 19685.625 | 2.109 | 13.35 | 0.38175 | 7/15/2014 | 45:12.3 | 0 |
| 104.245 | 19685.611 | 2.113 | 13.339 | 0.366323 | 7/15/2014 | 45:12.4 | 0 |
| 104.476 | 19685.604 | 2.115 | 13.337 | 0.370917 | 7/15/2014 | 45:12.5 | 0 |
| 104.708 | 19685.596 | 2.119 | 13.339 | 0.348588 | 7/15/2014 | 45:12.6 | 0 |
| 104.997 | 19685.582 | 2.119 | 13.347 | 0.341864 | 7/15/2014 | 45:12.7 | 0 |
| 105.114 | 19685.579 | 2.119 | 13.35 | 0.362054 | 7/15/2014 | 45:12.8 | 0 |
| 105.348 | 19685.571 | 2.119 | 13.365 | 0.304206 | 7/15/2014 | 45:12.9 | 0 |
| 105.582 | 19685.563 | 2.119 | 13.384 | 0.317587 | 7/15/2014 | 45:13.0 | 0 |
| 105.817 | 19685.555 | 2.121 | 13.405 | 0.313819 | 7/15/2014 | 45:13.1 | 0 |
| 106.05 | 19685.547 | 2.121 | 13.427 | 0.317083 | 7/15/2014 | 45:13.2 | 0 |
| 106.284 | 19685.539 | 2.119 | 13.448 | 0.292883 | 7/15/2014 | 45:13.3 | 0 |
| 106.69 | 19685.523 | 2.113 | 13.473 | 0.291221 | 7/15/2014 | 45:13.4 | 0 |
| 106.921 | 19685.517 | 2.113 | 13.487 | 0.264838 | 7/15/2014 | 45:13.5 | 0 |
| 107.152 | 19685.508 | 2.113 | 13.497 | 0.272866 | 7/15/2014 | 45:13.6 | 0 |
| 107.442 | 19685.498 | 2.111 | 13.504 | 0.26255 | 7/15/2014 | 45:13.7 | 0 |
| 107.558 | 19685.494 | 2.107 | 13.508 | 0.25526 | 7/15/2014 | 45:13.8 | 0 |
| 107.791 | 19685.486 | 2.11 | 13.509 | 0.253189 | 7/15/2014 | 45:13.9 | 0 |
| 108.022 | 19685.479 | 2.11 | 13.507 | 0.26484 | 7/15/2014 | 45:14.0 | 0 |
| 108.257 | 19685.471 | 2.119 | 13.502 | 0.253472 | 7/15/2014 | 45:14.1 | 0 |
| 108.489 | 19685.463 | 2.114 | 13.495 | 0.252022 | 7/15/2014 | 45:14.2 | 0 |
| 108.722 | 19685.455 | 2.117 | 13.487 | 0.233396 | 7/15/2014 | 45:14.3 | 0 |
| 109.128 | 19685.442 | 2.117 | 13.472 | 0.232004 | 7/15/2014 | 45:14.4 | 0 |
| 109.359 | 19685.436 | 2.117 | 13.462 | 0.219257 | 7/15/2014 | 45:14.5 | 0 |
| 109.591 | 19685.428 | 2.115 | 13.451 | 0.217658 | 7/15/2014 | 45:14.6 | 0 |
| 109.881 | 19685.418 | 2.115 | 13.439 | 0.208454 | 7/15/2014 | 45:14.7 | 0 |
| 109.998 | 19685.414 | 2.111 | 13.433 | 0.206113 | 7/15/2014 | 45:14.8 | 0 |
| 110.234 | 19685.406 | 2.119 | 13.42 | 0.216195 | 7/15/2014 | 45:14.9 | 0 |
| 110.469 | 19685.399 | 2.117 | 13.406 | 0.22477 | 7/15/2014 | 45:15.0 | 0 |
| 110.708 | 19685.392 | 2.122 | 13.391 | 0.222035 | 7/15/2014 | 45:15.1 | 0 |
| 110.943 | 19685.385 | 2.119 | 13.374 | 0.235354 | 7/15/2014 | 45:15.2 | 0 |
| 111.182 | 19685.377 | 2.124 | 13.356 | 0.23149 | 7/15/2014 | 45:15.3 | 0 |
| 111.599 | 19685.364 | 2.123 | 13.324 | 0.241462 | 7/15/2014 | 45:15.4 | 0 |
| 111.839 | 19685.356 | 2.125 | 13.302 | 0.230349 | 7/15/2014 | 45:15.5 | 0 |
| 112.078 | 19685.35 | 2.125 | 13.278 | 0.232395 | 7/15/2014 | 45:15.6 | 0 |
| 112.381 | 19685.34 | 2.13 | 13.254 | 0.223926 | 7/15/2014 | 45:15.7 | 0 |

| | | | | | | | |
|---|---|---|---|---|---|---|---|
| 112.501 | 19685.336 | 2.129 | 13.242 | 0.217577 | 7/15/2014 | 45:15.8 | 0 |
| 112.745 | 19685.328 | 2.131 | 13.217 | 0.211512 | 7/15/2014 | 45:15.9 | 0 |
| 112.99 | 19685.321 | 2.135 | 13.192 | 0.209257 | 7/15/2014 | 45:16.0 | 0 |
| 113.236 | 19685.313 | 2.14 | 13.167 | 0.213858 | 7/15/2014 | 45:16.1 | 0 |
| 113.481 | 19685.307 | 2.14 | 13.145 | 0.212548 | 7/15/2014 | 45:16.2 | 0 |
| 113.726 | 19685.301 | 2.142 | 13.123 | 0.215659 | 7/15/2014 | 45:16.3 | 0 |
| 114.154 | 19685.287 | 2.141 | 13.097 | 0.223536 | 7/15/2014 | 45:16.4 | 0 |
| 114.398 | 19685.28 | 2.141 | 13.082 | 0.224554 | 7/15/2014 | 45:16.5 | 0 |
| 114.64 | 19685.273 | 2.145 | 13.07 | 0.227528 | 7/15/2014 | 45:16.6 | 0 |
| 114.942 | 19685.264 | 2.141 | 13.062 | 0.22884 | 7/15/2014 | 45:16.7 | 0 |
| 115.067 | 19685.261 | 2.142 | 13.057 | 0.230867 | 7/15/2014 | 45:16.8 | 0 |
| 115.309 | 19685.254 | 2.142 | 13.055 | 0.225313 | 7/15/2014 | 45:16.9 | 0 |
| 115.552 | 19685.248 | 2.142 | 13.057 | 0.2228 | 7/15/2014 | 45:17.0 | 0 |
| 115.793 | 19685.24 | 2.142 | 13.063 | 0.219098 | 7/15/2014 | 45:17.1 | 0 |
| 116.034 | 19685.234 | 2.136 | 13.074 | 0.218819 | 7/15/2014 | 45:17.2 | 0 |
| 116.273 | 19685.229 | 2.136 | 13.087 | 0.216083 | 7/15/2014 | 45:17.3 | 0 |
| 116.688 | 19685.215 | 2.129 | 13.113 | 0.257702 | 7/15/2014 | 45:17.4 | 0 |
| 116.924 | 19685.209 | 2.129 | 13.13 | 0.24615 | 7/15/2014 | 45:17.5 | 0 |
| 117.159 | 19685.203 | 2.127 | 13.147 | 0.279515 | 7/15/2014 | 45:17.6 | 0 |
| 117.453 | 19685.193 | 2.124 | 13.163 | 0.277466 | 7/15/2014 | 45:17.7 | 0 |
| 117.573 | 19685.19 | 2.123 | 13.172 | 0.285708 | 7/15/2014 | 45:17.8 | 0 |
| 117.809 | 19685.185 | 2.125 | 13.184 | 0.30012 | 7/15/2014 | 45:17.9 | 0 |
| 118.046 | 19685.178 | 2.125 | 13.194 | 0.303107 | 7/15/2014 | 45:18.0 | 0 |
| 118.283 | 19685.17 | 2.128 | 13.2 | 0.308676 | 7/15/2014 | 45:18.1 | 0 |
| 118.52 | 19685.165 | 2.13 | 13.203 | 0.31468 | 7/15/2014 | 45:18.2 | 0 |
| 118.757 | 19685.158 | 2.134 | 13.203 | 0.31184 | 7/15/2014 | 45:18.3 | 0 |
| 119.168 | 19685.145 | 2.136 | 13.197 | 0.313988 | 7/15/2014 | 45:18.4 | 0 |
| 119.403 | 19685.14 | 2.138 | 13.19 | 0.321864 | 7/15/2014 | 45:18.5 | 0 |
| 119.638 | 19685.133 | 2.138 | 13.182 | 0.320647 | 7/15/2014 | 45:18.6 | 0 |
| 119.932 | 19685.123 | 2.138 | 13.171 | 0.324853 | 7/15/2014 | 45:18.7 | 0 |
| 120.052 | 19685.12 | 2.139 | 13.166 | 0.330483 | 7/15/2014 | 45:18.8 | 0 |
| 120.29 | 19685.114 | 2.139 | 13.152 | 0.334963 | 7/15/2014 | 45:18.9 | 0 |
| 120.527 | 19685.107 | 2.135 | 13.135 | 0.342104 | 7/15/2014 | 45:19.0 | 0 |
| 120.767 | 19685.1 | 2.142 | 13.117 | 0.352461 | 7/15/2014 | 45:19.1 | 0 |
| 121.004 | 19685.094 | 2.146 | 13.097 | 0.367714 | 7/15/2014 | 45:19.2 | 0 |
| 121.244 | 19685.086 | 2.153 | 13.075 | 0.372418 | 7/15/2014 | 45:19.3 | 0 |
| 121.661 | 19685.074 | 2.16 | 13.041 | 0.386065 | 7/15/2014 | 45:19.4 | 0 |
| 121.898 | 19685.067 | 2.163 | 13.017 | 0.389786 | 7/15/2014 | 45:19.5 | 0 |
| 122.138 | 19685.061 | 2.169 | 12.994 | 0.403252 | 7/15/2014 | 45:19.6 | 0 |
| 122.438 | 19685.051 | 2.176 | 12.971 | 0.401497 | 7/15/2014 | 45:19.7 | 0 |
| 122.557 | 19685.047 | 2.174 | 12.959 | 0.399474 | 7/15/2014 | 45:19.8 | 0 |
| 122.799 | 19685.039 | 2.178 | 12.937 | 0.392225 | 7/15/2014 | 45:19.9 | 0 |
| 123.038 | 19685.035 | 2.174 | 12.915 | 0.390107 | 7/15/2014 | 45:20.0 | 0 |
| 123.282 | 19685.026 | 2.178 | 12.895 | 0.390795 | 7/15/2014 | 45:20.1 | 0 |
| 123.52 | 19685.019 | 2.172 | 12.876 | 0.385656 | 7/15/2014 | 45:20.2 | 0 |
| 123.763 | 19685.012 | 2.175 | 12.857 | 0.375862 | 7/15/2014 | 45:20.3 | 0 |
| 124.18 | 19684.998 | 2.164 | 12.83 | 0.378303 | 7/15/2014 | 45:20.4 | 0 |

| | | | | | | | |
|---|---|---|---|---|---|---|---|
| 124.418 | 19684.99 | 2.158 | 12.812 | 0.380843 | 7/15/2014 | 45:20.5 | 0 |
| 124.656 | 19684.984 | 2.158 | 12.795 | 0.38761 | 7/15/2014 | 45:20.6 | 0 |
| 124.952 | 19684.974 | 2.154 | 12.777 | 0.387818 | 7/15/2014 | 45:20.7 | 0 |
| 125.072 | 19684.97 | 2.153 | 12.769 | 0.390098 | 7/15/2014 | 45:20.8 | 0 |
| 125.312 | 19684.964 | 2.153 | 12.751 | 0.395356 | 7/15/2014 | 45:20.9 | 0 |
| 125.55 | 19684.956 | 2.146 | 12.734 | 0.393277 | 7/15/2014 | 45:21.0 | 0 |
| 125.791 | 19684.947 | 2.156 | 12.718 | 0.396845 | 7/15/2014 | 45:21.1 | 0 |
| 126.029 | 19684.94 | 2.153 | 12.702 | 0.408482 | 7/15/2014 | 45:21.2 | 0 |
| 126.269 | 19684.934 | 2.153 | 12.686 | 0.404048 | 7/15/2014 | 45:21.3 | 0 |
| 126.686 | 19684.92 | 2.148 | 12.666 | 0.412491 | 7/15/2014 | 45:21.4 | 0 |
| 126.923 | 19684.913 | 2.148 | 12.654 | 0.416854 | 7/15/2014 | 45:21.5 | 0 |
| 127.161 | 19684.905 | 2.145 | 12.642 | 0.424241 | 7/15/2014 | 45:21.6 | 0 |
| 127.462 | 19684.895 | 2.143 | 12.633 | 0.424793 | 7/15/2014 | 45:21.7 | 0 |
| 127.581 | 19684.892 | 2.134 | 12.627 | 0.423203 | 7/15/2014 | 45:21.8 | 0 |
| 127.822 | 19684.885 | 2.134 | 12.619 | 0.422989 | 7/15/2014 | 45:21.9 | 0 |
| 128.064 | 19684.876 | 2.131 | 12.612 | 0.433167 | 7/15/2014 | 45:22.0 | 0 |
| 128.307 | 19684.869 | 2.131 | 12.606 | 0.443056 | 7/15/2014 | 45:22.1 | 0 |
| 128.548 | 19684.861 | 2.128 | 12.6 | 0.442211 | 7/15/2014 | 45:22.2 | 0 |
| 128.79 | 19684.854 | 2.128 | 12.595 | 0.452901 | 7/15/2014 | 45:22.3 | 0 |
| 129.213 | 19684.84 | 2.127 | 12.588 | 0.463587 | 7/15/2014 | 45:22.4 | 0 |
| 129.454 | 19684.832 | 2.129 | 12.583 | 0.476308 | 7/15/2014 | 45:22.5 | 0 |
| 129.696 | 19684.824 | 2.134 | 12.578 | 0.463266 | 7/15/2014 | 45:22.6 | 0 |
| 129.995 | 19684.814 | 2.134 | 12.573 | 0.449602 | 7/15/2014 | 45:22.7 | 0 |
| 130.118 | 19684.811 | 2.134 | 12.571 | 0.456581 | 7/15/2014 | 45:22.8 | 0 |
| 130.36 | 19684.803 | 2.134 | 12.566 | 0.475199 | 7/15/2014 | 45:22.9 | 0 |
| 130.602 | 19684.795 | 2.131 | 12.56 | 0.485705 | 7/15/2014 | 45:23.0 | 0 |
| 130.846 | 19684.787 | 2.131 | 12.554 | 0.491941 | 7/15/2014 | 45:23.1 | 0 |
| 131.089 | 19684.779 | 2.129 | 12.547 | 0.49424 | 7/15/2014 | 45:23.2 | 0 |
| 131.332 | 19684.771 | 2.129 | 12.54 | 0.511249 | 7/15/2014 | 45:23.3 | 0 |
| 131.754 | 19684.758 | 2.122 | 12.527 | 0.544406 | 7/15/2014 | 45:23.4 | 0 |
| 131.995 | 19684.75 | 2.124 | 12.518 | 0.504725 | 7/15/2014 | 45:23.5 | 0 |
| 132.236 | 19684.742 | 2.124 | 12.508 | 0.512367 | 7/15/2014 | 45:23.6 | 0 |
| 132.54 | 19684.732 | 2.124 | 12.497 | 0.502844 | 7/15/2014 | 45:23.7 | 0 |
| 132.662 | 19684.729 | 2.125 | 12.492 | 0.502409 | 7/15/2014 | 45:23.8 | 0 |
| 132.905 | 19684.721 | 2.128 | 12.481 | 0.494154 | 7/15/2014 | 45:23.9 | 0 |
| 133.151 | 19684.713 | 2.125 | 12.47 | 0.505593 | 7/15/2014 | 45:24.0 | 0 |
| 133.396 | 19684.703 | 2.128 | 12.458 | 0.504198 | 7/15/2014 | 45:24.1 | 0 |
| 133.641 | 19684.695 | 2.128 | 12.446 | 0.527486 | 7/15/2014 | 45:24.2 | 0 |
| 133.885 | 19684.688 | 2.128 | 12.434 | 0.506164 | 7/15/2014 | 45:24.3 | 0 |
| 134.312 | 19684.674 | 2.127 | 12.414 | 0.52028 | 7/15/2014 | 45:24.4 | 0 |
| 134.554 | 19684.665 | 2.127 | 12.402 | 0.52849 | 7/15/2014 | 45:24.5 | 0 |
| 134.798 | 19684.658 | 2.127 | 12.389 | 0.504821 | 7/15/2014 | 45:24.6 | 0 |
| 135.099 | 19684.646 | 2.127 | 12.377 | 0.483145 | 7/15/2014 | 45:24.7 | 0 |
| 135.223 | 19684.642 | 2.125 | 12.37 | 0.471225 | 7/15/2014 | 45:24.8 | 0 |
| 135.468 | 19684.633 | 2.128 | 12.359 | 0.457001 | 7/15/2014 | 45:24.9 | 0 |
| 135.71 | 19684.625 | 2.125 | 12.348 | 0.438235 | 7/15/2014 | 45:25.0 | 0 |
| 135.952 | 19684.617 | 2.125 | 12.338 | 0.412161 | 7/15/2014 | 45:25.1 | 0 |

| | | | | | | | |
|---|---|---|---|---|---|---|---|
| 136.193 | 19684.608 | 2.123 | 12.328 | 0.399054 | 7/15/2014 | 45:25.2 | 0 |
| 136.434 | 19684.602 | 2.12 | 12.319 | 0.374552 | 7/15/2014 | 45:25.3 | 0 |
| 136.851 | 19684.586 | 2.114 | 12.307 | 0.386325 | 7/15/2014 | 45:25.4 | 0 |
| 137.09 | 19684.578 | 2.112 | 12.299 | 0.385716 | 7/15/2014 | 45:25.5 | 0 |
| 137.327 | 19684.568 | 2.107 | 12.29 | 0.392697 | 7/15/2014 | 45:25.6 | 0 |
| 137.624 | 19684.559 | 2.105 | 12.282 | 0.377348 | 7/15/2014 | 45:25.7 | 0 |
| 137.743 | 19684.555 | 2.101 | 12.278 | 0.366185 | 7/15/2014 | 45:25.8 | 0 |
| 137.982 | 19684.547 | 2.11 | 12.269 | 0.395969 | 7/15/2014 | 45:25.9 | 0 |
| 138.22 | 19684.539 | 2.11 | 12.26 | 0.367454 | 7/15/2014 | 45:26.0 | 0 |
| 138.459 | 19684.53 | 2.112 | 12.251 | 0.390929 | 7/15/2014 | 45:26.1 | 0 |
| 138.698 | 19684.523 | 2.112 | 12.242 | 0.413461 | 7/15/2014 | 45:26.2 | 0 |
| 138.935 | 19684.514 | 2.114 | 12.233 | 0.385775 | 7/15/2014 | 45:26.3 | 0 |
| 139.351 | 19684.5 | 2.112 | 12.22 | 0.426895 | 7/15/2014 | 45:26.4 | 0 |
| 139.588 | 19684.492 | 2.109 | 12.211 | 0.427281 | 7/15/2014 | 45:26.5 | 0 |
| 139.826 | 19684.484 | 2.104 | 12.203 | 0.433777 | 7/15/2014 | 45:26.6 | 0 |
| 140.122 | 19684.475 | 2.103 | 12.196 | 0.397977 | 7/15/2014 | 45:26.7 | 0 |
| 140.242 | 19684.471 | 2.1 | 12.192 | 0.378269 | 7/15/2014 | 45:26.8 | 0 |
| 140.483 | 19684.463 | 2.103 | 12.185 | 0.404977 | 7/15/2014 | 45:26.9 | 0 |
| 140.722 | 19684.455 | 2.096 | 12.179 | 0.369992 | 7/15/2014 | 45:27.0 | 0 |
| 140.965 | 19684.447 | 2.099 | 12.173 | 0.367696 | 7/15/2014 | 45:27.1 | 0 |
| 141.205 | 19684.439 | 2.101 | 12.168 | 0.338807 | 7/15/2014 | 45:27.2 | 0 |
| 141.447 | 19684.432 | 2.105 | 12.163 | 0.361257 | 7/15/2014 | 45:27.3 | 0 |
| 141.867 | 19684.418 | 2.099 | 12.156 | 0.393548 | 7/15/2014 | 45:27.4 | 0 |
| 142.107 | 19684.41 | 2.097 | 12.151 | 0.397993 | 7/15/2014 | 45:27.5 | 0 |
| 142.348 | 19684.401 | 2.095 | 12.147 | 0.423295 | 7/15/2014 | 45:27.6 | 0 |
| 142.649 | 19684.393 | 2.098 | 12.142 | 0.385995 | 7/15/2014 | 45:27.7 | 0 |
| 142.771 | 19684.389 | 2.095 | 12.14 | 0.407885 | 7/15/2014 | 45:27.8 | 0 |
| 143.013 | 19684.379 | 2.095 | 12.135 | 0.394212 | 7/15/2014 | 45:27.9 | 0 |
| 143.256 | 19684.372 | 2.092 | 12.131 | 0.399883 | 7/15/2014 | 45:28.0 | 0 |
| 143.499 | 19684.363 | 2.088 | 12.125 | 0.384999 | 7/15/2014 | 45:28.1 | 0 |
| 143.739 | 19684.355 | 2.088 | 12.12 | 0.370093 | 7/15/2014 | 45:28.2 | 0 |
| 143.982 | 19684.347 | 2.088 | 12.113 | 0.353622 | 7/15/2014 | 45:28.3 | 0 |
| 144.402 | 19684.334 | 2.083 | 12.103 | 0.394455 | 7/15/2014 | 45:28.4 | 0 |
| 144.639 | 19684.324 | 2.077 | 12.095 | 0.379477 | 7/15/2014 | 45:28.5 | 0 |
| 144.88 | 19684.317 | 2.07 | 12.087 | 0.384396 | 7/15/2014 | 45:28.6 | 0 |
| 145.177 | 19684.307 | 2.064 | 12.079 | 0.367543 | 7/15/2014 | 45:28.7 | 0 |
| 145.298 | 19684.303 | 2.063 | 12.075 | 0.366782 | 7/15/2014 | 45:28.8 | 0 |
| 145.54 | 19684.295 | 2.063 | 12.066 | 0.385861 | 7/15/2014 | 45:28.9 | 0 |
| 145.778 | 19684.287 | 2.059 | 12.057 | 0.353542 | 7/15/2014 | 45:29.0 | 0 |
| 146.021 | 19684.278 | 2.063 | 12.047 | 0.370475 | 7/15/2014 | 45:29.1 | 0 |
| 146.259 | 19684.271 | 2.057 | 12.037 | 0.38269 | 7/15/2014 | 45:29.2 | 0 |
| 146.5 | 19684.262 | 2.06 | 12.026 | 0.34263 | 7/15/2014 | 45:29.3 | 0 |
| 146.915 | 19684.249 | 2.057 | 12.009 | 0.389754 | 7/15/2014 | 45:29.4 | 0 |
| 147.153 | 19684.24 | 2.057 | 11.997 | 0.388339 | 7/15/2014 | 45:29.5 | 0 |
| 147.389 | 19684.233 | 2.057 | 11.985 | 0.396398 | 7/15/2014 | 45:29.6 | 0 |
| 147.685 | 19684.224 | 2.055 | 11.973 | 0.365224 | 7/15/2014 | 45:29.7 | 0 |
| 147.805 | 19684.221 | 2.052 | 11.967 | 0.355343 | 7/15/2014 | 45:29.8 | 0 |

| | | | | | | | |
|---|---|---|---|---|---|---|---|
| 148.044 | 19684.211 | 2.054 | 11.954 | 0.388568 | 7/15/2014 | 45:29.9 | 0 |
| 148.279 | 19684.204 | 2.048 | 11.942 | 0.312327 | 7/15/2014 | 45:30.0 | 0 |
| 148.518 | 19684.196 | 2.051 | 11.931 | 0.334909 | 7/15/2014 | 45:30.1 | 0 |
| 148.754 | 19684.188 | 2.047 | 11.92 | 0.300521 | 7/15/2014 | 45:30.2 | 0 |
| 148.992 | 19684.181 | 2.053 | 11.909 | 0.288998 | 7/15/2014 | 45:30.3 | 0 |
| 149.402 | 19684.168 | 2.049 | 11.893 | 0.319101 | 7/15/2014 | 45:30.4 | 0 |
| 149.637 | 19684.16 | 2.049 | 11.883 | 0.30872 | 7/15/2014 | 45:30.5 | 0 |
| 149.871 | 19684.152 | 2.047 | 11.874 | 0.320767 | 7/15/2014 | 45:30.6 | 0 |
| 150.164 | 19684.144 | 2.049 | 11.865 | 0.305962 | 7/15/2014 | 45:30.7 | 0 |
| 150.282 | 19684.14 | 2.049 | 11.86 | 0.320774 | 7/15/2014 | 45:30.8 | 0 |
| 150.521 | 19684.132 | 2.053 | 11.851 | 0.34753 | 7/15/2014 | 45:30.9 | 0 |
| 150.756 | 19684.125 | 2.047 | 11.843 | 0.320433 | 7/15/2014 | 45:31.0 | 0 |
| 150.996 | 19684.117 | 2.051 | 11.835 | 0.348432 | 7/15/2014 | 45:31.1 | 0 |
| 151.232 | 19684.109 | 2.048 | 11.828 | 0.333605 | 7/15/2014 | 45:31.2 | 0 |
| 151.472 | 19684.102 | 2.045 | 11.821 | 0.32823 | 7/15/2014 | 45:31.3 | 0 |
| 151.888 | 19684.089 | 2.04 | 11.814 | 0.327026 | 7/15/2014 | 45:31.4 | 0 |
| 152.128 | 19684.081 | 2.038 | 11.809 | 0.29735 | 7/15/2014 | 45:31.5 | 0 |
| 152.367 | 19684.074 | 2.031 | 11.806 | 0.295547 | 7/15/2014 | 45:31.6 | 0 |
| 152.67 | 19684.065 | 2.028 | 11.804 | 0.263963 | 7/15/2014 | 45:31.7 | 0 |
| 152.789 | 19684.062 | 2.022 | 11.802 | 0.270103 | 7/15/2014 | 45:31.8 | 0 |
| 153.033 | 19684.054 | 2.016 | 11.801 | 0.268524 | 7/15/2014 | 45:31.9 | 0 |
| 153.277 | 19684.046 | 2.013 | 11.801 | 0.27631 | 7/15/2014 | 45:32.0 | 0 |
| 153.524 | 19684.039 | 2.017 | 11.801 | 0.252502 | 7/15/2014 | 45:32.1 | 0 |
| 153.769 | 19684.031 | 2.013 | 11.801 | 0.254989 | 7/15/2014 | 45:32.2 | 0 |
| 154.014 | 19684.022 | 2.007 | 11.8 | 0.239614 | 7/15/2014 | 45:32.3 | 0 |
| 154.445 | 19684.011 | 2.008 | 11.796 | 0.219539 | 7/15/2014 | 45:32.4 | 0 |
| 154.689 | 19684.003 | 2.006 | 11.792 | 0.204141 | 7/15/2014 | 45:32.5 | 0 |
| 154.934 | 19683.995 | 2.006 | 11.787 | 0.183781 | 7/15/2014 | 45:32.6 | 0 |
| 155.242 | 19683.986 | 2.006 | 11.78 | 0.180564 | 7/15/2014 | 45:32.7 | 0 |
| 155.366 | 19683.982 | 1.999 | 11.777 | 0.172574 | 7/15/2014 | 45:32.8 | 0 |
| 155.613 | 19683.975 | 1.999 | 11.769 | 0.176671 | 7/15/2014 | 45:32.9 | 0 |
| 155.86 | 19683.968 | 2.001 | 11.759 | 0.178673 | 7/15/2014 | 45:33.0 | 0 |
| 156.107 | 19683.96 | 2.001 | 11.749 | 0.176567 | 7/15/2014 | 45:33.1 | 0 |
| 156.352 | 19683.953 | 1.997 | 11.738 | 0.168056 | 7/15/2014 | 45:33.2 | 0 |
| 156.598 | 19683.945 | 2 | 11.726 | 0.17419 | 7/15/2014 | 45:33.3 | 0 |
| 157.025 | 19683.932 | 2.001 | 11.71 | 0.192007 | 7/15/2014 | 45:33.4 | 0 |
| 157.269 | 19683.926 | 2.003 | 11.699 | 0.174915 | 7/15/2014 | 45:33.5 | 0 |
| 157.512 | 19683.919 | 2.003 | 11.689 | 0.197289 | 7/15/2014 | 45:33.6 | 0 |
| 157.815 | 19683.909 | 1.999 | 11.679 | 0.192681 | 7/15/2014 | 45:33.7 | 0 |
| 157.938 | 19683.906 | 2 | 11.674 | 0.206832 | 7/15/2014 | 45:33.8 | 0 |
| 158.184 | 19683.898 | 1.997 | 11.665 | 0.206098 | 7/15/2014 | 45:33.9 | 0 |
| 158.428 | 19683.892 | 1.995 | 11.656 | 0.193544 | 7/15/2014 | 45:34.0 | 0 |
| 158.674 | 19683.884 | 1.997 | 11.648 | 0.214359 | 7/15/2014 | 45:34.1 | 0 |
| 158.918 | 19683.878 | 1.989 | 11.64 | 0.217113 | 7/15/2014 | 45:34.2 | 0 |
| 159.162 | 19683.871 | 1.978 | 11.632 | 0.180281 | 7/15/2014 | 45:34.3 | 0 |
| 159.589 | 19683.858 | 1.965 | 11.619 | 0.19294 | 7/15/2014 | 45:34.4 | 0 |
| 159.833 | 19683.852 | 1.961 | 11.61 | 0.180025 | 7/15/2014 | 45:34.5 | 0 |

| | | | | | | | |
|---|---|---|---|---|---|---|---|
| 160.077 | 19683.845 | 1.954 | 11.602 | 0.173698 | 7/15/2014 | 45:34.6 | 0 |
| 160.383 | 19683.835 | 1.947 | 11.593 | 0.16846 | 7/15/2014 | 45:34.7 | 0 |
| 160.506 | 19683.832 | 1.938 | 11.589 | 0.168026 | 7/15/2014 | 45:34.8 | 0 |
| 160.753 | 19683.825 | 1.938 | 11.58 | 0.178814 | 7/15/2014 | 45:34.9 | 0 |
| 161 | 19683.817 | 1.94 | 11.572 | 0.170166 | 7/15/2014 | 45:35.0 | 0 |
| 161.249 | 19683.812 | 1.943 | 11.564 | 0.159258 | 7/15/2014 | 45:35.1 | 0 |
| 161.499 | 19683.805 | 1.94 | 11.556 | 0.169698 | 7/15/2014 | 45:35.2 | 0 |
| 161.746 | 19683.797 | 1.934 | 11.549 | 0.180953 | 7/15/2014 | 45:35.3 | 0 |
| 162.057 | 19683.787 | 1.931 | 11.542 | 0.175697 | 7/15/2014 | 45:35.4 | 0 |
| 162.308 | 19683.78 | 1.929 | 11.535 | 0.174851 | 7/15/2014 | 45:35.5 | 0 |
| 162.56 | 19683.772 | 1.921 | 11.528 | 0.177566 | 7/15/2014 | 45:35.6 | 0 |
| 162.812 | 19683.768 | 1.917 | 11.522 | 0.176951 | 7/15/2014 | 45:35.7 | 0 |
| 163.131 | 19683.758 | 1.911 | 11.515 | 0.170575 | 7/15/2014 | 45:35.8 | 0 |
| 163.386 | 19683.75 | 1.907 | 11.508 | 0.164312 | 7/15/2014 | 45:35.9 | 0 |
| 163.644 | 19683.742 | 1.905 | 11.501 | 0.152375 | 7/15/2014 | 45:36.0 | 0 |
| 163.904 | 19683.734 | 1.906 | 11.493 | 0.159291 | 7/15/2014 | 45:36.1 | 0 |
| 164.162 | 19683.727 | 1.904 | 11.485 | 0.154384 | 7/15/2014 | 45:36.2 | 0 |
| 164.422 | 19683.721 | 1.906 | 11.477 | 0.14618 | 7/15/2014 | 45:36.3 | 0 |
| 164.746 | 19683.712 | 1.898 | 11.468 | 0.145744 | 7/15/2014 | 45:36.4 | 0 |
| 165.008 | 19683.704 | 1.892 | 11.46 | 0.135928 | 7/15/2014 | 45:36.5 | 0 |
| 165.269 | 19683.697 | 1.887 | 11.451 | 0.147393 | 7/15/2014 | 45:36.6 | 0 |
| 165.534 | 19683.689 | 1.887 | 11.444 | 0.139392 | 7/15/2014 | 45:36.7 | 0 |
| 165.862 | 19683.68 | 1.877 | 11.436 | 0.150265 | 7/15/2014 | 45:36.8 | 0 |
| 166.129 | 19683.672 | 1.875 | 11.429 | 0.147223 | 7/15/2014 | 45:36.9 | 0 |
| 166.394 | 19683.667 | 1.87 | 11.422 | 0.157606 | 7/15/2014 | 45:37.0 | 0 |
| 166.662 | 19683.659 | 1.873 | 11.416 | 0.148067 | 7/15/2014 | 45:37.1 | 0 |
| 166.928 | 19683.652 | 1.869 | 11.41 | 0.138785 | 7/15/2014 | 45:37.2 | 0 |
| 167.196 | 19683.645 | 1.865 | 11.403 | 0.147849 | 7/15/2014 | 45:37.3 | 0 |
| 167.528 | 19683.635 | 1.856 | 11.396 | 0.135531 | 7/15/2014 | 45:37.4 | 0 |
| 167.797 | 19683.629 | 1.863 | 11.388 | 0.140142 | 7/15/2014 | 45:37.5 | 0 |
| 168.063 | 19683.623 | 1.856 | 11.38 | 0.130357 | 7/15/2014 | 45:37.6 | 0 |
| 168.331 | 19683.615 | 1.85 | 11.371 | 0.14878 | 7/15/2014 | 45:37.7 | 0 |
| 168.664 | 19683.606 | 1.848 | 11.361 | 0.14811 | 7/15/2014 | 45:37.8 | 0 |
| 168.933 | 19683.6 | 1.854 | 11.35 | 0.137035 | 7/15/2014 | 45:37.9 | 0 |
| 169.201 | 19683.592 | 1.854 | 11.339 | 0.144815 | 7/15/2014 | 45:38.0 | 0 |
| 169.47 | 19683.586 | 1.854 | 11.329 | 0.134551 | 7/15/2014 | 45:38.1 | 0 |
| 169.737 | 19683.579 | 1.848 | 11.318 | 0.150866 | 7/15/2014 | 45:38.2 | 0 |
| 170.004 | 19683.572 | 1.854 | 11.307 | 0.144708 | 7/15/2014 | 45:38.3 | 0 |
| 170.335 | 19683.563 | 1.854 | 11.296 | 0.152065 | 7/15/2014 | 45:38.4 | 0 |
| 170.601 | 19683.557 | 1.85 | 11.285 | 0.15719 | 7/15/2014 | 45:38.5 | 0 |
| 170.866 | 19683.55 | 1.844 | 11.274 | 0.165432 | 7/15/2014 | 45:38.6 | 0 |
| 171.131 | 19683.542 | 1.844 | 11.263 | 0.192713 | 7/15/2014 | 45:38.7 | 0 |
| 171.461 | 19683.534 | 1.844 | 11.251 | 0.180068 | 7/15/2014 | 45:38.8 | 0 |
| 171.726 | 19683.527 | 1.844 | 11.239 | 0.200847 | 7/15/2014 | 45:38.9 | 0 |
| 171.991 | 19683.521 | 1.83 | 11.225 | 0.210233 | 7/15/2014 | 45:39.0 | 0 |
| 172.258 | 19683.514 | 1.832 | 11.21 | 0.234337 | 7/15/2014 | 45:39.1 | 0 |
| 172.523 | 19683.507 | 1.827 | 11.194 | 0.233009 | 7/15/2014 | 45:39.2 | 0 |

| | | | | | | | |
|---|---|---|---|---|---|---|---|
| 172.789 | 19683.499 | 1.827 | 11.176 | 0.227621 | 7/15/2014 | 45:39.3 | 0 |
| 173.117 | 19683.49 | 1.825 | 11.156 | 0.243588 | 7/15/2014 | 45:39.4 | 0 |
| 173.382 | 19683.482 | 1.822 | 11.133 | 0.243556 | 7/15/2014 | 45:39.5 | 0 |
| 173.646 | 19683.476 | 1.814 | 11.108 | 0.251139 | 7/15/2014 | 45:39.6 | 0 |
| 173.913 | 19683.471 | 1.816 | 11.08 | 0.254432 | 7/15/2014 | 45:39.7 | 0 |
| 174.243 | 19683.461 | 1.811 | 11.048 | 0.228423 | 7/15/2014 | 45:39.8 | 0 |
| 174.511 | 19683.453 | 1.814 | 11.014 | 0.239282 | 7/15/2014 | 45:39.9 | 0 |
| 174.776 | 19683.445 | 1.805 | 10.978 | 0.233054 | 7/15/2014 | 45:40.0 | 0 |
| 175.045 | 19683.438 | 1.801 | 10.941 | 0.250729 | 7/15/2014 | 45:40.1 | 0 |
| 175.312 | 19683.43 | 1.797 | 10.904 | 0.241898 | 7/15/2014 | 45:40.2 | 0 |
| 175.581 | 19683.424 | 1.803 | 10.868 | 0.24389 | 7/15/2014 | 45:40.3 | 0 |
| 175.912 | 19683.414 | 1.798 | 10.835 | 0.255363 | 7/15/2014 | 45:40.4 | 0 |
| 176.18 | 19683.406 | 1.798 | 10.805 | 0.258859 | 7/15/2014 | 45:40.5 | 0 |
| 176.446 | 19683.398 | 1.796 | 10.78 | 0.267966 | 7/15/2014 | 45:40.6 | 0 |
| 176.715 | 19683.391 | 1.798 | 10.759 | 0.250226 | 7/15/2014 | 45:40.7 | 0 |
| 177.048 | 19683.381 | 1.8 | 10.742 | 0.252131 | 7/15/2014 | 45:40.8 | 0 |
| 177.317 | 19683.373 | 1.803 | 10.728 | 0.251748 | 7/15/2014 | 45:40.9 | 0 |
| 177.585 | 19683.365 | 1.795 | 10.718 | 0.250689 | 7/15/2014 | 45:41.0 | 0 |
| 177.855 | 19683.356 | 1.797 | 10.71 | 0.251446 | 7/15/2014 | 45:41.1 | 0 |
| 178.124 | 19683.348 | 1.787 | 10.704 | 0.252908 | 7/15/2014 | 45:41.2 | 0 |
| 178.392 | 19683.34 | 1.78 | 10.699 | 0.255272 | 7/15/2014 | 45:41.3 | 0 |
| 178.726 | 19683.33 | 1.774 | 10.694 | 0.262768 | 7/15/2014 | 45:41.4 | 0 |
| 178.994 | 19683.322 | 1.774 | 10.69 | 0.274529 | 7/15/2014 | 45:41.5 | 0 |
| 179.262 | 19683.314 | 1.764 | 10.686 | 0.27432 | 7/15/2014 | 45:41.6 | 0 |
| 179.531 | 19683.304 | 1.77 | 10.681 | 0.271716 | 7/15/2014 | 45:41.7 | 0 |
| 179.865 | 19683.293 | 1.767 | 10.676 | 0.272766 | 7/15/2014 | 45:41.8 | 0 |
| 180.134 | 19683.285 | 1.77 | 10.67 | 0.266757 | 7/15/2014 | 45:41.9 | 0 |
| 180.403 | 19683.277 | 1.768 | 10.663 | 0.251667 | 7/15/2014 | 45:42.0 | 0 |
| 180.674 | 19683.269 | 1.774 | 10.655 | 0.253343 | 7/15/2014 | 45:42.1 | 0 |
| 180.942 | 19683.26 | 1.764 | 10.646 | 0.265141 | 7/15/2014 | 45:42.2 | 0 |
| 181.212 | 19683.25 | 1.764 | 10.636 | 0.280668 | 7/15/2014 | 45:42.3 | 0 |
| 181.546 | 19683.24 | 1.757 | 10.625 | 0.269229 | 7/15/2014 | 45:42.4 | 0 |
| 181.816 | 19683.231 | 1.76 | 10.614 | 0.295327 | 7/15/2014 | 45:42.5 | 0 |
| 182.081 | 19683.223 | 1.753 | 10.601 | 0.324062 | 7/15/2014 | 45:42.6 | 0 |
| 182.348 | 19683.214 | 1.75 | 10.587 | 0.308676 | 7/15/2014 | 45:42.7 | 0 |
| 182.681 | 19683.203 | 1.743 | 10.573 | 0.283377 | 7/15/2014 | 45:42.8 | 0 |
| 182.948 | 19683.194 | 1.746 | 10.557 | 0.31665 | 7/15/2014 | 45:42.9 | 0 |
| 183.216 | 19683.186 | 1.742 | 10.54 | 0.362235 | 7/15/2014 | 45:43.0 | 0 |
| 170.628 | 19520.615 | 1.714 | 13.698 | 0.387011 | 7/15/2014 | 53:46.7 | 1 |
| 170.316 | 19520.783 | 1.722 | 13.683 | 0.385316 | 7/15/2014 | 53:46.8 | 1 |
| 170.191 | 19520.852 | 1.726 | 13.674 | 0.383009 | 7/15/2014 | 53:46.8 | 1 |
| 169.94 | 19520.984 | 1.709 | 13.667 | 0.393423 | 7/15/2014 | 53:46.9 | 1 |
| 169.687 | 19521.118 | 1.705 | 13.667 | 0.394756 | 7/15/2014 | 53:47.0 | 1 |
| 169.433 | 19521.251 | 1.712 | 13.674 | 0.417953 | 7/15/2014 | 53:47.1 | 1 |
| 169.178 | 19521.385 | 1.709 | 13.688 | 0.423205 | 7/15/2014 | 53:47.2 | 1 |
| 168.73 | 19521.615 | 1.702 | 13.721 | 0.449547 | 7/15/2014 | 53:47.4 | 1 |
| 168.474 | 19521.747 | 1.699 | 13.747 | 0.445362 | 7/15/2014 | 53:47.5 | 1 |

| | | | | | | | |
|---|---|---|---|---|---|---|---|
| 168.216 | 19521.878 | 1.699 | 13.776 | 0.456412 | 7/15/2014 | 53:47.6 | 1 |
| 167.958 | 19522.008 | 1.715 | 13.807 | 0.446993 | 7/15/2014 | 53:47.7 | 1 |
| 167.635 | 19522.17 | 1.709 | 13.838 | 0.445315 | 7/15/2014 | 53:47.8 | 1 |
| 167.505 | 19522.235 | 1.704 | 13.854 | 0.446657 | 7/15/2014 | 53:47.8 | 1 |
| 167.243 | 19522.364 | 1.701 | 13.885 | 0.429675 | 7/15/2014 | 53:47.9 | 1 |
| 166.983 | 19522.494 | 1.701 | 13.912 | 0.421043 | 7/15/2014 | 53:48.0 | 1 |
| 166.719 | 19522.623 | 1.691 | 13.937 | 0.435314 | 7/15/2014 | 53:48.1 | 1 |
| 166.457 | 19522.749 | 1.701 | 13.957 | 0.426519 | 7/15/2014 | 53:48.2 | 1 |
| 165.995 | 19522.971 | 1.7 | 13.978 | 0.438518 | 7/15/2014 | 53:48.4 | 1 |
| 165.73 | 19523.097 | 1.71 | 13.987 | 0.431945 | 7/15/2014 | 53:48.5 | 1 |
| 165.465 | 19523.223 | 1.704 | 13.992 | 0.444955 | 7/15/2014 | 53:48.6 | 1 |
| 165.197 | 19523.35 | 1.708 | 13.992 | 0.446706 | 7/15/2014 | 53:48.7 | 1 |
| 164.863 | 19523.504 | 1.716 | 13.988 | 0.449794 | 7/15/2014 | 53:48.8 | 1 |
| 164.728 | 19523.568 | 1.716 | 13.986 | 0.451875 | 7/15/2014 | 53:48.8 | 1 |
| 164.457 | 19523.693 | 1.712 | 13.977 | 0.446086 | 7/15/2014 | 53:48.9 | 1 |
| 164.187 | 19523.816 | 1.716 | 13.965 | 0.445671 | 7/15/2014 | 53:49.0 | 1 |
| 163.914 | 19523.941 | 1.719 | 13.953 | 0.453499 | 7/15/2014 | 53:49.1 | 1 |
| 163.639 | 19524.066 | 1.715 | 13.943 | 0.453427 | 7/15/2014 | 53:49.2 | 1 |
| 163.299 | 19524.219 | 1.718 | 13.936 | 0.452205 | 7/15/2014 | 53:49.3 | 1 |
| 163.022 | 19524.344 | 1.704 | 13.935 | 0.450916 | 7/15/2014 | 53:49.4 | 1 |
| 162.746 | 19524.465 | 1.697 | 13.94 | 0.447043 | 7/15/2014 | 53:49.5 | 1 |
| 162.468 | 19524.588 | 1.675 | 13.954 | 0.444914 | 7/15/2014 | 53:49.6 | 1 |
| 162.189 | 19524.711 | 1.682 | 13.974 | 0.461187 | 7/15/2014 | 53:49.7 | 1 |
| 161.841 | 19524.86 | 1.682 | 14.002 | 0.444687 | 7/15/2014 | 53:49.8 | 1 |
| 161.56 | 19524.982 | 1.675 | 14.034 | 0.448578 | 7/15/2014 | 53:49.9 | 1 |
| 161.28 | 19525.103 | 1.663 | 14.071 | 0.44763 | 7/15/2014 | 53:50.0 | 1 |
| 160.997 | 19525.224 | 1.666 | 14.109 | 0.450269 | 7/15/2014 | 53:50.1 | 1 |
| 160.714 | 19525.344 | 1.673 | 14.148 | 0.447188 | 7/15/2014 | 53:50.2 | 1 |
| 160.361 | 19525.493 | 1.663 | 14.185 | 0.434282 | 7/15/2014 | 53:50.3 | 1 |
| 160.077 | 19525.611 | 1.654 | 14.221 | 0.4273 | 7/15/2014 | 53:50.4 | 1 |
| 159.793 | 19525.729 | 1.644 | 14.253 | 0.418643 | 7/15/2014 | 53:50.5 | 1 |
| 159.507 | 19525.849 | 1.657 | 14.282 | 0.424326 | 7/15/2014 | 53:50.6 | 1 |
| 159.222 | 19525.966 | 1.657 | 14.308 | 0.402949 | 7/15/2014 | 53:50.7 | 1 |
| 158.868 | 19526.11 | 1.657 | 14.33 | 0.416759 | 7/15/2014 | 53:50.8 | 1 |
| 158.585 | 19526.225 | 1.626 | 14.348 | 0.394574 | 7/15/2014 | 53:50.9 | 1 |
| 158.3 | 19526.34 | 1.629 | 14.361 | 0.37945 | 7/15/2014 | 53:51.0 | 1 |
| 158.017 | 19526.454 | 1.627 | 14.369 | 0.385421 | 7/15/2014 | 53:51.1 | 1 |
| 157.734 | 19526.567 | 1.62 | 14.372 | 0.387423 | 7/15/2014 | 53:51.2 | 1 |
| 157.382 | 19526.706 | 1.589 | 14.37 | 0.392052 | 7/15/2014 | 53:51.3 | 1 |
| 157.101 | 19526.817 | 1.565 | 14.363 | 0.396861 | 7/15/2014 | 53:51.4 | 1 |
| 156.818 | 19526.929 | 1.554 | 14.351 | 0.435849 | 7/15/2014 | 53:51.5 | 1 |
| 156.537 | 19527.038 | 1.553 | 14.336 | 0.390253 | 7/15/2014 | 53:51.6 | 1 |
| 156.255 | 19527.146 | 1.541 | 14.319 | 0.432331 | 7/15/2014 | 53:51.7 | 1 |
| 155.903 | 19527.281 | 1.531 | 14.302 | 0.416837 | 7/15/2014 | 53:51.8 | 1 |
| 155.621 | 19527.39 | 1.528 | 14.286 | 0.415673 | 7/15/2014 | 53:51.9 | 1 |
| 155.339 | 19527.496 | 1.549 | 14.272 | 0.403244 | 7/15/2014 | 53:52.0 | 1 |
| 155.057 | 19527.603 | 1.539 | 14.262 | 0.434568 | 7/15/2014 | 53:52.1 | 1 |

| | | | | | | | |
|---|---|---|---|---|---|---|---|
| 154.773 | 19527.708 | 1.535 | 14.255 | 0.411877 | 7/15/2014 | 53:52.2 | 1 |
| 154.421 | 19527.84 | 1.525 | 14.252 | 0.417013 | 7/15/2014 | 53:52.3 | 1 |
| 154.14 | 19527.945 | 1.505 | 14.253 | 0.412714 | 7/15/2014 | 53:52.4 | 1 |
| 153.857 | 19528.048 | 1.51 | 14.257 | 0.427173 | 7/15/2014 | 53:52.5 | 1 |
| 153.575 | 19528.151 | 1.503 | 14.265 | 0.42202 | 7/15/2014 | 53:52.6 | 1 |
| 153.088 | 19528.327 | 1.517 | 14.283 | 0.423857 | 7/15/2014 | 53:52.8 | 1 |
| 152.945 | 19528.379 | 1.508 | 14.288 | 0.420968 | 7/15/2014 | 53:52.8 | 1 |
| 152.666 | 19528.48 | 1.497 | 14.303 | 0.428516 | 7/15/2014 | 53:52.9 | 1 |
| 152.387 | 19528.579 | 1.514 | 14.319 | 0.442249 | 7/15/2014 | 53:53.0 | 1 |
| 152.11 | 19528.678 | 1.507 | 14.337 | 0.44323 | 7/15/2014 | 53:53.1 | 1 |
| 151.834 | 19528.777 | 1.494 | 14.355 | 0.454884 | 7/15/2014 | 53:53.2 | 1 |
| 151.354 | 19528.944 | 1.504 | 14.384 | 0.481208 | 7/15/2014 | 53:53.4 | 1 |
| 151.081 | 19529.04 | 1.492 | 14.405 | 0.473157 | 7/15/2014 | 53:53.5 | 1 |
| 150.809 | 19529.134 | 1.505 | 14.426 | 0.480201 | 7/15/2014 | 53:53.6 | 1 |
| 150.536 | 19529.227 | 1.499 | 14.447 | 0.471774 | 7/15/2014 | 53:53.7 | 1 |
| 150.197 | 19529.343 | 1.487 | 14.47 | 0.461008 | 7/15/2014 | 53:53.8 | 1 |
| 150.059 | 19529.39 | 1.489 | 14.481 | 0.476014 | 7/15/2014 | 53:53.8 | 1 |
| 149.787 | 19529.483 | 1.479 | 14.504 | 0.457078 | 7/15/2014 | 53:53.9 | 1 |
| 149.515 | 19529.575 | 1.481 | 14.527 | 0.453507 | 7/15/2014 | 53:54.0 | 1 |
| 149.241 | 19529.666 | 1.501 | 14.551 | 0.457542 | 7/15/2014 | 53:54.1 | 1 |
| 148.968 | 19529.756 | 1.504 | 14.574 | 0.444272 | 7/15/2014 | 53:54.2 | 1 |
| 148.489 | 19529.913 | 1.505 | 14.606 | 0.434108 | 7/15/2014 | 53:54.4 | 1 |
| 148.216 | 19530.003 | 1.529 | 14.628 | 0.445154 | 7/15/2014 | 53:54.5 | 1 |
| 147.945 | 19530.091 | 1.525 | 14.648 | 0.459441 | 7/15/2014 | 53:54.6 | 1 |
| 147.67 | 19530.179 | 1.517 | 14.668 | 0.409581 | 7/15/2014 | 53:54.7 | 1 |
| 147.33 | 19530.287 | 1.502 | 14.688 | 0.409844 | 7/15/2014 | 53:54.8 | 1 |
| 147.191 | 19530.333 | 1.496 | 14.697 | 0.406851 | 7/15/2014 | 53:54.8 | 1 |
| 146.917 | 19530.419 | 1.483 | 14.716 | 0.417501 | 7/15/2014 | 53:54.9 | 1 |
| 146.645 | 19530.504 | 1.49 | 14.733 | 0.400829 | 7/15/2014 | 53:55.0 | 1 |
| 146.372 | 19530.589 | 1.459 | 14.748 | 0.385542 | 7/15/2014 | 53:55.1 | 1 |
| 146.101 | 19530.673 | 1.435 | 14.762 | 0.410548 | 7/15/2014 | 53:55.2 | 1 |
| 145.628 | 19530.817 | 1.44 | 14.778 | 0.411516 | 7/15/2014 | 53:55.4 | 1 |
| 145.358 | 19530.898 | 1.448 | 14.787 | 0.410319 | 7/15/2014 | 53:55.5 | 1 |
| 145.09 | 19530.979 | 1.448 | 14.793 | 0.403371 | 7/15/2014 | 53:55.6 | 1 |
| 144.821 | 19531.059 | 1.44 | 14.797 | 0.420649 | 7/15/2014 | 53:55.7 | 1 |
| 144.491 | 19531.156 | 1.448 | 14.799 | 0.388774 | 7/15/2014 | 53:55.8 | 1 |
| 144.354 | 19531.197 | 1.472 | 14.8 | 0.397552 | 7/15/2014 | 53:55.8 | 1 |
| 144.088 | 19531.274 | 1.465 | 14.799 | 0.398062 | 7/15/2014 | 53:55.9 | 1 |
| 143.824 | 19531.351 | 1.448 | 14.798 | 0.392488 | 7/15/2014 | 53:56.0 | 1 |
| 143.557 | 19531.427 | 1.431 | 14.796 | 0.39975 | 7/15/2014 | 53:56.1 | 1 |
| 143.293 | 19531.5 | 1.416 | 14.794 | 0.385005 | 7/15/2014 | 53:56.2 | 1 |
| 142.833 | 19531.629 | 1.422 | 14.796 | 0.401844 | 7/15/2014 | 53:56.4 | 1 |
| 142.568 | 19531.701 | 1.412 | 14.799 | 0.405222 | 7/15/2014 | 53:56.5 | 1 |
| 142.306 | 19531.773 | 1.404 | 14.804 | 0.406894 | 7/15/2014 | 53:56.6 | 1 |
| 142.041 | 19531.845 | 1.406 | 14.812 | 0.38004 | 7/15/2014 | 53:56.7 | 1 |
| 141.71 | 19531.932 | 1.402 | 14.822 | 0.37435 | 7/15/2014 | 53:56.8 | 1 |
| 141.577 | 19531.969 | 1.405 | 14.827 | 0.358852 | 7/15/2014 | 53:56.8 | 1 |

| | | | | | | | |
|---|---|---|---|---|---|---|---|
| 141.311 | 19532.038 | 1.403 | 14.841 | 0.367937 | 7/15/2014 | 53:56.9 | 1 |
| 141.043 | 19532.107 | 1.396 | 14.858 | 0.346693 | 7/15/2014 | 53:57.0 | 1 |
| 140.774 | 19532.178 | 1.4 | 14.876 | 0.362352 | 7/15/2014 | 53:57.1 | 1 |
| 140.504 | 19532.246 | 1.403 | 14.895 | 0.379624 | 7/15/2014 | 53:57.2 | 1 |
| 140.029 | 19532.364 | 1.397 | 14.927 | 0.384857 | 7/15/2014 | 53:57.4 | 1 |
| 139.757 | 19532.433 | 1.383 | 14.949 | 0.387279 | 7/15/2014 | 53:57.5 | 1 |
| 139.484 | 19532.5 | 1.385 | 14.972 | 0.394293 | 7/15/2014 | 53:57.6 | 1 |
| 139.211 | 19532.566 | 1.389 | 14.995 | 0.364958 | 7/15/2014 | 53:57.7 | 1 |
| 138.868 | 19532.647 | 1.38 | 15.02 | 0.34646 | 7/15/2014 | 53:57.8 | 1 |
| 138.729 | 19532.682 | 1.383 | 15.032 | 0.341091 | 7/15/2014 | 53:57.8 | 1 |
| 138.453 | 19532.747 | 1.408 | 15.058 | 0.351475 | 7/15/2014 | 53:57.9 | 1 |
| 138.177 | 19532.813 | 1.397 | 15.083 | 0.353257 | 7/15/2014 | 53:58.0 | 1 |
| 137.898 | 19532.877 | 1.401 | 15.107 | 0.313504 | 7/15/2014 | 53:58.1 | 1 |
| 137.622 | 19532.939 | 1.384 | 15.129 | 0.353962 | 7/15/2014 | 53:58.2 | 1 |
| 137.134 | 19533.05 | 1.385 | 15.154 | 0.366328 | 7/15/2014 | 53:58.4 | 1 |
| 136.855 | 19533.113 | 1.391 | 15.166 | 0.322049 | 7/15/2014 | 53:58.5 | 1 |
| 136.578 | 19533.174 | 1.371 | 15.173 | 0.319861 | 7/15/2014 | 53:58.6 | 1 |
| 136.298 | 19533.235 | 1.36 | 15.175 | 0.341308 | 7/15/2014 | 53:58.7 | 1 |
| 135.95 | 19533.311 | 1.366 | 15.17 | 0.312912 | 7/15/2014 | 53:58.8 | 1 |
| 135.807 | 19533.342 | 1.366 | 15.168 | 0.316403 | 7/15/2014 | 53:58.8 | 1 |
| 135.527 | 19533.402 | 1.356 | 15.154 | 0.31012 | 7/15/2014 | 53:58.9 | 1 |
| 135.246 | 19533.463 | 1.359 | 15.134 | 0.311954 | 7/15/2014 | 53:59.0 | 1 |
| 134.964 | 19533.521 | 1.347 | 15.109 | 0.307213 | 7/15/2014 | 53:59.1 | 1 |
| 134.684 | 19533.58 | 1.339 | 15.081 | 0.303641 | 7/15/2014 | 53:59.2 | 1 |
| 134.19 | 19533.682 | 1.336 | 15.036 | 0.288849 | 7/15/2014 | 53:59.4 | 1 |
| 133.909 | 19533.738 | 1.336 | 15.007 | 0.310511 | 7/15/2014 | 53:59.5 | 1 |
| 133.628 | 19533.795 | 1.339 | 14.98 | 0.290276 | 7/15/2014 | 53:59.6 | 1 |
| 133.346 | 19533.852 | 1.339 | 14.957 | 0.303966 | 7/15/2014 | 53:59.7 | 1 |
| 132.996 | 19533.92 | 1.349 | 14.939 | 0.270452 | 7/15/2014 | 53:59.8 | 1 |
| 132.853 | 19533.948 | 1.357 | 14.929 | 0.260376 | 7/15/2014 | 53:59.8 | 1 |
| 132.571 | 19534.004 | 1.378 | 14.919 | 0.271007 | 7/15/2014 | 53:59.9 | 1 |
| 132.289 | 19534.059 | 1.392 | 14.916 | 0.259158 | 7/15/2014 | 54:00.0 | 1 |
| 132.008 | 19534.111 | 1.388 | 14.92 | 0.277112 | 7/15/2014 | 54:00.1 | 1 |
| 131.727 | 19534.165 | 1.388 | 14.93 | 0.228398 | 7/15/2014 | 54:00.2 | 1 |
| 131.234 | 19534.257 | 1.376 | 14.961 | 0.264944 | 7/15/2014 | 54:00.4 | 1 |
| 130.953 | 19534.309 | 1.379 | 14.986 | 0.27 | 7/15/2014 | 54:00.5 | 1 |
| 130.673 | 19534.36 | 1.361 | 15.016 | 0.250359 | 7/15/2014 | 54:00.6 | 1 |
| 130.392 | 19534.41 | 1.358 | 15.049 | 0.236577 | 7/15/2014 | 54:00.7 | 1 |
| 130.043 | 19534.473 | 1.362 | 15.085 | 0.241902 | 7/15/2014 | 54:00.8 | 1 |
| 129.9 | 19534.499 | 1.361 | 15.103 | 0.203745 | 7/15/2014 | 54:00.8 | 1 |
| 129.619 | 19534.549 | 1.357 | 15.141 | 0.217643 | 7/15/2014 | 54:00.9 | 1 |
| 129.338 | 19534.598 | 1.368 | 15.179 | 0.16938 | 7/15/2014 | 54:01.0 | 1 |
| 129.056 | 19534.647 | 1.354 | 15.215 | 0.135658 | 7/15/2014 | 54:01.1 | 1 |
| 128.776 | 19534.695 | 1.364 | 15.247 | 0.216696 | 7/15/2014 | 54:01.2 | 1 |
| 128.285 | 19534.777 | 1.362 | 15.283 | 0.194583 | 7/15/2014 | 54:01.4 | 1 |
| 128.005 | 19534.824 | 1.368 | 15.3 | 0.205377 | 7/15/2014 | 54:01.5 | 1 |
| 127.727 | 19534.871 | 1.356 | 15.311 | 0.212274 | 7/15/2014 | 54:01.6 | 1 |

| | | | | | | | |
|---|---|---|---|---|---|---|---|
| 127.446 | 19534.918 | 1.341 | 15.315 | 0.239197 | 7/15/2014 | 54:01.7 | 1 |
| 127.101 | 19534.975 | 1.344 | 15.313 | 0.15347 | 7/15/2014 | 54:01.8 | 1 |
| 126.958 | 19534.998 | 1.34 | 15.313 | 0.201718 | 7/15/2014 | 54:01.8 | 1 |
| 126.679 | 19535.042 | 1.35 | 15.302 | 0.18105 | 7/15/2014 | 54:01.9 | 1 |
| 126.401 | 19535.086 | 1.339 | 15.287 | 0.134848 | 7/15/2014 | 54:02.0 | 1 |
| 126.123 | 19535.131 | 1.325 | 15.269 | 0.135173 | 7/15/2014 | 54:02.1 | 1 |
| 125.845 | 19535.174 | 1.335 | 15.252 | 0.140431 | 7/15/2014 | 54:02.2 | 1 |
| 125.36 | 19535.248 | 1.344 | 15.233 | 0.117545 | 7/15/2014 | 54:02.4 | 1 |
| 125.081 | 19535.291 | 1.338 | 15.224 | 0.130279 | 7/15/2014 | 54:02.5 | 1 |
| 124.804 | 19535.333 | 1.326 | 15.22 | 0.153126 | 7/15/2014 | 54:02.6 | 1 |
| 124.524 | 19535.375 | 1.326 | 15.221 | 0.147938 | 7/15/2014 | 54:02.7 | 1 |
| 124.177 | 19535.426 | 1.334 | 15.227 | 0.134507 | 7/15/2014 | 54:02.8 | 1 |
| 124.036 | 19535.447 | 1.333 | 15.23 | 0.141363 | 7/15/2014 | 54:02.8 | 1 |
| 123.755 | 19535.487 | 1.347 | 15.243 | 0.176693 | 7/15/2014 | 54:02.9 | 1 |
| 123.473 | 19535.529 | 1.337 | 15.258 | 0.162868 | 7/15/2014 | 54:03.0 | 1 |
| 123.19 | 19535.569 | 1.346 | 15.273 | 0.161779 | 7/15/2014 | 54:03.1 | 1 |
| 122.906 | 19535.609 | 1.353 | 15.286 | 0.181575 | 7/15/2014 | 54:03.2 | 1 |
| 122.408 | 19535.678 | 1.364 | 15.298 | 0.188708 | 7/15/2014 | 54:03.4 | 1 |
| 122.124 | 19535.716 | 1.355 | 15.301 | 0.157144 | 7/15/2014 | 54:03.5 | 1 |
| 121.84 | 19535.756 | 1.351 | 15.3 | 0.16577 | 7/15/2014 | 54:03.6 | 1 |
| 121.554 | 19535.793 | 1.369 | 15.295 | 0.174743 | 7/15/2014 | 54:03.7 | 1 |
| 121.198 | 19535.84 | 1.368 | 15.285 | 0.158873 | 7/15/2014 | 54:03.8 | 1 |
| 121.053 | 19535.859 | 1.368 | 15.281 | 0.172183 | 7/15/2014 | 54:03.8 | 1 |
| 120.767 | 19535.896 | 1.35 | 15.268 | 0.152636 | 7/15/2014 | 54:03.9 | 1 |
| 120.481 | 19535.932 | 1.357 | 15.255 | 0.188541 | 7/15/2014 | 54:04.0 | 1 |
| 120.195 | 19535.969 | 1.361 | 15.243 | 0.18894 | 7/15/2014 | 54:04.1 | 1 |
| 119.909 | 19536.004 | 1.356 | 15.236 | 0.146781 | 7/15/2014 | 54:04.2 | 1 |
| 119.409 | 19536.064 | 1.332 | 15.237 | 0.15261 | 7/15/2014 | 54:04.4 | 1 |
| 119.123 | 19536.098 | 1.329 | 15.244 | 0.180988 | 7/15/2014 | 54:04.5 | 1 |
| 118.84 | 19536.133 | 1.35 | 15.257 | 0.130203 | 7/15/2014 | 54:04.6 | 1 |
| 118.556 | 19536.166 | 1.346 | 15.275 | 0.121696 | 7/15/2014 | 54:04.7 | 1 |
| 118.203 | 19536.206 | 1.352 | 15.299 | 0.153494 | 7/15/2014 | 54:04.8 | 1 |
| 118.059 | 19536.222 | 1.354 | 15.31 | 0.133837 | 7/15/2014 | 54:04.8 | 1 |
| 117.776 | 19536.255 | 1.376 | 15.341 | 0.128441 | 7/15/2014 | 54:04.9 | 1 |
| 117.493 | 19536.285 | 1.38 | 15.374 | 0.107151 | 7/15/2014 | 54:05.0 | 1 |
| 117.213 | 19536.318 | 1.376 | 15.408 | 0.115858 | 7/15/2014 | 54:05.1 | 1 |
| 116.933 | 19536.348 | 1.373 | 15.443 | 0.109602 | 7/15/2014 | 54:05.2 | 1 |
| 116.445 | 19536.399 | 1.352 | 15.494 | 0.0824143 | 7/15/2014 | 54:05.4 | 1 |
| 116.168 | 19536.429 | 1.352 | 15.528 | 0.109828 | 7/15/2014 | 54:05.5 | 1 |
| 115.89 | 19536.457 | 1.338 | 15.56 | 0.0788347 | 7/15/2014 | 54:05.6 | 1 |
| 115.613 | 19536.486 | 1.335 | 15.592 | 0.0994194 | 7/15/2014 | 54:05.7 | 1 |
| 115.268 | 19536.52 | 1.335 | 15.622 | 0.102067 | 7/15/2014 | 54:05.8 | 1 |
| 115.127 | 19536.534 | 1.331 | 15.638 | 0.128874 | 7/15/2014 | 54:05.8 | 1 |
| 114.85 | 19536.563 | 1.346 | 15.667 | 0.107722 | 7/15/2014 | 54:05.9 | 1 |
| 114.572 | 19536.589 | 1.342 | 15.697 | 0.104268 | 7/15/2014 | 54:06.0 | 1 |
| 114.292 | 19536.615 | 1.339 | 15.727 | 0.136753 | 7/15/2014 | 54:06.1 | 1 |
| 114.013 | 19536.643 | 1.353 | 15.756 | 0.108082 | 7/15/2014 | 54:06.2 | 1 |

| | | | | | | | |
|---|---|---|---|---|---|---|---|
| 113.525 | 19536.688 | 1.347 | 15.8 | 0.110237 | 7/15/2014 | 54:06.4 | 1 |
| 113.244 | 19536.713 | 1.345 | 15.828 | 0.134274 | 7/15/2014 | 54:06.5 | 1 |
| 112.963 | 19536.737 | 1.349 | 15.856 | 0.141493 | 7/15/2014 | 54:06.6 | 1 |
| 112.681 | 19536.762 | 1.338 | 15.882 | 0.126164 | 7/15/2014 | 54:06.7 | 1 |
| 112.33 | 19536.793 | 1.317 | 15.908 | 0.171652 | 7/15/2014 | 54:06.8 | 1 |
| 112.188 | 19536.805 | 1.302 | 15.921 | 0.175108 | 7/15/2014 | 54:06.8 | 1 |
| 111.904 | 19536.83 | 1.307 | 15.943 | 0.139779 | 7/15/2014 | 54:06.9 | 1 |
| 111.62 | 19536.854 | 1.294 | 15.965 | 0.131008 | 7/15/2014 | 54:07.0 | 1 |
| 111.335 | 19536.877 | 1.291 | 15.985 | 0.13411 | 7/15/2014 | 54:07.1 | 1 |
| 111.051 | 19536.9 | 1.253 | 16.003 | 0.154882 | 7/15/2014 | 54:07.2 | 1 |
| 110.553 | 19536.939 | 1.26 | 16.029 | 0.126465 | 7/15/2014 | 54:07.4 | 1 |
| 110.268 | 19536.962 | 1.274 | 16.045 | 0.138256 | 7/15/2014 | 54:07.5 | 1 |
| 109.984 | 19536.984 | 1.259 | 16.062 | 0.175516 | 7/15/2014 | 54:07.6 | 1 |
| 109.699 | 19537.006 | 1.27 | 16.078 | 0.159203 | 7/15/2014 | 54:07.7 | 1 |
| 109.344 | 19537.032 | 1.268 | 16.095 | 0.176907 | 7/15/2014 | 54:07.8 | 1 |
| 109.2 | 19537.043 | 1.283 | 16.103 | 0.207109 | 7/15/2014 | 54:07.8 | 1 |
| 108.914 | 19537.063 | 1.283 | 16.12 | 0.188649 | 7/15/2014 | 54:07.9 | 1 |
| 108.628 | 19537.084 | 1.25 | 16.137 | 0.219341 | 7/15/2014 | 54:08.0 | 1 |
| 108.342 | 19537.104 | 1.237 | 16.154 | 0.22979 | 7/15/2014 | 54:08.1 | 1 |
| 108.056 | 19537.123 | 1.226 | 16.171 | 0.236081 | 7/15/2014 | 54:08.2 | 1 |
| 107.556 | 19537.155 | 1.211 | 16.195 | 0.221117 | 7/15/2014 | 54:08.4 | 1 |
| 107.27 | 19537.175 | 1.211 | 16.21 | 0.186513 | 7/15/2014 | 54:08.5 | 1 |
| 106.987 | 19537.192 | 1.198 | 16.223 | 0.185502 | 7/15/2014 | 54:08.6 | 1 |
| 106.701 | 19537.211 | 1.223 | 16.236 | 0.145807 | 7/15/2014 | 54:08.7 | 1 |
| 106.347 | 19537.232 | 1.219 | 16.246 | 0.150998 | 7/15/2014 | 54:08.8 | 1 |
| 106.201 | 19537.242 | 1.223 | 16.252 | 0.146631 | 7/15/2014 | 54:08.8 | 1 |
| 105.918 | 19537.257 | 1.223 | 16.26 | 0.148481 | 7/15/2014 | 54:08.9 | 1 |
| 105.634 | 19537.275 | 1.212 | 16.266 | 0.130721 | 7/15/2014 | 54:09.0 | 1 |
| 105.351 | 19537.29 | 1.193 | 16.27 | 0.129465 | 7/15/2014 | 54:09.1 | 1 |
| 105.07 | 19537.306 | 1.179 | 16.272 | 0.123552 | 7/15/2014 | 54:09.2 | 1 |
| 104.58 | 19537.334 | 1.164 | 16.269 | 0.130167 | 7/15/2014 | 54:09.4 | 1 |
| 104.3 | 19537.347 | 1.156 | 16.265 | 0.0843906 | 7/15/2014 | 54:09.5 | 1 |
| 104.022 | 19537.363 | 1.132 | 16.259 | 0.0933228 | 7/15/2014 | 54:09.6 | 1 |
| 103.744 | 19537.376 | 1.133 | 16.251 | 0.079548 | 7/15/2014 | 54:09.7 | 1 |
| 103.399 | 19537.393 | 1.135 | 16.243 | 0.07321 | 7/15/2014 | 54:09.8 | 1 |
| 103.258 | 19537.4 | 1.142 | 16.238 | 0.0702991 | 7/15/2014 | 54:09.8 | 1 |
| 102.981 | 19537.414 | 1.128 | 16.228 | 0.0646983 | 7/15/2014 | 54:09.9 | 1 |
| 102.703 | 19537.426 | 1.11 | 16.218 | 0.0669426 | 7/15/2014 | 54:10.0 | 1 |
| 102.426 | 19537.439 | 1.086 | 16.209 | 0.054911 | 7/15/2014 | 54:10.1 | 1 |
| 102.148 | 19537.451 | 1.086 | 16.2 | 0.0578569 | 7/15/2014 | 54:10.2 | 1 |
| 101.663 | 19537.471 | 1.05 | 16.188 | 0.0695778 | 7/15/2014 | 54:10.4 | 1 |
| 101.385 | 19537.482 | 1.044 | 16.181 | 0.0549243 | 7/15/2014 | 54:10.5 | 1 |
| 101.108 | 19537.494 | 1.038 | 16.175 | 0.0493211 | 7/15/2014 | 54:10.6 | 1 |
| 100.83 | 19537.504 | 1.054 | 16.17 | 0.0468638 | 7/15/2014 | 54:10.7 | 1 |
| 100.481 | 19537.517 | 1.06 | 16.164 | 0.0612902 | 7/15/2014 | 54:10.8 | 1 |
| 100.341 | 19537.521 | 1.062 | 16.162 | 0.0939124 | 7/15/2014 | 54:10.8 | 1 |
| 100.06 | 19537.53 | 1.071 | 16.156 | 0.0739667 | 7/15/2014 | 54:10.9 | 1 |

| | | | | | | | |
|---|---|---|---|---|---|---|---|
| 99.778 | 19537.541 | 1.083 | 16.149 | 0.090668 | 7/15/2014 | 54:11.0 | 1 |
| 99.496 | 19537.551 | 1.073 | 16.14 | 0.0731895 | 7/15/2014 | 54:11.1 | 1 |
| 99.213 | 19537.559 | 1.053 | 16.129 | 0.113997 | 7/15/2014 | 54:11.2 | 1 |
| 98.717 | 19537.572 | 1.048 | 16.109 | 0.0908207 | 7/15/2014 | 54:11.4 | 1 |
| 98.433 | 19537.579 | 1.054 | 16.094 | 0.0943835 | 7/15/2014 | 54:11.5 | 1 |
| 98.15 | 19537.588 | 1.049 | 16.078 | 0.090083 | 7/15/2014 | 54:11.6 | 1 |
| 97.865 | 19537.595 | 1.043 | 16.061 | 0.0961021 | 7/15/2014 | 54:11.7 | 1 |
| 97.512 | 19537.603 | 1.052 | 16.044 | 0.0638701 | 7/15/2014 | 54:11.8 | 1 |
| 97.368 | 19537.606 | 1.065 | 16.036 | 0.0501929 | 7/15/2014 | 54:11.8 | 1 |
| 97.083 | 19537.612 | 1.07 | 16.019 | 0.0412613 | 7/15/2014 | 54:11.9 | 1 |
| 96.798 | 19537.617 | 1.08 | 16.003 | 0.0231452 | 7/15/2014 | 54:12.0 | 1 |
| 96.514 | 19537.623 | 1.087 | 15.988 | 0.0363525 | 7/15/2014 | 54:12.1 | 1 |
| 96.23 | 19537.628 | 1.104 | 15.975 | 0.0495226 | 7/15/2014 | 54:12.2 | 1 |
| 95.734 | 19537.636 | 1.109 | 15.96 | 0.120891 | 7/15/2014 | 54:12.4 | 1 |
| 95.45 | 19537.64 | 1.103 | 15.953 | 0.109122 | 7/15/2014 | 54:12.5 | 1 |
| 95.168 | 19537.643 | 1.097 | 15.948 | 0.0977744 | 7/15/2014 | 54:12.6 | 1 |
| 94.884 | 19537.646 | 1.085 | 15.946 | 0.124267 | 7/15/2014 | 54:12.7 | 1 |
| 94.531 | 19537.648 | 1.072 | 15.946 | 0.0987803 | 7/15/2014 | 54:12.8 | 1 |
| 94.387 | 19537.65 | 1.065 | 15.946 | 0.0985606 | 7/15/2014 | 54:12.8 | 1 |
| 94.103 | 19537.653 | 1.043 | 15.951 | 0.11278 | 7/15/2014 | 54:12.9 | 1 |
| 93.819 | 19537.654 | 1.049 | 15.957 | 0.114393 | 7/15/2014 | 54:13.0 | 1 |
| 93.534 | 19537.656 | 1.042 | 15.965 | 0.139424 | 7/15/2014 | 54:13.1 | 1 |
| 93.25 | 19537.656 | 1.032 | 15.973 | 0.187423 | 7/15/2014 | 54:13.2 | 1 |
| 92.752 | 19537.656 | 1.061 | 15.987 | 0.214733 | 7/15/2014 | 54:13.4 | 1 |
| 92.467 | 19537.656 | 1.067 | 15.996 | 0.208895 | 7/15/2014 | 54:13.5 | 1 |
| 92.184 | 19537.655 | 1.069 | 16.004 | 0.227268 | 7/15/2014 | 54:13.6 | 1 |
| 91.897 | 19537.654 | 1.058 | 16.012 | 0.234669 | 7/15/2014 | 54:13.7 | 1 |
| 91.542 | 19537.651 | 1.049 | 16.018 | 0.14836 | 7/15/2014 | 54:13.8 | 1 |
| 91.398 | 19537.65 | 1.054 | 16.022 | 0.147597 | 7/15/2014 | 54:13.8 | 1 |
| 91.111 | 19537.648 | 1.03 | 16.027 | 0.116053 | 7/15/2014 | 54:13.9 | 1 |
| 90.824 | 19537.646 | 1.014 | 16.032 | 0.0993226 | 7/15/2014 | 54:14.0 | 1 |
| 90.538 | 19537.641 | 1.011 | 16.036 | 0.104122 | 7/15/2014 | 54:14.1 | 1 |
| 90.25 | 19537.637 | 1.029 | 16.039 | 0.104955 | 7/15/2014 | 54:14.2 | 1 |
| 89.748 | 19537.629 | 1.008 | 16.042 | 0.0872532 | 7/15/2014 | 54:14.4 | 1 |
| 89.459 | 19537.623 | 1.015 | 16.042 | 0.0898455 | 7/15/2014 | 54:14.5 | 1 |
| 89.174 | 19537.618 | 1.003 | 16.041 | 0.0868503 | 7/15/2014 | 54:14.6 | 1 |
| 88.887 | 19537.609 | 1.015 | 16.04 | 0.0797921 | 7/15/2014 | 54:14.7 | 1 |
| 88.531 | 19537.602 | 1.023 | 16.037 | 0.0853697 | 7/15/2014 | 54:14.8 | 1 |
| 88.385 | 19537.599 | 1.008 | 16.035 | 0.0721365 | 7/15/2014 | 54:14.8 | 1 |
| 88.099 | 19537.592 | 1.022 | 16.031 | 0.0717171 | 7/15/2014 | 54:14.9 | 1 |
| 87.813 | 19537.583 | 1.036 | 16.026 | 0.0620205 | 7/15/2014 | 54:15.0 | 1 |
| 87.529 | 19537.575 | 1.046 | 16.02 | 0.0609016 | 7/15/2014 | 54:15.1 | 1 |
| 87.245 | 19537.565 | 1.053 | 16.016 | 0.0767942 | 7/15/2014 | 54:15.2 | 1 |
| 86.75 | 19537.549 | 1.031 | 16.012 | 0.0544655 | 7/15/2014 | 54:15.4 | 1 |
| 86.469 | 19537.539 | 1.012 | 16.011 | 0.0661181 | 7/15/2014 | 54:15.5 | 1 |
| 86.189 | 19537.528 | 1.009 | 16.012 | 0.0507234 | 7/15/2014 | 54:15.6 | 1 |
| 85.91 | 19537.518 | 1.003 | 16.015 | 0.0378231 | 7/15/2014 | 54:15.7 | 1 |

| | | | | | | | |
|---|---|---|---|---|---|---|---|
| 85.564 | 19537.503 | 1 | 16.02 | 0.0365525 | 7/15/2014 | 54:15.8 | 1 |
| 85.422 | 19537.498 | 1.002 | 16.023 | 0.0282902 | 7/15/2014 | 54:15.8 | 1 |
| 85.144 | 19537.484 | 1.009 | 16.032 | 0.0533895 | 7/15/2014 | 54:15.9 | 1 |
| 84.868 | 19537.473 | 1.018 | 16.043 | 0.0795266 | 7/15/2014 | 54:16.0 | 1 |
| 84.592 | 19537.459 | 1.011 | 16.056 | 0.0973516 | 7/15/2014 | 54:16.1 | 1 |
| 84.317 | 19537.445 | 0.995 | 16.07 | 0.136956 | 7/15/2014 | 54:16.2 | 1 |
| 83.839 | 19537.421 | 0.994 | 16.092 | 0.167576 | 7/15/2014 | 54:16.4 | 1 |
| 83.565 | 19537.404 | 0.978 | 16.106 | 0.185566 | 7/15/2014 | 54:16.5 | 1 |
| 83.293 | 19537.391 | 0.968 | 16.12 | 0.219702 | 7/15/2014 | 54:16.6 | 1 |
| 83.021 | 19537.374 | 0.96 | 16.132 | 0.223137 | 7/15/2014 | 54:16.7 | 1 |
| 82.681 | 19537.353 | 0.96 | 16.144 | 0.229379 | 7/15/2014 | 54:16.8 | 1 |
| 82.406 | 19537.336 | 0.977 | 16.153 | 0.222857 | 7/15/2014 | 54:16.9 | 1 |
| 82.132 | 19537.319 | 0.984 | 16.161 | 0.236196 | 7/15/2014 | 54:17.0 | 1 |
| 81.857 | 19537.301 | 0.978 | 16.166 | 0.213308 | 7/15/2014 | 54:17.1 | 1 |
| 81.721 | 19537.293 | 0.984 | 16.169 | 0.204463 | 7/15/2014 | 54:17.1 | 1 |
| 81.446 | 19537.273 | 0.979 | 16.17 | 0.190326 | 7/15/2014 | 54:17.2 | 1 |
| 80.964 | 19537.237 | 1 | 16.166 | 0.155152 | 7/15/2014 | 54:17.4 | 1 |
| 80.688 | 19537.219 | 1.006 | 16.161 | 0.16642 | 7/15/2014 | 54:17.5 | 1 |
| 80.412 | 19537.197 | 1.004 | 16.154 | 0.120906 | 7/15/2014 | 54:17.6 | 1 |
| 80.135 | 19537.176 | 1.006 | 16.144 | 0.125034 | 7/15/2014 | 54:17.7 | 1 |
| 79.787 | 19537.147 | 1.027 | 16.133 | 0.0991798 | 7/15/2014 | 54:17.8 | 1 |
| 79.647 | 19537.137 | 1.04 | 16.128 | 0.100027 | 7/15/2014 | 54:17.8 | 1 |
| 79.368 | 19537.111 | 1.054 | 16.114 | 0.106005 | 7/15/2014 | 54:17.9 | 1 |
| 79.087 | 19537.09 | 1.033 | 16.1 | 0.118255 | 7/15/2014 | 54:18.0 | 1 |
| 78.805 | 19537.064 | 1.029 | 16.086 | 0.139334 | 7/15/2014 | 54:18.1 | 1 |
| 78.524 | 19537.039 | 1.022 | 16.071 | 0.134141 | 7/15/2014 | 54:18.2 | 1 |
| 78.028 | 19536.994 | 1.039 | 16.051 | 0.14542 | 7/15/2014 | 54:18.4 | 1 |
| 77.745 | 19536.966 | 1.033 | 16.038 | 0.143156 | 7/15/2014 | 54:18.5 | 1 |
| 77.462 | 19536.939 | 1.027 | 16.027 | 0.116042 | 7/15/2014 | 54:18.6 | 1 |
| 77.176 | 19536.91 | 1.035 | 16.016 | 0.104516 | 7/15/2014 | 54:18.7 | 1 |
| 76.821 | 19536.875 | 1.057 | 16.007 | 0.097362 | 7/15/2014 | 54:18.8 | 1 |
| 76.676 | 19536.861 | 1.071 | 16.002 | 0.0944262 | 7/15/2014 | 54:18.8 | 1 |
| 76.391 | 19536.831 | 1.077 | 15.995 | 0.0930495 | 7/15/2014 | 54:18.9 | 1 |
| 76.104 | 19536.8 | 1.087 | 15.989 | 0.107598 | 7/15/2014 | 54:19.0 | 1 |
| 75.817 | 19536.77 | 1.094 | 15.983 | 0.140803 | 7/15/2014 | 54:19.1 | 1 |
| 75.53 | 19536.736 | 1.098 | 15.978 | 0.133201 | 7/15/2014 | 54:19.2 | 1 |
| 75.029 | 19536.682 | 1.125 | 15.971 | 0.177816 | 7/15/2014 | 54:19.4 | 1 |
| 74.744 | 19536.647 | 1.129 | 15.967 | 0.181325 | 7/15/2014 | 54:19.5 | 1 |
| 74.459 | 19536.613 | 1.118 | 15.963 | 0.182482 | 7/15/2014 | 54:19.6 | 1 |
| 74.173 | 19536.579 | 1.138 | 15.959 | 0.184195 | 7/15/2014 | 54:19.7 | 1 |
| 73.817 | 19536.535 | 1.138 | 15.955 | 0.20151 | 7/15/2014 | 54:19.8 | 1 |
| 73.673 | 19536.52 | 1.152 | 15.953 | 0.20872 | 7/15/2014 | 54:19.8 | 1 |
| 73.388 | 19536.483 | 1.162 | 15.948 | 0.185643 | 7/15/2014 | 54:19.9 | 1 |
| 73.103 | 19536.447 | 1.162 | 15.944 | 0.207005 | 7/15/2014 | 54:20.0 | 1 |
| 72.817 | 19536.41 | 1.171 | 15.938 | 0.208189 | 7/15/2014 | 54:20.1 | 1 |
| 72.532 | 19536.372 | 1.188 | 15.932 | 0.203643 | 7/15/2014 | 54:20.2 | 1 |
| 72.033 | 19536.306 | 1.202 | 15.92 | 0.193432 | 7/15/2014 | 54:20.4 | 1 |

| | | | | | | | |
|---|---|---|---|---|---|---|---|
| 71.748 | 19536.267 | 1.228 | 15.91 | 0.19283 | 7/15/2014 | 54:20.5 | 1 |
| 71.463 | 19536.228 | 1.229 | 15.9 | 0.174057 | 7/15/2014 | 54:20.6 | 1 |
| 71.178 | 19536.188 | 1.249 | 15.888 | 0.165002 | 7/15/2014 | 54:20.7 | 1 |
| 70.824 | 19536.137 | 1.265 | 15.875 | 0.15924 | 7/15/2014 | 54:20.8 | 1 |
| 70.679 | 19536.117 | 1.28 | 15.869 | 0.153309 | 7/15/2014 | 54:20.8 | 1 |
| 70.395 | 19536.076 | 1.291 | 15.854 | 0.147502 | 7/15/2014 | 54:20.9 | 1 |
| 70.11 | 19536.035 | 1.302 | 15.84 | 0.143449 | 7/15/2014 | 54:21.0 | 1 |
| 69.827 | 19535.993 | 1.301 | 15.826 | 0.142055 | 7/15/2014 | 54:21.1 | 1 |
| 69.544 | 19535.949 | 1.332 | 15.813 | 0.143468 | 7/15/2014 | 54:21.2 | 1 |
| 69.051 | 19535.875 | 1.349 | 15.794 | 0.149572 | 7/15/2014 | 54:21.4 | 1 |
| 68.77 | 19535.831 | 1.345 | 15.782 | 0.150146 | 7/15/2014 | 54:21.5 | 1 |
| 68.491 | 19535.788 | 1.335 | 15.772 | 0.155557 | 7/15/2014 | 54:21.6 | 1 |
| 68.21 | 19535.744 | 1.339 | 15.762 | 0.158958 | 7/15/2014 | 54:21.7 | 1 |
| 67.861 | 19535.687 | 1.351 | 15.754 | 0.162895 | 7/15/2014 | 54:21.8 | 1 |
| 67.719 | 19535.665 | 1.342 | 15.75 | 0.163663 | 7/15/2014 | 54:21.8 | 1 |
| 67.438 | 19535.62 | 1.346 | 15.744 | 0.167736 | 7/15/2014 | 54:21.9 | 1 |
| 67.156 | 19535.572 | 1.348 | 15.739 | 0.177342 | 7/15/2014 | 54:22.0 | 1 |
| 66.875 | 19535.526 | 1.362 | 15.735 | 0.190343 | 7/15/2014 | 54:22.1 | 1 |
| 66.593 | 19535.48 | 1.388 | 15.733 | 0.197774 | 7/15/2014 | 54:22.2 | 1 |
| 66.1 | 19535.396 | 1.398 | 15.732 | 0.213108 | 7/15/2014 | 54:22.4 | 1 |
| 65.817 | 19535.348 | 1.396 | 15.732 | 0.21884 | 7/15/2014 | 54:22.5 | 1 |
| 65.534 | 19535.298 | 1.402 | 15.733 | 0.231967 | 7/15/2014 | 54:22.6 | 1 |
| 65.251 | 19535.249 | 1.416 | 15.734 | 0.23816 | 7/15/2014 | 54:22.7 | 1 |
| 64.897 | 19535.188 | 1.43 | 15.736 | 0.233749 | 7/15/2014 | 54:22.8 | 1 |
| 64.753 | 19535.162 | 1.427 | 15.737 | 0.246703 | 7/15/2014 | 54:22.8 | 1 |
| 64.468 | 19535.111 | 1.429 | 15.739 | 0.258831 | 7/15/2014 | 54:22.9 | 1 |
| 64.182 | 19535.062 | 1.432 | 15.741 | 0.270469 | 7/15/2014 | 54:23.0 | 1 |
| 63.896 | 19535.009 | 1.453 | 15.742 | 0.273919 | 7/15/2014 | 54:23.1 | 1 |
| 63.611 | 19534.956 | 1.457 | 15.744 | 0.303768 | 7/15/2014 | 54:23.2 | 1 |
| 63.111 | 19534.865 | 1.457 | 15.745 | 0.318553 | 7/15/2014 | 54:23.4 | 1 |
| 62.824 | 19534.812 | 1.457 | 15.746 | 0.31503 | 7/15/2014 | 54:23.5 | 1 |
| 62.54 | 19534.76 | 1.468 | 15.746 | 0.311134 | 7/15/2014 | 54:23.6 | 1 |
| 62.253 | 19534.705 | 1.482 | 15.746 | 0.30863 | 7/15/2014 | 54:23.7 | 1 |
| 61.898 | 19534.637 | 1.485 | 15.746 | 0.271927 | 7/15/2014 | 54:23.8 | 1 |
| 61.754 | 19534.61 | 1.487 | 15.746 | 0.27332 | 7/15/2014 | 54:23.8 | 1 |
| 61.467 | 19534.557 | 1.473 | 15.747 | 0.236236 | 7/15/2014 | 54:23.9 | 1 |
| 61.18 | 19534.499 | 1.487 | 15.747 | 0.236905 | 7/15/2014 | 54:24.0 | 1 |
| 60.894 | 19534.445 | 1.484 | 15.748 | 0.244515 | 7/15/2014 | 54:24.1 | 1 |
| 60.608 | 19534.389 | 1.481 | 15.749 | 0.199316 | 7/15/2014 | 54:24.2 | 1 |
| 60.108 | 19534.289 | 1.479 | 15.753 | 0.233549 | 7/15/2014 | 54:24.4 | 1 |
| 59.822 | 19534.232 | 1.479 | 15.757 | 0.239012 | 7/15/2014 | 54:24.5 | 1 |
| 59.538 | 19534.175 | 1.479 | 15.761 | 0.240539 | 7/15/2014 | 54:24.6 | 1 |
| 59.253 | 19534.118 | 1.49 | 15.766 | 0.225814 | 7/15/2014 | 54:24.7 | 1 |
| 58.9 | 19534.045 | 1.49 | 15.773 | 0.239722 | 7/15/2014 | 54:24.8 | 1 |
| 58.755 | 19534.016 | 1.49 | 15.776 | 0.231023 | 7/15/2014 | 54:24.8 | 1 |
| 58.471 | 19533.958 | 1.494 | 15.784 | 0.232804 | 7/15/2014 | 54:24.9 | 1 |
| 58.188 | 19533.898 | 1.501 | 15.793 | 0.236709 | 7/15/2014 | 54:25.0 | 1 |

| | | | | | | | |
|---|---|---|---|---|---|---|---|
| 57.907 | 19533.841 | 1.501 | 15.802 | 0.227479 | 7/15/2014 | 54:25.1 | 1 |
| 57.626 | 19533.782 | 1.501 | 15.812 | 0.236322 | 7/15/2014 | 54:25.2 | 1 |
| 57.137 | 19533.678 | 1.485 | 15.826 | 0.252209 | 7/15/2014 | 54:25.4 | 1 |
| 56.858 | 19533.619 | 1.496 | 15.835 | 0.263399 | 7/15/2014 | 54:25.5 | 1 |
| 56.581 | 19533.559 | 1.484 | 15.844 | 0.26659 | 7/15/2014 | 54:25.6 | 1 |
| 56.305 | 19533.5 | 1.486 | 15.852 | 0.262805 | 7/15/2014 | 54:25.7 | 1 |
| 55.962 | 19533.426 | 1.474 | 15.859 | 0.263199 | 7/15/2014 | 54:25.8 | 1 |
| 55.821 | 19533.395 | 1.479 | 15.862 | 0.258188 | 7/15/2014 | 54:25.8 | 1 |
| 55.546 | 19533.335 | 1.472 | 15.869 | 0.260829 | 7/15/2014 | 54:25.9 | 1 |
| 55.272 | 19533.274 | 1.479 | 15.875 | 0.224038 | 7/15/2014 | 54:26.0 | 1 |
| 54.997 | 19533.213 | 1.476 | 15.881 | 0.211057 | 7/15/2014 | 54:26.1 | 1 |
| 54.725 | 19533.152 | 1.47 | 15.888 | 0.20711 | 7/15/2014 | 54:26.2 | 1 |
| 54.248 | 19533.045 | 1.457 | 15.898 | 0.214143 | 7/15/2014 | 54:26.4 | 1 |
| 53.977 | 19532.983 | 1.457 | 15.905 | 0.242424 | 7/15/2014 | 54:26.5 | 1 |
| 53.706 | 19532.922 | 1.463 | 15.913 | 0.243411 | 7/15/2014 | 54:26.6 | 1 |
| 53.435 | 19532.859 | 1.457 | 15.922 | 0.255175 | 7/15/2014 | 54:26.7 | 1 |
| 53.099 | 19532.781 | 1.449 | 15.931 | 0.26848 | 7/15/2014 | 54:26.8 | 1 |
| 52.961 | 19532.75 | 1.452 | 15.936 | 0.279787 | 7/15/2014 | 54:26.8 | 1 |
| 52.69 | 19532.688 | 1.456 | 15.946 | 0.285891 | 7/15/2014 | 54:26.9 | 1 |
| 52.419 | 19532.623 | 1.453 | 15.957 | 0.307093 | 7/15/2014 | 54:27.0 | 1 |
| 52.148 | 19532.561 | 1.47 | 15.968 | 0.282789 | 7/15/2014 | 54:27.1 | 1 |
| 51.879 | 19532.496 | 1.457 | 15.979 | 0.284135 | 7/15/2014 | 54:27.2 | 1 |
| 51.405 | 19532.383 | 1.452 | 15.995 | 0.280672 | 7/15/2014 | 54:27.4 | 1 |
| 51.134 | 19532.318 | 1.455 | 16.006 | 0.266176 | 7/15/2014 | 54:27.5 | 1 |
| 50.864 | 19532.252 | 1.451 | 16.017 | 0.271108 | 7/15/2014 | 54:27.6 | 1 |
| 50.593 | 19532.188 | 1.46 | 16.028 | 0.267393 | 7/15/2014 | 54:27.7 | 1 |
| 50.255 | 19532.104 | 1.449 | 16.038 | 0.259092 | 7/15/2014 | 54:27.8 | 1 |
| 50.118 | 19532.07 | 1.452 | 16.043 | 0.257396 | 7/15/2014 | 54:27.8 | 1 |
| 49.846 | 19532.003 | 1.452 | 16.053 | 0.266649 | 7/15/2014 | 54:27.9 | 1 |
| 49.574 | 19531.936 | 1.446 | 16.063 | 0.260068 | 7/15/2014 | 54:28.0 | 1 |
| 49.3 | 19531.867 | 1.446 | 16.073 | 0.273078 | 7/15/2014 | 54:28.1 | 1 |
| 49.027 | 19531.799 | 1.436 | 16.083 | 0.256023 | 7/15/2014 | 54:28.2 | 1 |
| 48.55 | 19531.677 | 1.426 | 16.099 | 0.269283 | 7/15/2014 | 54:28.4 | 1 |
| 48.276 | 19531.606 | 1.42 | 16.11 | 0.282899 | 7/15/2014 | 54:28.5 | 1 |
| 48.004 | 19531.537 | 1.414 | 16.121 | 0.292232 | 7/15/2014 | 54:28.6 | 1 |
| 47.731 | 19531.467 | 1.413 | 16.132 | 0.287522 | 7/15/2014 | 54:28.7 | 1 |
| 47.392 | 19531.381 | 1.408 | 16.143 | 0.288818 | 7/15/2014 | 54:28.8 | 1 |
| 47.254 | 19531.345 | 1.408 | 16.148 | 0.294063 | 7/15/2014 | 54:28.8 | 1 |
| 46.983 | 19531.274 | 1.391 | 16.159 | 0.307403 | 7/15/2014 | 54:28.9 | 1 |
| 46.71 | 19531.202 | 1.398 | 16.168 | 0.286639 | 7/15/2014 | 54:29.0 | 1 |
| 46.439 | 19531.131 | 1.398 | 16.176 | 0.302962 | 7/15/2014 | 54:29.1 | 1 |
| 46.169 | 19531.061 | 1.395 | 16.182 | 0.275746 | 7/15/2014 | 54:29.2 | 1 |
| 45.699 | 19530.938 | 1.4 | 16.189 | 0.286865 | 7/15/2014 | 54:29.4 | 1 |
| 45.432 | 19530.867 | 1.394 | 16.193 | 0.265187 | 7/15/2014 | 54:29.5 | 1 |
| 45.164 | 19530.795 | 1.397 | 16.195 | 0.287805 | 7/15/2014 | 54:29.6 | 1 |
| 44.896 | 19530.724 | 1.405 | 16.196 | 0.261426 | 7/15/2014 | 54:29.7 | 1 |
| 44.565 | 19530.635 | 1.399 | 16.197 | 0.279817 | 7/15/2014 | 54:29.8 | 1 |

| | | | | | | | |
|---|---|---|---|---|---|---|---|
| 44.43 | 19530.598 | 1.401 | 16.197 | 0.257991 | 7/15/2014 | 54:29.8 | 1 |
| 44.163 | 19530.527 | 1.405 | 16.197 | 0.268117 | 7/15/2014 | 54:29.9 | 1 |
| 43.898 | 19530.457 | 1.407 | 16.197 | 0.280805 | 7/15/2014 | 54:30.0 | 1 |
| 43.631 | 19530.384 | 1.41 | 16.197 | 0.252278 | 7/15/2014 | 54:30.1 | 1 |
| 43.366 | 19530.313 | 1.411 | 16.198 | 0.249607 | 7/15/2014 | 54:30.2 | 1 |
| 42.904 | 19530.187 | 1.396 | 16.197 | 0.248362 | 7/15/2014 | 54:30.4 | 1 |
| 42.639 | 19530.115 | 1.399 | 16.198 | 0.240534 | 7/15/2014 | 54:30.5 | 1 |
| 42.376 | 19530.045 | 1.393 | 16.198 | 0.227792 | 7/15/2014 | 54:30.6 | 1 |
| 42.112 | 19529.971 | 1.39 | 16.199 | 0.225315 | 7/15/2014 | 54:30.7 | 1 |
| 41.784 | 19529.883 | 1.39 | 16.2 | 0.227682 | 7/15/2014 | 54:30.8 | 1 |
| 41.519 | 19529.811 | 1.383 | 16.202 | 0.227606 | 7/15/2014 | 54:30.9 | 1 |
| 41.256 | 19529.738 | 1.387 | 16.205 | 0.242569 | 7/15/2014 | 54:31.0 | 1 |
| 40.992 | 19529.665 | 1.397 | 16.21 | 0.241143 | 7/15/2014 | 54:31.1 | 1 |
| 40.729 | 19529.594 | 1.397 | 16.214 | 0.254632 | 7/15/2014 | 54:31.2 | 1 |
| 40.467 | 19529.521 | 1.394 | 16.22 | 0.26999 | 7/15/2014 | 54:31.3 | 1 |
| 40.142 | 19529.432 | 1.398 | 16.226 | 0.278508 | 7/15/2014 | 54:31.4 | 1 |
| 39.881 | 19529.361 | 1.392 | 16.232 | 0.270565 | 7/15/2014 | 54:31.5 | 1 |
| 39.623 | 19529.29 | 1.398 | 16.238 | 0.262283 | 7/15/2014 | 54:31.6 | 1 |
| 39.364 | 19529.218 | 1.4 | 16.244 | 0.251179 | 7/15/2014 | 54:31.7 | 1 |
| 39.045 | 19529.129 | 1.381 | 16.25 | 0.253658 | 7/15/2014 | 54:31.8 | 1 |
| 38.787 | 19529.059 | 1.381 | 16.256 | 0.243561 | 7/15/2014 | 54:31.9 | 1 |
| 38.531 | 19528.986 | 1.377 | 16.262 | 0.236083 | 7/15/2014 | 54:32.0 | 1 |
| 38.275 | 19528.916 | 1.375 | 16.268 | 0.246912 | 7/15/2014 | 54:32.1 | 1 |
| 38.022 | 19528.847 | 1.371 | 16.274 | 0.240617 | 7/15/2014 | 54:32.2 | 1 |
| 37.768 | 19528.775 | 1.371 | 16.281 | 0.243578 | 7/15/2014 | 54:32.3 | 1 |
| 37.455 | 19528.688 | 1.363 | 16.288 | 0.244938 | 7/15/2014 | 54:32.4 | 1 |
| 37.204 | 19528.618 | 1.376 | 16.295 | 0.259446 | 7/15/2014 | 54:32.5 | 1 |
| 36.955 | 19528.549 | 1.375 | 16.302 | 0.26306 | 7/15/2014 | 54:32.6 | 1 |
| 36.706 | 19528.479 | 1.378 | 16.309 | 0.271035 | 7/15/2014 | 54:32.7 | 1 |
| 36.4 | 19528.395 | 1.369 | 16.315 | 0.285476 | 7/15/2014 | 54:32.8 | 1 |
| 36.151 | 19528.324 | 1.376 | 16.322 | 0.302213 | 7/15/2014 | 54:32.9 | 1 |
| 35.904 | 19528.256 | 1.376 | 16.329 | 0.314155 | 7/15/2014 | 54:33.0 | 1 |
| 35.659 | 19528.187 | 1.366 | 16.335 | 0.324084 | 7/15/2014 | 54:33.1 | 1 |
| 35.414 | 19528.119 | 1.36 | 16.342 | 0.327782 | 7/15/2014 | 54:33.2 | 1 |
| 35.17 | 19528.05 | 1.358 | 16.349 | 0.327487 | 7/15/2014 | 54:33.3 | 1 |
| 34.87 | 19527.966 | 1.351 | 16.356 | 0.324338 | 7/15/2014 | 54:33.4 | 1 |
| 34.628 | 19527.898 | 1.36 | 16.363 | 0.315898 | 7/15/2014 | 54:33.5 | 1 |
| 34.272 | 19527.799 | 1.35 | 16.372 | 0.305348 | 7/15/2014 | 54:33.6 | 1 |
| 33.977 | 19527.715 | 1.343 | 16.377 | 0.305195 | 7/15/2014 | 54:33.7 | 1 |
| 33.855 | 19527.682 | 1.347 | 16.38 | 0.288345 | 7/15/2014 | 54:33.8 | 1 |
| 33.616 | 19527.613 | 1.355 | 16.383 | 0.285534 | 7/15/2014 | 54:33.9 | 1 |
| 33.38 | 19527.547 | 1.349 | 16.386 | 0.275176 | 7/15/2014 | 54:34.0 | 1 |
| 33.141 | 19527.48 | 1.353 | 16.388 | 0.269254 | 7/15/2014 | 54:34.1 | 1 |
| 32.907 | 19527.414 | 1.346 | 16.388 | 0.25357 | 7/15/2014 | 54:34.2 | 1 |
| 32.67 | 19527.348 | 1.354 | 16.387 | 0.252699 | 7/15/2014 | 54:34.3 | 1 |
| 32.26 | 19527.232 | 1.35 | 16.384 | 0.238182 | 7/15/2014 | 54:34.4 | 1 |
| 32.027 | 19527.166 | 1.35 | 16.381 | 0.236423 | 7/15/2014 | 54:34.5 | 1 |

| | | | | | | | |
|---|---|---|---|---|---|---|---|
| 31.794 | 19527.1 | 1.344 | 16.378 | 0.232486 | 7/15/2014 | 54:34.6 | 1 |
| 31.504 | 19527.018 | 1.347 | 16.375 | 0.231213 | 7/15/2014 | 54:34.7 | 1 |
| 31.387 | 19526.984 | 1.344 | 16.373 | 0.233801 | 7/15/2014 | 54:34.8 | 1 |
| 31.153 | 19526.92 | 1.348 | 16.369 | 0.243089 | 7/15/2014 | 54:34.9 | 1 |
| 30.919 | 19526.854 | 1.342 | 16.366 | 0.248215 | 7/15/2014 | 54:35.0 | 1 |
| 30.684 | 19526.787 | 1.344 | 16.362 | 0.25392 | 7/15/2014 | 54:35.1 | 1 |
| 30.452 | 19526.722 | 1.341 | 16.359 | 0.257993 | 7/15/2014 | 54:35.2 | 1 |
| 30.219 | 19526.656 | 1.341 | 16.356 | 0.256645 | 7/15/2014 | 54:35.3 | 1 |
| 29.815 | 19526.542 | 1.337 | 16.352 | 0.247591 | 7/15/2014 | 54:35.4 | 1 |
| 29.586 | 19526.479 | 1.337 | 16.35 | 0.244856 | 7/15/2014 | 54:35.5 | 1 |
| 29.356 | 19526.414 | 1.337 | 16.348 | 0.233406 | 7/15/2014 | 54:35.6 | 1 |
| 29.07 | 19526.334 | 1.335 | 16.347 | 0.230437 | 7/15/2014 | 54:35.7 | 1 |
| 28.954 | 19526.302 | 1.335 | 16.347 | 0.22507 | 7/15/2014 | 54:35.8 | 1 |
| 28.725 | 19526.238 | 1.337 | 16.347 | 0.214849 | 7/15/2014 | 54:35.9 | 1 |
| 28.495 | 19526.174 | 1.337 | 16.348 | 0.216963 | 7/15/2014 | 54:36.0 | 1 |
| 28.266 | 19526.11 | 1.335 | 16.349 | 0.227788 | 7/15/2014 | 54:36.1 | 1 |
| 28.037 | 19526.046 | 1.338 | 16.351 | 0.232626 | 7/15/2014 | 54:36.2 | 1 |
| 27.81 | 19525.983 | 1.344 | 16.353 | 0.245774 | 7/15/2014 | 54:36.3 | 1 |
| 27.413 | 19525.873 | 1.338 | 16.356 | 0.262084 | 7/15/2014 | 54:36.4 | 1 |
| 27.188 | 19525.812 | 1.34 | 16.357 | 0.267882 | 7/15/2014 | 54:36.5 | 1 |
| 26.962 | 19525.75 | 1.34 | 16.358 | 0.2749 | 7/15/2014 | 54:36.6 | 1 |
| 26.681 | 19525.672 | 1.338 | 16.358 | 0.27681 | 7/15/2014 | 54:36.7 | 1 |
| 26.566 | 19525.64 | 1.341 | 16.358 | 0.269493 | 7/15/2014 | 54:36.8 | 1 |
| 26.338 | 19525.577 | 1.341 | 16.357 | 0.258245 | 7/15/2014 | 54:36.9 | 1 |
| 26.112 | 19525.515 | 1.332 | 16.354 | 0.254898 | 7/15/2014 | 54:37.0 | 1 |
| 25.883 | 19525.451 | 1.338 | 16.351 | 0.221868 | 7/15/2014 | 54:37.1 | 1 |
| 25.655 | 19525.39 | 1.336 | 16.347 | 0.210021 | 7/15/2014 | 54:37.2 | 1 |
| 25.428 | 19525.328 | 1.334 | 16.341 | 0.202362 | 7/15/2014 | 54:37.3 | 1 |
| 25.032 | 19525.221 | 1.332 | 16.332 | 0.1847 | 7/15/2014 | 54:37.4 | 1 |
| 24.806 | 19525.159 | 1.332 | 16.326 | 0.18141 | 7/15/2014 | 54:37.5 | 1 |
| 24.58 | 19525.098 | 1.329 | 16.32 | 0.183931 | 7/15/2014 | 54:37.6 | 1 |
| 24.298 | 19525.021 | 1.332 | 16.314 | 0.187626 | 7/15/2014 | 54:37.7 | 1 |
| 24.184 | 19524.99 | 1.327 | 16.311 | 0.198304 | 7/15/2014 | 54:37.8 | 1 |
| 23.956 | 19524.93 | 1.334 | 16.305 | 0.202396 | 7/15/2014 | 54:37.9 | 1 |
| 23.73 | 19524.87 | 1.331 | 16.301 | 0.248996 | 7/15/2014 | 54:38.0 | 1 |
| 23.503 | 19524.809 | 1.331 | 16.297 | 0.245757 | 7/15/2014 | 54:38.1 | 1 |
| 23.278 | 19524.749 | 1.332 | 16.293 | 0.256133 | 7/15/2014 | 54:38.2 | 1 |
| 23.052 | 19524.688 | 1.335 | 16.29 | 0.261415 | 7/15/2014 | 54:38.3 | 1 |
| 22.662 | 19524.585 | 1.339 | 16.288 | 0.262064 | 7/15/2014 | 54:38.4 | 1 |
| 22.439 | 19524.526 | 1.336 | 16.286 | 0.270184 | 7/15/2014 | 54:38.5 | 1 |
| 22.218 | 19524.468 | 1.333 | 16.285 | 0.242211 | 7/15/2014 | 54:38.6 | 1 |
| 21.943 | 19524.395 | 1.335 | 16.285 | 0.261972 | 7/15/2014 | 54:38.7 | 1 |
| 21.721 | 19524.338 | 1.34 | 16.285 | 0.261703 | 7/15/2014 | 54:38.8 | 1 |
| 21.499 | 19524.28 | 1.34 | 16.285 | 0.249526 | 7/15/2014 | 54:38.9 | 1 |
| 21.278 | 19524.222 | 1.346 | 16.286 | 0.236977 | 7/15/2014 | 54:39.0 | 1 |
| 21.057 | 19524.165 | 1.348 | 16.286 | 0.242183 | 7/15/2014 | 54:39.1 | 1 |
| 20.838 | 19524.107 | 1.345 | 16.287 | 0.22318 | 7/15/2014 | 54:39.2 | 1 |

| | | | | | | | |
|---|---|---|---|---|---|---|---|
| 20.619 | 19524.052 | 1.35 | 16.286 | 0.226946 | 7/15/2014 | 54:39.3 | 1 |
| 20.348 | 19523.98 | 1.357 | 16.285 | 0.219416 | 7/15/2014 | 54:39.4 | 1 |
| 20.131 | 19523.926 | 1.365 | 16.284 | 0.218417 | 7/15/2014 | 54:39.5 | 1 |
| 19.914 | 19523.872 | 1.37 | 16.281 | 0.222967 | 7/15/2014 | 54:39.6 | 1 |
| 19.641 | 19523.801 | 1.374 | 16.279 | 0.216065 | 7/15/2014 | 54:39.7 | 1 |
| 19.421 | 19523.746 | 1.377 | 16.276 | 0.199141 | 7/15/2014 | 54:39.8 | 1 |
| 19.2 | 19523.69 | 1.38 | 16.274 | 0.193201 | 7/15/2014 | 54:39.9 | 1 |
| 18.978 | 19523.635 | 1.385 | 16.273 | 0.17701 | 7/15/2014 | 54:40.0 | 1 |
| 18.756 | 19523.578 | 1.387 | 16.273 | 0.164513 | 7/15/2014 | 54:40.1 | 1 |
| 18.533 | 19523.523 | 1.392 | 16.273 | 0.147889 | 7/15/2014 | 54:40.2 | 1 |
| 18.31 | 19523.469 | 1.401 | 16.276 | 0.147713 | 7/15/2014 | 54:40.3 | 1 |
| 18.033 | 19523.399 | 1.409 | 16.28 | 0.136314 | 7/15/2014 | 54:40.4 | 1 |
| 17.809 | 19523.345 | 1.416 | 16.285 | 0.142851 | 7/15/2014 | 54:40.5 | 1 |
| 17.587 | 19523.29 | 1.419 | 16.292 | 0.159926 | 7/15/2014 | 54:40.6 | 1 |
| 17.307 | 19523.221 | 1.425 | 16.301 | 0.170983 | 7/15/2014 | 54:40.7 | 1 |
| 17.193 | 19523.192 | 1.432 | 16.305 | 0.178717 | 7/15/2014 | 54:40.8 | 1 |
| 16.966 | 19523.139 | 1.448 | 16.316 | 0.21456 | 7/15/2014 | 54:40.9 | 1 |
| 16.741 | 19523.083 | 1.451 | 16.328 | 0.220933 | 7/15/2014 | 54:41.0 | 1 |
| 16.514 | 19523.028 | 1.457 | 16.341 | 0.253457 | 7/15/2014 | 54:41.1 | 1 |
| 16.289 | 19522.976 | 1.454 | 16.354 | 0.279807 | 7/15/2014 | 54:41.2 | 1 |
| 16.063 | 19522.921 | 1.465 | 16.367 | 0.283442 | 7/15/2014 | 54:41.3 | 1 |
| 15.671 | 19522.826 | 1.466 | 16.386 | 0.307188 | 7/15/2014 | 54:41.4 | 1 |
| 15.447 | 19522.773 | 1.469 | 16.398 | 0.3069 | 7/15/2014 | 54:41.5 | 1 |
| 15.225 | 19522.721 | 1.465 | 16.409 | 0.298806 | 7/15/2014 | 54:41.6 | 1 |
| 14.947 | 19522.655 | 1.464 | 16.42 | 0.297335 | 7/15/2014 | 54:41.7 | 1 |
| 14.724 | 19522.604 | 1.464 | 16.429 | 0.283768 | 7/15/2014 | 54:41.8 | 1 |
| 14.5 | 19522.552 | 1.47 | 16.437 | 0.281384 | 7/15/2014 | 54:41.9 | 1 |
| 14.276 | 19522.5 | 1.473 | 16.444 | 0.290001 | 7/15/2014 | 54:42.0 | 1 |
| 14.053 | 19522.448 | 1.484 | 16.45 | 0.269298 | 7/15/2014 | 54:42.1 | 1 |
| 13.831 | 19522.396 | 1.492 | 16.455 | 0.256402 | 7/15/2014 | 54:42.2 | 1 |
| 13.61 | 19522.346 | 1.503 | 16.458 | 0.262653 | 7/15/2014 | 54:42.3 | 1 |
| 13.336 | 19522.284 | 1.513 | 16.459 | 0.23851 | 7/15/2014 | 54:42.4 | 1 |
| 13.117 | 19522.234 | 1.524 | 16.46 | 0.220452 | 7/15/2014 | 54:42.5 | 1 |
| 12.898 | 19522.185 | 1.534 | 16.459 | 0.221664 | 7/15/2014 | 54:42.6 | 1 |
| 12.625 | 19522.125 | 1.539 | 16.457 | 0.183856 | 7/15/2014 | 54:42.7 | 1 |
| 12.405 | 19522.075 | 1.543 | 16.455 | 0.184327 | 7/15/2014 | 54:42.8 | 1 |
| 12.185 | 19522.026 | 1.551 | 16.451 | 0.183957 | 7/15/2014 | 54:42.9 | 1 |
| 11.965 | 19521.977 | 1.558 | 16.446 | 0.183557 | 7/15/2014 | 54:43.0 | 1 |
| 11.745 | 19521.93 | 1.565 | 16.441 | 0.165525 | 7/15/2014 | 54:43.1 | 1 |
| 11.525 | 19521.882 | 1.571 | 16.436 | 0.174356 | 7/15/2014 | 54:43.2 | 1 |
| 11.306 | 19521.834 | 1.573 | 16.43 | 0.184051 | 7/15/2014 | 54:43.3 | 1 |
| 11.034 | 19521.775 | 1.58 | 16.424 | 0.187431 | 7/15/2014 | 54:43.4 | 1 |
| 10.816 | 19521.728 | 1.588 | 16.419 | 0.190458 | 7/15/2014 | 54:43.5 | 1 |
| 10.597 | 19521.682 | 1.593 | 16.413 | 0.191112 | 7/15/2014 | 54:43.6 | 1 |
| 10.323 | 19521.623 | 1.598 | 16.408 | 0.168609 | 7/15/2014 | 54:43.7 | 1 |
| 10.102 | 19521.577 | 1.602 | 16.404 | 0.205974 | 7/15/2014 | 54:43.8 | 1 |
| 9.881 | 19521.53 | 1.604 | 16.4 | 0.203126 | 7/15/2014 | 54:43.9 | 1 |

| | | | | | | | |
|---|---|---|---|---|---|---|---|
| 9.658 | 19521.484 | 1.612 | 16.396 | 0.178689 | 7/15/2014 | 54:44.0 | 1 |
| 9.436 | 19521.438 | 1.619 | 16.393 | 0.202874 | 7/15/2014 | 54:44.1 | 1 |
| 9.215 | 19521.392 | 1.622 | 16.391 | 0.19383 | 7/15/2014 | 54:44.2 | 1 |
| 8.993 | 19521.347 | 1.63 | 16.389 | 0.201295 | 7/15/2014 | 54:44.3 | 1 |
| 8.719 | 19521.289 | 1.637 | 16.388 | 0.246487 | 7/15/2014 | 54:44.4 | 1 |
| 8.498 | 19521.245 | 1.636 | 16.387 | 0.245539 | 7/15/2014 | 54:44.5 | 1 |
| 8.278 | 19521.2 | 1.639 | 16.387 | 0.24352 | 7/15/2014 | 54:44.6 | 1 |
| 8.005 | 19521.146 | 1.642 | 16.387 | 0.272511 | 7/15/2014 | 54:44.7 | 1 |
| 7.784 | 19521.102 | 1.647 | 16.388 | 0.28407 | 7/15/2014 | 54:44.8 | 1 |
| 7.565 | 19521.058 | 1.65 | 16.39 | 0.278699 | 7/15/2014 | 54:44.9 | 1 |
| 7.344 | 19521.015 | 1.653 | 16.392 | 0.30792 | 7/15/2014 | 54:45.0 | 1 |
| 7.125 | 19520.971 | 1.657 | 16.395 | 0.29006 | 7/15/2014 | 54:45.1 | 1 |
| 6.907 | 19520.929 | 1.66 | 16.398 | 0.282565 | 7/15/2014 | 54:45.2 | 1 |
| 6.691 | 19520.887 | 1.66 | 16.401 | 0.295832 | 7/15/2014 | 54:45.3 | 1 |
| 6.422 | 19520.836 | 1.663 | 16.404 | 0.298902 | 7/15/2014 | 54:45.4 | 1 |
| 6.208 | 19520.795 | 1.666 | 16.406 | 0.291739 | 7/15/2014 | 54:45.5 | 1 |
| 5.994 | 19520.752 | 1.666 | 16.408 | 0.300988 | 7/15/2014 | 54:45.6 | 1 |
| 5.728 | 19520.703 | 1.666 | 16.41 | 0.269192 | 7/15/2014 | 54:45.7 | 1 |
| 5.512 | 19520.663 | 1.668 | 16.41 | 0.243115 | 7/15/2014 | 54:45.8 | 1 |
| 5.298 | 19520.623 | 1.665 | 16.409 | 0.285865 | 7/15/2014 | 54:45.9 | 1 |
| 5.084 | 19520.583 | 1.663 | 16.408 | 0.274423 | 7/15/2014 | 54:46.0 | 1 |
| 4.868 | 19520.544 | 1.67 | 16.406 | 0.264747 | 7/15/2014 | 54:46.1 | 1 |
| 4.655 | 19520.505 | 1.67 | 16.403 | 0.274346 | 7/15/2014 | 54:46.2 | 1 |
| 4.441 | 19520.465 | 1.67 | 16.399 | 0.325178 | 7/15/2014 | 54:46.3 | 1 |
| 4.177 | 19520.417 | 1.67 | 16.396 | 0.315994 | 7/15/2014 | 54:46.4 | 1 |
| 3.965 | 19520.38 | 1.67 | 16.392 | 0.364531 | 7/15/2014 | 54:46.5 | 1 |
| 3.753 | 19520.341 | 1.668 | 16.388 | 0.336479 | 7/15/2014 | 54:46.6 | 1 |
| 3.489 | 19520.295 | 1.671 | 16.384 | 0.358799 | 7/15/2014 | 54:46.7 | 1 |
| 3.277 | 19520.257 | 1.671 | 16.381 | 0.38609 | 7/15/2014 | 54:46.8 | 1 |
| 3.064 | 19520.22 | 1.667 | 16.377 | 0.373343 | 7/15/2014 | 54:46.9 | 1 |
| 2.851 | 19520.183 | 1.667 | 16.373 | 0.351659 | 7/15/2014 | 54:47.0 | 1 |
| 2.638 | 19520.145 | 1.667 | 16.37 | 0.347234 | 7/15/2014 | 54:47.1 | 1 |
| 2.426 | 19520.109 | 1.665 | 16.366 | 0.337257 | 7/15/2014 | 54:47.2 | 1 |
| 2.216 | 19520.072 | 1.665 | 16.362 | 0.331536 | 7/15/2014 | 54:47.3 | 1 |
| 1.952 | 19520.028 | 1.664 | 16.359 | 0.314565 | 7/15/2014 | 54:47.4 | 1 |
| 1.743 | 19519.992 | 1.661 | 16.356 | 0.2992 | 7/15/2014 | 54:47.5 | 1 |
| 1.531 | 19519.957 | 1.663 | 16.353 | 0.292557 | 7/15/2014 | 54:47.6 | 1 |
| 1.268 | 19519.912 | 1.663 | 16.35 | 0.279694 | 7/15/2014 | 54:47.7 | 1 |
| 1.055 | 19519.876 | 1.663 | 16.348 | 0.288195 | 7/15/2014 | 54:47.8 | 1 |
| 0.843 | 19519.842 | 1.66 | 16.346 | 0.282525 | 7/15/2014 | 54:47.9 | 1 |
| 0.629 | 19519.807 | 1.657 | 16.344 | 0.289253 | 7/15/2014 | 54:48.0 | 1 |
| 0.415 | 19519.771 | 1.657 | 16.342 | 0.284981 | 7/15/2014 | 54:48.1 | 1 |
| 0.202 | 19519.736 | 1.659 | 16.34 | 0.282772 | 7/15/2014 | 54:48.2 | 1 |
| -0.012 | 19519.701 | 1.659 | 16.338 | 0.281173 | 7/15/2014 | 54:48.3 | 1 |
| -0.277 | 19519.657 | 1.657 | 16.335 | 0.276099 | 7/15/2014 | 54:48.4 | 1 |
| -0.49 | 19519.623 | 1.66 | 16.332 | 0.279574 | 7/15/2014 | 54:48.5 | 1 |
| -0.702 | 19519.59 | 1.662 | 16.328 | 0.271037 | 7/15/2014 | 54:48.6 | 1 |

| | | | | | | | |
|---|---|---|---|---|---|---|---|
| -0.97 | 19519.547 | 1.665 | 16.323 | 0.266338 | 7/15/2014 | 54:48.7 | 1 |
| -1.185 | 19519.512 | 1.669 | 16.318 | 0.263513 | 7/15/2014 | 54:48.8 | 1 |
| -1.401 | 19519.478 | 1.674 | 16.313 | 0.258916 | 7/15/2014 | 54:48.9 | 1 |
| -1.618 | 19519.443 | 1.674 | 16.307 | 0.255598 | 7/15/2014 | 54:49.0 | 1 |
| -1.834 | 19519.408 | 1.677 | 16.301 | 0.252854 | 7/15/2014 | 54:49.1 | 1 |
| -2.05 | 19519.375 | 1.679 | 16.294 | 0.255941 | 7/15/2014 | 54:49.2 | 1 |
| -2.266 | 19519.342 | 1.679 | 16.288 | 0.238879 | 7/15/2014 | 54:49.3 | 1 |
| -2.533 | 19519.299 | 1.681 | 16.281 | 0.23625 | 7/15/2014 | 54:49.4 | 1 |
| -2.746 | 19519.266 | 1.689 | 16.274 | 0.232168 | 7/15/2014 | 54:49.5 | 1 |
| -2.96 | 19519.232 | 1.696 | 16.267 | 0.222458 | 7/15/2014 | 54:49.6 | 1 |
| -3.225 | 19519.191 | 1.694 | 16.26 | 0.215011 | 7/15/2014 | 54:49.7 | 1 |
| -3.438 | 19519.158 | 1.696 | 16.254 | 0.210257 | 7/15/2014 | 54:49.8 | 1 |
| -3.652 | 19519.126 | 1.704 | 16.248 | 0.195589 | 7/15/2014 | 54:49.9 | 1 |
| -3.865 | 19519.093 | 1.709 | 16.242 | 0.204594 | 7/15/2014 | 54:50.0 | 1 |
| -4.078 | 19519.059 | 1.716 | 16.237 | 0.186608 | 7/15/2014 | 54:50.1 | 1 |
| -4.289 | 19519.027 | 1.722 | 16.233 | 0.184609 | 7/15/2014 | 54:50.2 | 1 |
| -4.499 | 19518.995 | 1.725 | 16.23 | 0.18917 | 7/15/2014 | 54:50.3 | 1 |
| -4.861 | 19518.939 | 1.732 | 16.226 | 0.181112 | 7/15/2014 | 54:50.5 | 1 |
| -5.069 | 19518.908 | 1.745 | 16.224 | 0.17801 | 7/15/2014 | 54:50.6 | 1 |
| -5.325 | 19518.87 | 1.749 | 16.223 | 0.161119 | 7/15/2014 | 54:50.7 | 1 |
| -5.43 | 19518.854 | 1.751 | 16.222 | 0.157589 | 7/15/2014 | 54:50.7 | 1 |
| -5.636 | 19518.822 | 1.756 | 16.221 | 0.159612 | 7/15/2014 | 54:50.8 | 1 |
| -5.844 | 19518.791 | 1.758 | 16.221 | 0.162945 | 7/15/2014 | 54:50.9 | 1 |
| -6.052 | 19518.76 | 1.763 | 16.22 | 0.166203 | 7/15/2014 | 54:51.0 | 1 |
| -6.259 | 19518.729 | 1.774 | 16.218 | 0.170453 | 7/15/2014 | 54:51.1 | 1 |
| -6.466 | 19518.697 | 1.777 | 16.217 | 0.177877 | 7/15/2014 | 54:51.2 | 1 |
| -6.673 | 19518.666 | 1.782 | 16.214 | 0.182505 | 7/15/2014 | 54:51.3 | 1 |
| -7.031 | 19518.612 | 1.783 | 16.208 | 0.184516 | 7/15/2014 | 54:51.5 | 1 |
| -7.236 | 19518.583 | 1.788 | 16.203 | 0.187332 | 7/15/2014 | 54:51.6 | 1 |
| -7.494 | 19518.545 | 1.791 | 16.197 | 0.185597 | 7/15/2014 | 54:51.7 | 1 |
| -7.598 | 19518.529 | 1.796 | 16.195 | 0.185435 | 7/15/2014 | 54:51.7 | 1 |
| -7.806 | 19518.498 | 1.799 | 16.187 | 0.184679 | 7/15/2014 | 54:51.8 | 1 |
| -8.015 | 19518.467 | 1.796 | 16.179 | 0.181721 | 7/15/2014 | 54:51.9 | 1 |
| -8.225 | 19518.434 | 1.799 | 16.169 | 0.181533 | 7/15/2014 | 54:52.0 | 1 |
| -8.435 | 19518.402 | 1.802 | 16.16 | 0.178113 | 7/15/2014 | 54:52.1 | 1 |
| -8.645 | 19518.371 | 1.81 | 16.15 | 0.173898 | 7/15/2014 | 54:52.2 | 1 |
| -8.855 | 19518.341 | 1.815 | 16.14 | 0.172105 | 7/15/2014 | 54:52.3 | 1 |
| -9.116 | 19518.303 | 1.822 | 16.131 | 0.176551 | 7/15/2014 | 54:52.4 | 1 |
| -9.326 | 19518.271 | 1.828 | 16.123 | 0.17212 | 7/15/2014 | 54:52.5 | 1 |
| -9.537 | 19518.238 | 1.832 | 16.115 | 0.170659 | 7/15/2014 | 54:52.6 | 1 |
| -9.8 | 19518.197 | 1.835 | 16.108 | 0.175464 | 7/15/2014 | 54:52.7 | 1 |
| -10.014 | 19518.166 | 1.842 | 16.102 | 0.173156 | 7/15/2014 | 54:52.8 | 1 |
| -10.228 | 19518.135 | 1.842 | 16.097 | 0.173313 | 7/15/2014 | 54:52.9 | 1 |
| -10.444 | 19518.102 | 1.842 | 16.093 | 0.179199 | 7/15/2014 | 54:53.0 | 1 |
| -10.659 | 19518.07 | 1.845 | 16.089 | 0.178927 | 7/15/2014 | 54:53.1 | 1 |
| -10.873 | 19518.037 | 1.848 | 16.085 | 0.180234 | 7/15/2014 | 54:53.2 | 1 |
| -11.088 | 19518.004 | 1.85 | 16.082 | 0.184188 | 7/15/2014 | 54:53.3 | 1 |

| | | | | | | | |
|---|---|---|---|---|---|---|---|
| -11.355 | 19517.965 | 1.856 | 16.079 | 0.190194 | 7/15/2014 | 54:53.4 | 1 |
| -11.568 | 19517.933 | 1.864 | 16.076 | 0.187475 | 7/15/2014 | 54:53.5 | 1 |
| -11.783 | 19517.9 | 1.867 | 16.074 | 0.188366 | 7/15/2014 | 54:53.6 | 1 |
| -12.05 | 19517.859 | 1.871 | 16.072 | 0.200846 | 7/15/2014 | 54:53.7 | 1 |
| -12.267 | 19517.827 | 1.874 | 16.071 | 0.189843 | 7/15/2014 | 54:53.8 | 1 |
| -12.482 | 19517.794 | 1.874 | 16.07 | 0.189882 | 7/15/2014 | 54:53.9 | 1 |
| -12.698 | 19517.762 | 1.874 | 16.069 | 0.193333 | 7/15/2014 | 54:54.0 | 1 |
| -12.916 | 19517.729 | 1.874 | 16.069 | 0.18255 | 7/15/2014 | 54:54.1 | 1 |
| -13.131 | 19517.696 | 1.871 | 16.069 | 0.188387 | 7/15/2014 | 54:54.2 | 1 |
| -13.346 | 19517.664 | 1.871 | 16.07 | 0.183655 | 7/15/2014 | 54:54.3 | 1 |
| -13.613 | 19517.623 | 1.866 | 16.071 | 0.18545 | 7/15/2014 | 54:54.4 | 1 |
| -13.827 | 19517.591 | 1.869 | 16.073 | 0.192183 | 7/15/2014 | 54:54.5 | 1 |
| -14.04 | 19517.559 | 1.872 | 16.075 | 0.202639 | 7/15/2014 | 54:54.6 | 1 |
| -14.307 | 19517.518 | 1.879 | 16.077 | 0.186935 | 7/15/2014 | 54:54.7 | 1 |
| -14.521 | 19517.484 | 1.879 | 16.079 | 0.197357 | 7/15/2014 | 54:54.8 | 1 |
| -14.738 | 19517.451 | 1.882 | 16.08 | 0.195016 | 7/15/2014 | 54:54.9 | 1 |
| -14.953 | 19517.42 | 1.887 | 16.082 | 0.198586 | 7/15/2014 | 54:55.0 | 1 |
| -15.169 | 19517.387 | 1.891 | 16.083 | 0.201482 | 7/15/2014 | 54:55.1 | 1 |
| -15.384 | 19517.354 | 1.893 | 16.084 | 0.203503 | 7/15/2014 | 54:55.2 | 1 |
| -15.599 | 19517.32 | 1.896 | 16.083 | 0.208142 | 7/15/2014 | 54:55.3 | 1 |
| -15.865 | 19517.279 | 1.896 | 16.082 | 0.212805 | 7/15/2014 | 54:55.4 | 1 |
| -16.079 | 19517.248 | 1.896 | 16.081 | 0.21922 | 7/15/2014 | 54:55.5 | 1 |
| -16.294 | 19517.213 | 1.901 | 16.079 | 0.224437 | 7/15/2014 | 54:55.6 | 1 |
| -16.562 | 19517.172 | 1.901 | 16.076 | 0.224987 | 7/15/2014 | 54:55.7 | 1 |
| -16.778 | 19517.141 | 1.899 | 16.072 | 0.229022 | 7/15/2014 | 54:55.8 | 1 |
| -16.995 | 19517.105 | 1.899 | 16.069 | 0.22464 | 7/15/2014 | 54:55.9 | 1 |
| -17.213 | 19517.071 | 1.906 | 16.065 | 0.232179 | 7/15/2014 | 54:56.0 | 1 |
| -17.43 | 19517.039 | 1.906 | 16.061 | 0.225117 | 7/15/2014 | 54:56.1 | 1 |
| -17.648 | 19517.006 | 1.91 | 16.056 | 0.226795 | 7/15/2014 | 54:56.2 | 1 |
| -17.865 | 19516.972 | 1.914 | 16.053 | 0.229082 | 7/15/2014 | 54:56.3 | 1 |
| -18.133 | 19516.93 | 1.914 | 16.049 | 0.222579 | 7/15/2014 | 54:56.4 | 1 |
| -18.348 | 19516.896 | 1.919 | 16.045 | 0.218169 | 7/15/2014 | 54:56.5 | 1 |
| -18.564 | 19516.862 | 1.924 | 16.042 | 0.229021 | 7/15/2014 | 54:56.6 | 1 |
| -18.832 | 19516.821 | 1.928 | 16.038 | 0.219306 | 7/15/2014 | 54:56.7 | 1 |
| -19.047 | 19516.788 | 1.928 | 16.035 | 0.214089 | 7/15/2014 | 54:56.8 | 1 |
| -19.264 | 19516.754 | 1.93 | 16.031 | 0.219819 | 7/15/2014 | 54:56.9 | 1 |
| -19.479 | 19516.721 | 1.93 | 16.027 | 0.213224 | 7/15/2014 | 54:57.0 | 1 |
| -19.694 | 19516.688 | 1.93 | 16.023 | 0.214624 | 7/15/2014 | 54:57.1 | 1 |
| -19.908 | 19516.653 | 1.932 | 16.019 | 0.22113 | 7/15/2014 | 54:57.2 | 1 |
| -20.12 | 19516.62 | 1.934 | 16.015 | 0.218201 | 7/15/2014 | 54:57.3 | 1 |
| -20.385 | 19516.578 | 1.936 | 16.011 | 0.218627 | 7/15/2014 | 54:57.4 | 1 |
| -20.697 | 19516.53 | 1.938 | 16.006 | 0.229777 | 7/15/2014 | 54:57.6 | 1 |
| -20.956 | 19516.488 | 1.941 | 16.003 | 0.230005 | 7/15/2014 | 54:57.7 | 1 |
| -21.062 | 19516.472 | 1.944 | 16.001 | 0.231568 | 7/15/2014 | 54:57.7 | 1 |
| -21.271 | 19516.439 | 1.949 | 15.998 | 0.238084 | 7/15/2014 | 54:57.8 | 1 |
| -21.478 | 19516.407 | 1.949 | 15.996 | 0.240615 | 7/15/2014 | 54:57.9 | 1 |
| -21.687 | 19516.373 | 1.954 | 15.995 | 0.242308 | 7/15/2014 | 54:58.0 | 1 |

| | | | | | | | |
|---|---|---|---|---|---|---|---|
| -21.894 | 19516.34 | 1.96 | 15.993 | 0.244447 | 7/15/2014 | 54:58.1 | 1 |
| -22.099 | 19516.308 | 1.963 | 15.993 | 0.246169 | 7/15/2014 | 54:58.2 | 1 |
| -22.304 | 19516.276 | 1.966 | 15.992 | 0.245885 | 7/15/2014 | 54:58.3 | 1 |
| -22.659 | 19516.219 | 1.968 | 15.992 | 0.250794 | 7/15/2014 | 54:58.5 | 1 |
| -22.863 | 19516.187 | 1.972 | 15.993 | 0.250926 | 7/15/2014 | 54:58.6 | 1 |
| -23.116 | 19516.146 | 1.972 | 15.993 | 0.250406 | 7/15/2014 | 54:58.7 | 1 |
| -23.219 | 19516.129 | 1.976 | 15.993 | 0.248524 | 7/15/2014 | 54:58.7 | 1 |
| -23.425 | 19516.096 | 1.982 | 15.994 | 0.254538 | 7/15/2014 | 54:58.8 | 1 |
| -23.63 | 19516.063 | 1.984 | 15.994 | 0.246566 | 7/15/2014 | 54:58.9 | 1 |
| -23.838 | 19516.03 | 1.992 | 15.994 | 0.249226 | 7/15/2014 | 54:59.0 | 1 |
| -24.046 | 19515.996 | 2 | 15.994 | 0.247812 | 7/15/2014 | 54:59.1 | 1 |
| -24.254 | 19515.963 | 2.005 | 15.993 | 0.245564 | 7/15/2014 | 54:59.2 | 1 |
| -24.461 | 19515.929 | 2.01 | 15.992 | 0.249581 | 7/15/2014 | 54:59.3 | 1 |
| -24.722 | 19515.887 | 2.016 | 15.99 | 0.243063 | 7/15/2014 | 54:59.4 | 1 |
| -24.933 | 19515.852 | 2.019 | 15.988 | 0.24412 | 7/15/2014 | 54:59.5 | 1 |
| -25.142 | 19515.818 | 2.017 | 15.986 | 0.246215 | 7/15/2014 | 54:59.6 | 1 |
| -25.409 | 19515.775 | 2.02 | 15.984 | 0.24431 | 7/15/2014 | 54:59.7 | 1 |
| -25.623 | 19515.74 | 2.017 | 15.983 | 0.24338 | 7/15/2014 | 54:59.8 | 1 |
| -25.839 | 19515.705 | 2.017 | 15.981 | 0.24425 | 7/15/2014 | 54:59.9 | 1 |
| -26.055 | 19515.668 | 2.019 | 15.981 | 0.242166 | 7/15/2014 | 55:00.0 | 1 |
| -26.273 | 19515.633 | 2.023 | 15.981 | 0.252954 | 7/15/2014 | 55:00.1 | 1 |
| -26.49 | 19515.598 | 2.025 | 15.982 | 0.246873 | 7/15/2014 | 55:00.2 | 1 |
| -26.705 | 19515.563 | 2.027 | 15.984 | 0.254205 | 7/15/2014 | 55:00.3 | 1 |
| -26.975 | 19515.518 | 2.036 | 15.987 | 0.259696 | 7/15/2014 | 55:00.4 | 1 |
| -27.19 | 19515.482 | 2.04 | 15.991 | 0.273983 | 7/15/2014 | 55:00.5 | 1 |
| -27.406 | 19515.447 | 2.045 | 15.996 | 0.267643 | 7/15/2014 | 55:00.6 | 1 |
| -27.674 | 19515.402 | 2.048 | 16.001 | 0.270268 | 7/15/2014 | 55:00.7 | 1 |
| -27.89 | 19515.367 | 2.051 | 16.008 | 0.26966 | 7/15/2014 | 55:00.8 | 1 |
| -28.106 | 19515.332 | 2.051 | 16.014 | 0.274684 | 7/15/2014 | 55:00.9 | 1 |
| -28.321 | 19515.296 | 2.051 | 16.021 | 0.273176 | 7/15/2014 | 55:01.0 | 1 |
| -28.536 | 19515.26 | 2.051 | 16.028 | 0.268623 | 7/15/2014 | 55:01.1 | 1 |
| -28.749 | 19515.226 | 2.049 | 16.034 | 0.266873 | 7/15/2014 | 55:01.2 | 1 |
| -28.962 | 19515.19 | 2.051 | 16.04 | 0.271807 | 7/15/2014 | 55:01.3 | 1 |
| -29.329 | 19515.131 | 2.05 | 16.045 | 0.271544 | 7/15/2014 | 55:01.5 | 1 |
| -29.538 | 19515.096 | 2.052 | 16.048 | 0.27302 | 7/15/2014 | 55:01.6 | 1 |
| -29.797 | 19515.054 | 2.047 | 16.049 | 0.275399 | 7/15/2014 | 55:01.7 | 1 |
| -29.903 | 19515.036 | 2.05 | 16.05 | 0.277148 | 7/15/2014 | 55:01.7 | 1 |
| -30.113 | 19515.002 | 2.05 | 16.05 | 0.274091 | 7/15/2014 | 55:01.8 | 1 |
| -30.323 | 19514.968 | 2.055 | 16.048 | 0.277106 | 7/15/2014 | 55:01.9 | 1 |
| -30.533 | 19514.933 | 2.055 | 16.046 | 0.275687 | 7/15/2014 | 55:02.0 | 1 |
| -30.742 | 19514.899 | 2.055 | 16.043 | 0.270244 | 7/15/2014 | 55:02.1 | 1 |
| -30.952 | 19514.865 | 2.059 | 16.039 | 0.27146 | 7/15/2014 | 55:02.2 | 1 |
| -31.16 | 19514.83 | 2.065 | 16.036 | 0.270696 | 7/15/2014 | 55:02.3 | 1 |
| -31.521 | 19514.772 | 2.074 | 16.031 | 0.270003 | 7/15/2014 | 55:02.5 | 1 |
| -31.728 | 19514.739 | 2.084 | 16.028 | 0.267975 | 7/15/2014 | 55:02.6 | 1 |
| -31.985 | 19514.697 | 2.086 | 16.025 | 0.272282 | 7/15/2014 | 55:02.7 | 1 |
| -32.089 | 19514.68 | 2.088 | 16.023 | 0.271202 | 7/15/2014 | 55:02.7 | 1 |

| | | | | | | | |
|---|---|---|---|---|---|---|---|
| -32.298 | 19514.646 | 2.092 | 16.02 | 0.275884 | 7/15/2014 | 55:02.8 | 1 |
| -32.507 | 19514.612 | 2.092 | 16.017 | 0.281966 | 7/15/2014 | 55:02.9 | 1 |
| -32.718 | 19514.578 | 2.094 | 16.014 | 0.279321 | 7/15/2014 | 55:03.0 | 1 |
| -32.927 | 19514.544 | 2.096 | 16.01 | 0.281654 | 7/15/2014 | 55:03.1 | 1 |
| -33.135 | 19514.512 | 2.096 | 16.004 | 0.284766 | 7/15/2014 | 55:03.2 | 1 |
| -33.343 | 19514.478 | 2.096 | 15.998 | 0.282888 | 7/15/2014 | 55:03.3 | 1 |
| -33.705 | 19514.42 | 2.099 | 15.985 | 0.272554 | 7/15/2014 | 55:03.5 | 1 |
| -33.913 | 19514.387 | 2.104 | 15.975 | 0.26158 | 7/15/2014 | 55:03.6 | 1 |
| -34.17 | 19514.345 | 2.106 | 15.965 | 0.254184 | 7/15/2014 | 55:03.7 | 1 |
| -34.276 | 19514.328 | 2.111 | 15.96 | 0.250502 | 7/15/2014 | 55:03.7 | 1 |
| -34.485 | 19514.295 | 2.113 | 15.947 | 0.244533 | 7/15/2014 | 55:03.8 | 1 |
| -34.694 | 19514.264 | 2.115 | 15.933 | 0.239635 | 7/15/2014 | 55:03.9 | 1 |
| -34.904 | 19514.229 | 2.117 | 15.919 | 0.241764 | 7/15/2014 | 55:04.0 | 1 |
| -35.116 | 19514.196 | 2.121 | 15.905 | 0.239125 | 7/15/2014 | 55:04.1 | 1 |
| -35.324 | 19514.164 | 2.123 | 15.89 | 0.238626 | 7/15/2014 | 55:04.2 | 1 |
| -35.532 | 19514.132 | 2.128 | 15.876 | 0.235627 | 7/15/2014 | 55:04.3 | 1 |
| -35.897 | 19514.074 | 2.131 | 15.855 | 0.240478 | 7/15/2014 | 55:04.5 | 1 |
| -36.105 | 19514.043 | 2.138 | 15.842 | 0.241202 | 7/15/2014 | 55:04.6 | 1 |
| -36.367 | 19514.004 | 2.142 | 15.829 | 0.24181 | 7/15/2014 | 55:04.7 | 1 |
| -36.471 | 19513.987 | 2.145 | 15.822 | 0.241185 | 7/15/2014 | 55:04.7 | 1 |
| -36.684 | 19513.955 | 2.147 | 15.811 | 0.243535 | 7/15/2014 | 55:04.8 | 1 |
| -36.896 | 19513.922 | 2.147 | 15.8 | 0.240813 | 7/15/2014 | 55:04.9 | 1 |
| -37.109 | 19513.89 | 2.15 | 15.791 | 0.244131 | 7/15/2014 | 55:05.0 | 1 |
| -37.322 | 19513.857 | 2.152 | 15.782 | 0.244234 | 7/15/2014 | 55:05.1 | 1 |
| -37.535 | 19513.826 | 2.155 | 15.773 | 0.247085 | 7/15/2014 | 55:05.2 | 1 |
| -37.748 | 19513.795 | 2.155 | 15.766 | 0.246111 | 7/15/2014 | 55:05.3 | 1 |
| -38.014 | 19513.755 | 2.155 | 15.759 | 0.250321 | 7/15/2014 | 55:05.4 | 1 |
| -38.228 | 19513.724 | 2.155 | 15.754 | 0.247823 | 7/15/2014 | 55:05.5 | 1 |
| -38.444 | 19513.69 | 2.164 | 15.748 | 0.256549 | 7/15/2014 | 55:05.6 | 1 |
| -38.713 | 19513.652 | 2.167 | 15.744 | 0.244313 | 7/15/2014 | 55:05.7 | 1 |
| -38.931 | 19513.621 | 2.17 | 15.74 | 0.246471 | 7/15/2014 | 55:05.8 | 1 |
| -39.148 | 19513.59 | 2.167 | 15.737 | 0.244082 | 7/15/2014 | 55:05.9 | 1 |
| -39.368 | 19513.559 | 2.167 | 15.735 | 0.235059 | 7/15/2014 | 55:06.0 | 1 |
| -39.586 | 19513.526 | 2.167 | 15.733 | 0.233824 | 7/15/2014 | 55:06.1 | 1 |
| -39.806 | 19513.495 | 2.17 | 15.732 | 0.232052 | 7/15/2014 | 55:06.2 | 1 |
| -40.024 | 19513.466 | 2.17 | 15.732 | 0.234289 | 7/15/2014 | 55:06.3 | 1 |
| -40.296 | 19513.428 | 2.168 | 15.732 | 0.23326 | 7/15/2014 | 55:06.4 | 1 |
| -40.516 | 19513.396 | 2.168 | 15.733 | 0.230381 | 7/15/2014 | 55:06.5 | 1 |
| -40.734 | 19513.367 | 2.165 | 15.734 | 0.235906 | 7/15/2014 | 55:06.6 | 1 |
| -41.007 | 19513.33 | 2.165 | 15.736 | 0.238658 | 7/15/2014 | 55:06.7 | 1 |
| -41.227 | 19513.299 | 2.17 | 15.739 | 0.24527 | 7/15/2014 | 55:06.8 | 1 |
| -41.449 | 19513.27 | 2.174 | 15.742 | 0.249668 | 7/15/2014 | 55:06.9 | 1 |
| -41.669 | 19513.241 | 2.172 | 15.745 | 0.243948 | 7/15/2014 | 55:07.0 | 1 |
| -41.892 | 19513.211 | 2.172 | 15.748 | 0.256984 | 7/15/2014 | 55:07.1 | 1 |
| -42.114 | 19513.182 | 2.172 | 15.752 | 0.277586 | 7/15/2014 | 55:07.2 | 1 |
| -42.333 | 19513.154 | 2.169 | 15.757 | 0.253351 | 7/15/2014 | 55:07.3 | 1 |
| -42.607 | 19513.118 | 2.169 | 15.761 | 0.261974 | 7/15/2014 | 55:07.4 | 1 |

| | | | | | | | |
|---|---|---|---|---|---|---|---|
| -42.827 | 19513.088 | 2.169 | 15.765 | 0.254525 | 7/15/2014 | 55:07.5 | 1 |
| -43.047 | 19513.062 | 2.167 | 15.769 | 0.257843 | 7/15/2014 | 55:07.6 | 1 |
| -43.323 | 19513.026 | 2.167 | 15.773 | 0.256206 | 7/15/2014 | 55:07.7 | 1 |
| -43.545 | 19512.998 | 2.168 | 15.777 | 0.255931 | 7/15/2014 | 55:07.8 | 1 |
| -43.769 | 19512.971 | 2.165 | 15.78 | 0.249199 | 7/15/2014 | 55:07.9 | 1 |
| -43.993 | 19512.943 | 2.165 | 15.783 | 0.258564 | 7/15/2014 | 55:08.0 | 1 |
| -44.217 | 19512.915 | 2.168 | 15.786 | 0.257209 | 7/15/2014 | 55:08.1 | 1 |
| -44.441 | 19512.887 | 2.171 | 15.788 | 0.267763 | 7/15/2014 | 55:08.2 | 1 |
| -44.666 | 19512.86 | 2.175 | 15.791 | 0.2651 | 7/15/2014 | 55:08.3 | 1 |
| -44.944 | 19512.826 | 2.168 | 15.793 | 0.261864 | 7/15/2014 | 55:08.4 | 1 |
| -45.168 | 19512.801 | 2.168 | 15.795 | 0.265537 | 7/15/2014 | 55:08.5 | 1 |
| -45.391 | 19512.773 | 2.162 | 15.797 | 0.279032 | 7/15/2014 | 55:08.6 | 1 |
| -45.676 | 19512.74 | 2.16 | 15.799 | 0.243963 | 7/15/2014 | 55:08.7 | 1 |
| -45.904 | 19512.713 | 2.157 | 15.801 | 0.264579 | 7/15/2014 | 55:08.8 | 1 |
| -46.134 | 19512.688 | 2.149 | 15.802 | 0.242519 | 7/15/2014 | 55:08.9 | 1 |
| -46.366 | 19512.66 | 2.142 | 15.803 | 0.255057 | 7/15/2014 | 55:09.0 | 1 |
| -46.478 | 19512.646 | 2.138 | 15.804 | 0.256078 | 7/15/2014 | 55:09.1 | 1 |
| -46.708 | 19512.623 | 2.138 | 15.805 | 0.259122 | 7/15/2014 | 55:09.2 | 1 |
| -46.941 | 19512.596 | 2.136 | 15.805 | 0.24258 | 7/15/2014 | 55:09.3 | 1 |
| -47.346 | 19512.552 | 2.132 | 15.804 | 0.273022 | 7/15/2014 | 55:09.4 | 1 |
| -47.579 | 19512.525 | 2.132 | 15.804 | 0.260705 | 7/15/2014 | 55:09.5 | 1 |
| -47.811 | 19512.502 | 2.127 | 15.803 | 0.278672 | 7/15/2014 | 55:09.6 | 1 |
| -48.104 | 19512.471 | 2.125 | 15.802 | 0.266143 | 7/15/2014 | 55:09.7 | 1 |
| -48.221 | 19512.459 | 2.124 | 15.802 | 0.266892 | 7/15/2014 | 55:09.8 | 1 |
| -48.459 | 19512.434 | 2.121 | 15.801 | 0.268737 | 7/15/2014 | 55:09.9 | 1 |
| -48.694 | 19512.409 | 2.115 | 15.8 | 0.252017 | 7/15/2014 | 55:10.0 | 1 |
| -48.932 | 19512.385 | 2.115 | 15.8 | 0.239504 | 7/15/2014 | 55:10.1 | 1 |
| -49.167 | 19512.36 | 2.11 | 15.8 | 0.242489 | 7/15/2014 | 55:10.2 | 1 |
| -49.405 | 19512.337 | 2.108 | 15.8 | 0.228694 | 7/15/2014 | 55:10.3 | 1 |
| -49.818 | 19512.297 | 2.1 | 15.802 | 0.22455 | 7/15/2014 | 55:10.4 | 1 |
| -50.053 | 19512.274 | 2.097 | 15.804 | 0.229901 | 7/15/2014 | 55:10.5 | 1 |
| -50.289 | 19512.251 | 2.09 | 15.807 | 0.231663 | 7/15/2014 | 55:10.6 | 1 |
| -50.584 | 19512.223 | 2.082 | 15.81 | 0.246321 | 7/15/2014 | 55:10.7 | 1 |
| -50.702 | 19512.211 | 2.076 | 15.811 | 0.244217 | 7/15/2014 | 55:10.8 | 1 |
| -50.941 | 19512.188 | 2.079 | 15.815 | 0.24764 | 7/15/2014 | 55:10.9 | 1 |
| -51.178 | 19512.168 | 2.07 | 15.819 | 0.249818 | 7/15/2014 | 55:11.0 | 1 |
| -51.417 | 19512.145 | 2.073 | 15.824 | 0.263732 | 7/15/2014 | 55:11.1 | 1 |
| -51.654 | 19512.124 | 2.061 | 15.828 | 0.256931 | 7/15/2014 | 55:11.2 | 1 |
| -51.891 | 19512.104 | 2.058 | 15.832 | 0.276019 | 7/15/2014 | 55:11.3 | 1 |
| -52.306 | 19512.066 | 2.054 | 15.839 | 0.250942 | 7/15/2014 | 55:11.4 | 1 |
| -52.544 | 19512.046 | 2.054 | 15.843 | 0.257446 | 7/15/2014 | 55:11.5 | 1 |
| -52.781 | 19512.025 | 2.046 | 15.847 | 0.264835 | 7/15/2014 | 55:11.6 | 1 |
| -53.079 | 19512 | 2.043 | 15.852 | 0.247279 | 7/15/2014 | 55:11.7 | 1 |
| -53.199 | 19511.99 | 2.038 | 15.854 | 0.247662 | 7/15/2014 | 55:11.8 | 1 |
| -53.438 | 19511.971 | 2.041 | 15.858 | 0.247025 | 7/15/2014 | 55:11.9 | 1 |
| -53.679 | 19511.95 | 2.038 | 15.863 | 0.253773 | 7/15/2014 | 55:12.0 | 1 |
| -53.92 | 19511.933 | 2.035 | 15.869 | 0.248313 | 7/15/2014 | 55:12.1 | 1 |

| | | | | | | | |
|---|---|---|---|---|---|---|---|
| -54.161 | 19511.913 | 2.035 | 15.874 | 0.241257 | 7/15/2014 | 55:12.2 | 1 |
| -54.401 | 19511.893 | 2.032 | 15.88 | 0.235959 | 7/15/2014 | 55:12.3 | 1 |
| -54.823 | 19511.861 | 2.03 | 15.889 | 0.243594 | 7/15/2014 | 55:12.4 | 1 |
| -55.062 | 19511.843 | 2.027 | 15.895 | 0.236601 | 7/15/2014 | 55:12.5 | 1 |
| -55.303 | 19511.824 | 2.024 | 15.901 | 0.243189 | 7/15/2014 | 55:12.6 | 1 |
| -55.603 | 19511.805 | 2.022 | 15.908 | 0.232806 | 7/15/2014 | 55:12.7 | 1 |
| -55.725 | 19511.795 | 2.018 | 15.911 | 0.233881 | 7/15/2014 | 55:12.8 | 1 |
| -55.966 | 19511.776 | 2.022 | 15.917 | 0.2295 | 7/15/2014 | 55:12.9 | 1 |
| -56.207 | 19511.76 | 2.018 | 15.924 | 0.225603 | 7/15/2014 | 55:13.0 | 1 |
| -56.45 | 19511.742 | 2.018 | 15.931 | 0.223899 | 7/15/2014 | 55:13.1 | 1 |
| -56.689 | 19511.726 | 2.008 | 15.938 | 0.226108 | 7/15/2014 | 55:13.2 | 1 |
| -56.932 | 19511.709 | 2.011 | 15.946 | 0.215094 | 7/15/2014 | 55:13.3 | 1 |
| -57.348 | 19511.683 | 2.004 | 15.958 | 0.227433 | 7/15/2014 | 55:13.4 | 1 |
| -57.585 | 19511.666 | 2.003 | 15.966 | 0.221128 | 7/15/2014 | 55:13.5 | 1 |
| -57.821 | 19511.651 | 1.997 | 15.974 | 0.233062 | 7/15/2014 | 55:13.6 | 1 |
| -58.116 | 19511.632 | 1.995 | 15.981 | 0.240617 | 7/15/2014 | 55:13.7 | 1 |
| -58.235 | 19511.624 | 1.991 | 15.985 | 0.246721 | 7/15/2014 | 55:13.8 | 1 |
| -58.473 | 19511.609 | 1.991 | 15.992 | 0.22582 | 7/15/2014 | 55:13.9 | 1 |
| -58.707 | 19511.595 | 1.985 | 15.997 | 0.239966 | 7/15/2014 | 55:14.0 | 1 |
| -58.942 | 19511.581 | 1.987 | 16 | 0.221262 | 7/15/2014 | 55:14.1 | 1 |
| -59.176 | 19511.566 | 1.985 | 16.002 | 0.232129 | 7/15/2014 | 55:14.2 | 1 |
| -59.411 | 19511.553 | 1.988 | 16.002 | 0.216567 | 7/15/2014 | 55:14.3 | 1 |
| -59.815 | 19511.53 | 1.985 | 15.998 | 0.194328 | 7/15/2014 | 55:14.4 | 1 |
| -60.046 | 19511.517 | 1.985 | 15.993 | 0.201134 | 7/15/2014 | 55:14.5 | 1 |
| -60.279 | 19511.503 | 1.988 | 15.988 | 0.186512 | 7/15/2014 | 55:14.6 | 1 |
| -60.57 | 19511.487 | 1.985 | 15.982 | 0.184765 | 7/15/2014 | 55:14.7 | 1 |
| -60.686 | 19511.48 | 1.984 | 15.979 | 0.173603 | 7/15/2014 | 55:14.8 | 1 |
| -60.922 | 19511.467 | 1.984 | 15.971 | 0.175728 | 7/15/2014 | 55:14.9 | 1 |
| -61.156 | 19511.455 | 1.975 | 15.964 | 0.182806 | 7/15/2014 | 55:15.0 | 1 |
| -61.394 | 19511.443 | 1.978 | 15.957 | 0.180489 | 7/15/2014 | 55:15.1 | 1 |
| -61.633 | 19511.43 | 1.978 | 15.951 | 0.19133 | 7/15/2014 | 55:15.2 | 1 |
| -61.872 | 19511.416 | 1.978 | 15.944 | 0.184392 | 7/15/2014 | 55:15.3 | 1 |
| -62.29 | 19511.396 | 1.971 | 15.936 | 0.184747 | 7/15/2014 | 55:15.4 | 1 |
| -62.53 | 19511.383 | 1.971 | 15.93 | 0.189901 | 7/15/2014 | 55:15.5 | 1 |
| -62.77 | 19511.371 | 1.968 | 15.924 | 0.186303 | 7/15/2014 | 55:15.6 | 1 |
| -63.07 | 19511.355 | 1.968 | 15.919 | 0.195871 | 7/15/2014 | 55:15.7 | 1 |
| -63.193 | 19511.349 | 1.964 | 15.916 | 0.185916 | 7/15/2014 | 55:15.8 | 1 |
| -63.436 | 19511.336 | 1.964 | 15.912 | 0.200489 | 7/15/2014 | 55:15.9 | 1 |
| -63.679 | 19511.324 | 1.956 | 15.907 | 0.207551 | 7/15/2014 | 55:16.0 | 1 |
| -63.923 | 19511.313 | 1.956 | 15.903 | 0.212299 | 7/15/2014 | 55:16.1 | 1 |
| -64.166 | 19511.302 | 1.952 | 15.9 | 0.209887 | 7/15/2014 | 55:16.2 | 1 |
| -64.408 | 19511.291 | 1.952 | 15.897 | 0.211263 | 7/15/2014 | 55:16.3 | 1 |
| -64.83 | 19511.27 | 1.951 | 15.893 | 0.225913 | 7/15/2014 | 55:16.4 | 1 |
| -65.071 | 19511.258 | 1.948 | 15.891 | 0.197833 | 7/15/2014 | 55:16.5 | 1 |
| -65.312 | 19511.247 | 1.945 | 15.889 | 0.212186 | 7/15/2014 | 55:16.6 | 1 |
| -65.613 | 19511.232 | 1.945 | 15.887 | 0.20064 | 7/15/2014 | 55:16.7 | 1 |
| -65.735 | 19511.228 | 1.944 | 15.886 | 0.196264 | 7/15/2014 | 55:16.8 | 1 |

| | | | | | | | |
|---|---|---|---|---|---|---|---|
| -65.978 | 19511.219 | 1.944 | 15.883 | 0.199578 | 7/15/2014 | 55:16.9 | 1 |
| -66.219 | 19511.207 | 1.945 | 15.88 | 0.195565 | 7/15/2014 | 55:17.0 | 1 |
| -66.464 | 19511.196 | 1.956 | 15.876 | 0.170722 | 7/15/2014 | 55:17.1 | 1 |
| -66.705 | 19511.186 | 1.947 | 15.872 | 0.199259 | 7/15/2014 | 55:17.2 | 1 |
| -66.949 | 19511.175 | 1.947 | 15.867 | 0.180831 | 7/15/2014 | 55:17.3 | 1 |
| -67.372 | 19511.156 | 1.949 | 15.857 | 0.167684 | 7/15/2014 | 55:17.4 | 1 |
| -67.613 | 19511.146 | 1.951 | 15.851 | 0.163543 | 7/15/2014 | 55:17.5 | 1 |
| -67.855 | 19511.137 | 1.951 | 15.845 | 0.160585 | 7/15/2014 | 55:17.6 | 1 |
| -68.157 | 19511.124 | 1.951 | 15.839 | 0.152757 | 7/15/2014 | 55:17.7 | 1 |
| -68.279 | 19511.119 | 1.945 | 15.835 | 0.139466 | 7/15/2014 | 55:17.8 | 1 |
| -68.524 | 19511.109 | 1.953 | 15.829 | 0.14347 | 7/15/2014 | 55:17.9 | 1 |
| -68.767 | 19511.099 | 1.953 | 15.824 | 0.117286 | 7/15/2014 | 55:18.0 | 1 |
| -69.012 | 19511.09 | 1.959 | 15.82 | 0.114914 | 7/15/2014 | 55:18.1 | 1 |
| -69.254 | 19511.078 | 1.957 | 15.816 | 0.120293 | 7/15/2014 | 55:18.2 | 1 |
| -69.499 | 19511.069 | 1.963 | 15.812 | 0.110269 | 7/15/2014 | 55:18.3 | 1 |
| -69.923 | 19511.053 | 1.965 | 15.81 | 0.115211 | 7/15/2014 | 55:18.4 | 1 |
| -70.165 | 19511.043 | 1.965 | 15.808 | 0.0944197 | 7/15/2014 | 55:18.5 | 1 |
| -70.407 | 19511.033 | 1.965 | 15.808 | 0.121157 | 7/15/2014 | 55:18.6 | 1 |
| -70.709 | 19511.021 | 1.97 | 15.809 | 0.122825 | 7/15/2014 | 55:18.7 | 1 |
| -70.831 | 19511.016 | 1.969 | 15.809 | 0.10591 | 7/15/2014 | 55:18.8 | 1 |
| -71.075 | 19511.007 | 1.974 | 15.811 | 0.111766 | 7/15/2014 | 55:18.9 | 1 |
| -71.318 | 19510.996 | 1.971 | 15.815 | 0.0955319 | 7/15/2014 | 55:19.0 | 1 |
| -71.562 | 19510.988 | 1.978 | 15.82 | 0.100135 | 7/15/2014 | 55:19.1 | 1 |
| -71.805 | 19510.977 | 1.98 | 15.826 | 0.116885 | 7/15/2014 | 55:19.2 | 1 |
| -72.048 | 19510.969 | 1.985 | 15.832 | 0.111532 | 7/15/2014 | 55:19.3 | 1 |
| -72.472 | 19510.951 | 1.989 | 15.844 | 0.119278 | 7/15/2014 | 55:19.4 | 1 |
| -72.715 | 19510.943 | 1.986 | 15.851 | 0.123537 | 7/15/2014 | 55:19.5 | 1 |
| -72.957 | 19510.932 | 1.986 | 15.859 | 0.133776 | 7/15/2014 | 55:19.6 | 1 |
| -73.262 | 19510.922 | 1.991 | 15.865 | 0.126682 | 7/15/2014 | 55:19.7 | 1 |
| -73.384 | 19510.916 | 1.991 | 15.869 | 0.126731 | 7/15/2014 | 55:19.8 | 1 |
| -73.631 | 19510.906 | 1.994 | 15.874 | 0.127097 | 7/15/2014 | 55:19.9 | 1 |
| -73.877 | 19510.898 | 1.992 | 15.878 | 0.129781 | 7/15/2014 | 55:20.0 | 1 |
| -74.126 | 19510.887 | 1.994 | 15.881 | 0.119305 | 7/15/2014 | 55:20.1 | 1 |
| -74.374 | 19510.879 | 1.991 | 15.883 | 0.119176 | 7/15/2014 | 55:20.2 | 1 |
| -74.624 | 19510.867 | 2.004 | 15.883 | 0.140641 | 7/15/2014 | 55:20.3 | 1 |
| -74.931 | 19510.857 | 1.999 | 15.882 | 0.126459 | 7/15/2014 | 55:20.4 | 1 |
| -75.182 | 19510.846 | 2.002 | 15.881 | 0.139631 | 7/15/2014 | 55:20.5 | 1 |
| -75.432 | 19510.838 | 1.998 | 15.879 | 0.100326 | 7/15/2014 | 55:20.6 | 1 |
| -75.683 | 19510.827 | 2.004 | 15.876 | 0.117832 | 7/15/2014 | 55:20.7 | 1 |
| -75.996 | 19510.816 | 2.008 | 15.873 | 0.106228 | 7/15/2014 | 55:20.8 | 1 |
| -76.25 | 19510.806 | 2.005 | 15.869 | 0.0881431 | 7/15/2014 | 55:20.9 | 1 |
| -76.501 | 19510.797 | 2.002 | 15.864 | 0.100632 | 7/15/2014 | 55:21.0 | 1 |
| -76.756 | 19510.787 | 2.01 | 15.857 | 0.0963398 | 7/15/2014 | 55:21.1 | 1 |
| -77.008 | 19510.777 | 2.006 | 15.849 | 0.104332 | 7/15/2014 | 55:21.2 | 1 |
| -77.261 | 19510.769 | 2.006 | 15.84 | 0.107805 | 7/15/2014 | 55:21.3 | 1 |
| -77.575 | 19510.756 | 2 | 15.83 | 0.118524 | 7/15/2014 | 55:21.4 | 1 |
| -77.827 | 19510.747 | 1.996 | 15.818 | 0.129713 | 7/15/2014 | 55:21.5 | 1 |

| | | | | | | | |
|---|---|---|---|---|---|---|---|
| -78.078 | 19510.736 | 1.996 | 15.807 | 0.145018 | 7/15/2014 | 55:21.6 | 1 |
| -78.332 | 19510.729 | 1.992 | 15.796 | 0.138792 | 7/15/2014 | 55:21.7 | 1 |
| -78.645 | 19510.715 | 1.986 | 15.785 | 0.124331 | 7/15/2014 | 55:21.8 | 1 |
| -78.899 | 19510.707 | 1.99 | 15.775 | 0.120472 | 7/15/2014 | 55:21.9 | 1 |
| -79.15 | 19510.695 | 1.99 | 15.766 | 0.113618 | 7/15/2014 | 55:22.0 | 1 |
| -79.404 | 19510.688 | 1.993 | 15.759 | 0.104187 | 7/15/2014 | 55:22.1 | 1 |
| -79.654 | 19510.676 | 1.993 | 15.753 | 0.098178 | 7/15/2014 | 55:22.2 | 1 |
| -79.907 | 19510.668 | 2.003 | 15.749 | 0.093691 | 7/15/2014 | 55:22.3 | 1 |
| -80.219 | 19510.654 | 2.003 | 15.746 | 0.0980822 | 7/15/2014 | 55:22.4 | 1 |
| -80.47 | 19510.646 | 2.006 | 15.745 | 0.0963228 | 7/15/2014 | 55:22.5 | 1 |
| -80.719 | 19510.635 | 2.002 | 15.746 | 0.0991012 | 7/15/2014 | 55:22.6 | 1 |
| -80.97 | 19510.627 | 2.002 | 15.748 | 0.0968494 | 7/15/2014 | 55:22.7 | 1 |
| -81.28 | 19510.613 | 2.004 | 15.75 | 0.0989155 | 7/15/2014 | 55:22.8 | 1 |
| -81.533 | 19510.604 | 2.008 | 15.754 | 0.0971954 | 7/15/2014 | 55:22.9 | 1 |
| -81.781 | 19510.594 | 1.998 | 15.758 | 0.0979634 | 7/15/2014 | 55:23.0 | 1 |
| -82.032 | 19510.584 | 2.002 | 15.762 | 0.102782 | 7/15/2014 | 55:23.1 | 1 |
| -82.281 | 19510.574 | 2.002 | 15.765 | 0.0906528 | 7/15/2014 | 55:23.2 | 1 |
| -82.531 | 19510.564 | 2.006 | 15.769 | 0.111291 | 7/15/2014 | 55:23.3 | 1 |
| -82.965 | 19510.547 | 2.01 | 15.772 | 0.106965 | 7/15/2014 | 55:23.4 | 1 |
| -83.211 | 19510.535 | 2.007 | 15.774 | 0.110569 | 7/15/2014 | 55:23.5 | 1 |
| -83.457 | 19510.526 | 2.007 | 15.776 | 0.109475 | 7/15/2014 | 55:23.6 | 1 |
| -83.766 | 19510.514 | 2.015 | 15.777 | 0.102294 | 7/15/2014 | 55:23.7 | 1 |
| -83.89 | 19510.51 | 2.014 | 15.777 | 0.108171 | 7/15/2014 | 55:23.8 | 1 |
| -84.139 | 19510.498 | 2.021 | 15.778 | 0.0937173 | 7/15/2014 | 55:23.9 | 1 |
| -84.385 | 19510.487 | 2.021 | 15.778 | 0.108999 | 7/15/2014 | 55:24.0 | 1 |
| -84.633 | 19510.478 | 2.025 | 15.777 | 0.117029 | 7/15/2014 | 55:24.1 | 1 |
| -84.881 | 19510.467 | 2.021 | 15.777 | 0.110651 | 7/15/2014 | 55:24.2 | 1 |
| -85.127 | 19510.456 | 2.031 | 15.777 | 0.142233 | 7/15/2014 | 55:24.3 | 1 |
| -85.557 | 19510.438 | 2.024 | 15.777 | 0.155328 | 7/15/2014 | 55:24.4 | 1 |
| -85.803 | 19510.427 | 2.022 | 15.778 | 0.160042 | 7/15/2014 | 55:24.5 | 1 |
| -86.049 | 19510.416 | 2.019 | 15.779 | 0.178997 | 7/15/2014 | 55:24.6 | 1 |
| -86.357 | 19510.401 | 2.013 | 15.779 | 0.166955 | 7/15/2014 | 55:24.7 | 1 |
| -86.482 | 19510.396 | 2.013 | 15.78 | 0.189102 | 7/15/2014 | 55:24.8 | 1 |
| -86.731 | 19510.385 | 2.009 | 15.781 | 0.151502 | 7/15/2014 | 55:24.9 | 1 |
| -86.98 | 19510.373 | 1.999 | 15.782 | 0.172547 | 7/15/2014 | 55:25.0 | 1 |
| -87.234 | 19510.361 | 2.007 | 15.783 | 0.159993 | 7/15/2014 | 55:25.1 | 1 |
| -87.483 | 19510.351 | 2.005 | 15.785 | 0.15072 | 7/15/2014 | 55:25.2 | 1 |
| -87.737 | 19510.34 | 2.006 | 15.785 | 0.14798 | 7/15/2014 | 55:25.3 | 1 |
| -88.048 | 19510.323 | 2.005 | 15.786 | 0.150552 | 7/15/2014 | 55:25.4 | 1 |
| -88.302 | 19510.311 | 2.015 | 15.787 | 0.167993 | 7/15/2014 | 55:25.5 | 1 |
| -88.553 | 19510.299 | 2.019 | 15.787 | 0.155027 | 7/15/2014 | 55:25.6 | 1 |
| -88.809 | 19510.286 | 2.026 | 15.787 | 0.152955 | 7/15/2014 | 55:25.7 | 1 |
| -89.124 | 19510.271 | 2.019 | 15.787 | 0.156564 | 7/15/2014 | 55:25.8 | 1 |
| -89.381 | 19510.258 | 2.026 | 15.787 | 0.130899 | 7/15/2014 | 55:25.9 | 1 |
| -89.634 | 19510.245 | 2.026 | 15.788 | 0.13167 | 7/15/2014 | 55:26.0 | 1 |
| -89.892 | 19510.23 | 2.029 | 15.788 | 0.133949 | 7/15/2014 | 55:26.1 | 1 |
| -90.145 | 19510.219 | 2.026 | 15.789 | 0.120675 | 7/15/2014 | 55:26.2 | 1 |

| | | | | | | | |
|---|---|---|---|---|---|---|---|
| -90.401 | 19510.204 | 2.022 | 15.789 | 0.128688 | 7/15/2014 | 55:26.3 | 1 |
| -90.716 | 19510.189 | 2.015 | 15.79 | 0.119505 | 7/15/2014 | 55:26.4 | 1 |
| -90.973 | 19510.174 | 2.019 | 15.79 | 0.138389 | 7/15/2014 | 55:26.5 | 1 |
| -91.224 | 19510.162 | 2.009 | 15.79 | 0.134285 | 7/15/2014 | 55:26.6 | 1 |
| -91.479 | 19510.147 | 2.005 | 15.789 | 0.105903 | 7/15/2014 | 55:26.7 | 1 |
| -91.794 | 19510.131 | 1.996 | 15.787 | 0.130048 | 7/15/2014 | 55:26.8 | 1 |
| -92.048 | 19510.116 | 1.996 | 15.782 | 0.103994 | 7/15/2014 | 55:26.9 | 1 |
| -92.303 | 19510.104 | 1.993 | 15.776 | 0.118968 | 7/15/2014 | 55:27.0 | 1 |
| -92.56 | 19510.088 | 1.995 | 15.768 | 0.133705 | 7/15/2014 | 55:27.1 | 1 |
| -92.812 | 19510.075 | 1.991 | 15.757 | 0.106722 | 7/15/2014 | 55:27.2 | 1 |
| -93.065 | 19510.062 | 1.993 | 15.744 | 0.108397 | 7/15/2014 | 55:27.3 | 1 |
| -93.38 | 19510.043 | 1.993 | 15.729 | 0.114415 | 7/15/2014 | 55:27.4 | 1 |
| -93.632 | 19510.028 | 1.996 | 15.712 | 0.0825755 | 7/15/2014 | 55:27.5 | 1 |
| -93.884 | 19510.015 | 1.992 | 15.694 | 0.115716 | 7/15/2014 | 55:27.6 | 1 |
| -94.137 | 19510 | 1.995 | 15.675 | 0.0772782 | 7/15/2014 | 55:27.7 | 1 |
| -94.45 | 19509.981 | 1.991 | 15.656 | 0.0902571 | 7/15/2014 | 55:27.8 | 1 |
| -94.701 | 19509.968 | 1.991 | 15.637 | 0.106067 | 7/15/2014 | 55:27.9 | 1 |
| -94.953 | 19509.952 | 1.991 | 15.619 | 0.0970142 | 7/15/2014 | 55:28.0 | 1 |
| -95.204 | 19509.938 | 1.991 | 15.602 | 0.132065 | 7/15/2014 | 55:28.1 | 1 |
| -95.454 | 19509.922 | 1.991 | 15.586 | 0.108448 | 7/15/2014 | 55:28.2 | 1 |
| -95.703 | 19509.908 | 1.991 | 15.572 | 0.121721 | 7/15/2014 | 55:28.3 | 1 |
| -96.136 | 19509.881 | 1.989 | 15.553 | 0.137722 | 7/15/2014 | 55:28.4 | 1 |
| -96.383 | 19509.866 | 1.986 | 15.542 | 0.13675 | 7/15/2014 | 55:28.5 | 1 |
| -96.629 | 19509.851 | 1.986 | 15.533 | 0.154993 | 7/15/2014 | 55:28.6 | 1 |
| -96.935 | 19509.832 | 1.984 | 15.525 | 0.146145 | 7/15/2014 | 55:28.7 | 1 |
| -97.06 | 19509.823 | 1.983 | 15.52 | 0.134419 | 7/15/2014 | 55:28.8 | 1 |
| -97.307 | 19509.809 | 1.983 | 15.515 | 0.141606 | 7/15/2014 | 55:28.9 | 1 |
| -97.553 | 19509.793 | 1.974 | 15.511 | 0.169698 | 7/15/2014 | 55:29.0 | 1 |
| -97.8 | 19509.776 | 1.966 | 15.509 | 0.158104 | 7/15/2014 | 55:29.1 | 1 |
| -98.047 | 19509.761 | 1.966 | 15.509 | 0.157658 | 7/15/2014 | 55:29.2 | 1 |
| -98.294 | 19509.744 | 1.969 | 15.512 | 0.128986 | 7/15/2014 | 55:29.3 | 1 |
| -98.723 | 19509.716 | 1.957 | 15.52 | 0.145239 | 7/15/2014 | 55:29.4 | 1 |
| -98.968 | 19509.699 | 1.955 | 15.527 | 0.14162 | 7/15/2014 | 55:29.5 | 1 |
| -99.213 | 19509.684 | 1.953 | 15.535 | 0.111653 | 7/15/2014 | 55:29.6 | 1 |
| -99.519 | 19509.661 | 1.956 | 15.544 | 0.117398 | 7/15/2014 | 55:29.7 | 1 |
| -99.643 | 19509.653 | 1.959 | 15.548 | 0.11806 | 7/15/2014 | 55:29.8 | 1 |
| -99.892 | 19509.637 | 1.951 | 15.558 | 0.136317 | 7/15/2014 | 55:29.9 | 1 |
| -100.139 | 19509.62 | 1.942 | 15.568 | 0.13074 | 7/15/2014 | 55:30.0 | 1 |
| -100.386 | 19509.603 | 1.946 | 15.578 | 0.102562 | 7/15/2014 | 55:30.1 | 1 |
| -100.633 | 19509.584 | 1.942 | 15.588 | 0.130672 | 7/15/2014 | 55:30.2 | 1 |
| -100.882 | 19509.568 | 1.93 | 15.598 | 0.121598 | 7/15/2014 | 55:30.3 | 1 |
| -101.312 | 19509.536 | 1.922 | 15.612 | 0.115632 | 7/15/2014 | 55:30.4 | 1 |
| -101.557 | 19509.52 | 1.914 | 15.62 | 0.107286 | 7/15/2014 | 55:30.5 | 1 |
| -101.804 | 19509.501 | 1.916 | 15.628 | 0.121881 | 7/15/2014 | 55:30.6 | 1 |
| -102.109 | 19509.479 | 1.91 | 15.636 | 0.125547 | 7/15/2014 | 55:30.7 | 1 |
| -102.233 | 19509.469 | 1.903 | 15.64 | 0.0993679 | 7/15/2014 | 55:30.8 | 1 |
| -102.482 | 19509.452 | 1.9 | 15.647 | 0.108137 | 7/15/2014 | 55:30.9 | 1 |

| | | | | | | | |
|---|---|---|---|---|---|---|---|
| -102.729 | 19509.433 | 1.902 | 15.653 | 0.104249 | 7/15/2014 | 55:31.0 | 1 |
| -102.978 | 19509.414 | 1.899 | 15.66 | 0.104313 | 7/15/2014 | 55:31.1 | 1 |
| -103.224 | 19509.396 | 1.889 | 15.666 | 0.0958188 | 7/15/2014 | 55:31.2 | 1 |
| -103.472 | 19509.377 | 1.889 | 15.673 | 0.105767 | 7/15/2014 | 55:31.3 | 1 |
| -103.903 | 19509.344 | 1.881 | 15.684 | 0.116621 | 7/15/2014 | 55:31.4 | 1 |
| -104.147 | 19509.325 | 1.881 | 15.691 | 0.12982 | 7/15/2014 | 55:31.5 | 1 |
| -104.391 | 19509.306 | 1.877 | 15.698 | 0.136681 | 7/15/2014 | 55:31.6 | 1 |
| -104.696 | 19509.281 | 1.874 | 15.706 | 0.139556 | 7/15/2014 | 55:31.7 | 1 |
| -104.821 | 19509.272 | 1.872 | 15.71 | 0.145725 | 7/15/2014 | 55:31.8 | 1 |
| -105.066 | 19509.253 | 1.872 | 15.719 | 0.13232 | 7/15/2014 | 55:31.9 | 1 |
| -105.311 | 19509.234 | 1.868 | 15.727 | 0.147351 | 7/15/2014 | 55:32.0 | 1 |
| -105.555 | 19509.214 | 1.863 | 15.736 | 0.158373 | 7/15/2014 | 55:32.1 | 1 |
| -105.798 | 19509.195 | 1.855 | 15.745 | 0.143737 | 7/15/2014 | 55:32.2 | 1 |
| -106.04 | 19509.176 | 1.853 | 15.754 | 0.154587 | 7/15/2014 | 55:32.3 | 1 |
| -106.458 | 19509.142 | 1.848 | 15.768 | 0.172091 | 7/15/2014 | 55:32.4 | 1 |
| -106.696 | 19509.122 | 1.844 | 15.778 | 0.168942 | 7/15/2014 | 55:32.5 | 1 |
| -106.934 | 19509.104 | 1.839 | 15.787 | 0.166183 | 7/15/2014 | 55:32.6 | 1 |
| -107.229 | 19509.079 | 1.835 | 15.796 | 0.159177 | 7/15/2014 | 55:32.7 | 1 |
| -107.351 | 19509.07 | 1.836 | 15.8 | 0.162002 | 7/15/2014 | 55:32.8 | 1 |
| -107.588 | 19509.051 | 1.833 | 15.809 | 0.174481 | 7/15/2014 | 55:32.9 | 1 |
| -107.825 | 19509.031 | 1.829 | 15.817 | 0.202614 | 7/15/2014 | 55:33.0 | 1 |
| -108.063 | 19509.012 | 1.824 | 15.825 | 0.158116 | 7/15/2014 | 55:33.1 | 1 |
| -108.3 | 19508.992 | 1.824 | 15.832 | 0.164318 | 7/15/2014 | 55:33.2 | 1 |
| -108.536 | 19508.973 | 1.821 | 15.838 | 0.175223 | 7/15/2014 | 55:33.3 | 1 |
| -108.95 | 19508.939 | 1.813 | 15.847 | 0.171848 | 7/15/2014 | 55:33.4 | 1 |
| -109.185 | 19508.92 | 1.809 | 15.853 | 0.160254 | 7/15/2014 | 55:33.5 | 1 |
| -109.423 | 19508.899 | 1.809 | 15.858 | 0.143761 | 7/15/2014 | 55:33.6 | 1 |
| -109.72 | 19508.875 | 1.803 | 15.863 | 0.153752 | 7/15/2014 | 55:33.7 | 1 |
| -109.839 | 19508.865 | 1.802 | 15.865 | 0.147 | 7/15/2014 | 55:33.8 | 1 |
| -110.08 | 19508.846 | 1.796 | 15.869 | 0.142309 | 7/15/2014 | 55:33.9 | 1 |
| -110.32 | 19508.826 | 1.79 | 15.873 | 0.139454 | 7/15/2014 | 55:34.0 | 1 |
| -110.563 | 19508.805 | 1.793 | 15.877 | 0.140206 | 7/15/2014 | 55:34.1 | 1 |
| -110.804 | 19508.785 | 1.787 | 15.881 | 0.144615 | 7/15/2014 | 55:34.2 | 1 |
| -111.049 | 19508.765 | 1.787 | 15.885 | 0.148504 | 7/15/2014 | 55:34.3 | 1 |
| -111.474 | 19508.729 | 1.778 | 15.89 | 0.153397 | 7/15/2014 | 55:34.4 | 1 |
| -111.717 | 19508.709 | 1.772 | 15.894 | 0.161289 | 7/15/2014 | 55:34.5 | 1 |
| -111.961 | 19508.689 | 1.77 | 15.899 | 0.166793 | 7/15/2014 | 55:34.6 | 1 |
| -112.265 | 19508.662 | 1.765 | 15.903 | 0.168316 | 7/15/2014 | 55:34.7 | 1 |
| -112.388 | 19508.652 | 1.76 | 15.906 | 0.171854 | 7/15/2014 | 55:34.8 | 1 |
| -112.636 | 19508.633 | 1.758 | 15.911 | 0.178314 | 7/15/2014 | 55:34.9 | 1 |
| -112.881 | 19508.612 | 1.748 | 15.916 | 0.169259 | 7/15/2014 | 55:35.0 | 1 |
| -113.128 | 19508.592 | 1.745 | 15.922 | 0.173396 | 7/15/2014 | 55:35.1 | 1 |
| -113.371 | 19508.572 | 1.735 | 15.927 | 0.174804 | 7/15/2014 | 55:35.2 | 1 |
| -113.618 | 19508.553 | 1.735 | 15.932 | 0.174218 | 7/15/2014 | 55:35.3 | 1 |
| -114.042 | 19508.518 | 1.725 | 15.94 | 0.16997 | 7/15/2014 | 55:35.4 | 1 |
| -114.285 | 19508.498 | 1.72 | 15.945 | 0.170285 | 7/15/2014 | 55:35.5 | 1 |
| -114.527 | 19508.479 | 1.717 | 15.95 | 0.170813 | 7/15/2014 | 55:35.6 | 1 |

| | | | | | | | |
|---|---|---|---|---|---|---|---|
| -114.826 | 19508.455 | 1.712 | 15.955 | 0.171604 | 7/15/2014 | 55:35.7 | 1 |
| -114.948 | 19508.445 | 1.709 | 15.958 | 0.167239 | 7/15/2014 | 55:35.8 | 1 |
| -115.191 | 19508.426 | 1.707 | 15.963 | 0.165646 | 7/15/2014 | 55:35.9 | 1 |
| -115.431 | 19508.406 | 1.697 | 15.967 | 0.163483 | 7/15/2014 | 55:36.0 | 1 |
| -115.671 | 19508.389 | 1.697 | 15.972 | 0.161987 | 7/15/2014 | 55:36.1 | 1 |
| -115.91 | 19508.369 | 1.692 | 15.976 | 0.159471 | 7/15/2014 | 55:36.2 | 1 |
| -116.149 | 19508.35 | 1.692 | 15.98 | 0.1631 | 7/15/2014 | 55:36.3 | 1 |
| -116.56 | 19508.318 | 1.684 | 15.986 | 0.161978 | 7/15/2014 | 55:36.4 | 1 |
| -116.795 | 19508.301 | 1.682 | 15.989 | 0.163364 | 7/15/2014 | 55:36.5 | 1 |
| -117.03 | 19508.281 | 1.682 | 15.992 | 0.165146 | 7/15/2014 | 55:36.6 | 1 |
| -117.322 | 19508.262 | 1.683 | 15.994 | 0.168659 | 7/15/2014 | 55:36.7 | 1 |
| -117.44 | 19508.252 | 1.676 | 15.996 | 0.169973 | 7/15/2014 | 55:36.8 | 1 |
| -117.676 | 19508.232 | 1.673 | 15.998 | 0.166617 | 7/15/2014 | 55:36.9 | 1 |
| -117.911 | 19508.217 | 1.667 | 16.001 | 0.172582 | 7/15/2014 | 55:37.0 | 1 |
| -118.149 | 19508.197 | 1.663 | 16.004 | 0.174166 | 7/15/2014 | 55:37.1 | 1 |
| -118.385 | 19508.182 | 1.657 | 16.007 | 0.174804 | 7/15/2014 | 55:37.2 | 1 |
| -118.622 | 19508.163 | 1.657 | 16.011 | 0.16434 | 7/15/2014 | 55:37.3 | 1 |
| -119.034 | 19508.135 | 1.648 | 16.02 | 0.168848 | 7/15/2014 | 55:37.4 | 1 |
| -119.269 | 19508.116 | 1.645 | 16.026 | 0.171993 | 7/15/2014 | 55:37.5 | 1 |
| -119.506 | 19508.101 | 1.648 | 16.033 | 0.174521 | 7/15/2014 | 55:37.6 | 1 |
| -119.804 | 19508.078 | 1.643 | 16.04 | 0.177064 | 7/15/2014 | 55:37.7 | 1 |
| -119.923 | 19508.07 | 1.643 | 16.044 | 0.176198 | 7/15/2014 | 55:37.8 | 1 |
| -120.164 | 19508.054 | 1.643 | 16.053 | 0.183586 | 7/15/2014 | 55:37.9 | 1 |
| -120.404 | 19508.037 | 1.633 | 16.061 | 0.193119 | 7/15/2014 | 55:38.0 | 1 |
| -120.646 | 19508.021 | 1.632 | 16.069 | 0.196028 | 7/15/2014 | 55:38.1 | 1 |
| -120.887 | 19508.004 | 1.628 | 16.077 | 0.201092 | 7/15/2014 | 55:38.2 | 1 |
| -121.13 | 19507.988 | 1.624 | 16.084 | 0.195139 | 7/15/2014 | 55:38.3 | 1 |
| -121.551 | 19507.96 | 1.611 | 16.093 | 0.190069 | 7/15/2014 | 55:38.4 | 1 |
| -121.793 | 19507.944 | 1.608 | 16.098 | 0.177185 | 7/15/2014 | 55:38.5 | 1 |
| -122.033 | 19507.93 | 1.603 | 16.102 | 0.172507 | 7/15/2014 | 55:38.6 | 1 |
| -122.332 | 19507.909 | 1.601 | 16.105 | 0.170888 | 7/15/2014 | 55:38.7 | 1 |
| -122.455 | 19507.901 | 1.598 | 16.107 | 0.169756 | 7/15/2014 | 55:38.8 | 1 |
| -122.698 | 19507.887 | 1.596 | 16.109 | 0.171032 | 7/15/2014 | 55:38.9 | 1 |
| -122.938 | 19507.872 | 1.591 | 16.111 | 0.172144 | 7/15/2014 | 55:39.0 | 1 |
| -123.182 | 19507.857 | 1.585 | 16.112 | 0.174592 | 7/15/2014 | 55:39.1 | 1 |
| -123.422 | 19507.843 | 1.579 | 16.113 | 0.180527 | 7/15/2014 | 55:39.2 | 1 |
| -123.662 | 19507.828 | 1.581 | 16.113 | 0.187869 | 7/15/2014 | 55:39.3 | 1 |
| -124.08 | 19507.803 | 1.577 | 16.114 | 0.194656 | 7/15/2014 | 55:39.4 | 1 |
| -124.318 | 19507.79 | 1.572 | 16.114 | 0.195698 | 7/15/2014 | 55:39.5 | 1 |
| -124.556 | 19507.777 | 1.57 | 16.113 | 0.191247 | 7/15/2014 | 55:39.6 | 1 |
| -124.853 | 19507.761 | 1.57 | 16.113 | 0.192082 | 7/15/2014 | 55:39.7 | 1 |
| -124.973 | 19507.754 | 1.571 | 16.112 | 0.203261 | 7/15/2014 | 55:39.8 | 1 |
| -125.213 | 19507.74 | 1.58 | 16.111 | 0.199601 | 7/15/2014 | 55:39.9 | 1 |
| -125.451 | 19507.728 | 1.577 | 16.109 | 0.178817 | 7/15/2014 | 55:40.0 | 1 |
| -125.692 | 19507.715 | 1.582 | 16.106 | 0.175386 | 7/15/2014 | 55:40.1 | 1 |
| -125.93 | 19507.701 | 1.58 | 16.103 | 0.14825 | 7/15/2014 | 55:40.2 | 1 |
| -126.17 | 19507.69 | 1.586 | 16.1 | 0.149818 | 7/15/2014 | 55:40.3 | 1 |

| | | | | | | | |
|---|---|---|---|---|---|---|---|
| -126.586 | 19507.67 | 1.582 | 16.095 | 0.130358 | 7/15/2014 | 55:40.4 | 1 |
| -126.824 | 19507.658 | 1.58 | 16.092 | 0.125847 | 7/15/2014 | 55:40.5 | 1 |
| -127.061 | 19507.646 | 1.576 | 16.089 | 0.122679 | 7/15/2014 | 55:40.6 | 1 |
| -127.359 | 19507.633 | 1.574 | 16.087 | 0.121793 | 7/15/2014 | 55:40.7 | 1 |
| -127.479 | 19507.625 | 1.57 | 16.086 | 0.12865 | 7/15/2014 | 55:40.8 | 1 |
| -127.72 | 19507.616 | 1.573 | 16.085 | 0.139611 | 7/15/2014 | 55:40.9 | 1 |
| -127.96 | 19507.604 | 1.573 | 16.084 | 0.12506 | 7/15/2014 | 55:41.0 | 1 |
| -128.202 | 19507.596 | 1.579 | 16.084 | 0.151942 | 7/15/2014 | 55:41.1 | 1 |
| -128.442 | 19507.584 | 1.574 | 16.085 | 0.137359 | 7/15/2014 | 55:41.2 | 1 |
| -128.683 | 19507.574 | 1.574 | 16.085 | 0.151801 | 7/15/2014 | 55:41.3 | 1 |
| -129.105 | 19507.557 | 1.577 | 16.085 | 0.150813 | 7/15/2014 | 55:41.4 | 1 |
| -129.347 | 19507.549 | 1.579 | 16.084 | 0.138543 | 7/15/2014 | 55:41.5 | 1 |
| -129.588 | 19507.539 | 1.577 | 16.084 | 0.149454 | 7/15/2014 | 55:41.6 | 1 |
| -129.891 | 19507.529 | 1.575 | 16.082 | 0.138103 | 7/15/2014 | 55:41.7 | 1 |
| -130.014 | 19507.523 | 1.574 | 16.082 | 0.153082 | 7/15/2014 | 55:41.8 | 1 |
| -130.258 | 19507.516 | 1.579 | 16.08 | 0.135436 | 7/15/2014 | 55:41.9 | 1 |
| -130.502 | 19507.509 | 1.577 | 16.077 | 0.132013 | 7/15/2014 | 55:42.0 | 1 |
| -130.749 | 19507.498 | 1.582 | 16.073 | 0.121763 | 7/15/2014 | 55:42.1 | 1 |
| -130.994 | 19507.492 | 1.577 | 16.069 | 0.122996 | 7/15/2014 | 55:42.2 | 1 |
| -131.24 | 19507.484 | 1.575 | 16.063 | 0.111748 | 7/15/2014 | 55:42.3 | 1 |
| -131.668 | 19507.473 | 1.582 | 16.052 | 0.109744 | 7/15/2014 | 55:42.4 | 1 |
| -131.911 | 19507.466 | 1.581 | 16.043 | 0.106787 | 7/15/2014 | 55:42.5 | 1 |
| -132.155 | 19507.46 | 1.579 | 16.033 | 0.111473 | 7/15/2014 | 55:42.6 | 1 |
| -132.46 | 19507.451 | 1.582 | 16.021 | 0.110213 | 7/15/2014 | 55:42.7 | 1 |
| -132.584 | 19507.449 | 1.58 | 16.016 | 0.109516 | 7/15/2014 | 55:42.8 | 1 |
| -132.828 | 19507.443 | 1.588 | 16.002 | 0.103369 | 7/15/2014 | 55:42.9 | 1 |
| -133.073 | 19507.438 | 1.597 | 15.988 | 0.0870923 | 7/15/2014 | 55:43.0 | 1 |
| -133.317 | 19507.433 | 1.597 | 15.973 | 0.0856481 | 7/15/2014 | 55:43.1 | 1 |
| -133.559 | 19507.429 | 1.597 | 15.958 | 0.0937274 | 7/15/2014 | 55:43.2 | 1 |
| -133.803 | 19507.424 | 1.603 | 15.942 | 0.0848674 | 7/15/2014 | 55:43.3 | 1 |
| -134.225 | 19507.416 | 1.603 | 15.918 | 0.0729694 | 7/15/2014 | 55:43.4 | 1 |
| -134.465 | 19507.413 | 1.605 | 15.903 | 0.0795555 | 7/15/2014 | 55:43.5 | 1 |
| -134.705 | 19507.41 | 1.609 | 15.888 | 0.0794549 | 7/15/2014 | 55:43.6 | 1 |
| -135.003 | 19507.405 | 1.611 | 15.873 | 0.0708164 | 7/15/2014 | 55:43.7 | 1 |
| -135.124 | 19507.404 | 1.613 | 15.866 | 0.0697633 | 7/15/2014 | 55:43.8 | 1 |
| -135.366 | 19507.402 | 1.621 | 15.852 | 0.0612933 | 7/15/2014 | 55:43.9 | 1 |
| -135.606 | 19507.399 | 1.629 | 15.838 | 0.0553377 | 7/15/2014 | 55:44.0 | 1 |
| -135.847 | 19507.396 | 1.634 | 15.825 | 0.0737353 | 7/15/2014 | 55:44.1 | 1 |
| -136.087 | 19507.396 | 1.64 | 15.812 | 0.0669135 | 7/15/2014 | 55:44.2 | 1 |
| -136.329 | 19507.395 | 1.652 | 15.799 | 0.0697386 | 7/15/2014 | 55:44.3 | 1 |
| -136.748 | 19507.393 | 1.662 | 15.781 | 0.0833054 | 7/15/2014 | 55:44.4 | 1 |
| -136.989 | 19507.393 | 1.662 | 15.77 | 0.0843508 | 7/15/2014 | 55:44.5 | 1 |
| -137.23 | 19507.392 | 1.662 | 15.759 | 0.0713311 | 7/15/2014 | 55:44.6 | 1 |
| -137.533 | 19507.392 | 1.665 | 15.749 | 0.0937455 | 7/15/2014 | 55:44.7 | 1 |
| -137.654 | 19507.392 | 1.672 | 15.744 | 0.0749473 | 7/15/2014 | 55:44.8 | 1 |
| -137.901 | 19507.392 | 1.687 | 15.735 | 0.07541 | 7/15/2014 | 55:44.9 | 1 |
| -138.147 | 19507.392 | 1.687 | 15.727 | 0.0980017 | 7/15/2014 | 55:45.0 | 1 |

| | | | | | | | |
|---|---|---|---|---|---|---|---|
| -138.396 | 19507.394 | 1.689 | 15.72 | 0.0834727 | 7/15/2014 | 55:45.1 | 1 |
| -138.644 | 19507.395 | 1.689 | 15.714 | 0.0876342 | 7/15/2014 | 55:45.2 | 1 |
| -138.893 | 19507.396 | 1.695 | 15.71 | 0.099515 | 7/15/2014 | 55:45.3 | 1 |
| -139.204 | 19507.399 | 1.698 | 15.706 | 0.104635 | 7/15/2014 | 55:45.4 | 1 |
| -139.457 | 19507.4 | 1.704 | 15.703 | 0.108909 | 7/15/2014 | 55:45.5 | 1 |
| -139.708 | 19507.403 | 1.698 | 15.702 | 0.122706 | 7/15/2014 | 55:45.6 | 1 |
| -139.96 | 19507.406 | 1.708 | 15.7 | 0.115293 | 7/15/2014 | 55:45.7 | 1 |
| -140.27 | 19507.41 | 1.707 | 15.699 | 0.119915 | 7/15/2014 | 55:45.8 | 1 |
| -140.523 | 19507.414 | 1.71 | 15.698 | 0.134474 | 7/15/2014 | 55:45.9 | 1 |
| -140.774 | 19507.418 | 1.714 | 15.698 | 0.122009 | 7/15/2014 | 55:46.0 | 1 |
| -141.025 | 19507.423 | 1.72 | 15.697 | 0.115661 | 7/15/2014 | 55:46.1 | 1 |
| -141.275 | 19507.427 | 1.718 | 15.697 | 0.126876 | 7/15/2014 | 55:46.2 | 1 |
| -141.522 | 19507.432 | 1.72 | 15.697 | 0.119679 | 7/15/2014 | 55:46.3 | 1 |
| -141.951 | 19507.441 | 1.713 | 15.698 | 0.133538 | 7/15/2014 | 55:46.4 | 1 |
| -142.196 | 19507.445 | 1.71 | 15.699 | 0.113895 | 7/15/2014 | 55:46.5 | 1 |
| -142.439 | 19507.451 | 1.713 | 15.701 | 0.111288 | 7/15/2014 | 55:46.6 | 1 |
| -142.742 | 19507.461 | 1.713 | 15.704 | 0.105366 | 7/15/2014 | 55:46.7 | 1 |
| -142.865 | 19507.463 | 1.71 | 15.705 | 0.0948161 | 7/15/2014 | 55:46.8 | 1 |
| -143.11 | 19507.47 | 1.715 | 15.708 | 0.093545 | 7/15/2014 | 55:46.9 | 1 |
| -143.351 | 19507.476 | 1.709 | 15.712 | 0.0753647 | 7/15/2014 | 55:47.0 | 1 |
| -143.595 | 19507.484 | 1.715 | 15.717 | 0.0728607 | 7/15/2014 | 55:47.1 | 1 |
| -143.838 | 19507.49 | 1.715 | 15.722 | 0.0746985 | 7/15/2014 | 55:47.2 | 1 |
| -144.08 | 19507.498 | 1.721 | 15.727 | 0.0771569 | 7/15/2014 | 55:47.3 | 1 |
| -144.504 | 19507.514 | 1.718 | 15.734 | 0.0733526 | 7/15/2014 | 55:47.4 | 1 |
| -144.745 | 19507.521 | 1.72 | 15.739 | 0.0722022 | 7/15/2014 | 55:47.5 | 1 |
| -144.985 | 19507.528 | 1.716 | 15.744 | 0.0700693 | 7/15/2014 | 55:47.6 | 1 |
| -145.289 | 19507.541 | 1.719 | 15.749 | 0.071672 | 7/15/2014 | 55:47.7 | 1 |
| -145.411 | 19507.545 | 1.718 | 15.751 | 0.0825679 | 7/15/2014 | 55:47.8 | 1 |
| -145.656 | 19507.555 | 1.718 | 15.755 | 0.0831945 | 7/15/2014 | 55:47.9 | 1 |
| -145.901 | 19507.563 | 1.712 | 15.759 | 0.109869 | 7/15/2014 | 55:48.0 | 1 |
| -146.15 | 19507.574 | 1.716 | 15.763 | 0.0963478 | 7/15/2014 | 55:48.1 | 1 |
| -146.397 | 19507.584 | 1.711 | 15.766 | 0.100408 | 7/15/2014 | 55:48.2 | 1 |
| -146.645 | 19507.594 | 1.713 | 15.768 | 0.133438 | 7/15/2014 | 55:48.3 | 1 |
| -146.953 | 19507.607 | 1.71 | 15.771 | 0.133892 | 7/15/2014 | 55:48.4 | 1 |
| -147.201 | 19507.619 | 1.71 | 15.773 | 0.12646 | 7/15/2014 | 55:48.5 | 1 |
| -147.449 | 19507.629 | 1.71 | 15.775 | 0.130915 | 7/15/2014 | 55:48.6 | 1 |
| -147.699 | 19507.642 | 1.716 | 15.777 | 0.152531 | 7/15/2014 | 55:48.7 | 1 |
| -148.01 | 19507.656 | 1.71 | 15.779 | 0.136859 | 7/15/2014 | 55:48.8 | 1 |
| -148.262 | 19507.669 | 1.712 | 15.78 | 0.132642 | 7/15/2014 | 55:48.9 | 1 |
| -148.511 | 19507.682 | 1.707 | 15.782 | 0.126722 | 7/15/2014 | 55:49.0 | 1 |
| -148.761 | 19507.694 | 1.711 | 15.783 | 0.131826 | 7/15/2014 | 55:49.1 | 1 |
| -149.011 | 19507.707 | 1.705 | 15.784 | 0.134084 | 7/15/2014 | 55:49.2 | 1 |
| -149.261 | 19507.721 | 1.702 | 15.785 | 0.156093 | 7/15/2014 | 55:49.3 | 1 |
| -149.57 | 19507.736 | 1.7 | 15.786 | 0.162819 | 7/15/2014 | 55:49.4 | 1 |
| -149.942 | 19507.759 | 1.703 | 15.788 | 0.162051 | 7/15/2014 | 55:49.5 | 1 |
| -150.187 | 19507.772 | 1.698 | 15.789 | 0.168526 | 7/15/2014 | 55:49.6 | 1 |
| -150.494 | 19507.79 | 1.698 | 15.791 | 0.152586 | 7/15/2014 | 55:49.7 | 1 |

| | | | | | | | |
|---|---|---|---|---|---|---|---|
| -150.619 | 19507.797 | 1.696 | 15.792 | 0.136076 | 7/15/2014 | 55:49.8 | 1 |
| -150.867 | 19507.812 | 1.701 | 15.794 | 0.0901218 | 7/15/2014 | 55:49.9 | 1 |
| -151.113 | 19507.826 | 1.701 | 15.796 | 0.139199 | 7/15/2014 | 55:50.0 | 1 |
| -151.36 | 19507.842 | 1.697 | 15.798 | 0.108415 | 7/15/2014 | 55:50.1 | 1 |
| -151.606 | 19507.857 | 1.694 | 15.8 | 0.0967967 | 7/15/2014 | 55:50.2 | 1 |
| -151.85 | 19507.873 | 1.697 | 15.803 | 0.1032 | 7/15/2014 | 55:50.3 | 1 |
| -152.277 | 19507.9 | 1.701 | 15.805 | 0.132717 | 7/15/2014 | 55:50.4 | 1 |
| -152.519 | 19507.916 | 1.701 | 15.806 | 0.159116 | 7/15/2014 | 55:50.5 | 1 |
| -152.76 | 19507.933 | 1.702 | 15.807 | 0.108362 | 7/15/2014 | 55:50.6 | 1 |
| -153.061 | 19507.953 | 1.699 | 15.807 | 0.123501 | 7/15/2014 | 55:50.7 | 1 |
| -153.184 | 19507.961 | 1.701 | 15.807 | 0.131731 | 7/15/2014 | 55:50.8 | 1 |
| -153.427 | 19507.979 | 1.701 | 15.806 | 0.104032 | 7/15/2014 | 55:50.9 | 1 |
| -153.67 | 19507.996 | 1.697 | 15.805 | 0.0950572 | 7/15/2014 | 55:51.0 | 1 |
| -153.914 | 19508.014 | 1.694 | 15.804 | 0.102235 | 7/15/2014 | 55:51.1 | 1 |
| -154.154 | 19508.029 | 1.691 | 15.803 | 0.101109 | 7/15/2014 | 55:51.2 | 1 |
| -154.396 | 19508.048 | 1.688 | 15.802 | 0.0970993 | 7/15/2014 | 55:51.3 | 1 |
| -154.817 | 19508.08 | 1.689 | 15.801 | 0.0845829 | 7/15/2014 | 55:51.4 | 1 |
| -155.056 | 19508.098 | 1.689 | 15.801 | 0.105204 | 7/15/2014 | 55:51.5 | 1 |
| -155.296 | 19508.115 | 1.689 | 15.8 | 0.0853805 | 7/15/2014 | 55:51.6 | 1 |
| -155.595 | 19508.141 | 1.687 | 15.8 | 0.0833907 | 7/15/2014 | 55:51.7 | 1 |
| -155.715 | 19508.148 | 1.688 | 15.801 | 0.0836988 | 7/15/2014 | 55:51.8 | 1 |
| -155.957 | 19508.168 | 1.688 | 15.801 | 0.0845431 | 7/15/2014 | 55:51.9 | 1 |
| -156.197 | 19508.187 | 1.688 | 15.801 | 0.0879325 | 7/15/2014 | 55:52.0 | 1 |
| -156.44 | 19508.207 | 1.688 | 15.8 | 0.0979913 | 7/15/2014 | 55:52.1 | 1 |
| -156.68 | 19508.225 | 1.682 | 15.8 | 0.102368 | 7/15/2014 | 55:52.2 | 1 |
| -156.921 | 19508.244 | 1.682 | 15.799 | 0.103965 | 7/15/2014 | 55:52.3 | 1 |
| -157.341 | 19508.281 | 1.682 | 15.798 | 0.120873 | 7/15/2014 | 55:52.4 | 1 |
| -157.581 | 19508.301 | 1.687 | 15.797 | 0.114388 | 7/15/2014 | 55:52.5 | 1 |
| -157.819 | 19508.319 | 1.69 | 15.796 | 0.129969 | 7/15/2014 | 55:52.6 | 1 |
| -158.12 | 19508.346 | 1.687 | 15.795 | 0.126908 | 7/15/2014 | 55:52.7 | 1 |
| -158.241 | 19508.355 | 1.688 | 15.795 | 0.117749 | 7/15/2014 | 55:52.8 | 1 |
| -158.485 | 19508.376 | 1.688 | 15.795 | 0.112507 | 7/15/2014 | 55:52.9 | 1 |
| -158.727 | 19508.396 | 1.691 | 15.795 | 0.113586 | 7/15/2014 | 55:53.0 | 1 |
| -158.971 | 19508.42 | 1.699 | 15.796 | 0.0989774 | 7/15/2014 | 55:53.1 | 1 |
| -159.215 | 19508.439 | 1.695 | 15.797 | 0.100666 | 7/15/2014 | 55:53.2 | 1 |
| -159.459 | 19508.461 | 1.691 | 15.8 | 0.0859982 | 7/15/2014 | 55:53.3 | 1 |
| -159.885 | 19508.5 | 1.694 | 15.805 | 0.071478 | 7/15/2014 | 55:53.4 | 1 |
| -160.13 | 19508.522 | 1.697 | 15.81 | 0.0951939 | 7/15/2014 | 55:53.5 | 1 |
| -160.375 | 19508.545 | 1.699 | 15.815 | 0.0630627 | 7/15/2014 | 55:53.6 | 1 |
| -160.68 | 19508.573 | 1.701 | 15.821 | 0.072402 | 7/15/2014 | 55:53.7 | 1 |
| -160.804 | 19508.583 | 1.7 | 15.824 | 0.0844613 | 7/15/2014 | 55:53.8 | 1 |
| -161.051 | 19508.606 | 1.706 | 15.83 | 0.0722739 | 7/15/2014 | 55:53.9 | 1 |
| -161.298 | 19508.63 | 1.703 | 15.837 | 0.0663617 | 7/15/2014 | 55:54.0 | 1 |
| -161.546 | 19508.654 | 1.7 | 15.843 | 0.0758449 | 7/15/2014 | 55:54.1 | 1 |
| -161.794 | 19508.678 | 1.696 | 15.848 | 0.0665032 | 7/15/2014 | 55:54.2 | 1 |
| -162.042 | 19508.701 | 1.702 | 15.853 | 0.0853404 | 7/15/2014 | 55:54.3 | 1 |
| -162.472 | 19508.742 | 1.698 | 15.856 | 0.0795391 | 7/15/2014 | 55:54.4 | 1 |

| | | | | | | | |
|---|---|---|---|---|---|---|---|
| -162.717 | 19508.765 | 1.696 | 15.857 | 0.0985067 | 7/15/2014 | 55:54.5 | 1 |
| -162.961 | 19508.789 | 1.696 | 15.856 | 0.121576 | 7/15/2014 | 55:54.6 | 1 |
| -163.264 | 19508.819 | 1.701 | 15.853 | 0.120027 | 7/15/2014 | 55:54.7 | 1 |
| -163.388 | 19508.831 | 1.703 | 15.852 | 0.11783 | 7/15/2014 | 55:54.8 | 1 |
| -163.633 | 19508.854 | 1.709 | 15.848 | 0.13724 | 7/15/2014 | 55:54.9 | 1 |
| -163.877 | 19508.879 | 1.707 | 15.843 | 0.142671 | 7/15/2014 | 55:55.0 | 1 |
| -164.121 | 19508.903 | 1.716 | 15.837 | 0.138849 | 7/15/2014 | 55:55.1 | 1 |
| -164.363 | 19508.926 | 1.717 | 15.832 | 0.140594 | 7/15/2014 | 55:55.2 | 1 |
| -164.605 | 19508.951 | 1.722 | 15.827 | 0.137133 | 7/15/2014 | 55:55.3 | 1 |
| -165.024 | 19508.993 | 1.722 | 15.821 | 0.140013 | 7/15/2014 | 55:55.4 | 1 |
| -165.262 | 19509.018 | 1.724 | 15.818 | 0.135708 | 7/15/2014 | 55:55.5 | 1 |
| -165.501 | 19509.043 | 1.726 | 15.814 | 0.137435 | 7/15/2014 | 55:55.6 | 1 |
| -165.8 | 19509.072 | 1.728 | 15.811 | 0.148114 | 7/15/2014 | 55:55.7 | 1 |
| -165.92 | 19509.084 | 1.728 | 15.809 | 0.138279 | 7/15/2014 | 55:55.8 | 1 |
| -166.162 | 19509.109 | 1.733 | 15.805 | 0.132827 | 7/15/2014 | 55:55.9 | 1 |
| -166.402 | 19509.133 | 1.733 | 15.801 | 0.143691 | 7/15/2014 | 55:56.0 | 1 |
| -166.644 | 19509.158 | 1.741 | 15.796 | 0.142419 | 7/15/2014 | 55:56.1 | 1 |
| -166.885 | 19509.185 | 1.741 | 15.791 | 0.142088 | 7/15/2014 | 55:56.2 | 1 |
| -167.126 | 19509.209 | 1.747 | 15.783 | 0.144219 | 7/15/2014 | 55:56.3 | 1 |
| -167.547 | 19509.252 | 1.755 | 15.769 | 0.144454 | 7/15/2014 | 55:56.4 | 1 |
| -167.788 | 19509.277 | 1.761 | 15.759 | 0.155119 | 7/15/2014 | 55:56.5 | 1 |
| -168.029 | 19509.302 | 1.764 | 15.747 | 0.159609 | 7/15/2014 | 55:56.6 | 1 |
| -168.332 | 19509.334 | 1.769 | 15.734 | 0.166728 | 7/15/2014 | 55:56.7 | 1 |
| -168.454 | 19509.348 | 1.769 | 15.727 | 0.169617 | 7/15/2014 | 55:56.8 | 1 |
| -168.698 | 19509.371 | 1.779 | 15.712 | 0.172162 | 7/15/2014 | 55:56.9 | 1 |
| -168.942 | 19509.398 | 1.779 | 15.697 | 0.171981 | 7/15/2014 | 55:57.0 | 1 |
| -169.188 | 19509.424 | 1.783 | 15.681 | 0.170691 | 7/15/2014 | 55:57.1 | 1 |
| -169.433 | 19509.449 | 1.786 | 15.666 | 0.168089 | 7/15/2014 | 55:57.2 | 1 |
| -169.677 | 19509.475 | 1.799 | 15.651 | 0.168053 | 7/15/2014 | 55:57.3 | 1 |
| -170.106 | 19509.521 | 1.81 | 15.63 | 0.164537 | 7/15/2014 | 55:57.4 | 1 |
| -170.352 | 19509.545 | 1.813 | 15.616 | 0.164751 | 7/15/2014 | 55:57.5 | 1 |
| -170.596 | 19509.572 | 1.818 | 15.602 | 0.162382 | 7/15/2014 | 55:57.6 | 1 |
| -170.904 | 19509.605 | 1.826 | 15.589 | 0.164932 | 7/15/2014 | 55:57.7 | 1 |
| -171.028 | 19509.618 | 1.83 | 15.583 | 0.163142 | 7/15/2014 | 55:57.8 | 1 |
| -171.276 | 19509.644 | 1.832 | 15.571 | 0.163608 | 7/15/2014 | 55:57.9 | 1 |
| -171.524 | 19509.67 | 1.832 | 15.559 | 0.160864 | 7/15/2014 | 55:58.0 | 1 |
| -171.771 | 19509.697 | 1.838 | 15.548 | 0.156711 | 7/15/2014 | 55:58.1 | 1 |
| -172.02 | 19509.723 | 1.841 | 15.538 | 0.154086 | 7/15/2014 | 55:58.2 | 1 |
| -172.267 | 19509.749 | 1.849 | 15.529 | 0.156971 | 7/15/2014 | 55:58.3 | 1 |
| -172.698 | 19509.795 | 1.856 | 15.517 | 0.15887 | 7/15/2014 | 55:58.4 | 1 |
| -172.943 | 19509.821 | 1.859 | 15.51 | 0.152609 | 7/15/2014 | 55:58.5 | 1 |
| -173.19 | 19509.848 | 1.865 | 15.504 | 0.147993 | 7/15/2014 | 55:58.6 | 1 |
| -173.498 | 19509.881 | 1.867 | 15.498 | 0.145845 | 7/15/2014 | 55:58.7 | 1 |
| -173.623 | 19509.895 | 1.866 | 15.495 | 0.148078 | 7/15/2014 | 55:58.8 | 1 |
| -173.871 | 19509.921 | 1.872 | 15.49 | 0.145352 | 7/15/2014 | 55:58.9 | 1 |
| -174.12 | 19509.948 | 1.872 | 15.485 | 0.140207 | 7/15/2014 | 55:59.0 | 1 |
| -174.369 | 19509.973 | 1.875 | 15.48 | 0.13517 | 7/15/2014 | 55:59.1 | 1 |

| | | | | | | | |
|---|---|---|---|---|---|---|---|
| -174.618 | 19510 | 1.875 | 15.475 | 0.131273 | 7/15/2014 | 55:59.2 | 1 |
| -174.867 | 19510.027 | 1.886 | 15.471 | 0.131026 | 7/15/2014 | 55:59.3 | 1 |
| -175.177 | 19510.061 | 1.894 | 15.467 | 0.131579 | 7/15/2014 | 55:59.4 | 1 |
| -175.426 | 19510.088 | 1.897 | 15.463 | 0.133998 | 7/15/2014 | 55:59.5 | 1 |
| -175.675 | 19510.115 | 1.897 | 15.46 | 0.127936 | 7/15/2014 | 55:59.6 | 1 |
| -175.925 | 19510.141 | 1.908 | 15.458 | 0.153216 | 7/15/2014 | 55:59.7 | 1 |
| -176.238 | 19510.174 | 1.908 | 15.457 | 0.141365 | 7/15/2014 | 55:59.8 | 1 |
| -176.489 | 19510.201 | 1.91 | 15.456 | 0.141297 | 7/15/2014 | 55:59.9 | 1 |
| -176.741 | 19510.229 | 1.91 | 15.456 | 0.147187 | 7/15/2014 | 56:00.0 | 1 |
| -176.993 | 19510.256 | 1.91 | 15.457 | 0.144589 | 7/15/2014 | 56:00.1 | 1 |
| -177.245 | 19510.283 | 1.916 | 15.458 | 0.171502 | 7/15/2014 | 56:00.2 | 1 |
| -177.497 | 19510.311 | 1.928 | 15.459 | 0.140696 | 7/15/2014 | 56:00.3 | 1 |
| -177.807 | 19510.344 | 1.928 | 15.46 | 0.153247 | 7/15/2014 | 56:00.4 | 1 |
| -178.059 | 19510.371 | 1.934 | 15.461 | 0.162775 | 7/15/2014 | 56:00.5 | 1 |
| -178.309 | 19510.397 | 1.934 | 15.461 | 0.163486 | 7/15/2014 | 56:00.6 | 1 |
| -178.56 | 19510.424 | 1.941 | 15.462 | 0.173513 | 7/15/2014 | 56:00.7 | 1 |
| -178.872 | 19510.458 | 1.947 | 15.464 | 0.153505 | 7/15/2014 | 56:00.8 | 1 |
| -179.124 | 19510.485 | 1.953 | 15.465 | 0.162814 | 7/15/2014 | 56:00.9 | 1 |
| -179.374 | 19510.512 | 1.95 | 15.467 | 0.164078 | 7/15/2014 | 56:01.0 | 1 |
| -179.626 | 19510.539 | 1.954 | 15.47 | 0.158021 | 7/15/2014 | 56:01.1 | 1 |
| -179.876 | 19510.565 | 1.96 | 15.473 | 0.170658 | 7/15/2014 | 56:01.2 | 1 |
| -180.128 | 19510.592 | 1.97 | 15.477 | 0.139574 | 7/15/2014 | 56:01.3 | 1 |
| -180.44 | 19510.626 | 1.968 | 15.482 | 0.161288 | 7/15/2014 | 56:01.4 | 1 |
| -180.69 | 19510.653 | 1.968 | 15.488 | 0.146557 | 7/15/2014 | 56:01.5 | 1 |
| -180.94 | 19510.68 | 1.974 | 15.495 | 0.144822 | 7/15/2014 | 56:01.6 | 1 |
| -181.191 | 19510.707 | 1.982 | 15.503 | 0.137433 | 7/15/2014 | 56:01.7 | 1 |
| -181.504 | 19510.74 | 1.982 | 15.512 | 0.104988 | 7/15/2014 | 56:01.8 | 1 |
| -181.757 | 19510.768 | 1.984 | 15.521 | 0.120272 | 7/15/2014 | 56:01.9 | 1 |
| -182.009 | 19510.795 | 1.984 | 15.531 | 0.0939334 | 7/15/2014 | 56:02.0 | 1 |
| -182.262 | 19510.822 | 1.993 | 15.541 | 0.089262 | 7/15/2014 | 56:02.1 | 1 |
| -182.515 | 19510.85 | 1.996 | 15.551 | 0.067245 | 7/15/2014 | 56:02.2 | 1 |
| -182.768 | 19510.877 | 1.996 | 15.562 | 0.0767759 | 7/15/2014 | 56:02.3 | 1 |
| -183.082 | 19510.91 | 1.988 | 15.572 | 0.0580369 | 7/15/2014 | 56:02.4 | 1 |
| -183.336 | 19510.938 | 1.988 | 15.581 | 0.057315 | 7/15/2014 | 56:02.5 | 1 |
| -183.588 | 19510.965 | 1.986 | 15.59 | 0.0726526 | 7/15/2014 | 56:02.6 | 1 |
| -183.841 | 19510.991 | 1.989 | 15.597 | 0.0768088 | 7/15/2014 | 56:02.7 | 1 |
| -184.157 | 19511.024 | 1.981 | 15.604 | 0.0963025 | 7/15/2014 | 56:02.8 | 1 |
| -184.412 | 19511.052 | 1.977 | 15.609 | 0.0754306 | 7/15/2014 | 56:02.9 | 1 |
| -184.666 | 19511.078 | 1.971 | 15.613 | 0.113266 | 7/15/2014 | 56:03.0 | 1 |
| -184.921 | 19511.104 | 1.974 | 15.616 | 0.0972147 | 7/15/2014 | 56:03.1 | 1 |
| -185.175 | 19511.132 | 1.971 | 15.618 | 0.0729964 | 7/15/2014 | 56:03.2 | 1 |
| -185.429 | 19511.157 | 1.971 | 15.619 | 0.0800088 | 7/15/2014 | 56:03.3 | 1 |
| -185.745 | 19511.19 | 1.962 | 15.62 | 0.0770105 | 7/15/2014 | 56:03.4 | 1 |
| -185.998 | 19511.216 | 1.962 | 15.62 | 0.0616372 | 7/15/2014 | 56:03.5 | 1 |
| -186.251 | 19511.242 | 1.962 | 15.62 | 0.058118 | 7/15/2014 | 56:03.6 | 1 |
| -186.504 | 19511.268 | 1.959 | 15.621 | 0.0615295 | 7/15/2014 | 56:03.7 | 1 |
| -186.82 | 19511.299 | 1.953 | 15.621 | 0.0803242 | 7/15/2014 | 56:03.8 | 1 |

| | | | | | | | |
|---|---|---|---|---|---|---|---|
| -187.074 | 19511.324 | 1.953 | 15.622 | 0.0721625 | 7/15/2014 | 56:03.9 | 1 |
| -187.328 | 19511.351 | 1.957 | 15.623 | 0.0746411 | 7/15/2014 | 56:04.0 | 1 |
| -187.582 | 19511.375 | 1.963 | 15.624 | 0.0795557 | 7/15/2014 | 56:04.1 | 1 |
| -187.835 | 19511.4 | 1.959 | 15.626 | 0.0800553 | 7/15/2014 | 56:04.2 | 1 |
| -188.087 | 19511.426 | 1.956 | 15.628 | 0.0860832 | 7/15/2014 | 56:04.3 | 1 |
| -188.399 | 19511.455 | 1.958 | 15.631 | 0.10524 | 7/15/2014 | 56:04.4 | 1 |
| -188.652 | 19511.48 | 1.964 | 15.634 | 0.0984479 | 7/15/2014 | 56:04.5 | 1 |
| -188.902 | 19511.505 | 1.964 | 15.637 | 0.0969959 | 7/15/2014 | 56:04.6 | 1 |
| -189.153 | 19511.529 | 1.96 | 15.641 | 0.109819 | 7/15/2014 | 56:04.7 | 1 |
| -189.464 | 19511.559 | 1.954 | 15.645 | 0.102252 | 7/15/2014 | 56:04.8 | 1 |
| -189.714 | 19511.583 | 1.96 | 15.649 | 0.0938615 | 7/15/2014 | 56:04.9 | 1 |
| -189.963 | 19511.606 | 1.957 | 15.653 | 0.099498 | 7/15/2014 | 56:05.0 | 1 |
| -190.216 | 19511.631 | 1.954 | 15.658 | 0.0810109 | 7/15/2014 | 56:05.1 | 1 |
| -190.465 | 19511.654 | 1.939 | 15.663 | 0.107228 | 7/15/2014 | 56:05.2 | 1 |
| -190.714 | 19511.678 | 1.935 | 15.669 | 0.09163 | 7/15/2014 | 56:05.3 | 1 |
| -191.025 | 19511.706 | 1.925 | 15.675 | 0.11087 | 7/15/2014 | 56:05.4 | 1 |
| -191.276 | 19511.729 | 1.925 | 15.681 | 0.121805 | 7/15/2014 | 56:05.5 | 1 |
| -191.523 | 19511.752 | 1.919 | 15.689 | 0.104769 | 7/15/2014 | 56:05.6 | 1 |
| -191.775 | 19511.775 | 1.919 | 15.696 | 0.121348 | 7/15/2014 | 56:05.7 | 1 |
| -192.088 | 19511.803 | 1.912 | 15.704 | 0.0807497 | 7/15/2014 | 56:05.8 | 1 |
| -192.341 | 19511.826 | 1.915 | 15.712 | 0.115165 | 7/15/2014 | 56:05.9 | 1 |
| -192.594 | 19511.847 | 1.909 | 15.72 | 0.088818 | 7/15/2014 | 56:06.0 | 1 |
| -192.85 | 19511.869 | 1.907 | 15.727 | 0.0902949 | 7/15/2014 | 56:06.1 | 1 |
| -193.103 | 19511.892 | 1.901 | 15.734 | 0.0899106 | 7/15/2014 | 56:06.2 | 1 |
| -193.358 | 19511.914 | 1.891 | 15.741 | 0.105687 | 7/15/2014 | 56:06.3 | 1 |
| -193.674 | 19511.941 | 1.885 | 15.747 | 0.115756 | 7/15/2014 | 56:06.4 | 1 |
| -193.928 | 19511.963 | 1.882 | 15.752 | 0.113666 | 7/15/2014 | 56:06.5 | 1 |
| -194.183 | 19511.982 | 1.879 | 15.757 | 0.15186 | 7/15/2014 | 56:06.6 | 1 |
| -194.437 | 19512.004 | 1.875 | 15.761 | 0.131674 | 7/15/2014 | 56:06.7 | 1 |
| -194.753 | 19512.03 | 1.873 | 15.764 | 0.148869 | 7/15/2014 | 56:06.8 | 1 |
| -195.007 | 19512.051 | 1.875 | 15.767 | 0.153638 | 7/15/2014 | 56:06.9 | 1 |
| -195.261 | 19512.073 | 1.87 | 15.769 | 0.166531 | 7/15/2014 | 56:07.0 | 1 |
| -195.516 | 19512.094 | 1.862 | 15.771 | 0.170547 | 7/15/2014 | 56:07.1 | 1 |
| -195.769 | 19512.113 | 1.856 | 15.772 | 0.174931 | 7/15/2014 | 56:07.2 | 1 |
| -196.022 | 19512.133 | 1.853 | 15.773 | 0.205238 | 7/15/2014 | 56:07.3 | 1 |
| -196.336 | 19512.158 | 1.846 | 15.774 | 0.182602 | 7/15/2014 | 56:07.4 | 1 |
| -196.589 | 19512.178 | 1.845 | 15.774 | 0.212584 | 7/15/2014 | 56:07.5 | 1 |
| -196.839 | 19512.198 | 1.836 | 15.774 | 0.197809 | 7/15/2014 | 56:07.6 | 1 |
| -197.091 | 19512.219 | 1.839 | 15.773 | 0.215396 | 7/15/2014 | 56:07.7 | 1 |
| -197.404 | 19512.242 | 1.845 | 15.773 | 0.191533 | 7/15/2014 | 56:07.8 | 1 |
| -197.656 | 19512.262 | 1.845 | 15.772 | 0.179174 | 7/15/2014 | 56:07.9 | 1 |
| -197.909 | 19512.28 | 1.845 | 15.771 | 0.194575 | 7/15/2014 | 56:08.0 | 1 |
| -198.162 | 19512.299 | 1.853 | 15.77 | 0.191256 | 7/15/2014 | 56:08.1 | 1 |
| -198.413 | 19512.318 | 1.848 | 15.769 | 0.193101 | 7/15/2014 | 56:08.2 | 1 |
| -198.664 | 19512.338 | 1.848 | 15.769 | 0.171716 | 7/15/2014 | 56:08.3 | 1 |
| -198.977 | 19512.36 | 1.848 | 15.769 | 0.166392 | 7/15/2014 | 56:08.4 | 1 |
| -199.228 | 19512.379 | 1.845 | 15.77 | 0.182157 | 7/15/2014 | 56:08.5 | 1 |

| | | | | | | | |
|---|---|---|---|---|---|---|---|
| -199.478 | 19512.396 | 1.84 | 15.772 | 0.173627 | 7/15/2014 | 56:08.6 | 1 |
| -199.731 | 19512.414 | 1.838 | 15.775 | 0.132955 | 7/15/2014 | 56:08.7 | 1 |
| -200.046 | 19512.438 | 1.832 | 15.78 | 0.130135 | 7/15/2014 | 56:08.8 | 1 |
| -200.301 | 19512.455 | 1.832 | 15.785 | 0.111775 | 7/15/2014 | 56:08.9 | 1 |
| -200.556 | 19512.473 | 1.827 | 15.792 | 0.112945 | 7/15/2014 | 56:09.0 | 1 |
| -200.812 | 19512.49 | 1.825 | 15.799 | 0.1076 | 7/15/2014 | 56:09.1 | 1 |
| -201.067 | 19512.508 | 1.82 | 15.808 | 0.0896701 | 7/15/2014 | 56:09.2 | 1 |
| -201.326 | 19512.525 | 1.82 | 15.818 | 0.0973913 | 7/15/2014 | 56:09.3 | 1 |
| -201.646 | 19512.548 | 1.817 | 15.828 | 0.106712 | 7/15/2014 | 56:09.4 | 1 |
| -201.905 | 19512.563 | 1.815 | 15.839 | 0.112976 | 7/15/2014 | 56:09.5 | 1 |
| -202.164 | 19512.581 | 1.812 | 15.851 | 0.11554 | 7/15/2014 | 56:09.6 | 1 |
| -202.424 | 19512.599 | 1.812 | 15.863 | 0.0989198 | 7/15/2014 | 56:09.7 | 1 |
| -202.75 | 19512.62 | 1.808 | 15.876 | 0.106304 | 7/15/2014 | 56:09.8 | 1 |
| -203.014 | 19512.637 | 1.805 | 15.89 | 0.104394 | 7/15/2014 | 56:09.9 | 1 |
| -203.278 | 19512.653 | 1.803 | 15.904 | 0.107119 | 7/15/2014 | 56:10.0 | 1 |
| -203.543 | 19512.669 | 1.8 | 15.919 | 0.120388 | 7/15/2014 | 56:10.1 | 1 |
| -203.806 | 19512.687 | 1.798 | 15.933 | 0.111888 | 7/15/2014 | 56:10.2 | 1 |
| -204.071 | 19512.703 | 1.798 | 15.948 | 0.120645 | 7/15/2014 | 56:10.3 | 1 |
| -204.399 | 19512.725 | 1.795 | 15.962 | 0.121991 | 7/15/2014 | 56:10.4 | 1 |
| -204.664 | 19512.739 | 1.798 | 15.976 | 0.119401 | 7/15/2014 | 56:10.5 | 1 |
| -204.927 | 19512.756 | 1.792 | 15.988 | 0.129059 | 7/15/2014 | 56:10.6 | 1 |
| -205.191 | 19512.771 | 1.791 | 15.999 | 0.102452 | 7/15/2014 | 56:10.7 | 1 |
| -205.518 | 19512.79 | 1.787 | 16.009 | 0.0968966 | 7/15/2014 | 56:10.8 | 1 |
| -205.783 | 19512.806 | 1.781 | 16.019 | 0.0971525 | 7/15/2014 | 56:10.9 | 1 |
| -206.045 | 19512.822 | 1.772 | 16.028 | 0.112317 | 7/15/2014 | 56:11.0 | 1 |
| -206.31 | 19512.836 | 1.769 | 16.036 | 0.10729 | 7/15/2014 | 56:11.1 | 1 |
| -206.572 | 19512.852 | 1.76 | 16.044 | 0.0959196 | 7/15/2014 | 56:11.2 | 1 |
| -206.834 | 19512.866 | 1.766 | 16.052 | 0.0955157 | 7/15/2014 | 56:11.3 | 1 |
| -207.157 | 19512.885 | 1.758 | 16.061 | 0.120603 | 7/15/2014 | 56:11.4 | 1 |
| -207.418 | 19512.898 | 1.755 | 16.068 | 0.109678 | 7/15/2014 | 56:11.5 | 1 |
| -207.677 | 19512.914 | 1.755 | 16.076 | 0.0894576 | 7/15/2014 | 56:11.6 | 1 |
| -207.936 | 19512.928 | 1.761 | 16.083 | 0.0866889 | 7/15/2014 | 56:11.7 | 1 |
| -208.257 | 19512.945 | 1.758 | 16.09 | 0.0813793 | 7/15/2014 | 56:11.8 | 1 |
| -208.517 | 19512.96 | 1.76 | 16.095 | 0.0826149 | 7/15/2014 | 56:11.9 | 1 |
| -208.776 | 19512.975 | 1.754 | 16.101 | 0.0822887 | 7/15/2014 | 56:12.0 | 1 |
| -209.035 | 19512.986 | 1.758 | 16.106 | 0.0809266 | 7/15/2014 | 56:12.1 | 1 |
| -209.292 | 19513.001 | 1.75 | 16.111 | 0.0823355 | 7/15/2014 | 56:12.2 | 1 |
| -209.55 | 19513.015 | 1.753 | 16.116 | 0.0784957 | 7/15/2014 | 56:12.3 | 1 |
| -209.868 | 19513.032 | 1.743 | 16.12 | 0.081225 | 7/15/2014 | 56:12.4 | 1 |
| -210.126 | 19513.046 | 1.744 | 16.125 | 0.0876118 | 7/15/2014 | 56:12.5 | 1 |
| -210.382 | 19513.059 | 1.744 | 16.131 | 0.106741 | 7/15/2014 | 56:12.6 | 1 |
| -210.636 | 19513.072 | 1.74 | 16.136 | 0.0806684 | 7/15/2014 | 56:12.7 | 1 |
| -210.956 | 19513.089 | 1.73 | 16.141 | 0.0908978 | 7/15/2014 | 56:12.8 | 1 |
| -211.215 | 19513.102 | 1.74 | 16.145 | 0.100348 | 7/15/2014 | 56:12.9 | 1 |
| -211.471 | 19513.114 | 1.736 | 16.149 | 0.0983091 | 7/15/2014 | 56:13.0 | 1 |
| -211.733 | 19513.128 | 1.74 | 16.153 | 0.102429 | 7/15/2014 | 56:13.1 | 1 |
| -211.991 | 19513.141 | 1.732 | 16.156 | 0.0957934 | 7/15/2014 | 56:13.2 | 1 |

| | | | | | | | |
|---|---|---|---|---|---|---|---|
| -212.249 | 19513.154 | 1.729 | 16.158 | 0.116622 | 7/15/2014 | 56:13.3 | 1 |
| -212.572 | 19513.171 | 1.729 | 16.16 | 0.0907597 | 7/15/2014 | 56:13.4 | 1 |
| -212.833 | 19513.184 | 1.741 | 16.161 | 0.0967499 | 7/15/2014 | 56:13.5 | 1 |
| -213.091 | 19513.195 | 1.731 | 16.161 | 0.109103 | 7/15/2014 | 56:13.6 | 1 |
| -213.353 | 19513.208 | 1.729 | 16.162 | 0.091 | 7/15/2014 | 56:13.7 | 1 |
| -213.678 | 19513.225 | 1.727 | 16.162 | 0.0891791 | 7/15/2014 | 56:13.8 | 1 |
| -213.941 | 19513.236 | 1.731 | 16.161 | 0.0871066 | 7/15/2014 | 56:13.9 | 1 |
| -214.204 | 19513.249 | 1.725 | 16.16 | 0.110394 | 7/15/2014 | 56:14.0 | 1 |
| -214.47 | 19513.262 | 1.725 | 16.159 | 0.0830036 | 7/15/2014 | 56:14.1 | 1 |
| -214.731 | 19513.273 | 1.715 | 16.158 | 0.0823389 | 7/15/2014 | 56:14.2 | 1 |
| -214.993 | 19513.285 | 1.721 | 16.155 | 0.0778256 | 7/15/2014 | 56:14.3 | 1 |
| -215.321 | 19513.301 | 1.715 | 16.152 | 0.0798133 | 7/15/2014 | 56:14.4 | 1 |
| -215.583 | 19513.313 | 1.715 | 16.148 | 0.0747074 | 7/15/2014 | 56:14.5 | 1 |
| -215.845 | 19513.324 | 1.715 | 16.144 | 0.06856 | 7/15/2014 | 56:14.6 | 1 |
| -216.109 | 19513.336 | 1.721 | 16.139 | 0.104469 | 7/15/2014 | 56:14.7 | 1 |
| -216.435 | 19513.35 | 1.724 | 16.134 | 0.0801367 | 7/15/2014 | 56:14.8 | 1 |
| -216.698 | 19513.361 | 1.727 | 16.128 | 0.084133 | 7/15/2014 | 56:14.9 | 1 |
| -216.961 | 19513.373 | 1.719 | 16.123 | 0.094671 | 7/15/2014 | 56:15.0 | 1 |
| -217.226 | 19513.385 | 1.731 | 16.118 | 0.110625 | 7/15/2014 | 56:15.1 | 1 |
| -217.489 | 19513.396 | 1.725 | 16.114 | 0.104782 | 7/15/2014 | 56:15.2 | 1 |
| -217.752 | 19513.406 | 1.721 | 16.111 | 0.104688 | 7/15/2014 | 56:15.3 | 1 |
| -218.079 | 19513.42 | 1.721 | 16.108 | 0.111787 | 7/15/2014 | 56:15.4 | 1 |
| -218.341 | 19513.432 | 1.719 | 16.106 | 0.125415 | 7/15/2014 | 56:15.5 | 1 |
| -218.602 | 19513.441 | 1.719 | 16.104 | 0.11159 | 7/15/2014 | 56:15.6 | 1 |
| -218.865 | 19513.453 | 1.721 | 16.103 | 0.116095 | 7/15/2014 | 56:15.7 | 1 |
| -219.191 | 19513.466 | 1.715 | 16.102 | 0.104534 | 7/15/2014 | 56:15.8 | 1 |
| -219.453 | 19513.477 | 1.719 | 16.101 | 0.096085 | 7/15/2014 | 56:15.9 | 1 |
| -219.715 | 19513.487 | 1.719 | 16.1 | 0.0883474 | 7/15/2014 | 56:16.0 | 1 |
| -219.976 | 19513.498 | 1.716 | 16.1 | 0.0932409 | 7/15/2014 | 56:16.1 | 1 |
| -220.237 | 19513.508 | 1.71 | 16.1 | 0.0871956 | 7/15/2014 | 56:16.2 | 1 |
| -220.497 | 19513.518 | 1.71 | 16.101 | 0.100554 | 7/15/2014 | 56:16.3 | 1 |
| -220.82 | 19513.531 | 1.714 | 16.103 | 0.100213 | 7/15/2014 | 56:16.4 | 1 |
| -221.078 | 19513.543 | 1.716 | 16.106 | 0.107464 | 7/15/2014 | 56:16.5 | 1 |
| -221.336 | 19513.552 | 1.71 | 16.111 | 0.113994 | 7/15/2014 | 56:16.6 | 1 |
| -221.594 | 19513.563 | 1.714 | 16.117 | 0.124847 | 7/15/2014 | 56:16.7 | 1 |
| -221.913 | 19513.574 | 1.714 | 16.124 | 0.133735 | 7/15/2014 | 56:16.8 | 1 |
| -222.173 | 19513.584 | 1.716 | 16.132 | 0.146107 | 7/15/2014 | 56:16.9 | 1 |
| -222.43 | 19513.596 | 1.708 | 16.14 | 0.161669 | 7/15/2014 | 56:17.0 | 1 |
| -222.69 | 19513.605 | 1.708 | 16.149 | 0.184495 | 7/15/2014 | 56:17.1 | 1 |
| -222.948 | 19513.615 | 1.703 | 16.157 | 0.167933 | 7/15/2014 | 56:17.2 | 1 |
| -223.207 | 19513.625 | 1.705 | 16.165 | 0.198471 | 7/15/2014 | 56:17.3 | 1 |
| -223.526 | 19513.637 | 1.697 | 16.172 | 0.194078 | 7/15/2014 | 56:17.4 | 1 |
| -223.785 | 19513.648 | 1.691 | 16.177 | 0.201868 | 7/15/2014 | 56:17.5 | 1 |
| -224.043 | 19513.658 | 1.688 | 16.182 | 0.203451 | 7/15/2014 | 56:17.6 | 1 |
| -224.302 | 19513.668 | 1.691 | 16.186 | 0.188758 | 7/15/2014 | 56:17.7 | 1 |
| -224.625 | 19513.681 | 1.685 | 16.189 | 0.219157 | 7/15/2014 | 56:17.8 | 1 |
| -224.887 | 19513.689 | 1.683 | 16.191 | 0.189376 | 7/15/2014 | 56:17.9 | 1 |

| | | | | | | | |
|---|---|---|---|---|---|---|---|
| -225.147 | 19513.701 | 1.679 | 16.192 | 0.198634 | 7/15/2014 | 56:18.0 | 1 |
| -225.409 | 19513.711 | 1.683 | 16.194 | 0.188848 | 7/15/2014 | 56:18.1 | 1 |
| -225.669 | 19513.721 | 1.68 | 16.195 | 0.203106 | 7/15/2014 | 56:18.2 | 1 |
| -225.931 | 19513.732 | 1.68 | 16.197 | 0.19892 | 7/15/2014 | 56:18.3 | 1 |
| -226.257 | 19513.743 | 1.677 | 16.2 | 0.182124 | 7/15/2014 | 56:18.4 | 1 |
| -226.519 | 19513.754 | 1.677 | 16.203 | 0.200565 | 7/15/2014 | 56:18.5 | 1 |
| -226.778 | 19513.766 | 1.675 | 16.207 | 0.202068 | 7/15/2014 | 56:18.6 | 1 |
| -227.04 | 19513.773 | 1.671 | 16.21 | 0.203427 | 7/15/2014 | 56:18.7 | 1 |
| -227.363 | 19513.787 | 1.66 | 16.214 | 0.192806 | 7/15/2014 | 56:18.8 | 1 |
| -227.625 | 19513.799 | 1.662 | 16.217 | 0.201374 | 7/15/2014 | 56:18.9 | 1 |
| -227.886 | 19513.807 | 1.662 | 16.22 | 0.197999 | 7/15/2014 | 56:19.0 | 1 |
| -228.148 | 19513.818 | 1.656 | 16.223 | 0.197487 | 7/15/2014 | 56:19.1 | 1 |
| -228.406 | 19513.83 | 1.644 | 16.225 | 0.205332 | 7/15/2014 | 56:19.2 | 1 |
| -228.666 | 19513.838 | 1.64 | 16.226 | 0.197777 | 7/15/2014 | 56:19.3 | 1 |
| -228.988 | 19513.852 | 1.636 | 16.228 | 0.211028 | 7/15/2014 | 56:19.4 | 1 |
| -229.245 | 19513.863 | 1.63 | 16.229 | 0.207602 | 7/15/2014 | 56:19.5 | 1 |
| -229.502 | 19513.871 | 1.623 | 16.23 | 0.20591 | 7/15/2014 | 56:19.6 | 1 |
| -229.759 | 19513.883 | 1.613 | 16.232 | 0.209458 | 7/15/2014 | 56:19.7 | 1 |
| -230.077 | 19513.896 | 1.607 | 16.233 | 0.212042 | 7/15/2014 | 56:19.8 | 1 |
| -230.333 | 19513.906 | 1.61 | 16.235 | 0.22806 | 7/15/2014 | 56:19.9 | 1 |
| -230.589 | 19513.918 | 1.599 | 16.237 | 0.22608 | 7/15/2014 | 56:20.0 | 1 |
| -230.846 | 19513.93 | 1.596 | 16.239 | 0.224602 | 7/15/2014 | 56:20.1 | 1 |
| -231.101 | 19513.939 | 1.601 | 16.24 | 0.227739 | 7/15/2014 | 56:20.2 | 1 |
| -231.355 | 19513.95 | 1.605 | 16.241 | 0.232287 | 7/15/2014 | 56:20.3 | 1 |
| -231.671 | 19513.963 | 1.598 | 16.241 | 0.223116 | 7/15/2014 | 56:20.4 | 1 |
| -231.925 | 19513.975 | 1.602 | 16.24 | 0.215359 | 7/15/2014 | 56:20.5 | 1 |
| -232.176 | 19513.986 | 1.6 | 16.24 | 0.208331 | 7/15/2014 | 56:20.6 | 1 |
| -232.43 | 19513.998 | 1.602 | 16.239 | 0.219808 | 7/15/2014 | 56:20.7 | 1 |
| -232.745 | 19514.012 | 1.594 | 16.239 | 0.229775 | 7/15/2014 | 56:20.8 | 1 |
| -232.998 | 19514.022 | 1.583 | 16.239 | 0.224321 | 7/15/2014 | 56:20.9 | 1 |
| -233.251 | 19514.033 | 1.572 | 16.24 | 0.230007 | 7/15/2014 | 56:21.0 | 1 |
| -233.507 | 19514.045 | 1.577 | 16.242 | 0.228569 | 7/15/2014 | 56:21.1 | 1 |
| -233.759 | 19514.057 | 1.566 | 16.244 | 0.220503 | 7/15/2014 | 56:21.2 | 1 |
| -234.012 | 19514.068 | 1.561 | 16.246 | 0.205205 | 7/15/2014 | 56:21.3 | 1 |
| -234.327 | 19514.082 | 1.552 | 16.249 | 0.188563 | 7/15/2014 | 56:21.4 | 1 |
| -234.579 | 19514.094 | 1.552 | 16.252 | 0.186412 | 7/15/2014 | 56:21.5 | 1 |
| -234.83 | 19514.105 | 1.548 | 16.256 | 0.174974 | 7/15/2014 | 56:21.6 | 1 |
| -235.084 | 19514.117 | 1.548 | 16.26 | 0.150967 | 7/15/2014 | 56:21.7 | 1 |
| -235.397 | 19514.132 | 1.536 | 16.265 | 0.160228 | 7/15/2014 | 56:21.8 | 1 |
| -235.65 | 19514.144 | 1.54 | 16.27 | 0.149635 | 7/15/2014 | 56:21.9 | 1 |
| -235.902 | 19514.155 | 1.532 | 16.276 | 0.15828 | 7/15/2014 | 56:22.0 | 1 |
| -236.156 | 19514.168 | 1.538 | 16.282 | 0.157436 | 7/15/2014 | 56:22.1 | 1 |
| -236.406 | 19514.18 | 1.538 | 16.288 | 0.162791 | 7/15/2014 | 56:22.2 | 1 |
| -236.66 | 19514.191 | 1.548 | 16.296 | 0.173417 | 7/15/2014 | 56:22.3 | 1 |
| -236.971 | 19514.206 | 1.541 | 16.303 | 0.181469 | 7/15/2014 | 56:22.4 | 1 |
| -237.224 | 19514.219 | 1.55 | 16.311 | 0.196565 | 7/15/2014 | 56:22.5 | 1 |
| -237.473 | 19514.23 | 1.549 | 16.319 | 0.188778 | 7/15/2014 | 56:22.6 | 1 |

| | | | | | | | |
|---|---|---|---|---|---|---|---|
| -237.724 | 19514.244 | 1.553 | 16.326 | 0.189102 | 7/15/2014 | 56:22.7 | 1 |
| -238.036 | 19514.26 | 1.541 | 16.332 | 0.193208 | 7/15/2014 | 56:22.8 | 1 |
| -238.289 | 19514.271 | 1.537 | 16.336 | 0.188559 | 7/15/2014 | 56:22.9 | 1 |
| -238.539 | 19514.284 | 1.531 | 16.339 | 0.191362 | 7/15/2014 | 56:23.0 | 1 |
| -238.792 | 19514.298 | 1.534 | 16.34 | 0.175821 | 7/15/2014 | 56:23.1 | 1 |
| -239.043 | 19514.31 | 1.525 | 16.34 | 0.168189 | 7/15/2014 | 56:23.2 | 1 |
| -239.293 | 19514.324 | 1.518 | 16.337 | 0.161177 | 7/15/2014 | 56:23.3 | 1 |
| -239.604 | 19514.34 | 1.512 | 16.333 | 0.156847 | 7/15/2014 | 56:23.4 | 1 |
| -239.855 | 19514.354 | 1.518 | 16.328 | 0.137711 | 7/15/2014 | 56:23.5 | 1 |
| -240.103 | 19514.366 | 1.516 | 16.322 | 0.177605 | 7/15/2014 | 56:23.6 | 1 |
| -240.352 | 19514.38 | 1.512 | 16.315 | 0.143239 | 7/15/2014 | 56:23.7 | 1 |
| -240.661 | 19514.396 | 1.506 | 16.308 | 0.142697 | 7/15/2014 | 56:23.8 | 1 |
| -240.91 | 19514.411 | 1.51 | 16.301 | 0.143598 | 7/15/2014 | 56:23.9 | 1 |
| -241.157 | 19514.424 | 1.511 | 16.295 | 0.146746 | 7/15/2014 | 56:24.0 | 1 |
| -241.407 | 19514.439 | 1.52 | 16.288 | 0.147524 | 7/15/2014 | 56:24.1 | 1 |
| -241.653 | 19514.451 | 1.517 | 16.283 | 0.128435 | 7/15/2014 | 56:24.2 | 1 |
| -241.899 | 19514.466 | 1.517 | 16.278 | 0.165372 | 7/15/2014 | 56:24.3 | 1 |
| -242.328 | 19514.491 | 1.523 | 16.273 | 0.156592 | 7/15/2014 | 56:24.4 | 1 |
| -242.572 | 19514.505 | 1.526 | 16.271 | 0.159781 | 7/15/2014 | 56:24.5 | 1 |
| -242.816 | 19514.52 | 1.526 | 16.27 | 0.160632 | 7/15/2014 | 56:24.6 | 1 |
| -243.122 | 19514.539 | 1.523 | 16.269 | 0.166468 | 7/15/2014 | 56:24.7 | 1 |
| -243.245 | 19514.545 | 1.518 | 16.268 | 0.165232 | 7/15/2014 | 56:24.8 | 1 |
| -243.491 | 19514.56 | 1.518 | 16.269 | 0.170105 | 7/15/2014 | 56:24.9 | 1 |
| -243.738 | 19514.573 | 1.522 | 16.269 | 0.170234 | 7/15/2014 | 56:25.0 | 1 |
| -243.986 | 19514.59 | 1.522 | 16.27 | 0.176079 | 7/15/2014 | 56:25.1 | 1 |
| -244.233 | 19514.604 | 1.517 | 16.272 | 0.181815 | 7/15/2014 | 56:25.2 | 1 |
| -244.481 | 19514.62 | 1.516 | 16.274 | 0.189817 | 7/15/2014 | 56:25.3 | 1 |
| -244.913 | 19514.647 | 1.511 | 16.278 | 0.196803 | 7/15/2014 | 56:25.4 | 1 |
| -245.035 | 19514.654 | 1.514 | 16.279 | 0.213786 | 7/15/2014 | 56:25.5 | 1 |
| -245.404 | 19514.678 | 1.513 | 16.285 | 0.232482 | 7/15/2014 | 56:25.6 | 1 |
| -245.711 | 19514.698 | 1.508 | 16.289 | 0.212763 | 7/15/2014 | 56:25.7 | 1 |
| -245.836 | 19514.706 | 1.506 | 16.291 | 0.209036 | 7/15/2014 | 56:25.8 | 1 |
| -246.084 | 19514.723 | 1.505 | 16.296 | 0.231189 | 7/15/2014 | 56:25.9 | 1 |
| -246.33 | 19514.738 | 1.503 | 16.301 | 0.22988 | 7/15/2014 | 56:26.0 | 1 |
| -246.578 | 19514.755 | 1.505 | 16.306 | 0.23747 | 7/15/2014 | 56:26.1 | 1 |
| -246.823 | 19514.771 | 1.499 | 16.31 | 0.237695 | 7/15/2014 | 56:26.2 | 1 |
| -247.069 | 19514.788 | 1.505 | 16.314 | 0.236272 | 7/15/2014 | 56:26.3 | 1 |
| -247.498 | 19514.817 | 1.5 | 16.317 | 0.236476 | 7/15/2014 | 56:26.4 | 1 |
| -247.742 | 19514.835 | 1.506 | 16.318 | 0.230993 | 7/15/2014 | 56:26.5 | 1 |
| -247.989 | 19514.852 | 1.511 | 16.318 | 0.225814 | 7/15/2014 | 56:26.6 | 1 |
| -248.299 | 19514.873 | 1.514 | 16.317 | 0.218215 | 7/15/2014 | 56:26.7 | 1 |
| -248.421 | 19514.881 | 1.517 | 16.316 | 0.216327 | 7/15/2014 | 56:26.8 | 1 |
| -248.673 | 19514.899 | 1.52 | 16.314 | 0.201897 | 7/15/2014 | 56:26.9 | 1 |
| -248.925 | 19514.917 | 1.517 | 16.31 | 0.191885 | 7/15/2014 | 56:27.0 | 1 |
| -249.181 | 19514.936 | 1.526 | 16.306 | 0.183804 | 7/15/2014 | 56:27.1 | 1 |
| -249.435 | 19514.954 | 1.517 | 16.301 | 0.172757 | 7/15/2014 | 56:27.2 | 1 |
| -249.692 | 19514.972 | 1.514 | 16.296 | 0.16958 | 7/15/2014 | 56:27.3 | 1 |

| | | | | | | | |
|---|---|---|---|---|---|---|---|
| -250.012 | 19514.994 | 1.509 | 16.291 | 0.160576 | 7/15/2014 | 56:27.4 | 1 |
| -250.271 | 19515.014 | 1.505 | 16.287 | 0.163754 | 7/15/2014 | 56:27.5 | 1 |
| -250.531 | 19515.032 | 1.5 | 16.283 | 0.160345 | 7/15/2014 | 56:27.6 | 1 |
| -250.792 | 19515.052 | 1.503 | 16.281 | 0.15471 | 7/15/2014 | 56:27.7 | 1 |
| -251.116 | 19515.075 | 1.494 | 16.28 | 0.157336 | 7/15/2014 | 56:27.8 | 1 |
| -251.378 | 19515.094 | 1.5 | 16.28 | 0.161906 | 7/15/2014 | 56:27.9 | 1 |
| -251.64 | 19515.113 | 1.504 | 16.281 | 0.167298 | 7/15/2014 | 56:28.0 | 1 |
| -251.902 | 19515.134 | 1.503 | 16.283 | 0.177603 | 7/15/2014 | 56:28.1 | 1 |
| -252.162 | 19515.153 | 1.503 | 16.286 | 0.186377 | 7/15/2014 | 56:28.2 | 1 |
| -252.423 | 19515.174 | 1.511 | 16.289 | 0.196105 | 7/15/2014 | 56:28.3 | 1 |
| -252.744 | 19515.197 | 1.509 | 16.293 | 0.211953 | 7/15/2014 | 56:28.4 | 1 |
| -253.004 | 19515.217 | 1.511 | 16.297 | 0.218879 | 7/15/2014 | 56:28.5 | 1 |
| -253.26 | 19515.236 | 1.515 | 16.299 | 0.224845 | 7/15/2014 | 56:28.6 | 1 |
| -253.517 | 19515.257 | 1.511 | 16.301 | 0.228036 | 7/15/2014 | 56:28.7 | 1 |
| -253.835 | 19515.282 | 1.506 | 16.302 | 0.227322 | 7/15/2014 | 56:28.8 | 1 |
| -254.093 | 19515.303 | 1.509 | 16.302 | 0.230833 | 7/15/2014 | 56:28.9 | 1 |
| -254.348 | 19515.322 | 1.503 | 16.3 | 0.22767 | 7/15/2014 | 56:29.0 | 1 |
| -254.605 | 19515.342 | 1.497 | 16.297 | 0.218799 | 7/15/2014 | 56:29.1 | 1 |
| -254.859 | 19515.363 | 1.494 | 16.292 | 0.210842 | 7/15/2014 | 56:29.2 | 1 |
| -255.113 | 19515.383 | 1.494 | 16.287 | 0.198711 | 7/15/2014 | 56:29.3 | 1 |
| -255.427 | 19515.408 | 1.492 | 16.281 | 0.193449 | 7/15/2014 | 56:29.4 | 1 |
| -255.679 | 19515.428 | 1.492 | 16.274 | 0.18355 | 7/15/2014 | 56:29.5 | 1 |
| -255.929 | 19515.447 | 1.489 | 16.267 | 0.177602 | 7/15/2014 | 56:29.6 | 1 |
| -256.179 | 19515.468 | 1.492 | 16.26 | 0.175452 | 7/15/2014 | 56:29.7 | 1 |
| -256.487 | 19515.494 | 1.494 | 16.254 | 0.175413 | 7/15/2014 | 56:29.8 | 1 |
| -256.735 | 19515.514 | 1.498 | 16.248 | 0.172809 | 7/15/2014 | 56:29.9 | 1 |
| -256.982 | 19515.533 | 1.494 | 16.242 | 0.164944 | 7/15/2014 | 56:30.0 | 1 |
| -257.229 | 19515.553 | 1.5 | 16.238 | 0.17572 | 7/15/2014 | 56:30.1 | 1 |
| -257.474 | 19515.572 | 1.5 | 16.233 | 0.171675 | 7/15/2014 | 56:30.2 | 1 |
| -257.718 | 19515.593 | 1.499 | 16.229 | 0.17232 | 7/15/2014 | 56:30.3 | 1 |
| -258.139 | 19515.627 | 1.499 | 16.223 | 0.168863 | 7/15/2014 | 56:30.4 | 1 |
| -258.379 | 19515.646 | 1.499 | 16.219 | 0.164627 | 7/15/2014 | 56:30.5 | 1 |
| -258.619 | 19515.666 | 1.502 | 16.215 | 0.16692 | 7/15/2014 | 56:30.6 | 1 |
| -258.916 | 19515.689 | 1.505 | 16.211 | 0.165902 | 7/15/2014 | 56:30.7 | 1 |
| -259.038 | 19515.699 | 1.503 | 16.209 | 0.161111 | 7/15/2014 | 56:30.8 | 1 |
| -259.277 | 19515.719 | 1.511 | 16.206 | 0.161155 | 7/15/2014 | 56:30.9 | 1 |
| -259.517 | 19515.738 | 1.518 | 16.204 | 0.177126 | 7/15/2014 | 56:31.0 | 1 |
| -259.756 | 19515.758 | 1.524 | 16.202 | 0.158925 | 7/15/2014 | 56:31.1 | 1 |
| -259.995 | 19515.777 | 1.524 | 16.203 | 0.173097 | 7/15/2014 | 56:31.2 | 1 |
| -260.234 | 19515.797 | 1.53 | 16.205 | 0.164354 | 7/15/2014 | 56:31.3 | 1 |
| -260.65 | 19515.83 | 1.53 | 16.215 | 0.167914 | 7/15/2014 | 56:31.4 | 1 |
| -260.888 | 19515.851 | 1.532 | 16.222 | 0.158045 | 7/15/2014 | 56:31.5 | 1 |
| -261.126 | 19515.871 | 1.532 | 16.232 | 0.148728 | 7/15/2014 | 56:31.6 | 1 |
| -261.424 | 19515.895 | 1.527 | 16.243 | 0.148514 | 7/15/2014 | 56:31.7 | 1 |
| -261.544 | 19515.904 | 1.526 | 16.248 | 0.155554 | 7/15/2014 | 56:31.8 | 1 |
| -261.786 | 19515.924 | 1.531 | 16.261 | 0.15342 | 7/15/2014 | 56:31.9 | 1 |
| -262.028 | 19515.943 | 1.531 | 16.275 | 0.14229 | 7/15/2014 | 56:32.0 | 1 |

| | | | | | | | |
|---|---|---|---|---|---|---|---|
| -262.271 | 19515.965 | 1.531 | 16.288 | 0.147822 | 7/15/2014 | 56:32.1 | 1 |
| -262.514 | 19515.985 | 1.531 | 16.301 | 0.149746 | 7/15/2014 | 56:32.2 | 1 |
| -262.758 | 19516.006 | 1.533 | 16.313 | 0.157444 | 7/15/2014 | 56:32.3 | 1 |
| -263.184 | 19516.041 | 1.533 | 16.329 | 0.175026 | 7/15/2014 | 56:32.4 | 1 |
| -263.429 | 19516.061 | 1.541 | 16.338 | 0.185781 | 7/15/2014 | 56:32.5 | 1 |
| -263.673 | 19516.08 | 1.541 | 16.348 | 0.196154 | 7/15/2014 | 56:32.6 | 1 |
| -263.98 | 19516.107 | 1.538 | 16.356 | 0.206851 | 7/15/2014 | 56:32.7 | 1 |
| -264.104 | 19516.117 | 1.538 | 16.36 | 0.212436 | 7/15/2014 | 56:32.8 | 1 |
| -264.351 | 19516.137 | 1.538 | 16.368 | 0.220406 | 7/15/2014 | 56:32.9 | 1 |
| -264.599 | 19516.156 | 1.532 | 16.376 | 0.228309 | 7/15/2014 | 56:33.0 | 1 |
| -264.847 | 19516.18 | 1.532 | 16.384 | 0.229945 | 7/15/2014 | 56:33.1 | 1 |
| -265.095 | 19516.199 | 1.528 | 16.393 | 0.22864 | 7/15/2014 | 56:33.2 | 1 |
| -265.343 | 19516.219 | 1.522 | 16.401 | 0.231492 | 7/15/2014 | 56:33.3 | 1 |
| -265.652 | 19516.246 | 1.522 | 16.409 | 0.218034 | 7/15/2014 | 56:33.4 | 1 |
| -265.899 | 19516.266 | 1.522 | 16.417 | 0.223914 | 7/15/2014 | 56:33.5 | 1 |
| -266.146 | 19516.285 | 1.514 | 16.425 | 0.212067 | 7/15/2014 | 56:33.6 | 1 |
| -266.577 | 19516.322 | 1.511 | 16.435 | 0.200822 | 7/15/2014 | 56:33.7 | 1 |
| -266.702 | 19516.332 | 1.508 | 16.439 | 0.203159 | 7/15/2014 | 56:33.8 | 1 |
| -266.95 | 19516.353 | 1.505 | 16.445 | 0.190776 | 7/15/2014 | 56:33.9 | 1 |
| -267.198 | 19516.375 | 1.503 | 16.45 | 0.18992 | 7/15/2014 | 56:34.0 | 1 |
| -267.446 | 19516.395 | 1.501 | 16.454 | 0.189625 | 7/15/2014 | 56:34.1 | 1 |
| -267.693 | 19516.414 | 1.498 | 16.457 | 0.172712 | 7/15/2014 | 56:34.2 | 1 |
| -267.941 | 19516.435 | 1.501 | 16.458 | 0.177973 | 7/15/2014 | 56:34.3 | 1 |
| -268.37 | 19516.471 | 1.495 | 16.456 | 0.161996 | 7/15/2014 | 56:34.4 | 1 |
| -268.615 | 19516.491 | 1.495 | 16.453 | 0.158589 | 7/15/2014 | 56:34.5 | 1 |
| -268.86 | 19516.513 | 1.495 | 16.449 | 0.155638 | 7/15/2014 | 56:34.6 | 1 |
| -269.166 | 19516.537 | 1.495 | 16.445 | 0.153374 | 7/15/2014 | 56:34.7 | 1 |
| -269.289 | 19516.547 | 1.49 | 16.442 | 0.150444 | 7/15/2014 | 56:34.8 | 1 |
| -269.536 | 19516.567 | 1.484 | 16.436 | 0.161342 | 7/15/2014 | 56:34.9 | 1 |
| -269.782 | 19516.59 | 1.479 | 16.43 | 0.157154 | 7/15/2014 | 56:35.0 | 1 |
| -270.029 | 19516.609 | 1.479 | 16.425 | 0.168276 | 7/15/2014 | 56:35.1 | 1 |
| -270.275 | 19516.629 | 1.476 | 16.42 | 0.177599 | 7/15/2014 | 56:35.2 | 1 |
| -270.522 | 19516.649 | 1.474 | 16.415 | 0.184589 | 7/15/2014 | 56:35.3 | 1 |
| -270.95 | 19516.684 | 1.467 | 16.41 | 0.196548 | 7/15/2014 | 56:35.4 | 1 |
| -271.194 | 19516.703 | 1.457 | 16.406 | 0.204759 | 7/15/2014 | 56:35.5 | 1 |
| -271.438 | 19516.725 | 1.457 | 16.404 | 0.203967 | 7/15/2014 | 56:35.6 | 1 |
| -271.742 | 19516.75 | 1.457 | 16.401 | 0.201839 | 7/15/2014 | 56:35.7 | 1 |
| -271.866 | 19516.76 | 1.455 | 16.4 | 0.201032 | 7/15/2014 | 56:35.8 | 1 |
| -272.112 | 19516.779 | 1.451 | 16.399 | 0.196063 | 7/15/2014 | 56:35.9 | 1 |
| -272.356 | 19516.799 | 1.445 | 16.398 | 0.190456 | 7/15/2014 | 56:36.0 | 1 |
| -272.601 | 19516.818 | 1.445 | 16.397 | 0.178376 | 7/15/2014 | 56:36.1 | 1 |
| -272.846 | 19516.838 | 1.437 | 16.398 | 0.17379 | 7/15/2014 | 56:36.2 | 1 |
| -273.09 | 19516.857 | 1.443 | 16.4 | 0.158136 | 7/15/2014 | 56:36.3 | 1 |
| -273.513 | 19516.892 | 1.437 | 16.406 | 0.143313 | 7/15/2014 | 56:36.4 | 1 |
| -273.755 | 19516.91 | 1.429 | 16.41 | 0.136777 | 7/15/2014 | 56:36.5 | 1 |
| -273.996 | 19516.93 | 1.424 | 16.414 | 0.134947 | 7/15/2014 | 56:36.6 | 1 |
| -274.295 | 19516.953 | 1.421 | 16.418 | 0.127655 | 7/15/2014 | 56:36.7 | 1 |

| | | | | | | | |
|---|---|---|---|---|---|---|---|
| -274.417 | 19516.963 | 1.42 | 16.421 | 0.122131 | 7/15/2014 | 56:36.8 | 1 |
| -274.659 | 19516.982 | 1.422 | 16.424 | 0.118364 | 7/15/2014 | 56:36.9 | 1 |
| -274.899 | 19517.002 | 1.409 | 16.427 | 0.105928 | 7/15/2014 | 56:37.0 | 1 |
| -275.141 | 19517.019 | 1.406 | 16.428 | 0.108431 | 7/15/2014 | 56:37.1 | 1 |
| -275.378 | 19517.037 | 1.403 | 16.428 | 0.107438 | 7/15/2014 | 56:37.2 | 1 |
| -275.619 | 19517.057 | 1.409 | 16.427 | 0.0864827 | 7/15/2014 | 56:37.3 | 1 |
| -276.031 | 19517.088 | 1.403 | 16.425 | 0.0747706 | 7/15/2014 | 56:37.4 | 1 |
| -276.268 | 19517.107 | 1.403 | 16.422 | 0.0711036 | 7/15/2014 | 56:37.5 | 1 |
| -276.504 | 19517.125 | 1.401 | 16.419 | 0.0730147 | 7/15/2014 | 56:37.6 | 1 |
| -276.798 | 19517.146 | 1.406 | 16.416 | 0.0609388 | 7/15/2014 | 56:37.7 | 1 |
| -276.916 | 19517.156 | 1.405 | 16.415 | 0.0660439 | 7/15/2014 | 56:37.8 | 1 |
| -277.154 | 19517.175 | 1.405 | 16.412 | 0.0540018 | 7/15/2014 | 56:37.9 | 1 |
| -277.39 | 19517.192 | 1.401 | 16.408 | 0.0540163 | 7/15/2014 | 56:38.0 | 1 |
| -277.627 | 19517.21 | 1.404 | 16.406 | 0.0527252 | 7/15/2014 | 56:38.1 | 1 |
| -277.864 | 19517.229 | 1.404 | 16.403 | 0.0552125 | 7/15/2014 | 56:38.2 | 1 |
| -278.101 | 19517.244 | 1.404 | 16.4 | 0.0616803 | 7/15/2014 | 56:38.3 | 1 |
| -278.513 | 19517.275 | 1.398 | 16.396 | 0.0951543 | 7/15/2014 | 56:38.4 | 1 |
| -278.749 | 19517.294 | 1.397 | 16.392 | 0.0952185 | 7/15/2014 | 56:38.5 | 1 |
| -278.985 | 19517.311 | 1.403 | 16.389 | 0.114014 | 7/15/2014 | 56:38.6 | 1 |
| -279.281 | 19517.333 | 1.402 | 16.384 | 0.112696 | 7/15/2014 | 56:38.7 | 1 |
| -279.4 | 19517.342 | 1.402 | 16.382 | 0.111691 | 7/15/2014 | 56:38.8 | 1 |
| -279.639 | 19517.357 | 1.408 | 16.376 | 0.109452 | 7/15/2014 | 56:38.9 | 1 |
| -279.878 | 19517.376 | 1.408 | 16.37 | 0.110823 | 7/15/2014 | 56:39.0 | 1 |
| -280.118 | 19517.393 | 1.414 | 16.362 | 0.0869329 | 7/15/2014 | 56:39.1 | 1 |
| -280.358 | 19517.41 | 1.411 | 16.354 | 0.0750639 | 7/15/2014 | 56:39.2 | 1 |
| -280.597 | 19517.428 | 1.411 | 16.344 | 0.0523509 | 7/15/2014 | 56:39.3 | 1 |
| -281.016 | 19517.457 | 1.408 | 16.329 | 0.0566616 | 7/15/2014 | 56:39.4 | 1 |
| -281.255 | 19517.474 | 1.412 | 16.319 | 0.0530875 | 7/15/2014 | 56:39.5 | 1 |
| -281.494 | 19517.491 | 1.412 | 16.309 | 0.0720404 | 7/15/2014 | 56:39.6 | 1 |
| -281.792 | 19517.512 | 1.412 | 16.298 | 0.0528778 | 7/15/2014 | 56:39.7 | 1 |
| -281.914 | 19517.52 | 1.413 | 16.293 | 0.027353 | 7/15/2014 | 56:39.8 | 1 |
| -282.154 | 19517.537 | 1.417 | 16.282 | 0.0428639 | 7/15/2014 | 56:39.9 | 1 |
| -282.395 | 19517.553 | 1.414 | 16.271 | 0.0265996 | 7/15/2014 | 56:40.0 | 1 |
| -282.637 | 19517.569 | 1.417 | 16.261 | 0.0348495 | 7/15/2014 | 56:40.1 | 1 |
| -282.878 | 19517.586 | 1.423 | 16.25 | 0.0301798 | 7/15/2014 | 56:40.2 | 1 |
| -283.118 | 19517.603 | 1.42 | 16.239 | 0.0559388 | 7/15/2014 | 56:40.3 | 1 |
| -283.539 | 19517.63 | 1.413 | 16.224 | 0.0741454 | 7/15/2014 | 56:40.4 | 1 |
| -283.777 | 19517.646 | 1.413 | 16.214 | 0.0902266 | 7/15/2014 | 56:40.5 | 1 |
| -284.018 | 19517.661 | 1.415 | 16.205 | 0.108332 | 7/15/2014 | 56:40.6 | 1 |
| -284.319 | 19517.681 | 1.419 | 16.197 | 0.089235 | 7/15/2014 | 56:40.7 | 1 |
| -284.44 | 19517.688 | 1.417 | 16.192 | 0.10355 | 7/15/2014 | 56:40.8 | 1 |
| -284.683 | 19517.704 | 1.417 | 16.184 | 0.0912289 | 7/15/2014 | 56:40.9 | 1 |
| -284.927 | 19517.72 | 1.419 | 16.178 | 0.0755623 | 7/15/2014 | 56:41.0 | 1 |
| -285.171 | 19517.735 | 1.428 | 16.172 | 0.104416 | 7/15/2014 | 56:41.1 | 1 |
| -285.413 | 19517.75 | 1.429 | 16.167 | 0.0901362 | 7/15/2014 | 56:41.2 | 1 |
| -285.657 | 19517.766 | 1.434 | 16.163 | 0.0789935 | 7/15/2014 | 56:41.3 | 1 |
| -286.082 | 19517.791 | 1.436 | 16.159 | 0.100186 | 7/15/2014 | 56:41.4 | 1 |

| | | | | | | | |
|---|---|---|---|---|---|---|---|
| -286.324 | 19517.807 | 1.436 | 16.157 | 0.100196 | 7/15/2014 | 56:41.5 | 1 |
| -286.568 | 19517.82 | 1.433 | 16.155 | 0.1076 | 7/15/2014 | 56:41.6 | 1 |
| -286.871 | 19517.84 | 1.433 | 16.153 | 0.0911761 | 7/15/2014 | 56:41.7 | 1 |
| -286.994 | 19517.847 | 1.431 | 16.153 | 0.102038 | 7/15/2014 | 56:41.8 | 1 |
| -287.239 | 19517.86 | 1.434 | 16.151 | 0.10419 | 7/15/2014 | 56:41.9 | 1 |
| -287.484 | 19517.875 | 1.428 | 16.149 | 0.0897614 | 7/15/2014 | 56:42.0 | 1 |
| -287.729 | 19517.89 | 1.428 | 16.147 | 0.0812768 | 7/15/2014 | 56:42.1 | 1 |
| -287.972 | 19517.904 | 1.424 | 16.144 | 0.0900096 | 7/15/2014 | 56:42.2 | 1 |
| -288.216 | 19517.917 | 1.429 | 16.14 | 0.0730903 | 7/15/2014 | 56:42.3 | 1 |
| -288.641 | 19517.941 | 1.434 | 16.133 | 0.0662187 | 7/15/2014 | 56:42.4 | 1 |
| -288.882 | 19517.955 | 1.436 | 16.127 | 0.0632613 | 7/15/2014 | 56:42.5 | 1 |
| -289.123 | 19517.969 | 1.438 | 16.121 | 0.0678521 | 7/15/2014 | 56:42.6 | 1 |
| -289.422 | 19517.984 | 1.446 | 16.114 | 0.0819304 | 7/15/2014 | 56:42.7 | 1 |
| -289.545 | 19517.991 | 1.446 | 16.111 | 0.0711788 | 7/15/2014 | 56:42.8 | 1 |
| -289.786 | 19518.005 | 1.462 | 16.103 | 0.103503 | 7/15/2014 | 56:42.9 | 1 |
| -290.026 | 19518.018 | 1.465 | 16.095 | 0.095382 | 7/15/2014 | 56:43.0 | 1 |
| -290.265 | 19518.029 | 1.465 | 16.086 | 0.0841534 | 7/15/2014 | 56:43.1 | 1 |
| -290.504 | 19518.043 | 1.463 | 16.077 | 0.105645 | 7/15/2014 | 56:43.2 | 1 |
| -290.742 | 19518.055 | 1.467 | 16.068 | 0.0986905 | 7/15/2014 | 56:43.3 | 1 |
| -291.154 | 19518.076 | 1.468 | 16.055 | 0.117101 | 7/15/2014 | 56:43.4 | 1 |
| -291.388 | 19518.088 | 1.468 | 16.047 | 0.0860428 | 7/15/2014 | 56:43.5 | 1 |
| -291.622 | 19518.1 | 1.462 | 16.04 | 0.111231 | 7/15/2014 | 56:43.6 | 1 |
| -291.912 | 19518.114 | 1.459 | 16.035 | 0.117344 | 7/15/2014 | 56:43.7 | 1 |
| -292.03 | 19518.12 | 1.461 | 16.031 | 0.118362 | 7/15/2014 | 56:43.8 | 1 |
| -292.266 | 19518.132 | 1.467 | 16.029 | 0.101125 | 7/15/2014 | 56:43.9 | 1 |
| -292.499 | 19518.144 | 1.463 | 16.029 | 0.0975313 | 7/15/2014 | 56:44.0 | 1 |
| -292.736 | 19518.155 | 1.467 | 16.031 | 0.0840951 | 7/15/2014 | 56:44.1 | 1 |
| -292.968 | 19518.166 | 1.463 | 16.037 | 0.0945193 | 7/15/2014 | 56:44.2 | 1 |
| -293.203 | 19518.178 | 1.467 | 16.044 | 0.0792166 | 7/15/2014 | 56:44.3 | 1 |
| -293.61 | 19518.196 | 1.468 | 16.06 | 0.0788544 | 7/15/2014 | 56:44.4 | 1 |
| -293.842 | 19518.208 | 1.468 | 16.071 | 0.0804464 | 7/15/2014 | 56:44.5 | 1 |
| -294.074 | 19518.219 | 1.471 | 16.083 | 0.0788842 | 7/15/2014 | 56:44.6 | 1 |
| -294.364 | 19518.232 | 1.471 | 16.095 | 0.0841658 | 7/15/2014 | 56:44.7 | 1 |
| -294.481 | 19518.238 | 1.47 | 16.101 | 0.0797363 | 7/15/2014 | 56:44.8 | 1 |
| -294.714 | 19518.248 | 1.47 | 16.112 | 0.0952118 | 7/15/2014 | 56:44.9 | 1 |
| -294.947 | 19518.26 | 1.467 | 16.122 | 0.108071 | 7/15/2014 | 56:45.0 | 1 |
| -295.297 | 19518.273 | 1.471 | 16.13 | 0.105997 | 7/15/2014 | 56:45.1 | 1 |
| -295.411 | 19518.279 | 1.467 | 16.134 | 0.113116 | 7/15/2014 | 56:45.2 | 1 |
| -295.759 | 19518.295 | 1.471 | 16.134 | 0.135023 | 7/15/2014 | 56:45.3 | 1 |
| -296.046 | 19518.307 | 1.471 | 16.131 | 0.163787 | 7/15/2014 | 56:45.4 | 1 |
| -296.274 | 19518.315 | 1.471 | 16.125 | 0.166657 | 7/15/2014 | 56:45.5 | 1 |
| -296.503 | 19518.326 | 1.474 | 16.117 | 0.163432 | 7/15/2014 | 56:45.6 | 1 |
| -296.789 | 19518.336 | 1.471 | 16.105 | 0.176097 | 7/15/2014 | 56:45.7 | 1 |
| -297.019 | 19518.348 | 1.471 | 16.09 | 0.169111 | 7/15/2014 | 56:45.8 | 1 |
| -297.249 | 19518.355 | 1.471 | 16.074 | 0.1732 | 7/15/2014 | 56:45.9 | 1 |
| -297.481 | 19518.366 | 1.476 | 16.055 | 0.1734 | 7/15/2014 | 56:46.0 | 1 |
| -297.711 | 19518.375 | 1.476 | 16.036 | 0.161855 | 7/15/2014 | 56:46.1 | 1 |

| | | | | | | | |
|---|---|---|---|---|---|---|---|
| -297.941 | 19518.384 | 1.475 | 16.015 | 0.154824 | 7/15/2014 | 56:46.2 | 1 |
| -298.17 | 19518.393 | 1.48 | 15.995 | 0.155569 | 7/15/2014 | 56:46.3 | 1 |
| -298.455 | 19518.404 | 1.48 | 15.976 | 0.148383 | 7/15/2014 | 56:46.4 | 1 |
| -298.683 | 19518.414 | 1.486 | 15.958 | 0.150204 | 7/15/2014 | 56:46.5 | 1 |
| -298.912 | 19518.422 | 1.491 | 15.942 | 0.144826 | 7/15/2014 | 56:46.6 | 1 |
| -299.199 | 19518.433 | 1.493 | 15.929 | 0.146862 | 7/15/2014 | 56:46.7 | 1 |
| -299.429 | 19518.441 | 1.493 | 15.919 | 0.145852 | 7/15/2014 | 56:46.8 | 1 |
| -299.661 | 19518.449 | 1.498 | 15.913 | 0.151232 | 7/15/2014 | 56:46.9 | 1 |
| -299.892 | 19518.457 | 1.501 | 15.911 | 0.15173 | 7/15/2014 | 56:47.0 | 1 |
| -300.124 | 19518.469 | 1.504 | 15.914 | 0.149758 | 7/15/2014 | 56:47.1 | 1 |
| -300.356 | 19518.477 | 1.51 | 15.92 | 0.152905 | 7/15/2014 | 56:47.2 | 1 |
| -300.471 | 19518.48 | 1.512 | 15.922 | 0.164443 | 7/15/2014 | 56:47.3 | 1 |
| -300.873 | 19518.494 | 1.512 | 15.943 | 0.164513 | 7/15/2014 | 56:47.4 | 1 |
| -301.103 | 19518.502 | 1.518 | 15.958 | 0.158535 | 7/15/2014 | 56:47.5 | 1 |
| -301.333 | 19518.51 | 1.52 | 15.975 | 0.144425 | 7/15/2014 | 56:47.6 | 1 |
| -301.618 | 19518.52 | 1.518 | 15.992 | 0.137839 | 7/15/2014 | 56:47.7 | 1 |
| -301.85 | 19518.529 | 1.522 | 16.008 | 0.132903 | 7/15/2014 | 56:47.8 | 1 |
| -302.08 | 19518.536 | 1.522 | 16.022 | 0.129534 | 7/15/2014 | 56:47.9 | 1 |
| -302.312 | 19518.543 | 1.526 | 16.034 | 0.130266 | 7/15/2014 | 56:48.0 | 1 |
| -302.543 | 19518.551 | 1.528 | 16.042 | 0.128386 | 7/15/2014 | 56:48.1 | 1 |
| -302.773 | 19518.559 | 1.53 | 16.047 | 0.130226 | 7/15/2014 | 56:48.2 | 1 |
| -303.003 | 19518.566 | 1.53 | 16.049 | 0.128476 | 7/15/2014 | 56:48.3 | 1 |
| -303.287 | 19518.575 | 1.533 | 16.047 | 0.128712 | 7/15/2014 | 56:48.4 | 1 |
| -303.515 | 19518.582 | 1.539 | 16.042 | 0.130555 | 7/15/2014 | 56:48.5 | 1 |
| -303.742 | 19518.589 | 1.544 | 16.034 | 0.131501 | 7/15/2014 | 56:48.6 | 1 |
| -304.026 | 19518.598 | 1.548 | 16.022 | 0.133416 | 7/15/2014 | 56:48.7 | 1 |
| -304.255 | 19518.605 | 1.551 | 16.008 | 0.137573 | 7/15/2014 | 56:48.8 | 1 |
| -304.484 | 19518.611 | 1.555 | 15.992 | 0.139833 | 7/15/2014 | 56:48.9 | 1 |
| -304.714 | 19518.619 | 1.56 | 15.973 | 0.143113 | 7/15/2014 | 56:49.0 | 1 |
| -304.942 | 19518.625 | 1.562 | 15.953 | 0.146615 | 7/15/2014 | 56:49.1 | 1 |
| -305.171 | 19518.631 | 1.57 | 15.931 | 0.156206 | 7/15/2014 | 56:49.2 | 1 |
| -305.398 | 19518.639 | 1.575 | 15.908 | 0.160515 | 7/15/2014 | 56:49.3 | 1 |
| -305.681 | 19518.648 | 1.577 | 15.885 | 0.158261 | 7/15/2014 | 56:49.4 | 1 |
| -305.907 | 19518.654 | 1.584 | 15.861 | 0.164817 | 7/15/2014 | 56:49.5 | 1 |
| -306.133 | 19518.66 | 1.593 | 15.838 | 0.160217 | 7/15/2014 | 56:49.6 | 1 |
| -306.417 | 19518.669 | 1.6 | 15.816 | 0.163692 | 7/15/2014 | 56:49.7 | 1 |
| -306.645 | 19518.675 | 1.605 | 15.796 | 0.158072 | 7/15/2014 | 56:49.8 | 1 |
| -306.874 | 19518.682 | 1.613 | 15.777 | 0.160173 | 7/15/2014 | 56:49.9 | 1 |
| -307.104 | 19518.688 | 1.616 | 15.76 | 0.155987 | 7/15/2014 | 56:50.0 | 1 |
| -307.335 | 19518.695 | 1.625 | 15.745 | 0.165112 | 7/15/2014 | 56:50.1 | 1 |
| -307.563 | 19518.7 | 1.63 | 15.732 | 0.181497 | 7/15/2014 | 56:50.2 | 1 |
| -307.794 | 19518.707 | 1.633 | 15.721 | 0.167418 | 7/15/2014 | 56:50.3 | 1 |
| -308.079 | 19518.716 | 1.633 | 15.712 | 0.174844 | 7/15/2014 | 56:50.4 | 1 |
| -308.31 | 19518.722 | 1.636 | 15.706 | 0.157412 | 7/15/2014 | 56:50.5 | 1 |
| -308.54 | 19518.729 | 1.636 | 15.702 | 0.152171 | 7/15/2014 | 56:50.6 | 1 |
| -308.83 | 19518.735 | 1.64 | 15.7 | 0.163014 | 7/15/2014 | 56:50.7 | 1 |
| -308.947 | 19518.739 | 1.641 | 15.698 | 0.155093 | 7/15/2014 | 56:50.8 | 1 |

| | | | | | | | |
|---|---|---|---|---|---|---|---|
| -309.181 | 19518.745 | 1.641 | 15.699 | 0.135819 | 7/15/2014 | 56:50.9 | 1 |
| -309.417 | 19518.752 | 1.642 | 15.701 | 0.135523 | 7/15/2014 | 56:51.0 | 1 |
| -309.654 | 19518.759 | 1.648 | 15.705 | 0.141238 | 7/15/2014 | 56:51.1 | 1 |
| -309.889 | 19518.765 | 1.648 | 15.709 | 0.140924 | 7/15/2014 | 56:51.2 | 1 |
| -310.126 | 19518.771 | 1.654 | 15.713 | 0.141482 | 7/15/2014 | 56:51.3 | 1 |
| -310.54 | 19518.782 | 1.665 | 15.718 | 0.135203 | 7/15/2014 | 56:51.4 | 1 |
| -310.775 | 19518.789 | 1.669 | 15.72 | 0.157929 | 7/15/2014 | 56:51.5 | 1 |
| -311.011 | 19518.797 | 1.671 | 15.721 | 0.144874 | 7/15/2014 | 56:51.6 | 1 |
| -311.307 | 19518.804 | 1.675 | 15.721 | 0.139446 | 7/15/2014 | 56:51.7 | 1 |
| -311.426 | 19518.807 | 1.675 | 15.721 | 0.138841 | 7/15/2014 | 56:51.8 | 1 |
| -311.665 | 19518.814 | 1.683 | 15.719 | 0.145974 | 7/15/2014 | 56:51.9 | 1 |
| -311.902 | 19518.82 | 1.685 | 15.714 | 0.1281 | 7/15/2014 | 56:52.0 | 1 |
| -312.141 | 19518.826 | 1.694 | 15.708 | 0.146311 | 7/15/2014 | 56:52.1 | 1 |
| -312.377 | 19518.834 | 1.688 | 15.701 | 0.143858 | 7/15/2014 | 56:52.2 | 1 |
| -312.615 | 19518.842 | 1.696 | 15.692 | 0.151219 | 7/15/2014 | 56:52.3 | 1 |
| -313.028 | 19518.853 | 1.705 | 15.675 | 0.155491 | 7/15/2014 | 56:52.4 | 1 |
| -313.262 | 19518.859 | 1.708 | 15.663 | 0.161997 | 7/15/2014 | 56:52.5 | 1 |
| -313.499 | 19518.866 | 1.716 | 15.65 | 0.176456 | 7/15/2014 | 56:52.6 | 1 |
| -313.794 | 19518.875 | 1.725 | 15.637 | 0.185706 | 7/15/2014 | 56:52.7 | 1 |
| -313.912 | 19518.879 | 1.726 | 15.63 | 0.185526 | 7/15/2014 | 56:52.8 | 1 |
| -314.15 | 19518.887 | 1.738 | 15.615 | 0.193011 | 7/15/2014 | 56:52.9 | 1 |
| -314.386 | 19518.894 | 1.741 | 15.601 | 0.202727 | 7/15/2014 | 56:53.0 | 1 |
| -314.626 | 19518.9 | 1.75 | 15.586 | 0.206847 | 7/15/2014 | 56:53.1 | 1 |
| -314.862 | 19518.907 | 1.756 | 15.572 | 0.225825 | 7/15/2014 | 56:53.2 | 1 |
| -315.1 | 19518.915 | 1.768 | 15.559 | 0.219225 | 7/15/2014 | 56:53.3 | 1 |
| -315.516 | 19518.929 | 1.769 | 15.543 | 0.222341 | 7/15/2014 | 56:53.4 | 1 |
| -315.752 | 19518.938 | 1.769 | 15.533 | 0.219338 | 7/15/2014 | 56:53.5 | 1 |
| -315.989 | 19518.944 | 1.774 | 15.524 | 0.220327 | 7/15/2014 | 56:53.6 | 1 |
| -316.286 | 19518.954 | 1.776 | 15.516 | 0.211417 | 7/15/2014 | 56:53.7 | 1 |
| -316.405 | 19518.958 | 1.775 | 15.512 | 0.20791 | 7/15/2014 | 56:53.8 | 1 |
| -316.645 | 19518.966 | 1.78 | 15.505 | 0.203182 | 7/15/2014 | 56:53.9 | 1 |
| -316.885 | 19518.974 | 1.779 | 15.498 | 0.197009 | 7/15/2014 | 56:54.0 | 1 |
| -317.126 | 19518.981 | 1.787 | 15.491 | 0.175749 | 7/15/2014 | 56:54.1 | 1 |
| -317.366 | 19518.99 | 1.79 | 15.483 | 0.177091 | 7/15/2014 | 56:54.2 | 1 |
| -317.607 | 19518.999 | 1.798 | 15.475 | 0.166546 | 7/15/2014 | 56:54.3 | 1 |
| -318.026 | 19519.014 | 1.799 | 15.46 | 0.173109 | 7/15/2014 | 56:54.4 | 1 |
| -318.265 | 19519.023 | 1.803 | 15.45 | 0.163708 | 7/15/2014 | 56:54.5 | 1 |
| -318.505 | 19519.031 | 1.81 | 15.44 | 0.163681 | 7/15/2014 | 56:54.6 | 1 |
| -318.803 | 19519.043 | 1.81 | 15.431 | 0.171876 | 7/15/2014 | 56:54.7 | 1 |
| -318.925 | 19519.047 | 1.808 | 15.426 | 0.160299 | 7/15/2014 | 56:54.8 | 1 |
| -319.167 | 19519.057 | 1.815 | 15.418 | 0.162919 | 7/15/2014 | 56:54.9 | 1 |
| -319.407 | 19519.065 | 1.815 | 15.412 | 0.157774 | 7/15/2014 | 56:55.0 | 1 |
| -319.648 | 19519.074 | 1.825 | 15.408 | 0.153223 | 7/15/2014 | 56:55.1 | 1 |
| -319.886 | 19519.086 | 1.825 | 15.406 | 0.157939 | 7/15/2014 | 56:55.2 | 1 |
| -320.126 | 19519.094 | 1.829 | 15.405 | 0.161452 | 7/15/2014 | 56:55.3 | 1 |
| -320.539 | 19519.112 | 1.825 | 15.409 | 0.166069 | 7/15/2014 | 56:55.4 | 1 |
| -320.776 | 19519.121 | 1.831 | 15.413 | 0.174235 | 7/15/2014 | 56:55.5 | 1 |

| | | | | | | | |
|---|---|---|---|---|---|---|---|
| -321.011 | 19519.132 | 1.831 | 15.416 | 0.17309 | 7/15/2014 | 56:55.6 | 1 |
| -321.303 | 19519.145 | 1.834 | 15.42 | 0.176955 | 7/15/2014 | 56:55.7 | 1 |
| -321.423 | 19519.15 | 1.832 | 15.422 | 0.184317 | 7/15/2014 | 56:55.8 | 1 |
| -321.659 | 19519.16 | 1.838 | 15.425 | 0.183199 | 7/15/2014 | 56:55.9 | 1 |
| -321.893 | 19519.171 | 1.841 | 15.427 | 0.194037 | 7/15/2014 | 56:56.0 | 1 |
| -322.129 | 19519.181 | 1.85 | 15.426 | 0.195567 | 7/15/2014 | 56:56.1 | 1 |
| -322.363 | 19519.191 | 1.85 | 15.424 | 0.20459 | 7/15/2014 | 56:56.2 | 1 |
| -322.597 | 19519.203 | 1.852 | 15.418 | 0.203117 | 7/15/2014 | 56:56.3 | 1 |
| -323.007 | 19519.223 | 1.856 | 15.404 | 0.218194 | 7/15/2014 | 56:56.4 | 1 |
| -323.24 | 19519.234 | 1.86 | 15.393 | 0.238451 | 7/15/2014 | 56:56.5 | 1 |
| -323.472 | 19519.245 | 1.862 | 15.381 | 0.228564 | 7/15/2014 | 56:56.6 | 1 |
| -323.764 | 19519.26 | 1.866 | 15.367 | 0.234233 | 7/15/2014 | 56:56.7 | 1 |
| -323.881 | 19519.266 | 1.864 | 15.361 | 0.235691 | 7/15/2014 | 56:56.8 | 1 |
| -324.116 | 19519.277 | 1.876 | 15.347 | 0.237798 | 7/15/2014 | 56:56.9 | 1 |
| -324.351 | 19519.289 | 1.879 | 15.334 | 0.23834 | 7/15/2014 | 56:57.0 | 1 |
| -324.588 | 19519.301 | 1.884 | 15.321 | 0.245175 | 7/15/2014 | 56:57.1 | 1 |
| -324.823 | 19519.313 | 1.887 | 15.311 | 0.246311 | 7/15/2014 | 56:57.2 | 1 |
| -325.058 | 19519.325 | 1.887 | 15.301 | 0.249736 | 7/15/2014 | 56:57.3 | 1 |
| -325.469 | 19519.348 | 1.892 | 15.29 | 0.245463 | 7/15/2014 | 56:57.4 | 1 |
| -325.702 | 19519.359 | 1.892 | 15.283 | 0.240473 | 7/15/2014 | 56:57.5 | 1 |
| -325.937 | 19519.373 | 1.894 | 15.276 | 0.246366 | 7/15/2014 | 56:57.6 | 1 |
| -326.23 | 19519.389 | 1.894 | 15.27 | 0.242405 | 7/15/2014 | 56:57.7 | 1 |
| -326.348 | 19519.395 | 1.896 | 15.267 | 0.230055 | 7/15/2014 | 56:57.8 | 1 |
| -326.586 | 19519.408 | 1.898 | 15.261 | 0.216768 | 7/15/2014 | 56:57.9 | 1 |
| -326.823 | 19519.422 | 1.904 | 15.254 | 0.225878 | 7/15/2014 | 56:58.0 | 1 |
| -327.063 | 19519.435 | 1.91 | 15.248 | 0.211597 | 7/15/2014 | 56:58.1 | 1 |
| -327.301 | 19519.447 | 1.907 | 15.241 | 0.216068 | 7/15/2014 | 56:58.2 | 1 |
| -327.54 | 19519.463 | 1.913 | 15.234 | 0.197104 | 7/15/2014 | 56:58.3 | 1 |
| -327.957 | 19519.486 | 1.908 | 15.224 | 0.197127 | 7/15/2014 | 56:58.4 | 1 |
| -328.196 | 19519.501 | 1.911 | 15.219 | 0.199731 | 7/15/2014 | 56:58.5 | 1 |
| -328.436 | 19519.516 | 1.911 | 15.215 | 0.20161 | 7/15/2014 | 56:58.6 | 1 |
| -328.738 | 19519.533 | 1.908 | 15.213 | 0.203478 | 7/15/2014 | 56:58.7 | 1 |
| -328.859 | 19519.54 | 1.906 | 15.211 | 0.207068 | 7/15/2014 | 56:58.8 | 1 |
| -329.101 | 19519.556 | 1.906 | 15.212 | 0.214434 | 7/15/2014 | 56:58.9 | 1 |
| -329.345 | 19519.57 | 1.902 | 15.214 | 0.214393 | 7/15/2014 | 56:59.0 | 1 |
| -329.59 | 19519.585 | 1.902 | 15.219 | 0.22462 | 7/15/2014 | 56:59.1 | 1 |
| -329.834 | 19519.601 | 1.906 | 15.227 | 0.226998 | 7/15/2014 | 56:59.2 | 1 |
| -330.079 | 19519.616 | 1.902 | 15.237 | 0.244126 | 7/15/2014 | 56:59.3 | 1 |
| -330.507 | 19519.643 | 1.896 | 15.257 | 0.237158 | 7/15/2014 | 56:59.4 | 1 |
| -330.752 | 19519.659 | 1.896 | 15.272 | 0.242012 | 7/15/2014 | 56:59.5 | 1 |
| -330.998 | 19519.675 | 1.899 | 15.288 | 0.229794 | 7/15/2014 | 56:59.6 | 1 |
| -331.304 | 19519.694 | 1.897 | 15.304 | 0.227357 | 7/15/2014 | 56:59.7 | 1 |
| -331.428 | 19519.702 | 1.897 | 15.312 | 0.225655 | 7/15/2014 | 56:59.8 | 1 |
| -331.676 | 19519.719 | 1.891 | 15.328 | 0.215038 | 7/15/2014 | 56:59.9 | 1 |
| -331.924 | 19519.735 | 1.891 | 15.342 | 0.217403 | 7/15/2014 | 57:00.0 | 1 |
| -332.173 | 19519.751 | 1.891 | 15.355 | 0.198627 | 7/15/2014 | 57:00.1 | 1 |
| -332.422 | 19519.768 | 1.886 | 15.367 | 0.177385 | 7/15/2014 | 57:00.2 | 1 |

| | | | | | | | |
|---|---|---|---|---|---|---|---|
| -332.672 | 19519.784 | 1.886 | 15.376 | 0.209022 | 7/15/2014 | 57:00.3 | 1 |
| -332.978 | 19519.805 | 1.88 | 15.383 | 0.205133 | 7/15/2014 | 57:00.4 | 1 |
| -333.228 | 19519.821 | 1.882 | 15.388 | 0.179344 | 7/15/2014 | 57:00.5 | 1 |
| -333.475 | 19519.838 | 1.88 | 15.391 | 0.188633 | 7/15/2014 | 57:00.6 | 1 |
| -333.906 | 19519.868 | 1.876 | 15.388 | 0.16248 | 7/15/2014 | 57:00.7 | 1 |
| -334.031 | 19519.876 | 1.875 | 15.388 | 0.167807 | 7/15/2014 | 57:00.8 | 1 |
| -334.28 | 19519.894 | 1.878 | 15.383 | 0.181243 | 7/15/2014 | 57:00.9 | 1 |
| -334.527 | 19519.911 | 1.869 | 15.375 | 0.183808 | 7/15/2014 | 57:01.0 | 1 |
| -334.776 | 19519.928 | 1.867 | 15.365 | 0.20563 | 7/15/2014 | 57:01.1 | 1 |
| -335.022 | 19519.944 | 1.856 | 15.353 | 0.213775 | 7/15/2014 | 57:01.2 | 1 |
| -335.268 | 19519.963 | 1.852 | 15.341 | 0.229414 | 7/15/2014 | 57:01.3 | 1 |
| -335.697 | 19519.993 | 1.85 | 15.323 | 0.249204 | 7/15/2014 | 57:01.4 | 1 |
| -335.941 | 19520.011 | 1.845 | 15.312 | 0.244415 | 7/15/2014 | 57:01.5 | 1 |
| -336.185 | 19520.028 | 1.84 | 15.303 | 0.256487 | 7/15/2014 | 57:01.6 | 1 |
| -336.487 | 19520.051 | 1.84 | 15.296 | 0.239197 | 7/15/2014 | 57:01.7 | 1 |
| -336.611 | 19520.059 | 1.837 | 15.291 | 0.229565 | 7/15/2014 | 57:01.8 | 1 |
| -336.855 | 19520.078 | 1.839 | 15.289 | 0.236191 | 7/15/2014 | 57:01.9 | 1 |
| -337.099 | 19520.096 | 1.837 | 15.289 | 0.225094 | 7/15/2014 | 57:02.0 | 1 |
| -337.344 | 19520.113 | 1.834 | 15.291 | 0.231396 | 7/15/2014 | 57:02.1 | 1 |
| -337.587 | 19520.132 | 1.831 | 15.297 | 0.227591 | 7/15/2014 | 57:02.2 | 1 |
| -337.831 | 19520.15 | 1.831 | 15.304 | 0.232735 | 7/15/2014 | 57:02.3 | 1 |
| -338.253 | 19520.183 | 1.829 | 15.321 | 0.238977 | 7/15/2014 | 57:02.4 | 1 |
| -338.494 | 19520.202 | 1.827 | 15.333 | 0.249905 | 7/15/2014 | 57:02.5 | 1 |
| -338.734 | 19520.221 | 1.825 | 15.346 | 0.24365 | 7/15/2014 | 57:02.6 | 1 |
| -339.034 | 19520.244 | 1.821 | 15.36 | 0.250365 | 7/15/2014 | 57:02.7 | 1 |
| -339.156 | 19520.254 | 1.821 | 15.368 | 0.255037 | 7/15/2014 | 57:02.8 | 1 |
| -339.399 | 19520.273 | 1.821 | 15.383 | 0.262668 | 7/15/2014 | 57:02.9 | 1 |
| -339.639 | 19520.292 | 1.816 | 15.399 | 0.260676 | 7/15/2014 | 57:03.0 | 1 |
| -339.882 | 19520.312 | 1.814 | 15.415 | 0.264842 | 7/15/2014 | 57:03.1 | 1 |
| -340.122 | 19520.331 | 1.809 | 15.431 | 0.250578 | 7/15/2014 | 57:03.2 | 1 |
| -340.363 | 19520.351 | 1.809 | 15.445 | 0.276877 | 7/15/2014 | 57:03.3 | 1 |
| -340.779 | 19520.385 | 1.806 | 15.463 | 0.268815 | 7/15/2014 | 57:03.4 | 1 |
| -341.017 | 19520.404 | 1.806 | 15.472 | 0.263156 | 7/15/2014 | 57:03.5 | 1 |
| -341.253 | 19520.423 | 1.804 | 15.478 | 0.271478 | 7/15/2014 | 57:03.6 | 1 |
| -341.548 | 19520.449 | 1.804 | 15.481 | 0.280362 | 7/15/2014 | 57:03.7 | 1 |
| -341.668 | 19520.459 | 1.804 | 15.484 | 0.293749 | 7/15/2014 | 57:03.8 | 1 |
| -341.907 | 19520.479 | 1.802 | 15.482 | 0.258444 | 7/15/2014 | 57:03.9 | 1 |
| -342.144 | 19520.499 | 1.793 | 15.476 | 0.266329 | 7/15/2014 | 57:04.0 | 1 |
| -342.383 | 19520.52 | 1.789 | 15.468 | 0.275889 | 7/15/2014 | 57:04.1 | 1 |
| -342.62 | 19520.54 | 1.784 | 15.457 | 0.26568 | 7/15/2014 | 57:04.2 | 1 |
| -342.86 | 19520.561 | 1.786 | 15.445 | 0.269901 | 7/15/2014 | 57:04.3 | 1 |
| -343.28 | 19520.6 | 1.776 | 15.427 | 0.248836 | 7/15/2014 | 57:04.4 | 1 |
| -343.522 | 19520.621 | 1.772 | 15.416 | 0.254276 | 7/15/2014 | 57:04.5 | 1 |
| -343.766 | 19520.644 | 1.772 | 15.407 | 0.255358 | 7/15/2014 | 57:04.6 | 1 |
| -344.011 | 19520.666 | 1.77 | 15.4 | 0.239271 | 7/15/2014 | 57:04.7 | 1 |
| -344.258 | 19520.689 | 1.77 | 15.397 | 0.251739 | 7/15/2014 | 57:04.8 | 1 |
| -344.379 | 19520.7 | 1.768 | 15.394 | 0.242783 | 7/15/2014 | 57:04.9 | 1 |

| | | | | | | | |
|---|---|---|---|---|---|---|---|
| -344.629 | 19520.724 | 1.762 | 15.396 | 0.250169 | 7/15/2014 | 57:05.0 | 1 |
| -344.879 | 19520.748 | 1.759 | 15.402 | 0.243764 | 7/15/2014 | 57:05.1 | 1 |
| -345.128 | 19520.772 | 1.756 | 15.413 | 0.246249 | 7/15/2014 | 57:05.2 | 1 |
| -345.38 | 19520.796 | 1.753 | 15.427 | 0.240633 | 7/15/2014 | 57:05.3 | 1 |
| -345.628 | 19520.82 | 1.743 | 15.445 | 0.245513 | 7/15/2014 | 57:05.4 | 1 |
| -345.877 | 19520.846 | 1.746 | 15.466 | 0.228847 | 7/15/2014 | 57:05.5 | 1 |
| -346.241 | 19520.882 | 1.739 | 15.504 | 0.24128 | 7/15/2014 | 57:05.6 | 1 |
| -346.543 | 19520.913 | 1.734 | 15.53 | 0.242299 | 7/15/2014 | 57:05.7 | 1 |
| -346.668 | 19520.926 | 1.732 | 15.543 | 0.239067 | 7/15/2014 | 57:05.8 | 1 |
| -346.912 | 19520.95 | 1.734 | 15.569 | 0.242754 | 7/15/2014 | 57:05.9 | 1 |
| -347.151 | 19520.975 | 1.73 | 15.593 | 0.245277 | 7/15/2014 | 57:06.0 | 1 |
| -347.393 | 19521 | 1.73 | 15.615 | 0.279382 | 7/15/2014 | 57:06.1 | 1 |
| -347.631 | 19521.024 | 1.724 | 15.633 | 0.265238 | 7/15/2014 | 57:06.2 | 1 |
| -347.871 | 19521.051 | 1.721 | 15.647 | 0.290427 | 7/15/2014 | 57:06.3 | 1 |
| -348.291 | 19521.097 | 1.724 | 15.654 | 0.307673 | 7/15/2014 | 57:06.4 | 1 |
| -348.529 | 19521.123 | 1.724 | 15.654 | 0.316729 | 7/15/2014 | 57:06.5 | 1 |
| -348.769 | 19521.148 | 1.729 | 15.648 | 0.328159 | 7/15/2014 | 57:06.6 | 1 |
| -349.008 | 19521.175 | 1.729 | 15.638 | 0.331614 | 7/15/2014 | 57:06.7 | 1 |
| -349.249 | 19521.201 | 1.729 | 15.623 | 0.350855 | 7/15/2014 | 57:06.8 | 1 |
| -349.489 | 19521.229 | 1.729 | 15.604 | 0.355768 | 7/15/2014 | 57:06.9 | 1 |
| -349.729 | 19521.256 | 1.731 | 15.581 | 0.365388 | 7/15/2014 | 57:07.0 | 1 |
| -349.969 | 19521.282 | 1.731 | 15.557 | 0.386833 | 7/15/2014 | 57:07.1 | 1 |
| -350.206 | 19521.31 | 1.73 | 15.531 | 0.403635 | 7/15/2014 | 57:07.2 | 1 |
| -350.444 | 19521.337 | 1.73 | 15.506 | 0.412126 | 7/15/2014 | 57:07.3 | 1 |
| -350.678 | 19521.364 | 1.724 | 15.482 | 0.413372 | 7/15/2014 | 57:07.4 | 1 |
| -350.911 | 19521.392 | 1.726 | 15.459 | 0.423145 | 7/15/2014 | 57:07.5 | 1 |
| -351.143 | 19521.419 | 1.728 | 15.439 | 0.433792 | 7/15/2014 | 57:07.6 | 1 |
| -351.428 | 19521.453 | 1.728 | 15.423 | 0.412593 | 7/15/2014 | 57:07.7 | 1 |
| -351.657 | 19521.479 | 1.726 | 15.411 | 0.403326 | 7/15/2014 | 57:07.8 | 1 |
| -351.888 | 19521.507 | 1.728 | 15.402 | 0.405243 | 7/15/2014 | 57:07.9 | 1 |
| -352.117 | 19521.536 | 1.726 | 15.399 | 0.392085 | 7/15/2014 | 57:08.0 | 1 |
| -352.346 | 19521.563 | 1.728 | 15.399 | 0.400674 | 7/15/2014 | 57:08.1 | 1 |
| -352.574 | 19521.592 | 1.728 | 15.403 | 0.382915 | 7/15/2014 | 57:08.2 | 1 |
| -352.801 | 19521.62 | 1.726 | 15.41 | 0.38382 | 7/15/2014 | 57:08.3 | 1 |
| -353.083 | 19521.656 | 1.724 | 15.419 | 0.389255 | 7/15/2014 | 57:08.4 | 1 |
| -353.31 | 19521.684 | 1.722 | 15.431 | 0.381081 | 7/15/2014 | 57:08.5 | 1 |
| -353.538 | 19521.713 | 1.722 | 15.444 | 0.385966 | 7/15/2014 | 57:08.6 | 1 |
| -353.824 | 19521.751 | 1.714 | 15.457 | 0.385313 | 7/15/2014 | 57:08.7 | 1 |
| -353.939 | 19521.765 | 1.714 | 15.463 | 0.371511 | 7/15/2014 | 57:08.8 | 1 |
| -354.17 | 19521.794 | 1.714 | 15.476 | 0.386714 | 7/15/2014 | 57:08.9 | 1 |
| -354.402 | 19521.824 | 1.711 | 15.488 | 0.389698 | 7/15/2014 | 57:09.0 | 1 |
| -354.635 | 19521.855 | 1.709 | 15.497 | 0.400521 | 7/15/2014 | 57:09.1 | 1 |
| -354.866 | 19521.886 | 1.707 | 15.505 | 0.417063 | 7/15/2014 | 57:09.2 | 1 |
| -355.1 | 19521.918 | 1.709 | 15.51 | 0.386686 | 7/15/2014 | 57:09.3 | 1 |
| -355.504 | 19521.972 | 1.71 | 15.51 | 0.407572 | 7/15/2014 | 57:09.4 | 1 |
| -355.735 | 19522.002 | 1.715 | 15.507 | 0.400065 | 7/15/2014 | 57:09.5 | 1 |
| -355.965 | 19522.034 | 1.711 | 15.501 | 0.396334 | 7/15/2014 | 57:09.6 | 1 |

| | | | | | | | |
|---|---|---|---|---|---|---|---|
| -356.251 | 19522.074 | 1.709 | 15.491 | 0.39149 | 7/15/2014 | 57:09.7 | 1 |
| -356.368 | 19522.091 | 1.71 | 15.487 | 0.388797 | 7/15/2014 | 57:09.8 | 1 |
| -356.6 | 19522.123 | 1.709 | 15.473 | 0.414807 | 7/15/2014 | 57:09.9 | 1 |
| -356.942 | 19522.172 | 1.706 | 15.445 | 0.410354 | 7/15/2014 | 57:10.0 | 1 |
| -357.172 | 19522.204 | 1.706 | 15.425 | 0.404808 | 7/15/2014 | 57:10.1 | 1 |
| -357.4 | 19522.236 | 1.704 | 15.404 | 0.420666 | 7/15/2014 | 57:10.2 | 1 |
| -357.627 | 19522.271 | 1.698 | 15.382 | 0.424077 | 7/15/2014 | 57:10.3 | 1 |
| -357.909 | 19522.311 | 1.698 | 15.361 | 0.425534 | 7/15/2014 | 57:10.4 | 1 |
| -358.134 | 19522.344 | 1.695 | 15.342 | 0.424275 | 7/15/2014 | 57:10.5 | 1 |
| -358.359 | 19522.378 | 1.693 | 15.324 | 0.423703 | 7/15/2014 | 57:10.6 | 1 |
| -358.639 | 19522.42 | 1.687 | 15.309 | 0.411763 | 7/15/2014 | 57:10.7 | 1 |
| -358.867 | 19522.454 | 1.687 | 15.296 | 0.408821 | 7/15/2014 | 57:10.8 | 1 |
| -359.093 | 19522.488 | 1.68 | 15.287 | 0.398056 | 7/15/2014 | 57:10.9 | 1 |
| -359.321 | 19522.524 | 1.672 | 15.281 | 0.373008 | 7/15/2014 | 57:11.0 | 1 |
| -359.549 | 19522.559 | 1.669 | 15.278 | 0.391617 | 7/15/2014 | 57:11.1 | 1 |
| -359.775 | 19522.595 | 1.662 | 15.279 | 0.375969 | 7/15/2014 | 57:11.2 | 1 |
| -360.002 | 19522.63 | 1.659 | 15.283 | 0.365399 | 7/15/2014 | 57:11.3 | 1 |
| -360.284 | 19522.674 | 1.659 | 15.291 | 0.362128 | 7/15/2014 | 57:11.4 | 1 |
| -360.51 | 19522.711 | 1.657 | 15.302 | 0.36829 | 7/15/2014 | 57:11.5 | 1 |
| -360.733 | 19522.746 | 1.651 | 15.317 | 0.352743 | 7/15/2014 | 57:11.6 | 1 |
| -361.014 | 19522.792 | 1.651 | 15.334 | 0.363617 | 7/15/2014 | 57:11.7 | 1 |
| -361.24 | 19522.83 | 1.648 | 15.354 | 0.35451 | 7/15/2014 | 57:11.8 | 1 |
| -361.466 | 19522.866 | 1.643 | 15.377 | 0.349717 | 7/15/2014 | 57:11.9 | 1 |
| -361.693 | 19522.903 | 1.638 | 15.402 | 0.347731 | 7/15/2014 | 57:12.0 | 1 |
| -361.92 | 19522.941 | 1.633 | 15.428 | 0.348291 | 7/15/2014 | 57:12.1 | 1 |
| -362.144 | 19522.98 | 1.629 | 15.454 | 0.34966 | 7/15/2014 | 57:12.2 | 1 |
| -362.367 | 19523.016 | 1.629 | 15.479 | 0.344976 | 7/15/2014 | 57:12.3 | 1 |
| -362.645 | 19523.063 | 1.627 | 15.502 | 0.35605 | 7/15/2014 | 57:12.4 | 1 |
| -362.868 | 19523.103 | 1.625 | 15.521 | 0.344425 | 7/15/2014 | 57:12.5 | 1 |
| -363.09 | 19523.139 | 1.625 | 15.537 | 0.35017 | 7/15/2014 | 57:12.6 | 1 |
| -363.369 | 19523.188 | 1.625 | 15.547 | 0.353997 | 7/15/2014 | 57:12.7 | 1 |
| -363.593 | 19523.228 | 1.63 | 15.55 | 0.35232 | 7/15/2014 | 57:12.8 | 1 |
| -363.817 | 19523.267 | 1.628 | 15.547 | 0.357761 | 7/15/2014 | 57:12.9 | 1 |
| -364.043 | 19523.305 | 1.626 | 15.538 | 0.364844 | 7/15/2014 | 57:13.0 | 1 |
| -364.267 | 19523.346 | 1.62 | 15.523 | 0.375838 | 7/15/2014 | 57:13.1 | 1 |
| -364.493 | 19523.386 | 1.62 | 15.502 | 0.380066 | 7/15/2014 | 57:13.2 | 1 |
| -364.715 | 19523.425 | 1.615 | 15.479 | 0.390674 | 7/15/2014 | 57:13.3 | 1 |
| -364.994 | 19523.475 | 1.617 | 15.454 | 0.401668 | 7/15/2014 | 57:13.4 | 1 |
| -365.217 | 19523.515 | 1.618 | 15.429 | 0.410365 | 7/15/2014 | 57:13.5 | 1 |
| -365.439 | 19523.556 | 1.618 | 15.407 | 0.422495 | 7/15/2014 | 57:13.6 | 1 |
| -365.716 | 19523.605 | 1.618 | 15.39 | 0.434546 | 7/15/2014 | 57:13.7 | 1 |
| -365.939 | 19523.647 | 1.618 | 15.379 | 0.438578 | 7/15/2014 | 57:13.8 | 1 |
| -366.162 | 19523.688 | 1.618 | 15.373 | 0.447386 | 7/15/2014 | 57:13.9 | 1 |
| -366.385 | 19523.729 | 1.616 | 15.375 | 0.452951 | 7/15/2014 | 57:14.0 | 1 |
| -366.61 | 19523.771 | 1.621 | 15.381 | 0.452207 | 7/15/2014 | 57:14.1 | 1 |
| -366.831 | 19523.812 | 1.619 | 15.391 | 0.453964 | 7/15/2014 | 57:14.2 | 1 |
| -367.053 | 19523.854 | 1.619 | 15.404 | 0.457054 | 7/15/2014 | 57:14.3 | 1 |

| | | | | | | | |
|---|---|---|---|---|---|---|---|
| -367.327 | 19523.904 | 1.617 | 15.417 | 0.444666 | 7/15/2014 | 57:14.4 | 1 |
| -367.548 | 19523.946 | 1.62 | 15.43 | 0.446086 | 7/15/2014 | 57:14.5 | 1 |
| -367.768 | 19523.986 | 1.62 | 15.44 | 0.445156 | 7/15/2014 | 57:14.6 | 1 |
| -368.043 | 19524.04 | 1.623 | 15.445 | 0.447568 | 7/15/2014 | 57:14.7 | 1 |
| -368.266 | 19524.082 | 1.625 | 15.446 | 0.439219 | 7/15/2014 | 57:14.8 | 1 |
| -368.489 | 19524.125 | 1.625 | 15.442 | 0.436877 | 7/15/2014 | 57:14.9 | 1 |
| -368.712 | 19524.167 | 1.63 | 15.433 | 0.427347 | 7/15/2014 | 57:15.0 | 1 |
| -368.936 | 19524.211 | 1.633 | 15.419 | 0.43611 | 7/15/2014 | 57:15.1 | 1 |
| -369.159 | 19524.253 | 1.635 | 15.401 | 0.435353 | 7/15/2014 | 57:15.2 | 1 |
| -369.382 | 19524.295 | 1.639 | 15.38 | 0.44435 | 7/15/2014 | 57:15.3 | 1 |
| -369.659 | 19524.35 | 1.644 | 15.356 | 0.446619 | 7/15/2014 | 57:15.4 | 1 |
| -369.881 | 19524.394 | 1.646 | 15.332 | 0.46387 | 7/15/2014 | 57:15.5 | 1 |
| -370.104 | 19524.437 | 1.649 | 15.308 | 0.474404 | 7/15/2014 | 57:15.6 | 1 |
| -370.382 | 19524.491 | 1.646 | 15.284 | 0.491005 | 7/15/2014 | 57:15.7 | 1 |
| -370.606 | 19524.535 | 1.641 | 15.263 | 0.499958 | 7/15/2014 | 57:15.8 | 1 |
| -370.831 | 19524.578 | 1.646 | 15.244 | 0.510324 | 7/15/2014 | 57:15.9 | 1 |
| -371.055 | 19524.621 | 1.65 | 15.229 | 0.52906 | 7/15/2014 | 57:16.0 | 1 |
| -371.279 | 19524.667 | 1.651 | 15.217 | 0.534382 | 7/15/2014 | 57:16.1 | 1 |
| -371.503 | 19524.711 | 1.647 | 15.21 | 0.533733 | 7/15/2014 | 57:16.2 | 1 |
| -371.726 | 19524.756 | 1.65 | 15.207 | 0.537994 | 7/15/2014 | 57:16.3 | 1 |
| -372.002 | 19524.811 | 1.65 | 15.208 | 0.538641 | 7/15/2014 | 57:16.4 | 1 |
| -372.224 | 19524.854 | 1.657 | 15.212 | 0.534073 | 7/15/2014 | 57:16.5 | 1 |
| -372.446 | 19524.896 | 1.662 | 15.22 | 0.527756 | 7/15/2014 | 57:16.6 | 1 |
| -372.722 | 19524.951 | 1.657 | 15.231 | 0.518909 | 7/15/2014 | 57:16.7 | 1 |
| -372.945 | 19524.996 | 1.657 | 15.244 | 0.502485 | 7/15/2014 | 57:16.8 | 1 |
| -373.169 | 19525.041 | 1.659 | 15.257 | 0.489032 | 7/15/2014 | 57:16.9 | 1 |
| -373.393 | 19525.086 | 1.664 | 15.271 | 0.476537 | 7/15/2014 | 57:17.0 | 1 |
| -373.617 | 19525.131 | 1.664 | 15.283 | 0.45639 | 7/15/2014 | 57:17.1 | 1 |
| -373.84 | 19525.174 | 1.668 | 15.294 | 0.446251 | 7/15/2014 | 57:17.2 | 1 |
| -374.063 | 19525.217 | 1.668 | 15.301 | 0.431506 | 7/15/2014 | 57:17.3 | 1 |
| -374.34 | 19525.273 | 1.668 | 15.305 | 0.422972 | 7/15/2014 | 57:17.4 | 1 |
| -374.563 | 19525.316 | 1.673 | 15.306 | 0.41708 | 7/15/2014 | 57:17.5 | 1 |
| -374.787 | 19525.363 | 1.673 | 15.302 | 0.418376 | 7/15/2014 | 57:17.6 | 1 |
| -375.068 | 19525.418 | 1.673 | 15.295 | 0.423828 | 7/15/2014 | 57:17.7 | 1 |
| -375.295 | 19525.465 | 1.676 | 15.284 | 0.430854 | 7/15/2014 | 57:17.8 | 1 |
| -375.522 | 19525.508 | 1.679 | 15.27 | 0.440632 | 7/15/2014 | 57:17.9 | 1 |
| -375.752 | 19525.553 | 1.685 | 15.254 | 0.454486 | 7/15/2014 | 57:18.0 | 1 |
| -375.865 | 19525.576 | 1.686 | 15.247 | 0.462207 | 7/15/2014 | 57:18.1 | 1 |
| -376.093 | 19525.621 | 1.686 | 15.228 | 0.465909 | 7/15/2014 | 57:18.2 | 1 |
| -376.322 | 19525.668 | 1.686 | 15.208 | 0.480895 | 7/15/2014 | 57:18.3 | 1 |
| -376.725 | 19525.746 | 1.688 | 15.177 | 0.483746 | 7/15/2014 | 57:18.4 | 1 |
| -376.955 | 19525.793 | 1.688 | 15.156 | 0.483907 | 7/15/2014 | 57:18.5 | 1 |
| -377.186 | 19525.838 | 1.688 | 15.136 | 0.492207 | 7/15/2014 | 57:18.6 | 1 |
| -377.474 | 19525.895 | 1.69 | 15.115 | 0.489782 | 7/15/2014 | 57:18.7 | 1 |
| -377.59 | 19525.918 | 1.686 | 15.105 | 0.490831 | 7/15/2014 | 57:18.8 | 1 |
| -377.824 | 19525.964 | 1.692 | 15.085 | 0.488267 | 7/15/2014 | 57:18.9 | 1 |
| -378.056 | 19526.01 | 1.692 | 15.066 | 0.490137 | 7/15/2014 | 57:19.0 | 1 |

| | | | | | | | |
|---|---|---|---|---|---|---|---|
| -378.291 | 19526.055 | 1.698 | 15.047 | 0.492988 | 7/15/2014 | 57:19.1 | 1 |
| -378.521 | 19526.101 | 1.695 | 15.029 | 0.495993 | 7/15/2014 | 57:19.2 | 1 |
| -378.755 | 19526.146 | 1.698 | 15.013 | 0.495836 | 7/15/2014 | 57:19.3 | 1 |
| -379.157 | 19526.225 | 1.692 | 14.994 | 0.491833 | 7/15/2014 | 57:19.4 | 1 |
| -379.387 | 19526.27 | 1.692 | 14.985 | 0.493291 | 7/15/2014 | 57:19.5 | 1 |
| -379.616 | 19526.314 | 1.695 | 14.978 | 0.499434 | 7/15/2014 | 57:19.6 | 1 |
| -379.9 | 19526.37 | 1.697 | 14.975 | 0.498089 | 7/15/2014 | 57:19.7 | 1 |
| -380.015 | 19526.393 | 1.695 | 14.972 | 0.492013 | 7/15/2014 | 57:19.8 | 1 |
| -380.246 | 19526.437 | 1.704 | 14.974 | 0.497803 | 7/15/2014 | 57:19.9 | 1 |
| -380.585 | 19526.503 | 1.713 | 14.983 | 0.501495 | 7/15/2014 | 57:20.0 | 1 |
| -380.811 | 19526.546 | 1.721 | 14.989 | 0.499211 | 7/15/2014 | 57:20.1 | 1 |
| -381.038 | 19526.59 | 1.721 | 14.996 | 0.524222 | 7/15/2014 | 57:20.2 | 1 |
| -381.265 | 19526.634 | 1.724 | 15.003 | 0.517986 | 7/15/2014 | 57:20.3 | 1 |
| -381.543 | 19526.688 | 1.726 | 15.008 | 0.529832 | 7/15/2014 | 57:20.4 | 1 |
| -381.767 | 19526.73 | 1.729 | 15.012 | 0.531203 | 7/15/2014 | 57:20.5 | 1 |
| -381.991 | 19526.773 | 1.731 | 15.014 | 0.543739 | 7/15/2014 | 57:20.6 | 1 |
| -382.273 | 19526.827 | 1.737 | 15.016 | 0.540898 | 7/15/2014 | 57:20.7 | 1 |
| -382.5 | 19526.87 | 1.737 | 15.017 | 0.542345 | 7/15/2014 | 57:20.8 | 1 |
| -382.727 | 19526.913 | 1.74 | 15.017 | 0.543419 | 7/15/2014 | 57:20.9 | 1 |
| -382.957 | 19526.956 | 1.748 | 15.018 | 0.540927 | 7/15/2014 | 57:21.0 | 1 |
| -383.07 | 19526.978 | 1.753 | 15.018 | 0.540579 | 7/15/2014 | 57:21.1 | 1 |
| -383.298 | 19527.022 | 1.758 | 15.021 | 0.541675 | 7/15/2014 | 57:21.2 | 1 |
| -383.529 | 19527.065 | 1.768 | 15.026 | 0.535858 | 7/15/2014 | 57:21.3 | 1 |
| -383.933 | 19527.141 | 1.782 | 15.038 | 0.534139 | 7/15/2014 | 57:21.4 | 1 |
| -384.165 | 19527.184 | 1.789 | 15.047 | 0.528555 | 7/15/2014 | 57:21.5 | 1 |
| -384.395 | 19527.227 | 1.796 | 15.058 | 0.521585 | 7/15/2014 | 57:21.6 | 1 |
| -384.687 | 19527.279 | 1.8 | 15.07 | 0.512949 | 7/15/2014 | 57:21.7 | 1 |
| -384.804 | 19527.303 | 1.806 | 15.076 | 0.508365 | 7/15/2014 | 57:21.8 | 1 |
| -385.04 | 19527.346 | 1.81 | 15.09 | 0.511097 | 7/15/2014 | 57:21.9 | 1 |
| -385.274 | 19527.389 | 1.81 | 15.102 | 0.50491 | 7/15/2014 | 57:22.0 | 1 |
| -385.511 | 19527.432 | 1.814 | 15.114 | 0.506002 | 7/15/2014 | 57:22.1 | 1 |
| -385.746 | 19527.475 | 1.81 | 15.124 | 0.506356 | 7/15/2014 | 57:22.2 | 1 |
| -385.982 | 19527.518 | 1.816 | 15.132 | 0.513136 | 7/15/2014 | 57:22.3 | 1 |
| -386.394 | 19527.592 | 1.828 | 15.134 | 0.5165 | 7/15/2014 | 57:22.4 | 1 |
| -386.63 | 19527.634 | 1.84 | 15.132 | 0.513173 | 7/15/2014 | 57:22.5 | 1 |
| -386.864 | 19527.676 | 1.85 | 15.125 | 0.511098 | 7/15/2014 | 57:22.6 | 1 |
| -387.158 | 19527.729 | 1.862 | 15.114 | 0.510324 | 7/15/2014 | 57:22.7 | 1 |
| -387.276 | 19527.75 | 1.865 | 15.11 | 0.507232 | 7/15/2014 | 57:22.8 | 1 |
| -387.515 | 19527.792 | 1.881 | 15.092 | 0.499924 | 7/15/2014 | 57:22.9 | 1 |
| -387.753 | 19527.834 | 1.883 | 15.071 | 0.494424 | 7/15/2014 | 57:23.0 | 1 |
| -387.991 | 19527.877 | 1.894 | 15.047 | 0.486026 | 7/15/2014 | 57:23.1 | 1 |
| -388.229 | 19527.917 | 1.896 | 15.021 | 0.477385 | 7/15/2014 | 57:23.2 | 1 |
| -388.468 | 19527.959 | 1.899 | 14.993 | 0.466926 | 7/15/2014 | 57:23.3 | 1 |
| -388.884 | 19528.031 | 1.901 | 14.954 | 0.455595 | 7/15/2014 | 57:23.4 | 1 |
| -389.121 | 19528.073 | 1.901 | 14.929 | 0.449326 | 7/15/2014 | 57:23.5 | 1 |
| -389.36 | 19528.113 | 1.901 | 14.907 | 0.440479 | 7/15/2014 | 57:23.6 | 1 |
| -389.658 | 19528.164 | 1.899 | 14.888 | 0.437959 | 7/15/2014 | 57:23.7 | 1 |

| | | | | | | | |
|---|---|---|---|---|---|---|---|
| -389.777 | 19528.184 | 1.896 | 14.877 | 0.434962 | 7/15/2014 | 57:23.8 | 1 |
| -390.019 | 19528.227 | 1.893 | 14.863 | 0.429678 | 7/15/2014 | 57:23.9 | 1 |
| -390.259 | 19528.268 | 1.887 | 14.854 | 0.426529 | 7/15/2014 | 57:24.0 | 1 |
| -390.502 | 19528.307 | 1.883 | 14.849 | 0.423388 | 7/15/2014 | 57:24.1 | 1 |
| -390.743 | 19528.35 | 1.883 | 14.849 | 0.419437 | 7/15/2014 | 57:24.2 | 1 |
| -390.985 | 19528.389 | 1.888 | 14.851 | 0.414834 | 7/15/2014 | 57:24.3 | 1 |
| -391.404 | 19528.457 | 1.89 | 14.863 | 0.416637 | 7/15/2014 | 57:24.4 | 1 |
| -391.645 | 19528.498 | 1.897 | 14.873 | 0.407527 | 7/15/2014 | 57:24.5 | 1 |
| -391.883 | 19528.536 | 1.905 | 14.884 | 0.413796 | 7/15/2014 | 57:24.6 | 1 |
| -392.18 | 19528.584 | 1.91 | 14.897 | 0.407909 | 7/15/2014 | 57:24.7 | 1 |
| -392.302 | 19528.605 | 1.915 | 14.903 | 0.409617 | 7/15/2014 | 57:24.8 | 1 |
| -392.543 | 19528.645 | 1.915 | 14.917 | 0.408891 | 7/15/2014 | 57:24.9 | 1 |
| -392.78 | 19528.682 | 1.909 | 14.931 | 0.41409 | 7/15/2014 | 57:25.0 | 1 |
| -393.019 | 19528.72 | 1.913 | 14.944 | 0.409495 | 7/15/2014 | 57:25.1 | 1 |
| -393.258 | 19528.758 | 1.918 | 14.955 | 0.406408 | 7/15/2014 | 57:25.2 | 1 |
| -393.494 | 19528.795 | 1.921 | 14.965 | 0.40109 | 7/15/2014 | 57:25.3 | 1 |
| -393.903 | 19528.859 | 1.915 | 14.974 | 0.396844 | 7/15/2014 | 57:25.4 | 1 |
| -394.136 | 19528.896 | 1.912 | 14.977 | 0.393062 | 7/15/2014 | 57:25.5 | 1 |
| -394.368 | 19528.932 | 1.909 | 14.978 | 0.39349 | 7/15/2014 | 57:25.6 | 1 |
| -394.658 | 19528.976 | 1.909 | 14.974 | 0.387989 | 7/15/2014 | 57:25.7 | 1 |
| -394.776 | 19528.994 | 1.905 | 14.974 | 0.391512 | 7/15/2014 | 57:25.8 | 1 |
| -395.01 | 19529.03 | 1.902 | 14.965 | 0.389056 | 7/15/2014 | 57:25.9 | 1 |
| -395.242 | 19529.066 | 1.896 | 14.954 | 0.38959 | 7/15/2014 | 57:26.0 | 1 |
| -395.475 | 19529.101 | 1.9 | 14.94 | 0.38157 | 7/15/2014 | 57:26.1 | 1 |
| -395.706 | 19529.135 | 1.896 | 14.924 | 0.382086 | 7/15/2014 | 57:26.2 | 1 |
| -395.938 | 19529.17 | 1.902 | 14.907 | 0.381926 | 7/15/2014 | 57:26.3 | 1 |
| -396.342 | 19529.229 | 1.91 | 14.881 | 0.385837 | 7/15/2014 | 57:26.4 | 1 |
| -396.574 | 19529.263 | 1.912 | 14.866 | 0.38424 | 7/15/2014 | 57:26.5 | 1 |
| -396.805 | 19529.296 | 1.914 | 14.853 | 0.384269 | 7/15/2014 | 57:26.6 | 1 |
| -397.097 | 19529.337 | 1.917 | 14.842 | 0.381608 | 7/15/2014 | 57:26.7 | 1 |
| -397.213 | 19529.354 | 1.917 | 14.836 | 0.381814 | 7/15/2014 | 57:26.8 | 1 |
| -397.449 | 19529.389 | 1.924 | 14.83 | 0.379797 | 7/15/2014 | 57:26.9 | 1 |
| -397.685 | 19529.421 | 1.924 | 14.829 | 0.378328 | 7/15/2014 | 57:27.0 | 1 |
| -397.922 | 19529.453 | 1.927 | 14.832 | 0.371733 | 7/15/2014 | 57:27.1 | 1 |
| -398.161 | 19529.487 | 1.924 | 14.839 | 0.367428 | 7/15/2014 | 57:27.2 | 1 |
| -398.401 | 19529.519 | 1.932 | 14.848 | 0.369116 | 7/15/2014 | 57:27.3 | 1 |
| -398.821 | 19529.576 | 1.928 | 14.868 | 0.372487 | 7/15/2014 | 57:27.4 | 1 |
| -399.062 | 19529.609 | 1.928 | 14.882 | 0.380628 | 7/15/2014 | 57:27.5 | 1 |
| -399.301 | 19529.641 | 1.928 | 14.895 | 0.383891 | 7/15/2014 | 57:27.6 | 1 |
| -399.603 | 19529.68 | 1.93 | 14.907 | 0.389457 | 7/15/2014 | 57:27.7 | 1 |
| -399.725 | 19529.697 | 1.929 | 14.914 | 0.392426 | 7/15/2014 | 57:27.8 | 1 |
| -399.972 | 19529.729 | 1.936 | 14.924 | 0.397264 | 7/15/2014 | 57:27.9 | 1 |
| -400.215 | 19529.761 | 1.932 | 14.932 | 0.40229 | 7/15/2014 | 57:28.0 | 1 |
| -400.462 | 19529.793 | 1.938 | 14.936 | 0.404712 | 7/15/2014 | 57:28.1 | 1 |
| -400.71 | 19529.823 | 1.938 | 14.937 | 0.412046 | 7/15/2014 | 57:28.2 | 1 |
| -400.955 | 19529.854 | 1.944 | 14.936 | 0.412054 | 7/15/2014 | 57:28.3 | 1 |
| -401.384 | 19529.908 | 1.948 | 14.928 | 0.415856 | 7/15/2014 | 57:28.4 | 1 |

| | | | | | | | |
|---|---|---|---|---|---|---|---|
| -401.629 | 19529.938 | 1.952 | 14.921 | 0.415402 | 7/15/2014 | 57:28.5 | 1 |
| -401.751 | 19529.954 | 1.95 | 14.917 | 0.415454 | 7/15/2014 | 57:28.6 | 1 |
| -402.179 | 19530.006 | 1.955 | 14.901 | 0.406557 | 7/15/2014 | 57:28.7 | 1 |
| -402.305 | 19530.021 | 1.958 | 14.896 | 0.398278 | 7/15/2014 | 57:28.8 | 1 |
| -402.551 | 19530.05 | 1.96 | 14.884 | 0.392687 | 7/15/2014 | 57:28.9 | 1 |
| -402.798 | 19530.079 | 1.96 | 14.871 | 0.385373 | 7/15/2014 | 57:29.0 | 1 |
| -403.045 | 19530.109 | 1.96 | 14.856 | 0.367261 | 7/15/2014 | 57:29.1 | 1 |
| -403.291 | 19530.138 | 1.958 | 14.841 | 0.354554 | 7/15/2014 | 57:29.2 | 1 |
| -403.536 | 19530.166 | 1.958 | 14.824 | 0.344525 | 7/15/2014 | 57:29.3 | 1 |
| -403.964 | 19530.215 | 1.956 | 14.796 | 0.341406 | 7/15/2014 | 57:29.4 | 1 |
| -404.208 | 19530.242 | 1.958 | 14.776 | 0.342484 | 7/15/2014 | 57:29.5 | 1 |
| -404.452 | 19530.27 | 1.961 | 14.755 | 0.33743 | 7/15/2014 | 57:29.6 | 1 |
| -404.756 | 19530.304 | 1.961 | 14.734 | 0.338969 | 7/15/2014 | 57:29.7 | 1 |
| -404.879 | 19530.317 | 1.961 | 14.723 | 0.341242 | 7/15/2014 | 57:29.8 | 1 |
| -405.124 | 19530.344 | 1.963 | 14.701 | 0.337168 | 7/15/2014 | 57:29.9 | 1 |
| -405.368 | 19530.37 | 1.959 | 14.68 | 0.336718 | 7/15/2014 | 57:30.0 | 1 |
| -405.614 | 19530.396 | 1.957 | 14.66 | 0.335307 | 7/15/2014 | 57:30.1 | 1 |
| -405.857 | 19530.424 | 1.956 | 14.643 | 0.334077 | 7/15/2014 | 57:30.2 | 1 |
| -406.102 | 19530.448 | 1.953 | 14.629 | 0.328824 | 7/15/2014 | 57:30.3 | 1 |
| -406.527 | 19530.493 | 1.952 | 14.617 | 0.338128 | 7/15/2014 | 57:30.4 | 1 |
| -406.769 | 19530.518 | 1.95 | 14.612 | 0.337065 | 7/15/2014 | 57:30.5 | 1 |
| -407.009 | 19530.542 | 1.95 | 14.61 | 0.340932 | 7/15/2014 | 57:30.6 | 1 |
| -407.31 | 19530.572 | 1.953 | 14.611 | 0.342797 | 7/15/2014 | 57:30.7 | 1 |
| -407.432 | 19530.586 | 1.954 | 14.611 | 0.34533 | 7/15/2014 | 57:30.8 | 1 |
| -407.675 | 19530.609 | 1.961 | 14.616 | 0.351909 | 7/15/2014 | 57:30.9 | 1 |
| -407.916 | 19530.634 | 1.961 | 14.622 | 0.360005 | 7/15/2014 | 57:31.0 | 1 |
| -408.157 | 19530.658 | 1.967 | 14.628 | 0.363686 | 7/15/2014 | 57:31.1 | 1 |
| -408.398 | 19530.682 | 1.961 | 14.635 | 0.351576 | 7/15/2014 | 57:31.2 | 1 |
| -408.637 | 19530.704 | 1.964 | 14.641 | 0.360844 | 7/15/2014 | 57:31.3 | 1 |
| -409.053 | 19530.744 | 1.959 | 14.646 | 0.346286 | 7/15/2014 | 57:31.4 | 1 |
| -409.289 | 19530.766 | 1.954 | 14.649 | 0.34155 | 7/15/2014 | 57:31.5 | 1 |
| -409.526 | 19530.789 | 1.949 | 14.65 | 0.326324 | 7/15/2014 | 57:31.6 | 1 |
| -409.82 | 19530.816 | 1.945 | 14.649 | 0.317775 | 7/15/2014 | 57:31.7 | 1 |
| -409.94 | 19530.828 | 1.94 | 14.649 | 0.312036 | 7/15/2014 | 57:31.8 | 1 |
| -410.179 | 19530.85 | 1.936 | 14.645 | 0.315951 | 7/15/2014 | 57:31.9 | 1 |
| -410.416 | 19530.871 | 1.93 | 14.638 | 0.301007 | 7/15/2014 | 57:32.0 | 1 |
| -410.656 | 19530.894 | 1.932 | 14.628 | 0.2983 | 7/15/2014 | 57:32.1 | 1 |
| -410.894 | 19530.914 | 1.929 | 14.615 | 0.290154 | 7/15/2014 | 57:32.2 | 1 |
| -411.134 | 19530.936 | 1.929 | 14.599 | 0.292896 | 7/15/2014 | 57:32.3 | 1 |
| -411.55 | 19530.973 | 1.921 | 14.566 | 0.295374 | 7/15/2014 | 57:32.4 | 1 |
| -411.789 | 19530.993 | 1.918 | 14.543 | 0.293864 | 7/15/2014 | 57:32.5 | 1 |
| -412.028 | 19531.014 | 1.921 | 14.519 | 0.291088 | 7/15/2014 | 57:32.6 | 1 |
| -412.329 | 19531.04 | 1.921 | 14.494 | 0.292795 | 7/15/2014 | 57:32.7 | 1 |
| -412.449 | 19531.051 | 1.915 | 14.481 | 0.293493 | 7/15/2014 | 57:32.8 | 1 |
| -412.693 | 19531.07 | 1.911 | 14.457 | 0.296813 | 7/15/2014 | 57:32.9 | 1 |
| -412.936 | 19531.09 | 1.908 | 14.438 | 0.299757 | 7/15/2014 | 57:33.0 | 1 |
| -413.182 | 19531.111 | 1.911 | 14.422 | 0.302037 | 7/15/2014 | 57:33.1 | 1 |

| | | | | | | | |
|---|---|---|---|---|---|---|---|
| -413.425 | 19531.131 | 1.905 | 14.413 | 0.300026 | 7/15/2014 | 57:33.2 | 1 |
| -413.671 | 19531.15 | 1.905 | 14.409 | 0.303352 | 7/15/2014 | 57:33.3 | 1 |
| -414.098 | 19531.185 | 1.895 | 14.418 | 0.286054 | 7/15/2014 | 57:33.4 | 1 |
| -414.343 | 19531.203 | 1.892 | 14.428 | 0.280708 | 7/15/2014 | 57:33.5 | 1 |
| -414.587 | 19531.223 | 1.892 | 14.443 | 0.272877 | 7/15/2014 | 57:33.6 | 1 |
| -414.894 | 19531.246 | 1.892 | 14.461 | 0.259016 | 7/15/2014 | 57:33.7 | 1 |
| -415.017 | 19531.256 | 1.888 | 14.471 | 0.255693 | 7/15/2014 | 57:33.8 | 1 |
| -415.265 | 19531.273 | 1.888 | 14.493 | 0.247517 | 7/15/2014 | 57:33.9 | 1 |
| -415.512 | 19531.292 | 1.886 | 14.516 | 0.246974 | 7/15/2014 | 57:34.0 | 1 |
| -415.759 | 19531.31 | 1.883 | 14.539 | 0.240657 | 7/15/2014 | 57:34.1 | 1 |
| -416.007 | 19531.327 | 1.886 | 14.559 | 0.23753 | 7/15/2014 | 57:34.2 | 1 |
| -416.253 | 19531.346 | 1.883 | 14.576 | 0.228052 | 7/15/2014 | 57:34.3 | 1 |
| -416.685 | 19531.376 | 1.877 | 14.592 | 0.2231 | 7/15/2014 | 57:34.4 | 1 |
| -416.931 | 19531.393 | 1.872 | 14.599 | 0.226117 | 7/15/2014 | 57:34.5 | 1 |
| -417.178 | 19531.409 | 1.866 | 14.601 | 0.219722 | 7/15/2014 | 57:34.6 | 1 |
| -417.486 | 19531.43 | 1.859 | 14.599 | 0.214594 | 7/15/2014 | 57:34.7 | 1 |
| -417.611 | 19531.438 | 1.857 | 14.599 | 0.21541 | 7/15/2014 | 57:34.8 | 1 |
| -417.861 | 19531.455 | 1.848 | 14.592 | 0.213312 | 7/15/2014 | 57:34.9 | 1 |
| -418.11 | 19531.472 | 1.84 | 14.582 | 0.213342 | 7/15/2014 | 57:35.0 | 1 |
| -418.361 | 19531.487 | 1.84 | 14.571 | 0.206646 | 7/15/2014 | 57:35.1 | 1 |
| -418.611 | 19531.505 | 1.831 | 14.56 | 0.203415 | 7/15/2014 | 57:35.2 | 1 |
| -418.862 | 19531.52 | 1.827 | 14.55 | 0.195029 | 7/15/2014 | 57:35.3 | 1 |
| -419.173 | 19531.539 | 1.824 | 14.543 | 0.194064 | 7/15/2014 | 57:35.4 | 1 |
| -419.425 | 19531.555 | 1.827 | 14.538 | 0.195092 | 7/15/2014 | 57:35.5 | 1 |
| -419.675 | 19531.571 | 1.822 | 14.538 | 0.182472 | 7/15/2014 | 57:35.6 | 1 |
| -419.928 | 19531.585 | 1.822 | 14.542 | 0.189357 | 7/15/2014 | 57:35.7 | 1 |
| -420.24 | 19531.604 | 1.812 | 14.55 | 0.184712 | 7/15/2014 | 57:35.8 | 1 |
| -420.493 | 19531.618 | 1.81 | 14.561 | 0.18486 | 7/15/2014 | 57:35.9 | 1 |
| -420.747 | 19531.633 | 1.807 | 14.577 | 0.192635 | 7/15/2014 | 57:36.0 | 1 |
| -421 | 19531.646 | 1.807 | 14.595 | 0.190034 | 7/15/2014 | 57:36.1 | 1 |
| -421.252 | 19531.662 | 1.805 | 14.615 | 0.195012 | 7/15/2014 | 57:36.2 | 1 |
| -421.504 | 19531.676 | 1.807 | 14.638 | 0.191997 | 7/15/2014 | 57:36.3 | 1 |
| -421.815 | 19531.693 | 1.802 | 14.661 | 0.197433 | 7/15/2014 | 57:36.4 | 1 |
| -422.067 | 19531.706 | 1.805 | 14.686 | 0.198533 | 7/15/2014 | 57:36.5 | 1 |
| -422.315 | 19531.72 | 1.802 | 14.71 | 0.197376 | 7/15/2014 | 57:36.6 | 1 |
| -422.742 | 19531.74 | 1.802 | 14.747 | 0.185996 | 7/15/2014 | 57:36.7 | 1 |
| -422.869 | 19531.748 | 1.8 | 14.76 | 0.18695 | 7/15/2014 | 57:36.8 | 1 |
| -423.115 | 19531.761 | 1.798 | 14.784 | 0.196378 | 7/15/2014 | 57:36.9 | 1 |
| -423.358 | 19531.775 | 1.793 | 14.807 | 0.19557 | 7/15/2014 | 57:37.0 | 1 |
| -423.602 | 19531.787 | 1.793 | 14.829 | 0.204655 | 7/15/2014 | 57:37.1 | 1 |
| -423.843 | 19531.8 | 1.787 | 14.85 | 0.20586 | 7/15/2014 | 57:37.2 | 1 |
| -424.083 | 19531.813 | 1.789 | 14.869 | 0.202957 | 7/15/2014 | 57:37.3 | 1 |
| -424.496 | 19531.832 | 1.781 | 14.892 | 0.210256 | 7/15/2014 | 57:37.4 | 1 |
| -424.732 | 19531.844 | 1.778 | 14.905 | 0.203152 | 7/15/2014 | 57:37.5 | 1 |
| -424.965 | 19531.857 | 1.776 | 14.916 | 0.197836 | 7/15/2014 | 57:37.6 | 1 |
| -425.257 | 19531.871 | 1.772 | 14.923 | 0.193365 | 7/15/2014 | 57:37.7 | 1 |
| -425.375 | 19531.877 | 1.771 | 14.928 | 0.191973 | 7/15/2014 | 57:37.8 | 1 |

| | | | | | | | |
|---|---|---|---|---|---|---|---|
| -425.611 | 19531.889 | 1.777 | 14.929 | 0.184243 | 7/15/2014 | 57:37.9 | 1 |
| -425.844 | 19531.9 | 1.774 | 14.928 | 0.175866 | 7/15/2014 | 57:38.0 | 1 |
| -426.08 | 19531.912 | 1.777 | 14.923 | 0.169429 | 7/15/2014 | 57:38.1 | 1 |
| -426.313 | 19531.924 | 1.777 | 14.914 | 0.157821 | 7/15/2014 | 57:38.2 | 1 |
| -426.547 | 19531.936 | 1.78 | 14.904 | 0.140897 | 7/15/2014 | 57:38.3 | 1 |
| -426.956 | 19531.955 | 1.774 | 14.886 | 0.12751 | 7/15/2014 | 57:38.4 | 1 |
| -427.191 | 19531.967 | 1.774 | 14.875 | 0.120053 | 7/15/2014 | 57:38.5 | 1 |
| -427.426 | 19531.979 | 1.771 | 14.865 | 0.113904 | 7/15/2014 | 57:38.6 | 1 |
| -427.721 | 19531.993 | 1.771 | 14.856 | 0.11997 | 7/15/2014 | 57:38.7 | 1 |
| -427.839 | 19531.999 | 1.766 | 14.851 | 0.115383 | 7/15/2014 | 57:38.8 | 1 |
| -428.077 | 19532.011 | 1.77 | 14.846 | 0.102232 | 7/15/2014 | 57:38.9 | 1 |
| -428.317 | 19532.022 | 1.764 | 14.844 | 0.108007 | 7/15/2014 | 57:39.0 | 1 |
| -428.557 | 19532.034 | 1.761 | 14.846 | 0.117468 | 7/15/2014 | 57:39.1 | 1 |
| -428.798 | 19532.046 | 1.761 | 14.852 | 0.126575 | 7/15/2014 | 57:39.2 | 1 |
| -429.041 | 19532.058 | 1.761 | 14.861 | 0.123984 | 7/15/2014 | 57:39.3 | 1 |
| -429.463 | 19532.079 | 1.762 | 14.883 | 0.134962 | 7/15/2014 | 57:39.4 | 1 |
| -429.705 | 19532.09 | 1.759 | 14.899 | 0.141561 | 7/15/2014 | 57:39.5 | 1 |
| -429.948 | 19532.102 | 1.755 | 14.916 | 0.159788 | 7/15/2014 | 57:39.6 | 1 |
| -430.252 | 19532.116 | 1.758 | 14.935 | 0.168724 | 7/15/2014 | 57:39.7 | 1 |
| -430.375 | 19532.122 | 1.76 | 14.945 | 0.173562 | 7/15/2014 | 57:39.8 | 1 |
| -430.62 | 19532.134 | 1.764 | 14.963 | 0.188399 | 7/15/2014 | 57:39.9 | 1 |
| -430.866 | 19532.146 | 1.761 | 14.98 | 0.19391 | 7/15/2014 | 57:40.0 | 1 |
| -431.112 | 19532.158 | 1.767 | 14.995 | 0.198266 | 7/15/2014 | 57:40.1 | 1 |
| -431.359 | 19532.169 | 1.764 | 15.008 | 0.209235 | 7/15/2014 | 57:40.2 | 1 |
| -431.604 | 19532.181 | 1.77 | 15.017 | 0.209634 | 7/15/2014 | 57:40.3 | 1 |
| -432.032 | 19532.199 | 1.773 | 15.023 | 0.205063 | 7/15/2014 | 57:40.4 | 1 |
| -432.275 | 19532.211 | 1.773 | 15.025 | 0.201841 | 7/15/2014 | 57:40.5 | 1 |
| -432.521 | 19532.223 | 1.773 | 15.024 | 0.193755 | 7/15/2014 | 57:40.6 | 1 |
| -432.826 | 19532.236 | 1.776 | 15.022 | 0.192387 | 7/15/2014 | 57:40.7 | 1 |
| -432.95 | 19532.242 | 1.772 | 15.021 | 0.19239 | 7/15/2014 | 57:40.8 | 1 |
| -433.196 | 19532.254 | 1.778 | 15.016 | 0.183696 | 7/15/2014 | 57:40.9 | 1 |
| -433.442 | 19532.265 | 1.778 | 15.009 | 0.173136 | 7/15/2014 | 57:41.0 | 1 |
| -433.688 | 19532.275 | 1.784 | 15.002 | 0.162641 | 7/15/2014 | 57:41.1 | 1 |
| -433.933 | 19532.287 | 1.782 | 14.992 | 0.157585 | 7/15/2014 | 57:41.2 | 1 |
| -434.179 | 19532.299 | 1.786 | 14.982 | 0.151524 | 7/15/2014 | 57:41.3 | 1 |
| -434.606 | 19532.318 | 1.787 | 14.959 | 0.153864 | 7/15/2014 | 57:41.4 | 1 |
| -434.849 | 19532.329 | 1.79 | 14.942 | 0.152276 | 7/15/2014 | 57:41.5 | 1 |
| -435.092 | 19532.34 | 1.788 | 14.922 | 0.157172 | 7/15/2014 | 57:41.6 | 1 |
| -435.393 | 19532.354 | 1.783 | 14.899 | 0.152782 | 7/15/2014 | 57:41.7 | 1 |
| -435.517 | 19532.359 | 1.781 | 14.888 | 0.150771 | 7/15/2014 | 57:41.8 | 1 |
| -435.762 | 19532.371 | 1.783 | 14.861 | 0.138816 | 7/15/2014 | 57:41.9 | 1 |
| -436.005 | 19532.383 | 1.783 | 14.832 | 0.137584 | 7/15/2014 | 57:42.0 | 1 |
| -436.248 | 19532.395 | 1.777 | 14.803 | 0.132845 | 7/15/2014 | 57:42.1 | 1 |
| -436.49 | 19532.403 | 1.773 | 14.773 | 0.128237 | 7/15/2014 | 57:42.2 | 1 |
| -436.732 | 19532.414 | 1.772 | 14.743 | 0.120328 | 7/15/2014 | 57:42.3 | 1 |
| -437.15 | 19532.434 | 1.773 | 14.703 | 0.116283 | 7/15/2014 | 57:42.4 | 1 |
| -437.39 | 19532.444 | 1.777 | 14.678 | 0.116323 | 7/15/2014 | 57:42.5 | 1 |

| | | | | | | | |
|---|---|---|---|---|---|---|---|
| -437.628 | 19532.455 | 1.775 | 14.655 | 0.117233 | 7/15/2014 | 57:42.6 | 1 |
| -437.927 | 19532.469 | 1.771 | 14.634 | 0.125128 | 7/15/2014 | 57:42.7 | 1 |
| -438.048 | 19532.473 | 1.774 | 14.623 | 0.127202 | 7/15/2014 | 57:42.8 | 1 |
| -438.289 | 19532.484 | 1.78 | 14.606 | 0.135569 | 7/15/2014 | 57:42.9 | 1 |
| -438.528 | 19532.496 | 1.777 | 14.592 | 0.146256 | 7/15/2014 | 57:43.0 | 1 |
| -438.768 | 19532.508 | 1.777 | 14.58 | 0.15893 | 7/15/2014 | 57:43.1 | 1 |
| -439.008 | 19532.517 | 1.777 | 14.57 | 0.159151 | 7/15/2014 | 57:43.2 | 1 |
| -439.248 | 19532.527 | 1.777 | 14.563 | 0.167047 | 7/15/2014 | 57:43.3 | 1 |
| -439.665 | 19532.547 | 1.775 | 14.557 | 0.150406 | 7/15/2014 | 57:43.4 | 1 |
| -439.903 | 19532.559 | 1.775 | 14.555 | 0.133488 | 7/15/2014 | 57:43.5 | 1 |
| -440.14 | 19532.568 | 1.775 | 14.554 | 0.123581 | 7/15/2014 | 57:43.6 | 1 |
| -440.436 | 19532.583 | 1.778 | 14.556 | 0.118713 | 7/15/2014 | 57:43.7 | 1 |
| -440.557 | 19532.587 | 1.777 | 14.557 | 0.100392 | 7/15/2014 | 57:43.8 | 1 |
| -440.796 | 19532.598 | 1.777 | 14.561 | 0.0801386 | 7/15/2014 | 57:43.9 | 1 |
| -441.034 | 19532.609 | 1.773 | 14.567 | 0.081569 | 7/15/2014 | 57:44.0 | 1 |
| -441.273 | 19532.621 | 1.773 | 14.573 | 0.0655662 | 7/15/2014 | 57:44.1 | 1 |
| -441.512 | 19532.633 | 1.767 | 14.581 | 0.0762003 | 7/15/2014 | 57:44.2 | 1 |
| -441.749 | 19532.643 | 1.767 | 14.589 | 0.073685 | 7/15/2014 | 57:44.3 | 1 |
| -442.163 | 19532.663 | 1.758 | 14.602 | 0.0797165 | 7/15/2014 | 57:44.4 | 1 |
| -442.398 | 19532.672 | 1.749 | 14.61 | 0.0855587 | 7/15/2014 | 57:44.5 | 1 |
| -442.634 | 19532.684 | 1.746 | 14.619 | 0.0915996 | 7/15/2014 | 57:44.6 | 1 |
| -442.928 | 19532.698 | 1.744 | 14.626 | 0.0999834 | 7/15/2014 | 57:44.7 | 1 |
| -443.047 | 19532.704 | 1.742 | 14.631 | 0.101558 | 7/15/2014 | 57:44.8 | 1 |
| -443.284 | 19532.715 | 1.744 | 14.637 | 0.104465 | 7/15/2014 | 57:44.9 | 1 |
| -443.519 | 19532.727 | 1.738 | 14.643 | 0.109496 | 7/15/2014 | 57:45.0 | 1 |
| -443.758 | 19532.738 | 1.742 | 14.647 | 0.107748 | 7/15/2014 | 57:45.1 | 1 |
| -443.992 | 19532.748 | 1.738 | 14.648 | 0.118577 | 7/15/2014 | 57:45.2 | 1 |
| -444.229 | 19532.76 | 1.738 | 14.647 | 0.114686 | 7/15/2014 | 57:45.3 | 1 |
| -444.641 | 19532.78 | 1.735 | 14.637 | 0.137948 | 7/15/2014 | 57:45.4 | 1 |
| -444.876 | 19532.791 | 1.729 | 14.627 | 0.129472 | 7/15/2014 | 57:45.5 | 1 |
| -445.11 | 19532.803 | 1.723 | 14.616 | 0.143782 | 7/15/2014 | 57:45.6 | 1 |
| -445.407 | 19532.818 | 1.723 | 14.602 | 0.144814 | 7/15/2014 | 57:45.7 | 1 |
| -445.525 | 19532.824 | 1.718 | 14.595 | 0.173247 | 7/15/2014 | 57:45.8 | 1 |
| -445.764 | 19532.836 | 1.72 | 14.578 | 0.145547 | 7/15/2014 | 57:45.9 | 1 |
| -446.002 | 19532.848 | 1.72 | 14.561 | 0.163229 | 7/15/2014 | 57:46.0 | 1 |
| -446.243 | 19532.859 | 1.726 | 14.545 | 0.134528 | 7/15/2014 | 57:46.1 | 1 |
| -446.483 | 19532.871 | 1.72 | 14.53 | 0.135484 | 7/15/2014 | 57:46.2 | 1 |
| -446.724 | 19532.883 | 1.73 | 14.518 | 0.13259 | 7/15/2014 | 57:46.3 | 1 |
| -447.147 | 19532.906 | 1.728 | 14.508 | 0.129709 | 7/15/2014 | 57:46.4 | 1 |
| -447.388 | 19532.918 | 1.724 | 14.505 | 0.133164 | 7/15/2014 | 57:46.5 | 1 |
| -447.629 | 19532.93 | 1.721 | 14.506 | 0.13466 | 7/15/2014 | 57:46.6 | 1 |
| -447.935 | 19532.945 | 1.721 | 14.511 | 0.116933 | 7/15/2014 | 57:46.7 | 1 |
| -448.056 | 19532.951 | 1.716 | 14.512 | 0.110135 | 7/15/2014 | 57:46.8 | 1 |
| -448.302 | 19532.963 | 1.716 | 14.524 | 0.106396 | 7/15/2014 | 57:46.9 | 1 |
| -448.548 | 19532.977 | 1.706 | 14.538 | 0.119322 | 7/15/2014 | 57:47.0 | 1 |
| -448.796 | 19532.988 | 1.712 | 14.556 | 0.111103 | 7/15/2014 | 57:47.1 | 1 |
| -449.042 | 19533.001 | 1.708 | 14.576 | 0.0987308 | 7/15/2014 | 57:47.2 | 1 |

| | | | | | | | |
|---|---|---|---|---|---|---|---|
| -449.289 | 19533.014 | 1.714 | 14.598 | 0.0895825 | 7/15/2014 | 57:47.3 | 1 |
| -449.721 | 19533.035 | 1.714 | 14.634 | 0.102489 | 7/15/2014 | 57:47.4 | 1 |
| -449.966 | 19533.047 | 1.714 | 14.657 | 0.105618 | 7/15/2014 | 57:47.5 | 1 |
| -450.212 | 19533.059 | 1.714 | 14.681 | 0.104113 | 7/15/2014 | 57:47.6 | 1 |
| -450.519 | 19533.074 | 1.715 | 14.704 | 0.111686 | 7/15/2014 | 57:47.7 | 1 |
| -450.644 | 19533.081 | 1.712 | 14.716 | 0.119006 | 7/15/2014 | 57:47.8 | 1 |
| -450.892 | 19533.094 | 1.712 | 14.736 | 0.124467 | 7/15/2014 | 57:47.9 | 1 |
| -451.139 | 19533.105 | 1.704 | 14.755 | 0.123339 | 7/15/2014 | 57:48.0 | 1 |
| -451.386 | 19533.117 | 1.7 | 14.771 | 0.120191 | 7/15/2014 | 57:48.1 | 1 |
| -451.633 | 19533.129 | 1.7 | 14.783 | 0.127566 | 7/15/2014 | 57:48.2 | 1 |
| -451.879 | 19533.141 | 1.7 | 14.791 | 0.12535 | 7/15/2014 | 57:48.3 | 1 |
| -452.307 | 19533.162 | 1.695 | 14.792 | 0.12275 | 7/15/2014 | 57:48.4 | 1 |
| -452.552 | 19533.174 | 1.698 | 14.79 | 0.123128 | 7/15/2014 | 57:48.5 | 1 |
| -452.797 | 19533.186 | 1.7 | 14.783 | 0.118538 | 7/15/2014 | 57:48.6 | 1 |
| -453.101 | 19533.199 | 1.705 | 14.773 | 0.122177 | 7/15/2014 | 57:48.7 | 1 |
| -453.225 | 19533.205 | 1.708 | 14.768 | 0.118765 | 7/15/2014 | 57:48.8 | 1 |
| -453.472 | 19533.217 | 1.704 | 14.752 | 0.129759 | 7/15/2014 | 57:48.9 | 1 |
| -453.718 | 19533.229 | 1.702 | 14.733 | 0.119375 | 7/15/2014 | 57:49.0 | 1 |
| -453.965 | 19533.24 | 1.708 | 14.712 | 0.129132 | 7/15/2014 | 57:49.1 | 1 |
| -454.211 | 19533.252 | 1.711 | 14.69 | 0.135844 | 7/15/2014 | 57:49.2 | 1 |
| -454.458 | 19533.264 | 1.714 | 14.667 | 0.133978 | 7/15/2014 | 57:49.3 | 1 |
| -454.887 | 19533.283 | 1.708 | 14.631 | 0.13793 | 7/15/2014 | 57:49.4 | 1 |
| -455.133 | 19533.295 | 1.703 | 14.608 | 0.139827 | 7/15/2014 | 57:49.5 | 1 |
| -455.378 | 19533.307 | 1.708 | 14.585 | 0.146997 | 7/15/2014 | 57:49.6 | 1 |
| -455.685 | 19533.32 | 1.708 | 14.563 | 0.149578 | 7/15/2014 | 57:49.7 | 1 |
| -455.809 | 19533.326 | 1.705 | 14.551 | 0.155946 | 7/15/2014 | 57:49.8 | 1 |
| -456.058 | 19533.338 | 1.705 | 14.53 | 0.16132 | 7/15/2014 | 57:49.9 | 1 |
| -456.307 | 19533.349 | 1.703 | 14.511 | 0.157346 | 7/15/2014 | 57:50.0 | 1 |
| -456.555 | 19533.361 | 1.703 | 14.492 | 0.156549 | 7/15/2014 | 57:50.1 | 1 |
| -456.804 | 19533.371 | 1.706 | 14.475 | 0.144812 | 7/15/2014 | 57:50.2 | 1 |
| -457.052 | 19533.383 | 1.712 | 14.459 | 0.138337 | 7/15/2014 | 57:50.3 | 1 |
| -457.36 | 19533.396 | 1.71 | 14.444 | 0.124328 | 7/15/2014 | 57:50.4 | 1 |
| -457.61 | 19533.408 | 1.715 | 14.431 | 0.112437 | 7/15/2014 | 57:50.5 | 1 |
| -457.857 | 19533.419 | 1.715 | 14.42 | 0.1029 | 7/15/2014 | 57:50.6 | 1 |
| -458.288 | 19533.439 | 1.72 | 14.406 | 0.115966 | 7/15/2014 | 57:50.7 | 1 |
| -458.414 | 19533.443 | 1.721 | 14.401 | 0.0885239 | 7/15/2014 | 57:50.8 | 1 |
| -458.661 | 19533.454 | 1.721 | 14.393 | 0.103783 | 7/15/2014 | 57:50.9 | 1 |
| -458.908 | 19533.465 | 1.721 | 14.386 | 0.104703 | 7/15/2014 | 57:51.0 | 1 |
| -459.156 | 19533.477 | 1.721 | 14.381 | 0.131753 | 7/15/2014 | 57:51.1 | 1 |
| -459.401 | 19533.488 | 1.719 | 14.377 | 0.0968223 | 7/15/2014 | 57:51.2 | 1 |
| -459.648 | 19533.5 | 1.719 | 14.375 | 0.125826 | 7/15/2014 | 57:51.3 | 1 |
| -460.073 | 19533.518 | 1.717 | 14.377 | 0.0943721 | 7/15/2014 | 57:51.4 | 1 |
| -460.315 | 19533.528 | 1.717 | 14.381 | 0.127383 | 7/15/2014 | 57:51.5 | 1 |
| -460.557 | 19533.538 | 1.717 | 14.387 | 0.140057 | 7/15/2014 | 57:51.6 | 1 |
| -460.859 | 19533.552 | 1.719 | 14.396 | 0.146267 | 7/15/2014 | 57:51.7 | 1 |
| -460.98 | 19533.558 | 1.72 | 14.399 | 0.156678 | 7/15/2014 | 57:51.8 | 1 |
| -461.225 | 19533.568 | 1.726 | 14.413 | 0.166395 | 7/15/2014 | 57:51.9 | 1 |

| | | | | | | | |
|---|---|---|---|---|---|---|---|
| -461.467 | 19533.578 | 1.728 | 14.429 | 0.166432 | 7/15/2014 | 57:52.0 | 1 |
| -461.711 | 19533.59 | 1.734 | 14.449 | 0.173747 | 7/15/2014 | 57:52.1 | 1 |
| -461.953 | 19533.601 | 1.732 | 14.472 | 0.158603 | 7/15/2014 | 57:52.2 | 1 |
| -462.195 | 19533.61 | 1.732 | 14.497 | 0.148555 | 7/15/2014 | 57:52.3 | 1 |
| -462.619 | 19533.63 | 1.734 | 14.54 | 0.158657 | 7/15/2014 | 57:52.4 | 1 |
| -462.861 | 19533.64 | 1.732 | 14.569 | 0.129421 | 7/15/2014 | 57:52.5 | 1 |
| -463.102 | 19533.65 | 1.728 | 14.599 | 0.146631 | 7/15/2014 | 57:52.6 | 1 |
| -463.406 | 19533.664 | 1.728 | 14.628 | 0.105461 | 7/15/2014 | 57:52.7 | 1 |
| -463.527 | 19533.669 | 1.725 | 14.644 | 0.106486 | 7/15/2014 | 57:52.8 | 1 |
| -463.772 | 19533.68 | 1.727 | 14.672 | 0.112915 | 7/15/2014 | 57:52.9 | 1 |
| -464.017 | 19533.691 | 1.727 | 14.698 | 0.114218 | 7/15/2014 | 57:53.0 | 1 |
| -464.262 | 19533.702 | 1.727 | 14.722 | 0.106046 | 7/15/2014 | 57:53.1 | 1 |
| -464.508 | 19533.712 | 1.725 | 14.742 | 0.114153 | 7/15/2014 | 57:53.2 | 1 |
| -464.754 | 19533.723 | 1.728 | 14.759 | 0.104984 | 7/15/2014 | 57:53.3 | 1 |
| -465.18 | 19533.742 | 1.73 | 14.773 | 0.117989 | 7/15/2014 | 57:53.4 | 1 |
| -465.424 | 19533.753 | 1.733 | 14.781 | 0.12115 | 7/15/2014 | 57:53.5 | 1 |
| -465.668 | 19533.764 | 1.739 | 14.785 | 0.115951 | 7/15/2014 | 57:53.6 | 1 |
| -465.971 | 19533.776 | 1.739 | 14.786 | 0.118204 | 7/15/2014 | 57:53.7 | 1 |
| -466.095 | 19533.781 | 1.741 | 14.787 | 0.130702 | 7/15/2014 | 57:53.8 | 1 |
| -466.34 | 19533.793 | 1.743 | 14.785 | 0.13275 | 7/15/2014 | 57:53.9 | 1 |
| -466.583 | 19533.805 | 1.745 | 14.781 | 0.132932 | 7/15/2014 | 57:54.0 | 1 |
| -466.828 | 19533.814 | 1.755 | 14.778 | 0.147258 | 7/15/2014 | 57:54.1 | 1 |
| -467.07 | 19533.825 | 1.752 | 14.774 | 0.126046 | 7/15/2014 | 57:54.2 | 1 |
| -467.315 | 19533.836 | 1.752 | 14.77 | 0.142672 | 7/15/2014 | 57:54.3 | 1 |
| -467.734 | 19533.854 | 1.742 | 14.764 | 0.133715 | 7/15/2014 | 57:54.4 | 1 |
| -467.973 | 19533.865 | 1.74 | 14.76 | 0.138704 | 7/15/2014 | 57:54.5 | 1 |
| -468.212 | 19533.877 | 1.74 | 14.755 | 0.155452 | 7/15/2014 | 57:54.6 | 1 |
| -468.511 | 19533.889 | 1.737 | 14.75 | 0.104876 | 7/15/2014 | 57:54.7 | 1 |
| -468.631 | 19533.895 | 1.731 | 14.747 | 0.108115 | 7/15/2014 | 57:54.8 | 1 |
| -468.871 | 19533.906 | 1.731 | 14.74 | 0.110259 | 7/15/2014 | 57:54.9 | 1 |
| -469.109 | 19533.917 | 1.727 | 14.731 | 0.142707 | 7/15/2014 | 57:55.0 | 1 |
| -469.349 | 19533.926 | 1.731 | 14.72 | 0.0794131 | 7/15/2014 | 57:55.1 | 1 |
| -469.586 | 19533.938 | 1.731 | 14.705 | 0.0858512 | 7/15/2014 | 57:55.2 | 1 |
| -469.822 | 19533.949 | 1.727 | 14.688 | 0.100452 | 7/15/2014 | 57:55.3 | 1 |
| -470.234 | 19533.967 | 1.72 | 14.653 | 0.122212 | 7/15/2014 | 57:55.4 | 1 |
| -470.468 | 19533.979 | 1.72 | 14.629 | 0.0984495 | 7/15/2014 | 57:55.5 | 1 |
| -470.701 | 19533.988 | 1.72 | 14.602 | 0.0885947 | 7/15/2014 | 57:55.6 | 1 |
| -470.994 | 19534.001 | 1.719 | 14.574 | 0.107179 | 7/15/2014 | 57:55.7 | 1 |
| -471.112 | 19534.007 | 1.714 | 14.56 | 0.0992736 | 7/15/2014 | 57:55.8 | 1 |
| -471.347 | 19534.018 | 1.714 | 14.531 | 0.0748644 | 7/15/2014 | 57:55.9 | 1 |
| -471.582 | 19534.027 | 1.718 | 14.503 | 0.101422 | 7/15/2014 | 57:56.0 | 1 |
| -471.817 | 19534.039 | 1.718 | 14.477 | 0.0852724 | 7/15/2014 | 57:56.1 | 1 |
| -472.052 | 19534.049 | 1.718 | 14.454 | 0.10002 | 7/15/2014 | 57:56.2 | 1 |
| -472.288 | 19534.06 | 1.721 | 14.434 | 0.0957478 | 7/15/2014 | 57:56.3 | 1 |
| -472.697 | 19534.079 | 1.72 | 14.415 | 0.104733 | 7/15/2014 | 57:56.4 | 1 |
| -472.932 | 19534.088 | 1.72 | 14.405 | 0.0959399 | 7/15/2014 | 57:56.5 | 1 |
| -473.166 | 19534.1 | 1.718 | 14.4 | 0.105945 | 7/15/2014 | 57:56.6 | 1 |

| | | | | | | | |
|---|---|---|---|---|---|---|---|
| -473.46 | 19534.113 | 1.715 | 14.398 | 0.0699745 | 7/15/2014 | 57:56.7 | 1 |
| -473.579 | 19534.117 | 1.719 | 14.397 | 0.0637547 | 7/15/2014 | 57:56.8 | 1 |
| -473.817 | 19534.129 | 1.719 | 14.4 | 0.0756212 | 7/15/2014 | 57:56.9 | 1 |
| -474.054 | 19534.141 | 1.713 | 14.406 | 0.0746828 | 7/15/2014 | 57:57.0 | 1 |
| -474.292 | 19534.152 | 1.715 | 14.414 | 0.0760459 | 7/15/2014 | 57:57.1 | 1 |
| -474.53 | 19534.161 | 1.709 | 14.422 | 0.091025 | 7/15/2014 | 57:57.2 | 1 |
| -474.767 | 19534.172 | 1.707 | 14.43 | 0.071918 | 7/15/2014 | 57:57.3 | 1 |
| -475.182 | 19534.191 | 1.704 | 14.438 | 0.0950862 | 7/15/2014 | 57:57.4 | 1 |
| -475.418 | 19534.203 | 1.707 | 14.44 | 0.0797742 | 7/15/2014 | 57:57.5 | 1 |
| -475.655 | 19534.214 | 1.709 | 14.439 | 0.0869467 | 7/15/2014 | 57:57.6 | 1 |
| -475.95 | 19534.229 | 1.709 | 14.435 | 0.0804806 | 7/15/2014 | 57:57.7 | 1 |
| -476.07 | 19534.233 | 1.707 | 14.434 | 0.0674 | 7/15/2014 | 57:57.8 | 1 |
| -476.309 | 19534.243 | 1.71 | 14.423 | 0.0722842 | 7/15/2014 | 57:57.9 | 1 |
| -476.547 | 19534.255 | 1.71 | 14.409 | 0.101553 | 7/15/2014 | 57:58.0 | 1 |
| -476.786 | 19534.266 | 1.716 | 14.391 | 0.0739441 | 7/15/2014 | 57:58.1 | 1 |
| -477.024 | 19534.277 | 1.714 | 14.37 | 0.082509 | 7/15/2014 | 57:58.2 | 1 |
| -477.261 | 19534.288 | 1.716 | 14.348 | 0.0865149 | 7/15/2014 | 57:58.3 | 1 |
| -477.676 | 19534.309 | 1.71 | 14.316 | 0.0926314 | 7/15/2014 | 57:58.4 | 1 |
| -477.912 | 19534.317 | 1.705 | 14.297 | 0.091716 | 7/15/2014 | 57:58.5 | 1 |
| -478.15 | 19534.329 | 1.705 | 14.28 | 0.0883576 | 7/15/2014 | 57:58.6 | 1 |
| -478.445 | 19534.343 | 1.703 | 14.266 | 0.0927473 | 7/15/2014 | 57:58.7 | 1 |
| -478.565 | 19534.349 | 1.702 | 14.258 | 0.087056 | 7/15/2014 | 57:58.8 | 1 |
| -478.804 | 19534.359 | 1.696 | 14.249 | 0.0895664 | 7/15/2014 | 57:58.9 | 1 |
| -479.043 | 19534.371 | 1.687 | 14.244 | 0.0965914 | 7/15/2014 | 57:59.0 | 1 |
| -479.283 | 19534.383 | 1.687 | 14.242 | 0.0815688 | 7/15/2014 | 57:59.1 | 1 |
| -479.52 | 19534.393 | 1.688 | 14.244 | 0.0901886 | 7/15/2014 | 57:59.2 | 1 |
| -479.76 | 19534.404 | 1.701 | 14.248 | 0.109635 | 7/15/2014 | 57:59.3 | 1 |
| -480.176 | 19534.425 | 1.704 | 14.26 | 0.112163 | 7/15/2014 | 57:59.4 | 1 |
| -480.413 | 19534.436 | 1.706 | 14.269 | 0.127753 | 7/15/2014 | 57:59.5 | 1 |
| -480.651 | 19534.447 | 1.712 | 14.279 | 0.124571 | 7/15/2014 | 57:59.6 | 1 |
| -480.948 | 19534.461 | 1.717 | 14.29 | 0.127777 | 7/15/2014 | 57:59.7 | 1 |
| -481.068 | 19534.467 | 1.718 | 14.295 | 0.136395 | 7/15/2014 | 57:59.8 | 1 |
| -481.307 | 19534.479 | 1.72 | 14.308 | 0.111234 | 7/15/2014 | 57:59.9 | 1 |
| -481.546 | 19534.49 | 1.711 | 14.32 | 0.123139 | 7/15/2014 | 58:00.0 | 1 |
| -481.787 | 19534.502 | 1.711 | 14.333 | 0.135115 | 7/15/2014 | 58:00.1 | 1 |
| -482.026 | 19534.514 | 1.709 | 14.346 | 0.134445 | 7/15/2014 | 58:00.2 | 1 |
| -482.264 | 19534.525 | 1.709 | 14.36 | 0.143712 | 7/15/2014 | 58:00.3 | 1 |
| -482.68 | 19534.546 | 1.714 | 14.382 | 0.153158 | 7/15/2014 | 58:00.4 | 1 |
| -482.916 | 19534.559 | 1.714 | 14.397 | 0.156652 | 7/15/2014 | 58:00.5 | 1 |
| -483.153 | 19534.57 | 1.716 | 14.412 | 0.157536 | 7/15/2014 | 58:00.6 | 1 |
| -483.45 | 19534.585 | 1.72 | 14.427 | 0.16852 | 7/15/2014 | 58:00.7 | 1 |
| -483.57 | 19534.591 | 1.721 | 14.435 | 0.165512 | 7/15/2014 | 58:00.8 | 1 |
| -483.808 | 19534.603 | 1.724 | 14.45 | 0.154202 | 7/15/2014 | 58:00.9 | 1 |
| -484.047 | 19534.615 | 1.73 | 14.463 | 0.186219 | 7/15/2014 | 58:01.0 | 1 |
| -484.285 | 19534.628 | 1.733 | 14.474 | 0.140412 | 7/15/2014 | 58:01.1 | 1 |
| -484.523 | 19534.641 | 1.733 | 14.481 | 0.170295 | 7/15/2014 | 58:01.2 | 1 |
| -484.762 | 19534.653 | 1.737 | 14.485 | 0.148178 | 7/15/2014 | 58:01.3 | 1 |

| | | | | | | | |
|---|---|---|---|---|---|---|---|
| -485.176 | 19534.675 | 1.742 | 14.477 | 0.175926 | 7/15/2014 | 58:01.4 | 1 |
| -485.411 | 19534.688 | 1.746 | 14.468 | 0.189759 | 7/15/2014 | 58:01.5 | 1 |
| -485.648 | 19534.701 | 1.752 | 14.453 | 0.184001 | 7/15/2014 | 58:01.6 | 1 |
| -485.943 | 19534.717 | 1.752 | 14.433 | 0.194455 | 7/15/2014 | 58:01.7 | 1 |
| -486.062 | 19534.723 | 1.752 | 14.424 | 0.183915 | 7/15/2014 | 58:01.8 | 1 |
| -486.303 | 19534.735 | 1.762 | 14.396 | 0.192829 | 7/15/2014 | 58:01.9 | 1 |
| -486.54 | 19534.75 | 1.768 | 14.365 | 0.175394 | 7/15/2014 | 58:02.0 | 1 |
| -486.781 | 19534.762 | 1.771 | 14.331 | 0.157678 | 7/15/2014 | 58:02.1 | 1 |
| -487.02 | 19534.775 | 1.771 | 14.297 | 0.174852 | 7/15/2014 | 58:02.2 | 1 |
| -487.26 | 19534.789 | 1.78 | 14.263 | 0.152735 | 7/15/2014 | 58:02.3 | 1 |
| -487.679 | 19534.813 | 1.794 | 14.219 | 0.181374 | 7/15/2014 | 58:02.4 | 1 |
| -487.918 | 19534.826 | 1.801 | 14.193 | 0.183275 | 7/15/2014 | 58:02.5 | 1 |
| -488.157 | 19534.84 | 1.803 | 14.17 | 0.188327 | 7/15/2014 | 58:02.6 | 1 |
| -488.456 | 19534.856 | 1.803 | 14.152 | 0.193105 | 7/15/2014 | 58:02.7 | 1 |
| -488.578 | 19534.863 | 1.81 | 14.142 | 0.191207 | 7/15/2014 | 58:02.8 | 1 |
| -488.819 | 19534.877 | 1.818 | 14.13 | 0.191185 | 7/15/2014 | 58:02.9 | 1 |
| -489.06 | 19534.892 | 1.818 | 14.121 | 0.177289 | 7/15/2014 | 58:03.0 | 1 |
| -489.302 | 19534.904 | 1.821 | 14.116 | 0.176099 | 7/15/2014 | 58:03.1 | 1 |
| -489.542 | 19534.92 | 1.815 | 14.113 | 0.161872 | 7/15/2014 | 58:03.2 | 1 |
| -489.782 | 19534.933 | 1.817 | 14.112 | 0.149302 | 7/15/2014 | 58:03.3 | 1 |
| -490.199 | 19534.958 | 1.817 | 14.114 | 0.152607 | 7/15/2014 | 58:03.4 | 1 |
| -490.436 | 19534.972 | 1.821 | 14.117 | 0.15599 | 7/15/2014 | 58:03.5 | 1 |
| -490.675 | 19534.986 | 1.82 | 14.12 | 0.179021 | 7/15/2014 | 58:03.6 | 1 |
| -490.971 | 19535.004 | 1.818 | 14.124 | 0.130188 | 7/15/2014 | 58:03.7 | 1 |
| -491.091 | 19535.012 | 1.816 | 14.126 | 0.140088 | 7/15/2014 | 58:03.8 | 1 |
| -491.331 | 19535.025 | 1.818 | 14.13 | 0.165061 | 7/15/2014 | 58:03.9 | 1 |
| -491.568 | 19535.039 | 1.814 | 14.133 | 0.138081 | 7/15/2014 | 58:04.0 | 1 |
| -491.809 | 19535.055 | 1.82 | 14.134 | 0.15684 | 7/15/2014 | 58:04.1 | 1 |
| -492.046 | 19535.069 | 1.823 | 14.132 | 0.164666 | 7/15/2014 | 58:04.2 | 1 |
| -492.286 | 19535.083 | 1.829 | 14.126 | 0.143913 | 7/15/2014 | 58:04.3 | 1 |
| -492.701 | 19535.11 | 1.829 | 14.103 | 0.230444 | 7/15/2014 | 58:04.4 | 1 |
| -492.938 | 19535.125 | 1.829 | 14.083 | 0.204395 | 7/15/2014 | 58:04.5 | 1 |
| -493.175 | 19535.139 | 1.829 | 14.057 | 0.209748 | 7/15/2014 | 58:04.6 | 1 |
| -493.472 | 19535.158 | 1.829 | 14.026 | 0.192811 | 7/15/2014 | 58:04.7 | 1 |
| -493.591 | 19535.166 | 1.825 | 14.011 | 0.198619 | 7/15/2014 | 58:04.8 | 1 |
| -493.832 | 19535.181 | 1.824 | 13.973 | 0.207195 | 7/15/2014 | 58:04.9 | 1 |
| -494.07 | 19535.196 | 1.815 | 13.934 | 0.182763 | 7/15/2014 | 58:05.0 | 1 |
| -494.31 | 19535.212 | 1.815 | 13.895 | 0.137327 | 7/15/2014 | 58:05.1 | 1 |
| -494.547 | 19535.228 | 1.808 | 13.858 | 0.138213 | 7/15/2014 | 58:05.2 | 1 |
| -494.786 | 19535.242 | 1.81 | 13.826 | 0.145625 | 7/15/2014 | 58:05.3 | 1 |
| -495.201 | 19535.27 | 1.809 | 13.792 | 0.142757 | 7/15/2014 | 58:05.4 | 1 |
| -495.437 | 19535.286 | 1.809 | 13.776 | 0.138373 | 7/15/2014 | 58:05.5 | 1 |
| -495.673 | 19535.302 | 1.804 | 13.766 | 0.151135 | 7/15/2014 | 58:05.6 | 1 |
| -495.969 | 19535.321 | 1.804 | 13.765 | 0.16657 | 7/15/2014 | 58:05.7 | 1 |
| -496.089 | 19535.329 | 1.801 | 13.762 | 0.177617 | 7/15/2014 | 58:05.8 | 1 |
| -496.328 | 19535.346 | 1.803 | 13.77 | 0.173623 | 7/15/2014 | 58:05.9 | 1 |
| -496.566 | 19535.362 | 1.798 | 13.785 | 0.182813 | 7/15/2014 | 58:06.0 | 1 |

| | | | | | | | |
|---|---|---|---|---|---|---|---|
| -496.805 | 19535.377 | 1.796 | 13.804 | 0.201438 | 7/15/2014 | 58:06.1 | 1 |
| -497.043 | 19535.395 | 1.791 | 13.826 | 0.21852 | 7/15/2014 | 58:06.2 | 1 |
| -497.281 | 19535.411 | 1.787 | 13.849 | 0.197559 | 7/15/2014 | 58:06.3 | 1 |
| -497.699 | 19535.439 | 1.786 | 13.883 | 0.215483 | 7/15/2014 | 58:06.4 | 1 |
| -497.936 | 19535.457 | 1.784 | 13.905 | 0.224327 | 7/15/2014 | 58:06.5 | 1 |
| -498.173 | 19535.474 | 1.786 | 13.923 | 0.211852 | 7/15/2014 | 58:06.6 | 1 |
| -498.468 | 19535.494 | 1.783 | 13.939 | 0.190852 | 7/15/2014 | 58:06.7 | 1 |
| -498.589 | 19535.503 | 1.781 | 13.948 | 0.179217 | 7/15/2014 | 58:06.8 | 1 |
| -498.831 | 19535.52 | 1.786 | 13.958 | 0.250434 | 7/15/2014 | 58:06.9 | 1 |
| -499.069 | 19535.537 | 1.784 | 13.964 | 0.200568 | 7/15/2014 | 58:07.0 | 1 |
| -499.309 | 19535.554 | 1.78 | 13.967 | 0.198037 | 7/15/2014 | 58:07.1 | 1 |
| -499.548 | 19535.571 | 1.778 | 13.967 | 0.189104 | 7/15/2014 | 58:07.2 | 1 |
| -499.786 | 19535.589 | 1.774 | 13.964 | 0.218644 | 7/15/2014 | 58:07.3 | 1 |
| -500.199 | 19535.619 | 1.765 | 13.956 | 0.224019 | 7/15/2014 | 58:07.4 | 1 |
| -500.437 | 19535.637 | 1.765 | 13.95 | 0.166468 | 7/15/2014 | 58:07.5 | 1 |
| -500.672 | 19535.654 | 1.758 | 13.944 | 0.184683 | 7/15/2014 | 58:07.6 | 1 |
| -500.966 | 19535.676 | 1.752 | 13.939 | 0.17693 | 7/15/2014 | 58:07.7 | 1 |
| -501.086 | 19535.685 | 1.752 | 13.936 | 0.176045 | 7/15/2014 | 58:07.8 | 1 |
| -501.324 | 19535.703 | 1.749 | 13.933 | 0.203517 | 7/15/2014 | 58:07.9 | 1 |
| -501.56 | 19535.721 | 1.748 | 13.931 | 0.190396 | 7/15/2014 | 58:08.0 | 1 |
| -501.798 | 19535.738 | 1.755 | 13.932 | 0.188536 | 7/15/2014 | 58:08.1 | 1 |
| -502.036 | 19535.758 | 1.755 | 13.935 | 0.236499 | 7/15/2014 | 58:08.2 | 1 |
| -502.272 | 19535.774 | 1.752 | 13.94 | 0.238446 | 7/15/2014 | 58:08.3 | 1 |
| -502.685 | 19535.807 | 1.75 | 13.952 | 0.239955 | 7/15/2014 | 58:08.4 | 1 |
| -502.921 | 19535.826 | 1.752 | 13.961 | 0.184166 | 7/15/2014 | 58:08.5 | 1 |
| -503.158 | 19535.844 | 1.752 | 13.971 | 0.202911 | 7/15/2014 | 58:08.6 | 1 |
| -503.454 | 19535.867 | 1.749 | 13.982 | 0.227928 | 7/15/2014 | 58:08.7 | 1 |
| -503.574 | 19535.877 | 1.745 | 13.987 | 0.201028 | 7/15/2014 | 58:08.8 | 1 |
| -503.814 | 19535.896 | 1.741 | 13.999 | 0.187002 | 7/15/2014 | 58:08.9 | 1 |
| -504.053 | 19535.913 | 1.744 | 14.011 | 0.196689 | 7/15/2014 | 58:09.0 | 1 |
| -504.295 | 19535.934 | 1.75 | 14.023 | 0.219808 | 7/15/2014 | 58:09.1 | 1 |
| -504.533 | 19535.953 | 1.744 | 14.035 | 0.257593 | 7/15/2014 | 58:09.2 | 1 |
| -504.775 | 19535.973 | 1.747 | 14.047 | 0.240052 | 7/15/2014 | 58:09.3 | 1 |
| -505.194 | 19536.006 | 1.746 | 14.065 | 0.249931 | 7/15/2014 | 58:09.4 | 1 |
| -505.433 | 19536.025 | 1.744 | 14.078 | 0.258659 | 7/15/2014 | 58:09.5 | 1 |
| -505.672 | 19536.044 | 1.744 | 14.091 | 0.267611 | 7/15/2014 | 58:09.6 | 1 |
| -505.971 | 19536.07 | 1.738 | 14.105 | 0.250858 | 7/15/2014 | 58:09.7 | 1 |
| -506.091 | 19536.08 | 1.733 | 14.111 | 0.242857 | 7/15/2014 | 58:09.8 | 1 |
| -506.333 | 19536.1 | 1.735 | 14.125 | 0.258462 | 7/15/2014 | 58:09.9 | 1 |
| -506.573 | 19536.119 | 1.731 | 14.139 | 0.226757 | 7/15/2014 | 58:10.0 | 1 |
| -506.815 | 19536.139 | 1.731 | 14.154 | 0.215186 | 7/15/2014 | 58:10.1 | 1 |
| -507.052 | 19536.158 | 1.725 | 14.167 | 0.231569 | 7/15/2014 | 58:10.2 | 1 |
| -507.293 | 19536.18 | 1.731 | 14.181 | 0.211974 | 7/15/2014 | 58:10.3 | 1 |
| -507.709 | 19536.215 | 1.731 | 14.199 | 0.207907 | 7/15/2014 | 58:10.4 | 1 |
| -507.944 | 19536.234 | 1.736 | 14.21 | 0.20296 | 7/15/2014 | 58:10.5 | 1 |
| -508.182 | 19536.255 | 1.743 | 14.219 | 0.232174 | 7/15/2014 | 58:10.6 | 1 |
| -508.479 | 19536.279 | 1.744 | 14.227 | 0.19839 | 7/15/2014 | 58:10.7 | 1 |

| | | | | | | | |
|---|---|---|---|---|---|---|---|
| -508.597 | 19536.289 | 1.745 | 14.232 | 0.176484 | 7/15/2014 | 58:10.8 | 1 |
| -508.836 | 19536.311 | 1.755 | 14.237 | 0.201996 | 7/15/2014 | 58:10.9 | 1 |
| -509.072 | 19536.332 | 1.755 | 14.239 | 0.20423 | 7/15/2014 | 58:11.0 | 1 |
| -509.311 | 19536.352 | 1.762 | 14.239 | 0.205134 | 7/15/2014 | 58:11.1 | 1 |
| -509.547 | 19536.371 | 1.766 | 14.235 | 0.186371 | 7/15/2014 | 58:11.2 | 1 |
| -509.785 | 19536.393 | 1.766 | 14.225 | 0.167129 | 7/15/2014 | 58:11.3 | 1 |
| -510.198 | 19536.428 | 1.764 | 14.197 | 0.178688 | 7/15/2014 | 58:11.4 | 1 |
| -510.433 | 19536.447 | 1.766 | 14.173 | 0.183516 | 7/15/2014 | 58:11.5 | 1 |
| -510.669 | 19536.47 | 1.763 | 14.143 | 0.152232 | 7/15/2014 | 58:11.6 | 1 |
| -510.965 | 19536.494 | 1.763 | 14.107 | 0.161887 | 7/15/2014 | 58:11.7 | 1 |
| -511.084 | 19536.504 | 1.761 | 14.09 | 0.169113 | 7/15/2014 | 58:11.8 | 1 |
| -511.322 | 19536.526 | 1.757 | 14.046 | 0.152282 | 7/15/2014 | 58:11.9 | 1 |
| -511.56 | 19536.547 | 1.753 | 13.999 | 0.17151 | 7/15/2014 | 58:12.0 | 1 |
| -511.801 | 19536.566 | 1.757 | 13.95 | 0.177598 | 7/15/2014 | 58:12.1 | 1 |
| -512.041 | 19536.587 | 1.757 | 13.903 | 0.14643 | 7/15/2014 | 58:12.2 | 1 |
| -512.281 | 19536.608 | 1.761 | 13.857 | 0.172235 | 7/15/2014 | 58:12.3 | 1 |
| -512.702 | 19536.645 | 1.763 | 13.8 | 0.15747 | 7/15/2014 | 58:12.4 | 1 |
| -512.943 | 19536.666 | 1.766 | 13.766 | 0.164076 | 7/15/2014 | 58:12.5 | 1 |
| -513.182 | 19536.688 | 1.769 | 13.738 | 0.167173 | 7/15/2014 | 58:12.6 | 1 |
| -513.486 | 19536.713 | 1.775 | 13.716 | 0.150814 | 7/15/2014 | 58:12.7 | 1 |
| -513.608 | 19536.724 | 1.775 | 13.703 | 0.166119 | 7/15/2014 | 58:12.8 | 1 |
| -513.853 | 19536.744 | 1.777 | 13.689 | 0.175258 | 7/15/2014 | 58:12.9 | 1 |
| -514.097 | 19536.767 | 1.782 | 13.681 | 0.184667 | 7/15/2014 | 58:13.0 | 1 |
| -514.343 | 19536.787 | 1.784 | 13.676 | 0.160922 | 7/15/2014 | 58:13.1 | 1 |
| -514.588 | 19536.809 | 1.784 | 13.675 | 0.161632 | 7/15/2014 | 58:13.2 | 1 |
| -514.834 | 19536.83 | 1.789 | 13.676 | 0.173841 | 7/15/2014 | 58:13.3 | 1 |
| -515.264 | 19536.867 | 1.786 | 13.68 | 0.168745 | 7/15/2014 | 58:13.4 | 1 |
| -515.51 | 19536.889 | 1.786 | 13.684 | 0.172817 | 7/15/2014 | 58:13.5 | 1 |
| -515.757 | 19536.91 | 1.791 | 13.687 | 0.179731 | 7/15/2014 | 58:13.6 | 1 |
| -516.067 | 19536.936 | 1.789 | 13.69 | 0.180205 | 7/15/2014 | 58:13.7 | 1 |
| -516.19 | 19536.947 | 1.791 | 13.692 | 0.186804 | 7/15/2014 | 58:13.8 | 1 |
| -516.441 | 19536.968 | 1.794 | 13.694 | 0.182389 | 7/15/2014 | 58:13.9 | 1 |
| -516.691 | 19536.99 | 1.791 | 13.697 | 0.1892 | 7/15/2014 | 58:14.0 | 1 |
| -516.942 | 19537.012 | 1.8 | 13.701 | 0.193885 | 7/15/2014 | 58:14.1 | 1 |
| -517.192 | 19537.033 | 1.8 | 13.705 | 0.186639 | 7/15/2014 | 58:14.2 | 1 |
| -517.444 | 19537.054 | 1.805 | 13.71 | 0.153132 | 7/15/2014 | 58:14.3 | 1 |
| -517.755 | 19537.08 | 1.797 | 13.716 | 0.174643 | 7/15/2014 | 58:14.4 | 1 |
| -518.007 | 19537.102 | 1.797 | 13.723 | 0.158774 | 7/15/2014 | 58:14.5 | 1 |
| -518.255 | 19537.123 | 1.795 | 13.732 | 0.15048 | 7/15/2014 | 58:14.6 | 1 |
| -518.506 | 19537.145 | 1.797 | 13.741 | 0.158376 | 7/15/2014 | 58:14.7 | 1 |
| -518.813 | 19537.17 | 1.793 | 13.753 | 0.149616 | 7/15/2014 | 58:14.8 | 1 |
| -519.062 | 19537.19 | 1.793 | 13.765 | 0.162484 | 7/15/2014 | 58:14.9 | 1 |
| -519.309 | 19537.211 | 1.793 | 13.778 | 0.157258 | 7/15/2014 | 58:15.0 | 1 |
| -519.555 | 19537.231 | 1.797 | 13.792 | 0.173812 | 7/15/2014 | 58:15.1 | 1 |
| -519.798 | 19537.253 | 1.797 | 13.807 | 0.169001 | 7/15/2014 | 58:15.2 | 1 |
| -520.043 | 19537.273 | 1.802 | 13.823 | 0.157711 | 7/15/2014 | 58:15.3 | 1 |
| -520.463 | 19537.309 | 1.801 | 13.846 | 0.168394 | 7/15/2014 | 58:15.4 | 1 |

| | | | | | | | |
|---|---|---|---|---|---|---|---|
| -520.703 | 19537.328 | 1.803 | 13.861 | 0.169837 | 7/15/2014 | 58:15.5 | 1 |
| -520.943 | 19537.348 | 1.806 | 13.876 | 0.17628 | 7/15/2014 | 58:15.6 | 1 |
| -521.24 | 19537.371 | 1.803 | 13.89 | 0.169306 | 7/15/2014 | 58:15.7 | 1 |
| -521.36 | 19537.382 | 1.802 | 13.898 | 0.174508 | 7/15/2014 | 58:15.8 | 1 |
| -521.603 | 19537.402 | 1.807 | 13.911 | 0.179388 | 7/15/2014 | 58:15.9 | 1 |
| -521.842 | 19537.422 | 1.807 | 13.923 | 0.173684 | 7/15/2014 | 58:16.0 | 1 |
| -522.084 | 19537.441 | 1.81 | 13.934 | 0.185443 | 7/15/2014 | 58:16.1 | 1 |
| -522.324 | 19537.461 | 1.81 | 13.943 | 0.189687 | 7/15/2014 | 58:16.2 | 1 |
| -522.565 | 19537.48 | 1.812 | 13.95 | 0.184143 | 7/15/2014 | 58:16.3 | 1 |
| -522.986 | 19537.514 | 1.816 | 13.952 | 0.191527 | 7/15/2014 | 58:16.4 | 1 |
| -523.227 | 19537.533 | 1.82 | 13.95 | 0.181555 | 7/15/2014 | 58:16.5 | 1 |
| -523.467 | 19537.552 | 1.82 | 13.944 | 0.175028 | 7/15/2014 | 58:16.6 | 1 |
| -523.768 | 19537.575 | 1.822 | 13.932 | 0.168938 | 7/15/2014 | 58:16.7 | 1 |
| -523.89 | 19537.585 | 1.824 | 13.928 | 0.17015 | 7/15/2014 | 58:16.8 | 1 |
| -524.133 | 19537.605 | 1.832 | 13.907 | 0.166443 | 7/15/2014 | 58:16.9 | 1 |
| -524.375 | 19537.623 | 1.838 | 13.88 | 0.163822 | 7/15/2014 | 58:17.0 | 1 |
| -524.619 | 19537.643 | 1.843 | 13.847 | 0.161554 | 7/15/2014 | 58:17.1 | 1 |
| -524.86 | 19537.662 | 1.838 | 13.807 | 0.177889 | 7/15/2014 | 58:17.2 | 1 |
| -525.102 | 19537.681 | 1.841 | 13.763 | 0.160557 | 7/15/2014 | 58:17.3 | 1 |
| -525.524 | 19537.713 | 1.844 | 13.691 | 0.169092 | 7/15/2014 | 58:17.4 | 1 |
| -525.764 | 19537.732 | 1.85 | 13.643 | 0.171491 | 7/15/2014 | 58:17.5 | 1 |
| -526.005 | 19537.75 | 1.85 | 13.596 | 0.167968 | 7/15/2014 | 58:17.6 | 1 |
| -526.307 | 19537.773 | 1.849 | 13.552 | 0.157667 | 7/15/2014 | 58:17.7 | 1 |
| -526.428 | 19537.783 | 1.849 | 13.527 | 0.154474 | 7/15/2014 | 58:17.8 | 1 |
| -526.671 | 19537.803 | 1.858 | 13.488 | 0.169411 | 7/15/2014 | 58:17.9 | 1 |
| -526.913 | 19537.819 | 1.858 | 13.454 | 0.157219 | 7/15/2014 | 58:18.0 | 1 |
| -527.157 | 19537.839 | 1.864 | 13.427 | 0.157718 | 7/15/2014 | 58:18.1 | 1 |
| -527.398 | 19537.856 | 1.858 | 13.406 | 0.162508 | 7/15/2014 | 58:18.2 | 1 |
| -527.642 | 19537.876 | 1.858 | 13.392 | 0.164088 | 7/15/2014 | 58:18.3 | 1 |
| -528.064 | 19537.907 | 1.859 | 13.388 | 0.16117 | 7/15/2014 | 58:18.4 | 1 |
| -528.305 | 19537.926 | 1.859 | 13.39 | 0.161183 | 7/15/2014 | 58:18.5 | 1 |
| -528.545 | 19537.943 | 1.856 | 13.398 | 0.164592 | 7/15/2014 | 58:18.6 | 1 |
| -528.845 | 19537.965 | 1.856 | 13.411 | 0.158965 | 7/15/2014 | 58:18.7 | 1 |
| -528.967 | 19537.974 | 1.854 | 13.416 | 0.154616 | 7/15/2014 | 58:18.8 | 1 |
| -529.209 | 19537.993 | 1.86 | 13.437 | 0.15643 | 7/15/2014 | 58:18.9 | 1 |
| -529.451 | 19538.01 | 1.86 | 13.46 | 0.16655 | 7/15/2014 | 58:19.0 | 1 |
| -529.692 | 19538.028 | 1.86 | 13.485 | 0.18498 | 7/15/2014 | 58:19.1 | 1 |
| -529.932 | 19538.045 | 1.856 | 13.512 | 0.164843 | 7/15/2014 | 58:19.2 | 1 |
| -530.171 | 19538.063 | 1.86 | 13.538 | 0.167137 | 7/15/2014 | 58:19.3 | 1 |
| -530.587 | 19538.093 | 1.861 | 13.573 | 0.187399 | 7/15/2014 | 58:19.4 | 1 |
| -530.823 | 19538.111 | 1.862 | 13.594 | 0.1765 | 7/15/2014 | 58:19.5 | 1 |
| -531.06 | 19538.127 | 1.859 | 13.612 | 0.170631 | 7/15/2014 | 58:19.6 | 1 |
| -531.354 | 19538.149 | 1.854 | 13.626 | 0.159344 | 7/15/2014 | 58:19.7 | 1 |
| -531.474 | 19538.157 | 1.852 | 13.634 | 0.169306 | 7/15/2014 | 58:19.8 | 1 |
| -531.711 | 19538.175 | 1.852 | 13.641 | 0.157765 | 7/15/2014 | 58:19.9 | 1 |
| -531.948 | 19538.191 | 1.846 | 13.643 | 0.169204 | 7/15/2014 | 58:20.0 | 1 |
| -532.185 | 19538.209 | 1.849 | 13.638 | 0.150872 | 7/15/2014 | 58:20.1 | 1 |

| | | | | | | | |
|---|---|---|---|---|---|---|---|
| -532.419 | 19538.227 | 1.843 | 13.627 | 0.148769 | 7/15/2014 | 58:20.2 | 1 |
| -532.655 | 19538.242 | 1.842 | 13.61 | 0.148412 | 7/15/2014 | 58:20.3 | 1 |
| -533.064 | 19538.271 | 1.838 | 13.57 | 0.154391 | 7/15/2014 | 58:20.4 | 1 |
| -533.297 | 19538.288 | 1.836 | 13.54 | 0.165787 | 7/15/2014 | 58:20.5 | 1 |
| -533.531 | 19538.305 | 1.836 | 13.508 | 0.162572 | 7/15/2014 | 58:20.6 | 1 |
| -533.822 | 19538.325 | 1.834 | 13.474 | 0.165683 | 7/15/2014 | 58:20.7 | 1 |
| -533.94 | 19538.334 | 1.829 | 13.456 | 0.175103 | 7/15/2014 | 58:20.8 | 1 |
| -534.176 | 19538.35 | 1.831 | 13.421 | 0.177043 | 7/15/2014 | 58:20.9 | 1 |
| -534.41 | 19538.366 | 1.829 | 13.39 | 0.170116 | 7/15/2014 | 58:21.0 | 1 |
| -534.647 | 19538.383 | 1.831 | 13.363 | 0.168617 | 7/15/2014 | 58:21.1 | 1 |
| -534.88 | 19538.399 | 1.826 | 13.342 | 0.164102 | 7/15/2014 | 58:21.2 | 1 |
| -535.115 | 19538.416 | 1.826 | 13.327 | 0.166245 | 7/15/2014 | 58:21.3 | 1 |
| -535.524 | 19538.443 | 1.82 | 13.319 | 0.163905 | 7/15/2014 | 58:21.4 | 1 |
| -535.758 | 19538.459 | 1.82 | 13.317 | 0.165824 | 7/15/2014 | 58:21.5 | 1 |
| -535.991 | 19538.476 | 1.818 | 13.322 | 0.160913 | 7/15/2014 | 58:21.6 | 1 |
| -536.282 | 19538.495 | 1.815 | 13.331 | 0.156668 | 7/15/2014 | 58:21.7 | 1 |
| -536.399 | 19538.504 | 1.813 | 13.335 | 0.153414 | 7/15/2014 | 58:21.8 | 1 |
| -536.634 | 19538.521 | 1.811 | 13.35 | 0.15186 | 7/15/2014 | 58:21.9 | 1 |
| -536.868 | 19538.535 | 1.806 | 13.368 | 0.151925 | 7/15/2014 | 58:22.0 | 1 |
| -537.104 | 19538.552 | 1.806 | 13.388 | 0.151435 | 7/15/2014 | 58:22.1 | 1 |
| -537.337 | 19538.567 | 1.795 | 13.408 | 0.150386 | 7/15/2014 | 58:22.2 | 1 |
| -537.572 | 19538.583 | 1.789 | 13.43 | 0.149851 | 7/15/2014 | 58:22.3 | 1 |
| -537.978 | 19538.61 | 1.778 | 13.46 | 0.155421 | 7/15/2014 | 58:22.4 | 1 |
| -538.209 | 19538.626 | 1.771 | 13.479 | 0.157972 | 7/15/2014 | 58:22.5 | 1 |
| -538.44 | 19538.641 | 1.769 | 13.496 | 0.156066 | 7/15/2014 | 58:22.6 | 1 |
| -538.732 | 19538.661 | 1.767 | 13.512 | 0.155329 | 7/15/2014 | 58:22.7 | 1 |
| -538.848 | 19538.669 | 1.764 | 13.521 | 0.156139 | 7/15/2014 | 58:22.8 | 1 |
| -539.082 | 19538.684 | 1.766 | 13.534 | 0.155022 | 7/15/2014 | 58:22.9 | 1 |
| -539.314 | 19538.699 | 1.764 | 13.546 | 0.165637 | 7/15/2014 | 58:23.0 | 1 |
| -539.55 | 19538.715 | 1.769 | 13.556 | 0.145025 | 7/15/2014 | 58:23.1 | 1 |
| -539.781 | 19538.729 | 1.762 | 13.565 | 0.156201 | 7/15/2014 | 58:23.2 | 1 |
| -540.014 | 19538.744 | 1.766 | 13.573 | 0.147707 | 7/15/2014 | 58:23.3 | 1 |
| -540.422 | 19538.771 | 1.759 | 13.585 | 0.142571 | 7/15/2014 | 58:23.4 | 1 |
| -540.654 | 19538.785 | 1.755 | 13.593 | 0.152816 | 7/15/2014 | 58:23.5 | 1 |
| -540.885 | 19538.8 | 1.75 | 13.602 | 0.143619 | 7/15/2014 | 58:23.6 | 1 |
| -541.176 | 19538.819 | 1.748 | 13.611 | 0.153747 | 7/15/2014 | 58:23.7 | 1 |
| -541.293 | 19538.827 | 1.745 | 13.615 | 0.145758 | 7/15/2014 | 58:23.8 | 1 |
| -541.527 | 19538.841 | 1.745 | 13.625 | 0.15752 | 7/15/2014 | 58:23.9 | 1 |
| -541.761 | 19538.855 | 1.738 | 13.636 | 0.168822 | 7/15/2014 | 58:24.0 | 1 |
| -541.997 | 19538.87 | 1.738 | 13.647 | 0.18662 | 7/15/2014 | 58:24.1 | 1 |
| -542.232 | 19538.884 | 1.738 | 13.66 | 0.18375 | 7/15/2014 | 58:24.2 | 1 |
| -542.466 | 19538.898 | 1.744 | 13.673 | 0.181534 | 7/15/2014 | 58:24.3 | 1 |
| -542.877 | 19538.924 | 1.744 | 13.696 | 0.185785 | 7/15/2014 | 58:24.4 | 1 |
| -543.11 | 19538.938 | 1.737 | 13.712 | 0.175134 | 7/15/2014 | 58:24.5 | 1 |
| -543.344 | 19538.952 | 1.732 | 13.73 | 0.172966 | 7/15/2014 | 58:24.6 | 1 |
| -543.639 | 19538.969 | 1.73 | 13.749 | 0.170983 | 7/15/2014 | 58:24.7 | 1 |
| -543.758 | 19538.976 | 1.727 | 13.758 | 0.168429 | 7/15/2014 | 58:24.8 | 1 |

| | | | | | | | |
|---|---|---|---|---|---|---|---|
| -543.997 | 19538.99 | 1.727 | 13.779 | 0.181457 | 7/15/2014 | 58:24.9 | 1 |
| -544.234 | 19539.005 | 1.713 | 13.8 | 0.177655 | 7/15/2014 | 58:25.0 | 1 |
| -544.475 | 19539.018 | 1.717 | 13.822 | 0.193303 | 7/15/2014 | 58:25.1 | 1 |
| -544.712 | 19539.033 | 1.711 | 13.844 | 0.209999 | 7/15/2014 | 58:25.2 | 1 |
| -544.952 | 19539.047 | 1.717 | 13.866 | 0.203877 | 7/15/2014 | 58:25.3 | 1 |
| -545.372 | 19539.07 | 1.719 | 13.895 | 0.231513 | 7/15/2014 | 58:25.4 | 1 |
| -545.612 | 19539.084 | 1.714 | 13.914 | 0.216842 | 7/15/2014 | 58:25.5 | 1 |
| -545.85 | 19539.099 | 1.711 | 13.931 | 0.226263 | 7/15/2014 | 58:25.6 | 1 |
| -546.151 | 19539.114 | 1.712 | 13.945 | 0.203734 | 7/15/2014 | 58:25.7 | 1 |
| -546.272 | 19539.122 | 1.706 | 13.953 | 0.20255 | 7/15/2014 | 58:25.8 | 1 |
| -546.516 | 19539.136 | 1.71 | 13.963 | 0.213069 | 7/15/2014 | 58:25.9 | 1 |
| -546.758 | 19539.149 | 1.701 | 13.97 | 0.208724 | 7/15/2014 | 58:26.0 | 1 |
| -547.002 | 19539.163 | 1.695 | 13.973 | 0.208749 | 7/15/2014 | 58:26.1 | 1 |
| -547.244 | 19539.176 | 1.685 | 13.97 | 0.209573 | 7/15/2014 | 58:26.2 | 1 |
| -547.486 | 19539.19 | 1.688 | 13.961 | 0.212745 | 7/15/2014 | 58:26.3 | 1 |
| -547.908 | 19539.214 | 1.687 | 13.929 | 0.213333 | 7/15/2014 | 58:26.4 | 1 |
| -548.149 | 19539.227 | 1.687 | 13.901 | 0.212774 | 7/15/2014 | 58:26.5 | 1 |
| -548.388 | 19539.24 | 1.681 | 13.866 | 0.212957 | 7/15/2014 | 58:26.6 | 1 |
| -548.687 | 19539.258 | 1.683 | 13.825 | 0.208657 | 7/15/2014 | 58:26.7 | 1 |
| -548.809 | 19539.264 | 1.685 | 13.806 | 0.209658 | 7/15/2014 | 58:26.8 | 1 |
| -549.049 | 19539.277 | 1.688 | 13.756 | 0.206771 | 7/15/2014 | 58:26.9 | 1 |
| -549.287 | 19539.29 | 1.683 | 13.703 | 0.20164 | 7/15/2014 | 58:27.0 | 1 |
| -549.527 | 19539.305 | 1.681 | 13.65 | 0.197258 | 7/15/2014 | 58:27.1 | 1 |
| -549.763 | 19539.316 | 1.674 | 13.597 | 0.19585 | 7/15/2014 | 58:27.2 | 1 |
| -550 | 19539.331 | 1.677 | 13.547 | 0.196738 | 7/15/2014 | 58:27.3 | 1 |
| -550.41 | 19539.354 | 1.681 | 13.482 | 0.192465 | 7/15/2014 | 58:27.4 | 1 |
| -550.643 | 19539.367 | 1.684 | 13.442 | 0.191586 | 7/15/2014 | 58:27.5 | 1 |
| -550.874 | 19539.38 | 1.687 | 13.408 | 0.188875 | 7/15/2014 | 58:27.6 | 1 |
| -551.164 | 19539.396 | 1.695 | 13.379 | 0.190262 | 7/15/2014 | 58:27.7 | 1 |
| -551.282 | 19539.402 | 1.699 | 13.364 | 0.192097 | 7/15/2014 | 58:27.8 | 1 |
| -551.515 | 19539.418 | 1.701 | 13.343 | 0.17854 | 7/15/2014 | 58:27.9 | 1 |
| -551.863 | 19539.437 | 1.702 | 13.324 | 0.182478 | 7/15/2014 | 58:28.0 | 1 |
| -552.096 | 19539.45 | 1.702 | 13.315 | 0.175212 | 7/15/2014 | 58:28.1 | 1 |
| -552.328 | 19539.463 | 1.707 | 13.309 | 0.179453 | 7/15/2014 | 58:28.2 | 1 |
| -552.443 | 19539.469 | 1.707 | 13.306 | 0.179194 | 7/15/2014 | 58:28.3 | 1 |
| -552.847 | 19539.492 | 1.704 | 13.307 | 0.171154 | 7/15/2014 | 58:28.4 | 1 |
| -553.075 | 19539.508 | 1.701 | 13.31 | 0.183939 | 7/15/2014 | 58:28.5 | 1 |
| -553.306 | 19539.52 | 1.704 | 13.316 | 0.188623 | 7/15/2014 | 58:28.6 | 1 |
| -553.594 | 19539.536 | 1.704 | 13.323 | 0.189486 | 7/15/2014 | 58:28.7 | 1 |
| -553.71 | 19539.543 | 1.704 | 13.327 | 0.197731 | 7/15/2014 | 58:28.8 | 1 |
| -553.944 | 19539.557 | 1.711 | 13.337 | 0.194403 | 7/15/2014 | 58:28.9 | 1 |
| -554.175 | 19539.57 | 1.705 | 13.349 | 0.208084 | 7/15/2014 | 58:29.0 | 1 |
| -554.409 | 19539.584 | 1.709 | 13.362 | 0.207235 | 7/15/2014 | 58:29.1 | 1 |
| -554.642 | 19539.599 | 1.709 | 13.377 | 0.194857 | 7/15/2014 | 58:29.2 | 1 |
| -554.875 | 19539.612 | 1.712 | 13.392 | 0.187338 | 7/15/2014 | 58:29.3 | 1 |
| -555.284 | 19539.637 | 1.712 | 13.415 | 0.174271 | 7/15/2014 | 58:29.4 | 1 |
| -555.519 | 19539.65 | 1.712 | 13.43 | 0.174078 | 7/15/2014 | 58:29.5 | 1 |

| | | | | | | | |
|---|---|---|---|---|---|---|---|
| -555.753 | 19539.664 | 1.714 | 13.445 | 0.173312 | 7/15/2014 | 58:29.6 | 1 |
| -556.045 | 19539.683 | 1.714 | 13.46 | 0.169092 | 7/15/2014 | 58:29.7 | 1 |
| -556.164 | 19539.689 | 1.714 | 13.467 | 0.164249 | 7/15/2014 | 58:29.8 | 1 |
| -556.402 | 19539.704 | 1.72 | 13.48 | 0.166168 | 7/15/2014 | 58:29.9 | 1 |
| -556.638 | 19539.719 | 1.723 | 13.492 | 0.168563 | 7/15/2014 | 58:30.0 | 1 |
| -556.876 | 19539.734 | 1.725 | 13.502 | 0.170134 | 7/15/2014 | 58:30.1 | 1 |
| -557.113 | 19539.747 | 1.725 | 13.512 | 0.168104 | 7/15/2014 | 58:30.2 | 1 |
| -557.353 | 19539.763 | 1.725 | 13.521 | 0.173549 | 7/15/2014 | 58:30.3 | 1 |
| -557.766 | 19539.788 | 1.72 | 13.532 | 0.165878 | 7/15/2014 | 58:30.4 | 1 |
| -558.002 | 19539.803 | 1.715 | 13.539 | 0.163779 | 7/15/2014 | 58:30.5 | 1 |
| -558.238 | 19539.817 | 1.71 | 13.546 | 0.161796 | 7/15/2014 | 58:30.6 | 1 |
| -558.535 | 19539.837 | 1.708 | 13.552 | 0.157509 | 7/15/2014 | 58:30.7 | 1 |
| -558.655 | 19539.844 | 1.705 | 13.555 | 0.153062 | 7/15/2014 | 58:30.8 | 1 |
| -558.896 | 19539.858 | 1.709 | 13.561 | 0.154431 | 7/15/2014 | 58:30.9 | 1 |
| -559.133 | 19539.873 | 1.705 | 13.566 | 0.1548 | 7/15/2014 | 58:31.0 | 1 |
| -559.375 | 19539.889 | 1.709 | 13.57 | 0.144987 | 7/15/2014 | 58:31.1 | 1 |
| -559.612 | 19539.904 | 1.705 | 13.574 | 0.139869 | 7/15/2014 | 58:31.2 | 1 |
| -559.853 | 19539.919 | 1.715 | 13.576 | 0.140102 | 7/15/2014 | 58:31.3 | 1 |
| -560.273 | 19539.946 | 1.714 | 13.577 | 0.146234 | 7/15/2014 | 58:31.4 | 1 |
| -560.512 | 19539.961 | 1.716 | 13.577 | 0.134039 | 7/15/2014 | 58:31.5 | 1 |
| -560.752 | 19539.977 | 1.716 | 13.574 | 0.138205 | 7/15/2014 | 58:31.6 | 1 |
| -561.052 | 19539.996 | 1.716 | 13.57 | 0.134189 | 7/15/2014 | 58:31.7 | 1 |
| -561.172 | 19540.004 | 1.714 | 13.568 | 0.134313 | 7/15/2014 | 58:31.8 | 1 |
| -561.417 | 19540.021 | 1.714 | 13.561 | 0.126252 | 7/15/2014 | 58:31.9 | 1 |
| -561.657 | 19540.036 | 1.71 | 13.551 | 0.132596 | 7/15/2014 | 58:32.0 | 1 |
| -561.902 | 19540.052 | 1.724 | 13.537 | 0.120181 | 7/15/2014 | 58:32.1 | 1 |
| -562.142 | 19540.068 | 1.722 | 13.52 | 0.11443 | 7/15/2014 | 58:32.2 | 1 |
| -562.385 | 19540.084 | 1.722 | 13.5 | 0.108847 | 7/15/2014 | 58:32.3 | 1 |
| -562.805 | 19540.111 | 1.714 | 13.459 | 0.110175 | 7/15/2014 | 58:32.4 | 1 |
| -563.046 | 19540.128 | 1.719 | 13.43 | 0.107616 | 7/15/2014 | 58:32.5 | 1 |
| -563.286 | 19540.145 | 1.722 | 13.398 | 0.106337 | 7/15/2014 | 58:32.6 | 1 |
| -563.584 | 19540.164 | 1.725 | 13.365 | 0.108791 | 7/15/2014 | 58:32.7 | 1 |
| -563.704 | 19540.173 | 1.717 | 13.348 | 0.113418 | 7/15/2014 | 58:32.8 | 1 |
| -563.945 | 19540.188 | 1.725 | 13.315 | 0.109103 | 7/15/2014 | 58:32.9 | 1 |
| -564.183 | 19540.205 | 1.725 | 13.283 | 0.110218 | 7/15/2014 | 58:33.0 | 1 |
| -564.424 | 19540.221 | 1.734 | 13.253 | 0.11224 | 7/15/2014 | 58:33.1 | 1 |
| -564.66 | 19540.236 | 1.74 | 13.227 | 0.110917 | 7/15/2014 | 58:33.2 | 1 |
| -564.9 | 19540.253 | 1.744 | 13.204 | 0.114546 | 7/15/2014 | 58:33.3 | 1 |
| -565.311 | 19540.281 | 1.744 | 13.18 | 0.1169 | 7/15/2014 | 58:33.4 | 1 |
| -565.546 | 19540.298 | 1.742 | 13.167 | 0.121657 | 7/15/2014 | 58:33.5 | 1 |
| -565.78 | 19540.314 | 1.739 | 13.158 | 0.117437 | 7/15/2014 | 58:33.6 | 1 |
| -566.073 | 19540.334 | 1.739 | 13.152 | 0.118386 | 7/15/2014 | 58:33.7 | 1 |
| -566.191 | 19540.342 | 1.733 | 13.148 | 0.118971 | 7/15/2014 | 58:33.8 | 1 |
| -566.429 | 19540.358 | 1.741 | 13.148 | 0.116739 | 7/15/2014 | 58:33.9 | 1 |
| -566.663 | 19540.374 | 1.741 | 13.15 | 0.120055 | 7/15/2014 | 58:34.0 | 1 |
| -566.9 | 19540.392 | 1.748 | 13.154 | 0.117887 | 7/15/2014 | 58:34.1 | 1 |
| -567.133 | 19540.409 | 1.742 | 13.161 | 0.115383 | 7/15/2014 | 58:34.2 | 1 |

| | | | | | | | |
|---|---|---|---|---|---|---|---|
| -567.37 | 19540.426 | 1.752 | 13.169 | 0.118977 | 7/15/2014 | 58:34.3 | 1 |
| -567.779 | 19540.454 | 1.749 | 13.186 | 0.124396 | 7/15/2014 | 58:34.4 | 1 |
| -568.014 | 19540.472 | 1.756 | 13.197 | 0.116393 | 7/15/2014 | 58:34.5 | 1 |
| -568.248 | 19540.488 | 1.756 | 13.211 | 0.120472 | 7/15/2014 | 58:34.6 | 1 |
| -568.542 | 19540.51 | 1.756 | 13.225 | 0.116092 | 7/15/2014 | 58:34.7 | 1 |
| -568.659 | 19540.519 | 1.753 | 13.232 | 0.116073 | 7/15/2014 | 58:34.8 | 1 |
| -568.897 | 19540.536 | 1.759 | 13.247 | 0.117381 | 7/15/2014 | 58:34.9 | 1 |
| -569.134 | 19540.553 | 1.749 | 13.264 | 0.130729 | 7/15/2014 | 58:35.0 | 1 |
| -569.372 | 19540.57 | 1.752 | 13.281 | 0.128381 | 7/15/2014 | 58:35.1 | 1 |
| -569.609 | 19540.588 | 1.748 | 13.298 | 0.124399 | 7/15/2014 | 58:35.2 | 1 |
| -569.848 | 19540.606 | 1.748 | 13.316 | 0.124636 | 7/15/2014 | 58:35.3 | 1 |
| -570.265 | 19540.637 | 1.737 | 13.342 | 0.126105 | 7/15/2014 | 58:35.4 | 1 |
| -570.502 | 19540.654 | 1.736 | 13.359 | 0.120829 | 7/15/2014 | 58:35.5 | 1 |
| -570.74 | 19540.672 | 1.736 | 13.376 | 0.101453 | 7/15/2014 | 58:35.6 | 1 |
| -571.039 | 19540.694 | 1.733 | 13.391 | 0.0978249 | 7/15/2014 | 58:35.7 | 1 |
| -571.159 | 19540.703 | 1.731 | 13.399 | 0.103949 | 7/15/2014 | 58:35.8 | 1 |
| -571.401 | 19540.722 | 1.728 | 13.413 | 0.108691 | 7/15/2014 | 58:35.9 | 1 |
| -571.641 | 19540.74 | 1.718 | 13.427 | 0.0970043 | 7/15/2014 | 58:36.0 | 1 |
| -571.884 | 19540.758 | 1.728 | 13.439 | 0.0992185 | 7/15/2014 | 58:36.1 | 1 |
| -572.123 | 19540.775 | 1.725 | 13.451 | 0.10933 | 7/15/2014 | 58:36.2 | 1 |
| -572.365 | 19540.793 | 1.725 | 13.463 | 0.101212 | 7/15/2014 | 58:36.3 | 1 |
| -572.784 | 19540.826 | 1.723 | 13.481 | 0.103997 | 7/15/2014 | 58:36.4 | 1 |
| -573.022 | 19540.844 | 1.731 | 13.493 | 0.101471 | 7/15/2014 | 58:36.5 | 1 |
| -573.262 | 19540.862 | 1.736 | 13.506 | 0.105422 | 7/15/2014 | 58:36.6 | 1 |
| -573.56 | 19540.885 | 1.739 | 13.519 | 0.108587 | 7/15/2014 | 58:36.7 | 1 |
| -573.68 | 19540.894 | 1.737 | 13.525 | 0.113482 | 7/15/2014 | 58:36.8 | 1 |
| -573.922 | 19540.912 | 1.741 | 13.539 | 0.115055 | 7/15/2014 | 58:36.9 | 1 |
| -574.159 | 19540.93 | 1.731 | 13.552 | 0.121254 | 7/15/2014 | 58:37.0 | 1 |
| -574.4 | 19540.949 | 1.737 | 13.564 | 0.13127 | 7/15/2014 | 58:37.1 | 1 |
| -574.637 | 19540.968 | 1.737 | 13.575 | 0.132824 | 7/15/2014 | 58:37.2 | 1 |
| -574.876 | 19540.985 | 1.734 | 13.583 | 0.135249 | 7/15/2014 | 58:37.3 | 1 |
| -575.288 | 19541.018 | 1.731 | 13.586 | 0.137768 | 7/15/2014 | 58:37.4 | 1 |
| -575.523 | 19541.036 | 1.729 | 13.583 | 0.138934 | 7/15/2014 | 58:37.5 | 1 |
| -575.758 | 19541.053 | 1.729 | 13.574 | 0.149125 | 7/15/2014 | 58:37.6 | 1 |
| -576.05 | 19541.076 | 1.732 | 13.559 | 0.149698 | 7/15/2014 | 58:37.7 | 1 |
| -576.168 | 19541.086 | 1.73 | 13.554 | 0.144952 | 7/15/2014 | 58:37.8 | 1 |
| -576.405 | 19541.104 | 1.727 | 13.528 | 0.144767 | 7/15/2014 | 58:37.9 | 1 |
| -576.637 | 19541.121 | 1.723 | 13.496 | 0.135131 | 7/15/2014 | 58:38.0 | 1 |
| -576.872 | 19541.141 | 1.723 | 13.458 | 0.133658 | 7/15/2014 | 58:38.1 | 1 |
| -577.105 | 19541.158 | 1.717 | 13.416 | 0.134021 | 7/15/2014 | 58:38.2 | 1 |
| -577.338 | 19541.176 | 1.72 | 13.372 | 0.127014 | 7/15/2014 | 58:38.3 | 1 |
| -577.744 | 19541.208 | 1.716 | 13.306 | 0.12836 | 7/15/2014 | 58:38.4 | 1 |
| -577.975 | 19541.226 | 1.716 | 13.265 | 0.1297 | 7/15/2014 | 58:38.5 | 1 |
| -578.204 | 19541.244 | 1.714 | 13.228 | 0.129254 | 7/15/2014 | 58:38.6 | 1 |
| -578.493 | 19541.268 | 1.714 | 13.195 | 0.122473 | 7/15/2014 | 58:38.7 | 1 |
| -578.61 | 19541.276 | 1.715 | 13.176 | 0.121364 | 7/15/2014 | 58:38.8 | 1 |
| -578.843 | 19541.295 | 1.715 | 13.153 | 0.126434 | 7/15/2014 | 58:38.9 | 1 |

| | | | | | | | |
|---|---|---|---|---|---|---|---|
| -579.076 | 19541.313 | 1.713 | 13.137 | 0.12435 | 7/15/2014 | 58:39.0 | 1 |
| -579.312 | 19541.331 | 1.719 | 13.128 | 0.126047 | 7/15/2014 | 58:39.1 | 1 |
| -579.544 | 19541.35 | 1.721 | 13.128 | 0.117567 | 7/15/2014 | 58:39.2 | 1 |
| -579.777 | 19541.369 | 1.724 | 13.136 | 0.122894 | 7/15/2014 | 58:39.3 | 1 |
| -580.185 | 19541.401 | 1.713 | 13.165 | 0.131609 | 7/15/2014 | 58:39.4 | 1 |
| -580.419 | 19541.42 | 1.708 | 13.19 | 0.135754 | 7/15/2014 | 58:39.5 | 1 |
| -580.652 | 19541.438 | 1.708 | 13.22 | 0.145029 | 7/15/2014 | 58:39.6 | 1 |
| -580.946 | 19541.461 | 1.708 | 13.254 | 0.153388 | 7/15/2014 | 58:39.7 | 1 |
| -581.063 | 19541.471 | 1.702 | 13.271 | 0.161065 | 7/15/2014 | 58:39.8 | 1 |
| -581.299 | 19541.49 | 1.696 | 13.31 | 0.169028 | 7/15/2014 | 58:39.9 | 1 |
| -581.535 | 19541.51 | 1.689 | 13.352 | 0.176272 | 7/15/2014 | 58:40.0 | 1 |
| -581.772 | 19541.528 | 1.689 | 13.395 | 0.185739 | 7/15/2014 | 58:40.1 | 1 |
| -582.008 | 19541.547 | 1.689 | 13.438 | 0.193156 | 7/15/2014 | 58:40.2 | 1 |
| -582.244 | 19541.566 | 1.692 | 13.481 | 0.192564 | 7/15/2014 | 58:40.3 | 1 |
| -582.655 | 19541.6 | 1.686 | 13.544 | 0.193511 | 7/15/2014 | 58:40.4 | 1 |
| -582.89 | 19541.619 | 1.676 | 13.585 | 0.192135 | 7/15/2014 | 58:40.5 | 1 |
| -583.126 | 19541.639 | 1.673 | 13.625 | 0.184761 | 7/15/2014 | 58:40.6 | 1 |
| -583.421 | 19541.662 | 1.668 | 13.663 | 0.173889 | 7/15/2014 | 58:40.7 | 1 |
| -583.54 | 19541.672 | 1.667 | 13.682 | 0.170706 | 7/15/2014 | 58:40.8 | 1 |
| -583.778 | 19541.691 | 1.664 | 13.717 | 0.163564 | 7/15/2014 | 58:40.9 | 1 |
| -584.016 | 19541.711 | 1.656 | 13.75 | 0.173288 | 7/15/2014 | 58:41.0 | 1 |
| -584.254 | 19541.73 | 1.658 | 13.78 | 0.152916 | 7/15/2014 | 58:41.1 | 1 |
| -584.492 | 19541.75 | 1.648 | 13.807 | 0.172022 | 7/15/2014 | 58:41.2 | 1 |
| -584.731 | 19541.77 | 1.648 | 13.829 | 0.15582 | 7/15/2014 | 58:41.3 | 1 |
| -585.149 | 19541.805 | 1.643 | 13.851 | 0.169616 | 7/15/2014 | 58:41.4 | 1 |
| -585.387 | 19541.824 | 1.638 | 13.86 | 0.170077 | 7/15/2014 | 58:41.5 | 1 |
| -585.626 | 19541.844 | 1.64 | 13.863 | 0.1754 | 7/15/2014 | 58:41.6 | 1 |
| -585.924 | 19541.867 | 1.64 | 13.859 | 0.168439 | 7/15/2014 | 58:41.7 | 1 |
| -586.044 | 19541.877 | 1.634 | 13.859 | 0.163009 | 7/15/2014 | 58:41.8 | 1 |
| -586.285 | 19541.896 | 1.636 | 13.845 | 0.176374 | 7/15/2014 | 58:41.9 | 1 |
| -586.526 | 19541.916 | 1.636 | 13.824 | 0.177903 | 7/15/2014 | 58:42.0 | 1 |
| -586.768 | 19541.939 | 1.63 | 13.796 | 0.173305 | 7/15/2014 | 58:42.1 | 1 |
| -587.009 | 19541.959 | 1.626 | 13.762 | 0.179192 | 7/15/2014 | 58:42.2 | 1 |
| -587.251 | 19541.979 | 1.626 | 13.722 | 0.163929 | 7/15/2014 | 58:42.3 | 1 |
| -587.671 | 19542.012 | 1.624 | 13.649 | 0.17585 | 7/15/2014 | 58:42.4 | 1 |
| -587.911 | 19542.031 | 1.622 | 13.598 | 0.170762 | 7/15/2014 | 58:42.5 | 1 |
| -588.15 | 19542.051 | 1.62 | 13.543 | 0.157829 | 7/15/2014 | 58:42.6 | 1 |
| -588.449 | 19542.078 | 1.62 | 13.487 | 0.171763 | 7/15/2014 | 58:42.7 | 1 |
| -588.57 | 19542.088 | 1.622 | 13.459 | 0.169952 | 7/15/2014 | 58:42.8 | 1 |
| -588.811 | 19542.107 | 1.622 | 13.401 | 0.156001 | 7/15/2014 | 58:42.9 | 1 |
| -589.051 | 19542.127 | 1.616 | 13.345 | 0.171795 | 7/15/2014 | 58:43.0 | 1 |
| -589.293 | 19542.146 | 1.612 | 13.293 | 0.166956 | 7/15/2014 | 58:43.1 | 1 |
| -589.532 | 19542.166 | 1.603 | 13.245 | 0.165246 | 7/15/2014 | 58:43.2 | 1 |
| -589.772 | 19542.188 | 1.603 | 13.203 | 0.162327 | 7/15/2014 | 58:43.3 | 1 |
| -590.188 | 19542.223 | 1.602 | 13.156 | 0.154749 | 7/15/2014 | 58:43.4 | 1 |
| -590.425 | 19542.242 | 1.602 | 13.13 | 0.169085 | 7/15/2014 | 58:43.5 | 1 |
| -590.66 | 19542.262 | 1.604 | 13.11 | 0.161613 | 7/15/2014 | 58:43.6 | 1 |

| | | | | | | | |
|---|---|---|---|---|---|---|---|
| -590.955 | 19542.287 | 1.604 | 13.096 | 0.156978 | 7/15/2014 | 58:43.7 | 1 |
| -591.074 | 19542.297 | 1.601 | 13.087 | 0.15662 | 7/15/2014 | 58:43.8 | 1 |
| -591.313 | 19542.318 | 1.605 | 13.082 | 0.187364 | 7/15/2014 | 58:43.9 | 1 |
| -591.548 | 19542.338 | 1.601 | 13.081 | 0.196808 | 7/15/2014 | 58:44.0 | 1 |
| -591.786 | 19542.357 | 1.609 | 13.085 | 0.174229 | 7/15/2014 | 58:44.1 | 1 |
| -592.022 | 19542.377 | 1.603 | 13.092 | 0.189409 | 7/15/2014 | 58:44.2 | 1 |
| -592.258 | 19542.396 | 1.6 | 13.101 | 0.166945 | 7/15/2014 | 58:44.3 | 1 |
| -592.666 | 19542.432 | 1.598 | 13.117 | 0.210915 | 7/15/2014 | 58:44.4 | 1 |
| -592.899 | 19542.451 | 1.6 | 13.128 | 0.179695 | 7/15/2014 | 58:44.5 | 1 |
| -593.132 | 19542.471 | 1.596 | 13.138 | 0.216164 | 7/15/2014 | 58:44.6 | 1 |
| -593.425 | 19542.496 | 1.599 | 13.148 | 0.205552 | 7/15/2014 | 58:44.7 | 1 |
| -593.542 | 19542.506 | 1.592 | 13.154 | 0.192867 | 7/15/2014 | 58:44.8 | 1 |
| -593.78 | 19542.525 | 1.601 | 13.163 | 0.242004 | 7/15/2014 | 58:44.9 | 1 |
| -594.014 | 19542.545 | 1.6 | 13.171 | 0.193648 | 7/15/2014 | 58:45.0 | 1 |
| -594.254 | 19542.564 | 1.604 | 13.179 | 0.188631 | 7/15/2014 | 58:45.1 | 1 |
| -594.49 | 19542.584 | 1.6 | 13.187 | 0.183336 | 7/15/2014 | 58:45.2 | 1 |
| -594.731 | 19542.605 | 1.616 | 13.196 | 0.167171 | 7/15/2014 | 58:45.3 | 1 |
| -595.153 | 19542.641 | 1.62 | 13.212 | 0.158013 | 7/15/2014 | 58:45.4 | 1 |
| -595.395 | 19542.662 | 1.62 | 13.223 | 0.139934 | 7/15/2014 | 58:45.5 | 1 |
| -595.639 | 19542.682 | 1.617 | 13.235 | 0.14508 | 7/15/2014 | 58:45.6 | 1 |
| -595.884 | 19542.703 | 1.617 | 13.248 | 0.153442 | 7/15/2014 | 58:45.7 | 1 |
| -596.13 | 19542.723 | 1.622 | 13.26 | 0.140022 | 7/15/2014 | 58:45.8 | 1 |
| -596.377 | 19542.744 | 1.625 | 13.272 | 0.14108 | 7/15/2014 | 58:45.9 | 1 |
| -596.623 | 19542.765 | 1.62 | 13.283 | 0.146621 | 7/15/2014 | 58:46.0 | 1 |
| -596.869 | 19542.785 | 1.622 | 13.291 | 0.168516 | 7/15/2014 | 58:46.1 | 1 |
| -597.113 | 19542.807 | 1.622 | 13.297 | 0.151061 | 7/15/2014 | 58:46.2 | 1 |
| -597.358 | 19542.826 | 1.628 | 13.298 | 0.162424 | 7/15/2014 | 58:46.3 | 1 |
| -597.599 | 19542.849 | 1.631 | 13.296 | 0.169711 | 7/15/2014 | 58:46.4 | 1 |
| -597.84 | 19542.869 | 1.635 | 13.289 | 0.184522 | 7/15/2014 | 58:46.5 | 1 |
| -598.078 | 19542.889 | 1.631 | 13.278 | 0.171578 | 7/15/2014 | 58:46.6 | 1 |
| -598.373 | 19542.913 | 1.631 | 13.264 | 0.178777 | 7/15/2014 | 58:46.7 | 1 |
| -598.494 | 19542.924 | 1.629 | 13.257 | 0.173616 | 7/15/2014 | 58:46.8 | 1 |
| -598.731 | 19542.944 | 1.633 | 13.237 | 0.187963 | 7/15/2014 | 58:46.9 | 1 |
| -598.967 | 19542.965 | 1.632 | 13.215 | 0.176295 | 7/15/2014 | 58:47.0 | 1 |
| -599.203 | 19542.985 | 1.635 | 13.193 | 0.162343 | 7/15/2014 | 58:47.1 | 1 |
| -599.439 | 19543.006 | 1.629 | 13.172 | 0.158929 | 7/15/2014 | 58:47.2 | 1 |
| -599.675 | 19543.026 | 1.628 | 13.154 | 0.15708 | 7/15/2014 | 58:47.3 | 1 |
| -600.092 | 19543.061 | 1.638 | 13.137 | 0.134 | 7/15/2014 | 58:47.4 | 1 |
| -600.331 | 19543.083 | 1.641 | 13.13 | 0.145587 | 7/15/2014 | 58:47.5 | 1 |
| -600.572 | 19543.104 | 1.641 | 13.129 | 0.135871 | 7/15/2014 | 58:47.6 | 1 |
| -600.814 | 19543.125 | 1.644 | 13.134 | 0.133996 | 7/15/2014 | 58:47.7 | 1 |
| -601.058 | 19543.145 | 1.644 | 13.144 | 0.119426 | 7/15/2014 | 58:47.8 | 1 |
| -601.302 | 19543.165 | 1.649 | 13.161 | 0.0921467 | 7/15/2014 | 58:47.9 | 1 |
| -601.547 | 19543.188 | 1.652 | 13.184 | 0.111651 | 7/15/2014 | 58:48.0 | 1 |
| -601.791 | 19543.207 | 1.65 | 13.21 | 0.132555 | 7/15/2014 | 58:48.1 | 1 |
| -602.036 | 19543.23 | 1.652 | 13.241 | 0.0941918 | 7/15/2014 | 58:48.2 | 1 |
| -602.28 | 19543.25 | 1.652 | 13.273 | 0.102774 | 7/15/2014 | 58:48.3 | 1 |

| | | | | | | | |
|---|---|---|---|---|---|---|---|
| -602.523 | 19543.271 | 1.655 | 13.304 | 0.0902844 | 7/15/2014 | 58:48.4 | 1 |
| -602.766 | 19543.293 | 1.655 | 13.334 | 0.105276 | 7/15/2014 | 58:48.5 | 1 |
| -603.006 | 19543.313 | 1.652 | 13.361 | 0.145172 | 7/15/2014 | 58:48.6 | 1 |
| -603.307 | 19543.34 | 1.652 | 13.381 | 0.0721984 | 7/15/2014 | 58:48.7 | 1 |
| -603.429 | 19543.35 | 1.653 | 13.394 | 0.0579771 | 7/15/2014 | 58:48.8 | 1 |
| -603.671 | 19543.371 | 1.656 | 13.404 | 0.092409 | 7/15/2014 | 58:48.9 | 1 |
| -603.912 | 19543.391 | 1.647 | 13.406 | 0.089995 | 7/15/2014 | 58:49.0 | 1 |
| -604.155 | 19543.412 | 1.65 | 13.401 | 0.0980228 | 7/15/2014 | 58:49.1 | 1 |
| -604.397 | 19543.434 | 1.647 | 13.388 | 0.0720589 | 7/15/2014 | 58:49.2 | 1 |
| -604.641 | 19543.455 | 1.65 | 13.37 | 0.0842001 | 7/15/2014 | 58:49.3 | 1 |
| -605.072 | 19543.492 | 1.645 | 13.337 | 0.11385 | 7/15/2014 | 58:49.4 | 1 |
| -605.192 | 19543.502 | 1.645 | 13.326 | 0.104107 | 7/15/2014 | 58:49.5 | 1 |
| -605.44 | 19543.523 | 1.643 | 13.303 | 0.104278 | 7/15/2014 | 58:49.6 | 1 |
| -605.691 | 19543.547 | 1.649 | 13.283 | 0.0935035 | 7/15/2014 | 58:49.7 | 1 |
| -605.942 | 19543.567 | 1.643 | 13.265 | 0.132652 | 7/15/2014 | 58:49.8 | 1 |
| -606.196 | 19543.59 | 1.645 | 13.251 | 0.0986166 | 7/15/2014 | 58:49.9 | 1 |
| -606.448 | 19543.613 | 1.645 | 13.239 | 0.131528 | 7/15/2014 | 58:50.0 | 1 |
| -606.701 | 19543.634 | 1.649 | 13.231 | 0.114836 | 7/15/2014 | 58:50.1 | 1 |
| -606.953 | 19543.656 | 1.641 | 13.226 | 0.121353 | 7/15/2014 | 58:50.2 | 1 |
| -607.205 | 19543.68 | 1.646 | 13.223 | 0.13525 | 7/15/2014 | 58:50.3 | 1 |
| -607.454 | 19543.7 | 1.64 | 13.223 | 0.107424 | 7/15/2014 | 58:50.4 | 1 |
| -607.823 | 19543.732 | 1.638 | 13.229 | 0.107101 | 7/15/2014 | 58:50.5 | 1 |
| -608.068 | 19543.754 | 1.636 | 13.235 | 0.119075 | 7/15/2014 | 58:50.6 | 1 |
| -608.371 | 19543.78 | 1.63 | 13.243 | 0.0973804 | 7/15/2014 | 58:50.7 | 1 |
| -608.496 | 19543.791 | 1.628 | 13.246 | 0.0864523 | 7/15/2014 | 58:50.8 | 1 |
| -608.74 | 19543.814 | 1.637 | 13.257 | 0.0757437 | 7/15/2014 | 58:50.9 | 1 |
| -608.981 | 19543.834 | 1.628 | 13.269 | 0.106069 | 7/15/2014 | 58:51.0 | 1 |
| -609.225 | 19543.856 | 1.626 | 13.283 | 0.0624352 | 7/15/2014 | 58:51.1 | 1 |
| -609.466 | 19543.877 | 1.623 | 13.297 | 0.083404 | 7/15/2014 | 58:51.2 | 1 |
| -609.707 | 19543.896 | 1.629 | 13.312 | 0.0613132 | 7/15/2014 | 58:51.3 | 1 |
| -610.128 | 19543.934 | 1.623 | 13.335 | 0.102903 | 7/15/2014 | 58:51.4 | 1 |
| -610.368 | 19543.957 | 1.621 | 13.35 | 0.111103 | 7/15/2014 | 58:51.5 | 1 |
| -610.609 | 19543.977 | 1.623 | 13.366 | 0.0734911 | 7/15/2014 | 58:51.6 | 1 |
| -610.911 | 19544.004 | 1.621 | 13.382 | 0.0840289 | 7/15/2014 | 58:51.7 | 1 |
| -611.033 | 19544.014 | 1.623 | 13.389 | 0.0796984 | 7/15/2014 | 58:51.8 | 1 |
| -611.276 | 19544.035 | 1.623 | 13.406 | 0.0953833 | 7/15/2014 | 58:51.9 | 1 |
| -611.52 | 19544.057 | 1.626 | 13.423 | 0.0728999 | 7/15/2014 | 58:52.0 | 1 |
| -611.765 | 19544.078 | 1.632 | 13.442 | 0.0708747 | 7/15/2014 | 58:52.1 | 1 |
| -612.008 | 19544.1 | 1.629 | 13.462 | 0.0770395 | 7/15/2014 | 58:52.2 | 1 |
| -612.253 | 19544.121 | 1.635 | 13.484 | 0.0745052 | 7/15/2014 | 58:52.3 | 1 |
| -612.678 | 19544.158 | 1.633 | 13.518 | 0.0748375 | 7/15/2014 | 58:52.4 | 1 |
| -612.92 | 19544.18 | 1.636 | 13.541 | 0.0764323 | 7/15/2014 | 58:52.5 | 1 |
| -613.162 | 19544.201 | 1.639 | 13.563 | 0.0916132 | 7/15/2014 | 58:52.6 | 1 |
| -613.464 | 19544.229 | 1.633 | 13.584 | 0.0900718 | 7/15/2014 | 58:52.7 | 1 |
| -613.587 | 19544.238 | 1.632 | 13.595 | 0.0988883 | 7/15/2014 | 58:52.8 | 1 |
| -613.831 | 19544.26 | 1.638 | 13.613 | 0.116963 | 7/15/2014 | 58:52.9 | 1 |
| -614.072 | 19544.279 | 1.634 | 13.628 | 0.107508 | 7/15/2014 | 58:53.0 | 1 |

| | | | | | | | |
|---|---|---|---|---|---|---|---|
| -614.317 | 19544.303 | 1.638 | 13.639 | 0.124095 | 7/15/2014 | 58:53.1 | 1 |
| -614.56 | 19544.322 | 1.632 | 13.646 | 0.13934 | 7/15/2014 | 58:53.2 | 1 |
| -614.801 | 19544.345 | 1.635 | 13.648 | 0.144027 | 7/15/2014 | 58:53.3 | 1 |
| -615.223 | 19544.381 | 1.637 | 13.637 | 0.174418 | 7/15/2014 | 58:53.4 | 1 |
| -615.463 | 19544.402 | 1.637 | 13.626 | 0.166271 | 7/15/2014 | 58:53.5 | 1 |
| -615.703 | 19544.422 | 1.637 | 13.609 | 0.18493 | 7/15/2014 | 58:53.6 | 1 |
| -616.005 | 19544.449 | 1.642 | 13.587 | 0.200141 | 7/15/2014 | 58:53.7 | 1 |
| -616.127 | 19544.459 | 1.641 | 13.577 | 0.181017 | 7/15/2014 | 58:53.8 | 1 |
| -616.368 | 19544.48 | 1.641 | 13.547 | 0.154509 | 7/15/2014 | 58:53.9 | 1 |
| -616.611 | 19544.502 | 1.641 | 13.514 | 0.151199 | 7/15/2014 | 58:54.0 | 1 |
| -616.853 | 19544.521 | 1.641 | 13.477 | 0.137173 | 7/15/2014 | 58:54.1 | 1 |
| -617.094 | 19544.545 | 1.641 | 13.438 | 0.142795 | 7/15/2014 | 58:54.2 | 1 |
| -617.336 | 19544.564 | 1.641 | 13.397 | 0.134452 | 7/15/2014 | 58:54.3 | 1 |
| -617.756 | 19544.602 | 1.636 | 13.334 | 0.115118 | 7/15/2014 | 58:54.4 | 1 |
| -617.993 | 19544.623 | 1.632 | 13.293 | 0.14919 | 7/15/2014 | 58:54.5 | 1 |
| -618.232 | 19544.645 | 1.632 | 13.253 | 0.142845 | 7/15/2014 | 58:54.6 | 1 |
| -618.529 | 19544.67 | 1.629 | 13.215 | 0.136891 | 7/15/2014 | 58:54.7 | 1 |
| -618.65 | 19544.682 | 1.627 | 13.195 | 0.11865 | 7/15/2014 | 58:54.8 | 1 |
| -618.888 | 19544.701 | 1.627 | 13.16 | 0.140345 | 7/15/2014 | 58:54.9 | 1 |
| -619.125 | 19544.723 | 1.627 | 13.129 | 0.135606 | 7/15/2014 | 58:55.0 | 1 |
| -619.365 | 19544.744 | 1.634 | 13.101 | 0.139626 | 7/15/2014 | 58:55.1 | 1 |
| -619.601 | 19544.765 | 1.628 | 13.078 | 0.142468 | 7/15/2014 | 58:55.2 | 1 |
| -619.839 | 19544.785 | 1.626 | 13.057 | 0.157161 | 7/15/2014 | 58:55.3 | 1 |
| -620.251 | 19544.822 | 1.622 | 13.035 | 0.161263 | 7/15/2014 | 58:55.4 | 1 |
| -620.486 | 19544.843 | 1.619 | 13.023 | 0.189614 | 7/15/2014 | 58:55.5 | 1 |
| -620.721 | 19544.863 | 1.615 | 13.013 | 0.190984 | 7/15/2014 | 58:55.6 | 1 |
| -621.016 | 19544.891 | 1.61 | 13.005 | 0.18233 | 7/15/2014 | 58:55.7 | 1 |
| -621.136 | 19544.9 | 1.605 | 13.001 | 0.176338 | 7/15/2014 | 58:55.8 | 1 |
| -621.374 | 19544.921 | 1.602 | 12.997 | 0.171347 | 7/15/2014 | 58:55.9 | 1 |
| -621.611 | 19544.943 | 1.598 | 12.994 | 0.142235 | 7/15/2014 | 58:56.0 | 1 |
| -621.851 | 19544.963 | 1.602 | 12.994 | 0.165662 | 7/15/2014 | 58:56.1 | 1 |
| -622.088 | 19544.984 | 1.593 | 12.995 | 0.139635 | 7/15/2014 | 58:56.2 | 1 |
| -622.327 | 19545.006 | 1.594 | 12.999 | 0.136457 | 7/15/2014 | 58:56.3 | 1 |
| -622.742 | 19545.043 | 1.592 | 13.01 | 0.15475 | 7/15/2014 | 58:56.4 | 1 |
| -622.978 | 19545.063 | 1.592 | 13.021 | 0.138378 | 7/15/2014 | 58:56.5 | 1 |
| -623.214 | 19545.085 | 1.594 | 13.035 | 0.134693 | 7/15/2014 | 58:56.6 | 1 |
| -623.506 | 19545.109 | 1.592 | 13.052 | 0.133503 | 7/15/2014 | 58:56.7 | 1 |
| -623.625 | 19545.12 | 1.588 | 13.06 | 0.114602 | 7/15/2014 | 58:56.8 | 1 |
| -623.862 | 19545.141 | 1.592 | 13.082 | 0.102742 | 7/15/2014 | 58:56.9 | 1 |
| -624.095 | 19545.162 | 1.586 | 13.108 | 0.0958337 | 7/15/2014 | 58:57.0 | 1 |
| -624.33 | 19545.184 | 1.588 | 13.137 | 0.0911435 | 7/15/2014 | 58:57.1 | 1 |
| -624.562 | 19545.203 | 1.585 | 13.168 | 0.103905 | 7/15/2014 | 58:57.2 | 1 |
| -624.796 | 19545.224 | 1.588 | 13.2 | 0.0886261 | 7/15/2014 | 58:57.3 | 1 |
| -625.196 | 19545.259 | 1.579 | 13.248 | 0.102984 | 7/15/2014 | 58:57.4 | 1 |
| -625.426 | 19545.279 | 1.576 | 13.279 | 0.119384 | 7/15/2014 | 58:57.5 | 1 |
| -625.654 | 19545.3 | 1.573 | 13.307 | 0.103358 | 7/15/2014 | 58:57.6 | 1 |
| -625.941 | 19545.326 | 1.573 | 13.332 | 0.120217 | 7/15/2014 | 58:57.7 | 1 |

| | | | | | | | |
|---|---|---|---|---|---|---|---|
| -626.171 | 19545.346 | 1.573 | 13.353 | 0.10382 | 7/15/2014 | 58:57.8 | 1 |
| -626.404 | 19545.365 | 1.569 | 13.369 | 0.138015 | 7/15/2014 | 58:57.9 | 1 |
| -626.635 | 19545.385 | 1.564 | 13.38 | 0.179502 | 7/15/2014 | 58:58.0 | 1 |
| -626.75 | 19545.396 | 1.568 | 13.387 | 0.181826 | 7/15/2014 | 58:58.1 | 1 |
| -626.982 | 19545.418 | 1.565 | 13.391 | 0.123145 | 7/15/2014 | 58:58.2 | 1 |
| -627.213 | 19545.438 | 1.568 | 13.39 | 0.135557 | 7/15/2014 | 58:58.3 | 1 |
| -627.62 | 19545.473 | 1.57 | 13.382 | 0.124549 | 7/15/2014 | 58:58.4 | 1 |
| -627.851 | 19545.493 | 1.57 | 13.376 | 0.151864 | 7/15/2014 | 58:58.5 | 1 |
| -628.083 | 19545.514 | 1.566 | 13.37 | 0.142307 | 7/15/2014 | 58:58.6 | 1 |
| -628.374 | 19545.537 | 1.56 | 13.367 | 0.100156 | 7/15/2014 | 58:58.7 | 1 |
| -628.492 | 19545.549 | 1.56 | 13.363 | 0.161946 | 7/15/2014 | 58:58.8 | 1 |
| -628.729 | 19545.569 | 1.56 | 13.363 | 0.147642 | 7/15/2014 | 58:58.9 | 1 |
| -628.965 | 19545.59 | 1.563 | 13.366 | 0.126379 | 7/15/2014 | 58:59.0 | 1 |
| -629.203 | 19545.609 | 1.565 | 13.373 | 0.183062 | 7/15/2014 | 58:59.1 | 1 |
| -629.44 | 19545.63 | 1.559 | 13.382 | 0.141109 | 7/15/2014 | 58:59.2 | 1 |
| -629.678 | 19545.652 | 1.562 | 13.394 | 0.159339 | 7/15/2014 | 58:59.3 | 1 |
| -630.095 | 19545.686 | 1.564 | 13.415 | 0.112364 | 7/15/2014 | 58:59.4 | 1 |
| -630.333 | 19545.706 | 1.566 | 13.427 | 0.124093 | 7/15/2014 | 58:59.5 | 1 |
| -630.572 | 19545.727 | 1.566 | 13.438 | 0.106913 | 7/15/2014 | 58:59.6 | 1 |
| -630.871 | 19545.752 | 1.564 | 13.448 | 0.0806424 | 7/15/2014 | 58:59.7 | 1 |
| -630.992 | 19545.762 | 1.562 | 13.453 | 0.100409 | 7/15/2014 | 58:59.8 | 1 |
| -631.234 | 19545.784 | 1.564 | 13.458 | 0.0751749 | 7/15/2014 | 58:59.9 | 1 |
| -631.475 | 19545.805 | 1.562 | 13.459 | 0.112269 | 7/15/2014 | 59:00.0 | 1 |
| -631.718 | 19545.824 | 1.562 | 13.456 | 0.123491 | 7/15/2014 | 59:00.1 | 1 |
| -631.959 | 19545.844 | 1.557 | 13.449 | 0.111611 | 7/15/2014 | 59:00.2 | 1 |
| -632.201 | 19545.865 | 1.563 | 13.439 | 0.156646 | 7/15/2014 | 59:00.3 | 1 |
| -632.62 | 19545.9 | 1.565 | 13.416 | 0.116224 | 7/15/2014 | 59:00.4 | 1 |
| -632.859 | 19545.921 | 1.567 | 13.398 | 0.121259 | 7/15/2014 | 59:00.5 | 1 |
| -633.097 | 19545.941 | 1.574 | 13.376 | 0.132759 | 7/15/2014 | 59:00.6 | 1 |
| -633.392 | 19545.967 | 1.574 | 13.352 | 0.0884218 | 7/15/2014 | 59:00.7 | 1 |
| -633.513 | 19545.977 | 1.576 | 13.341 | 0.0855346 | 7/15/2014 | 59:00.8 | 1 |
| -633.752 | 19545.998 | 1.585 | 13.312 | 0.0641592 | 7/15/2014 | 59:00.9 | 1 |
| -633.987 | 19546.018 | 1.585 | 13.281 | 0.100957 | 7/15/2014 | 59:01.0 | 1 |
| -634.224 | 19546.037 | 1.587 | 13.248 | 0.0837808 | 7/15/2014 | 59:01.1 | 1 |
| -634.458 | 19546.055 | 1.592 | 13.212 | 0.0991649 | 7/15/2014 | 59:01.2 | 1 |
| -634.693 | 19546.074 | 1.592 | 13.176 | 0.111058 | 7/15/2014 | 59:01.3 | 1 |
| -635.096 | 19546.109 | 1.585 | 13.12 | 0.119544 | 7/15/2014 | 59:01.4 | 1 |
| -635.327 | 19546.129 | 1.587 | 13.084 | 0.149045 | 7/15/2014 | 59:01.5 | 1 |
| -635.556 | 19546.148 | 1.587 | 13.048 | 0.141993 | 7/15/2014 | 59:01.6 | 1 |
| -635.84 | 19546.172 | 1.592 | 13.015 | 0.159836 | 7/15/2014 | 59:01.7 | 1 |
| -636.068 | 19546.188 | 1.596 | 12.983 | 0.152569 | 7/15/2014 | 59:01.8 | 1 |
| -636.295 | 19546.207 | 1.596 | 12.954 | 0.159792 | 7/15/2014 | 59:01.9 | 1 |
| -636.524 | 19546.227 | 1.604 | 12.928 | 0.150506 | 7/15/2014 | 59:02.0 | 1 |
| -636.75 | 19546.244 | 1.609 | 12.905 | 0.167609 | 7/15/2014 | 59:02.1 | 1 |
| -636.977 | 19546.264 | 1.618 | 12.886 | 0.114303 | 7/15/2014 | 59:02.2 | 1 |
| -637.203 | 19546.283 | 1.628 | 12.869 | 0.1041 | 7/15/2014 | 59:02.3 | 1 |
| -637.482 | 19546.307 | 1.638 | 12.856 | 0.088813 | 7/15/2014 | 59:02.4 | 1 |

| | | | | | | | |
|---|---|---|---|---|---|---|---|
| -637.704 | 19546.322 | 1.638 | 12.845 | 0.0728207 | 7/15/2014 | 59:02.5 | 1 |
| -637.926 | 19546.342 | 1.643 | 12.838 | 0.0271416 | 7/15/2014 | 59:02.6 | 1 |
| -638.206 | 19546.365 | 1.649 | 12.832 | 0.0414121 | 7/15/2014 | 59:02.7 | 1 |
| -638.43 | 19546.382 | 1.649 | 12.828 | 0.0192697 | 7/15/2014 | 59:02.8 | 1 |
| -638.657 | 19546.4 | 1.655 | 12.826 | 0.0226467 | 7/15/2014 | 59:02.9 | 1 |
| -638.884 | 19546.42 | 1.659 | 12.824 | 0.0312321 | 7/15/2014 | 59:03.0 | 1 |
| -639.111 | 19546.439 | 1.657 | 12.823 | 0.0306129 | 7/15/2014 | 59:03.1 | 1 |
| -639.339 | 19546.457 | 1.657 | 12.823 | 0.0611191 | 7/15/2014 | 59:03.2 | 1 |
| -639.565 | 19546.477 | 1.662 | 12.822 | 0.0767349 | 7/15/2014 | 59:03.3 | 1 |
| -639.848 | 19546.498 | 1.662 | 12.822 | 0.0968278 | 7/15/2014 | 59:03.4 | 1 |
| -640.077 | 19546.517 | 1.664 | 12.822 | 0.107595 | 7/15/2014 | 59:03.5 | 1 |
| -640.308 | 19546.535 | 1.666 | 12.822 | 0.120526 | 7/15/2014 | 59:03.6 | 1 |
| -640.598 | 19546.561 | 1.666 | 12.822 | 0.136083 | 7/15/2014 | 59:03.7 | 1 |
| -640.713 | 19546.57 | 1.664 | 12.822 | 0.150578 | 7/15/2014 | 59:03.8 | 1 |
| -640.949 | 19546.59 | 1.679 | 12.823 | 0.1279 | 7/15/2014 | 59:03.9 | 1 |
| -641.185 | 19546.609 | 1.683 | 12.824 | 0.137431 | 7/15/2014 | 59:04.0 | 1 |
| -641.423 | 19546.627 | 1.685 | 12.826 | 0.115987 | 7/15/2014 | 59:04.1 | 1 |
| -641.661 | 19546.646 | 1.691 | 12.829 | 0.124355 | 7/15/2014 | 59:04.2 | 1 |
| -641.9 | 19546.666 | 1.693 | 12.833 | 0.107717 | 7/15/2014 | 59:04.3 | 1 |
| -642.319 | 19546.701 | 1.703 | 12.84 | 0.101067 | 7/15/2014 | 59:04.4 | 1 |
| -642.557 | 19546.721 | 1.708 | 12.846 | 0.0950023 | 7/15/2014 | 59:04.5 | 1 |
| -642.796 | 19546.74 | 1.699 | 12.853 | 0.0957248 | 7/15/2014 | 59:04.6 | 1 |
| -643.094 | 19546.766 | 1.699 | 12.862 | 0.0934018 | 7/15/2014 | 59:04.7 | 1 |
| -643.216 | 19546.775 | 1.702 | 12.865 | 0.0928566 | 7/15/2014 | 59:04.8 | 1 |
| -643.457 | 19546.795 | 1.702 | 12.875 | 0.076484 | 7/15/2014 | 59:04.9 | 1 |
| -643.698 | 19546.814 | 1.706 | 12.886 | 0.100167 | 7/15/2014 | 59:05.0 | 1 |
| -643.942 | 19546.834 | 1.712 | 12.897 | 0.057702 | 7/15/2014 | 59:05.1 | 1 |
| -644.179 | 19546.854 | 1.702 | 12.909 | 0.0665424 | 7/15/2014 | 59:05.2 | 1 |
| -644.42 | 19546.875 | 1.706 | 12.92 | 0.0353743 | 7/15/2014 | 59:05.3 | 1 |
| -644.835 | 19546.91 | 1.709 | 12.935 | 0.0336881 | 7/15/2014 | 59:05.4 | 1 |
| -645.071 | 19546.93 | 1.709 | 12.944 | 0.0463744 | 7/15/2014 | 59:05.5 | 1 |
| -645.309 | 19546.949 | 1.712 | 12.952 | 0.029704 | 7/15/2014 | 59:05.6 | 1 |
| -645.604 | 19546.973 | 1.718 | 12.96 | 0.0274636 | 7/15/2014 | 59:05.7 | 1 |
| -645.723 | 19546.982 | 1.713 | 12.964 | 0.0238447 | 7/15/2014 | 59:05.8 | 1 |
| -645.962 | 19547.005 | 1.723 | 12.969 | 0.057802 | 7/15/2014 | 59:05.9 | 1 |
| -646.196 | 19547.025 | 1.716 | 12.974 | 0.0476225 | 7/15/2014 | 59:06.0 | 1 |
| -646.434 | 19547.045 | 1.716 | 12.979 | 0.0767902 | 7/15/2014 | 59:06.1 | 1 |
| -646.669 | 19547.064 | 1.718 | 12.984 | 0.0777151 | 7/15/2014 | 59:06.2 | 1 |
| -646.906 | 19547.084 | 1.725 | 12.99 | 0.100599 | 7/15/2014 | 59:06.3 | 1 |
| -647.315 | 19547.117 | 1.719 | 13.003 | 0.124773 | 7/15/2014 | 59:06.4 | 1 |
| -647.548 | 19547.139 | 1.719 | 13.013 | 0.12528 | 7/15/2014 | 59:06.5 | 1 |
| -647.779 | 19547.16 | 1.719 | 13.027 | 0.117972 | 7/15/2014 | 59:06.6 | 1 |
| -648.07 | 19547.184 | 1.722 | 13.043 | 0.10287 | 7/15/2014 | 59:06.7 | 1 |
| -648.187 | 19547.193 | 1.724 | 13.05 | 0.112301 | 7/15/2014 | 59:06.8 | 1 |
| -648.422 | 19547.213 | 1.728 | 13.072 | 0.0950114 | 7/15/2014 | 59:06.9 | 1 |
| -648.655 | 19547.233 | 1.728 | 13.097 | 0.0723088 | 7/15/2014 | 59:07.0 | 1 |
| -648.889 | 19547.254 | 1.738 | 13.125 | 0.0647031 | 7/15/2014 | 59:07.1 | 1 |

| | | | | | | | |
|---|---|---|---|---|---|---|---|
| -649.122 | 19547.274 | 1.74 | 13.157 | 0.0529906 | 7/15/2014 | 59:07.2 | 1 |
| -649.354 | 19547.295 | 1.744 | 13.193 | 0.0484176 | 7/15/2014 | 59:07.3 | 1 |
| -649.758 | 19547.328 | 1.742 | 13.252 | 0.0573206 | 7/15/2014 | 59:07.4 | 1 |
| -649.988 | 19547.348 | 1.742 | 13.294 | 0.0417402 | 7/15/2014 | 59:07.5 | 1 |
| -650.218 | 19547.368 | 1.742 | 13.337 | 0.0418795 | 7/15/2014 | 59:07.6 | 1 |
| -650.505 | 19547.395 | 1.742 | 13.38 | 0.0193045 | 7/15/2014 | 59:07.7 | 1 |
| -650.622 | 19547.404 | 1.742 | 13.402 | 0.0202639 | 7/15/2014 | 59:07.8 | 1 |
| -650.966 | 19547.434 | 1.742 | 13.467 | 0.0288726 | 7/15/2014 | 59:07.9 | 1 |
| -651.197 | 19547.453 | 1.741 | 13.508 | 0.0676795 | 7/15/2014 | 59:08.0 | 1 |
| -651.426 | 19547.473 | 1.741 | 13.545 | 0.0453577 | 7/15/2014 | 59:08.1 | 1 |
| -651.655 | 19547.492 | 1.746 | 13.577 | 0.0402527 | 7/15/2014 | 59:08.2 | 1 |
| -651.883 | 19547.512 | 1.75 | 13.603 | 0.0447004 | 7/15/2014 | 59:08.3 | 1 |
| -652.167 | 19547.537 | 1.75 | 13.621 | 0.0874605 | 7/15/2014 | 59:08.4 | 1 |
| -652.392 | 19547.557 | 1.748 | 13.63 | 0.0348714 | 7/15/2014 | 59:08.5 | 1 |
| -652.619 | 19547.576 | 1.75 | 13.629 | 0.0782328 | 7/15/2014 | 59:08.6 | 1 |
| -652.901 | 19547.6 | 1.748 | 13.617 | 0.0557703 | 7/15/2014 | 59:08.7 | 1 |
| -653.128 | 19547.619 | 1.748 | 13.594 | 0.0906682 | 7/15/2014 | 59:08.8 | 1 |
| -653.357 | 19547.639 | 1.75 | 13.561 | 0.0859646 | 7/15/2014 | 59:08.9 | 1 |
| -653.585 | 19547.658 | 1.744 | 13.518 | 0.0946266 | 7/15/2014 | 59:09.0 | 1 |
| -653.815 | 19547.678 | 1.747 | 13.466 | 0.067708 | 7/15/2014 | 59:09.1 | 1 |
| -654.043 | 19547.698 | 1.752 | 13.408 | 0.100226 | 7/15/2014 | 59:09.2 | 1 |
| -654.272 | 19547.719 | 1.754 | 13.344 | 0.090742 | 7/15/2014 | 59:09.3 | 1 |
| -654.556 | 19547.742 | 1.752 | 13.278 | 0.0821099 | 7/15/2014 | 59:09.4 | 1 |
| -654.785 | 19547.762 | 1.754 | 13.212 | 0.0765062 | 7/15/2014 | 59:09.5 | 1 |
| -655.016 | 19547.781 | 1.757 | 13.147 | 0.103426 | 7/15/2014 | 59:09.6 | 1 |
| -655.305 | 19547.807 | 1.755 | 13.085 | 0.0879194 | 7/15/2014 | 59:09.7 | 1 |
| -655.421 | 19547.816 | 1.756 | 13.052 | 0.100853 | 7/15/2014 | 59:09.8 | 1 |
| -655.657 | 19547.837 | 1.762 | 12.997 | 0.113503 | 7/15/2014 | 59:09.9 | 1 |
| -655.89 | 19547.857 | 1.756 | 12.947 | 0.112054 | 7/15/2014 | 59:10.0 | 1 |
| -656.127 | 19547.877 | 1.756 | 12.903 | 0.118373 | 7/15/2014 | 59:10.1 | 1 |
| -656.364 | 19547.898 | 1.754 | 12.865 | 0.136303 | 7/15/2014 | 59:10.2 | 1 |
| -656.602 | 19547.918 | 1.756 | 12.834 | 0.122863 | 7/15/2014 | 59:10.3 | 1 |
| -657.018 | 19547.955 | 1.752 | 12.799 | 0.143187 | 7/15/2014 | 59:10.4 | 1 |
| -657.256 | 19547.975 | 1.752 | 12.78 | 0.109693 | 7/15/2014 | 59:10.5 | 1 |
| -657.495 | 19547.994 | 1.752 | 12.766 | 0.102526 | 7/15/2014 | 59:10.6 | 1 |
| -657.794 | 19548.02 | 1.752 | 12.755 | 0.108844 | 7/15/2014 | 59:10.7 | 1 |
| -657.915 | 19548.031 | 1.753 | 12.749 | 0.0888091 | 7/15/2014 | 59:10.8 | 1 |
| -658.157 | 19548.052 | 1.759 | 12.745 | 0.0693767 | 7/15/2014 | 59:10.9 | 1 |
| -658.399 | 19548.073 | 1.756 | 12.742 | 0.0660052 | 7/15/2014 | 59:11.0 | 1 |
| -658.644 | 19548.094 | 1.759 | 12.743 | 0.0745649 | 7/15/2014 | 59:11.1 | 1 |
| -658.886 | 19548.113 | 1.756 | 12.745 | 0.075295 | 7/15/2014 | 59:11.2 | 1 |
| -659.129 | 19548.135 | 1.762 | 12.749 | 0.0487462 | 7/15/2014 | 59:11.3 | 1 |
| -659.554 | 19548.17 | 1.755 | 12.759 | 0.0869009 | 7/15/2014 | 59:11.4 | 1 |
| -659.796 | 19548.192 | 1.753 | 12.767 | 0.104186 | 7/15/2014 | 59:11.5 | 1 |
| -660.04 | 19548.213 | 1.752 | 12.776 | 0.117279 | 7/15/2014 | 59:11.6 | 1 |
| -660.344 | 19548.236 | 1.755 | 12.787 | 0.0736328 | 7/15/2014 | 59:11.7 | 1 |
| -660.466 | 19548.246 | 1.754 | 12.792 | 0.0842323 | 7/15/2014 | 59:11.8 | 1 |

| | | | | | | | |
|---|---|---|---|---|---|---|---|
| -660.711 | 19548.27 | 1.756 | 12.805 | 0.104383 | 7/15/2014 | 59:11.9 | 1 |
| -660.956 | 19548.289 | 1.75 | 12.819 | 0.100238 | 7/15/2014 | 59:12.0 | 1 |
| -661.203 | 19548.309 | 1.753 | 12.834 | 0.0943619 | 7/15/2014 | 59:12.1 | 1 |
| -661.446 | 19548.33 | 1.75 | 12.849 | 0.069395 | 7/15/2014 | 59:12.2 | 1 |
| -661.692 | 19548.352 | 1.753 | 12.866 | 0.0918981 | 7/15/2014 | 59:12.3 | 1 |
| -662.118 | 19548.385 | 1.748 | 12.891 | 0.0947269 | 7/15/2014 | 59:12.4 | 1 |
| -662.361 | 19548.406 | 1.744 | 12.909 | 0.115012 | 7/15/2014 | 59:12.5 | 1 |
| -662.604 | 19548.426 | 1.737 | 12.926 | 0.0804236 | 7/15/2014 | 59:12.6 | 1 |
| -662.909 | 19548.451 | 1.737 | 12.944 | 0.0911929 | 7/15/2014 | 59:12.7 | 1 |
| -663.032 | 19548.461 | 1.735 | 12.953 | 0.106652 | 7/15/2014 | 59:12.8 | 1 |
| -663.279 | 19548.482 | 1.737 | 12.971 | 0.081899 | 7/15/2014 | 59:12.9 | 1 |
| -663.524 | 19548.502 | 1.735 | 12.988 | 0.103484 | 7/15/2014 | 59:13.0 | 1 |
| -663.771 | 19548.521 | 1.737 | 13.005 | 0.0916659 | 7/15/2014 | 59:13.1 | 1 |
| -664.017 | 19548.541 | 1.735 | 13.022 | 0.0612627 | 7/15/2014 | 59:13.2 | 1 |
| -664.264 | 19548.561 | 1.731 | 13.039 | 0.0813627 | 7/15/2014 | 59:13.3 | 1 |
| -664.696 | 19548.596 | 1.738 | 13.062 | 0.0550255 | 7/15/2014 | 59:13.4 | 1 |
| -664.819 | 19548.605 | 1.739 | 13.07 | 0.0522052 | 7/15/2014 | 59:13.5 | 1 |
| -665.065 | 19548.625 | 1.735 | 13.084 | 0.0414899 | 7/15/2014 | 59:13.6 | 1 |
| -665.313 | 19548.645 | 1.739 | 13.097 | 0.0754555 | 7/15/2014 | 59:13.7 | 1 |
| -665.622 | 19548.668 | 1.731 | 13.11 | 0.0697282 | 7/15/2014 | 59:13.8 | 1 |
| -665.872 | 19548.688 | 1.733 | 13.121 | 0.045378 | 7/15/2014 | 59:13.9 | 1 |
| -666.121 | 19548.707 | 1.735 | 13.131 | 0.0741443 | 7/15/2014 | 59:14.0 | 1 |
| -666.372 | 19548.726 | 1.735 | 13.141 | 0.0615968 | 7/15/2014 | 59:14.1 | 1 |
| -666.621 | 19548.744 | 1.726 | 13.151 | 0.066588 | 7/15/2014 | 59:14.2 | 1 |
| -666.871 | 19548.764 | 1.726 | 13.159 | 0.0590821 | 7/15/2014 | 59:14.3 | 1 |
| -667.18 | 19548.787 | 1.721 | 13.168 | 0.0704452 | 7/15/2014 | 59:14.4 | 1 |
| -667.429 | 19548.807 | 1.727 | 13.177 | 0.0825273 | 7/15/2014 | 59:14.5 | 1 |
| -667.677 | 19548.824 | 1.725 | 13.186 | 0.0447042 | 7/15/2014 | 59:14.6 | 1 |
| -668.107 | 19548.856 | 1.72 | 13.202 | 0.068451 | 7/15/2014 | 59:14.7 | 1 |
| -668.232 | 19548.865 | 1.719 | 13.207 | 0.0635461 | 7/15/2014 | 59:14.8 | 1 |
| -668.48 | 19548.884 | 1.719 | 13.219 | 0.0607346 | 7/15/2014 | 59:14.9 | 1 |
| -668.726 | 19548.902 | 1.717 | 13.231 | 0.0794965 | 7/15/2014 | 59:15.0 | 1 |
| -668.975 | 19548.92 | 1.717 | 13.245 | 0.0712036 | 7/15/2014 | 59:15.1 | 1 |
| -669.221 | 19548.938 | 1.711 | 13.259 | 0.0825915 | 7/15/2014 | 59:15.2 | 1 |
| -669.467 | 19548.956 | 1.708 | 13.274 | 0.0450359 | 7/15/2014 | 59:15.3 | 1 |
| -669.893 | 19548.987 | 1.705 | 13.294 | 0.0828078 | 7/15/2014 | 59:15.4 | 1 |
| -670.136 | 19549.005 | 1.707 | 13.307 | 0.0668148 | 7/15/2014 | 59:15.5 | 1 |
| -670.377 | 19549.021 | 1.707 | 13.319 | 0.0758979 | 7/15/2014 | 59:15.6 | 1 |
| -670.68 | 19549.042 | 1.703 | 13.331 | 0.0544009 | 7/15/2014 | 59:15.7 | 1 |
| -670.803 | 19549.052 | 1.699 | 13.336 | 0.0545114 | 7/15/2014 | 59:15.8 | 1 |
| -671.047 | 19549.069 | 1.702 | 13.347 | 0.0479994 | 7/15/2014 | 59:15.9 | 1 |
| -671.289 | 19549.086 | 1.702 | 13.356 | 0.0341684 | 7/15/2014 | 59:16.0 | 1 |
| -671.532 | 19549.104 | 1.704 | 13.366 | 0.0177175 | 7/15/2014 | 59:16.1 | 1 |
| -671.775 | 19549.12 | 1.701 | 13.376 | 0.0192589 | 7/15/2014 | 59:16.2 | 1 |
| -672.016 | 19549.137 | 1.701 | 13.387 | 0.0303876 | 7/15/2014 | 59:16.3 | 1 |
| -672.437 | 19549.165 | 1.695 | 13.403 | 0.043488 | 7/15/2014 | 59:16.4 | 1 |
| -672.676 | 19549.182 | 1.701 | 13.414 | 0.0856019 | 7/15/2014 | 59:16.5 | 1 |

| | | | | | | | |
|---|---|---|---|---|---|---|---|
| -672.916 | 19549.197 | 1.701 | 13.424 | 0.0757154 | 7/15/2014 | 59:16.6 | 1 |
| -673.214 | 19549.217 | 1.695 | 13.433 | 0.0836236 | 7/15/2014 | 59:16.7 | 1 |
| -673.335 | 19549.225 | 1.694 | 13.438 | 0.0842878 | 7/15/2014 | 59:16.8 | 1 |
| -673.577 | 19549.241 | 1.694 | 13.444 | 0.0712991 | 7/15/2014 | 59:16.9 | 1 |
| -673.818 | 19549.257 | 1.694 | 13.446 | 0.0400582 | 7/15/2014 | 59:17.0 | 1 |
| -674.061 | 19549.272 | 1.7 | 13.444 | 0.0694975 | 7/15/2014 | 59:17.1 | 1 |
| -674.302 | 19549.287 | 1.694 | 13.437 | 0.0840236 | 7/15/2014 | 59:17.2 | 1 |
| -674.544 | 19549.303 | 1.688 | 13.424 | 0.0923855 | 7/15/2014 | 59:17.3 | 1 |
| -674.965 | 19549.328 | 1.685 | 13.39 | 0.0977436 | 7/15/2014 | 59:17.4 | 1 |
| -675.206 | 19549.343 | 1.685 | 13.364 | 0.0929253 | 7/15/2014 | 59:17.5 | 1 |
| -675.448 | 19549.358 | 1.683 | 13.334 | 0.136531 | 7/15/2014 | 59:17.6 | 1 |
| -675.75 | 19549.376 | 1.679 | 13.301 | 0.0920355 | 7/15/2014 | 59:17.7 | 1 |
| -675.871 | 19549.383 | 1.676 | 13.284 | 0.0690469 | 7/15/2014 | 59:17.8 | 1 |
| -676.117 | 19549.398 | 1.68 | 13.248 | 0.0839237 | 7/15/2014 | 59:17.9 | 1 |
| -676.36 | 19549.412 | 1.672 | 13.213 | 0.12596 | 7/15/2014 | 59:18.0 | 1 |
| -676.608 | 19549.427 | 1.676 | 13.18 | 0.088446 | 7/15/2014 | 59:18.1 | 1 |
| -676.851 | 19549.439 | 1.67 | 13.15 | 0.0804319 | 7/15/2014 | 59:18.2 | 1 |
| -677.098 | 19549.455 | 1.666 | 13.124 | 0.090687 | 7/15/2014 | 59:18.3 | 1 |
| -677.526 | 19549.478 | 1.654 | 13.097 | 0.111068 | 7/15/2014 | 59:18.4 | 1 |
| -677.772 | 19549.492 | 1.649 | 13.082 | 0.105735 | 7/15/2014 | 59:18.5 | 1 |
| -678.016 | 19549.504 | 1.642 | 13.072 | 0.109679 | 7/15/2014 | 59:18.6 | 1 |
| -678.325 | 19549.52 | 1.642 | 13.066 | 0.109226 | 7/15/2014 | 59:18.7 | 1 |
| -678.448 | 19549.525 | 1.636 | 13.062 | 0.123186 | 7/15/2014 | 59:18.8 | 1 |
| -678.696 | 19549.54 | 1.633 | 13.063 | 0.116709 | 7/15/2014 | 59:18.9 | 1 |
| -678.945 | 19549.552 | 1.63 | 13.067 | 0.12016 | 7/15/2014 | 59:19.0 | 1 |
| -679.194 | 19549.564 | 1.633 | 13.073 | 0.126313 | 7/15/2014 | 59:19.1 | 1 |
| -679.442 | 19549.578 | 1.63 | 13.082 | 0.116417 | 7/15/2014 | 59:19.2 | 1 |
| -679.689 | 19549.59 | 1.628 | 13.092 | 0.122492 | 7/15/2014 | 59:19.3 | 1 |
| -680.123 | 19549.607 | 1.622 | 13.112 | 0.111308 | 7/15/2014 | 59:19.4 | 1 |
| -680.369 | 19549.62 | 1.618 | 13.125 | 0.121453 | 7/15/2014 | 59:19.5 | 1 |
| -680.616 | 19549.632 | 1.618 | 13.14 | 0.109948 | 7/15/2014 | 59:19.6 | 1 |
| -680.922 | 19549.645 | 1.616 | 13.155 | 0.109976 | 7/15/2014 | 59:19.7 | 1 |
| -681.047 | 19549.651 | 1.613 | 13.162 | 0.120541 | 7/15/2014 | 59:19.8 | 1 |
| -681.294 | 19549.662 | 1.608 | 13.178 | 0.0849956 | 7/15/2014 | 59:19.9 | 1 |
| -681.541 | 19549.672 | 1.606 | 13.194 | 0.124103 | 7/15/2014 | 59:20.0 | 1 |
| -681.789 | 19549.682 | 1.606 | 13.21 | 0.091552 | 7/15/2014 | 59:20.1 | 1 |
| -682.035 | 19549.693 | 1.603 | 13.225 | 0.103599 | 7/15/2014 | 59:20.2 | 1 |
| -682.281 | 19549.703 | 1.601 | 13.241 | 0.0709972 | 7/15/2014 | 59:20.3 | 1 |
| -682.708 | 19549.72 | 1.599 | 13.262 | 0.0592316 | 7/15/2014 | 59:20.4 | 1 |
| -682.951 | 19549.729 | 1.597 | 13.276 | 0.131716 | 7/15/2014 | 59:20.5 | 1 |
| -683.194 | 19549.74 | 1.597 | 13.29 | 0.0801831 | 7/15/2014 | 59:20.6 | 1 |
| -683.498 | 19549.75 | 1.597 | 13.303 | 0.102848 | 7/15/2014 | 59:20.7 | 1 |
| -683.621 | 19549.756 | 1.594 | 13.31 | 0.0731665 | 7/15/2014 | 59:20.8 | 1 |
| -683.867 | 19549.764 | 1.594 | 13.323 | 0.115383 | 7/15/2014 | 59:20.9 | 1 |
| -684.112 | 19549.773 | 1.594 | 13.337 | 0.0970924 | 7/15/2014 | 59:21.0 | 1 |
| -684.358 | 19549.782 | 1.589 | 13.35 | 0.152476 | 7/15/2014 | 59:21.1 | 1 |
| -684.604 | 19549.792 | 1.589 | 13.363 | 0.0922169 | 7/15/2014 | 59:21.2 | 1 |

| | | | | | | | |
|---|---|---|---|---|---|---|---|
| -684.849 | 19549.801 | 1.591 | 13.376 | 0.144832 | 7/15/2014 | 59:21.3 | 1 |
| -685.279 | 19549.814 | 1.586 | 13.389 | 0.166087 | 7/15/2014 | 59:21.4 | 1 |
| -685.522 | 19549.822 | 1.58 | 13.395 | 0.160039 | 7/15/2014 | 59:21.5 | 1 |
| -685.769 | 19549.83 | 1.582 | 13.398 | 0.16811 | 7/15/2014 | 59:21.6 | 1 |
| -686.077 | 19549.841 | 1.584 | 13.398 | 0.155013 | 7/15/2014 | 59:21.7 | 1 |
| -686.201 | 19549.846 | 1.584 | 13.4 | 0.168994 | 7/15/2014 | 59:21.8 | 1 |
| -686.449 | 19549.854 | 1.582 | 13.395 | 0.165676 | 7/15/2014 | 59:21.9 | 1 |
| -686.695 | 19549.859 | 1.578 | 13.387 | 0.174651 | 7/15/2014 | 59:22.0 | 1 |
| -686.946 | 19549.867 | 1.578 | 13.376 | 0.163842 | 7/15/2014 | 59:22.1 | 1 |
| -687.194 | 19549.875 | 1.568 | 13.364 | 0.175847 | 7/15/2014 | 59:22.2 | 1 |
| -687.444 | 19549.883 | 1.568 | 13.351 | 0.156064 | 7/15/2014 | 59:22.3 | 1 |
| -687.753 | 19549.893 | 1.559 | 13.337 | 0.150811 | 7/15/2014 | 59:22.4 | 1 |
| -688.003 | 19549.899 | 1.556 | 13.323 | 0.140161 | 7/15/2014 | 59:22.5 | 1 |
| -688.25 | 19549.904 | 1.553 | 13.309 | 0.136197 | 7/15/2014 | 59:22.6 | 1 |
| -688.501 | 19549.912 | 1.554 | 13.295 | 0.119287 | 7/15/2014 | 59:22.7 | 1 |
| -688.812 | 19549.92 | 1.558 | 13.283 | 0.114648 | 7/15/2014 | 59:22.8 | 1 |
| -689.064 | 19549.928 | 1.561 | 13.272 | 0.133034 | 7/15/2014 | 59:22.9 | 1 |
| -689.315 | 19549.934 | 1.558 | 13.263 | 0.11129 | 7/15/2014 | 59:23.0 | 1 |
| -689.569 | 19549.94 | 1.558 | 13.256 | 0.10663 | 7/15/2014 | 59:23.1 | 1 |
| -689.82 | 19549.945 | 1.55 | 13.25 | 0.102239 | 7/15/2014 | 59:23.2 | 1 |
| -690.073 | 19549.953 | 1.553 | 13.247 | 0.113596 | 7/15/2014 | 59:23.3 | 1 |
| -690.386 | 19549.96 | 1.545 | 13.246 | 0.0833683 | 7/15/2014 | 59:23.4 | 1 |
| -690.639 | 19549.967 | 1.548 | 13.246 | 0.07797 | 7/15/2014 | 59:23.5 | 1 |
| -690.891 | 19549.972 | 1.545 | 13.248 | 0.0609998 | 7/15/2014 | 59:23.6 | 1 |
| -691.144 | 19549.979 | 1.551 | 13.252 | 0.0504824 | 7/15/2014 | 59:23.7 | 1 |
| -691.459 | 19549.984 | 1.551 | 13.257 | 0.0551505 | 7/15/2014 | 59:23.8 | 1 |
| -691.713 | 19549.99 | 1.559 | 13.264 | 0.0344847 | 7/15/2014 | 59:23.9 | 1 |
| -691.965 | 19549.997 | 1.553 | 13.273 | 0.0370874 | 7/15/2014 | 59:24.0 | 1 |
| -692.218 | 19550.002 | 1.556 | 13.285 | 0.0279365 | 7/15/2014 | 59:24.1 | 1 |
| -692.47 | 19550.008 | 1.55 | 13.299 | 0.0573149 | 7/15/2014 | 59:24.2 | 1 |
| -692.721 | 19550.014 | 1.547 | 13.316 | 0.0575918 | 7/15/2014 | 59:24.3 | 1 |
| -693.033 | 19550.021 | 1.536 | 13.336 | 0.117364 | 7/15/2014 | 59:24.4 | 1 |
| -693.284 | 19550.025 | 1.536 | 13.358 | 0.113734 | 7/15/2014 | 59:24.5 | 1 |
| -693.532 | 19550.031 | 1.528 | 13.381 | 0.119616 | 7/15/2014 | 59:24.6 | 1 |
| -693.962 | 19550.039 | 1.529 | 13.417 | 0.154634 | 7/15/2014 | 59:24.7 | 1 |
| -694.088 | 19550.042 | 1.525 | 13.429 | 0.163923 | 7/15/2014 | 59:24.8 | 1 |
| -694.336 | 19550.048 | 1.525 | 13.451 | 0.165736 | 7/15/2014 | 59:24.9 | 1 |
| -694.582 | 19550.054 | 1.525 | 13.471 | 0.165848 | 7/15/2014 | 59:25.0 | 1 |
| -694.831 | 19550.059 | 1.533 | 13.487 | 0.162026 | 7/15/2014 | 59:25.1 | 1 |
| -695.077 | 19550.063 | 1.531 | 13.499 | 0.163027 | 7/15/2014 | 59:25.2 | 1 |
| -695.323 | 19550.068 | 1.534 | 13.508 | 0.142456 | 7/15/2014 | 59:25.3 | 1 |
| -695.75 | 19550.075 | 1.539 | 13.511 | 0.162703 | 7/15/2014 | 59:25.4 | 1 |
| -695.994 | 19550.081 | 1.539 | 13.511 | 0.193618 | 7/15/2014 | 59:25.5 | 1 |
| -696.237 | 19550.086 | 1.539 | 13.508 | 0.202298 | 7/15/2014 | 59:25.6 | 1 |
| -696.541 | 19550.091 | 1.545 | 13.503 | 0.198561 | 7/15/2014 | 59:25.7 | 1 |
| -696.664 | 19550.093 | 1.543 | 13.5 | 0.213544 | 7/15/2014 | 59:25.8 | 1 |
| -696.909 | 19550.098 | 1.549 | 13.492 | 0.213382 | 7/15/2014 | 59:25.9 | 1 |

| | | | | | | | |
|---|---|---|---|---|---|---|---|
| -697.154 | 19550.103 | 1.543 | 13.481 | 0.194378 | 7/15/2014 | 59:26.0 | 1 |
| -697.399 | 19550.106 | 1.537 | 13.469 | 0.154698 | 7/15/2014 | 59:26.1 | 1 |
| -697.643 | 19550.111 | 1.534 | 13.455 | 0.113363 | 7/15/2014 | 59:26.2 | 1 |
| -697.887 | 19550.115 | 1.538 | 13.439 | 0.10534 | 7/15/2014 | 59:26.3 | 1 |
| -698.312 | 19550.122 | 1.544 | 13.411 | 0.108037 | 7/15/2014 | 59:26.4 | 1 |
| -698.553 | 19550.126 | 1.542 | 13.39 | 0.11336 | 7/15/2014 | 59:26.5 | 1 |
| -698.795 | 19550.132 | 1.544 | 13.367 | 0.122436 | 7/15/2014 | 59:26.6 | 1 |
| -699.094 | 19550.137 | 1.547 | 13.342 | 0.130321 | 7/15/2014 | 59:26.7 | 1 |
| -699.217 | 19550.139 | 1.549 | 13.331 | 0.134853 | 7/15/2014 | 59:26.8 | 1 |
| -699.459 | 19550.143 | 1.555 | 13.303 | 0.127844 | 7/15/2014 | 59:26.9 | 1 |
| -699.701 | 19550.146 | 1.555 | 13.275 | 0.119442 | 7/15/2014 | 59:27.0 | 1 |
| -699.942 | 19550.151 | 1.551 | 13.245 | 0.11227 | 7/15/2014 | 59:27.1 | 1 |
| -700.18 | 19550.155 | 1.551 | 13.216 | 0.0866289 | 7/15/2014 | 59:27.2 | 1 |
| -700.421 | 19550.161 | 1.556 | 13.187 | 0.0686964 | 7/15/2014 | 59:27.3 | 1 |
| -700.836 | 19550.168 | 1.562 | 13.147 | 0.0762839 | 7/15/2014 | 59:27.4 | 1 |
| -701.071 | 19550.173 | 1.566 | 13.122 | 0.0695713 | 7/15/2014 | 59:27.5 | 1 |
| -701.307 | 19550.178 | 1.577 | 13.099 | 0.0842405 | 7/15/2014 | 59:27.6 | 1 |
| -701.599 | 19550.184 | 1.581 | 13.078 | 0.0965702 | 7/15/2014 | 59:27.7 | 1 |
| -701.718 | 19550.186 | 1.588 | 13.067 | 0.109273 | 7/15/2014 | 59:27.8 | 1 |
| -701.956 | 19550.19 | 1.6 | 13.05 | 0.118063 | 7/15/2014 | 59:27.9 | 1 |
| -702.19 | 19550.195 | 1.602 | 13.036 | 0.131326 | 7/15/2014 | 59:28.0 | 1 |
| -702.427 | 19550.199 | 1.608 | 13.024 | 0.142809 | 7/15/2014 | 59:28.1 | 1 |
| -702.659 | 19550.205 | 1.607 | 13.014 | 0.155005 | 7/15/2014 | 59:28.2 | 1 |
| -702.894 | 19550.211 | 1.617 | 13.005 | 0.137421 | 7/15/2014 | 59:28.3 | 1 |
| -703.299 | 19550.218 | 1.616 | 12.994 | 0.169949 | 7/15/2014 | 59:28.4 | 1 |
| -703.532 | 19550.224 | 1.619 | 12.986 | 0.152183 | 7/15/2014 | 59:28.5 | 1 |
| -703.764 | 19550.229 | 1.621 | 12.979 | 0.166856 | 7/15/2014 | 59:28.6 | 1 |
| -704.056 | 19550.234 | 1.621 | 12.972 | 0.136605 | 7/15/2014 | 59:28.7 | 1 |
| -704.171 | 19550.237 | 1.622 | 12.969 | 0.137438 | 7/15/2014 | 59:28.8 | 1 |
| -704.408 | 19550.244 | 1.629 | 12.962 | 0.147645 | 7/15/2014 | 59:28.9 | 1 |
| -704.642 | 19550.248 | 1.625 | 12.957 | 0.143505 | 7/15/2014 | 59:29.0 | 1 |
| -704.88 | 19550.254 | 1.629 | 12.952 | 0.115419 | 7/15/2014 | 59:29.1 | 1 |
| -705.117 | 19550.262 | 1.63 | 12.948 | 0.130761 | 7/15/2014 | 59:29.2 | 1 |
| -705.356 | 19550.267 | 1.638 | 12.946 | 0.119267 | 7/15/2014 | 59:29.3 | 1 |
| -705.772 | 19550.277 | 1.644 | 12.945 | 0.122737 | 7/15/2014 | 59:29.4 | 1 |
| -706.01 | 19550.284 | 1.653 | 12.946 | 0.131503 | 7/15/2014 | 59:29.5 | 1 |
| -706.247 | 19550.289 | 1.655 | 12.947 | 0.112753 | 7/15/2014 | 59:29.6 | 1 |
| -706.547 | 19550.299 | 1.66 | 12.949 | 0.108609 | 7/15/2014 | 59:29.7 | 1 |
| -706.668 | 19550.303 | 1.663 | 12.95 | 0.106659 | 7/15/2014 | 59:29.8 | 1 |
| -706.91 | 19550.31 | 1.673 | 12.954 | 0.0987423 | 7/15/2014 | 59:29.9 | 1 |
| -707.15 | 19550.316 | 1.674 | 12.957 | 0.104031 | 7/15/2014 | 59:30.0 | 1 |
| -707.394 | 19550.324 | 1.677 | 12.96 | 0.102714 | 7/15/2014 | 59:30.1 | 1 |
| -707.634 | 19550.331 | 1.68 | 12.962 | 0.10018 | 7/15/2014 | 59:30.2 | 1 |
| -707.876 | 19550.338 | 1.689 | 12.963 | 0.0992807 | 7/15/2014 | 59:30.3 | 1 |
| -708.297 | 19550.352 | 1.695 | 12.959 | 0.0950889 | 7/15/2014 | 59:30.4 | 1 |
| -708.539 | 19550.359 | 1.701 | 12.956 | 0.0922453 | 7/15/2014 | 59:30.5 | 1 |
| -708.779 | 19550.367 | 1.701 | 12.949 | 0.0879877 | 7/15/2014 | 59:30.6 | 1 |

| | | | | | | | |
|---|---|---|---|---|---|---|---|
| -709.081 | 19550.378 | 1.703 | 12.942 | 0.0842194 | 7/15/2014 | 59:30.7 | 1 |
| -709.202 | 19550.382 | 1.703 | 12.938 | 0.083353 | 7/15/2014 | 59:30.8 | 1 |
| -709.446 | 19550.391 | 1.708 | 12.928 | 0.0830577 | 7/15/2014 | 59:30.9 | 1 |
| -709.691 | 19550.398 | 1.705 | 12.917 | 0.0801973 | 7/15/2014 | 59:31.0 | 1 |
| -709.936 | 19550.407 | 1.711 | 12.906 | 0.0789838 | 7/15/2014 | 59:31.1 | 1 |
| -710.181 | 19550.416 | 1.718 | 12.896 | 0.0884015 | 7/15/2014 | 59:31.2 | 1 |
| -710.427 | 19550.425 | 1.724 | 12.887 | 0.0805272 | 7/15/2014 | 59:31.3 | 1 |
| -710.856 | 19550.44 | 1.729 | 12.881 | 0.0825015 | 7/15/2014 | 59:31.4 | 1 |
| -711.099 | 19550.451 | 1.738 | 12.879 | 0.0840626 | 7/15/2014 | 59:31.5 | 1 |
| -711.343 | 19550.459 | 1.744 | 12.881 | 0.0888956 | 7/15/2014 | 59:31.6 | 1 |
| -711.647 | 19550.472 | 1.753 | 12.887 | 0.0904568 | 7/15/2014 | 59:31.7 | 1 |
| -711.772 | 19550.477 | 1.754 | 12.888 | 0.0938373 | 7/15/2014 | 59:31.8 | 1 |
| -712.018 | 19550.487 | 1.76 | 12.899 | 0.0979678 | 7/15/2014 | 59:31.9 | 1 |
| -712.262 | 19550.497 | 1.76 | 12.914 | 0.103967 | 7/15/2014 | 59:32.0 | 1 |
| -712.506 | 19550.506 | 1.764 | 12.931 | 0.105876 | 7/15/2014 | 59:32.1 | 1 |
| -712.748 | 19550.518 | 1.764 | 12.949 | 0.103512 | 7/15/2014 | 59:32.2 | 1 |
| -712.991 | 19550.527 | 1.764 | 12.968 | 0.10473 | 7/15/2014 | 59:32.3 | 1 |
| -713.411 | 19550.545 | 1.762 | 12.992 | 0.106568 | 7/15/2014 | 59:32.4 | 1 |
| -713.652 | 19550.557 | 1.758 | 13.007 | 0.0971795 | 7/15/2014 | 59:32.5 | 1 |
| -713.89 | 19550.567 | 1.758 | 13.018 | 0.0924445 | 7/15/2014 | 59:32.6 | 1 |
| -714.189 | 19550.58 | 1.762 | 13.025 | 0.0897359 | 7/15/2014 | 59:32.7 | 1 |
| -714.31 | 19550.586 | 1.76 | 13.029 | 0.0893519 | 7/15/2014 | 59:32.8 | 1 |
| -714.551 | 19550.598 | 1.767 | 13.03 | 0.0850488 | 7/15/2014 | 59:32.9 | 1 |
| -714.793 | 19550.607 | 1.767 | 13.024 | 0.0961111 | 7/15/2014 | 59:33.0 | 1 |
| -715.035 | 19550.619 | 1.773 | 13.014 | 0.0939432 | 7/15/2014 | 59:33.1 | 1 |
| -715.277 | 19550.632 | 1.769 | 12.999 | 0.0984389 | 7/15/2014 | 59:33.2 | 1 |
| -715.518 | 19550.644 | 1.773 | 12.98 | 0.112453 | 7/15/2014 | 59:33.3 | 1 |
| -715.941 | 19550.664 | 1.767 | 12.944 | 0.100072 | 7/15/2014 | 59:33.4 | 1 |
| -716.182 | 19550.676 | 1.767 | 12.92 | 0.101106 | 7/15/2014 | 59:33.5 | 1 |
| -716.423 | 19550.688 | 1.765 | 12.895 | 0.088164 | 7/15/2014 | 59:33.6 | 1 |
| -716.724 | 19550.704 | 1.765 | 12.871 | 0.097697 | 7/15/2014 | 59:33.7 | 1 |
| -716.846 | 19550.711 | 1.762 | 12.858 | 0.0915059 | 7/15/2014 | 59:33.8 | 1 |
| -717.089 | 19550.724 | 1.765 | 12.837 | 0.091458 | 7/15/2014 | 59:33.9 | 1 |
| -717.332 | 19550.736 | 1.759 | 12.819 | 0.100686 | 7/15/2014 | 59:34.0 | 1 |
| -717.575 | 19550.75 | 1.765 | 12.804 | 0.0984264 | 7/15/2014 | 59:34.1 | 1 |
| -717.817 | 19550.762 | 1.765 | 12.793 | 0.102986 | 7/15/2014 | 59:34.2 | 1 |
| -718.06 | 19550.776 | 1.768 | 12.786 | 0.10456 | 7/15/2014 | 59:34.3 | 1 |
| -718.48 | 19550.8 | 1.764 | 12.785 | 0.114503 | 7/15/2014 | 59:34.4 | 1 |
| -718.718 | 19550.813 | 1.764 | 12.786 | 0.113496 | 7/15/2014 | 59:34.5 | 1 |
| -718.957 | 19550.826 | 1.76 | 12.791 | 0.11024 | 7/15/2014 | 59:34.6 | 1 |
| -719.255 | 19550.844 | 1.763 | 12.796 | 0.11698 | 7/15/2014 | 59:34.7 | 1 |
| -719.376 | 19550.85 | 1.759 | 12.8 | 0.123295 | 7/15/2014 | 59:34.8 | 1 |
| -719.616 | 19550.865 | 1.759 | 12.807 | 0.133836 | 7/15/2014 | 59:34.9 | 1 |
| -719.854 | 19550.878 | 1.757 | 12.815 | 0.124198 | 7/15/2014 | 59:35.0 | 1 |
| -720.093 | 19550.894 | 1.757 | 12.822 | 0.133427 | 7/15/2014 | 59:35.1 | 1 |
| -720.33 | 19550.906 | 1.748 | 12.828 | 0.143708 | 7/15/2014 | 59:35.2 | 1 |
| -720.568 | 19550.922 | 1.748 | 12.833 | 0.1435 | 7/15/2014 | 59:35.3 | 1 |

| | | | | | | | |
|---|---|---|---|---|---|---|---|
| -720.981 | 19550.946 | 1.742 | 12.839 | 0.14688 | 7/15/2014 | 59:35.4 | 1 |
| -721.216 | 19550.961 | 1.737 | 12.843 | 0.142503 | 7/15/2014 | 59:35.5 | 1 |
| -721.454 | 19550.977 | 1.734 | 12.847 | 0.14097 | 7/15/2014 | 59:35.6 | 1 |
| -721.751 | 19550.995 | 1.731 | 12.852 | 0.134638 | 7/15/2014 | 59:35.7 | 1 |
| -721.869 | 19551.002 | 1.729 | 12.854 | 0.132822 | 7/15/2014 | 59:35.8 | 1 |
| -722.109 | 19551.018 | 1.735 | 12.861 | 0.138 | 7/15/2014 | 59:35.9 | 1 |
| -722.349 | 19551.033 | 1.729 | 12.869 | 0.135964 | 7/15/2014 | 59:36.0 | 1 |
| -722.591 | 19551.049 | 1.73 | 12.879 | 0.130544 | 7/15/2014 | 59:36.1 | 1 |
| -722.83 | 19551.064 | 1.729 | 12.891 | 0.141341 | 7/15/2014 | 59:36.2 | 1 |
| -723.073 | 19551.081 | 1.735 | 12.903 | 0.128091 | 7/15/2014 | 59:36.3 | 1 |
| -723.495 | 19551.108 | 1.728 | 12.924 | 0.150768 | 7/15/2014 | 59:36.4 | 1 |
| -723.735 | 19551.124 | 1.725 | 12.938 | 0.142254 | 7/15/2014 | 59:36.5 | 1 |
| -723.976 | 19551.142 | 1.725 | 12.951 | 0.143882 | 7/15/2014 | 59:36.6 | 1 |
| -724.274 | 19551.161 | 1.722 | 12.964 | 0.149791 | 7/15/2014 | 59:36.7 | 1 |
| -724.396 | 19551.17 | 1.721 | 12.971 | 0.141953 | 7/15/2014 | 59:36.8 | 1 |
| -724.639 | 19551.187 | 1.719 | 12.984 | 0.171521 | 7/15/2014 | 59:36.9 | 1 |
| -724.877 | 19551.202 | 1.713 | 12.996 | 0.154581 | 7/15/2014 | 59:37.0 | 1 |
| -725.118 | 19551.22 | 1.715 | 13.007 | 0.162245 | 7/15/2014 | 59:37.1 | 1 |
| -725.356 | 19551.235 | 1.712 | 13.017 | 0.157714 | 7/15/2014 | 59:37.2 | 1 |
| -725.595 | 19551.253 | 1.713 | 13.028 | 0.157881 | 7/15/2014 | 59:37.3 | 1 |
| -726.006 | 19551.281 | 1.711 | 13.043 | 0.15247 | 7/15/2014 | 59:37.4 | 1 |
| -726.241 | 19551.298 | 1.711 | 13.052 | 0.147564 | 7/15/2014 | 59:37.5 | 1 |
| -726.475 | 19551.315 | 1.717 | 13.062 | 0.143426 | 7/15/2014 | 59:37.6 | 1 |
| -726.766 | 19551.337 | 1.713 | 13.071 | 0.140191 | 7/15/2014 | 59:37.7 | 1 |
| -726.885 | 19551.345 | 1.713 | 13.076 | 0.136892 | 7/15/2014 | 59:37.8 | 1 |
| -727.122 | 19551.362 | 1.717 | 13.084 | 0.134294 | 7/15/2014 | 59:37.9 | 1 |
| -727.356 | 19551.379 | 1.715 | 13.092 | 0.132455 | 7/15/2014 | 59:38.0 | 1 |
| -727.595 | 19551.396 | 1.719 | 13.1 | 0.131373 | 7/15/2014 | 59:38.1 | 1 |
| -727.829 | 19551.413 | 1.715 | 13.107 | 0.126175 | 7/15/2014 | 59:38.2 | 1 |
| -728.067 | 19551.432 | 1.721 | 13.115 | 0.144923 | 7/15/2014 | 59:38.3 | 1 |
| -728.479 | 19551.464 | 1.721 | 13.125 | 0.138087 | 7/15/2014 | 59:38.4 | 1 |
| -728.714 | 19551.481 | 1.726 | 13.131 | 0.137251 | 7/15/2014 | 59:38.5 | 1 |
| -728.95 | 19551.498 | 1.728 | 13.136 | 0.14015 | 7/15/2014 | 59:38.6 | 1 |
| -729.247 | 19551.522 | 1.726 | 13.139 | 0.142249 | 7/15/2014 | 59:38.7 | 1 |
| -729.365 | 19551.531 | 1.723 | 13.142 | 0.144067 | 7/15/2014 | 59:38.8 | 1 |
| -729.606 | 19551.549 | 1.729 | 13.141 | 0.159026 | 7/15/2014 | 59:38.9 | 1 |
| -729.844 | 19551.568 | 1.727 | 13.137 | 0.133602 | 7/15/2014 | 59:39.0 | 1 |
| -730.086 | 19551.588 | 1.73 | 13.13 | 0.126719 | 7/15/2014 | 59:39.1 | 1 |
| -730.324 | 19551.606 | 1.73 | 13.118 | 0.119194 | 7/15/2014 | 59:39.2 | 1 |
| -730.565 | 19551.625 | 1.734 | 13.103 | 0.107092 | 7/15/2014 | 59:39.3 | 1 |
| -730.984 | 19551.659 | 1.74 | 13.076 | 0.102213 | 7/15/2014 | 59:39.4 | 1 |
| -731.223 | 19551.678 | 1.746 | 13.057 | 0.105683 | 7/15/2014 | 59:39.5 | 1 |
| -731.461 | 19551.697 | 1.744 | 13.039 | 0.102895 | 7/15/2014 | 59:39.6 | 1 |
| -731.759 | 19551.723 | 1.744 | 13.021 | 0.101193 | 7/15/2014 | 59:39.7 | 1 |
| -731.881 | 19551.732 | 1.744 | 13.011 | 0.103865 | 7/15/2014 | 59:39.8 | 1 |
| -732.121 | 19551.752 | 1.747 | 12.996 | 0.110984 | 7/15/2014 | 59:39.9 | 1 |
| -732.36 | 19551.771 | 1.744 | 12.982 | 0.109707 | 7/15/2014 | 59:40.0 | 1 |

| | | | | | | | |
|---|---|---|---|---|---|---|---|
| -732.602 | 19551.791 | 1.744 | 12.97 | 0.107916 | 7/15/2014 | 59:40.1 | 1 |
| -732.841 | 19551.811 | 1.738 | 12.961 | 0.109469 | 7/15/2014 | 59:40.2 | 1 |
| -733.078 | 19551.832 | 1.738 | 12.953 | 0.122211 | 7/15/2014 | 59:40.3 | 1 |
| -733.494 | 19551.867 | 1.749 | 12.948 | 0.107937 | 7/15/2014 | 59:40.4 | 1 |
| -733.73 | 19551.887 | 1.754 | 12.946 | 0.119764 | 7/15/2014 | 59:40.5 | 1 |
| -733.966 | 19551.906 | 1.756 | 12.947 | 0.127538 | 7/15/2014 | 59:40.6 | 1 |
| -734.261 | 19551.932 | 1.757 | 12.95 | 0.117675 | 7/15/2014 | 59:40.7 | 1 |
| -734.381 | 19551.941 | 1.76 | 12.952 | 0.123356 | 7/15/2014 | 59:40.8 | 1 |
| -734.618 | 19551.963 | 1.76 | 12.959 | 0.129209 | 7/15/2014 | 59:40.9 | 1 |
| -734.857 | 19551.982 | 1.76 | 12.97 | 0.138153 | 7/15/2014 | 59:41.0 | 1 |
| -735.095 | 19552.004 | 1.76 | 12.984 | 0.170594 | 7/15/2014 | 59:41.1 | 1 |
| -735.333 | 19552.023 | 1.753 | 13.001 | 0.180615 | 7/15/2014 | 59:41.2 | 1 |
| -735.572 | 19552.045 | 1.75 | 13.02 | 0.180982 | 7/15/2014 | 59:41.3 | 1 |
| -735.987 | 19552.08 | 1.739 | 13.056 | 0.225627 | 7/15/2014 | 59:41.4 | 1 |
| -736.224 | 19552.102 | 1.739 | 13.08 | 0.206484 | 7/15/2014 | 59:41.5 | 1 |
| -736.463 | 19552.121 | 1.739 | 13.106 | 0.193311 | 7/15/2014 | 59:41.6 | 1 |
| -736.76 | 19552.148 | 1.737 | 13.133 | 0.204733 | 7/15/2014 | 59:41.7 | 1 |
| -736.881 | 19552.158 | 1.733 | 13.147 | 0.209696 | 7/15/2014 | 59:41.8 | 1 |
| -737.121 | 19552.182 | 1.73 | 13.174 | 0.187777 | 7/15/2014 | 59:41.9 | 1 |
| -737.362 | 19552.201 | 1.724 | 13.201 | 0.189439 | 7/15/2014 | 59:42.0 | 1 |
| -737.603 | 19552.223 | 1.727 | 13.227 | 0.178748 | 7/15/2014 | 59:42.1 | 1 |
| -737.843 | 19552.244 | 1.717 | 13.25 | 0.17331 | 7/15/2014 | 59:42.2 | 1 |
| -738.085 | 19552.266 | 1.713 | 13.27 | 0.181893 | 7/15/2014 | 59:42.3 | 1 |
| -738.505 | 19552.303 | 1.706 | 13.29 | 0.170663 | 7/15/2014 | 59:42.4 | 1 |
| -738.743 | 19552.324 | 1.703 | 13.3 | 0.162346 | 7/15/2014 | 59:42.5 | 1 |
| -738.984 | 19552.346 | 1.703 | 13.305 | 0.165003 | 7/15/2014 | 59:42.6 | 1 |
| -739.283 | 19552.373 | 1.703 | 13.306 | 0.154615 | 7/15/2014 | 59:42.7 | 1 |
| -739.403 | 19552.383 | 1.697 | 13.307 | 0.136181 | 7/15/2014 | 59:42.8 | 1 |
| -739.647 | 19552.405 | 1.704 | 13.301 | 0.149454 | 7/15/2014 | 59:42.9 | 1 |
| -739.888 | 19552.427 | 1.703 | 13.292 | 0.129876 | 7/15/2014 | 59:43.0 | 1 |
| -740.131 | 19552.449 | 1.707 | 13.28 | 0.107653 | 7/15/2014 | 59:43.1 | 1 |
| -740.371 | 19552.469 | 1.701 | 13.266 | 0.106883 | 7/15/2014 | 59:43.2 | 1 |
| -740.614 | 19552.492 | 1.701 | 13.251 | 0.0890795 | 7/15/2014 | 59:43.3 | 1 |
| -741.035 | 19552.529 | 1.698 | 13.231 | 0.0784705 | 7/15/2014 | 59:43.4 | 1 |
| -741.276 | 19552.552 | 1.694 | 13.22 | 0.0786993 | 7/15/2014 | 59:43.5 | 1 |
| -741.515 | 19552.573 | 1.691 | 13.21 | 0.0739418 | 7/15/2014 | 59:43.6 | 1 |
| -741.817 | 19552.602 | 1.688 | 13.203 | 0.0736098 | 7/15/2014 | 59:43.7 | 1 |
| -741.939 | 19552.611 | 1.683 | 13.199 | 0.0879958 | 7/15/2014 | 59:43.8 | 1 |
| -742.182 | 19552.635 | 1.685 | 13.197 | 0.0991355 | 7/15/2014 | 59:43.9 | 1 |
| -742.424 | 19552.656 | 1.685 | 13.199 | 0.074144 | 7/15/2014 | 59:44.0 | 1 |
| -742.668 | 19552.678 | 1.685 | 13.204 | 0.0886213 | 7/15/2014 | 59:44.1 | 1 |
| -742.909 | 19552.701 | 1.678 | 13.212 | 0.0581561 | 7/15/2014 | 59:44.2 | 1 |
| -743.15 | 19552.722 | 1.678 | 13.222 | 0.0546582 | 7/15/2014 | 59:44.3 | 1 |
| -743.571 | 19552.761 | 1.684 | 13.239 | 0.0490157 | 7/15/2014 | 59:44.4 | 1 |
| -743.811 | 19552.783 | 1.687 | 13.25 | 0.0530227 | 7/15/2014 | 59:44.5 | 1 |
| -744.05 | 19552.805 | 1.687 | 13.259 | 0.0484957 | 7/15/2014 | 59:44.6 | 1 |
| -744.348 | 19552.832 | 1.684 | 13.268 | 0.0624136 | 7/15/2014 | 59:44.7 | 1 |

| | | | | | | | |
|---|---|---|---|---|---|---|---|
| -744.47 | 19552.844 | 1.685 | 13.273 | 0.0601626 | 7/15/2014 | 59:44.8 | 1 |
| -744.71 | 19552.864 | 1.689 | 13.278 | 0.0447515 | 7/15/2014 | 59:44.9 | 1 |
| -744.949 | 19552.887 | 1.689 | 13.281 | 0.0584091 | 7/15/2014 | 59:45.0 | 1 |
| -745.189 | 19552.908 | 1.692 | 13.283 | 0.0607668 | 7/15/2014 | 59:45.1 | 1 |
| -745.426 | 19552.93 | 1.692 | 13.283 | 0.0633347 | 7/15/2014 | 59:45.2 | 1 |
| -745.665 | 19552.953 | 1.696 | 13.282 | 0.0713733 | 7/15/2014 | 59:45.3 | 1 |
| -746.078 | 19552.99 | 1.7 | 13.279 | 0.0702081 | 7/15/2014 | 59:45.4 | 1 |
| -746.315 | 19553.012 | 1.7 | 13.277 | 0.0747175 | 7/15/2014 | 59:45.5 | 1 |
| -746.551 | 19553.033 | 1.703 | 13.274 | 0.0678834 | 7/15/2014 | 59:45.6 | 1 |
| -746.846 | 19553.061 | 1.706 | 13.27 | 0.0621367 | 7/15/2014 | 59:45.7 | 1 |
| -746.964 | 19553.071 | 1.703 | 13.268 | 0.0640542 | 7/15/2014 | 59:45.8 | 1 |
| -747.204 | 19553.094 | 1.709 | 13.262 | 0.0595202 | 7/15/2014 | 59:45.9 | 1 |
| -747.44 | 19553.115 | 1.708 | 13.254 | 0.0547034 | 7/15/2014 | 59:46.0 | 1 |
| -747.679 | 19553.138 | 1.718 | 13.244 | 0.0527978 | 7/15/2014 | 59:46.1 | 1 |
| -747.918 | 19553.159 | 1.728 | 13.231 | 0.0521496 | 7/15/2014 | 59:46.2 | 1 |
| -748.157 | 19553.182 | 1.741 | 13.215 | 0.0527331 | 7/15/2014 | 59:46.3 | 1 |
| -748.573 | 19553.222 | 1.747 | 13.185 | 0.0554766 | 7/15/2014 | 59:46.4 | 1 |
| -748.812 | 19553.242 | 1.755 | 13.164 | 0.0598097 | 7/15/2014 | 59:46.5 | 1 |
| -749.05 | 19553.266 | 1.758 | 13.14 | 0.0719458 | 7/15/2014 | 59:46.6 | 1 |
| -749.351 | 19553.294 | 1.762 | 13.116 | 0.0694362 | 7/15/2014 | 59:46.7 | 1 |
| -749.471 | 19553.305 | 1.761 | 13.103 | 0.0776896 | 7/15/2014 | 59:46.8 | 1 |
| -749.713 | 19553.327 | 1.757 | 13.078 | 0.0736216 | 7/15/2014 | 59:46.9 | 1 |
| -749.956 | 19553.35 | 1.757 | 13.052 | 0.0910261 | 7/15/2014 | 59:47.0 | 1 |
| -750.199 | 19553.373 | 1.76 | 13.029 | 0.0862459 | 7/15/2014 | 59:47.1 | 1 |
| -750.441 | 19553.395 | 1.76 | 13.006 | 0.0908828 | 7/15/2014 | 59:47.2 | 1 |
| -750.684 | 19553.418 | 1.762 | 12.986 | 0.0914307 | 7/15/2014 | 59:47.3 | 1 |
| -751.109 | 19553.459 | 1.771 | 12.962 | 0.0913308 | 7/15/2014 | 59:47.4 | 1 |
| -751.35 | 19553.481 | 1.779 | 12.948 | 0.0887772 | 7/15/2014 | 59:47.5 | 1 |
| -751.592 | 19553.503 | 1.791 | 12.936 | 0.0884724 | 7/15/2014 | 59:47.6 | 1 |
| -751.894 | 19553.531 | 1.795 | 12.925 | 0.0849437 | 7/15/2014 | 59:47.7 | 1 |
| -752.016 | 19553.543 | 1.799 | 12.92 | 0.0830727 | 7/15/2014 | 59:47.8 | 1 |
| -752.261 | 19553.566 | 1.807 | 12.912 | 0.0849978 | 7/15/2014 | 59:47.9 | 1 |
| -752.504 | 19553.59 | 1.811 | 12.905 | 0.0802884 | 7/15/2014 | 59:48.0 | 1 |
| -752.749 | 19553.613 | 1.816 | 12.9 | 0.0783081 | 7/15/2014 | 59:48.1 | 1 |
| -752.991 | 19553.636 | 1.814 | 12.896 | 0.0788235 | 7/15/2014 | 59:48.2 | 1 |
| -753.235 | 19553.659 | 1.819 | 12.894 | 0.0759197 | 7/15/2014 | 59:48.3 | 1 |
| -753.656 | 19553.698 | 1.817 | 12.895 | 0.0736886 | 7/15/2014 | 59:48.4 | 1 |
| -753.899 | 19553.721 | 1.817 | 12.898 | 0.0786331 | 7/15/2014 | 59:48.5 | 1 |
| -754.141 | 19553.744 | 1.817 | 12.903 | 0.0762969 | 7/15/2014 | 59:48.6 | 1 |
| -754.446 | 19553.773 | 1.817 | 12.91 | 0.0838128 | 7/15/2014 | 59:48.7 | 1 |
| -754.568 | 19553.784 | 1.816 | 12.913 | 0.0788317 | 7/15/2014 | 59:48.8 | 1 |
| -754.814 | 19553.809 | 1.822 | 12.924 | 0.071302 | 7/15/2014 | 59:48.9 | 1 |
| -755.06 | 19553.831 | 1.82 | 12.937 | 0.0712124 | 7/15/2014 | 59:49.0 | 1 |
| -755.308 | 19553.854 | 1.828 | 12.952 | 0.0686341 | 7/15/2014 | 59:49.1 | 1 |
| -755.555 | 19553.877 | 1.825 | 12.967 | 0.0713104 | 7/15/2014 | 59:49.2 | 1 |
| -755.803 | 19553.9 | 1.836 | 12.983 | 0.073965 | 7/15/2014 | 59:49.3 | 1 |
| -756.111 | 19553.93 | 1.839 | 12.998 | 0.0907355 | 7/15/2014 | 59:49.4 | 1 |

| | | | | | | | |
|---|---|---|---|---|---|---|---|
| -756.36 | 19553.953 | 1.848 | 13.011 | 0.0729053 | 7/15/2014 | 59:49.5 | 1 |
| -756.609 | 19553.977 | 1.853 | 13.023 | 0.089439 | 7/15/2014 | 59:49.6 | 1 |
| -756.859 | 19554 | 1.859 | 13.033 | 0.0852915 | 7/15/2014 | 59:49.7 | 1 |
| -757.169 | 19554.029 | 1.859 | 13.04 | 0.0921777 | 7/15/2014 | 59:49.8 | 1 |
| -757.42 | 19554.053 | 1.867 | 13.044 | 0.0998227 | 7/15/2014 | 59:49.9 | 1 |
| -757.67 | 19554.076 | 1.867 | 13.046 | 0.106098 | 7/15/2014 | 59:50.0 | 1 |
| -757.922 | 19554.1 | 1.87 | 13.047 | 0.106946 | 7/15/2014 | 59:50.1 | 1 |
| -758.172 | 19554.122 | 1.873 | 13.046 | 0.106576 | 7/15/2014 | 59:50.2 | 1 |
| -758.42 | 19554.145 | 1.873 | 13.044 | 0.106815 | 7/15/2014 | 59:50.3 | 1 |
| -758.73 | 19554.174 | 1.876 | 13.041 | 0.107993 | 7/15/2014 | 59:50.4 | 1 |
| -758.978 | 19554.197 | 1.878 | 13.038 | 0.107648 | 7/15/2014 | 59:50.5 | 1 |
| -759.225 | 19554.221 | 1.878 | 13.034 | 0.117242 | 7/15/2014 | 59:50.6 | 1 |
| -759.652 | 19554.26 | 1.884 | 13.029 | 0.102268 | 7/15/2014 | 59:50.7 | 1 |
| -759.778 | 19554.271 | 1.888 | 13.027 | 0.0993493 | 7/15/2014 | 59:50.8 | 1 |
| -760.024 | 19554.291 | 1.892 | 13.023 | 0.126084 | 7/15/2014 | 59:50.9 | 1 |
| -760.269 | 19554.314 | 1.899 | 13.018 | 0.106825 | 7/15/2014 | 59:51.0 | 1 |
| -760.514 | 19554.336 | 1.909 | 13.011 | 0.102367 | 7/15/2014 | 59:51.1 | 1 |
| -760.757 | 19554.358 | 1.919 | 13.004 | 0.093454 | 7/15/2014 | 59:51.2 | 1 |
| -761 | 19554.379 | 1.928 | 12.995 | 0.0845335 | 7/15/2014 | 59:51.3 | 1 |
| -761.42 | 19554.418 | 1.922 | 12.977 | 0.0871107 | 7/15/2014 | 59:51.4 | 1 |
| -761.66 | 19554.438 | 1.928 | 12.964 | 0.0672756 | 7/15/2014 | 59:51.5 | 1 |
| -761.9 | 19554.459 | 1.928 | 12.949 | 0.0746769 | 7/15/2014 | 59:51.6 | 1 |
| -762.198 | 19554.484 | 1.925 | 12.934 | 0.0694823 | 7/15/2014 | 59:51.7 | 1 |
| -762.319 | 19554.495 | 1.922 | 12.926 | 0.0781223 | 7/15/2014 | 59:51.8 | 1 |
| -762.561 | 19554.516 | 1.92 | 12.909 | 0.0649726 | 7/15/2014 | 59:51.9 | 1 |
| -762.802 | 19554.535 | 1.914 | 12.893 | 0.0684939 | 7/15/2014 | 59:52.0 | 1 |
| -763.045 | 19554.557 | 1.921 | 12.879 | 0.0729378 | 7/15/2014 | 59:52.1 | 1 |
| -763.287 | 19554.578 | 1.921 | 12.866 | 0.0536085 | 7/15/2014 | 59:52.2 | 1 |
| -763.53 | 19554.598 | 1.924 | 12.855 | 0.0527783 | 7/15/2014 | 59:52.3 | 1 |
| -763.951 | 19554.633 | 1.924 | 12.848 | 0.0460231 | 7/15/2014 | 59:52.4 | 1 |
| -764.194 | 19554.652 | 1.924 | 12.845 | 0.0550251 | 7/15/2014 | 59:52.5 | 1 |
| -764.435 | 19554.672 | 1.921 | 12.846 | 0.0481478 | 7/15/2014 | 59:52.6 | 1 |
| -764.738 | 19554.697 | 1.921 | 12.85 | 0.0491782 | 7/15/2014 | 59:52.7 | 1 |
| -764.86 | 19554.709 | 1.92 | 12.851 | 0.0554835 | 7/15/2014 | 59:52.8 | 1 |
| -765.105 | 19554.729 | 1.922 | 12.859 | 0.0520402 | 7/15/2014 | 59:52.9 | 1 |
| -765.348 | 19554.748 | 1.916 | 12.869 | 0.0522057 | 7/15/2014 | 59:53.0 | 1 |
| -765.592 | 19554.768 | 1.916 | 12.882 | 0.0621649 | 7/15/2014 | 59:53.1 | 1 |
| -765.835 | 19554.787 | 1.907 | 12.895 | 0.0543431 | 7/15/2014 | 59:53.2 | 1 |
| -766.079 | 19554.807 | 1.907 | 12.91 | 0.0526512 | 7/15/2014 | 59:53.3 | 1 |
| -766.503 | 19554.842 | 1.906 | 12.933 | 0.0560745 | 7/15/2014 | 59:53.4 | 1 |
| -766.744 | 19554.86 | 1.906 | 12.947 | 0.0572067 | 7/15/2014 | 59:53.5 | 1 |
| -766.985 | 19554.879 | 1.901 | 12.96 | 0.0730099 | 7/15/2014 | 59:53.6 | 1 |
| -767.285 | 19554.902 | 1.901 | 12.972 | 0.0586448 | 7/15/2014 | 59:53.7 | 1 |
| -767.406 | 19554.912 | 1.897 | 12.979 | 0.079203 | 7/15/2014 | 59:53.8 | 1 |
| -767.649 | 19554.932 | 1.903 | 12.987 | 0.0979965 | 7/15/2014 | 59:53.9 | 1 |
| -767.889 | 19554.951 | 1.899 | 12.993 | 0.0678176 | 7/15/2014 | 59:54.0 | 1 |
| -768.131 | 19554.97 | 1.899 | 12.996 | 0.0705661 | 7/15/2014 | 59:54.1 | 1 |

| | | | | | | | |
|---|---|---|---|---|---|---|---|
| -768.372 | 19554.987 | 1.903 | 12.995 | 0.0749686 | 7/15/2014 | 59:54.2 | 1 |
| -768.611 | 19555.006 | 1.903 | 12.992 | 0.0775393 | 7/15/2014 | 59:54.3 | 1 |
| -769.027 | 19555.037 | 1.898 | 12.985 | 0.0774684 | 7/15/2014 | 59:54.4 | 1 |
| -769.264 | 19555.057 | 1.892 | 12.979 | 0.0671152 | 7/15/2014 | 59:54.5 | 1 |
| -769.501 | 19555.075 | 1.892 | 12.975 | 0.0749935 | 7/15/2014 | 59:54.6 | 1 |
| -769.799 | 19555.097 | 1.89 | 12.972 | 0.0777729 | 7/15/2014 | 59:54.7 | 1 |
| -769.919 | 19555.105 | 1.888 | 12.969 | 0.0809081 | 7/15/2014 | 59:54.8 | 1 |
| -770.159 | 19555.125 | 1.878 | 12.969 | 0.0742117 | 7/15/2014 | 59:54.9 | 1 |
| -770.399 | 19555.144 | 1.872 | 12.973 | 0.0749095 | 7/15/2014 | 59:55.0 | 1 |
| -770.642 | 19555.16 | 1.878 | 12.98 | 0.0670714 | 7/15/2014 | 59:55.1 | 1 |
| -770.881 | 19555.18 | 1.875 | 12.991 | 0.0662629 | 7/15/2014 | 59:55.2 | 1 |
| -771.123 | 19555.196 | 1.883 | 13.005 | 0.0600507 | 7/15/2014 | 59:55.3 | 1 |
| -771.546 | 19555.227 | 1.877 | 13.033 | 0.0565564 | 7/15/2014 | 59:55.4 | 1 |
| -771.787 | 19555.245 | 1.874 | 13.054 | 0.0515945 | 7/15/2014 | 59:55.5 | 1 |
| -772.03 | 19555.263 | 1.873 | 13.076 | 0.0467378 | 7/15/2014 | 59:55.6 | 1 |
| -772.334 | 19555.284 | 1.871 | 13.099 | 0.0541942 | 7/15/2014 | 59:55.7 | 1 |
| -772.455 | 19555.294 | 1.867 | 13.111 | 0.0507244 | 7/15/2014 | 59:55.8 | 1 |
| -772.701 | 19555.311 | 1.867 | 13.134 | 0.0644311 | 7/15/2014 | 59:55.9 | 1 |
| -772.947 | 19555.329 | 1.852 | 13.157 | 0.061869 | 7/15/2014 | 59:56.0 | 1 |
| -773.193 | 19555.346 | 1.849 | 13.178 | 0.0728419 | 7/15/2014 | 59:56.1 | 1 |
| -773.44 | 19555.362 | 1.843 | 13.197 | 0.0717392 | 7/15/2014 | 59:56.2 | 1 |
| -773.686 | 19555.38 | 1.847 | 13.213 | 0.0957511 | 7/15/2014 | 59:56.3 | 1 |
| -774.116 | 19555.409 | 1.842 | 13.228 | 0.109292 | 7/15/2014 | 59:56.4 | 1 |
| -774.362 | 19555.426 | 1.841 | 13.237 | 0.0904598 | 7/15/2014 | 59:56.5 | 1 |
| -774.606 | 19555.443 | 1.841 | 13.242 | 0.098788 | 7/15/2014 | 59:56.6 | 1 |
| -774.913 | 19555.464 | 1.846 | 13.245 | 0.08753 | 7/15/2014 | 59:56.7 | 1 |
| -775.038 | 19555.472 | 1.847 | 13.247 | 0.0960553 | 7/15/2014 | 59:56.8 | 1 |
| -775.285 | 19555.488 | 1.847 | 13.245 | 0.0912461 | 7/15/2014 | 59:56.9 | 1 |
| -775.531 | 19555.505 | 1.838 | 13.242 | 0.0772113 | 7/15/2014 | 59:57.0 | 1 |
| -775.777 | 19555.521 | 1.84 | 13.235 | 0.0694285 | 7/15/2014 | 59:57.1 | 1 |
| -776.024 | 19555.537 | 1.837 | 13.227 | 0.0917693 | 7/15/2014 | 59:57.2 | 1 |
| -776.27 | 19555.554 | 1.837 | 13.216 | 0.0689063 | 7/15/2014 | 59:57.3 | 1 |
| -776.698 | 19555.581 | 1.829 | 13.195 | 0.0733881 | 7/15/2014 | 59:57.4 | 1 |
| -776.942 | 19555.597 | 1.826 | 13.181 | 0.0624718 | 7/15/2014 | 59:57.5 | 1 |
| -777.186 | 19555.611 | 1.826 | 13.167 | 0.061255 | 7/15/2014 | 59:57.6 | 1 |
| -777.491 | 19555.632 | 1.83 | 13.154 | 0.0595929 | 7/15/2014 | 59:57.7 | 1 |
| -777.615 | 19555.64 | 1.829 | 13.146 | 0.0573606 | 7/15/2014 | 59:57.8 | 1 |
| -777.861 | 19555.654 | 1.831 | 13.134 | 0.0656269 | 7/15/2014 | 59:57.9 | 1 |
| -778.108 | 19555.67 | 1.829 | 13.124 | 0.0451644 | 7/15/2014 | 59:58.0 | 1 |
| -778.356 | 19555.686 | 1.831 | 13.116 | 0.0368572 | 7/15/2014 | 59:58.1 | 1 |
| -778.602 | 19555.701 | 1.832 | 13.11 | 0.0414435 | 7/15/2014 | 59:58.2 | 1 |
| -778.85 | 19555.715 | 1.836 | 13.106 | 0.0583662 | 7/15/2014 | 59:58.3 | 1 |
| -779.283 | 19555.741 | 1.842 | 13.104 | 0.0484242 | 7/15/2014 | 59:58.4 | 1 |
| -779.529 | 19555.756 | 1.84 | 13.104 | 0.0298502 | 7/15/2014 | 59:58.5 | 1 |
| -779.652 | 19555.764 | 1.841 | 13.104 | 0.0610902 | 7/15/2014 | 59:58.6 | 1 |
| -780.085 | 19555.789 | 1.845 | 13.107 | 0.0615432 | 7/15/2014 | 59:58.7 | 1 |
| -780.21 | 19555.797 | 1.847 | 13.108 | 0.0525821 | 7/15/2014 | 59:58.8 | 1 |

| | | | | | | | |
|---|---|---|---|---|---|---|---|
| -780.46 | 19555.811 | 1.846 | 13.112 | 0.0468232 | 7/15/2014 | 59:58.9 | 1 |
| -780.71 | 19555.825 | 1.846 | 13.115 | 0.040681 | 7/15/2014 | 59:59.0 | 1 |
| -780.96 | 19555.839 | 1.846 | 13.12 | 0.0494457 | 7/15/2014 | 59:59.1 | 1 |
| -781.21 | 19555.854 | 1.846 | 13.124 | 0.0626362 | 7/15/2014 | 59:59.2 | 1 |
| -781.461 | 19555.867 | 1.858 | 13.128 | 0.0720378 | 7/15/2014 | 59:59.3 | 1 |
| -781.77 | 19555.886 | 1.854 | 13.133 | 0.0828533 | 7/15/2014 | 59:59.4 | 1 |
| -782.02 | 19555.899 | 1.86 | 13.137 | 0.0878136 | 7/15/2014 | 59:59.5 | 1 |
| -782.269 | 19555.912 | 1.864 | 13.141 | 0.0929873 | 7/15/2014 | 59:59.6 | 1 |
| -782.52 | 19555.927 | 1.868 | 13.145 | 0.0935844 | 7/15/2014 | 59:59.7 | 1 |
| -782.83 | 19555.943 | 1.864 | 13.15 | 0.0942315 | 7/15/2014 | 59:59.8 | 1 |
| -783.082 | 19555.959 | 1.864 | 13.156 | 0.0851286 | 7/15/2014 | 59:59.9 | 1 |
| -783.33 | 19555.972 | 1.858 | 13.163 | 0.0808014 | 7/15/2014 | 00:00.0 | 1 |
| -783.582 | 19555.984 | 1.862 | 13.171 | 0.0646923 | 7/15/2014 | 00:00.1 | 1 |
| -783.831 | 19555.998 | 1.856 | 13.181 | 0.0528321 | 7/15/2014 | 00:00.2 | 1 |
| -784.083 | 19556.012 | 1.856 | 13.192 | 0.0522572 | 7/15/2014 | 00:00.3 | 1 |
| -784.391 | 19556.028 | 1.848 | 13.204 | 0.0430544 | 7/15/2014 | 00:00.4 | 1 |
| -784.642 | 19556.043 | 1.856 | 13.218 | 0.0278409 | 7/15/2014 | 00:00.5 | 1 |
| -784.888 | 19556.055 | 1.856 | 13.233 | 0.0328821 | 7/15/2014 | 00:00.6 | 1 |
| -785.139 | 19556.068 | 1.859 | 13.25 | 0.0421234 | 7/15/2014 | 00:00.7 | 1 |
| -785.447 | 19556.085 | 1.856 | 13.267 | 0.0574604 | 7/15/2014 | 00:00.8 | 1 |
| -785.699 | 19556.098 | 1.863 | 13.285 | 0.0665211 | 7/15/2014 | 00:00.9 | 1 |
| -785.947 | 19556.11 | 1.859 | 13.303 | 0.06773 | 7/15/2014 | 00:01.0 | 1 |
| -786.199 | 19556.123 | 1.863 | 13.321 | 0.0788206 | 7/15/2014 | 00:01.1 | 1 |
| -786.447 | 19556.137 | 1.863 | 13.338 | 0.0819986 | 7/15/2014 | 00:01.2 | 1 |
| -786.697 | 19556.15 | 1.867 | 13.353 | 0.0736617 | 7/15/2014 | 00:01.3 | 1 |
| -787.005 | 19556.165 | 1.867 | 13.365 | 0.0727156 | 7/15/2014 | 00:01.4 | 1 |
| -787.254 | 19556.178 | 1.874 | 13.373 | 0.0653841 | 7/15/2014 | 00:01.5 | 1 |
| -787.623 | 19556.197 | 1.872 | 13.373 | 0.0541184 | 7/15/2014 | 00:01.6 | 1 |
| -787.932 | 19556.212 | 1.881 | 13.37 | 0.025434 | 7/15/2014 | 00:01.7 | 1 |
| -788.057 | 19556.22 | 1.884 | 13.37 | 0.0210511 | 7/15/2014 | 00:01.8 | 1 |
| -788.305 | 19556.232 | 1.89 | 13.36 | 0.0191875 | 7/15/2014 | 00:01.9 | 1 |
| -788.551 | 19556.244 | 1.882 | 13.345 | 0.0276813 | 7/15/2014 | 00:02.0 | 1 |
| -788.8 | 19556.256 | 1.882 | 13.328 | 0.0150502 | 7/15/2014 | 00:02.1 | 1 |
| -789.046 | 19556.271 | 1.881 | 13.309 | 0.030131 | 7/15/2014 | 00:02.2 | 1 |
| -789.293 | 19556.283 | 1.881 | 13.289 | 0.0286784 | 7/15/2014 | 00:02.3 | 1 |
| -789.724 | 19556.305 | 1.882 | 13.26 | 0.0278905 | 7/15/2014 | 00:02.4 | 1 |
| -789.969 | 19556.318 | 1.876 | 13.242 | 0.0259057 | 7/15/2014 | 00:02.5 | 1 |
| -790.214 | 19556.33 | 1.873 | 13.225 | 0.0529566 | 7/15/2014 | 00:02.6 | 1 |
| -790.524 | 19556.346 | 1.873 | 13.211 | 0.0171982 | 7/15/2014 | 00:02.7 | 1 |
| -790.647 | 19556.353 | 1.871 | 13.203 | 0.0258929 | 7/15/2014 | 00:02.8 | 1 |
| -790.898 | 19556.365 | 1.869 | 13.191 | 0.0143267 | 7/15/2014 | 00:02.9 | 1 |
| -791.148 | 19556.379 | 1.865 | 13.182 | 0.0173681 | 7/15/2014 | 00:03.0 | 1 |
| -791.399 | 19556.392 | 1.872 | 13.175 | 0.0111891 | 7/15/2014 | 00:03.1 | 1 |
| -791.651 | 19556.406 | 1.874 | 13.168 | 0.0178713 | 7/15/2014 | 00:03.2 | 1 |
| -791.903 | 19556.418 | 1.877 | 13.163 | 0.0371282 | 7/15/2014 | 00:03.3 | 1 |
| -792.216 | 19556.435 | 1.875 | 13.158 | 0.0371694 | 7/15/2014 | 00:03.4 | 1 |
| -792.471 | 19556.447 | 1.882 | 13.153 | 0.0461638 | 7/15/2014 | 00:03.5 | 1 |

| | | | | | | | |
|---|---|---|---|---|---|---|---|
| -792.725 | 19556.463 | 1.886 | 13.149 | 0.0634184 | 7/15/2014 | 00:03.6 | 1 |
| -792.98 | 19556.475 | 1.889 | 13.145 | 0.0613506 | 7/15/2014 | 00:03.7 | 1 |
| -793.3 | 19556.493 | 1.887 | 13.142 | 0.0704408 | 7/15/2014 | 00:03.8 | 1 |
| -793.557 | 19556.506 | 1.886 | 13.14 | 0.0668865 | 7/15/2014 | 00:03.9 | 1 |
| -793.816 | 19556.52 | 1.889 | 13.138 | 0.0701647 | 7/15/2014 | 00:04.0 | 1 |
| -794.075 | 19556.534 | 1.889 | 13.138 | 0.061215 | 7/15/2014 | 00:04.1 | 1 |
| -794.334 | 19556.549 | 1.889 | 13.138 | 0.0440446 | 7/15/2014 | 00:04.2 | 1 |
| -794.592 | 19556.563 | 1.891 | 13.141 | 0.0426038 | 7/15/2014 | 00:04.3 | 1 |
| -794.915 | 19556.581 | 1.891 | 13.144 | 0.0171025 | 7/15/2014 | 00:04.4 | 1 |
| -795.174 | 19556.595 | 1.896 | 13.149 | 0.0231998 | 7/15/2014 | 00:04.5 | 1 |
| -795.431 | 19556.609 | 1.894 | 13.154 | 0.0168299 | 7/15/2014 | 00:04.6 | 1 |
| -795.689 | 19556.625 | 1.894 | 13.161 | 0.0193985 | 7/15/2014 | 00:04.7 | 1 |
| -796.01 | 19556.643 | 1.896 | 13.169 | 0.0241475 | 7/15/2014 | 00:04.8 | 1 |
| -796.268 | 19556.659 | 1.899 | 13.178 | 0.0234791 | 7/15/2014 | 00:04.9 | 1 |
| -796.526 | 19556.674 | 1.896 | 13.186 | 0.0289749 | 7/15/2014 | 00:05.0 | 1 |
| -796.783 | 19556.689 | 1.899 | 13.194 | 0.0280582 | 7/15/2014 | 00:05.1 | 1 |
| -797.038 | 19556.704 | 1.896 | 13.202 | 0.0283122 | 7/15/2014 | 00:05.2 | 1 |
| -797.293 | 19556.721 | 1.902 | 13.208 | 0.0257014 | 7/15/2014 | 00:05.3 | 1 |
| -797.609 | 19556.739 | 1.906 | 13.213 | 0.0210927 | 7/15/2014 | 00:05.4 | 1 |
| -797.862 | 19556.756 | 1.908 | 13.216 | 0.0259251 | 7/15/2014 | 00:05.5 | 1 |
| -798.112 | 19556.771 | 1.906 | 13.217 | 0.0398087 | 7/15/2014 | 00:05.6 | 1 |
| -798.362 | 19556.788 | 1.908 | 13.217 | 0.0339142 | 7/15/2014 | 00:05.7 | 1 |
| -798.672 | 19556.807 | 1.908 | 13.216 | 0.0353604 | 7/15/2014 | 00:05.8 | 1 |
| -798.921 | 19556.824 | 1.911 | 13.213 | 0.0485463 | 7/15/2014 | 00:05.9 | 1 |
| -799.169 | 19556.84 | 1.911 | 13.21 | 0.0502035 | 7/15/2014 | 00:06.0 | 1 |
| -799.418 | 19556.856 | 1.91 | 13.207 | 0.0534778 | 7/15/2014 | 00:06.1 | 1 |
| -799.662 | 19556.873 | 1.907 | 13.204 | 0.0657526 | 7/15/2014 | 00:06.2 | 1 |
| -799.909 | 19556.89 | 1.916 | 13.203 | 0.0544309 | 7/15/2014 | 00:06.3 | 1 |
| -800.335 | 19556.918 | 1.914 | 13.205 | 0.0609688 | 7/15/2014 | 00:06.4 | 1 |
| -800.577 | 19556.936 | 1.912 | 13.207 | 0.0558313 | 7/15/2014 | 00:06.5 | 1 |
| -800.819 | 19556.952 | 1.912 | 13.212 | 0.0575266 | 7/15/2014 | 00:06.6 | 1 |
| -801.122 | 19556.974 | 1.908 | 13.219 | 0.0532613 | 7/15/2014 | 00:06.7 | 1 |
| -801.243 | 19556.981 | 1.907 | 13.222 | 0.0537473 | 7/15/2014 | 00:06.8 | 1 |
| -801.488 | 19556.999 | 1.909 | 13.231 | 0.0539715 | 7/15/2014 | 00:06.9 | 1 |
| -801.731 | 19557.018 | 1.906 | 13.242 | 0.0568426 | 7/15/2014 | 00:07.0 | 1 |
| -801.976 | 19557.033 | 1.908 | 13.254 | 0.0596578 | 7/15/2014 | 00:07.1 | 1 |
| -802.218 | 19557.052 | 1.903 | 13.265 | 0.0575336 | 7/15/2014 | 00:07.2 | 1 |
| -802.463 | 19557.07 | 1.912 | 13.277 | 0.0571217 | 7/15/2014 | 00:07.3 | 1 |
| -802.886 | 19557.101 | 1.919 | 13.29 | 0.0517142 | 7/15/2014 | 00:07.4 | 1 |
| -803.129 | 19557.119 | 1.919 | 13.298 | 0.0541575 | 7/15/2014 | 00:07.5 | 1 |
| -803.372 | 19557.139 | 1.917 | 13.304 | 0.0494405 | 7/15/2014 | 00:07.6 | 1 |
| -803.674 | 19557.16 | 1.917 | 13.307 | 0.0432001 | 7/15/2014 | 00:07.7 | 1 |
| -803.796 | 19557.17 | 1.919 | 13.309 | 0.0475016 | 7/15/2014 | 00:07.8 | 1 |
| -804.041 | 19557.189 | 1.924 | 13.308 | 0.0322794 | 7/15/2014 | 00:07.9 | 1 |
| -804.284 | 19557.208 | 1.918 | 13.305 | 0.0339435 | 7/15/2014 | 00:08.0 | 1 |
| -804.529 | 19557.227 | 1.918 | 13.299 | 0.0325088 | 7/15/2014 | 00:08.1 | 1 |
| -804.772 | 19557.246 | 1.912 | 13.291 | 0.0241495 | 7/15/2014 | 00:08.2 | 1 |

| | | | | | | | |
|---|---|---|---|---|---|---|---|
| -805.017 | 19557.266 | 1.918 | 13.283 | 0.0362195 | 7/15/2014 | 00:08.3 | 1 |
| -805.442 | 19557.301 | 1.926 | 13.27 | 0.0229902 | 7/15/2014 | 00:08.4 | 1 |
| -805.684 | 19557.32 | 1.93 | 13.261 | 0.0244925 | 7/15/2014 | 00:08.5 | 1 |
| -805.927 | 19557.34 | 1.938 | 13.253 | 0.0323472 | 7/15/2014 | 00:08.6 | 1 |
| -806.231 | 19557.365 | 1.94 | 13.246 | 0.0430519 | 7/15/2014 | 00:08.7 | 1 |
| -806.353 | 19557.375 | 1.94 | 13.242 | 0.0584297 | 7/15/2014 | 00:08.8 | 1 |
| -806.597 | 19557.395 | 1.946 | 13.236 | 0.0659976 | 7/15/2014 | 00:08.9 | 1 |
| -806.842 | 19557.416 | 1.946 | 13.23 | 0.0871515 | 7/15/2014 | 00:09.0 | 1 |
| -807.087 | 19557.438 | 1.948 | 13.224 | 0.0858834 | 7/15/2014 | 00:09.1 | 1 |
| -807.332 | 19557.459 | 1.951 | 13.22 | 0.0788853 | 7/15/2014 | 00:09.2 | 1 |
| -807.577 | 19557.479 | 1.953 | 13.216 | 0.073905 | 7/15/2014 | 00:09.3 | 1 |
| -808 | 19557.517 | 1.946 | 13.213 | 0.0498692 | 7/15/2014 | 00:09.4 | 1 |
| -808.242 | 19557.537 | 1.949 | 13.212 | 0.0502652 | 7/15/2014 | 00:09.5 | 1 |
| -808.482 | 19557.561 | 1.951 | 13.212 | 0.0467198 | 7/15/2014 | 00:09.6 | 1 |
| -808.782 | 19557.586 | 1.953 | 13.213 | 0.0486832 | 7/15/2014 | 00:09.7 | 1 |
| -808.905 | 19557.598 | 1.952 | 13.213 | 0.0493753 | 7/15/2014 | 00:09.8 | 1 |
| -809.147 | 19557.62 | 1.956 | 13.216 | 0.0637064 | 7/15/2014 | 00:09.9 | 1 |
| -809.386 | 19557.641 | 1.953 | 13.219 | 0.0504328 | 7/15/2014 | 00:10.0 | 1 |
| -809.627 | 19557.663 | 1.963 | 13.224 | 0.0506629 | 7/15/2014 | 00:10.1 | 1 |
| -809.866 | 19557.686 | 1.97 | 13.229 | 0.0496708 | 7/15/2014 | 00:10.2 | 1 |
| -810.103 | 19557.709 | 1.98 | 13.235 | 0.0495159 | 7/15/2014 | 00:10.3 | 1 |
| -810.517 | 19557.746 | 1.983 | 13.246 | 0.049162 | 7/15/2014 | 00:10.4 | 1 |
| -810.753 | 19557.77 | 1.981 | 13.254 | 0.0473026 | 7/15/2014 | 00:10.5 | 1 |
| -810.988 | 19557.791 | 1.981 | 13.262 | 0.0574132 | 7/15/2014 | 00:10.6 | 1 |
| -811.285 | 19557.819 | 1.986 | 13.271 | 0.0674996 | 7/15/2014 | 00:10.7 | 1 |
| -811.404 | 19557.831 | 1.986 | 13.276 | 0.0749923 | 7/15/2014 | 00:10.8 | 1 |
| -811.642 | 19557.855 | 1.983 | 13.286 | 0.0546666 | 7/15/2014 | 00:10.9 | 1 |
| -811.878 | 19557.876 | 1.973 | 13.296 | 0.0729138 | 7/15/2014 | 00:11.0 | 1 |
| -812.119 | 19557.9 | 1.98 | 13.306 | 0.0693801 | 7/15/2014 | 00:11.1 | 1 |
| -812.357 | 19557.924 | 1.976 | 13.317 | 0.0683293 | 7/15/2014 | 00:11.2 | 1 |
| -812.597 | 19557.948 | 1.983 | 13.326 | 0.0801494 | 7/15/2014 | 00:11.3 | 1 |
| -813.015 | 19557.989 | 1.981 | 13.338 | 0.0482674 | 7/15/2014 | 00:11.4 | 1 |
| -813.254 | 19558.014 | 1.975 | 13.345 | 0.0673972 | 7/15/2014 | 00:11.5 | 1 |
| -813.492 | 19558.037 | 1.97 | 13.349 | 0.0391033 | 7/15/2014 | 00:11.6 | 1 |
| -813.791 | 19558.068 | 1.967 | 13.351 | 0.0337962 | 7/15/2014 | 00:11.7 | 1 |
| -813.912 | 19558.08 | 1.961 | 13.354 | 0.0168318 | 7/15/2014 | 00:11.8 | 1 |
| -814.154 | 19558.106 | 1.958 | 13.352 | 0.0176182 | 7/15/2014 | 00:11.9 | 1 |
| -814.395 | 19558.13 | 1.956 | 13.349 | 0.03299 | 7/15/2014 | 00:12.0 | 1 |
| -814.638 | 19558.156 | 1.95 | 13.343 | 0.0298277 | 7/15/2014 | 00:12.1 | 1 |
| -814.879 | 19558.181 | 1.946 | 13.335 | 0.0348683 | 7/15/2014 | 00:12.2 | 1 |
| -815.121 | 19558.206 | 1.946 | 13.327 | 0.044336 | 7/15/2014 | 00:12.3 | 1 |
| -815.543 | 19558.252 | 1.949 | 13.316 | 0.0415159 | 7/15/2014 | 00:12.4 | 1 |
| -815.786 | 19558.279 | 1.955 | 13.31 | 0.0715784 | 7/15/2014 | 00:12.5 | 1 |
| -816.029 | 19558.305 | 1.952 | 13.305 | 0.0502463 | 7/15/2014 | 00:12.6 | 1 |
| -816.336 | 19558.338 | 1.947 | 13.302 | 0.0509913 | 7/15/2014 | 00:12.7 | 1 |
| -816.458 | 19558.351 | 1.947 | 13.299 | 0.0555893 | 7/15/2014 | 00:12.8 | 1 |
| -816.705 | 19558.377 | 1.945 | 13.299 | 0.0572067 | 7/15/2014 | 00:12.9 | 1 |

| | | | | | | | |
|---|---|---|---|---|---|---|---|
| -816.955 | 19558.406 | 1.945 | 13.301 | 0.0373357 | 7/15/2014 | 00:13.0 | 1 |
| -817.205 | 19558.434 | 1.949 | 13.305 | 0.0705182 | 7/15/2014 | 00:13.1 | 1 |
| -817.456 | 19558.461 | 1.949 | 13.311 | 0.0373728 | 7/15/2014 | 00:13.2 | 1 |
| -817.707 | 19558.49 | 1.949 | 13.319 | 0.039145 | 7/15/2014 | 00:13.3 | 1 |
| -818.019 | 19558.523 | 1.945 | 13.329 | 0.0471352 | 7/15/2014 | 00:13.4 | 1 |
| -818.273 | 19558.553 | 1.951 | 13.34 | 0.0441416 | 7/15/2014 | 00:13.5 | 1 |
| -818.526 | 19558.582 | 1.957 | 13.353 | 0.048327 | 7/15/2014 | 00:13.6 | 1 |
| -818.778 | 19558.61 | 1.957 | 13.366 | 0.0506524 | 7/15/2014 | 00:13.7 | 1 |
| -819.09 | 19558.646 | 1.95 | 13.379 | 0.0552332 | 7/15/2014 | 00:13.8 | 1 |
| -819.342 | 19558.676 | 1.946 | 13.392 | 0.0570825 | 7/15/2014 | 00:13.9 | 1 |
| -819.59 | 19558.703 | 1.936 | 13.405 | 0.0564292 | 7/15/2014 | 00:14.0 | 1 |
| -819.842 | 19558.733 | 1.94 | 13.418 | 0.0548511 | 7/15/2014 | 00:14.1 | 1 |
| -820.089 | 19558.762 | 1.93 | 13.43 | 0.0584954 | 7/15/2014 | 00:14.2 | 1 |
| -820.336 | 19558.791 | 1.924 | 13.442 | 0.0577678 | 7/15/2014 | 00:14.3 | 1 |
| -820.761 | 19558.841 | 1.919 | 13.46 | 0.0577359 | 7/15/2014 | 00:14.4 | 1 |
| -821.004 | 19558.87 | 1.92 | 13.473 | 0.057143 | 7/15/2014 | 00:14.5 | 1 |
| -821.244 | 19558.898 | 1.923 | 13.484 | 0.0572552 | 7/15/2014 | 00:14.6 | 1 |
| -821.544 | 19558.935 | 1.929 | 13.496 | 0.0594117 | 7/15/2014 | 00:14.7 | 1 |
| -821.665 | 19558.949 | 1.927 | 13.502 | 0.0689004 | 7/15/2014 | 00:14.8 | 1 |
| -821.906 | 19558.979 | 1.933 | 13.513 | 0.0876831 | 7/15/2014 | 00:14.9 | 1 |
| -822.145 | 19559.008 | 1.935 | 13.523 | 0.0777884 | 7/15/2014 | 00:15.0 | 1 |
| -822.385 | 19559.036 | 1.938 | 13.53 | 0.0888463 | 7/15/2014 | 00:15.1 | 1 |
| -822.622 | 19559.065 | 1.938 | 13.534 | 0.098364 | 7/15/2014 | 00:15.2 | 1 |
| -822.86 | 19559.095 | 1.94 | 13.535 | 0.0743357 | 7/15/2014 | 00:15.3 | 1 |
| -823.273 | 19559.146 | 1.93 | 13.527 | 0.0683265 | 7/15/2014 | 00:15.4 | 1 |
| -823.508 | 19559.175 | 1.924 | 13.519 | 0.050824 | 7/15/2014 | 00:15.5 | 1 |
| -823.743 | 19559.204 | 1.922 | 13.508 | 0.0544024 | 7/15/2014 | 00:15.6 | 1 |
| -824.035 | 19559.241 | 1.917 | 13.495 | 0.0485888 | 7/15/2014 | 00:15.7 | 1 |
| -824.152 | 19559.256 | 1.913 | 13.489 | 0.042402 | 7/15/2014 | 00:15.8 | 1 |
| -824.391 | 19559.285 | 1.919 | 13.473 | 0.0441407 | 7/15/2014 | 00:15.9 | 1 |
| -824.625 | 19559.314 | 1.91 | 13.458 | 0.0451895 | 7/15/2014 | 00:16.0 | 1 |
| -824.864 | 19559.345 | 1.913 | 13.444 | 0.0456837 | 7/15/2014 | 00:16.1 | 1 |
| -825.1 | 19559.374 | 1.906 | 13.431 | 0.0487219 | 7/15/2014 | 00:16.2 | 1 |
| -825.337 | 19559.403 | 1.912 | 13.421 | 0.0416978 | 7/15/2014 | 00:16.3 | 1 |
| -825.749 | 19559.457 | 1.911 | 13.412 | 0.0500185 | 7/15/2014 | 00:16.4 | 1 |
| -825.986 | 19559.487 | 1.907 | 13.407 | 0.0710999 | 7/15/2014 | 00:16.5 | 1 |
| -826.221 | 19559.518 | 1.902 | 13.404 | 0.0622548 | 7/15/2014 | 00:16.6 | 1 |
| -826.52 | 19559.557 | 1.902 | 13.403 | 0.0468826 | 7/15/2014 | 00:16.7 | 1 |
| -826.638 | 19559.571 | 1.899 | 13.403 | 0.0540957 | 7/15/2014 | 00:16.8 | 1 |
| -826.881 | 19559.602 | 1.902 | 13.404 | 0.0766125 | 7/15/2014 | 00:16.9 | 1 |
| -827.119 | 19559.633 | 1.894 | 13.407 | 0.0653146 | 7/15/2014 | 00:17.0 | 1 |
| -827.365 | 19559.664 | 1.894 | 13.411 | 0.0550133 | 7/15/2014 | 00:17.1 | 1 |
| -827.605 | 19559.696 | 1.887 | 13.415 | 0.0413901 | 7/15/2014 | 00:17.2 | 1 |
| -827.849 | 19559.729 | 1.894 | 13.421 | 0.0462078 | 7/15/2014 | 00:17.3 | 1 |
| -828.274 | 19559.785 | 1.894 | 13.431 | 0.0635432 | 7/15/2014 | 00:17.4 | 1 |
| -828.518 | 19559.816 | 1.894 | 13.438 | 0.0475749 | 7/15/2014 | 00:17.5 | 1 |
| -828.761 | 19559.85 | 1.891 | 13.447 | 0.0505265 | 7/15/2014 | 00:17.6 | 1 |

| | | | | | | | |
|---|---|---|---|---|---|---|---|
| -829.069 | 19559.89 | 1.888 | 13.457 | 0.046766 | 7/15/2014 | 00:17.7 | 1 |
| -829.191 | 19559.906 | 1.886 | 13.461 | 0.046612 | 7/15/2014 | 00:17.8 | 1 |
| -829.441 | 19559.939 | 1.886 | 13.472 | 0.0516866 | 7/15/2014 | 00:17.9 | 1 |
| -829.687 | 19559.973 | 1.876 | 13.484 | 0.0539507 | 7/15/2014 | 00:18.0 | 1 |
| -829.938 | 19560.007 | 1.876 | 13.495 | 0.0460312 | 7/15/2014 | 00:18.1 | 1 |
| -830.186 | 19560.039 | 1.872 | 13.506 | 0.0620332 | 7/15/2014 | 00:18.2 | 1 |
| -830.434 | 19560.074 | 1.876 | 13.515 | 0.057615 | 7/15/2014 | 00:18.3 | 1 |
| -830.743 | 19560.116 | 1.876 | 13.522 | 0.066221 | 7/15/2014 | 00:18.4 | 1 |
| -830.994 | 19560.148 | 1.882 | 13.527 | 0.0770657 | 7/15/2014 | 00:18.5 | 1 |
| -831.239 | 19560.184 | 1.882 | 13.528 | 0.050063 | 7/15/2014 | 00:18.6 | 1 |
| -831.489 | 19560.216 | 1.884 | 13.526 | 0.0537158 | 7/15/2014 | 00:18.7 | 1 |
| -831.796 | 19560.258 | 1.884 | 13.522 | 0.0586048 | 7/15/2014 | 00:18.8 | 1 |
| -832.044 | 19560.293 | 1.892 | 13.515 | 0.0650013 | 7/15/2014 | 00:18.9 | 1 |
| -832.29 | 19560.326 | 1.888 | 13.508 | 0.062967 | 7/15/2014 | 00:19.0 | 1 |
| -832.539 | 19560.359 | 1.892 | 13.5 | 0.0375798 | 7/15/2014 | 00:19.1 | 1 |
| -832.782 | 19560.393 | 1.892 | 13.493 | 0.0749623 | 7/15/2014 | 00:19.2 | 1 |
| -833.026 | 19560.426 | 1.895 | 13.488 | 0.0771406 | 7/15/2014 | 00:19.3 | 1 |
| -833.45 | 19560.484 | 1.896 | 13.486 | 0.0545638 | 7/15/2014 | 00:19.4 | 1 |
| -833.693 | 19560.518 | 1.896 | 13.486 | 0.0595189 | 7/15/2014 | 00:19.5 | 1 |
| -833.933 | 19560.55 | 1.893 | 13.49 | 0.0311998 | 7/15/2014 | 00:19.6 | 1 |
| -834.233 | 19560.592 | 1.896 | 13.495 | 0.0523642 | 7/15/2014 | 00:19.7 | 1 |
| -834.354 | 19560.607 | 1.895 | 13.498 | 0.0217946 | 7/15/2014 | 00:19.8 | 1 |
| -834.596 | 19560.641 | 1.899 | 13.507 | 0.0249177 | 7/15/2014 | 00:19.9 | 1 |
| -834.836 | 19560.674 | 1.899 | 13.518 | 0.0370776 | 7/15/2014 | 00:20.0 | 1 |
| -835.078 | 19560.707 | 1.903 | 13.531 | 0.0458363 | 7/15/2014 | 00:20.1 | 1 |
| -835.317 | 19560.739 | 1.903 | 13.544 | 0.0692119 | 7/15/2014 | 00:20.2 | 1 |
| -835.558 | 19560.772 | 1.905 | 13.559 | 0.0513781 | 7/15/2014 | 00:20.3 | 1 |
| -835.976 | 19560.83 | 1.898 | 13.581 | 0.0739027 | 7/15/2014 | 00:20.4 | 1 |
| -836.216 | 19560.862 | 1.898 | 13.595 | 0.0726519 | 7/15/2014 | 00:20.5 | 1 |
| -836.454 | 19560.896 | 1.897 | 13.61 | 0.0627481 | 7/15/2014 | 00:20.6 | 1 |
| -836.755 | 19560.938 | 1.894 | 13.625 | 0.0713696 | 7/15/2014 | 00:20.7 | 1 |
| -836.875 | 19560.954 | 1.893 | 13.632 | 0.0699203 | 7/15/2014 | 00:20.8 | 1 |
| -837.118 | 19560.987 | 1.897 | 13.646 | 0.0647242 | 7/15/2014 | 00:20.9 | 1 |
| -837.359 | 19561.021 | 1.894 | 13.66 | 0.069449 | 7/15/2014 | 00:21.0 | 1 |
| -837.602 | 19561.055 | 1.901 | 13.673 | 0.0642438 | 7/15/2014 | 00:21.1 | 1 |
| -837.844 | 19561.087 | 1.905 | 13.685 | 0.0718651 | 7/15/2014 | 00:21.2 | 1 |
| -838.088 | 19561.121 | 1.905 | 13.696 | 0.0697933 | 7/15/2014 | 00:21.3 | 1 |
| -838.512 | 19561.18 | 1.904 | 13.71 | 0.0936998 | 7/15/2014 | 00:21.4 | 1 |
| -838.754 | 19561.214 | 1.907 | 13.718 | 0.0872905 | 7/15/2014 | 00:21.5 | 1 |
| -838.995 | 19561.246 | 1.91 | 13.724 | 0.0860026 | 7/15/2014 | 00:21.6 | 1 |
| -839.297 | 19561.288 | 1.91 | 13.728 | 0.0817809 | 7/15/2014 | 00:21.7 | 1 |
| -839.419 | 19561.304 | 1.907 | 13.731 | 0.0869005 | 7/15/2014 | 00:21.8 | 1 |
| -839.664 | 19561.338 | 1.911 | 13.731 | 0.0854474 | 7/15/2014 | 00:21.9 | 1 |
| -839.905 | 19561.37 | 1.911 | 13.729 | 0.0841685 | 7/15/2014 | 00:22.0 | 1 |
| -840.149 | 19561.404 | 1.921 | 13.725 | 0.0687508 | 7/15/2014 | 00:22.1 | 1 |
| -840.389 | 19561.438 | 1.915 | 13.719 | 0.0622561 | 7/15/2014 | 00:22.2 | 1 |
| -840.632 | 19561.469 | 1.921 | 13.712 | 0.0669433 | 7/15/2014 | 00:22.3 | 1 |

| | | | | | | | |
|---|---|---|---|---|---|---|---|
| -841.05 | 19561.526 | 1.92 | 13.703 | 0.0586384 | 7/15/2014 | 00:22.4 | 1 |
| -841.288 | 19561.56 | 1.92 | 13.698 | 0.0548063 | 7/15/2014 | 00:22.5 | 1 |
| -841.528 | 19561.592 | 1.922 | 13.694 | 0.047936 | 7/15/2014 | 00:22.6 | 1 |
| -841.825 | 19561.633 | 1.926 | 13.691 | 0.0584968 | 7/15/2014 | 00:22.7 | 1 |
| -841.945 | 19561.648 | 1.923 | 13.689 | 0.0571943 | 7/15/2014 | 00:22.8 | 1 |
| -842.186 | 19561.68 | 1.929 | 13.689 | 0.0560407 | 7/15/2014 | 00:22.9 | 1 |
| -842.424 | 19561.713 | 1.929 | 13.69 | 0.0649989 | 7/15/2014 | 00:23.0 | 1 |
| -842.665 | 19561.746 | 1.933 | 13.693 | 0.0710391 | 7/15/2014 | 00:23.1 | 1 |
| -842.903 | 19561.778 | 1.937 | 13.696 | 0.0741564 | 7/15/2014 | 00:23.2 | 1 |
| -843.143 | 19561.811 | 1.943 | 13.701 | 0.078246 | 7/15/2014 | 00:23.3 | 1 |
| -843.56 | 19561.867 | 1.937 | 13.708 | 0.0789826 | 7/15/2014 | 00:23.4 | 1 |
| -843.798 | 19561.898 | 1.934 | 13.712 | 0.0777923 | 7/15/2014 | 00:23.5 | 1 |
| -844.036 | 19561.93 | 1.934 | 13.716 | 0.0890025 | 7/15/2014 | 00:23.6 | 1 |
| -844.334 | 19561.971 | 1.934 | 13.72 | 0.0685388 | 7/15/2014 | 00:23.7 | 1 |
| -844.454 | 19561.986 | 1.933 | 13.723 | 0.0743693 | 7/15/2014 | 00:23.8 | 1 |
| -844.696 | 19562.018 | 1.932 | 13.726 | 0.0747651 | 7/15/2014 | 00:23.9 | 1 |
| -844.935 | 19562.049 | 1.926 | 13.73 | 0.0567879 | 7/15/2014 | 00:24.0 | 1 |
| -845.177 | 19562.084 | 1.929 | 13.735 | 0.0758998 | 7/15/2014 | 00:24.1 | 1 |
| -845.418 | 19562.115 | 1.929 | 13.74 | 0.0655299 | 7/15/2014 | 00:24.2 | 1 |
| -845.659 | 19562.146 | 1.933 | 13.745 | 0.0649532 | 7/15/2014 | 00:24.3 | 1 |
| -846.079 | 19562.201 | 1.932 | 13.754 | 0.0792734 | 7/15/2014 | 00:24.4 | 1 |
| -846.32 | 19562.232 | 1.93 | 13.76 | 0.0783824 | 7/15/2014 | 00:24.5 | 1 |
| -846.559 | 19562.264 | 1.931 | 13.764 | 0.0818425 | 7/15/2014 | 00:24.6 | 1 |
| -846.859 | 19562.305 | 1.936 | 13.768 | 0.0885707 | 7/15/2014 | 00:24.7 | 1 |
| -846.981 | 19562.32 | 1.935 | 13.77 | 0.0909299 | 7/15/2014 | 00:24.8 | 1 |
| -847.222 | 19562.352 | 1.937 | 13.771 | 0.0942303 | 7/15/2014 | 00:24.9 | 1 |
| -847.464 | 19562.383 | 1.937 | 13.77 | 0.0909943 | 7/15/2014 | 00:25.0 | 1 |
| -847.706 | 19562.414 | 1.939 | 13.768 | 0.0964793 | 7/15/2014 | 00:25.1 | 1 |
| -847.948 | 19562.445 | 1.937 | 13.764 | 0.0914189 | 7/15/2014 | 00:25.2 | 1 |
| -848.189 | 19562.477 | 1.939 | 13.759 | 0.0927176 | 7/15/2014 | 00:25.3 | 1 |
| -848.612 | 19562.531 | 1.932 | 13.754 | 0.0748051 | 7/15/2014 | 00:25.4 | 1 |
| -848.851 | 19562.563 | 1.927 | 13.752 | 0.0587968 | 7/15/2014 | 00:25.5 | 1 |
| -849.093 | 19562.593 | 1.925 | 13.752 | 0.0551432 | 7/15/2014 | 00:25.6 | 1 |
| -849.396 | 19562.632 | 1.927 | 13.754 | 0.0572182 | 7/15/2014 | 00:25.7 | 1 |
| -849.518 | 19562.647 | 1.925 | 13.755 | 0.05508 | 7/15/2014 | 00:25.8 | 1 |
| -849.763 | 19562.68 | 1.927 | 13.761 | 0.0446873 | 7/15/2014 | 00:25.9 | 1 |
| -850.009 | 19562.711 | 1.931 | 13.771 | 0.0826142 | 7/15/2014 | 00:26.0 | 1 |
| -850.257 | 19562.742 | 1.936 | 13.782 | 0.0869181 | 7/15/2014 | 00:26.1 | 1 |
| -850.503 | 19562.773 | 1.936 | 13.796 | 0.0806672 | 7/15/2014 | 00:26.2 | 1 |
| -850.754 | 19562.805 | 1.942 | 13.81 | 0.118775 | 7/15/2014 | 00:26.3 | 1 |
| -851.065 | 19562.845 | 1.944 | 13.823 | 0.0995049 | 7/15/2014 | 00:26.4 | 1 |
| -851.318 | 19562.878 | 1.947 | 13.835 | 0.115788 | 7/15/2014 | 00:26.5 | 1 |
| -851.572 | 19562.91 | 1.941 | 13.844 | 0.0804121 | 7/15/2014 | 00:26.6 | 1 |
| -851.829 | 19562.941 | 1.931 | 13.849 | 0.0949923 | 7/15/2014 | 00:26.7 | 1 |
| -852.084 | 19562.974 | 1.922 | 13.85 | 0.104106 | 7/15/2014 | 00:26.8 | 1 |
| -852.343 | 19563.007 | 1.922 | 13.846 | 0.0885166 | 7/15/2014 | 00:26.9 | 1 |
| -852.601 | 19563.039 | 1.92 | 13.839 | 0.0883721 | 7/15/2014 | 00:27.0 | 1 |

| | | | | | | | |
|---|---|---|---|---|---|---|---|
| -852.859 | 19563.071 | 1.92 | 13.829 | 0.0920984 | 7/15/2014 | 00:27.1 | 1 |
| -853.115 | 19563.104 | 1.915 | 13.816 | 0.108799 | 7/15/2014 | 00:27.2 | 1 |
| -853.371 | 19563.135 | 1.915 | 13.803 | 0.100612 | 7/15/2014 | 00:27.3 | 1 |
| -853.624 | 19563.167 | 1.915 | 13.79 | 0.100084 | 7/15/2014 | 00:27.4 | 1 |
| -853.877 | 19563.199 | 1.919 | 13.779 | 0.107136 | 7/15/2014 | 00:27.5 | 1 |
| -854.125 | 19563.229 | 1.911 | 13.77 | 0.11087 | 7/15/2014 | 00:27.6 | 1 |
| -854.552 | 19563.281 | 1.909 | 13.766 | 0.114636 | 7/15/2014 | 00:27.7 | 1 |
| -854.679 | 19563.297 | 1.904 | 13.763 | 0.116015 | 7/15/2014 | 00:27.8 | 1 |
| -854.926 | 19563.327 | 1.904 | 13.766 | 0.12155 | 7/15/2014 | 00:27.9 | 1 |
| -855.17 | 19563.357 | 1.904 | 13.772 | 0.113195 | 7/15/2014 | 00:28.0 | 1 |
| -855.415 | 19563.389 | 1.908 | 13.782 | 0.117224 | 7/15/2014 | 00:28.1 | 1 |
| -855.657 | 19563.417 | 1.904 | 13.795 | 0.111626 | 7/15/2014 | 00:28.2 | 1 |
| -855.902 | 19563.447 | 1.908 | 13.81 | 0.107504 | 7/15/2014 | 00:28.3 | 1 |
| -856.332 | 19563.5 | 1.904 | 13.836 | 0.100384 | 7/15/2014 | 00:28.4 | 1 |
| -856.578 | 19563.53 | 1.904 | 13.854 | 0.0896422 | 7/15/2014 | 00:28.5 | 1 |
| -856.698 | 19563.545 | 1.902 | 13.863 | 0.0917635 | 7/15/2014 | 00:28.6 | 1 |
| -856.948 | 19563.576 | 1.902 | 13.881 | 0.0891269 | 7/15/2014 | 00:28.7 | 1 |
| -857.196 | 19563.605 | 1.894 | 13.899 | 0.0954763 | 7/15/2014 | 00:28.8 | 1 |
| -857.447 | 19563.636 | 1.891 | 13.915 | 0.0877213 | 7/15/2014 | 00:28.9 | 1 |
| -857.699 | 19563.667 | 1.887 | 13.931 | 0.0915111 | 7/15/2014 | 00:29.0 | 1 |
| -857.954 | 19563.698 | 1.891 | 13.945 | 0.095004 | 7/15/2014 | 00:29.1 | 1 |
| -858.203 | 19563.729 | 1.887 | 13.957 | 0.0938564 | 7/15/2014 | 00:29.2 | 1 |
| -858.458 | 19563.76 | 1.891 | 13.967 | 0.0967502 | 7/15/2014 | 00:29.3 | 1 |
| -858.706 | 19563.789 | 1.885 | 13.974 | 0.0932107 | 7/15/2014 | 00:29.4 | 1 |
| -858.957 | 19563.819 | 1.892 | 13.978 | 0.10482 | 7/15/2014 | 00:29.5 | 1 |
| -859.205 | 19563.85 | 1.896 | 13.979 | 0.0951557 | 7/15/2014 | 00:29.6 | 1 |
| -859.636 | 19563.901 | 1.894 | 13.972 | 0.0914826 | 7/15/2014 | 00:29.7 | 1 |
| -859.76 | 19563.916 | 1.89 | 13.971 | 0.0897231 | 7/15/2014 | 00:29.8 | 1 |
| -860.01 | 19563.946 | 1.89 | 13.962 | 0.0897835 | 7/15/2014 | 00:29.9 | 1 |
| -860.256 | 19563.976 | 1.89 | 13.952 | 0.0884354 | 7/15/2014 | 00:30.0 | 1 |
| -860.506 | 19564.004 | 1.896 | 13.941 | 0.0920279 | 7/15/2014 | 00:30.1 | 1 |
| -860.754 | 19564.034 | 1.888 | 13.929 | 0.0925063 | 7/15/2014 | 00:30.2 | 1 |
| -861.004 | 19564.064 | 1.885 | 13.918 | 0.10483 | 7/15/2014 | 00:30.3 | 1 |
| -861.314 | 19564.101 | 1.879 | 13.908 | 0.0959347 | 7/15/2014 | 00:30.4 | 1 |
| -861.569 | 19564.131 | 1.879 | 13.9 | 0.106929 | 7/15/2014 | 00:30.5 | 1 |
| -861.822 | 19564.16 | 1.87 | 13.895 | 0.102596 | 7/15/2014 | 00:30.6 | 1 |
| -862.079 | 19564.189 | 1.867 | 13.892 | 0.109687 | 7/15/2014 | 00:30.7 | 1 |
| -862.337 | 19564.221 | 1.863 | 13.891 | 0.0995503 | 7/15/2014 | 00:30.8 | 1 |
| -862.596 | 19564.252 | 1.861 | 13.893 | 0.117168 | 7/15/2014 | 00:30.9 | 1 |
| -862.855 | 19564.28 | 1.856 | 13.898 | 0.10461 | 7/15/2014 | 00:31.0 | 1 |
| -863.117 | 19564.312 | 1.856 | 13.904 | 0.111891 | 7/15/2014 | 00:31.1 | 1 |
| -863.377 | 19564.342 | 1.856 | 13.912 | 0.123431 | 7/15/2014 | 00:31.2 | 1 |
| -863.637 | 19564.371 | 1.858 | 13.92 | 0.114614 | 7/15/2014 | 00:31.3 | 1 |
| -863.896 | 19564.402 | 1.856 | 13.929 | 0.121443 | 7/15/2014 | 00:31.4 | 1 |
| -864.153 | 19564.432 | 1.849 | 13.938 | 0.113165 | 7/15/2014 | 00:31.5 | 1 |
| -864.409 | 19564.46 | 1.844 | 13.946 | 0.110171 | 7/15/2014 | 00:31.6 | 1 |
| -864.664 | 19564.49 | 1.843 | 13.954 | 0.119989 | 7/15/2014 | 00:31.7 | 1 |

| | | | | | | | |
|---|---|---|---|---|---|---|---|
| -864.981 | 19564.527 | 1.841 | 13.963 | 0.106309 | 7/15/2014 | 00:31.8 | 1 |
| -865.237 | 19564.555 | 1.841 | 13.971 | 0.103179 | 7/15/2014 | 00:31.9 | 1 |
| -865.491 | 19564.584 | 1.836 | 13.98 | 0.102402 | 7/15/2014 | 00:32.0 | 1 |
| -865.746 | 19564.611 | 1.833 | 13.989 | 0.0998999 | 7/15/2014 | 00:32.1 | 1 |
| -866 | 19564.642 | 1.833 | 13.998 | 0.0989975 | 7/15/2014 | 00:32.2 | 1 |
| -866.256 | 19564.67 | 1.838 | 14.008 | 0.102801 | 7/15/2014 | 00:32.3 | 1 |
| -866.573 | 19564.706 | 1.838 | 14.016 | 0.0996001 | 7/15/2014 | 00:32.4 | 1 |
| -866.831 | 19564.734 | 1.84 | 14.023 | 0.100892 | 7/15/2014 | 00:32.5 | 1 |
| -867.087 | 19564.762 | 1.838 | 14.028 | 0.109462 | 7/15/2014 | 00:32.6 | 1 |
| -867.348 | 19564.79 | 1.84 | 14.03 | 0.104405 | 7/15/2014 | 00:32.7 | 1 |
| -867.605 | 19564.82 | 1.843 | 14.028 | 0.124042 | 7/15/2014 | 00:32.8 | 1 |
| -867.865 | 19564.848 | 1.845 | 14.023 | 0.12801 | 7/15/2014 | 00:32.9 | 1 |
| -868.122 | 19564.876 | 1.843 | 14.015 | 0.131647 | 7/15/2014 | 00:33.0 | 1 |
| -868.381 | 19564.904 | 1.848 | 14.002 | 0.149593 | 7/15/2014 | 00:33.1 | 1 |
| -868.636 | 19564.932 | 1.845 | 13.985 | 0.13662 | 7/15/2014 | 00:33.2 | 1 |
| -868.892 | 19564.959 | 1.848 | 13.966 | 0.158763 | 7/15/2014 | 00:33.3 | 1 |
| -869.142 | 19564.986 | 1.848 | 13.944 | 0.151205 | 7/15/2014 | 00:33.4 | 1 |
| -869.392 | 19565.013 | 1.848 | 13.921 | 0.140801 | 7/15/2014 | 00:33.5 | 1 |
| -869.757 | 19565.052 | 1.851 | 13.886 | 0.144166 | 7/15/2014 | 00:33.6 | 1 |
| -870.06 | 19565.084 | 1.851 | 13.865 | 0.142023 | 7/15/2014 | 00:33.7 | 1 |
| -870.184 | 19565.098 | 1.848 | 13.853 | 0.1525 | 7/15/2014 | 00:33.8 | 1 |
| -870.427 | 19565.122 | 1.85 | 13.836 | 0.154112 | 7/15/2014 | 00:33.9 | 1 |
| -870.667 | 19565.147 | 1.85 | 13.822 | 0.145848 | 7/15/2014 | 00:34.0 | 1 |
| -870.909 | 19565.173 | 1.855 | 13.812 | 0.159117 | 7/15/2014 | 00:34.1 | 1 |
| -871.147 | 19565.198 | 1.852 | 13.807 | 0.173202 | 7/15/2014 | 00:34.2 | 1 |
| -871.387 | 19565.223 | 1.857 | 13.808 | 0.180967 | 7/15/2014 | 00:34.3 | 1 |
| -871.804 | 19565.267 | 1.855 | 13.82 | 0.196432 | 7/15/2014 | 00:34.4 | 1 |
| -872.042 | 19565.29 | 1.855 | 13.833 | 0.182742 | 7/15/2014 | 00:34.5 | 1 |
| -872.282 | 19565.315 | 1.857 | 13.849 | 0.194616 | 7/15/2014 | 00:34.6 | 1 |
| -872.584 | 19565.347 | 1.855 | 13.87 | 0.182525 | 7/15/2014 | 00:34.7 | 1 |
| -872.703 | 19565.359 | 1.852 | 13.88 | 0.181707 | 7/15/2014 | 00:34.8 | 1 |
| -872.949 | 19565.383 | 1.855 | 13.906 | 0.159882 | 7/15/2014 | 00:34.9 | 1 |
| -873.192 | 19565.408 | 1.848 | 13.935 | 0.166471 | 7/15/2014 | 00:35.0 | 1 |
| -873.44 | 19565.434 | 1.856 | 13.966 | 0.143085 | 7/15/2014 | 00:35.1 | 1 |
| -873.686 | 19565.458 | 1.856 | 13.999 | 0.152712 | 7/15/2014 | 00:35.2 | 1 |
| -873.934 | 19565.482 | 1.867 | 14.033 | 0.150336 | 7/15/2014 | 00:35.3 | 1 |
| -874.241 | 19565.514 | 1.862 | 14.066 | 0.159564 | 7/15/2014 | 00:35.4 | 1 |
| -874.492 | 19565.538 | 1.867 | 14.099 | 0.159634 | 7/15/2014 | 00:35.5 | 1 |
| -874.74 | 19565.563 | 1.867 | 14.129 | 0.165509 | 7/15/2014 | 00:35.6 | 1 |
| -874.991 | 19565.588 | 1.869 | 14.156 | 0.16417 | 7/15/2014 | 00:35.7 | 1 |
| -875.303 | 19565.619 | 1.858 | 14.18 | 0.173121 | 7/15/2014 | 00:35.8 | 1 |
| -875.557 | 19565.643 | 1.856 | 14.2 | 0.16065 | 7/15/2014 | 00:35.9 | 1 |
| -875.808 | 19565.668 | 1.845 | 14.215 | 0.1653 | 7/15/2014 | 00:36.0 | 1 |
| -876.063 | 19565.693 | 1.854 | 14.225 | 0.158737 | 7/15/2014 | 00:36.1 | 1 |
| -876.317 | 19565.717 | 1.851 | 14.229 | 0.161924 | 7/15/2014 | 00:36.2 | 1 |
| -876.57 | 19565.742 | 1.849 | 14.229 | 0.157841 | 7/15/2014 | 00:36.3 | 1 |
| -876.884 | 19565.773 | 1.851 | 14.223 | 0.158167 | 7/15/2014 | 00:36.4 | 1 |

| | | | | | | | |
|---|---|---|---|---|---|---|---|
| -877.138 | 19565.797 | 1.856 | 14.213 | 0.154995 | 7/15/2014 | 00:36.5 | 1 |
| -877.39 | 19565.822 | 1.856 | 14.199 | 0.162381 | 7/15/2014 | 00:36.6 | 1 |
| -877.643 | 19565.847 | 1.856 | 14.182 | 0.150415 | 7/15/2014 | 00:36.7 | 1 |
| -877.958 | 19565.876 | 1.845 | 14.162 | 0.151744 | 7/15/2014 | 00:36.8 | 1 |
| -878.211 | 19565.9 | 1.843 | 14.141 | 0.15616 | 7/15/2014 | 00:36.9 | 1 |
| -878.463 | 19565.926 | 1.843 | 14.12 | 0.147572 | 7/15/2014 | 00:37.0 | 1 |
| -878.716 | 19565.949 | 1.843 | 14.1 | 0.155194 | 7/15/2014 | 00:37.1 | 1 |
| -878.967 | 19565.974 | 1.834 | 14.081 | 0.143025 | 7/15/2014 | 00:37.2 | 1 |
| -879.218 | 19565.997 | 1.832 | 14.064 | 0.145711 | 7/15/2014 | 00:37.3 | 1 |
| -879.527 | 19566.026 | 1.829 | 14.049 | 0.151171 | 7/15/2014 | 00:37.4 | 1 |
| -879.776 | 19566.05 | 1.832 | 14.036 | 0.149823 | 7/15/2014 | 00:37.5 | 1 |
| -880.141 | 19566.084 | 1.833 | 14.021 | 0.152676 | 7/15/2014 | 00:37.6 | 1 |
| -880.444 | 19566.113 | 1.83 | 14.012 | 0.155582 | 7/15/2014 | 00:37.7 | 1 |
| -880.569 | 19566.125 | 1.827 | 14.008 | 0.156883 | 7/15/2014 | 00:37.8 | 1 |
| -880.814 | 19566.147 | 1.833 | 14.002 | 0.16235 | 7/15/2014 | 00:37.9 | 1 |
| -881.056 | 19566.17 | 1.831 | 13.996 | 0.169528 | 7/15/2014 | 00:38.0 | 1 |
| -881.299 | 19566.193 | 1.833 | 13.992 | 0.18049 | 7/15/2014 | 00:38.1 | 1 |
| -881.54 | 19566.215 | 1.831 | 13.987 | 0.182939 | 7/15/2014 | 00:38.2 | 1 |
| -881.78 | 19566.237 | 1.833 | 13.983 | 0.194413 | 7/15/2014 | 00:38.3 | 1 |
| -882.196 | 19566.277 | 1.824 | 13.975 | 0.220643 | 7/15/2014 | 00:38.4 | 1 |
| -882.435 | 19566.298 | 1.826 | 13.968 | 0.230068 | 7/15/2014 | 00:38.5 | 1 |
| -882.671 | 19566.32 | 1.828 | 13.961 | 0.228261 | 7/15/2014 | 00:38.6 | 1 |
| -882.967 | 19566.348 | 1.83 | 13.952 | 0.234822 | 7/15/2014 | 00:38.7 | 1 |
| -883.087 | 19566.358 | 1.829 | 13.948 | 0.238136 | 7/15/2014 | 00:38.8 | 1 |
| -883.326 | 19566.382 | 1.836 | 13.936 | 0.243047 | 7/15/2014 | 00:38.9 | 1 |
| -883.564 | 19566.402 | 1.836 | 13.923 | 0.23412 | 7/15/2014 | 00:39.0 | 1 |
| -883.803 | 19566.426 | 1.842 | 13.908 | 0.229785 | 7/15/2014 | 00:39.1 | 1 |
| -884.042 | 19566.446 | 1.844 | 13.892 | 0.2214 | 7/15/2014 | 00:39.2 | 1 |
| -884.282 | 19566.469 | 1.849 | 13.874 | 0.208107 | 7/15/2014 | 00:39.3 | 1 |
| -884.697 | 19566.506 | 1.85 | 13.848 | 0.206056 | 7/15/2014 | 00:39.4 | 1 |
| -884.936 | 19566.529 | 1.847 | 13.832 | 0.210165 | 7/15/2014 | 00:39.5 | 1 |
| -885.174 | 19566.549 | 1.845 | 13.816 | 0.202208 | 7/15/2014 | 00:39.6 | 1 |
| -885.472 | 19566.577 | 1.845 | 13.801 | 0.203767 | 7/15/2014 | 00:39.7 | 1 |
| -885.592 | 19566.588 | 1.846 | 13.794 | 0.200371 | 7/15/2014 | 00:39.8 | 1 |
| -885.834 | 19566.609 | 1.844 | 13.782 | 0.209054 | 7/15/2014 | 00:39.9 | 1 |
| -886.075 | 19566.633 | 1.837 | 13.772 | 0.21337 | 7/15/2014 | 00:40.0 | 1 |
| -886.318 | 19566.652 | 1.839 | 13.765 | 0.220682 | 7/15/2014 | 00:40.1 | 1 |
| -886.559 | 19566.676 | 1.837 | 13.759 | 0.235282 | 7/15/2014 | 00:40.2 | 1 |
| -886.803 | 19566.696 | 1.842 | 13.754 | 0.228119 | 7/15/2014 | 00:40.3 | 1 |
| -887.225 | 19566.734 | 1.837 | 13.746 | 0.259699 | 7/15/2014 | 00:40.4 | 1 |
| -887.465 | 19566.756 | 1.835 | 13.741 | 0.260292 | 7/15/2014 | 00:40.5 | 1 |
| -887.707 | 19566.778 | 1.839 | 13.733 | 0.287074 | 7/15/2014 | 00:40.6 | 1 |
| -888.008 | 19566.805 | 1.841 | 13.724 | 0.265619 | 7/15/2014 | 00:40.7 | 1 |
| -888.13 | 19566.816 | 1.837 | 13.72 | 0.261748 | 7/15/2014 | 00:40.8 | 1 |
| -888.373 | 19566.837 | 1.837 | 13.707 | 0.267521 | 7/15/2014 | 00:40.9 | 1 |
| -888.614 | 19566.857 | 1.832 | 13.69 | 0.244046 | 7/15/2014 | 00:41.0 | 1 |
| -888.856 | 19566.88 | 1.83 | 13.672 | 0.229364 | 7/15/2014 | 00:41.1 | 1 |

| | | | | | | | |
|---|---|---|---|---|---|---|---|
| -889.096 | 19566.9 | 1.832 | 13.651 | 0.229652 | 7/15/2014 | 00:41.2 | 1 |
| -889.337 | 19566.921 | 1.832 | 13.629 | 0.212558 | 7/15/2014 | 00:41.3 | 1 |
| -889.753 | 19566.959 | 1.829 | 13.598 | 0.211353 | 7/15/2014 | 00:41.4 | 1 |
| -889.991 | 19566.979 | 1.826 | 13.579 | 0.196346 | 7/15/2014 | 00:41.5 | 1 |
| -890.23 | 19567 | 1.826 | 13.562 | 0.187921 | 7/15/2014 | 00:41.6 | 1 |
| -890.528 | 19567.027 | 1.831 | 13.547 | 0.189823 | 7/15/2014 | 00:41.7 | 1 |
| -890.648 | 19567.037 | 1.832 | 13.539 | 0.174273 | 7/15/2014 | 00:41.8 | 1 |
| -890.889 | 19567.057 | 1.838 | 13.529 | 0.148554 | 7/15/2014 | 00:41.9 | 1 |
| -891.131 | 19567.08 | 1.843 | 13.521 | 0.149386 | 7/15/2014 | 00:42.0 | 1 |
| -891.374 | 19567.1 | 1.854 | 13.517 | 0.144208 | 7/15/2014 | 00:42.1 | 1 |
| -891.617 | 19567.122 | 1.856 | 13.514 | 0.150531 | 7/15/2014 | 00:42.2 | 1 |
| -891.861 | 19567.143 | 1.863 | 13.514 | 0.152997 | 7/15/2014 | 00:42.3 | 1 |
| -892.289 | 19567.18 | 1.87 | 13.513 | 0.16373 | 7/15/2014 | 00:42.4 | 1 |
| -892.534 | 19567.201 | 1.87 | 13.512 | 0.16895 | 7/15/2014 | 00:42.5 | 1 |
| -892.781 | 19567.223 | 1.869 | 13.51 | 0.184606 | 7/15/2014 | 00:42.6 | 1 |
| -893.091 | 19567.249 | 1.869 | 13.507 | 0.196773 | 7/15/2014 | 00:42.7 | 1 |
| -893.215 | 19567.261 | 1.866 | 13.507 | 0.206516 | 7/15/2014 | 00:42.8 | 1 |
| -893.466 | 19567.281 | 1.866 | 13.502 | 0.195659 | 7/15/2014 | 00:42.9 | 1 |
| -893.717 | 19567.304 | 1.861 | 13.495 | 0.222199 | 7/15/2014 | 00:43.0 | 1 |
| -893.97 | 19567.324 | 1.857 | 13.489 | 0.213547 | 7/15/2014 | 00:43.1 | 1 |
| -894.224 | 19567.348 | 1.857 | 13.482 | 0.237949 | 7/15/2014 | 00:43.2 | 1 |
| -894.478 | 19567.367 | 1.86 | 13.476 | 0.243636 | 7/15/2014 | 00:43.3 | 1 |
| -894.791 | 19567.396 | 1.855 | 13.47 | 0.218889 | 7/15/2014 | 00:43.4 | 1 |
| -895.046 | 19567.416 | 1.861 | 13.465 | 0.222303 | 7/15/2014 | 00:43.5 | 1 |
| -895.298 | 19567.438 | 1.861 | 13.462 | 0.217594 | 7/15/2014 | 00:43.6 | 1 |
| -895.551 | 19567.459 | 1.869 | 13.459 | 0.18116 | 7/15/2014 | 00:43.7 | 1 |
| -895.864 | 19567.486 | 1.867 | 13.456 | 0.156696 | 7/15/2014 | 00:43.8 | 1 |
| -896.117 | 19567.506 | 1.866 | 13.453 | 0.14729 | 7/15/2014 | 00:43.9 | 1 |
| -896.365 | 19567.527 | 1.86 | 13.45 | 0.145578 | 7/15/2014 | 00:44.0 | 1 |
| -896.618 | 19567.547 | 1.868 | 13.446 | 0.121028 | 7/15/2014 | 00:44.1 | 1 |
| -896.865 | 19567.568 | 1.866 | 13.441 | 0.119784 | 7/15/2014 | 00:44.2 | 1 |
| -897.113 | 19567.588 | 1.869 | 13.435 | 0.129949 | 7/15/2014 | 00:44.3 | 1 |
| -897.542 | 19567.623 | 1.867 | 13.426 | 0.162874 | 7/15/2014 | 00:44.4 | 1 |
| -897.785 | 19567.643 | 1.869 | 13.42 | 0.15198 | 7/15/2014 | 00:44.5 | 1 |
| -898.026 | 19567.662 | 1.873 | 13.414 | 0.154425 | 7/15/2014 | 00:44.6 | 1 |
| -898.328 | 19567.686 | 1.879 | 13.409 | 0.165626 | 7/15/2014 | 00:44.7 | 1 |
| -898.45 | 19567.695 | 1.877 | 13.406 | 0.162197 | 7/15/2014 | 00:44.8 | 1 |
| -898.692 | 19567.714 | 1.879 | 13.403 | 0.174232 | 7/15/2014 | 00:44.9 | 1 |
| -898.933 | 19567.732 | 1.875 | 13.4 | 0.183506 | 7/15/2014 | 00:45.0 | 1 |
| -899.176 | 19567.752 | 1.881 | 13.397 | 0.154288 | 7/15/2014 | 00:45.1 | 1 |
| -899.414 | 19567.771 | 1.875 | 13.395 | 0.162588 | 7/15/2014 | 00:45.2 | 1 |
| -899.654 | 19567.789 | 1.875 | 13.393 | 0.154154 | 7/15/2014 | 00:45.3 | 1 |
| -900.072 | 19567.821 | 1.875 | 13.389 | 0.170671 | 7/15/2014 | 00:45.4 | 1 |
| -900.31 | 19567.839 | 1.871 | 13.386 | 0.170607 | 7/15/2014 | 00:45.5 | 1 |
| -900.549 | 19567.857 | 1.868 | 13.383 | 0.156797 | 7/15/2014 | 00:45.6 | 1 |
| -900.85 | 19567.881 | 1.871 | 13.381 | 0.16721 | 7/15/2014 | 00:45.7 | 1 |
| -900.969 | 19567.89 | 1.868 | 13.379 | 0.166877 | 7/15/2014 | 00:45.8 | 1 |

| | | | | | | | | |
|---|---|---|---|---|---|---|---|---|
| -901.213 | 19567.906 | 1.874 | 13.377 | 0.17914 | 7/15/2014 | 00:45.9 | 1 |
| -901.456 | 19567.926 | 1.873 | 13.377 | 0.179919 | 7/15/2014 | 00:46.0 | 1 |
| -901.703 | 19567.944 | 1.879 | 13.377 | 0.181472 | 7/15/2014 | 00:46.1 | 1 |
| -901.948 | 19567.962 | 1.871 | 13.379 | 0.156939 | 7/15/2014 | 00:46.2 | 1 |
| -902.197 | 19567.98 | 1.879 | 13.383 | 0.173667 | 7/15/2014 | 00:46.3 | 1 |
| -902.504 | 19568.004 | 1.885 | 13.389 | 0.160835 | 7/15/2014 | 00:46.4 | 1 |
| -902.755 | 19568.021 | 1.888 | 13.397 | 0.158057 | 7/15/2014 | 00:46.5 | 1 |
| -903.005 | 19568.039 | 1.888 | 13.407 | 0.150326 | 7/15/2014 | 00:46.6 | 1 |
| -903.258 | 19568.059 | 1.894 | 13.419 | 0.145488 | 7/15/2014 | 00:46.7 | 1 |
| -903.573 | 19568.08 | 1.896 | 13.433 | 0.157672 | 7/15/2014 | 00:46.8 | 1 |
| -903.828 | 19568.098 | 1.902 | 13.447 | 0.155659 | 7/15/2014 | 00:46.9 | 1 |
| -904.082 | 19568.117 | 1.894 | 13.462 | 0.170256 | 7/15/2014 | 00:47.0 | 1 |
| -904.339 | 19568.135 | 1.888 | 13.477 | 0.16159 | 7/15/2014 | 00:47.1 | 1 |
| -904.593 | 19568.152 | 1.882 | 13.49 | 0.178755 | 7/15/2014 | 00:47.2 | 1 |
| -904.849 | 19568.171 | 1.882 | 13.5 | 0.165859 | 7/15/2014 | 00:47.3 | 1 |
| -905.165 | 19568.192 | 1.879 | 13.508 | 0.156949 | 7/15/2014 | 00:47.4 | 1 |
| -905.42 | 19568.211 | 1.873 | 13.513 | 0.15347 | 7/15/2014 | 00:47.5 | 1 |
| -905.672 | 19568.229 | 1.871 | 13.514 | 0.141898 | 7/15/2014 | 00:47.6 | 1 |
| -905.926 | 19568.246 | 1.877 | 13.511 | 0.144058 | 7/15/2014 | 00:47.7 | 1 |
| -906.239 | 19568.268 | 1.872 | 13.506 | 0.103093 | 7/15/2014 | 00:47.8 | 1 |
| -906.492 | 19568.285 | 1.874 | 13.499 | 0.118027 | 7/15/2014 | 00:47.9 | 1 |
| -906.742 | 19568.303 | 1.868 | 13.49 | 0.135511 | 7/15/2014 | 00:48.0 | 1 |
| -906.994 | 19568.32 | 1.871 | 13.481 | 0.111032 | 7/15/2014 | 00:48.1 | 1 |
| -907.242 | 19568.337 | 1.864 | 13.473 | 0.124459 | 7/15/2014 | 00:48.2 | 1 |
| -907.491 | 19568.354 | 1.86 | 13.466 | 0.0857501 | 7/15/2014 | 00:48.3 | 1 |
| -907.922 | 19568.384 | 1.852 | 13.46 | 0.130464 | 7/15/2014 | 00:48.4 | 1 |
| -908.166 | 19568.4 | 1.846 | 13.456 | 0.0915052 | 7/15/2014 | 00:48.5 | 1 |
| -908.412 | 19568.417 | 1.84 | 13.454 | 0.0675131 | 7/15/2014 | 00:48.6 | 1 |
| -908.719 | 19568.438 | 1.838 | 13.453 | 0.0400773 | 7/15/2014 | 00:48.7 | 1 |
| -908.843 | 19568.446 | 1.83 | 13.453 | 0.0297624 | 7/15/2014 | 00:48.8 | 1 |
| -909.089 | 19568.462 | 1.832 | 13.454 | 0.0524245 | 7/15/2014 | 00:48.9 | 1 |
| -909.336 | 19568.479 | 1.826 | 13.456 | 0.0594403 | 7/15/2014 | 00:49.0 | 1 |
| -909.586 | 19568.496 | 1.828 | 13.459 | 0.0681729 | 7/15/2014 | 00:49.1 | 1 |
| -909.832 | 19568.513 | 1.826 | 13.462 | 0.0771411 | 7/15/2014 | 00:49.2 | 1 |
| -910.08 | 19568.528 | 1.832 | 13.467 | 0.0825267 | 7/15/2014 | 00:49.3 | 1 |
| -910.389 | 19568.549 | 1.832 | 13.473 | 0.0798033 | 7/15/2014 | 00:49.4 | 1 |
| -910.636 | 19568.565 | 1.832 | 13.48 | 0.0828055 | 7/15/2014 | 00:49.5 | 1 |
| -910.882 | 19568.582 | 1.826 | 13.487 | 0.0728211 | 7/15/2014 | 00:49.6 | 1 |
| -911.314 | 19568.609 | 1.82 | 13.499 | 0.0633835 | 7/15/2014 | 00:49.7 | 1 |
| -911.439 | 19568.618 | 1.815 | 13.503 | 0.0679238 | 7/15/2014 | 00:49.8 | 1 |
| -911.688 | 19568.633 | 1.809 | 13.512 | 0.0727549 | 7/15/2014 | 00:49.9 | 1 |
| -911.936 | 19568.648 | 1.799 | 13.521 | 0.0570871 | 7/15/2014 | 00:50.0 | 1 |
| -912.184 | 19568.665 | 1.796 | 13.531 | 0.0920493 | 7/15/2014 | 00:50.1 | 1 |
| -912.431 | 19568.681 | 1.786 | 13.542 | 0.0768898 | 7/15/2014 | 00:50.2 | 1 |
| -912.678 | 19568.696 | 1.786 | 13.553 | 0.0813784 | 7/15/2014 | 00:50.3 | 1 |
| -913.107 | 19568.723 | 1.783 | 13.569 | 0.0868019 | 7/15/2014 | 00:50.4 | 1 |
| -913.351 | 19568.736 | 1.78 | 13.58 | 0.0829097 | 7/15/2014 | 00:50.5 | 1 |

| | | | | | | | |
|---|---|---|---|---|---|---|---|
| -913.595 | 19568.752 | 1.78 | 13.59 | 0.106129 | 7/15/2014 | 00:50.6 | 1 |
| -913.898 | 19568.771 | 1.783 | 13.599 | 0.093742 | 7/15/2014 | 00:50.7 | 1 |
| -914.022 | 19568.778 | 1.782 | 13.604 | 0.102216 | 7/15/2014 | 00:50.8 | 1 |
| -914.266 | 19568.793 | 1.786 | 13.612 | 0.119284 | 7/15/2014 | 00:50.9 | 1 |
| -914.51 | 19568.808 | 1.786 | 13.62 | 0.118185 | 7/15/2014 | 00:51.0 | 1 |
| -914.754 | 19568.821 | 1.792 | 13.626 | 0.13108 | 7/15/2014 | 00:51.1 | 1 |
| -914.997 | 19568.836 | 1.788 | 13.632 | 0.112038 | 7/15/2014 | 00:51.2 | 1 |
| -915.239 | 19568.85 | 1.792 | 13.637 | 0.119723 | 7/15/2014 | 00:51.3 | 1 |
| -915.661 | 19568.874 | 1.787 | 13.646 | 0.138122 | 7/15/2014 | 00:51.4 | 1 |
| -915.901 | 19568.889 | 1.781 | 13.653 | 0.134403 | 7/15/2014 | 00:51.5 | 1 |
| -916.143 | 19568.901 | 1.776 | 13.661 | 0.153949 | 7/15/2014 | 00:51.6 | 1 |
| -916.446 | 19568.918 | 1.771 | 13.67 | 0.133266 | 7/15/2014 | 00:51.7 | 1 |
| -916.567 | 19568.926 | 1.765 | 13.673 | 0.153167 | 7/15/2014 | 00:51.8 | 1 |
| -916.813 | 19568.939 | 1.763 | 13.684 | 0.135935 | 7/15/2014 | 00:51.9 | 1 |
| -917.057 | 19568.953 | 1.757 | 13.696 | 0.149451 | 7/15/2014 | 00:52.0 | 1 |
| -917.305 | 19568.966 | 1.763 | 13.709 | 0.109746 | 7/15/2014 | 00:52.1 | 1 |
| -917.551 | 19568.98 | 1.754 | 13.723 | 0.1222 | 7/15/2014 | 00:52.2 | 1 |
| -917.798 | 19568.993 | 1.752 | 13.737 | 0.102207 | 7/15/2014 | 00:52.3 | 1 |
| -918.107 | 19569.01 | 1.749 | 13.752 | 0.082713 | 7/15/2014 | 00:52.4 | 1 |
| -918.357 | 19569.023 | 1.749 | 13.766 | 0.100077 | 7/15/2014 | 00:52.5 | 1 |
| -918.606 | 19569.035 | 1.747 | 13.78 | 0.0501665 | 7/15/2014 | 00:52.6 | 1 |
| -918.857 | 19569.05 | 1.744 | 13.793 | 0.06269 | 7/15/2014 | 00:52.7 | 1 |
| -919.169 | 19569.064 | 1.736 | 13.806 | 0.0593242 | 7/15/2014 | 00:52.8 | 1 |
| -919.42 | 19569.078 | 1.735 | 13.818 | 0.0696349 | 7/15/2014 | 00:52.9 | 1 |
| -919.673 | 19569.092 | 1.732 | 13.83 | 0.0778795 | 7/15/2014 | 00:53.0 | 1 |
| -919.925 | 19569.104 | 1.732 | 13.841 | 0.0900091 | 7/15/2014 | 00:53.1 | 1 |
| -920.178 | 19569.115 | 1.725 | 13.852 | 0.0970553 | 7/15/2014 | 00:53.2 | 1 |
| -920.429 | 19569.129 | 1.725 | 13.862 | 0.108416 | 7/15/2014 | 00:53.3 | 1 |
| -920.742 | 19569.143 | 1.722 | 13.872 | 0.114253 | 7/15/2014 | 00:53.4 | 1 |
| -920.993 | 19569.155 | 1.722 | 13.881 | 0.114688 | 7/15/2014 | 00:53.5 | 1 |
| -921.243 | 19569.168 | 1.722 | 13.89 | 0.123413 | 7/15/2014 | 00:53.6 | 1 |
| -921.492 | 19569.18 | 1.728 | 13.898 | 0.114975 | 7/15/2014 | 00:53.7 | 1 |
| -921.802 | 19569.193 | 1.728 | 13.904 | 0.124059 | 7/15/2014 | 00:53.8 | 1 |
| -922.051 | 19569.205 | 1.731 | 13.909 | 0.120298 | 7/15/2014 | 00:53.9 | 1 |
| -922.3 | 19569.216 | 1.731 | 13.912 | 0.104552 | 7/15/2014 | 00:54.0 | 1 |
| -922.549 | 19569.227 | 1.737 | 13.911 | 0.127464 | 7/15/2014 | 00:54.1 | 1 |
| -922.795 | 19569.238 | 1.731 | 13.907 | 0.121156 | 7/15/2014 | 00:54.2 | 1 |
| -923.041 | 19569.249 | 1.734 | 13.899 | 0.119028 | 7/15/2014 | 00:54.3 | 1 |
| -923.469 | 19569.268 | 1.736 | 13.878 | 0.146796 | 7/15/2014 | 00:54.4 | 1 |
| -923.712 | 19569.277 | 1.738 | 13.863 | 0.152398 | 7/15/2014 | 00:54.5 | 1 |
| -923.954 | 19569.288 | 1.743 | 13.846 | 0.160999 | 7/15/2014 | 00:54.6 | 1 |
| -924.257 | 19569.299 | 1.745 | 13.827 | 0.144365 | 7/15/2014 | 00:54.7 | 1 |
| -924.38 | 19569.305 | 1.745 | 13.817 | 0.138551 | 7/15/2014 | 00:54.8 | 1 |
| -924.624 | 19569.315 | 1.753 | 13.799 | 0.15686 | 7/15/2014 | 00:54.9 | 1 |
| -924.868 | 19569.324 | 1.751 | 13.781 | 0.142977 | 7/15/2014 | 00:55.0 | 1 |
| -925.112 | 19569.336 | 1.751 | 13.766 | 0.149801 | 7/15/2014 | 00:55.1 | 1 |
| -925.356 | 19569.344 | 1.751 | 13.754 | 0.131632 | 7/15/2014 | 00:55.2 | 1 |

| | | | | | | | |
|---|---|---|---|---|---|---|---|
| -925.6 | 19569.355 | 1.748 | 13.745 | 0.158319 | 7/15/2014 | 00:55.3 | 1 |
| -926.026 | 19569.371 | 1.745 | 13.743 | 0.140612 | 7/15/2014 | 00:55.4 | 1 |
| -926.268 | 19569.381 | 1.743 | 13.745 | 0.144317 | 7/15/2014 | 00:55.5 | 1 |
| -926.512 | 19569.389 | 1.741 | 13.752 | 0.19089 | 7/15/2014 | 00:55.6 | 1 |
| -926.819 | 19569.402 | 1.743 | 13.764 | 0.14257 | 7/15/2014 | 00:55.7 | 1 |
| -926.942 | 19569.406 | 1.742 | 13.769 | 0.175234 | 7/15/2014 | 00:55.8 | 1 |
| -927.189 | 19569.416 | 1.742 | 13.787 | 0.144675 | 7/15/2014 | 00:55.9 | 1 |
| -927.436 | 19569.424 | 1.745 | 13.81 | 0.176805 | 7/15/2014 | 00:56.0 | 1 |
| -927.685 | 19569.436 | 1.745 | 13.836 | 0.193912 | 7/15/2014 | 00:56.1 | 1 |
| -927.933 | 19569.443 | 1.745 | 13.865 | 0.171869 | 7/15/2014 | 00:56.2 | 1 |
| -928.183 | 19569.451 | 1.745 | 13.895 | 0.154342 | 7/15/2014 | 00:56.3 | 1 |
| -928.493 | 19569.465 | 1.745 | 13.925 | 0.190802 | 7/15/2014 | 00:56.4 | 1 |
| -928.742 | 19569.473 | 1.745 | 13.955 | 0.179253 | 7/15/2014 | 00:56.5 | 1 |
| -928.991 | 19569.48 | 1.742 | 13.983 | 0.156359 | 7/15/2014 | 00:56.6 | 1 |
| -929.241 | 19569.492 | 1.742 | 14.008 | 0.136609 | 7/15/2014 | 00:56.7 | 1 |
| -929.553 | 19569.502 | 1.74 | 14.03 | 0.163165 | 7/15/2014 | 00:56.8 | 1 |
| -929.803 | 19569.51 | 1.74 | 14.047 | 0.116913 | 7/15/2014 | 00:56.9 | 1 |
| -930.053 | 19569.52 | 1.737 | 14.059 | 0.146523 | 7/15/2014 | 00:57.0 | 1 |
| -930.305 | 19569.529 | 1.737 | 14.066 | 0.132153 | 7/15/2014 | 00:57.1 | 1 |
| -930.554 | 19569.537 | 1.732 | 14.069 | 0.114992 | 7/15/2014 | 00:57.2 | 1 |
| -930.805 | 19569.547 | 1.735 | 14.067 | 0.14909 | 7/15/2014 | 00:57.3 | 1 |
| -931.114 | 19569.558 | 1.73 | 14.063 | 0.133452 | 7/15/2014 | 00:57.4 | 1 |
| -931.363 | 19569.566 | 1.73 | 14.058 | 0.153944 | 7/15/2014 | 00:57.5 | 1 |
| -931.61 | 19569.574 | 1.725 | 14.052 | 0.135005 | 7/15/2014 | 00:57.6 | 1 |
| -932.043 | 19569.591 | 1.718 | 14.048 | 0.144398 | 7/15/2014 | 00:57.7 | 1 |
| -932.168 | 19569.595 | 1.715 | 14.045 | 0.172495 | 7/15/2014 | 00:57.8 | 1 |
| -932.419 | 19569.604 | 1.718 | 14.046 | 0.109079 | 7/15/2014 | 00:57.9 | 1 |
| -932.667 | 19569.612 | 1.715 | 14.051 | 0.118966 | 7/15/2014 | 00:58.0 | 1 |
| -932.916 | 19569.621 | 1.722 | 14.06 | 0.111445 | 7/15/2014 | 00:58.1 | 1 |
| -933.165 | 19569.63 | 1.719 | 14.071 | 0.107981 | 7/15/2014 | 00:58.2 | 1 |
| -933.413 | 19569.639 | 1.722 | 14.084 | 0.125123 | 7/15/2014 | 00:58.3 | 1 |
| -933.849 | 19569.653 | 1.723 | 14.106 | 0.133068 | 7/15/2014 | 00:58.4 | 1 |
| -933.973 | 19569.658 | 1.728 | 14.114 | 0.161917 | 7/15/2014 | 00:58.5 | 1 |
| -934.22 | 19569.668 | 1.722 | 14.128 | 0.155927 | 7/15/2014 | 00:58.6 | 1 |
| -934.469 | 19569.676 | 1.722 | 14.141 | 0.183916 | 7/15/2014 | 00:58.7 | 1 |
| -934.779 | 19569.687 | 1.712 | 14.152 | 0.142655 | 7/15/2014 | 00:58.8 | 1 |
| -935.03 | 19569.697 | 1.712 | 14.161 | 0.153329 | 7/15/2014 | 00:58.9 | 1 |
| -935.281 | 19569.705 | 1.709 | 14.168 | 0.173924 | 7/15/2014 | 00:59.0 | 1 |
| -935.534 | 19569.713 | 1.709 | 14.174 | 0.161418 | 7/15/2014 | 00:59.1 | 1 |
| -935.785 | 19569.725 | 1.703 | 14.179 | 0.154561 | 7/15/2014 | 00:59.2 | 1 |
| -936.036 | 19569.732 | 1.707 | 14.183 | 0.152019 | 7/15/2014 | 00:59.3 | 1 |
| -936.348 | 19569.744 | 1.701 | 14.187 | 0.191114 | 7/15/2014 | 00:59.4 | 1 |
| -936.599 | 19569.754 | 1.705 | 14.192 | 0.169036 | 7/15/2014 | 00:59.5 | 1 |
| -936.849 | 19569.763 | 1.701 | 14.196 | 0.127382 | 7/15/2014 | 00:59.6 | 1 |
| -937.099 | 19569.771 | 1.701 | 14.202 | 0.181962 | 7/15/2014 | 00:59.7 | 1 |
| -937.41 | 19569.783 | 1.704 | 14.209 | 0.182023 | 7/15/2014 | 00:59.8 | 1 |
| -937.659 | 19569.793 | 1.704 | 14.217 | 0.190122 | 7/15/2014 | 00:59.9 | 1 |

| | | | | | | | |
|---|---|---|---|---|---|---|---|
| -937.908 | 19569.803 | 1.691 | 14.225 | 0.211167 | 7/15/2014 | 01:00.0 | 1 |
| -938.158 | 19569.813 | 1.691 | 14.236 | 0.220392 | 7/15/2014 | 01:00.1 | 1 |
| -938.405 | 19569.823 | 1.677 | 14.248 | 0.272989 | 7/15/2014 | 01:00.2 | 1 |
| -938.653 | 19569.832 | 1.677 | 14.262 | 0.250862 | 7/15/2014 | 01:00.3 | 1 |
| -939.082 | 19569.848 | 1.668 | 14.288 | 0.292444 | 7/15/2014 | 01:00.4 | 1 |
| -939.326 | 19569.859 | 1.659 | 14.306 | 0.290922 | 7/15/2014 | 01:00.5 | 1 |
| -939.571 | 19569.867 | 1.657 | 14.325 | 0.298388 | 7/15/2014 | 01:00.6 | 1 |
| -939.878 | 19569.881 | 1.66 | 14.345 | 0.290567 | 7/15/2014 | 01:00.7 | 1 |
| -940.001 | 19569.887 | 1.656 | 14.356 | 0.277558 | 7/15/2014 | 01:00.8 | 1 |
| -940.25 | 19569.896 | 1.666 | 14.375 | 0.268326 | 7/15/2014 | 01:00.9 | 1 |
| -940.496 | 19569.905 | 1.656 | 14.392 | 0.343969 | 7/15/2014 | 01:01.0 | 1 |
| -940.746 | 19569.916 | 1.656 | 14.405 | 0.29284 | 7/15/2014 | 01:01.1 | 1 |
| -940.994 | 19569.928 | 1.652 | 14.414 | 0.310287 | 7/15/2014 | 01:01.2 | 1 |
| -941.245 | 19569.938 | 1.658 | 14.418 | 0.286899 | 7/15/2014 | 01:01.3 | 1 |
| -941.553 | 19569.951 | 1.657 | 14.417 | 0.306119 | 7/15/2014 | 01:01.4 | 1 |
| -941.805 | 19569.963 | 1.663 | 14.412 | 0.298799 | 7/15/2014 | 01:01.5 | 1 |
| -942.054 | 19569.972 | 1.66 | 14.402 | 0.237479 | 7/15/2014 | 01:01.6 | 1 |
| -942.307 | 19569.982 | 1.669 | 14.39 | 0.226738 | 7/15/2014 | 01:01.7 | 1 |
| -942.621 | 19569.998 | 1.675 | 14.377 | 0.205623 | 7/15/2014 | 01:01.8 | 1 |
| -942.876 | 19570.009 | 1.681 | 14.363 | 0.194396 | 7/15/2014 | 01:01.9 | 1 |
| -943.13 | 19570.021 | 1.675 | 14.352 | 0.18397 | 7/15/2014 | 01:02.0 | 1 |
| -943.388 | 19570.033 | 1.677 | 14.343 | 0.172175 | 7/15/2014 | 01:02.1 | 1 |
| -943.641 | 19570.043 | 1.668 | 14.337 | 0.164179 | 7/15/2014 | 01:02.2 | 1 |
| -943.9 | 19570.055 | 1.675 | 14.334 | 0.162093 | 7/15/2014 | 01:02.3 | 1 |
| -944.217 | 19570.07 | 1.672 | 14.336 | 0.155876 | 7/15/2014 | 01:02.4 | 1 |
| -944.474 | 19570.081 | 1.668 | 14.34 | 0.149832 | 7/15/2014 | 01:02.5 | 1 |
| -944.73 | 19570.094 | 1.664 | 14.347 | 0.145897 | 7/15/2014 | 01:02.6 | 1 |
| -944.99 | 19570.107 | 1.674 | 14.356 | 0.149486 | 7/15/2014 | 01:02.7 | 1 |
| -945.308 | 19570.123 | 1.679 | 14.366 | 0.150084 | 7/15/2014 | 01:02.8 | 1 |
| -945.568 | 19570.135 | 1.691 | 14.376 | 0.137478 | 7/15/2014 | 01:02.9 | 1 |
| -945.823 | 19570.146 | 1.684 | 14.386 | 0.129828 | 7/15/2014 | 01:03.0 | 1 |
| -946.082 | 19570.16 | 1.688 | 14.395 | 0.133577 | 7/15/2014 | 01:03.1 | 1 |
| -946.337 | 19570.173 | 1.688 | 14.402 | 0.127255 | 7/15/2014 | 01:03.2 | 1 |
| -946.595 | 19570.187 | 1.691 | 14.408 | 0.119356 | 7/15/2014 | 01:03.3 | 1 |
| -946.911 | 19570.203 | 1.688 | 14.411 | 0.126588 | 7/15/2014 | 01:03.4 | 1 |
| -947.167 | 19570.216 | 1.689 | 14.413 | 0.11845 | 7/15/2014 | 01:03.5 | 1 |
| -947.417 | 19570.229 | 1.685 | 14.413 | 0.136974 | 7/15/2014 | 01:03.6 | 1 |
| -947.672 | 19570.243 | 1.686 | 14.412 | 0.143464 | 7/15/2014 | 01:03.7 | 1 |
| -947.983 | 19570.26 | 1.683 | 14.409 | 0.137542 | 7/15/2014 | 01:03.8 | 1 |
| -948.237 | 19570.272 | 1.686 | 14.405 | 0.171457 | 7/15/2014 | 01:03.9 | 1 |
| -948.487 | 19570.287 | 1.683 | 14.401 | 0.16649 | 7/15/2014 | 01:04.0 | 1 |
| -948.74 | 19570.301 | 1.683 | 14.397 | 0.182631 | 7/15/2014 | 01:04.1 | 1 |
| -948.989 | 19570.314 | 1.679 | 14.393 | 0.179494 | 7/15/2014 | 01:04.2 | 1 |
| -949.24 | 19570.329 | 1.683 | 14.39 | 0.161295 | 7/15/2014 | 01:04.3 | 1 |
| -949.674 | 19570.354 | 1.68 | 14.389 | 0.149764 | 7/15/2014 | 01:04.4 | 1 |
| -949.798 | 19570.36 | 1.683 | 14.388 | 0.138392 | 7/15/2014 | 01:04.5 | 1 |
| -950.168 | 19570.383 | 1.683 | 14.394 | 0.146391 | 7/15/2014 | 01:04.6 | 1 |

| | | | | | | | |
|---|---|---|---|---|---|---|---|
| -950.478 | 19570.401 | 1.692 | 14.399 | 0.146764 | 7/15/2014 | 01:04.7 | 1 |
| -950.602 | 19570.408 | 1.692 | 14.401 | 0.138685 | 7/15/2014 | 01:04.8 | 1 |
| -950.852 | 19570.423 | 1.696 | 14.409 | 0.160264 | 7/15/2014 | 01:04.9 | 1 |
| -951.099 | 19570.438 | 1.689 | 14.418 | 0.158783 | 7/15/2014 | 01:05.0 | 1 |
| -951.35 | 19570.453 | 1.69 | 14.427 | 0.186879 | 7/15/2014 | 01:05.1 | 1 |
| -951.597 | 19570.468 | 1.686 | 14.435 | 0.165189 | 7/15/2014 | 01:05.2 | 1 |
| -951.847 | 19570.484 | 1.69 | 14.442 | 0.150507 | 7/15/2014 | 01:05.3 | 1 |
| -952.281 | 19570.512 | 1.685 | 14.448 | 0.13597 | 7/15/2014 | 01:05.4 | 1 |
| -952.404 | 19570.519 | 1.678 | 14.45 | 0.136211 | 7/15/2014 | 01:05.5 | 1 |
| -952.773 | 19570.542 | 1.671 | 14.452 | 0.142224 | 7/15/2014 | 01:05.6 | 1 |
| -953.082 | 19570.563 | 1.672 | 14.451 | 0.151498 | 7/15/2014 | 01:05.7 | 1 |
| -953.206 | 19570.57 | 1.672 | 14.451 | 0.154975 | 7/15/2014 | 01:05.8 | 1 |
| -953.455 | 19570.588 | 1.678 | 14.45 | 0.162911 | 7/15/2014 | 01:05.9 | 1 |
| -953.702 | 19570.604 | 1.675 | 14.447 | 0.165981 | 7/15/2014 | 01:06.0 | 1 |
| -953.951 | 19570.619 | 1.678 | 14.444 | 0.138966 | 7/15/2014 | 01:06.1 | 1 |
| -954.198 | 19570.636 | 1.674 | 14.441 | 0.116501 | 7/15/2014 | 01:06.2 | 1 |
| -954.447 | 19570.652 | 1.686 | 14.437 | 0.10742 | 7/15/2014 | 01:06.3 | 1 |
| -954.878 | 19570.683 | 1.681 | 14.429 | 0.0928356 | 7/15/2014 | 01:06.4 | 1 |
| -955.123 | 19570.699 | 1.683 | 14.424 | 0.0871147 | 7/15/2014 | 01:06.5 | 1 |
| -955.368 | 19570.716 | 1.683 | 14.419 | 0.0834073 | 7/15/2014 | 01:06.6 | 1 |
| -955.671 | 19570.736 | 1.685 | 14.413 | 0.0782511 | 7/15/2014 | 01:06.7 | 1 |
| -955.796 | 19570.745 | 1.689 | 14.411 | 0.0807241 | 7/15/2014 | 01:06.8 | 1 |
| -956.044 | 19570.763 | 1.692 | 14.405 | 0.0798187 | 7/15/2014 | 01:06.9 | 1 |
| -956.289 | 19570.78 | 1.692 | 14.398 | 0.0796025 | 7/15/2014 | 01:07.0 | 1 |
| -956.536 | 19570.798 | 1.699 | 14.392 | 0.0777683 | 7/15/2014 | 01:07.1 | 1 |
| -956.782 | 19570.814 | 1.702 | 14.387 | 0.0882664 | 7/15/2014 | 01:07.2 | 1 |
| -957.026 | 19570.832 | 1.705 | 14.381 | 0.120012 | 7/15/2014 | 01:07.3 | 1 |
| -957.451 | 19570.863 | 1.709 | 14.374 | 0.156196 | 7/15/2014 | 01:07.4 | 1 |
| -957.695 | 19570.881 | 1.712 | 14.371 | 0.121712 | 7/15/2014 | 01:07.5 | 1 |
| -957.938 | 19570.898 | 1.709 | 14.367 | 0.115727 | 7/15/2014 | 01:07.6 | 1 |
| -958.241 | 19570.921 | 1.701 | 14.365 | 0.109688 | 7/15/2014 | 01:07.7 | 1 |
| -958.363 | 19570.93 | 1.699 | 14.363 | 0.112525 | 7/15/2014 | 01:07.8 | 1 |
| -958.609 | 19570.948 | 1.696 | 14.362 | 0.108516 | 7/15/2014 | 01:07.9 | 1 |
| -958.855 | 19570.967 | 1.693 | 14.361 | 0.104928 | 7/15/2014 | 01:08.0 | 1 |
| -959.101 | 19570.986 | 1.693 | 14.36 | 0.0979566 | 7/15/2014 | 01:08.1 | 1 |
| -959.346 | 19571.005 | 1.689 | 14.36 | 0.101999 | 7/15/2014 | 01:08.2 | 1 |
| -959.593 | 19571.023 | 1.689 | 14.361 | 0.112697 | 7/15/2014 | 01:08.3 | 1 |
| -960.02 | 19571.057 | 1.692 | 14.363 | 0.103536 | 7/15/2014 | 01:08.4 | 1 |
| -960.266 | 19571.076 | 1.695 | 14.364 | 0.102952 | 7/15/2014 | 01:08.5 | 1 |
| -960.51 | 19571.096 | 1.701 | 14.367 | 0.100672 | 7/15/2014 | 01:08.6 | 1 |
| -960.814 | 19571.119 | 1.701 | 14.369 | 0.0987335 | 7/15/2014 | 01:08.7 | 1 |
| -960.937 | 19571.129 | 1.699 | 14.371 | 0.101921 | 7/15/2014 | 01:08.8 | 1 |
| -961.183 | 19571.148 | 1.703 | 14.374 | 0.0982063 | 7/15/2014 | 01:08.9 | 1 |
| -961.427 | 19571.168 | 1.696 | 14.379 | 0.0970824 | 7/15/2014 | 01:09.0 | 1 |
| -961.671 | 19571.188 | 1.696 | 14.384 | 0.0967249 | 7/15/2014 | 01:09.1 | 1 |
| -961.914 | 19571.208 | 1.696 | 14.388 | 0.102686 | 7/15/2014 | 01:09.2 | 1 |
| -962.156 | 19571.229 | 1.696 | 14.393 | 0.0960199 | 7/15/2014 | 01:09.3 | 1 |

| | | | | | | | |
|---|---|---|---|---|---|---|---|
| -962.576 | 19571.262 | 1.692 | 14.397 | 0.098056 | 7/15/2014 | 01:09.4 | 1 |
| -962.815 | 19571.281 | 1.695 | 14.398 | 0.0991214 | 7/15/2014 | 01:09.5 | 1 |
| -963.054 | 19571.301 | 1.692 | 14.398 | 0.0906594 | 7/15/2014 | 01:09.6 | 1 |
| -963.351 | 19571.326 | 1.692 | 14.396 | 0.0934625 | 7/15/2014 | 01:09.7 | 1 |
| -963.472 | 19571.337 | 1.689 | 14.396 | 0.0888817 | 7/15/2014 | 01:09.8 | 1 |
| -963.712 | 19571.358 | 1.693 | 14.391 | 0.0866532 | 7/15/2014 | 01:09.9 | 1 |
| -963.952 | 19571.379 | 1.69 | 14.384 | 0.0844843 | 7/15/2014 | 01:10.0 | 1 |
| -964.193 | 19571.398 | 1.699 | 14.376 | 0.0809487 | 7/15/2014 | 01:10.1 | 1 |
| -964.432 | 19571.42 | 1.705 | 14.367 | 0.0868487 | 7/15/2014 | 01:10.2 | 1 |
| -964.673 | 19571.44 | 1.704 | 14.359 | 0.089209 | 7/15/2014 | 01:10.3 | 1 |
| -965.093 | 19571.479 | 1.705 | 14.348 | 0.108342 | 7/15/2014 | 01:10.4 | 1 |
| -965.333 | 19571.5 | 1.71 | 14.342 | 0.115931 | 7/15/2014 | 01:10.5 | 1 |
| -965.574 | 19571.521 | 1.712 | 14.338 | 0.126409 | 7/15/2014 | 01:10.6 | 1 |
| -965.88 | 19571.549 | 1.72 | 14.335 | 0.134588 | 7/15/2014 | 01:10.7 | 1 |
| -966 | 19571.561 | 1.716 | 14.333 | 0.119745 | 7/15/2014 | 01:10.8 | 1 |
| -966.247 | 19571.583 | 1.722 | 14.333 | 0.156456 | 7/15/2014 | 01:10.9 | 1 |
| -966.493 | 19571.605 | 1.72 | 14.335 | 0.12166 | 7/15/2014 | 01:11.0 | 1 |
| -966.743 | 19571.629 | 1.728 | 14.337 | 0.106873 | 7/15/2014 | 01:11.1 | 1 |
| -966.991 | 19571.652 | 1.728 | 14.341 | 0.114155 | 7/15/2014 | 01:11.2 | 1 |
| -967.241 | 19571.676 | 1.73 | 14.346 | 0.0922894 | 7/15/2014 | 01:11.3 | 1 |
| -967.552 | 19571.705 | 1.737 | 14.351 | 0.110069 | 7/15/2014 | 01:11.4 | 1 |
| -967.804 | 19571.729 | 1.739 | 14.355 | 0.076621 | 7/15/2014 | 01:11.5 | 1 |
| -968.054 | 19571.753 | 1.733 | 14.36 | 0.0786223 | 7/15/2014 | 01:11.6 | 1 |
| -968.306 | 19571.776 | 1.741 | 14.363 | 0.0791432 | 7/15/2014 | 01:11.7 | 1 |
| -968.617 | 19571.807 | 1.737 | 14.366 | 0.0642266 | 7/15/2014 | 01:11.8 | 1 |
| -968.869 | 19571.831 | 1.741 | 14.367 | 0.0666478 | 7/15/2014 | 01:11.9 | 1 |
| -969.12 | 19571.855 | 1.744 | 14.366 | 0.0774003 | 7/15/2014 | 01:12.0 | 1 |
| -969.37 | 19571.88 | 1.74 | 14.363 | 0.0628959 | 7/15/2014 | 01:12.1 | 1 |
| -969.62 | 19571.904 | 1.738 | 14.358 | 0.0638812 | 7/15/2014 | 01:12.2 | 1 |
| -969.868 | 19571.929 | 1.74 | 14.352 | 0.0816334 | 7/15/2014 | 01:12.3 | 1 |
| -970.298 | 19571.971 | 1.737 | 14.341 | 0.0831107 | 7/15/2014 | 01:12.4 | 1 |
| -970.542 | 19571.994 | 1.739 | 14.334 | 0.0483472 | 7/15/2014 | 01:12.5 | 1 |
| -970.787 | 19572.02 | 1.737 | 14.328 | 0.067456 | 7/15/2014 | 01:12.6 | 1 |
| -971.09 | 19572.05 | 1.737 | 14.323 | 0.0745669 | 7/15/2014 | 01:12.7 | 1 |
| -971.214 | 19572.062 | 1.735 | 14.32 | 0.0785 | 7/15/2014 | 01:12.8 | 1 |
| -971.457 | 19572.087 | 1.741 | 14.317 | 0.0916387 | 7/15/2014 | 01:12.9 | 1 |
| -971.699 | 19572.111 | 1.739 | 14.316 | 0.098275 | 7/15/2014 | 01:13.0 | 1 |
| -971.943 | 19572.136 | 1.739 | 14.317 | 0.10581 | 7/15/2014 | 01:13.1 | 1 |
| -972.184 | 19572.16 | 1.73 | 14.319 | 0.121492 | 7/15/2014 | 01:13.2 | 1 |
| -972.426 | 19572.186 | 1.73 | 14.322 | 0.120466 | 7/15/2014 | 01:13.3 | 1 |
| -972.845 | 19572.229 | 1.73 | 14.327 | 0.10593 | 7/15/2014 | 01:13.4 | 1 |
| -973.082 | 19572.253 | 1.728 | 14.331 | 0.10379 | 7/15/2014 | 01:13.5 | 1 |
| -973.321 | 19572.278 | 1.725 | 14.335 | 0.105244 | 7/15/2014 | 01:13.6 | 1 |
| -973.619 | 19572.309 | 1.723 | 14.339 | 0.0970159 | 7/15/2014 | 01:13.7 | 1 |
| -973.739 | 19572.321 | 1.724 | 14.341 | 0.102657 | 7/15/2014 | 01:13.8 | 1 |
| -973.982 | 19572.348 | 1.733 | 14.346 | 0.0853817 | 7/15/2014 | 01:13.9 | 1 |
| -974.222 | 19572.373 | 1.73 | 14.351 | 0.0875147 | 7/15/2014 | 01:14.0 | 1 |

| | | | | | | | |
|---|---|---|---|---|---|---|---|
| -974.465 | 19572.399 | 1.73 | 14.358 | 0.0866231 | 7/15/2014 | 01:14.1 | 1 |
| -974.707 | 19572.424 | 1.724 | 14.365 | 0.107667 | 7/15/2014 | 01:14.2 | 1 |
| -974.951 | 19572.45 | 1.726 | 14.374 | 0.105873 | 7/15/2014 | 01:14.3 | 1 |
| -975.375 | 19572.495 | 1.722 | 14.387 | 0.0972377 | 7/15/2014 | 01:14.4 | 1 |
| -975.618 | 19572.521 | 1.719 | 14.396 | 0.103903 | 7/15/2014 | 01:14.5 | 1 |
| -975.861 | 19572.549 | 1.711 | 14.405 | 0.092015 | 7/15/2014 | 01:14.6 | 1 |
| -976.167 | 19572.581 | 1.711 | 14.414 | 0.100611 | 7/15/2014 | 01:14.7 | 1 |
| -976.29 | 19572.594 | 1.709 | 14.419 | 0.0699201 | 7/15/2014 | 01:14.8 | 1 |
| -976.537 | 19572.621 | 1.709 | 14.427 | 0.060164 | 7/15/2014 | 01:14.9 | 1 |
| -976.783 | 19572.648 | 1.7 | 14.435 | 0.0599975 | 7/15/2014 | 01:15.0 | 1 |
| -977.032 | 19572.675 | 1.703 | 14.443 | 0.0511861 | 7/15/2014 | 01:15.1 | 1 |
| -977.278 | 19572.701 | 1.697 | 14.451 | 0.0526999 | 7/15/2014 | 01:15.2 | 1 |
| -977.524 | 19572.729 | 1.7 | 14.458 | 0.0492727 | 7/15/2014 | 01:15.3 | 1 |
| -977.954 | 19572.775 | 1.697 | 14.47 | 0.0518638 | 7/15/2014 | 01:15.4 | 1 |
| -978.197 | 19572.803 | 1.69 | 14.477 | 0.0748974 | 7/15/2014 | 01:15.5 | 1 |
| -978.442 | 19572.83 | 1.69 | 14.484 | 0.0766111 | 7/15/2014 | 01:15.6 | 1 |
| -978.745 | 19572.865 | 1.685 | 14.491 | 0.0832893 | 7/15/2014 | 01:15.7 | 1 |
| -978.869 | 19572.879 | 1.678 | 14.494 | 0.0798207 | 7/15/2014 | 01:15.8 | 1 |
| -979.114 | 19572.906 | 1.678 | 14.501 | 0.0907836 | 7/15/2014 | 01:15.9 | 1 |
| -979.357 | 19572.933 | 1.671 | 14.506 | 0.116061 | 7/15/2014 | 01:16.0 | 1 |
| -979.602 | 19572.961 | 1.673 | 14.511 | 0.106722 | 7/15/2014 | 01:16.1 | 1 |
| -979.842 | 19572.988 | 1.667 | 14.514 | 0.0988377 | 7/15/2014 | 01:16.2 | 1 |
| -980.086 | 19573.016 | 1.669 | 14.515 | 0.0859563 | 7/15/2014 | 01:16.3 | 1 |
| -980.506 | 19573.064 | 1.666 | 14.514 | 0.0940532 | 7/15/2014 | 01:16.4 | 1 |
| -980.745 | 19573.092 | 1.666 | 14.513 | 0.103271 | 7/15/2014 | 01:16.5 | 1 |
| -980.985 | 19573.119 | 1.666 | 14.51 | 0.10655 | 7/15/2014 | 01:16.6 | 1 |
| -981.285 | 19573.154 | 1.663 | 14.506 | 0.0961381 | 7/15/2014 | 01:16.7 | 1 |
| -981.405 | 19573.168 | 1.66 | 14.504 | 0.080804 | 7/15/2014 | 01:16.8 | 1 |
| -981.649 | 19573.196 | 1.664 | 14.499 | 0.103196 | 7/15/2014 | 01:16.9 | 1 |
| -981.889 | 19573.225 | 1.66 | 14.494 | 0.0870854 | 7/15/2014 | 01:17.0 | 1 |
| -982.135 | 19573.252 | 1.661 | 14.489 | 0.147024 | 7/15/2014 | 01:17.1 | 1 |
| -982.376 | 19573.28 | 1.658 | 14.485 | 0.125127 | 7/15/2014 | 01:17.2 | 1 |
| -982.62 | 19573.31 | 1.658 | 14.48 | 0.146133 | 7/15/2014 | 01:17.3 | 1 |
| -983.045 | 19573.359 | 1.65 | 14.474 | 0.157164 | 7/15/2014 | 01:17.4 | 1 |
| -983.29 | 19573.388 | 1.65 | 14.47 | 0.152088 | 7/15/2014 | 01:17.5 | 1 |
| -983.532 | 19573.417 | 1.647 | 14.467 | 0.148072 | 7/15/2014 | 01:17.6 | 1 |
| -983.84 | 19573.453 | 1.647 | 14.464 | 0.129704 | 7/15/2014 | 01:17.7 | 1 |
| -983.963 | 19573.468 | 1.644 | 14.463 | 0.150034 | 7/15/2014 | 01:17.8 | 1 |
| -984.21 | 19573.496 | 1.644 | 14.462 | 0.145543 | 7/15/2014 | 01:17.9 | 1 |
| -984.459 | 19573.526 | 1.644 | 14.462 | 0.15108 | 7/15/2014 | 01:18.0 | 1 |
| -984.707 | 19573.556 | 1.638 | 14.465 | 0.169297 | 7/15/2014 | 01:18.1 | 1 |
| -984.957 | 19573.584 | 1.632 | 14.468 | 0.147972 | 7/15/2014 | 01:18.2 | 1 |
| -985.206 | 19573.614 | 1.63 | 14.473 | 0.173018 | 7/15/2014 | 01:18.3 | 1 |
| -985.517 | 19573.652 | 1.63 | 14.48 | 0.181363 | 7/15/2014 | 01:18.4 | 1 |
| -985.768 | 19573.68 | 1.632 | 14.488 | 0.145082 | 7/15/2014 | 01:18.5 | 1 |
| -986.017 | 19573.711 | 1.63 | 14.496 | 0.161872 | 7/15/2014 | 01:18.6 | 1 |
| -986.268 | 19573.74 | 1.63 | 14.505 | 0.136579 | 7/15/2014 | 01:18.7 | 1 |

| | | | | | | | |
|---|---|---|---|---|---|---|---|
| -986.581 | 19573.777 | 1.633 | 14.513 | 0.152445 | 7/15/2014 | 01:18.8 | 1 |
| -986.832 | 19573.807 | 1.639 | 14.522 | 0.130159 | 7/15/2014 | 01:18.9 | 1 |
| -987.084 | 19573.838 | 1.636 | 14.529 | 0.127462 | 7/15/2014 | 01:19.0 | 1 |
| -987.338 | 19573.866 | 1.632 | 14.536 | 0.125771 | 7/15/2014 | 01:19.1 | 1 |
| -987.588 | 19573.896 | 1.624 | 14.542 | 0.12874 | 7/15/2014 | 01:19.2 | 1 |
| -987.84 | 19573.926 | 1.628 | 14.546 | 0.130132 | 7/15/2014 | 01:19.3 | 1 |
| -988.151 | 19573.963 | 1.622 | 14.551 | 0.125928 | 7/15/2014 | 01:19.4 | 1 |
| -988.4 | 19573.992 | 1.619 | 14.555 | 0.117461 | 7/15/2014 | 01:19.5 | 1 |
| -988.649 | 19574.021 | 1.613 | 14.559 | 0.100651 | 7/15/2014 | 01:19.6 | 1 |
| -988.9 | 19574.051 | 1.619 | 14.565 | 0.10203 | 7/15/2014 | 01:19.7 | 1 |
| -989.209 | 19574.088 | 1.62 | 14.571 | 0.0946086 | 7/15/2014 | 01:19.8 | 1 |
| -989.46 | 19574.116 | 1.62 | 14.578 | 0.0890872 | 7/15/2014 | 01:19.9 | 1 |
| -989.709 | 19574.145 | 1.612 | 14.586 | 0.0869387 | 7/15/2014 | 01:20.0 | 1 |
| -989.959 | 19574.176 | 1.612 | 14.595 | 0.0844146 | 7/15/2014 | 01:20.1 | 1 |
| -990.208 | 19574.203 | 1.608 | 14.603 | 0.087016 | 7/15/2014 | 01:20.2 | 1 |
| -990.459 | 19574.232 | 1.611 | 14.612 | 0.0753405 | 7/15/2014 | 01:20.3 | 1 |
| -990.767 | 19574.27 | 1.605 | 14.619 | 0.0760198 | 7/15/2014 | 01:20.4 | 1 |
| -991.017 | 19574.298 | 1.605 | 14.625 | 0.0640714 | 7/15/2014 | 01:20.5 | 1 |
| -991.264 | 19574.326 | 1.602 | 14.631 | 0.0628653 | 7/15/2014 | 01:20.6 | 1 |
| -991.513 | 19574.355 | 1.599 | 14.635 | 0.0613528 | 7/15/2014 | 01:20.7 | 1 |
| -991.821 | 19574.391 | 1.596 | 14.638 | 0.0583207 | 7/15/2014 | 01:20.8 | 1 |
| -992.071 | 19574.419 | 1.599 | 14.64 | 0.0573468 | 7/15/2014 | 01:20.9 | 1 |
| -992.319 | 19574.447 | 1.592 | 14.641 | 0.0574257 | 7/15/2014 | 01:21.0 | 1 |
| -992.57 | 19574.476 | 1.592 | 14.641 | 0.0669279 | 7/15/2014 | 01:21.1 | 1 |
| -992.817 | 19574.504 | 1.586 | 14.641 | 0.0681499 | 7/15/2014 | 01:21.2 | 1 |
| -993.065 | 19574.533 | 1.587 | 14.641 | 0.0774027 | 7/15/2014 | 01:21.3 | 1 |
| -993.492 | 19574.582 | 1.584 | 14.642 | 0.0682837 | 7/15/2014 | 01:21.4 | 1 |
| -993.737 | 19574.608 | 1.587 | 14.643 | 0.0789047 | 7/15/2014 | 01:21.5 | 1 |
| -993.979 | 19574.635 | 1.584 | 14.644 | 0.0868717 | 7/15/2014 | 01:21.6 | 1 |
| -994.282 | 19574.668 | 1.584 | 14.645 | 0.074107 | 7/15/2014 | 01:21.7 | 1 |
| -994.406 | 19574.682 | 1.582 | 14.646 | 0.0799971 | 7/15/2014 | 01:21.8 | 1 |
| -994.65 | 19574.711 | 1.584 | 14.648 | 0.0707204 | 7/15/2014 | 01:21.9 | 1 |
| -994.891 | 19574.738 | 1.583 | 14.651 | 0.0879375 | 7/15/2014 | 01:22.0 | 1 |
| -995.134 | 19574.765 | 1.59 | 14.655 | 0.0836758 | 7/15/2014 | 01:22.1 | 1 |
| -995.375 | 19574.79 | 1.594 | 14.659 | 0.0866622 | 7/15/2014 | 01:22.2 | 1 |
| -995.615 | 19574.816 | 1.597 | 14.664 | 0.095615 | 7/15/2014 | 01:22.3 | 1 |
| -996.032 | 19574.861 | 1.604 | 14.672 | 0.0977092 | 7/15/2014 | 01:22.4 | 1 |
| -996.271 | 19574.888 | 1.61 | 14.677 | 0.114154 | 7/15/2014 | 01:22.5 | 1 |
| -996.508 | 19574.914 | 1.613 | 14.682 | 0.106342 | 7/15/2014 | 01:22.6 | 1 |
| -996.807 | 19574.944 | 1.616 | 14.686 | 0.105957 | 7/15/2014 | 01:22.7 | 1 |
| -996.926 | 19574.957 | 1.615 | 14.688 | 0.102954 | 7/15/2014 | 01:22.8 | 1 |
| -997.167 | 19574.984 | 1.621 | 14.69 | 0.102949 | 7/15/2014 | 01:22.9 | 1 |
| -997.406 | 19575.008 | 1.621 | 14.69 | 0.105125 | 7/15/2014 | 01:23.0 | 1 |
| -997.648 | 19575.035 | 1.625 | 14.688 | 0.105115 | 7/15/2014 | 01:23.1 | 1 |
| -997.889 | 19575.06 | 1.625 | 14.684 | 0.104786 | 7/15/2014 | 01:23.2 | 1 |
| -998.129 | 19575.085 | 1.627 | 14.678 | 0.101956 | 7/15/2014 | 01:23.3 | 1 |
| -998.552 | 19575.128 | 1.635 | 14.669 | 0.106967 | 7/15/2014 | 01:23.4 | 1 |

| | | | | | | | |
|---|---|---|---|---|---|---|---|
| -998.794 | 19575.154 | 1.64 | 14.664 | 0.101024 | 7/15/2014 | 01:23.5 | 1 |
| -999.037 | 19575.178 | 1.643 | 14.658 | 0.100276 | 7/15/2014 | 01:23.6 | 1 |
| -999.343 | 19575.21 | 1.645 | 14.653 | 0.100065 | 7/15/2014 | 01:23.7 | 1 |
| -999.465 | 19575.222 | 1.648 | 14.651 | 0.0986358 | 7/15/2014 | 01:23.8 | 1 |
| -999.713 | 19575.247 | 1.654 | 14.647 | 0.0973473 | 7/15/2014 | 01:23.9 | 1 |
| -999.962 | 19575.271 | 1.654 | 14.644 | 0.0990198 | 7/15/2014 | 01:24.0 | 1 |
| -1000.212 | 19575.297 | 1.658 | 14.641 | 0.100744 | 7/15/2014 | 01:24.1 | 1 |
| -1000.464 | 19575.322 | 1.658 | 14.637 | 0.102668 | 7/15/2014 | 01:24.2 | 1 |
| -1000.716 | 19575.347 | 1.661 | 14.634 | 0.108752 | 7/15/2014 | 01:24.3 | 1 |
| -1001.03 | 19575.378 | 1.658 | 14.63 | 0.112365 | 7/15/2014 | 01:24.4 | 1 |
| -1001.285 | 19575.403 | 1.658 | 14.626 | 0.119419 | 7/15/2014 | 01:24.5 | 1 |
| -1001.539 | 19575.427 | 1.654 | 14.622 | 0.102036 | 7/15/2014 | 01:24.6 | 1 |
| -1001.796 | 19575.452 | 1.666 | 14.618 | 0.107393 | 7/15/2014 | 01:24.7 | 1 |
| -1002.114 | 19575.482 | 1.666 | 14.614 | 0.100269 | 7/15/2014 | 01:24.8 | 1 |
| -1002.371 | 19575.507 | 1.666 | 14.611 | 0.101713 | 7/15/2014 | 01:24.9 | 1 |
| -1002.629 | 19575.531 | 1.662 | 14.607 | 0.0878518 | 7/15/2014 | 01:25.0 | 1 |
| -1002.887 | 19575.557 | 1.665 | 14.603 | 0.0805435 | 7/15/2014 | 01:25.1 | 1 |
| -1003.143 | 19575.581 | 1.665 | 14.599 | 0.0894168 | 7/15/2014 | 01:25.2 | 1 |
| -1003.4 | 19575.605 | 1.671 | 14.594 | 0.0847924 | 7/15/2014 | 01:25.3 | 1 |
| -1003.717 | 19575.635 | 1.662 | 14.589 | 0.0918461 | 7/15/2014 | 01:25.4 | 1 |
| -1003.972 | 19575.657 | 1.665 | 14.585 | 0.106736 | 7/15/2014 | 01:25.5 | 1 |
| -1004.225 | 19575.68 | 1.665 | 14.58 | 0.090718 | 7/15/2014 | 01:25.6 | 1 |
| -1004.479 | 19575.704 | 1.671 | 14.575 | 0.0988723 | 7/15/2014 | 01:25.7 | 1 |
| -1004.792 | 19575.732 | 1.672 | 14.571 | 0.098454 | 7/15/2014 | 01:25.8 | 1 |
| -1005.045 | 19575.756 | 1.676 | 14.567 | 0.112055 | 7/15/2014 | 01:25.9 | 1 |
| -1005.295 | 19575.777 | 1.676 | 14.564 | 0.117393 | 7/15/2014 | 01:26.0 | 1 |
| -1005.547 | 19575.8 | 1.678 | 14.562 | 0.096169 | 7/15/2014 | 01:26.1 | 1 |
| -1005.795 | 19575.82 | 1.678 | 14.56 | 0.10541 | 7/15/2014 | 01:26.2 | 1 |
| -1006.044 | 19575.843 | 1.681 | 14.559 | 0.111048 | 7/15/2014 | 01:26.3 | 1 |
| -1006.473 | 19575.879 | 1.681 | 14.559 | 0.104965 | 7/15/2014 | 01:26.4 | 1 |
| -1006.716 | 19575.9 | 1.681 | 14.56 | 0.10235 | 7/15/2014 | 01:26.5 | 1 |
| -1006.96 | 19575.92 | 1.686 | 14.563 | 0.101594 | 7/15/2014 | 01:26.6 | 1 |
| -1007.263 | 19575.947 | 1.686 | 14.566 | 0.104194 | 7/15/2014 | 01:26.7 | 1 |
| -1007.386 | 19575.957 | 1.686 | 14.567 | 0.103297 | 7/15/2014 | 01:26.8 | 1 |
| -1007.629 | 19575.978 | 1.689 | 14.572 | 0.1089 | 7/15/2014 | 01:26.9 | 1 |
| -1007.872 | 19575.998 | 1.689 | 14.577 | 0.104783 | 7/15/2014 | 01:27.0 | 1 |
| -1008.115 | 19576.018 | 1.692 | 14.584 | 0.105702 | 7/15/2014 | 01:27.1 | 1 |
| -1008.357 | 19576.037 | 1.687 | 14.591 | 0.1147 | 7/15/2014 | 01:27.2 | 1 |
| -1008.599 | 19576.057 | 1.695 | 14.6 | 0.0968643 | 7/15/2014 | 01:27.3 | 1 |
| -1009.021 | 19576.09 | 1.692 | 14.617 | 0.0892355 | 7/15/2014 | 01:27.4 | 1 |
| -1009.261 | 19576.109 | 1.69 | 14.629 | 0.0832178 | 7/15/2014 | 01:27.5 | 1 |
| -1009.504 | 19576.129 | 1.689 | 14.643 | 0.0751514 | 7/15/2014 | 01:27.6 | 1 |
| -1009.807 | 19576.152 | 1.687 | 14.658 | 0.0684597 | 7/15/2014 | 01:27.7 | 1 |
| -1009.929 | 19576.162 | 1.687 | 14.665 | 0.0665611 | 7/15/2014 | 01:27.8 | 1 |
| -1010.175 | 19576.182 | 1.695 | 14.682 | 0.063785 | 7/15/2014 | 01:27.9 | 1 |
| -1010.42 | 19576.199 | 1.687 | 14.699 | 0.0751794 | 7/15/2014 | 01:28.0 | 1 |
| -1010.669 | 19576.217 | 1.689 | 14.717 | 0.0708447 | 7/15/2014 | 01:28.1 | 1 |

| | | | | | | | |
|---|---|---|---|---|---|---|---|
| -1010.916 | 19576.236 | 1.684 | 14.734 | 0.0857228 | 7/15/2014 | 01:28.2 | 1 |
| -1011.167 | 19576.256 | 1.689 | 14.749 | 0.117211 | 7/15/2014 | 01:28.3 | 1 |
| -1011.476 | 19576.278 | 1.68 | 14.764 | 0.116817 | 7/15/2014 | 01:28.4 | 1 |
| -1011.728 | 19576.297 | 1.682 | 14.776 | 0.117258 | 7/15/2014 | 01:28.5 | 1 |
| -1011.978 | 19576.314 | 1.674 | 14.787 | 0.11063 | 7/15/2014 | 01:28.6 | 1 |
| -1012.231 | 19576.332 | 1.676 | 14.794 | 0.113188 | 7/15/2014 | 01:28.7 | 1 |
| -1012.547 | 19576.354 | 1.67 | 14.8 | 0.0964214 | 7/15/2014 | 01:28.8 | 1 |
| -1012.803 | 19576.373 | 1.672 | 14.803 | 0.0971606 | 7/15/2014 | 01:28.9 | 1 |
| -1013.057 | 19576.391 | 1.664 | 14.804 | 0.0828094 | 7/15/2014 | 01:29.0 | 1 |
| -1013.314 | 19576.408 | 1.667 | 14.804 | 0.0785771 | 7/15/2014 | 01:29.1 | 1 |
| -1013.569 | 19576.425 | 1.665 | 14.803 | 0.0738141 | 7/15/2014 | 01:29.2 | 1 |
| -1013.826 | 19576.442 | 1.661 | 14.801 | 0.0738648 | 7/15/2014 | 01:29.3 | 1 |
| -1014.144 | 19576.463 | 1.655 | 14.8 | 0.0723692 | 7/15/2014 | 01:29.4 | 1 |
| -1014.4 | 19576.48 | 1.655 | 14.799 | 0.0727493 | 7/15/2014 | 01:29.5 | 1 |
| -1014.655 | 19576.497 | 1.647 | 14.8 | 0.0728175 | 7/15/2014 | 01:29.6 | 1 |
| -1014.91 | 19576.514 | 1.647 | 14.801 | 0.0701908 | 7/15/2014 | 01:29.7 | 1 |
| -1015.228 | 19576.533 | 1.643 | 14.803 | 0.0747017 | 7/15/2014 | 01:29.8 | 1 |
| -1015.483 | 19576.55 | 1.637 | 14.805 | 0.0755365 | 7/15/2014 | 01:29.9 | 1 |
| -1015.737 | 19576.564 | 1.631 | 14.807 | 0.0857029 | 7/15/2014 | 01:30.0 | 1 |
| -1015.992 | 19576.581 | 1.625 | 14.809 | 0.0899351 | 7/15/2014 | 01:30.1 | 1 |
| -1016.245 | 19576.596 | 1.619 | 14.811 | 0.101365 | 7/15/2014 | 01:30.2 | 1 |
| -1016.499 | 19576.611 | 1.615 | 14.812 | 0.11613 | 7/15/2014 | 01:30.3 | 1 |
| -1016.811 | 19576.631 | 1.609 | 14.813 | 0.123532 | 7/15/2014 | 01:30.4 | 1 |
| -1017.063 | 19576.646 | 1.607 | 14.813 | 0.137973 | 7/15/2014 | 01:30.5 | 1 |
| -1017.311 | 19576.659 | 1.6 | 14.813 | 0.156678 | 7/15/2014 | 01:30.6 | 1 |
| -1017.561 | 19576.674 | 1.6 | 14.812 | 0.164171 | 7/15/2014 | 01:30.7 | 1 |
| -1017.871 | 19576.691 | 1.6 | 14.81 | 0.159044 | 7/15/2014 | 01:30.8 | 1 |
| -1018.123 | 19576.706 | 1.6 | 14.807 | 0.159479 | 7/15/2014 | 01:30.9 | 1 |
| -1018.372 | 19576.721 | 1.6 | 14.804 | 0.148974 | 7/15/2014 | 01:31.0 | 1 |
| -1018.624 | 19576.734 | 1.603 | 14.8 | 0.158979 | 7/15/2014 | 01:31.1 | 1 |
| -1018.872 | 19576.748 | 1.596 | 14.796 | 0.137125 | 7/15/2014 | 01:31.2 | 1 |
| -1019.121 | 19576.761 | 1.6 | 14.792 | 0.128533 | 7/15/2014 | 01:31.3 | 1 |
| -1019.431 | 19576.779 | 1.596 | 14.788 | 0.124337 | 7/15/2014 | 01:31.4 | 1 |
| -1019.68 | 19576.792 | 1.592 | 14.784 | 0.124384 | 7/15/2014 | 01:31.5 | 1 |
| -1019.93 | 19576.805 | 1.589 | 14.781 | 0.118199 | 7/15/2014 | 01:31.6 | 1 |
| -1020.18 | 19576.818 | 1.589 | 14.778 | 0.114458 | 7/15/2014 | 01:31.7 | 1 |
| -1020.492 | 19576.836 | 1.579 | 14.777 | 0.1075 | 7/15/2014 | 01:31.8 | 1 |
| -1020.744 | 19576.848 | 1.583 | 14.777 | 0.1006 | 7/15/2014 | 01:31.9 | 1 |
| -1020.997 | 19576.861 | 1.579 | 14.778 | 0.100979 | 7/15/2014 | 01:32.0 | 1 |
| -1021.251 | 19576.873 | 1.583 | 14.78 | 0.0933604 | 7/15/2014 | 01:32.1 | 1 |
| -1021.505 | 19576.886 | 1.583 | 14.783 | 0.0928449 | 7/15/2014 | 01:32.2 | 1 |
| -1021.759 | 19576.899 | 1.583 | 14.786 | 0.0975451 | 7/15/2014 | 01:32.3 | 1 |
| -1022.075 | 19576.916 | 1.577 | 14.791 | 0.110916 | 7/15/2014 | 01:32.4 | 1 |
| -1022.331 | 19576.928 | 1.583 | 14.796 | 0.118567 | 7/15/2014 | 01:32.5 | 1 |
| -1022.586 | 19576.939 | 1.579 | 14.802 | 0.0945856 | 7/15/2014 | 01:32.6 | 1 |
| -1022.842 | 19576.951 | 1.579 | 14.809 | 0.111272 | 7/15/2014 | 01:32.7 | 1 |
| -1023.162 | 19576.968 | 1.576 | 14.817 | 0.104731 | 7/15/2014 | 01:32.8 | 1 |

| | | | | | | | |
|---|---|---|---|---|---|---|---|
| -1023.419 | 19576.98 | 1.576 | 14.826 | 0.110701 | 7/15/2014 | 01:32.9 | 1 |
| -1023.676 | 19576.992 | 1.576 | 14.836 | 0.112876 | 7/15/2014 | 01:33.0 | 1 |
| -1023.935 | 19577.004 | 1.578 | 14.847 | 0.119213 | 7/15/2014 | 01:33.1 | 1 |
| -1024.192 | 19577.015 | 1.57 | 14.858 | 0.123696 | 7/15/2014 | 01:33.2 | 1 |
| -1024.449 | 19577.025 | 1.57 | 14.867 | 0.120898 | 7/15/2014 | 01:33.3 | 1 |
| -1024.768 | 19577.041 | 1.565 | 14.876 | 0.126091 | 7/15/2014 | 01:33.4 | 1 |
| -1025.024 | 19577.053 | 1.562 | 14.883 | 0.123588 | 7/15/2014 | 01:33.5 | 1 |
| -1025.279 | 19577.064 | 1.558 | 14.887 | 0.116478 | 7/15/2014 | 01:33.6 | 1 |
| -1025.534 | 19577.076 | 1.553 | 14.889 | 0.108508 | 7/15/2014 | 01:33.7 | 1 |
| -1025.851 | 19577.089 | 1.55 | 14.889 | 0.102986 | 7/15/2014 | 01:33.8 | 1 |
| -1026.108 | 19577.098 | 1.559 | 14.887 | 0.0933554 | 7/15/2014 | 01:33.9 | 1 |
| -1026.363 | 19577.109 | 1.559 | 14.884 | 0.0897474 | 7/15/2014 | 01:34.0 | 1 |
| -1026.619 | 19577.121 | 1.567 | 14.88 | 0.0832851 | 7/15/2014 | 01:34.1 | 1 |
| -1026.873 | 19577.131 | 1.57 | 14.876 | 0.0797037 | 7/15/2014 | 01:34.2 | 1 |
| -1027.127 | 19577.141 | 1.578 | 14.872 | 0.0691663 | 7/15/2014 | 01:34.3 | 1 |
| -1027.444 | 19577.154 | 1.583 | 14.868 | 0.0664613 | 7/15/2014 | 01:34.4 | 1 |
| -1027.697 | 19577.162 | 1.585 | 14.865 | 0.0654861 | 7/15/2014 | 01:34.5 | 1 |
| -1027.95 | 19577.174 | 1.582 | 14.862 | 0.061663 | 7/15/2014 | 01:34.6 | 1 |
| -1028.203 | 19577.184 | 1.588 | 14.858 | 0.0508422 | 7/15/2014 | 01:34.7 | 1 |
| -1028.519 | 19577.195 | 1.588 | 14.855 | 0.0541843 | 7/15/2014 | 01:34.8 | 1 |
| -1028.773 | 19577.203 | 1.591 | 14.852 | 0.0631954 | 7/15/2014 | 01:34.9 | 1 |
| -1029.028 | 19577.213 | 1.593 | 14.847 | 0.0761606 | 7/15/2014 | 01:35.0 | 1 |
| -1029.282 | 19577.221 | 1.599 | 14.843 | 0.0833524 | 7/15/2014 | 01:35.1 | 1 |
| -1029.536 | 19577.232 | 1.603 | 14.838 | 0.110782 | 7/15/2014 | 01:35.2 | 1 |
| -1029.791 | 19577.24 | 1.609 | 14.833 | 0.107915 | 7/15/2014 | 01:35.3 | 1 |
| -1030.107 | 19577.252 | 1.606 | 14.829 | 0.119564 | 7/15/2014 | 01:35.4 | 1 |
| -1030.361 | 19577.26 | 1.61 | 14.824 | 0.121043 | 7/15/2014 | 01:35.5 | 1 |
| -1030.613 | 19577.27 | 1.609 | 14.818 | 0.110049 | 7/15/2014 | 01:35.6 | 1 |
| -1030.867 | 19577.277 | 1.613 | 14.813 | 0.112238 | 7/15/2014 | 01:35.7 | 1 |
| -1031.182 | 19577.289 | 1.613 | 14.808 | 0.114969 | 7/15/2014 | 01:35.8 | 1 |
| -1031.438 | 19577.297 | 1.619 | 14.802 | 0.117335 | 7/15/2014 | 01:35.9 | 1 |
| -1031.692 | 19577.305 | 1.619 | 14.795 | 0.120896 | 7/15/2014 | 01:36.0 | 1 |
| -1031.948 | 19577.313 | 1.629 | 14.787 | 0.124832 | 7/15/2014 | 01:36.1 | 1 |
| -1032.201 | 19577.322 | 1.631 | 14.779 | 0.135659 | 7/15/2014 | 01:36.2 | 1 |
| -1032.456 | 19577.33 | 1.637 | 14.771 | 0.131635 | 7/15/2014 | 01:36.3 | 1 |
| -1032.771 | 19577.34 | 1.637 | 14.763 | 0.139595 | 7/15/2014 | 01:36.4 | 1 |
| -1033.026 | 19577.348 | 1.647 | 14.756 | 0.130335 | 7/15/2014 | 01:36.5 | 1 |
| -1033.277 | 19577.354 | 1.647 | 14.751 | 0.142223 | 7/15/2014 | 01:36.6 | 1 |
| -1033.533 | 19577.361 | 1.654 | 14.747 | 0.12625 | 7/15/2014 | 01:36.7 | 1 |
| -1033.848 | 19577.371 | 1.647 | 14.746 | 0.124647 | 7/15/2014 | 01:36.8 | 1 |
| -1034.104 | 19577.378 | 1.651 | 14.747 | 0.129115 | 7/15/2014 | 01:36.9 | 1 |
| -1034.359 | 19577.385 | 1.651 | 14.75 | 0.127527 | 7/15/2014 | 01:37.0 | 1 |
| -1034.615 | 19577.393 | 1.654 | 14.753 | 0.130876 | 7/15/2014 | 01:37.1 | 1 |
| -1034.869 | 19577.398 | 1.652 | 14.757 | 0.130001 | 7/15/2014 | 01:37.2 | 1 |
| -1035.125 | 19577.404 | 1.658 | 14.76 | 0.135207 | 7/15/2014 | 01:37.3 | 1 |
| -1035.44 | 19577.414 | 1.66 | 14.762 | 0.125755 | 7/15/2014 | 01:37.4 | 1 |
| -1035.695 | 19577.421 | 1.676 | 14.762 | 0.126446 | 7/15/2014 | 01:37.5 | 1 |

| | | | | | | | |
|---|---|---|---|---|---|---|---|
| -1035.946 | 19577.426 | 1.679 | 14.761 | 0.117616 | 7/15/2014 | 01:37.6 | 1 |
| -1036.201 | 19577.434 | 1.687 | 14.758 | 0.112821 | 7/15/2014 | 01:37.7 | 1 |
| -1036.515 | 19577.441 | 1.69 | 14.753 | 0.107449 | 7/15/2014 | 01:37.8 | 1 |
| -1036.768 | 19577.447 | 1.694 | 14.748 | 0.107477 | 7/15/2014 | 01:37.9 | 1 |
| -1037.02 | 19577.454 | 1.697 | 14.742 | 0.113625 | 7/15/2014 | 01:38.0 | 1 |
| -1037.272 | 19577.461 | 1.699 | 14.736 | 0.115203 | 7/15/2014 | 01:38.1 | 1 |
| -1037.522 | 19577.467 | 1.693 | 14.73 | 0.120127 | 7/15/2014 | 01:38.2 | 1 |
| -1037.773 | 19577.473 | 1.699 | 14.725 | 0.130637 | 7/15/2014 | 01:38.3 | 1 |
| -1038.208 | 19577.482 | 1.706 | 14.72 | 0.132599 | 7/15/2014 | 01:38.4 | 1 |
| -1038.457 | 19577.49 | 1.708 | 14.717 | 0.132419 | 7/15/2014 | 01:38.5 | 1 |
| -1038.706 | 19577.496 | 1.711 | 14.715 | 0.133365 | 7/15/2014 | 01:38.6 | 1 |
| -1039.018 | 19577.504 | 1.711 | 14.715 | 0.139665 | 7/15/2014 | 01:38.7 | 1 |
| -1039.143 | 19577.508 | 1.713 | 14.714 | 0.14014 | 7/15/2014 | 01:38.8 | 1 |
| -1039.395 | 19577.512 | 1.721 | 14.715 | 0.140815 | 7/15/2014 | 01:38.9 | 1 |
| -1039.648 | 19577.519 | 1.721 | 14.717 | 0.145403 | 7/15/2014 | 01:39.0 | 1 |
| -1039.902 | 19577.524 | 1.726 | 14.721 | 0.153541 | 7/15/2014 | 01:39.1 | 1 |
| -1040.155 | 19577.531 | 1.726 | 14.725 | 0.166255 | 7/15/2014 | 01:39.2 | 1 |
| -1040.409 | 19577.538 | 1.732 | 14.73 | 0.173486 | 7/15/2014 | 01:39.3 | 1 |
| -1040.726 | 19577.546 | 1.728 | 14.736 | 0.178564 | 7/15/2014 | 01:39.4 | 1 |
| -1040.981 | 19577.551 | 1.734 | 14.742 | 0.182175 | 7/15/2014 | 01:39.5 | 1 |
| -1041.237 | 19577.559 | 1.732 | 14.748 | 0.192117 | 7/15/2014 | 01:39.6 | 1 |
| -1041.493 | 19577.564 | 1.738 | 14.755 | 0.194302 | 7/15/2014 | 01:39.7 | 1 |
| -1041.81 | 19577.572 | 1.733 | 14.761 | 0.192755 | 7/15/2014 | 01:39.8 | 1 |
| -1042.067 | 19577.579 | 1.737 | 14.767 | 0.196885 | 7/15/2014 | 01:39.9 | 1 |
| -1042.322 | 19577.585 | 1.731 | 14.773 | 0.204508 | 7/15/2014 | 01:40.0 | 1 |
| -1042.579 | 19577.592 | 1.741 | 14.778 | 0.206609 | 7/15/2014 | 01:40.1 | 1 |
| -1042.833 | 19577.598 | 1.738 | 14.783 | 0.218361 | 7/15/2014 | 01:40.2 | 1 |
| -1043.088 | 19577.604 | 1.738 | 14.788 | 0.22259 | 7/15/2014 | 01:40.3 | 1 |
| -1043.402 | 19577.613 | 1.732 | 14.794 | 0.221016 | 7/15/2014 | 01:40.4 | 1 |
| -1043.656 | 19577.619 | 1.736 | 14.8 | 0.229781 | 7/15/2014 | 01:40.5 | 1 |
| -1043.906 | 19577.625 | 1.735 | 14.807 | 0.231981 | 7/15/2014 | 01:40.6 | 1 |
| -1044.159 | 19577.632 | 1.742 | 14.815 | 0.235673 | 7/15/2014 | 01:40.7 | 1 |
| -1044.47 | 19577.639 | 1.74 | 14.824 | 0.23667 | 7/15/2014 | 01:40.8 | 1 |
| -1044.721 | 19577.646 | 1.743 | 14.835 | 0.236074 | 7/15/2014 | 01:40.9 | 1 |
| -1044.971 | 19577.654 | 1.746 | 14.847 | 0.240728 | 7/15/2014 | 01:41.0 | 1 |
| -1045.224 | 19577.659 | 1.749 | 14.859 | 0.244044 | 7/15/2014 | 01:41.1 | 1 |
| -1045.473 | 19577.666 | 1.749 | 14.872 | 0.242474 | 7/15/2014 | 01:41.2 | 1 |
| -1045.723 | 19577.673 | 1.751 | 14.885 | 0.244188 | 7/15/2014 | 01:41.3 | 1 |
| -1046.156 | 19577.684 | 1.752 | 14.902 | 0.231324 | 7/15/2014 | 01:41.4 | 1 |
| -1046.404 | 19577.691 | 1.752 | 14.913 | 0.234663 | 7/15/2014 | 01:41.5 | 1 |
| -1046.65 | 19577.699 | 1.752 | 14.922 | 0.235775 | 7/15/2014 | 01:41.6 | 1 |
| -1046.958 | 19577.705 | 1.75 | 14.93 | 0.241107 | 7/15/2014 | 01:41.7 | 1 |
| -1047.084 | 19577.709 | 1.75 | 14.935 | 0.233088 | 7/15/2014 | 01:41.8 | 1 |
| -1047.331 | 19577.717 | 1.752 | 14.941 | 0.231228 | 7/15/2014 | 01:41.9 | 1 |
| -1047.577 | 19577.724 | 1.751 | 14.945 | 0.241622 | 7/15/2014 | 01:42.0 | 1 |
| -1047.824 | 19577.73 | 1.751 | 14.949 | 0.245066 | 7/15/2014 | 01:42.1 | 1 |
| -1048.071 | 19577.737 | 1.752 | 14.951 | 0.236501 | 7/15/2014 | 01:42.2 | 1 |

| | | | | | | | |
|---|---|---|---|---|---|---|---|
| -1048.317 | 19577.744 | 1.752 | 14.953 | 0.237273 | 7/15/2014 | 01:42.3 | 1 |
| -1048.744 | 19577.756 | 1.748 | 14.953 | 0.215018 | 7/15/2014 | 01:42.4 | 1 |
| -1048.987 | 19577.764 | 1.751 | 14.953 | 0.222295 | 7/15/2014 | 01:42.5 | 1 |
| -1049.229 | 19577.771 | 1.751 | 14.953 | 0.218165 | 7/15/2014 | 01:42.6 | 1 |
| -1049.533 | 19577.781 | 1.752 | 14.952 | 0.212842 | 7/15/2014 | 01:42.7 | 1 |
| -1049.656 | 19577.784 | 1.75 | 14.952 | 0.21164 | 7/15/2014 | 01:42.8 | 1 |
| -1049.9 | 19577.789 | 1.752 | 14.951 | 0.210031 | 7/15/2014 | 01:42.9 | 1 |
| -1050.142 | 19577.797 | 1.748 | 14.951 | 0.21213 | 7/15/2014 | 01:43.0 | 1 |
| -1050.389 | 19577.806 | 1.752 | 14.951 | 0.216845 | 7/15/2014 | 01:43.1 | 1 |
| -1050.633 | 19577.813 | 1.755 | 14.951 | 0.213759 | 7/15/2014 | 01:43.2 | 1 |
| -1050.877 | 19577.821 | 1.759 | 14.951 | 0.213245 | 7/15/2014 | 01:43.3 | 1 |
| -1051.302 | 19577.835 | 1.758 | 14.952 | 0.206775 | 7/15/2014 | 01:43.4 | 1 |
| -1051.545 | 19577.842 | 1.76 | 14.952 | 0.20674 | 7/15/2014 | 01:43.5 | 1 |
| -1051.787 | 19577.85 | 1.76 | 14.953 | 0.203435 | 7/15/2014 | 01:43.6 | 1 |
| -1052.094 | 19577.86 | 1.764 | 14.953 | 0.200431 | 7/15/2014 | 01:43.7 | 1 |
| -1052.217 | 19577.863 | 1.766 | 14.954 | 0.196545 | 7/15/2014 | 01:43.8 | 1 |
| -1052.462 | 19577.872 | 1.763 | 14.954 | 0.195878 | 7/15/2014 | 01:43.9 | 1 |
| -1052.708 | 19577.88 | 1.761 | 14.955 | 0.194306 | 7/15/2014 | 01:44.0 | 1 |
| -1052.955 | 19577.889 | 1.763 | 14.954 | 0.19572 | 7/15/2014 | 01:44.1 | 1 |
| -1053.201 | 19577.898 | 1.763 | 14.954 | 0.201524 | 7/15/2014 | 01:44.2 | 1 |
| -1053.447 | 19577.906 | 1.763 | 14.953 | 0.203295 | 7/15/2014 | 01:44.3 | 1 |
| -1053.878 | 19577.922 | 1.762 | 14.951 | 0.206379 | 7/15/2014 | 01:44.4 | 1 |
| -1054.124 | 19577.931 | 1.762 | 14.95 | 0.211359 | 7/15/2014 | 01:44.5 | 1 |
| -1054.37 | 19577.939 | 1.768 | 14.949 | 0.218258 | 7/15/2014 | 01:44.6 | 1 |
| -1054.677 | 19577.952 | 1.77 | 14.949 | 0.227616 | 7/15/2014 | 01:44.7 | 1 |
| -1054.801 | 19577.957 | 1.77 | 14.949 | 0.231339 | 7/15/2014 | 01:44.8 | 1 |
| -1055.049 | 19577.965 | 1.77 | 14.949 | 0.233631 | 7/15/2014 | 01:44.9 | 1 |
| -1055.297 | 19577.977 | 1.773 | 14.951 | 0.237301 | 7/15/2014 | 01:45.0 | 1 |
| -1055.545 | 19577.986 | 1.777 | 14.953 | 0.245369 | 7/15/2014 | 01:45.1 | 1 |
| -1055.793 | 19577.997 | 1.775 | 14.957 | 0.248381 | 7/15/2014 | 01:45.2 | 1 |
| -1056.041 | 19578.006 | 1.777 | 14.961 | 0.25036 | 7/15/2014 | 01:45.3 | 1 |
| -1056.472 | 19578.025 | 1.772 | 14.968 | 0.243029 | 7/15/2014 | 01:45.4 | 1 |
| -1056.717 | 19578.035 | 1.772 | 14.973 | 0.238053 | 7/15/2014 | 01:45.5 | 1 |
| -1056.964 | 19578.047 | 1.776 | 14.978 | 0.244178 | 7/15/2014 | 01:45.6 | 1 |
| -1057.271 | 19578.061 | 1.774 | 14.983 | 0.23131 | 7/15/2014 | 01:45.7 | 1 |
| -1057.396 | 19578.066 | 1.773 | 14.986 | 0.231235 | 7/15/2014 | 01:45.8 | 1 |
| -1057.644 | 19578.076 | 1.775 | 14.991 | 0.221739 | 7/15/2014 | 01:45.9 | 1 |
| -1057.891 | 19578.088 | 1.773 | 14.996 | 0.22108 | 7/15/2014 | 01:46.0 | 1 |
| -1058.14 | 19578.1 | 1.78 | 15.001 | 0.216014 | 7/15/2014 | 01:46.1 | 1 |
| -1058.387 | 19578.111 | 1.78 | 15.005 | 0.219202 | 7/15/2014 | 01:46.2 | 1 |
| -1058.635 | 19578.124 | 1.784 | 15.009 | 0.209033 | 7/15/2014 | 01:46.3 | 1 |
| -1059.064 | 19578.145 | 1.78 | 15.014 | 0.215345 | 7/15/2014 | 01:46.4 | 1 |
| -1059.309 | 19578.156 | 1.78 | 15.016 | 0.224296 | 7/15/2014 | 01:46.5 | 1 |
| -1059.553 | 19578.169 | 1.787 | 15.018 | 0.212884 | 7/15/2014 | 01:46.6 | 1 |
| -1059.858 | 19578.184 | 1.793 | 15.019 | 0.212805 | 7/15/2014 | 01:46.7 | 1 |
| -1059.982 | 19578.189 | 1.792 | 15.02 | 0.205538 | 7/15/2014 | 01:46.8 | 1 |
| -1060.227 | 19578.204 | 1.798 | 15.019 | 0.213509 | 7/15/2014 | 01:46.9 | 1 |

| | | | | | | | |
|---|---|---|---|---|---|---|---|
| -1060.471 | 19578.217 | 1.795 | 15.019 | 0.199364 | 7/15/2014 | 01:47.0 | 1 |
| -1060.715 | 19578.229 | 1.797 | 15.018 | 0.211102 | 7/15/2014 | 01:47.1 | 1 |
| -1060.957 | 19578.242 | 1.803 | 15.017 | 0.209792 | 7/15/2014 | 01:47.2 | 1 |
| -1061.2 | 19578.256 | 1.808 | 15.017 | 0.208954 | 7/15/2014 | 01:47.3 | 1 |
| -1061.62 | 19578.278 | 1.807 | 15.016 | 0.215357 | 7/15/2014 | 01:47.4 | 1 |
| -1061.859 | 19578.293 | 1.805 | 15.016 | 0.220383 | 7/15/2014 | 01:47.5 | 1 |
| -1062.099 | 19578.305 | 1.807 | 15.015 | 0.232103 | 7/15/2014 | 01:47.6 | 1 |
| -1062.396 | 19578.321 | 1.812 | 15.014 | 0.21371 | 7/15/2014 | 01:47.7 | 1 |
| -1062.517 | 19578.329 | 1.814 | 15.014 | 0.221075 | 7/15/2014 | 01:47.8 | 1 |
| -1062.757 | 19578.343 | 1.819 | 15.012 | 0.225236 | 7/15/2014 | 01:47.9 | 1 |
| -1062.997 | 19578.357 | 1.819 | 15.01 | 0.20695 | 7/15/2014 | 01:48.0 | 1 |
| -1063.238 | 19578.372 | 1.821 | 15.007 | 0.218468 | 7/15/2014 | 01:48.1 | 1 |
| -1063.476 | 19578.385 | 1.825 | 15.005 | 0.215208 | 7/15/2014 | 01:48.2 | 1 |
| -1063.718 | 19578.398 | 1.836 | 15.003 | 0.202776 | 7/15/2014 | 01:48.3 | 1 |
| -1064.135 | 19578.424 | 1.831 | 15 | 0.209239 | 7/15/2014 | 01:48.4 | 1 |
| -1064.372 | 19578.438 | 1.828 | 14.999 | 0.201009 | 7/15/2014 | 01:48.5 | 1 |
| -1064.612 | 19578.453 | 1.828 | 14.999 | 0.206635 | 7/15/2014 | 01:48.6 | 1 |
| -1064.912 | 19578.472 | 1.831 | 14.999 | 0.196378 | 7/15/2014 | 01:48.7 | 1 |
| -1065.032 | 19578.478 | 1.829 | 14.999 | 0.19376 | 7/15/2014 | 01:48.8 | 1 |
| -1065.275 | 19578.493 | 1.834 | 15 | 0.19916 | 7/15/2014 | 01:48.9 | 1 |
| -1065.516 | 19578.509 | 1.832 | 15.001 | 0.196887 | 7/15/2014 | 01:49.0 | 1 |
| -1065.761 | 19578.523 | 1.84 | 15.003 | 0.215028 | 7/15/2014 | 01:49.1 | 1 |
| -1066.003 | 19578.538 | 1.843 | 15.006 | 0.225165 | 7/15/2014 | 01:49.2 | 1 |
| -1066.248 | 19578.554 | 1.851 | 15.01 | 0.235496 | 7/15/2014 | 01:49.3 | 1 |
| -1066.674 | 19578.58 | 1.85 | 15.016 | 0.25124 | 7/15/2014 | 01:49.4 | 1 |
| -1066.919 | 19578.596 | 1.852 | 15.021 | 0.26038 | 7/15/2014 | 01:49.5 | 1 |
| -1067.162 | 19578.612 | 1.85 | 15.025 | 0.26355 | 7/15/2014 | 01:49.6 | 1 |
| -1067.469 | 19578.632 | 1.847 | 15.029 | 0.266762 | 7/15/2014 | 01:49.7 | 1 |
| -1067.593 | 19578.64 | 1.845 | 15.031 | 0.272798 | 7/15/2014 | 01:49.8 | 1 |
| -1067.84 | 19578.655 | 1.843 | 15.033 | 0.264123 | 7/15/2014 | 01:49.9 | 1 |
| -1068.088 | 19578.672 | 1.838 | 15.035 | 0.269225 | 7/15/2014 | 01:50.0 | 1 |
| -1068.338 | 19578.688 | 1.834 | 15.034 | 0.251065 | 7/15/2014 | 01:50.1 | 1 |
| -1068.585 | 19578.704 | 1.832 | 15.033 | 0.252167 | 7/15/2014 | 01:50.2 | 1 |
| -1068.834 | 19578.721 | 1.834 | 15.03 | 0.236286 | 7/15/2014 | 01:50.3 | 1 |
| -1069.142 | 19578.741 | 1.834 | 15.028 | 0.225618 | 7/15/2014 | 01:50.4 | 1 |
| -1069.391 | 19578.758 | 1.843 | 15.025 | 0.234437 | 7/15/2014 | 01:50.5 | 1 |
| -1069.637 | 19578.774 | 1.843 | 15.024 | 0.231499 | 7/15/2014 | 01:50.6 | 1 |
| -1070.068 | 19578.804 | 1.84 | 15.025 | 0.244755 | 7/15/2014 | 01:50.7 | 1 |
| -1070.193 | 19578.813 | 1.843 | 15.024 | 0.242371 | 7/15/2014 | 01:50.8 | 1 |
| -1070.443 | 19578.828 | 1.845 | 15.027 | 0.256132 | 7/15/2014 | 01:50.9 | 1 |
| -1070.69 | 19578.846 | 1.837 | 15.032 | 0.271132 | 7/15/2014 | 01:51.0 | 1 |
| -1070.939 | 19578.862 | 1.833 | 15.037 | 0.264715 | 7/15/2014 | 01:51.1 | 1 |
| -1071.185 | 19578.879 | 1.821 | 15.043 | 0.276462 | 7/15/2014 | 01:51.2 | 1 |
| -1071.432 | 19578.896 | 1.817 | 15.049 | 0.270221 | 7/15/2014 | 01:51.3 | 1 |
| -1071.86 | 19578.927 | 1.81 | 15.057 | 0.272831 | 7/15/2014 | 01:51.4 | 1 |
| -1072.104 | 19578.943 | 1.81 | 15.062 | 0.2722 | 7/15/2014 | 01:51.5 | 1 |
| -1072.348 | 19578.96 | 1.807 | 15.066 | 0.27018 | 7/15/2014 | 01:51.6 | 1 |

| | | | | | | | |
|---|---|---|---|---|---|---|---|
| -1072.651 | 19578.981 | 1.81 | 15.069 | 0.268081 | 7/15/2014 | 01:51.7 | 1 |
| -1072.774 | 19578.99 | 1.809 | 15.071 | 0.271468 | 7/15/2014 | 01:51.8 | 1 |
| -1073.019 | 19579.007 | 1.815 | 15.073 | 0.273925 | 7/15/2014 | 01:51.9 | 1 |
| -1073.261 | 19579.025 | 1.81 | 15.075 | 0.274817 | 7/15/2014 | 01:52.0 | 1 |
| -1073.506 | 19579.042 | 1.813 | 15.076 | 0.263848 | 7/15/2014 | 01:52.1 | 1 |
| -1073.746 | 19579.061 | 1.808 | 15.076 | 0.269796 | 7/15/2014 | 01:52.2 | 1 |
| -1073.989 | 19579.076 | 1.808 | 15.077 | 0.266049 | 7/15/2014 | 01:52.3 | 1 |
| -1074.408 | 19579.107 | 1.808 | 15.079 | 0.27235 | 7/15/2014 | 01:52.4 | 1 |
| -1074.647 | 19579.124 | 1.805 | 15.08 | 0.268254 | 7/15/2014 | 01:52.5 | 1 |
| -1074.885 | 19579.143 | 1.8 | 15.083 | 0.264531 | 7/15/2014 | 01:52.6 | 1 |
| -1075.182 | 19579.164 | 1.8 | 15.086 | 0.271048 | 7/15/2014 | 01:52.7 | 1 |
| -1075.302 | 19579.174 | 1.8 | 15.087 | 0.265896 | 7/15/2014 | 01:52.8 | 1 |
| -1075.542 | 19579.189 | 1.802 | 15.092 | 0.255128 | 7/15/2014 | 01:52.9 | 1 |
| -1075.78 | 19579.208 | 1.8 | 15.099 | 0.264922 | 7/15/2014 | 01:53.0 | 1 |
| -1076.02 | 19579.225 | 1.802 | 15.107 | 0.246404 | 7/15/2014 | 01:53.1 | 1 |
| -1076.257 | 19579.243 | 1.796 | 15.117 | 0.243077 | 7/15/2014 | 01:53.2 | 1 |
| -1076.496 | 19579.261 | 1.796 | 15.128 | 0.234415 | 7/15/2014 | 01:53.3 | 1 |
| -1076.911 | 19579.293 | 1.795 | 15.148 | 0.237951 | 7/15/2014 | 01:53.4 | 1 |
| -1077.148 | 19579.31 | 1.795 | 15.162 | 0.241689 | 7/15/2014 | 01:53.5 | 1 |
| -1077.386 | 19579.328 | 1.797 | 15.178 | 0.248853 | 7/15/2014 | 01:53.6 | 1 |
| -1077.683 | 19579.35 | 1.794 | 15.195 | 0.256243 | 7/15/2014 | 01:53.7 | 1 |
| -1077.802 | 19579.359 | 1.794 | 15.203 | 0.263271 | 7/15/2014 | 01:53.8 | 1 |
| -1078.043 | 19579.378 | 1.789 | 15.221 | 0.266259 | 7/15/2014 | 01:53.9 | 1 |
| -1078.283 | 19579.396 | 1.786 | 15.24 | 0.275465 | 7/15/2014 | 01:54.0 | 1 |
| -1078.526 | 19579.415 | 1.786 | 15.26 | 0.273665 | 7/15/2014 | 01:54.1 | 1 |
| -1078.766 | 19579.434 | 1.779 | 15.281 | 0.27553 | 7/15/2014 | 01:54.2 | 1 |
| -1079.008 | 19579.452 | 1.773 | 15.302 | 0.273224 | 7/15/2014 | 01:54.3 | 1 |
| -1079.431 | 19579.484 | 1.763 | 15.335 | 0.273393 | 7/15/2014 | 01:54.4 | 1 |
| -1079.672 | 19579.504 | 1.763 | 15.357 | 0.280703 | 7/15/2014 | 01:54.5 | 1 |
| -1079.914 | 19579.524 | 1.765 | 15.378 | 0.282342 | 7/15/2014 | 01:54.6 | 1 |
| -1080.217 | 19579.548 | 1.765 | 15.399 | 0.285137 | 7/15/2014 | 01:54.7 | 1 |
| -1080.339 | 19579.558 | 1.762 | 15.41 | 0.288407 | 7/15/2014 | 01:54.8 | 1 |
| -1080.584 | 19579.577 | 1.765 | 15.428 | 0.304084 | 7/15/2014 | 01:54.9 | 1 |
| -1080.829 | 19579.596 | 1.768 | 15.445 | 0.305868 | 7/15/2014 | 01:55.0 | 1 |
| -1081.074 | 19579.617 | 1.772 | 15.459 | 0.30434 | 7/15/2014 | 01:55.1 | 1 |
| -1081.318 | 19579.637 | 1.766 | 15.47 | 0.30678 | 7/15/2014 | 01:55.2 | 1 |
| -1081.564 | 19579.657 | 1.762 | 15.476 | 0.293231 | 7/15/2014 | 01:55.3 | 1 |
| -1081.991 | 19579.691 | 1.755 | 15.472 | 0.286101 | 7/15/2014 | 01:55.4 | 1 |
| -1082.235 | 19579.713 | 1.753 | 15.466 | 0.276782 | 7/15/2014 | 01:55.5 | 1 |
| -1082.478 | 19579.732 | 1.748 | 15.455 | 0.268943 | 7/15/2014 | 01:55.6 | 1 |
| -1082.783 | 19579.76 | 1.748 | 15.44 | 0.271291 | 7/15/2014 | 01:55.7 | 1 |
| -1082.905 | 19579.77 | 1.745 | 15.433 | 0.259697 | 7/15/2014 | 01:55.8 | 1 |
| -1083.152 | 19579.789 | 1.748 | 15.412 | 0.250075 | 7/15/2014 | 01:55.9 | 1 |
| -1083.396 | 19579.811 | 1.745 | 15.388 | 0.24675 | 7/15/2014 | 01:56.0 | 1 |
| -1083.643 | 19579.83 | 1.751 | 15.364 | 0.243331 | 7/15/2014 | 01:56.1 | 1 |
| -1083.887 | 19579.852 | 1.749 | 15.34 | 0.245645 | 7/15/2014 | 01:56.2 | 1 |
| -1084.13 | 19579.873 | 1.748 | 15.318 | 0.240795 | 7/15/2014 | 01:56.3 | 1 |

| | | | | | | | |
|---|---|---|---|---|---|---|---|
| -1084.557 | 19579.91 | 1.747 | 15.291 | 0.221074 | 7/15/2014 | 01:56.4 | 1 |
| -1084.799 | 19579.93 | 1.745 | 15.276 | 0.241374 | 7/15/2014 | 01:56.5 | 1 |
| -1085.042 | 19579.952 | 1.75 | 15.265 | 0.224607 | 7/15/2014 | 01:56.6 | 1 |
| -1085.346 | 19579.979 | 1.753 | 15.257 | 0.231427 | 7/15/2014 | 01:56.7 | 1 |
| -1085.468 | 19579.988 | 1.751 | 15.252 | 0.22502 | 7/15/2014 | 01:56.8 | 1 |
| -1085.712 | 19580.01 | 1.751 | 15.251 | 0.226238 | 7/15/2014 | 01:56.9 | 1 |
| -1085.957 | 19580.031 | 1.749 | 15.252 | 0.242057 | 7/15/2014 | 01:57.0 | 1 |
| -1086.203 | 19580.053 | 1.753 | 15.256 | 0.24821 | 7/15/2014 | 01:57.1 | 1 |
| -1086.448 | 19580.074 | 1.754 | 15.262 | 0.218714 | 7/15/2014 | 01:57.2 | 1 |
| -1086.694 | 19580.096 | 1.754 | 15.27 | 0.22266 | 7/15/2014 | 01:57.3 | 1 |
| -1087.122 | 19580.135 | 1.746 | 15.283 | 0.209068 | 7/15/2014 | 01:57.4 | 1 |
| -1087.365 | 19580.155 | 1.743 | 15.292 | 0.210281 | 7/15/2014 | 01:57.5 | 1 |
| -1087.611 | 19580.178 | 1.739 | 15.301 | 0.206266 | 7/15/2014 | 01:57.6 | 1 |
| -1087.917 | 19580.204 | 1.739 | 15.31 | 0.197769 | 7/15/2014 | 01:57.7 | 1 |
| -1088.041 | 19580.216 | 1.738 | 15.314 | 0.202045 | 7/15/2014 | 01:57.8 | 1 |
| -1088.288 | 19580.237 | 1.738 | 15.323 | 0.209874 | 7/15/2014 | 01:57.9 | 1 |
| -1088.535 | 19580.26 | 1.738 | 15.332 | 0.212571 | 7/15/2014 | 01:58.0 | 1 |
| -1088.783 | 19580.283 | 1.74 | 15.34 | 0.224992 | 7/15/2014 | 01:58.1 | 1 |
| -1089.027 | 19580.306 | 1.74 | 15.349 | 0.228535 | 7/15/2014 | 01:58.2 | 1 |
| -1089.275 | 19580.328 | 1.747 | 15.357 | 0.22898 | 7/15/2014 | 01:58.3 | 1 |
| -1089.705 | 19580.367 | 1.751 | 15.367 | 0.232985 | 7/15/2014 | 01:58.4 | 1 |
| -1089.949 | 19580.389 | 1.748 | 15.372 | 0.233916 | 7/15/2014 | 01:58.5 | 1 |
| -1090.195 | 19580.412 | 1.75 | 15.376 | 0.23546 | 7/15/2014 | 01:58.6 | 1 |
| -1090.501 | 19580.441 | 1.75 | 15.378 | 0.235629 | 7/15/2014 | 01:58.7 | 1 |
| -1090.624 | 19580.453 | 1.748 | 15.38 | 0.235518 | 7/15/2014 | 01:58.8 | 1 |
| -1090.872 | 19580.476 | 1.745 | 15.379 | 0.233761 | 7/15/2014 | 01:58.9 | 1 |
| -1091.119 | 19580.499 | 1.743 | 15.377 | 0.237973 | 7/15/2014 | 01:59.0 | 1 |
| -1091.367 | 19580.522 | 1.749 | 15.373 | 0.235763 | 7/15/2014 | 01:59.1 | 1 |
| -1091.614 | 19580.545 | 1.747 | 15.369 | 0.237924 | 7/15/2014 | 01:59.2 | 1 |
| -1091.863 | 19580.568 | 1.753 | 15.365 | 0.236233 | 7/15/2014 | 01:59.3 | 1 |
| -1092.171 | 19580.599 | 1.75 | 15.363 | 0.234922 | 7/15/2014 | 01:59.4 | 1 |
| -1092.419 | 19580.621 | 1.749 | 15.363 | 0.227781 | 7/15/2014 | 01:59.5 | 1 |
| -1092.668 | 19580.646 | 1.751 | 15.366 | 0.22895 | 7/15/2014 | 01:59.6 | 1 |
| -1092.917 | 19580.67 | 1.748 | 15.372 | 0.223866 | 7/15/2014 | 01:59.7 | 1 |
| -1093.229 | 19580.7 | 1.742 | 15.382 | 0.234893 | 7/15/2014 | 01:59.8 | 1 |
| -1093.481 | 19580.725 | 1.739 | 15.395 | 0.227465 | 7/15/2014 | 01:59.9 | 1 |
| -1093.733 | 19580.75 | 1.731 | 15.41 | 0.229525 | 7/15/2014 | 02:00.0 | 1 |
| -1093.987 | 19580.774 | 1.727 | 15.429 | 0.236952 | 7/15/2014 | 02:00.1 | 1 |
| -1094.239 | 19580.8 | 1.725 | 15.449 | 0.243633 | 7/15/2014 | 02:00.2 | 1 |
| -1094.492 | 19580.825 | 1.725 | 15.472 | 0.247841 | 7/15/2014 | 02:00.3 | 1 |
| -1094.807 | 19580.856 | 1.728 | 15.495 | 0.263066 | 7/15/2014 | 02:00.4 | 1 |
| -1095.061 | 19580.882 | 1.728 | 15.518 | 0.274831 | 7/15/2014 | 02:00.5 | 1 |
| -1095.315 | 19580.907 | 1.722 | 15.54 | 0.289098 | 7/15/2014 | 02:00.6 | 1 |
| -1095.57 | 19580.934 | 1.725 | 15.562 | 0.308591 | 7/15/2014 | 02:00.7 | 1 |
| -1095.888 | 19580.966 | 1.725 | 15.582 | 0.293992 | 7/15/2014 | 02:00.8 | 1 |
| -1096.145 | 19580.993 | 1.719 | 15.6 | 0.309713 | 7/15/2014 | 02:00.9 | 1 |
| -1096.403 | 19581.02 | 1.716 | 15.616 | 0.303429 | 7/15/2014 | 02:01.0 | 1 |

| | | | | | | | |
|---|---|---|---|---|---|---|---|
| -1096.66 | 19581.046 | 1.713 | 15.629 | 0.307821 | 7/15/2014 | 02:01.1 | 1 |
| -1096.917 | 19581.072 | 1.703 | 15.639 | 0.297016 | 7/15/2014 | 02:01.2 | 1 |
| -1097.174 | 19581.1 | 1.695 | 15.647 | 0.299534 | 7/15/2014 | 02:01.3 | 1 |
| -1097.494 | 19581.133 | 1.689 | 15.652 | 0.33452 | 7/15/2014 | 02:01.4 | 1 |
| -1097.751 | 19581.16 | 1.689 | 15.655 | 0.336261 | 7/15/2014 | 02:01.5 | 1 |
| -1098.005 | 19581.188 | 1.686 | 15.656 | 0.33595 | 7/15/2014 | 02:01.6 | 1 |
| -1098.261 | 19581.215 | 1.679 | 15.655 | 0.356009 | 7/15/2014 | 02:01.7 | 1 |
| -1098.574 | 19581.249 | 1.67 | 15.651 | 0.327669 | 7/15/2014 | 02:01.8 | 1 |
| -1098.828 | 19581.276 | 1.679 | 15.646 | 0.331289 | 7/15/2014 | 02:01.9 | 1 |
| -1099.079 | 19581.303 | 1.683 | 15.639 | 0.332413 | 7/15/2014 | 02:02.0 | 1 |
| -1099.331 | 19581.33 | 1.683 | 15.631 | 0.329813 | 7/15/2014 | 02:02.1 | 1 |
| -1099.578 | 19581.357 | 1.673 | 15.622 | 0.364599 | 7/15/2014 | 02:02.2 | 1 |
| -1099.826 | 19581.385 | 1.677 | 15.613 | 0.318452 | 7/15/2014 | 02:02.3 | 1 |
| -1100.25 | 19581.433 | 1.682 | 15.603 | 0.342178 | 7/15/2014 | 02:02.4 | 1 |
| -1100.492 | 19581.46 | 1.687 | 15.597 | 0.326594 | 7/15/2014 | 02:02.5 | 1 |
| -1100.73 | 19581.485 | 1.682 | 15.593 | 0.301998 | 7/15/2014 | 02:02.6 | 1 |
| -1101.026 | 19581.52 | 1.682 | 15.591 | 0.260628 | 7/15/2014 | 02:02.7 | 1 |
| -1101.148 | 19581.533 | 1.682 | 15.589 | 0.247625 | 7/15/2014 | 02:02.8 | 1 |
| -1101.385 | 19581.561 | 1.682 | 15.59 | 0.233686 | 7/15/2014 | 02:02.9 | 1 |
| -1101.622 | 19581.588 | 1.682 | 15.593 | 0.217854 | 7/15/2014 | 02:03.0 | 1 |
| -1101.859 | 19581.614 | 1.691 | 15.597 | 0.21891 | 7/15/2014 | 02:03.1 | 1 |
| -1102.093 | 19581.642 | 1.691 | 15.603 | 0.217702 | 7/15/2014 | 02:03.2 | 1 |
| -1102.327 | 19581.668 | 1.685 | 15.61 | 0.219629 | 7/15/2014 | 02:03.3 | 1 |
| -1102.735 | 19581.716 | 1.693 | 15.619 | 0.232237 | 7/15/2014 | 02:03.4 | 1 |
| -1102.966 | 19581.743 | 1.693 | 15.625 | 0.231986 | 7/15/2014 | 02:03.5 | 1 |
| -1103.199 | 19581.771 | 1.699 | 15.63 | 0.239636 | 7/15/2014 | 02:03.6 | 1 |
| -1103.49 | 19581.804 | 1.699 | 15.634 | 0.229237 | 7/15/2014 | 02:03.7 | 1 |
| -1103.606 | 19581.817 | 1.692 | 15.637 | 0.233425 | 7/15/2014 | 02:03.8 | 1 |
| -1103.842 | 19581.846 | 1.695 | 15.639 | 0.236429 | 7/15/2014 | 02:03.9 | 1 |
| -1104.079 | 19581.874 | 1.705 | 15.642 | 0.226866 | 7/15/2014 | 02:04.0 | 1 |
| -1104.316 | 19581.902 | 1.705 | 15.643 | 0.222012 | 7/15/2014 | 02:04.1 | 1 |
| -1104.552 | 19581.931 | 1.698 | 15.646 | 0.216875 | 7/15/2014 | 02:04.2 | 1 |
| -1104.792 | 19581.959 | 1.704 | 15.648 | 0.217514 | 7/15/2014 | 02:04.3 | 1 |
| -1105.209 | 19582.009 | 1.714 | 15.655 | 0.215627 | 7/15/2014 | 02:04.4 | 1 |
| -1105.447 | 19582.038 | 1.716 | 15.661 | 0.226954 | 7/15/2014 | 02:04.5 | 1 |
| -1105.686 | 19582.067 | 1.716 | 15.668 | 0.222094 | 7/15/2014 | 02:04.6 | 1 |
| -1105.984 | 19582.104 | 1.713 | 15.677 | 0.226947 | 7/15/2014 | 02:04.7 | 1 |
| -1106.105 | 19582.118 | 1.717 | 15.681 | 0.23388 | 7/15/2014 | 02:04.8 | 1 |
| -1106.349 | 19582.147 | 1.719 | 15.693 | 0.251068 | 7/15/2014 | 02:04.9 | 1 |
| -1106.589 | 19582.177 | 1.715 | 15.707 | 0.246916 | 7/15/2014 | 02:05.0 | 1 |
| -1106.835 | 19582.207 | 1.719 | 15.722 | 0.255202 | 7/15/2014 | 02:05.1 | 1 |
| -1107.077 | 19582.237 | 1.709 | 15.738 | 0.252926 | 7/15/2014 | 02:05.2 | 1 |
| -1107.323 | 19582.267 | 1.716 | 15.754 | 0.268681 | 7/15/2014 | 02:05.3 | 1 |
| -1107.753 | 19582.319 | 1.718 | 15.777 | 0.287228 | 7/15/2014 | 02:05.4 | 1 |
| -1107.874 | 19582.334 | 1.721 | 15.785 | 0.284094 | 7/15/2014 | 02:05.5 | 1 |
| -1108.121 | 19582.365 | 1.717 | 15.798 | 0.302475 | 7/15/2014 | 02:05.6 | 1 |
| -1108.372 | 19582.396 | 1.721 | 15.809 | 0.289435 | 7/15/2014 | 02:05.7 | 1 |

| | | | | | | | |
|---|---|---|---|---|---|---|---|
| -1108.622 | 19582.428 | 1.718 | 15.817 | 0.311505 | 7/15/2014 | 02:05.8 | 1 |
| -1108.876 | 19582.459 | 1.721 | 15.823 | 0.285857 | 7/15/2014 | 02:05.9 | 1 |
| -1109.128 | 19582.491 | 1.708 | 15.827 | 0.292476 | 7/15/2014 | 02:06.0 | 1 |
| -1109.382 | 19582.521 | 1.702 | 15.829 | 0.273575 | 7/15/2014 | 02:06.1 | 1 |
| -1109.634 | 19582.554 | 1.698 | 15.83 | 0.263564 | 7/15/2014 | 02:06.2 | 1 |
| -1109.888 | 19582.586 | 1.708 | 15.831 | 0.26625 | 7/15/2014 | 02:06.3 | 1 |
| -1110.139 | 19582.616 | 1.708 | 15.832 | 0.253568 | 7/15/2014 | 02:06.4 | 1 |
| -1110.389 | 19582.648 | 1.705 | 15.833 | 0.26391 | 7/15/2014 | 02:06.5 | 1 |
| -1110.635 | 19582.679 | 1.699 | 15.835 | 0.246404 | 7/15/2014 | 02:06.6 | 1 |
| -1111.062 | 19582.732 | 1.714 | 15.84 | 0.235036 | 7/15/2014 | 02:06.7 | 1 |
| -1111.187 | 19582.748 | 1.715 | 15.841 | 0.233863 | 7/15/2014 | 02:06.8 | 1 |
| -1111.434 | 19582.78 | 1.715 | 15.845 | 0.228024 | 7/15/2014 | 02:06.9 | 1 |
| -1111.678 | 19582.81 | 1.702 | 15.85 | 0.227407 | 7/15/2014 | 02:07.0 | 1 |
| -1111.923 | 19582.84 | 1.695 | 15.854 | 0.217581 | 7/15/2014 | 02:07.1 | 1 |
| -1112.167 | 19582.871 | 1.688 | 15.858 | 0.218857 | 7/15/2014 | 02:07.2 | 1 |
| -1112.414 | 19582.902 | 1.692 | 15.863 | 0.212925 | 7/15/2014 | 02:07.3 | 1 |
| -1112.844 | 19582.957 | 1.679 | 15.869 | 0.217075 | 7/15/2014 | 02:07.4 | 1 |
| -1112.965 | 19582.973 | 1.672 | 15.872 | 0.214537 | 7/15/2014 | 02:07.5 | 1 |
| -1113.212 | 19583.003 | 1.666 | 15.876 | 0.207229 | 7/15/2014 | 02:07.6 | 1 |
| -1113.462 | 19583.033 | 1.672 | 15.88 | 0.210199 | 7/15/2014 | 02:07.7 | 1 |
| -1113.713 | 19583.066 | 1.669 | 15.885 | 0.214536 | 7/15/2014 | 02:07.8 | 1 |
| -1113.968 | 19583.099 | 1.672 | 15.889 | 0.214677 | 7/15/2014 | 02:07.9 | 1 |
| -1114.218 | 19583.13 | 1.669 | 15.894 | 0.22402 | 7/15/2014 | 02:08.0 | 1 |
| -1114.471 | 19583.162 | 1.672 | 15.898 | 0.204585 | 7/15/2014 | 02:08.1 | 1 |
| -1114.722 | 19583.193 | 1.664 | 15.901 | 0.212285 | 7/15/2014 | 02:08.2 | 1 |
| -1114.974 | 19583.226 | 1.666 | 15.904 | 0.221442 | 7/15/2014 | 02:08.3 | 1 |
| -1115.224 | 19583.259 | 1.658 | 15.906 | 0.215488 | 7/15/2014 | 02:08.4 | 1 |
| -1115.474 | 19583.291 | 1.658 | 15.907 | 0.205957 | 7/15/2014 | 02:08.5 | 1 |
| -1115.839 | 19583.338 | 1.653 | 15.906 | 0.197125 | 7/15/2014 | 02:08.6 | 1 |
| -1116.143 | 19583.375 | 1.645 | 15.904 | 0.206646 | 7/15/2014 | 02:08.7 | 1 |
| -1116.268 | 19583.391 | 1.641 | 15.904 | 0.206593 | 7/15/2014 | 02:08.8 | 1 |
| -1116.513 | 19583.423 | 1.639 | 15.902 | 0.188594 | 7/15/2014 | 02:08.9 | 1 |
| -1116.757 | 19583.455 | 1.635 | 15.9 | 0.192687 | 7/15/2014 | 02:09.0 | 1 |
| -1117.001 | 19583.486 | 1.637 | 15.899 | 0.182097 | 7/15/2014 | 02:09.1 | 1 |
| -1117.245 | 19583.52 | 1.641 | 15.9 | 0.189299 | 7/15/2014 | 02:09.2 | 1 |
| -1117.49 | 19583.551 | 1.646 | 15.901 | 0.176624 | 7/15/2014 | 02:09.3 | 1 |
| -1117.92 | 19583.604 | 1.641 | 15.907 | 0.169505 | 7/15/2014 | 02:09.4 | 1 |
| -1118.041 | 19583.62 | 1.644 | 15.908 | 0.178644 | 7/15/2014 | 02:09.5 | 1 |
| -1118.289 | 19583.652 | 1.65 | 15.914 | 0.156008 | 7/15/2014 | 02:09.6 | 1 |
| -1118.539 | 19583.684 | 1.661 | 15.921 | 0.159014 | 7/15/2014 | 02:09.7 | 1 |
| -1118.791 | 19583.718 | 1.665 | 15.928 | 0.163148 | 7/15/2014 | 02:09.8 | 1 |
| -1119.043 | 19583.75 | 1.661 | 15.936 | 0.164687 | 7/15/2014 | 02:09.9 | 1 |
| -1119.296 | 19583.781 | 1.665 | 15.945 | 0.165922 | 7/15/2014 | 02:10.0 | 1 |
| -1119.549 | 19583.815 | 1.673 | 15.953 | 0.168767 | 7/15/2014 | 02:10.1 | 1 |
| -1119.801 | 19583.848 | 1.673 | 15.962 | 0.17771 | 7/15/2014 | 02:10.2 | 1 |
| -1120.053 | 19583.879 | 1.673 | 15.97 | 0.179131 | 7/15/2014 | 02:10.3 | 1 |
| -1120.304 | 19583.911 | 1.667 | 15.979 | 0.177854 | 7/15/2014 | 02:10.4 | 1 |

| | | | | | | | |
|---|---|---|---|---|---|---|---|
| -1120.553 | 19583.943 | 1.667 | 15.987 | 0.180314 | 7/15/2014 | 02:10.5 | 1 |
| -1120.919 | 19583.99 | 1.671 | 16.001 | 0.171253 | 7/15/2014 | 02:10.6 | 1 |
| -1121.223 | 19584.03 | 1.668 | 16.009 | 0.17008 | 7/15/2014 | 02:10.7 | 1 |
| -1121.349 | 19584.046 | 1.664 | 16.013 | 0.170398 | 7/15/2014 | 02:10.8 | 1 |
| -1121.593 | 19584.076 | 1.668 | 16.021 | 0.164765 | 7/15/2014 | 02:10.9 | 1 |
| -1121.837 | 19584.107 | 1.671 | 16.027 | 0.181065 | 7/15/2014 | 02:11.0 | 1 |
| -1122.082 | 19584.139 | 1.679 | 16.033 | 0.159881 | 7/15/2014 | 02:11.1 | 1 |
| -1122.324 | 19584.17 | 1.681 | 16.037 | 0.169206 | 7/15/2014 | 02:11.2 | 1 |
| -1122.569 | 19584.201 | 1.683 | 16.041 | 0.177439 | 7/15/2014 | 02:11.3 | 1 |
| -1123 | 19584.255 | 1.683 | 16.044 | 0.161163 | 7/15/2014 | 02:11.4 | 1 |
| -1123.12 | 19584.27 | 1.687 | 16.045 | 0.166696 | 7/15/2014 | 02:11.5 | 1 |
| -1123.367 | 19584.301 | 1.69 | 16.046 | 0.153276 | 7/15/2014 | 02:11.6 | 1 |
| -1123.619 | 19584.332 | 1.696 | 16.047 | 0.153034 | 7/15/2014 | 02:11.7 | 1 |
| -1123.869 | 19584.363 | 1.692 | 16.049 | 0.143769 | 7/15/2014 | 02:11.8 | 1 |
| -1124.123 | 19584.395 | 1.693 | 16.051 | 0.144974 | 7/15/2014 | 02:11.9 | 1 |
| -1124.375 | 19584.426 | 1.691 | 16.054 | 0.140924 | 7/15/2014 | 02:12.0 | 1 |
| -1124.632 | 19584.457 | 1.701 | 16.057 | 0.140891 | 7/15/2014 | 02:12.1 | 1 |
| -1124.884 | 19584.487 | 1.697 | 16.061 | 0.133756 | 7/15/2014 | 02:12.2 | 1 |
| -1125.14 | 19584.519 | 1.701 | 16.065 | 0.143887 | 7/15/2014 | 02:12.3 | 1 |
| -1125.391 | 19584.549 | 1.697 | 16.068 | 0.139051 | 7/15/2014 | 02:12.4 | 1 |
| -1125.645 | 19584.579 | 1.703 | 16.071 | 0.137546 | 7/15/2014 | 02:12.5 | 1 |
| -1125.894 | 19584.609 | 1.705 | 16.073 | 0.127099 | 7/15/2014 | 02:12.6 | 1 |
| -1126.324 | 19584.66 | 1.707 | 16.071 | 0.137837 | 7/15/2014 | 02:12.7 | 1 |
| -1126.45 | 19584.675 | 1.705 | 16.071 | 0.138968 | 7/15/2014 | 02:12.8 | 1 |
| -1126.701 | 19584.705 | 1.712 | 16.068 | 0.126489 | 7/15/2014 | 02:12.9 | 1 |
| -1126.947 | 19584.734 | 1.712 | 16.064 | 0.132145 | 7/15/2014 | 02:13.0 | 1 |
| -1127.198 | 19584.764 | 1.719 | 16.059 | 0.123675 | 7/15/2014 | 02:13.1 | 1 |
| -1127.446 | 19584.793 | 1.715 | 16.054 | 0.125701 | 7/15/2014 | 02:13.2 | 1 |
| -1127.696 | 19584.82 | 1.715 | 16.049 | 0.117468 | 7/15/2014 | 02:13.3 | 1 |
| -1128.006 | 19584.857 | 1.715 | 16.044 | 0.121635 | 7/15/2014 | 02:13.4 | 1 |
| -1128.261 | 19584.888 | 1.725 | 16.04 | 0.116465 | 7/15/2014 | 02:13.5 | 1 |
| -1128.515 | 19584.917 | 1.719 | 16.038 | 0.120103 | 7/15/2014 | 02:13.6 | 1 |
| -1128.775 | 19584.946 | 1.719 | 16.036 | 0.129476 | 7/15/2014 | 02:13.7 | 1 |
| -1129.034 | 19584.977 | 1.711 | 16.036 | 0.133728 | 7/15/2014 | 02:13.8 | 1 |
| -1129.299 | 19585.006 | 1.715 | 16.037 | 0.146034 | 7/15/2014 | 02:13.9 | 1 |
| -1129.562 | 19585.035 | 1.709 | 16.04 | 0.130682 | 7/15/2014 | 02:14.0 | 1 |
| -1129.83 | 19585.066 | 1.707 | 16.044 | 0.147719 | 7/15/2014 | 02:14.1 | 1 |
| -1130.094 | 19585.097 | 1.697 | 16.051 | 0.156675 | 7/15/2014 | 02:14.2 | 1 |
| -1130.362 | 19585.125 | 1.707 | 16.059 | 0.138936 | 7/15/2014 | 02:14.3 | 1 |
| -1130.626 | 19585.156 | 1.703 | 16.068 | 0.141268 | 7/15/2014 | 02:14.4 | 1 |
| -1130.892 | 19585.186 | 1.71 | 16.08 | 0.144733 | 7/15/2014 | 02:14.5 | 1 |
| -1131.153 | 19585.213 | 1.707 | 16.094 | 0.132045 | 7/15/2014 | 02:14.6 | 1 |
| -1131.415 | 19585.242 | 1.713 | 16.109 | 0.126339 | 7/15/2014 | 02:14.7 | 1 |
| -1131.734 | 19585.277 | 1.709 | 16.125 | 0.12093 | 7/15/2014 | 02:14.8 | 1 |
| -1131.994 | 19585.305 | 1.719 | 16.143 | 0.119357 | 7/15/2014 | 02:14.9 | 1 |
| -1132.249 | 19585.334 | 1.709 | 16.161 | 0.109864 | 7/15/2014 | 02:15.0 | 1 |
| -1132.507 | 19585.36 | 1.71 | 16.18 | 0.110635 | 7/15/2014 | 02:15.1 | 1 |

| | | | | | | | |
|---|---|---|---|---|---|---|---|
| -1132.761 | 19585.388 | 1.707 | 16.198 | 0.124572 | 7/15/2014 | 02:15.2 | 1 |
| -1133.016 | 19585.414 | 1.71 | 16.215 | 0.112725 | 7/15/2014 | 02:15.3 | 1 |
| -1133.33 | 19585.447 | 1.71 | 16.231 | 0.120544 | 7/15/2014 | 02:15.4 | 1 |
| -1133.585 | 19585.475 | 1.717 | 16.246 | 0.111792 | 7/15/2014 | 02:15.5 | 1 |
| -1133.838 | 19585.502 | 1.713 | 16.259 | 0.125284 | 7/15/2014 | 02:15.6 | 1 |
| -1134.094 | 19585.528 | 1.723 | 16.27 | 0.103142 | 7/15/2014 | 02:15.7 | 1 |
| -1134.35 | 19585.555 | 1.725 | 16.279 | 0.0888127 | 7/15/2014 | 02:15.8 | 1 |
| -1134.606 | 19585.581 | 1.725 | 16.285 | 0.108795 | 7/15/2014 | 02:15.9 | 1 |
| -1134.862 | 19585.607 | 1.725 | 16.288 | 0.0840248 | 7/15/2014 | 02:16.0 | 1 |
| -1135.117 | 19585.634 | 1.731 | 16.288 | 0.080383 | 7/15/2014 | 02:16.1 | 1 |
| -1135.372 | 19585.66 | 1.729 | 16.285 | 0.0752188 | 7/15/2014 | 02:16.2 | 1 |
| -1135.625 | 19585.687 | 1.729 | 16.279 | 0.0855213 | 7/15/2014 | 02:16.3 | 1 |
| -1135.876 | 19585.711 | 1.725 | 16.27 | 0.0887801 | 7/15/2014 | 02:16.4 | 1 |
| -1136.125 | 19585.737 | 1.731 | 16.26 | 0.0755742 | 7/15/2014 | 02:16.5 | 1 |
| -1136.491 | 19585.773 | 1.73 | 16.241 | 0.0883581 | 7/15/2014 | 02:16.6 | 1 |
| -1136.794 | 19585.804 | 1.73 | 16.228 | 0.086378 | 7/15/2014 | 02:16.7 | 1 |
| -1136.92 | 19585.816 | 1.725 | 16.221 | 0.0982051 | 7/15/2014 | 02:16.8 | 1 |
| -1137.163 | 19585.841 | 1.731 | 16.208 | 0.0961547 | 7/15/2014 | 02:16.9 | 1 |
| -1137.405 | 19585.864 | 1.735 | 16.195 | 0.103389 | 7/15/2014 | 02:17.0 | 1 |
| -1137.646 | 19585.888 | 1.738 | 16.183 | 0.119821 | 7/15/2014 | 02:17.1 | 1 |
| -1137.886 | 19585.912 | 1.736 | 16.173 | 0.115985 | 7/15/2014 | 02:17.2 | 1 |
| -1138.126 | 19585.935 | 1.74 | 16.164 | 0.107356 | 7/15/2014 | 02:17.3 | 1 |
| -1138.546 | 19585.975 | 1.746 | 16.155 | 0.105502 | 7/15/2014 | 02:17.4 | 1 |
| -1138.785 | 19585.998 | 1.749 | 16.15 | 0.0885253 | 7/15/2014 | 02:17.5 | 1 |
| -1139.025 | 19586.021 | 1.753 | 16.147 | 0.0912032 | 7/15/2014 | 02:17.6 | 1 |
| -1139.326 | 19586.048 | 1.753 | 16.145 | 0.0703274 | 7/15/2014 | 02:17.7 | 1 |
| -1139.447 | 19586.061 | 1.754 | 16.144 | 0.0631808 | 7/15/2014 | 02:17.8 | 1 |
| -1139.691 | 19586.083 | 1.758 | 16.145 | 0.086636 | 7/15/2014 | 02:17.9 | 1 |
| -1139.935 | 19586.104 | 1.761 | 16.148 | 0.057791 | 7/15/2014 | 02:18.0 | 1 |
| -1140.182 | 19586.127 | 1.765 | 16.152 | 0.0734261 | 7/15/2014 | 02:18.1 | 1 |
| -1140.427 | 19586.15 | 1.763 | 16.156 | 0.117329 | 7/15/2014 | 02:18.2 | 1 |
| -1140.674 | 19586.171 | 1.768 | 16.162 | 0.0977518 | 7/15/2014 | 02:18.3 | 1 |
| -1140.98 | 19586.199 | 1.765 | 16.169 | 0.108619 | 7/15/2014 | 02:18.4 | 1 |
| -1141.229 | 19586.223 | 1.773 | 16.176 | 0.072393 | 7/15/2014 | 02:18.5 | 1 |
| -1141.476 | 19586.242 | 1.768 | 16.184 | 0.0993405 | 7/15/2014 | 02:18.6 | 1 |
| -1141.726 | 19586.265 | 1.77 | 16.192 | 0.100575 | 7/15/2014 | 02:18.7 | 1 |
| -1142.037 | 19586.291 | 1.768 | 16.202 | 0.0836039 | 7/15/2014 | 02:18.8 | 1 |
| -1142.289 | 19586.313 | 1.773 | 16.214 | 0.0982735 | 7/15/2014 | 02:18.9 | 1 |
| -1142.54 | 19586.334 | 1.762 | 16.227 | 0.0864555 | 7/15/2014 | 02:19.0 | 1 |
| -1142.795 | 19586.357 | 1.762 | 16.242 | 0.137539 | 7/15/2014 | 02:19.1 | 1 |
| -1143.046 | 19586.377 | 1.757 | 16.259 | 0.0934961 | 7/15/2014 | 02:19.2 | 1 |
| -1143.3 | 19586.397 | 1.762 | 16.279 | 0.103988 | 7/15/2014 | 02:19.3 | 1 |
| -1143.614 | 19586.422 | 1.757 | 16.301 | 0.0879345 | 7/15/2014 | 02:19.4 | 1 |
| -1143.867 | 19586.445 | 1.754 | 16.324 | 0.0839221 | 7/15/2014 | 02:19.5 | 1 |
| -1144.12 | 19586.465 | 1.75 | 16.349 | 0.0796185 | 7/15/2014 | 02:19.6 | 1 |
| -1144.375 | 19586.486 | 1.758 | 16.374 | 0.0851289 | 7/15/2014 | 02:19.7 | 1 |
| -1144.692 | 19586.51 | 1.756 | 16.401 | 0.0846038 | 7/15/2014 | 02:19.8 | 1 |

| | | | | | | | |
|---|---|---|---|---|---|---|---|
| -1144.948 | 19586.529 | 1.756 | 16.427 | 0.0804434 | 7/15/2014 | 02:19.9 | 1 |
| -1145.202 | 19586.549 | 1.747 | 16.452 | 0.0770603 | 7/15/2014 | 02:20.0 | 1 |
| -1145.458 | 19586.57 | 1.752 | 16.475 | 0.0715933 | 7/15/2014 | 02:20.1 | 1 |
| -1145.712 | 19586.59 | 1.755 | 16.495 | 0.068783 | 7/15/2014 | 02:20.2 | 1 |
| -1145.967 | 19586.608 | 1.759 | 16.512 | 0.0684906 | 7/15/2014 | 02:20.3 | 1 |
| -1146.282 | 19586.631 | 1.757 | 16.524 | 0.0800413 | 7/15/2014 | 02:20.4 | 1 |
| -1146.535 | 19586.65 | 1.756 | 16.532 | 0.0752444 | 7/15/2014 | 02:20.5 | 1 |
| -1146.785 | 19586.668 | 1.753 | 16.536 | 0.0775652 | 7/15/2014 | 02:20.6 | 1 |
| -1147.038 | 19586.687 | 1.759 | 16.534 | 0.0750858 | 7/15/2014 | 02:20.7 | 1 |
| -1147.348 | 19586.709 | 1.751 | 16.529 | 0.0757334 | 7/15/2014 | 02:20.8 | 1 |
| -1147.599 | 19586.726 | 1.751 | 16.52 | 0.0723861 | 7/15/2014 | 02:20.9 | 1 |
| -1147.847 | 19586.743 | 1.748 | 16.508 | 0.0685164 | 7/15/2014 | 02:21.0 | 1 |
| -1148.097 | 19586.761 | 1.748 | 16.494 | 0.065814 | 7/15/2014 | 02:21.1 | 1 |
| -1148.344 | 19586.776 | 1.75 | 16.478 | 0.0616548 | 7/15/2014 | 02:21.2 | 1 |
| -1148.593 | 19586.793 | 1.75 | 16.461 | 0.0577697 | 7/15/2014 | 02:21.3 | 1 |
| -1149.021 | 19586.821 | 1.746 | 16.435 | 0.0534758 | 7/15/2014 | 02:21.4 | 1 |
| -1149.267 | 19586.837 | 1.749 | 16.418 | 0.0590292 | 7/15/2014 | 02:21.5 | 1 |
| -1149.511 | 19586.853 | 1.747 | 16.402 | 0.0542864 | 7/15/2014 | 02:21.6 | 1 |
| -1149.816 | 19586.872 | 1.745 | 16.387 | 0.0566356 | 7/15/2014 | 02:21.7 | 1 |
| -1149.939 | 19586.88 | 1.745 | 16.379 | 0.0566985 | 7/15/2014 | 02:21.8 | 1 |
| -1150.186 | 19586.895 | 1.747 | 16.367 | 0.0548222 | 7/15/2014 | 02:21.9 | 1 |
| -1150.431 | 19586.909 | 1.75 | 16.357 | 0.0507023 | 7/15/2014 | 02:22.0 | 1 |
| -1150.677 | 19586.925 | 1.753 | 16.35 | 0.0531377 | 7/15/2014 | 02:22.1 | 1 |
| -1150.923 | 19586.939 | 1.751 | 16.346 | 0.0505394 | 7/15/2014 | 02:22.2 | 1 |
| -1151.169 | 19586.953 | 1.756 | 16.347 | 0.0528514 | 7/15/2014 | 02:22.3 | 1 |
| -1151.598 | 19586.979 | 1.751 | 16.359 | 0.0543271 | 7/15/2014 | 02:22.4 | 1 |
| -1151.844 | 19586.992 | 1.748 | 16.371 | 0.0612713 | 7/15/2014 | 02:22.5 | 1 |
| -1152.089 | 19587.006 | 1.748 | 16.387 | 0.0597198 | 7/15/2014 | 02:22.6 | 1 |
| -1152.397 | 19587.022 | 1.743 | 16.407 | 0.0686419 | 7/15/2014 | 02:22.7 | 1 |
| -1152.521 | 19587.029 | 1.742 | 16.416 | 0.0805137 | 7/15/2014 | 02:22.8 | 1 |
| -1152.771 | 19587.043 | 1.742 | 16.441 | 0.0638564 | 7/15/2014 | 02:22.9 | 1 |
| -1153.02 | 19587.056 | 1.734 | 16.467 | 0.0524566 | 7/15/2014 | 02:23.0 | 1 |
| -1153.271 | 19587.068 | 1.73 | 16.493 | 0.0609818 | 7/15/2014 | 02:23.1 | 1 |
| -1153.523 | 19587.081 | 1.734 | 16.519 | 0.066768 | 7/15/2014 | 02:23.2 | 1 |
| -1153.775 | 19587.094 | 1.737 | 16.544 | 0.0716891 | 7/15/2014 | 02:23.3 | 1 |
| -1154.087 | 19587.108 | 1.731 | 16.566 | 0.0804659 | 7/15/2014 | 02:23.4 | 1 |
| -1154.343 | 19587.121 | 1.734 | 16.586 | 0.0797569 | 7/15/2014 | 02:23.5 | 1 |
| -1154.596 | 19587.133 | 1.734 | 16.603 | 0.0838107 | 7/15/2014 | 02:23.6 | 1 |
| -1154.85 | 19587.145 | 1.737 | 16.618 | 0.0881699 | 7/15/2014 | 02:23.7 | 1 |
| -1155.169 | 19587.158 | 1.741 | 16.631 | 0.0929613 | 7/15/2014 | 02:23.8 | 1 |
| -1155.425 | 19587.17 | 1.744 | 16.643 | 0.117106 | 7/15/2014 | 02:23.9 | 1 |
| -1155.679 | 19587.182 | 1.734 | 16.654 | 0.10538 | 7/15/2014 | 02:24.0 | 1 |
| -1155.939 | 19587.191 | 1.734 | 16.666 | 0.112114 | 7/15/2014 | 02:24.1 | 1 |
| -1156.193 | 19587.203 | 1.725 | 16.678 | 0.114962 | 7/15/2014 | 02:24.2 | 1 |
| -1156.45 | 19587.213 | 1.722 | 16.693 | 0.109388 | 7/15/2014 | 02:24.3 | 1 |
| -1156.766 | 19587.227 | 1.714 | 16.708 | 0.116391 | 7/15/2014 | 02:24.4 | 1 |
| -1157.021 | 19587.236 | 1.71 | 16.725 | 0.119029 | 7/15/2014 | 02:24.5 | 1 |

| | | | | | | | |
|---|---|---|---|---|---|---|---|
| -1157.272 | 19587.246 | 1.698 | 16.742 | 0.115637 | 7/15/2014 | 02:24.6 | 1 |
| -1157.528 | 19587.255 | 1.702 | 16.76 | 0.120638 | 7/15/2014 | 02:24.7 | 1 |
| -1157.841 | 19587.268 | 1.702 | 16.778 | 0.103609 | 7/15/2014 | 02:24.8 | 1 |
| -1158.095 | 19587.275 | 1.711 | 16.794 | 0.115204 | 7/15/2014 | 02:24.9 | 1 |
| -1158.346 | 19587.285 | 1.711 | 16.808 | 0.104375 | 7/15/2014 | 02:25.0 | 1 |
| -1158.599 | 19587.293 | 1.715 | 16.82 | 0.121048 | 7/15/2014 | 02:25.1 | 1 |
| -1158.848 | 19587.301 | 1.705 | 16.829 | 0.108606 | 7/15/2014 | 02:25.2 | 1 |
| -1159.1 | 19587.311 | 1.711 | 16.835 | 0.107683 | 7/15/2014 | 02:25.3 | 1 |
| -1159.411 | 19587.32 | 1.711 | 16.839 | 0.12793 | 7/15/2014 | 02:25.4 | 1 |
| -1159.781 | 19587.332 | 1.703 | 16.84 | 0.112384 | 7/15/2014 | 02:25.5 | 1 |
| -1160.028 | 19587.34 | 1.698 | 16.84 | 0.114731 | 7/15/2014 | 02:25.6 | 1 |
| -1160.333 | 19587.349 | 1.69 | 16.839 | 0.0855761 | 7/15/2014 | 02:25.7 | 1 |
| -1160.458 | 19587.353 | 1.685 | 16.838 | 0.0951734 | 7/15/2014 | 02:25.8 | 1 |
| -1160.706 | 19587.36 | 1.693 | 16.837 | 0.0904038 | 7/15/2014 | 02:25.9 | 1 |
| -1160.951 | 19587.367 | 1.686 | 16.836 | 0.0900638 | 7/15/2014 | 02:26.0 | 1 |
| -1161.196 | 19587.374 | 1.686 | 16.834 | 0.0856041 | 7/15/2014 | 02:26.1 | 1 |
| -1161.439 | 19587.381 | 1.684 | 16.833 | 0.079949 | 7/15/2014 | 02:26.2 | 1 |
| -1161.684 | 19587.389 | 1.684 | 16.831 | 0.0826937 | 7/15/2014 | 02:26.3 | 1 |
| -1162.107 | 19587.399 | 1.68 | 16.828 | 0.0783659 | 7/15/2014 | 02:26.4 | 1 |
| -1162.349 | 19587.404 | 1.68 | 16.826 | 0.0775325 | 7/15/2014 | 02:26.5 | 1 |
| -1162.591 | 19587.412 | 1.68 | 16.823 | 0.0729573 | 7/15/2014 | 02:26.6 | 1 |
| -1162.895 | 19587.419 | 1.677 | 16.822 | 0.0629037 | 7/15/2014 | 02:26.7 | 1 |
| -1163.015 | 19587.422 | 1.673 | 16.82 | 0.0621 | 7/15/2014 | 02:26.8 | 1 |
| -1163.262 | 19587.428 | 1.673 | 16.82 | 0.0532207 | 7/15/2014 | 02:26.9 | 1 |
| -1163.507 | 19587.432 | 1.673 | 16.821 | 0.0491923 | 7/15/2014 | 02:27.0 | 1 |
| -1163.756 | 19587.438 | 1.677 | 16.824 | 0.0443384 | 7/15/2014 | 02:27.1 | 1 |
| -1164.004 | 19587.443 | 1.675 | 16.829 | 0.0379578 | 7/15/2014 | 02:27.2 | 1 |
| -1164.254 | 19587.449 | 1.679 | 16.836 | 0.0535319 | 7/15/2014 | 02:27.3 | 1 |
| -1164.564 | 19587.455 | 1.675 | 16.845 | 0.039368 | 7/15/2014 | 02:27.4 | 1 |
| -1164.815 | 19587.459 | 1.675 | 16.856 | 0.0580809 | 7/15/2014 | 02:27.5 | 1 |
| -1165.068 | 19587.464 | 1.673 | 16.868 | 0.0442534 | 7/15/2014 | 02:27.6 | 1 |
| -1165.322 | 19587.469 | 1.673 | 16.881 | 0.055066 | 7/15/2014 | 02:27.7 | 1 |
| -1165.64 | 19587.475 | 1.669 | 16.896 | 0.0540211 | 7/15/2014 | 02:27.8 | 1 |
| -1165.896 | 19587.479 | 1.667 | 16.912 | 0.0589255 | 7/15/2014 | 02:27.9 | 1 |
| -1166.152 | 19587.482 | 1.661 | 16.929 | 0.0645519 | 7/15/2014 | 02:28.0 | 1 |
| -1166.41 | 19587.485 | 1.655 | 16.949 | 0.0714023 | 7/15/2014 | 02:28.1 | 1 |
| -1166.667 | 19587.49 | 1.656 | 16.97 | 0.0731447 | 7/15/2014 | 02:28.2 | 1 |
| -1166.925 | 19587.493 | 1.655 | 16.993 | 0.0753382 | 7/15/2014 | 02:28.3 | 1 |
| -1167.245 | 19587.498 | 1.655 | 17.019 | 0.0740447 | 7/15/2014 | 02:28.4 | 1 |
| -1167.501 | 19587.502 | 1.652 | 17.045 | 0.0671626 | 7/15/2014 | 02:28.5 | 1 |
| -1167.756 | 19587.505 | 1.646 | 17.072 | 0.0576943 | 7/15/2014 | 02:28.6 | 1 |
| -1168.013 | 19587.508 | 1.655 | 17.099 | 0.0525345 | 7/15/2014 | 02:28.7 | 1 |
| -1168.332 | 19587.511 | 1.655 | 17.126 | 0.0440465 | 7/15/2014 | 02:28.8 | 1 |
| -1168.59 | 19587.514 | 1.651 | 17.151 | 0.0381864 | 7/15/2014 | 02:28.9 | 1 |
| -1168.848 | 19587.517 | 1.646 | 17.174 | 0.0257386 | 7/15/2014 | 02:29.0 | 1 |
| -1169.107 | 19587.52 | 1.646 | 17.196 | 0.0369725 | 7/15/2014 | 02:29.1 | 1 |
| -1169.363 | 19587.522 | 1.646 | 17.215 | 0.029398 | 7/15/2014 | 02:29.2 | 1 |

| | | | | | | | |
|---|---|---|---|---|---|---|---|
| -1169.621 | 19587.525 | 1.646 | 17.233 | 0.0184567 | 7/15/2014 | 02:29.3 | 1 |
| -1169.939 | 19587.527 | 1.638 | 17.248 | 0.00930742 | 7/15/2014 | 02:29.4 | 1 |
| -1170.195 | 19587.53 | 1.641 | 17.262 | 0.0292227 | 7/15/2014 | 02:29.5 | 1 |
| -1170.451 | 19587.532 | 1.641 | 17.273 | 0.0157249 | 7/15/2014 | 02:29.6 | 1 |
| -1170.708 | 19587.534 | 1.644 | 17.281 | 0.0301518 | 7/15/2014 | 02:29.7 | 1 |
| -1171.025 | 19587.538 | 1.638 | 17.285 | 0.0166411 | 7/15/2014 | 02:29.8 | 1 |
| -1171.282 | 19587.54 | 1.64 | 17.285 | 0.0285656 | 7/15/2014 | 02:29.9 | 1 |
| -1171.537 | 19587.541 | 1.638 | 17.28 | 0.0290315 | 7/15/2014 | 02:30.0 | 1 |
| -1171.793 | 19587.544 | 1.64 | 17.271 | 0.0423421 | 7/15/2014 | 02:30.1 | 1 |
| -1172.047 | 19587.545 | 1.64 | 17.256 | 0.0431538 | 7/15/2014 | 02:30.2 | 1 |
| -1172.301 | 19587.548 | 1.644 | 17.237 | 0.0469758 | 7/15/2014 | 02:30.3 | 1 |
| -1172.616 | 19587.55 | 1.638 | 17.213 | 0.0498914 | 7/15/2014 | 02:30.4 | 1 |
| -1172.869 | 19587.552 | 1.644 | 17.187 | 0.0453289 | 7/15/2014 | 02:30.5 | 1 |
| -1173.119 | 19587.553 | 1.641 | 17.159 | 0.0700047 | 7/15/2014 | 02:30.6 | 1 |
| -1173.37 | 19587.555 | 1.641 | 17.13 | 0.0422743 | 7/15/2014 | 02:30.7 | 1 |
| -1173.68 | 19587.557 | 1.633 | 17.1 | 0.0641992 | 7/15/2014 | 02:30.8 | 1 |
| -1173.932 | 19587.559 | 1.627 | 17.072 | 0.0616518 | 7/15/2014 | 02:30.9 | 1 |
| -1174.182 | 19587.56 | 1.623 | 17.046 | 0.0331154 | 7/15/2014 | 02:31.0 | 1 |
| -1174.433 | 19587.562 | 1.629 | 17.021 | 0.0467486 | 7/15/2014 | 02:31.1 | 1 |
| -1174.682 | 19587.563 | 1.615 | 16.999 | 0.0381901 | 7/15/2014 | 02:31.2 | 1 |
| -1174.93 | 19587.564 | 1.609 | 16.98 | 0.0490378 | 7/15/2014 | 02:31.3 | 1 |
| -1175.362 | 19587.566 | 1.608 | 16.956 | 0.0689024 | 7/15/2014 | 02:31.4 | 1 |
| -1175.608 | 19587.568 | 1.605 | 16.942 | 0.0785117 | 7/15/2014 | 02:31.5 | 1 |
| -1175.855 | 19587.569 | 1.607 | 16.93 | 0.0856204 | 7/15/2014 | 02:31.6 | 1 |
| -1176.163 | 19587.572 | 1.602 | 16.918 | 0.0896984 | 7/15/2014 | 02:31.7 | 1 |
| -1176.288 | 19587.572 | 1.597 | 16.913 | 0.0969238 | 7/15/2014 | 02:31.8 | 1 |
| -1176.534 | 19587.574 | 1.596 | 16.903 | 0.0937999 | 7/15/2014 | 02:31.9 | 1 |
| -1176.783 | 19587.575 | 1.596 | 16.893 | 0.102243 | 7/15/2014 | 02:32.0 | 1 |
| -1177.031 | 19587.576 | 1.592 | 16.884 | 0.0895506 | 7/15/2014 | 02:32.1 | 1 |
| -1177.279 | 19587.579 | 1.582 | 16.875 | 0.0823086 | 7/15/2014 | 02:32.2 | 1 |
| -1177.528 | 19587.58 | 1.59 | 16.867 | 0.0802504 | 7/15/2014 | 02:32.3 | 1 |
| -1177.96 | 19587.584 | 1.581 | 16.858 | 0.0815469 | 7/15/2014 | 02:32.4 | 1 |
| -1178.205 | 19587.586 | 1.578 | 16.854 | 0.0682302 | 7/15/2014 | 02:32.5 | 1 |
| -1178.453 | 19587.588 | 1.581 | 16.851 | 0.0916436 | 7/15/2014 | 02:32.6 | 1 |
| -1178.759 | 19587.59 | 1.578 | 16.85 | 0.0619269 | 7/15/2014 | 02:32.7 | 1 |
| -1178.885 | 19587.591 | 1.58 | 16.849 | 0.0737746 | 7/15/2014 | 02:32.8 | 1 |
| -1179.133 | 19587.594 | 1.58 | 16.852 | 0.0616279 | 7/15/2014 | 02:32.9 | 1 |
| -1179.381 | 19587.597 | 1.573 | 16.857 | 0.0601751 | 7/15/2014 | 02:33.0 | 1 |
| -1179.629 | 19587.598 | 1.569 | 16.864 | 0.0285477 | 7/15/2014 | 02:33.1 | 1 |
| -1179.876 | 19587.602 | 1.573 | 16.874 | 0.0507668 | 7/15/2014 | 02:33.2 | 1 |
| -1180.124 | 19587.604 | 1.576 | 16.886 | 0.0354041 | 7/15/2014 | 02:33.3 | 1 |
| -1180.553 | 19587.607 | 1.572 | 16.909 | 0.0495215 | 7/15/2014 | 02:33.4 | 1 |
| -1180.798 | 19587.611 | 1.572 | 16.925 | 0.0563841 | 7/15/2014 | 02:33.5 | 1 |
| -1181.043 | 19587.614 | 1.572 | 16.943 | 0.0538955 | 7/15/2014 | 02:33.6 | 1 |
| -1181.349 | 19587.617 | 1.572 | 16.963 | 0.0792191 | 7/15/2014 | 02:33.7 | 1 |
| -1181.474 | 19587.619 | 1.573 | 16.972 | 0.0606134 | 7/15/2014 | 02:33.8 | 1 |
| -1181.72 | 19587.622 | 1.567 | 16.993 | 0.0616236 | 7/15/2014 | 02:33.9 | 1 |

| | | | | | | | |
|---|---|---|---|---|---|---|---|
| -1181.965 | 19587.625 | 1.561 | 17.015 | 0.0826581 | 7/15/2014 | 02:34.0 | 1 |
| -1182.213 | 19587.629 | 1.561 | 17.036 | 0.0570008 | 7/15/2014 | 02:34.1 | 1 |
| -1182.458 | 19587.633 | 1.558 | 17.057 | 0.0806871 | 7/15/2014 | 02:34.2 | 1 |
| -1182.704 | 19587.637 | 1.558 | 17.077 | 0.0706005 | 7/15/2014 | 02:34.3 | 1 |
| -1183.132 | 19587.643 | 1.56 | 17.103 | 0.067613 | 7/15/2014 | 02:34.4 | 1 |
| -1183.375 | 19587.647 | 1.556 | 17.119 | 0.0858026 | 7/15/2014 | 02:34.5 | 1 |
| -1183.62 | 19587.652 | 1.558 | 17.132 | 0.0488259 | 7/15/2014 | 02:34.6 | 1 |
| -1183.926 | 19587.658 | 1.556 | 17.143 | 0.0638236 | 7/15/2014 | 02:34.7 | 1 |
| -1184.049 | 19587.66 | 1.555 | 17.149 | 0.0330187 | 7/15/2014 | 02:34.8 | 1 |
| -1184.298 | 19587.664 | 1.557 | 17.155 | 0.0523672 | 7/15/2014 | 02:34.9 | 1 |
| -1184.545 | 19587.668 | 1.558 | 17.158 | 0.0551566 | 7/15/2014 | 02:35.0 | 1 |
| -1184.795 | 19587.674 | 1.561 | 17.159 | 0.0640996 | 7/15/2014 | 02:35.1 | 1 |
| -1185.042 | 19587.679 | 1.552 | 17.157 | 0.0590278 | 7/15/2014 | 02:35.2 | 1 |
| -1185.293 | 19587.685 | 1.557 | 17.152 | 0.0529608 | 7/15/2014 | 02:35.3 | 1 |
| -1185.602 | 19587.691 | 1.555 | 17.145 | 0.103779 | 7/15/2014 | 02:35.4 | 1 |
| -1185.853 | 19587.696 | 1.557 | 17.138 | 0.0448525 | 7/15/2014 | 02:35.5 | 1 |
| -1186.103 | 19587.701 | 1.55 | 17.13 | 0.0794329 | 7/15/2014 | 02:35.6 | 1 |
| -1186.355 | 19587.709 | 1.544 | 17.122 | 0.0660247 | 7/15/2014 | 02:35.7 | 1 |
| -1186.67 | 19587.715 | 1.536 | 17.115 | 0.0427985 | 7/15/2014 | 02:35.8 | 1 |
| -1186.925 | 19587.722 | 1.541 | 17.109 | 0.0635938 | 7/15/2014 | 02:35.9 | 1 |
| -1187.179 | 19587.729 | 1.534 | 17.106 | 0.0339354 | 7/15/2014 | 02:36.0 | 1 |
| -1187.437 | 19587.735 | 1.534 | 17.104 | 0.0423992 | 7/15/2014 | 02:36.1 | 1 |
| -1187.691 | 19587.742 | 1.532 | 17.104 | 0.0306356 | 7/15/2014 | 02:36.2 | 1 |
| -1187.946 | 19587.75 | 1.536 | 17.106 | 0.035811 | 7/15/2014 | 02:36.3 | 1 |
| -1188.264 | 19587.758 | 1.534 | 17.109 | 0.0409129 | 7/15/2014 | 02:36.4 | 1 |
| -1188.52 | 19587.766 | 1.534 | 17.114 | 0.0279904 | 7/15/2014 | 02:36.5 | 1 |
| -1188.773 | 19587.773 | 1.525 | 17.119 | 0.0407652 | 7/15/2014 | 02:36.6 | 1 |
| -1189.029 | 19587.781 | 1.525 | 17.126 | 0.0315457 | 7/15/2014 | 02:36.7 | 1 |
| -1189.343 | 19587.793 | 1.513 | 17.133 | 0.0498125 | 7/15/2014 | 02:36.8 | 1 |
| -1189.597 | 19587.801 | 1.513 | 17.142 | 0.0293248 | 7/15/2014 | 02:36.9 | 1 |
| -1189.849 | 19587.809 | 1.505 | 17.152 | 0.0271761 | 7/15/2014 | 02:37.0 | 1 |
| -1190.101 | 19587.818 | 1.507 | 17.163 | 0.0274367 | 7/15/2014 | 02:37.1 | 1 |
| -1190.35 | 19587.826 | 1.506 | 17.177 | 0.0272215 | 7/15/2014 | 02:37.2 | 1 |
| -1190.601 | 19587.836 | 1.509 | 17.192 | 0.0233363 | 7/15/2014 | 02:37.3 | 1 |
| -1191.031 | 19587.854 | 1.507 | 17.222 | 0.033754 | 7/15/2014 | 02:37.4 | 1 |
| -1191.276 | 19587.861 | 1.51 | 17.243 | 0.0170558 | 7/15/2014 | 02:37.5 | 1 |
| -1191.521 | 19587.871 | 1.508 | 17.268 | 0.0141016 | 7/15/2014 | 02:37.6 | 1 |
| -1191.826 | 19587.885 | 1.51 | 17.295 | 0.0279969 | 7/15/2014 | 02:37.7 | 1 |
| -1191.949 | 19587.889 | 1.507 | 17.308 | 0.0240677 | 7/15/2014 | 02:37.8 | 1 |
| -1192.195 | 19587.899 | 1.507 | 17.339 | 0.0199033 | 7/15/2014 | 02:37.9 | 1 |
| -1192.438 | 19587.908 | 1.501 | 17.372 | 0.0233352 | 7/15/2014 | 02:38.0 | 1 |
| -1192.685 | 19587.92 | 1.507 | 17.405 | 0.0201672 | 7/15/2014 | 02:38.1 | 1 |
| -1192.927 | 19587.93 | 1.506 | 17.439 | 0.0257864 | 7/15/2014 | 02:38.2 | 1 |
| -1193.171 | 19587.941 | 1.51 | 17.47 | 0.0232886 | 7/15/2014 | 02:38.3 | 1 |
| -1193.595 | 19587.961 | 1.512 | 17.51 | 0.00252114 | 7/15/2014 | 02:38.4 | 1 |
| -1193.835 | 19587.973 | 1.514 | 17.532 | 0.020449 | 7/15/2014 | 02:38.5 | 1 |
| -1194.076 | 19587.984 | 1.514 | 17.549 | 0.0231836 | 7/15/2014 | 02:38.6 | 1 |

| | | | | | | | |
|---|---|---|---|---|---|---|---|
| -1194.376 | 19587.999 | 1.514 | 17.561 | 0.0372315 | 7/15/2014 | 02:38.7 | 1 |
| -1194.497 | 19588.005 | 1.511 | 17.569 | 0.043911 | 7/15/2014 | 02:38.8 | 1 |
| -1194.74 | 19588.018 | 1.511 | 17.572 | 0.0703737 | 7/15/2014 | 02:38.9 | 1 |
| -1194.981 | 19588.03 | 1.509 | 17.571 | 0.0797808 | 7/15/2014 | 02:39.0 | 1 |
| -1195.224 | 19588.043 | 1.508 | 17.564 | 0.0964976 | 7/15/2014 | 02:39.1 | 1 |
| -1195.464 | 19588.057 | 1.502 | 17.554 | 0.102718 | 7/15/2014 | 02:39.2 | 1 |
| -1195.703 | 19588.07 | 1.5 | 17.542 | 0.125275 | 7/15/2014 | 02:39.3 | 1 |
| -1196.121 | 19588.094 | 1.5 | 17.521 | 0.123493 | 7/15/2014 | 02:39.4 | 1 |
| -1196.36 | 19588.108 | 1.5 | 17.508 | 0.107451 | 7/15/2014 | 02:39.5 | 1 |
| -1196.598 | 19588.123 | 1.5 | 17.495 | 0.111842 | 7/15/2014 | 02:39.6 | 1 |
| -1196.895 | 19588.141 | 1.498 | 17.484 | 0.0924379 | 7/15/2014 | 02:39.7 | 1 |
| -1197.015 | 19588.147 | 1.497 | 17.477 | 0.0870439 | 7/15/2014 | 02:39.8 | 1 |
| -1197.254 | 19588.163 | 1.502 | 17.469 | 0.0501597 | 7/15/2014 | 02:39.9 | 1 |
| -1197.493 | 19588.179 | 1.497 | 17.462 | 0.0307977 | 7/15/2014 | 02:40.0 | 1 |
| -1197.733 | 19588.193 | 1.497 | 17.458 | 0.0167522 | 7/15/2014 | 02:40.1 | 1 |
| -1197.97 | 19588.21 | 1.492 | 17.456 | 0.0229946 | 7/15/2014 | 02:40.2 | 1 |
| -1198.208 | 19588.226 | 1.492 | 17.454 | 0.0413399 | 7/15/2014 | 02:40.3 | 1 |
| -1198.62 | 19588.254 | 1.484 | 17.454 | 0.0408002 | 7/15/2014 | 02:40.4 | 1 |
| -1198.854 | 19588.271 | 1.479 | 17.454 | 0.0494969 | 7/15/2014 | 02:40.5 | 1 |
| -1199.087 | 19588.288 | 1.475 | 17.454 | 0.0519756 | 7/15/2014 | 02:40.6 | 1 |
| -1199.377 | 19588.309 | 1.477 | 17.454 | 0.0514909 | 7/15/2014 | 02:40.7 | 1 |
| -1199.495 | 19588.317 | 1.473 | 17.455 | 0.0621271 | 7/15/2014 | 02:40.8 | 1 |
| -1199.729 | 19588.334 | 1.475 | 17.454 | 0.0422727 | 7/15/2014 | 02:40.9 | 1 |
| -1200.075 | 19588.36 | 1.475 | 17.453 | 0.043745 | 7/15/2014 | 02:41.0 | 1 |
| -1200.307 | 19588.379 | 1.478 | 17.452 | 0.0688304 | 7/15/2014 | 02:41.1 | 1 |
| -1200.539 | 19588.397 | 1.478 | 17.449 | 0.0540378 | 7/15/2014 | 02:41.2 | 1 |
| -1200.769 | 19588.416 | 1.481 | 17.446 | 0.121527 | 7/15/2014 | 02:41.3 | 1 |
| -1201.055 | 19588.438 | 1.481 | 17.442 | 0.0658697 | 7/15/2014 | 02:41.4 | 1 |
| -1201.284 | 19588.455 | 1.476 | 17.438 | 0.0944149 | 7/15/2014 | 02:41.5 | 1 |
| -1201.513 | 19588.475 | 1.474 | 17.432 | 0.0712052 | 7/15/2014 | 02:41.6 | 1 |
| -1201.803 | 19588.498 | 1.466 | 17.426 | 0.0741027 | 7/15/2014 | 02:41.7 | 1 |
| -1201.917 | 19588.508 | 1.463 | 17.423 | 0.0524403 | 7/15/2014 | 02:41.8 | 1 |
| -1202.152 | 19588.527 | 1.46 | 17.417 | 0.0561127 | 7/15/2014 | 02:41.9 | 1 |
| -1202.388 | 19588.549 | 1.457 | 17.41 | 0.0373488 | 7/15/2014 | 02:42.0 | 1 |
| -1202.627 | 19588.568 | 1.456 | 17.403 | 0.0361564 | 7/15/2014 | 02:42.1 | 1 |
| -1202.864 | 19588.588 | 1.45 | 17.396 | 0.0364634 | 7/15/2014 | 02:42.2 | 1 |
| -1203.103 | 19588.609 | 1.45 | 17.391 | 0.0420489 | 7/15/2014 | 02:42.3 | 1 |
| -1203.525 | 19588.647 | 1.449 | 17.386 | 0.0490791 | 7/15/2014 | 02:42.4 | 1 |
| -1203.768 | 19588.668 | 1.453 | 17.385 | 0.0360509 | 7/15/2014 | 02:42.5 | 1 |
| -1204.011 | 19588.692 | 1.457 | 17.387 | 0.0463093 | 7/15/2014 | 02:42.6 | 1 |
| -1204.319 | 19588.72 | 1.453 | 17.391 | 0.0302652 | 7/15/2014 | 02:42.7 | 1 |
| -1204.442 | 19588.73 | 1.452 | 17.392 | 0.0330018 | 7/15/2014 | 02:42.8 | 1 |
| -1204.69 | 19588.755 | 1.456 | 17.4 | 0.0308669 | 7/15/2014 | 02:42.9 | 1 |
| -1204.94 | 19588.777 | 1.456 | 17.41 | 0.00989321 | 7/15/2014 | 02:43.0 | 1 |
| -1205.191 | 19588.802 | 1.456 | 17.423 | 0.0168984 | 7/15/2014 | 02:43.1 | 1 |
| -1205.441 | 19588.825 | 1.447 | 17.438 | 0.013854 | 7/15/2014 | 02:43.2 | 1 |
| -1205.692 | 19588.851 | 1.447 | 17.453 | 0.015575 | 7/15/2014 | 02:43.3 | 1 |

| | | | | | | | |
|---|---|---|---|---|---|---|---|
| -1206.005 | 19588.88 | 1.441 | 17.469 | 0.0102195 | 7/15/2014 | 02:43.4 | 1 |
| -1206.256 | 19588.905 | 1.439 | 17.484 | 0.0149866 | 7/15/2014 | 02:43.5 | 1 |
| -1206.507 | 19588.93 | 1.43 | 17.499 | 0.0177289 | 7/15/2014 | 02:43.6 | 1 |
| -1206.758 | 19588.955 | 1.43 | 17.513 | 0.0292664 | 7/15/2014 | 02:43.7 | 1 |
| -1207.069 | 19588.986 | 1.426 | 17.525 | 0.0263165 | 7/15/2014 | 02:43.8 | 1 |
| -1207.321 | 19589.012 | 1.424 | 17.535 | 0.0276182 | 7/15/2014 | 02:43.9 | 1 |
| -1207.57 | 19589.038 | 1.42 | 17.542 | 0.0299873 | 7/15/2014 | 02:44.0 | 1 |
| -1207.82 | 19589.063 | 1.422 | 17.546 | 0.0199659 | 7/15/2014 | 02:44.1 | 1 |
| -1208.068 | 19589.089 | 1.422 | 17.549 | 0.0110232 | 7/15/2014 | 02:44.2 | 1 |
| -1208.316 | 19589.115 | 1.427 | 17.548 | 0.0503777 | 7/15/2014 | 02:44.3 | 1 |
| -1208.743 | 19589.159 | 1.423 | 17.545 | 0.0301524 | 7/15/2014 | 02:44.4 | 1 |
| -1208.987 | 19589.186 | 1.425 | 17.542 | 0.0451263 | 7/15/2014 | 02:44.5 | 1 |
| -1209.229 | 19589.213 | 1.426 | 17.538 | 0.0342207 | 7/15/2014 | 02:44.6 | 1 |
| -1209.532 | 19589.245 | 1.421 | 17.536 | 0.0558255 | 7/15/2014 | 02:44.7 | 1 |
| -1209.654 | 19589.258 | 1.417 | 17.534 | 0.0490575 | 7/15/2014 | 02:44.8 | 1 |
| -1209.898 | 19589.284 | 1.417 | 17.532 | 0.0419675 | 7/15/2014 | 02:44.9 | 1 |
| -1210.14 | 19589.31 | 1.412 | 17.532 | 0.0285598 | 7/15/2014 | 02:45.0 | 1 |
| -1210.383 | 19589.336 | 1.415 | 17.533 | 0.0467472 | 7/15/2014 | 02:45.1 | 1 |
| -1210.624 | 19589.363 | 1.415 | 17.536 | 0.0255709 | 7/15/2014 | 02:45.2 | 1 |
| -1210.865 | 19589.391 | 1.409 | 17.54 | 0.0222149 | 7/15/2014 | 02:45.3 | 1 |
| -1211.286 | 19589.438 | 1.401 | 17.549 | 0.0227112 | 7/15/2014 | 02:45.4 | 1 |
| -1211.527 | 19589.464 | 1.406 | 17.557 | 0.00696831 | 7/15/2014 | 02:45.5 | 1 |
| -1211.767 | 19589.491 | 1.404 | 17.566 | 0.038753 | 7/15/2014 | 02:45.6 | 1 |
| -1212.069 | 19589.526 | 1.403 | 17.577 | 0.0158149 | 7/15/2014 | 02:45.7 | 1 |
| -1212.19 | 19589.54 | 1.397 | 17.582 | 0.0270927 | 7/15/2014 | 02:45.8 | 1 |
| -1212.433 | 19589.568 | 1.397 | 17.596 | 0.0358837 | 7/15/2014 | 02:45.9 | 1 |
| -1212.678 | 19589.596 | 1.401 | 17.611 | 0.00743318 | 7/15/2014 | 02:46.0 | 1 |
| -1212.923 | 19589.624 | 1.403 | 17.627 | 0.0717816 | 7/15/2014 | 02:46.1 | 1 |
| -1213.168 | 19589.652 | 1.403 | 17.643 | 0.0283487 | 7/15/2014 | 02:46.2 | 1 |
| -1213.414 | 19589.683 | 1.405 | 17.659 | 0.0371916 | 7/15/2014 | 02:46.3 | 1 |
| -1213.845 | 19589.734 | 1.4 | 17.678 | 0.0405438 | 7/15/2014 | 02:46.4 | 1 |
| -1213.966 | 19589.749 | 1.398 | 17.685 | 0.038541 | 7/15/2014 | 02:46.5 | 1 |
| -1214.213 | 19589.777 | 1.398 | 17.694 | 0.0586664 | 7/15/2014 | 02:46.6 | 1 |
| -1214.461 | 19589.808 | 1.395 | 17.699 | 0.010018 | 7/15/2014 | 02:46.7 | 1 |
| -1214.77 | 19589.845 | 1.395 | 17.701 | 0.0440244 | 7/15/2014 | 02:46.8 | 1 |
| -1215.02 | 19589.876 | 1.407 | 17.698 | 0.0470097 | 7/15/2014 | 02:46.9 | 1 |
| -1215.268 | 19589.906 | 1.404 | 17.691 | 0.0624115 | 7/15/2014 | 02:47.0 | 1 |
| -1215.519 | 19589.938 | 1.409 | 17.68 | 0.0872042 | 7/15/2014 | 02:47.1 | 1 |
| -1215.768 | 19589.969 | 1.413 | 17.667 | 0.102283 | 7/15/2014 | 02:47.2 | 1 |
| -1216.017 | 19590 | 1.418 | 17.652 | 0.112682 | 7/15/2014 | 02:47.3 | 1 |
| -1216.325 | 19590.038 | 1.418 | 17.635 | 0.106519 | 7/15/2014 | 02:47.4 | 1 |
| -1216.574 | 19590.07 | 1.424 | 17.618 | 0.150613 | 7/15/2014 | 02:47.5 | 1 |
| -1216.82 | 19590.103 | 1.411 | 17.602 | 0.109033 | 7/15/2014 | 02:47.6 | 1 |
| -1217.068 | 19590.134 | 1.416 | 17.588 | 0.0954929 | 7/15/2014 | 02:47.7 | 1 |
| -1217.374 | 19590.174 | 1.42 | 17.577 | 0.0791685 | 7/15/2014 | 02:47.8 | 1 |
| -1217.621 | 19590.205 | 1.422 | 17.568 | 0.059516 | 7/15/2014 | 02:47.9 | 1 |
| -1217.867 | 19590.236 | 1.42 | 17.561 | 0.0493277 | 7/15/2014 | 02:48.0 | 1 |

| | | | | | | | |
|---|---|---|---|---|---|---|---|
| -1218.113 | 19590.271 | 1.426 | 17.557 | 0.0566726 | 7/15/2014 | 02:48.1 | 1 |
| -1218.356 | 19590.303 | 1.426 | 17.554 | 0.0380837 | 7/15/2014 | 02:48.2 | 1 |
| -1218.6 | 19590.334 | 1.434 | 17.55 | 0.0624889 | 7/15/2014 | 02:48.3 | 1 |
| -1219.022 | 19590.391 | 1.436 | 17.541 | 0.0794122 | 7/15/2014 | 02:48.4 | 1 |
| -1219.262 | 19590.422 | 1.436 | 17.533 | 0.0832141 | 7/15/2014 | 02:48.5 | 1 |
| -1219.502 | 19590.455 | 1.439 | 17.521 | 0.0785148 | 7/15/2014 | 02:48.6 | 1 |
| -1219.801 | 19590.496 | 1.441 | 17.506 | 0.101195 | 7/15/2014 | 02:48.7 | 1 |
| -1219.922 | 19590.512 | 1.443 | 17.499 | 0.0959186 | 7/15/2014 | 02:48.8 | 1 |
| -1220.163 | 19590.543 | 1.445 | 17.479 | 0.0941969 | 7/15/2014 | 02:48.9 | 1 |
| -1220.404 | 19590.578 | 1.449 | 17.457 | 0.105708 | 7/15/2014 | 02:49.0 | 1 |
| -1220.644 | 19590.61 | 1.455 | 17.433 | 0.107115 | 7/15/2014 | 02:49.1 | 1 |
| -1220.884 | 19590.643 | 1.455 | 17.408 | 0.112876 | 7/15/2014 | 02:49.2 | 1 |
| -1221.124 | 19590.678 | 1.461 | 17.384 | 0.123212 | 7/15/2014 | 02:49.3 | 1 |
| -1221.541 | 19590.734 | 1.466 | 17.351 | 0.117577 | 7/15/2014 | 02:49.4 | 1 |
| -1221.779 | 19590.768 | 1.472 | 17.331 | 0.118272 | 7/15/2014 | 02:49.5 | 1 |
| -1222.017 | 19590.801 | 1.479 | 17.312 | 0.121795 | 7/15/2014 | 02:49.6 | 1 |
| -1222.312 | 19590.843 | 1.479 | 17.296 | 0.11322 | 7/15/2014 | 02:49.7 | 1 |
| -1222.433 | 19590.859 | 1.478 | 17.287 | 0.111957 | 7/15/2014 | 02:49.8 | 1 |
| -1222.672 | 19590.895 | 1.487 | 17.274 | 0.113167 | 7/15/2014 | 02:49.9 | 1 |
| -1222.91 | 19590.928 | 1.495 | 17.263 | 0.111873 | 7/15/2014 | 02:50.0 | 1 |
| -1223.149 | 19590.962 | 1.5 | 17.254 | 0.101745 | 7/15/2014 | 02:50.1 | 1 |
| -1223.386 | 19590.996 | 1.5 | 17.246 | 0.100048 | 7/15/2014 | 02:50.2 | 1 |
| -1223.624 | 19591.029 | 1.505 | 17.239 | 0.0970427 | 7/15/2014 | 02:50.3 | 1 |
| -1224.036 | 19591.09 | 1.51 | 17.23 | 0.12296 | 7/15/2014 | 02:50.4 | 1 |
| -1224.273 | 19591.125 | 1.514 | 17.223 | 0.114414 | 7/15/2014 | 02:50.5 | 1 |
| -1224.509 | 19591.158 | 1.519 | 17.215 | 0.123429 | 7/15/2014 | 02:50.6 | 1 |
| -1224.806 | 19591.202 | 1.521 | 17.206 | 0.137628 | 7/15/2014 | 02:50.7 | 1 |
| -1224.924 | 19591.219 | 1.521 | 17.202 | 0.145656 | 7/15/2014 | 02:50.8 | 1 |
| -1225.165 | 19591.254 | 1.534 | 17.191 | 0.144267 | 7/15/2014 | 02:50.9 | 1 |
| -1225.405 | 19591.289 | 1.537 | 17.179 | 0.138136 | 7/15/2014 | 02:51.0 | 1 |
| -1225.647 | 19591.326 | 1.547 | 17.166 | 0.15473 | 7/15/2014 | 02:51.1 | 1 |
| -1225.889 | 19591.361 | 1.547 | 17.152 | 0.134718 | 7/15/2014 | 02:51.2 | 1 |
| -1226.13 | 19591.396 | 1.559 | 17.138 | 0.107334 | 7/15/2014 | 02:51.3 | 1 |
| -1226.556 | 19591.459 | 1.56 | 17.116 | 0.12675 | 7/15/2014 | 02:51.4 | 1 |
| -1226.799 | 19591.496 | 1.565 | 17.102 | 0.0993262 | 7/15/2014 | 02:51.5 | 1 |
| -1227.042 | 19591.531 | 1.567 | 17.088 | 0.0984367 | 7/15/2014 | 02:51.6 | 1 |
| -1227.349 | 19591.578 | 1.571 | 17.074 | 0.0775189 | 7/15/2014 | 02:51.7 | 1 |
| -1227.472 | 19591.596 | 1.57 | 17.067 | 0.0861546 | 7/15/2014 | 02:51.8 | 1 |
| -1227.718 | 19591.634 | 1.576 | 17.054 | 0.0883401 | 7/15/2014 | 02:51.9 | 1 |
| -1227.965 | 19591.67 | 1.58 | 17.042 | 0.0761805 | 7/15/2014 | 02:52.0 | 1 |
| -1228.214 | 19591.707 | 1.59 | 17.03 | 0.0544655 | 7/15/2014 | 02:52.1 | 1 |
| -1228.461 | 19591.746 | 1.584 | 17.018 | 0.0718086 | 7/15/2014 | 02:52.2 | 1 |
| -1228.709 | 19591.781 | 1.584 | 17.006 | 0.0863307 | 7/15/2014 | 02:52.3 | 1 |
| -1229.017 | 19591.828 | 1.581 | 16.994 | 0.0788428 | 7/15/2014 | 02:52.4 | 1 |
| -1229.264 | 19591.867 | 1.584 | 16.982 | 0.087681 | 7/15/2014 | 02:52.5 | 1 |
| -1229.512 | 19591.902 | 1.581 | 16.969 | 0.102982 | 7/15/2014 | 02:52.6 | 1 |
| -1229.759 | 19591.941 | 1.575 | 16.956 | 0.111581 | 7/15/2014 | 02:52.7 | 1 |

| | | | | | | | |
|---|---|---|---|---|---|---|---|
| -1230.069 | 19591.988 | 1.575 | 16.943 | 0.115602 | 7/15/2014 | 02:52.8 | 1 |
| -1230.318 | 19592.025 | 1.584 | 16.929 | 0.124237 | 7/15/2014 | 02:52.9 | 1 |
| -1230.568 | 19592.064 | 1.584 | 16.915 | 0.129265 | 7/15/2014 | 02:53.0 | 1 |
| -1230.819 | 19592.104 | 1.587 | 16.901 | 0.141281 | 7/15/2014 | 02:53.1 | 1 |
| -1231.069 | 19592.139 | 1.586 | 16.887 | 0.15847 | 7/15/2014 | 02:53.2 | 1 |
| -1231.319 | 19592.178 | 1.595 | 16.874 | 0.152174 | 7/15/2014 | 02:53.3 | 1 |
| -1231.629 | 19592.225 | 1.595 | 16.861 | 0.159585 | 7/15/2014 | 02:53.4 | 1 |
| -1231.879 | 19592.264 | 1.598 | 16.848 | 0.143941 | 7/15/2014 | 02:53.5 | 1 |
| -1232.129 | 19592.303 | 1.598 | 16.835 | 0.141715 | 7/15/2014 | 02:53.6 | 1 |
| -1232.38 | 19592.342 | 1.604 | 16.823 | 0.129808 | 7/15/2014 | 02:53.7 | 1 |
| -1232.691 | 19592.387 | 1.602 | 16.81 | 0.109168 | 7/15/2014 | 02:53.8 | 1 |
| -1232.942 | 19592.426 | 1.604 | 16.798 | 0.107419 | 7/15/2014 | 02:53.9 | 1 |
| -1233.191 | 19592.463 | 1.598 | 16.785 | 0.095797 | 7/15/2014 | 02:54.0 | 1 |
| -1233.443 | 19592.502 | 1.603 | 16.771 | 0.0940819 | 7/15/2014 | 02:54.1 | 1 |
| -1233.693 | 19592.541 | 1.602 | 16.757 | 0.0936002 | 7/15/2014 | 02:54.2 | 1 |
| -1233.942 | 19592.58 | 1.604 | 16.743 | 0.0975957 | 7/15/2014 | 02:54.3 | 1 |
| -1234.252 | 19592.627 | 1.604 | 16.728 | 0.106764 | 7/15/2014 | 02:54.4 | 1 |
| -1234.5 | 19592.664 | 1.609 | 16.713 | 0.105122 | 7/15/2014 | 02:54.5 | 1 |
| -1234.746 | 19592.701 | 1.607 | 16.698 | 0.121299 | 7/15/2014 | 02:54.6 | 1 |
| -1234.993 | 19592.74 | 1.609 | 16.682 | 0.131544 | 7/15/2014 | 02:54.7 | 1 |
| -1235.3 | 19592.787 | 1.609 | 16.666 | 0.138145 | 7/15/2014 | 02:54.8 | 1 |
| -1235.547 | 19592.825 | 1.615 | 16.65 | 0.14686 | 7/15/2014 | 02:54.9 | 1 |
| -1235.794 | 19592.863 | 1.612 | 16.634 | 0.146192 | 7/15/2014 | 02:55.0 | 1 |
| -1236.041 | 19592.9 | 1.612 | 16.617 | 0.158629 | 7/15/2014 | 02:55.1 | 1 |
| -1236.287 | 19592.939 | 1.615 | 16.602 | 0.140229 | 7/15/2014 | 02:55.2 | 1 |
| -1236.533 | 19592.978 | 1.624 | 16.587 | 0.134017 | 7/15/2014 | 02:55.3 | 1 |
| -1236.962 | 19593.043 | 1.631 | 16.569 | 0.132645 | 7/15/2014 | 02:55.4 | 1 |
| -1237.084 | 19593.062 | 1.635 | 16.562 | 0.126034 | 7/15/2014 | 02:55.5 | 1 |
| -1237.45 | 19593.119 | 1.643 | 16.55 | 0.125736 | 7/15/2014 | 02:55.6 | 1 |
| -1237.754 | 19593.166 | 1.646 | 16.544 | 0.131167 | 7/15/2014 | 02:55.7 | 1 |
| -1237.878 | 19593.186 | 1.648 | 16.54 | 0.124091 | 7/15/2014 | 02:55.8 | 1 |
| -1238.124 | 19593.224 | 1.654 | 16.538 | 0.131178 | 7/15/2014 | 02:55.9 | 1 |
| -1238.369 | 19593.261 | 1.658 | 16.538 | 0.128804 | 7/15/2014 | 02:56.0 | 1 |
| -1238.615 | 19593.299 | 1.664 | 16.539 | 0.130003 | 7/15/2014 | 02:56.1 | 1 |
| -1238.858 | 19593.338 | 1.663 | 16.542 | 0.122868 | 7/15/2014 | 02:56.2 | 1 |
| -1239.104 | 19593.377 | 1.67 | 16.546 | 0.115863 | 7/15/2014 | 02:56.3 | 1 |
| -1239.528 | 19593.442 | 1.667 | 16.553 | 0.11147 | 7/15/2014 | 02:56.4 | 1 |
| -1239.772 | 19593.481 | 1.67 | 16.557 | 0.100061 | 7/15/2014 | 02:56.5 | 1 |
| -1240.015 | 19593.518 | 1.676 | 16.56 | 0.113091 | 7/15/2014 | 02:56.6 | 1 |
| -1240.318 | 19593.566 | 1.679 | 16.563 | 0.0937047 | 7/15/2014 | 02:56.7 | 1 |
| -1240.44 | 19593.586 | 1.68 | 16.565 | 0.0943391 | 7/15/2014 | 02:56.8 | 1 |
| -1240.686 | 19593.625 | 1.69 | 16.566 | 0.102745 | 7/15/2014 | 02:56.9 | 1 |
| -1240.931 | 19593.664 | 1.686 | 16.566 | 0.110028 | 7/15/2014 | 02:57.0 | 1 |
| -1241.177 | 19593.702 | 1.688 | 16.566 | 0.101301 | 7/15/2014 | 02:57.1 | 1 |
| -1241.424 | 19593.741 | 1.697 | 16.566 | 0.107869 | 7/15/2014 | 02:57.2 | 1 |
| -1241.67 | 19593.78 | 1.708 | 16.566 | 0.105366 | 7/15/2014 | 02:57.3 | 1 |
| -1241.975 | 19593.83 | 1.705 | 16.566 | 0.107222 | 7/15/2014 | 02:57.4 | 1 |

| | | | | | | | |
|---|---|---|---|---|---|---|---|
| -1242.223 | 19593.869 | 1.704 | 16.567 | 0.113056 | 7/15/2014 | 02:57.5 | 1 |
| -1242.469 | 19593.909 | 1.704 | 16.568 | 0.117834 | 7/15/2014 | 02:57.6 | 1 |
| -1242.717 | 19593.948 | 1.713 | 16.568 | 0.128411 | 7/15/2014 | 02:57.7 | 1 |
| -1243.026 | 19593.997 | 1.716 | 16.569 | 0.143183 | 7/15/2014 | 02:57.8 | 1 |
| -1243.275 | 19594.037 | 1.714 | 16.569 | 0.148299 | 7/15/2014 | 02:57.9 | 1 |
| -1243.523 | 19594.078 | 1.714 | 16.569 | 0.150588 | 7/15/2014 | 02:58.0 | 1 |
| -1243.774 | 19594.118 | 1.725 | 16.57 | 0.145558 | 7/15/2014 | 02:58.1 | 1 |
| -1244.022 | 19594.158 | 1.733 | 16.57 | 0.147515 | 7/15/2014 | 02:58.2 | 1 |
| -1244.271 | 19594.198 | 1.742 | 16.572 | 0.1458 | 7/15/2014 | 02:58.3 | 1 |
| -1244.579 | 19594.247 | 1.742 | 16.575 | 0.141435 | 7/15/2014 | 02:58.4 | 1 |
| -1244.829 | 19594.287 | 1.748 | 16.579 | 0.138044 | 7/15/2014 | 02:58.5 | 1 |
| -1245.076 | 19594.327 | 1.756 | 16.585 | 0.137564 | 7/15/2014 | 02:58.6 | 1 |
| -1245.326 | 19594.368 | 1.766 | 16.593 | 0.139805 | 7/15/2014 | 02:58.7 | 1 |
| -1245.634 | 19594.417 | 1.766 | 16.602 | 0.139762 | 7/15/2014 | 02:58.8 | 1 |
| -1245.886 | 19594.458 | 1.77 | 16.612 | 0.136923 | 7/15/2014 | 02:58.9 | 1 |
| -1246.133 | 19594.497 | 1.766 | 16.622 | 0.141677 | 7/15/2014 | 02:59.0 | 1 |
| -1246.385 | 19594.538 | 1.776 | 16.632 | 0.143259 | 7/15/2014 | 02:59.1 | 1 |
| -1246.636 | 19594.577 | 1.778 | 16.642 | 0.156829 | 7/15/2014 | 02:59.2 | 1 |
| -1246.886 | 19594.619 | 1.781 | 16.649 | 0.143783 | 7/15/2014 | 02:59.3 | 1 |
| -1247.197 | 19594.669 | 1.781 | 16.654 | 0.147122 | 7/15/2014 | 02:59.4 | 1 |
| -1247.448 | 19594.709 | 1.781 | 16.656 | 0.13966 | 7/15/2014 | 02:59.5 | 1 |
| -1247.697 | 19594.749 | 1.781 | 16.654 | 0.142955 | 7/15/2014 | 02:59.6 | 1 |
| -1247.95 | 19594.79 | 1.787 | 16.649 | 0.13896 | 7/15/2014 | 02:59.7 | 1 |
| -1248.263 | 19594.839 | 1.783 | 16.639 | 0.131695 | 7/15/2014 | 02:59.8 | 1 |
| -1248.518 | 19594.881 | 1.789 | 16.627 | 0.13085 | 7/15/2014 | 02:59.9 | 1 |
| -1248.77 | 19594.922 | 1.784 | 16.611 | 0.128646 | 7/15/2014 | 03:00.0 | 1 |
| -1249.024 | 19594.962 | 1.786 | 16.593 | 0.132193 | 7/15/2014 | 03:00.1 | 1 |
| -1249.277 | 19595.002 | 1.792 | 16.573 | 0.125023 | 7/15/2014 | 03:00.2 | 1 |
| -1249.53 | 19595.043 | 1.794 | 16.553 | 0.116756 | 7/15/2014 | 03:00.3 | 1 |
| -1249.844 | 19595.093 | 1.798 | 16.532 | 0.11595 | 7/15/2014 | 03:00.4 | 1 |
| -1250.097 | 19595.134 | 1.804 | 16.513 | 0.119285 | 7/15/2014 | 03:00.5 | 1 |
| -1250.348 | 19595.174 | 1.804 | 16.494 | 0.119025 | 7/15/2014 | 03:00.6 | 1 |
| -1250.6 | 19595.214 | 1.812 | 16.477 | 0.113601 | 7/15/2014 | 03:00.7 | 1 |
| -1250.913 | 19595.264 | 1.809 | 16.463 | 0.114683 | 7/15/2014 | 03:00.8 | 1 |
| -1251.165 | 19595.304 | 1.805 | 16.451 | 0.125225 | 7/15/2014 | 03:00.9 | 1 |
| -1251.416 | 19595.344 | 1.803 | 16.442 | 0.125007 | 7/15/2014 | 03:01.0 | 1 |
| -1251.669 | 19595.384 | 1.805 | 16.435 | 0.126962 | 7/15/2014 | 03:01.1 | 1 |
| -1251.919 | 19595.424 | 1.805 | 16.43 | 0.138035 | 7/15/2014 | 03:01.2 | 1 |
| -1252.169 | 19595.463 | 1.805 | 16.427 | 0.133208 | 7/15/2014 | 03:01.3 | 1 |
| -1252.48 | 19595.514 | 1.799 | 16.425 | 0.139925 | 7/15/2014 | 03:01.4 | 1 |
| -1252.73 | 19595.553 | 1.805 | 16.424 | 0.141396 | 7/15/2014 | 03:01.5 | 1 |
| -1252.977 | 19595.592 | 1.805 | 16.423 | 0.145473 | 7/15/2014 | 03:01.6 | 1 |
| -1253.227 | 19595.631 | 1.814 | 16.422 | 0.151223 | 7/15/2014 | 03:01.7 | 1 |
| -1253.535 | 19595.68 | 1.814 | 16.42 | 0.154622 | 7/15/2014 | 03:01.8 | 1 |
| -1253.784 | 19595.72 | 1.818 | 16.417 | 0.163443 | 7/15/2014 | 03:01.9 | 1 |
| -1254.031 | 19595.759 | 1.818 | 16.413 | 0.170731 | 7/15/2014 | 03:02.0 | 1 |
| -1254.281 | 19595.799 | 1.829 | 16.408 | 0.172983 | 7/15/2014 | 03:02.1 | 1 |

| | | | | | | | |
|---|---|---|---|---|---|---|---|
| -1254.527 | 19595.837 | 1.833 | 16.403 | 0.176286 | 7/15/2014 | 03:02.2 | 1 |
| -1254.776 | 19595.877 | 1.842 | 16.396 | 0.172667 | 7/15/2014 | 03:02.3 | 1 |
| -1255.204 | 19595.943 | 1.844 | 16.385 | 0.172502 | 7/15/2014 | 03:02.4 | 1 |
| -1255.448 | 19595.982 | 1.842 | 16.379 | 0.166465 | 7/15/2014 | 03:02.5 | 1 |
| -1255.692 | 19596.021 | 1.842 | 16.373 | 0.172566 | 7/15/2014 | 03:02.6 | 1 |
| -1255.996 | 19596.069 | 1.844 | 16.368 | 0.170603 | 7/15/2014 | 03:02.7 | 1 |
| -1256.119 | 19596.088 | 1.843 | 16.365 | 0.169002 | 7/15/2014 | 03:02.8 | 1 |
| -1256.366 | 19596.128 | 1.842 | 16.362 | 0.169798 | 7/15/2014 | 03:02.9 | 1 |
| -1256.61 | 19596.165 | 1.839 | 16.361 | 0.173505 | 7/15/2014 | 03:03.0 | 1 |
| -1256.856 | 19596.204 | 1.848 | 16.363 | 0.177229 | 7/15/2014 | 03:03.1 | 1 |
| -1257.099 | 19596.242 | 1.842 | 16.367 | 0.172554 | 7/15/2014 | 03:03.2 | 1 |
| -1257.344 | 19596.28 | 1.846 | 16.375 | 0.171195 | 7/15/2014 | 03:03.3 | 1 |
| -1257.767 | 19596.347 | 1.843 | 16.396 | 0.15647 | 7/15/2014 | 03:03.4 | 1 |
| -1258.009 | 19596.384 | 1.843 | 16.414 | 0.14152 | 7/15/2014 | 03:03.5 | 1 |
| -1258.25 | 19596.422 | 1.843 | 16.435 | 0.138488 | 7/15/2014 | 03:03.6 | 1 |
| -1258.549 | 19596.469 | 1.843 | 16.46 | 0.121853 | 7/15/2014 | 03:03.7 | 1 |
| -1258.671 | 19596.488 | 1.838 | 16.471 | 0.115295 | 7/15/2014 | 03:03.8 | 1 |
| -1258.912 | 19596.524 | 1.838 | 16.501 | 0.110837 | 7/15/2014 | 03:03.9 | 1 |
| -1259.152 | 19596.561 | 1.829 | 16.532 | 0.108456 | 7/15/2014 | 03:04.0 | 1 |
| -1259.393 | 19596.6 | 1.832 | 16.565 | 0.109479 | 7/15/2014 | 03:04.1 | 1 |
| -1259.632 | 19596.635 | 1.829 | 16.597 | 0.106146 | 7/15/2014 | 03:04.2 | 1 |
| -1259.871 | 19596.672 | 1.832 | 16.629 | 0.122871 | 7/15/2014 | 03:04.3 | 1 |
| -1260.286 | 19596.734 | 1.832 | 16.672 | 0.126958 | 7/15/2014 | 03:04.4 | 1 |
| -1260.522 | 19596.77 | 1.832 | 16.698 | 0.12737 | 7/15/2014 | 03:04.5 | 1 |
| -1260.758 | 19596.806 | 1.832 | 16.722 | 0.129438 | 7/15/2014 | 03:04.6 | 1 |
| -1261.055 | 19596.852 | 1.835 | 16.744 | 0.135263 | 7/15/2014 | 03:04.7 | 1 |
| -1261.174 | 19596.869 | 1.835 | 16.755 | 0.137222 | 7/15/2014 | 03:04.8 | 1 |
| -1261.414 | 19596.904 | 1.838 | 16.773 | 0.141017 | 7/15/2014 | 03:04.9 | 1 |
| -1261.653 | 19596.939 | 1.829 | 16.788 | 0.140787 | 7/15/2014 | 03:05.0 | 1 |
| -1261.894 | 19596.976 | 1.829 | 16.803 | 0.141074 | 7/15/2014 | 03:05.1 | 1 |
| -1262.135 | 19597.012 | 1.823 | 16.816 | 0.142384 | 7/15/2014 | 03:05.2 | 1 |
| -1262.377 | 19597.048 | 1.823 | 16.828 | 0.149308 | 7/15/2014 | 03:05.3 | 1 |
| -1262.8 | 19597.111 | 1.822 | 16.846 | 0.160636 | 7/15/2014 | 03:05.4 | 1 |
| -1263.042 | 19597.146 | 1.822 | 16.858 | 0.14661 | 7/15/2014 | 03:05.5 | 1 |
| -1263.285 | 19597.182 | 1.817 | 16.87 | 0.161081 | 7/15/2014 | 03:05.6 | 1 |
| -1263.59 | 19597.228 | 1.817 | 16.881 | 0.149636 | 7/15/2014 | 03:05.7 | 1 |
| -1263.712 | 19597.246 | 1.812 | 16.887 | 0.150304 | 7/15/2014 | 03:05.8 | 1 |
| -1263.959 | 19597.281 | 1.812 | 16.896 | 0.140478 | 7/15/2014 | 03:05.9 | 1 |
| -1264.206 | 19597.319 | 1.806 | 16.902 | 0.132571 | 7/15/2014 | 03:06.0 | 1 |
| -1264.456 | 19597.355 | 1.805 | 16.905 | 0.129173 | 7/15/2014 | 03:06.1 | 1 |
| -1264.703 | 19597.391 | 1.799 | 16.903 | 0.11983 | 7/15/2014 | 03:06.2 | 1 |
| -1264.951 | 19597.426 | 1.793 | 16.896 | 0.114395 | 7/15/2014 | 03:06.3 | 1 |
| -1265.259 | 19597.472 | 1.788 | 16.883 | 0.111722 | 7/15/2014 | 03:06.4 | 1 |
| -1265.508 | 19597.508 | 1.791 | 16.865 | 0.111624 | 7/15/2014 | 03:06.5 | 1 |
| -1265.755 | 19597.544 | 1.788 | 16.841 | 0.115812 | 7/15/2014 | 03:06.6 | 1 |
| -1266.004 | 19597.58 | 1.786 | 16.813 | 0.116737 | 7/15/2014 | 03:06.7 | 1 |
| -1266.313 | 19597.623 | 1.781 | 16.782 | 0.121525 | 7/15/2014 | 03:06.8 | 1 |

| | | | | | | | |
|---|---|---|---|---|---|---|---|
| -1266.563 | 19597.658 | 1.781 | 16.749 | 0.116335 | 7/15/2014 | 03:06.9 | 1 |
| -1266.811 | 19597.694 | 1.781 | 16.715 | 0.119294 | 7/15/2014 | 03:07.0 | 1 |
| -1267.061 | 19597.73 | 1.783 | 16.683 | 0.120746 | 7/15/2014 | 03:07.1 | 1 |
| -1267.309 | 19597.766 | 1.778 | 16.652 | 0.118903 | 7/15/2014 | 03:07.2 | 1 |
| -1267.557 | 19597.801 | 1.778 | 16.624 | 0.125239 | 7/15/2014 | 03:07.3 | 1 |
| -1267.864 | 19597.844 | 1.773 | 16.6 | 0.111321 | 7/15/2014 | 03:07.4 | 1 |
| -1268.113 | 19597.879 | 1.773 | 16.579 | 0.108558 | 7/15/2014 | 03:07.5 | 1 |
| -1268.358 | 19597.914 | 1.77 | 16.563 | 0.117471 | 7/15/2014 | 03:07.6 | 1 |
| -1268.606 | 19597.948 | 1.77 | 16.552 | 0.125566 | 7/15/2014 | 03:07.7 | 1 |
| -1268.913 | 19597.991 | 1.763 | 16.544 | 0.117716 | 7/15/2014 | 03:07.8 | 1 |
| -1269.16 | 19598.025 | 1.763 | 16.541 | 0.114521 | 7/15/2014 | 03:07.9 | 1 |
| -1269.407 | 19598.061 | 1.758 | 16.541 | 0.111935 | 7/15/2014 | 03:08.0 | 1 |
| -1269.656 | 19598.095 | 1.761 | 16.544 | 0.117429 | 7/15/2014 | 03:08.1 | 1 |
| -1269.902 | 19598.13 | 1.758 | 16.548 | 0.108829 | 7/15/2014 | 03:08.2 | 1 |
| -1270.149 | 19598.163 | 1.754 | 16.552 | 0.100152 | 7/15/2014 | 03:08.3 | 1 |
| -1270.578 | 19598.222 | 1.741 | 16.555 | 0.0991177 | 7/15/2014 | 03:08.4 | 1 |
| -1270.822 | 19598.256 | 1.738 | 16.554 | 0.101624 | 7/15/2014 | 03:08.5 | 1 |
| -1271.067 | 19598.29 | 1.733 | 16.55 | 0.100244 | 7/15/2014 | 03:08.6 | 1 |
| -1271.371 | 19598.331 | 1.727 | 16.542 | 0.107907 | 7/15/2014 | 03:08.7 | 1 |
| -1271.495 | 19598.349 | 1.723 | 16.54 | 0.103687 | 7/15/2014 | 03:08.8 | 1 |
| -1271.741 | 19598.381 | 1.717 | 16.526 | 0.113853 | 7/15/2014 | 03:08.9 | 1 |
| -1271.986 | 19598.416 | 1.717 | 16.51 | 0.12505 | 7/15/2014 | 03:09.0 | 1 |
| -1272.232 | 19598.447 | 1.715 | 16.491 | 0.136407 | 7/15/2014 | 03:09.1 | 1 |
| -1272.475 | 19598.481 | 1.711 | 16.472 | 0.138794 | 7/15/2014 | 03:09.2 | 1 |
| -1272.721 | 19598.515 | 1.715 | 16.453 | 0.145518 | 7/15/2014 | 03:09.3 | 1 |
| -1273.147 | 19598.572 | 1.707 | 16.431 | 0.15036 | 7/15/2014 | 03:09.4 | 1 |
| -1273.391 | 19598.604 | 1.712 | 16.418 | 0.153644 | 7/15/2014 | 03:09.5 | 1 |
| -1273.634 | 19598.637 | 1.712 | 16.409 | 0.148567 | 7/15/2014 | 03:09.6 | 1 |
| -1273.938 | 19598.676 | 1.712 | 16.405 | 0.143104 | 7/15/2014 | 03:09.7 | 1 |
| -1274.06 | 19598.692 | 1.715 | 16.401 | 0.144484 | 7/15/2014 | 03:09.8 | 1 |
| -1274.307 | 19598.725 | 1.716 | 16.403 | 0.138798 | 7/15/2014 | 03:09.9 | 1 |
| -1274.551 | 19598.757 | 1.712 | 16.409 | 0.141011 | 7/15/2014 | 03:10.0 | 1 |
| -1274.798 | 19598.79 | 1.722 | 16.419 | 0.139288 | 7/15/2014 | 03:10.1 | 1 |
| -1275.043 | 19598.821 | 1.718 | 16.433 | 0.125307 | 7/15/2014 | 03:10.2 | 1 |
| -1275.29 | 19598.854 | 1.715 | 16.451 | 0.121827 | 7/15/2014 | 03:10.3 | 1 |
| -1275.719 | 19598.908 | 1.707 | 16.484 | 0.114974 | 7/15/2014 | 03:10.4 | 1 |
| -1275.963 | 19598.939 | 1.695 | 16.507 | 0.0986186 | 7/15/2014 | 03:10.5 | 1 |
| -1276.209 | 19598.972 | 1.694 | 16.531 | 0.101006 | 7/15/2014 | 03:10.6 | 1 |
| -1276.517 | 19599.011 | 1.691 | 16.556 | 0.0987814 | 7/15/2014 | 03:10.7 | 1 |
| -1276.64 | 19599.026 | 1.684 | 16.568 | 0.0944438 | 7/15/2014 | 03:10.8 | 1 |
| -1276.889 | 19599.059 | 1.685 | 16.592 | 0.0705217 | 7/15/2014 | 03:10.9 | 1 |
| -1277.137 | 19599.09 | 1.682 | 16.613 | 0.0692355 | 7/15/2014 | 03:11.0 | 1 |
| -1277.389 | 19599.121 | 1.682 | 16.631 | 0.0861986 | 7/15/2014 | 03:11.1 | 1 |
| -1277.636 | 19599.152 | 1.678 | 16.647 | 0.0824774 | 7/15/2014 | 03:11.2 | 1 |
| -1277.887 | 19599.184 | 1.688 | 16.66 | 0.0866873 | 7/15/2014 | 03:11.3 | 1 |
| -1278.197 | 19599.223 | 1.682 | 16.671 | 0.0912153 | 7/15/2014 | 03:11.4 | 1 |
| -1278.448 | 19599.254 | 1.679 | 16.68 | 0.104612 | 7/15/2014 | 03:11.5 | 1 |

| | | | | | | | |
|---|---|---|---|---|---|---|---|
| -1278.696 | 19599.285 | 1.676 | 16.686 | 0.107769 | 7/15/2014 | 03:11.6 | 1 |
| -1278.948 | 19599.315 | 1.677 | 16.692 | 0.117198 | 7/15/2014 | 03:11.7 | 1 |
| -1279.256 | 19599.354 | 1.673 | 16.696 | 0.123183 | 7/15/2014 | 03:11.8 | 1 |
| -1279.507 | 19599.385 | 1.673 | 16.699 | 0.125637 | 7/15/2014 | 03:11.9 | 1 |
| -1279.754 | 19599.415 | 1.669 | 16.7 | 0.13491 | 7/15/2014 | 03:12.0 | 1 |
| -1280.003 | 19599.445 | 1.673 | 16.701 | 0.142191 | 7/15/2014 | 03:12.1 | 1 |
| -1280.249 | 19599.477 | 1.673 | 16.7 | 0.150245 | 7/15/2014 | 03:12.2 | 1 |
| -1280.495 | 19599.505 | 1.673 | 16.697 | 0.155617 | 7/15/2014 | 03:12.3 | 1 |
| -1280.92 | 19599.558 | 1.675 | 16.69 | 0.16402 | 7/15/2014 | 03:12.4 | 1 |
| -1281.16 | 19599.587 | 1.678 | 16.683 | 0.171273 | 7/15/2014 | 03:12.5 | 1 |
| -1281.399 | 19599.616 | 1.681 | 16.674 | 0.174736 | 7/15/2014 | 03:12.6 | 1 |
| -1281.693 | 19599.651 | 1.676 | 16.663 | 0.180621 | 7/15/2014 | 03:12.7 | 1 |
| -1281.816 | 19599.667 | 1.677 | 16.658 | 0.183158 | 7/15/2014 | 03:12.8 | 1 |
| -1282.053 | 19599.695 | 1.679 | 16.642 | 0.185954 | 7/15/2014 | 03:12.9 | 1 |
| -1282.289 | 19599.725 | 1.679 | 16.624 | 0.188347 | 7/15/2014 | 03:13.0 | 1 |
| -1282.525 | 19599.753 | 1.682 | 16.603 | 0.189014 | 7/15/2014 | 03:13.1 | 1 |
| -1282.757 | 19599.78 | 1.678 | 16.58 | 0.185609 | 7/15/2014 | 03:13.2 | 1 |
| -1282.99 | 19599.81 | 1.684 | 16.556 | 0.184058 | 7/15/2014 | 03:13.3 | 1 |
| -1283.387 | 19599.857 | 1.683 | 16.518 | 0.172831 | 7/15/2014 | 03:13.4 | 1 |
| -1283.614 | 19599.886 | 1.686 | 16.493 | 0.165528 | 7/15/2014 | 03:13.5 | 1 |
| -1283.84 | 19599.912 | 1.684 | 16.469 | 0.158988 | 7/15/2014 | 03:13.6 | 1 |
| -1284.119 | 19599.946 | 1.686 | 16.447 | 0.145282 | 7/15/2014 | 03:13.7 | 1 |
| -1284.343 | 19599.973 | 1.684 | 16.426 | 0.14105 | 7/15/2014 | 03:13.8 | 1 |
| -1284.569 | 19600.001 | 1.682 | 16.407 | 0.136562 | 7/15/2014 | 03:13.9 | 1 |
| -1284.793 | 19600.028 | 1.685 | 16.39 | 0.115313 | 7/15/2014 | 03:14.0 | 1 |
| -1285.016 | 19600.055 | 1.688 | 16.374 | 0.141235 | 7/15/2014 | 03:14.1 | 1 |
| -1285.24 | 19600.083 | 1.691 | 16.36 | 0.117199 | 7/15/2014 | 03:14.2 | 1 |
| -1285.461 | 19600.108 | 1.691 | 16.348 | 0.101814 | 7/15/2014 | 03:14.3 | 1 |
| -1285.739 | 19600.143 | 1.696 | 16.336 | 0.124986 | 7/15/2014 | 03:14.4 | 1 |
| -1285.96 | 19600.168 | 1.696 | 16.326 | 0.110605 | 7/15/2014 | 03:14.5 | 1 |
| -1286.184 | 19600.196 | 1.702 | 16.316 | 0.108126 | 7/15/2014 | 03:14.6 | 1 |
| -1286.468 | 19600.23 | 1.702 | 16.305 | 0.128714 | 7/15/2014 | 03:14.7 | 1 |
| -1286.697 | 19600.257 | 1.696 | 16.294 | 0.130162 | 7/15/2014 | 03:14.8 | 1 |
| -1286.928 | 19600.287 | 1.691 | 16.283 | 0.143042 | 7/15/2014 | 03:14.9 | 1 |
| -1287.039 | 19600.3 | 1.688 | 16.277 | 0.154524 | 7/15/2014 | 03:15.0 | 1 |
| -1287.275 | 19600.327 | 1.698 | 16.264 | 0.160464 | 7/15/2014 | 03:15.1 | 1 |
| -1287.509 | 19600.355 | 1.701 | 16.249 | 0.168184 | 7/15/2014 | 03:15.2 | 1 |
| -1287.747 | 19600.385 | 1.705 | 16.234 | 0.168438 | 7/15/2014 | 03:15.3 | 1 |
| -1288.166 | 19600.435 | 1.701 | 16.209 | 0.16752 | 7/15/2014 | 03:15.4 | 1 |
| -1288.406 | 19600.463 | 1.706 | 16.192 | 0.164599 | 7/15/2014 | 03:15.5 | 1 |
| -1288.647 | 19600.493 | 1.708 | 16.174 | 0.161196 | 7/15/2014 | 03:15.6 | 1 |
| -1288.95 | 19600.527 | 1.713 | 16.158 | 0.154588 | 7/15/2014 | 03:15.7 | 1 |
| -1289.073 | 19600.543 | 1.71 | 16.149 | 0.151389 | 7/15/2014 | 03:15.8 | 1 |
| -1289.318 | 19600.573 | 1.706 | 16.133 | 0.147588 | 7/15/2014 | 03:15.9 | 1 |
| -1289.561 | 19600.602 | 1.702 | 16.118 | 0.142684 | 7/15/2014 | 03:16.0 | 1 |
| -1289.807 | 19600.632 | 1.706 | 16.104 | 0.150067 | 7/15/2014 | 03:16.1 | 1 |
| -1290.05 | 19600.66 | 1.706 | 16.092 | 0.160631 | 7/15/2014 | 03:16.2 | 1 |

| | | | | | | | |
|---|---|---|---|---|---|---|---|
| -1290.293 | 19600.689 | 1.706 | 16.083 | 0.146148 | 7/15/2014 | 03:16.3 | 1 |
| -1290.713 | 19600.738 | 1.704 | 16.077 | 0.150806 | 7/15/2014 | 03:16.4 | 1 |
| -1290.952 | 19600.768 | 1.701 | 16.075 | 0.148165 | 7/15/2014 | 03:16.5 | 1 |
| -1291.19 | 19600.795 | 1.704 | 16.075 | 0.155565 | 7/15/2014 | 03:16.6 | 1 |
| -1291.487 | 19600.832 | 1.707 | 16.079 | 0.146967 | 7/15/2014 | 03:16.7 | 1 |
| -1291.608 | 19600.846 | 1.709 | 16.08 | 0.146873 | 7/15/2014 | 03:16.8 | 1 |
| -1291.846 | 19600.874 | 1.712 | 16.086 | 0.144024 | 7/15/2014 | 03:16.9 | 1 |
| -1292.082 | 19600.902 | 1.716 | 16.092 | 0.148456 | 7/15/2014 | 03:17.0 | 1 |
| -1292.32 | 19600.93 | 1.726 | 16.097 | 0.135304 | 7/15/2014 | 03:17.1 | 1 |
| -1292.554 | 19600.957 | 1.726 | 16.101 | 0.1386 | 7/15/2014 | 03:17.2 | 1 |
| -1292.789 | 19600.984 | 1.736 | 16.101 | 0.133929 | 7/15/2014 | 03:17.3 | 1 |
| -1293.198 | 19601.034 | 1.738 | 16.091 | 0.128868 | 7/15/2014 | 03:17.4 | 1 |
| -1293.429 | 19601.062 | 1.741 | 16.081 | 0.127001 | 7/15/2014 | 03:17.5 | 1 |
| -1293.661 | 19601.089 | 1.741 | 16.066 | 0.122436 | 7/15/2014 | 03:17.6 | 1 |
| -1293.952 | 19601.123 | 1.738 | 16.047 | 0.122471 | 7/15/2014 | 03:17.7 | 1 |
| -1294.069 | 19601.137 | 1.736 | 16.038 | 0.122958 | 7/15/2014 | 03:17.8 | 1 |
| -1294.303 | 19601.164 | 1.739 | 16.013 | 0.124077 | 7/15/2014 | 03:17.9 | 1 |
| -1294.538 | 19601.191 | 1.733 | 15.986 | 0.12246 | 7/15/2014 | 03:18.0 | 1 |
| -1294.775 | 19601.22 | 1.732 | 15.957 | 0.125608 | 7/15/2014 | 03:18.1 | 1 |
| -1295.009 | 19601.247 | 1.729 | 15.928 | 0.127981 | 7/15/2014 | 03:18.2 | 1 |
| -1295.246 | 19601.274 | 1.732 | 15.899 | 0.124098 | 7/15/2014 | 03:18.3 | 1 |
| -1295.659 | 19601.322 | 1.735 | 15.858 | 0.117221 | 7/15/2014 | 03:18.4 | 1 |
| -1295.896 | 19601.35 | 1.74 | 15.831 | 0.118741 | 7/15/2014 | 03:18.5 | 1 |
| -1296.134 | 19601.377 | 1.74 | 15.806 | 0.113216 | 7/15/2014 | 03:18.6 | 1 |
| -1296.435 | 19601.412 | 1.743 | 15.782 | 0.111497 | 7/15/2014 | 03:18.7 | 1 |
| -1296.553 | 19601.426 | 1.745 | 15.77 | 0.10858 | 7/15/2014 | 03:18.8 | 1 |
| -1296.797 | 19601.453 | 1.748 | 15.747 | 0.103977 | 7/15/2014 | 03:18.9 | 1 |
| -1297.039 | 19601.48 | 1.748 | 15.725 | 0.102233 | 7/15/2014 | 03:19.0 | 1 |
| -1297.284 | 19601.508 | 1.753 | 15.702 | 0.108682 | 7/15/2014 | 03:19.1 | 1 |
| -1297.529 | 19601.535 | 1.75 | 15.679 | 0.114208 | 7/15/2014 | 03:19.2 | 1 |
| -1297.774 | 19601.563 | 1.744 | 15.655 | 0.118258 | 7/15/2014 | 03:19.3 | 1 |
| -1298.08 | 19601.6 | 1.744 | 15.63 | 0.133279 | 7/15/2014 | 03:19.4 | 1 |
| -1298.328 | 19601.627 | 1.749 | 15.603 | 0.143325 | 7/15/2014 | 03:19.5 | 1 |
| -1298.574 | 19601.654 | 1.749 | 15.576 | 0.15035 | 7/15/2014 | 03:19.6 | 1 |
| -1298.823 | 19601.682 | 1.751 | 15.548 | 0.158388 | 7/15/2014 | 03:19.7 | 1 |
| -1299.13 | 19601.715 | 1.749 | 15.521 | 0.166331 | 7/15/2014 | 03:19.8 | 1 |
| -1299.38 | 19601.743 | 1.76 | 15.496 | 0.156667 | 7/15/2014 | 03:19.9 | 1 |
| -1299.628 | 19601.771 | 1.766 | 15.473 | 0.158849 | 7/15/2014 | 03:20.0 | 1 |
| -1299.878 | 19601.799 | 1.768 | 15.453 | 0.144608 | 7/15/2014 | 03:20.1 | 1 |
| -1300.125 | 19601.826 | 1.766 | 15.438 | 0.137962 | 7/15/2014 | 03:20.2 | 1 |
| -1300.374 | 19601.854 | 1.771 | 15.426 | 0.131881 | 7/15/2014 | 03:20.3 | 1 |
| -1300.804 | 19601.898 | 1.778 | 15.42 | 0.124604 | 7/15/2014 | 03:20.4 | 1 |
| -1301.051 | 19601.926 | 1.782 | 15.42 | 0.122457 | 7/15/2014 | 03:20.5 | 1 |
| -1301.296 | 19601.953 | 1.779 | 15.423 | 0.114128 | 7/15/2014 | 03:20.6 | 1 |
| -1301.604 | 19601.986 | 1.783 | 15.429 | 0.113361 | 7/15/2014 | 03:20.7 | 1 |
| -1301.727 | 19602 | 1.787 | 15.432 | 0.116149 | 7/15/2014 | 03:20.8 | 1 |
| -1301.976 | 19602.027 | 1.796 | 15.443 | 0.123231 | 7/15/2014 | 03:20.9 | 1 |

| | | | | | | | |
|---|---|---|---|---|---|---|---|
| -1302.223 | 19602.053 | 1.793 | 15.457 | 0.120207 | 7/15/2014 | 03:21.0 | 1 |
| -1302.471 | 19602.078 | 1.791 | 15.471 | 0.127441 | 7/15/2014 | 03:21.1 | 1 |
| -1302.718 | 19602.105 | 1.787 | 15.487 | 0.132373 | 7/15/2014 | 03:21.2 | 1 |
| -1302.964 | 19602.132 | 1.79 | 15.502 | 0.136785 | 7/15/2014 | 03:21.3 | 1 |
| -1303.395 | 19602.178 | 1.795 | 15.52 | 0.141005 | 7/15/2014 | 03:21.4 | 1 |
| -1303.639 | 19602.203 | 1.793 | 15.53 | 0.133473 | 7/15/2014 | 03:21.5 | 1 |
| -1303.885 | 19602.229 | 1.792 | 15.538 | 0.13147 | 7/15/2014 | 03:21.6 | 1 |
| -1304.19 | 19602.261 | 1.794 | 15.542 | 0.133296 | 7/15/2014 | 03:21.7 | 1 |
| -1304.315 | 19602.274 | 1.797 | 15.545 | 0.128996 | 7/15/2014 | 03:21.8 | 1 |
| -1304.562 | 19602.3 | 1.803 | 15.544 | 0.129332 | 7/15/2014 | 03:21.9 | 1 |
| -1304.807 | 19602.325 | 1.806 | 15.54 | 0.129275 | 7/15/2014 | 03:22.0 | 1 |
| -1305.055 | 19602.352 | 1.812 | 15.533 | 0.136047 | 7/15/2014 | 03:22.1 | 1 |
| -1305.299 | 19602.376 | 1.809 | 15.523 | 0.140207 | 7/15/2014 | 03:22.2 | 1 |
| -1305.546 | 19602.402 | 1.82 | 15.512 | 0.144443 | 7/15/2014 | 03:22.3 | 1 |
| -1305.974 | 19602.446 | 1.827 | 15.492 | 0.147457 | 7/15/2014 | 03:22.4 | 1 |
| -1306.219 | 19602.472 | 1.832 | 15.478 | 0.148358 | 7/15/2014 | 03:22.5 | 1 |
| -1306.463 | 19602.497 | 1.834 | 15.463 | 0.151252 | 7/15/2014 | 03:22.6 | 1 |
| -1306.77 | 19602.527 | 1.836 | 15.449 | 0.145676 | 7/15/2014 | 03:22.7 | 1 |
| -1306.893 | 19602.54 | 1.837 | 15.441 | 0.15014 | 7/15/2014 | 03:22.8 | 1 |
| -1307.141 | 19602.565 | 1.846 | 15.426 | 0.142878 | 7/15/2014 | 03:22.9 | 1 |
| -1307.388 | 19602.591 | 1.843 | 15.41 | 0.144221 | 7/15/2014 | 03:23.0 | 1 |
| -1307.637 | 19602.617 | 1.84 | 15.394 | 0.142174 | 7/15/2014 | 03:23.1 | 1 |
| -1307.885 | 19602.642 | 1.837 | 15.377 | 0.136915 | 7/15/2014 | 03:23.2 | 1 |
| -1308.134 | 19602.667 | 1.84 | 15.36 | 0.134108 | 7/15/2014 | 03:23.3 | 1 |
| -1308.444 | 19602.697 | 1.84 | 15.343 | 0.136275 | 7/15/2014 | 03:23.4 | 1 |
| -1308.693 | 19602.723 | 1.841 | 15.325 | 0.136664 | 7/15/2014 | 03:23.5 | 1 |
| -1308.941 | 19602.748 | 1.835 | 15.306 | 0.116964 | 7/15/2014 | 03:23.6 | 1 |
| -1309.192 | 19602.774 | 1.841 | 15.287 | 0.107938 | 7/15/2014 | 03:23.7 | 1 |
| -1309.503 | 19602.805 | 1.847 | 15.267 | 0.105829 | 7/15/2014 | 03:23.8 | 1 |
| -1309.754 | 19602.829 | 1.853 | 15.246 | 0.0842401 | 7/15/2014 | 03:23.9 | 1 |
| -1310.005 | 19602.854 | 1.853 | 15.225 | 0.0736118 | 7/15/2014 | 03:24.0 | 1 |
| -1310.259 | 19602.88 | 1.861 | 15.202 | 0.0647702 | 7/15/2014 | 03:24.1 | 1 |
| -1310.509 | 19602.904 | 1.859 | 15.18 | 0.0607907 | 7/15/2014 | 03:24.2 | 1 |
| -1310.763 | 19602.93 | 1.865 | 15.157 | 0.07394 | 7/15/2014 | 03:24.3 | 1 |
| -1311.074 | 19602.961 | 1.859 | 15.135 | 0.0583692 | 7/15/2014 | 03:24.4 | 1 |
| -1311.326 | 19602.985 | 1.861 | 15.113 | 0.0602959 | 7/15/2014 | 03:24.5 | 1 |
| -1311.577 | 19603.01 | 1.855 | 15.091 | 0.0645548 | 7/15/2014 | 03:24.6 | 1 |
| -1311.831 | 19603.035 | 1.858 | 15.07 | 0.0703194 | 7/15/2014 | 03:24.7 | 1 |
| -1312.143 | 19603.064 | 1.851 | 15.049 | 0.0718106 | 7/15/2014 | 03:24.8 | 1 |
| -1312.399 | 19603.09 | 1.855 | 15.028 | 0.0799767 | 7/15/2014 | 03:24.9 | 1 |
| -1312.651 | 19603.114 | 1.855 | 15.008 | 0.0838508 | 7/15/2014 | 03:25.0 | 1 |
| -1312.906 | 19603.14 | 1.857 | 14.988 | 0.0801919 | 7/15/2014 | 03:25.1 | 1 |
| -1313.159 | 19603.163 | 1.856 | 14.97 | 0.0855724 | 7/15/2014 | 03:25.2 | 1 |
| -1313.415 | 19603.188 | 1.862 | 14.952 | 0.0918094 | 7/15/2014 | 03:25.3 | 1 |
| -1313.729 | 19603.219 | 1.858 | 14.936 | 0.0940117 | 7/15/2014 | 03:25.4 | 1 |
| -1313.984 | 19603.243 | 1.865 | 14.922 | 0.0996526 | 7/15/2014 | 03:25.5 | 1 |
| -1314.236 | 19603.267 | 1.859 | 14.911 | 0.105672 | 7/15/2014 | 03:25.6 | 1 |

| | | | | | | | |
|---|---|---|---|---|---|---|---|
| -1314.491 | 19603.291 | 1.857 | 14.903 | 0.104566 | 7/15/2014 | 03:25.7 | 1 |
| -1314.807 | 19603.32 | 1.855 | 14.899 | 0.105186 | 7/15/2014 | 03:25.8 | 1 |
| -1315.065 | 19603.346 | 1.858 | 14.898 | 0.102465 | 7/15/2014 | 03:25.9 | 1 |
| -1315.318 | 19603.37 | 1.855 | 14.902 | 0.09841 | 7/15/2014 | 03:26.0 | 1 |
| -1315.576 | 19603.395 | 1.848 | 14.909 | 0.0948568 | 7/15/2014 | 03:26.1 | 1 |
| -1315.831 | 19603.418 | 1.841 | 14.919 | 0.0943938 | 7/15/2014 | 03:26.2 | 1 |
| -1316.087 | 19603.441 | 1.844 | 14.932 | 0.0943336 | 7/15/2014 | 03:26.3 | 1 |
| -1316.405 | 19603.471 | 1.836 | 14.947 | 0.0957878 | 7/15/2014 | 03:26.4 | 1 |
| -1316.662 | 19603.495 | 1.836 | 14.962 | 0.0994826 | 7/15/2014 | 03:26.5 | 1 |
| -1316.917 | 19603.519 | 1.832 | 14.978 | 0.111018 | 7/15/2014 | 03:26.6 | 1 |
| -1317.173 | 19603.543 | 1.832 | 14.992 | 0.111432 | 7/15/2014 | 03:26.7 | 1 |
| -1317.494 | 19603.572 | 1.832 | 15.006 | 0.123251 | 7/15/2014 | 03:26.8 | 1 |
| -1317.752 | 19603.596 | 1.83 | 15.018 | 0.119648 | 7/15/2014 | 03:26.9 | 1 |
| -1318.01 | 19603.619 | 1.821 | 15.028 | 0.131485 | 7/15/2014 | 03:27.0 | 1 |
| -1318.27 | 19603.643 | 1.825 | 15.037 | 0.125175 | 7/15/2014 | 03:27.1 | 1 |
| -1318.529 | 19603.666 | 1.821 | 15.044 | 0.112793 | 7/15/2014 | 03:27.2 | 1 |
| -1318.788 | 19603.689 | 1.819 | 15.05 | 0.125243 | 7/15/2014 | 03:27.3 | 1 |
| -1319.11 | 19603.719 | 1.813 | 15.056 | 0.101874 | 7/15/2014 | 03:27.4 | 1 |
| -1319.368 | 19603.742 | 1.813 | 15.06 | 0.115064 | 7/15/2014 | 03:27.5 | 1 |
| -1319.625 | 19603.766 | 1.809 | 15.063 | 0.0938019 | 7/15/2014 | 03:27.6 | 1 |
| -1319.884 | 19603.787 | 1.818 | 15.064 | 0.0914382 | 7/15/2014 | 03:27.7 | 1 |
| -1320.205 | 19603.816 | 1.809 | 15.063 | 0.0919071 | 7/15/2014 | 03:27.8 | 1 |
| -1320.464 | 19603.84 | 1.809 | 15.06 | 0.0957906 | 7/15/2014 | 03:27.9 | 1 |
| -1320.721 | 19603.861 | 1.807 | 15.054 | 0.0953998 | 7/15/2014 | 03:28.0 | 1 |
| -1320.979 | 19603.885 | 1.81 | 15.045 | 0.0846594 | 7/15/2014 | 03:28.1 | 1 |
| -1321.235 | 19603.908 | 1.808 | 15.033 | 0.0922809 | 7/15/2014 | 03:28.2 | 1 |
| -1321.492 | 19603.928 | 1.808 | 15.019 | 0.0826298 | 7/15/2014 | 03:28.3 | 1 |
| -1321.808 | 19603.957 | 1.801 | 15.002 | 0.0819709 | 7/15/2014 | 03:28.4 | 1 |
| -1322.063 | 19603.98 | 1.81 | 14.983 | 0.069101 | 7/15/2014 | 03:28.5 | 1 |
| -1322.314 | 19604 | 1.81 | 14.962 | 0.0672019 | 7/15/2014 | 03:28.6 | 1 |
| -1322.567 | 19604.021 | 1.814 | 14.941 | 0.0766916 | 7/15/2014 | 03:28.7 | 1 |
| -1322.879 | 19604.049 | 1.814 | 14.918 | 0.0592262 | 7/15/2014 | 03:28.8 | 1 |
| -1323.131 | 19604.07 | 1.821 | 14.896 | 0.066618 | 7/15/2014 | 03:28.9 | 1 |
| -1323.382 | 19604.092 | 1.824 | 14.874 | 0.0682572 | 7/15/2014 | 03:29.0 | 1 |
| -1323.633 | 19604.115 | 1.828 | 14.853 | 0.0605484 | 7/15/2014 | 03:29.1 | 1 |
| -1323.881 | 19604.135 | 1.824 | 14.835 | 0.0727108 | 7/15/2014 | 03:29.2 | 1 |
| -1324.129 | 19604.154 | 1.828 | 14.819 | 0.0547504 | 7/15/2014 | 03:29.3 | 1 |
| -1324.562 | 19604.191 | 1.834 | 14.802 | 0.0694384 | 7/15/2014 | 03:29.4 | 1 |
| -1324.806 | 19604.213 | 1.831 | 14.792 | 0.0598597 | 7/15/2014 | 03:29.5 | 1 |
| -1325.052 | 19604.232 | 1.828 | 14.786 | 0.0538877 | 7/15/2014 | 03:29.6 | 1 |
| -1325.36 | 19604.26 | 1.828 | 14.781 | 0.0644042 | 7/15/2014 | 03:29.7 | 1 |
| -1325.483 | 19604.27 | 1.828 | 14.779 | 0.0510638 | 7/15/2014 | 03:29.8 | 1 |
| -1325.732 | 19604.289 | 1.835 | 14.777 | 0.0687502 | 7/15/2014 | 03:29.9 | 1 |
| -1325.979 | 19604.309 | 1.832 | 14.777 | 0.0405699 | 7/15/2014 | 03:30.0 | 1 |
| -1326.229 | 19604.33 | 1.832 | 14.777 | 0.0533245 | 7/15/2014 | 03:30.1 | 1 |
| -1326.475 | 19604.35 | 1.816 | 14.778 | 0.0646338 | 7/15/2014 | 03:30.2 | 1 |
| -1326.723 | 19604.369 | 1.82 | 14.779 | 0.0777996 | 7/15/2014 | 03:30.3 | 1 |

| | | | | | | | |
|---|---|---|---|---|---|---|---|
| -1327.031 | 19604.396 | 1.823 | 14.78 | 0.087392 | 7/15/2014 | 03:30.4 | 1 |
| -1327.28 | 19604.416 | 1.82 | 14.782 | 0.0993571 | 7/15/2014 | 03:30.5 | 1 |
| -1327.526 | 19604.436 | 1.812 | 14.784 | 0.147761 | 7/15/2014 | 03:30.6 | 1 |
| -1327.957 | 19604.469 | 1.818 | 14.789 | 0.130222 | 7/15/2014 | 03:30.7 | 1 |
| -1328.081 | 19604.479 | 1.816 | 14.79 | 0.117328 | 7/15/2014 | 03:30.8 | 1 |
| -1328.33 | 19604.498 | 1.826 | 14.795 | 0.0934129 | 7/15/2014 | 03:30.9 | 1 |
| -1328.576 | 19604.52 | 1.822 | 14.8 | 0.0770604 | 7/15/2014 | 03:31.0 | 1 |
| -1328.824 | 19604.539 | 1.826 | 14.807 | 0.0779937 | 7/15/2014 | 03:31.1 | 1 |
| -1329.068 | 19604.559 | 1.827 | 14.816 | 0.0615834 | 7/15/2014 | 03:31.2 | 1 |
| -1329.315 | 19604.578 | 1.827 | 14.826 | 0.0631288 | 7/15/2014 | 03:31.3 | 1 |
| -1329.741 | 19604.611 | 1.817 | 14.846 | 0.0604651 | 7/15/2014 | 03:31.4 | 1 |
| -1329.984 | 19604.63 | 1.815 | 14.862 | 0.0561773 | 7/15/2014 | 03:31.5 | 1 |
| -1330.227 | 19604.648 | 1.809 | 14.878 | 0.0799065 | 7/15/2014 | 03:31.6 | 1 |
| -1330.533 | 19604.672 | 1.809 | 14.896 | 0.0678584 | 7/15/2014 | 03:31.7 | 1 |
| -1330.654 | 19604.682 | 1.802 | 14.905 | 0.0658528 | 7/15/2014 | 03:31.8 | 1 |
| -1330.9 | 19604.701 | 1.801 | 14.925 | 0.0557137 | 7/15/2014 | 03:31.9 | 1 |
| -1331.146 | 19604.721 | 1.801 | 14.944 | 0.072943 | 7/15/2014 | 03:32.0 | 1 |
| -1331.394 | 19604.74 | 1.805 | 14.962 | 0.0677855 | 7/15/2014 | 03:32.1 | 1 |
| -1331.638 | 19604.759 | 1.805 | 14.977 | 0.117949 | 7/15/2014 | 03:32.2 | 1 |
| -1331.886 | 19604.776 | 1.807 | 14.99 | 0.0739011 | 7/15/2014 | 03:32.3 | 1 |
| -1332.318 | 19604.809 | 1.805 | 14.998 | 0.114636 | 7/15/2014 | 03:32.4 | 1 |
| -1332.44 | 19604.818 | 1.805 | 15.003 | 0.124083 | 7/15/2014 | 03:32.5 | 1 |
| -1332.688 | 19604.838 | 1.8 | 15.003 | 0.108472 | 7/15/2014 | 03:32.6 | 1 |
| -1332.938 | 19604.856 | 1.8 | 14.998 | 0.128887 | 7/15/2014 | 03:32.7 | 1 |
| -1333.248 | 19604.88 | 1.794 | 14.99 | 0.109147 | 7/15/2014 | 03:32.8 | 1 |
| -1333.498 | 19604.897 | 1.789 | 14.978 | 0.122254 | 7/15/2014 | 03:32.9 | 1 |
| -1333.749 | 19604.916 | 1.784 | 14.964 | 0.0712355 | 7/15/2014 | 03:33.0 | 1 |
| -1334.002 | 19604.935 | 1.782 | 14.947 | 0.078417 | 7/15/2014 | 03:33.1 | 1 |
| -1334.252 | 19604.953 | 1.78 | 14.929 | 0.0837707 | 7/15/2014 | 03:33.2 | 1 |
| -1334.503 | 19604.972 | 1.782 | 14.91 | 0.0567601 | 7/15/2014 | 03:33.3 | 1 |
| -1334.815 | 19604.993 | 1.775 | 14.89 | 0.0509517 | 7/15/2014 | 03:33.4 | 1 |
| -1335.066 | 19605.011 | 1.777 | 14.871 | 0.0539609 | 7/15/2014 | 03:33.5 | 1 |
| -1335.315 | 19605.029 | 1.777 | 14.852 | 0.0603977 | 7/15/2014 | 03:33.6 | 1 |
| -1335.566 | 19605.047 | 1.782 | 14.833 | 0.0536041 | 7/15/2014 | 03:33.7 | 1 |
| -1335.876 | 19605.068 | 1.78 | 14.817 | 0.0553541 | 7/15/2014 | 03:33.8 | 1 |
| -1336.126 | 19605.087 | 1.78 | 14.802 | 0.057692 | 7/15/2014 | 03:33.9 | 1 |
| -1336.375 | 19605.104 | 1.777 | 14.79 | 0.0564747 | 7/15/2014 | 03:34.0 | 1 |
| -1336.625 | 19605.12 | 1.78 | 14.782 | 0.0576408 | 7/15/2014 | 03:34.1 | 1 |
| -1336.873 | 19605.138 | 1.772 | 14.777 | 0.0561603 | 7/15/2014 | 03:34.2 | 1 |
| -1337.121 | 19605.155 | 1.77 | 14.777 | 0.0702984 | 7/15/2014 | 03:34.3 | 1 |
| -1337.551 | 19605.186 | 1.768 | 14.788 | 0.0583074 | 7/15/2014 | 03:34.4 | 1 |
| -1337.796 | 19605.202 | 1.768 | 14.797 | 0.0556363 | 7/15/2014 | 03:34.5 | 1 |
| -1338.041 | 19605.219 | 1.77 | 14.809 | 0.0600363 | 7/15/2014 | 03:34.6 | 1 |
| -1338.346 | 19605.239 | 1.768 | 14.824 | 0.0563113 | 7/15/2014 | 03:34.7 | 1 |
| -1338.47 | 19605.248 | 1.768 | 14.831 | 0.0603258 | 7/15/2014 | 03:34.8 | 1 |
| -1338.716 | 19605.266 | 1.768 | 14.847 | 0.0575907 | 7/15/2014 | 03:34.9 | 1 |
| -1338.963 | 19605.282 | 1.77 | 14.861 | 0.0558631 | 7/15/2014 | 03:35.0 | 1 |

| | | | | | | | |
|---|---|---|---|---|---|---|---|
| -1339.21 | 19605.299 | 1.77 | 14.873 | 0.0639169 | 7/15/2014 | 03:35.1 | 1 |
| -1339.456 | 19605.315 | 1.765 | 14.882 | 0.0714437 | 7/15/2014 | 03:35.2 | 1 |
| -1339.703 | 19605.331 | 1.767 | 14.888 | 0.0785652 | 7/15/2014 | 03:35.3 | 1 |
| -1340.13 | 19605.36 | 1.758 | 14.888 | 0.0831587 | 7/15/2014 | 03:35.4 | 1 |
| -1340.374 | 19605.376 | 1.752 | 14.884 | 0.0893942 | 7/15/2014 | 03:35.5 | 1 |
| -1340.621 | 19605.393 | 1.756 | 14.878 | 0.0997771 | 7/15/2014 | 03:35.6 | 1 |
| -1340.926 | 19605.412 | 1.756 | 14.87 | 0.103025 | 7/15/2014 | 03:35.7 | 1 |
| -1341.05 | 19605.421 | 1.754 | 14.866 | 0.100757 | 7/15/2014 | 03:35.8 | 1 |
| -1341.298 | 19605.437 | 1.757 | 14.854 | 0.101181 | 7/15/2014 | 03:35.9 | 1 |
| -1341.545 | 19605.451 | 1.746 | 14.84 | 0.10838 | 7/15/2014 | 03:36.0 | 1 |
| -1341.793 | 19605.469 | 1.752 | 14.826 | 0.109414 | 7/15/2014 | 03:36.1 | 1 |
| -1342.04 | 19605.482 | 1.75 | 14.81 | 0.102439 | 7/15/2014 | 03:36.2 | 1 |
| -1342.289 | 19605.5 | 1.752 | 14.794 | 0.115367 | 7/15/2014 | 03:36.3 | 1 |
| -1342.721 | 19605.527 | 1.741 | 14.768 | 0.102561 | 7/15/2014 | 03:36.4 | 1 |
| -1342.844 | 19605.535 | 1.737 | 14.759 | 0.103505 | 7/15/2014 | 03:36.5 | 1 |
| -1343.092 | 19605.55 | 1.733 | 14.741 | 0.106664 | 7/15/2014 | 03:36.6 | 1 |
| -1343.341 | 19605.565 | 1.739 | 14.723 | 0.0876375 | 7/15/2014 | 03:36.7 | 1 |
| -1343.651 | 19605.584 | 1.733 | 14.704 | 0.0832784 | 7/15/2014 | 03:36.8 | 1 |
| -1343.902 | 19605.6 | 1.733 | 14.685 | 0.0823605 | 7/15/2014 | 03:36.9 | 1 |
| -1344.153 | 19605.615 | 1.733 | 14.665 | 0.0769236 | 7/15/2014 | 03:37.0 | 1 |
| -1344.404 | 19605.631 | 1.739 | 14.645 | 0.0760131 | 7/15/2014 | 03:37.1 | 1 |
| -1344.653 | 19605.646 | 1.742 | 14.626 | 0.0731766 | 7/15/2014 | 03:37.2 | 1 |
| -1344.905 | 19605.661 | 1.745 | 14.606 | 0.0675825 | 7/15/2014 | 03:37.3 | 1 |
| -1345.216 | 19605.68 | 1.739 | 14.588 | 0.0679241 | 7/15/2014 | 03:37.4 | 1 |
| -1345.467 | 19605.693 | 1.745 | 14.571 | 0.0689755 | 7/15/2014 | 03:37.5 | 1 |
| -1345.716 | 19605.709 | 1.742 | 14.557 | 0.069802 | 7/15/2014 | 03:37.6 | 1 |
| -1345.966 | 19605.724 | 1.74 | 14.545 | 0.0745353 | 7/15/2014 | 03:37.7 | 1 |
| -1346.277 | 19605.742 | 1.735 | 14.537 | 0.0704722 | 7/15/2014 | 03:37.8 | 1 |
| -1346.528 | 19605.758 | 1.735 | 14.532 | 0.0778265 | 7/15/2014 | 03:37.9 | 1 |
| -1346.778 | 19605.771 | 1.733 | 14.531 | 0.0886085 | 7/15/2014 | 03:38.0 | 1 |
| -1347.031 | 19605.785 | 1.739 | 14.533 | 0.0974288 | 7/15/2014 | 03:38.1 | 1 |
| -1347.279 | 19605.801 | 1.736 | 14.538 | 0.102279 | 7/15/2014 | 03:38.2 | 1 |
| -1347.529 | 19605.813 | 1.739 | 14.546 | 0.10006 | 7/15/2014 | 03:38.3 | 1 |
| -1347.838 | 19605.832 | 1.742 | 14.556 | 0.092937 | 7/15/2014 | 03:38.4 | 1 |
| -1348.087 | 19605.847 | 1.739 | 14.567 | 0.0945529 | 7/15/2014 | 03:38.5 | 1 |
| -1348.335 | 19605.86 | 1.73 | 14.578 | 0.0888699 | 7/15/2014 | 03:38.6 | 1 |
| -1348.586 | 19605.873 | 1.733 | 14.589 | 0.0923676 | 7/15/2014 | 03:38.7 | 1 |
| -1348.897 | 19605.892 | 1.727 | 14.598 | 0.0945232 | 7/15/2014 | 03:38.8 | 1 |
| -1349.148 | 19605.904 | 1.723 | 14.605 | 0.0952721 | 7/15/2014 | 03:38.9 | 1 |
| -1349.399 | 19605.918 | 1.714 | 14.609 | 0.0998531 | 7/15/2014 | 03:39.0 | 1 |
| -1349.651 | 19605.933 | 1.717 | 14.609 | 0.100471 | 7/15/2014 | 03:39.1 | 1 |
| -1349.903 | 19605.945 | 1.72 | 14.605 | 0.0930764 | 7/15/2014 | 03:39.2 | 1 |
| -1350.155 | 19605.957 | 1.727 | 14.597 | 0.0979623 | 7/15/2014 | 03:39.3 | 1 |
| -1350.468 | 19605.976 | 1.72 | 14.586 | 0.0843005 | 7/15/2014 | 03:39.4 | 1 |
| -1350.721 | 19605.988 | 1.727 | 14.572 | 0.0820476 | 7/15/2014 | 03:39.5 | 1 |
| -1350.972 | 19606 | 1.727 | 14.556 | 0.0764411 | 7/15/2014 | 03:39.6 | 1 |
| -1351.225 | 19606.015 | 1.733 | 14.541 | 0.0735577 | 7/15/2014 | 03:39.7 | 1 |

| | | | | | | | |
|---|---|---|---|---|---|---|---|
| -1351.538 | 19606.029 | 1.724 | 14.526 | 0.071505 | 7/15/2014 | 03:39.8 | 1 |
| -1351.79 | 19606.041 | 1.72 | 14.514 | 0.0802593 | 7/15/2014 | 03:39.9 | 1 |
| -1352.043 | 19606.056 | 1.717 | 14.504 | 0.0772919 | 7/15/2014 | 03:40.0 | 1 |
| -1352.296 | 19606.068 | 1.717 | 14.497 | 0.0759822 | 7/15/2014 | 03:40.1 | 1 |
| -1352.546 | 19606.08 | 1.711 | 14.493 | 0.0748432 | 7/15/2014 | 03:40.2 | 1 |
| -1352.798 | 19606.093 | 1.714 | 14.492 | 0.0676892 | 7/15/2014 | 03:40.3 | 1 |
| -1353.11 | 19606.107 | 1.711 | 14.492 | 0.0771911 | 7/15/2014 | 03:40.4 | 1 |
| -1353.36 | 19606.119 | 1.719 | 14.493 | 0.0841454 | 7/15/2014 | 03:40.5 | 1 |
| -1353.609 | 19606.132 | 1.723 | 14.493 | 0.0699467 | 7/15/2014 | 03:40.6 | 1 |
| -1353.858 | 19606.145 | 1.726 | 14.492 | 0.0779209 | 7/15/2014 | 03:40.7 | 1 |
| -1354.169 | 19606.16 | 1.724 | 14.488 | 0.0754742 | 7/15/2014 | 03:40.8 | 1 |
| -1354.421 | 19606.172 | 1.724 | 14.482 | 0.0695542 | 7/15/2014 | 03:40.9 | 1 |
| -1354.672 | 19606.184 | 1.72 | 14.472 | 0.0627296 | 7/15/2014 | 03:41.0 | 1 |
| -1354.924 | 19606.195 | 1.724 | 14.458 | 0.070455 | 7/15/2014 | 03:41.1 | 1 |
| -1355.174 | 19606.207 | 1.714 | 14.442 | 0.057864 | 7/15/2014 | 03:41.2 | 1 |
| -1355.425 | 19606.219 | 1.711 | 14.424 | 0.0634297 | 7/15/2014 | 03:41.3 | 1 |
| -1355.737 | 19606.234 | 1.705 | 14.404 | 0.0771187 | 7/15/2014 | 03:41.4 | 1 |
| -1355.987 | 19606.246 | 1.707 | 14.385 | 0.0703864 | 7/15/2014 | 03:41.5 | 1 |
| -1356.237 | 19606.258 | 1.711 | 14.365 | 0.0959027 | 7/15/2014 | 03:41.6 | 1 |
| -1356.488 | 19606.27 | 1.717 | 14.348 | 0.0845948 | 7/15/2014 | 03:41.7 | 1 |
| -1356.798 | 19606.283 | 1.713 | 14.332 | 0.0898373 | 7/15/2014 | 03:41.8 | 1 |
| -1357.05 | 19606.295 | 1.719 | 14.319 | 0.0977061 | 7/15/2014 | 03:41.9 | 1 |
| -1357.3 | 19606.307 | 1.719 | 14.309 | 0.0942604 | 7/15/2014 | 03:42.0 | 1 |
| -1357.551 | 19606.318 | 1.725 | 14.301 | 0.102273 | 7/15/2014 | 03:42.1 | 1 |
| -1357.802 | 19606.33 | 1.729 | 14.297 | 0.104789 | 7/15/2014 | 03:42.2 | 1 |
| -1358.052 | 19606.342 | 1.729 | 14.295 | 0.104993 | 7/15/2014 | 03:42.3 | 1 |
| -1358.362 | 19606.355 | 1.72 | 14.296 | 0.100724 | 7/15/2014 | 03:42.4 | 1 |
| -1358.612 | 19606.366 | 1.723 | 14.3 | 0.0937625 | 7/15/2014 | 03:42.5 | 1 |
| -1358.86 | 19606.377 | 1.721 | 14.307 | 0.0954826 | 7/15/2014 | 03:42.6 | 1 |
| -1359.11 | 19606.389 | 1.724 | 14.316 | 0.0834385 | 7/15/2014 | 03:42.7 | 1 |
| -1359.422 | 19606.402 | 1.721 | 14.327 | 0.0772036 | 7/15/2014 | 03:42.8 | 1 |
| -1359.673 | 19606.414 | 1.721 | 14.34 | 0.0735267 | 7/15/2014 | 03:42.9 | 1 |
| -1359.925 | 19606.426 | 1.721 | 14.355 | 0.0665635 | 7/15/2014 | 03:43.0 | 1 |
| -1360.177 | 19606.437 | 1.729 | 14.371 | 0.0656609 | 7/15/2014 | 03:43.1 | 1 |
| -1360.429 | 19606.447 | 1.729 | 14.388 | 0.0702651 | 7/15/2014 | 03:43.2 | 1 |
| -1360.681 | 19606.458 | 1.729 | 14.405 | 0.0656858 | 7/15/2014 | 03:43.3 | 1 |
| -1360.994 | 19606.471 | 1.729 | 14.421 | 0.0805283 | 7/15/2014 | 03:43.4 | 1 |
| -1361.247 | 19606.482 | 1.738 | 14.435 | 0.0662187 | 7/15/2014 | 03:43.5 | 1 |
| -1361.498 | 19606.494 | 1.738 | 14.446 | 0.0792075 | 7/15/2014 | 03:43.6 | 1 |
| -1361.75 | 19606.506 | 1.74 | 14.455 | 0.0731004 | 7/15/2014 | 03:43.7 | 1 |
| -1362.064 | 19606.52 | 1.734 | 14.459 | 0.0653597 | 7/15/2014 | 03:43.8 | 1 |
| -1362.318 | 19606.528 | 1.733 | 14.459 | 0.0590795 | 7/15/2014 | 03:43.9 | 1 |
| -1362.57 | 19606.539 | 1.736 | 14.456 | 0.0628618 | 7/15/2014 | 03:44.0 | 1 |
| -1362.824 | 19606.551 | 1.736 | 14.448 | 0.0634148 | 7/15/2014 | 03:44.1 | 1 |
| -1363.076 | 19606.561 | 1.732 | 14.436 | 0.0752942 | 7/15/2014 | 03:44.2 | 1 |
| -1363.329 | 19606.572 | 1.734 | 14.42 | 0.0749879 | 7/15/2014 | 03:44.3 | 1 |
| -1363.641 | 19606.585 | 1.734 | 14.401 | 0.0779642 | 7/15/2014 | 03:44.4 | 1 |

| | | | | | | | |
|---|---|---|---|---|---|---|---|
| -1363.893 | 19606.594 | 1.74 | 14.378 | 0.0817815 | 7/15/2014 | 03:44.5 | 1 |
| -1364.142 | 19606.605 | 1.744 | 14.353 | 0.0914657 | 7/15/2014 | 03:44.6 | 1 |
| -1364.393 | 19606.615 | 1.746 | 14.326 | 0.0883296 | 7/15/2014 | 03:44.7 | 1 |
| -1364.703 | 19606.627 | 1.746 | 14.298 | 0.0998327 | 7/15/2014 | 03:44.8 | 1 |
| -1364.954 | 19606.639 | 1.749 | 14.27 | 0.0941666 | 7/15/2014 | 03:44.9 | 1 |
| -1365.202 | 19606.647 | 1.751 | 14.243 | 0.10223 | 7/15/2014 | 03:45.0 | 1 |
| -1365.451 | 19606.658 | 1.753 | 14.217 | 0.0948429 | 7/15/2014 | 03:45.1 | 1 |
| -1365.698 | 19606.668 | 1.753 | 14.193 | 0.105603 | 7/15/2014 | 03:45.2 | 1 |
| -1365.945 | 19606.677 | 1.753 | 14.172 | 0.0896026 | 7/15/2014 | 03:45.3 | 1 |
| -1366.372 | 19606.693 | 1.753 | 14.147 | 0.0996629 | 7/15/2014 | 03:45.4 | 1 |
| -1366.615 | 19606.705 | 1.755 | 14.132 | 0.102985 | 7/15/2014 | 03:45.5 | 1 |
| -1366.859 | 19606.713 | 1.759 | 14.12 | 0.110026 | 7/15/2014 | 03:45.6 | 1 |
| -1367.163 | 19606.727 | 1.755 | 14.11 | 0.106295 | 7/15/2014 | 03:45.7 | 1 |
| -1367.285 | 19606.73 | 1.753 | 14.104 | 0.108583 | 7/15/2014 | 03:45.8 | 1 |
| -1367.532 | 19606.741 | 1.756 | 14.097 | 0.108554 | 7/15/2014 | 03:45.9 | 1 |
| -1367.777 | 19606.75 | 1.758 | 14.091 | 0.112156 | 7/15/2014 | 03:46.0 | 1 |
| -1368.025 | 19606.759 | 1.763 | 14.086 | 0.104576 | 7/15/2014 | 03:46.1 | 1 |
| -1368.272 | 19606.77 | 1.761 | 14.081 | 0.104333 | 7/15/2014 | 03:46.2 | 1 |
| -1368.523 | 19606.779 | 1.763 | 14.078 | 0.0984463 | 7/15/2014 | 03:46.3 | 1 |
| -1368.834 | 19606.791 | 1.761 | 14.076 | 0.0891351 | 7/15/2014 | 03:46.4 | 1 |
| -1369.088 | 19606.803 | 1.766 | 14.075 | 0.107996 | 7/15/2014 | 03:46.5 | 1 |
| -1369.344 | 19606.813 | 1.766 | 14.076 | 0.119111 | 7/15/2014 | 03:46.6 | 1 |
| -1369.603 | 19606.821 | 1.763 | 14.079 | 0.114421 | 7/15/2014 | 03:46.7 | 1 |
| -1369.864 | 19606.832 | 1.761 | 14.083 | 0.101035 | 7/15/2014 | 03:46.8 | 1 |
| -1370.128 | 19606.842 | 1.763 | 14.09 | 0.111017 | 7/15/2014 | 03:46.9 | 1 |
| -1370.393 | 19606.852 | 1.763 | 14.099 | 0.145189 | 7/15/2014 | 03:47.0 | 1 |
| -1370.658 | 19606.863 | 1.766 | 14.108 | 0.102811 | 7/15/2014 | 03:47.1 | 1 |
| -1370.925 | 19606.875 | 1.768 | 14.119 | 0.0950579 | 7/15/2014 | 03:47.2 | 1 |
| -1371.192 | 19606.883 | 1.768 | 14.131 | 0.0967473 | 7/15/2014 | 03:47.3 | 1 |
| -1371.458 | 19606.895 | 1.773 | 14.143 | 0.0912689 | 7/15/2014 | 03:47.4 | 1 |
| -1371.724 | 19606.902 | 1.783 | 14.154 | 0.0949962 | 7/15/2014 | 03:47.5 | 1 |
| -1371.988 | 19606.914 | 1.786 | 14.166 | 0.0779687 | 7/15/2014 | 03:47.6 | 1 |
| -1372.252 | 19606.926 | 1.786 | 14.176 | 0.104348 | 7/15/2014 | 03:47.7 | 1 |
| -1372.578 | 19606.938 | 1.783 | 14.186 | 0.130149 | 7/15/2014 | 03:47.8 | 1 |
| -1372.841 | 19606.947 | 1.783 | 14.196 | 0.0954124 | 7/15/2014 | 03:47.9 | 1 |
| -1373.103 | 19606.959 | 1.783 | 14.204 | 0.0996508 | 7/15/2014 | 03:48.0 | 1 |
| -1373.368 | 19606.967 | 1.783 | 14.212 | 0.105901 | 7/15/2014 | 03:48.1 | 1 |
| -1373.629 | 19606.979 | 1.773 | 14.219 | 0.0844715 | 7/15/2014 | 03:48.2 | 1 |
| -1373.893 | 19606.99 | 1.77 | 14.225 | 0.0778496 | 7/15/2014 | 03:48.3 | 1 |
| -1374.22 | 19607.002 | 1.77 | 14.232 | 0.0742497 | 7/15/2014 | 03:48.4 | 1 |
| -1374.485 | 19607.012 | 1.77 | 14.238 | 0.076665 | 7/15/2014 | 03:48.5 | 1 |
| -1374.751 | 19607.023 | 1.768 | 14.244 | 0.0778453 | 7/15/2014 | 03:48.6 | 1 |
| -1375.021 | 19607.033 | 1.77 | 14.25 | 0.075401 | 7/15/2014 | 03:48.7 | 1 |
| -1375.289 | 19607.045 | 1.768 | 14.256 | 0.0774699 | 7/15/2014 | 03:48.8 | 1 |
| -1375.56 | 19607.057 | 1.773 | 14.261 | 0.0847247 | 7/15/2014 | 03:48.9 | 1 |
| -1375.828 | 19607.068 | 1.77 | 14.267 | 0.0874895 | 7/15/2014 | 03:49.0 | 1 |
| -1376.099 | 19607.077 | 1.77 | 14.271 | 0.0865891 | 7/15/2014 | 03:49.1 | 1 |

| | | | | | | | |
|---|---|---|---|---|---|---|---|
| -1376.367 | 19607.089 | 1.77 | 14.274 | 0.101844 | 7/15/2014 | 03:49.2 | 1 |
| -1376.636 | 19607.1 | 1.768 | 14.277 | 0.0995582 | 7/15/2014 | 03:49.3 | 1 |
| -1376.901 | 19607.111 | 1.763 | 14.277 | 0.104597 | 7/15/2014 | 03:49.4 | 1 |
| -1377.165 | 19607.123 | 1.763 | 14.275 | 0.109813 | 7/15/2014 | 03:49.5 | 1 |
| -1377.425 | 19607.135 | 1.76 | 14.271 | 0.107948 | 7/15/2014 | 03:49.6 | 1 |
| -1377.683 | 19607.144 | 1.762 | 14.265 | 0.105077 | 7/15/2014 | 03:49.7 | 1 |
| -1378.002 | 19607.159 | 1.762 | 14.255 | 0.103175 | 7/15/2014 | 03:49.8 | 1 |
| -1378.258 | 19607.168 | 1.762 | 14.244 | 0.0938652 | 7/15/2014 | 03:49.9 | 1 |
| -1378.514 | 19607.18 | 1.765 | 14.23 | 0.0956174 | 7/15/2014 | 03:50.0 | 1 |
| -1378.768 | 19607.191 | 1.769 | 14.213 | 0.100442 | 7/15/2014 | 03:50.1 | 1 |
| -1379.023 | 19607.203 | 1.769 | 14.195 | 0.11142 | 7/15/2014 | 03:50.2 | 1 |
| -1379.277 | 19607.213 | 1.769 | 14.174 | 0.111176 | 7/15/2014 | 03:50.3 | 1 |
| -1379.594 | 19607.229 | 1.767 | 14.152 | 0.137678 | 7/15/2014 | 03:50.4 | 1 |
| -1379.85 | 19607.238 | 1.767 | 14.129 | 0.1439 | 7/15/2014 | 03:50.5 | 1 |
| -1380.109 | 19607.25 | 1.767 | 14.105 | 0.120252 | 7/15/2014 | 03:50.6 | 1 |
| -1380.37 | 19607.264 | 1.769 | 14.081 | 0.120858 | 7/15/2014 | 03:50.7 | 1 |
| -1380.631 | 19607.275 | 1.765 | 14.058 | 0.106509 | 7/15/2014 | 03:50.8 | 1 |
| -1380.896 | 19607.287 | 1.765 | 14.036 | 0.110581 | 7/15/2014 | 03:50.9 | 1 |
| -1381.16 | 19607.299 | 1.765 | 14.018 | 0.0977284 | 7/15/2014 | 03:51.0 | 1 |
| -1381.427 | 19607.311 | 1.769 | 14.003 | 0.101724 | 7/15/2014 | 03:51.1 | 1 |
| -1381.693 | 19607.322 | 1.772 | 13.993 | 0.109741 | 7/15/2014 | 03:51.2 | 1 |
| -1381.959 | 19607.336 | 1.774 | 13.988 | 0.100759 | 7/15/2014 | 03:51.3 | 1 |
| -1382.224 | 19607.349 | 1.774 | 13.988 | 0.104645 | 7/15/2014 | 03:51.4 | 1 |
| -1382.489 | 19607.361 | 1.779 | 13.993 | 0.108793 | 7/15/2014 | 03:51.5 | 1 |
| -1382.751 | 19607.373 | 1.779 | 14.003 | 0.105544 | 7/15/2014 | 03:51.6 | 1 |
| -1383.014 | 19607.385 | 1.781 | 14.016 | 0.106248 | 7/15/2014 | 03:51.7 | 1 |
| -1383.338 | 19607.4 | 1.781 | 14.033 | 0.108083 | 7/15/2014 | 03:51.8 | 1 |
| -1383.6 | 19607.412 | 1.784 | 14.05 | 0.111243 | 7/15/2014 | 03:51.9 | 1 |
| -1383.86 | 19607.425 | 1.781 | 14.068 | 0.113512 | 7/15/2014 | 03:52.0 | 1 |
| -1384.122 | 19607.438 | 1.784 | 14.084 | 0.113191 | 7/15/2014 | 03:52.1 | 1 |
| -1384.383 | 19607.451 | 1.781 | 14.098 | 0.114254 | 7/15/2014 | 03:52.2 | 1 |
| -1384.644 | 19607.463 | 1.784 | 14.11 | 0.11544 | 7/15/2014 | 03:52.3 | 1 |
| -1384.969 | 19607.479 | 1.784 | 14.119 | 0.116291 | 7/15/2014 | 03:52.4 | 1 |
| -1385.234 | 19607.492 | 1.786 | 14.125 | 0.11698 | 7/15/2014 | 03:52.5 | 1 |
| -1385.498 | 19607.504 | 1.784 | 14.128 | 0.117792 | 7/15/2014 | 03:52.6 | 1 |
| -1385.766 | 19607.518 | 1.795 | 14.128 | 0.117928 | 7/15/2014 | 03:52.7 | 1 |
| -1386.032 | 19607.531 | 1.792 | 14.126 | 0.121527 | 7/15/2014 | 03:52.8 | 1 |
| -1386.301 | 19607.545 | 1.795 | 14.122 | 0.119429 | 7/15/2014 | 03:52.9 | 1 |
| -1386.568 | 19607.557 | 1.795 | 14.117 | 0.125749 | 7/15/2014 | 03:53.0 | 1 |
| -1386.837 | 19607.572 | 1.801 | 14.111 | 0.131617 | 7/15/2014 | 03:53.1 | 1 |
| -1387.104 | 19607.584 | 1.796 | 14.105 | 0.129996 | 7/15/2014 | 03:53.2 | 1 |
| -1387.371 | 19607.599 | 1.796 | 14.1 | 0.119267 | 7/15/2014 | 03:53.3 | 1 |
| -1387.635 | 19607.611 | 1.785 | 14.096 | 0.122365 | 7/15/2014 | 03:53.4 | 1 |
| -1387.899 | 19607.625 | 1.791 | 14.095 | 0.136046 | 7/15/2014 | 03:53.5 | 1 |
| -1388.159 | 19607.639 | 1.791 | 14.097 | 0.120475 | 7/15/2014 | 03:53.6 | 1 |
| -1388.418 | 19607.652 | 1.793 | 14.103 | 0.115439 | 7/15/2014 | 03:53.7 | 1 |
| -1388.739 | 19607.668 | 1.787 | 14.112 | 0.122338 | 7/15/2014 | 03:53.8 | 1 |

| | | | | | | | |
|---|---|---|---|---|---|---|---|
| -1388.997 | 19607.681 | 1.792 | 14.125 | 0.123716 | 7/15/2014 | 03:53.9 | 1 |
| -1389.253 | 19607.695 | 1.792 | 14.14 | 0.137091 | 7/15/2014 | 03:54.0 | 1 |
| -1389.512 | 19607.707 | 1.801 | 14.157 | 0.118431 | 7/15/2014 | 03:54.1 | 1 |
| -1389.769 | 19607.723 | 1.797 | 14.174 | 0.126193 | 7/15/2014 | 03:54.2 | 1 |
| -1390.027 | 19607.735 | 1.799 | 14.191 | 0.132508 | 7/15/2014 | 03:54.3 | 1 |
| -1390.347 | 19607.752 | 1.801 | 14.207 | 0.124579 | 7/15/2014 | 03:54.4 | 1 |
| -1390.608 | 19607.766 | 1.801 | 14.221 | 0.135347 | 7/15/2014 | 03:54.5 | 1 |
| -1390.868 | 19607.78 | 1.801 | 14.232 | 0.130196 | 7/15/2014 | 03:54.6 | 1 |
| -1391.132 | 19607.793 | 1.803 | 14.242 | 0.141249 | 7/15/2014 | 03:54.7 | 1 |
| -1391.395 | 19607.809 | 1.797 | 14.248 | 0.138349 | 7/15/2014 | 03:54.8 | 1 |
| -1391.66 | 19607.823 | 1.797 | 14.252 | 0.141046 | 7/15/2014 | 03:54.9 | 1 |
| -1391.923 | 19607.836 | 1.795 | 14.252 | 0.138745 | 7/15/2014 | 03:55.0 | 1 |
| -1392.189 | 19607.852 | 1.797 | 14.25 | 0.127763 | 7/15/2014 | 03:55.1 | 1 |
| -1392.453 | 19607.866 | 1.803 | 14.246 | 0.128703 | 7/15/2014 | 03:55.2 | 1 |
| -1392.714 | 19607.881 | 1.807 | 14.239 | 0.132619 | 7/15/2014 | 03:55.3 | 1 |
| -1392.974 | 19607.895 | 1.807 | 14.23 | 0.136373 | 7/15/2014 | 03:55.4 | 1 |
| -1393.233 | 19607.91 | 1.813 | 14.22 | 0.151325 | 7/15/2014 | 03:55.5 | 1 |
| -1393.488 | 19607.922 | 1.813 | 14.209 | 0.141648 | 7/15/2014 | 03:55.6 | 1 |
| -1393.743 | 19607.938 | 1.815 | 14.197 | 0.156289 | 7/15/2014 | 03:55.7 | 1 |
| -1394.055 | 19607.956 | 1.809 | 14.187 | 0.146235 | 7/15/2014 | 03:55.8 | 1 |
| -1394.306 | 19607.971 | 1.805 | 14.178 | 0.108859 | 7/15/2014 | 03:55.9 | 1 |
| -1394.556 | 19607.984 | 1.802 | 14.171 | 0.137721 | 7/15/2014 | 03:56.0 | 1 |
| -1394.806 | 19607.999 | 1.802 | 14.167 | 0.11837 | 7/15/2014 | 03:56.1 | 1 |
| -1395.054 | 19608.012 | 1.793 | 14.167 | 0.12044 | 7/15/2014 | 03:56.2 | 1 |
| -1395.302 | 19608.027 | 1.791 | 14.17 | 0.128344 | 7/15/2014 | 03:56.3 | 1 |
| -1395.74 | 19608.054 | 1.787 | 14.184 | 0.155819 | 7/15/2014 | 03:56.4 | 1 |
| -1395.862 | 19608.061 | 1.788 | 14.188 | 0.2007 | 7/15/2014 | 03:56.5 | 1 |
| -1396.112 | 19608.076 | 1.783 | 14.201 | 0.157607 | 7/15/2014 | 03:56.6 | 1 |
| -1396.365 | 19608.091 | 1.78 | 14.217 | 0.169521 | 7/15/2014 | 03:56.7 | 1 |
| -1396.616 | 19608.105 | 1.776 | 14.235 | 0.225436 | 7/15/2014 | 03:56.8 | 1 |
| -1396.871 | 19608.121 | 1.778 | 14.252 | 0.205027 | 7/15/2014 | 03:56.9 | 1 |
| -1397.124 | 19608.137 | 1.776 | 14.268 | 0.184259 | 7/15/2014 | 03:57.0 | 1 |
| -1397.38 | 19608.152 | 1.776 | 14.281 | 0.1611 | 7/15/2014 | 03:57.1 | 1 |
| -1397.633 | 19608.169 | 1.765 | 14.29 | 0.168561 | 7/15/2014 | 03:57.2 | 1 |
| -1397.888 | 19608.185 | 1.765 | 14.294 | 0.180908 | 7/15/2014 | 03:57.3 | 1 |
| -1398.139 | 19608.2 | 1.762 | 14.293 | 0.140806 | 7/15/2014 | 03:57.4 | 1 |
| -1398.392 | 19608.216 | 1.762 | 14.287 | 0.120425 | 7/15/2014 | 03:57.5 | 1 |
| -1398.64 | 19608.231 | 1.754 | 14.277 | 0.106145 | 7/15/2014 | 03:57.6 | 1 |
| -1399.073 | 19608.259 | 1.745 | 14.257 | 0.143692 | 7/15/2014 | 03:57.7 | 1 |
| -1399.2 | 19608.268 | 1.742 | 14.251 | 0.144484 | 7/15/2014 | 03:57.8 | 1 |
| -1399.45 | 19608.284 | 1.745 | 14.237 | 0.128869 | 7/15/2014 | 03:57.9 | 1 |
| -1399.699 | 19608.3 | 1.743 | 14.226 | 0.133928 | 7/15/2014 | 03:58.0 | 1 |
| -1399.951 | 19608.315 | 1.739 | 14.216 | 0.177927 | 7/15/2014 | 03:58.1 | 1 |
| -1400.201 | 19608.332 | 1.737 | 14.211 | 0.183334 | 7/15/2014 | 03:58.2 | 1 |
| -1400.453 | 19608.349 | 1.74 | 14.208 | 0.177212 | 7/15/2014 | 03:58.3 | 1 |
| -1400.769 | 19608.369 | 1.74 | 14.209 | 0.172377 | 7/15/2014 | 03:58.4 | 1 |
| -1401.024 | 19608.387 | 1.744 | 14.213 | 0.205491 | 7/15/2014 | 03:58.5 | 1 |

| | | | | | | | |
|---|---|---|---|---|---|---|---|
| -1401.282 | 19608.403 | 1.74 | 14.218 | 0.211158 | 7/15/2014 | 03:58.6 | 1 |
| -1401.542 | 19608.42 | 1.74 | 14.225 | 0.174504 | 7/15/2014 | 03:58.7 | 1 |
| -1401.802 | 19608.438 | 1.736 | 14.232 | 0.175145 | 7/15/2014 | 03:58.8 | 1 |
| -1402.064 | 19608.455 | 1.732 | 14.24 | 0.168861 | 7/15/2014 | 03:58.9 | 1 |
| -1402.327 | 19608.474 | 1.722 | 14.247 | 0.17834 | 7/15/2014 | 03:59.0 | 1 |
| -1402.589 | 19608.491 | 1.722 | 14.253 | 0.122397 | 7/15/2014 | 03:59.1 | 1 |
| -1402.85 | 19608.509 | 1.719 | 14.258 | 0.119445 | 7/15/2014 | 03:59.2 | 1 |
| -1403.11 | 19608.526 | 1.722 | 14.262 | 0.129981 | 7/15/2014 | 03:59.3 | 1 |
| -1403.367 | 19608.544 | 1.722 | 14.264 | 0.125988 | 7/15/2014 | 03:59.4 | 1 |
| -1403.625 | 19608.563 | 1.728 | 14.264 | 0.123591 | 7/15/2014 | 03:59.5 | 1 |
| -1403.879 | 19608.58 | 1.723 | 14.263 | 0.115414 | 7/15/2014 | 03:59.6 | 1 |
| -1404.131 | 19608.599 | 1.731 | 14.26 | 0.156207 | 7/15/2014 | 03:59.7 | 1 |
| -1404.442 | 19608.62 | 1.734 | 14.256 | 0.166166 | 7/15/2014 | 03:59.8 | 1 |
| -1404.692 | 19608.638 | 1.74 | 14.252 | 0.161712 | 7/15/2014 | 03:59.9 | 1 |
| -1404.939 | 19608.656 | 1.738 | 14.247 | 0.167725 | 7/15/2014 | 04:00.0 | 1 |
| -1405.188 | 19608.675 | 1.738 | 14.242 | 0.15681 | 7/15/2014 | 04:00.1 | 1 |
| -1405.434 | 19608.691 | 1.738 | 14.236 | 0.193565 | 7/15/2014 | 04:00.2 | 1 |
| -1405.68 | 19608.711 | 1.747 | 14.232 | 0.186983 | 7/15/2014 | 04:00.3 | 1 |
| -1406.108 | 19608.742 | 1.74 | 14.225 | 0.171205 | 7/15/2014 | 04:00.4 | 1 |
| -1406.352 | 19608.76 | 1.74 | 14.221 | 0.198951 | 7/15/2014 | 04:00.5 | 1 |
| -1406.595 | 19608.779 | 1.736 | 14.217 | 0.191445 | 7/15/2014 | 04:00.6 | 1 |
| -1406.902 | 19608.802 | 1.733 | 14.215 | 0.19417 | 7/15/2014 | 04:00.7 | 1 |
| -1407.025 | 19608.811 | 1.731 | 14.213 | 0.189474 | 7/15/2014 | 04:00.8 | 1 |
| -1407.273 | 19608.83 | 1.731 | 14.211 | 0.18455 | 7/15/2014 | 04:00.9 | 1 |
| -1407.519 | 19608.85 | 1.725 | 14.211 | 0.184734 | 7/15/2014 | 04:01.0 | 1 |
| -1407.767 | 19608.868 | 1.727 | 14.212 | 0.189544 | 7/15/2014 | 04:01.1 | 1 |
| -1408.014 | 19608.887 | 1.731 | 14.213 | 0.16648 | 7/15/2014 | 04:01.2 | 1 |
| -1408.261 | 19608.906 | 1.731 | 14.216 | 0.192561 | 7/15/2014 | 04:01.3 | 1 |
| -1408.693 | 19608.939 | 1.737 | 14.221 | 0.174098 | 7/15/2014 | 04:01.4 | 1 |
| -1408.939 | 19608.959 | 1.737 | 14.224 | 0.187278 | 7/15/2014 | 04:01.5 | 1 |
| -1409.186 | 19608.979 | 1.737 | 14.227 | 0.180506 | 7/15/2014 | 04:01.6 | 1 |
| -1409.494 | 19609.002 | 1.74 | 14.229 | 0.165887 | 7/15/2014 | 04:01.7 | 1 |
| -1409.619 | 19609.012 | 1.743 | 14.231 | 0.167802 | 7/15/2014 | 04:01.8 | 1 |
| -1409.868 | 19609.031 | 1.743 | 14.231 | 0.127253 | 7/15/2014 | 04:01.9 | 1 |
| -1410.116 | 19609.051 | 1.737 | 14.23 | 0.130812 | 7/15/2014 | 04:02.0 | 1 |
| -1410.366 | 19609.072 | 1.734 | 14.227 | 0.133518 | 7/15/2014 | 04:02.1 | 1 |
| -1410.613 | 19609.092 | 1.732 | 14.222 | 0.146547 | 7/15/2014 | 04:02.2 | 1 |
| -1410.863 | 19609.111 | 1.734 | 14.214 | 0.161598 | 7/15/2014 | 04:02.3 | 1 |
| -1411.172 | 19609.135 | 1.731 | 14.204 | 0.147446 | 7/15/2014 | 04:02.4 | 1 |
| -1411.421 | 19609.156 | 1.733 | 14.193 | 0.188588 | 7/15/2014 | 04:02.5 | 1 |
| -1411.669 | 19609.176 | 1.733 | 14.182 | 0.170424 | 7/15/2014 | 04:02.6 | 1 |
| -1411.919 | 19609.197 | 1.739 | 14.171 | 0.173501 | 7/15/2014 | 04:02.7 | 1 |
| -1412.23 | 19609.223 | 1.733 | 14.161 | 0.134229 | 7/15/2014 | 04:02.8 | 1 |
| -1412.483 | 19609.244 | 1.736 | 14.153 | 0.136064 | 7/15/2014 | 04:02.9 | 1 |
| -1412.734 | 19609.264 | 1.733 | 14.147 | 0.133392 | 7/15/2014 | 04:03.0 | 1 |
| -1412.988 | 19609.285 | 1.737 | 14.144 | 0.128692 | 7/15/2014 | 04:03.1 | 1 |
| -1413.24 | 19609.307 | 1.731 | 14.144 | 0.154533 | 7/15/2014 | 04:03.2 | 1 |

| | | | | | | | |
|---|---|---|---|---|---|---|---|
| -1413.494 | 19609.328 | 1.727 | 14.146 | 0.120909 | 7/15/2014 | 04:03.3 | 1 |
| -1413.807 | 19609.354 | 1.724 | 14.15 | 0.147618 | 7/15/2014 | 04:03.4 | 1 |
| -1414.061 | 19609.377 | 1.728 | 14.155 | 0.146975 | 7/15/2014 | 04:03.5 | 1 |
| -1414.313 | 19609.396 | 1.722 | 14.162 | 0.14637 | 7/15/2014 | 04:03.6 | 1 |
| -1414.567 | 19609.418 | 1.722 | 14.169 | 0.171461 | 7/15/2014 | 04:03.7 | 1 |
| -1414.881 | 19609.445 | 1.715 | 14.175 | 0.138831 | 7/15/2014 | 04:03.8 | 1 |
| -1415.135 | 19609.467 | 1.715 | 14.179 | 0.177593 | 7/15/2014 | 04:03.9 | 1 |
| -1415.387 | 19609.488 | 1.707 | 14.182 | 0.159456 | 7/15/2014 | 04:04.0 | 1 |
| -1415.641 | 19609.511 | 1.701 | 14.183 | 0.161451 | 7/15/2014 | 04:04.1 | 1 |
| -1415.894 | 19609.531 | 1.697 | 14.181 | 0.170542 | 7/15/2014 | 04:04.2 | 1 |
| -1416.146 | 19609.555 | 1.697 | 14.177 | 0.150066 | 7/15/2014 | 04:04.3 | 1 |
| -1416.458 | 19609.58 | 1.701 | 14.171 | 0.165712 | 7/15/2014 | 04:04.4 | 1 |
| -1416.708 | 19609.602 | 1.701 | 14.163 | 0.158911 | 7/15/2014 | 04:04.5 | 1 |
| -1416.955 | 19609.623 | 1.694 | 14.154 | 0.161071 | 7/15/2014 | 04:04.6 | 1 |
| -1417.206 | 19609.646 | 1.701 | 14.144 | 0.181645 | 7/15/2014 | 04:04.7 | 1 |
| -1417.514 | 19609.672 | 1.703 | 14.134 | 0.175408 | 7/15/2014 | 04:04.8 | 1 |
| -1417.762 | 19609.693 | 1.707 | 14.124 | 0.1972 | 7/15/2014 | 04:04.9 | 1 |
| -1418.009 | 19609.714 | 1.701 | 14.115 | 0.174478 | 7/15/2014 | 04:05.0 | 1 |
| -1418.256 | 19609.736 | 1.701 | 14.107 | 0.173089 | 7/15/2014 | 04:05.1 | 1 |
| -1418.501 | 19609.758 | 1.698 | 14.1 | 0.192329 | 7/15/2014 | 04:05.2 | 1 |
| -1418.746 | 19609.779 | 1.702 | 14.096 | 0.178551 | 7/15/2014 | 04:05.3 | 1 |
| -1419.17 | 19609.816 | 1.697 | 14.095 | 0.181918 | 7/15/2014 | 04:05.4 | 1 |
| -1419.41 | 19609.836 | 1.694 | 14.097 | 0.174904 | 7/15/2014 | 04:05.5 | 1 |
| -1419.65 | 19609.857 | 1.694 | 14.102 | 0.166866 | 7/15/2014 | 04:05.6 | 1 |
| -1419.951 | 19609.884 | 1.702 | 14.11 | 0.198915 | 7/15/2014 | 04:05.7 | 1 |
| -1420.073 | 19609.896 | 1.698 | 14.113 | 0.192314 | 7/15/2014 | 04:05.8 | 1 |
| -1420.314 | 19609.916 | 1.701 | 14.123 | 0.169686 | 7/15/2014 | 04:05.9 | 1 |
| -1420.554 | 19609.937 | 1.707 | 14.134 | 0.1818 | 7/15/2014 | 04:06.0 | 1 |
| -1420.796 | 19609.959 | 1.713 | 14.145 | 0.178064 | 7/15/2014 | 04:06.1 | 1 |
| -1421.035 | 19609.979 | 1.716 | 14.154 | 0.177186 | 7/15/2014 | 04:06.2 | 1 |
| -1421.276 | 19610.001 | 1.724 | 14.16 | 0.201662 | 7/15/2014 | 04:06.3 | 1 |
| -1421.695 | 19610.037 | 1.718 | 14.161 | 0.201006 | 7/15/2014 | 04:06.4 | 1 |
| -1421.934 | 19610.059 | 1.715 | 14.157 | 0.204443 | 7/15/2014 | 04:06.5 | 1 |
| -1422.174 | 19610.078 | 1.718 | 14.15 | 0.2151 | 7/15/2014 | 04:06.6 | 1 |
| -1422.472 | 19610.105 | 1.715 | 14.138 | 0.216022 | 7/15/2014 | 04:06.7 | 1 |
| -1422.593 | 19610.115 | 1.716 | 14.133 | 0.22481 | 7/15/2014 | 04:06.8 | 1 |
| -1422.835 | 19610.138 | 1.719 | 14.115 | 0.231832 | 7/15/2014 | 04:06.9 | 1 |
| -1423.075 | 19610.158 | 1.715 | 14.094 | 0.236814 | 7/15/2014 | 04:07.0 | 1 |
| -1423.32 | 19610.18 | 1.725 | 14.072 | 0.238632 | 7/15/2014 | 04:07.1 | 1 |
| -1423.562 | 19610.201 | 1.727 | 14.048 | 0.240642 | 7/15/2014 | 04:07.2 | 1 |
| -1423.805 | 19610.222 | 1.736 | 14.026 | 0.241601 | 7/15/2014 | 04:07.3 | 1 |
| -1424.226 | 19610.258 | 1.746 | 13.998 | 0.236621 | 7/15/2014 | 04:07.4 | 1 |
| -1424.468 | 19610.279 | 1.751 | 13.982 | 0.235519 | 7/15/2014 | 04:07.5 | 1 |
| -1424.709 | 19610.301 | 1.753 | 13.969 | 0.228638 | 7/15/2014 | 04:07.6 | 1 |
| -1425.012 | 19610.327 | 1.761 | 13.959 | 0.213585 | 7/15/2014 | 04:07.7 | 1 |
| -1425.134 | 19610.338 | 1.762 | 13.954 | 0.211483 | 7/15/2014 | 04:07.8 | 1 |
| -1425.38 | 19610.359 | 1.767 | 13.95 | 0.197077 | 7/15/2014 | 04:07.9 | 1 |

| | | | | | | | |
|---|---|---|---|---|---|---|---|
| -1425.623 | 19610.381 | 1.765 | 13.95 | 0.176661 | 7/15/2014 | 04:08.0 | 1 |
| -1425.87 | 19610.401 | 1.767 | 13.953 | 0.167689 | 7/15/2014 | 04:08.1 | 1 |
| -1426.114 | 19610.423 | 1.762 | 13.958 | 0.152575 | 7/15/2014 | 04:08.2 | 1 |
| -1426.36 | 19610.443 | 1.767 | 13.966 | 0.138825 | 7/15/2014 | 04:08.3 | 1 |
| -1426.789 | 19610.48 | 1.767 | 13.983 | 0.138786 | 7/15/2014 | 04:08.4 | 1 |
| -1427.035 | 19610.503 | 1.769 | 13.994 | 0.14061 | 7/15/2014 | 04:08.5 | 1 |
| -1427.28 | 19610.523 | 1.772 | 14.005 | 0.15695 | 7/15/2014 | 04:08.6 | 1 |
| -1427.59 | 19610.55 | 1.774 | 14.016 | 0.145491 | 7/15/2014 | 04:08.7 | 1 |
| -1427.714 | 19610.561 | 1.773 | 14.022 | 0.148679 | 7/15/2014 | 04:08.8 | 1 |
| -1427.963 | 19610.582 | 1.776 | 14.032 | 0.156371 | 7/15/2014 | 04:08.9 | 1 |
| -1428.213 | 19610.604 | 1.779 | 14.04 | 0.158234 | 7/15/2014 | 04:09.0 | 1 |
| -1428.463 | 19610.625 | 1.785 | 14.046 | 0.159072 | 7/15/2014 | 04:09.1 | 1 |
| -1428.713 | 19610.646 | 1.788 | 14.05 | 0.158121 | 7/15/2014 | 04:09.2 | 1 |
| -1428.963 | 19610.668 | 1.788 | 14.052 | 0.16582 | 7/15/2014 | 04:09.3 | 1 |
| -1429.274 | 19610.693 | 1.782 | 14.054 | 0.15721 | 7/15/2014 | 04:09.4 | 1 |
| -1429.524 | 19610.717 | 1.784 | 14.055 | 0.152168 | 7/15/2014 | 04:09.5 | 1 |
| -1429.774 | 19610.736 | 1.786 | 14.055 | 0.146197 | 7/15/2014 | 04:09.6 | 1 |
| -1430.024 | 19610.758 | 1.783 | 14.055 | 0.146339 | 7/15/2014 | 04:09.7 | 1 |
| -1430.335 | 19610.783 | 1.783 | 14.055 | 0.140503 | 7/15/2014 | 04:09.8 | 1 |
| -1430.586 | 19610.807 | 1.789 | 14.055 | 0.138132 | 7/15/2014 | 04:09.9 | 1 |
| -1430.836 | 19610.826 | 1.789 | 14.055 | 0.135231 | 7/15/2014 | 04:10.0 | 1 |
| -1431.087 | 19610.848 | 1.795 | 14.054 | 0.128051 | 7/15/2014 | 04:10.1 | 1 |
| -1431.337 | 19610.869 | 1.795 | 14.053 | 0.124721 | 7/15/2014 | 04:10.2 | 1 |
| -1431.587 | 19610.889 | 1.795 | 14.05 | 0.120387 | 7/15/2014 | 04:10.3 | 1 |
| -1431.896 | 19610.916 | 1.795 | 14.047 | 0.115464 | 7/15/2014 | 04:10.4 | 1 |
| -1432.144 | 19610.938 | 1.791 | 14.043 | 0.114014 | 7/15/2014 | 04:10.5 | 1 |
| -1432.393 | 19610.959 | 1.782 | 14.039 | 0.107928 | 7/15/2014 | 04:10.6 | 1 |
| -1432.642 | 19610.979 | 1.785 | 14.034 | 0.109985 | 7/15/2014 | 04:10.7 | 1 |
| -1432.953 | 19611.006 | 1.781 | 14.028 | 0.114041 | 7/15/2014 | 04:10.8 | 1 |
| -1433.205 | 19611.025 | 1.783 | 14.023 | 0.114638 | 7/15/2014 | 04:10.9 | 1 |
| -1433.454 | 19611.049 | 1.781 | 14.018 | 0.111939 | 7/15/2014 | 04:11.0 | 1 |
| -1433.707 | 19611.068 | 1.783 | 14.013 | 0.121688 | 7/15/2014 | 04:11.1 | 1 |
| -1433.957 | 19611.092 | 1.783 | 14.008 | 0.119421 | 7/15/2014 | 04:11.2 | 1 |
| -1434.209 | 19611.111 | 1.783 | 14.002 | 0.122645 | 7/15/2014 | 04:11.3 | 1 |
| -1434.521 | 19611.139 | 1.778 | 13.996 | 0.110231 | 7/15/2014 | 04:11.4 | 1 |
| -1434.774 | 19611.158 | 1.778 | 13.988 | 0.11077 | 7/15/2014 | 04:11.5 | 1 |
| -1435.023 | 19611.18 | 1.771 | 13.979 | 0.104717 | 7/15/2014 | 04:11.6 | 1 |
| -1435.276 | 19611.201 | 1.771 | 13.968 | 0.102878 | 7/15/2014 | 04:11.7 | 1 |
| -1435.588 | 19611.229 | 1.766 | 13.956 | 0.0988279 | 7/15/2014 | 04:11.8 | 1 |
| -1435.841 | 19611.248 | 1.766 | 13.941 | 0.096885 | 7/15/2014 | 04:11.9 | 1 |
| -1436.093 | 19611.27 | 1.766 | 13.925 | 0.0933137 | 7/15/2014 | 04:12.0 | 1 |
| -1436.347 | 19611.291 | 1.766 | 13.908 | 0.0961079 | 7/15/2014 | 04:12.1 | 1 |
| -1436.598 | 19611.311 | 1.764 | 13.891 | 0.0949323 | 7/15/2014 | 04:12.2 | 1 |
| -1436.85 | 19611.332 | 1.769 | 13.874 | 0.108315 | 7/15/2014 | 04:12.3 | 1 |
| -1437.164 | 19611.359 | 1.769 | 13.86 | 0.101358 | 7/15/2014 | 04:12.4 | 1 |
| -1437.415 | 19611.379 | 1.766 | 13.849 | 0.106729 | 7/15/2014 | 04:12.5 | 1 |
| -1437.667 | 19611.399 | 1.761 | 13.84 | 0.108943 | 7/15/2014 | 04:12.6 | 1 |

| | | | | | | | |
|---|---|---|---|---|---|---|---|
| -1437.92 | 19611.422 | 1.764 | 13.836 | 0.0853406 | 7/15/2014 | 04:12.7 | 1 |
| -1438.236 | 19611.445 | 1.761 | 13.836 | 0.116106 | 7/15/2014 | 04:12.8 | 1 |
| -1438.491 | 19611.467 | 1.759 | 13.841 | 0.097139 | 7/15/2014 | 04:12.9 | 1 |
| -1379.265 | 19405.961 | 2.564 | 12.668 | 0.0953245 | 7/15/2014 | 10:24.8 | 2 |
| -1379.048 | 19405.946 | 2.567 | 12.683 | 0.10356 | 7/15/2014 | 10:24.9 | 2 |
| -1378.829 | 19405.932 | 2.573 | 12.704 | 0.106211 | 7/15/2014 | 10:25.0 | 2 |
| -1378.611 | 19405.917 | 2.576 | 12.733 | 0.120623 | 7/15/2014 | 10:25.1 | 2 |
| -1378.393 | 19405.902 | 2.578 | 12.757 | 0.109835 | 7/15/2014 | 10:25.2 | 2 |
| -1378.175 | 19405.888 | 2.578 | 12.783 | 0.103093 | 7/15/2014 | 10:25.3 | 2 |
| -1377.904 | 19405.87 | 2.58 | 12.813 | 0.111264 | 7/15/2014 | 10:25.4 | 2 |
| -1377.687 | 19405.855 | 2.58 | 12.849 | 0.112582 | 7/15/2014 | 10:25.5 | 2 |
| -1377.467 | 19405.841 | 2.583 | 12.879 | 0.111516 | 7/15/2014 | 10:25.6 | 2 |
| -1377.193 | 19405.823 | 2.583 | 12.899 | 0.115012 | 7/15/2014 | 10:25.7 | 2 |
| -1376.972 | 19405.808 | 2.581 | 12.916 | 0.111411 | 7/15/2014 | 10:25.8 | 2 |
| -1376.751 | 19405.793 | 2.581 | 12.923 | 0.111495 | 7/15/2014 | 10:25.9 | 2 |
| -1376.53 | 19405.777 | 2.578 | 12.929 | 0.111135 | 7/15/2014 | 10:26.0 | 2 |
| -1376.308 | 19405.763 | 2.578 | 12.933 | 0.110546 | 7/15/2014 | 10:26.1 | 2 |
| -1376.089 | 19405.747 | 2.573 | 12.933 | 0.11039 | 7/15/2014 | 10:26.2 | 2 |
| -1375.867 | 19405.733 | 2.575 | 12.925 | 0.110545 | 7/15/2014 | 10:26.3 | 2 |
| -1375.594 | 19405.715 | 2.573 | 12.922 | 0.11077 | 7/15/2014 | 10:26.4 | 2 |
| -1375.376 | 19405.699 | 2.573 | 12.918 | 0.111008 | 7/15/2014 | 10:26.5 | 2 |
| -1375.159 | 19405.686 | 2.575 | 12.911 | 0.112569 | 7/15/2014 | 10:26.6 | 2 |
| -1374.889 | 19405.666 | 2.575 | 12.906 | 0.113549 | 7/15/2014 | 10:26.7 | 2 |
| -1374.67 | 19405.65 | 2.578 | 12.903 | 0.112712 | 7/15/2014 | 10:26.8 | 2 |
| -1374.454 | 19405.635 | 2.58 | 12.904 | 0.115339 | 7/15/2014 | 10:26.9 | 2 |
| -1374.235 | 19405.621 | 2.583 | 12.903 | 0.114304 | 7/15/2014 | 10:27.0 | 2 |
| -1374.017 | 19405.605 | 2.581 | 12.902 | 0.11663 | 7/15/2014 | 10:27.1 | 2 |
| -1373.802 | 19405.59 | 2.583 | 12.904 | 0.11523 | 7/15/2014 | 10:27.2 | 2 |
| -1373.588 | 19405.576 | 2.585 | 12.911 | 0.116488 | 7/15/2014 | 10:27.3 | 2 |
| -1373.321 | 19405.557 | 2.585 | 12.897 | 0.116628 | 7/15/2014 | 10:27.4 | 2 |
| -1373.003 | 19405.533 | 2.586 | 12.844 | 0.122671 | 7/15/2014 | 10:27.6 | 2 |
| -1372.743 | 19405.514 | 2.581 | 12.814 | 0.118434 | 7/15/2014 | 10:27.7 | 2 |
| -1372.634 | 19405.507 | 2.581 | 12.802 | 0.119066 | 7/15/2014 | 10:27.7 | 2 |
| -1372.423 | 19405.491 | 2.581 | 12.764 | 0.119424 | 7/15/2014 | 10:27.8 | 2 |
| -1372.212 | 19405.477 | 2.581 | 12.724 | 0.119836 | 7/15/2014 | 10:27.9 | 2 |
| -1372 | 19405.462 | 2.579 | 12.679 | 0.12013 | 7/15/2014 | 10:28.0 | 2 |
| -1371.789 | 19405.446 | 2.577 | 12.635 | 0.120992 | 7/15/2014 | 10:28.1 | 2 |
| -1371.579 | 19405.431 | 2.575 | 12.615 | 0.121088 | 7/15/2014 | 10:28.2 | 2 |
| -1371.371 | 19405.415 | 2.577 | 12.606 | 0.121916 | 7/15/2014 | 10:28.3 | 2 |
| -1371.01 | 19405.389 | 2.579 | 12.614 | 0.125802 | 7/15/2014 | 10:28.5 | 2 |
| -1370.8 | 19405.373 | 2.584 | 12.631 | 0.125986 | 7/15/2014 | 10:28.6 | 2 |
| -1370.544 | 19405.354 | 2.586 | 12.637 | 0.126218 | 7/15/2014 | 10:28.7 | 2 |
| -1370.438 | 19405.347 | 2.589 | 12.645 | 0.126864 | 7/15/2014 | 10:28.7 | 2 |
| -1370.231 | 19405.331 | 2.591 | 12.668 | 0.133143 | 7/15/2014 | 10:28.8 | 2 |
| -1370.023 | 19405.314 | 2.593 | 12.677 | 0.131357 | 7/15/2014 | 10:28.9 | 2 |
| -1369.814 | 19405.298 | 2.595 | 12.706 | 0.131234 | 7/15/2014 | 10:29.0 | 2 |
| -1369.604 | 19405.284 | 2.593 | 12.738 | 0.13325 | 7/15/2014 | 10:29.1 | 2 |

| | | | | | | | |
|---|---|---|---|---|---|---|---|
| -1369.396 | 19405.268 | 2.593 | 12.76 | 0.132718 | 7/15/2014 | 10:29.2 | 2 |
| -1369.188 | 19405.252 | 2.593 | 12.785 | 0.132693 | 7/15/2014 | 10:29.3 | 2 |
| -1368.825 | 19405.223 | 2.589 | 12.799 | 0.1327 | 7/15/2014 | 10:29.5 | 2 |
| -1368.616 | 19405.207 | 2.588 | 12.818 | 0.140862 | 7/15/2014 | 10:29.6 | 2 |
| -1368.357 | 19405.186 | 2.586 | 12.827 | 0.135746 | 7/15/2014 | 10:29.7 | 2 |
| -1368.251 | 19405.177 | 2.586 | 12.845 | 0.136296 | 7/15/2014 | 10:29.7 | 2 |
| -1368.042 | 19405.161 | 2.586 | 12.847 | 0.136359 | 7/15/2014 | 10:29.8 | 2 |
| -1367.828 | 19405.145 | 2.591 | 12.848 | 0.135115 | 7/15/2014 | 10:29.9 | 2 |
| -1367.617 | 19405.129 | 2.591 | 12.856 | 0.135822 | 7/15/2014 | 10:30.0 | 2 |
| -1367.404 | 19405.111 | 2.589 | 12.868 | 0.131945 | 7/15/2014 | 10:30.1 | 2 |
| -1367.192 | 19405.094 | 2.587 | 12.883 | 0.146629 | 7/15/2014 | 10:30.2 | 2 |
| -1366.979 | 19405.076 | 2.589 | 12.898 | 0.142057 | 7/15/2014 | 10:30.3 | 2 |
| -1366.714 | 19405.055 | 2.588 | 12.912 | 0.156173 | 7/15/2014 | 10:30.4 | 2 |
| -1366.502 | 19405.037 | 2.584 | 12.93 | 0.145327 | 7/15/2014 | 10:30.5 | 2 |
| -1366.286 | 19405.02 | 2.582 | 12.953 | 0.147847 | 7/15/2014 | 10:30.6 | 2 |
| -1366.018 | 19404.998 | 2.582 | 12.974 | 0.154674 | 7/15/2014 | 10:30.7 | 2 |
| -1365.803 | 19404.98 | 2.585 | 12.992 | 0.163814 | 7/15/2014 | 10:30.8 | 2 |
| -1365.584 | 19404.963 | 2.589 | 12.998 | 0.13311 | 7/15/2014 | 10:30.9 | 2 |
| -1365.368 | 19404.943 | 2.589 | 13.005 | 0.119592 | 7/15/2014 | 10:31.0 | 2 |
| -1365.15 | 19404.926 | 2.59 | 13.018 | 0.125022 | 7/15/2014 | 10:31.1 | 2 |
| -1364.932 | 19404.908 | 2.591 | 13.027 | 0.125055 | 7/15/2014 | 10:31.2 | 2 |
| -1364.716 | 19404.889 | 2.591 | 13.032 | 0.124661 | 7/15/2014 | 10:31.3 | 2 |
| -1364.445 | 19404.865 | 2.591 | 13.03 | 0.121812 | 7/15/2014 | 10:31.4 | 2 |
| -1364.231 | 19404.848 | 2.585 | 13.032 | 0.118238 | 7/15/2014 | 10:31.5 | 2 |
| -1364.014 | 19404.83 | 2.582 | 13.033 | 0.114372 | 7/15/2014 | 10:31.6 | 2 |
| -1363.747 | 19404.807 | 2.581 | 13.032 | 0.118942 | 7/15/2014 | 10:31.7 | 2 |
| -1363.529 | 19404.788 | 2.586 | 13.034 | 0.115679 | 7/15/2014 | 10:31.8 | 2 |
| -1363.314 | 19404.77 | 2.585 | 13.028 | 0.093525 | 7/15/2014 | 10:31.9 | 2 |
| -1363.097 | 19404.752 | 2.585 | 13.024 | 0.101786 | 7/15/2014 | 10:32.0 | 2 |
| -1362.88 | 19404.732 | 2.585 | 13.024 | 0.104934 | 7/15/2014 | 10:32.1 | 2 |
| -1362.664 | 19404.713 | 2.585 | 13.022 | 0.101781 | 7/15/2014 | 10:32.2 | 2 |
| -1362.447 | 19404.695 | 2.585 | 13.018 | 0.104076 | 7/15/2014 | 10:32.3 | 2 |
| -1362.178 | 19404.672 | 2.585 | 13.015 | 0.0910995 | 7/15/2014 | 10:32.4 | 2 |
| -1361.962 | 19404.652 | 2.58 | 13.017 | 0.0837619 | 7/15/2014 | 10:32.5 | 2 |
| -1361.744 | 19404.633 | 2.572 | 13.016 | 0.0991594 | 7/15/2014 | 10:32.6 | 2 |
| -1361.527 | 19404.615 | 2.566 | 13.016 | 0.0954351 | 7/15/2014 | 10:32.7 | 2 |
| -1361.31 | 19404.597 | 2.563 | 13.017 | 0.0910247 | 7/15/2014 | 10:32.8 | 2 |
| -1361.09 | 19404.577 | 2.56 | 13.014 | 0.082309 | 7/15/2014 | 10:32.9 | 2 |
| -1360.871 | 19404.559 | 2.56 | 13.013 | 0.0944617 | 7/15/2014 | 10:33.0 | 2 |
| -1360.654 | 19404.539 | 2.56 | 13.006 | 0.0931777 | 7/15/2014 | 10:33.1 | 2 |
| -1360.438 | 19404.52 | 2.555 | 12.998 | 0.101936 | 7/15/2014 | 10:33.2 | 2 |
| -1360.219 | 19404.501 | 2.561 | 12.983 | 0.0929548 | 7/15/2014 | 10:33.4 | 2 |
| -1360.006 | 19404.482 | 2.564 | 12.97 | 0.0830088 | 7/15/2014 | 10:33.5 | 2 |
| -1359.691 | 19404.456 | 2.564 | 12.945 | 0.0834776 | 7/15/2014 | 10:33.6 | 2 |
| -1359.426 | 19404.433 | 2.561 | 12.928 | 0.100742 | 7/15/2014 | 10:33.7 | 2 |
| -1359.316 | 19404.424 | 2.564 | 12.918 | 0.0865811 | 7/15/2014 | 10:33.7 | 2 |
| -1359.104 | 19404.404 | 2.564 | 12.9 | 0.0910029 | 7/15/2014 | 10:33.8 | 2 |

| | | | | | | | |
|---|---|---|---|---|---|---|---|
| -1358.892 | 19404.385 | 2.564 | 12.886 | 0.102128 | 7/15/2014 | 10:33.9 | 2 |
| -1358.677 | 19404.366 | 2.564 | 12.875 | 0.0957466 | 7/15/2014 | 10:34.0 | 2 |
| -1358.464 | 19404.35 | 2.558 | 12.864 | 0.117462 | 7/15/2014 | 10:34.1 | 2 |
| -1358.249 | 19404.33 | 2.558 | 12.858 | 0.101761 | 7/15/2014 | 10:34.2 | 2 |
| -1358.036 | 19404.313 | 2.561 | 12.853 | 0.103116 | 7/15/2014 | 10:34.3 | 2 |
| -1357.767 | 19404.289 | 2.564 | 12.848 | 0.0931845 | 7/15/2014 | 10:34.4 | 2 |
| -1357.549 | 19404.269 | 2.564 | 12.845 | 0.10434 | 7/15/2014 | 10:34.5 | 2 |
| -1357.33 | 19404.249 | 2.561 | 12.846 | 0.0823449 | 7/15/2014 | 10:34.6 | 2 |
| -1357.108 | 19404.229 | 2.561 | 12.854 | 0.0889745 | 7/15/2014 | 10:34.7 | 2 |
| -1356.885 | 19404.211 | 2.561 | 12.858 | 0.0740787 | 7/15/2014 | 10:34.8 | 2 |
| -1356.659 | 19404.191 | 2.564 | 12.872 | 0.07756 | 7/15/2014 | 10:34.9 | 2 |
| -1356.435 | 19404.172 | 2.563 | 12.907 | 0.0736206 | 7/15/2014 | 10:35.0 | 2 |
| -1356.209 | 19404.152 | 2.563 | 12.955 | 0.0797895 | 7/15/2014 | 10:35.1 | 2 |
| -1355.985 | 19404.132 | 2.56 | 13.003 | 0.0795262 | 7/15/2014 | 10:35.2 | 2 |
| -1355.762 | 19404.112 | 2.56 | 13.054 | 0.082995 | 7/15/2014 | 10:35.3 | 2 |
| -1355.538 | 19404.094 | 2.557 | 13.11 | 0.0821903 | 7/15/2014 | 10:35.4 | 2 |
| -1355.316 | 19404.073 | 2.557 | 13.162 | 0.0866628 | 7/15/2014 | 10:35.5 | 2 |
| -1355.096 | 19404.055 | 2.554 | 13.221 | 0.0881074 | 7/15/2014 | 10:35.6 | 2 |
| -1354.823 | 19404.031 | 2.549 | 13.271 | 0.0905323 | 7/15/2014 | 10:35.7 | 2 |
| -1354.602 | 19404.012 | 2.546 | 13.297 | 0.0931599 | 7/15/2014 | 10:35.8 | 2 |
| -1354.382 | 19403.992 | 2.543 | 13.311 | 0.0905687 | 7/15/2014 | 10:35.9 | 2 |
| -1354.161 | 19403.973 | 2.54 | 13.328 | 0.098527 | 7/15/2014 | 10:36.0 | 2 |
| -1353.941 | 19403.954 | 2.54 | 13.344 | 0.0974018 | 7/15/2014 | 10:36.1 | 2 |
| -1353.721 | 19403.937 | 2.54 | 13.353 | 0.0957659 | 7/15/2014 | 10:36.2 | 2 |
| -1353.5 | 19403.918 | 2.537 | 13.374 | 0.0978002 | 7/15/2014 | 10:36.3 | 2 |
| -1353.227 | 19403.895 | 2.54 | 13.378 | 0.101409 | 7/15/2014 | 10:36.4 | 2 |
| -1353.003 | 19403.873 | 2.542 | 13.383 | 0.0814041 | 7/15/2014 | 10:36.5 | 2 |
| -1352.779 | 19403.854 | 2.542 | 13.39 | 0.0806628 | 7/15/2014 | 10:36.6 | 2 |
| -1352.553 | 19403.834 | 2.542 | 13.403 | 0.0771372 | 7/15/2014 | 10:36.7 | 2 |
| -1352.326 | 19403.814 | 2.542 | 13.406 | 0.0801218 | 7/15/2014 | 10:36.8 | 2 |
| -1352.098 | 19403.797 | 2.542 | 13.403 | 0.0783995 | 7/15/2014 | 10:36.9 | 2 |
| -1351.87 | 19403.779 | 2.542 | 13.395 | 0.0802969 | 7/15/2014 | 10:37.0 | 2 |
| -1351.643 | 19403.758 | 2.538 | 13.381 | 0.0780208 | 7/15/2014 | 10:37.1 | 2 |
| -1351.414 | 19403.738 | 2.537 | 13.366 | 0.0808177 | 7/15/2014 | 10:37.2 | 2 |
| -1351.186 | 19403.719 | 2.537 | 13.355 | 0.0792888 | 7/15/2014 | 10:37.3 | 2 |
| -1350.961 | 19403.699 | 2.534 | 13.337 | 0.0851622 | 7/15/2014 | 10:37.4 | 2 |
| -1350.737 | 19403.68 | 2.534 | 13.311 | 0.0800248 | 7/15/2014 | 10:37.5 | 2 |
| -1350.514 | 19403.662 | 2.534 | 13.292 | 0.0769788 | 7/15/2014 | 10:37.6 | 2 |
| -1350.241 | 19403.641 | 2.534 | 13.275 | 0.0738886 | 7/15/2014 | 10:37.7 | 2 |
| -1350.022 | 19403.619 | 2.534 | 13.259 | 0.0765538 | 7/15/2014 | 10:37.8 | 2 |
| -1349.803 | 19403.604 | 2.536 | 13.24 | 0.0773456 | 7/15/2014 | 10:37.9 | 2 |
| -1349.583 | 19403.584 | 2.533 | 13.231 | 0.0772259 | 7/15/2014 | 10:38.0 | 2 |
| -1349.365 | 19403.566 | 2.533 | 13.218 | 0.0761578 | 7/15/2014 | 10:38.1 | 2 |
| -1349.148 | 19403.547 | 2.533 | 13.205 | 0.0781983 | 7/15/2014 | 10:38.2 | 2 |
| -1348.932 | 19403.529 | 2.531 | 13.202 | 0.0814776 | 7/15/2014 | 10:38.3 | 2 |
| -1348.662 | 19403.508 | 2.534 | 13.195 | 0.0785109 | 7/15/2014 | 10:38.4 | 2 |
| -1348.446 | 19403.488 | 2.532 | 13.195 | 0.0774599 | 7/15/2014 | 10:38.5 | 2 |

| | | | | | | | |
|---|---|---|---|---|---|---|---|
| -1348.229 | 19403.471 | 2.535 | 13.195 | 0.0788901 | 7/15/2014 | 10:38.6 | 2 |
| -1348.01 | 19403.453 | 2.535 | 13.202 | 0.0805975 | 7/15/2014 | 10:38.7 | 2 |
| -1347.789 | 19403.436 | 2.535 | 13.232 | 0.0923803 | 7/15/2014 | 10:38.8 | 2 |
| -1347.57 | 19403.416 | 2.536 | 13.277 | 0.0910034 | 7/15/2014 | 10:38.9 | 2 |
| -1347.349 | 19403.398 | 2.539 | 13.326 | 0.0986788 | 7/15/2014 | 10:39.0 | 2 |
| -1347.129 | 19403.381 | 2.542 | 13.371 | 0.0996371 | 7/15/2014 | 10:39.1 | 2 |
| -1346.908 | 19403.363 | 2.542 | 13.423 | 0.101876 | 7/15/2014 | 10:39.2 | 2 |
| -1346.689 | 19403.344 | 2.54 | 13.472 | 0.104926 | 7/15/2014 | 10:39.3 | 2 |
| -1346.469 | 19403.328 | 2.537 | 13.53 | 0.100721 | 7/15/2014 | 10:39.4 | 2 |
| -1346.251 | 19403.309 | 2.537 | 13.584 | 0.104754 | 7/15/2014 | 10:39.5 | 2 |
| -1346.033 | 19403.291 | 2.537 | 13.613 | 0.106563 | 7/15/2014 | 10:39.6 | 2 |
| -1345.762 | 19403.27 | 2.534 | 13.633 | 0.111769 | 7/15/2014 | 10:39.7 | 2 |
| -1345.543 | 19403.252 | 2.531 | 13.657 | 0.10766 | 7/15/2014 | 10:39.8 | 2 |
| -1345.323 | 19403.235 | 2.527 | 13.682 | 0.10863 | 7/15/2014 | 10:39.9 | 2 |
| -1345.103 | 19403.217 | 2.524 | 13.709 | 0.116423 | 7/15/2014 | 10:40.0 | 2 |
| -1344.881 | 19403.2 | 2.529 | 13.731 | 0.108622 | 7/15/2014 | 10:40.1 | 2 |
| -1344.661 | 19403.182 | 2.532 | 13.745 | 0.1072 | 7/15/2014 | 10:40.2 | 2 |
| -1344.438 | 19403.164 | 2.537 | 13.753 | 0.107407 | 7/15/2014 | 10:40.3 | 2 |
| -1344.162 | 19403.141 | 2.54 | 13.765 | 0.109186 | 7/15/2014 | 10:40.4 | 2 |
| -1343.937 | 19403.124 | 2.543 | 13.776 | 0.112277 | 7/15/2014 | 10:40.5 | 2 |
| -1343.711 | 19403.106 | 2.543 | 13.778 | 0.110946 | 7/15/2014 | 10:40.6 | 2 |
| -1343.482 | 19403.088 | 2.545 | 13.781 | 0.110164 | 7/15/2014 | 10:40.7 | 2 |
| -1343.252 | 19403.069 | 2.548 | 13.777 | 0.111195 | 7/15/2014 | 10:40.8 | 2 |
| -1343.023 | 19403.052 | 2.548 | 13.777 | 0.13288 | 7/15/2014 | 10:40.9 | 2 |
| -1342.792 | 19403.033 | 2.545 | 13.779 | 0.123739 | 7/15/2014 | 10:41.0 | 2 |
| -1342.564 | 19403.014 | 2.54 | 13.778 | 0.131864 | 7/15/2014 | 10:41.1 | 2 |
| -1342.335 | 19402.997 | 2.54 | 13.776 | 0.122623 | 7/15/2014 | 10:41.2 | 2 |
| -1342.107 | 19402.979 | 2.537 | 13.773 | 0.13465 | 7/15/2014 | 10:41.3 | 2 |
| -1341.88 | 19402.961 | 2.54 | 13.77 | 0.132646 | 7/15/2014 | 10:41.4 | 2 |
| -1341.657 | 19402.943 | 2.543 | 13.763 | 0.124945 | 7/15/2014 | 10:41.5 | 2 |
| -1341.435 | 19402.926 | 2.54 | 13.758 | 0.117679 | 7/15/2014 | 10:41.6 | 2 |
| -1341.164 | 19402.904 | 2.541 | 13.754 | 0.105378 | 7/15/2014 | 10:41.7 | 2 |
| -1340.945 | 19402.887 | 2.544 | 13.75 | 0.121925 | 7/15/2014 | 10:41.8 | 2 |
| -1340.727 | 19402.87 | 2.546 | 13.749 | 0.112333 | 7/15/2014 | 10:41.9 | 2 |
| -1340.511 | 19402.854 | 2.543 | 13.752 | 0.111059 | 7/15/2014 | 10:42.0 | 2 |
| -1340.295 | 19402.837 | 2.543 | 13.759 | 0.110362 | 7/15/2014 | 10:42.1 | 2 |
| -1340.079 | 19402.82 | 2.545 | 13.77 | 0.110122 | 7/15/2014 | 10:42.2 | 2 |
| -1339.866 | 19402.804 | 2.545 | 13.785 | 0.120583 | 7/15/2014 | 10:42.3 | 2 |
| -1339.493 | 19402.773 | 2.542 | 13.81 | 0.135817 | 7/15/2014 | 10:42.5 | 2 |
| -1339.388 | 19402.766 | 2.542 | 13.818 | 0.1104 | 7/15/2014 | 10:42.5 | 2 |
| -1339.172 | 19402.749 | 2.542 | 13.832 | 0.125736 | 7/15/2014 | 10:42.6 | 2 |
| -1338.958 | 19402.734 | 2.542 | 13.847 | 0.10736 | 7/15/2014 | 10:42.7 | 2 |
| -1338.743 | 19402.718 | 2.545 | 13.855 | 0.115819 | 7/15/2014 | 10:42.8 | 2 |
| -1338.525 | 19402.7 | 2.551 | 13.859 | 0.098911 | 7/15/2014 | 10:42.9 | 2 |
| -1338.307 | 19402.684 | 2.552 | 13.855 | 0.0850218 | 7/15/2014 | 10:43.0 | 2 |
| -1338.092 | 19402.667 | 2.55 | 13.848 | 0.0827822 | 7/15/2014 | 10:43.1 | 2 |
| -1337.875 | 19402.65 | 2.55 | 13.837 | 0.0767566 | 7/15/2014 | 10:43.2 | 2 |

| | | | | | | | |
|---|---|---|---|---|---|---|---|
| -1337.659 | 19402.634 | 2.55 | 13.822 | 0.0928475 | 7/15/2014 | 10:43.3 | 2 |
| -1337.442 | 19402.618 | 2.55 | 13.805 | 0.106116 | 7/15/2014 | 10:43.4 | 2 |
| -1337.226 | 19402.603 | 2.553 | 13.787 | 0.0862283 | 7/15/2014 | 10:43.5 | 2 |
| -1337.011 | 19402.585 | 2.55 | 13.765 | 0.0906863 | 7/15/2014 | 10:43.6 | 2 |
| -1336.74 | 19402.564 | 2.546 | 13.74 | 0.102803 | 7/15/2014 | 10:43.7 | 2 |
| -1336.526 | 19402.549 | 2.543 | 13.715 | 0.100821 | 7/15/2014 | 10:43.8 | 2 |
| -1336.307 | 19402.533 | 2.542 | 13.691 | 0.0869197 | 7/15/2014 | 10:43.9 | 2 |
| -1336.089 | 19402.516 | 2.545 | 13.664 | 0.0728594 | 7/15/2014 | 10:44.0 | 2 |
| -1335.868 | 19402.499 | 2.55 | 13.639 | 0.0627021 | 7/15/2014 | 10:44.1 | 2 |
| -1335.648 | 19402.483 | 2.55 | 13.613 | 0.0665785 | 7/15/2014 | 10:44.2 | 2 |
| -1335.427 | 19402.466 | 2.55 | 13.588 | 0.0637649 | 7/15/2014 | 10:44.3 | 2 |
| -1335.151 | 19402.445 | 2.553 | 13.568 | 0.0647093 | 7/15/2014 | 10:44.4 | 2 |
| -1334.928 | 19402.43 | 2.551 | 13.554 | 0.067856 | 7/15/2014 | 10:44.5 | 2 |
| -1334.701 | 19402.413 | 2.551 | 13.542 | 0.0750858 | 7/15/2014 | 10:44.6 | 2 |
| -1334.474 | 19402.396 | 2.551 | 13.529 | 0.0712272 | 7/15/2014 | 10:44.7 | 2 |
| -1334.247 | 19402.379 | 2.551 | 13.524 | 0.0772808 | 7/15/2014 | 10:44.8 | 2 |
| -1334.019 | 19402.363 | 2.545 | 13.522 | 0.0739738 | 7/15/2014 | 10:44.9 | 2 |
| -1333.79 | 19402.347 | 2.545 | 13.524 | 0.0831697 | 7/15/2014 | 10:45.0 | 2 |
| -1333.563 | 19402.33 | 2.54 | 13.528 | 0.0755288 | 7/15/2014 | 10:45.1 | 2 |
| -1333.336 | 19402.313 | 2.54 | 13.536 | 0.0667467 | 7/15/2014 | 10:45.2 | 2 |
| -1333.11 | 19402.298 | 2.542 | 13.54 | 0.0648694 | 7/15/2014 | 10:45.3 | 2 |
| -1332.886 | 19402.281 | 2.544 | 13.546 | 0.068064 | 7/15/2014 | 10:45.4 | 2 |
| -1332.665 | 19402.266 | 2.548 | 13.557 | 0.0705983 | 7/15/2014 | 10:45.5 | 2 |
| -1332.446 | 19402.25 | 2.55 | 13.567 | 0.071928 | 7/15/2014 | 10:45.6 | 2 |
| -1332.173 | 19402.231 | 2.552 | 13.582 | 0.0823813 | 7/15/2014 | 10:45.7 | 2 |
| -1331.956 | 19402.216 | 2.554 | 13.595 | 0.0827898 | 7/15/2014 | 10:45.8 | 2 |
| -1331.736 | 19402.2 | 2.556 | 13.613 | 0.0840161 | 7/15/2014 | 10:45.9 | 2 |
| -1331.518 | 19402.186 | 2.558 | 13.627 | 0.0905782 | 7/15/2014 | 10:46.0 | 2 |
| -1331.3 | 19402.17 | 2.558 | 13.642 | 0.0897432 | 7/15/2014 | 10:46.1 | 2 |
| -1331.083 | 19402.154 | 2.556 | 13.657 | 0.090839 | 7/15/2014 | 10:46.2 | 2 |
| -1330.866 | 19402.139 | 2.554 | 13.67 | 0.0929423 | 7/15/2014 | 10:46.3 | 2 |
| -1330.595 | 19402.12 | 2.552 | 13.677 | 0.0952618 | 7/15/2014 | 10:46.4 | 2 |
| -1330.376 | 19402.104 | 2.552 | 13.679 | 0.0923593 | 7/15/2014 | 10:46.5 | 2 |
| -1330.156 | 19402.09 | 2.55 | 13.682 | 0.0998903 | 7/15/2014 | 10:46.6 | 2 |
| -1329.935 | 19402.074 | 2.553 | 13.678 | 0.0989908 | 7/15/2014 | 10:46.7 | 2 |
| -1329.714 | 19402.059 | 2.551 | 13.68 | 0.103786 | 7/15/2014 | 10:46.8 | 2 |
| -1329.492 | 19402.043 | 2.554 | 13.682 | 0.117509 | 7/15/2014 | 10:46.9 | 2 |
| -1329.271 | 19402.027 | 2.554 | 13.682 | 0.105857 | 7/15/2014 | 10:47.0 | 2 |
| -1329.048 | 19402.013 | 2.553 | 13.682 | 0.102633 | 7/15/2014 | 10:47.1 | 2 |
| -1328.827 | 19401.997 | 2.555 | 13.687 | 0.111274 | 7/15/2014 | 10:47.2 | 2 |
| -1328.607 | 19401.981 | 2.556 | 13.693 | 0.116496 | 7/15/2014 | 10:47.3 | 2 |
| -1328.387 | 19401.966 | 2.556 | 13.701 | 0.113431 | 7/15/2014 | 10:47.4 | 2 |
| -1328.169 | 19401.951 | 2.556 | 13.703 | 0.130553 | 7/15/2014 | 10:47.5 | 2 |
| -1327.951 | 19401.936 | 2.556 | 13.7 | 0.127286 | 7/15/2014 | 10:47.6 | 2 |
| -1327.682 | 19401.916 | 2.556 | 13.705 | 0.134757 | 7/15/2014 | 10:47.7 | 2 |
| -1327.465 | 19401.901 | 2.556 | 13.721 | 0.146739 | 7/15/2014 | 10:47.8 | 2 |
| -1327.247 | 19401.886 | 2.559 | 13.732 | 0.14524 | 7/15/2014 | 10:47.9 | 2 |

| | | | | | | | |
|---|---|---|---|---|---|---|---|
| -1327.03 | 19401.87 | 2.559 | 13.748 | 0.14154 | 7/15/2014 | 10:48.0 | 2 |
| -1326.812 | 19401.854 | 2.559 | 13.774 | 0.152519 | 7/15/2014 | 10:48.1 | 2 |
| -1326.593 | 19401.84 | 2.561 | 13.802 | 0.142447 | 7/15/2014 | 10:48.2 | 2 |
| -1326.375 | 19401.824 | 2.559 | 13.839 | 0.148003 | 7/15/2014 | 10:48.3 | 2 |
| -1326.102 | 19401.804 | 2.559 | 13.883 | 0.149907 | 7/15/2014 | 10:48.4 | 2 |
| -1325.881 | 19401.787 | 2.559 | 13.916 | 0.149143 | 7/15/2014 | 10:48.5 | 2 |
| -1325.659 | 19401.771 | 2.559 | 13.94 | 0.1496 | 7/15/2014 | 10:48.6 | 2 |
| -1325.435 | 19401.756 | 2.556 | 13.968 | 0.151281 | 7/15/2014 | 10:48.7 | 2 |
| -1325.212 | 19401.739 | 2.554 | 13.994 | 0.15457 | 7/15/2014 | 10:48.8 | 2 |
| -1324.986 | 19401.724 | 2.553 | 14.01 | 0.156131 | 7/15/2014 | 10:48.9 | 2 |
| -1324.761 | 19401.707 | 2.551 | 14.021 | 0.154775 | 7/15/2014 | 10:49.0 | 2 |
| -1324.534 | 19401.69 | 2.55 | 14.034 | 0.154006 | 7/15/2014 | 10:49.1 | 2 |
| -1324.31 | 19401.674 | 2.55 | 14.04 | 0.152387 | 7/15/2014 | 10:49.2 | 2 |
| -1324.086 | 19401.658 | 2.55 | 14.059 | 0.152159 | 7/15/2014 | 10:49.3 | 2 |
| -1323.862 | 19401.643 | 2.553 | 14.07 | 0.151396 | 7/15/2014 | 10:49.4 | 2 |
| -1323.643 | 19401.626 | 2.556 | 14.082 | 0.151623 | 7/15/2014 | 10:49.5 | 2 |
| -1323.421 | 19401.609 | 2.561 | 14.093 | 0.152627 | 7/15/2014 | 10:49.6 | 2 |
| -1323.151 | 19401.59 | 2.564 | 14.098 | 0.153418 | 7/15/2014 | 10:49.7 | 2 |
| -1322.932 | 19401.574 | 2.567 | 14.108 | 0.153904 | 7/15/2014 | 10:49.8 | 2 |
| -1322.714 | 19401.559 | 2.57 | 14.114 | 0.158249 | 7/15/2014 | 10:49.9 | 2 |
| -1322.494 | 19401.543 | 2.575 | 14.125 | 0.156866 | 7/15/2014 | 10:50.0 | 2 |
| -1322.275 | 19401.526 | 2.577 | 14.12 | 0.15861 | 7/15/2014 | 10:50.1 | 2 |
| -1322.056 | 19401.511 | 2.577 | 14.123 | 0.159744 | 7/15/2014 | 10:50.2 | 2 |
| -1321.838 | 19401.493 | 2.579 | 14.126 | 0.167313 | 7/15/2014 | 10:50.3 | 2 |
| -1321.566 | 19401.475 | 2.579 | 14.128 | 0.165488 | 7/15/2014 | 10:50.4 | 2 |
| -1321.348 | 19401.459 | 2.579 | 14.136 | 0.17296 | 7/15/2014 | 10:50.5 | 2 |
| -1321.125 | 19401.443 | 2.582 | 14.143 | 0.165438 | 7/15/2014 | 10:50.6 | 2 |
| -1320.903 | 19401.427 | 2.585 | 14.141 | 0.173718 | 7/15/2014 | 10:50.7 | 2 |
| -1320.682 | 19401.409 | 2.581 | 14.137 | 0.168051 | 7/15/2014 | 10:50.8 | 2 |
| -1320.457 | 19401.394 | 2.581 | 14.134 | 0.169997 | 7/15/2014 | 10:50.9 | 2 |
| -1320.234 | 19401.377 | 2.58 | 14.132 | 0.170951 | 7/15/2014 | 10:51.0 | 2 |
| -1320.009 | 19401.361 | 2.578 | 14.127 | 0.167596 | 7/15/2014 | 10:51.1 | 2 |
| -1319.787 | 19401.345 | 2.578 | 14.121 | 0.178032 | 7/15/2014 | 10:51.2 | 2 |
| -1319.565 | 19401.329 | 2.578 | 14.112 | 0.173984 | 7/15/2014 | 10:51.3 | 2 |
| -1319.346 | 19401.313 | 2.575 | 14.103 | 0.169478 | 7/15/2014 | 10:51.4 | 2 |
| -1319.127 | 19401.296 | 2.572 | 14.1 | 0.192714 | 7/15/2014 | 10:51.5 | 2 |
| -1318.909 | 19401.28 | 2.575 | 14.102 | 0.181434 | 7/15/2014 | 10:51.6 | 2 |
| -1318.642 | 19401.262 | 2.575 | 14.111 | 0.173379 | 7/15/2014 | 10:51.7 | 2 |
| -1318.425 | 19401.245 | 2.578 | 14.118 | 0.176552 | 7/15/2014 | 10:51.8 | 2 |
| -1318.209 | 19401.229 | 2.584 | 14.133 | 0.170381 | 7/15/2014 | 10:51.9 | 2 |
| -1317.997 | 19401.213 | 2.58 | 14.143 | 0.177056 | 7/15/2014 | 10:52.0 | 2 |
| -1317.781 | 19401.198 | 2.586 | 14.154 | 0.167311 | 7/15/2014 | 10:52.1 | 2 |
| -1317.566 | 19401.181 | 2.589 | 14.165 | 0.166967 | 7/15/2014 | 10:52.2 | 2 |
| -1317.353 | 19401.165 | 2.589 | 14.178 | 0.174626 | 7/15/2014 | 10:52.3 | 2 |
| -1317.087 | 19401.146 | 2.589 | 14.186 | 0.176338 | 7/15/2014 | 10:52.4 | 2 |
| -1316.873 | 19401.129 | 2.589 | 14.188 | 0.187274 | 7/15/2014 | 10:52.5 | 2 |
| -1316.655 | 19401.112 | 2.589 | 14.193 | 0.181537 | 7/15/2014 | 10:52.6 | 2 |

| | | | | | | | |
|---|---|---|---|---|---|---|---|
| -1316.44 | 19401.097 | 2.589 | 14.193 | 0.188237 | 7/15/2014 | 10:52.7 | 2 |
| -1316.22 | 19401.08 | 2.595 | 14.196 | 0.181647 | 7/15/2014 | 10:52.8 | 2 |
| -1316.001 | 19401.063 | 2.595 | 14.2 | 0.177269 | 7/15/2014 | 10:52.9 | 2 |
| -1315.783 | 19401.047 | 2.598 | 14.2 | 0.178042 | 7/15/2014 | 10:53.0 | 2 |
| -1315.564 | 19401.031 | 2.6 | 14.191 | 0.179341 | 7/15/2014 | 10:53.1 | 2 |
| -1315.345 | 19401.015 | 2.604 | 14.179 | 0.175448 | 7/15/2014 | 10:53.2 | 2 |
| -1315.126 | 19400.997 | 2.607 | 14.17 | 0.182299 | 7/15/2014 | 10:53.3 | 2 |
| -1314.907 | 19400.981 | 2.61 | 14.156 | 0.188709 | 7/15/2014 | 10:53.4 | 2 |
| -1314.69 | 19400.965 | 2.61 | 14.144 | 0.199887 | 7/15/2014 | 10:53.5 | 2 |
| -1314.474 | 19400.949 | 2.61 | 14.125 | 0.200133 | 7/15/2014 | 10:53.6 | 2 |
| -1314.205 | 19400.928 | 2.61 | 14.104 | 0.206953 | 7/15/2014 | 10:53.7 | 2 |
| -1313.99 | 19400.912 | 2.61 | 14.086 | 0.221034 | 7/15/2014 | 10:53.8 | 2 |
| -1313.772 | 19400.896 | 2.616 | 14.075 | 0.239184 | 7/15/2014 | 10:53.9 | 2 |
| -1313.556 | 19400.88 | 2.613 | 14.06 | 0.232884 | 7/15/2014 | 10:54.0 | 2 |
| -1313.337 | 19400.863 | 2.616 | 14.039 | 0.211574 | 7/15/2014 | 10:54.1 | 2 |
| -1313.119 | 19400.848 | 2.616 | 14.027 | 0.232555 | 7/15/2014 | 10:54.2 | 2 |
| -1312.902 | 19400.831 | 2.616 | 14.013 | 0.222063 | 7/15/2014 | 10:54.3 | 2 |
| -1312.631 | 19400.811 | 2.616 | 13.997 | 0.239779 | 7/15/2014 | 10:54.4 | 2 |
| -1312.412 | 19400.793 | 2.62 | 13.982 | 0.219502 | 7/15/2014 | 10:54.5 | 2 |
| -1312.192 | 19400.777 | 2.62 | 13.964 | 0.218956 | 7/15/2014 | 10:54.6 | 2 |
| -1311.972 | 19400.761 | 2.616 | 13.957 | 0.214609 | 7/15/2014 | 10:54.7 | 2 |
| -1311.749 | 19400.743 | 2.62 | 13.953 | 0.217624 | 7/15/2014 | 10:54.8 | 2 |
| -1311.527 | 19400.728 | 2.616 | 13.999 | 0.206587 | 7/15/2014 | 10:54.9 | 2 |
| -1311.306 | 19400.71 | 2.616 | 14.007 | 0.224139 | 7/15/2014 | 10:55.0 | 2 |
| -1311.086 | 19400.694 | 2.613 | 14.049 | 0.205493 | 7/15/2014 | 10:55.1 | 2 |
| -1310.865 | 19400.676 | 2.613 | 14.08 | 0.205366 | 7/15/2014 | 10:55.2 | 2 |
| -1310.647 | 19400.66 | 2.61 | 14.114 | 0.204839 | 7/15/2014 | 10:55.3 | 2 |
| -1310.431 | 19400.644 | 2.612 | 14.156 | 0.192918 | 7/15/2014 | 10:55.4 | 2 |
| -1310.216 | 19400.627 | 2.612 | 14.181 | 0.20624 | 7/15/2014 | 10:55.5 | 2 |
| -1309.852 | 19400.6 | 2.616 | 14.208 | 0.203165 | 7/15/2014 | 10:55.7 | 2 |
| -1309.742 | 19400.591 | 2.618 | 14.192 | 0.190187 | 7/15/2014 | 10:55.7 | 2 |
| -1309.533 | 19400.575 | 2.622 | 14.21 | 0.182248 | 7/15/2014 | 10:55.8 | 2 |
| -1309.323 | 19400.559 | 2.63 | 14.21 | 0.197451 | 7/15/2014 | 10:55.9 | 2 |
| -1309.113 | 19400.543 | 2.633 | 14.225 | 0.180091 | 7/15/2014 | 10:56.0 | 2 |
| -1308.905 | 19400.527 | 2.636 | 14.235 | 0.216655 | 7/15/2014 | 10:56.1 | 2 |
| -1308.699 | 19400.512 | 2.636 | 14.238 | 0.212875 | 7/15/2014 | 10:56.2 | 2 |
| -1308.493 | 19400.496 | 2.636 | 14.247 | 0.227277 | 7/15/2014 | 10:56.3 | 2 |
| -1308.137 | 19400.469 | 2.635 | 14.262 | 0.222611 | 7/15/2014 | 10:56.5 | 2 |
| -1307.932 | 19400.453 | 2.635 | 14.271 | 0.242459 | 7/15/2014 | 10:56.6 | 2 |
| -1307.677 | 19400.434 | 2.631 | 14.281 | 0.219735 | 7/15/2014 | 10:56.7 | 2 |
| -1307.573 | 19400.426 | 2.629 | 14.275 | 0.234092 | 7/15/2014 | 10:56.7 | 2 |
| -1307.368 | 19400.409 | 2.629 | 14.274 | 0.21609 | 7/15/2014 | 10:56.8 | 2 |
| -1307.159 | 19400.394 | 2.629 | 14.277 | 0.224867 | 7/15/2014 | 10:56.9 | 2 |
| -1306.951 | 19400.377 | 2.629 | 14.28 | 0.202825 | 7/15/2014 | 10:57.0 | 2 |
| -1306.742 | 19400.361 | 2.632 | 14.283 | 0.199582 | 7/15/2014 | 10:57.1 | 2 |
| -1306.534 | 19400.346 | 2.632 | 14.284 | 0.201056 | 7/15/2014 | 10:57.2 | 2 |
| -1306.328 | 19400.329 | 2.632 | 14.283 | 0.200077 | 7/15/2014 | 10:57.3 | 2 |

| | | | | | | | |
|---|---|---|---|---|---|---|---|
| -1305.965 | 19400.301 | 2.634 | 14.281 | 0.206143 | 7/15/2014 | 10:57.5 | 2 |
| -1305.758 | 19400.284 | 2.634 | 14.281 | 0.218037 | 7/15/2014 | 10:57.6 | 2 |
| -1305.499 | 19400.265 | 2.629 | 14.28 | 0.19901 | 7/15/2014 | 10:57.7 | 2 |
| -1305.393 | 19400.256 | 2.629 | 14.278 | 0.208196 | 7/15/2014 | 10:57.7 | 2 |
| -1305.183 | 19400.239 | 2.629 | 14.28 | 0.224952 | 7/15/2014 | 10:57.8 | 2 |
| -1304.972 | 19400.223 | 2.629 | 14.287 | 0.225106 | 7/15/2014 | 10:57.9 | 2 |
| -1304.763 | 19400.206 | 2.629 | 14.296 | 0.216221 | 7/15/2014 | 10:58.0 | 2 |
| -1304.55 | 19400.189 | 2.629 | 14.312 | 0.240996 | 7/15/2014 | 10:58.1 | 2 |
| -1304.34 | 19400.174 | 2.629 | 14.332 | 0.259005 | 7/15/2014 | 10:58.2 | 2 |
| -1304.132 | 19400.157 | 2.629 | 14.35 | 0.265569 | 7/15/2014 | 10:58.3 | 2 |
| -1303.765 | 19400.129 | 2.628 | 14.371 | 0.348869 | 7/15/2014 | 10:58.5 | 2 |
| -1303.554 | 19400.112 | 2.633 | 14.384 | 0.369778 | 7/15/2014 | 10:58.6 | 2 |
| -1303.294 | 19400.091 | 2.628 | 14.389 | 0.367676 | 7/15/2014 | 10:58.7 | 2 |
| -1303.188 | 19400.083 | 2.628 | 14.396 | 0.396884 | 7/15/2014 | 10:58.7 | 2 |
| -1302.976 | 19400.066 | 2.628 | 14.398 | 0.400607 | 7/15/2014 | 10:58.8 | 2 |
| -1302.764 | 19400.051 | 2.628 | 14.395 | 0.40397 | 7/15/2014 | 10:58.9 | 2 |
| -1302.551 | 19400.034 | 2.628 | 14.385 | 0.405368 | 7/15/2014 | 10:59.0 | 2 |
| -1302.338 | 19400.017 | 2.627 | 14.373 | 0.388355 | 7/15/2014 | 10:59.1 | 2 |
| -1302.125 | 19400 | 2.63 | 14.364 | 0.372116 | 7/15/2014 | 10:59.2 | 2 |
| -1301.913 | 19399.984 | 2.635 | 14.354 | 0.344831 | 7/15/2014 | 10:59.3 | 2 |
| -1301.546 | 19399.956 | 2.636 | 14.34 | 0.383042 | 7/15/2014 | 10:59.5 | 2 |
| -1301.333 | 19399.939 | 2.64 | 14.341 | 0.369493 | 7/15/2014 | 10:59.6 | 2 |
| -1301.071 | 19399.919 | 2.636 | 14.345 | 0.360841 | 7/15/2014 | 10:59.7 | 2 |
| -1300.963 | 19399.911 | 2.637 | 14.341 | 0.354425 | 7/15/2014 | 10:59.7 | 2 |
| -1300.751 | 19399.894 | 2.637 | 14.342 | 0.362132 | 7/15/2014 | 10:59.8 | 2 |
| -1300.537 | 19399.877 | 2.637 | 14.343 | 0.345804 | 7/15/2014 | 10:59.9 | 2 |
| -1300.322 | 19399.861 | 2.635 | 14.346 | 0.354973 | 7/15/2014 | 11:00.0 | 2 |
| -1300.109 | 19399.846 | 2.63 | 14.348 | 0.340281 | 7/15/2014 | 11:00.1 | 2 |
| -1299.896 | 19399.828 | 2.627 | 14.352 | 0.327312 | 7/15/2014 | 11:00.2 | 2 |
| -1299.683 | 19399.813 | 2.627 | 14.361 | 0.300197 | 7/15/2014 | 11:00.3 | 2 |
| -1299.419 | 19399.792 | 2.627 | 14.36 | 0.26972 | 7/15/2014 | 11:00.4 | 2 |
| -1299.102 | 19399.768 | 2.634 | 14.352 | 0.235511 | 7/15/2014 | 11:00.6 | 2 |
| -1298.841 | 19399.747 | 2.636 | 14.347 | 0.201091 | 7/15/2014 | 11:00.7 | 2 |
| -1298.733 | 19399.739 | 2.637 | 14.348 | 0.177589 | 7/15/2014 | 11:00.7 | 2 |
| -1298.521 | 19399.725 | 2.642 | 14.346 | 0.145046 | 7/15/2014 | 11:00.8 | 2 |
| -1298.31 | 19399.707 | 2.65 | 14.349 | 0.123168 | 7/15/2014 | 11:00.9 | 2 |
| -1298.099 | 19399.69 | 2.652 | 14.355 | 0.127759 | 7/15/2014 | 11:01.0 | 2 |
| -1297.888 | 19399.675 | 2.652 | 14.365 | 0.132474 | 7/15/2014 | 11:01.1 | 2 |
| -1297.679 | 19399.659 | 2.654 | 14.372 | 0.12383 | 7/15/2014 | 11:01.2 | 2 |
| -1297.469 | 19399.644 | 2.658 | 14.38 | 0.129575 | 7/15/2014 | 11:01.3 | 2 |
| -1297.107 | 19399.615 | 2.658 | 14.406 | 0.13843 | 7/15/2014 | 11:01.5 | 2 |
| -1296.899 | 19399.602 | 2.658 | 14.425 | 0.155342 | 7/15/2014 | 11:01.6 | 2 |
| -1296.644 | 19399.582 | 2.652 | 14.454 | 0.144914 | 7/15/2014 | 11:01.7 | 2 |
| -1296.54 | 19399.573 | 2.651 | 14.465 | 0.139715 | 7/15/2014 | 11:01.7 | 2 |
| -1296.332 | 19399.558 | 2.654 | 14.492 | 0.169582 | 7/15/2014 | 11:01.8 | 2 |
| -1296.122 | 19399.542 | 2.656 | 14.517 | 0.159938 | 7/15/2014 | 11:01.9 | 2 |
| -1295.913 | 19399.526 | 2.656 | 14.551 | 0.167749 | 7/15/2014 | 11:02.0 | 2 |

| | | | | | | | |
|---|---|---|---|---|---|---|---|
| -1295.704 | 19399.511 | 2.656 | 14.584 | 0.171076 | 7/15/2014 | 11:02.1 | 2 |
| -1295.495 | 19399.495 | 2.656 | 14.627 | 0.185362 | 7/15/2014 | 11:02.2 | 2 |
| -1295.286 | 19399.48 | 2.656 | 14.678 | 0.191969 | 7/15/2014 | 11:02.3 | 2 |
| -1294.923 | 19399.453 | 2.657 | 14.731 | 0.228685 | 7/15/2014 | 11:02.5 | 2 |
| -1294.713 | 19399.438 | 2.663 | 14.756 | 0.227882 | 7/15/2014 | 11:02.6 | 2 |
| -1294.453 | 19399.418 | 2.663 | 14.778 | 0.220438 | 7/15/2014 | 11:02.7 | 2 |
| -1294.348 | 19399.41 | 2.666 | 14.794 | 0.228949 | 7/15/2014 | 11:02.7 | 2 |
| -1294.135 | 19399.394 | 2.669 | 14.81 | 0.219882 | 7/15/2014 | 11:02.8 | 2 |
| -1293.926 | 19399.378 | 2.669 | 14.826 | 0.260722 | 7/15/2014 | 11:02.9 | 2 |
| -1293.712 | 19399.361 | 2.672 | 14.823 | 0.257237 | 7/15/2014 | 11:03.0 | 2 |
| -1293.501 | 19399.345 | 2.672 | 14.813 | 0.248803 | 7/15/2014 | 11:03.1 | 2 |
| -1293.289 | 19399.33 | 2.672 | 14.808 | 0.259598 | 7/15/2014 | 11:03.2 | 2 |
| -1293.076 | 19399.314 | 2.672 | 14.801 | 0.227096 | 7/15/2014 | 11:03.3 | 2 |
| -1292.709 | 19399.287 | 2.671 | 14.783 | 0.267944 | 7/15/2014 | 11:03.5 | 2 |
| -1292.498 | 19399.271 | 2.668 | 14.776 | 0.302863 | 7/15/2014 | 11:03.6 | 2 |
| -1292.235 | 19399.251 | 2.665 | 14.767 | 0.244753 | 7/15/2014 | 11:03.7 | 2 |
| -1292.128 | 19399.242 | 2.666 | 14.761 | 0.25171 | 7/15/2014 | 11:03.7 | 2 |
| -1291.915 | 19399.227 | 2.666 | 14.755 | 0.267007 | 7/15/2014 | 11:03.8 | 2 |
| -1291.703 | 19399.21 | 2.666 | 14.751 | 0.266252 | 7/15/2014 | 11:03.9 | 2 |
| -1291.488 | 19399.194 | 2.666 | 14.745 | 0.290953 | 7/15/2014 | 11:04.0 | 2 |
| -1291.272 | 19399.179 | 2.666 | 14.745 | 0.297107 | 7/15/2014 | 11:04.1 | 2 |
| -1291.059 | 19399.162 | 2.661 | 14.751 | 0.295752 | 7/15/2014 | 11:04.2 | 2 |
| -1290.846 | 19399.146 | 2.661 | 14.757 | 0.287146 | 7/15/2014 | 11:04.3 | 2 |
| -1290.58 | 19399.127 | 2.661 | 14.762 | 0.309721 | 7/15/2014 | 11:04.4 | 2 |
| -1290.365 | 19399.111 | 2.661 | 14.77 | 0.321947 | 7/15/2014 | 11:04.5 | 2 |
| -1290.153 | 19399.095 | 2.661 | 14.781 | 0.307713 | 7/15/2014 | 11:04.6 | 2 |
| -1289.886 | 19399.074 | 2.661 | 14.791 | 0.301898 | 7/15/2014 | 11:04.7 | 2 |
| -1289.671 | 19399.059 | 2.661 | 14.805 | 0.289465 | 7/15/2014 | 11:04.8 | 2 |
| -1289.455 | 19399.042 | 2.661 | 14.819 | 0.293575 | 7/15/2014 | 11:04.9 | 2 |
| -1289.238 | 19399.025 | 2.663 | 14.835 | 0.3074 | 7/15/2014 | 11:05.0 | 2 |
| -1289.022 | 19399.01 | 2.663 | 14.848 | 0.324538 | 7/15/2014 | 11:05.1 | 2 |
| -1288.807 | 19398.994 | 2.663 | 14.861 | 0.321412 | 7/15/2014 | 11:05.2 | 2 |
| -1288.593 | 19398.978 | 2.663 | 14.876 | 0.333618 | 7/15/2014 | 11:05.3 | 2 |
| -1288.326 | 19398.958 | 2.66 | 14.901 | 0.367859 | 7/15/2014 | 11:05.4 | 2 |
| -1288.113 | 19398.941 | 2.657 | 14.916 | 0.377646 | 7/15/2014 | 11:05.5 | 2 |
| -1287.901 | 19398.925 | 2.657 | 14.936 | 0.425979 | 7/15/2014 | 11:05.6 | 2 |
| -1287.635 | 19398.904 | 2.655 | 14.955 | 0.388364 | 7/15/2014 | 11:05.7 | 2 |
| -1287.422 | 19398.889 | 2.655 | 14.967 | 0.395043 | 7/15/2014 | 11:05.8 | 2 |
| -1287.208 | 19398.873 | 2.653 | 14.983 | 0.410724 | 7/15/2014 | 11:05.9 | 2 |
| -1286.993 | 19398.856 | 2.65 | 15.002 | 0.417783 | 7/15/2014 | 11:06.0 | 2 |
| -1286.78 | 19398.84 | 2.648 | 15.016 | 0.420536 | 7/15/2014 | 11:06.1 | 2 |
| -1286.566 | 19398.823 | 2.646 | 15.017 | 0.424489 | 7/15/2014 | 11:06.2 | 2 |
| -1286.356 | 19398.807 | 2.64 | 15.031 | 0.424416 | 7/15/2014 | 11:06.3 | 2 |
| -1285.987 | 19398.779 | 2.636 | 15.038 | 0.456246 | 7/15/2014 | 11:06.5 | 2 |
| -1285.775 | 19398.763 | 2.636 | 15.04 | 0.46954 | 7/15/2014 | 11:06.6 | 2 |
| -1285.512 | 19398.742 | 2.631 | 15.043 | 0.44116 | 7/15/2014 | 11:06.7 | 2 |
| -1285.405 | 19398.734 | 2.63 | 15.046 | 0.453552 | 7/15/2014 | 11:06.7 | 2 |

| | | | | | | | |
|---|---|---|---|---|---|---|---|
| -1285.193 | 19398.717 | 2.63 | 15.05 | 0.436205 | 7/15/2014 | 11:06.8 | 2 |
| -1284.982 | 19398.701 | 2.624 | 15.049 | 0.425409 | 7/15/2014 | 11:06.9 | 2 |
| -1284.769 | 19398.685 | 2.624 | 15.048 | 0.425463 | 7/15/2014 | 11:07.0 | 2 |
| -1284.556 | 19398.668 | 2.624 | 15.042 | 0.422752 | 7/15/2014 | 11:07.1 | 2 |
| -1284.345 | 19398.652 | 2.617 | 15.037 | 0.402513 | 7/15/2014 | 11:07.2 | 2 |
| -1284.133 | 19398.636 | 2.617 | 15.033 | 0.406124 | 7/15/2014 | 11:07.3 | 2 |
| -1283.766 | 19398.605 | 2.612 | 15.026 | 0.422831 | 7/15/2014 | 11:07.5 | 2 |
| -1283.558 | 19398.59 | 2.61 | 15.022 | 0.409648 | 7/15/2014 | 11:07.6 | 2 |
| -1283.298 | 19398.57 | 2.603 | 15.015 | 0.41269 | 7/15/2014 | 11:07.7 | 2 |
| -1283.191 | 19398.562 | 2.604 | 15.014 | 0.393592 | 7/15/2014 | 11:07.7 | 2 |
| -1282.981 | 19398.545 | 2.601 | 15.013 | 0.396355 | 7/15/2014 | 11:07.8 | 2 |
| -1282.771 | 19398.529 | 2.599 | 15.013 | 0.398555 | 7/15/2014 | 11:07.9 | 2 |
| -1282.56 | 19398.513 | 2.599 | 15.014 | 0.373649 | 7/15/2014 | 11:08.0 | 2 |
| -1282.349 | 19398.495 | 2.597 | 15.011 | 0.363328 | 7/15/2014 | 11:08.1 | 2 |
| -1282.139 | 19398.479 | 2.594 | 15.013 | 0.346545 | 7/15/2014 | 11:08.2 | 2 |
| -1281.931 | 19398.463 | 2.592 | 15.018 | 0.353816 | 7/15/2014 | 11:08.3 | 2 |
| -1281.567 | 19398.434 | 2.589 | 15.022 | 0.332792 | 7/15/2014 | 11:08.5 | 2 |
| -1281.357 | 19398.418 | 2.591 | 15.024 | 0.321268 | 7/15/2014 | 11:08.6 | 2 |
| -1281.099 | 19398.398 | 2.587 | 15.028 | 0.313737 | 7/15/2014 | 11:08.7 | 2 |
| -1280.994 | 19398.39 | 2.588 | 15.03 | 0.312993 | 7/15/2014 | 11:08.7 | 2 |
| -1280.784 | 19398.372 | 2.588 | 15.031 | 0.305525 | 7/15/2014 | 11:08.8 | 2 |
| -1280.573 | 19398.355 | 2.585 | 15.04 | 0.3062 | 7/15/2014 | 11:08.9 | 2 |
| -1280.361 | 19398.34 | 2.585 | 15.042 | 0.302326 | 7/15/2014 | 11:09.0 | 2 |
| -1280.149 | 19398.321 | 2.585 | 15.042 | 0.312686 | 7/15/2014 | 11:09.1 | 2 |
| -1279.937 | 19398.305 | 2.585 | 15.042 | 0.319541 | 7/15/2014 | 11:09.2 | 2 |
| -1279.724 | 19398.288 | 2.585 | 15.044 | 0.312681 | 7/15/2014 | 11:09.3 | 2 |
| -1279.356 | 19398.26 | 2.582 | 15.047 | 0.326947 | 7/15/2014 | 11:09.5 | 2 |
| -1279.251 | 19398.251 | 2.583 | 15.045 | 0.327789 | 7/15/2014 | 11:09.5 | 2 |
| -1279.04 | 19398.232 | 2.583 | 15.047 | 0.322696 | 7/15/2014 | 11:09.6 | 2 |
| -1278.774 | 19398.213 | 2.583 | 15.041 | 0.316307 | 7/15/2014 | 11:09.7 | 2 |
| -1278.559 | 19398.193 | 2.577 | 15.038 | 0.299706 | 7/15/2014 | 11:09.8 | 2 |
| -1278.343 | 19398.178 | 2.574 | 15.036 | 0.304921 | 7/15/2014 | 11:09.9 | 2 |
| -1278.125 | 19398.159 | 2.574 | 15.032 | 0.309195 | 7/15/2014 | 11:10.0 | 2 |
| -1277.907 | 19398.143 | 2.576 | 15.028 | 0.297361 | 7/15/2014 | 11:10.1 | 2 |
| -1277.691 | 19398.125 | 2.578 | 15.026 | 0.283238 | 7/15/2014 | 11:10.2 | 2 |
| -1277.476 | 19398.105 | 2.575 | 15.018 | 0.284575 | 7/15/2014 | 11:10.3 | 2 |
| -1277.207 | 19398.085 | 2.573 | 15.015 | 0.290545 | 7/15/2014 | 11:10.4 | 2 |
| -1276.992 | 19398.066 | 2.573 | 15.009 | 0.297809 | 7/15/2014 | 11:10.5 | 2 |
| -1276.773 | 19398.049 | 2.578 | 15 | 0.286929 | 7/15/2014 | 11:10.6 | 2 |
| -1276.501 | 19398.027 | 2.582 | 14.988 | 0.283608 | 7/15/2014 | 11:10.7 | 2 |
| -1276.282 | 19398.01 | 2.582 | 14.977 | 0.299943 | 7/15/2014 | 11:10.8 | 2 |
| -1276.064 | 19397.99 | 2.576 | 14.961 | 0.306238 | 7/15/2014 | 11:10.9 | 2 |
| -1275.845 | 19397.973 | 2.574 | 14.944 | 0.31132 | 7/15/2014 | 11:11.0 | 2 |
| -1275.624 | 19397.954 | 2.574 | 14.926 | 0.323307 | 7/15/2014 | 11:11.1 | 2 |
| -1275.407 | 19397.936 | 2.574 | 14.905 | 0.312078 | 7/15/2014 | 11:11.2 | 2 |
| -1275.189 | 19397.917 | 2.574 | 14.89 | 0.313499 | 7/15/2014 | 11:11.3 | 2 |
| -1274.918 | 19397.896 | 2.572 | 14.874 | 0.325867 | 7/15/2014 | 11:11.4 | 2 |

| | | | | | | | |
|---|---|---|---|---|---|---|---|
| -1274.703 | 19397.877 | 2.569 | 14.858 | 0.306269 | 7/15/2014 | 11:11.5 | 2 |
| -1274.49 | 19397.859 | 2.569 | 14.843 | 0.305721 | 7/15/2014 | 11:11.6 | 2 |
| -1274.223 | 19397.838 | 2.566 | 14.828 | 0.287565 | 7/15/2014 | 11:11.7 | 2 |
| -1274.008 | 19397.818 | 2.563 | 14.814 | 0.278312 | 7/15/2014 | 11:11.8 | 2 |
| -1273.793 | 19397.801 | 2.563 | 14.801 | 0.284911 | 7/15/2014 | 11:11.9 | 2 |
| -1273.579 | 19397.782 | 2.556 | 14.79 | 0.279806 | 7/15/2014 | 11:12.0 | 2 |
| -1273.364 | 19397.764 | 2.556 | 14.778 | 0.276712 | 7/15/2014 | 11:12.1 | 2 |
| -1273.149 | 19397.746 | 2.553 | 14.77 | 0.28376 | 7/15/2014 | 11:12.2 | 2 |
| -1272.939 | 19397.729 | 2.55 | 14.764 | 0.279423 | 7/15/2014 | 11:12.3 | 2 |
| -1272.572 | 19397.697 | 2.548 | 14.757 | 0.289968 | 7/15/2014 | 11:12.5 | 2 |
| -1272.362 | 19397.678 | 2.551 | 14.759 | 0.285933 | 7/15/2014 | 11:12.6 | 2 |
| -1272.098 | 19397.656 | 2.548 | 14.758 | 0.286478 | 7/15/2014 | 11:12.7 | 2 |
| -1271.993 | 19397.646 | 2.547 | 14.759 | 0.286895 | 7/15/2014 | 11:12.7 | 2 |
| -1271.778 | 19397.627 | 2.55 | 14.764 | 0.280348 | 7/15/2014 | 11:12.8 | 2 |
| -1271.565 | 19397.611 | 2.547 | 14.766 | 0.276063 | 7/15/2014 | 11:12.9 | 2 |
| -1271.352 | 19397.592 | 2.543 | 14.771 | 0.279907 | 7/15/2014 | 11:13.0 | 2 |
| -1271.136 | 19397.572 | 2.543 | 14.777 | 0.274196 | 7/15/2014 | 11:13.1 | 2 |
| -1270.921 | 19397.555 | 2.54 | 14.781 | 0.271257 | 7/15/2014 | 11:13.2 | 2 |
| -1270.708 | 19397.535 | 2.54 | 14.788 | 0.265805 | 7/15/2014 | 11:13.3 | 2 |
| -1270.441 | 19397.512 | 2.537 | 14.793 | 0.267557 | 7/15/2014 | 11:13.4 | 2 |
| -1270.225 | 19397.492 | 2.536 | 14.798 | 0.266357 | 7/15/2014 | 11:13.5 | 2 |
| -1270.011 | 19397.476 | 2.533 | 14.804 | 0.27474 | 7/15/2014 | 11:13.6 | 2 |
| -1269.74 | 19397.451 | 2.533 | 14.815 | 0.262654 | 7/15/2014 | 11:13.7 | 2 |
| -1269.522 | 19397.432 | 2.533 | 14.821 | 0.25755 | 7/15/2014 | 11:13.8 | 2 |
| -1269.301 | 19397.412 | 2.533 | 14.832 | 0.260221 | 7/15/2014 | 11:13.9 | 2 |
| -1269.081 | 19397.393 | 2.533 | 14.842 | 0.260929 | 7/15/2014 | 11:14.0 | 2 |
| -1268.862 | 19397.373 | 2.533 | 14.851 | 0.257351 | 7/15/2014 | 11:14.1 | 2 |
| -1268.64 | 19397.354 | 2.536 | 14.861 | 0.249444 | 7/15/2014 | 11:14.2 | 2 |
| -1268.422 | 19397.335 | 2.534 | 14.872 | 0.249694 | 7/15/2014 | 11:14.3 | 2 |
| -1268.149 | 19397.31 | 2.534 | 14.883 | 0.250314 | 7/15/2014 | 11:14.4 | 2 |
| -1267.93 | 19397.29 | 2.534 | 14.894 | 0.247175 | 7/15/2014 | 11:14.5 | 2 |
| -1267.71 | 19397.271 | 2.534 | 14.912 | 0.245127 | 7/15/2014 | 11:14.6 | 2 |
| -1267.434 | 19397.246 | 2.534 | 14.927 | 0.24231 | 7/15/2014 | 11:14.7 | 2 |
| -1267.215 | 19397.227 | 2.529 | 14.944 | 0.238397 | 7/15/2014 | 11:14.8 | 2 |
| -1266.992 | 19397.207 | 2.529 | 14.961 | 0.236254 | 7/15/2014 | 11:14.9 | 2 |
| -1266.772 | 19397.188 | 2.529 | 14.977 | 0.235962 | 7/15/2014 | 11:15.0 | 2 |
| -1266.551 | 19397.167 | 2.531 | 14.988 | 0.235062 | 7/15/2014 | 11:15.1 | 2 |
| -1266.331 | 19397.147 | 2.531 | 14.996 | 0.226232 | 7/15/2014 | 11:15.2 | 2 |
| -1266.112 | 19397.127 | 2.531 | 14.994 | 0.222014 | 7/15/2014 | 11:15.3 | 2 |
| -1265.839 | 19397.103 | 2.531 | 14.987 | 0.219143 | 7/15/2014 | 11:15.4 | 2 |
| -1265.622 | 19397.083 | 2.531 | 14.975 | 0.213385 | 7/15/2014 | 11:15.5 | 2 |
| -1265.404 | 19397.063 | 2.536 | 14.961 | 0.206685 | 7/15/2014 | 11:15.6 | 2 |
| -1265.135 | 19397.039 | 2.536 | 14.943 | 0.203456 | 7/15/2014 | 11:15.7 | 2 |
| -1264.918 | 19397.02 | 2.533 | 14.924 | 0.203385 | 7/15/2014 | 11:15.8 | 2 |
| -1264.7 | 19397 | 2.531 | 14.908 | 0.203573 | 7/15/2014 | 11:15.9 | 2 |
| -1264.482 | 19396.979 | 2.531 | 14.891 | 0.206252 | 7/15/2014 | 11:16.0 | 2 |
| -1264.265 | 19396.96 | 2.531 | 14.884 | 0.207859 | 7/15/2014 | 11:16.1 | 2 |

| | | | | | | | |
|---|---|---|---|---|---|---|---|
| -1264.048 | 19396.94 | 2.531 | 14.876 | 0.213066 | 7/15/2014 | 11:16.2 | 2 |
| -1263.831 | 19396.921 | 2.531 | 14.867 | 0.210224 | 7/15/2014 | 11:16.3 | 2 |
| -1263.564 | 19396.896 | 2.526 | 14.859 | 0.211652 | 7/15/2014 | 11:16.4 | 2 |
| -1263.351 | 19396.877 | 2.524 | 14.853 | 0.213218 | 7/15/2014 | 11:16.5 | 2 |
| -1263.135 | 19396.856 | 2.524 | 14.845 | 0.2109 | 7/15/2014 | 11:16.6 | 2 |
| -1262.867 | 19396.832 | 2.526 | 14.836 | 0.210307 | 7/15/2014 | 11:16.7 | 2 |
| -1262.651 | 19396.813 | 2.526 | 14.828 | 0.209536 | 7/15/2014 | 11:16.8 | 2 |
| -1262.434 | 19396.793 | 2.526 | 14.812 | 0.208887 | 7/15/2014 | 11:16.9 | 2 |
| -1262.216 | 19396.772 | 2.526 | 14.803 | 0.213168 | 7/15/2014 | 11:17.0 | 2 |
| -1262 | 19396.752 | 2.526 | 14.793 | 0.211676 | 7/15/2014 | 11:17.1 | 2 |
| -1261.784 | 19396.732 | 2.528 | 14.788 | 0.213426 | 7/15/2014 | 11:17.2 | 2 |
| -1261.568 | 19396.712 | 2.53 | 14.779 | 0.217242 | 7/15/2014 | 11:17.3 | 2 |
| -1261.3 | 19396.687 | 2.53 | 14.771 | 0.215761 | 7/15/2014 | 11:17.4 | 2 |
| -1261.086 | 19396.667 | 2.528 | 14.763 | 0.216753 | 7/15/2014 | 11:17.5 | 2 |
| -1260.872 | 19396.646 | 2.528 | 14.758 | 0.218399 | 7/15/2014 | 11:17.6 | 2 |
| -1260.605 | 19396.621 | 2.528 | 14.758 | 0.218905 | 7/15/2014 | 11:17.7 | 2 |
| -1260.39 | 19396.601 | 2.528 | 14.758 | 0.221128 | 7/15/2014 | 11:17.8 | 2 |
| -1260.174 | 19396.58 | 2.528 | 14.765 | 0.221756 | 7/15/2014 | 11:17.9 | 2 |
| -1259.959 | 19396.561 | 2.531 | 14.766 | 0.223096 | 7/15/2014 | 11:18.0 | 2 |
| -1259.744 | 19396.539 | 2.529 | 14.776 | 0.229254 | 7/15/2014 | 11:18.1 | 2 |
| -1259.53 | 19396.52 | 2.529 | 14.78 | 0.220924 | 7/15/2014 | 11:18.2 | 2 |
| -1259.318 | 19396.498 | 2.529 | 14.786 | 0.225131 | 7/15/2014 | 11:18.3 | 2 |
| -1259.052 | 19396.474 | 2.529 | 14.795 | 0.228991 | 7/15/2014 | 11:18.4 | 2 |
| -1258.84 | 19396.453 | 2.527 | 14.797 | 0.223446 | 7/15/2014 | 11:18.5 | 2 |
| -1258.472 | 19396.417 | 2.525 | 14.796 | 0.228084 | 7/15/2014 | 11:18.7 | 2 |
| -1258.365 | 19396.407 | 2.525 | 14.793 | 0.227931 | 7/15/2014 | 11:18.7 | 2 |
| -1258.152 | 19396.387 | 2.521 | 14.795 | 0.222646 | 7/15/2014 | 11:18.8 | 2 |
| -1257.938 | 19396.366 | 2.524 | 14.791 | 0.234655 | 7/15/2014 | 11:18.9 | 2 |
| -1257.726 | 19396.346 | 2.524 | 14.799 | 0.231993 | 7/15/2014 | 11:19.0 | 2 |
| -1257.512 | 19396.324 | 2.529 | 14.803 | 0.223418 | 7/15/2014 | 11:19.1 | 2 |
| -1257.299 | 19396.304 | 2.529 | 14.8 | 0.238604 | 7/15/2014 | 11:19.2 | 2 |
| -1257.088 | 19396.284 | 2.529 | 14.805 | 0.221726 | 7/15/2014 | 11:19.3 | 2 |
| -1256.72 | 19396.247 | 2.525 | 14.813 | 0.237142 | 7/15/2014 | 11:19.5 | 2 |
| -1256.507 | 19396.227 | 2.527 | 14.821 | 0.233551 | 7/15/2014 | 11:19.6 | 2 |
| -1256.241 | 19396.2 | 2.525 | 14.826 | 0.222859 | 7/15/2014 | 11:19.7 | 2 |
| -1256.134 | 19396.19 | 2.525 | 14.83 | 0.218224 | 7/15/2014 | 11:19.7 | 2 |
| -1255.918 | 19396.168 | 2.528 | 14.836 | 0.208008 | 7/15/2014 | 11:19.8 | 2 |
| -1255.704 | 19396.147 | 2.525 | 14.843 | 0.207097 | 7/15/2014 | 11:19.9 | 2 |
| -1255.485 | 19396.127 | 2.528 | 14.854 | 0.214881 | 7/15/2014 | 11:20.0 | 2 |
| -1255.269 | 19396.104 | 2.533 | 14.864 | 0.205698 | 7/15/2014 | 11:20.1 | 2 |
| -1255.053 | 19396.082 | 2.533 | 14.876 | 0.1993 | 7/15/2014 | 11:20.2 | 2 |
| -1254.835 | 19396.061 | 2.537 | 14.884 | 0.213253 | 7/15/2014 | 11:20.3 | 2 |
| -1254.564 | 19396.034 | 2.537 | 14.888 | 0.211284 | 7/15/2014 | 11:20.4 | 2 |
| -1254.345 | 19396.013 | 2.539 | 14.9 | 0.227267 | 7/15/2014 | 11:20.5 | 2 |
| -1254.129 | 19395.99 | 2.536 | 14.913 | 0.227982 | 7/15/2014 | 11:20.6 | 2 |
| -1253.855 | 19395.964 | 2.535 | 14.929 | 0.227787 | 7/15/2014 | 11:20.7 | 2 |
| -1253.638 | 19395.941 | 2.532 | 14.999 | 0.228226 | 7/15/2014 | 11:20.8 | 2 |

| | | | | | | | |
|---|---|---|---|---|---|---|---|
| -1253.419 | 19395.918 | 2.527 | 15.07 | 0.244759 | 7/15/2014 | 11:20.9 | 2 |
| -1253.198 | 19395.896 | 2.525 | 15.153 | 0.217009 | 7/15/2014 | 11:21.0 | 2 |
| -1252.98 | 19395.875 | 2.521 | 15.233 | 0.226557 | 7/15/2014 | 11:21.1 | 2 |
| -1252.76 | 19395.853 | 2.521 | 15.317 | 0.239774 | 7/15/2014 | 11:21.2 | 2 |
| -1252.543 | 19395.83 | 2.521 | 15.391 | 0.231414 | 7/15/2014 | 11:21.3 | 2 |
| -1252.273 | 19395.804 | 2.521 | 15.459 | 0.217766 | 7/15/2014 | 11:21.4 | 2 |
| -1252.058 | 19395.782 | 2.521 | 15.527 | 0.238301 | 7/15/2014 | 11:21.5 | 2 |
| -1251.844 | 19395.76 | 2.521 | 15.539 | 0.230234 | 7/15/2014 | 11:21.6 | 2 |
| -1251.574 | 19395.732 | 2.523 | 15.55 | 0.22997 | 7/15/2014 | 11:21.7 | 2 |
| -1251.359 | 19395.711 | 2.525 | 15.549 | 0.23598 | 7/15/2014 | 11:21.8 | 2 |
| -1251.141 | 19395.688 | 2.527 | 15.56 | 0.232385 | 7/15/2014 | 11:21.9 | 2 |
| -1250.924 | 19395.667 | 2.525 | 15.56 | 0.226296 | 7/15/2014 | 11:22.0 | 2 |
| -1250.707 | 19395.645 | 2.525 | 15.565 | 0.249398 | 7/15/2014 | 11:22.1 | 2 |
| -1250.491 | 19395.624 | 2.525 | 15.573 | 0.236043 | 7/15/2014 | 11:22.2 | 2 |
| -1250.275 | 19395.602 | 2.523 | 15.579 | 0.224793 | 7/15/2014 | 11:22.3 | 2 |
| -1250.008 | 19395.573 | 2.523 | 15.588 | 0.229444 | 7/15/2014 | 11:22.4 | 2 |
| -1249.793 | 19395.553 | 2.521 | 15.596 | 0.238644 | 7/15/2014 | 11:22.5 | 2 |
| -1249.578 | 19395.53 | 2.521 | 15.601 | 0.245254 | 7/15/2014 | 11:22.6 | 2 |
| -1249.31 | 19395.504 | 2.523 | 15.601 | 0.230723 | 7/15/2014 | 11:22.7 | 2 |
| -1249.094 | 19395.481 | 2.523 | 15.606 | 0.241497 | 7/15/2014 | 11:22.8 | 2 |
| -1248.878 | 19395.46 | 2.518 | 15.613 | 0.231124 | 7/15/2014 | 11:22.9 | 2 |
| -1248.66 | 19395.438 | 2.518 | 15.62 | 0.238338 | 7/15/2014 | 11:23.0 | 2 |
| -1248.443 | 19395.415 | 2.521 | 15.632 | 0.26715 | 7/15/2014 | 11:23.1 | 2 |
| -1248.227 | 19395.394 | 2.521 | 15.646 | 0.251929 | 7/15/2014 | 11:23.2 | 2 |
| -1248.012 | 19395.371 | 2.521 | 15.656 | 0.248658 | 7/15/2014 | 11:23.3 | 2 |
| -1247.744 | 19395.345 | 2.521 | 15.66 | 0.271194 | 7/15/2014 | 11:23.4 | 2 |
| -1247.531 | 19395.323 | 2.518 | 15.671 | 0.275957 | 7/15/2014 | 11:23.5 | 2 |
| -1247.319 | 19395.3 | 2.521 | 15.679 | 0.29709 | 7/15/2014 | 11:23.6 | 2 |
| -1247.053 | 19395.273 | 2.521 | 15.686 | 0.270512 | 7/15/2014 | 11:23.7 | 2 |
| -1246.838 | 19395.253 | 2.521 | 15.688 | 0.294889 | 7/15/2014 | 11:23.8 | 2 |
| -1246.624 | 19395.229 | 2.521 | 15.672 | 0.272467 | 7/15/2014 | 11:23.9 | 2 |
| -1246.41 | 19395.208 | 2.521 | 15.655 | 0.276877 | 7/15/2014 | 11:24.0 | 2 |
| -1246.196 | 19395.187 | 2.521 | 15.629 | 0.270913 | 7/15/2014 | 11:24.1 | 2 |
| -1245.983 | 19395.165 | 2.521 | 15.607 | 0.255588 | 7/15/2014 | 11:24.2 | 2 |
| -1245.773 | 19395.143 | 2.521 | 15.579 | 0.253029 | 7/15/2014 | 11:24.3 | 2 |
| -1245.405 | 19395.105 | 2.518 | 15.533 | 0.258863 | 7/15/2014 | 11:24.5 | 2 |
| -1245.194 | 19395.084 | 2.521 | 15.501 | 0.240888 | 7/15/2014 | 11:24.6 | 2 |
| -1244.933 | 19395.057 | 2.52 | 15.47 | 0.259045 | 7/15/2014 | 11:24.7 | 2 |
| -1244.827 | 19395.046 | 2.518 | 15.458 | 0.243441 | 7/15/2014 | 11:24.7 | 2 |
| -1244.613 | 19395.025 | 2.522 | 15.432 | 0.256525 | 7/15/2014 | 11:24.8 | 2 |
| -1244.402 | 19395.003 | 2.524 | 15.414 | 0.265379 | 7/15/2014 | 11:24.9 | 2 |
| -1244.191 | 19394.981 | 2.527 | 15.392 | 0.276334 | 7/15/2014 | 11:25.0 | 2 |
| -1243.977 | 19394.959 | 2.529 | 15.369 | 0.278581 | 7/15/2014 | 11:25.1 | 2 |
| -1243.764 | 19394.938 | 2.534 | 15.356 | 0.282233 | 7/15/2014 | 11:25.2 | 2 |
| -1243.552 | 19394.915 | 2.536 | 15.341 | 0.291172 | 7/15/2014 | 11:25.3 | 2 |
| -1243.29 | 19394.889 | 2.536 | 15.323 | 0.314776 | 7/15/2014 | 11:25.4 | 2 |
| -1243.075 | 19394.868 | 2.538 | 15.301 | 0.303757 | 7/15/2014 | 11:25.5 | 2 |

| | | | | | | | |
|---|---|---|---|---|---|---|---|
| -1242.864 | 19394.845 | 2.538 | 15.278 | 0.295651 | 7/15/2014 | 11:25.6 | 2 |
| -1242.597 | 19394.817 | 2.538 | 15.254 | 0.270258 | 7/15/2014 | 11:25.7 | 2 |
| -1242.381 | 19394.796 | 2.535 | 15.237 | 0.259038 | 7/15/2014 | 11:25.8 | 2 |
| -1242.165 | 19394.773 | 2.531 | 15.218 | 0.266935 | 7/15/2014 | 11:25.9 | 2 |
| -1241.946 | 19394.751 | 2.528 | 15.195 | 0.260395 | 7/15/2014 | 11:26.0 | 2 |
| -1241.728 | 19394.729 | 2.528 | 15.177 | 0.254908 | 7/15/2014 | 11:26.1 | 2 |
| -1241.511 | 19394.706 | 2.531 | 15.161 | 0.271189 | 7/15/2014 | 11:26.2 | 2 |
| -1241.294 | 19394.683 | 2.531 | 15.147 | 0.262428 | 7/15/2014 | 11:26.3 | 2 |
| -1241.022 | 19394.654 | 2.534 | 15.137 | 0.278135 | 7/15/2014 | 11:26.4 | 2 |
| -1240.807 | 19394.633 | 2.534 | 15.128 | 0.29616 | 7/15/2014 | 11:26.5 | 2 |
| -1240.592 | 19394.611 | 2.537 | 15.117 | 0.29214 | 7/15/2014 | 11:26.6 | 2 |
| -1240.322 | 19394.583 | 2.545 | 15.11 | 0.266333 | 7/15/2014 | 11:26.7 | 2 |
| -1240.104 | 19394.56 | 2.551 | 15.1 | 0.272105 | 7/15/2014 | 11:26.8 | 2 |
| -1239.89 | 19394.537 | 2.551 | 15.089 | 0.269711 | 7/15/2014 | 11:26.9 | 2 |
| -1239.672 | 19394.515 | 2.554 | 15.083 | 0.268909 | 7/15/2014 | 11:27.0 | 2 |
| -1239.458 | 19394.492 | 2.554 | 15.073 | 0.262841 | 7/15/2014 | 11:27.1 | 2 |
| -1239.246 | 19394.471 | 2.551 | 15.066 | 0.252593 | 7/15/2014 | 11:27.2 | 2 |
| -1239.035 | 19394.449 | 2.551 | 15.059 | 0.226617 | 7/15/2014 | 11:27.3 | 2 |
| -1238.668 | 19394.411 | 2.551 | 15.049 | 0.249331 | 7/15/2014 | 11:27.5 | 2 |
| -1238.457 | 19394.389 | 2.554 | 15.045 | 0.236481 | 7/15/2014 | 11:27.6 | 2 |
| -1238.199 | 19394.362 | 2.548 | 15.045 | 0.223064 | 7/15/2014 | 11:27.7 | 2 |
| -1238.093 | 19394.352 | 2.55 | 15.043 | 0.22122 | 7/15/2014 | 11:27.7 | 2 |
| -1237.883 | 19394.329 | 2.55 | 15.041 | 0.230415 | 7/15/2014 | 11:27.8 | 2 |
| -1237.674 | 19394.308 | 2.553 | 15.048 | 0.24002 | 7/15/2014 | 11:27.9 | 2 |
| -1237.463 | 19394.286 | 2.553 | 15.052 | 0.254035 | 7/15/2014 | 11:28.0 | 2 |
| -1237.25 | 19394.265 | 2.556 | 15.056 | 0.246939 | 7/15/2014 | 11:28.1 | 2 |
| -1237.041 | 19394.243 | 2.553 | 15.067 | 0.237299 | 7/15/2014 | 11:28.2 | 2 |
| -1236.829 | 19394.222 | 2.556 | 15.074 | 0.248065 | 7/15/2014 | 11:28.3 | 2 |
| -1236.462 | 19394.184 | 2.558 | 15.093 | 0.244348 | 7/15/2014 | 11:28.5 | 2 |
| -1236.25 | 19394.161 | 2.561 | 15.103 | 0.230323 | 7/15/2014 | 11:28.6 | 2 |
| -1236.147 | 19394.15 | 2.558 | 15.109 | 0.221584 | 7/15/2014 | 11:28.6 | 2 |
| -1235.881 | 19394.123 | 2.555 | 15.114 | 0.221736 | 7/15/2014 | 11:28.7 | 2 |
| -1235.666 | 19394.102 | 2.555 | 15.124 | 0.225604 | 7/15/2014 | 11:28.8 | 2 |
| -1235.452 | 19394.08 | 2.552 | 15.134 | 0.221097 | 7/15/2014 | 11:28.9 | 2 |
| -1235.236 | 19394.058 | 2.552 | 15.139 | 0.233347 | 7/15/2014 | 11:29.0 | 2 |
| -1235.019 | 19394.034 | 2.552 | 15.149 | 0.23958 | 7/15/2014 | 11:29.1 | 2 |
| -1234.803 | 19394.013 | 2.55 | 15.149 | 0.266938 | 7/15/2014 | 11:29.2 | 2 |
| -1234.59 | 19393.99 | 2.55 | 15.159 | 0.265105 | 7/15/2014 | 11:29.3 | 2 |
| -1234.321 | 19393.963 | 2.555 | 15.166 | 0.280248 | 7/15/2014 | 11:29.4 | 2 |
| -1234.107 | 19393.941 | 2.558 | 15.169 | 0.288231 | 7/15/2014 | 11:29.5 | 2 |
| -1233.893 | 19393.92 | 2.564 | 15.181 | 0.300758 | 7/15/2014 | 11:29.6 | 2 |
| -1233.627 | 19393.892 | 2.567 | 15.185 | 0.269603 | 7/15/2014 | 11:29.7 | 2 |
| -1233.411 | 19393.869 | 2.57 | 15.196 | 0.267466 | 7/15/2014 | 11:29.8 | 2 |
| -1233.196 | 19393.848 | 2.57 | 15.218 | 0.248589 | 7/15/2014 | 11:29.9 | 2 |
| -1232.982 | 19393.826 | 2.567 | 15.237 | 0.269174 | 7/15/2014 | 11:30.0 | 2 |
| -1232.766 | 19393.804 | 2.567 | 15.305 | 0.261996 | 7/15/2014 | 11:30.1 | 2 |
| -1232.552 | 19393.782 | 2.565 | 15.386 | 0.2526 | 7/15/2014 | 11:30.2 | 2 |

| | | | | | | | |
|---|---|---|---|---|---|---|---|
| -1232.34 | 19393.761 | 2.565 | 15.478 | 0.250357 | 7/15/2014 | 11:30.3 | 2 |
| -1231.973 | 19393.724 | 2.563 | 15.6 | 0.289645 | 7/15/2014 | 11:30.5 | 2 |
| -1231.76 | 19393.702 | 2.568 | 15.678 | 0.299481 | 7/15/2014 | 11:30.6 | 2 |
| -1231.5 | 19393.677 | 2.565 | 15.752 | 0.299039 | 7/15/2014 | 11:30.7 | 2 |
| -1231.394 | 19393.667 | 2.567 | 15.804 | 0.292652 | 7/15/2014 | 11:30.7 | 2 |
| -1231.181 | 19393.645 | 2.572 | 15.874 | 0.309022 | 7/15/2014 | 11:30.8 | 2 |
| -1230.968 | 19393.624 | 2.572 | 15.895 | 0.325734 | 7/15/2014 | 11:30.9 | 2 |
| -1230.756 | 19393.603 | 2.577 | 15.905 | 0.32718 | 7/15/2014 | 11:31.0 | 2 |
| -1230.542 | 19393.582 | 2.581 | 15.907 | 0.338507 | 7/15/2014 | 11:31.1 | 2 |
| -1230.332 | 19393.562 | 2.581 | 15.904 | 0.333384 | 7/15/2014 | 11:31.2 | 2 |
| -1230.12 | 19393.541 | 2.586 | 15.909 | 0.341605 | 7/15/2014 | 11:31.3 | 2 |
| -1229.754 | 19393.505 | 2.587 | 15.915 | 0.372907 | 7/15/2014 | 11:31.5 | 2 |
| -1229.543 | 19393.484 | 2.592 | 15.917 | 0.352265 | 7/15/2014 | 11:31.6 | 2 |
| -1229.284 | 19393.461 | 2.589 | 15.921 | 0.331006 | 7/15/2014 | 11:31.7 | 2 |
| -1229.177 | 19393.45 | 2.593 | 15.922 | 0.342633 | 7/15/2014 | 11:31.7 | 2 |
| -1228.964 | 19393.429 | 2.59 | 15.923 | 0.318308 | 7/15/2014 | 11:31.8 | 2 |
| -1228.751 | 19393.409 | 2.592 | 15.925 | 0.319498 | 7/15/2014 | 11:31.9 | 2 |
| -1228.539 | 19393.389 | 2.592 | 15.927 | 0.320619 | 7/15/2014 | 11:32.0 | 2 |
| -1228.327 | 19393.368 | 2.592 | 15.93 | 0.304547 | 7/15/2014 | 11:32.1 | 2 |
| -1228.115 | 19393.348 | 2.592 | 15.93 | 0.283969 | 7/15/2014 | 11:32.2 | 2 |
| -1227.905 | 19393.328 | 2.592 | 15.929 | 0.314735 | 7/15/2014 | 11:32.3 | 2 |
| -1227.539 | 19393.295 | 2.591 | 15.932 | 0.298256 | 7/15/2014 | 11:32.5 | 2 |
| -1227.328 | 19393.274 | 2.594 | 15.933 | 0.301498 | 7/15/2014 | 11:32.6 | 2 |
| -1227.065 | 19393.25 | 2.594 | 15.93 | 0.288026 | 7/15/2014 | 11:32.7 | 2 |
| -1226.959 | 19393.239 | 2.593 | 15.932 | 0.273626 | 7/15/2014 | 11:32.7 | 2 |
| -1226.748 | 19393.22 | 2.593 | 15.933 | 0.270202 | 7/15/2014 | 11:32.8 | 2 |
| -1226.537 | 19393.201 | 2.59 | 15.927 | 0.269447 | 7/15/2014 | 11:32.9 | 2 |
| -1226.323 | 19393.181 | 2.59 | 15.933 | 0.258074 | 7/15/2014 | 11:33.0 | 2 |
| -1226.11 | 19393.162 | 2.59 | 15.935 | 0.263173 | 7/15/2014 | 11:33.1 | 2 |
| -1225.898 | 19393.143 | 2.59 | 15.929 | 0.25442 | 7/15/2014 | 11:33.2 | 2 |
| -1225.688 | 19393.123 | 2.592 | 15.929 | 0.250393 | 7/15/2014 | 11:33.3 | 2 |
| -1225.32 | 19393.089 | 2.593 | 15.932 | 0.272288 | 7/15/2014 | 11:33.5 | 2 |
| -1225.111 | 19393.07 | 2.593 | 15.933 | 0.266886 | 7/15/2014 | 11:33.6 | 2 |
| -1224.85 | 19393.049 | 2.591 | 15.938 | 0.269234 | 7/15/2014 | 11:33.7 | 2 |
| -1224.743 | 19393.038 | 2.593 | 15.941 | 0.260231 | 7/15/2014 | 11:33.7 | 2 |
| -1224.532 | 19393.02 | 2.593 | 15.948 | 0.272619 | 7/15/2014 | 11:33.8 | 2 |
| -1224.32 | 19393 | 2.593 | 15.95 | 0.26438 | 7/15/2014 | 11:33.9 | 2 |
| -1224.108 | 19392.98 | 2.593 | 15.956 | 0.263618 | 7/15/2014 | 11:34.0 | 2 |
| -1223.896 | 19392.963 | 2.597 | 15.968 | 0.278262 | 7/15/2014 | 11:34.1 | 2 |
| -1223.684 | 19392.943 | 2.597 | 15.974 | 0.262626 | 7/15/2014 | 11:34.2 | 2 |
| -1223.475 | 19392.926 | 2.591 | 15.983 | 0.264506 | 7/15/2014 | 11:34.3 | 2 |
| -1223.108 | 19392.895 | 2.587 | 15.969 | 0.254441 | 7/15/2014 | 11:34.5 | 2 |
| -1222.899 | 19392.877 | 2.584 | 15.941 | 0.242425 | 7/15/2014 | 11:34.6 | 2 |
| -1222.637 | 19392.854 | 2.579 | 15.908 | 0.246302 | 7/15/2014 | 11:34.7 | 2 |
| -1222.531 | 19392.844 | 2.577 | 15.901 | 0.240511 | 7/15/2014 | 11:34.7 | 2 |
| -1222.318 | 19392.826 | 2.575 | 15.859 | 0.222249 | 7/15/2014 | 11:34.8 | 2 |
| -1222.108 | 19392.809 | 2.566 | 15.815 | 0.222168 | 7/15/2014 | 11:34.9 | 2 |

| | | | | | | | |
|---|---|---|---|---|---|---|---|
| -1221.895 | 19392.79 | 2.564 | 15.764 | 0.219898 | 7/15/2014 | 11:35.0 | 2 |
| -1221.683 | 19392.773 | 2.564 | 15.71 | 0.215697 | 7/15/2014 | 11:35.1 | 2 |
| -1221.471 | 19392.754 | 2.562 | 15.649 | 0.219479 | 7/15/2014 | 11:35.2 | 2 |
| -1221.261 | 19392.738 | 2.564 | 15.621 | 0.226114 | 7/15/2014 | 11:35.3 | 2 |
| -1220.894 | 19392.707 | 2.564 | 15.589 | 0.217868 | 7/15/2014 | 11:35.5 | 2 |
| -1220.682 | 19392.689 | 2.569 | 15.565 | 0.224128 | 7/15/2014 | 11:35.6 | 2 |
| -1220.425 | 19392.67 | 2.563 | 15.544 | 0.216805 | 7/15/2014 | 11:35.7 | 2 |
| -1220.317 | 19392.66 | 2.565 | 15.53 | 0.217567 | 7/15/2014 | 11:35.7 | 2 |
| -1220.108 | 19392.644 | 2.562 | 15.515 | 0.221061 | 7/15/2014 | 11:35.8 | 2 |
| -1219.896 | 19392.625 | 2.562 | 15.497 | 0.214716 | 7/15/2014 | 11:35.9 | 2 |
| -1219.686 | 19392.609 | 2.559 | 15.479 | 0.20928 | 7/15/2014 | 11:36.0 | 2 |
| -1219.475 | 19392.593 | 2.556 | 15.464 | 0.20936 | 7/15/2014 | 11:36.1 | 2 |
| -1219.267 | 19392.576 | 2.548 | 15.44 | 0.205423 | 7/15/2014 | 11:36.2 | 2 |
| -1219.057 | 19392.561 | 2.551 | 15.426 | 0.20244 | 7/15/2014 | 11:36.3 | 2 |
| -1218.697 | 19392.532 | 2.544 | 15.399 | 0.202514 | 7/15/2014 | 11:36.5 | 2 |
| -1218.491 | 19392.516 | 2.546 | 15.386 | 0.194619 | 7/15/2014 | 11:36.6 | 2 |
| -1218.235 | 19392.496 | 2.545 | 15.381 | 0.195646 | 7/15/2014 | 11:36.7 | 2 |
| -1218.13 | 19392.488 | 2.544 | 15.374 | 0.194448 | 7/15/2014 | 11:36.7 | 2 |
| -1217.921 | 19392.473 | 2.549 | 15.364 | 0.191349 | 7/15/2014 | 11:36.8 | 2 |
| -1217.715 | 19392.457 | 2.549 | 15.354 | 0.185064 | 7/15/2014 | 11:36.9 | 2 |
| -1217.508 | 19392.441 | 2.552 | 15.354 | 0.177701 | 7/15/2014 | 11:37.0 | 2 |
| -1217.301 | 19392.426 | 2.549 | 15.358 | 0.174062 | 7/15/2014 | 11:37.1 | 2 |
| -1217.095 | 19392.409 | 2.549 | 15.366 | 0.170985 | 7/15/2014 | 11:37.2 | 2 |
| -1216.89 | 19392.395 | 2.549 | 15.383 | 0.168511 | 7/15/2014 | 11:37.3 | 2 |
| -1216.534 | 19392.369 | 2.547 | 15.407 | 0.163578 | 7/15/2014 | 11:37.5 | 2 |
| -1216.329 | 19392.354 | 2.546 | 15.412 | 0.162821 | 7/15/2014 | 11:37.6 | 2 |
| -1216.076 | 19392.336 | 2.538 | 15.423 | 0.159768 | 7/15/2014 | 11:37.7 | 2 |
| -1215.973 | 19392.328 | 2.536 | 15.436 | 0.160287 | 7/15/2014 | 11:37.7 | 2 |
| -1215.768 | 19392.313 | 2.533 | 15.448 | 0.158202 | 7/15/2014 | 11:37.8 | 2 |
| -1215.563 | 19392.299 | 2.533 | 15.466 | 0.160854 | 7/15/2014 | 11:37.9 | 2 |
| -1215.357 | 19392.285 | 2.53 | 15.479 | 0.164909 | 7/15/2014 | 11:38.0 | 2 |
| -1215.151 | 19392.271 | 2.53 | 15.513 | 0.161016 | 7/15/2014 | 11:38.1 | 2 |
| -1214.944 | 19392.256 | 2.533 | 15.561 | 0.160943 | 7/15/2014 | 11:38.2 | 2 |
| -1214.741 | 19392.242 | 2.533 | 15.641 | 0.166259 | 7/15/2014 | 11:38.3 | 2 |
| -1214.383 | 19392.219 | 2.535 | 15.765 | 0.167996 | 7/15/2014 | 11:38.5 | 2 |
| -1214.177 | 19392.204 | 2.541 | 15.842 | 0.164309 | 7/15/2014 | 11:38.6 | 2 |
| -1213.923 | 19392.188 | 2.541 | 15.912 | 0.160563 | 7/15/2014 | 11:38.7 | 2 |
| -1213.819 | 19392.181 | 2.543 | 15.957 | 0.158704 | 7/15/2014 | 11:38.7 | 2 |
| -1213.613 | 19392.167 | 2.546 | 16.031 | 0.166114 | 7/15/2014 | 11:38.8 | 2 |
| -1213.403 | 19392.154 | 2.546 | 16.078 | 0.161181 | 7/15/2014 | 11:38.9 | 2 |
| -1213.197 | 19392.141 | 2.543 | 16.11 | 0.160506 | 7/15/2014 | 11:39.0 | 2 |
| -1212.989 | 19392.127 | 2.546 | 16.116 | 0.155904 | 7/15/2014 | 11:39.1 | 2 |
| -1212.782 | 19392.115 | 2.543 | 16.112 | 0.153167 | 7/15/2014 | 11:39.2 | 2 |
| -1212.574 | 19392.102 | 2.543 | 16.124 | 0.155074 | 7/15/2014 | 11:39.3 | 2 |
| -1212.216 | 19392.079 | 2.538 | 16.135 | 0.152667 | 7/15/2014 | 11:39.5 | 2 |
| -1212.01 | 19392.066 | 2.541 | 16.142 | 0.154684 | 7/15/2014 | 11:39.6 | 2 |
| -1211.753 | 19392.052 | 2.541 | 16.146 | 0.153174 | 7/15/2014 | 11:39.7 | 2 |

| | | | | | | | |
|---|---|---|---|---|---|---|---|
| -1211.649 | 19392.045 | 2.543 | 16.149 | 0.154006 | 7/15/2014 | 11:39.7 | 2 |
| -1211.441 | 19392.032 | 2.543 | 16.151 | 0.153646 | 7/15/2014 | 11:39.8 | 2 |
| -1211.233 | 19392.021 | 2.543 | 16.15 | 0.154075 | 7/15/2014 | 11:39.9 | 2 |
| -1211.022 | 19392.009 | 2.549 | 16.149 | 0.15692 | 7/15/2014 | 11:40.0 | 2 |
| -1210.815 | 19391.996 | 2.552 | 16.15 | 0.153564 | 7/15/2014 | 11:40.1 | 2 |
| -1210.605 | 19391.983 | 2.555 | 16.144 | 0.154812 | 7/15/2014 | 11:40.2 | 2 |
| -1210.399 | 19391.971 | 2.555 | 16.14 | 0.152259 | 7/15/2014 | 11:40.3 | 2 |
| -1210.037 | 19391.951 | 2.552 | 16.132 | 0.149956 | 7/15/2014 | 11:40.5 | 2 |
| -1209.829 | 19391.939 | 2.555 | 16.132 | 0.145411 | 7/15/2014 | 11:40.6 | 2 |
| -1209.572 | 19391.926 | 2.552 | 16.132 | 0.145108 | 7/15/2014 | 11:40.7 | 2 |
| -1209.466 | 19391.92 | 2.552 | 16.132 | 0.143389 | 7/15/2014 | 11:40.7 | 2 |
| -1209.259 | 19391.908 | 2.546 | 16.129 | 0.142561 | 7/15/2014 | 11:40.8 | 2 |
| -1209.048 | 19391.896 | 2.546 | 16.129 | 0.138175 | 7/15/2014 | 11:40.9 | 2 |
| -1208.84 | 19391.885 | 2.543 | 16.122 | 0.136279 | 7/15/2014 | 11:41.0 | 2 |
| -1208.631 | 19391.874 | 2.546 | 16.116 | 0.138215 | 7/15/2014 | 11:41.1 | 2 |
| -1208.421 | 19391.863 | 2.546 | 16.11 | 0.137318 | 7/15/2014 | 11:41.2 | 2 |
| -1208.215 | 19391.853 | 2.544 | 16.102 | 0.134509 | 7/15/2014 | 11:41.3 | 2 |
| -1207.855 | 19391.833 | 2.533 | 16.097 | 0.1384 | 7/15/2014 | 11:41.5 | 2 |
| -1207.648 | 19391.824 | 2.535 | 16.09 | 0.136468 | 7/15/2014 | 11:41.6 | 2 |
| -1207.391 | 19391.811 | 2.533 | 16.088 | 0.136491 | 7/15/2014 | 11:41.7 | 2 |
| -1207.286 | 19391.805 | 2.531 | 16.084 | 0.139459 | 7/15/2014 | 11:41.7 | 2 |
| -1207.077 | 19391.793 | 2.526 | 16.084 | 0.140143 | 7/15/2014 | 11:41.8 | 2 |
| -1206.866 | 19391.784 | 2.52 | 16.079 | 0.130344 | 7/15/2014 | 11:41.9 | 2 |
| -1206.659 | 19391.773 | 2.517 | 16.08 | 0.136837 | 7/15/2014 | 11:42.0 | 2 |
| -1206.448 | 19391.762 | 2.519 | 16.082 | 0.124016 | 7/15/2014 | 11:42.1 | 2 |
| -1206.239 | 19391.752 | 2.519 | 16.082 | 0.117442 | 7/15/2014 | 11:42.2 | 2 |
| -1206.03 | 19391.742 | 2.521 | 16.081 | 0.121311 | 7/15/2014 | 11:42.3 | 2 |
| -1205.666 | 19391.725 | 2.518 | 16.085 | 0.113351 | 7/15/2014 | 11:42.5 | 2 |
| -1205.457 | 19391.715 | 2.52 | 16.085 | 0.112349 | 7/15/2014 | 11:42.6 | 2 |
| -1205.197 | 19391.703 | 2.518 | 16.092 | 0.117731 | 7/15/2014 | 11:42.7 | 2 |
| -1205.092 | 19391.697 | 2.52 | 16.096 | 0.11383 | 7/15/2014 | 11:42.7 | 2 |
| -1204.881 | 19391.688 | 2.52 | 16.105 | 0.111291 | 7/15/2014 | 11:42.8 | 2 |
| -1204.67 | 19391.678 | 2.52 | 16.104 | 0.115121 | 7/15/2014 | 11:42.9 | 2 |
| -1204.46 | 19391.668 | 2.518 | 16.095 | 0.111183 | 7/15/2014 | 11:43.0 | 2 |
| -1204.25 | 19391.66 | 2.515 | 16.085 | 0.133548 | 7/15/2014 | 11:43.1 | 2 |
| -1204.04 | 19391.648 | 2.515 | 16.082 | 0.12461 | 7/15/2014 | 11:43.2 | 2 |
| -1203.831 | 19391.641 | 2.515 | 16.07 | 0.116598 | 7/15/2014 | 11:43.3 | 2 |
| -1203.471 | 19391.625 | 2.517 | 16.059 | 0.108788 | 7/15/2014 | 11:43.5 | 2 |
| -1203.264 | 19391.615 | 2.52 | 16.046 | 0.105202 | 7/15/2014 | 11:43.6 | 2 |
| -1203.007 | 19391.604 | 2.52 | 16.031 | 0.10647 | 7/15/2014 | 11:43.7 | 2 |
| -1202.903 | 19391.6 | 2.52 | 16.025 | 0.105137 | 7/15/2014 | 11:43.7 | 2 |
| -1202.697 | 19391.592 | 2.525 | 16.038 | 0.099675 | 7/15/2014 | 11:43.8 | 2 |
| -1202.489 | 19391.582 | 2.529 | 16.045 | 0.0961791 | 7/15/2014 | 11:43.9 | 2 |
| -1202.281 | 19391.574 | 2.532 | 16.021 | 0.0930587 | 7/15/2014 | 11:44.0 | 2 |
| -1202.077 | 19391.566 | 2.53 | 16.008 | 0.105033 | 7/15/2014 | 11:44.1 | 2 |
| -1201.87 | 19391.558 | 2.53 | 15.983 | 0.0938192 | 7/15/2014 | 11:44.2 | 2 |
| -1201.666 | 19391.548 | 2.53 | 15.945 | 0.102579 | 7/15/2014 | 11:44.3 | 2 |

| | | | | | | | |
|---|---|---|---|---|---|---|---|
| -1201.309 | 19391.535 | 2.531 | 15.895 | 0.090893 | 7/15/2014 | 11:44.5 | 2 |
| -1201.105 | 19391.527 | 2.533 | 15.866 | 0.0919317 | 7/15/2014 | 11:44.6 | 2 |
| -1200.849 | 19391.516 | 2.533 | 15.816 | 0.0942807 | 7/15/2014 | 11:44.7 | 2 |
| -1200.745 | 19391.512 | 2.534 | 15.786 | 0.100578 | 7/15/2014 | 11:44.7 | 2 |
| -1200.54 | 19391.504 | 2.531 | 15.747 | 0.0881886 | 7/15/2014 | 11:44.8 | 2 |
| -1200.331 | 19391.496 | 2.533 | 15.706 | 0.0845668 | 7/15/2014 | 11:44.9 | 2 |
| -1200.122 | 19391.489 | 2.533 | 15.671 | 0.084021 | 7/15/2014 | 11:45.0 | 2 |
| -1199.913 | 19391.482 | 2.533 | 15.64 | 0.0838112 | 7/15/2014 | 11:45.1 | 2 |
| -1199.704 | 19391.473 | 2.533 | 15.596 | 0.0869815 | 7/15/2014 | 11:45.2 | 2 |
| -1199.496 | 19391.465 | 2.535 | 15.557 | 0.0821514 | 7/15/2014 | 11:45.3 | 2 |
| -1199.13 | 19391.45 | 2.532 | 15.499 | 0.0825327 | 7/15/2014 | 11:45.5 | 2 |
| -1198.923 | 19391.443 | 2.532 | 15.463 | 0.0817172 | 7/15/2014 | 11:45.6 | 2 |
| -1198.662 | 19391.435 | 2.532 | 15.44 | 0.0854213 | 7/15/2014 | 11:45.7 | 2 |
| -1198.555 | 19391.432 | 2.532 | 15.421 | 0.0838934 | 7/15/2014 | 11:45.7 | 2 |
| -1198.345 | 19391.424 | 2.532 | 15.401 | 0.0849809 | 7/15/2014 | 11:45.8 | 2 |
| -1198.133 | 19391.414 | 2.532 | 15.383 | 0.0862835 | 7/15/2014 | 11:45.9 | 2 |
| -1197.922 | 19391.406 | 2.529 | 15.374 | 0.0859778 | 7/15/2014 | 11:46.0 | 2 |
| -1197.71 | 19391.398 | 2.529 | 15.369 | 0.0899188 | 7/15/2014 | 11:46.1 | 2 |
| -1197.499 | 19391.393 | 2.529 | 15.367 | 0.097083 | 7/15/2014 | 11:46.2 | 2 |
| -1197.29 | 19391.387 | 2.527 | 15.37 | 0.0928228 | 7/15/2014 | 11:46.3 | 2 |
| -1196.924 | 19391.373 | 2.524 | 15.383 | 0.102078 | 7/15/2014 | 11:46.5 | 2 |
| -1196.716 | 19391.366 | 2.524 | 15.391 | 0.102614 | 7/15/2014 | 11:46.6 | 2 |
| -1196.455 | 19391.357 | 2.524 | 15.403 | 0.105832 | 7/15/2014 | 11:46.7 | 2 |
| -1196.349 | 19391.354 | 2.524 | 15.409 | 0.10639 | 7/15/2014 | 11:46.7 | 2 |
| -1196.139 | 19391.346 | 2.524 | 15.422 | 0.111483 | 7/15/2014 | 11:46.8 | 2 |
| -1195.928 | 19391.34 | 2.524 | 15.435 | 0.113543 | 7/15/2014 | 11:46.9 | 2 |
| -1195.717 | 19391.334 | 2.524 | 15.443 | 0.120222 | 7/15/2014 | 11:47.0 | 2 |
| -1195.506 | 19391.326 | 2.524 | 15.45 | 0.117496 | 7/15/2014 | 11:47.1 | 2 |
| -1195.294 | 19391.319 | 2.524 | 15.461 | 0.120007 | 7/15/2014 | 11:47.2 | 2 |
| -1195.086 | 19391.313 | 2.525 | 15.467 | 0.129127 | 7/15/2014 | 11:47.3 | 2 |
| -1194.72 | 19391.302 | 2.521 | 15.481 | 0.120722 | 7/15/2014 | 11:47.5 | 2 |
| -1194.51 | 19391.295 | 2.524 | 15.484 | 0.120761 | 7/15/2014 | 11:47.6 | 2 |
| -1194.25 | 19391.286 | 2.522 | 15.492 | 0.121708 | 7/15/2014 | 11:47.7 | 2 |
| -1194.144 | 19391.283 | 2.522 | 15.496 | 0.124277 | 7/15/2014 | 11:47.7 | 2 |
| -1193.934 | 19391.277 | 2.522 | 15.501 | 0.121547 | 7/15/2014 | 11:47.8 | 2 |
| -1193.722 | 19391.271 | 2.519 | 15.507 | 0.12331 | 7/15/2014 | 11:47.9 | 2 |
| -1193.512 | 19391.265 | 2.517 | 15.512 | 0.124685 | 7/15/2014 | 11:48.0 | 2 |
| -1193.299 | 19391.258 | 2.512 | 15.52 | 0.12729 | 7/15/2014 | 11:48.1 | 2 |
| -1193.088 | 19391.251 | 2.512 | 15.522 | 0.122536 | 7/15/2014 | 11:48.2 | 2 |
| -1192.878 | 19391.245 | 2.512 | 15.532 | 0.129587 | 7/15/2014 | 11:48.3 | 2 |
| -1192.512 | 19391.232 | 2.509 | 15.546 | 0.126279 | 7/15/2014 | 11:48.5 | 2 |
| -1192.301 | 19391.229 | 2.511 | 15.554 | 0.129256 | 7/15/2014 | 11:48.6 | 2 |
| -1192.04 | 19391.219 | 2.509 | 15.571 | 0.129047 | 7/15/2014 | 11:48.7 | 2 |
| -1191.934 | 19391.216 | 2.511 | 15.578 | 0.132615 | 7/15/2014 | 11:48.7 | 2 |
| -1191.722 | 19391.211 | 2.513 | 15.59 | 0.128354 | 7/15/2014 | 11:48.8 | 2 |
| -1191.508 | 19391.203 | 2.517 | 15.607 | 0.128511 | 7/15/2014 | 11:48.9 | 2 |
| -1191.296 | 19391.197 | 2.52 | 15.622 | 0.130824 | 7/15/2014 | 11:49.0 | 2 |

| | | | | | | | |
|---|---|---|---|---|---|---|---|
| -1191.082 | 19391.191 | 2.525 | 15.64 | 0.128048 | 7/15/2014 | 11:49.1 | 2 |
| -1190.868 | 19391.184 | 2.524 | 15.66 | 0.129532 | 7/15/2014 | 11:49.2 | 2 |
| -1190.656 | 19391.18 | 2.524 | 15.675 | 0.127999 | 7/15/2014 | 11:49.3 | 2 |
| -1190.285 | 19391.167 | 2.524 | 15.688 | 0.134066 | 7/15/2014 | 11:49.5 | 2 |
| -1190.18 | 19391.165 | 2.526 | 15.692 | 0.137591 | 7/15/2014 | 11:49.5 | 2 |
| -1189.809 | 19391.153 | 2.529 | 15.692 | 0.13359 | 7/15/2014 | 11:49.7 | 2 |
| -1189.702 | 19391.15 | 2.529 | 15.695 | 0.131354 | 7/15/2014 | 11:49.7 | 2 |
| -1189.487 | 19391.145 | 2.526 | 15.691 | 0.139394 | 7/15/2014 | 11:49.8 | 2 |
| -1189.272 | 19391.137 | 2.524 | 15.68 | 0.137714 | 7/15/2014 | 11:49.9 | 2 |
| -1189.057 | 19391.132 | 2.527 | 15.668 | 0.142872 | 7/15/2014 | 11:50.0 | 2 |
| -1188.842 | 19391.125 | 2.529 | 15.657 | 0.13976 | 7/15/2014 | 11:50.1 | 2 |
| -1188.627 | 19391.118 | 2.529 | 15.641 | 0.142117 | 7/15/2014 | 11:50.2 | 2 |
| -1188.414 | 19391.112 | 2.529 | 15.628 | 0.154009 | 7/15/2014 | 11:50.3 | 2 |
| -1188.148 | 19391.104 | 2.532 | 15.614 | 0.143502 | 7/15/2014 | 11:50.4 | 2 |
| -1187.935 | 19391.098 | 2.529 | 15.601 | 0.151584 | 7/15/2014 | 11:50.5 | 2 |
| -1187.722 | 19391.091 | 2.532 | 15.595 | 0.15958 | 7/15/2014 | 11:50.6 | 2 |
| -1187.456 | 19391.083 | 2.532 | 15.584 | 0.151367 | 7/15/2014 | 11:50.7 | 2 |
| -1187.242 | 19391.076 | 2.529 | 15.575 | 0.144682 | 7/15/2014 | 11:50.8 | 2 |
| -1187.028 | 19391.07 | 2.529 | 15.565 | 0.143035 | 7/15/2014 | 11:50.9 | 2 |
| -1186.813 | 19391.063 | 2.529 | 15.56 | 0.152573 | 7/15/2014 | 11:51.0 | 2 |
| -1186.599 | 19391.057 | 2.529 | 15.554 | 0.152928 | 7/15/2014 | 11:51.1 | 2 |
| -1186.386 | 19391.05 | 2.526 | 15.552 | 0.151462 | 7/15/2014 | 11:51.2 | 2 |
| -1186.174 | 19391.044 | 2.526 | 15.553 | 0.141251 | 7/15/2014 | 11:51.3 | 2 |
| -1185.805 | 19391.032 | 2.524 | 15.543 | 0.150031 | 7/15/2014 | 11:51.5 | 2 |
| -1185.595 | 19391.025 | 2.529 | 15.542 | 0.143607 | 7/15/2014 | 11:51.6 | 2 |
| -1185.335 | 19391.019 | 2.527 | 15.539 | 0.131102 | 7/15/2014 | 11:51.7 | 2 |
| -1185.229 | 19391.015 | 2.527 | 15.539 | 0.122195 | 7/15/2014 | 11:51.7 | 2 |
| -1185.017 | 19391.008 | 2.53 | 15.537 | 0.139025 | 7/15/2014 | 11:51.8 | 2 |
| -1184.806 | 19391.001 | 2.53 | 15.539 | 0.120418 | 7/15/2014 | 11:51.9 | 2 |
| -1184.594 | 19390.995 | 2.533 | 15.54 | 0.125184 | 7/15/2014 | 11:52.0 | 2 |
| -1184.384 | 19390.988 | 2.533 | 15.541 | 0.129919 | 7/15/2014 | 11:52.1 | 2 |
| -1184.174 | 19390.981 | 2.533 | 15.548 | 0.121835 | 7/15/2014 | 11:52.2 | 2 |
| -1183.965 | 19390.976 | 2.535 | 15.551 | 0.135077 | 7/15/2014 | 11:52.3 | 2 |
| -1183.603 | 19390.964 | 2.532 | 15.563 | 0.149556 | 7/15/2014 | 11:52.5 | 2 |
| -1183.393 | 19390.957 | 2.534 | 15.569 | 0.139573 | 7/15/2014 | 11:52.6 | 2 |
| -1183.133 | 19390.949 | 2.531 | 15.572 | 0.147127 | 7/15/2014 | 11:52.7 | 2 |
| -1183.028 | 19390.946 | 2.532 | 15.574 | 0.142209 | 7/15/2014 | 11:52.7 | 2 |
| -1182.818 | 19390.939 | 2.532 | 15.58 | 0.163877 | 7/15/2014 | 11:52.8 | 2 |
| -1182.606 | 19390.932 | 2.532 | 15.574 | 0.172648 | 7/15/2014 | 11:52.9 | 2 |
| -1182.395 | 19390.927 | 2.529 | 15.566 | 0.160448 | 7/15/2014 | 11:53.0 | 2 |
| -1182.183 | 19390.92 | 2.532 | 15.562 | 0.167165 | 7/15/2014 | 11:53.1 | 2 |
| -1181.972 | 19390.912 | 2.532 | 15.551 | 0.148997 | 7/15/2014 | 11:53.2 | 2 |
| -1181.761 | 19390.905 | 2.531 | 15.539 | 0.164226 | 7/15/2014 | 11:53.3 | 2 |
| -1181.392 | 19390.895 | 2.533 | 15.521 | 0.145179 | 7/15/2014 | 11:53.5 | 2 |
| -1181.181 | 19390.887 | 2.539 | 15.511 | 0.142481 | 7/15/2014 | 11:53.6 | 2 |
| -1180.92 | 19390.879 | 2.536 | 15.497 | 0.126222 | 7/15/2014 | 11:53.7 | 2 |
| -1180.813 | 19390.877 | 2.538 | 15.494 | 0.122451 | 7/15/2014 | 11:53.7 | 2 |

| | | | | | | | |
|---|---|---|---|---|---|---|---|
| -1180.601 | 19390.869 | 2.541 | 15.489 | 0.121761 | 7/15/2014 | 11:53.8 | 2 |
| -1180.387 | 19390.861 | 2.541 | 15.48 | 0.125319 | 7/15/2014 | 11:53.9 | 2 |
| -1180.174 | 19390.855 | 2.544 | 15.472 | 0.11545 | 7/15/2014 | 11:54.0 | 2 |
| -1179.961 | 19390.85 | 2.544 | 15.466 | 0.139764 | 7/15/2014 | 11:54.1 | 2 |
| -1179.749 | 19390.842 | 2.544 | 15.459 | 0.151988 | 7/15/2014 | 11:54.2 | 2 |
| -1179.536 | 19390.834 | 2.544 | 15.453 | 0.138851 | 7/15/2014 | 11:54.3 | 2 |
| -1179.168 | 19390.822 | 2.537 | 15.445 | 0.146487 | 7/15/2014 | 11:54.5 | 2 |
| -1178.956 | 19390.814 | 2.542 | 15.439 | 0.144672 | 7/15/2014 | 11:54.6 | 2 |
| -1178.693 | 19390.807 | 2.54 | 15.435 | 0.137515 | 7/15/2014 | 11:54.7 | 2 |
| -1178.587 | 19390.804 | 2.538 | 15.433 | 0.136897 | 7/15/2014 | 11:54.7 | 2 |
| -1178.372 | 19390.797 | 2.538 | 15.427 | 0.128108 | 7/15/2014 | 11:54.8 | 2 |
| -1178.158 | 19390.789 | 2.542 | 15.424 | 0.10273 | 7/15/2014 | 11:54.9 | 2 |
| -1177.944 | 19390.781 | 2.544 | 15.421 | 0.106639 | 7/15/2014 | 11:55.0 | 2 |
| -1177.729 | 19390.773 | 2.547 | 15.42 | 0.101717 | 7/15/2014 | 11:55.1 | 2 |
| -1177.515 | 19390.766 | 2.547 | 15.418 | 0.104235 | 7/15/2014 | 11:55.2 | 2 |
| -1177.3 | 19390.758 | 2.549 | 15.416 | 0.109101 | 7/15/2014 | 11:55.3 | 2 |
| -1177.035 | 19390.751 | 2.547 | 15.415 | 0.0956478 | 7/15/2014 | 11:55.4 | 2 |
| -1176.821 | 19390.744 | 2.549 | 15.412 | 0.105635 | 7/15/2014 | 11:55.5 | 2 |
| -1176.607 | 19390.736 | 2.546 | 15.409 | 0.12224 | 7/15/2014 | 11:55.6 | 2 |
| -1176.34 | 19390.727 | 2.546 | 15.403 | 0.112273 | 7/15/2014 | 11:55.7 | 2 |
| -1176.125 | 19390.719 | 2.544 | 15.395 | 0.128592 | 7/15/2014 | 11:55.8 | 2 |
| -1175.909 | 19390.711 | 2.541 | 15.388 | 0.120814 | 7/15/2014 | 11:55.9 | 2 |
| -1175.694 | 19390.703 | 2.541 | 15.378 | 0.129836 | 7/15/2014 | 11:56.0 | 2 |
| -1175.479 | 19390.696 | 2.538 | 15.368 | 0.149113 | 7/15/2014 | 11:56.1 | 2 |
| -1175.263 | 19390.689 | 2.535 | 15.357 | 0.149488 | 7/15/2014 | 11:56.2 | 2 |
| -1175.049 | 19390.682 | 2.532 | 15.344 | 0.131126 | 7/15/2014 | 11:56.3 | 2 |
| -1174.783 | 19390.672 | 2.532 | 15.331 | 0.145251 | 7/15/2014 | 11:56.4 | 2 |
| -1174.571 | 19390.664 | 2.529 | 15.322 | 0.130898 | 7/15/2014 | 11:56.5 | 2 |
| -1174.204 | 19390.652 | 2.529 | 15.306 | 0.137638 | 7/15/2014 | 11:56.7 | 2 |
| -1174.096 | 19390.648 | 2.529 | 15.3 | 0.12366 | 7/15/2014 | 11:56.7 | 2 |
| -1173.884 | 19390.641 | 2.526 | 15.291 | 0.136978 | 7/15/2014 | 11:56.8 | 2 |
| -1173.671 | 19390.633 | 2.531 | 15.281 | 0.123262 | 7/15/2014 | 11:56.9 | 2 |
| -1173.458 | 19390.625 | 2.531 | 15.27 | 0.131819 | 7/15/2014 | 11:57.0 | 2 |
| -1173.247 | 19390.617 | 2.531 | 15.262 | 0.134714 | 7/15/2014 | 11:57.1 | 2 |
| -1173.036 | 19390.609 | 2.531 | 15.253 | 0.148928 | 7/15/2014 | 11:57.2 | 2 |
| -1172.826 | 19390.603 | 2.531 | 15.243 | 0.148089 | 7/15/2014 | 11:57.3 | 2 |
| -1172.463 | 19390.59 | 2.53 | 15.228 | 0.162374 | 7/15/2014 | 11:57.5 | 2 |
| -1172.253 | 19390.582 | 2.53 | 15.22 | 0.173222 | 7/15/2014 | 11:57.6 | 2 |
| -1171.993 | 19390.572 | 2.528 | 15.213 | 0.172778 | 7/15/2014 | 11:57.7 | 2 |
| -1171.888 | 19390.568 | 2.525 | 15.208 | 0.17646 | 7/15/2014 | 11:57.7 | 2 |
| -1171.677 | 19390.561 | 2.528 | 15.198 | 0.176393 | 7/15/2014 | 11:57.8 | 2 |
| -1171.465 | 19390.553 | 2.526 | 15.191 | 0.178594 | 7/15/2014 | 11:57.9 | 2 |
| -1171.253 | 19390.545 | 2.524 | 15.186 | 0.15911 | 7/15/2014 | 11:58.0 | 2 |
| -1171.041 | 19390.535 | 2.522 | 15.186 | 0.154279 | 7/15/2014 | 11:58.1 | 2 |
| -1170.828 | 19390.527 | 2.517 | 15.184 | 0.16464 | 7/15/2014 | 11:58.2 | 2 |
| -1170.616 | 19390.52 | 2.517 | 15.185 | 0.128641 | 7/15/2014 | 11:58.3 | 2 |
| -1170.353 | 19390.51 | 2.517 | 15.184 | 0.155929 | 7/15/2014 | 11:58.4 | 2 |

| | | | | | | | |
|---|---|---|---|---|---|---|---|
| -1170.14 | 19390.5 | 2.519 | 15.179 | 0.134157 | 7/15/2014 | 11:58.5 | 2 |
| -1169.927 | 19390.492 | 2.513 | 15.181 | 0.126847 | 7/15/2014 | 11:58.6 | 2 |
| -1169.663 | 19390.482 | 2.513 | 15.179 | 0.12979 | 7/15/2014 | 11:58.7 | 2 |
| -1169.449 | 19390.473 | 2.513 | 15.18 | 0.12717 | 7/15/2014 | 11:58.8 | 2 |
| -1169.235 | 19390.465 | 2.511 | 15.176 | 0.120141 | 7/15/2014 | 11:58.9 | 2 |
| -1169.02 | 19390.455 | 2.511 | 15.173 | 0.144347 | 7/15/2014 | 11:59.0 | 2 |
| -1168.807 | 19390.447 | 2.507 | 15.168 | 0.130614 | 7/15/2014 | 11:59.1 | 2 |
| -1168.593 | 19390.438 | 2.507 | 15.164 | 0.163185 | 7/15/2014 | 11:59.2 | 2 |
| -1168.382 | 19390.428 | 2.503 | 15.16 | 0.161084 | 7/15/2014 | 11:59.3 | 2 |
| -1168.015 | 19390.414 | 2.503 | 15.15 | 0.136527 | 7/15/2014 | 11:59.5 | 2 |
| -1167.803 | 19390.404 | 2.505 | 15.146 | 0.144118 | 7/15/2014 | 11:59.6 | 2 |
| -1167.542 | 19390.395 | 2.503 | 15.138 | 0.145035 | 7/15/2014 | 11:59.7 | 2 |
| -1167.435 | 19390.389 | 2.505 | 15.134 | 0.137994 | 7/15/2014 | 11:59.7 | 2 |
| -1167.224 | 19390.38 | 2.507 | 15.128 | 0.135755 | 7/15/2014 | 11:59.8 | 2 |
| -1167.012 | 19390.371 | 2.509 | 15.125 | 0.128931 | 7/15/2014 | 11:59.9 | 2 |
| -1166.8 | 19390.361 | 2.507 | 15.123 | 0.127285 | 7/15/2014 | 12:00.0 | 2 |
| -1166.588 | 19390.352 | 2.507 | 15.125 | 0.159589 | 7/15/2014 | 12:00.1 | 2 |
| -1166.376 | 19390.344 | 2.505 | 15.127 | 0.156554 | 7/15/2014 | 12:00.2 | 2 |
| -1166.165 | 19390.332 | 2.502 | 15.129 | 0.135107 | 7/15/2014 | 12:00.3 | 2 |
| -1165.799 | 19390.316 | 2.498 | 15.131 | 0.158363 | 7/15/2014 | 12:00.5 | 2 |
| -1165.589 | 19390.308 | 2.498 | 15.136 | 0.159409 | 7/15/2014 | 12:00.6 | 2 |
| -1165.327 | 19390.295 | 2.494 | 15.139 | 0.137506 | 7/15/2014 | 12:00.7 | 2 |
| -1165.221 | 19390.291 | 2.492 | 15.14 | 0.140436 | 7/15/2014 | 12:00.7 | 2 |
| -1165.008 | 19390.279 | 2.494 | 15.144 | 0.127139 | 7/15/2014 | 12:00.8 | 2 |
| -1164.795 | 19390.271 | 2.494 | 15.143 | 0.134225 | 7/15/2014 | 12:00.9 | 2 |
| -1164.582 | 19390.26 | 2.496 | 15.144 | 0.142568 | 7/15/2014 | 12:01.0 | 2 |
| -1164.369 | 19390.251 | 2.5 | 15.144 | 0.117305 | 7/15/2014 | 12:01.1 | 2 |
| -1164.156 | 19390.24 | 2.5 | 15.144 | 0.104261 | 7/15/2014 | 12:01.2 | 2 |
| -1163.945 | 19390.23 | 2.505 | 15.142 | 0.107021 | 7/15/2014 | 12:01.3 | 2 |
| -1163.68 | 19390.217 | 2.509 | 15.135 | 0.124011 | 7/15/2014 | 12:01.4 | 2 |
| -1163.362 | 19390.201 | 2.511 | 15.123 | 0.126907 | 7/15/2014 | 12:01.6 | 2 |
| -1163.1 | 19390.189 | 2.509 | 15.113 | 0.0910368 | 7/15/2014 | 12:01.7 | 2 |
| -1162.992 | 19390.184 | 2.511 | 15.111 | 0.0870518 | 7/15/2014 | 12:01.7 | 2 |
| -1162.779 | 19390.173 | 2.511 | 15.1 | 0.100262 | 7/15/2014 | 12:01.8 | 2 |
| -1162.566 | 19390.162 | 2.513 | 15.09 | 0.0971952 | 7/15/2014 | 12:01.9 | 2 |
| -1162.352 | 19390.151 | 2.513 | 15.086 | 0.0827784 | 7/15/2014 | 12:02.0 | 2 |
| -1162.139 | 19390.141 | 2.513 | 15.077 | 0.0893297 | 7/15/2014 | 12:02.1 | 2 |
| -1161.928 | 19390.131 | 2.511 | 15.071 | 0.0947932 | 7/15/2014 | 12:02.2 | 2 |
| -1161.715 | 19390.119 | 2.513 | 15.067 | 0.0904599 | 7/15/2014 | 12:02.3 | 2 |
| -1161.346 | 19390.101 | 2.511 | 15.064 | 0.142562 | 7/15/2014 | 12:02.5 | 2 |
| -1161.133 | 19390.09 | 2.511 | 15.063 | 0.150204 | 7/15/2014 | 12:02.6 | 2 |
| -1160.87 | 19390.075 | 2.507 | 15.065 | 0.149634 | 7/15/2014 | 12:02.7 | 2 |
| -1160.763 | 19390.07 | 2.507 | 15.065 | 0.160898 | 7/15/2014 | 12:02.7 | 2 |
| -1160.55 | 19390.059 | 2.507 | 15.061 | 0.146465 | 7/15/2014 | 12:02.8 | 2 |
| -1160.336 | 19390.048 | 2.507 | 15.058 | 0.131624 | 7/15/2014 | 12:02.9 | 2 |
| -1160.119 | 19390.036 | 2.509 | 15.053 | 0.136349 | 7/15/2014 | 12:03.0 | 2 |
| -1159.904 | 19390.024 | 2.509 | 15.046 | 0.131889 | 7/15/2014 | 12:03.1 | 2 |

| | | | | | | | |
|---|---|---|---|---|---|---|---|
| -1159.688 | 19390.013 | 2.512 | 15.037 | 0.106607 | 7/15/2014 | 12:03.2 | 2 |
| -1159.473 | 19390.002 | 2.514 | 15.027 | 0.104165 | 7/15/2014 | 12:03.3 | 2 |
| -1159.205 | 19389.987 | 2.517 | 15.016 | 0.0903897 | 7/15/2014 | 12:03.4 | 2 |
| -1158.989 | 19389.975 | 2.522 | 15.001 | 0.0760297 | 7/15/2014 | 12:03.5 | 2 |
| -1158.773 | 19389.964 | 2.525 | 14.991 | 0.099679 | 7/15/2014 | 12:03.6 | 2 |
| -1158.503 | 19389.948 | 2.525 | 14.981 | 0.0796209 | 7/15/2014 | 12:03.7 | 2 |
| -1158.286 | 19389.937 | 2.525 | 14.97 | 0.0803598 | 7/15/2014 | 12:03.8 | 2 |
| -1158.066 | 19389.924 | 2.527 | 14.959 | 0.106003 | 7/15/2014 | 12:03.9 | 2 |
| -1157.849 | 19389.91 | 2.522 | 14.954 | 0.0842014 | 7/15/2014 | 12:04.0 | 2 |
| -1157.629 | 19389.898 | 2.525 | 14.949 | 0.103921 | 7/15/2014 | 12:04.1 | 2 |
| -1157.411 | 19389.887 | 2.53 | 14.944 | 0.105728 | 7/15/2014 | 12:04.2 | 2 |
| -1157.193 | 19389.873 | 2.532 | 14.935 | 0.109947 | 7/15/2014 | 12:04.3 | 2 |
| -1156.924 | 19389.857 | 2.532 | 14.925 | 0.122452 | 7/15/2014 | 12:04.4 | 2 |
| -1156.708 | 19389.845 | 2.534 | 14.913 | 0.0992268 | 7/15/2014 | 12:04.5 | 2 |
| -1156.491 | 19389.833 | 2.539 | 14.904 | 0.106775 | 7/15/2014 | 12:04.6 | 2 |
| -1156.223 | 19389.814 | 2.542 | 14.892 | 0.0817832 | 7/15/2014 | 12:04.7 | 2 |
| -1156.006 | 19389.803 | 2.549 | 14.876 | 0.0808716 | 7/15/2014 | 12:04.8 | 2 |
| -1155.788 | 19389.79 | 2.551 | 14.859 | 0.0865169 | 7/15/2014 | 12:04.9 | 2 |
| -1155.571 | 19389.775 | 2.549 | 14.842 | 0.0695596 | 7/15/2014 | 12:05.0 | 2 |
| -1155.355 | 19389.763 | 2.547 | 14.833 | 0.075634 | 7/15/2014 | 12:05.1 | 2 |
| -1155.139 | 19389.749 | 2.547 | 14.819 | 0.0751422 | 7/15/2014 | 12:05.2 | 2 |
| -1154.924 | 19389.736 | 2.547 | 14.81 | 0.0685093 | 7/15/2014 | 12:05.3 | 2 |
| -1154.658 | 19389.719 | 2.545 | 14.805 | 0.0962138 | 7/15/2014 | 12:05.4 | 2 |
| -1154.446 | 19389.706 | 2.543 | 14.806 | 0.111117 | 7/15/2014 | 12:05.5 | 2 |
| -1154.075 | 19389.683 | 2.539 | 14.808 | 0.0826145 | 7/15/2014 | 12:05.7 | 2 |
| -1153.967 | 19389.676 | 2.537 | 14.809 | 0.0835825 | 7/15/2014 | 12:05.7 | 2 |
| -1153.755 | 19389.662 | 2.537 | 14.817 | 0.0961018 | 7/15/2014 | 12:05.8 | 2 |
| -1153.54 | 19389.648 | 2.536 | 14.823 | 0.112087 | 7/15/2014 | 12:05.9 | 2 |
| -1153.326 | 19389.635 | 2.536 | 14.833 | 0.0987897 | 7/15/2014 | 12:06.0 | 2 |
| -1153.112 | 19389.621 | 2.54 | 14.846 | 0.118733 | 7/15/2014 | 12:06.1 | 2 |
| -1152.901 | 19389.606 | 2.536 | 14.855 | 0.0949162 | 7/15/2014 | 12:06.2 | 2 |
| -1152.687 | 19389.593 | 2.535 | 14.867 | 0.151423 | 7/15/2014 | 12:06.3 | 2 |
| -1152.318 | 19389.568 | 2.534 | 14.892 | 0.179857 | 7/15/2014 | 12:06.5 | 2 |
| -1152.106 | 19389.555 | 2.537 | 14.913 | 0.165233 | 7/15/2014 | 12:06.6 | 2 |
| -1151.841 | 19389.537 | 2.534 | 14.946 | 0.158159 | 7/15/2014 | 12:06.7 | 2 |
| -1151.734 | 19389.53 | 2.535 | 14.956 | 0.1616 | 7/15/2014 | 12:06.7 | 2 |
| -1151.52 | 19389.517 | 2.535 | 14.976 | 0.171252 | 7/15/2014 | 12:06.8 | 2 |
| -1151.305 | 19389.501 | 2.532 | 15.018 | 0.1913 | 7/15/2014 | 12:06.9 | 2 |
| -1151.089 | 19389.486 | 2.535 | 15.065 | 0.142693 | 7/15/2014 | 12:07.0 | 2 |
| -1150.874 | 19389.473 | 2.539 | 15.105 | 0.140647 | 7/15/2014 | 12:07.1 | 2 |
| -1150.658 | 19389.457 | 2.539 | 15.139 | 0.100911 | 7/15/2014 | 12:07.2 | 2 |
| -1150.443 | 19389.442 | 2.542 | 15.17 | 0.105671 | 7/15/2014 | 12:07.3 | 2 |
| -1150.175 | 19389.424 | 2.542 | 15.189 | 0.108073 | 7/15/2014 | 12:07.4 | 2 |
| -1149.96 | 19389.409 | 2.545 | 15.191 | 0.0889056 | 7/15/2014 | 12:07.5 | 2 |
| -1149.745 | 19389.394 | 2.547 | 15.205 | 0.0717152 | 7/15/2014 | 12:07.6 | 2 |
| -1149.476 | 19389.374 | 2.55 | 15.196 | 0.0808302 | 7/15/2014 | 12:07.7 | 2 |
| -1149.259 | 19389.36 | 2.552 | 15.178 | 0.058708 | 7/15/2014 | 12:07.8 | 2 |

| | | | | | | | |
|---|---|---|---|---|---|---|---|
| -1149.042 | 19389.345 | 2.554 | 15.159 | 0.0893488 | 7/15/2014 | 12:07.9 | 2 |
| -1148.824 | 19389.328 | 2.554 | 15.142 | 0.0712044 | 7/15/2014 | 12:08.0 | 2 |
| -1148.607 | 19389.313 | 2.557 | 15.124 | 0.0861373 | 7/15/2014 | 12:08.1 | 2 |
| -1148.391 | 19389.297 | 2.559 | 15.108 | 0.0751247 | 7/15/2014 | 12:08.2 | 2 |
| -1148.175 | 19389.281 | 2.559 | 15.086 | 0.0680551 | 7/15/2014 | 12:08.3 | 2 |
| -1147.908 | 19389.262 | 2.559 | 15.064 | 0.0754222 | 7/15/2014 | 12:08.4 | 2 |
| -1147.694 | 19389.247 | 2.559 | 15.041 | 0.0818406 | 7/15/2014 | 12:08.5 | 2 |
| -1147.48 | 19389.231 | 2.559 | 15.014 | 0.0510788 | 7/15/2014 | 12:08.6 | 2 |
| -1147.213 | 19389.211 | 2.559 | 14.988 | 0.0332091 | 7/15/2014 | 12:08.7 | 2 |
| -1146.998 | 19389.195 | 2.561 | 14.963 | 0.0159139 | 7/15/2014 | 12:08.8 | 2 |
| -1146.783 | 19389.179 | 2.561 | 14.935 | 0.0133331 | 7/15/2014 | 12:08.9 | 2 |
| -1146.565 | 19389.163 | 2.564 | 14.908 | 0.0119469 | 7/15/2014 | 12:09.0 | 2 |
| -1146.351 | 19389.146 | 2.566 | 14.884 | 0.0142456 | 7/15/2014 | 12:09.1 | 2 |
| -1146.135 | 19389.131 | 2.572 | 14.86 | 0.0340512 | 7/15/2014 | 12:09.2 | 2 |
| -1145.922 | 19389.114 | 2.572 | 14.836 | 0.0314934 | 7/15/2014 | 12:09.3 | 2 |
| -1145.653 | 19389.094 | 2.576 | 14.814 | 0.0110782 | 7/15/2014 | 12:09.4 | 2 |
| -1145.441 | 19389.078 | 2.574 | 14.79 | 0.021477 | 7/15/2014 | 12:09.5 | 2 |
| -1145.224 | 19389.06 | 2.58 | 14.764 | 0.0248594 | 7/15/2014 | 12:09.6 | 2 |
| -1144.955 | 19389.04 | 2.58 | 14.74 | 0.0330763 | 7/15/2014 | 12:09.7 | 2 |
| -1144.737 | 19389.021 | 2.58 | 14.714 | 0.0467053 | 7/15/2014 | 12:09.8 | 2 |
| -1144.519 | 19389.006 | 2.578 | 14.69 | 0.0687854 | 7/15/2014 | 12:09.9 | 2 |
| -1144.299 | 19388.987 | 2.575 | 14.666 | 0.0709118 | 7/15/2014 | 12:10.0 | 2 |
| -1144.08 | 19388.971 | 2.575 | 14.642 | 0.0615722 | 7/15/2014 | 12:10.1 | 2 |
| -1143.861 | 19388.953 | 2.575 | 14.622 | 0.0549845 | 7/15/2014 | 12:10.2 | 2 |
| -1143.641 | 19388.934 | 2.575 | 14.608 | 0.0608989 | 7/15/2014 | 12:10.3 | 2 |
| -1143.369 | 19388.913 | 2.571 | 14.593 | 0.0690477 | 7/15/2014 | 12:10.4 | 2 |
| -1143.15 | 19388.895 | 2.569 | 14.583 | 0.0495603 | 7/15/2014 | 12:10.5 | 2 |
| -1142.931 | 19388.877 | 2.569 | 14.575 | 0.057297 | 7/15/2014 | 12:10.6 | 2 |
| -1142.658 | 19388.855 | 2.569 | 14.571 | 0.0483953 | 7/15/2014 | 12:10.7 | 2 |
| -1142.439 | 19388.838 | 2.566 | 14.565 | 0.0371791 | 7/15/2014 | 12:10.8 | 2 |
| -1142.217 | 19388.819 | 2.566 | 14.567 | 0.0624605 | 7/15/2014 | 12:10.9 | 2 |
| -1141.996 | 19388.801 | 2.566 | 14.562 | 0.0673919 | 7/15/2014 | 12:11.0 | 2 |
| -1141.776 | 19388.782 | 2.566 | 14.562 | 0.0511543 | 7/15/2014 | 12:11.1 | 2 |
| -1141.557 | 19388.764 | 2.564 | 14.564 | 0.0697019 | 7/15/2014 | 12:11.2 | 2 |
| -1141.339 | 19388.744 | 2.564 | 14.569 | 0.0769249 | 7/15/2014 | 12:11.3 | 2 |
| -1141.069 | 19388.725 | 2.56 | 14.577 | 0.0894041 | 7/15/2014 | 12:11.4 | 2 |
| -1140.853 | 19388.705 | 2.56 | 14.582 | 0.0972396 | 7/15/2014 | 12:11.5 | 2 |
| -1140.637 | 19388.687 | 2.558 | 14.594 | 0.0789222 | 7/15/2014 | 12:11.6 | 2 |
| -1140.365 | 19388.664 | 2.558 | 14.602 | 0.054884 | 7/15/2014 | 12:11.7 | 2 |
| -1140.15 | 19388.645 | 2.552 | 14.617 | 0.070244 | 7/15/2014 | 12:11.8 | 2 |
| -1139.93 | 19388.627 | 2.552 | 14.628 | 0.053323 | 7/15/2014 | 12:11.9 | 2 |
| -1139.713 | 19388.607 | 2.552 | 14.638 | 0.0419906 | 7/15/2014 | 12:12.0 | 2 |
| -1139.497 | 19388.59 | 2.552 | 14.644 | 0.0527671 | 7/15/2014 | 12:12.1 | 2 |
| -1139.281 | 19388.57 | 2.55 | 14.655 | 0.0436713 | 7/15/2014 | 12:12.2 | 2 |
| -1139.066 | 19388.551 | 2.547 | 14.656 | 0.0416455 | 7/15/2014 | 12:12.3 | 2 |
| -1138.798 | 19388.527 | 2.547 | 14.655 | 0.0676643 | 7/15/2014 | 12:12.4 | 2 |
| -1138.584 | 19388.508 | 2.547 | 14.651 | 0.0651751 | 7/15/2014 | 12:12.5 | 2 |

| | | | | | | | |
|---|---|---|---|---|---|---|---|
| -1138.37 | 19388.491 | 2.544 | 14.643 | 0.07269 | 7/15/2014 | 12:12.6 | 2 |
| -1138.102 | 19388.467 | 2.541 | 14.634 | 0.0659181 | 7/15/2014 | 12:12.7 | 2 |
| -1137.888 | 19388.447 | 2.538 | 14.62 | 0.0489043 | 7/15/2014 | 12:12.8 | 2 |
| -1137.673 | 19388.429 | 2.535 | 14.605 | 0.0731341 | 7/15/2014 | 12:12.9 | 2 |
| -1137.456 | 19388.408 | 2.535 | 14.591 | 0.0633743 | 7/15/2014 | 12:13.0 | 2 |
| -1137.24 | 19388.389 | 2.533 | 14.576 | 0.0643028 | 7/15/2014 | 12:13.1 | 2 |
| -1137.022 | 19388.369 | 2.533 | 14.565 | 0.0602091 | 7/15/2014 | 12:13.2 | 2 |
| -1136.805 | 19388.35 | 2.528 | 14.554 | 0.0644544 | 7/15/2014 | 12:13.3 | 2 |
| -1136.533 | 19388.324 | 2.528 | 14.544 | 0.0731285 | 7/15/2014 | 12:13.4 | 2 |
| -1136.314 | 19388.305 | 2.526 | 14.531 | 0.0793526 | 7/15/2014 | 12:13.5 | 2 |
| -1136.09 | 19388.284 | 2.523 | 14.523 | 0.0637632 | 7/15/2014 | 12:13.6 | 2 |
| -1135.865 | 19388.264 | 2.518 | 14.517 | 0.0729928 | 7/15/2014 | 12:13.7 | 2 |
| -1135.638 | 19388.242 | 2.514 | 14.502 | 0.0719236 | 7/15/2014 | 12:13.8 | 2 |
| -1135.41 | 19388.221 | 2.514 | 14.496 | 0.0711447 | 7/15/2014 | 12:13.9 | 2 |
| -1135.181 | 19388.199 | 2.509 | 14.487 | 0.0750733 | 7/15/2014 | 12:14.0 | 2 |
| -1134.949 | 19388.178 | 2.509 | 14.478 | 0.0599097 | 7/15/2014 | 12:14.1 | 2 |
| -1134.718 | 19388.156 | 2.509 | 14.475 | 0.0716411 | 7/15/2014 | 12:14.2 | 2 |
| -1134.487 | 19388.133 | 2.512 | 14.471 | 0.0678714 | 7/15/2014 | 12:14.3 | 2 |
| -1134.256 | 19388.112 | 2.515 | 14.467 | 0.068184 | 7/15/2014 | 12:14.4 | 2 |
| -1134.025 | 19388.09 | 2.515 | 14.465 | 0.0746646 | 7/15/2014 | 12:14.5 | 2 |
| -1133.797 | 19388.066 | 2.513 | 14.461 | 0.0568476 | 7/15/2014 | 12:14.6 | 2 |
| -1133.512 | 19388.04 | 2.519 | 14.459 | 0.0702305 | 7/15/2014 | 12:14.7 | 2 |
| -1133.284 | 19388.019 | 2.519 | 14.461 | 0.0699964 | 7/15/2014 | 12:14.8 | 2 |
| -1133.055 | 19387.996 | 2.516 | 14.457 | 0.0740585 | 7/15/2014 | 12:14.9 | 2 |
| -1132.828 | 19387.974 | 2.514 | 14.46 | 0.0929912 | 7/15/2014 | 12:15.0 | 2 |
| -1132.6 | 19387.951 | 2.509 | 14.47 | 0.10622 | 7/15/2014 | 12:15.1 | 2 |
| -1132.374 | 19387.93 | 2.506 | 14.479 | 0.099946 | 7/15/2014 | 12:15.2 | 2 |
| -1132.15 | 19387.906 | 2.508 | 14.492 | 0.140473 | 7/15/2014 | 12:15.3 | 2 |
| -1131.868 | 19387.88 | 2.51 | 14.507 | 0.137082 | 7/15/2014 | 12:15.4 | 2 |
| -1131.644 | 19387.857 | 2.508 | 14.51 | 0.156789 | 7/15/2014 | 12:15.5 | 2 |
| -1131.419 | 19387.836 | 2.513 | 14.511 | 0.15006 | 7/15/2014 | 12:15.6 | 2 |
| -1131.194 | 19387.813 | 2.518 | 14.51 | 0.121447 | 7/15/2014 | 12:15.7 | 2 |
| -1130.968 | 19387.791 | 2.52 | 14.512 | 0.130201 | 7/15/2014 | 12:15.8 | 2 |
| -1130.744 | 19387.77 | 2.525 | 14.5 | 0.147183 | 7/15/2014 | 12:15.9 | 2 |
| -1130.519 | 19387.747 | 2.528 | 14.502 | 0.142542 | 7/15/2014 | 12:16.0 | 2 |
| -1130.295 | 19387.725 | 2.526 | 14.499 | 0.1357 | 7/15/2014 | 12:16.1 | 2 |
| -1130.073 | 19387.703 | 2.529 | 14.515 | 0.158304 | 7/15/2014 | 12:16.2 | 2 |
| -1129.852 | 19387.681 | 2.526 | 14.534 | 0.195764 | 7/15/2014 | 12:16.3 | 2 |
| -1129.631 | 19387.659 | 2.523 | 14.546 | 0.198978 | 7/15/2014 | 12:16.4 | 2 |
| -1129.414 | 19387.638 | 2.521 | 14.559 | 0.195722 | 7/15/2014 | 12:16.5 | 2 |
| -1129.198 | 19387.616 | 2.519 | 14.554 | 0.170128 | 7/15/2014 | 12:16.6 | 2 |
| -1128.927 | 19387.59 | 2.515 | 14.553 | 0.154694 | 7/15/2014 | 12:16.7 | 2 |
| -1128.71 | 19387.566 | 2.515 | 14.541 | 0.163946 | 7/15/2014 | 12:16.8 | 2 |
| -1128.492 | 19387.546 | 2.521 | 14.524 | 0.128755 | 7/15/2014 | 12:16.9 | 2 |
| -1128.273 | 19387.522 | 2.521 | 14.489 | 0.0661401 | 7/15/2014 | 12:17.0 | 2 |
| -1128.053 | 19387.502 | 2.521 | 14.46 | 0.116333 | 7/15/2014 | 12:17.1 | 2 |
| -1127.834 | 19387.479 | 2.524 | 14.435 | 0.0910974 | 7/15/2014 | 12:17.2 | 2 |

| | | | | | | | |
|---|---|---|---|---|---|---|---|
| -1127.614 | 19387.457 | 2.524 | 14.413 | 0.1092 | 7/15/2014 | 12:17.3 | 2 |
| -1127.338 | 19387.43 | 2.526 | 14.398 | 0.102406 | 7/15/2014 | 12:17.4 | 2 |
| -1127.114 | 19387.406 | 2.526 | 14.385 | 0.106034 | 7/15/2014 | 12:17.5 | 2 |
| -1126.888 | 19387.384 | 2.521 | 14.375 | 0.101039 | 7/15/2014 | 12:17.6 | 2 |
| -1126.66 | 19387.36 | 2.518 | 14.368 | 0.14507 | 7/15/2014 | 12:17.7 | 2 |
| -1126.431 | 19387.338 | 2.518 | 14.364 | 0.0995892 | 7/15/2014 | 12:17.8 | 2 |
| -1126.201 | 19387.313 | 2.515 | 14.357 | 0.125318 | 7/15/2014 | 12:17.9 | 2 |
| -1125.972 | 19387.291 | 2.51 | 14.354 | 0.131386 | 7/15/2014 | 12:18.0 | 2 |
| -1125.742 | 19387.268 | 2.51 | 14.353 | 0.151661 | 7/15/2014 | 12:18.1 | 2 |
| -1125.514 | 19387.244 | 2.508 | 14.352 | 0.128696 | 7/15/2014 | 12:18.2 | 2 |
| -1125.287 | 19387.222 | 2.508 | 14.353 | 0.158909 | 7/15/2014 | 12:18.3 | 2 |
| -1125.06 | 19387.198 | 2.51 | 14.355 | 0.128847 | 7/15/2014 | 12:18.4 | 2 |
| -1124.836 | 19387.176 | 2.51 | 14.356 | 0.143865 | 7/15/2014 | 12:18.5 | 2 |
| -1124.614 | 19387.152 | 2.505 | 14.356 | 0.102267 | 7/15/2014 | 12:18.6 | 2 |
| -1124.34 | 19387.125 | 2.507 | 14.356 | 0.0926735 | 7/15/2014 | 12:18.7 | 2 |
| -1124.12 | 19387.103 | 2.507 | 14.357 | 0.0767778 | 7/15/2014 | 12:18.8 | 2 |
| -1123.9 | 19387.079 | 2.505 | 14.359 | 0.0852935 | 7/15/2014 | 12:18.9 | 2 |
| -1123.68 | 19387.058 | 2.507 | 14.362 | 0.0930497 | 7/15/2014 | 12:19.0 | 2 |
| -1123.462 | 19387.035 | 2.507 | 14.371 | 0.107606 | 7/15/2014 | 12:19.1 | 2 |
| -1123.245 | 19387.013 | 2.507 | 14.387 | 0.090747 | 7/15/2014 | 12:19.2 | 2 |
| -1123.028 | 19386.99 | 2.506 | 14.394 | 0.107371 | 7/15/2014 | 12:19.3 | 2 |
| -1122.758 | 19386.963 | 2.506 | 14.405 | 0.123323 | 7/15/2014 | 12:19.4 | 2 |
| -1122.542 | 19386.94 | 2.504 | 14.413 | 0.119455 | 7/15/2014 | 12:19.5 | 2 |
| -1122.323 | 19386.917 | 2.504 | 14.416 | 0.124754 | 7/15/2014 | 12:19.6 | 2 |
| -1122.103 | 19386.895 | 2.506 | 14.416 | 0.125815 | 7/15/2014 | 12:19.7 | 2 |
| -1121.885 | 19386.872 | 2.504 | 14.413 | 0.110394 | 7/15/2014 | 12:19.8 | 2 |
| -1121.664 | 19386.85 | 2.504 | 14.405 | 0.109086 | 7/15/2014 | 12:19.9 | 2 |
| -1121.446 | 19386.826 | 2.5 | 14.386 | 0.130348 | 7/15/2014 | 12:20.0 | 2 |
| -1121.228 | 19386.805 | 2.496 | 14.372 | 0.107392 | 7/15/2014 | 12:20.1 | 2 |
| -1121.009 | 19386.781 | 2.496 | 14.358 | 0.118012 | 7/15/2014 | 12:20.2 | 2 |
| -1120.792 | 19386.759 | 2.496 | 14.348 | 0.110797 | 7/15/2014 | 12:20.3 | 2 |
| -1120.575 | 19386.735 | 2.496 | 14.339 | 0.105903 | 7/15/2014 | 12:20.4 | 2 |
| -1120.361 | 19386.713 | 2.496 | 14.334 | 0.11453 | 7/15/2014 | 12:20.5 | 2 |
| -1119.994 | 19386.675 | 2.495 | 14.334 | 0.107598 | 7/15/2014 | 12:20.7 | 2 |
| -1119.884 | 19386.663 | 2.493 | 14.331 | 0.100302 | 7/15/2014 | 12:20.7 | 2 |
| -1119.672 | 19386.642 | 2.498 | 14.332 | 0.0951216 | 7/15/2014 | 12:20.8 | 2 |
| -1119.46 | 19386.618 | 2.5 | 14.33 | 0.0987658 | 7/15/2014 | 12:20.9 | 2 |
| -1119.248 | 19386.598 | 2.5 | 14.327 | 0.0732224 | 7/15/2014 | 12:21.0 | 2 |
| -1119.036 | 19386.574 | 2.502 | 14.322 | 0.0690912 | 7/15/2014 | 12:21.1 | 2 |
| -1118.823 | 19386.552 | 2.504 | 14.316 | 0.0579145 | 7/15/2014 | 12:21.2 | 2 |
| -1118.613 | 19386.529 | 2.5 | 14.306 | 0.0536796 | 7/15/2014 | 12:21.3 | 2 |
| -1118.348 | 19386.501 | 2.502 | 14.292 | 0.0615459 | 7/15/2014 | 12:21.4 | 2 |
| -1118.132 | 19386.479 | 2.502 | 14.278 | 0.0839503 | 7/15/2014 | 12:21.5 | 2 |
| -1117.918 | 19386.454 | 2.5 | 14.261 | 0.0574538 | 7/15/2014 | 12:21.6 | 2 |
| -1117.701 | 19386.434 | 2.498 | 14.249 | 0.0863453 | 7/15/2014 | 12:21.7 | 2 |
| -1117.481 | 19386.408 | 2.498 | 14.239 | 0.0733209 | 7/15/2014 | 12:21.8 | 2 |
| -1117.26 | 19386.385 | 2.498 | 14.228 | 0.0902092 | 7/15/2014 | 12:21.9 | 2 |

| | | | | | | | |
|---|---|---|---|---|---|---|---|
| -1117.04 | 19386.361 | 2.498 | 14.219 | 0.105519 | 7/15/2014 | 12:22.0 | 2 |
| -1116.82 | 19386.338 | 2.502 | 14.212 | 0.0937221 | 7/15/2014 | 12:22.1 | 2 |
| -1116.599 | 19386.314 | 2.502 | 14.207 | 0.0644792 | 7/15/2014 | 12:22.2 | 2 |
| -1116.379 | 19386.29 | 2.502 | 14.204 | 0.0805133 | 7/15/2014 | 12:22.3 | 2 |
| -1116.161 | 19386.268 | 2.502 | 14.2 | 0.0829595 | 7/15/2014 | 12:22.4 | 2 |
| -1115.941 | 19386.242 | 2.506 | 14.195 | 0.0918933 | 7/15/2014 | 12:22.5 | 2 |
| -1115.724 | 19386.219 | 2.506 | 14.189 | 0.0814158 | 7/15/2014 | 12:22.6 | 2 |
| -1115.453 | 19386.189 | 2.506 | 14.184 | 0.0785906 | 7/15/2014 | 12:22.7 | 2 |
| -1115.236 | 19386.166 | 2.506 | 14.184 | 0.0880005 | 7/15/2014 | 12:22.8 | 2 |
| -1115.02 | 19386.143 | 2.506 | 14.182 | 0.0910732 | 7/15/2014 | 12:22.9 | 2 |
| -1114.802 | 19386.119 | 2.508 | 14.181 | 0.101647 | 7/15/2014 | 12:23.0 | 2 |
| -1114.586 | 19386.096 | 2.508 | 14.184 | 0.110509 | 7/15/2014 | 12:23.1 | 2 |
| -1114.367 | 19386.071 | 2.51 | 14.186 | 0.102556 | 7/15/2014 | 12:23.2 | 2 |
| -1114.151 | 19386.047 | 2.51 | 14.196 | 0.109975 | 7/15/2014 | 12:23.3 | 2 |
| -1113.881 | 19386.018 | 2.51 | 14.21 | 0.124012 | 7/15/2014 | 12:23.4 | 2 |
| -1113.661 | 19385.992 | 2.51 | 14.226 | 0.10334 | 7/15/2014 | 12:23.5 | 2 |
| -1113.441 | 19385.969 | 2.51 | 14.236 | 0.089371 | 7/15/2014 | 12:23.6 | 2 |
| -1113.22 | 19385.945 | 2.51 | 14.249 | 0.118499 | 7/15/2014 | 12:23.7 | 2 |
| -1112.998 | 19385.92 | 2.514 | 14.255 | 0.104081 | 7/15/2014 | 12:23.8 | 2 |
| -1112.774 | 19385.896 | 2.514 | 14.265 | 0.104452 | 7/15/2014 | 12:23.9 | 2 |
| -1112.552 | 19385.871 | 2.515 | 14.269 | 0.114144 | 7/15/2014 | 12:24.0 | 2 |
| -1112.329 | 19385.846 | 2.518 | 14.265 | 0.0865609 | 7/15/2014 | 12:24.1 | 2 |
| -1112.105 | 19385.82 | 2.523 | 14.26 | 0.124566 | 7/15/2014 | 12:24.2 | 2 |
| -1111.883 | 19385.797 | 2.526 | 14.254 | 0.138002 | 7/15/2014 | 12:24.3 | 2 |
| -1111.663 | 19385.773 | 2.524 | 14.247 | 0.120997 | 7/15/2014 | 12:24.4 | 2 |
| -1111.443 | 19385.746 | 2.524 | 14.241 | 0.12645 | 7/15/2014 | 12:24.5 | 2 |
| -1111.225 | 19385.723 | 2.522 | 14.238 | 0.108331 | 7/15/2014 | 12:24.6 | 2 |
| -1110.955 | 19385.693 | 2.522 | 14.229 | 0.0964775 | 7/15/2014 | 12:24.7 | 2 |
| -1110.736 | 19385.668 | 2.526 | 14.226 | 0.123014 | 7/15/2014 | 12:24.8 | 2 |
| -1110.518 | 19385.645 | 2.523 | 14.217 | 0.0927831 | 7/15/2014 | 12:24.9 | 2 |
| -1110.301 | 19385.621 | 2.523 | 14.203 | 0.102601 | 7/15/2014 | 12:25.0 | 2 |
| -1110.085 | 19385.596 | 2.52 | 14.185 | 0.104097 | 7/15/2014 | 12:25.1 | 2 |
| -1109.867 | 19385.57 | 2.525 | 14.164 | 0.102802 | 7/15/2014 | 12:25.2 | 2 |
| -1109.651 | 19385.545 | 2.528 | 14.14 | 0.119484 | 7/15/2014 | 12:25.3 | 2 |
| -1109.381 | 19385.516 | 2.53 | 14.117 | 0.125526 | 7/15/2014 | 12:25.4 | 2 |
| -1109.162 | 19385.491 | 2.532 | 14.093 | 0.0939236 | 7/15/2014 | 12:25.5 | 2 |
| -1108.944 | 19385.467 | 2.53 | 14.067 | 0.113698 | 7/15/2014 | 12:25.6 | 2 |
| -1108.725 | 19385.441 | 2.533 | 14.05 | 0.1267 | 7/15/2014 | 12:25.7 | 2 |
| -1108.505 | 19385.415 | 2.533 | 14.03 | 0.117502 | 7/15/2014 | 12:25.8 | 2 |
| -1108.284 | 19385.391 | 2.537 | 14.017 | 0.115201 | 7/15/2014 | 12:25.9 | 2 |
| -1108.064 | 19385.365 | 2.539 | 14.004 | 0.118954 | 7/15/2014 | 12:26.0 | 2 |
| -1107.843 | 19385.34 | 2.541 | 13.992 | 0.118842 | 7/15/2014 | 12:26.1 | 2 |
| -1107.625 | 19385.315 | 2.539 | 13.983 | 0.123583 | 7/15/2014 | 12:26.2 | 2 |
| -1107.407 | 19385.291 | 2.537 | 13.971 | 0.146577 | 7/15/2014 | 12:26.3 | 2 |
| -1107.19 | 19385.264 | 2.537 | 13.961 | 0.13272 | 7/15/2014 | 12:26.4 | 2 |
| -1106.975 | 19385.239 | 2.533 | 13.951 | 0.137473 | 7/15/2014 | 12:26.5 | 2 |
| -1106.762 | 19385.215 | 2.531 | 13.95 | 0.116693 | 7/15/2014 | 12:26.6 | 2 |

| | | | | | | | |
|---|---|---|---|---|---|---|---|
| -1106.497 | 19385.186 | 2.531 | 13.943 | 0.139416 | 7/15/2014 | 12:26.7 | 2 |
| -1106.283 | 19385.16 | 2.529 | 13.935 | 0.1274 | 7/15/2014 | 12:26.8 | 2 |
| -1106.072 | 19385.137 | 2.526 | 13.928 | 0.139676 | 7/15/2014 | 12:26.9 | 2 |
| -1105.858 | 19385.111 | 2.528 | 13.915 | 0.120057 | 7/15/2014 | 12:27.0 | 2 |
| -1105.646 | 19385.086 | 2.528 | 13.904 | 0.0987773 | 7/15/2014 | 12:27.1 | 2 |
| -1105.433 | 19385.062 | 2.528 | 13.889 | 0.0809475 | 7/15/2014 | 12:27.2 | 2 |
| -1105.219 | 19385.037 | 2.533 | 13.873 | 0.0898447 | 7/15/2014 | 12:27.3 | 2 |
| -1104.955 | 19385.008 | 2.533 | 13.849 | 0.0726364 | 7/15/2014 | 12:27.4 | 2 |
| -1104.737 | 19384.982 | 2.53 | 13.828 | 0.0694219 | 7/15/2014 | 12:27.5 | 2 |
| -1104.519 | 19384.957 | 2.53 | 13.809 | 0.0659601 | 7/15/2014 | 12:27.6 | 2 |
| -1104.299 | 19384.931 | 2.527 | 13.792 | 0.0695261 | 7/15/2014 | 12:27.7 | 2 |
| -1104.077 | 19384.905 | 2.527 | 13.773 | 0.059019 | 7/15/2014 | 12:27.8 | 2 |
| -1103.853 | 19384.879 | 2.527 | 13.756 | 0.0563824 | 7/15/2014 | 12:27.9 | 2 |
| -1103.627 | 19384.853 | 2.527 | 13.742 | 0.0556263 | 7/15/2014 | 12:28.0 | 2 |
| -1103.403 | 19384.828 | 2.524 | 13.729 | 0.0663937 | 7/15/2014 | 12:28.1 | 2 |
| -1103.177 | 19384.801 | 2.528 | 13.717 | 0.0451512 | 7/15/2014 | 12:28.2 | 2 |
| -1102.95 | 19384.773 | 2.531 | 13.706 | 0.0686013 | 7/15/2014 | 12:28.3 | 2 |
| -1102.724 | 19384.747 | 2.531 | 13.701 | 0.063182 | 7/15/2014 | 12:28.4 | 2 |
| -1102.498 | 19384.721 | 2.534 | 13.695 | 0.0562426 | 7/15/2014 | 12:28.5 | 2 |
| -1102.274 | 19384.694 | 2.534 | 13.692 | 0.055835 | 7/15/2014 | 12:28.6 | 2 |
| -1101.993 | 19384.661 | 2.534 | 13.694 | 0.0305312 | 7/15/2014 | 12:28.7 | 2 |
| -1101.766 | 19384.635 | 2.531 | 13.699 | 0.0127883 | 7/15/2014 | 12:28.8 | 2 |
| -1101.541 | 19384.607 | 2.532 | 13.71 | 0.0133341 | 7/15/2014 | 12:28.9 | 2 |
| -1101.314 | 19384.58 | 2.527 | 13.717 | 0.0190689 | 7/15/2014 | 12:29.0 | 2 |
| -1101.087 | 19384.553 | 2.524 | 13.734 | 0.0413013 | 7/15/2014 | 12:29.1 | 2 |
| -1100.86 | 19384.526 | 2.521 | 13.76 | 0.0487653 | 7/15/2014 | 12:29.2 | 2 |
| -1100.633 | 19384.499 | 2.518 | 13.775 | 0.0732307 | 7/15/2014 | 12:29.3 | 2 |
| -1100.35 | 19384.466 | 2.518 | 13.811 | 0.0707417 | 7/15/2014 | 12:29.4 | 2 |
| -1100.121 | 19384.438 | 2.518 | 13.842 | 0.0833961 | 7/15/2014 | 12:29.5 | 2 |
| -1099.891 | 19384.41 | 2.521 | 13.869 | 0.0850902 | 7/15/2014 | 12:29.6 | 2 |
| -1099.66 | 19384.383 | 2.518 | 13.886 | 0.0909073 | 7/15/2014 | 12:29.7 | 2 |
| -1099.428 | 19384.354 | 2.521 | 13.903 | 0.0970479 | 7/15/2014 | 12:29.8 | 2 |
| -1099.195 | 19384.326 | 2.521 | 13.905 | 0.0872869 | 7/15/2014 | 12:29.9 | 2 |
| -1098.963 | 19384.298 | 2.519 | 13.893 | 0.103248 | 7/15/2014 | 12:30.0 | 2 |
| -1098.729 | 19384.271 | 2.519 | 13.89 | 0.109316 | 7/15/2014 | 12:30.1 | 2 |
| -1098.498 | 19384.242 | 2.514 | 13.865 | 0.0895422 | 7/15/2014 | 12:30.2 | 2 |
| -1098.267 | 19384.215 | 2.511 | 13.839 | 0.119722 | 7/15/2014 | 12:30.3 | 2 |
| -1098.037 | 19384.188 | 2.506 | 13.815 | 0.129473 | 7/15/2014 | 12:30.4 | 2 |
| -1097.808 | 19384.159 | 2.504 | 13.797 | 0.122947 | 7/15/2014 | 12:30.5 | 2 |
| -1097.581 | 19384.132 | 2.499 | 13.783 | 0.134858 | 7/15/2014 | 12:30.6 | 2 |
| -1097.3 | 19384.098 | 2.497 | 13.77 | 0.116945 | 7/15/2014 | 12:30.7 | 2 |
| -1097.073 | 19384.071 | 2.497 | 13.757 | 0.107137 | 7/15/2014 | 12:30.8 | 2 |
| -1096.846 | 19384.044 | 2.495 | 13.746 | 0.151018 | 7/15/2014 | 12:30.9 | 2 |
| -1096.619 | 19384.017 | 2.492 | 13.734 | 0.102151 | 7/15/2014 | 12:31.0 | 2 |
| -1096.391 | 19383.988 | 2.487 | 13.721 | 0.10922 | 7/15/2014 | 12:31.1 | 2 |
| -1096.162 | 19383.961 | 2.485 | 13.706 | 0.106287 | 7/15/2014 | 12:31.2 | 2 |
| -1095.935 | 19383.934 | 2.483 | 13.688 | 0.10001 | 7/15/2014 | 12:31.3 | 2 |

| | | | | | | | |
|---|---|---|---|---|---|---|---|
| -1095.65 | 19383.898 | 2.478 | 13.669 | 0.107967 | 7/15/2014 | 12:31.4 | 2 |
| -1095.42 | 19383.872 | 2.472 | 13.651 | 0.136159 | 7/15/2014 | 12:31.5 | 2 |
| -1095.186 | 19383.843 | 2.47 | 13.64 | 0.0954992 | 7/15/2014 | 12:31.6 | 2 |
| -1094.952 | 19383.815 | 2.465 | 13.626 | 0.0993449 | 7/15/2014 | 12:31.7 | 2 |
| -1094.716 | 19383.785 | 2.465 | 13.611 | 0.0977638 | 7/15/2014 | 12:31.8 | 2 |
| -1094.479 | 19383.757 | 2.465 | 13.596 | 0.103505 | 7/15/2014 | 12:31.9 | 2 |
| -1094.241 | 19383.728 | 2.462 | 13.582 | 0.0966967 | 7/15/2014 | 12:32.0 | 2 |
| -1094.003 | 19383.698 | 2.458 | 13.57 | 0.111255 | 7/15/2014 | 12:32.1 | 2 |
| -1093.765 | 19383.67 | 2.454 | 13.568 | 0.0835168 | 7/15/2014 | 12:32.2 | 2 |
| -1093.526 | 19383.64 | 2.452 | 13.561 | 0.0921314 | 7/15/2014 | 12:32.3 | 2 |
| -1093.29 | 19383.611 | 2.445 | 13.552 | 0.116621 | 7/15/2014 | 12:32.4 | 2 |
| -1093.052 | 19383.581 | 2.445 | 13.554 | 0.107534 | 7/15/2014 | 12:32.5 | 2 |
| -1092.816 | 19383.553 | 2.437 | 13.565 | 0.112478 | 7/15/2014 | 12:32.6 | 2 |
| -1092.522 | 19383.516 | 2.432 | 13.583 | 0.110518 | 7/15/2014 | 12:32.7 | 2 |
| -1092.404 | 19383.501 | 2.428 | 13.591 | 0.0985363 | 7/15/2014 | 12:32.8 | 2 |
| -1092.166 | 19383.472 | 2.425 | 13.616 | 0.110292 | 7/15/2014 | 12:32.9 | 2 |
| -1091.93 | 19383.442 | 2.419 | 13.638 | 0.0941131 | 7/15/2014 | 12:33.0 | 2 |
| -1091.69 | 19383.412 | 2.417 | 13.666 | 0.100083 | 7/15/2014 | 12:33.1 | 2 |
| -1091.452 | 19383.383 | 2.41 | 13.698 | 0.0907597 | 7/15/2014 | 12:33.2 | 2 |
| -1091.212 | 19383.354 | 2.404 | 13.716 | 0.0899252 | 7/15/2014 | 12:33.3 | 2 |
| -1090.786 | 19383.299 | 2.396 | 13.726 | 0.11116 | 7/15/2014 | 12:33.4 | 2 |
| -1090.539 | 19383.269 | 2.394 | 13.728 | 0.102209 | 7/15/2014 | 12:33.5 | 2 |
| -1090.421 | 19383.254 | 2.39 | 13.733 | 0.122506 | 7/15/2014 | 12:33.6 | 2 |
| -1090.17 | 19383.222 | 2.39 | 13.723 | 0.0969078 | 7/15/2014 | 12:33.7 | 2 |
| -1089.92 | 19383.19 | 2.38 | 13.715 | 0.121801 | 7/15/2014 | 12:33.8 | 2 |
| -1089.666 | 19383.159 | 2.38 | 13.699 | 0.104263 | 7/15/2014 | 12:33.9 | 2 |
| -1089.412 | 19383.128 | 2.371 | 13.689 | 0.113439 | 7/15/2014 | 12:34.0 | 2 |
| -1089.155 | 19383.094 | 2.371 | 13.684 | 0.108142 | 7/15/2014 | 12:34.1 | 2 |
| -1088.9 | 19383.062 | 2.365 | 13.677 | 0.106452 | 7/15/2014 | 12:34.2 | 2 |
| -1088.642 | 19383.028 | 2.362 | 13.676 | 0.100191 | 7/15/2014 | 12:34.3 | 2 |
| -1088.389 | 19382.996 | 2.355 | 13.681 | 0.0924213 | 7/15/2014 | 12:34.4 | 2 |
| -1088.133 | 19382.964 | 2.358 | 13.689 | 0.0968862 | 7/15/2014 | 12:34.5 | 2 |
| -1087.881 | 19382.934 | 2.352 | 13.703 | 0.0893514 | 7/15/2014 | 12:34.6 | 2 |
| -1087.628 | 19382.899 | 2.354 | 13.72 | 0.0808753 | 7/15/2014 | 12:34.7 | 2 |
| -1087.318 | 19382.858 | 2.348 | 13.737 | 0.080207 | 7/15/2014 | 12:34.8 | 2 |
| -1087.066 | 19382.826 | 2.348 | 13.75 | 0.0887854 | 7/15/2014 | 12:34.9 | 2 |
| -1086.817 | 19382.795 | 2.343 | 13.768 | 0.0917442 | 7/15/2014 | 12:35.0 | 2 |
| -1086.566 | 19382.763 | 2.34 | 13.774 | 0.101218 | 7/15/2014 | 12:35.1 | 2 |
| -1086.318 | 19382.732 | 2.328 | 13.78 | 0.114041 | 7/15/2014 | 12:35.2 | 2 |
| -1086.068 | 19382.698 | 2.322 | 13.782 | 0.117324 | 7/15/2014 | 12:35.3 | 2 |
| -1085.757 | 19382.658 | 2.314 | 13.782 | 0.114453 | 7/15/2014 | 12:35.4 | 2 |
| -1085.505 | 19382.626 | 2.311 | 13.776 | 0.11867 | 7/15/2014 | 12:35.5 | 2 |
| -1085.255 | 19382.594 | 2.302 | 13.776 | 0.121633 | 7/15/2014 | 12:35.6 | 2 |
| -1085 | 19382.563 | 2.298 | 13.765 | 0.0921829 | 7/15/2014 | 12:35.7 | 2 |
| -1084.748 | 19382.53 | 2.294 | 13.761 | 0.107512 | 7/15/2014 | 12:35.8 | 2 |
| -1084.494 | 19382.496 | 2.302 | 13.762 | 0.0922844 | 7/15/2014 | 12:35.9 | 2 |
| -1084.241 | 19382.465 | 2.298 | 13.757 | 0.105709 | 7/15/2014 | 12:36.0 | 2 |

| | | | | | | | |
|---|---|---|---|---|---|---|---|
| -1083.985 | 19382.432 | 2.302 | 13.751 | 0.094431 | 7/15/2014 | 12:36.1 | 2 |
| -1083.736 | 19382.4 | 2.292 | 13.749 | 0.0923182 | 7/15/2014 | 12:36.2 | 2 |
| -1083.483 | 19382.368 | 2.292 | 13.746 | 0.128171 | 7/15/2014 | 12:36.3 | 2 |
| -1083.235 | 19382.337 | 2.283 | 13.755 | 0.115097 | 7/15/2014 | 12:36.4 | 2 |
| -1082.867 | 19382.289 | 2.274 | 13.767 | 0.130668 | 7/15/2014 | 12:36.5 | 2 |
| -1082.621 | 19382.258 | 2.266 | 13.769 | 0.121642 | 7/15/2014 | 12:36.6 | 2 |
| -1082.316 | 19382.218 | 2.262 | 13.778 | 0.139658 | 7/15/2014 | 12:36.7 | 2 |
| -1082.193 | 19382.203 | 2.253 | 13.783 | 0.138626 | 7/15/2014 | 12:36.8 | 2 |
| -1081.948 | 19382.172 | 2.253 | 13.79 | 0.11577 | 7/15/2014 | 12:36.9 | 2 |
| -1081.704 | 19382.141 | 2.244 | 13.799 | 0.111247 | 7/15/2014 | 12:37.0 | 2 |
| -1081.457 | 19382.108 | 2.247 | 13.798 | 0.112418 | 7/15/2014 | 12:37.1 | 2 |
| -1081.213 | 19382.078 | 2.238 | 13.801 | 0.10958 | 7/15/2014 | 12:37.2 | 2 |
| -1080.965 | 19382.047 | 2.238 | 13.8 | 0.10407 | 7/15/2014 | 12:37.3 | 2 |
| -1080.658 | 19382.006 | 2.236 | 13.801 | 0.10065 | 7/15/2014 | 12:37.4 | 2 |
| -1080.407 | 19381.976 | 2.232 | 13.801 | 0.100574 | 7/15/2014 | 12:37.5 | 2 |
| -1080.156 | 19381.942 | 2.226 | 13.799 | 0.0961709 | 7/15/2014 | 12:37.6 | 2 |
| -1079.902 | 19381.91 | 2.226 | 13.801 | 0.103744 | 7/15/2014 | 12:37.7 | 2 |
| -1079.649 | 19381.879 | 2.214 | 13.803 | 0.104155 | 7/15/2014 | 12:37.8 | 2 |
| -1079.393 | 19381.846 | 2.214 | 13.799 | 0.114094 | 7/15/2014 | 12:37.9 | 2 |
| -1079.139 | 19381.813 | 2.2 | 13.801 | 0.103803 | 7/15/2014 | 12:38.0 | 2 |
| -1078.882 | 19381.78 | 2.197 | 13.801 | 0.113063 | 7/15/2014 | 12:38.1 | 2 |
| -1078.629 | 19381.748 | 2.191 | 13.802 | 0.116217 | 7/15/2014 | 12:38.2 | 2 |
| -1078.376 | 19381.715 | 2.187 | 13.806 | 0.113726 | 7/15/2014 | 12:38.3 | 2 |
| -1078.127 | 19381.685 | 2.183 | 13.805 | 0.111244 | 7/15/2014 | 12:38.4 | 2 |
| -1077.875 | 19381.651 | 2.187 | 13.807 | 0.107854 | 7/15/2014 | 12:38.5 | 2 |
| -1077.512 | 19381.605 | 2.18 | 13.823 | 0.104114 | 7/15/2014 | 12:38.6 | 2 |
| -1077.208 | 19381.566 | 2.18 | 13.834 | 0.105423 | 7/15/2014 | 12:38.7 | 2 |
| -1077.084 | 19381.551 | 2.175 | 13.84 | 0.105674 | 7/15/2014 | 12:38.8 | 2 |
| -1076.842 | 19381.52 | 2.171 | 13.875 | 0.100707 | 7/15/2014 | 12:38.9 | 2 |
| -1076.601 | 19381.49 | 2.166 | 13.904 | 0.0994686 | 7/15/2014 | 12:39.0 | 2 |
| -1076.358 | 19381.459 | 2.164 | 13.931 | 0.0979305 | 7/15/2014 | 12:39.1 | 2 |
| -1076.119 | 19381.43 | 2.152 | 13.955 | 0.0913146 | 7/15/2014 | 12:39.2 | 2 |
| -1075.879 | 19381.398 | 2.152 | 13.969 | 0.116145 | 7/15/2014 | 12:39.3 | 2 |
| -1075.457 | 19381.345 | 2.148 | 13.978 | 0.0985684 | 7/15/2014 | 12:39.4 | 2 |
| -1075.216 | 19381.314 | 2.146 | 13.982 | 0.103458 | 7/15/2014 | 12:39.5 | 2 |
| -1074.975 | 19381.284 | 2.146 | 13.963 | 0.101063 | 7/15/2014 | 12:39.6 | 2 |
| -1074.732 | 19381.253 | 2.143 | 13.953 | 0.103303 | 7/15/2014 | 12:39.7 | 2 |
| -1074.489 | 19381.224 | 2.14 | 13.94 | 0.107129 | 7/15/2014 | 12:39.8 | 2 |
| -1074.247 | 19381.193 | 2.142 | 13.927 | 0.0959418 | 7/15/2014 | 12:39.9 | 2 |
| -1074.004 | 19381.162 | 2.14 | 13.918 | 0.114725 | 7/15/2014 | 12:40.0 | 2 |
| -1073.763 | 19381.132 | 2.134 | 13.912 | 0.105451 | 7/15/2014 | 12:40.1 | 2 |
| -1073.521 | 19381.102 | 2.132 | 13.906 | 0.106502 | 7/15/2014 | 12:40.2 | 2 |
| -1073.281 | 19381.071 | 2.13 | 13.901 | 0.10801 | 7/15/2014 | 12:40.3 | 2 |
| -1073.045 | 19381.041 | 2.126 | 13.896 | 0.107886 | 7/15/2014 | 12:40.4 | 2 |
| -1072.808 | 19381.013 | 2.122 | 13.89 | 0.107464 | 7/15/2014 | 12:40.5 | 2 |
| -1072.576 | 19380.983 | 2.117 | 13.889 | 0.106977 | 7/15/2014 | 12:40.6 | 2 |
| -1072.289 | 19380.947 | 2.11 | 13.886 | 0.111017 | 7/15/2014 | 12:40.7 | 2 |

| | | | | | | | |
|---|---|---|---|---|---|---|---|
| -1072.058 | 19380.919 | 2.105 | 13.904 | 0.107553 | 7/15/2014 | 12:40.8 | 2 |
| -1071.829 | 19380.891 | 2.103 | 13.917 | 0.104343 | 7/15/2014 | 12:40.9 | 2 |
| -1071.598 | 19380.862 | 2.1 | 13.936 | 0.112666 | 7/15/2014 | 12:41.0 | 2 |
| -1071.37 | 19380.833 | 2.098 | 13.957 | 0.100614 | 7/15/2014 | 12:41.1 | 2 |
| -1071.143 | 19380.806 | 2.096 | 13.98 | 0.0903369 | 7/15/2014 | 12:41.2 | 2 |
| -1070.919 | 19380.777 | 2.095 | 14.007 | 0.0865731 | 7/15/2014 | 12:41.3 | 2 |
| -1070.64 | 19380.743 | 2.095 | 14.035 | 0.0846078 | 7/15/2014 | 12:41.4 | 2 |
| -1070.417 | 19380.716 | 2.095 | 14.064 | 0.0787418 | 7/15/2014 | 12:41.5 | 2 |
| -1070.196 | 19380.689 | 2.092 | 14.069 | 0.0771152 | 7/15/2014 | 12:41.6 | 2 |
| -1069.915 | 19380.655 | 2.092 | 14.075 | 0.0776299 | 7/15/2014 | 12:41.7 | 2 |
| -1069.69 | 19380.627 | 2.092 | 14.073 | 0.0771763 | 7/15/2014 | 12:41.8 | 2 |
| -1069.467 | 19380.601 | 2.088 | 14.07 | 0.0777887 | 7/15/2014 | 12:41.9 | 2 |
| -1069.243 | 19380.573 | 2.087 | 14.061 | 0.0784255 | 7/15/2014 | 12:42.0 | 2 |
| -1069.016 | 19380.546 | 2.087 | 14.043 | 0.0769762 | 7/15/2014 | 12:42.1 | 2 |
| -1068.794 | 19380.519 | 2.084 | 14.02 | 0.0770761 | 7/15/2014 | 12:42.2 | 2 |
| -1068.57 | 19380.492 | 2.086 | 13.996 | 0.0829604 | 7/15/2014 | 12:42.3 | 2 |
| -1068.292 | 19380.458 | 2.086 | 13.978 | 0.0768163 | 7/15/2014 | 12:42.4 | 2 |
| -1068.068 | 19380.432 | 2.083 | 13.959 | 0.0764741 | 7/15/2014 | 12:42.5 | 2 |
| -1067.846 | 19380.406 | 2.078 | 13.941 | 0.0855228 | 7/15/2014 | 12:42.6 | 2 |
| -1067.566 | 19380.372 | 2.078 | 13.924 | 0.078357 | 7/15/2014 | 12:42.7 | 2 |
| -1067.34 | 19380.345 | 2.078 | 13.911 | 0.0711652 | 7/15/2014 | 12:42.8 | 2 |
| -1067.114 | 19380.317 | 2.074 | 13.901 | 0.067691 | 7/15/2014 | 12:42.9 | 2 |
| -1066.886 | 19380.29 | 2.072 | 13.897 | 0.0664576 | 7/15/2014 | 12:43.0 | 2 |
| -1066.658 | 19380.263 | 2.067 | 13.897 | 0.0690164 | 7/15/2014 | 12:43.1 | 2 |
| -1066.43 | 19380.236 | 2.067 | 13.891 | 0.0709347 | 7/15/2014 | 12:43.2 | 2 |
| -1066.203 | 19380.211 | 2.065 | 13.895 | 0.0673839 | 7/15/2014 | 12:43.3 | 2 |
| -1065.92 | 19380.177 | 2.065 | 13.895 | 0.0716392 | 7/15/2014 | 12:43.4 | 2 |
| -1065.694 | 19380.149 | 2.063 | 13.893 | 0.0916737 | 7/15/2014 | 12:43.5 | 2 |
| -1065.468 | 19380.122 | 2.059 | 13.89 | 0.0776826 | 7/15/2014 | 12:43.6 | 2 |
| -1065.184 | 19380.089 | 2.055 | 13.888 | 0.0680738 | 7/15/2014 | 12:43.7 | 2 |
| -1064.958 | 19380.063 | 2.055 | 13.884 | 0.0629215 | 7/15/2014 | 12:43.8 | 2 |
| -1064.729 | 19380.036 | 2.055 | 13.877 | 0.0536404 | 7/15/2014 | 12:43.9 | 2 |
| -1064.502 | 19380.01 | 2.051 | 13.868 | 0.0513901 | 7/15/2014 | 12:44.0 | 2 |
| -1064.274 | 19379.984 | 2.048 | 13.857 | 0.0525395 | 7/15/2014 | 12:44.1 | 2 |
| -1064.049 | 19379.957 | 2.043 | 13.85 | 0.0524404 | 7/15/2014 | 12:44.2 | 2 |
| -1063.825 | 19379.932 | 2.039 | 13.848 | 0.0513971 | 7/15/2014 | 12:44.3 | 2 |
| -1063.546 | 19379.898 | 2.036 | 13.851 | 0.0488896 | 7/15/2014 | 12:44.4 | 2 |
| -1063.324 | 19379.875 | 2.037 | 13.851 | 0.0479505 | 7/15/2014 | 12:44.5 | 2 |
| -1063.1 | 19379.848 | 2.035 | 13.854 | 0.0578401 | 7/15/2014 | 12:44.6 | 2 |
| -1062.822 | 19379.816 | 2.03 | 13.857 | 0.0516697 | 7/15/2014 | 12:44.7 | 2 |
| -1062.596 | 19379.791 | 2.03 | 13.865 | 0.0552985 | 7/15/2014 | 12:44.8 | 2 |
| -1062.37 | 19379.766 | 2.026 | 13.875 | 0.0595912 | 7/15/2014 | 12:44.9 | 2 |
| -1062.142 | 19379.74 | 2.026 | 13.911 | 0.0573455 | 7/15/2014 | 12:45.0 | 2 |
| -1061.915 | 19379.714 | 2.021 | 13.939 | 0.0542938 | 7/15/2014 | 12:45.1 | 2 |
| -1061.688 | 19379.689 | 2.019 | 13.969 | 0.0537775 | 7/15/2014 | 12:45.2 | 2 |
| -1061.46 | 19379.662 | 2.014 | 14.001 | 0.0465246 | 7/15/2014 | 12:45.3 | 2 |
| -1061.176 | 19379.633 | 2.009 | 14.033 | 0.0436621 | 7/15/2014 | 12:45.4 | 2 |

| | | | | | | | |
|---|---|---|---|---|---|---|---|
| -1060.947 | 19379.605 | 2.003 | 14.064 | 0.0430465 | 7/15/2014 | 12:45.5 | 2 |
| -1060.718 | 19379.582 | 1.998 | 14.089 | 0.0416878 | 7/15/2014 | 12:45.6 | 2 |
| -1060.427 | 19379.549 | 1.995 | 14.11 | 0.0406432 | 7/15/2014 | 12:45.7 | 2 |
| -1060.312 | 19379.536 | 1.991 | 14.108 | 0.0440483 | 7/15/2014 | 12:45.8 | 2 |
| -1060.078 | 19379.512 | 1.991 | 14.111 | 0.0429891 | 7/15/2014 | 12:45.9 | 2 |
| -1059.845 | 19379.484 | 1.985 | 14.106 | 0.0441853 | 7/15/2014 | 12:46.0 | 2 |
| -1059.607 | 19379.461 | 1.985 | 14.099 | 0.0563605 | 7/15/2014 | 12:46.1 | 2 |
| -1059.374 | 19379.435 | 1.975 | 14.089 | 0.0483426 | 7/15/2014 | 12:46.2 | 2 |
| -1059.139 | 19379.41 | 1.978 | 14.08 | 0.0511315 | 7/15/2014 | 12:46.3 | 2 |
| -1058.729 | 19379.365 | 1.972 | 14.076 | 0.0444875 | 7/15/2014 | 12:46.4 | 2 |
| -1058.498 | 19379.343 | 1.971 | 14.072 | 0.0501093 | 7/15/2014 | 12:46.5 | 2 |
| -1058.266 | 19379.316 | 1.968 | 14.066 | 0.0596469 | 7/15/2014 | 12:46.6 | 2 |
| -1057.978 | 19379.288 | 1.965 | 14.06 | 0.0415857 | 7/15/2014 | 12:46.7 | 2 |
| -1057.86 | 19379.275 | 1.962 | 14.057 | 0.039644 | 7/15/2014 | 12:46.8 | 2 |
| -1057.626 | 19379.251 | 1.965 | 14.049 | 0.0417903 | 7/15/2014 | 12:46.9 | 2 |
| -1057.282 | 19379.216 | 1.962 | 14.03 | 0.0386326 | 7/15/2014 | 12:47.0 | 2 |
| -1057.052 | 19379.191 | 1.965 | 14.013 | 0.040801 | 7/15/2014 | 12:47.1 | 2 |
| -1056.824 | 19379.169 | 1.964 | 14 | 0.0414851 | 7/15/2014 | 12:47.2 | 2 |
| -1056.595 | 19379.145 | 1.964 | 13.982 | 0.0558997 | 7/15/2014 | 12:47.3 | 2 |
| -1056.314 | 19379.117 | 1.964 | 13.964 | 0.0529929 | 7/15/2014 | 12:47.4 | 2 |
| -1056.087 | 19379.095 | 1.967 | 13.944 | 0.0673403 | 7/15/2014 | 12:47.5 | 2 |
| -1055.863 | 19379.073 | 1.967 | 13.927 | 0.0619558 | 7/15/2014 | 12:47.6 | 2 |
| -1055.58 | 19379.043 | 1.964 | 13.912 | 0.0572808 | 7/15/2014 | 12:47.7 | 2 |
| -1055.355 | 19379.021 | 1.964 | 13.9 | 0.0470349 | 7/15/2014 | 12:47.8 | 2 |
| -1055.128 | 19379 | 1.961 | 13.888 | 0.0605298 | 7/15/2014 | 12:47.9 | 2 |
| -1054.9 | 19378.977 | 1.959 | 13.877 | 0.0484488 | 7/15/2014 | 12:48.0 | 2 |
| -1054.673 | 19378.955 | 1.956 | 13.869 | 0.0512668 | 7/15/2014 | 12:48.1 | 2 |
| -1054.446 | 19378.934 | 1.952 | 13.866 | 0.0351731 | 7/15/2014 | 12:48.2 | 2 |
| -1054.22 | 19378.911 | 1.945 | 13.861 | 0.0463929 | 7/15/2014 | 12:48.3 | 2 |
| -1053.938 | 19378.883 | 1.943 | 13.858 | 0.0391849 | 7/15/2014 | 12:48.4 | 2 |
| -1053.711 | 19378.861 | 1.943 | 13.851 | 0.0528518 | 7/15/2014 | 12:48.5 | 2 |
| -1053.485 | 19378.842 | 1.938 | 13.841 | 0.03897 | 7/15/2014 | 12:48.6 | 2 |
| -1053.202 | 19378.813 | 1.933 | 13.834 | 0.0472091 | 7/15/2014 | 12:48.7 | 2 |
| -1052.973 | 19378.792 | 1.93 | 13.825 | 0.0492864 | 7/15/2014 | 12:48.8 | 2 |
| -1052.744 | 19378.771 | 1.928 | 13.815 | 0.053601 | 7/15/2014 | 12:48.9 | 2 |
| -1052.514 | 19378.748 | 1.931 | 13.805 | 0.0499778 | 7/15/2014 | 12:49.0 | 2 |
| -1052.284 | 19378.728 | 1.937 | 13.797 | 0.0564336 | 7/15/2014 | 12:49.1 | 2 |
| -1052.054 | 19378.706 | 1.937 | 13.791 | 0.0493757 | 7/15/2014 | 12:49.2 | 2 |
| -1051.825 | 19378.686 | 1.934 | 13.791 | 0.0440571 | 7/15/2014 | 12:49.3 | 2 |
| -1051.539 | 19378.658 | 1.937 | 13.792 | 0.0504712 | 7/15/2014 | 12:49.4 | 2 |
| -1051.31 | 19378.637 | 1.939 | 13.803 | 0.0428269 | 7/15/2014 | 12:49.5 | 2 |
| -1051.081 | 19378.617 | 1.938 | 13.809 | 0.0428312 | 7/15/2014 | 12:49.6 | 2 |
| -1050.796 | 19378.59 | 1.933 | 13.825 | 0.0434765 | 7/15/2014 | 12:49.7 | 2 |
| -1050.68 | 19378.58 | 1.928 | 13.83 | 0.0459174 | 7/15/2014 | 12:49.8 | 2 |
| -1050.447 | 19378.559 | 1.925 | 13.843 | 0.047327 | 7/15/2014 | 12:49.9 | 2 |
| -1050.215 | 19378.537 | 1.915 | 13.867 | 0.0548422 | 7/15/2014 | 12:50.0 | 2 |
| -1049.982 | 19378.517 | 1.912 | 13.891 | 0.0553864 | 7/15/2014 | 12:50.1 | 2 |

| | | | | | | | |
|---|---|---|---|---|---|---|---|
| -1049.752 | 19378.494 | 1.904 | 13.927 | 0.0594295 | 7/15/2014 | 12:50.2 | 2 |
| -1049.519 | 19378.475 | 1.901 | 13.948 | 0.0626677 | 7/15/2014 | 12:50.3 | 2 |
| -1049.117 | 19378.438 | 1.896 | 13.982 | 0.0760732 | 7/15/2014 | 12:50.4 | 2 |
| -1048.885 | 19378.417 | 1.9 | 14 | 0.0939464 | 7/15/2014 | 12:50.5 | 2 |
| -1048.655 | 19378.395 | 1.897 | 14.018 | 0.0813413 | 7/15/2014 | 12:50.6 | 2 |
| -1048.369 | 19378.37 | 1.897 | 14.028 | 0.0837737 | 7/15/2014 | 12:50.7 | 2 |
| -1048.253 | 19378.358 | 1.896 | 14.035 | 0.0786619 | 7/15/2014 | 12:50.8 | 2 |
| -1048.019 | 19378.338 | 1.9 | 14.032 | 0.0834382 | 7/15/2014 | 12:50.9 | 2 |
| -1047.789 | 19378.317 | 1.896 | 14.036 | 0.0608648 | 7/15/2014 | 12:51.0 | 2 |
| -1047.439 | 19378.285 | 1.896 | 14.041 | 0.0596818 | 7/15/2014 | 12:51.1 | 2 |
| -1047.324 | 19378.275 | 1.893 | 14.042 | 0.0515349 | 7/15/2014 | 12:51.2 | 2 |
| -1047.092 | 19378.254 | 1.891 | 14.042 | 0.0536139 | 7/15/2014 | 12:51.3 | 2 |
| -1046.691 | 19378.218 | 1.887 | 14.039 | 0.0613919 | 7/15/2014 | 12:51.4 | 2 |
| -1046.46 | 19378.197 | 1.887 | 14.034 | 0.0600832 | 7/15/2014 | 12:51.5 | 2 |
| -1046.232 | 19378.178 | 1.883 | 14.027 | 0.0727017 | 7/15/2014 | 12:51.6 | 2 |
| -1045.945 | 19378.151 | 1.88 | 14.02 | 0.0610343 | 7/15/2014 | 12:51.7 | 2 |
| -1045.829 | 19378.141 | 1.878 | 14.015 | 0.05845 | 7/15/2014 | 12:51.8 | 2 |
| -1045.484 | 19378.111 | 1.872 | 13.999 | 0.0503849 | 7/15/2014 | 12:51.9 | 2 |
| -1045.253 | 19378.092 | 1.866 | 13.99 | 0.050203 | 7/15/2014 | 12:52.0 | 2 |
| -1045.023 | 19378.07 | 1.861 | 13.981 | 0.0483007 | 7/15/2014 | 12:52.1 | 2 |
| -1044.792 | 19378.05 | 1.858 | 13.971 | 0.0500935 | 7/15/2014 | 12:52.2 | 2 |
| -1044.565 | 19378.029 | 1.852 | 13.967 | 0.0615915 | 7/15/2014 | 12:52.3 | 2 |
| -1044.279 | 19378.004 | 1.849 | 13.964 | 0.0577549 | 7/15/2014 | 12:52.4 | 2 |
| -1044.053 | 19377.984 | 1.847 | 13.958 | 0.0692416 | 7/15/2014 | 12:52.5 | 2 |
| -1043.825 | 19377.965 | 1.847 | 13.956 | 0.0671322 | 7/15/2014 | 12:52.6 | 2 |
| -1043.542 | 19377.939 | 1.843 | 13.954 | 0.0843932 | 7/15/2014 | 12:52.7 | 2 |
| -1043.314 | 19377.92 | 1.843 | 13.951 | 0.0790395 | 7/15/2014 | 12:52.8 | 2 |
| -1043.085 | 19377.898 | 1.841 | 13.949 | 0.0886561 | 7/15/2014 | 12:52.9 | 2 |
| -1042.856 | 19377.877 | 1.839 | 13.949 | 0.09776 | 7/15/2014 | 12:53.0 | 2 |
| -1042.626 | 19377.857 | 1.839 | 13.949 | 0.107986 | 7/15/2014 | 12:53.1 | 2 |
| -1042.397 | 19377.837 | 1.839 | 13.945 | 0.101378 | 7/15/2014 | 12:53.2 | 2 |
| -1042.169 | 19377.816 | 1.836 | 13.941 | 0.104361 | 7/15/2014 | 12:53.3 | 2 |
| -1041.886 | 19377.791 | 1.836 | 13.936 | 0.0990711 | 7/15/2014 | 12:53.4 | 2 |
| -1041.659 | 19377.771 | 1.836 | 13.929 | 0.100382 | 7/15/2014 | 12:53.5 | 2 |
| -1041.433 | 19377.75 | 1.832 | 13.923 | 0.0917989 | 7/15/2014 | 12:53.6 | 2 |
| -1041.15 | 19377.724 | 1.829 | 13.916 | 0.0828195 | 7/15/2014 | 12:53.7 | 2 |
| -1040.921 | 19377.703 | 1.827 | 13.91 | 0.0786833 | 7/15/2014 | 12:53.8 | 2 |
| -1040.692 | 19377.683 | 1.825 | 13.898 | 0.0807577 | 7/15/2014 | 12:53.9 | 2 |
| -1040.464 | 19377.662 | 1.821 | 13.898 | 0.072527 | 7/15/2014 | 12:54.0 | 2 |
| -1040.234 | 19377.642 | 1.817 | 13.894 | 0.0797748 | 7/15/2014 | 12:54.1 | 2 |
| -1040.007 | 19377.62 | 1.815 | 13.893 | 0.0750865 | 7/15/2014 | 12:54.2 | 2 |
| -1039.78 | 19377.601 | 1.815 | 13.893 | 0.0784164 | 7/15/2014 | 12:54.3 | 2 |
| -1039.499 | 19377.574 | 1.815 | 13.892 | 0.0824823 | 7/15/2014 | 12:54.4 | 2 |
| -1039.273 | 19377.553 | 1.818 | 13.89 | 0.090009 | 7/15/2014 | 12:54.5 | 2 |
| -1039.049 | 19377.533 | 1.818 | 13.887 | 0.0975849 | 7/15/2014 | 12:54.6 | 2 |
| -1038.77 | 19377.508 | 1.815 | 13.886 | 0.109141 | 7/15/2014 | 12:54.7 | 2 |
| -1038.542 | 19377.487 | 1.819 | 13.88 | 0.112958 | 7/15/2014 | 12:54.8 | 2 |

| | | | | | | | |
|---|---|---|---|---|---|---|---|
| -1038.317 | 19377.467 | 1.819 | 13.877 | 0.119536 | 7/15/2014 | 12:54.9 | 2 |
| -1038.091 | 19377.445 | 1.817 | 13.87 | 0.12049 | 7/15/2014 | 12:55.0 | 2 |
| -1037.867 | 19377.425 | 1.814 | 13.866 | 0.122709 | 7/15/2014 | 12:55.1 | 2 |
| -1037.641 | 19377.404 | 1.816 | 13.86 | 0.120292 | 7/15/2014 | 12:55.2 | 2 |
| -1037.418 | 19377.384 | 1.816 | 13.855 | 0.122947 | 7/15/2014 | 12:55.3 | 2 |
| -1037.141 | 19377.357 | 1.818 | 13.854 | 0.118864 | 7/15/2014 | 12:55.4 | 2 |
| -1036.918 | 19377.338 | 1.822 | 13.85 | 0.126393 | 7/15/2014 | 12:55.5 | 2 |
| -1036.697 | 19377.317 | 1.819 | 13.845 | 0.126463 | 7/15/2014 | 12:55.6 | 2 |
| -1036.421 | 19377.291 | 1.823 | 13.843 | 0.128479 | 7/15/2014 | 12:55.7 | 2 |
| -1036.2 | 19377.271 | 1.825 | 13.84 | 0.129451 | 7/15/2014 | 12:55.8 | 2 |
| -1035.977 | 19377.251 | 1.828 | 13.833 | 0.137534 | 7/15/2014 | 12:55.9 | 2 |
| -1035.754 | 19377.229 | 1.828 | 13.833 | 0.14468 | 7/15/2014 | 12:56.0 | 2 |
| -1035.531 | 19377.208 | 1.83 | 13.83 | 0.145783 | 7/15/2014 | 12:56.1 | 2 |
| -1035.31 | 19377.188 | 1.83 | 13.824 | 0.148519 | 7/15/2014 | 12:56.2 | 2 |
| -1035.09 | 19377.167 | 1.832 | 13.821 | 0.152081 | 7/15/2014 | 12:56.3 | 2 |
| -1034.816 | 19377.142 | 1.835 | 13.817 | 0.150545 | 7/15/2014 | 12:56.4 | 2 |
| -1034.597 | 19377.12 | 1.838 | 13.812 | 0.148411 | 7/15/2014 | 12:56.5 | 2 |
| -1034.377 | 19377.101 | 1.843 | 13.809 | 0.15071 | 7/15/2014 | 12:56.6 | 2 |
| -1034.104 | 19377.074 | 1.845 | 13.811 | 0.149402 | 7/15/2014 | 12:56.7 | 2 |
| -1033.883 | 19377.054 | 1.85 | 13.809 | 0.14724 | 7/15/2014 | 12:56.8 | 2 |
| -1033.662 | 19377.031 | 1.85 | 13.818 | 0.142467 | 7/15/2014 | 12:56.9 | 2 |
| -1033.439 | 19377.012 | 1.856 | 13.82 | 0.140532 | 7/15/2014 | 12:57.0 | 2 |
| -1033.218 | 19376.99 | 1.855 | 13.82 | 0.138075 | 7/15/2014 | 12:57.1 | 2 |
| -1032.997 | 19376.969 | 1.855 | 13.82 | 0.139341 | 7/15/2014 | 12:57.2 | 2 |
| -1032.775 | 19376.948 | 1.858 | 13.822 | 0.142166 | 7/15/2014 | 12:57.3 | 2 |
| -1032.5 | 19376.922 | 1.858 | 13.821 | 0.145872 | 7/15/2014 | 12:57.4 | 2 |
| -1032.28 | 19376.901 | 1.858 | 13.817 | 0.144501 | 7/15/2014 | 12:57.5 | 2 |
| -1032.059 | 19376.879 | 1.858 | 13.812 | 0.149903 | 7/15/2014 | 12:57.6 | 2 |
| -1031.782 | 19376.854 | 1.858 | 13.801 | 0.154604 | 7/15/2014 | 12:57.7 | 2 |
| -1031.559 | 19376.832 | 1.858 | 13.796 | 0.153694 | 7/15/2014 | 12:57.8 | 2 |
| -1031.336 | 19376.81 | 1.858 | 13.788 | 0.159622 | 7/15/2014 | 12:57.9 | 2 |
| -1031.111 | 19376.788 | 1.858 | 13.778 | 0.164521 | 7/15/2014 | 12:58.0 | 2 |
| -1030.886 | 19376.767 | 1.858 | 13.77 | 0.168902 | 7/15/2014 | 12:58.1 | 2 |
| -1030.662 | 19376.745 | 1.858 | 13.762 | 0.162822 | 7/15/2014 | 12:58.2 | 2 |
| -1030.439 | 19376.723 | 1.863 | 13.753 | 0.162052 | 7/15/2014 | 12:58.3 | 2 |
| -1030.161 | 19376.696 | 1.863 | 13.745 | 0.164495 | 7/15/2014 | 12:58.4 | 2 |
| -1029.938 | 19376.675 | 1.866 | 13.737 | 0.162328 | 7/15/2014 | 12:58.5 | 2 |
| -1029.715 | 19376.651 | 1.866 | 13.732 | 0.166266 | 7/15/2014 | 12:58.6 | 2 |
| -1029.435 | 19376.625 | 1.869 | 13.732 | 0.164098 | 7/15/2014 | 12:58.7 | 2 |
| -1029.208 | 19376.603 | 1.872 | 13.734 | 0.163746 | 7/15/2014 | 12:58.8 | 2 |
| -1028.981 | 19376.58 | 1.872 | 13.73 | 0.157858 | 7/15/2014 | 12:58.9 | 2 |
| -1028.753 | 19376.558 | 1.872 | 13.728 | 0.159922 | 7/15/2014 | 12:59.0 | 2 |
| -1028.525 | 19376.535 | 1.872 | 13.723 | 0.161252 | 7/15/2014 | 12:59.1 | 2 |
| -1028.298 | 19376.513 | 1.874 | 13.72 | 0.1549 | 7/15/2014 | 12:59.2 | 2 |
| -1028.071 | 19376.49 | 1.874 | 13.716 | 0.160086 | 7/15/2014 | 12:59.3 | 2 |
| -1027.789 | 19376.463 | 1.872 | 13.711 | 0.14651 | 7/15/2014 | 12:59.4 | 2 |
| -1027.562 | 19376.44 | 1.874 | 13.702 | 0.145562 | 7/15/2014 | 12:59.5 | 2 |

| | | | | | | | |
|---|---|---|---|---|---|---|---|
| -1027.335 | 19376.417 | 1.874 | 13.689 | 0.158014 | 7/15/2014 | 12:59.6 | 2 |
| -1027.051 | 19376.39 | 1.874 | 13.68 | 0.154618 | 7/15/2014 | 12:59.7 | 2 |
| -1026.823 | 19376.367 | 1.876 | 13.676 | 0.150057 | 7/15/2014 | 12:59.8 | 2 |
| -1026.594 | 19376.344 | 1.876 | 13.677 | 0.151279 | 7/15/2014 | 12:59.9 | 2 |
| -1026.366 | 19376.322 | 1.874 | 13.676 | 0.152874 | 7/15/2014 | 13:00.0 | 2 |
| -1026.138 | 19376.299 | 1.874 | 13.674 | 0.16834 | 7/15/2014 | 13:00.1 | 2 |
| -1025.91 | 19376.277 | 1.878 | 13.669 | 0.175963 | 7/15/2014 | 13:00.2 | 2 |
| -1025.684 | 19376.254 | 1.88 | 13.666 | 0.192069 | 7/15/2014 | 13:00.3 | 2 |
| -1025.404 | 19376.227 | 1.882 | 13.663 | 0.197689 | 7/15/2014 | 13:00.4 | 2 |
| -1025.18 | 19376.205 | 1.886 | 13.66 | 0.205528 | 7/15/2014 | 13:00.5 | 2 |
| -1024.958 | 19376.183 | 1.89 | 13.649 | 0.206086 | 7/15/2014 | 13:00.6 | 2 |
| -1024.682 | 19376.156 | 1.892 | 13.634 | 0.203282 | 7/15/2014 | 13:00.7 | 2 |
| -1024.46 | 19376.133 | 1.9 | 13.619 | 0.205609 | 7/15/2014 | 13:00.8 | 2 |
| -1024.239 | 19376.112 | 1.908 | 13.602 | 0.20013 | 7/15/2014 | 13:00.9 | 2 |
| -1024.018 | 19376.09 | 1.911 | 13.59 | 0.193094 | 7/15/2014 | 13:01.0 | 2 |
| -1023.798 | 19376.068 | 1.914 | 13.578 | 0.189924 | 7/15/2014 | 13:01.1 | 2 |
| -1023.579 | 19376.046 | 1.917 | 13.569 | 0.17822 | 7/15/2014 | 13:01.2 | 2 |
| -1023.362 | 19376.025 | 1.92 | 13.56 | 0.179164 | 7/15/2014 | 13:01.3 | 2 |
| -1023.091 | 19375.998 | 1.923 | 13.554 | 0.176476 | 7/15/2014 | 13:01.4 | 2 |
| -1022.875 | 19375.977 | 1.926 | 13.549 | 0.169869 | 7/15/2014 | 13:01.5 | 2 |
| -1022.659 | 19375.955 | 1.926 | 13.547 | 0.165822 | 7/15/2014 | 13:01.6 | 2 |
| -1022.391 | 19375.929 | 1.929 | 13.552 | 0.17582 | 7/15/2014 | 13:01.7 | 2 |
| -1022.171 | 19375.907 | 1.934 | 13.555 | 0.169161 | 7/15/2014 | 13:01.8 | 2 |
| -1021.952 | 19375.884 | 1.939 | 13.561 | 0.178976 | 7/15/2014 | 13:01.9 | 2 |
| -1021.732 | 19375.863 | 1.945 | 13.564 | 0.182422 | 7/15/2014 | 13:02.0 | 2 |
| -1021.511 | 19375.84 | 1.947 | 13.566 | 0.193874 | 7/15/2014 | 13:02.1 | 2 |
| -1021.289 | 19375.818 | 1.951 | 13.571 | 0.199098 | 7/15/2014 | 13:02.2 | 2 |
| -1021.068 | 19375.796 | 1.96 | 13.58 | 0.209702 | 7/15/2014 | 13:02.3 | 2 |
| -1020.794 | 19375.769 | 1.967 | 13.587 | 0.206725 | 7/15/2014 | 13:02.4 | 2 |
| -1020.571 | 19375.747 | 1.974 | 13.603 | 0.215827 | 7/15/2014 | 13:02.5 | 2 |
| -1020.348 | 19375.724 | 1.977 | 13.614 | 0.209325 | 7/15/2014 | 13:02.6 | 2 |
| -1020.066 | 19375.695 | 1.977 | 13.616 | 0.20546 | 7/15/2014 | 13:02.7 | 2 |
| -1019.84 | 19375.672 | 1.984 | 13.616 | 0.207642 | 7/15/2014 | 13:02.8 | 2 |
| -1019.61 | 19375.648 | 1.987 | 13.617 | 0.212865 | 7/15/2014 | 13:02.9 | 2 |
| -1019.381 | 19375.626 | 1.995 | 13.609 | 0.19402 | 7/15/2014 | 13:03.0 | 2 |
| -1019.15 | 19375.603 | 1.997 | 13.595 | 0.187759 | 7/15/2014 | 13:03.1 | 2 |
| -1018.921 | 19375.579 | 1.999 | 13.581 | 0.181826 | 7/15/2014 | 13:03.2 | 2 |
| -1018.808 | 19375.567 | 2.001 | 13.562 | 0.201034 | 7/15/2014 | 13:03.3 | 2 |
| -1018.405 | 19375.526 | 2.006 | 13.525 | 0.193077 | 7/15/2014 | 13:03.4 | 2 |
| -1018.174 | 19375.502 | 2.013 | 13.505 | 0.198853 | 7/15/2014 | 13:03.5 | 2 |
| -1017.944 | 19375.479 | 2.013 | 13.488 | 0.169638 | 7/15/2014 | 13:03.6 | 2 |
| -1017.654 | 19375.449 | 2.02 | 13.471 | 0.166781 | 7/15/2014 | 13:03.7 | 2 |
| -1017.537 | 19375.438 | 2.018 | 13.464 | 0.172956 | 7/15/2014 | 13:03.8 | 2 |
| -1017.302 | 19375.412 | 2.024 | 13.455 | 0.169132 | 7/15/2014 | 13:03.9 | 2 |
| -1017.069 | 19375.389 | 2.024 | 13.455 | 0.17968 | 7/15/2014 | 13:04.0 | 2 |
| -1016.834 | 19375.364 | 2.03 | 13.451 | 0.167553 | 7/15/2014 | 13:04.1 | 2 |
| -1016.601 | 19375.34 | 2.032 | 13.447 | 0.161558 | 7/15/2014 | 13:04.2 | 2 |

| | | | | | | | |
|---|---|---|---|---|---|---|---|
| -1016.366 | 19375.315 | 2.036 | 13.443 | 0.176942 | 7/15/2014 | 13:04.3 | 2 |
| -1015.959 | 19375.273 | 2.042 | 13.443 | 0.174094 | 7/15/2014 | 13:04.4 | 2 |
| -1015.727 | 19375.249 | 2.044 | 13.443 | 0.173338 | 7/15/2014 | 13:04.5 | 2 |
| -1015.496 | 19375.225 | 2.046 | 13.438 | 0.173792 | 7/15/2014 | 13:04.6 | 2 |
| -1015.207 | 19375.194 | 2.049 | 13.431 | 0.168397 | 7/15/2014 | 13:04.7 | 2 |
| -1015.09 | 19375.183 | 2.049 | 13.431 | 0.168058 | 7/15/2014 | 13:04.8 | 2 |
| -1014.857 | 19375.157 | 2.05 | 13.422 | 0.158186 | 7/15/2014 | 13:04.9 | 2 |
| -1014.626 | 19375.134 | 2.053 | 13.411 | 0.156038 | 7/15/2014 | 13:05.0 | 2 |
| -1014.281 | 19375.098 | 2.052 | 13.38 | 0.150646 | 7/15/2014 | 13:05.1 | 2 |
| -1014.051 | 19375.074 | 2.052 | 13.364 | 0.143356 | 7/15/2014 | 13:05.2 | 2 |
| -1013.824 | 19375.05 | 2.049 | 13.347 | 0.132833 | 7/15/2014 | 13:05.3 | 2 |
| -1013.539 | 19375.02 | 2.055 | 13.331 | 0.126473 | 7/15/2014 | 13:05.4 | 2 |
| -1013.314 | 19374.995 | 2.057 | 13.309 | 0.140551 | 7/15/2014 | 13:05.5 | 2 |
| -1013.089 | 19374.971 | 2.061 | 13.3 | 0.13359 | 7/15/2014 | 13:05.6 | 2 |
| -1012.811 | 19374.942 | 2.059 | 13.294 | 0.129634 | 7/15/2014 | 13:05.7 | 2 |
| -1012.588 | 19374.919 | 2.064 | 13.289 | 0.137121 | 7/15/2014 | 13:05.8 | 2 |
| -1012.363 | 19374.896 | 2.067 | 13.298 | 0.132114 | 7/15/2014 | 13:05.9 | 2 |
| -1012.14 | 19374.873 | 2.067 | 13.295 | 0.144568 | 7/15/2014 | 13:06.0 | 2 |
| -1011.915 | 19374.849 | 2.072 | 13.293 | 0.156807 | 7/15/2014 | 13:06.1 | 2 |
| -1011.692 | 19374.825 | 2.069 | 13.289 | 0.156288 | 7/15/2014 | 13:06.2 | 2 |
| -1011.472 | 19374.801 | 2.069 | 13.293 | 0.163529 | 7/15/2014 | 13:06.3 | 2 |
| -1011.198 | 19374.772 | 2.072 | 13.303 | 0.17421 | 7/15/2014 | 13:06.4 | 2 |
| -1010.98 | 19374.749 | 2.074 | 13.3 | 0.175603 | 7/15/2014 | 13:06.5 | 2 |
| -1010.76 | 19374.727 | 2.073 | 13.297 | 0.192112 | 7/15/2014 | 13:06.6 | 2 |
| -1010.487 | 19374.697 | 2.077 | 13.283 | 0.183341 | 7/15/2014 | 13:06.7 | 2 |
| -1010.266 | 19374.675 | 2.082 | 13.281 | 0.194203 | 7/15/2014 | 13:06.8 | 2 |
| -1010.045 | 19374.651 | 2.085 | 13.279 | 0.183846 | 7/15/2014 | 13:06.9 | 2 |
| -1009.824 | 19374.629 | 2.086 | 13.285 | 0.196645 | 7/15/2014 | 13:07.0 | 2 |
| -1009.6 | 19374.604 | 2.091 | 13.283 | 0.18877 | 7/15/2014 | 13:07.1 | 2 |
| -1009.379 | 19374.581 | 2.091 | 13.265 | 0.16873 | 7/15/2014 | 13:07.2 | 2 |
| -1009.158 | 19374.559 | 2.096 | 13.26 | 0.170112 | 7/15/2014 | 13:07.3 | 2 |
| -1008.883 | 19374.529 | 2.1 | 13.276 | 0.171657 | 7/15/2014 | 13:07.4 | 2 |
| -1008.663 | 19374.507 | 2.096 | 13.266 | 0.148376 | 7/15/2014 | 13:07.5 | 2 |
| -1008.441 | 19374.483 | 2.099 | 13.252 | 0.147331 | 7/15/2014 | 13:07.6 | 2 |
| -1008.164 | 19374.457 | 2.099 | 13.241 | 0.130883 | 7/15/2014 | 13:07.7 | 2 |
| -1007.938 | 19374.433 | 2.103 | 13.224 | 0.130417 | 7/15/2014 | 13:07.8 | 2 |
| -1007.713 | 19374.409 | 2.101 | 13.212 | 0.133284 | 7/15/2014 | 13:07.9 | 2 |
| -1007.486 | 19374.387 | 2.103 | 13.202 | 0.133692 | 7/15/2014 | 13:08.0 | 2 |
| -1007.261 | 19374.363 | 2.1 | 13.193 | 0.130803 | 7/15/2014 | 13:08.1 | 2 |
| -1007.036 | 19374.34 | 2.1 | 13.162 | 0.13699 | 7/15/2014 | 13:08.2 | 2 |
| -1006.811 | 19374.316 | 2.107 | 13.156 | 0.139579 | 7/15/2014 | 13:08.3 | 2 |
| -1006.532 | 19374.289 | 2.107 | 13.154 | 0.142299 | 7/15/2014 | 13:08.4 | 2 |
| -1006.308 | 19374.267 | 2.111 | 13.148 | 0.145439 | 7/15/2014 | 13:08.5 | 2 |
| -1006.085 | 19374.244 | 2.111 | 13.143 | 0.146617 | 7/15/2014 | 13:08.6 | 2 |
| -1005.804 | 19374.215 | 2.114 | 13.14 | 0.138097 | 7/15/2014 | 13:08.7 | 2 |
| -1005.58 | 19374.192 | 2.111 | 13.135 | 0.139775 | 7/15/2014 | 13:08.8 | 2 |
| -1005.354 | 19374.17 | 2.117 | 13.128 | 0.14643 | 7/15/2014 | 13:08.9 | 2 |

| | | | | | | | |
|---|---|---|---|---|---|---|---|
| -1005.129 | 19374.146 | 2.117 | 13.129 | 0.144342 | 7/15/2014 | 13:09.0 | 2 |
| -1004.903 | 19374.124 | 2.111 | 13.124 | 0.138007 | 7/15/2014 | 13:09.1 | 2 |
| -1004.68 | 19374.102 | 2.111 | 13.124 | 0.134604 | 7/15/2014 | 13:09.2 | 2 |
| -1004.458 | 19374.08 | 2.109 | 13.125 | 0.135274 | 7/15/2014 | 13:09.3 | 2 |
| -1004.18 | 19374.051 | 2.111 | 13.121 | 0.139736 | 7/15/2014 | 13:09.4 | 2 |
| -1003.959 | 19374.029 | 2.111 | 13.116 | 0.136746 | 7/15/2014 | 13:09.5 | 2 |
| -1003.738 | 19374.008 | 2.114 | 13.113 | 0.136735 | 7/15/2014 | 13:09.6 | 2 |
| -1003.462 | 19373.979 | 2.111 | 13.115 | 0.136624 | 7/15/2014 | 13:09.7 | 2 |
| -1003.239 | 19373.958 | 2.116 | 13.109 | 0.132875 | 7/15/2014 | 13:09.8 | 2 |
| -1003.017 | 19373.936 | 2.119 | 13.105 | 0.132735 | 7/15/2014 | 13:09.9 | 2 |
| -1002.793 | 19373.913 | 2.121 | 13.097 | 0.127079 | 7/15/2014 | 13:10.0 | 2 |
| -1002.57 | 19373.891 | 2.123 | 13.09 | 0.12219 | 7/15/2014 | 13:10.1 | 2 |
| -1002.348 | 19373.869 | 2.119 | 13.089 | 0.117903 | 7/15/2014 | 13:10.2 | 2 |
| -1002.127 | 19373.848 | 2.121 | 13.09 | 0.118515 | 7/15/2014 | 13:10.3 | 2 |
| -1001.85 | 19373.819 | 2.123 | 13.09 | 0.113144 | 7/15/2014 | 13:10.4 | 2 |
| -1001.63 | 19373.799 | 2.123 | 13.091 | 0.116268 | 7/15/2014 | 13:10.5 | 2 |
| -1001.409 | 19373.776 | 2.123 | 13.087 | 0.130242 | 7/15/2014 | 13:10.6 | 2 |
| -1001.133 | 19373.749 | 2.123 | 13.092 | 0.125705 | 7/15/2014 | 13:10.7 | 2 |
| -1000.911 | 19373.728 | 2.123 | 13.091 | 0.127042 | 7/15/2014 | 13:10.8 | 2 |
| -1000.689 | 19373.706 | 2.123 | 13.09 | 0.124354 | 7/15/2014 | 13:10.9 | 2 |
| -1000.465 | 19373.685 | 2.131 | 13.085 | 0.13571 | 7/15/2014 | 13:11.0 | 2 |
| -1000.241 | 19373.662 | 2.133 | 13.081 | 0.134821 | 7/15/2014 | 13:11.1 | 2 |
| -1000.019 | 19373.642 | 2.136 | 13.079 | 0.13954 | 7/15/2014 | 13:11.2 | 2 |
| -999.797 | 19373.619 | 2.138 | 13.065 | 0.13422 | 7/15/2014 | 13:11.3 | 2 |
| -999.522 | 19373.593 | 2.141 | 13.058 | 0.139082 | 7/15/2014 | 13:11.4 | 2 |
| -999.3 | 19373.571 | 2.146 | 13.05 | 0.134617 | 7/15/2014 | 13:11.5 | 2 |
| -999.081 | 19373.55 | 2.149 | 13.054 | 0.13666 | 7/15/2014 | 13:11.6 | 2 |
| -998.804 | 19373.523 | 2.154 | 13.053 | 0.132509 | 7/15/2014 | 13:11.7 | 2 |
| -998.582 | 19373.502 | 2.154 | 13.055 | 0.126794 | 7/15/2014 | 13:11.8 | 2 |
| -998.361 | 19373.481 | 2.152 | 13.054 | 0.120099 | 7/15/2014 | 13:11.9 | 2 |
| -998.137 | 19373.459 | 2.152 | 13.051 | 0.102462 | 7/15/2014 | 13:12.0 | 2 |
| -997.915 | 19373.438 | 2.154 | 13.058 | 0.103225 | 7/15/2014 | 13:12.1 | 2 |
| -997.693 | 19373.417 | 2.151 | 13.065 | 0.103076 | 7/15/2014 | 13:12.2 | 2 |
| -997.474 | 19373.396 | 2.149 | 13.07 | 0.0940154 | 7/15/2014 | 13:12.3 | 2 |
| -997.199 | 19373.37 | 2.146 | 13.062 | 0.0906005 | 7/15/2014 | 13:12.4 | 2 |
| -996.98 | 19373.349 | 2.142 | 13.061 | 0.0844159 | 7/15/2014 | 13:12.5 | 2 |
| -996.762 | 19373.329 | 2.142 | 13.055 | 0.0914313 | 7/15/2014 | 13:12.6 | 2 |
| -996.488 | 19373.302 | 2.144 | 13.049 | 0.0919156 | 7/15/2014 | 13:12.7 | 2 |
| -996.268 | 19373.282 | 2.144 | 13.042 | 0.0860559 | 7/15/2014 | 13:12.8 | 2 |
| -996.046 | 19373.261 | 2.144 | 13.038 | 0.0900612 | 7/15/2014 | 13:12.9 | 2 |
| -995.826 | 19373.24 | 2.147 | 13.029 | 0.0924351 | 7/15/2014 | 13:13.0 | 2 |
| -995.604 | 19373.221 | 2.152 | 13.019 | 0.0796269 | 7/15/2014 | 13:13.1 | 2 |
| -995.382 | 19373.198 | 2.155 | 13.016 | 0.086032 | 7/15/2014 | 13:13.2 | 2 |
| -995.162 | 19373.179 | 2.159 | 13.01 | 0.0804854 | 7/15/2014 | 13:13.3 | 2 |
| -994.888 | 19373.153 | 2.159 | 13.003 | 0.0780079 | 7/15/2014 | 13:13.4 | 2 |
| -994.668 | 19373.133 | 2.159 | 12.996 | 0.0769849 | 7/15/2014 | 13:13.5 | 2 |
| -994.448 | 19373.111 | 2.159 | 12.987 | 0.0821248 | 7/15/2014 | 13:13.6 | 2 |

| | | | | | | | |
|---|---|---|---|---|---|---|---|
| -994.172 | 19373.087 | 2.159 | 12.974 | 0.0834741 | 7/15/2014 | 13:13.7 | 2 |
| -993.949 | 19373.067 | 2.159 | 12.962 | 0.0863686 | 7/15/2014 | 13:13.8 | 2 |
| -993.726 | 19373.045 | 2.155 | 12.952 | 0.0870848 | 7/15/2014 | 13:13.9 | 2 |
| -993.5 | 19373.025 | 2.159 | 12.942 | 0.0861946 | 7/15/2014 | 13:14.0 | 2 |
| -993.277 | 19373.006 | 2.155 | 12.935 | 0.0880478 | 7/15/2014 | 13:14.1 | 2 |
| -993.054 | 19372.984 | 2.152 | 12.934 | 0.0867759 | 7/15/2014 | 13:14.2 | 2 |
| -992.831 | 19372.963 | 2.152 | 12.933 | 0.0900568 | 7/15/2014 | 13:14.3 | 2 |
| -992.552 | 19372.938 | 2.155 | 12.935 | 0.0890872 | 7/15/2014 | 13:14.4 | 2 |
| -992.331 | 19372.919 | 2.159 | 12.939 | 0.0792115 | 7/15/2014 | 13:14.5 | 2 |
| -992.108 | 19372.898 | 2.155 | 12.944 | 0.0844527 | 7/15/2014 | 13:14.6 | 2 |
| -991.828 | 19372.874 | 2.155 | 12.947 | 0.0905352 | 7/15/2014 | 13:14.7 | 2 |
| -991.605 | 19372.855 | 2.152 | 12.95 | 0.0854236 | 7/15/2014 | 13:14.8 | 2 |
| -991.38 | 19372.834 | 2.152 | 12.954 | 0.0887156 | 7/15/2014 | 13:14.9 | 2 |
| -991.154 | 19372.813 | 2.152 | 12.952 | 0.102585 | 7/15/2014 | 13:15.0 | 2 |
| -990.927 | 19372.793 | 2.152 | 12.954 | 0.103758 | 7/15/2014 | 13:15.1 | 2 |
| -990.704 | 19372.773 | 2.149 | 12.952 | 0.0952446 | 7/15/2014 | 13:15.2 | 2 |
| -990.48 | 19372.755 | 2.144 | 12.948 | 0.0915897 | 7/15/2014 | 13:15.3 | 2 |
| -990.202 | 19372.731 | 2.144 | 12.946 | 0.122506 | 7/15/2014 | 13:15.4 | 2 |
| -989.98 | 19372.712 | 2.139 | 12.947 | 0.0996917 | 7/15/2014 | 13:15.5 | 2 |
| -989.756 | 19372.692 | 2.134 | 12.951 | 0.10599 | 7/15/2014 | 13:15.6 | 2 |
| -989.478 | 19372.668 | 2.134 | 12.95 | 0.0745954 | 7/15/2014 | 13:15.7 | 2 |
| -989.254 | 19372.648 | 2.134 | 12.951 | 0.066636 | 7/15/2014 | 13:15.8 | 2 |
| -989.029 | 19372.629 | 2.132 | 12.952 | 0.062056 | 7/15/2014 | 13:15.9 | 2 |
| -988.805 | 19372.61 | 2.129 | 12.954 | 0.0583481 | 7/15/2014 | 13:16.0 | 2 |
| -988.581 | 19372.592 | 2.129 | 12.954 | 0.0492697 | 7/15/2014 | 13:16.1 | 2 |
| -988.358 | 19372.573 | 2.129 | 12.957 | 0.0580667 | 7/15/2014 | 13:16.2 | 2 |
| -988.135 | 19372.555 | 2.131 | 12.955 | 0.0565065 | 7/15/2014 | 13:16.3 | 2 |
| -987.859 | 19372.531 | 2.131 | 12.952 | 0.0487051 | 7/15/2014 | 13:16.4 | 2 |
| -987.639 | 19372.514 | 2.127 | 12.952 | 0.0656576 | 7/15/2014 | 13:16.5 | 2 |
| -987.418 | 19372.494 | 2.125 | 12.952 | 0.0635758 | 7/15/2014 | 13:16.6 | 2 |
| -987.144 | 19372.471 | 2.122 | 12.95 | 0.0646942 | 7/15/2014 | 13:16.7 | 2 |
| -986.921 | 19372.451 | 2.12 | 12.95 | 0.0656985 | 7/15/2014 | 13:16.8 | 2 |
| -986.7 | 19372.436 | 2.115 | 12.947 | 0.0718365 | 7/15/2014 | 13:16.9 | 2 |
| -986.477 | 19372.416 | 2.111 | 12.947 | 0.0747944 | 7/15/2014 | 13:17.0 | 2 |
| -986.254 | 19372.398 | 2.111 | 12.946 | 0.0834125 | 7/15/2014 | 13:17.1 | 2 |
| -986.033 | 19372.381 | 2.111 | 12.94 | 0.0916355 | 7/15/2014 | 13:17.2 | 2 |
| -985.811 | 19372.361 | 2.111 | 12.938 | 0.0842364 | 7/15/2014 | 13:17.3 | 2 |
| -985.536 | 19372.34 | 2.108 | 12.942 | 0.0890224 | 7/15/2014 | 13:17.4 | 2 |
| -985.314 | 19372.32 | 2.108 | 12.942 | 0.0840589 | 7/15/2014 | 13:17.5 | 2 |
| -985.092 | 19372.305 | 2.108 | 12.946 | 0.0729932 | 7/15/2014 | 13:17.6 | 2 |
| -984.814 | 19372.281 | 2.111 | 12.954 | 0.0769827 | 7/15/2014 | 13:17.7 | 2 |
| -984.59 | 19372.264 | 2.111 | 12.958 | 0.0617225 | 7/15/2014 | 13:17.8 | 2 |
| -984.364 | 19372.246 | 2.106 | 12.961 | 0.0692415 | 7/15/2014 | 13:17.9 | 2 |
| -984.138 | 19372.227 | 2.106 | 12.974 | 0.0711344 | 7/15/2014 | 13:18.0 | 2 |
| -983.912 | 19372.211 | 2.103 | 12.978 | 0.0738182 | 7/15/2014 | 13:18.1 | 2 |
| -983.686 | 19372.191 | 2.105 | 12.977 | 0.0812808 | 7/15/2014 | 13:18.2 | 2 |
| -983.461 | 19372.175 | 2.102 | 12.982 | 0.089641 | 7/15/2014 | 13:18.3 | 2 |

| | | | | | | | |
|---|---|---|---|---|---|---|---|
| -983.18 | 19372.152 | 2.102 | 12.977 | 0.0838462 | 7/15/2014 | 13:18.4 | 2 |
| -982.958 | 19372.135 | 2.097 | 12.976 | 0.0834259 | 7/15/2014 | 13:18.5 | 2 |
| -982.732 | 19372.118 | 2.097 | 12.974 | 0.0682933 | 7/15/2014 | 13:18.6 | 2 |
| -982.452 | 19372.096 | 2.097 | 12.974 | 0.0676975 | 7/15/2014 | 13:18.7 | 2 |
| -982.227 | 19372.079 | 2.092 | 12.97 | 0.0585861 | 7/15/2014 | 13:18.8 | 2 |
| -982.002 | 19372.061 | 2.09 | 12.971 | 0.0466632 | 7/15/2014 | 13:18.9 | 2 |
| -981.777 | 19372.044 | 2.084 | 12.977 | 0.0428909 | 7/15/2014 | 13:19.0 | 2 |
| -981.551 | 19372.027 | 2.082 | 12.978 | 0.0443058 | 7/15/2014 | 13:19.1 | 2 |
| -981.329 | 19372.011 | 2.077 | 12.985 | 0.0491201 | 7/15/2014 | 13:19.2 | 2 |
| -981.107 | 19371.993 | 2.075 | 12.991 | 0.0418129 | 7/15/2014 | 13:19.3 | 2 |
| -980.83 | 19371.973 | 2.072 | 13 | 0.0454715 | 7/15/2014 | 13:19.4 | 2 |
| -980.61 | 19371.956 | 2.069 | 13.007 | 0.0591645 | 7/15/2014 | 13:19.5 | 2 |
| -980.39 | 19371.939 | 2.069 | 13.011 | 0.056557 | 7/15/2014 | 13:19.6 | 2 |
| -980.116 | 19371.919 | 2.072 | 13.012 | 0.0612177 | 7/15/2014 | 13:19.7 | 2 |
| -979.895 | 19371.904 | 2.072 | 13.007 | 0.0717413 | 7/15/2014 | 13:19.8 | 2 |
| -979.676 | 19371.888 | 2.069 | 13.001 | 0.0687464 | 7/15/2014 | 13:19.9 | 2 |
| -979.456 | 19371.871 | 2.067 | 12.993 | 0.0696146 | 7/15/2014 | 13:20.0 | 2 |
| -979.235 | 19371.855 | 2.065 | 12.981 | 0.061458 | 7/15/2014 | 13:20.1 | 2 |
| -979.016 | 19371.84 | 2.065 | 12.969 | 0.0553804 | 7/15/2014 | 13:20.2 | 2 |
| -978.798 | 19371.824 | 2.063 | 12.955 | 0.0666831 | 7/15/2014 | 13:20.3 | 2 |
| -978.527 | 19371.804 | 2.06 | 12.94 | 0.0794368 | 7/15/2014 | 13:20.4 | 2 |
| -978.311 | 19371.79 | 2.053 | 12.929 | 0.0473513 | 7/15/2014 | 13:20.5 | 2 |
| -978.094 | 19371.774 | 2.048 | 12.92 | 0.0736863 | 7/15/2014 | 13:20.6 | 2 |
| -977.823 | 19371.756 | 2.048 | 12.912 | 0.0523432 | 7/15/2014 | 13:20.7 | 2 |
| -977.604 | 19371.741 | 2.046 | 12.909 | 0.0781264 | 7/15/2014 | 13:20.8 | 2 |
| -977.385 | 19371.726 | 2.045 | 12.907 | 0.0529124 | 7/15/2014 | 13:20.9 | 2 |
| -977.164 | 19371.711 | 2.041 | 12.907 | 0.0494399 | 7/15/2014 | 13:21.0 | 2 |
| -976.945 | 19371.695 | 2.038 | 12.915 | 0.0534616 | 7/15/2014 | 13:21.1 | 2 |
| -976.725 | 19371.681 | 2.035 | 12.926 | 0.0514393 | 7/15/2014 | 13:21.2 | 2 |
| -976.505 | 19371.666 | 2.033 | 12.938 | 0.0533374 | 7/15/2014 | 13:21.3 | 2 |
| -976.231 | 19371.648 | 2.033 | 12.946 | 0.060142 | 7/15/2014 | 13:21.4 | 2 |
| -976.012 | 19371.635 | 2.031 | 12.953 | 0.0780714 | 7/15/2014 | 13:21.5 | 2 |
| -975.794 | 19371.62 | 2.031 | 12.955 | 0.0639549 | 7/15/2014 | 13:21.6 | 2 |
| -975.519 | 19371.604 | 2.029 | 12.957 | 0.0856775 | 7/15/2014 | 13:21.7 | 2 |
| -975.301 | 19371.588 | 2.031 | 12.957 | 0.0708508 | 7/15/2014 | 13:21.8 | 2 |
| -975.079 | 19371.574 | 2.034 | 12.948 | 0.0670432 | 7/15/2014 | 13:21.9 | 2 |
| -974.86 | 19371.56 | 2.034 | 12.939 | 0.0730402 | 7/15/2014 | 13:22.0 | 2 |
| -974.639 | 19371.547 | 2.04 | 12.926 | 0.0711194 | 7/15/2014 | 13:22.1 | 2 |
| -974.42 | 19371.533 | 2.04 | 12.917 | 0.0584836 | 7/15/2014 | 13:22.2 | 2 |
| -974.203 | 19371.52 | 2.036 | 12.91 | 0.0570612 | 7/15/2014 | 13:22.3 | 2 |
| -973.931 | 19371.503 | 2.034 | 12.903 | 0.0583864 | 7/15/2014 | 13:22.4 | 2 |
| -973.716 | 19371.489 | 2.034 | 12.898 | 0.0561164 | 7/15/2014 | 13:22.5 | 2 |
| -973.499 | 19371.477 | 2.03 | 12.891 | 0.0692495 | 7/15/2014 | 13:22.6 | 2 |
| -973.23 | 19371.459 | 2.025 | 12.886 | 0.062532 | 7/15/2014 | 13:22.7 | 2 |
| -973.013 | 19371.447 | 2.022 | 12.881 | 0.0693172 | 7/15/2014 | 13:22.8 | 2 |
| -972.795 | 19371.434 | 2.016 | 12.88 | 0.0676119 | 7/15/2014 | 13:22.9 | 2 |
| -972.578 | 19371.42 | 2.013 | 12.881 | 0.0634484 | 7/15/2014 | 13:23.0 | 2 |

| | | | | | | | |
|---|---|---|---|---|---|---|---|
| -972.358 | 19371.407 | 2.013 | 12.883 | 0.0778219 | 7/15/2014 | 13:23.1 | 2 |
| -972.139 | 19371.396 | 2.015 | 12.885 | 0.0684851 | 7/15/2014 | 13:23.2 | 2 |
| -971.922 | 19371.382 | 2.015 | 12.886 | 0.0717186 | 7/15/2014 | 13:23.3 | 2 |
| -971.651 | 19371.365 | 2.015 | 12.895 | 0.0867096 | 7/15/2014 | 13:23.4 | 2 |
| -971.435 | 19371.354 | 2.012 | 12.901 | 0.0681432 | 7/15/2014 | 13:23.5 | 2 |
| -971.217 | 19371.341 | 2.013 | 12.909 | 0.0756799 | 7/15/2014 | 13:23.6 | 2 |
| -970.945 | 19371.324 | 2.013 | 12.914 | 0.0813344 | 7/15/2014 | 13:23.7 | 2 |
| -970.725 | 19371.313 | 2.013 | 12.914 | 0.0875602 | 7/15/2014 | 13:23.8 | 2 |
| -970.503 | 19371.301 | 2.013 | 12.912 | 0.0944671 | 7/15/2014 | 13:23.9 | 2 |
| -970.283 | 19371.288 | 2.008 | 12.914 | 0.0874293 | 7/15/2014 | 13:24.0 | 2 |
| -970.06 | 19371.275 | 2.008 | 12.915 | 0.0931998 | 7/15/2014 | 13:24.1 | 2 |
| -969.841 | 19371.263 | 2.007 | 12.907 | 0.099864 | 7/15/2014 | 13:24.2 | 2 |
| -969.619 | 19371.251 | 2.01 | 12.903 | 0.102364 | 7/15/2014 | 13:24.3 | 2 |
| -969.345 | 19371.235 | 2.012 | 12.893 | 0.0937473 | 7/15/2014 | 13:24.4 | 2 |
| -969.127 | 19371.223 | 2.012 | 12.886 | 0.0886344 | 7/15/2014 | 13:24.5 | 2 |
| -968.907 | 19371.211 | 2.01 | 12.88 | 0.0831573 | 7/15/2014 | 13:24.6 | 2 |
| -968.632 | 19371.197 | 2.01 | 12.875 | 0.0772503 | 7/15/2014 | 13:24.7 | 2 |
| -968.41 | 19371.186 | 2.012 | 12.87 | 0.0771387 | 7/15/2014 | 13:24.8 | 2 |
| -968.188 | 19371.173 | 2.012 | 12.864 | 0.0742138 | 7/15/2014 | 13:24.9 | 2 |
| -967.964 | 19371.161 | 2.015 | 12.863 | 0.0656442 | 7/15/2014 | 13:25.0 | 2 |
| -967.741 | 19371.149 | 2.015 | 12.865 | 0.0712648 | 7/15/2014 | 13:25.1 | 2 |
| -967.519 | 19371.137 | 2.013 | 12.868 | 0.0727359 | 7/15/2014 | 13:25.2 | 2 |
| -967.299 | 19371.125 | 2.013 | 12.869 | 0.0806847 | 7/15/2014 | 13:25.3 | 2 |
| -967.025 | 19371.111 | 2.015 | 12.873 | 0.096301 | 7/15/2014 | 13:25.4 | 2 |
| -966.805 | 19371.1 | 2.015 | 12.875 | 0.0901896 | 7/15/2014 | 13:25.5 | 2 |
| -966.584 | 19371.09 | 2.015 | 12.883 | 0.0928831 | 7/15/2014 | 13:25.6 | 2 |
| -966.308 | 19371.075 | 2.015 | 12.887 | 0.110303 | 7/15/2014 | 13:25.7 | 2 |
| -966.088 | 19371.064 | 2.012 | 12.893 | 0.100757 | 7/15/2014 | 13:25.8 | 2 |
| -965.865 | 19371.053 | 2.012 | 12.893 | 0.112327 | 7/15/2014 | 13:25.9 | 2 |
| -965.645 | 19371.041 | 2.009 | 12.896 | 0.116498 | 7/15/2014 | 13:26.0 | 2 |
| -965.423 | 19371.03 | 2.009 | 12.896 | 0.0950396 | 7/15/2014 | 13:26.1 | 2 |
| -965.201 | 19371.02 | 2.01 | 12.893 | 0.0952201 | 7/15/2014 | 13:26.2 | 2 |
| -964.982 | 19371.01 | 2.007 | 12.891 | 0.0903547 | 7/15/2014 | 13:26.3 | 2 |
| -964.708 | 19370.997 | 2.007 | 12.883 | 0.0814608 | 7/15/2014 | 13:26.4 | 2 |
| -964.49 | 19370.986 | 2.007 | 12.878 | 0.0829969 | 7/15/2014 | 13:26.5 | 2 |
| -964.27 | 19370.975 | 2.007 | 12.874 | 0.062437 | 7/15/2014 | 13:26.6 | 2 |
| -963.996 | 19370.962 | 2.009 | 12.87 | 0.0614421 | 7/15/2014 | 13:26.7 | 2 |
| -963.773 | 19370.952 | 2.014 | 12.866 | 0.0688584 | 7/15/2014 | 13:26.8 | 2 |
| -963.551 | 19370.941 | 2.016 | 12.863 | 0.059851 | 7/15/2014 | 13:26.9 | 2 |
| -963.329 | 19370.931 | 2.015 | 12.863 | 0.0641105 | 7/15/2014 | 13:27.0 | 2 |
| -963.106 | 19370.922 | 2.018 | 12.865 | 0.0788514 | 7/15/2014 | 13:27.1 | 2 |
| -962.883 | 19370.91 | 2.018 | 12.865 | 0.0718034 | 7/15/2014 | 13:27.2 | 2 |
| -962.661 | 19370.9 | 2.018 | 12.863 | 0.0865701 | 7/15/2014 | 13:27.3 | 2 |
| -962.383 | 19370.888 | 2.022 | 12.859 | 0.0923085 | 7/15/2014 | 13:27.4 | 2 |
| -962.16 | 19370.877 | 2.022 | 12.857 | 0.104703 | 7/15/2014 | 13:27.5 | 2 |
| -961.939 | 19370.867 | 2.016 | 12.859 | 0.111118 | 7/15/2014 | 13:27.6 | 2 |
| -961.659 | 19370.854 | 2.014 | 12.858 | 0.106961 | 7/15/2014 | 13:27.7 | 2 |

| | | | | | | | |
|---|---|---|---|---|---|---|---|
| -961.433 | 19370.844 | 2.015 | 12.861 | 0.101476 | 7/15/2014 | 13:27.8 | 2 |
| -961.209 | 19370.834 | 2.015 | 12.856 | 0.132125 | 7/15/2014 | 13:27.9 | 2 |
| -960.981 | 19370.824 | 2.02 | 12.853 | 0.111729 | 7/15/2014 | 13:28.0 | 2 |
| -960.755 | 19370.814 | 2.022 | 12.85 | 0.125411 | 7/15/2014 | 13:28.1 | 2 |
| -960.531 | 19370.803 | 2.021 | 12.852 | 0.112249 | 7/15/2014 | 13:28.2 | 2 |
| -960.307 | 19370.794 | 2.023 | 12.852 | 0.106443 | 7/15/2014 | 13:28.3 | 2 |
| -960.027 | 19370.781 | 2.026 | 12.838 | 0.1102 | 7/15/2014 | 13:28.4 | 2 |
| -959.803 | 19370.771 | 2.028 | 12.83 | 0.113059 | 7/15/2014 | 13:28.5 | 2 |
| -959.58 | 19370.762 | 2.032 | 12.819 | 0.110078 | 7/15/2014 | 13:28.6 | 2 |
| -959.3 | 19370.748 | 2.038 | 12.812 | 0.106621 | 7/15/2014 | 13:28.7 | 2 |
| -959.076 | 19370.74 | 2.038 | 12.804 | 0.103034 | 7/15/2014 | 13:28.8 | 2 |
| -958.851 | 19370.729 | 2.041 | 12.797 | 0.105233 | 7/15/2014 | 13:28.9 | 2 |
| -958.627 | 19370.72 | 2.041 | 12.783 | 0.107793 | 7/15/2014 | 13:29.0 | 2 |
| -958.399 | 19370.709 | 2.044 | 12.774 | 0.0989532 | 7/15/2014 | 13:29.1 | 2 |
| -958.175 | 19370.7 | 2.048 | 12.771 | 0.0941085 | 7/15/2014 | 13:29.2 | 2 |
| -957.95 | 19370.689 | 2.054 | 12.773 | 0.0735429 | 7/15/2014 | 13:29.3 | 2 |
| -957.671 | 19370.678 | 2.054 | 12.766 | 0.10322 | 7/15/2014 | 13:29.4 | 2 |
| -957.448 | 19370.668 | 2.054 | 12.762 | 0.0853579 | 7/15/2014 | 13:29.5 | 2 |
| -957.224 | 19370.658 | 2.054 | 12.76 | 0.0946568 | 7/15/2014 | 13:29.6 | 2 |
| -956.946 | 19370.646 | 2.05 | 12.762 | 0.1053 | 7/15/2014 | 13:29.7 | 2 |
| -956.718 | 19370.635 | 2.051 | 12.768 | 0.109697 | 7/15/2014 | 13:29.8 | 2 |
| -956.491 | 19370.627 | 2.049 | 12.772 | 0.114232 | 7/15/2014 | 13:29.9 | 2 |
| -956.263 | 19370.615 | 2.047 | 12.773 | 0.13572 | 7/15/2014 | 13:30.0 | 2 |
| -956.036 | 19370.606 | 2.045 | 12.77 | 0.104619 | 7/15/2014 | 13:30.1 | 2 |
| -955.809 | 19370.596 | 2.045 | 12.772 | 0.115797 | 7/15/2014 | 13:30.2 | 2 |
| -955.583 | 19370.586 | 2.047 | 12.771 | 0.115284 | 7/15/2014 | 13:30.3 | 2 |
| -955.303 | 19370.574 | 2.047 | 12.771 | 0.109297 | 7/15/2014 | 13:30.4 | 2 |
| -955.077 | 19370.563 | 2.053 | 12.771 | 0.127148 | 7/15/2014 | 13:30.5 | 2 |
| -954.852 | 19370.555 | 2.055 | 12.766 | 0.126506 | 7/15/2014 | 13:30.6 | 2 |
| -954.572 | 19370.542 | 2.057 | 12.758 | 0.117565 | 7/15/2014 | 13:30.7 | 2 |
| -954.346 | 19370.532 | 2.062 | 12.754 | 0.117733 | 7/15/2014 | 13:30.8 | 2 |
| -954.12 | 19370.522 | 2.064 | 12.748 | 0.117075 | 7/15/2014 | 13:30.9 | 2 |
| -953.894 | 19370.512 | 2.064 | 12.746 | 0.113106 | 7/15/2014 | 13:31.0 | 2 |
| -953.67 | 19370.504 | 2.059 | 12.746 | 0.120574 | 7/15/2014 | 13:31.1 | 2 |
| -953.444 | 19370.492 | 2.062 | 12.742 | 0.0946897 | 7/15/2014 | 13:31.2 | 2 |
| -953.221 | 19370.484 | 2.06 | 12.735 | 0.094891 | 7/15/2014 | 13:31.3 | 2 |
| -952.944 | 19370.472 | 2.06 | 12.732 | 0.124604 | 7/15/2014 | 13:31.4 | 2 |
| -952.722 | 19370.463 | 2.06 | 12.727 | 0.119208 | 7/15/2014 | 13:31.5 | 2 |
| -952.5 | 19370.454 | 2.057 | 12.721 | 0.1102 | 7/15/2014 | 13:31.6 | 2 |
| -952.223 | 19370.441 | 2.057 | 12.716 | 0.11134 | 7/15/2014 | 13:31.7 | 2 |
| -952.001 | 19370.434 | 2.057 | 12.714 | 0.10119 | 7/15/2014 | 13:31.8 | 2 |
| -951.776 | 19370.422 | 2.062 | 12.707 | 0.113208 | 7/15/2014 | 13:31.9 | 2 |
| -951.55 | 19370.414 | 2.064 | 12.705 | 0.104596 | 7/15/2014 | 13:32.0 | 2 |
| -951.327 | 19370.402 | 2.066 | 12.703 | 0.0799568 | 7/15/2014 | 13:32.1 | 2 |
| -951.103 | 19370.395 | 2.068 | 12.699 | 0.089007 | 7/15/2014 | 13:32.2 | 2 |
| -950.881 | 19370.385 | 2.068 | 12.703 | 0.0899739 | 7/15/2014 | 13:32.3 | 2 |
| -950.603 | 19370.373 | 2.068 | 12.701 | 0.100867 | 7/15/2014 | 13:32.4 | 2 |

| | | | | | | | |
|---|---|---|---|---|---|---|---|
| -950.381 | 19370.363 | 2.071 | 12.702 | 0.106049 | 7/15/2014 | 13:32.5 | 2 |
| -950.158 | 19370.354 | 2.071 | 12.694 | 0.0989739 | 7/15/2014 | 13:32.6 | 2 |
| -949.878 | 19370.342 | 2.071 | 12.689 | 0.0842708 | 7/15/2014 | 13:32.7 | 2 |
| -949.653 | 19370.332 | 2.068 | 12.682 | 0.0959172 | 7/15/2014 | 13:32.8 | 2 |
| -949.426 | 19370.324 | 2.071 | 12.673 | 0.0750694 | 7/15/2014 | 13:32.9 | 2 |
| -949.2 | 19370.313 | 2.071 | 12.664 | 0.0816748 | 7/15/2014 | 13:33.0 | 2 |
| -948.974 | 19370.305 | 2.071 | 12.652 | 0.0812574 | 7/15/2014 | 13:33.1 | 2 |
| -948.747 | 19370.293 | 2.074 | 12.647 | 0.0709948 | 7/15/2014 | 13:33.2 | 2 |
| -948.522 | 19370.285 | 2.07 | 12.637 | 0.0771308 | 7/15/2014 | 13:33.3 | 2 |
| -948.239 | 19370.271 | 2.076 | 12.637 | 0.110647 | 7/15/2014 | 13:33.4 | 2 |
| -948.012 | 19370.264 | 2.076 | 12.631 | 0.0970604 | 7/15/2014 | 13:33.5 | 2 |
| -947.786 | 19370.255 | 2.074 | 12.626 | 0.126158 | 7/15/2014 | 13:33.6 | 2 |
| -947.499 | 19370.242 | 2.07 | 12.623 | 0.109827 | 7/15/2014 | 13:33.7 | 2 |
| -947.269 | 19370.232 | 2.07 | 12.622 | 0.122506 | 7/15/2014 | 13:33.8 | 2 |
| -947.036 | 19370.223 | 2.073 | 12.623 | 0.121778 | 7/15/2014 | 13:33.9 | 2 |
| -946.804 | 19370.213 | 2.076 | 12.623 | 0.116239 | 7/15/2014 | 13:34.0 | 2 |
| -946.688 | 19370.207 | 2.078 | 12.624 | 0.1101 | 7/15/2014 | 13:34.1 | 2 |
| -946.458 | 19370.199 | 2.072 | 12.627 | 0.127649 | 7/15/2014 | 13:34.2 | 2 |
| -946.224 | 19370.188 | 2.074 | 12.636 | 0.0974579 | 7/15/2014 | 13:34.3 | 2 |
| -945.818 | 19370.172 | 2.081 | 12.647 | 0.0962326 | 7/15/2014 | 13:34.4 | 2 |
| -945.586 | 19370.16 | 2.084 | 12.656 | 0.0895745 | 7/15/2014 | 13:34.5 | 2 |
| -945.353 | 19370.152 | 2.087 | 12.671 | 0.0953916 | 7/15/2014 | 13:34.6 | 2 |
| -945.064 | 19370.139 | 2.085 | 12.675 | 0.0865659 | 7/15/2014 | 13:34.7 | 2 |
| -944.946 | 19370.135 | 2.083 | 12.68 | 0.0756319 | 7/15/2014 | 13:34.8 | 2 |
| -944.712 | 19370.125 | 2.086 | 12.679 | 0.094144 | 7/15/2014 | 13:34.9 | 2 |
| -944.479 | 19370.115 | 2.084 | 12.676 | 0.0716287 | 7/15/2014 | 13:35.0 | 2 |
| -944.245 | 19370.104 | 2.084 | 12.669 | 0.0881998 | 7/15/2014 | 13:35.1 | 2 |
| -944.013 | 19370.094 | 2.078 | 12.666 | 0.0795216 | 7/15/2014 | 13:35.2 | 2 |
| -943.779 | 19370.084 | 2.08 | 12.658 | 0.0789933 | 7/15/2014 | 13:35.3 | 2 |
| -943.376 | 19370.066 | 2.081 | 12.644 | 0.09316 | 7/15/2014 | 13:35.4 | 2 |
| -943.147 | 19370.057 | 2.081 | 12.644 | 0.0866219 | 7/15/2014 | 13:35.5 | 2 |
| -942.916 | 19370.047 | 2.083 | 12.646 | 0.093786 | 7/15/2014 | 13:35.6 | 2 |
| -942.63 | 19370.034 | 2.082 | 12.653 | 0.0994387 | 7/15/2014 | 13:35.7 | 2 |
| -942.399 | 19370.023 | 2.084 | 12.659 | 0.110723 | 7/15/2014 | 13:35.8 | 2 |
| -942.168 | 19370.012 | 2.082 | 12.666 | 0.124319 | 7/15/2014 | 13:35.9 | 2 |
| -941.937 | 19370.004 | 2.082 | 12.671 | 0.14443 | 7/15/2014 | 13:36.0 | 2 |
| -941.707 | 19369.992 | 2.079 | 12.677 | 0.1488 | 7/15/2014 | 13:36.1 | 2 |
| -941.477 | 19369.98 | 2.079 | 12.678 | 0.167412 | 7/15/2014 | 13:36.2 | 2 |
| -941.249 | 19369.972 | 2.077 | 12.678 | 0.159034 | 7/15/2014 | 13:36.3 | 2 |
| -940.966 | 19369.959 | 2.072 | 12.673 | 0.187602 | 7/15/2014 | 13:36.4 | 2 |
| -940.739 | 19369.947 | 2.072 | 12.671 | 0.185604 | 7/15/2014 | 13:36.5 | 2 |
| -940.512 | 19369.936 | 2.068 | 12.67 | 0.201963 | 7/15/2014 | 13:36.6 | 2 |
| -940.229 | 19369.924 | 2.071 | 12.668 | 0.160006 | 7/15/2014 | 13:36.7 | 2 |
| -940.001 | 19369.912 | 2.067 | 12.669 | 0.169939 | 7/15/2014 | 13:36.8 | 2 |
| -939.773 | 19369.9 | 2.067 | 12.668 | 0.155059 | 7/15/2014 | 13:36.9 | 2 |
| -939.545 | 19369.89 | 2.062 | 12.67 | 0.177216 | 7/15/2014 | 13:37.0 | 2 |
| -939.316 | 19369.88 | 2.064 | 12.67 | 0.164391 | 7/15/2014 | 13:37.1 | 2 |

| | | | | | | | |
|---|---|---|---|---|---|---|---|
| -939.088 | 19369.868 | 2.066 | 12.674 | 0.160621 | 7/15/2014 | 13:37.2 | 2 |
| -938.861 | 19369.857 | 2.064 | 12.67 | 0.141697 | 7/15/2014 | 13:37.3 | 2 |
| -938.579 | 19369.843 | 2.06 | 12.664 | 0.204291 | 7/15/2014 | 13:37.4 | 2 |
| -938.353 | 19369.832 | 2.058 | 12.665 | 0.174613 | 7/15/2014 | 13:37.5 | 2 |
| -938.128 | 19369.82 | 2.056 | 12.661 | 0.186024 | 7/15/2014 | 13:37.6 | 2 |
| -937.846 | 19369.805 | 2.054 | 12.657 | 0.167013 | 7/15/2014 | 13:37.7 | 2 |
| -937.619 | 19369.794 | 2.054 | 12.657 | 0.165024 | 7/15/2014 | 13:37.8 | 2 |
| -937.391 | 19369.782 | 2.052 | 12.657 | 0.171783 | 7/15/2014 | 13:37.9 | 2 |
| -937.165 | 19369.771 | 2.047 | 12.655 | 0.186871 | 7/15/2014 | 13:38.0 | 2 |
| -936.938 | 19369.759 | 2.047 | 12.655 | 0.143212 | 7/15/2014 | 13:38.1 | 2 |
| -936.712 | 19369.748 | 2.049 | 12.662 | 0.153927 | 7/15/2014 | 13:38.2 | 2 |
| -936.487 | 19369.736 | 2.049 | 12.663 | 0.146927 | 7/15/2014 | 13:38.3 | 2 |
| -936.207 | 19369.721 | 2.049 | 12.671 | 0.141133 | 7/15/2014 | 13:38.4 | 2 |
| -935.984 | 19369.709 | 2.049 | 12.684 | 0.142178 | 7/15/2014 | 13:38.5 | 2 |
| -935.76 | 19369.696 | 2.047 | 12.694 | 0.140502 | 7/15/2014 | 13:38.6 | 2 |
| -935.482 | 19369.682 | 2.044 | 12.707 | 0.137473 | 7/15/2014 | 13:38.7 | 2 |
| -935.256 | 19369.67 | 2.046 | 12.719 | 0.137087 | 7/15/2014 | 13:38.8 | 2 |
| -935.03 | 19369.656 | 2.046 | 12.731 | 0.140887 | 7/15/2014 | 13:38.9 | 2 |
| -934.803 | 19369.645 | 2.044 | 12.739 | 0.139818 | 7/15/2014 | 13:39.0 | 2 |
| -934.578 | 19369.632 | 2.044 | 12.747 | 0.142228 | 7/15/2014 | 13:39.1 | 2 |
| -934.352 | 19369.62 | 2.04 | 12.752 | 0.1472 | 7/15/2014 | 13:39.2 | 2 |
| -934.127 | 19369.606 | 2.04 | 12.753 | 0.144687 | 7/15/2014 | 13:39.3 | 2 |
| -933.848 | 19369.591 | 2.04 | 12.757 | 0.143152 | 7/15/2014 | 13:39.4 | 2 |
| -933.622 | 19369.578 | 2.043 | 12.761 | 0.1503 | 7/15/2014 | 13:39.5 | 2 |
| -933.397 | 19369.563 | 2.041 | 12.764 | 0.152638 | 7/15/2014 | 13:39.6 | 2 |
| -933.114 | 19369.547 | 2.041 | 12.768 | 0.158259 | 7/15/2014 | 13:39.7 | 2 |
| -932.887 | 19369.534 | 2.041 | 12.773 | 0.15428 | 7/15/2014 | 13:39.8 | 2 |
| -932.659 | 19369.521 | 2.04 | 12.778 | 0.16022 | 7/15/2014 | 13:39.9 | 2 |
| -932.428 | 19369.507 | 2.044 | 12.78 | 0.154342 | 7/15/2014 | 13:40.0 | 2 |
| -932.199 | 19369.493 | 2.044 | 12.78 | 0.164541 | 7/15/2014 | 13:40.1 | 2 |
| -931.969 | 19369.479 | 2.046 | 12.774 | 0.161241 | 7/15/2014 | 13:40.2 | 2 |
| -931.741 | 19369.465 | 2.048 | 12.765 | 0.156152 | 7/15/2014 | 13:40.3 | 2 |
| -931.456 | 19369.447 | 2.05 | 12.761 | 0.155965 | 7/15/2014 | 13:40.4 | 2 |
| -931.228 | 19369.435 | 2.05 | 12.754 | 0.15007 | 7/15/2014 | 13:40.5 | 2 |
| -931 | 19369.42 | 2.053 | 12.747 | 0.145924 | 7/15/2014 | 13:40.6 | 2 |
| -930.712 | 19369.401 | 2.056 | 12.74 | 0.142028 | 7/15/2014 | 13:40.7 | 2 |
| -930.481 | 19369.387 | 2.056 | 12.735 | 0.137085 | 7/15/2014 | 13:40.8 | 2 |
| -930.249 | 19369.371 | 2.056 | 12.729 | 0.129829 | 7/15/2014 | 13:40.9 | 2 |
| -930.017 | 19369.357 | 2.056 | 12.723 | 0.128322 | 7/15/2014 | 13:41.0 | 2 |
| -929.786 | 19369.343 | 2.053 | 12.712 | 0.131122 | 7/15/2014 | 13:41.1 | 2 |
| -929.553 | 19369.328 | 2.053 | 12.701 | 0.120977 | 7/15/2014 | 13:41.2 | 2 |
| -929.439 | 19369.32 | 2.054 | 12.696 | 0.122038 | 7/15/2014 | 13:41.3 | 2 |
| -929.035 | 19369.294 | 2.06 | 12.672 | 0.129831 | 7/15/2014 | 13:41.4 | 2 |
| -928.806 | 19369.278 | 2.06 | 12.655 | 0.124514 | 7/15/2014 | 13:41.5 | 2 |
| -928.575 | 19369.264 | 2.06 | 12.641 | 0.122186 | 7/15/2014 | 13:41.6 | 2 |
| -928.287 | 19369.245 | 2.062 | 12.628 | 0.120394 | 7/15/2014 | 13:41.7 | 2 |
| -928.172 | 19369.237 | 2.062 | 12.62 | 0.119401 | 7/15/2014 | 13:41.8 | 2 |

| | | | | | | | |
|---|---|---|---|---|---|---|---|
| -927.938 | 19369.221 | 2.068 | 12.613 | 0.118964 | 7/15/2014 | 13:41.9 | 2 |
| -927.707 | 19369.205 | 2.068 | 12.607 | 0.123707 | 7/15/2014 | 13:42.0 | 2 |
| -927.473 | 19369.188 | 2.074 | 12.605 | 0.130575 | 7/15/2014 | 13:42.1 | 2 |
| -927.243 | 19369.173 | 2.068 | 12.607 | 0.121739 | 7/15/2014 | 13:42.2 | 2 |
| -927.01 | 19369.158 | 2.074 | 12.616 | 0.12577 | 7/15/2014 | 13:42.3 | 2 |
| -926.607 | 19369.13 | 2.08 | 12.62 | 0.127181 | 7/15/2014 | 13:42.4 | 2 |
| -926.377 | 19369.113 | 2.08 | 12.628 | 0.124422 | 7/15/2014 | 13:42.5 | 2 |
| -926.148 | 19369.097 | 2.081 | 12.634 | 0.119444 | 7/15/2014 | 13:42.6 | 2 |
| -925.859 | 19369.076 | 2.084 | 12.639 | 0.117529 | 7/15/2014 | 13:42.7 | 2 |
| -925.744 | 19369.068 | 2.082 | 12.643 | 0.121809 | 7/15/2014 | 13:42.8 | 2 |
| -925.395 | 19369.043 | 2.082 | 12.643 | 0.113808 | 7/15/2014 | 13:42.9 | 2 |
| -925.162 | 19369.027 | 2.079 | 12.642 | 0.111739 | 7/15/2014 | 13:43.0 | 2 |
| -925.045 | 19369.019 | 2.082 | 12.645 | 0.107352 | 7/15/2014 | 13:43.1 | 2 |
| -924.815 | 19369.001 | 2.073 | 12.651 | 0.106105 | 7/15/2014 | 13:43.2 | 2 |
| -924.582 | 19368.984 | 2.079 | 12.65 | 0.108175 | 7/15/2014 | 13:43.3 | 2 |
| -924.177 | 19368.955 | 2.082 | 12.65 | 0.106391 | 7/15/2014 | 13:43.4 | 2 |
| -923.948 | 19368.938 | 2.081 | 12.646 | 0.107984 | 7/15/2014 | 13:43.5 | 2 |
| -923.716 | 19368.921 | 2.083 | 12.64 | 0.105943 | 7/15/2014 | 13:43.6 | 2 |
| -923.428 | 19368.9 | 2.083 | 12.639 | 0.112451 | 7/15/2014 | 13:43.7 | 2 |
| -923.312 | 19368.891 | 2.084 | 12.636 | 0.111102 | 7/15/2014 | 13:43.8 | 2 |
| -923.078 | 19368.875 | 2.087 | 12.629 | 0.116664 | 7/15/2014 | 13:43.9 | 2 |
| -922.733 | 19368.85 | 2.084 | 12.623 | 0.112671 | 7/15/2014 | 13:44.0 | 2 |
| -922.501 | 19368.831 | 2.081 | 12.622 | 0.125614 | 7/15/2014 | 13:44.1 | 2 |
| -922.271 | 19368.814 | 2.078 | 12.618 | 0.125905 | 7/15/2014 | 13:44.2 | 2 |
| -922.041 | 19368.798 | 2.075 | 12.623 | 0.126906 | 7/15/2014 | 13:44.3 | 2 |
| -921.756 | 19368.776 | 2.072 | 12.642 | 0.124871 | 7/15/2014 | 13:44.4 | 2 |
| -921.528 | 19368.76 | 2.072 | 12.66 | 0.126901 | 7/15/2014 | 13:44.5 | 2 |
| -921.302 | 19368.741 | 2.07 | 12.674 | 0.128814 | 7/15/2014 | 13:44.6 | 2 |
| -921.017 | 19368.721 | 2.07 | 12.684 | 0.133129 | 7/15/2014 | 13:44.7 | 2 |
| -920.787 | 19368.704 | 2.07 | 12.695 | 0.129262 | 7/15/2014 | 13:44.8 | 2 |
| -920.555 | 19368.686 | 2.07 | 12.701 | 0.126682 | 7/15/2014 | 13:44.9 | 2 |
| -920.325 | 19368.669 | 2.067 | 12.705 | 0.134607 | 7/15/2014 | 13:45.0 | 2 |
| -920.093 | 19368.651 | 2.067 | 12.703 | 0.136394 | 7/15/2014 | 13:45.1 | 2 |
| -919.862 | 19368.634 | 2.071 | 12.686 | 0.137685 | 7/15/2014 | 13:45.2 | 2 |
| -919.749 | 19368.625 | 2.072 | 12.675 | 0.140965 | 7/15/2014 | 13:45.3 | 2 |
| -919.346 | 19368.594 | 2.079 | 12.652 | 0.134997 | 7/15/2014 | 13:45.4 | 2 |
| -919.115 | 19368.578 | 2.081 | 12.641 | 0.134984 | 7/15/2014 | 13:45.5 | 2 |
| -918.885 | 19368.56 | 2.083 | 12.631 | 0.132524 | 7/15/2014 | 13:45.6 | 2 |
| -918.597 | 19368.537 | 2.083 | 12.628 | 0.133071 | 7/15/2014 | 13:45.7 | 2 |
| -918.479 | 19368.528 | 2.087 | 12.622 | 0.133914 | 7/15/2014 | 13:45.8 | 2 |
| -918.246 | 19368.511 | 2.087 | 12.622 | 0.138672 | 7/15/2014 | 13:45.9 | 2 |
| -918.014 | 19368.493 | 2.081 | 12.628 | 0.139496 | 7/15/2014 | 13:46.0 | 2 |
| -917.779 | 19368.475 | 2.079 | 12.635 | 0.135999 | 7/15/2014 | 13:46.1 | 2 |
| -917.546 | 19368.458 | 2.071 | 12.644 | 0.132238 | 7/15/2014 | 13:46.2 | 2 |
| -917.313 | 19368.44 | 2.067 | 12.653 | 0.129751 | 7/15/2014 | 13:46.3 | 2 |
| -916.907 | 19368.409 | 2.066 | 12.658 | 0.14804 | 7/15/2014 | 13:46.4 | 2 |
| -916.676 | 19368.391 | 2.066 | 12.658 | 0.135811 | 7/15/2014 | 13:46.5 | 2 |

| | | | | | | | |
|---|---|---|---|---|---|---|---|
| -916.444 | 19368.374 | 2.072 | 12.659 | 0.134523 | 7/15/2014 | 13:46.6 | 2 |
| -916.155 | 19368.352 | 2.075 | 12.659 | 0.133631 | 7/15/2014 | 13:46.7 | 2 |
| -916.038 | 19368.343 | 2.075 | 12.659 | 0.133769 | 7/15/2014 | 13:46.8 | 2 |
| -915.805 | 19368.325 | 2.083 | 12.657 | 0.126098 | 7/15/2014 | 13:46.9 | 2 |
| -915.576 | 19368.307 | 2.081 | 12.652 | 0.116017 | 7/15/2014 | 13:47.0 | 2 |
| -915.237 | 19368.282 | 2.085 | 12.663 | 0.118887 | 7/15/2014 | 13:47.1 | 2 |
| -915.017 | 19368.265 | 2.087 | 12.665 | 0.115261 | 7/15/2014 | 13:47.2 | 2 |
| -914.804 | 19368.249 | 2.087 | 12.67 | 0.112191 | 7/15/2014 | 13:47.3 | 2 |
| -914.456 | 19368.223 | 2.089 | 12.675 | 0.112786 | 7/15/2014 | 13:47.5 | 2 |
| -914.265 | 19368.208 | 2.086 | 12.674 | 0.126209 | 7/15/2014 | 13:47.6 | 2 |
| -914.045 | 19368.19 | 2.085 | 12.665 | 0.142523 | 7/15/2014 | 13:47.7 | 2 |
| -913.822 | 19368.175 | 2.084 | 12.655 | 0.131652 | 7/15/2014 | 13:47.8 | 2 |
| -913.629 | 19368.159 | 2.082 | 12.646 | 0.138506 | 7/15/2014 | 13:47.9 | 2 |
| -913.424 | 19368.144 | 2.084 | 12.635 | 0.174079 | 7/15/2014 | 13:47.9 | 2 |
| -912.539 | 19368.077 | 2.082 | 12.63 | 0.11984 | 7/15/2014 | 13:48.0 | 2 |
| -912.446 | 19368.07 | 2.084 | 12.627 | 0.120546 | 7/15/2014 | 13:48.1 | 2 |
| -912.363 | 19368.064 | 2.083 | 12.624 | 0.118078 | 7/15/2014 | 13:48.1 | 2 |
| -912.287 | 19368.059 | 2.081 | 12.617 | 0.116155 | 7/15/2014 | 13:48.1 | 2 |
| -912.214 | 19368.053 | 2.083 | 12.615 | 0.122649 | 7/15/2014 | 13:48.1 | 2 |
| -912.179 | 19368.05 | 2.084 | 12.613 | 0.109856 | 7/15/2014 | 13:48.1 | 2 |
| -912.145 | 19368.047 | 2.082 | 12.613 | 0.11071 | 7/15/2014 | 13:48.1 | 2 |
| -912.107 | 19368.045 | 2.088 | 12.611 | 0.106564 | 7/15/2014 | 13:48.1 | 2 |
| -912.069 | 19368.042 | 2.086 | 12.61 | 0.107584 | 7/15/2014 | 13:48.2 | 2 |
| -912.028 | 19368.039 | 2.088 | 12.608 | 0.109966 | 7/15/2014 | 13:48.2 | 2 |
| -911.984 | 19368.035 | 2.084 | 12.607 | 0.101606 | 7/15/2014 | 13:48.3 | 2 |
| -911.935 | 19368.032 | 2.093 | 12.609 | 0.102094 | 7/15/2014 | 13:48.3 | 2 |
| -911.883 | 19368.027 | 2.089 | 12.613 | 0.100442 | 7/15/2014 | 13:48.5 | 2 |
| -910.346 | 19367.914 | 2.08 | 12.649 | 0.113827 | 7/15/2014 | 13:49.2 | 2 |
| -909.888 | 19367.881 | 2.076 | 12.673 | 0.114819 | 7/15/2014 | 13:49.4 | 2 |
| -909.522 | 19367.854 | 2.081 | 12.676 | 0.122542 | 7/15/2014 | 13:49.5 | 2 |
| -909.278 | 19367.834 | 2.078 | 12.679 | 0.12102 | 7/15/2014 | 13:49.6 | 2 |
| -908.985 | 19367.813 | 2.078 | 12.68 | 0.124006 | 7/15/2014 | 13:49.7 | 2 |
| -908.752 | 19367.797 | 2.078 | 12.684 | 0.125691 | 7/15/2014 | 13:49.8 | 2 |
| -908.521 | 19367.779 | 2.078 | 12.697 | 0.133171 | 7/15/2014 | 13:49.9 | 2 |
| -908.293 | 19367.763 | 2.078 | 12.716 | 0.134341 | 7/15/2014 | 13:50.0 | 2 |
| -908.067 | 19367.745 | 2.078 | 12.72 | 0.139157 | 7/15/2014 | 13:50.1 | 2 |
| -907.843 | 19367.729 | 2.075 | 12.74 | 0.1535 | 7/15/2014 | 13:50.2 | 2 |
| -907.622 | 19367.713 | 2.071 | 12.757 | 0.163579 | 7/15/2014 | 13:50.3 | 2 |
| -907.346 | 19367.692 | 2.071 | 12.775 | 0.184422 | 7/15/2014 | 13:50.4 | 2 |
| -907.124 | 19367.676 | 2.071 | 12.791 | 0.218791 | 7/15/2014 | 13:50.5 | 2 |
| -906.903 | 19367.66 | 2.071 | 12.805 | 0.249749 | 7/15/2014 | 13:50.6 | 2 |
| -906.625 | 19367.64 | 2.071 | 12.802 | 0.278593 | 7/15/2014 | 13:50.7 | 2 |
| -906.4 | 19367.622 | 2.07 | 12.799 | 0.285872 | 7/15/2014 | 13:50.8 | 2 |
| -906.175 | 19367.605 | 2.068 | 12.792 | 0.314177 | 7/15/2014 | 13:50.9 | 2 |
| -905.95 | 19367.59 | 2.068 | 12.782 | 0.34517 | 7/15/2014 | 13:51.0 | 2 |
| -905.724 | 19367.573 | 2.068 | 12.776 | 0.357745 | 7/15/2014 | 13:51.1 | 2 |
| -905.5 | 19367.556 | 2.064 | 12.773 | 0.373674 | 7/15/2014 | 13:51.2 | 2 |

| | | | | | | | |
|---|---|---|---|---|---|---|---|
| -905.275 | 19367.539 | 2.061 | 12.77 | 0.3659 | 7/15/2014 | 13:51.3 | 2 |
| -904.996 | 19367.52 | 2.054 | 12.769 | 0.399433 | 7/15/2014 | 13:51.4 | 2 |
| -904.771 | 19367.502 | 2.05 | 12.769 | 0.411629 | 7/15/2014 | 13:51.5 | 2 |
| -904.547 | 19367.485 | 2.045 | 12.765 | 0.384958 | 7/15/2014 | 13:51.6 | 2 |
| -904.265 | 19367.465 | 2.041 | 12.766 | 0.387865 | 7/15/2014 | 13:51.7 | 2 |
| -904.037 | 19367.448 | 2.041 | 12.766 | 0.368343 | 7/15/2014 | 13:51.8 | 2 |
| -903.81 | 19367.431 | 2.039 | 12.767 | 0.35218 | 7/15/2014 | 13:51.9 | 2 |
| -903.58 | 19367.414 | 2.039 | 12.766 | 0.352675 | 7/15/2014 | 13:52.0 | 2 |
| -903.35 | 19367.396 | 2.034 | 12.763 | 0.32262 | 7/15/2014 | 13:52.1 | 2 |
| -903.118 | 19367.38 | 2.028 | 12.762 | 0.305287 | 7/15/2014 | 13:52.2 | 2 |
| -903.006 | 19367.372 | 2.029 | 12.76 | 0.301392 | 7/15/2014 | 13:52.3 | 2 |
| -902.597 | 19367.341 | 2.02 | 12.766 | 0.27281 | 7/15/2014 | 13:52.4 | 2 |
| -902.361 | 19367.323 | 2.02 | 12.775 | 0.271626 | 7/15/2014 | 13:52.5 | 2 |
| -902.126 | 19367.305 | 2.013 | 12.79 | 0.24815 | 7/15/2014 | 13:52.6 | 2 |
| -901.89 | 19367.289 | 2.01 | 12.806 | 0.223585 | 7/15/2014 | 13:52.7 | 2 |
| -901.652 | 19367.27 | 2.005 | 12.822 | 0.201136 | 7/15/2014 | 13:52.8 | 2 |
| -901.411 | 19367.254 | 2.005 | 12.838 | 0.193809 | 7/15/2014 | 13:52.9 | 2 |
| -901.172 | 19367.234 | 2.008 | 12.852 | 0.175356 | 7/15/2014 | 13:53.0 | 2 |
| -900.932 | 19367.217 | 2.011 | 12.865 | 0.167323 | 7/15/2014 | 13:53.1 | 2 |
| -900.691 | 19367.199 | 2.011 | 12.872 | 0.158853 | 7/15/2014 | 13:53.2 | 2 |
| -900.454 | 19367.181 | 2.005 | 12.873 | 0.152378 | 7/15/2014 | 13:53.3 | 2 |
| -900.218 | 19367.164 | 1.998 | 12.87 | 0.148369 | 7/15/2014 | 13:53.4 | 2 |
| -899.983 | 19367.146 | 1.995 | 12.864 | 0.144544 | 7/15/2014 | 13:53.5 | 2 |
| -899.748 | 19367.13 | 1.992 | 12.857 | 0.144265 | 7/15/2014 | 13:53.6 | 2 |
| -899.456 | 19367.107 | 1.989 | 12.851 | 0.13932 | 7/15/2014 | 13:53.7 | 2 |
| -899.338 | 19367.099 | 1.984 | 12.846 | 0.138105 | 7/15/2014 | 13:53.8 | 2 |
| -898.988 | 19367.072 | 1.979 | 12.842 | 0.137395 | 7/15/2014 | 13:53.9 | 2 |
| -898.755 | 19367.056 | 1.973 | 12.843 | 0.127126 | 7/15/2014 | 13:54.0 | 2 |
| -898.521 | 19367.037 | 1.973 | 12.847 | 0.11904 | 7/15/2014 | 13:54.1 | 2 |
| -898.406 | 19367.028 | 1.973 | 12.848 | 0.11288 | 7/15/2014 | 13:54.2 | 2 |
| -898.173 | 19367.012 | 1.971 | 12.852 | 0.105247 | 7/15/2014 | 13:54.3 | 2 |
| -897.762 | 19366.981 | 1.967 | 12.842 | 0.105791 | 7/15/2014 | 13:54.4 | 2 |
| -897.526 | 19366.964 | 1.962 | 12.834 | 0.102534 | 7/15/2014 | 13:54.5 | 2 |
| -897.288 | 19366.945 | 1.962 | 12.829 | 0.0995409 | 7/15/2014 | 13:54.6 | 2 |
| -897.051 | 19366.929 | 1.958 | 12.818 | 0.0967564 | 7/15/2014 | 13:54.7 | 2 |
| -896.811 | 19366.91 | 1.956 | 12.808 | 0.101907 | 7/15/2014 | 13:54.8 | 2 |
| -896.572 | 19366.893 | 1.952 | 12.801 | 0.100752 | 7/15/2014 | 13:54.9 | 2 |
| -896.332 | 19366.874 | 1.952 | 12.797 | 0.0946845 | 7/15/2014 | 13:55.0 | 2 |
| -896.094 | 19366.856 | 1.952 | 12.798 | 0.0892969 | 7/15/2014 | 13:55.1 | 2 |
| -895.855 | 19366.84 | 1.95 | 12.804 | 0.0858064 | 7/15/2014 | 13:55.2 | 2 |
| -895.617 | 19366.82 | 1.947 | 12.815 | 0.0823019 | 7/15/2014 | 13:55.3 | 2 |
| -895.382 | 19366.804 | 1.942 | 12.827 | 0.0807486 | 7/15/2014 | 13:55.4 | 2 |
| -895.147 | 19366.786 | 1.936 | 12.838 | 0.0747911 | 7/15/2014 | 13:55.5 | 2 |
| -894.913 | 19366.769 | 1.931 | 12.848 | 0.0722681 | 7/15/2014 | 13:55.6 | 2 |
| -894.625 | 19366.747 | 1.924 | 12.855 | 0.0619838 | 7/15/2014 | 13:55.7 | 2 |
| -894.392 | 19366.729 | 1.919 | 12.86 | 0.063345 | 7/15/2014 | 13:55.8 | 2 |
| -894.159 | 19366.713 | 1.914 | 12.877 | 0.0513021 | 7/15/2014 | 13:55.9 | 2 |

| | | | | | | | |
|---|---|---|---|---|---|---|---|
| -893.926 | 19366.694 | 1.911 | 12.883 | 0.0510918 | 7/15/2014 | 13:56.0 | 2 |
| -893.695 | 19366.678 | 1.907 | 12.892 | 0.0479803 | 7/15/2014 | 13:56.1 | 2 |
| -893.462 | 19366.66 | 1.904 | 12.921 | 0.0537893 | 7/15/2014 | 13:56.2 | 2 |
| -893.347 | 19366.651 | 1.907 | 12.929 | 0.0512845 | 7/15/2014 | 13:56.3 | 2 |
| -892.94 | 19366.621 | 1.904 | 12.974 | 0.0559424 | 7/15/2014 | 13:56.4 | 2 |
| -892.706 | 19366.604 | 1.902 | 12.993 | 0.0458569 | 7/15/2014 | 13:56.5 | 2 |
| -892.47 | 19366.586 | 1.899 | 13.016 | 0.0607453 | 7/15/2014 | 13:56.6 | 2 |
| -892.233 | 19366.568 | 1.894 | 13.02 | 0.0539255 | 7/15/2014 | 13:56.7 | 2 |
| -891.997 | 19366.551 | 1.89 | 13.05 | 0.0537643 | 7/15/2014 | 13:56.8 | 2 |
| -891.758 | 19366.532 | 1.888 | 13.074 | 0.0394265 | 7/15/2014 | 13:56.9 | 2 |
| -891.52 | 19366.516 | 1.886 | 13.079 | 0.0429273 | 7/15/2014 | 13:57.0 | 2 |
| -891.283 | 19366.497 | 1.882 | 13.084 | 0.0436391 | 7/15/2014 | 13:57.1 | 2 |
| -891.046 | 19366.48 | 1.876 | 13.093 | 0.0390659 | 7/15/2014 | 13:57.2 | 2 |
| -890.81 | 19366.462 | 1.873 | 13.105 | 0.0298851 | 7/15/2014 | 13:57.3 | 2 |
| -890.575 | 19366.444 | 1.871 | 13.114 | 0.032494 | 7/15/2014 | 13:57.4 | 2 |
| -890.343 | 19366.427 | 1.869 | 13.128 | 0.0454093 | 7/15/2014 | 13:57.5 | 2 |
| -890.11 | 19366.408 | 1.867 | 13.128 | 0.0517774 | 7/15/2014 | 13:57.6 | 2 |
| -889.822 | 19366.389 | 1.862 | 13.126 | 0.0565905 | 7/15/2014 | 13:57.7 | 2 |
| -889.592 | 19366.37 | 1.858 | 13.124 | 0.0743017 | 7/15/2014 | 13:57.8 | 2 |
| -889.36 | 19366.354 | 1.853 | 13.123 | 0.0912493 | 7/15/2014 | 13:57.9 | 2 |
| -889.127 | 19366.335 | 1.85 | 13.12 | 0.102565 | 7/15/2014 | 13:58.0 | 2 |
| -888.896 | 19366.318 | 1.848 | 13.114 | 0.100117 | 7/15/2014 | 13:58.1 | 2 |
| -888.664 | 19366.3 | 1.844 | 13.112 | 0.0960776 | 7/15/2014 | 13:58.2 | 2 |
| -888.55 | 19366.291 | 1.843 | 13.112 | 0.106685 | 7/15/2014 | 13:58.3 | 2 |
| -888.143 | 19366.261 | 1.834 | 13.093 | 0.0963548 | 7/15/2014 | 13:58.4 | 2 |
| -887.908 | 19366.242 | 1.831 | 13.092 | 0.0774691 | 7/15/2014 | 13:58.5 | 2 |
| -887.672 | 19366.225 | 1.826 | 13.081 | 0.0565134 | 7/15/2014 | 13:58.6 | 2 |
| -887.433 | 19366.206 | 1.828 | 13.074 | 0.0398919 | 7/15/2014 | 13:58.7 | 2 |
| -887.196 | 19366.188 | 1.826 | 13.068 | 0.0385501 | 7/15/2014 | 13:58.8 | 2 |
| -886.955 | 19366.169 | 1.826 | 13.062 | 0.029663 | 7/15/2014 | 13:58.9 | 2 |
| -886.715 | 19366.15 | 1.821 | 13.056 | 0.0268258 | 7/15/2014 | 13:59.0 | 2 |
| -886.475 | 19366.132 | 1.823 | 13.058 | 0.0294117 | 7/15/2014 | 13:59.1 | 2 |
| -886.236 | 19366.114 | 1.821 | 13.057 | 0.0307172 | 7/15/2014 | 13:59.2 | 2 |
| -885.997 | 19366.095 | 1.821 | 13.049 | 0.0296534 | 7/15/2014 | 13:59.3 | 2 |
| -885.759 | 19366.077 | 1.821 | 13.046 | 0.0308152 | 7/15/2014 | 13:59.4 | 2 |
| -885.524 | 19366.059 | 1.816 | 13.043 | 0.0320085 | 7/15/2014 | 13:59.5 | 2 |
| -885.288 | 19366.04 | 1.816 | 13.046 | 0.0302251 | 7/15/2014 | 13:59.6 | 2 |
| -884.998 | 19366.018 | 1.814 | 13.051 | 0.0294258 | 7/15/2014 | 13:59.7 | 2 |
| -884.879 | 19366.008 | 1.814 | 13.051 | 0.0340911 | 7/15/2014 | 13:59.8 | 2 |
| -884.532 | 19365.981 | 1.814 | 13.058 | 0.0436267 | 7/15/2014 | 13:59.9 | 2 |
| -884.299 | 19365.962 | 1.816 | 13.073 | 0.0393681 | 7/15/2014 | 14:00.0 | 2 |
| -884.067 | 19365.943 | 1.813 | 13.086 | 0.0394736 | 7/15/2014 | 14:00.1 | 2 |
| -883.836 | 19365.925 | 1.807 | 13.1 | 0.0671979 | 7/15/2014 | 14:00.2 | 2 |
| -883.602 | 19365.906 | 1.804 | 13.113 | 0.0439062 | 7/15/2014 | 14:00.3 | 2 |
| -883.312 | 19365.884 | 1.802 | 13.121 | 0.0536835 | 7/15/2014 | 14:00.4 | 2 |
| -883.08 | 19365.865 | 1.796 | 13.128 | 0.0512697 | 7/15/2014 | 14:00.5 | 2 |
| -882.844 | 19365.848 | 1.794 | 13.137 | 0.0427376 | 7/15/2014 | 14:00.6 | 2 |

| | | | | | | | |
|---|---|---|---|---|---|---|---|
| -882.609 | 19365.828 | 1.79 | 13.139 | 0.0455686 | 7/15/2014 | 14:00.7 | 2 |
| -882.374 | 19365.809 | 1.785 | 13.134 | 0.0454142 | 7/15/2014 | 14:00.8 | 2 |
| -882.136 | 19365.787 | 1.782 | 13.13 | 0.0337321 | 7/15/2014 | 14:00.9 | 2 |
| -881.898 | 19365.768 | 1.782 | 13.126 | 0.0445764 | 7/15/2014 | 14:01.0 | 2 |
| -881.659 | 19365.749 | 1.785 | 13.12 | 0.0493079 | 7/15/2014 | 14:01.1 | 2 |
| -881.424 | 19365.73 | 1.782 | 13.114 | 0.0416408 | 7/15/2014 | 14:01.2 | 2 |
| -881.186 | 19365.711 | 1.785 | 13.107 | 0.0281307 | 7/15/2014 | 14:01.3 | 2 |
| -880.951 | 19365.691 | 1.785 | 13.096 | 0.0235138 | 7/15/2014 | 14:01.4 | 2 |
| -880.718 | 19365.672 | 1.785 | 13.088 | 0.0214443 | 7/15/2014 | 14:01.5 | 2 |
| -880.483 | 19365.65 | 1.788 | 13.084 | 0.0197896 | 7/15/2014 | 14:01.6 | 2 |
| -880.196 | 19365.627 | 1.788 | 13.076 | 0.021765 | 7/15/2014 | 14:01.7 | 2 |
| -879.962 | 19365.607 | 1.791 | 13.065 | 0.0414367 | 7/15/2014 | 14:01.8 | 2 |
| -879.73 | 19365.588 | 1.791 | 13.062 | 0.0465653 | 7/15/2014 | 14:01.9 | 2 |
| -879.497 | 19365.566 | 1.794 | 13.052 | 0.03946 | 7/15/2014 | 14:02.0 | 2 |
| -879.262 | 19365.547 | 1.794 | 13.043 | 0.0483698 | 7/15/2014 | 14:02.1 | 2 |
| -879.149 | 19365.537 | 1.791 | 13.038 | 0.0512247 | 7/15/2014 | 14:02.2 | 2 |
| -878.914 | 19365.518 | 1.795 | 13.028 | 0.0556735 | 7/15/2014 | 14:02.3 | 2 |
| -878.505 | 19365.482 | 1.792 | 13.01 | 0.0512528 | 7/15/2014 | 14:02.4 | 2 |
| -878.268 | 19365.463 | 1.796 | 13.005 | 0.0422019 | 7/15/2014 | 14:02.5 | 2 |
| -878.03 | 19365.441 | 1.798 | 13.001 | 0.0365943 | 7/15/2014 | 14:02.6 | 2 |
| -877.793 | 19365.42 | 1.798 | 12.994 | 0.039532 | 7/15/2014 | 14:02.7 | 2 |
| -877.553 | 19365.398 | 1.798 | 12.997 | 0.0359877 | 7/15/2014 | 14:02.8 | 2 |
| -877.312 | 19365.379 | 1.798 | 13.005 | 0.0281493 | 7/15/2014 | 14:02.9 | 2 |
| -877.072 | 19365.355 | 1.8 | 13.011 | 0.0299228 | 7/15/2014 | 14:03.0 | 2 |
| -876.832 | 19365.336 | 1.802 | 13.016 | 0.039157 | 7/15/2014 | 14:03.1 | 2 |
| -876.591 | 19365.314 | 1.805 | 13.017 | 0.0387818 | 7/15/2014 | 14:03.2 | 2 |
| -876.35 | 19365.293 | 1.81 | 13.018 | 0.0400797 | 7/15/2014 | 14:03.3 | 2 |
| -876.114 | 19365.273 | 1.81 | 13.016 | 0.04575 | 7/15/2014 | 14:03.4 | 2 |
| -875.879 | 19365.25 | 1.813 | 13.017 | 0.0480952 | 7/15/2014 | 14:03.5 | 2 |
| -875.643 | 19365.23 | 1.816 | 13.008 | 0.0537408 | 7/15/2014 | 14:03.6 | 2 |
| -875.353 | 19365.203 | 1.816 | 12.999 | 0.0606835 | 7/15/2014 | 14:03.7 | 2 |
| -875.234 | 19365.193 | 1.814 | 12.994 | 0.0555938 | 7/15/2014 | 14:03.8 | 2 |
| -874.884 | 19365.16 | 1.824 | 12.986 | 0.0809842 | 7/15/2014 | 14:03.9 | 2 |
| -874.651 | 19365.14 | 1.827 | 12.997 | 0.0723877 | 7/15/2014 | 14:04.0 | 2 |
| -874.42 | 19365.119 | 1.827 | 13 | 0.0795534 | 7/15/2014 | 14:04.1 | 2 |
| -874.189 | 19365.096 | 1.831 | 13.015 | 0.0918764 | 7/15/2014 | 14:04.2 | 2 |
| -874.074 | 19365.086 | 1.835 | 13.013 | 0.0864645 | 7/15/2014 | 14:04.3 | 2 |
| -873.674 | 19365.049 | 1.84 | 13.043 | 0.0741197 | 7/15/2014 | 14:04.4 | 2 |
| -873.444 | 19365.027 | 1.849 | 13.062 | 0.0784317 | 7/15/2014 | 14:04.5 | 2 |
| -873.16 | 19365 | 1.846 | 13.078 | 0.0769135 | 7/15/2014 | 14:04.6 | 2 |
| -873.044 | 19364.989 | 1.848 | 13.091 | 0.0821865 | 7/15/2014 | 14:04.7 | 2 |
| -872.702 | 19364.957 | 1.851 | 13.102 | 0.0727188 | 7/15/2014 | 14:04.8 | 2 |
| -872.472 | 19364.934 | 1.848 | 13.108 | 0.0683692 | 7/15/2014 | 14:04.9 | 2 |
| -872.243 | 19364.913 | 1.844 | 13.103 | 0.0625983 | 7/15/2014 | 14:05.0 | 2 |
| -872.014 | 19364.891 | 1.838 | 13.088 | 0.0634836 | 7/15/2014 | 14:05.1 | 2 |
| -871.784 | 19364.868 | 1.838 | 13.084 | 0.0622473 | 7/15/2014 | 14:05.2 | 2 |
| -871.504 | 19364.841 | 1.832 | 13.079 | 0.057357 | 7/15/2014 | 14:05.3 | 2 |

| | | | | | | | |
|---|---|---|---|---|---|---|---|
| -871.276 | 19364.818 | 1.833 | 13.079 | 0.0624117 | 7/15/2014 | 14:05.4 | 2 |
| -871.052 | 19364.797 | 1.83 | 13.077 | 0.059765 | 7/15/2014 | 14:05.5 | 2 |
| -870.828 | 19364.774 | 1.83 | 13.078 | 0.0593679 | 7/15/2014 | 14:05.6 | 2 |
| -870.55 | 19364.746 | 1.833 | 13.088 | 0.0581679 | 7/15/2014 | 14:05.7 | 2 |
| -870.323 | 19364.725 | 1.836 | 13.101 | 0.0694769 | 7/15/2014 | 14:05.8 | 2 |
| -870.097 | 19364.701 | 1.836 | 13.11 | 0.0627394 | 7/15/2014 | 14:05.9 | 2 |
| -869.87 | 19364.679 | 1.833 | 13.118 | 0.0473302 | 7/15/2014 | 14:06.0 | 2 |
| -869.643 | 19364.655 | 1.836 | 13.121 | 0.0486803 | 7/15/2014 | 14:06.1 | 2 |
| -869.415 | 19364.633 | 1.836 | 13.12 | 0.0463991 | 7/15/2014 | 14:06.2 | 2 |
| -869.188 | 19364.61 | 1.835 | 13.121 | 0.0481691 | 7/15/2014 | 14:06.3 | 2 |
| -868.904 | 19364.58 | 1.83 | 13.107 | 0.047738 | 7/15/2014 | 14:06.4 | 2 |
| -868.673 | 19364.557 | 1.83 | 13.094 | 0.0706279 | 7/15/2014 | 14:06.5 | 2 |
| -868.442 | 19364.533 | 1.827 | 13.079 | 0.068007 | 7/15/2014 | 14:06.6 | 2 |
| -868.207 | 19364.508 | 1.83 | 13.07 | 0.0535426 | 7/15/2014 | 14:06.7 | 2 |
| -867.971 | 19364.484 | 1.833 | 13.061 | 0.0840007 | 7/15/2014 | 14:06.8 | 2 |
| -867.734 | 19364.459 | 1.83 | 13.052 | 0.0928026 | 7/15/2014 | 14:06.9 | 2 |
| -867.496 | 19364.436 | 1.83 | 13.043 | 0.0768251 | 7/15/2014 | 14:07.0 | 2 |
| -867.257 | 19364.409 | 1.83 | 13.035 | 0.0685408 | 7/15/2014 | 14:07.1 | 2 |
| -867.02 | 19364.384 | 1.83 | 13.028 | 0.0581724 | 7/15/2014 | 14:07.2 | 2 |
| -866.781 | 19364.359 | 1.826 | 13.019 | 0.0599166 | 7/15/2014 | 14:07.3 | 2 |
| -866.542 | 19364.333 | 1.83 | 13.013 | 0.0685878 | 7/15/2014 | 14:07.4 | 2 |
| -866.304 | 19364.309 | 1.83 | 13.008 | 0.0425817 | 7/15/2014 | 14:07.5 | 2 |
| -866.067 | 19364.283 | 1.83 | 13.002 | 0.0410154 | 7/15/2014 | 14:07.6 | 2 |
| -865.773 | 19364.252 | 1.833 | 12.996 | 0.0358373 | 7/15/2014 | 14:07.7 | 2 |
| -865.654 | 19364.238 | 1.83 | 12.991 | 0.0326647 | 7/15/2014 | 14:07.8 | 2 |
| -865.417 | 19364.213 | 1.833 | 12.991 | 0.0327486 | 7/15/2014 | 14:07.9 | 2 |
| -865.181 | 19364.188 | 1.833 | 12.987 | 0.0371224 | 7/15/2014 | 14:08.0 | 2 |
| -864.942 | 19364.162 | 1.84 | 12.993 | 0.0416871 | 7/15/2014 | 14:08.1 | 2 |
| -864.707 | 19364.137 | 1.836 | 12.989 | 0.0579529 | 7/15/2014 | 14:08.2 | 2 |
| -864.468 | 19364.111 | 1.842 | 12.983 | 0.0624932 | 7/15/2014 | 14:08.3 | 2 |
| -864.051 | 19364.064 | 1.842 | 12.971 | 0.0580278 | 7/15/2014 | 14:08.4 | 2 |
| -863.811 | 19364.038 | 1.848 | 12.966 | 0.0639001 | 7/15/2014 | 14:08.5 | 2 |
| -863.57 | 19364.012 | 1.851 | 12.957 | 0.0636256 | 7/15/2014 | 14:08.6 | 2 |
| -863.327 | 19363.985 | 1.856 | 12.959 | 0.0669277 | 7/15/2014 | 14:08.7 | 2 |
| -863.085 | 19363.959 | 1.856 | 12.953 | 0.0636107 | 7/15/2014 | 14:08.8 | 2 |
| -862.841 | 19363.932 | 1.858 | 12.955 | 0.0541417 | 7/15/2014 | 14:08.9 | 2 |
| -862.598 | 19363.904 | 1.858 | 12.952 | 0.048308 | 7/15/2014 | 14:09.0 | 2 |
| -862.354 | 19363.877 | 1.862 | 12.948 | 0.0509893 | 7/15/2014 | 14:09.1 | 2 |
| -862.111 | 19363.85 | 1.862 | 12.949 | 0.0480273 | 7/15/2014 | 14:09.2 | 2 |
| -861.87 | 19363.823 | 1.862 | 12.951 | 0.0521002 | 7/15/2014 | 14:09.3 | 2 |
| -861.631 | 19363.797 | 1.865 | 12.944 | 0.0528477 | 7/15/2014 | 14:09.4 | 2 |
| -861.394 | 19363.77 | 1.861 | 12.933 | 0.0622912 | 7/15/2014 | 14:09.5 | 2 |
| -861.158 | 19363.743 | 1.867 | 12.922 | 0.0789751 | 7/15/2014 | 14:09.6 | 2 |
| -860.864 | 19363.71 | 1.87 | 12.911 | 0.0701907 | 7/15/2014 | 14:09.7 | 2 |
| -860.744 | 19363.696 | 1.87 | 12.908 | 0.0751538 | 7/15/2014 | 14:09.8 | 2 |
| -860.51 | 19363.67 | 1.872 | 12.901 | 0.0758414 | 7/15/2014 | 14:09.9 | 2 |
| -860.275 | 19363.644 | 1.87 | 12.891 | 0.0860519 | 7/15/2014 | 14:10.0 | 2 |

| | | | | | | | |
|---|---|---|---|---|---|---|---|
| -860.042 | 19363.617 | 1.87 | 12.88 | 0.0894626 | 7/15/2014 | 14:10.1 | 2 |
| -859.809 | 19363.59 | 1.866 | 12.876 | 0.0803044 | 7/15/2014 | 14:10.2 | 2 |
| -859.573 | 19363.563 | 1.874 | 12.873 | 0.0652285 | 7/15/2014 | 14:10.3 | 2 |
| -859.161 | 19363.516 | 1.873 | 12.876 | 0.0700339 | 7/15/2014 | 14:10.4 | 2 |
| -858.926 | 19363.488 | 1.873 | 12.882 | 0.0655985 | 7/15/2014 | 14:10.5 | 2 |
| -858.689 | 19363.461 | 1.873 | 12.893 | 0.0530307 | 7/15/2014 | 14:10.6 | 2 |
| -858.452 | 19363.434 | 1.875 | 12.912 | 0.0517754 | 7/15/2014 | 14:10.7 | 2 |
| -858.214 | 19363.406 | 1.878 | 12.929 | 0.0589271 | 7/15/2014 | 14:10.8 | 2 |
| -857.977 | 19363.379 | 1.881 | 12.952 | 0.0513611 | 7/15/2014 | 14:10.9 | 2 |
| -857.741 | 19363.352 | 1.878 | 12.977 | 0.0474119 | 7/15/2014 | 14:11.0 | 2 |
| -857.505 | 19363.324 | 1.875 | 13.006 | 0.0476911 | 7/15/2014 | 14:11.1 | 2 |
| -857.269 | 19363.297 | 1.872 | 13.024 | 0.0597194 | 7/15/2014 | 14:11.2 | 2 |
| -857.036 | 19363.27 | 1.872 | 13.05 | 0.0455469 | 7/15/2014 | 14:11.3 | 2 |
| -856.804 | 19363.242 | 1.874 | 13.07 | 0.0591017 | 7/15/2014 | 14:11.4 | 2 |
| -856.576 | 19363.217 | 1.874 | 13.077 | 0.0722798 | 7/15/2014 | 14:11.5 | 2 |
| -856.349 | 19363.189 | 1.874 | 13.084 | 0.0569283 | 7/15/2014 | 14:11.6 | 2 |
| -856.068 | 19363.156 | 1.871 | 13.084 | 0.0658847 | 7/15/2014 | 14:11.7 | 2 |
| -855.844 | 19363.132 | 1.878 | 13.078 | 0.0709626 | 7/15/2014 | 14:11.8 | 2 |
| -855.618 | 19363.105 | 1.882 | 13.065 | 0.0725145 | 7/15/2014 | 14:11.9 | 2 |
| -855.393 | 19363.078 | 1.885 | 13.056 | 0.0814398 | 7/15/2014 | 14:12.0 | 2 |
| -855.168 | 19363.052 | 1.89 | 13.033 | 0.0800065 | 7/15/2014 | 14:12.1 | 2 |
| -854.943 | 19363.026 | 1.895 | 13.013 | 0.0733205 | 7/15/2014 | 14:12.2 | 2 |
| -854.719 | 19362.999 | 1.898 | 12.991 | 0.0680932 | 7/15/2014 | 14:12.3 | 2 |
| -854.439 | 19362.966 | 1.903 | 12.974 | 0.0661914 | 7/15/2014 | 14:12.4 | 2 |
| -854.212 | 19362.939 | 1.906 | 12.957 | 0.0606409 | 7/15/2014 | 14:12.5 | 2 |
| -853.984 | 19362.912 | 1.901 | 12.938 | 0.062146 | 7/15/2014 | 14:12.6 | 2 |
| -853.754 | 19362.885 | 1.905 | 12.924 | 0.0530028 | 7/15/2014 | 14:12.7 | 2 |
| -853.521 | 19362.858 | 1.907 | 12.913 | 0.0493414 | 7/15/2014 | 14:12.8 | 2 |
| -853.288 | 19362.831 | 1.905 | 12.906 | 0.049063 | 7/15/2014 | 14:12.9 | 2 |
| -853.055 | 19362.803 | 1.9 | 12.902 | 0.0568459 | 7/15/2014 | 14:13.0 | 2 |
| -852.82 | 19362.774 | 1.9 | 12.902 | 0.0558158 | 7/15/2014 | 14:13.1 | 2 |
| -852.586 | 19362.746 | 1.9 | 12.903 | 0.0563721 | 7/15/2014 | 14:13.2 | 2 |
| -852.353 | 19362.719 | 1.9 | 12.909 | 0.0587889 | 7/15/2014 | 14:13.3 | 2 |
| -852.12 | 19362.691 | 1.902 | 12.919 | 0.0564755 | 7/15/2014 | 14:13.4 | 2 |
| -851.888 | 19362.665 | 1.905 | 12.93 | 0.0532042 | 7/15/2014 | 14:13.5 | 2 |
| -851.658 | 19362.639 | 1.905 | 12.945 | 0.0564358 | 7/15/2014 | 14:13.6 | 2 |
| -851.373 | 19362.604 | 1.904 | 12.957 | 0.057262 | 7/15/2014 | 14:13.7 | 2 |
| -851.144 | 19362.576 | 1.912 | 12.979 | 0.0530118 | 7/15/2014 | 14:13.8 | 2 |
| -850.915 | 19362.549 | 1.912 | 12.988 | 0.0620133 | 7/15/2014 | 14:13.9 | 2 |
| -850.687 | 19362.521 | 1.907 | 13.01 | 0.0588805 | 7/15/2014 | 14:14.0 | 2 |
| -850.459 | 19362.494 | 1.907 | 13.012 | 0.0642317 | 7/15/2014 | 14:14.1 | 2 |
| -850.23 | 19362.468 | 1.909 | 13.01 | 0.0730905 | 7/15/2014 | 14:14.2 | 2 |
| -850.003 | 19362.442 | 1.909 | 13.009 | 0.0736847 | 7/15/2014 | 14:14.3 | 2 |
| -849.72 | 19362.407 | 1.911 | 13.015 | 0.081232 | 7/15/2014 | 14:14.4 | 2 |
| -849.49 | 19362.381 | 1.915 | 13.012 | 0.0954177 | 7/15/2014 | 14:14.5 | 2 |
| -849.261 | 19362.354 | 1.91 | 12.995 | 0.0781294 | 7/15/2014 | 14:14.6 | 2 |
| -849.029 | 19362.326 | 1.914 | 12.992 | 0.0750383 | 7/15/2014 | 14:14.7 | 2 |

| | | | | | | | |
|---|---|---|---|---|---|---|---|
| -848.796 | 19362.299 | 1.918 | 12.972 | 0.0751733 | 7/15/2014 | 14:14.8 | 2 |
| -848.562 | 19362.271 | 1.921 | 12.976 | 0.0715146 | 7/15/2014 | 14:14.9 | 2 |
| -848.327 | 19362.244 | 1.921 | 12.972 | 0.0835805 | 7/15/2014 | 14:15.0 | 2 |
| -848.091 | 19362.215 | 1.923 | 12.966 | 0.0769243 | 7/15/2014 | 14:15.1 | 2 |
| -847.854 | 19362.188 | 1.926 | 12.948 | 0.0557656 | 7/15/2014 | 14:15.2 | 2 |
| -847.62 | 19362.16 | 1.926 | 12.942 | 0.0746624 | 7/15/2014 | 14:15.3 | 2 |
| -847.386 | 19362.133 | 1.928 | 12.937 | 0.060326 | 7/15/2014 | 14:15.4 | 2 |
| -847.152 | 19362.105 | 1.928 | 12.936 | 0.0557349 | 7/15/2014 | 14:15.5 | 2 |
| -846.919 | 19362.078 | 1.928 | 12.934 | 0.0590535 | 7/15/2014 | 14:15.6 | 2 |
| -846.629 | 19362.044 | 1.93 | 12.929 | 0.0598161 | 7/15/2014 | 14:15.7 | 2 |
| -846.511 | 19362.029 | 1.931 | 12.929 | 0.0604836 | 7/15/2014 | 14:15.8 | 2 |
| -846.277 | 19362.003 | 1.931 | 12.931 | 0.0606311 | 7/15/2014 | 14:15.9 | 2 |
| -845.927 | 19361.963 | 1.928 | 12.93 | 0.0555236 | 7/15/2014 | 14:16.0 | 2 |
| -845.812 | 19361.949 | 1.928 | 12.932 | 0.0563695 | 7/15/2014 | 14:16.1 | 2 |
| -845.581 | 19361.922 | 1.928 | 12.93 | 0.058468 | 7/15/2014 | 14:16.2 | 2 |
| -845.347 | 19361.896 | 1.932 | 12.921 | 0.0554335 | 7/15/2014 | 14:16.3 | 2 |
| -844.94 | 19361.848 | 1.929 | 12.912 | 0.0578914 | 7/15/2014 | 14:16.4 | 2 |
| -844.706 | 19361.821 | 1.929 | 12.901 | 0.0570788 | 7/15/2014 | 14:16.5 | 2 |
| -844.472 | 19361.795 | 1.929 | 12.891 | 0.06388 | 7/15/2014 | 14:16.6 | 2 |
| -844.235 | 19361.768 | 1.931 | 12.879 | 0.0637517 | 7/15/2014 | 14:16.7 | 2 |
| -844.002 | 19361.74 | 1.931 | 12.87 | 0.0622253 | 7/15/2014 | 14:16.8 | 2 |
| -843.765 | 19361.714 | 1.931 | 12.861 | 0.0612698 | 7/15/2014 | 14:16.9 | 2 |
| -843.531 | 19361.688 | 1.928 | 12.853 | 0.0564087 | 7/15/2014 | 14:17.0 | 2 |
| -843.297 | 19361.66 | 1.928 | 12.844 | 0.0548178 | 7/15/2014 | 14:17.1 | 2 |
| -843.063 | 19361.635 | 1.93 | 12.837 | 0.0525517 | 7/15/2014 | 14:17.2 | 2 |
| -842.832 | 19361.607 | 1.928 | 12.835 | 0.0539131 | 7/15/2014 | 14:17.3 | 2 |
| -842.602 | 19361.582 | 1.926 | 12.833 | 0.05168 | 7/15/2014 | 14:17.4 | 2 |
| -842.375 | 19361.557 | 1.922 | 12.83 | 0.0508115 | 7/15/2014 | 14:17.5 | 2 |
| -842.148 | 19361.53 | 1.918 | 12.824 | 0.0507611 | 7/15/2014 | 14:17.6 | 2 |
| -841.868 | 19361.5 | 1.918 | 12.818 | 0.0635294 | 7/15/2014 | 14:17.7 | 2 |
| -841.643 | 19361.475 | 1.916 | 12.814 | 0.0566556 | 7/15/2014 | 14:17.8 | 2 |
| -841.419 | 19361.451 | 1.916 | 12.812 | 0.0537924 | 7/15/2014 | 14:17.9 | 2 |
| -841.196 | 19361.424 | 1.916 | 12.808 | 0.0539766 | 7/15/2014 | 14:18.0 | 2 |
| -840.973 | 19361.4 | 1.916 | 12.806 | 0.0535795 | 7/15/2014 | 14:18.1 | 2 |
| -840.751 | 19361.377 | 1.918 | 12.803 | 0.0575787 | 7/15/2014 | 14:18.2 | 2 |
| -840.53 | 19361.352 | 1.921 | 12.803 | 0.0520782 | 7/15/2014 | 14:18.3 | 2 |
| -840.255 | 19361.322 | 1.921 | 12.804 | 0.0523847 | 7/15/2014 | 14:18.4 | 2 |
| -840.036 | 19361.299 | 1.913 | 12.808 | 0.0489095 | 7/15/2014 | 14:18.5 | 2 |
| -839.815 | 19361.274 | 1.915 | 12.811 | 0.0505831 | 7/15/2014 | 14:18.6 | 2 |
| -839.539 | 19361.242 | 1.913 | 12.81 | 0.0486263 | 7/15/2014 | 14:18.7 | 2 |
| -839.316 | 19361.219 | 1.911 | 12.812 | 0.0506179 | 7/15/2014 | 14:18.8 | 2 |
| -839.093 | 19361.195 | 1.911 | 12.81 | 0.0538752 | 7/15/2014 | 14:18.9 | 2 |
| -838.869 | 19361.172 | 1.911 | 12.811 | 0.0566558 | 7/15/2014 | 14:19.0 | 2 |
| -838.646 | 19361.148 | 1.909 | 12.816 | 0.0654319 | 7/15/2014 | 14:19.1 | 2 |
| -838.423 | 19361.125 | 1.909 | 12.823 | 0.0791042 | 7/15/2014 | 14:19.2 | 2 |
| -838.2 | 19361.101 | 1.914 | 12.838 | 0.0759419 | 7/15/2014 | 14:19.3 | 2 |
| -837.924 | 19361.07 | 1.914 | 12.847 | 0.091011 | 7/15/2014 | 14:19.4 | 2 |

| | | | | | | | |
|---|---|---|---|---|---|---|---|
| -837.703 | 19361.047 | 1.912 | 12.857 | 0.0793517 | 7/15/2014 | 14:19.5 | 2 |
| -837.482 | 19361.023 | 1.912 | 12.875 | 0.0817322 | 7/15/2014 | 14:19.6 | 2 |
| -837.207 | 19360.995 | 1.912 | 12.878 | 0.07687 | 7/15/2014 | 14:19.7 | 2 |
| -836.986 | 19360.973 | 1.91 | 12.881 | 0.0689425 | 7/15/2014 | 14:19.8 | 2 |
| -836.763 | 19360.949 | 1.912 | 12.881 | 0.0680447 | 7/15/2014 | 14:19.9 | 2 |
| -836.54 | 19360.926 | 1.91 | 12.874 | 0.0656867 | 7/15/2014 | 14:20.0 | 2 |
| -836.318 | 19360.903 | 1.908 | 12.86 | 0.0526337 | 7/15/2014 | 14:20.1 | 2 |
| -836.095 | 19360.88 | 1.906 | 12.849 | 0.054879 | 7/15/2014 | 14:20.2 | 2 |
| -835.872 | 19360.857 | 1.903 | 12.846 | 0.0650618 | 7/15/2014 | 14:20.3 | 2 |
| -835.595 | 19360.829 | 1.897 | 12.831 | 0.0522341 | 7/15/2014 | 14:20.4 | 2 |
| -835.371 | 19360.807 | 1.89 | 12.834 | 0.0605028 | 7/15/2014 | 14:20.5 | 2 |
| -835.145 | 19360.784 | 1.887 | 12.839 | 0.0480995 | 7/15/2014 | 14:20.6 | 2 |
| -834.919 | 19360.761 | 1.882 | 12.843 | 0.0433354 | 7/15/2014 | 14:20.7 | 2 |
| -834.691 | 19360.738 | 1.882 | 12.853 | 0.0451397 | 7/15/2014 | 14:20.8 | 2 |
| -834.463 | 19360.716 | 1.879 | 12.86 | 0.0498976 | 7/15/2014 | 14:20.9 | 2 |
| -834.233 | 19360.693 | 1.879 | 12.866 | 0.0538743 | 7/15/2014 | 14:21.0 | 2 |
| -834.004 | 19360.67 | 1.879 | 12.865 | 0.0599385 | 7/15/2014 | 14:21.1 | 2 |
| -833.773 | 19360.647 | 1.884 | 12.867 | 0.0591959 | 7/15/2014 | 14:21.2 | 2 |
| -833.544 | 19360.626 | 1.886 | 12.865 | 0.0733467 | 7/15/2014 | 14:21.3 | 2 |
| -833.316 | 19360.604 | 1.884 | 12.859 | 0.0551818 | 7/15/2014 | 14:21.4 | 2 |
| -833.087 | 19360.581 | 1.883 | 12.854 | 0.0532187 | 7/15/2014 | 14:21.5 | 2 |
| -832.859 | 19360.561 | 1.88 | 12.846 | 0.056234 | 7/15/2014 | 14:21.6 | 2 |
| -832.575 | 19360.533 | 1.875 | 12.839 | 0.0463219 | 7/15/2014 | 14:21.7 | 2 |
| -832.348 | 19360.511 | 1.869 | 12.836 | 0.0361576 | 7/15/2014 | 14:21.8 | 2 |
| -832.118 | 19360.489 | 1.867 | 12.837 | 0.0361494 | 7/15/2014 | 14:21.9 | 2 |
| -831.89 | 19360.468 | 1.862 | 12.836 | 0.0216982 | 7/15/2014 | 14:22.0 | 2 |
| -831.662 | 19360.446 | 1.86 | 12.84 | 0.0297903 | 7/15/2014 | 14:22.1 | 2 |
| -831.434 | 19360.426 | 1.857 | 12.847 | 0.0251527 | 7/15/2014 | 14:22.2 | 2 |
| -831.206 | 19360.405 | 1.857 | 12.857 | 0.0342439 | 7/15/2014 | 14:22.3 | 2 |
| -830.925 | 19360.379 | 1.855 | 12.867 | 0.0230874 | 7/15/2014 | 14:22.4 | 2 |
| -830.698 | 19360.358 | 1.859 | 12.883 | 0.0234846 | 7/15/2014 | 14:22.5 | 2 |
| -830.415 | 19360.334 | 1.859 | 12.902 | 0.0265809 | 7/15/2014 | 14:22.6 | 2 |
| -830.188 | 19360.313 | 1.859 | 12.925 | 0.0250525 | 7/15/2014 | 14:22.7 | 2 |
| -829.961 | 19360.293 | 1.856 | 12.949 | 0.037161 | 7/15/2014 | 14:22.8 | 2 |
| -829.733 | 19360.273 | 1.85 | 12.972 | 0.0343543 | 7/15/2014 | 14:22.9 | 2 |
| -829.504 | 19360.253 | 1.85 | 12.987 | 0.0555018 | 7/15/2014 | 14:23.0 | 2 |
| -829.277 | 19360.232 | 1.845 | 13.003 | 0.0534309 | 7/15/2014 | 14:23.1 | 2 |
| -829.049 | 19360.213 | 1.843 | 13.031 | 0.0658727 | 7/15/2014 | 14:23.2 | 2 |
| -828.769 | 19360.191 | 1.836 | 13.036 | 0.0500951 | 7/15/2014 | 14:23.3 | 2 |
| -828.544 | 19360.172 | 1.836 | 13.033 | 0.0774888 | 7/15/2014 | 14:23.4 | 2 |
| -828.323 | 19360.152 | 1.83 | 13.028 | 0.0729189 | 7/15/2014 | 14:23.5 | 2 |
| -828.1 | 19360.135 | 1.83 | 13.019 | 0.0726529 | 7/15/2014 | 14:23.6 | 2 |
| -827.823 | 19360.111 | 1.83 | 13.004 | 0.0837375 | 7/15/2014 | 14:23.7 | 2 |
| -827.599 | 19360.093 | 1.832 | 12.991 | 0.0606718 | 7/15/2014 | 14:23.8 | 2 |
| -827.375 | 19360.074 | 1.829 | 12.976 | 0.06245 | 7/15/2014 | 14:23.9 | 2 |
| -827.151 | 19360.058 | 1.826 | 12.946 | 0.0718448 | 7/15/2014 | 14:24.0 | 2 |
| -826.924 | 19360.039 | 1.826 | 12.932 | 0.0509777 | 7/15/2014 | 14:24.1 | 2 |

| | | | | | | | |
|---|---|---|---|---|---|---|---|
| -826.698 | 19360.021 | 1.82 | 12.921 | 0.0474013 | 7/15/2014 | 14:24.2 | 2 |
| -826.472 | 19360.004 | 1.82 | 12.906 | 0.0505339 | 7/15/2014 | 14:24.3 | 2 |
| -826.191 | 19359.982 | 1.817 | 12.892 | 0.045752 | 7/15/2014 | 14:24.4 | 2 |
| -825.963 | 19359.964 | 1.815 | 12.884 | 0.0484839 | 7/15/2014 | 14:24.5 | 2 |
| -825.735 | 19359.947 | 1.806 | 12.873 | 0.043868 | 7/15/2014 | 14:24.6 | 2 |
| -825.504 | 19359.93 | 1.806 | 12.865 | 0.0420639 | 7/15/2014 | 14:24.7 | 2 |
| -825.272 | 19359.912 | 1.803 | 12.859 | 0.046598 | 7/15/2014 | 14:24.8 | 2 |
| -825.037 | 19359.896 | 1.806 | 12.855 | 0.047821 | 7/15/2014 | 14:24.9 | 2 |
| -824.804 | 19359.878 | 1.806 | 12.855 | 0.0434695 | 7/15/2014 | 14:25.0 | 2 |
| -824.57 | 19359.862 | 1.804 | 12.855 | 0.0440086 | 7/15/2014 | 14:25.1 | 2 |
| -824.338 | 19359.844 | 1.801 | 12.857 | 0.0466448 | 7/15/2014 | 14:25.2 | 2 |
| -824.105 | 19359.828 | 1.801 | 12.857 | 0.0448675 | 7/15/2014 | 14:25.3 | 2 |
| -823.873 | 19359.812 | 1.799 | 12.867 | 0.0451755 | 7/15/2014 | 14:25.4 | 2 |
| -823.643 | 19359.796 | 1.795 | 12.871 | 0.0499162 | 7/15/2014 | 14:25.5 | 2 |
| -823.412 | 19359.78 | 1.793 | 12.881 | 0.0505363 | 7/15/2014 | 14:25.6 | 2 |
| -823.125 | 19359.761 | 1.785 | 12.889 | 0.0494085 | 7/15/2014 | 14:25.7 | 2 |
| -822.896 | 19359.745 | 1.78 | 12.92 | 0.0509965 | 7/15/2014 | 14:25.8 | 2 |
| -822.666 | 19359.729 | 1.778 | 12.947 | 0.0433148 | 7/15/2014 | 14:25.9 | 2 |
| -822.434 | 19359.714 | 1.778 | 12.971 | 0.0447723 | 7/15/2014 | 14:26.0 | 2 |
| -822.206 | 19359.698 | 1.772 | 12.995 | 0.0514028 | 7/15/2014 | 14:26.1 | 2 |
| -821.977 | 19359.685 | 1.772 | 13.011 | 0.0500411 | 7/15/2014 | 14:26.2 | 2 |
| -821.748 | 19359.669 | 1.767 | 13.027 | 0.0464269 | 7/15/2014 | 14:26.3 | 2 |
| -821.464 | 19359.651 | 1.767 | 13.039 | 0.0477977 | 7/15/2014 | 14:26.4 | 2 |
| -821.236 | 19359.637 | 1.767 | 13.054 | 0.0356015 | 7/15/2014 | 14:26.5 | 2 |
| -821.008 | 19359.622 | 1.769 | 13.034 | 0.0365067 | 7/15/2014 | 14:26.6 | 2 |
| -820.721 | 19359.604 | 1.769 | 13.017 | 0.0538036 | 7/15/2014 | 14:26.7 | 2 |
| -820.492 | 19359.59 | 1.765 | 13 | 0.0365755 | 7/15/2014 | 14:26.8 | 2 |
| -820.26 | 19359.576 | 1.768 | 12.995 | 0.0479769 | 7/15/2014 | 14:26.9 | 2 |
| -820.028 | 19359.562 | 1.77 | 12.99 | 0.0521766 | 7/15/2014 | 14:27.0 | 2 |
| -819.797 | 19359.548 | 1.778 | 12.995 | 0.042861 | 7/15/2014 | 14:27.1 | 2 |
| -819.567 | 19359.535 | 1.776 | 12.998 | 0.0453335 | 7/15/2014 | 14:27.2 | 2 |
| -819.338 | 19359.521 | 1.772 | 13 | 0.0493658 | 7/15/2014 | 14:27.3 | 2 |
| -819.053 | 19359.504 | 1.77 | 13.009 | 0.0342516 | 7/15/2014 | 14:27.4 | 2 |
| -818.824 | 19359.491 | 1.77 | 13.018 | 0.0421668 | 7/15/2014 | 14:27.5 | 2 |
| -818.597 | 19359.479 | 1.77 | 13.03 | 0.0421468 | 7/15/2014 | 14:27.6 | 2 |
| -818.313 | 19359.462 | 1.767 | 13.03 | 0.050238 | 7/15/2014 | 14:27.7 | 2 |
| -818.084 | 19359.449 | 1.763 | 13.03 | 0.0647408 | 7/15/2014 | 14:27.8 | 2 |
| -817.855 | 19359.438 | 1.761 | 13.026 | 0.0588611 | 7/15/2014 | 14:27.9 | 2 |
| -817.627 | 19359.425 | 1.763 | 13.025 | 0.0660287 | 7/15/2014 | 14:28.0 | 2 |
| -817.397 | 19359.412 | 1.766 | 13.02 | 0.0679486 | 7/15/2014 | 14:28.1 | 2 |
| -817.169 | 19359.4 | 1.764 | 13.018 | 0.058302 | 7/15/2014 | 14:28.2 | 2 |
| -816.943 | 19359.388 | 1.761 | 13.018 | 0.0886355 | 7/15/2014 | 14:28.3 | 2 |
| -816.661 | 19359.372 | 1.761 | 13.015 | 0.0861494 | 7/15/2014 | 14:28.4 | 2 |
| -816.434 | 19359.361 | 1.761 | 13.012 | 0.102391 | 7/15/2014 | 14:28.5 | 2 |
| -816.208 | 19359.35 | 1.754 | 13.009 | 0.12174 | 7/15/2014 | 14:28.6 | 2 |
| -815.925 | 19359.334 | 1.751 | 13.002 | 0.0885194 | 7/15/2014 | 14:28.7 | 2 |
| -815.695 | 19359.322 | 1.746 | 12.991 | 0.0848778 | 7/15/2014 | 14:28.8 | 2 |

| | | | | | | | |
|---|---|---|---|---|---|---|---|
| -815.468 | 19359.311 | 1.739 | 12.98 | 0.0875243 | 7/15/2014 | 14:28.9 | 2 |
| -815.238 | 19359.298 | 1.739 | 12.965 | 0.0756984 | 7/15/2014 | 14:29.0 | 2 |
| -815.008 | 19359.287 | 1.736 | 12.949 | 0.0870401 | 7/15/2014 | 14:29.1 | 2 |
| -814.777 | 19359.276 | 1.733 | 12.933 | 0.0521892 | 7/15/2014 | 14:29.2 | 2 |
| -814.551 | 19359.266 | 1.729 | 12.918 | 0.0475873 | 7/15/2014 | 14:29.3 | 2 |
| -814.265 | 19359.25 | 1.732 | 12.9 | 0.0685074 | 7/15/2014 | 14:29.4 | 2 |
| -814.038 | 19359.238 | 1.732 | 12.887 | 0.0972032 | 7/15/2014 | 14:29.5 | 2 |
| -813.81 | 19359.227 | 1.735 | 12.877 | 0.0785805 | 7/15/2014 | 14:29.6 | 2 |
| -813.526 | 19359.213 | 1.735 | 12.866 | 0.0905226 | 7/15/2014 | 14:29.7 | 2 |
| -813.298 | 19359.202 | 1.732 | 12.857 | 0.0667868 | 7/15/2014 | 14:29.8 | 2 |
| -813.069 | 19359.191 | 1.735 | 12.85 | 0.0795394 | 7/15/2014 | 14:29.9 | 2 |
| -812.841 | 19359.182 | 1.732 | 12.842 | 0.0831608 | 7/15/2014 | 14:30.0 | 2 |
| -812.61 | 19359.17 | 1.735 | 12.835 | 0.0953794 | 7/15/2014 | 14:30.1 | 2 |
| -812.384 | 19359.158 | 1.735 | 12.834 | 0.0714477 | 7/15/2014 | 14:30.2 | 2 |
| -812.158 | 19359.146 | 1.735 | 12.831 | 0.0895661 | 7/15/2014 | 14:30.3 | 2 |
| -811.877 | 19359.135 | 1.735 | 12.824 | 0.0701304 | 7/15/2014 | 14:30.4 | 2 |
| -811.652 | 19359.123 | 1.735 | 12.818 | 0.0935097 | 7/15/2014 | 14:30.5 | 2 |
| -811.427 | 19359.112 | 1.738 | 12.816 | 0.0975554 | 7/15/2014 | 14:30.6 | 2 |
| -811.147 | 19359.102 | 1.741 | 12.813 | 0.0884792 | 7/15/2014 | 14:30.7 | 2 |
| -810.92 | 19359.09 | 1.748 | 12.815 | 0.0845758 | 7/15/2014 | 14:30.8 | 2 |
| -810.695 | 19359.078 | 1.748 | 12.817 | 0.0990059 | 7/15/2014 | 14:30.9 | 2 |
| -810.468 | 19359.068 | 1.751 | 12.82 | 0.104739 | 7/15/2014 | 14:31.0 | 2 |
| -810.242 | 19359.059 | 1.748 | 12.829 | 0.10085 | 7/15/2014 | 14:31.1 | 2 |
| -810.017 | 19359.049 | 1.748 | 12.844 | 0.0936399 | 7/15/2014 | 14:31.2 | 2 |
| -809.792 | 19359.037 | 1.748 | 12.863 | 0.10361 | 7/15/2014 | 14:31.3 | 2 |
| -809.512 | 19359.026 | 1.745 | 12.884 | 0.122125 | 7/15/2014 | 14:31.4 | 2 |
| -809.29 | 19359.016 | 1.741 | 12.906 | 0.0971893 | 7/15/2014 | 14:31.5 | 2 |
| -809.068 | 19359.006 | 1.741 | 12.929 | 0.09259 | 7/15/2014 | 14:31.6 | 2 |
| -808.789 | 19358.994 | 1.738 | 12.955 | 0.0827204 | 7/15/2014 | 14:31.7 | 2 |
| -808.565 | 19358.983 | 1.74 | 12.969 | 0.104415 | 7/15/2014 | 14:31.8 | 2 |
| -808.341 | 19358.975 | 1.74 | 12.977 | 0.0795446 | 7/15/2014 | 14:31.9 | 2 |
| -808.117 | 19358.965 | 1.734 | 12.978 | 0.0862727 | 7/15/2014 | 14:32.0 | 2 |
| -807.891 | 19358.954 | 1.737 | 12.977 | 0.0702219 | 7/15/2014 | 14:32.1 | 2 |
| -807.667 | 19358.945 | 1.74 | 12.97 | 0.0856269 | 7/15/2014 | 14:32.2 | 2 |
| -807.444 | 19358.936 | 1.737 | 12.96 | 0.0832903 | 7/15/2014 | 14:32.3 | 2 |
| -807.168 | 19358.924 | 1.734 | 12.944 | 0.0910846 | 7/15/2014 | 14:32.4 | 2 |
| -806.944 | 19358.914 | 1.734 | 12.926 | 0.0808225 | 7/15/2014 | 14:32.5 | 2 |
| -806.721 | 19358.904 | 1.729 | 12.914 | 0.104288 | 7/15/2014 | 14:32.6 | 2 |
| -806.442 | 19358.894 | 1.729 | 12.904 | 0.0933136 | 7/15/2014 | 14:32.7 | 2 |
| -806.216 | 19358.883 | 1.732 | 12.895 | 0.114475 | 7/15/2014 | 14:32.8 | 2 |
| -805.989 | 19358.875 | 1.727 | 12.885 | 0.105897 | 7/15/2014 | 14:32.9 | 2 |
| -805.762 | 19358.865 | 1.723 | 12.879 | 0.112492 | 7/15/2014 | 14:33.0 | 2 |
| -805.536 | 19358.855 | 1.723 | 12.876 | 0.109402 | 7/15/2014 | 14:33.1 | 2 |
| -805.31 | 19358.847 | 1.72 | 12.875 | 0.139189 | 7/15/2014 | 14:33.2 | 2 |
| -805.082 | 19358.836 | 1.718 | 12.872 | 0.113005 | 7/15/2014 | 14:33.3 | 2 |
| -804.802 | 19358.825 | 1.715 | 12.869 | 0.119513 | 7/15/2014 | 14:33.4 | 2 |
| -804.575 | 19358.816 | 1.715 | 12.872 | 0.107317 | 7/15/2014 | 14:33.5 | 2 |

| | | | | | | | |
|---|---|---|---|---|---|---|---|
| -804.35 | 19358.809 | 1.71 | 12.877 | 0.111486 | 7/15/2014 | 14:33.6 | 2 |
| -804.066 | 19358.797 | 1.71 | 12.887 | 0.107232 | 7/15/2014 | 14:33.7 | 2 |
| -803.839 | 19358.787 | 1.706 | 12.895 | 0.105613 | 7/15/2014 | 14:33.8 | 2 |
| -803.611 | 19358.779 | 1.702 | 12.903 | 0.0964906 | 7/15/2014 | 14:33.9 | 2 |
| -803.381 | 19358.769 | 1.702 | 12.915 | 0.0930066 | 7/15/2014 | 14:34.0 | 2 |
| -803.152 | 19358.76 | 1.7 | 12.933 | 0.0891454 | 7/15/2014 | 14:34.1 | 2 |
| -802.922 | 19358.752 | 1.698 | 12.95 | 0.0913268 | 7/15/2014 | 14:34.2 | 2 |
| -802.694 | 19358.744 | 1.694 | 12.975 | 0.0928136 | 7/15/2014 | 14:34.3 | 2 |
| -802.409 | 19358.732 | 1.696 | 13 | 0.0895245 | 7/15/2014 | 14:34.4 | 2 |
| -802.181 | 19358.723 | 1.694 | 13.018 | 0.0946245 | 7/15/2014 | 14:34.5 | 2 |
| -801.952 | 19358.715 | 1.692 | 13.032 | 0.0812633 | 7/15/2014 | 14:34.6 | 2 |
| -801.665 | 19358.704 | 1.694 | 13.044 | 0.08017 | 7/15/2014 | 14:34.7 | 2 |
| -801.549 | 19358.699 | 1.691 | 13.055 | 0.0830725 | 7/15/2014 | 14:34.8 | 2 |
| -801.202 | 19358.688 | 1.689 | 13.053 | 0.0802848 | 7/15/2014 | 14:34.9 | 2 |
| -800.969 | 19358.679 | 1.689 | 13.06 | 0.0762848 | 7/15/2014 | 14:35.0 | 2 |
| -800.736 | 19358.67 | 1.689 | 13.053 | 0.0798974 | 7/15/2014 | 14:35.1 | 2 |
| -800.622 | 19358.664 | 1.686 | 13.045 | 0.0790699 | 7/15/2014 | 14:35.2 | 2 |
| -800.389 | 19358.656 | 1.691 | 13.034 | 0.0774532 | 7/15/2014 | 14:35.3 | 2 |
| -799.985 | 19358.643 | 1.689 | 13.027 | 0.0835116 | 7/15/2014 | 14:35.4 | 2 |
| -799.757 | 19358.634 | 1.687 | 13.018 | 0.0727525 | 7/15/2014 | 14:35.5 | 2 |
| -799.526 | 19358.625 | 1.687 | 13.007 | 0.0710609 | 7/15/2014 | 14:35.6 | 2 |
| -799.238 | 19358.614 | 1.69 | 12.997 | 0.0754302 | 7/15/2014 | 14:35.7 | 2 |
| -799.007 | 19358.605 | 1.687 | 12.98 | 0.0711745 | 7/15/2014 | 14:35.8 | 2 |
| -798.775 | 19358.598 | 1.683 | 12.967 | 0.0718117 | 7/15/2014 | 14:35.9 | 2 |
| -798.543 | 19358.589 | 1.679 | 12.952 | 0.0723118 | 7/15/2014 | 14:36.0 | 2 |
| -798.31 | 19358.581 | 1.679 | 12.932 | 0.0705526 | 7/15/2014 | 14:36.1 | 2 |
| -798.08 | 19358.571 | 1.677 | 12.917 | 0.081257 | 7/15/2014 | 14:36.2 | 2 |
| -797.85 | 19358.563 | 1.677 | 12.903 | 0.0755023 | 7/15/2014 | 14:36.3 | 2 |
| -797.563 | 19358.553 | 1.675 | 12.891 | 0.0694426 | 7/15/2014 | 14:36.4 | 2 |
| -797.334 | 19358.545 | 1.675 | 12.878 | 0.0725822 | 7/15/2014 | 14:36.5 | 2 |
| -797.106 | 19358.537 | 1.677 | 12.867 | 0.0847284 | 7/15/2014 | 14:36.6 | 2 |
| -796.818 | 19358.524 | 1.681 | 12.855 | 0.0771268 | 7/15/2014 | 14:36.7 | 2 |
| -796.588 | 19358.517 | 1.681 | 12.848 | 0.0801128 | 7/15/2014 | 14:36.8 | 2 |
| -796.357 | 19358.508 | 1.681 | 12.844 | 0.0803962 | 7/15/2014 | 14:36.9 | 2 |
| -796.125 | 19358.5 | 1.681 | 12.84 | 0.0765854 | 7/15/2014 | 14:37.0 | 2 |
| -795.894 | 19358.492 | 1.678 | 12.839 | 0.0840259 | 7/15/2014 | 14:37.1 | 2 |
| -795.663 | 19358.484 | 1.682 | 12.838 | 0.0928287 | 7/15/2014 | 14:37.2 | 2 |
| -795.434 | 19358.476 | 1.682 | 12.828 | 0.0834861 | 7/15/2014 | 14:37.3 | 2 |
| -795.148 | 19358.463 | 1.684 | 12.829 | 0.0910875 | 7/15/2014 | 14:37.4 | 2 |
| -794.921 | 19358.455 | 1.686 | 12.833 | 0.0900018 | 7/15/2014 | 14:37.5 | 2 |
| -794.693 | 19358.447 | 1.692 | 12.84 | 0.0962656 | 7/15/2014 | 14:37.6 | 2 |
| -794.408 | 19358.438 | 1.694 | 12.846 | 0.106844 | 7/15/2014 | 14:37.7 | 2 |
| -794.179 | 19358.43 | 1.701 | 12.855 | 0.0879439 | 7/15/2014 | 14:37.8 | 2 |
| -793.948 | 19358.422 | 1.707 | 12.865 | 0.083933 | 7/15/2014 | 14:37.9 | 2 |
| -793.719 | 19358.413 | 1.711 | 12.876 | 0.0994357 | 7/15/2014 | 14:38.0 | 2 |
| -793.49 | 19358.403 | 1.713 | 12.899 | 0.0992108 | 7/15/2014 | 14:38.1 | 2 |
| -793.261 | 19358.396 | 1.711 | 12.912 | 0.138786 | 7/15/2014 | 14:38.2 | 2 |

| | | | | | | | |
|---|---|---|---|---|---|---|---|
| -793.033 | 19358.387 | 1.711 | 12.919 | 0.120297 | 7/15/2014 | 14:38.3 | 2 |
| -792.749 | 19358.377 | 1.713 | 12.92 | 0.14529 | 7/15/2014 | 14:38.4 | 2 |
| -792.523 | 19358.369 | 1.713 | 12.915 | 0.159794 | 7/15/2014 | 14:38.5 | 2 |
| -792.295 | 19358.361 | 1.713 | 12.908 | 0.170445 | 7/15/2014 | 14:38.6 | 2 |
| -792.012 | 19358.352 | 1.713 | 12.89 | 0.155809 | 7/15/2014 | 14:38.7 | 2 |
| -791.784 | 19358.343 | 1.715 | 12.871 | 0.190601 | 7/15/2014 | 14:38.8 | 2 |
| -791.554 | 19358.333 | 1.717 | 12.845 | 0.155172 | 7/15/2014 | 14:38.9 | 2 |
| -791.325 | 19358.325 | 1.722 | 12.812 | 0.170178 | 7/15/2014 | 14:39.0 | 2 |
| -791.095 | 19358.316 | 1.722 | 12.78 | 0.146026 | 7/15/2014 | 14:39.1 | 2 |
| -790.866 | 19358.309 | 1.722 | 12.75 | 0.162765 | 7/15/2014 | 14:39.2 | 2 |
| -790.638 | 19358.301 | 1.727 | 12.721 | 0.147889 | 7/15/2014 | 14:39.3 | 2 |
| -790.353 | 19358.291 | 1.73 | 12.694 | 0.154263 | 7/15/2014 | 14:39.4 | 2 |
| -790.125 | 19358.282 | 1.733 | 12.674 | 0.113852 | 7/15/2014 | 14:39.5 | 2 |
| -789.897 | 19358.271 | 1.731 | 12.656 | 0.119643 | 7/15/2014 | 14:39.6 | 2 |
| -789.61 | 19358.263 | 1.734 | 12.644 | 0.107509 | 7/15/2014 | 14:39.7 | 2 |
| -789.38 | 19358.254 | 1.736 | 12.638 | 0.117569 | 7/15/2014 | 14:39.8 | 2 |
| -789.147 | 19358.244 | 1.743 | 12.638 | 0.0895668 | 7/15/2014 | 14:39.9 | 2 |
| -788.914 | 19358.236 | 1.747 | 12.638 | 0.0841559 | 7/15/2014 | 14:40.0 | 2 |
| -788.798 | 19358.232 | 1.748 | 12.64 | 0.0877823 | 7/15/2014 | 14:40.1 | 2 |
| -788.567 | 19358.224 | 1.748 | 12.643 | 0.0881837 | 7/15/2014 | 14:40.2 | 2 |
| -788.333 | 19358.215 | 1.754 | 12.647 | 0.0867024 | 7/15/2014 | 14:40.3 | 2 |
| -787.926 | 19358.199 | 1.754 | 12.652 | 0.078557 | 7/15/2014 | 14:40.4 | 2 |
| -787.695 | 19358.191 | 1.754 | 12.656 | 0.0817704 | 7/15/2014 | 14:40.5 | 2 |
| -787.464 | 19358.18 | 1.752 | 12.658 | 0.0771925 | 7/15/2014 | 14:40.6 | 2 |
| -787.172 | 19358.17 | 1.75 | 12.658 | 0.0773778 | 7/15/2014 | 14:40.7 | 2 |
| -787.055 | 19358.166 | 1.748 | 12.66 | 0.0810891 | 7/15/2014 | 14:40.8 | 2 |
| -786.82 | 19358.158 | 1.748 | 12.667 | 0.078337 | 7/15/2014 | 14:40.9 | 2 |
| -786.587 | 19358.146 | 1.738 | 12.671 | 0.0754751 | 7/15/2014 | 14:41.0 | 2 |
| -786.35 | 19358.139 | 1.748 | 12.675 | 0.0715777 | 7/15/2014 | 14:41.1 | 2 |
| -786.119 | 19358.129 | 1.738 | 12.677 | 0.0904339 | 7/15/2014 | 14:41.2 | 2 |
| -785.886 | 19358.119 | 1.738 | 12.681 | 0.0837668 | 7/15/2014 | 14:41.3 | 2 |
| -785.48 | 19358.105 | 1.746 | 12.699 | 0.0913482 | 7/15/2014 | 14:41.4 | 2 |
| -785.251 | 19358.096 | 1.746 | 12.705 | 0.0907949 | 7/15/2014 | 14:41.5 | 2 |
| -785.021 | 19358.086 | 1.74 | 12.707 | 0.09257 | 7/15/2014 | 14:41.6 | 2 |
| -784.733 | 19358.074 | 1.744 | 12.715 | 0.0914116 | 7/15/2014 | 14:41.7 | 2 |
| -784.503 | 19358.064 | 1.744 | 12.729 | 0.0844728 | 7/15/2014 | 14:41.8 | 2 |
| -784.274 | 19358.057 | 1.747 | 12.738 | 0.0857575 | 7/15/2014 | 14:41.9 | 2 |
| -784.042 | 19358.047 | 1.752 | 12.745 | 0.0826139 | 7/15/2014 | 14:42.0 | 2 |
| -783.812 | 19358.039 | 1.75 | 12.751 | 0.0756231 | 7/15/2014 | 14:42.1 | 2 |
| -783.585 | 19358.028 | 1.747 | 12.747 | 0.0708462 | 7/15/2014 | 14:42.2 | 2 |
| -783.358 | 19358.02 | 1.747 | 12.752 | 0.0703436 | 7/15/2014 | 14:42.3 | 2 |
| -783.075 | 19358.008 | 1.75 | 12.755 | 0.06393 | 7/15/2014 | 14:42.4 | 2 |
| -782.849 | 19358 | 1.75 | 12.751 | 0.0689475 | 7/15/2014 | 14:42.5 | 2 |
| -782.625 | 19357.99 | 1.747 | 12.741 | 0.0722655 | 7/15/2014 | 14:42.6 | 2 |
| -782.343 | 19357.979 | 1.747 | 12.735 | 0.0712764 | 7/15/2014 | 14:42.7 | 2 |
| -782.118 | 19357.969 | 1.741 | 12.726 | 0.0636092 | 7/15/2014 | 14:42.8 | 2 |
| -781.891 | 19357.96 | 1.741 | 12.714 | 0.0594869 | 7/15/2014 | 14:42.9 | 2 |

| | | | | | | | |
|---|---|---|---|---|---|---|---|
| -781.663 | 19357.949 | 1.745 | 12.71 | 0.057074 | 7/15/2014 | 14:43.0 | 2 |
| -781.437 | 19357.941 | 1.747 | 12.699 | 0.0583391 | 7/15/2014 | 14:43.1 | 2 |
| -781.211 | 19357.932 | 1.747 | 12.693 | 0.0571263 | 7/15/2014 | 14:43.2 | 2 |
| -780.985 | 19357.922 | 1.747 | 12.689 | 0.0423282 | 7/15/2014 | 14:43.3 | 2 |
| -780.704 | 19357.91 | 1.75 | 12.683 | 0.0387585 | 7/15/2014 | 14:43.4 | 2 |
| -780.481 | 19357.9 | 1.752 | 12.68 | 0.0393347 | 7/15/2014 | 14:43.5 | 2 |
| -780.257 | 19357.892 | 1.754 | 12.677 | 0.0516481 | 7/15/2014 | 14:43.6 | 2 |
| -779.975 | 19357.879 | 1.756 | 12.674 | 0.0459397 | 7/15/2014 | 14:43.7 | 2 |
| -779.748 | 19357.869 | 1.756 | 12.669 | 0.0548941 | 7/15/2014 | 14:43.8 | 2 |
| -779.523 | 19357.857 | 1.754 | 12.665 | 0.0678081 | 7/15/2014 | 14:43.9 | 2 |
| -779.294 | 19357.85 | 1.76 | 12.658 | 0.0739247 | 7/15/2014 | 14:44.0 | 2 |
| -779.067 | 19357.838 | 1.762 | 12.651 | 0.0809747 | 7/15/2014 | 14:44.1 | 2 |
| -778.839 | 19357.828 | 1.762 | 12.644 | 0.0869147 | 7/15/2014 | 14:44.2 | 2 |
| -778.613 | 19357.819 | 1.765 | 12.639 | 0.0989098 | 7/15/2014 | 14:44.3 | 2 |
| -778.332 | 19357.807 | 1.768 | 12.63 | 0.0935182 | 7/15/2014 | 14:44.4 | 2 |
| -778.105 | 19357.795 | 1.771 | 12.622 | 0.0873481 | 7/15/2014 | 14:44.5 | 2 |
| -777.879 | 19357.784 | 1.774 | 12.615 | 0.0815249 | 7/15/2014 | 14:44.6 | 2 |
| -777.594 | 19357.771 | 1.776 | 12.608 | 0.0826756 | 7/15/2014 | 14:44.7 | 2 |
| -777.364 | 19357.76 | 1.776 | 12.602 | 0.0844732 | 7/15/2014 | 14:44.8 | 2 |
| -777.132 | 19357.748 | 1.777 | 12.596 | 0.0875681 | 7/15/2014 | 14:44.9 | 2 |
| -776.9 | 19357.738 | 1.78 | 12.59 | 0.0872568 | 7/15/2014 | 14:45.0 | 2 |
| -776.786 | 19357.732 | 1.78 | 12.584 | 0.0933121 | 7/15/2014 | 14:45.1 | 2 |
| -776.554 | 19357.721 | 1.78 | 12.579 | 0.100798 | 7/15/2014 | 14:45.2 | 2 |
| -776.322 | 19357.71 | 1.784 | 12.574 | 0.115106 | 7/15/2014 | 14:45.3 | 2 |
| -775.917 | 19357.689 | 1.785 | 12.56 | 0.120166 | 7/15/2014 | 14:45.4 | 2 |
| -775.684 | 19357.678 | 1.794 | 12.554 | 0.117209 | 7/15/2014 | 14:45.5 | 2 |
| -775.452 | 19357.665 | 1.799 | 12.546 | 0.120374 | 7/15/2014 | 14:45.6 | 2 |
| -775.163 | 19357.65 | 1.805 | 12.542 | 0.121453 | 7/15/2014 | 14:45.7 | 2 |
| -775.045 | 19357.645 | 1.805 | 12.538 | 0.12452 | 7/15/2014 | 14:45.8 | 2 |
| -774.812 | 19357.632 | 1.813 | 12.544 | 0.119465 | 7/15/2014 | 14:45.9 | 2 |
| -774.579 | 19357.62 | 1.815 | 12.547 | 0.112586 | 7/15/2014 | 14:46.0 | 2 |
| -774.231 | 19357.602 | 1.822 | 12.572 | 0.107926 | 7/15/2014 | 14:46.1 | 2 |
| -773.998 | 19357.589 | 1.822 | 12.577 | 0.110636 | 7/15/2014 | 14:46.2 | 2 |
| -773.884 | 19357.582 | 1.822 | 12.584 | 0.108793 | 7/15/2014 | 14:46.3 | 2 |
| -773.483 | 19357.56 | 1.822 | 12.575 | 0.10593 | 7/15/2014 | 14:46.4 | 2 |
| -773.255 | 19357.547 | 1.822 | 12.566 | 0.0985931 | 7/15/2014 | 14:46.5 | 2 |
| -773.028 | 19357.534 | 1.822 | 12.551 | 0.110935 | 7/15/2014 | 14:46.6 | 2 |
| -772.745 | 19357.518 | 1.822 | 12.537 | 0.0983015 | 7/15/2014 | 14:46.7 | 2 |
| -772.517 | 19357.505 | 1.827 | 12.521 | 0.117332 | 7/15/2014 | 14:46.8 | 2 |
| -772.288 | 19357.491 | 1.829 | 12.491 | 0.100701 | 7/15/2014 | 14:46.9 | 2 |
| -772.061 | 19357.478 | 1.831 | 12.481 | 0.121691 | 7/15/2014 | 14:47.0 | 2 |
| -771.834 | 19357.464 | 1.833 | 12.479 | 0.11558 | 7/15/2014 | 14:47.1 | 2 |
| -771.608 | 19357.45 | 1.836 | 12.485 | 0.116847 | 7/15/2014 | 14:47.2 | 2 |
| -771.383 | 19357.437 | 1.836 | 12.509 | 0.112701 | 7/15/2014 | 14:47.3 | 2 |
| -771.104 | 19357.42 | 1.834 | 12.524 | 0.140599 | 7/15/2014 | 14:47.4 | 2 |
| -770.879 | 19357.406 | 1.834 | 12.555 | 0.127378 | 7/15/2014 | 14:47.5 | 2 |
| -770.656 | 19357.392 | 1.834 | 12.584 | 0.137153 | 7/15/2014 | 14:47.6 | 2 |

| | | | | | | | |
|---|---|---|---|---|---|---|---|
| -770.377 | 19357.374 | 1.832 | 12.619 | 0.12936 | 7/15/2014 | 14:47.7 | 2 |
| -770.152 | 19357.359 | 1.832 | 12.651 | 0.127219 | 7/15/2014 | 14:47.8 | 2 |
| -769.925 | 19357.344 | 1.832 | 12.678 | 0.116109 | 7/15/2014 | 14:47.9 | 2 |
| -769.699 | 19357.329 | 1.832 | 12.697 | 0.119655 | 7/15/2014 | 14:48.0 | 2 |
| -769.471 | 19357.314 | 1.836 | 12.686 | 0.11717 | 7/15/2014 | 14:48.1 | 2 |
| -769.243 | 19357.3 | 1.842 | 12.678 | 0.106015 | 7/15/2014 | 14:48.2 | 2 |
| -769.018 | 19357.284 | 1.844 | 12.651 | 0.10232 | 7/15/2014 | 14:48.3 | 2 |
| -768.736 | 19357.266 | 1.849 | 12.625 | 0.104239 | 7/15/2014 | 14:48.4 | 2 |
| -768.51 | 19357.25 | 1.854 | 12.591 | 0.102572 | 7/15/2014 | 14:48.5 | 2 |
| -768.282 | 19357.233 | 1.863 | 12.553 | 0.100213 | 7/15/2014 | 14:48.6 | 2 |
| -767.997 | 19357.213 | 1.868 | 12.52 | 0.102148 | 7/15/2014 | 14:48.7 | 2 |
| -767.765 | 19357.197 | 1.871 | 12.49 | 0.0987909 | 7/15/2014 | 14:48.8 | 2 |
| -767.534 | 19357.18 | 1.874 | 12.472 | 0.104785 | 7/15/2014 | 14:48.9 | 2 |
| -767.303 | 19357.164 | 1.876 | 12.46 | 0.107077 | 7/15/2014 | 14:49.0 | 2 |
| -767.07 | 19357.146 | 1.879 | 12.456 | 0.114541 | 7/15/2014 | 14:49.1 | 2 |
| -766.957 | 19357.139 | 1.876 | 12.452 | 0.116008 | 7/15/2014 | 14:49.2 | 2 |
| -766.723 | 19357.121 | 1.882 | 12.45 | 0.130202 | 7/15/2014 | 14:49.3 | 2 |
| -766.319 | 19357.091 | 1.879 | 12.441 | 0.117038 | 7/15/2014 | 14:49.4 | 2 |
| -766.087 | 19357.074 | 1.883 | 12.434 | 0.125077 | 7/15/2014 | 14:49.5 | 2 |
| -765.857 | 19357.056 | 1.885 | 12.423 | 0.121279 | 7/15/2014 | 14:49.6 | 2 |
| -765.568 | 19357.034 | 1.888 | 12.417 | 0.109552 | 7/15/2014 | 14:49.7 | 2 |
| -765.451 | 19357.024 | 1.889 | 12.413 | 0.111923 | 7/15/2014 | 14:49.8 | 2 |
| -765.217 | 19357.007 | 1.899 | 12.4 | 0.107869 | 7/15/2014 | 14:49.9 | 2 |
| -764.986 | 19356.988 | 1.896 | 12.384 | 0.107751 | 7/15/2014 | 14:50.0 | 2 |
| -764.751 | 19356.97 | 1.9 | 12.373 | 0.10624 | 7/15/2014 | 14:50.1 | 2 |
| -764.519 | 19356.952 | 1.9 | 12.36 | 0.107366 | 7/15/2014 | 14:50.2 | 2 |
| -764.286 | 19356.935 | 1.905 | 12.345 | 0.101093 | 7/15/2014 | 14:50.3 | 2 |
| -763.882 | 19356.901 | 1.905 | 12.324 | 0.0988488 | 7/15/2014 | 14:50.4 | 2 |
| -763.651 | 19356.883 | 1.907 | 12.309 | 0.0940975 | 7/15/2014 | 14:50.5 | 2 |
| -763.42 | 19356.863 | 1.904 | 12.296 | 0.0919493 | 7/15/2014 | 14:50.6 | 2 |
| -763.132 | 19356.84 | 1.906 | 12.288 | 0.0884953 | 7/15/2014 | 14:50.7 | 2 |
| -763.015 | 19356.831 | 1.908 | 12.279 | 0.0865123 | 7/15/2014 | 14:50.8 | 2 |
| -762.781 | 19356.813 | 1.914 | 12.275 | 0.0768875 | 7/15/2014 | 14:50.9 | 2 |
| -762.55 | 19356.793 | 1.91 | 12.271 | 0.077903 | 7/15/2014 | 14:51.0 | 2 |
| -762.317 | 19356.772 | 1.91 | 12.267 | 0.0664665 | 7/15/2014 | 14:51.1 | 2 |
| -762.085 | 19356.752 | 1.91 | 12.264 | 0.0631036 | 7/15/2014 | 14:51.2 | 2 |
| -761.854 | 19356.732 | 1.91 | 12.261 | 0.0557587 | 7/15/2014 | 14:51.3 | 2 |
| -761.45 | 19356.699 | 1.909 | 12.258 | 0.0501086 | 7/15/2014 | 14:51.4 | 2 |
| -761.22 | 19356.68 | 1.907 | 12.257 | 0.0454329 | 7/15/2014 | 14:51.5 | 2 |
| -760.992 | 19356.66 | 1.9 | 12.25 | 0.0449288 | 7/15/2014 | 14:51.6 | 2 |
| -760.705 | 19356.634 | 1.9 | 12.249 | 0.0482202 | 7/15/2014 | 14:51.7 | 2 |
| -760.475 | 19356.613 | 1.9 | 12.251 | 0.0478572 | 7/15/2014 | 14:51.8 | 2 |
| -760.243 | 19356.594 | 1.897 | 12.251 | 0.0537514 | 7/15/2014 | 14:51.9 | 2 |
| -760.011 | 19356.572 | 1.899 | 12.29 | 0.0571347 | 7/15/2014 | 14:52.0 | 2 |
| -759.781 | 19356.553 | 1.899 | 12.329 | 0.0603735 | 7/15/2014 | 14:52.1 | 2 |
| -759.55 | 19356.532 | 1.899 | 12.342 | 0.0723205 | 7/15/2014 | 14:52.2 | 2 |
| -759.32 | 19356.512 | 1.899 | 12.35 | 0.0670325 | 7/15/2014 | 14:52.3 | 2 |

| | | | | | | | |
|---|---|---|---|---|---|---|---|
| -759.034 | 19356.485 | 1.903 | 12.357 | 0.0722777 | 7/15/2014 | 14:52.4 | 2 |
| -758.805 | 19356.465 | 1.905 | 12.356 | 0.0828901 | 7/15/2014 | 14:52.5 | 2 |
| -758.575 | 19356.443 | 1.905 | 12.355 | 0.104471 | 7/15/2014 | 14:52.6 | 2 |
| -758.286 | 19356.416 | 1.907 | 12.353 | 0.0882677 | 7/15/2014 | 14:52.7 | 2 |
| -758.171 | 19356.406 | 1.905 | 12.323 | 0.0983614 | 7/15/2014 | 14:52.8 | 2 |
| -757.938 | 19356.385 | 1.907 | 12.273 | 0.108116 | 7/15/2014 | 14:52.9 | 2 |
| -757.705 | 19356.362 | 1.91 | 12.252 | 0.0997059 | 7/15/2014 | 14:53.0 | 2 |
| -757.47 | 19356.341 | 1.915 | 12.233 | 0.117767 | 7/15/2014 | 14:53.1 | 2 |
| -757.237 | 19356.318 | 1.915 | 12.221 | 0.10613 | 7/15/2014 | 14:53.2 | 2 |
| -757.003 | 19356.295 | 1.917 | 12.214 | 0.076889 | 7/15/2014 | 14:53.3 | 2 |
| -756.594 | 19356.256 | 1.918 | 12.211 | 0.0884675 | 7/15/2014 | 14:53.4 | 2 |
| -756.36 | 19356.234 | 1.92 | 12.209 | 0.093909 | 7/15/2014 | 14:53.5 | 2 |
| -756.127 | 19356.211 | 1.923 | 12.21 | 0.076364 | 7/15/2014 | 14:53.6 | 2 |
| -755.834 | 19356.183 | 1.923 | 12.21 | 0.0673813 | 7/15/2014 | 14:53.7 | 2 |
| -755.715 | 19356.171 | 1.923 | 12.211 | 0.0599597 | 7/15/2014 | 14:53.8 | 2 |
| -755.48 | 19356.147 | 1.923 | 12.212 | 0.0795968 | 7/15/2014 | 14:53.9 | 2 |
| -755.244 | 19356.124 | 1.923 | 12.212 | 0.0543435 | 7/15/2014 | 14:54.0 | 2 |
| -755.007 | 19356.101 | 1.926 | 12.213 | 0.0642516 | 7/15/2014 | 14:54.1 | 2 |
| -754.771 | 19356.077 | 1.923 | 12.22 | 0.0563691 | 7/15/2014 | 14:54.2 | 2 |
| -754.535 | 19356.054 | 1.927 | 12.23 | 0.0462218 | 7/15/2014 | 14:54.3 | 2 |
| -754.124 | 19356.012 | 1.924 | 12.24 | 0.0487974 | 7/15/2014 | 14:54.4 | 2 |
| -753.89 | 19355.988 | 1.924 | 12.244 | 0.0418142 | 7/15/2014 | 14:54.5 | 2 |
| -753.656 | 19355.965 | 1.927 | 12.244 | 0.0420474 | 7/15/2014 | 14:54.6 | 2 |
| -753.365 | 19355.936 | 1.925 | 12.246 | 0.0499656 | 7/15/2014 | 14:54.7 | 2 |
| -753.246 | 19355.923 | 1.926 | 12.249 | 0.0571399 | 7/15/2014 | 14:54.8 | 2 |
| -753.01 | 19355.898 | 1.932 | 12.244 | 0.0675914 | 7/15/2014 | 14:54.9 | 2 |
| -752.775 | 19355.875 | 1.932 | 12.232 | 0.0859583 | 7/15/2014 | 14:55.0 | 2 |
| -752.538 | 19355.849 | 1.938 | 12.222 | 0.0652263 | 7/15/2014 | 14:55.1 | 2 |
| -752.304 | 19355.825 | 1.934 | 12.212 | 0.0742966 | 7/15/2014 | 14:55.2 | 2 |
| -752.069 | 19355.802 | 1.938 | 12.21 | 0.0793141 | 7/15/2014 | 14:55.3 | 2 |
| -751.661 | 19355.759 | 1.936 | 12.219 | 0.102504 | 7/15/2014 | 14:55.4 | 2 |
| -751.427 | 19355.732 | 1.936 | 12.216 | 0.109925 | 7/15/2014 | 14:55.5 | 2 |
| -751.194 | 19355.709 | 1.936 | 12.208 | 0.10858 | 7/15/2014 | 14:55.6 | 2 |
| -750.901 | 19355.676 | 1.933 | 12.199 | 0.119528 | 7/15/2014 | 14:55.7 | 2 |
| -750.783 | 19355.664 | 1.93 | 12.2 | 0.128884 | 7/15/2014 | 14:55.8 | 2 |
| -750.547 | 19355.64 | 1.934 | 12.189 | 0.132053 | 7/15/2014 | 14:55.9 | 2 |
| -750.31 | 19355.613 | 1.934 | 12.172 | 0.107012 | 7/15/2014 | 14:56.0 | 2 |
| -750.073 | 19355.588 | 1.938 | 12.149 | 0.107541 | 7/15/2014 | 14:56.1 | 2 |
| -749.837 | 19355.561 | 1.935 | 12.131 | 0.124359 | 7/15/2014 | 14:56.2 | 2 |
| -749.6 | 19355.535 | 1.938 | 12.116 | 0.117553 | 7/15/2014 | 14:56.3 | 2 |
| -749.185 | 19355.49 | 1.939 | 12.102 | 0.132006 | 7/15/2014 | 14:56.4 | 2 |
| -748.952 | 19355.464 | 1.937 | 12.092 | 0.128968 | 7/15/2014 | 14:56.5 | 2 |
| -748.716 | 19355.438 | 1.934 | 12.083 | 0.144191 | 7/15/2014 | 14:56.6 | 2 |
| -748.422 | 19355.404 | 1.931 | 12.075 | 0.112874 | 7/15/2014 | 14:56.7 | 2 |
| -748.302 | 19355.391 | 1.928 | 12.071 | 0.102915 | 7/15/2014 | 14:56.8 | 2 |
| -748.064 | 19355.363 | 1.928 | 12.066 | 0.116144 | 7/15/2014 | 14:56.9 | 2 |
| -747.828 | 19355.34 | 1.926 | 12.059 | 0.124029 | 7/15/2014 | 14:57.0 | 2 |

| | | | | | | | |
|---|---|---|---|---|---|---|---|
| -747.59 | 19355.313 | 1.926 | 12.057 | 0.115392 | 7/15/2014 | 14:57.1 | 2 |
| -747.357 | 19355.285 | 1.922 | 12.056 | 0.11288 | 7/15/2014 | 14:57.2 | 2 |
| -747.122 | 19355.258 | 1.924 | 12.054 | 0.101287 | 7/15/2014 | 14:57.3 | 2 |
| -746.713 | 19355.211 | 1.924 | 12.054 | 0.106398 | 7/15/2014 | 14:57.4 | 2 |
| -746.48 | 19355.185 | 1.922 | 12.057 | 0.0991397 | 7/15/2014 | 14:57.5 | 2 |
| -746.25 | 19355.16 | 1.924 | 12.056 | 0.0967998 | 7/15/2014 | 14:57.6 | 2 |
| -745.962 | 19355.127 | 1.926 | 12.054 | 0.0955319 | 7/15/2014 | 14:57.7 | 2 |
| -745.844 | 19355.113 | 1.923 | 12.052 | 0.0928008 | 7/15/2014 | 14:57.8 | 2 |
| -745.613 | 19355.086 | 1.923 | 12.045 | 0.0880721 | 7/15/2014 | 14:57.9 | 2 |
| -745.266 | 19355.047 | 1.926 | 12.036 | 0.082879 | 7/15/2014 | 14:58.0 | 2 |
| -745.036 | 19355.02 | 1.929 | 12.035 | 0.0850908 | 7/15/2014 | 14:58.1 | 2 |
| -744.806 | 19354.994 | 1.929 | 12.033 | 0.0825208 | 7/15/2014 | 14:58.2 | 2 |
| -744.578 | 19354.968 | 1.932 | 12.03 | 0.0824259 | 7/15/2014 | 14:58.3 | 2 |
| -744.293 | 19354.936 | 1.932 | 12.025 | 0.0838181 | 7/15/2014 | 14:58.4 | 2 |
| -744.066 | 19354.908 | 1.929 | 12.031 | 0.0861746 | 7/15/2014 | 14:58.5 | 2 |
| -743.837 | 19354.882 | 1.933 | 12.031 | 0.0865244 | 7/15/2014 | 14:58.6 | 2 |
| -743.549 | 19354.85 | 1.933 | 12.035 | 0.0850932 | 7/15/2014 | 14:58.7 | 2 |
| -743.435 | 19354.836 | 1.93 | 12.037 | 0.084129 | 7/15/2014 | 14:58.8 | 2 |
| -743.204 | 19354.811 | 1.924 | 12.044 | 0.0822913 | 7/15/2014 | 14:58.9 | 2 |
| -742.971 | 19354.782 | 1.924 | 12.04 | 0.0769942 | 7/15/2014 | 14:59.0 | 2 |
| -742.737 | 19354.756 | 1.928 | 12.035 | 0.0753877 | 7/15/2014 | 14:59.1 | 2 |
| -742.504 | 19354.729 | 1.928 | 12.037 | 0.0744905 | 7/15/2014 | 14:59.2 | 2 |
| -742.268 | 19354.702 | 1.93 | 12.033 | 0.0724956 | 7/15/2014 | 14:59.3 | 2 |
| -741.855 | 19354.654 | 1.929 | 12.067 | 0.0709771 | 7/15/2014 | 14:59.4 | 2 |
| -741.62 | 19354.628 | 1.929 | 12.085 | 0.0708008 | 7/15/2014 | 14:59.5 | 2 |
| -741.384 | 19354.6 | 1.932 | 12.096 | 0.0810343 | 7/15/2014 | 14:59.6 | 2 |
| -741.088 | 19354.566 | 1.929 | 12.115 | 0.0743111 | 7/15/2014 | 14:59.7 | 2 |
| -740.968 | 19354.553 | 1.927 | 12.13 | 0.0781069 | 7/15/2014 | 14:59.8 | 2 |
| -740.728 | 19354.524 | 1.923 | 12.147 | 0.0759531 | 7/15/2014 | 14:59.9 | 2 |
| -740.489 | 19354.497 | 1.923 | 12.16 | 0.0772134 | 7/15/2014 | 15:00.0 | 2 |
| -740.249 | 19354.469 | 1.923 | 12.156 | 0.0749395 | 7/15/2014 | 15:00.1 | 2 |
| -740.01 | 19354.441 | 1.919 | 12.148 | 0.0839858 | 7/15/2014 | 15:00.2 | 2 |
| -739.771 | 19354.414 | 1.919 | 12.137 | 0.0785234 | 7/15/2014 | 15:00.3 | 2 |
| -739.357 | 19354.367 | 1.92 | 12.122 | 0.092075 | 7/15/2014 | 15:00.4 | 2 |
| -739.12 | 19354.339 | 1.922 | 12.115 | 0.0780862 | 7/15/2014 | 15:00.5 | 2 |
| -738.885 | 19354.312 | 1.922 | 12.11 | 0.0907667 | 7/15/2014 | 15:00.6 | 2 |
| -738.592 | 19354.277 | 1.922 | 12.11 | 0.0904284 | 7/15/2014 | 15:00.7 | 2 |
| -738.471 | 19354.264 | 1.922 | 12.107 | 0.0857256 | 7/15/2014 | 15:00.8 | 2 |
| -738.236 | 19354.235 | 1.922 | 12.112 | 0.086704 | 7/15/2014 | 15:00.9 | 2 |
| -738.002 | 19354.209 | 1.922 | 12.122 | 0.0935271 | 7/15/2014 | 15:01.0 | 2 |
| -737.767 | 19354.182 | 1.925 | 12.135 | 0.0847003 | 7/15/2014 | 15:01.1 | 2 |
| -737.534 | 19354.154 | 1.921 | 12.155 | 0.0911523 | 7/15/2014 | 15:01.2 | 2 |
| -737.302 | 19354.127 | 1.921 | 12.181 | 0.085397 | 7/15/2014 | 15:01.3 | 2 |
| -736.899 | 19354.08 | 1.921 | 12.21 | 0.0858534 | 7/15/2014 | 15:01.4 | 2 |
| -736.671 | 19354.053 | 1.917 | 12.221 | 0.0850574 | 7/15/2014 | 15:01.5 | 2 |
| -736.441 | 19354.027 | 1.915 | 12.229 | 0.0826438 | 7/15/2014 | 15:01.6 | 2 |
| -736.156 | 19353.993 | 1.915 | 12.231 | 0.0873359 | 7/15/2014 | 15:01.7 | 2 |

| | | | | | | | |
|---|---|---|---|---|---|---|---|
| -735.927 | 19353.967 | 1.915 | 12.233 | 0.0934342 | 7/15/2014 | 15:01.8 | 2 |
| -735.697 | 19353.939 | 1.913 | 12.229 | 0.0953336 | 7/15/2014 | 15:01.9 | 2 |
| -735.467 | 19353.912 | 1.913 | 12.218 | 0.0986034 | 7/15/2014 | 15:02.0 | 2 |
| -735.237 | 19353.885 | 1.913 | 12.205 | 0.104667 | 7/15/2014 | 15:02.1 | 2 |
| -735.008 | 19353.858 | 1.913 | 12.203 | 0.105444 | 7/15/2014 | 15:02.2 | 2 |
| -734.779 | 19353.832 | 1.92 | 12.198 | 0.106047 | 7/15/2014 | 15:02.3 | 2 |
| -734.494 | 19353.798 | 1.92 | 12.194 | 0.112978 | 7/15/2014 | 15:02.4 | 2 |
| -734.267 | 19353.773 | 1.916 | 12.192 | 0.0947568 | 7/15/2014 | 15:02.5 | 2 |
| -734.038 | 19353.746 | 1.916 | 12.186 | 0.0981086 | 7/15/2014 | 15:02.6 | 2 |
| -733.752 | 19353.712 | 1.914 | 12.18 | 0.0962418 | 7/15/2014 | 15:02.7 | 2 |
| -733.637 | 19353.698 | 1.913 | 12.178 | 0.0899124 | 7/15/2014 | 15:02.8 | 2 |
| -733.405 | 19353.671 | 1.91 | 12.177 | 0.0941959 | 7/15/2014 | 15:02.9 | 2 |
| -733.175 | 19353.644 | 1.905 | 12.17 | 0.0946091 | 7/15/2014 | 15:03.0 | 2 |
| -732.943 | 19353.616 | 1.899 | 12.188 | 0.0945818 | 7/15/2014 | 15:03.1 | 2 |
| -732.712 | 19353.591 | 1.893 | 12.21 | 0.0984378 | 7/15/2014 | 15:03.2 | 2 |
| -732.478 | 19353.563 | 1.896 | 12.222 | 0.104376 | 7/15/2014 | 15:03.3 | 2 |
| -732.073 | 19353.516 | 1.895 | 12.208 | 0.104044 | 7/15/2014 | 15:03.4 | 2 |
| -731.842 | 19353.489 | 1.895 | 12.203 | 0.107344 | 7/15/2014 | 15:03.5 | 2 |
| -731.61 | 19353.462 | 1.897 | 12.192 | 0.105924 | 7/15/2014 | 15:03.6 | 2 |
| -731.321 | 19353.428 | 1.894 | 12.181 | 0.111728 | 7/15/2014 | 15:03.7 | 2 |
| -731.204 | 19353.414 | 1.892 | 12.162 | 0.108889 | 7/15/2014 | 15:03.8 | 2 |
| -730.969 | 19353.387 | 1.896 | 12.118 | 0.108822 | 7/15/2014 | 15:03.9 | 2 |
| -730.735 | 19353.36 | 1.89 | 12.086 | 0.112552 | 7/15/2014 | 15:04.0 | 2 |
| -730.501 | 19353.333 | 1.886 | 12.064 | 0.104183 | 7/15/2014 | 15:04.1 | 2 |
| -730.268 | 19353.306 | 1.88 | 12.049 | 0.10604 | 7/15/2014 | 15:04.2 | 2 |
| -730.035 | 19353.278 | 1.88 | 12.034 | 0.102477 | 7/15/2014 | 15:04.3 | 2 |
| -729.628 | 19353.23 | 1.877 | 12.024 | 0.106309 | 7/15/2014 | 15:04.4 | 2 |
| -729.397 | 19353.203 | 1.877 | 12.018 | 0.111552 | 7/15/2014 | 15:04.5 | 2 |
| -729.164 | 19353.178 | 1.874 | 12.018 | 0.116804 | 7/15/2014 | 15:04.6 | 2 |
| -728.874 | 19353.143 | 1.874 | 12.02 | 0.126381 | 7/15/2014 | 15:04.7 | 2 |
| -728.757 | 19353.128 | 1.873 | 12.022 | 0.13065 | 7/15/2014 | 15:04.8 | 2 |
| -728.523 | 19353.101 | 1.873 | 12.028 | 0.129861 | 7/15/2014 | 15:04.9 | 2 |
| -728.291 | 19353.073 | 1.865 | 12.032 | 0.140085 | 7/15/2014 | 15:05.0 | 2 |
| -728.056 | 19353.047 | 1.862 | 12.035 | 0.13543 | 7/15/2014 | 15:05.1 | 2 |
| -727.824 | 19353.02 | 1.855 | 12.038 | 0.134751 | 7/15/2014 | 15:05.2 | 2 |
| -727.59 | 19352.993 | 1.858 | 12.048 | 0.138442 | 7/15/2014 | 15:05.3 | 2 |
| -727.185 | 19352.944 | 1.859 | 12.085 | 0.134327 | 7/15/2014 | 15:05.4 | 2 |
| -726.956 | 19352.918 | 1.853 | 12.112 | 0.131206 | 7/15/2014 | 15:05.5 | 2 |
| -726.726 | 19352.892 | 1.857 | 12.141 | 0.14165 | 7/15/2014 | 15:05.6 | 2 |
| -726.438 | 19352.858 | 1.854 | 12.174 | 0.135712 | 7/15/2014 | 15:05.7 | 2 |
| -726.322 | 19352.845 | 1.853 | 12.193 | 0.138034 | 7/15/2014 | 15:05.8 | 2 |
| -726.089 | 19352.817 | 1.857 | 12.226 | 0.1478 | 7/15/2014 | 15:05.9 | 2 |
| -725.742 | 19352.778 | 1.849 | 12.265 | 0.152039 | 7/15/2014 | 15:06.0 | 2 |
| -725.512 | 19352.75 | 1.844 | 12.29 | 0.152776 | 7/15/2014 | 15:06.1 | 2 |
| -725.281 | 19352.724 | 1.844 | 12.291 | 0.152611 | 7/15/2014 | 15:06.2 | 2 |
| -725.166 | 19352.71 | 1.847 | 12.295 | 0.153104 | 7/15/2014 | 15:06.3 | 2 |
| -724.767 | 19352.664 | 1.84 | 12.287 | 0.145457 | 7/15/2014 | 15:06.4 | 2 |

| | | | | | | | |
|---|---|---|---|---|---|---|---|
| -724.537 | 19352.638 | 1.84 | 12.276 | 0.135232 | 7/15/2014 | 15:06.5 | 2 |
| -724.309 | 19352.61 | 1.84 | 12.263 | 0.130808 | 7/15/2014 | 15:06.6 | 2 |
| -724.023 | 19352.578 | 1.84 | 12.248 | 0.121012 | 7/15/2014 | 15:06.7 | 2 |
| -723.793 | 19352.552 | 1.84 | 12.226 | 0.116998 | 7/15/2014 | 15:06.8 | 2 |
| -723.562 | 19352.524 | 1.84 | 12.204 | 0.110701 | 7/15/2014 | 15:06.9 | 2 |
| -723.332 | 19352.499 | 1.834 | 12.183 | 0.111429 | 7/15/2014 | 15:07.0 | 2 |
| -723.101 | 19352.473 | 1.834 | 12.164 | 0.106827 | 7/15/2014 | 15:07.1 | 2 |
| -722.871 | 19352.446 | 1.834 | 12.141 | 0.11193 | 7/15/2014 | 15:07.2 | 2 |
| -722.64 | 19352.419 | 1.834 | 12.125 | 0.130416 | 7/15/2014 | 15:07.3 | 2 |
| -722.356 | 19352.388 | 1.829 | 12.112 | 0.13749 | 7/15/2014 | 15:07.4 | 2 |
| -722.125 | 19352.361 | 1.831 | 12.097 | 0.140683 | 7/15/2014 | 15:07.5 | 2 |
| -721.897 | 19352.334 | 1.827 | 12.081 | 0.144988 | 7/15/2014 | 15:07.6 | 2 |
| -721.608 | 19352.303 | 1.829 | 12.067 | 0.153631 | 7/15/2014 | 15:07.7 | 2 |
| -721.492 | 19352.29 | 1.831 | 12.061 | 0.168983 | 7/15/2014 | 15:07.8 | 2 |
| -721.258 | 19352.264 | 1.834 | 12.053 | 0.164668 | 7/15/2014 | 15:07.9 | 2 |
| -721.025 | 19352.236 | 1.832 | 12.044 | 0.175977 | 7/15/2014 | 15:08.0 | 2 |
| -720.789 | 19352.211 | 1.838 | 12.034 | 0.180688 | 7/15/2014 | 15:08.1 | 2 |
| -720.555 | 19352.184 | 1.834 | 12.022 | 0.168535 | 7/15/2014 | 15:08.2 | 2 |
| -720.319 | 19352.158 | 1.838 | 12.02 | 0.165252 | 7/15/2014 | 15:08.3 | 2 |
| -719.906 | 19352.112 | 1.839 | 12.037 | 0.148366 | 7/15/2014 | 15:08.4 | 2 |
| -719.671 | 19352.086 | 1.837 | 12.062 | 0.150961 | 7/15/2014 | 15:08.5 | 2 |
| -719.435 | 19352.061 | 1.837 | 12.087 | 0.146783 | 7/15/2014 | 15:08.6 | 2 |
| -719.14 | 19352.027 | 1.839 | 12.11 | 0.13546 | 7/15/2014 | 15:08.7 | 2 |
| -719.022 | 19352.014 | 1.835 | 12.125 | 0.138209 | 7/15/2014 | 15:08.8 | 2 |
| -718.782 | 19351.99 | 1.845 | 12.15 | 0.14047 | 7/15/2014 | 15:08.9 | 2 |
| -718.546 | 19351.963 | 1.839 | 12.169 | 0.134114 | 7/15/2014 | 15:09.0 | 2 |
| -718.306 | 19351.937 | 1.845 | 12.179 | 0.144024 | 7/15/2014 | 15:09.1 | 2 |
| -718.071 | 19351.91 | 1.839 | 12.175 | 0.143107 | 7/15/2014 | 15:09.2 | 2 |
| -717.833 | 19351.887 | 1.845 | 12.153 | 0.150481 | 7/15/2014 | 15:09.3 | 2 |
| -717.426 | 19351.842 | 1.839 | 12.125 | 0.155758 | 7/15/2014 | 15:09.4 | 2 |
| -717.191 | 19351.818 | 1.839 | 12.111 | 0.15062 | 7/15/2014 | 15:09.5 | 2 |
| -716.959 | 19351.793 | 1.839 | 12.096 | 0.148444 | 7/15/2014 | 15:09.6 | 2 |
| -716.669 | 19351.762 | 1.841 | 12.079 | 0.149072 | 7/15/2014 | 15:09.7 | 2 |
| -716.551 | 19351.749 | 1.838 | 12.073 | 0.147652 | 7/15/2014 | 15:09.8 | 2 |
| -716.316 | 19351.726 | 1.848 | 12.063 | 0.136477 | 7/15/2014 | 15:09.9 | 2 |
| -716.085 | 19351.7 | 1.848 | 12.064 | 0.149372 | 7/15/2014 | 15:10.0 | 2 |
| -715.852 | 19351.677 | 1.857 | 12.089 | 0.133373 | 7/15/2014 | 15:10.1 | 2 |
| -715.621 | 19351.653 | 1.861 | 12.124 | 0.139116 | 7/15/2014 | 15:10.2 | 2 |
| -715.388 | 19351.63 | 1.868 | 12.148 | 0.148602 | 7/15/2014 | 15:10.3 | 2 |
| -714.987 | 19351.588 | 1.87 | 12.188 | 0.139445 | 7/15/2014 | 15:10.4 | 2 |
| -714.757 | 19351.564 | 1.87 | 12.193 | 0.138628 | 7/15/2014 | 15:10.5 | 2 |
| -714.527 | 19351.542 | 1.862 | 12.189 | 0.150611 | 7/15/2014 | 15:10.6 | 2 |
| -714.238 | 19351.513 | 1.856 | 12.179 | 0.154937 | 7/15/2014 | 15:10.7 | 2 |
| -714.123 | 19351.501 | 1.852 | 12.164 | 0.150092 | 7/15/2014 | 15:10.8 | 2 |
| -713.89 | 19351.479 | 1.846 | 12.113 | 0.143384 | 7/15/2014 | 15:10.9 | 2 |
| -713.658 | 19351.455 | 1.839 | 12.067 | 0.141306 | 7/15/2014 | 15:11.0 | 2 |
| -713.422 | 19351.432 | 1.835 | 12.024 | 0.14885 | 7/15/2014 | 15:11.1 | 2 |

| | | | | | | | |
|---|---|---|---|---|---|---|---|
| -713.19 | 19351.408 | 1.828 | 11.982 | 0.142002 | 7/15/2014 | 15:11.2 | 2 |
| -712.956 | 19351.387 | 1.828 | 11.962 | 0.145129 | 7/15/2014 | 15:11.3 | 2 |
| -712.545 | 19351.346 | 1.832 | 11.944 | 0.135434 | 7/15/2014 | 15:11.4 | 2 |
| -712.313 | 19351.324 | 1.832 | 11.937 | 0.142085 | 7/15/2014 | 15:11.5 | 2 |
| -712.078 | 19351.302 | 1.835 | 11.955 | 0.139766 | 7/15/2014 | 15:11.6 | 2 |
| -711.787 | 19351.273 | 1.835 | 11.96 | 0.144482 | 7/15/2014 | 15:11.7 | 2 |
| -711.668 | 19351.262 | 1.835 | 11.957 | 0.145253 | 7/15/2014 | 15:11.8 | 2 |
| -711.431 | 19351.24 | 1.839 | 11.979 | 0.14819 | 7/15/2014 | 15:11.9 | 2 |
| -711.196 | 19351.217 | 1.837 | 12.009 | 0.149841 | 7/15/2014 | 15:12.0 | 2 |
| -710.958 | 19351.194 | 1.839 | 12.043 | 0.154089 | 7/15/2014 | 15:12.1 | 2 |
| -710.722 | 19351.173 | 1.833 | 12.079 | 0.159881 | 7/15/2014 | 15:12.2 | 2 |
| -710.486 | 19351.151 | 1.835 | 12.109 | 0.154281 | 7/15/2014 | 15:12.3 | 2 |
| -710.073 | 19351.112 | 1.828 | 12.118 | 0.158411 | 7/15/2014 | 15:12.4 | 2 |
| -709.837 | 19351.091 | 1.821 | 12.135 | 0.159933 | 7/15/2014 | 15:12.5 | 2 |
| -709.601 | 19351.068 | 1.817 | 12.138 | 0.159061 | 7/15/2014 | 15:12.6 | 2 |
| -709.303 | 19351.042 | 1.817 | 12.133 | 0.160477 | 7/15/2014 | 15:12.7 | 2 |
| -709.184 | 19351.031 | 1.815 | 12.131 | 0.161148 | 7/15/2014 | 15:12.8 | 2 |
| -708.944 | 19351.009 | 1.815 | 12.122 | 0.157468 | 7/15/2014 | 15:12.9 | 2 |
| -708.704 | 19350.988 | 1.812 | 12.112 | 0.155415 | 7/15/2014 | 15:13.0 | 2 |
| -708.461 | 19350.965 | 1.815 | 12.101 | 0.148358 | 7/15/2014 | 15:13.1 | 2 |
| -708.22 | 19350.944 | 1.811 | 12.093 | 0.140004 | 7/15/2014 | 15:13.2 | 2 |
| -707.977 | 19350.922 | 1.817 | 12.086 | 0.131767 | 7/15/2014 | 15:13.3 | 2 |
| -707.553 | 19350.885 | 1.81 | 12.072 | 0.128383 | 7/15/2014 | 15:13.4 | 2 |
| -707.313 | 19350.862 | 1.805 | 12.063 | 0.124457 | 7/15/2014 | 15:13.5 | 2 |
| -707.07 | 19350.842 | 1.801 | 12.052 | 0.126616 | 7/15/2014 | 15:13.6 | 2 |
| -706.768 | 19350.814 | 1.796 | 12.043 | 0.128963 | 7/15/2014 | 15:13.7 | 2 |
| -706.644 | 19350.805 | 1.793 | 12.041 | 0.131182 | 7/15/2014 | 15:13.8 | 2 |
| -706.399 | 19350.783 | 1.791 | 12.035 | 0.139275 | 7/15/2014 | 15:13.9 | 2 |
| -706.157 | 19350.761 | 1.785 | 12.036 | 0.150309 | 7/15/2014 | 15:14.0 | 2 |
| -705.91 | 19350.74 | 1.783 | 12.033 | 0.155255 | 7/15/2014 | 15:14.1 | 2 |
| -705.668 | 19350.72 | 1.778 | 12.032 | 0.161777 | 7/15/2014 | 15:14.2 | 2 |
| -705.424 | 19350.697 | 1.781 | 12.034 | 0.164059 | 7/15/2014 | 15:14.3 | 2 |
| -705.002 | 19350.662 | 1.771 | 12.037 | 0.157684 | 7/15/2014 | 15:14.4 | 2 |
| -704.761 | 19350.643 | 1.769 | 12.04 | 0.154157 | 7/15/2014 | 15:14.5 | 2 |
| -704.522 | 19350.621 | 1.769 | 12.051 | 0.145092 | 7/15/2014 | 15:14.6 | 2 |
| -704.223 | 19350.596 | 1.766 | 12.052 | 0.138077 | 7/15/2014 | 15:14.7 | 2 |
| -704.102 | 19350.586 | 1.763 | 12.054 | 0.135587 | 7/15/2014 | 15:14.8 | 2 |
| -703.862 | 19350.564 | 1.765 | 12.066 | 0.124238 | 7/15/2014 | 15:14.9 | 2 |
| -703.625 | 19350.546 | 1.76 | 12.081 | 0.121419 | 7/15/2014 | 15:15.0 | 2 |
| -703.387 | 19350.525 | 1.766 | 12.097 | 0.115818 | 7/15/2014 | 15:15.1 | 2 |
| -703.152 | 19350.508 | 1.759 | 12.109 | 0.114843 | 7/15/2014 | 15:15.2 | 2 |
| -702.916 | 19350.488 | 1.755 | 12.122 | 0.112079 | 7/15/2014 | 15:15.3 | 2 |
| -702.509 | 19350.453 | 1.743 | 12.126 | 0.118918 | 7/15/2014 | 15:15.4 | 2 |
| -702.277 | 19350.434 | 1.737 | 12.137 | 0.122447 | 7/15/2014 | 15:15.5 | 2 |
| -702.044 | 19350.417 | 1.731 | 12.14 | 0.117092 | 7/15/2014 | 15:15.6 | 2 |
| -701.754 | 19350.393 | 1.725 | 12.14 | 0.126314 | 7/15/2014 | 15:15.7 | 2 |
| -701.639 | 19350.385 | 1.718 | 12.14 | 0.124187 | 7/15/2014 | 15:15.8 | 2 |

| | | | | | | | |
|---|---|---|---|---|---|---|---|
| -701.403 | 19350.365 | 1.718 | 12.14 | 0.127481 | 7/15/2014 | 15:15.9 | 2 |
| -701.169 | 19350.346 | 1.718 | 12.14 | 0.131728 | 7/15/2014 | 15:16.0 | 2 |
| -700.933 | 19350.326 | 1.721 | 12.142 | 0.127403 | 7/15/2014 | 15:16.1 | 2 |
| -700.698 | 19350.308 | 1.718 | 12.141 | 0.125284 | 7/15/2014 | 15:16.2 | 2 |
| -700.46 | 19350.291 | 1.721 | 12.136 | 0.136857 | 7/15/2014 | 15:16.3 | 2 |
| -700.048 | 19350.258 | 1.717 | 12.136 | 0.122557 | 7/15/2014 | 15:16.4 | 2 |
| -699.812 | 19350.238 | 1.715 | 12.133 | 0.128549 | 7/15/2014 | 15:16.5 | 2 |
| -699.575 | 19350.22 | 1.715 | 12.133 | 0.123996 | 7/15/2014 | 15:16.6 | 2 |
| -699.278 | 19350.195 | 1.71 | 12.132 | 0.128807 | 7/15/2014 | 15:16.7 | 2 |
| -699.159 | 19350.187 | 1.707 | 12.13 | 0.122598 | 7/15/2014 | 15:16.8 | 2 |
| -698.918 | 19350.169 | 1.704 | 12.129 | 0.118727 | 7/15/2014 | 15:16.9 | 2 |
| -698.681 | 19350.15 | 1.698 | 12.127 | 0.115066 | 7/15/2014 | 15:17.0 | 2 |
| -698.441 | 19350.131 | 1.701 | 12.123 | 0.114341 | 7/15/2014 | 15:17.1 | 2 |
| -698.203 | 19350.111 | 1.697 | 12.116 | 0.112085 | 7/15/2014 | 15:17.2 | 2 |
| -697.965 | 19350.095 | 1.699 | 12.111 | 0.109476 | 7/15/2014 | 15:17.3 | 2 |
| -697.552 | 19350.061 | 1.7 | 12.107 | 0.0996574 | 7/15/2014 | 15:17.4 | 2 |
| -697.318 | 19350.044 | 1.702 | 12.106 | 0.107466 | 7/15/2014 | 15:17.5 | 2 |
| -697.084 | 19350.025 | 1.704 | 12.108 | 0.127198 | 7/15/2014 | 15:17.6 | 2 |
| -696.795 | 19350.003 | 1.704 | 12.11 | 0.126918 | 7/15/2014 | 15:17.7 | 2 |
| -696.675 | 19349.993 | 1.704 | 12.111 | 0.12226 | 7/15/2014 | 15:17.8 | 2 |
| -696.442 | 19349.977 | 1.708 | 12.112 | 0.128169 | 7/15/2014 | 15:17.9 | 2 |
| -696.096 | 19349.949 | 1.708 | 12.103 | 0.123744 | 7/15/2014 | 15:18.0 | 2 |
| -695.865 | 19349.931 | 1.703 | 12.097 | 0.133234 | 7/15/2014 | 15:18.1 | 2 |
| -695.634 | 19349.914 | 1.703 | 12.086 | 0.143039 | 7/15/2014 | 15:18.2 | 2 |
| -695.405 | 19349.896 | 1.701 | 12.072 | 0.128987 | 7/15/2014 | 15:18.3 | 2 |
| -695.123 | 19349.875 | 1.701 | 12.059 | 0.125961 | 7/15/2014 | 15:18.4 | 2 |
| -694.897 | 19349.857 | 1.701 | 12.044 | 0.11552 | 7/15/2014 | 15:18.5 | 2 |
| -694.67 | 19349.841 | 1.698 | 12.033 | 0.127977 | 7/15/2014 | 15:18.6 | 2 |
| -694.388 | 19349.818 | 1.698 | 12.026 | 0.123329 | 7/15/2014 | 15:18.7 | 2 |
| -694.162 | 19349.802 | 1.701 | 12.024 | 0.107451 | 7/15/2014 | 15:18.8 | 2 |
| -693.934 | 19349.783 | 1.706 | 12.022 | 0.104944 | 7/15/2014 | 15:18.9 | 2 |
| -693.705 | 19349.768 | 1.709 | 12.024 | 0.0966446 | 7/15/2014 | 15:19.0 | 2 |
| -693.477 | 19349.749 | 1.714 | 12.025 | 0.0904838 | 7/15/2014 | 15:19.1 | 2 |
| -693.25 | 19349.732 | 1.717 | 12.026 | 0.10392 | 7/15/2014 | 15:19.2 | 2 |
| -693.021 | 19349.715 | 1.722 | 12.034 | 0.0898603 | 7/15/2014 | 15:19.3 | 2 |
| -692.738 | 19349.693 | 1.726 | 12.04 | 0.0834266 | 7/15/2014 | 15:19.4 | 2 |
| -692.51 | 19349.678 | 1.726 | 12.045 | 0.0931255 | 7/15/2014 | 15:19.5 | 2 |
| -692.281 | 19349.658 | 1.722 | 12.047 | 0.0804895 | 7/15/2014 | 15:19.6 | 2 |
| -691.993 | 19349.637 | 1.726 | 12.076 | 0.0996591 | 7/15/2014 | 15:19.7 | 2 |
| -691.877 | 19349.627 | 1.725 | 12.082 | 0.0976913 | 7/15/2014 | 15:19.8 | 2 |
| -691.645 | 19349.611 | 1.721 | 12.096 | 0.0760209 | 7/15/2014 | 15:19.9 | 2 |
| -691.412 | 19349.593 | 1.715 | 12.13 | 0.0767702 | 7/15/2014 | 15:20.0 | 2 |
| -691.177 | 19349.576 | 1.715 | 12.169 | 0.0902091 | 7/15/2014 | 15:20.1 | 2 |
| -690.943 | 19349.559 | 1.715 | 12.21 | 0.096186 | 7/15/2014 | 15:20.2 | 2 |
| -690.708 | 19349.54 | 1.719 | 12.253 | 0.0979919 | 7/15/2014 | 15:20.3 | 2 |
| -690.297 | 19349.508 | 1.717 | 12.298 | 0.0994754 | 7/15/2014 | 15:20.4 | 2 |
| -690.062 | 19349.491 | 1.714 | 12.304 | 0.104493 | 7/15/2014 | 15:20.5 | 2 |

| | | | | | | | |
|---|---|---|---|---|---|---|---|
| -689.828 | 19349.474 | 1.714 | 12.328 | 0.109704 | 7/15/2014 | 15:20.6 | 2 |
| -689.532 | 19349.451 | 1.716 | 12.334 | 0.111676 | 7/15/2014 | 15:20.7 | 2 |
| -689.413 | 19349.441 | 1.714 | 12.333 | 0.106781 | 7/15/2014 | 15:20.8 | 2 |
| -689.175 | 19349.423 | 1.714 | 12.328 | 0.118343 | 7/15/2014 | 15:20.9 | 2 |
| -688.937 | 19349.406 | 1.714 | 12.323 | 0.125998 | 7/15/2014 | 15:21.0 | 2 |
| -688.699 | 19349.387 | 1.714 | 12.32 | 0.117183 | 7/15/2014 | 15:21.1 | 2 |
| -688.462 | 19349.368 | 1.717 | 12.314 | 0.122876 | 7/15/2014 | 15:21.2 | 2 |
| -688.225 | 19349.352 | 1.717 | 12.312 | 0.114997 | 7/15/2014 | 15:21.3 | 2 |
| -687.813 | 19349.318 | 1.716 | 12.303 | 0.107184 | 7/15/2014 | 15:21.4 | 2 |
| -687.579 | 19349.301 | 1.716 | 12.293 | 0.103334 | 7/15/2014 | 15:21.5 | 2 |
| -687.345 | 19349.283 | 1.713 | 12.283 | 0.10185 | 7/15/2014 | 15:21.6 | 2 |
| -687.053 | 19349.261 | 1.71 | 12.275 | 0.102689 | 7/15/2014 | 15:21.7 | 2 |
| -686.934 | 19349.251 | 1.71 | 12.27 | 0.100953 | 7/15/2014 | 15:21.8 | 2 |
| -686.7 | 19349.234 | 1.707 | 12.266 | 0.121257 | 7/15/2014 | 15:21.9 | 2 |
| -686.466 | 19349.215 | 1.707 | 12.263 | 0.0917613 | 7/15/2014 | 15:22.0 | 2 |
| -686.232 | 19349.197 | 1.71 | 12.262 | 0.107051 | 7/15/2014 | 15:22.1 | 2 |
| -685.999 | 19349.18 | 1.71 | 12.268 | 0.106547 | 7/15/2014 | 15:22.2 | 2 |
| -685.767 | 19349.161 | 1.71 | 12.274 | 0.109644 | 7/15/2014 | 15:22.3 | 2 |
| -685.363 | 19349.13 | 1.708 | 12.282 | 0.112041 | 7/15/2014 | 15:22.4 | 2 |
| -685.134 | 19349.113 | 1.706 | 12.282 | 0.10476 | 7/15/2014 | 15:22.5 | 2 |
| -684.903 | 19349.094 | 1.709 | 12.281 | 0.112474 | 7/15/2014 | 15:22.6 | 2 |
| -684.616 | 19349.073 | 1.706 | 12.275 | 0.105282 | 7/15/2014 | 15:22.7 | 2 |
| -684.499 | 19349.063 | 1.703 | 12.274 | 0.109167 | 7/15/2014 | 15:22.8 | 2 |
| -684.267 | 19349.046 | 1.706 | 12.262 | 0.110622 | 7/15/2014 | 15:22.9 | 2 |
| -683.918 | 19349.02 | 1.704 | 12.243 | 0.107277 | 7/15/2014 | 15:23.0 | 2 |
| -683.802 | 19349.01 | 1.706 | 12.236 | 0.113393 | 7/15/2014 | 15:23.1 | 2 |
| -683.572 | 19348.992 | 1.703 | 12.223 | 0.101661 | 7/15/2014 | 15:23.2 | 2 |
| -683.339 | 19348.973 | 1.709 | 12.212 | 0.107109 | 7/15/2014 | 15:23.3 | 2 |
| -682.934 | 19348.942 | 1.707 | 12.193 | 0.116972 | 7/15/2014 | 15:23.4 | 2 |
| -682.703 | 19348.924 | 1.707 | 12.182 | 0.112751 | 7/15/2014 | 15:23.5 | 2 |
| -682.473 | 19348.906 | 1.701 | 12.171 | 0.116384 | 7/15/2014 | 15:23.6 | 2 |
| -682.183 | 19348.885 | 1.701 | 12.16 | 0.11859 | 7/15/2014 | 15:23.7 | 2 |
| -682.067 | 19348.875 | 1.701 | 12.154 | 0.129224 | 7/15/2014 | 15:23.8 | 2 |
| -681.833 | 19348.855 | 1.703 | 12.144 | 0.116172 | 7/15/2014 | 15:23.9 | 2 |
| -681.6 | 19348.84 | 1.697 | 12.135 | 0.117012 | 7/15/2014 | 15:24.0 | 2 |
| -681.366 | 19348.82 | 1.697 | 12.129 | 0.104605 | 7/15/2014 | 15:24.1 | 2 |
| -681.134 | 19348.801 | 1.691 | 12.126 | 0.10401 | 7/15/2014 | 15:24.2 | 2 |
| -680.9 | 19348.784 | 1.693 | 12.121 | 0.103874 | 7/15/2014 | 15:24.3 | 2 |
| -680.493 | 19348.752 | 1.693 | 12.117 | 0.0895453 | 7/15/2014 | 15:24.4 | 2 |
| -680.26 | 19348.732 | 1.693 | 12.121 | 0.105473 | 7/15/2014 | 15:24.5 | 2 |
| -680.028 | 19348.717 | 1.689 | 12.126 | 0.0905094 | 7/15/2014 | 15:24.6 | 2 |
| -679.736 | 19348.693 | 1.687 | 12.135 | 0.0938724 | 7/15/2014 | 15:24.7 | 2 |
| -679.619 | 19348.684 | 1.686 | 12.138 | 0.0948917 | 7/15/2014 | 15:24.8 | 2 |
| -679.382 | 19348.665 | 1.689 | 12.149 | 0.0981684 | 7/15/2014 | 15:24.9 | 2 |
| -679.148 | 19348.646 | 1.684 | 12.167 | 0.0954874 | 7/15/2014 | 15:25.0 | 2 |
| -678.911 | 19348.627 | 1.689 | 12.186 | 0.0960831 | 7/15/2014 | 15:25.1 | 2 |
| -678.676 | 19348.609 | 1.684 | 12.208 | 0.0896859 | 7/15/2014 | 15:25.2 | 2 |

| | | | | | | | | |
|---|---|---|---|---|---|---|---|---|
| -678.441 | 19348.591 | 1.682 | 12.22 | 0.0854689 | 7/15/2014 | 15:25.3 | 2 |
| -678.028 | 19348.559 | 1.685 | 12.227 | 0.0835651 | 7/15/2014 | 15:25.4 | 2 |
| -677.793 | 19348.539 | 1.685 | 12.227 | 0.0825299 | 7/15/2014 | 15:25.5 | 2 |
| -677.559 | 19348.521 | 1.68 | 12.222 | 0.0786733 | 7/15/2014 | 15:25.6 | 2 |
| -677.264 | 19348.496 | 1.678 | 12.215 | 0.0820516 | 7/15/2014 | 15:25.7 | 2 |
| -677.146 | 19348.488 | 1.674 | 12.211 | 0.081965 | 7/15/2014 | 15:25.8 | 2 |
| -676.909 | 19348.469 | 1.674 | 12.195 | 0.084559 | 7/15/2014 | 15:25.9 | 2 |
| -676.672 | 19348.451 | 1.674 | 12.181 | 0.0926978 | 7/15/2014 | 15:26.0 | 2 |
| -676.433 | 19348.432 | 1.677 | 12.168 | 0.10235 | 7/15/2014 | 15:26.1 | 2 |
| -676.197 | 19348.412 | 1.672 | 12.166 | 0.117189 | 7/15/2014 | 15:26.2 | 2 |
| -675.961 | 19348.394 | 1.674 | 12.164 | 0.122671 | 7/15/2014 | 15:26.3 | 2 |
| -675.547 | 19348.359 | 1.67 | 12.167 | 0.135678 | 7/15/2014 | 15:26.4 | 2 |
| -675.312 | 19348.341 | 1.672 | 12.164 | 0.134629 | 7/15/2014 | 15:26.5 | 2 |
| -675.077 | 19348.323 | 1.672 | 12.163 | 0.129642 | 7/15/2014 | 15:26.6 | 2 |
| -674.785 | 19348.299 | 1.67 | 12.166 | 0.139654 | 7/15/2014 | 15:26.7 | 2 |
| -674.665 | 19348.289 | 1.667 | 12.17 | 0.134272 | 7/15/2014 | 15:26.8 | 2 |
| -674.429 | 19348.27 | 1.667 | 12.167 | 0.130364 | 7/15/2014 | 15:26.9 | 2 |
| -674.194 | 19348.251 | 1.662 | 12.168 | 0.125209 | 7/15/2014 | 15:27.0 | 2 |
| -673.958 | 19348.232 | 1.667 | 12.165 | 0.119546 | 7/15/2014 | 15:27.1 | 2 |
| -673.724 | 19348.213 | 1.665 | 12.161 | 0.120662 | 7/15/2014 | 15:27.2 | 2 |
| -673.49 | 19348.194 | 1.665 | 12.159 | 0.116759 | 7/15/2014 | 15:27.3 | 2 |
| -673.085 | 19348.16 | 1.664 | 12.155 | 0.0962659 | 7/15/2014 | 15:27.4 | 2 |
| -672.854 | 19348.141 | 1.661 | 12.153 | 0.104321 | 7/15/2014 | 15:27.5 | 2 |
| -672.623 | 19348.122 | 1.661 | 12.151 | 0.116979 | 7/15/2014 | 15:27.6 | 2 |
| -672.336 | 19348.1 | 1.664 | 12.148 | 0.119437 | 7/15/2014 | 15:27.7 | 2 |
| -672.22 | 19348.09 | 1.662 | 12.146 | 0.121695 | 7/15/2014 | 15:27.8 | 2 |
| -671.874 | 19348.063 | 1.663 | 12.15 | 0.130995 | 7/15/2014 | 15:27.9 | 2 |
| -671.643 | 19348.043 | 1.663 | 12.153 | 0.150861 | 7/15/2014 | 15:28.0 | 2 |
| -671.411 | 19348.023 | 1.666 | 12.166 | 0.171335 | 7/15/2014 | 15:28.1 | 2 |
| -671.182 | 19348.004 | 1.666 | 12.173 | 0.164114 | 7/15/2014 | 15:28.2 | 2 |
| -670.952 | 19347.984 | 1.668 | 12.18 | 0.170259 | 7/15/2014 | 15:28.3 | 2 |
| -670.667 | 19347.961 | 1.668 | 12.191 | 0.161597 | 7/15/2014 | 15:28.4 | 2 |
| -670.439 | 19347.945 | 1.668 | 12.209 | 0.175401 | 7/15/2014 | 15:28.5 | 2 |
| -670.211 | 19347.926 | 1.67 | 12.226 | 0.182185 | 7/15/2014 | 15:28.6 | 2 |
| -669.928 | 19347.902 | 1.67 | 12.246 | 0.174625 | 7/15/2014 | 15:28.7 | 2 |
| -669.698 | 19347.883 | 1.672 | 12.266 | 0.155464 | 7/15/2014 | 15:28.8 | 2 |
| -669.468 | 19347.863 | 1.672 | 12.278 | 0.166401 | 7/15/2014 | 15:28.9 | 2 |
| -669.238 | 19347.844 | 1.672 | 12.297 | 0.141531 | 7/15/2014 | 15:29.0 | 2 |
| -669.01 | 19347.824 | 1.674 | 12.317 | 0.170457 | 7/15/2014 | 15:29.1 | 2 |
| -668.78 | 19347.809 | 1.676 | 12.335 | 0.170121 | 7/15/2014 | 15:29.2 | 2 |
| -668.553 | 19347.789 | 1.676 | 12.34 | 0.163173 | 7/15/2014 | 15:29.3 | 2 |
| -668.27 | 19347.766 | 1.674 | 12.342 | 0.129917 | 7/15/2014 | 15:29.4 | 2 |
| -668.043 | 19347.746 | 1.678 | 12.34 | 0.161986 | 7/15/2014 | 15:29.5 | 2 |
| -667.817 | 19347.727 | 1.678 | 12.334 | 0.160702 | 7/15/2014 | 15:29.6 | 2 |
| -667.534 | 19347.703 | 1.678 | 12.324 | 0.153694 | 7/15/2014 | 15:29.7 | 2 |
| -667.308 | 19347.685 | 1.68 | 12.312 | 0.132584 | 7/15/2014 | 15:29.8 | 2 |
| -667.079 | 19347.668 | 1.68 | 12.294 | 0.123898 | 7/15/2014 | 15:29.9 | 2 |

| | | | | | | | |
|---|---|---|---|---|---|---|---|
| -666.85 | 19347.648 | 1.682 | 12.278 | 0.117232 | 7/15/2014 | 15:30.0 | 2 |
| -666.623 | 19347.629 | 1.684 | 12.259 | 0.127117 | 7/15/2014 | 15:30.1 | 2 |
| -666.396 | 19347.609 | 1.686 | 12.24 | 0.117648 | 7/15/2014 | 15:30.2 | 2 |
| -666.17 | 19347.592 | 1.686 | 12.221 | 0.106144 | 7/15/2014 | 15:30.3 | 2 |
| -665.888 | 19347.568 | 1.686 | 12.202 | 0.103531 | 7/15/2014 | 15:30.4 | 2 |
| -665.662 | 19347.55 | 1.688 | 12.183 | 0.117832 | 7/15/2014 | 15:30.5 | 2 |
| -665.435 | 19347.531 | 1.69 | 12.163 | 0.110176 | 7/15/2014 | 15:30.6 | 2 |
| -665.152 | 19347.508 | 1.694 | 12.143 | 0.105715 | 7/15/2014 | 15:30.7 | 2 |
| -664.925 | 19347.488 | 1.699 | 12.117 | 0.131818 | 7/15/2014 | 15:30.8 | 2 |
| -664.697 | 19347.471 | 1.702 | 12.095 | 0.138227 | 7/15/2014 | 15:30.9 | 2 |
| -664.468 | 19347.453 | 1.704 | 12.076 | 0.142549 | 7/15/2014 | 15:31.0 | 2 |
| -664.239 | 19347.434 | 1.707 | 12.06 | 0.170652 | 7/15/2014 | 15:31.1 | 2 |
| -664.01 | 19347.414 | 1.712 | 12.041 | 0.174986 | 7/15/2014 | 15:31.2 | 2 |
| -663.781 | 19347.395 | 1.715 | 12.023 | 0.182671 | 7/15/2014 | 15:31.3 | 2 |
| -663.496 | 19347.373 | 1.72 | 12.008 | 0.218038 | 7/15/2014 | 15:31.4 | 2 |
| -663.267 | 19347.354 | 1.726 | 11.993 | 0.186247 | 7/15/2014 | 15:31.5 | 2 |
| -663.039 | 19347.336 | 1.724 | 11.979 | 0.181569 | 7/15/2014 | 15:31.6 | 2 |
| -662.75 | 19347.313 | 1.726 | 11.969 | 0.164358 | 7/15/2014 | 15:31.7 | 2 |
| -662.634 | 19347.303 | 1.729 | 11.962 | 0.151472 | 7/15/2014 | 15:31.8 | 2 |
| -662.401 | 19347.283 | 1.732 | 11.952 | 0.140367 | 7/15/2014 | 15:31.9 | 2 |
| -662.169 | 19347.264 | 1.732 | 11.941 | 0.141172 | 7/15/2014 | 15:32.0 | 2 |
| -661.934 | 19347.246 | 1.735 | 11.932 | 0.132447 | 7/15/2014 | 15:32.1 | 2 |
| -661.701 | 19347.228 | 1.736 | 11.922 | 0.143334 | 7/15/2014 | 15:32.2 | 2 |
| -661.466 | 19347.209 | 1.736 | 11.919 | 0.143697 | 7/15/2014 | 15:32.3 | 2 |
| -661.056 | 19347.176 | 1.74 | 11.922 | 0.167673 | 7/15/2014 | 15:32.4 | 2 |
| -660.822 | 19347.156 | 1.74 | 11.931 | 0.169674 | 7/15/2014 | 15:32.5 | 2 |
| -660.587 | 19347.137 | 1.745 | 11.936 | 0.168356 | 7/15/2014 | 15:32.6 | 2 |
| -660.295 | 19347.113 | 1.745 | 11.948 | 0.181545 | 7/15/2014 | 15:32.7 | 2 |
| -660.177 | 19347.104 | 1.747 | 11.955 | 0.176978 | 7/15/2014 | 15:32.8 | 2 |
| -659.941 | 19347.086 | 1.749 | 11.974 | 0.176818 | 7/15/2014 | 15:32.9 | 2 |
| -659.705 | 19347.067 | 1.747 | 11.986 | 0.164022 | 7/15/2014 | 15:33.0 | 2 |
| -659.469 | 19347.049 | 1.747 | 11.995 | 0.167172 | 7/15/2014 | 15:33.1 | 2 |
| -659.233 | 19347.029 | 1.747 | 12 | 0.158677 | 7/15/2014 | 15:33.2 | 2 |
| -658.996 | 19347.01 | 1.753 | 11.999 | 0.162543 | 7/15/2014 | 15:33.3 | 2 |
| -658.588 | 19346.977 | 1.753 | 11.999 | 0.167233 | 7/15/2014 | 15:33.4 | 2 |
| -658.355 | 19346.958 | 1.759 | 12.001 | 0.173595 | 7/15/2014 | 15:33.5 | 2 |
| -658.122 | 19346.94 | 1.762 | 12.005 | 0.184868 | 7/15/2014 | 15:33.6 | 2 |
| -657.833 | 19346.918 | 1.764 | 12.005 | 0.170287 | 7/15/2014 | 15:33.7 | 2 |
| -657.714 | 19346.908 | 1.767 | 12.008 | 0.185302 | 7/15/2014 | 15:33.8 | 2 |
| -657.48 | 19346.889 | 1.767 | 12.008 | 0.1755 | 7/15/2014 | 15:33.9 | 2 |
| -657.247 | 19346.869 | 1.767 | 12.013 | 0.164696 | 7/15/2014 | 15:34.0 | 2 |
| -657.012 | 19346.85 | 1.773 | 12.013 | 0.161417 | 7/15/2014 | 15:34.1 | 2 |
| -656.781 | 19346.833 | 1.769 | 12.007 | 0.155132 | 7/15/2014 | 15:34.2 | 2 |
| -656.549 | 19346.814 | 1.765 | 11.997 | 0.151346 | 7/15/2014 | 15:34.3 | 2 |
| -656.145 | 19346.782 | 1.76 | 11.973 | 0.14466 | 7/15/2014 | 15:34.4 | 2 |
| -655.915 | 19346.764 | 1.763 | 11.962 | 0.139256 | 7/15/2014 | 15:34.5 | 2 |
| -655.685 | 19346.744 | 1.766 | 11.953 | 0.135609 | 7/15/2014 | 15:34.6 | 2 |

| | | | | | | | |
|---|---|---|---|---|---|---|---|
| -655.398 | 19346.721 | 1.77 | 11.938 | 0.13106 | 7/15/2014 | 15:34.7 | 2 |
| -655.283 | 19346.713 | 1.769 | 11.931 | 0.129826 | 7/15/2014 | 15:34.8 | 2 |
| -654.936 | 19346.686 | 1.776 | 11.924 | 0.127408 | 7/15/2014 | 15:34.9 | 2 |
| -654.703 | 19346.666 | 1.785 | 11.92 | 0.124608 | 7/15/2014 | 15:35.0 | 2 |
| -654.47 | 19346.647 | 1.791 | 11.918 | 0.124153 | 7/15/2014 | 15:35.1 | 2 |
| -654.355 | 19346.639 | 1.794 | 11.917 | 0.122294 | 7/15/2014 | 15:35.2 | 2 |
| -654.122 | 19346.621 | 1.794 | 11.916 | 0.124287 | 7/15/2014 | 15:35.3 | 2 |
| -653.712 | 19346.588 | 1.791 | 11.938 | 0.123252 | 7/15/2014 | 15:35.4 | 2 |
| -653.478 | 19346.57 | 1.788 | 11.943 | 0.12272 | 7/15/2014 | 15:35.5 | 2 |
| -653.24 | 19346.551 | 1.788 | 11.97 | 0.121648 | 7/15/2014 | 15:35.7 | 2 |
| -653 | 19346.531 | 1.785 | 12.007 | 0.120306 | 7/15/2014 | 15:35.7 | 2 |
| -652.762 | 19346.512 | 1.782 | 12.046 | 0.120363 | 7/15/2014 | 15:35.8 | 2 |
| -652.521 | 19346.494 | 1.779 | 12.09 | 0.120007 | 7/15/2014 | 15:35.9 | 2 |
| -652.28 | 19346.475 | 1.782 | 12.137 | 0.115482 | 7/15/2014 | 15:36.0 | 2 |
| -652.037 | 19346.455 | 1.786 | 12.181 | 0.114781 | 7/15/2014 | 15:36.1 | 2 |
| -651.796 | 19346.436 | 1.788 | 12.221 | 0.11403 | 7/15/2014 | 15:36.2 | 2 |
| -651.555 | 19346.416 | 1.791 | 12.268 | 0.115612 | 7/15/2014 | 15:36.3 | 2 |
| -651.314 | 19346.396 | 1.791 | 12.294 | 0.123611 | 7/15/2014 | 15:36.4 | 2 |
| -651.075 | 19346.377 | 1.794 | 12.304 | 0.119368 | 7/15/2014 | 15:36.5 | 2 |
| -650.838 | 19346.357 | 1.794 | 12.315 | 0.119208 | 7/15/2014 | 15:36.6 | 2 |
| -650.542 | 19346.334 | 1.794 | 12.319 | 0.122502 | 7/15/2014 | 15:36.7 | 2 |
| -650.42 | 19346.324 | 1.794 | 12.323 | 0.125722 | 7/15/2014 | 15:36.8 | 2 |
| -650.182 | 19346.305 | 1.8 | 12.307 | 0.128327 | 7/15/2014 | 15:36.9 | 2 |
| -649.947 | 19346.285 | 1.794 | 12.284 | 0.124721 | 7/15/2014 | 15:37.0 | 2 |
| -649.709 | 19346.267 | 1.796 | 12.259 | 0.115177 | 7/15/2014 | 15:37.1 | 2 |
| -649.474 | 19346.248 | 1.792 | 12.234 | 0.115582 | 7/15/2014 | 15:37.2 | 2 |
| -649.238 | 19346.229 | 1.792 | 12.21 | 0.113781 | 7/15/2014 | 15:37.3 | 2 |
| -648.821 | 19346.195 | 1.787 | 12.169 | 0.103971 | 7/15/2014 | 15:37.4 | 2 |
| -648.582 | 19346.175 | 1.79 | 12.142 | 0.104682 | 7/15/2014 | 15:37.5 | 2 |
| -648.343 | 19346.154 | 1.79 | 12.123 | 0.102034 | 7/15/2014 | 15:37.6 | 2 |
| -648.102 | 19346.136 | 1.79 | 12.108 | 0.109731 | 7/15/2014 | 15:37.7 | 2 |
| -647.862 | 19346.117 | 1.788 | 12.098 | 0.100874 | 7/15/2014 | 15:37.8 | 2 |
| -647.62 | 19346.098 | 1.788 | 12.086 | 0.0999395 | 7/15/2014 | 15:37.9 | 2 |
| -647.378 | 19346.078 | 1.788 | 12.077 | 0.100849 | 7/15/2014 | 15:38.0 | 2 |
| -647.136 | 19346.059 | 1.788 | 12.069 | 0.10421 | 7/15/2014 | 15:38.1 | 2 |
| -646.895 | 19346.038 | 1.787 | 12.06 | 0.101994 | 7/15/2014 | 15:38.2 | 2 |
| -646.654 | 19346.018 | 1.787 | 12.052 | 0.102539 | 7/15/2014 | 15:38.3 | 2 |
| -646.415 | 19345.998 | 1.782 | 12.045 | 0.10479 | 7/15/2014 | 15:38.4 | 2 |
| -646.178 | 19345.979 | 1.782 | 12.037 | 0.104124 | 7/15/2014 | 15:38.5 | 2 |
| -645.942 | 19345.96 | 1.778 | 12.025 | 0.10658 | 7/15/2014 | 15:38.6 | 2 |
| -645.651 | 19345.936 | 1.775 | 12.016 | 0.111123 | 7/15/2014 | 15:38.7 | 2 |
| -645.531 | 19345.926 | 1.772 | 12.009 | 0.106226 | 7/15/2014 | 15:38.8 | 2 |
| -645.295 | 19345.906 | 1.772 | 11.999 | 0.107676 | 7/15/2014 | 15:38.9 | 2 |
| -645.062 | 19345.888 | 1.766 | 11.987 | 0.116519 | 7/15/2014 | 15:39.0 | 2 |
| -644.826 | 19345.869 | 1.762 | 11.974 | 0.112707 | 7/15/2014 | 15:39.1 | 2 |
| -644.593 | 19345.85 | 1.752 | 11.96 | 0.114656 | 7/15/2014 | 15:39.2 | 2 |
| -644.357 | 19345.831 | 1.75 | 11.949 | 0.126118 | 7/15/2014 | 15:39.3 | 2 |

| | | | | | | | |
|---|---|---|---|---|---|---|---|
| -643.944 | 19345.795 | 1.745 | 11.932 | 0.127052 | 7/15/2014 | 15:39.4 | 2 |
| -643.707 | 19345.775 | 1.745 | 11.921 | 0.133784 | 7/15/2014 | 15:39.5 | 2 |
| -643.468 | 19345.757 | 1.745 | 11.913 | 0.134015 | 7/15/2014 | 15:39.6 | 2 |
| -643.227 | 19345.738 | 1.751 | 11.906 | 0.145544 | 7/15/2014 | 15:39.7 | 2 |
| -642.989 | 19345.719 | 1.747 | 11.903 | 0.144103 | 7/15/2014 | 15:39.8 | 2 |
| -642.745 | 19345.699 | 1.754 | 11.905 | 0.136932 | 7/15/2014 | 15:39.9 | 2 |
| -642.505 | 19345.679 | 1.754 | 11.91 | 0.142119 | 7/15/2014 | 15:40.0 | 2 |
| -642.264 | 19345.66 | 1.751 | 11.923 | 0.149625 | 7/15/2014 | 15:40.1 | 2 |
| -642.025 | 19345.641 | 1.747 | 11.936 | 0.166222 | 7/15/2014 | 15:40.2 | 2 |
| -641.785 | 19345.621 | 1.742 | 11.955 | 0.180202 | 7/15/2014 | 15:40.3 | 2 |
| -641.545 | 19345.602 | 1.737 | 11.975 | 0.180651 | 7/15/2014 | 15:40.4 | 2 |
| -641.311 | 19345.584 | 1.728 | 11.996 | 0.193806 | 7/15/2014 | 15:40.5 | 2 |
| -641.075 | 19345.564 | 1.726 | 12.013 | 0.209266 | 7/15/2014 | 15:40.6 | 2 |
| -640.783 | 19345.542 | 1.72 | 12.018 | 0.195931 | 7/15/2014 | 15:40.7 | 2 |
| -640.664 | 19345.533 | 1.715 | 12.028 | 0.182563 | 7/15/2014 | 15:40.8 | 2 |
| -640.429 | 19345.515 | 1.722 | 12.019 | 0.182403 | 7/15/2014 | 15:40.9 | 2 |
| -640.195 | 19345.496 | 1.718 | 12.006 | 0.135432 | 7/15/2014 | 15:41.0 | 2 |
| -639.958 | 19345.477 | 1.726 | 11.984 | 0.118678 | 7/15/2014 | 15:41.1 | 2 |
| -639.725 | 19345.459 | 1.726 | 11.957 | 0.105054 | 7/15/2014 | 15:41.2 | 2 |
| -639.488 | 19345.439 | 1.733 | 11.926 | 0.0920354 | 7/15/2014 | 15:41.3 | 2 |
| -639.069 | 19345.406 | 1.74 | 11.882 | 0.0790056 | 7/15/2014 | 15:41.4 | 2 |
| -638.828 | 19345.389 | 1.743 | 11.858 | 0.0851587 | 7/15/2014 | 15:41.5 | 2 |
| -638.586 | 19345.37 | 1.74 | 11.839 | 0.102507 | 7/15/2014 | 15:41.6 | 2 |
| -638.34 | 19345.351 | 1.743 | 11.819 | 0.0974367 | 7/15/2014 | 15:41.7 | 2 |
| -638.095 | 19345.331 | 1.74 | 11.8 | 0.0857772 | 7/15/2014 | 15:41.8 | 2 |
| -637.846 | 19345.313 | 1.74 | 11.784 | 0.0842291 | 7/15/2014 | 15:41.9 | 2 |
| -637.724 | 19345.304 | 1.738 | 11.776 | 0.0826892 | 7/15/2014 | 15:42.0 | 2 |
| -637.472 | 19345.283 | 1.739 | 11.763 | 0.0863894 | 7/15/2014 | 15:42.1 | 2 |
| -637.225 | 19345.265 | 1.733 | 11.753 | 0.0749641 | 7/15/2014 | 15:42.2 | 2 |
| -636.974 | 19345.245 | 1.736 | 11.744 | 0.0738487 | 7/15/2014 | 15:42.3 | 2 |
| -636.727 | 19345.227 | 1.733 | 11.739 | 0.0780148 | 7/15/2014 | 15:42.4 | 2 |
| -636.36 | 19345.198 | 1.725 | 11.734 | 0.0764524 | 7/15/2014 | 15:42.5 | 2 |
| -636.113 | 19345.181 | 1.725 | 11.736 | 0.0709939 | 7/15/2014 | 15:42.6 | 2 |
| -635.808 | 19345.157 | 1.722 | 11.738 | 0.066969 | 7/15/2014 | 15:42.7 | 2 |
| -635.684 | 19345.147 | 1.717 | 11.74 | 0.0690522 | 7/15/2014 | 15:42.8 | 2 |
| -635.439 | 19345.129 | 1.713 | 11.741 | 0.0661524 | 7/15/2014 | 15:42.9 | 2 |
| -635.196 | 19345.111 | 1.706 | 11.746 | 0.0568288 | 7/15/2014 | 15:43.0 | 2 |
| -634.952 | 19345.094 | 1.706 | 11.749 | 0.0564202 | 7/15/2014 | 15:43.1 | 2 |
| -634.709 | 19345.074 | 1.7 | 11.755 | 0.0457337 | 7/15/2014 | 15:43.2 | 2 |
| -634.464 | 19345.057 | 1.705 | 11.763 | 0.0462622 | 7/15/2014 | 15:43.3 | 2 |
| -634.034 | 19345.024 | 1.702 | 11.774 | 0.0508576 | 7/15/2014 | 15:43.4 | 2 |
| -633.789 | 19345.006 | 1.704 | 11.783 | 0.057043 | 7/15/2014 | 15:43.5 | 2 |
| -633.542 | 19344.988 | 1.704 | 11.806 | 0.0514621 | 7/15/2014 | 15:43.6 | 2 |
| -633.42 | 19344.979 | 1.704 | 11.812 | 0.0568707 | 7/15/2014 | 15:43.7 | 2 |
| -633.172 | 19344.961 | 1.699 | 11.847 | 0.0606681 | 7/15/2014 | 15:43.8 | 2 |
| -632.922 | 19344.942 | 1.701 | 11.885 | 0.064116 | 7/15/2014 | 15:43.9 | 2 |
| -632.675 | 19344.926 | 1.699 | 11.923 | 0.0721022 | 7/15/2014 | 15:44.0 | 2 |

| | | | | | | | |
|---|---|---|---|---|---|---|---|
| -632.305 | 19344.897 | 1.698 | 11.979 | 0.0735626 | 7/15/2014 | 15:44.1 | 2 |
| -632.06 | 19344.879 | 1.693 | 12.019 | 0.0705614 | 7/15/2014 | 15:44.2 | 2 |
| -631.815 | 19344.862 | 1.688 | 12.061 | 0.0733926 | 7/15/2014 | 15:44.3 | 2 |
| -631.573 | 19344.845 | 1.688 | 12.095 | 0.0760593 | 7/15/2014 | 15:44.4 | 2 |
| -631.332 | 19344.826 | 1.695 | 12.121 | 0.0759883 | 7/15/2014 | 15:44.5 | 2 |
| -631.094 | 19344.811 | 1.695 | 12.143 | 0.0770702 | 7/15/2014 | 15:44.6 | 2 |
| -630.8 | 19344.789 | 1.695 | 12.161 | 0.0558775 | 7/15/2014 | 15:44.7 | 2 |
| -630.679 | 19344.78 | 1.693 | 12.173 | 0.0653368 | 7/15/2014 | 15:44.8 | 2 |
| -630.442 | 19344.763 | 1.701 | 12.185 | 0.0837544 | 7/15/2014 | 15:44.9 | 2 |
| -630.207 | 19344.746 | 1.705 | 12.186 | 0.0906313 | 7/15/2014 | 15:45.0 | 2 |
| -629.971 | 19344.729 | 1.713 | 12.179 | 0.0472456 | 7/15/2014 | 15:45.1 | 2 |
| -629.736 | 19344.712 | 1.711 | 12.165 | 0.0606597 | 7/15/2014 | 15:45.2 | 2 |
| -629.5 | 19344.695 | 1.713 | 12.145 | 0.0568526 | 7/15/2014 | 15:45.3 | 2 |
| -629.085 | 19344.666 | 1.715 | 12.11 | 0.0598918 | 7/15/2014 | 15:45.4 | 2 |
| -628.847 | 19344.649 | 1.715 | 12.085 | 0.0683666 | 7/15/2014 | 15:45.5 | 2 |
| -628.607 | 19344.631 | 1.715 | 12.063 | 0.0701566 | 7/15/2014 | 15:45.6 | 2 |
| -628.366 | 19344.615 | 1.715 | 12.043 | 0.0661582 | 7/15/2014 | 15:45.7 | 2 |
| -628.123 | 19344.597 | 1.712 | 12.026 | 0.0618975 | 7/15/2014 | 15:45.8 | 2 |
| -627.88 | 19344.58 | 1.711 | 12.007 | 0.0598848 | 7/15/2014 | 15:45.9 | 2 |
| -627.637 | 19344.562 | 1.709 | 11.986 | 0.0519671 | 7/15/2014 | 15:46.0 | 2 |
| -627.393 | 19344.545 | 1.712 | 11.966 | 0.0689998 | 7/15/2014 | 15:46.1 | 2 |
| -627.151 | 19344.528 | 1.707 | 11.95 | 0.053386 | 7/15/2014 | 15:46.2 | 2 |
| -626.908 | 19344.51 | 1.707 | 11.936 | 0.0715252 | 7/15/2014 | 15:46.3 | 2 |
| -626.668 | 19344.494 | 1.704 | 11.925 | 0.0777511 | 7/15/2014 | 15:46.5 | 2 |
| -626.428 | 19344.477 | 1.7 | 11.915 | 0.0895494 | 7/15/2014 | 15:46.5 | 2 |
| -626.19 | 19344.46 | 1.7 | 11.909 | 0.0689731 | 7/15/2014 | 15:46.6 | 2 |
| -625.894 | 19344.438 | 1.706 | 11.91 | 0.0733116 | 7/15/2014 | 15:46.7 | 2 |
| -625.773 | 19344.43 | 1.702 | 11.909 | 0.0655928 | 7/15/2014 | 15:46.8 | 2 |
| -625.536 | 19344.413 | 1.705 | 11.915 | 0.0798849 | 7/15/2014 | 15:46.9 | 2 |
| -625.301 | 19344.396 | 1.702 | 11.919 | 0.057904 | 7/15/2014 | 15:47.0 | 2 |
| -625.063 | 19344.379 | 1.709 | 11.922 | 0.0709688 | 7/15/2014 | 15:47.1 | 2 |
| -624.827 | 19344.363 | 1.709 | 11.924 | 0.0553775 | 7/15/2014 | 15:47.2 | 2 |
| -624.589 | 19344.346 | 1.715 | 11.921 | 0.0515498 | 7/15/2014 | 15:47.3 | 2 |
| -624.17 | 19344.315 | 1.711 | 11.914 | 0.0507952 | 7/15/2014 | 15:47.4 | 2 |
| -623.929 | 19344.299 | 1.708 | 11.906 | 0.0288277 | 7/15/2014 | 15:47.5 | 2 |
| -623.687 | 19344.281 | 1.708 | 11.9 | 0.0227538 | 7/15/2014 | 15:47.6 | 2 |
| -623.442 | 19344.264 | 1.71 | 11.894 | 0.0232172 | 7/15/2014 | 15:47.7 | 2 |
| -623.196 | 19344.246 | 1.71 | 11.887 | 0.0242945 | 7/15/2014 | 15:47.8 | 2 |
| -622.949 | 19344.229 | 1.71 | 11.88 | 0.0289509 | 7/15/2014 | 15:47.9 | 2 |
| -622.826 | 19344.221 | 1.707 | 11.878 | 0.0314162 | 7/15/2014 | 15:48.0 | 2 |
| -622.578 | 19344.202 | 1.71 | 11.87 | 0.0554768 | 7/15/2014 | 15:48.1 | 2 |
| -622.33 | 19344.186 | 1.707 | 11.863 | 0.0380315 | 7/15/2014 | 15:48.2 | 2 |
| -622.081 | 19344.167 | 1.707 | 11.857 | 0.0449028 | 7/15/2014 | 15:48.3 | 2 |
| -621.713 | 19344.142 | 1.701 | 11.852 | 0.0671666 | 7/15/2014 | 15:48.4 | 2 |
| -621.47 | 19344.123 | 1.697 | 11.849 | 0.0538398 | 7/15/2014 | 15:48.5 | 2 |
| -621.226 | 19344.107 | 1.697 | 11.845 | 0.0666632 | 7/15/2014 | 15:48.6 | 2 |
| -620.928 | 19344.085 | 1.695 | 11.842 | 0.0642192 | 7/15/2014 | 15:48.7 | 2 |

| | | | | | | | |
|---|---|---|---|---|---|---|---|
| -620.804 | 19344.077 | 1.694 | 11.841 | 0.076102 | 7/15/2014 | 15:48.8 | 2 |
| -620.563 | 19344.06 | 1.697 | 11.841 | 0.0644292 | 7/15/2014 | 15:48.9 | 2 |
| -620.324 | 19344.043 | 1.692 | 11.842 | 0.0699958 | 7/15/2014 | 15:49.0 | 2 |
| -620.085 | 19344.026 | 1.692 | 11.844 | 0.0680852 | 7/15/2014 | 15:49.1 | 2 |
| -619.848 | 19344.009 | 1.685 | 11.844 | 0.0745311 | 7/15/2014 | 15:49.2 | 2 |
| -619.608 | 19343.992 | 1.689 | 11.843 | 0.0742506 | 7/15/2014 | 15:49.3 | 2 |
| -619.19 | 19343.963 | 1.686 | 11.846 | 0.0759661 | 7/15/2014 | 15:49.4 | 2 |
| -618.95 | 19343.946 | 1.686 | 11.848 | 0.0764031 | 7/15/2014 | 15:49.5 | 2 |
| -618.708 | 19343.929 | 1.686 | 11.847 | 0.075139 | 7/15/2014 | 15:49.6 | 2 |
| -618.465 | 19343.912 | 1.686 | 11.845 | 0.0813522 | 7/15/2014 | 15:49.7 | 2 |
| -618.221 | 19343.895 | 1.688 | 11.84 | 0.0807444 | 7/15/2014 | 15:49.8 | 2 |
| -617.976 | 19343.877 | 1.692 | 11.834 | 0.0808824 | 7/15/2014 | 15:49.9 | 2 |
| -617.731 | 19343.86 | 1.692 | 11.83 | 0.0980433 | 7/15/2014 | 15:50.0 | 2 |
| -617.485 | 19343.843 | 1.69 | 11.824 | 0.0932173 | 7/15/2014 | 15:50.1 | 2 |
| -617.24 | 19343.826 | 1.693 | 11.822 | 0.0950523 | 7/15/2014 | 15:50.2 | 2 |
| -616.995 | 19343.809 | 1.691 | 11.82 | 0.116222 | 7/15/2014 | 15:50.3 | 2 |
| -616.751 | 19343.791 | 1.691 | 11.816 | 0.102997 | 7/15/2014 | 15:50.4 | 2 |
| -616.508 | 19343.774 | 1.691 | 11.814 | 0.0961955 | 7/15/2014 | 15:50.5 | 2 |
| -616.267 | 19343.758 | 1.689 | 11.81 | 0.096881 | 7/15/2014 | 15:50.6 | 2 |
| -615.965 | 19343.736 | 1.689 | 11.806 | 0.0810831 | 7/15/2014 | 15:50.7 | 2 |
| -615.843 | 19343.728 | 1.687 | 11.806 | 0.074989 | 7/15/2014 | 15:50.8 | 2 |
| -615.601 | 19343.711 | 1.692 | 11.803 | 0.08616 | 7/15/2014 | 15:50.9 | 2 |
| -615.36 | 19343.694 | 1.686 | 11.796 | 0.0673004 | 7/15/2014 | 15:51.0 | 2 |
| -615.117 | 19343.676 | 1.69 | 11.791 | 0.0646125 | 7/15/2014 | 15:51.1 | 2 |
| -614.875 | 19343.66 | 1.69 | 11.788 | 0.08905 | 7/15/2014 | 15:51.2 | 2 |
| -614.632 | 19343.644 | 1.699 | 11.785 | 0.0704742 | 7/15/2014 | 15:51.3 | 2 |
| -614.203 | 19343.613 | 1.699 | 11.783 | 0.0934241 | 7/15/2014 | 15:51.4 | 2 |
| -613.955 | 19343.596 | 1.697 | 11.783 | 0.0723304 | 7/15/2014 | 15:51.5 | 2 |
| -613.835 | 19343.588 | 1.696 | 11.782 | 0.0748587 | 7/15/2014 | 15:51.6 | 2 |
| -613.584 | 19343.568 | 1.696 | 11.784 | 0.0688359 | 7/15/2014 | 15:51.7 | 2 |
| -613.332 | 19343.553 | 1.692 | 11.785 | 0.0698123 | 7/15/2014 | 15:51.8 | 2 |
| -613.078 | 19343.534 | 1.692 | 11.789 | 0.0898993 | 7/15/2014 | 15:51.9 | 2 |
| -612.825 | 19343.517 | 1.686 | 11.793 | 0.0854855 | 7/15/2014 | 15:52.0 | 2 |
| -612.571 | 19343.499 | 1.689 | 11.798 | 0.075734 | 7/15/2014 | 15:52.1 | 2 |
| -612.318 | 19343.482 | 1.689 | 11.804 | 0.10146 | 7/15/2014 | 15:52.2 | 2 |
| -612.064 | 19343.463 | 1.698 | 11.813 | 0.106202 | 7/15/2014 | 15:52.3 | 2 |
| -611.813 | 19343.447 | 1.698 | 11.821 | 0.0960379 | 7/15/2014 | 15:52.4 | 2 |
| -611.562 | 19343.429 | 1.702 | 11.83 | 0.0928779 | 7/15/2014 | 15:52.5 | 2 |
| -611.196 | 19343.403 | 1.7 | 11.853 | 0.0934336 | 7/15/2014 | 15:52.6 | 2 |
| -610.892 | 19343.383 | 1.702 | 11.868 | 0.0923897 | 7/15/2014 | 15:52.7 | 2 |
| -610.767 | 19343.375 | 1.699 | 11.876 | 0.08455 | 7/15/2014 | 15:52.8 | 2 |
| -610.523 | 19343.357 | 1.705 | 11.907 | 0.091754 | 7/15/2014 | 15:52.9 | 2 |
| -610.283 | 19343.342 | 1.696 | 11.951 | 0.104427 | 7/15/2014 | 15:53.0 | 2 |
| -610.041 | 19343.325 | 1.699 | 12.003 | 0.0950603 | 7/15/2014 | 15:53.1 | 2 |
| -609.802 | 19343.309 | 1.699 | 12.056 | 0.102151 | 7/15/2014 | 15:53.2 | 2 |
| -609.561 | 19343.292 | 1.705 | 12.099 | 0.125883 | 7/15/2014 | 15:53.3 | 2 |
| -609.141 | 19343.264 | 1.705 | 12.149 | 0.12048 | 7/15/2014 | 15:53.4 | 2 |

| | | | | | | | |
|---|---|---|---|---|---|---|---|
| -608.902 | 19343.248 | 1.703 | 12.182 | 0.11777 | 7/15/2014 | 15:53.5 | 2 |
| -608.661 | 19343.232 | 1.705 | 12.2 | 0.0844719 | 7/15/2014 | 15:53.6 | 2 |
| -608.419 | 19343.217 | 1.709 | 12.205 | 0.0793674 | 7/15/2014 | 15:53.7 | 2 |
| -608.177 | 19343.2 | 1.712 | 12.201 | 0.0721867 | 7/15/2014 | 15:53.8 | 2 |
| -607.935 | 19343.184 | 1.712 | 12.191 | 0.0708643 | 7/15/2014 | 15:53.9 | 2 |
| -607.693 | 19343.168 | 1.715 | 12.178 | 0.0731599 | 7/15/2014 | 15:54.0 | 2 |
| -607.452 | 19343.151 | 1.718 | 12.163 | 0.055616 | 7/15/2014 | 15:54.1 | 2 |
| -607.212 | 19343.136 | 1.714 | 12.145 | 0.0494273 | 7/15/2014 | 15:54.2 | 2 |
| -606.972 | 19343.121 | 1.714 | 12.125 | 0.0746706 | 7/15/2014 | 15:54.3 | 2 |
| -606.733 | 19343.105 | 1.712 | 12.103 | 0.0626369 | 7/15/2014 | 15:54.4 | 2 |
| -606.496 | 19343.09 | 1.712 | 12.078 | 0.0589486 | 7/15/2014 | 15:54.5 | 2 |
| -606.261 | 19343.074 | 1.712 | 12.054 | 0.0560309 | 7/15/2014 | 15:54.6 | 2 |
| -605.968 | 19343.055 | 1.71 | 12.03 | 0.0612276 | 7/15/2014 | 15:54.7 | 2 |
| -605.85 | 19343.047 | 1.704 | 12.019 | 0.0649434 | 7/15/2014 | 15:54.8 | 2 |
| -605.615 | 19343.031 | 1.708 | 11.996 | 0.0613332 | 7/15/2014 | 15:54.9 | 2 |
| -605.381 | 19343.018 | 1.711 | 11.971 | 0.0612633 | 7/15/2014 | 15:55.0 | 2 |
| -605.146 | 19343.002 | 1.717 | 11.946 | 0.0681529 | 7/15/2014 | 15:55.1 | 2 |
| -604.91 | 19342.986 | 1.714 | 11.923 | 0.0662845 | 7/15/2014 | 15:55.2 | 2 |
| -604.675 | 19342.972 | 1.717 | 11.902 | 0.0724611 | 7/15/2014 | 15:55.3 | 2 |
| -604.257 | 19342.943 | 1.716 | 11.871 | 0.0657227 | 7/15/2014 | 15:55.4 | 2 |
| -604.016 | 19342.928 | 1.719 | 11.857 | 0.0765703 | 7/15/2014 | 15:55.5 | 2 |
| -603.774 | 19342.912 | 1.715 | 11.848 | 0.0813105 | 7/15/2014 | 15:55.6 | 2 |
| -603.53 | 19342.896 | 1.71 | 11.844 | 0.0884778 | 7/15/2014 | 15:55.7 | 2 |
| -603.283 | 19342.881 | 1.707 | 11.848 | 0.0958509 | 7/15/2014 | 15:55.8 | 2 |
| -603.037 | 19342.864 | 1.705 | 11.848 | 0.0968791 | 7/15/2014 | 15:55.9 | 2 |
| -602.915 | 19342.855 | 1.702 | 11.848 | 0.102608 | 7/15/2014 | 15:56.0 | 2 |
| -602.664 | 19342.84 | 1.7 | 11.863 | 0.105933 | 7/15/2014 | 15:56.1 | 2 |
| -602.415 | 19342.823 | 1.694 | 11.886 | 0.10512 | 7/15/2014 | 15:56.2 | 2 |
| -602.165 | 19342.807 | 1.692 | 11.897 | 0.106918 | 7/15/2014 | 15:56.3 | 2 |
| -601.918 | 19342.791 | 1.687 | 11.915 | 0.103134 | 7/15/2014 | 15:56.4 | 2 |
| -601.55 | 19342.768 | 1.689 | 11.948 | 0.111671 | 7/15/2014 | 15:56.5 | 2 |
| -601.305 | 19342.751 | 1.689 | 11.964 | 0.12258 | 7/15/2014 | 15:56.6 | 2 |
| -601.002 | 19342.731 | 1.687 | 11.986 | 0.134667 | 7/15/2014 | 15:56.7 | 2 |
| -600.878 | 19342.723 | 1.685 | 11.993 | 0.143422 | 7/15/2014 | 15:56.8 | 2 |
| -600.633 | 19342.707 | 1.685 | 11.998 | 0.13935 | 7/15/2014 | 15:56.9 | 2 |
| -600.391 | 19342.692 | 1.682 | 12.01 | 0.153129 | 7/15/2014 | 15:57.0 | 2 |
| -600.146 | 19342.676 | 1.687 | 12.016 | 0.157315 | 7/15/2014 | 15:57.1 | 2 |
| -599.905 | 19342.66 | 1.685 | 12.016 | 0.162743 | 7/15/2014 | 15:57.2 | 2 |
| -599.661 | 19342.645 | 1.685 | 12.014 | 0.166662 | 7/15/2014 | 15:57.3 | 2 |
| -599.233 | 19342.616 | 1.681 | 12.006 | 0.177578 | 7/15/2014 | 15:57.4 | 2 |
| -598.988 | 19342.601 | 1.681 | 11.999 | 0.197078 | 7/15/2014 | 15:57.5 | 2 |
| -598.742 | 19342.585 | 1.683 | 11.993 | 0.193698 | 7/15/2014 | 15:57.6 | 2 |
| -598.619 | 19342.576 | 1.685 | 11.992 | 0.20258 | 7/15/2014 | 15:57.7 | 2 |
| -598.372 | 19342.561 | 1.68 | 11.985 | 0.190944 | 7/15/2014 | 15:57.8 | 2 |
| -598.123 | 19342.545 | 1.68 | 11.977 | 0.181056 | 7/15/2014 | 15:57.9 | 2 |
| -597.872 | 19342.529 | 1.678 | 11.968 | 0.15417 | 7/15/2014 | 15:58.0 | 2 |
| -597.621 | 19342.512 | 1.68 | 11.961 | 0.166737 | 7/15/2014 | 15:58.1 | 2 |

| | | | | | | | |
|---|---|---|---|---|---|---|---|
| -597.373 | 19342.496 | 1.678 | 11.954 | 0.156979 | 7/15/2014 | 15:58.2 | 2 |
| -597.003 | 19342.472 | 1.676 | 11.947 | 0.151951 | 7/15/2014 | 15:58.3 | 2 |
| -596.757 | 19342.455 | 1.676 | 11.94 | 0.142544 | 7/15/2014 | 15:58.4 | 2 |
| -596.514 | 19342.44 | 1.676 | 11.931 | 0.169106 | 7/15/2014 | 15:58.5 | 2 |
| -596.271 | 19342.425 | 1.676 | 11.921 | 0.14233 | 7/15/2014 | 15:58.6 | 2 |
| -595.971 | 19342.404 | 1.679 | 11.91 | 0.119011 | 7/15/2014 | 15:58.7 | 2 |
| -595.848 | 19342.396 | 1.678 | 11.906 | 0.105637 | 7/15/2014 | 15:58.8 | 2 |
| -595.608 | 19342.382 | 1.682 | 11.893 | 0.120621 | 7/15/2014 | 15:58.9 | 2 |
| -595.37 | 19342.366 | 1.685 | 11.879 | 0.133398 | 7/15/2014 | 15:59.0 | 2 |
| -595.13 | 19342.35 | 1.691 | 11.866 | 0.145371 | 7/15/2014 | 15:59.1 | 2 |
| -594.893 | 19342.335 | 1.686 | 11.854 | 0.113101 | 7/15/2014 | 15:59.2 | 2 |
| -594.654 | 19342.319 | 1.688 | 11.842 | 0.164258 | 7/15/2014 | 15:59.3 | 2 |
| -594.235 | 19342.292 | 1.684 | 11.822 | 0.174625 | 7/15/2014 | 15:59.4 | 2 |
| -593.995 | 19342.277 | 1.684 | 11.809 | 0.163135 | 7/15/2014 | 15:59.5 | 2 |
| -593.752 | 19342.262 | 1.682 | 11.797 | 0.150405 | 7/15/2014 | 15:59.6 | 2 |
| -593.51 | 19342.245 | 1.677 | 11.787 | 0.149135 | 7/15/2014 | 15:59.7 | 2 |
| -593.265 | 19342.229 | 1.676 | 11.776 | 0.15257 | 7/15/2014 | 15:59.8 | 2 |
| -593.02 | 19342.214 | 1.676 | 11.768 | 0.133986 | 7/15/2014 | 15:59.9 | 2 |
| -592.774 | 19342.197 | 1.676 | 11.76 | 0.151817 | 7/15/2014 | 16:00.0 | 2 |
| -592.528 | 19342.183 | 1.678 | 11.754 | 0.141921 | 7/15/2014 | 16:00.1 | 2 |
| -592.283 | 19342.166 | 1.68 | 11.751 | 0.148324 | 7/15/2014 | 16:00.2 | 2 |
| -592.037 | 19342.15 | 1.678 | 11.748 | 0.127936 | 7/15/2014 | 16:00.3 | 2 |
| -591.792 | 19342.134 | 1.68 | 11.746 | 0.148311 | 7/15/2014 | 16:00.4 | 2 |
| -591.549 | 19342.118 | 1.688 | 11.746 | 0.130423 | 7/15/2014 | 16:00.5 | 2 |
| -591.307 | 19342.103 | 1.686 | 11.75 | 0.137724 | 7/15/2014 | 16:00.6 | 2 |
| -591.006 | 19342.082 | 1.686 | 11.752 | 0.11351 | 7/15/2014 | 16:00.7 | 2 |
| -590.883 | 19342.075 | 1.686 | 11.754 | 0.117362 | 7/15/2014 | 16:00.8 | 2 |
| -590.642 | 19342.059 | 1.686 | 11.757 | 0.108395 | 7/15/2014 | 16:00.9 | 2 |
| -590.402 | 19342.043 | 1.686 | 11.76 | 0.132396 | 7/15/2014 | 16:01.0 | 2 |
| -590.161 | 19342.027 | 1.688 | 11.771 | 0.103408 | 7/15/2014 | 16:01.1 | 2 |
| -589.922 | 19342.012 | 1.683 | 11.798 | 0.108718 | 7/15/2014 | 16:01.2 | 2 |
| -589.684 | 19341.996 | 1.679 | 11.849 | 0.128068 | 7/15/2014 | 16:01.3 | 2 |
| -589.268 | 19341.969 | 1.685 | 11.92 | 0.120578 | 7/15/2014 | 16:01.4 | 2 |
| -589.033 | 19341.953 | 1.687 | 11.965 | 0.113026 | 7/15/2014 | 16:01.5 | 2 |
| -588.797 | 19341.938 | 1.689 | 12.013 | 0.123071 | 7/15/2014 | 16:01.6 | 2 |
| -588.505 | 19341.919 | 1.691 | 12.058 | 0.119742 | 7/15/2014 | 16:01.7 | 2 |
| -588.385 | 19341.911 | 1.692 | 12.093 | 0.12905 | 7/15/2014 | 16:01.8 | 2 |
| -588.151 | 19341.896 | 1.692 | 12.126 | 0.117524 | 7/15/2014 | 16:01.9 | 2 |
| -587.917 | 19341.881 | 1.69 | 12.135 | 0.148288 | 7/15/2014 | 16:02.0 | 2 |
| -587.681 | 19341.865 | 1.692 | 12.148 | 0.136756 | 7/15/2014 | 16:02.1 | 2 |
| -587.335 | 19341.843 | 1.686 | 12.166 | 0.149759 | 7/15/2014 | 16:02.2 | 2 |
| -587.104 | 19341.827 | 1.686 | 12.174 | 0.128202 | 7/15/2014 | 16:02.3 | 2 |
| -586.817 | 19341.81 | 1.686 | 12.178 | 0.1495 | 7/15/2014 | 16:02.4 | 2 |
| -586.59 | 19341.794 | 1.684 | 12.18 | 0.138082 | 7/15/2014 | 16:02.5 | 2 |
| -586.361 | 19341.78 | 1.686 | 12.178 | 0.133633 | 7/15/2014 | 16:02.6 | 2 |
| -586.074 | 19341.762 | 1.688 | 12.174 | 0.140727 | 7/15/2014 | 16:02.7 | 2 |
| -585.843 | 19341.746 | 1.688 | 12.166 | 0.138446 | 7/15/2014 | 16:02.8 | 2 |

| | | | | | | | |
|---|---|---|---|---|---|---|---|
| -585.611 | 19341.731 | 1.69 | 12.15 | 0.153762 | 7/15/2014 | 16:02.9 | 2 |
| -585.377 | 19341.717 | 1.69 | 12.136 | 0.157961 | 7/15/2014 | 16:03.0 | 2 |
| -585.262 | 19341.71 | 1.691 | 12.129 | 0.152639 | 7/15/2014 | 16:03.1 | 2 |
| -585.029 | 19341.694 | 1.686 | 12.112 | 0.142848 | 7/15/2014 | 16:03.2 | 2 |
| -584.794 | 19341.679 | 1.688 | 12.094 | 0.155977 | 7/15/2014 | 16:03.3 | 2 |
| -584.377 | 19341.652 | 1.688 | 12.067 | 0.14546 | 7/15/2014 | 16:03.4 | 2 |
| -584.137 | 19341.637 | 1.686 | 12.048 | 0.150634 | 7/15/2014 | 16:03.5 | 2 |
| -583.895 | 19341.621 | 1.684 | 12.028 | 0.15001 | 7/15/2014 | 16:03.6 | 2 |
| -583.651 | 19341.606 | 1.682 | 12.011 | 0.167712 | 7/15/2014 | 16:03.7 | 2 |
| -583.406 | 19341.591 | 1.679 | 11.994 | 0.164274 | 7/15/2014 | 16:03.8 | 2 |
| -583.16 | 19341.573 | 1.682 | 11.978 | 0.165386 | 7/15/2014 | 16:03.9 | 2 |
| -582.915 | 19341.559 | 1.679 | 11.965 | 0.171716 | 7/15/2014 | 16:04.0 | 2 |
| -582.668 | 19341.542 | 1.677 | 11.952 | 0.177718 | 7/15/2014 | 16:04.1 | 2 |
| -582.421 | 19341.525 | 1.675 | 11.941 | 0.166426 | 7/15/2014 | 16:04.2 | 2 |
| -582.175 | 19341.511 | 1.673 | 11.931 | 0.159738 | 7/15/2014 | 16:04.3 | 2 |
| -581.931 | 19341.495 | 1.671 | 11.921 | 0.149682 | 7/15/2014 | 16:04.4 | 2 |
| -581.687 | 19341.479 | 1.671 | 11.913 | 0.152755 | 7/15/2014 | 16:04.5 | 2 |
| -581.445 | 19341.463 | 1.669 | 11.903 | 0.144999 | 7/15/2014 | 16:04.6 | 2 |
| -581.145 | 19341.443 | 1.665 | 11.89 | 0.14553 | 7/15/2014 | 16:04.7 | 2 |
| -581.021 | 19341.436 | 1.662 | 11.884 | 0.145084 | 7/15/2014 | 16:04.8 | 2 |
| -580.78 | 19341.419 | 1.662 | 11.867 | 0.150103 | 7/15/2014 | 16:04.9 | 2 |
| -580.54 | 19341.404 | 1.66 | 11.849 | 0.143265 | 7/15/2014 | 16:05.0 | 2 |
| -580.299 | 19341.389 | 1.664 | 11.831 | 0.151276 | 7/15/2014 | 16:05.1 | 2 |
| -580.059 | 19341.372 | 1.662 | 11.814 | 0.144464 | 7/15/2014 | 16:05.2 | 2 |
| -579.818 | 19341.356 | 1.664 | 11.796 | 0.144707 | 7/15/2014 | 16:05.3 | 2 |
| -579.394 | 19341.329 | 1.667 | 11.776 | 0.141575 | 7/15/2014 | 16:05.4 | 2 |
| -579.151 | 19341.313 | 1.669 | 11.764 | 0.143741 | 7/15/2014 | 16:05.5 | 2 |
| -578.906 | 19341.297 | 1.669 | 11.759 | 0.150942 | 7/15/2014 | 16:05.6 | 2 |
| -578.661 | 19341.28 | 1.671 | 11.749 | 0.147006 | 7/15/2014 | 16:05.7 | 2 |
| -578.415 | 19341.265 | 1.669 | 11.742 | 0.147589 | 7/15/2014 | 16:05.8 | 2 |
| -578.167 | 19341.247 | 1.671 | 11.735 | 0.153011 | 7/15/2014 | 16:05.9 | 2 |
| -578.043 | 19341.239 | 1.669 | 11.731 | 0.154401 | 7/15/2014 | 16:06.0 | 2 |
| -577.794 | 19341.223 | 1.672 | 11.728 | 0.147588 | 7/15/2014 | 16:06.1 | 2 |
| -577.424 | 19341.198 | 1.665 | 11.719 | 0.149894 | 7/15/2014 | 16:06.2 | 2 |
| -577.177 | 19341.182 | 1.665 | 11.712 | 0.143741 | 7/15/2014 | 16:06.3 | 2 |
| -576.932 | 19341.165 | 1.667 | 11.704 | 0.143404 | 7/15/2014 | 16:06.4 | 2 |
| -576.688 | 19341.149 | 1.667 | 11.699 | 0.141024 | 7/15/2014 | 16:06.5 | 2 |
| -576.445 | 19341.133 | 1.669 | 11.693 | 0.1372 | 7/15/2014 | 16:06.6 | 2 |
| -576.144 | 19341.111 | 1.669 | 11.688 | 0.135786 | 7/15/2014 | 16:06.7 | 2 |
| -576.021 | 19341.104 | 1.667 | 11.683 | 0.13585 | 7/15/2014 | 16:06.8 | 2 |
| -575.778 | 19341.089 | 1.672 | 11.676 | 0.143311 | 7/15/2014 | 16:06.9 | 2 |
| -575.536 | 19341.071 | 1.669 | 11.671 | 0.142114 | 7/15/2014 | 16:07.0 | 2 |
| -575.294 | 19341.056 | 1.669 | 11.664 | 0.14132 | 7/15/2014 | 16:07.1 | 2 |
| -575.052 | 19341.039 | 1.667 | 11.657 | 0.138635 | 7/15/2014 | 16:07.2 | 2 |
| -574.81 | 19341.022 | 1.667 | 11.651 | 0.135574 | 7/15/2014 | 16:07.3 | 2 |
| -574.382 | 19340.993 | 1.667 | 11.637 | 0.139418 | 7/15/2014 | 16:07.4 | 2 |
| -574.136 | 19340.978 | 1.667 | 11.627 | 0.128759 | 7/15/2014 | 16:07.5 | 2 |

| | | | | | | | |
|---|---|---|---|---|---|---|---|
| -573.888 | 19340.96 | 1.667 | 11.619 | 0.125823 | 7/15/2014 | 16:07.6 | 2 |
| -573.767 | 19340.952 | 1.669 | 11.615 | 0.131729 | 7/15/2014 | 16:07.7 | 2 |
| -573.517 | 19340.935 | 1.672 | 11.606 | 0.121651 | 7/15/2014 | 16:07.8 | 2 |
| -573.266 | 19340.917 | 1.678 | 11.596 | 0.121662 | 7/15/2014 | 16:07.9 | 2 |
| -573.014 | 19340.9 | 1.68 | 11.586 | 0.0997065 | 7/15/2014 | 16:08.0 | 2 |
| -572.762 | 19340.883 | 1.686 | 11.581 | 0.0976425 | 7/15/2014 | 16:08.1 | 2 |
| -572.51 | 19340.865 | 1.684 | 11.572 | 0.0901181 | 7/15/2014 | 16:08.2 | 2 |
| -572.258 | 19340.848 | 1.692 | 11.564 | 0.0886489 | 7/15/2014 | 16:08.3 | 2 |
| -572.009 | 19340.83 | 1.692 | 11.565 | 0.0807776 | 7/15/2014 | 16:08.4 | 2 |
| -571.759 | 19340.813 | 1.695 | 11.605 | 0.0735615 | 7/15/2014 | 16:08.5 | 2 |
| -571.392 | 19340.787 | 1.69 | 11.693 | 0.0741277 | 7/15/2014 | 16:08.6 | 2 |
| -571.086 | 19340.766 | 1.69 | 11.747 | 0.070261 | 7/15/2014 | 16:08.7 | 2 |
| -570.961 | 19340.757 | 1.687 | 11.772 | 0.0706466 | 7/15/2014 | 16:08.8 | 2 |
| -570.714 | 19340.739 | 1.695 | 11.842 | 0.0707687 | 7/15/2014 | 16:08.9 | 2 |
| -570.471 | 19340.722 | 1.691 | 11.921 | 0.0725863 | 7/15/2014 | 16:09.0 | 2 |
| -570.226 | 19340.705 | 1.694 | 11.993 | 0.0757306 | 7/15/2014 | 16:09.1 | 2 |
| -569.984 | 19340.687 | 1.691 | 12.028 | 0.0746772 | 7/15/2014 | 16:09.2 | 2 |
| -569.74 | 19340.67 | 1.699 | 12.058 | 0.0723471 | 7/15/2014 | 16:09.3 | 2 |
| -569.317 | 19340.64 | 1.703 | 12.081 | 0.0749138 | 7/15/2014 | 16:09.4 | 2 |
| -569.075 | 19340.622 | 1.706 | 12.097 | 0.0711791 | 7/15/2014 | 16:09.5 | 2 |
| -568.832 | 19340.604 | 1.708 | 12.109 | 0.0704562 | 7/15/2014 | 16:09.6 | 2 |
| -568.531 | 19340.582 | 1.709 | 12.111 | 0.0774705 | 7/15/2014 | 16:09.7 | 2 |
| -568.409 | 19340.573 | 1.713 | 12.112 | 0.0793827 | 7/15/2014 | 16:09.8 | 2 |
| -568.164 | 19340.557 | 1.719 | 12.114 | 0.0714203 | 7/15/2014 | 16:09.9 | 2 |
| -567.921 | 19340.539 | 1.71 | 12.117 | 0.0901391 | 7/15/2014 | 16:10.0 | 2 |
| -567.676 | 19340.521 | 1.713 | 12.117 | 0.0756892 | 7/15/2014 | 16:10.1 | 2 |
| -567.434 | 19340.503 | 1.707 | 12.107 | 0.0783149 | 7/15/2014 | 16:10.2 | 2 |
| -567.191 | 19340.485 | 1.703 | 12.095 | 0.069969 | 7/15/2014 | 16:10.3 | 2 |
| -566.768 | 19340.453 | 1.704 | 12.053 | 0.0763079 | 7/15/2014 | 16:10.4 | 2 |
| -566.526 | 19340.437 | 1.703 | 12.025 | 0.0683108 | 7/15/2014 | 16:10.5 | 2 |
| -566.286 | 19340.419 | 1.703 | 11.992 | 0.0719982 | 7/15/2014 | 16:10.6 | 2 |
| -565.985 | 19340.396 | 1.708 | 11.955 | 0.0722456 | 7/15/2014 | 16:10.7 | 2 |
| -565.864 | 19340.387 | 1.709 | 11.932 | 0.0726697 | 7/15/2014 | 16:10.8 | 2 |
| -565.622 | 19340.369 | 1.715 | 11.901 | 0.0722574 | 7/15/2014 | 16:10.9 | 2 |
| -565.379 | 19340.352 | 1.721 | 11.867 | 0.0732654 | 7/15/2014 | 16:11.0 | 2 |
| -565.137 | 19340.332 | 1.724 | 11.837 | 0.07402 | 7/15/2014 | 16:11.1 | 2 |
| -564.897 | 19340.313 | 1.721 | 11.812 | 0.0837696 | 7/15/2014 | 16:11.2 | 2 |
| -564.655 | 19340.295 | 1.725 | 11.783 | 0.0850686 | 7/15/2014 | 16:11.3 | 2 |
| -564.235 | 19340.264 | 1.725 | 11.749 | 0.0880664 | 7/15/2014 | 16:11.4 | 2 |
| -563.996 | 19340.245 | 1.723 | 11.727 | 0.0849048 | 7/15/2014 | 16:11.5 | 2 |
| -563.755 | 19340.227 | 1.725 | 11.703 | 0.0862974 | 7/15/2014 | 16:11.6 | 2 |
| -563.456 | 19340.203 | 1.723 | 11.683 | 0.0886834 | 7/15/2014 | 16:11.7 | 2 |
| -563.335 | 19340.193 | 1.72 | 11.672 | 0.0909083 | 7/15/2014 | 16:11.8 | 2 |
| -563.092 | 19340.174 | 1.723 | 11.657 | 0.0920855 | 7/15/2014 | 16:11.9 | 2 |
| -562.849 | 19340.157 | 1.723 | 11.644 | 0.0912019 | 7/15/2014 | 16:12.0 | 2 |
| -562.607 | 19340.139 | 1.723 | 11.632 | 0.0934345 | 7/15/2014 | 16:12.1 | 2 |
| -562.366 | 19340.118 | 1.716 | 11.621 | 0.0941126 | 7/15/2014 | 16:12.2 | 2 |

| | | | | | | | |
|---|---|---|---|---|---|---|---|
| -562.124 | 19340.099 | 1.719 | 11.616 | 0.0925589 | 7/15/2014 | 16:12.3 | 2 |
| -561.703 | 19340.066 | 1.72 | 11.61 | 0.0925779 | 7/15/2014 | 16:12.4 | 2 |
| -561.464 | 19340.047 | 1.722 | 11.61 | 0.106156 | 7/15/2014 | 16:12.5 | 2 |
| -561.224 | 19340.028 | 1.722 | 11.606 | 0.101786 | 7/15/2014 | 16:12.6 | 2 |
| -560.926 | 19340.006 | 1.722 | 11.604 | 0.118581 | 7/15/2014 | 16:12.7 | 2 |
| -560.805 | 19339.995 | 1.721 | 11.604 | 0.114614 | 7/15/2014 | 16:12.8 | 2 |
| -560.564 | 19339.976 | 1.727 | 11.607 | 0.107455 | 7/15/2014 | 16:12.9 | 2 |
| -560.326 | 19339.957 | 1.725 | 11.61 | 0.105855 | 7/15/2014 | 16:13.0 | 2 |
| -560.085 | 19339.938 | 1.727 | 11.62 | 0.103886 | 7/15/2014 | 16:13.1 | 2 |
| -559.847 | 19339.92 | 1.721 | 11.626 | 0.110751 | 7/15/2014 | 16:13.2 | 2 |
| -559.609 | 19339.9 | 1.721 | 11.628 | 0.116268 | 7/15/2014 | 16:13.3 | 2 |
| -559.194 | 19339.867 | 1.722 | 11.66 | 0.0942423 | 7/15/2014 | 16:13.4 | 2 |
| -558.959 | 19339.848 | 1.719 | 11.685 | 0.108105 | 7/15/2014 | 16:13.5 | 2 |
| -558.723 | 19339.828 | 1.719 | 11.718 | 0.101988 | 7/15/2014 | 16:13.6 | 2 |
| -558.428 | 19339.806 | 1.719 | 11.745 | 0.119815 | 7/15/2014 | 16:13.7 | 2 |
| -558.309 | 19339.797 | 1.715 | 11.756 | 0.0912768 | 7/15/2014 | 16:13.8 | 2 |
| -558.07 | 19339.777 | 1.721 | 11.789 | 0.110342 | 7/15/2014 | 16:13.9 | 2 |
| -557.832 | 19339.757 | 1.721 | 11.825 | 0.119518 | 7/15/2014 | 16:14.0 | 2 |
| -557.593 | 19339.736 | 1.726 | 11.855 | 0.116018 | 7/15/2014 | 16:14.1 | 2 |
| -557.355 | 19339.717 | 1.721 | 11.894 | 0.0831314 | 7/15/2014 | 16:14.2 | 2 |
| -557.117 | 19339.697 | 1.725 | 11.923 | 0.109446 | 7/15/2014 | 16:14.3 | 2 |
| -556.702 | 19339.664 | 1.725 | 11.955 | 0.0648183 | 7/15/2014 | 16:14.4 | 2 |
| -556.463 | 19339.645 | 1.727 | 11.98 | 0.0612505 | 7/15/2014 | 16:14.5 | 2 |
| -556.225 | 19339.624 | 1.727 | 12.003 | 0.0661321 | 7/15/2014 | 16:14.6 | 2 |
| -555.928 | 19339.598 | 1.727 | 12.024 | 0.0473688 | 7/15/2014 | 16:14.7 | 2 |
| -555.809 | 19339.588 | 1.725 | 12.031 | 0.0720769 | 7/15/2014 | 16:14.8 | 2 |
| -555.568 | 19339.568 | 1.727 | 12.046 | 0.0270003 | 7/15/2014 | 16:14.9 | 2 |
| -555.327 | 19339.549 | 1.73 | 12.059 | 0.0458715 | 7/15/2014 | 16:15.0 | 2 |
| -555.086 | 19339.529 | 1.732 | 12.072 | 0.06545 | 7/15/2014 | 16:15.1 | 2 |
| -554.846 | 19339.51 | 1.73 | 12.078 | 0.0600217 | 7/15/2014 | 16:15.2 | 2 |
| -554.604 | 19339.488 | 1.732 | 12.082 | 0.0695838 | 7/15/2014 | 16:15.3 | 2 |
| -554.182 | 19339.451 | 1.729 | 12.087 | 0.070162 | 7/15/2014 | 16:15.4 | 2 |
| -553.942 | 19339.432 | 1.731 | 12.092 | 0.0967519 | 7/15/2014 | 16:15.5 | 2 |
| -553.701 | 19339.412 | 1.731 | 12.092 | 0.0602217 | 7/15/2014 | 16:15.6 | 2 |
| -553.399 | 19339.387 | 1.729 | 12.092 | 0.0636858 | 7/15/2014 | 16:15.7 | 2 |
| -553.278 | 19339.377 | 1.727 | 12.092 | 0.0660052 | 7/15/2014 | 16:15.8 | 2 |
| -553.034 | 19339.354 | 1.732 | 12.09 | 0.0504647 | 7/15/2014 | 16:15.9 | 2 |
| -552.792 | 19339.334 | 1.73 | 12.09 | 0.0392714 | 7/15/2014 | 16:16.0 | 2 |
| -552.547 | 19339.312 | 1.735 | 12.084 | 0.057889 | 7/15/2014 | 16:16.1 | 2 |
| -552.305 | 19339.291 | 1.73 | 12.084 | 0.03467 | 7/15/2014 | 16:16.2 | 2 |
| -552.062 | 19339.271 | 1.73 | 12.075 | 0.0672987 | 7/15/2014 | 16:16.3 | 2 |
| -551.639 | 19339.234 | 1.733 | 12.065 | 0.0615319 | 7/15/2014 | 16:16.4 | 2 |
| -551.399 | 19339.212 | 1.735 | 12.054 | 0.0399936 | 7/15/2014 | 16:16.5 | 2 |
| -551.16 | 19339.191 | 1.735 | 12.044 | 0.043297 | 7/15/2014 | 16:16.6 | 2 |
| -550.86 | 19339.165 | 1.737 | 12.032 | 0.0286683 | 7/15/2014 | 16:16.7 | 2 |
| -550.739 | 19339.154 | 1.736 | 12.03 | 0.0389474 | 7/15/2014 | 16:16.8 | 2 |
| -550.498 | 19339.134 | 1.741 | 12.015 | 0.0185403 | 7/15/2014 | 16:16.9 | 2 |

| | | | | | | | |
|---|---|---|---|---|---|---|---|
| -550.257 | 19339.111 | 1.739 | 12.005 | 0.0213206 | 7/15/2014 | 16:17.0 | 2 |
| -550.016 | 19339.092 | 1.739 | 11.995 | 0.0183033 | 7/15/2014 | 16:17.1 | 2 |
| -549.776 | 19339.068 | 1.739 | 11.987 | 0.0187369 | 7/15/2014 | 16:17.2 | 2 |
| -549.537 | 19339.049 | 1.739 | 11.982 | 0.0199949 | 7/15/2014 | 16:17.3 | 2 |
| -549.118 | 19339.012 | 1.742 | 11.98 | 0.0124429 | 7/15/2014 | 16:17.4 | 2 |
| -548.882 | 19338.991 | 1.742 | 11.981 | 0.0181532 | 7/15/2014 | 16:17.5 | 2 |
| -548.645 | 19338.969 | 1.742 | 11.982 | 0.0325647 | 7/15/2014 | 16:17.6 | 2 |
| -548.348 | 19338.943 | 1.747 | 11.982 | 0.0586604 | 7/15/2014 | 16:17.7 | 2 |
| -548.227 | 19338.932 | 1.748 | 11.983 | 0.0616826 | 7/15/2014 | 16:17.8 | 2 |
| -547.988 | 19338.912 | 1.748 | 11.983 | 0.0537659 | 7/15/2014 | 16:17.9 | 2 |
| -547.749 | 19338.892 | 1.746 | 11.985 | 0.0735022 | 7/15/2014 | 16:18.0 | 2 |
| -547.507 | 19338.869 | 1.75 | 11.987 | 0.0771173 | 7/15/2014 | 16:18.1 | 2 |
| -547.271 | 19338.85 | 1.75 | 11.987 | 0.0719918 | 7/15/2014 | 16:18.2 | 2 |
| -547.032 | 19338.827 | 1.757 | 11.983 | 0.0909462 | 7/15/2014 | 16:18.3 | 2 |
| -546.616 | 19338.79 | 1.765 | 11.973 | 0.0641048 | 7/15/2014 | 16:18.4 | 2 |
| -546.381 | 19338.77 | 1.765 | 11.965 | 0.102666 | 7/15/2014 | 16:18.5 | 2 |
| -546.144 | 19338.75 | 1.765 | 11.952 | 0.0944241 | 7/15/2014 | 16:18.6 | 2 |
| -545.848 | 19338.723 | 1.767 | 11.937 | 0.0985962 | 7/15/2014 | 16:18.7 | 2 |
| -545.729 | 19338.713 | 1.767 | 11.93 | 0.0989186 | 7/15/2014 | 16:18.8 | 2 |
| -545.489 | 19338.691 | 1.77 | 11.91 | 0.108294 | 7/15/2014 | 16:18.9 | 2 |
| -545.253 | 19338.67 | 1.763 | 11.893 | 0.11095 | 7/15/2014 | 16:19.0 | 2 |
| -545.012 | 19338.649 | 1.763 | 11.874 | 0.134974 | 7/15/2014 | 16:19.1 | 2 |
| -544.776 | 19338.627 | 1.759 | 11.853 | 0.125508 | 7/15/2014 | 16:19.2 | 2 |
| -544.537 | 19338.607 | 1.763 | 11.833 | 0.140741 | 7/15/2014 | 16:19.3 | 2 |
| -544.121 | 19338.57 | 1.769 | 11.813 | 0.136121 | 7/15/2014 | 16:19.4 | 2 |
| -543.884 | 19338.55 | 1.772 | 11.795 | 0.126112 | 7/15/2014 | 16:19.5 | 2 |
| -543.648 | 19338.527 | 1.772 | 11.781 | 0.130677 | 7/15/2014 | 16:19.6 | 2 |
| -543.35 | 19338.502 | 1.779 | 11.763 | 0.117894 | 7/15/2014 | 16:19.7 | 2 |
| -543.231 | 19338.49 | 1.78 | 11.754 | 0.117491 | 7/15/2014 | 16:19.8 | 2 |
| -542.991 | 19338.471 | 1.784 | 11.747 | 0.117369 | 7/15/2014 | 16:19.9 | 2 |
| -542.753 | 19338.45 | 1.784 | 11.735 | 0.116921 | 7/15/2014 | 16:20.0 | 2 |
| -542.513 | 19338.428 | 1.791 | 11.718 | 0.116177 | 7/15/2014 | 16:20.1 | 2 |
| -542.275 | 19338.408 | 1.785 | 11.705 | 0.119231 | 7/15/2014 | 16:20.2 | 2 |
| -542.036 | 19338.387 | 1.789 | 11.694 | 0.120297 | 7/15/2014 | 16:20.3 | 2 |
| -541.62 | 19338.35 | 1.786 | 11.67 | 0.122816 | 7/15/2014 | 16:20.4 | 2 |
| -541.385 | 19338.33 | 1.783 | 11.656 | 0.125312 | 7/15/2014 | 16:20.5 | 2 |
| -541.147 | 19338.309 | 1.783 | 11.635 | 0.127971 | 7/15/2014 | 16:20.6 | 2 |
| -540.851 | 19338.281 | 1.783 | 11.619 | 0.129678 | 7/15/2014 | 16:20.7 | 2 |
| -540.732 | 19338.271 | 1.781 | 11.611 | 0.133175 | 7/15/2014 | 16:20.8 | 2 |
| -540.492 | 19338.252 | 1.785 | 11.598 | 0.133703 | 7/15/2014 | 16:20.9 | 2 |
| -540.255 | 19338.23 | 1.781 | 11.588 | 0.134816 | 7/15/2014 | 16:21.0 | 2 |
| -540.015 | 19338.209 | 1.785 | 11.581 | 0.130082 | 7/15/2014 | 16:21.1 | 2 |
| -539.778 | 19338.189 | 1.787 | 11.573 | 0.142295 | 7/15/2014 | 16:21.2 | 2 |
| -539.539 | 19338.167 | 1.791 | 11.565 | 0.145472 | 7/15/2014 | 16:21.3 | 2 |
| -539.125 | 19338.131 | 1.788 | 11.559 | 0.145384 | 7/15/2014 | 16:21.4 | 2 |
| -538.889 | 19338.111 | 1.788 | 11.556 | 0.176896 | 7/15/2014 | 16:21.5 | 2 |
| -538.655 | 19338.09 | 1.79 | 11.555 | 0.166152 | 7/15/2014 | 16:21.6 | 2 |

| | | | | | | | |
|---|---|---|---|---|---|---|---|
| -538.361 | 19338.063 | 1.793 | 11.55 | 0.189989 | 7/15/2014 | 16:21.7 | 2 |
| -538.241 | 19338.054 | 1.794 | 11.547 | 0.175222 | 7/15/2014 | 16:21.8 | 2 |
| -538.005 | 19338.032 | 1.794 | 11.548 | 0.190762 | 7/15/2014 | 16:21.9 | 2 |
| -537.772 | 19338.012 | 1.79 | 11.549 | 0.186901 | 7/15/2014 | 16:22.0 | 2 |
| -537.535 | 19337.992 | 1.794 | 11.548 | 0.197991 | 7/15/2014 | 16:22.1 | 2 |
| -537.301 | 19337.972 | 1.794 | 11.554 | 0.176339 | 7/15/2014 | 16:22.2 | 2 |
| -537.068 | 19337.949 | 1.794 | 11.554 | 0.202276 | 7/15/2014 | 16:22.3 | 2 |
| -536.663 | 19337.915 | 1.793 | 11.551 | 0.199892 | 7/15/2014 | 16:22.4 | 2 |
| -536.432 | 19337.894 | 1.793 | 11.553 | 0.194438 | 7/15/2014 | 16:22.5 | 2 |
| -536.203 | 19337.873 | 1.791 | 11.548 | 0.184939 | 7/15/2014 | 16:22.6 | 2 |
| -535.914 | 19337.849 | 1.795 | 11.541 | 0.171405 | 7/15/2014 | 16:22.7 | 2 |
| -535.798 | 19337.838 | 1.795 | 11.541 | 0.165606 | 7/15/2014 | 16:22.8 | 2 |
| -535.566 | 19337.816 | 1.795 | 11.528 | 0.151514 | 7/15/2014 | 16:22.9 | 2 |
| -535.333 | 19337.797 | 1.797 | 11.514 | 0.130325 | 7/15/2014 | 16:23.0 | 2 |
| -535.1 | 19337.777 | 1.803 | 11.498 | 0.130637 | 7/15/2014 | 16:23.1 | 2 |
| -534.867 | 19337.755 | 1.801 | 11.482 | 0.113645 | 7/15/2014 | 16:23.2 | 2 |
| -534.635 | 19337.734 | 1.803 | 11.461 | 0.114034 | 7/15/2014 | 16:23.3 | 2 |
| -534.227 | 19337.699 | 1.804 | 11.432 | 0.120481 | 7/15/2014 | 16:23.4 | 2 |
| -533.996 | 19337.678 | 1.801 | 11.415 | 0.120855 | 7/15/2014 | 16:23.5 | 2 |
| -533.761 | 19337.658 | 1.801 | 11.396 | 0.120412 | 7/15/2014 | 16:23.6 | 2 |
| -533.469 | 19337.633 | 1.795 | 11.382 | 0.0944563 | 7/15/2014 | 16:23.7 | 2 |
| -533.351 | 19337.621 | 1.79 | 11.373 | 0.0897495 | 7/15/2014 | 16:23.8 | 2 |
| -533.114 | 19337.601 | 1.79 | 11.359 | 0.0888969 | 7/15/2014 | 16:23.9 | 2 |
| -532.876 | 19337.58 | 1.787 | 11.346 | 0.0637227 | 7/15/2014 | 16:24.0 | 2 |
| -532.638 | 19337.559 | 1.787 | 11.338 | 0.0470554 | 7/15/2014 | 16:24.1 | 2 |
| -532.402 | 19337.537 | 1.785 | 11.334 | 0.0494843 | 7/15/2014 | 16:24.2 | 2 |
| -532.163 | 19337.518 | 1.791 | 11.329 | 0.0565549 | 7/15/2014 | 16:24.3 | 2 |
| -531.75 | 19337.48 | 1.791 | 11.331 | 0.0436688 | 7/15/2014 | 16:24.4 | 2 |
| -531.512 | 19337.459 | 1.794 | 11.332 | 0.0529577 | 7/15/2014 | 16:24.5 | 2 |
| -531.276 | 19337.438 | 1.794 | 11.338 | 0.0507857 | 7/15/2014 | 16:24.6 | 2 |
| -530.982 | 19337.411 | 1.79 | 11.344 | 0.0534447 | 7/15/2014 | 16:24.7 | 2 |
| -530.862 | 19337.4 | 1.79 | 11.347 | 0.059803 | 7/15/2014 | 16:24.8 | 2 |
| -530.624 | 19337.381 | 1.79 | 11.354 | 0.0806826 | 7/15/2014 | 16:24.9 | 2 |
| -530.386 | 19337.359 | 1.788 | 11.366 | 0.0756933 | 7/15/2014 | 16:25.0 | 2 |
| -530.149 | 19337.338 | 1.788 | 11.383 | 0.0828909 | 7/15/2014 | 16:25.1 | 2 |
| -529.913 | 19337.317 | 1.78 | 11.396 | 0.104011 | 7/15/2014 | 16:25.2 | 2 |
| -529.676 | 19337.297 | 1.777 | 11.417 | 0.0946294 | 7/15/2014 | 16:25.3 | 2 |
| -529.265 | 19337.26 | 1.779 | 11.461 | 0.122803 | 7/15/2014 | 16:25.4 | 2 |
| -529.03 | 19337.24 | 1.779 | 11.514 | 0.09905 | 7/15/2014 | 16:25.5 | 2 |
| -528.794 | 19337.22 | 1.779 | 11.57 | 0.108571 | 7/15/2014 | 16:25.6 | 2 |
| -528.499 | 19337.193 | 1.775 | 11.625 | 0.109812 | 7/15/2014 | 16:25.7 | 2 |
| -528.381 | 19337.184 | 1.773 | 11.651 | 0.10459 | 7/15/2014 | 16:25.8 | 2 |
| -528.142 | 19337.162 | 1.781 | 11.716 | 0.112226 | 7/15/2014 | 16:25.9 | 2 |
| -527.903 | 19337.141 | 1.784 | 11.778 | 0.117637 | 7/15/2014 | 16:26.0 | 2 |
| -527.663 | 19337.121 | 1.784 | 11.832 | 0.0900095 | 7/15/2014 | 16:26.1 | 2 |
| -527.426 | 19337.1 | 1.78 | 11.89 | 0.108663 | 7/15/2014 | 16:26.2 | 2 |
| -527.185 | 19337.079 | 1.787 | 11.917 | 0.11047 | 7/15/2014 | 16:26.3 | 2 |

| | | | | | | | |
|---|---|---|---|---|---|---|---|
| -526.766 | 19337.043 | 1.784 | 11.946 | 0.115732 | 7/15/2014 | 16:26.4 | 2 |
| -526.528 | 19337.021 | 1.786 | 11.961 | 0.12919 | 7/15/2014 | 16:26.5 | 2 |
| -526.287 | 19337.001 | 1.786 | 11.966 | 0.104439 | 7/15/2014 | 16:26.6 | 2 |
| -525.987 | 19336.977 | 1.786 | 11.963 | 0.103813 | 7/15/2014 | 16:26.7 | 2 |
| -525.867 | 19336.965 | 1.785 | 11.966 | 0.0981947 | 7/15/2014 | 16:26.8 | 2 |
| -525.624 | 19336.943 | 1.791 | 11.95 | 0.0872066 | 7/15/2014 | 16:26.9 | 2 |
| -525.382 | 19336.923 | 1.785 | 11.931 | 0.106883 | 7/15/2014 | 16:27.0 | 2 |
| -525.14 | 19336.902 | 1.785 | 11.905 | 0.069078 | 7/15/2014 | 16:27.1 | 2 |
| -524.899 | 19336.881 | 1.782 | 11.88 | 0.0753103 | 7/15/2014 | 16:27.2 | 2 |
| -524.657 | 19336.86 | 1.779 | 11.854 | 0.0685411 | 7/15/2014 | 16:27.3 | 2 |
| -524.236 | 19336.824 | 1.776 | 11.823 | 0.0792234 | 7/15/2014 | 16:27.4 | 2 |
| -523.996 | 19336.803 | 1.772 | 11.802 | 0.0967447 | 7/15/2014 | 16:27.5 | 2 |
| -523.757 | 19336.783 | 1.767 | 11.784 | 0.0837941 | 7/15/2014 | 16:27.6 | 2 |
| -523.459 | 19336.758 | 1.764 | 11.766 | 0.0936665 | 7/15/2014 | 16:27.7 | 2 |
| -523.337 | 19336.746 | 1.764 | 11.757 | 0.080862 | 7/15/2014 | 16:27.8 | 2 |
| -523.095 | 19336.727 | 1.77 | 11.745 | 0.0967842 | 7/15/2014 | 16:27.9 | 2 |
| -522.856 | 19336.707 | 1.77 | 11.733 | 0.097745 | 7/15/2014 | 16:28.0 | 2 |
| -522.615 | 19336.686 | 1.772 | 11.725 | 0.124796 | 7/15/2014 | 16:28.1 | 2 |
| -522.375 | 19336.666 | 1.765 | 11.718 | 0.0992956 | 7/15/2014 | 16:28.2 | 2 |
| -522.135 | 19336.645 | 1.768 | 11.712 | 0.0949181 | 7/15/2014 | 16:28.3 | 2 |
| -521.72 | 19336.609 | 1.763 | 11.718 | 0.0774883 | 7/15/2014 | 16:28.4 | 2 |
| -521.484 | 19336.59 | 1.763 | 11.721 | 0.0780063 | 7/15/2014 | 16:28.5 | 2 |
| -521.247 | 19336.57 | 1.76 | 11.736 | 0.0493772 | 7/15/2014 | 16:28.6 | 2 |
| -520.953 | 19336.545 | 1.757 | 11.775 | 0.0369474 | 7/15/2014 | 16:28.7 | 2 |
| -520.833 | 19336.535 | 1.756 | 11.784 | 0.038704 | 7/15/2014 | 16:28.8 | 2 |
| -520.593 | 19336.514 | 1.76 | 11.837 | 0.0499934 | 7/15/2014 | 16:28.9 | 2 |
| -520.358 | 19336.494 | 1.75 | 11.895 | 0.0580619 | 7/15/2014 | 16:29.0 | 2 |
| -520.118 | 19336.475 | 1.76 | 11.955 | 0.0630778 | 7/15/2014 | 16:29.1 | 2 |
| -519.883 | 19336.455 | 1.76 | 12.007 | 0.0681955 | 7/15/2014 | 16:29.2 | 2 |
| -519.645 | 19336.436 | 1.763 | 12.068 | 0.0851785 | 7/15/2014 | 16:29.3 | 2 |
| -519.232 | 19336.4 | 1.757 | 12.134 | 0.0934142 | 7/15/2014 | 16:29.4 | 2 |
| -518.996 | 19336.381 | 1.755 | 12.162 | 0.0916863 | 7/15/2014 | 16:29.5 | 2 |
| -518.76 | 19336.361 | 1.755 | 12.183 | 0.0821373 | 7/15/2014 | 16:29.6 | 2 |
| -518.466 | 19336.336 | 1.757 | 12.202 | 0.109736 | 7/15/2014 | 16:29.7 | 2 |
| -518.347 | 19336.326 | 1.752 | 12.212 | 0.106592 | 7/15/2014 | 16:29.8 | 2 |
| -518.108 | 19336.307 | 1.756 | 12.228 | 0.0975809 | 7/15/2014 | 16:29.9 | 2 |
| -517.872 | 19336.287 | 1.748 | 12.239 | 0.107681 | 7/15/2014 | 16:30.0 | 2 |
| -517.632 | 19336.268 | 1.745 | 12.244 | 0.116316 | 7/15/2014 | 16:30.1 | 2 |
| -517.395 | 19336.248 | 1.739 | 12.241 | 0.137085 | 7/15/2014 | 16:30.2 | 2 |
| -517.156 | 19336.229 | 1.741 | 12.235 | 0.146986 | 7/15/2014 | 16:30.3 | 2 |
| -516.741 | 19336.193 | 1.736 | 12.216 | 0.16265 | 7/15/2014 | 16:30.4 | 2 |
| -516.503 | 19336.174 | 1.734 | 12.206 | 0.197818 | 7/15/2014 | 16:30.5 | 2 |
| -516.268 | 19336.154 | 1.725 | 12.192 | 0.189059 | 7/15/2014 | 16:30.6 | 2 |
| -515.97 | 19336.129 | 1.727 | 12.176 | 0.191165 | 7/15/2014 | 16:30.7 | 2 |
| -515.85 | 19336.119 | 1.728 | 12.167 | 0.184118 | 7/15/2014 | 16:30.8 | 2 |
| -515.611 | 19336.1 | 1.728 | 12.153 | 0.206339 | 7/15/2014 | 16:30.9 | 2 |
| -515.37 | 19336.08 | 1.722 | 12.134 | 0.203049 | 7/15/2014 | 16:31.0 | 2 |

| | | | | | | | |
|---|---|---|---|---|---|---|---|
| -515.13 | 19336.061 | 1.722 | 12.12 | 0.196457 | 7/15/2014 | 16:31.1 | 2 |
| -514.89 | 19336.041 | 1.722 | 12.102 | 0.196289 | 7/15/2014 | 16:31.2 | 2 |
| -514.65 | 19336.021 | 1.722 | 12.085 | 0.216066 | 7/15/2014 | 16:31.3 | 2 |
| -514.23 | 19335.986 | 1.724 | 12.07 | 0.205677 | 7/15/2014 | 16:31.4 | 2 |
| -513.99 | 19335.967 | 1.719 | 12.054 | 0.212428 | 7/15/2014 | 16:31.5 | 2 |
| -513.75 | 19335.947 | 1.719 | 12.039 | 0.197331 | 7/15/2014 | 16:31.6 | 2 |
| -513.45 | 19335.922 | 1.719 | 12.026 | 0.168973 | 7/15/2014 | 16:31.7 | 2 |
| -513.329 | 19335.912 | 1.718 | 12.018 | 0.160981 | 7/15/2014 | 16:31.8 | 2 |
| -513.087 | 19335.893 | 1.709 | 12.005 | 0.173285 | 7/15/2014 | 16:31.9 | 2 |
| -512.844 | 19335.873 | 1.709 | 11.988 | 0.160056 | 7/15/2014 | 16:32.0 | 2 |
| -512.601 | 19335.854 | 1.707 | 11.975 | 0.170792 | 7/15/2014 | 16:32.1 | 2 |
| -512.359 | 19335.834 | 1.7 | 11.955 | 0.135726 | 7/15/2014 | 16:32.2 | 2 |
| -512.116 | 19335.814 | 1.709 | 11.943 | 0.150136 | 7/15/2014 | 16:32.3 | 2 |
| -511.694 | 19335.779 | 1.712 | 11.927 | 0.154357 | 7/15/2014 | 16:32.4 | 2 |
| -511.454 | 19335.76 | 1.716 | 11.919 | 0.127963 | 7/15/2014 | 16:32.5 | 2 |
| -511.212 | 19335.74 | 1.723 | 11.917 | 0.130105 | 7/15/2014 | 16:32.6 | 2 |
| -510.912 | 19335.717 | 1.721 | 11.961 | 0.120362 | 7/15/2014 | 16:32.7 | 2 |
| -510.791 | 19335.707 | 1.721 | 11.964 | 0.129552 | 7/15/2014 | 16:32.8 | 2 |
| -510.547 | 19335.688 | 1.723 | 12.021 | 0.120588 | 7/15/2014 | 16:32.9 | 2 |
| -510.305 | 19335.668 | 1.715 | 12.081 | 0.120274 | 7/15/2014 | 16:33.0 | 2 |
| -510.061 | 19335.648 | 1.712 | 12.149 | 0.114304 | 7/15/2014 | 16:33.1 | 2 |
| -509.82 | 19335.629 | 1.706 | 12.226 | 0.124036 | 7/15/2014 | 16:33.2 | 2 |
| -509.578 | 19335.609 | 1.706 | 12.304 | 0.110476 | 7/15/2014 | 16:33.3 | 2 |
| -509.157 | 19335.576 | 1.703 | 12.381 | 0.114641 | 7/15/2014 | 16:33.4 | 2 |
| -508.917 | 19335.557 | 1.703 | 12.397 | 0.1103 | 7/15/2014 | 16:33.5 | 2 |
| -508.677 | 19335.537 | 1.708 | 12.405 | 0.107552 | 7/15/2014 | 16:33.6 | 2 |
| -508.376 | 19335.514 | 1.716 | 12.412 | 0.123347 | 7/15/2014 | 16:33.7 | 2 |
| -508.255 | 19335.504 | 1.715 | 12.417 | 0.116835 | 7/15/2014 | 16:33.8 | 2 |
| -508.013 | 19335.484 | 1.723 | 12.4 | 0.122037 | 7/15/2014 | 16:33.9 | 2 |
| -507.77 | 19335.465 | 1.723 | 12.382 | 0.116359 | 7/15/2014 | 16:34.0 | 2 |
| -507.528 | 19335.445 | 1.727 | 12.361 | 0.128434 | 7/15/2014 | 16:34.1 | 2 |
| -507.287 | 19335.426 | 1.727 | 12.34 | 0.12527 | 7/15/2014 | 16:34.2 | 2 |
| -507.046 | 19335.406 | 1.729 | 12.315 | 0.12362 | 7/15/2014 | 16:34.3 | 2 |
| -506.625 | 19335.373 | 1.726 | 12.285 | 0.122286 | 7/15/2014 | 16:34.4 | 2 |
| -506.385 | 19335.354 | 1.724 | 12.266 | 0.127726 | 7/15/2014 | 16:34.5 | 2 |
| -506.146 | 19335.334 | 1.722 | 12.25 | 0.132605 | 7/15/2014 | 16:34.6 | 2 |
| -505.847 | 19335.311 | 1.719 | 12.232 | 0.121164 | 7/15/2014 | 16:34.7 | 2 |
| -505.725 | 19335.301 | 1.718 | 12.225 | 0.125038 | 7/15/2014 | 16:34.8 | 2 |
| -505.485 | 19335.282 | 1.715 | 12.21 | 0.141056 | 7/15/2014 | 16:34.9 | 2 |
| -505.243 | 19335.263 | 1.715 | 12.197 | 0.150747 | 7/15/2014 | 16:35.0 | 2 |
| -505.001 | 19335.243 | 1.715 | 12.186 | 0.134998 | 7/15/2014 | 16:35.1 | 2 |
| -504.761 | 19335.225 | 1.712 | 12.172 | 0.127123 | 7/15/2014 | 16:35.2 | 2 |
| -504.52 | 19335.205 | 1.712 | 12.156 | 0.130377 | 7/15/2014 | 16:35.3 | 2 |
| -504.101 | 19335.172 | 1.708 | 12.135 | 0.129501 | 7/15/2014 | 16:35.4 | 2 |
| -503.862 | 19335.153 | 1.709 | 12.118 | 0.126838 | 7/15/2014 | 16:35.5 | 2 |
| -503.622 | 19335.134 | 1.712 | 12.101 | 0.129327 | 7/15/2014 | 16:35.6 | 2 |
| -503.323 | 19335.11 | 1.71 | 12.084 | 0.127116 | 7/15/2014 | 16:35.7 | 2 |

| | | | | | | | |
|---|---|---|---|---|---|---|---|
| -503.203 | 19335.102 | 1.709 | 12.073 | 0.127045 | 7/15/2014 | 16:35.8 | 2 |
| -502.959 | 19335.082 | 1.711 | 12.056 | 0.126889 | 7/15/2014 | 16:35.9 | 2 |
| -502.718 | 19335.063 | 1.711 | 12.035 | 0.130822 | 7/15/2014 | 16:36.0 | 2 |
| -502.475 | 19335.044 | 1.717 | 12.02 | 0.137261 | 7/15/2014 | 16:36.1 | 2 |
| -502.234 | 19335.024 | 1.717 | 12.004 | 0.137782 | 7/15/2014 | 16:36.2 | 2 |
| -501.992 | 19335.006 | 1.72 | 11.992 | 0.125995 | 7/15/2014 | 16:36.3 | 2 |
| -501.57 | 19334.972 | 1.715 | 11.987 | 0.140013 | 7/15/2014 | 16:36.4 | 2 |
| -501.329 | 19334.953 | 1.715 | 11.978 | 0.150206 | 7/15/2014 | 16:36.5 | 2 |
| -501.088 | 19334.934 | 1.715 | 11.974 | 0.139896 | 7/15/2014 | 16:36.6 | 2 |
| -500.786 | 19334.91 | 1.711 | 11.971 | 0.124597 | 7/15/2014 | 16:36.7 | 2 |
| -500.664 | 19334.9 | 1.71 | 11.97 | 0.129521 | 7/15/2014 | 16:36.8 | 2 |
| -500.422 | 19334.883 | 1.71 | 11.962 | 0.14903 | 7/15/2014 | 16:36.9 | 2 |
| -500.18 | 19334.863 | 1.704 | 11.96 | 0.133608 | 7/15/2014 | 16:37.0 | 2 |
| -499.935 | 19334.843 | 1.71 | 11.946 | 0.123298 | 7/15/2014 | 16:37.1 | 2 |
| -499.695 | 19334.824 | 1.707 | 11.943 | 0.116027 | 7/15/2014 | 16:37.2 | 2 |
| -499.451 | 19334.804 | 1.71 | 11.942 | 0.121484 | 7/15/2014 | 16:37.3 | 2 |
| -499.03 | 19334.771 | 1.71 | 11.981 | 0.109699 | 7/15/2014 | 16:37.4 | 2 |
| -498.79 | 19334.752 | 1.711 | 12.018 | 0.103405 | 7/15/2014 | 16:37.5 | 2 |
| -498.551 | 19334.732 | 1.708 | 12.065 | 0.0918563 | 7/15/2014 | 16:37.6 | 2 |
| -498.251 | 19334.709 | 1.708 | 12.107 | 0.0828022 | 7/15/2014 | 16:37.7 | 2 |
| -498.13 | 19334.699 | 1.706 | 12.132 | 0.0761282 | 7/15/2014 | 16:37.8 | 2 |
| -497.888 | 19334.679 | 1.709 | 12.189 | 0.0639289 | 7/15/2014 | 16:37.9 | 2 |
| -497.647 | 19334.659 | 1.705 | 12.24 | 0.0748266 | 7/15/2014 | 16:38.0 | 2 |
| -497.406 | 19334.641 | 1.709 | 12.284 | 0.0731983 | 7/15/2014 | 16:38.1 | 2 |
| -497.167 | 19334.621 | 1.704 | 12.295 | 0.0826483 | 7/15/2014 | 16:38.2 | 2 |
| -496.926 | 19334.602 | 1.709 | 12.307 | 0.0699512 | 7/15/2014 | 16:38.3 | 2 |
| -496.508 | 19334.568 | 1.71 | 12.316 | 0.0831044 | 7/15/2014 | 16:38.4 | 2 |
| -496.269 | 19334.549 | 1.708 | 12.331 | 0.0854613 | 7/15/2014 | 16:38.5 | 2 |
| -496.03 | 19334.529 | 1.706 | 12.344 | 0.0621891 | 7/15/2014 | 16:38.6 | 2 |
| -495.73 | 19334.506 | 1.707 | 12.361 | 0.0842606 | 7/15/2014 | 16:38.7 | 2 |
| -495.61 | 19334.496 | 1.705 | 12.375 | 0.0802665 | 7/15/2014 | 16:38.8 | 2 |
| -495.366 | 19334.477 | 1.707 | 12.393 | 0.0907528 | 7/15/2014 | 16:38.9 | 2 |
| -495.123 | 19334.457 | 1.705 | 12.419 | 0.0597467 | 7/15/2014 | 16:39.0 | 2 |
| -494.879 | 19334.438 | 1.705 | 12.448 | 0.0734714 | 7/15/2014 | 16:39.1 | 2 |
| -494.636 | 19334.418 | 1.701 | 12.479 | 0.0801181 | 7/15/2014 | 16:39.2 | 2 |
| -494.391 | 19334.398 | 1.706 | 12.505 | 0.0827476 | 7/15/2014 | 16:39.3 | 2 |
| -493.963 | 19334.364 | 1.705 | 12.547 | 0.059027 | 7/15/2014 | 16:39.4 | 2 |
| -493.719 | 19334.345 | 1.704 | 12.579 | 0.0579913 | 7/15/2014 | 16:39.5 | 2 |
| -493.474 | 19334.325 | 1.701 | 12.608 | 0.060172 | 7/15/2014 | 16:39.6 | 2 |
| -493.169 | 19334.301 | 1.696 | 12.635 | 0.0559985 | 7/15/2014 | 16:39.7 | 2 |
| -493.045 | 19334.291 | 1.696 | 12.649 | 0.0515137 | 7/15/2014 | 16:39.8 | 2 |
| -492.798 | 19334.271 | 1.696 | 12.667 | 0.0493144 | 7/15/2014 | 16:39.9 | 2 |
| -492.553 | 19334.251 | 1.693 | 12.683 | 0.0463028 | 7/15/2014 | 16:40.0 | 2 |
| -492.305 | 19334.231 | 1.696 | 12.688 | 0.0470815 | 7/15/2014 | 16:40.1 | 2 |
| -492.061 | 19334.212 | 1.689 | 12.686 | 0.040966 | 7/15/2014 | 16:40.2 | 2 |
| -491.814 | 19334.192 | 1.688 | 12.681 | 0.0541878 | 7/15/2014 | 16:40.3 | 2 |
| -491.389 | 19334.158 | 1.687 | 12.663 | 0.0459689 | 7/15/2014 | 16:40.4 | 2 |

| | | | | | | | |
|---|---|---|---|---|---|---|---|
| -491.146 | 19334.139 | 1.687 | 12.648 | 0.0419598 | 7/15/2014 | 16:40.5 | 2 |
| -490.904 | 19334.119 | 1.687 | 12.638 | 0.0475483 | 7/15/2014 | 16:40.6 | 2 |
| -490.602 | 19334.095 | 1.685 | 12.626 | 0.0488397 | 7/15/2014 | 16:40.7 | 2 |
| -490.479 | 19334.085 | 1.683 | 12.622 | 0.0511334 | 7/15/2014 | 16:40.8 | 2 |
| -490.234 | 19334.064 | 1.686 | 12.617 | 0.0600611 | 7/15/2014 | 16:40.9 | 2 |
| -489.991 | 19334.046 | 1.683 | 12.612 | 0.0712642 | 7/15/2014 | 16:41.0 | 2 |
| -489.745 | 19334.025 | 1.689 | 12.609 | 0.0776111 | 7/15/2014 | 16:41.1 | 2 |
| -489.503 | 19334.006 | 1.691 | 12.601 | 0.0939439 | 7/15/2014 | 16:41.2 | 2 |
| -489.26 | 19333.986 | 1.694 | 12.593 | 0.0980192 | 7/15/2014 | 16:41.3 | 2 |
| -488.838 | 19333.952 | 1.688 | 12.578 | 0.08772 | 7/15/2014 | 16:41.4 | 2 |
| -488.595 | 19333.932 | 1.688 | 12.573 | 0.0945947 | 7/15/2014 | 16:41.5 | 2 |
| -488.354 | 19333.913 | 1.684 | 12.563 | 0.0840997 | 7/15/2014 | 16:41.6 | 2 |
| -488.054 | 19333.889 | 1.68 | 12.551 | 0.0906291 | 7/15/2014 | 16:41.7 | 2 |
| -487.932 | 19333.879 | 1.674 | 12.545 | 0.0744618 | 7/15/2014 | 16:41.8 | 2 |
| -487.689 | 19333.859 | 1.668 | 12.545 | 0.063163 | 7/15/2014 | 16:41.9 | 2 |
| -487.447 | 19333.84 | 1.662 | 12.544 | 0.0785136 | 7/15/2014 | 16:42.0 | 2 |
| -487.205 | 19333.82 | 1.662 | 12.545 | 0.0882668 | 7/15/2014 | 16:42.1 | 2 |
| -486.963 | 19333.801 | 1.662 | 12.547 | 0.0665364 | 7/15/2014 | 16:42.2 | 2 |
| -486.723 | 19333.781 | 1.664 | 12.551 | 0.0519473 | 7/15/2014 | 16:42.3 | 2 |
| -486.307 | 19333.748 | 1.664 | 12.558 | 0.0790447 | 7/15/2014 | 16:42.4 | 2 |
| -486.069 | 19333.729 | 1.666 | 12.562 | 0.0754395 | 7/15/2014 | 16:42.5 | 2 |
| -485.832 | 19333.709 | 1.668 | 12.562 | 0.0704413 | 7/15/2014 | 16:42.6 | 2 |
| -485.538 | 19333.686 | 1.67 | 12.563 | 0.0685772 | 7/15/2014 | 16:42.7 | 2 |
| -485.417 | 19333.676 | 1.671 | 12.566 | 0.0824565 | 7/15/2014 | 16:42.8 | 2 |
| -485.181 | 19333.656 | 1.671 | 12.564 | 0.0968975 | 7/15/2014 | 16:42.9 | 2 |
| -484.945 | 19333.638 | 1.671 | 12.557 | 0.100391 | 7/15/2014 | 16:43.0 | 2 |
| -484.709 | 19333.617 | 1.676 | 12.552 | 0.0966912 | 7/15/2014 | 16:43.1 | 2 |
| -484.473 | 19333.599 | 1.676 | 12.55 | 0.1018 | 7/15/2014 | 16:43.2 | 2 |
| -484.239 | 19333.58 | 1.678 | 12.552 | 0.133818 | 7/15/2014 | 16:43.3 | 2 |
| -483.832 | 19333.547 | 1.676 | 12.547 | 0.144325 | 7/15/2014 | 16:43.4 | 2 |
| -483.601 | 19333.527 | 1.67 | 12.545 | 0.144125 | 7/15/2014 | 16:43.5 | 2 |
| -483.368 | 19333.509 | 1.668 | 12.543 | 0.142078 | 7/15/2014 | 16:43.6 | 2 |
| -483.079 | 19333.484 | 1.662 | 12.539 | 0.1321 | 7/15/2014 | 16:43.7 | 2 |
| -482.962 | 19333.476 | 1.659 | 12.539 | 0.143876 | 7/15/2014 | 16:43.8 | 2 |
| -482.727 | 19333.457 | 1.657 | 12.535 | 0.109436 | 7/15/2014 | 16:43.9 | 2 |
| -482.493 | 19333.438 | 1.655 | 12.524 | 0.0995768 | 7/15/2014 | 16:44.0 | 2 |
| -482.26 | 19333.418 | 1.655 | 12.515 | 0.0874869 | 7/15/2014 | 16:44.1 | 2 |
| -482.026 | 19333.398 | 1.65 | 12.504 | 0.0816676 | 7/15/2014 | 16:44.2 | 2 |
| -481.79 | 19333.379 | 1.655 | 12.491 | 0.0679255 | 7/15/2014 | 16:44.3 | 2 |
| -481.382 | 19333.346 | 1.658 | 12.468 | 0.0660776 | 7/15/2014 | 16:44.4 | 2 |
| -481.148 | 19333.326 | 1.66 | 12.459 | 0.0652095 | 7/15/2014 | 16:44.5 | 2 |
| -480.913 | 19333.307 | 1.662 | 12.443 | 0.064401 | 7/15/2014 | 16:44.6 | 2 |
| -480.622 | 19333.281 | 1.662 | 12.43 | 0.0637029 | 7/15/2014 | 16:44.7 | 2 |
| -480.503 | 19333.271 | 1.662 | 12.423 | 0.0633529 | 7/15/2014 | 16:44.8 | 2 |
| -480.267 | 19333.252 | 1.662 | 12.414 | 0.0660422 | 7/15/2014 | 16:44.9 | 2 |
| -480.031 | 19333.232 | 1.659 | 12.406 | 0.0684481 | 7/15/2014 | 16:45.0 | 2 |
| -479.792 | 19333.213 | 1.659 | 12.407 | 0.0668517 | 7/15/2014 | 16:45.1 | 2 |

| | | | | | | | |
|---|---|---|---|---|---|---|---|
| -479.557 | 19333.193 | 1.655 | 12.41 | 0.0750207 | 7/15/2014 | 16:45.2 | 2 |
| -479.32 | 19333.173 | 1.655 | 12.43 | 0.0800611 | 7/15/2014 | 16:45.3 | 2 |
| -478.908 | 19333.137 | 1.649 | 12.536 | 0.0928415 | 7/15/2014 | 16:45.4 | 2 |
| -478.672 | 19333.117 | 1.647 | 12.602 | 0.0960109 | 7/15/2014 | 16:45.5 | 2 |
| -478.436 | 19333.098 | 1.649 | 12.671 | 0.101029 | 7/15/2014 | 16:45.6 | 2 |
| -478.141 | 19333.072 | 1.649 | 12.745 | 0.111018 | 7/15/2014 | 16:45.7 | 2 |
| -478.023 | 19333.063 | 1.647 | 12.791 | 0.113533 | 7/15/2014 | 16:45.8 | 2 |
| -477.785 | 19333.042 | 1.65 | 12.871 | 0.120747 | 7/15/2014 | 16:45.9 | 2 |
| -477.548 | 19333.02 | 1.647 | 12.933 | 0.117996 | 7/15/2014 | 16:46.0 | 2 |
| -477.31 | 19333 | 1.645 | 12.96 | 0.113976 | 7/15/2014 | 16:46.1 | 2 |
| -477.073 | 19332.98 | 1.645 | 12.981 | 0.118813 | 7/15/2014 | 16:46.2 | 2 |
| -476.834 | 19332.959 | 1.645 | 12.993 | 0.11737 | 7/15/2014 | 16:46.3 | 2 |
| -476.423 | 19332.924 | 1.643 | 12.998 | 0.116145 | 7/15/2014 | 16:46.4 | 2 |
| -476.188 | 19332.904 | 1.641 | 12.992 | 0.112695 | 7/15/2014 | 16:46.5 | 2 |
| -475.954 | 19332.882 | 1.639 | 12.985 | 0.111217 | 7/15/2014 | 16:46.6 | 2 |
| -475.662 | 19332.857 | 1.641 | 12.975 | 0.106692 | 7/15/2014 | 16:46.7 | 2 |
| -475.542 | 19332.848 | 1.643 | 12.97 | 0.105294 | 7/15/2014 | 16:46.8 | 2 |
| -475.306 | 19332.826 | 1.645 | 12.96 | 0.100797 | 7/15/2014 | 16:46.9 | 2 |
| -475.072 | 19332.806 | 1.645 | 12.949 | 0.0976237 | 7/15/2014 | 16:47.0 | 2 |
| -474.836 | 19332.785 | 1.647 | 12.94 | 0.094442 | 7/15/2014 | 16:47.1 | 2 |
| -474.603 | 19332.766 | 1.645 | 12.927 | 0.0929827 | 7/15/2014 | 16:47.2 | 2 |
| -474.37 | 19332.745 | 1.644 | 12.921 | 0.0910445 | 7/15/2014 | 16:47.3 | 2 |
| -473.963 | 19332.71 | 1.639 | 12.91 | 0.0940809 | 7/15/2014 | 16:47.4 | 2 |
| -473.732 | 19332.689 | 1.633 | 12.901 | 0.0898421 | 7/15/2014 | 16:47.5 | 2 |
| -473.501 | 19332.669 | 1.629 | 12.893 | 0.0959403 | 7/15/2014 | 16:47.6 | 2 |
| -473.212 | 19332.643 | 1.627 | 12.885 | 0.0981242 | 7/15/2014 | 16:47.7 | 2 |
| -473.095 | 19332.633 | 1.622 | 12.882 | 0.106147 | 7/15/2014 | 16:47.8 | 2 |
| -472.863 | 19332.613 | 1.625 | 12.872 | 0.094671 | 7/15/2014 | 16:47.9 | 2 |
| -472.631 | 19332.594 | 1.621 | 12.86 | 0.0916897 | 7/15/2014 | 16:48.0 | 2 |
| -472.398 | 19332.573 | 1.627 | 12.848 | 0.0934846 | 7/15/2014 | 16:48.1 | 2 |
| -472.167 | 19332.553 | 1.627 | 12.836 | 0.0957119 | 7/15/2014 | 16:48.2 | 2 |
| -471.936 | 19332.533 | 1.635 | 12.825 | 0.0961064 | 7/15/2014 | 16:48.3 | 2 |
| -471.532 | 19332.498 | 1.639 | 12.807 | 0.0975306 | 7/15/2014 | 16:48.4 | 2 |
| -471.303 | 19332.479 | 1.639 | 12.794 | 0.103525 | 7/15/2014 | 16:48.5 | 2 |
| -471.072 | 19332.459 | 1.637 | 12.781 | 0.103543 | 7/15/2014 | 16:48.6 | 2 |
| -470.786 | 19332.434 | 1.636 | 12.771 | 0.0984811 | 7/15/2014 | 16:48.7 | 2 |
| -470.669 | 19332.424 | 1.635 | 12.766 | 0.0986321 | 7/15/2014 | 16:48.8 | 2 |
| -470.436 | 19332.404 | 1.633 | 12.759 | 0.103152 | 7/15/2014 | 16:48.9 | 2 |
| -470.203 | 19332.385 | 1.631 | 12.747 | 0.0912433 | 7/15/2014 | 16:49.0 | 2 |
| -469.97 | 19332.364 | 1.631 | 12.734 | 0.0847758 | 7/15/2014 | 16:49.1 | 2 |
| -469.737 | 19332.344 | 1.628 | 12.722 | 0.0899663 | 7/15/2014 | 16:49.2 | 2 |
| -469.504 | 19332.324 | 1.631 | 12.712 | 0.0833507 | 7/15/2014 | 16:49.3 | 2 |
| -469.097 | 19332.289 | 1.627 | 12.697 | 0.088824 | 7/15/2014 | 16:49.4 | 2 |
| -468.862 | 19332.27 | 1.632 | 12.682 | 0.091007 | 7/15/2014 | 16:49.5 | 2 |
| -468.628 | 19332.248 | 1.634 | 12.667 | 0.0908986 | 7/15/2014 | 16:49.6 | 2 |
| -468.336 | 19332.224 | 1.631 | 12.659 | 0.0894586 | 7/15/2014 | 16:49.7 | 2 |
| -468.218 | 19332.213 | 1.629 | 12.657 | 0.0904856 | 7/15/2014 | 16:49.8 | 2 |

| | | | | | | | |
|---|---|---|---|---|---|---|---|
| -467.98 | 19332.193 | 1.632 | 12.65 | 0.0926892 | 7/15/2014 | 16:49.9 | 2 |
| -467.744 | 19332.172 | 1.632 | 12.643 | 0.0959999 | 7/15/2014 | 16:50.0 | 2 |
| -467.505 | 19332.152 | 1.635 | 12.64 | 0.102755 | 7/15/2014 | 16:50.1 | 2 |
| -467.267 | 19332.133 | 1.635 | 12.639 | 0.101133 | 7/15/2014 | 16:50.2 | 2 |
| -467.03 | 19332.111 | 1.635 | 12.644 | 0.0995772 | 7/15/2014 | 16:50.3 | 2 |
| -466.617 | 19332.076 | 1.637 | 12.73 | 0.0968313 | 7/15/2014 | 16:50.4 | 2 |
| -466.379 | 19332.057 | 1.642 | 12.786 | 0.0950719 | 7/15/2014 | 16:50.5 | 2 |
| -466.143 | 19332.035 | 1.644 | 12.843 | 0.0977186 | 7/15/2014 | 16:50.6 | 2 |
| -465.848 | 19332.01 | 1.644 | 12.904 | 0.0973461 | 7/15/2014 | 16:50.7 | 2 |
| -465.728 | 19332 | 1.646 | 12.942 | 0.0972553 | 7/15/2014 | 16:50.8 | 2 |
| -465.491 | 19331.98 | 1.651 | 13.007 | 0.0974568 | 7/15/2014 | 16:50.9 | 2 |
| -465.254 | 19331.959 | 1.651 | 13.071 | 0.10833 | 7/15/2014 | 16:51.0 | 2 |
| -465.017 | 19331.939 | 1.651 | 13.093 | 0.112369 | 7/15/2014 | 16:51.1 | 2 |
| -464.78 | 19331.92 | 1.651 | 13.113 | 0.116745 | 7/15/2014 | 16:51.2 | 2 |
| -464.544 | 19331.899 | 1.649 | 13.121 | 0.116964 | 7/15/2014 | 16:51.3 | 2 |
| -464.133 | 19331.863 | 1.65 | 13.133 | 0.113318 | 7/15/2014 | 16:51.4 | 2 |
| -463.9 | 19331.844 | 1.65 | 13.136 | 0.120375 | 7/15/2014 | 16:51.5 | 2 |
| -463.666 | 19331.824 | 1.648 | 13.14 | 0.116202 | 7/15/2014 | 16:51.6 | 2 |
| -463.374 | 19331.8 | 1.648 | 13.141 | 0.118148 | 7/15/2014 | 16:51.7 | 2 |
| -463.256 | 19331.79 | 1.649 | 13.143 | 0.113594 | 7/15/2014 | 16:51.8 | 2 |
| -463.019 | 19331.769 | 1.654 | 13.136 | 0.127121 | 7/15/2014 | 16:51.9 | 2 |
| -462.784 | 19331.748 | 1.654 | 13.126 | 0.128958 | 7/15/2014 | 16:52.0 | 2 |
| -462.547 | 19331.729 | 1.656 | 13.11 | 0.134042 | 7/15/2014 | 16:52.1 | 2 |
| -462.313 | 19331.709 | 1.66 | 13.088 | 0.138156 | 7/15/2014 | 16:52.2 | 2 |
| -462.077 | 19331.689 | 1.664 | 13.067 | 0.131134 | 7/15/2014 | 16:52.3 | 2 |
| -461.665 | 19331.655 | 1.669 | 13.03 | 0.138105 | 7/15/2014 | 16:52.4 | 2 |
| -461.43 | 19331.635 | 1.671 | 13.007 | 0.136765 | 7/15/2014 | 16:52.5 | 2 |
| -461.194 | 19331.613 | 1.669 | 12.989 | 0.142291 | 7/15/2014 | 16:52.6 | 2 |
| -460.897 | 19331.59 | 1.671 | 12.967 | 0.137531 | 7/15/2014 | 16:52.7 | 2 |
| -460.779 | 19331.58 | 1.67 | 12.957 | 0.128289 | 7/15/2014 | 16:52.8 | 2 |
| -460.539 | 19331.561 | 1.667 | 12.938 | 0.143072 | 7/15/2014 | 16:52.9 | 2 |
| -460.298 | 19331.539 | 1.667 | 12.917 | 0.119366 | 7/15/2014 | 16:53.0 | 2 |
| -460.055 | 19331.52 | 1.672 | 12.903 | 0.113695 | 7/15/2014 | 16:53.1 | 2 |
| -459.814 | 19331.5 | 1.665 | 12.884 | 0.103582 | 7/15/2014 | 16:53.2 | 2 |
| -459.57 | 19331.477 | 1.668 | 12.868 | 0.0984243 | 7/15/2014 | 16:53.3 | 2 |
| -459.144 | 19331.443 | 1.671 | 12.843 | 0.0826829 | 7/15/2014 | 16:53.4 | 2 |
| -458.902 | 19331.424 | 1.671 | 12.828 | 0.0935599 | 7/15/2014 | 16:53.5 | 2 |
| -458.656 | 19331.402 | 1.673 | 12.816 | 0.0808951 | 7/15/2014 | 16:53.6 | 2 |
| -458.351 | 19331.377 | 1.671 | 12.809 | 0.0845258 | 7/15/2014 | 16:53.7 | 2 |
| -458.227 | 19331.367 | 1.67 | 12.801 | 0.0820225 | 7/15/2014 | 16:53.8 | 2 |
| -457.98 | 19331.346 | 1.676 | 12.793 | 0.0864614 | 7/15/2014 | 16:53.9 | 2 |
| -457.733 | 19331.324 | 1.674 | 12.786 | 0.0842473 | 7/15/2014 | 16:54.0 | 2 |
| -457.486 | 19331.305 | 1.674 | 12.784 | 0.086143 | 7/15/2014 | 16:54.1 | 2 |
| -457.241 | 19331.285 | 1.672 | 12.786 | 0.0710763 | 7/15/2014 | 16:54.2 | 2 |
| -456.993 | 19331.265 | 1.674 | 12.797 | 0.0958619 | 7/15/2014 | 16:54.3 | 2 |
| -456.566 | 19331.229 | 1.675 | 12.856 | 0.105902 | 7/15/2014 | 16:54.4 | 2 |
| -456.323 | 19331.209 | 1.675 | 12.919 | 0.0916353 | 7/15/2014 | 16:54.5 | 2 |

| | | | | | | | |
|---|---|---|---|---|---|---|---|
| -456.08 | 19331.188 | 1.672 | 12.985 | 0.0896869 | 7/15/2014 | 16:54.6 | 2 |
| -455.777 | 19331.162 | 1.677 | 13.054 | 0.0809269 | 7/15/2014 | 16:54.7 | 2 |
| -455.654 | 19331.152 | 1.677 | 13.092 | 0.0765591 | 7/15/2014 | 16:54.8 | 2 |
| -455.41 | 19331.132 | 1.686 | 13.171 | 0.055201 | 7/15/2014 | 16:54.9 | 2 |
| -455.167 | 19331.111 | 1.685 | 13.247 | 0.0538218 | 7/15/2014 | 16:55.0 | 2 |
| -454.924 | 19331.092 | 1.688 | 13.321 | 0.060233 | 7/15/2014 | 16:55.1 | 2 |
| -454.683 | 19331.072 | 1.689 | 13.348 | 0.05444 | 7/15/2014 | 16:55.2 | 2 |
| -454.44 | 19331.053 | 1.695 | 13.367 | 0.0730116 | 7/15/2014 | 16:55.3 | 2 |
| -454.019 | 19331.018 | 1.696 | 13.376 | 0.0735974 | 7/15/2014 | 16:55.4 | 2 |
| -453.778 | 19330.998 | 1.692 | 13.378 | 0.0898911 | 7/15/2014 | 16:55.5 | 2 |
| -453.538 | 19330.979 | 1.69 | 13.378 | 0.0826858 | 7/15/2014 | 16:55.6 | 2 |
| -453.235 | 19330.953 | 1.69 | 13.378 | 0.0726831 | 7/15/2014 | 16:55.7 | 2 |
| -453.115 | 19330.943 | 1.689 | 13.377 | 0.0817916 | 7/15/2014 | 16:55.8 | 2 |
| -452.871 | 19330.924 | 1.691 | 13.374 | 0.0681501 | 7/15/2014 | 16:55.9 | 2 |
| -452.626 | 19330.904 | 1.687 | 13.36 | 0.0604291 | 7/15/2014 | 16:56.0 | 2 |
| -452.382 | 19330.885 | 1.687 | 13.354 | 0.0693579 | 7/15/2014 | 16:56.1 | 2 |
| -452.137 | 19330.865 | 1.681 | 13.342 | 0.037506 | 7/15/2014 | 16:56.2 | 2 |
| -451.892 | 19330.844 | 1.685 | 13.33 | 0.0328673 | 7/15/2014 | 16:56.3 | 2 |
| -451.462 | 19330.809 | 1.681 | 13.302 | 0.0297058 | 7/15/2014 | 16:56.4 | 2 |
| -451.217 | 19330.789 | 1.681 | 13.283 | 0.0480436 | 7/15/2014 | 16:56.5 | 2 |
| -450.971 | 19330.768 | 1.678 | 13.263 | 0.0367802 | 7/15/2014 | 16:56.6 | 2 |
| -450.664 | 19330.744 | 1.675 | 13.244 | 0.0470859 | 7/15/2014 | 16:56.7 | 2 |
| -450.54 | 19330.734 | 1.675 | 13.232 | 0.046262 | 7/15/2014 | 16:56.8 | 2 |
| -450.29 | 19330.711 | 1.681 | 13.215 | 0.0651421 | 7/15/2014 | 16:56.9 | 2 |
| -450.042 | 19330.691 | 1.675 | 13.194 | 0.0552553 | 7/15/2014 | 16:57.0 | 2 |
| -449.791 | 19330.672 | 1.675 | 13.176 | 0.0276791 | 7/15/2014 | 16:57.1 | 2 |
| -449.542 | 19330.652 | 1.673 | 13.163 | 0.0159679 | 7/15/2014 | 16:57.2 | 2 |
| -449.293 | 19330.629 | 1.677 | 13.146 | 0.0208075 | 7/15/2014 | 16:57.3 | 2 |
| -448.983 | 19330.605 | 1.677 | 13.13 | 0.0316698 | 7/15/2014 | 16:57.4 | 2 |
| -448.733 | 19330.584 | 1.679 | 13.115 | 0.0477305 | 7/15/2014 | 16:57.5 | 2 |
| -448.485 | 19330.563 | 1.677 | 13.103 | 0.0449199 | 7/15/2014 | 16:57.6 | 2 |
| -448.234 | 19330.543 | 1.679 | 13.094 | 0.0402596 | 7/15/2014 | 16:57.7 | 2 |
| -447.923 | 19330.518 | 1.677 | 13.087 | 0.0424797 | 7/15/2014 | 16:57.8 | 2 |
| -447.672 | 19330.496 | 1.683 | 13.078 | 0.0436229 | 7/15/2014 | 16:57.9 | 2 |
| -447.421 | 19330.477 | 1.683 | 13.072 | 0.0482368 | 7/15/2014 | 16:58.0 | 2 |
| -447.17 | 19330.455 | 1.686 | 13.07 | 0.0461142 | 7/15/2014 | 16:58.1 | 2 |
| -446.922 | 19330.434 | 1.687 | 13.072 | 0.0407854 | 7/15/2014 | 16:58.2 | 2 |
| -446.671 | 19330.414 | 1.69 | 13.074 | 0.0495697 | 7/15/2014 | 16:58.3 | 2 |
| -446.361 | 19330.389 | 1.694 | 13.075 | 0.0467695 | 7/15/2014 | 16:58.4 | 2 |
| -446.112 | 19330.367 | 1.7 | 13.079 | 0.0436762 | 7/15/2014 | 16:58.5 | 2 |
| -445.864 | 19330.348 | 1.7 | 13.082 | 0.0493677 | 7/15/2014 | 16:58.6 | 2 |
| -445.433 | 19330.312 | 1.7 | 13.088 | 0.0465182 | 7/15/2014 | 16:58.7 | 2 |
| -445.308 | 19330.302 | 1.7 | 13.09 | 0.0474071 | 7/15/2014 | 16:58.8 | 2 |
| -445.059 | 19330.281 | 1.702 | 13.088 | 0.0524146 | 7/15/2014 | 16:58.9 | 2 |
| -444.811 | 19330.262 | 1.698 | 13.088 | 0.0551485 | 7/15/2014 | 16:59.0 | 2 |
| -444.562 | 19330.24 | 1.698 | 13.081 | 0.0640611 | 7/15/2014 | 16:59.1 | 2 |
| -444.314 | 19330.221 | 1.695 | 13.071 | 0.0585795 | 7/15/2014 | 16:59.2 | 2 |

| | | | | | | | |
|---|---|---|---|---|---|---|---|
| -444.067 | 19330.201 | 1.691 | 13.06 | 0.0649145 | 7/15/2014 | 16:59.3 | 2 |
| -443.635 | 19330.166 | 1.689 | 13.042 | 0.072364 | 7/15/2014 | 16:59.4 | 2 |
| -443.389 | 19330.145 | 1.689 | 13.032 | 0.069919 | 7/15/2014 | 16:59.5 | 2 |
| -443.142 | 19330.125 | 1.689 | 13.023 | 0.0758705 | 7/15/2014 | 16:59.6 | 2 |
| -442.833 | 19330.102 | 1.692 | 13.012 | 0.0803248 | 7/15/2014 | 16:59.7 | 2 |
| -442.708 | 19330.092 | 1.691 | 13.009 | 0.0793149 | 7/15/2014 | 16:59.8 | 2 |
| -442.459 | 19330.071 | 1.697 | 13.002 | 0.0851884 | 7/15/2014 | 16:59.9 | 2 |
| -442.209 | 19330.051 | 1.701 | 12.996 | 0.0892193 | 7/15/2014 | 17:00.0 | 2 |
| -441.959 | 19330.03 | 1.703 | 12.986 | 0.100113 | 7/15/2014 | 17:00.1 | 2 |
| -441.709 | 19330.01 | 1.707 | 12.981 | 0.105804 | 7/15/2014 | 17:00.2 | 2 |
| -441.459 | 19329.99 | 1.707 | 12.975 | 0.104022 | 7/15/2014 | 17:00.3 | 2 |
| -441.148 | 19329.966 | 1.709 | 12.979 | 0.103217 | 7/15/2014 | 17:00.4 | 2 |
| -440.898 | 19329.944 | 1.712 | 12.983 | 0.110867 | 7/15/2014 | 17:00.5 | 2 |
| -440.648 | 19329.924 | 1.712 | 12.99 | 0.116599 | 7/15/2014 | 17:00.6 | 2 |
| -440.398 | 19329.904 | 1.714 | 12.996 | 0.107039 | 7/15/2014 | 17:00.7 | 2 |
| -440.086 | 19329.879 | 1.714 | 13.004 | 0.101282 | 7/15/2014 | 17:00.8 | 2 |
| -439.834 | 19329.859 | 1.719 | 13.01 | 0.106455 | 7/15/2014 | 17:00.9 | 2 |
| -439.583 | 19329.839 | 1.724 | 13.016 | 0.11631 | 7/15/2014 | 17:01.0 | 2 |
| -439.332 | 19329.816 | 1.73 | 13.013 | 0.111719 | 7/15/2014 | 17:01.1 | 2 |
| -439.081 | 19329.797 | 1.727 | 13.009 | 0.111844 | 7/15/2014 | 17:01.2 | 2 |
| -438.83 | 19329.777 | 1.73 | 13.003 | 0.120188 | 7/15/2014 | 17:01.3 | 2 |
| -438.518 | 19329.752 | 1.736 | 13.001 | 0.112301 | 7/15/2014 | 17:01.4 | 2 |
| -438.268 | 19329.732 | 1.742 | 12.999 | 0.119679 | 7/15/2014 | 17:01.5 | 2 |
| -438.019 | 19329.711 | 1.742 | 13 | 0.112898 | 7/15/2014 | 17:01.6 | 2 |
| -437.77 | 19329.689 | 1.746 | 13.002 | 0.119947 | 7/15/2014 | 17:01.7 | 2 |
| -437.461 | 19329.666 | 1.743 | 13.009 | 0.109051 | 7/15/2014 | 17:01.8 | 2 |
| -437.21 | 19329.646 | 1.754 | 13.019 | 0.106456 | 7/15/2014 | 17:01.9 | 2 |
| -436.96 | 19329.625 | 1.754 | 13.031 | 0.119485 | 7/15/2014 | 17:02.0 | 2 |
| -436.711 | 19329.605 | 1.758 | 13.047 | 0.106229 | 7/15/2014 | 17:02.1 | 2 |
| -436.464 | 19329.584 | 1.758 | 13.065 | 0.116971 | 7/15/2014 | 17:02.2 | 2 |
| -436.216 | 19329.564 | 1.765 | 13.095 | 0.123126 | 7/15/2014 | 17:02.3 | 2 |
| -435.787 | 19329.529 | 1.768 | 13.172 | 0.126754 | 7/15/2014 | 17:02.4 | 2 |
| -435.544 | 19329.509 | 1.767 | 13.209 | 0.147995 | 7/15/2014 | 17:02.5 | 2 |
| -435.299 | 19329.488 | 1.764 | 13.243 | 0.156377 | 7/15/2014 | 17:02.6 | 2 |
| -434.996 | 19329.465 | 1.765 | 13.276 | 0.144865 | 7/15/2014 | 17:02.7 | 2 |
| -434.873 | 19329.455 | 1.762 | 13.3 | 0.145798 | 7/15/2014 | 17:02.8 | 2 |
| -434.627 | 19329.434 | 1.762 | 13.328 | 0.12613 | 7/15/2014 | 17:02.9 | 2 |
| -434.385 | 19329.413 | 1.758 | 13.341 | 0.123651 | 7/15/2014 | 17:03.0 | 2 |
| -434.139 | 19329.393 | 1.758 | 13.332 | 0.136439 | 7/15/2014 | 17:03.1 | 2 |
| -433.897 | 19329.373 | 1.755 | 13.322 | 0.122659 | 7/15/2014 | 17:03.2 | 2 |
| -433.653 | 19329.352 | 1.764 | 13.312 | 0.113881 | 7/15/2014 | 17:03.3 | 2 |
| -433.234 | 19329.318 | 1.761 | 13.304 | 0.141875 | 7/15/2014 | 17:03.4 | 2 |
| -432.993 | 19329.297 | 1.763 | 13.295 | 0.154239 | 7/15/2014 | 17:03.5 | 2 |
| -432.752 | 19329.277 | 1.766 | 13.289 | 0.154063 | 7/15/2014 | 17:03.6 | 2 |
| -432.454 | 19329.252 | 1.766 | 13.28 | 0.172936 | 7/15/2014 | 17:03.7 | 2 |
| -432.332 | 19329.242 | 1.767 | 13.277 | 0.169321 | 7/15/2014 | 17:03.8 | 2 |
| -432.089 | 19329.221 | 1.77 | 13.269 | 0.173514 | 7/15/2014 | 17:03.9 | 2 |

| | | | | | | | |
|---|---|---|---|---|---|---|---|
| -431.851 | 19329.201 | 1.764 | 13.256 | 0.17252 | 7/15/2014 | 17:04.0 | 2 |
| -431.609 | 19329.182 | 1.772 | 13.238 | 0.182845 | 7/15/2014 | 17:04.1 | 2 |
| -431.37 | 19329.16 | 1.772 | 13.208 | 0.178922 | 7/15/2014 | 17:04.2 | 2 |
| -431.13 | 19329.141 | 1.772 | 13.183 | 0.200686 | 7/15/2014 | 17:04.3 | 2 |
| -430.713 | 19329.105 | 1.764 | 13.141 | 0.170734 | 7/15/2014 | 17:04.4 | 2 |
| -430.475 | 19329.084 | 1.764 | 13.114 | 0.184659 | 7/15/2014 | 17:04.5 | 2 |
| -430.237 | 19329.064 | 1.766 | 13.087 | 0.192126 | 7/15/2014 | 17:04.6 | 2 |
| -429.938 | 19329.039 | 1.766 | 13.07 | 0.144365 | 7/15/2014 | 17:04.7 | 2 |
| -429.819 | 19329.027 | 1.763 | 13.059 | 0.150552 | 7/15/2014 | 17:04.8 | 2 |
| -429.579 | 19329.008 | 1.769 | 13.037 | 0.129756 | 7/15/2014 | 17:04.9 | 2 |
| -429.339 | 19328.988 | 1.769 | 13.025 | 0.135993 | 7/15/2014 | 17:05.0 | 2 |
| -429.095 | 19328.967 | 1.779 | 13.007 | 0.125232 | 7/15/2014 | 17:05.1 | 2 |
| -428.856 | 19328.945 | 1.785 | 12.995 | 0.0845605 | 7/15/2014 | 17:05.2 | 2 |
| -428.612 | 19328.926 | 1.789 | 12.984 | 0.0861904 | 7/15/2014 | 17:05.3 | 2 |
| -428.189 | 19328.889 | 1.783 | 13.022 | 0.108541 | 7/15/2014 | 17:05.4 | 2 |
| -427.949 | 19328.867 | 1.785 | 13.055 | 0.0862174 | 7/15/2014 | 17:05.5 | 2 |
| -427.708 | 19328.848 | 1.785 | 13.11 | 0.0871554 | 7/15/2014 | 17:05.6 | 2 |
| -427.405 | 19328.82 | 1.785 | 13.162 | 0.0942761 | 7/15/2014 | 17:05.7 | 2 |
| -427.283 | 19328.811 | 1.783 | 13.193 | 0.115173 | 7/15/2014 | 17:05.8 | 2 |
| -427.037 | 19328.789 | 1.78 | 13.253 | 0.0801698 | 7/15/2014 | 17:05.9 | 2 |
| -426.794 | 19328.768 | 1.779 | 13.315 | 0.0893497 | 7/15/2014 | 17:06.0 | 2 |
| -426.547 | 19328.747 | 1.785 | 13.374 | 0.0727189 | 7/15/2014 | 17:06.1 | 2 |
| -426.303 | 19328.725 | 1.785 | 13.374 | 0.102294 | 7/15/2014 | 17:06.2 | 2 |
| -426.058 | 19328.703 | 1.789 | 13.374 | 0.0927399 | 7/15/2014 | 17:06.3 | 2 |
| -425.632 | 19328.666 | 1.785 | 13.357 | 0.0819521 | 7/15/2014 | 17:06.4 | 2 |
| -425.387 | 19328.645 | 1.789 | 13.342 | 0.0964264 | 7/15/2014 | 17:06.5 | 2 |
| -425.144 | 19328.623 | 1.791 | 13.33 | 0.102473 | 7/15/2014 | 17:06.6 | 2 |
| -424.839 | 19328.597 | 1.789 | 13.318 | 0.105026 | 7/15/2014 | 17:06.7 | 2 |
| -424.716 | 19328.586 | 1.786 | 13.312 | 0.0884639 | 7/15/2014 | 17:06.8 | 2 |
| -424.468 | 19328.564 | 1.786 | 13.305 | 0.128912 | 7/15/2014 | 17:06.9 | 2 |
| -424.223 | 19328.543 | 1.784 | 13.306 | 0.105682 | 7/15/2014 | 17:07.0 | 2 |
| -423.975 | 19328.52 | 1.79 | 13.298 | 0.119618 | 7/15/2014 | 17:07.1 | 2 |
| -423.73 | 19328.5 | 1.784 | 13.289 | 0.147245 | 7/15/2014 | 17:07.2 | 2 |
| -423.483 | 19328.477 | 1.784 | 13.286 | 0.129392 | 7/15/2014 | 17:07.3 | 2 |
| -423.054 | 19328.439 | 1.784 | 13.275 | 0.179445 | 7/15/2014 | 17:07.4 | 2 |
| -422.808 | 19328.418 | 1.79 | 13.269 | 0.175468 | 7/15/2014 | 17:07.5 | 2 |
| -422.563 | 19328.395 | 1.792 | 13.259 | 0.196466 | 7/15/2014 | 17:07.6 | 2 |
| -422.257 | 19328.367 | 1.792 | 13.247 | 0.179419 | 7/15/2014 | 17:07.7 | 2 |
| -422.134 | 19328.357 | 1.79 | 13.245 | 0.160822 | 7/15/2014 | 17:07.8 | 2 |
| -421.887 | 19328.334 | 1.796 | 13.236 | 0.192381 | 7/15/2014 | 17:07.9 | 2 |
| -421.641 | 19328.313 | 1.799 | 13.23 | 0.155446 | 7/15/2014 | 17:08.0 | 2 |
| -421.396 | 19328.291 | 1.799 | 13.222 | 0.151892 | 7/15/2014 | 17:08.1 | 2 |
| -421.151 | 19328.268 | 1.796 | 13.21 | 0.152081 | 7/15/2014 | 17:08.2 | 2 |
| -420.907 | 19328.246 | 1.796 | 13.202 | 0.160628 | 7/15/2014 | 17:08.3 | 2 |
| -420.483 | 19328.207 | 1.795 | 13.181 | 0.181784 | 7/15/2014 | 17:08.4 | 2 |
| -420.242 | 19328.186 | 1.795 | 13.169 | 0.159194 | 7/15/2014 | 17:08.5 | 2 |
| -420.002 | 19328.164 | 1.793 | 13.158 | 0.175313 | 7/15/2014 | 17:08.6 | 2 |

| | | | | | | | |
|---|---|---|---|---|---|---|---|
| -419.703 | 19328.137 | 1.793 | 13.14 | 0.152421 | 7/15/2014 | 17:08.7 | 2 |
| -419.581 | 19328.125 | 1.794 | 13.132 | 0.141113 | 7/15/2014 | 17:08.8 | 2 |
| -419.34 | 19328.104 | 1.796 | 13.118 | 0.132943 | 7/15/2014 | 17:08.9 | 2 |
| -419.1 | 19328.082 | 1.796 | 13.098 | 0.129245 | 7/15/2014 | 17:09.0 | 2 |
| -418.86 | 19328.059 | 1.799 | 13.084 | 0.105318 | 7/15/2014 | 17:09.1 | 2 |
| -418.621 | 19328.038 | 1.796 | 13.066 | 0.110789 | 7/15/2014 | 17:09.2 | 2 |
| -418.383 | 19328.015 | 1.799 | 13.05 | 0.119404 | 7/15/2014 | 17:09.3 | 2 |
| -417.966 | 19327.977 | 1.799 | 13.03 | 0.115519 | 7/15/2014 | 17:09.4 | 2 |
| -417.731 | 19327.954 | 1.794 | 13.022 | 0.119521 | 7/15/2014 | 17:09.5 | 2 |
| -417.492 | 19327.934 | 1.792 | 13.01 | 0.127217 | 7/15/2014 | 17:09.6 | 2 |
| -417.195 | 19327.904 | 1.792 | 13.007 | 0.131899 | 7/15/2014 | 17:09.7 | 2 |
| -417.075 | 19327.894 | 1.79 | 13.004 | 0.122777 | 7/15/2014 | 17:09.8 | 2 |
| -416.836 | 19327.871 | 1.793 | 13 | 0.111406 | 7/15/2014 | 17:09.9 | 2 |
| -416.596 | 19327.85 | 1.786 | 13.004 | 0.126215 | 7/15/2014 | 17:10.0 | 2 |
| -416.354 | 19327.826 | 1.786 | 13.01 | 0.125914 | 7/15/2014 | 17:10.1 | 2 |
| -416.114 | 19327.804 | 1.782 | 13.044 | 0.106749 | 7/15/2014 | 17:10.2 | 2 |
| -415.872 | 19327.781 | 1.788 | 13.083 | 0.0891199 | 7/15/2014 | 17:10.3 | 2 |
| -415.45 | 19327.74 | 1.785 | 13.154 | 0.0900541 | 7/15/2014 | 17:10.4 | 2 |
| -415.21 | 19327.718 | 1.782 | 13.187 | 0.0828495 | 7/15/2014 | 17:10.5 | 2 |
| -414.971 | 19327.696 | 1.782 | 13.227 | 0.076629 | 7/15/2014 | 17:10.6 | 2 |
| -414.671 | 19327.668 | 1.785 | 13.255 | 0.0763394 | 7/15/2014 | 17:10.7 | 2 |
| -414.548 | 19327.656 | 1.783 | 13.286 | 0.0761676 | 7/15/2014 | 17:10.8 | 2 |
| -414.306 | 19327.633 | 1.787 | 13.289 | 0.077265 | 7/15/2014 | 17:10.9 | 2 |
| -414.066 | 19327.609 | 1.784 | 13.282 | 0.0817698 | 7/15/2014 | 17:11.0 | 2 |
| -413.824 | 19327.586 | 1.788 | 13.271 | 0.081225 | 7/15/2014 | 17:11.1 | 2 |
| -413.585 | 19327.563 | 1.781 | 13.257 | 0.0860869 | 7/15/2014 | 17:11.2 | 2 |
| -413.344 | 19327.54 | 1.781 | 13.242 | 0.085861 | 7/15/2014 | 17:11.3 | 2 |
| -412.927 | 19327.5 | 1.781 | 13.223 | 0.094701 | 7/15/2014 | 17:11.4 | 2 |
| -412.691 | 19327.478 | 1.778 | 13.211 | 0.101975 | 7/15/2014 | 17:11.5 | 2 |
| -412.453 | 19327.454 | 1.78 | 13.192 | 0.109893 | 7/15/2014 | 17:11.6 | 2 |
| -412.159 | 19327.426 | 1.779 | 13.173 | 0.114707 | 7/15/2014 | 17:11.7 | 2 |
| -412.04 | 19327.414 | 1.778 | 13.165 | 0.10309 | 7/15/2014 | 17:11.8 | 2 |
| -411.799 | 19327.391 | 1.788 | 13.145 | 0.124383 | 7/15/2014 | 17:11.9 | 2 |
| -411.561 | 19327.368 | 1.788 | 13.123 | 0.12466 | 7/15/2014 | 17:12.0 | 2 |
| -411.323 | 19327.344 | 1.79 | 13.103 | 0.121096 | 7/15/2014 | 17:12.1 | 2 |
| -411.087 | 19327.322 | 1.788 | 13.076 | 0.100741 | 7/15/2014 | 17:12.2 | 2 |
| -410.849 | 19327.298 | 1.791 | 13.049 | 0.114273 | 7/15/2014 | 17:12.3 | 2 |
| -410.437 | 19327.259 | 1.793 | 13.017 | 0.120349 | 7/15/2014 | 17:12.4 | 2 |
| -410.2 | 19327.235 | 1.794 | 12.997 | 0.108969 | 7/15/2014 | 17:12.5 | 2 |
| -409.965 | 19327.212 | 1.792 | 12.977 | 0.122063 | 7/15/2014 | 17:12.6 | 2 |
| -409.67 | 19327.184 | 1.792 | 12.959 | 0.120713 | 7/15/2014 | 17:12.7 | 2 |
| -409.551 | 19327.173 | 1.79 | 12.943 | 0.150433 | 7/15/2014 | 17:12.8 | 2 |
| -409.313 | 19327.148 | 1.793 | 12.929 | 0.130438 | 7/15/2014 | 17:12.9 | 2 |
| -409.075 | 19327.125 | 1.795 | 12.918 | 0.123641 | 7/15/2014 | 17:13.0 | 2 |
| -408.836 | 19327.103 | 1.795 | 12.906 | 0.131909 | 7/15/2014 | 17:13.1 | 2 |
| -408.598 | 19327.079 | 1.793 | 12.897 | 0.163596 | 7/15/2014 | 17:13.2 | 2 |
| -408.36 | 19327.056 | 1.795 | 12.887 | 0.143416 | 7/15/2014 | 17:13.3 | 2 |

| | | | | | | | |
|---|---|---|---|---|---|---|---|
| -407.944 | 19327.016 | 1.794 | 12.882 | 0.162959 | 7/15/2014 | 17:13.4 | 2 |
| -407.707 | 19326.992 | 1.794 | 12.884 | 0.153862 | 7/15/2014 | 17:13.5 | 2 |
| -407.469 | 19326.969 | 1.792 | 12.886 | 0.165814 | 7/15/2014 | 17:13.6 | 2 |
| -407.173 | 19326.939 | 1.792 | 12.89 | 0.164543 | 7/15/2014 | 17:13.7 | 2 |
| -407.052 | 19326.928 | 1.79 | 12.893 | 0.153379 | 7/15/2014 | 17:13.8 | 2 |
| -406.814 | 19326.904 | 1.79 | 12.913 | 0.165828 | 7/15/2014 | 17:13.9 | 2 |
| -406.575 | 19326.881 | 1.788 | 12.958 | 0.144861 | 7/15/2014 | 17:14.0 | 2 |
| -406.336 | 19326.857 | 1.79 | 13.006 | 0.142589 | 7/15/2014 | 17:14.1 | 2 |
| -406.098 | 19326.834 | 1.79 | 13.051 | 0.15307 | 7/15/2014 | 17:14.2 | 2 |
| -405.86 | 19326.811 | 1.793 | 13.093 | 0.14774 | 7/15/2014 | 17:14.3 | 2 |
| -405.447 | 19326.77 | 1.796 | 13.152 | 0.143953 | 7/15/2014 | 17:14.4 | 2 |
| -405.211 | 19326.747 | 1.796 | 13.187 | 0.139624 | 7/15/2014 | 17:14.5 | 2 |
| -404.975 | 19326.724 | 1.798 | 13.213 | 0.14071 | 7/15/2014 | 17:14.6 | 2 |
| -404.68 | 19326.694 | 1.798 | 13.219 | 0.136479 | 7/15/2014 | 17:14.7 | 2 |
| -404.561 | 19326.683 | 1.798 | 13.221 | 0.136138 | 7/15/2014 | 17:14.8 | 2 |
| -404.323 | 19326.659 | 1.8 | 13.221 | 0.135741 | 7/15/2014 | 17:14.9 | 2 |
| -404.085 | 19326.638 | 1.798 | 13.227 | 0.138131 | 7/15/2014 | 17:15.0 | 2 |
| -403.846 | 19326.612 | 1.798 | 13.229 | 0.140825 | 7/15/2014 | 17:15.1 | 2 |
| -403.608 | 19326.59 | 1.798 | 13.227 | 0.146183 | 7/15/2014 | 17:15.2 | 2 |
| -403.369 | 19326.566 | 1.795 | 13.222 | 0.149001 | 7/15/2014 | 17:15.3 | 2 |
| -402.951 | 19326.525 | 1.794 | 13.215 | 0.150252 | 7/15/2014 | 17:15.4 | 2 |
| -402.712 | 19326.502 | 1.794 | 13.209 | 0.153975 | 7/15/2014 | 17:15.5 | 2 |
| -402.473 | 19326.48 | 1.792 | 13.206 | 0.152443 | 7/15/2014 | 17:15.6 | 2 |
| -402.173 | 19326.449 | 1.789 | 13.195 | 0.153142 | 7/15/2014 | 17:15.7 | 2 |
| -402.052 | 19326.438 | 1.787 | 13.189 | 0.152413 | 7/15/2014 | 17:15.8 | 2 |
| -401.81 | 19326.415 | 1.787 | 13.172 | 0.14722 | 7/15/2014 | 17:15.9 | 2 |
| -401.567 | 19326.392 | 1.787 | 13.161 | 0.145669 | 7/15/2014 | 17:16.0 | 2 |
| -401.323 | 19326.368 | 1.789 | 13.146 | 0.144944 | 7/15/2014 | 17:16.1 | 2 |
| -401.078 | 19326.344 | 1.787 | 13.128 | 0.147529 | 7/15/2014 | 17:16.2 | 2 |
| -400.833 | 19326.321 | 1.789 | 13.11 | 0.147008 | 7/15/2014 | 17:16.3 | 2 |
| -400.4 | 19326.278 | 1.788 | 13.081 | 0.147093 | 7/15/2014 | 17:16.4 | 2 |
| -400.279 | 19326.268 | 1.792 | 13.071 | 0.15002 | 7/15/2014 | 17:16.5 | 2 |
| -400.03 | 19326.244 | 1.789 | 13.059 | 0.153821 | 7/15/2014 | 17:16.6 | 2 |
| -399.779 | 19326.221 | 1.792 | 13.046 | 0.154154 | 7/15/2014 | 17:16.7 | 2 |
| -399.528 | 19326.196 | 1.785 | 13.036 | 0.153573 | 7/15/2014 | 17:16.8 | 2 |
| -399.274 | 19326.172 | 1.788 | 13.029 | 0.155128 | 7/15/2014 | 17:16.9 | 2 |
| -399.021 | 19326.148 | 1.785 | 13.029 | 0.15397 | 7/15/2014 | 17:17.0 | 2 |
| -398.768 | 19326.124 | 1.791 | 13.029 | 0.155729 | 7/15/2014 | 17:17.1 | 2 |
| -398.515 | 19326.101 | 1.791 | 13.038 | 0.153023 | 7/15/2014 | 17:17.2 | 2 |
| -398.262 | 19326.077 | 1.794 | 13.078 | 0.14966 | 7/15/2014 | 17:17.3 | 2 |
| -398.013 | 19326.055 | 1.79 | 13.116 | 0.139369 | 7/15/2014 | 17:17.4 | 2 |
| -397.762 | 19326.03 | 1.796 | 13.153 | 0.14556 | 7/15/2014 | 17:17.5 | 2 |
| -397.394 | 19325.995 | 1.8 | 13.202 | 0.145695 | 7/15/2014 | 17:17.6 | 2 |
| -397.088 | 19325.968 | 1.8 | 13.23 | 0.140515 | 7/15/2014 | 17:17.7 | 2 |
| -396.963 | 19325.956 | 1.799 | 13.257 | 0.138207 | 7/15/2014 | 17:17.8 | 2 |
| -396.718 | 19325.934 | 1.799 | 13.284 | 0.136257 | 7/15/2014 | 17:17.9 | 2 |
| -396.474 | 19325.912 | 1.799 | 13.274 | 0.160786 | 7/15/2014 | 17:18.0 | 2 |

| | | | | | | | |
|---|---|---|---|---|---|---|---|
| -396.229 | 19325.889 | 1.803 | 13.265 | 0.148384 | 7/15/2014 | 17:18.1 | 2 |
| -395.984 | 19325.865 | 1.803 | 13.255 | 0.137017 | 7/15/2014 | 17:18.2 | 2 |
| -395.74 | 19325.843 | 1.799 | 13.245 | 0.146101 | 7/15/2014 | 17:18.3 | 2 |
| -395.309 | 19325.805 | 1.796 | 13.232 | 0.154261 | 7/15/2014 | 17:18.4 | 2 |
| -395.062 | 19325.782 | 1.796 | 13.222 | 0.146782 | 7/15/2014 | 17:18.5 | 2 |
| -394.941 | 19325.771 | 1.795 | 13.216 | 0.134582 | 7/15/2014 | 17:18.6 | 2 |
| -394.691 | 19325.748 | 1.793 | 13.207 | 0.128051 | 7/15/2014 | 17:18.7 | 2 |
| -394.44 | 19325.725 | 1.793 | 13.196 | 0.134384 | 7/15/2014 | 17:18.8 | 2 |
| -394.188 | 19325.704 | 1.793 | 13.189 | 0.122572 | 7/15/2014 | 17:18.9 | 2 |
| -393.938 | 19325.682 | 1.787 | 13.18 | 0.13365 | 7/15/2014 | 17:19.0 | 2 |
| -393.684 | 19325.658 | 1.794 | 13.174 | 0.115986 | 7/15/2014 | 17:19.1 | 2 |
| -393.432 | 19325.637 | 1.792 | 13.164 | 0.121158 | 7/15/2014 | 17:19.2 | 2 |
| -393.182 | 19325.613 | 1.794 | 13.154 | 0.123422 | 7/15/2014 | 17:19.3 | 2 |
| -392.932 | 19325.592 | 1.796 | 13.145 | 0.11793 | 7/15/2014 | 17:19.4 | 2 |
| -392.565 | 19325.561 | 1.797 | 13.135 | 0.124284 | 7/15/2014 | 17:19.5 | 2 |
| -392.322 | 19325.539 | 1.8 | 13.125 | 0.115302 | 7/15/2014 | 17:19.6 | 2 |
| -392.018 | 19325.512 | 1.803 | 13.117 | 0.11409 | 7/15/2014 | 17:19.7 | 2 |
| -391.893 | 19325.502 | 1.8 | 13.107 | 0.11249 | 7/15/2014 | 17:19.8 | 2 |
| -391.652 | 19325.48 | 1.8 | 13.1 | 0.112591 | 7/15/2014 | 17:19.9 | 2 |
| -391.41 | 19325.461 | 1.798 | 13.095 | 0.110268 | 7/15/2014 | 17:20.0 | 2 |
| -391.168 | 19325.44 | 1.8 | 13.093 | 0.101928 | 7/15/2014 | 17:20.1 | 2 |
| -390.929 | 19325.418 | 1.8 | 13.1 | 0.0937974 | 7/15/2014 | 17:20.2 | 2 |
| -390.688 | 19325.398 | 1.804 | 13.104 | 0.0896618 | 7/15/2014 | 17:20.3 | 2 |
| -390.264 | 19325.363 | 1.798 | 13.117 | 0.0778538 | 7/15/2014 | 17:20.4 | 2 |
| -390.022 | 19325.342 | 1.798 | 13.139 | 0.0770653 | 7/15/2014 | 17:20.5 | 2 |
| -389.778 | 19325.322 | 1.798 | 13.168 | 0.0888505 | 7/15/2014 | 17:20.6 | 2 |
| -389.532 | 19325.303 | 1.798 | 13.192 | 0.0781208 | 7/15/2014 | 17:20.7 | 2 |
| -389.287 | 19325.28 | 1.794 | 13.217 | 0.0927394 | 7/15/2014 | 17:20.8 | 2 |
| -389.04 | 19325.26 | 1.792 | 13.237 | 0.0963567 | 7/15/2014 | 17:20.9 | 2 |
| -388.793 | 19325.24 | 1.792 | 13.261 | 0.112143 | 7/15/2014 | 17:21.0 | 2 |
| -388.546 | 19325.221 | 1.792 | 13.287 | 0.125062 | 7/15/2014 | 17:21.1 | 2 |
| -388.299 | 19325.199 | 1.792 | 13.313 | 0.129884 | 7/15/2014 | 17:21.2 | 2 |
| -388.054 | 19325.18 | 1.792 | 13.327 | 0.136712 | 7/15/2014 | 17:21.3 | 2 |
| -387.809 | 19325.16 | 1.794 | 13.333 | 0.136599 | 7/15/2014 | 17:21.4 | 2 |
| -387.566 | 19325.14 | 1.794 | 13.343 | 0.135776 | 7/15/2014 | 17:21.5 | 2 |
| -387.324 | 19325.119 | 1.796 | 13.353 | 0.135258 | 7/15/2014 | 17:21.6 | 2 |
| -387.024 | 19325.096 | 1.798 | 13.351 | 0.129408 | 7/15/2014 | 17:21.7 | 2 |
| -386.9 | 19325.086 | 1.794 | 13.357 | 0.120282 | 7/15/2014 | 17:21.8 | 2 |
| -386.659 | 19325.066 | 1.799 | 13.35 | 0.112112 | 7/15/2014 | 17:21.9 | 2 |
| -386.419 | 19325.048 | 1.793 | 13.34 | 0.10906 | 7/15/2014 | 17:22.0 | 2 |
| -386.179 | 19325.029 | 1.793 | 13.327 | 0.103099 | 7/15/2014 | 17:22.1 | 2 |
| -385.938 | 19325.01 | 1.791 | 13.319 | 0.0965997 | 7/15/2014 | 17:22.2 | 2 |
| -385.697 | 19324.99 | 1.791 | 13.309 | 0.0944657 | 7/15/2014 | 17:22.3 | 2 |
| -385.271 | 19324.957 | 1.788 | 13.298 | 0.0873475 | 7/15/2014 | 17:22.4 | 2 |
| -385.026 | 19324.938 | 1.786 | 13.289 | 0.0825391 | 7/15/2014 | 17:22.5 | 2 |
| -384.78 | 19324.919 | 1.781 | 13.279 | 0.0777282 | 7/15/2014 | 17:22.6 | 2 |
| -384.66 | 19324.91 | 1.785 | 13.277 | 0.0759909 | 7/15/2014 | 17:22.7 | 2 |

| | | | | | | | |
|---|---|---|---|---|---|---|---|
| -384.412 | 19324.892 | 1.781 | 13.269 | 0.081151 | 7/15/2014 | 17:22.8 | 2 |
| -384.16 | 19324.872 | 1.781 | 13.26 | 0.0895693 | 7/15/2014 | 17:22.9 | 2 |
| -383.91 | 19324.854 | 1.773 | 13.252 | 0.0854743 | 7/15/2014 | 17:23.0 | 2 |
| -383.657 | 19324.834 | 1.773 | 13.245 | 0.0974399 | 7/15/2014 | 17:23.1 | 2 |
| -383.405 | 19324.814 | 1.776 | 13.235 | 0.0989957 | 7/15/2014 | 17:23.2 | 2 |
| -383.154 | 19324.795 | 1.778 | 13.233 | 0.10393 | 7/15/2014 | 17:23.3 | 2 |
| -382.904 | 19324.778 | 1.775 | 13.233 | 0.103932 | 7/15/2014 | 17:23.4 | 2 |
| -382.654 | 19324.759 | 1.781 | 13.23 | 0.104192 | 7/15/2014 | 17:23.5 | 2 |
| -382.287 | 19324.732 | 1.775 | 13.228 | 0.10184 | 7/15/2014 | 17:23.6 | 2 |
| -381.981 | 19324.711 | 1.773 | 13.228 | 0.0991661 | 7/15/2014 | 17:23.7 | 2 |
| -381.856 | 19324.701 | 1.771 | 13.228 | 0.0975844 | 7/15/2014 | 17:23.8 | 2 |
| -381.609 | 19324.683 | 1.769 | 13.265 | 0.0970078 | 7/15/2014 | 17:23.9 | 2 |
| -381.366 | 19324.666 | 1.759 | 13.301 | 0.098125 | 7/15/2014 | 17:24.0 | 2 |
| -381.121 | 19324.647 | 1.755 | 13.33 | 0.0930506 | 7/15/2014 | 17:24.1 | 2 |
| -380.877 | 19324.632 | 1.745 | 13.367 | 0.0954928 | 7/15/2014 | 17:24.2 | 2 |
| -380.631 | 19324.613 | 1.753 | 13.407 | 0.0983369 | 7/15/2014 | 17:24.3 | 2 |
| -380.203 | 19324.584 | 1.753 | 13.453 | 0.100569 | 7/15/2014 | 17:24.4 | 2 |
| -379.959 | 19324.567 | 1.759 | 13.491 | 0.101596 | 7/15/2014 | 17:24.5 | 2 |
| -379.714 | 19324.55 | 1.759 | 13.495 | 0.100375 | 7/15/2014 | 17:24.6 | 2 |
| -379.469 | 19324.533 | 1.762 | 13.503 | 0.109041 | 7/15/2014 | 17:24.7 | 2 |
| -379.223 | 19324.518 | 1.768 | 13.518 | 0.103528 | 7/15/2014 | 17:24.8 | 2 |
| -378.978 | 19324.501 | 1.768 | 13.529 | 0.113695 | 7/15/2014 | 17:24.9 | 2 |
| -378.735 | 19324.484 | 1.762 | 13.539 | 0.102048 | 7/15/2014 | 17:25.0 | 2 |
| -378.491 | 19324.468 | 1.754 | 13.555 | 0.0993334 | 7/15/2014 | 17:25.1 | 2 |
| -378.249 | 19324.453 | 1.754 | 13.568 | 0.0923331 | 7/15/2014 | 17:25.2 | 2 |
| -378.009 | 19324.437 | 1.749 | 13.579 | 0.0915801 | 7/15/2014 | 17:25.3 | 2 |
| -377.772 | 19324.421 | 1.742 | 13.588 | 0.0872358 | 7/15/2014 | 17:25.4 | 2 |
| -377.535 | 19324.407 | 1.737 | 13.597 | 0.0860074 | 7/15/2014 | 17:25.5 | 2 |
| -377.301 | 19324.393 | 1.73 | 13.606 | 0.085776 | 7/15/2014 | 17:25.6 | 2 |
| -377.008 | 19324.374 | 1.73 | 13.613 | 0.0876117 | 7/15/2014 | 17:25.7 | 2 |
| -376.891 | 19324.366 | 1.73 | 13.62 | 0.0888119 | 7/15/2014 | 17:25.8 | 2 |
| -376.654 | 19324.352 | 1.742 | 13.622 | 0.084616 | 7/15/2014 | 17:25.9 | 2 |
| -376.421 | 19324.338 | 1.738 | 13.625 | 0.086565 | 7/15/2014 | 17:26.0 | 2 |
| -376.185 | 19324.322 | 1.741 | 13.625 | 0.0809701 | 7/15/2014 | 17:26.1 | 2 |
| -375.948 | 19324.31 | 1.741 | 13.629 | 0.0915273 | 7/15/2014 | 17:26.2 | 2 |
| -375.709 | 19324.296 | 1.747 | 13.632 | 0.100808 | 7/15/2014 | 17:26.3 | 2 |
| -375.287 | 19324.271 | 1.751 | 13.639 | 0.0859808 | 7/15/2014 | 17:26.4 | 2 |
| -375.044 | 19324.256 | 1.751 | 13.644 | 0.081424 | 7/15/2014 | 17:26.5 | 2 |
| -374.799 | 19324.242 | 1.751 | 13.646 | 0.0685581 | 7/15/2014 | 17:26.6 | 2 |
| -374.554 | 19324.229 | 1.746 | 13.651 | 0.0703356 | 7/15/2014 | 17:26.7 | 2 |
| -374.432 | 19324.222 | 1.748 | 13.655 | 0.0672608 | 7/15/2014 | 17:26.8 | 2 |
| -374.182 | 19324.208 | 1.748 | 13.669 | 0.0630435 | 7/15/2014 | 17:26.9 | 2 |
| -373.933 | 19324.193 | 1.744 | 13.684 | 0.063918 | 7/15/2014 | 17:27.0 | 2 |
| -373.681 | 19324.18 | 1.746 | 13.702 | 0.0654151 | 7/15/2014 | 17:27.1 | 2 |
| -373.435 | 19324.166 | 1.738 | 13.715 | 0.0673711 | 7/15/2014 | 17:27.2 | 2 |
| -373.066 | 19324.146 | 1.737 | 13.74 | 0.0807303 | 7/15/2014 | 17:27.3 | 2 |
| -372.823 | 19324.135 | 1.731 | 13.757 | 0.0739494 | 7/15/2014 | 17:27.4 | 2 |

| | | | | | | | |
|---|---|---|---|---|---|---|---|
| -372.581 | 19324.122 | 1.733 | 13.769 | 0.0772092 | 7/15/2014 | 17:27.5 | 2 |
| -372.34 | 19324.109 | 1.733 | 13.77 | 0.0812331 | 7/15/2014 | 17:27.6 | 2 |
| -372.043 | 19324.095 | 1.733 | 13.768 | 0.0907804 | 7/15/2014 | 17:27.7 | 2 |
| -371.922 | 19324.088 | 1.732 | 13.769 | 0.0813546 | 7/15/2014 | 17:27.8 | 2 |
| -371.685 | 19324.076 | 1.736 | 13.765 | 0.087278 | 7/15/2014 | 17:27.9 | 2 |
| -371.45 | 19324.064 | 1.736 | 13.749 | 0.104225 | 7/15/2014 | 17:28.0 | 2 |
| -371.213 | 19324.053 | 1.743 | 13.735 | 0.102608 | 7/15/2014 | 17:28.1 | 2 |
| -370.981 | 19324.043 | 1.747 | 13.718 | 0.0936914 | 7/15/2014 | 17:28.2 | 2 |
| -370.745 | 19324.031 | 1.75 | 13.702 | 0.104432 | 7/15/2014 | 17:28.3 | 2 |
| -370.335 | 19324.012 | 1.746 | 13.684 | 0.0901415 | 7/15/2014 | 17:28.4 | 2 |
| -370.099 | 19324.001 | 1.741 | 13.674 | 0.104804 | 7/15/2014 | 17:28.5 | 2 |
| -369.861 | 19323.99 | 1.732 | 13.661 | 0.0796568 | 7/15/2014 | 17:28.6 | 2 |
| -369.62 | 19323.979 | 1.727 | 13.653 | 0.082401 | 7/15/2014 | 17:28.7 | 2 |
| -369.38 | 19323.969 | 1.715 | 13.643 | 0.0809722 | 7/15/2014 | 17:28.8 | 2 |
| -369.137 | 19323.959 | 1.707 | 13.637 | 0.0640685 | 7/15/2014 | 17:28.9 | 2 |
| -368.894 | 19323.947 | 1.704 | 13.632 | 0.0702033 | 7/15/2014 | 17:29.0 | 2 |
| -368.651 | 19323.937 | 1.701 | 13.623 | 0.0732309 | 7/15/2014 | 17:29.1 | 2 |
| -368.406 | 19323.927 | 1.699 | 13.614 | 0.0622866 | 7/15/2014 | 17:29.2 | 2 |
| -368.163 | 19323.916 | 1.697 | 13.605 | 0.0603701 | 7/15/2014 | 17:29.3 | 2 |
| -367.92 | 19323.906 | 1.697 | 13.601 | 0.0762856 | 7/15/2014 | 17:29.4 | 2 |
| -367.677 | 19323.896 | 1.697 | 13.599 | 0.0616661 | 7/15/2014 | 17:29.5 | 2 |
| -367.433 | 19323.887 | 1.7 | 13.627 | 0.087927 | 7/15/2014 | 17:29.6 | 2 |
| -367.131 | 19323.875 | 1.7 | 13.651 | 0.0831867 | 7/15/2014 | 17:29.7 | 2 |
| -367.009 | 19323.87 | 1.695 | 13.656 | 0.0798781 | 7/15/2014 | 17:29.8 | 2 |
| -366.764 | 19323.859 | 1.698 | 13.693 | 0.0959843 | 7/15/2014 | 17:29.9 | 2 |
| -366.523 | 19323.85 | 1.692 | 13.731 | 0.0876688 | 7/15/2014 | 17:30.0 | 2 |
| -366.278 | 19323.841 | 1.702 | 13.768 | 0.077992 | 7/15/2014 | 17:30.1 | 2 |
| -366.036 | 19323.831 | 1.702 | 13.801 | 0.0899563 | 7/15/2014 | 17:30.2 | 2 |
| -365.79 | 19323.822 | 1.702 | 13.834 | 0.0644634 | 7/15/2014 | 17:30.3 | 2 |
| -365.358 | 19323.807 | 1.7 | 13.829 | 0.063688 | 7/15/2014 | 17:30.4 | 2 |
| -365.11 | 19323.797 | 1.7 | 13.832 | 0.0705465 | 7/15/2014 | 17:30.5 | 2 |
| -364.989 | 19323.793 | 1.698 | 13.831 | 0.07507 | 7/15/2014 | 17:30.6 | 2 |
| -364.738 | 19323.784 | 1.701 | 13.827 | 0.0669107 | 7/15/2014 | 17:30.7 | 2 |
| -364.488 | 19323.775 | 1.691 | 13.821 | 0.0695961 | 7/15/2014 | 17:30.8 | 2 |
| -364.235 | 19323.768 | 1.694 | 13.812 | 0.0834241 | 7/15/2014 | 17:30.9 | 2 |
| -363.983 | 19323.757 | 1.694 | 13.8 | 0.0917905 | 7/15/2014 | 17:31.0 | 2 |
| -363.729 | 19323.75 | 1.692 | 13.785 | 0.0778524 | 7/15/2014 | 17:31.1 | 2 |
| -363.479 | 19323.742 | 1.686 | 13.768 | 0.0741139 | 7/15/2014 | 17:31.2 | 2 |
| -363.228 | 19323.734 | 1.686 | 13.743 | 0.0750644 | 7/15/2014 | 17:31.3 | 2 |
| -362.859 | 19323.723 | 1.684 | 13.717 | 0.0670911 | 7/15/2014 | 17:31.4 | 2 |
| -362.613 | 19323.717 | 1.684 | 13.7 | 0.0575614 | 7/15/2014 | 17:31.5 | 2 |
| -362.369 | 19323.708 | 1.681 | 13.691 | 0.05748 | 7/15/2014 | 17:31.6 | 2 |
| -362.067 | 19323.7 | 1.679 | 13.679 | 0.0787368 | 7/15/2014 | 17:31.7 | 2 |
| -361.943 | 19323.696 | 1.68 | 13.674 | 0.0801659 | 7/15/2014 | 17:31.8 | 2 |
| -361.701 | 19323.689 | 1.682 | 13.673 | 0.0745612 | 7/15/2014 | 17:31.9 | 2 |
| -361.459 | 19323.684 | 1.682 | 13.67 | 0.0862247 | 7/15/2014 | 17:32.0 | 2 |
| -361.217 | 19323.676 | 1.685 | 13.671 | 0.0845065 | 7/15/2014 | 17:32.1 | 2 |

| | | | | | | | |
|---|---|---|---|---|---|---|---|
| -360.976 | 19323.671 | 1.685 | 13.706 | 0.0901351 | 7/15/2014 | 17:32.2 | 2 |
| -360.733 | 19323.664 | 1.691 | 13.713 | 0.0846147 | 7/15/2014 | 17:32.3 | 2 |
| -360.307 | 19323.653 | 1.689 | 13.747 | 0.0685464 | 7/15/2014 | 17:32.4 | 2 |
| -360.062 | 19323.647 | 1.691 | 13.777 | 0.0703418 | 7/15/2014 | 17:32.5 | 2 |
| -359.816 | 19323.643 | 1.691 | 13.803 | 0.0764932 | 7/15/2014 | 17:32.6 | 2 |
| -359.568 | 19323.637 | 1.691 | 13.835 | 0.0692761 | 7/15/2014 | 17:32.7 | 2 |
| -359.445 | 19323.634 | 1.691 | 13.857 | 0.0633891 | 7/15/2014 | 17:32.8 | 2 |
| -359.194 | 19323.629 | 1.694 | 13.854 | 0.0687357 | 7/15/2014 | 17:32.9 | 2 |
| -358.945 | 19323.623 | 1.69 | 13.879 | 0.072886 | 7/15/2014 | 17:33.0 | 2 |
| -358.694 | 19323.617 | 1.694 | 13.898 | 0.0704519 | 7/15/2014 | 17:33.1 | 2 |
| -358.445 | 19323.612 | 1.692 | 13.891 | 0.0600461 | 7/15/2014 | 17:33.2 | 2 |
| -358.195 | 19323.607 | 1.695 | 13.886 | 0.0531392 | 7/15/2014 | 17:33.3 | 2 |
| -357.827 | 19323.601 | 1.695 | 13.878 | 0.06023 | 7/15/2014 | 17:33.5 | 2 |
| -357.583 | 19323.597 | 1.693 | 13.869 | 0.0644441 | 7/15/2014 | 17:33.6 | 2 |
| -357.339 | 19323.592 | 1.688 | 13.859 | 0.063485 | 7/15/2014 | 17:33.6 | 2 |
| -357.036 | 19323.586 | 1.686 | 13.848 | 0.0593325 | 7/15/2014 | 17:33.7 | 2 |
| -356.913 | 19323.584 | 1.684 | 13.843 | 0.0638264 | 7/15/2014 | 17:33.8 | 2 |
| -356.669 | 19323.58 | 1.69 | 13.828 | 0.068755 | 7/15/2014 | 17:33.9 | 2 |
| -356.43 | 19323.575 | 1.687 | 13.814 | 0.0581059 | 7/15/2014 | 17:34.0 | 2 |
| -356.186 | 19323.572 | 1.696 | 13.797 | 0.0565684 | 7/15/2014 | 17:34.1 | 2 |
| -355.947 | 19323.569 | 1.693 | 13.774 | 0.0539534 | 7/15/2014 | 17:34.2 | 2 |
| -355.705 | 19323.565 | 1.699 | 13.755 | 0.0506534 | 7/15/2014 | 17:34.3 | 2 |
| -355.285 | 19323.56 | 1.701 | 13.735 | 0.0461131 | 7/15/2014 | 17:34.4 | 2 |
| -355.043 | 19323.557 | 1.706 | 13.726 | 0.042906 | 7/15/2014 | 17:34.5 | 2 |
| -354.745 | 19323.552 | 1.709 | 13.719 | 0.0473009 | 7/15/2014 | 17:34.6 | 2 |
| -354.622 | 19323.551 | 1.712 | 13.713 | 0.0399417 | 7/15/2014 | 17:34.7 | 2 |
| -354.381 | 19323.549 | 1.708 | 13.714 | 0.0415108 | 7/15/2014 | 17:34.8 | 2 |
| -354.138 | 19323.546 | 1.712 | 13.715 | 0.0382819 | 7/15/2014 | 17:34.9 | 2 |
| -353.896 | 19323.543 | 1.712 | 13.735 | 0.0411672 | 7/15/2014 | 17:35.0 | 2 |
| -353.652 | 19323.541 | 1.719 | 13.747 | 0.0202299 | 7/15/2014 | 17:35.1 | 2 |
| -353.409 | 19323.539 | 1.719 | 13.761 | 0.0244222 | 7/15/2014 | 17:35.2 | 2 |
| -352.987 | 19323.534 | 1.72 | 13.794 | 0.0124055 | 7/15/2014 | 17:35.3 | 2 |
| -352.746 | 19323.533 | 1.72 | 13.824 | 0.0152728 | 7/15/2014 | 17:35.4 | 2 |
| -352.507 | 19323.531 | 1.718 | 13.842 | 0.0211085 | 7/15/2014 | 17:35.5 | 2 |
| -352.267 | 19323.529 | 1.718 | 13.867 | 0.0225921 | 7/15/2014 | 17:35.6 | 2 |
| -351.968 | 19323.528 | 1.713 | 13.874 | 0.0257662 | 7/15/2014 | 17:35.7 | 2 |
| -351.847 | 19323.527 | 1.709 | 13.888 | 0.0503765 | 7/15/2014 | 17:35.8 | 2 |
| -351.604 | 19323.526 | 1.709 | 13.903 | 0.037582 | 7/15/2014 | 17:35.9 | 2 |
| -351.362 | 19323.523 | 1.703 | 13.908 | 0.0440373 | 7/15/2014 | 17:36.0 | 2 |
| -351.12 | 19323.523 | 1.705 | 13.916 | 0.0415281 | 7/15/2014 | 17:36.1 | 2 |
| -350.878 | 19323.522 | 1.698 | 13.91 | 0.0433399 | 7/15/2014 | 17:36.2 | 2 |
| -350.634 | 19323.521 | 1.7 | 13.917 | 0.0386264 | 7/15/2014 | 17:36.3 | 2 |
| -350.207 | 19323.521 | 1.695 | 13.931 | 0.0328969 | 7/15/2014 | 17:36.4 | 2 |
| -349.959 | 19323.521 | 1.697 | 13.937 | 0.0264989 | 7/15/2014 | 17:36.5 | 2 |
| -349.711 | 19323.52 | 1.696 | 13.941 | 0.0290042 | 7/15/2014 | 17:36.6 | 2 |
| -349.589 | 19323.52 | 1.696 | 13.948 | 0.0220526 | 7/15/2014 | 17:36.7 | 2 |
| -349.339 | 19323.52 | 1.696 | 13.949 | 0.0279728 | 7/15/2014 | 17:36.8 | 2 |

| | | | | | | | |
|---|---|---|---|---|---|---|---|
| -349.086 | 19323.52 | 1.703 | 13.956 | 0.0480789 | 7/15/2014 | 17:36.9 | 2 |
| -348.836 | 19323.52 | 1.697 | 13.961 | 0.0495699 | 7/15/2014 | 17:37.0 | 2 |
| -348.582 | 19323.521 | 1.705 | 13.961 | 0.0509071 | 7/15/2014 | 17:37.1 | 2 |
| -348.329 | 19323.521 | 1.706 | 13.964 | 0.0606468 | 7/15/2014 | 17:37.2 | 2 |
| -348.077 | 19323.521 | 1.713 | 13.965 | 0.0634901 | 7/15/2014 | 17:37.3 | 2 |
| -347.826 | 19323.521 | 1.713 | 13.974 | 0.0665923 | 7/15/2014 | 17:37.4 | 2 |
| -347.575 | 19323.521 | 1.717 | 13.98 | 0.0581604 | 7/15/2014 | 17:37.5 | 2 |
| -347.206 | 19323.522 | 1.71 | 13.986 | 0.05079 | 7/15/2014 | 17:37.6 | 2 |
| -346.898 | 19323.524 | 1.71 | 13.993 | 0.047975 | 7/15/2014 | 17:37.7 | 2 |
| -346.772 | 19323.524 | 1.706 | 13.995 | 0.04688 | 7/15/2014 | 17:37.8 | 2 |
| -346.523 | 19323.525 | 1.708 | 13.997 | 0.0396022 | 7/15/2014 | 17:37.9 | 2 |
| -346.277 | 19323.527 | 1.705 | 14.004 | 0.0434523 | 7/15/2014 | 17:38.0 | 2 |
| -346.028 | 19323.527 | 1.712 | 14.006 | 0.0467598 | 7/15/2014 | 17:38.1 | 2 |
| -345.782 | 19323.529 | 1.705 | 14.009 | 0.0369665 | 7/15/2014 | 17:38.2 | 2 |
| -345.53 | 19323.53 | 1.708 | 14.014 | 0.0464794 | 7/15/2014 | 17:38.3 | 2 |
| -345.222 | 19323.532 | 1.705 | 14.016 | 0.0526866 | 7/15/2014 | 17:38.4 | 2 |
| -344.967 | 19323.534 | 1.714 | 14.019 | 0.0508914 | 7/15/2014 | 17:38.5 | 2 |
| -344.713 | 19323.536 | 1.714 | 14.024 | 0.0601176 | 7/15/2014 | 17:38.6 | 2 |
| -344.457 | 19323.537 | 1.714 | 14.022 | 0.0502535 | 7/15/2014 | 17:38.7 | 2 |
| -344.2 | 19323.539 | 1.711 | 14.022 | 0.0531384 | 7/15/2014 | 17:38.8 | 2 |
| -343.94 | 19323.541 | 1.714 | 14.021 | 0.0609497 | 7/15/2014 | 17:38.9 | 2 |
| -343.682 | 19323.544 | 1.714 | 14.02 | 0.0475477 | 7/15/2014 | 17:39.0 | 2 |
| -343.42 | 19323.546 | 1.718 | 14.021 | 0.0340317 | 7/15/2014 | 17:39.1 | 2 |
| -343.161 | 19323.548 | 1.715 | 14.016 | 0.0331519 | 7/15/2014 | 17:39.2 | 2 |
| -342.902 | 19323.55 | 1.715 | 14.011 | 0.0486755 | 7/15/2014 | 17:39.3 | 2 |
| -342.644 | 19323.552 | 1.715 | 14.009 | 0.0498601 | 7/15/2014 | 17:39.4 | 2 |
| -342.388 | 19323.555 | 1.718 | 14.001 | 0.0483945 | 7/15/2014 | 17:39.5 | 2 |
| -342.134 | 19323.558 | 1.718 | 13.992 | 0.0344773 | 7/15/2014 | 17:39.6 | 2 |
| -341.881 | 19323.56 | 1.718 | 13.987 | 0.0345563 | 7/15/2014 | 17:39.7 | 2 |
| -341.569 | 19323.563 | 1.714 | 13.981 | 0.0330532 | 7/15/2014 | 17:39.8 | 2 |
| -341.316 | 19323.565 | 1.718 | 13.971 | 0.0263367 | 7/15/2014 | 17:39.9 | 2 |
| -341.067 | 19323.568 | 1.712 | 13.962 | 0.0274099 | 7/15/2014 | 17:40.0 | 2 |
| -340.814 | 19323.57 | 1.712 | 13.964 | 0.0386796 | 7/15/2014 | 17:40.1 | 2 |
| -340.565 | 19323.573 | 1.702 | 13.964 | 0.0509782 | 7/15/2014 | 17:40.2 | 2 |
| -340.313 | 19323.576 | 1.702 | 13.965 | 0.053792 | 7/15/2014 | 17:40.3 | 2 |
| -340.001 | 19323.58 | 1.702 | 13.987 | 0.0615902 | 7/15/2014 | 17:40.4 | 2 |
| -339.748 | 19323.583 | 1.706 | 14.007 | 0.0658027 | 7/15/2014 | 17:40.5 | 2 |
| -339.496 | 19323.586 | 1.699 | 14.035 | 0.0740797 | 7/15/2014 | 17:40.6 | 2 |
| -339.239 | 19323.588 | 1.708 | 14.061 | 0.0784085 | 7/15/2014 | 17:40.7 | 2 |
| -338.984 | 19323.592 | 1.708 | 14.081 | 0.0581836 | 7/15/2014 | 17:40.8 | 2 |
| -338.725 | 19323.596 | 1.714 | 14.105 | 0.0700901 | 7/15/2014 | 17:40.9 | 2 |
| -338.469 | 19323.599 | 1.708 | 14.128 | 0.0693182 | 7/15/2014 | 17:41.0 | 2 |
| -338.211 | 19323.601 | 1.708 | 14.131 | 0.0687701 | 7/15/2014 | 17:41.1 | 2 |
| -337.956 | 19323.604 | 1.705 | 14.132 | 0.0514154 | 7/15/2014 | 17:41.2 | 2 |
| -337.699 | 19323.607 | 1.709 | 14.127 | 0.0585448 | 7/15/2014 | 17:41.3 | 2 |
| -337.447 | 19323.611 | 1.701 | 14.127 | 0.061332 | 7/15/2014 | 17:41.4 | 2 |
| -337.193 | 19323.614 | 1.701 | 14.122 | 0.0609831 | 7/15/2014 | 17:41.5 | 2 |

| | | | | | | | |
|---|---|---|---|---|---|---|---|
| -336.945 | 19323.617 | 1.701 | 14.117 | 0.0440354 | 7/15/2014 | 17:41.6 | 2 |
| -336.515 | 19323.622 | 1.708 | 14.114 | 0.0384306 | 7/15/2014 | 17:41.7 | 2 |
| -336.388 | 19323.624 | 1.707 | 14.11 | 0.0356609 | 7/15/2014 | 17:41.8 | 2 |
| -336.142 | 19323.628 | 1.71 | 14.105 | 0.0389951 | 7/15/2014 | 17:41.9 | 2 |
| -335.899 | 19323.632 | 1.706 | 14.105 | 0.0437751 | 7/15/2014 | 17:42.0 | 2 |
| -335.65 | 19323.635 | 1.707 | 14.104 | 0.0531727 | 7/15/2014 | 17:42.1 | 2 |
| -335.406 | 19323.638 | 1.704 | 14.102 | 0.0761611 | 7/15/2014 | 17:42.2 | 2 |
| -335.16 | 19323.642 | 1.704 | 14.1 | 0.0893277 | 7/15/2014 | 17:42.3 | 2 |
| -334.727 | 19323.647 | 1.705 | 14.095 | 0.0922279 | 7/15/2014 | 17:42.4 | 2 |
| -334.481 | 19323.651 | 1.705 | 14.092 | 0.0986466 | 7/15/2014 | 17:42.5 | 2 |
| -334.361 | 19323.653 | 1.704 | 14.092 | 0.0979275 | 7/15/2014 | 17:42.6 | 2 |
| -334.108 | 19323.657 | 1.711 | 14.088 | 0.0930793 | 7/15/2014 | 17:42.7 | 2 |
| -333.858 | 19323.66 | 1.717 | 14.08 | 0.093371 | 7/15/2014 | 17:42.8 | 2 |
| -333.605 | 19323.665 | 1.721 | 14.072 | 0.0863615 | 7/15/2014 | 17:42.9 | 2 |
| -333.353 | 19323.668 | 1.721 | 14.063 | 0.0840891 | 7/15/2014 | 17:43.0 | 2 |
| -333.098 | 19323.672 | 1.728 | 14.049 | 0.0849013 | 7/15/2014 | 17:43.1 | 2 |
| -332.846 | 19323.677 | 1.724 | 14.038 | 0.0700171 | 7/15/2014 | 17:43.2 | 2 |
| -332.593 | 19323.681 | 1.728 | 14.026 | 0.0705896 | 7/15/2014 | 17:43.3 | 2 |
| -332.341 | 19323.686 | 1.722 | 14.014 | 0.0689351 | 7/15/2014 | 17:43.4 | 2 |
| -332.091 | 19323.69 | 1.718 | 14.003 | 0.0607038 | 7/15/2014 | 17:43.5 | 2 |
| -331.718 | 19323.695 | 1.718 | 14.001 | 0.0775963 | 7/15/2014 | 17:43.6 | 2 |
| -331.407 | 19323.701 | 1.715 | 14.004 | 0.0634002 | 7/15/2014 | 17:43.7 | 2 |
| -331.281 | 19323.704 | 1.711 | 14.003 | 0.0761389 | 7/15/2014 | 17:43.8 | 2 |
| -331.03 | 19323.708 | 1.711 | 14.016 | 0.0878583 | 7/15/2014 | 17:43.9 | 2 |
| -330.778 | 19323.713 | 1.707 | 14.039 | 0.075438 | 7/15/2014 | 17:44.0 | 2 |
| -330.528 | 19323.717 | 1.71 | 14.061 | 0.0944692 | 7/15/2014 | 17:44.1 | 2 |
| -330.275 | 19323.721 | 1.706 | 14.087 | 0.0880654 | 7/15/2014 | 17:44.2 | 2 |
| -330.02 | 19323.726 | 1.71 | 14.113 | 0.0947757 | 7/15/2014 | 17:44.3 | 2 |
| -329.705 | 19323.732 | 1.706 | 14.133 | 0.114694 | 7/15/2014 | 17:44.4 | 2 |
| -329.446 | 19323.736 | 1.713 | 14.151 | 0.0892756 | 7/15/2014 | 17:44.5 | 2 |
| -329.186 | 19323.74 | 1.713 | 14.16 | 0.0904159 | 7/15/2014 | 17:44.6 | 2 |
| -328.925 | 19323.746 | 1.713 | 14.163 | 0.0965615 | 7/15/2014 | 17:44.7 | 2 |
| -328.664 | 19323.75 | 1.716 | 14.164 | 0.0955045 | 7/15/2014 | 17:44.8 | 2 |
| -328.398 | 19323.755 | 1.719 | 14.16 | 0.0908265 | 7/15/2014 | 17:44.9 | 2 |
| -328.137 | 19323.761 | 1.716 | 14.153 | 0.0926977 | 7/15/2014 | 17:45.0 | 2 |
| -327.872 | 19323.766 | 1.723 | 14.147 | 0.091589 | 7/15/2014 | 17:45.1 | 2 |
| -327.61 | 19323.77 | 1.719 | 14.136 | 0.0932025 | 7/15/2014 | 17:45.2 | 2 |
| -327.349 | 19323.776 | 1.723 | 14.125 | 0.0962173 | 7/15/2014 | 17:45.3 | 2 |
| -327.091 | 19323.78 | 1.729 | 14.111 | 0.0993505 | 7/15/2014 | 17:45.4 | 2 |
| -326.832 | 19323.785 | 1.73 | 14.095 | 0.0983008 | 7/15/2014 | 17:45.5 | 2 |
| -326.579 | 19323.79 | 1.734 | 14.083 | 0.098932 | 7/15/2014 | 17:45.6 | 2 |
| -326.324 | 19323.796 | 1.74 | 14.068 | 0.10552 | 7/15/2014 | 17:45.7 | 2 |
| -326.013 | 19323.801 | 1.74 | 14.055 | 0.107099 | 7/15/2014 | 17:45.8 | 2 |
| -325.762 | 19323.806 | 1.744 | 14.043 | 0.107195 | 7/15/2014 | 17:45.9 | 2 |
| -325.513 | 19323.811 | 1.743 | 14.03 | 0.11131 | 7/15/2014 | 17:46.0 | 2 |
| -325.263 | 19323.816 | 1.743 | 14.022 | 0.113985 | 7/15/2014 | 17:46.1 | 2 |
| -325.014 | 19323.82 | 1.743 | 14.013 | 0.115863 | 7/15/2014 | 17:46.2 | 2 |

| | | | | | | | |
|---|---|---|---|---|---|---|---|
| -324.765 | 19323.826 | 1.745 | 14.005 | 0.119887 | 7/15/2014 | 17:46.3 | 2 |
| -324.328 | 19323.834 | 1.746 | 14.002 | 0.119706 | 7/15/2014 | 17:46.4 | 2 |
| -324.206 | 19323.836 | 1.747 | 13.998 | 0.126364 | 7/15/2014 | 17:46.5 | 2 |
| -323.958 | 19323.842 | 1.744 | 14.016 | 0.123484 | 7/15/2014 | 17:46.6 | 2 |
| -323.705 | 19323.847 | 1.745 | 14.036 | 0.124708 | 7/15/2014 | 17:46.7 | 2 |
| -323.452 | 19323.852 | 1.745 | 14.051 | 0.12018 | 7/15/2014 | 17:46.8 | 2 |
| -323.199 | 19323.856 | 1.748 | 14.071 | 0.121338 | 7/15/2014 | 17:46.9 | 2 |
| -322.947 | 19323.863 | 1.745 | 14.089 | 0.118114 | 7/15/2014 | 17:47.0 | 2 |
| -322.693 | 19323.867 | 1.748 | 14.109 | 0.150436 | 7/15/2014 | 17:47.1 | 2 |
| -322.442 | 19323.872 | 1.745 | 14.122 | 0.154749 | 7/15/2014 | 17:47.2 | 2 |
| -322.192 | 19323.876 | 1.749 | 14.116 | 0.145978 | 7/15/2014 | 17:47.3 | 2 |
| -321.822 | 19323.885 | 1.75 | 14.105 | 0.161786 | 7/15/2014 | 17:47.4 | 2 |
| -321.574 | 19323.889 | 1.748 | 14.1 | 0.174672 | 7/15/2014 | 17:47.5 | 2 |
| -321.329 | 19323.895 | 1.748 | 14.095 | 0.157159 | 7/15/2014 | 17:47.6 | 2 |
| -321.026 | 19323.9 | 1.75 | 14.087 | 0.144813 | 7/15/2014 | 17:47.7 | 2 |
| -320.902 | 19323.902 | 1.746 | 14.081 | 0.136831 | 7/15/2014 | 17:47.8 | 2 |
| -320.659 | 19323.906 | 1.749 | 14.074 | 0.14926 | 7/15/2014 | 17:47.9 | 2 |
| -320.417 | 19323.911 | 1.749 | 14.073 | 0.119774 | 7/15/2014 | 17:48.0 | 2 |
| -320.173 | 19323.916 | 1.754 | 14.066 | 0.12881 | 7/15/2014 | 17:48.1 | 2 |
| -319.93 | 19323.921 | 1.748 | 14.065 | 0.124668 | 7/15/2014 | 17:48.2 | 2 |
| -319.686 | 19323.925 | 1.75 | 14.064 | 0.121998 | 7/15/2014 | 17:48.3 | 2 |
| -319.258 | 19323.934 | 1.75 | 14.063 | 0.125014 | 7/15/2014 | 17:48.4 | 2 |
| -319.011 | 19323.938 | 1.752 | 14.064 | 0.129157 | 7/15/2014 | 17:48.5 | 2 |
| -318.765 | 19323.941 | 1.752 | 14.065 | 0.14727 | 7/15/2014 | 17:48.6 | 2 |
| -318.642 | 19323.943 | 1.754 | 14.063 | 0.132355 | 7/15/2014 | 17:48.7 | 2 |
| -318.395 | 19323.948 | 1.745 | 14.063 | 0.128432 | 7/15/2014 | 17:48.8 | 2 |
| -318.143 | 19323.952 | 1.751 | 14.065 | 0.143131 | 7/15/2014 | 17:48.9 | 2 |
| -317.895 | 19323.957 | 1.749 | 14.063 | 0.148198 | 7/15/2014 | 17:49.0 | 2 |
| -317.644 | 19323.961 | 1.751 | 14.059 | 0.132606 | 7/15/2014 | 17:49.1 | 2 |
| -317.273 | 19323.967 | 1.752 | 14.054 | 0.117878 | 7/15/2014 | 17:49.2 | 2 |
| -317.027 | 19323.971 | 1.75 | 14.056 | 0.119358 | 7/15/2014 | 17:49.3 | 2 |
| -316.782 | 19323.975 | 1.75 | 14.054 | 0.133731 | 7/15/2014 | 17:49.4 | 2 |
| -316.537 | 19323.979 | 1.755 | 14.054 | 0.125277 | 7/15/2014 | 17:49.5 | 2 |
| -316.296 | 19323.982 | 1.755 | 14.05 | 0.100861 | 7/15/2014 | 17:49.6 | 2 |
| -315.996 | 19323.986 | 1.755 | 14.057 | 0.11646 | 7/15/2014 | 17:49.7 | 2 |
| -315.872 | 19323.988 | 1.755 | 14.059 | 0.10809 | 7/15/2014 | 17:49.8 | 2 |
| -315.631 | 19323.992 | 1.755 | 14.059 | 0.0994581 | 7/15/2014 | 17:49.9 | 2 |
| -315.391 | 19323.996 | 1.755 | 14.073 | 0.098324 | 7/15/2014 | 17:50.0 | 2 |
| -315.148 | 19324 | 1.757 | 14.076 | 0.0725473 | 7/15/2014 | 17:50.1 | 2 |
| -314.907 | 19324.003 | 1.757 | 14.087 | 0.0942225 | 7/15/2014 | 17:50.2 | 2 |
| -314.665 | 19324.007 | 1.76 | 14.094 | 0.0827591 | 7/15/2014 | 17:50.3 | 2 |
| -314.237 | 19324.012 | 1.761 | 14.099 | 0.0586576 | 7/15/2014 | 17:50.4 | 2 |
| -313.99 | 19324.015 | 1.764 | 14.099 | 0.0534896 | 7/15/2014 | 17:50.5 | 2 |
| -313.742 | 19324.019 | 1.766 | 14.109 | 0.0488079 | 7/15/2014 | 17:50.6 | 2 |
| -313.62 | 19324.021 | 1.765 | 14.105 | 0.0542986 | 7/15/2014 | 17:50.7 | 2 |
| -313.369 | 19324.022 | 1.759 | 14.109 | 0.0508993 | 7/15/2014 | 17:50.8 | 2 |
| -313.116 | 19324.026 | 1.761 | 14.104 | 0.0547289 | 7/15/2014 | 17:50.9 | 2 |

| | | | | | | | |
|---|---|---|---|---|---|---|---|
| -312.863 | 19324.028 | 1.756 | 14.102 | 0.0602336 | 7/15/2014 | 17:51.0 | 2 |
| -312.607 | 19324.032 | 1.759 | 14.1 | 0.063719 | 7/15/2014 | 17:51.1 | 2 |
| -312.352 | 19324.034 | 1.756 | 14.098 | 0.0718856 | 7/15/2014 | 17:51.2 | 2 |
| -312.098 | 19324.038 | 1.756 | 14.094 | 0.0744073 | 7/15/2014 | 17:51.3 | 2 |
| -311.844 | 19324.04 | 1.754 | 14.085 | 0.0711509 | 7/15/2014 | 17:51.4 | 2 |
| -311.591 | 19324.043 | 1.76 | 14.075 | 0.0823789 | 7/15/2014 | 17:51.5 | 2 |
| -311.341 | 19324.045 | 1.757 | 14.08 | 0.0751227 | 7/15/2014 | 17:51.6 | 2 |
| -310.91 | 19324.05 | 1.761 | 14.069 | 0.0875634 | 7/15/2014 | 17:51.7 | 2 |
| -310.782 | 19324.051 | 1.76 | 14.068 | 0.0875707 | 7/15/2014 | 17:51.8 | 2 |
| -310.535 | 19324.055 | 1.762 | 14.07 | 0.0726019 | 7/15/2014 | 17:51.9 | 2 |
| -310.289 | 19324.057 | 1.76 | 14.071 | 0.0683839 | 7/15/2014 | 17:52.0 | 2 |
| -310.043 | 19324.059 | 1.76 | 14.072 | 0.0586892 | 7/15/2014 | 17:52.1 | 2 |
| -309.798 | 19324.061 | 1.757 | 14.083 | 0.0599464 | 7/15/2014 | 17:52.2 | 2 |
| -309.555 | 19324.063 | 1.76 | 14.088 | 0.0556097 | 7/15/2014 | 17:52.3 | 2 |
| -309.132 | 19324.066 | 1.761 | 14.109 | 0.0530965 | 7/15/2014 | 17:52.4 | 2 |
| -308.892 | 19324.069 | 1.759 | 14.126 | 0.0605827 | 7/15/2014 | 17:52.5 | 2 |
| -308.652 | 19324.071 | 1.759 | 14.134 | 0.0629338 | 7/15/2014 | 17:52.6 | 2 |
| -308.352 | 19324.074 | 1.761 | 14.14 | 0.0703779 | 7/15/2014 | 17:52.7 | 2 |
| -308.23 | 19324.075 | 1.762 | 14.149 | 0.0790744 | 7/15/2014 | 17:52.8 | 2 |
| -307.988 | 19324.077 | 1.768 | 14.148 | 0.0821918 | 7/15/2014 | 17:52.9 | 2 |
| -307.749 | 19324.079 | 1.768 | 14.141 | 0.0938618 | 7/15/2014 | 17:53.0 | 2 |
| -307.508 | 19324.081 | 1.768 | 14.139 | 0.107762 | 7/15/2014 | 17:53.1 | 2 |
| -307.27 | 19324.083 | 1.766 | 14.13 | 0.110082 | 7/15/2014 | 17:53.2 | 2 |
| -307.029 | 19324.085 | 1.774 | 14.12 | 0.126936 | 7/15/2014 | 17:53.3 | 2 |
| -306.614 | 19324.089 | 1.779 | 14.108 | 0.118084 | 7/15/2014 | 17:53.4 | 2 |
| -306.376 | 19324.091 | 1.785 | 14.102 | 0.134447 | 7/15/2014 | 17:53.5 | 2 |
| -306.14 | 19324.092 | 1.782 | 14.098 | 0.142683 | 7/15/2014 | 17:53.6 | 2 |
| -305.843 | 19324.095 | 1.779 | 14.094 | 0.106258 | 7/15/2014 | 17:53.7 | 2 |
| -305.723 | 19324.096 | 1.781 | 14.089 | 0.108586 | 7/15/2014 | 17:53.8 | 2 |
| -305.481 | 19324.098 | 1.787 | 14.084 | 0.122368 | 7/15/2014 | 17:53.9 | 2 |
| -305.243 | 19324.099 | 1.783 | 14.076 | 0.123842 | 7/15/2014 | 17:54.0 | 2 |
| -305.002 | 19324.101 | 1.783 | 14.073 | 0.134288 | 7/15/2014 | 17:54.1 | 2 |
| -304.76 | 19324.102 | 1.777 | 14.069 | 0.134018 | 7/15/2014 | 17:54.2 | 2 |
| -304.518 | 19324.104 | 1.781 | 14.066 | 0.120203 | 7/15/2014 | 17:54.3 | 2 |
| -304.095 | 19324.106 | 1.786 | 14.065 | 0.145919 | 7/15/2014 | 17:54.4 | 2 |
| -303.853 | 19324.107 | 1.788 | 14.065 | 0.139357 | 7/15/2014 | 17:54.5 | 2 |
| -303.612 | 19324.108 | 1.791 | 14.07 | 0.161332 | 7/15/2014 | 17:54.6 | 2 |
| -303.309 | 19324.109 | 1.794 | 14.089 | 0.159265 | 7/15/2014 | 17:54.7 | 2 |
| -303.187 | 19324.109 | 1.798 | 14.092 | 0.165066 | 7/15/2014 | 17:54.8 | 2 |
| -302.942 | 19324.111 | 1.805 | 14.106 | 0.186072 | 7/15/2014 | 17:54.9 | 2 |
| -302.699 | 19324.111 | 1.805 | 14.124 | 0.154879 | 7/15/2014 | 17:55.0 | 2 |
| -302.454 | 19324.113 | 1.812 | 14.14 | 0.1861 | 7/15/2014 | 17:55.1 | 2 |
| -302.214 | 19324.113 | 1.805 | 14.158 | 0.164748 | 7/15/2014 | 17:55.2 | 2 |
| -301.97 | 19324.115 | 1.808 | 14.176 | 0.167866 | 7/15/2014 | 17:55.3 | 2 |
| -301.55 | 19324.115 | 1.807 | 14.211 | 0.158055 | 7/15/2014 | 17:55.4 | 2 |
| -301.313 | 19324.115 | 1.804 | 14.217 | 0.142725 | 7/15/2014 | 17:55.5 | 2 |
| -301.075 | 19324.115 | 1.798 | 14.231 | 0.159018 | 7/15/2014 | 17:55.6 | 2 |

| | | | | | | | |
|---|---|---|---|---|---|---|---|
| -300.778 | 19324.116 | 1.791 | 14.241 | 0.143149 | 7/15/2014 | 17:55.7 | 2 |
| -300.658 | 19324.116 | 1.787 | 14.248 | 0.145372 | 7/15/2014 | 17:55.8 | 2 |
| -300.417 | 19324.117 | 1.794 | 14.258 | 0.14776 | 7/15/2014 | 17:55.9 | 2 |
| -300.18 | 19324.117 | 1.794 | 14.27 | 0.136145 | 7/15/2014 | 17:56.0 | 2 |
| -299.941 | 19324.117 | 1.799 | 14.269 | 0.14631 | 7/15/2014 | 17:56.1 | 2 |
| -299.705 | 19324.117 | 1.792 | 14.275 | 0.15261 | 7/15/2014 | 17:56.2 | 2 |
| -299.467 | 19324.117 | 1.799 | 14.287 | 0.14218 | 7/15/2014 | 17:56.3 | 2 |
| -299.052 | 19324.116 | 1.799 | 14.299 | 0.14324 | 7/15/2014 | 17:56.4 | 2 |
| -298.816 | 19324.115 | 1.799 | 14.305 | 0.143802 | 7/15/2014 | 17:56.5 | 2 |
| -298.58 | 19324.115 | 1.795 | 14.312 | 0.156225 | 7/15/2014 | 17:56.6 | 2 |
| -298.282 | 19324.114 | 1.795 | 14.314 | 0.146009 | 7/15/2014 | 17:56.7 | 2 |
| -298.163 | 19324.114 | 1.789 | 14.318 | 0.141349 | 7/15/2014 | 17:56.8 | 2 |
| -297.921 | 19324.113 | 1.796 | 14.32 | 0.135295 | 7/15/2014 | 17:56.9 | 2 |
| -297.683 | 19324.112 | 1.796 | 14.315 | 0.132726 | 7/15/2014 | 17:57.0 | 2 |
| -297.441 | 19324.111 | 1.804 | 14.313 | 0.13915 | 7/15/2014 | 17:57.1 | 2 |
| -297.201 | 19324.109 | 1.804 | 14.314 | 0.134007 | 7/15/2014 | 17:57.2 | 2 |
| -296.961 | 19324.108 | 1.811 | 14.319 | 0.120232 | 7/15/2014 | 17:57.3 | 2 |
| -296.54 | 19324.106 | 1.812 | 14.328 | 0.141033 | 7/15/2014 | 17:57.4 | 2 |
| -296.301 | 19324.104 | 1.809 | 14.338 | 0.141778 | 7/15/2014 | 17:57.5 | 2 |
| -296.06 | 19324.104 | 1.809 | 14.346 | 0.152416 | 7/15/2014 | 17:57.6 | 2 |
| -295.763 | 19324.101 | 1.803 | 14.357 | 0.143023 | 7/15/2014 | 17:57.7 | 2 |
| -295.641 | 19324.1 | 1.8 | 14.361 | 0.139974 | 7/15/2014 | 17:57.8 | 2 |
| -295.398 | 19324.097 | 1.804 | 14.371 | 0.13203 | 7/15/2014 | 17:57.9 | 2 |
| -295.158 | 19324.095 | 1.804 | 14.375 | 0.120838 | 7/15/2014 | 17:58.0 | 2 |
| -294.915 | 19324.094 | 1.804 | 14.378 | 0.128508 | 7/15/2014 | 17:58.1 | 2 |
| -294.675 | 19324.09 | 1.804 | 14.383 | 0.128212 | 7/15/2014 | 17:58.2 | 2 |
| -294.433 | 19324.088 | 1.811 | 14.383 | 0.125591 | 7/15/2014 | 17:58.3 | 2 |
| -294.013 | 19324.084 | 1.803 | 14.389 | 0.119183 | 7/15/2014 | 17:58.4 | 2 |
| -293.773 | 19324.08 | 1.801 | 14.395 | 0.131832 | 7/15/2014 | 17:58.5 | 2 |
| -293.532 | 19324.077 | 1.801 | 14.399 | 0.122084 | 7/15/2014 | 17:58.6 | 2 |
| -293.231 | 19324.074 | 1.792 | 14.407 | 0.12239 | 7/15/2014 | 17:58.7 | 2 |
| -293.109 | 19324.072 | 1.792 | 14.412 | 0.122811 | 7/15/2014 | 17:58.8 | 2 |
| -292.865 | 19324.068 | 1.786 | 14.416 | 0.115501 | 7/15/2014 | 17:58.9 | 2 |
| -292.623 | 19324.065 | 1.778 | 14.42 | 0.102804 | 7/15/2014 | 17:59.0 | 2 |
| -292.376 | 19324.063 | 1.782 | 14.42 | 0.126926 | 7/15/2014 | 17:59.1 | 2 |
| -292.134 | 19324.058 | 1.778 | 14.42 | 0.112032 | 7/15/2014 | 17:59.2 | 2 |
| -291.887 | 19324.054 | 1.784 | 14.425 | 0.120477 | 7/15/2014 | 17:59.3 | 2 |
| -291.461 | 19324.047 | 1.779 | 14.423 | 0.11423 | 7/15/2014 | 17:59.4 | 2 |
| -291.217 | 19324.043 | 1.782 | 14.427 | 0.115343 | 7/15/2014 | 17:59.5 | 2 |
| -290.974 | 19324.038 | 1.784 | 14.429 | 0.112253 | 7/15/2014 | 17:59.6 | 2 |
| -290.67 | 19324.031 | 1.784 | 14.433 | 0.106301 | 7/15/2014 | 17:59.7 | 2 |
| -290.547 | 19324.028 | 1.782 | 14.438 | 0.102256 | 7/15/2014 | 17:59.8 | 2 |
| -290.301 | 19324.024 | 1.779 | 14.44 | 0.0997121 | 7/15/2014 | 17:59.9 | 2 |
| -290.058 | 19324.018 | 1.773 | 14.442 | 0.0957648 | 7/15/2014 | 18:00.0 | 2 |
| -289.811 | 19324.014 | 1.777 | 14.443 | 0.0895856 | 7/15/2014 | 18:00.1 | 2 |
| -289.57 | 19324.008 | 1.771 | 14.442 | 0.088352 | 7/15/2014 | 18:00.2 | 2 |
| -289.325 | 19324.002 | 1.775 | 14.431 | 0.0863162 | 7/15/2014 | 18:00.3 | 2 |

| | | | | | | | |
|---|---|---|---|---|---|---|---|
| -288.904 | 19323.99 | 1.772 | 14.425 | 0.0888127 | 7/15/2014 | 18:00.4 | 2 |
| -288.664 | 19323.985 | 1.772 | 14.421 | 0.0912863 | 7/15/2014 | 18:00.5 | 2 |
| -288.424 | 19323.979 | 1.774 | 14.424 | 0.0918342 | 7/15/2014 | 18:00.6 | 2 |
| -288.123 | 19323.971 | 1.776 | 14.418 | 0.0946145 | 7/15/2014 | 18:00.7 | 2 |
| -288.002 | 19323.967 | 1.773 | 14.417 | 0.0947026 | 7/15/2014 | 18:00.8 | 2 |
| -287.758 | 19323.96 | 1.783 | 14.417 | 0.0909138 | 7/15/2014 | 18:00.9 | 2 |
| -287.518 | 19323.953 | 1.779 | 14.42 | 0.0891322 | 7/15/2014 | 18:01.0 | 2 |
| -287.275 | 19323.945 | 1.782 | 14.426 | 0.0957491 | 7/15/2014 | 18:01.1 | 2 |
| -287.035 | 19323.938 | 1.778 | 14.431 | 0.0932329 | 7/15/2014 | 18:01.2 | 2 |
| -286.793 | 19323.932 | 1.782 | 14.435 | 0.0895514 | 7/15/2014 | 18:01.3 | 2 |
| -286.373 | 19323.918 | 1.781 | 14.44 | 0.093638 | 7/15/2014 | 18:01.4 | 2 |
| -286.132 | 19323.91 | 1.781 | 14.456 | 0.106784 | 7/15/2014 | 18:01.5 | 2 |
| -285.892 | 19323.9 | 1.777 | 14.47 | 0.126875 | 7/15/2014 | 18:01.6 | 2 |
| -285.594 | 19323.891 | 1.775 | 14.479 | 0.130048 | 7/15/2014 | 18:01.7 | 2 |
| -285.472 | 19323.887 | 1.776 | 14.483 | 0.138035 | 7/15/2014 | 18:01.8 | 2 |
| -285.23 | 19323.878 | 1.776 | 14.488 | 0.133836 | 7/15/2014 | 18:01.9 | 2 |
| -284.989 | 19323.869 | 1.772 | 14.496 | 0.171445 | 7/15/2014 | 18:02.0 | 2 |
| -284.746 | 19323.861 | 1.776 | 14.5 | 0.155983 | 7/15/2014 | 18:02.1 | 2 |
| -284.506 | 19323.852 | 1.776 | 14.5 | 0.152937 | 7/15/2014 | 18:02.2 | 2 |
| -284.262 | 19323.84 | 1.783 | 14.491 | 0.157529 | 7/15/2014 | 18:02.3 | 2 |
| -283.84 | 19323.824 | 1.785 | 14.49 | 0.141771 | 7/15/2014 | 18:02.4 | 2 |
| -283.6 | 19323.813 | 1.787 | 14.491 | 0.14454 | 7/15/2014 | 18:02.5 | 2 |
| -283.356 | 19323.803 | 1.793 | 14.497 | 0.141362 | 7/15/2014 | 18:02.6 | 2 |
| -283.05 | 19323.791 | 1.793 | 14.5 | 0.133283 | 7/15/2014 | 18:02.7 | 2 |
| -282.929 | 19323.785 | 1.793 | 14.505 | 0.131713 | 7/15/2014 | 18:02.8 | 2 |
| -282.68 | 19323.773 | 1.8 | 14.511 | 0.120987 | 7/15/2014 | 18:02.9 | 2 |
| -282.434 | 19323.763 | 1.796 | 14.515 | 0.110079 | 7/15/2014 | 18:03.0 | 2 |
| -282.183 | 19323.75 | 1.804 | 14.513 | 0.11251 | 7/15/2014 | 18:03.1 | 2 |
| -281.935 | 19323.738 | 1.796 | 14.515 | 0.123276 | 7/15/2014 | 18:03.2 | 2 |
| -281.683 | 19323.726 | 1.806 | 14.516 | 0.122142 | 7/15/2014 | 18:03.3 | 2 |
| -281.373 | 19323.711 | 1.803 | 14.511 | 0.125655 | 7/15/2014 | 18:03.4 | 2 |
| -281.119 | 19323.699 | 1.81 | 14.509 | 0.11991 | 7/15/2014 | 18:03.5 | 2 |
| -280.868 | 19323.685 | 1.812 | 14.499 | 0.123718 | 7/15/2014 | 18:03.6 | 2 |
| -280.614 | 19323.672 | 1.812 | 14.493 | 0.119968 | 7/15/2014 | 18:03.7 | 2 |
| -280.301 | 19323.654 | 1.811 | 14.489 | 0.107121 | 7/15/2014 | 18:03.8 | 2 |
| -280.045 | 19323.641 | 1.815 | 14.486 | 0.103998 | 7/15/2014 | 18:03.9 | 2 |
| -279.791 | 19323.627 | 1.809 | 14.481 | 0.0957301 | 7/15/2014 | 18:04.0 | 2 |
| -279.534 | 19323.613 | 1.815 | 14.475 | 0.108233 | 7/15/2014 | 18:04.1 | 2 |
| -279.279 | 19323.598 | 1.817 | 14.47 | 0.11515 | 7/15/2014 | 18:04.2 | 2 |
| -279.024 | 19323.584 | 1.817 | 14.466 | 0.112576 | 7/15/2014 | 18:04.3 | 2 |
| -278.71 | 19323.564 | 1.817 | 14.471 | 0.123656 | 7/15/2014 | 18:04.4 | 2 |
| -278.454 | 19323.55 | 1.82 | 14.467 | 0.124928 | 7/15/2014 | 18:04.5 | 2 |
| -278.2 | 19323.534 | 1.822 | 14.467 | 0.0973594 | 7/15/2014 | 18:04.6 | 2 |
| -277.943 | 19323.519 | 1.826 | 14.464 | 0.123307 | 7/15/2014 | 18:04.7 | 2 |
| -277.624 | 19323.498 | 1.82 | 14.465 | 0.112594 | 7/15/2014 | 18:04.8 | 2 |
| -277.368 | 19323.482 | 1.817 | 14.463 | 0.112339 | 7/15/2014 | 18:04.9 | 2 |
| -277.112 | 19323.466 | 1.817 | 14.461 | 0.135839 | 7/15/2014 | 18:05.0 | 2 |

| | | | | | | | |
|---|---|---|---|---|---|---|---|
| -276.855 | 19323.449 | 1.823 | 14.45 | 0.143073 | 7/15/2014 | 18:05.1 | 2 |
| -276.6 | 19323.432 | 1.821 | 14.444 | 0.160918 | 7/15/2014 | 18:05.2 | 2 |
| -276.344 | 19323.415 | 1.825 | 14.433 | 0.133575 | 7/15/2014 | 18:05.3 | 2 |
| -276.026 | 19323.393 | 1.823 | 14.428 | 0.13838 | 7/15/2014 | 18:05.4 | 2 |
| -275.771 | 19323.376 | 1.829 | 14.416 | 0.170641 | 7/15/2014 | 18:05.5 | 2 |
| -275.517 | 19323.358 | 1.829 | 14.407 | 0.148871 | 7/15/2014 | 18:05.6 | 2 |
| -275.263 | 19323.341 | 1.832 | 14.405 | 0.142439 | 7/15/2014 | 18:05.7 | 2 |
| -274.947 | 19323.318 | 1.835 | 14.397 | 0.156657 | 7/15/2014 | 18:05.8 | 2 |
| -274.693 | 19323.301 | 1.841 | 14.397 | 0.17199 | 7/15/2014 | 18:05.9 | 2 |
| -274.441 | 19323.281 | 1.835 | 14.401 | 0.196505 | 7/15/2014 | 18:06.0 | 2 |
| -274.184 | 19323.263 | 1.839 | 14.402 | 0.178873 | 7/15/2014 | 18:06.1 | 2 |
| -273.931 | 19323.244 | 1.833 | 14.406 | 0.202236 | 7/15/2014 | 18:06.2 | 2 |
| -273.677 | 19323.226 | 1.835 | 14.408 | 0.190477 | 7/15/2014 | 18:06.3 | 2 |
| -273.363 | 19323.202 | 1.833 | 14.404 | 0.188537 | 7/15/2014 | 18:06.4 | 2 |
| -273.112 | 19323.184 | 1.829 | 14.407 | 0.192218 | 7/15/2014 | 18:06.5 | 2 |
| -272.861 | 19323.164 | 1.826 | 14.405 | 0.16923 | 7/15/2014 | 18:06.6 | 2 |
| -272.607 | 19323.145 | 1.832 | 14.402 | 0.172602 | 7/15/2014 | 18:06.7 | 2 |
| -272.295 | 19323.119 | 1.832 | 14.398 | 0.161234 | 7/15/2014 | 18:06.8 | 2 |
| -272.044 | 19323.1 | 1.838 | 14.394 | 0.171919 | 7/15/2014 | 18:06.9 | 2 |
| -271.791 | 19323.08 | 1.838 | 14.391 | 0.175639 | 7/15/2014 | 18:07.0 | 2 |
| -271.539 | 19323.061 | 1.844 | 14.391 | 0.165695 | 7/15/2014 | 18:07.1 | 2 |
| -271.289 | 19323.04 | 1.844 | 14.391 | 0.172105 | 7/15/2014 | 18:07.2 | 2 |
| -271.039 | 19323.02 | 1.842 | 14.389 | 0.168508 | 7/15/2014 | 18:07.3 | 2 |
| -270.729 | 19322.994 | 1.835 | 14.387 | 0.176076 | 7/15/2014 | 18:07.4 | 2 |
| -270.479 | 19322.973 | 1.835 | 14.394 | 0.171943 | 7/15/2014 | 18:07.5 | 2 |
| -270.23 | 19322.953 | 1.835 | 14.405 | 0.171166 | 7/15/2014 | 18:07.6 | 2 |
| -269.981 | 19322.934 | 1.829 | 14.414 | 0.174713 | 7/15/2014 | 18:07.7 | 2 |
| -269.671 | 19322.907 | 1.817 | 14.423 | 0.168856 | 7/15/2014 | 18:07.8 | 2 |
| -269.421 | 19322.885 | 1.815 | 14.436 | 0.170358 | 7/15/2014 | 18:07.9 | 2 |
| -269.172 | 19322.863 | 1.811 | 14.459 | 0.16729 | 7/15/2014 | 18:08.0 | 2 |
| -268.919 | 19322.842 | 1.817 | 14.479 | 0.165943 | 7/15/2014 | 18:08.1 | 2 |
| -268.668 | 19322.82 | 1.817 | 14.496 | 0.165411 | 7/15/2014 | 18:08.2 | 2 |
| -268.418 | 19322.799 | 1.815 | 14.508 | 0.163299 | 7/15/2014 | 18:08.3 | 2 |
| -268.106 | 19322.771 | 1.812 | 14.523 | 0.16167 | 7/15/2014 | 18:08.4 | 2 |
| -267.855 | 19322.748 | 1.816 | 14.54 | 0.164144 | 7/15/2014 | 18:08.5 | 2 |
| -267.605 | 19322.725 | 1.816 | 14.546 | 0.159242 | 7/15/2014 | 18:08.6 | 2 |
| -267.352 | 19322.704 | 1.82 | 14.549 | 0.160474 | 7/15/2014 | 18:08.7 | 2 |
| -267.037 | 19322.675 | 1.814 | 14.546 | 0.160497 | 7/15/2014 | 18:08.8 | 2 |
| -266.783 | 19322.651 | 1.804 | 14.538 | 0.160558 | 7/15/2014 | 18:08.9 | 2 |
| -266.528 | 19322.627 | 1.797 | 14.531 | 0.162043 | 7/15/2014 | 18:09.0 | 2 |
| -266.271 | 19322.604 | 1.799 | 14.523 | 0.167048 | 7/15/2014 | 18:09.1 | 2 |
| -266.016 | 19322.58 | 1.795 | 14.512 | 0.165407 | 7/15/2014 | 18:09.2 | 2 |
| -265.761 | 19322.556 | 1.788 | 14.503 | 0.172262 | 7/15/2014 | 18:09.3 | 2 |
| -265.443 | 19322.526 | 1.781 | 14.487 | 0.177345 | 7/15/2014 | 18:09.4 | 2 |
| -265.186 | 19322.501 | 1.785 | 14.477 | 0.171297 | 7/15/2014 | 18:09.5 | 2 |
| -264.932 | 19322.477 | 1.785 | 14.468 | 0.173155 | 7/15/2014 | 18:09.6 | 2 |
| -264.675 | 19322.452 | 1.786 | 14.464 | 0.173409 | 7/15/2014 | 18:09.7 | 2 |

| | | | | | | | |
|---|---|---|---|---|---|---|---|
| -264.361 | 19322.423 | 1.78 | 14.457 | 0.174414 | 7/15/2014 | 18:09.8 | 2 |
| -264.105 | 19322.396 | 1.78 | 14.447 | 0.176052 | 7/15/2014 | 18:09.9 | 2 |
| -263.852 | 19322.372 | 1.78 | 14.442 | 0.177464 | 7/15/2014 | 18:10.0 | 2 |
| -263.597 | 19322.346 | 1.787 | 14.44 | 0.178046 | 7/15/2014 | 18:10.1 | 2 |
| -263.347 | 19322.322 | 1.78 | 14.435 | 0.176844 | 7/15/2014 | 18:10.2 | 2 |
| -263.094 | 19322.296 | 1.783 | 14.432 | 0.177959 | 7/15/2014 | 18:10.3 | 2 |
| -262.785 | 19322.267 | 1.782 | 14.424 | 0.178122 | 7/15/2014 | 18:10.4 | 2 |
| -262.534 | 19322.241 | 1.792 | 14.43 | 0.174479 | 7/15/2014 | 18:10.5 | 2 |
| -262.289 | 19322.216 | 1.786 | 14.428 | 0.176701 | 7/15/2014 | 18:10.6 | 2 |
| -261.858 | 19322.173 | 1.785 | 14.433 | 0.176658 | 7/15/2014 | 18:10.7 | 2 |
| -261.733 | 19322.161 | 1.78 | 14.432 | 0.170255 | 7/15/2014 | 18:10.8 | 2 |
| -261.483 | 19322.135 | 1.783 | 14.433 | 0.169897 | 7/15/2014 | 18:10.9 | 2 |
| -261.238 | 19322.109 | 1.777 | 14.435 | 0.170893 | 7/15/2014 | 18:11.0 | 2 |
| -260.988 | 19322.085 | 1.781 | 14.44 | 0.173339 | 7/15/2014 | 18:11.1 | 2 |
| -260.743 | 19322.06 | 1.777 | 14.445 | 0.174633 | 7/15/2014 | 18:11.2 | 2 |
| -260.494 | 19322.034 | 1.783 | 14.452 | 0.162761 | 7/15/2014 | 18:11.3 | 2 |
| -260.065 | 19321.991 | 1.785 | 14.464 | 0.172287 | 7/15/2014 | 18:11.4 | 2 |
| -259.821 | 19321.965 | 1.785 | 14.467 | 0.170633 | 7/15/2014 | 18:11.5 | 2 |
| -259.577 | 19321.939 | 1.785 | 14.477 | 0.166052 | 7/15/2014 | 18:11.6 | 2 |
| -259.271 | 19321.908 | 1.787 | 14.481 | 0.168762 | 7/15/2014 | 18:11.7 | 2 |
| -259.148 | 19321.896 | 1.786 | 14.486 | 0.162833 | 7/15/2014 | 18:11.8 | 2 |
| -258.9 | 19321.871 | 1.789 | 14.48 | 0.17026 | 7/15/2014 | 18:11.9 | 2 |
| -258.658 | 19321.845 | 1.786 | 14.485 | 0.171756 | 7/15/2014 | 18:12.0 | 2 |
| -258.41 | 19321.82 | 1.789 | 14.482 | 0.165538 | 7/15/2014 | 18:12.1 | 2 |
| -258.169 | 19321.793 | 1.786 | 14.477 | 0.156163 | 7/15/2014 | 18:12.2 | 2 |
| -257.925 | 19321.769 | 1.798 | 14.473 | 0.159426 | 7/15/2014 | 18:12.3 | 2 |
| -257.504 | 19321.725 | 1.806 | 14.471 | 0.153762 | 7/15/2014 | 18:12.4 | 2 |
| -257.264 | 19321.7 | 1.812 | 14.474 | 0.14031 | 7/15/2014 | 18:12.5 | 2 |
| -257.026 | 19321.675 | 1.815 | 14.477 | 0.144299 | 7/15/2014 | 18:12.6 | 2 |
| -256.728 | 19321.645 | 1.821 | 14.482 | 0.127405 | 7/15/2014 | 18:12.7 | 2 |
| -256.608 | 19321.631 | 1.825 | 14.485 | 0.128853 | 7/15/2014 | 18:12.8 | 2 |
| -256.368 | 19321.606 | 1.828 | 14.499 | 0.127361 | 7/15/2014 | 18:12.9 | 2 |
| -256.131 | 19321.582 | 1.822 | 14.515 | 0.134564 | 7/15/2014 | 18:13.0 | 2 |
| -255.893 | 19321.557 | 1.822 | 14.537 | 0.13674 | 7/15/2014 | 18:13.1 | 2 |
| -255.657 | 19321.532 | 1.819 | 14.547 | 0.160464 | 7/15/2014 | 18:13.2 | 2 |
| -255.422 | 19321.507 | 1.821 | 14.559 | 0.151774 | 7/15/2014 | 18:13.3 | 2 |
| -255.013 | 19321.465 | 1.821 | 14.576 | 0.167758 | 7/15/2014 | 18:13.4 | 2 |
| -254.783 | 19321.441 | 1.818 | 14.583 | 0.178213 | 7/15/2014 | 18:13.5 | 2 |
| -254.552 | 19321.418 | 1.82 | 14.587 | 0.148307 | 7/15/2014 | 18:13.6 | 2 |
| -254.264 | 19321.387 | 1.825 | 14.587 | 0.147328 | 7/15/2014 | 18:13.7 | 2 |
| -254.146 | 19321.375 | 1.826 | 14.583 | 0.135815 | 7/15/2014 | 18:13.8 | 2 |
| -253.802 | 19321.34 | 1.832 | 14.586 | 0.132634 | 7/15/2014 | 18:13.9 | 2 |
| -253.571 | 19321.315 | 1.832 | 14.59 | 0.138521 | 7/15/2014 | 18:14.0 | 2 |
| -253.341 | 19321.292 | 1.834 | 14.589 | 0.132089 | 7/15/2014 | 18:14.1 | 2 |
| -253.114 | 19321.269 | 1.836 | 14.594 | 0.127631 | 7/15/2014 | 18:14.2 | 2 |
| -252.886 | 19321.245 | 1.841 | 14.592 | 0.132852 | 7/15/2014 | 18:14.3 | 2 |
| -252.604 | 19321.216 | 1.847 | 14.592 | 0.12403 | 7/15/2014 | 18:14.4 | 2 |

| | | | | | | | |
|---|---|---|---|---|---|---|---|
| -252.379 | 19321.193 | 1.847 | 14.592 | 0.126696 | 7/15/2014 | 18:14.5 | 2 |
| -252.154 | 19321.171 | 1.85 | 14.587 | 0.123782 | 7/15/2014 | 18:14.6 | 2 |
| -251.875 | 19321.141 | 1.85 | 14.589 | 0.129744 | 7/15/2014 | 18:14.7 | 2 |
| -251.649 | 19321.118 | 1.852 | 14.58 | 0.122598 | 7/15/2014 | 18:14.8 | 2 |
| -251.423 | 19321.095 | 1.852 | 14.572 | 0.125648 | 7/15/2014 | 18:14.9 | 2 |
| -251.197 | 19321.07 | 1.852 | 14.561 | 0.133014 | 7/15/2014 | 18:15.0 | 2 |
| -250.971 | 19321.048 | 1.852 | 14.55 | 0.132276 | 7/15/2014 | 18:15.1 | 2 |
| -250.745 | 19321.024 | 1.852 | 14.539 | 0.13424 | 7/15/2014 | 18:15.2 | 2 |
| -250.521 | 19321.002 | 1.855 | 14.527 | 0.131927 | 7/15/2014 | 18:15.3 | 2 |
| -250.243 | 19320.973 | 1.855 | 14.516 | 0.125752 | 7/15/2014 | 18:15.4 | 2 |
| -250.019 | 19320.949 | 1.857 | 14.5 | 0.130109 | 7/15/2014 | 18:15.5 | 2 |
| -249.797 | 19320.927 | 1.862 | 14.49 | 0.136712 | 7/15/2014 | 18:15.6 | 2 |
| -249.519 | 19320.897 | 1.868 | 14.48 | 0.138153 | 7/15/2014 | 18:15.7 | 2 |
| -249.296 | 19320.875 | 1.871 | 14.47 | 0.133968 | 7/15/2014 | 18:15.8 | 2 |
| -249.071 | 19320.852 | 1.876 | 14.464 | 0.147736 | 7/15/2014 | 18:15.9 | 2 |
| -248.848 | 19320.828 | 1.876 | 14.46 | 0.143722 | 7/15/2014 | 18:16.0 | 2 |
| -248.624 | 19320.805 | 1.878 | 14.454 | 0.14608 | 7/15/2014 | 18:16.1 | 2 |
| -248.402 | 19320.781 | 1.878 | 14.45 | 0.159503 | 7/15/2014 | 18:16.2 | 2 |
| -248.181 | 19320.76 | 1.881 | 14.444 | 0.138711 | 7/15/2014 | 18:16.3 | 2 |
| -247.906 | 19320.73 | 1.881 | 14.44 | 0.142341 | 7/15/2014 | 18:16.4 | 2 |
| -247.687 | 19320.709 | 1.886 | 14.433 | 0.148843 | 7/15/2014 | 18:16.5 | 2 |
| -247.467 | 19320.686 | 1.892 | 14.425 | 0.144118 | 7/15/2014 | 18:16.6 | 2 |
| -247.194 | 19320.657 | 1.894 | 14.416 | 0.13746 | 7/15/2014 | 18:16.7 | 2 |
| -246.972 | 19320.634 | 1.899 | 14.416 | 0.135803 | 7/15/2014 | 18:16.8 | 2 |
| -246.752 | 19320.61 | 1.901 | 14.407 | 0.131207 | 7/15/2014 | 18:16.9 | 2 |
| -246.529 | 19320.589 | 1.907 | 14.399 | 0.135707 | 7/15/2014 | 18:17.0 | 2 |
| -246.307 | 19320.565 | 1.912 | 14.395 | 0.118023 | 7/15/2014 | 18:17.1 | 2 |
| -246.087 | 19320.543 | 1.919 | 14.387 | 0.124316 | 7/15/2014 | 18:17.2 | 2 |
| -245.866 | 19320.52 | 1.915 | 14.387 | 0.115195 | 7/15/2014 | 18:17.3 | 2 |
| -245.592 | 19320.491 | 1.919 | 14.385 | 0.122666 | 7/15/2014 | 18:17.4 | 2 |
| -245.371 | 19320.468 | 1.922 | 14.391 | 0.125327 | 7/15/2014 | 18:17.5 | 2 |
| -245.151 | 19320.444 | 1.922 | 14.385 | 0.126061 | 7/15/2014 | 18:17.6 | 2 |
| -244.875 | 19320.417 | 1.922 | 14.388 | 0.12432 | 7/15/2014 | 18:17.7 | 2 |
| -244.65 | 19320.393 | 1.925 | 14.391 | 0.138485 | 7/15/2014 | 18:17.8 | 2 |
| -244.427 | 19320.369 | 1.928 | 14.394 | 0.136773 | 7/15/2014 | 18:17.9 | 2 |
| -244.203 | 19320.346 | 1.928 | 14.401 | 0.133904 | 7/15/2014 | 18:18.0 | 2 |
| -243.977 | 19320.322 | 1.935 | 14.401 | 0.141641 | 7/15/2014 | 18:18.1 | 2 |
| -243.753 | 19320.299 | 1.937 | 14.405 | 0.138092 | 7/15/2014 | 18:18.2 | 2 |
| -243.529 | 19320.275 | 1.94 | 14.406 | 0.128307 | 7/15/2014 | 18:18.3 | 2 |
| -243.25 | 19320.247 | 1.947 | 14.405 | 0.132306 | 7/15/2014 | 18:18.4 | 2 |
| -243.026 | 19320.223 | 1.954 | 14.405 | 0.133506 | 7/15/2014 | 18:18.5 | 2 |
| -242.801 | 19320.199 | 1.956 | 14.404 | 0.12978 | 7/15/2014 | 18:18.6 | 2 |
| -242.521 | 19320.17 | 1.956 | 14.404 | 0.128494 | 7/15/2014 | 18:18.7 | 2 |
| -242.294 | 19320.146 | 1.96 | 14.402 | 0.115958 | 7/15/2014 | 18:18.8 | 2 |
| -242.067 | 19320.122 | 1.96 | 14.404 | 0.120011 | 7/15/2014 | 18:18.9 | 2 |
| -241.838 | 19320.098 | 1.962 | 14.4 | 0.115308 | 7/15/2014 | 18:19.0 | 2 |
| -241.611 | 19320.074 | 1.964 | 14.397 | 0.12322 | 7/15/2014 | 18:19.1 | 2 |

| | | | | | | | |
|---|---|---|---|---|---|---|---|
| -241.384 | 19320.051 | 1.964 | 14.395 | 0.123144 | 7/15/2014 | 18:19.2 | 2 |
| -241.157 | 19320.027 | 1.967 | 14.395 | 0.125416 | 7/15/2014 | 18:19.3 | 2 |
| -240.876 | 19319.998 | 1.971 | 14.399 | 0.130665 | 7/15/2014 | 18:19.4 | 2 |
| -240.649 | 19319.974 | 1.975 | 14.399 | 0.140356 | 7/15/2014 | 18:19.5 | 2 |
| -240.424 | 19319.95 | 1.977 | 14.401 | 0.141453 | 7/15/2014 | 18:19.6 | 2 |
| -240.14 | 19319.919 | 1.981 | 14.402 | 0.154186 | 7/15/2014 | 18:19.7 | 2 |
| -239.913 | 19319.896 | 1.984 | 14.407 | 0.159055 | 7/15/2014 | 18:19.8 | 2 |
| -239.684 | 19319.872 | 1.986 | 14.408 | 0.172792 | 7/15/2014 | 18:19.9 | 2 |
| -239.454 | 19319.848 | 1.991 | 14.411 | 0.170567 | 7/15/2014 | 18:20.0 | 2 |
| -239.225 | 19319.824 | 1.995 | 14.415 | 0.184518 | 7/15/2014 | 18:20.1 | 2 |
| -238.996 | 19319.801 | 1.995 | 14.415 | 0.175656 | 7/15/2014 | 18:20.2 | 2 |
| -238.769 | 19319.777 | 1.995 | 14.42 | 0.180685 | 7/15/2014 | 18:20.3 | 2 |
| -238.484 | 19319.747 | 1.996 | 14.422 | 0.185925 | 7/15/2014 | 18:20.4 | 2 |
| -238.257 | 19319.725 | 1.996 | 14.426 | 0.176237 | 7/15/2014 | 18:20.5 | 2 |
| -238.028 | 19319.699 | 2 | 14.432 | 0.178847 | 7/15/2014 | 18:20.6 | 2 |
| -237.742 | 19319.669 | 2.002 | 14.437 | 0.174253 | 7/15/2014 | 18:20.7 | 2 |
| -237.512 | 19319.646 | 2.002 | 14.442 | 0.166418 | 7/15/2014 | 18:20.8 | 2 |
| -237.282 | 19319.622 | 2.002 | 14.444 | 0.173608 | 7/15/2014 | 18:20.9 | 2 |
| -237.05 | 19319.598 | 2.005 | 14.45 | 0.170276 | 7/15/2014 | 18:21.0 | 2 |
| -236.819 | 19319.574 | 2.005 | 14.452 | 0.16874 | 7/15/2014 | 18:21.1 | 2 |
| -236.706 | 19319.563 | 2.005 | 14.454 | 0.177853 | 7/15/2014 | 18:21.2 | 2 |
| -236.473 | 19319.538 | 2.014 | 14.457 | 0.159559 | 7/15/2014 | 18:21.3 | 2 |
| -236.073 | 19319.497 | 2.013 | 14.462 | 0.173875 | 7/15/2014 | 18:21.4 | 2 |
| -235.843 | 19319.474 | 2.017 | 14.464 | 0.177106 | 7/15/2014 | 18:21.5 | 2 |
| -235.615 | 19319.45 | 2.02 | 14.468 | 0.177581 | 7/15/2014 | 18:21.6 | 2 |
| -235.328 | 19319.42 | 2.023 | 14.472 | 0.180462 | 7/15/2014 | 18:21.7 | 2 |
| -235.213 | 19319.408 | 2.022 | 14.474 | 0.180325 | 7/15/2014 | 18:21.8 | 2 |
| -234.868 | 19319.373 | 2.027 | 14.482 | 0.18042 | 7/15/2014 | 18:21.9 | 2 |
| -234.635 | 19319.35 | 2.026 | 14.485 | 0.191645 | 7/15/2014 | 18:22.0 | 2 |
| -234.405 | 19319.326 | 2.025 | 14.488 | 0.18238 | 7/15/2014 | 18:22.1 | 2 |
| -234.174 | 19319.302 | 2.028 | 14.491 | 0.179493 | 7/15/2014 | 18:22.2 | 2 |
| -234.06 | 19319.29 | 2.029 | 14.494 | 0.177294 | 7/15/2014 | 18:22.3 | 2 |
| -233.659 | 19319.249 | 2.028 | 14.502 | 0.176296 | 7/15/2014 | 18:22.4 | 2 |
| -233.429 | 19319.226 | 2.028 | 14.506 | 0.173549 | 7/15/2014 | 18:22.5 | 2 |
| -233.203 | 19319.202 | 2.026 | 14.509 | 0.171357 | 7/15/2014 | 18:22.6 | 2 |
| -232.915 | 19319.173 | 2.032 | 14.51 | 0.162086 | 7/15/2014 | 18:22.7 | 2 |
| -232.801 | 19319.161 | 2.03 | 14.511 | 0.163319 | 7/15/2014 | 18:22.8 | 2 |
| -232.454 | 19319.125 | 2.043 | 14.516 | 0.165544 | 7/15/2014 | 18:22.9 | 2 |
| -232.222 | 19319.102 | 2.043 | 14.52 | 0.154124 | 7/15/2014 | 18:23.0 | 2 |
| -231.991 | 19319.078 | 2.043 | 14.522 | 0.164325 | 7/15/2014 | 18:23.1 | 2 |
| -231.76 | 19319.055 | 2.043 | 14.531 | 0.168352 | 7/15/2014 | 18:23.2 | 2 |
| -231.646 | 19319.043 | 2.046 | 14.532 | 0.171162 | 7/15/2014 | 18:23.3 | 2 |
| -231.244 | 19319.002 | 2.051 | 14.545 | 0.169728 | 7/15/2014 | 18:23.4 | 2 |
| -231.015 | 19318.979 | 2.051 | 14.554 | 0.174404 | 7/15/2014 | 18:23.5 | 2 |
| -230.787 | 19318.955 | 2.051 | 14.559 | 0.175143 | 7/15/2014 | 18:23.6 | 2 |
| -230.501 | 19318.927 | 2.049 | 14.572 | 0.171581 | 7/15/2014 | 18:23.7 | 2 |
| -230.269 | 19318.902 | 2.051 | 14.582 | 0.172353 | 7/15/2014 | 18:23.8 | 2 |

| | | | | | | | |
|---|---|---|---|---|---|---|---|
| -230.038 | 19318.879 | 2.054 | 14.589 | 0.176649 | 7/15/2014 | 18:23.9 | 2 |
| -229.806 | 19318.855 | 2.054 | 14.594 | 0.182212 | 7/15/2014 | 18:24.0 | 2 |
| -229.572 | 19318.832 | 2.053 | 14.596 | 0.185616 | 7/15/2014 | 18:24.1 | 2 |
| -229.459 | 19318.82 | 2.052 | 14.603 | 0.190947 | 7/15/2014 | 18:24.2 | 2 |
| -229.227 | 19318.796 | 2.054 | 14.61 | 0.198172 | 7/15/2014 | 18:24.3 | 2 |
| -228.822 | 19318.756 | 2.053 | 14.625 | 0.208992 | 7/15/2014 | 18:24.4 | 2 |
| -228.59 | 19318.731 | 2.053 | 14.635 | 0.208074 | 7/15/2014 | 18:24.5 | 2 |
| -228.358 | 19318.708 | 2.053 | 14.638 | 0.217583 | 7/15/2014 | 18:24.6 | 2 |
| -228.066 | 19318.678 | 2.053 | 14.643 | 0.219094 | 7/15/2014 | 18:24.7 | 2 |
| -227.948 | 19318.666 | 2.054 | 14.648 | 0.224095 | 7/15/2014 | 18:24.8 | 2 |
| -227.712 | 19318.641 | 2.054 | 14.652 | 0.231783 | 7/15/2014 | 18:24.9 | 2 |
| -227.476 | 19318.617 | 2.048 | 14.655 | 0.229568 | 7/15/2014 | 18:25.0 | 2 |
| -227.237 | 19318.594 | 2.051 | 14.656 | 0.228498 | 7/15/2014 | 18:25.1 | 2 |
| -227.001 | 19318.569 | 2.045 | 14.652 | 0.237414 | 7/15/2014 | 18:25.2 | 2 |
| -226.761 | 19318.545 | 2.047 | 14.64 | 0.237181 | 7/15/2014 | 18:25.3 | 2 |
| -226.345 | 19318.502 | 2.039 | 14.647 | 0.246931 | 7/15/2014 | 18:25.4 | 2 |
| -226.106 | 19318.477 | 2.036 | 14.649 | 0.235742 | 7/15/2014 | 18:25.5 | 2 |
| -225.869 | 19318.453 | 2.034 | 14.653 | 0.255186 | 7/15/2014 | 18:25.6 | 2 |
| -225.568 | 19318.422 | 2.034 | 14.658 | 0.241746 | 7/15/2014 | 18:25.7 | 2 |
| -225.448 | 19318.409 | 2.029 | 14.661 | 0.241744 | 7/15/2014 | 18:25.8 | 2 |
| -225.206 | 19318.385 | 2.029 | 14.669 | 0.230743 | 7/15/2014 | 18:25.9 | 2 |
| -224.965 | 19318.361 | 2.018 | 14.679 | 0.226091 | 7/15/2014 | 18:26.0 | 2 |
| -224.721 | 19318.335 | 2.021 | 14.69 | 0.227975 | 7/15/2014 | 18:26.1 | 2 |
| -224.48 | 19318.31 | 2.021 | 14.696 | 0.242938 | 7/15/2014 | 18:26.2 | 2 |
| -224.237 | 19318.285 | 2.027 | 14.706 | 0.221332 | 7/15/2014 | 18:26.3 | 2 |
| -223.815 | 19318.24 | 2.024 | 14.718 | 0.245059 | 7/15/2014 | 18:26.4 | 2 |
| -223.574 | 19318.217 | 2.022 | 14.725 | 0.256966 | 7/15/2014 | 18:26.5 | 2 |
| -223.332 | 19318.191 | 2.02 | 14.735 | 0.25834 | 7/15/2014 | 18:26.6 | 2 |
| -223.029 | 19318.16 | 2.022 | 14.741 | 0.248936 | 7/15/2014 | 18:26.7 | 2 |
| -222.908 | 19318.147 | 2.019 | 14.743 | 0.239871 | 7/15/2014 | 18:26.8 | 2 |
| -222.663 | 19318.121 | 2.019 | 14.746 | 0.251125 | 7/15/2014 | 18:26.9 | 2 |
| -222.419 | 19318.097 | 2.008 | 14.753 | 0.227595 | 7/15/2014 | 18:27.0 | 2 |
| -222.175 | 19318.07 | 2.008 | 14.757 | 0.233737 | 7/15/2014 | 18:27.1 | 2 |
| -221.931 | 19318.045 | 2.004 | 14.761 | 0.222192 | 7/15/2014 | 18:27.2 | 2 |
| -221.687 | 19318.02 | 2.004 | 14.767 | 0.219051 | 7/15/2014 | 18:27.3 | 2 |
| -221.26 | 19317.975 | 2.006 | 14.777 | 0.223949 | 7/15/2014 | 18:27.4 | 2 |
| -221.018 | 19317.949 | 2.001 | 14.785 | 0.218684 | 7/15/2014 | 18:27.5 | 2 |
| -220.772 | 19317.923 | 1.999 | 14.793 | 0.216369 | 7/15/2014 | 18:27.6 | 2 |
| -220.465 | 19317.891 | 2.001 | 14.802 | 0.208577 | 7/15/2014 | 18:27.7 | 2 |
| -220.342 | 19317.877 | 2 | 14.808 | 0.216958 | 7/15/2014 | 18:27.8 | 2 |
| -220.095 | 19317.852 | 2.005 | 14.82 | 0.209661 | 7/15/2014 | 18:27.9 | 2 |
| -219.847 | 19317.824 | 2.007 | 14.83 | 0.212918 | 7/15/2014 | 18:28.0 | 2 |
| -219.597 | 19317.797 | 2.005 | 14.833 | 0.208067 | 7/15/2014 | 18:28.1 | 2 |
| -219.35 | 19317.771 | 1.999 | 14.835 | 0.212329 | 7/15/2014 | 18:28.2 | 2 |
| -219.097 | 19317.744 | 2.005 | 14.838 | 0.223225 | 7/15/2014 | 18:28.3 | 2 |
| -218.786 | 19317.711 | 2.001 | 14.842 | 0.236198 | 7/15/2014 | 18:28.4 | 2 |
| -218.535 | 19317.684 | 1.998 | 14.84 | 0.240474 | 7/15/2014 | 18:28.5 | 2 |

| | | | | | | | |
|---|---|---|---|---|---|---|---|
| -218.283 | 19317.656 | 1.989 | 14.837 | 0.261716 | 7/15/2014 | 18:28.6 | 2 |
| -218.031 | 19317.629 | 1.983 | 14.836 | 0.258843 | 7/15/2014 | 18:28.7 | 2 |
| -217.716 | 19317.595 | 1.979 | 14.839 | 0.271536 | 7/15/2014 | 18:28.8 | 2 |
| -217.459 | 19317.566 | 1.98 | 14.839 | 0.251864 | 7/15/2014 | 18:28.9 | 2 |
| -217.206 | 19317.539 | 1.971 | 14.838 | 0.256445 | 7/15/2014 | 18:29.0 | 2 |
| -216.949 | 19317.512 | 1.972 | 14.833 | 0.253128 | 7/15/2014 | 18:29.1 | 2 |
| -216.695 | 19317.483 | 1.97 | 14.827 | 0.240966 | 7/15/2014 | 18:29.2 | 2 |
| -216.439 | 19317.455 | 1.973 | 14.823 | 0.264741 | 7/15/2014 | 18:29.3 | 2 |
| -216.123 | 19317.421 | 1.967 | 14.813 | 0.25179 | 7/15/2014 | 18:29.4 | 2 |
| -215.867 | 19317.393 | 1.967 | 14.803 | 0.269663 | 7/15/2014 | 18:29.5 | 2 |
| -215.614 | 19317.363 | 1.963 | 14.793 | 0.28963 | 7/15/2014 | 18:29.6 | 2 |
| -215.357 | 19317.336 | 1.963 | 14.784 | 0.302796 | 7/15/2014 | 18:29.7 | 2 |
| -215.039 | 19317.3 | 1.954 | 14.777 | 0.29889 | 7/15/2014 | 18:29.8 | 2 |
| -214.781 | 19317.271 | 1.948 | 14.773 | 0.310544 | 7/15/2014 | 18:29.9 | 2 |
| -214.524 | 19317.242 | 1.934 | 14.768 | 0.324553 | 7/15/2014 | 18:30.0 | 2 |
| -214.265 | 19317.214 | 1.932 | 14.77 | 0.326548 | 7/15/2014 | 18:30.1 | 2 |
| -214.007 | 19317.184 | 1.926 | 14.774 | 0.326034 | 7/15/2014 | 18:30.2 | 2 |
| -213.749 | 19317.154 | 1.917 | 14.779 | 0.324013 | 7/15/2014 | 18:30.3 | 2 |
| -213.427 | 19317.118 | 1.909 | 14.786 | 0.340144 | 7/15/2014 | 18:30.4 | 2 |
| -213.167 | 19317.089 | 1.905 | 14.796 | 0.311957 | 7/15/2014 | 18:30.5 | 2 |
| -212.908 | 19317.059 | 1.899 | 14.811 | 0.322311 | 7/15/2014 | 18:30.6 | 2 |
| -212.648 | 19317.03 | 1.905 | 14.828 | 0.307945 | 7/15/2014 | 18:30.7 | 2 |
| -212.324 | 19316.993 | 1.905 | 14.838 | 0.295098 | 7/15/2014 | 18:30.8 | 2 |
| -212.064 | 19316.963 | 1.899 | 14.847 | 0.281075 | 7/15/2014 | 18:30.9 | 2 |
| -211.802 | 19316.933 | 1.895 | 14.861 | 0.290627 | 7/15/2014 | 18:31.0 | 2 |
| -211.54 | 19316.902 | 1.895 | 14.871 | 0.295363 | 7/15/2014 | 18:31.1 | 2 |
| -211.28 | 19316.871 | 1.892 | 14.883 | 0.286552 | 7/15/2014 | 18:31.2 | 2 |
| -211.019 | 19316.844 | 1.892 | 14.891 | 0.284506 | 7/15/2014 | 18:31.3 | 2 |
| -210.697 | 19316.806 | 1.884 | 14.896 | 0.295526 | 7/15/2014 | 18:31.4 | 2 |
| -210.439 | 19316.775 | 1.876 | 14.909 | 0.303584 | 7/15/2014 | 18:31.5 | 2 |
| -210.182 | 19316.745 | 1.869 | 14.923 | 0.283783 | 7/15/2014 | 18:31.6 | 2 |
| -209.923 | 19316.715 | 1.873 | 14.928 | 0.303125 | 7/15/2014 | 18:31.7 | 2 |
| -209.604 | 19316.678 | 1.869 | 14.936 | 0.25132 | 7/15/2014 | 18:31.8 | 2 |
| -209.346 | 19316.648 | 1.867 | 14.946 | 0.284153 | 7/15/2014 | 18:31.9 | 2 |
| -209.091 | 19316.618 | 1.861 | 14.949 | 0.264824 | 7/15/2014 | 18:32.0 | 2 |
| -208.833 | 19316.588 | 1.861 | 14.953 | 0.258069 | 7/15/2014 | 18:32.1 | 2 |
| -208.58 | 19316.558 | 1.863 | 14.954 | 0.266835 | 7/15/2014 | 18:32.2 | 2 |
| -208.324 | 19316.527 | 1.869 | 14.955 | 0.252106 | 7/15/2014 | 18:32.3 | 2 |
| -208.01 | 19316.491 | 1.853 | 14.96 | 0.283381 | 7/15/2014 | 18:32.4 | 2 |
| -207.754 | 19316.46 | 1.857 | 14.968 | 0.263281 | 7/15/2014 | 18:32.5 | 2 |
| -207.504 | 19316.431 | 1.85 | 14.967 | 0.267129 | 7/15/2014 | 18:32.6 | 2 |
| -207.249 | 19316.4 | 1.856 | 14.975 | 0.278905 | 7/15/2014 | 18:32.7 | 2 |
| -206.935 | 19316.362 | 1.85 | 14.983 | 0.282217 | 7/15/2014 | 18:32.8 | 2 |
| -206.68 | 19316.332 | 1.84 | 14.992 | 0.274927 | 7/15/2014 | 18:32.9 | 2 |
| -206.426 | 19316.302 | 1.834 | 14.998 | 0.265919 | 7/15/2014 | 18:33.0 | 2 |
| -206.169 | 19316.271 | 1.842 | 15.008 | 0.261023 | 7/15/2014 | 18:33.1 | 2 |
| -205.915 | 19316.24 | 1.836 | 15.015 | 0.268887 | 7/15/2014 | 18:33.2 | 2 |

| | | | | | | | |
|---|---|---|---|---|---|---|---|
| -205.66 | 19316.209 | 1.842 | 15.022 | 0.256554 | 7/15/2014 | 18:33.3 | 2 |
| -205.343 | 19316.171 | 1.836 | 15.036 | 0.253306 | 7/15/2014 | 18:33.4 | 2 |
| -205.088 | 19316.141 | 1.839 | 15.048 | 0.248613 | 7/15/2014 | 18:33.5 | 2 |
| -204.832 | 19316.109 | 1.848 | 15.058 | 0.253629 | 7/15/2014 | 18:33.6 | 2 |
| -204.577 | 19316.078 | 1.851 | 15.07 | 0.252947 | 7/15/2014 | 18:33.7 | 2 |
| -204.257 | 19316.039 | 1.848 | 15.078 | 0.253733 | 7/15/2014 | 18:33.8 | 2 |
| -204 | 19316.008 | 1.845 | 15.087 | 0.242879 | 7/15/2014 | 18:33.9 | 2 |
| -203.744 | 19315.977 | 1.836 | 15.096 | 0.243485 | 7/15/2014 | 18:34.0 | 2 |
| -203.485 | 19315.944 | 1.839 | 15.1 | 0.236733 | 7/15/2014 | 18:34.1 | 2 |
| -203.228 | 19315.913 | 1.836 | 15.105 | 0.228303 | 7/15/2014 | 18:34.2 | 2 |
| -202.971 | 19315.881 | 1.833 | 15.109 | 0.227864 | 7/15/2014 | 18:34.3 | 2 |
| -202.652 | 19315.842 | 1.83 | 15.113 | 0.216426 | 7/15/2014 | 18:34.4 | 2 |
| -202.395 | 19315.811 | 1.83 | 15.117 | 0.223941 | 7/15/2014 | 18:34.5 | 2 |
| -202.141 | 19315.778 | 1.828 | 15.122 | 0.219213 | 7/15/2014 | 18:34.6 | 2 |
| -201.884 | 19315.746 | 1.834 | 15.123 | 0.229107 | 7/15/2014 | 18:34.7 | 2 |
| -201.564 | 19315.707 | 1.829 | 15.122 | 0.226885 | 7/15/2014 | 18:34.8 | 2 |
| -201.306 | 19315.674 | 1.831 | 15.122 | 0.237298 | 7/15/2014 | 18:34.9 | 2 |
| -201.051 | 19315.643 | 1.827 | 15.121 | 0.237677 | 7/15/2014 | 18:35.0 | 2 |
| -200.792 | 19315.61 | 1.827 | 15.12 | 0.245781 | 7/15/2014 | 18:35.1 | 2 |
| -200.536 | 19315.577 | 1.824 | 15.117 | 0.231532 | 7/15/2014 | 18:35.2 | 2 |
| -200.28 | 19315.546 | 1.83 | 15.111 | 0.235839 | 7/15/2014 | 18:35.3 | 2 |
| -199.962 | 19315.506 | 1.824 | 15.108 | 0.237685 | 7/15/2014 | 18:35.4 | 2 |
| -199.706 | 19315.473 | 1.824 | 15.105 | 0.2369 | 7/15/2014 | 18:35.5 | 2 |
| -199.455 | 19315.441 | 1.81 | 15.102 | 0.236479 | 7/15/2014 | 18:35.6 | 2 |
| -199.199 | 19315.409 | 1.81 | 15.1 | 0.227477 | 7/15/2014 | 18:35.7 | 2 |
| -198.886 | 19315.368 | 1.807 | 15.095 | 0.227364 | 7/15/2014 | 18:35.8 | 2 |
| -198.632 | 19315.337 | 1.807 | 15.094 | 0.221905 | 7/15/2014 | 18:35.9 | 2 |
| -198.382 | 19315.304 | 1.797 | 15.093 | 0.220013 | 7/15/2014 | 18:36.0 | 2 |
| -198.129 | 19315.271 | 1.801 | 15.094 | 0.220359 | 7/15/2014 | 18:36.1 | 2 |
| -197.881 | 19315.24 | 1.794 | 15.099 | 0.2197 | 7/15/2014 | 18:36.2 | 2 |
| -197.629 | 19315.208 | 1.806 | 15.102 | 0.21751 | 7/15/2014 | 18:36.3 | 2 |
| -197.319 | 19315.167 | 1.8 | 15.105 | 0.219197 | 7/15/2014 | 18:36.4 | 2 |
| -197.07 | 19315.136 | 1.796 | 15.114 | 0.214341 | 7/15/2014 | 18:36.5 | 2 |
| -196.824 | 19315.103 | 1.789 | 15.123 | 0.218727 | 7/15/2014 | 18:36.6 | 2 |
| -196.573 | 19315.07 | 1.796 | 15.137 | 0.221452 | 7/15/2014 | 18:36.7 | 2 |
| -196.262 | 19315.03 | 1.793 | 15.144 | 0.225262 | 7/15/2014 | 18:36.8 | 2 |
| -196.007 | 19314.997 | 1.796 | 15.146 | 0.224871 | 7/15/2014 | 18:36.9 | 2 |
| -195.754 | 19314.963 | 1.79 | 15.149 | 0.224273 | 7/15/2014 | 18:37.0 | 2 |
| -195.5 | 19314.93 | 1.793 | 15.159 | 0.225137 | 7/15/2014 | 18:37.1 | 2 |
| -195.248 | 19314.896 | 1.789 | 15.162 | 0.229692 | 7/15/2014 | 18:37.2 | 2 |
| -194.99 | 19314.863 | 1.793 | 15.168 | 0.224264 | 7/15/2014 | 18:37.3 | 2 |
| -194.675 | 19314.821 | 1.783 | 15.166 | 0.223631 | 7/15/2014 | 18:37.4 | 2 |
| -194.417 | 19314.787 | 1.78 | 15.169 | 0.224909 | 7/15/2014 | 18:37.5 | 2 |
| -194.162 | 19314.752 | 1.774 | 15.175 | 0.238976 | 7/15/2014 | 18:37.6 | 2 |
| -193.905 | 19314.717 | 1.772 | 15.182 | 0.224193 | 7/15/2014 | 18:37.7 | 2 |
| -193.587 | 19314.675 | 1.768 | 15.177 | 0.220665 | 7/15/2014 | 18:37.8 | 2 |
| -193.328 | 19314.643 | 1.772 | 15.179 | 0.225061 | 7/15/2014 | 18:37.9 | 2 |

| | | | | | | | |
|---|---|---|---|---|---|---|---|
| -193.072 | 19314.607 | 1.766 | 15.172 | 0.231382 | 7/15/2014 | 18:38.0 | 2 |
| -192.813 | 19314.572 | 1.766 | 15.176 | 0.216013 | 7/15/2014 | 18:38.1 | 2 |
| -192.557 | 19314.537 | 1.762 | 15.177 | 0.215097 | 7/15/2014 | 18:38.2 | 2 |
| -192.298 | 19314.501 | 1.768 | 15.182 | 0.224935 | 7/15/2014 | 18:38.3 | 2 |
| -191.981 | 19314.458 | 1.765 | 15.186 | 0.222533 | 7/15/2014 | 18:38.4 | 2 |
| -191.724 | 19314.425 | 1.762 | 15.189 | 0.224575 | 7/15/2014 | 18:38.5 | 2 |
| -191.468 | 19314.391 | 1.752 | 15.186 | 0.220299 | 7/15/2014 | 18:38.6 | 2 |
| -191.212 | 19314.355 | 1.755 | 15.194 | 0.222234 | 7/15/2014 | 18:38.7 | 2 |
| -190.892 | 19314.311 | 1.755 | 15.193 | 0.219458 | 7/15/2014 | 18:38.8 | 2 |
| -190.632 | 19314.274 | 1.761 | 15.201 | 0.214878 | 7/15/2014 | 18:38.9 | 2 |
| -190.377 | 19314.239 | 1.755 | 15.2 | 0.215416 | 7/15/2014 | 18:39.0 | 2 |
| -190.12 | 19314.204 | 1.755 | 15.2 | 0.213581 | 7/15/2014 | 18:39.1 | 2 |
| -189.863 | 19314.17 | 1.757 | 15.204 | 0.215522 | 7/15/2014 | 18:39.2 | 2 |
| -189.607 | 19314.134 | 1.763 | 15.216 | 0.229054 | 7/15/2014 | 18:39.3 | 2 |
| -189.288 | 19314.089 | 1.761 | 15.22 | 0.233478 | 7/15/2014 | 18:39.4 | 2 |
| -189.032 | 19314.054 | 1.757 | 15.226 | 0.239102 | 7/15/2014 | 18:39.5 | 2 |
| -188.779 | 19314.018 | 1.751 | 15.232 | 0.248632 | 7/15/2014 | 18:39.6 | 2 |
| -188.524 | 19313.982 | 1.751 | 15.24 | 0.252254 | 7/15/2014 | 18:39.7 | 2 |
| -188.207 | 19313.938 | 1.747 | 15.252 | 0.248103 | 7/15/2014 | 18:39.8 | 2 |
| -187.951 | 19313.902 | 1.744 | 15.264 | 0.240636 | 7/15/2014 | 18:39.9 | 2 |
| -187.695 | 19313.866 | 1.738 | 15.263 | 0.23705 | 7/15/2014 | 18:40.0 | 2 |
| -187.439 | 19313.831 | 1.738 | 15.265 | 0.23631 | 7/15/2014 | 18:40.1 | 2 |
| -187.183 | 19313.793 | 1.742 | 15.267 | 0.247666 | 7/15/2014 | 18:40.2 | 2 |
| -186.927 | 19313.758 | 1.742 | 15.265 | 0.244792 | 7/15/2014 | 18:40.3 | 2 |
| -186.609 | 19313.713 | 1.738 | 15.266 | 0.263125 | 7/15/2014 | 18:40.4 | 2 |
| -186.353 | 19313.677 | 1.738 | 15.265 | 0.264897 | 7/15/2014 | 18:40.5 | 2 |
| -186.097 | 19313.64 | 1.741 | 15.262 | 0.262612 | 7/15/2014 | 18:40.6 | 2 |
| -185.84 | 19313.604 | 1.744 | 15.265 | 0.266561 | 7/15/2014 | 18:40.7 | 2 |
| -185.519 | 19313.556 | 1.736 | 15.268 | 0.244657 | 7/15/2014 | 18:40.8 | 2 |
| -185.26 | 19313.52 | 1.736 | 15.27 | 0.231545 | 7/15/2014 | 18:40.9 | 2 |
| -185 | 19313.482 | 1.732 | 15.269 | 0.23358 | 7/15/2014 | 18:41.0 | 2 |
| -184.74 | 19313.444 | 1.732 | 15.276 | 0.228 | 7/15/2014 | 18:41.1 | 2 |
| -184.48 | 19313.407 | 1.73 | 15.273 | 0.221346 | 7/15/2014 | 18:41.2 | 2 |
| -184.22 | 19313.369 | 1.727 | 15.27 | 0.227419 | 7/15/2014 | 18:41.3 | 2 |
| -183.897 | 19313.321 | 1.725 | 15.266 | 0.240816 | 7/15/2014 | 18:41.4 | 2 |
| -183.635 | 19313.284 | 1.733 | 15.262 | 0.230306 | 7/15/2014 | 18:41.5 | 2 |
| -183.375 | 19313.246 | 1.727 | 15.261 | 0.246152 | 7/15/2014 | 18:41.6 | 2 |
| -183.114 | 19313.208 | 1.725 | 15.263 | 0.23162 | 7/15/2014 | 18:41.7 | 2 |
| -182.788 | 19313.16 | 1.722 | 15.255 | 0.24505 | 7/15/2014 | 18:41.8 | 2 |
| -182.523 | 19313.121 | 1.725 | 15.257 | 0.238972 | 7/15/2014 | 18:41.9 | 2 |
| -182.262 | 19313.082 | 1.713 | 15.265 | 0.238341 | 7/15/2014 | 18:42.0 | 2 |
| -181.996 | 19313.044 | 1.704 | 15.275 | 0.246937 | 7/15/2014 | 18:42.1 | 2 |
| -181.733 | 19313.005 | 1.692 | 15.284 | 0.242467 | 7/15/2014 | 18:42.2 | 2 |
| -181.468 | 19312.967 | 1.69 | 15.29 | 0.249027 | 7/15/2014 | 18:42.3 | 2 |
| -181.139 | 19312.918 | 1.687 | 15.295 | 0.241885 | 7/15/2014 | 18:42.4 | 2 |
| -180.874 | 19312.878 | 1.685 | 15.302 | 0.251009 | 7/15/2014 | 18:42.5 | 2 |
| -180.613 | 19312.84 | 1.676 | 15.313 | 0.245176 | 7/15/2014 | 18:42.6 | 2 |

| | | | | | | | |
|---|---|---|---|---|---|---|---|
| -180.347 | 19312.801 | 1.683 | 15.312 | 0.257519 | 7/15/2014 | 18:42.7 | 2 |
| -180.021 | 19312.752 | 1.681 | 15.319 | 0.251336 | 7/15/2014 | 18:42.8 | 2 |
| -179.755 | 19312.713 | 1.685 | 15.319 | 0.262771 | 7/15/2014 | 18:42.9 | 2 |
| -179.493 | 19312.674 | 1.681 | 15.325 | 0.263944 | 7/15/2014 | 18:43.0 | 2 |
| -179.229 | 19312.634 | 1.675 | 15.327 | 0.265696 | 7/15/2014 | 18:43.1 | 2 |
| -178.969 | 19312.596 | 1.669 | 15.327 | 0.268028 | 7/15/2014 | 18:43.2 | 2 |
| -178.707 | 19312.557 | 1.671 | 15.325 | 0.242593 | 7/15/2014 | 18:43.3 | 2 |
| -178.385 | 19312.508 | 1.663 | 15.328 | 0.250597 | 7/15/2014 | 18:43.4 | 2 |
| -178.126 | 19312.47 | 1.665 | 15.326 | 0.260802 | 7/15/2014 | 18:43.5 | 2 |
| -177.872 | 19312.432 | 1.659 | 15.332 | 0.244403 | 7/15/2014 | 18:43.6 | 2 |
| -177.616 | 19312.393 | 1.659 | 15.329 | 0.239863 | 7/15/2014 | 18:43.7 | 2 |
| -177.301 | 19312.346 | 1.659 | 15.33 | 0.245943 | 7/15/2014 | 18:43.8 | 2 |
| -177.048 | 19312.307 | 1.659 | 15.332 | 0.244613 | 7/15/2014 | 18:43.9 | 2 |
| -176.796 | 19312.27 | 1.646 | 15.331 | 0.266444 | 7/15/2014 | 18:44.0 | 2 |
| -176.545 | 19312.233 | 1.646 | 15.329 | 0.236767 | 7/15/2014 | 18:44.1 | 2 |
| -176.297 | 19312.195 | 1.639 | 15.326 | 0.218971 | 7/15/2014 | 18:44.2 | 2 |
| -176.048 | 19312.156 | 1.639 | 15.321 | 0.232932 | 7/15/2014 | 18:44.3 | 2 |
| -175.618 | 19312.092 | 1.627 | 15.318 | 0.230496 | 7/15/2014 | 18:44.4 | 2 |
| -175.372 | 19312.055 | 1.622 | 15.313 | 0.233159 | 7/15/2014 | 18:44.5 | 2 |
| -175.127 | 19312.019 | 1.619 | 15.31 | 0.22684 | 7/15/2014 | 18:44.6 | 2 |
| -174.819 | 19311.973 | 1.623 | 15.307 | 0.220575 | 7/15/2014 | 18:44.7 | 2 |
| -174.696 | 19311.953 | 1.619 | 15.307 | 0.238734 | 7/15/2014 | 18:44.8 | 2 |
| -174.448 | 19311.914 | 1.617 | 15.303 | 0.231545 | 7/15/2014 | 18:44.9 | 2 |
| -174.199 | 19311.879 | 1.608 | 15.302 | 0.226786 | 7/15/2014 | 18:45.0 | 2 |
| -173.948 | 19311.839 | 1.606 | 15.302 | 0.230187 | 7/15/2014 | 18:45.1 | 2 |
| -173.697 | 19311.803 | 1.603 | 15.303 | 0.22598 | 7/15/2014 | 18:45.2 | 2 |
| -173.446 | 19311.765 | 1.607 | 15.306 | 0.229196 | 7/15/2014 | 18:45.3 | 2 |
| -173.135 | 19311.717 | 1.597 | 15.309 | 0.230409 | 7/15/2014 | 18:45.4 | 2 |
| -172.881 | 19311.678 | 1.591 | 15.31 | 0.231225 | 7/15/2014 | 18:45.5 | 2 |
| -172.627 | 19311.641 | 1.585 | 15.315 | 0.221858 | 7/15/2014 | 18:45.6 | 2 |
| -172.372 | 19311.601 | 1.589 | 15.318 | 0.24198 | 7/15/2014 | 18:45.7 | 2 |
| -172.054 | 19311.553 | 1.585 | 15.322 | 0.236255 | 7/15/2014 | 18:45.8 | 2 |
| -171.794 | 19311.513 | 1.585 | 15.326 | 0.221671 | 7/15/2014 | 18:45.9 | 2 |
| -171.538 | 19311.473 | 1.573 | 15.329 | 0.232149 | 7/15/2014 | 18:46.0 | 2 |
| -171.276 | 19311.434 | 1.574 | 15.335 | 0.219822 | 7/15/2014 | 18:46.1 | 2 |
| -171.018 | 19311.394 | 1.57 | 15.34 | 0.228477 | 7/15/2014 | 18:46.2 | 2 |
| -170.758 | 19311.354 | 1.573 | 15.343 | 0.214341 | 7/15/2014 | 18:46.3 | 2 |
| -170.435 | 19311.305 | 1.563 | 15.35 | 0.214794 | 7/15/2014 | 18:46.4 | 2 |
| -170.173 | 19311.263 | 1.564 | 15.35 | 0.213561 | 7/15/2014 | 18:46.5 | 2 |
| -169.916 | 19311.223 | 1.556 | 15.351 | 0.214505 | 7/15/2014 | 18:46.6 | 2 |
| -169.654 | 19311.184 | 1.556 | 15.352 | 0.209848 | 7/15/2014 | 18:46.7 | 2 |
| -169.331 | 19311.134 | 1.552 | 15.351 | 0.213222 | 7/15/2014 | 18:46.8 | 2 |
| -169.071 | 19311.094 | 1.548 | 15.353 | 0.210171 | 7/15/2014 | 18:46.9 | 2 |
| -168.811 | 19311.055 | 1.538 | 15.359 | 0.215622 | 7/15/2014 | 18:47.0 | 2 |
| -168.55 | 19311.012 | 1.535 | 15.359 | 0.219061 | 7/15/2014 | 18:47.1 | 2 |
| -168.294 | 19310.973 | 1.523 | 15.368 | 0.225827 | 7/15/2014 | 18:47.2 | 2 |
| -168.035 | 19310.934 | 1.521 | 15.374 | 0.231365 | 7/15/2014 | 18:47.3 | 2 |

| | | | | | | | |
|---|---|---|---|---|---|---|---|
| -167.715 | 19310.885 | 1.515 | 15.38 | 0.236063 | 7/15/2014 | 18:47.4 | 2 |
| -167.459 | 19310.845 | 1.512 | 15.391 | 0.227933 | 7/15/2014 | 18:47.5 | 2 |
| -167.204 | 19310.806 | 1.507 | 15.401 | 0.233769 | 7/15/2014 | 18:47.6 | 2 |
| -166.948 | 19310.764 | 1.502 | 15.405 | 0.224829 | 7/15/2014 | 18:47.7 | 2 |
| -166.632 | 19310.715 | 1.49 | 15.42 | 0.223112 | 7/15/2014 | 18:47.8 | 2 |
| -166.374 | 19310.676 | 1.49 | 15.428 | 0.207844 | 7/15/2014 | 18:47.9 | 2 |
| -166.119 | 19310.637 | 1.485 | 15.44 | 0.203788 | 7/15/2014 | 18:48.0 | 2 |
| -165.862 | 19310.597 | 1.482 | 15.455 | 0.198298 | 7/15/2014 | 18:48.1 | 2 |
| -165.606 | 19310.556 | 1.477 | 15.462 | 0.195494 | 7/15/2014 | 18:48.2 | 2 |
| -165.351 | 19310.516 | 1.48 | 15.475 | 0.188252 | 7/15/2014 | 18:48.3 | 2 |
| -165.034 | 19310.466 | 1.48 | 15.485 | 0.187628 | 7/15/2014 | 18:48.4 | 2 |
| -164.779 | 19310.426 | 1.483 | 15.494 | 0.185888 | 7/15/2014 | 18:48.5 | 2 |
| -164.524 | 19310.387 | 1.476 | 15.504 | 0.185381 | 7/15/2014 | 18:48.6 | 2 |
| -164.269 | 19310.348 | 1.472 | 15.511 | 0.181563 | 7/15/2014 | 18:48.7 | 2 |
| -163.949 | 19310.297 | 1.465 | 15.515 | 0.179328 | 7/15/2014 | 18:48.8 | 2 |
| -163.693 | 19310.257 | 1.461 | 15.523 | 0.175276 | 7/15/2014 | 18:48.9 | 2 |
| -163.436 | 19310.216 | 1.449 | 15.524 | 0.171022 | 7/15/2014 | 18:49.0 | 2 |
| -163.181 | 19310.176 | 1.445 | 15.534 | 0.166121 | 7/15/2014 | 18:49.1 | 2 |
| -162.924 | 19310.136 | 1.442 | 15.532 | 0.162653 | 7/15/2014 | 18:49.2 | 2 |
| -162.668 | 19310.097 | 1.439 | 15.537 | 0.161789 | 7/15/2014 | 18:49.3 | 2 |
| -162.351 | 19310.047 | 1.433 | 15.537 | 0.162336 | 7/15/2014 | 18:49.4 | 2 |
| -162.095 | 19310.007 | 1.433 | 15.535 | 0.165939 | 7/15/2014 | 18:49.5 | 2 |
| -161.841 | 19309.966 | 1.427 | 15.532 | 0.172694 | 7/15/2014 | 18:49.6 | 2 |
| -161.585 | 19309.926 | 1.43 | 15.534 | 0.176139 | 7/15/2014 | 18:49.7 | 2 |
| -161.266 | 19309.876 | 1.421 | 15.529 | 0.181403 | 7/15/2014 | 18:49.8 | 2 |
| -161.01 | 19309.837 | 1.425 | 15.527 | 0.185914 | 7/15/2014 | 18:49.9 | 2 |
| -160.755 | 19309.796 | 1.425 | 15.521 | 0.185855 | 7/15/2014 | 18:50.0 | 2 |
| -160.497 | 19309.756 | 1.427 | 15.519 | 0.186569 | 7/15/2014 | 18:50.1 | 2 |
| -160.241 | 19309.716 | 1.425 | 15.517 | 0.185933 | 7/15/2014 | 18:50.2 | 2 |
| -159.984 | 19309.675 | 1.427 | 15.513 | 0.185245 | 7/15/2014 | 18:50.3 | 2 |
| -159.666 | 19309.625 | 1.422 | 15.505 | 0.182671 | 7/15/2014 | 18:50.4 | 2 |
| -159.41 | 19309.586 | 1.431 | 15.501 | 0.181894 | 7/15/2014 | 18:50.5 | 2 |
| -159.155 | 19309.546 | 1.429 | 15.495 | 0.175543 | 7/15/2014 | 18:50.6 | 2 |
| -158.899 | 19309.506 | 1.431 | 15.495 | 0.170098 | 7/15/2014 | 18:50.7 | 2 |
| -158.583 | 19309.457 | 1.431 | 15.491 | 0.162011 | 7/15/2014 | 18:50.8 | 2 |
| -158.327 | 19309.417 | 1.431 | 15.494 | 0.154551 | 7/15/2014 | 18:50.9 | 2 |
| -158.074 | 19309.377 | 1.428 | 15.493 | 0.150274 | 7/15/2014 | 18:51.0 | 2 |
| -157.818 | 19309.338 | 1.433 | 15.492 | 0.147866 | 7/15/2014 | 18:51.1 | 2 |
| -157.567 | 19309.299 | 1.427 | 15.489 | 0.139371 | 7/15/2014 | 18:51.2 | 2 |
| -157.314 | 19309.26 | 1.424 | 15.49 | 0.137098 | 7/15/2014 | 18:51.3 | 2 |
| -157.002 | 19309.211 | 1.419 | 15.483 | 0.134503 | 7/15/2014 | 18:51.4 | 2 |
| -156.752 | 19309.172 | 1.419 | 15.48 | 0.135709 | 7/15/2014 | 18:51.5 | 2 |
| -156.505 | 19309.135 | 1.419 | 15.469 | 0.130555 | 7/15/2014 | 18:51.6 | 2 |
| -156.255 | 19309.096 | 1.425 | 15.467 | 0.128907 | 7/15/2014 | 18:51.7 | 2 |
| -155.948 | 19309.049 | 1.423 | 15.461 | 0.125601 | 7/15/2014 | 18:51.8 | 2 |
| -155.7 | 19309.01 | 1.425 | 15.453 | 0.121488 | 7/15/2014 | 18:51.9 | 2 |
| -155.452 | 19308.972 | 1.423 | 15.448 | 0.128726 | 7/15/2014 | 18:52.0 | 2 |

| | | | | | | | |
|---|---|---|---|---|---|---|---|
| -155.204 | 19308.934 | 1.425 | 15.442 | 0.129518 | 7/15/2014 | 18:52.1 | 2 |
| -154.957 | 19308.896 | 1.418 | 15.442 | 0.134997 | 7/15/2014 | 18:52.2 | 2 |
| -154.711 | 19308.857 | 1.418 | 15.44 | 0.136573 | 7/15/2014 | 18:52.3 | 2 |
| -154.405 | 19308.813 | 1.409 | 15.435 | 0.141345 | 7/15/2014 | 18:52.4 | 2 |
| -154.158 | 19308.773 | 1.415 | 15.432 | 0.147711 | 7/15/2014 | 18:52.5 | 2 |
| -153.914 | 19308.735 | 1.413 | 15.429 | 0.14355 | 7/15/2014 | 18:52.6 | 2 |
| -153.488 | 19308.672 | 1.415 | 15.421 | 0.145943 | 7/15/2014 | 18:52.7 | 2 |
| -153.363 | 19308.652 | 1.415 | 15.421 | 0.147014 | 7/15/2014 | 18:52.8 | 2 |
| -153.117 | 19308.613 | 1.423 | 15.413 | 0.144982 | 7/15/2014 | 18:52.9 | 2 |
| -152.873 | 19308.578 | 1.423 | 15.411 | 0.139961 | 7/15/2014 | 18:53.0 | 2 |
| -152.626 | 19308.541 | 1.427 | 15.409 | 0.140691 | 7/15/2014 | 18:53.1 | 2 |
| -152.383 | 19308.502 | 1.425 | 15.405 | 0.129534 | 7/15/2014 | 18:53.2 | 2 |
| -152.138 | 19308.467 | 1.427 | 15.402 | 0.129031 | 7/15/2014 | 18:53.3 | 2 |
| -151.716 | 19308.402 | 1.43 | 15.401 | 0.115763 | 7/15/2014 | 18:53.4 | 2 |
| -151.476 | 19308.365 | 1.43 | 15.404 | 0.116932 | 7/15/2014 | 18:53.5 | 2 |
| -151.236 | 19308.33 | 1.43 | 15.401 | 0.110187 | 7/15/2014 | 18:53.6 | 2 |
| -150.938 | 19308.287 | 1.43 | 15.398 | 0.103438 | 7/15/2014 | 18:53.7 | 2 |
| -150.817 | 19308.268 | 1.43 | 15.396 | 0.102712 | 7/15/2014 | 18:53.8 | 2 |
| -150.576 | 19308.232 | 1.429 | 15.396 | 0.105166 | 7/15/2014 | 18:53.9 | 2 |
| -150.338 | 19308.195 | 1.426 | 15.396 | 0.0992023 | 7/15/2014 | 18:54.0 | 2 |
| -150.1 | 19308.16 | 1.424 | 15.395 | 0.113603 | 7/15/2014 | 18:54.1 | 2 |
| -149.864 | 19308.126 | 1.421 | 15.397 | 0.0977239 | 7/15/2014 | 18:54.2 | 2 |
| -149.627 | 19308.091 | 1.424 | 15.393 | 0.113045 | 7/15/2014 | 18:54.3 | 2 |
| -149.219 | 19308.031 | 1.421 | 15.402 | 0.106258 | 7/15/2014 | 18:54.4 | 2 |
| -148.986 | 19307.996 | 1.424 | 15.403 | 0.11008 | 7/15/2014 | 18:54.5 | 2 |
| -148.753 | 19307.963 | 1.424 | 15.404 | 0.102345 | 7/15/2014 | 18:54.6 | 2 |
| -148.463 | 19307.92 | 1.427 | 15.404 | 0.097771 | 7/15/2014 | 18:54.7 | 2 |
| -148.346 | 19307.903 | 1.429 | 15.405 | 0.101123 | 7/15/2014 | 18:54.8 | 2 |
| -148.111 | 19307.869 | 1.431 | 15.404 | 0.0818521 | 7/15/2014 | 18:54.9 | 2 |
| -147.878 | 19307.836 | 1.432 | 15.398 | 0.0861661 | 7/15/2014 | 18:55.0 | 2 |
| -147.642 | 19307.801 | 1.44 | 15.388 | 0.0795005 | 7/15/2014 | 18:55.1 | 2 |
| -147.41 | 19307.767 | 1.442 | 15.377 | 0.0726053 | 7/15/2014 | 18:55.2 | 2 |
| -147.175 | 19307.734 | 1.444 | 15.368 | 0.0870257 | 7/15/2014 | 18:55.3 | 2 |
| -146.767 | 19307.675 | 1.445 | 15.362 | 0.0855169 | 7/15/2014 | 18:55.4 | 2 |
| -146.532 | 19307.642 | 1.445 | 15.357 | 0.103316 | 7/15/2014 | 18:55.5 | 2 |
| -146.298 | 19307.609 | 1.448 | 15.351 | 0.102685 | 7/15/2014 | 18:55.6 | 2 |
| -146.004 | 19307.566 | 1.445 | 15.353 | 0.108224 | 7/15/2014 | 18:55.7 | 2 |
| -145.887 | 19307.55 | 1.443 | 15.353 | 0.112173 | 7/15/2014 | 18:55.8 | 2 |
| -145.648 | 19307.517 | 1.445 | 15.355 | 0.119475 | 7/15/2014 | 18:55.9 | 2 |
| -145.41 | 19307.483 | 1.445 | 15.358 | 0.120332 | 7/15/2014 | 18:56.0 | 2 |
| -145.171 | 19307.448 | 1.448 | 15.359 | 0.115736 | 7/15/2014 | 18:56.1 | 2 |
| -144.933 | 19307.416 | 1.448 | 15.357 | 0.102358 | 7/15/2014 | 18:56.2 | 2 |
| -144.693 | 19307.383 | 1.452 | 15.358 | 0.116671 | 7/15/2014 | 18:56.3 | 2 |
| -144.275 | 19307.324 | 1.453 | 15.357 | 0.118224 | 7/15/2014 | 18:56.4 | 2 |
| -144.036 | 19307.292 | 1.458 | 15.352 | 0.106063 | 7/15/2014 | 18:56.5 | 2 |
| -143.797 | 19307.26 | 1.461 | 15.349 | 0.10436 | 7/15/2014 | 18:56.6 | 2 |
| -143.499 | 19307.219 | 1.458 | 15.349 | 0.127589 | 7/15/2014 | 18:56.7 | 2 |

| | | | | | | | |
|---|---|---|---|---|---|---|---|
| -143.378 | 19307.203 | 1.458 | 15.348 | 0.129457 | 7/15/2014 | 18:56.8 | 2 |
| -143.136 | 19307.169 | 1.461 | 15.344 | 0.120968 | 7/15/2014 | 18:56.9 | 2 |
| -142.896 | 19307.137 | 1.456 | 15.339 | 0.113386 | 7/15/2014 | 18:57.0 | 2 |
| -142.654 | 19307.104 | 1.46 | 15.336 | 0.126914 | 7/15/2014 | 18:57.1 | 2 |
| -142.413 | 19307.072 | 1.455 | 15.331 | 0.121136 | 7/15/2014 | 18:57.2 | 2 |
| -142.171 | 19307.041 | 1.455 | 15.328 | 0.122935 | 7/15/2014 | 18:57.3 | 2 |
| -141.745 | 19306.984 | 1.453 | 15.322 | 0.103226 | 7/15/2014 | 18:57.4 | 2 |
| -141.5 | 19306.952 | 1.455 | 15.315 | 0.100738 | 7/15/2014 | 18:57.5 | 2 |
| -141.254 | 19306.918 | 1.457 | 15.309 | 0.105759 | 7/15/2014 | 18:57.6 | 2 |
| -141.132 | 19306.902 | 1.46 | 15.307 | 0.0970025 | 7/15/2014 | 18:57.7 | 2 |
| -140.884 | 19306.871 | 1.46 | 15.301 | 0.091679 | 7/15/2014 | 18:57.8 | 2 |
| -140.633 | 19306.839 | 1.468 | 15.293 | 0.090093 | 7/15/2014 | 18:57.9 | 2 |
| -140.383 | 19306.805 | 1.474 | 15.286 | 0.100413 | 7/15/2014 | 18:58.0 | 2 |
| -140.13 | 19306.773 | 1.483 | 15.283 | 0.0923589 | 7/15/2014 | 18:58.1 | 2 |
| -139.879 | 19306.742 | 1.481 | 15.276 | 0.0950789 | 7/15/2014 | 18:58.2 | 2 |
| -139.626 | 19306.709 | 1.487 | 15.271 | 0.0979183 | 7/15/2014 | 18:58.3 | 2 |
| -139.374 | 19306.678 | 1.487 | 15.268 | 0.101194 | 7/15/2014 | 18:58.4 | 2 |
| -139.123 | 19306.646 | 1.487 | 15.267 | 0.105129 | 7/15/2014 | 18:58.5 | 2 |
| -138.872 | 19306.613 | 1.483 | 15.268 | 0.10941 | 7/15/2014 | 18:58.6 | 2 |
| -138.623 | 19306.582 | 1.477 | 15.269 | 0.10408 | 7/15/2014 | 18:58.7 | 2 |
| -138.313 | 19306.544 | 1.471 | 15.267 | 0.100932 | 7/15/2014 | 18:58.8 | 2 |
| -138.061 | 19306.513 | 1.479 | 15.27 | 0.0981146 | 7/15/2014 | 18:58.9 | 2 |
| -137.81 | 19306.481 | 1.479 | 15.271 | 0.0916888 | 7/15/2014 | 18:59.0 | 2 |
| -137.558 | 19306.45 | 1.483 | 15.278 | 0.0893325 | 7/15/2014 | 18:59.1 | 2 |
| -137.305 | 19306.419 | 1.483 | 15.281 | 0.0848933 | 7/15/2014 | 18:59.2 | 2 |
| -137.052 | 19306.388 | 1.483 | 15.285 | 0.0822736 | 7/15/2014 | 18:59.3 | 2 |
| -136.736 | 19306.35 | 1.485 | 15.29 | 0.0796655 | 7/15/2014 | 18:59.4 | 2 |
| -136.479 | 19306.317 | 1.492 | 15.293 | 0.0808181 | 7/15/2014 | 18:59.5 | 2 |
| -136.221 | 19306.286 | 1.483 | 15.297 | 0.0790563 | 7/15/2014 | 18:59.6 | 2 |
| -135.961 | 19306.255 | 1.479 | 15.3 | 0.07956 | 7/15/2014 | 18:59.7 | 2 |
| -135.701 | 19306.224 | 1.473 | 15.3 | 0.0824718 | 7/15/2014 | 18:59.8 | 2 |
| -135.44 | 19306.192 | 1.473 | 15.302 | 0.0841259 | 7/15/2014 | 18:59.9 | 2 |
| -135.18 | 19306.161 | 1.471 | 15.301 | 0.0810751 | 7/15/2014 | 19:00.0 | 2 |
| -134.92 | 19306.131 | 1.471 | 15.303 | 0.0767201 | 7/15/2014 | 19:00.1 | 2 |
| -134.661 | 19306.1 | 1.467 | 15.305 | 0.0851308 | 7/15/2014 | 19:00.2 | 2 |
| -134.404 | 19306.069 | 1.47 | 15.306 | 0.073112 | 7/15/2014 | 19:00.3 | 2 |
| -134.149 | 19306.039 | 1.467 | 15.306 | 0.0714876 | 7/15/2014 | 19:00.4 | 2 |
| -133.896 | 19306.01 | 1.473 | 15.307 | 0.0627774 | 7/15/2014 | 19:00.5 | 2 |
| -133.647 | 19305.98 | 1.471 | 15.307 | 0.0602728 | 7/15/2014 | 19:00.6 | 2 |
| -133.222 | 19305.932 | 1.469 | 15.306 | 0.0537957 | 7/15/2014 | 19:00.7 | 2 |
| -133.095 | 19305.916 | 1.468 | 15.305 | 0.0512426 | 7/15/2014 | 19:00.8 | 2 |
| -132.849 | 19305.888 | 1.468 | 15.305 | 0.0467189 | 7/15/2014 | 19:00.9 | 2 |
| -132.605 | 19305.859 | 1.466 | 15.302 | 0.0440495 | 7/15/2014 | 19:01.0 | 2 |
| -132.36 | 19305.832 | 1.468 | 15.3 | 0.0438687 | 7/15/2014 | 19:01.1 | 2 |
| -132.118 | 19305.805 | 1.462 | 15.299 | 0.0424016 | 7/15/2014 | 19:01.2 | 2 |
| -131.875 | 19305.777 | 1.465 | 15.299 | 0.0436372 | 7/15/2014 | 19:01.3 | 2 |
| -131.448 | 19305.729 | 1.466 | 15.302 | 0.0437626 | 7/15/2014 | 19:01.4 | 2 |

| | | | | | | | |
|---|---|---|---|---|---|---|---|
| -131.202 | 19305.7 | 1.47 | 15.304 | 0.0482827 | 7/15/2014 | 19:01.5 | 2 |
| -130.956 | 19305.673 | 1.473 | 15.304 | 0.0485706 | 7/15/2014 | 19:01.6 | 2 |
| -130.835 | 19305.659 | 1.476 | 15.305 | 0.0490122 | 7/15/2014 | 19:01.7 | 2 |
| -130.587 | 19305.631 | 1.476 | 15.311 | 0.0478433 | 7/15/2014 | 19:01.8 | 2 |
| -130.338 | 19305.604 | 1.482 | 15.313 | 0.0452478 | 7/15/2014 | 19:01.9 | 2 |
| -130.089 | 19305.576 | 1.483 | 15.316 | 0.041968 | 7/15/2014 | 19:02.0 | 2 |
| -129.839 | 19305.549 | 1.483 | 15.319 | 0.0429441 | 7/15/2014 | 19:02.1 | 2 |
| -129.591 | 19305.521 | 1.481 | 15.319 | 0.0409374 | 7/15/2014 | 19:02.2 | 2 |
| -129.223 | 19305.481 | 1.48 | 15.319 | 0.0413367 | 7/15/2014 | 19:02.3 | 2 |
| -128.978 | 19305.453 | 1.477 | 15.32 | 0.0427605 | 7/15/2014 | 19:02.4 | 2 |
| -128.735 | 19305.427 | 1.477 | 15.322 | 0.042337 | 7/15/2014 | 19:02.5 | 2 |
| -128.495 | 19305.4 | 1.473 | 15.327 | 0.0548063 | 7/15/2014 | 19:02.6 | 2 |
| -128.196 | 19305.367 | 1.472 | 15.337 | 0.0465645 | 7/15/2014 | 19:02.7 | 2 |
| -128.072 | 19305.354 | 1.472 | 15.339 | 0.0489276 | 7/15/2014 | 19:02.8 | 2 |
| -127.833 | 19305.328 | 1.478 | 15.34 | 0.0512708 | 7/15/2014 | 19:02.9 | 2 |
| -127.595 | 19305.305 | 1.482 | 15.338 | 0.0426412 | 7/15/2014 | 19:03.0 | 2 |
| -127.357 | 19305.277 | 1.488 | 15.339 | 0.0432259 | 7/15/2014 | 19:03.1 | 2 |
| -127.119 | 19305.252 | 1.492 | 15.339 | 0.0413925 | 7/15/2014 | 19:03.2 | 2 |
| -126.88 | 19305.227 | 1.495 | 15.334 | 0.0345377 | 7/15/2014 | 19:03.3 | 2 |
| -126.46 | 19305.182 | 1.499 | 15.326 | 0.0406447 | 7/15/2014 | 19:03.4 | 2 |
| -126.219 | 19305.156 | 1.499 | 15.314 | 0.0416672 | 7/15/2014 | 19:03.5 | 2 |
| -125.977 | 19305.132 | 1.499 | 15.313 | 0.0476024 | 7/15/2014 | 19:03.6 | 2 |
| -125.735 | 19305.105 | 1.494 | 15.315 | 0.0500014 | 7/15/2014 | 19:03.7 | 2 |
| -125.49 | 19305.079 | 1.488 | 15.317 | 0.0593089 | 7/15/2014 | 19:03.8 | 2 |
| -125.244 | 19305.055 | 1.483 | 15.316 | 0.0514571 | 7/15/2014 | 19:03.9 | 2 |
| -124.999 | 19305.028 | 1.483 | 15.317 | 0.0467321 | 7/15/2014 | 19:04.0 | 2 |
| -124.754 | 19305.004 | 1.486 | 15.32 | 0.0454943 | 7/15/2014 | 19:04.1 | 2 |
| -124.51 | 19304.979 | 1.483 | 15.321 | 0.0499283 | 7/15/2014 | 19:04.2 | 2 |
| -124.266 | 19304.953 | 1.483 | 15.325 | 0.0397755 | 7/15/2014 | 19:04.3 | 2 |
| -124.024 | 19304.928 | 1.483 | 15.323 | 0.0570798 | 7/15/2014 | 19:04.4 | 2 |
| -123.783 | 19304.903 | 1.485 | 15.325 | 0.0348579 | 7/15/2014 | 19:04.5 | 2 |
| -123.543 | 19304.879 | 1.488 | 15.322 | 0.0508585 | 7/15/2014 | 19:04.6 | 2 |
| -123.246 | 19304.85 | 1.49 | 15.322 | 0.0421628 | 7/15/2014 | 19:04.7 | 2 |
| -123.124 | 19304.837 | 1.489 | 15.323 | 0.0457159 | 7/15/2014 | 19:04.8 | 2 |
| -122.885 | 19304.813 | 1.489 | 15.323 | 0.0331614 | 7/15/2014 | 19:04.9 | 2 |
| -122.649 | 19304.788 | 1.485 | 15.323 | 0.036475 | 7/15/2014 | 19:05.0 | 2 |
| -122.411 | 19304.764 | 1.493 | 15.322 | 0.0346955 | 7/15/2014 | 19:05.1 | 2 |
| -122.175 | 19304.741 | 1.489 | 15.324 | 0.0345618 | 7/15/2014 | 19:05.2 | 2 |
| -121.936 | 19304.716 | 1.489 | 15.318 | 0.0394157 | 7/15/2014 | 19:05.3 | 2 |
| -121.519 | 19304.676 | 1.487 | 15.315 | 0.0352806 | 7/15/2014 | 19:05.4 | 2 |
| -121.277 | 19304.651 | 1.494 | 15.313 | 0.0338572 | 7/15/2014 | 19:05.5 | 2 |
| -121.036 | 19304.627 | 1.493 | 15.309 | 0.0310836 | 7/15/2014 | 19:05.6 | 2 |
| -120.793 | 19304.604 | 1.496 | 15.303 | 0.0312041 | 7/15/2014 | 19:05.7 | 2 |
| -120.55 | 19304.58 | 1.496 | 15.299 | 0.0292221 | 7/15/2014 | 19:05.8 | 2 |
| -120.306 | 19304.555 | 1.503 | 15.292 | 0.0325789 | 7/15/2014 | 19:05.9 | 2 |
| -120.061 | 19304.531 | 1.509 | 15.285 | 0.0263462 | 7/15/2014 | 19:06.0 | 2 |
| -119.817 | 19304.508 | 1.512 | 15.277 | 0.0268383 | 7/15/2014 | 19:06.1 | 2 |

| | | | | | | | |
|---|---|---|---|---|---|---|---|
| -119.572 | 19304.485 | 1.52 | 15.271 | 0.0273032 | 7/15/2014 | 19:06.2 | 2 |
| -119.328 | 19304.461 | 1.517 | 15.263 | 0.0241961 | 7/15/2014 | 19:06.3 | 2 |
| -119.086 | 19304.438 | 1.52 | 15.258 | 0.0390837 | 7/15/2014 | 19:06.4 | 2 |
| -118.845 | 19304.414 | 1.52 | 15.254 | 0.0298729 | 7/15/2014 | 19:06.5 | 2 |
| -118.605 | 19304.391 | 1.52 | 15.25 | 0.0405646 | 7/15/2014 | 19:06.6 | 2 |
| -118.307 | 19304.362 | 1.52 | 15.249 | 0.0440677 | 7/15/2014 | 19:06.7 | 2 |
| -118.185 | 19304.351 | 1.52 | 15.249 | 0.0658742 | 7/15/2014 | 19:06.8 | 2 |
| -117.945 | 19304.328 | 1.522 | 15.248 | 0.0593116 | 7/15/2014 | 19:06.9 | 2 |
| -117.706 | 19304.306 | 1.522 | 15.245 | 0.0777434 | 7/15/2014 | 19:07.0 | 2 |
| -117.467 | 19304.282 | 1.532 | 15.243 | 0.0538233 | 7/15/2014 | 19:07.1 | 2 |
| -117.227 | 19304.26 | 1.532 | 15.236 | 0.0775008 | 7/15/2014 | 19:07.2 | 2 |
| -116.986 | 19304.237 | 1.54 | 15.232 | 0.0793898 | 7/15/2014 | 19:07.3 | 2 |
| -116.561 | 19304.197 | 1.548 | 15.229 | 0.0607389 | 7/15/2014 | 19:07.4 | 2 |
| -116.318 | 19304.174 | 1.548 | 15.221 | 0.0560975 | 7/15/2014 | 19:07.5 | 2 |
| -116.072 | 19304.151 | 1.55 | 15.217 | 0.0614705 | 7/15/2014 | 19:07.6 | 2 |
| -115.951 | 19304.14 | 1.551 | 15.215 | 0.0675207 | 7/15/2014 | 19:07.7 | 2 |
| -115.704 | 19304.117 | 1.548 | 15.21 | 0.0541418 | 7/15/2014 | 19:07.8 | 2 |
| -115.454 | 19304.094 | 1.552 | 15.206 | 0.0585924 | 7/15/2014 | 19:07.9 | 2 |
| -115.205 | 19304.07 | 1.55 | 15.203 | 0.0643664 | 7/15/2014 | 19:08.0 | 2 |
| -114.953 | 19304.047 | 1.552 | 15.193 | 0.0685732 | 7/15/2014 | 19:08.1 | 2 |
| -114.704 | 19304.023 | 1.552 | 15.188 | 0.0681568 | 7/15/2014 | 19:08.2 | 2 |
| -114.455 | 19304 | 1.563 | 15.181 | 0.0476779 | 7/15/2014 | 19:08.3 | 2 |
| -114.088 | 19303.967 | 1.571 | 15.165 | 0.0428719 | 7/15/2014 | 19:08.4 | 2 |
| -113.843 | 19303.943 | 1.577 | 15.159 | 0.0482503 | 7/15/2014 | 19:08.5 | 2 |
| -113.6 | 19303.922 | 1.582 | 15.149 | 0.0373323 | 7/15/2014 | 19:08.6 | 2 |
| -113.299 | 19303.895 | 1.587 | 15.14 | 0.045377 | 7/15/2014 | 19:08.7 | 2 |
| -113.175 | 19303.883 | 1.591 | 15.134 | 0.0530919 | 7/15/2014 | 19:08.8 | 2 |
| -112.935 | 19303.86 | 1.588 | 15.126 | 0.0426674 | 7/15/2014 | 19:08.9 | 2 |
| -112.696 | 19303.839 | 1.585 | 15.121 | 0.0314524 | 7/15/2014 | 19:09.0 | 2 |
| -112.455 | 19303.817 | 1.589 | 15.116 | 0.0287254 | 7/15/2014 | 19:09.1 | 2 |
| -112.218 | 19303.795 | 1.586 | 15.113 | 0.028671 | 7/15/2014 | 19:09.2 | 2 |
| -111.979 | 19303.775 | 1.586 | 15.108 | 0.0311412 | 7/15/2014 | 19:09.3 | 2 |
| -111.56 | 19303.738 | 1.589 | 15.103 | 0.0290669 | 7/15/2014 | 19:09.4 | 2 |
| -111.322 | 19303.716 | 1.592 | 15.097 | 0.0337874 | 7/15/2014 | 19:09.5 | 2 |
| -111.081 | 19303.695 | 1.6 | 15.091 | 0.0369245 | 7/15/2014 | 19:09.6 | 2 |
| -110.841 | 19303.673 | 1.6 | 15.087 | 0.0365897 | 7/15/2014 | 19:09.7 | 2 |
| -110.601 | 19303.652 | 1.599 | 15.085 | 0.0347639 | 7/15/2014 | 19:09.8 | 2 |
| -110.361 | 19303.632 | 1.601 | 15.081 | 0.0292903 | 7/15/2014 | 19:09.9 | 2 |
| -110.119 | 19303.609 | 1.607 | 15.08 | 0.0371287 | 7/15/2014 | 19:10.0 | 2 |
| -109.88 | 19303.59 | 1.607 | 15.081 | 0.0402551 | 7/15/2014 | 19:10.1 | 2 |
| -109.641 | 19303.568 | 1.607 | 15.084 | 0.0325385 | 7/15/2014 | 19:10.2 | 2 |
| -109.404 | 19303.549 | 1.607 | 15.084 | 0.0298888 | 7/15/2014 | 19:10.3 | 2 |
| -109.17 | 19303.527 | 1.607 | 15.082 | 0.0314542 | 7/15/2014 | 19:10.4 | 2 |
| -108.937 | 19303.508 | 1.612 | 15.081 | 0.0282195 | 7/15/2014 | 19:10.5 | 2 |
| -108.706 | 19303.487 | 1.617 | 15.082 | 0.0248728 | 7/15/2014 | 19:10.6 | 2 |
| -108.422 | 19303.463 | 1.617 | 15.078 | 0.0325083 | 7/15/2014 | 19:10.7 | 2 |
| -108.193 | 19303.443 | 1.617 | 15.077 | 0.0309817 | 7/15/2014 | 19:10.8 | 2 |

| | | | | | | | |
|---|---|---|---|---|---|---|---|
| -107.965 | 19303.424 | 1.619 | 15.068 | 0.0374839 | 7/15/2014 | 19:10.9 | 2 |
| -107.736 | 19303.404 | 1.625 | 15.061 | 0.0407679 | 7/15/2014 | 19:11.0 | 2 |
| -107.507 | 19303.385 | 1.628 | 15.055 | 0.0377698 | 7/15/2014 | 19:11.1 | 2 |
| -107.28 | 19303.365 | 1.63 | 15.048 | 0.0451947 | 7/15/2014 | 19:11.2 | 2 |
| -107.052 | 19303.346 | 1.632 | 15.041 | 0.0393137 | 7/15/2014 | 19:11.3 | 2 |
| -106.769 | 19303.322 | 1.634 | 15.028 | 0.055556 | 7/15/2014 | 19:11.4 | 2 |
| -106.539 | 19303.303 | 1.639 | 15.02 | 0.0453125 | 7/15/2014 | 19:11.5 | 2 |
| -106.308 | 19303.283 | 1.644 | 15.005 | 0.0700923 | 7/15/2014 | 19:11.6 | 2 |
| -106.074 | 19303.264 | 1.652 | 14.999 | 0.0668597 | 7/15/2014 | 19:11.7 | 2 |
| -105.838 | 19303.244 | 1.654 | 14.985 | 0.0947862 | 7/15/2014 | 19:11.8 | 2 |
| -105.601 | 19303.225 | 1.662 | 14.978 | 0.0870699 | 7/15/2014 | 19:11.9 | 2 |
| -105.364 | 19303.203 | 1.667 | 14.972 | 0.114631 | 7/15/2014 | 19:12.0 | 2 |
| -105.126 | 19303.184 | 1.67 | 14.963 | 0.114944 | 7/15/2014 | 19:12.1 | 2 |
| -104.888 | 19303.164 | 1.673 | 14.955 | 0.112419 | 7/15/2014 | 19:12.2 | 2 |
| -104.65 | 19303.145 | 1.681 | 14.951 | 0.12263 | 7/15/2014 | 19:12.3 | 2 |
| -104.414 | 19303.125 | 1.686 | 14.942 | 0.120851 | 7/15/2014 | 19:12.4 | 2 |
| -104.177 | 19303.107 | 1.688 | 14.935 | 0.109951 | 7/15/2014 | 19:12.5 | 2 |
| -103.942 | 19303.088 | 1.693 | 14.93 | 0.126828 | 7/15/2014 | 19:12.6 | 2 |
| -103.653 | 19303.063 | 1.696 | 14.917 | 0.124376 | 7/15/2014 | 19:12.7 | 2 |
| -103.533 | 19303.053 | 1.697 | 14.912 | 0.126807 | 7/15/2014 | 19:12.8 | 2 |
| -103.186 | 19303.023 | 1.704 | 14.894 | 0.133481 | 7/15/2014 | 19:12.9 | 2 |
| -102.953 | 19303.004 | 1.709 | 14.889 | 0.129549 | 7/15/2014 | 19:13.0 | 2 |
| -102.719 | 19302.984 | 1.715 | 14.878 | 0.132001 | 7/15/2014 | 19:13.1 | 2 |
| -102.487 | 19302.967 | 1.72 | 14.871 | 0.136012 | 7/15/2014 | 19:13.2 | 2 |
| -102.257 | 19302.949 | 1.725 | 14.863 | 0.109249 | 7/15/2014 | 19:13.3 | 2 |
| -101.969 | 19302.926 | 1.73 | 14.856 | 0.116326 | 7/15/2014 | 19:13.4 | 2 |
| -101.737 | 19302.905 | 1.735 | 14.854 | 0.109751 | 7/15/2014 | 19:13.5 | 2 |
| -101.504 | 19302.887 | 1.743 | 14.85 | 0.103765 | 7/15/2014 | 19:13.6 | 2 |
| -101.274 | 19302.867 | 1.751 | 14.846 | 0.103464 | 7/15/2014 | 19:13.7 | 2 |
| -101.04 | 19302.848 | 1.758 | 14.838 | 0.0938508 | 7/15/2014 | 19:13.8 | 2 |
| -100.808 | 19302.829 | 1.76 | 14.835 | 0.0849885 | 7/15/2014 | 19:13.9 | 2 |
| -100.577 | 19302.813 | 1.763 | 14.834 | 0.0884491 | 7/15/2014 | 19:14.0 | 2 |
| -100.346 | 19302.793 | 1.767 | 14.832 | 0.0771969 | 7/15/2014 | 19:14.1 | 2 |
| -100.115 | 19302.773 | 1.77 | 14.832 | 0.0825651 | 7/15/2014 | 19:14.2 | 2 |
| -99.886 | 19302.756 | 1.772 | 14.828 | 0.0658551 | 7/15/2014 | 19:14.3 | 2 |
| -99.659 | 19302.736 | 1.772 | 14.83 | 0.0719564 | 7/15/2014 | 19:14.4 | 2 |
| -99.434 | 19302.719 | 1.772 | 14.833 | 0.0783102 | 7/15/2014 | 19:14.5 | 2 |
| -99.209 | 19302.701 | 1.772 | 14.835 | 0.0850882 | 7/15/2014 | 19:14.6 | 2 |
| -98.931 | 19302.68 | 1.772 | 14.832 | 0.0990844 | 7/15/2014 | 19:14.7 | 2 |
| -98.706 | 19302.66 | 1.775 | 14.828 | 0.10802 | 7/15/2014 | 19:14.8 | 2 |
| -98.48 | 19302.643 | 1.778 | 14.824 | 0.100826 | 7/15/2014 | 19:14.9 | 2 |
| -98.255 | 19302.625 | 1.781 | 14.82 | 0.114769 | 7/15/2014 | 19:15.0 | 2 |
| -98.027 | 19302.605 | 1.784 | 14.818 | 0.102709 | 7/15/2014 | 19:15.1 | 2 |
| -97.802 | 19302.588 | 1.787 | 14.814 | 0.107174 | 7/15/2014 | 19:15.2 | 2 |
| -97.573 | 19302.571 | 1.791 | 14.808 | 0.092398 | 7/15/2014 | 19:15.3 | 2 |
| -97.29 | 19302.549 | 1.794 | 14.806 | 0.0956738 | 7/15/2014 | 19:15.4 | 2 |
| -97.058 | 19302.529 | 1.794 | 14.806 | 0.0832606 | 7/15/2014 | 19:15.5 | 2 |

| | | | | | | | |
|---|---|---|---|---|---|---|---|
| -96.824 | 19302.512 | 1.794 | 14.805 | 0.0859632 | 7/15/2014 | 19:15.6 | 2 |
| -96.588 | 19302.494 | 1.794 | 14.803 | 0.0822783 | 7/15/2014 | 19:15.7 | 2 |
| -96.351 | 19302.474 | 1.794 | 14.798 | 0.0917014 | 7/15/2014 | 19:15.8 | 2 |
| -96.112 | 19302.455 | 1.794 | 14.796 | 0.0847754 | 7/15/2014 | 19:15.9 | 2 |
| -95.873 | 19302.438 | 1.791 | 14.794 | 0.0907683 | 7/15/2014 | 19:16.0 | 2 |
| -95.635 | 19302.419 | 1.787 | 14.789 | 0.0760319 | 7/15/2014 | 19:16.1 | 2 |
| -95.396 | 19302.4 | 1.787 | 14.787 | 0.075573 | 7/15/2014 | 19:16.2 | 2 |
| -95.157 | 19302.382 | 1.794 | 14.781 | 0.0792421 | 7/15/2014 | 19:16.3 | 2 |
| -94.919 | 19302.364 | 1.799 | 14.777 | 0.075071 | 7/15/2014 | 19:16.4 | 2 |
| -94.684 | 19302.345 | 1.802 | 14.775 | 0.0737193 | 7/15/2014 | 19:16.5 | 2 |
| -94.449 | 19302.327 | 1.799 | 14.772 | 0.0718076 | 7/15/2014 | 19:16.6 | 2 |
| -94.158 | 19302.305 | 1.802 | 14.768 | 0.071597 | 7/15/2014 | 19:16.7 | 2 |
| -94.039 | 19302.296 | 1.804 | 14.766 | 0.0836712 | 7/15/2014 | 19:16.8 | 2 |
| -93.69 | 19302.27 | 1.812 | 14.747 | 0.0823878 | 7/15/2014 | 19:16.9 | 2 |
| -93.456 | 19302.251 | 1.811 | 14.739 | 0.0801191 | 7/15/2014 | 19:17.0 | 2 |
| -93.221 | 19302.233 | 1.813 | 14.735 | 0.0790225 | 7/15/2014 | 19:17.1 | 2 |
| -93.107 | 19302.225 | 1.81 | 14.731 | 0.0733401 | 7/15/2014 | 19:17.2 | 2 |
| -92.875 | 19302.206 | 1.813 | 14.724 | 0.092925 | 7/15/2014 | 19:17.3 | 2 |
| -92.465 | 19302.176 | 1.82 | 14.722 | 0.0820507 | 7/15/2014 | 19:17.4 | 2 |
| -92.231 | 19302.158 | 1.822 | 14.719 | 0.104364 | 7/15/2014 | 19:17.5 | 2 |
| -91.995 | 19302.141 | 1.826 | 14.713 | 0.0814901 | 7/15/2014 | 19:17.6 | 2 |
| -91.759 | 19302.122 | 1.829 | 14.726 | 0.0855481 | 7/15/2014 | 19:17.7 | 2 |
| -91.52 | 19302.104 | 1.834 | 14.725 | 0.0990506 | 7/15/2014 | 19:17.8 | 2 |
| -91.283 | 19302.086 | 1.834 | 14.727 | 0.0880046 | 7/15/2014 | 19:17.9 | 2 |
| -91.045 | 19302.068 | 1.834 | 14.73 | 0.0850821 | 7/15/2014 | 19:18.0 | 2 |
| -90.807 | 19302.051 | 1.834 | 14.734 | 0.0859547 | 7/15/2014 | 19:18.1 | 2 |
| -90.571 | 19302.033 | 1.831 | 14.736 | 0.0791016 | 7/15/2014 | 19:18.2 | 2 |
| -90.335 | 19302.016 | 1.833 | 14.74 | 0.0805495 | 7/15/2014 | 19:18.3 | 2 |
| -90.102 | 19301.998 | 1.836 | 14.745 | 0.0805421 | 7/15/2014 | 19:18.4 | 2 |
| -89.868 | 19301.981 | 1.836 | 14.749 | 0.0819978 | 7/15/2014 | 19:18.5 | 2 |
| -89.637 | 19301.964 | 1.836 | 14.752 | 0.0769404 | 7/15/2014 | 19:18.6 | 2 |
| -89.35 | 19301.942 | 1.836 | 14.752 | 0.0779166 | 7/15/2014 | 19:18.7 | 2 |
| -89.12 | 19301.926 | 1.838 | 14.755 | 0.0712117 | 7/15/2014 | 19:18.8 | 2 |
| -88.889 | 19301.908 | 1.835 | 14.754 | 0.0717063 | 7/15/2014 | 19:18.9 | 2 |
| -88.658 | 19301.892 | 1.838 | 14.756 | 0.0691628 | 7/15/2014 | 19:19.0 | 2 |
| -88.427 | 19301.875 | 1.833 | 14.762 | 0.0638353 | 7/15/2014 | 19:19.1 | 2 |
| -88.195 | 19301.858 | 1.833 | 14.763 | 0.0619851 | 7/15/2014 | 19:19.2 | 2 |
| -87.965 | 19301.841 | 1.83 | 14.765 | 0.066664 | 7/15/2014 | 19:19.3 | 2 |
| -87.676 | 19301.819 | 1.828 | 14.765 | 0.0590175 | 7/15/2014 | 19:19.4 | 2 |
| -87.443 | 19301.803 | 1.825 | 14.766 | 0.0595999 | 7/15/2014 | 19:19.5 | 2 |
| -87.207 | 19301.786 | 1.822 | 14.764 | 0.0588062 | 7/15/2014 | 19:19.6 | 2 |
| -86.97 | 19301.769 | 1.822 | 14.764 | 0.0569725 | 7/15/2014 | 19:19.7 | 2 |
| -86.73 | 19301.751 | 1.822 | 14.765 | 0.0559504 | 7/15/2014 | 19:19.8 | 2 |
| -86.491 | 19301.733 | 1.82 | 14.757 | 0.0564874 | 7/15/2014 | 19:19.9 | 2 |
| -86.251 | 19301.716 | 1.815 | 14.756 | 0.0601126 | 7/15/2014 | 19:20.0 | 2 |
| -86.01 | 19301.698 | 1.815 | 14.756 | 0.0594347 | 7/15/2014 | 19:20.1 | 2 |
| -85.771 | 19301.682 | 1.815 | 14.756 | 0.0610906 | 7/15/2014 | 19:20.2 | 2 |

| | | | | | | | |
|---|---|---|---|---|---|---|---|
| -85.531 | 19301.665 | 1.815 | 14.756 | 0.0691772 | 7/15/2014 | 19:20.3 | 2 |
| -85.291 | 19301.646 | 1.818 | 14.76 | 0.0639468 | 7/15/2014 | 19:20.4 | 2 |
| -85.053 | 19301.63 | 1.818 | 14.762 | 0.063802 | 7/15/2014 | 19:20.5 | 2 |
| -84.816 | 19301.612 | 1.823 | 14.764 | 0.0667703 | 7/15/2014 | 19:20.6 | 2 |
| -84.522 | 19301.592 | 1.829 | 14.764 | 0.0729031 | 7/15/2014 | 19:20.7 | 2 |
| -84.403 | 19301.583 | 1.829 | 14.765 | 0.072252 | 7/15/2014 | 19:20.8 | 2 |
| -84.166 | 19301.566 | 1.832 | 14.765 | 0.074946 | 7/15/2014 | 19:20.9 | 2 |
| -83.929 | 19301.549 | 1.829 | 14.766 | 0.0815122 | 7/15/2014 | 19:21.0 | 2 |
| -83.691 | 19301.531 | 1.832 | 14.767 | 0.0959254 | 7/15/2014 | 19:21.1 | 2 |
| -83.455 | 19301.516 | 1.825 | 14.767 | 0.0906453 | 7/15/2014 | 19:21.2 | 2 |
| -83.216 | 19301.498 | 1.828 | 14.765 | 0.0854921 | 7/15/2014 | 19:21.3 | 2 |
| -82.795 | 19301.469 | 1.826 | 14.764 | 0.0769898 | 7/15/2014 | 19:21.4 | 2 |
| -82.555 | 19301.451 | 1.826 | 14.769 | 0.0846917 | 7/15/2014 | 19:21.5 | 2 |
| -82.311 | 19301.435 | 1.83 | 14.767 | 0.0882805 | 7/15/2014 | 19:21.6 | 2 |
| -82.067 | 19301.416 | 1.83 | 14.767 | 0.101588 | 7/15/2014 | 19:21.7 | 2 |
| -81.822 | 19301.4 | 1.83 | 14.767 | 0.0954733 | 7/15/2014 | 19:21.8 | 2 |
| -81.576 | 19301.382 | 1.828 | 14.764 | 0.100313 | 7/15/2014 | 19:21.9 | 2 |
| -81.33 | 19301.365 | 1.828 | 14.768 | 0.0943667 | 7/15/2014 | 19:22.0 | 2 |
| -81.082 | 19301.349 | 1.824 | 14.771 | 0.0855041 | 7/15/2014 | 19:22.1 | 2 |
| -80.838 | 19301.331 | 1.821 | 14.771 | 0.0652 | 7/15/2014 | 19:22.2 | 2 |
| -80.594 | 19301.314 | 1.818 | 14.775 | 0.0628983 | 7/15/2014 | 19:22.3 | 2 |
| -80.352 | 19301.297 | 1.812 | 14.789 | 0.0578842 | 7/15/2014 | 19:22.4 | 2 |
| -80.112 | 19301.281 | 1.81 | 14.796 | 0.0561934 | 7/15/2014 | 19:22.5 | 2 |
| -79.873 | 19301.264 | 1.807 | 14.81 | 0.0672565 | 7/15/2014 | 19:22.6 | 2 |
| -79.578 | 19301.244 | 1.809 | 14.819 | 0.0543025 | 7/15/2014 | 19:22.7 | 2 |
| -79.456 | 19301.236 | 1.806 | 14.825 | 0.0615807 | 7/15/2014 | 19:22.8 | 2 |
| -79.219 | 19301.22 | 1.803 | 14.838 | 0.0662493 | 7/15/2014 | 19:22.9 | 2 |
| -78.985 | 19301.203 | 1.797 | 14.849 | 0.0857205 | 7/15/2014 | 19:23.0 | 2 |
| -78.751 | 19301.188 | 1.797 | 14.859 | 0.0761603 | 7/15/2014 | 19:23.1 | 2 |
| -78.517 | 19301.172 | 1.797 | 14.865 | 0.0836352 | 7/15/2014 | 19:23.2 | 2 |
| -78.283 | 19301.155 | 1.797 | 14.877 | 0.068905 | 7/15/2014 | 19:23.3 | 2 |
| -77.87 | 19301.128 | 1.799 | 14.883 | 0.0445806 | 7/15/2014 | 19:23.4 | 2 |
| -77.635 | 19301.112 | 1.802 | 14.89 | 0.0392771 | 7/15/2014 | 19:23.5 | 2 |
| -77.398 | 19301.097 | 1.804 | 14.892 | 0.0356423 | 7/15/2014 | 19:23.6 | 2 |
| -77.161 | 19301.08 | 1.801 | 14.904 | 0.0376314 | 7/15/2014 | 19:23.7 | 2 |
| -76.92 | 19301.064 | 1.799 | 14.911 | 0.0488695 | 7/15/2014 | 19:23.8 | 2 |
| -76.68 | 19301.048 | 1.797 | 14.911 | 0.0638405 | 7/15/2014 | 19:23.9 | 2 |
| -76.439 | 19301.031 | 1.802 | 14.913 | 0.0678353 | 7/15/2014 | 19:24.0 | 2 |
| -76.196 | 19301.016 | 1.804 | 14.917 | 0.0452228 | 7/15/2014 | 19:24.1 | 2 |
| -75.956 | 19301 | 1.801 | 14.918 | 0.0493431 | 7/15/2014 | 19:24.2 | 2 |
| -75.715 | 19300.984 | 1.801 | 14.922 | 0.0480338 | 7/15/2014 | 19:24.3 | 2 |
| -75.476 | 19300.968 | 1.801 | 14.927 | 0.0556889 | 7/15/2014 | 19:24.4 | 2 |
| -75.236 | 19300.952 | 1.801 | 14.925 | 0.0580774 | 7/15/2014 | 19:24.5 | 2 |
| -74.997 | 19300.937 | 1.803 | 14.925 | 0.0629591 | 7/15/2014 | 19:24.6 | 2 |
| -74.7 | 19300.917 | 1.803 | 14.928 | 0.0726584 | 7/15/2014 | 19:24.7 | 2 |
| -74.579 | 19300.909 | 1.8 | 14.929 | 0.0581447 | 7/15/2014 | 19:24.8 | 2 |
| -74.338 | 19300.893 | 1.809 | 14.932 | 0.0659846 | 7/15/2014 | 19:24.9 | 2 |

| | | | | | | | |
|---|---|---|---|---|---|---|---|
| -74.1 | 19300.877 | 1.809 | 14.939 | 0.060023 | 7/15/2014 | 19:25.0 | 2 |
| -73.86 | 19300.861 | 1.809 | 14.941 | 0.0627208 | 7/15/2014 | 19:25.1 | 2 |
| -73.622 | 19300.846 | 1.809 | 14.943 | 0.0576076 | 7/15/2014 | 19:25.2 | 2 |
| -73.38 | 19300.829 | 1.818 | 14.946 | 0.0630274 | 7/15/2014 | 19:25.3 | 2 |
| -72.955 | 19300.803 | 1.826 | 14.947 | 0.0601897 | 7/15/2014 | 19:25.4 | 2 |
| -72.711 | 19300.786 | 1.831 | 14.949 | 0.0489209 | 7/15/2014 | 19:25.5 | 2 |
| -72.466 | 19300.771 | 1.826 | 14.95 | 0.0405501 | 7/15/2014 | 19:25.6 | 2 |
| -72.221 | 19300.754 | 1.829 | 14.946 | 0.0484607 | 7/15/2014 | 19:25.7 | 2 |
| -71.977 | 19300.739 | 1.825 | 14.948 | 0.0540156 | 7/15/2014 | 19:25.8 | 2 |
| -71.73 | 19300.722 | 1.829 | 14.948 | 0.058577 | 7/15/2014 | 19:25.9 | 2 |
| -71.484 | 19300.707 | 1.832 | 14.947 | 0.0639603 | 7/15/2014 | 19:26.0 | 2 |
| -71.238 | 19300.691 | 1.834 | 14.951 | 0.0776412 | 7/15/2014 | 19:26.1 | 2 |
| -70.994 | 19300.676 | 1.836 | 14.954 | 0.0713307 | 7/15/2014 | 19:26.2 | 2 |
| -70.752 | 19300.66 | 1.836 | 14.957 | 0.0700275 | 7/15/2014 | 19:26.3 | 2 |
| -70.513 | 19300.645 | 1.833 | 14.962 | 0.0676928 | 7/15/2014 | 19:26.4 | 2 |
| -70.274 | 19300.631 | 1.833 | 14.969 | 0.0680021 | 7/15/2014 | 19:26.5 | 2 |
| -70.036 | 19300.614 | 1.839 | 14.973 | 0.0677961 | 7/15/2014 | 19:26.6 | 2 |
| -69.742 | 19300.597 | 1.834 | 14.98 | 0.0583459 | 7/15/2014 | 19:26.7 | 2 |
| -69.623 | 19300.589 | 1.831 | 14.986 | 0.0559191 | 7/15/2014 | 19:26.8 | 2 |
| -69.386 | 19300.574 | 1.832 | 14.989 | 0.0615118 | 7/15/2014 | 19:26.9 | 2 |
| -69.153 | 19300.559 | 1.823 | 14.996 | 0.0603073 | 7/15/2014 | 19:27.0 | 2 |
| -68.917 | 19300.545 | 1.829 | 15.003 | 0.0640141 | 7/15/2014 | 19:27.1 | 2 |
| -68.684 | 19300.531 | 1.832 | 15.008 | 0.075315 | 7/15/2014 | 19:27.2 | 2 |
| -68.446 | 19300.516 | 1.836 | 15.01 | 0.0717522 | 7/15/2014 | 19:27.3 | 2 |
| -68.031 | 19300.489 | 1.832 | 15.019 | 0.0607162 | 7/15/2014 | 19:27.4 | 2 |
| -67.793 | 19300.476 | 1.839 | 15.023 | 0.0555511 | 7/15/2014 | 19:27.5 | 2 |
| -67.553 | 19300.46 | 1.839 | 15.03 | 0.0515217 | 7/15/2014 | 19:27.6 | 2 |
| -67.312 | 19300.445 | 1.839 | 15.032 | 0.053368 | 7/15/2014 | 19:27.7 | 2 |
| -67.069 | 19300.431 | 1.834 | 15.033 | 0.0448711 | 7/15/2014 | 19:27.8 | 2 |
| -66.826 | 19300.416 | 1.831 | 15.034 | 0.039379 | 7/15/2014 | 19:27.9 | 2 |
| -66.583 | 19300.4 | 1.828 | 15.036 | 0.0409479 | 7/15/2014 | 19:28.0 | 2 |
| -66.339 | 19300.386 | 1.828 | 15.036 | 0.0480436 | 7/15/2014 | 19:28.1 | 2 |
| -66.096 | 19300.371 | 1.825 | 15.034 | 0.0762486 | 7/15/2014 | 19:28.2 | 2 |
| -65.854 | 19300.356 | 1.819 | 15.038 | 0.0518357 | 7/15/2014 | 19:28.3 | 2 |
| -65.614 | 19300.343 | 1.816 | 15.032 | 0.0541314 | 7/15/2014 | 19:28.4 | 2 |
| -65.375 | 19300.327 | 1.819 | 15.029 | 0.0632122 | 7/15/2014 | 19:28.5 | 2 |
| -65.137 | 19300.314 | 1.822 | 15.028 | 0.0662201 | 7/15/2014 | 19:28.6 | 2 |
| -64.844 | 19300.296 | 1.826 | 15.029 | 0.0653141 | 7/15/2014 | 19:28.7 | 2 |
| -64.724 | 19300.289 | 1.825 | 15.03 | 0.076017 | 7/15/2014 | 19:28.8 | 2 |
| -64.486 | 19300.274 | 1.835 | 15.027 | 0.0639208 | 7/15/2014 | 19:28.9 | 2 |
| -64.252 | 19300.262 | 1.838 | 15.027 | 0.0759415 | 7/15/2014 | 19:29.0 | 2 |
| -64.016 | 19300.248 | 1.846 | 15.021 | 0.0647546 | 7/15/2014 | 19:29.1 | 2 |
| -63.783 | 19300.234 | 1.845 | 15.021 | 0.0600915 | 7/15/2014 | 19:29.2 | 2 |
| -63.547 | 19300.221 | 1.842 | 15.021 | 0.0588283 | 7/15/2014 | 19:29.3 | 2 |
| -63.132 | 19300.197 | 1.834 | 15.023 | 0.0513544 | 7/15/2014 | 19:29.4 | 2 |
| -62.894 | 19300.184 | 1.829 | 15.021 | 0.0507146 | 7/15/2014 | 19:29.5 | 2 |
| -62.653 | 19300.17 | 1.821 | 15.019 | 0.0538675 | 7/15/2014 | 19:29.6 | 2 |

| | | | | | | | |
|---|---|---|---|---|---|---|---|
| -62.413 | 19300.156 | 1.815 | 15.023 | 0.0587914 | 7/15/2014 | 19:29.7 | 2 |
| -62.17 | 19300.143 | 1.815 | 15.021 | 0.0648316 | 7/15/2014 | 19:29.8 | 2 |
| -61.928 | 19300.129 | 1.817 | 15.02 | 0.0695329 | 7/15/2014 | 19:29.9 | 2 |
| -61.682 | 19300.116 | 1.823 | 15.02 | 0.0692637 | 7/15/2014 | 19:30.0 | 2 |
| -61.437 | 19300.103 | 1.823 | 15.018 | 0.0738734 | 7/15/2014 | 19:30.1 | 2 |
| -61.193 | 19300.089 | 1.829 | 15.019 | 0.0725614 | 7/15/2014 | 19:30.2 | 2 |
| -60.949 | 19300.074 | 1.829 | 15.024 | 0.0685834 | 7/15/2014 | 19:30.3 | 2 |
| -60.706 | 19300.063 | 1.831 | 15.029 | 0.0669077 | 7/15/2014 | 19:30.4 | 2 |
| -60.464 | 19300.049 | 1.831 | 15.031 | 0.0688886 | 7/15/2014 | 19:30.5 | 2 |
| -60.223 | 19300.035 | 1.828 | 15.034 | 0.0617186 | 7/15/2014 | 19:30.6 | 2 |
| -59.925 | 19300.02 | 1.82 | 15.036 | 0.0684373 | 7/15/2014 | 19:30.7 | 2 |
| -59.803 | 19300.013 | 1.816 | 15.039 | 0.0711383 | 7/15/2014 | 19:30.8 | 2 |
| -59.562 | 19299.999 | 1.819 | 15.039 | 0.077918 | 7/15/2014 | 19:30.9 | 2 |
| -59.324 | 19299.986 | 1.817 | 15.038 | 0.0910712 | 7/15/2014 | 19:31.0 | 2 |
| -59.085 | 19299.975 | 1.823 | 15.034 | 0.0776076 | 7/15/2014 | 19:31.1 | 2 |
| -58.85 | 19299.963 | 1.819 | 15.028 | 0.0804514 | 7/15/2014 | 19:31.2 | 2 |
| -58.611 | 19299.947 | 1.829 | 15.025 | 0.0893447 | 7/15/2014 | 19:31.3 | 2 |
| -58.2 | 19299.927 | 1.829 | 15.027 | 0.0830742 | 7/15/2014 | 19:31.4 | 2 |
| -57.966 | 19299.914 | 1.829 | 15.028 | 0.0947544 | 7/15/2014 | 19:31.5 | 2 |
| -57.674 | 19299.899 | 1.834 | 15.028 | 0.0884868 | 7/15/2014 | 19:31.6 | 2 |
| -57.555 | 19299.894 | 1.831 | 15.029 | 0.10545 | 7/15/2014 | 19:31.7 | 2 |
| -57.322 | 19299.881 | 1.835 | 15.031 | 0.112337 | 7/15/2014 | 19:31.8 | 2 |
| -57.087 | 19299.867 | 1.842 | 15.034 | 0.0854468 | 7/15/2014 | 19:31.9 | 2 |
| -56.854 | 19299.856 | 1.833 | 15.046 | 0.118834 | 7/15/2014 | 19:32.0 | 2 |
| -56.619 | 19299.845 | 1.837 | 15.05 | 0.103183 | 7/15/2014 | 19:32.1 | 2 |
| -56.388 | 19299.833 | 1.833 | 15.056 | 0.0854595 | 7/15/2014 | 19:32.2 | 2 |
| -55.983 | 19299.813 | 1.833 | 15.065 | 0.0985602 | 7/15/2014 | 19:32.3 | 2 |
| -55.754 | 19299.801 | 1.828 | 15.075 | 0.0987104 | 7/15/2014 | 19:32.4 | 2 |
| -55.526 | 19299.789 | 1.822 | 15.082 | 0.120831 | 7/15/2014 | 19:32.5 | 2 |
| -55.296 | 19299.778 | 1.818 | 15.091 | 0.132609 | 7/15/2014 | 19:32.6 | 2 |
| -55.01 | 19299.765 | 1.817 | 15.089 | 0.115111 | 7/15/2014 | 19:32.7 | 2 |
| -54.778 | 19299.753 | 1.82 | 15.096 | 0.118602 | 7/15/2014 | 19:32.8 | 2 |
| -54.546 | 19299.742 | 1.82 | 15.099 | 0.119533 | 7/15/2014 | 19:32.9 | 2 |
| -54.312 | 19299.73 | 1.826 | 15.102 | 0.116699 | 7/15/2014 | 19:33.0 | 2 |
| -54.198 | 19299.725 | 1.828 | 15.104 | 0.119433 | 7/15/2014 | 19:33.1 | 2 |
| -53.965 | 19299.713 | 1.831 | 15.103 | 0.103614 | 7/15/2014 | 19:33.2 | 2 |
| -53.729 | 19299.701 | 1.841 | 15.104 | 0.104421 | 7/15/2014 | 19:33.3 | 2 |
| -53.313 | 19299.682 | 1.846 | 15.097 | 0.108189 | 7/15/2014 | 19:33.4 | 2 |
| -53.073 | 19299.67 | 1.846 | 15.095 | 0.103499 | 7/15/2014 | 19:33.5 | 2 |
| -52.83 | 19299.658 | 1.846 | 15.091 | 0.111686 | 7/15/2014 | 19:33.6 | 2 |
| -52.585 | 19299.646 | 1.851 | 15.089 | 0.107322 | 7/15/2014 | 19:33.7 | 2 |
| -52.339 | 19299.635 | 1.853 | 15.086 | 0.123763 | 7/15/2014 | 19:33.8 | 2 |
| -52.091 | 19299.623 | 1.855 | 15.084 | 0.107682 | 7/15/2014 | 19:33.9 | 2 |
| -51.969 | 19299.617 | 1.853 | 15.082 | 0.106761 | 7/15/2014 | 19:34.0 | 2 |
| -51.719 | 19299.605 | 1.853 | 15.08 | 0.113837 | 7/15/2014 | 19:34.1 | 2 |
| -51.47 | 19299.594 | 1.853 | 15.077 | 0.111778 | 7/15/2014 | 19:34.2 | 2 |
| -51.218 | 19299.582 | 1.865 | 15.073 | 0.112499 | 7/15/2014 | 19:34.3 | 2 |

| | | | | | | | |
|---|---|---|---|---|---|---|---|
| -50.97 | 19299.57 | 1.868 | 15.072 | 0.106581 | 7/15/2014 | 19:34.4 | 2 |
| -50.601 | 19299.555 | 1.868 | 15.068 | 0.0953576 | 7/15/2014 | 19:34.5 | 2 |
| -50.357 | 19299.543 | 1.868 | 15.067 | 0.0843475 | 7/15/2014 | 19:34.6 | 2 |
| -50.054 | 19299.529 | 1.866 | 15.07 | 0.0824502 | 7/15/2014 | 19:34.7 | 2 |
| -49.929 | 19299.523 | 1.868 | 15.07 | 0.079495 | 7/15/2014 | 19:34.8 | 2 |
| -49.686 | 19299.512 | 1.868 | 15.071 | 0.0758995 | 7/15/2014 | 19:34.9 | 2 |
| -49.445 | 19299.502 | 1.863 | 15.073 | 0.0825946 | 7/15/2014 | 19:35.0 | 2 |
| -49.204 | 19299.491 | 1.86 | 15.076 | 0.0919394 | 7/15/2014 | 19:35.1 | 2 |
| -48.964 | 19299.48 | 1.858 | 15.085 | 0.102595 | 7/15/2014 | 19:35.2 | 2 |
| -48.725 | 19299.469 | 1.855 | 15.089 | 0.109238 | 7/15/2014 | 19:35.3 | 2 |
| -48.304 | 19299.451 | 1.852 | 15.092 | 0.100211 | 7/15/2014 | 19:35.4 | 2 |
| -48.064 | 19299.439 | 1.852 | 15.09 | 0.111269 | 7/15/2014 | 19:35.5 | 2 |
| -47.822 | 19299.432 | 1.852 | 15.091 | 0.0985372 | 7/15/2014 | 19:35.6 | 2 |
| -47.58 | 19299.42 | 1.854 | 15.091 | 0.0985618 | 7/15/2014 | 19:35.7 | 2 |
| -47.336 | 19299.408 | 1.856 | 15.09 | 0.0975961 | 7/15/2014 | 19:35.8 | 2 |
| -47.092 | 19299.398 | 1.853 | 15.087 | 0.0961924 | 7/15/2014 | 19:35.9 | 2 |
| -46.847 | 19299.389 | 1.853 | 15.086 | 0.096851 | 7/15/2014 | 19:36.0 | 2 |
| -46.602 | 19299.378 | 1.856 | 15.085 | 0.0927798 | 7/15/2014 | 19:36.1 | 2 |
| -46.358 | 19299.367 | 1.858 | 15.084 | 0.109566 | 7/15/2014 | 19:36.2 | 2 |
| -46.115 | 19299.357 | 1.858 | 15.084 | 0.0955083 | 7/15/2014 | 19:36.3 | 2 |
| -45.873 | 19299.348 | 1.86 | 15.087 | 0.0962171 | 7/15/2014 | 19:36.4 | 2 |
| -45.632 | 19299.336 | 1.86 | 15.091 | 0.10116 | 7/15/2014 | 19:36.5 | 2 |
| -45.393 | 19299.328 | 1.858 | 15.096 | 0.0856879 | 7/15/2014 | 19:36.6 | 2 |
| -45.095 | 19299.314 | 1.864 | 15.098 | 0.0958873 | 7/15/2014 | 19:36.7 | 2 |
| -44.973 | 19299.31 | 1.865 | 15.099 | 0.093396 | 7/15/2014 | 19:36.8 | 2 |
| -44.733 | 19299.301 | 1.87 | 15.103 | 0.083139 | 7/15/2014 | 19:36.9 | 2 |
| -44.497 | 19299.29 | 1.868 | 15.113 | 0.0936814 | 7/15/2014 | 19:37.0 | 2 |
| -44.259 | 19299.281 | 1.868 | 15.117 | 0.0944617 | 7/15/2014 | 19:37.1 | 2 |
| -44.022 | 19299.271 | 1.865 | 15.125 | 0.0746719 | 7/15/2014 | 19:37.2 | 2 |
| -43.785 | 19299.262 | 1.865 | 15.129 | 0.0817782 | 7/15/2014 | 19:37.3 | 2 |
| -43.371 | 19299.245 | 1.87 | 15.134 | 0.0846091 | 7/15/2014 | 19:37.4 | 2 |
| -43.135 | 19299.236 | 1.87 | 15.138 | 0.0822046 | 7/15/2014 | 19:37.5 | 2 |
| -42.898 | 19299.228 | 1.873 | 15.143 | 0.076215 | 7/15/2014 | 19:37.6 | 2 |
| -42.603 | 19299.215 | 1.868 | 15.148 | 0.0691358 | 7/15/2014 | 19:37.7 | 2 |
| -42.483 | 19299.211 | 1.87 | 15.154 | 0.0824604 | 7/15/2014 | 19:37.8 | 2 |
| -42.243 | 19299.201 | 1.873 | 15.158 | 0.0724682 | 7/15/2014 | 19:37.9 | 2 |
| -42.004 | 19299.193 | 1.87 | 15.166 | 0.0630228 | 7/15/2014 | 19:38.0 | 2 |
| -41.763 | 19299.183 | 1.868 | 15.177 | 0.0539796 | 7/15/2014 | 19:38.1 | 2 |
| -41.523 | 19299.174 | 1.865 | 15.197 | 0.0535349 | 7/15/2014 | 19:38.2 | 2 |
| -41.282 | 19299.166 | 1.865 | 15.216 | 0.054434 | 7/15/2014 | 19:38.3 | 2 |
| -40.864 | 19299.148 | 1.858 | 15.241 | 0.0857712 | 7/15/2014 | 19:38.4 | 2 |
| -40.626 | 19299.141 | 1.861 | 15.25 | 0.0798834 | 7/15/2014 | 19:38.5 | 2 |
| -40.389 | 19299.131 | 1.861 | 15.264 | 0.0964018 | 7/15/2014 | 19:38.6 | 2 |
| -40.093 | 19299.119 | 1.864 | 15.273 | 0.0910835 | 7/15/2014 | 19:38.7 | 2 |
| -39.972 | 19299.115 | 1.865 | 15.276 | 0.0943542 | 7/15/2014 | 19:38.8 | 2 |
| -39.733 | 19299.107 | 1.868 | 15.278 | 0.0988318 | 7/15/2014 | 19:38.9 | 2 |
| -39.497 | 19299.097 | 1.868 | 15.279 | 0.0880645 | 7/15/2014 | 19:39.0 | 2 |

| | | | | | | | |
|---|---|---|---|---|---|---|---|
| -39.261 | 19299.088 | 1.87 | 15.283 | 0.0790987 | 7/15/2014 | 19:39.1 | 2 |
| -39.026 | 19299.08 | 1.868 | 15.285 | 0.0754982 | 7/15/2014 | 19:39.2 | 2 |
| -38.791 | 19299.07 | 1.872 | 15.295 | 0.0730328 | 7/15/2014 | 19:39.3 | 2 |
| -38.385 | 19299.057 | 1.874 | 15.313 | 0.103318 | 7/15/2014 | 19:39.4 | 2 |
| -38.154 | 19299.047 | 1.874 | 15.329 | 0.106707 | 7/15/2014 | 19:39.5 | 2 |
| -37.924 | 19299.038 | 1.874 | 15.342 | 0.121308 | 7/15/2014 | 19:39.6 | 2 |
| -37.638 | 19299.027 | 1.874 | 15.36 | 0.0904723 | 7/15/2014 | 19:39.7 | 2 |
| -37.405 | 19299.02 | 1.876 | 15.374 | 0.0886474 | 7/15/2014 | 19:39.8 | 2 |
| -37.173 | 19299.012 | 1.876 | 15.384 | 0.08869 | 7/15/2014 | 19:39.9 | 2 |
| -36.942 | 19299.004 | 1.876 | 15.393 | 0.0700033 | 7/15/2014 | 19:40.0 | 2 |
| -36.71 | 19298.995 | 1.88 | 15.407 | 0.0684227 | 7/15/2014 | 19:40.1 | 2 |
| -36.479 | 19298.985 | 1.88 | 15.407 | 0.0743287 | 7/15/2014 | 19:40.2 | 2 |
| -36.248 | 19298.977 | 1.882 | 15.409 | 0.0673578 | 7/15/2014 | 19:40.3 | 2 |
| -35.962 | 19298.967 | 1.885 | 15.41 | 0.0748777 | 7/15/2014 | 19:40.4 | 2 |
| -35.732 | 19298.959 | 1.887 | 15.412 | 0.0817851 | 7/15/2014 | 19:40.5 | 2 |
| -35.503 | 19298.951 | 1.887 | 15.414 | 0.0766005 | 7/15/2014 | 19:40.6 | 2 |
| -35.215 | 19298.941 | 1.889 | 15.415 | 0.0878933 | 7/15/2014 | 19:40.7 | 2 |
| -34.984 | 19298.933 | 1.892 | 15.416 | 0.0890848 | 7/15/2014 | 19:40.8 | 2 |
| -34.75 | 19298.925 | 1.889 | 15.411 | 0.0774475 | 7/15/2014 | 19:40.9 | 2 |
| -34.517 | 19298.914 | 1.892 | 15.412 | 0.0841911 | 7/15/2014 | 19:41.0 | 2 |
| -34.401 | 19298.91 | 1.892 | 15.412 | 0.0836075 | 7/15/2014 | 19:41.1 | 2 |
| -34.168 | 19298.902 | 1.889 | 15.422 | 0.0782076 | 7/15/2014 | 19:41.2 | 2 |
| -33.936 | 19298.895 | 1.892 | 15.423 | 0.0881035 | 7/15/2014 | 19:41.3 | 2 |
| -33.53 | 19298.881 | 1.889 | 15.419 | 0.0808271 | 7/15/2014 | 19:41.4 | 2 |
| -33.297 | 19298.871 | 1.891 | 15.417 | 0.0836967 | 7/15/2014 | 19:41.5 | 2 |
| -33.064 | 19298.863 | 1.891 | 15.417 | 0.0866918 | 7/15/2014 | 19:41.6 | 2 |
| -32.774 | 19298.852 | 1.897 | 15.412 | 0.0920098 | 7/15/2014 | 19:41.7 | 2 |
| -32.656 | 19298.848 | 1.895 | 15.413 | 0.102493 | 7/15/2014 | 19:41.8 | 2 |
| -32.421 | 19298.84 | 1.898 | 15.397 | 0.0902383 | 7/15/2014 | 19:41.9 | 2 |
| -32.186 | 19298.83 | 1.895 | 15.385 | 0.102137 | 7/15/2014 | 19:42.0 | 2 |
| -31.95 | 19298.822 | 1.901 | 15.372 | 0.0968864 | 7/15/2014 | 19:42.1 | 2 |
| -31.716 | 19298.813 | 1.904 | 15.365 | 0.11081 | 7/15/2014 | 19:42.2 | 2 |
| -31.48 | 19298.803 | 1.908 | 15.359 | 0.106949 | 7/15/2014 | 19:42.3 | 2 |
| -31.072 | 19298.789 | 1.908 | 15.341 | 0.099582 | 7/15/2014 | 19:42.4 | 2 |
| -30.838 | 19298.779 | 1.908 | 15.334 | 0.103782 | 7/15/2014 | 19:42.5 | 2 |
| -30.603 | 19298.77 | 1.912 | 15.328 | 0.112391 | 7/15/2014 | 19:42.6 | 2 |
| -30.312 | 19298.759 | 1.917 | 15.321 | 0.106515 | 7/15/2014 | 19:42.7 | 2 |
| -30.194 | 19298.755 | 1.918 | 15.319 | 0.101189 | 7/15/2014 | 19:42.8 | 2 |
| -29.957 | 19298.744 | 1.924 | 15.313 | 0.115449 | 7/15/2014 | 19:42.9 | 2 |
| -29.723 | 19298.736 | 1.924 | 15.305 | 0.106058 | 7/15/2014 | 19:43.0 | 2 |
| -29.487 | 19298.727 | 1.924 | 15.303 | 0.126029 | 7/15/2014 | 19:43.1 | 2 |
| -29.251 | 19298.717 | 1.924 | 15.301 | 0.128601 | 7/15/2014 | 19:43.2 | 2 |
| -29.014 | 19298.707 | 1.924 | 15.298 | 0.115718 | 7/15/2014 | 19:43.3 | 2 |
| -28.601 | 19298.691 | 1.922 | 15.296 | 0.126396 | 7/15/2014 | 19:43.4 | 2 |
| -28.366 | 19298.682 | 1.92 | 15.293 | 0.132028 | 7/15/2014 | 19:43.5 | 2 |
| -28.13 | 19298.673 | 1.92 | 15.291 | 0.135185 | 7/15/2014 | 19:43.6 | 2 |
| -27.833 | 19298.66 | 1.928 | 15.291 | 0.110395 | 7/15/2014 | 19:43.7 | 2 |

| | | | | | | | |
|---|---|---|---|---|---|---|---|
| -27.714 | 19298.656 | 1.93 | 15.289 | 0.116011 | 7/15/2014 | 19:43.8 | 2 |
| -27.475 | 19298.645 | 1.936 | 15.289 | 0.134024 | 7/15/2014 | 19:43.9 | 2 |
| -27.236 | 19298.635 | 1.938 | 15.292 | 0.128131 | 7/15/2014 | 19:44.0 | 2 |
| -26.993 | 19298.626 | 1.947 | 15.295 | 0.124974 | 7/15/2014 | 19:44.1 | 2 |
| -26.753 | 19298.615 | 1.949 | 15.298 | 0.106023 | 7/15/2014 | 19:44.2 | 2 |
| -26.511 | 19298.604 | 1.958 | 15.304 | 0.115825 | 7/15/2014 | 19:44.3 | 2 |
| -26.091 | 19298.588 | 1.956 | 15.316 | 0.118387 | 7/15/2014 | 19:44.4 | 2 |
| -25.852 | 19298.576 | 1.954 | 15.323 | 0.109661 | 7/15/2014 | 19:44.5 | 2 |
| -25.614 | 19298.564 | 1.954 | 15.329 | 0.108367 | 7/15/2014 | 19:44.6 | 2 |
| -25.314 | 19298.553 | 1.955 | 15.333 | 0.108952 | 7/15/2014 | 19:44.7 | 2 |
| -25.192 | 19298.547 | 1.953 | 15.335 | 0.1158 | 7/15/2014 | 19:44.8 | 2 |
| -24.951 | 19298.535 | 1.953 | 15.339 | 0.111026 | 7/15/2014 | 19:44.9 | 2 |
| -24.713 | 19298.526 | 1.951 | 15.342 | 0.116694 | 7/15/2014 | 19:45.0 | 2 |
| -24.471 | 19298.516 | 1.954 | 15.342 | 0.112112 | 7/15/2014 | 19:45.1 | 2 |
| -24.231 | 19298.504 | 1.95 | 15.343 | 0.107422 | 7/15/2014 | 19:45.2 | 2 |
| -23.993 | 19298.492 | 1.95 | 15.345 | 0.107914 | 7/15/2014 | 19:45.3 | 2 |
| -23.577 | 19298.473 | 1.952 | 15.346 | 0.108955 | 7/15/2014 | 19:45.4 | 2 |
| -23.342 | 19298.463 | 1.954 | 15.35 | 0.110763 | 7/15/2014 | 19:45.5 | 2 |
| -23.107 | 19298.453 | 1.954 | 15.352 | 0.10351 | 7/15/2014 | 19:45.6 | 2 |
| -22.814 | 19298.438 | 1.951 | 15.355 | 0.100432 | 7/15/2014 | 19:45.7 | 2 |
| -22.694 | 19298.432 | 1.951 | 15.358 | 0.103812 | 7/15/2014 | 19:45.8 | 2 |
| -22.457 | 19298.421 | 1.953 | 15.361 | 0.0982119 | 7/15/2014 | 19:45.9 | 2 |
| -22.221 | 19298.409 | 1.953 | 15.366 | 0.0942471 | 7/15/2014 | 19:46.0 | 2 |
| -21.984 | 19298.398 | 1.953 | 15.37 | 0.0905907 | 7/15/2014 | 19:46.1 | 2 |
| -21.75 | 19298.389 | 1.951 | 15.375 | 0.0830891 | 7/15/2014 | 19:46.2 | 2 |
| -21.516 | 19298.377 | 1.953 | 15.377 | 0.0758922 | 7/15/2014 | 19:46.3 | 2 |
| -21.111 | 19298.355 | 1.951 | 15.384 | 0.0729023 | 7/15/2014 | 19:46.4 | 2 |
| -20.877 | 19298.345 | 1.954 | 15.389 | 0.0781608 | 7/15/2014 | 19:46.5 | 2 |
| -20.647 | 19298.333 | 1.952 | 15.394 | 0.0724066 | 7/15/2014 | 19:46.6 | 2 |
| -20.358 | 19298.319 | 1.95 | 15.4 | 0.0792699 | 7/15/2014 | 19:46.7 | 2 |
| -20.24 | 19298.313 | 1.95 | 15.402 | 0.076536 | 7/15/2014 | 19:46.8 | 2 |
| -20.007 | 19298.302 | 1.952 | 15.407 | 0.0773792 | 7/15/2014 | 19:46.9 | 2 |
| -19.775 | 19298.29 | 1.947 | 15.412 | 0.0817046 | 7/15/2014 | 19:47.0 | 2 |
| -19.542 | 19298.277 | 1.947 | 15.418 | 0.084648 | 7/15/2014 | 19:47.1 | 2 |
| -19.308 | 19298.266 | 1.945 | 15.425 | 0.0767033 | 7/15/2014 | 19:47.2 | 2 |
| -19.076 | 19298.254 | 1.939 | 15.428 | 0.0783856 | 7/15/2014 | 19:47.3 | 2 |
| -18.67 | 19298.233 | 1.939 | 15.436 | 0.0797906 | 7/15/2014 | 19:47.4 | 2 |
| -18.438 | 19298.222 | 1.935 | 15.439 | 0.0941458 | 7/15/2014 | 19:47.5 | 2 |
| -18.207 | 19298.21 | 1.933 | 15.444 | 0.0709994 | 7/15/2014 | 19:47.6 | 2 |
| -17.918 | 19298.193 | 1.933 | 15.447 | 0.07745 | 7/15/2014 | 19:47.7 | 2 |
| -17.8 | 19298.188 | 1.933 | 15.449 | 0.0887955 | 7/15/2014 | 19:47.8 | 2 |
| -17.567 | 19298.176 | 1.933 | 15.451 | 0.0868467 | 7/15/2014 | 19:47.9 | 2 |
| -17.333 | 19298.164 | 1.936 | 15.456 | 0.076003 | 7/15/2014 | 19:48.0 | 2 |
| -17.1 | 19298.152 | 1.936 | 15.454 | 0.0863619 | 7/15/2014 | 19:48.1 | 2 |
| -16.867 | 19298.141 | 1.936 | 15.455 | 0.0774464 | 7/15/2014 | 19:48.2 | 2 |
| -16.635 | 19298.128 | 1.94 | 15.46 | 0.0860192 | 7/15/2014 | 19:48.3 | 2 |
| -16.23 | 19298.105 | 1.934 | 15.465 | 0.0610807 | 7/15/2014 | 19:48.4 | 2 |

| | | | | | | | |
|---|---|---|---|---|---|---|---|
| -16 | 19298.094 | 1.934 | 15.468 | 0.0597466 | 7/15/2014 | 19:48.5 | 2 |
| -15.77 | 19298.082 | 1.936 | 15.47 | 0.0544935 | 7/15/2014 | 19:48.6 | 2 |
| -15.482 | 19298.066 | 1.936 | 15.472 | 0.052892 | 7/15/2014 | 19:48.7 | 2 |
| -15.366 | 19298.061 | 1.936 | 15.476 | 0.0512547 | 7/15/2014 | 19:48.8 | 2 |
| -15.018 | 19298.041 | 1.933 | 15.479 | 0.0424481 | 7/15/2014 | 19:48.9 | 2 |
| -14.785 | 19298.028 | 1.93 | 15.478 | 0.0418699 | 7/15/2014 | 19:49.0 | 2 |
| -14.553 | 19298.015 | 1.93 | 15.48 | 0.0486827 | 7/15/2014 | 19:49.1 | 2 |
| -14.438 | 19298.009 | 1.929 | 15.481 | 0.0590278 | 7/15/2014 | 19:49.2 | 2 |
| -14.206 | 19297.996 | 1.929 | 15.483 | 0.0610418 | 7/15/2014 | 19:49.3 | 2 |
| -13.801 | 19297.973 | 1.928 | 15.483 | 0.0549293 | 7/15/2014 | 19:49.4 | 2 |
| -13.57 | 19297.96 | 1.924 | 15.483 | 0.0967846 | 7/15/2014 | 19:49.5 | 2 |
| -13.34 | 19297.948 | 1.921 | 15.483 | 0.0779179 | 7/15/2014 | 19:49.6 | 2 |
| -13.051 | 19297.931 | 1.918 | 15.483 | 0.101112 | 7/15/2014 | 19:49.7 | 2 |
| -12.934 | 19297.924 | 1.919 | 15.483 | 0.110414 | 7/15/2014 | 19:49.8 | 2 |
| -12.699 | 19297.91 | 1.925 | 15.482 | 0.0969623 | 7/15/2014 | 19:49.9 | 2 |
| -12.467 | 19297.897 | 1.915 | 15.482 | 0.109567 | 7/15/2014 | 19:50.0 | 2 |
| -12.231 | 19297.883 | 1.911 | 15.484 | 0.144961 | 7/15/2014 | 19:50.1 | 2 |
| -11.998 | 19297.87 | 1.903 | 15.486 | 0.112234 | 7/15/2014 | 19:50.2 | 2 |
| -11.764 | 19297.857 | 1.903 | 15.488 | 0.151185 | 7/15/2014 | 19:50.3 | 2 |
| -11.358 | 19297.832 | 1.901 | 15.493 | 0.1138 | 7/15/2014 | 19:50.4 | 2 |
| -11.127 | 19297.818 | 1.901 | 15.494 | 0.138453 | 7/15/2014 | 19:50.5 | 2 |
| -10.895 | 19297.805 | 1.899 | 15.498 | 0.145357 | 7/15/2014 | 19:50.6 | 2 |
| -10.607 | 19297.787 | 1.901 | 15.501 | 0.115368 | 7/15/2014 | 19:50.7 | 2 |
| -10.49 | 19297.78 | 1.9 | 15.502 | 0.115253 | 7/15/2014 | 19:50.8 | 2 |
| -10.143 | 19297.761 | 1.91 | 15.506 | 0.113574 | 7/15/2014 | 19:50.9 | 2 |
| -9.912 | 19297.745 | 1.913 | 15.51 | 0.121917 | 7/15/2014 | 19:51.0 | 2 |
| -9.68 | 19297.731 | 1.913 | 15.516 | 0.119378 | 7/15/2014 | 19:51.1 | 2 |
| -9.45 | 19297.717 | 1.913 | 15.519 | 0.114668 | 7/15/2014 | 19:51.2 | 2 |
| -9.222 | 19297.703 | 1.913 | 15.525 | 0.105669 | 7/15/2014 | 19:51.3 | 2 |
| -8.939 | 19297.686 | 1.91 | 15.53 | 0.119866 | 7/15/2014 | 19:51.4 | 2 |
| -8.712 | 19297.672 | 1.91 | 15.534 | 0.087057 | 7/15/2014 | 19:51.5 | 2 |
| -8.487 | 19297.658 | 1.904 | 15.538 | 0.114375 | 7/15/2014 | 19:51.6 | 2 |
| -8.205 | 19297.64 | 1.901 | 15.544 | 0.100898 | 7/15/2014 | 19:51.7 | 2 |
| -7.978 | 19297.626 | 1.901 | 15.547 | 0.0983881 | 7/15/2014 | 19:51.8 | 2 |
| -7.75 | 19297.61 | 1.908 | 15.548 | 0.0942884 | 7/15/2014 | 19:51.9 | 2 |
| -7.524 | 19297.597 | 1.911 | 15.55 | 0.105144 | 7/15/2014 | 19:52.0 | 2 |
| -7.297 | 19297.582 | 1.911 | 15.55 | 0.102946 | 7/15/2014 | 19:52.1 | 2 |
| -7.071 | 19297.566 | 1.914 | 15.551 | 0.121715 | 7/15/2014 | 19:52.2 | 2 |
| -6.846 | 19297.552 | 1.914 | 15.553 | 0.118545 | 7/15/2014 | 19:52.3 | 2 |
| -6.566 | 19297.533 | 1.917 | 15.552 | 0.12476 | 7/15/2014 | 19:52.4 | 2 |
| -6.342 | 19297.52 | 1.917 | 15.55 | 0.148255 | 7/15/2014 | 19:52.5 | 2 |
| -6.117 | 19297.504 | 1.917 | 15.55 | 0.166541 | 7/15/2014 | 19:52.6 | 2 |
| -5.839 | 19297.486 | 1.914 | 15.552 | 0.154655 | 7/15/2014 | 19:52.7 | 2 |
| -5.614 | 19297.47 | 1.914 | 15.55 | 0.159245 | 7/15/2014 | 19:52.8 | 2 |
| -5.388 | 19297.455 | 1.914 | 15.549 | 0.173036 | 7/15/2014 | 19:52.9 | 2 |
| -5.162 | 19297.439 | 1.911 | 15.548 | 0.163499 | 7/15/2014 | 19:53.0 | 2 |
| -4.935 | 19297.423 | 1.914 | 15.545 | 0.171263 | 7/15/2014 | 19:53.1 | 2 |

| | | | | | | | |
|---|---|---|---|---|---|---|---|
| -4.71 | 19297.407 | 1.914 | 15.542 | 0.180742 | 7/15/2014 | 19:53.2 | 2 |
| -4.486 | 19297.392 | 1.914 | 15.541 | 0.169754 | 7/15/2014 | 19:53.3 | 2 |
| -4.206 | 19297.373 | 1.911 | 15.539 | 0.196537 | 7/15/2014 | 19:53.4 | 2 |
| -3.981 | 19297.356 | 1.905 | 15.531 | 0.203782 | 7/15/2014 | 19:53.5 | 2 |
| -3.756 | 19297.341 | 1.905 | 15.527 | 0.2053 | 7/15/2014 | 19:53.6 | 2 |
| -3.472 | 19297.32 | 1.905 | 15.522 | 0.208801 | 7/15/2014 | 19:53.7 | 2 |
| -3.243 | 19297.304 | 1.905 | 15.516 | 0.218457 | 7/15/2014 | 19:53.8 | 2 |
| -3.013 | 19297.285 | 1.902 | 15.513 | 0.217756 | 7/15/2014 | 19:53.9 | 2 |
| -2.784 | 19297.27 | 1.899 | 15.509 | 0.233564 | 7/15/2014 | 19:54.0 | 2 |
| -2.55 | 19297.253 | 1.902 | 15.502 | 0.223799 | 7/15/2014 | 19:54.1 | 2 |
| -2.438 | 19297.245 | 1.901 | 15.498 | 0.222321 | 7/15/2014 | 19:54.2 | 2 |
| -2.206 | 19297.228 | 1.913 | 15.494 | 0.219425 | 7/15/2014 | 19:54.3 | 2 |
| -1.802 | 19297.197 | 1.913 | 15.487 | 0.231481 | 7/15/2014 | 19:54.4 | 2 |
| -1.571 | 19297.181 | 1.919 | 15.481 | 0.211853 | 7/15/2014 | 19:54.5 | 2 |
| -1.342 | 19297.162 | 1.925 | 15.478 | 0.222399 | 7/15/2014 | 19:54.6 | 2 |
| -1.055 | 19297.142 | 1.931 | 15.473 | 0.214471 | 7/15/2014 | 19:54.7 | 2 |
| -0.936 | 19297.133 | 1.934 | 15.47 | 0.218738 | 7/15/2014 | 19:54.8 | 2 |
| -0.593 | 19297.106 | 1.936 | 15.463 | 0.229206 | 7/15/2014 | 19:54.9 | 2 |
| -0.361 | 19297.089 | 1.934 | 15.46 | 0.234018 | 7/15/2014 | 19:55.0 | 2 |
| -0.132 | 19297.071 | 1.931 | 15.457 | 0.230437 | 7/15/2014 | 19:55.1 | 2 |
| 0.097 | 19297.054 | 1.928 | 15.456 | 0.247248 | 7/15/2014 | 19:55.2 | 2 |
| 0.325 | 19297.037 | 1.921 | 15.454 | 0.237871 | 7/15/2014 | 19:55.3 | 2 |
| 0.609 | 19297.015 | 1.919 | 15.451 | 0.239871 | 7/15/2014 | 19:55.4 | 2 |
| 0.835 | 19296.997 | 1.916 | 15.457 | 0.232215 | 7/15/2014 | 19:55.5 | 2 |
| 1.061 | 19296.979 | 1.916 | 15.458 | 0.242001 | 7/15/2014 | 19:55.6 | 2 |
| 1.347 | 19296.957 | 1.911 | 15.467 | 0.232461 | 7/15/2014 | 19:55.7 | 2 |
| 1.576 | 19296.938 | 1.911 | 15.47 | 0.233993 | 7/15/2014 | 19:55.8 | 2 |
| 1.807 | 19296.92 | 1.911 | 15.478 | 0.233959 | 7/15/2014 | 19:55.9 | 2 |
| 2.04 | 19296.902 | 1.911 | 15.484 | 0.236237 | 7/15/2014 | 19:56.0 | 2 |
| 2.155 | 19296.893 | 1.914 | 15.491 | 0.236621 | 7/15/2014 | 19:56.1 | 2 |
| 2.385 | 19296.874 | 1.907 | 15.499 | 0.243076 | 7/15/2014 | 19:56.2 | 2 |
| 2.62 | 19296.855 | 1.907 | 15.501 | 0.250195 | 7/15/2014 | 19:56.3 | 2 |
| 3.025 | 19296.822 | 1.896 | 15.507 | 0.248561 | 7/15/2014 | 19:56.4 | 2 |
| 3.259 | 19296.805 | 1.896 | 15.506 | 0.248207 | 7/15/2014 | 19:56.5 | 2 |
| 3.49 | 19296.785 | 1.889 | 15.515 | 0.245805 | 7/15/2014 | 19:56.6 | 2 |
| 3.781 | 19296.76 | 1.889 | 15.513 | 0.242731 | 7/15/2014 | 19:56.7 | 2 |
| 3.898 | 19296.75 | 1.886 | 15.515 | 0.240753 | 7/15/2014 | 19:56.8 | 2 |
| 4.133 | 19296.73 | 1.886 | 15.515 | 0.232554 | 7/15/2014 | 19:56.9 | 2 |
| 4.364 | 19296.711 | 1.883 | 15.518 | 0.22672 | 7/15/2014 | 19:57.0 | 2 |
| 4.711 | 19296.682 | 1.881 | 15.523 | 0.222945 | 7/15/2014 | 19:57.1 | 2 |
| 4.942 | 19296.664 | 1.881 | 15.529 | 0.217071 | 7/15/2014 | 19:57.2 | 2 |
| 5.171 | 19296.645 | 1.879 | 15.539 | 0.220703 | 7/15/2014 | 19:57.3 | 2 |
| 5.457 | 19296.619 | 1.879 | 15.54 | 0.211793 | 7/15/2014 | 19:57.4 | 2 |
| 5.684 | 19296.6 | 1.879 | 15.547 | 0.207181 | 7/15/2014 | 19:57.5 | 2 |
| 5.912 | 19296.58 | 1.877 | 15.554 | 0.198832 | 7/15/2014 | 19:57.6 | 2 |
| 6.196 | 19296.555 | 1.874 | 15.562 | 0.190566 | 7/15/2014 | 19:57.7 | 2 |
| 6.425 | 19296.535 | 1.877 | 15.573 | 0.182497 | 7/15/2014 | 19:57.8 | 2 |

| | | | | | | | |
|---|---|---|---|---|---|---|---|
| 6.655 | 19296.516 | 1.882 | 15.581 | 0.180245 | 7/15/2014 | 19:57.9 | 2 |
| 6.886 | 19296.495 | 1.885 | 15.588 | 0.170858 | 7/15/2014 | 19:58.0 | 2 |
| 7.117 | 19296.475 | 1.885 | 15.588 | 0.166419 | 7/15/2014 | 19:58.1 | 2 |
| 7.348 | 19296.455 | 1.883 | 15.597 | 0.164612 | 7/15/2014 | 19:58.2 | 2 |
| 7.461 | 19296.445 | 1.882 | 15.602 | 0.162624 | 7/15/2014 | 19:58.3 | 2 |
| 7.863 | 19296.408 | 1.88 | 15.604 | 0.163422 | 7/15/2014 | 19:58.4 | 2 |
| 8.093 | 19296.389 | 1.882 | 15.604 | 0.159527 | 7/15/2014 | 19:58.5 | 2 |
| 8.323 | 19296.367 | 1.882 | 15.603 | 0.156599 | 7/15/2014 | 19:58.6 | 2 |
| 8.61 | 19296.341 | 1.877 | 15.605 | 0.15665 | 7/15/2014 | 19:58.7 | 2 |
| 8.727 | 19296.33 | 1.872 | 15.605 | 0.158645 | 7/15/2014 | 19:58.8 | 2 |
| 9.073 | 19296.299 | 1.876 | 15.603 | 0.160665 | 7/15/2014 | 19:58.9 | 2 |
| 9.304 | 19296.276 | 1.878 | 15.598 | 0.159664 | 7/15/2014 | 19:59.0 | 2 |
| 9.535 | 19296.256 | 1.88 | 15.596 | 0.154647 | 7/15/2014 | 19:59.1 | 2 |
| 9.767 | 19296.235 | 1.878 | 15.593 | 0.165037 | 7/15/2014 | 19:59.2 | 2 |
| 9.881 | 19296.224 | 1.877 | 15.593 | 0.17963 | 7/15/2014 | 19:59.3 | 2 |
| 10.282 | 19296.187 | 1.881 | 15.589 | 0.168392 | 7/15/2014 | 19:59.4 | 2 |
| 10.51 | 19296.165 | 1.883 | 15.584 | 0.175856 | 7/15/2014 | 19:59.5 | 2 |
| 10.738 | 19296.143 | 1.883 | 15.586 | 0.17995 | 7/15/2014 | 19:59.6 | 2 |
| 11.023 | 19296.115 | 1.881 | 15.583 | 0.172892 | 7/15/2014 | 19:59.7 | 2 |
| 11.251 | 19296.094 | 1.881 | 15.584 | 0.181967 | 7/15/2014 | 19:59.8 | 2 |
| 11.482 | 19296.071 | 1.881 | 15.582 | 0.158151 | 7/15/2014 | 19:59.9 | 2 |
| 11.711 | 19296.05 | 1.881 | 15.579 | 0.175909 | 7/15/2014 | 20:00.0 | 2 |
| 11.94 | 19296.027 | 1.879 | 15.575 | 0.1718 | 7/15/2014 | 20:00.1 | 2 |
| 12.17 | 19296.004 | 1.876 | 15.572 | 0.165894 | 7/15/2014 | 20:00.2 | 2 |
| 12.397 | 19295.981 | 1.877 | 15.566 | 0.136257 | 7/15/2014 | 20:00.3 | 2 |
| 12.681 | 19295.954 | 1.877 | 15.561 | 0.134626 | 7/15/2014 | 20:00.4 | 2 |
| 12.908 | 19295.932 | 1.879 | 15.561 | 0.125403 | 7/15/2014 | 20:00.5 | 2 |
| 13.135 | 19295.908 | 1.879 | 15.555 | 0.137553 | 7/15/2014 | 20:00.6 | 2 |
| 13.42 | 19295.88 | 1.879 | 15.553 | 0.102042 | 7/15/2014 | 20:00.7 | 2 |
| 13.648 | 19295.856 | 1.881 | 15.551 | 0.0996917 | 7/15/2014 | 20:00.8 | 2 |
| 13.88 | 19295.833 | 1.881 | 15.551 | 0.102508 | 7/15/2014 | 20:00.9 | 2 |
| 14.11 | 19295.81 | 1.884 | 15.548 | 0.11882 | 7/15/2014 | 20:01.0 | 2 |
| 14.342 | 19295.787 | 1.881 | 15.547 | 0.133976 | 7/15/2014 | 20:01.1 | 2 |
| 14.456 | 19295.775 | 1.88 | 15.546 | 0.130195 | 7/15/2014 | 20:01.2 | 2 |
| 14.686 | 19295.751 | 1.878 | 15.536 | 0.116069 | 7/15/2014 | 20:01.3 | 2 |
| 15.09 | 19295.709 | 1.877 | 15.531 | 0.155043 | 7/15/2014 | 20:01.4 | 2 |
| 15.319 | 19295.686 | 1.882 | 15.526 | 0.14145 | 7/15/2014 | 20:01.5 | 2 |
| 15.55 | 19295.661 | 1.882 | 15.521 | 0.12566 | 7/15/2014 | 20:01.6 | 2 |
| 15.837 | 19295.631 | 1.884 | 15.515 | 0.0963663 | 7/15/2014 | 20:01.7 | 2 |
| 15.954 | 19295.619 | 1.88 | 15.513 | 0.0761809 | 7/15/2014 | 20:01.8 | 2 |
| 16.186 | 19295.595 | 1.883 | 15.512 | 0.0825364 | 7/15/2014 | 20:01.9 | 2 |
| 16.533 | 19295.557 | 1.883 | 15.509 | 0.0548848 | 7/15/2014 | 20:02.0 | 2 |
| 16.765 | 19295.532 | 1.883 | 15.511 | 0.0611654 | 7/15/2014 | 20:02.1 | 2 |
| 16.995 | 19295.508 | 1.883 | 15.51 | 0.0605771 | 7/15/2014 | 20:02.2 | 2 |
| 17.11 | 19295.495 | 1.879 | 15.51 | 0.0619765 | 7/15/2014 | 20:02.3 | 2 |
| 17.51 | 19295.451 | 1.878 | 15.506 | 0.0938128 | 7/15/2014 | 20:02.4 | 2 |
| 17.738 | 19295.428 | 1.878 | 15.506 | 0.0892157 | 7/15/2014 | 20:02.5 | 2 |

| | | | | | | | |
|---|---|---|---|---|---|---|---|
| 17.966 | 19295.401 | 1.875 | 15.502 | 0.114692 | 7/15/2014 | 20:02.6 | 2 |
| 18.249 | 19295.369 | 1.873 | 15.5 | 0.115468 | 7/15/2014 | 20:02.7 | 2 |
| 18.479 | 19295.345 | 1.873 | 15.498 | 0.107367 | 7/15/2014 | 20:02.8 | 2 |
| 18.708 | 19295.319 | 1.871 | 15.493 | 0.125884 | 7/15/2014 | 20:02.9 | 2 |
| 18.939 | 19295.294 | 1.871 | 15.489 | 0.109743 | 7/15/2014 | 20:03.0 | 2 |
| 19.167 | 19295.268 | 1.869 | 15.487 | 0.09312 | 7/15/2014 | 20:03.1 | 2 |
| 19.397 | 19295.244 | 1.869 | 15.487 | 0.0965424 | 7/15/2014 | 20:03.2 | 2 |
| 19.623 | 19295.217 | 1.862 | 15.485 | 0.0854674 | 7/15/2014 | 20:03.3 | 2 |
| 19.905 | 19295.184 | 1.862 | 15.484 | 0.0772622 | 7/15/2014 | 20:03.4 | 2 |
| 20.132 | 19295.16 | 1.859 | 15.484 | 0.0812423 | 7/15/2014 | 20:03.5 | 2 |
| 20.357 | 19295.134 | 1.857 | 15.485 | 0.072552 | 7/15/2014 | 20:03.6 | 2 |
| 20.638 | 19295.102 | 1.859 | 15.484 | 0.080373 | 7/15/2014 | 20:03.7 | 2 |
| 20.863 | 19295.076 | 1.859 | 15.485 | 0.0845004 | 7/15/2014 | 20:03.8 | 2 |
| 21.089 | 19295.052 | 1.861 | 15.485 | 0.0906032 | 7/15/2014 | 20:03.9 | 2 |
| 21.314 | 19295.024 | 1.861 | 15.484 | 0.0975166 | 7/15/2014 | 20:04.0 | 2 |
| 21.54 | 19294.998 | 1.866 | 15.484 | 0.0949282 | 7/15/2014 | 20:04.1 | 2 |
| 21.764 | 19294.974 | 1.866 | 15.482 | 0.098381 | 7/15/2014 | 20:04.2 | 2 |
| 21.987 | 19294.947 | 1.869 | 15.482 | 0.100229 | 7/15/2014 | 20:04.3 | 2 |
| 22.264 | 19294.914 | 1.867 | 15.482 | 0.0956398 | 7/15/2014 | 20:04.4 | 2 |
| 22.485 | 19294.89 | 1.865 | 15.48 | 0.111279 | 7/15/2014 | 20:04.5 | 2 |
| 22.706 | 19294.864 | 1.865 | 15.48 | 0.0916928 | 7/15/2014 | 20:04.6 | 2 |
| 22.983 | 19294.831 | 1.863 | 15.478 | 0.0934244 | 7/15/2014 | 20:04.7 | 2 |
| 23.207 | 19294.805 | 1.861 | 15.474 | 0.0999321 | 7/15/2014 | 20:04.8 | 2 |
| 23.431 | 19294.778 | 1.859 | 15.47 | 0.0941699 | 7/15/2014 | 20:04.9 | 2 |
| 23.657 | 19294.752 | 1.864 | 15.47 | 0.0950741 | 7/15/2014 | 20:05.0 | 2 |
| 23.882 | 19294.725 | 1.866 | 15.465 | 0.0979922 | 7/15/2014 | 20:05.1 | 2 |
| 24.108 | 19294.698 | 1.873 | 15.458 | 0.104451 | 7/15/2014 | 20:05.2 | 2 |
| 24.333 | 19294.672 | 1.878 | 15.455 | 0.108013 | 7/15/2014 | 20:05.3 | 2 |
| 24.613 | 19294.639 | 1.881 | 15.447 | 0.112683 | 7/15/2014 | 20:05.4 | 2 |
| 24.839 | 19294.611 | 1.885 | 15.447 | 0.113854 | 7/15/2014 | 20:05.5 | 2 |
| 25.065 | 19294.584 | 1.887 | 15.446 | 0.109408 | 7/15/2014 | 20:05.6 | 2 |
| 25.349 | 19294.55 | 1.895 | 15.444 | 0.115014 | 7/15/2014 | 20:05.7 | 2 |
| 25.578 | 19294.521 | 1.891 | 15.438 | 0.121767 | 7/15/2014 | 20:05.8 | 2 |
| 25.809 | 19294.494 | 1.894 | 15.438 | 0.104726 | 7/15/2014 | 20:05.9 | 2 |
| 26.04 | 19294.467 | 1.894 | 15.44 | 0.108594 | 7/15/2014 | 20:06.0 | 2 |
| 26.27 | 19294.438 | 1.894 | 15.441 | 0.095197 | 7/15/2014 | 20:06.1 | 2 |
| 26.383 | 19294.425 | 1.891 | 15.442 | 0.0927261 | 7/15/2014 | 20:06.2 | 2 |
| 26.614 | 19294.397 | 1.895 | 15.444 | 0.0984339 | 7/15/2014 | 20:06.3 | 2 |
| 27.014 | 19294.349 | 1.903 | 15.447 | 0.103515 | 7/15/2014 | 20:06.4 | 2 |
| 27.24 | 19294.319 | 1.903 | 15.449 | 0.105744 | 7/15/2014 | 20:06.5 | 2 |
| 27.469 | 19294.292 | 1.91 | 15.453 | 0.112908 | 7/15/2014 | 20:06.6 | 2 |
| 27.752 | 19294.258 | 1.913 | 15.457 | 0.116239 | 7/15/2014 | 20:06.7 | 2 |
| 27.98 | 19294.229 | 1.916 | 15.456 | 0.12029 | 7/15/2014 | 20:06.8 | 2 |
| 28.208 | 19294.202 | 1.916 | 15.456 | 0.126187 | 7/15/2014 | 20:06.9 | 2 |
| 28.434 | 19294.174 | 1.916 | 15.451 | 0.132475 | 7/15/2014 | 20:07.0 | 2 |
| 28.663 | 19294.146 | 1.916 | 15.451 | 0.131869 | 7/15/2014 | 20:07.1 | 2 |
| 28.888 | 19294.117 | 1.916 | 15.449 | 0.143325 | 7/15/2014 | 20:07.2 | 2 |

| | | | | | | | |
|---|---|---|---|---|---|---|---|
| 29.113 | 19294.09 | 1.912 | 15.448 | 0.136361 | 7/15/2014 | 20:07.3 | 2 |
| 29.392 | 19294.055 | 1.912 | 15.446 | 0.135026 | 7/15/2014 | 20:07.4 | 2 |
| 29.615 | 19294.027 | 1.909 | 15.442 | 0.127663 | 7/15/2014 | 20:07.5 | 2 |
| 29.838 | 19294 | 1.909 | 15.442 | 0.124078 | 7/15/2014 | 20:07.6 | 2 |
| 30.115 | 19293.966 | 1.912 | 15.444 | 0.119919 | 7/15/2014 | 20:07.7 | 2 |
| 30.341 | 19293.938 | 1.917 | 15.446 | 0.114618 | 7/15/2014 | 20:07.8 | 2 |
| 30.567 | 19293.909 | 1.923 | 15.446 | 0.110049 | 7/15/2014 | 20:07.9 | 2 |
| 30.792 | 19293.882 | 1.925 | 15.445 | 0.108778 | 7/15/2014 | 20:08.0 | 2 |
| 31.018 | 19293.854 | 1.925 | 15.45 | 0.10589 | 7/15/2014 | 20:08.1 | 2 |
| 31.242 | 19293.825 | 1.922 | 15.455 | 0.106024 | 7/15/2014 | 20:08.2 | 2 |
| 31.468 | 19293.798 | 1.922 | 15.46 | 0.105269 | 7/15/2014 | 20:08.3 | 2 |
| 31.748 | 19293.763 | 1.918 | 15.467 | 0.101731 | 7/15/2014 | 20:08.4 | 2 |
| 31.973 | 19293.735 | 1.915 | 15.474 | 0.101413 | 7/15/2014 | 20:08.5 | 2 |
| 32.197 | 19293.707 | 1.907 | 15.479 | 0.115092 | 7/15/2014 | 20:08.6 | 2 |
| 32.479 | 19293.672 | 1.898 | 15.484 | 0.105867 | 7/15/2014 | 20:08.7 | 2 |
| 32.706 | 19293.645 | 1.896 | 15.492 | 0.101714 | 7/15/2014 | 20:08.8 | 2 |
| 32.932 | 19293.616 | 1.896 | 15.497 | 0.112183 | 7/15/2014 | 20:08.9 | 2 |
| 33.161 | 19293.588 | 1.899 | 15.501 | 0.106772 | 7/15/2014 | 20:09.0 | 2 |
| 33.389 | 19293.559 | 1.897 | 15.507 | 0.107597 | 7/15/2014 | 20:09.1 | 2 |
| 33.615 | 19293.531 | 1.894 | 15.511 | 0.10383 | 7/15/2014 | 20:09.2 | 2 |
| 33.842 | 19293.503 | 1.892 | 15.513 | 0.106227 | 7/15/2014 | 20:09.3 | 2 |
| 34.124 | 19293.467 | 1.895 | 15.519 | 0.107711 | 7/15/2014 | 20:09.4 | 2 |
| 34.35 | 19293.439 | 1.898 | 15.529 | 0.108622 | 7/15/2014 | 20:09.5 | 2 |
| 34.575 | 19293.412 | 1.895 | 15.536 | 0.0992951 | 7/15/2014 | 20:09.6 | 2 |
| 34.856 | 19293.377 | 1.898 | 15.542 | 0.1084 | 7/15/2014 | 20:09.7 | 2 |
| 35.083 | 19293.348 | 1.898 | 15.546 | 0.106688 | 7/15/2014 | 20:09.8 | 2 |
| 35.309 | 19293.32 | 1.903 | 15.551 | 0.108515 | 7/15/2014 | 20:09.9 | 2 |
| 35.537 | 19293.292 | 1.91 | 15.558 | 0.0886402 | 7/15/2014 | 20:10.0 | 2 |
| 35.764 | 19293.264 | 1.912 | 15.562 | 0.0919927 | 7/15/2014 | 20:10.1 | 2 |
| 35.99 | 19293.235 | 1.915 | 15.564 | 0.0920711 | 7/15/2014 | 20:10.2 | 2 |
| 36.216 | 19293.208 | 1.912 | 15.567 | 0.0964499 | 7/15/2014 | 20:10.3 | 2 |
| 36.495 | 19293.173 | 1.917 | 15.571 | 0.0819251 | 7/15/2014 | 20:10.4 | 2 |
| 36.719 | 19293.145 | 1.917 | 15.575 | 0.0856433 | 7/15/2014 | 20:10.5 | 2 |
| 36.944 | 19293.117 | 1.917 | 15.578 | 0.0834567 | 7/15/2014 | 20:10.6 | 2 |
| 37.224 | 19293.083 | 1.914 | 15.584 | 0.0799906 | 7/15/2014 | 20:10.7 | 2 |
| 37.449 | 19293.055 | 1.908 | 15.584 | 0.0817431 | 7/15/2014 | 20:10.8 | 2 |
| 37.678 | 19293.026 | 1.911 | 15.589 | 0.0847401 | 7/15/2014 | 20:10.9 | 2 |
| 37.905 | 19292.998 | 1.911 | 15.591 | 0.0902754 | 7/15/2014 | 20:11.0 | 2 |
| 38.133 | 19292.97 | 1.911 | 15.592 | 0.088778 | 7/15/2014 | 20:11.1 | 2 |
| 38.36 | 19292.941 | 1.911 | 15.591 | 0.0948939 | 7/15/2014 | 20:11.2 | 2 |
| 38.587 | 19292.914 | 1.908 | 15.59 | 0.0937933 | 7/15/2014 | 20:11.3 | 2 |
| 38.868 | 19292.879 | 1.904 | 15.592 | 0.0926465 | 7/15/2014 | 20:11.4 | 2 |
| 39.094 | 19292.851 | 1.908 | 15.589 | 0.0886469 | 7/15/2014 | 20:11.5 | 2 |
| 39.323 | 19292.822 | 1.914 | 15.591 | 0.0856441 | 7/15/2014 | 20:11.6 | 2 |
| 39.609 | 19292.786 | 1.911 | 15.589 | 0.0842862 | 7/15/2014 | 20:11.7 | 2 |
| 39.723 | 19292.772 | 1.907 | 15.589 | 0.0785198 | 7/15/2014 | 20:11.8 | 2 |
| 39.956 | 19292.743 | 1.915 | 15.591 | 0.0822131 | 7/15/2014 | 20:11.9 | 2 |

| | | | | | | | |
|---|---|---|---|---|---|---|---|
| 40.185 | 19292.716 | 1.909 | 15.597 | 0.085844 | 7/15/2014 | 20:12.0 | 2 |
| 40.418 | 19292.687 | 1.916 | 15.601 | 0.0889093 | 7/15/2014 | 20:12.1 | 2 |
| 40.649 | 19292.657 | 1.916 | 15.603 | 0.0878901 | 7/15/2014 | 20:12.2 | 2 |
| 40.882 | 19292.628 | 1.916 | 15.606 | 0.0961125 | 7/15/2014 | 20:12.3 | 2 |
| 41.29 | 19292.577 | 1.915 | 15.611 | 0.0961184 | 7/15/2014 | 20:12.4 | 2 |
| 41.523 | 19292.548 | 1.918 | 15.616 | 0.101374 | 7/15/2014 | 20:12.5 | 2 |
| 41.756 | 19292.519 | 1.92 | 15.615 | 0.0968056 | 7/15/2014 | 20:12.6 | 2 |
| 42.05 | 19292.482 | 1.92 | 15.613 | 0.0934334 | 7/15/2014 | 20:12.7 | 2 |
| 42.166 | 19292.467 | 1.918 | 15.614 | 0.097118 | 7/15/2014 | 20:12.8 | 2 |
| 42.403 | 19292.437 | 1.921 | 15.615 | 0.0956578 | 7/15/2014 | 20:12.9 | 2 |
| 42.641 | 19292.407 | 1.921 | 15.616 | 0.0938561 | 7/15/2014 | 20:13.0 | 2 |
| 42.882 | 19292.378 | 1.925 | 15.615 | 0.0963729 | 7/15/2014 | 20:13.1 | 2 |
| 43.118 | 19292.348 | 1.921 | 15.611 | 0.0998051 | 7/15/2014 | 20:13.2 | 2 |
| 43.358 | 19292.316 | 1.923 | 15.611 | 0.0986895 | 7/15/2014 | 20:13.3 | 2 |
| 43.775 | 19292.265 | 1.918 | 15.611 | 0.104572 | 7/15/2014 | 20:13.4 | 2 |
| 44.014 | 19292.234 | 1.92 | 15.61 | 0.111241 | 7/15/2014 | 20:13.5 | 2 |
| 44.254 | 19292.204 | 1.924 | 15.608 | 0.110404 | 7/15/2014 | 20:13.6 | 2 |
| 44.554 | 19292.167 | 1.929 | 15.604 | 0.133736 | 7/15/2014 | 20:13.7 | 2 |
| 44.674 | 19292.151 | 1.928 | 15.603 | 0.124197 | 7/15/2014 | 20:13.8 | 2 |
| 44.915 | 19292.121 | 1.934 | 15.599 | 0.135782 | 7/15/2014 | 20:13.9 | 2 |
| 45.155 | 19292.09 | 1.932 | 15.594 | 0.168327 | 7/15/2014 | 20:14.0 | 2 |
| 45.398 | 19292.06 | 1.94 | 15.587 | 0.176726 | 7/15/2014 | 20:14.1 | 2 |
| 45.638 | 19292.03 | 1.947 | 15.581 | 0.188685 | 7/15/2014 | 20:14.2 | 2 |
| 45.879 | 19291.999 | 1.95 | 15.575 | 0.182734 | 7/15/2014 | 20:14.3 | 2 |
| 46.297 | 19291.946 | 1.948 | 15.57 | 0.20496 | 7/15/2014 | 20:14.4 | 2 |
| 46.533 | 19291.916 | 1.945 | 15.57 | 0.197089 | 7/15/2014 | 20:14.5 | 2 |
| 46.771 | 19291.886 | 1.947 | 15.569 | 0.170708 | 7/15/2014 | 20:14.6 | 2 |
| 47.068 | 19291.848 | 1.953 | 15.568 | 0.17147 | 7/15/2014 | 20:14.7 | 2 |
| 47.189 | 19291.833 | 1.95 | 15.568 | 0.167345 | 7/15/2014 | 20:14.8 | 2 |
| 47.427 | 19291.803 | 1.954 | 15.569 | 0.175408 | 7/15/2014 | 20:14.9 | 2 |
| 47.664 | 19291.772 | 1.95 | 15.571 | 0.161998 | 7/15/2014 | 20:15.0 | 2 |
| 47.903 | 19291.742 | 1.953 | 15.571 | 0.124941 | 7/15/2014 | 20:15.1 | 2 |
| 48.139 | 19291.712 | 1.953 | 15.571 | 0.146284 | 7/15/2014 | 20:15.2 | 2 |
| 48.377 | 19291.683 | 1.956 | 15.569 | 0.142155 | 7/15/2014 | 20:15.3 | 2 |
| 48.79 | 19291.629 | 1.95 | 15.57 | 0.142882 | 7/15/2014 | 20:15.4 | 2 |
| 49.024 | 19291.601 | 1.947 | 15.568 | 0.112319 | 7/15/2014 | 20:15.5 | 2 |
| 49.259 | 19291.57 | 1.944 | 15.567 | 0.111365 | 7/15/2014 | 20:15.6 | 2 |
| 49.555 | 19291.531 | 1.947 | 15.569 | 0.10771 | 7/15/2014 | 20:15.7 | 2 |
| 49.674 | 19291.517 | 1.947 | 15.569 | 0.096209 | 7/15/2014 | 20:15.8 | 2 |
| 49.911 | 19291.487 | 1.949 | 15.569 | 0.0912199 | 7/15/2014 | 20:15.9 | 2 |
| 50.146 | 19291.457 | 1.945 | 15.571 | 0.0838194 | 7/15/2014 | 20:16.0 | 2 |
| 50.387 | 19291.426 | 1.951 | 15.574 | 0.050036 | 7/15/2014 | 20:16.1 | 2 |
| 50.623 | 19291.397 | 1.948 | 15.573 | 0.0753675 | 7/15/2014 | 20:16.2 | 2 |
| 50.861 | 19291.366 | 1.954 | 15.58 | 0.0694471 | 7/15/2014 | 20:16.3 | 2 |
| 51.274 | 19291.313 | 1.952 | 15.584 | 0.0826814 | 7/15/2014 | 20:16.4 | 2 |
| 51.509 | 19291.282 | 1.951 | 15.589 | 0.0597356 | 7/15/2014 | 20:16.5 | 2 |
| 51.743 | 19291.254 | 1.951 | 15.596 | 0.0823986 | 7/15/2014 | 20:16.6 | 2 |

| | | | | | | | |
|---|---|---|---|---|---|---|---|
| 52.036 | 19291.216 | 1.953 | 15.599 | 0.0854532 | 7/15/2014 | 20:16.7 | 2 |
| 52.156 | 19291.201 | 1.953 | 15.602 | 0.0711341 | 7/15/2014 | 20:16.8 | 2 |
| 52.391 | 19291.171 | 1.955 | 15.604 | 0.0710253 | 7/15/2014 | 20:16.9 | 2 |
| 52.626 | 19291.142 | 1.953 | 15.604 | 0.0501275 | 7/15/2014 | 20:17.0 | 2 |
| 52.862 | 19291.11 | 1.958 | 15.618 | 0.0508457 | 7/15/2014 | 20:17.1 | 2 |
| 53.095 | 19291.08 | 1.958 | 15.621 | 0.0700463 | 7/15/2014 | 20:17.2 | 2 |
| 53.329 | 19291.052 | 1.958 | 15.624 | 0.0429651 | 7/15/2014 | 20:17.3 | 2 |
| 53.734 | 19291 | 1.953 | 15.625 | 0.0531696 | 7/15/2014 | 20:17.4 | 2 |
| 53.965 | 19290.971 | 1.955 | 15.628 | 0.0493516 | 7/15/2014 | 20:17.5 | 2 |
| 54.196 | 19290.941 | 1.953 | 15.631 | 0.0775557 | 7/15/2014 | 20:17.6 | 2 |
| 54.485 | 19290.904 | 1.953 | 15.637 | 0.0716703 | 7/15/2014 | 20:17.7 | 2 |
| 54.601 | 19290.89 | 1.948 | 15.63 | 0.0605997 | 7/15/2014 | 20:17.8 | 2 |
| 54.833 | 19290.86 | 1.948 | 15.634 | 0.0817244 | 7/15/2014 | 20:17.9 | 2 |
| 55.065 | 19290.831 | 1.948 | 15.635 | 0.107064 | 7/15/2014 | 20:18.0 | 2 |
| 55.298 | 19290.802 | 1.953 | 15.64 | 0.116053 | 7/15/2014 | 20:18.1 | 2 |
| 55.528 | 19290.772 | 1.949 | 15.643 | 0.11969 | 7/15/2014 | 20:18.2 | 2 |
| 55.762 | 19290.743 | 1.954 | 15.646 | 0.109226 | 7/15/2014 | 20:18.3 | 2 |
| 56.164 | 19290.692 | 1.958 | 15.646 | 0.146831 | 7/15/2014 | 20:18.4 | 2 |
| 56.393 | 19290.663 | 1.958 | 15.647 | 0.143183 | 7/15/2014 | 20:18.5 | 2 |
| 56.623 | 19290.634 | 1.958 | 15.652 | 0.116203 | 7/15/2014 | 20:18.6 | 2 |
| 56.911 | 19290.598 | 1.958 | 15.653 | 0.100728 | 7/15/2014 | 20:18.7 | 2 |
| 57.025 | 19290.583 | 1.956 | 15.654 | 0.0999854 | 7/15/2014 | 20:18.8 | 2 |
| 57.26 | 19290.554 | 1.96 | 15.658 | 0.0907478 | 7/15/2014 | 20:18.9 | 2 |
| 57.49 | 19290.524 | 1.953 | 15.661 | 0.0836276 | 7/15/2014 | 20:19.0 | 2 |
| 57.723 | 19290.495 | 1.953 | 15.666 | 0.0843727 | 7/15/2014 | 20:19.1 | 2 |
| 57.954 | 19290.466 | 1.946 | 15.678 | 0.0926127 | 7/15/2014 | 20:19.2 | 2 |
| 58.186 | 19290.437 | 1.946 | 15.682 | 0.0859268 | 7/15/2014 | 20:19.3 | 2 |
| 58.591 | 19290.385 | 1.946 | 15.683 | 0.0879271 | 7/15/2014 | 20:19.4 | 2 |
| 58.823 | 19290.356 | 1.946 | 15.688 | 0.0846601 | 7/15/2014 | 20:19.5 | 2 |
| 59.054 | 19290.327 | 1.946 | 15.69 | 0.0847669 | 7/15/2014 | 20:19.6 | 2 |
| 59.342 | 19290.291 | 1.945 | 15.691 | 0.0970444 | 7/15/2014 | 20:19.7 | 2 |
| 59.459 | 19290.276 | 1.944 | 15.695 | 0.0846764 | 7/15/2014 | 20:19.8 | 2 |
| 59.694 | 19290.247 | 1.947 | 15.688 | 0.0764058 | 7/15/2014 | 20:19.9 | 2 |
| 59.928 | 19290.218 | 1.949 | 15.687 | 0.0889913 | 7/15/2014 | 20:20.0 | 2 |
| 60.161 | 19290.188 | 1.949 | 15.687 | 0.0748636 | 7/15/2014 | 20:20.1 | 2 |
| 60.394 | 19290.16 | 1.943 | 15.685 | 0.0920983 | 7/15/2014 | 20:20.2 | 2 |
| 60.628 | 19290.131 | 1.943 | 15.681 | 0.0877783 | 7/15/2014 | 20:20.3 | 2 |
| 61.032 | 19290.081 | 1.939 | 15.672 | 0.0695819 | 7/15/2014 | 20:20.4 | 2 |
| 61.265 | 19290.052 | 1.942 | 15.666 | 0.0810986 | 7/15/2014 | 20:20.5 | 2 |
| 61.496 | 19290.022 | 1.942 | 15.659 | 0.0744588 | 7/15/2014 | 20:20.6 | 2 |
| 61.784 | 19289.988 | 1.938 | 15.651 | 0.0762313 | 7/15/2014 | 20:20.7 | 2 |
| 61.9 | 19289.975 | 1.936 | 15.646 | 0.0567383 | 7/15/2014 | 20:20.8 | 2 |
| 62.133 | 19289.945 | 1.94 | 15.634 | 0.0465125 | 7/15/2014 | 20:20.9 | 2 |
| 62.366 | 19289.917 | 1.937 | 15.62 | 0.0650798 | 7/15/2014 | 20:21.0 | 2 |
| 62.598 | 19289.89 | 1.943 | 15.607 | 0.0695816 | 7/15/2014 | 20:21.1 | 2 |
| 62.83 | 19289.861 | 1.937 | 15.591 | 0.0638156 | 7/15/2014 | 20:21.2 | 2 |
| 63.061 | 19289.832 | 1.935 | 15.578 | 0.0683599 | 7/15/2014 | 20:21.3 | 2 |

| | | | | | | | |
|---|---|---|---|---|---|---|---|
| 63.464 | 19289.785 | 1.932 | 15.559 | 0.0757487 | 7/15/2014 | 20:21.4 | 2 |
| 63.694 | 19289.757 | 1.932 | 15.547 | 0.0967438 | 7/15/2014 | 20:21.5 | 2 |
| 63.923 | 19289.729 | 1.935 | 15.538 | 0.0871947 | 7/15/2014 | 20:21.6 | 2 |
| 64.21 | 19289.696 | 1.931 | 15.534 | 0.0874827 | 7/15/2014 | 20:21.7 | 2 |
| 64.326 | 19289.683 | 1.929 | 15.531 | 0.0904331 | 7/15/2014 | 20:21.8 | 2 |
| 64.557 | 19289.654 | 1.932 | 15.528 | 0.0821803 | 7/15/2014 | 20:21.9 | 2 |
| 64.904 | 19289.615 | 1.923 | 15.532 | 0.0821772 | 7/15/2014 | 20:22.0 | 2 |
| 65.134 | 19289.588 | 1.92 | 15.535 | 0.0884294 | 7/15/2014 | 20:22.1 | 2 |
| 65.366 | 19289.561 | 1.92 | 15.547 | 0.0904997 | 7/15/2014 | 20:22.2 | 2 |
| 65.48 | 19289.547 | 1.918 | 15.553 | 0.0952704 | 7/15/2014 | 20:22.3 | 2 |
| 65.88 | 19289.502 | 1.914 | 15.562 | 0.109607 | 7/15/2014 | 20:22.4 | 2 |
| 66.106 | 19289.476 | 1.911 | 15.566 | 0.108684 | 7/15/2014 | 20:22.5 | 2 |
| 66.332 | 19289.451 | 1.905 | 15.573 | 0.13292 | 7/15/2014 | 20:22.6 | 2 |
| 66.615 | 19289.419 | 1.908 | 15.579 | 0.116144 | 7/15/2014 | 20:22.7 | 2 |
| 66.841 | 19289.393 | 1.908 | 15.58 | 0.121177 | 7/15/2014 | 20:22.8 | 2 |
| 67.068 | 19289.367 | 1.905 | 15.589 | 0.116165 | 7/15/2014 | 20:22.9 | 2 |
| 67.293 | 19289.341 | 1.901 | 15.585 | 0.120187 | 7/15/2014 | 20:23.0 | 2 |
| 67.518 | 19289.317 | 1.898 | 15.587 | 0.109447 | 7/15/2014 | 20:23.1 | 2 |
| 67.741 | 19289.292 | 1.892 | 15.588 | 0.105576 | 7/15/2014 | 20:23.2 | 2 |
| 67.963 | 19289.268 | 1.892 | 15.591 | 0.100385 | 7/15/2014 | 20:23.3 | 2 |
| 68.239 | 19289.237 | 1.892 | 15.59 | 0.100806 | 7/15/2014 | 20:23.4 | 2 |
| 68.46 | 19289.213 | 1.886 | 15.591 | 0.100422 | 7/15/2014 | 20:23.5 | 2 |
| 68.677 | 19289.189 | 1.883 | 15.599 | 0.104513 | 7/15/2014 | 20:23.6 | 2 |
| 68.952 | 19289.159 | 1.881 | 15.595 | 0.103715 | 7/15/2014 | 20:23.7 | 2 |
| 69.172 | 19289.136 | 1.879 | 15.595 | 0.103937 | 7/15/2014 | 20:23.8 | 2 |
| 69.391 | 19289.111 | 1.877 | 15.595 | 0.100283 | 7/15/2014 | 20:23.9 | 2 |
| 69.614 | 19289.088 | 1.883 | 15.592 | 0.0965944 | 7/15/2014 | 20:24.0 | 2 |
| 69.833 | 19289.064 | 1.883 | 15.592 | 0.10182 | 7/15/2014 | 20:24.1 | 2 |
| 70.053 | 19289.042 | 1.883 | 15.592 | 0.100894 | 7/15/2014 | 20:24.2 | 2 |
| 70.273 | 19289.019 | 1.885 | 15.593 | 0.0930324 | 7/15/2014 | 20:24.3 | 2 |
| 70.546 | 19288.989 | 1.885 | 15.589 | 0.113345 | 7/15/2014 | 20:24.4 | 2 |
| 70.767 | 19288.967 | 1.885 | 15.591 | 0.0995156 | 7/15/2014 | 20:24.5 | 2 |
| 70.985 | 19288.943 | 1.884 | 15.594 | 0.109131 | 7/15/2014 | 20:24.6 | 2 |
| 71.263 | 19288.915 | 1.884 | 15.598 | 0.114668 | 7/15/2014 | 20:24.7 | 2 |
| 71.483 | 19288.892 | 1.88 | 15.605 | 0.11254 | 7/15/2014 | 20:24.8 | 2 |
| 71.707 | 19288.87 | 1.88 | 15.611 | 0.117494 | 7/15/2014 | 20:24.9 | 2 |
| 71.931 | 19288.847 | 1.88 | 15.618 | 0.112767 | 7/15/2014 | 20:25.0 | 2 |
| 72.155 | 19288.824 | 1.878 | 15.624 | 0.114362 | 7/15/2014 | 20:25.1 | 2 |
| 72.379 | 19288.802 | 1.878 | 15.632 | 0.111602 | 7/15/2014 | 20:25.2 | 2 |
| 72.602 | 19288.779 | 1.878 | 15.641 | 0.111463 | 7/15/2014 | 20:25.3 | 2 |
| 72.882 | 19288.751 | 1.878 | 15.649 | 0.108163 | 7/15/2014 | 20:25.4 | 2 |
| 73.106 | 19288.729 | 1.878 | 15.656 | 0.103163 | 7/15/2014 | 20:25.5 | 2 |
| 73.332 | 19288.707 | 1.878 | 15.664 | 0.101478 | 7/15/2014 | 20:25.6 | 2 |
| 73.612 | 19288.679 | 1.878 | 15.67 | 0.10484 | 7/15/2014 | 20:25.7 | 2 |
| 73.839 | 19288.657 | 1.878 | 15.678 | 0.102237 | 7/15/2014 | 20:25.8 | 2 |
| 74.067 | 19288.636 | 1.88 | 15.683 | 0.104373 | 7/15/2014 | 20:25.9 | 2 |
| 74.294 | 19288.613 | 1.88 | 15.687 | 0.103701 | 7/15/2014 | 20:26.0 | 2 |

| | | | | | | | |
|---|---|---|---|---|---|---|---|
| 74.524 | 19288.591 | 1.882 | 15.692 | 0.108293 | 7/15/2014 | 20:26.1 | 2 |
| 74.752 | 19288.569 | 1.882 | 15.7 | 0.113879 | 7/15/2014 | 20:26.2 | 2 |
| 74.98 | 19288.549 | 1.884 | 15.705 | 0.132429 | 7/15/2014 | 20:26.3 | 2 |
| 75.262 | 19288.521 | 1.881 | 15.709 | 0.120439 | 7/15/2014 | 20:26.4 | 2 |
| 75.489 | 19288.5 | 1.879 | 15.72 | 0.12827 | 7/15/2014 | 20:26.5 | 2 |
| 75.716 | 19288.48 | 1.879 | 15.725 | 0.129942 | 7/15/2014 | 20:26.6 | 2 |
| 75.999 | 19288.453 | 1.877 | 15.734 | 0.142727 | 7/15/2014 | 20:26.7 | 2 |
| 76.228 | 19288.433 | 1.877 | 15.745 | 0.139936 | 7/15/2014 | 20:26.8 | 2 |
| 76.456 | 19288.412 | 1.877 | 15.752 | 0.134573 | 7/15/2014 | 20:26.9 | 2 |
| 76.686 | 19288.392 | 1.877 | 15.76 | 0.133341 | 7/15/2014 | 20:27.0 | 2 |
| 76.915 | 19288.37 | 1.877 | 15.769 | 0.12982 | 7/15/2014 | 20:27.1 | 2 |
| 77.143 | 19288.35 | 1.877 | 15.784 | 0.133154 | 7/15/2014 | 20:27.2 | 2 |
| 77.37 | 19288.33 | 1.877 | 15.795 | 0.139013 | 7/15/2014 | 20:27.3 | 2 |
| 77.653 | 19288.305 | 1.875 | 15.806 | 0.132143 | 7/15/2014 | 20:27.4 | 2 |
| 77.878 | 19288.285 | 1.872 | 15.82 | 0.13577 | 7/15/2014 | 20:27.5 | 2 |
| 78.106 | 19288.266 | 1.872 | 15.832 | 0.156086 | 7/15/2014 | 20:27.6 | 2 |
| 78.389 | 19288.241 | 1.87 | 15.842 | 0.147791 | 7/15/2014 | 20:27.7 | 2 |
| 78.616 | 19288.222 | 1.868 | 15.857 | 0.156536 | 7/15/2014 | 20:27.8 | 2 |
| 78.844 | 19288.201 | 1.864 | 15.869 | 0.149926 | 7/15/2014 | 20:27.9 | 2 |
| 79.074 | 19288.182 | 1.862 | 15.873 | 0.164403 | 7/15/2014 | 20:28.0 | 2 |
| 79.303 | 19288.164 | 1.862 | 15.876 | 0.170027 | 7/15/2014 | 20:28.1 | 2 |
| 79.53 | 19288.145 | 1.864 | 15.885 | 0.169362 | 7/15/2014 | 20:28.2 | 2 |
| 79.758 | 19288.125 | 1.866 | 15.889 | 0.166518 | 7/15/2014 | 20:28.3 | 2 |
| 80.041 | 19288.103 | 1.864 | 15.891 | 0.168522 | 7/15/2014 | 20:28.4 | 2 |
| 80.268 | 19288.084 | 1.864 | 15.895 | 0.19929 | 7/15/2014 | 20:28.5 | 2 |
| 80.494 | 19288.064 | 1.864 | 15.896 | 0.199166 | 7/15/2014 | 20:28.6 | 2 |
| 80.779 | 19288.042 | 1.864 | 15.897 | 0.204841 | 7/15/2014 | 20:28.7 | 2 |
| 81.009 | 19288.025 | 1.864 | 15.902 | 0.19187 | 7/15/2014 | 20:28.8 | 2 |
| 81.238 | 19288.006 | 1.859 | 15.907 | 0.223994 | 7/15/2014 | 20:28.9 | 2 |
| 81.47 | 19287.988 | 1.857 | 15.905 | 0.214441 | 7/15/2014 | 20:29.0 | 2 |
| 81.701 | 19287.97 | 1.857 | 15.906 | 0.241947 | 7/15/2014 | 20:29.1 | 2 |
| 81.931 | 19287.952 | 1.854 | 15.904 | 0.204191 | 7/15/2014 | 20:29.2 | 2 |
| 82.045 | 19287.942 | 1.853 | 15.906 | 0.214214 | 7/15/2014 | 20:29.3 | 2 |
| 82.449 | 19287.912 | 1.847 | 15.904 | 0.22151 | 7/15/2014 | 20:29.4 | 2 |
| 82.679 | 19287.895 | 1.844 | 15.903 | 0.222495 | 7/15/2014 | 20:29.5 | 2 |
| 82.91 | 19287.876 | 1.841 | 15.897 | 0.209445 | 7/15/2014 | 20:29.6 | 2 |
| 83.199 | 19287.855 | 1.844 | 15.89 | 0.204694 | 7/15/2014 | 20:29.7 | 2 |
| 83.316 | 19287.846 | 1.841 | 15.89 | 0.197451 | 7/15/2014 | 20:29.8 | 2 |
| 83.549 | 19287.829 | 1.839 | 15.884 | 0.205188 | 7/15/2014 | 20:29.9 | 2 |
| 83.783 | 19287.812 | 1.839 | 15.883 | 0.202475 | 7/15/2014 | 20:30.0 | 2 |
| 84.019 | 19287.794 | 1.844 | 15.879 | 0.201731 | 7/15/2014 | 20:30.1 | 2 |
| 84.25 | 19287.777 | 1.838 | 15.876 | 0.19985 | 7/15/2014 | 20:30.2 | 2 |
| 84.484 | 19287.76 | 1.841 | 15.875 | 0.198184 | 7/15/2014 | 20:30.3 | 2 |
| 84.889 | 19287.73 | 1.837 | 15.879 | 0.199631 | 7/15/2014 | 20:30.4 | 2 |
| 85.12 | 19287.715 | 1.837 | 15.879 | 0.201448 | 7/15/2014 | 20:30.5 | 2 |
| 85.351 | 19287.699 | 1.834 | 15.886 | 0.197854 | 7/15/2014 | 20:30.6 | 2 |
| 85.638 | 19287.679 | 1.828 | 15.886 | 0.19458 | 7/15/2014 | 20:30.7 | 2 |

| | | | | | | | |
|---|---|---|---|---|---|---|---|
| 85.868 | 19287.663 | 1.826 | 15.886 | 0.199712 | 7/15/2014 | 20:30.8 | 2 |
| 86.098 | 19287.647 | 1.824 | 15.887 | 0.195028 | 7/15/2014 | 20:30.9 | 2 |
| 86.329 | 19287.632 | 1.819 | 15.887 | 0.196493 | 7/15/2014 | 20:31.0 | 2 |
| 86.558 | 19287.615 | 1.819 | 15.882 | 0.201506 | 7/15/2014 | 20:31.1 | 2 |
| 86.787 | 19287.601 | 1.819 | 15.882 | 0.191746 | 7/15/2014 | 20:31.2 | 2 |
| 87.013 | 19287.586 | 1.816 | 15.882 | 0.194602 | 7/15/2014 | 20:31.3 | 2 |
| 87.295 | 19287.567 | 1.816 | 15.875 | 0.195958 | 7/15/2014 | 20:31.4 | 2 |
| 87.52 | 19287.552 | 1.818 | 15.875 | 0.192346 | 7/15/2014 | 20:31.5 | 2 |
| 87.744 | 19287.538 | 1.818 | 15.876 | 0.206239 | 7/15/2014 | 20:31.6 | 2 |
| 88.023 | 19287.52 | 1.817 | 15.874 | 0.197013 | 7/15/2014 | 20:31.7 | 2 |
| 88.249 | 19287.506 | 1.819 | 15.878 | 0.191168 | 7/15/2014 | 20:31.8 | 2 |
| 88.475 | 19287.491 | 1.819 | 15.882 | 0.208431 | 7/15/2014 | 20:31.9 | 2 |
| 88.701 | 19287.477 | 1.822 | 15.885 | 0.199503 | 7/15/2014 | 20:32.0 | 2 |
| 88.926 | 19287.463 | 1.822 | 15.887 | 0.189351 | 7/15/2014 | 20:32.1 | 2 |
| 89.152 | 19287.448 | 1.825 | 15.892 | 0.204727 | 7/15/2014 | 20:32.2 | 2 |
| 89.376 | 19287.435 | 1.825 | 15.898 | 0.197116 | 7/15/2014 | 20:32.3 | 2 |
| 89.653 | 19287.417 | 1.828 | 15.903 | 0.203392 | 7/15/2014 | 20:32.4 | 2 |
| 89.878 | 19287.404 | 1.831 | 15.909 | 0.206633 | 7/15/2014 | 20:32.5 | 2 |
| 90.103 | 19287.39 | 1.834 | 15.912 | 0.202477 | 7/15/2014 | 20:32.6 | 2 |
| 90.384 | 19287.374 | 1.831 | 15.919 | 0.208652 | 7/15/2014 | 20:32.7 | 2 |
| 90.61 | 19287.359 | 1.828 | 15.926 | 0.220534 | 7/15/2014 | 20:32.8 | 2 |
| 90.838 | 19287.347 | 1.826 | 15.934 | 0.215982 | 7/15/2014 | 20:32.9 | 2 |
| 91.067 | 19287.332 | 1.823 | 15.943 | 0.218471 | 7/15/2014 | 20:33.0 | 2 |
| 91.296 | 19287.319 | 1.823 | 15.953 | 0.217761 | 7/15/2014 | 20:33.1 | 2 |
| 91.525 | 19287.305 | 1.82 | 15.964 | 0.214553 | 7/15/2014 | 20:33.2 | 2 |
| 91.753 | 19287.292 | 1.82 | 15.973 | 0.21598 | 7/15/2014 | 20:33.3 | 2 |
| 92.037 | 19287.277 | 1.818 | 15.985 | 0.215898 | 7/15/2014 | 20:33.4 | 2 |
| 92.265 | 19287.262 | 1.817 | 15.998 | 0.210449 | 7/15/2014 | 20:33.5 | 2 |
| 92.494 | 19287.25 | 1.82 | 16.01 | 0.20896 | 7/15/2014 | 20:33.6 | 2 |
| 92.78 | 19287.232 | 1.826 | 16.018 | 0.208515 | 7/15/2014 | 20:33.7 | 2 |
| 93.01 | 19287.221 | 1.831 | 16.021 | 0.201356 | 7/15/2014 | 20:33.8 | 2 |
| 93.241 | 19287.207 | 1.828 | 16.018 | 0.206571 | 7/15/2014 | 20:33.9 | 2 |
| 93.472 | 19287.194 | 1.828 | 16.012 | 0.208105 | 7/15/2014 | 20:34.0 | 2 |
| 93.703 | 19287.182 | 1.828 | 16.003 | 0.200779 | 7/15/2014 | 20:34.1 | 2 |
| 93.933 | 19287.168 | 1.828 | 15.991 | 0.212164 | 7/15/2014 | 20:34.2 | 2 |
| 94.161 | 19287.156 | 1.83 | 15.974 | 0.213109 | 7/15/2014 | 20:34.3 | 2 |
| 94.445 | 19287.141 | 1.825 | 15.959 | 0.217127 | 7/15/2014 | 20:34.4 | 2 |
| 94.672 | 19287.129 | 1.822 | 15.947 | 0.236644 | 7/15/2014 | 20:34.5 | 2 |
| 94.898 | 19287.117 | 1.817 | 15.935 | 0.252479 | 7/15/2014 | 20:34.6 | 2 |
| 95.181 | 19287.101 | 1.819 | 15.924 | 0.231588 | 7/15/2014 | 20:34.7 | 2 |
| 95.409 | 19287.088 | 1.821 | 15.914 | 0.230849 | 7/15/2014 | 20:34.8 | 2 |
| 95.636 | 19287.076 | 1.821 | 15.906 | 0.216775 | 7/15/2014 | 20:34.9 | 2 |
| 95.865 | 19287.063 | 1.826 | 15.898 | 0.220575 | 7/15/2014 | 20:35.0 | 2 |
| 96.092 | 19287.053 | 1.829 | 15.891 | 0.196045 | 7/15/2014 | 20:35.1 | 2 |
| 96.319 | 19287.041 | 1.831 | 15.881 | 0.19803 | 7/15/2014 | 20:35.2 | 2 |
| 96.544 | 19287.029 | 1.831 | 15.873 | 0.171387 | 7/15/2014 | 20:35.3 | 2 |
| 96.825 | 19287.014 | 1.831 | 15.865 | 0.175604 | 7/15/2014 | 20:35.4 | 2 |

| | | | | | | | |
|---|---|---|---|---|---|---|---|
| 97.051 | 19287.001 | 1.831 | 15.858 | 0.176095 | 7/15/2014 | 20:35.5 | 2 |
| 97.275 | 19286.99 | 1.835 | 15.852 | 0.171879 | 7/15/2014 | 20:35.6 | 2 |
| 97.556 | 19286.974 | 1.835 | 15.844 | 0.171504 | 7/15/2014 | 20:35.7 | 2 |
| 97.781 | 19286.964 | 1.829 | 15.838 | 0.178069 | 7/15/2014 | 20:35.8 | 2 |
| 98.007 | 19286.952 | 1.823 | 15.832 | 0.184876 | 7/15/2014 | 20:35.9 | 2 |
| 98.236 | 19286.941 | 1.828 | 15.824 | 0.189337 | 7/15/2014 | 20:36.0 | 2 |
| 98.462 | 19286.929 | 1.828 | 15.822 | 0.206394 | 7/15/2014 | 20:36.1 | 2 |
| 98.688 | 19286.917 | 1.831 | 15.819 | 0.21466 | 7/15/2014 | 20:36.2 | 2 |
| 98.915 | 19286.906 | 1.831 | 15.815 | 0.21089 | 7/15/2014 | 20:36.3 | 2 |
| 99.195 | 19286.891 | 1.826 | 15.81 | 0.21817 | 7/15/2014 | 20:36.4 | 2 |
| 99.419 | 19286.88 | 1.823 | 15.807 | 0.210728 | 7/15/2014 | 20:36.5 | 2 |
| 99.645 | 19286.868 | 1.826 | 15.806 | 0.198534 | 7/15/2014 | 20:36.6 | 2 |
| 99.931 | 19286.854 | 1.832 | 15.804 | 0.178096 | 7/15/2014 | 20:36.7 | 2 |
| 100.159 | 19286.842 | 1.83 | 15.799 | 0.150968 | 7/15/2014 | 20:36.8 | 2 |
| 100.39 | 19286.832 | 1.83 | 15.787 | 0.147968 | 7/15/2014 | 20:36.9 | 2 |
| 100.621 | 19286.82 | 1.83 | 15.779 | 0.149864 | 7/15/2014 | 20:37.0 | 2 |
| 100.851 | 19286.809 | 1.83 | 15.77 | 0.154725 | 7/15/2014 | 20:37.1 | 2 |
| 100.964 | 19286.803 | 1.832 | 15.767 | 0.169238 | 7/15/2014 | 20:37.2 | 2 |
| 101.197 | 19286.79 | 1.838 | 15.755 | 0.184366 | 7/15/2014 | 20:37.3 | 2 |
| 101.6 | 19286.77 | 1.832 | 15.734 | 0.182607 | 7/15/2014 | 20:37.4 | 2 |
| 101.83 | 19286.758 | 1.829 | 15.72 | 0.179771 | 7/15/2014 | 20:37.5 | 2 |
| 102.061 | 19286.748 | 1.829 | 15.709 | 0.163963 | 7/15/2014 | 20:37.6 | 2 |
| 102.35 | 19286.732 | 1.829 | 15.698 | 0.15365 | 7/15/2014 | 20:37.7 | 2 |
| 102.467 | 19286.727 | 1.827 | 15.69 | 0.157318 | 7/15/2014 | 20:37.8 | 2 |
| 102.701 | 19286.715 | 1.831 | 15.678 | 0.158978 | 7/15/2014 | 20:37.9 | 2 |
| 103.05 | 19286.699 | 1.83 | 15.659 | 0.175157 | 7/15/2014 | 20:38.0 | 2 |
| 103.166 | 19286.692 | 1.832 | 15.652 | 0.19149 | 7/15/2014 | 20:38.1 | 2 |
| 103.399 | 19286.68 | 1.832 | 15.638 | 0.19259 | 7/15/2014 | 20:38.2 | 2 |
| 103.633 | 19286.669 | 1.835 | 15.625 | 0.206788 | 7/15/2014 | 20:38.3 | 2 |
| 104.044 | 19286.648 | 1.837 | 15.603 | 0.231784 | 7/15/2014 | 20:38.4 | 2 |
| 104.28 | 19286.638 | 1.84 | 15.589 | 0.259571 | 7/15/2014 | 20:38.5 | 2 |
| 104.516 | 19286.626 | 1.84 | 15.576 | 0.240206 | 7/15/2014 | 20:38.6 | 2 |
| 104.753 | 19286.615 | 1.843 | 15.564 | 0.217134 | 7/15/2014 | 20:38.7 | 2 |
| 104.991 | 19286.603 | 1.845 | 15.551 | 0.23433 | 7/15/2014 | 20:38.8 | 2 |
| 105.229 | 19286.591 | 1.848 | 15.542 | 0.241412 | 7/15/2014 | 20:38.9 | 2 |
| 105.468 | 19286.58 | 1.847 | 15.529 | 0.248864 | 7/15/2014 | 20:39.0 | 2 |
| 105.704 | 19286.568 | 1.844 | 15.521 | 0.25555 | 7/15/2014 | 20:39.1 | 2 |
| 105.941 | 19286.557 | 1.841 | 15.511 | 0.249861 | 7/15/2014 | 20:39.2 | 2 |
| 106.176 | 19286.545 | 1.838 | 15.504 | 0.261364 | 7/15/2014 | 20:39.3 | 2 |
| 106.41 | 19286.535 | 1.838 | 15.498 | 0.275879 | 7/15/2014 | 20:39.4 | 2 |
| 106.643 | 19286.523 | 1.838 | 15.49 | 0.25529 | 7/15/2014 | 20:39.5 | 2 |
| 106.874 | 19286.512 | 1.836 | 15.486 | 0.225042 | 7/15/2014 | 20:39.6 | 2 |
| 107.161 | 19286.498 | 1.832 | 15.476 | 0.210284 | 7/15/2014 | 20:39.7 | 2 |
| 107.39 | 19286.486 | 1.833 | 15.47 | 0.18715 | 7/15/2014 | 20:39.8 | 2 |
| 107.621 | 19286.475 | 1.837 | 15.464 | 0.186294 | 7/15/2014 | 20:39.9 | 2 |
| 107.851 | 19286.464 | 1.837 | 15.462 | 0.171186 | 7/15/2014 | 20:40.0 | 2 |
| 108.082 | 19286.454 | 1.84 | 15.456 | 0.170674 | 7/15/2014 | 20:40.1 | 2 |

| | | | | | | | |
|---|---|---|---|---|---|---|---|
| 108.313 | 19286.443 | 1.842 | 15.449 | 0.17366 | 7/15/2014 | 20:40.2 | 2 |
| 108.543 | 19286.432 | 1.839 | 15.451 | 0.179984 | 7/15/2014 | 20:40.3 | 2 |
| 108.831 | 19286.418 | 1.839 | 15.448 | 0.185819 | 7/15/2014 | 20:40.4 | 2 |
| 109.064 | 19286.406 | 1.839 | 15.448 | 0.179607 | 7/15/2014 | 20:40.5 | 2 |
| 109.299 | 19286.395 | 1.839 | 15.448 | 0.175293 | 7/15/2014 | 20:40.6 | 2 |
| 109.535 | 19286.384 | 1.839 | 15.447 | 0.184732 | 7/15/2014 | 20:40.7 | 2 |
| 109.773 | 19286.372 | 1.842 | 15.441 | 0.179829 | 7/15/2014 | 20:40.8 | 2 |
| 110.012 | 19286.361 | 1.839 | 15.439 | 0.187204 | 7/15/2014 | 20:40.9 | 2 |
| 110.251 | 19286.35 | 1.839 | 15.437 | 0.174533 | 7/15/2014 | 20:41.0 | 2 |
| 110.487 | 19286.338 | 1.836 | 15.431 | 0.174125 | 7/15/2014 | 20:41.1 | 2 |
| 110.727 | 19286.326 | 1.832 | 15.429 | 0.174176 | 7/15/2014 | 20:41.2 | 2 |
| 110.964 | 19286.314 | 1.832 | 15.427 | 0.176443 | 7/15/2014 | 20:41.3 | 2 |
| 111.2 | 19286.303 | 1.832 | 15.422 | 0.175962 | 7/15/2014 | 20:41.4 | 2 |
| 111.435 | 19286.292 | 1.829 | 15.417 | 0.175313 | 7/15/2014 | 20:41.5 | 2 |
| 111.669 | 19286.281 | 1.824 | 15.417 | 0.173782 | 7/15/2014 | 20:41.6 | 2 |
| 111.958 | 19286.267 | 1.821 | 15.418 | 0.174616 | 7/15/2014 | 20:41.7 | 2 |
| 112.191 | 19286.255 | 1.821 | 15.419 | 0.178625 | 7/15/2014 | 20:41.8 | 2 |
| 112.425 | 19286.244 | 1.824 | 15.42 | 0.179454 | 7/15/2014 | 20:41.9 | 2 |
| 112.659 | 19286.232 | 1.827 | 15.422 | 0.179629 | 7/15/2014 | 20:42.0 | 2 |
| 112.892 | 19286.221 | 1.827 | 15.423 | 0.18028 | 7/15/2014 | 20:42.1 | 2 |
| 113.125 | 19286.21 | 1.827 | 15.43 | 0.178544 | 7/15/2014 | 20:42.2 | 2 |
| 113.239 | 19286.205 | 1.828 | 15.434 | 0.180609 | 7/15/2014 | 20:42.3 | 2 |
| 113.649 | 19286.184 | 1.825 | 15.443 | 0.17216 | 7/15/2014 | 20:42.4 | 2 |
| 113.886 | 19286.172 | 1.828 | 15.449 | 0.167923 | 7/15/2014 | 20:42.5 | 2 |
| 114.123 | 19286.16 | 1.825 | 15.45 | 0.161282 | 7/15/2014 | 20:42.6 | 2 |
| 114.36 | 19286.148 | 1.822 | 15.449 | 0.156243 | 7/15/2014 | 20:42.7 | 2 |
| 114.602 | 19286.137 | 1.819 | 15.448 | 0.147831 | 7/15/2014 | 20:42.8 | 2 |
| 114.842 | 19286.125 | 1.819 | 15.449 | 0.145203 | 7/15/2014 | 20:42.9 | 2 |
| 115.084 | 19286.113 | 1.816 | 15.445 | 0.142065 | 7/15/2014 | 20:43.0 | 2 |
| 115.323 | 19286.101 | 1.815 | 15.437 | 0.143556 | 7/15/2014 | 20:43.1 | 2 |
| 115.565 | 19286.089 | 1.817 | 15.436 | 0.146835 | 7/15/2014 | 20:43.2 | 2 |
| 115.805 | 19286.076 | 1.817 | 15.427 | 0.149174 | 7/15/2014 | 20:43.3 | 2 |
| 116.042 | 19286.063 | 1.814 | 15.424 | 0.151897 | 7/15/2014 | 20:43.4 | 2 |
| 116.28 | 19286.052 | 1.817 | 15.424 | 0.153992 | 7/15/2014 | 20:43.5 | 2 |
| 116.516 | 19286.041 | 1.817 | 15.419 | 0.155985 | 7/15/2014 | 20:43.6 | 2 |
| 116.808 | 19286.025 | 1.817 | 15.412 | 0.16129 | 7/15/2014 | 20:43.7 | 2 |
| 116.927 | 19286.02 | 1.816 | 15.411 | 0.163232 | 7/15/2014 | 20:43.8 | 2 |
| 117.162 | 19286.008 | 1.82 | 15.405 | 0.159601 | 7/15/2014 | 20:43.9 | 2 |
| 117.513 | 19285.99 | 1.823 | 15.395 | 0.152621 | 7/15/2014 | 20:44.0 | 2 |
| 117.748 | 19285.978 | 1.823 | 15.392 | 0.150395 | 7/15/2014 | 20:44.1 | 2 |
| 117.863 | 19285.972 | 1.824 | 15.389 | 0.152595 | 7/15/2014 | 20:44.2 | 2 |
| 118.097 | 19285.961 | 1.824 | 15.376 | 0.150209 | 7/15/2014 | 20:44.3 | 2 |
| 118.509 | 19285.939 | 1.826 | 15.363 | 0.150623 | 7/15/2014 | 20:44.4 | 2 |
| 118.744 | 19285.928 | 1.826 | 15.355 | 0.14393 | 7/15/2014 | 20:44.5 | 2 |
| 118.981 | 19285.916 | 1.826 | 15.346 | 0.139524 | 7/15/2014 | 20:44.6 | 2 |
| 119.219 | 19285.904 | 1.826 | 15.338 | 0.142469 | 7/15/2014 | 20:44.7 | 2 |
| 119.458 | 19285.893 | 1.823 | 15.331 | 0.146641 | 7/15/2014 | 20:44.8 | 2 |

| | | | | | | | |
|---|---|---|---|---|---|---|---|
| 119.698 | 19285.88 | 1.823 | 15.322 | 0.142152 | 7/15/2014 | 20:44.9 | 2 |
| 119.937 | 19285.868 | 1.826 | 15.318 | 0.145268 | 7/15/2014 | 20:45.0 | 2 |
| 120.176 | 19285.855 | 1.826 | 15.316 | 0.147625 | 7/15/2014 | 20:45.1 | 2 |
| 120.414 | 19285.844 | 1.826 | 15.31 | 0.149682 | 7/15/2014 | 20:45.2 | 2 |
| 120.652 | 19285.832 | 1.826 | 15.308 | 0.15084 | 7/15/2014 | 20:45.3 | 2 |
| 120.888 | 19285.821 | 1.829 | 15.306 | 0.145964 | 7/15/2014 | 20:45.4 | 2 |
| 121.123 | 19285.81 | 1.829 | 15.303 | 0.148087 | 7/15/2014 | 20:45.5 | 2 |
| 121.356 | 19285.798 | 1.829 | 15.301 | 0.141503 | 7/15/2014 | 20:45.6 | 2 |
| 121.646 | 19285.782 | 1.833 | 15.298 | 0.143541 | 7/15/2014 | 20:45.7 | 2 |
| 121.879 | 19285.77 | 1.833 | 15.293 | 0.134784 | 7/15/2014 | 20:45.8 | 2 |
| 122.113 | 19285.758 | 1.833 | 15.285 | 0.134753 | 7/15/2014 | 20:45.9 | 2 |
| 122.347 | 19285.746 | 1.829 | 15.28 | 0.133526 | 7/15/2014 | 20:46.0 | 2 |
| 122.579 | 19285.735 | 1.826 | 15.272 | 0.134942 | 7/15/2014 | 20:46.1 | 2 |
| 122.694 | 19285.729 | 1.824 | 15.269 | 0.137322 | 7/15/2014 | 20:46.2 | 2 |
| 122.928 | 19285.719 | 1.827 | 15.264 | 0.14429 | 7/15/2014 | 20:46.3 | 2 |
| 123.34 | 19285.697 | 1.82 | 15.255 | 0.165125 | 7/15/2014 | 20:46.4 | 2 |
| 123.577 | 19285.686 | 1.815 | 15.251 | 0.15979 | 7/15/2014 | 20:46.5 | 2 |
| 123.815 | 19285.674 | 1.811 | 15.244 | 0.162657 | 7/15/2014 | 20:46.6 | 2 |
| 124.055 | 19285.662 | 1.809 | 15.24 | 0.154494 | 7/15/2014 | 20:46.7 | 2 |
| 124.294 | 19285.65 | 1.807 | 15.234 | 0.158951 | 7/15/2014 | 20:46.8 | 2 |
| 124.535 | 19285.639 | 1.805 | 15.229 | 0.157006 | 7/15/2014 | 20:46.9 | 2 |
| 124.777 | 19285.627 | 1.802 | 15.223 | 0.155993 | 7/15/2014 | 20:47.0 | 2 |
| 125.018 | 19285.615 | 1.805 | 15.213 | 0.156153 | 7/15/2014 | 20:47.1 | 2 |
| 125.259 | 19285.604 | 1.806 | 15.211 | 0.155914 | 7/15/2014 | 20:47.2 | 2 |
| 125.498 | 19285.593 | 1.805 | 15.207 | 0.159622 | 7/15/2014 | 20:47.3 | 2 |
| 125.736 | 19285.582 | 1.805 | 15.207 | 0.158332 | 7/15/2014 | 20:47.4 | 2 |
| 125.972 | 19285.57 | 1.802 | 15.203 | 0.160187 | 7/15/2014 | 20:47.5 | 2 |
| 126.207 | 19285.559 | 1.804 | 15.202 | 0.162795 | 7/15/2014 | 20:47.6 | 2 |
| 126.497 | 19285.545 | 1.806 | 15.204 | 0.161838 | 7/15/2014 | 20:47.7 | 2 |
| 126.732 | 19285.533 | 1.806 | 15.204 | 0.164206 | 7/15/2014 | 20:47.8 | 2 |
| 126.965 | 19285.521 | 1.801 | 15.202 | 0.169659 | 7/15/2014 | 20:47.9 | 2 |
| 127.199 | 19285.511 | 1.803 | 15.201 | 0.165864 | 7/15/2014 | 20:48.0 | 2 |
| 127.432 | 19285.501 | 1.801 | 15.197 | 0.160196 | 7/15/2014 | 20:48.1 | 2 |
| 127.666 | 19285.489 | 1.801 | 15.197 | 0.159868 | 7/15/2014 | 20:48.2 | 2 |
| 127.781 | 19285.483 | 1.804 | 15.198 | 0.160179 | 7/15/2014 | 20:48.3 | 2 |
| 128.189 | 19285.465 | 1.803 | 15.186 | 0.146832 | 7/15/2014 | 20:48.4 | 2 |
| 128.424 | 19285.453 | 1.803 | 15.181 | 0.143257 | 7/15/2014 | 20:48.5 | 2 |
| 128.66 | 19285.441 | 1.801 | 15.175 | 0.141825 | 7/15/2014 | 20:48.6 | 2 |
| 128.898 | 19285.43 | 1.806 | 15.168 | 0.133796 | 7/15/2014 | 20:48.7 | 2 |
| 129.134 | 19285.419 | 1.804 | 15.159 | 0.135085 | 7/15/2014 | 20:48.8 | 2 |
| 129.372 | 19285.409 | 1.804 | 15.151 | 0.133731 | 7/15/2014 | 20:48.9 | 2 |
| 129.61 | 19285.398 | 1.807 | 15.14 | 0.134379 | 7/15/2014 | 20:49.0 | 2 |
| 129.849 | 19285.387 | 1.81 | 15.137 | 0.140209 | 7/15/2014 | 20:49.1 | 2 |
| 130.085 | 19285.375 | 1.814 | 15.132 | 0.145921 | 7/15/2014 | 20:49.2 | 2 |
| 130.322 | 19285.364 | 1.817 | 15.124 | 0.153072 | 7/15/2014 | 20:49.3 | 2 |
| 130.557 | 19285.354 | 1.819 | 15.12 | 0.158625 | 7/15/2014 | 20:49.4 | 2 |
| 130.789 | 19285.342 | 1.816 | 15.118 | 0.162233 | 7/15/2014 | 20:49.5 | 2 |

| | | | | | | | |
|---|---|---|---|---|---|---|---|
| 131.021 | 19285.334 | 1.818 | 15.117 | 0.168016 | 7/15/2014 | 20:49.6 | 2 |
| 131.308 | 19285.32 | 1.82 | 15.116 | 0.169198 | 7/15/2014 | 20:49.7 | 2 |
| 131.54 | 19285.309 | 1.817 | 15.115 | 0.166694 | 7/15/2014 | 20:49.8 | 2 |
| 131.772 | 19285.298 | 1.815 | 15.117 | 0.16229 | 7/15/2014 | 20:49.9 | 2 |
| 132.003 | 19285.288 | 1.812 | 15.114 | 0.162392 | 7/15/2014 | 20:50.0 | 2 |
| 132.234 | 19285.277 | 1.806 | 15.115 | 0.157568 | 7/15/2014 | 20:50.1 | 2 |
| 132.466 | 19285.267 | 1.809 | 15.112 | 0.155068 | 7/15/2014 | 20:50.2 | 2 |
| 132.699 | 19285.258 | 1.81 | 15.11 | 0.155243 | 7/15/2014 | 20:50.3 | 2 |
| 132.986 | 19285.244 | 1.81 | 15.105 | 0.152807 | 7/15/2014 | 20:50.4 | 2 |
| 133.221 | 19285.232 | 1.808 | 15.103 | 0.156308 | 7/15/2014 | 20:50.5 | 2 |
| 133.458 | 19285.224 | 1.808 | 15.099 | 0.161116 | 7/15/2014 | 20:50.6 | 2 |
| 133.694 | 19285.213 | 1.805 | 15.094 | 0.162299 | 7/15/2014 | 20:50.7 | 2 |
| 133.933 | 19285.201 | 1.805 | 15.092 | 0.162707 | 7/15/2014 | 20:50.8 | 2 |
| 134.172 | 19285.191 | 1.805 | 15.087 | 0.169247 | 7/15/2014 | 20:50.9 | 2 |
| 134.413 | 19285.182 | 1.805 | 15.083 | 0.170342 | 7/15/2014 | 20:51.0 | 2 |
| 134.651 | 19285.17 | 1.804 | 15.083 | 0.172892 | 7/15/2014 | 20:51.1 | 2 |
| 134.891 | 19285.16 | 1.804 | 15.081 | 0.166515 | 7/15/2014 | 20:51.2 | 2 |
| 135.129 | 19285.15 | 1.812 | 15.075 | 0.166161 | 7/15/2014 | 20:51.3 | 2 |
| 135.365 | 19285.14 | 1.812 | 15.07 | 0.152077 | 7/15/2014 | 20:51.4 | 2 |
| 135.601 | 19285.129 | 1.817 | 15.063 | 0.166365 | 7/15/2014 | 20:51.5 | 2 |
| 135.833 | 19285.12 | 1.82 | 15.054 | 0.15141 | 7/15/2014 | 20:51.6 | 2 |
| 136.123 | 19285.107 | 1.823 | 15.044 | 0.147872 | 7/15/2014 | 20:51.7 | 2 |
| 136.354 | 19285.098 | 1.825 | 15.04 | 0.154141 | 7/15/2014 | 20:51.8 | 2 |
| 136.587 | 19285.088 | 1.826 | 15.027 | 0.158915 | 7/15/2014 | 20:51.9 | 2 |
| 136.819 | 19285.078 | 1.829 | 15.017 | 0.162785 | 7/15/2014 | 20:52.0 | 2 |
| 137.05 | 19285.069 | 1.826 | 15.007 | 0.179405 | 7/15/2014 | 20:52.1 | 2 |
| 137.281 | 19285.059 | 1.826 | 15 | 0.185323 | 7/15/2014 | 20:52.2 | 2 |
| 137.511 | 19285.051 | 1.823 | 15 | 0.195279 | 7/15/2014 | 20:52.3 | 2 |
| 137.8 | 19285.038 | 1.82 | 14.998 | 0.20453 | 7/15/2014 | 20:52.4 | 2 |
| 138.033 | 19285.029 | 1.822 | 14.994 | 0.211031 | 7/15/2014 | 20:52.5 | 2 |
| 138.267 | 19285.02 | 1.817 | 14.991 | 0.211582 | 7/15/2014 | 20:52.6 | 2 |
| 138.503 | 19285.01 | 1.817 | 14.987 | 0.211995 | 7/15/2014 | 20:52.7 | 2 |
| 138.741 | 19285.002 | 1.817 | 14.985 | 0.211095 | 7/15/2014 | 20:52.8 | 2 |
| 138.978 | 19284.99 | 1.814 | 14.983 | 0.205711 | 7/15/2014 | 20:52.9 | 2 |
| 139.217 | 19284.982 | 1.814 | 14.98 | 0.200817 | 7/15/2014 | 20:53.0 | 2 |
| 139.458 | 19284.974 | 1.817 | 14.969 | 0.202935 | 7/15/2014 | 20:53.1 | 2 |
| 139.697 | 19284.963 | 1.82 | 14.964 | 0.205086 | 7/15/2014 | 20:53.2 | 2 |
| 139.936 | 19284.955 | 1.82 | 14.963 | 0.203611 | 7/15/2014 | 20:53.3 | 2 |
| 140.175 | 19284.945 | 1.82 | 14.956 | 0.219903 | 7/15/2014 | 20:53.4 | 2 |
| 140.412 | 19284.937 | 1.822 | 14.955 | 0.209892 | 7/15/2014 | 20:53.5 | 2 |
| 140.647 | 19284.926 | 1.819 | 14.951 | 0.231437 | 7/15/2014 | 20:53.6 | 2 |
| 140.941 | 19284.916 | 1.819 | 14.948 | 0.220716 | 7/15/2014 | 20:53.7 | 2 |
| 141.061 | 19284.912 | 1.819 | 14.946 | 0.228925 | 7/15/2014 | 20:53.8 | 2 |
| 141.297 | 19284.902 | 1.822 | 14.945 | 0.238103 | 7/15/2014 | 20:53.9 | 2 |
| 141.531 | 19284.895 | 1.815 | 14.94 | 0.242826 | 7/15/2014 | 20:54.0 | 2 |
| 141.767 | 19284.886 | 1.815 | 14.932 | 0.23894 | 7/15/2014 | 20:54.1 | 2 |
| 142.001 | 19284.876 | 1.812 | 14.926 | 0.241301 | 7/15/2014 | 20:54.2 | 2 |

| | | | | | | | |
|---|---|---|---|---|---|---|---|
| 142.237 | 19284.867 | 1.815 | 14.918 | 0.238587 | 7/15/2014 | 20:54.3 | 2 |
| 142.649 | 19284.854 | 1.814 | 14.904 | 0.259329 | 7/15/2014 | 20:54.4 | 2 |
| 142.887 | 19284.844 | 1.817 | 14.896 | 0.247518 | 7/15/2014 | 20:54.5 | 2 |
| 143.124 | 19284.836 | 1.819 | 14.886 | 0.25713 | 7/15/2014 | 20:54.6 | 2 |
| 143.362 | 19284.827 | 1.822 | 14.876 | 0.258288 | 7/15/2014 | 20:54.7 | 2 |
| 143.601 | 19284.818 | 1.824 | 14.872 | 0.266454 | 7/15/2014 | 20:54.8 | 2 |
| 143.84 | 19284.811 | 1.83 | 14.865 | 0.259467 | 7/15/2014 | 20:54.9 | 2 |
| 144.079 | 19284.803 | 1.833 | 14.859 | 0.275818 | 7/15/2014 | 20:55.0 | 2 |
| 144.316 | 19284.795 | 1.835 | 14.854 | 0.27276 | 7/15/2014 | 20:55.1 | 2 |
| 144.553 | 19284.785 | 1.84 | 14.85 | 0.288397 | 7/15/2014 | 20:55.2 | 2 |
| 144.789 | 19284.777 | 1.843 | 14.841 | 0.281324 | 7/15/2014 | 20:55.3 | 2 |
| 145.023 | 19284.77 | 1.843 | 14.834 | 0.284721 | 7/15/2014 | 20:55.4 | 2 |
| 145.256 | 19284.762 | 1.843 | 14.826 | 0.295721 | 7/15/2014 | 20:55.5 | 2 |
| 145.487 | 19284.754 | 1.846 | 14.821 | 0.288477 | 7/15/2014 | 20:55.6 | 2 |
| 145.774 | 19284.744 | 1.846 | 14.817 | 0.285045 | 7/15/2014 | 20:55.7 | 2 |
| 146.004 | 19284.737 | 1.846 | 14.814 | 0.279811 | 7/15/2014 | 20:55.8 | 2 |
| 146.234 | 19284.73 | 1.846 | 14.806 | 0.276093 | 7/15/2014 | 20:55.9 | 2 |
| 146.466 | 19284.723 | 1.846 | 14.798 | 0.270365 | 7/15/2014 | 20:56.0 | 2 |
| 146.697 | 19284.715 | 1.846 | 14.795 | 0.277933 | 7/15/2014 | 20:56.1 | 2 |
| 146.928 | 19284.707 | 1.848 | 14.794 | 0.271695 | 7/15/2014 | 20:56.2 | 2 |
| 147.161 | 19284.699 | 1.854 | 14.791 | 0.277523 | 7/15/2014 | 20:56.3 | 2 |
| 147.449 | 19284.689 | 1.856 | 14.783 | 0.270652 | 7/15/2014 | 20:56.4 | 2 |
| 147.683 | 19284.683 | 1.858 | 14.778 | 0.278717 | 7/15/2014 | 20:56.5 | 2 |
| 147.918 | 19284.676 | 1.86 | 14.772 | 0.271183 | 7/15/2014 | 20:56.6 | 2 |
| 148.156 | 19284.668 | 1.867 | 14.774 | 0.27157 | 7/15/2014 | 20:56.7 | 2 |
| 148.394 | 19284.661 | 1.87 | 14.772 | 0.264459 | 7/15/2014 | 20:56.8 | 2 |
| 148.633 | 19284.653 | 1.872 | 14.766 | 0.263207 | 7/15/2014 | 20:56.9 | 2 |
| 148.873 | 19284.646 | 1.875 | 14.762 | 0.262459 | 7/15/2014 | 20:57.0 | 2 |
| 149.112 | 19284.639 | 1.877 | 14.755 | 0.265755 | 7/15/2014 | 20:57.1 | 2 |
| 149.351 | 19284.631 | 1.875 | 14.748 | 0.258439 | 7/15/2014 | 20:57.2 | 2 |
| 149.589 | 19284.623 | 1.875 | 14.737 | 0.266519 | 7/15/2014 | 20:57.3 | 2 |
| 149.826 | 19284.616 | 1.878 | 14.727 | 0.273673 | 7/15/2014 | 20:57.4 | 2 |
| 150.061 | 19284.61 | 1.876 | 14.712 | 0.26856 | 7/15/2014 | 20:57.5 | 2 |
| 150.296 | 19284.603 | 1.876 | 14.704 | 0.276906 | 7/15/2014 | 20:57.6 | 2 |
| 150.586 | 19284.595 | 1.874 | 14.703 | 0.285997 | 7/15/2014 | 20:57.7 | 2 |
| 150.705 | 19284.592 | 1.87 | 14.697 | 0.287074 | 7/15/2014 | 20:57.8 | 2 |
| 151.056 | 19284.58 | 1.871 | 14.694 | 0.293122 | 7/15/2014 | 20:57.9 | 2 |
| 151.291 | 19284.572 | 1.873 | 14.692 | 0.299204 | 7/15/2014 | 20:58.0 | 2 |
| 151.526 | 19284.566 | 1.875 | 14.694 | 0.301607 | 7/15/2014 | 20:58.1 | 2 |
| 151.64 | 19284.563 | 1.871 | 14.697 | 0.309076 | 7/15/2014 | 20:58.2 | 2 |
| 151.875 | 19284.557 | 1.873 | 14.704 | 0.309531 | 7/15/2014 | 20:58.3 | 2 |
| 152.288 | 19284.545 | 1.874 | 14.71 | 0.306543 | 7/15/2014 | 20:58.4 | 2 |
| 152.526 | 19284.538 | 1.881 | 14.712 | 0.306006 | 7/15/2014 | 20:58.5 | 2 |
| 152.765 | 19284.531 | 1.879 | 14.714 | 0.306252 | 7/15/2014 | 20:58.6 | 2 |
| 153.004 | 19284.523 | 1.877 | 14.716 | 0.305773 | 7/15/2014 | 20:58.7 | 2 |
| 153.244 | 19284.518 | 1.877 | 14.716 | 0.30555 | 7/15/2014 | 20:58.8 | 2 |
| 153.485 | 19284.512 | 1.875 | 14.714 | 0.305715 | 7/15/2014 | 20:58.9 | 2 |

| | | | | | | | |
|---|---|---|---|---|---|---|---|
| 153.726 | 19284.504 | 1.875 | 14.712 | 0.307992 | 7/15/2014 | 20:59.0 | 2 |
| 153.966 | 19284.498 | 1.873 | 14.708 | 0.308558 | 7/15/2014 | 20:59.1 | 2 |
| 154.205 | 19284.491 | 1.873 | 14.697 | 0.309323 | 7/15/2014 | 20:59.2 | 2 |
| 154.443 | 19284.485 | 1.867 | 14.688 | 0.3077 | 7/15/2014 | 20:59.3 | 2 |
| 154.679 | 19284.479 | 1.87 | 14.682 | 0.307705 | 7/15/2014 | 20:59.4 | 2 |
| 154.913 | 19284.472 | 1.87 | 14.673 | 0.303977 | 7/15/2014 | 20:59.5 | 2 |
| 155.146 | 19284.467 | 1.867 | 14.661 | 0.297006 | 7/15/2014 | 20:59.6 | 2 |
| 155.435 | 19284.459 | 1.867 | 14.648 | 0.294247 | 7/15/2014 | 20:59.7 | 2 |
| 155.667 | 19284.453 | 1.865 | 14.635 | 0.290921 | 7/15/2014 | 20:59.8 | 2 |
| 155.899 | 19284.447 | 1.865 | 14.622 | 0.285566 | 7/15/2014 | 20:59.9 | 2 |
| 156.13 | 19284.44 | 1.865 | 14.611 | 0.282819 | 7/15/2014 | 21:00.0 | 2 |
| 156.362 | 19284.436 | 1.865 | 14.601 | 0.278862 | 7/15/2014 | 21:00.1 | 2 |
| 156.595 | 19284.429 | 1.865 | 14.586 | 0.277863 | 7/15/2014 | 21:00.2 | 2 |
| 156.826 | 19284.424 | 1.864 | 14.577 | 0.277079 | 7/15/2014 | 21:00.3 | 2 |
| 157.115 | 19284.417 | 1.866 | 14.569 | 0.277662 | 7/15/2014 | 21:00.4 | 2 |
| 157.349 | 19284.41 | 1.866 | 14.565 | 0.277607 | 7/15/2014 | 21:00.5 | 2 |
| 157.584 | 19284.405 | 1.868 | 14.56 | 0.279542 | 7/15/2014 | 21:00.6 | 2 |
| 157.821 | 19284.398 | 1.868 | 14.559 | 0.277747 | 7/15/2014 | 21:00.7 | 2 |
| 158.058 | 19284.395 | 1.866 | 14.555 | 0.277096 | 7/15/2014 | 21:00.8 | 2 |
| 158.295 | 19284.389 | 1.861 | 14.554 | 0.278062 | 7/15/2014 | 21:00.9 | 2 |
| 158.534 | 19284.383 | 1.861 | 14.552 | 0.2728 | 7/15/2014 | 21:01.0 | 2 |
| 158.772 | 19284.378 | 1.858 | 14.55 | 0.269409 | 7/15/2014 | 21:01.1 | 2 |
| 159.01 | 19284.371 | 1.856 | 14.55 | 0.266049 | 7/15/2014 | 21:01.2 | 2 |
| 159.246 | 19284.366 | 1.856 | 14.558 | 0.264101 | 7/15/2014 | 21:01.3 | 2 |
| 159.482 | 19284.361 | 1.854 | 14.544 | 0.265667 | 7/15/2014 | 21:01.4 | 2 |
| 159.716 | 19284.355 | 1.854 | 14.531 | 0.259105 | 7/15/2014 | 21:01.5 | 2 |
| 159.948 | 19284.351 | 1.854 | 14.544 | 0.263152 | 7/15/2014 | 21:01.6 | 2 |
| 160.238 | 19284.344 | 1.859 | 14.54 | 0.25438 | 7/15/2014 | 21:01.7 | 2 |
| 160.471 | 19284.339 | 1.857 | 14.546 | 0.261212 | 7/15/2014 | 21:01.8 | 2 |
| 160.704 | 19284.332 | 1.862 | 14.555 | 0.256453 | 7/15/2014 | 21:01.9 | 2 |
| 160.938 | 19284.328 | 1.859 | 14.56 | 0.251103 | 7/15/2014 | 21:02.0 | 2 |
| 161.171 | 19284.323 | 1.859 | 14.56 | 0.248045 | 7/15/2014 | 21:02.1 | 2 |
| 161.285 | 19284.319 | 1.859 | 14.574 | 0.247859 | 7/15/2014 | 21:02.2 | 2 |
| 161.519 | 19284.314 | 1.855 | 14.592 | 0.242655 | 7/15/2014 | 21:02.3 | 2 |
| 161.931 | 19284.306 | 1.856 | 14.581 | 0.243443 | 7/15/2014 | 21:02.4 | 2 |
| 162.166 | 19284.3 | 1.854 | 14.584 | 0.236271 | 7/15/2014 | 21:02.5 | 2 |
| 162.405 | 19284.295 | 1.854 | 14.576 | 0.226576 | 7/15/2014 | 21:02.6 | 2 |
| 162.644 | 19284.29 | 1.846 | 14.565 | 0.232412 | 7/15/2014 | 21:02.7 | 2 |
| 162.885 | 19284.284 | 1.85 | 14.558 | 0.226893 | 7/15/2014 | 21:02.8 | 2 |
| 163.126 | 19284.279 | 1.846 | 14.547 | 0.220321 | 7/15/2014 | 21:02.9 | 2 |
| 163.368 | 19284.273 | 1.85 | 14.538 | 0.223604 | 7/15/2014 | 21:03.0 | 2 |
| 163.608 | 19284.269 | 1.847 | 14.525 | 0.221187 | 7/15/2014 | 21:03.1 | 2 |
| 163.849 | 19284.263 | 1.847 | 14.519 | 0.21884 | 7/15/2014 | 21:03.2 | 2 |
| 164.089 | 19284.258 | 1.847 | 14.513 | 0.226432 | 7/15/2014 | 21:03.3 | 2 |
| 164.328 | 19284.253 | 1.847 | 14.509 | 0.226215 | 7/15/2014 | 21:03.4 | 2 |
| 164.566 | 19284.247 | 1.851 | 14.514 | 0.228826 | 7/15/2014 | 21:03.5 | 2 |
| 164.803 | 19284.242 | 1.851 | 14.512 | 0.236578 | 7/15/2014 | 21:03.6 | 2 |

| | | | | | | | |
|---|---|---|---|---|---|---|---|
| 165.096 | 19284.235 | 1.852 | 14.512 | 0.226608 | 7/15/2014 | 21:03.7 | 2 |
| 165.215 | 19284.233 | 1.85 | 14.511 | 0.226196 | 7/15/2014 | 21:03.8 | 2 |
| 165.453 | 19284.228 | 1.853 | 14.517 | 0.226118 | 7/15/2014 | 21:03.9 | 2 |
| 165.687 | 19284.223 | 1.85 | 14.523 | 0.204447 | 7/15/2014 | 21:04.0 | 2 |
| 165.924 | 19284.218 | 1.853 | 14.53 | 0.205866 | 7/15/2014 | 21:04.1 | 2 |
| 166.159 | 19284.212 | 1.85 | 14.534 | 0.207831 | 7/15/2014 | 21:04.2 | 2 |
| 166.397 | 19284.206 | 1.855 | 14.531 | 0.191784 | 7/15/2014 | 21:04.3 | 2 |
| 166.815 | 19284.198 | 1.856 | 14.522 | 0.179266 | 7/15/2014 | 21:04.4 | 2 |
| 167.055 | 19284.191 | 1.853 | 14.512 | 0.166285 | 7/15/2014 | 21:04.5 | 2 |
| 167.296 | 19284.188 | 1.855 | 14.499 | 0.18095 | 7/15/2014 | 21:04.6 | 2 |
| 167.539 | 19284.182 | 1.857 | 14.484 | 0.162219 | 7/15/2014 | 21:04.7 | 2 |
| 167.783 | 19284.176 | 1.859 | 14.468 | 0.153425 | 7/15/2014 | 21:04.8 | 2 |
| 168.028 | 19284.171 | 1.859 | 14.45 | 0.149363 | 7/15/2014 | 21:04.9 | 2 |
| 168.274 | 19284.165 | 1.859 | 14.432 | 0.153854 | 7/15/2014 | 21:05.0 | 2 |
| 168.518 | 19284.16 | 1.859 | 14.418 | 0.14838 | 7/15/2014 | 21:05.1 | 2 |
| 168.762 | 19284.154 | 1.861 | 14.409 | 0.151076 | 7/15/2014 | 21:05.2 | 2 |
| 169.005 | 19284.15 | 1.861 | 14.403 | 0.145974 | 7/15/2014 | 21:05.3 | 2 |
| 169.247 | 19284.144 | 1.859 | 14.401 | 0.152503 | 7/15/2014 | 21:05.4 | 2 |
| 169.488 | 19284.139 | 1.859 | 14.4 | 0.143551 | 7/15/2014 | 21:05.5 | 2 |
| 169.725 | 19284.134 | 1.859 | 14.398 | 0.165268 | 7/15/2014 | 21:05.6 | 2 |
| 170.021 | 19284.128 | 1.859 | 14.397 | 0.143016 | 7/15/2014 | 21:05.7 | 2 |
| 170.142 | 19284.125 | 1.856 | 14.399 | 0.146869 | 7/15/2014 | 21:05.8 | 2 |
| 170.38 | 19284.12 | 1.859 | 14.398 | 0.144992 | 7/15/2014 | 21:05.9 | 2 |
| 170.615 | 19284.115 | 1.856 | 14.394 | 0.133781 | 7/15/2014 | 21:06.0 | 2 |
| 170.852 | 19284.109 | 1.865 | 14.392 | 0.142763 | 7/15/2014 | 21:06.1 | 2 |
| 171.087 | 19284.105 | 1.862 | 14.384 | 0.139196 | 7/15/2014 | 21:06.2 | 2 |
| 171.324 | 19284.1 | 1.862 | 14.373 | 0.120215 | 7/15/2014 | 21:06.3 | 2 |
| 171.74 | 19284.092 | 1.857 | 14.364 | 0.116003 | 7/15/2014 | 21:06.4 | 2 |
| 171.98 | 19284.086 | 1.856 | 14.361 | 0.116944 | 7/15/2014 | 21:06.5 | 2 |
| 172.22 | 19284.08 | 1.854 | 14.358 | 0.113076 | 7/15/2014 | 21:06.6 | 2 |
| 172.461 | 19284.076 | 1.854 | 14.364 | 0.0997834 | 7/15/2014 | 21:06.7 | 2 |
| 172.703 | 19284.071 | 1.852 | 14.361 | 0.0983452 | 7/15/2014 | 21:06.8 | 2 |
| 172.948 | 19284.065 | 1.854 | 14.361 | 0.0969997 | 7/15/2014 | 21:06.9 | 2 |
| 173.191 | 19284.061 | 1.854 | 14.36 | 0.0955118 | 7/15/2014 | 21:07.0 | 2 |
| 173.435 | 19284.056 | 1.854 | 14.359 | 0.0973252 | 7/15/2014 | 21:07.1 | 2 |
| 173.678 | 19284.05 | 1.859 | 14.357 | 0.098184 | 7/15/2014 | 21:07.2 | 2 |
| 173.92 | 19284.045 | 1.86 | 14.356 | 0.101148 | 7/15/2014 | 21:07.3 | 2 |
| 174.161 | 19284.041 | 1.86 | 14.357 | 0.104949 | 7/15/2014 | 21:07.4 | 2 |
| 174.401 | 19284.035 | 1.86 | 14.357 | 0.106444 | 7/15/2014 | 21:07.5 | 2 |
| 174.64 | 19284.029 | 1.862 | 14.365 | 0.109547 | 7/15/2014 | 21:07.6 | 2 |
| 174.935 | 19284.024 | 1.857 | 14.377 | 0.113803 | 7/15/2014 | 21:07.7 | 2 |
| 175.056 | 19284.021 | 1.856 | 14.383 | 0.116815 | 7/15/2014 | 21:07.8 | 2 |
| 175.293 | 19284.016 | 1.859 | 14.399 | 0.116832 | 7/15/2014 | 21:07.9 | 2 |
| 175.529 | 19284.012 | 1.854 | 14.417 | 0.118932 | 7/15/2014 | 21:08.0 | 2 |
| 175.767 | 19284.007 | 1.856 | 14.43 | 0.119962 | 7/15/2014 | 21:08.1 | 2 |
| 176.004 | 19284.001 | 1.853 | 14.44 | 0.129599 | 7/15/2014 | 21:08.2 | 2 |
| 176.24 | 19283.996 | 1.85 | 14.441 | 0.127221 | 7/15/2014 | 21:08.3 | 2 |

| | | | | | | | |
|---|---|---|---|---|---|---|---|
| 176.657 | 19283.987 | 1.841 | 14.452 | 0.126985 | 7/15/2014 | 21:08.4 | 2 |
| 176.896 | 19283.982 | 1.839 | 14.455 | 0.137208 | 7/15/2014 | 21:08.5 | 2 |
| 177.135 | 19283.978 | 1.831 | 14.454 | 0.131768 | 7/15/2014 | 21:08.6 | 2 |
| 177.376 | 19283.972 | 1.828 | 14.454 | 0.134768 | 7/15/2014 | 21:08.7 | 2 |
| 177.618 | 19283.967 | 1.828 | 14.454 | 0.132147 | 7/15/2014 | 21:08.8 | 2 |
| 177.859 | 19283.962 | 1.829 | 14.456 | 0.131293 | 7/15/2014 | 21:08.9 | 2 |
| 178.101 | 19283.956 | 1.828 | 14.455 | 0.136097 | 7/15/2014 | 21:09.0 | 2 |
| 178.341 | 19283.951 | 1.828 | 14.438 | 0.138743 | 7/15/2014 | 21:09.1 | 2 |
| 178.581 | 19283.946 | 1.828 | 14.427 | 0.139074 | 7/15/2014 | 21:09.2 | 2 |
| 178.819 | 19283.94 | 1.83 | 14.411 | 0.142751 | 7/15/2014 | 21:09.3 | 2 |
| 179.057 | 19283.936 | 1.835 | 14.397 | 0.150933 | 7/15/2014 | 21:09.4 | 2 |
| 179.292 | 19283.931 | 1.837 | 14.376 | 0.147822 | 7/15/2014 | 21:09.5 | 2 |
| 179.524 | 19283.925 | 1.832 | 14.358 | 0.163545 | 7/15/2014 | 21:09.6 | 2 |
| 179.814 | 19283.918 | 1.832 | 14.338 | 0.156173 | 7/15/2014 | 21:09.7 | 2 |
| 180.046 | 19283.914 | 1.832 | 14.33 | 0.159931 | 7/15/2014 | 21:09.8 | 2 |
| 180.277 | 19283.909 | 1.832 | 14.323 | 0.165825 | 7/15/2014 | 21:09.9 | 2 |
| 180.509 | 19283.903 | 1.832 | 14.317 | 0.169386 | 7/15/2014 | 21:10.0 | 2 |
| 180.741 | 19283.898 | 1.832 | 14.313 | 0.161003 | 7/15/2014 | 21:10.1 | 2 |
| 180.971 | 19283.894 | 1.827 | 14.311 | 0.168026 | 7/15/2014 | 21:10.2 | 2 |
| 181.201 | 19283.888 | 1.828 | 14.315 | 0.163044 | 7/15/2014 | 21:10.3 | 2 |
| 181.489 | 19283.881 | 1.83 | 14.319 | 0.161104 | 7/15/2014 | 21:10.4 | 2 |
| 181.721 | 19283.877 | 1.829 | 14.329 | 0.177816 | 7/15/2014 | 21:10.5 | 2 |
| 181.952 | 19283.871 | 1.829 | 14.337 | 0.174895 | 7/15/2014 | 21:10.6 | 2 |
| 182.185 | 19283.866 | 1.829 | 14.343 | 0.18783 | 7/15/2014 | 21:10.7 | 2 |
| 182.419 | 19283.861 | 1.832 | 14.349 | 0.184238 | 7/15/2014 | 21:10.8 | 2 |
| 182.651 | 19283.856 | 1.832 | 14.353 | 0.195244 | 7/15/2014 | 21:10.9 | 2 |
| 182.885 | 19283.851 | 1.837 | 14.356 | 0.191628 | 7/15/2014 | 21:11.0 | 2 |
| 183.117 | 19283.846 | 1.834 | 14.356 | 0.225606 | 7/15/2014 | 21:11.1 | 2 |
| 183.35 | 19283.841 | 1.837 | 14.354 | 0.193038 | 7/15/2014 | 21:11.2 | 2 |
| 183.579 | 19283.834 | 1.835 | 14.349 | 0.209039 | 7/15/2014 | 21:11.3 | 2 |
| 183.807 | 19283.83 | 1.836 | 14.333 | 0.20642 | 7/15/2014 | 21:11.4 | 2 |
| 184.035 | 19283.825 | 1.839 | 14.322 | 0.212427 | 7/15/2014 | 21:11.5 | 2 |
| 184.26 | 19283.819 | 1.836 | 14.307 | 0.223458 | 7/15/2014 | 21:11.6 | 2 |
| 184.54 | 19283.813 | 1.836 | 14.293 | 0.218186 | 7/15/2014 | 21:11.7 | 2 |
| 184.765 | 19283.809 | 1.836 | 14.279 | 0.210848 | 7/15/2014 | 21:11.8 | 2 |
| 184.99 | 19283.804 | 1.836 | 14.265 | 0.25772 | 7/15/2014 | 21:11.9 | 2 |
| 185.215 | 19283.798 | 1.834 | 14.255 | 0.248935 | 7/15/2014 | 21:12.0 | 2 |
| 185.442 | 19283.793 | 1.836 | 14.244 | 0.241698 | 7/15/2014 | 21:12.1 | 2 |
| 185.668 | 19283.788 | 1.838 | 14.238 | 0.266302 | 7/15/2014 | 21:12.2 | 2 |
| 185.895 | 19283.782 | 1.835 | 14.236 | 0.228259 | 7/15/2014 | 21:12.3 | 2 |
| 186.177 | 19283.776 | 1.84 | 14.238 | 0.243583 | 7/15/2014 | 21:12.4 | 2 |
| 186.406 | 19283.771 | 1.843 | 14.245 | 0.219249 | 7/15/2014 | 21:12.5 | 2 |
| 186.637 | 19283.766 | 1.845 | 14.249 | 0.183714 | 7/15/2014 | 21:12.6 | 2 |
| 186.869 | 19283.761 | 1.845 | 14.251 | 0.164249 | 7/15/2014 | 21:12.7 | 2 |
| 187.103 | 19283.755 | 1.847 | 14.255 | 0.151288 | 7/15/2014 | 21:12.8 | 2 |
| 187.337 | 19283.75 | 1.848 | 14.256 | 0.143862 | 7/15/2014 | 21:12.9 | 2 |
| 187.572 | 19283.744 | 1.845 | 14.26 | 0.156205 | 7/15/2014 | 21:13.0 | 2 |

| | | | | | | | |
|---|---|---|---|---|---|---|---|
| 187.808 | 19283.738 | 1.847 | 14.259 | 0.148806 | 7/15/2014 | 21:13.1 | 2 |
| 188.041 | 19283.734 | 1.847 | 14.259 | 0.155218 | 7/15/2014 | 21:13.2 | 2 |
| 188.273 | 19283.728 | 1.847 | 14.251 | 0.1623 | 7/15/2014 | 21:13.3 | 2 |
| 188.506 | 19283.723 | 1.845 | 14.249 | 0.175161 | 7/15/2014 | 21:13.4 | 2 |
| 188.736 | 19283.718 | 1.851 | 14.246 | 0.183763 | 7/15/2014 | 21:13.5 | 2 |
| 188.964 | 19283.713 | 1.853 | 14.237 | 0.196069 | 7/15/2014 | 21:13.6 | 2 |
| 189.245 | 19283.706 | 1.853 | 14.235 | 0.18803 | 7/15/2014 | 21:13.7 | 2 |
| 189.472 | 19283.7 | 1.856 | 14.224 | 0.195944 | 7/15/2014 | 21:13.8 | 2 |
| 189.697 | 19283.695 | 1.854 | 14.218 | 0.198722 | 7/15/2014 | 21:13.9 | 2 |
| 189.922 | 19283.69 | 1.854 | 14.21 | 0.190664 | 7/15/2014 | 21:14.0 | 2 |
| 190.147 | 19283.686 | 1.851 | 14.204 | 0.185586 | 7/15/2014 | 21:14.1 | 2 |
| 190.372 | 19283.682 | 1.851 | 14.196 | 0.186182 | 7/15/2014 | 21:14.2 | 2 |
| 190.596 | 19283.677 | 1.848 | 14.195 | 0.16951 | 7/15/2014 | 21:14.3 | 2 |
| 190.874 | 19283.67 | 1.846 | 14.192 | 0.176227 | 7/15/2014 | 21:14.4 | 2 |
| 191.101 | 19283.665 | 1.845 | 14.185 | 0.17022 | 7/15/2014 | 21:14.5 | 2 |
| 191.327 | 19283.66 | 1.845 | 14.18 | 0.176685 | 7/15/2014 | 21:14.6 | 2 |
| 191.557 | 19283.654 | 1.845 | 14.175 | 0.174469 | 7/15/2014 | 21:14.7 | 2 |
| 191.788 | 19283.65 | 1.845 | 14.17 | 0.171648 | 7/15/2014 | 21:14.8 | 2 |
| 192.02 | 19283.646 | 1.845 | 14.169 | 0.177017 | 7/15/2014 | 21:14.9 | 2 |
| 192.254 | 19283.641 | 1.845 | 14.165 | 0.185512 | 7/15/2014 | 21:15.0 | 2 |
| 192.488 | 19283.636 | 1.845 | 14.152 | 0.185312 | 7/15/2014 | 21:15.1 | 2 |
| 192.723 | 19283.631 | 1.845 | 14.141 | 0.198663 | 7/15/2014 | 21:15.2 | 2 |
| 192.957 | 19283.627 | 1.843 | 14.125 | 0.19847 | 7/15/2014 | 21:15.3 | 2 |
| 193.191 | 19283.621 | 1.845 | 14.108 | 0.209609 | 7/15/2014 | 21:15.4 | 2 |
| 193.426 | 19283.617 | 1.853 | 14.086 | 0.222424 | 7/15/2014 | 21:15.5 | 2 |
| 193.658 | 19283.613 | 1.853 | 14.061 | 0.227466 | 7/15/2014 | 21:15.6 | 2 |
| 193.948 | 19283.606 | 1.858 | 14.031 | 0.226686 | 7/15/2014 | 21:15.7 | 2 |
| 194.066 | 19283.604 | 1.858 | 14.016 | 0.226443 | 7/15/2014 | 21:15.8 | 2 |
| 194.413 | 19283.599 | 1.862 | 13.976 | 0.224351 | 7/15/2014 | 21:15.9 | 2 |
| 194.645 | 19283.595 | 1.864 | 13.95 | 0.226669 | 7/15/2014 | 21:16.0 | 2 |
| 194.879 | 19283.59 | 1.872 | 13.928 | 0.24472 | 7/15/2014 | 21:16.1 | 2 |
| 195.111 | 19283.586 | 1.872 | 13.912 | 0.243239 | 7/15/2014 | 21:16.2 | 2 |
| 195.343 | 19283.582 | 1.872 | 13.894 | 0.242845 | 7/15/2014 | 21:16.3 | 2 |
| 195.633 | 19283.576 | 1.875 | 13.883 | 0.254862 | 7/15/2014 | 21:16.4 | 2 |
| 195.864 | 19283.573 | 1.877 | 13.878 | 0.264545 | 7/15/2014 | 21:16.5 | 2 |
| 196.096 | 19283.569 | 1.877 | 13.875 | 0.262485 | 7/15/2014 | 21:16.6 | 2 |
| 196.331 | 19283.565 | 1.877 | 13.875 | 0.264945 | 7/15/2014 | 21:16.7 | 2 |
| 196.566 | 19283.561 | 1.877 | 13.885 | 0.249812 | 7/15/2014 | 21:16.8 | 2 |
| 196.801 | 19283.557 | 1.88 | 13.899 | 0.231859 | 7/15/2014 | 21:16.9 | 2 |
| 197.037 | 19283.553 | 1.887 | 13.912 | 0.216638 | 7/15/2014 | 21:17.0 | 2 |
| 197.271 | 19283.549 | 1.887 | 13.93 | 0.222655 | 7/15/2014 | 21:17.1 | 2 |
| 197.505 | 19283.545 | 1.887 | 13.943 | 0.198584 | 7/15/2014 | 21:17.2 | 2 |
| 197.736 | 19283.541 | 1.89 | 13.955 | 0.176971 | 7/15/2014 | 21:17.3 | 2 |
| 197.968 | 19283.537 | 1.893 | 13.965 | 0.161491 | 7/15/2014 | 21:17.4 | 2 |
| 198.198 | 19283.533 | 1.895 | 13.972 | 0.158777 | 7/15/2014 | 21:17.5 | 2 |
| 198.427 | 19283.529 | 1.898 | 13.972 | 0.145243 | 7/15/2014 | 21:17.6 | 2 |
| 198.713 | 19283.525 | 1.892 | 13.965 | 0.144143 | 7/15/2014 | 21:17.7 | 2 |

| | | | | | | | |
|---|---|---|---|---|---|---|---|
| 198.942 | 19283.521 | 1.884 | 13.956 | 0.144129 | 7/15/2014 | 21:17.8 | 2 |
| 199.174 | 19283.518 | 1.884 | 13.946 | 0.143613 | 7/15/2014 | 21:17.9 | 2 |
| 199.405 | 19283.514 | 1.884 | 13.937 | 0.154343 | 7/15/2014 | 21:18.0 | 2 |
| 199.635 | 19283.51 | 1.88 | 13.929 | 0.153168 | 7/15/2014 | 21:18.1 | 2 |
| 199.867 | 19283.505 | 1.877 | 13.923 | 0.156095 | 7/15/2014 | 21:18.2 | 2 |
| 199.979 | 19283.503 | 1.878 | 13.919 | 0.171667 | 7/15/2014 | 21:18.3 | 2 |
| 200.393 | 19283.497 | 1.87 | 13.914 | 0.176739 | 7/15/2014 | 21:18.4 | 2 |
| 200.627 | 19283.493 | 1.868 | 13.91 | 0.175639 | 7/15/2014 | 21:18.5 | 2 |
| 200.868 | 19283.488 | 1.868 | 13.907 | 0.176932 | 7/15/2014 | 21:18.6 | 2 |
| 201.11 | 19283.484 | 1.863 | 13.903 | 0.172459 | 7/15/2014 | 21:18.7 | 2 |
| 201.353 | 19283.48 | 1.859 | 13.899 | 0.176372 | 7/15/2014 | 21:18.8 | 2 |
| 201.598 | 19283.476 | 1.854 | 13.888 | 0.175293 | 7/15/2014 | 21:18.9 | 2 |
| 201.844 | 19283.471 | 1.852 | 13.876 | 0.182874 | 7/15/2014 | 21:19.0 | 2 |
| 202.092 | 19283.466 | 1.853 | 13.863 | 0.184056 | 7/15/2014 | 21:19.1 | 2 |
| 202.337 | 19283.462 | 1.85 | 13.841 | 0.179054 | 7/15/2014 | 21:19.2 | 2 |
| 202.585 | 19283.456 | 1.852 | 13.817 | 0.190331 | 7/15/2014 | 21:19.3 | 2 |
| 202.832 | 19283.451 | 1.849 | 13.802 | 0.178304 | 7/15/2014 | 21:19.4 | 2 |
| 203.078 | 19283.447 | 1.844 | 13.774 | 0.18264 | 7/15/2014 | 21:19.5 | 2 |
| 203.324 | 19283.442 | 1.839 | 13.742 | 0.171314 | 7/15/2014 | 21:19.6 | 2 |
| 203.63 | 19283.436 | 1.837 | 13.714 | 0.172245 | 7/15/2014 | 21:19.7 | 2 |
| 203.754 | 19283.433 | 1.834 | 13.699 | 0.172631 | 7/15/2014 | 21:19.8 | 2 |
| 204 | 19283.428 | 1.834 | 13.663 | 0.162435 | 7/15/2014 | 21:19.9 | 2 |
| 204.247 | 19283.423 | 1.826 | 13.641 | 0.160302 | 7/15/2014 | 21:20.0 | 2 |
| 204.494 | 19283.417 | 1.822 | 13.629 | 0.163464 | 7/15/2014 | 21:20.1 | 2 |
| 204.738 | 19283.412 | 1.82 | 13.602 | 0.170652 | 7/15/2014 | 21:20.2 | 2 |
| 204.985 | 19283.405 | 1.824 | 13.592 | 0.176052 | 7/15/2014 | 21:20.3 | 2 |
| 205.415 | 19283.396 | 1.823 | 13.586 | 0.186212 | 7/15/2014 | 21:20.4 | 2 |
| 205.66 | 19283.391 | 1.825 | 13.584 | 0.186833 | 7/15/2014 | 21:20.5 | 2 |
| 205.905 | 19283.385 | 1.824 | 13.582 | 0.195485 | 7/15/2014 | 21:20.6 | 2 |
| 206.214 | 19283.377 | 1.827 | 13.58 | 0.202717 | 7/15/2014 | 21:20.7 | 2 |
| 206.338 | 19283.374 | 1.826 | 13.575 | 0.201781 | 7/15/2014 | 21:20.8 | 2 |
| 206.586 | 19283.367 | 1.828 | 13.572 | 0.21322 | 7/15/2014 | 21:20.9 | 2 |
| 206.833 | 19283.361 | 1.829 | 13.572 | 0.221601 | 7/15/2014 | 21:21.0 | 2 |
| 207.083 | 19283.355 | 1.829 | 13.57 | 0.225724 | 7/15/2014 | 21:21.1 | 2 |
| 207.33 | 19283.35 | 1.824 | 13.566 | 0.239182 | 7/15/2014 | 21:21.2 | 2 |
| 207.578 | 19283.342 | 1.824 | 13.564 | 0.229173 | 7/15/2014 | 21:21.3 | 2 |
| 208.01 | 19283.329 | 1.823 | 13.566 | 0.257879 | 7/15/2014 | 21:21.4 | 2 |
| 208.255 | 19283.323 | 1.823 | 13.565 | 0.259534 | 7/15/2014 | 21:21.5 | 2 |
| 208.501 | 19283.316 | 1.821 | 13.566 | 0.268931 | 7/15/2014 | 21:21.6 | 2 |
| 208.808 | 19283.309 | 1.816 | 13.564 | 0.241929 | 7/15/2014 | 21:21.7 | 2 |
| 208.932 | 19283.305 | 1.816 | 13.562 | 0.240232 | 7/15/2014 | 21:21.8 | 2 |
| 209.182 | 19283.297 | 1.818 | 13.562 | 0.24016 | 7/15/2014 | 21:21.9 | 2 |
| 209.429 | 19283.289 | 1.814 | 13.565 | 0.234948 | 7/15/2014 | 21:22.0 | 2 |
| 209.679 | 19283.281 | 1.816 | 13.56 | 0.237314 | 7/15/2014 | 21:22.1 | 2 |
| 209.926 | 19283.274 | 1.814 | 13.562 | 0.236948 | 7/15/2014 | 21:22.2 | 2 |
| 210.176 | 19283.267 | 1.816 | 13.567 | 0.238911 | 7/15/2014 | 21:22.3 | 2 |
| 210.484 | 19283.257 | 1.816 | 13.572 | 0.265901 | 7/15/2014 | 21:22.4 | 2 |

| | | | | | | | |
|---|---|---|---|---|---|---|---|
| 210.734 | 19283.248 | 1.818 | 13.576 | 0.254037 | 7/15/2014 | 21:22.5 | 2 |
| 210.982 | 19283.24 | 1.816 | 13.583 | 0.275449 | 7/15/2014 | 21:22.6 | 2 |
| 211.232 | 19283.232 | 1.818 | 13.586 | 0.257121 | 7/15/2014 | 21:22.7 | 2 |
| 211.544 | 19283.221 | 1.814 | 13.593 | 0.273344 | 7/15/2014 | 21:22.8 | 2 |
| 211.797 | 19283.212 | 1.81 | 13.609 | 0.264444 | 7/15/2014 | 21:22.9 | 2 |
| 212.048 | 19283.204 | 1.807 | 13.619 | 0.255189 | 7/15/2014 | 21:23.0 | 2 |
| 212.302 | 19283.193 | 1.813 | 13.637 | 0.287477 | 7/15/2014 | 21:23.1 | 2 |
| 212.552 | 19283.186 | 1.807 | 13.659 | 0.269442 | 7/15/2014 | 21:23.2 | 2 |
| 212.805 | 19283.176 | 1.804 | 13.69 | 0.277279 | 7/15/2014 | 21:23.3 | 2 |
| 213.12 | 19283.163 | 1.801 | 13.732 | 0.277383 | 7/15/2014 | 21:23.4 | 2 |
| 213.373 | 19283.154 | 1.806 | 13.78 | 0.281191 | 7/15/2014 | 21:23.5 | 2 |
| 213.623 | 19283.146 | 1.807 | 13.811 | 0.285025 | 7/15/2014 | 21:23.6 | 2 |
| 213.876 | 19283.135 | 1.807 | 13.849 | 0.283428 | 7/15/2014 | 21:23.7 | 2 |
| 214.187 | 19283.123 | 1.799 | 13.881 | 0.270713 | 7/15/2014 | 21:23.8 | 2 |
| 214.44 | 19283.111 | 1.805 | 13.904 | 0.287303 | 7/15/2014 | 21:23.9 | 2 |
| 214.693 | 19283.102 | 1.809 | 13.916 | 0.2812 | 7/15/2014 | 21:24.0 | 2 |
| 214.946 | 19283.092 | 1.809 | 13.922 | 0.285521 | 7/15/2014 | 21:24.1 | 2 |
| 215.195 | 19283.081 | 1.797 | 13.918 | 0.293427 | 7/15/2014 | 21:24.2 | 2 |
| 215.447 | 19283.07 | 1.799 | 13.921 | 0.300389 | 7/15/2014 | 21:24.3 | 2 |
| 215.759 | 19283.057 | 1.799 | 13.918 | 0.34258 | 7/15/2014 | 21:24.4 | 2 |
| 216.009 | 19283.046 | 1.799 | 13.913 | 0.342074 | 7/15/2014 | 21:24.5 | 2 |
| 216.259 | 19283.035 | 1.79 | 13.91 | 0.353175 | 7/15/2014 | 21:24.6 | 2 |
| 216.509 | 19283.025 | 1.793 | 13.908 | 0.365293 | 7/15/2014 | 21:24.7 | 2 |
| 216.819 | 19283.01 | 1.789 | 13.904 | 0.350304 | 7/15/2014 | 21:24.8 | 2 |
| 367.179 | 19109.793 | 2.044 | 12.589 | 0.456984 | 7/15/2014 | 23:38.6 | 3 |
| 366.879 | 19109.758 | 2.044 | 12.597 | 0.443519 | 7/15/2014 | 23:38.7 | 3 |
| 366.758 | 19109.744 | 2.04 | 12.599 | 0.440999 | 7/15/2014 | 23:38.8 | 3 |
| 366.515 | 19109.717 | 2.042 | 12.615 | 0.438178 | 7/15/2014 | 23:38.9 | 3 |
| 366.274 | 19109.689 | 2.036 | 12.624 | 0.429815 | 7/15/2014 | 23:39.0 | 3 |
| 366.03 | 19109.664 | 2.036 | 12.637 | 0.415836 | 7/15/2014 | 23:39.1 | 3 |
| 365.789 | 19109.637 | 2.034 | 12.646 | 0.426765 | 7/15/2014 | 23:39.2 | 3 |
| 365.545 | 19109.611 | 2.034 | 12.661 | 0.395793 | 7/15/2014 | 23:39.3 | 3 |
| 365.124 | 19109.563 | 2.029 | 12.677 | 0.381525 | 7/15/2014 | 23:39.4 | 3 |
| 364.884 | 19109.537 | 2.027 | 12.694 | 0.361726 | 7/15/2014 | 23:39.5 | 3 |
| 364.644 | 19109.51 | 2.025 | 12.703 | 0.343734 | 7/15/2014 | 23:39.6 | 3 |
| 364.344 | 19109.479 | 2.023 | 12.717 | 0.349552 | 7/15/2014 | 23:39.7 | 3 |
| 364.222 | 19109.465 | 2.022 | 12.724 | 0.33584 | 7/15/2014 | 23:39.8 | 3 |
| 363.98 | 19109.438 | 2.019 | 12.737 | 0.326109 | 7/15/2014 | 23:39.9 | 3 |
| 363.741 | 19109.412 | 2.013 | 12.746 | 0.321371 | 7/15/2014 | 23:40.0 | 3 |
| 363.498 | 19109.385 | 2.01 | 12.764 | 0.311889 | 7/15/2014 | 23:40.1 | 3 |
| 363.261 | 19109.36 | 2 | 12.779 | 0.320624 | 7/15/2014 | 23:40.2 | 3 |
| 363.02 | 19109.334 | 1.996 | 12.794 | 0.310168 | 7/15/2014 | 23:40.3 | 3 |
| 362.604 | 19109.289 | 1.985 | 12.818 | 0.31464 | 7/15/2014 | 23:40.4 | 3 |
| 362.369 | 19109.263 | 1.978 | 12.837 | 0.301887 | 7/15/2014 | 23:40.5 | 3 |
| 362.132 | 19109.238 | 1.975 | 12.852 | 0.303739 | 7/15/2014 | 23:40.6 | 3 |
| 361.836 | 19109.205 | 1.972 | 12.869 | 0.298849 | 7/15/2014 | 23:40.7 | 3 |
| 361.717 | 19109.192 | 1.97 | 12.876 | 0.28466 | 7/15/2014 | 23:40.8 | 3 |

| | | | | | | | |
|---|---|---|---|---|---|---|---|
| 361.477 | 19109.168 | 1.977 | 12.889 | 0.298705 | 7/15/2014 | 23:40.9 | 3 |
| 361.24 | 19109.142 | 1.971 | 12.897 | 0.298102 | 7/15/2014 | 23:41.0 | 3 |
| 360.999 | 19109.116 | 1.971 | 12.906 | 0.280587 | 7/15/2014 | 23:41.1 | 3 |
| 360.762 | 19109.09 | 1.967 | 12.919 | 0.300264 | 7/15/2014 | 23:41.2 | 3 |
| 360.522 | 19109.065 | 1.967 | 12.925 | 0.295959 | 7/15/2014 | 23:41.3 | 3 |
| 360.106 | 19109.021 | 1.964 | 12.945 | 0.305912 | 7/15/2014 | 23:41.4 | 3 |
| 359.867 | 19108.996 | 1.959 | 12.954 | 0.310533 | 7/15/2014 | 23:41.5 | 3 |
| 359.63 | 19108.971 | 1.953 | 12.963 | 0.311399 | 7/15/2014 | 23:41.6 | 3 |
| 359.332 | 19108.938 | 1.953 | 12.973 | 0.316817 | 7/15/2014 | 23:41.7 | 3 |
| 359.211 | 19108.926 | 1.952 | 12.978 | 0.312029 | 7/15/2014 | 23:41.8 | 3 |
| 358.97 | 19108.901 | 1.952 | 12.989 | 0.319314 | 7/15/2014 | 23:41.9 | 3 |
| 358.731 | 19108.876 | 1.944 | 13.001 | 0.317405 | 7/15/2014 | 23:42.0 | 3 |
| 358.49 | 19108.851 | 1.942 | 13.011 | 0.314186 | 7/15/2014 | 23:42.1 | 3 |
| 358.25 | 19108.826 | 1.937 | 13.015 | 0.321649 | 7/15/2014 | 23:42.2 | 3 |
| 358.011 | 19108.802 | 1.94 | 13.024 | 0.315842 | 7/15/2014 | 23:42.3 | 3 |
| 357.594 | 19108.758 | 1.938 | 13.036 | 0.322471 | 7/15/2014 | 23:42.4 | 3 |
| 357.358 | 19108.734 | 1.934 | 13.045 | 0.314275 | 7/15/2014 | 23:42.5 | 3 |
| 357.121 | 19108.71 | 1.934 | 13.05 | 0.324839 | 7/15/2014 | 23:42.6 | 3 |
| 356.826 | 19108.68 | 1.934 | 13.053 | 0.31012 | 7/15/2014 | 23:42.7 | 3 |
| 356.706 | 19108.668 | 1.932 | 13.056 | 0.309306 | 7/15/2014 | 23:42.8 | 3 |
| 356.468 | 19108.644 | 1.932 | 13.06 | 0.307055 | 7/15/2014 | 23:42.9 | 3 |
| 356.232 | 19108.618 | 1.928 | 13.068 | 0.307079 | 7/15/2014 | 23:43.0 | 3 |
| 355.994 | 19108.595 | 1.925 | 13.077 | 0.299173 | 7/15/2014 | 23:43.1 | 3 |
| 355.758 | 19108.571 | 1.92 | 13.08 | 0.294941 | 7/15/2014 | 23:43.2 | 3 |
| 355.521 | 19108.548 | 1.923 | 13.086 | 0.293328 | 7/15/2014 | 23:43.3 | 3 |
| 355.112 | 19108.506 | 1.917 | 13.096 | 0.302928 | 7/15/2014 | 23:43.4 | 3 |
| 354.878 | 19108.482 | 1.917 | 13.103 | 0.294802 | 7/15/2014 | 23:43.5 | 3 |
| 354.644 | 19108.459 | 1.919 | 13.112 | 0.295001 | 7/15/2014 | 23:43.6 | 3 |
| 354.352 | 19108.43 | 1.919 | 13.114 | 0.295039 | 7/15/2014 | 23:43.7 | 3 |
| 354.233 | 19108.418 | 1.918 | 13.116 | 0.299643 | 7/15/2014 | 23:43.8 | 3 |
| 353.997 | 19108.396 | 1.918 | 13.122 | 0.283806 | 7/15/2014 | 23:43.9 | 3 |
| 353.762 | 19108.371 | 1.915 | 13.126 | 0.300951 | 7/15/2014 | 23:44.0 | 3 |
| 353.526 | 19108.348 | 1.918 | 13.133 | 0.287734 | 7/15/2014 | 23:44.1 | 3 |
| 353.29 | 19108.324 | 1.918 | 13.135 | 0.301694 | 7/15/2014 | 23:44.2 | 3 |
| 353.055 | 19108.302 | 1.918 | 13.143 | 0.293475 | 7/15/2014 | 23:44.3 | 3 |
| 352.645 | 19108.262 | 1.912 | 13.146 | 0.303463 | 7/15/2014 | 23:44.4 | 3 |
| 352.412 | 19108.24 | 1.908 | 13.153 | 0.307526 | 7/15/2014 | 23:44.5 | 3 |
| 352.177 | 19108.218 | 1.905 | 13.158 | 0.308089 | 7/15/2014 | 23:44.6 | 3 |
| 351.885 | 19108.189 | 1.902 | 13.167 | 0.318156 | 7/15/2014 | 23:44.7 | 3 |
| 351.766 | 19108.178 | 1.898 | 13.168 | 0.315917 | 7/15/2014 | 23:44.8 | 3 |
| 351.532 | 19108.154 | 1.895 | 13.174 | 0.315732 | 7/15/2014 | 23:44.9 | 3 |
| 351.296 | 19108.133 | 1.887 | 13.177 | 0.32437 | 7/15/2014 | 23:45.0 | 3 |
| 351.057 | 19108.11 | 1.885 | 13.183 | 0.330676 | 7/15/2014 | 23:45.1 | 3 |
| 350.82 | 19108.088 | 1.879 | 13.186 | 0.339196 | 7/15/2014 | 23:45.2 | 3 |
| 350.584 | 19108.066 | 1.879 | 13.191 | 0.337292 | 7/15/2014 | 23:45.3 | 3 |
| 350.17 | 19108.027 | 1.876 | 13.192 | 0.335627 | 7/15/2014 | 23:45.4 | 3 |
| 349.936 | 19108.005 | 1.876 | 13.192 | 0.325469 | 7/15/2014 | 23:45.5 | 3 |

| | | | | | | | |
|---|---|---|---|---|---|---|---|
| 349.7 | 19107.983 | 1.876 | 13.194 | 0.325484 | 7/15/2014 | 23:45.6 | 3 |
| 349.404 | 19107.957 | 1.88 | 13.197 | 0.315615 | 7/15/2014 | 23:45.7 | 3 |
| 349.284 | 19107.945 | 1.879 | 13.197 | 0.308867 | 7/15/2014 | 23:45.8 | 3 |
| 349.047 | 19107.925 | 1.878 | 13.201 | 0.297018 | 7/15/2014 | 23:45.9 | 3 |
| 348.809 | 19107.902 | 1.878 | 13.198 | 0.307058 | 7/15/2014 | 23:46.0 | 3 |
| 348.57 | 19107.881 | 1.881 | 13.202 | 0.295937 | 7/15/2014 | 23:46.1 | 3 |
| 348.333 | 19107.858 | 1.881 | 13.206 | 0.304697 | 7/15/2014 | 23:46.2 | 3 |
| 348.095 | 19107.838 | 1.885 | 13.21 | 0.306767 | 7/15/2014 | 23:46.3 | 3 |
| 347.682 | 19107.801 | 1.89 | 13.209 | 0.306452 | 7/15/2014 | 23:46.4 | 3 |
| 347.449 | 19107.781 | 1.89 | 13.21 | 0.314302 | 7/15/2014 | 23:46.5 | 3 |
| 347.212 | 19107.76 | 1.896 | 13.213 | 0.307516 | 7/15/2014 | 23:46.6 | 3 |
| 346.919 | 19107.734 | 1.899 | 13.213 | 0.296339 | 7/15/2014 | 23:46.7 | 3 |
| 346.801 | 19107.723 | 1.902 | 13.214 | 0.300253 | 7/15/2014 | 23:46.8 | 3 |
| 346.564 | 19107.703 | 1.905 | 13.211 | 0.297608 | 7/15/2014 | 23:46.9 | 3 |
| 346.328 | 19107.682 | 1.905 | 13.205 | 0.306343 | 7/15/2014 | 23:47.0 | 3 |
| 346.093 | 19107.662 | 1.908 | 13.203 | 0.305153 | 7/15/2014 | 23:47.1 | 3 |
| 345.859 | 19107.643 | 1.902 | 13.201 | 0.311544 | 7/15/2014 | 23:47.2 | 3 |
| 345.624 | 19107.623 | 1.908 | 13.197 | 0.325627 | 7/15/2014 | 23:47.3 | 3 |
| 345.216 | 19107.588 | 1.908 | 13.191 | 0.300023 | 7/15/2014 | 23:47.4 | 3 |
| 344.983 | 19107.569 | 1.908 | 13.189 | 0.321923 | 7/15/2014 | 23:47.5 | 3 |
| 344.75 | 19107.55 | 1.913 | 13.186 | 0.32181 | 7/15/2014 | 23:47.6 | 3 |
| 344.459 | 19107.526 | 1.913 | 13.184 | 0.307592 | 7/15/2014 | 23:47.7 | 3 |
| 344.342 | 19107.517 | 1.913 | 13.183 | 0.308041 | 7/15/2014 | 23:47.8 | 3 |
| 344.107 | 19107.498 | 1.919 | 13.177 | 0.295415 | 7/15/2014 | 23:47.9 | 3 |
| 343.872 | 19107.479 | 1.919 | 13.171 | 0.288209 | 7/15/2014 | 23:48.0 | 3 |
| 343.636 | 19107.459 | 1.924 | 13.165 | 0.293154 | 7/15/2014 | 23:48.1 | 3 |
| 343.402 | 19107.442 | 1.924 | 13.158 | 0.281081 | 7/15/2014 | 23:48.2 | 3 |
| 343.165 | 19107.423 | 1.929 | 13.154 | 0.263929 | 7/15/2014 | 23:48.3 | 3 |
| 342.755 | 19107.392 | 1.929 | 13.15 | 0.258432 | 7/15/2014 | 23:48.4 | 3 |
| 342.522 | 19107.373 | 1.931 | 13.145 | 0.262184 | 7/15/2014 | 23:48.5 | 3 |
| 342.286 | 19107.354 | 1.935 | 13.141 | 0.271282 | 7/15/2014 | 23:48.6 | 3 |
| 341.991 | 19107.332 | 1.939 | 13.138 | 0.282297 | 7/15/2014 | 23:48.7 | 3 |
| 341.873 | 19107.324 | 1.939 | 13.137 | 0.294913 | 7/15/2014 | 23:48.8 | 3 |
| 341.635 | 19107.305 | 1.946 | 13.135 | 0.296752 | 7/15/2014 | 23:48.9 | 3 |
| 341.397 | 19107.287 | 1.948 | 13.13 | 0.303702 | 7/15/2014 | 23:49.0 | 3 |
| 341.158 | 19107.27 | 1.953 | 13.131 | 0.318943 | 7/15/2014 | 23:49.1 | 3 |
| 340.921 | 19107.252 | 1.951 | 13.127 | 0.35284 | 7/15/2014 | 23:49.2 | 3 |
| 340.682 | 19107.233 | 1.959 | 13.129 | 0.350694 | 7/15/2014 | 23:49.3 | 3 |
| 340.265 | 19107.203 | 1.957 | 13.13 | 0.356987 | 7/15/2014 | 23:49.4 | 3 |
| 340.028 | 19107.186 | 1.957 | 13.13 | 0.371181 | 7/15/2014 | 23:49.5 | 3 |
| 339.79 | 19107.17 | 1.955 | 13.13 | 0.378187 | 7/15/2014 | 23:49.6 | 3 |
| 339.494 | 19107.147 | 1.953 | 13.132 | 0.35566 | 7/15/2014 | 23:49.7 | 3 |
| 339.374 | 19107.14 | 1.952 | 13.13 | 0.339468 | 7/15/2014 | 23:49.8 | 3 |
| 339.135 | 19107.121 | 1.954 | 13.129 | 0.349373 | 7/15/2014 | 23:49.9 | 3 |
| 338.896 | 19107.105 | 1.948 | 13.127 | 0.339686 | 7/15/2014 | 23:50.0 | 3 |
| 338.658 | 19107.088 | 1.948 | 13.128 | 0.326566 | 7/15/2014 | 23:50.1 | 3 |
| 338.421 | 19107.071 | 1.946 | 13.128 | 0.312261 | 7/15/2014 | 23:50.2 | 3 |

| | | | | | | | |
|---|---|---|---|---|---|---|---|
| 338.184 | 19107.056 | 1.95 | 13.129 | 0.311204 | 7/15/2014 | 23:50.3 | 3 |
| 337.773 | 19107.027 | 1.954 | 13.134 | 0.298896 | 7/15/2014 | 23:50.4 | 3 |
| 337.539 | 19107.011 | 1.956 | 13.135 | 0.278043 | 7/15/2014 | 23:50.5 | 3 |
| 337.305 | 19106.995 | 1.961 | 13.141 | 0.282234 | 7/15/2014 | 23:50.6 | 3 |
| 337.015 | 19106.977 | 1.964 | 13.144 | 0.270404 | 7/15/2014 | 23:50.7 | 3 |
| 336.896 | 19106.968 | 1.966 | 13.146 | 0.270856 | 7/15/2014 | 23:50.8 | 3 |
| 336.662 | 19106.952 | 1.969 | 13.147 | 0.261792 | 7/15/2014 | 23:50.9 | 3 |
| 336.428 | 19106.937 | 1.969 | 13.147 | 0.26418 | 7/15/2014 | 23:51.0 | 3 |
| 336.193 | 19106.922 | 1.978 | 13.143 | 0.257193 | 7/15/2014 | 23:51.1 | 3 |
| 335.962 | 19106.906 | 1.972 | 13.142 | 0.255145 | 7/15/2014 | 23:51.2 | 3 |
| 335.73 | 19106.892 | 1.976 | 13.139 | 0.25262 | 7/15/2014 | 23:51.3 | 3 |
| 335.324 | 19106.866 | 1.974 | 13.132 | 0.245589 | 7/15/2014 | 23:51.4 | 3 |
| 335.094 | 19106.852 | 1.974 | 13.13 | 0.244286 | 7/15/2014 | 23:51.5 | 3 |
| 334.864 | 19106.836 | 1.979 | 13.128 | 0.247197 | 7/15/2014 | 23:51.6 | 3 |
| 334.573 | 19106.819 | 1.976 | 13.128 | 0.245439 | 7/15/2014 | 23:51.7 | 3 |
| 334.458 | 19106.812 | 1.971 | 13.129 | 0.244786 | 7/15/2014 | 23:51.8 | 3 |
| 334.225 | 19106.797 | 1.977 | 13.131 | 0.241248 | 7/15/2014 | 23:51.9 | 3 |
| 333.992 | 19106.782 | 1.977 | 13.133 | 0.24291 | 7/15/2014 | 23:52.0 | 3 |
| 333.756 | 19106.769 | 1.987 | 13.136 | 0.242851 | 7/15/2014 | 23:52.1 | 3 |
| 333.525 | 19106.755 | 1.987 | 13.136 | 0.243712 | 7/15/2014 | 23:52.2 | 3 |
| 333.29 | 19106.74 | 1.994 | 13.136 | 0.242972 | 7/15/2014 | 23:52.3 | 3 |
| 332.883 | 19106.717 | 1.997 | 13.14 | 0.238677 | 7/15/2014 | 23:52.4 | 3 |
| 332.65 | 19106.701 | 2 | 13.141 | 0.243444 | 7/15/2014 | 23:52.5 | 3 |
| 332.418 | 19106.688 | 2.006 | 13.144 | 0.248712 | 7/15/2014 | 23:52.6 | 3 |
| 332.125 | 19106.672 | 2.009 | 13.145 | 0.256676 | 7/15/2014 | 23:52.7 | 3 |
| 332.008 | 19106.665 | 2.006 | 13.146 | 0.258735 | 7/15/2014 | 23:52.8 | 3 |
| 331.773 | 19106.65 | 2.012 | 13.148 | 0.268553 | 7/15/2014 | 23:52.9 | 3 |
| 331.539 | 19106.637 | 2.011 | 13.15 | 0.272585 | 7/15/2014 | 23:53.0 | 3 |
| 331.303 | 19106.624 | 2.014 | 13.148 | 0.278343 | 7/15/2014 | 23:53.1 | 3 |
| 331.069 | 19106.609 | 2.014 | 13.149 | 0.280065 | 7/15/2014 | 23:53.2 | 3 |
| 330.836 | 19106.597 | 2.02 | 13.151 | 0.277802 | 7/15/2014 | 23:53.3 | 3 |
| 330.429 | 19106.574 | 2.013 | 13.146 | 0.265651 | 7/15/2014 | 23:53.4 | 3 |
| 330.2 | 19106.561 | 2.01 | 13.148 | 0.258613 | 7/15/2014 | 23:53.5 | 3 |
| 329.969 | 19106.548 | 2.01 | 13.15 | 0.253258 | 7/15/2014 | 23:53.6 | 3 |
| 329.681 | 19106.532 | 2.011 | 13.149 | 0.247101 | 7/15/2014 | 23:53.7 | 3 |
| 329.451 | 19106.519 | 2.011 | 13.15 | 0.235228 | 7/15/2014 | 23:53.8 | 3 |
| 329.221 | 19106.505 | 2.011 | 13.148 | 0.231371 | 7/15/2014 | 23:53.9 | 3 |
| 328.991 | 19106.492 | 2.011 | 13.145 | 0.220007 | 7/15/2014 | 23:54.0 | 3 |
| 328.762 | 19106.48 | 2.014 | 13.146 | 0.219662 | 7/15/2014 | 23:54.1 | 3 |
| 328.535 | 19106.469 | 2.018 | 13.14 | 0.214755 | 7/15/2014 | 23:54.2 | 3 |
| 328.308 | 19106.456 | 2.022 | 13.133 | 0.211599 | 7/15/2014 | 23:54.3 | 3 |
| 328.027 | 19106.441 | 2.025 | 13.125 | 0.20765 | 7/15/2014 | 23:54.4 | 3 |
| 327.804 | 19106.427 | 2.027 | 13.118 | 0.206114 | 7/15/2014 | 23:54.5 | 3 |
| 327.58 | 19106.415 | 2.032 | 13.111 | 0.20553 | 7/15/2014 | 23:54.6 | 3 |
| 327.301 | 19106.4 | 2.036 | 13.105 | 0.204754 | 7/15/2014 | 23:54.7 | 3 |
| 327.078 | 19106.388 | 2.042 | 13.099 | 0.20518 | 7/15/2014 | 23:54.8 | 3 |
| 326.853 | 19106.377 | 2.047 | 13.09 | 0.204247 | 7/15/2014 | 23:54.9 | 3 |

| | | | | | | | |
|---|---|---|---|---|---|---|---|
| 326.629 | 19106.364 | 2.047 | 13.087 | 0.205501 | 7/15/2014 | 23:55.0 | 3 |
| 326.405 | 19106.352 | 2.049 | 13.081 | 0.205907 | 7/15/2014 | 23:55.1 | 3 |
| 326.181 | 19106.338 | 2.051 | 13.074 | 0.210581 | 7/15/2014 | 23:55.2 | 3 |
| 325.959 | 19106.327 | 2.051 | 13.068 | 0.204205 | 7/15/2014 | 23:55.3 | 3 |
| 325.681 | 19106.313 | 2.051 | 13.063 | 0.206098 | 7/15/2014 | 23:55.4 | 3 |
| 325.46 | 19106.299 | 2.051 | 13.057 | 0.199589 | 7/15/2014 | 23:55.5 | 3 |
| 325.239 | 19106.287 | 2.051 | 13.05 | 0.199647 | 7/15/2014 | 23:55.6 | 3 |
| 324.961 | 19106.271 | 2.053 | 13.047 | 0.208933 | 7/15/2014 | 23:55.7 | 3 |
| 324.738 | 19106.258 | 2.056 | 13.042 | 0.201661 | 7/15/2014 | 23:55.8 | 3 |
| 324.513 | 19106.246 | 2.061 | 13.036 | 0.204378 | 7/15/2014 | 23:55.9 | 3 |
| 324.29 | 19106.234 | 2.061 | 13.032 | 0.207773 | 7/15/2014 | 23:56.0 | 3 |
| 324.065 | 19106.222 | 2.066 | 13.03 | 0.208188 | 7/15/2014 | 23:56.1 | 3 |
| 323.842 | 19106.208 | 2.066 | 13.024 | 0.216237 | 7/15/2014 | 23:56.2 | 3 |
| 323.62 | 19106.194 | 2.07 | 13.019 | 0.217119 | 7/15/2014 | 23:56.3 | 3 |
| 323.344 | 19106.179 | 2.07 | 13.014 | 0.21372 | 7/15/2014 | 23:56.4 | 3 |
| 323.123 | 19106.166 | 2.07 | 13.005 | 0.211812 | 7/15/2014 | 23:56.5 | 3 |
| 322.902 | 19106.153 | 2.072 | 12.998 | 0.207332 | 7/15/2014 | 23:56.6 | 3 |
| 322.627 | 19106.138 | 2.072 | 12.992 | 0.201293 | 7/15/2014 | 23:56.7 | 3 |
| 322.405 | 19106.124 | 2.076 | 12.985 | 0.207373 | 7/15/2014 | 23:56.8 | 3 |
| 322.184 | 19106.111 | 2.078 | 12.975 | 0.19606 | 7/15/2014 | 23:56.9 | 3 |
| 321.962 | 19106.097 | 2.082 | 12.969 | 0.194449 | 7/15/2014 | 23:57.0 | 3 |
| 321.742 | 19106.083 | 2.082 | 12.961 | 0.193561 | 7/15/2014 | 23:57.1 | 3 |
| 321.521 | 19106.07 | 2.087 | 12.953 | 0.193754 | 7/15/2014 | 23:57.2 | 3 |
| 321.304 | 19106.057 | 2.089 | 12.947 | 0.196304 | 7/15/2014 | 23:57.3 | 3 |
| 321.032 | 19106.04 | 2.094 | 12.938 | 0.199415 | 7/15/2014 | 23:57.4 | 3 |
| 320.816 | 19106.026 | 2.096 | 12.934 | 0.198506 | 7/15/2014 | 23:57.5 | 3 |
| 320.601 | 19106.013 | 2.1 | 12.929 | 0.199262 | 7/15/2014 | 23:57.6 | 3 |
| 320.331 | 19105.995 | 2.102 | 12.927 | 0.192687 | 7/15/2014 | 23:57.7 | 3 |
| 320.116 | 19105.981 | 2.105 | 12.921 | 0.190914 | 7/15/2014 | 23:57.8 | 3 |
| 319.9 | 19105.967 | 2.107 | 12.917 | 0.187121 | 7/15/2014 | 23:57.9 | 3 |
| 319.683 | 19105.953 | 2.112 | 12.912 | 0.185405 | 7/15/2014 | 23:58.0 | 3 |
| 319.466 | 19105.938 | 2.115 | 12.906 | 0.18281 | 7/15/2014 | 23:58.1 | 3 |
| 319.25 | 19105.924 | 2.117 | 12.905 | 0.180474 | 7/15/2014 | 23:58.2 | 3 |
| 319.034 | 19105.908 | 2.122 | 12.898 | 0.175871 | 7/15/2014 | 23:58.3 | 3 |
| 318.764 | 19105.891 | 2.125 | 12.893 | 0.183292 | 7/15/2014 | 23:58.4 | 3 |
| 318.549 | 19105.876 | 2.128 | 12.887 | 0.183446 | 7/15/2014 | 23:58.5 | 3 |
| 318.331 | 19105.86 | 2.134 | 12.881 | 0.189496 | 7/15/2014 | 23:58.6 | 3 |
| 318.114 | 19105.845 | 2.141 | 12.876 | 0.185281 | 7/15/2014 | 23:58.7 | 3 |
| 317.895 | 19105.829 | 2.144 | 12.871 | 0.187519 | 7/15/2014 | 23:58.8 | 3 |
| 317.676 | 19105.813 | 2.151 | 12.872 | 0.187044 | 7/15/2014 | 23:58.9 | 3 |
| 317.458 | 19105.797 | 2.154 | 12.864 | 0.187172 | 7/15/2014 | 23:59.0 | 3 |
| 317.24 | 19105.78 | 2.154 | 12.861 | 0.192641 | 7/15/2014 | 23:59.1 | 3 |
| 317.023 | 19105.765 | 2.156 | 12.856 | 0.19284 | 7/15/2014 | 23:59.2 | 3 |
| 316.805 | 19105.749 | 2.161 | 12.853 | 0.19488 | 7/15/2014 | 23:59.3 | 3 |
| 316.592 | 19105.73 | 2.161 | 12.851 | 0.190936 | 7/15/2014 | 23:59.4 | 3 |
| 316.275 | 19105.707 | 2.165 | 12.85 | 0.194744 | 7/15/2014 | 23:59.6 | 3 |
| 316.013 | 19105.686 | 2.165 | 12.851 | 0.200637 | 7/15/2014 | 23:59.7 | 3 |

| | | | | | | | |
|---|---|---|---|---|---|---|---|
| 315.904 | 19105.677 | 2.167 | 12.847 | 0.195845 | 7/15/2014 | 23:59.7 | 3 |
| 315.695 | 19105.66 | 2.17 | 12.85 | 0.206559 | 7/15/2014 | 23:59.8 | 3 |
| 315.484 | 19105.643 | 2.175 | 12.848 | 0.199701 | 7/15/2014 | 23:59.9 | 3 |
| 315.273 | 19105.627 | 2.177 | 12.849 | 0.214768 | 7/15/2014 | 24:00.0 | 3 |
| 315.065 | 19105.607 | 2.177 | 12.849 | 0.21629 | 7/15/2014 | 24:00.1 | 3 |
| 314.855 | 19105.59 | 2.183 | 12.849 | 0.22699 | 7/15/2014 | 24:00.2 | 3 |
| 314.647 | 19105.572 | 2.183 | 12.848 | 0.216551 | 7/15/2014 | 24:00.3 | 3 |
| 314.28 | 19105.539 | 2.191 | 12.848 | 0.245494 | 7/15/2014 | 24:00.5 | 3 |
| 314.07 | 19105.521 | 2.197 | 12.846 | 0.227788 | 7/15/2014 | 24:00.6 | 3 |
| 313.86 | 19105.501 | 2.199 | 12.844 | 0.234615 | 7/15/2014 | 24:00.7 | 3 |
| 313.649 | 19105.483 | 2.206 | 12.846 | 0.238954 | 7/15/2014 | 24:00.8 | 3 |
| 313.439 | 19105.463 | 2.21 | 12.847 | 0.255493 | 7/15/2014 | 24:00.9 | 3 |
| 313.227 | 19105.442 | 2.217 | 12.846 | 0.244757 | 7/15/2014 | 24:01.0 | 3 |
| 313.017 | 19105.423 | 2.217 | 12.845 | 0.261658 | 7/15/2014 | 24:01.1 | 3 |
| 312.804 | 19105.402 | 2.226 | 12.845 | 0.266848 | 7/15/2014 | 24:01.2 | 3 |
| 312.596 | 19105.382 | 2.228 | 12.844 | 0.24364 | 7/15/2014 | 24:01.3 | 3 |
| 312.386 | 19105.361 | 2.233 | 12.84 | 0.246626 | 7/15/2014 | 24:01.4 | 3 |
| 312.18 | 19105.341 | 2.233 | 12.836 | 0.252778 | 7/15/2014 | 24:01.5 | 3 |
| 311.973 | 19105.319 | 2.238 | 12.833 | 0.231568 | 7/15/2014 | 24:01.6 | 3 |
| 311.72 | 19105.293 | 2.238 | 12.826 | 0.211554 | 7/15/2014 | 24:01.7 | 3 |
| 311.615 | 19105.282 | 2.238 | 12.823 | 0.205368 | 7/15/2014 | 24:01.7 | 3 |
| 311.409 | 19105.261 | 2.244 | 12.818 | 0.203927 | 7/15/2014 | 24:01.8 | 3 |
| 311.204 | 19105.24 | 2.251 | 12.812 | 0.18715 | 7/15/2014 | 24:01.9 | 3 |
| 311.001 | 19105.218 | 2.253 | 12.803 | 0.193934 | 7/15/2014 | 24:02.0 | 3 |
| 310.797 | 19105.195 | 2.259 | 12.799 | 0.188911 | 7/15/2014 | 24:02.1 | 3 |
| 310.595 | 19105.174 | 2.264 | 12.793 | 0.188252 | 7/15/2014 | 24:02.2 | 3 |
| 310.391 | 19105.15 | 2.269 | 12.79 | 0.188524 | 7/15/2014 | 24:02.4 | 3 |
| 310.036 | 19105.109 | 2.276 | 12.782 | 0.19115 | 7/15/2014 | 24:02.5 | 3 |
| 309.83 | 19105.086 | 2.286 | 12.779 | 0.186883 | 7/15/2014 | 24:02.6 | 3 |
| 309.625 | 19105.063 | 2.291 | 12.78 | 0.190802 | 7/15/2014 | 24:02.7 | 3 |
| 309.419 | 19105.039 | 2.295 | 12.777 | 0.183809 | 7/15/2014 | 24:02.8 | 3 |
| 309.214 | 19105.014 | 2.3 | 12.777 | 0.186888 | 7/15/2014 | 24:02.9 | 3 |
| 309.007 | 19104.989 | 2.305 | 12.775 | 0.18926 | 7/15/2014 | 24:03.0 | 3 |
| 308.802 | 19104.965 | 2.307 | 12.776 | 0.188843 | 7/15/2014 | 24:03.1 | 3 |
| 308.595 | 19104.938 | 2.312 | 12.775 | 0.18606 | 7/15/2014 | 24:03.2 | 3 |
| 308.391 | 19104.912 | 2.314 | 12.775 | 0.191236 | 7/15/2014 | 24:03.3 | 3 |
| 308.186 | 19104.889 | 2.32 | 12.778 | 0.188627 | 7/15/2014 | 24:03.4 | 3 |
| 307.982 | 19104.861 | 2.32 | 12.78 | 0.18792 | 7/15/2014 | 24:03.5 | 3 |
| 307.781 | 19104.836 | 2.322 | 12.778 | 0.198551 | 7/15/2014 | 24:03.6 | 3 |
| 307.532 | 19104.803 | 2.322 | 12.781 | 0.193364 | 7/15/2014 | 24:03.7 | 3 |
| 307.428 | 19104.79 | 2.323 | 12.782 | 0.194904 | 7/15/2014 | 24:03.7 | 3 |
| 307.226 | 19104.764 | 2.328 | 12.784 | 0.197775 | 7/15/2014 | 24:03.8 | 3 |
| 306.924 | 19104.723 | 2.333 | 12.789 | 0.202315 | 7/15/2014 | 24:04.0 | 3 |
| 306.724 | 19104.694 | 2.335 | 12.795 | 0.206323 | 7/15/2014 | 24:04.1 | 3 |
| 306.521 | 19104.667 | 2.34 | 12.798 | 0.214245 | 7/15/2014 | 24:04.2 | 3 |
| 306.422 | 19104.653 | 2.341 | 12.801 | 0.215025 | 7/15/2014 | 24:04.3 | 3 |
| 306.224 | 19104.625 | 2.343 | 12.803 | 0.215468 | 7/15/2014 | 24:04.4 | 3 |

| | | | | | | | |
|---|---|---|---|---|---|---|---|
| 305.872 | 19104.576 | 2.345 | 12.808 | 0.223559 | 7/15/2014 | 24:04.5 | 3 |
| 305.666 | 19104.546 | 2.352 | 12.812 | 0.222009 | 7/15/2014 | 24:04.6 | 3 |
| 305.465 | 19104.516 | 2.352 | 12.817 | 0.224111 | 7/15/2014 | 24:04.7 | 3 |
| 305.26 | 19104.486 | 2.358 | 12.823 | 0.225521 | 7/15/2014 | 24:04.8 | 3 |
| 305.058 | 19104.456 | 2.358 | 12.827 | 0.224531 | 7/15/2014 | 24:04.9 | 3 |
| 304.852 | 19104.424 | 2.363 | 12.826 | 0.232504 | 7/15/2014 | 24:05.0 | 3 |
| 304.649 | 19104.394 | 2.361 | 12.829 | 0.234343 | 7/15/2014 | 24:05.1 | 3 |
| 304.444 | 19104.362 | 2.366 | 12.83 | 0.239891 | 7/15/2014 | 24:05.2 | 3 |
| 304.242 | 19104.331 | 2.366 | 12.832 | 0.23809 | 7/15/2014 | 24:05.3 | 3 |
| 304.038 | 19104.298 | 2.372 | 12.831 | 0.238635 | 7/15/2014 | 24:05.4 | 3 |
| 303.837 | 19104.266 | 2.369 | 12.83 | 0.243209 | 7/15/2014 | 24:05.5 | 3 |
| 303.636 | 19104.234 | 2.374 | 12.826 | 0.243558 | 7/15/2014 | 24:05.6 | 3 |
| 303.388 | 19104.193 | 2.371 | 12.821 | 0.241079 | 7/15/2014 | 24:05.7 | 3 |
| 303.286 | 19104.177 | 2.373 | 12.819 | 0.241948 | 7/15/2014 | 24:05.7 | 3 |
| 302.989 | 19104.127 | 2.378 | 12.82 | 0.241736 | 7/15/2014 | 24:05.9 | 3 |
| 302.789 | 19104.094 | 2.38 | 12.821 | 0.243043 | 7/15/2014 | 24:06.0 | 3 |
| 302.589 | 19104.059 | 2.38 | 12.822 | 0.255356 | 7/15/2014 | 24:06.1 | 3 |
| 302.389 | 19104.024 | 2.385 | 12.823 | 0.249185 | 7/15/2014 | 24:06.2 | 3 |
| 302.19 | 19103.99 | 2.385 | 12.826 | 0.253079 | 7/15/2014 | 24:06.3 | 3 |
| 302.093 | 19103.975 | 2.388 | 12.826 | 0.252657 | 7/15/2014 | 24:06.3 | 3 |
| 301.745 | 19103.912 | 2.388 | 12.833 | 0.272339 | 7/15/2014 | 24:06.5 | 3 |
| 301.545 | 19103.875 | 2.394 | 12.837 | 0.263637 | 7/15/2014 | 24:06.6 | 3 |
| 301.347 | 19103.839 | 2.392 | 12.84 | 0.26658 | 7/15/2014 | 24:06.7 | 3 |
| 301.147 | 19103.804 | 2.394 | 12.839 | 0.27055 | 7/15/2014 | 24:06.8 | 3 |
| 300.947 | 19103.767 | 2.396 | 12.838 | 0.259224 | 7/15/2014 | 24:06.9 | 3 |
| 300.747 | 19103.729 | 2.4 | 12.836 | 0.26957 | 7/15/2014 | 24:07.0 | 3 |
| 300.548 | 19103.691 | 2.4 | 12.832 | 0.276503 | 7/15/2014 | 24:07.1 | 3 |
| 300.348 | 19103.652 | 2.404 | 12.826 | 0.268473 | 7/15/2014 | 24:07.2 | 3 |
| 300.151 | 19103.615 | 2.406 | 12.817 | 0.273775 | 7/15/2014 | 24:07.3 | 3 |
| 299.954 | 19103.578 | 2.409 | 12.81 | 0.280253 | 7/15/2014 | 24:07.4 | 3 |
| 299.759 | 19103.539 | 2.409 | 12.8 | 0.286824 | 7/15/2014 | 24:07.5 | 3 |
| 299.566 | 19103.501 | 2.411 | 12.79 | 0.284092 | 7/15/2014 | 24:07.6 | 3 |
| 299.327 | 19103.454 | 2.411 | 12.784 | 0.29722 | 7/15/2014 | 24:07.7 | 3 |
| 299.134 | 19103.415 | 2.416 | 12.774 | 0.297364 | 7/15/2014 | 24:07.8 | 3 |
| 298.943 | 19103.377 | 2.416 | 12.767 | 0.299488 | 7/15/2014 | 24:07.9 | 3 |
| 298.75 | 19103.336 | 2.421 | 12.763 | 0.307189 | 7/15/2014 | 24:08.0 | 3 |
| 298.56 | 19103.297 | 2.421 | 12.759 | 0.312389 | 7/15/2014 | 24:08.1 | 3 |
| 298.366 | 19103.258 | 2.424 | 12.757 | 0.316623 | 7/15/2014 | 24:08.2 | 3 |
| 298.177 | 19103.217 | 2.424 | 12.757 | 0.325512 | 7/15/2014 | 24:08.3 | 3 |
| 297.984 | 19103.176 | 2.427 | 12.76 | 0.330204 | 7/15/2014 | 24:08.4 | 3 |
| 297.749 | 19103.126 | 2.425 | 12.767 | 0.33271 | 7/15/2014 | 24:08.5 | 3 |
| 297.556 | 19103.085 | 2.427 | 12.769 | 0.336037 | 7/15/2014 | 24:08.6 | 3 |
| 297.364 | 19103.043 | 2.424 | 12.778 | 0.332342 | 7/15/2014 | 24:08.7 | 3 |
| 297.168 | 19103 | 2.427 | 12.787 | 0.341912 | 7/15/2014 | 24:08.8 | 3 |
| 296.976 | 19102.957 | 2.424 | 12.794 | 0.327476 | 7/15/2014 | 24:08.9 | 3 |
| 296.778 | 19102.912 | 2.427 | 12.804 | 0.330341 | 7/15/2014 | 24:09.0 | 3 |
| 296.585 | 19102.868 | 2.427 | 12.817 | 0.329756 | 7/15/2014 | 24:09.1 | 3 |

| | | | | | | | |
|---|---|---|---|---|---|---|---|
| 296.388 | 19102.824 | 2.43 | 12.828 | 0.329472 | 7/15/2014 | 24:09.2 | 3 |
| 296.196 | 19102.779 | 2.427 | 12.834 | 0.332205 | 7/15/2014 | 24:09.3 | 3 |
| 296 | 19102.735 | 2.431 | 12.845 | 0.335188 | 7/15/2014 | 24:09.4 | 3 |
| 295.807 | 19102.689 | 2.43 | 12.853 | 0.338964 | 7/15/2014 | 24:09.5 | 3 |
| 295.611 | 19102.645 | 2.432 | 12.856 | 0.340873 | 7/15/2014 | 24:09.6 | 3 |
| 295.371 | 19102.587 | 2.432 | 12.861 | 0.344499 | 7/15/2014 | 24:09.7 | 3 |
| 295.175 | 19102.539 | 2.435 | 12.871 | 0.344509 | 7/15/2014 | 24:09.8 | 3 |
| 294.98 | 19102.493 | 2.435 | 12.873 | 0.346539 | 7/15/2014 | 24:09.9 | 3 |
| 294.784 | 19102.445 | 2.435 | 12.875 | 0.352744 | 7/15/2014 | 24:10.0 | 3 |
| 294.589 | 19102.397 | 2.433 | 12.875 | 0.349712 | 7/15/2014 | 24:10.1 | 3 |
| 294.391 | 19102.349 | 2.436 | 12.879 | 0.355677 | 7/15/2014 | 24:10.2 | 3 |
| 294.297 | 19102.326 | 2.435 | 12.876 | 0.356863 | 7/15/2014 | 24:10.2 | 3 |
| 294.1 | 19102.277 | 2.441 | 12.879 | 0.353232 | 7/15/2014 | 24:10.3 | 3 |
| 293.753 | 19102.19 | 2.439 | 12.885 | 0.354669 | 7/15/2014 | 24:10.5 | 3 |
| 293.554 | 19102.139 | 2.439 | 12.89 | 0.355472 | 7/15/2014 | 24:10.6 | 3 |
| 293.354 | 19102.089 | 2.437 | 12.889 | 0.354065 | 7/15/2014 | 24:10.7 | 3 |
| 293.154 | 19102.037 | 2.437 | 12.896 | 0.356366 | 7/15/2014 | 24:10.8 | 3 |
| 292.952 | 19101.985 | 2.435 | 12.899 | 0.354726 | 7/15/2014 | 24:10.9 | 3 |
| 292.751 | 19101.932 | 2.435 | 12.909 | 0.35144 | 7/15/2014 | 24:11.0 | 3 |
| 292.55 | 19101.881 | 2.431 | 12.912 | 0.352459 | 7/15/2014 | 24:11.1 | 3 |
| 292.348 | 19101.827 | 2.431 | 12.92 | 0.35361 | 7/15/2014 | 24:11.2 | 3 |
| 292.148 | 19101.773 | 2.431 | 12.926 | 0.352173 | 7/15/2014 | 24:11.3 | 3 |
| 291.948 | 19101.721 | 2.431 | 12.937 | 0.355216 | 7/15/2014 | 24:11.4 | 3 |
| 291.75 | 19101.668 | 2.431 | 12.94 | 0.350553 | 7/15/2014 | 24:11.5 | 3 |
| 291.553 | 19101.615 | 2.433 | 12.942 | 0.353717 | 7/15/2014 | 24:11.6 | 3 |
| 291.309 | 19101.548 | 2.431 | 12.944 | 0.351775 | 7/15/2014 | 24:11.7 | 3 |
| 291.208 | 19101.521 | 2.431 | 12.948 | 0.352625 | 7/15/2014 | 24:11.7 | 3 |
| 291.013 | 19101.469 | 2.433 | 12.941 | 0.353021 | 7/15/2014 | 24:11.8 | 3 |
| 290.717 | 19101.387 | 2.437 | 12.933 | 0.355553 | 7/15/2014 | 24:12.0 | 3 |
| 290.52 | 19101.33 | 2.435 | 12.921 | 0.361865 | 7/15/2014 | 24:12.1 | 3 |
| 290.423 | 19101.305 | 2.434 | 12.918 | 0.366398 | 7/15/2014 | 24:12.1 | 3 |
| 290.227 | 19101.251 | 2.436 | 12.902 | 0.364302 | 7/15/2014 | 24:12.2 | 3 |
| 290.03 | 19101.195 | 2.434 | 12.888 | 0.369494 | 7/15/2014 | 24:12.3 | 3 |
| 289.683 | 19101.097 | 2.433 | 12.872 | 0.369063 | 7/15/2014 | 24:12.5 | 3 |
| 289.484 | 19101.041 | 2.435 | 12.861 | 0.376215 | 7/15/2014 | 24:12.6 | 3 |
| 289.284 | 19100.984 | 2.433 | 12.858 | 0.380596 | 7/15/2014 | 24:12.7 | 3 |
| 289.084 | 19100.928 | 2.434 | 12.849 | 0.382315 | 7/15/2014 | 24:12.8 | 3 |
| 288.881 | 19100.869 | 2.433 | 12.847 | 0.385655 | 7/15/2014 | 24:12.9 | 3 |
| 288.679 | 19100.811 | 2.434 | 12.854 | 0.389001 | 7/15/2014 | 24:13.0 | 3 |
| 288.476 | 19100.752 | 2.433 | 12.862 | 0.395462 | 7/15/2014 | 24:13.1 | 3 |
| 288.272 | 19100.693 | 2.433 | 12.87 | 0.394837 | 7/15/2014 | 24:13.2 | 3 |
| 288.071 | 19100.635 | 2.431 | 12.881 | 0.399805 | 7/15/2014 | 24:13.3 | 3 |
| 287.868 | 19100.576 | 2.433 | 12.894 | 0.404358 | 7/15/2014 | 24:13.4 | 3 |
| 287.667 | 19100.518 | 2.428 | 12.906 | 0.404789 | 7/15/2014 | 24:13.5 | 3 |
| 287.462 | 19100.458 | 2.432 | 12.92 | 0.411741 | 7/15/2014 | 24:13.6 | 3 |
| 287.209 | 19100.385 | 2.43 | 12.938 | 0.408105 | 7/15/2014 | 24:13.7 | 3 |
| 287.107 | 19100.354 | 2.43 | 12.947 | 0.410965 | 7/15/2014 | 24:13.7 | 3 |

| | | | | | | | |
|---|---|---|---|---|---|---|---|
| 286.902 | 19100.293 | 2.432 | 12.961 | 0.405276 | 7/15/2014 | 24:13.8 | 3 |
| 286.698 | 19100.232 | 2.432 | 12.977 | 0.414238 | 7/15/2014 | 24:13.9 | 3 |
| 286.49 | 19100.172 | 2.437 | 12.998 | 0.412465 | 7/15/2014 | 24:14.0 | 3 |
| 286.284 | 19100.11 | 2.439 | 13.022 | 0.411954 | 7/15/2014 | 24:14.1 | 3 |
| 286.076 | 19100.049 | 2.441 | 13.035 | 0.416899 | 7/15/2014 | 24:14.2 | 3 |
| 285.866 | 19099.985 | 2.446 | 13.048 | 0.420753 | 7/15/2014 | 24:14.3 | 3 |
| 285.606 | 19099.908 | 2.444 | 13.053 | 0.434979 | 7/15/2014 | 24:14.4 | 3 |
| 285.393 | 19099.844 | 2.444 | 13.062 | 0.447658 | 7/15/2014 | 24:14.5 | 3 |
| 285.179 | 19099.779 | 2.444 | 13.073 | 0.454205 | 7/15/2014 | 24:14.6 | 3 |
| 284.965 | 19099.715 | 2.446 | 13.071 | 0.458669 | 7/15/2014 | 24:14.7 | 3 |
| 284.751 | 19099.651 | 2.442 | 13.068 | 0.4751 | 7/15/2014 | 24:14.8 | 3 |
| 284.534 | 19099.586 | 2.44 | 13.07 | 0.469132 | 7/15/2014 | 24:14.9 | 3 |
| 284.32 | 19099.521 | 2.436 | 13.073 | 0.481029 | 7/15/2014 | 24:15.0 | 3 |
| 284.106 | 19099.457 | 2.433 | 13.073 | 0.480748 | 7/15/2014 | 24:15.1 | 3 |
| 283.893 | 19099.392 | 2.431 | 13.079 | 0.489722 | 7/15/2014 | 24:15.2 | 3 |
| 283.681 | 19099.328 | 2.431 | 13.089 | 0.489312 | 7/15/2014 | 24:15.3 | 3 |
| 283.473 | 19099.264 | 2.427 | 13.094 | 0.499222 | 7/15/2014 | 24:15.4 | 3 |
| 283.266 | 19099.203 | 2.425 | 13.11 | 0.501943 | 7/15/2014 | 24:15.5 | 3 |
| 282.914 | 19099.095 | 2.429 | 13.134 | 0.494185 | 7/15/2014 | 24:15.7 | 3 |
| 282.808 | 19099.063 | 2.429 | 13.134 | 0.48864 | 7/15/2014 | 24:15.7 | 3 |
| 282.605 | 19099.002 | 2.431 | 13.151 | 0.503074 | 7/15/2014 | 24:15.8 | 3 |
| 282.402 | 19098.94 | 2.431 | 13.174 | 0.503401 | 7/15/2014 | 24:15.9 | 3 |
| 282.201 | 19098.879 | 2.431 | 13.189 | 0.504501 | 7/15/2014 | 24:16.0 | 3 |
| 281.999 | 19098.818 | 2.428 | 13.205 | 0.509551 | 7/15/2014 | 24:16.1 | 3 |
| 281.797 | 19098.758 | 2.43 | 13.22 | 0.509632 | 7/15/2014 | 24:16.2 | 3 |
| 281.596 | 19098.695 | 2.427 | 13.227 | 0.509173 | 7/15/2014 | 24:16.3 | 3 |
| 281.245 | 19098.589 | 2.417 | 13.238 | 0.530425 | 7/15/2014 | 24:16.5 | 3 |
| 281.042 | 19098.527 | 2.414 | 13.24 | 0.521724 | 7/15/2014 | 24:16.6 | 3 |
| 280.839 | 19098.467 | 2.409 | 13.243 | 0.528844 | 7/15/2014 | 24:16.7 | 3 |
| 280.739 | 19098.436 | 2.407 | 13.244 | 0.527489 | 7/15/2014 | 24:16.7 | 3 |
| 280.535 | 19098.375 | 2.404 | 13.245 | 0.522928 | 7/15/2014 | 24:16.8 | 3 |
| 280.331 | 19098.313 | 2.401 | 13.242 | 0.527366 | 7/15/2014 | 24:16.9 | 3 |
| 280.126 | 19098.251 | 2.396 | 13.239 | 0.524746 | 7/15/2014 | 24:17.0 | 3 |
| 279.82 | 19098.159 | 2.396 | 13.24 | 0.520753 | 7/15/2014 | 24:17.2 | 3 |
| 279.62 | 19098.098 | 2.392 | 13.239 | 0.516568 | 7/15/2014 | 24:17.3 | 3 |
| 279.417 | 19098.037 | 2.395 | 13.238 | 0.519139 | 7/15/2014 | 24:17.4 | 3 |
| 279.217 | 19097.977 | 2.393 | 13.242 | 0.513848 | 7/15/2014 | 24:17.5 | 3 |
| 279.018 | 19097.917 | 2.393 | 13.242 | 0.519794 | 7/15/2014 | 24:17.6 | 3 |
| 278.772 | 19097.843 | 2.391 | 13.244 | 0.519211 | 7/15/2014 | 24:17.7 | 3 |
| 278.669 | 19097.813 | 2.394 | 13.244 | 0.517924 | 7/15/2014 | 24:17.7 | 3 |
| 278.471 | 19097.754 | 2.391 | 13.248 | 0.516282 | 7/15/2014 | 24:17.8 | 3 |
| 278.275 | 19097.695 | 2.386 | 13.252 | 0.530832 | 7/15/2014 | 24:17.9 | 3 |
| 278.076 | 19097.635 | 2.384 | 13.254 | 0.53856 | 7/15/2014 | 24:18.0 | 3 |
| 277.878 | 19097.576 | 2.384 | 13.26 | 0.543435 | 7/15/2014 | 24:18.1 | 3 |
| 277.681 | 19097.518 | 2.384 | 13.265 | 0.55521 | 7/15/2014 | 24:18.2 | 3 |
| 277.486 | 19097.46 | 2.381 | 13.271 | 0.549268 | 7/15/2014 | 24:18.3 | 3 |
| 277.139 | 19097.358 | 2.375 | 13.282 | 0.55812 | 7/15/2014 | 24:18.5 | 3 |

| | | | | | | | |
|---|---|---|---|---|---|---|---|
| 276.94 | 19097.299 | 2.375 | 13.292 | 0.569053 | 7/15/2014 | 24:18.6 | 3 |
| 276.744 | 19097.241 | 2.372 | 13.296 | 0.561649 | 7/15/2014 | 24:18.7 | 3 |
| 276.541 | 19097.182 | 2.377 | 13.302 | 0.561186 | 7/15/2014 | 24:18.8 | 3 |
| 276.343 | 19097.124 | 2.374 | 13.306 | 0.564484 | 7/15/2014 | 24:18.9 | 3 |
| 276.143 | 19097.066 | 2.371 | 13.312 | 0.564778 | 7/15/2014 | 24:19.0 | 3 |
| 275.944 | 19097.008 | 2.367 | 13.315 | 0.560132 | 7/15/2014 | 24:19.1 | 3 |
| 275.743 | 19096.951 | 2.369 | 13.316 | 0.565489 | 7/15/2014 | 24:19.2 | 3 |
| 275.546 | 19096.895 | 2.368 | 13.316 | 0.560172 | 7/15/2014 | 24:19.3 | 3 |
| 275.346 | 19096.835 | 2.372 | 13.317 | 0.559675 | 7/15/2014 | 24:19.4 | 3 |
| 275.152 | 19096.779 | 2.369 | 13.315 | 0.560936 | 7/15/2014 | 24:19.5 | 3 |
| 274.955 | 19096.723 | 2.372 | 13.319 | 0.572429 | 7/15/2014 | 24:19.6 | 3 |
| 274.716 | 19096.656 | 2.368 | 13.319 | 0.573459 | 7/15/2014 | 24:19.7 | 3 |
| 274.521 | 19096.602 | 2.372 | 13.323 | 0.57806 | 7/15/2014 | 24:19.8 | 3 |
| 274.328 | 19096.546 | 2.369 | 13.325 | 0.606805 | 7/15/2014 | 24:19.9 | 3 |
| 274.131 | 19096.492 | 2.372 | 13.326 | 0.620567 | 7/15/2014 | 24:20.0 | 3 |
| 273.939 | 19096.438 | 2.372 | 13.331 | 0.623067 | 7/15/2014 | 24:20.1 | 3 |
| 273.744 | 19096.384 | 2.375 | 13.335 | 0.645633 | 7/15/2014 | 24:20.2 | 3 |
| 273.554 | 19096.33 | 2.37 | 13.346 | 0.656495 | 7/15/2014 | 24:20.3 | 3 |
| 273.36 | 19096.277 | 2.37 | 13.352 | 0.652429 | 7/15/2014 | 24:20.4 | 3 |
| 273.122 | 19096.212 | 2.364 | 13.359 | 0.653602 | 7/15/2014 | 24:20.5 | 3 |
| 272.927 | 19096.159 | 2.366 | 13.365 | 0.661581 | 7/15/2014 | 24:20.6 | 3 |
| 272.734 | 19096.106 | 2.361 | 13.371 | 0.647227 | 7/15/2014 | 24:20.7 | 3 |
| 272.537 | 19096.054 | 2.369 | 13.375 | 0.621333 | 7/15/2014 | 24:20.8 | 3 |
| 272.343 | 19096.001 | 2.366 | 13.379 | 0.629642 | 7/15/2014 | 24:20.9 | 3 |
| 272.147 | 19095.949 | 2.366 | 13.38 | 0.610671 | 7/15/2014 | 24:21.0 | 3 |
| 271.954 | 19095.896 | 2.365 | 13.378 | 0.60205 | 7/15/2014 | 24:21.1 | 3 |
| 271.758 | 19095.846 | 2.369 | 13.369 | 0.596175 | 7/15/2014 | 24:21.2 | 3 |
| 271.565 | 19095.795 | 2.369 | 13.369 | 0.594068 | 7/15/2014 | 24:21.3 | 3 |
| 271.37 | 19095.743 | 2.377 | 13.357 | 0.58591 | 7/15/2014 | 24:21.4 | 3 |
| 271.177 | 19095.693 | 2.38 | 13.35 | 0.583114 | 7/15/2014 | 24:21.5 | 3 |
| 270.983 | 19095.644 | 2.385 | 13.339 | 0.582309 | 7/15/2014 | 24:21.6 | 3 |
| 270.746 | 19095.582 | 2.385 | 13.33 | 0.581353 | 7/15/2014 | 24:21.7 | 3 |
| 270.553 | 19095.533 | 2.392 | 13.322 | 0.58455 | 7/15/2014 | 24:21.8 | 3 |
| 270.362 | 19095.483 | 2.394 | 13.318 | 0.585792 | 7/15/2014 | 24:21.9 | 3 |
| 270.166 | 19095.434 | 2.402 | 13.319 | 0.585759 | 7/15/2014 | 24:22.0 | 3 |
| 269.975 | 19095.387 | 2.402 | 13.323 | 0.588637 | 7/15/2014 | 24:22.1 | 3 |
| 269.78 | 19095.337 | 2.409 | 13.326 | 0.588895 | 7/15/2014 | 24:22.2 | 3 |
| 269.587 | 19095.289 | 2.409 | 13.329 | 0.587833 | 7/15/2014 | 24:22.3 | 3 |
| 269.392 | 19095.241 | 2.414 | 13.332 | 0.588409 | 7/15/2014 | 24:22.4 | 3 |
| 269.153 | 19095.182 | 2.417 | 13.342 | 0.588293 | 7/15/2014 | 24:22.5 | 3 |
| 268.956 | 19095.135 | 2.421 | 13.349 | 0.587808 | 7/15/2014 | 24:22.6 | 3 |
| 268.76 | 19095.087 | 2.423 | 13.355 | 0.583717 | 7/15/2014 | 24:22.7 | 3 |
| 268.561 | 19095.039 | 2.43 | 13.355 | 0.583414 | 7/15/2014 | 24:22.8 | 3 |
| 268.365 | 19094.992 | 2.428 | 13.354 | 0.580994 | 7/15/2014 | 24:22.9 | 3 |
| 268.165 | 19094.944 | 2.433 | 13.36 | 0.580625 | 7/15/2014 | 24:23.0 | 3 |
| 267.967 | 19094.897 | 2.434 | 13.363 | 0.577439 | 7/15/2014 | 24:23.1 | 3 |
| 267.766 | 19094.851 | 2.441 | 13.366 | 0.583988 | 7/15/2014 | 24:23.2 | 3 |

| | | | | | | | |
|---|---|---|---|---|---|---|---|
| 267.57 | 19094.805 | 2.439 | 13.367 | 0.585105 | 7/15/2014 | 24:23.3 | 3 |
| 267.37 | 19094.759 | 2.443 | 13.37 | 0.592201 | 7/15/2014 | 24:23.4 | 3 |
| 267.174 | 19094.715 | 2.443 | 13.369 | 0.587945 | 7/15/2014 | 24:23.5 | 3 |
| 266.978 | 19094.67 | 2.445 | 13.372 | 0.595324 | 7/15/2014 | 24:23.6 | 3 |
| 266.736 | 19094.615 | 2.443 | 13.369 | 0.5912 | 7/15/2014 | 24:23.7 | 3 |
| 266.538 | 19094.57 | 2.451 | 13.367 | 0.595698 | 7/15/2014 | 24:23.8 | 3 |
| 266.344 | 19094.528 | 2.453 | 13.351 | 0.589537 | 7/15/2014 | 24:23.9 | 3 |
| 266.146 | 19094.484 | 2.458 | 13.335 | 0.586752 | 7/15/2014 | 24:24.0 | 3 |
| 265.95 | 19094.443 | 2.458 | 13.324 | 0.58614 | 7/15/2014 | 24:24.1 | 3 |
| 265.752 | 19094.399 | 2.465 | 13.311 | 0.581496 | 7/15/2014 | 24:24.2 | 3 |
| 265.556 | 19094.357 | 2.467 | 13.293 | 0.579789 | 7/15/2014 | 24:24.3 | 3 |
| 265.461 | 19094.336 | 2.471 | 13.285 | 0.563226 | 7/15/2014 | 24:24.3 | 3 |
| 265.114 | 19094.263 | 2.479 | 13.252 | 0.574778 | 7/15/2014 | 24:24.5 | 3 |
| 264.914 | 19094.221 | 2.487 | 13.234 | 0.556117 | 7/15/2014 | 24:24.6 | 3 |
| 264.716 | 19094.182 | 2.487 | 13.215 | 0.550999 | 7/15/2014 | 24:24.7 | 3 |
| 264.515 | 19094.139 | 2.494 | 13.2 | 0.546789 | 7/15/2014 | 24:24.8 | 3 |
| 264.313 | 19094.1 | 2.498 | 13.183 | 0.536444 | 7/15/2014 | 24:24.9 | 3 |
| 264.112 | 19094.059 | 2.504 | 13.168 | 0.518752 | 7/15/2014 | 24:25.0 | 3 |
| 263.909 | 19094.018 | 2.508 | 13.153 | 0.520861 | 7/15/2014 | 24:25.1 | 3 |
| 263.705 | 19093.977 | 2.517 | 13.138 | 0.519192 | 7/15/2014 | 24:25.2 | 3 |
| 263.506 | 19093.938 | 2.517 | 13.128 | 0.507275 | 7/15/2014 | 24:25.3 | 3 |
| 263.305 | 19093.898 | 2.526 | 13.125 | 0.509579 | 7/15/2014 | 24:25.4 | 3 |
| 263.107 | 19093.861 | 2.529 | 13.118 | 0.501251 | 7/15/2014 | 24:25.5 | 3 |
| 262.906 | 19093.822 | 2.536 | 13.111 | 0.49876 | 7/15/2014 | 24:25.6 | 3 |
| 262.661 | 19093.777 | 2.542 | 13.104 | 0.499128 | 7/15/2014 | 24:25.7 | 3 |
| 262.463 | 19093.74 | 2.549 | 13.097 | 0.503999 | 7/15/2014 | 24:25.8 | 3 |
| 262.266 | 19093.703 | 2.557 | 13.092 | 0.501631 | 7/15/2014 | 24:25.9 | 3 |
| 262.067 | 19093.667 | 2.565 | 13.087 | 0.491913 | 7/15/2014 | 24:26.0 | 3 |
| 261.871 | 19093.632 | 2.566 | 13.085 | 0.499125 | 7/15/2014 | 24:26.1 | 3 |
| 261.673 | 19093.596 | 2.578 | 13.08 | 0.498451 | 7/15/2014 | 24:26.2 | 3 |
| 261.476 | 19093.561 | 2.582 | 13.074 | 0.491383 | 7/15/2014 | 24:26.3 | 3 |
| 261.277 | 19093.526 | 2.591 | 13.075 | 0.4982 | 7/15/2014 | 24:26.4 | 3 |
| 261.033 | 19093.484 | 2.595 | 13.078 | 0.494214 | 7/15/2014 | 24:26.5 | 3 |
| 260.833 | 19093.45 | 2.6 | 13.081 | 0.493455 | 7/15/2014 | 24:26.6 | 3 |
| 260.633 | 19093.416 | 2.604 | 13.085 | 0.491534 | 7/15/2014 | 24:26.7 | 3 |
| 260.433 | 19093.383 | 2.608 | 13.087 | 0.4978 | 7/15/2014 | 24:26.8 | 3 |
| 260.23 | 19093.35 | 2.612 | 13.093 | 0.489037 | 7/15/2014 | 24:26.9 | 3 |
| 260.026 | 19093.316 | 2.618 | 13.095 | 0.503668 | 7/15/2014 | 24:27.0 | 3 |
| 259.824 | 19093.283 | 2.618 | 13.099 | 0.498456 | 7/15/2014 | 24:27.1 | 3 |
| 259.619 | 19093.251 | 2.623 | 13.099 | 0.505528 | 7/15/2014 | 24:27.2 | 3 |
| 259.416 | 19093.219 | 2.623 | 13.097 | 0.512471 | 7/15/2014 | 24:27.3 | 3 |
| 259.209 | 19093.187 | 2.631 | 13.093 | 0.507383 | 7/15/2014 | 24:27.4 | 3 |
| 259.007 | 19093.156 | 2.631 | 13.086 | 0.510747 | 7/15/2014 | 24:27.5 | 3 |
| 258.802 | 19093.125 | 2.637 | 13.077 | 0.503874 | 7/15/2014 | 24:27.6 | 3 |
| 258.549 | 19093.087 | 2.639 | 13.067 | 0.509953 | 7/15/2014 | 24:27.7 | 3 |
| 258.445 | 19093.071 | 2.642 | 13.062 | 0.502078 | 7/15/2014 | 24:27.7 | 3 |
| 258.239 | 19093.041 | 2.645 | 13.049 | 0.49939 | 7/15/2014 | 24:27.8 | 3 |

| | | | | | | | |
|---|---|---|---|---|---|---|---|
| 258.035 | 19093.012 | 2.645 | 13.034 | 0.494068 | 7/15/2014 | 24:27.9 | 3 |
| 257.827 | 19092.981 | 2.648 | 13.019 | 0.491786 | 7/15/2014 | 24:28.0 | 3 |
| 257.619 | 19092.951 | 2.651 | 13.003 | 0.495542 | 7/15/2014 | 24:28.1 | 3 |
| 257.412 | 19092.924 | 2.654 | 12.988 | 0.480288 | 7/15/2014 | 24:28.2 | 3 |
| 257.203 | 19092.896 | 2.657 | 12.976 | 0.480027 | 7/15/2014 | 24:28.3 | 3 |
| 256.835 | 19092.847 | 2.655 | 12.962 | 0.475428 | 7/15/2014 | 24:28.5 | 3 |
| 256.732 | 19092.832 | 2.656 | 12.956 | 0.48195 | 7/15/2014 | 24:28.5 | 3 |
| 256.519 | 19092.804 | 2.656 | 12.948 | 0.47182 | 7/15/2014 | 24:28.6 | 3 |
| 256.304 | 19092.776 | 2.659 | 12.943 | 0.461906 | 7/15/2014 | 24:28.7 | 3 |
| 256.088 | 19092.748 | 2.659 | 12.937 | 0.453075 | 7/15/2014 | 24:28.8 | 3 |
| 255.869 | 19092.721 | 2.662 | 12.936 | 0.459773 | 7/15/2014 | 24:28.9 | 3 |
| 255.653 | 19092.695 | 2.66 | 12.938 | 0.45429 | 7/15/2014 | 24:29.0 | 3 |
| 255.434 | 19092.669 | 2.663 | 12.942 | 0.457895 | 7/15/2014 | 24:29.1 | 3 |
| 255.217 | 19092.643 | 2.665 | 12.947 | 0.453423 | 7/15/2014 | 24:29.2 | 3 |
| 255 | 19092.616 | 2.668 | 12.955 | 0.445713 | 7/15/2014 | 24:29.3 | 3 |
| 254.785 | 19092.592 | 2.673 | 12.959 | 0.443165 | 7/15/2014 | 24:29.4 | 3 |
| 254.571 | 19092.568 | 2.676 | 12.969 | 0.449329 | 7/15/2014 | 24:29.5 | 3 |
| 254.203 | 19092.527 | 2.679 | 12.982 | 0.441576 | 7/15/2014 | 24:29.7 | 3 |
| 254.094 | 19092.516 | 2.68 | 12.986 | 0.43942 | 7/15/2014 | 24:29.7 | 3 |
| 253.883 | 19092.492 | 2.68 | 12.99 | 0.438886 | 7/15/2014 | 24:29.8 | 3 |
| 253.67 | 19092.469 | 2.68 | 12.99 | 0.435597 | 7/15/2014 | 24:29.9 | 3 |
| 253.458 | 19092.447 | 2.682 | 12.989 | 0.43751 | 7/15/2014 | 24:30.0 | 3 |
| 253.247 | 19092.426 | 2.682 | 12.987 | 0.4476 | 7/15/2014 | 24:30.1 | 3 |
| 253.036 | 19092.405 | 2.68 | 12.987 | 0.440983 | 7/15/2014 | 24:30.2 | 3 |
| 252.824 | 19092.384 | 2.68 | 12.985 | 0.440476 | 7/15/2014 | 24:30.3 | 3 |
| 252.453 | 19092.348 | 2.679 | 12.991 | 0.431385 | 7/15/2014 | 24:30.5 | 3 |
| 252.35 | 19092.337 | 2.68 | 12.991 | 0.429429 | 7/15/2014 | 24:30.5 | 3 |
| 252.135 | 19092.316 | 2.682 | 12.993 | 0.431661 | 7/15/2014 | 24:30.6 | 3 |
| 251.921 | 19092.297 | 2.684 | 13.007 | 0.424269 | 7/15/2014 | 24:30.7 | 3 |
| 251.704 | 19092.278 | 2.682 | 13.02 | 0.409984 | 7/15/2014 | 24:30.8 | 3 |
| 251.488 | 19092.259 | 2.682 | 13.034 | 0.407229 | 7/15/2014 | 24:30.9 | 3 |
| 251.272 | 19092.239 | 2.68 | 13.055 | 0.397842 | 7/15/2014 | 24:31.0 | 3 |
| 251.053 | 19092.221 | 2.68 | 13.066 | 0.399154 | 7/15/2014 | 24:31.1 | 3 |
| 250.836 | 19092.201 | 2.678 | 13.089 | 0.3934 | 7/15/2014 | 24:31.2 | 3 |
| 250.62 | 19092.184 | 2.678 | 13.101 | 0.397829 | 7/15/2014 | 24:31.3 | 3 |
| 250.405 | 19092.166 | 2.676 | 13.122 | 0.391521 | 7/15/2014 | 24:31.4 | 3 |
| 250.191 | 19092.149 | 2.667 | 13.132 | 0.396081 | 7/15/2014 | 24:31.5 | 3 |
| 249.975 | 19092.133 | 2.667 | 13.154 | 0.399912 | 7/15/2014 | 24:31.6 | 3 |
| 249.71 | 19092.111 | 2.667 | 13.174 | 0.401474 | 7/15/2014 | 24:31.7 | 3 |
| 249.495 | 19092.097 | 2.667 | 13.194 | 0.396912 | 7/15/2014 | 24:31.8 | 3 |
| 249.279 | 19092.079 | 2.667 | 13.22 | 0.402185 | 7/15/2014 | 24:31.9 | 3 |
| 249.064 | 19092.063 | 2.665 | 13.229 | 0.393551 | 7/15/2014 | 24:32.0 | 3 |
| 248.847 | 19092.049 | 2.665 | 13.243 | 0.400105 | 7/15/2014 | 24:32.1 | 3 |
| 248.631 | 19092.033 | 2.667 | 13.256 | 0.397758 | 7/15/2014 | 24:32.2 | 3 |
| 248.412 | 19092.019 | 2.671 | 13.265 | 0.393459 | 7/15/2014 | 24:32.3 | 3 |
| 248.142 | 19092 | 2.668 | 13.266 | 0.393356 | 7/15/2014 | 24:32.4 | 3 |
| 247.921 | 19091.985 | 2.665 | 13.264 | 0.392388 | 7/15/2014 | 24:32.5 | 3 |

| | | | | | | | |
|---|---|---|---|---|---|---|---|
| 247.698 | 19091.971 | 2.662 | 13.253 | 0.387055 | 7/15/2014 | 24:32.6 | 3 |
| 247.473 | 19091.956 | 2.659 | 13.241 | 0.397631 | 7/15/2014 | 24:32.7 | 3 |
| 247.247 | 19091.943 | 2.656 | 13.23 | 0.390766 | 7/15/2014 | 24:32.8 | 3 |
| 247.019 | 19091.928 | 2.65 | 13.218 | 0.392688 | 7/15/2014 | 24:32.9 | 3 |
| 246.792 | 19091.915 | 2.645 | 13.205 | 0.395096 | 7/15/2014 | 24:33.0 | 3 |
| 246.564 | 19091.902 | 2.643 | 13.198 | 0.391607 | 7/15/2014 | 24:33.1 | 3 |
| 246.336 | 19091.889 | 2.638 | 13.19 | 0.386831 | 7/15/2014 | 24:33.2 | 3 |
| 246.11 | 19091.876 | 2.632 | 13.187 | 0.387997 | 7/15/2014 | 24:33.3 | 3 |
| 245.882 | 19091.863 | 2.63 | 13.19 | 0.379716 | 7/15/2014 | 24:33.4 | 3 |
| 245.657 | 19091.852 | 2.623 | 13.202 | 0.372123 | 7/15/2014 | 24:33.5 | 3 |
| 245.431 | 19091.84 | 2.619 | 13.212 | 0.373948 | 7/15/2014 | 24:33.6 | 3 |
| 245.154 | 19091.826 | 2.615 | 13.229 | 0.357062 | 7/15/2014 | 24:33.7 | 3 |
| 244.929 | 19091.814 | 2.611 | 13.247 | 0.353359 | 7/15/2014 | 24:33.8 | 3 |
| 244.706 | 19091.804 | 2.608 | 13.26 | 0.340347 | 7/15/2014 | 24:33.9 | 3 |
| 244.483 | 19091.793 | 2.603 | 13.279 | 0.336495 | 7/15/2014 | 24:34.0 | 3 |
| 244.257 | 19091.783 | 2.598 | 13.296 | 0.336518 | 7/15/2014 | 24:34.1 | 3 |
| 244.033 | 19091.771 | 2.595 | 13.313 | 0.326249 | 7/15/2014 | 24:34.2 | 3 |
| 243.809 | 19091.764 | 2.59 | 13.32 | 0.328197 | 7/15/2014 | 24:34.3 | 3 |
| 243.53 | 19091.75 | 2.584 | 13.332 | 0.330108 | 7/15/2014 | 24:34.4 | 3 |
| 243.303 | 19091.74 | 2.578 | 13.345 | 0.321223 | 7/15/2014 | 24:34.5 | 3 |
| 243.077 | 19091.731 | 2.572 | 13.354 | 0.333462 | 7/15/2014 | 24:34.6 | 3 |
| 242.849 | 19091.723 | 2.565 | 13.367 | 0.323937 | 7/15/2014 | 24:34.7 | 3 |
| 242.618 | 19091.715 | 2.565 | 13.378 | 0.30388 | 7/15/2014 | 24:34.8 | 3 |
| 242.387 | 19091.705 | 2.563 | 13.388 | 0.328684 | 7/15/2014 | 24:34.9 | 3 |
| 242.159 | 19091.697 | 2.554 | 13.393 | 0.29534 | 7/15/2014 | 24:35.0 | 3 |
| 241.928 | 19091.688 | 2.554 | 13.408 | 0.308853 | 7/15/2014 | 24:35.1 | 3 |
| 241.697 | 19091.68 | 2.557 | 13.427 | 0.308118 | 7/15/2014 | 24:35.2 | 3 |
| 241.469 | 19091.672 | 2.553 | 13.441 | 0.312424 | 7/15/2014 | 24:35.3 | 3 |
| 241.241 | 19091.665 | 2.553 | 13.454 | 0.303395 | 7/15/2014 | 24:35.4 | 3 |
| 241.015 | 19091.658 | 2.551 | 13.472 | 0.315093 | 7/15/2014 | 24:35.5 | 3 |
| 240.79 | 19091.65 | 2.548 | 13.485 | 0.322904 | 7/15/2014 | 24:35.6 | 3 |
| 240.513 | 19091.643 | 2.545 | 13.499 | 0.322951 | 7/15/2014 | 24:35.7 | 3 |
| 240.287 | 19091.636 | 2.548 | 13.514 | 0.299264 | 7/15/2014 | 24:35.8 | 3 |
| 240.063 | 19091.629 | 2.545 | 13.521 | 0.314895 | 7/15/2014 | 24:35.9 | 3 |
| 239.84 | 19091.622 | 2.538 | 13.527 | 0.307589 | 7/15/2014 | 24:36.0 | 3 |
| 239.615 | 19091.616 | 2.538 | 13.535 | 0.318423 | 7/15/2014 | 24:36.1 | 3 |
| 239.392 | 19091.611 | 2.534 | 13.54 | 0.314612 | 7/15/2014 | 24:36.2 | 3 |
| 239.168 | 19091.605 | 2.527 | 13.541 | 0.283734 | 7/15/2014 | 24:36.3 | 3 |
| 238.889 | 19091.599 | 2.525 | 13.545 | 0.283846 | 7/15/2014 | 24:36.4 | 3 |
| 238.662 | 19091.593 | 2.522 | 13.55 | 0.300856 | 7/15/2014 | 24:36.5 | 3 |
| 238.438 | 19091.588 | 2.514 | 13.558 | 0.284145 | 7/15/2014 | 24:36.6 | 3 |
| 238.209 | 19091.583 | 2.508 | 13.564 | 0.279485 | 7/15/2014 | 24:36.7 | 3 |
| 237.978 | 19091.578 | 2.508 | 13.57 | 0.270297 | 7/15/2014 | 24:36.8 | 3 |
| 237.749 | 19091.574 | 2.501 | 13.573 | 0.273672 | 7/15/2014 | 24:36.9 | 3 |
| 237.518 | 19091.57 | 2.498 | 13.582 | 0.270782 | 7/15/2014 | 24:37.0 | 3 |
| 237.287 | 19091.565 | 2.498 | 13.589 | 0.262015 | 7/15/2014 | 24:37.1 | 3 |
| 237.057 | 19091.561 | 2.489 | 13.594 | 0.27145 | 7/15/2014 | 24:37.2 | 3 |

| | | | | | | | |
|---|---|---|---|---|---|---|---|
| 236.829 | 19091.557 | 2.484 | 13.6 | 0.248456 | 7/15/2014 | 24:37.3 | 3 |
| 236.6 | 19091.554 | 2.478 | 13.603 | 0.254085 | 7/15/2014 | 24:37.4 | 3 |
| 236.372 | 19091.551 | 2.474 | 13.615 | 0.259344 | 7/15/2014 | 24:37.5 | 3 |
| 236.147 | 19091.547 | 2.466 | 13.621 | 0.243097 | 7/15/2014 | 24:37.6 | 3 |
| 235.866 | 19091.543 | 2.463 | 13.627 | 0.246078 | 7/15/2014 | 24:37.7 | 3 |
| 235.642 | 19091.539 | 2.458 | 13.633 | 0.256747 | 7/15/2014 | 24:37.8 | 3 |
| 235.419 | 19091.536 | 2.451 | 13.638 | 0.247715 | 7/15/2014 | 24:37.9 | 3 |
| 235.193 | 19091.533 | 2.445 | 13.641 | 0.250837 | 7/15/2014 | 24:38.0 | 3 |
| 234.97 | 19091.531 | 2.438 | 13.649 | 0.246032 | 7/15/2014 | 24:38.1 | 3 |
| 234.748 | 19091.529 | 2.433 | 13.652 | 0.235376 | 7/15/2014 | 24:38.2 | 3 |
| 234.524 | 19091.526 | 2.43 | 13.652 | 0.240208 | 7/15/2014 | 24:38.3 | 3 |
| 234.246 | 19091.523 | 2.427 | 13.656 | 0.2434 | 7/15/2014 | 24:38.4 | 3 |
| 234.024 | 19091.521 | 2.417 | 13.658 | 0.268415 | 7/15/2014 | 24:38.5 | 3 |
| 233.801 | 19091.519 | 2.41 | 13.66 | 0.255654 | 7/15/2014 | 24:38.6 | 3 |
| 233.576 | 19091.518 | 2.408 | 13.662 | 0.256676 | 7/15/2014 | 24:38.7 | 3 |
| 233.353 | 19091.516 | 2.403 | 13.668 | 0.28066 | 7/15/2014 | 24:38.8 | 3 |
| 233.13 | 19091.514 | 2.401 | 13.671 | 0.26841 | 7/15/2014 | 24:38.9 | 3 |
| 232.905 | 19091.513 | 2.401 | 13.675 | 0.251321 | 7/15/2014 | 24:39.0 | 3 |
| 232.682 | 19091.512 | 2.394 | 13.677 | 0.274095 | 7/15/2014 | 24:39.1 | 3 |
| 232.461 | 19091.51 | 2.39 | 13.678 | 0.263406 | 7/15/2014 | 24:39.2 | 3 |
| 232.241 | 19091.509 | 2.386 | 13.684 | 0.256582 | 7/15/2014 | 24:39.3 | 3 |
| 232.022 | 19091.508 | 2.384 | 13.688 | 0.261201 | 7/15/2014 | 24:39.4 | 3 |
| 231.805 | 19091.507 | 2.382 | 13.694 | 0.253773 | 7/15/2014 | 24:39.5 | 3 |
| 231.591 | 19091.506 | 2.377 | 13.699 | 0.245656 | 7/15/2014 | 24:39.6 | 3 |
| 231.323 | 19091.506 | 2.377 | 13.704 | 0.25221 | 7/15/2014 | 24:39.7 | 3 |
| 231.109 | 19091.505 | 2.371 | 13.713 | 0.238926 | 7/15/2014 | 24:39.8 | 3 |
| 230.894 | 19091.504 | 2.371 | 13.722 | 0.213628 | 7/15/2014 | 24:39.9 | 3 |
| 230.68 | 19091.504 | 2.366 | 13.732 | 0.217111 | 7/15/2014 | 24:40.0 | 3 |
| 230.465 | 19091.503 | 2.369 | 13.738 | 0.22858 | 7/15/2014 | 24:40.1 | 3 |
| 230.249 | 19091.502 | 2.366 | 13.746 | 0.21609 | 7/15/2014 | 24:40.2 | 3 |
| 230.034 | 19091.502 | 2.363 | 13.756 | 0.229286 | 7/15/2014 | 24:40.3 | 3 |
| 229.766 | 19091.502 | 2.363 | 13.77 | 0.240614 | 7/15/2014 | 24:40.4 | 3 |
| 229.547 | 19091.502 | 2.358 | 13.778 | 0.232936 | 7/15/2014 | 24:40.5 | 3 |
| 229.328 | 19091.501 | 2.353 | 13.786 | 0.227057 | 7/15/2014 | 24:40.6 | 3 |
| 229.109 | 19091.501 | 2.35 | 13.795 | 0.239925 | 7/15/2014 | 24:40.7 | 3 |
| 228.887 | 19091.501 | 2.347 | 13.809 | 0.228922 | 7/15/2014 | 24:40.8 | 3 |
| 228.664 | 19091.5 | 2.338 | 13.823 | 0.221133 | 7/15/2014 | 24:40.9 | 3 |
| 228.44 | 19091.5 | 2.336 | 13.836 | 0.220186 | 7/15/2014 | 24:41.0 | 3 |
| 228.218 | 19091.5 | 2.331 | 13.849 | 0.234167 | 7/15/2014 | 24:41.1 | 3 |
| 227.994 | 19091.501 | 2.329 | 13.86 | 0.228527 | 7/15/2014 | 24:41.2 | 3 |
| 227.774 | 19091.501 | 2.325 | 13.875 | 0.208982 | 7/15/2014 | 24:41.3 | 3 |
| 227.554 | 19091.501 | 2.322 | 13.887 | 0.212623 | 7/15/2014 | 24:41.4 | 3 |
| 227.335 | 19091.5 | 2.32 | 13.903 | 0.181217 | 7/15/2014 | 24:41.5 | 3 |
| 227.117 | 19091.501 | 2.316 | 13.918 | 0.213793 | 7/15/2014 | 24:41.6 | 3 |
| 226.846 | 19091.501 | 2.312 | 13.93 | 0.192868 | 7/15/2014 | 24:41.7 | 3 |
| 226.629 | 19091.501 | 2.306 | 13.943 | 0.173138 | 7/15/2014 | 24:41.8 | 3 |
| 226.411 | 19091.501 | 2.304 | 13.958 | 0.180558 | 7/15/2014 | 24:41.9 | 3 |

| | | | | | | | |
|---|---|---|---|---|---|---|---|
| 226.195 | 19091.502 | 2.296 | 13.969 | 0.181078 | 7/15/2014 | 24:42.0 | 3 |
| 225.979 | 19091.503 | 2.288 | 13.983 | 0.173057 | 7/15/2014 | 24:42.1 | 3 |
| 225.762 | 19091.502 | 2.283 | 13.996 | 0.184248 | 7/15/2014 | 24:42.2 | 3 |
| 225.547 | 19091.503 | 2.276 | 14.004 | 0.158987 | 7/15/2014 | 24:42.3 | 3 |
| 225.278 | 19091.503 | 2.273 | 14.01 | 0.163221 | 7/15/2014 | 24:42.4 | 3 |
| 225.061 | 19091.503 | 2.269 | 14.016 | 0.158012 | 7/15/2014 | 24:42.5 | 3 |
| 224.843 | 19091.503 | 2.264 | 14.022 | 0.154461 | 7/15/2014 | 24:42.6 | 3 |
| 224.624 | 19091.503 | 2.262 | 14.027 | 0.153961 | 7/15/2014 | 24:42.7 | 3 |
| 224.406 | 19091.504 | 2.26 | 14.03 | 0.151111 | 7/15/2014 | 24:42.8 | 3 |
| 224.186 | 19091.504 | 2.257 | 14.03 | 0.141303 | 7/15/2014 | 24:42.9 | 3 |
| 223.965 | 19091.504 | 2.257 | 14.032 | 0.143288 | 7/15/2014 | 24:43.0 | 3 |
| 223.748 | 19091.504 | 2.254 | 14.039 | 0.138899 | 7/15/2014 | 24:43.1 | 3 |
| 223.529 | 19091.505 | 2.251 | 14.044 | 0.136657 | 7/15/2014 | 24:43.2 | 3 |
| 223.312 | 19091.505 | 2.251 | 14.046 | 0.131011 | 7/15/2014 | 24:43.3 | 3 |
| 223.096 | 19091.505 | 2.251 | 14.05 | 0.124555 | 7/15/2014 | 24:43.4 | 3 |
| 222.779 | 19091.505 | 2.246 | 14.053 | 0.120689 | 7/15/2014 | 24:43.6 | 3 |
| 222.517 | 19091.505 | 2.241 | 14.058 | 0.119222 | 7/15/2014 | 24:43.7 | 3 |
| 222.406 | 19091.505 | 2.243 | 14.06 | 0.118577 | 7/15/2014 | 24:43.7 | 3 |
| 222.194 | 19091.506 | 2.241 | 14.066 | 0.120487 | 7/15/2014 | 24:43.8 | 3 |
| 221.982 | 19091.506 | 2.239 | 14.069 | 0.124585 | 7/15/2014 | 24:43.9 | 3 |
| 221.768 | 19091.506 | 2.237 | 14.072 | 0.123114 | 7/15/2014 | 24:44.0 | 3 |
| 221.556 | 19091.506 | 2.237 | 14.083 | 0.125234 | 7/15/2014 | 24:44.1 | 3 |
| 221.345 | 19091.507 | 2.237 | 14.089 | 0.128957 | 7/15/2014 | 24:44.2 | 3 |
| 221.133 | 19091.507 | 2.237 | 14.094 | 0.134934 | 7/15/2014 | 24:44.3 | 3 |
| 220.869 | 19091.506 | 2.234 | 14.101 | 0.128203 | 7/15/2014 | 24:44.4 | 3 |
| 220.654 | 19091.507 | 2.234 | 14.107 | 0.128985 | 7/15/2014 | 24:44.5 | 3 |
| 220.439 | 19091.506 | 2.232 | 14.109 | 0.124299 | 7/15/2014 | 24:44.6 | 3 |
| 220.222 | 19091.506 | 2.232 | 14.115 | 0.119147 | 7/15/2014 | 24:44.7 | 3 |
| 220.003 | 19091.506 | 2.232 | 14.121 | 0.124123 | 7/15/2014 | 24:44.8 | 3 |
| 219.784 | 19091.507 | 2.232 | 14.119 | 0.117143 | 7/15/2014 | 24:44.9 | 3 |
| 219.567 | 19091.507 | 2.232 | 14.122 | 0.11328 | 7/15/2014 | 24:45.0 | 3 |
| 219.348 | 19091.505 | 2.229 | 14.124 | 0.11458 | 7/15/2014 | 24:45.1 | 3 |
| 219.129 | 19091.505 | 2.231 | 14.126 | 0.104905 | 7/15/2014 | 24:45.2 | 3 |
| 218.911 | 19091.505 | 2.231 | 14.128 | 0.105454 | 7/15/2014 | 24:45.3 | 3 |
| 218.696 | 19091.505 | 2.23 | 14.13 | 0.108886 | 7/15/2014 | 24:45.4 | 3 |
| 218.478 | 19091.505 | 2.23 | 14.134 | 0.113276 | 7/15/2014 | 24:45.5 | 3 |
| 218.263 | 19091.504 | 2.229 | 14.136 | 0.106122 | 7/15/2014 | 24:45.6 | 3 |
| 217.995 | 19091.503 | 2.224 | 14.138 | 0.0924409 | 7/15/2014 | 24:45.7 | 3 |
| 217.78 | 19091.502 | 2.224 | 14.14 | 0.0946162 | 7/15/2014 | 24:45.8 | 3 |
| 217.564 | 19091.502 | 2.226 | 14.142 | 0.0848304 | 7/15/2014 | 24:45.9 | 3 |
| 217.348 | 19091.501 | 2.226 | 14.144 | 0.0992604 | 7/15/2014 | 24:46.0 | 3 |
| 217.134 | 19091.5 | 2.228 | 14.147 | 0.0880997 | 7/15/2014 | 24:46.1 | 3 |
| 216.917 | 19091.5 | 2.231 | 14.15 | 0.096468 | 7/15/2014 | 24:46.2 | 3 |
| 216.704 | 19091.499 | 2.231 | 14.147 | 0.0917871 | 7/15/2014 | 24:46.3 | 3 |
| 216.436 | 19091.496 | 2.234 | 14.146 | 0.131381 | 7/15/2014 | 24:46.4 | 3 |
| 216.222 | 19091.496 | 2.234 | 14.148 | 0.113257 | 7/15/2014 | 24:46.5 | 3 |
| 216.007 | 19091.496 | 2.234 | 14.149 | 0.203841 | 7/15/2014 | 24:46.6 | 3 |

| | | | | | | | |
|---|---|---|---|---|---|---|---|
| 215.738 | 19091.493 | 2.234 | 14.153 | 0.153305 | 7/15/2014 | 24:46.7 | 3 |
| 215.521 | 19091.492 | 2.234 | 14.157 | 0.185017 | 7/15/2014 | 24:46.8 | 3 |
| 215.304 | 19091.491 | 2.234 | 14.158 | 0.192294 | 7/15/2014 | 24:46.9 | 3 |
| 215.086 | 19091.49 | 2.234 | 14.161 | 0.212866 | 7/15/2014 | 24:47.0 | 3 |
| 214.869 | 19091.487 | 2.237 | 14.164 | 0.195733 | 7/15/2014 | 24:47.1 | 3 |
| 214.652 | 19091.486 | 2.237 | 14.169 | 0.201876 | 7/15/2014 | 24:47.2 | 3 |
| 214.437 | 19091.486 | 2.239 | 14.172 | 0.161992 | 7/15/2014 | 24:47.3 | 3 |
| 214.169 | 19091.483 | 2.239 | 14.174 | 0.195753 | 7/15/2014 | 24:47.4 | 3 |
| 213.955 | 19091.482 | 2.239 | 14.176 | 0.173141 | 7/15/2014 | 24:47.5 | 3 |
| 213.742 | 19091.48 | 2.236 | 14.181 | 0.164821 | 7/15/2014 | 24:47.6 | 3 |
| 213.474 | 19091.478 | 2.236 | 14.183 | 0.114058 | 7/15/2014 | 24:47.7 | 3 |
| 213.259 | 19091.476 | 2.239 | 14.188 | 0.125406 | 7/15/2014 | 24:47.8 | 3 |
| 213.044 | 19091.474 | 2.239 | 14.192 | 0.114537 | 7/15/2014 | 24:47.9 | 3 |
| 212.83 | 19091.473 | 2.239 | 14.198 | 0.10723 | 7/15/2014 | 24:48.0 | 3 |
| 212.614 | 19091.471 | 2.239 | 14.206 | 0.105938 | 7/15/2014 | 24:48.1 | 3 |
| 212.4 | 19091.469 | 2.242 | 14.22 | 0.107653 | 7/15/2014 | 24:48.2 | 3 |
| 212.186 | 19091.466 | 2.245 | 14.227 | 0.110776 | 7/15/2014 | 24:48.3 | 3 |
| 211.921 | 19091.463 | 2.251 | 14.233 | 0.111954 | 7/15/2014 | 24:48.4 | 3 |
| 211.601 | 19091.459 | 2.257 | 14.245 | 0.113304 | 7/15/2014 | 24:48.6 | 3 |
| 211.496 | 19091.458 | 2.254 | 14.249 | 0.108434 | 7/15/2014 | 24:48.6 | 3 |
| 211.231 | 19091.455 | 2.261 | 14.257 | 0.106671 | 7/15/2014 | 24:48.7 | 3 |
| 211.017 | 19091.453 | 2.264 | 14.265 | 0.103511 | 7/15/2014 | 24:48.8 | 3 |
| 210.802 | 19091.45 | 2.264 | 14.27 | 0.113209 | 7/15/2014 | 24:48.9 | 3 |
| 210.586 | 19091.447 | 2.264 | 14.273 | 0.113017 | 7/15/2014 | 24:49.0 | 3 |
| 210.373 | 19091.444 | 2.261 | 14.281 | 0.0974872 | 7/15/2014 | 24:49.1 | 3 |
| 210.159 | 19091.441 | 2.258 | 14.289 | 0.0970491 | 7/15/2014 | 24:49.2 | 3 |
| 209.944 | 19091.438 | 2.261 | 14.296 | 0.0858173 | 7/15/2014 | 24:49.3 | 3 |
| 209.678 | 19091.435 | 2.261 | 14.305 | 0.0807013 | 7/15/2014 | 24:49.4 | 3 |
| 209.466 | 19091.432 | 2.261 | 14.314 | 0.0813373 | 7/15/2014 | 24:49.5 | 3 |
| 209.254 | 19091.428 | 2.261 | 14.32 | 0.069662 | 7/15/2014 | 24:49.6 | 3 |
| 208.985 | 19091.425 | 2.267 | 14.328 | 0.068858 | 7/15/2014 | 24:49.7 | 3 |
| 208.772 | 19091.421 | 2.267 | 14.336 | 0.0714456 | 7/15/2014 | 24:49.8 | 3 |
| 208.555 | 19091.416 | 2.275 | 14.341 | 0.0730097 | 7/15/2014 | 24:49.9 | 3 |
| 208.336 | 19091.413 | 2.283 | 14.343 | 0.0778446 | 7/15/2014 | 24:50.0 | 3 |
| 208.12 | 19091.41 | 2.283 | 14.347 | 0.0864538 | 7/15/2014 | 24:50.1 | 3 |
| 207.903 | 19091.405 | 2.283 | 14.349 | 0.101549 | 7/15/2014 | 24:50.2 | 3 |
| 207.687 | 19091.402 | 2.283 | 14.348 | 0.105644 | 7/15/2014 | 24:50.3 | 3 |
| 207.418 | 19091.398 | 2.285 | 14.349 | 0.129376 | 7/15/2014 | 24:50.4 | 3 |
| 207.202 | 19091.394 | 2.285 | 14.351 | 0.147907 | 7/15/2014 | 24:50.5 | 3 |
| 206.986 | 19091.39 | 2.287 | 14.352 | 0.154093 | 7/15/2014 | 24:50.6 | 3 |
| 206.714 | 19091.386 | 2.287 | 14.352 | 0.144573 | 7/15/2014 | 24:50.7 | 3 |
| 206.498 | 19091.381 | 2.287 | 14.351 | 0.155828 | 7/15/2014 | 24:50.8 | 3 |
| 206.279 | 19091.376 | 2.289 | 14.351 | 0.173311 | 7/15/2014 | 24:50.9 | 3 |
| 206.06 | 19091.373 | 2.296 | 14.35 | 0.192723 | 7/15/2014 | 24:51.0 | 3 |
| 205.842 | 19091.368 | 2.298 | 14.351 | 0.181445 | 7/15/2014 | 24:51.1 | 3 |
| 205.624 | 19091.363 | 2.302 | 14.351 | 0.191296 | 7/15/2014 | 24:51.2 | 3 |
| 205.408 | 19091.36 | 2.306 | 14.35 | 0.191841 | 7/15/2014 | 24:51.3 | 3 |

| | | | | | | | |
|---|---|---|---|---|---|---|---|
| 205.138 | 19091.355 | 2.311 | 14.354 | 0.196229 | 7/15/2014 | 24:51.4 | 3 |
| 204.924 | 19091.351 | 2.313 | 14.354 | 0.199897 | 7/15/2014 | 24:51.5 | 3 |
| 204.708 | 19091.347 | 2.315 | 14.354 | 0.196884 | 7/15/2014 | 24:51.6 | 3 |
| 204.438 | 19091.341 | 2.317 | 14.356 | 0.200223 | 7/15/2014 | 24:51.7 | 3 |
| 204.222 | 19091.336 | 2.32 | 14.357 | 0.196861 | 7/15/2014 | 24:51.8 | 3 |
| 204.004 | 19091.331 | 2.322 | 14.357 | 0.204988 | 7/15/2014 | 24:51.9 | 3 |
| 203.786 | 19091.326 | 2.325 | 14.36 | 0.205113 | 7/15/2014 | 24:52.0 | 3 |
| 203.567 | 19091.322 | 2.331 | 14.358 | 0.209038 | 7/15/2014 | 24:52.1 | 3 |
| 203.349 | 19091.318 | 2.337 | 14.35 | 0.200485 | 7/15/2014 | 24:52.2 | 3 |
| 203.133 | 19091.314 | 2.342 | 14.348 | 0.2028 | 7/15/2014 | 24:52.3 | 3 |
| 202.864 | 19091.309 | 2.346 | 14.35 | 0.207134 | 7/15/2014 | 24:52.4 | 3 |
| 202.646 | 19091.304 | 2.351 | 14.346 | 0.215784 | 7/15/2014 | 24:52.5 | 3 |
| 202.428 | 19091.298 | 2.354 | 14.343 | 0.213037 | 7/15/2014 | 24:52.6 | 3 |
| 202.155 | 19091.291 | 2.36 | 14.342 | 0.209564 | 7/15/2014 | 24:52.7 | 3 |
| 201.934 | 19091.287 | 2.365 | 14.337 | 0.21059 | 7/15/2014 | 24:52.8 | 3 |
| 201.713 | 19091.283 | 2.365 | 14.335 | 0.230686 | 7/15/2014 | 24:52.9 | 3 |
| 201.492 | 19091.277 | 2.37 | 14.334 | 0.242412 | 7/15/2014 | 24:53.0 | 3 |
| 201.27 | 19091.273 | 2.375 | 14.333 | 0.224102 | 7/15/2014 | 24:53.1 | 3 |
| 201.049 | 19091.269 | 2.383 | 14.33 | 0.24734 | 7/15/2014 | 24:53.2 | 3 |
| 200.827 | 19091.263 | 2.389 | 14.33 | 0.25055 | 7/15/2014 | 24:53.3 | 3 |
| 200.554 | 19091.256 | 2.394 | 14.33 | 0.270941 | 7/15/2014 | 24:53.4 | 3 |
| 200.332 | 19091.252 | 2.399 | 14.326 | 0.280444 | 7/15/2014 | 24:53.5 | 3 |
| 200.113 | 19091.248 | 2.402 | 14.325 | 0.295683 | 7/15/2014 | 24:53.6 | 3 |
| 199.836 | 19091.24 | 2.408 | 14.329 | 0.263729 | 7/15/2014 | 24:53.7 | 3 |
| 199.612 | 19091.235 | 2.41 | 14.325 | 0.305295 | 7/15/2014 | 24:53.8 | 3 |
| 199.39 | 19091.23 | 2.41 | 14.323 | 0.269056 | 7/15/2014 | 24:53.9 | 3 |
| 199.165 | 19091.226 | 2.412 | 14.316 | 0.288529 | 7/15/2014 | 24:54.0 | 3 |
| 198.942 | 19091.221 | 2.415 | 14.314 | 0.280585 | 7/15/2014 | 24:54.1 | 3 |
| 198.719 | 19091.216 | 2.412 | 14.314 | 0.261203 | 7/15/2014 | 24:54.2 | 3 |
| 198.496 | 19091.212 | 2.415 | 14.312 | 0.290954 | 7/15/2014 | 24:54.3 | 3 |
| 198.218 | 19091.205 | 2.417 | 14.313 | 0.295361 | 7/15/2014 | 24:54.4 | 3 |
| 197.996 | 19091.2 | 2.417 | 14.31 | 0.256563 | 7/15/2014 | 24:54.5 | 3 |
| 197.774 | 19091.194 | 2.417 | 14.314 | 0.296987 | 7/15/2014 | 24:54.6 | 3 |
| 197.494 | 19091.189 | 2.422 | 14.313 | 0.263854 | 7/15/2014 | 24:54.7 | 3 |
| 197.271 | 19091.185 | 2.424 | 14.316 | 0.276779 | 7/15/2014 | 24:54.8 | 3 |
| 197.046 | 19091.18 | 2.427 | 14.314 | 0.293523 | 7/15/2014 | 24:54.9 | 3 |
| 196.821 | 19091.175 | 2.432 | 14.311 | 0.259606 | 7/15/2014 | 24:55.0 | 3 |
| 196.595 | 19091.17 | 2.434 | 14.311 | 0.266483 | 7/15/2014 | 24:55.1 | 3 |
| 196.372 | 19091.166 | 2.436 | 14.311 | 0.282868 | 7/15/2014 | 24:55.2 | 3 |
| 196.149 | 19091.161 | 2.439 | 14.308 | 0.256881 | 7/15/2014 | 24:55.3 | 3 |
| 195.87 | 19091.156 | 2.443 | 14.306 | 0.273981 | 7/15/2014 | 24:55.4 | 3 |
| 195.65 | 19091.15 | 2.443 | 14.307 | 0.268197 | 7/15/2014 | 24:55.5 | 3 |
| 195.427 | 19091.146 | 2.443 | 14.307 | 0.286614 | 7/15/2014 | 24:55.6 | 3 |
| 195.153 | 19091.141 | 2.443 | 14.309 | 0.241812 | 7/15/2014 | 24:55.7 | 3 |
| 194.931 | 19091.137 | 2.443 | 14.312 | 0.250857 | 7/15/2014 | 24:55.8 | 3 |
| 194.709 | 19091.133 | 2.443 | 14.316 | 0.2631 | 7/15/2014 | 24:55.9 | 3 |
| 194.487 | 19091.129 | 2.446 | 14.316 | 0.276662 | 7/15/2014 | 24:56.0 | 3 |

| | | | | | | | |
|---|---|---|---|---|---|---|---|
| 194.264 | 19091.125 | 2.448 | 14.316 | 0.26754 | 7/15/2014 | 24:56.1 | 3 |
| 194.044 | 19091.12 | 2.448 | 14.318 | 0.250551 | 7/15/2014 | 24:56.2 | 3 |
| 193.824 | 19091.116 | 2.448 | 14.32 | 0.228444 | 7/15/2014 | 24:56.3 | 3 |
| 193.551 | 19091.111 | 2.45 | 14.323 | 0.2839 | 7/15/2014 | 24:56.4 | 3 |
| 193.333 | 19091.107 | 2.45 | 14.325 | 0.270054 | 7/15/2014 | 24:56.5 | 3 |
| 193.114 | 19091.104 | 2.454 | 14.327 | 0.26113 | 7/15/2014 | 24:56.6 | 3 |
| 192.84 | 19091.099 | 2.458 | 14.332 | 0.232299 | 7/15/2014 | 24:56.7 | 3 |
| 192.621 | 19091.096 | 2.458 | 14.333 | 0.233882 | 7/15/2014 | 24:56.8 | 3 |
| 192.398 | 19091.092 | 2.462 | 14.335 | 0.247554 | 7/15/2014 | 24:56.9 | 3 |
| 192.178 | 19091.088 | 2.462 | 14.335 | 0.244546 | 7/15/2014 | 24:57.0 | 3 |
| 191.955 | 19091.084 | 2.467 | 14.338 | 0.232596 | 7/15/2014 | 24:57.1 | 3 |
| 191.735 | 19091.08 | 2.467 | 14.338 | 0.242026 | 7/15/2014 | 24:57.2 | 3 |
| 191.514 | 19091.076 | 2.471 | 14.34 | 0.231583 | 7/15/2014 | 24:57.3 | 3 |
| 191.24 | 19091.072 | 2.471 | 14.343 | 0.252957 | 7/15/2014 | 24:57.4 | 3 |
| 191.021 | 19091.069 | 2.469 | 14.344 | 0.260613 | 7/15/2014 | 24:57.5 | 3 |
| 190.799 | 19091.066 | 2.469 | 14.345 | 0.283444 | 7/15/2014 | 24:57.6 | 3 |
| 190.524 | 19091.062 | 2.467 | 14.348 | 0.261069 | 7/15/2014 | 24:57.7 | 3 |
| 190.301 | 19091.059 | 2.467 | 14.353 | 0.250227 | 7/15/2014 | 24:57.8 | 3 |
| 190.077 | 19091.055 | 2.467 | 14.354 | 0.243939 | 7/15/2014 | 24:57.9 | 3 |
| 189.853 | 19091.051 | 2.467 | 14.356 | 0.26269 | 7/15/2014 | 24:58.0 | 3 |
| 189.63 | 19091.048 | 2.465 | 14.356 | 0.230716 | 7/15/2014 | 24:58.1 | 3 |
| 189.407 | 19091.044 | 2.465 | 14.354 | 0.230959 | 7/15/2014 | 24:58.2 | 3 |
| 189.183 | 19091.042 | 2.467 | 14.354 | 0.214153 | 7/15/2014 | 24:58.3 | 3 |
| 188.907 | 19091.038 | 2.467 | 14.356 | 0.260605 | 7/15/2014 | 24:58.4 | 3 |
| 188.684 | 19091.035 | 2.467 | 14.357 | 0.260692 | 7/15/2014 | 24:58.5 | 3 |
| 188.462 | 19091.031 | 2.467 | 14.352 | 0.255509 | 7/15/2014 | 24:58.6 | 3 |
| 188.185 | 19091.027 | 2.465 | 14.352 | 0.231288 | 7/15/2014 | 24:58.7 | 3 |
| 187.962 | 19091.024 | 2.465 | 14.352 | 0.252161 | 7/15/2014 | 24:58.8 | 3 |
| 187.737 | 19091.021 | 2.467 | 14.353 | 0.230759 | 7/15/2014 | 24:58.9 | 3 |
| 187.514 | 19091.019 | 2.467 | 14.353 | 0.221884 | 7/15/2014 | 24:59.0 | 3 |
| 187.291 | 19091.016 | 2.467 | 14.35 | 0.185664 | 7/15/2014 | 24:59.1 | 3 |
| 187.069 | 19091.013 | 2.467 | 14.346 | 0.172416 | 7/15/2014 | 24:59.2 | 3 |
| 186.848 | 19091.01 | 2.469 | 14.341 | 0.192283 | 7/15/2014 | 24:59.3 | 3 |
| 186.574 | 19091.006 | 2.472 | 14.341 | 0.188429 | 7/15/2014 | 24:59.5 | 3 |
| 186.356 | 19091.004 | 2.474 | 14.334 | 0.200803 | 7/15/2014 | 24:59.5 | 3 |
| 186.139 | 19091 | 2.477 | 14.329 | 0.190952 | 7/15/2014 | 24:59.7 | 3 |
| 185.868 | 19090.998 | 2.481 | 14.323 | 0.198869 | 7/15/2014 | 24:59.8 | 3 |
| 185.649 | 19090.994 | 2.487 | 14.318 | 0.209086 | 7/15/2014 | 24:59.8 | 3 |
| 185.432 | 19090.992 | 2.49 | 14.309 | 0.233203 | 7/15/2014 | 24:59.9 | 3 |
| 185.213 | 19090.989 | 2.499 | 14.301 | 0.228488 | 7/15/2014 | 25:00.0 | 3 |
| 184.995 | 19090.986 | 2.505 | 14.289 | 0.232981 | 7/15/2014 | 25:00.1 | 3 |
| 184.781 | 19090.983 | 2.51 | 14.276 | 0.218664 | 7/15/2014 | 25:00.2 | 3 |
| 184.566 | 19090.981 | 2.517 | 14.266 | 0.245356 | 7/15/2014 | 25:00.3 | 3 |
| 184.299 | 19090.979 | 2.525 | 14.253 | 0.24873 | 7/15/2014 | 25:00.4 | 3 |
| 183.98 | 19090.974 | 2.529 | 14.236 | 0.221123 | 7/15/2014 | 25:00.6 | 3 |
| 183.874 | 19090.973 | 2.529 | 14.226 | 0.217453 | 7/15/2014 | 25:00.6 | 3 |
| 183.607 | 19090.97 | 2.532 | 14.217 | 0.197088 | 7/15/2014 | 25:00.7 | 3 |

| | | | | | | | |
|---|---|---|---|---|---|---|---|
| 183.391 | 19090.967 | 2.532 | 14.208 | 0.201791 | 7/15/2014 | 25:00.8 | 3 |
| 183.176 | 19090.964 | 2.534 | 14.208 | 0.203606 | 7/15/2014 | 25:00.9 | 3 |
| 182.959 | 19090.963 | 2.539 | 14.203 | 0.191485 | 7/15/2014 | 25:01.0 | 3 |
| 182.745 | 19090.959 | 2.541 | 14.196 | 0.196666 | 7/15/2014 | 25:01.1 | 3 |
| 182.531 | 19090.957 | 2.543 | 14.194 | 0.211756 | 7/15/2014 | 25:01.2 | 3 |
| 182.316 | 19090.954 | 2.549 | 14.19 | 0.214326 | 7/15/2014 | 25:01.3 | 3 |
| 182.051 | 19090.951 | 2.552 | 14.188 | 0.206368 | 7/15/2014 | 25:01.4 | 3 |
| 181.834 | 19090.948 | 2.558 | 14.184 | 0.21024 | 7/15/2014 | 25:01.5 | 3 |
| 181.62 | 19090.946 | 2.563 | 14.189 | 0.204327 | 7/15/2014 | 25:01.6 | 3 |
| 181.351 | 19090.942 | 2.568 | 14.183 | 0.198678 | 7/15/2014 | 25:01.7 | 3 |
| 181.136 | 19090.939 | 2.565 | 14.182 | 0.202187 | 7/15/2014 | 25:01.8 | 3 |
| 180.919 | 19090.937 | 2.568 | 14.184 | 0.190347 | 7/15/2014 | 25:01.9 | 3 |
| 180.701 | 19090.936 | 2.568 | 14.183 | 0.190265 | 7/15/2014 | 25:02.0 | 3 |
| 180.483 | 19090.932 | 2.57 | 14.185 | 0.188049 | 7/15/2014 | 25:02.1 | 3 |
| 180.265 | 19090.929 | 2.574 | 14.185 | 0.180902 | 7/15/2014 | 25:02.2 | 3 |
| 180.049 | 19090.927 | 2.578 | 14.186 | 0.182426 | 7/15/2014 | 25:02.3 | 3 |
| 179.78 | 19090.923 | 2.58 | 14.18 | 0.183273 | 7/15/2014 | 25:02.4 | 3 |
| 179.565 | 19090.92 | 2.58 | 14.18 | 0.171356 | 7/15/2014 | 25:02.5 | 3 |
| 179.35 | 19090.916 | 2.585 | 14.183 | 0.177922 | 7/15/2014 | 25:02.6 | 3 |
| 179.078 | 19090.915 | 2.589 | 14.187 | 0.174342 | 7/15/2014 | 25:02.7 | 3 |
| 178.861 | 19090.912 | 2.591 | 14.189 | 0.173066 | 7/15/2014 | 25:02.8 | 3 |
| 178.643 | 19090.908 | 2.588 | 14.19 | 0.181028 | 7/15/2014 | 25:02.9 | 3 |
| 178.423 | 19090.906 | 2.591 | 14.2 | 0.174735 | 7/15/2014 | 25:03.0 | 3 |
| 178.207 | 19090.903 | 2.591 | 14.217 | 0.174419 | 7/15/2014 | 25:03.1 | 3 |
| 177.99 | 19090.9 | 2.591 | 14.226 | 0.179165 | 7/15/2014 | 25:03.2 | 3 |
| 177.773 | 19090.896 | 2.593 | 14.238 | 0.180501 | 7/15/2014 | 25:03.3 | 3 |
| 177.505 | 19090.895 | 2.591 | 14.243 | 0.18717 | 7/15/2014 | 25:03.4 | 3 |
| 177.29 | 19090.893 | 2.589 | 14.247 | 0.182866 | 7/15/2014 | 25:03.5 | 3 |
| 177.074 | 19090.889 | 2.592 | 14.258 | 0.172737 | 7/15/2014 | 25:03.6 | 3 |
| 176.806 | 19090.886 | 2.598 | 14.266 | 0.168631 | 7/15/2014 | 25:03.7 | 3 |
| 176.592 | 19090.883 | 2.594 | 14.269 | 0.167238 | 7/15/2014 | 25:03.8 | 3 |
| 176.375 | 19090.881 | 2.597 | 14.267 | 0.167111 | 7/15/2014 | 25:03.9 | 3 |
| 176.157 | 19090.877 | 2.6 | 14.272 | 0.171482 | 7/15/2014 | 25:04.0 | 3 |
| 175.943 | 19090.875 | 2.6 | 14.271 | 0.162612 | 7/15/2014 | 25:04.1 | 3 |
| 175.728 | 19090.873 | 2.602 | 14.277 | 0.16655 | 7/15/2014 | 25:04.2 | 3 |
| 175.511 | 19090.869 | 2.607 | 14.279 | 0.163782 | 7/15/2014 | 25:04.3 | 3 |
| 175.246 | 19090.866 | 2.609 | 14.281 | 0.181199 | 7/15/2014 | 25:04.4 | 3 |
| 175.034 | 19090.863 | 2.609 | 14.281 | 0.170203 | 7/15/2014 | 25:04.5 | 3 |
| 174.82 | 19090.861 | 2.615 | 14.283 | 0.169939 | 7/15/2014 | 25:04.6 | 3 |
| 174.553 | 19090.857 | 2.618 | 14.283 | 0.16126 | 7/15/2014 | 25:04.7 | 3 |
| 174.34 | 19090.855 | 2.62 | 14.287 | 0.159609 | 7/15/2014 | 25:04.8 | 3 |
| 174.126 | 19090.853 | 2.625 | 14.291 | 0.16074 | 7/15/2014 | 25:04.9 | 3 |
| 173.91 | 19090.85 | 2.63 | 14.3 | 0.156001 | 7/15/2014 | 25:05.0 | 3 |
| 173.696 | 19090.847 | 2.633 | 14.303 | 0.152703 | 7/15/2014 | 25:05.1 | 3 |
| 173.484 | 19090.845 | 2.633 | 14.306 | 0.150279 | 7/15/2014 | 25:05.2 | 3 |
| 173.27 | 19090.842 | 2.636 | 14.308 | 0.145128 | 7/15/2014 | 25:05.3 | 3 |
| 172.903 | 19090.838 | 2.633 | 14.31 | 0.14546 | 7/15/2014 | 25:05.5 | 3 |

| | | | | | | | |
|---|---|---|---|---|---|---|---|
| 172.691 | 19090.835 | 2.635 | 14.31 | 0.141232 | 7/15/2014 | 25:05.6 | 3 |
| 172.428 | 19090.831 | 2.635 | 14.306 | 0.140279 | 7/15/2014 | 25:05.7 | 3 |
| 172.321 | 19090.83 | 2.635 | 14.308 | 0.13966 | 7/15/2014 | 25:05.7 | 3 |
| 172.11 | 19090.827 | 2.635 | 14.301 | 0.141052 | 7/15/2014 | 25:05.8 | 3 |
| 171.897 | 19090.826 | 2.635 | 14.3 | 0.142005 | 7/15/2014 | 25:05.9 | 3 |
| 171.685 | 19090.822 | 2.635 | 14.301 | 0.142498 | 7/15/2014 | 25:06.0 | 3 |
| 171.474 | 19090.82 | 2.634 | 14.301 | 0.143047 | 7/15/2014 | 25:06.1 | 3 |
| 171.262 | 19090.818 | 2.634 | 14.302 | 0.151776 | 7/15/2014 | 25:06.2 | 3 |
| 171.05 | 19090.814 | 2.637 | 14.3 | 0.147831 | 7/15/2014 | 25:06.3 | 3 |
| 170.684 | 19090.811 | 2.637 | 14.302 | 0.151394 | 7/15/2014 | 25:06.5 | 3 |
| 170.473 | 19090.809 | 2.637 | 14.304 | 0.156685 | 7/15/2014 | 25:06.6 | 3 |
| 170.21 | 19090.805 | 2.637 | 14.307 | 0.152453 | 7/15/2014 | 25:06.7 | 3 |
| 170.104 | 19090.803 | 2.637 | 14.309 | 0.153755 | 7/15/2014 | 25:06.7 | 3 |
| 169.894 | 19090.802 | 2.637 | 14.309 | 0.146157 | 7/15/2014 | 25:06.8 | 3 |
| 169.68 | 19090.799 | 2.64 | 14.312 | 0.142281 | 7/15/2014 | 25:06.9 | 3 |
| 169.467 | 19090.796 | 2.643 | 14.311 | 0.135735 | 7/15/2014 | 25:07.0 | 3 |
| 169.255 | 19090.795 | 2.643 | 14.314 | 0.130685 | 7/15/2014 | 25:07.1 | 3 |
| 169.044 | 19090.791 | 2.64 | 14.316 | 0.128851 | 7/15/2014 | 25:07.2 | 3 |
| 168.831 | 19090.79 | 2.643 | 14.319 | 0.122588 | 7/15/2014 | 25:07.3 | 3 |
| 168.463 | 19090.784 | 2.648 | 14.321 | 0.123576 | 7/15/2014 | 25:07.5 | 3 |
| 168.253 | 19090.783 | 2.648 | 14.321 | 0.118925 | 7/15/2014 | 25:07.6 | 3 |
| 167.991 | 19090.779 | 2.648 | 14.322 | 0.116469 | 7/15/2014 | 25:07.7 | 3 |
| 167.885 | 19090.778 | 2.649 | 14.321 | 0.112722 | 7/15/2014 | 25:07.7 | 3 |
| 167.672 | 19090.775 | 2.647 | 14.321 | 0.125035 | 7/15/2014 | 25:07.8 | 3 |
| 167.461 | 19090.772 | 2.647 | 14.32 | 0.112857 | 7/15/2014 | 25:07.9 | 3 |
| 167.249 | 19090.771 | 2.651 | 14.318 | 0.113898 | 7/15/2014 | 25:08.0 | 3 |
| 167.036 | 19090.769 | 2.651 | 14.314 | 0.117946 | 7/15/2014 | 25:08.1 | 3 |
| 166.826 | 19090.767 | 2.645 | 14.31 | 0.117919 | 7/15/2014 | 25:08.2 | 3 |
| 166.615 | 19090.764 | 2.645 | 14.306 | 0.129095 | 7/15/2014 | 25:08.3 | 3 |
| 166.248 | 19090.76 | 2.648 | 14.302 | 0.125085 | 7/15/2014 | 25:08.5 | 3 |
| 166.037 | 19090.757 | 2.65 | 14.299 | 0.116209 | 7/15/2014 | 25:08.6 | 3 |
| 165.775 | 19090.754 | 2.651 | 14.299 | 0.130428 | 7/15/2014 | 25:08.7 | 3 |
| 165.669 | 19090.753 | 2.653 | 14.298 | 0.127375 | 7/15/2014 | 25:08.7 | 3 |
| 165.456 | 19090.751 | 2.65 | 14.298 | 0.108601 | 7/15/2014 | 25:08.8 | 3 |
| 165.244 | 19090.748 | 2.65 | 14.298 | 0.112004 | 7/15/2014 | 25:08.9 | 3 |
| 165.028 | 19090.745 | 2.65 | 14.297 | 0.106007 | 7/15/2014 | 25:09.0 | 3 |
| 164.813 | 19090.743 | 2.653 | 14.295 | 0.125879 | 7/15/2014 | 25:09.1 | 3 |
| 164.599 | 19090.74 | 2.653 | 14.294 | 0.113807 | 7/15/2014 | 25:09.2 | 3 |
| 164.385 | 19090.737 | 2.653 | 14.291 | 0.112924 | 7/15/2014 | 25:09.3 | 3 |
| 164.119 | 19090.735 | 2.652 | 14.287 | 0.118461 | 7/15/2014 | 25:09.4 | 3 |
| 163.907 | 19090.732 | 2.646 | 14.283 | 0.126275 | 7/15/2014 | 25:09.5 | 3 |
| 163.692 | 19090.729 | 2.651 | 14.277 | 0.122727 | 7/15/2014 | 25:09.6 | 3 |
| 163.424 | 19090.727 | 2.651 | 14.27 | 0.134463 | 7/15/2014 | 25:09.7 | 3 |
| 163.208 | 19090.725 | 2.656 | 14.267 | 0.122717 | 7/15/2014 | 25:09.8 | 3 |
| 162.992 | 19090.721 | 2.653 | 14.264 | 0.132534 | 7/15/2014 | 25:09.9 | 3 |
| 162.775 | 19090.718 | 2.652 | 14.258 | 0.12845 | 7/15/2014 | 25:10.0 | 3 |
| 162.56 | 19090.717 | 2.652 | 14.254 | 0.128497 | 7/15/2014 | 25:10.1 | 3 |

| | | | | | | | |
|---|---|---|---|---|---|---|---|
| 162.346 | 19090.713 | 2.654 | 14.253 | 0.128849 | 7/15/2014 | 25:10.2 | 3 |
| 162.13 | 19090.711 | 2.657 | 14.247 | 0.117066 | 7/15/2014 | 25:10.3 | 3 |
| 161.865 | 19090.708 | 2.657 | 14.243 | 0.114748 | 7/15/2014 | 25:10.4 | 3 |
| 161.546 | 19090.704 | 2.66 | 14.238 | 0.108897 | 7/15/2014 | 25:10.6 | 3 |
| 161.283 | 19090.701 | 2.655 | 14.232 | 0.103915 | 7/15/2014 | 25:10.7 | 3 |
| 161.174 | 19090.7 | 2.657 | 14.229 | 0.10088 | 7/15/2014 | 25:10.7 | 3 |
| 160.962 | 19090.697 | 2.658 | 14.226 | 0.0960593 | 7/15/2014 | 25:10.8 | 3 |
| 160.746 | 19090.694 | 2.658 | 14.222 | 0.0959791 | 7/15/2014 | 25:10.9 | 3 |
| 160.531 | 19090.692 | 2.658 | 14.218 | 0.108776 | 7/15/2014 | 25:11.0 | 3 |
| 160.316 | 19090.689 | 2.663 | 14.218 | 0.112393 | 7/15/2014 | 25:11.1 | 3 |
| 160.104 | 19090.688 | 2.663 | 14.222 | 0.0965663 | 7/15/2014 | 25:11.2 | 3 |
| 159.891 | 19090.685 | 2.663 | 14.222 | 0.104926 | 7/15/2014 | 25:11.3 | 3 |
| 159.626 | 19090.683 | 2.663 | 14.222 | 0.118629 | 7/15/2014 | 25:11.4 | 3 |
| 159.31 | 19090.679 | 2.665 | 14.221 | 0.107664 | 7/15/2014 | 25:11.6 | 3 |
| 159.046 | 19090.676 | 2.665 | 14.221 | 0.101402 | 7/15/2014 | 25:11.7 | 3 |
| 158.937 | 19090.675 | 2.668 | 14.222 | 0.0890264 | 7/15/2014 | 25:11.7 | 3 |
| 158.724 | 19090.672 | 2.668 | 14.218 | 0.0918923 | 7/15/2014 | 25:11.8 | 3 |
| 158.512 | 19090.671 | 2.666 | 14.216 | 0.0991346 | 7/15/2014 | 25:11.9 | 3 |
| 158.299 | 19090.668 | 2.666 | 14.211 | 0.120493 | 7/15/2014 | 25:12.0 | 3 |
| 158.085 | 19090.666 | 2.671 | 14.211 | 0.118709 | 7/15/2014 | 25:12.1 | 3 |
| 157.873 | 19090.664 | 2.671 | 14.211 | 0.145245 | 7/15/2014 | 25:12.2 | 3 |
| 157.662 | 19090.661 | 2.674 | 14.211 | 0.18095 | 7/15/2014 | 25:12.3 | 3 |
| 157.293 | 19090.658 | 2.675 | 14.215 | 0.17625 | 7/15/2014 | 25:12.5 | 3 |
| 157.085 | 19090.656 | 2.678 | 14.216 | 0.141285 | 7/15/2014 | 25:12.6 | 3 |
| 156.824 | 19090.654 | 2.678 | 14.22 | 0.145538 | 7/15/2014 | 25:12.7 | 3 |
| 156.717 | 19090.653 | 2.679 | 14.222 | 0.12805 | 7/15/2014 | 25:12.7 | 3 |
| 156.506 | 19090.651 | 2.682 | 14.223 | 0.113672 | 7/15/2014 | 25:12.8 | 3 |
| 156.294 | 19090.65 | 2.682 | 14.224 | 0.0692909 | 7/15/2014 | 25:12.9 | 3 |
| 156.084 | 19090.646 | 2.682 | 14.223 | 0.0639462 | 7/15/2014 | 25:13.0 | 3 |
| 155.872 | 19090.646 | 2.684 | 14.222 | 0.0625887 | 7/15/2014 | 25:13.1 | 3 |
| 155.661 | 19090.645 | 2.686 | 14.223 | 0.0668217 | 7/15/2014 | 25:13.2 | 3 |
| 155.452 | 19090.643 | 2.688 | 14.221 | 0.0629436 | 7/15/2014 | 25:13.3 | 3 |
| 155.089 | 19090.642 | 2.689 | 14.222 | 0.0611955 | 7/15/2014 | 25:13.5 | 3 |
| 154.883 | 19090.639 | 2.689 | 14.221 | 0.0614051 | 7/15/2014 | 25:13.6 | 3 |
| 154.626 | 19090.639 | 2.689 | 14.221 | 0.0562351 | 7/15/2014 | 25:13.7 | 3 |
| 154.521 | 19090.637 | 2.69 | 14.221 | 0.0534303 | 7/15/2014 | 25:13.7 | 3 |
| 154.313 | 19090.637 | 2.695 | 14.222 | 0.0624993 | 7/15/2014 | 25:13.8 | 3 |
| 154.107 | 19090.635 | 2.697 | 14.222 | 0.0575941 | 7/15/2014 | 25:13.9 | 3 |
| 153.901 | 19090.634 | 2.697 | 14.22 | 0.0636271 | 7/15/2014 | 25:14.0 | 3 |
| 153.695 | 19090.633 | 2.697 | 14.22 | 0.0618277 | 7/15/2014 | 25:14.1 | 3 |
| 153.49 | 19090.633 | 2.697 | 14.22 | 0.0777133 | 7/15/2014 | 25:14.2 | 3 |
| 153.287 | 19090.631 | 2.7 | 14.219 | 0.0729506 | 7/15/2014 | 25:14.3 | 3 |
| 152.933 | 19090.631 | 2.704 | 14.216 | 0.0692985 | 7/15/2014 | 25:14.5 | 3 |
| 152.728 | 19090.631 | 2.707 | 14.215 | 0.0709362 | 7/15/2014 | 25:14.6 | 3 |
| 152.479 | 19090.629 | 2.704 | 14.214 | 0.0827833 | 7/15/2014 | 25:14.7 | 3 |
| 152.377 | 19090.629 | 2.705 | 14.214 | 0.0794761 | 7/15/2014 | 25:14.7 | 3 |
| 152.171 | 19090.628 | 2.708 | 14.212 | 0.0910422 | 7/15/2014 | 25:14.8 | 3 |

| | | | | | | | |
|---|---|---|---|---|---|---|---|
| 151.966 | 19090.628 | 2.714 | 14.209 | 0.0782715 | 7/15/2014 | 25:14.9 | 3 |
| 151.762 | 19090.629 | 2.718 | 14.205 | 0.0835169 | 7/15/2014 | 25:15.0 | 3 |
| 151.556 | 19090.629 | 2.718 | 14.204 | 0.0842645 | 7/15/2014 | 25:15.1 | 3 |
| 151.348 | 19090.629 | 2.721 | 14.203 | 0.0896846 | 7/15/2014 | 25:15.2 | 3 |
| 151.142 | 19090.629 | 2.721 | 14.203 | 0.0908243 | 7/15/2014 | 25:15.3 | 3 |
| 150.783 | 19090.628 | 2.718 | 14.201 | 0.0939526 | 7/15/2014 | 25:15.5 | 3 |
| 150.573 | 19090.629 | 2.724 | 14.204 | 0.0943959 | 7/15/2014 | 25:15.6 | 3 |
| 150.314 | 19090.63 | 2.724 | 14.204 | 0.090343 | 7/15/2014 | 25:15.7 | 3 |
| 150.211 | 19090.631 | 2.721 | 14.205 | 0.0922734 | 7/15/2014 | 25:15.7 | 3 |
| 150.002 | 19090.631 | 2.724 | 14.204 | 0.0896141 | 7/15/2014 | 25:15.8 | 3 |
| 149.789 | 19090.631 | 2.73 | 14.203 | 0.0878214 | 7/15/2014 | 25:15.9 | 3 |
| 149.579 | 19090.632 | 2.734 | 14.202 | 0.0848845 | 7/15/2014 | 25:16.0 | 3 |
| 149.366 | 19090.633 | 2.737 | 14.203 | 0.0869786 | 7/15/2014 | 25:16.1 | 3 |
| 149.157 | 19090.633 | 2.74 | 14.204 | 0.0874117 | 7/15/2014 | 25:16.2 | 3 |
| 148.947 | 19090.635 | 2.743 | 14.204 | 0.0888601 | 7/15/2014 | 25:16.3 | 3 |
| 148.578 | 19090.638 | 2.748 | 14.203 | 0.0849312 | 7/15/2014 | 25:16.5 | 3 |
| 148.368 | 19090.639 | 2.752 | 14.203 | 0.0846082 | 7/15/2014 | 25:16.6 | 3 |
| 148.108 | 19090.64 | 2.752 | 14.205 | 0.0829902 | 7/15/2014 | 25:16.7 | 3 |
| 148.002 | 19090.641 | 2.753 | 14.207 | 0.0833481 | 7/15/2014 | 25:16.7 | 3 |
| 147.79 | 19090.643 | 2.757 | 14.208 | 0.0856636 | 7/15/2014 | 25:16.8 | 3 |
| 147.579 | 19090.644 | 2.757 | 14.211 | 0.0805124 | 7/15/2014 | 25:16.9 | 3 |
| 147.369 | 19090.646 | 2.759 | 14.212 | 0.0801249 | 7/15/2014 | 25:17.0 | 3 |
| 147.154 | 19090.648 | 2.762 | 14.217 | 0.0818145 | 7/15/2014 | 25:17.1 | 3 |
| 146.943 | 19090.649 | 2.76 | 14.219 | 0.0913474 | 7/15/2014 | 25:17.2 | 3 |
| 146.734 | 19090.652 | 2.76 | 14.22 | 0.0862056 | 7/15/2014 | 25:17.3 | 3 |
| 146.37 | 19090.655 | 2.763 | 14.226 | 0.0898734 | 7/15/2014 | 25:17.5 | 3 |
| 146.16 | 19090.658 | 2.766 | 14.229 | 0.096394 | 7/15/2014 | 25:17.6 | 3 |
| 145.902 | 19090.661 | 2.764 | 14.233 | 0.0964917 | 7/15/2014 | 25:17.7 | 3 |
| 145.797 | 19090.662 | 2.766 | 14.235 | 0.0974101 | 7/15/2014 | 25:17.7 | 3 |
| 145.586 | 19090.666 | 2.768 | 14.234 | 0.0862889 | 7/15/2014 | 25:17.8 | 3 |
| 145.376 | 19090.668 | 2.77 | 14.233 | 0.101383 | 7/15/2014 | 25:17.9 | 3 |
| 145.166 | 19090.671 | 2.775 | 14.233 | 0.0888884 | 7/15/2014 | 25:18.0 | 3 |
| 144.955 | 19090.673 | 2.78 | 14.235 | 0.0933652 | 7/15/2014 | 25:18.1 | 3 |
| 144.746 | 19090.677 | 2.783 | 14.235 | 0.0900062 | 7/15/2014 | 25:18.2 | 3 |
| 144.537 | 19090.68 | 2.785 | 14.229 | 0.0936649 | 7/15/2014 | 25:18.3 | 3 |
| 144.173 | 19090.686 | 2.79 | 14.223 | 0.10346 | 7/15/2014 | 25:18.5 | 3 |
| 143.964 | 19090.689 | 2.795 | 14.217 | 0.116914 | 7/15/2014 | 25:18.6 | 3 |
| 143.704 | 19090.695 | 2.798 | 14.214 | 0.137385 | 7/15/2014 | 25:18.7 | 3 |
| 143.598 | 19090.696 | 2.802 | 14.212 | 0.138064 | 7/15/2014 | 25:18.7 | 3 |
| 143.385 | 19090.699 | 2.805 | 14.209 | 0.162889 | 7/15/2014 | 25:18.8 | 3 |
| 143.174 | 19090.704 | 2.808 | 14.202 | 0.158189 | 7/15/2014 | 25:18.9 | 3 |
| 142.959 | 19090.708 | 2.811 | 14.197 | 0.155208 | 7/15/2014 | 25:19.0 | 3 |
| 142.747 | 19090.712 | 2.814 | 14.192 | 0.155767 | 7/15/2014 | 25:19.1 | 3 |
| 142.532 | 19090.717 | 2.818 | 14.188 | 0.170675 | 7/15/2014 | 25:19.2 | 3 |
| 142.32 | 19090.721 | 2.818 | 14.188 | 0.13293 | 7/15/2014 | 25:19.3 | 3 |
| 142.053 | 19090.728 | 2.823 | 14.184 | 0.140894 | 7/15/2014 | 25:19.4 | 3 |
| 141.84 | 19090.732 | 2.825 | 14.181 | 0.12605 | 7/15/2014 | 25:19.5 | 3 |

| | | | | | | | |
|---|---|---|---|---|---|---|---|
| 141.626 | 19090.737 | 2.827 | 14.175 | 0.132046 | 7/15/2014 | 25:19.6 | 3 |
| 141.357 | 19090.745 | 2.83 | 14.172 | 0.128697 | 7/15/2014 | 25:19.7 | 3 |
| 141.144 | 19090.749 | 2.83 | 14.173 | 0.123276 | 7/15/2014 | 25:19.8 | 3 |
| 140.927 | 19090.756 | 2.836 | 14.173 | 0.123603 | 7/15/2014 | 25:19.9 | 3 |
| 140.712 | 19090.761 | 2.838 | 14.171 | 0.127517 | 7/15/2014 | 25:20.0 | 3 |
| 140.494 | 19090.767 | 2.841 | 14.167 | 0.123922 | 7/15/2014 | 25:20.1 | 3 |
| 140.28 | 19090.773 | 2.841 | 14.17 | 0.124343 | 7/15/2014 | 25:20.2 | 3 |
| 140.065 | 19090.779 | 2.844 | 14.171 | 0.119236 | 7/15/2014 | 25:20.3 | 3 |
| 139.799 | 19090.787 | 2.844 | 14.173 | 0.116809 | 7/15/2014 | 25:20.4 | 3 |
| 139.587 | 19090.793 | 2.844 | 14.179 | 0.119927 | 7/15/2014 | 25:20.5 | 3 |
| 139.218 | 19090.807 | 2.845 | 14.181 | 0.107081 | 7/15/2014 | 25:20.7 | 3 |
| 139.11 | 19090.81 | 2.844 | 14.182 | 0.108518 | 7/15/2014 | 25:20.7 | 3 |
| 138.896 | 19090.816 | 2.849 | 14.185 | 0.109786 | 7/15/2014 | 25:20.8 | 3 |
| 138.684 | 19090.824 | 2.852 | 14.189 | 0.109686 | 7/15/2014 | 25:20.9 | 3 |
| 138.471 | 19090.83 | 2.857 | 14.192 | 0.109403 | 7/15/2014 | 25:21.0 | 3 |
| 138.26 | 19090.838 | 2.857 | 14.196 | 0.113095 | 7/15/2014 | 25:21.1 | 3 |
| 138.048 | 19090.846 | 2.859 | 14.199 | 0.111571 | 7/15/2014 | 25:21.2 | 3 |
| 137.839 | 19090.854 | 2.861 | 14.197 | 0.114167 | 7/15/2014 | 25:21.3 | 3 |
| 137.474 | 19090.867 | 2.862 | 14.192 | 0.115628 | 7/15/2014 | 25:21.5 | 3 |
| 137.263 | 19090.876 | 2.866 | 14.192 | 0.12025 | 7/15/2014 | 25:21.6 | 3 |
| 137.003 | 19090.887 | 2.863 | 14.191 | 0.120235 | 7/15/2014 | 25:21.7 | 3 |
| 136.897 | 19090.89 | 2.864 | 14.188 | 0.124816 | 7/15/2014 | 25:21.7 | 3 |
| 136.687 | 19090.898 | 2.861 | 14.182 | 0.131694 | 7/15/2014 | 25:21.8 | 3 |
| 136.476 | 19090.907 | 2.861 | 14.177 | 0.126297 | 7/15/2014 | 25:21.9 | 3 |
| 136.263 | 19090.916 | 2.864 | 14.171 | 0.128647 | 7/15/2014 | 25:22.0 | 3 |
| 136.05 | 19090.925 | 2.87 | 14.172 | 0.125113 | 7/15/2014 | 25:22.1 | 3 |
| 135.839 | 19090.934 | 2.873 | 14.169 | 0.126994 | 7/15/2014 | 25:22.2 | 3 |
| 135.626 | 19090.943 | 2.878 | 14.164 | 0.130703 | 7/15/2014 | 25:22.3 | 3 |
| 135.257 | 19090.961 | 2.88 | 14.154 | 0.136748 | 7/15/2014 | 25:22.5 | 3 |
| 135.045 | 19090.97 | 2.883 | 14.148 | 0.138262 | 7/15/2014 | 25:22.6 | 3 |
| 134.94 | 19090.975 | 2.884 | 14.145 | 0.143133 | 7/15/2014 | 25:22.6 | 3 |
| 134.673 | 19090.986 | 2.89 | 14.14 | 0.159073 | 7/15/2014 | 25:22.7 | 3 |
| 134.458 | 19090.997 | 2.892 | 14.135 | 0.145879 | 7/15/2014 | 25:22.8 | 3 |
| 134.244 | 19091.008 | 2.892 | 14.128 | 0.14112 | 7/15/2014 | 25:22.9 | 3 |
| 134.027 | 19091.019 | 2.894 | 14.124 | 0.138174 | 7/15/2014 | 25:23.0 | 3 |
| 133.812 | 19091.029 | 2.894 | 14.119 | 0.138545 | 7/15/2014 | 25:23.1 | 3 |
| 133.597 | 19091.041 | 2.894 | 14.114 | 0.132961 | 7/15/2014 | 25:23.2 | 3 |
| 133.383 | 19091.052 | 2.897 | 14.11 | 0.131367 | 7/15/2014 | 25:23.3 | 3 |
| 133.115 | 19091.063 | 2.902 | 14.108 | 0.11501 | 7/15/2014 | 25:23.4 | 3 |
| 132.902 | 19091.076 | 2.902 | 14.104 | 0.12941 | 7/15/2014 | 25:23.5 | 3 |
| 132.688 | 19091.088 | 2.904 | 14.102 | 0.119446 | 7/15/2014 | 25:23.6 | 3 |
| 132.42 | 19091.102 | 2.906 | 14.098 | 0.116794 | 7/15/2014 | 25:23.7 | 3 |
| 132.207 | 19091.114 | 2.908 | 14.095 | 0.10754 | 7/15/2014 | 25:23.8 | 3 |
| 131.992 | 19091.127 | 2.91 | 14.093 | 0.107155 | 7/15/2014 | 25:23.9 | 3 |
| 131.777 | 19091.139 | 2.914 | 14.092 | 0.107613 | 7/15/2014 | 25:24.0 | 3 |
| 131.562 | 19091.149 | 2.914 | 14.09 | 0.102604 | 7/15/2014 | 25:24.1 | 3 |
| 131.347 | 19091.163 | 2.914 | 14.088 | 0.100767 | 7/15/2014 | 25:24.2 | 3 |

| | | | | | | | |
|---|---|---|---|---|---|---|---|
| 131.134 | 19091.176 | 2.914 | 14.085 | 0.101163 | 7/15/2014 | 25:24.3 | 3 |
| 130.762 | 19091.197 | 2.914 | 14.083 | 0.0976174 | 7/15/2014 | 25:24.5 | 3 |
| 130.551 | 19091.211 | 2.916 | 14.084 | 0.0966012 | 7/15/2014 | 25:24.6 | 3 |
| 130.288 | 19091.228 | 2.912 | 14.083 | 0.0927509 | 7/15/2014 | 25:24.7 | 3 |
| 130.181 | 19091.233 | 2.912 | 14.083 | 0.091625 | 7/15/2014 | 25:24.7 | 3 |
| 129.967 | 19091.246 | 2.912 | 14.084 | 0.0856135 | 7/15/2014 | 25:24.8 | 3 |
| 129.753 | 19091.26 | 2.912 | 14.085 | 0.0976997 | 7/15/2014 | 25:24.9 | 3 |
| 129.539 | 19091.274 | 2.91 | 14.088 | 0.0941951 | 7/15/2014 | 25:25.0 | 3 |
| 129.324 | 19091.288 | 2.912 | 14.094 | 0.0875115 | 7/15/2014 | 25:25.1 | 3 |
| 129.11 | 19091.303 | 2.91 | 14.097 | 0.0893886 | 7/15/2014 | 25:25.2 | 3 |
| 128.897 | 19091.317 | 2.912 | 14.097 | 0.0960652 | 7/15/2014 | 25:25.3 | 3 |
| 128.633 | 19091.334 | 2.91 | 14.096 | 0.113861 | 7/15/2014 | 25:25.4 | 3 |
| 128.315 | 19091.355 | 2.91 | 14.104 | 0.114443 | 7/15/2014 | 25:25.6 | 3 |
| 128.209 | 19091.362 | 2.908 | 14.106 | 0.11084 | 7/15/2014 | 25:25.6 | 3 |
| 127.944 | 19091.382 | 2.908 | 14.11 | 0.118378 | 7/15/2014 | 25:25.7 | 3 |
| 127.73 | 19091.396 | 2.907 | 14.111 | 0.148387 | 7/15/2014 | 25:25.8 | 3 |
| 127.516 | 19091.412 | 2.905 | 14.111 | 0.126804 | 7/15/2014 | 25:25.9 | 3 |
| 127.3 | 19091.428 | 2.907 | 14.113 | 0.144887 | 7/15/2014 | 25:26.0 | 3 |
| 127.085 | 19091.442 | 2.905 | 14.117 | 0.130873 | 7/15/2014 | 25:26.1 | 3 |
| 126.873 | 19091.457 | 2.907 | 14.12 | 0.117578 | 7/15/2014 | 25:26.2 | 3 |
| 126.658 | 19091.473 | 2.909 | 14.124 | 0.123912 | 7/15/2014 | 25:26.3 | 3 |
| 126.394 | 19091.493 | 2.911 | 14.121 | 0.125777 | 7/15/2014 | 25:26.4 | 3 |
| 126.181 | 19091.51 | 2.913 | 14.121 | 0.112763 | 7/15/2014 | 25:26.5 | 3 |
| 125.969 | 19091.525 | 2.915 | 14.126 | 0.134739 | 7/15/2014 | 25:26.6 | 3 |
| 125.701 | 19091.545 | 2.92 | 14.127 | 0.117616 | 7/15/2014 | 25:26.7 | 3 |
| 125.487 | 19091.562 | 2.92 | 14.128 | 0.135118 | 7/15/2014 | 25:26.8 | 3 |
| 125.272 | 19091.578 | 2.922 | 14.128 | 0.137164 | 7/15/2014 | 25:26.9 | 3 |
| 125.057 | 19091.594 | 2.922 | 14.131 | 0.157885 | 7/15/2014 | 25:27.0 | 3 |
| 124.843 | 19091.611 | 2.924 | 14.131 | 0.147307 | 7/15/2014 | 25:27.1 | 3 |
| 124.631 | 19091.629 | 2.924 | 14.132 | 0.149084 | 7/15/2014 | 25:27.2 | 3 |
| 124.417 | 19091.645 | 2.928 | 14.136 | 0.144829 | 7/15/2014 | 25:27.3 | 3 |
| 124.047 | 19091.674 | 2.928 | 14.131 | 0.14831 | 7/15/2014 | 25:27.5 | 3 |
| 123.835 | 19091.692 | 2.931 | 14.131 | 0.147134 | 7/15/2014 | 25:27.6 | 3 |
| 123.574 | 19091.715 | 2.931 | 14.131 | 0.143961 | 7/15/2014 | 25:27.7 | 3 |
| 123.467 | 19091.723 | 2.931 | 14.131 | 0.136692 | 7/15/2014 | 25:27.7 | 3 |
| 123.254 | 19091.739 | 2.935 | 14.129 | 0.152957 | 7/15/2014 | 25:27.8 | 3 |
| 123.044 | 19091.758 | 2.935 | 14.132 | 0.151777 | 7/15/2014 | 25:27.9 | 3 |
| 122.832 | 19091.773 | 2.937 | 14.134 | 0.156972 | 7/15/2014 | 25:28.0 | 3 |
| 122.62 | 19091.793 | 2.935 | 14.134 | 0.156226 | 7/15/2014 | 25:28.1 | 3 |
| 122.409 | 19091.811 | 2.935 | 14.138 | 0.164988 | 7/15/2014 | 25:28.2 | 3 |
| 122.197 | 19091.828 | 2.932 | 14.142 | 0.164421 | 7/15/2014 | 25:28.3 | 3 |
| 121.835 | 19091.861 | 2.932 | 14.146 | 0.179178 | 7/15/2014 | 25:28.5 | 3 |
| 121.623 | 19091.877 | 2.937 | 14.15 | 0.169017 | 7/15/2014 | 25:28.6 | 3 |
| 121.367 | 19091.9 | 2.934 | 14.154 | 0.173986 | 7/15/2014 | 25:28.7 | 3 |
| 121.262 | 19091.908 | 2.935 | 14.154 | 0.173633 | 7/15/2014 | 25:28.7 | 3 |
| 121.05 | 19091.928 | 2.936 | 14.159 | 0.174407 | 7/15/2014 | 25:28.8 | 3 |
| 120.842 | 19091.947 | 2.936 | 14.168 | 0.171777 | 7/15/2014 | 25:28.9 | 3 |

| | | | | | | | |
|---|---|---|---|---|---|---|---|
| 120.63 | 19091.964 | 2.939 | 14.175 | 0.174007 | 7/15/2014 | 25:29.0 | 3 |
| 120.42 | 19091.982 | 2.941 | 14.181 | 0.175289 | 7/15/2014 | 25:29.1 | 3 |
| 120.211 | 19092.002 | 2.941 | 14.186 | 0.176486 | 7/15/2014 | 25:29.2 | 3 |
| 120.003 | 19092.02 | 2.941 | 14.192 | 0.17606 | 7/15/2014 | 25:29.3 | 3 |
| 119.641 | 19092.051 | 2.938 | 14.199 | 0.186617 | 7/15/2014 | 25:29.5 | 3 |
| 119.431 | 19092.07 | 2.942 | 14.203 | 0.185165 | 7/15/2014 | 25:29.6 | 3 |
| 119.175 | 19092.094 | 2.939 | 14.209 | 0.185842 | 7/15/2014 | 25:29.7 | 3 |
| 119.068 | 19092.103 | 2.941 | 14.207 | 0.187371 | 7/15/2014 | 25:29.7 | 3 |
| 118.859 | 19092.121 | 2.943 | 14.212 | 0.18875 | 7/15/2014 | 25:29.8 | 3 |
| 118.648 | 19092.141 | 2.947 | 14.213 | 0.187189 | 7/15/2014 | 25:29.9 | 3 |
| 118.439 | 19092.16 | 2.947 | 14.213 | 0.182953 | 7/15/2014 | 25:30.0 | 3 |
| 118.227 | 19092.18 | 2.95 | 14.211 | 0.182659 | 7/15/2014 | 25:30.1 | 3 |
| 118.019 | 19092.197 | 2.95 | 14.212 | 0.178999 | 7/15/2014 | 25:30.2 | 3 |
| 117.808 | 19092.216 | 2.953 | 14.209 | 0.172576 | 7/15/2014 | 25:30.3 | 3 |
| 117.445 | 19092.248 | 2.951 | 14.212 | 0.168109 | 7/15/2014 | 25:30.5 | 3 |
| 117.236 | 19092.268 | 2.954 | 14.21 | 0.169767 | 7/15/2014 | 25:30.6 | 3 |
| 116.976 | 19092.291 | 2.954 | 14.206 | 0.167262 | 7/15/2014 | 25:30.7 | 3 |
| 116.871 | 19092.301 | 2.954 | 14.205 | 0.164907 | 7/15/2014 | 25:30.7 | 3 |
| 116.661 | 19092.32 | 2.956 | 14.211 | 0.165682 | 7/15/2014 | 25:30.8 | 3 |
| 116.451 | 19092.34 | 2.956 | 14.215 | 0.162939 | 7/15/2014 | 25:30.9 | 3 |
| 116.241 | 19092.358 | 2.956 | 14.218 | 0.163088 | 7/15/2014 | 25:31.0 | 3 |
| 116.031 | 19092.377 | 2.956 | 14.221 | 0.165361 | 7/15/2014 | 25:31.1 | 3 |
| 115.822 | 19092.396 | 2.956 | 14.223 | 0.166946 | 7/15/2014 | 25:31.2 | 3 |
| 115.616 | 19092.414 | 2.953 | 14.22 | 0.175595 | 7/15/2014 | 25:31.3 | 3 |
| 115.255 | 19092.447 | 2.952 | 14.22 | 0.175724 | 7/15/2014 | 25:31.5 | 3 |
| 115.049 | 19092.467 | 2.95 | 14.22 | 0.18 | 7/15/2014 | 25:31.6 | 3 |
| 114.792 | 19092.49 | 2.948 | 14.215 | 0.181028 | 7/15/2014 | 25:31.7 | 3 |
| 114.688 | 19092.5 | 2.948 | 14.215 | 0.184173 | 7/15/2014 | 25:31.7 | 3 |
| 114.482 | 19092.52 | 2.948 | 14.211 | 0.188653 | 7/15/2014 | 25:31.8 | 3 |
| 114.277 | 19092.537 | 2.944 | 14.209 | 0.189906 | 7/15/2014 | 25:31.9 | 3 |
| 114.071 | 19092.555 | 2.947 | 14.204 | 0.191526 | 7/15/2014 | 25:32.0 | 3 |
| 113.865 | 19092.574 | 2.947 | 14.202 | 0.195323 | 7/15/2014 | 25:32.1 | 3 |
| 113.659 | 19092.594 | 2.947 | 14.197 | 0.195542 | 7/15/2014 | 25:32.2 | 3 |
| 113.454 | 19092.613 | 2.95 | 14.193 | 0.198064 | 7/15/2014 | 25:32.3 | 3 |
| 113.098 | 19092.646 | 2.95 | 14.191 | 0.196129 | 7/15/2014 | 25:32.5 | 3 |
| 112.895 | 19092.664 | 2.95 | 14.192 | 0.192528 | 7/15/2014 | 25:32.6 | 3 |
| 112.643 | 19092.688 | 2.95 | 14.189 | 0.194385 | 7/15/2014 | 25:32.7 | 3 |
| 112.539 | 19092.696 | 2.953 | 14.188 | 0.194329 | 7/15/2014 | 25:32.7 | 3 |
| 112.336 | 19092.715 | 2.951 | 14.184 | 0.189253 | 7/15/2014 | 25:32.8 | 3 |
| 112.127 | 19092.734 | 2.956 | 14.181 | 0.191017 | 7/15/2014 | 25:32.9 | 3 |
| 111.92 | 19092.754 | 2.958 | 14.177 | 0.188217 | 7/15/2014 | 25:33.0 | 3 |
| 111.714 | 19092.773 | 2.958 | 14.174 | 0.185764 | 7/15/2014 | 25:33.1 | 3 |
| 111.506 | 19092.792 | 2.961 | 14.173 | 0.186431 | 7/15/2014 | 25:33.2 | 3 |
| 111.302 | 19092.811 | 2.963 | 14.171 | 0.184178 | 7/15/2014 | 25:33.3 | 3 |
| 110.942 | 19092.843 | 2.963 | 14.166 | 0.191091 | 7/15/2014 | 25:33.5 | 3 |
| 110.736 | 19092.861 | 2.965 | 14.166 | 0.18957 | 7/15/2014 | 25:33.6 | 3 |
| 110.478 | 19092.885 | 2.965 | 14.169 | 0.199448 | 7/15/2014 | 25:33.7 | 3 |

| | | | | | | | |
|---|---|---|---|---|---|---|---|
| 110.373 | 19092.895 | 2.965 | 14.169 | 0.198276 | 7/15/2014 | 25:33.7 | 3 |
| 110.166 | 19092.914 | 2.96 | 14.175 | 0.190196 | 7/15/2014 | 25:33.8 | 3 |
| 109.956 | 19092.934 | 2.96 | 14.179 | 0.200406 | 7/15/2014 | 25:33.9 | 3 |
| 109.747 | 19092.953 | 2.958 | 14.179 | 0.204735 | 7/15/2014 | 25:34.0 | 3 |
| 109.537 | 19092.973 | 2.956 | 14.184 | 0.2083 | 7/15/2014 | 25:34.1 | 3 |
| 109.328 | 19092.989 | 2.955 | 14.189 | 0.206268 | 7/15/2014 | 25:34.2 | 3 |
| 109.122 | 19093.008 | 2.953 | 14.195 | 0.213517 | 7/15/2014 | 25:34.3 | 3 |
| 108.76 | 19093.041 | 2.949 | 14.205 | 0.201675 | 7/15/2014 | 25:34.5 | 3 |
| 108.554 | 19093.06 | 2.949 | 14.21 | 0.203983 | 7/15/2014 | 25:34.6 | 3 |
| 108.298 | 19093.082 | 2.947 | 14.214 | 0.204171 | 7/15/2014 | 25:34.7 | 3 |
| 108.193 | 19093.092 | 2.947 | 14.217 | 0.204163 | 7/15/2014 | 25:34.7 | 3 |
| 107.986 | 19093.111 | 2.947 | 14.224 | 0.198563 | 7/15/2014 | 25:34.8 | 3 |
| 107.777 | 19093.128 | 2.945 | 14.225 | 0.197945 | 7/15/2014 | 25:34.9 | 3 |
| 107.571 | 19093.146 | 2.94 | 14.227 | 0.198105 | 7/15/2014 | 25:35.0 | 3 |
| 107.367 | 19093.165 | 2.937 | 14.229 | 0.209698 | 7/15/2014 | 25:35.1 | 3 |
| 107.16 | 19093.184 | 2.935 | 14.229 | 0.223963 | 7/15/2014 | 25:35.2 | 3 |
| 106.955 | 19093.202 | 2.932 | 14.234 | 0.224736 | 7/15/2014 | 25:35.3 | 3 |
| 106.602 | 19093.232 | 2.925 | 14.24 | 0.236518 | 7/15/2014 | 25:35.5 | 3 |
| 106.4 | 19093.25 | 2.922 | 14.242 | 0.247558 | 7/15/2014 | 25:35.6 | 3 |
| 106.152 | 19093.27 | 2.914 | 14.243 | 0.257564 | 7/15/2014 | 25:35.7 | 3 |
| 106.049 | 19093.279 | 2.912 | 14.245 | 0.266369 | 7/15/2014 | 25:35.7 | 3 |
| 105.847 | 19093.298 | 2.912 | 14.256 | 0.242338 | 7/15/2014 | 25:35.8 | 3 |
| 105.646 | 19093.314 | 2.908 | 14.26 | 0.249418 | 7/15/2014 | 25:35.9 | 3 |
| 105.442 | 19093.332 | 2.908 | 14.262 | 0.266428 | 7/15/2014 | 25:36.0 | 3 |
| 105.139 | 19093.357 | 2.908 | 14.264 | 0.266732 | 7/15/2014 | 25:36.2 | 3 |
| 105.039 | 19093.367 | 2.908 | 14.267 | 0.271035 | 7/15/2014 | 25:36.2 | 3 |
| 104.74 | 19093.391 | 2.905 | 14.271 | 0.290412 | 7/15/2014 | 25:36.4 | 3 |
| 104.494 | 19093.41 | 2.905 | 14.273 | 0.286398 | 7/15/2014 | 25:36.5 | 3 |
| 104.294 | 19093.429 | 2.908 | 14.275 | 0.301973 | 7/15/2014 | 25:36.6 | 3 |
| 104.05 | 19093.447 | 2.901 | 14.273 | 0.299287 | 7/15/2014 | 25:36.7 | 3 |
| 103.847 | 19093.465 | 2.902 | 14.275 | 0.287624 | 7/15/2014 | 25:36.8 | 3 |
| 103.748 | 19093.473 | 2.898 | 14.277 | 0.286952 | 7/15/2014 | 25:36.8 | 3 |
| 103.445 | 19093.497 | 2.895 | 14.288 | 0.296913 | 7/15/2014 | 25:37.0 | 3 |
| 103.347 | 19093.505 | 2.89 | 14.289 | 0.290104 | 7/15/2014 | 25:37.0 | 3 |
| 103.043 | 19093.529 | 2.889 | 14.297 | 0.274762 | 7/15/2014 | 25:37.2 | 3 |
| 102.944 | 19093.537 | 2.886 | 14.298 | 0.279972 | 7/15/2014 | 25:37.2 | 3 |
| 102.747 | 19093.554 | 2.881 | 14.305 | 0.270172 | 7/15/2014 | 25:37.3 | 3 |
| 102.401 | 19093.578 | 2.874 | 14.31 | 0.270462 | 7/15/2014 | 25:37.5 | 3 |
| 102.201 | 19093.596 | 2.874 | 14.315 | 0.265351 | 7/15/2014 | 25:37.6 | 3 |
| 101.956 | 19093.613 | 2.871 | 14.319 | 0.267847 | 7/15/2014 | 25:37.7 | 3 |
| 101.754 | 19093.63 | 2.868 | 14.324 | 0.255527 | 7/15/2014 | 25:37.8 | 3 |
| 101.556 | 19093.646 | 2.863 | 14.326 | 0.262831 | 7/15/2014 | 25:37.9 | 3 |
| 101.353 | 19093.66 | 2.863 | 14.329 | 0.265653 | 7/15/2014 | 25:38.0 | 3 |
| 101.155 | 19093.676 | 2.855 | 14.33 | 0.258639 | 7/15/2014 | 25:38.1 | 3 |
| 100.954 | 19093.69 | 2.852 | 14.334 | 0.254279 | 7/15/2014 | 25:38.2 | 3 |
| 100.755 | 19093.704 | 2.848 | 14.338 | 0.251226 | 7/15/2014 | 25:38.3 | 3 |
| 100.555 | 19093.719 | 2.848 | 14.339 | 0.25259 | 7/15/2014 | 25:38.4 | 3 |

| | | | | | | | |
|---|---|---|---|---|---|---|---|
| 100.309 | 19093.738 | 2.845 | 14.342 | 0.254026 | 7/15/2014 | 25:38.5 | 3 |
| 100.111 | 19093.753 | 2.842 | 14.343 | 0.255486 | 7/15/2014 | 25:38.6 | 3 |
| 99.864 | 19093.771 | 2.84 | 14.343 | 0.257027 | 7/15/2014 | 25:38.7 | 3 |
| 99.663 | 19093.784 | 2.843 | 14.346 | 0.256756 | 7/15/2014 | 25:38.8 | 3 |
| 99.464 | 19093.798 | 2.839 | 14.35 | 0.263208 | 7/15/2014 | 25:38.9 | 3 |
| 99.26 | 19093.813 | 2.84 | 14.354 | 0.277349 | 7/15/2014 | 25:39.0 | 3 |
| 99.16 | 19093.82 | 2.84 | 14.355 | 0.279316 | 7/15/2014 | 25:39.1 | 3 |
| 98.956 | 19093.834 | 2.843 | 14.36 | 0.284988 | 7/15/2014 | 25:39.2 | 3 |
| 98.753 | 19093.849 | 2.839 | 14.367 | 0.283171 | 7/15/2014 | 25:39.3 | 3 |
| 98.551 | 19093.862 | 2.837 | 14.372 | 0.297537 | 7/15/2014 | 25:39.4 | 3 |
| 98.191 | 19093.886 | 2.834 | 14.386 | 0.302571 | 7/15/2014 | 25:39.5 | 3 |
| 97.982 | 19093.9 | 2.836 | 14.398 | 0.306942 | 7/15/2014 | 25:39.6 | 3 |
| 97.777 | 19093.913 | 2.831 | 14.405 | 0.317888 | 7/15/2014 | 25:39.7 | 3 |
| 97.565 | 19093.927 | 2.833 | 14.418 | 0.313831 | 7/15/2014 | 25:39.8 | 3 |
| 97.356 | 19093.94 | 2.831 | 14.43 | 0.333426 | 7/15/2014 | 25:39.9 | 3 |
| 97.143 | 19093.954 | 2.831 | 14.437 | 0.336606 | 7/15/2014 | 25:40.0 | 3 |
| 97.04 | 19093.961 | 2.83 | 14.443 | 0.335914 | 7/15/2014 | 25:40.0 | 3 |
| 96.721 | 19093.979 | 2.827 | 14.453 | 0.351072 | 7/15/2014 | 25:40.2 | 3 |
| 96.616 | 19093.986 | 2.826 | 14.457 | 0.355066 | 7/15/2014 | 25:40.2 | 3 |
| 96.299 | 19094.006 | 2.825 | 14.467 | 0.369124 | 7/15/2014 | 25:40.4 | 3 |
| 96.091 | 19094.02 | 2.822 | 14.471 | 0.372866 | 7/15/2014 | 25:40.5 | 3 |
| 95.88 | 19094.031 | 2.822 | 14.481 | 0.386865 | 7/15/2014 | 25:40.6 | 3 |
| 95.622 | 19094.046 | 2.818 | 14.488 | 0.378722 | 7/15/2014 | 25:40.7 | 3 |
| 95.513 | 19094.053 | 2.817 | 14.487 | 0.380795 | 7/15/2014 | 25:40.7 | 3 |
| 95.306 | 19094.064 | 2.815 | 14.495 | 0.395067 | 7/15/2014 | 25:40.8 | 3 |
| 95.097 | 19094.076 | 2.815 | 14.509 | 0.398211 | 7/15/2014 | 25:40.9 | 3 |
| 94.888 | 19094.089 | 2.813 | 14.516 | 0.408066 | 7/15/2014 | 25:41.1 | 3 |
| 94.68 | 19094.1 | 2.813 | 14.522 | 0.406693 | 7/15/2014 | 25:41.1 | 3 |
| 94.471 | 19094.111 | 2.811 | 14.532 | 0.420426 | 7/15/2014 | 25:41.3 | 3 |
| 94.263 | 19094.124 | 2.811 | 14.543 | 0.420368 | 7/15/2014 | 25:41.3 | 3 |
| 93.898 | 19094.143 | 2.804 | 14.547 | 0.444765 | 7/15/2014 | 25:41.5 | 3 |
| 93.688 | 19094.154 | 2.804 | 14.548 | 0.440216 | 7/15/2014 | 25:41.6 | 3 |
| 93.479 | 19094.165 | 2.8 | 14.552 | 0.453443 | 7/15/2014 | 25:41.7 | 3 |
| 93.267 | 19094.176 | 2.8 | 14.549 | 0.454253 | 7/15/2014 | 25:41.8 | 3 |
| 93.056 | 19094.187 | 2.798 | 14.552 | 0.470926 | 7/15/2014 | 25:41.9 | 3 |
| 92.843 | 19094.196 | 2.798 | 14.556 | 0.464935 | 7/15/2014 | 25:42.0 | 3 |
| 92.631 | 19094.208 | 2.795 | 14.555 | 0.466942 | 7/15/2014 | 25:42.1 | 3 |
| 92.42 | 19094.219 | 2.795 | 14.554 | 0.47668 | 7/15/2014 | 25:42.2 | 3 |
| 92.211 | 19094.229 | 2.79 | 14.556 | 0.488461 | 7/15/2014 | 25:42.3 | 3 |
| 91.999 | 19094.238 | 2.794 | 14.564 | 0.492714 | 7/15/2014 | 25:42.4 | 3 |
| 91.792 | 19094.249 | 2.79 | 14.568 | 0.498154 | 7/15/2014 | 25:42.5 | 3 |
| 91.582 | 19094.258 | 2.789 | 14.572 | 0.505678 | 7/15/2014 | 25:42.6 | 3 |
| 91.325 | 19094.27 | 2.787 | 14.577 | 0.500361 | 7/15/2014 | 25:42.7 | 3 |
| 91.218 | 19094.274 | 2.786 | 14.581 | 0.504885 | 7/15/2014 | 25:42.7 | 3 |
| 91.009 | 19094.285 | 2.786 | 14.585 | 0.516212 | 7/15/2014 | 25:42.8 | 3 |
| 90.802 | 19094.295 | 2.784 | 14.593 | 0.514517 | 7/15/2014 | 25:42.9 | 3 |
| 90.594 | 19094.303 | 2.781 | 14.597 | 0.51374 | 7/15/2014 | 25:43.0 | 3 |

| | | | | | | | |
|---|---|---|---|---|---|---|---|
| 90.386 | 19094.311 | 2.781 | 14.6 | 0.519334 | 7/15/2014 | 25:43.1 | 3 |
| 90.178 | 19094.32 | 2.779 | 14.599 | 0.522145 | 7/15/2014 | 25:43.2 | 3 |
| 89.968 | 19094.33 | 2.779 | 14.603 | 0.530529 | 7/15/2014 | 25:43.3 | 3 |
| 89.601 | 19094.344 | 2.777 | 14.605 | 0.543442 | 7/15/2014 | 25:43.5 | 3 |
| 89.389 | 19094.354 | 2.774 | 14.608 | 0.551877 | 7/15/2014 | 25:43.6 | 3 |
| 89.287 | 19094.357 | 2.77 | 14.61 | 0.560815 | 7/15/2014 | 25:43.6 | 3 |
| 89.071 | 19094.365 | 2.77 | 14.607 | 0.572211 | 7/15/2014 | 25:43.7 | 3 |
| 88.858 | 19094.373 | 2.768 | 14.609 | 0.591414 | 7/15/2014 | 25:43.8 | 3 |
| 88.642 | 19094.381 | 2.768 | 14.613 | 0.615694 | 7/15/2014 | 25:43.9 | 3 |
| 88.426 | 19094.391 | 2.765 | 14.612 | 0.620955 | 7/15/2014 | 25:44.0 | 3 |
| 88.209 | 19094.397 | 2.763 | 14.613 | 0.635696 | 7/15/2014 | 25:44.1 | 3 |
| 87.993 | 19094.404 | 2.763 | 14.62 | 0.648714 | 7/15/2014 | 25:44.2 | 3 |
| 87.779 | 19094.412 | 2.763 | 14.622 | 0.654066 | 7/15/2014 | 25:44.3 | 3 |
| 87.565 | 19094.42 | 2.763 | 14.623 | 0.650979 | 7/15/2014 | 25:44.4 | 3 |
| 87.25 | 19094.43 | 2.763 | 14.625 | 0.653868 | 7/15/2014 | 25:44.6 | 3 |
| 86.987 | 19094.438 | 2.761 | 14.626 | 0.657119 | 7/15/2014 | 25:44.7 | 3 |
| 86.878 | 19094.441 | 2.761 | 14.626 | 0.658116 | 7/15/2014 | 25:44.7 | 3 |
| 86.666 | 19094.446 | 2.763 | 14.626 | 0.665943 | 7/15/2014 | 25:44.8 | 3 |
| 86.456 | 19094.453 | 2.765 | 14.625 | 0.669796 | 7/15/2014 | 25:44.9 | 3 |
| 86.246 | 19094.459 | 2.763 | 14.618 | 0.67847 | 7/15/2014 | 25:45.0 | 3 |
| 86.036 | 19094.466 | 2.763 | 14.618 | 0.691202 | 7/15/2014 | 25:45.1 | 3 |
| 85.826 | 19094.471 | 2.763 | 14.617 | 0.700033 | 7/15/2014 | 25:45.2 | 3 |
| 85.616 | 19094.477 | 2.761 | 14.615 | 0.713769 | 7/15/2014 | 25:45.3 | 3 |
| 85.247 | 19094.485 | 2.763 | 14.611 | 0.725341 | 7/15/2014 | 25:45.5 | 3 |
| 85.035 | 19094.49 | 2.768 | 14.608 | 0.726094 | 7/15/2014 | 25:45.6 | 3 |
| 84.824 | 19094.496 | 2.764 | 14.606 | 0.734556 | 7/15/2014 | 25:45.7 | 3 |
| 84.719 | 19094.498 | 2.764 | 14.605 | 0.734413 | 7/15/2014 | 25:45.7 | 3 |
| 84.504 | 19094.503 | 2.766 | 14.604 | 0.739069 | 7/15/2014 | 25:45.8 | 3 |
| 84.291 | 19094.508 | 2.766 | 14.597 | 0.740104 | 7/15/2014 | 25:45.9 | 3 |
| 84.076 | 19094.512 | 2.769 | 14.589 | 0.745252 | 7/15/2014 | 25:46.0 | 3 |
| 83.861 | 19094.516 | 2.771 | 14.586 | 0.753787 | 7/15/2014 | 25:46.1 | 3 |
| 83.648 | 19094.521 | 2.775 | 14.585 | 0.75455 | 7/15/2014 | 25:46.2 | 3 |
| 83.332 | 19094.527 | 2.773 | 14.576 | 0.762476 | 7/15/2014 | 25:46.4 | 3 |
| 83.122 | 19094.531 | 2.771 | 14.57 | 0.766586 | 7/15/2014 | 25:46.5 | 3 |
| 82.91 | 19094.534 | 2.772 | 14.562 | 0.765785 | 7/15/2014 | 25:46.6 | 3 |
| 82.65 | 19094.539 | 2.771 | 14.554 | 0.771404 | 7/15/2014 | 25:46.7 | 3 |
| 82.543 | 19094.541 | 2.771 | 14.552 | 0.775333 | 7/15/2014 | 25:46.7 | 3 |
| 82.333 | 19094.544 | 2.771 | 14.547 | 0.773507 | 7/15/2014 | 25:46.8 | 3 |
| 82.122 | 19094.547 | 2.771 | 14.538 | 0.777169 | 7/15/2014 | 25:46.9 | 3 |
| 81.913 | 19094.551 | 2.769 | 14.53 | 0.779822 | 7/15/2014 | 25:47.0 | 3 |
| 81.702 | 19094.554 | 2.769 | 14.526 | 0.787599 | 7/15/2014 | 25:47.1 | 3 |
| 81.492 | 19094.558 | 2.773 | 14.517 | 0.791887 | 7/15/2014 | 25:47.2 | 3 |
| 81.28 | 19094.56 | 2.773 | 14.514 | 0.80123 | 7/15/2014 | 25:47.3 | 3 |
| 80.907 | 19094.564 | 2.771 | 14.51 | 0.803562 | 7/15/2014 | 25:47.5 | 3 |
| 80.803 | 19094.565 | 2.773 | 14.509 | 0.808909 | 7/15/2014 | 25:47.5 | 3 |
| 80.587 | 19094.567 | 2.769 | 14.506 | 0.809217 | 7/15/2014 | 25:47.6 | 3 |
| 80.371 | 19094.57 | 2.767 | 14.502 | 0.810822 | 7/15/2014 | 25:47.7 | 3 |

| | | | | | | | |
|---|---|---|---|---|---|---|---|
| 80.152 | 19094.572 | 2.764 | 14.498 | 0.80582 | 7/15/2014 | 25:47.8 | 3 |
| 79.933 | 19094.575 | 2.762 | 14.495 | 0.799739 | 7/15/2014 | 25:47.9 | 3 |
| 79.713 | 19094.577 | 2.759 | 14.494 | 0.791013 | 7/15/2014 | 25:48.0 | 3 |
| 79.493 | 19094.579 | 2.757 | 14.491 | 0.793539 | 7/15/2014 | 25:48.1 | 3 |
| 79.272 | 19094.58 | 2.759 | 14.486 | 0.78659 | 7/15/2014 | 25:48.2 | 3 |
| 79.052 | 19094.582 | 2.759 | 14.484 | 0.780544 | 7/15/2014 | 25:48.3 | 3 |
| 78.834 | 19094.584 | 2.764 | 14.481 | 0.779865 | 7/15/2014 | 25:48.4 | 3 |
| 78.617 | 19094.585 | 2.766 | 14.478 | 0.776762 | 7/15/2014 | 25:48.5 | 3 |
| 78.4 | 19094.587 | 2.771 | 14.475 | 0.7793 | 7/15/2014 | 25:48.6 | 3 |
| 78.132 | 19094.588 | 2.771 | 14.47 | 0.773901 | 7/15/2014 | 25:48.7 | 3 |
| 77.915 | 19094.59 | 2.774 | 14.469 | 0.768814 | 7/15/2014 | 25:48.8 | 3 |
| 77.698 | 19094.59 | 2.776 | 14.464 | 0.763362 | 7/15/2014 | 25:48.9 | 3 |
| 77.482 | 19094.591 | 2.774 | 14.46 | 0.766983 | 7/15/2014 | 25:49.0 | 3 |
| 77.266 | 19094.593 | 2.774 | 14.456 | 0.756192 | 7/15/2014 | 25:49.1 | 3 |
| 77.05 | 19094.592 | 2.77 | 14.452 | 0.759747 | 7/15/2014 | 25:49.2 | 3 |
| 76.834 | 19094.594 | 2.768 | 14.448 | 0.747094 | 7/15/2014 | 25:49.3 | 3 |
| 76.566 | 19094.594 | 2.763 | 14.442 | 0.752894 | 7/15/2014 | 25:49.4 | 3 |
| 76.348 | 19094.596 | 2.765 | 14.44 | 0.747314 | 7/15/2014 | 25:49.5 | 3 |
| 76.13 | 19094.596 | 2.765 | 14.432 | 0.75048 | 7/15/2014 | 25:49.6 | 3 |
| 75.91 | 19094.596 | 2.765 | 14.434 | 0.746426 | 7/15/2014 | 25:49.7 | 3 |
| 75.689 | 19094.597 | 2.767 | 14.429 | 0.742478 | 7/15/2014 | 25:49.8 | 3 |
| 75.468 | 19094.596 | 2.767 | 14.429 | 0.733713 | 7/15/2014 | 25:49.9 | 3 |
| 75.247 | 19094.597 | 2.767 | 14.424 | 0.731177 | 7/15/2014 | 25:50.0 | 3 |
| 75.026 | 19094.597 | 2.769 | 14.42 | 0.730821 | 7/15/2014 | 25:50.1 | 3 |
| 74.804 | 19094.597 | 2.772 | 14.417 | 0.72054 | 7/15/2014 | 25:50.2 | 3 |
| 74.583 | 19094.597 | 2.772 | 14.41 | 0.711708 | 7/15/2014 | 25:50.3 | 3 |
| 74.363 | 19094.597 | 2.772 | 14.404 | 0.706225 | 7/15/2014 | 25:50.4 | 3 |
| 74.144 | 19094.597 | 2.772 | 14.394 | 0.710831 | 7/15/2014 | 25:50.5 | 3 |
| 73.926 | 19094.596 | 2.772 | 14.387 | 0.709262 | 7/15/2014 | 25:50.6 | 3 |
| 73.656 | 19094.597 | 2.772 | 14.378 | 0.70377 | 7/15/2014 | 25:50.7 | 3 |
| 73.438 | 19094.596 | 2.772 | 14.371 | 0.691079 | 7/15/2014 | 25:50.8 | 3 |
| 73.221 | 19094.595 | 2.772 | 14.362 | 0.704538 | 7/15/2014 | 25:50.9 | 3 |
| 73.002 | 19094.595 | 2.774 | 14.355 | 0.70071 | 7/15/2014 | 25:51.0 | 3 |
| 72.784 | 19094.595 | 2.774 | 14.345 | 0.704859 | 7/15/2014 | 25:51.1 | 3 |
| 72.568 | 19094.594 | 2.772 | 14.336 | 0.698664 | 7/15/2014 | 25:51.2 | 3 |
| 72.349 | 19094.593 | 2.772 | 14.327 | 0.699517 | 7/15/2014 | 25:51.3 | 3 |
| 72.079 | 19094.593 | 2.773 | 14.319 | 0.711636 | 7/15/2014 | 25:51.4 | 3 |
| 71.86 | 19094.592 | 2.773 | 14.311 | 0.701459 | 7/15/2014 | 25:51.5 | 3 |
| 71.642 | 19094.592 | 2.771 | 14.303 | 0.696731 | 7/15/2014 | 25:51.6 | 3 |
| 71.421 | 19094.591 | 2.771 | 14.296 | 0.693783 | 7/15/2014 | 25:51.7 | 3 |
| 71.201 | 19094.59 | 2.767 | 14.29 | 0.67664 | 7/15/2014 | 25:51.8 | 3 |
| 70.981 | 19094.589 | 2.771 | 14.282 | 0.672198 | 7/15/2014 | 25:51.9 | 3 |
| 70.76 | 19094.589 | 2.773 | 14.276 | 0.669037 | 7/15/2014 | 25:52.0 | 3 |
| 70.542 | 19094.587 | 2.773 | 14.271 | 0.65851 | 7/15/2014 | 25:52.1 | 3 |
| 70.323 | 19094.587 | 2.769 | 14.263 | 0.659434 | 7/15/2014 | 25:52.2 | 3 |
| 70.106 | 19094.586 | 2.769 | 14.255 | 0.653143 | 7/15/2014 | 25:52.3 | 3 |
| 69.891 | 19094.585 | 2.769 | 14.248 | 0.644653 | 7/15/2014 | 25:52.4 | 3 |

| | | | | | | | |
|---|---|---|---|---|---|---|---|
| 69.572 | 19094.583 | 2.767 | 14.237 | 0.652437 | 7/15/2014 | 25:52.6 | 3 |
| 69.309 | 19094.582 | 2.763 | 14.23 | 0.645098 | 7/15/2014 | 25:52.7 | 3 |
| 69.198 | 19094.582 | 2.76 | 14.228 | 0.638113 | 7/15/2014 | 25:52.7 | 3 |
| 68.986 | 19094.581 | 2.76 | 14.224 | 0.635081 | 7/15/2014 | 25:52.8 | 3 |
| 68.775 | 19094.581 | 2.76 | 14.214 | 0.640644 | 7/15/2014 | 25:52.9 | 3 |
| 68.561 | 19094.58 | 2.762 | 14.209 | 0.638132 | 7/15/2014 | 25:53.0 | 3 |
| 68.35 | 19094.579 | 2.76 | 14.205 | 0.618613 | 7/15/2014 | 25:53.1 | 3 |
| 68.137 | 19094.579 | 2.76 | 14.201 | 0.60566 | 7/15/2014 | 25:53.2 | 3 |
| 67.925 | 19094.578 | 2.76 | 14.198 | 0.592167 | 7/15/2014 | 25:53.3 | 3 |
| 67.659 | 19094.577 | 2.76 | 14.193 | 0.596821 | 7/15/2014 | 25:53.4 | 3 |
| 67.443 | 19094.576 | 2.76 | 14.189 | 0.588155 | 7/15/2014 | 25:53.5 | 3 |
| 67.227 | 19094.576 | 2.76 | 14.183 | 0.564374 | 7/15/2014 | 25:53.6 | 3 |
| 67.01 | 19094.576 | 2.755 | 14.181 | 0.555362 | 7/15/2014 | 25:53.7 | 3 |
| 66.791 | 19094.575 | 2.75 | 14.179 | 0.55639 | 7/15/2014 | 25:53.8 | 3 |
| 66.573 | 19094.575 | 2.747 | 14.177 | 0.541907 | 7/15/2014 | 25:53.9 | 3 |
| 66.352 | 19094.575 | 2.745 | 14.177 | 0.549385 | 7/15/2014 | 25:54.0 | 3 |
| 66.132 | 19094.575 | 2.745 | 14.178 | 0.528557 | 7/15/2014 | 25:54.1 | 3 |
| 65.913 | 19094.574 | 2.743 | 14.184 | 0.533687 | 7/15/2014 | 25:54.2 | 3 |
| 65.695 | 19094.575 | 2.74 | 14.188 | 0.532599 | 7/15/2014 | 25:54.3 | 3 |
| 65.479 | 19094.575 | 2.735 | 14.201 | 0.533451 | 7/15/2014 | 25:54.4 | 3 |
| 65.262 | 19094.575 | 2.739 | 14.211 | 0.538252 | 7/15/2014 | 25:54.5 | 3 |
| 64.899 | 19094.576 | 2.736 | 14.221 | 0.534285 | 7/15/2014 | 25:54.7 | 3 |
| 64.787 | 19094.576 | 2.739 | 14.225 | 0.524722 | 7/15/2014 | 25:54.7 | 3 |
| 64.574 | 19094.576 | 2.741 | 14.234 | 0.531254 | 7/15/2014 | 25:54.8 | 3 |
| 64.362 | 19094.577 | 2.741 | 14.24 | 0.515699 | 7/15/2014 | 25:54.9 | 3 |
| 64.154 | 19094.579 | 2.741 | 14.246 | 0.506748 | 7/15/2014 | 25:55.0 | 3 |
| 63.944 | 19094.579 | 2.741 | 14.256 | 0.492626 | 7/15/2014 | 25:55.1 | 3 |
| 63.733 | 19094.58 | 2.741 | 14.257 | 0.47543 | 7/15/2014 | 25:55.2 | 3 |
| 63.524 | 19094.581 | 2.741 | 14.261 | 0.476619 | 7/15/2014 | 25:55.3 | 3 |
| 63.16 | 19094.584 | 2.738 | 14.271 | 0.455945 | 7/15/2014 | 25:55.5 | 3 |
| 62.949 | 19094.584 | 2.743 | 14.279 | 0.456573 | 7/15/2014 | 25:55.6 | 3 |
| 62.739 | 19094.587 | 2.741 | 14.282 | 0.459253 | 7/15/2014 | 25:55.7 | 3 |
| 62.529 | 19094.588 | 2.741 | 14.288 | 0.454 | 7/15/2014 | 25:55.8 | 3 |
| 62.319 | 19094.59 | 2.743 | 14.291 | 0.450913 | 7/15/2014 | 25:55.9 | 3 |
| 62.106 | 19094.592 | 2.748 | 14.295 | 0.438403 | 7/15/2014 | 25:56.0 | 3 |
| 61.894 | 19094.595 | 2.75 | 14.294 | 0.42319 | 7/15/2014 | 25:56.1 | 3 |
| 61.683 | 19094.596 | 2.75 | 14.293 | 0.414394 | 7/15/2014 | 25:56.2 | 3 |
| 61.472 | 19094.6 | 2.752 | 14.289 | 0.399692 | 7/15/2014 | 25:56.3 | 3 |
| 61.261 | 19094.601 | 2.754 | 14.289 | 0.383589 | 7/15/2014 | 25:56.4 | 3 |
| 61.051 | 19094.604 | 2.753 | 14.285 | 0.366257 | 7/15/2014 | 25:56.5 | 3 |
| 60.842 | 19094.605 | 2.754 | 14.284 | 0.351572 | 7/15/2014 | 25:56.6 | 3 |
| 60.581 | 19094.611 | 2.756 | 14.28 | 0.348721 | 7/15/2014 | 25:56.7 | 3 |
| 60.475 | 19094.612 | 2.756 | 14.278 | 0.349752 | 7/15/2014 | 25:56.7 | 3 |
| 60.265 | 19094.615 | 2.754 | 14.273 | 0.35305 | 7/15/2014 | 25:56.8 | 3 |
| 60.055 | 19094.618 | 2.751 | 14.273 | 0.344575 | 7/15/2014 | 25:56.9 | 3 |
| 59.844 | 19094.623 | 2.756 | 14.273 | 0.348086 | 7/15/2014 | 25:57.0 | 3 |
| 59.634 | 19094.626 | 2.756 | 14.274 | 0.360945 | 7/15/2014 | 25:57.1 | 3 |

| | | | | | | | |
|---|---|---|---|---|---|---|---|
| 59.423 | 19094.63 | 2.76 | 14.274 | 0.362385 | 7/15/2014 | 25:57.2 | 3 |
| 59.213 | 19094.633 | 2.762 | 14.276 | 0.387082 | 7/15/2014 | 25:57.3 | 3 |
| 58.842 | 19094.643 | 2.764 | 14.271 | 0.381344 | 7/15/2014 | 25:57.5 | 3 |
| 58.738 | 19094.644 | 2.764 | 14.272 | 0.37138 | 7/15/2014 | 25:57.5 | 3 |
| 58.525 | 19094.647 | 2.766 | 14.267 | 0.379653 | 7/15/2014 | 25:57.6 | 3 |
| 58.309 | 19094.653 | 2.772 | 14.271 | 0.399264 | 7/15/2014 | 25:57.7 | 3 |
| 58.094 | 19094.658 | 2.775 | 14.271 | 0.399015 | 7/15/2014 | 25:57.8 | 3 |
| 57.878 | 19094.662 | 2.78 | 14.263 | 0.41312 | 7/15/2014 | 25:57.9 | 3 |
| 57.664 | 19094.668 | 2.783 | 14.261 | 0.431172 | 7/15/2014 | 25:58.0 | 3 |
| 57.45 | 19094.672 | 2.786 | 14.259 | 0.467327 | 7/15/2014 | 25:58.1 | 3 |
| 57.237 | 19094.68 | 2.79 | 14.253 | 0.501895 | 7/15/2014 | 25:58.2 | 3 |
| 56.922 | 19094.688 | 2.794 | 14.249 | 0.511546 | 7/15/2014 | 25:58.4 | 3 |
| 56.712 | 19094.694 | 2.796 | 14.249 | 0.527575 | 7/15/2014 | 25:58.5 | 3 |
| 56.503 | 19094.699 | 2.797 | 14.24 | 0.51581 | 7/15/2014 | 25:58.6 | 3 |
| 56.245 | 19094.707 | 2.795 | 14.233 | 0.499973 | 7/15/2014 | 25:58.7 | 3 |
| 56.138 | 19094.711 | 2.793 | 14.236 | 0.47974 | 7/15/2014 | 25:58.7 | 3 |
| 55.932 | 19094.717 | 2.793 | 14.227 | 0.436473 | 7/15/2014 | 25:58.8 | 3 |
| 55.724 | 19094.725 | 2.795 | 14.219 | 0.417657 | 7/15/2014 | 25:58.9 | 3 |
| 55.518 | 19094.731 | 2.797 | 14.214 | 0.417336 | 7/15/2014 | 25:59.0 | 3 |
| 55.309 | 19094.737 | 2.799 | 14.206 | 0.414752 | 7/15/2014 | 25:59.1 | 3 |
| 55.101 | 19094.746 | 2.804 | 14.203 | 0.349875 | 7/15/2014 | 25:59.2 | 3 |
| 54.894 | 19094.752 | 2.809 | 14.198 | 0.353021 | 7/15/2014 | 25:59.3 | 3 |
| 54.526 | 19094.766 | 2.812 | 14.197 | 0.351049 | 7/15/2014 | 25:59.5 | 3 |
| 54.314 | 19094.775 | 2.819 | 14.194 | 0.363895 | 7/15/2014 | 25:59.6 | 3 |
| 54.212 | 19094.779 | 2.821 | 14.192 | 0.357005 | 7/15/2014 | 25:59.6 | 3 |
| 53.999 | 19094.787 | 2.821 | 14.19 | 0.351976 | 7/15/2014 | 25:59.7 | 3 |
| 53.783 | 19094.795 | 2.823 | 14.193 | 0.348093 | 7/15/2014 | 25:59.8 | 3 |
| 53.568 | 19094.805 | 2.825 | 14.193 | 0.398278 | 7/15/2014 | 25:59.9 | 3 |
| 53.351 | 19094.814 | 2.825 | 14.193 | 0.402399 | 7/15/2014 | 26:00.0 | 3 |
| 53.135 | 19094.822 | 2.825 | 14.193 | 0.43511 | 7/15/2014 | 26:00.1 | 3 |
| 52.918 | 19094.832 | 2.825 | 14.191 | 0.471972 | 7/15/2014 | 26:00.2 | 3 |
| 52.703 | 19094.842 | 2.827 | 14.185 | 0.525118 | 7/15/2014 | 26:00.3 | 3 |
| 52.488 | 19094.852 | 2.828 | 14.185 | 0.570119 | 7/15/2014 | 26:00.4 | 3 |
| 52.273 | 19094.862 | 2.83 | 14.187 | 0.596843 | 7/15/2014 | 26:00.5 | 3 |
| 51.904 | 19094.881 | 2.832 | 14.179 | 0.648086 | 7/15/2014 | 26:00.7 | 3 |
| 51.794 | 19094.887 | 2.833 | 14.18 | 0.672279 | 7/15/2014 | 26:00.7 | 3 |
| 51.581 | 19094.896 | 2.833 | 14.18 | 0.728568 | 7/15/2014 | 26:00.8 | 3 |
| 51.366 | 19094.908 | 2.836 | 14.175 | 0.74252 | 7/15/2014 | 26:00.9 | 3 |
| 51.151 | 19094.918 | 2.839 | 14.173 | 0.767737 | 7/15/2014 | 26:01.0 | 3 |
| 50.936 | 19094.93 | 2.845 | 14.17 | 0.820697 | 7/15/2014 | 26:01.1 | 3 |
| 50.722 | 19094.943 | 2.849 | 14.168 | 0.837191 | 7/15/2014 | 26:01.2 | 3 |
| 50.506 | 19094.955 | 2.852 | 14.162 | 0.886648 | 7/15/2014 | 26:01.3 | 3 |
| 50.238 | 19094.97 | 2.857 | 14.158 | 0.92418 | 7/15/2014 | 26:01.4 | 3 |
| 50.02 | 19094.982 | 2.857 | 14.155 | 0.918371 | 7/15/2014 | 26:01.5 | 3 |
| 49.799 | 19094.995 | 2.869 | 14.15 | 0.901205 | 7/15/2014 | 26:01.6 | 3 |
| 49.579 | 19095.008 | 2.873 | 14.145 | 0.853032 | 7/15/2014 | 26:01.7 | 3 |
| 49.355 | 19095.021 | 2.882 | 14.143 | 0.788444 | 7/15/2014 | 26:01.8 | 3 |

| | | | | | | | |
|---|---|---|---|---|---|---|---|
| 49.134 | 19095.036 | 2.884 | 14.138 | 0.752094 | 7/15/2014 | 26:01.9 | 3 |
| 48.912 | 19095.05 | 2.887 | 14.134 | 0.669624 | 7/15/2014 | 26:02.0 | 3 |
| 48.687 | 19095.063 | 2.891 | 14.128 | 0.612019 | 7/15/2014 | 26:02.1 | 3 |
| 48.463 | 19095.078 | 2.898 | 14.123 | 0.563095 | 7/15/2014 | 26:02.2 | 3 |
| 48.238 | 19095.093 | 2.908 | 14.122 | 0.524355 | 7/15/2014 | 26:02.3 | 3 |
| 48.019 | 19095.107 | 2.908 | 14.116 | 0.502447 | 7/15/2014 | 26:02.4 | 3 |
| 47.796 | 19095.123 | 2.915 | 14.113 | 0.487354 | 7/15/2014 | 26:02.5 | 3 |
| 47.578 | 19095.137 | 2.919 | 14.105 | 0.466416 | 7/15/2014 | 26:02.6 | 3 |
| 47.308 | 19095.156 | 2.926 | 14.101 | 0.44817 | 7/15/2014 | 26:02.7 | 3 |
| 47.088 | 19095.172 | 2.933 | 14.096 | 0.444451 | 7/15/2014 | 26:02.8 | 3 |
| 46.869 | 19095.188 | 2.939 | 14.093 | 0.436405 | 7/15/2014 | 26:02.9 | 3 |
| 46.649 | 19095.203 | 2.944 | 14.09 | 0.426754 | 7/15/2014 | 26:03.0 | 3 |
| 46.431 | 19095.22 | 2.944 | 14.084 | 0.416063 | 7/15/2014 | 26:03.1 | 3 |
| 46.212 | 19095.236 | 2.948 | 14.078 | 0.408824 | 7/15/2014 | 26:03.2 | 3 |
| 45.994 | 19095.251 | 2.95 | 14.072 | 0.397561 | 7/15/2014 | 26:03.3 | 3 |
| 45.722 | 19095.272 | 2.95 | 14.069 | 0.393123 | 7/15/2014 | 26:03.4 | 3 |
| 45.502 | 19095.289 | 2.952 | 14.065 | 0.382678 | 7/15/2014 | 26:03.5 | 3 |
| 45.281 | 19095.307 | 2.954 | 14.06 | 0.37377 | 7/15/2014 | 26:03.6 | 3 |
| 45.057 | 19095.324 | 2.959 | 14.056 | 0.367855 | 7/15/2014 | 26:03.7 | 3 |
| 44.835 | 19095.342 | 2.959 | 14.052 | 0.363504 | 7/15/2014 | 26:03.8 | 3 |
| 44.611 | 19095.36 | 2.963 | 14.049 | 0.351824 | 7/15/2014 | 26:03.9 | 3 |
| 44.386 | 19095.377 | 2.965 | 14.044 | 0.349774 | 7/15/2014 | 26:04.0 | 3 |
| 44.162 | 19095.396 | 2.967 | 14.039 | 0.345993 | 7/15/2014 | 26:04.1 | 3 |
| 43.938 | 19095.414 | 2.969 | 14.033 | 0.34177 | 7/15/2014 | 26:04.2 | 3 |
| 43.713 | 19095.434 | 2.973 | 14.028 | 0.333476 | 7/15/2014 | 26:04.3 | 3 |
| 43.491 | 19095.453 | 2.973 | 14.025 | 0.330238 | 7/15/2014 | 26:04.4 | 3 |
| 43.27 | 19095.47 | 2.976 | 14.021 | 0.327407 | 7/15/2014 | 26:04.5 | 3 |
| 43.049 | 19095.489 | 2.981 | 14.013 | 0.325451 | 7/15/2014 | 26:04.6 | 3 |
| 42.776 | 19095.514 | 2.983 | 14.004 | 0.32266 | 7/15/2014 | 26:04.7 | 3 |
| 42.558 | 19095.532 | 2.983 | 13.999 | 0.318603 | 7/15/2014 | 26:04.8 | 3 |
| 42.337 | 19095.552 | 2.983 | 13.995 | 0.317552 | 7/15/2014 | 26:04.9 | 3 |
| 42.114 | 19095.572 | 2.986 | 13.988 | 0.331081 | 7/15/2014 | 26:05.0 | 3 |
| 41.895 | 19095.592 | 2.986 | 13.981 | 0.325315 | 7/15/2014 | 26:05.1 | 3 |
| 41.673 | 19095.61 | 2.993 | 13.975 | 0.324585 | 7/15/2014 | 26:05.2 | 3 |
| 41.453 | 19095.631 | 2.993 | 13.965 | 0.331302 | 7/15/2014 | 26:05.3 | 3 |
| 41.178 | 19095.657 | 2.996 | 13.962 | 0.332975 | 7/15/2014 | 26:05.4 | 3 |
| 40.957 | 19095.677 | 2.996 | 13.955 | 0.335533 | 7/15/2014 | 26:05.5 | 3 |
| 40.731 | 19095.698 | 3.002 | 13.947 | 0.340434 | 7/15/2014 | 26:05.6 | 3 |
| 40.507 | 19095.719 | 3.007 | 13.941 | 0.333815 | 7/15/2014 | 26:05.7 | 3 |
| 40.28 | 19095.74 | 3.01 | 13.94 | 0.334756 | 7/15/2014 | 26:05.8 | 3 |
| 40.052 | 19095.762 | 3.017 | 13.933 | 0.334565 | 7/15/2014 | 26:05.9 | 3 |
| 39.826 | 19095.784 | 3.017 | 13.923 | 0.333129 | 7/15/2014 | 26:06.0 | 3 |
| 39.6 | 19095.806 | 3.02 | 13.918 | 0.335056 | 7/15/2014 | 26:06.1 | 3 |
| 39.373 | 19095.828 | 3.02 | 13.907 | 0.335698 | 7/15/2014 | 26:06.2 | 3 |
| 39.146 | 19095.851 | 3.023 | 13.9 | 0.336991 | 7/15/2014 | 26:06.3 | 3 |
| 38.924 | 19095.871 | 3.023 | 13.896 | 0.33696 | 7/15/2014 | 26:06.4 | 3 |
| 38.701 | 19095.894 | 3.023 | 13.888 | 0.339595 | 7/15/2014 | 26:06.5 | 3 |

| | | | | | | | |
|---|---|---|---|---|---|---|---|
| 38.478 | 19095.916 | 3.023 | 13.876 | 0.339679 | 7/15/2014 | 26:06.6 | 3 |
| 38.204 | 19095.944 | 3.019 | 13.87 | 0.34117 | 7/15/2014 | 26:06.7 | 3 |
| 37.983 | 19095.967 | 3.019 | 13.862 | 0.341371 | 7/15/2014 | 26:06.8 | 3 |
| 37.759 | 19095.989 | 3.019 | 13.854 | 0.343571 | 7/15/2014 | 26:06.9 | 3 |
| 37.537 | 19096.012 | 3.019 | 13.849 | 0.341634 | 7/15/2014 | 26:07.0 | 3 |
| 37.315 | 19096.035 | 3.022 | 13.843 | 0.340456 | 7/15/2014 | 26:07.1 | 3 |
| 37.094 | 19096.058 | 3.022 | 13.829 | 0.339723 | 7/15/2014 | 26:07.2 | 3 |
| 36.87 | 19096.081 | 3.025 | 13.822 | 0.336336 | 7/15/2014 | 26:07.3 | 3 |
| 36.595 | 19096.11 | 3.025 | 13.813 | 0.334296 | 7/15/2014 | 26:07.4 | 3 |
| 36.369 | 19096.135 | 3.028 | 13.804 | 0.352594 | 7/15/2014 | 26:07.5 | 3 |
| 36.143 | 19096.157 | 3.028 | 13.796 | 0.397994 | 7/15/2014 | 26:07.6 | 3 |
| 35.918 | 19096.183 | 3.025 | 13.786 | 0.470234 | 7/15/2014 | 26:07.7 | 3 |
| 35.689 | 19096.207 | 3.025 | 13.776 | 0.518895 | 7/15/2014 | 26:07.8 | 3 |
| 35.46 | 19096.232 | 3.025 | 13.771 | 0.534923 | 7/15/2014 | 26:07.9 | 3 |
| 35.232 | 19096.256 | 3.025 | 13.765 | 0.590292 | 7/15/2014 | 26:08.0 | 3 |
| 35.004 | 19096.282 | 3.025 | 13.754 | 0.573704 | 7/15/2014 | 26:08.1 | 3 |
| 34.778 | 19096.307 | 3.025 | 13.746 | 0.53231 | 7/15/2014 | 26:08.2 | 3 |
| 34.554 | 19096.332 | 3.019 | 13.739 | 0.466358 | 7/15/2014 | 26:08.3 | 3 |
| 34.329 | 19096.357 | 3.016 | 13.734 | 0.370328 | 7/15/2014 | 26:08.4 | 3 |
| 34.104 | 19096.382 | 3.019 | 13.729 | 0.330962 | 7/15/2014 | 26:08.5 | 3 |
| 33.883 | 19096.408 | 3.019 | 13.723 | 0.306947 | 7/15/2014 | 26:08.6 | 3 |
| 33.609 | 19096.438 | 3.016 | 13.71 | 0.240907 | 7/15/2014 | 26:08.7 | 3 |
| 33.39 | 19096.465 | 3.01 | 13.701 | 0.235155 | 7/15/2014 | 26:08.8 | 3 |
| 33.17 | 19096.489 | 3.003 | 13.696 | 0.222604 | 7/15/2014 | 26:08.9 | 3 |
| 32.949 | 19096.516 | 3.003 | 13.69 | 0.195744 | 7/15/2014 | 26:09.0 | 3 |
| 32.727 | 19096.541 | 3.006 | 13.687 | 0.205701 | 7/15/2014 | 26:09.1 | 3 |
| 32.506 | 19096.567 | 3.006 | 13.678 | 0.203876 | 7/15/2014 | 26:09.2 | 3 |
| 32.286 | 19096.594 | 3.004 | 13.671 | 0.191311 | 7/15/2014 | 26:09.3 | 3 |
| 32.011 | 19096.626 | 3.001 | 13.665 | 0.214982 | 7/15/2014 | 26:09.4 | 3 |
| 31.792 | 19096.652 | 2.998 | 13.661 | 0.213416 | 7/15/2014 | 26:09.5 | 3 |
| 31.567 | 19096.679 | 3.001 | 13.659 | 0.203634 | 7/15/2014 | 26:09.6 | 3 |
| 31.342 | 19096.705 | 3.001 | 13.653 | 0.219558 | 7/15/2014 | 26:09.7 | 3 |
| 31.114 | 19096.732 | 2.996 | 13.651 | 0.183136 | 7/15/2014 | 26:09.8 | 3 |
| 30.889 | 19096.761 | 2.991 | 13.645 | 0.163763 | 7/15/2014 | 26:09.9 | 3 |
| 30.66 | 19096.788 | 2.988 | 13.641 | 0.175239 | 7/15/2014 | 26:10.0 | 3 |
| 30.435 | 19096.815 | 2.985 | 13.639 | 0.135378 | 7/15/2014 | 26:10.1 | 3 |
| 30.211 | 19096.844 | 2.98 | 13.637 | 0.140571 | 7/15/2014 | 26:10.2 | 3 |
| 29.984 | 19096.871 | 2.978 | 13.63 | 0.121121 | 7/15/2014 | 26:10.3 | 3 |
| 29.761 | 19096.898 | 2.976 | 13.621 | 0.124034 | 7/15/2014 | 26:10.4 | 3 |
| 29.538 | 19096.926 | 2.974 | 13.617 | 0.12671 | 7/15/2014 | 26:10.5 | 3 |
| 29.318 | 19096.953 | 2.974 | 13.608 | 0.138767 | 7/15/2014 | 26:10.6 | 3 |
| 29.045 | 19096.987 | 2.974 | 13.601 | 0.128187 | 7/15/2014 | 26:10.7 | 3 |
| 28.828 | 19097.014 | 2.968 | 13.598 | 0.145259 | 7/15/2014 | 26:10.8 | 3 |
| 28.609 | 19097.043 | 2.966 | 13.597 | 0.143473 | 7/15/2014 | 26:10.9 | 3 |
| 28.391 | 19097.07 | 2.964 | 13.593 | 0.149564 | 7/15/2014 | 26:11.0 | 3 |
| 28.171 | 19097.098 | 2.962 | 13.595 | 0.143078 | 7/15/2014 | 26:11.1 | 3 |
| 27.955 | 19097.125 | 2.957 | 13.596 | 0.157736 | 7/15/2014 | 26:11.2 | 3 |

| | | | | | | | |
|---|---|---|---|---|---|---|---|
| 27.736 | 19097.152 | 2.951 | 13.6 | 0.145974 | 7/15/2014 | 26:11.3 | 3 |
| 27.467 | 19097.188 | 2.946 | 13.603 | 0.155262 | 7/15/2014 | 26:11.4 | 3 |
| 27.249 | 19097.214 | 2.944 | 13.601 | 0.141833 | 7/15/2014 | 26:11.5 | 3 |
| 27.029 | 19097.243 | 2.946 | 13.6 | 0.153769 | 7/15/2014 | 26:11.6 | 3 |
| 26.809 | 19097.271 | 2.943 | 13.596 | 0.153318 | 7/15/2014 | 26:11.7 | 3 |
| 26.588 | 19097.3 | 2.943 | 13.594 | 0.171735 | 7/15/2014 | 26:11.8 | 3 |
| 26.367 | 19097.328 | 2.943 | 13.587 | 0.166274 | 7/15/2014 | 26:11.9 | 3 |
| 26.145 | 19097.356 | 2.943 | 13.583 | 0.164671 | 7/15/2014 | 26:12.0 | 3 |
| 25.925 | 19097.386 | 2.941 | 13.574 | 0.188833 | 7/15/2014 | 26:12.1 | 3 |
| 25.703 | 19097.414 | 2.941 | 13.572 | 0.203603 | 7/15/2014 | 26:12.2 | 3 |
| 25.484 | 19097.442 | 2.941 | 13.57 | 0.176914 | 7/15/2014 | 26:12.3 | 3 |
| 25.266 | 19097.471 | 2.939 | 13.565 | 0.196796 | 7/15/2014 | 26:12.4 | 3 |
| 25.05 | 19097.499 | 2.94 | 13.559 | 0.165327 | 7/15/2014 | 26:12.5 | 3 |
| 24.834 | 19097.527 | 2.936 | 13.554 | 0.18832 | 7/15/2014 | 26:12.6 | 3 |
| 24.568 | 19097.561 | 2.936 | 13.551 | 0.188439 | 7/15/2014 | 26:12.7 | 3 |
| 24.355 | 19097.589 | 2.933 | 13.544 | 0.155757 | 7/15/2014 | 26:12.8 | 3 |
| 24.141 | 19097.618 | 2.933 | 13.538 | 0.161228 | 7/15/2014 | 26:12.9 | 3 |
| 23.927 | 19097.646 | 2.933 | 13.53 | 0.18724 | 7/15/2014 | 26:13.0 | 3 |
| 23.714 | 19097.673 | 2.933 | 13.521 | 0.163785 | 7/15/2014 | 26:13.1 | 3 |
| 23.502 | 19097.701 | 2.933 | 13.512 | 0.173627 | 7/15/2014 | 26:13.2 | 3 |
| 23.29 | 19097.729 | 2.933 | 13.5 | 0.163621 | 7/15/2014 | 26:13.3 | 3 |
| 23.025 | 19097.762 | 2.936 | 13.491 | 0.165954 | 7/15/2014 | 26:13.4 | 3 |
| 22.812 | 19097.79 | 2.933 | 13.481 | 0.164002 | 7/15/2014 | 26:13.5 | 3 |
| 22.599 | 19097.819 | 2.936 | 13.472 | 0.179008 | 7/15/2014 | 26:13.6 | 3 |
| 22.383 | 19097.847 | 2.939 | 13.461 | 0.134584 | 7/15/2014 | 26:13.7 | 3 |
| 22.168 | 19097.875 | 2.939 | 13.446 | 0.173522 | 7/15/2014 | 26:13.8 | 3 |
| 21.952 | 19097.904 | 2.939 | 13.433 | 0.16296 | 7/15/2014 | 26:13.9 | 3 |
| 21.738 | 19097.932 | 2.939 | 13.423 | 0.148536 | 7/15/2014 | 26:14.0 | 3 |
| 21.522 | 19097.959 | 2.941 | 13.417 | 0.163157 | 7/15/2014 | 26:14.1 | 3 |
| 21.308 | 19097.987 | 2.941 | 13.404 | 0.170232 | 7/15/2014 | 26:14.2 | 3 |
| 21.095 | 19098.016 | 2.944 | 13.392 | 0.150209 | 7/15/2014 | 26:14.3 | 3 |
| 20.781 | 19098.055 | 2.946 | 13.375 | 0.164267 | 7/15/2014 | 26:14.5 | 3 |
| 20.571 | 19098.083 | 2.954 | 13.364 | 0.150331 | 7/15/2014 | 26:14.6 | 3 |
| 20.313 | 19098.117 | 2.954 | 13.355 | 0.151415 | 7/15/2014 | 26:14.7 | 3 |
| 20.207 | 19098.131 | 2.954 | 13.351 | 0.153848 | 7/15/2014 | 26:14.7 | 3 |
| 19.999 | 19098.158 | 2.956 | 13.34 | 0.160932 | 7/15/2014 | 26:14.8 | 3 |
| 19.792 | 19098.186 | 2.956 | 13.33 | 0.169639 | 7/15/2014 | 26:14.9 | 3 |
| 19.584 | 19098.212 | 2.958 | 13.324 | 0.166111 | 7/15/2014 | 26:15.0 | 3 |
| 19.377 | 19098.24 | 2.958 | 13.316 | 0.171454 | 7/15/2014 | 26:15.1 | 3 |
| 19.17 | 19098.266 | 2.958 | 13.31 | 0.187413 | 7/15/2014 | 26:15.2 | 3 |
| 18.961 | 19098.293 | 2.958 | 13.305 | 0.171469 | 7/15/2014 | 26:15.3 | 3 |
| 18.596 | 19098.34 | 2.954 | 13.295 | 0.158681 | 7/15/2014 | 26:15.5 | 3 |
| 18.384 | 19098.367 | 2.958 | 13.288 | 0.125666 | 7/15/2014 | 26:15.6 | 3 |
| 18.282 | 19098.381 | 2.958 | 13.287 | 0.113249 | 7/15/2014 | 26:15.6 | 3 |
| 18.071 | 19098.408 | 2.958 | 13.279 | 0.108214 | 7/15/2014 | 26:15.7 | 3 |
| 17.855 | 19098.438 | 2.961 | 13.271 | 0.106064 | 7/15/2014 | 26:15.8 | 3 |
| 17.642 | 19098.465 | 2.961 | 13.269 | 0.0843066 | 7/15/2014 | 26:15.9 | 3 |

| | | | | | | | |
|---|---|---|---|---|---|---|---|
| 17.424 | 19098.493 | 2.966 | 13.262 | 0.0855608 | 7/15/2014 | 26:16.0 | 3 |
| 17.21 | 19098.521 | 2.968 | 13.255 | 0.0829561 | 7/15/2014 | 26:16.1 | 3 |
| 16.993 | 19098.548 | 2.968 | 13.252 | 0.0834182 | 7/15/2014 | 26:16.2 | 3 |
| 16.775 | 19098.576 | 2.97 | 13.242 | 0.0913761 | 7/15/2014 | 26:16.3 | 3 |
| 16.559 | 19098.604 | 2.97 | 13.233 | 0.0903831 | 7/15/2014 | 26:16.4 | 3 |
| 16.343 | 19098.633 | 2.97 | 13.222 | 0.093543 | 7/15/2014 | 26:16.5 | 3 |
| 16.129 | 19098.66 | 2.965 | 13.216 | 0.0968279 | 7/15/2014 | 26:16.6 | 3 |
| 15.856 | 19098.694 | 2.965 | 13.202 | 0.0979577 | 7/15/2014 | 26:16.7 | 3 |
| 15.639 | 19098.724 | 2.958 | 13.192 | 0.113239 | 7/15/2014 | 26:16.8 | 3 |
| 15.419 | 19098.751 | 2.956 | 13.178 | 0.0952474 | 7/15/2014 | 26:16.9 | 3 |
| 15.197 | 19098.779 | 2.953 | 13.164 | 0.105006 | 7/15/2014 | 26:17.0 | 3 |
| 14.973 | 19098.809 | 2.951 | 13.155 | 0.107132 | 7/15/2014 | 26:17.1 | 3 |
| 14.751 | 19098.837 | 2.946 | 13.144 | 0.10264 | 7/15/2014 | 26:17.2 | 3 |
| 14.526 | 19098.865 | 2.943 | 13.136 | 0.106826 | 7/15/2014 | 26:17.3 | 3 |
| 14.245 | 19098.9 | 2.94 | 13.125 | 0.107889 | 7/15/2014 | 26:17.4 | 3 |
| 14.017 | 19098.931 | 2.934 | 13.117 | 0.127389 | 7/15/2014 | 26:17.5 | 3 |
| 13.784 | 19098.959 | 2.929 | 13.109 | 0.139368 | 7/15/2014 | 26:17.6 | 3 |
| 13.55 | 19098.988 | 2.922 | 13.101 | 0.150693 | 7/15/2014 | 26:17.7 | 3 |
| 13.312 | 19099.02 | 2.918 | 13.097 | 0.141284 | 7/15/2014 | 26:17.8 | 3 |
| 13.075 | 19099.05 | 2.913 | 13.094 | 0.146666 | 7/15/2014 | 26:17.9 | 3 |
| 12.835 | 19099.079 | 2.91 | 13.092 | 0.14742 | 7/15/2014 | 26:18.0 | 3 |
| 12.598 | 19099.109 | 2.908 | 13.09 | 0.168194 | 7/15/2014 | 26:18.1 | 3 |
| 12.36 | 19099.14 | 2.903 | 13.09 | 0.156834 | 7/15/2014 | 26:18.2 | 3 |
| 12.122 | 19099.169 | 2.901 | 13.093 | 0.15905 | 7/15/2014 | 26:18.3 | 3 |
| 11.884 | 19099.199 | 2.901 | 13.096 | 0.141101 | 7/15/2014 | 26:18.4 | 3 |
| 11.649 | 19099.229 | 2.895 | 13.101 | 0.156149 | 7/15/2014 | 26:18.5 | 3 |
| 11.412 | 19099.259 | 2.891 | 13.107 | 0.171896 | 7/15/2014 | 26:18.6 | 3 |
| 11.12 | 19099.295 | 2.885 | 13.109 | 0.177919 | 7/15/2014 | 26:18.7 | 3 |
| 11.001 | 19099.31 | 2.88 | 13.112 | 0.170327 | 7/15/2014 | 26:18.8 | 3 |
| 10.765 | 19099.34 | 2.878 | 13.113 | 0.17717 | 7/15/2014 | 26:18.9 | 3 |
| 10.531 | 19099.369 | 2.871 | 13.116 | 0.202525 | 7/15/2014 | 26:19.0 | 3 |
| 10.294 | 19099.398 | 2.867 | 13.116 | 0.197538 | 7/15/2014 | 26:19.1 | 3 |
| 10.062 | 19099.428 | 2.855 | 13.115 | 0.203866 | 7/15/2014 | 26:19.2 | 3 |
| 9.824 | 19099.456 | 2.855 | 13.115 | 0.207811 | 7/15/2014 | 26:19.3 | 3 |
| 9.406 | 19099.509 | 2.842 | 13.115 | 0.204141 | 7/15/2014 | 26:19.4 | 3 |
| 9.165 | 19099.538 | 2.834 | 13.117 | 0.197862 | 7/15/2014 | 26:19.5 | 3 |
| 8.923 | 19099.568 | 2.826 | 13.125 | 0.198217 | 7/15/2014 | 26:19.6 | 3 |
| 8.675 | 19099.599 | 2.821 | 13.128 | 0.203757 | 7/15/2014 | 26:19.7 | 3 |
| 8.557 | 19099.613 | 2.815 | 13.128 | 0.203451 | 7/15/2014 | 26:19.8 | 3 |
| 8.308 | 19099.643 | 2.811 | 13.135 | 0.204521 | 7/15/2014 | 26:19.9 | 3 |
| 8.059 | 19099.674 | 2.8 | 13.139 | 0.200806 | 7/15/2014 | 26:20.0 | 3 |
| 7.807 | 19099.705 | 2.797 | 13.138 | 0.206354 | 7/15/2014 | 26:20.1 | 3 |
| 7.559 | 19099.735 | 2.788 | 13.141 | 0.184767 | 7/15/2014 | 26:20.2 | 3 |
| 7.305 | 19099.766 | 2.782 | 13.143 | 0.1873 | 7/15/2014 | 26:20.3 | 3 |
| 7.052 | 19099.797 | 2.774 | 13.14 | 0.19221 | 7/15/2014 | 26:20.4 | 3 |
| 6.801 | 19099.826 | 2.765 | 13.142 | 0.184159 | 7/15/2014 | 26:20.5 | 3 |
| 6.552 | 19099.855 | 2.758 | 13.151 | 0.181449 | 7/15/2014 | 26:20.6 | 3 |

| | | | | | | | |
|---|---|---|---|---|---|---|---|
| 6.299 | 19099.887 | 2.758 | 13.161 | 0.182662 | 7/15/2014 | 26:20.7 | 3 |
| 5.99 | 19099.924 | 2.747 | 13.17 | 0.190702 | 7/15/2014 | 26:20.8 | 3 |
| 5.737 | 19099.954 | 2.738 | 13.18 | 0.176033 | 7/15/2014 | 26:20.9 | 3 |
| 5.485 | 19099.984 | 2.727 | 13.192 | 0.168233 | 7/15/2014 | 26:21.0 | 3 |
| 5.231 | 19100.014 | 2.719 | 13.203 | 0.166781 | 7/15/2014 | 26:21.1 | 3 |
| 4.978 | 19100.043 | 2.712 | 13.213 | 0.160436 | 7/15/2014 | 26:21.2 | 3 |
| 4.722 | 19100.074 | 2.703 | 13.218 | 0.159965 | 7/15/2014 | 26:21.3 | 3 |
| 4.407 | 19100.111 | 2.689 | 13.221 | 0.163759 | 7/15/2014 | 26:21.4 | 3 |
| 4.147 | 19100.143 | 2.684 | 13.228 | 0.157873 | 7/15/2014 | 26:21.5 | 3 |
| 3.887 | 19100.172 | 2.678 | 13.235 | 0.166818 | 7/15/2014 | 26:21.6 | 3 |
| 3.625 | 19100.203 | 2.674 | 13.238 | 0.180174 | 7/15/2014 | 26:21.7 | 3 |
| 3.362 | 19100.233 | 2.662 | 13.245 | 0.194797 | 7/15/2014 | 26:21.8 | 3 |
| 3.096 | 19100.265 | 2.655 | 13.252 | 0.182746 | 7/15/2014 | 26:21.9 | 3 |
| 2.833 | 19100.295 | 2.643 | 13.259 | 0.174074 | 7/15/2014 | 26:22.0 | 3 |
| 2.566 | 19100.326 | 2.634 | 13.273 | 0.192713 | 7/15/2014 | 26:22.1 | 3 |
| 2.302 | 19100.356 | 2.622 | 13.285 | 0.19018 | 7/15/2014 | 26:22.2 | 3 |
| 2.039 | 19100.388 | 2.613 | 13.296 | 0.171073 | 7/15/2014 | 26:22.3 | 3 |
| 1.777 | 19100.417 | 2.605 | 13.312 | 0.175147 | 7/15/2014 | 26:22.4 | 3 |
| 1.515 | 19100.448 | 2.602 | 13.325 | 0.174959 | 7/15/2014 | 26:22.5 | 3 |
| 1.259 | 19100.477 | 2.59 | 13.338 | 0.179201 | 7/15/2014 | 26:22.6 | 3 |
| 1.001 | 19100.506 | 2.587 | 13.352 | 0.179001 | 7/15/2014 | 26:22.7 | 3 |
| 0.686 | 19100.543 | 2.577 | 13.361 | 0.177635 | 7/15/2014 | 26:22.8 | 3 |
| 0.431 | 19100.571 | 2.569 | 13.371 | 0.19038 | 7/15/2014 | 26:22.9 | 3 |
| 0.177 | 19100.602 | 2.556 | 13.382 | 0.18245 | 7/15/2014 | 26:23.0 | 3 |
| -0.077 | 19100.631 | 2.546 | 13.395 | 0.185881 | 7/15/2014 | 26:23.1 | 3 |
| -0.328 | 19100.658 | 2.53 | 13.407 | 0.189839 | 7/15/2014 | 26:23.2 | 3 |
| -0.583 | 19100.688 | 2.53 | 13.419 | 0.185591 | 7/15/2014 | 26:23.3 | 3 |
| -0.896 | 19100.723 | 2.522 | 13.429 | 0.183419 | 7/15/2014 | 26:23.4 | 3 |
| -1.152 | 19100.752 | 2.518 | 13.443 | 0.185492 | 7/15/2014 | 26:23.5 | 3 |
| -1.408 | 19100.783 | 2.509 | 13.453 | 0.183255 | 7/15/2014 | 26:23.6 | 3 |
| -1.666 | 19100.811 | 2.505 | 13.463 | 0.18306 | 7/15/2014 | 26:23.7 | 3 |
| -1.925 | 19100.84 | 2.496 | 13.469 | 0.183165 | 7/15/2014 | 26:23.8 | 3 |
| -2.187 | 19100.869 | 2.494 | 13.479 | 0.183696 | 7/15/2014 | 26:23.9 | 3 |
| -2.446 | 19100.896 | 2.479 | 13.489 | 0.181156 | 7/15/2014 | 26:24.0 | 3 |
| -2.71 | 19100.928 | 2.473 | 13.499 | 0.182749 | 7/15/2014 | 26:24.1 | 3 |
| -2.97 | 19100.958 | 2.465 | 13.503 | 0.18468 | 7/15/2014 | 26:24.2 | 3 |
| -3.231 | 19100.986 | 2.459 | 13.51 | 0.18412 | 7/15/2014 | 26:24.3 | 3 |
| -3.492 | 19101.016 | 2.451 | 13.514 | 0.183049 | 7/15/2014 | 26:24.4 | 3 |
| -3.751 | 19101.043 | 2.45 | 13.523 | 0.184843 | 7/15/2014 | 26:24.5 | 3 |
| -4.007 | 19101.072 | 2.446 | 13.53 | 0.185028 | 7/15/2014 | 26:24.6 | 3 |
| -4.265 | 19101.102 | 2.443 | 13.535 | 0.199017 | 7/15/2014 | 26:24.7 | 3 |
| -4.581 | 19101.136 | 2.433 | 13.535 | 0.178196 | 7/15/2014 | 26:24.8 | 3 |
| -4.837 | 19101.164 | 2.429 | 13.541 | 0.171827 | 7/15/2014 | 26:24.9 | 3 |
| -5.091 | 19101.191 | 2.423 | 13.546 | 0.175847 | 7/15/2014 | 26:25.0 | 3 |
| -5.348 | 19101.221 | 2.423 | 13.554 | 0.16936 | 7/15/2014 | 26:25.1 | 3 |
| -5.603 | 19101.25 | 2.41 | 13.555 | 0.169546 | 7/15/2014 | 26:25.2 | 3 |
| -5.86 | 19101.277 | 2.404 | 13.555 | 0.160039 | 7/15/2014 | 26:25.3 | 3 |

| | | | | | | | |
|---|---|---|---|---|---|---|---|
| -6.177 | 19101.312 | 2.401 | 13.56 | 0.161213 | 7/15/2014 | 26:25.4 | 3 |
| -6.437 | 19101.34 | 2.401 | 13.565 | 0.154043 | 7/15/2014 | 26:25.5 | 3 |
| -6.694 | 19101.367 | 2.396 | 13.572 | 0.157253 | 7/15/2014 | 26:25.6 | 3 |
| -6.957 | 19101.398 | 2.396 | 13.575 | 0.153917 | 7/15/2014 | 26:25.7 | 3 |
| -7.217 | 19101.426 | 2.391 | 13.579 | 0.145666 | 7/15/2014 | 26:25.8 | 3 |
| -7.48 | 19101.453 | 2.396 | 13.582 | 0.141996 | 7/15/2014 | 26:25.9 | 3 |
| -7.742 | 19101.481 | 2.394 | 13.589 | 0.148363 | 7/15/2014 | 26:26.0 | 3 |
| -8.005 | 19101.512 | 2.394 | 13.591 | 0.143994 | 7/15/2014 | 26:26.1 | 3 |
| -8.263 | 19101.539 | 2.385 | 13.598 | 0.149513 | 7/15/2014 | 26:26.2 | 3 |
| -8.526 | 19101.567 | 2.383 | 13.599 | 0.141001 | 7/15/2014 | 26:26.3 | 3 |
| -8.782 | 19101.595 | 2.374 | 13.613 | 0.137337 | 7/15/2014 | 26:26.4 | 3 |
| -9.038 | 19101.621 | 2.368 | 13.62 | 0.16 | 7/15/2014 | 26:26.5 | 3 |
| -9.292 | 19101.648 | 2.356 | 13.629 | 0.137935 | 7/15/2014 | 26:26.6 | 3 |
| -9.545 | 19101.678 | 2.35 | 13.634 | 0.178004 | 7/15/2014 | 26:26.7 | 3 |
| -9.857 | 19101.711 | 2.338 | 13.639 | 0.148258 | 7/15/2014 | 26:26.8 | 3 |
| -10.11 | 19101.737 | 2.338 | 13.645 | 0.153154 | 7/15/2014 | 26:26.9 | 3 |
| -10.361 | 19101.765 | 2.334 | 13.657 | 0.172338 | 7/15/2014 | 26:27.0 | 3 |
| -10.613 | 19101.789 | 2.334 | 13.657 | 0.121764 | 7/15/2014 | 26:27.1 | 3 |
| -10.866 | 19101.816 | 2.334 | 13.664 | 0.12621 | 7/15/2014 | 26:27.2 | 3 |
| -11.119 | 19101.844 | 2.334 | 13.671 | 0.104753 | 7/15/2014 | 26:27.3 | 3 |
| -11.435 | 19101.878 | 2.331 | 13.676 | 0.102688 | 7/15/2014 | 26:27.4 | 3 |
| -11.691 | 19101.905 | 2.331 | 13.681 | 0.0906926 | 7/15/2014 | 26:27.5 | 3 |
| -11.949 | 19101.933 | 2.325 | 13.684 | 0.0829456 | 7/15/2014 | 26:27.6 | 3 |
| -12.209 | 19101.959 | 2.322 | 13.681 | 0.0923416 | 7/15/2014 | 26:27.7 | 3 |
| -12.472 | 19101.986 | 2.319 | 13.681 | 0.0993022 | 7/15/2014 | 26:27.8 | 3 |
| -12.735 | 19102.014 | 2.319 | 13.678 | 0.0960861 | 7/15/2014 | 26:27.9 | 3 |
| -12.998 | 19102.042 | 2.316 | 13.676 | 0.0983339 | 7/15/2014 | 26:28.0 | 3 |
| -13.262 | 19102.069 | 2.314 | 13.679 | 0.10825 | 7/15/2014 | 26:28.1 | 3 |
| -13.525 | 19102.097 | 2.312 | 13.68 | 0.123358 | 7/15/2014 | 26:28.2 | 3 |
| -13.788 | 19102.125 | 2.318 | 13.682 | 0.121902 | 7/15/2014 | 26:28.3 | 3 |
| -14.049 | 19102.151 | 2.314 | 13.683 | 0.128559 | 7/15/2014 | 26:28.4 | 3 |
| -14.308 | 19102.178 | 2.315 | 13.683 | 0.120881 | 7/15/2014 | 26:28.5 | 3 |
| -14.564 | 19102.204 | 2.309 | 13.687 | 0.120586 | 7/15/2014 | 26:28.6 | 3 |
| -14.819 | 19102.23 | 2.313 | 13.689 | 0.13025 | 7/15/2014 | 26:28.7 | 3 |
| -15.131 | 19102.263 | 2.31 | 13.686 | 0.117863 | 7/15/2014 | 26:28.8 | 3 |
| -15.384 | 19102.289 | 2.314 | 13.687 | 0.122952 | 7/15/2014 | 26:28.9 | 3 |
| -15.632 | 19102.313 | 2.308 | 13.688 | 0.102444 | 7/15/2014 | 26:29.0 | 3 |
| -15.881 | 19102.339 | 2.308 | 13.691 | 0.0985978 | 7/15/2014 | 26:29.1 | 3 |
| -16.126 | 19102.364 | 2.299 | 13.692 | 0.0984069 | 7/15/2014 | 26:29.2 | 3 |
| -16.373 | 19102.389 | 2.299 | 13.693 | 0.0955221 | 7/15/2014 | 26:29.3 | 3 |
| -16.798 | 19102.433 | 2.292 | 13.699 | 0.111829 | 7/15/2014 | 26:29.4 | 3 |
| -17.04 | 19102.457 | 2.29 | 13.703 | 0.0910916 | 7/15/2014 | 26:29.5 | 3 |
| -17.282 | 19102.48 | 2.285 | 13.707 | 0.123419 | 7/15/2014 | 26:29.6 | 3 |
| -17.583 | 19102.512 | 2.28 | 13.71 | 0.119107 | 7/15/2014 | 26:29.7 | 3 |
| -17.704 | 19102.524 | 2.278 | 13.711 | 0.120565 | 7/15/2014 | 26:29.8 | 3 |
| -17.948 | 19102.548 | 2.28 | 13.715 | 0.117296 | 7/15/2014 | 26:29.9 | 3 |
| -18.189 | 19102.572 | 2.275 | 13.721 | 0.138939 | 7/15/2014 | 26:30.0 | 3 |

| | | | | | | | |
|---|---|---|---|---|---|---|---|
| -18.432 | 19102.598 | 2.278 | 13.727 | 0.110164 | 7/15/2014 | 26:30.1 | 3 |
| -18.673 | 19102.621 | 2.273 | 13.733 | 0.123511 | 7/15/2014 | 26:30.2 | 3 |
| -18.915 | 19102.645 | 2.275 | 13.739 | 0.0888999 | 7/15/2014 | 26:30.3 | 3 |
| -19.333 | 19102.688 | 2.274 | 13.749 | 0.0969601 | 7/15/2014 | 26:30.4 | 3 |
| -19.572 | 19102.711 | 2.274 | 13.754 | 0.107621 | 7/15/2014 | 26:30.5 | 3 |
| -19.811 | 19102.734 | 2.274 | 13.76 | 0.107477 | 7/15/2014 | 26:30.6 | 3 |
| -20.109 | 19102.765 | 2.276 | 13.765 | 0.0968685 | 7/15/2014 | 26:30.7 | 3 |
| -20.23 | 19102.776 | 2.275 | 13.764 | 0.102624 | 7/15/2014 | 26:30.8 | 3 |
| -20.469 | 19102.799 | 2.274 | 13.771 | 0.127664 | 7/15/2014 | 26:30.9 | 3 |
| -20.71 | 19102.822 | 2.274 | 13.772 | 0.123766 | 7/15/2014 | 26:31.0 | 3 |
| -20.951 | 19102.847 | 2.276 | 13.773 | 0.124441 | 7/15/2014 | 26:31.1 | 3 |
| -21.191 | 19102.87 | 2.274 | 13.776 | 0.110246 | 7/15/2014 | 26:31.2 | 3 |
| -21.431 | 19102.894 | 2.276 | 13.778 | 0.107471 | 7/15/2014 | 26:31.3 | 3 |
| -21.85 | 19102.935 | 2.274 | 13.782 | 0.132211 | 7/15/2014 | 26:31.4 | 3 |
| -22.087 | 19102.957 | 2.274 | 13.786 | 0.116637 | 7/15/2014 | 26:31.5 | 3 |
| -22.327 | 19102.979 | 2.28 | 13.788 | 0.121532 | 7/15/2014 | 26:31.6 | 3 |
| -22.624 | 19103.008 | 2.272 | 13.794 | 0.126072 | 7/15/2014 | 26:31.7 | 3 |
| -22.745 | 19103.02 | 2.27 | 13.796 | 0.130432 | 7/15/2014 | 26:31.8 | 3 |
| -22.987 | 19103.043 | 2.27 | 13.809 | 0.140835 | 7/15/2014 | 26:31.9 | 3 |
| -23.226 | 19103.065 | 2.264 | 13.821 | 0.145721 | 7/15/2014 | 26:32.0 | 3 |
| -23.468 | 19103.089 | 2.264 | 13.831 | 0.155634 | 7/15/2014 | 26:32.1 | 3 |
| -23.708 | 19103.111 | 2.262 | 13.84 | 0.176063 | 7/15/2014 | 26:32.2 | 3 |
| -23.948 | 19103.134 | 2.262 | 13.849 | 0.182823 | 7/15/2014 | 26:32.3 | 3 |
| -24.363 | 19103.172 | 2.256 | 13.861 | 0.181329 | 7/15/2014 | 26:32.4 | 3 |
| -24.6 | 19103.195 | 2.259 | 13.871 | 0.176324 | 7/15/2014 | 26:32.5 | 3 |
| -24.836 | 19103.216 | 2.258 | 13.876 | 0.196926 | 7/15/2014 | 26:32.6 | 3 |
| -25.134 | 19103.244 | 2.258 | 13.881 | 0.171456 | 7/15/2014 | 26:32.7 | 3 |
| -25.254 | 19103.255 | 2.257 | 13.885 | 0.159522 | 7/15/2014 | 26:32.8 | 3 |
| -25.495 | 19103.277 | 2.257 | 13.89 | 0.13784 | 7/15/2014 | 26:32.9 | 3 |
| -25.73 | 19103.3 | 2.253 | 13.893 | 0.15569 | 7/15/2014 | 26:33.0 | 3 |
| -25.971 | 19103.32 | 2.253 | 13.895 | 0.167267 | 7/15/2014 | 26:33.1 | 3 |
| -26.208 | 19103.344 | 2.249 | 13.901 | 0.138001 | 7/15/2014 | 26:33.2 | 3 |
| -26.447 | 19103.364 | 2.253 | 13.902 | 0.127217 | 7/15/2014 | 26:33.3 | 3 |
| -26.861 | 19103.402 | 2.248 | 13.901 | 0.125087 | 7/15/2014 | 26:33.4 | 3 |
| -27.099 | 19103.424 | 2.248 | 13.9 | 0.13701 | 7/15/2014 | 26:33.5 | 3 |
| -27.335 | 19103.445 | 2.25 | 13.902 | 0.131013 | 7/15/2014 | 26:33.6 | 3 |
| -27.627 | 19103.471 | 2.25 | 13.905 | 0.12294 | 7/15/2014 | 26:33.7 | 3 |
| -27.747 | 19103.482 | 2.25 | 13.907 | 0.139378 | 7/15/2014 | 26:33.8 | 3 |
| -27.987 | 19103.503 | 2.256 | 13.909 | 0.147232 | 7/15/2014 | 26:33.9 | 3 |
| -28.221 | 19103.524 | 2.252 | 13.911 | 0.158019 | 7/15/2014 | 26:34.0 | 3 |
| -28.458 | 19103.546 | 2.262 | 13.912 | 0.16847 | 7/15/2014 | 26:34.1 | 3 |
| -28.692 | 19103.566 | 2.259 | 13.919 | 0.191747 | 7/15/2014 | 26:34.2 | 3 |
| -28.928 | 19103.586 | 2.259 | 13.925 | 0.170586 | 7/15/2014 | 26:34.3 | 3 |
| -29.334 | 19103.623 | 2.253 | 13.937 | 0.183939 | 7/15/2014 | 26:34.4 | 3 |
| -29.567 | 19103.643 | 2.251 | 13.943 | 0.15848 | 7/15/2014 | 26:34.5 | 3 |
| -29.797 | 19103.663 | 2.248 | 13.948 | 0.133627 | 7/15/2014 | 26:34.6 | 3 |
| -30.088 | 19103.688 | 2.248 | 13.956 | 0.152175 | 7/15/2014 | 26:34.7 | 3 |

| | | | | | | | |
|---|---|---|---|---|---|---|---|
| -30.204 | 19103.698 | 2.243 | 13.961 | 0.141427 | 7/15/2014 | 26:34.8 | 3 |
| -30.436 | 19103.718 | 2.246 | 13.968 | 0.130995 | 7/15/2014 | 26:34.9 | 3 |
| -30.669 | 19103.738 | 2.248 | 13.976 | 0.125539 | 7/15/2014 | 26:35.0 | 3 |
| -30.902 | 19103.76 | 2.248 | 13.985 | 0.121774 | 7/15/2014 | 26:35.1 | 3 |
| -31.135 | 19103.779 | 2.252 | 13.99 | 0.112133 | 7/15/2014 | 26:35.2 | 3 |
| -31.366 | 19103.799 | 2.254 | 13.997 | 0.130798 | 7/15/2014 | 26:35.3 | 3 |
| -31.772 | 19103.833 | 2.252 | 14.013 | 0.0941477 | 7/15/2014 | 26:35.4 | 3 |
| -32.002 | 19103.853 | 2.252 | 14.022 | 0.119391 | 7/15/2014 | 26:35.5 | 3 |
| -32.234 | 19103.872 | 2.252 | 14.033 | 0.108284 | 7/15/2014 | 26:35.6 | 3 |
| -32.522 | 19103.896 | 2.25 | 14.042 | 0.085692 | 7/15/2014 | 26:35.7 | 3 |
| -32.64 | 19103.906 | 2.252 | 14.045 | 0.0971647 | 7/15/2014 | 26:35.8 | 3 |
| -32.872 | 19103.926 | 2.248 | 14.058 | 0.0912542 | 7/15/2014 | 26:35.9 | 3 |
| -33.105 | 19103.945 | 2.246 | 14.068 | 0.102175 | 7/15/2014 | 26:36.0 | 3 |
| -33.338 | 19103.965 | 2.248 | 14.08 | 0.113249 | 7/15/2014 | 26:36.1 | 3 |
| -33.568 | 19103.984 | 2.24 | 14.087 | 0.104428 | 7/15/2014 | 26:36.2 | 3 |
| -33.802 | 19104.004 | 2.242 | 14.095 | 0.122026 | 7/15/2014 | 26:36.3 | 3 |
| -34.206 | 19104.035 | 2.24 | 14.111 | 0.14929 | 7/15/2014 | 26:36.4 | 3 |
| -34.435 | 19104.055 | 2.237 | 14.124 | 0.132797 | 7/15/2014 | 26:36.5 | 3 |
| -34.666 | 19104.074 | 2.237 | 14.133 | 0.12936 | 7/15/2014 | 26:36.6 | 3 |
| -34.953 | 19104.098 | 2.237 | 14.151 | 0.111417 | 7/15/2014 | 26:36.7 | 3 |
| -35.069 | 19104.107 | 2.235 | 14.155 | 0.104236 | 7/15/2014 | 26:36.8 | 3 |
| -35.416 | 19104.136 | 2.241 | 14.172 | 0.103617 | 7/15/2014 | 26:36.9 | 3 |
| -35.649 | 19104.152 | 2.238 | 14.185 | 0.105347 | 7/15/2014 | 26:37.0 | 3 |
| -35.882 | 19104.172 | 2.241 | 14.193 | 0.0954789 | 7/15/2014 | 26:37.1 | 3 |
| -35.996 | 19104.182 | 2.238 | 14.199 | 0.101229 | 7/15/2014 | 26:37.2 | 3 |
| -36.228 | 19104.201 | 2.243 | 14.205 | 0.0893653 | 7/15/2014 | 26:37.3 | 3 |
| -36.632 | 19104.232 | 2.243 | 14.214 | 0.0916568 | 7/15/2014 | 26:37.4 | 3 |
| -36.862 | 19104.252 | 2.241 | 14.214 | 0.107746 | 7/15/2014 | 26:37.5 | 3 |
| -37.092 | 19104.269 | 2.241 | 14.22 | 0.099043 | 7/15/2014 | 26:37.6 | 3 |
| -37.382 | 19104.291 | 2.243 | 14.226 | 0.0904613 | 7/15/2014 | 26:37.7 | 3 |
| -37.499 | 19104.301 | 2.243 | 14.227 | 0.0847443 | 7/15/2014 | 26:37.8 | 3 |
| -37.732 | 19104.32 | 2.246 | 14.229 | 0.0946003 | 7/15/2014 | 26:37.9 | 3 |
| -37.966 | 19104.339 | 2.243 | 14.232 | 0.108058 | 7/15/2014 | 26:38.0 | 3 |
| -38.2 | 19104.355 | 2.246 | 14.237 | 0.13087 | 7/15/2014 | 26:38.1 | 3 |
| -38.433 | 19104.375 | 2.246 | 14.241 | 0.121692 | 7/15/2014 | 26:38.2 | 3 |
| -38.668 | 19104.391 | 2.251 | 14.243 | 0.14723 | 7/15/2014 | 26:38.3 | 3 |
| -39.076 | 19104.424 | 2.247 | 14.248 | 0.148711 | 7/15/2014 | 26:38.4 | 3 |
| -39.309 | 19104.441 | 2.249 | 14.249 | 0.147515 | 7/15/2014 | 26:38.5 | 3 |
| -39.542 | 19104.461 | 2.249 | 14.252 | 0.130718 | 7/15/2014 | 26:38.6 | 3 |
| -39.833 | 19104.48 | 2.249 | 14.254 | 0.136777 | 7/15/2014 | 26:38.7 | 3 |
| -39.951 | 19104.49 | 2.248 | 14.253 | 0.114405 | 7/15/2014 | 26:38.8 | 3 |
| -40.186 | 19104.508 | 2.246 | 14.254 | 0.123179 | 7/15/2014 | 26:38.9 | 3 |
| -40.42 | 19104.526 | 2.241 | 14.259 | 0.139901 | 7/15/2014 | 26:39.0 | 3 |
| -40.657 | 19104.545 | 2.243 | 14.261 | 0.134209 | 7/15/2014 | 26:39.1 | 3 |
| -40.892 | 19104.562 | 2.241 | 14.265 | 0.146991 | 7/15/2014 | 26:39.2 | 3 |
| -41.127 | 19104.58 | 2.241 | 14.268 | 0.171913 | 7/15/2014 | 26:39.3 | 3 |
| -41.536 | 19104.609 | 2.235 | 14.281 | 0.155502 | 7/15/2014 | 26:39.4 | 3 |

| | | | | | | | |
|---|---|---|---|---|---|---|---|
| -41.769 | 19104.628 | 2.233 | 14.287 | 0.162262 | 7/15/2014 | 26:39.5 | 3 |
| -42.003 | 19104.646 | 2.233 | 14.293 | 0.172487 | 7/15/2014 | 26:39.6 | 3 |
| -42.297 | 19104.667 | 2.229 | 14.298 | 0.155689 | 7/15/2014 | 26:39.7 | 3 |
| -42.415 | 19104.676 | 2.228 | 14.301 | 0.157573 | 7/15/2014 | 26:39.8 | 3 |
| -42.652 | 19104.693 | 2.23 | 14.307 | 0.128197 | 7/15/2014 | 26:39.9 | 3 |
| -42.887 | 19104.712 | 2.222 | 14.311 | 0.124387 | 7/15/2014 | 26:40.0 | 3 |
| -43.125 | 19104.729 | 2.228 | 14.316 | 0.13928 | 7/15/2014 | 26:40.1 | 3 |
| -43.36 | 19104.746 | 2.219 | 14.317 | 0.108997 | 7/15/2014 | 26:40.2 | 3 |
| -43.598 | 19104.764 | 2.218 | 14.326 | 0.116302 | 7/15/2014 | 26:40.3 | 3 |
| -44.009 | 19104.793 | 2.216 | 14.334 | 0.0899916 | 7/15/2014 | 26:40.4 | 3 |
| -44.245 | 19104.811 | 2.216 | 14.341 | 0.096395 | 7/15/2014 | 26:40.5 | 3 |
| -44.479 | 19104.827 | 2.212 | 14.348 | 0.077622 | 7/15/2014 | 26:40.6 | 3 |
| -44.774 | 19104.849 | 2.215 | 14.354 | 0.0664866 | 7/15/2014 | 26:40.7 | 3 |
| -44.892 | 19104.857 | 2.212 | 14.359 | 0.0722708 | 7/15/2014 | 26:40.8 | 3 |
| -45.131 | 19104.875 | 2.214 | 14.364 | 0.0564408 | 7/15/2014 | 26:40.9 | 3 |
| -45.365 | 19104.891 | 2.212 | 14.368 | 0.0597768 | 7/15/2014 | 26:41.0 | 3 |
| -45.602 | 19104.908 | 2.212 | 14.375 | 0.0896258 | 7/15/2014 | 26:41.1 | 3 |
| -45.838 | 19104.926 | 2.212 | 14.38 | 0.0584268 | 7/15/2014 | 26:41.2 | 3 |
| -46.074 | 19104.941 | 2.216 | 14.384 | 0.0805534 | 7/15/2014 | 26:41.3 | 3 |
| -46.485 | 19104.972 | 2.21 | 14.402 | 0.0918309 | 7/15/2014 | 26:41.4 | 3 |
| -46.72 | 19104.988 | 2.207 | 14.41 | 0.0778402 | 7/15/2014 | 26:41.5 | 3 |
| -46.953 | 19105.005 | 2.208 | 14.417 | 0.104074 | 7/15/2014 | 26:41.6 | 3 |
| -47.246 | 19105.026 | 2.211 | 14.423 | 0.070037 | 7/15/2014 | 26:41.7 | 3 |
| -47.365 | 19105.035 | 2.21 | 14.427 | 0.0926923 | 7/15/2014 | 26:41.8 | 3 |
| -47.601 | 19105.051 | 2.206 | 14.433 | 0.0824929 | 7/15/2014 | 26:41.9 | 3 |
| -47.837 | 19105.067 | 2.202 | 14.44 | 0.063012 | 7/15/2014 | 26:42.0 | 3 |
| -48.073 | 19105.085 | 2.206 | 14.433 | 0.0627605 | 7/15/2014 | 26:42.1 | 3 |
| -48.309 | 19105.102 | 2.206 | 14.437 | 0.0932393 | 7/15/2014 | 26:42.2 | 3 |
| -48.545 | 19105.119 | 2.212 | 14.439 | 0.0953775 | 7/15/2014 | 26:42.3 | 3 |
| -48.954 | 19105.148 | 2.203 | 14.441 | 0.116937 | 7/15/2014 | 26:42.4 | 3 |
| -49.186 | 19105.164 | 2.197 | 14.442 | 0.108274 | 7/15/2014 | 26:42.5 | 3 |
| -49.42 | 19105.182 | 2.197 | 14.441 | 0.14308 | 7/15/2014 | 26:42.6 | 3 |
| -49.711 | 19105.202 | 2.197 | 14.443 | 0.143712 | 7/15/2014 | 26:42.7 | 3 |
| -49.829 | 19105.211 | 2.194 | 14.444 | 0.13691 | 7/15/2014 | 26:42.8 | 3 |
| -50.064 | 19105.228 | 2.197 | 14.444 | 0.146397 | 7/15/2014 | 26:42.9 | 3 |
| -50.294 | 19105.244 | 2.188 | 14.447 | 0.149897 | 7/15/2014 | 26:43.0 | 3 |
| -50.531 | 19105.262 | 2.195 | 14.454 | 0.122528 | 7/15/2014 | 26:43.1 | 3 |
| -50.763 | 19105.277 | 2.195 | 14.459 | 0.133773 | 7/15/2014 | 26:43.2 | 3 |
| -50.996 | 19105.296 | 2.199 | 14.466 | 0.152981 | 7/15/2014 | 26:43.3 | 3 |
| -51.402 | 19105.325 | 2.198 | 14.478 | 0.154122 | 7/15/2014 | 26:43.4 | 3 |
| -51.633 | 19105.341 | 2.198 | 14.482 | 0.115157 | 7/15/2014 | 26:43.5 | 3 |
| -51.863 | 19105.358 | 2.198 | 14.49 | 0.127858 | 7/15/2014 | 26:43.6 | 3 |
| -52.152 | 19105.379 | 2.201 | 14.493 | 0.144608 | 7/15/2014 | 26:43.7 | 3 |
| -52.269 | 19105.387 | 2.199 | 14.494 | 0.15057 | 7/15/2014 | 26:43.8 | 3 |
| -52.504 | 19105.405 | 2.202 | 14.498 | 0.117008 | 7/15/2014 | 26:43.9 | 3 |
| -52.736 | 19105.422 | 2.199 | 14.503 | 0.137534 | 7/15/2014 | 26:44.0 | 3 |
| -52.972 | 19105.439 | 2.199 | 14.509 | 0.119117 | 7/15/2014 | 26:44.1 | 3 |

| | | | | | | | |
|---|---|---|---|---|---|---|---|
| -53.206 | 19105.456 | 2.199 | 14.521 | 0.101693 | 7/15/2014 | 26:44.2 | 3 |
| -53.439 | 19105.474 | 2.199 | 14.526 | 0.118846 | 7/15/2014 | 26:44.3 | 3 |
| -53.846 | 19105.502 | 2.198 | 14.532 | 0.103324 | 7/15/2014 | 26:44.4 | 3 |
| -54.081 | 19105.521 | 2.201 | 14.54 | 0.0768766 | 7/15/2014 | 26:44.5 | 3 |
| -54.313 | 19105.537 | 2.198 | 14.547 | 0.0701448 | 7/15/2014 | 26:44.6 | 3 |
| -54.607 | 19105.559 | 2.201 | 14.552 | 0.0666887 | 7/15/2014 | 26:44.7 | 3 |
| -54.725 | 19105.567 | 2.202 | 14.554 | 0.0868106 | 7/15/2014 | 26:44.8 | 3 |
| -54.961 | 19105.585 | 2.202 | 14.551 | 0.0701534 | 7/15/2014 | 26:44.9 | 3 |
| -55.196 | 19105.603 | 2.206 | 14.558 | 0.0665215 | 7/15/2014 | 26:45.0 | 3 |
| -55.434 | 19105.619 | 2.206 | 14.564 | 0.0597449 | 7/15/2014 | 26:45.1 | 3 |
| -55.671 | 19105.638 | 2.202 | 14.567 | 0.082846 | 7/15/2014 | 26:45.2 | 3 |
| -55.908 | 19105.654 | 2.202 | 14.572 | 0.0899169 | 7/15/2014 | 26:45.3 | 3 |
| -56.321 | 19105.685 | 2.205 | 14.576 | 0.0902634 | 7/15/2014 | 26:45.4 | 3 |
| -56.555 | 19105.702 | 2.201 | 14.58 | 0.0882448 | 7/15/2014 | 26:45.5 | 3 |
| -56.792 | 19105.72 | 2.205 | 14.583 | 0.09433 | 7/15/2014 | 26:45.6 | 3 |
| -57.087 | 19105.741 | 2.201 | 14.581 | 0.0849929 | 7/15/2014 | 26:45.7 | 3 |
| -57.207 | 19105.75 | 2.202 | 14.582 | 0.0852254 | 7/15/2014 | 26:45.8 | 3 |
| -57.445 | 19105.768 | 2.206 | 14.588 | 0.0853208 | 7/15/2014 | 26:45.9 | 3 |
| -57.683 | 19105.785 | 2.206 | 14.584 | 0.0798207 | 7/15/2014 | 26:46.0 | 3 |
| -57.922 | 19105.803 | 2.214 | 14.584 | 0.0975941 | 7/15/2014 | 26:46.1 | 3 |
| -58.157 | 19105.82 | 2.211 | 14.582 | 0.0843706 | 7/15/2014 | 26:46.2 | 3 |
| -58.397 | 19105.838 | 2.223 | 14.58 | 0.0772514 | 7/15/2014 | 26:46.3 | 3 |
| -58.809 | 19105.868 | 2.225 | 14.581 | 0.11875 | 7/15/2014 | 26:46.4 | 3 |
| -59.043 | 19105.887 | 2.228 | 14.582 | 0.116802 | 7/15/2014 | 26:46.5 | 3 |
| -59.279 | 19105.903 | 2.23 | 14.575 | 0.118929 | 7/15/2014 | 26:46.6 | 3 |
| -59.571 | 19105.926 | 2.23 | 14.575 | 0.111386 | 7/15/2014 | 26:46.7 | 3 |
| -59.69 | 19105.935 | 2.227 | 14.574 | 0.128816 | 7/15/2014 | 26:46.8 | 3 |
| -59.925 | 19105.951 | 2.233 | 14.585 | 0.133252 | 7/15/2014 | 26:46.9 | 3 |
| -60.159 | 19105.97 | 2.228 | 14.582 | 0.118581 | 7/15/2014 | 26:47.0 | 3 |
| -60.393 | 19105.986 | 2.228 | 14.577 | 0.1168 | 7/15/2014 | 26:47.1 | 3 |
| -60.626 | 19106.005 | 2.224 | 14.572 | 0.107571 | 7/15/2014 | 26:47.2 | 3 |
| -60.86 | 19106.021 | 2.227 | 14.568 | 0.11411 | 7/15/2014 | 26:47.3 | 3 |
| -61.262 | 19106.053 | 2.226 | 14.567 | 0.112831 | 7/15/2014 | 26:47.4 | 3 |
| -61.492 | 19106.07 | 2.226 | 14.565 | 0.119034 | 7/15/2014 | 26:47.5 | 3 |
| -61.722 | 19106.088 | 2.225 | 14.554 | 0.0996615 | 7/15/2014 | 26:47.6 | 3 |
| -62.008 | 19106.109 | 2.225 | 14.55 | 0.0944484 | 7/15/2014 | 26:47.7 | 3 |
| -62.239 | 19106.126 | 2.223 | 14.548 | 0.0957968 | 7/15/2014 | 26:47.8 | 3 |
| -62.47 | 19106.145 | 2.228 | 14.548 | 0.11275 | 7/15/2014 | 26:47.9 | 3 |
| -62.703 | 19106.162 | 2.233 | 14.546 | 0.120027 | 7/15/2014 | 26:48.0 | 3 |
| -62.934 | 19106.18 | 2.233 | 14.543 | 0.102305 | 7/15/2014 | 26:48.1 | 3 |
| -63.165 | 19106.199 | 2.233 | 14.541 | 0.0979407 | 7/15/2014 | 26:48.2 | 3 |
| -63.278 | 19106.207 | 2.235 | 14.54 | 0.101462 | 7/15/2014 | 26:48.3 | 3 |
| -63.682 | 19106.238 | 2.241 | 14.541 | 0.0987904 | 7/15/2014 | 26:48.4 | 3 |
| -63.911 | 19106.256 | 2.246 | 14.544 | 0.106027 | 7/15/2014 | 26:48.5 | 3 |
| -64.143 | 19106.275 | 2.248 | 14.546 | 0.101857 | 7/15/2014 | 26:48.6 | 3 |
| -64.432 | 19106.298 | 2.248 | 14.545 | 0.0958978 | 7/15/2014 | 26:48.7 | 3 |
| -64.547 | 19106.307 | 2.244 | 14.547 | 0.0974754 | 7/15/2014 | 26:48.8 | 3 |

| | | | | | | | |
|---|---|---|---|---|---|---|---|
| -64.782 | 19106.324 | 2.248 | 14.551 | 0.113592 | 7/15/2014 | 26:48.9 | 3 |
| -65.015 | 19106.344 | 2.248 | 14.555 | 0.107897 | 7/15/2014 | 26:49.0 | 3 |
| -65.25 | 19106.361 | 2.252 | 14.561 | 0.127566 | 7/15/2014 | 26:49.1 | 3 |
| -65.484 | 19106.381 | 2.246 | 14.561 | 0.131328 | 7/15/2014 | 26:49.2 | 3 |
| -65.721 | 19106.4 | 2.245 | 14.563 | 0.105514 | 7/15/2014 | 26:49.3 | 3 |
| -66.129 | 19106.433 | 2.247 | 14.569 | 0.119754 | 7/15/2014 | 26:49.4 | 3 |
| -66.364 | 19106.45 | 2.247 | 14.579 | 0.106314 | 7/15/2014 | 26:49.5 | 3 |
| -66.597 | 19106.469 | 2.248 | 14.583 | 0.109746 | 7/15/2014 | 26:49.6 | 3 |
| -66.889 | 19106.493 | 2.248 | 14.583 | 0.0992203 | 7/15/2014 | 26:49.7 | 3 |
| -67.008 | 19106.503 | 2.246 | 14.585 | 0.0891685 | 7/15/2014 | 26:49.8 | 3 |
| -67.245 | 19106.521 | 2.25 | 14.589 | 0.105739 | 7/15/2014 | 26:49.9 | 3 |
| -67.48 | 19106.541 | 2.247 | 14.592 | 0.102571 | 7/15/2014 | 26:50.0 | 3 |
| -67.716 | 19106.56 | 2.25 | 14.596 | 0.0928126 | 7/15/2014 | 26:50.1 | 3 |
| -67.95 | 19106.579 | 2.246 | 14.595 | 0.0817347 | 7/15/2014 | 26:50.2 | 3 |
| -68.184 | 19106.598 | 2.252 | 14.586 | 0.0775253 | 7/15/2014 | 26:50.3 | 3 |
| -68.592 | 19106.632 | 2.252 | 14.587 | 0.0679797 | 7/15/2014 | 26:50.4 | 3 |
| -68.823 | 19106.65 | 2.257 | 14.589 | 0.066865 | 7/15/2014 | 26:50.5 | 3 |
| -69.054 | 19106.67 | 2.26 | 14.587 | 0.0762978 | 7/15/2014 | 26:50.6 | 3 |
| -69.341 | 19106.693 | 2.26 | 14.593 | 0.0667818 | 7/15/2014 | 26:50.7 | 3 |
| -69.458 | 19106.703 | 2.26 | 14.591 | 0.0610763 | 7/15/2014 | 26:50.8 | 3 |
| -69.802 | 19106.73 | 2.26 | 14.588 | 0.058884 | 7/15/2014 | 26:50.9 | 3 |
| -70.034 | 19106.75 | 2.26 | 14.592 | 0.0449365 | 7/15/2014 | 26:51.0 | 3 |
| -70.265 | 19106.77 | 2.265 | 14.594 | 0.0437862 | 7/15/2014 | 26:51.1 | 3 |
| -70.494 | 19106.789 | 2.265 | 14.592 | 0.0750609 | 7/15/2014 | 26:51.2 | 3 |
| -70.721 | 19106.809 | 2.263 | 14.594 | 0.0948284 | 7/15/2014 | 26:51.3 | 3 |
| -71.004 | 19106.832 | 2.265 | 14.594 | 0.12215 | 7/15/2014 | 26:51.4 | 3 |
| -71.23 | 19106.852 | 2.267 | 14.591 | 0.150648 | 7/15/2014 | 26:51.5 | 3 |
| -71.456 | 19106.869 | 2.27 | 14.599 | 0.168165 | 7/15/2014 | 26:51.6 | 3 |
| -71.739 | 19106.893 | 2.275 | 14.601 | 0.176 | 7/15/2014 | 26:51.7 | 3 |
| -71.967 | 19106.912 | 2.275 | 14.601 | 0.19781 | 7/15/2014 | 26:51.8 | 3 |
| -72.196 | 19106.932 | 2.275 | 14.604 | 0.173665 | 7/15/2014 | 26:51.9 | 3 |
| -72.426 | 19106.951 | 2.281 | 14.609 | 0.194573 | 7/15/2014 | 26:52.0 | 3 |
| -72.655 | 19106.971 | 2.283 | 14.61 | 0.182516 | 7/15/2014 | 26:52.1 | 3 |
| -72.884 | 19106.99 | 2.281 | 14.615 | 0.156116 | 7/15/2014 | 26:52.2 | 3 |
| -73.113 | 19107.01 | 2.284 | 14.617 | 0.174546 | 7/15/2014 | 26:52.3 | 3 |
| -73.399 | 19107.033 | 2.287 | 14.618 | 0.144738 | 7/15/2014 | 26:52.4 | 3 |
| -73.626 | 19107.053 | 2.287 | 14.626 | 0.162265 | 7/15/2014 | 26:52.5 | 3 |
| -73.857 | 19107.072 | 2.29 | 14.633 | 0.150537 | 7/15/2014 | 26:52.6 | 3 |
| -74.145 | 19107.098 | 2.29 | 14.639 | 0.133388 | 7/15/2014 | 26:52.7 | 3 |
| -74.26 | 19107.107 | 2.286 | 14.642 | 0.129662 | 7/15/2014 | 26:52.8 | 3 |
| -74.494 | 19107.127 | 2.292 | 14.649 | 0.142983 | 7/15/2014 | 26:52.9 | 3 |
| -74.725 | 19107.146 | 2.292 | 14.656 | 0.127299 | 7/15/2014 | 26:53.0 | 3 |
| -74.962 | 19107.166 | 2.296 | 14.665 | 0.125968 | 7/15/2014 | 26:53.1 | 3 |
| -75.192 | 19107.186 | 2.292 | 14.675 | 0.121194 | 7/15/2014 | 26:53.2 | 3 |
| -75.429 | 19107.205 | 2.296 | 14.684 | 0.119942 | 7/15/2014 | 26:53.3 | 3 |
| -75.836 | 19107.242 | 2.29 | 14.692 | 0.118417 | 7/15/2014 | 26:53.4 | 3 |
| -76.07 | 19107.262 | 2.29 | 14.696 | 0.11714 | 7/15/2014 | 26:53.5 | 3 |

| | | | | | | | |
|---|---|---|---|---|---|---|---|
| -76.303 | 19107.281 | 2.285 | 14.701 | 0.11453 | 7/15/2014 | 26:53.6 | 3 |
| -76.598 | 19107.305 | 2.285 | 14.705 | 0.109453 | 7/15/2014 | 26:53.7 | 3 |
| -76.715 | 19107.314 | 2.282 | 14.707 | 0.109507 | 7/15/2014 | 26:53.8 | 3 |
| -76.955 | 19107.336 | 2.286 | 14.709 | 0.111834 | 7/15/2014 | 26:53.9 | 3 |
| -77.189 | 19107.355 | 2.276 | 14.711 | 0.106196 | 7/15/2014 | 26:54.0 | 3 |
| -77.429 | 19107.377 | 2.276 | 14.712 | 0.133494 | 7/15/2014 | 26:54.1 | 3 |
| -77.666 | 19107.396 | 2.276 | 14.714 | 0.12416 | 7/15/2014 | 26:54.2 | 3 |
| -77.904 | 19107.416 | 2.276 | 14.72 | 0.118571 | 7/15/2014 | 26:54.3 | 3 |
| -78.319 | 19107.451 | 2.276 | 14.731 | 0.114542 | 7/15/2014 | 26:54.4 | 3 |
| -78.557 | 19107.473 | 2.273 | 14.735 | 0.110255 | 7/15/2014 | 26:54.5 | 3 |
| -78.791 | 19107.492 | 2.27 | 14.742 | 0.092102 | 7/15/2014 | 26:54.6 | 3 |
| -79.086 | 19107.516 | 2.27 | 14.751 | 0.0908059 | 7/15/2014 | 26:54.7 | 3 |
| -79.207 | 19107.525 | 2.269 | 14.756 | 0.0925439 | 7/15/2014 | 26:54.8 | 3 |
| -79.445 | 19107.545 | 2.265 | 14.77 | 0.0768755 | 7/15/2014 | 26:54.9 | 3 |
| -79.682 | 19107.566 | 2.259 | 14.779 | 0.0856173 | 7/15/2014 | 26:55.0 | 3 |
| -79.921 | 19107.588 | 2.259 | 14.789 | 0.0667648 | 7/15/2014 | 26:55.1 | 3 |
| -80.156 | 19107.607 | 2.251 | 14.799 | 0.0709155 | 7/15/2014 | 26:55.2 | 3 |
| -80.392 | 19107.627 | 2.251 | 14.813 | 0.0721448 | 7/15/2014 | 26:55.3 | 3 |
| -80.801 | 19107.66 | 2.251 | 14.83 | 0.0463727 | 7/15/2014 | 26:55.4 | 3 |
| -81.034 | 19107.68 | 2.251 | 14.839 | 0.0584645 | 7/15/2014 | 26:55.5 | 3 |
| -81.266 | 19107.699 | 2.251 | 14.846 | 0.0265539 | 7/15/2014 | 26:55.6 | 3 |
| -81.555 | 19107.725 | 2.249 | 14.86 | 0.0603029 | 7/15/2014 | 26:55.7 | 3 |
| -81.672 | 19107.734 | 2.245 | 14.865 | 0.0416698 | 7/15/2014 | 26:55.8 | 3 |
| -81.906 | 19107.754 | 2.249 | 14.874 | 0.0305316 | 7/15/2014 | 26:55.9 | 3 |
| -82.252 | 19107.783 | 2.253 | 14.874 | 0.0342957 | 7/15/2014 | 26:56.0 | 3 |
| -82.483 | 19107.803 | 2.253 | 14.878 | 0.0284524 | 7/15/2014 | 26:56.1 | 3 |
| -82.712 | 19107.822 | 2.249 | 14.877 | 0.0242894 | 7/15/2014 | 26:56.2 | 3 |
| -82.941 | 19107.842 | 2.249 | 14.877 | 0.0123215 | 7/15/2014 | 26:56.3 | 3 |
| -83.225 | 19107.865 | 2.249 | 14.879 | 0.0102009 | 7/15/2014 | 26:56.4 | 3 |
| -83.451 | 19107.885 | 2.251 | 14.874 | 0.0196514 | 7/15/2014 | 26:56.5 | 3 |
| -83.679 | 19107.902 | 2.254 | 14.875 | 0.0216801 | 7/15/2014 | 26:56.6 | 3 |
| -83.962 | 19107.928 | 2.249 | 14.878 | 0.0391779 | 7/15/2014 | 26:56.7 | 3 |
| -84.191 | 19107.946 | 2.249 | 14.884 | 0.0497632 | 7/15/2014 | 26:56.8 | 3 |
| -84.42 | 19107.965 | 2.247 | 14.895 | 0.0907077 | 7/15/2014 | 26:56.9 | 3 |
| -84.651 | 19107.984 | 2.25 | 14.901 | 0.0505265 | 7/15/2014 | 26:57.0 | 3 |
| -84.881 | 19108.004 | 2.25 | 14.908 | 0.082103 | 7/15/2014 | 26:57.1 | 3 |
| -85.111 | 19108.023 | 2.25 | 14.913 | 0.0938097 | 7/15/2014 | 26:57.2 | 3 |
| -85.225 | 19108.031 | 2.252 | 14.917 | 0.0985864 | 7/15/2014 | 26:57.3 | 3 |
| -85.626 | 19108.065 | 2.25 | 14.923 | 0.0888682 | 7/15/2014 | 26:57.4 | 3 |
| -85.855 | 19108.083 | 2.253 | 14.929 | 0.0742841 | 7/15/2014 | 26:57.5 | 3 |
| -86.085 | 19108.102 | 2.257 | 14.929 | 0.0510314 | 7/15/2014 | 26:57.6 | 3 |
| -86.371 | 19108.124 | 2.26 | 14.923 | 0.0744729 | 7/15/2014 | 26:57.7 | 3 |
| -86.487 | 19108.134 | 2.257 | 14.924 | 0.0641395 | 7/15/2014 | 26:57.8 | 3 |
| -86.834 | 19108.162 | 2.26 | 14.919 | 0.0420259 | 7/15/2014 | 26:57.9 | 3 |
| -87.066 | 19108.18 | 2.263 | 14.914 | 0.0534049 | 7/15/2014 | 26:58.0 | 3 |
| -87.297 | 19108.198 | 2.263 | 14.912 | 0.0602636 | 7/15/2014 | 26:58.1 | 3 |
| -87.528 | 19108.216 | 2.263 | 14.904 | 0.075872 | 7/15/2014 | 26:58.2 | 3 |

| | | | | | | | |
|---|---|---|---|---|---|---|---|
| -87.757 | 19108.234 | 2.26 | 14.899 | 0.0777435 | 7/15/2014 | 26:58.3 | 3 |
| -88.042 | 19108.256 | 2.26 | 14.892 | 0.106677 | 7/15/2014 | 26:58.4 | 3 |
| -88.269 | 19108.272 | 2.254 | 14.887 | 0.0841591 | 7/15/2014 | 26:58.5 | 3 |
| -88.496 | 19108.291 | 2.254 | 14.884 | 0.126876 | 7/15/2014 | 26:58.6 | 3 |
| -88.777 | 19108.311 | 2.254 | 14.879 | 0.113695 | 7/15/2014 | 26:58.7 | 3 |
| -89.002 | 19108.328 | 2.252 | 14.878 | 0.114563 | 7/15/2014 | 26:58.8 | 3 |
| -89.229 | 19108.346 | 2.252 | 14.876 | 0.10818 | 7/15/2014 | 26:58.9 | 3 |
| -89.455 | 19108.363 | 2.252 | 14.874 | 0.126549 | 7/15/2014 | 26:59.0 | 3 |
| -89.68 | 19108.38 | 2.255 | 14.873 | 0.108476 | 7/15/2014 | 26:59.1 | 3 |
| -89.904 | 19108.396 | 2.255 | 14.879 | 0.14109 | 7/15/2014 | 26:59.2 | 3 |
| -90.125 | 19108.412 | 2.258 | 14.879 | 0.117962 | 7/15/2014 | 26:59.3 | 3 |
| -90.4 | 19108.432 | 2.258 | 14.882 | 0.153982 | 7/15/2014 | 26:59.4 | 3 |
| -90.618 | 19108.447 | 2.258 | 14.892 | 0.148935 | 7/15/2014 | 26:59.5 | 3 |
| -90.836 | 19108.464 | 2.26 | 14.901 | 0.171645 | 7/15/2014 | 26:59.6 | 3 |
| -91.108 | 19108.483 | 2.265 | 14.907 | 0.140542 | 7/15/2014 | 26:59.7 | 3 |
| -91.324 | 19108.499 | 2.265 | 14.917 | 0.158898 | 7/15/2014 | 26:59.8 | 3 |
| -91.542 | 19108.514 | 2.264 | 14.92 | 0.149754 | 7/15/2014 | 26:59.9 | 3 |
| -91.757 | 19108.529 | 2.264 | 14.921 | 0.144096 | 7/15/2014 | 27:00.0 | 3 |
| -91.974 | 19108.545 | 2.269 | 14.925 | 0.141739 | 7/15/2014 | 27:00.1 | 3 |
| -92.187 | 19108.56 | 2.271 | 14.923 | 0.148909 | 7/15/2014 | 27:00.2 | 3 |
| -92.401 | 19108.574 | 2.277 | 14.921 | 0.126831 | 7/15/2014 | 27:00.3 | 3 |
| -92.769 | 19108.599 | 2.277 | 14.914 | 0.132568 | 7/15/2014 | 27:00.5 | 3 |
| -92.98 | 19108.614 | 2.28 | 14.91 | 0.135931 | 7/15/2014 | 27:00.6 | 3 |
| -93.241 | 19108.631 | 2.282 | 14.903 | 0.128533 | 7/15/2014 | 27:00.7 | 3 |
| -93.348 | 19108.639 | 2.286 | 14.901 | 0.131077 | 7/15/2014 | 27:00.7 | 3 |
| -93.559 | 19108.653 | 2.288 | 14.896 | 0.12874 | 7/15/2014 | 27:00.8 | 3 |
| -93.77 | 19108.667 | 2.29 | 14.891 | 0.130953 | 7/15/2014 | 27:00.9 | 3 |
| -93.983 | 19108.681 | 2.294 | 14.886 | 0.132361 | 7/15/2014 | 27:01.0 | 3 |
| -94.193 | 19108.694 | 2.298 | 14.881 | 0.120644 | 7/15/2014 | 27:01.1 | 3 |
| -94.404 | 19108.708 | 2.303 | 14.872 | 0.128468 | 7/15/2014 | 27:01.2 | 3 |
| -94.612 | 19108.722 | 2.307 | 14.865 | 0.117175 | 7/15/2014 | 27:01.3 | 3 |
| -94.979 | 19108.745 | 2.314 | 14.858 | 0.1171 | 7/15/2014 | 27:01.5 | 3 |
| -95.187 | 19108.759 | 2.318 | 14.856 | 0.115951 | 7/15/2014 | 27:01.6 | 3 |
| -95.447 | 19108.774 | 2.323 | 14.852 | 0.116541 | 7/15/2014 | 27:01.7 | 3 |
| -95.554 | 19108.781 | 2.327 | 14.85 | 0.114126 | 7/15/2014 | 27:01.7 | 3 |
| -95.762 | 19108.794 | 2.332 | 14.848 | 0.115533 | 7/15/2014 | 27:01.8 | 3 |
| -95.973 | 19108.808 | 2.335 | 14.841 | 0.118191 | 7/15/2014 | 27:01.9 | 3 |
| -96.182 | 19108.819 | 2.335 | 14.838 | 0.112373 | 7/15/2014 | 27:02.0 | 3 |
| -96.394 | 19108.832 | 2.344 | 14.835 | 0.114302 | 7/15/2014 | 27:02.1 | 3 |
| -96.603 | 19108.845 | 2.346 | 14.83 | 0.120484 | 7/15/2014 | 27:02.2 | 3 |
| -96.81 | 19108.857 | 2.346 | 14.824 | 0.118866 | 7/15/2014 | 27:02.3 | 3 |
| -97.171 | 19108.878 | 2.351 | 14.812 | 0.119848 | 7/15/2014 | 27:02.5 | 3 |
| -97.376 | 19108.89 | 2.354 | 14.808 | 0.107864 | 7/15/2014 | 27:02.6 | 3 |
| -97.632 | 19108.904 | 2.354 | 14.807 | 0.107324 | 7/15/2014 | 27:02.7 | 3 |
| -97.737 | 19108.91 | 2.356 | 14.805 | 0.10502 | 7/15/2014 | 27:02.7 | 3 |
| -97.944 | 19108.922 | 2.361 | 14.797 | 0.104047 | 7/15/2014 | 27:02.8 | 3 |
| -98.15 | 19108.934 | 2.361 | 14.791 | 0.105757 | 7/15/2014 | 27:02.9 | 3 |

| | | | | | | | |
|---|---|---|---|---|---|---|---|
| -98.357 | 19108.944 | 2.366 | 14.781 | 0.106787 | 7/15/2014 | 27:03.0 | 3 |
| -98.563 | 19108.955 | 2.372 | 14.775 | 0.10644 | 7/15/2014 | 27:03.1 | 3 |
| -98.768 | 19108.967 | 2.379 | 14.764 | 0.110478 | 7/15/2014 | 27:03.2 | 3 |
| -98.972 | 19108.979 | 2.383 | 14.749 | 0.114089 | 7/15/2014 | 27:03.3 | 3 |
| -99.326 | 19108.996 | 2.388 | 14.721 | 0.115456 | 7/15/2014 | 27:03.5 | 3 |
| -99.53 | 19109.007 | 2.392 | 14.698 | 0.123395 | 7/15/2014 | 27:03.6 | 3 |
| -99.782 | 19109.02 | 2.394 | 14.684 | 0.123921 | 7/15/2014 | 27:03.7 | 3 |
| -99.885 | 19109.024 | 2.398 | 14.674 | 0.125411 | 7/15/2014 | 27:03.7 | 3 |
| -100.09 | 19109.035 | 2.403 | 14.659 | 0.128024 | 7/15/2014 | 27:03.8 | 3 |
| -100.294 | 19109.045 | 2.405 | 14.642 | 0.116078 | 7/15/2014 | 27:03.9 | 3 |
| -100.499 | 19109.057 | 2.405 | 14.627 | 0.125576 | 7/15/2014 | 27:04.0 | 3 |
| -100.705 | 19109.065 | 2.405 | 14.616 | 0.116602 | 7/15/2014 | 27:04.1 | 3 |
| -100.909 | 19109.076 | 2.408 | 14.602 | 0.114316 | 7/15/2014 | 27:04.2 | 3 |
| -101.113 | 19109.085 | 2.413 | 14.595 | 0.108982 | 7/15/2014 | 27:04.3 | 3 |
| -101.469 | 19109.102 | 2.418 | 14.584 | 0.107536 | 7/15/2014 | 27:04.5 | 3 |
| -101.674 | 19109.111 | 2.425 | 14.576 | 0.108411 | 7/15/2014 | 27:04.6 | 3 |
| -101.927 | 19109.121 | 2.425 | 14.567 | 0.101102 | 7/15/2014 | 27:04.7 | 3 |
| -102.03 | 19109.127 | 2.428 | 14.563 | 0.0940558 | 7/15/2014 | 27:04.7 | 3 |
| -102.235 | 19109.135 | 2.433 | 14.554 | 0.099606 | 7/15/2014 | 27:04.8 | 3 |
| -102.44 | 19109.146 | 2.44 | 14.543 | 0.0977034 | 7/15/2014 | 27:04.9 | 3 |
| -102.648 | 19109.154 | 2.448 | 14.537 | 0.0966318 | 7/15/2014 | 27:05.0 | 3 |
| -102.853 | 19109.163 | 2.453 | 14.531 | 0.0932201 | 7/15/2014 | 27:05.1 | 3 |
| -103.058 | 19109.172 | 2.458 | 14.521 | 0.0892151 | 7/15/2014 | 27:05.2 | 3 |
| -103.262 | 19109.18 | 2.462 | 14.516 | 0.0888188 | 7/15/2014 | 27:05.3 | 3 |
| -103.618 | 19109.195 | 2.464 | 14.507 | 0.0765229 | 7/15/2014 | 27:05.5 | 3 |
| -103.821 | 19109.202 | 2.472 | 14.502 | 0.0744527 | 7/15/2014 | 27:05.6 | 3 |
| -104.073 | 19109.213 | 2.472 | 14.499 | 0.0700172 | 7/15/2014 | 27:05.7 | 3 |
| -104.177 | 19109.217 | 2.474 | 14.498 | 0.066571 | 7/15/2014 | 27:05.7 | 3 |
| -104.38 | 19109.225 | 2.474 | 14.496 | 0.0687483 | 7/15/2014 | 27:05.8 | 3 |
| -104.587 | 19109.232 | 2.471 | 14.49 | 0.0623808 | 7/15/2014 | 27:05.9 | 3 |
| -104.792 | 19109.24 | 2.474 | 14.486 | 0.0575143 | 7/15/2014 | 27:06.0 | 3 |
| -104.999 | 19109.248 | 2.477 | 14.483 | 0.0550463 | 7/15/2014 | 27:06.1 | 3 |
| -105.202 | 19109.258 | 2.48 | 14.48 | 0.0572937 | 7/15/2014 | 27:06.2 | 3 |
| -105.406 | 19109.265 | 2.484 | 14.475 | 0.0567724 | 7/15/2014 | 27:06.3 | 3 |
| -105.758 | 19109.276 | 2.486 | 14.475 | 0.0532351 | 7/15/2014 | 27:06.5 | 3 |
| -105.96 | 19109.283 | 2.492 | 14.472 | 0.0519859 | 7/15/2014 | 27:06.6 | 3 |
| -106.212 | 19109.293 | 2.495 | 14.467 | 0.0528541 | 7/15/2014 | 27:06.7 | 3 |
| -106.315 | 19109.297 | 2.5 | 14.466 | 0.0568619 | 7/15/2014 | 27:06.7 | 3 |
| -106.52 | 19109.304 | 2.502 | 14.465 | 0.0669684 | 7/15/2014 | 27:06.8 | 3 |
| -106.722 | 19109.311 | 2.505 | 14.462 | 0.0635966 | 7/15/2014 | 27:06.9 | 3 |
| -106.926 | 19109.318 | 2.505 | 14.462 | 0.0822308 | 7/15/2014 | 27:07.0 | 3 |
| -107.13 | 19109.325 | 2.508 | 14.46 | 0.0821557 | 7/15/2014 | 27:07.1 | 3 |
| -107.332 | 19109.331 | 2.51 | 14.457 | 0.102089 | 7/15/2014 | 27:07.2 | 3 |
| -107.534 | 19109.338 | 2.507 | 14.453 | 0.0897773 | 7/15/2014 | 27:07.3 | 3 |
| -107.885 | 19109.35 | 2.509 | 14.453 | 0.0930766 | 7/15/2014 | 27:07.5 | 3 |
| -108.087 | 19109.356 | 2.513 | 14.455 | 0.0782234 | 7/15/2014 | 27:07.6 | 3 |
| -108.334 | 19109.363 | 2.511 | 14.453 | 0.0764052 | 7/15/2014 | 27:07.7 | 3 |

| | | | | | | | |
|---|---|---|---|---|---|---|---|
| -108.435 | 19109.367 | 2.511 | 14.453 | 0.058706 | 7/15/2014 | 27:07.7 | 3 |
| -108.637 | 19109.372 | 2.516 | 14.449 | 0.086288 | 7/15/2014 | 27:07.8 | 3 |
| -108.939 | 19109.381 | 2.518 | 14.445 | 0.0655785 | 7/15/2014 | 27:08.0 | 3 |
| -109.14 | 19109.389 | 2.519 | 14.44 | 0.10918 | 7/15/2014 | 27:08.1 | 3 |
| -109.343 | 19109.394 | 2.525 | 14.434 | 0.109666 | 7/15/2014 | 27:08.2 | 3 |
| -109.541 | 19109.399 | 2.525 | 14.428 | 0.10923 | 7/15/2014 | 27:08.3 | 3 |
| -109.741 | 19109.406 | 2.529 | 14.426 | 0.152932 | 7/15/2014 | 27:08.4 | 3 |
| -109.987 | 19109.412 | 2.529 | 14.42 | 0.182614 | 7/15/2014 | 27:08.5 | 3 |
| -110.185 | 19109.418 | 2.533 | 14.418 | 0.189986 | 7/15/2014 | 27:08.6 | 3 |
| -110.43 | 19109.426 | 2.531 | 14.412 | 0.179895 | 7/15/2014 | 27:08.7 | 3 |
| -110.631 | 19109.43 | 2.535 | 14.409 | 0.233223 | 7/15/2014 | 27:08.8 | 3 |
| -110.831 | 19109.438 | 2.535 | 14.404 | 0.189265 | 7/15/2014 | 27:08.9 | 3 |
| -111.032 | 19109.441 | 2.537 | 14.401 | 0.195458 | 7/15/2014 | 27:09.0 | 3 |
| -111.233 | 19109.447 | 2.537 | 14.399 | 0.190332 | 7/15/2014 | 27:09.1 | 3 |
| -111.434 | 19109.453 | 2.539 | 14.396 | 0.150251 | 7/15/2014 | 27:09.2 | 3 |
| -111.634 | 19109.458 | 2.539 | 14.39 | 0.149449 | 7/15/2014 | 27:09.3 | 3 |
| -111.732 | 19109.462 | 2.538 | 14.387 | 0.132855 | 7/15/2014 | 27:09.3 | 3 |
| -112.08 | 19109.471 | 2.539 | 14.381 | 0.113499 | 7/15/2014 | 27:09.5 | 3 |
| -112.279 | 19109.477 | 2.54 | 14.382 | 0.0991815 | 7/15/2014 | 27:09.6 | 3 |
| -112.529 | 19109.484 | 2.54 | 14.381 | 0.109043 | 7/15/2014 | 27:09.7 | 3 |
| -112.629 | 19109.486 | 2.54 | 14.38 | 0.110409 | 7/15/2014 | 27:09.7 | 3 |
| -112.831 | 19109.491 | 2.54 | 14.38 | 0.0863039 | 7/15/2014 | 27:09.8 | 3 |
| -113.033 | 19109.498 | 2.54 | 14.38 | 0.0957754 | 7/15/2014 | 27:09.9 | 3 |
| -113.235 | 19109.504 | 2.54 | 14.382 | 0.087965 | 7/15/2014 | 27:10.0 | 3 |
| -113.437 | 19109.51 | 2.54 | 14.391 | 0.0869616 | 7/15/2014 | 27:10.1 | 3 |
| -113.639 | 19109.514 | 2.544 | 14.39 | 0.0866497 | 7/15/2014 | 27:10.2 | 3 |
| -113.839 | 19109.521 | 2.544 | 14.39 | 0.0864012 | 7/15/2014 | 27:10.3 | 3 |
| -114.188 | 19109.531 | 2.546 | 14.388 | 0.0893412 | 7/15/2014 | 27:10.5 | 3 |
| -114.386 | 19109.535 | 2.546 | 14.388 | 0.101601 | 7/15/2014 | 27:10.6 | 3 |
| -114.634 | 19109.545 | 2.546 | 14.385 | 0.0958534 | 7/15/2014 | 27:10.7 | 3 |
| -114.835 | 19109.55 | 2.552 | 14.383 | 0.110701 | 7/15/2014 | 27:10.8 | 3 |
| -115.033 | 19109.557 | 2.555 | 14.382 | 0.112812 | 7/15/2014 | 27:10.9 | 3 |
| -115.235 | 19109.561 | 2.56 | 14.373 | 0.106869 | 7/15/2014 | 27:11.0 | 3 |
| -115.434 | 19109.568 | 2.557 | 14.371 | 0.0933466 | 7/15/2014 | 27:11.1 | 3 |
| -115.635 | 19109.572 | 2.563 | 14.37 | 0.0991668 | 7/15/2014 | 27:11.2 | 3 |
| -115.832 | 19109.58 | 2.563 | 14.369 | 0.0897911 | 7/15/2014 | 27:11.3 | 3 |
| -116.03 | 19109.584 | 2.563 | 14.365 | 0.088741 | 7/15/2014 | 27:11.4 | 3 |
| -116.276 | 19109.594 | 2.563 | 14.36 | 0.0929896 | 7/15/2014 | 27:11.5 | 3 |
| -116.475 | 19109.6 | 2.569 | 14.358 | 0.0805717 | 7/15/2014 | 27:11.6 | 3 |
| -116.719 | 19109.607 | 2.569 | 14.355 | 0.0831898 | 7/15/2014 | 27:11.7 | 3 |
| -116.919 | 19109.613 | 2.572 | 14.35 | 0.0891735 | 7/15/2014 | 27:11.8 | 3 |
| -117.117 | 19109.619 | 2.572 | 14.347 | 0.0994421 | 7/15/2014 | 27:11.9 | 3 |
| -117.318 | 19109.625 | 2.577 | 14.34 | 0.107917 | 7/15/2014 | 27:12.0 | 3 |
| -117.516 | 19109.633 | 2.577 | 14.335 | 0.119591 | 7/15/2014 | 27:12.1 | 3 |
| -117.716 | 19109.637 | 2.584 | 14.327 | 0.132864 | 7/15/2014 | 27:12.2 | 3 |
| -117.913 | 19109.645 | 2.587 | 14.32 | 0.129117 | 7/15/2014 | 27:12.3 | 3 |
| -118.112 | 19109.652 | 2.595 | 14.315 | 0.130176 | 7/15/2014 | 27:12.4 | 3 |

| | | | | | | | |
|---|---|---|---|---|---|---|---|
| -118.358 | 19109.658 | 2.594 | 14.31 | 0.137921 | 7/15/2014 | 27:12.5 | 3 |
| -118.556 | 19109.666 | 2.598 | 14.306 | 0.136287 | 7/15/2014 | 27:12.6 | 3 |
| -118.801 | 19109.674 | 2.598 | 14.301 | 0.120404 | 7/15/2014 | 27:12.7 | 3 |
| -118.999 | 19109.68 | 2.6 | 14.296 | 0.0985402 | 7/15/2014 | 27:12.8 | 3 |
| -119.198 | 19109.688 | 2.6 | 14.293 | 0.107525 | 7/15/2014 | 27:12.9 | 3 |
| -119.4 | 19109.693 | 2.603 | 14.291 | 0.101527 | 7/15/2014 | 27:13.0 | 3 |
| -119.597 | 19109.701 | 2.603 | 14.289 | 0.102167 | 7/15/2014 | 27:13.1 | 3 |
| -119.798 | 19109.709 | 2.607 | 14.285 | 0.111118 | 7/15/2014 | 27:13.2 | 3 |
| -119.995 | 19109.715 | 2.607 | 14.279 | 0.105215 | 7/15/2014 | 27:13.3 | 3 |
| -120.194 | 19109.723 | 2.611 | 14.273 | 0.10801 | 7/15/2014 | 27:13.4 | 3 |
| -120.437 | 19109.73 | 2.609 | 14.264 | 0.112893 | 7/15/2014 | 27:13.5 | 3 |
| -120.635 | 19109.736 | 2.613 | 14.257 | 0.112935 | 7/15/2014 | 27:13.6 | 3 |
| -120.881 | 19109.746 | 2.615 | 14.25 | 0.110201 | 7/15/2014 | 27:13.7 | 3 |
| -121.081 | 19109.754 | 2.618 | 14.242 | 0.110569 | 7/15/2014 | 27:13.8 | 3 |
| -121.278 | 19109.762 | 2.616 | 14.231 | 0.110481 | 7/15/2014 | 27:13.9 | 3 |
| -121.48 | 19109.77 | 2.62 | 14.221 | 0.109482 | 7/15/2014 | 27:14.0 | 3 |
| -121.68 | 19109.777 | 2.62 | 14.214 | 0.109844 | 7/15/2014 | 27:14.1 | 3 |
| -121.881 | 19109.786 | 2.624 | 14.207 | 0.108654 | 7/15/2014 | 27:14.2 | 3 |
| -122.079 | 19109.793 | 2.626 | 14.201 | 0.10635 | 7/15/2014 | 27:14.3 | 3 |
| -122.279 | 19109.801 | 2.63 | 14.194 | 0.114257 | 7/15/2014 | 27:14.4 | 3 |
| -122.526 | 19109.811 | 2.632 | 14.187 | 0.114376 | 7/15/2014 | 27:14.5 | 3 |
| -122.724 | 19109.818 | 2.633 | 14.18 | 0.116234 | 7/15/2014 | 27:14.6 | 3 |
| -122.97 | 19109.83 | 2.633 | 14.173 | 0.132863 | 7/15/2014 | 27:14.7 | 3 |
| -123.173 | 19109.838 | 2.64 | 14.167 | 0.113459 | 7/15/2014 | 27:14.8 | 3 |
| -123.274 | 19109.842 | 2.64 | 14.163 | 0.11462 | 7/15/2014 | 27:14.8 | 3 |
| -123.575 | 19109.856 | 2.645 | 14.156 | 0.110999 | 7/15/2014 | 27:15.0 | 3 |
| -123.775 | 19109.865 | 2.645 | 14.149 | 0.112849 | 7/15/2014 | 27:15.1 | 3 |
| -123.978 | 19109.875 | 2.65 | 14.14 | 0.113996 | 7/15/2014 | 27:15.2 | 3 |
| -124.078 | 19109.879 | 2.652 | 14.138 | 0.114538 | 7/15/2014 | 27:15.2 | 3 |
| -124.276 | 19109.887 | 2.655 | 14.134 | 0.116463 | 7/15/2014 | 27:15.3 | 3 |
| -124.623 | 19109.904 | 2.662 | 14.116 | 0.117973 | 7/15/2014 | 27:15.5 | 3 |
| -124.825 | 19109.914 | 2.669 | 14.105 | 0.117165 | 7/15/2014 | 27:15.6 | 3 |
| -125.07 | 19109.925 | 2.669 | 14.093 | 0.116801 | 7/15/2014 | 27:15.7 | 3 |
| -125.274 | 19109.936 | 2.675 | 14.08 | 0.114262 | 7/15/2014 | 27:15.8 | 3 |
| -125.373 | 19109.939 | 2.676 | 14.071 | 0.114526 | 7/15/2014 | 27:15.8 | 3 |
| -125.676 | 19109.955 | 2.685 | 14.047 | 0.115042 | 7/15/2014 | 27:16.0 | 3 |
| -125.876 | 19109.965 | 2.687 | 14.033 | 0.118532 | 7/15/2014 | 27:16.1 | 3 |
| -126.079 | 19109.976 | 2.694 | 14.01 | 0.113845 | 7/15/2014 | 27:16.2 | 3 |
| -126.177 | 19109.979 | 2.694 | 14.001 | 0.114922 | 7/15/2014 | 27:16.2 | 3 |
| -126.378 | 19109.99 | 2.7 | 13.985 | 0.116848 | 7/15/2014 | 27:16.3 | 3 |
| -126.724 | 19110.008 | 2.707 | 13.956 | 0.117202 | 7/15/2014 | 27:16.5 | 3 |
| -126.926 | 19110.019 | 2.717 | 13.939 | 0.118669 | 7/15/2014 | 27:16.6 | 3 |
| -127.173 | 19110.032 | 2.721 | 13.919 | 0.121972 | 7/15/2014 | 27:16.7 | 3 |
| -127.274 | 19110.036 | 2.725 | 13.909 | 0.11754 | 7/15/2014 | 27:16.7 | 3 |
| -127.476 | 19110.049 | 2.731 | 13.892 | 0.123427 | 7/15/2014 | 27:16.8 | 3 |
| -127.78 | 19110.064 | 2.738 | 13.863 | 0.170591 | 7/15/2014 | 27:17.0 | 3 |
| -127.88 | 19110.069 | 2.739 | 13.856 | 0.168785 | 7/15/2014 | 27:17.0 | 3 |

| | | | | | | | |
|---|---|---|---|---|---|---|---|
| -128.083 | 19110.08 | 2.744 | 13.837 | 0.202375 | 7/15/2014 | 27:17.1 | 3 |
| -128.283 | 19110.093 | 2.747 | 13.821 | 0.231029 | 7/15/2014 | 27:17.2 | 3 |
| -128.485 | 19110.104 | 2.752 | 13.801 | 0.22205 | 7/15/2014 | 27:17.3 | 3 |
| -128.836 | 19110.123 | 2.756 | 13.768 | 0.209359 | 7/15/2014 | 27:17.5 | 3 |
| -129.039 | 19110.136 | 2.762 | 13.751 | 0.199718 | 7/15/2014 | 27:17.6 | 3 |
| -129.29 | 19110.15 | 2.767 | 13.729 | 0.176306 | 7/15/2014 | 27:17.7 | 3 |
| -129.392 | 19110.156 | 2.771 | 13.717 | 0.1601 | 7/15/2014 | 27:17.7 | 3 |
| -129.596 | 19110.167 | 2.777 | 13.698 | 0.126137 | 7/15/2014 | 27:17.8 | 3 |
| -129.8 | 19110.181 | 2.786 | 13.677 | 0.122381 | 7/15/2014 | 27:17.9 | 3 |
| -130.005 | 19110.192 | 2.792 | 13.66 | 0.0871973 | 7/15/2014 | 27:18.0 | 3 |
| -130.209 | 19110.204 | 2.801 | 13.646 | 0.088411 | 7/15/2014 | 27:18.1 | 3 |
| -130.414 | 19110.217 | 2.807 | 13.631 | 0.0709529 | 7/15/2014 | 27:18.2 | 3 |
| -130.619 | 19110.229 | 2.813 | 13.618 | 0.0649211 | 7/15/2014 | 27:18.3 | 3 |
| -130.973 | 19110.252 | 2.817 | 13.592 | 0.0488945 | 7/15/2014 | 27:18.5 | 3 |
| -131.227 | 19110.268 | 2.821 | 13.576 | 0.0524622 | 7/15/2014 | 27:18.6 | 3 |
| -131.329 | 19110.274 | 2.822 | 13.57 | 0.0497685 | 7/15/2014 | 27:18.6 | 3 |
| -131.531 | 19110.287 | 2.822 | 13.554 | 0.0489224 | 7/15/2014 | 27:18.7 | 3 |
| -131.737 | 19110.3 | 2.829 | 13.535 | 0.0502693 | 7/15/2014 | 27:18.8 | 3 |
| -131.94 | 19110.313 | 2.829 | 13.517 | 0.0603054 | 7/15/2014 | 27:18.9 | 3 |
| -132.146 | 19110.327 | 2.833 | 13.503 | 0.0536403 | 7/15/2014 | 27:19.0 | 3 |
| -132.35 | 19110.341 | 2.838 | 13.491 | 0.0581906 | 7/15/2014 | 27:19.1 | 3 |
| -132.554 | 19110.354 | 2.842 | 13.48 | 0.0663064 | 7/15/2014 | 27:19.2 | 3 |
| -132.909 | 19110.378 | 2.847 | 13.466 | 0.0768554 | 7/15/2014 | 27:19.4 | 3 |
| -133.108 | 19110.392 | 2.849 | 13.457 | 0.0817179 | 7/15/2014 | 27:19.5 | 3 |
| -133.31 | 19110.405 | 2.853 | 13.451 | 0.0671776 | 7/15/2014 | 27:19.6 | 3 |
| -133.558 | 19110.424 | 2.85 | 13.444 | 0.070708 | 7/15/2014 | 27:19.7 | 3 |
| -133.661 | 19110.431 | 2.854 | 13.441 | 0.079301 | 7/15/2014 | 27:19.7 | 3 |
| -133.864 | 19110.444 | 2.859 | 13.432 | 0.0753378 | 7/15/2014 | 27:19.8 | 3 |
| -134.065 | 19110.459 | 2.859 | 13.425 | 0.0871123 | 7/15/2014 | 27:19.9 | 3 |
| -134.267 | 19110.474 | 2.862 | 13.416 | 0.0883802 | 7/15/2014 | 27:20.0 | 3 |
| -134.469 | 19110.488 | 2.865 | 13.403 | 0.0879219 | 7/15/2014 | 27:20.1 | 3 |
| -134.671 | 19110.503 | 2.87 | 13.392 | 0.114882 | 7/15/2014 | 27:20.2 | 3 |
| -134.87 | 19110.517 | 2.87 | 13.381 | 0.172787 | 7/15/2014 | 27:20.3 | 3 |
| -135.218 | 19110.544 | 2.881 | 13.362 | 0.167021 | 7/15/2014 | 27:20.5 | 3 |
| -135.467 | 19110.562 | 2.888 | 13.35 | 0.156368 | 7/15/2014 | 27:20.6 | 3 |
| -135.566 | 19110.568 | 2.887 | 13.345 | 0.145763 | 7/15/2014 | 27:20.6 | 3 |
| -135.766 | 19110.584 | 2.892 | 13.325 | 0.169492 | 7/15/2014 | 27:20.7 | 3 |
| -135.966 | 19110.601 | 2.896 | 13.313 | 0.132519 | 7/15/2014 | 27:20.8 | 3 |
| -136.271 | 19110.625 | 2.903 | 13.299 | 0.120906 | 7/15/2014 | 27:21.0 | 3 |
| -136.37 | 19110.632 | 2.904 | 13.294 | 0.109022 | 7/15/2014 | 27:21.0 | 3 |
| -136.57 | 19110.647 | 2.909 | 13.284 | 0.0942343 | 7/15/2014 | 27:21.1 | 3 |
| -136.772 | 19110.664 | 2.912 | 13.27 | 0.0825055 | 7/15/2014 | 27:21.2 | 3 |
| -137.123 | 19110.691 | 2.913 | 13.257 | 0.111979 | 7/15/2014 | 27:21.4 | 3 |
| -137.32 | 19110.707 | 2.913 | 13.247 | 0.0715187 | 7/15/2014 | 27:21.5 | 3 |
| -137.521 | 19110.726 | 2.918 | 13.239 | 0.0983243 | 7/15/2014 | 27:21.6 | 3 |
| -137.771 | 19110.746 | 2.921 | 13.231 | 0.0754756 | 7/15/2014 | 27:21.7 | 3 |
| -137.873 | 19110.754 | 2.924 | 13.228 | 0.0691855 | 7/15/2014 | 27:21.7 | 3 |

| | | | | | | | |
|---|---|---|---|---|---|---|---|
| -138.073 | 19110.771 | 2.926 | 13.215 | 0.0425074 | 7/15/2014 | 27:21.8 | 3 |
| -138.276 | 19110.788 | 2.928 | 13.212 | 0.0703989 | 7/15/2014 | 27:21.9 | 3 |
| -138.479 | 19110.805 | 2.93 | 13.203 | 0.0715706 | 7/15/2014 | 27:22.0 | 3 |
| -138.685 | 19110.823 | 2.936 | 13.194 | 0.0617609 | 7/15/2014 | 27:22.1 | 3 |
| -138.887 | 19110.84 | 2.938 | 13.183 | 0.0667929 | 7/15/2014 | 27:22.2 | 3 |
| -139.093 | 19110.859 | 2.943 | 13.174 | 0.0739063 | 7/15/2014 | 27:22.3 | 3 |
| -139.453 | 19110.891 | 2.946 | 13.16 | 0.0735371 | 7/15/2014 | 27:22.5 | 3 |
| -139.658 | 19110.91 | 2.948 | 13.151 | 0.0952085 | 7/15/2014 | 27:22.6 | 3 |
| -139.863 | 19110.928 | 2.948 | 13.145 | 0.107481 | 7/15/2014 | 27:22.7 | 3 |
| -140.072 | 19110.947 | 2.955 | 13.132 | 0.0925639 | 7/15/2014 | 27:22.8 | 3 |
| -140.278 | 19110.966 | 2.955 | 13.124 | 0.0953986 | 7/15/2014 | 27:22.9 | 3 |
| -140.487 | 19110.984 | 2.959 | 13.114 | 0.108725 | 7/15/2014 | 27:23.0 | 3 |
| -140.693 | 19111.004 | 2.959 | 13.105 | 0.0796093 | 7/15/2014 | 27:23.1 | 3 |
| -140.9 | 19111.023 | 2.963 | 13.096 | 0.102963 | 7/15/2014 | 27:23.2 | 3 |
| -141.104 | 19111.043 | 2.963 | 13.084 | 0.0938926 | 7/15/2014 | 27:23.3 | 3 |
| -141.31 | 19111.063 | 2.968 | 13.075 | 0.0865095 | 7/15/2014 | 27:23.4 | 3 |
| -141.513 | 19111.082 | 2.973 | 13.062 | 0.0569543 | 7/15/2014 | 27:23.5 | 3 |
| -141.716 | 19111.102 | 2.977 | 13.049 | 0.0665269 | 7/15/2014 | 27:23.6 | 3 |
| -141.964 | 19111.126 | 2.978 | 13.037 | 0.0641456 | 7/15/2014 | 27:23.7 | 3 |
| -142.168 | 19111.146 | 2.983 | 13.032 | 0.0483199 | 7/15/2014 | 27:23.8 | 3 |
| -142.369 | 19111.166 | 2.986 | 13.025 | 0.0696178 | 7/15/2014 | 27:23.9 | 3 |
| -142.572 | 19111.186 | 2.99 | 13.017 | 0.0492303 | 7/15/2014 | 27:24.0 | 3 |
| -142.773 | 19111.206 | 2.99 | 13.008 | 0.0569774 | 7/15/2014 | 27:24.1 | 3 |
| -142.977 | 19111.227 | 2.997 | 13 | 0.0796693 | 7/15/2014 | 27:24.2 | 3 |
| -143.075 | 19111.236 | 2.997 | 12.996 | 0.0759478 | 7/15/2014 | 27:24.2 | 3 |
| -143.277 | 19111.257 | 2.997 | 12.993 | 0.0996028 | 7/15/2014 | 27:24.3 | 3 |
| -143.632 | 19111.294 | 3 | 12.982 | 0.103545 | 7/15/2014 | 27:24.5 | 3 |
| -143.838 | 19111.314 | 3.006 | 12.976 | 0.136175 | 7/15/2014 | 27:24.6 | 3 |
| -144.043 | 19111.336 | 3.009 | 12.963 | 0.111489 | 7/15/2014 | 27:24.7 | 3 |
| -144.249 | 19111.357 | 3.014 | 12.952 | 0.14468 | 7/15/2014 | 27:24.8 | 3 |
| -144.456 | 19111.38 | 3.019 | 12.946 | 0.138946 | 7/15/2014 | 27:24.9 | 3 |
| -144.664 | 19111.402 | 3.023 | 12.939 | 0.123474 | 7/15/2014 | 27:25.0 | 3 |
| -144.872 | 19111.425 | 3.026 | 12.932 | 0.119207 | 7/15/2014 | 27:25.1 | 3 |
| -145.081 | 19111.446 | 3.031 | 12.921 | 0.103507 | 7/15/2014 | 27:25.2 | 3 |
| -145.286 | 19111.469 | 3.033 | 12.916 | 0.101229 | 7/15/2014 | 27:25.3 | 3 |
| -145.494 | 19111.491 | 3.04 | 12.911 | 0.0740722 | 7/15/2014 | 27:25.4 | 3 |
| -145.7 | 19111.514 | 3.04 | 12.909 | 0.0866538 | 7/15/2014 | 27:25.5 | 3 |
| -145.904 | 19111.537 | 3.042 | 12.906 | 0.0725468 | 7/15/2014 | 27:25.6 | 3 |
| -146.157 | 19111.564 | 3.042 | 12.901 | 0.0729443 | 7/15/2014 | 27:25.7 | 3 |
| -146.262 | 19111.576 | 3.043 | 12.897 | 0.0734949 | 7/15/2014 | 27:25.7 | 3 |
| -146.467 | 19111.6 | 3.045 | 12.893 | 0.0782564 | 7/15/2014 | 27:25.8 | 3 |
| -146.668 | 19111.622 | 3.045 | 12.886 | 0.0701598 | 7/15/2014 | 27:25.9 | 3 |
| -146.875 | 19111.645 | 3.05 | 12.885 | 0.0939108 | 7/15/2014 | 27:26.0 | 3 |
| -147.078 | 19111.668 | 3.05 | 12.88 | 0.0712884 | 7/15/2014 | 27:26.1 | 3 |
| -147.283 | 19111.691 | 3.052 | 12.872 | 0.093264 | 7/15/2014 | 27:26.2 | 3 |
| -147.487 | 19111.715 | 3.058 | 12.859 | 0.0871937 | 7/15/2014 | 27:26.3 | 3 |
| -147.847 | 19111.756 | 3.061 | 12.846 | 0.103872 | 7/15/2014 | 27:26.5 | 3 |

| | | | | | | | |
|---|---|---|---|---|---|---|---|
| -148.056 | 19111.78 | 3.065 | 12.843 | 0.114771 | 7/15/2014 | 27:26.6 | 3 |
| -148.261 | 19111.805 | 3.063 | 12.835 | 0.131454 | 7/15/2014 | 27:26.7 | 3 |
| -148.475 | 19111.829 | 3.068 | 12.829 | 0.0895673 | 7/15/2014 | 27:26.8 | 3 |
| -148.577 | 19111.841 | 3.066 | 12.823 | 0.102223 | 7/15/2014 | 27:26.8 | 3 |
| -148.789 | 19111.865 | 3.066 | 12.814 | 0.0962116 | 7/15/2014 | 27:26.9 | 3 |
| -149.003 | 19111.891 | 3.066 | 12.806 | 0.0989416 | 7/15/2014 | 27:27.0 | 3 |
| -149.215 | 19111.917 | 3.061 | 12.799 | 0.0894088 | 7/15/2014 | 27:27.1 | 3 |
| -149.428 | 19111.941 | 3.061 | 12.793 | 0.0823865 | 7/15/2014 | 27:27.2 | 3 |
| -149.642 | 19111.967 | 3.066 | 12.785 | 0.0749166 | 7/15/2014 | 27:27.3 | 3 |
| -149.854 | 19111.992 | 3.066 | 12.773 | 0.0680537 | 7/15/2014 | 27:27.4 | 3 |
| -150.067 | 19112.018 | 3.069 | 12.773 | 0.0799414 | 7/15/2014 | 27:27.5 | 3 |
| -150.432 | 19112.063 | 3.067 | 12.759 | 0.0691647 | 7/15/2014 | 27:27.7 | 3 |
| -150.541 | 19112.076 | 3.071 | 12.755 | 0.0674526 | 7/15/2014 | 27:27.7 | 3 |
| -150.752 | 19112.102 | 3.071 | 12.751 | 0.0639568 | 7/15/2014 | 27:27.8 | 3 |
| -150.965 | 19112.127 | 3.073 | 12.744 | 0.0636184 | 7/15/2014 | 27:27.9 | 3 |
| -151.177 | 19112.153 | 3.073 | 12.739 | 0.0618872 | 7/15/2014 | 27:28.0 | 3 |
| -151.389 | 19112.18 | 3.073 | 12.732 | 0.0588856 | 7/15/2014 | 27:28.1 | 3 |
| -151.601 | 19112.206 | 3.073 | 12.726 | 0.0526094 | 7/15/2014 | 27:28.2 | 3 |
| -151.812 | 19112.231 | 3.074 | 12.711 | 0.0607729 | 7/15/2014 | 27:28.3 | 3 |
| -152.077 | 19112.266 | 3.074 | 12.703 | 0.051199 | 7/15/2014 | 27:28.4 | 3 |
| -152.291 | 19112.292 | 3.077 | 12.695 | 0.0578279 | 7/15/2014 | 27:28.5 | 3 |
| -152.508 | 19112.318 | 3.08 | 12.684 | 0.0743942 | 7/15/2014 | 27:28.6 | 3 |
| -152.721 | 19112.346 | 3.083 | 12.679 | 0.0807225 | 7/15/2014 | 27:28.7 | 3 |
| -152.939 | 19112.373 | 3.086 | 12.666 | 0.0525677 | 7/15/2014 | 27:28.8 | 3 |
| -153.154 | 19112.4 | 3.086 | 12.657 | 0.0736283 | 7/15/2014 | 27:28.9 | 3 |
| -153.371 | 19112.429 | 3.089 | 12.647 | 0.0504897 | 7/15/2014 | 27:29.0 | 3 |
| -153.586 | 19112.455 | 3.091 | 12.636 | 0.0445157 | 7/15/2014 | 27:29.1 | 3 |
| -153.801 | 19112.483 | 3.091 | 12.627 | 0.0533232 | 7/15/2014 | 27:29.2 | 3 |
| -154.017 | 19112.511 | 3.091 | 12.618 | 0.0389111 | 7/15/2014 | 27:29.3 | 3 |
| -154.23 | 19112.537 | 3.091 | 12.609 | 0.0387078 | 7/15/2014 | 27:29.4 | 3 |
| -154.543 | 19112.578 | 3.093 | 12.596 | 0.0601485 | 7/15/2014 | 27:29.6 | 3 |
| -154.798 | 19112.611 | 3.09 | 12.592 | 0.0361395 | 7/15/2014 | 27:29.7 | 3 |
| -154.907 | 19112.627 | 3.091 | 12.587 | 0.0400333 | 7/15/2014 | 27:29.7 | 3 |
| -155.113 | 19112.652 | 3.09 | 12.582 | 0.0629602 | 7/15/2014 | 27:29.8 | 3 |
| -155.319 | 19112.68 | 3.09 | 12.579 | 0.0345803 | 7/15/2014 | 27:29.9 | 3 |
| -155.526 | 19112.707 | 3.09 | 12.576 | 0.0387005 | 7/15/2014 | 27:30.0 | 3 |
| -155.734 | 19112.733 | 3.09 | 12.575 | 0.0406005 | 7/15/2014 | 27:30.1 | 3 |
| -155.94 | 19112.762 | 3.09 | 12.576 | 0.0492496 | 7/15/2014 | 27:30.2 | 3 |
| -156.145 | 19112.789 | 3.087 | 12.573 | 0.0613405 | 7/15/2014 | 27:30.3 | 3 |
| -156.504 | 19112.836 | 3.078 | 12.575 | 0.051922 | 7/15/2014 | 27:30.5 | 3 |
| -156.712 | 19112.864 | 3.078 | 12.575 | 0.0635278 | 7/15/2014 | 27:30.6 | 3 |
| -156.917 | 19112.892 | 3.073 | 12.577 | 0.0540746 | 7/15/2014 | 27:30.7 | 3 |
| -157.127 | 19112.919 | 3.076 | 12.576 | 0.0574777 | 7/15/2014 | 27:30.8 | 3 |
| -157.334 | 19112.946 | 3.073 | 12.573 | 0.0582005 | 7/15/2014 | 27:30.9 | 3 |
| -157.547 | 19112.976 | 3.076 | 12.569 | 0.0498023 | 7/15/2014 | 27:31.0 | 3 |
| -157.752 | 19113.004 | 3.07 | 12.564 | 0.0613814 | 7/15/2014 | 27:31.1 | 3 |
| -157.963 | 19113.031 | 3.076 | 12.563 | 0.0549617 | 7/15/2014 | 27:31.2 | 3 |

| | | | | | | | |
|---|---|---|---|---|---|---|---|
| -158.17 | 19113.059 | 3.076 | 12.559 | 0.0714333 | 7/15/2014 | 27:31.3 | 3 |
| -158.382 | 19113.087 | 3.082 | 12.557 | 0.0672626 | 7/15/2014 | 27:31.4 | 3 |
| -158.585 | 19113.115 | 3.079 | 12.557 | 0.0698532 | 7/15/2014 | 27:31.5 | 3 |
| -158.793 | 19113.143 | 3.079 | 12.551 | 0.070126 | 7/15/2014 | 27:31.6 | 3 |
| -159.048 | 19113.176 | 3.073 | 12.548 | 0.0729544 | 7/15/2014 | 27:31.7 | 3 |
| -159.155 | 19113.19 | 3.075 | 12.548 | 0.0803891 | 7/15/2014 | 27:31.7 | 3 |
| -159.361 | 19113.221 | 3.072 | 12.546 | 0.077922 | 7/15/2014 | 27:31.8 | 3 |
| -159.567 | 19113.247 | 3.072 | 12.542 | 0.0619082 | 7/15/2014 | 27:31.9 | 3 |
| -159.773 | 19113.275 | 3.069 | 12.534 | 0.0522166 | 7/15/2014 | 27:32.0 | 3 |
| -159.98 | 19113.303 | 3.069 | 12.528 | 0.051261 | 7/15/2014 | 27:32.1 | 3 |
| -160.187 | 19113.332 | 3.066 | 12.521 | 0.0453164 | 7/15/2014 | 27:32.2 | 3 |
| -160.392 | 19113.359 | 3.066 | 12.51 | 0.0520756 | 7/15/2014 | 27:32.3 | 3 |
| -160.758 | 19113.408 | 3.067 | 12.5 | 0.0773559 | 7/15/2014 | 27:32.5 | 3 |
| -160.972 | 19113.438 | 3.07 | 12.491 | 0.108621 | 7/15/2014 | 27:32.6 | 3 |
| -161.072 | 19113.451 | 3.069 | 12.487 | 0.114919 | 7/15/2014 | 27:32.6 | 3 |
| -161.285 | 19113.48 | 3.069 | 12.481 | 0.122586 | 7/15/2014 | 27:32.7 | 3 |
| -161.498 | 19113.51 | 3.072 | 12.478 | 0.148945 | 7/15/2014 | 27:32.8 | 3 |
| -161.713 | 19113.539 | 3.072 | 12.474 | 0.177546 | 7/15/2014 | 27:32.9 | 3 |
| -161.928 | 19113.568 | 3.075 | 12.47 | 0.186442 | 7/15/2014 | 27:33.0 | 3 |
| -162.145 | 19113.597 | 3.078 | 12.468 | 0.183848 | 7/15/2014 | 27:33.1 | 3 |
| -162.358 | 19113.627 | 3.082 | 12.47 | 0.205545 | 7/15/2014 | 27:33.2 | 3 |
| -162.574 | 19113.655 | 3.085 | 12.468 | 0.21717 | 7/15/2014 | 27:33.3 | 3 |
| -162.784 | 19113.684 | 3.082 | 12.468 | 0.213398 | 7/15/2014 | 27:33.4 | 3 |
| -162.998 | 19113.713 | 3.082 | 12.47 | 0.211156 | 7/15/2014 | 27:33.5 | 3 |
| -163.358 | 19113.761 | 3.076 | 12.468 | 0.1837 | 7/15/2014 | 27:33.7 | 3 |
| -163.468 | 19113.776 | 3.078 | 12.467 | 0.188344 | 7/15/2014 | 27:33.7 | 3 |
| -163.679 | 19113.804 | 3.075 | 12.461 | 0.218138 | 7/15/2014 | 27:33.8 | 3 |
| -163.888 | 19113.833 | 3.075 | 12.457 | 0.174458 | 7/15/2014 | 27:33.9 | 3 |
| -164.096 | 19113.86 | 3.072 | 12.447 | 0.160505 | 7/15/2014 | 27:34.0 | 3 |
| -164.3 | 19113.888 | 3.069 | 12.44 | 0.149772 | 7/15/2014 | 27:34.1 | 3 |
| -164.509 | 19113.916 | 3.075 | 12.435 | 0.135552 | 7/15/2014 | 27:34.2 | 3 |
| -164.716 | 19113.942 | 3.075 | 12.425 | 0.121964 | 7/15/2014 | 27:34.3 | 3 |
| -165.076 | 19113.99 | 3.073 | 12.41 | 0.119681 | 7/15/2014 | 27:34.5 | 3 |
| -165.285 | 19114.019 | 3.074 | 12.404 | 0.131425 | 7/15/2014 | 27:34.6 | 3 |
| -165.49 | 19114.046 | 3.069 | 12.397 | 0.138228 | 7/15/2014 | 27:34.7 | 3 |
| -165.698 | 19114.073 | 3.067 | 12.387 | 0.178739 | 7/15/2014 | 27:34.8 | 3 |
| -165.901 | 19114.1 | 3.063 | 12.381 | 0.191176 | 7/15/2014 | 27:34.9 | 3 |
| -166.111 | 19114.127 | 3.065 | 12.376 | 0.211396 | 7/15/2014 | 27:35.0 | 3 |
| -166.313 | 19114.153 | 3.058 | 12.369 | 0.230245 | 7/15/2014 | 27:35.1 | 3 |
| -166.522 | 19114.182 | 3.061 | 12.366 | 0.295354 | 7/15/2014 | 27:35.2 | 3 |
| -166.723 | 19114.206 | 3.056 | 12.365 | 0.284134 | 7/15/2014 | 27:35.3 | 3 |
| -166.93 | 19114.234 | 3.059 | 12.369 | 0.284682 | 7/15/2014 | 27:35.4 | 3 |
| -167.129 | 19114.26 | 3.056 | 12.373 | 0.283218 | 7/15/2014 | 27:35.5 | 3 |
| -167.334 | 19114.285 | 3.061 | 12.374 | 0.243377 | 7/15/2014 | 27:35.6 | 3 |
| -167.58 | 19114.316 | 3.055 | 12.381 | 0.202328 | 7/15/2014 | 27:35.7 | 3 |
| -167.785 | 19114.342 | 3.058 | 12.387 | 0.202431 | 7/15/2014 | 27:35.8 | 3 |
| -167.983 | 19114.368 | 3.053 | 12.397 | 0.173058 | 7/15/2014 | 27:35.9 | 3 |

| | | | | | | | |
|---|---|---|---|---|---|---|---|
| -168.187 | 19114.395 | 3.055 | 12.402 | 0.126056 | 7/15/2014 | 27:36.0 | 3 |
| -168.387 | 19114.419 | 3.05 | 12.408 | 0.106564 | 7/15/2014 | 27:36.1 | 3 |
| -168.593 | 19114.445 | 3.053 | 12.41 | 0.0802176 | 7/15/2014 | 27:36.2 | 3 |
| -168.689 | 19114.457 | 3.05 | 12.41 | 0.0687114 | 7/15/2014 | 27:36.2 | 3 |
| -168.891 | 19114.481 | 3.05 | 12.411 | 0.0782561 | 7/15/2014 | 27:36.3 | 3 |
| -169.247 | 19114.525 | 3.047 | 12.42 | 0.0577588 | 7/15/2014 | 27:36.5 | 3 |
| -169.453 | 19114.551 | 3.049 | 12.423 | 0.0387221 | 7/15/2014 | 27:36.6 | 3 |
| -169.657 | 19114.576 | 3.046 | 12.425 | 0.0534279 | 7/15/2014 | 27:36.7 | 3 |
| -169.866 | 19114.602 | 3.049 | 12.424 | 0.0512278 | 7/15/2014 | 27:36.8 | 3 |
| -170.072 | 19114.627 | 3.047 | 12.43 | 0.0490113 | 7/15/2014 | 27:36.9 | 3 |
| -170.281 | 19114.652 | 3.047 | 12.432 | 0.0549355 | 7/15/2014 | 27:37.0 | 3 |
| -170.487 | 19114.677 | 3.044 | 12.435 | 0.0650591 | 7/15/2014 | 27:37.1 | 3 |
| -170.696 | 19114.701 | 3.044 | 12.441 | 0.0956477 | 7/15/2014 | 27:37.2 | 3 |
| -170.901 | 19114.726 | 3.041 | 12.442 | 0.0991206 | 7/15/2014 | 27:37.3 | 3 |
| -171.107 | 19114.75 | 3.043 | 12.442 | 0.0909163 | 7/15/2014 | 27:37.4 | 3 |
| -171.312 | 19114.773 | 3.04 | 12.442 | 0.0969298 | 7/15/2014 | 27:37.5 | 3 |
| -171.518 | 19114.797 | 3.045 | 12.442 | 0.0950766 | 7/15/2014 | 27:37.6 | 3 |
| -171.766 | 19114.826 | 3.042 | 12.442 | 0.080649 | 7/15/2014 | 27:37.7 | 3 |
| -171.87 | 19114.839 | 3.042 | 12.441 | 0.0858597 | 7/15/2014 | 27:37.7 | 3 |
| -172.073 | 19114.861 | 3.045 | 12.441 | 0.0868638 | 7/15/2014 | 27:37.8 | 3 |
| -172.369 | 19114.895 | 3.045 | 12.441 | 0.0766723 | 7/15/2014 | 27:38.0 | 3 |
| -172.57 | 19114.918 | 3.045 | 12.437 | 0.0855293 | 7/15/2014 | 27:38.1 | 3 |
| -172.77 | 19114.939 | 3.047 | 12.435 | 0.0770211 | 7/15/2014 | 27:38.2 | 3 |
| -172.966 | 19114.962 | 3.046 | 12.435 | 0.0809063 | 7/15/2014 | 27:38.3 | 3 |
| -173.165 | 19114.983 | 3.051 | 12.433 | 0.0930992 | 7/15/2014 | 27:38.4 | 3 |
| -173.405 | 19115.01 | 3.046 | 12.431 | 0.124124 | 7/15/2014 | 27:38.5 | 3 |
| -173.604 | 19115.031 | 3.049 | 12.428 | 0.125587 | 7/15/2014 | 27:38.6 | 3 |
| -173.843 | 19115.059 | 3.046 | 12.426 | 0.127488 | 7/15/2014 | 27:38.7 | 3 |
| -174.041 | 19115.078 | 3.049 | 12.426 | 0.118312 | 7/15/2014 | 27:38.8 | 3 |
| -174.236 | 19115.1 | 3.047 | 12.423 | 0.129977 | 7/15/2014 | 27:38.9 | 3 |
| -174.435 | 19115.119 | 3.047 | 12.421 | 0.162409 | 7/15/2014 | 27:39.0 | 3 |
| -174.631 | 19115.142 | 3.044 | 12.419 | 0.164625 | 7/15/2014 | 27:39.1 | 3 |
| -174.829 | 19115.162 | 3.044 | 12.416 | 0.167092 | 7/15/2014 | 27:39.2 | 3 |
| -175.024 | 19115.182 | 3.041 | 12.416 | 0.177506 | 7/15/2014 | 27:39.3 | 3 |
| -175.223 | 19115.202 | 3.044 | 12.415 | 0.170405 | 7/15/2014 | 27:39.4 | 3 |
| -175.463 | 19115.228 | 3.041 | 12.415 | 0.159308 | 7/15/2014 | 27:39.5 | 3 |
| -175.66 | 19115.247 | 3.041 | 12.414 | 0.179347 | 7/15/2014 | 27:39.6 | 3 |
| -175.907 | 19115.271 | 3.041 | 12.409 | 0.163149 | 7/15/2014 | 27:39.7 | 3 |
| -176.106 | 19115.291 | 3.048 | 12.407 | 0.1629 | 7/15/2014 | 27:39.8 | 3 |
| -176.305 | 19115.312 | 3.048 | 12.403 | 0.152588 | 7/15/2014 | 27:39.9 | 3 |
| -176.509 | 19115.331 | 3.051 | 12.403 | 0.156192 | 7/15/2014 | 27:40.0 | 3 |
| -176.607 | 19115.341 | 3.053 | 12.402 | 0.152814 | 7/15/2014 | 27:40.0 | 3 |
| -176.811 | 19115.36 | 3.053 | 12.404 | 0.141589 | 7/15/2014 | 27:40.1 | 3 |
| -177.015 | 19115.38 | 3.055 | 12.404 | 0.141096 | 7/15/2014 | 27:40.2 | 3 |
| -177.219 | 19115.399 | 3.055 | 12.404 | 0.139356 | 7/15/2014 | 27:40.3 | 3 |
| -177.587 | 19115.434 | 3.058 | 12.407 | 0.139035 | 7/15/2014 | 27:40.5 | 3 |
| -177.8 | 19115.453 | 3.06 | 12.412 | 0.144001 | 7/15/2014 | 27:40.6 | 3 |

| | | | | | | | |
|---|---|---|---|---|---|---|---|
| -177.9 | 19115.463 | 3.057 | 12.415 | 0.145743 | 7/15/2014 | 27:40.6 | 3 |
| -178.115 | 19115.481 | 3.057 | 12.422 | 0.144426 | 7/15/2014 | 27:40.7 | 3 |
| -178.331 | 19115.5 | 3.059 | 12.429 | 0.140865 | 7/15/2014 | 27:40.8 | 3 |
| -178.549 | 19115.52 | 3.059 | 12.433 | 0.139377 | 7/15/2014 | 27:40.9 | 3 |
| -178.768 | 19115.539 | 3.057 | 12.439 | 0.13597 | 7/15/2014 | 27:41.0 | 3 |
| -178.987 | 19115.559 | 3.057 | 12.447 | 0.138584 | 7/15/2014 | 27:41.1 | 3 |
| -179.207 | 19115.576 | 3.055 | 12.46 | 0.154542 | 7/15/2014 | 27:41.2 | 3 |
| -179.426 | 19115.596 | 3.055 | 12.468 | 0.167519 | 7/15/2014 | 27:41.3 | 3 |
| -179.645 | 19115.612 | 3.053 | 12.475 | 0.183547 | 7/15/2014 | 27:41.4 | 3 |
| -179.863 | 19115.63 | 3.053 | 12.483 | 0.188592 | 7/15/2014 | 27:41.5 | 3 |
| -180.081 | 19115.648 | 3.051 | 12.49 | 0.187154 | 7/15/2014 | 27:41.6 | 3 |
| -180.35 | 19115.67 | 3.051 | 12.498 | 0.196399 | 7/15/2014 | 27:41.7 | 3 |
| -180.567 | 19115.688 | 3.051 | 12.507 | 0.205405 | 7/15/2014 | 27:41.8 | 3 |
| -180.784 | 19115.703 | 3.051 | 12.515 | 0.211713 | 7/15/2014 | 27:41.9 | 3 |
| -181 | 19115.721 | 3.044 | 12.519 | 0.208859 | 7/15/2014 | 27:42.0 | 3 |
| -181.219 | 19115.736 | 3.041 | 12.529 | 0.221367 | 7/15/2014 | 27:42.1 | 3 |
| -181.436 | 19115.753 | 3.041 | 12.537 | 0.195044 | 7/15/2014 | 27:42.2 | 3 |
| -181.653 | 19115.77 | 3.038 | 12.542 | 0.225159 | 7/15/2014 | 27:42.3 | 3 |
| -181.921 | 19115.789 | 3.032 | 12.546 | 0.23022 | 7/15/2014 | 27:42.4 | 3 |
| -182.14 | 19115.805 | 3.027 | 12.553 | 0.246933 | 7/15/2014 | 27:42.5 | 3 |
| -182.36 | 19115.82 | 3.02 | 12.559 | 0.183319 | 7/15/2014 | 27:42.6 | 3 |
| -182.58 | 19115.836 | 3.015 | 12.564 | 0.190403 | 7/15/2014 | 27:42.7 | 3 |
| -182.804 | 19115.852 | 3.015 | 12.566 | 0.186767 | 7/15/2014 | 27:42.8 | 3 |
| -183.03 | 19115.866 | 3.013 | 12.566 | 0.200669 | 7/15/2014 | 27:42.9 | 3 |
| -183.253 | 19115.881 | 3.004 | 12.566 | 0.161724 | 7/15/2014 | 27:43.0 | 3 |
| -183.478 | 19115.896 | 3.002 | 12.57 | 0.155514 | 7/15/2014 | 27:43.1 | 3 |
| -183.701 | 19115.911 | 3 | 12.572 | 0.108811 | 7/15/2014 | 27:43.2 | 3 |
| -183.926 | 19115.926 | 3 | 12.572 | 0.13704 | 7/15/2014 | 27:43.3 | 3 |
| -184.15 | 19115.939 | 2.998 | 12.573 | 0.136294 | 7/15/2014 | 27:43.4 | 3 |
| -184.373 | 19115.954 | 2.994 | 12.573 | 0.107442 | 7/15/2014 | 27:43.5 | 3 |
| -184.598 | 19115.967 | 2.992 | 12.573 | 0.0994946 | 7/15/2014 | 27:43.6 | 3 |
| -184.875 | 19115.983 | 2.987 | 12.576 | 0.0965407 | 7/15/2014 | 27:43.7 | 3 |
| -185.1 | 19115.997 | 2.985 | 12.578 | 0.107197 | 7/15/2014 | 27:43.8 | 3 |
| -185.323 | 19116.011 | 2.978 | 12.579 | 0.0805303 | 7/15/2014 | 27:43.9 | 3 |
| -185.549 | 19116.023 | 2.972 | 12.579 | 0.093609 | 7/15/2014 | 27:44.0 | 3 |
| -185.775 | 19116.035 | 2.965 | 12.577 | 0.0774733 | 7/15/2014 | 27:44.1 | 3 |
| -186.002 | 19116.048 | 2.96 | 12.576 | 0.0902528 | 7/15/2014 | 27:44.2 | 3 |
| -186.23 | 19116.06 | 2.956 | 12.577 | 0.0924343 | 7/15/2014 | 27:44.3 | 3 |
| -186.512 | 19116.075 | 2.949 | 12.577 | 0.132891 | 7/15/2014 | 27:44.4 | 3 |
| -186.743 | 19116.087 | 2.946 | 12.579 | 0.139298 | 7/15/2014 | 27:44.5 | 3 |
| -186.974 | 19116.099 | 2.94 | 12.586 | 0.184781 | 7/15/2014 | 27:44.6 | 3 |
| -187.21 | 19116.109 | 2.938 | 12.588 | 0.1899 | 7/15/2014 | 27:44.7 | 3 |
| -187.444 | 19116.121 | 2.936 | 12.592 | 0.195932 | 7/15/2014 | 27:44.8 | 3 |
| -187.682 | 19116.133 | 2.934 | 12.6 | 0.210491 | 7/15/2014 | 27:44.9 | 3 |
| -187.916 | 19116.143 | 2.926 | 12.611 | 0.191161 | 7/15/2014 | 27:45.0 | 3 |
| -188.153 | 19116.154 | 2.92 | 12.615 | 0.17655 | 7/15/2014 | 27:45.1 | 3 |
| -188.389 | 19116.164 | 2.915 | 12.621 | 0.18201 | 7/15/2014 | 27:45.2 | 3 |

| | | | | | | | |
|---|---|---|---|---|---|---|---|
| -188.624 | 19116.175 | 2.906 | 12.627 | 0.155921 | 7/15/2014 | 27:45.3 | 3 |
| -188.861 | 19116.184 | 2.904 | 12.633 | 0.144545 | 7/15/2014 | 27:45.4 | 3 |
| -189.092 | 19116.193 | 2.898 | 12.643 | 0.139354 | 7/15/2014 | 27:45.5 | 3 |
| -189.325 | 19116.203 | 2.889 | 12.647 | 0.141611 | 7/15/2014 | 27:45.6 | 3 |
| -189.61 | 19116.213 | 2.883 | 12.65 | 0.135108 | 7/15/2014 | 27:45.7 | 3 |
| -189.842 | 19116.225 | 2.88 | 12.65 | 0.126373 | 7/15/2014 | 27:45.8 | 3 |
| -190.073 | 19116.232 | 2.877 | 12.656 | 0.102412 | 7/15/2014 | 27:45.9 | 3 |
| -190.304 | 19116.242 | 2.874 | 12.659 | 0.112005 | 7/15/2014 | 27:46.0 | 3 |
| -190.536 | 19116.25 | 2.87 | 12.662 | 0.101863 | 7/15/2014 | 27:46.1 | 3 |
| -190.767 | 19116.258 | 2.863 | 12.662 | 0.103958 | 7/15/2014 | 27:46.2 | 3 |
| -190.996 | 19116.264 | 2.859 | 12.664 | 0.0931076 | 7/15/2014 | 27:46.3 | 3 |
| -191.285 | 19116.273 | 2.857 | 12.67 | 0.102148 | 7/15/2014 | 27:46.4 | 3 |
| -191.516 | 19116.283 | 2.853 | 12.676 | 0.0987776 | 7/15/2014 | 27:46.5 | 3 |
| -191.75 | 19116.291 | 2.848 | 12.676 | 0.101012 | 7/15/2014 | 27:46.6 | 3 |
| -191.985 | 19116.298 | 2.844 | 12.678 | 0.0905303 | 7/15/2014 | 27:46.7 | 3 |
| -192.221 | 19116.305 | 2.842 | 12.682 | 0.0927261 | 7/15/2014 | 27:46.8 | 3 |
| -192.457 | 19116.313 | 2.839 | 12.684 | 0.0815385 | 7/15/2014 | 27:46.9 | 3 |
| -192.693 | 19116.318 | 2.835 | 12.684 | 0.0782639 | 7/15/2014 | 27:47.0 | 3 |
| -192.929 | 19116.324 | 2.831 | 12.683 | 0.0850184 | 7/15/2014 | 27:47.1 | 3 |
| -193.165 | 19116.332 | 2.829 | 12.683 | 0.0942411 | 7/15/2014 | 27:47.2 | 3 |
| -193.401 | 19116.338 | 2.826 | 12.682 | 0.111696 | 7/15/2014 | 27:47.3 | 3 |
| -193.635 | 19116.344 | 2.823 | 12.684 | 0.109181 | 7/15/2014 | 27:47.4 | 3 |
| -193.867 | 19116.35 | 2.816 | 12.685 | 0.120153 | 7/15/2014 | 27:47.5 | 3 |
| -194.099 | 19116.355 | 2.811 | 12.685 | 0.116267 | 7/15/2014 | 27:47.6 | 3 |
| -194.384 | 19116.363 | 2.808 | 12.686 | 0.0971022 | 7/15/2014 | 27:47.7 | 3 |
| -194.615 | 19116.367 | 2.805 | 12.687 | 0.10796 | 7/15/2014 | 27:47.8 | 3 |
| -194.845 | 19116.373 | 2.801 | 12.686 | 0.0958768 | 7/15/2014 | 27:47.9 | 3 |
| -195.077 | 19116.378 | 2.798 | 12.685 | 0.0901369 | 7/15/2014 | 27:48.0 | 3 |
| -195.304 | 19116.384 | 2.793 | 12.684 | 0.0719309 | 7/15/2014 | 27:48.1 | 3 |
| -195.531 | 19116.389 | 2.79 | 12.685 | 0.0629339 | 7/15/2014 | 27:48.2 | 3 |
| -195.759 | 19116.393 | 2.787 | 12.687 | 0.0681561 | 7/15/2014 | 27:48.3 | 3 |
| -196.039 | 19116.398 | 2.782 | 12.689 | 0.0575227 | 7/15/2014 | 27:48.4 | 3 |
| -196.267 | 19116.403 | 2.78 | 12.69 | 0.0453233 | 7/15/2014 | 27:48.5 | 3 |
| -196.49 | 19116.407 | 2.777 | 12.691 | 0.0459303 | 7/15/2014 | 27:48.6 | 3 |
| -196.774 | 19116.413 | 2.775 | 12.697 | 0.0495133 | 7/15/2014 | 27:48.7 | 3 |
| -196.999 | 19116.418 | 2.769 | 12.703 | 0.0550327 | 7/15/2014 | 27:48.8 | 3 |
| -197.224 | 19116.422 | 2.764 | 12.707 | 0.0590353 | 7/15/2014 | 27:48.9 | 3 |
| -197.453 | 19116.426 | 2.762 | 12.71 | 0.0601072 | 7/15/2014 | 27:49.0 | 3 |
| -197.678 | 19116.43 | 2.76 | 12.714 | 0.0682096 | 7/15/2014 | 27:49.1 | 3 |
| -197.905 | 19116.435 | 2.756 | 12.716 | 0.0807341 | 7/15/2014 | 27:49.2 | 3 |
| -198.128 | 19116.438 | 2.751 | 12.723 | 0.0570029 | 7/15/2014 | 27:49.3 | 3 |
| -198.408 | 19116.443 | 2.746 | 12.728 | 0.0870916 | 7/15/2014 | 27:49.4 | 3 |
| -198.629 | 19116.447 | 2.744 | 12.727 | 0.0699101 | 7/15/2014 | 27:49.5 | 3 |
| -198.851 | 19116.451 | 2.744 | 12.728 | 0.0651755 | 7/15/2014 | 27:49.6 | 3 |
| -199.128 | 19116.455 | 2.744 | 12.727 | 0.0655709 | 7/15/2014 | 27:49.7 | 3 |
| -199.349 | 19116.459 | 2.742 | 12.729 | 0.0689074 | 7/15/2014 | 27:49.8 | 3 |
| -199.573 | 19116.463 | 2.738 | 12.729 | 0.0615036 | 7/15/2014 | 27:49.9 | 3 |

| | | | | | | | |
|---|---|---|---|---|---|---|---|
| -199.796 | 19116.467 | 2.736 | 12.728 | 0.0887602 | 7/15/2014 | 27:50.0 | 3 |
| -200.021 | 19116.471 | 2.736 | 12.722 | 0.0645584 | 7/15/2014 | 27:50.1 | 3 |
| -200.243 | 19116.474 | 2.734 | 12.727 | 0.0695614 | 7/15/2014 | 27:50.2 | 3 |
| -200.466 | 19116.478 | 2.727 | 12.726 | 0.0756394 | 7/15/2014 | 27:50.3 | 3 |
| -200.744 | 19116.481 | 2.725 | 12.723 | 0.105127 | 7/15/2014 | 27:50.4 | 3 |
| -200.968 | 19116.484 | 2.725 | 12.722 | 0.113698 | 7/15/2014 | 27:50.5 | 3 |
| -201.193 | 19116.488 | 2.721 | 12.719 | 0.122433 | 7/15/2014 | 27:50.6 | 3 |
| -201.418 | 19116.492 | 2.721 | 12.715 | 0.119635 | 7/15/2014 | 27:50.7 | 3 |
| -201.644 | 19116.496 | 2.721 | 12.715 | 0.155297 | 7/15/2014 | 27:50.8 | 3 |
| -201.871 | 19116.499 | 2.721 | 12.713 | 0.176228 | 7/15/2014 | 27:50.9 | 3 |
| -202.096 | 19116.503 | 2.721 | 12.706 | 0.205056 | 7/15/2014 | 27:51.0 | 3 |
| -202.322 | 19116.507 | 2.724 | 12.705 | 0.204591 | 7/15/2014 | 27:51.1 | 3 |
| -202.547 | 19116.509 | 2.72 | 12.702 | 0.197775 | 7/15/2014 | 27:51.2 | 3 |
| -202.77 | 19116.513 | 2.716 | 12.7 | 0.184712 | 7/15/2014 | 27:51.3 | 3 |
| -202.993 | 19116.517 | 2.716 | 12.697 | 0.180005 | 7/15/2014 | 27:51.4 | 3 |
| -203.214 | 19116.52 | 2.714 | 12.691 | 0.181276 | 7/15/2014 | 27:51.5 | 3 |
| -203.434 | 19116.523 | 2.714 | 12.683 | 0.183874 | 7/15/2014 | 27:51.6 | 3 |
| -203.705 | 19116.528 | 2.71 | 12.676 | 0.155928 | 7/15/2014 | 27:51.7 | 3 |
| -203.925 | 19116.531 | 2.71 | 12.664 | 0.166074 | 7/15/2014 | 27:51.8 | 3 |
| -204.144 | 19116.535 | 2.708 | 12.656 | 0.189678 | 7/15/2014 | 27:51.9 | 3 |
| -204.363 | 19116.538 | 2.708 | 12.652 | 0.1851 | 7/15/2014 | 27:52.0 | 3 |
| -204.584 | 19116.542 | 2.712 | 12.646 | 0.198451 | 7/15/2014 | 27:52.1 | 3 |
| -204.803 | 19116.545 | 2.712 | 12.643 | 0.193897 | 7/15/2014 | 27:52.2 | 3 |
| -205.022 | 19116.549 | 2.71 | 12.642 | 0.166928 | 7/15/2014 | 27:52.3 | 3 |
| -205.296 | 19116.554 | 2.71 | 12.644 | 0.206926 | 7/15/2014 | 27:52.4 | 3 |
| -205.519 | 19116.557 | 2.71 | 12.657 | 0.170198 | 7/15/2014 | 27:52.5 | 3 |
| -205.743 | 19116.562 | 2.71 | 12.665 | 0.137444 | 7/15/2014 | 27:52.6 | 3 |
| -205.968 | 19116.564 | 2.71 | 12.674 | 0.166491 | 7/15/2014 | 27:52.7 | 3 |
| -206.196 | 19116.568 | 2.71 | 12.681 | 0.147745 | 7/15/2014 | 27:52.8 | 3 |
| -206.422 | 19116.572 | 2.705 | 12.69 | 0.158534 | 7/15/2014 | 27:52.9 | 3 |
| -206.65 | 19116.576 | 2.705 | 12.699 | 0.154862 | 7/15/2014 | 27:53.0 | 3 |
| -206.878 | 19116.58 | 2.707 | 12.71 | 0.12059 | 7/15/2014 | 27:53.1 | 3 |
| -207.105 | 19116.584 | 2.707 | 12.721 | 0.16035 | 7/15/2014 | 27:53.2 | 3 |
| -207.333 | 19116.588 | 2.709 | 12.72 | 0.15301 | 7/15/2014 | 27:53.3 | 3 |
| -207.558 | 19116.592 | 2.709 | 12.726 | 0.126872 | 7/15/2014 | 27:53.4 | 3 |
| -207.782 | 19116.596 | 2.711 | 12.731 | 0.150449 | 7/15/2014 | 27:53.5 | 3 |
| -208.004 | 19116.6 | 2.711 | 12.735 | 0.137047 | 7/15/2014 | 27:53.6 | 3 |
| -208.279 | 19116.605 | 2.711 | 12.737 | 0.126189 | 7/15/2014 | 27:53.7 | 3 |
| -208.5 | 19116.609 | 2.709 | 12.735 | 0.139778 | 7/15/2014 | 27:53.8 | 3 |
| -208.722 | 19116.613 | 2.707 | 12.742 | 0.119327 | 7/15/2014 | 27:53.9 | 3 |
| -208.943 | 19116.617 | 2.704 | 12.744 | 0.11847 | 7/15/2014 | 27:54.0 | 3 |
| -209.164 | 19116.621 | 2.702 | 12.745 | 0.123759 | 7/15/2014 | 27:54.1 | 3 |
| -209.383 | 19116.625 | 2.702 | 12.741 | 0.118184 | 7/15/2014 | 27:54.2 | 3 |
| -209.603 | 19116.629 | 2.702 | 12.736 | 0.113478 | 7/15/2014 | 27:54.3 | 3 |
| -209.875 | 19116.636 | 2.702 | 12.734 | 0.114547 | 7/15/2014 | 27:54.4 | 3 |
| -210.094 | 19116.64 | 2.704 | 12.73 | 0.108237 | 7/15/2014 | 27:54.5 | 3 |
| -210.316 | 19116.644 | 2.707 | 12.726 | 0.108731 | 7/15/2014 | 27:54.6 | 3 |

| | | | | | | | |
|---|---|---|---|---|---|---|---|
| -210.537 | 19116.648 | 2.707 | 12.714 | 0.112934 | 7/15/2014 | 27:54.7 | 3 |
| -210.76 | 19116.652 | 2.707 | 12.706 | 0.112304 | 7/15/2014 | 27:54.8 | 3 |
| -210.982 | 19116.657 | 2.707 | 12.701 | 0.108002 | 7/15/2014 | 27:54.9 | 3 |
| -211.203 | 19116.661 | 2.702 | 12.703 | 0.105191 | 7/15/2014 | 27:55.0 | 3 |
| -211.427 | 19116.667 | 2.702 | 12.702 | 0.110698 | 7/15/2014 | 27:55.1 | 3 |
| -211.647 | 19116.671 | 2.7 | 12.702 | 0.105252 | 7/15/2014 | 27:55.2 | 3 |
| -211.868 | 19116.675 | 2.698 | 12.7 | 0.113822 | 7/15/2014 | 27:55.3 | 3 |
| -212.086 | 19116.682 | 2.698 | 12.702 | 0.0994169 | 7/15/2014 | 27:55.4 | 3 |
| -212.304 | 19116.686 | 2.7 | 12.701 | 0.106933 | 7/15/2014 | 27:55.5 | 3 |
| -212.52 | 19116.69 | 2.702 | 12.696 | 0.111621 | 7/15/2014 | 27:55.6 | 3 |
| -212.786 | 19116.696 | 2.704 | 12.693 | 0.099285 | 7/15/2014 | 27:55.7 | 3 |
| -213.002 | 19116.702 | 2.709 | 12.683 | 0.0952649 | 7/15/2014 | 27:55.8 | 3 |
| -213.217 | 19116.707 | 2.709 | 12.681 | 0.114086 | 7/15/2014 | 27:55.9 | 3 |
| -213.433 | 19116.711 | 2.711 | 12.672 | 0.0998055 | 7/15/2014 | 27:56.0 | 3 |
| -213.648 | 19116.719 | 2.713 | 12.665 | 0.123106 | 7/15/2014 | 27:56.1 | 3 |
| -213.863 | 19116.724 | 2.713 | 12.656 | 0.118964 | 7/15/2014 | 27:56.2 | 3 |
| -214.078 | 19116.729 | 2.711 | 12.648 | 0.10949 | 7/15/2014 | 27:56.3 | 3 |
| -214.345 | 19116.735 | 2.714 | 12.65 | 0.122528 | 7/15/2014 | 27:56.4 | 3 |
| -214.562 | 19116.741 | 2.712 | 12.648 | 0.116123 | 7/15/2014 | 27:56.5 | 3 |
| -214.778 | 19116.748 | 2.71 | 12.646 | 0.101065 | 7/15/2014 | 27:56.6 | 3 |
| -214.999 | 19116.753 | 2.71 | 12.644 | 0.100637 | 7/15/2014 | 27:56.7 | 3 |
| -215.218 | 19116.76 | 2.71 | 12.644 | 0.101054 | 7/15/2014 | 27:56.8 | 3 |
| -215.439 | 19116.767 | 2.71 | 12.648 | 0.0930574 | 7/15/2014 | 27:56.9 | 3 |
| -215.659 | 19116.773 | 2.71 | 12.654 | 0.091289 | 7/15/2014 | 27:57.0 | 3 |
| -215.879 | 19116.78 | 2.712 | 12.657 | 0.0925782 | 7/15/2014 | 27:57.1 | 3 |
| -216.099 | 19116.786 | 2.712 | 12.652 | 0.0935351 | 7/15/2014 | 27:57.2 | 3 |
| -216.317 | 19116.793 | 2.714 | 12.653 | 0.0988151 | 7/15/2014 | 27:57.3 | 3 |
| -216.535 | 19116.8 | 2.716 | 12.656 | 0.0920739 | 7/15/2014 | 27:57.4 | 3 |
| -216.751 | 19116.807 | 2.716 | 12.652 | 0.0894698 | 7/15/2014 | 27:57.5 | 3 |
| -216.965 | 19116.814 | 2.716 | 12.654 | 0.0894154 | 7/15/2014 | 27:57.6 | 3 |
| -217.23 | 19116.824 | 2.716 | 12.654 | 0.08454 | 7/15/2014 | 27:57.7 | 3 |
| -217.442 | 19116.832 | 2.714 | 12.648 | 0.101394 | 7/15/2014 | 27:57.8 | 3 |
| -217.656 | 19116.839 | 2.714 | 12.646 | 0.0842905 | 7/15/2014 | 27:57.9 | 3 |
| -217.868 | 19116.846 | 2.712 | 12.643 | 0.0801248 | 7/15/2014 | 27:58.0 | 3 |
| -218.081 | 19116.855 | 2.712 | 12.637 | 0.0831441 | 7/15/2014 | 27:58.1 | 3 |
| -218.293 | 19116.863 | 2.715 | 12.63 | 0.0733929 | 7/15/2014 | 27:58.2 | 3 |
| -218.505 | 19116.871 | 2.715 | 12.627 | 0.0722652 | 7/15/2014 | 27:58.3 | 3 |
| -218.876 | 19116.887 | 2.715 | 12.616 | 0.0707264 | 7/15/2014 | 27:58.5 | 3 |
| -218.98 | 19116.891 | 2.716 | 12.61 | 0.0745772 | 7/15/2014 | 27:58.5 | 3 |
| -219.194 | 19116.898 | 2.719 | 12.602 | 0.0695009 | 7/15/2014 | 27:58.6 | 3 |
| -219.408 | 19116.908 | 2.719 | 12.591 | 0.0723713 | 7/15/2014 | 27:58.7 | 3 |
| -219.624 | 19116.919 | 2.721 | 12.584 | 0.0734782 | 7/15/2014 | 27:58.8 | 3 |
| -219.84 | 19116.928 | 2.725 | 12.573 | 0.0702343 | 7/15/2014 | 27:58.9 | 3 |
| -220.055 | 19116.938 | 2.725 | 12.565 | 0.0821237 | 7/15/2014 | 27:59.0 | 3 |
| -220.271 | 19116.947 | 2.73 | 12.556 | 0.0761432 | 7/15/2014 | 27:59.1 | 3 |
| -220.485 | 19116.957 | 2.732 | 12.549 | 0.107566 | 7/15/2014 | 27:59.2 | 3 |
| -220.801 | 19116.972 | 2.737 | 12.54 | 0.120686 | 7/15/2014 | 27:59.4 | 3 |

| | | | | | | | |
|---|---|---|---|---|---|---|---|
| -221.012 | 19116.982 | 2.738 | 12.534 | 0.149878 | 7/15/2014 | 27:59.5 | 3 |
| -221.222 | 19116.993 | 2.743 | 12.529 | 0.164602 | 7/15/2014 | 27:59.6 | 3 |
| -221.479 | 19117.006 | 2.741 | 12.523 | 0.163456 | 7/15/2014 | 27:59.7 | 3 |
| -221.588 | 19117.012 | 2.743 | 12.521 | 0.162027 | 7/15/2014 | 27:59.7 | 3 |
| -221.797 | 19117.022 | 2.745 | 12.514 | 0.161997 | 7/15/2014 | 27:59.8 | 3 |
| -222.005 | 19117.033 | 2.745 | 12.51 | 0.148035 | 7/15/2014 | 27:59.9 | 3 |
| -222.212 | 19117.045 | 2.745 | 12.505 | 0.128858 | 7/15/2014 | 28:00.0 | 3 |
| -222.421 | 19117.056 | 2.747 | 12.499 | 0.132231 | 7/15/2014 | 28:00.1 | 3 |
| -222.628 | 19117.066 | 2.749 | 12.494 | 0.118533 | 7/15/2014 | 28:00.2 | 3 |
| -222.836 | 19117.079 | 2.751 | 12.49 | 0.1122 | 7/15/2014 | 28:00.3 | 3 |
| -223.201 | 19117.101 | 2.757 | 12.48 | 0.10767 | 7/15/2014 | 28:00.5 | 3 |
| -223.413 | 19117.112 | 2.766 | 12.473 | 0.10704 | 7/15/2014 | 28:00.6 | 3 |
| -223.622 | 19117.125 | 2.766 | 12.468 | 0.106876 | 7/15/2014 | 28:00.7 | 3 |
| -223.835 | 19117.138 | 2.772 | 12.459 | 0.104991 | 7/15/2014 | 28:00.8 | 3 |
| -223.94 | 19117.144 | 2.776 | 12.454 | 0.106164 | 7/15/2014 | 28:00.8 | 3 |
| -224.152 | 19117.156 | 2.781 | 12.445 | 0.105215 | 7/15/2014 | 28:00.9 | 3 |
| -224.367 | 19117.17 | 2.786 | 12.438 | 0.101915 | 7/15/2014 | 28:01.0 | 3 |
| -224.579 | 19117.184 | 2.789 | 12.433 | 0.11296 | 7/15/2014 | 28:01.1 | 3 |
| -224.792 | 19117.197 | 2.789 | 12.429 | 0.102935 | 7/15/2014 | 28:01.2 | 3 |
| -225.111 | 19117.218 | 2.796 | 12.424 | 0.105765 | 7/15/2014 | 28:01.4 | 3 |
| -225.319 | 19117.231 | 2.794 | 12.418 | 0.10342 | 7/15/2014 | 28:01.5 | 3 |
| -225.531 | 19117.245 | 2.8 | 12.412 | 0.10591 | 7/15/2014 | 28:01.6 | 3 |
| -225.791 | 19117.264 | 2.8 | 12.405 | 0.107598 | 7/15/2014 | 28:01.7 | 3 |
| -225.899 | 19117.271 | 2.801 | 12.403 | 0.108749 | 7/15/2014 | 28:01.7 | 3 |
| -226.108 | 19117.284 | 2.803 | 12.394 | 0.106672 | 7/15/2014 | 28:01.8 | 3 |
| -226.317 | 19117.298 | 2.803 | 12.382 | 0.121419 | 7/15/2014 | 28:01.9 | 3 |
| -226.53 | 19117.314 | 2.808 | 12.369 | 0.113225 | 7/15/2014 | 28:02.0 | 3 |
| -226.739 | 19117.329 | 2.808 | 12.355 | 0.126003 | 7/15/2014 | 28:02.1 | 3 |
| -226.95 | 19117.344 | 2.81 | 12.344 | 0.133626 | 7/15/2014 | 28:02.2 | 3 |
| -227.163 | 19117.359 | 2.814 | 12.337 | 0.139958 | 7/15/2014 | 28:02.3 | 3 |
| -227.426 | 19117.379 | 2.814 | 12.325 | 0.15246 | 7/15/2014 | 28:02.4 | 3 |
| -227.639 | 19117.394 | 2.816 | 12.316 | 0.158347 | 7/15/2014 | 28:02.5 | 3 |
| -227.854 | 19117.41 | 2.823 | 12.308 | 0.138239 | 7/15/2014 | 28:02.6 | 3 |
| -228.069 | 19117.427 | 2.827 | 12.3 | 0.173413 | 7/15/2014 | 28:02.7 | 3 |
| -228.284 | 19117.443 | 2.827 | 12.293 | 0.164916 | 7/15/2014 | 28:02.8 | 3 |
| -228.502 | 19117.46 | 2.829 | 12.284 | 0.145718 | 7/15/2014 | 28:02.9 | 3 |
| -228.721 | 19117.479 | 2.832 | 12.276 | 0.144322 | 7/15/2014 | 28:03.0 | 3 |
| -228.936 | 19117.494 | 2.832 | 12.265 | 0.141144 | 7/15/2014 | 28:03.1 | 3 |
| -229.152 | 19117.512 | 2.835 | 12.261 | 0.142143 | 7/15/2014 | 28:03.2 | 3 |
| -229.369 | 19117.529 | 2.835 | 12.254 | 0.142139 | 7/15/2014 | 28:03.3 | 3 |
| -229.581 | 19117.547 | 2.832 | 12.248 | 0.142981 | 7/15/2014 | 28:03.4 | 3 |
| -229.898 | 19117.573 | 2.837 | 12.242 | 0.145303 | 7/15/2014 | 28:03.6 | 3 |
| -230.159 | 19117.594 | 2.837 | 12.238 | 0.143665 | 7/15/2014 | 28:03.7 | 3 |
| -230.269 | 19117.604 | 2.838 | 12.238 | 0.143646 | 7/15/2014 | 28:03.7 | 3 |
| -230.481 | 19117.621 | 2.842 | 12.234 | 0.144356 | 7/15/2014 | 28:03.8 | 3 |
| -230.693 | 19117.641 | 2.845 | 12.23 | 0.143972 | 7/15/2014 | 28:03.9 | 3 |
| -230.905 | 19117.658 | 2.848 | 12.226 | 0.14385 | 7/15/2014 | 28:04.0 | 3 |

| | | | | | | | |
|---|---|---|---|---|---|---|---|
| -231.116 | 19117.678 | 2.851 | 12.224 | 0.145395 | 7/15/2014 | 28:04.1 | 3 |
| -231.331 | 19117.695 | 2.854 | 12.225 | 0.150655 | 7/15/2014 | 28:04.2 | 3 |
| -231.544 | 19117.715 | 2.854 | 12.226 | 0.144678 | 7/15/2014 | 28:04.3 | 3 |
| -231.81 | 19117.736 | 2.854 | 12.227 | 0.149483 | 7/15/2014 | 28:04.4 | 3 |
| -232.025 | 19117.757 | 2.854 | 12.229 | 0.145915 | 7/15/2014 | 28:04.5 | 3 |
| -232.245 | 19117.777 | 2.851 | 12.228 | 0.155547 | 7/15/2014 | 28:04.6 | 3 |
| -232.463 | 19117.797 | 2.851 | 12.226 | 0.151089 | 7/15/2014 | 28:04.7 | 3 |
| -232.683 | 19117.817 | 2.851 | 12.224 | 0.145074 | 7/15/2014 | 28:04.8 | 3 |
| -232.907 | 19117.838 | 2.851 | 12.221 | 0.142091 | 7/15/2014 | 28:04.9 | 3 |
| -233.128 | 19117.858 | 2.851 | 12.214 | 0.152719 | 7/15/2014 | 28:05.0 | 3 |
| -233.352 | 19117.879 | 2.855 | 12.206 | 0.148767 | 7/15/2014 | 28:05.1 | 3 |
| -233.573 | 19117.9 | 2.855 | 12.201 | 0.143437 | 7/15/2014 | 28:05.2 | 3 |
| -233.796 | 19117.922 | 2.861 | 12.193 | 0.145521 | 7/15/2014 | 28:05.3 | 3 |
| -234.015 | 19117.942 | 2.864 | 12.191 | 0.157021 | 7/15/2014 | 28:05.4 | 3 |
| -234.235 | 19117.964 | 2.861 | 12.186 | 0.170825 | 7/15/2014 | 28:05.5 | 3 |
| -234.456 | 19117.984 | 2.861 | 12.191 | 0.169161 | 7/15/2014 | 28:05.6 | 3 |
| -234.726 | 19118.011 | 2.858 | 12.186 | 0.175262 | 7/15/2014 | 28:05.7 | 3 |
| -234.947 | 19118.033 | 2.858 | 12.185 | 0.171088 | 7/15/2014 | 28:05.8 | 3 |
| -235.166 | 19118.055 | 2.858 | 12.179 | 0.164207 | 7/15/2014 | 28:05.9 | 3 |
| -235.385 | 19118.076 | 2.858 | 12.17 | 0.152936 | 7/15/2014 | 28:06.0 | 3 |
| -235.604 | 19118.099 | 2.858 | 12.164 | 0.134313 | 7/15/2014 | 28:06.1 | 3 |
| -235.821 | 19118.121 | 2.858 | 12.153 | 0.144269 | 7/15/2014 | 28:06.2 | 3 |
| -236.042 | 19118.143 | 2.864 | 12.149 | 0.129517 | 7/15/2014 | 28:06.3 | 3 |
| -236.312 | 19118.17 | 2.864 | 12.136 | 0.129523 | 7/15/2014 | 28:06.4 | 3 |
| -236.534 | 19118.193 | 2.87 | 12.13 | 0.13956 | 7/15/2014 | 28:06.5 | 3 |
| -236.755 | 19118.216 | 2.87 | 12.121 | 0.159218 | 7/15/2014 | 28:06.6 | 3 |
| -236.977 | 19118.239 | 2.873 | 12.119 | 0.149289 | 7/15/2014 | 28:06.7 | 3 |
| -237.199 | 19118.262 | 2.876 | 12.113 | 0.165087 | 7/15/2014 | 28:06.8 | 3 |
| -237.422 | 19118.287 | 2.876 | 12.106 | 0.16936 | 7/15/2014 | 28:06.9 | 3 |
| -237.645 | 19118.311 | 2.878 | 12.102 | 0.185374 | 7/15/2014 | 28:07.0 | 3 |
| -237.867 | 19118.334 | 2.878 | 12.097 | 0.178876 | 7/15/2014 | 28:07.1 | 3 |
| -238.088 | 19118.357 | 2.878 | 12.093 | 0.186691 | 7/15/2014 | 28:07.2 | 3 |
| -238.306 | 19118.381 | 2.874 | 12.089 | 0.15623 | 7/15/2014 | 28:07.3 | 3 |
| -238.526 | 19118.405 | 2.874 | 12.089 | 0.185219 | 7/15/2014 | 28:07.4 | 3 |
| -238.743 | 19118.428 | 2.874 | 12.085 | 0.14642 | 7/15/2014 | 28:07.5 | 3 |
| -238.959 | 19118.453 | 2.872 | 12.085 | 0.122483 | 7/15/2014 | 28:07.6 | 3 |
| -239.227 | 19118.482 | 2.869 | 12.085 | 0.103639 | 7/15/2014 | 28:07.7 | 3 |
| -239.441 | 19118.506 | 2.867 | 12.084 | 0.0738151 | 7/15/2014 | 28:07.8 | 3 |
| -239.657 | 19118.529 | 2.867 | 12.08 | 0.0380133 | 7/15/2014 | 28:07.9 | 3 |
| -239.871 | 19118.553 | 2.867 | 12.074 | 0.0805001 | 7/15/2014 | 28:08.0 | 3 |
| -240.088 | 19118.578 | 2.87 | 12.072 | 0.0473973 | 7/15/2014 | 28:08.1 | 3 |
| -240.305 | 19118.602 | 2.873 | 12.066 | 0.0625568 | 7/15/2014 | 28:08.2 | 3 |
| -240.519 | 19118.626 | 2.869 | 12.065 | 0.055215 | 7/15/2014 | 28:08.3 | 3 |
| -240.788 | 19118.656 | 2.869 | 12.066 | 0.0415893 | 7/15/2014 | 28:08.4 | 3 |
| -241.006 | 19118.682 | 2.873 | 12.073 | 0.0482298 | 7/15/2014 | 28:08.5 | 3 |
| -241.225 | 19118.706 | 2.873 | 12.074 | 0.0519576 | 7/15/2014 | 28:08.6 | 3 |
| -241.443 | 19118.73 | 2.869 | 12.074 | 0.0385199 | 7/15/2014 | 28:08.7 | 3 |

| | | | | | | | |
|---|---|---|---|---|---|---|---|
| -241.667 | 19118.758 | 2.866 | 12.075 | 0.0464647 | 7/15/2014 | 28:08.8 | 3 |
| -241.89 | 19118.782 | 2.866 | 12.077 | 0.033306 | 7/15/2014 | 28:08.9 | 3 |
| -242.111 | 19118.809 | 2.864 | 12.079 | 0.0330176 | 7/15/2014 | 28:09.0 | 3 |
| -242.332 | 19118.834 | 2.861 | 12.081 | 0.0471957 | 7/15/2014 | 28:09.1 | 3 |
| -242.555 | 19118.861 | 2.861 | 12.08 | 0.0390422 | 7/15/2014 | 28:09.2 | 3 |
| -242.776 | 19118.887 | 2.857 | 12.078 | 0.0402381 | 7/15/2014 | 28:09.3 | 3 |
| -242.993 | 19118.912 | 2.854 | 12.079 | 0.0380689 | 7/15/2014 | 28:09.4 | 3 |
| -243.213 | 19118.938 | 2.854 | 12.081 | 0.0359547 | 7/15/2014 | 28:09.5 | 3 |
| -243.43 | 19118.964 | 2.857 | 12.081 | 0.0318906 | 7/15/2014 | 28:09.6 | 3 |
| -243.698 | 19118.996 | 2.851 | 12.072 | 0.0395502 | 7/15/2014 | 28:09.7 | 3 |
| -243.914 | 19119.022 | 2.85 | 12.066 | 0.0385433 | 7/15/2014 | 28:09.8 | 3 |
| -244.132 | 19119.049 | 2.854 | 12.055 | 0.0408765 | 7/15/2014 | 28:09.9 | 3 |
| -244.349 | 19119.075 | 2.85 | 12.044 | 0.040986 | 7/15/2014 | 28:10.0 | 3 |
| -244.566 | 19119.102 | 2.85 | 12.028 | 0.0461001 | 7/15/2014 | 28:10.1 | 3 |
| -244.782 | 19119.128 | 2.85 | 12.018 | 0.047935 | 7/15/2014 | 28:10.2 | 3 |
| -244.998 | 19119.154 | 2.85 | 12.005 | 0.054784 | 7/15/2014 | 28:10.3 | 3 |
| -245.265 | 19119.187 | 2.847 | 11.99 | 0.0726694 | 7/15/2014 | 28:10.4 | 3 |
| -245.483 | 19119.214 | 2.847 | 11.982 | 0.0600079 | 7/15/2014 | 28:10.5 | 3 |
| -245.703 | 19119.24 | 2.844 | 11.973 | 0.0645606 | 7/15/2014 | 28:10.6 | 3 |
| -245.92 | 19119.268 | 2.839 | 11.97 | 0.0650672 | 7/15/2014 | 28:10.7 | 3 |
| -246.139 | 19119.296 | 2.836 | 11.966 | 0.0662021 | 7/15/2014 | 28:10.8 | 3 |
| -246.359 | 19119.323 | 2.832 | 11.966 | 0.0672304 | 7/15/2014 | 28:10.9 | 3 |
| -246.579 | 19119.352 | 2.832 | 11.963 | 0.0671091 | 7/15/2014 | 28:11.0 | 3 |
| -246.796 | 19119.379 | 2.827 | 11.967 | 0.0711756 | 7/15/2014 | 28:11.1 | 3 |
| -247.012 | 19119.406 | 2.822 | 11.974 | 0.067787 | 7/15/2014 | 28:11.2 | 3 |
| -247.23 | 19119.433 | 2.822 | 11.977 | 0.0694654 | 7/15/2014 | 28:11.3 | 3 |
| -247.444 | 19119.46 | 2.822 | 11.979 | 0.0710898 | 7/15/2014 | 28:11.4 | 3 |
| -247.756 | 19119.501 | 2.822 | 11.976 | 0.0724615 | 7/15/2014 | 28:11.6 | 3 |
| -248.017 | 19119.534 | 2.82 | 11.976 | 0.0825393 | 7/15/2014 | 28:11.7 | 3 |
| -248.127 | 19119.549 | 2.819 | 11.976 | 0.0825021 | 7/15/2014 | 28:11.7 | 3 |
| -248.335 | 19119.576 | 2.816 | 11.974 | 0.0835016 | 7/15/2014 | 28:11.8 | 3 |
| -248.544 | 19119.604 | 2.816 | 11.97 | 0.0836338 | 7/15/2014 | 28:11.9 | 3 |
| -248.755 | 19119.63 | 2.822 | 11.967 | 0.0836359 | 7/15/2014 | 28:12.0 | 3 |
| -248.964 | 19119.657 | 2.819 | 11.968 | 0.0845478 | 7/15/2014 | 28:12.1 | 3 |
| -249.173 | 19119.684 | 2.817 | 11.974 | 0.0845904 | 7/15/2014 | 28:12.2 | 3 |
| -249.379 | 19119.711 | 2.814 | 11.978 | 0.0858387 | 7/15/2014 | 28:12.3 | 3 |
| -249.746 | 19119.76 | 2.808 | 12 | 0.0947443 | 7/15/2014 | 28:12.5 | 3 |
| -249.955 | 19119.787 | 2.81 | 12.01 | 0.0932116 | 7/15/2014 | 28:12.6 | 3 |
| -250.165 | 19119.814 | 2.804 | 12.019 | 0.0945999 | 7/15/2014 | 28:12.7 | 3 |
| -250.27 | 19119.828 | 2.804 | 12.024 | 0.0983024 | 7/15/2014 | 28:12.7 | 3 |
| -250.482 | 19119.857 | 2.798 | 12.032 | 0.101382 | 7/15/2014 | 28:12.8 | 3 |
| -250.696 | 19119.885 | 2.795 | 12.036 | 0.104705 | 7/15/2014 | 28:12.9 | 3 |
| -250.906 | 19119.912 | 2.79 | 12.042 | 0.107262 | 7/15/2014 | 28:13.0 | 3 |
| -251.119 | 19119.941 | 2.787 | 12.047 | 0.107864 | 7/15/2014 | 28:13.1 | 3 |
| -251.331 | 19119.971 | 2.784 | 12.04 | 0.111682 | 7/15/2014 | 28:13.2 | 3 |
| -251.644 | 19120.012 | 2.782 | 12.037 | 0.103402 | 7/15/2014 | 28:13.4 | 3 |
| -251.85 | 19120.039 | 2.775 | 12.033 | 0.108937 | 7/15/2014 | 28:13.5 | 3 |

| | | | | | | | |
|---|---|---|---|---|---|---|---|
| -252.058 | 19120.067 | 2.775 | 12.032 | 0.101402 | 7/15/2014 | 28:13.6 | 3 |
| -252.314 | 19120.102 | 2.77 | 12.028 | 0.106227 | 7/15/2014 | 28:13.7 | 3 |
| -252.419 | 19120.115 | 2.769 | 12.029 | 0.103404 | 7/15/2014 | 28:13.7 | 3 |
| -252.624 | 19120.144 | 2.766 | 12.024 | 0.110974 | 7/15/2014 | 28:13.8 | 3 |
| -252.828 | 19120.171 | 2.764 | 12.022 | 0.101022 | 7/15/2014 | 28:13.9 | 3 |
| -253.032 | 19120.197 | 2.762 | 12.023 | 0.100862 | 7/15/2014 | 28:14.0 | 3 |
| -253.237 | 19120.225 | 2.76 | 12.025 | 0.106201 | 7/15/2014 | 28:14.1 | 3 |
| -253.438 | 19120.252 | 2.758 | 12.032 | 0.10422 | 7/15/2014 | 28:14.2 | 3 |
| -253.639 | 19120.278 | 2.758 | 12.033 | 0.111803 | 7/15/2014 | 28:14.3 | 3 |
| -253.99 | 19120.326 | 2.754 | 12.037 | 0.109772 | 7/15/2014 | 28:14.5 | 3 |
| -254.191 | 19120.354 | 2.753 | 12.043 | 0.113247 | 7/15/2014 | 28:14.6 | 3 |
| -254.441 | 19120.386 | 2.748 | 12.052 | 0.118073 | 7/15/2014 | 28:14.7 | 3 |
| -254.543 | 19120.398 | 2.748 | 12.057 | 0.121553 | 7/15/2014 | 28:14.7 | 3 |
| -254.747 | 19120.427 | 2.746 | 12.062 | 0.11455 | 7/15/2014 | 28:14.8 | 3 |
| -254.95 | 19120.454 | 2.741 | 12.066 | 0.148101 | 7/15/2014 | 28:14.9 | 3 |
| -255.157 | 19120.482 | 2.739 | 12.063 | 0.124573 | 7/15/2014 | 28:15.0 | 3 |
| -255.361 | 19120.51 | 2.731 | 12.067 | 0.127338 | 7/15/2014 | 28:15.1 | 3 |
| -255.566 | 19120.537 | 2.729 | 12.068 | 0.132439 | 7/15/2014 | 28:15.2 | 3 |
| -255.771 | 19120.564 | 2.727 | 12.066 | 0.121876 | 7/15/2014 | 28:15.3 | 3 |
| -256.128 | 19120.611 | 2.727 | 12.067 | 0.125725 | 7/15/2014 | 28:15.5 | 3 |
| -256.333 | 19120.64 | 2.73 | 12.064 | 0.13822 | 7/15/2014 | 28:15.6 | 3 |
| -256.588 | 19120.675 | 2.73 | 12.065 | 0.127374 | 7/15/2014 | 28:15.7 | 3 |
| -256.693 | 19120.689 | 2.733 | 12.064 | 0.132781 | 7/15/2014 | 28:15.7 | 3 |
| -256.897 | 19120.717 | 2.733 | 12.065 | 0.137717 | 7/15/2014 | 28:15.8 | 3 |
| -257.103 | 19120.742 | 2.733 | 12.07 | 0.137695 | 7/15/2014 | 28:15.9 | 3 |
| -257.31 | 19120.771 | 2.733 | 12.072 | 0.140961 | 7/15/2014 | 28:16.0 | 3 |
| -257.517 | 19120.798 | 2.73 | 12.079 | 0.146061 | 7/15/2014 | 28:16.1 | 3 |
| -257.721 | 19120.825 | 2.725 | 12.081 | 0.146015 | 7/15/2014 | 28:16.2 | 3 |
| -257.927 | 19120.852 | 2.722 | 12.079 | 0.12993 | 7/15/2014 | 28:16.3 | 3 |
| -258.283 | 19120.9 | 2.717 | 12.073 | 0.135558 | 7/15/2014 | 28:16.5 | 3 |
| -258.485 | 19120.928 | 2.712 | 12.068 | 0.133732 | 7/15/2014 | 28:16.6 | 3 |
| -258.738 | 19120.959 | 2.707 | 12.061 | 0.127762 | 7/15/2014 | 28:16.7 | 3 |
| -258.84 | 19120.974 | 2.708 | 12.057 | 0.126561 | 7/15/2014 | 28:16.7 | 3 |
| -259.046 | 19121.001 | 2.707 | 12.047 | 0.125821 | 7/15/2014 | 28:16.8 | 3 |
| -259.25 | 19121.028 | 2.707 | 12.036 | 0.127132 | 7/15/2014 | 28:16.9 | 3 |
| -259.455 | 19121.055 | 2.707 | 12.026 | 0.132128 | 7/15/2014 | 28:17.0 | 3 |
| -259.66 | 19121.083 | 2.707 | 12.017 | 0.130533 | 7/15/2014 | 28:17.1 | 3 |
| -259.864 | 19121.108 | 2.705 | 12.012 | 0.129395 | 7/15/2014 | 28:17.2 | 3 |
| -260.066 | 19121.135 | 2.701 | 12.002 | 0.130013 | 7/15/2014 | 28:17.3 | 3 |
| -260.421 | 19121.181 | 2.697 | 12.004 | 0.145608 | 7/15/2014 | 28:17.5 | 3 |
| -260.626 | 19121.208 | 2.697 | 12.001 | 0.14044 | 7/15/2014 | 28:17.6 | 3 |
| -260.878 | 19121.24 | 2.69 | 12.001 | 0.165247 | 7/15/2014 | 28:17.7 | 3 |
| -260.981 | 19121.254 | 2.688 | 12 | 0.171701 | 7/15/2014 | 28:17.7 | 3 |
| -261.188 | 19121.281 | 2.688 | 12.004 | 0.146628 | 7/15/2014 | 28:17.8 | 3 |
| -261.394 | 19121.308 | 2.686 | 12.016 | 0.152667 | 7/15/2014 | 28:17.9 | 3 |
| -261.6 | 19121.334 | 2.686 | 12.02 | 0.16465 | 7/15/2014 | 28:18.0 | 3 |
| -261.808 | 19121.36 | 2.683 | 12.026 | 0.158249 | 7/15/2014 | 28:18.1 | 3 |

| | | | | | | | |
|---|---|---|---|---|---|---|---|
| -262.015 | 19121.387 | 2.683 | 12.034 | 0.155047 | 7/15/2014 | 28:18.2 | 3 |
| -262.223 | 19121.413 | 2.687 | 12.032 | 0.161197 | 7/15/2014 | 28:18.3 | 3 |
| -262.582 | 19121.458 | 2.681 | 12.036 | 0.143042 | 7/15/2014 | 28:18.5 | 3 |
| -262.793 | 19121.484 | 2.684 | 12.036 | 0.163387 | 7/15/2014 | 28:18.6 | 3 |
| -263.051 | 19121.518 | 2.679 | 12.042 | 0.156055 | 7/15/2014 | 28:18.7 | 3 |
| -263.156 | 19121.531 | 2.678 | 12.036 | 0.150508 | 7/15/2014 | 28:18.7 | 3 |
| -263.366 | 19121.558 | 2.676 | 12.037 | 0.142238 | 7/15/2014 | 28:18.8 | 3 |
| -263.578 | 19121.584 | 2.676 | 12.038 | 0.159431 | 7/15/2014 | 28:18.9 | 3 |
| -263.792 | 19121.609 | 2.674 | 12.037 | 0.137117 | 7/15/2014 | 28:19.0 | 3 |
| -264.001 | 19121.637 | 2.671 | 12.039 | 0.15267 | 7/15/2014 | 28:19.1 | 3 |
| -264.214 | 19121.663 | 2.672 | 12.044 | 0.142171 | 7/15/2014 | 28:19.2 | 3 |
| -264.425 | 19121.688 | 2.672 | 12.049 | 0.140925 | 7/15/2014 | 28:19.3 | 3 |
| -264.686 | 19121.721 | 2.669 | 12.058 | 0.140154 | 7/15/2014 | 28:19.4 | 3 |
| -265.004 | 19121.76 | 2.67 | 12.061 | 0.144829 | 7/15/2014 | 28:19.6 | 3 |
| -265.262 | 19121.79 | 2.668 | 12.067 | 0.132531 | 7/15/2014 | 28:19.7 | 3 |
| -265.37 | 19121.803 | 2.669 | 12.07 | 0.143843 | 7/15/2014 | 28:19.7 | 3 |
| -265.581 | 19121.83 | 2.669 | 12.075 | 0.124076 | 7/15/2014 | 28:19.8 | 3 |
| -265.792 | 19121.855 | 2.669 | 12.081 | 0.141612 | 7/15/2014 | 28:19.9 | 3 |
| -266.001 | 19121.88 | 2.665 | 12.086 | 0.146207 | 7/15/2014 | 28:20.0 | 3 |
| -266.211 | 19121.906 | 2.665 | 12.092 | 0.144695 | 7/15/2014 | 28:20.1 | 3 |
| -266.422 | 19121.931 | 2.671 | 12.098 | 0.141017 | 7/15/2014 | 28:20.2 | 3 |
| -266.63 | 19121.956 | 2.671 | 12.102 | 0.153482 | 7/15/2014 | 28:20.3 | 3 |
| -266.99 | 19121.998 | 2.669 | 12.105 | 0.141764 | 7/15/2014 | 28:20.5 | 3 |
| -267.197 | 19122.023 | 2.669 | 12.109 | 0.146779 | 7/15/2014 | 28:20.6 | 3 |
| -267.452 | 19122.053 | 2.667 | 12.106 | 0.13793 | 7/15/2014 | 28:20.7 | 3 |
| -267.556 | 19122.065 | 2.668 | 12.107 | 0.133631 | 7/15/2014 | 28:20.7 | 3 |
| -267.763 | 19122.09 | 2.668 | 12.104 | 0.123635 | 7/15/2014 | 28:20.8 | 3 |
| -267.972 | 19122.113 | 2.668 | 12.092 | 0.128728 | 7/15/2014 | 28:20.9 | 3 |
| -268.179 | 19122.138 | 2.665 | 12.073 | 0.114243 | 7/15/2014 | 28:21.0 | 3 |
| -268.386 | 19122.162 | 2.665 | 12.057 | 0.128199 | 7/15/2014 | 28:21.1 | 3 |
| -268.592 | 19122.188 | 2.668 | 12.037 | 0.11906 | 7/15/2014 | 28:21.2 | 3 |
| -268.796 | 19122.211 | 2.668 | 12.011 | 0.123489 | 7/15/2014 | 28:21.3 | 3 |
| -269.154 | 19122.25 | 2.673 | 11.976 | 0.126264 | 7/15/2014 | 28:21.5 | 3 |
| -269.36 | 19122.273 | 2.678 | 11.956 | 0.139229 | 7/15/2014 | 28:21.6 | 3 |
| -269.614 | 19122.303 | 2.68 | 11.931 | 0.147818 | 7/15/2014 | 28:21.7 | 3 |
| -269.718 | 19122.314 | 2.682 | 11.921 | 0.156715 | 7/15/2014 | 28:21.7 | 3 |
| -269.923 | 19122.338 | 2.681 | 11.909 | 0.158642 | 7/15/2014 | 28:21.8 | 3 |
| -270.132 | 19122.361 | 2.681 | 11.896 | 0.161835 | 7/15/2014 | 28:21.9 | 3 |
| -270.34 | 19122.385 | 2.681 | 11.888 | 0.163721 | 7/15/2014 | 28:22.0 | 3 |
| -270.549 | 19122.406 | 2.687 | 11.882 | 0.162696 | 7/15/2014 | 28:22.1 | 3 |
| -270.755 | 19122.43 | 2.687 | 11.876 | 0.159285 | 7/15/2014 | 28:22.2 | 3 |
| -270.962 | 19122.452 | 2.687 | 11.871 | 0.149707 | 7/15/2014 | 28:22.3 | 3 |
| -271.321 | 19122.492 | 2.685 | 11.868 | 0.147584 | 7/15/2014 | 28:22.5 | 3 |
| -271.529 | 19122.515 | 2.688 | 11.869 | 0.146154 | 7/15/2014 | 28:22.6 | 3 |
| -271.788 | 19122.541 | 2.694 | 11.87 | 0.151893 | 7/15/2014 | 28:22.7 | 3 |
| -271.894 | 19122.553 | 2.698 | 11.87 | 0.151799 | 7/15/2014 | 28:22.7 | 3 |
| -272.103 | 19122.574 | 2.701 | 11.868 | 0.163411 | 7/15/2014 | 28:22.8 | 3 |

| | | | | | | | |
|---|---|---|---|---|---|---|---|
| -272.312 | 19122.598 | 2.698 | 11.876 | 0.149937 | 7/15/2014 | 28:22.9 | 3 |
| -272.522 | 19122.618 | 2.701 | 11.88 | 0.167937 | 7/15/2014 | 28:23.0 | 3 |
| -272.734 | 19122.642 | 2.708 | 11.883 | 0.163722 | 7/15/2014 | 28:23.1 | 3 |
| -272.943 | 19122.662 | 2.713 | 11.887 | 0.165509 | 7/15/2014 | 28:23.2 | 3 |
| -273.153 | 19122.684 | 2.72 | 11.891 | 0.168051 | 7/15/2014 | 28:23.3 | 3 |
| -273.517 | 19122.721 | 2.725 | 11.899 | 0.161277 | 7/15/2014 | 28:23.5 | 3 |
| -273.726 | 19122.741 | 2.729 | 11.905 | 0.138847 | 7/15/2014 | 28:23.6 | 3 |
| -273.982 | 19122.768 | 2.729 | 11.912 | 0.13966 | 7/15/2014 | 28:23.7 | 3 |
| -274.089 | 19122.777 | 2.736 | 11.91 | 0.129334 | 7/15/2014 | 28:23.7 | 3 |
| -274.3 | 19122.799 | 2.738 | 11.914 | 0.121332 | 7/15/2014 | 28:23.8 | 3 |
| -274.51 | 19122.818 | 2.738 | 11.923 | 0.11343 | 7/15/2014 | 28:23.9 | 3 |
| -274.719 | 19122.839 | 2.738 | 11.929 | 0.115447 | 7/15/2014 | 28:24.0 | 3 |
| -274.93 | 19122.859 | 2.738 | 11.934 | 0.118656 | 7/15/2014 | 28:24.1 | 3 |
| -275.138 | 19122.879 | 2.738 | 11.939 | 0.121842 | 7/15/2014 | 28:24.2 | 3 |
| -275.347 | 19122.899 | 2.74 | 11.943 | 0.134213 | 7/15/2014 | 28:24.3 | 3 |
| -275.709 | 19122.932 | 2.738 | 11.943 | 0.139568 | 7/15/2014 | 28:24.5 | 3 |
| -275.919 | 19122.951 | 2.742 | 11.945 | 0.139406 | 7/15/2014 | 28:24.6 | 3 |
| -276.177 | 19122.975 | 2.742 | 11.943 | 0.123225 | 7/15/2014 | 28:24.7 | 3 |
| -276.282 | 19122.984 | 2.744 | 11.94 | 0.115064 | 7/15/2014 | 28:24.7 | 3 |
| -276.492 | 19123.002 | 2.746 | 11.932 | 0.11983 | 7/15/2014 | 28:24.8 | 3 |
| -276.702 | 19123.021 | 2.748 | 11.923 | 0.110184 | 7/15/2014 | 28:24.9 | 3 |
| -276.914 | 19123.039 | 2.75 | 11.91 | 0.104848 | 7/15/2014 | 28:25.0 | 3 |
| -277.126 | 19123.058 | 2.753 | 11.894 | 0.110718 | 7/15/2014 | 28:25.1 | 3 |
| -277.336 | 19123.075 | 2.753 | 11.873 | 0.113825 | 7/15/2014 | 28:25.2 | 3 |
| -277.547 | 19123.094 | 2.757 | 11.851 | 0.110822 | 7/15/2014 | 28:25.3 | 3 |
| -277.914 | 19123.124 | 2.757 | 11.813 | 0.128116 | 7/15/2014 | 28:25.5 | 3 |
| -278.123 | 19123.141 | 2.762 | 11.79 | 0.12688 | 7/15/2014 | 28:25.6 | 3 |
| -278.384 | 19123.162 | 2.765 | 11.768 | 0.140263 | 7/15/2014 | 28:25.7 | 3 |
| -278.49 | 19123.17 | 2.767 | 11.755 | 0.144626 | 7/15/2014 | 28:25.7 | 3 |
| -278.701 | 19123.188 | 2.77 | 11.738 | 0.127201 | 7/15/2014 | 28:25.8 | 3 |
| -278.913 | 19123.204 | 2.773 | 11.718 | 0.133 | 7/15/2014 | 28:25.9 | 3 |
| -279.124 | 19123.22 | 2.776 | 11.706 | 0.126752 | 7/15/2014 | 28:26.0 | 3 |
| -279.336 | 19123.235 | 2.776 | 11.695 | 0.133361 | 7/15/2014 | 28:26.1 | 3 |
| -279.547 | 19123.252 | 2.781 | 11.686 | 0.13034 | 7/15/2014 | 28:26.2 | 3 |
| -279.755 | 19123.268 | 2.784 | 11.68 | 0.1344 | 7/15/2014 | 28:26.3 | 3 |
| -280.118 | 19123.294 | 2.786 | 11.675 | 0.133734 | 7/15/2014 | 28:26.5 | 3 |
| -280.325 | 19123.309 | 2.79 | 11.677 | 0.145816 | 7/15/2014 | 28:26.6 | 3 |
| -280.581 | 19123.326 | 2.79 | 11.679 | 0.147687 | 7/15/2014 | 28:26.7 | 3 |
| -280.686 | 19123.333 | 2.791 | 11.68 | 0.159964 | 7/15/2014 | 28:26.7 | 3 |
| -280.895 | 19123.349 | 2.795 | 11.686 | 0.154037 | 7/15/2014 | 28:26.8 | 3 |
| -281.104 | 19123.362 | 2.8 | 11.69 | 0.164526 | 7/15/2014 | 28:26.9 | 3 |
| -281.31 | 19123.376 | 2.803 | 11.699 | 0.151699 | 7/15/2014 | 28:27.0 | 3 |
| -281.517 | 19123.39 | 2.803 | 11.704 | 0.144896 | 7/15/2014 | 28:27.1 | 3 |
| -281.723 | 19123.403 | 2.805 | 11.711 | 0.145507 | 7/15/2014 | 28:27.2 | 3 |
| -281.929 | 19123.416 | 2.809 | 11.721 | 0.138029 | 7/15/2014 | 28:27.3 | 3 |
| -282.285 | 19123.438 | 2.808 | 11.73 | 0.11686 | 7/15/2014 | 28:27.5 | 3 |
| -282.489 | 19123.452 | 2.812 | 11.735 | 0.111432 | 7/15/2014 | 28:27.6 | 3 |

| | | | | | | | |
|---|---|---|---|---|---|---|---|
| -282.743 | 19123.467 | 2.808 | 11.74 | 0.105705 | 7/15/2014 | 28:27.7 | 3 |
| -282.846 | 19123.474 | 2.809 | 11.745 | 0.107164 | 7/15/2014 | 28:27.7 | 3 |
| -283.052 | 19123.485 | 2.809 | 11.75 | 0.111024 | 7/15/2014 | 28:27.8 | 3 |
| -283.258 | 19123.498 | 2.811 | 11.761 | 0.0973511 | 7/15/2014 | 28:27.9 | 3 |
| -283.466 | 19123.509 | 2.811 | 11.769 | 0.108656 | 7/15/2014 | 28:28.0 | 3 |
| -283.674 | 19123.52 | 2.809 | 11.775 | 0.0833052 | 7/15/2014 | 28:28.1 | 3 |
| -283.88 | 19123.532 | 2.809 | 11.782 | 0.087056 | 7/15/2014 | 28:28.2 | 3 |
| -284.086 | 19123.544 | 2.811 | 11.792 | 0.0928863 | 7/15/2014 | 28:28.3 | 3 |
| -284.446 | 19123.563 | 2.812 | 11.796 | 0.0919062 | 7/15/2014 | 28:28.5 | 3 |
| -284.653 | 19123.574 | 2.816 | 11.801 | 0.0781449 | 7/15/2014 | 28:28.6 | 3 |
| -284.91 | 19123.588 | 2.814 | 11.803 | 0.0750136 | 7/15/2014 | 28:28.7 | 3 |
| -285.015 | 19123.593 | 2.815 | 11.802 | 0.0707702 | 7/15/2014 | 28:28.7 | 3 |
| -285.224 | 19123.602 | 2.818 | 11.805 | 0.0801065 | 7/15/2014 | 28:28.8 | 3 |
| -285.434 | 19123.612 | 2.821 | 11.804 | 0.0686433 | 7/15/2014 | 28:28.9 | 3 |
| -285.644 | 19123.623 | 2.821 | 11.805 | 0.0638756 | 7/15/2014 | 28:29.0 | 3 |
| -285.854 | 19123.633 | 2.818 | 11.802 | 0.0642196 | 7/15/2014 | 28:29.1 | 3 |
| -286.064 | 19123.643 | 2.818 | 11.801 | 0.0614175 | 7/15/2014 | 28:29.2 | 3 |
| -286.273 | 19123.652 | 2.818 | 11.803 | 0.0662605 | 7/15/2014 | 28:29.3 | 3 |
| -286.638 | 19123.668 | 2.817 | 11.799 | 0.0737429 | 7/15/2014 | 28:29.5 | 3 |
| -286.846 | 19123.676 | 2.817 | 11.8 | 0.0770055 | 7/15/2014 | 28:29.6 | 3 |
| -287.107 | 19123.688 | 2.812 | 11.801 | 0.0830328 | 7/15/2014 | 28:29.7 | 3 |
| -287.213 | 19123.693 | 2.811 | 11.804 | 0.0911098 | 7/15/2014 | 28:29.7 | 3 |
| -287.425 | 19123.7 | 2.811 | 11.804 | 0.0743071 | 7/15/2014 | 28:29.8 | 3 |
| -287.637 | 19123.709 | 2.811 | 11.806 | 0.0721152 | 7/15/2014 | 28:29.9 | 3 |
| -287.851 | 19123.719 | 2.814 | 11.807 | 0.0778239 | 7/15/2014 | 28:30.0 | 3 |
| -288.064 | 19123.727 | 2.812 | 11.809 | 0.0639966 | 7/15/2014 | 28:30.1 | 3 |
| -288.277 | 19123.734 | 2.812 | 11.806 | 0.0703836 | 7/15/2014 | 28:30.2 | 3 |
| -288.489 | 19123.741 | 2.812 | 11.803 | 0.0701135 | 7/15/2014 | 28:30.3 | 3 |
| -288.753 | 19123.752 | 2.814 | 11.796 | 0.0643368 | 7/15/2014 | 28:30.4 | 3 |
| -288.964 | 19123.759 | 2.812 | 11.788 | 0.0672055 | 7/15/2014 | 28:30.5 | 3 |
| -289.177 | 19123.766 | 2.81 | 11.778 | 0.069431 | 7/15/2014 | 28:30.6 | 3 |
| -289.444 | 19123.776 | 2.81 | 11.769 | 0.0649892 | 7/15/2014 | 28:30.7 | 3 |
| -289.657 | 19123.783 | 2.805 | 11.757 | 0.0671011 | 7/15/2014 | 28:30.8 | 3 |
| -289.872 | 19123.791 | 2.801 | 11.747 | 0.0638399 | 7/15/2014 | 28:30.9 | 3 |
| -290.087 | 19123.797 | 2.799 | 11.737 | 0.0680664 | 7/15/2014 | 28:31.0 | 3 |
| -290.301 | 19123.803 | 2.797 | 11.73 | 0.0638755 | 7/15/2014 | 28:31.1 | 3 |
| -290.513 | 19123.811 | 2.793 | 11.727 | 0.0669055 | 7/15/2014 | 28:31.2 | 3 |
| -290.727 | 19123.816 | 2.793 | 11.725 | 0.0589868 | 7/15/2014 | 28:31.3 | 3 |
| -291.096 | 19123.828 | 2.792 | 11.728 | 0.0594523 | 7/15/2014 | 28:31.5 | 3 |
| -291.307 | 19123.834 | 2.792 | 11.725 | 0.0559719 | 7/15/2014 | 28:31.6 | 3 |
| -291.568 | 19123.842 | 2.787 | 11.729 | 0.0575343 | 7/15/2014 | 28:31.7 | 3 |
| -291.676 | 19123.845 | 2.789 | 11.729 | 0.0546791 | 7/15/2014 | 28:31.7 | 3 |
| -291.887 | 19123.85 | 2.789 | 11.739 | 0.0708667 | 7/15/2014 | 28:31.8 | 3 |
| -292.097 | 19123.857 | 2.785 | 11.753 | 0.049596 | 7/15/2014 | 28:31.9 | 3 |
| -292.31 | 19123.861 | 2.788 | 11.767 | 0.0541959 | 7/15/2014 | 28:32.0 | 3 |
| -292.522 | 19123.869 | 2.784 | 11.776 | 0.0476835 | 7/15/2014 | 28:32.1 | 3 |
| -292.731 | 19123.874 | 2.782 | 11.787 | 0.0614211 | 7/15/2014 | 28:32.2 | 3 |

| | | | | | | | |
|---|---|---|---|---|---|---|---|
| -292.941 | 19123.879 | 2.779 | 11.8 | 0.0547499 | 7/15/2014 | 28:32.3 | 3 |
| -293.304 | 19123.889 | 2.772 | 11.816 | 0.0423792 | 7/15/2014 | 28:32.5 | 3 |
| -293.514 | 19123.893 | 2.774 | 11.828 | 0.0374241 | 7/15/2014 | 28:32.6 | 3 |
| -293.772 | 19123.9 | 2.769 | 11.837 | 0.038381 | 7/15/2014 | 28:32.7 | 3 |
| -293.878 | 19123.902 | 2.771 | 11.838 | 0.0427499 | 7/15/2014 | 28:32.7 | 3 |
| -294.088 | 19123.908 | 2.767 | 11.844 | 0.0352563 | 7/15/2014 | 28:32.8 | 3 |
| -294.297 | 19123.912 | 2.767 | 11.853 | 0.0376587 | 7/15/2014 | 28:32.9 | 3 |
| -294.509 | 19123.918 | 2.77 | 11.859 | 0.0464093 | 7/15/2014 | 28:33.0 | 3 |
| -294.719 | 19123.923 | 2.77 | 11.859 | 0.0476021 | 7/15/2014 | 28:33.1 | 3 |
| -294.929 | 19123.928 | 2.773 | 11.861 | 0.0386778 | 7/15/2014 | 28:33.2 | 3 |
| -295.139 | 19123.932 | 2.776 | 11.865 | 0.0466186 | 7/15/2014 | 28:33.3 | 3 |
| -295.503 | 19123.94 | 2.772 | 11.874 | 0.0555282 | 7/15/2014 | 28:33.5 | 3 |
| -295.713 | 19123.944 | 2.777 | 11.876 | 0.0898952 | 7/15/2014 | 28:33.6 | 3 |
| -295.972 | 19123.949 | 2.773 | 11.877 | 0.0621913 | 7/15/2014 | 28:33.7 | 3 |
| -296.079 | 19123.953 | 2.775 | 11.878 | 0.0941382 | 7/15/2014 | 28:33.7 | 3 |
| -296.288 | 19123.958 | 2.769 | 11.879 | 0.0683624 | 7/15/2014 | 28:33.8 | 3 |
| -296.5 | 19123.962 | 2.769 | 11.88 | 0.0711144 | 7/15/2014 | 28:33.9 | 3 |
| -296.712 | 19123.966 | 2.766 | 11.878 | 0.078978 | 7/15/2014 | 28:34.0 | 3 |
| -296.924 | 19123.972 | 2.763 | 11.876 | 0.071474 | 7/15/2014 | 28:34.1 | 3 |
| -297.136 | 19123.976 | 2.765 | 11.87 | 0.0634477 | 7/15/2014 | 28:34.2 | 3 |
| -297.347 | 19123.979 | 2.762 | 11.862 | 0.0602301 | 7/15/2014 | 28:34.3 | 3 |
| -297.714 | 19123.986 | 2.755 | 11.848 | 0.0720819 | 7/15/2014 | 28:34.5 | 3 |
| -297.924 | 19123.992 | 2.757 | 11.84 | 0.0687315 | 7/15/2014 | 28:34.6 | 3 |
| -298.188 | 19123.997 | 2.755 | 11.83 | 0.0652519 | 7/15/2014 | 28:34.7 | 3 |
| -298.294 | 19123.999 | 2.755 | 11.826 | 0.0645798 | 7/15/2014 | 28:34.7 | 3 |
| -298.507 | 19124.004 | 2.755 | 11.818 | 0.0723273 | 7/15/2014 | 28:34.8 | 3 |
| -298.722 | 19124.009 | 2.755 | 11.812 | 0.0716321 | 7/15/2014 | 28:34.9 | 3 |
| -298.934 | 19124.013 | 2.753 | 11.803 | 0.0667687 | 7/15/2014 | 28:35.0 | 3 |
| -299.148 | 19124.017 | 2.753 | 11.792 | 0.066113 | 7/15/2014 | 28:35.1 | 3 |
| -299.362 | 19124.021 | 2.751 | 11.785 | 0.0641151 | 7/15/2014 | 28:35.2 | 3 |
| -299.573 | 19124.026 | 2.745 | 11.777 | 0.0587801 | 7/15/2014 | 28:35.3 | 3 |
| -299.944 | 19124.034 | 2.741 | 11.764 | 0.062406 | 7/15/2014 | 28:35.5 | 3 |
| -300.157 | 19124.039 | 2.741 | 11.758 | 0.0595525 | 7/15/2014 | 28:35.6 | 3 |
| -300.421 | 19124.046 | 2.737 | 11.75 | 0.0599925 | 7/15/2014 | 28:35.7 | 3 |
| -300.528 | 19124.048 | 2.735 | 11.744 | 0.0591025 | 7/15/2014 | 28:35.7 | 3 |
| -300.742 | 19124.052 | 2.73 | 11.737 | 0.0586358 | 7/15/2014 | 28:35.8 | 3 |
| -300.957 | 19124.057 | 2.728 | 11.737 | 0.0601563 | 7/15/2014 | 28:35.9 | 3 |
| -301.173 | 19124.062 | 2.728 | 11.738 | 0.0658391 | 7/15/2014 | 28:36.0 | 3 |
| -301.389 | 19124.067 | 2.731 | 11.731 | 0.0702644 | 7/15/2014 | 28:36.1 | 3 |
| -301.604 | 19124.071 | 2.731 | 11.728 | 0.0656749 | 7/15/2014 | 28:36.2 | 3 |
| -301.818 | 19124.076 | 2.728 | 11.722 | 0.0655695 | 7/15/2014 | 28:36.3 | 3 |
| -302.084 | 19124.084 | 2.726 | 11.72 | 0.0635237 | 7/15/2014 | 28:36.4 | 3 |
| -302.298 | 19124.088 | 2.723 | 11.726 | 0.0740417 | 7/15/2014 | 28:36.5 | 3 |
| -302.513 | 19124.094 | 2.722 | 11.724 | 0.0649915 | 7/15/2014 | 28:36.6 | 3 |
[truncated: 478,842 more chars]
